# Immune landscape in liver of neonatal mice with phlebotomy-induced anemia

**DOI:** 10.1038/s41390-025-04361-x

**Published:** 2025-09-17

**Authors:** Balamurugan Ramatchandirin, Wenjia Wang, Marie Amalie Balamurugan, Yasemin Alnahhas, Suneetha Desiraju, Arjun Subrramanya, Juanitaa George Raj, Zainab D. Lawal, Megan Ferris, George Tseng, Liza Konnikova, Krishnan MohanKumar

**Affiliations:** 1 Department of Biochemistry and Molecular Biology, University of Nebraska Medical Center, Omaha, NE USA; 2 Department of Pediatrics, Division of Neonatal-Perinatal Medicine, University of Texas Southwestern Medical Center, Dallas, TX USA; 3 Department of Biostatistics, University of Pittsburgh, Pittsburgh, PA USA; 4 Department of Pediatrics, Johns Hopkins University School of Medicine, Baltimore, MD USA; 5 Child Health Research Institute, Omaha, NE USA; 6 Departments of Pediatrics, Immunobiology, and Obstetrics, Gynecology and Reproductive Sciences, Yale University School of Medicine, New Haven, CT USA; 7 Department of Pediatrics, University of Nebraska Medical Center, Omaha, NE USA

## Abstract

**Background:**

Severe anemia is a common comorbidity in preterm infants in the neonatal intensive care unit, which is caused by phlebotomy, low erythropoietin levels, low red blood cell (RBC) lifespan, and exacerbated by the underlying erythropoietic immaturity. Anemia causes tissue hypoxia, which may alter the hematopoiesis niche in the liver. This study utilized our preclinical mouse model of phlebotomy-induced anemia (PIA) to investigate the immune cell atlas in the liver.

**Methods:**

C57BL/6 mice were subjected to timed phlebotomy between postnatal days 2–10 to induce severe anemia. Immune cells in anemic liver were characterized by Single-cell (sc) RNA-sequencing and a flow cytometry approach.

**Results:**

The scRNA-seq analysis revealed that PIA is associated with an altered immune landscape of the neonatal murine liver. We identified increased numbers of Ly6C2^+^monocytes and Gypa^+^erythroid cells and decreased numbers of lymphocytes (CD20^+^ [MS4a1]-B cells and Tcells) in the anemic liver. Further analysis of monocytes revealed a pro-inflammatory and highly chemotactic phenotype, while erythroid cells displayed a downregulation of inflammatory markers and maturational deficits. Lymphocytes (B and T cells) exhibited suppressed lipid metabolism processes, including those of steroids and hormones.

**Conclusion:**

PIA in neonatal mouse pups is associated with myelopoiesis (specifically monopoiesis) and erythropoiesis while suppressing lymphopoiesis in the liver.

**Impact:**

- Anemia is nearly universal in preterm infants and is associated with increased morbidity and mortality worldwide, investigation of immune cell response in settings of preclinical anemia may be an index of therapeutic targets to modulate the response in anemia-related comorbidity.
- Our findings showed that phlebotomy-induced anemia in murine pup alters liver hematopoiesis including myelopoiesis and stressed erythropoiesis with suppressed lymphopoiesis.
- This study sheds light on emergency myelopoiesis, stressed erythropoiesis, and deficiency of lymphocytes in anemic liver, which may provide novel insight into the development of therapeutics to treat anemia in preterm infants and neonates.

## Introduction

Anemia is nearly universal in preterm infants and is associated with increased morbidity and mortality worldwide.^1–10^ Anemia is caused primarily by phlebotomy essential for medical care, and exacerbated by a variety of factors inherent to immaturity in the ex-utero environment (e.g., low erythropoietin levels, high postnatal oxygen tension, and many others).^7,11–15^ Infant hematopoiesis is gradually activated over the first 6 to 8 weeks of life after an initial period of physiological suppression due to relative hyperoxia ex utero. During this period normal red blood cell (RBC) turnover leads to progressive anemia, providing the chemical and hormonal catalysts for hematopoiesis. In preterm infants, phlebotomy losses inherent to intensive care treatments often result in steeper anemia that overwhelms physiologic processes, leading to the need for one or more RBC transfusions.^2,4,7,9,16^

Anemia, known to affect organ function due to inadequate oxygen delivery, can contribute to tissue hypoxia.^17–19^ We and others showed that phlebotomy-induced severe anemia causes a high degree of hypoxia in the developing intestine thus leading to intestinal barrier leakage with the recruitment of circulating monocytes.^5,6^ Singh et al.,^20^ also showed that PIA causes brain tissue hypoxia and iron deficiency in murine neonates, leading to long-term sex-dependent neurobehavioral abnormalities.^20^ Unlike these organs, the liver is the primary site of hematopoiesis in the human fetus/preterm neonate until 22 weeks gestation and during the first 2 postnatal weeks in the mouse.^21–23^ Although it has been shown that hypoxia has a major role in leukocyte development in fetal/neonatal liver, the effect of PIA on immune cell development in the liver of murine neonates is unknown. Thus, we used single-cell (sc) RNA sequencing to investigate whether PIA alters the immune cell development in the neonatal murine liver.

## Methods

### Animals

All experimental procedures were approved in advance by the Institutional Animal Care and Use Committees of Johns Hopkins University (JHU) and University of Nebraska Medical Center (UNMC) and complied with the National Institutes of Health Guide for the Care and Use of Laboratory Animals for testing and research. Neonatal C57BL/6J mice pups (postnatal day [P] 1 to 11) were used for the experiments. Littermates were derived from C57BL/6J breeder mice (JAX no. 000664) that were housed in JHU and UNMC animal facilities under standard conditions.

### Animal model

C57BL/6 mice of both sexes (equal numbers) were randomly assigned to two study groups: (a) naive control, and (b) phlebotomy-induced anemia was performed as described previously.^5,6,24–26^ Briefly, PIA was performed in mouse pups by facial vein phlebotomy to remove 20 µL blood/g body weight on P2, P4, P6, P8, and P10 to reduce the hematocrit (Hct) index from an initial 45–60% range, down to 20–24%. Hematocrits and RBC indices were measured at each phlebotomy; 5 µL of blood was diluted 1:20 in Cellpak reagent (Sysmex America, Mundelein, IL; catalog #DCL-310A) and analyzed in the Sysmex XT-2000iV veterinary hematology analyzer. Non-phlebotomized control animals were handled similarly to phlebotomized animals with a daily non-phlebotomizing needle stick. Pups were weighed daily to determine the volume of blood to be drawn. The animals were euthanized 24 h (P11) later, and the liver tissue was processed for scRNA sequencing.

### Liver tissue dissociation

The mouse liver tissues were surgically removed and first washed with Dulbecco’s phosphate-buffered saline (DPBS) without calcium and magnesium to wash away blood and preservation medium, transferred into a petri dish and diced into small pieces (approximately 1 cm^2^ for resections and 0.25 cm^2^ for biopsies) using a scalpel. The liver tissue was dissociated for single-cell preparation using the mouse liver dissociation kit (Cat #130-105-807, Miltenyi Biotech, Auburn, CA) and gentleMACS Dissociator (Cat #130-093-235, Miltenyi Biotech, Auburn, CA). In detail, the surgically removed liver tissue was distributed evenly into four gentleMacs C Tubes, then each tube received 5 ml of Liberase enzymatic digestion solution (0.2 Wünsch/ml) reconstituted in HepatoZYME media (without growth factors, which may influence the downstream scRNA-seq) containing DNAse I (2000 U/mL). The mixture was warmed to 37 °C for optimal enzymatic activity. The enzymatic digestion occurred in an incubating shaker at 37 °C and 200 RPM for 30 min. The partially degraded extracellular tissue matrix in each C tube was mechanically dissociated by running two “B” cycles in the gentleMACS Dissociator. A 1:1 ratio of 20% FBS (Fetal Bovine Serum) to 80% DPBS was added to neutralize the enzymatic reaction, and the cell suspensions were filtered through 70 μm filters to remove large pieces of debris, gently mashing pieces through the filter. Cells dissociated from liver tissues were purified by centrifuging the cell suspensions at 50 × *g*, 4 °C for 5 min to pelletize the cells; dissociated single cells were then stained for viability assessment using 0.05% trypan blue (Cat #T6146, Millipore Sigma, St. Louis, MA) in PBS on a Cellometer AutoT4 cell counter (Nexcelom Bioscience, Waltham, MA).

#### Single-cell RNA library preparation and sequencing

Single-cell RNA-sequencing was performed on dissociated liver single cells using the Chromium Next GEM Single-Cell 3ʹ Reagent Kits v3.1 (10× Genomics) at the Single Cell Transcriptome core, Johns Hopkins University. Briefly, liver cells (∼20,000 cells per sample) were loaded into the 10× Chromium controller, and downstream scRNA-seq libraries were generated and indexed by following the manufacturer’s instructions. Libraries were pooled and sequenced on an Illumina NovaSeq 6000, targeting 50,000 reads per cell.

#### Analysis of RNA-sequencing data

The 10x library indexing system tags each FASTA sequence read with a barcode that identifies the specific cell being sequenced so that the cells can be compared individually or by biological class. Using 10x software, these sequence reads were aligned to the current mouse transcriptome, and the expression level was calculated for each sequenced cell for each gene transcript and assembled into a matrix. Then, the generated cell-by-gene count matrix was used as the input for downstream analysis. The count matrix was analyzed using the Seurat R package.^27–31^ Cells that had <500 unique molecular identifiers (UMIs) or >50,000 UMIs, along with cells that had >50% mitochondrial content, were filtered out. The single-cell gene expressions were normalized by the global scaling normalization functions of NormalizeData and ScaleData in Seurat, which used total expression level to adjust for the variation of single-cell library sizes scaled and log-transformed the expression measurements. Furthermore, we selected a subset of 2000 highly variable genes by cell-to-cell variation for downstream clustering and visualization analysis.

#### Data integration and clustering

The cell data sets were integrated by a comprehensive integration method^29^ (*IntegrateData* function in Seurat). In this way, batch effects between samples were controlled. The expression measurement of each gene was standardized with a mean of 0 and a standard deviation of 1 across cells, and principal components were then calculated based on the top 2000 variable features. The cells were clustered by a KNN graph-based clustering approach^32^ implemented by the *FindClusters* function on the top 20 principal components. The non-linear dimension reduction method UMAP^33^ was used for visualizing the cells and resulting clusters in 2D space. The clusters were manually annotated with cell types based on the markers (differentially expressed genes) identified by the Wilcoxon rank-sum test between clusters.^34–37^ To further zoom in on the immune cells of our interest, we re-clustered the subset of immune cells, and again manually annotated the sub-clusters based on the markers identified by the Wilcoxon rank-sum test.

#### Differential expression analysis

To investigate the gene expression differences within each immune cell type between naive control mice and mice with phlebotomy-induced anemia, we used pseudo-bulk aggregation of gene expression profiles. Many have shown that pseudo-bulk-based case-control differential expression analyses robustly detect gene-level differences with lower false discovery due to repeated measures from single cells of the same individual.^38–40^ The raw UMI (unique molecular identifiers) counts were added together from the same individual sample and cell type to create the pseudo bulk profiles, resulting in 13,534 genes and 96 (8 cell types × 12 individual samples) pseudobulk profiles after filtering out mitochondrial, ribosomal, and low-expressing genes with less than 5 average UMI counts. We then applied the voom-limma method^41^ to the pseudobulk profiles for differential gene expression analyses between the control group and the anemia group for each cell type through a linear regression model incorporating the interaction effect between sample groups and cell types. In addition to calculating p-values of group effects for all genes, we used the swfdr R package^42^ to compute the FDR correction within each cell type comparison and between all cell type comparisons. Considering the multiple axes of meaningful biological variation to be addressed, we estimated quality weights for adjusting for cell type proportions with the function *voomWithQualityWeights* in the limma R package.^43^ We used the normalized counts per million (CPM) of each pseudobulk profile corrected for the batch effects. For heatmap visualizations, we also normalized the corrected expression profile of each gene grouped by cell type, since gene expression is highly cell-type specific. Volcano plots were generated by the EnhancedVolcano R package^44^ to visualize the differentially expressed genes whose absolute value of log fold change (logFC) is larger than 2 and whose adjusted *p*-value within cell type is smaller than 0.05. The genes with *p*-values smaller than 0.01 are also shown in heatmaps for each cell type using the *heatmap.2* function in the gplots R package.^45^

#### Gene set enrichment analysis

We identified pathways that are differentially altered between the control group and the anemia group for each cell type using gene set enrichment analyses (GSEA)^46^ with the Gene Ontology (GO) database^47^ including gene sets of three orthogonal ontologies, i.e., molecular function (MF), biological process (BP), and cellular component (CC). Ranking all genes based on their phenotypes, GSEA aggregates the per-gene statistics across genes within a gene set and determines whether the members of the gene set are randomly distributed throughout the ranked gene list or primarily found at the top or bottom. We implemented GSEA using gene ontology by the function *gseGO* in clusterProfiler R package^48^ and visualized the top 20 significantly enriched/activated pathways and suppressed pathways in each cell type (ranked in adjusted p-values by the Benjamini-Hochberg procedure), using the ggplot2 R package.^49^ The running enrichment scores of some interesting pathways were illustrated using the function *gseaplot* in enrichplot R package^50^ to visualize the distribution of the gene sets.

### Flow cytometry

For the validation of scRNA sequence analysis findings in anemic liver, we used a multicolor flow cytometry approach as previously described.^6,26^ Briefly, dissociated cells from each liver were pelleted (300 × *g*, 5 min, 4°C) and resuspended in 0.5 ml of ice-cold cell staining buffer (BioLegend; Cat No. 420201). Cells were incubated with 100 μl of FcR blocking reagent (Miltenyi Biotec no. 130-092-575) for 10 min at 4 °C and then labeled with each of the following flow antibodies with L-D viability dye (Thermo Fisher; catalog #L34957): CD45 (dilution 1:25; clone #30-F11), CD11b (dilution 1:25; clone #M1/70), Ly6C (dilution 1:25; clone #HK1.4), F4/80 (dilution 1:25; clone #BM8), Ly6G (dilution 1:25; clone #1A8), CD11c (dilution 1:25; clone #N418), CD3 (dilution 1:25; clone #17A2), CD20 (dilution 1:25; clone 2H7) and CD49B (dilution 1:25; clone #HMα2; all antibodies rom BioLegend, San Diego, CA). Data was acquired on a BD LSR-II flow cytometer and analyzed using the software package FlowJo version 10.5.3 (Becton Dickinson, Franklin Lakes, NJ).

#### Statistical analysis

The comparisons are based on the Student *t-*test and nonparametric test (i.e., Mann-Whitney *t* test) with *p*-values less than 0.05 considered to be significant. Statistical analysis was performed using GraphPad Prism 10 software. (GraphPad, La Jolla, CA).

## Results

### Immune cell identification in murine liver

**Fig. 1:**
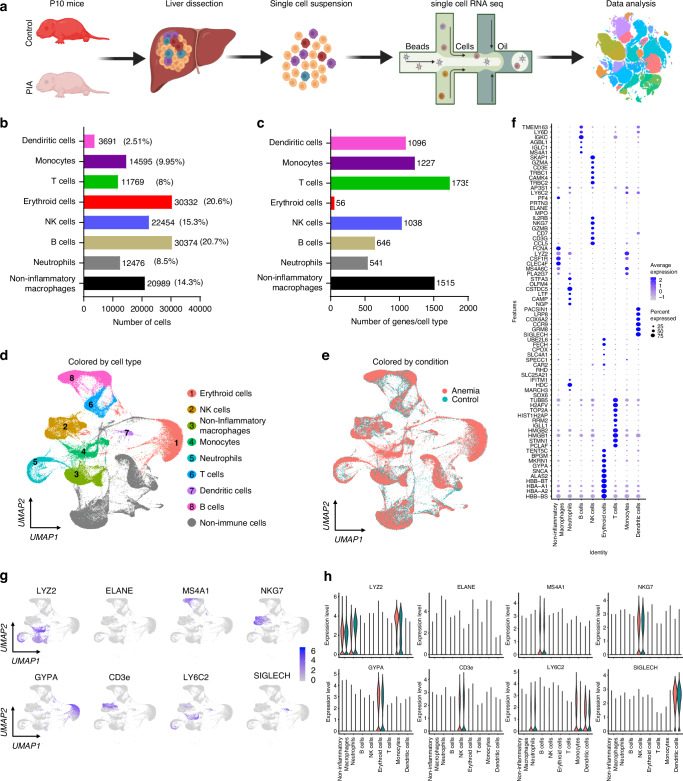
Immune cell identification in murine liver. **a** Workflow of liver analysis from postnatal day (P)10 of control and anemic groups. **b** Bar plot showing the total number of detected immune cells and **c** total number of detected genes per cell type. **d** UMAP visualization identified eight major clusters of immune cells (1-Erythroid cells, 2-NK cells, 3-non-inflammatory macrophages, 4-monocytes, 5-neutrophils, 6-T-cells, 7-dendritic cells, and 8-B cells). Cell clusters were color-coded and annotated post hoc based on their transcriptional profile identities. **e** UMAP visualization of identified immune cell clusters in conditions of control and anemia. **f** Plot showing the identification of marker genes for each cell cluster, with the size of the dot corresponding to the percentage of cells within the cell population expressing the gene. The brightness of the color represents the average expression level across all cells within the cluster. **g** Feature plots showing the expression of the key marker genes of the eight immune cell types. **h** Violin plots depicting expression levels of key marker genes of each cluster in eight types of immune cells.

To investigate the effect of PIA on neonatal murine liver immune cell regulation, we performed scRNA-seq to profile the reprogramming of the liver immune cells from control and anemic mice (*n* = 6 each/group). Individual cells from the liver tissue were isolated and a cDNA library was built and sequenced by 10X Genomics (Fig. 1a). After integration and filtering of low-quality data (Fig. S1), we obtained 146,680 immune cells from the liver of control and anemic mice. The aggregated 146,680 cells comprised non-inflammatory macrophages (14.3%), neutrophils (8.5%), B cells (20.7%) NK Cells (15.3%), erythroid cells (20.6%), T cells (8%), monocytes (9.95%) and dendritic cells (2.51%) (Fig. 1b, c), based on the marker genes of cell populations. We used UMAP to visualize the overall cell clusters. We observed eight immune cell clusters based on the expression of specific markers and the grey-colored non-immune cells that were excluded from this study (Fig. 1d) and showed excellent integration of the two different conditions (control and anemia, Fig. 1e). The cell types were annotated based on the top expression of specific markers (Fig. 1f and Table S1) with distinct expression profiles: non-inflammatory macrophages (Lyz2), neutrophils (Elane), B cells (MS4a1), NK cells (NKG7), erythroid cells (Gypa), T cells (CD3E), monocytes (Ly6C2) and dendritic cells (Siglech) were identified (Fig. 1g, h).

### PIA alters the immune cell profile of the murine liver

**Fig. 2:**
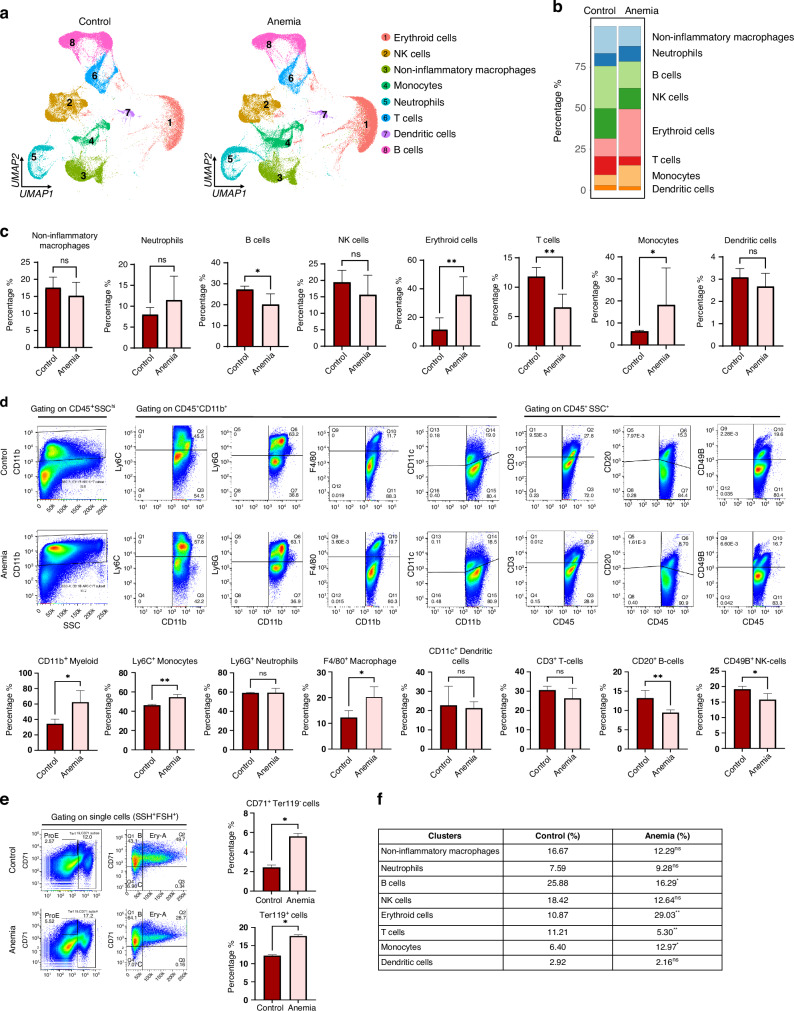
PIA alters the immune cell atlas in the liver. **a** UMAP visualization of the comparison of immune cell clusters between control and anemic liver. **b** The median percentage of 8 immune cell clusters is color-coded to correspond with cells derived from the control and anemic liver. **c** Bar diagrams (mean ± SE) show the percentage of each identified immune cluster in control and anemic liver, *n* = 6 mice/per group; Mann-Whitney multiple comparison test; *p < 0.05, **p < 0.01, and ***p < 0.001 *vs* anemia. **d** *top:* Representative flow cytometry scatter plots from the liver of control anemic mice. The CD45^+^/SSC^hi^ cells were gated from single cells, and then the CD11b^+^ myeloid cell population was further gated from CD45^+^/SSC^hi^ cells to identify the myeloid cell populations of Ly6C-monocytes, Ly6G-neutrophils, F4/80-macrophages, and CD11c-dendritic cells with CD11b. The B cells, T cells, and NK cells were gated on CD45^+^/SSC^hi^ cells using CD3, CD20, and CD49B, respectively. *Below:* Bar diagrams (mean ± SE) represent the percentage of each flow scatter plot based on the surface marker expression (details in methods), *n* = 6 mice/per group; Mann-Whitney multiple comparison test; *p < 0.05 and **p < 0.01 *vs* anemia. **e** Flow cytometry scatter *p*lots show the liver single-cell population from control and anemic groups gated against CD71 and Ter119 for the discretion of mature and immature erythroid cells, proE-proerythroblasts (CD71^high^Ter119^intermediate^). Further analysis of Ter119^high^ cells with gating of CD71 *vs* FSC shows CD71high cells are subdivided into less mature, large ‘EryA’ erythroblasts (CD71^high^Ter119^high^FSC^high^) and smaller, more mature ‘EryB’ erythroblasts (CD71^high^Ter119^high^FSC^low^). into different stages of erythroid cells and the mature erythroblast subset is ‘EryC’ (CD71^low^Ter119^high^FSC^low^). Bar diagrams (mean ± SE) represent the percentage of CD71^+^Ter119^−^ and CD71^+^Ter119^+^ erythroid cells in control and anemic liver, *n*^=^6 mice/per group; Mann-Whitney multiple comparison test; *p < 0.05 *vs* anemia. **f** The table summarizes the total percentage of cells identified in the liver of control and anemic groups by scRNA analysis *n* = 6 mice/per group; Mann-Whitney multiple comparison test; *p < 0.05 and **p < 0.01 *vs* anemia.

To differentiate the cellular identity in the anemic liver from that of control, the UMAP was split by condition (Fig. 2a). The monocytes and erythroid cells were significantly enriched in the anemic liver compared with control mice, and both lymphocyte populations (B cells and T cells) were markedly decreased in the anemic liver (Fig. 2b and 2c). In addition, there weren’t any significant changes in the percentage of non-inflammatory macrophages (Lyz2), neutrophils (Elane), NK cells (NKg7), and dendritic cells (Siglech) in the anemic liver relative to control (*n* = 6 each per group) (Fig. 2c). The identified individual percentages of cell clusters are listed in Fig. 2f.

To validate scRNAseq findings, we performed flow cytometry analysis (Fig. 2d) on a single-cell suspension from control or anemic livers. Consistent with the scRNAseq data, the leukocyte population in the anemic liver was enriched for CD11b^+^ myeloid cells, compared with the control liver. Further analysis of the CD45^+^CD11b^+^ cell population showed an increased proportion of Ly6C-monocytes and F4/80-macrophages with minimal change in Ly6G-neutrophils and CD11c-dendritic cells, consistent with the scRNAseq data (Fig. 2a, b). Similarly, CD20-B cells significantly decreased in anemic liver versus control, mirroring the scRNA-seq results (Fig. 2a, b). However, the flow data did not demonstrate a change in T cell numbers between the conditions. CD49b^+^ -NK cell counts decreased in anemic liver versus control, contrasting with the scRNA-seq results (Fig. 2a, b). Flow analysis of erythroid cells using CD71 and TER119 to determine the different stages of erythroid cell development showed that the frequencies (%) of CD71^+^ erythroid cells (ProErythroblast [Pro-E]) were significantly increased in the anemic liver. Flow gating with TER119 indicates the greater presence of immature erythrocytes than mature RBCs, showing that neonatal anemic hypoxia may trigger stressed erythropoiesis with a maturation deficiency (Fig. 2e). Additional analysis of TER119^+^ cells with CD71 *vs* FSC showed decreased Ery-A^+^ cells and increased Ery-B^+^ cells with no changes in Ery-C^+^ cells.

These results indicate that only the monocyte, erythroid cell, and B cell populations were significantly altered during anemia in the liver by both the scRNA-seq study and flow cytometry, whereas T cell populations were altered only by scRNA-seq. Therefore, we further analyzed these monocytes, erythroid cells, B cells, and T cells for their gene expression and function in this study.

### PIA increases monocytes that are pro-inflammatory with chemotactic in neonatal murine liver

**Fig. 3:**
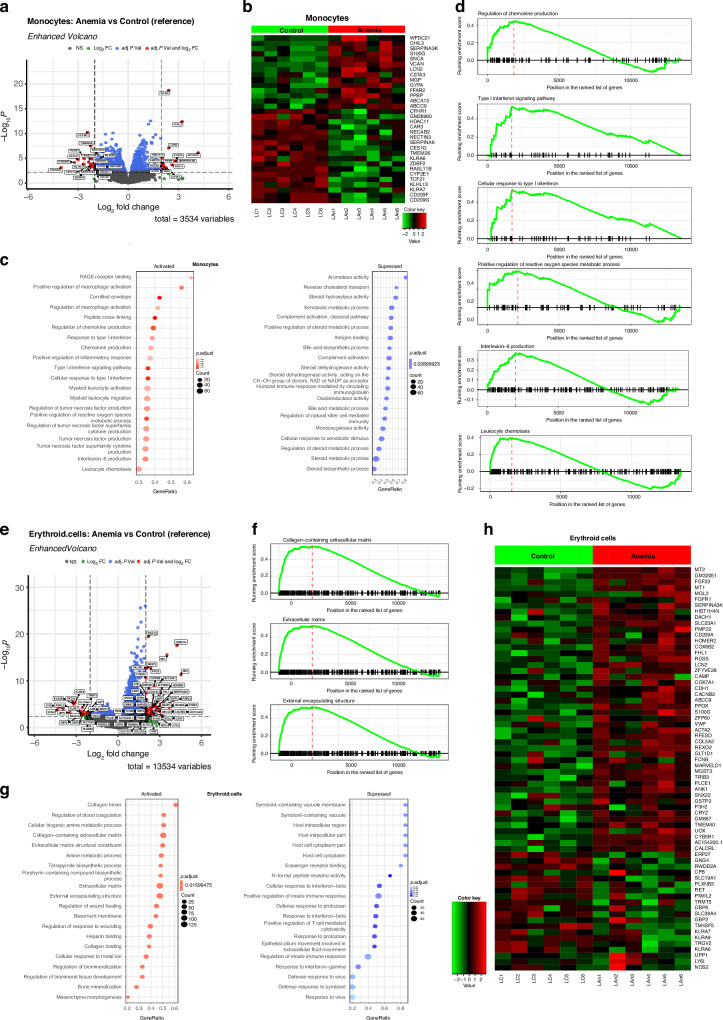
PIA increases monocytes that are pro-inflammatory with chemotactic and causes rapid erythropoiesis in neonatal murine liver. **a** Volcano plot showing differentially expressed genes (DEGs) of monocyte clusters derived from the liver between control and anemic groups. Genes with average log2 fold change >0.2 and −log10 (*P* value-adjust) >2 are plotted in red; others are blue. **b** Heat map showing the comparison between the top 15 up- and downregulated genes in the monocyte cluster between control and anemia. **c** Gene ontology enrichment analysis of monocytes showed significantly enriched activated and suppressed pathways. The vertical items are the names of gene set enrichment analysis (GSEA) terms, and the length of the horizontal graph represents the gene ratio. The depth of the color represents the adjusted *p*-value. The area of the circle in the graph correlates with gene counts. **d** GSEA enrichment score curves of major pathways in monocyte clusters. In each graph, probes on the far left (red dot line) correlated with the most upregulated anemia probes. The vertical black lines indicate the position of each probe of the studied gene set in the ordered, non-redundant data set. The green curve denotes the ES (enrichment score) curve, the running sum of the weighted enrichment score in GSEA. **e** Volcano plot showing DEGs of erythroid cluster derived from the liver between the control and anemic groups. Genes with average log2 fold change >0.2 and −log10 (*P* value-adjust) >2 are plotted in red; others are blue. **f** GSEA enrichment score curves of major pathways in erythroid clusters. **g** Gene ontology enrichment analysis of the erythroid cluster showed significantly enriched activated and suppressed pathways. **h** Heat map showing the comparison between top-up- and downregulated genes in an erythroid cluster between control and anemia. Specific details about figures e-h are presented for monocyte clusters in (**a**–**d**).

To further characterize if gene expression was also altered in monocytes in addition to their increased proportions, we compared the differentially expressed genes (DEG) between the two groups. DEG analysis revealed that the monocyte cluster in the anemic liver was transcriptionally distinct from that observed in the control livers. As shown in Fig. 3a, b, the monocyte cluster highly expressed *Vcan, Chil3, Lcn2, S100G, Snca, Abca13, Abcc9*, and *Serpina3K* genes with low expression of the *CD209G, CD209F, Klhl13, Tcf21, Cyp2e1, Tmem26 and Klra6* genes. The complete list of upregulated and downregulated genes in monocyte clusters of anemic liver is presented in Table S2. To determine which pathways were altered in the anemic liver-associated monocytes, the GSEA pathway analysis was performed on the upregulated genes in the anemic liver-derived monocytes. The GSEA pathway analysis showed that the leukocyte chemotaxis, IL-6 production, positive regulation of reactive oxygen species (ROS) metabolism, chemokine production, and the interferon (IFN) pathway were highly activated in the anemic-liver derived monocytes (Fig. 3c). Consistent with this observation, the GSEA running enrichment score (ES) reflects that the pathways involved in the regulation of chemokine production, type I interferon signaling pathway, cellular response to type I interferon, positive regulation of reactive oxygen species (ROS) metabolism, IL-6 production and leukocyte chemotaxis were enriched in anemic liver-derived monocytes than control (Fig. 3d). The mediators involved in these activated pathways – chemotaxis (*Cxcl2, S100A8, Ccr2, Syk, Fcer1g*, and *Csf3r*), IL6 production (*IL-1β, IL17RA, Tyrobp*, and *Cebpb*), ROS *(Tspo, Sod2, Itgam, Gstp1, Xdh* and *Fpr2*), chemokine production (*Mif, Lilrb4a, Gstp1, Lrp1, IL17RA, Arg2)* and type I IFN signaling (*Ifitm3, Samhd1, Ifnar2, Zbp1*) – are presented in Fig. S2. In addition, a similar pattern of inflammatory and leukocyte migration pathways was observed in clusters of non-inflammatory macrophages, neutrophils, and dendritic cells (Figs. S3, S4, S5). The complete list of upregulated and downregulated genes in non-inflammatory macrophages, neutrophils, and dendritic cells in the anemic liver is presented in Tables S3, S4, and S5, respectively.

### PIA causes rapid erythropoiesis in neonatal murine liver

As demonstrated in Fig. 1f-h, based on the high expression of Gypa, we were able to identify erythroid cells in the scRNAseq dataset. To better characterize this population, we also performed a DEG analysis between anemic and control livers. We found that a large proportion of genes in erythroid cells were altered between the conditions (a total of 13,534 genes) (Fig. 3e, h). The complete list of upregulated and downregulated genes in erythroid cell clusters of anemic liver is presented in Table S6. Functional enrichment analysis of the DEGs between the control and anemic groups showed pathways related to the collagen trimer, blood coagulation regulation, metabolic processes, extracellular matrix, and cellular response to iron were activated and cellular response to IFN-beta and positive regulation of innate immune response were suppressed in the anemic condition (Fig. 3g, h). Consistent with these findings, we found upregulated genes (*Mt1, Mt2, Mgst3, CD209A, Serpina3k*, and *Slc23A1*) (Fig. S6). Together, these results suggest that anemia may induce stressed erythropoiesis that might be associated with the upregulation of extracellular matrix functional genes and the downregulation of inflammatory genes.

### Defective lymphopoiesis in the murine neonatal liver during PIA

**Fig. 4:**
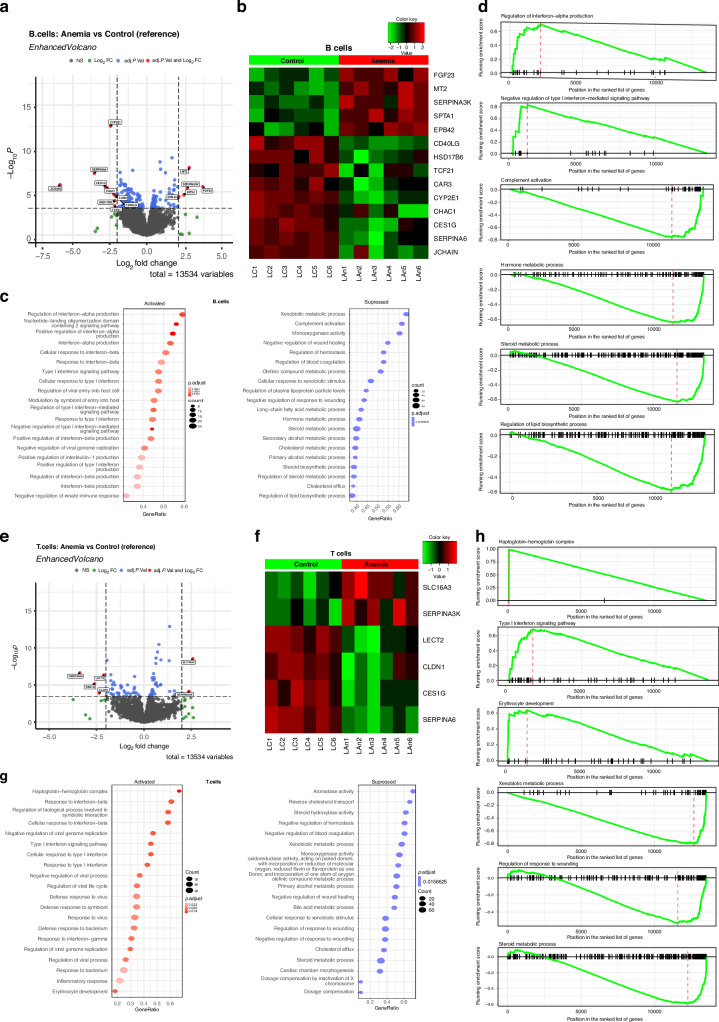
Defective lymphopoiesis in the murine neonatal liver during PIA. **a** Volcano plot showing differentially expressed genes (DEGs) of B-cell clusters derived from the liver between control and anemic groups. Genes with average log2 fold change >0.2 and −log10 (*P* value-adjust) >2 are plotted in red; others are blue. **b** Heat map showing the comparison between top-up- and downregulated genes in the B cell cluster between control and anemia. **c** Gene ontology enrichment analysis of B cells showed significantly enriched activated and suppressed pathways. **d** GSEA enrichment score curves of major suppressed pathways in anemic liver-derived B cell clusters. **e** Volcano plot showing DEGs of T-cell cluster derived from the liver between the control and anemic groups. Genes with average log2 fold change >0.2 and −log10 (*P* value-adjust) >2 are plotted in red; others are blue. **f** Heat map showing the comparison between top up- and downregulated genes in the T-cell cluster between control and anemia. **g** Gene ontology enrichment analysis of the T-cell cluster showed significantly enriched activated and suppressed pathways. **h** GSEA enrichment score curves of major pathways in the T-cell cluster derived from anemic liver. Specific details about (**a**–**h**) are as presented for monocyte clusters in Fig. 3a-d.

We then performed DEG analysis of the B and T cell clusters (Fig. 4a, b). We found decreased B and T cells in the anemic group compared to the control; meanwhile, the genes of *Fgf23, Mt2, Serpina3k, Spta1* and *Epb42* were upregulated and genes of *Jchain, Cyp2e1, Tcf2*1 and *CD40lg* were downregulated in anemic-liver derived B cells. Pathway analysis demonstrated that the interferon-signaling pathway and positive regulation of the interleukin-1 production pathway were also highly activated in these cells in the anemic liver (Fig. 4c). Interestingly, the pathways responsible for the hormone and steroid metabolic processes, complement activation, and regulation of lipid biosynthetic processes were significantly suppressed in anemic-liver derived B cells (Fig. 4d) and their markers including steroids (*ApoA2, ApoA1, ApoE* and *ApoC1*), hormones (*Ttr and Rbp4*), and lipid biosynthetic (*ApoA1, ApoE* and *ApoC1*) are listed in Fig. S7. In addition, the pathway involved in complement activation was suppressed in these cells with a significant reduction in the expression of the *Iglc2* gene.

The genes *Slc16a3 and Serpina3k* were upregulated and *Lect2, Cldn1, Ces1g* and *Serpina6* were downregulated in T cells of anemic liver (Fig. 4e, f). Similar to B cells, T-cells were enriched for pathways of interferon signaling and inflammatory responses and suppressed for metabolic processes (Fig. 4g). The complete lists of upregulated and downregulated genes in B- and T-cell clusters of anemic liver are presented in Tables S7 and S8, respectively. Similar to our findings in B cells, GSEA pathway analysis of T cells from the anemic liver revealed suppressed steroid metabolic processes (Fig. 4h) and we also observed activation of the haptoglobin-hemoglobin complex, type I interferon signaling pathway, (Fig. 4h) with markers representing steroid metabolic processes (*ApoA2, ApoA1, ApoE* and *ApoC1*) being downregulated (Fig. S8). These results indicate that adaptive immune cell pathways are highly suppressed in the anemic group compared to the control. A similar pattern of activation of interferon signaling and suppression of metabolic processes was also noted in NK cells (Figure S9), and the complete list of upregulated and downregulated genes in NK cells is presented in Table S9.

## Discussion

Anemia in preterm infants is increasingly prevalent and strongly associated with short- and long-term complications, even including death.^1,10,51–56^ Since the liver is functionally positioned between the gastrointestinal tract and the systemic circulation, it plays a major role in immunity and metabolism, enabling infants and adults to process detoxification. In particular, the liver is the primary site of hematopoiesis in the human preterm neonate until 22 weeks gestation (and the first 2 postnatal weeks in the mouse), a developmental stage when the newborn encounters many physiological and non-physiological (e.g., phlebotomy) challenges from the first moments after birth. In the mouse fetal liver, hematopoietic stem cells (HSCs) seed the liver and migrate to the bone marrow during birth. Under stress conditions, either liver-resident HSCs or egress from the bone marrow may take residence in the liver to initiate extramedullary hematopoiesis (EMH).^57^ We recently showed that severe anemia leads to a “leaky gut” and allows for bacterial translocation into circulation,^5^ which could presumably lead to the direct uptake of bacterial products into the neonatal liver thus activating the hepatic leukocyte responses by EMH. To address the severe PIA that alters the liver immune pool, we report a map of the immune cellular landscape and transcriptome coverage of the mouse pup’s liver using single-cell RNA sequencing.

To date, no specific transcriptional signature has been associated with livers in anemic premature infants. Our recent findings identified the diversity and profile of immune cells from the blood of murine pups with PIA, suggesting that the rate of immune cell alteration is influenced by PIA.^25^ Consistent with this finding, our present results on neonatal livers also demonstrate that PIA alters the immune composition and transcriptional signature of the liver. Profiling transcriptomes from PIA and control livers, our study uncovered differences in immune cell profiles in the liver of anemic and control murine neonates. These data add to the notion that neonatal anemic liver cells may be biologically and/or immunologically fundamentally distinct.

We previously described^25^ that monocyte counts in the blood were slightly increased on days 4 and 6 in response to phlebotomy, followed by a downtrend bringing the counts back down to a level similar to age-matched controls (day 10). Our previous findings well support these monocyte dynamics in the blood that severe anemic conditions lead to low-grade inflammation in the intestine by paracellular permeability^5^ and hypoxia, likely recruiting the circulating monocytes from blood to the intestine. The present findings suggest that the liver similarly contains a higher number of monocytes with inflammatory and highly chemotactic phenotypes, which appear similar to the monocytes we found in earlier studies.^6,25,26^ These profiles strongly suggest that the liver, a major hematopoietic organ in neonates, contributes to the inflammatory response during neonatal anemia. A higher number of erythrocyte progenitors in anemic liver was found in the setting of anemia, suggestive of stressed erythropoiesis. Several existing studies reported that anemia induced by phlebotomy,^58^ iron deficiency,^59^ phenylhydrazine,^60–63^ inflammation,^64^ and sepsis^65,66^ causes stressed erythropoiesis in bone marrow and extramedullary hematopoietic organs like the spleen. However, this study suggests that PIA-induced stressed erythropoiesis in the neonatal liver includes increased production of erythroid cells with activation of extracellular matrix (ECM), which helps orchestrate hematopoietic niche architecture and regulates the cellular function of erythroid cells by colony formation, differentiation, progenitor proliferation, and regulation of its fitness. Although the activation of ECM pathways indicates the enhancement of erythroid production from proerythroblasts, the maturation deficits might lead to increased release of nucleated erythroid cells into the circulation.^67^ Consistent with these findings, we recently reported that PIA pups showed a significant elevation of reticulocyte percentage from day 6 to day 10,^25^ correlated with higher reticulocyte counts, in preterm human infants with anemia of prematurity. Additionally, the pathway analysis suggests that these anemic erythroid cells suppress the inflammatory response by downregulating interferon and innate immune response signaling, indicating that erythroid progenitors might be resistant to endotoxins due to the absence of toll-like receptor-4 (TLR4^68^).

Overall, our study proposes that anemia dysregulates the development and function of immune cell populations in the liver. A wide range of functional alterations of immune cells with inflammatory potentials, like the monocytes, may induce emergency myelopoiesis and cause erythroid cells with noninflammatory phenotypes to undergo stressed erythropoiesis.

When we analyzed the adaptive immune cells, B cells, and T cells in the anemic liver, we found evidence that adaptive immunity was highly suppressed by activating inflammatory pathways and/or reducing metabolic processes. Our previous study showed that only no/minimal changes in lymphocyte populations in the blood during PIA,^25^ yet this study shows a reduction in counts of B cells and T cells in anemic liver. Together, these findings indicate that lymphocytopenia might occur, with possible mechanisms including (i) decreased production of these cells by the neonatal liver; (ii) increased trafficking to anemia-associated hypoxic tissues, and (iii) increased destruction due to activation of these cells by anemia-associated endotoxin exposure. In addition to endotoxin, hypoxia is also possibly triggered by anemia in the liver, and hypoxia has been shown to inhibit lymphocyte proliferation through HIF-1α signaling.^69^ The HIF signaling pathway also increases glycolytic metabolism in germinal center (GC) B cells, which affects B cell proliferation.^70^ Hypoxia can cause T cell exhaustion and negatively impact the proliferation of naïve and memory T cells through metabolic stress.^71^ Moreover, nucleated erythroid cells are known to be immunosuppressive, including suppression of B and T lymphocytes.^72,73^ Consistent with this, the increased production of erythroid cells found in our study might suppress the activity of both B-cell- and T-cell-mediated immune responses. The likely explanation for suppressed lymphopoiesis in these mice is that endotoxin or hypoxia may have dysregulated the production of these immune cells. In addition, the suppressed lipid biosynthesis and steroid pathways in anemic liver-derived lymphocytes might be due to anemia-associated nutritional imbalance that may significantly influence lipid biosynthesis in lymphocytes, impacting their function and survival, also crucial for adaptive immunity. Their mechanism(s) in neonatal hematopoiesis and metabolic remodeling remain a subject of further investigation.

In conclusion, we identified diverse immune cell populations with increased numbers of monocytes with proinflammatory phenotype and stressed erythroid cells and suppressed lymphocytes (B and T cells) numbers, with altered metabolic pathways were detected in the anemic liver. This study sheds light on emergency myelopoiesis, stressed erythropoiesis, and deficiency of lymphocytes in anemic liver, which may provide novel insight for the development of therapeutics to treat anemia in preterm infants and neonates.

## Supplementary information

Supplementary Figures

Table S2

Table S3

Table S4

Table S5

Table S6

Table S7

Table S8

Table S9

## Data Availability

Single-cell data related to all control and anemic mouse liver cells were deposited at Gene Expression Omnibus under accession number GSE285887.
