# Supplementary material for: Immune landscape in liver of neonatal mice with phlebotomy-induced anemia
Source: Pediatr Res. 2025 Sep 17;99(4):1602–12. doi: 10.1038/s41390-025-04361-x (PMC12659965; doi:10.1038/s41390-025-04361-x)
Supplement: Supplementary file 1 — Supplementary Figures [file 41390_2025_4361_MOESM1_ESM.pdf]

Figure S1

Quality Control

- Besides filtering out the cells with the total number of RNAs less than 500, we further filter out the cells with mitochondrial content <50%. The remaining number of cells in each sample:

LAn1	LAn2	LAn3	LAn4	LAn5	LAn6	LC1	LC2	LC3	LC4	LC5	LC6
16471	17840	21186	18872	18007	15707	15513	18154	14185	15561	16554	18119

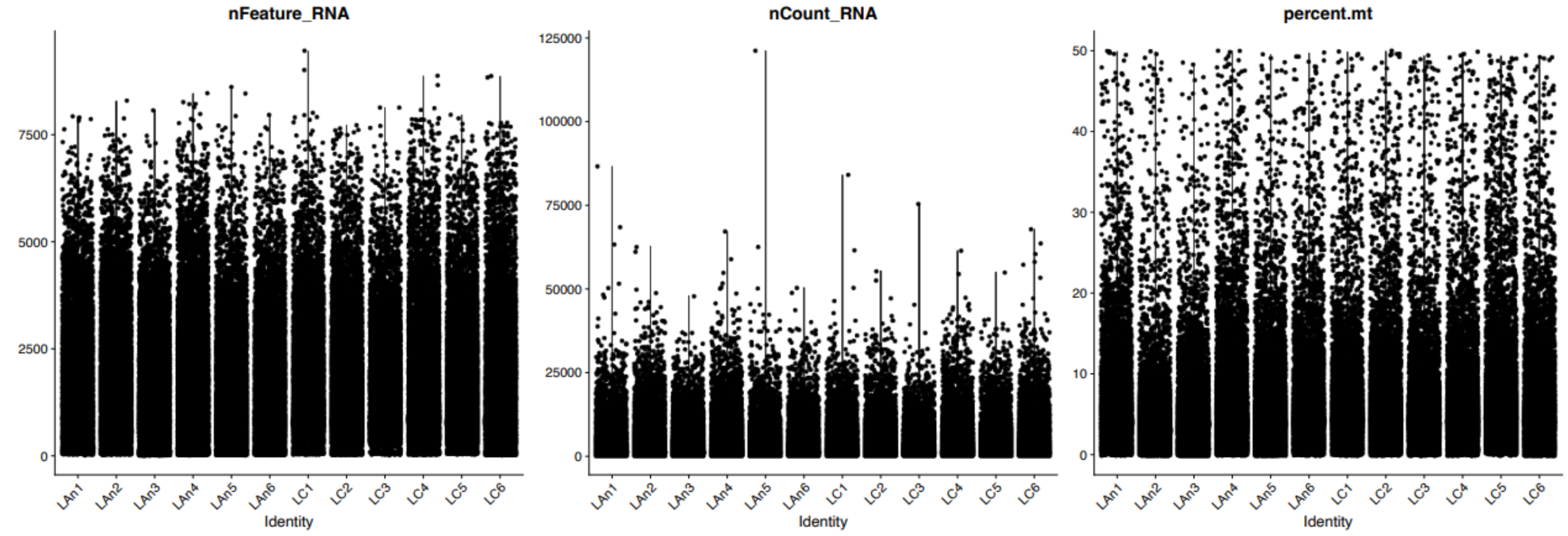

Fig S1. Uniform and data-driven quality control (QC) thresholds for scRNA-seq data. Cells (N = 146,680) from murine liver tissue sample with the number of unique genes detected (x-axis) and percent of cell counts mapping to mitochondrial (mtDNA) genes (y-axis). Illustration of removing cells with a uniform QC threshold of greater than 10% cell counts mapping to mtDNA genes (solid black line) and a more data-driven threshold of greater than 3 median absolute deviations (MADs) of the percent of counts mapping to mtDNA genes (dotted black line).

Table S1

Top marker genes specifically expressed in 8 immune cell clusters

Clusters	Cluster identity genes
Non-inflammatory macrophages	Lyz2, C1QC, C1QB, CD5L, MARCO, C1QA, SLC40A1, CTSB, FCNA
Neutrophils	S100A8, Elane, NGP, RETNLG, LTF
B Cells	MS4a1, IGKC, IGHM, EBF1, CD79B, CD79A, BACH2, Ly6D
NK Cells	Nkg7, GZMA, GZMB, ITK, PTPN22
Erythroid Cells	GYPA, SNCA, ALAS2, HBB-BS, HBA-A2, HBA-A1, HBB-BT
T Cells	CD3e, STMN1, HMGB2, IGLL1, RRM2, TOP2A
Monocytes	Ly6C2, PLAC8, MS4A6C, PID1, TRPS1
Dendritic cells	Siglech, CCR9, GRM8, TCF4

Supplementary Table 1. shows the high-specificity marker genes that were used as a validation of correct cell type assignments from the 8 clusters.

Figure S2

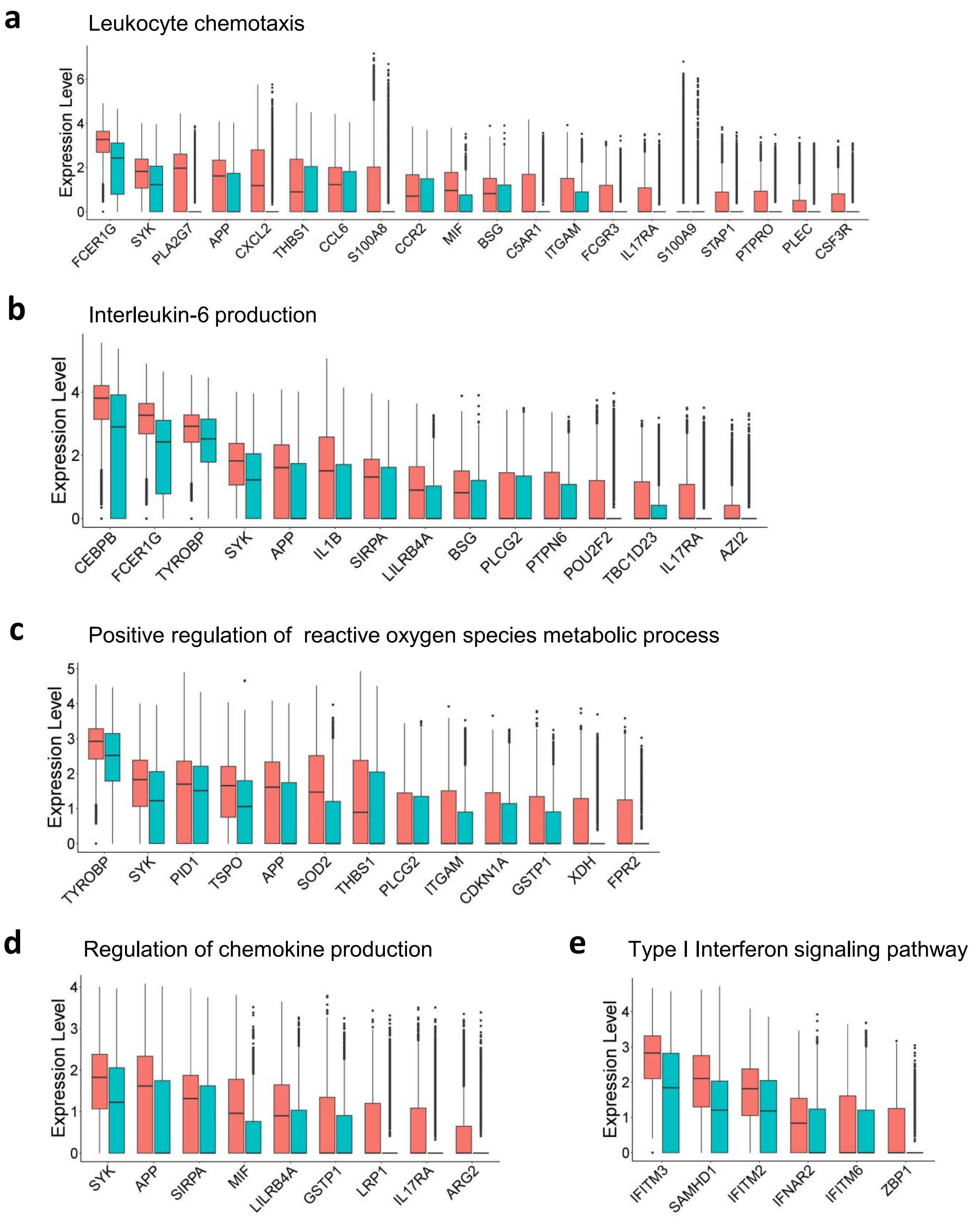

Fig. S2. Bar diagram represents the expression level of upregulated and downregulated genes of respective pathways that activated in anemic-liver derived monocyte cluster.

Figure S3

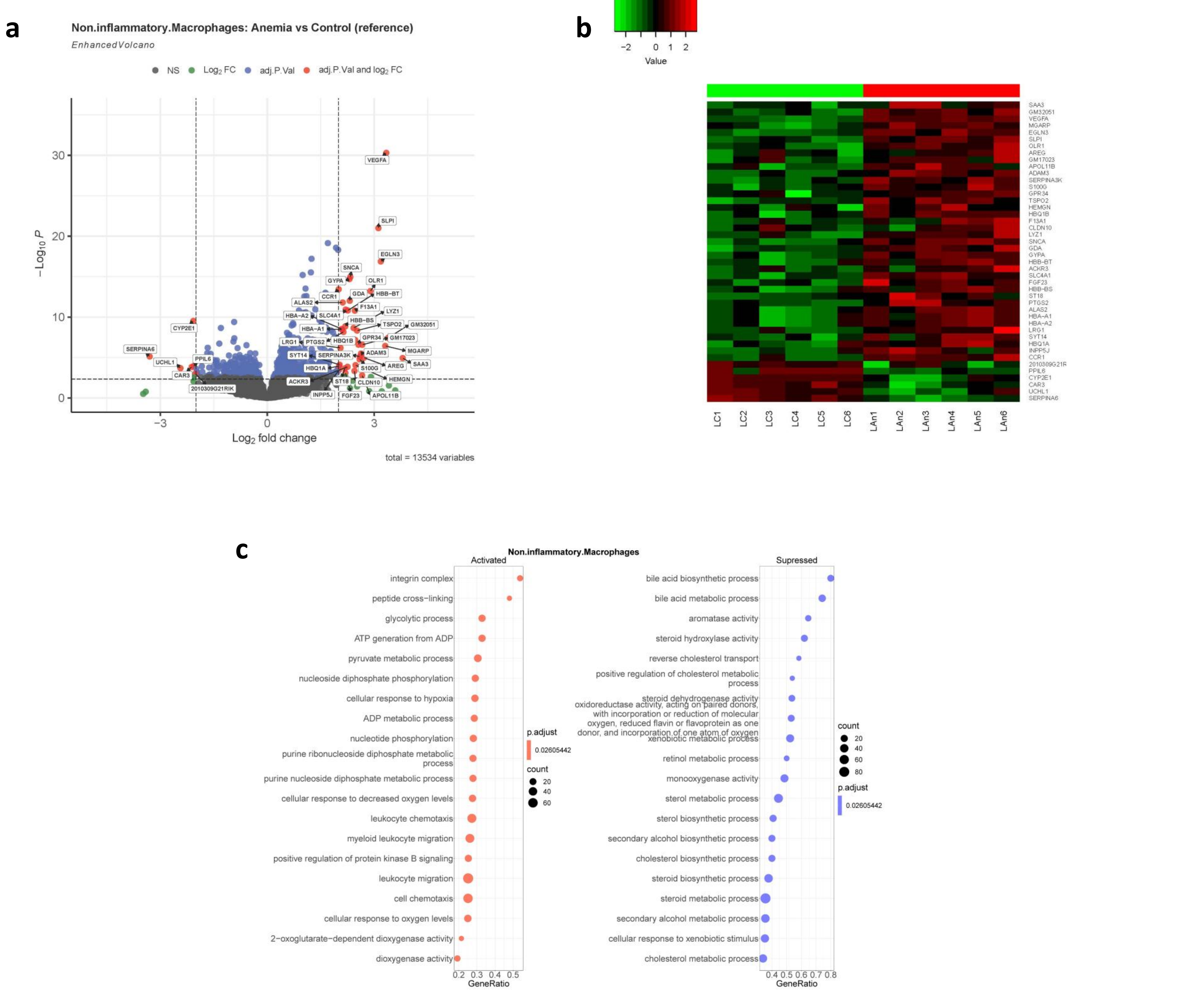

Fig S3. (a) Volcano plot showing differentially expressed genes (DEGs) of non-inflammatory macrophage clusters derived from the liver between control and anemic groups. Average_log2 fold change > 0.2 & -log10 (P value-adjust) > 2 are red; others are blue. (b) Heat map showing the comparison between top 15 up- and downregulated genes in non-inflammatory macrophage cluster between control and anemia. (c) Gene ontology enrichment analysis of non-inflammatory macrophages showed significantly enriched activated and suppressed pathways. The vertical items are the names of GSEA terms, and the length of the horizontal graph represents the gene ratio. The depth of the color represents the adjusted p-value. The area of the circle in the graph means gene counts.

Figure S4

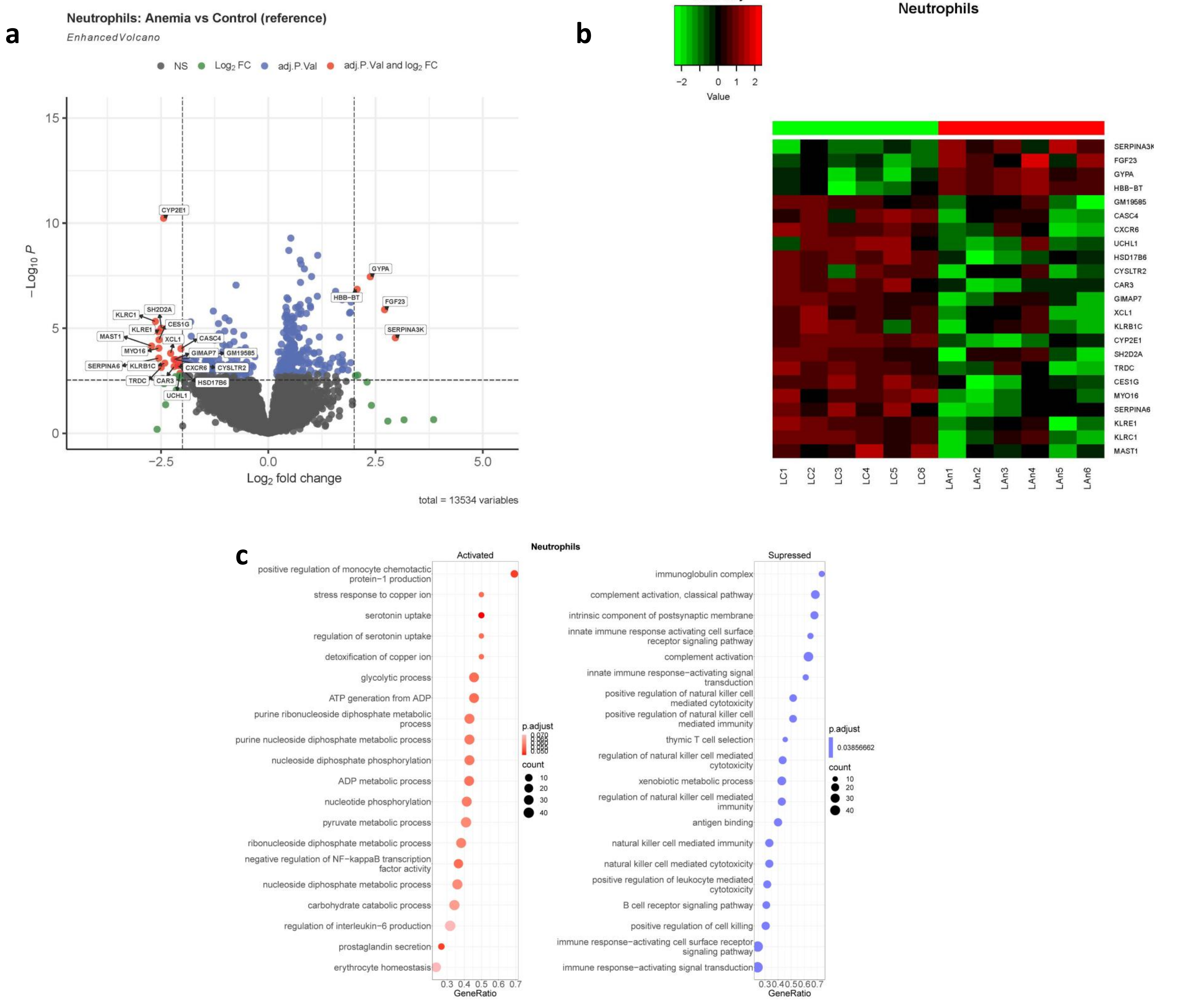

Fig S4. (a) Volcano plot showing differentially expressed genes (DEGs) of neutrophil clusters derived from the liver between control and anemic groups. Average_log2 fold change > 0.2 & -log10 (P value-adjust) > 2 are red; others are blue. (b) Heat map showing the comparison between top 15 up- and downregulated genes in a neutrophil cluster between control and anemia. (c) Gene ontology enrichment analysis of neutrophils showed significantly enriched activated and suppressed pathways. The vertical items are the names of GSEA terms, and the length of the horizontal graph represents the gene ratio. The depth of the color represents the adjusted p-value. The area of the circle in the graph means gene counts.

Figure S5

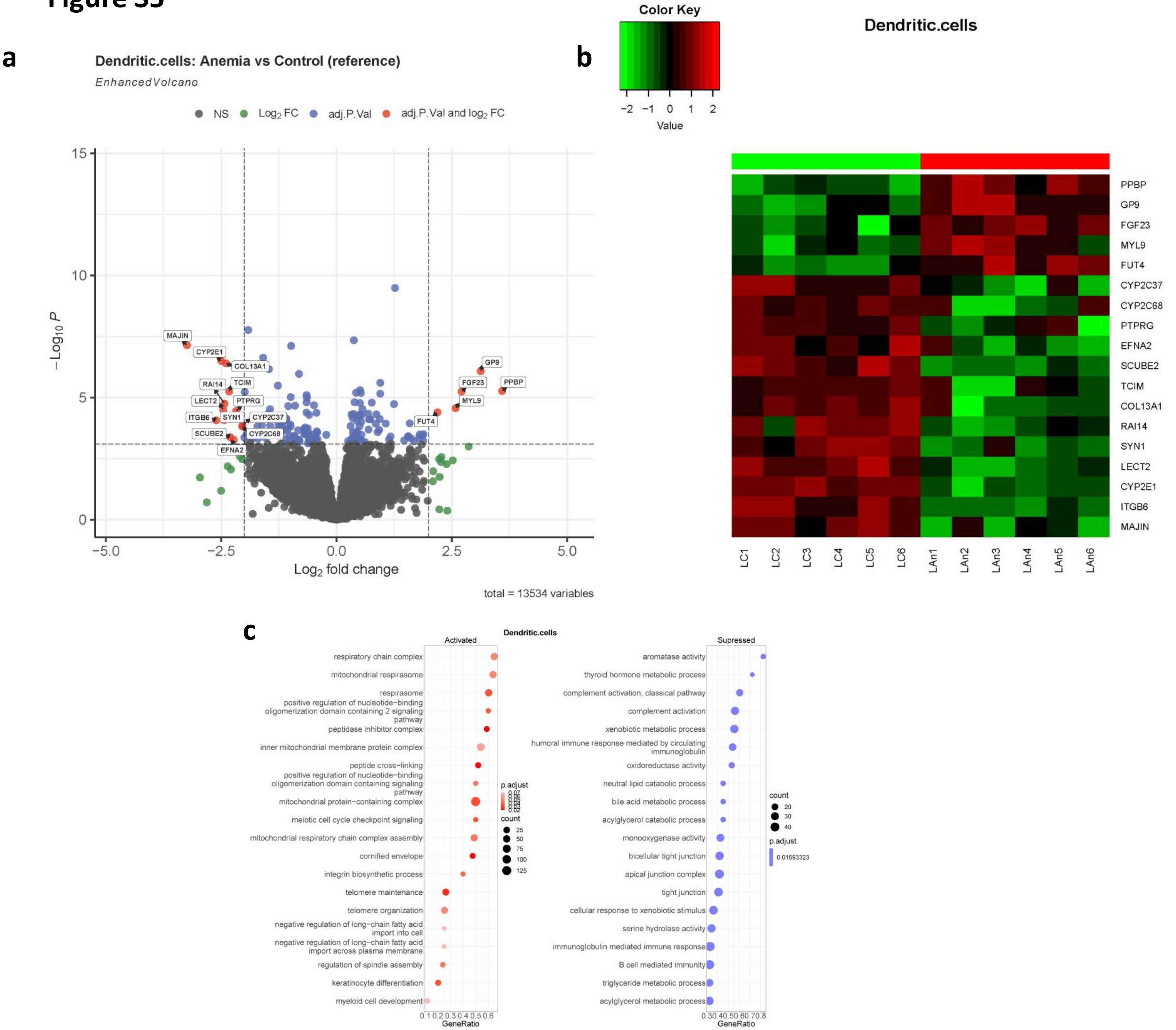

Fig S5. (a) Volcano plot showing differentially expressed genes (DEGs) of dendritic cell clusters derived from the liver between control and anemic groups. Average_log2 fold change > 0.2 & -log10 (P value-adjust) > 2 are red; others are blue. (b) Heat map showing the comparison between top 15 up- and downregulated genes in dendritic cell cluster between control and anemia. (c) Gene ontology enrichment analysis of dendritic cells showed significantly enriched activated and suppressed pathways. The vertical items are the names of GSEA terms, and the length of the horizontal graph represents the gene ratio. The depth of the color represents the adjusted p-value. The area of the circle in the graph means gene counts.

Figure S6

a

Collagen-containing extracellular matrix,
External encapsulating structure
Extracellular matrix

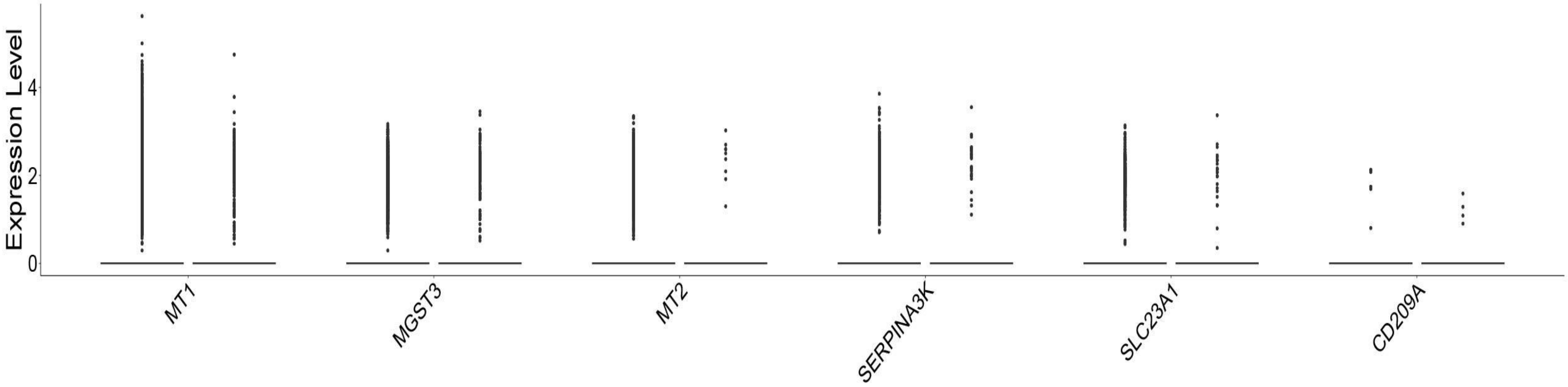

Fig. S6. Bar diagram represents the expression level of upregulated genes of respective pathways that activated in anemic-liver derived erythroid cell cluster.

Figure S7

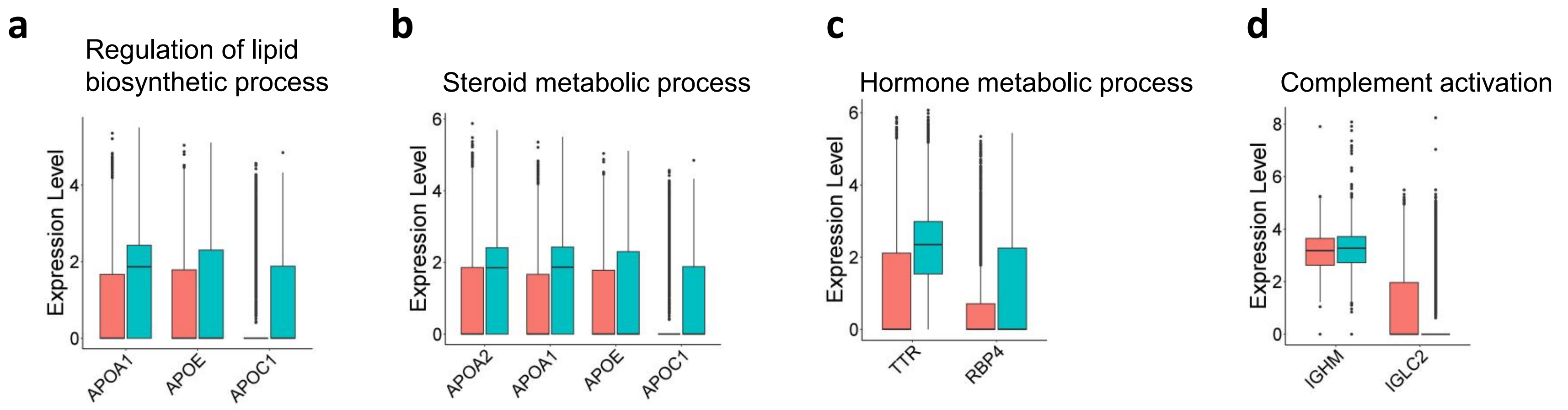

Fig. S7. Bar diagram represents the expression level of upregulated and downregulated genes of respective pathways that activated in anemic-liver derived B cell cluster.

Figure S8

a

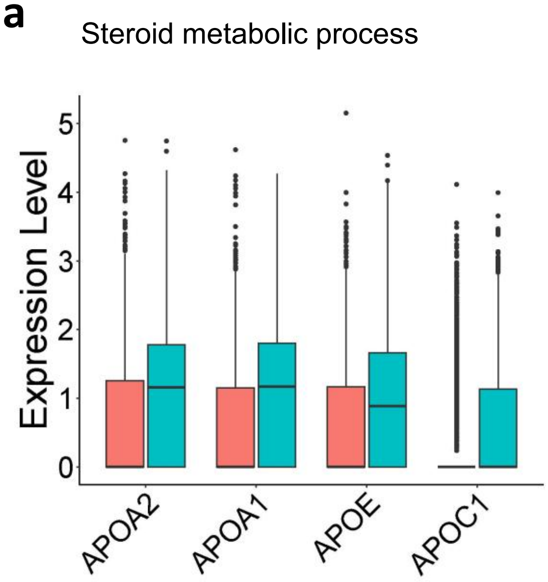

Fig. S8. Bar diagram represents the expression level of upregulated and downregulated genes of respective pathways that activated in anemic-liver derived T cell cluster.

Figure S9

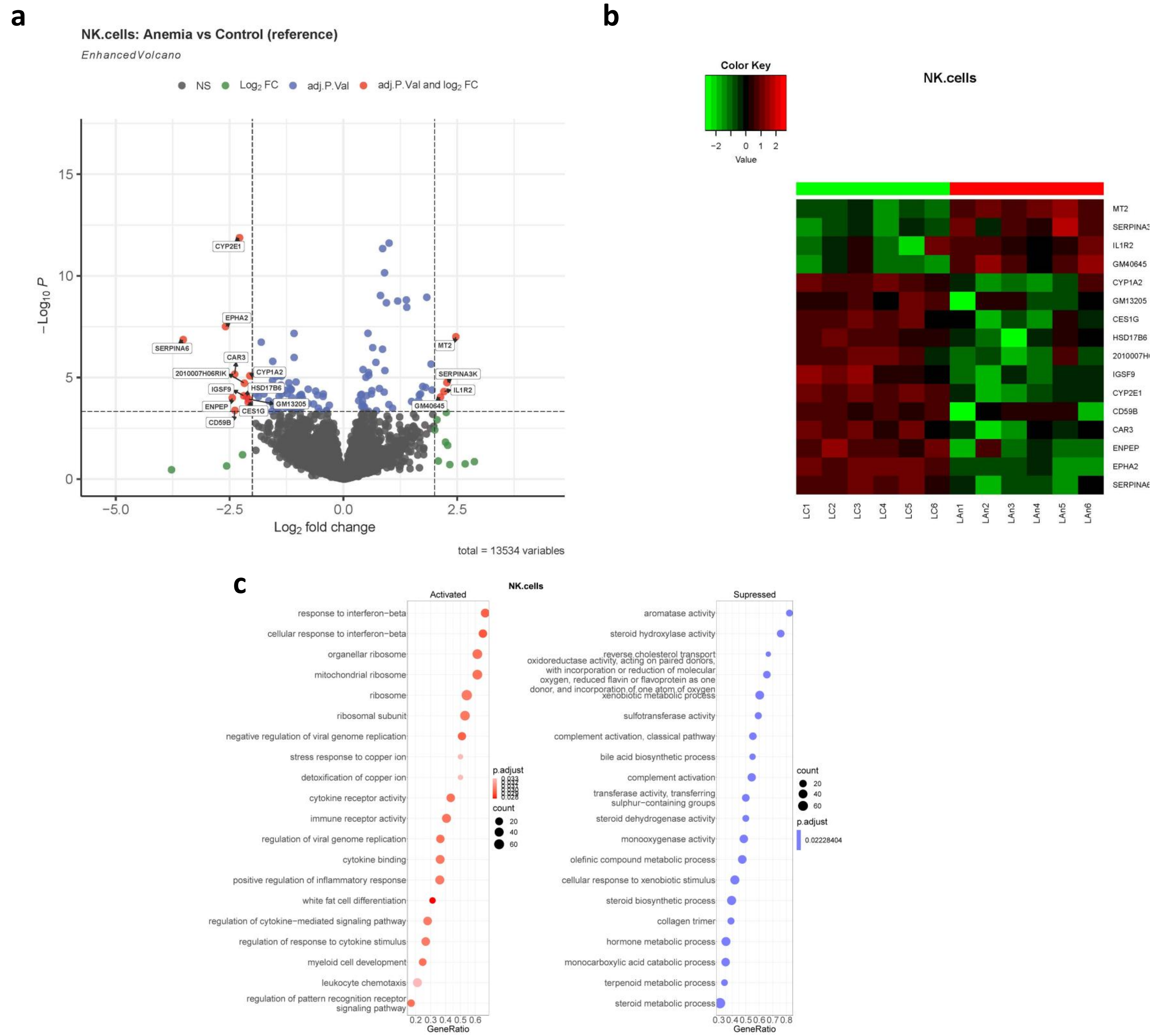

Fig S9. (a) Volcano plot showing differentially expressed genes (DEGs) of NK cell clusters derived from the liver between control and anemic groups. Average_log2 fold change > 0.2 & -log10 (P value-adjust) > 2 are red; others are blue. (b) Heat map showing the comparison between top 15 up- and downregulated genes in NK cell cluster between control and anemia. (c) Gene ontology enrichment analysis of NK cell clusters showed significantly enriched activated and suppressed pathways. The vertical items are the names of GSEA terms, and the length of the horizontal graph represents the gene ratio. The depth of the color represents the adjusted p-value. The area of the circle in the graph means gene counts.
