# Supplementary material for: Immune landscape in liver of neonatal mice with phlebotomy-induced anemia
Source: Pediatr Res. 2025 Sep 17;99(4):1602–12. doi: 10.1038/s41390-025-04361-x (PMC12659965; doi:10.1038/s41390-025-04361-x)
Supplement: Supplementary file 2 — Table S2 [file 41390_2025_4361_MOESM2_ESM.pdf]

immunecell	gene	logFC	AveExpr	t	P.Value	B	adj.P.Val. Within	adj.P.Val. Between
Monocytes	VCAN	2.430203	0.76602	11.60999	1.80E-19	32.27813	1.99E-15	1.72E-15
Monocytes	GDA	1.323818	3.902395	8.590866	2.76E-13	19.88408	1.25E-09	4.79E-10
Monocytes	CHIL3	3.226028	2.447415	8.529897	3.69E-13	19.10051	1.30E-09	6.47E-10
Monocytes	CCL9	1.210223	2.831707	8.260982	1.32E-12	18.182	3.45E-09	1.95E-09
Monocytes	CYP2E1	-2.42461	4.291137	-7.47267	5.34E-11	13.66716	1.07E-07	4.87E-08
Monocytes	NUDT9	-0.9121	4.362001	-7.39502	7.66E-11	14.323	1.28E-07	6.57E-08
Monocytes	SERPINB9	-1.35941	3.483496	-7.26436	1.40E-10	13.73639	2.06E-07	1.17E-07
Monocytes	MMP8	1.628814	1.517071	7.227077	1.67E-10	13.43901	2.27E-07	1.42E-07
Monocytes	GSR	0.685841	6.441975	7.167081	2.20E-10	13.36135	2.31E-07	1.60E-07
Monocytes	SLPI	1.487127	4.206306	6.95839	5.75E-10	12.43662	5.79E-07	3.97E-07
Monocytes	MS4A4A	1.936228	0.617812	6.837	1.00E-09	10.27011	1.02E-06	7.13E-07
Monocytes	PIANP	-0.97382	-0.06426	-6.76696	1.38E-09	11.43674	1.31E-06	9.57E-07
Monocytes	GAB1	0.919945	4.473058	6.700672	1.86E-09	11.34719	1.43E-06	1.09E-06
Monocytes	HOPX	0.846558	4.034194	6.580339	3.21E-09	10.83518	2.32E-06	1.71E-06
Monocytes	FABP4	1.374724	6.966529	6.461896	5.47E-09	10.32768	3.40E-06	2.38E-06
Monocytes	SELL	1.020452	5.472373	6.447842	5.83E-09	10.24472	3.54E-06	2.60E-06
Monocytes	SERPINB6B	-1.33734	2.936635	-6.4021	7.16E-09	9.95658	4.39E-06	3.31E-06
Monocytes	GM9733	1.080385	0.76406	6.389419	7.57E-09	10.0054	4.67E-06	3.68E-06
Monocytes	ARHGAP24	0.822954	6.825889	6.321627	1.03E-08	9.713981	4.67E-06	4.04E-06
Monocytes	SLC16A3	0.840275	3.935418	6.320046	1.03E-08	9.724006	5.06E-06	4.39E-06
Monocytes	PTGS2	1.40356	2.215449	6.316669	1.05E-08	9.702265	5.32E-06	4.63E-06
Monocytes	CCL6	0.796163	5.306299	6.278404	1.24E-08	9.464724	5.51E-06	4.96E-06
Monocytes	GM14548	1.065762	1.47249	6.245133	1.44E-08	9.231669	6.81E-06	6.34E-06
Monocytes	CD81	-0.72237	7.604661	-6.22929	1.55E-08	9.266884	5.89E-06	5.61E-06
Monocytes	GM10076	0.771684	6.671496	6.220198	1.61E-08	9.280998	6.05E-06	5.96E-06
Monocytes	DMPK	-1.61858	2.786398	-6.19891	1.77E-08	8.568535	7.13E-06	7.14E-06
Monocytes	IL17RA	0.703626	5.907729	6.11302	2.58E-08	8.846822	9.19E-06	9.30E-06
Monocytes	ACTN1	-0.71752	5.757216	-6.09162	2.84E-08	8.761147	9.77E-06	1.02E-05
Monocytes	NRG1	0.929424	2.541088	6.064775	3.19E-08	8.624548	1.16E-05	1.24E-05
Monocytes	TMEM26	-2.23769	2.190341	-6.03218	3.68E-08	6.809163	1.31E-05	1.38E-05
Monocytes	TANC2	-1.0379	5.521032	-5.96274	4.99E-08	8.19858	1.56E-05	1.62E-05
Monocytes	MS4A6B	1.019428	4.588359	5.897909	6.62E-08	7.948792	2.06E-05	2.15E-05
Monocytes	LCN2	2.410828	3.398879	5.837922	8.59E-08	7.436113	2.68E-05	2.79E-05
Monocytes	STAP1	0.609293	4.891894	5.827534	8.98E-08	7.661649	2.61E-05	2.78E-05
Monocytes	SLC40A1	-1.22471	5.835325	-5.79316	1.04E-07	7.53026	2.86E-05	3.09E-05
Monocytes	PAKAP.1	-1.30018	4.53559	-5.77056	1.15E-07	7.127939	3.18E-05	3.49E-05
Monocytes	DNASE1L3	-0.89995	6.30437	-5.76223	1.19E-07	7.319959	3.04E-05	3.43E-05
Monocytes	SMOX	0.784076	5.116761	5.757376	1.22E-07	7.346237	3.14E-05	3.60E-05
Monocytes	WNK1	-0.46677	9.07644	-5.73598	1.33E-07	7.012731	3.01E-05	3.51E-05
Monocytes	APOC2	0.866514	4.203003	5.72047	1.43E-07	7.225114	3.59E-05	4.24E-05
Monocytes	LY75	-0.94003	3.332572	-5.64056	2.01E-07	6.909555	5.05E-05	5.88E-05
Monocytes	WDR91	-0.60713	4.750458	-5.6275	2.12E-07	6.856738	5.01E-05	5.94E-05
Monocytes	IL2RB	-1.35458	3.130268	-5.60417	2.34E-07	6.661874	5.66E-05	6.71E-05
Monocytes	MREG	-1.20184	3.199109	-5.57506	2.65E-07	6.614528	6.24E-05	7.45E-05

Monocytes	THBS1	0.98808	4.967156	5.567253	2.74E-07	6.393634	6.00E-05	7.29E-05
Monocytes	FSCN1	-1.05672	3.378992	-5.54728	2.98E-07	6.526285	6.69E-05	8.12E-05
Monocytes	CRTC3	0.537933	5.658838	5.518757	3.37E-07	6.398853	6.69E-05	8.32E-05
Monocytes	TMEM38B	0.68661	4.174266	5.518736	3.37E-07	6.414796	6.97E-05	8.67E-05
Monocytes	SVIL	0.471429	6.860609	5.517134	3.39E-07	6.321544	6.47E-05	8.05E-05
Monocytes	KDM2B	-0.82877	7.133876	-5.50584	3.56E-07	6.265403	6.48E-05	8.26E-05
Monocytes	4930523C07RIk	-0.48515	6.126408	-5.50415	3.58E-07	6.327025	6.67E-05	8.50E-05
Monocytes	TGFBI	0.664902	4.412001	5.500781	3.63E-07	6.248879	6.99E-05	9.02E-05
Monocytes	ATRNL1	0.5719	6.820202	5.496065	3.71E-07	6.213233	6.54E-05	8.56E-05
Monocytes	CHST11	-0.79022	6.242747	-5.46091	4.30E-07	6.094893	7.57E-05	9.78E-05
Monocytes	IFITM6	0.831129	3.518134	5.443249	4.63E-07	6.052868	8.64E-05	0.000113
Monocytes	GM11808	-0.56679	7.262783	-5.42326	5.03E-07	5.932564	8.31E-05	0.00011
Monocytes	THRB	-1.53319	3.815098	-5.40546	5.42E-07	5.743642	9.69E-05	0.000128
Monocytes	F13A1	1.142867	2.512117	5.380287	6.03E-07	5.819904	0.00011	0.000147
Monocytes	WFDC21	4.187274	2.947804	5.342859	7.05E-07	4.695685	0.000125	0.000165
Monocytes	MIF	0.778859	7.650894	5.334403	7.30E-07	5.584906	0.000111	0.000149
Monocytes	HP	0.837902	7.365035	5.310352	8.07E-07	5.410029	0.000122	0.000165
Monocytes	HPSE	0.923578	3.426146	5.286225	8.92E-07	5.434682	0.000148	0.0002
Monocytes	TPD52	0.525561	8.000639	5.280203	9.14E-07	5.192847	0.000131	0.00018
Monocytes	IFITM2	0.545324	7.227901	5.265733	9.71E-07	5.20126	0.00014	0.000193
Monocytes	APPL1	-0.51592	5.974209	-5.23627	1.10E-06	5.259992	0.000162	0.000218
Monocytes	CCR7	-0.95412	4.535564	-5.22946	1.13E-06	5.280357	0.000171	0.000232
Monocytes	CCND3	0.640371	7.994145	5.210715	1.22E-06	5.032105	0.000163	0.000224
Monocytes	NCOA7	-0.50132	5.299685	-5.208	1.23E-06	5.136628	0.000176	0.000243
Monocytes	1-Sep	-0.81036	6.037433	-5.20625	1.24E-06	5.174277	0.000172	0.00024
Monocytes	CORO1B	0.671265	4.252422	5.197517	1.29E-06	5.17327	0.000181	0.000259
Monocytes	HFE	-0.55711	3.452689	-5.19745	1.29E-06	5.137885	0.000186	0.000264
Monocytes	MSRB1	0.491482	5.958368	5.19523	1.30E-06	5.039093	0.000173	0.000248
Monocytes	ENO1	0.549502	8.066335	5.189752	1.33E-06	4.914789	0.000165	0.000238
Monocytes	TMX4	0.725226	4.420261	5.180938	1.38E-06	5.106714	0.000184	0.000272
Monocytes	GCLC	-0.6284	6.841762	-5.17972	1.38E-06	5.00043	0.000171	0.000255
Monocytes	ETV3	-0.57641	4.950898	-5.17818	1.39E-06	4.9813	0.000181	0.00027
Monocytes	LAMP2	0.389989	7.069003	5.168046	1.45E-06	4.894912	0.000175	0.000262
Monocytes	KLRA5	-1.80354	2.005641	-5.15294	1.55E-06	4.678958	0.000212	0.000319
Monocytes	FBXL5	0.728878	6.173273	5.092665	1.98E-06	4.732004	0.000239	0.000345
Monocytes	PPP1R16B	-0.82579	6.504104	-5.05667	2.29E-06	4.635251	0.000268	0.000387
Monocytes	ZDHHC14	-0.85409	7.022709	-5.05608	2.29E-06	4.537928	0.000264	0.000382
Monocytes	MAB21L3	-1.3212	-0.30498	-5.04559	2.39E-06	4.373846	0.000334	0.000486
Monocytes	CD300LF	0.934883	3.385271	5.03485	2.50E-06	4.552068	0.000311	0.000454
Monocytes	AP1S2	0.46206	5.081901	5.026257	2.59E-06	4.459857	0.000303	0.000445
Monocytes	PPARD	0.529815	5.613407	5.022537	2.63E-06	4.465252	0.000299	0.000444
Monocytes	TRPS1	0.468457	7.683996	5.008411	2.78E-06	4.044355	0.000296	0.000438
Monocytes	PLEC	0.517927	4.805597	4.996126	2.93E-06	4.374134	0.000333	0.000493
Monocytes	LECT2	-1.9014	3.632928	-4.97497	3.19E-06	3.94492	0.000371	0.000541
Monocytes	IL12B	-1.18151	-0.29714	-4.96492	3.32E-06	4.291021	0.000426	0.00062
Monocytes	EEPD1	0.646692	4.881821	4.951262	3.51E-06	4.221496	0.000385	0.000561
Monocytes	TRIM35	-0.48901	5.607952	-4.94196	3.64E-06	4.086594	0.000387	0.000565

Monocytes	CYP8B1	-1.17931	1.074088	-4.93923	3.68E-06	3.901795	0.00044	0.000645
Monocytes	PLA2G7	0.971236	4.021195	4.930304	3.81E-06	4.024761	0.000415	0.000614
Monocytes	FABP2	-1.69794	3.257265	-4.91131	4.11E-06	3.57902	0.00045	0.000668
Monocytes	MS4A8A	1.421356	0.670009	4.908061	4.17E-06	3.837961	0.000484	0.000723
Monocytes	CYP4F18	0.673872	3.551567	4.907773	4.17E-06	4.080715	0.000446	0.000667
Monocytes	GALNT7	-0.47956	6.060844	-4.89463	4.40E-06	3.910872	0.000434	0.00065
Monocytes	BEND4	1.147807	3.557561	4.888168	4.51E-06	3.821162	0.000472	0.000713
Monocytes	RRAS2	-0.72673	5.198697	-4.87837	4.69E-06	3.973006	0.000463	0.000702
Monocytes	GPX4	0.363193	7.153816	4.876386	4.73E-06	3.802638	0.000439	0.000669
Monocytes	BCL2A1B	-0.66981	5.877283	-4.86954	4.86E-06	3.717876	0.000463	0.000707
Monocytes	ZC3H12C	-0.66839	4.431325	-4.85713	5.11E-06	3.749892	0.000501	0.000762
Monocytes	LCK	-1.1334	3.728178	-4.8151	6.04E-06	3.476759	0.000598	0.000885
Monocytes	TENT5C	-0.86997	5.86321	-4.80745	6.22E-06	3.584315	0.000572	0.000855
Monocytes	PON1	-1.5564	4.304512	-4.80516	6.28E-06	3.58843	0.000597	0.0009
Monocytes	NKG7	-1.10897	4.013022	-4.80425	6.30E-06	3.701459	0.000602	0.000907
Monocytes	LIMA1	-0.79336	4.507655	-4.79583	6.52E-06	3.634694	0.000608	0.000916
Monocytes	RTL8A	-0.51388	3.632481	-4.79234	6.61E-06	3.59171	0.000626	0.000946
Monocytes	RNF149	0.600984	5.786169	4.78713	6.74E-06	3.478118	0.000593	0.000906
Monocytes	LAPTM4B	-0.83925	3.501356	-4.78588	6.78E-06	3.635205	0.000632	0.000969
Monocytes	PPFIBP2	-0.65129	4.285147	-4.78255	6.87E-06	3.499299	0.000618	0.000958
Monocytes	STOM	0.658228	3.274711	4.781613	6.89E-06	3.617129	0.000636	0.000987
Monocytes	CCL22	-1.15055	-0.37257	-4.77937	6.95E-06	3.610061	0.000705	0.001098
Monocytes	S100A4	0.682747	2.87429	4.772746	7.14E-06	3.482496	0.000655	0.001021
Monocytes	KLRC1	-1.60616	1.444197	-4.7666	7.31E-06	2.957054	0.000692	0.001081
Monocytes	LDLRAD3	0.567394	4.591753	4.758601	7.55E-06	3.454817	0.000648	0.001014
Monocytes	CTLA2A	1.047049	4.28991	4.756524	7.61E-06	3.524919	0.000653	0.001025
Monocytes	GM15987	0.798553	2.537923	4.734651	8.29E-06	3.449353	0.000742	0.001151
Monocytes	ITGA9	-0.93295	5.58915	-4.72958	8.46E-06	3.409406	0.000689	0.001074
Monocytes	SLC12A2	-0.766	3.544469	-4.71998	8.79E-06	3.396822	0.000751	0.001166
Monocytes	CAPG	0.685298	5.264343	4.716061	8.92E-06	3.30149	0.000719	0.001122
Monocytes	ABCA13	2.037861	0.73813	4.714435	8.98E-06	2.720527	0.000817	0.001279
Monocytes	RABGAP1L	-0.48104	7.908574	-4.70087	9.47E-06	3.083819	0.000699	0.001102
Monocytes	NEDD4	-0.72458	5.933587	-4.6795	1.03E-05	3.246576	0.000794	0.001238
Monocytes	TNIK	-1.084	4.035038	-4.67733	1.04E-05	3.190247	0.000837	0.00131
Monocytes	NPEPPS	0.42347	7.184287	4.67495	1.05E-05	3.033014	0.000767	0.001206
Monocytes	RNF19B	-0.49951	5.758355	-4.67425	1.05E-05	3.111331	0.000798	0.001255
Monocytes	ARHGAP21	-0.51932	5.511128	-4.66263	1.10E-05	3.138671	0.000824	0.001316
Monocytes	BAIAP2	-0.68355	5.269985	-4.66233	1.10E-05	3.165573	0.000829	0.001325
Monocytes	CST3	-0.48918	9.157123	-4.66177	1.10E-05	2.34968	0.000744	0.00119
Monocytes	BNIP3	0.913121	5.086642	4.641294	1.19E-05	3.110295	0.000896	0.001417
Monocytes	VSIG4	-1.23044	4.823941	-4.62873	1.25E-05	3.055137	0.000937	0.00149
Monocytes	TCF21	-2.63753	0.477537	-4.62656	1.27E-05	0.741533	0.001059	0.001686
Monocytes	AGPAT3	-0.51721	5.164345	-4.62589	1.27E-05	3.017424	0.000928	0.00148
Monocytes	MS4A6C	0.668037	5.165825	4.613547	1.33E-05	2.719383	0.00096	0.001546
Monocytes	CASK	0.453442	5.500078	4.613428	1.33E-05	2.849774	0.000951	0.001531
Monocytes	FOLR2	-1.23808	3.655517	-4.60666	1.37E-05	2.975505	0.00102	0.001648
Monocytes	NECTIN3	-2.19326	1.220532	-4.6032	1.39E-05	0.890777	0.0011	0.001782

Monocytes	ABCB9	-1.03624	3.155352	-4.59885	1.41E-05	2.793368	0.001051	0.001708
Monocytes	EGLN3	1.631026	3.294296	4.585774	1.48E-05	2.460104	0.001093	0.001778
Monocytes	CDH5	-1.15835	4.480665	-4.58332	1.50E-05	2.894867	0.00106	0.00173
Monocytes	KCNJ10	-1.85934	-0.1202	-4.56831	1.58E-05	1.093456	0.001264	0.002066
Monocytes	KNG1	-1.03625	6.039457	-4.56788	1.59E-05	2.815539	0.001062	0.001742
Monocytes	CD3E	-1.34591	3.278256	-4.56492	1.61E-05	2.806894	0.001153	0.001895
Monocytes	XLR	-1.02903	2.01867	-4.55835	1.65E-05	2.808197	0.001217	0.002002
Monocytes	CD33	1.031208	2.518088	4.549063	1.71E-05	2.765729	0.001233	0.002017
Monocytes	TNFRSF13B	0.557103	4.518479	4.547688	1.72E-05	2.777368	0.001165	0.001908
Monocytes	ATP1A3	0.759902	1.709416	4.539876	1.77E-05	2.756432	0.001284	0.002118
Monocytes	VAMP4	0.367861	6.154005	4.538748	1.78E-05	2.595048	0.001133	0.001875
Monocytes	TMTC2	-0.75791	4.574445	-4.53679	1.79E-05	2.570128	0.001185	0.001969
Monocytes	CD5L	-1.16767	6.068559	-4.53587	1.80E-05	2.612981	0.001136	0.001889
Monocytes	PAQR9	-1.17178	4.029832	-4.53331	1.81E-05	2.631541	0.001203	0.002005
Monocytes	NR4A3	-0.5786	6.012984	-4.53261	1.82E-05	2.433947	0.001138	0.0019
Monocytes	ETS1	-0.64487	7.418769	-4.5288	1.85E-05	2.674413	0.001103	0.001847
Monocytes	KLRA7	-2.99667	0.646446	-4.52174	1.90E-05	1.580277	0.001362	0.002274
Monocytes	SBF2	-0.59388	5.368073	-4.5041	2.03E-05	2.583621	0.001267	0.002107
Monocytes	GM47662	-1.94146	-0.79475	-4.50149	2.05E-05	0.976384	0.001514	0.002523
Monocytes	PACSIN1	-1.1872	2.953403	-4.48236	2.20E-05	2.222943	0.001455	0.002416
Monocytes	APP	0.50642	5.938302	4.477286	2.25E-05	2.232848	0.001349	0.002262
Monocytes	PTPN1	0.43101	7.078745	4.476875	2.25E-05	2.303802	0.001307	0.002192
Monocytes	GPR35	0.675259	2.516821	4.474274	2.27E-05	2.497217	0.001491	0.002507
Monocytes	MAN2A1	0.461867	7.183028	4.464234	2.36E-05	2.266951	0.001351	0.002277
Monocytes	KLF13	0.359398	8.183848	4.460837	2.39E-05	2.157832	0.001323	0.002236
Monocytes	CCR1	0.748791	2.32125	4.457393	2.42E-05	2.437439	0.00157	0.002663
Monocytes	TARM1	1.352771	-0.18149	4.434769	2.64E-05	2.087227	0.001821	0.003074
Monocytes	PTMS	-0.40892	6.015415	-4.43336	2.66E-05	2.145945	0.001529	0.002592
Monocytes	PGK1	0.504043	7.765914	4.432157	2.67E-05	2.061784	0.001456	0.002472
Monocytes	ADAM22	-1.00363	0.796933	-4.42823	2.71E-05	2.311808	0.001777	0.003027
Monocytes	BBX	-0.44828	6.494425	-4.42823	2.71E-05	2.178292	0.001513	0.002583
Monocytes	CLEC4A2	0.570771	3.416946	4.421172	2.78E-05	2.232892	0.001685	0.002864
Monocytes	TBRG1	-0.50363	5.618457	-4.4196	2.80E-05	2.250743	0.001584	0.002706
Monocytes	ALDOA	0.602889	8.900702	4.412897	2.87E-05	1.851558	0.001474	0.002525
Monocytes	GSTA3	-1.71945	4.0016	-4.40928	2.91E-05	2.211387	0.001703	0.00293
Monocytes	XCR1	-0.62388	0.412393	-4.39738	3.04E-05	2.181135	0.001961	0.003364
Monocytes	TPI1	0.527738	6.690385	4.393273	3.09E-05	2.069345	0.001658	0.002859
Monocytes	DAND5	-1.32985	3.96611	-4.39193	3.11E-05	2.172614	0.00179	0.003093
Monocytes	PCNX	0.473529	5.518161	4.386846	3.17E-05	2.13711	0.001737	0.003009
Monocytes	CD3G	-1.13134	3.343766	-4.38336	3.21E-05	2.213666	0.00186	0.003228
Monocytes	RAMP1	0.380184	4.507186	4.373289	3.33E-05	2.095639	0.001857	0.003235
Monocytes	TACC2	-1.35499	3.088667	-4.37157	3.35E-05	1.598593	0.001933	0.00338
Monocytes	SERPINB6A	-0.66929	5.570318	-4.36919	3.38E-05	2.134446	0.001803	0.003172
Monocytes	SQSTM1	-0.4109	7.466775	-4.36773	3.40E-05	1.83303	0.00171	0.003019
Monocytes	PRORSD1	0.585932	4.586531	4.367658	3.40E-05	2.160957	0.001853	0.003271
Monocytes	S100G	2.786106	0.605317	4.360999	3.49E-05	0.384394	0.002115	0.003726
Monocytes	TBC1D23	0.374774	5.852371	4.359468	3.51E-05	1.992927	0.001825	0.003231

Monocytes	CCR2	0.551203	3.092746	4.354041	3.58E-05	1.852038	0.002002	0.003542
Monocytes	ST7	0.600361	5.144851	4.349401	3.65E-05	2.077502	0.001904	0.003382
Monocytes	ARG2	0.754564	3.174154	4.349166	3.65E-05	2.073868	0.002012	0.003573
Monocytes	C5AR1	0.810511	4.179898	4.346794	3.68E-05	1.957583	0.001963	0.003496
Monocytes	TSPAN5	0.510169	5.373256	4.342581	3.74E-05	1.995228	0.001913	0.003428
Monocytes	APOA1	-1.22274	8.277086	-4.34201	3.75E-05	1.773835	0.001764	0.003165
Monocytes	F10	0.875164	4.54109	4.339706	3.78E-05	1.911018	0.001964	0.003526
Monocytes	FRRS1	0.449763	4.851486	4.334998	3.85E-05	1.954448	0.001966	0.00354
Monocytes	SERPINA3K	2.825545	1.878609	4.334292	3.86E-05	1.192507	0.002138	0.003852
Monocytes	IGF1	-1.02715	5.292109	-4.33267	3.88E-05	2.01112	0.001942	0.003508
Monocytes	BCL2L1	-0.69606	6.15449	-4.3316	3.90E-05	1.957208	0.001896	0.003432
Monocytes	PNKP	0.563565	4.959191	4.319356	4.08E-05	1.968349	0.002028	0.003671
Monocytes	TRBC2	-1.38006	3.943953	-4.31843	4.09E-05	1.993339	0.002087	0.003781
Monocytes	VCAM1	-1.18471	4.334733	-4.31807	4.10E-05	1.979029	0.002064	0.003742
Monocytes	MAST4	-0.79585	6.39259	-4.31715	4.11E-05	1.889077	0.001948	0.003538
Monocytes	GM11837	-1.34016	0.292319	-4.31578	4.13E-05	1.712664	0.002314	0.004209
Monocytes	ATP7A	0.384422	5.8288	4.312647	4.18E-05	1.822125	0.001993	0.003643
Monocytes	TMCC3	-0.72797	5.475701	-4.31051	4.22E-05	1.866656	0.002019	0.003697
Monocytes	H2AFJ	0.328014	7.521162	4.30758	4.26E-05	1.677505	0.001918	0.003506
Monocytes	ADAM3	1.509791	-0.17326	4.296915	4.44E-05	1.083048	0.002467	0.004467
Monocytes	SERPINF1	-1.06241	3.83888	-4.28979	4.56E-05	1.886377	0.002247	0.004087
Monocytes	EMILIN2	0.638466	4.009199	4.288871	4.57E-05	1.637108	0.002236	0.004074
Monocytes	RAB24	0.468284	5.118704	4.280779	4.71E-05	1.853135	0.002223	0.004049
Monocytes	PTGS2OS2	1.569031	-0.48663	4.270162	4.90E-05	1.039071	0.002675	0.004898
Monocytes	BCL2A1D	-0.62576	4.122114	-4.26978	4.91E-05	1.6003	0.002348	0.00431
Monocytes	HSPA5	-0.3363	8.355671	-4.26945	4.91E-05	1.429049	0.002086	0.003832
Monocytes	UBA52	0.389335	10.38927	4.267422	4.95E-05	1.140752	0.001972	0.00364
Monocytes	PTPRE	0.442945	5.539856	4.267271	4.95E-05	1.640064	0.002256	0.004166
Monocytes	CRIM1	-0.84957	5.191827	-4.2629	5.03E-05	1.783933	0.002304	0.004258
Monocytes	DLC1	-1.21822	4.570858	-4.24995	5.28E-05	1.758022	0.002449	0.004496
Monocytes	SUSD3	0.558588	3.170727	4.246154	5.36E-05	1.742971	0.002571	0.004727
Monocytes	TMEM39A	-0.45685	4.395391	-4.24469	5.39E-05	1.710991	0.002486	0.004581
Monocytes	SOAT1	0.456069	5.176779	4.23906	5.50E-05	1.533579	0.002465	0.004544
Monocytes	CCL24	-1.4637	3.596362	-4.2386	5.51E-05	1.721305	0.002577	0.004749
Monocytes	SLAIN1	-1.05796	3.6132	-4.23668	5.55E-05	1.295514	0.002582	0.004771
Monocytes	DGKG	0.530084	2.506906	4.229679	5.69E-05	1.59512	0.002722	0.005018
Monocytes	ITGAM	0.637209	4.619395	4.226296	5.77E-05	1.496137	0.002585	0.004773
Monocytes	RASL11B	-2.36587	0.665224	-4.21565	6.00E-05	-0.38752	0.002993	0.005496
Monocytes	RGL1	-0.71888	5.711711	-4.21223	6.07E-05	1.597159	0.002617	0.004825
Monocytes	CDC42SE2	-0.32482	7.179375	-4.20685	6.19E-05	1.355431	0.002546	0.004691
Monocytes	PSD3	-0.79291	5.794857	-4.20585	6.22E-05	1.602696	0.002646	0.00488
Monocytes	SNAPIN	0.548572	4.083623	4.202061	6.30E-05	1.606604	0.002776	0.005158
Monocytes	CIAO2A	0.363775	6.679979	4.201443	6.32E-05	1.351283	0.002581	0.004805
Monocytes	LAMB3	-0.80689	4.067884	-4.20079	6.33E-05	1.601222	0.002777	0.005172
Monocytes	F5	0.770941	4.094976	4.200263	6.35E-05	1.487474	0.002775	0.005168
Monocytes	PFKL	0.524934	5.330071	4.200163	6.35E-05	1.548367	0.00268	0.004993
Monocytes	VPS13C	0.53196	4.373385	4.199085	6.37E-05	1.565746	0.002753	0.005144

Monocytes	SOCS3	0.725811	4.698376	4.19715	6.42E-05	1.512915	0.002736	0.005129
Monocytes	PCOLCE2	-1.62889	1.678726	-4.19092	6.57E-05	1.178153	0.003035	0.005659
Monocytes	CD209F	-3.21569	2.767776	-4.188	6.64E-05	1.263933	0.002952	0.005535
Monocytes	LRRC32	-1.22814	1.241998	-4.18776	6.64E-05	1.22365	0.003082	0.005775
Monocytes	MARCKS	-0.41855	7.242888	-4.18668	6.67E-05	1.183121	0.002603	0.004902
Monocytes	S100A6	0.709782	7.580063	4.181801	6.79E-05	0.97583	0.002615	0.004937
Monocytes	TBC1D4	-0.56201	4.706501	-4.17468	6.97E-05	1.313854	0.002896	0.005452
Monocytes	H2-Q10	-1.16494	4.038149	-4.17218	7.03E-05	1.426435	0.002959	0.005585
Monocytes	MS4A6D	0.786781	2.866447	4.170548	7.08E-05	1.436829	0.003059	0.005794
Monocytes	APOBEC1	0.571605	6.087599	4.170058	7.09E-05	1.285489	0.002794	0.005302
Monocytes	ABCD2	0.750426	2.633823	4.169389	7.11E-05	1.498886	0.003079	0.005843
Monocytes	BIN1	-0.4474	5.230633	-4.16773	7.15E-05	1.430595	0.002864	0.005454
Monocytes	PRKCA	-0.74866	7.401898	-4.16693	7.17E-05	1.417368	0.002696	0.005138
Monocytes	FCRLA	-0.82521	5.107141	-4.16493	7.22E-05	1.476534	0.002883	0.005512
Monocytes	CKB	-0.51109	4.578345	-4.16269	7.28E-05	1.258524	0.002939	0.005625
Monocytes	PLCB1	0.52067	4.622522	4.158116	7.41E-05	1.113389	0.00297	0.005686
Monocytes	CUX1	0.328879	7.953864	4.157256	7.43E-05	1.118132	0.002706	0.005191
Monocytes	GPR141	0.679252	2.440611	4.1558	7.47E-05	1.229236	0.003162	0.006078
Monocytes	ERO1L	0.609424	5.171152	4.152039	7.57E-05	1.385364	0.002957	0.005678
Monocytes	ODC1	-0.49609	5.188947	-4.14375	7.81E-05	1.346571	0.003034	0.005809
Monocytes	IL13RA1	0.585013	3.558786	4.141979	7.86E-05	1.314102	0.003182	0.00611
Monocytes	CLIC4	-0.50451	7.449524	-4.14114	7.88E-05	0.989952	0.002853	0.005485
Monocytes	RARRES2	-1.39546	3.7462	-4.13863	7.95E-05	1.318186	0.003182	0.00611
Monocytes	APRT	0.433338	6.416788	4.133502	8.10E-05	1.091916	0.002996	0.005759
Monocytes	ADSSL1	0.47949	4.426295	4.131525	8.16E-05	1.336119	0.003178	0.006126
Monocytes	EEF1AKNMT	0.994461	2.318289	4.129051	8.24E-05	1.101062	0.00339	0.006534
Monocytes	CXCL9	-1.36702	2.080321	-4.1274	8.29E-05	1.356216	0.00342	0.006589
Monocytes	TCF7L2	-0.57549	7.34951	-4.12303	8.42E-05	1.030241	0.002986	0.005745
Monocytes	IFITM3	0.638846	7.709419	4.116991	8.60E-05	0.763139	0.00301	0.005765
Monocytes	SORL1	0.544164	5.66068	4.114518	8.68E-05	1.096843	0.003204	0.006137
Monocytes	CLEC4F	-1.4583	5.458864	-4.11115	8.79E-05	1.171918	0.003249	0.006228
Monocytes	CPNE8	-1.1492	2.649239	-4.0961	9.28E-05	1.145974	0.0037	0.007028
Monocytes	GPR171	-0.65015	4.027921	-4.09308	9.39E-05	1.165917	0.003584	0.006804
Monocytes	NCF2	0.391036	6.576577	4.086102	9.63E-05	0.882961	0.00341	0.006452
Monocytes	SERPIND1	-1.49901	2.832849	-4.08109	9.80E-05	0.90497	0.003841	0.007274
Monocytes	FRMD4B	-0.64984	5.267906	-4.08019	9.83E-05	1.03391	0.003587	0.006813
Monocytes	RRP1B	0.577293	4.132089	4.079047	9.87E-05	1.167971	0.003704	0.007055
Monocytes	HMGN1	-0.46624	7.26929	-4.07725	9.94E-05	0.951452	0.003402	0.006481
Monocytes	MINDY1	0.516955	3.987424	4.071554	0.000101	1.170952	0.003793	0.007214
Monocytes	PTPRCAP	-0.81956	5.807512	-4.06851	0.000103	1.166918	0.003631	0.006905
Monocytes	AI839979	0.757494	0.274557	4.065977	0.000104	1.161329	0.004267	0.008101
Monocytes	CLEC16A	-0.47431	4.763553	-4.06429	0.000104	1.098821	0.003768	0.007165
Monocytes	TRBC1	-1.01292	3.183542	-4.0617	0.000105	1.149183	0.003962	0.007552
Monocytes	DR1	0.370866	4.953577	4.054873	0.000108	1.044611	0.00385	0.007327
Monocytes	BCL2L14	-0.90672	0.459361	-4.03801	0.000114	1.06998	0.004616	0.008663
Monocytes	EGR3	-0.94518	3.533307	-4.03771	0.000115	1.021394	0.004231	0.007955
Monocytes	FAM102A	-0.66756	4.182226	-4.03621	0.000115	1.066805	0.004162	0.007849

Monocytes	CD3D	-1.22711	2.678526	-4.03307	0.000117	0.952566	0.004376	0.008232
Monocytes	GM28960	-2.01882	-0.82667	-4.03186	0.000117	-0.54909	0.004837	0.009099
Monocytes	RFFL	0.43527	5.915131	4.028036	0.000119	0.819502	0.004039	0.007619
Monocytes	LMNA	-0.57204	4.617164	-4.02692	0.000119	0.898823	0.004191	0.007918
Monocytes	CSTA2	1.567831	1.96893	4.025707	0.00012	0.96302	0.004519	0.008548
Monocytes	PPBP	2.068695	0.842206	4.020259	0.000122	0.655084	0.004726	0.008961
Monocytes	GIGYF2	0.432247	5.464029	4.020206	0.000122	0.933645	0.004148	0.00788
Monocytes	GM8797	-1.33139	2.19543	-4.01713	0.000123	0.312512	0.004582	0.008706
Monocytes	ABCC9	2.028986	1.277502	4.015429	0.000124	-0.08427	0.004715	0.008979
Monocytes	EHD3	-1.28769	3.517645	-4.01201	0.000126	0.727732	0.004465	0.008514
Monocytes	ZFP971	0.716974	1.911606	4.010836	0.000126	0.952593	0.004676	0.008908
Monocytes	SNTB2	0.496699	5.607555	4.004393	0.000129	0.804727	0.004297	0.008161
Monocytes	CTSW	-1.33072	2.120663	-3.99943	0.000131	0.790814	0.004809	0.009103
Monocytes	CAPN7	-0.37689	5.340998	-3.99676	0.000133	0.868345	0.004419	0.00838
Monocytes	ITGAX	-0.499	3.098724	-3.99493	0.000134	0.782315	0.004721	0.00895
Monocytes	MXD1	0.438833	6.036212	3.992717	0.000135	0.745285	0.004367	0.008289
Monocytes	BCL2A1A	-0.6481	2.399522	-3.99091	0.000136	0.735464	0.00485	0.009204
Monocytes	KLHL13	-2.65934	0.360663	-3.98977	0.000136	-0.76846	0.005139	0.009766
Monocytes	LAMTOR4	0.395125	6.205335	3.988686	0.000137	0.693235	0.004358	0.008326
Monocytes	DDI2	0.380058	5.943537	3.987825	0.000137	0.740342	0.00439	0.008401
Monocytes	PTPRM	-0.92766	5.186994	-3.98724	0.000137	0.842758	0.004484	0.008591
Monocytes	SH2B2	0.483583	4.550186	3.985187	0.000138	0.799391	0.004578	0.008783
Monocytes	PRUNE1	0.63529	3.600837	3.984602	0.000139	0.898633	0.004702	0.009024
Monocytes	IL1R2	0.807836	3.320344	3.978517	0.000142	0.782125	0.004827	0.00926
Monocytes	TSPAN15	-0.96788	2.388192	-3.97581	0.000143	0.710085	0.004987	0.009581
Monocytes	KLRA6	-2.244	-0.71608	-3.96904	0.000147	-0.56567	0.005558	0.010659
Monocytes	GM14029	-1.84818	0.297438	-3.96828	0.000147	-0.19972	0.0054	0.010377
Monocytes	CES1C	-1.45125	4.023969	-3.96437	0.000149	0.809491	0.004912	0.009466
Monocytes	BE692007	1.17359	2.816357	3.962826	0.00015	0.733048	0.005093	0.009829
Monocytes	PIK3R5	0.472019	4.45137	3.961715	0.00015	0.73725	0.004867	0.009415
Monocytes	PKP4	-0.55961	5.958306	-3.95935	0.000152	0.670152	0.004682	0.009072
Monocytes	DUSP3	0.324447	5.553941	3.958932	0.000152	0.551523	0.004736	0.009181
Monocytes	6-Sep	-0.4593	5.624977	-3.94266	0.000161	0.591852	0.004991	0.009631
Monocytes	EPHX1	-1.29442	3.053436	-3.93684	0.000164	0.426241	0.005458	0.010539
Monocytes	TMEM131	-0.29802	6.606387	-3.93537	0.000165	0.375301	0.00494	0.009564
Monocytes	GPR146	0.816742	3.278936	3.935115	0.000165	0.656721	0.005423	0.010492
Monocytes	TTR	-1.14106	9.202897	-3.92949	0.000169	0.230571	0.004674	0.009037
Monocytes	UBE2E3	-0.28483	7.066891	-3.92541	0.000171	0.434673	0.005016	0.009696
Monocytes	LRMDA	0.566342	7.760594	3.922723	0.000173	0.188033	0.004951	0.009544
Monocytes	CADM1	-0.59522	5.440386	-3.92043	0.000174	0.231832	0.005308	0.010253
Monocytes	IMPA2	0.473994	4.492173	3.916682	0.000176	0.626274	0.005507	0.010645
Monocytes	RTL8B	-0.45453	3.297356	-3.91114	0.00018	0.528535	0.005789	0.011149
Monocytes	RELB	-0.41467	6.088989	-3.89956	0.000187	0.410445	0.005559	0.010644
Monocytes	CLASP2	-0.3054	6.927855	-3.89488	0.00019	0.263312	0.005503	0.010531
Monocytes	2310022A10RIK	0.781632	2.998253	3.893683	0.000191	0.543816	0.006147	0.011778
Monocytes	OXCT1	-0.32767	5.719852	-3.893	0.000192	0.402605	0.005694	0.01093
Monocytes	CD7	-1.07082	4.199176	-3.88866	0.000195	0.570267	0.006015	0.011546

Monocytes	ABLIM1	-0.70994	6.121137	-3.88581	0.000197	0.590479	0.00574	0.011026
Monocytes	CAMKK2	0.334998	5.188179	3.884184	0.000198	0.414364	0.005906	0.011364
Monocytes	SULT2A5	-1.81078	2.02078	-3.88112	0.0002	0.01337	0.006457	0.012494
Monocytes	FAAH	-1.50235	1.451163	-3.88066	0.0002	-0.13782	0.006562	0.012706
Monocytes	ZBTB46	-0.49967	3.021217	-3.88009	0.000201	0.478196	0.006277	0.012179
Monocytes	EMB	0.529827	5.68619	3.879857	0.000201	0.237804	0.005825	0.01131
Monocytes	CD24A	-0.63896	7.730753	-3.87957	0.000201	0.227512	0.005502	0.010688
Monocytes	DMXL2	0.974447	2.146865	3.878576	0.000202	0.561277	0.006434	0.01252
Monocytes	TRAC	-1.43989	2.266621	-3.8782	0.000202	0.341023	0.006413	0.012486
Monocytes	SERPINF2	-1.26469	3.929119	-3.8757	0.000204	0.511207	0.006141	0.012004
Monocytes	MTMR3	0.300224	7.886466	3.874808	0.000204	0.178513	0.005497	0.010754
Monocytes	CSTDC4	1.668243	3.661016	3.874656	0.000205	0.554923	0.006187	0.012097
Monocytes	CLEC9A	-0.50772	2.519945	-3.87179	0.000207	0.326429	0.006405	0.012577
Monocytes	CHKA	-0.40859	6.940391	-3.87164	0.000207	0.287265	0.005658	0.011121
Monocytes	GPR183	0.62285	3.488292	3.87145	0.000207	0.462856	0.006232	0.012243
Monocytes	RNF125	-1.01819	3.852901	-3.8698	0.000208	0.480407	0.006186	0.01218
Monocytes	SNCA	2.608133	4.248926	3.866863	0.00021	0.150307	0.006154	0.012148
Monocytes	CXCL16	-0.55205	3.468405	-3.86644	0.00021	0.3616	0.006291	0.012425
Monocytes	FER	-0.91808	3.489418	-3.85806	0.000217	0.451352	0.006443	0.012703
Monocytes	UBL3	0.248794	7.408809	3.857773	0.000217	0.113405	0.005774	0.011394
Monocytes	KPTN	-0.74324	4.098478	-3.85671	0.000218	0.502644	0.006339	0.012501
Monocytes	CD180	-0.43647	5.032672	-3.8531	0.000221	0.267906	0.006222	0.012265
Monocytes	IL21R	-0.63564	5.150614	-3.85287	0.000221	0.407206	0.006202	0.012225
Monocytes	ECE1	-0.44759	5.613294	-3.84614	0.000226	0.181381	0.00625	0.012304
Monocytes	TSPAN4	-0.80341	3.356981	-3.84419	0.000227	0.391862	0.006685	0.013165
Monocytes	CFI	-1.36354	3.743943	-3.83825	0.000232	0.396043	0.006732	0.013255
Monocytes	LIMS1	0.33162	7.288787	3.835982	0.000234	0.097732	0.006126	0.012073
Monocytes	CES1D	-1.84661	1.90006	-3.83519	0.000235	-0.22264	0.007127	0.014056
Monocytes	SULT2B1	-0.91101	2.700547	-3.83395	0.000236	0.382805	0.006978	0.01378
Monocytes	DYNLL2	-0.49067	5.082989	-3.83144	0.000238	0.300314	0.006536	0.012991
Monocytes	CYP3A11	-1.6072	3.352158	-3.83115	0.000238	0.257762	0.006861	0.013632
Monocytes	NECAB2	-2.04836	0.442881	-3.83109	0.000238	-0.67698	0.00745	0.01478
Monocytes	TBX21	-1.0467	2.437202	-3.82536	0.000243	0.387543	0.007149	0.014192
Monocytes	SH3KBP1	0.297735	7.863843	3.8251	0.000243	0.012564	0.006141	0.012206
Monocytes	MIR155HG	-0.7045	4.086438	-3.82415	0.000244	0.269084	0.006828	0.013595
Monocytes	CES1G	-2.22794	0.85155	-3.82308	0.000245	-0.70836	0.007489	0.014921
Monocytes	IGSF5	-1.56975	1.922281	-3.82181	0.000246	0.015726	0.007277	0.01452
Monocytes	FAM111A	0.398122	5.742953	3.819047	0.000248	0.193651	0.00658	0.013139
Monocytes	LYZ2	0.695877	7.170673	3.814669	0.000252	-0.41412	0.006402	0.01278
Monocytes	ANAPC16	0.358506	5.656735	3.812922	0.000254	0.262175	0.006701	0.013395
Monocytes	PPT1	-0.32385	6.003515	-3.80888	0.000257	-0.02054	0.006706	0.013411
Monocytes	HSD17B2	-1.35015	2.446494	-3.80835	0.000258	-0.02531	0.007411	0.014824
Monocytes	PTGS1	-0.66953	3.986721	-3.80385	0.000262	0.319418	0.007188	0.014363
Monocytes	PHF11A	-0.70335	1.004104	-3.79923	0.000266	0.3115	0.007922	0.015748
Monocytes	SH2D1A	-1.71008	0.051779	-3.79806	0.000267	-0.53583	0.008139	0.016226
Monocytes	IRF2BP2	0.31834	7.59206	3.797773	0.000267	-0.03823	0.006584	0.01316
Monocytes	NMD3	-0.37182	4.999909	-3.79419	0.000271	0.266517	0.007147	0.014277

Monocytes	GM32089	1.357241	-1.09921	3.792553	0.000272	-0.3135	0.008518	0.016979
Monocytes	NFE2	0.695281	1.59738	3.791072	0.000274	0.303228	0.007909	0.015816
Monocytes	CP	-1.00731	4.588111	-3.78713	0.000277	0.291278	0.00735	0.014699
Monocytes	NREP	-1.62604	1.234795	-3.78342	0.000281	-0.50461	0.008162	0.016254
Monocytes	ATRN	0.399022	5.965865	3.773286	0.000291	0.006726	0.007378	0.01464
Monocytes	HDAC9	-0.52797	7.563843	-3.76996	0.000294	-0.21801	0.00712	0.01413
Monocytes	FFAR2	2.085837	-0.53401	3.768979	0.000295	-0.83818	0.00895	0.017727
Monocytes	CHD3	-0.65093	4.533669	-3.76497	0.000299	0.218506	0.007842	0.015552
Monocytes	UAP1	-0.3854	5.112787	-3.76243	0.000302	0.040704	0.007743	0.015405
Monocytes	HBB-BS	1.740859	11.91128	3.762411	0.000302	-0.49543	0.006414	0.012751
Monocytes	FRY	0.404771	6.378637	3.757883	0.000307	-0.11754	0.007571	0.015039
Monocytes	ITIH2	-1.42253	3.79432	-3.75695	0.000308	0.172271	0.008145	0.016187
Monocytes	FNBP1	-0.32594	8.248905	-3.75503	0.00031	-0.42038	0.007221	0.014368
Monocytes	EMILIN1	0.731504	2.608435	3.747559	0.000318	0.112058	0.008654	0.017124
Monocytes	C1QC	-0.82618	6.384084	-3.74561	0.00032	-0.15935	0.007815	0.015475
Monocytes	GM16556	1.149275	1.676001	3.743556	0.000322	0.01434	0.008927	0.017689
Monocytes	ETHE1	0.43901	4.997365	3.743547	0.000322	0.072836	0.008129	0.016127
Monocytes	LILRB4A	0.47451	4.163584	3.742173	0.000324	-0.13041	0.008322	0.016538
Monocytes	PLEKHF2	0.356047	5.470642	3.742034	0.000324	0.020147	0.008022	0.015947
Monocytes	NFIL3	0.485087	4.515288	3.741692	0.000324	-0.13905	0.00824	0.016386
Monocytes	CKAP4	0.575099	4.030233	3.73993	0.000326	0.087098	0.008365	0.016678
Monocytes	CD36	-0.59148	5.712063	-3.7398	0.000326	-0.18755	0.00798	0.015915
Monocytes	FUT10	1.868891	-0.01434	3.737173	0.000329	-1.02008	0.00944	0.018755
Monocytes	TNFRSF1B	0.426309	4.903674	3.736215	0.000331	-0.10258	0.008222	0.016402
Monocytes	DCAKD	0.646235	4.076447	3.732887	0.000334	0.12775	0.00849	0.016947
Monocytes	ASB2	-0.93031	2.906427	-3.72981	0.000338	0.118231	0.008845	0.017612
Monocytes	PDE1C	-1.51041	3.989093	-3.72724	0.000341	0.008767	0.008615	0.017203
Monocytes	CCDC138	-0.69868	4.628632	-3.72714	0.000341	0.086149	0.008462	0.016899
Monocytes	MYO5A	0.49004	5.016572	3.72532	0.000343	-0.04293	0.008402	0.016768
Monocytes	RAPGEFL1	-1.60901	2.784487	-3.72256	0.000346	-0.60698	0.009009	0.018001
Monocytes	NAT2	0.966534	2.532818	3.71962	0.00035	-0.07661	0.009142	0.018269
Monocytes	KSR2	-1.22769	2.695294	-3.71548	0.000355	0.013812	0.009185	0.018342
Monocytes	CDC14A	-0.46187	5.803629	-3.71546	0.000355	-0.15102	0.008417	0.016822
Monocytes	MYB	-0.9035	5.137938	-3.71321	0.000358	0.062745	0.00862	0.01722
Monocytes	VEGFB	-0.74879	3.802219	-3.71084	0.000361	0.033296	0.008973	0.017946
Monocytes	GM14221	-1.12259	1.910062	-3.71037	0.000361	0.020758	0.009465	0.01893
Monocytes	AUH	0.341712	5.641378	3.710292	0.000361	-0.18643	0.008522	0.017063
Monocytes	SEMA6D	-0.92674	3.713919	-3.7057	0.000367	0.034897	0.009115	0.018205
Monocytes	HIST3H2BA	1.415081	0.997637	3.701202	0.000373	-0.76013	0.009949	0.019837
Monocytes	SDHAF1	0.5588	4.132974	3.698702	0.000376	0.0037	0.009106	0.018267
Monocytes	GAPDH	0.408218	10.59184	3.6986	0.000376	-0.8078	0.007609	0.015262
Monocytes	IKZF2	-0.88189	4.781138	-3.69816	0.000376	-0.09059	0.008942	0.017947
Monocytes	GRASP	-0.43349	3.781204	-3.69722	0.000378	-0.15638	0.009197	0.018486
Monocytes	CD164	-0.41744	6.379209	-3.69677	0.000378	-0.12184	0.008551	0.017202
Monocytes	1200007C13RIK	1.777375	-0.38465	3.696243	0.000379	-0.72219	0.010348	0.020763
Monocytes	TTC39A	-0.65972	2.027911	-3.6962	0.000379	-0.01143	0.009663	0.019417
Monocytes	LIFR	0.70739	3.842745	3.696179	0.000379	-0.0217	0.009181	0.018461

Monocytes	CAR3	-2.03736	3.476221	-3.68972	0.000387	-0.07345	0.009441	0.018902
Monocytes	ZDBF2	-2.27506	-0.23932	-3.6896	0.000388	-1.0371	0.010489	0.020958
Monocytes	TNFRSF13C	-0.89146	3.92598	-3.68228	0.000397	-0.06837	0.009505	0.018966
Monocytes	GNPTG	-0.70365	3.368723	-3.68218	0.000397	-0.07739	0.009656	0.019263
Monocytes	RBBP7	-0.34912	6.2357	-3.68179	0.000398	-0.26724	0.008909	0.017799
Monocytes	PROC	-1.2764	2.923674	-3.68038	0.0004	-0.19873	0.0098	0.019601
Monocytes	GGH	0.445044	4.555539	3.679331	0.000401	-0.25178	0.00936	0.018775
Monocytes	SP3	0.294196	6.891028	3.679053	0.000402	-0.32461	0.008768	0.017593
Monocytes	GOT1	-0.41593	6.081478	-3.6748	0.000408	-0.35211	0.009078	0.018195
Monocytes	PLCG2	0.274436	7.02313	3.673534	0.000409	-0.41534	0.00886	0.017773
Monocytes	MGP	2.12474	0.463003	3.667829	0.000417	-0.92395	0.010839	0.021606
Monocytes	GNB4	-0.68579	2.895468	-3.66539	0.000421	-0.07314	0.010178	0.020301
Monocytes	AKR1C6	-1.30625	4.841164	-3.6647	0.000422	-0.08985	0.009636	0.019263
Monocytes	GRK5	0.472815	5.747033	3.662459	0.000425	-0.47959	0.009444	0.018896
Monocytes	GP9	1.582193	0.659096	3.659801	0.000429	-0.3148	0.010947	0.021942
Monocytes	GM14634	-1.18134	1.834434	-3.65947	0.000429	-0.34731	0.010588	0.021249
Monocytes	IER3	0.551575	6.13903	3.659181	0.00043	-0.52119	0.009381	0.018859
Monocytes	SLC30A9	0.396444	5.265307	3.657225	0.000433	-0.17248	0.009655	0.019369
Monocytes	OSM	0.634533	2.305551	3.654626	0.000436	-0.25408	0.010562	0.021139
Monocytes	GRAP2	-0.54557	4.975054	-3.64724	0.000447	-0.33981	0.01002	0.019969
Monocytes	CSRP1	-0.47242	5.881401	-3.64663	0.000448	-0.28047	0.009769	0.019481
Monocytes	NCK2	-0.45705	4.870391	-3.64153	0.000456	-0.33184	0.010189	0.020273
Monocytes	NAAA	-0.34727	3.389339	-3.64119	0.000457	-0.48753	0.010623	0.021129
Monocytes	NXF7	-1.93143	-1.34285	-3.63667	0.000464	-1.64329	0.012307	0.024391
Monocytes	PXK	0.357559	6.270189	3.632072	0.000471	-0.41088	0.01005	0.019881
Monocytes	GRSF1	0.318781	5.185543	3.631631	0.000472	-0.3109	0.01036	0.020501
Monocytes	GM13986	0.903222	1.376917	3.630385	0.000474	-0.21573	0.011534	0.022824
Monocytes	OSBPL9	0.257016	7.511715	3.629376	0.000475	-0.57103	0.009708	0.01927
Monocytes	WFDC18	1.355748	-0.88323	3.629117	0.000476	-0.92821	0.012299	0.024337
Monocytes	TNFAIP8L2	0.507384	3.852483	3.628998	0.000476	-0.21456	0.010755	0.021336
Monocytes	RIPK2	0.469463	4.205503	3.625402	0.000482	-0.31558	0.010736	0.021306
Monocytes	C1D	0.295023	5.843961	3.625264	0.000482	-0.39739	0.010254	0.020356
Monocytes	TRAF4	-0.93514	4.196822	-3.6245	0.000483	-0.19465	0.010742	0.021333
Monocytes	TAP2	-0.52943	5.110651	-3.61718	0.000495	-0.36435	0.010707	0.021118
Monocytes	SLFN2	0.479628	6.335056	3.616185	0.000497	-0.58328	0.010358	0.020452
Monocytes	DSG2	-1.94437	0.740688	-3.61556	0.000498	-1.22312	0.012125	0.023898
Monocytes	GM19585	-1.2384	2.331819	-3.61441	0.0005	-0.26733	0.01161	0.022942
Monocytes	ADTRP	-1.58534	1.835711	-3.6111	0.000505	-0.82543	0.01188	0.023484
Monocytes	IFNAR2	0.320442	7.128736	3.607599	0.000511	-0.62103	0.010337	0.020477
Monocytes	SAP30	0.400165	5.143228	3.606854	0.000513	-0.42709	0.010931	0.021673
Monocytes	RFX7	-0.35391	7.368214	-3.60352	0.000518	-0.64897	0.010365	0.020549
Monocytes	ITK	-0.88074	4.224474	-3.59863	0.000527	-0.36948	0.011476	0.022663
Monocytes	MERTK	-0.85867	3.774359	-3.59782	0.000528	-0.30321	0.011622	0.023
Monocytes	ERC1	-0.56497	4.872109	-3.59716	0.00053	-0.31085	0.011269	0.022335
Monocytes	BATF	0.488284	4.183315	3.596809	0.00053	-0.32982	0.011489	0.022783
Monocytes	EMID1	-1.15831	3.280299	-3.59513	0.000533	-0.63851	0.01182	0.023479
Monocytes	TCRG-C1	-1.90487	0.880703	-3.59463	0.000534	-0.74237	0.01265	0.02512

Monocytes	PLAUR	0.468834	6.837467	3.593079	0.000537	-0.81179	0.010731	0.021352
Monocytes	ELMSAN1	-0.37945	6.924696	-3.59165	0.000539	-0.7367	0.010733	0.02136
Monocytes	KRT80	0.540164	0.161146	3.589445	0.000543	-0.30365	0.013028	0.025928
Monocytes	NAGK	-0.50354	3.956557	-3.58939	0.000543	-0.32634	0.011702	0.023331
Monocytes	CDKN1A	0.415233	5.267608	3.58756	0.000547	-0.63308	0.011324	0.022565
Monocytes	TSPAN3	-0.45565	4.739337	-3.58625	0.000549	-0.44401	0.011519	0.022968
Monocytes	KTN1	-0.31973	5.826158	-3.58435	0.000553	-0.4969	0.011208	0.02237
Monocytes	SHTN1	-0.38668	3.187667	-3.58407	0.000553	-0.59681	0.01207	0.024086
Monocytes	DOCK4	0.370188	6.427831	3.579755	0.000561	-0.79886	0.01115	0.022252
Monocytes	PITPNA	0.222188	7.992513	3.578671	0.000563	-0.83507	0.010675	0.02135
Monocytes	TREM1	0.646333	1.639916	3.578532	0.000564	-0.37631	0.012757	0.025478
Monocytes	AEBP2	-0.34556	5.787827	-3.57809	0.000564	-0.5846	0.011351	0.022702
Monocytes	CDKN1C	-1.29503	3.302414	-3.57624	0.000568	-0.41625	0.012221	0.024431
Monocytes	SLC25A12	0.374736	5.092687	3.573887	0.000572	-0.44819	0.011689	0.023382
Monocytes	GSTP1	0.41374	6.928594	3.572605	0.000575	-0.68177	0.011109	0.022278
Monocytes	KLK8	-1.08094	1.605933	-3.57251	0.000575	-0.61311	0.012901	0.025835
Monocytes	VEGFA	0.570586	4.223513	3.571449	0.000577	-0.42559	0.011983	0.024074
Monocytes	GRAMD3	-0.52414	6.080829	-3.57124	0.000577	-0.5241	0.011375	0.022861
Monocytes	PRKAR2A	-0.45633	5.222556	-3.57058	0.000579	-0.74524	0.011652	0.023439
Monocytes	ATP11B	0.335889	7.035822	3.570021	0.00058	-0.73276	0.011076	0.022309
Monocytes	ABHD17B	0.314836	6.542955	3.565978	0.000588	-0.58017	0.011358	0.022832
Monocytes	PGAM1	0.363001	6.892134	3.565227	0.000589	-0.76977	0.011254	0.02265
Monocytes	MSH2	-0.59786	4.128132	-3.56452	0.000591	-0.36599	0.012163	0.024496
Monocytes	XYLT1	0.356435	7.355246	3.563585	0.000592	-0.94723	0.011125	0.022429
Monocytes	CRPPA	-0.82878	2.552625	-3.56292	0.000594	-0.39057	0.012731	0.025674
Monocytes	PYGL	0.470333	5.16283	3.559169	0.000601	-0.65406	0.011954	0.024102
Monocytes	AHSG	-0.95856	9.130873	-3.558	0.000603	-0.95858	0.010705	0.021623
Monocytes	SERPINC1	-0.8749	5.285455	-3.55739	0.000605	-0.46092	0.011914	0.024089
Monocytes	SERPINA6	-2.20247	1.127419	-3.55731	0.000605	-1.0891	0.013396	0.027043
Monocytes	DDX28	0.983668	1.558733	3.556408	0.000607	-0.65311	0.013246	0.02675
Monocytes	MAPK3	0.380816	4.874121	3.550952	0.000618	-0.57085	0.01226	0.024687
Monocytes	BC035044	-0.56485	5.146379	-3.54786	0.000624	-0.5954	0.012257	0.024662
Monocytes	S100A8	0.876481	6.633628	3.547146	0.000626	-1.06592	0.011757	0.0237
Monocytes	POR	0.388788	5.6252	3.546134	0.000628	-0.6602	0.012093	0.024427
Monocytes	G3BP1	-0.30271	6.728766	-3.54603	0.000628	-0.7758	0.011726	0.023688
Monocytes	TMEM123	-0.3699	6.484305	-3.54573	0.000629	-0.70553	0.011806	0.023855
Monocytes	ZBTB4	-0.54625	3.655496	-3.53571	0.00065	-0.46043	0.013186	0.026431
Monocytes	4930486L24RIK	-1.71289	0.484692	-3.53493	0.000651	-1.22243	0.014432	0.028901
Monocytes	PLAAT3	-0.59081	5.19136	-3.53191	0.000658	-0.62937	0.012739	0.02553
Monocytes	AGMO	-0.68155	3.650343	-3.52905	0.000664	-0.5103	0.013402	0.026753
Monocytes	KLRA2	0.663738	2.524963	3.528222	0.000666	-0.63247	0.013845	0.027655
Monocytes	RASAL2	-0.61952	5.468995	-3.52586	0.000671	-0.59287	0.012819	0.025632
Monocytes	HDAC11	-2.02994	0.5008	-3.52527	0.000673	-1.52475	0.014748	0.029461
Monocytes	DNAJC3	-0.30735	6.725717	-3.52319	0.000677	-0.86968	0.012387	0.024877
Monocytes	PLAC8	0.700165	8.2646	3.523181	0.000677	-1.17858	0.011868	0.023834
Monocytes	HLX	0.636945	3.117664	3.522859	0.000678	-0.48488	0.013706	0.027506
Monocytes	SLC27A2	-1.18554	3.949769	-3.52267	0.000678	-0.49126	0.013388	0.026876

Monocytes	TM4SF5	-1.17633	0.261552	-3.5212	0.000682	-0.59054	0.014904	0.029869
Monocytes	ATXN1	-0.45902	6.919224	-3.51864	0.000688	-0.82559	0.012436	0.024963
Monocytes	CYP2C68	-1.42019	2.791797	-3.51757	0.00069	-0.70293	0.013962	0.028032
Monocytes	MGST3	0.60872	3.846236	3.5175	0.00069	-0.55489	0.013553	0.027221
Monocytes	AMBP	-0.94539	4.730423	-3.51538	0.000695	-0.53294	0.013268	0.026722
Monocytes	CTPS2	0.414439	5.066744	3.51491	0.000696	-0.60198	0.013143	0.026488
Monocytes	CD209G	-4.01202	1.089148	-3.51446	0.000697	-1.16742	0.014704	0.029587
Monocytes	CYFIP1	0.359561	5.64309	3.514084	0.000698	-0.80049	0.012933	0.026081
Monocytes	B3GNT5	0.713358	2.546338	3.510619	0.000706	-0.52192	0.014245	0.028678
Monocytes	WTIP	-1.05136	1.051984	-3.50789	0.000712	-0.749	0.014964	0.030096
Monocytes	ATPIF1	-0.30843	7.745006	-3.50736	0.000714	-1.08303	0.012399	0.024983
Monocytes	TLR12	-0.99209	2.045397	-3.50593	0.000717	-0.54727	0.014589	0.02939
Monocytes	DUSP22	0.424871	4.486105	3.503569	0.000723	-0.69976	0.013699	0.027625
Monocytes	SPIDR	0.378892	5.228567	3.502569	0.000725	-0.6972	0.013428	0.027137
Monocytes	CYP2C23	-1.78714	0.860959	-3.50217	0.000726	-1.33323	0.015188	0.030643
Monocytes	SYPL	0.339045	5.860238	3.501598	0.000727	-0.78143	0.013193	0.02669
Monocytes	IFNAR1	0.371004	5.246213	3.5001	0.000731	-0.71564	0.013454	0.027261
Monocytes	VSIR	0.406169	4.468275	3.499714	0.000732	-0.76026	0.013751	0.027866
Monocytes	GNA13	-0.31542	7.201206	-3.49898	0.000734	-1.00882	0.012746	0.025853
Monocytes	LRP1	0.401873	4.178873	3.495246	0.000743	-0.8278	0.014016	0.02836
Monocytes	SYK	0.33536	8.336181	3.493726	0.000746	-1.1428	0.012519	0.025365
Monocytes	CSTA3	2.261753	0.791872	3.492098	0.00075	-1.11235	0.015525	0.031393
Monocytes	BTBD11	-0.91142	4.309098	-3.49115	0.000753	-0.59141	0.014075	0.02854
Monocytes	ZFP984	0.494969	3.878402	3.489697	0.000756	-0.58793	0.014288	0.028972
Monocytes	ODF2	0.322618	5.483537	3.488668	0.000759	-0.74784	0.013679	0.027751
Monocytes	CEBPB	0.494695	9.155958	3.487125	0.000763	-1.48236	0.01239	0.025127
Monocytes	GM10371	-1.73582	-0.68835	-3.48597	0.000766	-1.60745	0.016369	0.033102
Monocytes	HACD4	0.49111	3.532004	3.477141	0.000788	-0.82086	0.014924	0.030088
Monocytes	MAFF	-0.53536	3.876198	-3.47522	0.000793	-0.74613	0.014846	0.02993
Monocytes	STK39	-0.98592	3.517793	-3.47386	0.000797	-0.62394	0.015025	0.030277
Monocytes	APOA5	-1.70893	1.793969	-3.47352	0.000798	-1.0664	0.015774	0.031764
Monocytes	CABLES1	-1.16553	4.513404	-3.46858	0.000811	-0.63808	0.014821	0.029827
Monocytes	EIF4E	-0.32095	6.950147	-3.46723	0.000814	-1.11175	0.013877	0.027927
Monocytes	APOL7C	-1.69079	-0.99321	-3.46671	0.000815	-1.03822	0.017361	0.034839
Monocytes	RETNLG	1.961299	3.313557	3.46577	0.000818	-0.79471	0.015366	0.030979
Monocytes	EIF4EBP1	0.320178	5.454955	3.465631	0.000818	-0.89357	0.014468	0.029188
Monocytes	WWOX	0.336809	6.961551	3.463428	0.000824	-1.01193	0.013947	0.028107
Monocytes	CALHM2	0.572826	3.088827	3.462415	0.000827	-0.6552	0.015552	0.031371
Monocytes	SNX18	0.370156	6.77742	3.462228	0.000828	-1.04383	0.014023	0.02831
Monocytes	CD247	-0.91226	3.01096	-3.46084	0.000831	-0.67014	0.015616	0.031547
Monocytes	STRIP2	-0.63465	1.016356	-3.46024	0.000833	-0.69776	0.016523	0.03337
Monocytes	PRELID1	0.287067	7.529242	3.459969	0.000834	-1.12198	0.01376	0.02784
Monocytes	SMAGP	-0.61273	3.61538	-3.45737	0.000841	-0.6825	0.015456	0.03123
Monocytes	CCSER2	-0.44182	4.778998	-3.45542	0.000846	-0.7848	0.015027	0.030377
Monocytes	PKM	0.380722	8.60779	3.45133	0.000858	-1.32795	0.013662	0.02758
Monocytes	ARID1B	-0.2486	8.198201	-3.44783	0.000867	-1.20979	0.013952	0.02811
Monocytes	GAB2	0.378294	7.176286	3.446139	0.000872	-1.25991	0.014407	0.028997

Monocytes	HBA-A1	1.684701	10.46682	3.443375	0.00088	-1.30697	0.013239	0.026622
Monocytes	4632427E13RIK	0.540497	4.404143	3.443069	0.000881	-0.72178	0.015669	0.03152
Monocytes	YWHAQ	-0.23883	7.746706	-3.44202	0.000884	-1.16665	0.014296	0.028782
Monocytes	MTHFSL	-0.32422	5.211119	-3.44054	0.000888	-0.97809	0.015391	0.031011
Monocytes	TLR7	0.710954	2.963787	3.437467	0.000897	-0.74389	0.01653	0.033262
Monocytes	ITPR3	-0.80993	4.288295	-3.43667	0.000899	-0.73994	0.015938	0.032115
Monocytes	ZEB2	0.416662	9.36649	3.435759	0.000902	-1.55056	0.013836	0.027919
Monocytes	DCLRE1B	0.649374	2.770832	3.435444	0.000903	-0.75289	0.016636	0.033551
Monocytes	PKIG	0.383607	7.198634	3.435016	0.000904	-1.01062	0.014694	0.02969
Monocytes	FBXW11	-0.31646	7.259166	-3.43256	0.000912	-1.2304	0.014761	0.029816
Monocytes	DENND6A	0.371811	5.102952	3.431658	0.000914	-0.87076	0.015681	0.031728
Monocytes	SSH1	-0.50715	3.775324	-3.4314	0.000915	-0.75847	0.016277	0.032936
Monocytes	MAML3	0.459705	6.974808	3.429763	0.00092	-1.12398	0.014923	0.030227
Monocytes	CXCR6	-1.27385	1.255073	-3.42947	0.000921	-0.92297	0.017527	0.035448
Monocytes	NDUFB1-PS	0.218612	8.902498	3.427794	0.000926	-1.41851	0.014181	0.028742
Monocytes	GM28192	0.921909	0.064757	3.427629	0.000926	-0.87254	0.018175	0.036755
Monocytes	PEX10	1.506004	0.82136	3.426427	0.00093	-1.42351	0.017827	0.036102
Monocytes	ACPP	0.934151	2.739914	3.424939	0.000934	-0.76285	0.016908	0.034338
Monocytes	CSF3R	0.534467	2.933462	3.424931	0.000934	-0.91529	0.016816	0.034153
Monocytes	FBXO28	0.403994	4.523471	3.423822	0.000938	-0.89676	0.01611	0.032713
Monocytes	CARD19	0.352634	5.613475	3.42085	0.000947	-1.10686	0.01575	0.031952
Monocytes	SFPQ	-0.24475	8.189863	-3.42014	0.000949	-1.28014	0.014667	0.029773
Monocytes	RCN2	-0.44219	5.075901	-3.4179	0.000956	-0.91195	0.016087	0.032641
Monocytes	MBNL2	-0.26904	8.406165	-3.41687	0.000959	-1.4676	0.01466	0.029838
Monocytes	DLST	0.281045	5.852364	3.416738	0.00096	-1.01061	0.015741	0.032037
Monocytes	FAM167A	-1.49337	1.837572	-3.41622	0.000961	-1.08964	0.017623	0.035853
Monocytes	TOX	-0.8771	4.351569	-3.4158	0.000963	-0.84176	0.016417	0.033451
Monocytes	UCK2	0.375473	5.87449	3.414915	0.000965	-1.20602	0.015749	0.032142
Monocytes	GZMB	-0.95615	3.971899	-3.41337	0.00097	-0.91767	0.016668	0.03398
Monocytes	PI4K2A	-0.32908	5.639245	-3.41178	0.000975	-1.16745	0.015961	0.032518
Monocytes	CD27	-0.89801	3.318035	-3.40934	0.000983	-0.84252	0.017143	0.034862
Monocytes	ARHGDIB	0.346273	8.995183	3.408736	0.000985	-1.48241	0.014633	0.029813
Monocytes	UOX	1.006958	5.010934	3.407043	0.00099	-0.88029	0.016412	0.033452
Monocytes	GPAT3	0.475276	5.178486	3.404897	0.000997	-0.92829	0.016421	0.033475
Monocytes	CREB3L3	-1.46122	1.62088	-3.40052	0.001011	-1.28032	0.018381	0.037293
Monocytes	DYRK2	-0.42971	4.884934	-3.39869	0.001017	-0.90221	0.016836	0.034198
Monocytes	C6	-1.11518	2.802964	-3.39781	0.00102	-0.83425	0.017865	0.036293
Monocytes	BANK1	-0.70803	6.09331	-3.39708	0.001023	-1.05026	0.016287	0.033125
Monocytes	DSTYK	0.424745	3.925134	3.396846	0.001023	-0.88216	0.017308	0.035186
Monocytes	CLEC4A1	0.551807	3.158732	3.396401	0.001025	-1.12819	0.017686	0.035982
Monocytes	MED14	-0.3622	6.321429	-3.39444	0.001031	-1.21308	0.01626	0.033089
Monocytes	NCEH1	0.435463	4.906119	3.393544	0.001034	-1.07978	0.016938	0.034497
Monocytes	ASAP2	-0.48245	3.068411	-3.39236	0.001038	-0.9815	0.017875	0.036386
Monocytes	COBLL1	-0.66672	5.069977	-3.38888	0.00105	-0.86783	0.01705	0.034665
Monocytes	NUP210L	-0.63105	6.407383	-3.38853	0.001051	-1.20185	0.016424	0.033398
Monocytes	CALM1	-0.20994	9.782329	-3.38667	0.001058	-1.68588	0.015021	0.030533
Monocytes	KLHL2	0.330686	5.570335	3.383758	0.001067	-1.17436	0.016998	0.034585

Monocytes	MTHFS	-0.53373	5.861718	-3.38335	0.001069	-1.24854	0.01686	0.034337
Monocytes	GEM	-0.67764	5.207345	-3.38272	0.001071	-0.93692	0.017171	0.035013
Monocytes	KANK1	-1.36508	1.172928	-3.38235	0.001072	-1.30203	0.01924	0.039187
Monocytes	CES2E	-1.51333	1.228112	-3.38207	0.001073	-1.35298	0.01921	0.039137
Monocytes	MT1	0.65284	6.314344	3.380321	0.001079	-1.32436	0.016715	0.034121
Monocytes	CLDN5	-1.91475	1.006603	-3.37888	0.001084	-1.69954	0.019454	0.039658
Monocytes	ZFYVE9	0.458671	2.603777	3.378584	0.001085	-1.1214	0.018594	0.037957
Monocytes	SCIN	-1.17402	0.090871	-3.37673	0.001092	-0.94906	0.020027	0.04083
Monocytes	TRGV2	-1.85983	-0.51519	-3.37611	0.001094	-1.55827	0.020375	0.041523
Monocytes	SNN	-1.00784	3.649853	-3.37606	0.001094	-0.98496	0.018108	0.036984
Monocytes	DST	-0.75465	4.335211	-3.37563	0.001096	-0.90462	0.017762	0.036308
Monocytes	AZI2	0.292091	5.839646	3.37242	0.001107	-1.13186	0.017177	0.035099
Monocytes	ATP6AP1	0.310216	5.990589	3.371751	0.00111	-1.18501	0.017114	0.035
Monocytes	GYPA	2.117165	3.611578	3.369003	0.001119	-1.16233	0.018421	0.037585
Monocytes	CYBB	0.497657	7.155496	3.368361	0.001122	-1.53251	0.016681	0.034111
Monocytes	HBEGF	-1.06613	2.789221	-3.36778	0.001124	-0.92095	0.018853	0.038562
Monocytes	SAMD4	-1.27525	3.187151	-3.36762	0.001124	-0.92417	0.018643	0.038142
Monocytes	NECAP1	0.351156	4.774239	3.366761	0.001127	-1.05441	0.017842	0.036581
Monocytes	ACVR1B	0.682425	2.543644	3.366398	0.001129	-0.93182	0.018999	0.038955
Monocytes	BSG	0.36199	7.716678	3.365426	0.001132	-1.39796	0.01646	0.033811
Monocytes	FGD4	0.420032	4.134435	3.363851	0.001138	-1.27316	0.018215	0.037489
Monocytes	KCNA2	-1.78189	0.96787	-3.36328	0.00114	-1.27398	0.019919	0.040969
Monocytes	SRSF10	-0.2587	6.4448	-3.3627	0.001142	-1.2052	0.017073	0.035222
Monocytes	BCL2L13	0.450513	5.133362	3.362336	0.001144	-1.03041	0.017711	0.036537
Monocytes	CERS6	0.35921	7.330325	3.362115	0.001144	-1.55026	0.016657	0.034369
Monocytes	PIRB	0.496856	4.941687	3.362101	0.001144	-1.28011	0.017806	0.036732
Monocytes	IGF1R	0.430979	6.357541	3.358893	0.001156	-1.24042	0.017264	0.035588
Monocytes	SLC3A2	-0.34341	6.772545	-3.3574	0.001162	-1.37637	0.017121	0.035309
Monocytes	PDE4C	-1.4403	3.131277	-3.35673	0.001164	-0.97528	0.018972	0.039115
Monocytes	GM20528	0.992198	-0.01428	3.348479	0.001195	-1.0565	0.021209	0.043472
Monocytes	GSE1	-0.37583	5.664856	-3.34832	0.001196	-1.13652	0.018068	0.037121
Monocytes	LTF	1.624008	2.942911	3.348277	0.001196	-1.10244	0.019504	0.040042
Monocytes	SLC39A4	-1.53676	1.149584	-3.34642	0.001203	-1.58842	0.020582	0.042282
Monocytes	DSCAML1	-1.22142	-0.596	-3.34636	0.001204	-1.2181	0.021628	0.044381
Monocytes	ZAP70	-1.18086	1.982594	-3.3429	0.001217	-1.10491	0.020262	0.041619
Monocytes	RGS1	-0.54382	5.563816	-3.3429	0.001217	-1.37317	0.018319	0.037672
Monocytes	CCDC148	-1.79761	1.762244	-3.34181	0.001221	-1.12661	0.020428	0.041928
Monocytes	CYP2C37	-1.30365	1.772648	-3.33977	0.001229	-1.33288	0.020524	0.042081
Monocytes	TUSC1	0.443405	3.124075	3.337789	0.001237	-1.07181	0.019849	0.040711
Monocytes	TOR1AIP2	-0.26344	6.580878	-3.33544	0.001246	-1.34268	0.018079	0.03717
Monocytes	TCRG-C2	-1.88764	0.685448	-3.33474	0.001249	-1.25995	0.021343	0.043864
Monocytes	PZP	-1.0528	4.39586	-3.33431	0.001251	-1.01818	0.019219	0.039593
Monocytes	BEX3	-0.40196	4.990762	-3.33422	0.001251	-1.18662	0.018901	0.038943
Monocytes	ENPP1	-1.22392	2.31349	-3.33416	0.001251	-1.15559	0.020381	0.041955
Monocytes	AMOTL2	1.80276	0.684568	3.333594	0.001254	-1.60492	0.021343	0.043921
Monocytes	UBASH3B	-0.37664	5.794781	-3.33331	0.001255	-1.55322	0.01848	0.038101
Monocytes	ATG9A	0.477289	3.85849	3.332445	0.001258	-1.04861	0.019536	0.040292

Monocytes	FCNA	-1.06977	4.555419	-3.32903	0.001272	-1.17332	0.019329	0.039766
Monocytes	SLC35G1	-1.7776	1.026313	-3.3285	0.001274	-1.65781	0.021354	0.043867
Monocytes	SPINT2	-0.40568	4.563442	-3.3282	0.001275	-1.15018	0.019325	0.039771
Monocytes	TSSC4	-0.52901	3.902903	-3.32749	0.001278	-1.04435	0.019701	0.040542
Monocytes	LILRA6	0.596417	1.213739	3.326605	0.001282	-1.08859	0.021284	0.043735
Monocytes	CLEC4D	0.70004	2.949351	3.324249	0.001292	-1.29333	0.020386	0.041892
Monocytes	TMEM117	-1.94921	-0.04691	-3.32319	0.001296	-1.66917	0.022233	0.045613
Monocytes	NFKBIB	-0.35025	5.622292	-3.31979	0.00131	-1.34008	0.019123	0.039251
Monocytes	GYG	0.338308	6.126163	3.318493	0.001315	-1.51118	0.018905	0.038823
Monocytes	DNAJB2	-0.72787	2.766414	-3.31634	0.001324	-1.07039	0.020887	0.042859
Monocytes	HBB-BT	1.733648	8.105747	3.314267	0.001333	-1.33993	0.018079	0.037119
Monocytes	HSPA8	-0.24808	10.08692	-3.31289	0.001339	-1.94406	0.017164	0.035235
Monocytes	HERPUD1	-0.42066	7.853564	-3.31239	0.001341	-1.52023	0.01826	0.037511
Monocytes	4930417O13RII	-1.26775	0.963159	-3.31088	0.001348	-1.2676	0.022232	0.045575
Monocytes	SELENBP1	0.710991	3.795416	3.30851	0.001358	-1.09692	0.020646	0.042356
Monocytes	ATP13A3	0.22617	7.315105	3.30767	0.001361	-1.53887	0.018725	0.038487
Monocytes	NGP	1.301051	5.022684	3.307292	0.001363	-1.09452	0.019964	0.041053
Monocytes	ANKRD37	0.723186	3.981977	3.306784	0.001365	-1.09553	0.020559	0.042303
Monocytes	FCER1G	0.450501	8.628675	3.305778	0.00137	-1.90764	0.01808	0.03726
Monocytes	SFXN5	0.624599	3.363367	3.305323	0.001372	-1.09792	0.020946	0.043163
Monocytes	MT2	1.196742	2.001416	3.304981	0.001373	-1.11533	0.021768	0.044838
Monocytes	MBL2	-1.08549	4.246085	-3.30445	0.001375	-1.09832	0.020437	0.042156
Monocytes	TBCA	-0.25105	7.625569	-3.30382	0.001378	-1.64919	0.018604	0.038407
Monocytes	FGFBP3	-1.51399	0.028033	-3.303	0.001382	-1.42173	0.023063	0.047518
Monocytes	DAPP1	-0.26024	6.482475	-3.30185	0.001387	-1.56624	0.019248	0.039794
Monocytes	MCUB	0.496869	1.557619	3.301502	0.001388	-1.29421	0.022108	0.045663
Monocytes	STFA2L1	1.118938	2.448793	3.301266	0.001389	-1.09805	0.021557	0.044563
Monocytes	GPNMB	1.030709	0.898381	3.300506	0.001393	-1.10743	0.022546	0.046572
Monocytes	ANK2	-0.98484	2.998552	-3.29948	0.001397	-1.14146	0.021255	0.043939
Monocytes	SLC7A11	0.599807	4.278698	3.299446	0.001397	-1.56681	0.020502	0.042402
Monocytes	PTGS2OS	1.685167	-1.09526	3.297847	0.001405	-1.6267	0.023958	0.049412
Monocytes	STAT5A	-0.43257	3.992851	-3.29653	0.00141	-1.20701	0.020799	0.043046
Monocytes	UBTD2	-0.55758	3.37685	-3.29332	0.001425	-1.14753	0.021319	0.044089
Monocytes	HRG	-1.16027	3.61876	-3.29329	0.001425	-1.12124	0.021174	0.043793
Monocytes	SYAP1	0.335462	5.053275	3.291307	0.001434	-1.25803	0.020436	0.042285
Monocytes	1-Mar	0.602148	4.477654	3.289638	0.001441	-1.44859	0.020843	0.04312
Monocytes	WASHC4	0.290748	5.792598	3.289249	0.001443	-1.36727	0.020089	0.041582
Monocytes	FXYD5	0.360017	6.974009	3.28794	0.001449	-1.73912	0.019471	0.040373
Monocytes	PMEPA1	-0.50629	4.361383	-3.28779	0.00145	-1.37656	0.020948	0.04342
Monocytes	PDE6D	0.462124	3.812865	3.285767	0.001459	-1.1458	0.021379	0.044297
Monocytes	ARID5B	0.493582	7.244193	3.285083	0.001462	-1.55726	0.019435	0.040335
Monocytes	HBA-A2	1.604081	9.98555	3.283303	0.001471	-1.72677	0.01809	0.037506
Monocytes	STAP2	-1.70155	0.504981	-3.27795	0.001496	-1.81072	0.023958	0.049402
Monocytes	ARHGEF10L	0.415391	2.695098	3.273098	0.001519	-1.41616	0.022832	0.046992
Monocytes	PGD	0.306491	5.716274	3.271212	0.001528	-1.50112	0.021068	0.043371
Monocytes	GJB2	-1.25749	1.638244	-3.26737	0.001546	-1.39069	0.023885	0.048908
Monocytes	ATG4C	0.451736	3.11799	3.265195	0.001557	-1.2683	0.023012	0.047194

Monocytes	RELT	0.474209	3.728351	3.264721	0.001559	-1.26993	0.02262	0.046453
Monocytes	CNOT10	-0.32131	5.238486	-3.26423	0.001562	-1.40108	0.021681	0.044595
Monocytes	PIK3R1	-0.33318	7.694648	-3.26409	0.001562	-1.79239	0.020244	0.041649
Monocytes	AAGAB	-0.34061	4.862045	-3.26303	0.001568	-1.38944	0.021953	0.045159
Monocytes	DYNLT1C	1.274745	1.100031	3.258263	0.001591	-1.63104	0.024745	0.050675
Monocytes	CXCR2	1.699311	-0.00657	3.256437	0.001601	-1.77199	0.025634	0.05247
Monocytes	MAN1B1	-0.45974	4.927787	-3.25613	0.001602	-1.38811	0.022298	0.045742
Monocytes	GSTA4	-1.44359	1.583658	-3.2553	0.001606	-1.67612	0.024532	0.050264
Monocytes	DNA2	0.333642	3.583362	3.254769	0.001609	-1.4493	0.023191	0.047592
Monocytes	YPEL3	0.389603	7.069109	3.249898	0.001634	-1.62516	0.021328	0.04371
Monocytes	TMEM71	0.646567	3.858441	3.248938	0.001639	-1.25	0.023371	0.047845
Monocytes	TNFRSF4	-1.1193	0.30724	-3.24736	0.001647	-1.315	0.025919	0.052931
Monocytes	CTSF	-1.26522	1.860603	-3.24707	0.001648	-1.39793	0.024802	0.050707
Monocytes	CACNB3	-1.13679	-0.407	-3.24653	0.001651	-1.27518	0.026459	0.054059
Monocytes	TMEM40	1.751161	0.497552	3.24527	0.001658	-1.91428	0.025854	0.052888
Monocytes	CD163	-1.66008	1.13156	-3.24368	0.001666	-1.37322	0.025461	0.052091
Monocytes	CTNNA1	-0.28641	6.643473	-3.24352	0.001667	-1.78788	0.021804	0.044693
Monocytes	JDP2	-0.34586	5.304027	-3.24055	0.001682	-1.77407	0.022815	0.046715
Monocytes	SLC1A5	-0.35836	6.46392	-3.2383	0.001694	-1.60745	0.022212	0.045473
Monocytes	BFSP2	-1.43837	2.440103	-3.23724	0.0017	-1.5848	0.024918	0.050934
Monocytes	CNBP	-0.20434	8.132464	-3.2361	0.001706	-1.83892	0.021292	0.043523
Monocytes	RTL5	-0.89534	0.491888	-3.22889	0.001745	-1.38227	0.026954	0.054812
Monocytes	TANC1	-0.82916	3.838336	-3.22688	0.001756	-1.29187	0.024641	0.050143
Monocytes	GM42869	1.036687	1.342309	3.225827	0.001762	-1.5357	0.026491	0.053837
Monocytes	CCND1	-0.49929	4.140959	-3.2247	0.001768	-1.50252	0.02452	0.049948
Monocytes	FN1	0.683339	5.580946	3.224265	0.00177	-1.90646	0.023549	0.048019
Monocytes	CLEC4G	-0.99706	3.468481	-3.22397	0.001772	-1.33309	0.024989	0.050958
Monocytes	EMP1	0.954475	1.978152	3.220677	0.00179	-1.3082	0.026246	0.053542
Monocytes	APOF	-0.92942	3.847555	-3.22042	0.001792	-1.30983	0.024897	0.050839
Monocytes	MRC1	-0.85393	5.240936	-3.22041	0.001792	-1.53956	0.023942	0.048904
Monocytes	ZEB1	-0.45206	6.848689	-3.2197	0.001795	-1.70493	0.022909	0.046806
Monocytes	ZNHIT1	0.294689	5.422819	3.21799	0.001805	-1.58853	0.023935	0.048914
Monocytes	8030462N17RII	0.240489	6.073398	3.214862	0.001823	-1.61112	0.023701	0.048382
Monocytes	GM42556	1.311365	0.793712	3.214257	0.001826	-1.60303	0.027516	0.056098
Monocytes	ANXA11	0.297142	5.969579	3.211455	0.001842	-1.68231	0.023958	0.048804
Monocytes	LTB4R1	0.477089	2.689349	3.21082	0.001846	-1.60191	0.026288	0.053559
Monocytes	LARP7	-0.28645	5.919166	-3.21007	0.00185	-1.60337	0.02403	0.049037
Monocytes	2500002B13RIk	1.056107	1.915535	3.209332	0.001854	-1.58421	0.026921	0.054872
Monocytes	LRRC1	-0.54003	3.264954	-3.20849	0.001859	-1.42732	0.025934	0.052852
Monocytes	IL18BP	-0.97758	3.740937	-3.20822	0.001861	-1.37726	0.025588	0.05217
Monocytes	CSTDC5	1.168283	5.46156	3.207046	0.001868	-1.47176	0.024438	0.049824
Monocytes	PATJ	-1.05535	3.348038	-3.20544	0.001877	-1.35702	0.026027	0.053004
Monocytes	VGLL4	-0.3502	6.477378	-3.20336	0.001889	-1.61914	0.023963	0.048788
Monocytes	TMEM201	-1.20927	2.102835	-3.20107	0.001903	-1.75565	0.027254	0.055309
Monocytes	RGN	-1.04046	4.650255	-3.2005	0.001906	-1.40163	0.025377	0.051592
Monocytes	THAP11	0.46344	4.457867	3.199682	0.001911	-1.42343	0.025545	0.051935
Monocytes	UTP18	-0.32371	5.338906	-3.19761	0.001923	-1.60021	0.025049	0.050957

Monocytes	GIMAP9	-1.05319	3.560572	-3.19579	0.001934	-1.50958	0.026446	0.053743
Monocytes	ADAM19	-0.45955	5.568234	-3.19535	0.001937	-1.76806	0.024997	0.050827
Monocytes	0610012G03RII	0.320068	5.039442	3.194809	0.00194	-1.5565	0.025379	0.051652
Monocytes	CD14	0.581635	4.260683	3.193639	0.001947	-1.84463	0.026	0.052926
Monocytes	HABP2	-1.7418	0.622763	-3.19117	0.001962	-1.98939	0.028997	0.058827
Monocytes	MYO6	-0.79454	3.1857	-3.19044	0.001966	-1.40044	0.026994	0.05486
Monocytes	JAK2	-0.29729	6.674709	-3.18866	0.001977	-1.96386	0.024581	0.049974
Monocytes	C1QA	-0.73646	5.480946	-3.18767	0.001983	-1.72974	0.025451	0.051742
Monocytes	TUSC3	-0.3718	4.978446	-3.18682	0.001989	-1.58233	0.025811	0.052543
Monocytes	PNPLA7	0.390295	6.039516	3.186715	0.001989	-1.67616	0.025056	0.051014
Monocytes	RNF130	0.251956	7.282781	3.186107	0.001993	-1.93281	0.024202	0.049341
Monocytes	GIMAP4	-0.88592	4.091849	-3.18515	0.001999	-1.40205	0.026462	0.053993
Monocytes	WFDC17	0.818613	5.906168	3.185057	0.002	-1.95131	0.02515	0.051333
Monocytes	EMP3	0.303346	7.421524	3.184819	0.002001	-1.95665	0.024109	0.049213
Monocytes	ZFP280C	0.506948	4.164903	3.1848	0.002001	-1.419	0.026408	0.053883
Monocytes	TBL1XR1	0.27259	6.669065	3.184005	0.002006	-1.77874	0.024625	0.050291
Monocytes	GM10634	-0.73978	0.651485	-3.18388	0.002007	-1.40342	0.029171	0.059457
Monocytes	CD96	-1.73061	0.686804	-3.1816	0.002021	-1.76784	0.029245	0.059618
Monocytes	PLXNC1	-0.39166	5.427157	-3.18158	0.002021	-1.80089	0.025585	0.052253
Monocytes	CLEC1B	-0.72589	3.741016	-3.18124	0.002023	-1.47297	0.026825	0.054802
Monocytes	SUPT4A	0.297496	6.836136	3.181057	0.002024	-1.82475	0.024597	0.050277
Monocytes	LRRC8B	0.420449	3.358799	3.180603	0.002027	-1.4601	0.027119	0.055431
Monocytes	IFI205	-0.47096	2.758001	-3.17989	0.002032	-1.87693	0.027607	0.056438
Monocytes	RBP4	-0.81069	8.031076	-3.17874	0.002039	-1.94619	0.023861	0.048862
Monocytes	ABCC3	-0.94887	2.689429	-3.17831	0.002042	-1.41815	0.027711	0.056702
Monocytes	LRPAP1	0.359074	4.499631	3.178049	0.002043	-1.62491	0.026334	0.053916
Monocytes	DUSP5	-0.35084	6.834196	-3.17605	0.002056	-1.92657	0.02479	0.050691
Monocytes	GYPC	-0.73065	3.080733	-3.17156	0.002085	-1.4409	0.02789	0.056787
Monocytes	ENDOD1	-0.52925	2.901638	-3.17101	0.002088	-1.45453	0.028042	0.057103
Monocytes	SCAMP1	0.492298	2.970331	3.168081	0.002107	-1.55689	0.028206	0.057363
Monocytes	SLAMF7	-0.40277	5.151332	-3.16739	0.002112	-1.8292	0.026551	0.054051
Monocytes	CLEC4E	0.634547	2.622943	3.164976	0.002128	-1.8005	0.028684	0.058371
Monocytes	UGT2B36	-1.28985	2.198922	-3.16057	0.002157	-1.64514	0.029389	0.059601
Monocytes	RASA2	-0.41374	6.158678	-3.15932	0.002165	-1.75287	0.026361	0.053539
Monocytes	CMTM8	-0.74334	4.192364	-3.15703	0.00218	-1.48098	0.028012	0.056747
Monocytes	RARG	0.655713	1.622679	3.156309	0.002185	-1.48921	0.030119	0.060973
Monocytes	ADAM9	0.3649	5.178783	3.156246	0.002186	-1.69987	0.027247	0.055229
Monocytes	ZFP618	-1.70558	0.531603	-3.15405	0.0022	-2.15476	0.031197	0.063106
Monocytes	ARL4A	-0.43351	4.453783	-3.15345	0.002204	-1.57026	0.027925	0.056672
Monocytes	YARS	-0.48203	5.391202	-3.15266	0.00221	-1.6656	0.0272	0.05528
Monocytes	CFHR1	-2.0114	0.449486	-3.15235	0.002212	-2.15074	0.03127	0.063442
Monocytes	TOMM6	0.239328	7.550466	3.152225	0.002213	-2.03108	0.02561	0.052068
Monocytes	LAMP1	0.231367	7.757865	3.152189	0.002213	-2.15738	0.025462	0.051769
Monocytes	FOXA3	-1.34251	1.425712	-3.15199	0.002214	-1.82613	0.030416	0.06176
Monocytes	CXCL12	-1.01375	2.838235	-3.15112	0.00222	-1.5476	0.029236	0.059486
Monocytes	GM12596	-1.19813	2.627238	-3.15105	0.002221	-1.76923	0.02941	0.059837
Monocytes	P3H2	-0.52975	1.190669	-3.14392	0.00227	-1.84093	0.031239	0.062949

Monocytes	MFSD7A	0.931785	-0.44923	3.143831	0.002271	-1.54345	0.032729	0.065881
Monocytes	F2R	-0.80594	3.599158	-3.14186	0.002284	-1.52048	0.029324	0.059124
Monocytes	TBC1D10C	-0.619	4.736281	-3.13915	0.002303	-1.56381	0.028602	0.057559
Monocytes	S100A10	0.360152	6.601647	3.135822	0.002327	-2.06726	0.027391	0.055007
Monocytes	PID1	0.306275	5.484883	3.135086	0.002332	-2.20168	0.02827	0.056839
Monocytes	LNX2	-0.4446	3.592505	-3.13489	0.002334	-1.62206	0.029813	0.05992
Monocytes	ZBTB10	-0.70822	4.077959	-3.1337	0.002342	-1.53249	0.029479	0.059259
Monocytes	UST	-0.53326	6.028853	-3.13267	0.00235	-2.04469	0.027964	0.056271
Monocytes	CTSA	0.355009	6.259221	3.131986	0.002355	-1.95982	0.0278	0.055974
Monocytes	BHLHE40	-0.40847	6.323106	-3.13167	0.002357	-2.02801	0.027751	0.055902
Monocytes	EGR1	-0.64054	6.545199	-3.12293	0.002421	-1.9627	0.028291	0.056636
Monocytes	TCF7	-1.15074	2.524319	-3.12192	0.002428	-1.59896	0.031729	0.063456
Monocytes	GOLM1	0.557452	3.2957	3.121536	0.002431	-1.5751	0.031046	0.06212
Monocytes	CCSER1	-0.48582	1.697174	-3.11888	0.002451	-1.89723	0.032705	0.065211
Monocytes	IBTK	0.416894	4.642613	3.117089	0.002464	-1.72875	0.030203	0.060272
Monocytes	SBNO2	0.284025	5.566821	3.116268	0.00247	-1.9103	0.029431	0.058847
Monocytes	TMEM230	-0.31966	4.742854	-3.11622	0.002471	-1.7286	0.030118	0.060213
Monocytes	POLD2	-0.72951	3.401936	-3.11612	0.002472	-1.57594	0.031276	0.062503
Monocytes	KATNAL1	-1.56836	0.973387	-3.11536	0.002477	-2.15359	0.033536	0.066912
Monocytes	ENDOG	0.624477	2.943039	3.113871	0.002489	-1.5843	0.031823	0.06349
Monocytes	IYD	-1.55107	0.499692	-3.11175	0.002505	-2.13394	0.034282	0.068334
Monocytes	MFHAS1	-0.64895	3.729832	-3.1103	0.002516	-1.60677	0.031389	0.062661
Monocytes	AIF1	-0.43226	4.896333	-3.10613	0.002548	-2.10166	0.030698	0.061064
Monocytes	SESN3	-0.48764	4.83647	-3.1056	0.002552	-1.67682	0.030749	0.061209
Monocytes	1110008P14RIK	0.345206	5.226277	3.105537	0.002553	-1.82189	0.030415	0.060549
Monocytes	PLPP3	-1.09246	4.693864	-3.10525	0.002555	-1.6239	0.030873	0.061485
Monocytes	HNRNPR	-0.2492	6.29021	-3.10386	0.002566	-1.93231	0.029612	0.058981
Monocytes	AMACR	-1.24841	2.326832	-3.10327	0.00257	-1.77991	0.033114	0.06591
Monocytes	HSPA1B	1.104039	4.531616	3.102933	0.002573	-1.88328	0.03112	0.062011
Monocytes	DLG4	-0.92176	3.571009	-3.10222	0.002578	-1.61833	0.032003	0.063795
Monocytes	PDE4A	-0.53026	3.81993	-3.09641	0.002624	-1.77777	0.032306	0.064071
Monocytes	B230219D22RII	0.217838	6.252623	3.095485	0.002632	-2.00054	0.030224	0.059977
Monocytes	AURKAIP1	0.284185	5.837172	3.094507	0.00264	-1.97046	0.030631	0.060754
Monocytes	CIB1	0.343653	5.732242	3.093247	0.00265	-1.87997	0.030802	0.061067
Monocytes	ANXA2	0.338848	5.910919	3.089203	0.002682	-2.18776	0.03099	0.061079
Monocytes	RAB31	0.343589	4.770711	3.087345	0.002698	-1.95863	0.032138	0.063277
Monocytes	SYTL2	-1.39414	1.257049	-3.08681	0.002702	-1.83352	0.035502	0.069831
Monocytes	H2-OA	-0.51979	3.19042	-3.08587	0.00271	-1.83049	0.033668	0.066291
Monocytes	ITGAE	-0.4931	2.127038	-3.08454	0.002721	-1.84935	0.034759	0.06846
Monocytes	INTS6L	0.380878	5.229737	3.084467	0.002721	-1.86818	0.031852	0.062797
Monocytes	STXBP6	0.557211	2.953526	3.083662	0.002728	-1.89681	0.033999	0.067049
Monocytes	ERBB4	-1.77813	0.909465	-3.08285	0.002735	-2.1083	0.036069	0.071125
Monocytes	MRPL33	0.274118	6.142277	3.0797	0.002761	-2.12246	0.031368	0.061826
Monocytes	RAB37	0.90709	2.340912	3.079518	0.002762	-1.75891	0.034905	0.068725
Monocytes	MAP3K20	0.466545	3.798494	3.077466	0.00278	-1.80754	0.033667	0.066226
Monocytes	TRP53INP2	0.475403	4.134264	3.075984	0.002792	-1.73061	0.033437	0.065828
Monocytes	AKR1B8	-1.24788	1.592252	-3.07583	0.002793	-1.86164	0.035924	0.070652

Monocytes	ADGRL3	-0.7975	3.47827	-3.07487	0.002802	-1.89724	0.034119	0.0672
Monocytes	SOS2	0.327888	5.600384	3.072282	0.002824	-1.97504	0.032359	0.063705
Monocytes	SP3OS	0.462236	4.205373	3.07184	0.002827	-1.74115	0.033655	0.06628
Monocytes	PLG	-1.03301	4.004656	-3.07013	0.002842	-1.69797	0.033955	0.066834
Monocytes	TREM2	0.903637	0.639882	3.068796	0.002853	-1.71757	0.037343	0.07357
Monocytes	ATF7IP	0.334934	6.540023	3.068788	0.002853	-2.03675	0.031627	0.062433
Monocytes	RHOG	0.303408	7.243088	3.06857	0.002855	-2.25955	0.031014	0.061223
Monocytes	RAPGEF2	-0.34597	7.429198	-3.06841	0.002857	-2.32008	0.030853	0.060907
Monocytes	DNAJC18	0.614863	3.682567	3.068344	0.002857	-1.6954	0.034265	0.067602
Monocytes	RAB10	-0.24753	7.670572	-3.06804	0.00286	-2.32908	0.030647	0.060521
Monocytes	PQLC3	0.412863	3.812077	3.066171	0.002876	-1.8581	0.034271	0.067584
Monocytes	CASC3	-0.33077	5.292913	-3.066	0.002878	-1.93474	0.032875	0.064871
Monocytes	APOA2	-0.7821	8.419445	-3.06126	0.002919	-2.32997	0.03053	0.059904
Monocytes	LST1	0.348572	5.23223	3.060651	0.002924	-2.2043	0.033388	0.065518
Monocytes	MCEMP1	0.588621	1.650063	3.058026	0.002948	-1.92536	0.03717	0.072703
Monocytes	TLNRD1	-0.46043	4.581796	-3.05777	0.00295	-1.87725	0.03422	0.067025
Monocytes	CAMK1D	-0.27044	8.628354	-3.05435	0.002981	-2.57631	0.030822	0.060362
Monocytes	PMF1	-0.39753	5.697613	-3.05425	0.002981	-2.01127	0.033442	0.065495
Monocytes	SPP2	-1.13462	3.372982	-3.05179	0.003004	-1.73889	0.035894	0.070235
Monocytes	YES1	-0.63623	4.740029	-3.05166	0.003005	-1.87468	0.03454	0.067613
Monocytes	G430095P16RII	0.633979	0.465518	3.049621	0.003023	-1.74729	0.03912	0.076466
Monocytes	SGMS2	0.400499	3.643674	3.049601	0.003023	-2.24656	0.035757	0.069999
Monocytes	RAB11A	-0.2009	7.145444	-3.04867	0.003032	-2.29495	0.032417	0.063604
Monocytes	RNFT1	0.320022	4.495375	3.048614	0.003032	-1.93958	0.034911	0.068474
Monocytes	CMC2	-0.51169	4.535117	-3.04799	0.003038	-1.94352	0.034872	0.068398
Monocytes	ZMYND15	-0.7961	0.109664	-3.04799	0.003038	-1.77974	0.039518	0.077354
Monocytes	SEM1	0.196146	9.100384	3.047711	0.003041	-2.57381	0.030706	0.060236
Monocytes	NMNAT2	-1.21684	2.111738	-3.04723	0.003045	-1.75787	0.037348	0.073256
Monocytes	FAM174A	0.32778	6.050716	3.04566	0.003059	-2.21647	0.033552	0.065897
Monocytes	HPGDS	-0.56611	3.647133	-3.04519	0.003064	-1.84807	0.035902	0.070497
Monocytes	ETV5	-0.79796	3.199847	-3.04392	0.003076	-1.75635	0.036425	0.071575
Monocytes	1810030O07RII	-0.32338	4.824335	-3.04381	0.003076	-1.98737	0.034798	0.068411
Monocytes	RHOU	1.083127	1.27725	3.043127	0.003083	-1.7822	0.038495	0.075534
Monocytes	TOPORS	-0.39626	5.749708	-3.04245	0.003089	-2.01149	0.033968	0.066737
Monocytes	SAT1	0.272318	8.474503	3.040556	0.003107	-2.60719	0.031599	0.062087
Monocytes	MAFB	0.437887	4.60884	3.040144	0.003111	-2.2042	0.035197	0.06916
Monocytes	PIRA2	0.461198	3.589246	3.039856	0.003113	-2.05061	0.03622	0.071194
Monocytes	CAMP	1.347945	4.463959	3.039754	0.003114	-1.77679	0.03534	0.069485
Monocytes	MICAL3	-0.63491	3.436889	-3.03854	0.003126	-1.77302	0.036467	0.071624
Monocytes	ITIH1	-1.28424	2.749811	-3.03804	0.00313	-1.83361	0.037195	0.07307
Monocytes	UBE2E1	-0.22243	6.229498	-3.03689	0.003141	-2.14797	0.03381	0.066498
Monocytes	GM16867	1.228251	2.522419	3.035243	0.003157	-1.91661	0.037627	0.073921
Monocytes	PCMTD2	0.61487	3.680281	3.034227	0.003166	-1.7829	0.036418	0.071712
Monocytes	CBFA2T3	-0.3382	5.507245	-3.03413	0.003167	-2.19702	0.034597	0.068156
Monocytes	PLEKHA2	-0.34753	7.455833	-3.03412	0.003167	-2.16125	0.032766	0.064558
Monocytes	RAB27A	0.386587	4.50985	3.034071	0.003168	-1.96974	0.035578	0.070075
Monocytes	CCPG1	0.328148	6.037912	3.032658	0.003181	-2.10873	0.034194	0.067327

Monocytes	ARF3	-0.22872	6.29303	-3.03161	0.003191	-2.21145	0.034019	0.066992
Monocytes	SLFN1	0.954129	2.335521	3.030332	0.003203	-1.86658	0.038124	0.074987
Monocytes	TTYH2	-0.74228	3.075271	-3.02935	0.003213	-1.7932	0.037397	0.073616
Monocytes	RAB3GAP1	0.252639	6.044858	3.029043	0.003216	-2.09207	0.034405	0.067821
Monocytes	NXF1	-0.33094	5.180351	-3.02786	0.003227	-1.98353	0.035333	0.069613
Monocytes	OSBPL8	0.288842	7.62098	3.026565	0.00324	-2.36594	0.033077	0.065193
Monocytes	TSPAN33	-0.4006	2.55857	-3.0264	0.003241	-2.11759	0.03812	0.075058
Monocytes	ID1	-0.45327	4.07039	-3.02436	0.003261	-2.09628	0.036712	0.072223
Monocytes	ABHD18	0.787569	3.182254	3.023468	0.00327	-1.81573	0.0377	0.07419
Monocytes	ZFP26	0.46665	3.995742	3.022373	0.003281	-1.8305	0.036926	0.072656
Monocytes	TUBB6	-0.3821	3.749371	-3.02029	0.003301	-2.17965	0.037374	0.073414
Monocytes	TMEM140	-0.74031	3.514222	-3.01832	0.003321	-1.83953	0.037803	0.074104
Monocytes	AHR	-0.38779	4.719836	-3.01699	0.003334	-2.33717	0.036647	0.071833
Monocytes	DENR	-0.26549	6.279481	-3.01323	0.003371	-2.1862	0.035438	0.069244
Monocytes	IFI27L2A	0.724166	7.373467	3.012352	0.00338	-2.44338	0.034405	0.067259
Monocytes	CDKN2D	0.330258	6.09877	3.012183	0.003382	-2.14748	0.03565	0.069689
Monocytes	GM2000	-0.4882	4.799183	-3.01044	0.0034	-2.02162	0.037062	0.07248
Monocytes	UBC	-0.28558	8.132317	-3.01041	0.0034	-2.51317	0.03377	0.066057
Monocytes	DCAF8	0.246653	5.699638	3.010253	0.003402	-2.10671	0.036139	0.070697
Monocytes	GPM6B	-1.27353	2.439081	-3.00979	0.003406	-2.04056	0.039621	0.077445
Monocytes	GTPBP4	-0.2597	5.956432	-3.0085	0.003419	-2.20104	0.035989	0.070355
Monocytes	SLC25A20	-0.2752	5.597573	-3.00243	0.003482	-2.4046	0.036975	0.071749
Monocytes	PTP4A2	-0.159	8.699413	-3.00094	0.003497	-2.60959	0.033973	0.06601
Monocytes	CEP290	-0.6731	2.468766	-3.00092	0.003497	-1.86813	0.040444	0.07851
Monocytes	IER3IP1	0.214388	6.363638	3.000417	0.003503	-2.28613	0.036252	0.07047
Monocytes	SH2D2A	-0.86229	2.764079	-3.0004	0.003503	-1.86172	0.040108	0.077897
Monocytes	SLC41A1	-0.49673	2.387756	-2.99935	0.003514	-1.91099	0.040619	0.078924
Monocytes	PADI4	1.47282	1.890862	2.998654	0.003521	-1.99528	0.041225	0.080108
Monocytes	ILF3	-0.2885	6.541502	-2.99836	0.003524	-2.25672	0.036173	0.070427
Monocytes	SMCO4	-0.49815	4.10439	-2.99669	0.003542	-1.94644	0.038846	0.075616
Monocytes	SSBP3	-0.33012	5.600863	-2.99661	0.003543	-2.13813	0.037249	0.072531
Monocytes	BLOC1S1	0.306702	6.24963	2.995535	0.003554	-2.29594	0.036657	0.071426
Monocytes	LYN	0.250605	10.03311	2.994285	0.003567	-2.83977	0.033092	0.064433
Monocytes	HNRNPH1	-0.29222	6.424984	-2.99356	0.003575	-2.24521	0.036614	0.071339
Monocytes	NDUFA12	-0.3264	5.362792	-2.99283	0.003583	-2.11525	0.037758	0.073568
Monocytes	LSM14B	0.445345	3.604671	2.989042	0.003623	-1.91652	0.040053	0.077942
Monocytes	PRMT1	-0.29473	5.803475	-2.98886	0.003625	-2.19806	0.037656	0.073318
Monocytes	TET2	0.248236	6.024646	2.987489	0.00364	-2.3802	0.037536	0.07305
Monocytes	NFYC	0.210217	6.202043	2.986749	0.003648	-2.26432	0.037378	0.072787
Monocytes	EPB41L3	-1.03992	2.334313	-2.98641	0.003652	-1.89551	0.041671	0.081063
Monocytes	KLF7	0.398807	6.159891	2.98615	0.003654	-2.25359	0.037422	0.072897
Monocytes	CEP78	0.645899	3.001386	2.985628	0.00366	-1.90765	0.040912	0.079661
Monocytes	RNF13	0.289661	6.065838	2.98467	0.003671	-2.26379	0.037605	0.073285
Monocytes	TRAF1	-0.45236	3.343827	-2.98254	0.003694	-2.27236	0.040805	0.079369
Monocytes	VCP	-0.21395	7.431716	-2.98002	0.003721	-2.46544	0.036624	0.071188
Monocytes	LY6C2	0.540303	5.53324	2.977429	0.00375	-2.58708	0.038838	0.075315
Monocytes	ADORA2B	0.627827	0.37813	2.977366	0.003751	-1.9694	0.044918	0.086926

Monocytes	CCDC122	-0.84606	1.315452	-2.9767	0.003758	-1.95093	0.043742	0.084757
Monocytes	ZFP821	0.5189	3.905173	2.976625	0.003759	-1.94437	0.040657	0.078866
Monocytes	SMAD3	-0.35689	6.315623	-2.97561	0.00377	-2.37393	0.038066	0.073917
Monocytes	TMEM173	0.415433	4.250581	2.975322	0.003774	-2.0582	0.040333	0.078339
Monocytes	SSNA1	0.285295	5.922585	2.974305	0.003785	-2.26026	0.038561	0.074911
Monocytes	NARF	0.40806	4.893244	2.97306	0.003799	-2.05991	0.039793	0.077297
Monocytes	NFAM1	0.284992	4.570884	2.972612	0.003804	-2.34104	0.040166	0.078063
Monocytes	GM26740	0.343381	7.068845	2.970176	0.003831	-2.43703	0.037686	0.073136
Monocytes	CCDC58	0.432091	4.247902	2.968189	0.003854	-2.01755	0.040909	0.079362
Monocytes	ANK	0.469626	3.446389	2.968132	0.003855	-2.1209	0.041842	0.081152
Monocytes	NUP107	-0.32788	5.353646	-2.96798	0.003856	-2.23118	0.039659	0.076956
Monocytes	FYN	-0.36643	7.843798	-2.96743	0.003863	-2.71438	0.036998	0.071851
Monocytes	TPM3	-0.15463	8.661561	-2.96734	0.003864	-2.69769	0.036168	0.070237
Monocytes	AADAC	-1.33329	2.701819	-2.96681	0.00387	-2.00738	0.042752	0.083
Monocytes	WDR62	0.519217	3.526858	2.96496	0.003891	-2.01587	0.041953	0.081368
Monocytes	UBXN7	0.330859	5.704222	2.963364	0.003909	-2.1895	0.03961	0.076763
Monocytes	SLC12A8	-1.33332	0.657727	-2.96232	0.003921	-2.25028	0.045745	0.088438
Monocytes	SLA	0.349197	5.280678	2.962068	0.003924	-2.3225	0.040152	0.077774
Monocytes	CRELD2	-0.4203	4.759514	-2.96133	0.003933	-2.14525	0.040769	0.078996
Monocytes	HSD17B11	-0.29787	4.796644	-2.96105	0.003936	-2.28146	0.040726	0.078915
Monocytes	CAMKMT	0.450443	4.501622	2.960788	0.003939	-2.08697	0.041065	0.079589
Monocytes	AIFM2	0.836897	1.347203	2.959114	0.003959	-1.97193	0.045063	0.087114
Monocytes	CD40	-0.81131	2.17854	-2.95792	0.003973	-1.98109	0.044056	0.085405
Monocytes	DGAT2	0.446099	3.862389	2.957752	0.003975	-2.12016	0.042012	0.081498
Monocytes	MRPS28	0.427063	6.885185	2.957739	0.003975	-2.57739	0.038602	0.07492
Monocytes	CD209A	0.55588	0.102824	2.956982	0.003984	-2.13835	0.046782	0.090619
Monocytes	NFATC1	-0.31444	5.77824	-2.95659	0.003988	-2.29111	0.039865	0.077439
Monocytes	ZSCAN26	0.379598	4.652438	2.952702	0.004034	-2.15032	0.041574	0.080487
Monocytes	S100A9	0.834263	6.814997	2.952026	0.004042	-2.61697	0.039171	0.075865
Monocytes	WDR37	0.286213	5.478401	2.951439	0.004049	-2.29671	0.040689	0.078841
Monocytes	GM33677	-1.89864	-1.06476	-2.94949	0.004072	-2.58279	0.049123	0.094892
Monocytes	TEF	-0.68026	3.230001	-2.94891	0.004079	-1.99372	0.043491	0.084274
Monocytes	SLC35D1	-0.42299	4.114625	-2.9489	0.00408	-2.07173	0.042421	0.082224
Monocytes	GLYCTK	-1.41593	0.979545	-2.94888	0.00408	-2.39741	0.046349	0.089716
Monocytes	ARHGAP15	0.233227	9.872031	2.948325	0.004086	-2.9527	0.036158	0.070117
Monocytes	IRF8	-0.35007	6.49623	-2.94788	0.004092	-2.59106	0.039718	0.07707
Monocytes	TAPBPL	-0.46219	4.125165	-2.94708	0.004101	-2.09644	0.042501	0.082383
Monocytes	FUBP1	-0.21162	6.678668	-2.94439	0.004134	-2.42962	0.039804	0.076978
Monocytes	VTN	-0.89274	4.267725	-2.94436	0.004134	-2.03464	0.042584	0.082322
Monocytes	APOH	-0.83768	4.983199	-2.94401	0.004139	-2.1335	0.041738	0.080742
Monocytes	TSTD1	-1.09204	2.589608	-2.94262	0.004156	-2.05407	0.044712	0.086419
Monocytes	BMP6	-1.82043	0.450706	-2.94256	0.004156	-2.62441	0.047505	0.091712
Monocytes	ITGB1	-0.22653	6.871704	-2.94247	0.004157	-2.57559	0.039649	0.076709
Monocytes	CREG1	-0.29083	7.514034	-2.94089	0.004177	-2.70267	0.039088	0.075564
Monocytes	NME2	0.295198	8.557653	2.93719	0.004222	-2.783	0.038347	0.073982
Monocytes	ZDHHC4	0.380515	4.474275	2.936261	0.004234	-2.15303	0.043048	0.083002
Monocytes	AFAP1	-0.49039	1.955044	-2.93498	0.00425	-2.06172	0.046344	0.089221

Monocytes	TOMM34	-0.2689	5.601463	-2.93449	0.004256	-2.34682	0.041841	0.080655
Monocytes	TNFRSF9	-0.82274	2.460851	-2.93234	0.004283	-2.03819	0.045886	0.088383
Monocytes	GOLPH3L	0.433369	4.39604	2.93197	0.004287	-2.14734	0.043451	0.083749
Monocytes	VAV2	-0.47487	4.977829	-2.93186	0.004289	-2.1838	0.042747	0.082404
Monocytes	LIMD2	0.293871	7.333227	2.931658	0.004291	-2.57593	0.040025	0.077202
Monocytes	PTPN6	0.306128	6.758702	2.931414	0.004294	-2.52253	0.04067	0.078484
Monocytes	CHIL1	1.564923	-0.07877	2.930327	0.004308	-2.52405	0.049351	0.095206
Monocytes	C1QB	-0.60466	6.51237	-2.93024	0.004309	-2.58128	0.040983	0.07926
Monocytes	LYZ1	0.952511	-0.68152	2.930036	0.004312	-2.04858	0.050205	0.09685
Monocytes	NCKAP1	-0.93395	2.425386	-2.92977	0.004315	-2.15647	0.045968	0.08885
Monocytes	IKBKB	0.237075	5.778617	2.929255	0.004322	-2.49664	0.041845	0.080997
Monocytes	SDCBP2	-0.7901	1.097863	-2.92897	0.004325	-2.03244	0.047744	0.092263
Monocytes	FCOR	0.696054	0.865885	2.927409	0.004345	-2.03634	0.048187	0.093088
Monocytes	GM43936	-1.76664	-1.34803	-2.92738	0.004345	-2.75797	0.051319	0.098985
Monocytes	TRP53BP2	0.410613	3.957204	2.92655	0.004356	-2.15853	0.044219	0.085547
Monocytes	ST6GALNAC3	-0.65256	5.127632	-2.92611	0.004362	-2.17426	0.042802	0.082819
Monocytes	GPI1	0.245258	7.844302	2.924964	0.004376	-2.72189	0.03977	0.076957
Monocytes	OLFM1	-0.33943	2.469141	-2.92103	0.004427	-2.45547	0.046724	0.090089
Monocytes	BCL6	0.387101	5.992679	2.920008	0.00444	-2.56334	0.042403	0.081875
Monocytes	EYA2	-1.18957	1.389743	-2.91749	0.004473	-2.2161	0.048578	0.093446
Monocytes	HSD17B13	-0.97945	2.671988	-2.9158	0.004495	-2.06868	0.047031	0.090416
Monocytes	WEE1	-0.65907	3.947727	-2.91484	0.004508	-2.07167	0.045423	0.087411
Monocytes	NIN	0.299383	5.920519	2.914719	0.004509	-2.48582	0.042978	0.082751
Monocytes	GM11099	-1.85068	-0.70994	-2.91429	0.004515	-2.68663	0.051841	0.099584
Monocytes	FASL	-1.04761	1.037842	-2.91105	0.004558	-2.12019	0.049747	0.095461
Monocytes	RNF43	-1.16374	1.563577	-2.91063	0.004563	-2.16437	0.049023	0.094101
Monocytes	CCDC192	-0.91542	-0.69514	-2.91013	0.00457	-2.09277	0.052295	0.100203
Monocytes	CRTAM	-0.83429	1.176627	-2.90745	0.004606	-2.10286	0.049816	0.09565
Monocytes	DHPS	-0.35998	4.486343	-2.90741	0.004606	-2.20866	0.045373	0.087237
Monocytes	SESTD1	0.479563	2.300083	2.907241	0.004609	-2.34019	0.048256	0.092712
Monocytes	NHSL2	0.594784	2.487556	2.907197	0.004609	-2.19649	0.048001	0.09223
Monocytes	GM19951	1.076613	3.23896	2.906838	0.004614	-2.09004	0.046997	0.090381
Monocytes	FGF23	2.074209	0.594781	2.905346	0.004634	-2.47111	0.050819	0.097566
Monocytes	RASA3	0.251341	6.702935	2.902727	0.00467	-2.61111	0.043049	0.082647
Monocytes	MACO1	-0.26409	6.27208	-2.9026	0.004671	-2.48374	0.04357	0.083651
Monocytes	RFX2	-0.58931	3.630115	-2.90049	0.0047	-2.12558	0.047163	0.090427
Monocytes	HSD17B6	-1.5563	0.767255	-2.89969	0.004711	-2.42815	0.051208	0.098054
Monocytes	FGA	-0.62957	5.727993	-2.89929	0.004717	-2.354	0.044534	0.085432
Monocytes	TPM1	-0.44008	5.177563	-2.89869	0.004725	-2.31398	0.04526	0.086846
Monocytes	H2-Q6	-1.01231	3.115257	-2.89803	0.004734	-2.15402	0.048008	0.092094
Monocytes	LAT	-0.87136	2.414617	-2.89731	0.004744	-2.16539	0.049021	0.093999
Monocytes	STARD3	0.416221	4.116736	2.895716	0.004766	-2.24354	0.046896	0.089874
Monocytes	TRIM25	0.310045	6.978283	2.894559	0.004782	-2.75454	0.043389	0.083164
Monocytes	NRG4	0.672373	2.017195	2.89259	0.004809	-2.17607	0.050113	0.09588
Monocytes	CISD3	-0.67744	3.000972	-2.89225	0.004814	-2.11925	0.04874	0.093344
Monocytes	CYP1A2	-1.34648	1.633956	-2.88994	0.004847	-2.33421	0.050952	0.097377
Monocytes	CARS	-0.54695	4.118134	-2.88815	0.004872	-2.19257	0.047703	0.091079

Monocytes	WDFY4	-0.30248	7.251372	-2.8871	0.004887	-2.85984	0.043762	0.083519
Monocytes	WDR3	0.340515	4.225869	2.886991	0.004888	-2.29613	0.047627	0.090858
Monocytes	AGPS	0.234898	6.813953	2.884367	0.004926	-2.64	0.044593	0.084951
Monocytes	ARHGAP27OS3	1.191354	0.465307	2.883131	0.004943	-2.44903	0.05346	0.101477
Monocytes	PELI2	0.585693	2.831902	2.882677	0.00495	-2.20851	0.050012	0.095034
Monocytes	ZFP691	0.732248	3.075803	2.882304	0.004955	-2.1447	0.049675	0.09445
Monocytes	ALB	-0.88544	9.441682	-2.88147	0.004967	-2.94947	0.041638	0.079221
Monocytes	GM43660	1.303777	0.127169	2.881157	0.004972	-2.33146	0.054068	0.102712
Monocytes	ADGB	-0.75541	2.335955	-2.88078	0.004977	-2.20005	0.050786	0.096636
Monocytes	CYTH1	0.270474	8.072227	2.880548	0.00498	-2.81905	0.043246	0.082395
Monocytes	ZFR2	0.993772	0.817601	2.879398	0.004997	-2.28665	0.053107	0.101055
Monocytes	GM15283	0.467616	4.261918	2.879306	0.004998	-2.29986	0.048183	0.091829
Monocytes	DIP2C	-0.54499	6.172429	-2.8779	0.005019	-2.45942	0.045814	0.087243
Monocytes	NRGN	0.974961	3.663682	2.874296	0.005071	-2.16601	0.04954	0.094074
Monocytes	3110082I17RIK	0.42966	4.199896	2.874234	0.005072	-2.35043	0.048798	0.092681
Monocytes	LPXN	0.30178	5.23201	2.874204	0.005073	-2.5694	0.047405	0.090056
Monocytes	BMPR1A	-1.2781	2.310682	-2.87349	0.005083	-2.29105	0.051525	0.097742
Monocytes	ADAMTS10	0.599573	3.015513	2.87136	0.005114	-2.16796	0.050773	0.096169
Monocytes	HSD3B7	-0.54383	3.537133	-2.86995	0.005135	-2.25537	0.050182	0.094845
Monocytes	PTGES	1.086332	0.477516	2.869498	0.005142	-2.18247	0.054719	0.103357
Monocytes	SYS1	0.227991	6.417243	2.869332	0.005145	-2.62073	0.046288	0.087639
Monocytes	5430427O19RII	0.472631	3.846602	2.867886	0.005166	-2.29177	0.049899	0.094349
Monocytes	BTG2	0.331891	7.421641	2.867617	0.00517	-2.76777	0.045148	0.085411
Monocytes	CWF19L2	0.351273	5.038452	2.866092	0.005193	-2.35483	0.048385	0.091557
Monocytes	MRPL20	0.263114	6.1388	2.866036	0.005194	-2.60225	0.046917	0.088795
Monocytes	PRDX5	0.408688	7.635313	2.864793	0.005212	-2.91646	0.04512	0.085358
Monocytes	SEMA7A	-0.76024	2.017527	-2.86314	0.005237	-2.18597	0.053006	0.100127
Monocytes	NFKB2	-0.4056	5.096214	-2.86293	0.00524	-2.47005	0.048604	0.091909
Monocytes	ARL15	0.265923	7.591924	2.860431	0.005278	-2.83053	0.045617	0.086104
Monocytes	CEACAM1	0.397332	3.675486	2.859325	0.005295	-2.35119	0.051015	0.0961
Monocytes	GM37982	0.507504	2.996043	2.858406	0.005309	-2.36929	0.05209	0.098095
Monocytes	NXPE4	0.552666	2.181788	2.856601	0.005337	-2.31055	0.053529	0.100654
Monocytes	PROCA1	-0.60657	2.988273	-2.85602	0.005346	-2.21438	0.052336	0.098496
Monocytes	PCYT1A	0.372209	5.255992	2.855691	0.005351	-2.4752	0.049102	0.09253
Monocytes	ITM2C	-0.28932	5.637577	-2.85554	0.005353	-2.68893	0.048581	0.091556
Monocytes	MEGF9	0.517121	4.103779	2.855045	0.005361	-2.33456	0.050717	0.095597
Monocytes	CYP2D10	-1.20208	1.738911	-2.85489	0.005363	-2.35999	0.054217	0.102111
Monocytes	MTBP	-0.66122	3.256156	-2.85274	0.005396	-2.20992	0.052215	0.098274
Monocytes	BRD2	-0.22408	7.085393	-2.85172	0.005412	-2.76858	0.046965	0.088463
Monocytes	FADS2	-1.07216	3.530927	-2.85163	0.005414	-2.22718	0.051882	0.097664
Monocytes	GM17749	-0.46829	4.634129	-2.85016	0.005437	-2.67718	0.05046	0.094976
Monocytes	XYLT2	0.840831	1.972195	2.849652	0.005445	-2.29532	0.054392	0.10233
Monocytes	MAPRE1	-0.18595	7.148653	-2.84953	0.005447	-2.78819	0.047034	0.088607
Monocytes	SLC25A28	-0.31773	4.806244	-2.84914	0.005453	-2.48965	0.050226	0.094632
Monocytes	SLC8A2	-1.22168	1.394752	-2.84571	0.005507	-2.25071	0.055795	0.104728
Monocytes	ABCB4	-1.00195	3.391454	-2.84405	0.005533	-2.27096	0.052936	0.099431
Monocytes	TRMT12	1.05159	1.30746	2.843279	0.005545	-2.41671	0.05622	0.105445

Monocytes	KCNQ1OT1	-0.37921	6.399833	-2.84223	0.005562	-2.61678	0.04881	0.091702
Monocytes	SFMBT1	-0.30594	6.08199	-2.84196	0.005566	-2.59999	0.049245	0.092552
Monocytes	CLEC4A3	0.435267	3.297848	2.839736	0.005602	-2.71642	0.053538	0.100375
Monocytes	GSTM5	-1.39197	1.19189	-2.83932	0.005609	-2.46285	0.056839	0.106504
Monocytes	ITIH3	-0.90078	3.636116	-2.8389	0.005615	-2.25161	0.05306	0.099584
Monocytes	GATA3	-1.18209	1.116393	-2.83825	0.005626	-2.37723	0.057029	0.106989
Monocytes	GFRA2	-0.86097	2.545769	-2.83792	0.005631	-2.24977	0.054769	0.102856
Monocytes	MS4A4C	0.613197	3.224383	2.837336	0.005641	-2.60728	0.053766	0.101032
Monocytes	CTLA2B	-0.84826	3.353706	-2.83694	0.005647	-2.30728	0.053571	0.100712
Monocytes	VPS16	0.316088	4.436967	2.836725	0.005651	-2.40341	0.051962	0.097743
Monocytes	FEM1B	-0.37837	4.728435	-2.83606	0.005661	-2.44117	0.051562	0.097059
Monocytes	H2-T24	1.215525	1.828462	2.835923	0.005664	-2.38758	0.055955	0.105215
Monocytes	LILR4B	0.502278	3.079256	2.835269	0.005674	-2.59427	0.054063	0.101747
Monocytes	CRYBG3	0.331111	4.474287	2.834479	0.005687	-2.56562	0.052052	0.097984
Monocytes	RTL4	-1.78319	0.402884	-2.83247	0.00572	-2.65624	0.058681	0.110137
Monocytes	1810020O05RII	-1.0517	-0.23349	-2.83169	0.005733	-2.28249	0.059832	0.112261
Monocytes	PI16	0.554435	1.135522	2.829832	0.005763	-2.32806	0.057803	0.10839
Monocytes	PERP	-1.39193	1.228461	-2.82858	0.005784	-2.52373	0.057752	0.108284
Monocytes	MDM4	0.260707	5.749077	2.828366	0.005787	-2.57698	0.050846	0.095525
Monocytes	GAS7	0.436688	6.417925	2.828036	0.005793	-2.76729	0.049905	0.093807
Monocytes	AMD2	-1.34435	0.044662	-2.82795	0.005794	-2.70227	0.059726	0.112001
Monocytes	MTMR14	-0.31441	5.427711	-2.82696	0.005811	-2.59333	0.051405	0.096581
Monocytes	SGF29	-0.32429	4.671195	-2.82663	0.005816	-2.44517	0.052508	0.098689
Monocytes	IFITM10	-0.92229	3.144158	-2.82588	0.005829	-2.30289	0.054881	0.103088
Monocytes	CPQ	-0.31676	5.056734	-2.82435	0.005854	-2.71136	0.052187	0.097963
Monocytes	LNCPINT	-0.3548	8.328661	-2.82298	0.005877	-3.13103	0.047782	0.089662
Monocytes	TBL1X	-0.26178	7.155308	-2.82236	0.005888	-2.79949	0.049406	0.092711
Monocytes	TMSB15B1	-0.73446	2.422831	-2.82177	0.005898	-2.29135	0.05646	0.105883
Monocytes	CD300C	-0.88925	0.72621	-2.8215	0.005902	-2.32167	0.05924	0.111038
Monocytes	CENPC1	0.273152	5.265605	2.819657	0.005933	-2.54273	0.052341	0.098227
Monocytes	ATAD2B	0.235936	6.740991	2.819376	0.005938	-2.82361	0.050226	0.094312
Monocytes	HIGD1A	0.269481	6.029655	2.817919	0.005963	-2.72748	0.051401	0.096359
Monocytes	FARP1	-0.71693	3.619271	-2.81604	0.005995	-2.29367	0.055244	0.103354
Monocytes	WFS1	0.918749	1.279726	2.815527	0.006004	-2.46303	0.059052	0.110346
Monocytes	XDH	0.514126	4.807401	2.81459	0.00602	-2.69137	0.053472	0.100229
Monocytes	SCIMP	-0.55021	3.702449	-2.81431	0.006024	-2.45039	0.055159	0.103413
Monocytes	INHBC	-1.60103	0.808252	-2.81427	0.006025	-2.67728	0.059862	0.112079
Monocytes	B230217O12RII	1.354754	1.757632	2.814181	0.006027	-2.56123	0.058272	0.109168
Monocytes	HMGA1	0.403332	4.670399	2.812311	0.006059	-2.53303	0.053916	0.101071
Monocytes	BCL6B	-1.50955	0.649778	-2.81189	0.006066	-2.77636	0.060417	0.113143
Monocytes	RHOC	-0.59945	4.059106	-2.81112	0.006079	-2.34172	0.054938	0.103034
Monocytes	UCP1	-1.02494	-1.00774	-2.80981	0.006102	-2.49928	0.063594	0.118917
Monocytes	USF2	0.252836	6.021725	2.809131	0.006114	-2.73628	0.052142	0.097875
Monocytes	USHBP1	-1.35532	1.085061	-2.80903	0.006116	-2.63204	0.05992	0.112285
Monocytes	ZFP326	-0.28015	5.21559	-2.80889	0.006118	-2.59458	0.053332	0.100096
Monocytes	A930024E05RIK	-1.1064	1.108621	-2.80771	0.006139	-2.41271	0.060028	0.112393
Monocytes	IGSF9	-1.01491	0.790152	-2.80694	0.006152	-2.45106	0.0606	0.11357

Monocytes	GOT2	-0.24145	6.235083	-2.80669	0.006157	-2.8148	0.051984	0.097605
Monocytes	TUBGCP5	0.50981	3.976604	2.806613	0.006158	-2.33359	0.055381	0.103939
Monocytes	ELP5	-0.3289	4.633093	-2.80591	0.00617	-2.5428	0.054429	0.102065
Monocytes	BACE2	-1.54518	1.120623	-2.80554	0.006177	-2.7126	0.060111	0.11255
Monocytes	OPRM1	0.709173	2.803916	2.80473	0.006191	-2.33614	0.057397	0.107549
Monocytes	NOTCH2	0.216686	7.16468	2.80116	0.006254	-3.06132	0.051263	0.095895
Monocytes	RUNDC3B	-0.67617	3.848947	-2.799	0.006293	-2.39104	0.056545	0.105511
Monocytes	FOXN3	0.25793	8.899803	2.796712	0.006334	-3.16681	0.049387	0.091933
Monocytes	SREBF2	-0.27454	5.9605	-2.79383	0.006386	-2.8258	0.053902	0.100291
Monocytes	ZFP827	-1.14225	2.85843	-2.79344	0.006393	-2.46197	0.058811	0.109416
Monocytes	LAD1	-1.08844	-0.99444	-2.79321	0.006397	-2.36307	0.065603	0.121798
Monocytes	AB124611	0.308302	5.452214	2.79316	0.006398	-2.74366	0.054674	0.101815
Monocytes	CACNA1E	-0.73754	4.907691	-2.79308	0.006399	-2.59411	0.055515	0.10337
Monocytes	PDE1B	-0.45255	3.114849	-2.7926	0.006408	-2.4324	0.058387	0.108687
Monocytes	PICALM	0.196502	8.404518	2.79252	0.00641	-3.23445	0.050357	0.093811
Monocytes	LRBA	-0.31535	6.139874	-2.78977	0.00646	-2.86732	0.053991	0.100393
Monocytes	NUAK1	-1.06293	3.219187	-2.78955	0.006464	-2.39261	0.058604	0.108941
Monocytes	MORC3	0.259778	5.857789	2.789093	0.006472	-2.76972	0.054442	0.101226
Monocytes	GM12248	-0.71731	2.619411	-2.78353	0.006575	-2.36615	0.060525	0.111881
Monocytes	SETBP1	-0.55685	4.621756	-2.78272	0.00659	-2.66115	0.057286	0.106018
Monocytes	LMTK2	-0.30337	5.616268	-2.78151	0.006613	-2.68331	0.055854	0.103323
Monocytes	KPNA1	-0.25145	6.778024	-2.7805	0.006632	-2.89055	0.054178	0.100209
Monocytes	NSD1	0.207552	7.273059	2.779309	0.006654	-2.94442	0.053541	0.099041
Monocytes	LPCAT3	0.332296	5.162712	2.779188	0.006656	-2.65384	0.056792	0.105045
Monocytes	SPRED1	-0.3887	4.710293	-2.77874	0.006665	-2.6832	0.05754	0.106384
Monocytes	TMEM167	0.201024	6.804318	2.777048	0.006697	-2.93701	0.054409	0.100584
Monocytes	1810009A15RIK	0.645365	2.243882	2.776988	0.006698	-2.39078	0.061842	0.114196
Monocytes	FYB	0.380883	6.869674	2.776591	0.006705	-3.0605	0.05431	0.100428
Monocytes	PAXBP1	-0.30807	5.312121	-2.77658	0.006705	-2.7633	0.056725	0.104878
Monocytes	JPT2	-0.52406	3.513173	-2.77609	0.006715	-2.44439	0.059697	0.110355
Monocytes	UBE2N	-0.18392	7.48412	-2.77564	0.006723	-3.0225	0.053438	0.098876
Monocytes	ID2	-0.26557	6.76154	-2.77432	0.006748	-3.16514	0.054681	0.101021
Monocytes	FOS	0.315988	8.489188	2.774005	0.006754	-3.3864	0.052119	0.096318
Monocytes	BIN2	0.295266	5.165118	2.773088	0.006772	-2.76691	0.057277	0.105823
Monocytes	UHRF1BP1L	0.268425	5.734451	2.772405	0.006785	-2.80556	0.056431	0.104264
Monocytes	PLEKHM3	0.298758	6.068485	2.771445	0.006803	-2.94735	0.05601	0.103503
Monocytes	SLC38A9	0.356458	5.16902	2.769577	0.006839	-2.6578	0.057691	0.106472
Monocytes	CDK6	0.449485	6.322112	2.768257	0.006865	-2.79782	0.05602	0.103347
Monocytes	ROGDI	-0.35967	4.014028	-2.76733	0.006883	-2.63043	0.059869	0.110347
Monocytes	GM26756	1.210837	0.354794	2.765963	0.006909	-2.48752	0.066594	0.122455
Monocytes	PAFAH1B3	-0.3964	5.814072	-2.76548	0.006919	-2.71159	0.057115	0.105306
Monocytes	CFAP43	0.440725	3.09234	2.76521	0.006924	-2.47864	0.061653	0.113644
Monocytes	TNFSF13OS	1.588157	0.055873	2.763837	0.006951	-2.69354	0.067392	0.12399
Monocytes	RHOV	1.551734	-0.80702	2.762997	0.006967	-2.65569	0.069172	0.127108
Monocytes	FOXP1	0.190008	9.650428	2.761947	0.006988	-3.31719	0.051682	0.09525
Monocytes	5430431A17RIK	0.972501	1.423829	2.761173	0.007003	-2.56912	0.065146	0.119935
Monocytes	OASL1	-0.75248	2.957835	-2.75916	0.007043	-2.62043	0.062681	0.115307

Monocytes	CMIP	0.242976	9.635584	2.757374	0.007078	-3.39195	0.052239	0.095954
Monocytes	SELENOK	-0.22027	7.584969	-2.75452	0.007135	-3.09835	0.055583	0.102195
Monocytes	LRG1	1.385477	2.460703	2.75452	0.007135	-2.48093	0.064171	0.117862
Monocytes	SKAP1	-0.57489	4.523681	-2.7544	0.007138	-2.70098	0.060548	0.111287
Monocytes	BSPRY	-1.43316	0.341542	-2.75429	0.00714	-2.7811	0.068142	0.125012
Monocytes	0610040J01RIK	0.521651	2.378015	2.752447	0.007177	-2.54175	0.064545	0.118532
Monocytes	NRROS	0.219951	7.067702	2.752446	0.007177	-3.12584	0.056585	0.104027
Monocytes	ATXN3	0.278644	5.054807	2.751067	0.007205	-2.72302	0.060041	0.11017
Monocytes	SNHG1	-0.33854	5.230493	-2.75065	0.007213	-2.68733	0.059748	0.109679
Monocytes	NCKIPSD	-0.46345	2.952621	-2.75045	0.007218	-2.47829	0.063701	0.116865
Monocytes	SERPINA1A	-0.74647	8.012506	-2.74944	0.007238	-3.10279	0.055397	0.101626
Monocytes	INPP5F	0.370548	4.366787	2.748547	0.007256	-2.70783	0.061438	0.112661
Monocytes	RALA	-0.22249	5.871704	-2.74501	0.007328	-2.98971	0.059437	0.108658
Monocytes	MRPL43	0.298394	5.608686	2.744145	0.007346	-2.80237	0.059938	0.109649
Monocytes	EPC1	0.255533	6.811099	2.74404	0.007348	-2.98116	0.057958	0.106047
Monocytes	SLC16A6	-0.39741	4.554893	-2.74357	0.007358	-2.75639	0.061765	0.113012
Monocytes	TUBB2A	-0.36852	5.517234	-2.74281	0.007374	-2.88361	0.060175	0.110179
Monocytes	KLRC2	-1.0061	1.332802	-2.74264	0.007377	-2.45883	0.067705	0.123819
Monocytes	CAND1	-0.26384	5.132169	-2.74212	0.007388	-2.75334	0.060865	0.111458
Monocytes	TIMD4	-0.59358	3.658253	-2.74133	0.007405	-2.6998	0.063525	0.116266
Monocytes	RESF1	0.295298	6.500817	2.741038	0.00741	-2.93196	0.058659	0.10743
Monocytes	GM21188	0.4887	1.578983	2.740686	0.007418	-2.77884	0.067377	0.123285
Monocytes	SERPINA1C	-0.78121	7.348457	-2.73731	0.007488	-3.04019	0.057794	0.105686
Monocytes	MSI2	-0.24445	7.509903	-2.73673	0.0075	-3.0636	0.057559	0.105291
Monocytes	AKAP13	-0.20906	8.500476	-2.73656	0.007504	-3.26336	0.055998	0.102438
Monocytes	FOSL2	0.294193	5.561041	2.735649	0.007523	-3.07925	0.06088	0.111301
Monocytes	PNISR	0.233327	5.773836	2.735182	0.007533	-2.82008	0.060548	0.11073
Monocytes	RAP2B	0.317284	4.614993	2.734367	0.00755	-2.74515	0.062637	0.114439
Monocytes	EIF2S1	-0.24894	6.052768	-2.73368	0.007565	-2.90902	0.06023	0.110063
Monocytes	TXNDC17	-0.259	6.116505	-2.73295	0.00758	-2.99441	0.060195	0.109976
Monocytes	NBR1	0.294835	5.455137	2.732506	0.00759	-2.73267	0.061344	0.112074
Monocytes	RASSF3	0.269946	6.834864	2.731689	0.007607	-3.15674	0.05911	0.10797
Monocytes	CLEC4B1	0.570311	-0.61033	2.728703	0.007671	-2.51827	0.073467	0.133475
Monocytes	FLNA	0.28715	6.097597	2.727949	0.007687	-3.10922	0.060872	0.110874
Monocytes	ARL5B	-0.33883	5.460574	-2.72703	0.007706	-2.90571	0.062073	0.113014
Monocytes	PODNL1	-1.38214	-1.02387	-2.72439	0.007763	-2.97171	0.07505	0.136029
Monocytes	CACNA1D	0.507965	3.396539	2.723625	0.00778	-2.62362	0.066298	0.120441
Monocytes	NOCT	0.365803	5.04776	2.723095	0.007792	-2.88537	0.063332	0.115141
Monocytes	DCLRE1C	0.266816	6.497189	2.722551	0.007803	-3.05284	0.060857	0.110678
Monocytes	CLDN1	-0.76617	1.493829	-2.72168	0.007822	-2.53901	0.070165	0.127398
Monocytes	PDCD1	-1.55544	0.743001	-2.71875	0.007887	-2.62017	0.072205	0.130948
Monocytes	CDC42BPB	-0.39932	3.262023	-2.71818	0.007899	-2.75638	0.06726	0.12223
Monocytes	KLRE1	-0.87519	1.626605	-2.71804	0.007902	-2.51636	0.070442	0.127948
Monocytes	TBC1D24	0.792073	1.799284	2.717031	0.007924	-2.52448	0.0702	0.127542
Monocytes	AI504432	0.408322	2.840905	2.716921	0.007927	-2.64004	0.068164	0.1239
Monocytes	KCTD20	0.286422	4.885027	2.714926	0.007971	-2.80137	0.064658	0.117427
Monocytes	SLC27A5	-1.28343	1.457834	-2.71443	0.007982	-2.6535	0.071259	0.129331

Monocytes	KLF6	-0.30705	7.581942	-2.71281	0.008018	-3.30293	0.060223	0.109313
Monocytes	CD300A	0.31588	4.623283	2.711668	0.008043	-3.08829	0.065527	0.118833
Monocytes	1110002J07RIK	-0.92252	-0.51304	-2.71159	0.008045	-2.53009	0.075772	0.137072
Monocytes	SLC7A5	-0.43268	6.118367	-2.71108	0.008057	-2.88057	0.062878	0.114112
Monocytes	TYROBP	0.271785	9.095091	2.709052	0.008102	-3.56173	0.058129	0.105415
Monocytes	NCOA6	0.24279	5.600455	2.70898	0.008104	-2.90144	0.064063	0.116187
Monocytes	SUSD6	0.251498	7.690279	2.708452	0.008116	-3.20747	0.060476	0.109706
Monocytes	CNR2	0.449136	3.101889	2.705953	0.008172	-2.63361	0.06919	0.125156
Monocytes	PANK3	0.320673	4.591077	2.705629	0.00818	-2.73041	0.066356	0.120132
Monocytes	GM4117	0.884891	0.660809	2.705188	0.00819	-2.58015	0.074176	0.134046
Monocytes	H2-OB	-0.42728	4.007971	-2.70477	0.008199	-2.93641	0.067504	0.122233
Monocytes	GRIK4	-1.86777	0.238216	-2.70407	0.008215	-2.94841	0.075182	0.13591
Monocytes	CSGALNACT2	0.262507	4.10884	2.70279	0.008244	-2.87531	0.067513	0.12219
Monocytes	PSMA3	-0.1721	7.755055	-2.70278	0.008245	-3.27133	0.06097	0.110396
Monocytes	UBB	-0.26241	10.85501	-2.70253	0.00825	-3.61882	0.055968	0.101311
Monocytes	COL13A1	-1.44445	0.62777	-2.70186	0.008266	-2.84737	0.074571	0.134758
Monocytes	LRRC8A	0.320995	5.427019	2.701042	0.008284	-2.80367	0.065224	0.118035
Monocytes	DEPDC5	0.343261	4.978079	2.698992	0.008332	-2.74934	0.066372	0.119998
Monocytes	ALOX5AP	0.37218	7.325815	2.698336	0.008347	-3.59067	0.062222	0.112481
Monocytes	NAPSA	0.256853	6.459379	2.696451	0.00839	-3.30883	0.064024	0.115611
Monocytes	TLR3	-0.42379	1.534056	-2.69469	0.008431	-2.87916	0.073822	0.13295
Monocytes	9030404E10RIK	-1.43003	-0.16276	-2.69444	0.008437	-2.83691	0.077465	0.139417
Monocytes	CYP2D26	-1.07976	3.247258	-2.69418	0.008443	-2.56337	0.070333	0.126835
Monocytes	ATRX	-0.20881	7.666179	-2.693	0.008471	-3.22994	0.062302	0.112447
Monocytes	4931413K12RIK	0.83987	1.943113	2.691883	0.008497	-2.61498	0.07332	0.132094
Monocytes	ZFP626	0.655082	2.562798	2.690412	0.008532	-2.57597	0.072282	0.130039
Monocytes	FAM83E	-1.18494	1.10583	-2.68987	0.008545	-2.78555	0.075379	0.135573
Monocytes	MRPS23	0.389957	4.411962	2.687141	0.008609	-2.75279	0.069125	0.124241
Monocytes	CGNL1	-1.05642	1.622928	-2.68595	0.008638	-2.60612	0.074927	0.134442
Monocytes	ZFP524	0.418474	3.672126	2.685355	0.008652	-2.74312	0.070714	0.127054
Monocytes	RUNX2	0.474814	4.240789	2.685309	0.008653	-2.98528	0.069591	0.125059
Monocytes	GM15563	1.204257	1.923249	2.685291	0.008653	-2.72737	0.074293	0.133388
Monocytes	CERS4	-0.62684	3.651416	-2.68498	0.008661	-2.58856	0.070759	0.127207
Monocytes	TGIF2	-0.61063	3.122183	-2.68433	0.008677	-2.58307	0.071895	0.129229
Monocytes	FRAT1	0.487226	3.463579	2.682226	0.008727	-2.60887	0.071563	0.128538
Monocytes	SH3BP1	-0.24886	5.21446	-2.68087	0.00876	-3.12819	0.068308	0.122748
Monocytes	CARD9	0.639155	0.997223	2.680689	0.008764	-2.61782	0.076935	0.138041
Monocytes	PTBP1	-0.25677	6.221744	-2.67962	0.00879	-3.03032	0.066552	0.119558
Monocytes	HIC1	-0.84297	2.158311	-2.67772	0.008836	-2.62032	0.074938	0.134421
Monocytes	FAM160A2	0.535357	2.604929	2.675252	0.008897	-2.61764	0.074353	0.133364
Monocytes	AI413582	0.311939	4.68032	2.675123	0.0089	-2.95745	0.070132	0.12588
Monocytes	PHF21A	0.277355	7.069098	2.675122	0.0089	-3.17547	0.065601	0.117781
Monocytes	PPP1R13B	-0.32871	5.30235	-2.67438	0.008918	-2.90963	0.069006	0.12387
Monocytes	HGF	0.468766	2.743326	2.672737	0.008958	-2.91317	0.074433	0.133464
Monocytes	GDE1	0.430103	4.883129	2.671326	0.008993	-2.87893	0.0703	0.125947
Monocytes	IFFO2	-0.51426	3.364648	-2.67091	0.009004	-2.67354	0.073393	0.131492
Monocytes	AMIGO2	-1.72086	0.934211	-2.67013	0.009023	-2.94766	0.078668	0.140841

Monocytes	CLDN3	-1.25177	1.461002	-2.67009	0.009024	-2.77039	0.077502	0.138795
Monocytes	POLG2	-0.29823	5.080463	-2.66939	0.009041	-2.95468	0.070013	0.125646
Monocytes	CD28	-0.89372	2.935145	-2.66937	0.009042	-2.61456	0.074368	0.133376
Monocytes	CD84	0.335567	5.366126	2.668546	0.009062	-3.03544	0.069554	0.12483
Monocytes	ZFYVE26	0.37571	5.03643	2.668277	0.009069	-2.80925	0.070199	0.126053
Monocytes	ABCB1A	-0.80236	2.010725	-2.66786	0.009079	-2.64876	0.076473	0.137227
Monocytes	RAD23B	-0.18447	6.360558	-2.66714	0.009097	-3.13508	0.067752	0.121713
Monocytes	TUBA1A	-0.37877	5.430541	-2.66579	0.009131	-3.04036	0.06971	0.125144
Monocytes	GM10125	-0.71114	2.003063	-2.66566	0.009134	-2.62381	0.076772	0.137682
Monocytes	ASNSD1	0.245613	5.360445	2.664923	0.009153	-3.01071	0.069934	0.125549
Monocytes	STRBP	-0.27216	7.195368	-2.66417	0.009172	-3.18943	0.066526	0.119441
Monocytes	ATOX1	-0.19192	8.244709	-2.66314	0.009198	-3.57885	0.064748	0.116239
Monocytes	A430093F15RIK	-0.85283	2.363356	-2.66065	0.009261	-2.65916	0.076761	0.137557
Monocytes	RHOA	0.127639	9.258828	2.660482	0.009265	-3.57181	0.0633	0.113536
Monocytes	CEBPD	0.34422	3.956744	2.660296	0.00927	-2.94695	0.073388	0.131616
Monocytes	DNAJA1	-0.18658	7.461326	-2.65801	0.009328	-3.25977	0.0669	0.119786
Monocytes	MAP2K4	0.273704	6.321239	2.656987	0.009355	-3.19975	0.069176	0.123793
Monocytes	FETUB	-0.98644	3.175824	-2.65683	0.009359	-2.6411	0.075559	0.135157
Monocytes	IQCH	-0.8742	0.250926	-2.65618	0.009375	-2.65169	0.082167	0.146773
Monocytes	CC2D2B	-1.10155	2.70829	-2.65573	0.009387	-2.67695	0.076654	0.137187
Monocytes	STK17B	0.22843	8.129238	2.655549	0.009392	-3.38208	0.065859	0.117973
Monocytes	PKD1L2	0.590767	1.173969	2.65179	0.009489	-2.74218	0.080821	0.143975
Monocytes	PRRC2B	-0.25739	6.226887	-2.65066	0.009518	-3.05554	0.070266	0.125343
Monocytes	GM19557	-0.949	-0.95554	-2.64966	0.009544	-2.84902	0.086203	0.153233
Monocytes	ZBTB20	-0.26421	8.246328	-2.6495	0.009548	-3.36827	0.066535	0.118635
Monocytes	SLC25A25	-0.32445	4.951297	-2.64856	0.009573	-2.92677	0.073072	0.130182
Monocytes	ZFP653	0.535679	3.131364	2.648146	0.009584	-2.67587	0.076937	0.137022
Monocytes	DISP1	0.485788	3.443949	2.647671	0.009596	-2.70247	0.076302	0.135934
Monocytes	H3F3A	0.181321	10.71708	2.64695	0.009615	-3.79319	0.062391	0.11116
Monocytes	SNAP23	0.233865	6.222753	2.646341	0.009631	-3.20715	0.070727	0.126136
Monocytes	MTOR	-0.29649	4.766611	-2.64575	0.009647	-2.92005	0.073733	0.131505
Monocytes	GIMAP5	-0.80931	3.319876	-2.64515	0.009663	-2.66602	0.076861	0.137
Monocytes	IL4I1	-0.58907	2.841673	-2.64375	0.0097	-2.81621	0.078145	0.139232
Monocytes	APOC4	-0.75103	5.632488	-2.64212	0.009743	-2.97659	0.072444	0.129096
Monocytes	PPFIBP1	-0.42582	3.366596	-2.64192	0.009748	-2.77864	0.077205	0.137536
Monocytes	ADAM8	-0.36614	2.602795	-2.64191	0.009749	-3.06337	0.078887	0.14049
Monocytes	MAST3	0.471501	4.169613	2.641016	0.009773	-2.7789	0.07554	0.134648
Monocytes	AGT	-0.94711	4.084746	-2.64096	0.009774	-2.73929	0.075721	0.134966
Monocytes	ROR1	-1.195	1.374979	-2.64077	0.009779	-2.77172	0.081742	0.145556
Monocytes	SYNJ2	-0.67213	2.324633	-2.64	0.0098	-2.67893	0.07968	0.141984
Monocytes	GBP8	-0.63924	3.65151	-2.63926	0.00982	-2.73886	0.076849	0.136965
Monocytes	FAM220A	1.13701	2.03012	2.638735	0.009834	-2.76099	0.080502	0.143385
Monocytes	DAPK2	-0.67414	2.818088	-2.63707	0.009879	-2.69736	0.079028	0.140558
Monocytes	MYL9	1.036545	1.95475	2.635696	0.009916	-2.68627	0.081168	0.144303
Monocytes	PFKFB4	0.482786	3.15878	2.635271	0.009927	-2.75142	0.078454	0.139557
Monocytes	TCP11L2	0.384563	6.196085	2.635052	0.009933	-3.09828	0.072042	0.128243
Monocytes	SERINC3	0.193864	9.707046	2.634942	0.009936	-3.54578	0.065353	0.116305

Monocytes	AGO3	-0.26821	5.940327	-2.63482	0.009939	-3.12326	0.072559	0.129159
Monocytes	ST3GAL1	0.347866	5.796993	2.634396	0.009951	-3.26914	0.072879	0.129702
Monocytes	GNE	-0.51289	4.467546	-2.63335	0.009979	-2.70693	0.075803	0.134848
Monocytes	CCNYL1	-0.2985	4.635175	-2.63266	0.009998	-3.01307	0.075532	0.13439
Monocytes	E230016K23RIK	0.895235	0.143576	2.632378	0.010006	-2.71454	0.085756	0.152314
Monocytes	NTNG2	0.5026	3.05713	2.631645	0.010026	-2.901	0.079062	0.140572
Monocytes	NOP58	-0.32781	5.838554	-2.63117	0.010039	-3.09781	0.073145	0.130144
Monocytes	PRODH2	-1.18157	2.255274	-2.63096	0.010044	-2.73003	0.080901	0.143853
Monocytes	TMEM229B	-0.4564	4.03146	-2.62809	0.010123	-2.7841	0.077474	0.137516
Monocytes	KCNJ2	0.876845	-0.01521	2.627905	0.010128	-2.80576	0.08687	0.15389
Monocytes	KLRA1	-1.80096	0.700337	-2.62735	0.010144	-2.91507	0.085187	0.151007
Monocytes	URGCP	0.403625	4.434418	2.626989	0.010154	-2.87797	0.076676	0.136128
Monocytes	FAM120A	0.193003	6.352078	2.626275	0.010173	-3.24483	0.072737	0.12922
Monocytes	ITCH	0.239592	7.100446	2.625478	0.010195	-3.3367	0.071235	0.126705
Monocytes	SLC38A1	-0.25102	7.937408	-2.62537	0.010199	-3.50019	0.069596	0.123787
Monocytes	GPR174	-1.12615	2.267506	-2.62501	0.010208	-2.82735	0.081584	0.145018
Monocytes	MAMLD1	-0.76185	0.110917	-2.625	0.010209	-2.70904	0.086728	0.153973
Monocytes	SLC17A2	-1.29321	1.156237	-2.62479	0.010215	-2.88076	0.084192	0.149568
Monocytes	FTH1	0.399895	12.5537	2.624698	0.010217	-4.12293	0.061295	0.108933
Monocytes	FCGR3	0.385081	4.189291	2.62422	0.01023	-3.15494	0.077308	0.137539
Monocytes	ZNRF1	0.268699	6.496008	2.624016	0.010236	-3.21924	0.072471	0.128992
Monocytes	IMMP2L	-0.39402	6.534254	-2.62311	0.010261	-3.24857	0.072519	0.129065
Monocytes	BCAT1	-1.80371	0.852062	-2.62205	0.010291	-3.08655	0.085264	0.151474
Monocytes	XPNPEP1	0.304115	4.365592	2.621838	0.010297	-2.95786	0.077209	0.137429
Monocytes	TSR1	-0.51194	3.584651	-2.62129	0.010312	-2.76841	0.078983	0.140567
Monocytes	SPEF1	-1.40535	0.343281	-2.62095	0.010322	-3.0411	0.086585	0.1539
Monocytes	CDK18	-1.54135	-0.22726	-2.62041	0.010337	-3.06457	0.088065	0.156538
Monocytes	CRCP	0.309145	4.449755	2.619673	0.010358	-2.89481	0.077251	0.137585
Monocytes	GM26812	-1.64439	0.020142	-2.6192	0.010371	-3.1247	0.087604	0.15569
Monocytes	4-Sep	-1.11227	1.735848	-2.6179	0.010407	-2.81089	0.083655	0.148783
Monocytes	ABTB2	-0.40626	7.412469	-2.61771	0.010413	-3.41453	0.071331	0.127089
Monocytes	XPR1	0.261747	6.866995	2.616488	0.010448	-3.3227	0.072609	0.129184
Monocytes	DIABLO	-0.36028	4.259159	-2.61529	0.010482	-2.88967	0.078273	0.139279
Monocytes	FKBP5	0.474203	5.30259	2.615158	0.010485	-2.97569	0.076014	0.135319
Monocytes	B930036N10RIK	-0.42241	5.605823	-2.6144	0.010507	-3.08788	0.075471	0.134327
Monocytes	RETREG1	0.255437	6.437217	2.614042	0.010517	-3.37169	0.073754	0.131314
Monocytes	ECPAS	0.23154	6.743157	2.612516	0.010561	-3.26155	0.073376	0.130539
Monocytes	ATP8B4	0.341379	4.781456	2.6108	0.01061	-3.3796	0.077819	0.138255
Monocytes	SIRPA	0.293216	5.832373	2.60987	0.010637	-3.39784	0.075652	0.134482
Monocytes	GTF2IRD1	-0.66421	3.292146	-2.60981	0.010638	-2.75588	0.081245	0.144335
Monocytes	GM15952	1.106648	1.028007	2.60942	0.01065	-2.86029	0.086618	0.153715
Monocytes	BORCS7	0.3877	3.716689	2.609267	0.010654	-2.85379	0.080279	0.142677
Monocytes	TEX261	0.248885	5.229001	2.608423	0.010679	-3.10418	0.07706	0.137038
Monocytes	KLHL6	0.362671	6.109159	2.608016	0.01069	-3.1416	0.075213	0.133782
Monocytes	FBXL20	0.407481	5.495646	2.607536	0.010704	-3.09228	0.076557	0.136233
Monocytes	SQOR	0.414691	3.496234	2.606828	0.010725	-2.97975	0.081024	0.144162
Monocytes	VPS53	0.271465	4.462382	2.606175	0.010744	-2.95297	0.078851	0.140461

Monocytes	GM14305	-0.57852	2.260659	-2.60609	0.010746	-2.74704	0.083899	0.149335
Monocytes	HPS6	-0.76646	1.22115	-2.606	0.010749	-2.75578	0.086405	0.153713
Monocytes	KLRA9	-1.53102	-0.30711	-2.60598	0.010749	-3.00541	0.090238	0.160376
Monocytes	SLC1A2	-0.57809	2.197177	-2.60549	0.010764	-2.78048	0.0841	0.149686
Monocytes	LIPA	-0.2872	5.64326	-2.6046	0.01079	-3.27489	0.076372	0.136257
Monocytes	ARSB	-0.39535	4.938464	-2.60447	0.010793	-3.24952	0.077895	0.138957
Monocytes	ANGPTL7	-1.66852	-0.05517	-2.60434	0.010797	-3.13388	0.089698	0.159681
Monocytes	SNX27	0.221282	5.803763	2.604197	0.010801	-3.22011	0.076029	0.135681
Monocytes	RBPJ	-0.31397	6.055966	-2.60327	0.010828	-3.45442	0.075574	0.134907
Monocytes	VPS51	0.383616	3.730024	2.60327	0.010829	-2.90669	0.08067	0.143931
Monocytes	PAK1	-0.38825	5.334473	-2.60264	0.010847	-3.38788	0.077192	0.137776
Monocytes	FNDC5	-0.53928	0.474524	-2.60072	0.010903	-2.85677	0.088932	0.158136
Monocytes	IL12RB2	-0.48942	3.672057	-2.60027	0.010917	-2.96838	0.081282	0.144705
Monocytes	NSMF	-0.65604	2.696993	-2.59961	0.010936	-2.76046	0.083559	0.148835
Monocytes	MILR1	0.30004	4.330073	2.599163	0.010949	-3.05817	0.079802	0.142311
Monocytes	ENPP2	-1.03715	2.411456	-2.59896	0.010955	-2.80214	0.084236	0.150151
Monocytes	MDH1	-0.22114	8.120076	-2.59866	0.010964	-3.53788	0.071787	0.128112
Monocytes	SUMO3	-0.25898	5.724941	-2.5986	0.010966	-3.19399	0.076741	0.136945
Monocytes	NT5DC2	0.642104	2.432435	2.598572	0.010967	-2.77217	0.084186	0.150087
Monocytes	COPG2	0.270411	5.004811	2.598185	0.010978	-3.15483	0.078305	0.139791
Monocytes	RAB43	-0.24834	6.948549	-2.59807	0.010982	-3.50301	0.074164	0.13244
Monocytes	KCNAB2	0.360802	4.287693	2.597621	0.010995	-3.0146	0.079936	0.142667
Monocytes	R74862	-1.0863	1.054776	-2.59681	0.011019	-2.81994	0.087705	0.156245
Monocytes	RSF1OS2	0.973061	1.663513	2.596378	0.011032	-2.82201	0.086244	0.153646
Monocytes	TSHZ3	-1.23787	2.187789	-2.5961	0.01104	-2.81645	0.084975	0.151462
Monocytes	SLC8B1	-0.24955	4.721857	-2.59576	0.011051	-3.342	0.079136	0.141158
Monocytes	RNF113A2	0.391036	4.271264	2.594859	0.011077	-2.90438	0.080281	0.143052
Monocytes	HSPA14	-0.31503	5.034968	-2.59431	0.011094	-3.09096	0.078638	0.140168
Monocytes	SNIP1	-0.40867	3.633076	-2.59317	0.011128	-2.82275	0.081991	0.146079
Monocytes	TRIM11	0.383841	5.06784	2.591975	0.011164	-2.92507	0.078935	0.140712
Monocytes	EPS8L1	-1.90035	0.408966	-2.59177	0.01117	-3.03639	0.09003	0.160185
Monocytes	HSPA1A	0.785249	3.985226	2.59124	0.011186	-3.18044	0.08143	0.145157
Monocytes	PPAN	-0.4859	4.072585	-2.59089	0.011197	-2.86689	0.081248	0.144897
Monocytes	MAPKAPK3	0.264833	5.306805	2.590606	0.011205	-3.2985	0.078485	0.140049
Monocytes	LRRC8C	-0.27988	6.412612	-2.5896	0.011236	-3.40599	0.076248	0.135974
Monocytes	DYSF	-1.07646	2.592245	-2.5877	0.011293	-2.78742	0.085258	0.151756
Monocytes	PLEKHN1	-0.64153	1.876111	-2.58522	0.011369	-2.79109	0.08746	0.155407
Monocytes	RBM48	0.544089	3.037834	2.585221	0.011369	-2.79288	0.084635	0.150466
Monocytes	GM26749	0.717757	2.222722	2.583418	0.011424	-2.79572	0.086965	0.154323
Monocytes	GIMAP7	-1.07013	1.458503	-2.58268	0.011447	-2.82866	0.088981	0.157884
Monocytes	ANKRD13C	0.281451	5.868682	2.582266	0.01146	-3.2073	0.078621	0.139681
Monocytes	ST3GAL5	-0.28575	6.23691	-2.58106	0.011497	-3.47526	0.078012	0.138502
Monocytes	INTS2	-0.48203	4.120259	-2.58048	0.011515	-2.91249	0.08285	0.146922
Monocytes	NDRG2	-0.90914	2.964877	-2.57886	0.011565	-2.8029	0.085865	0.152016
Monocytes	GLCCI1	-0.32691	7.348661	-2.57873	0.011569	-3.31986	0.075938	0.134573
Monocytes	MARK2	0.200892	6.879023	2.578502	0.011576	-3.37797	0.076937	0.136362
Monocytes	CAST	0.198621	6.266263	2.578078	0.01159	-3.38438	0.078297	0.138811

Monocytes	NUAK2	-0.24499	4.289152	-2.57744	0.01161	-3.30655	0.082785	0.146769
Monocytes	ARPC2	-0.12469	9.318766	-2.57733	0.011613	-3.86563	0.071957	0.127598
Monocytes	KLRC3	-1.17876	-1.20661	-2.57716	0.011618	-3.1701	0.096727	0.170987
Monocytes	MALAT1	-0.1973	14.72453	-2.57598	0.011655	-4.51473	0.062231	0.110101
Monocytes	AXL	-0.51523	3.708037	-2.57283	0.011754	-3.13898	0.084995	0.150209
Monocytes	TFDP2	-0.46109	5.591554	-2.57231	0.01177	-3.0587	0.080617	0.142653
Monocytes	GRB2	-0.19342	7.898248	-2.57221	0.011774	-3.62995	0.075596	0.133795
Monocytes	SOCS6	0.340464	3.829946	2.572117	0.011777	-2.96776	0.084704	0.149856
Monocytes	NADK2	-0.34516	4.994117	-2.56981	0.01185	-3.07594	0.082429	0.145684
Monocytes	NIPAL1	-1.12878	1.060638	-2.56882	0.011881	-2.92518	0.092281	0.162772
Monocytes	EML6	-0.49589	2.764743	-2.56847	0.011892	-3.09773	0.087957	0.155341
Monocytes	2610037D02RIK	0.321898	5.44268	2.567689	0.011917	-3.13205	0.081597	0.144361
Monocytes	ATG7	0.282461	5.786606	2.567621	0.011919	-3.31189	0.080815	0.142991
Monocytes	MANF	-0.2166	6.808848	-2.56738	0.011927	-3.40878	0.078541	0.138988
Monocytes	H2-EB2	-0.69388	0.547733	-2.56733	0.011929	-2.84142	0.093679	0.165435
Monocytes	1600010M07RI	0.345113	4.96425	2.565483	0.011988	-3.17041	0.08305	0.146818
Monocytes	TRAIP	-0.82866	2.119414	-2.56429	0.012026	-2.84381	0.090203	0.159246
Monocytes	ARHGAP26	0.226991	6.685583	2.563794	0.012042	-3.69338	0.079393	0.140367
Monocytes	ARHGAP10	-0.55276	5.105594	-2.5616	0.012113	-3.09069	0.083404	0.147246
Monocytes	SHISA5	0.299551	6.572889	2.561361	0.012121	-3.53439	0.080051	0.141423
Monocytes	SMIM12	0.328616	4.0506	2.560581	0.012146	-3.03616	0.086031	0.151832
Monocytes	NAMPT	-0.35864	6.301782	-2.56023	0.012158	-3.45864	0.08079	0.142701
Monocytes	SLCO5A1	-0.68551	-0.20706	-2.55923	0.01219	-2.85821	0.09721	0.171257
Monocytes	IL10RA	0.29599	4.971561	2.559164	0.012192	-3.31097	0.083976	0.148296
Monocytes	LACC1	-0.37425	3.279516	-2.554	0.012362	-3.15598	0.089231	0.156645
Monocytes	PPIP5K2	0.321022	4.496011	2.553584	0.012375	-3.06782	0.086262	0.151533
Monocytes	HPN	-0.86879	2.642028	-2.55196	0.012429	-2.86362	0.091178	0.159839
Monocytes	IFI44	-1.79116	0.579228	-2.55153	0.012443	-3.07832	0.096663	0.169271
Monocytes	RTN4	-0.16555	7.592315	-2.55151	0.012444	-3.66049	0.079365	0.139265
Monocytes	EFNA2	-1.28937	1.10216	-2.55134	0.01245	-2.91961	0.095239	0.16683
Monocytes	CD93	0.573635	3.89899	2.549937	0.012497	-2.91268	0.088273	0.154657
Monocytes	PLA2G4A	0.447495	3.294743	2.548733	0.012537	-3.10671	0.089994	0.157591
Monocytes	GM15832	0.586891	1.685015	2.548422	0.012547	-2.89143	0.094182	0.164807
Monocytes	KAT6A	0.23158	6.479855	2.548295	0.012551	-3.41419	0.082302	0.14422
Monocytes	PITPNM2	-0.69826	3.66119	-2.54759	0.012575	-2.86617	0.089146	0.156218
Monocytes	TACSTD2	1.253478	-0.35025	2.547453	0.01258	-3.02382	0.099869	0.174659
Monocytes	SYNGR2	-0.215	6.111593	-2.54711	0.012591	-3.52742	0.083243	0.146024
Monocytes	MIGA1	-0.54392	2.677211	-2.54685	0.0126	-2.86829	0.091675	0.160679
Monocytes	E330009J07RIK	0.689397	2.552592	2.546547	0.01261	-2.88333	0.092	0.161288
Monocytes	CD200R2	-1.08153	0.789771	-2.54632	0.012618	-2.87425	0.096709	0.169388
Monocytes	CYP2C70	-0.87112	4.144913	-2.54562	0.012641	-2.9619	0.088066	0.154355
Monocytes	IQCN	-1.08522	1.791223	-2.54532	0.012651	-2.91248	0.094124	0.164864
Monocytes	CBL	0.206789	7.22396	2.544871	0.012666	-3.56157	0.080836	0.141859
Monocytes	ZFAND2A	0.323405	3.630572	2.544232	0.012688	-3.08309	0.089394	0.156888
Monocytes	ASAP1	0.209331	7.200045	2.544075	0.012693	-3.65585	0.08089	0.142048
Monocytes	CDK2AP2	0.203951	6.467925	2.543985	0.012696	-3.52825	0.082558	0.144973
Monocytes	SLC2A1	0.333901	4.676925	2.543886	0.0127	-3.15821	0.086802	0.152385

Monocytes	XPC	0.492647	3.385386	2.543222	0.012722	-2.90191	0.090088	0.158109
Monocytes	SIRPB1C	0.438892	1.778958	2.542892	0.012733	-3.30608	0.09427	0.165385
Monocytes	HMGCL	0.294419	5.187114	2.542801	0.012736	-3.25818	0.085639	0.150422
Monocytes	SH3BGRL	0.200179	6.824052	2.541753	0.012772	-3.60884	0.081879	0.143977
Monocytes	ITGA2B	0.941654	0.938407	2.541471	0.012782	-2.94378	0.096624	0.169633
Monocytes	TNFSF13	0.590309	1.863945	2.541461	0.012782	-2.97279	0.094123	0.165326
Monocytes	TMEM214	-0.31874	4.338367	-2.54134	0.012786	-3.05821	0.087778	0.154352
Monocytes	AI182371	-1.00564	2.439191	-2.54109	0.012795	-2.88758	0.092604	0.162728
Monocytes	FAM118B	0.436813	3.464677	2.540938	0.0128	-2.94153	0.089963	0.158175
Monocytes	KLF3	0.244645	6.281206	2.540605	0.012811	-3.53229	0.083145	0.146351
Monocytes	APOB	-0.76878	4.836413	-2.54	0.012832	-3.08881	0.086658	0.152499
Monocytes	OTUD7B	-0.37476	5.17042	-2.5393	0.012856	-3.06111	0.08595	0.151154
Monocytes	TOP1	-0.22151	8.354587	-2.53773	0.012909	-3.72571	0.078903	0.138653
Monocytes	FHL2	-1.58532	0.017257	-2.53758	0.012915	-3.09322	0.099734	0.174848
Monocytes	SLC30A1	-0.48155	3.386735	-2.53727	0.012925	-3.01501	0.090672	0.159245
Monocytes	PLK3	-0.36148	4.483011	-2.53701	0.012934	-3.26996	0.087921	0.154484
Monocytes	NUP98	0.215103	7.461994	2.532828	0.013079	-3.6732	0.081743	0.143004
Monocytes	SNAPC5	0.295654	4.755447	2.530151	0.013172	-3.20823	0.088716	0.154932
Monocytes	SVIP	0.503873	1.34626	2.529747	0.013186	-2.97374	0.097671	0.170404
Monocytes	AKR1E1	-0.65767	2.622552	-2.52971	0.013187	-2.90296	0.094207	0.164463
Monocytes	GCNT1	0.635494	2.451925	2.529129	0.013208	-2.90402	0.094744	0.165289
Monocytes	POC5	0.623339	2.510705	2.528426	0.013232	-2.90444	0.094699	0.165063
Monocytes	TRP53	-0.25809	5.681235	-2.52806	0.013245	-3.30637	0.086648	0.151169
Monocytes	PTPRO	0.368128	2.363394	2.527338	0.013271	-3.3082	0.09524	0.165964
Monocytes	RAB29	0.387937	3.635461	2.526672	0.013294	-3.10975	0.091983	0.160302
Monocytes	GM36723	-0.39896	3.324777	-2.52541	0.013338	-3.48707	0.093029	0.162018
Monocytes	CHST8	-1.68303	-0.80735	-2.52497	0.013354	-3.20563	0.104588	0.181829
Monocytes	TNFRSF18	-0.50672	2.322619	-2.52494	0.013355	-2.9585	0.095698	0.166691
Monocytes	CYP2B9	-1.28205	1.9577	-2.52346	0.013408	-2.95576	0.097005	0.168686
Monocytes	MMP25	-0.60492	0.499533	-2.52185	0.013465	-2.97304	0.101465	0.176103
Monocytes	DMWD	-0.84067	2.228423	-2.52124	0.013487	-2.92598	0.096685	0.168013
Monocytes	PIK3CG	0.30146	4.936727	2.520875	0.0135	-3.26168	0.089584	0.155842
Monocytes	TUBB2B	-0.72812	2.9596	-2.52021	0.013524	-2.92195	0.094708	0.164708
Monocytes	SMAD2	-0.19594	6.426685	-2.52012	0.013527	-3.47887	0.085926	0.149561
Monocytes	CYP2J5	-1.32023	1.06057	-2.52004	0.013529	-3.07443	0.099936	0.173645
Monocytes	E230029C05RIK	0.375955	3.508508	2.520043	0.01353	-3.27521	0.093254	0.162218
Monocytes	TXNRD3	-0.78651	1.950944	-2.5192	0.01356	-2.94293	0.097548	0.169597
Monocytes	SMNDC1	-0.19134	6.059801	-2.51911	0.013563	-3.43552	0.086901	0.151272
Monocytes	AP1B1	-0.26508	5.190975	-2.51889	0.013571	-3.31591	0.08904	0.154997
Monocytes	MSR1	0.339678	3.816756	2.517494	0.013621	-3.38765	0.092824	0.16133
Monocytes	BCL9	-0.45587	3.399574	-2.51689	0.013643	-3.0222	0.093932	0.163388
Monocytes	BST1	0.539514	3.315218	2.516805	0.013646	-3.09314	0.094156	0.163786
Monocytes	BLZF1	0.452912	3.200504	2.516546	0.013655	-2.97756	0.094461	0.164349
Monocytes	PIN4	0.241793	4.949389	2.516438	0.013659	-3.28023	0.089927	0.15654
Monocytes	MARCO	-0.79506	5.795483	-2.51521	0.013704	-3.49106	0.088048	0.153217
Monocytes	ZDHHC15	1.177951	1.125862	2.512994	0.013784	-3.06538	0.10095	0.175145
Monocytes	EBPL	-0.39886	4.469152	-2.51136	0.013844	-3.10813	0.092193	0.15998

Monocytes	PLOD3	0.364942	3.277379	2.510797	0.013864	-3.18429	0.095417	0.16556
Monocytes	PRG4	2.405451	1.084056	2.509849	0.013899	-2.9752	0.101709	0.176266
Monocytes	MPLKIP	0.257789	4.793719	2.509381	0.013916	-3.22169	0.091653	0.159117
Monocytes	SLFN8	0.630482	3.326481	2.508679	0.013942	-3.07007	0.095628	0.165936
Monocytes	9430038I01RIK	0.276289	4.248537	2.507131	0.013999	-3.19057	0.093413	0.162132
Monocytes	RBBP8	-0.21286	6.609426	-2.50711	0.014	-3.59669	0.087437	0.151818
Monocytes	APOC1	-0.63042	7.949726	-2.50697	0.014005	-3.68003	0.084236	0.146281
Monocytes	RDH7	-0.98019	2.864625	-2.5057	0.014052	-2.94986	0.097376	0.168786
Monocytes	IPCEF1	0.404096	4.679593	2.50549	0.01406	-3.35023	0.092526	0.160538
Monocytes	GM31814	0.617116	-0.06084	2.504638	0.014091	-2.95565	0.10596	0.183398
Monocytes	GALNT12	-0.54119	2.991393	-2.50414	0.01411	-2.99226	0.097246	0.168593
Monocytes	FRMD5	-0.90453	2.390592	-2.50238	0.014175	-3.02942	0.099305	0.171959
Monocytes	IL1R1	1.003349	2.051839	2.501867	0.014194	-2.9595	0.100265	0.17371
Monocytes	GCNT2	0.311281	4.580489	2.501853	0.014195	-3.52585	0.093367	0.161908
Monocytes	GK	0.321864	4.764608	2.501453	0.01421	-3.42087	0.092886	0.161186
Monocytes	CDC23	0.552642	2.927739	2.501311	0.014215	-2.96088	0.097814	0.169656
Monocytes	CHP2	-1.64913	-0.09865	-2.50042	0.014249	-3.07387	0.106572	0.184761
Monocytes	CCDC90B	-0.43004	3.165156	-2.50041	0.014249	-3.05194	0.097161	0.16874
Monocytes	SPON1	1.188745	1.404705	2.500164	0.014258	-2.99532	0.102119	0.177244
Monocytes	GM27216	-1.38491	0.912751	-2.4997	0.014276	-3.12857	0.103554	0.179734
Monocytes	DOCK9	-0.57888	4.197961	-2.49967	0.014277	-3.05469	0.094376	0.16404
Monocytes	CERS2	0.25788	5.262709	2.49959	0.01428	-3.36586	0.091598	0.159262
Monocytes	HIPK1	0.259288	6.269668	2.4994	0.014287	-3.4943	0.089054	0.154875
Monocytes	FCHO2	0.216938	6.902577	2.49918	0.014295	-3.62188	0.087495	0.152206
Monocytes	TSPAN14	0.25701	6.835826	2.498764	0.014311	-3.49677	0.087658	0.152533
Monocytes	DHX38	0.308469	4.560036	2.498739	0.014312	-3.26596	0.093421	0.16251
Monocytes	PTPRC	0.218215	8.915439	2.49861	0.014317	-3.95444	0.08274	0.143956
Monocytes	ARHGEF12	-0.56421	3.963016	-2.49833	0.014327	-3.03546	0.095009	0.165306
Monocytes	NEURL2	1.036757	0.801683	2.497638	0.014353	-3.01111	0.10401	0.180564
Monocytes	2010109A12RIK	-0.75858	2.521995	-2.49723	0.014369	-2.97044	0.099107	0.172229
Monocytes	GLRX2	0.252359	5.111058	2.496355	0.014402	-3.32745	0.092293	0.160539
Monocytes	GM5086	0.781467	-0.01663	2.494803	0.014461	-2.98447	0.107043	0.185578
Monocytes	GM10847	1.301091	0.165978	2.494518	0.014472	-3.20889	0.106499	0.184686
Monocytes	NKRF	-0.57077	2.983842	-2.49359	0.014507	-2.98984	0.098411	0.171077
Monocytes	NDUFV3	0.237957	7.098589	2.493043	0.014528	-3.63569	0.087688	0.152628
Monocytes	OSTM1	0.287698	4.762239	2.492886	0.014534	-3.24886	0.093607	0.162885
Monocytes	PRKCQ	-0.84685	3.12892	-2.49288	0.014534	-2.97488	0.098009	0.170461
Monocytes	F630028O10RIK	0.662143	1.027185	2.492746	0.014539	-2.99191	0.104012	0.180725
Monocytes	GM17023	0.927798	-0.64235	2.49272	0.01454	-2.975	0.109066	0.189297
Monocytes	SPTY2D1	-0.229	5.765398	-2.49233	0.014555	-3.47502	0.091017	0.158503
Monocytes	ZDHHC17	0.361786	4.015064	2.492204	0.01456	-3.13317	0.095598	0.166418
Monocytes	GM16853	-0.68103	2.351019	-2.4915	0.014587	-2.97816	0.100312	0.174405
Monocytes	SLC25A37	0.537571	5.267981	2.490615	0.014621	-3.16891	0.09256	0.160981
Monocytes	ANAPC15	-0.31876	4.974484	-2.48967	0.014657	-3.27836	0.093443	0.162438
Monocytes	WDR6	-0.53001	3.277321	-2.48964	0.014658	-3.019	0.09801	0.170296
Monocytes	LACTB	-0.2434	5.139278	-2.48923	0.014674	-3.45957	0.09305	0.161696
Monocytes	IL1A	-0.73736	2.787687	-2.48852	0.014701	-3.06787	0.099529	0.172823

Monocytes	YAE1D1	0.519911	3.240113	2.488303	0.01471	-3.06967	0.098267	0.170715
Monocytes	GNB5	0.812684	0.819995	2.48789	0.014726	-3.01384	0.105239	0.182693
Monocytes	AFF3	-0.40165	7.930696	-2.48778	0.01473	-3.60988	0.086185	0.149897
Monocytes	DCTD	-1.13358	1.018621	-2.48687	0.014765	-3.18267	0.104831	0.181923
Monocytes	MYO1F	0.266982	5.259228	2.486243	0.014789	-3.67412	0.093108	0.161827
Monocytes	H2-AB1	-0.48631	6.764416	-2.48561	0.014814	-4.02467	0.089363	0.155301
Monocytes	SWAP70	-0.35334	5.61218	-2.48522	0.014829	-3.4328	0.092295	0.160459
Monocytes	GM12236	-0.82432	1.087964	-2.48508	0.014834	-2.99608	0.104845	0.182023
Monocytes	SCAND1	0.174839	7.018464	2.48475	0.014847	-3.7064	0.088761	0.154388
Monocytes	PDGFC	-1.09912	1.879122	-2.48424	0.014867	-2.99263	0.102617	0.178253
Monocytes	SIRPB1B	0.463861	1.445504	2.483267	0.014905	-3.32942	0.104041	0.180736
Monocytes	GM50399	-1.72878	0.166375	-2.48317	0.014909	-3.30372	0.107887	0.187272
Monocytes	GIMAP3	-1.10025	2.932139	-2.48134	0.01498	-3.01438	0.100162	0.173698
Monocytes	TNNI1	-1.68666	-0.907	-2.48074	0.015004	-3.33092	0.111684	0.193486
Monocytes	DUSP28	0.729487	1.99306	2.480545	0.015012	-3.00643	0.102855	0.178525
Monocytes	REL	-0.23139	8.12408	-2.48046	0.015015	-3.96277	0.086614	0.150519
Monocytes	MSN	0.196509	8.454473	2.48041	0.015017	-3.90741	0.085824	0.149142
Monocytes	FARP2	-0.52852	2.6131	-2.47929	0.015061	-3.06988	0.101299	0.175768
Monocytes	NAIP2	0.340899	3.006951	2.478763	0.015082	-3.33469	0.100254	0.174049
Monocytes	NDUFA2	0.193334	7.238115	2.478285	0.015101	-3.71297	0.089097	0.154811
Monocytes	1700025G04RII	-0.30554	4.957756	-2.47578	0.0152	-3.62024	0.095521	0.165613
Monocytes	THY1	-0.92942	1.573983	-2.47531	0.015219	-3.01434	0.105145	0.182113
Monocytes	PPIE	-0.29446	4.586932	-2.47471	0.015243	-3.2756	0.096669	0.167607
Monocytes	GIMAP6	-0.61721	5.672059	-2.47283	0.015318	-3.18909	0.094174	0.1631
Monocytes	SEPHS2	0.341135	6.39868	2.470165	0.015425	-3.57536	0.092832	0.160487
Monocytes	SPAG9	-0.23385	9.266573	-2.47006	0.015429	-4.17709	0.085725	0.148176
Monocytes	E2F6	-0.64168	2.266229	-2.46982	0.015439	-3.01937	0.104262	0.18007
Monocytes	STIM2	0.243423	6.210089	2.468346	0.015498	-3.62574	0.093622	0.161575
Monocytes	2410018L13RIK	-1.3307	0.727626	-2.46796	0.015514	-3.3019	0.109298	0.188254
Monocytes	TDP1	-0.53721	3.397629	-2.4673	0.015541	-3.06881	0.101459	0.174999
Monocytes	TIMM10B	0.215771	6.181583	2.466753	0.015563	-3.61749	0.093908	0.162079
Monocytes	SDC3	-0.40458	4.594842	-2.46618	0.015586	-3.55616	0.098261	0.16954
Monocytes	F7	1.074523	0.719823	2.464587	0.015651	-3.07515	0.11001	0.189334
Monocytes	RDH9	-1.33306	0.805989	-2.46404	0.015673	-3.18448	0.109802	0.188962
Monocytes	TREML4	-0.40034	2.758058	-2.46389	0.015679	-3.45011	0.103899	0.178981
Monocytes	TBC1D10B	0.273071	5.147294	2.463262	0.015705	-3.4001	0.097199	0.167588
Monocytes	BC030867	0.766238	2.099983	2.46319	0.015708	-3.04442	0.105908	0.182419
Monocytes	2610035D17RII	-0.46234	4.872984	-2.46276	0.015726	-3.35423	0.097983	0.168931
Monocytes	SMIM3	0.260026	4.840554	2.462165	0.01575	-3.53904	0.098072	0.169302
Monocytes	RMDN3	-0.41778	3.398108	-2.46192	0.01576	-3.08886	0.102132	0.176287
Monocytes	PABPC1L	0.539945	2.906471	2.461911	0.01576	-3.11813	0.103558	0.178715
Monocytes	SYNJ2BP	0.254575	5.520643	2.461849	0.015763	-3.38964	0.096221	0.166179
Monocytes	MRPL1	0.271869	4.619868	2.461092	0.015794	-3.27995	0.098814	0.170542
Monocytes	SH3BGRL2	-0.93228	2.700678	-2.46084	0.015804	-3.03677	0.104304	0.179904
Monocytes	CALR	-0.22156	7.35156	-2.46047	0.015819	-3.78806	0.091582	0.1581
Monocytes	MAPK14	0.185264	6.656022	2.459697	0.015852	-3.72121	0.093504	0.161406
Monocytes	DRG2	0.397224	3.507943	2.458985	0.015881	-3.11955	0.102257	0.176352

Monocytes	RSRC1	0.213541	6.605429	2.458708	0.015892	-3.65607	0.09376	0.161801
Monocytes	RRAD	-0.55127	2.552475	-2.45845	0.015903	-3.11246	0.105065	0.181159
Monocytes	ALAS2	1.460517	4.928547	2.457816	0.015929	-3.06996	0.0983	0.169638
Monocytes	ABHD14B	-0.89244	2.125702	-2.45781	0.01593	-3.08494	0.106376	0.183399
Monocytes	BMP2	-1.14764	1.613957	-2.45739	0.015947	-3.12839	0.107928	0.186164
Monocytes	JARID2	0.193641	8.144583	2.457254	0.015953	-4.00516	0.089865	0.155258
Monocytes	CTU1	1.201733	0.850388	2.457118	0.015958	-3.18918	0.11029	0.190237
Monocytes	FBXO38	0.225811	5.443447	2.456711	0.015975	-3.46894	0.096934	0.167514
Monocytes	CD160	-0.97688	1.916487	-2.45631	0.015992	-3.04894	0.107098	0.184834
Monocytes	NOS1AP	0.59044	3.163666	2.45606	0.016002	-3.15475	0.103394	0.178568
Monocytes	HMCES	-0.39218	4.456587	-2.45552	0.016025	-3.24014	0.099777	0.172455
Monocytes	GNL1	-0.30808	4.299889	-2.45399	0.016089	-3.30072	0.100556	0.173598
Monocytes	COLGALT2	-1.69833	-0.12576	-2.45361	0.016105	-3.35664	0.113981	0.196372
Monocytes	TIFAB	0.406389	2.73524	2.453449	0.016111	-3.37735	0.105103	0.181398
Monocytes	MFSD8	0.469278	2.979793	2.451967	0.016173	-3.06816	0.104717	0.180481
Monocytes	IL15RA	-0.61888	2.38833	-2.45144	0.016195	-3.0946	0.106561	0.183589
Monocytes	ARG1	0.994202	3.498182	2.451185	0.016206	-3.0782	0.10328	0.178064
Monocytes	MRPL23	0.193479	6.069484	2.449934	0.016259	-3.57242	0.096233	0.16599
Monocytes	OTUB2	-1.08985	1.885457	-2.44992	0.01626	-3.16603	0.108253	0.186488
Monocytes	UBE2A	0.179705	6.786705	2.449896	0.016261	-3.65271	0.094326	0.162708
Monocytes	PDLIM1	0.33883	4.944927	2.448901	0.016303	-3.41757	0.099452	0.17155
Monocytes	LRRC4	0.414992	3.774431	2.448874	0.016304	-3.2471	0.102777	0.177231
Monocytes	TNFSF14	0.781471	-0.05669	2.448632	0.016314	-3.09195	0.114548	0.197176
Monocytes	FBLIM1	-1.0812	0.928973	-2.44813	0.016335	-3.08466	0.111453	0.191984
Monocytes	GRINA	0.395354	6.205395	2.447923	0.016344	-3.56266	0.096063	0.165818
Monocytes	SGSM2	-0.39964	3.786605	-2.44722	0.016374	-3.2187	0.102929	0.177429
Monocytes	SUCLG1	-0.28797	5.422184	-2.44638	0.01641	-3.46871	0.098463	0.169734
Monocytes	MRTFA	-0.22809	7.084199	-2.44426	0.0165	-3.88039	0.094458	0.162574
Monocytes	B4GALT5	0.281912	5.975954	2.444004	0.016511	-3.69253	0.097428	0.167701
Monocytes	PIGN	0.372155	4.40092	2.443352	0.016539	-3.31712	0.101934	0.17538
Monocytes	LONRF1	0.438018	3.091293	2.44294	0.016557	-3.21806	0.105769	0.181923
Monocytes	BCL3	0.320744	5.236561	2.442855	0.01656	-3.52772	0.099578	0.171378
Monocytes	IFNG	-1.19665	1.241502	-2.44179	0.016606	-3.08446	0.111689	0.191659
Monocytes	B3GNTL1	0.573757	2.673331	2.441202	0.016631	-3.09962	0.107276	0.184379
Monocytes	PSMB9	-0.37348	6.228284	-2.44113	0.016635	-3.76544	0.097081	0.167008
Monocytes	HYKK	1.254156	0.618386	2.440999	0.01664	-3.28059	0.113704	0.195217
Monocytes	SERP1	0.189147	7.981742	2.439649	0.016698	-3.89547	0.092722	0.159437
Monocytes	ANKLE2	0.267256	5.177792	2.439171	0.016719	-3.48449	0.100328	0.172477
Monocytes	FCGR2B	0.338522	4.881357	2.43886	0.016732	-3.60531	0.101185	0.173933
Monocytes	PELI1	-0.19702	7.676753	-2.43643	0.016838	-3.86019	0.094072	0.161464
Monocytes	FGG	-0.50652	6.166651	-2.43639	0.01684	-3.54955	0.098114	0.168396
Monocytes	CLN6	0.362938	3.482895	2.435674	0.016871	-3.31996	0.105919	0.181664
Monocytes	SPATA5	-0.26682	5.905372	-2.43521	0.016891	-3.63166	0.099014	0.169933
Monocytes	TATDN1	0.408864	3.630234	2.433999	0.016944	-3.17994	0.105809	0.181314
Monocytes	DAB2IP	-1.00874	2.225574	-2.4329	0.016992	-3.16551	0.110287	0.188814
Monocytes	RLF	0.225219	6.699162	2.432266	0.01702	-3.6914	0.097264	0.166864
Monocytes	CD69	0.665768	5.620451	2.432256	0.017021	-3.30168	0.10024	0.171952

Monocytes	ART2B	-1.68542	0.097769	-2.43216	0.017025	-3.27876	0.117145	0.200499
Monocytes	FNDC3B	0.224945	6.549271	2.43212	0.017027	-3.80063	0.097672	0.167562
Monocytes	DPY19L4	0.299957	4.417697	2.431634	0.017048	-3.37127	0.103746	0.177945
Monocytes	USP40	0.551941	3.64171	2.430246	0.017109	-3.16672	0.106226	0.182247
Monocytes	PDIA3	-0.18232	8.546649	-2.43023	0.01711	-4.07087	0.092623	0.158988
Monocytes	GM44284	-1.23536	-0.05378	-2.43023	0.01711	-3.24506	0.11794	0.201963
Monocytes	WDR7	0.326838	5.188411	2.429794	0.017129	-3.4767	0.101762	0.174659
Monocytes	ATF4	-0.26391	6.443614	-2.42953	0.017141	-3.68316	0.098259	0.168734
Monocytes	GM15860	-1.34959	0.373729	-2.42916	0.017157	-3.38837	0.116603	0.19989
Monocytes	METTL1	-0.57174	3.431367	-2.42873	0.017176	-3.14422	0.106938	0.183739
Monocytes	TULP4	-0.239	5.928036	-2.42858	0.017183	-3.65587	0.0997	0.171451
Monocytes	CYSTM1	-0.58448	3.36709	-2.42853	0.017185	-3.32525	0.107132	0.184119
Monocytes	TCTEX1D2	0.348901	3.301638	2.42762	0.017226	-3.34201	0.107465	0.184741
Monocytes	GM34466	-1.22919	-1.10689	-2.42749	0.017232	-3.36635	0.121774	0.208833
Monocytes	SRD5A3	0.322925	4.511422	2.427256	0.017242	-3.43877	0.103868	0.178656
Monocytes	PSEN2	0.246468	5.03423	2.426938	0.017256	-3.49866	0.102355	0.176177
Monocytes	TAGAP1	-0.87494	2.214287	-2.42661	0.017271	-3.11196	0.110815	0.190653
Monocytes	CCDC136	-1.33525	0.119291	-2.42624	0.017287	-3.19896	0.117597	0.202146
Monocytes	SLIT1	-1.0518	1.464859	-2.42621	0.017288	-3.15057	0.113191	0.194731
Monocytes	UGT2B34	-1.09799	1.748323	-2.42617	0.01729	-3.15197	0.112286	0.193203
Monocytes	SGK3	0.244518	5.592861	2.425612	0.017315	-3.71377	0.100842	0.173683
Monocytes	DDX5	-0.13031	9.318404	-2.42542	0.017324	-4.10355	0.090918	0.156631
Monocytes	SLC41A2	-0.59876	2.900939	-2.4249	0.017347	-3.1854	0.108789	0.187389
Monocytes	ILDR1	-0.43521	1.791756	-2.42483	0.01735	-3.40686	0.112253	0.193259
Monocytes	KLRA17	0.818368	0.224958	2.424487	0.017365	-3.11844	0.117355	0.201961
Monocytes	AI597479	-0.77512	1.688645	-2.42424	0.017377	-3.1309	0.112582	0.193994
Monocytes	TTC3	-0.30788	5.355192	-2.42418	0.017379	-3.43191	0.101534	0.175183
Monocytes	EP400	-0.18762	6.870294	-2.42385	0.017394	-3.74515	0.097349	0.167968
Monocytes	TMEM29	-0.3279	4.706351	-2.42351	0.017409	-3.34234	0.103454	0.178447
Monocytes	SYTL3	-0.7499	2.803793	-2.42286	0.017438	-3.16852	0.109204	0.188285
Monocytes	PABPC4	-0.26553	5.897724	-2.42284	0.017439	-3.54706	0.100111	0.172749
Monocytes	CORO1A	0.215505	8.516071	2.421726	0.017489	-4.10598	0.093279	0.160924
Monocytes	AOPEP	0.251931	6.329542	2.421519	0.017499	-3.61159	0.099128	0.171064
Monocytes	MAPRE2	-0.20614	6.802434	-2.42117	0.017514	-3.84637	0.097856	0.168926
Monocytes	SLC25A46	0.247646	4.587825	2.420862	0.017528	-3.38561	0.104111	0.179719
Monocytes	GM44148	1.047982	1.90428	2.420642	0.017538	-3.16122	0.112294	0.193677
Monocytes	HMGN3	-0.53621	3.255903	-2.42048	0.017545	-3.23277	0.108087	0.18654
Monocytes	SLC12A3	-1.23814	1.874484	-2.42021	0.017558	-3.21614	0.112402	0.193916
Monocytes	ADAM15	0.413149	2.475369	2.418894	0.017617	-3.37436	0.110816	0.190871
Monocytes	FPR3	1.226017	-0.4789	2.418628	0.017629	-3.22789	0.120513	0.207299
Monocytes	GM44899	0.957868	0.774696	2.418234	0.017647	-3.18467	0.116343	0.200237
Monocytes	AQP1	-0.853	2.964821	-2.41572	0.017762	-3.12262	0.109995	0.188959
Monocytes	PRF1	-1.20837	0.377462	-2.41515	0.017788	-3.2055	0.11846	0.203203
Monocytes	ZFAND5	0.169077	7.203792	2.414648	0.017811	-3.8792	0.097825	0.168152
Monocytes	F2	-0.78903	4.237274	-2.41249	0.01791	-3.25562	0.106818	0.1833
Monocytes	1500004A13RIK	0.648872	2.078931	2.411795	0.017942	-3.13135	0.113616	0.194743
Monocytes	XRCC3	1.138112	0.159666	2.411338	0.017963	-3.3171	0.119971	0.205409

Monocytes	MED7	0.365337	3.583194	2.411279	0.017966	-3.26087	0.108892	0.186762
Monocytes	C5AR2	0.739238	1.231827	2.411243	0.017967	-3.13899	0.116374	0.199383
Monocytes	PPM1M	-0.24415	4.583696	-2.41071	0.017992	-3.61043	0.105883	0.181792
Monocytes	PSME1	-0.269	8.384368	-2.41052	0.018001	-4.1245	0.095229	0.16355
Monocytes	OSGEPL1	-0.65339	2.472736	-2.41051	0.018001	-3.13908	0.112371	0.192801
Monocytes	SLC45A3	-0.97941	1.111286	-2.41014	0.018018	-3.1462	0.116811	0.200354
Monocytes	IGFBP7	-0.505	5.173401	-2.40996	0.018026	-3.41188	0.104169	0.179007
Monocytes	IGSF3	-1.44112	0.826018	-2.40961	0.018043	-3.3762	0.11777	0.202129
Monocytes	HTR7	-0.37813	0.754108	-2.40947	0.018049	-3.76049	0.118011	0.202551
Monocytes	UNC119B	-0.36054	3.551645	-2.40353	0.018326	-3.35167	0.110403	0.189048
Monocytes	QTRT1	-0.39075	4.980734	-2.40332	0.018336	-3.51782	0.106056	0.181676
Monocytes	D1ERTD622E	0.229805	5.295192	2.403314	0.018337	-3.67309	0.105125	0.180092
Monocytes	TMEM63A	0.512896	3.49863	2.403299	0.018337	-3.24322	0.110568	0.189327
Monocytes	CRTC1	-0.65508	3.126748	-2.40325	0.01834	-3.15119	0.111733	0.191297
Monocytes	GM15336	0.481221	1.682133	2.403234	0.01834	-3.22035	0.116389	0.199139
Monocytes	HSPBAP1	0.415336	3.764205	2.400633	0.018463	-3.36765	0.110386	0.188616
Monocytes	UCHL3	-0.25775	6.235223	-2.4005	0.018469	-3.66682	0.102996	0.176078
Monocytes	SIVA1	0.293651	5.470666	2.399181	0.018532	-3.66117	0.105516	0.180328
Monocytes	1700123O20RII	0.298039	4.989927	2.39875	0.018552	-3.47193	0.107002	0.182896
Monocytes	GM9929	0.741354	1.684976	2.398434	0.018567	-3.16373	0.117475	0.200552
Monocytes	CNPY4	0.583578	2.433697	2.397418	0.018616	-3.16132	0.115241	0.196549
Monocytes	AKR1C20	-1.01705	2.839499	-2.39698	0.018637	-3.15837	0.113928	0.194377
Monocytes	AI847159	-1.23336	-0.25702	-2.39697	0.018637	-3.31757	0.124379	0.211842
Monocytes	CD83	-0.34195	5.198788	-2.39668	0.018651	-4.01176	0.106631	0.182032
Monocytes	PDE4D	-0.36332	6.428002	-2.39621	0.018673	-3.75783	0.103052	0.175938
Monocytes	TIPRL	-0.25504	5.525539	-2.39613	0.018677	-3.5408	0.105685	0.180419
Monocytes	LPL	-0.38503	5.132487	-2.3955	0.018707	-3.70494	0.106927	0.182567
Monocytes	C8G	-0.83784	3.782028	-2.39541	0.018711	-3.2421	0.111061	0.189566
Monocytes	MRNIP	-0.61822	2.538227	-2.395	0.018731	-3.19495	0.11508	0.196335
Monocytes	GM35154	0.455608	1.526848	2.394596	0.01875	-3.40824	0.118461	0.202071
Monocytes	SKINT3	0.449567	0.115469	2.394406	0.018759	-3.31944	0.123303	0.210203
Monocytes	EIF2D	-0.42907	3.5699	-2.39303	0.018825	-3.2368	0.112146	0.191321
Monocytes	5033421B08RIK	-1.33961	-0.4422	-2.39226	0.018862	-3.28476	0.125815	0.214086
Monocytes	THAP3	0.378114	3.799217	2.390408	0.018952	-3.3247	0.112044	0.190594
Monocytes	RNASEH2C	0.277489	4.998911	2.389788	0.018982	-3.54008	0.108408	0.184473
Monocytes	STARD9	0.351564	3.529605	2.389385	0.019002	-3.46886	0.112979	0.192263
Monocytes	SEMA4C	-0.57369	2.202853	-2.38926	0.019008	-3.19204	0.11729	0.199492
Monocytes	DNAJB5	0.881365	1.175316	2.389216	0.01901	-3.20811	0.120753	0.205272
Monocytes	ARIH2	-0.17564	7.527213	-2.38761	0.019088	-4.06838	0.101283	0.172351
Monocytes	CYB5R1	-0.32367	3.659051	-2.38756	0.01909	-3.43266	0.112863	0.191947
Monocytes	FLOT2	0.284969	4.443449	2.387511	0.019093	-3.50252	0.1104	0.187801
Monocytes	LCT	-1.35004	0.170332	-2.3869	0.019123	-3.39145	0.12466	0.211658
Monocytes	ASPA	-1.07217	1.375432	-2.38678	0.019128	-3.17849	0.120469	0.204703
Monocytes	EEF1B2	-0.17105	8.556525	-2.38497	0.019217	-4.18842	0.098898	0.16815
Monocytes	ATP1B3	0.302759	7.281647	2.383313	0.019298	-3.96233	0.102837	0.174744
Monocytes	SLCO1B2	-0.76221	4.25024	-2.38253	0.019337	-3.32301	0.112082	0.190257
Monocytes	INAFM2	0.531692	3.225336	2.382353	0.019345	-3.21227	0.115363	0.195801

Monocytes	TLR13	0.404351	1.881588	2.381536	0.019386	-3.48287	0.11996	0.203394
Monocytes	LIX1	-1.13829	0.723268	-2.38146	0.019389	-3.28254	0.123964	0.210047
Monocytes	HEPACAM2	-0.35907	0.779743	-2.38067	0.019428	-3.66133	0.123943	0.209984
Monocytes	SPRY2	-0.52664	5.158508	-2.38033	0.019445	-3.39824	0.109553	0.186007
Monocytes	WASHC1	-0.29477	4.182087	-2.38017	0.019453	-3.40647	0.112598	0.191168
Monocytes	TCEANC	0.929235	1.730178	2.379192	0.019502	-3.21919	0.120896	0.20499
Monocytes	ATXN7	-0.23553	6.23066	-2.37811	0.019555	-3.78621	0.106684	0.181199
Monocytes	MYD88	0.31192	4.609439	2.377913	0.019565	-3.54377	0.111639	0.189641
Monocytes	MDN1	-0.31674	5.788045	-2.37776	0.019573	-3.73126	0.108012	0.183528
Monocytes	BCKDHA	0.341892	4.820285	2.377688	0.019577	-3.48805	0.11098	0.18854
Monocytes	SLC22A18	-0.73348	1.143718	-2.37688	0.019617	-3.19589	0.123288	0.209121
Monocytes	ENO3	-0.42025	2.077848	-2.37633	0.019644	-3.47029	0.120069	0.203911
Monocytes	CALCA	-1.68758	-0.96013	-2.3758	0.019671	-3.44364	0.130887	0.222035
Monocytes	CD1D1	-0.46843	3.932282	-2.37564	0.019679	-3.4148	0.113949	0.19382
Monocytes	RWDD2A	0.845037	0.48641	2.375603	0.019681	-3.20706	0.12561	0.213309
Monocytes	SNX8	-0.41814	5.468943	-2.37541	0.019691	-3.61019	0.109138	0.185713
Monocytes	TXLNG	0.264012	5.107247	2.375323	0.019695	-3.59463	0.11025	0.187611
Monocytes	SLC25A38	-0.32277	3.517886	-2.37515	0.019703	-3.4555	0.115286	0.196116
Monocytes	SLC22A1	-1.17189	0.740269	-2.3751	0.019706	-3.28543	0.124708	0.211869
Monocytes	WDR90	-0.68601	1.959845	-2.3739	0.019766	-3.2001	0.120768	0.205149
Monocytes	HK1OS	0.981273	0.741866	2.373305	0.019796	-3.24564	0.125129	0.212411
Monocytes	ARHGAP42	-0.66884	1.752056	-2.37306	0.019808	-3.22758	0.121603	0.206578
Monocytes	AMN1	0.409929	3.42505	2.371922	0.019866	-3.31326	0.116259	0.197468
Monocytes	RNPEP	0.272732	5.72187	2.371298	0.019897	-3.80566	0.109065	0.185331
Monocytes	USB1	0.473064	3.245957	2.371233	0.0199	-3.29516	0.116919	0.198554
Monocytes	TKT	0.224414	6.932084	2.369947	0.019965	-3.94128	0.105726	0.179499
Monocytes	IL6	0.918656	1.106723	2.369612	0.019983	-3.3139	0.124582	0.211148
Monocytes	PAPOLG	0.523908	3.005898	2.369311	0.019998	-3.24577	0.118084	0.200396
Monocytes	CAMK2D	0.273851	7.214604	2.368912	0.020018	-4.01659	0.104944	0.178338
Monocytes	NBEA	-0.91452	2.602645	-2.36888	0.02002	-3.22551	0.119436	0.202766
Monocytes	PDK1	0.370509	4.164844	2.368069	0.020061	-3.37238	0.114447	0.19441
Monocytes	MRPS6	-0.2671	6.008603	-2.36789	0.02007	-3.67174	0.108681	0.184747
Monocytes	SLC9A9	-0.3288	7.120003	-2.36768	0.020081	-4.08021	0.105363	0.179154
Monocytes	GNA14	-1.56369	-0.0617	-2.36618	0.020157	-3.37658	0.129346	0.219141
Monocytes	CELA1	-0.78335	2.24977	-2.36611	0.020161	-3.21606	0.121139	0.205519
Monocytes	AARS	-0.31546	5.020707	-2.36534	0.0202	-3.52705	0.112204	0.190418
Monocytes	ATP6V1A	0.21284	6.534998	2.36472	0.020232	-3.91406	0.107658	0.182604
Monocytes	CASP8AP2	0.262177	5.182109	2.364393	0.020249	-3.59469	0.111839	0.189696
Monocytes	R3HCC1L	0.23957	5.540599	2.363379	0.020301	-3.71773	0.110932	0.188087
Monocytes	BC051226	0.50304	2.179737	2.363197	0.02031	-3.24384	0.121936	0.20658
Monocytes	PRAM1	0.448828	3.279629	2.360846	0.020432	-3.30408	0.118846	0.200926
Monocytes	MAML2	-0.3013	7.972039	-2.36037	0.020456	-3.96319	0.104288	0.176359
Monocytes	TSC22D4	0.225441	6.684783	2.358388	0.020559	-3.83	0.108519	0.183199
Monocytes	CXCL3	1.545937	-0.07498	2.358326	0.020562	-3.30444	0.131291	0.221096
Monocytes	PKIB	-0.32146	5.453591	-2.35815	0.020572	-4.09494	0.112317	0.189587
Monocytes	ERP44	-0.15596	6.074446	-2.35741	0.02061	-3.82628	0.110528	0.186586
Monocytes	PSME3	-0.30805	5.282258	-2.35607	0.02068	-3.62165	0.113284	0.191096

Monocytes	GM47167	0.501971	2.927116	2.355991	0.020684	-3.27759	0.121041	0.204043
Monocytes	D130043K22RII	-0.97605	1.054045	-2.35517	0.020727	-3.27257	0.127793	0.215241
Monocytes	SLC29A1	-0.3706	5.215066	-2.35504	0.020734	-3.53445	0.113644	0.191746
Monocytes	A530088E08RIK	1.058133	1.760356	2.354107	0.020783	-3.31739	0.125427	0.211238
Monocytes	BTBD8	-1.18792	1.572677	-2.35404	0.020786	-3.36986	0.126095	0.212343
Monocytes	H2-Q7	-0.73472	3.889878	-2.35385	0.020796	-3.50014	0.11811	0.199117
Monocytes	CCNE1	-0.55063	3.530373	-2.35317	0.020832	-3.31663	0.119448	0.201243
Monocytes	ZEB2OS	0.303763	4.678362	2.351108	0.020941	-3.68045	0.116179	0.195443
Monocytes	PEX6	0.424254	4.039128	2.350937	0.02095	-3.39533	0.118284	0.198959
Monocytes	AGTPBP1	0.258784	5.128653	2.350337	0.020982	-3.77678	0.11483	0.193174
Monocytes	VPS37B	-0.29924	7.797142	-2.34783	0.021115	-4.09394	0.107207	0.179831
Monocytes	PACS2	0.250816	4.036916	2.346977	0.02116	-3.63113	0.11928	0.199755
Monocytes	CD34	-0.53277	0.953495	-2.34666	0.021177	-3.55596	0.130169	0.217711
Monocytes	ARAP2	-0.35352	5.924266	-2.3462	0.021202	-3.71993	0.113226	0.189707
Monocytes	CRADD	0.263495	5.224096	2.345794	0.021223	-3.61634	0.115521	0.193557
Monocytes	PVT1	-0.46997	5.282726	-2.3446	0.021288	-3.6683	0.115593	0.193476
Monocytes	1700056E22RIK	-0.77851	1.466557	-2.34446	0.021295	-3.2547	0.128717	0.21515
Monocytes	SAMHD1	0.322849	7.45997	2.344229	0.021307	-4.16943	0.108778	0.182152
Monocytes	SLC31A1	0.267368	5.429507	2.343778	0.021332	-3.70545	0.115187	0.192851
Monocytes	GM44127	-1.55969	-0.52381	-2.34239	0.021406	-3.50231	0.136583	0.227906
Monocytes	TIMD2	-1.06233	0.986598	-2.34217	0.021418	-3.33177	0.130844	0.218548
Monocytes	BMPR2	0.278029	5.519027	2.342163	0.021419	-3.82972	0.115151	0.192658
Monocytes	PHC3	0.321531	4.499127	2.342029	0.021426	-3.5113	0.118492	0.198252
Monocytes	CACNA1I	-1.04825	-0.17458	-2.34159	0.021449	-3.48745	0.135306	0.225919
Monocytes	TNPO1	0.199623	7.082293	2.340789	0.021493	-3.95271	0.110394	0.184834
Monocytes	GM27201	-0.58557	2.321008	-2.34078	0.021493	-3.26048	0.126177	0.211026
Monocytes	ANGPTL8	-1.08032	1.825356	-2.34057	0.021505	-3.27813	0.127958	0.214004
Monocytes	COX7A1	0.795462	0.986976	2.340141	0.021528	-3.27686	0.131104	0.219238
Monocytes	CYP3A16	-1.20369	0.777697	-2.33976	0.021549	-3.36939	0.13194	0.220612
Monocytes	CDKN1B	0.226563	6.470899	2.336983	0.0217	-3.96562	0.113098	0.189017
Monocytes	FBF1	-1.08993	1.539498	-2.33676	0.021712	-3.28189	0.129924	0.21682
Monocytes	SIRPB1A	0.524071	-0.18195	2.336562	0.021723	-3.38023	0.136431	0.22747
Monocytes	GLIPR2	-0.35679	4.6029	-2.33641	0.021731	-3.77451	0.119169	0.19917
Monocytes	MARVELD2	-0.87991	1.369704	-2.33599	0.021754	-3.27633	0.130617	0.218074
Monocytes	SCARB1	0.239126	4.995715	2.335084	0.021803	-3.76998	0.118126	0.197346
Monocytes	IQCB1	-0.29049	4.824078	-2.33467	0.021826	-3.5853	0.118754	0.19841
Monocytes	NUDT5	-0.29967	5.148841	-2.33271	0.021934	-3.6316	0.118194	0.19722
Monocytes	HOXB4	0.687998	1.361865	2.332444	0.021949	-3.27563	0.131526	0.219233
Monocytes	EIF2AK2	0.355289	4.927436	2.331855	0.021981	-3.67406	0.119047	0.198711
Monocytes	SLC16A9	-1.20968	0.530927	-2.33166	0.021992	-3.29908	0.134779	0.224558
Monocytes	STFA2	0.955263	2.469209	2.331363	0.022008	-3.2795	0.127604	0.212803
Monocytes	BACH2OS	-0.91555	1.526179	-2.33056	0.022053	-3.31338	0.131248	0.218551
Monocytes	ZBTB38	-0.3074	5.068147	-2.33015	0.022075	-3.62099	0.118834	0.198143
Monocytes	CARD11	-0.46205	4.618562	-2.32851	0.022166	-3.56729	0.120629	0.201127
Monocytes	ZSWIM8	-0.25555	4.703109	-2.3284	0.022173	-3.62668	0.120343	0.200654
Monocytes	ARID3A	0.282362	5.513727	2.328375	0.022174	-3.71237	0.117639	0.196176
Monocytes	UBA6	0.237917	5.172539	2.328047	0.022192	-3.7581	0.118769	0.198126

Monocytes	NUP85	-0.39904	4.345133	-2.32804	0.022193	-3.50647	0.121559	0.202743
Monocytes	GM28501	1.031809	0.476037	2.327894	0.022201	-3.37886	0.135603	0.225813
Monocytes	KDM6A	-0.48332	6.914035	-2.32754	0.02222	-3.96886	0.113144	0.188921
Monocytes	ARMH2	-1.43965	0.301427	-2.32696	0.022253	-3.49968	0.136298	0.227251
Monocytes	DACH1	1.041491	1.877933	2.326722	0.022266	-3.28697	0.130339	0.217529
Monocytes	GM15448	0.663767	-0.00747	2.326528	0.022277	-3.28612	0.1375	0.229287
Monocytes	ALYREF2	0.47257	3.243584	2.326252	0.022292	-3.33944	0.125406	0.209527
Monocytes	THAP12	-0.26705	4.362104	-2.32617	0.022297	-3.5717	0.12152	0.203105
Monocytes	MLF2	-0.21413	5.85253	-2.32615	0.022298	-3.84994	0.116547	0.19485
Monocytes	GM15879	-1.1922	0.515986	-2.32595	0.022309	-3.41412	0.13547	0.226085
Monocytes	CTC1	0.420321	3.25694	2.325878	0.022313	-3.3551	0.125359	0.209498
Monocytes	AC154200.1	0.953966	0.660722	2.325405	0.02234	-3.37065	0.135001	0.225337
Monocytes	SLCO3A1	-0.31577	4.38978	-2.32508	0.022358	-3.92026	0.121537	0.203204
Monocytes	DNAH8	-0.61238	3.758702	-2.32322	0.022462	-3.33356	0.124225	0.207095
Monocytes	PIAS4	0.331974	4.224377	2.322127	0.022524	-3.47895	0.122876	0.204781
Monocytes	METTL23	0.205134	5.675126	2.321826	0.022541	-3.78655	0.118001	0.196741
Monocytes	BEX4	1.198188	0.397887	2.321499	0.022559	-3.45296	0.136978	0.227939
Monocytes	MEX3B	-0.66783	2.233947	-2.32055	0.022613	-3.3003	0.130173	0.216777
Monocytes	SMIM7	0.259244	4.932589	2.320424	0.02262	-3.64776	0.120645	0.201096
Monocytes	ECM1	0.733644	3.680244	2.320419	0.02262	-3.45118	0.124967	0.20823
Monocytes	RAB30	-0.49099	2.141089	-2.32006	0.02264	-3.47779	0.13056	0.217444
Monocytes	KCNK6	-0.43666	2.306704	-2.31927	0.022685	-3.41842	0.130139	0.216729
Monocytes	ANPEP	-0.45865	1.184937	-2.31859	0.022724	-3.58696	0.134495	0.223825
Monocytes	CXXC5	-0.4071	5.11182	-2.31772	0.022773	-3.44161	0.120506	0.200944
Monocytes	GM44067	-0.71944	1.717425	-2.31747	0.022788	-3.30241	0.132602	0.220847
Monocytes	USP48	-0.24318	5.391239	-2.31729	0.022798	-3.70564	0.119566	0.199386
Monocytes	CELF1	-0.15009	7.40812	-2.31724	0.022801	-4.06196	0.113021	0.1885
Monocytes	MITF	0.322768	4.369202	2.317147	0.022806	-3.83139	0.123044	0.205141
Monocytes	CDCA7L	-0.55584	3.766654	-2.31626	0.022857	-3.40547	0.125359	0.208883
Monocytes	SNU13	-0.1869	6.96176	-2.31601	0.022871	-4.01279	0.114635	0.191159
Monocytes	CLCN6	-0.51568	3.649755	-2.31529	0.022912	-3.36919	0.125941	0.209807
Monocytes	TRDC	-1.12171	1.265066	-2.31392	0.022991	-3.30859	0.13511	0.22466
Monocytes	TNR	1.114304	0.036927	2.312819	0.023054	-3.44738	0.140134	0.232759
Monocytes	LAMTOR5	0.222935	5.527925	2.312674	0.023063	-3.83248	0.120014	0.199802
Monocytes	SLCO4C1	1.199719	-1.1708	2.312291	0.023085	-3.51955	0.145036	0.240853
Monocytes	UTP11	-0.27739	4.845019	-2.31223	0.023088	-3.62594	0.122333	0.203771
Monocytes	RAD17	0.243391	5.023722	2.311982	0.023102	-3.69201	0.121722	0.20276
Monocytes	NDFIP1	-0.1817	7.521506	-2.31197	0.023103	-4.11153	0.113521	0.18914
Monocytes	ANAPC2	-0.32344	4.497598	-2.31165	0.023122	-3.57288	0.123566	0.205868
Monocytes	GM10874	1.159105	-0.06869	2.311239	0.023145	-3.43332	0.140656	0.233888
Monocytes	ZFP568	0.399589	4.08691	2.310859	0.023167	-3.48158	0.125056	0.208455
Monocytes	GDPD5	-0.59439	1.750804	-2.31083	0.023169	-3.35778	0.133578	0.222452
Monocytes	ORAI1	-0.20827	6.491763	-2.31041	0.023193	-4.02123	0.116966	0.195067
Monocytes	EXOC2	0.224646	5.578749	2.309936	0.023221	-3.80549	0.120068	0.200184
Monocytes	SLC7A6OS	-0.28998	4.789888	-2.30923	0.023261	-3.59792	0.122811	0.204836
Monocytes	HEG1	-0.30682	5.971346	-2.30903	0.023273	-3.82333	0.118813	0.198233
Monocytes	MED16	-0.34378	4.12299	-2.3089	0.023281	-3.47742	0.125133	0.208709

Monocytes	PPP2R5A	0.150863	7.861178	2.30889	0.023281	-4.19648	0.112719	0.188082
Monocytes	ARHGAP31	-0.27418	6.152696	-2.3086	0.023298	-4.0326	0.118236	0.197243
Monocytes	TMTC1	-1.05184	2.714836	-2.30833	0.023314	-3.31823	0.130233	0.21715
Monocytes	HAUS7	-0.4886	2.992661	-2.3081	0.023327	-3.35122	0.129215	0.215534
Monocytes	APBA1	0.272303	4.384633	2.307931	0.023337	-3.9838	0.124249	0.207378
Monocytes	B3GALT5	-1.10271	0.779445	-2.30614	0.023442	-3.33445	0.138111	0.229888
Monocytes	TMF1	0.193465	6.243916	2.305722	0.023466	-3.91674	0.11841	0.197506
Monocytes	USP1	0.264313	5.737466	2.305321	0.023489	-3.83591	0.120099	0.200311
Monocytes	KLHDC10	0.246944	5.434437	2.305125	0.023501	-3.81089	0.121122	0.202009
Monocytes	ZFP592	0.199447	5.89966	2.305076	0.023504	-3.83366	0.119555	0.199409
Monocytes	FLT4	-0.80253	2.321562	-2.30507	0.023504	-3.32419	0.13221	0.220289
Monocytes	ASGR1	-0.75285	3.372794	-2.30458	0.023533	-3.36086	0.128431	0.214089
Monocytes	MAP2K3	-0.2561	6.01457	-2.30417	0.023557	-3.99956	0.119312	0.199054
Monocytes	OTUB1	0.17403	5.728978	2.302916	0.023631	-3.87052	0.120581	0.201044
Monocytes	SYNCRIP	-0.18445	7.375906	-2.30188	0.023691	-4.08544	0.115306	0.192333
Monocytes	1700037H04RII	-0.42422	3.714085	-2.30175	0.023699	-3.44274	0.127751	0.213013
Monocytes	MRPL42	-0.25193	6.521235	-2.30148	0.023715	-4.01056	0.118084	0.197067
Monocytes	TRPC4AP	-0.19271	5.651799	-2.30147	0.023716	-3.86744	0.120988	0.201896
Monocytes	SCNN1A	0.796765	0.542515	2.3012	0.023732	-3.33691	0.139739	0.23276
Monocytes	ZFP609	0.213637	6.067152	2.301156	0.023734	-3.92631	0.119591	0.199601
Monocytes	CREB3	-0.30132	3.923009	-2.30027	0.023787	-3.56053	0.127216	0.212053
Monocytes	ICE1	-0.38143	4.081492	-2.29955	0.023829	-3.53371	0.126752	0.211354
Monocytes	MDFIC	0.335479	4.328783	2.299521	0.023831	-3.84825	0.125874	0.209904
Monocytes	TMEM50A	0.174923	7.711684	2.298991	0.023862	-4.1751	0.114609	0.19112
Monocytes	INHBA	1.213248	1.050066	2.297347	0.02396	-3.33825	0.138651	0.230567
Monocytes	CLMP	0.907554	1.140521	2.29724	0.023967	-3.34316	0.138296	0.230004
Monocytes	ENHO	-0.73196	2.481211	-2.29674	0.023996	-3.35159	0.13324	0.221726
Monocytes	BACE1	0.587163	2.654059	2.296378	0.024018	-3.34262	0.132599	0.220706
Monocytes	RASGEF1B	-0.31936	5.547786	-2.2963	0.024023	-4.09992	0.122236	0.203648
Monocytes	NSA2	-0.13716	7.622863	-2.29598	0.024042	-4.20081	0.115392	0.192349
Monocytes	PRKN	-0.5491	3.428362	-2.29526	0.024085	-3.50854	0.129931	0.216361
Monocytes	ACY1	-1.02998	1.799123	-2.29473	0.024117	-3.4005	0.136159	0.226566
Monocytes	H2-DMB1	-0.32887	4.478206	-2.2938	0.024173	-4.20533	0.126421	0.210537
Monocytes	DEF6	0.231465	5.457612	2.293765	0.024174	-3.84077	0.122996	0.204874
Monocytes	CAR8	-0.94084	2.375063	-2.29309	0.024215	-3.34867	0.134295	0.223394
Monocytes	OLFM4	1.544046	-0.01496	2.292842	0.02423	-3.48191	0.143723	0.238774
Monocytes	TMEM18	0.768971	1.593876	2.291674	0.0243	-3.35497	0.137635	0.228752
Monocytes	TCIRG1	0.266754	5.455039	2.290914	0.024346	-3.85812	0.123619	0.205693
Monocytes	TES	-0.2381	5.486918	-2.29053	0.024369	-3.99724	0.123563	0.205528
Monocytes	RMI1	-0.37537	3.629414	-2.2903	0.024383	-3.4474	0.130185	0.216529
Monocytes	5430427M07RI	1.029634	1.237849	2.290081	0.024396	-3.37558	0.13929	0.231522
Monocytes	1700096K18RIK	0.583128	2.613489	2.28839	0.024499	-3.37189	0.134418	0.223362
Monocytes	A230072C01RIK	-0.80955	1.545767	-2.28832	0.024503	-3.36856	0.13854	0.230092
Monocytes	EIF5B	-0.16616	7.158051	-2.28797	0.024525	-4.11722	0.118373	0.196915
Monocytes	TEC	0.283403	5.751113	2.287332	0.024563	-3.84541	0.123242	0.204858
Monocytes	CROT	-0.37088	5.259869	-2.28682	0.024595	-3.69119	0.124987	0.207817
Monocytes	GM33280	-1.50643	-0.66423	-2.28662	0.024606	-3.57959	0.147768	0.245068

Monocytes	NCF1	0.284918	5.301832	2.286434	0.024618	-3.92176	0.12484	0.207638
Monocytes	UBE2E2	-0.62415	3.465951	-2.28637	0.024622	-3.46878	0.131449	0.218525
Monocytes	MRPS21	0.162228	7.034242	2.286031	0.024643	-4.09807	0.118981	0.197903
Monocytes	FGB	-0.47592	5.797972	-2.28565	0.024666	-3.81263	0.123212	0.204931
Monocytes	SLC22A23	-0.55534	3.017213	-2.28543	0.024679	-3.5834	0.133225	0.221453
Monocytes	WDR77	-0.42357	3.895337	-2.28421	0.024754	-3.47434	0.130272	0.216567
Monocytes	NFRKB	-0.29946	4.478568	-2.28407	0.024762	-3.56079	0.128153	0.21313
Monocytes	TXK	-0.57134	3.287256	-2.28239	0.024866	-3.51802	0.132992	0.220996
Monocytes	AU022252	0.623308	2.452615	2.282032	0.024888	-3.36671	0.136162	0.226214
Monocytes	MEG3	-1.61271	1.040515	-2.28202	0.024889	-3.42952	0.141716	0.23527
Monocytes	ALKBH1	-0.25226	4.99535	-2.28025	0.024998	-3.7723	0.12719	0.211255
Monocytes	FAM110B	-1.14893	0.653098	-2.28022	0.025	-3.42764	0.143774	0.238374
Monocytes	RNH1	0.226616	5.988026	2.278854	0.025084	-4.08851	0.124057	0.205989
Monocytes	ARHGEF17	-1.34872	-0.37609	-2.27821	0.025124	-3.54116	0.148578	0.246042
Monocytes	EEF1D	-0.18379	7.27018	-2.27813	0.025129	-4.17116	0.11979	0.198925
Monocytes	CACNA2D1	-1.49048	0.864423	-2.27752	0.025167	-3.49804	0.143575	0.237885
Monocytes	ATP6V0A1	0.29294	4.202735	2.276938	0.025203	-3.72737	0.130773	0.216926
Monocytes	ITGA3	-0.57619	-0.28405	-2.27646	0.025233	-3.39255	0.148573	0.245822
Monocytes	GM45353	-1.19779	-0.0712	-2.27597	0.025264	-3.60747	0.147779	0.24455
Monocytes	TMEM127	0.284029	4.290896	2.275589	0.025287	-3.62825	0.130643	0.216723
Monocytes	TSEN34	0.37098	4.195681	2.275504	0.025293	-3.57873	0.130993	0.217309
Monocytes	PRKD3	0.254352	5.198034	2.27514	0.025316	-3.85137	0.127378	0.211373
Monocytes	DLGAP4	-0.25891	5.015503	-2.27503	0.025323	-3.90402	0.128031	0.21245
Monocytes	NCBP2	-0.27973	4.599992	-2.2742	0.025375	-3.70271	0.129716	0.215075
Monocytes	PRDX6	0.175524	7.110346	2.27404	0.025384	-4.20342	0.120922	0.200557
Monocytes	AXDND1	0.584766	1.557778	2.273806	0.025399	-3.48184	0.141347	0.234102
Monocytes	NDOR1	0.550417	3.086341	2.273431	0.025423	-3.4075	0.135403	0.224413
Monocytes	CYB5R4	0.203971	5.869945	2.273171	0.025439	-3.98392	0.12522	0.207753
Monocytes	YKT6	-0.28606	4.483546	-2.27308	0.025444	-3.65349	0.130181	0.215955
Monocytes	GM38115	-0.25355	3.861728	-2.27206	0.025509	-3.83345	0.132717	0.220041
Monocytes	CCDC85B	0.316883	3.779227	2.271937	0.025516	-3.58813	0.133025	0.220579
Monocytes	SPATC1	1.03683	-1.06407	2.271614	0.025537	-3.55541	0.152637	0.25242
Monocytes	NRF1	0.177639	6.474245	2.27133	0.025555	-4.01312	0.123405	0.204754
Monocytes	SLAIN2	0.210934	5.670672	2.270934	0.02558	-3.87622	0.126266	0.209468
Monocytes	PITHD1	-0.3052	4.744392	-2.27064	0.025598	-3.69734	0.129614	0.214991
Monocytes	MBNL3	-0.39717	4.597519	-2.27039	0.025614	-3.64608	0.130165	0.215872
Monocytes	ZFP182	0.395777	4.211679	2.269848	0.025648	-3.59216	0.131691	0.218329
Monocytes	GM1043	-1.30895	1.915295	-2.26949	0.025671	-3.44448	0.140558	0.232916
Monocytes	ATXN1L	0.443201	2.959513	2.268065	0.025761	-3.47304	0.136829	0.226587
Monocytes	INSR	0.322573	5.770425	2.268009	0.025765	-3.83574	0.126438	0.209535
Monocytes	SKIL	-0.20819	7.304614	-2.26759	0.025791	-4.30114	0.121203	0.200882
Monocytes	XLR4A	-0.99118	0.802973	-2.26731	0.025809	-3.45449	0.145544	0.240722
Monocytes	KHDRBS3	-1.01732	1.943216	-2.26695	0.025832	-3.40993	0.140927	0.233393
Monocytes	HNRNPLL	0.210569	4.231786	2.266862	0.025838	-4.03629	0.132118	0.219006
Monocytes	TSACC	0.933366	1.881856	2.266366	0.025869	-3.39315	0.141273	0.233909
Monocytes	SLC39A11	0.304915	4.419343	2.266016	0.025892	-3.79035	0.131563	0.218086
Monocytes	FAM168B	-0.16394	6.280422	-2.26551	0.025924	-4.02135	0.124972	0.207207

Monocytes	DNASE1L1	0.320801	3.048154	2.264105	0.026014	-3.70656	0.13716	0.227129
Monocytes	RCC1L	-0.54337	3.072184	-2.26392	0.026026	-3.43127	0.137067	0.227034
Monocytes	CLIP1	0.232452	5.440971	2.263692	0.02604	-3.95689	0.128238	0.212586
Monocytes	HIST1H3I	-1.05883	1.296054	-2.26362	0.026045	-3.42818	0.144127	0.238571
Monocytes	FIP1L1	-0.15657	6.639254	-2.26354	0.02605	-4.01792	0.124015	0.205611
Monocytes	GM1673	0.386259	2.42109	2.262002	0.026149	-3.61363	0.139996	0.231624
Monocytes	ARHGEF18	0.313012	5.591269	2.261846	0.026159	-3.83506	0.128053	0.212064
Monocytes	F11R	0.386528	3.432174	2.261801	0.026162	-3.71205	0.136058	0.2252
Monocytes	KIF21B	-0.29279	4.865707	-2.2612	0.0262	-3.77343	0.130768	0.216513
Monocytes	POU2F2	0.342136	5.772063	2.261127	0.026205	-4.04813	0.127489	0.211118
Monocytes	LOXL3	-1.11765	0.451305	-2.26068	0.026234	-3.43803	0.148215	0.244856
Monocytes	ACTR6	0.382379	3.522286	2.259322	0.026322	-3.55336	0.136267	0.225312
Monocytes	HEXB	0.22091	5.741415	2.258281	0.026389	-4.03607	0.128251	0.212095
Monocytes	GM17268	0.861602	0.383225	2.258233	0.026392	-3.41763	0.149173	0.246184
Monocytes	ABCA5	-1.32871	0.444818	-2.25741	0.026446	-3.56164	0.149096	0.246025
Monocytes	PRKCB	0.198528	8.511819	2.257121	0.026464	-4.26022	0.118877	0.196638
Monocytes	ST13	-0.17058	6.987135	-2.25695	0.026476	-4.12246	0.12402	0.205174
Monocytes	GM5089	1.118219	0.395428	2.256912	0.026478	-3.48941	0.149305	0.24647
Monocytes	SMPDL3A	0.275011	5.614644	2.256327	0.026516	-4.19727	0.128986	0.213166
Monocytes	SAMD3	-1.17945	0.184815	-2.2557	0.026557	-3.45698	0.150499	0.248108
Monocytes	IMMT	0.186119	6.049157	2.255428	0.026575	-4.00304	0.127581	0.210804
Monocytes	HPS4	-0.30504	4.16681	-2.25448	0.026636	-3.82734	0.134738	0.222397
Monocytes	TNFRSF19	-1.29285	0.886333	-2.25301	0.026733	-3.55223	0.148238	0.244003
Monocytes	KLRI1	-0.569	-0.19768	-2.25293	0.026738	-3.488	0.152874	0.251461
Monocytes	STOX2	-1.00182	3.196694	-2.25269	0.026754	-3.42799	0.138873	0.228908
Monocytes	CMKLR1	0.588791	2.151939	2.252065	0.026795	-3.59878	0.143139	0.235871
Monocytes	MLLT11	-0.4446	3.193389	-2.25197	0.026801	-3.46334	0.138991	0.229136
Monocytes	ACOX2	-1.26706	1.030725	-2.25086	0.026874	-3.50355	0.148086	0.243671
Monocytes	CRYBG2	-1.29479	0.051796	-2.25017	0.02692	-3.59923	0.152443	0.250604
Monocytes	TCAIM	0.699495	1.789758	2.249039	0.026994	-3.42201	0.145414	0.239045
Monocytes	HDAC5	0.329063	4.378269	2.2488	0.02701	-3.74176	0.135175	0.222495
Monocytes	BC024386	-1.02556	1.614053	-2.2487	0.027017	-3.44023	0.146139	0.240307
Monocytes	SPSB1	-0.72288	1.685361	-2.24803	0.027061	-3.43784	0.145991	0.239935
Monocytes	IQCG	-1.59412	0.050135	-2.24775	0.02708	-3.6274	0.152924	0.251159
Monocytes	SF3B5	0.197595	6.277364	2.247392	0.027104	-4.08838	0.128301	0.211311
Monocytes	GM43331	-0.56483	2.057137	-2.24734	0.027107	-3.42968	0.144463	0.237646
Monocytes	ST3GAL3	-0.21312	5.293649	-2.24727	0.027111	-4.04665	0.13188	0.217193
Monocytes	GM2245	0.692815	1.558462	2.246607	0.027156	-3.43846	0.146549	0.241134
Monocytes	GM5244	1.127149	-0.39473	2.246247	0.02718	-3.47994	0.154907	0.254573
Monocytes	TNF	-0.49801	4.405834	-2.24622	0.027182	-3.94981	0.135237	0.22277
Monocytes	ESYT2	-0.1944	6.802347	-2.24617	0.027185	-4.15128	0.126464	0.208392
Monocytes	PILRB2	0.459139	2.237136	2.246164	0.027185	-3.63995	0.143762	0.236627
Monocytes	APBB1IP	0.180754	7.638967	2.245742	0.027213	-4.29154	0.12361	0.203726
Monocytes	AARSD1	-0.3642	3.977143	-2.24557	0.027225	-3.64459	0.13694	0.225663
Monocytes	EHHADH	-0.95598	1.66442	-2.24405	0.027326	-3.44994	0.146648	0.241004
Monocytes	TNIP3	0.469425	1.037658	2.242923	0.027402	-3.95429	0.149616	0.245663
Monocytes	GM15788	-1.25409	0.240256	-2.24265	0.02742	-3.54232	0.153061	0.251234

Monocytes	TNFSF13B	0.744079	0.855901	2.242466	0.027432	-3.43909	0.150408	0.247042
Monocytes	SPG11	0.321354	4.094018	2.241759	0.02748	-3.73118	0.137428	0.226011
Monocytes	GM44752	0.402457	2.484975	2.241474	0.027499	-3.56876	0.143834	0.23639
Monocytes	GM43672	0.72545	1.700182	2.241179	0.027519	-3.43896	0.147096	0.241664
Monocytes	MPP1	0.252195	6.203718	2.240389	0.027572	-4.05498	0.129731	0.21349
Monocytes	PROCR	-1.06451	0.279761	-2.24034	0.027575	-3.43936	0.153308	0.251742
Monocytes	GM12979	-1.11293	0.049111	-2.24	0.027598	-3.52845	0.154339	0.253461
Monocytes	VWCE	-1.12036	0.865529	-2.23987	0.027607	-3.52628	0.150801	0.24778
Monocytes	PARVG	-0.24416	5.071901	-2.23963	0.027623	-3.92537	0.133938	0.22042
Monocytes	TPK1	-0.27072	4.865838	-2.23881	0.027678	-3.89538	0.134918	0.221947
Monocytes	BABAM2	0.175232	7.216423	2.237679	0.027755	-4.25384	0.126593	0.208247
Monocytes	SIGLEC1	-1.16636	0.449661	-2.23755	0.027764	-3.45669	0.153143	0.251441
Monocytes	DENND4B	-0.31008	3.747946	-2.23739	0.027775	-3.71512	0.139503	0.229466
Monocytes	DENND5B	-0.61894	4.198991	-2.23707	0.027796	-3.46225	0.137784	0.226746
Monocytes	KDSR	0.381149	3.963038	2.236541	0.027832	-3.57951	0.138811	0.22835
Monocytes	IP6K1	0.239726	6.598351	2.235771	0.027885	-4.03833	0.129111	0.212292
Monocytes	IL23R	-1.29369	-0.9447	-2.23419	0.027992	-3.58722	0.160271	0.262424
Monocytes	SMIM41	-1.19327	0.89661	-2.23114	0.028201	-3.57424	0.153157	0.250467
Monocytes	LYST	0.205947	6.729745	2.230564	0.028241	-4.26306	0.130091	0.213166
Monocytes	JUND	-0.25413	10.62781	-2.22946	0.028317	-4.64319	0.11704	0.19152
Monocytes	SNX1	0.168996	5.483558	2.229185	0.028336	-4.03645	0.135021	0.220999
Monocytes	CYP2D22	-1.09485	0.864397	-2.22867	0.028372	-3.5305	0.1539	0.251513
Monocytes	SEMA6B	0.876214	1.154578	2.228524	0.028382	-3.46783	0.152638	0.249523
Monocytes	BLVRB	-0.41579	7.092023	-2.2283	0.028397	-4.10169	0.129178	0.211582
Monocytes	GALNT2	-0.29679	5.565067	-2.22795	0.028421	-3.88493	0.134854	0.220791
Monocytes	STX18	0.286363	4.637177	2.226797	0.028501	-3.8066	0.13873	0.22685
Monocytes	TTC21B	-0.74061	1.614885	-2.22636	0.028532	-3.47254	0.15116	0.246874
Monocytes	C2	-0.85509	2.413631	-2.22519	0.028613	-3.47041	0.148132	0.241889
Monocytes	TSPO	0.25774	7.399271	2.224736	0.028645	-4.40953	0.12888	0.210628
Monocytes	RBMS1	0.155234	7.558573	2.224514	0.02866	-4.40222	0.128317	0.209728
Monocytes	MEX3A	-0.97457	1.577184	-2.22428	0.028676	-3.50652	0.151792	0.247734
Monocytes	R3HDM2	0.166494	6.193799	2.223697	0.028717	-4.09006	0.133426	0.217976
Monocytes	HMBOX1	0.272225	5.578159	2.222752	0.028783	-3.92472	0.135988	0.221976
Monocytes	CD74	-0.3947	11.27972	-2.22239	0.028808	-5.2235	0.116162	0.189547
Monocytes	GC	-0.65402	5.287073	-2.22206	0.028831	-3.86952	0.137197	0.224054
Monocytes	PDE2A	-0.47337	6.050057	-2.22163	0.028862	-3.78059	0.134357	0.219476
Monocytes	RASGRP2	0.235284	6.609517	2.221407	0.028877	-4.06478	0.132275	0.216161
Monocytes	GPX7	-1.28882	0.610424	-2.22124	0.028889	-3.57968	0.156616	0.255481
Monocytes	DDX43	-1.0657	0.153087	-2.22109	0.0289	-3.52381	0.158665	0.258818
Monocytes	MAP4	-0.18798	6.676158	-2.21997	0.028978	-4.20633	0.132225	0.21617
Monocytes	RTCB	-0.23402	5.290693	-2.21978	0.028992	-3.96491	0.137446	0.224699
Monocytes	EXOSC4	0.396675	3.784732	2.219612	0.029004	-3.63647	0.143383	0.234368
Monocytes	ASS1	0.409034	6.535258	2.219523	0.02901	-4.24683	0.132745	0.217099
Monocytes	SCAPER	-0.23226	6.110801	-2.21933	0.029023	-4.07934	0.134328	0.219704
Monocytes	1700120C14RIK	0.874673	1.482076	2.219278	0.029027	-3.52718	0.153018	0.249915
Monocytes	NCOA1	0.220799	7.07231	2.218333	0.029094	-4.25979	0.13101	0.214184
Monocytes	FARS2	0.160017	6.899126	2.21763	0.029144	-4.21454	0.131712	0.21537

Monocytes	EML3	-0.31471	4.139794	-2.21734	0.029164	-3.75719	0.14229	0.232631
Monocytes	EDEM1	0.212985	6.015715	2.217297	0.029167	-4.13748	0.135	0.220794
Monocytes	NR2F2	-0.81454	3.13223	-2.21724	0.029171	-3.49271	0.146385	0.239247
Monocytes	TTC36	-0.62358	5.028124	-2.21683	0.0292	-3.82785	0.138784	0.226949
Monocytes	NAB2	-0.43817	3.217989	-2.21676	0.029205	-3.64548	0.146032	0.238676
Monocytes	NCR1	-1.08577	1.170957	-2.21672	0.029208	-3.47661	0.154734	0.252656
Monocytes	RTL6	-1.34416	0.292529	-2.21647	0.029226	-3.63156	0.158661	0.259031
Monocytes	APAF1	0.226224	5.726771	2.215817	0.029272	-4.09186	0.136197	0.222798
Monocytes	PCNA	-0.27163	6.710032	-2.21577	0.029275	-4.18718	0.132507	0.216782
Monocytes	LPCAT1	-0.2521	4.107714	-2.21562	0.029286	-3.94146	0.142525	0.23307
Monocytes	GM20470	0.841632	1.403571	2.214752	0.029348	-3.48697	0.154047	0.2516
Monocytes	SLC16A2	-0.65995	2.626868	-2.21465	0.029355	-3.49596	0.148806	0.243187
Monocytes	RAB27B	-1.04258	0.615378	-2.21424	0.029385	-3.4967	0.157611	0.257291
Monocytes	IRF2BPL	0.402392	4.091669	2.212816	0.029486	-3.77281	0.143158	0.233977
Monocytes	CCL8	-1.20619	-1.46778	-2.2128	0.029487	-3.70092	0.167584	0.273054
Monocytes	FGR	0.363473	4.732993	2.212501	0.029508	-4.14365	0.140601	0.229854
Monocytes	ICOS	-0.7936	2.283057	-2.2125	0.029509	-3.49883	0.150648	0.24607
Monocytes	STX17	-0.31459	4.034327	-2.21239	0.029516	-3.83558	0.143389	0.234407
Monocytes	DIAPH1	0.159735	7.355352	2.211798	0.029559	-4.3718	0.130751	0.21382
Monocytes	KCTD2	0.40536	3.355478	2.211726	0.029564	-3.60472	0.146251	0.239021
Monocytes	PHF20L1	0.197973	7.046788	2.211129	0.029607	-4.21392	0.132006	0.215935
Monocytes	MTR	0.266	4.54186	2.210599	0.029645	-3.84683	0.141701	0.231787
Monocytes	CRIP2	-0.45595	4.565121	-2.21038	0.029661	-3.70154	0.141616	0.231697
Monocytes	HNRNPAB	-0.18763	8.05587	-2.20984	0.0297	-4.3906	0.12857	0.210442
Monocytes	2610507I01RIK	1.114529	0.583661	2.209647	0.029713	-3.53632	0.158601	0.259074
Monocytes	RAB5IF	0.169086	7.307335	2.209439	0.029728	-4.34482	0.131275	0.214903
Monocytes	GM27008	0.809918	1.012976	2.208941	0.029764	-3.50139	0.156687	0.256163
Monocytes	PGM1	0.311344	4.592397	2.208515	0.029795	-3.83917	0.141629	0.23193
Monocytes	FBXW9	-0.54859	2.418265	-2.20851	0.029795	-3.49781	0.150575	0.246395
Monocytes	SYDE1	-1.19267	0.308678	-2.2085	0.029796	-3.66741	0.159851	0.261271
Monocytes	STAB2	-0.77767	5.148934	-2.20844	0.0298	-3.7426	0.139435	0.228366
Monocytes	SMARCE1	-0.17239	6.302182	-2.20784	0.029843	-4.11704	0.13514	0.221283
Monocytes	ZFP445	0.244932	4.93802	2.207357	0.029878	-3.87822	0.140498	0.229945
Monocytes	HAUS3	-0.35396	4.199727	-2.20671	0.029925	-3.62695	0.143597	0.234981
Monocytes	GGTA1	0.244526	5.365487	2.206188	0.029963	-4.15629	0.139085	0.227587
Monocytes	NR4A2	-0.29497	6.380914	-2.20504	0.030046	-4.54561	0.135386	0.221706
Monocytes	BDH1	-0.66828	3.282333	-2.20478	0.030065	-3.52396	0.147682	0.241773
Monocytes	MYBPC2	-1.06977	2.760882	-2.20467	0.030073	-3.52999	0.149871	0.245316
Monocytes	LDHC	1.332582	0.610466	2.204614	0.030077	-3.56065	0.15928	0.260426
Monocytes	CNDP2	0.248316	5.051865	2.204611	0.030077	-4.03555	0.140515	0.230163
Monocytes	UBA5	-0.252	4.489487	-2.20367	0.030146	-3.78997	0.143006	0.234198
Monocytes	RALGAPA2	0.284886	6.409122	2.203245	0.030177	-4.16868	0.135598	0.222121
Monocytes	PSMB8	-0.27585	7.077604	-2.20294	0.030199	-4.38701	0.133127	0.218046
Monocytes	SLC2A2	-1.01443	1.627926	-2.20162	0.030296	-3.51595	0.15555	0.254318
Monocytes	SEMA6A	-1.30363	2.821738	-2.20148	0.030306	-3.50253	0.150387	0.246075
Monocytes	2200002D01RII	-1.07198	1.337585	-2.20092	0.030347	-3.51984	0.156834	0.256466
Monocytes	DDIAS	-0.63875	2.134705	-2.20081	0.030355	-3.5137	0.153335	0.250846

Monocytes	ZFP532	-1.01177	1.223667	-2.20076	0.030359	-3.51542	0.157342	0.257286
Monocytes	SEPSECS	-0.39624	3.143326	-2.20071	0.030362	-3.68741	0.149028	0.243917
Monocytes	DBN1	-0.59354	1.388658	-2.19965	0.03044	-3.60155	0.156936	0.256448
Monocytes	DRAM2	0.24042	4.847911	2.199428	0.030456	-4.0202	0.142357	0.232962
Monocytes	FAM124A	-0.92117	1.64482	-2.19876	0.030506	-3.54402	0.155954	0.25498
Monocytes	MYCL	-0.32826	1.305739	-2.19866	0.030513	-3.93636	0.157459	0.257406
Monocytes	NEK6	-0.28718	3.873937	-2.19821	0.030546	-3.88759	0.146531	0.239792
Monocytes	INO80	-0.20814	6.666636	-2.1961	0.030702	-4.23717	0.136105	0.222436
Monocytes	SNX32	0.468817	2.812618	2.195784	0.030725	-3.56336	0.151651	0.247636
Monocytes	GM43149	0.901004	0.774872	2.195685	0.030733	-3.53447	0.160656	0.262069
Monocytes	TEX30	-0.26028	5.223272	-2.19559	0.03074	-3.90452	0.141709	0.231566
Monocytes	FIG4	0.25552	4.603641	2.194925	0.030789	-3.80959	0.144342	0.235678
Monocytes	LAP3	-0.34815	4.901491	-2.19462	0.030811	-3.94852	0.14314	0.233776
Monocytes	MYO9A	-0.26626	6.336763	-2.19458	0.030815	-4.2697	0.137504	0.224619
Monocytes	DDAH2	-0.68225	3.503269	-2.19409	0.030851	-3.51857	0.148982	0.243222
Monocytes	ZKSCAN1	0.323432	4.068204	2.192567	0.030965	-3.71516	0.147103	0.239853
Monocytes	TUG1	0.207208	6.098897	2.190853	0.031093	-4.1219	0.139472	0.227265
Monocytes	SULT1D1	-0.89029	2.290136	-2.18976	0.031175	-3.52145	0.15557	0.253049
Monocytes	RORA	-0.41223	5.020685	-2.18948	0.031196	-3.91081	0.144087	0.234534
Monocytes	ZWINT	-0.24882	5.039001	-2.18851	0.031268	-3.92953	0.144195	0.234729
Monocytes	MBD3	-0.22225	5.696238	-2.18838	0.031278	-4.0473	0.141565	0.230479
Monocytes	RAB11FIP1	-0.25376	4.829945	-2.18824	0.031289	-4.38653	0.145043	0.236108
Monocytes	PYCR2	-0.3988	3.687049	-2.18758	0.031339	-3.6949	0.149778	0.243933
Monocytes	NEIL3	0.537839	4.375558	2.187203	0.031367	-3.73876	0.146905	0.239349
Monocytes	1700021F05RIK	0.409704	3.209032	2.187139	0.031372	-3.63848	0.151809	0.247247
Monocytes	B3GALNT2	0.391504	3.643954	2.187126	0.031373	-3.70303	0.14996	0.244273
Monocytes	GALNS	0.338852	3.757313	2.187116	0.031374	-3.76362	0.149482	0.243504
Monocytes	TAF4B	-0.34073	5.130162	-2.1869	0.03139	-4.05078	0.143827	0.234379
Monocytes	METTL9	0.236313	5.718204	2.186823	0.031396	-4.0803	0.141478	0.230594
Monocytes	CEBPG	0.205022	5.586056	2.186494	0.031421	-4.06636	0.142002	0.231455
Monocytes	ZFP638	0.192701	6.22747	2.186438	0.031425	-4.15894	0.139478	0.227358
Monocytes	BASP1	-0.31884	5.646818	-2.18642	0.031426	-4.38883	0.141761	0.231064
Monocytes	SELENON	0.460815	3.694822	2.18614	0.031447	-3.67935	0.149745	0.244057
Monocytes	CCDC71L	0.334008	4.455272	2.186036	0.031455	-3.85972	0.146577	0.238984
Monocytes	ATP6V1B2	0.169882	5.98524	2.184764	0.031551	-4.23267	0.14079	0.22926
Monocytes	FKBP11	-0.60158	1.995525	-2.1833	0.031663	-3.53339	0.15795	0.256652
Monocytes	SKA3	-0.55706	2.738324	-2.18297	0.031688	-3.57174	0.154669	0.251442
Monocytes	ARHGEF37	0.422086	0.616444	2.182636	0.031713	-4.0145	0.164247	0.266718
Monocytes	CYSLTR1	0.312659	2.70023	2.182603	0.031716	-4.01048	0.154835	0.251709
Monocytes	SAFB2	0.176125	5.993263	2.182509	0.031723	-4.13575	0.141151	0.229663
Monocytes	E030042O20RII	-1.23756	0.180529	-2.18244	0.031728	-3.60647	0.166293	0.269985
Monocytes	NFIX	0.325209	3.676603	2.181644	0.031789	-3.91872	0.150797	0.245171
Monocytes	MASP2	-1.05255	1.894509	-2.18159	0.031793	-3.54045	0.158578	0.257642
Monocytes	IDH3A	-0.30168	4.865883	-2.18129	0.031816	-3.87507	0.14584	0.237295
Monocytes	TMEM98	-1.28779	0.620631	-2.18121	0.031822	-3.68577	0.164409	0.267034
Monocytes	EIF1	-0.19193	9.721973	-2.18068	0.031863	-4.68334	0.127512	0.207336
Monocytes	CREM	-0.30422	6.0952	-2.18014	0.031904	-4.23903	0.141095	0.229397

Monocytes	AR	-1.28094	0.218656	-2.18007	0.031909	-3.58878	0.166521	0.270133
Monocytes	AQP9	-0.67357	2.330322	-2.17933	0.031966	-3.5693	0.15699	0.254883
Monocytes	TMEM80	-0.41213	3.276451	-2.1793	0.031968	-3.5965	0.152853	0.248262
Monocytes	ZFAND2B	0.306075	4.313639	2.178197	0.032053	-3.82332	0.148779	0.241533
Monocytes	KPNA3	-0.20061	6.307968	-2.17768	0.032092	-4.20682	0.140803	0.228661
Monocytes	KCNQ1	-0.98263	1.396097	-2.17735	0.032118	-3.54516	0.161728	0.262212
Monocytes	GADD45G	-0.4715	4.465474	-2.17711	0.032137	-3.83003	0.148327	0.240829
Monocytes	TMEM14C	0.191493	6.950542	2.176164	0.032209	-4.34555	0.138535	0.224849
Monocytes	ALAD	-0.47953	4.057214	-2.17607	0.032217	-3.69875	0.150224	0.243725
Monocytes	RB1	0.217089	7.240705	2.175745	0.032242	-4.30176	0.13742	0.223178
Monocytes	CXADR	-0.96412	1.645302	-2.17573	0.032243	-3.56968	0.160807	0.260783
Monocytes	RRAGD	-0.8421	2.299081	-2.17554	0.032258	-3.55315	0.157859	0.256134
Monocytes	ITIH5	-0.95962	1.943297	-2.17544	0.032265	-3.56776	0.159456	0.258687
Monocytes	RIN3	0.203697	5.640444	2.175066	0.032294	-4.22438	0.14376	0.233512
Monocytes	PRRC2A	-0.21623	5.517966	-2.1744	0.032346	-4.03041	0.144416	0.234507
Monocytes	MYCBP2	0.197711	7.768752	2.173777	0.032394	-4.49218	0.135771	0.220508
Monocytes	LAMA4	-1.33406	1.088771	-2.1727	0.032478	-3.61588	0.164135	0.265828
Monocytes	GPS1	-0.2426	4.905295	-2.17241	0.0325	-3.98268	0.147395	0.239111
Monocytes	NUPR1	0.468545	2.41246	2.17228	0.032511	-4.06317	0.158113	0.256307
Monocytes	ARID5A	0.332746	4.681741	2.171929	0.032538	-3.85492	0.148379	0.240721
Monocytes	PYCARD	0.233203	5.876191	2.171029	0.032608	-4.21442	0.143734	0.233068
Monocytes	PSMA5	-0.23742	5.884469	-2.17085	0.032622	-4.16969	0.1437	0.233072
Monocytes	INPP1	-0.29783	4.03687	-2.17012	0.032679	-3.95469	0.15154	0.245595
Monocytes	TXNDC15	-0.21664	4.911319	-2.1698	0.032704	-4.15744	0.147876	0.239809
Monocytes	EPRS	-0.26415	6.581496	-2.1697	0.032712	-4.31457	0.141126	0.228947
Monocytes	4933432I03RIK	0.968361	0.408312	2.168874	0.032777	-3.56871	0.168181	0.272101
Monocytes	CAP2	-1.28806	0.071585	-2.1686	0.032798	-3.65956	0.169833	0.274702
Monocytes	CC2D2A	0.974625	0.81427	2.167402	0.032892	-3.56896	0.166616	0.269536
Monocytes	B130055M24RI	0.702287	2.013314	2.167312	0.032899	-3.55738	0.16105	0.260731
Monocytes	AP3S1	0.169436	7.096627	2.167001	0.032924	-4.40343	0.139629	0.226407
Monocytes	TAF13	0.266001	4.566883	2.166971	0.032926	-3.97665	0.149865	0.242927
Monocytes	NOMO1	-0.40347	3.5373	-2.16682	0.032938	-3.67936	0.154268	0.250039
Monocytes	D830036C21RII	-0.79295	0.420218	-2.1662	0.032987	-3.55946	0.16857	0.272915
Monocytes	COX4I2	-0.88593	0.956141	-2.16599	0.033004	-3.58222	0.166024	0.268993
Monocytes	NUDT2	-0.35879	3.238769	-2.16581	0.033018	-3.71651	0.155644	0.252504
Monocytes	TAPT1	0.337063	5.802801	2.165805	0.033018	-3.91958	0.14483	0.235111
Monocytes	GIMAP1	-0.55202	4.06141	-2.16567	0.033029	-3.59484	0.15208	0.246789
Monocytes	KANSL2	0.25358	5.119718	2.165216	0.033064	-3.95818	0.147667	0.239679
Monocytes	TK1	-0.43727	4.882981	-2.16517	0.033068	-3.93742	0.14865	0.241264
Monocytes	ZBED3	-0.41716	3.559356	-2.16405	0.033156	-3.67589	0.154619	0.250615
Monocytes	LAIR1	0.407982	4.246709	2.163776	0.033178	-4.11297	0.151657	0.245967
Monocytes	SH3RF1	-0.32412	4.357213	-2.1637	0.033184	-3.92802	0.151186	0.245236
Monocytes	GIMAP8	-0.83619	2.465426	-2.16189	0.033328	-3.5665	0.160041	0.258975
Monocytes	ZFP106	0.203783	6.288522	2.161809	0.033334	-4.27184	0.143741	0.232833
Monocytes	UGT3A2	-1.03995	1.174889	-2.16133	0.033373	-3.60502	0.16611	0.268618
Monocytes	RABGEF1	0.250644	6.01057	2.160097	0.033471	-4.29879	0.145306	0.235202
Monocytes	GM28050	-1.0286	0.410517	-2.15996	0.033482	-3.62881	0.170156	0.274881

Monocytes	AMDHD1	-0.84618	2.26974	-2.15917	0.033545	-3.57096	0.161652	0.261366
Monocytes	RAF1	0.207654	5.986456	2.15854	0.033595	-4.2282	0.145764	0.235854
Monocytes	TMED2	0.151975	8.136615	2.158257	0.033618	-4.53316	0.137322	0.222189
Monocytes	STIMATE	0.297538	4.159047	2.15806	0.033634	-3.8989	0.153463	0.248242
Monocytes	TBC1D19	-0.68669	1.749793	-2.15764	0.033668	-3.57953	0.164314	0.26555
Monocytes	C87436	0.335135	3.905756	2.157538	0.033676	-3.80403	0.154613	0.250092
Monocytes	XBP1	0.191502	6.135195	2.157073	0.033713	-4.2625	0.145338	0.235243
Monocytes	PYGM	-0.43359	4.300481	-2.15641	0.033767	-3.86136	0.153171	0.247755
Monocytes	EIF4EBP2	0.151601	6.850744	2.155985	0.0338	-4.30331	0.142618	0.230825
Monocytes	ENTPD1	-0.25077	6.9129	-2.15581	0.033815	-4.46022	0.142372	0.230431
Monocytes	RTN1	-0.33593	2.561189	-2.15565	0.033828	-4.15053	0.160865	0.260181
Monocytes	GPD2	-0.23384	6.625661	-2.1556	0.033832	-4.59752	0.143516	0.232357
Monocytes	TRMT5	-0.94483	1.213824	-2.15548	0.033841	-3.60515	0.167117	0.270137
Monocytes	KIF19A	-0.89358	1.071139	-2.15514	0.033868	-3.61283	0.16785	0.271286
Monocytes	PPT2	-0.24277	4.199673	-2.15497	0.033882	-4.11113	0.153656	0.248713
Monocytes	KALRN	-0.90393	2.750236	-2.15468	0.033905	-3.57762	0.160097	0.259056
Monocytes	STON2	-0.38932	3.518101	-2.15408	0.033954	-3.87169	0.156769	0.253721
Monocytes	TBC1D17	0.378729	3.823308	2.154033	0.033958	-3.76169	0.155427	0.251575
Monocytes	RECK	-1.15573	1.480341	-2.15346	0.034004	-3.60445	0.16615	0.268604
Monocytes	CD55	-0.74766	4.863884	-2.15343	0.034007	-3.68051	0.151031	0.244473
Monocytes	SERTAD3	0.467261	4.131362	2.15263	0.034072	-3.7379	0.154325	0.249569
Monocytes	SIGLECH	1.407938	0.771994	2.152627	0.034072	-3.65055	0.169692	0.274008
Monocytes	SEC13	-0.22024	5.361757	-2.15141	0.03417	-4.08537	0.149451	0.241497
Monocytes	GM37529	-0.84228	1.962925	-2.15046	0.034248	-3.5847	0.164671	0.265826
Monocytes	RGS10	-0.19484	5.509055	-2.15043	0.03425	-4.31621	0.149026	0.240846
Monocytes	ZFP120	0.443557	2.536502	2.15031	0.03426	-3.64762	0.162022	0.261638
Monocytes	VBP1	0.236135	4.92592	2.149996	0.034286	-3.96981	0.151527	0.244859
Monocytes	PCIF1	0.185127	6.021144	2.149095	0.034359	-4.1875	0.147199	0.237815
Monocytes	PRKCE	0.233643	7.233184	2.148909	0.034374	-4.41256	0.142306	0.229937
Monocytes	EDC3	0.362006	3.628178	2.148477	0.03441	-3.73167	0.157513	0.254296
Monocytes	CCDC146	-0.41657	2.867299	-2.14803	0.034447	-3.87083	0.161029	0.25986
Monocytes	HSPH1	0.447364	4.050366	2.147378	0.0345	-3.91683	0.15592	0.251601
Monocytes	ARMC8	-0.23148	5.019279	-2.14685	0.034543	-3.9614	0.151825	0.245047
Monocytes	FAM49B	0.125182	9.086243	2.146752	0.034551	-4.7623	0.135568	0.218849
Monocytes	PTPN22	-0.29309	5.324165	-2.14639	0.034581	-4.26999	0.150597	0.243107
Monocytes	ADAM23	-0.52869	3.184267	-2.14591	0.03462	-3.82241	0.159996	0.258185
Monocytes	NFIB	-0.76349	3.880029	-2.14584	0.034626	-3.69744	0.156891	0.253234
Monocytes	CHORDC1	-0.2328	5.082056	-2.14535	0.034666	-4.01627	0.15179	0.245042
Monocytes	SASH1	-0.35144	5.303699	-2.14511	0.034686	-4.17541	0.150871	0.243637
Monocytes	ZFP273	1.040641	0.440289	2.14441	0.034744	-3.68229	0.173226	0.279185
Monocytes	HECTD3	0.458994	2.789878	2.144343	0.034749	-3.64458	0.162074	0.26155
Monocytes	FAM219A	-0.40327	4.171512	-2.14368	0.034804	-3.90413	0.155966	0.251863
Monocytes	LRRFIP2	0.164294	5.900501	2.143509	0.034818	-4.35383	0.148585	0.240027
Monocytes	ITIH4	-0.66409	3.988196	-2.14345	0.034823	-3.73072	0.156772	0.253152
Monocytes	SETX	0.240808	5.905752	2.143379	0.034829	-4.15455	0.148563	0.239992
Monocytes	HMGCS2	-0.69613	4.456909	-2.14252	0.0349	-3.8522	0.154891	0.250172
Monocytes	ATG12	0.279264	4.873947	2.142304	0.034918	-3.96659	0.153088	0.24737

Monocytes	PHF11B	-0.32439	4.313119	-2.14224	0.034923	-4.23353	0.155518	0.251278
Monocytes	SAT2	-1.48798	-0.24549	-2.14211	0.034934	-3.74175	0.176921	0.285272
Monocytes	6-Mar	0.170956	6.3538	2.141979	0.034945	-4.26294	0.146877	0.237455
Monocytes	SOCS2	-0.51643	3.962092	-2.14139	0.034994	-3.78137	0.157211	0.253944
Monocytes	VIPR1	-0.98987	0.964939	-2.14104	0.035023	-3.6197	0.17117	0.276178
Monocytes	KLHL24	0.242945	6.228518	2.14053	0.035065	-4.17081	0.147699	0.238617
Monocytes	DCBLD1	-0.80693	3.33755	-2.14026	0.035087	-3.60203	0.16018	0.258679
Monocytes	GM42567	-0.8207	1.12927	-2.14016	0.035095	-3.61412	0.1705	0.275081
Monocytes	ARHGAP33	-1.20921	0.549492	-2.13998	0.035111	-3.69569	0.173329	0.279579
Monocytes	GM29488	1.126187	0.172443	2.139692	0.035135	-3.6658	0.175238	0.282613
Monocytes	SKP1A	-0.17299	6.639256	-2.1392	0.035175	-4.30391	0.146111	0.236196
Monocytes	CEBPE	0.713145	0.2028	2.139145	0.03518	-3.61363	0.175159	0.282508
Monocytes	UBFD1	-0.2668	4.520398	-2.1385	0.035234	-3.98578	0.155198	0.250781
Monocytes	PAIP2	0.157913	7.620191	2.138338	0.035248	-4.39847	0.142321	0.230047
Monocytes	SARDH	-0.86989	2.900243	-2.13768	0.035302	-3.62464	0.162623	0.262648
Monocytes	COPZ1	0.170539	6.366584	2.136837	0.035373	-4.31157	0.147772	0.238849
Monocytes	NUDC	-0.20129	5.698731	-2.13665	0.035388	-4.20083	0.150556	0.243347
Monocytes	TMEM87B	0.222672	5.127611	2.13641	0.035409	-4.09415	0.153004	0.247278
Monocytes	GM32569	-1.41276	1.845691	-2.13605	0.035439	-3.67192	0.167865	0.271045
Monocytes	KCNQ5	-0.37271	7.024096	-2.13594	0.035448	-4.12713	0.145141	0.234705
Monocytes	B430010I23RIK	0.931955	-0.41456	2.134966	0.03553	-3.64329	0.179322	0.288866
Monocytes	ERI2	0.859031	1.310882	2.134741	0.035549	-3.61835	0.170761	0.275432
Monocytes	2310010J17RIK	-0.44367	3.692212	-2.1343	0.035586	-3.77558	0.159745	0.257939
Monocytes	BZW2	-0.23393	5.869307	-2.13407	0.035606	-4.19469	0.150297	0.242771
Monocytes	MAP4K3	0.354225	4.283134	2.133391	0.035663	-3.91014	0.157313	0.253949
Monocytes	SLC2A6	0.471722	1.781297	2.133111	0.035686	-3.78417	0.168831	0.272318
Monocytes	CRKL	0.239347	5.071994	2.132984	0.035697	-4.02019	0.153881	0.248492
Monocytes	ITSN2	-0.18242	6.989544	-2.13211	0.035771	-4.4071	0.14609	0.235732
Monocytes	PLCXD2	-0.48405	3.46805	-2.1318	0.035797	-3.72879	0.161288	0.260123
Monocytes	ZFP654	0.256588	6.120389	2.131373	0.035833	-4.20676	0.149758	0.24172
Monocytes	RABAC1	0.195021	5.988734	2.131313	0.035838	-4.26476	0.15031	0.242608
Monocytes	SLFN4	1.12462	0.48225	2.130981	0.035867	-3.61533	0.17555	0.282893
Monocytes	ZFP426	0.512927	2.769149	2.130947	0.035869	-3.64727	0.164541	0.265471
Monocytes	PSME2	-0.28212	7.278608	-2.13032	0.035923	-4.5491	0.145102	0.234239
Monocytes	A630001G21RII	0.272908	5.126842	2.130275	0.035926	-4.03157	0.154091	0.2487
Monocytes	BAHD1	0.507773	2.543391	2.129953	0.035954	-3.65318	0.165727	0.267294
Monocytes	D430040D24RII	-1.25343	-0.34621	-2.12986	0.035962	-3.72561	0.179879	0.289676
Monocytes	2610027K06RIK	0.899465	0.681173	2.12953	0.03599	-3.64472	0.174702	0.281566
Monocytes	FAM83D	-0.53952	2.774945	-2.1295	0.035992	-3.66373	0.164646	0.265647
Monocytes	GM39302	0.98817	0.880898	2.129056	0.03603	-3.64506	0.17382	0.280139
Monocytes	RABL3	0.597093	2.218563	2.128254	0.036098	-3.62905	0.167502	0.270116
Monocytes	DTNBP1	-0.14936	6.683213	-2.12821	0.036102	-4.34911	0.14776	0.238553
Monocytes	TM9SF3	0.132553	7.288714	2.128135	0.036108	-4.47781	0.145289	0.234565
Monocytes	MFSD14B	0.176766	5.841678	2.127369	0.036174	-4.34779	0.151482	0.244483
Monocytes	POFUT2	0.284607	4.257323	2.126874	0.036216	-3.97562	0.158479	0.255662
Monocytes	CAR5A	-1.13191	0.542195	-2.12614	0.036279	-3.67517	0.176243	0.283704
Monocytes	MALSU1	0.285985	4.843801	2.125973	0.036293	-4.00314	0.156086	0.25177

Monocytes	CD8A	-0.82686	1.360615	-2.12541	0.036341	-3.66264	0.172351	0.277597
Monocytes	TRP53I11	-0.55718	4.565294	-2.12483	0.036391	-3.67425	0.15758	0.254124
Monocytes	PDHB	-0.20157	5.703174	-2.1247	0.036403	-4.18361	0.152633	0.246259
Monocytes	TMED7	0.146333	6.308978	2.124487	0.036421	-4.35462	0.150079	0.242218
Monocytes	ZFHX2	-0.56188	2.765643	-2.12429	0.036438	-3.69732	0.165788	0.267346
Monocytes	IDI1	-0.34895	3.90584	-2.12404	0.036459	-3.84661	0.160568	0.259107
Monocytes	POT1B	0.263563	4.421725	2.123756	0.036484	-4.18832	0.158257	0.25552
Monocytes	NAA60	0.26333	4.697726	2.123512	0.036505	-4.00745	0.157035	0.253599
Monocytes	SMIM14	0.155387	7.74729	2.123403	0.036514	-4.51589	0.144214	0.23297
Monocytes	TRAPPC2L	0.201267	5.811456	2.123302	0.036523	-4.27195	0.15221	0.245908
Monocytes	EPS15	0.171159	6.183956	2.122833	0.036563	-4.3416	0.150637	0.243447
Monocytes	GM20732	0.2565	4.58993	2.122827	0.036564	-4.014	0.157514	0.254494
Monocytes	CX3CR1	0.342633	3.268892	2.122743	0.036571	-4.19013	0.163479	0.264025
Monocytes	H2-AA	-0.40423	8.331767	-2.12212	0.036624	-5.0945	0.141992	0.229495
Monocytes	CDK5R1	-0.78056	2.111423	-2.12201	0.036634	-3.63108	0.169024	0.272922
Monocytes	DCK	0.262278	5.832657	2.121895	0.036644	-4.21194	0.152225	0.24612
Monocytes	P4HA3	-1.1618	0.308298	-2.12173	0.036659	-3.727	0.177889	0.287
Monocytes	ZKSCAN16	-1.18651	-0.59074	-2.12129	0.036696	-3.78859	0.182606	0.294446
Monocytes	LEPR	-1.06182	1.823369	-2.12067	0.03675	-3.63521	0.170683	0.275586
Monocytes	SH2D1B1	0.423541	1.695197	2.120353	0.036777	-3.92576	0.171359	0.276656
Monocytes	GLS	-0.18859	7.7257	-2.12005	0.036804	-4.55558	0.144723	0.233995
Monocytes	ACO1	-0.32538	3.712371	-2.11944	0.036856	-3.84609	0.162078	0.261874
Monocytes	D730003I15RIK	0.4584	2.591958	2.118861	0.036907	-3.72269	0.167439	0.270285
Monocytes	SPATS2	-0.6345	2.720159	-2.11821	0.036963	-3.67978	0.167018	0.269468
Monocytes	AGXT	-0.77504	3.279368	-2.1177	0.037008	-3.71545	0.16451	0.265492
Monocytes	CTTN	-0.79639	1.717261	-2.11757	0.037019	-3.63831	0.171933	0.277329
Monocytes	0610005C13RIK	-0.78824	1.911141	-2.11553	0.037197	-3.64008	0.171742	0.276478
Monocytes	THAP1	-0.49979	2.879636	-2.1148	0.037261	-3.68287	0.167297	0.269469
Monocytes	EIF4B	-0.18094	6.136378	-2.11467	0.037272	-4.31458	0.152674	0.246137
Monocytes	CUEDC2	0.225703	5.245055	2.114023	0.037329	-4.16818	0.156702	0.252592
Monocytes	LATS2	0.180813	5.889771	2.113816	0.037347	-4.35908	0.153908	0.2481
Monocytes	TRP53INP1	0.417881	5.187354	2.113092	0.037411	-3.90016	0.157164	0.253254
Monocytes	MAT2B	0.217002	5.679223	2.112316	0.037479	-4.16598	0.15523	0.250099
Monocytes	RDH11	0.436289	3.215453	2.111666	0.037537	-3.73619	0.166537	0.267962
Monocytes	ATP1B1	-0.65937	5.798004	-2.11131	0.037568	-3.76398	0.154949	0.249433
Monocytes	NT5C3B	-0.44288	3.396774	-2.11034	0.037654	-3.71617	0.166022	0.267106
Monocytes	CPT1B	-0.62912	1.536488	-2.11026	0.037661	-3.64844	0.174983	0.281294
Monocytes	GNMT	0.649211	4.232803	2.109334	0.037743	-3.92041	0.162445	0.261348
Monocytes	AFMID	-0.27695	4.371552	-2.10831	0.037834	-4.11002	0.16207	0.26064
Monocytes	B230206L02RIK	-0.79589	1.02404	-2.10829	0.037835	-3.69513	0.178136	0.286073
Monocytes	RAB7B	-0.28229	2.804284	-2.10792	0.037868	-4.37068	0.169461	0.272353
Monocytes	PRNP	-0.60413	2.012971	-2.10701	0.037949	-3.67652	0.173559	0.278715
Monocytes	NAGA	-0.21741	4.159018	-2.10667	0.03798	-4.35393	0.163365	0.262578
Monocytes	FAM149A	-0.47367	-0.42534	-2.10654	0.037991	-3.78727	0.185995	0.298263
Monocytes	TOB1	-0.24685	5.050855	-2.10652	0.037993	-4.18636	0.159325	0.256139
Monocytes	GCDH	-0.66875	3.116618	-2.10635	0.038008	-3.70907	0.168232	0.270307
Monocytes	LSS	-0.99366	0.604735	-2.10612	0.038028	-3.70301	0.180627	0.289939

Monocytes	RET	-1.19332	0.727544	-2.10599	0.03804	-3.75546	0.179998	0.288994
Monocytes	NSG2	-1.07527	0.587521	-2.105	0.038129	-3.7352	0.18106	0.290485
Monocytes	CHP1	0.159818	7.01438	2.104387	0.038183	-4.54299	0.151258	0.243108
Monocytes	SF1	-0.14326	7.215593	-2.1039	0.038227	-4.48015	0.15052	0.241848
Monocytes	HCFC1R1	0.213577	5.33224	2.103278	0.038283	-4.24948	0.158812	0.255027
Monocytes	ZFP219	-0.5869	1.771622	-2.10247	0.038355	-3.66463	0.175822	0.281893
Monocytes	4930438A08RIK	0.912091	-0.84008	2.101609	0.038433	-3.73389	0.189663	0.303467
Monocytes	RARB	-0.95191	1.586463	-2.10109	0.038479	-3.66814	0.177121	0.283837
Monocytes	KLHL18	0.306281	4.310869	2.101025	0.038485	-3.97413	0.164015	0.263121
Monocytes	BTF3L4	0.267009	4.269235	2.10055	0.038528	-3.95066	0.16432	0.263556
Monocytes	ABCB11	-0.90648	1.681529	-2.1001	0.038569	-3.66737	0.176879	0.283305
Monocytes	IFNGR1	0.16939	7.03854	2.098928	0.038674	-4.68355	0.152516	0.244501
Monocytes	EWSR1	-0.14288	7.421625	-2.09847	0.038716	-4.52328	0.150997	0.242038
Monocytes	PSMD1	-0.17893	6.546401	-2.09783	0.038774	-4.40355	0.154848	0.24822
Monocytes	WDR24	0.69571	1.796393	2.097763	0.03878	-3.66724	0.176969	0.28331
Monocytes	RAB22A	-0.20012	5.60028	-2.09682	0.038866	-4.24345	0.159273	0.255088
Monocytes	EAR2	0.303882	3.55304	2.096658	0.03888	-4.35957	0.168697	0.270126
Monocytes	5830408C22RIK	-0.54123	2.989299	-2.09637	0.038906	-3.68556	0.171441	0.274454
Monocytes	NR6A1	-0.29509	5.241175	-2.09512	0.039021	-4.17817	0.16131	0.258054
Monocytes	SCD1	-0.76539	3.037555	-2.09498	0.039033	-3.7075	0.171621	0.274392
Monocytes	EPSTI1	-0.2831	7.192656	-2.09402	0.039121	-4.66107	0.153032	0.244589
Monocytes	ABHD11	0.357333	3.541149	2.093601	0.039159	-3.90584	0.169565	0.270763
Monocytes	MASP1	-1.03004	1.673868	-2.09333	0.039184	-3.69682	0.178748	0.285276
Monocytes	CLEC2D	-0.41204	4.7558	-2.09315	0.0392	-4.1481	0.163872	0.261866
Monocytes	NDUFAF8	0.209634	5.052226	2.093109	0.039204	-4.17897	0.162515	0.259714
Monocytes	ARSG	0.545756	1.867658	2.09298	0.039216	-3.684	0.17777	0.283797
Monocytes	DYNLT3	0.250188	4.662522	2.09275	0.039237	-4.04562	0.16432	0.262632
Monocytes	NUP210	-0.22506	5.041054	-2.09178	0.039326	-4.22617	0.162883	0.260285
Monocytes	TNFSF8	-1.24923	-0.51498	-2.09025	0.039466	-3.72761	0.191183	0.304021
Monocytes	ZC3H12B	-1.27353	1.084598	-2.08922	0.039562	-3.7959	0.18301	0.291189
Monocytes	RNF180	-0.44009	2.247716	-2.08914	0.039569	-3.95727	0.17708	0.281958
Monocytes	SLC35D2	-0.34942	4.232	-2.0884	0.039637	-4.0104	0.167663	0.267147
Monocytes	FERMT2	-0.59333	3.451103	-2.08702	0.039765	-3.7416	0.171867	0.273513
Monocytes	LCMT2	0.490969	2.687418	2.086728	0.039792	-3.72325	0.175657	0.279454
Monocytes	TRUB2	0.290136	4.526877	2.085499	0.039906	-4.01895	0.167141	0.265924
Monocytes	RAMP3	-0.58293	0.947281	-2.08546	0.03991	-3.79655	0.184915	0.293763
Monocytes	RPRD2	0.209503	5.916624	2.084332	0.040015	-4.30871	0.161113	0.256104
Monocytes	NCAM1	0.677209	1.275146	2.083746	0.04007	-3.84612	0.183784	0.291584
Monocytes	TAF9B	-0.92513	0.956005	-2.08315	0.040125	-3.70984	0.185635	0.294452
Monocytes	PALD1	-0.90144	1.292537	-2.08249	0.040186	-3.69363	0.184026	0.291773
Monocytes	SNRPB2	0.160486	6.282619	2.08243	0.040192	-4.43343	0.159914	0.253966
Monocytes	ZBTB11OS1	0.538075	2.065369	2.082011	0.040232	-3.70752	0.180142	0.285678
Monocytes	CCDC92	0.998018	-0.17821	2.081502	0.040279	-3.74222	0.192128	0.304239
Monocytes	MTHFD2	-0.28	5.486209	-2.08102	0.040325	-4.29734	0.163767	0.259994
Monocytes	MTMR1	-0.26162	4.622377	-2.08079	0.040346	-4.0923	0.167782	0.266342
Monocytes	THAP2	-0.3607	3.329281	-2.08071	0.040353	-3.93127	0.173998	0.276103
Monocytes	XCL1	-0.68815	2.335401	-2.08069	0.040356	-3.869	0.178949	0.283843

Monocytes	RECQL4	-1.1267	-0.06717	-2.07981	0.040438	-3.80775	0.191876	0.303732
Monocytes	APOM	-0.62283	4.854579	-2.07756	0.04065	-4.05023	0.16777	0.265661
Monocytes	APCS	0.457055	4.833138	2.077073	0.040696	-4.0497	0.167989	0.265997
Monocytes	POGLUT1	-0.36889	2.752407	-2.07672	0.04073	-3.88948	0.178196	0.281938
Monocytes	CDK14	-0.29928	5.455475	-2.0763	0.040769	-4.71372	0.165243	0.261633
Monocytes	I830077J02RIK	0.350629	2.758256	2.074796	0.040912	-4.09953	0.178813	0.28259
Monocytes	TRIM72	-0.94113	0.692552	-2.07409	0.04098	-3.71559	0.189817	0.299625
Monocytes	MICU2	0.249835	4.939969	2.073339	0.041051	-4.17653	0.168539	0.266477
Monocytes	AA467197	1.153379	0.801131	2.073229	0.041061	-3.70512	0.189421	0.299036
Monocytes	C030006K11RIK	-0.82866	1.498775	-2.07312	0.041071	-3.7082	0.18571	0.293321
Monocytes	SLC25A10	-0.41788	3.077126	-2.07266	0.041115	-3.86184	0.177655	0.280821
Monocytes	GADD45B	-0.30363	5.322838	-2.07246	0.041135	-4.3626	0.166785	0.263825
Monocytes	SETDB1	0.273127	4.832864	2.072367	0.041143	-4.09525	0.169092	0.267448
Monocytes	KIF2A	-0.18172	6.338703	-2.07218	0.041161	-4.39882	0.162114	0.256501
Monocytes	JAZF1	-0.433	3.086872	-2.07211	0.041168	-3.88787	0.177606	0.280807
Monocytes	RRN3	0.272094	4.400393	2.071754	0.041202	-4.02495	0.17121	0.270824
Monocytes	PHC2	-0.22633	5.798035	-2.07153	0.041224	-4.40039	0.164632	0.260548
Monocytes	PTGER4	-0.25858	4.681638	-2.07144	0.041232	-4.36657	0.169863	0.268807
Monocytes	VPS39	-0.39482	3.331542	-2.07106	0.041268	-3.82857	0.176459	0.279168
Monocytes	PHLPP1	-0.22107	7.671226	-2.07082	0.041291	-4.82151	0.156273	0.247426
Monocytes	PRXL2B	0.408636	2.256934	2.070799	0.041293	-3.83518	0.181894	0.287669
Monocytes	ARHGAP18	-0.25403	6.528672	-2.07052	0.04132	-4.60219	0.161324	0.25546
Monocytes	PRPF3	-0.25671	4.472577	-2.0705	0.041322	-4.00648	0.170883	0.270509
Monocytes	BNIP3L	0.240048	7.263429	2.070265	0.041344	-4.54116	0.158056	0.250332
Monocytes	CEBPA	0.404685	2.906324	2.070137	0.041357	-4.02412	0.178589	0.282643
Monocytes	GM12158	-1.28839	0.549233	-2.06969	0.0414	-3.80834	0.191009	0.301994
Monocytes	MINDY2	0.195265	6.610817	2.069565	0.041412	-4.37253	0.161036	0.255129
Monocytes	NRARP	-0.77789	1.780032	-2.06857	0.041507	-3.71529	0.184805	0.292173
Monocytes	TLR4	0.390346	3.122783	2.067905	0.041571	-4.02479	0.178128	0.281644
Monocytes	S100A1	0.280872	4.086611	2.06697	0.041661	-4.15983	0.173661	0.274478
Monocytes	SOGA1	-0.38805	3.807296	-2.06669	0.041688	-3.98813	0.175073	0.27669
Monocytes	IFNGR2	0.239546	5.782195	2.066446	0.041712	-4.47044	0.16566	0.261961
Monocytes	TBXA2R	-1.08633	2.36173	-2.06616	0.041739	-3.72936	0.182431	0.28822
Monocytes	GM20404	0.709897	1.661785	2.065717	0.041782	-3.71685	0.186195	0.294035
Monocytes	PSMG2	-0.38386	3.919974	-2.06552	0.041801	-3.91922	0.174702	0.276174
Monocytes	WDR1	-0.18113	7.335798	-2.06511	0.041841	-4.64366	0.158863	0.251273
Monocytes	CEMIP2	0.315771	5.494454	2.064896	0.041862	-4.1875	0.167255	0.264495
Monocytes	CREB5	0.592802	2.17534	2.064441	0.041906	-3.92625	0.183749	0.290243
Monocytes	PDF	0.803217	0.794298	2.062941	0.042052	-3.72456	0.191666	0.302192
Monocytes	OPA3	-0.22411	5.128547	-2.0623	0.042114	-4.2515	0.169786	0.268057
Monocytes	MMP12	0.821212	-0.05819	2.061484	0.042193	-3.78539	0.196853	0.309888
Monocytes	CFAP77	-1.12519	0.931954	-2.06133	0.042208	-3.7907	0.191394	0.301485
Monocytes	GADD45A	0.361858	4.876654	2.060651	0.042275	-4.13491	0.171432	0.270272
Monocytes	PPA1	-0.30006	4.797054	-2.06043	0.042297	-4.1786	0.171834	0.270883
Monocytes	GSPT1	-0.15537	6.707096	-2.05973	0.042364	-4.48564	0.163087	0.257042
Monocytes	PPP1R3B	0.574333	2.491892	2.058831	0.042453	-3.75024	0.183756	0.289376
Monocytes	WDR75	-0.369	3.575364	-2.0588	0.042456	-3.89924	0.178225	0.280783

Monocytes	HS6ST1	-0.29314	4.156867	-2.05878	0.042458	-4.09372	0.175332	0.276276
Monocytes	ZFP668	-0.34575	3.829078	-2.0566	0.042672	-3.90615	0.177725	0.279627
Monocytes	CSTDC6	0.923725	-1.16323	2.05652	0.04268	-3.83347	0.204732	0.321267
Monocytes	POLR2F	0.189585	5.381223	2.056369	0.042695	-4.31872	0.170146	0.267839
Monocytes	SMS	-0.18616	6.477089	-2.05593	0.042738	-4.42978	0.165054	0.259838
Monocytes	PDGFA	-0.69353	2.114128	-2.05591	0.042741	-3.77412	0.186583	0.293385
Monocytes	FAM83G	-0.88367	0.263823	-2.05531	0.042799	-3.76604	0.196754	0.30901
Monocytes	TBL2	-0.49269	2.392625	-2.0551	0.04282	-3.74194	0.185234	0.291264
Monocytes	CCNT1	0.153739	6.841664	2.055083	0.042822	-4.51205	0.163485	0.25734
Monocytes	UBN1	-0.14109	6.944041	-2.05493	0.042837	-4.55337	0.163019	0.25663
Monocytes	TXNIP	0.348918	5.791358	2.053607	0.042968	-4.20329	0.168795	0.265241
Monocytes	SMYD2	-0.44298	3.028651	-2.05263	0.043065	-3.90488	0.182636	0.28681
Monocytes	VOPP1	0.331157	3.965239	2.052619	0.043066	-4.10871	0.17788	0.279431
Monocytes	CCDC88C	0.251074	5.294187	2.05256	0.043072	-4.27546	0.171365	0.269299
Monocytes	FOXK1	0.23621	4.627409	2.051923	0.043135	-4.17575	0.174785	0.274513
Monocytes	CD200	-0.57093	1.910379	-2.05131	0.043196	-3.85963	0.188882	0.296346
Monocytes	CSPP1	0.2298	6.098729	2.051045	0.043222	-4.35747	0.167891	0.263825
Monocytes	ZFP169	0.561182	2.782441	2.050812	0.043246	-3.83209	0.184283	0.289417
Monocytes	KITL	-1.10752	1.956944	-2.0508	0.043247	-3.7481	0.188633	0.296138
Monocytes	TBCEL	0.319023	4.594507	2.05001	0.043326	-4.02833	0.175363	0.275453
Monocytes	JPT1	-0.17497	7.832843	-2.04917	0.04341	-4.69674	0.160381	0.252037
Monocytes	ZDHHC7	0.305198	3.834677	2.049161	0.04341	-4.02699	0.179354	0.28171
Monocytes	TPBGL	-1.26444	0.559425	-2.04865	0.043461	-3.81712	0.196837	0.308809
Monocytes	RAB3D	0.345482	3.11921	2.048632	0.043463	-4.05314	0.18308	0.287598
Monocytes	ZFP260	0.311946	4.245881	2.048123	0.043514	-3.93538	0.177499	0.27882
Monocytes	ADCY6	-0.6997	0.433361	-2.04726	0.0436	-3.75189	0.197925	0.310296
Monocytes	FGF1	-0.99925	0.63081	-2.04726	0.0436	-3.78906	0.196819	0.3086
Monocytes	MCTS1	0.163325	6.207891	2.046223	0.043705	-4.49781	0.168535	0.264607
Monocytes	CLTA	-0.13011	8.442682	-2.04526	0.043801	-4.82069	0.158661	0.248963
Monocytes	GM34471	-1.01618	0.232245	-2.04487	0.043841	-3.78543	0.199917	0.312852
Monocytes	I730030J21RIK	-1.03698	0.707146	-2.04459	0.04387	-3.78614	0.197243	0.308754
Monocytes	TCN2	0.305336	4.791081	2.044505	0.043878	-4.27413	0.175764	0.275601
Monocytes	AP2A2	0.177328	5.931782	2.043442	0.043985	-4.45553	0.170557	0.267339
Monocytes	SULT2A8	-1.21287	0.571296	-2.04334	0.043996	-3.78214	0.198377	0.310331
Monocytes	ULK1	0.387134	3.637915	2.042621	0.044068	-3.91774	0.182051	0.285141
Monocytes	0610030E20RIK	0.273877	4.591291	2.042442	0.044086	-4.10485	0.177235	0.277674
Monocytes	E130317F20RIK	-0.8467	0.77534	-2.04241	0.04409	-3.78331	0.197399	0.308765
Monocytes	FAM8A1	0.461535	3.231881	2.042265	0.044104	-3.81407	0.184146	0.288392
Monocytes	IGF2BP1	-1.25005	-0.96106	-2.04081	0.044252	-3.86411	0.208004	0.324565
Monocytes	CDON	-0.5174	1.952332	-2.03909	0.044427	-3.86914	0.192166	0.299821
Monocytes	IKZF3	-0.39509	5.866917	-2.03827	0.044511	-4.18954	0.17238	0.269049
Monocytes	CXCL10	-0.56769	4.788598	-2.03787	0.044551	-4.32089	0.177735	0.277318
Monocytes	ATP13A2	0.274262	5.610722	2.037746	0.044564	-4.33569	0.173687	0.271082
Monocytes	ZFP52	0.340648	3.859767	2.037248	0.044615	-4.03409	0.182518	0.284809
Monocytes	ARL4D	-0.77535	1.705958	-2.03703	0.044638	-3.76357	0.19396	0.302412
Monocytes	LDLRAP1	0.306852	4.099537	2.037023	0.044638	-4.15498	0.181291	0.282931
Monocytes	MAPK8IP3	-0.25727	4.521476	-2.03571	0.044773	-4.11192	0.179621	0.280171

Monocytes	HBQ1B	1.275892	-0.16343	2.035203	0.044825	-3.81969	0.20522	0.319302
Monocytes	KHDRBS1	-0.12968	7.151511	-2.03501	0.044844	-4.61577	0.167018	0.260586
Monocytes	HYAL3	-1.09654	0.035974	-2.03458	0.044889	-3.82666	0.204128	0.317754
Monocytes	GRHL1	-0.84056	1.025537	-2.03453	0.044894	-3.76272	0.198472	0.309142
Monocytes	CNKSR3	-0.38233	4.754523	-2.03428	0.04492	-4.1752	0.178677	0.278702
Monocytes	EZR	-0.16326	7.933064	-2.03341	0.045009	-4.68874	0.16374	0.255396
Monocytes	PNLDC1	-1.04771	0.924032	-2.03311	0.04504	-3.79946	0.199363	0.310487
Monocytes	H2-Q4	-0.43088	4.933329	-2.03292	0.04506	-4.16633	0.178038	0.277781
Monocytes	CHFR	0.190037	5.641975	2.032916	0.04506	-4.36582	0.174538	0.272355
Monocytes	FBXL7	-0.88591	3.621991	-2.03278	0.045074	-3.84583	0.184723	0.288156
Monocytes	NKIRAS2	0.267001	4.042562	2.031892	0.045166	-4.11572	0.182847	0.285115
Monocytes	0610009E02RIK	-0.82468	0.843747	-2.03142	0.045215	-3.78927	0.20023	0.311808
Monocytes	DENND4C	0.251386	5.068456	2.031357	0.045222	-4.24914	0.17773	0.277239
Monocytes	FES	0.241136	4.284767	2.030881	0.045271	-4.36518	0.181809	0.283547
Monocytes	TRAPPC2	0.309863	4.171105	2.030134	0.045348	-4.07907	0.182607	0.284686
Monocytes	TSPO2	1.170401	0.283153	2.02991	0.045372	-3.81759	0.203824	0.31728
Monocytes	CLEC5A	0.443835	0.512385	2.02984	0.045379	-4.0046	0.202501	0.315267
Monocytes	ATE1	0.270478	3.945361	2.029659	0.045398	-4.05662	0.183773	0.286565
Monocytes	ZFYVE27	0.303701	4.063949	2.0293	0.045435	-4.01748	0.183238	0.285666
Monocytes	TNPO3	-0.16094	6.365771	-2.02834	0.045535	-4.5593	0.172039	0.268325
Monocytes	PPP1R11	-0.23229	4.640461	-2.02832	0.045537	-4.32963	0.180553	0.281528
Monocytes	TGFBR2	-0.20537	7.292661	-2.02762	0.04561	-4.46918	0.167795	0.261742
Monocytes	RAB19	-0.49671	2.561986	-2.02759	0.045613	-3.88129	0.191602	0.298579
Monocytes	SMAD7	-0.31302	5.164939	-2.02743	0.04563	-4.18152	0.178068	0.277757
Monocytes	SEC14L2	-0.8894	1.70477	-2.02624	0.045754	-3.77588	0.196757	0.306131
Monocytes	BC052040	-0.38297	4.165796	-2.02508	0.045876	-4.04148	0.183973	0.286161
Monocytes	LRP1B	0.770877	1.219155	2.024526	0.045933	-3.77871	0.200107	0.310714
Monocytes	CIDEB	-0.75862	1.942054	-2.02332	0.04606	-3.77922	0.196446	0.304985
Monocytes	EVA1A	-0.83887	1.060527	-2.0233	0.046062	-3.78331	0.201414	0.312549
Monocytes	CTSS	0.273002	7.501211	2.023051	0.046089	-4.92927	0.168081	0.261321
Monocytes	SLC17A5	0.398352	3.572742	2.022924	0.046102	-3.97594	0.187618	0.291585
Monocytes	FCGRT	-0.29522	4.823452	-2.02231	0.046167	-4.46205	0.181195	0.281683
Monocytes	PEAR1	-0.69457	2.352213	-2.02221	0.046177	-3.7826	0.194256	0.301779
Monocytes	GPR137B	-0.22153	5.316315	-2.02212	0.046187	-4.55141	0.178709	0.277885
Monocytes	PHB2	-0.17294	6.570723	-2.02209	0.04619	-4.54767	0.17255	0.268348
Monocytes	AW011738	0.554214	2.812185	2.021834	0.046217	-3.92097	0.191782	0.298012
Monocytes	GM43914	-0.34101	0.384486	-2.02159	0.046243	-4.11195	0.205466	0.31889
Monocytes	GM42658	0.68896	1.544714	2.021281	0.046275	-3.78549	0.198874	0.308934
Monocytes	EPS8	-0.27572	4.963297	-2.02108	0.046296	-4.67088	0.180613	0.280953
Monocytes	ANXA11OS	0.697764	0.812081	2.019982	0.046412	-3.79705	0.203433	0.315391
Monocytes	SRC	-0.66871	0.980292	-2.01993	0.046418	-3.80006	0.202464	0.313921
Monocytes	OGFRL1	0.236685	5.082967	2.019754	0.046436	-4.45791	0.18034	0.280063
Monocytes	UROS	-0.43442	2.669254	-2.01947	0.046466	-3.83387	0.193063	0.299615
Monocytes	IGFBP4	-0.6674	5.679023	-2.019	0.046516	-4.24101	0.177512	0.275656
Monocytes	MRPL13	-0.2509	4.834872	-2.01884	0.046533	-4.21817	0.181761	0.282234
Monocytes	FAM45A	0.242664	3.979967	2.018132	0.046609	-4.15403	0.186408	0.289131
Monocytes	COG3	0.287664	4.292574	2.017434	0.046683	-4.13305	0.184852	0.286778

Monocytes	LDB2	-0.7811	3.160304	-2.01733	0.046694	-3.82113	0.19084	0.295989
Monocytes	ADD3	0.239832	6.047415	2.01728	0.046699	-4.61116	0.175981	0.273142
Monocytes	TMEM116	-0.64398	2.038927	-2.0171	0.046718	-3.81987	0.196981	0.305409
Monocytes	PPP1R3E	0.597208	1.031561	2.017055	0.046723	-3.78963	0.202684	0.31408
Monocytes	UHMK1	-0.2464	4.630018	-2.01691	0.046738	-4.16093	0.183108	0.284173
Monocytes	1110046J04RIK	-1.20308	0.031359	-2.01674	0.046756	-3.88202	0.208523	0.322947
Monocytes	PPP2CA	-0.103	7.762516	-2.01637	0.046797	-4.80161	0.167847	0.260563
Monocytes	KLRB1C	-0.74677	1.954564	-2.01609	0.046826	-3.88508	0.197591	0.306311
Monocytes	9330160F10RIK	0.627263	2.295032	2.015282	0.046912	-3.79967	0.19598	0.30374
Monocytes	GM15441	0.813172	1.344167	2.015057	0.046936	-3.80233	0.201351	0.311901
Monocytes	TRP53RKA	0.520036	2.472703	2.014554	0.04699	-3.81742	0.195169	0.302459
Monocytes	AKR7A5	-0.26537	4.13514	-2.0136	0.047092	-4.21315	0.186532	0.289149
Monocytes	ACSM1	-1.18043	1.026687	-2.0135	0.047102	-3.80373	0.203651	0.315306
Monocytes	RAB5B	0.272458	4.382557	2.013142	0.047141	-4.17411	0.18532	0.287273
Monocytes	ARRDC2	0.624695	1.79539	2.012896	0.047168	-3.81025	0.199382	0.30876
Monocytes	CLTB	-0.23516	4.396264	-2.01247	0.047214	-4.32402	0.185387	0.28733
Monocytes	MGAT4A	0.267616	5.666978	2.011964	0.047268	-4.30901	0.17903	0.27755
Monocytes	GM5150	0.416722	2.574091	2.011799	0.047286	-4.22373	0.195297	0.3025
Monocytes	CMPK1	0.132294	6.881961	2.011267	0.047343	-4.6902	0.173134	0.26844
Monocytes	RNF144B	-0.38528	2.562148	-2.01126	0.047343	-4.02632	0.195449	0.302738
Monocytes	SLC25A32	0.362084	3.120627	2.010734	0.0474	-3.96891	0.192483	0.298157
Monocytes	SRSF4	-0.16653	6.225555	-2.01071	0.047403	-4.49597	0.176417	0.27347
Monocytes	ETS2	0.244594	5.865691	2.010526	0.047423	-4.53991	0.178205	0.276237
Monocytes	GM2682	-0.62979	2.755703	-2.00961	0.047521	-4.00291	0.194809	0.301586
Monocytes	GMFG	0.205041	7.424355	2.009424	0.047542	-4.70791	0.170919	0.264873
Monocytes	CYP4F16	0.450339	1.441368	2.009219	0.047564	-3.99915	0.2022	0.312905
Monocytes	PHRF1	-0.1978	5.804424	-2.00901	0.047587	-4.3923	0.178823	0.27716
Monocytes	SUMO2	-0.13101	8.656817	-2.0089	0.047598	-4.88549	0.165176	0.256033
Monocytes	GM15417	0.413904	2.477041	2.008353	0.047657	-3.96593	0.196527	0.304262
Monocytes	NFKB1	-0.23025	8.743793	-2.00786	0.047711	-4.97073	0.164979	0.255719
Monocytes	ZMYM2	0.220894	5.970893	2.007849	0.047712	-4.41352	0.178209	0.276238
Monocytes	EVI5	0.255067	5.166502	2.007453	0.047755	-4.41444	0.18236	0.282669
Monocytes	PDK3	0.282166	4.945028	2.007026	0.047801	-4.35834	0.1836	0.284516
Monocytes	TIMM8B	0.247155	5.242586	2.00686	0.047819	-4.38767	0.182075	0.282224
Monocytes	GLRX	-0.21552	6.214402	-2.00667	0.04784	-4.6335	0.177191	0.274752
Monocytes	1600014C10RIK	0.420842	4.275444	2.00628	0.047882	-4.1755	0.187084	0.290077
Monocytes	PECAM1	-0.29057	7.734027	-2.00621	0.04789	-4.65617	0.169847	0.263466
Monocytes	TMEM129	0.687056	2.084833	2.006158	0.047896	-3.81284	0.199005	0.308325
Monocytes	C130050O18RII	0.384413	1.379067	2.006004	0.047912	-4.07348	0.203022	0.314479
Monocytes	PRLR	-0.89405	3.152323	-2.00558	0.047959	-3.83635	0.193184	0.299509
Monocytes	FOXO1	-0.21804	7.035015	-2.00546	0.047972	-4.52862	0.173262	0.268826
Monocytes	C2CD3	0.230732	4.657214	2.004677	0.048057	-4.20267	0.185431	0.287465
Monocytes	MFSD11	-0.33303	3.854162	-2.00365	0.048169	-4.10272	0.190006	0.294423
Monocytes	TMEM106C	-0.39502	3.205819	-2.00354	0.048181	-3.93045	0.193508	0.299783
Monocytes	MAP7D1	0.187803	5.36793	2.002831	0.048259	-4.42754	0.182322	0.282534
Monocytes	DDX58	0.38601	4.759373	2.002494	0.048295	-4.14871	0.18553	0.287458
Monocytes	SHLD1	0.39853	3.33904	2.002115	0.048337	-3.96508	0.193148	0.299145

Monocytes	RNF114	0.242916	5.645299	2.001972	0.048353	-4.41738	0.181033	0.280544
Monocytes	PIGX	0.204756	5.308726	2.001854	0.048366	-4.41527	0.182747	0.283185
Monocytes	GM28875	0.271293	4.612323	2.001434	0.048412	-4.35476	0.186457	0.288864
Monocytes	ZFPM1	-0.52577	4.248218	-2.00123	0.048434	-3.89271	0.188388	0.291887
Monocytes	FMNL2	-0.27002	7.100621	-2.001	0.048459	-4.89576	0.17396	0.269633
Monocytes	PTTG1	-0.22757	5.78	-2.00053	0.048511	-4.42076	0.180615	0.279875
Monocytes	GLB1L	0.467401	2.192734	2.000198	0.048548	-3.86898	0.199864	0.309339
Monocytes	NEK7	0.187915	6.476805	1.999514	0.048623	-4.55457	0.177403	0.274805
Monocytes	SHMT1	-0.4816	4.012421	-1.99934	0.048642	-3.95877	0.190087	0.294354
Monocytes	TSC22D2	-0.16597	6.616128	-1.99893	0.048687	-4.62148	0.176811	0.273944
Monocytes	SLC25A23	-0.69245	2.25448	-1.99836	0.04875	-3.81658	0.200039	0.30953
Monocytes	IFT88	-0.61731	1.778503	-1.99733	0.048864	-3.82031	0.203019	0.313964
Monocytes	BICC1	-1.07857	0.332453	-1.99714	0.048885	-3.83061	0.211518	0.326952
Monocytes	AMOTL1	-0.92307	1.696223	-1.99706	0.048894	-3.82502	0.203492	0.314825
Monocytes	TSPAN7	-0.58466	3.257326	-1.99699	0.048902	-3.87281	0.194711	0.301448
Monocytes	PPP2CB	0.178536	5.60171	1.996926	0.048909	-4.42885	0.182301	0.282405
Monocytes	PTPRK	-0.82873	3.178658	-1.99653	0.048953	-3.86045	0.19518	0.302177
Monocytes	CLCC1	0.222842	4.329275	1.996401	0.048967	-4.20097	0.188959	0.292689
Monocytes	TCP11L1	0.310019	2.689841	1.996167	0.048993	-4.17504	0.197891	0.306441
Monocytes	RELCH	0.165575	6.238997	1.995949	0.049017	-4.54351	0.179114	0.277616
Monocytes	GEMIN5	0.398661	3.385628	1.995869	0.049026	-3.95692	0.194044	0.300566
Monocytes	OPLAH	-0.80623	1.500611	-1.99583	0.049031	-3.82156	0.204661	0.316744
Monocytes	DGKA	0.325151	5.023185	1.995082	0.049113	-4.25403	0.185554	0.287368
Monocytes	GM49085	-0.59574	2.025817	-1.99479	0.049145	-3.82638	0.201957	0.312461
Monocytes	MGMT	-0.56439	3.273792	-1.99365	0.049273	-3.88317	0.195394	0.302273
Monocytes	A630089N07RII	-0.65339	2.672384	-1.99273	0.049375	-3.83414	0.199074	0.307778
Monocytes	NLRP12	1.084014	-0.12085	1.992227	0.049431	-3.85898	0.215638	0.332767
Monocytes	OAS1A	0.565764	2.355225	1.991716	0.049488	-4.05382	0.201172	0.310832
Monocytes	ZFP729B	0.319584	3.639068	1.991277	0.049537	-4.05998	0.194102	0.300085
Monocytes	ITSN1	-0.33525	5.300914	-1.99118	0.049548	-4.31627	0.185249	0.286528
Monocytes	EHBP1	-0.44992	3.52414	-1.99083	0.049587	-4.04993	0.194809	0.301177
Monocytes	GM30198	0.580896	1.865066	1.990539	0.04962	-3.96395	0.204159	0.315414
Monocytes	HTT	-0.19722	5.362069	-1.99048	0.049627	-4.45673	0.185011	0.286167
Monocytes	KRT222	-1.17314	-0.05515	-1.99025	0.049652	-3.90756	0.21562	0.332822
Monocytes	PAX5	-0.59034	5.141192	-1.98991	0.04969	-3.99522	0.186256	0.288144
Monocytes	RSL24D1	0.186325	5.674542	1.989323	0.049757	-4.48823	0.18367	0.284132
Monocytes	FAM76B	0.208146	5.401156	1.988846	0.04981	-4.32897	0.18521	0.286521
Monocytes	ACSF2	0.275419	4.286847	1.987458	0.049966	-4.29839	0.191618	0.295977
Monocytes	MARK3	-0.14774	6.304211	-1.98673	0.050048	-4.617	0.181295	0.279939
Monocytes	PIK3CD	0.244752	6.624599	1.98655	0.050069	-4.54966	0.179682	0.27751
Monocytes	CEP72	0.59161	2.208429	1.986444	0.050081	-3.84882	0.203414	0.313829
Monocytes	SNHG9	-0.55692	4.309776	-1.98611	0.050118	-4.12107	0.191783	0.296115
Monocytes	KMT5B	0.193063	5.91383	1.985794	0.050154	-4.47255	0.183413	0.283273
Monocytes	TLR2	0.246012	3.875493	1.985491	0.050189	-4.58507	0.194265	0.299972
Monocytes	GM13919	0.567346	3.111975	1.985025	0.050241	-3.88863	0.198622	0.306652
Monocytes	LGALS3	0.208761	6.142988	1.984568	0.050293	-4.99529	0.182536	0.281946
Monocytes	5930403N24RII	-0.95281	-0.48768	-1.98429	0.050325	-3.84549	0.220096	0.339101

Monocytes	EPB41L5	-0.61084	3.379051	-1.98423	0.050332	-3.87632	0.197258	0.304578
Monocytes	REXO4	-0.25539	4.291897	-1.98404	0.050353	-4.20664	0.192255	0.296999
Monocytes	SMPD5	1.067605	-0.18019	1.983896	0.050369	-3.92827	0.218178	0.336382
Monocytes	DAPL1	-1.06771	-1.31502	-1.98324	0.050444	-3.92803	0.225595	0.347256
Monocytes	ELK3	0.256569	5.288608	1.983053	0.050465	-4.3755	0.187166	0.289029
Monocytes	SCAMP5	-0.70894	1.229049	-1.98275	0.0505	-3.838	0.209924	0.323587
Monocytes	DYNLT1B	0.798398	1.188553	1.980453	0.050762	-3.84139	0.211161	0.32485
Monocytes	ZFP563	0.747789	0.800774	1.980309	0.050778	-3.85361	0.213497	0.328368
Monocytes	TRIM59	-0.34765	4.368452	-1.97966	0.050853	-4.2032	0.193242	0.297531
Monocytes	PSEN1	0.153142	6.297037	1.979005	0.050927	-4.64022	0.183278	0.28216
Monocytes	GM14963	-0.84811	0.898614	-1.97766	0.051081	-3.84991	0.213864	0.32826
Monocytes	MVK	-0.53038	2.634356	-1.97741	0.05111	-3.89893	0.203609	0.312861
Monocytes	RPN1	-0.1885	5.957169	-1.97733	0.051119	-4.58307	0.185456	0.285224
Monocytes	GM32051	1.216021	-0.14913	1.977301	0.051123	-3.88516	0.220326	0.338018
Monocytes	PDPN	0.954087	-0.21403	1.976254	0.051243	-3.85329	0.221102	0.339127
Monocytes	MEAF6	-0.1853	4.941914	-1.97622	0.051247	-4.40783	0.191124	0.293836
Monocytes	NUDT15	-0.57868	1.647281	-1.97546	0.051335	-3.86697	0.209979	0.3224
Monocytes	FOSB	-0.24872	6.636378	-1.97534	0.051348	-4.82914	0.182498	0.280641
Monocytes	NSF	0.186331	6.770124	1.974965	0.051392	-4.76503	0.181905	0.279724
Monocytes	PRICKLE1	-0.77284	3.386121	-1.97347	0.051564	-3.89907	0.200603	0.307967
Monocytes	GCHFR	-0.67532	3.389908	-1.97308	0.05161	-3.96942	0.200682	0.308023
Monocytes	GM36862	-0.92601	0.340355	-1.97267	0.051658	-3.85941	0.218813	0.33542
Monocytes	DPH7	-0.80974	1.586984	-1.97266	0.051659	-3.85172	0.21121	0.324003
Monocytes	MAPKAPK2	0.176081	7.207807	1.970935	0.051859	-4.83207	0.180917	0.277545
Monocytes	9430015G10RII	-0.63376	1.911053	-1.97092	0.051861	-3.85845	0.209926	0.321629
Monocytes	DDX41	0.271866	4.356626	1.970786	0.051876	-4.18729	0.195935	0.300511
Monocytes	GM48855	0.561793	1.463573	1.970335	0.051929	-3.86922	0.212738	0.325863
Monocytes	COLEC10	-0.90809	0.557157	-1.96924	0.052057	-3.87016	0.218734	0.334725
Monocytes	DCUN1D3	0.290179	4.424811	1.968231	0.052175	-4.24027	0.196396	0.300977
Monocytes	SPTBN4	0.979243	-0.18469	1.968215	0.052177	-3.8956	0.22374	0.342127
Monocytes	WDR26	0.142653	7.53429	1.967398	0.052272	-4.82261	0.180198	0.276134
Monocytes	M6PR	-0.16321	6.293265	-1.96738	0.052274	-4.72717	0.186538	0.28584
Monocytes	PLEKHA1	0.275516	4.707996	1.967338	0.052279	-4.47538	0.195003	0.298738
Monocytes	POGLUT3	-0.75939	0.979034	-1.96666	0.052359	-3.86011	0.216894	0.33169
Monocytes	NDEL1	0.15587	6.927832	1.966047	0.052431	-4.83018	0.183658	0.281233
Monocytes	IRAK2	0.232762	6.796286	1.965531	0.052491	-4.87645	0.184457	0.282418
Monocytes	RAPGEF6	-0.15979	8.049527	-1.96541	0.052505	-4.85866	0.178138	0.27275
Monocytes	TMEM37	-0.45892	3.751452	-1.96467	0.052593	-4.11429	0.201143	0.30759
Monocytes	TRIM5	-0.41897	3.589282	-1.96426	0.052641	-4.04658	0.20217	0.309035
Monocytes	ZSWIM4	0.232598	4.579223	1.963419	0.05274	-4.44218	0.196874	0.30087
Monocytes	FASN	-0.41371	3.14635	-1.96335	0.052748	-4.06067	0.204976	0.313115
Monocytes	NLRC4	0.30762	3.885651	1.962784	0.052815	-4.27192	0.200928	0.306854
Monocytes	LZTS2	-0.849	1.249256	-1.96262	0.052834	-3.86574	0.216462	0.330221
Monocytes	ABHD16A	-0.22593	4.808556	-1.96245	0.052854	-4.3771	0.195784	0.299148
Monocytes	SP1	0.178855	5.924217	1.962135	0.052892	-4.50806	0.189824	0.290053
Monocytes	LCAT	-0.75171	2.143834	-1.96192	0.052918	-3.87417	0.211119	0.322235
Monocytes	PHF3	0.149181	6.980017	1.961772	0.052935	-4.72073	0.184312	0.2817

Monocytes	CDYL	0.174676	5.659184	1.961635	0.052951	-4.53206	0.191236	0.292252
Monocytes	ZRSR2	0.175884	5.063714	1.961294	0.052992	-4.41414	0.194528	0.297225
Monocytes	SLC12A9	0.261767	4.242634	1.960714	0.05306	-4.4025	0.199248	0.304353
Monocytes	MTPAP	0.293247	4.284578	1.959619	0.05319	-4.22533	0.199427	0.3044
Monocytes	RBM19	-0.43963	2.925866	-1.95944	0.053212	-3.97313	0.207214	0.316185
Monocytes	H2-EB1	-0.37866	6.754688	-1.95905	0.053258	-5.21257	0.1862	0.28427
Monocytes	SRMS	-1.24586	-0.72874	-1.95841	0.053335	-3.95872	0.230199	0.350263
Monocytes	ARHGAP9	0.220852	5.14536	1.958047	0.053378	-4.50418	0.19506	0.29763
Monocytes	DYNC2H1	0.379441	3.842041	1.957253	0.053472	-4.08399	0.202617	0.308926
Monocytes	SLC25A40	-0.44145	3.227708	-1.95576	0.053651	-3.99497	0.206767	0.314976
Monocytes	ATP11C	-0.15539	6.869185	-1.95536	0.053698	-4.71466	0.186794	0.28477
Monocytes	MPZL3	0.649163	1.727945	1.954856	0.053759	-3.91034	0.215972	0.328737
Monocytes	KLF9	0.389612	3.693914	1.954729	0.053774	-4.19849	0.204309	0.311243
Monocytes	SLC43A2	0.29213	6.179865	1.954235	0.053834	-4.58382	0.190688	0.290564
Monocytes	YPEL1	-0.58113	2.4444	-1.95393	0.05387	-3.88918	0.211803	0.322536
Monocytes	RAB44	0.449292	1.487929	1.953549	0.053916	-4.19032	0.217613	0.331365
Monocytes	HNRNPA0	-0.1395	7.214278	-1.95335	0.05394	-4.77044	0.185275	0.282587
Monocytes	IGFBP2	-0.80311	4.701389	-1.95316	0.053963	-4.24067	0.198757	0.303098
Monocytes	C4BP	-0.90051	1.968581	-1.95311	0.05397	-3.88009	0.214672	0.327044
Monocytes	PIPOX	-0.8159	2.011866	-1.95286	0.053999	-3.88233	0.214409	0.326762
Monocytes	CUL1	-0.14222	6.562305	-1.95259	0.054032	-4.7132	0.188672	0.287995
Monocytes	NUDT13	0.500669	2.358115	1.952469	0.054046	-3.94206	0.21232	0.32376
Monocytes	POLN	-0.68709	3.191009	-1.95233	0.054063	-3.9294	0.207386	0.316383
Monocytes	WDR43	-0.19359	6.033494	-1.95227	0.05407	-4.62098	0.191478	0.292314
Monocytes	RASL11A	-1.02507	0.28073	-1.95223	0.054076	-3.90008	0.225197	0.34304
Monocytes	ADAR	0.305792	4.311216	1.95222	0.054076	-4.29012	0.200948	0.306671
Monocytes	CHPT1	0.290765	3.607446	1.951727	0.054136	-4.199	0.205112	0.312877
Monocytes	SOX4	-0.65746	5.508952	-1.95157	0.054155	-4.13358	0.194447	0.296761
Monocytes	GET4	-0.22504	5.110437	-1.95137	0.054179	-4.40671	0.196644	0.300076
Monocytes	STK32C	-0.72632	0.553606	-1.95116	0.054204	-3.88417	0.223653	0.340624
Monocytes	GM43560	-0.74604	0.578606	-1.95091	0.054234	-3.89749	0.223537	0.340388
Monocytes	MAMDC2	-0.85913	0.366618	-1.94965	0.054387	-3.88372	0.225438	0.342941
Monocytes	GM47200	-1.06733	0.11613	-1.94804	0.054582	-3.9229	0.227778	0.345824
Monocytes	TSC22D3	0.298798	5.71665	1.947573	0.054639	-4.56373	0.194605	0.296133
Monocytes	MSRB2	-0.93995	1.023579	-1.94743	0.054656	-3.89477	0.222121	0.337519
Monocytes	NDUFB3	0.185422	5.72376	1.946108	0.054818	-4.58	0.19507	0.296594
Monocytes	KBTBD8	0.909328	0.251382	1.945577	0.054882	-3.90807	0.227695	0.345566
Monocytes	ADK	-0.25606	6.968234	-1.94537	0.054908	-4.75655	0.188461	0.286727
Monocytes	GM15564	-0.66667	2.474453	-1.94531	0.054915	-3.92299	0.213793	0.324949
Monocytes	ACSL1	0.329607	5.758511	1.945193	0.054929	-4.47956	0.194934	0.296607
Monocytes	MEF2C	0.222912	7.538963	1.945086	0.054942	-4.86177	0.185491	0.282291
Monocytes	TAF1D	-0.17353	6.027224	-1.94487	0.054969	-4.6001	0.193475	0.294513
Monocytes	CAMK4	-0.54705	3.194525	-1.94456	0.055007	-4.21875	0.209491	0.318834
Monocytes	KIF9	0.564419	1.680578	1.944485	0.055016	-3.9365	0.218648	0.332578
Monocytes	GM5577	0.838917	0.20371	1.944305	0.055038	-3.89383	0.228004	0.34656
Monocytes	RIN2	0.258977	3.687666	1.944044	0.05507	-4.46481	0.2066	0.314569
Monocytes	GINS3	0.631012	2.330816	1.943995	0.055076	-3.90751	0.214662	0.326671

Monocytes	CMTR1	-0.21852	5.166737	-1.94387	0.055091	-4.69172	0.198191	0.301881
Monocytes	TONSL	-0.78377	1.456718	-1.94383	0.055096	-3.89211	0.220039	0.334706
Monocytes	H1F0	0.255938	6.001224	1.943493	0.055137	-4.68205	0.193691	0.29499
Monocytes	THOP1	-0.70338	2.236401	-1.94302	0.055196	-3.89937	0.215469	0.327742
Monocytes	TIFA	0.328323	6.262161	1.942377	0.055274	-4.53398	0.192519	0.293268
Monocytes	TPD52L2	0.176917	5.433659	1.942062	0.055313	-4.48938	0.197031	0.300263
Monocytes	PTTG1IP	-0.19154	4.790183	-1.9419	0.055334	-4.44695	0.200618	0.305715
Monocytes	GM29264	-0.94097	0.505466	-1.94189	0.055335	-3.92378	0.226421	0.344404
Monocytes	MAPKAPK5	0.491337	2.128701	1.941823	0.055343	-3.92863	0.21624	0.329215
Monocytes	XKR6	-0.87129	0.862903	-1.94179	0.055346	-3.90053	0.224135	0.341004
Monocytes	HIST1H2BN	-0.85747	1.240034	-1.94158	0.055372	-3.89863	0.221772	0.33752
Monocytes	ANTXR2	-0.30136	7.654982	-1.94119	0.055421	-5.15717	0.185305	0.282468
Monocytes	GM43707	-1.02995	0.407926	-1.94095	0.05545	-3.92561	0.227224	0.345569
Monocytes	GORAB	0.550345	1.673364	1.94068	0.055483	-3.93153	0.219265	0.333631
Monocytes	BRAF	0.196646	7.588384	1.940209	0.055541	-4.92432	0.185848	0.283039
Monocytes	RXRB	0.260988	4.393184	1.939582	0.055619	-4.31513	0.203418	0.309544
Monocytes	DMC1	-0.90463	0.152078	-1.93849	0.055753	-3.92002	0.229714	0.348615
Monocytes	DHX32	0.400682	3.380004	1.938279	0.05578	-4.00227	0.209654	0.318713
Monocytes	PKDCC	-0.49426	1.303453	-1.93821	0.055788	-4.01603	0.222328	0.337669
Monocytes	C3	0.363347	6.327835	1.938167	0.055794	-4.75837	0.193013	0.293612
Monocytes	1810006J02RIK	-0.91529	-0.01116	-1.93777	0.055843	-3.91164	0.230901	0.350342
Monocytes	DGKZ	0.217305	6.2774	1.937062	0.055931	-4.67491	0.193618	0.294473
Monocytes	C130026I21RIK	-0.39085	2.944263	-1.93667	0.05598	-4.20429	0.21271	0.32326
Monocytes	GM4673	0.512028	2.578427	1.936526	0.055997	-3.93413	0.214918	0.326627
Monocytes	GM8369	0.657251	3.324129	1.935153	0.056168	-3.9941	0.211008	0.320291
Monocytes	TUBA1C	-0.22482	7.576145	-1.93463	0.056233	-4.90939	0.187475	0.28471
Monocytes	FBXL2	0.388471	3.231347	1.934348	0.056268	-4.09428	0.211749	0.321426
Monocytes	NCOR1	-0.11988	8.098707	-1.93426	0.056279	-4.92245	0.184791	0.280725
Monocytes	PHYHD1	-0.63126	2.730001	-1.93395	0.056318	-3.9236	0.214826	0.326053
Monocytes	DAXX	0.310245	4.352332	1.933804	0.056336	-4.3126	0.20523	0.311722
Monocytes	PTPRJ	-0.15677	8.855581	-1.93359	0.056362	-5.14116	0.181025	0.275061
Monocytes	ALYREF	-0.19551	8.216984	-1.93286	0.056455	-4.99934	0.18449	0.280225
Monocytes	PHTF2	-0.22286	5.998759	-1.93217	0.056541	-4.58336	0.196437	0.29826
Monocytes	GM13889	-0.83622	1.14195	-1.93198	0.056564	-3.90995	0.225231	0.341415
Monocytes	TBC1D8B	0.378372	3.257326	1.931896	0.056575	-4.06523	0.212153	0.321912
Monocytes	ITGB2L	0.989235	-0.61932	1.931634	0.056608	-3.96874	0.23679	0.35857
Monocytes	ACAA1B	-0.921	3.318751	-1.93159	0.056613	-4.02598	0.211786	0.321405
Monocytes	ITPRID2	0.18003	5.08881	1.931297	0.05665	-4.71126	0.201565	0.306045
Monocytes	MICAL2	-0.49843	1.966497	-1.93097	0.056691	-4.08696	0.220143	0.333949
Monocytes	QRSL1	0.45028	3.18623	1.930736	0.05672	-4.03401	0.212687	0.322832
Monocytes	NEURL1A	-1.20808	-0.39095	-1.93071	0.056724	-3.96172	0.235376	0.356571
Monocytes	SLC18A1	-0.86519	0.497721	-1.93014	0.056795	-3.91177	0.229708	0.348078
Monocytes	ARHGEF40	-0.57636	0.550928	-1.92987	0.056829	-3.99221	0.229418	0.347665
Monocytes	RABGAP1	0.20182	5.920481	1.929542	0.05687	-4.56837	0.197264	0.29949
Monocytes	ROCK1	-0.14408	8.134703	-1.92903	0.056935	-4.95539	0.1855	0.281787
Monocytes	PSMB1	-0.15059	7.299863	-1.92902	0.056936	-4.85701	0.189853	0.288411
Monocytes	TSPAN17	-0.5564	1.115501	-1.92878	0.056966	-3.94795	0.225894	0.342644

Monocytes	PBX2	0.27347	4.930622	1.928697	0.056977	-4.38276	0.202845	0.30818
Monocytes	C1GALT1C1	0.212554	4.044817	1.928357	0.05702	-4.52209	0.207956	0.315979
Monocytes	TMEM108	-0.94169	5.137851	-1.92835	0.057021	-3.97707	0.20167	0.306509
Monocytes	CRYZL1	0.236612	4.570122	1.928317	0.057025	-4.38709	0.204908	0.311392
Monocytes	CATSPERD	-0.90553	1.329786	-1.92783	0.057087	-3.91363	0.224689	0.340883
Monocytes	NEBL	-1.1549	1.182115	-1.92757	0.057119	-3.91633	0.225638	0.342284
Monocytes	ARMC1	0.210851	4.827556	1.927488	0.057129	-4.40371	0.203586	0.309284
Monocytes	SLC48A1	-0.21045	5.074284	-1.92657	0.057246	-4.47378	0.202511	0.307468
Monocytes	GM11476	0.425012	2.536389	1.926362	0.057272	-4.09874	0.217506	0.329977
Monocytes	ZFP277	0.169937	5.309649	1.926265	0.057284	-4.60089	0.201179	0.305484
Monocytes	LPP	-0.22252	7.6885	-1.92587	0.057333	-4.88597	0.188318	0.28601
Monocytes	SPDEF	-1.08352	-0.4296	-1.9258	0.057343	-3.94219	0.236667	0.358479
Monocytes	CD244A	0.290166	3.429777	1.925544	0.057375	-4.46265	0.2122	0.322109
Monocytes	ARHGAP45	0.186777	6.710119	1.925327	0.057403	-4.77156	0.193579	0.294033
Monocytes	AMZ1	-0.30025	3.157688	-1.92507	0.057435	-4.51068	0.213905	0.324725
Monocytes	FAM89A	-0.60163	0.879902	-1.92484	0.057464	-3.92919	0.228174	0.346012
Monocytes	OSBPL11	0.248753	5.368512	1.924139	0.057554	-4.4753	0.201197	0.305617
Monocytes	TCEAL9	-0.25509	5.693961	-1.924	0.057571	-4.49216	0.199372	0.30286
Monocytes	NLN	0.277365	4.446951	1.923897	0.057585	-4.35903	0.206466	0.313559
Monocytes	RNF135	-0.56273	2.08421	-1.92385	0.05759	-3.95302	0.220687	0.334865
Monocytes	SLAMF8	-0.45332	0.718707	-1.92347	0.057639	-4.32519	0.229506	0.347957
Monocytes	LYPD6B	0.855561	0.004013	1.923147	0.05768	-3.92468	0.234297	0.354972
Monocytes	RHOF	-0.38492	3.558194	-1.9222	0.057801	-4.20701	0.21224	0.321868
Monocytes	XRCC4	0.218623	5.185209	1.920934	0.057962	-4.49476	0.203175	0.308114
Monocytes	SARAF	-0.20018	5.365255	-1.92092	0.057964	-4.57693	0.202153	0.306573
Monocytes	PLPP1	-0.32452	4.327383	-1.92061	0.058003	-4.48748	0.208126	0.315619
Monocytes	GNAS	-0.13619	8.931692	-1.92047	0.058022	-5.07629	0.183046	0.277675
Monocytes	CTH	0.691371	3.512521	1.920451	0.058024	-4.10419	0.212953	0.322884
Monocytes	FFAR1	-1.00153	-0.22509	-1.91974	0.058116	-3.99077	0.236923	0.358353
Monocytes	GNB1	-0.10053	8.848488	-1.91961	0.058131	-5.1056	0.18362	0.278436
Monocytes	CDK19	0.215951	6.390727	1.919589	0.058135	-4.64634	0.196601	0.298149
Monocytes	RUSC1	-0.29244	3.288288	-1.91924	0.058179	-4.33188	0.214567	0.325089
Monocytes	L3MBTL3	0.308961	5.067059	1.918956	0.058216	-4.33228	0.20415	0.309487
Monocytes	ARHGAP28	-0.54048	0.598833	-1.91874	0.058244	-4.12114	0.231589	0.350499
Monocytes	GSTP3	-0.5904	3.39573	-1.91865	0.058255	-4.07744	0.21397	0.324285
Monocytes	BC048403	0.564039	1.255612	1.918437	0.058283	-3.93045	0.227339	0.344227
Monocytes	AKR1C12	-0.90801	0.756271	-1.91814	0.05832	-3.94601	0.230601	0.349118
Monocytes	TCEANC2	0.246117	4.247598	1.91792	0.058349	-4.30406	0.208941	0.316875
Monocytes	DYNC1H1	-0.14704	6.255557	-1.91791	0.05835	-4.71093	0.19751	0.299648
Monocytes	SORBS2	-1.04088	1.878636	-1.91752	0.0584	-3.92738	0.223495	0.33863
Monocytes	CCDC125	0.367376	4.497843	1.916476	0.058535	-4.24852	0.207988	0.315096
Monocytes	HIKESHI	0.208275	5.228907	1.916133	0.058579	-4.49736	0.203847	0.308873
Monocytes	ELMOD3	-0.56579	3.437093	-1.91563	0.058644	-4.08179	0.214431	0.324793
Monocytes	PALLD	-0.43398	3.882236	-1.91561	0.058646	-4.20503	0.211759	0.320794
Monocytes	TLR1	0.664081	1.219121	1.915529	0.058657	-3.97577	0.228305	0.345457
Monocytes	SPTLC2	0.144148	6.245992	1.915309	0.058686	-4.74395	0.198207	0.300437
Monocytes	ALMS1	-0.30525	3.824261	-1.91469	0.058766	-4.31454	0.212288	0.321577

Monocytes	RCN1	-0.55061	3.060814	-1.91466	0.05877	-3.96511	0.216904	0.328482
Monocytes	CLNS1A	-0.17855	5.340846	-1.91357	0.058911	-4.54323	0.203849	0.308653
Monocytes	FAM189B	-0.63245	2.569109	-1.91341	0.058931	-3.96764	0.220381	0.33344
Monocytes	IRAK4	0.214387	4.419459	1.911673	0.059157	-4.48261	0.209841	0.317246
Monocytes	GAN	0.259087	4.031697	1.91161	0.059165	-4.45918	0.212141	0.320714
Monocytes	AKAP1	-0.73074	1.799595	-1.91151	0.059178	-3.93545	0.225934	0.341292
Monocytes	RHOBTB1	-0.44494	3.180961	-1.91089	0.059259	-4.21274	0.217357	0.328627
Monocytes	PIGL	0.392476	2.746364	1.910817	0.059269	-4.02903	0.220038	0.332625
Monocytes	SLC23A3	-0.90038	1.461516	-1.91081	0.059269	-3.94865	0.228182	0.344723
Monocytes	RNMT	0.223701	4.970618	1.910719	0.059281	-4.43883	0.206687	0.312704
Monocytes	ANGPTL2	-0.94562	0.84131	-1.91034	0.059331	-3.93757	0.232343	0.350929
Monocytes	GM16599	0.265529	4.782001	1.909883	0.05939	-4.36623	0.208019	0.314713
Monocytes	GCKR	-0.87353	1.581851	-1.90969	0.059415	-3.93796	0.227677	0.344042
Monocytes	HINT3	-0.21217	4.643869	-1.90906	0.059498	-4.55853	0.209058	0.316107
Monocytes	OSER1	-0.19187	6.59749	-1.90884	0.059527	-4.70336	0.197961	0.299455
Monocytes	UQCRQ	0.17557	7.858453	1.908539	0.059566	-4.98621	0.191192	0.289199
Monocytes	SERPINB2	1.201789	0.991964	1.908229	0.059607	-4.17458	0.23193	0.350218
Monocytes	EGFL6	-1.1944	0.548891	-1.90808	0.059627	-3.99607	0.234865	0.354629
Monocytes	NDUFAF6	-0.36646	2.973661	-1.90748	0.059705	-4.11935	0.219496	0.331767
Monocytes	SLC15A4	0.255594	4.804844	1.906955	0.059774	-4.45585	0.208637	0.315429
Monocytes	POU6F1	-0.64689	2.610644	-1.90673	0.059804	-3.95545	0.221965	0.335417
Monocytes	ANKRD17	-0.146	7.985576	-1.90565	0.059945	-4.97518	0.191324	0.289187
Monocytes	EIF2AK4	0.263964	4.653928	1.905344	0.059985	-4.44727	0.209987	0.317475
Monocytes	A3GALT2	-0.34595	2.675415	-1.90531	0.059991	-4.30972	0.222018	0.335446
Monocytes	TRMT10C	-0.2344	4.996252	-1.90492	0.060041	-4.41489	0.208061	0.314575
Monocytes	CDKL5	-0.69428	2.066071	-1.90481	0.060056	-3.97845	0.225963	0.341314
Monocytes	CAPN2	0.197979	4.14309	1.903928	0.060172	-4.56543	0.213375	0.32238
Monocytes	CTU2	0.364843	3.206943	1.90391	0.060174	-4.12672	0.219075	0.330889
Monocytes	CHIC2	0.130106	7.043743	1.90338	0.060244	-4.83332	0.196897	0.297509
Monocytes	ADH1	-0.70104	3.503888	-1.90271	0.060333	-4.10722	0.217637	0.3286
Monocytes	CHERP	-0.20234	5.016827	-1.90261	0.060346	-4.47406	0.208576	0.315085
Monocytes	CBR2	1.422261	-1.3198	1.901931	0.060436	-4.00529	0.249774	0.376069
Monocytes	EIF2S2	-0.14377	7.925804	-1.90186	0.060445	-4.98069	0.192497	0.290815
Monocytes	SPATS2L	-1.09938	0.9742	-1.90129	0.060521	-3.96188	0.234205	0.353067
Monocytes	NRXN2	-0.98112	2.095803	-1.9006	0.060613	-3.95597	0.227107	0.342415
Monocytes	DPM3	0.1666	6.309203	1.900423	0.060636	-4.74397	0.20174	0.304585
Monocytes	NAALADL2	-0.67589	1.964286	-1.90036	0.060645	-3.95787	0.227954	0.343737
Monocytes	PCID2	0.20154	4.736836	1.899679	0.060735	-4.47284	0.21102	0.318554
Monocytes	ITM2A	-0.68111	2.544803	-1.8996	0.060745	-3.98264	0.224457	0.338627
Monocytes	ARL5A	0.178367	5.831093	1.898477	0.060895	-4.73218	0.205082	0.309469
Monocytes	VIRMA	0.207399	5.71037	1.898014	0.060957	-4.60124	0.205912	0.310639
Monocytes	GM16541	0.395684	2.915994	1.897528	0.061022	-4.12522	0.222894	0.335934
Monocytes	CASP1	0.288988	3.51676	1.89715	0.061072	-4.42441	0.219255	0.330457
Monocytes	CUL3	0.126658	7.198802	1.896012	0.061225	-4.86585	0.198141	0.298605
Monocytes	MGST1	0.275147	7.132587	1.895873	0.061243	-4.97476	0.198507	0.299156
Monocytes	ZFP367	-0.28036	5.436634	-1.89581	0.061251	-4.588	0.208134	0.313619
Monocytes	ZFP709	0.501307	1.431477	1.895543	0.061287	-3.97778	0.233054	0.350629

Monocytes	GM3336	-0.63239	1.732452	-1.89512	0.061344	-4.05515	0.231208	0.347845
Monocytes	GM16104	-1.22308	-0.60575	-1.89462	0.061411	-3.99633	0.247247	0.37141
Monocytes	GKAP1	-0.36074	3.541031	-1.89449	0.061428	-4.2314	0.219843	0.331046
Monocytes	TIGD2	0.334817	3.85213	1.893256	0.061595	-4.16694	0.21837	0.328535
Monocytes	SPATA2L	0.944101	0.521707	1.893244	0.061596	-3.96609	0.239933	0.360411
Monocytes	CCNQ	0.401782	3.060749	1.892861	0.061648	-4.08825	0.223403	0.336004
Monocytes	CSPG5	-1.27378	0.28413	-1.89261	0.061682	-3.99403	0.241706	0.362989
Monocytes	ALDOB	-0.5732	4.949636	-1.89249	0.061698	-4.4085	0.211869	0.318853
Monocytes	SMAP2	0.155906	6.821529	1.892147	0.061744	-4.79595	0.201143	0.302709
Monocytes	LY6A	0.837614	4.339309	1.891497	0.061832	-4.38014	0.215846	0.324432
Monocytes	MRPS17	0.200096	5.224333	1.890541	0.061961	-4.55649	0.210918	0.317031
Monocytes	GPR84	0.776892	0.260814	1.890147	0.062014	-3.97519	0.242716	0.364071
Monocytes	SPATA6	0.234766	4.52297	1.890065	0.062025	-4.56887	0.215174	0.323381
Monocytes	UCHL1	-1.18258	0.862756	-1.88992	0.062045	-4.01045	0.238602	0.358036
Monocytes	ATP6V0E	-0.12684	7.713213	-1.88961	0.062087	-5.05364	0.196842	0.295981
Monocytes	4931428F04RIK	-0.93101	0.65018	-1.88955	0.062095	-3.98177	0.240066	0.360256
Monocytes	ITPRIP	0.294803	3.468077	1.889409	0.062114	-4.34652	0.221674	0.333173
Monocytes	F9	-0.89449	0.896192	-1.88895	0.062177	-3.96655	0.238499	0.358018
Monocytes	TINF2	-0.29151	4.054948	-1.88889	0.062184	-4.35749	0.218136	0.327909
Monocytes	SLC2A9	0.426801	2.879571	1.888527	0.062234	-4.18359	0.225572	0.338876
Monocytes	ABI3	0.399629	4.401261	1.888389	0.062253	-4.37249	0.21611	0.324883
Monocytes	SPIC	-0.67744	1.863637	-1.88551	0.062645	-4.15625	0.233423	0.349787
Monocytes	ZFP738	0.370925	2.843416	1.885367	0.062664	-4.11081	0.227047	0.340394
Monocytes	AOAH	0.375235	4.784961	1.885307	0.062673	-4.71748	0.214972	0.322509
Monocytes	CDR2	-0.68398	2.488604	-1.88523	0.062683	-3.99113	0.229334	0.343826
Monocytes	MACROD2	0.510942	2.251559	1.884597	0.06277	-4.11928	0.231104	0.346325
Monocytes	CIB2	0.437472	2.152566	1.884379	0.062799	-4.10307	0.231752	0.347328
Monocytes	AKR1C14	-0.87953	0.911524	-1.8843	0.06281	-3.97685	0.240044	0.359518
Monocytes	CARNS1	-0.81852	2.476067	-1.88298	0.062991	-3.97802	0.230223	0.344744
Monocytes	PTPN14	-1.15652	0.524362	-1.8826	0.063044	-4.01372	0.243431	0.364158
Monocytes	TRIM24	-0.25332	5.087563	-1.88191	0.063137	-4.44139	0.214188	0.321024
Monocytes	TUBB4B	-0.30837	6.422022	-1.8819	0.063139	-4.72013	0.20634	0.309316
Monocytes	MPZL1	-0.62479	2.78686	-1.88167	0.063171	-4.0736	0.22855	0.342367
Monocytes	TOMM20	-0.15887	6.725117	-1.88041	0.063344	-4.8435	0.205087	0.307291
Monocytes	TMEM177	-1.02605	0.336385	-1.88034	0.063354	-3.99889	0.245515	0.367093
Monocytes	KREMEN1	0.422227	3.039966	1.879956	0.063407	-4.07112	0.227535	0.340618
Monocytes	TRDV2-2	-0.7529	-1.43025	-1.87914	0.06352	-4.02763	0.258677	0.385958
Monocytes	NF1	-0.16333	6.550388	-1.87879	0.063568	-4.84592	0.206572	0.309248
Monocytes	ZCCHC4	-0.29973	3.455439	-1.8785	0.063608	-4.37064	0.22531	0.337149
Monocytes	GM15247	0.772266	0.995367	1.878316	0.063634	-4.00861	0.24154	0.361072
Monocytes	VPS54	-0.1866	6.506686	-1.8783	0.063637	-4.81145	0.206833	0.309739
Monocytes	HKDC1	-0.7842	0.37121	-1.87809	0.063665	-4.01444	0.245885	0.367443
Monocytes	SLC25A21	-1.19227	1.867089	-1.87789	0.063692	-3.98013	0.235691	0.352546
Monocytes	TCOF1	-0.19872	5.717375	-1.87758	0.063736	-4.79364	0.211556	0.316869
Monocytes	COLGALT1	-0.18736	5.889717	-1.87722	0.063786	-4.69087	0.210587	0.31543
Monocytes	HEATR1	0.258206	4.84445	1.877157	0.063794	-4.53542	0.216843	0.324767
Monocytes	E2F7	-0.59752	2.983824	-1.87675	0.06385	-4.08824	0.228612	0.342237

Monocytes	LSM5	0.204772	5.652829	1.876613	0.06387	-4.69046	0.212092	0.317783
Monocytes	SFI1	-0.20041	5.320089	-1.87614	0.063935	-4.71922	0.214222	0.320825
Monocytes	PDE6H	0.575594	2.093346	1.875922	0.063966	-4.0046	0.234627	0.351046
Monocytes	NFX1	0.20956	4.900546	1.875706	0.063996	-4.49478	0.216813	0.324762
Monocytes	1110059E24RIK	0.30263	4.393524	1.874594	0.06415	-4.28291	0.220366	0.329951
Monocytes	ACBD4	0.688919	1.764413	1.87446	0.064169	-3.98668	0.23733	0.355017
Monocytes	9530082P21RIK	0.754357	0.699259	1.873638	0.064284	-3.98632	0.244906	0.366101
Monocytes	SLC6A6	-0.1523	7.287991	-1.87358	0.064292	-5.04455	0.203482	0.304787
Monocytes	GM12802	0.713925	0.658153	1.8731	0.064359	-3.98778	0.245363	0.366738
Monocytes	CXCL2	0.542794	7.158496	1.872859	0.064392	-5.17327	0.204396	0.306138
Monocytes	RWDD2B	0.635765	1.849393	1.872549	0.064436	-4.00976	0.237321	0.355018
Monocytes	RGS12	-0.5038	2.240588	-1.87242	0.064453	-4.04542	0.234709	0.351211
Monocytes	HUWE1	-0.16469	7.155414	-1.87215	0.064491	-4.94091	0.204518	0.306398
Monocytes	OAS1C	0.73913	1.903745	1.871993	0.064514	-3.99993	0.237015	0.354671
Monocytes	NBEAL2	-0.34798	2.635242	-1.87127	0.064616	-4.16676	0.232408	0.347643
Monocytes	HSPA4L	0.286678	4.348791	1.871192	0.064626	-4.42273	0.221455	0.331455
Monocytes	SPDL1	-0.49223	2.833783	-1.87072	0.064692	-4.11012	0.231265	0.345968
Monocytes	EPHX2	-0.75377	2.477792	-1.86984	0.064816	-4.02722	0.233839	0.349749
Monocytes	PABPC1	-0.17719	10.47512	-1.86983	0.064817	-5.41582	0.187082	0.279965
Monocytes	CDC37	-0.13444	6.605127	-1.86978	0.064824	-4.86398	0.208249	0.311792
Monocytes	KPNA2	0.272971	4.937072	1.86962	0.064846	-4.61211	0.218198	0.326613
Monocytes	GM28694	-1.06971	-0.23311	-1.86946	0.064869	-4.02602	0.25253	0.377144
Monocytes	UNC45B	1.147058	0.423376	1.868944	0.064941	-4.00976	0.247998	0.370527
Monocytes	SAP130	0.197449	5.619105	1.868886	0.06495	-4.6776	0.21419	0.320678
Monocytes	ZFP704	-0.43698	3.792086	-1.8686	0.064989	-4.31144	0.225521	0.337441
Monocytes	AP3D1	-0.16292	4.873786	-1.86838	0.065021	-4.57648	0.218785	0.327454
Monocytes	NUB1	-0.17594	5.534309	-1.86826	0.065038	-4.64727	0.214773	0.321493
Monocytes	MAP3K4	-0.26549	4.074187	-1.86778	0.065105	-4.37937	0.22391	0.334873
Monocytes	VPS36	0.175806	5.315956	1.867018	0.065213	-4.64413	0.216504	0.323781
Monocytes	MIOS	0.332063	3.665658	1.866902	0.065229	-4.1876	0.226776	0.339028
Monocytes	CTNND1	-0.28531	4.294756	-1.86592	0.065369	-4.50008	0.223198	0.333484
Monocytes	CXXC1	-0.26881	4.307295	-1.86516	0.065476	-4.3703	0.223199	0.33364
Monocytes	CD300E	-0.80045	0.628868	-1.86498	0.065501	-4.12198	0.247644	0.369615
Monocytes	OSBP	0.209076	5.016429	1.864934	0.065508	-4.57291	0.2188	0.327159
Monocytes	GM41790	-1.12493	-0.09939	-1.86489	0.065515	-4.0123	0.252823	0.377188
Monocytes	TST	-0.64646	3.470042	-1.86482	0.065524	-4.15347	0.22852	0.341569
Monocytes	AMPD3	0.390333	2.578161	1.86481	0.065526	-4.28446	0.234343	0.350151
Monocytes	SOD1	-0.17416	5.993562	-1.86397	0.065645	-4.88542	0.213011	0.318599
Monocytes	RSAD1	-0.76069	1.298807	-1.8639	0.065654	-4.02173	0.243115	0.363058
Monocytes	NF2	0.243953	4.781261	1.863801	0.065669	-4.48452	0.220368	0.329536
Monocytes	NLRX1	0.51279	1.80919	1.863781	0.065672	-4.11492	0.239625	0.357945
Monocytes	VPS33A	0.232329	4.291318	1.863775	0.065672	-4.44682	0.223421	0.334061
Monocytes	TAP1	-0.37848	5.446269	-1.86235	0.065875	-4.79777	0.21689	0.324068
Monocytes	SUSD1	-0.38821	5.183728	-1.86186	0.065946	-4.28133	0.218653	0.326568
Monocytes	LANCL1	-0.32647	3.448045	-1.86148	0.065999	-4.15826	0.229692	0.342821
Monocytes	NSMCE1	-0.25379	4.829662	-1.86035	0.06616	-4.48675	0.221402	0.330275
Monocytes	SSB	-0.13422	6.803522	-1.86012	0.066194	-4.87959	0.209547	0.312651

Monocytes	GM31812	0.959878	0.191942	1.857762	0.066531	-4.01396	0.253567	0.376789
Monocytes	FCHSD2	-0.26152	6.823892	-1.85774	0.066535	-4.91969	0.210366	0.313312
Monocytes	ADAM30	-0.56237	0.62147	-1.85629	0.066743	-4.0813	0.251191	0.373089
Monocytes	PLBD1	-0.19616	5.389651	-1.85569	0.06683	-5.17199	0.219788	0.326954
Monocytes	PDIK1L	0.426578	3.065249	1.855441	0.066865	-4.12158	0.234635	0.348901
Monocytes	IFI203	0.276161	6.302394	1.855339	0.06688	-4.84566	0.214255	0.318837
Monocytes	SPARCL1	1.406878	0.3267	1.854976	0.066932	-4.01517	0.253553	0.376578
Monocytes	CYB5A	-0.29879	7.336341	-1.85494	0.066937	-5.03053	0.208168	0.309857
Monocytes	ASAH1	0.156281	6.301867	1.854895	0.066944	-4.98684	0.214258	0.318925
Monocytes	GM10143	0.720272	1.245831	1.853476	0.067149	-4.01214	0.247653	0.36773
Monocytes	GM39556	0.549684	3.384757	1.853402	0.06716	-4.12741	0.233123	0.346495
Monocytes	KMT2D	0.169852	5.945956	1.852429	0.067301	-4.71143	0.217289	0.322961
Monocytes	CXCR4	0.226834	6.146916	1.852366	0.06731	-4.92737	0.216072	0.321159
Monocytes	SMU1	-0.16912	5.608875	-1.85198	0.067366	-4.683	0.219456	0.326106
Monocytes	SLBP	-0.18946	7.147492	-1.85128	0.067467	-4.8975	0.21041	0.312622
Monocytes	WAS	0.167531	5.349391	1.851184	0.067482	-4.70034	0.221246	0.32867
Monocytes	GM16618	0.835465	0.733543	1.851114	0.067492	-4.01995	0.252011	0.3737
Monocytes	OCIAD1	-0.15481	5.885315	-1.85026	0.067617	-4.75446	0.218282	0.324158
Monocytes	ZFP266	0.273509	4.029092	1.849818	0.06768	-4.28455	0.230013	0.341495
Monocytes	CTSC	0.25275	7.322442	1.84981	0.067682	-5.18625	0.209765	0.311578
Monocytes	MPRIP	-0.20908	5.481388	-1.84938	0.067744	-4.69331	0.220862	0.328013
Monocytes	FAM76A	0.196308	5.119697	1.849365	0.067747	-4.61732	0.223111	0.331333
Monocytes	GNL3	-0.23568	5.420028	-1.84911	0.067783	-4.61994	0.221242	0.328639
Monocytes	RHOD	-0.83378	1.328394	-1.84911	0.067783	-4.01746	0.248281	0.368263
Monocytes	ABL2	-0.21909	6.21523	-1.84878	0.067832	-4.77712	0.216457	0.321579
Monocytes	ELOVL7	0.963001	-0.19718	1.848549	0.067865	-4.02509	0.259413	0.384418
Monocytes	EPB41L4B	-0.8596	1.976143	-1.84812	0.067929	-4.01755	0.244029	0.361951
Monocytes	EFNA5	1.159376	2.313548	1.847992	0.067947	-4.01812	0.241711	0.35862
Monocytes	4933412E12RIK	0.704341	1.908564	1.846958	0.068098	-4.03643	0.244958	0.363176
Monocytes	CD9	-0.22556	6.033746	-1.8464	0.068179	-5.06057	0.218252	0.323996
Monocytes	NCALD	-0.78184	2.377149	-1.84624	0.068203	-4.02275	0.24188	0.358737
Monocytes	C1QBP	-0.22592	6.055545	-1.84621	0.068208	-4.78561	0.218119	0.323833
Monocytes	GM11342	-1.08201	2.120734	-1.8458	0.068268	-4.03074	0.243773	0.361478
Monocytes	SPATA2	-0.29646	4.217056	-1.84537	0.068331	-4.43505	0.229916	0.341102
Monocytes	FAM217B	0.578565	0.616789	1.844698	0.06843	-4.08526	0.25482	0.377261
Monocytes	GRK4	-0.43752	3.108893	-1.84423	0.068498	-4.14997	0.237624	0.352136
Monocytes	ZFP110	0.311448	4.039873	1.844048	0.068525	-4.31651	0.231488	0.34321
Monocytes	CPNE3	0.199598	5.367342	1.842933	0.06869	-4.77809	0.223482	0.331192
Monocytes	PRXL2C	0.231327	4.932963	1.841943	0.068836	-4.52599	0.226588	0.335684
Monocytes	TPCN1	-0.23772	4.187411	-1.84186	0.068848	-4.73381	0.231382	0.342742
Monocytes	SNRPA	-0.18716	5.131803	-1.84169	0.068873	-4.61453	0.225335	0.333915
Monocytes	TMEM132E	-1.24567	0.984501	-1.84141	0.068914	-4.05237	0.253299	0.374846
Monocytes	SLC10A7	0.184127	5.908741	1.841384	0.068919	-4.74055	0.220487	0.326855
Monocytes	SORD	-0.42184	4.176195	-1.84117	0.068951	-4.32616	0.231493	0.343087
Monocytes	UBR7	0.28272	4.241439	1.840413	0.069062	-4.41169	0.231366	0.342656
Monocytes	MFSD4A	0.713236	2.041521	1.840012	0.069122	-4.03376	0.246304	0.364297
Monocytes	BMX	0.540876	0.02253	1.839856	0.069145	-4.18928	0.260821	0.385399

Monocytes	BRAP	0.207706	4.924367	1.839272	0.069232	-4.57321	0.2273	0.336398
Monocytes	GM16124	-0.40202	2.669782	-1.83875	0.069309	-4.30739	0.242382	0.358437
Monocytes	C230066G23RII	0.865765	0.025289	1.838537	0.069341	-4.02957	0.261279	0.385773
Monocytes	RAB28	0.253793	4.828065	1.838266	0.069381	-4.49329	0.22818	0.337612
Monocytes	PDHX	0.305574	3.395129	1.837865	0.069441	-4.23743	0.237688	0.351527
Monocytes	FANCD2	-0.51031	2.80256	-1.83704	0.069563	-4.10814	0.242039	0.357577
Monocytes	SELENOM	0.477645	2.384919	1.836479	0.069647	-4.23269	0.245126	0.361938
Monocytes	ERLEC1	0.284775	4.341715	1.836184	0.069691	-4.40611	0.232043	0.342868
Monocytes	LTO1	-0.32555	3.526077	-1.83577	0.069754	-4.2793	0.237564	0.350886
Monocytes	ARRDC3	0.373905	3.403751	1.834639	0.069922	-4.34803	0.238881	0.352419
Monocytes	GM15675	-0.86696	2.317552	-1.83405	0.07001	-4.03528	0.246497	0.36336
Monocytes	AFM	-0.6764	2.294567	-1.83399	0.070019	-4.07532	0.246657	0.363592
Monocytes	PTPN4	-0.23467	5.463527	-1.83374	0.070057	-4.67947	0.22566	0.332931
Monocytes	NEK10	-0.76091	1.324395	-1.83326	0.070129	-4.05863	0.253754	0.373705
Monocytes	GJB1	-0.90896	1.59761	-1.83285	0.070191	-4.03682	0.251935	0.371009
Monocytes	HSD3B3	-0.84882	2.014179	-1.83221	0.070287	-4.05288	0.249198	0.366905
Monocytes	HINT2	-0.25166	4.356845	-1.83206	0.070309	-4.48758	0.233268	0.343805
Monocytes	TNFRSF10B	0.986946	-0.0063	1.831654	0.07037	-4.05592	0.263897	0.388345
Monocytes	MLLT3	-0.23741	5.728512	-1.83164	0.070373	-4.84432	0.224467	0.331093
Monocytes	LSM10	0.338556	3.07773	1.831511	0.070392	-4.30193	0.241822	0.356503
Monocytes	WIPI1	0.613998	2.277802	1.831286	0.070426	-4.09644	0.247347	0.364618
Monocytes	KCNK5	-0.86676	1.47886	-1.83113	0.070449	-4.04014	0.253004	0.372841
Monocytes	1110020A21RIK	0.92028	0.468534	1.830945	0.070477	-4.04765	0.26036	0.383547
Monocytes	KLRK1	-0.31958	3.149025	-1.83081	0.070497	-4.7439	0.241337	0.356041
Monocytes	BPNT1	-0.30876	3.339718	-1.8308	0.070499	-4.29261	0.240042	0.354157
Monocytes	MYNN	0.247991	4.303762	1.830742	0.070508	-4.40049	0.233617	0.3448
Monocytes	RUFY3	0.195295	5.26954	1.830264	0.07058	-4.73688	0.227528	0.335769
Monocytes	TESK2	0.390204	4.072329	1.829525	0.070692	-4.35516	0.235599	0.347341
Monocytes	IGSF6	0.283948	3.689623	1.828325	0.070873	-4.80203	0.238683	0.351504
Monocytes	GM47917	0.733187	0.467794	1.827799	0.070953	-4.04308	0.261544	0.384666
Monocytes	GM11084	0.361855	3.368087	1.827668	0.070973	-4.22734	0.240936	0.354928
Monocytes	VAMP3	-0.15091	5.320751	-1.82764	0.070978	-4.75344	0.228072	0.336156
Monocytes	A930007I19RIK	0.381767	4.385631	1.827409	0.071012	-4.53509	0.234135	0.345099
Monocytes	MYCBP	0.255193	4.22905	1.827395	0.071014	-4.48776	0.235167	0.346606
Monocytes	CRK	0.166075	6.391053	1.826943	0.071083	-4.91301	0.221467	0.326513
Monocytes	C1S1	-0.68012	1.787052	-1.82683	0.071099	-4.06257	0.252078	0.371201
Monocytes	P4HA1	0.272015	5.786072	1.826259	0.071187	-4.82865	0.225445	0.332228
Monocytes	SAP18	0.13738	7.043708	1.826079	0.071214	-4.99581	0.217681	0.320875
Monocytes	F12	-0.85675	1.691224	-1.82562	0.071284	-4.05889	0.253137	0.372541
Monocytes	UNC119	-0.15919	5.47027	-1.82552	0.071298	-4.91735	0.227579	0.335405
Monocytes	5031425F14RIK	-1.08298	-0.17235	-1.82505	0.07137	-4.06744	0.266987	0.392545
Monocytes	CFP	-0.25373	5.518079	-1.82495	0.071386	-4.90228	0.22736	0.335126
Monocytes	6030468B19RIK	-0.834	0.279697	-1.82488	0.071396	-4.05726	0.263578	0.387699
Monocytes	HIST1H2BB	-0.79061	1.116611	-1.82433	0.07148	-4.04716	0.257608	0.37907
Monocytes	SERPINA7	-1.00053	0.531403	-1.82389	0.071547	-4.05211	0.262085	0.38543
Monocytes	PHACTR1	-0.71145	1.519212	-1.82304	0.071677	-4.06057	0.255221	0.375359
Monocytes	PGGT1B	0.209324	4.818529	1.822784	0.071716	-4.58707	0.232598	0.342568

Monocytes	CD300LB	0.379327	1.973752	1.822235	0.0718	-4.50092	0.252226	0.370905
Monocytes	CMTM3	-0.26223	3.593966	-1.82079	0.072021	-4.53869	0.241488	0.35516
Monocytes	MET	0.294302	1.991608	1.82075	0.072028	-4.69819	0.252663	0.371362
Monocytes	DNAJB6	-0.1305	8.310874	-1.82072	0.072032	-5.1781	0.211658	0.311469
Monocytes	VASP	-0.18945	6.541669	-1.82054	0.072059	-4.93713	0.222346	0.327207
Monocytes	DPM2	0.276841	3.936241	1.819954	0.07215	-4.4128	0.239388	0.351977
Monocytes	AKR1D1	-0.74598	2.587501	-1.81984	0.072167	-4.11598	0.248666	0.365437
Monocytes	4931406G06RII	-0.29422	0.347467	-1.81951	0.072219	-4.71274	0.264999	0.389041
Monocytes	GM15494	-0.98851	0.759331	-1.81946	0.072226	-4.05314	0.261918	0.384616
Monocytes	DNASE2A	-0.23673	4.427932	-1.819	0.072297	-4.58546	0.236241	0.347484
Monocytes	LEMD2	-0.20746	4.232797	-1.81895	0.072304	-4.59315	0.23754	0.349376
Monocytes	STAT4	-0.36484	5.177658	-1.81865	0.07235	-4.62888	0.231394	0.340351
Monocytes	DTL	-0.39812	4.961318	-1.81812	0.072432	-4.51343	0.232988	0.342528
Monocytes	MCRIP2	-0.50123	2.015335	-1.81767	0.072501	-4.11852	0.253311	0.37192
Monocytes	SPC25	-0.39354	4.372308	-1.81741	0.072541	-4.48232	0.237076	0.348428
Monocytes	HIF1A	0.183811	6.608469	1.817092	0.072591	-4.98136	0.222768	0.327473
Monocytes	JAKMIP1	-0.62011	3.981458	-1.81671	0.07265	-4.10581	0.239821	0.352417
Monocytes	TMED8	0.396088	2.954093	1.816709	0.07265	-4.18616	0.246864	0.362637
Monocytes	SLC22A17	-0.88073	-0.51359	-1.81618	0.072732	-4.05989	0.272583	0.399528
Monocytes	FPR2	0.559752	1.833327	1.815901	0.072775	-4.45227	0.255081	0.374325
Monocytes	RNF141	0.233318	4.511433	1.815477	0.072841	-4.51887	0.236644	0.347586
Monocytes	ANXA6	-0.17287	6.174347	-1.81536	0.072859	-5.0029	0.225874	0.331888
Monocytes	METTL7B	-0.87443	2.263028	-1.81452	0.07299	-4.10277	0.252482	0.370333
Monocytes	MAP4K5	-0.24246	4.52083	-1.81439	0.07301	-4.66854	0.236918	0.347839
Monocytes	DNAJC9	-0.27288	5.617283	-1.81368	0.073119	-4.69994	0.230016	0.337629
Monocytes	RERG	-1.17948	0.522461	-1.81263	0.073284	-4.08594	0.265987	0.389519
Monocytes	CAT	-0.26065	6.72379	-1.81249	0.073304	-4.92504	0.223374	0.327833
Monocytes	TSPAN31	0.237643	4.424978	1.812466	0.073308	-4.63173	0.238219	0.3495
Monocytes	MAN2B1	-0.18041	6.851978	-1.81209	0.073368	-5.15086	0.222685	0.32689
Monocytes	RBM4B	-0.18988	5.747385	-1.81179	0.073414	-4.77673	0.22973	0.337206
Monocytes	ANKDD1A	-0.46091	2.346094	-1.81144	0.073469	-4.56037	0.2529	0.370798
Monocytes	CAP1	0.137947	6.878742	1.811186	0.073508	-5.01745	0.222727	0.326977
Monocytes	PATL1	-0.19315	5.222719	-1.81098	0.07354	-4.71585	0.233303	0.342507
Monocytes	ZFP62	-0.24074	4.580193	-1.81058	0.073602	-4.52555	0.237597	0.348758
Monocytes	IFI204	0.300331	4.390085	1.810436	0.073625	-4.89943	0.238869	0.350609
Monocytes	HES1	-0.31589	5.161335	-1.8104	0.073631	-4.65864	0.233755	0.343161
Monocytes	ARSK	0.432499	2.705302	1.81027	0.073651	-4.25683	0.250479	0.367454
Monocytes	FOPNL	0.263892	4.468642	1.80987	0.073714	-4.45348	0.23836	0.349959
Monocytes	B630019A10RII	-0.73581	1.596371	-1.80976	0.073731	-4.07783	0.258478	0.37907
Monocytes	LYL1	0.359535	3.172684	1.809714	0.073738	-4.39405	0.247215	0.362811
Monocytes	PEX2	0.281748	3.914925	1.809626	0.073752	-4.49511	0.2421	0.355426
Monocytes	MLXIPL	-1.24981	0.355448	-1.80947	0.073776	-4.0779	0.267741	0.392466
Monocytes	SEC24D	-0.24991	4.470665	-1.80759	0.074071	-4.56417	0.239141	0.350672
Monocytes	CTSH	0.204822	5.779307	1.807457	0.074092	-5.00418	0.230528	0.338157
Monocytes	CLEC1A	0.511572	0.972503	1.807448	0.074093	-4.24676	0.263966	0.386542
Monocytes	LBR	0.138774	7.219631	1.80724	0.074126	-4.99542	0.221472	0.324919
Monocytes	ZFP617	0.59357	2.165547	1.806483	0.074245	-4.10773	0.255553	0.37429

Monocytes	PCYT2	-0.2282	4.635801	-1.80613	0.074301	-4.6829	0.238472	0.349538
Monocytes	NFATC2	-0.30337	2.489047	-1.80586	0.074344	-4.49513	0.253382	0.371186
Monocytes	TMEM62	-0.48627	2.375873	-1.80574	0.074363	-4.16054	0.254194	0.372356
Monocytes	KDM3B	0.150588	6.131762	1.805505	0.074399	-4.87369	0.228766	0.33544
Monocytes	FAM162A	0.27336	5.640039	1.804887	0.074497	-4.76987	0.232162	0.340284
Monocytes	GNG3	-0.71375	1.449153	-1.80452	0.074555	-4.08209	0.261356	0.382552
Monocytes	SLC25A11	-0.20451	5.162778	-1.80428	0.074592	-4.74282	0.235382	0.34506
Monocytes	KIF7	0.895023	-0.46987	1.803912	0.074651	-4.07414	0.275996	0.403559
Monocytes	FGD6	-0.2991	4.002414	-1.80382	0.074665	-4.50956	0.243178	0.356388
Monocytes	BRPF1	0.214407	4.929241	1.803821	0.074665	-4.69665	0.236928	0.347311
Monocytes	GAR1	-0.28639	4.430651	-1.80378	0.074671	-4.52273	0.240268	0.352165
Monocytes	CPEB3	-0.30977	4.234811	-1.80263	0.074853	-4.63491	0.241953	0.354412
Monocytes	ZFP385A	0.210376	3.938147	1.802553	0.074866	-4.73807	0.24398	0.357351
Monocytes	CAPZB	-0.10333	8.722615	-1.80248	0.074878	-5.33596	0.213476	0.312792
Monocytes	SCHIP1	-0.74777	1.591487	-1.80247	0.074878	-4.08249	0.260691	0.381456
Monocytes	KLRD1	-0.36714	3.767425	-1.80169	0.075002	-4.68038	0.245414	0.359332
Monocytes	ITPR2	0.186242	6.912603	1.801624	0.075013	-4.89977	0.224719	0.329208
Monocytes	CYP2D9	-0.90063	0.530177	-1.80152	0.07503	-4.08063	0.268942	0.393208
Monocytes	CENPH	-0.51774	3.059161	-1.80116	0.075087	-4.22953	0.250424	0.366611
Monocytes	ELF2	0.142506	7.39491	1.8011	0.075096	-5.10852	0.221777	0.324951
Monocytes	SERPINA3M	-0.57914	3.688152	-1.80041	0.075205	-4.28276	0.246302	0.360561
Monocytes	BGN	-0.6091	2.420901	-1.79985	0.075295	-4.12264	0.255453	0.37374
Monocytes	SNRPN	-1.22297	-0.6475	-1.79976	0.075309	-4.10466	0.278689	0.407
Monocytes	2210016F16RIK	-0.17509	4.954824	-1.7993	0.075383	-4.74423	0.23802	0.348549
Monocytes	PROX1	-0.91092	2.15604	-1.79915	0.075407	-4.08875	0.257544	0.376881
Monocytes	SNX33	0.777688	0.759185	1.798208	0.075557	-4.07925	0.268389	0.392132
Monocytes	ABI2	-0.28107	3.903545	-1.79693	0.075761	-4.54742	0.246148	0.359673
Monocytes	CIZ1	0.255542	3.940619	1.796229	0.075874	-4.47282	0.246178	0.359634
Monocytes	AU041133	-0.68511	1.264215	-1.79602	0.075907	-4.08209	0.265542	0.387536
Monocytes	CDS2	0.239663	4.708784	1.793651	0.076288	-4.54197	0.242081	0.352907
Monocytes	RRP9	-0.45022	2.59462	-1.79349	0.076314	-4.16249	0.256938	0.374341
Monocytes	GPATCH1	0.387276	2.73622	1.793118	0.076374	-4.22203	0.25603	0.37307
Monocytes	GM26510	0.347679	4.122817	1.792812	0.076423	-4.38296	0.246299	0.359065
Monocytes	DHRS4	0.275973	4.374568	1.792236	0.076516	-4.57034	0.244721	0.356694
Monocytes	FABP1	-0.50095	7.428716	-1.7922	0.076521	-5.04923	0.224691	0.32762
Monocytes	PLCB4	-0.35429	3.876337	-1.79183	0.076581	-4.50772	0.248208	0.361779
Monocytes	SAA3	1.877342	0.738679	1.791696	0.076603	-4.14869	0.271229	0.394762
Monocytes	MGL2	0.735284	-0.18973	1.791598	0.076619	-4.27663	0.278479	0.40507
Monocytes	SLC5A3	-0.46809	3.816287	-1.79153	0.07663	-4.3299	0.248628	0.362398
Monocytes	PRMT7	0.473717	3.247895	1.791294	0.076668	-4.2662	0.252645	0.368211
Monocytes	FUS	-0.1214	7.820328	-1.79122	0.07668	-5.12311	0.222294	0.324227
Monocytes	FKBP15	0.192832	5.239594	1.790549	0.076788	-4.73077	0.239152	0.348629
Monocytes	KDM7A	0.140959	7.129544	1.789958	0.076884	-5.23413	0.227065	0.330958
Monocytes	DIAPH2	0.151803	7.666379	1.789274	0.076995	-5.28872	0.223951	0.326254
Monocytes	B3GALT2	0.881958	0.771125	1.788704	0.077088	-4.0918	0.272078	0.395444
Monocytes	GCLM	-0.22669	6.090034	-1.7883	0.077154	-4.8481	0.234269	0.341083
Monocytes	SLC35E3	-0.56163	1.909396	-1.78795	0.07721	-4.15315	0.263502	0.383293

Monocytes	COPG1	-0.21083	5.141092	-1.78791	0.077217	-4.70351	0.240574	0.35033
Monocytes	2610301B20RIK	0.576874	1.613471	1.787684	0.077253	-4.11296	0.265719	0.386582
Monocytes	TFIP11	-0.30868	3.322157	-1.78763	0.077262	-4.38697	0.253195	0.368654
Monocytes	MYBPC3	-0.40996	1.123244	-1.78754	0.077276	-4.31774	0.269436	0.391899
Monocytes	NPR1	-0.85401	1.263007	-1.78754	0.077277	-4.09208	0.268371	0.39038
Monocytes	COX6B1	0.142823	7.821955	1.787292	0.077317	-5.20502	0.223278	0.3253
Monocytes	TRIM13	0.85991	0.46615	1.78616	0.077502	-4.09322	0.274937	0.399571
Monocytes	OTULINL	0.183438	5.347398	1.785894	0.077545	-5.0832	0.239561	0.348931
Monocytes	GM3550	-0.53458	1.752212	-1.78581	0.077558	-4.12202	0.265091	0.385643
Monocytes	RNF145	-0.23417	5.159727	-1.78581	0.077558	-4.67508	0.240824	0.350759
Monocytes	HIST1H4H	0.851527	0.513275	1.785696	0.077577	-4.09568	0.274569	0.399161
Monocytes	TMEM223	0.227887	4.652686	1.785478	0.077613	-4.58447	0.244274	0.355838
Monocytes	IL2RA	-0.95877	2.557963	-1.78546	0.077615	-4.11238	0.259118	0.377197
Monocytes	SMIM15	0.207516	4.858356	1.78544	0.077619	-4.61582	0.242868	0.353807
Monocytes	RALGPS1	0.325493	4.92479	1.784993	0.077692	-4.45274	0.242567	0.353291
Monocytes	TNKS	0.171592	6.066905	1.78482	0.07772	-5.01323	0.234944	0.342323
Monocytes	RSBN1	0.207111	5.326631	1.784381	0.077792	-4.72419	0.239997	0.349587
Monocytes	HAUS2	0.316227	3.755191	1.784251	0.077813	-4.40459	0.250825	0.365248
Monocytes	TMEM160	0.156714	5.666014	1.784062	0.077844	-4.91089	0.237746	0.346404
Monocytes	PLD2	-0.53698	1.042444	-1.78276	0.078058	-4.17619	0.271439	0.394193
Monocytes	RNF17	1.150511	-0.02892	1.782699	0.078068	-4.1048	0.279825	0.406104
Monocytes	SEMA4D	0.226405	6.446214	1.782	0.078183	-5.066	0.233376	0.339347
Monocytes	SNX21	0.365507	2.718125	1.781913	0.078197	-4.37797	0.259129	0.376461
Monocytes	CASP9	0.353962	2.447605	1.781765	0.078221	-4.25651	0.261117	0.379326
Monocytes	NADK	0.16518	6.266885	1.781376	0.078285	-5.05633	0.234665	0.341143
Monocytes	PNPT1	0.266383	4.198204	1.781032	0.078342	-4.52402	0.248677	0.361535
Monocytes	ACIN1	-0.11831	7.577859	-1.78099	0.078348	-5.1131	0.226254	0.32907
Monocytes	4833420G17RIK	0.243423	5.494494	1.780758	0.078387	-4.73553	0.239795	0.348759
Monocytes	HMOX1	-0.34661	4.807778	-1.78063	0.078408	-4.89873	0.244455	0.355491
Monocytes	STAT5B	0.185598	6.043742	1.780572	0.078417	-4.88538	0.236139	0.343471
Monocytes	ENKD1	0.684741	1.3576	1.780463	0.078435	-4.10499	0.26944	0.391356
Monocytes	ATP5D	0.126256	7.898894	1.780054	0.078503	-5.23571	0.224368	0.326383
Monocytes	NUP93	-0.22058	4.586111	-1.77967	0.078566	-4.64722	0.246236	0.358051
Monocytes	TRIM7	-0.51082	0.591087	-1.77939	0.078612	-4.21534	0.275721	0.400232
Monocytes	CSNK1E	0.221005	4.953409	1.778736	0.07872	-4.7256	0.243989	0.354728
Monocytes	SHISA8	-1.11837	-0.21439	-1.77843	0.078771	-4.11704	0.282354	0.409665
Monocytes	FYTTD1	-0.16697	5.267108	-1.7784	0.078776	-4.72866	0.241853	0.351749
Monocytes	MTSS2	-0.8649	0.842863	-1.77832	0.078788	-4.10248	0.273998	0.397831
Monocytes	SRRM2	-0.11967	8.62357	-1.77823	0.078804	-5.29763	0.220263	0.320416
Monocytes	AKT1	-0.17362	6.230392	-1.77712	0.078987	-4.97139	0.235674	0.342955
Monocytes	LRATD2	-0.52702	1.700967	-1.77706	0.078998	-4.15358	0.267699	0.389055
Monocytes	TGTP2	-1.30123	0.773598	-1.77704	0.079002	-4.10662	0.27483	0.399211
Monocytes	SMIM40	0.677626	0.301755	1.776898	0.079024	-4.12828	0.278537	0.404516
Monocytes	ASRGL1	0.403009	3.315534	1.776731	0.079052	-4.34666	0.255762	0.372077
Monocytes	SDHAF2	0.245582	4.655098	1.776679	0.079061	-4.55699	0.246303	0.35846
Monocytes	NINJ1	0.238723	5.750418	1.776673	0.079062	-4.93412	0.238857	0.347695
Monocytes	STAU2	-0.54398	2.900653	-1.77648	0.079094	-4.17658	0.258796	0.376452

Monocytes	DDX60	-0.61547	2.866859	-1.77602	0.079169	-4.29285	0.259209	0.376947
Monocytes	GM34983	-0.91531	0.754004	-1.77586	0.079197	-4.10709	0.275194	0.399845
Monocytes	CDH13	-1.13766	2.030566	-1.77562	0.079236	-4.11235	0.26544	0.385979
Monocytes	PFKP	-0.18887	5.538024	-1.77545	0.079265	-5.05698	0.240486	0.350194
Monocytes	USF1	0.232543	4.934416	1.77537	0.079278	-4.69614	0.244589	0.356149
Monocytes	TM9SF1	-0.20742	5.042685	-1.77458	0.079409	-4.73832	0.244127	0.355277
Monocytes	CAR2	-0.56977	5.171918	-1.77453	0.079418	-4.43633	0.243244	0.354001
Monocytes	GM15503	0.547425	0.81285	1.774358	0.079446	-4.13111	0.275084	0.399651
Monocytes	RBBP6	-0.13443	7.269431	-1.774	0.079506	-5.09642	0.229512	0.334111
Monocytes	ZFP280D	0.219767	5.625842	1.773578	0.079576	-4.71521	0.240422	0.349916
Monocytes	AC166172.1	0.776836	0.939575	1.772951	0.079681	-4.10976	0.274648	0.398789
Monocytes	FBL	-0.16977	6.091074	-1.77278	0.079709	-4.97655	0.237564	0.345592
Monocytes	NR1H3	-0.41738	3.325046	-1.77258	0.079743	-4.5264	0.256772	0.373333
Monocytes	CDK20	0.812712	0.593617	1.772192	0.079808	-4.11032	0.27754	0.402933
Monocytes	ACBD5	0.162497	6.231959	1.771713	0.079888	-4.91744	0.236877	0.344626
Monocytes	CDK7	-0.25308	4.595584	-1.77169	0.079892	-4.5927	0.247985	0.360689
Monocytes	TNRC6B	0.168625	8.234075	1.771309	0.079955	-5.16774	0.224115	0.325966
Monocytes	CUL4A	0.181924	5.002429	1.771212	0.079971	-4.73456	0.245253	0.356671
Monocytes	WIPF1	-0.16061	7.425545	-1.77108	0.079994	-5.24889	0.229204	0.333405
Monocytes	SIAH2	-0.18224	4.793136	-1.77036	0.080114	-4.77782	0.246946	0.359014
Monocytes	SPSB4	0.998944	0.417547	1.7703	0.080124	-4.11812	0.279428	0.405498
Monocytes	GSTP2	-0.72341	0.861498	-1.77002	0.080172	-4.11522	0.276005	0.400634
Monocytes	ADAM17	0.204532	6.061586	1.769649	0.080233	-5.01884	0.238504	0.346744
Monocytes	SGCB	-0.66158	2.212559	-1.76912	0.080322	-4.14448	0.265894	0.386023
Monocytes	ZFP516	0.188574	4.66128	1.769118	0.080323	-4.90532	0.248166	0.360596
Monocytes	NRIP1	-0.1882	6.353871	-1.7685	0.080427	-5.08864	0.236754	0.344175
Monocytes	CCDC47	0.188926	5.110399	1.768466	0.080432	-4.71801	0.245135	0.356298
Monocytes	COPB1	0.131789	6.220287	1.76844	0.080437	-4.97129	0.237639	0.345458
Monocytes	PEPD	-0.18306	5.308275	-1.76832	0.080457	-4.87028	0.243779	0.354396
Monocytes	ARF6	-0.1695	6.770128	-1.76791	0.080526	-5.06222	0.234021	0.340364
Monocytes	SLC25A18	0.583302	4.491338	1.767844	0.080537	-4.33049	0.24943	0.362657
Monocytes	CCNG2	0.271006	5.189545	1.767807	0.080543	-4.65878	0.244592	0.355676
Monocytes	RGS18	0.318258	3.108266	1.767782	0.080547	-4.54699	0.259332	0.376888
Monocytes	YPEL5	0.208098	6.583092	1.767284	0.080631	-4.91719	0.235407	0.342348
Monocytes	SELENOS	-0.15408	6.113957	-1.76702	0.080676	-4.97546	0.23851	0.346968
Monocytes	SLC36A4	0.37386	3.21113	1.766993	0.08068	-4.42086	0.25876	0.37617
Monocytes	YIF1B	-0.20278	4.886916	-1.76686	0.080703	-4.74461	0.246847	0.359035
Monocytes	B3GALT6	-0.65526	1.806345	-1.76585	0.080872	-4.15577	0.269693	0.391579
Monocytes	NAXE	-0.1991	5.283838	-1.76575	0.08089	-4.79804	0.244529	0.355503
Monocytes	AGPAT2	0.343497	4.355109	1.765343	0.080959	-4.55174	0.251122	0.364991
Monocytes	UTP3	0.193969	5.450096	1.764887	0.081036	-4.80405	0.243648	0.354225
Monocytes	SLC35A3	-0.17634	4.775291	-1.76471	0.081066	-4.72646	0.248301	0.361018
Monocytes	PIP4P1	0.136494	5.856707	1.764657	0.081075	-4.90438	0.240891	0.350315
Monocytes	RRP36	-0.25074	3.775241	-1.76396	0.081193	-4.45476	0.255673	0.371463
Monocytes	PIM2	-0.43708	3.150665	-1.76369	0.081239	-4.25258	0.260279	0.378131
Monocytes	PTK2	-0.36747	4.164046	-1.76337	0.081292	-4.43928	0.253023	0.367685
Monocytes	ARL11	0.405782	1.439587	1.763266	0.081311	-4.2878	0.273251	0.396633

Monocytes	UQCC2	0.183379	6.141014	1.762611	0.081422	-4.9976	0.239461	0.348116
Monocytes	IL1RN	0.494673	2.526967	1.762577	0.081428	-4.67173	0.265057	0.384962
Monocytes	GM20069	-0.93594	-0.52038	-1.76231	0.081473	-4.1229	0.288987	0.419002
Monocytes	SULT2A1	-0.68532	3.901678	-1.7623	0.081475	-4.39432	0.25498	0.370545
Monocytes	RNF41	0.208509	4.424549	1.762289	0.081477	-4.62759	0.251258	0.365192
Monocytes	EMC2	-0.14757	5.762132	-1.7622	0.081491	-4.93246	0.242011	0.351885
Monocytes	INTS5	0.388442	2.704825	1.762057	0.081516	-4.23871	0.263729	0.383191
Monocytes	GM44751	0.40783	1.310612	1.761929	0.081538	-4.33853	0.27434	0.39835
Monocytes	PTPRB	-0.70912	3.708559	-1.76155	0.081602	-4.29704	0.256424	0.372669
Monocytes	RIC8A	0.239588	3.980741	1.76125	0.081653	-4.56897	0.254467	0.36992
Monocytes	PARP2	0.187229	4.841287	1.761225	0.081658	-4.70569	0.248388	0.361164
Monocytes	BRI3	0.145605	7.328714	1.761058	0.081686	-5.19392	0.231711	0.337047
Monocytes	EXOC8	0.550022	1.951234	1.761008	0.081694	-4.1622	0.269464	0.391461
Monocytes	RAPGEF4	0.614666	2.563789	1.760943	0.081706	-4.1941	0.264838	0.384862
Monocytes	PSTPIP1	0.219771	4.472927	1.760436	0.081792	-4.82112	0.251158	0.365125
Monocytes	IFT57	0.318095	2.891644	1.760017	0.081864	-4.42927	0.262743	0.381601
Monocytes	BCL11B	-0.75697	1.080616	-1.75817	0.08218	-4.20956	0.27754	0.402098
Monocytes	FRMPD4	0.938451	0.351798	1.757749	0.082251	-4.12643	0.283501	0.410466
Monocytes	GATM	-0.23915	4.530012	-1.75746	0.0823	-4.89546	0.252002	0.365526
Monocytes	UBOX5	0.603793	1.390449	1.757322	0.082325	-4.15195	0.275344	0.398906
Monocytes	AI662270	-0.20012	6.090129	-1.75667	0.082436	-4.95567	0.241478	0.350244
Monocytes	R3HDM1	0.13514	6.683532	1.756519	0.082462	-5.04453	0.237513	0.344549
Monocytes	CYSLTR2	-0.69726	1.496466	-1.75626	0.082507	-4.18458	0.274808	0.398098
Monocytes	TMEM192	0.197696	4.694631	1.756225	0.082513	-4.74426	0.251105	0.364213
Monocytes	ABHD2	-0.19163	5.471153	-1.75597	0.082557	-4.76708	0.245753	0.356448
Monocytes	ALDH7A1	-0.46372	3.227154	-1.75553	0.082632	-4.2826	0.261906	0.379514
Monocytes	CBX7	-0.43282	2.651462	-1.75531	0.08267	-4.20348	0.266236	0.385688
Monocytes	TMEM9B	0.153222	5.558526	1.754615	0.08279	-4.89828	0.245618	0.355951
Monocytes	CPB2	-0.47767	3.209826	-1.75426	0.08285	-4.29854	0.262486	0.380193
Monocytes	SLC4A8	-0.6232	0.467759	-1.75346	0.082989	-4.21322	0.284041	0.410517
Monocytes	GHITM	-0.20982	7.4577	-1.75334	0.08301	-5.14019	0.233347	0.337977
Monocytes	UROD	0.275494	4.467162	1.752411	0.08317	-4.57064	0.254104	0.36778
Monocytes	MRPS24	0.145588	6.031221	1.752137	0.083217	-4.99156	0.243268	0.352165
Monocytes	NSUN3	0.372578	2.658689	1.751999	0.083241	-4.28161	0.267449	0.386889
Monocytes	MTUS1	0.304985	3.721745	1.751788	0.083278	-4.75567	0.259585	0.375715
Monocytes	FBXO21	0.334204	3.292952	1.751318	0.083359	-4.36636	0.262917	0.380355
Monocytes	MSL1	0.201341	5.419802	1.751098	0.083397	-4.80376	0.247702	0.358564
Monocytes	VKORC1	-0.29045	4.701765	-1.75019	0.083555	-4.66294	0.253139	0.366138
Monocytes	MBD5	0.157235	6.903175	1.749801	0.083622	-5.06632	0.238143	0.344527
Monocytes	MARCKSL1	-0.23967	7.792982	-1.74912	0.08374	-5.27169	0.232577	0.336383
Monocytes	SLC25A30	0.281	3.550303	1.748812	0.083794	-4.48448	0.261979	0.378701
Monocytes	RPE	0.22526	4.554255	1.748647	0.083823	-4.68678	0.254694	0.368334
Monocytes	ERCC6L2	-0.35853	3.372895	-1.74827	0.083889	-4.37113	0.263402	0.380824
Monocytes	MAGED1	-0.67197	2.409871	-1.74817	0.083906	-4.15773	0.270659	0.39119
Monocytes	PTPN12	0.180003	6.049828	1.747881	0.083956	-5.05207	0.244409	0.353605
Monocytes	SPG20	-0.45718	2.615271	-1.74771	0.083986	-4.21845	0.269188	0.389156
Monocytes	HNRNPK	-0.09952	8.860645	-1.74726	0.084065	-5.37566	0.226132	0.327182

Monocytes	HNRNPUL2	0.126358	6.468104	1.747228	0.08407	-5.07028	0.241677	0.349711
Monocytes	KIF21A	-0.90981	0.515306	-1.74689	0.084129	-4.1395	0.285841	0.412805
Monocytes	COTL1	0.154228	6.629821	1.746858	0.084135	-5.14826	0.240628	0.348236
Monocytes	ATG4D	0.263013	3.954415	1.746349	0.084224	-4.54869	0.259552	0.375317
Monocytes	9530068E07RIK	0.212097	4.851397	1.746188	0.084252	-4.77803	0.2531	0.366104
Monocytes	2410022M11RI	0.507494	1.591485	1.745325	0.084403	-4.2	0.277878	0.401273
Monocytes	CD63	-0.3319	4.105165	-1.74468	0.084516	-4.67819	0.259115	0.374447
Monocytes	LDLRAD4	0.225931	6.109073	1.744116	0.084614	-5.04991	0.245076	0.354339
Monocytes	ACER3	0.142805	6.311004	1.744075	0.084621	-5.22709	0.243697	0.352352
Monocytes	DSE	-0.27957	3.834005	-1.74397	0.084641	-4.77828	0.261222	0.37751
Monocytes	CYB5D1	-0.95557	0.775244	-1.74382	0.084665	-4.14447	0.284813	0.411051
Monocytes	ATP6V1F	0.122717	6.902317	1.743718	0.084684	-5.1503	0.239711	0.346661
Monocytes	RSL1	0.803252	1.039109	1.743521	0.084719	-4.14761	0.282719	0.408217
Monocytes	LGALS1	0.250375	6.036335	1.743199	0.084775	-5.09212	0.245614	0.355274
Monocytes	ZFP943	0.237788	4.280266	1.743154	0.084783	-4.51009	0.258006	0.373074
Monocytes	TGFB1I1	-0.88908	0.90381	-1.74305	0.084801	-4.14308	0.283822	0.409806
Monocytes	CHD8	0.158494	5.733086	1.742777	0.084849	-4.86896	0.247773	0.358375
Monocytes	HSPA12A	-0.88152	0.150275	-1.74251	0.084897	-4.14428	0.290114	0.418765
Monocytes	GTF2A2	0.159229	5.923594	1.742144	0.084961	-4.93457	0.246602	0.356701
Monocytes	JRKL	0.619062	1.537523	1.742054	0.084976	-4.18704	0.279009	0.40303
Monocytes	XPO6	0.16564	5.572883	1.741634	0.085051	-4.86728	0.249175	0.36037
Monocytes	UVRAG	-0.15162	8.569242	-1.74111	0.085143	-5.46182	0.229393	0.33166
Monocytes	NDUFAF2	-0.24877	4.440034	-1.74099	0.085164	-4.59419	0.257403	0.372096
Monocytes	N4BP1	0.20827	5.4056	1.740848	0.085189	-4.97169	0.250525	0.362259
Monocytes	TGFB1	0.130753	8.800362	1.740244	0.085296	-5.4142	0.228032	0.329875
Monocytes	PSMD8	0.155161	6.592353	1.74021	0.085302	-5.08576	0.242459	0.35079
Monocytes	CLYBL	0.258009	4.489599	1.740097	0.085322	-4.65814	0.257159	0.372005
Monocytes	GABBR1	-0.68423	2.766527	-1.74	0.085339	-4.2976	0.269946	0.390279
Monocytes	DOCK1	0.216634	4.079995	1.739904	0.085356	-4.86467	0.260137	0.3763
Monocytes	ADCY4	-0.54594	1.413611	-1.73958	0.085413	-4.34437	0.28058	0.405341
Monocytes	ENPP4	-0.3728	2.924155	-1.73922	0.085476	-4.39944	0.268964	0.388881
Monocytes	BRMS1	0.235235	4.236998	1.738878	0.085537	-4.64799	0.259257	0.375046
Monocytes	PRAG1	0.611826	1.923732	1.73874	0.085561	-4.19499	0.276737	0.400026
Monocytes	SENP2	-0.14851	6.656089	-1.73858	0.085589	-5.10645	0.242277	0.350728
Monocytes	EARS2	-0.50236	1.644083	-1.73853	0.085598	-4.20486	0.278937	0.403243
Monocytes	ATP6V0D1	0.105342	7.228201	1.73814	0.085668	-5.20707	0.238509	0.345307
Monocytes	HIST1H4I	0.378781	4.409355	1.738022	0.085688	-4.58392	0.258073	0.373502
Monocytes	DEFB1	-0.96689	0.86149	-1.73797	0.085698	-4.14989	0.285271	0.412237
Monocytes	RNF4	-0.13601	5.964365	-1.73772	0.085741	-4.9808	0.247119	0.357733
Monocytes	CUL5	-0.15019	5.877947	-1.73749	0.085783	-4.92082	0.24772	0.358642
Monocytes	NUDT6	-0.48586	2.183509	-1.73736	0.085807	-4.2392	0.274845	0.397538
Monocytes	HIVEP1	-0.18876	6.40877	-1.73728	0.08582	-5.0173	0.244073	0.353444
Monocytes	UGDH	0.225413	4.250685	1.736781	0.085909	-4.7257	0.259473	0.375509
Monocytes	GM43848	0.360122	2.767038	1.736494	0.08596	-4.35623	0.270628	0.391353
Monocytes	AKR1B10	-0.31807	3.494629	-1.73606	0.086036	-4.48828	0.265189	0.383618
Monocytes	SETD2	0.155236	6.922415	1.735922	0.086061	-5.08402	0.240902	0.34872
Monocytes	TRAK2	0.229475	5.153104	1.735647	0.08611	-4.71155	0.253113	0.366352

Monocytes	ATF7	0.17352	6.314357	1.735584	0.086121	-5.00655	0.245022	0.354707
Monocytes	MED13L	0.137867	7.196724	1.735505	0.086135	-5.21482	0.239068	0.346122
Monocytes	ALDH1L1	-0.56356	3.098676	-1.73543	0.086149	-4.27536	0.268166	0.387956
Monocytes	SLC39A9	0.209659	4.172579	1.735372	0.086159	-4.57229	0.260177	0.376533
Monocytes	SINHCAF	-0.21009	4.557551	-1.73448	0.086318	-4.73304	0.257774	0.372952
Monocytes	CCN1	-1.00625	1.098666	-1.73379	0.086441	-4.15697	0.284531	0.410834
Monocytes	SECTM1A	-1.06278	-1.13086	-1.73332	0.086526	-4.174	0.30336	0.437274
Monocytes	SLA2	-0.55658	2.746588	-1.73306	0.086572	-4.22724	0.27176	0.392696
Monocytes	KAT8	0.314944	3.624929	1.732748	0.086627	-4.43209	0.265111	0.383279
Monocytes	GON4L	0.188962	5.15234	1.732722	0.086632	-4.76064	0.253975	0.367325
Monocytes	0610010F05RIK	-0.24708	4.458879	-1.7327	0.086637	-4.72111	0.258965	0.374485
Monocytes	CNTLN	-0.2913	4.34763	-1.73258	0.086658	-4.66617	0.259776	0.375701
Monocytes	GM11613	-0.46941	2.020755	-1.73212	0.086739	-4.42637	0.277571	0.401106
Monocytes	GPR68	-0.40312	1.433054	-1.73107	0.086927	-4.51732	0.282587	0.408105
Monocytes	RDH16F2	-0.92132	1.257357	-1.73103	0.086934	-4.16482	0.283998	0.410115
Monocytes	MPP5	-0.21851	5.379551	-1.73098	0.086943	-4.76721	0.252846	0.365677
Monocytes	BANP	0.222813	4.601582	1.730925	0.086954	-4.62384	0.258423	0.373688
Monocytes	OFD1	-0.30655	3.31561	-1.7294	0.087228	-4.37701	0.26871	0.388028
Monocytes	ACSBG1	-0.92373	-0.5064	-1.72924	0.087256	-4.16346	0.299452	0.431559
Monocytes	EGLN1	0.185897	5.631732	1.729034	0.087293	-4.89867	0.251823	0.36388
Monocytes	TRIM28	-0.20988	5.460856	-1.7286	0.087371	-4.83513	0.253177	0.365807
Monocytes	RFX5	-0.50134	1.943489	-1.72846	0.087396	-4.2346	0.279532	0.403477
Monocytes	AKIRIN1	0.131578	6.287656	1.728094	0.087462	-5.01413	0.247505	0.357748
Monocytes	ELOC	-0.15028	7.093954	-1.72732	0.087601	-5.15462	0.242315	0.350014
Monocytes	LYPLAL1	-0.86815	1.634017	-1.72691	0.087676	-4.1664	0.282603	0.407614
Monocytes	KCTD14	0.42189	1.055743	1.72677	0.087701	-4.3513	0.287272	0.414234
Monocytes	D430020J02RIK	-0.94884	0.440007	-1.7266	0.087732	-4.16329	0.292337	0.421395
Monocytes	GM36199	0.681797	-0.13106	1.726546	0.087742	-4.19596	0.297121	0.428128
Monocytes	FAM89B	0.181696	5.277421	1.725597	0.087913	-4.94429	0.255455	0.368518
Monocytes	GM2788	0.780589	0.569462	1.724976	0.088026	-4.16669	0.292033	0.420393
Monocytes	E130309D02RIK	-0.2552	3.983364	-1.72483	0.088053	-4.57078	0.265169	0.382322
Monocytes	TRIT1	-0.22598	4.076961	-1.72451	0.08811	-4.6709	0.264565	0.381379
Monocytes	ADAT1	0.574768	2.003267	1.724239	0.08816	-4.21674	0.280578	0.404102
Monocytes	EXOSC8	-0.22212	5.033192	-1.72391	0.088219	-4.8418	0.257703	0.371538
Monocytes	RELN	-0.71588	3.647841	-1.72378	0.088243	-4.28494	0.267934	0.386154
Monocytes	ERLIN1	-0.17035	4.765624	-1.72342	0.088309	-4.98375	0.259645	0.374398
Monocytes	COLEC11	-0.67116	1.589372	-1.72338	0.088316	-4.1836	0.283973	0.408989
Monocytes	SLC30A7	-0.18031	5.62501	-1.72337	0.088318	-4.97258	0.253466	0.365547
Monocytes	ZC3HC1	0.23578	4.34425	1.72305	0.088375	-4.62196	0.262829	0.378941
Monocytes	SLC25A44	0.339711	3.239164	1.722314	0.088509	-4.41432	0.271463	0.391076
Monocytes	ZFP53	0.254568	4.791451	1.721808	0.088601	-4.65906	0.260058	0.374683
Monocytes	TRNT1	0.208643	4.772449	1.721399	0.088676	-4.70993	0.260308	0.374992
Monocytes	FAM57A	-0.79593	1.118395	-1.72131	0.088692	-4.17091	0.288579	0.415146
Monocytes	CFDP1	-0.16137	5.986865	-1.72104	0.088742	-4.96648	0.251671	0.362689
Monocytes	RRAGC	-0.16473	5.819399	-1.72071	0.088802	-4.9721	0.252876	0.364402
Monocytes	GIT1	-0.25392	3.282244	-1.7207	0.088803	-4.47136	0.27155	0.391068
Monocytes	SYNJ1	-0.1331	6.731012	-1.72034	0.08887	-5.31035	0.246632	0.355324

Monocytes	FAM129C	-0.54969	3.433215	-1.71991	0.088947	-4.22268	0.270676	0.389596
Monocytes	NSMAF	-0.21415	4.903953	-1.71957	0.08901	-4.86042	0.259791	0.373995
Monocytes	PHLDB3	0.446086	2.97973	1.719476	0.089027	-4.28295	0.274243	0.394586
Monocytes	ARMC3	0.566204	1.900651	1.719021	0.08911	-4.23261	0.282915	0.406738
Monocytes	ESCO2	-0.4901	3.734708	-1.71861	0.089185	-4.4536	0.268789	0.386653
Monocytes	DHX33	-0.3069	3.32737	-1.71811	0.089276	-4.45705	0.272087	0.391362
Monocytes	GM43256	-0.88359	0.309245	-1.71654	0.089564	-4.17378	0.297221	0.426274
Monocytes	ATP6V1E1	0.143853	7.59223	1.716202	0.089627	-5.24472	0.242229	0.348214
Monocytes	CYP7A1	-0.87374	0.739983	-1.71614	0.089639	-4.17602	0.293679	0.421356
Monocytes	MARF1	0.161077	6.147381	1.71561	0.089736	-4.96838	0.252379	0.362679
Monocytes	GM614	-0.94878	-0.15476	-1.71492	0.089863	-4.18556	0.301815	0.432408
Monocytes	EGR2	-0.47386	2.237621	-1.71434	0.08997	-4.49241	0.282269	0.404765
Monocytes	IVNS1ABP	-0.16959	6.027762	-1.71396	0.09004	-5.00321	0.253831	0.364307
Monocytes	GM42418	-0.3695	11.10924	-1.71387	0.090057	-5.72249	0.220511	0.316281
Monocytes	RGS19	0.191518	5.393744	1.712693	0.090274	-4.96873	0.25892	0.371259
Monocytes	MTCH2	0.154383	5.950226	1.712103	0.090383	-5.022	0.25503	0.365691
Monocytes	CCL5	-0.61286	7.931718	-1.7121	0.090383	-5.57191	0.241333	0.346066
Monocytes	1110059G10RIK	0.226751	3.928256	1.712051	0.090393	-4.63049	0.269911	0.386867
Monocytes	PIK3C2A	-0.15425	6.854141	-1.71145	0.090504	-5.23877	0.248872	0.356887
Monocytes	IGKV1-117	-0.8991	-0.39916	-1.71138	0.090517	-4.19821	0.305325	0.436659
Monocytes	RIC8B	-0.20823	4.502862	-1.71113	0.090564	-4.74794	0.265851	0.381143
Monocytes	LARGE1	-0.27243	5.482959	-1.71099	0.090589	-4.91169	0.258643	0.370961
Monocytes	B4GALT6	0.264467	2.991751	1.710651	0.090652	-4.77879	0.277482	0.397795
Monocytes	SMURF2	0.15519	6.82379	1.710567	0.090668	-5.1415	0.249204	0.357591
Monocytes	RREB1	0.232133	6.814971	1.709756	0.090819	-5.0984	0.249606	0.357976
Monocytes	4932438H23RIK	-0.36126	-0.27879	-1.70959	0.090849	-4.59028	0.304857	0.436115
Monocytes	WDFY3	-0.20282	5.666809	-1.70937	0.09089	-5.1653	0.257792	0.369734
Monocytes	PRPF6	0.170441	5.300623	1.708642	0.091026	-4.84663	0.260708	0.373819
Monocytes	ORC2	0.250171	4.279461	1.708393	0.091072	-4.59946	0.268287	0.384598
Monocytes	AVIL	-0.59196	2.092681	-1.70832	0.091085	-4.23888	0.285351	0.408725
Monocytes	A430090L17RIK	0.654133	0.628221	1.708309	0.091088	-4.18959	0.297443	0.425701
Monocytes	GDPD3	0.350088	1.793559	1.707858	0.091172	-4.57409	0.287958	0.412222
Monocytes	PBLD2	-0.72526	0.872415	-1.70734	0.091269	-4.1845	0.295803	0.423093
Monocytes	SRSF5	-0.12506	6.955445	-1.70691	0.091349	-5.18985	0.249423	0.357337
Monocytes	RBP1	-0.52697	3.578118	-1.70666	0.091395	-4.38339	0.274236	0.392686
Monocytes	RSPH3A	-0.40677	2.558451	-1.70577	0.091561	-4.38013	0.282645	0.40428
Monocytes	NAA15	-0.12606	6.743192	-1.70567	0.09158	-5.10653	0.251319	0.35986
Monocytes	PROSER1	-0.26018	4.213066	-1.70522	0.091665	-4.63537	0.269939	0.386329
Monocytes	UBE2D1	0.158501	5.377113	1.705073	0.091692	-4.88783	0.261264	0.37402
Monocytes	HEATR3	-0.24973	4.273336	-1.70472	0.091758	-4.68113	0.269578	0.385922
Monocytes	SPPL2B	0.497208	2.17345	1.704429	0.091813	-4.29344	0.286021	0.409253
Monocytes	MEFV	0.481483	-0.03339	1.704377	0.091823	-4.40044	0.304494	0.435147
Monocytes	GM47819	0.794665	-0.22199	1.704326	0.091832	-4.21619	0.306132	0.437434
Monocytes	FAM104A	-0.15112	6.153765	-1.70403	0.091888	-5.06829	0.25582	0.366489
Monocytes	FXYD4	-0.37131	3.001862	-1.70373	0.091944	-4.47432	0.279583	0.400244
Monocytes	GM45435	0.902627	0.920103	1.700065	0.092634	-4.19674	0.298684	0.425688
Monocytes	CYB5R3	-0.19079	4.85016	-1.69988	0.09267	-4.868	0.267354	0.381661

Monocytes	OTUD5	-0.1593	5.568497	-1.69939	0.092762	-4.9247	0.262211	0.374225
Monocytes	CHEK1	-0.47198	2.722134	-1.69909	0.092819	-4.33025	0.284148	0.405208
Monocytes	NAIP1	0.666259	-0.47909	1.698794	0.092874	-4.22563	0.311244	0.443075
Monocytes	ZFP36	0.183677	7.849088	1.698632	0.092905	-5.47395	0.246219	0.351501
Monocytes	TRAM2	0.228103	4.061754	1.698266	0.092974	-4.67226	0.27384	0.390614
Monocytes	COG5	0.163854	6.582266	1.698119	0.093002	-5.11987	0.255174	0.364171
Monocytes	SCLT1	-0.26365	4.474106	-1.69759	0.093102	-4.74343	0.270898	0.386466
Monocytes	HIRIP3	0.357805	3.169999	1.696719	0.093268	-4.45547	0.281445	0.401174
Monocytes	COL18A1	-0.66948	2.743841	-1.69632	0.093344	-4.28825	0.285	0.406034
Monocytes	GM7030	-0.74903	2.084686	-1.69593	0.093418	-4.24539	0.290381	0.413651
Monocytes	PMP22	-0.58574	1.847479	-1.69591	0.093422	-4.34578	0.292337	0.416388
Monocytes	ATP6V0A2	-0.1673	4.619482	-1.69584	0.093434	-4.928	0.270359	0.385481
Monocytes	LITAF	0.122137	8.523937	1.695708	0.09346	-5.48307	0.242461	0.345781
Monocytes	TSPAN6	-1.13437	1.103606	-1.69535	0.093528	-4.20474	0.298693	0.425086
Monocytes	COX6A2	-1.17618	2.070766	-1.69483	0.093627	-4.19862	0.29068	0.414056
Monocytes	SYNE2	0.281268	5.373755	1.694681	0.093656	-4.78672	0.264869	0.377711
Monocytes	6430590A07RII	-0.5311	1.647577	-1.6944	0.093709	-4.22041	0.294183	0.418975
Monocytes	TMTC3	-0.30146	2.514058	-1.69433	0.093723	-4.44288	0.28706	0.409001
Monocytes	PIGW	-0.63887	0.765108	-1.69431	0.093726	-4.20848	0.301636	0.429375
Monocytes	PURB	-0.12557	6.824936	-1.69431	0.093727	-5.18904	0.254344	0.362755
Monocytes	COMMD7	-0.13718	5.29863	-1.69425	0.093737	-4.93661	0.265427	0.378502
Monocytes	SNF8	0.147156	5.731869	1.693304	0.093918	-5.03651	0.262659	0.3743
Monocytes	AKAP12	-0.49813	6.169014	-1.69248	0.094076	-4.65422	0.259806	0.370267
Monocytes	GM16083	-0.95805	1.977218	-1.69225	0.09412	-4.2002	0.29231	0.416173
Monocytes	DEGS2	0.722469	0.674225	1.69203	0.094162	-4.2143	0.303307	0.431654
Monocytes	CCL7	0.752768	1.659903	1.691955	0.094176	-4.49789	0.294947	0.42
Monocytes	ACAP2	0.147033	7.320314	1.691952	0.094177	-5.32045	0.251599	0.358785
Monocytes	SAA2	1.563921	-1.02347	1.691734	0.094219	-4.21321	0.318363	0.452572
Monocytes	PPIL3	0.200453	4.605985	1.69118	0.094325	-4.7731	0.271644	0.387259
Monocytes	LSM6	-0.16831	6.681324	-1.69114	0.094332	-5.17655	0.256317	0.36553
Monocytes	LLPH	0.129058	6.447819	1.690954	0.094368	-5.16078	0.258016	0.368034
Monocytes	TIRAP	0.364084	3.005516	1.690706	0.094416	-4.40274	0.284215	0.405096
Monocytes	1300017J02RIK	-0.52238	3.006144	-1.69042	0.094471	-4.34272	0.28421	0.40521
Monocytes	A530076I17RIK	0.822825	-0.88246	1.690297	0.094494	-4.20104	0.317344	0.451432
Monocytes	IL6RA	0.18598	5.504339	1.690212	0.09451	-5.1916	0.264937	0.377991
Monocytes	1110017D15RII	-0.96273	0.201816	-1.69019	0.094514	-4.20179	0.307702	0.438056
Monocytes	TNRC6A	-0.14826	6.648204	-1.68993	0.094565	-5.13385	0.256618	0.366215
Monocytes	NPEPL1	-0.18227	5.049543	-1.68974	0.0946	-4.91612	0.268351	0.382883
Monocytes	MSS51	-0.51834	2.436698	-1.68974	0.094601	-4.27929	0.288834	0.411761
Monocytes	NARS2	0.256134	4.271035	1.689384	0.09467	-4.69869	0.274399	0.391418
Monocytes	CEP76	0.403707	2.990307	1.688842	0.094774	-4.37776	0.284708	0.4059
Monocytes	TDRD3	-0.26356	4.059195	-1.68867	0.094807	-4.61526	0.276279	0.394059
Monocytes	GM20406	0.775605	-0.53168	1.687876	0.09496	-4.20357	0.314862	0.447994
Monocytes	EPHA1	-0.8467	0.461905	-1.68787	0.09496	-4.20579	0.30609	0.43581
Monocytes	SHANK3	-0.90902	1.430051	-1.68786	0.094962	-4.20907	0.297798	0.424246
Monocytes	GM31522	0.604549	-1.00368	1.687177	0.095095	-4.20601	0.319477	0.454185
Monocytes	PAQR3	0.415109	1.469925	1.685954	0.095331	-4.35246	0.298444	0.424537

Monocytes	FAM168A	-0.15235	5.93211	-1.6857	0.095379	-5.09979	0.26327	0.375012
Monocytes	STK10	-0.1349	7.315855	-1.68503	0.095509	-5.35579	0.253574	0.361108
Monocytes	MYH10	0.441925	2.573809	1.68472	0.09557	-4.35359	0.289662	0.412113
Monocytes	BET1L	0.257028	3.860623	1.684715	0.095571	-4.60445	0.279341	0.397618
Monocytes	CNTROB	-0.56694	2.044211	-1.68422	0.095667	-4.26305	0.294244	0.418423
Monocytes	GM4952	-0.65129	2.667628	-1.68382	0.095744	-4.32494	0.289205	0.411373
Monocytes	BRPF3	-0.22539	4.451036	-1.68373	0.095761	-4.6327	0.275036	0.391489
Monocytes	RYR1	-0.3817	1.831874	-1.68349	0.095809	-4.55501	0.296121	0.421121
Monocytes	SIK1	0.153701	6.868808	1.683478	0.09581	-5.21761	0.257044	0.366039
Monocytes	ORC3	0.203389	4.958044	1.683207	0.095863	-4.77756	0.271221	0.386167
Monocytes	ATP6V0B	0.109287	8.046898	1.682861	0.09593	-5.44449	0.248861	0.354495
Monocytes	ACSF3	-0.47735	1.59243	-1.68285	0.095932	-4.30062	0.298257	0.42425
Monocytes	SFT2D2	0.229412	4.677531	1.682495	0.096001	-4.7922	0.273504	0.389453
Monocytes	PIK3C2B	-0.68247	2.667746	-1.68239	0.096022	-4.23567	0.289428	0.411867
Monocytes	DMAC2	0.379976	3.093682	1.681609	0.096174	-4.41668	0.286286	0.40735
Monocytes	UGT1A7C	0.579757	-0.45047	1.681499	0.096195	-4.27817	0.316536	0.449544
Monocytes	NRP2	-0.40983	3.300945	-1.68142	0.096211	-4.67614	0.284618	0.405042
Monocytes	ZFP36L1	-0.16815	7.742233	-1.68012	0.096465	-5.33822	0.251711	0.358321
Monocytes	E2F5	-0.33774	2.69996	-1.68005	0.096479	-4.42821	0.289907	0.412312
Monocytes	CPT1A	0.160157	5.446894	1.6799	0.096507	-4.97554	0.268354	0.381964
Monocytes	FLNB	-0.35004	5.398208	-1.67987	0.096513	-5.06265	0.26872	0.382482
Monocytes	ZZEF1	0.155333	5.867775	1.679843	0.096518	-5.02827	0.265212	0.377515
Monocytes	TRIM8	-0.15296	5.616506	-1.6798	0.096526	-5.09306	0.267082	0.380165
Monocytes	OSGIN1	-0.38324	3.869004	-1.67971	0.096544	-4.56974	0.280508	0.399153
Monocytes	NDUFA7	0.113417	7.909207	1.679445	0.096596	-5.37202	0.250562	0.356767
Monocytes	JMJD6	-0.21652	4.999369	-1.67928	0.096628	-4.8212	0.271758	0.386905
Monocytes	UBE2V1	-0.13032	6.983076	-1.6787	0.096741	-5.21137	0.2571	0.366299
Monocytes	NUCB2	0.19755	4.304904	1.678638	0.096754	-4.76425	0.277108	0.394675
Monocytes	MRPL30	0.129108	6.172662	1.678563	0.096768	-5.13409	0.262978	0.374683
Monocytes	MMP9	0.883875	1.381978	1.678561	0.096769	-4.24682	0.300939	0.428112
Monocytes	KPNB1	-0.16149	5.739818	-1.67847	0.096788	-4.98742	0.26618	0.379245
Monocytes	ZFP719	0.481405	2.119349	1.678438	0.096793	-4.26671	0.294722	0.419452
Monocytes	TMCO4	-0.26636	4.054604	-1.67841	0.096799	-4.7183	0.279065	0.397471
Monocytes	CMTM4	-0.40876	3.141567	-1.67815	0.09685	-4.39977	0.286363	0.407745
Monocytes	CPLANE2	0.834742	0.331465	1.678077	0.096864	-4.21458	0.310076	0.440872
Monocytes	SMYD5	-0.578	2.056268	-1.67709	0.097057	-4.29849	0.295781	0.42062
Monocytes	SMIM20	0.194403	4.914811	1.676856	0.097103	-4.84354	0.272914	0.388575
Monocytes	CCDC167	-0.22608	4.065969	-1.67678	0.097119	-4.80571	0.279499	0.397884
Monocytes	DNAJB11	-0.1443	6.073023	-1.67645	0.097184	-5.09115	0.264307	0.376359
Monocytes	ZKSCAN5	0.456502	2.402977	1.675924	0.097286	-4.33177	0.293254	0.41698
Monocytes	SLC25A34	-0.77577	-0.47643	-1.67571	0.097328	-4.21934	0.318259	0.451706
Monocytes	GGA1	0.170282	4.730426	1.675368	0.097395	-4.88536	0.27477	0.390844
Monocytes	ZDHHC18	-0.22947	5.57552	-1.67519	0.09743	-4.83846	0.268338	0.381772
Monocytes	GPSM3	0.151491	6.022347	1.675138	0.097441	-5.16368	0.265005	0.377057
Monocytes	CPLANE1	0.258573	4.78519	1.674701	0.097527	-4.79037	0.274433	0.390451
Monocytes	MTA3	0.185319	6.109959	1.674663	0.097534	-5.12042	0.264439	0.376323
Monocytes	MBD1	0.246335	4.580853	1.674563	0.097554	-4.68844	0.276011	0.392713

Monocytes	ZFP329	0.409231	2.511051	1.674413	0.097584	-4.31182	0.292588	0.416065
Monocytes	FASTKD2	0.306071	2.952537	1.674231	0.097619	-4.44632	0.288987	0.41108
Monocytes	PIGF	-0.26903	3.388301	-1.67391	0.097682	-4.68369	0.285559	0.40616
Monocytes	AKR1B3	-0.16446	5.402163	-1.67367	0.09773	-4.99813	0.269907	0.384081
Monocytes	MMP19	0.386482	1.213067	1.673439	0.097776	-4.62178	0.303789	0.431627
Monocytes	MBD4	-0.3685	3.212172	-1.67289	0.097883	-4.43858	0.287328	0.408498
Monocytes	BAZ2B	-0.1244	8.430417	-1.67205	0.09805	-5.47255	0.248641	0.353598
Monocytes	MYL4	-0.6741	4.638008	-1.67197	0.098067	-4.41118	0.27639	0.39301
Monocytes	TSHZ1	-0.2411	5.060403	-1.67143	0.098172	-4.89255	0.273349	0.38861
Monocytes	EIF3A	-0.1107	7.379667	-1.67096	0.098266	-5.31419	0.256377	0.364512
Monocytes	GZMA	-0.60448	4.671597	-1.67078	0.098302	-5.09817	0.276558	0.393091
Monocytes	ZFP874A	0.538951	2.146516	1.670561	0.098345	-4.27339	0.296997	0.421807
Monocytes	STXBP3	0.175144	5.278015	1.670323	0.098392	-5.02432	0.271988	0.386718
Monocytes	GM43813	-0.32439	4.535806	-1.6697	0.098516	-4.63487	0.277904	0.394894
Monocytes	HK1	0.192903	4.85354	1.669696	0.098517	-4.91801	0.275437	0.391416
Monocytes	PLXND1	0.24254	3.5086	1.669515	0.098553	-4.6949	0.286075	0.40647
Monocytes	PPP3CC	-0.4178	3.993482	-1.66898	0.098659	-4.47292	0.28239	0.401173
Monocytes	CNST	0.25639	3.943862	1.668853	0.098684	-4.56259	0.282785	0.401758
Monocytes	SERTAD4	-1.09783	-0.0023	-1.66875	0.098704	-4.2324	0.316186	0.448358
Monocytes	EIF2S3Y	3.254999	1.945318	1.668548	0.098745	-4.37041	0.29923	0.42475
Monocytes	MINK1	-0.20973	4.171023	-1.66793	0.098868	-4.82582	0.281214	0.399395
Monocytes	DGCR6	0.307676	3.650268	1.667862	0.098881	-4.52344	0.285365	0.405226
Monocytes	SRSF2	-0.1439	7.941182	-1.66779	0.098896	-5.36219	0.253095	0.359602
Monocytes	MSH5	-0.68995	2.9838	-1.66727	0.098998	-4.27174	0.290995	0.412961
Monocytes	BET1	0.24823	4.246255	1.665649	0.099323	-4.67498	0.281671	0.399278
Monocytes	BLNK	-0.3479	7.200732	-1.66536	0.09938	-4.87028	0.259403	0.367777
Monocytes	RHOH	-0.33188	6.471792	-1.6648	0.099493	-4.80986	0.264897	0.375534
Monocytes	WBP11	0.132881	6.346343	1.664763	0.0995	-5.16038	0.265826	0.376848
Monocytes	ATCAYOS	-0.97346	1.220314	-1.66413	0.099628	-4.23898	0.307388	0.434785
Monocytes	ANGPTL6	-0.79281	0.97577	-1.66386	0.09968	-4.23053	0.309534	0.437749
Monocytes	CFHR2	-0.6271	2.56401	-1.66375	0.099703	-4.33792	0.295924	0.418843
Monocytes	CYP4F13	0.361379	2.829707	1.663696	0.099714	-4.4696	0.293712	0.415759
Monocytes	QPCT	-0.2748	2.264701	-1.66323	0.099807	-4.76033	0.298632	0.422435
Monocytes	PLAGL2	0.20884	4.367209	1.662937	0.099866	-4.80757	0.28147	0.398526
Monocytes	TTC28	-0.35426	4.798912	-1.66288	0.099878	-4.65855	0.278079	0.393766
Monocytes	SIPA1	-0.16818	5.681522	-1.66277	0.099899	-4.97757	0.271285	0.384227
Monocytes	CUEDC1	-0.54476	2.113054	-1.66252	0.09995	-4.29798	0.29997	0.424438
Monocytes	GM11523	-1.08779	-0.95948	-1.66244	0.099965	-4.23156	0.327316	0.462272
Monocytes	MTO1	0.272149	3.553315	1.662117	0.100031	-4.57823	0.288068	0.407972
Monocytes	GM16794	-0.8401	0.41499	-1.66203	0.100049	-4.23256	0.314815	0.445182
Monocytes	PDE7B	0.338217	5.37371	1.661942	0.100066	-5.21026	0.273707	0.387828
Monocytes	GM26520	-0.50165	2.362216	-1.66149	0.100156	-4.48293	0.297915	0.421835
Monocytes	PGAM5	-0.25339	3.934831	-1.6614	0.100175	-4.62768	0.284991	0.403776
Monocytes	MAN1A2	-0.13652	6.392544	-1.66138	0.10018	-5.12368	0.266021	0.377073
Monocytes	B3GALNT1	-0.94345	0.351359	-1.66128	0.1002	-4.23221	0.315386	0.446111
Monocytes	LIG4	-0.7878	1.902715	-1.6611	0.100236	-4.24123	0.301812	0.427326
Monocytes	C1RA	-0.64669	1.359965	-1.66101	0.100254	-4.25193	0.306487	0.433827

Monocytes	RNASET2A	-0.23893	5.439463	-1.66096	0.100263	-4.99167	0.273205	0.387278
Monocytes	C9ORF72	-0.23872	3.893192	-1.66069	0.100319	-5.00398	0.285345	0.404412
Monocytes	TNFRSF21	0.21069	4.10783	1.660649	0.100326	-5.02216	0.283628	0.402004
Monocytes	PILRB1	0.385745	2.049283	1.660147	0.100428	-4.5958	0.300788	0.425869
Monocytes	NDUFA4	-0.16227	8.596899	-1.65994	0.100469	-5.50406	0.250393	0.354922
Monocytes	PEA15A	-0.25884	3.90143	-1.65989	0.100479	-4.71702	0.285473	0.404481
Monocytes	NKAIN2	0.843418	1.350359	1.659539	0.10055	-4.25677	0.306933	0.434294
Monocytes	ERC2	1.065334	0.862583	1.658904	0.100679	-4.25126	0.31149	0.440505
Monocytes	DPYSL3	-0.95276	0.753878	-1.65881	0.100698	-4.23489	0.312452	0.441897
Monocytes	CACFD1	0.292375	3.174111	1.658605	0.100739	-4.52215	0.291811	0.413239
Monocytes	ABCE1	-0.20791	5.007871	-1.65824	0.100814	-4.88212	0.277271	0.392825
Monocytes	METRNL	0.26976	3.205494	1.657574	0.100948	-4.96845	0.291993	0.413231
Monocytes	GRTP1	0.676483	0.35364	1.656271	0.101212	-4.24775	0.317283	0.447828
Monocytes	BRCC3	-0.19507	4.874732	-1.65584	0.101299	-4.86963	0.279414	0.395094
Monocytes	ATP2C1	0.143391	5.85373	1.655306	0.101408	-5.13996	0.272072	0.384607
Monocytes	GM29282	-0.38045	1.712401	-1.65515	0.10144	-4.54978	0.305719	0.431585
Monocytes	CSTF2T	0.349157	3.017029	1.65496	0.101478	-4.4286	0.294678	0.416262
Monocytes	TMEM51	0.193112	3.130512	1.654826	0.101506	-5.07734	0.293736	0.415024
Monocytes	IFI207	0.243062	4.721848	1.653844	0.101706	-5.31145	0.281349	0.397477
Monocytes	STYX	0.196381	4.526153	1.65369	0.101737	-4.79082	0.282907	0.399666
Monocytes	PGLYRP2	-0.65918	2.088786	-1.65333	0.101809	-4.28892	0.303159	0.427918
Monocytes	A430035B10RII	0.512091	3.071834	1.65317	0.101843	-4.4228	0.294872	0.416494
Monocytes	ESPL1	0.444505	2.563574	1.652884	0.101901	-4.39987	0.299221	0.422588
Monocytes	PSME2B	0.374517	2.815452	1.652545	0.101971	-4.53388	0.297219	0.41984
Monocytes	LRAT	-0.88488	-0.03713	-1.65195	0.102092	-4.24096	0.322536	0.454811
Monocytes	STXBP1	-0.38023	3.786972	-1.6518	0.102123	-4.52833	0.289461	0.408945
Monocytes	HEATR9	-0.70849	-0.41964	-1.65148	0.102189	-4.25332	0.326193	0.459795
Monocytes	MCOLN2	-0.4444	3.161941	-1.65082	0.102323	-4.35658	0.295016	0.416471
Monocytes	KDM5C	-0.20352	6.827805	-1.65062	0.102364	-5.25708	0.26623	0.3761
Monocytes	FOXO3	0.198813	6.397005	1.650427	0.102404	-5.10371	0.269474	0.380723
Monocytes	MYO7A	-0.37459	3.25231	-1.65029	0.102431	-4.44248	0.294331	0.415623
Monocytes	ZSWIM1	0.691865	1.4089	1.649859	0.10252	-4.28251	0.310258	0.437531
Monocytes	UNC13B	-0.81434	0.758993	-1.64953	0.102588	-4.24769	0.316152	0.445637
Monocytes	STX16	0.176015	5.756821	1.649205	0.102655	-5.07527	0.274708	0.387921
Monocytes	USP20	-0.33096	2.877475	-1.64906	0.102684	-4.45328	0.297864	0.420295
Monocytes	ANKRD42	-0.97184	0.346625	-1.64847	0.102805	-4.2446	0.320243	0.45106
Monocytes	GOLGA5	0.177004	4.913418	1.648318	0.102837	-4.90601	0.281495	0.397335
Monocytes	TERF2IP	-0.29388	3.58313	-1.64826	0.102849	-4.51396	0.29222	0.412338
Monocytes	CCDC51	0.501224	1.810129	1.648065	0.102889	-4.29909	0.307257	0.433191
Monocytes	CLCN7	0.28723	3.608444	1.64792	0.102919	-4.73013	0.292049	0.412081
Monocytes	GM16090	-1.05927	-0.96132	-1.64758	0.102988	-4.24651	0.33252	0.467994
Monocytes	NPAS2	-0.92151	0.089778	-1.6475	0.103004	-4.24548	0.322719	0.454576
Monocytes	UBE3B	0.202375	4.424846	1.647004	0.103107	-4.76972	0.285679	0.403065
Monocytes	GSN	0.192153	5.844895	1.646908	0.103127	-5.23153	0.274531	0.387441
Monocytes	GM36161	0.320063	0.563057	1.646735	0.103163	-4.81592	0.318633	0.448783
Monocytes	STRN	0.20454	5.530644	1.646577	0.103195	-4.93919	0.276986	0.390935
Monocytes	MTFR1L	0.194349	4.250609	1.645769	0.103362	-4.79312	0.287427	0.405363

Monocytes	ADGRL4	-0.60464	3.201942	-1.64575	0.103365	-4.36397	0.296039	0.417368
Monocytes	ANKRD13D	-0.43797	2.738056	-1.64561	0.103396	-4.38252	0.299945	0.422829
Monocytes	KRT8	-0.68245	1.975849	-1.64527	0.103465	-4.31721	0.306512	0.431992
Monocytes	GM48696	0.485391	2.476417	1.645176	0.103484	-4.3675	0.302204	0.426021
Monocytes	STXBP2	0.176522	5.31968	1.645134	0.103493	-5.00846	0.278968	0.393609
Monocytes	POPDC3	-1.05995	0.894439	-1.64499	0.103524	-4.24822	0.316055	0.445201
Monocytes	DDX39B	-0.14779	7.251694	-1.64416	0.103694	-5.29478	0.264685	0.37347
Monocytes	STX2	-0.21857	3.698442	-1.64396	0.103735	-4.71937	0.292406	0.412339
Monocytes	TMEM120A	0.269092	3.799392	1.642607	0.104016	-4.65276	0.292211	0.411612
Monocytes	GAS2	0.424885	2.456745	1.642595	0.104019	-4.3862	0.303489	0.427279
Monocytes	BACH2	-0.23845	8.964274	-1.64196	0.10415	-5.39961	0.253197	0.35672
Monocytes	PLEKHA5	-0.20277	4.693302	-1.6419	0.104164	-4.99269	0.2852	0.401785
Monocytes	LOXL2	0.756212	1.406795	1.641572	0.104231	-4.26108	0.312987	0.440329
Monocytes	CKAP2L	-0.28129	4.357364	-1.64149	0.104249	-4.83	0.287979	0.405636
Monocytes	ATP11A	0.251829	2.676915	1.641339	0.10428	-4.77426	0.301947	0.425066
Monocytes	SMN1	0.209464	4.851728	1.640868	0.104378	-4.89489	0.284205	0.400275
Monocytes	KLF4	-0.1675	6.447469	-1.64044	0.104467	-5.44384	0.271918	0.383025
Monocytes	WLS	0.246726	4.263574	1.640361	0.104483	-4.90943	0.289071	0.407044
Monocytes	UBXN2A	-0.16571	5.233503	-1.63946	0.104671	-4.99636	0.281663	0.396585
Monocytes	FAM53A	0.250017	3.797306	1.639321	0.1047	-4.67328	0.293257	0.412777
Monocytes	DUSP1	0.183873	7.412842	1.639315	0.104701	-5.56801	0.265036	0.373253
Monocytes	ZFP398	-0.2914	4.517006	-1.63812	0.104951	-4.71565	0.287989	0.405029
Monocytes	METAP1D	0.313362	3.768583	1.637579	0.105064	-4.60468	0.294348	0.413684
Monocytes	NET1	-0.25793	4.035875	-1.6373	0.105122	-4.84108	0.292223	0.410751
Monocytes	ABCC10	-0.66114	0.65899	-1.63695	0.105195	-4.26157	0.321572	0.451259
Monocytes	1110035H17RII	0.638189	1.216236	1.636897	0.105207	-4.29165	0.316528	0.444327
Monocytes	ARL3	-0.21437	4.04145	-1.63603	0.105389	-4.84535	0.292665	0.411161
Monocytes	TRAF7	0.187675	4.585124	1.635913	0.105413	-4.84276	0.288228	0.405025
Monocytes	DEGS1	0.163028	6.525466	1.635681	0.105462	-5.30579	0.272994	0.383811
Monocytes	HACD3	-0.19131	4.907611	-1.63563	0.105472	-4.8502	0.285634	0.401494
Monocytes	D8ERTD738E	0.110886	7.358725	1.635286	0.105545	-5.39183	0.266839	0.375077
Monocytes	GM4924	0.892755	0.278075	1.635051	0.105594	-4.25872	0.325729	0.456889
Monocytes	TMEM241	0.299093	4.310016	1.634809	0.105645	-4.71322	0.290652	0.408469
Monocytes	PIK3R6	0.331198	2.596377	1.634761	0.105655	-4.82743	0.30503	0.428418
Monocytes	MFSD3	-0.71622	0.642198	-1.63432	0.105747	-4.27023	0.32258	0.452388
Monocytes	SAR1A	-0.1646	5.508767	-1.63406	0.105802	-5.04662	0.281278	0.395113
Monocytes	SULT2A2	-0.58304	4.037802	-1.63367	0.105884	-4.61772	0.293164	0.411766
Monocytes	RTN4IP1	0.588787	1.927586	1.633606	0.105898	-4.32325	0.311149	0.436663
Monocytes	HAO2	-0.94855	1.393032	-1.63359	0.105901	-4.28146	0.315895	0.4432
Monocytes	SRP72	0.121358	6.657086	1.633211	0.105981	-5.2687	0.272544	0.382957
Monocytes	NCL	-0.18086	7.783001	-1.6331	0.106005	-5.37943	0.264135	0.371141
Monocytes	MRPL44	0.37613	2.945204	1.632646	0.1061	-4.45712	0.302637	0.424785
Monocytes	MBTD1	-0.14209	7.420536	-1.63253	0.106124	-5.43196	0.266966	0.3751
Monocytes	NEMF	-0.13739	5.95092	-1.63157	0.106328	-5.11969	0.278591	0.391149
Monocytes	3830403N18RII	-0.94909	0.704685	-1.63139	0.106365	-4.2838	0.322993	0.452679
Monocytes	ENTHD1	-0.55508	0.023444	-1.63127	0.10639	-4.41237	0.329306	0.461372
Monocytes	DNAAF5	-0.38336	2.988111	-1.63103	0.106441	-4.42305	0.302842	0.424982

Monocytes	BCL2L2	0.469964	1.276497	1.630582	0.106536	-4.34337	0.318059	0.445816
Monocytes	DANCR	0.622958	1.545559	1.630355	0.106584	-4.2923	0.315692	0.44259
Monocytes	ACTR2	0.110747	8.066808	1.630228	0.106611	-5.51033	0.262914	0.369134
Monocytes	HOOK1	-0.43176	2.667358	-1.63001	0.106657	-4.40224	0.30588	0.428998
Monocytes	GM36371	-0.59644	0.884673	-1.62956	0.106753	-4.27543	0.321908	0.450884
Monocytes	ZFP143	0.264855	3.815014	1.629432	0.106779	-4.61494	0.296321	0.4156
Monocytes	PXDC1	-0.63299	2.191629	-1.62888	0.106896	-4.38654	0.310461	0.435066
Monocytes	RFC3	-0.26417	4.31335	-1.62848	0.106982	-4.67623	0.29255	0.410198
Monocytes	FBXO3	0.179544	5.012921	1.628411	0.106996	-4.87758	0.286861	0.402304
Monocytes	GM50333	-0.51574	1.191453	-1.62813	0.107056	-4.33442	0.319596	0.447501
Monocytes	STRADA	0.212872	4.58715	1.627882	0.107108	-4.89625	0.290455	0.407302
Monocytes	HSDL2	-0.20196	4.684002	-1.62773	0.107141	-4.91289	0.289675	0.406231
Monocytes	NAA35	-0.18195	4.851078	-1.62726	0.10724	-4.91244	0.28844	0.404477
Monocytes	TAGLN	-0.76181	2.304796	-1.62699	0.107298	-4.38633	0.309886	0.434177
Monocytes	B430306N03RII	-0.40858	2.692804	-1.62683	0.107333	-4.5067	0.306507	0.429517
Monocytes	MRPL3	-0.23511	4.701467	-1.62681	0.107337	-4.83463	0.289654	0.406166
Monocytes	HMBS	0.290618	4.451319	1.626703	0.107359	-4.77894	0.291695	0.40904
Monocytes	CCDC86	0.165791	5.146452	1.626698	0.10736	-5.0914	0.286061	0.4012
Monocytes	TMEM135	0.171146	5.7512	1.626295	0.107446	-5.15784	0.281405	0.394677
Monocytes	TMEM19	-0.21622	3.958743	-1.62606	0.107496	-4.76959	0.295976	0.414902
Monocytes	FBXL18	-0.50981	2.985602	-1.6255	0.107616	-4.50063	0.304461	0.426501
Monocytes	NPHP4	0.757034	0.297045	1.625199	0.10768	-4.27764	0.328651	0.45971
Monocytes	GM4316	0.76644	0.707324	1.624412	0.107848	-4.28132	0.325263	0.454724
Monocytes	VPS37C	-0.19551	3.950923	-1.62421	0.107891	-4.82216	0.296805	0.41556
Monocytes	TSPAN2	-0.36706	3.159011	-1.62356	0.10803	-4.47559	0.303754	0.425106
Monocytes	BECN1	0.149966	5.896307	1.623516	0.108039	-5.13272	0.281278	0.393928
Monocytes	PDP2	-0.24271	3.724878	-1.62294	0.108162	-4.71344	0.299087	0.418722
Monocytes	GPT2	-0.59791	1.738624	-1.62291	0.108169	-4.33772	0.31634	0.442507
Monocytes	GARS	-0.16243	5.511566	-1.62287	0.108177	-5.09057	0.284454	0.398405
Monocytes	ASTL	-0.57515	1.424213	-1.6223	0.1083	-4.38178	0.319337	0.446537
Monocytes	SLC35C1	0.309469	2.749333	1.622243	0.108312	-4.60988	0.307589	0.430383
Monocytes	KLHL14	-0.78995	2.717642	-1.6222	0.108322	-4.30422	0.307864	0.430763
Monocytes	RAET1E	0.224227	4.113795	1.621345	0.108505	-4.91663	0.296407	0.414545
Monocytes	CYB561D2	-0.2822	3.329402	-1.62095	0.10859	-4.61203	0.303182	0.423908
Monocytes	H2-DMA	-0.21311	5.184297	-1.62076	0.108631	-5.35966	0.287787	0.402586
Monocytes	HK2	-0.20997	5.335441	-1.62066	0.108652	-5.24611	0.286571	0.40094
Monocytes	SELPLG	-0.15785	6.845492	-1.62029	0.108732	-5.35404	0.274811	0.384583
Monocytes	PF4	0.625306	4.278945	1.620216	0.108747	-4.99356	0.295279	0.413086
Monocytes	HOMER3	0.349812	3.103095	1.6201	0.108772	-4.45157	0.305219	0.426855
Monocytes	ZFP651	-0.55128	1.578642	-1.61997	0.108801	-4.32055	0.318659	0.445398
Monocytes	ZFP963	0.574444	1.15161	1.619368	0.10893	-4.30464	0.322754	0.450849
Monocytes	1600012H06RII	0.30007	2.771016	1.619327	0.108939	-4.52068	0.308299	0.431
Monocytes	MKRN2	-0.18988	4.074932	-1.61922	0.108962	-4.77235	0.297177	0.415642
Monocytes	CELF4	0.620283	0.6776	1.618998	0.10901	-4.31327	0.327136	0.456864
Monocytes	DNAJC2	-0.15133	5.81945	-1.61893	0.109024	-5.10839	0.282996	0.395973
Monocytes	FBXW2	0.130178	6.193608	1.61871	0.109072	-5.17104	0.280066	0.391923
Monocytes	P2RY13	-0.7309	1.100765	-1.61863	0.109089	-4.33304	0.323247	0.451617

Monocytes	MPC1	0.13807	7.569491	1.618371	0.109145	-5.45516	0.269597	0.377268
Monocytes	RXRA	-0.27428	3.1994	-1.61818	0.109187	-4.71897	0.304731	0.426109
Monocytes	E230016M11RI	-0.43644	2.465953	-1.61776	0.109277	-4.47844	0.311261	0.4349
Monocytes	SC5D	-0.29095	4.248539	-1.61765	0.1093	-4.6912	0.296009	0.413855
Monocytes	CCDC62	-0.53226	2.858429	-1.61752	0.109329	-4.41102	0.30783	0.430178
Monocytes	SIRT1	-0.16822	5.043074	-1.61731	0.109375	-4.94157	0.289521	0.404832
Monocytes	ITGA4	0.153684	7.657286	1.617076	0.109424	-5.5968	0.269194	0.376474
Monocytes	BRMS1L	0.229976	4.269409	1.616259	0.109601	-4.7355	0.296211	0.414015
Monocytes	9530077C05RIK	0.863062	1.213471	1.616212	0.109611	-4.2776	0.322903	0.450761
Monocytes	TBC1D2B	0.230809	4.091379	1.61616	0.109623	-4.89983	0.297697	0.416085
Monocytes	NIPA2	0.15235	6.042829	1.615941	0.10967	-5.19727	0.281847	0.394084
Monocytes	PRKCH	-0.22101	6.999653	-1.61592	0.109675	-5.52841	0.274422	0.383725
Monocytes	CPNE2	-0.32183	3.37335	-1.61548	0.109771	-4.7388	0.303961	0.424542
Monocytes	GM31763	-0.33837	3.257819	-1.61531	0.109806	-4.69414	0.30497	0.426
Monocytes	CEP97	0.467901	2.922474	1.615022	0.109869	-4.38232	0.307961	0.430022
Monocytes	PPP5C	-0.21894	4.346979	-1.61463	0.109955	-4.7899	0.296004	0.413396
Monocytes	CACNA1A	0.674268	1.424509	1.61445	0.109993	-4.30779	0.321478	0.448417
Monocytes	PAK4	0.346103	2.299468	1.614119	0.110065	-4.51092	0.313718	0.437754
Monocytes	DOP1A	0.24471	4.09244	1.614011	0.110089	-4.80838	0.298251	0.416452
Monocytes	SHANK2	-0.77831	1.410698	-1.61341	0.110219	-4.28598	0.322005	0.448991
Monocytes	GM47754	0.60602	0.667894	1.6129	0.11033	-4.36577	0.328965	0.458473
Monocytes	GM2629	0.439912	0.511245	1.612887	0.110333	-4.42684	0.330431	0.460471
Monocytes	NUP62	-0.32103	4.181003	-1.61268	0.110378	-4.74091	0.297876	0.415841
Monocytes	JOSD1	-0.22525	3.99961	-1.61248	0.110422	-4.7617	0.2994	0.418023
Monocytes	RAD51	-0.4022	3.891445	-1.61246	0.110425	-4.68506	0.300312	0.419283
Monocytes	ZCCHC9	0.150507	5.404138	1.612206	0.110481	-5.06587	0.287826	0.402054
Monocytes	HACL1	-0.42998	3.523051	-1.6122	0.110482	-4.52152	0.303443	0.423667
Monocytes	SYNPO	-0.6373	0.219102	-1.61206	0.110514	-4.33777	0.333185	0.464495
Monocytes	9130230L23RIK	-0.28108	3.687563	-1.61194	0.110538	-5.0972	0.302041	0.42182
Monocytes	SLC44A2	0.207913	6.242042	1.611893	0.110549	-5.09999	0.281159	0.392865
Monocytes	TENM3	-0.76569	1.688761	-1.61172	0.110587	-4.30776	0.319603	0.446056
Monocytes	MTHFD1	0.298839	3.79072	1.611472	0.110641	-4.64042	0.301254	0.42082
Monocytes	CREBRF	0.190232	6.98351	1.611196	0.110701	-5.24682	0.275562	0.385089
Monocytes	RHOBTB2	-0.39661	2.943311	-1.61105	0.110734	-4.4686	0.308628	0.430943
Monocytes	MAGOHB	0.233158	4.560419	1.610912	0.110763	-5.0054	0.294893	0.412029
Monocytes	MBOAT2	1.106137	0.666194	1.610465	0.110861	-4.28241	0.329379	0.459241
Monocytes	TMPRSS6	-0.75054	1.467899	-1.61029	0.1109	-4.31655	0.322002	0.449158
Monocytes	TNK2	-0.44498	3.209858	-1.60976	0.111015	-4.41206	0.306768	0.428049
Monocytes	DNAAF3	-0.75598	-0.6587	-1.60958	0.111055	-4.28972	0.342347	0.476751
Monocytes	SKA2	-0.40633	3.293834	-1.60944	0.111084	-4.49656	0.306069	0.427192
Monocytes	FMN2	-0.97124	2.359589	-1.60885	0.111215	-4.30123	0.314248	0.438572
Monocytes	CABIN1	0.172176	5.4487	1.608842	0.111216	-5.06373	0.288087	0.402443
Monocytes	TRAPPC6B	0.119073	6.134079	1.608803	0.111225	-5.21913	0.282617	0.394837
Monocytes	MAP3K5	0.157858	7.255072	1.608731	0.11124	-5.41659	0.27392	0.38271
Monocytes	ADCY9	0.349347	3.136828	1.60846	0.1113	-4.63909	0.307427	0.429298
Monocytes	ZFPM2	-0.73728	2.777013	-1.60845	0.111303	-4.41026	0.310564	0.433617
Monocytes	RGS16	-0.95149	1.351563	-1.60838	0.111317	-4.2852	0.323343	0.451172

Monocytes	IFI213	0.596032	3.327221	1.608345	0.111325	-4.69094	0.305781	0.427052
Monocytes	NAXD	0.215335	4.128354	1.608226	0.111351	-4.81018	0.298961	0.417647
Monocytes	SPECC1	-0.22646	4.276803	-1.60746	0.111518	-5.2732	0.298084	0.416061
Monocytes	JKAMP	0.219893	3.680975	1.606869	0.111649	-4.71072	0.303249	0.423296
Monocytes	ADSL	-0.25218	4.033205	-1.60673	0.11168	-4.71115	0.300258	0.419187
Monocytes	TMEM189	0.188034	6.387922	1.60667	0.111693	-5.28171	0.281084	0.392603
Monocytes	CYREN	-0.30595	3.374354	-1.60665	0.111698	-4.65764	0.30588	0.426959
Monocytes	MRPL36	0.166998	5.835469	1.606578	0.111713	-5.18042	0.285457	0.398688
Monocytes	CEP44	0.247314	3.531339	1.606426	0.111746	-4.61386	0.304539	0.425142
Monocytes	PITPNC1	-0.17536	8.799744	-1.60629	0.111776	-5.58149	0.262867	0.367186
Monocytes	BCL2	0.321783	5.016128	1.605868	0.111869	-5.01359	0.292149	0.408152
Monocytes	SSH2	0.179837	8.396317	1.605814	0.111881	-5.52333	0.265873	0.371499
Monocytes	GM16287	0.641984	-0.44109	1.605668	0.111913	-4.30423	0.340873	0.475023
Monocytes	GPX1	0.177329	11.17498	1.605651	0.111917	-6.05886	0.246268	0.343877
Monocytes	LRCH1	0.149179	7.432191	1.604906	0.112081	-5.38569	0.273409	0.381829
Monocytes	SLC25A4	-0.16089	7.14787	-1.60474	0.112117	-5.42543	0.275581	0.384951
Monocytes	TMEM179B	0.127995	5.864754	1.604543	0.112161	-5.19766	0.285626	0.39899
Monocytes	SERPINA10	-0.74678	0.966902	-1.60451	0.112168	-4.29747	0.327891	0.457246
Monocytes	MBOAT1	0.432732	0.682144	1.604103	0.112258	-4.59309	0.330729	0.46099
Monocytes	SLC4A7	0.179103	5.767425	1.603926	0.112297	-5.18986	0.286582	0.400207
Monocytes	B4GALT3	0.230523	3.874826	1.603385	0.112416	-4.69166	0.302453	0.422026
Monocytes	ACOXL	-0.50784	1.285897	-1.603	0.112502	-4.48532	0.325562	0.453706
Monocytes	CTBS	0.265889	2.934998	1.602738	0.112559	-4.67378	0.310802	0.433537
Monocytes	GM20186	0.410131	3.559404	1.602169	0.112685	-4.58457	0.30562	0.426382
Monocytes	ENDOU	-1.01075	0.913658	-1.60207	0.112708	-4.29055	0.329358	0.459008
Monocytes	ANKRD9	-0.56912	2.821177	-1.60145	0.112846	-4.37129	0.312346	0.435566
Monocytes	ZFP84	-0.2895	3.7569	-1.60116	0.112908	-4.69768	0.304303	0.424371
Monocytes	SDC2	-0.61954	2.267759	-1.60034	0.11309	-4.37283	0.317594	0.442587
Monocytes	KIF13A	0.206083	4.605824	1.60029	0.113102	-5.01646	0.297345	0.414712
Monocytes	PPARA	-0.61171	2.784821	-1.60027	0.113105	-4.43602	0.312988	0.43627
Monocytes	MMP27	-0.80644	-0.20195	-1.60025	0.113111	-4.29404	0.340639	0.474023
Monocytes	ST6GAL1	-0.28574	5.862113	-1.59969	0.113236	-5.16678	0.287302	0.400698
Monocytes	ACOT11	0.335675	1.943725	1.599412	0.113297	-4.54769	0.320876	0.446989
Monocytes	COPS4	-0.15057	5.581159	-1.59911	0.113363	-5.08613	0.289741	0.404039
Monocytes	SETD1B	0.19524	5.026478	1.598756	0.113443	-4.97603	0.294407	0.41051
Monocytes	PSMD3	-0.20426	4.642778	-1.59845	0.11351	-4.87909	0.297665	0.415068
Monocytes	SMG5	-0.16181	5.55464	-1.59832	0.11354	-5.05642	0.290152	0.404697
Monocytes	ZFP97	0.62897	1.263623	1.597631	0.113693	-4.32232	0.327444	0.456098
Monocytes	ABHD5	0.250699	3.453436	1.597606	0.113699	-4.79364	0.307791	0.429151
Monocytes	ZFP775	0.716885	0.81684	1.597557	0.11371	-4.30483	0.331619	0.461794
Monocytes	AK4	0.712002	1.048143	1.597527	0.113716	-4.33689	0.32945	0.458837
Monocytes	TGOLN1	-0.16922	5.839155	-1.59733	0.113761	-5.15606	0.287852	0.401599
Monocytes	JUP	-0.26035	3.562597	-1.59695	0.113846	-4.81853	0.306846	0.427949
Monocytes	NISCH	0.14892	6.194239	1.59691	0.113854	-5.18435	0.285009	0.397718
Monocytes	RAP2C	0.180617	4.944479	1.596754	0.113889	-4.98879	0.295156	0.411851
Monocytes	KIDINS220	0.167185	5.419693	1.596599	0.113923	-5.06522	0.291251	0.406453
Monocytes	IFT43	-0.53504	1.54627	-1.59658	0.113928	-4.34996	0.324831	0.452707

Monocytes	ZFP948	-0.43661	2.965976	-1.59656	0.113931	-4.5514	0.312051	0.435182
Monocytes	CD38	0.368396	5.882237	1.596435	0.11396	-5.01088	0.287505	0.401253
Monocytes	SLC38A4	-0.58111	3.153421	-1.59634	0.113982	-4.51208	0.310405	0.432919
Monocytes	RCAN1	-0.24388	3.708543	-1.59629	0.113992	-4.79193	0.305588	0.426283
Monocytes	UTP14A	0.180244	5.238791	1.596235	0.114005	-5.01496	0.292731	0.408506
Monocytes	AAK1	-0.17434	6.392459	-1.59622	0.114008	-5.20597	0.283436	0.395593
Monocytes	RGS9	-0.61664	0.03543	-1.59581	0.114099	-4.37759	0.339242	0.472242
Monocytes	GFPT1	-0.17379	5.095805	-1.59544	0.114183	-5.2888	0.294132	0.410418
Monocytes	PIGYL	0.218198	4.270837	1.595276	0.114219	-4.87056	0.301023	0.419952
Monocytes	MFSD1	0.151252	5.149534	1.595246	0.114226	-5.1752	0.29369	0.409804
Monocytes	ABHD15	0.413629	2.977881	1.595156	0.114246	-4.56043	0.312186	0.435338
Monocytes	GMEB1	0.200673	5.025748	1.594347	0.114427	-4.96242	0.295018	0.411557
Monocytes	IGF2BP3	0.170146	6.5516	1.594318	0.114434	-5.27091	0.282692	0.394446
Monocytes	CHRNB1	-0.77863	0.775917	-1.59422	0.114455	-4.30153	0.332607	0.463235
Monocytes	STAG2	0.148261	7.464582	1.59396	0.114514	-5.395	0.275663	0.384687
Monocytes	GM15886	0.481086	1.881502	1.59302	0.114725	-4.34573	0.322937	0.449616
Monocytes	DDB1	-0.17898	5.611857	-1.59276	0.114783	-5.09243	0.290802	0.405299
Monocytes	MSL2	0.140616	6.010868	1.592437	0.114856	-5.22504	0.287575	0.400938
Monocytes	ZPR1	0.203509	4.397865	1.592405	0.114863	-4.8472	0.300872	0.419359
Monocytes	AGXT2	-0.72995	1.612637	-1.59223	0.114902	-4.34336	0.325468	0.453173
Monocytes	DAGLA	-0.88176	-0.78197	-1.59199	0.114956	-4.2994	0.348377	0.484351
Monocytes	CPA6	0.934008	-0.07229	1.591895	0.114978	-4.29958	0.341411	0.47491
Monocytes	MBTPS2	0.253033	3.917498	1.591753	0.115009	-4.73251	0.304964	0.425054
Monocytes	OTC	-0.5838	3.438444	-1.59156	0.115052	-4.55902	0.309105	0.430795
Monocytes	JAM2	0.819277	1.449339	1.59145	0.115078	-4.33628	0.326977	0.45531
Monocytes	CDC27	0.164153	6.009717	1.591248	0.115123	-5.13236	0.287584	0.401139
Monocytes	MYC	-0.45255	3.533741	-1.59124	0.115126	-4.69421	0.308277	0.429763
Monocytes	CEP164	0.259672	4.052145	1.591198	0.115134	-4.71253	0.303811	0.423607
Monocytes	RASIP1	-0.59529	1.808725	-1.59118	0.115138	-4.37938	0.323666	0.450886
Monocytes	PKNOX1	0.262805	4.569323	1.5909	0.115201	-4.7896	0.299481	0.417625
Monocytes	HSF5	-0.71019	0.277217	-1.59082	0.115218	-4.32442	0.338096	0.47057
Monocytes	ARAP3	-0.35808	3.537048	-1.59065	0.115257	-4.65627	0.308326	0.429807
Monocytes	JADE3	-0.3113	3.471551	-1.59045	0.115304	-4.57278	0.308934	0.430658
Monocytes	PPP1R9A	-0.38296	3.383193	-1.59032	0.115332	-4.68647	0.309705	0.43173
Monocytes	BAG2	-0.65945	1.435408	-1.58942	0.115535	-4.32165	0.327438	0.456215
Monocytes	TADA2B	-0.33645	3.468615	-1.58938	0.115543	-4.57284	0.309156	0.431134
Monocytes	NUBPL	-0.34708	2.913085	-1.58938	0.115545	-4.52979	0.314038	0.43785
Monocytes	PARP16	-0.72749	1.117003	-1.58937	0.115548	-4.31929	0.330407	0.46027
Monocytes	TTC5	0.185591	4.64029	1.589331	0.115555	-4.89546	0.299134	0.417308
Monocytes	CYBA	0.131581	8.55691	1.58911	0.115605	-5.70484	0.268225	0.374286
Monocytes	CCDC162	-0.41185	2.876554	-1.58827	0.115795	-4.71173	0.314647	0.438757
Monocytes	GLG1	0.145661	6.951102	1.587943	0.115869	-5.32733	0.280667	0.391864
Monocytes	SSR3	0.133481	6.071068	1.587932	0.115872	-5.28377	0.287644	0.401583
Monocytes	LRRC42	-0.29738	3.272197	-1.58783	0.115894	-4.59806	0.311155	0.434158
Monocytes	FLOT1	0.19763	4.575358	1.587706	0.115923	-5.0181	0.299952	0.418724
Monocytes	ECHDC2	-0.74441	1.612612	-1.58759	0.11595	-4.33762	0.326095	0.454716
Monocytes	MTMR6	0.179415	4.718718	1.587568	0.115954	-5.05133	0.298748	0.417071

Monocytes	CLEC2G	0.569101	0.015976	1.587545	0.115959	-4.4022	0.34121	0.475335
Monocytes	TPRA1	-0.29022	2.802051	-1.58754	0.115961	-4.67512	0.31531	0.439922
Monocytes	PHF14	0.143604	6.331947	1.586562	0.116182	-5.21737	0.285844	0.398991
Monocytes	FGF13	-0.48963	3.379693	-1.58648	0.116201	-4.6479	0.310527	0.433166
Monocytes	ST8SIA4	-0.17224	7.324778	-1.58644	0.11621	-5.51063	0.278043	0.38813
Monocytes	4833417C18RIK	-0.71579	0.707506	-1.58639	0.116222	-4.31567	0.33491	0.46662
Monocytes	ACP5	0.293639	5.117131	1.586355	0.116229	-5.03471	0.295726	0.412761
Monocytes	7-Sep	-0.09521	8.277792	-1.58617	0.116272	-5.55988	0.270809	0.378044
Monocytes	GRAMD1B	-0.25223	5.536445	-1.58577	0.116361	-5.06691	0.292453	0.408172
Monocytes	ANGPT2	-0.83971	0.571482	-1.58551	0.116421	-4.30626	0.336489	0.468635
Monocytes	NSD2	-0.16709	6.699324	-1.58539	0.116448	-5.28459	0.283168	0.395223
Monocytes	UTP6	-0.21993	4.42275	-1.58485	0.116571	-4.86344	0.30204	0.421239
Monocytes	PFDN5	-0.09812	7.413663	-1.58448	0.116655	-5.49227	0.277891	0.387692
Monocytes	POU2F1	0.231749	5.39401	1.584439	0.116664	-5.0502	0.294006	0.410115
Monocytes	D230025D16RIK	0.227139	4.57771	1.584017	0.11676	-4.86536	0.30091	0.419623
Monocytes	ECHS1	0.204575	5.549982	1.58401	0.116762	-5.17581	0.292818	0.408417
Monocytes	GM48086	0.622002	1.511775	1.58377	0.116817	-4.33719	0.328162	0.457023
Monocytes	PGRMC2	-0.23377	3.628957	-1.58345	0.11689	-4.67616	0.309207	0.431036
Monocytes	CD79A	-0.35895	6.200185	-1.58335	0.116913	-4.87714	0.287691	0.40128
Monocytes	KLRB1F	0.671372	0.424065	1.582806	0.117036	-4.39814	0.338742	0.471327
Monocytes	RBM43	0.342494	2.961078	1.582793	0.11704	-4.57922	0.315262	0.439234
Monocytes	MFAP2	-0.95751	0.586159	-1.58214	0.117188	-4.30973	0.337465	0.469249
Monocytes	TRAPPC4	0.1505	5.137813	1.582032	0.117213	-5.12754	0.296785	0.413448
Monocytes	FXN	-0.24388	4.085717	-1.58197	0.117227	-4.74001	0.305682	0.425736
Monocytes	ARPC5	0.098097	8.144041	1.581798	0.117267	-5.59824	0.272939	0.38029
Monocytes	MCL1	0.134882	8.364811	1.581231	0.117396	-5.65866	0.271503	0.378077
Monocytes	ASB1	0.538769	1.712368	1.579782	0.117728	-4.36475	0.328001	0.455539
Monocytes	DFFA	-0.37462	2.939835	-1.57954	0.117783	-4.52014	0.316861	0.440269
Monocytes	DOCK6	-0.52354	1.914885	-1.57944	0.117806	-4.40662	0.326173	0.453045
Monocytes	TMEM154	0.374617	2.583653	1.579215	0.117858	-4.6229	0.320123	0.444839
Monocytes	USP3	0.151516	6.873279	1.578794	0.117955	-5.30019	0.283974	0.394888
Monocytes	LEF1	-0.50832	5.638758	-1.57851	0.118019	-4.80143	0.29402	0.40878
Monocytes	MFSD5	0.2196	4.282812	1.578179	0.118096	-4.92892	0.305537	0.424659
Monocytes	IRS1	-0.80735	0.821216	-1.57795	0.118147	-4.31813	0.336928	0.46764
Monocytes	SPICE1	0.448166	1.890422	1.577906	0.118159	-4.45177	0.326871	0.45395
Monocytes	CCR3	-1.51519	-0.02075	-1.5776	0.118228	-4.31606	0.345198	0.478752
Monocytes	HSD11B1	0.303482	4.593839	1.576337	0.11852	-4.84529	0.303653	0.421564
Monocytes	AK2	-0.17081	5.995761	-1.57584	0.118633	-5.17047	0.292162	0.405615
Monocytes	GM38832	-0.37092	0.763984	-1.5756	0.11869	-4.60166	0.338645	0.469292
Monocytes	ZFP652	0.194064	5.654413	1.575439	0.118727	-5.14719	0.295045	0.409675
Monocytes	SLC38A3	-0.70814	1.479322	-1.57494	0.118843	-4.35296	0.331887	0.460371
Monocytes	TICAM2	0.333252	1.274799	1.574808	0.118873	-4.63751	0.333816	0.462996
Monocytes	LIN37	0.276108	3.646925	1.574796	0.118875	-4.71216	0.312175	0.433437
Monocytes	MXI1	0.162054	7.063981	1.574714	0.118894	-5.55723	0.283683	0.394106
Monocytes	ZC3H15	-0.11606	7.266033	-1.57467	0.118904	-5.40456	0.282091	0.391896
Monocytes	FAP	-0.70767	0.744801	-1.57436	0.118977	-4.43476	0.338873	0.469957
Monocytes	GLIS3	0.458891	3.068689	1.574308	0.118988	-4.77268	0.317304	0.440556

Monocytes	MICALL1	-0.26658	2.97359	-1.57415	0.119025	-4.68037	0.318157	0.44181
Monocytes	KIF24	0.27769	3.667581	1.574081	0.119041	-4.68317	0.311993	0.433366
Monocytes	GNPAT	-0.23554	4.15461	-1.57408	0.119041	-4.79146	0.307747	0.427531
Monocytes	HELB	0.283812	3.704754	1.573817	0.119101	-4.72888	0.311745	0.433048
Monocytes	NRD1	0.140524	6.185357	1.57343	0.119191	-5.28844	0.290938	0.404274
Monocytes	EBI3	-0.18494	4.387447	-1.57304	0.119282	-5.17531	0.306124	0.425136
Monocytes	STK38	0.143307	6.531625	1.572789	0.11934	-5.33661	0.288329	0.400531
Monocytes	GM26917	-0.23727	5.74366	-1.57259	0.119385	-5.12544	0.294747	0.409455
Monocytes	FBXO30	-0.22032	5.032199	-1.57251	0.119404	-5.00345	0.300679	0.417677
Monocytes	VPS4A	-0.20521	4.241466	-1.57226	0.119463	-4.85558	0.307429	0.427095
Monocytes	PTPN13	-0.82817	0.415506	-1.57223	0.119469	-4.32866	0.342538	0.475038
Monocytes	FADS1	-0.4031	3.059286	-1.57217	0.119484	-4.52064	0.317835	0.441401
Monocytes	MECP2	0.1448	5.847525	1.571985	0.119526	-5.18179	0.293913	0.408491
Monocytes	SFMBT2	0.708532	0.60235	1.571867	0.119553	-4.34284	0.34075	0.472722
Monocytes	RAD23A	-0.18911	5.807004	-1.57164	0.119606	-5.1458	0.294299	0.409062
Monocytes	CAMK2G	0.210843	4.893224	1.57138	0.119666	-4.97605	0.301971	0.419677
Monocytes	THBD	0.295042	3.145231	1.571308	0.119683	-5.00504	0.31719	0.4406
Monocytes	CXCR3	-0.3622	1.731497	-1.57076	0.119811	-4.80088	0.330147	0.458507
Monocytes	LZTFL1	0.209217	5.075487	1.570559	0.119857	-5.10262	0.300458	0.417784
Monocytes	SRGAP1	-0.62699	2.559229	-1.57044	0.119885	-4.4054	0.322508	0.448089
Monocytes	GM38394	0.664053	0.848639	1.570322	0.119912	-4.35015	0.338513	0.46991
Monocytes	KCTD12	-0.15023	6.383969	-1.57024	0.119931	-5.40473	0.289658	0.402838
Monocytes	IL1B	0.272555	5.12575	1.570239	0.119932	-5.54875	0.300035	0.4172
Monocytes	COL23A1	-0.65454	1.376373	-1.57014	0.119955	-4.38579	0.333485	0.46307
Monocytes	LNPEP	0.126354	7.335044	1.570081	0.119968	-5.48283	0.282082	0.392312
Monocytes	IL18R1	-0.65786	0.978465	-1.57002	0.119983	-4.38342	0.337269	0.468218
Monocytes	NDUFA6	0.138369	6.56293	1.569947	0.119999	-5.38266	0.288215	0.400836
Monocytes	ARHGAP5	-0.23324	4.809305	-1.56928	0.120155	-5.14366	0.303026	0.421099
Monocytes	GSTM4	-0.65864	1.121715	-1.56896	0.12023	-4.36447	0.336375	0.466668
Monocytes	BOLL	0.868262	1.308178	1.568617	0.120309	-4.32685	0.334737	0.464411
Monocytes	4930414N06RII	0.410444	3.331114	1.56831	0.120381	-4.55841	0.316241	0.439106
Monocytes	GPSM1	-0.45841	2.672071	-1.5679	0.120477	-4.43398	0.322225	0.447349
Monocytes	RP9	0.11649	6.834563	1.567868	0.120484	-5.32311	0.286701	0.398416
Monocytes	CRYZ	-0.58072	1.288432	-1.56772	0.120519	-4.37108	0.335089	0.464962
Monocytes	5031439G07RII	-0.1523	5.696635	-1.56766	0.120534	-5.31721	0.295958	0.411292
Monocytes	YIPF1	0.143627	5.234712	1.567576	0.120552	-5.12894	0.299811	0.416616
Monocytes	ABCA8A	-0.91778	1.028779	-1.56735	0.120606	-4.34754	0.337598	0.468398
Monocytes	DNAJA3	0.307201	3.76841	1.567265	0.120625	-4.6553	0.312446	0.43407
Monocytes	LARP1B	-0.17216	5.599705	-1.56696	0.120696	-5.20198	0.296888	0.412603
Monocytes	NHSL1	-0.64656	1.728678	-1.56634	0.120842	-4.35764	0.331394	0.459943
Monocytes	PPP2R5B	0.664285	1.645269	1.565956	0.120931	-4.37883	0.332283	0.46101
Monocytes	IPO7	0.18843	5.656347	1.565912	0.120942	-5.18548	0.296796	0.412324
Monocytes	CD274	-0.43616	5.805413	-1.56454	0.121262	-5.35177	0.296184	0.411178
Monocytes	SUMO1	-0.07602	7.992553	-1.56453	0.121266	-5.54886	0.278673	0.38689
Monocytes	SGCZ	-0.66892	1.627249	-1.56443	0.121289	-4.38274	0.333153	0.461883
Monocytes	BMP1	-0.8616	0.911089	-1.56397	0.121396	-4.33321	0.340118	0.471375
Monocytes	SLC22A15	0.209439	3.077716	1.563968	0.121397	-4.97395	0.319895	0.443818

Monocytes	CAPRIN2	-0.47488	3.181523	-1.56383	0.12143	-4.55046	0.318963	0.442583
Monocytes	USP8	0.131956	5.372996	1.563415	0.121527	-5.25152	0.300015	0.416508
Monocytes	NDUFS3	0.157036	6.050235	1.563389	0.121534	-5.21654	0.294384	0.408729
Monocytes	ZFP292	0.156386	7.046023	1.5631	0.121601	-5.31743	0.286405	0.397619
Monocytes	TPCN2	-0.35463	2.597655	-1.56268	0.1217	-4.60706	0.324543	0.450121
Monocytes	ZFP622	0.17183	5.377893	1.562588	0.121722	-5.09875	0.300128	0.416606
Monocytes	CDS1	0.307076	2.788548	1.562558	0.121729	-4.88765	0.322799	0.447738
Monocytes	TDRKH	-0.55455	1.697145	-1.56247	0.12175	-4.4059	0.332915	0.461535
Monocytes	3-Mar	-0.24942	6.392222	-1.56219	0.121815	-5.35476	0.291816	0.405104
Monocytes	DOCK7	-0.1885	3.830588	-1.56185	0.121896	-5.12938	0.313671	0.435202
Monocytes	ABCC1	0.208831	4.467713	1.561593	0.121956	-5.09048	0.308177	0.427655
Monocytes	AREG	0.670918	1.634087	1.561037	0.122087	-4.80798	0.33401	0.462973
Monocytes	ANKRD33B	0.367515	4.882196	1.560963	0.122105	-5.04851	0.304784	0.422989
Monocytes	FTL1-PS1	-0.3299	3.902493	-1.56082	0.12214	-4.86596	0.31329	0.434736
Monocytes	ARPC1B	0.104859	8.892807	1.560773	0.12215	-5.81206	0.272559	0.378371
Monocytes	MBD6	0.244567	3.632499	1.560646	0.12218	-4.79087	0.315681	0.438105
Monocytes	4930581F22RIK	0.286552	3.634004	1.560074	0.122315	-4.68364	0.315937	0.438317
Monocytes	UNK	-0.2657	4.287183	-1.55964	0.122418	-4.81911	0.310366	0.430533
Monocytes	U2AF2	-0.14052	6.245648	-1.55937	0.122482	-5.27105	0.293834	0.407693
Monocytes	ATP8B1	-0.70137	0.813793	-1.55931	0.122496	-4.35992	0.342409	0.474204
Monocytes	GM156	-0.88647	-1.54566	-1.55899	0.122571	-4.33129	0.366221	0.506497
Monocytes	WTAP	0.114362	6.931297	1.558941	0.122583	-5.39071	0.288293	0.400171
Monocytes	ZBTB7B	0.322178	2.473173	1.558783	0.12262	-4.75607	0.326719	0.453065
Monocytes	QSOX2	-0.61343	0.948725	-1.55874	0.12263	-4.36754	0.34113	0.47268
Monocytes	OFCC1	0.879096	-0.63548	1.55823	0.122751	-4.3306	0.357103	0.494095
Monocytes	IFIT1BL2	0.966779	-0.46837	1.557625	0.122895	-4.3311	0.35555	0.491946
Monocytes	COQ9	0.309628	3.205352	1.557543	0.122914	-4.64604	0.320398	0.444206
Monocytes	SLC37A3	-0.2267	3.658041	-1.55754	0.122914	-4.8259	0.316337	0.438646
Monocytes	ROBO2	-1.02222	0.744725	-1.5574	0.122949	-4.33519	0.343499	0.475679
Monocytes	SECISBP2L	0.182174	4.964176	1.557182	0.123	-5.11003	0.304935	0.423028
Monocytes	SFSWAP	0.124723	5.786795	1.557156	0.123006	-5.18969	0.29799	0.41345
Monocytes	TMEM64	-0.25684	5.051385	-1.55711	0.123017	-4.95784	0.30419	0.422002
Monocytes	2610203C22RIK	-0.70084	0.354453	-1.55696	0.123053	-4.36169	0.347342	0.480962
Monocytes	PRDM9	0.667984	1.146615	1.556806	0.123089	-4.35537	0.339633	0.470588
Monocytes	2610008E11RIK	0.527734	2.466966	1.55644	0.123176	-4.4198	0.327248	0.453776
Monocytes	NOP10	0.13648	7.15867	1.556429	0.123179	-5.43914	0.286886	0.398223
Monocytes	VIPAS39	0.173462	4.131233	1.556285	0.123213	-4.94355	0.312258	0.433309
Monocytes	PGLYRP1	0.216018	4.959034	1.555859	0.123314	-5.39625	0.305256	0.423633
Monocytes	GRAMD4	0.228984	5.165751	1.55568	0.123357	-5.07616	0.303521	0.421223
Monocytes	PHF2	-0.17446	5.076145	-1.55525	0.123459	-4.96209	0.304331	0.422345
Monocytes	DCAF15	0.304122	3.456621	1.55523	0.123464	-4.67639	0.318504	0.441819
Monocytes	TNFAIP6	0.429479	1.129558	1.55521	0.123469	-4.72131	0.340162	0.471355
Monocytes	DDC	-0.76123	1.455602	-1.55505	0.123507	-4.34759	0.337053	0.467206
Monocytes	PUS7	-0.37709	2.95762	-1.5544	0.123661	-4.5418	0.323358	0.44829
Monocytes	GPATCH8	0.119484	7.002079	1.554169	0.123717	-5.39066	0.288746	0.400626
Monocytes	MAP3K3	0.158038	6.795781	1.553612	0.123849	-5.36408	0.290481	0.403144
Monocytes	2900026A02RIK	-0.54255	3.595436	-1.55358	0.123857	-4.55279	0.317737	0.440709

Monocytes	RNF181	-0.24321	4.477136	-1.55354	0.123866	-4.89198	0.309954	0.430026
Monocytes	SRI	-0.14146	6.74315	-1.5535	0.123876	-5.40143	0.290908	0.403735
Monocytes	MYLIP	-0.19414	5.991118	-1.55339	0.123902	-5.19768	0.29708	0.412299
Monocytes	POLR2H	-0.19383	4.512761	-1.55295	0.124008	-4.92815	0.309728	0.429717
Monocytes	FMO5	-0.36034	3.247756	-1.55291	0.124018	-4.64214	0.320952	0.445116
Monocytes	SACM1L	-0.16397	5.504292	-1.55274	0.124058	-5.16627	0.301235	0.418018
Monocytes	RPAP2	0.333104	3.02458	1.552733	0.124059	-4.5713	0.322979	0.447889
Monocytes	MAPK7	0.292543	3.195275	1.552567	0.124099	-4.73765	0.32145	0.445807
Monocytes	DDX6	-0.11914	8.314732	-1.55222	0.124182	-5.59315	0.278682	0.386633
Monocytes	ZFP354B	-0.75189	-0.81695	-1.55132	0.124397	-4.33945	0.360681	0.498768
Monocytes	ESAM	-0.54888	2.065322	-1.55124	0.124417	-4.43679	0.332342	0.460404
Monocytes	DCTN4	0.128254	6.423452	1.551088	0.124453	-5.30739	0.294027	0.407855
Monocytes	GM15345	-0.49051	2.98417	-1.55107	0.124458	-4.65298	0.323826	0.448832
Monocytes	CDK5RAP2	-0.22351	4.446752	-1.55106	0.12446	-4.84799	0.310762	0.430929
Monocytes	PRKAR1B	0.988119	-0.37005	1.550377	0.124623	-4.3373	0.356498	0.492864
Monocytes	GATAD2B	-0.13488	7.433227	-1.54999	0.124717	-5.47621	0.286317	0.396884
Monocytes	IFFO1	0.442332	2.880878	1.549661	0.124795	-4.58114	0.325352	0.450557
Monocytes	SENP1	0.162546	5.013417	1.549609	0.124808	-5.03558	0.306406	0.424602
Monocytes	TRMT2A	-0.28369	3.456488	-1.54936	0.124868	-4.66579	0.320188	0.443585
Monocytes	SIGLECG	-0.27598	4.076853	-1.54901	0.124953	-4.77135	0.314755	0.436123
Monocytes	SOD2	0.297338	6.929524	1.548889	0.124981	-5.48858	0.290595	0.402845
Monocytes	TIMM13	0.137448	6.732513	1.548788	0.125005	-5.37754	0.292195	0.405123
Monocytes	POMT1	-0.57023	2.21731	-1.54747	0.125323	-4.43589	0.332423	0.459805
Monocytes	RRP15	-0.25061	3.823389	-1.5474	0.125339	-4.78042	0.317696	0.439735
Monocytes	ALPK1	0.201166	4.802266	1.547114	0.125408	-5.29102	0.309169	0.427995
Monocytes	BRD3	0.184848	5.712362	1.546976	0.125441	-5.09049	0.301389	0.417326
Monocytes	TSFM	-0.21689	4.258618	-1.54677	0.12549	-4.87142	0.313974	0.434562
Monocytes	NDUFA1	0.13372	6.729501	1.546138	0.125643	-5.41572	0.293155	0.405885
Monocytes	PACS1	0.183443	5.972963	1.546112	0.12565	-5.26415	0.299412	0.414525
Monocytes	KDM8	-0.51739	1.814343	-1.54609	0.125655	-4.38981	0.336578	0.465383
Monocytes	TEN1	0.137906	5.852604	1.545622	0.125768	-5.24074	0.300617	0.41604
Monocytes	ZMIZ1	-0.11905	6.978265	-1.54528	0.125849	-5.50149	0.291389	0.403257
Monocytes	MYZAP	-0.55026	3.284402	-1.54524	0.125861	-4.42816	0.323175	0.446951
Monocytes	TNS3	-0.16349	5.235143	-1.54439	0.126065	-5.49031	0.306352	0.423763
Monocytes	NXPE2	0.833608	1.56591	1.544066	0.126144	-4.3513	0.339853	0.469421
Monocytes	A230083N12RI	-0.86259	0.546139	-1.54382	0.126204	-4.34789	0.349856	0.482941
Monocytes	GM45894	0.332789	2.290972	1.543666	0.126241	-4.54211	0.332979	0.460168
Monocytes	C1RL	0.480622	1.632594	1.543459	0.126291	-4.53919	0.339241	0.468706
Monocytes	TRIM41	-0.18765	4.185669	-1.54328	0.126336	-4.92262	0.315658	0.43669
Monocytes	IGFBP6	0.851994	-0.02144	1.543056	0.126389	-4.36028	0.355544	0.491022
Monocytes	PRPSAP1	-0.18643	4.34081	-1.54298	0.126408	-4.95158	0.314285	0.434953
Monocytes	ESD	0.152567	7.081837	1.54285	0.126439	-5.51541	0.291085	0.403022
Monocytes	RALGAPB	0.167621	4.920242	1.542836	0.126442	-5.09174	0.309213	0.428021
Monocytes	TMEM234	0.100569	7.040364	1.542801	0.126451	-5.46787	0.291421	0.403488
Monocytes	MXRA7	-0.60169	1.654737	-1.54273	0.126468	-4.4188	0.339028	0.468739
Monocytes	GM26590	0.51037	1.314817	1.542517	0.12652	-4.37935	0.342363	0.47329
Monocytes	GM15956	-0.73329	0.27605	-1.54214	0.126612	-4.35389	0.352684	0.487315

Monocytes	SGSM3	-0.23261	4.063557	-1.54211	0.126618	-4.98461	0.316864	0.438607
Monocytes	TM4SF4	-0.63178	2.168937	-1.54194	0.126659	-4.44601	0.334257	0.462364
Monocytes	ZSCAN21	0.348972	3.394527	1.541756	0.126705	-4.62984	0.322889	0.446969
Monocytes	GM13431	-0.66459	0.492651	-1.54176	0.126705	-4.3817	0.350521	0.484523
Monocytes	PDE3A	-1.01472	0.411945	-1.5416	0.126742	-4.35229	0.351342	0.4857
Monocytes	GM11655	-0.93395	0.316941	-1.54103	0.12688	-4.34681	0.352588	0.487084
Monocytes	GM17276	-0.52035	0.785789	-1.54062	0.126981	-4.51963	0.348116	0.480881
Monocytes	MRPL57	0.145612	5.995401	1.540411	0.127032	-5.29874	0.300635	0.4161
Monocytes	GM28809	-0.54142	-0.12461	-1.54018	0.127089	-4.44503	0.357364	0.493451
Monocytes	IL1F9	0.711944	-0.11555	1.54004	0.127122	-4.39095	0.357278	0.493398
Monocytes	9030622O22RII	-0.81882	1.05914	-1.53963	0.127223	-4.37704	0.345742	0.477675
Monocytes	WDR86	-0.42802	0.234245	-1.5395	0.127255	-4.46719	0.353936	0.488773
Monocytes	AGAP1	-0.22669	4.225875	-1.5393	0.127303	-5.1177	0.316195	0.437446
Monocytes	QPRT	-0.59806	2.660001	-1.53896	0.127386	-4.46555	0.330459	0.457068
Monocytes	SERPINA1B	-0.41971	7.994808	-1.53894	0.127392	-5.54938	0.284594	0.393996
Monocytes	AP2B1	-0.11818	6.179239	-1.53875	0.127438	-5.33595	0.299352	0.414457
Monocytes	CNN2	-0.16307	6.538205	-1.53863	0.127466	-5.53024	0.296367	0.410336
Monocytes	CCDC32	0.306127	3.31271	1.538393	0.127524	-4.666	0.324429	0.448969
Monocytes	MBOAT7	0.193525	4.278521	1.538214	0.127568	-5.01214	0.315728	0.437153
Monocytes	TMEM205	-0.32379	3.71868	-1.53809	0.127599	-4.81355	0.320739	0.44407
Monocytes	CEP41	-0.54126	1.886422	-1.53805	0.127607	-4.40852	0.337766	0.467291
Monocytes	SIPA1L2	0.202551	5.103799	1.538041	0.12761	-5.1579	0.308498	0.427274
Monocytes	UEVLD	0.381968	2.944551	1.538031	0.127612	-4.60221	0.327815	0.45374
Monocytes	TRA2B	-0.11149	7.832072	-1.5376	0.127718	-5.53977	0.286049	0.396131
Monocytes	G6PC	-0.78806	1.911843	-1.53733	0.127784	-4.42272	0.337811	0.467166
Monocytes	SLC24A3	-0.84141	1.280307	-1.53717	0.127823	-4.39375	0.343927	0.475485
Monocytes	CDK1	-0.32563	5.506863	-1.53695	0.127876	-5.19599	0.305364	0.422804
Monocytes	POMP	-0.11871	7.268227	-1.53667	0.127945	-5.59021	0.290752	0.402609
Monocytes	FAR1OS	0.482668	2.788806	1.536627	0.127956	-4.57955	0.329659	0.456073
Monocytes	BAIAP3	-0.84503	-0.32152	-1.5362	0.12806	-4.35481	0.360248	0.497442
Monocytes	RDX	-0.10688	6.872179	-1.53603	0.128102	-5.41455	0.294168	0.407294
Monocytes	GM27017	0.286645	3.311249	1.535626	0.128201	-4.82626	0.325147	0.449918
Monocytes	MCEE	0.225703	4.194477	1.535613	0.128204	-4.95345	0.317162	0.438994
Monocytes	GM43258	-0.73412	0.724157	-1.53548	0.128236	-4.35929	0.349838	0.483497
Monocytes	RASGRP3	-0.31762	3.586569	-1.53514	0.128321	-4.93217	0.322693	0.446657
Monocytes	MYL12B	-0.10172	8.183707	-1.53512	0.128325	-5.73186	0.283772	0.393005
Monocytes	PTP4A3	-0.2022	5.77385	-1.5349	0.128378	-5.01579	0.303476	0.420333
Monocytes	GLP2R	0.570686	0.940346	1.534877	0.128385	-4.42477	0.34776	0.480833
Monocytes	CHRNA9	-0.5945	2.214161	-1.53458	0.128458	-4.49178	0.335544	0.464225
Monocytes	BRD9	0.165739	5.185663	1.534253	0.128538	-5.10013	0.308732	0.427501
Monocytes	F630040K05RIK	0.446043	0.611295	1.534121	0.128571	-4.62078	0.351272	0.485572
Monocytes	MTX3	0.75757	0.591443	1.533728	0.128667	-4.36922	0.351633	0.486091
Monocytes	6430550D23RII	0.753517	1.142129	1.533597	0.128699	-4.36081	0.346181	0.478756
Monocytes	AGL	0.302549	3.963587	1.533494	0.128725	-4.83649	0.319661	0.442659
Monocytes	DNAJB4	0.263867	3.700143	1.533231	0.12879	-4.7452	0.322107	0.446018
Monocytes	CABCOCO1	-0.84834	0.315412	-1.53313	0.128815	-4.35228	0.354475	0.490046
Monocytes	DPP10	0.677776	0.411958	1.532818	0.128891	-4.43183	0.353627	0.488784

Monocytes	ARVCF	-0.83839	1.228411	-1.53235	0.129007	-4.35904	0.345701	0.477913
Monocytes	LRP6	-0.207	5.378913	-1.5323	0.129019	-5.09018	0.307535	0.425794
Monocytes	RHBDL3	-0.80355	0.747589	-1.53183	0.129136	-4.36782	0.350681	0.484546
Monocytes	LZIC	-0.27459	3.63991	-1.53065	0.129427	-4.72906	0.32379	0.44757
Monocytes	BTBD19	-0.3844	2.725168	-1.53019	0.12954	-4.54913	0.332379	0.459161
Monocytes	PRG3	-1.13636	1.171663	-1.52997	0.129593	-4.35765	0.347315	0.479494
Monocytes	HIVEP2	-0.19736	8.386816	-1.52997	0.129594	-5.73758	0.283673	0.39227
Monocytes	PDZD8	0.17269	5.970732	1.529935	0.129603	-5.25024	0.303408	0.419559
Monocytes	D5ERTD579E	-0.18279	5.221315	-1.52969	0.129663	-5.0971	0.309896	0.428469
Monocytes	PMPCB	-0.18596	4.974651	-1.52958	0.12969	-5.0854	0.312046	0.431448
Monocytes	PPCDC	-0.32264	3.567947	-1.52927	0.129768	-4.63862	0.324747	0.448806
Monocytes	GPS2	-0.1185	5.859455	-1.5289	0.12986	-5.25717	0.304587	0.421224
Monocytes	ABCA6	0.7573	0.705764	1.528844	0.129873	-4.40854	0.352207	0.486159
Monocytes	ATP8B2	-0.26879	2.774077	-1.52873	0.1299	-4.70013	0.332175	0.458997
Monocytes	HC	-0.59114	2.358321	-1.52857	0.129941	-4.50811	0.336099	0.464361
Monocytes	GM48796	0.713708	0.350478	1.528418	0.129979	-4.36548	0.355778	0.491017
Monocytes	KANTR	0.44405	2.529607	1.528382	0.129988	-4.5138	0.334476	0.462164
Monocytes	GHR	-0.5626	4.215001	-1.52808	0.130062	-4.80906	0.319065	0.441156
Monocytes	GAS6	-0.62446	1.545619	-1.52764	0.130173	-4.42447	0.344166	0.475326
Monocytes	COA5	-0.21745	4.468388	-1.52727	0.130265	-5.07066	0.316926	0.438419
Monocytes	SUV39H1	-0.25835	3.622039	-1.52722	0.130276	-4.76272	0.324561	0.44887
Monocytes	ANXA5	0.133805	6.137389	1.527202	0.130281	-5.57308	0.302448	0.418514
Monocytes	RGCC	0.210994	5.613883	1.527019	0.130326	-5.32266	0.306908	0.424659
Monocytes	ZFP157	0.449534	2.446699	1.527015	0.130327	-4.51447	0.335503	0.46379
Monocytes	POLR3F	0.252823	3.882542	1.526806	0.130379	-4.77328	0.322189	0.445669
Monocytes	KLHL5	0.139262	4.645979	1.526734	0.130397	-5.22061	0.315349	0.436298
Monocytes	GM14325	-0.31823	2.324076	-1.52673	0.130399	-4.58031	0.336668	0.465418
Monocytes	DNM1L	-0.15324	5.639142	-1.52601	0.130576	-5.22591	0.306865	0.424606
Monocytes	IRAK1BP1	-0.87037	0.288762	-1.52597	0.130586	-4.36584	0.356862	0.492738
Monocytes	COX16	0.112959	6.153691	1.52588	0.130609	-5.32	0.302481	0.418569
Monocytes	GRWD1	-0.41193	2.674334	-1.52581	0.130626	-4.53268	0.333541	0.461133
Monocytes	RB1CC1	0.156575	6.500368	1.525671	0.130661	-5.32469	0.299568	0.414549
Monocytes	GM43661	-0.30055	2.981343	-1.52567	0.130663	-4.94279	0.330663	0.457211
Monocytes	ZFP938	-0.54414	1.450658	-1.5255	0.130704	-4.41621	0.345314	0.477131
Monocytes	TAPBP	-0.19695	6.597076	-1.52536	0.13074	-5.46637	0.298792	0.413467
Monocytes	ZBTB11	0.132578	7.088839	1.525	0.130828	-5.47724	0.294786	0.40798
Monocytes	GM29966	-0.85963	1.411825	-1.52492	0.130848	-4.39381	0.345777	0.477807
Monocytes	SLMAP	0.145345	6.393027	1.524864	0.130862	-5.32909	0.30056	0.41597
Monocytes	VPS26B	0.221851	3.835261	1.524665	0.130912	-4.92215	0.322946	0.446717
Monocytes	DNHD1	0.631895	1.01891	1.524503	0.130952	-4.40472	0.349721	0.483213
Monocytes	FAM149B	0.326868	2.992688	1.524187	0.131031	-4.63188	0.330783	0.457446
Monocytes	TMA16	0.311888	3.964843	1.524014	0.131074	-4.83537	0.321846	0.445257
Monocytes	PAQR7	0.490754	1.552734	1.523847	0.131116	-4.51675	0.344527	0.476191
Monocytes	PARD6G	0.75443	1.981117	1.523821	0.131122	-4.4039	0.340374	0.470553
Monocytes	GM49439	-0.94046	-0.46286	-1.52378	0.131133	-4.36032	0.364823	0.503614
Monocytes	MMRN2	-0.72683	1.228883	-1.52347	0.131211	-4.374	0.347768	0.480557
Monocytes	TMED4	-0.26546	3.553347	-1.52343	0.131221	-4.76195	0.325656	0.450493

Monocytes	GM6377	-0.18097	3.091942	-1.52272	0.131397	-5.3367	0.330282	0.456608
Monocytes	METTL27	-1.01305	0.539053	-1.52234	0.131492	-4.36163	0.355205	0.490385
Monocytes	CEP120	-0.1584	5.494784	-1.52206	0.131563	-5.18113	0.308956	0.42739
Monocytes	CLCN5	0.208891	5.160349	1.521869	0.131611	-5.14413	0.311901	0.431388
Monocytes	MUC13	-0.78195	1.034673	-1.52101	0.131827	-4.39261	0.350879	0.484224
Monocytes	PATL2	-0.5365	2.023827	-1.52085	0.131866	-4.48793	0.341204	0.471108
Monocytes	GNA15	-0.28893	3.584316	-1.51997	0.132087	-4.85737	0.326917	0.451552
Monocytes	PRKX	0.137916	5.379346	1.519728	0.132148	-5.30881	0.310842	0.429643
Monocytes	GPR107	0.166535	5.057833	1.519709	0.132152	-5.10593	0.313655	0.433506
Monocytes	PURA	-0.1325	5.343404	-1.51933	0.132247	-5.19281	0.311155	0.43019
Monocytes	FMC1	0.256678	4.072806	1.519314	0.132252	-4.89268	0.322453	0.445678
Monocytes	CMAH	-0.50512	5.561409	-1.51931	0.132253	-4.67429	0.309261	0.427586
Monocytes	TMEM161A	-0.39168	2.853132	-1.51929	0.132258	-4.56975	0.333727	0.461064
Monocytes	NAT8L	0.481658	0.332665	1.519072	0.132312	-4.51231	0.35848	0.494543
Monocytes	SLC12A7	-0.34742	3.37429	-1.51879	0.132382	-4.69296	0.32893	0.454572
Monocytes	FKBP9	-0.75561	1.265181	-1.51879	0.132384	-4.39943	0.349137	0.482031
Monocytes	VTI1A	0.125076	7.233705	1.518326	0.1325	-5.53052	0.295412	0.408339
Monocytes	MAP3K14	-0.15934	5.189544	-1.51784	0.132622	-5.40467	0.312981	0.432414
Monocytes	PHLDB2	-0.58427	2.509693	-1.51745	0.13272	-4.49519	0.337666	0.466027
Monocytes	CERCAM	0.58643	0.652443	1.517297	0.132759	-4.41426	0.355918	0.490767
Monocytes	FOXO4	0.432472	2.699869	1.517179	0.132789	-4.52563	0.335871	0.463649
Monocytes	MOCS1	0.239761	3.367675	1.516879	0.132865	-4.8515	0.329711	0.455286
Monocytes	NEIL1	-0.53781	3.000738	-1.51657	0.132943	-4.4542	0.333255	0.460086
Monocytes	LYSMD4	0.302724	3.330154	1.516228	0.133029	-4.75828	0.330309	0.456114
Monocytes	CBLL1	-0.18222	4.917555	-1.5159	0.133113	-5.0233	0.315951	0.436475
Monocytes	DMTN	-0.77698	0.637339	-1.5158	0.133138	-4.37972	0.356527	0.491713
Monocytes	GM28379	0.795375	0.264238	1.515745	0.133151	-4.38022	0.360326	0.496834
Monocytes	NOSTRIN	-0.24413	3.426931	-1.51545	0.133225	-5.06916	0.329578	0.455069
Monocytes	ZCCHC18	-0.79216	1.04036	-1.51456	0.13345	-4.37291	0.353095	0.486534
Monocytes	BRD7	0.112228	6.307707	1.514343	0.133506	-5.38075	0.304484	0.420263
Monocytes	MANBAL	-0.16675	4.531107	-1.51406	0.133576	-5.01385	0.320042	0.44166
Monocytes	NCF4	0.16772	5.316123	1.51396	0.133603	-5.37444	0.313072	0.432132
Monocytes	TMEM251	-0.19074	4.619848	-1.51392	0.133613	-5.04092	0.319245	0.440602
Monocytes	RSL1D1	-0.1558	6.043414	-1.5135	0.133718	-5.30466	0.306934	0.423567
Monocytes	GM39469	0.303655	2.328802	1.513095	0.133822	-4.63147	0.340902	0.469805
Monocytes	GM36738	0.3198	3.095803	1.512555	0.133959	-4.71506	0.33377	0.460129
Monocytes	ZDHHC16	-0.27649	2.888345	-1.51247	0.133982	-4.6455	0.33573	0.462847
Monocytes	ZFP846	-0.45804	2.431015	-1.51244	0.133988	-4.48744	0.340094	0.468773
Monocytes	GAPVD1	-0.1216	6.832806	-1.51218	0.134055	-5.42948	0.300583	0.414801
Monocytes	TSPAN32	0.411761	2.972272	1.512153	0.134061	-4.61736	0.334959	0.46186
Monocytes	CDKL3	-0.46373	3.044103	-1.51187	0.134132	-4.57087	0.334377	0.460992
Monocytes	TCIM	-0.7897	1.634355	-1.51145	0.134239	-4.41742	0.348165	0.479553
Monocytes	MRPS18A	0.170899	5.09387	1.510533	0.134473	-5.13668	0.316279	0.43601
Monocytes	C8B	-0.7034	0.571894	-1.51044	0.134498	-4.38967	0.359334	0.494471
Monocytes	ZFP148	0.118068	6.830997	1.510284	0.134537	-5.41441	0.301303	0.415455
Monocytes	PXMP2	-0.45516	4.067436	-1.5097	0.134685	-4.84098	0.325762	0.448881
Monocytes	FKBP3	-0.18238	6.130579	-1.50936	0.134773	-5.29585	0.307455	0.423916

Monocytes	MLKL	0.405	2.335281	1.509294	0.134789	-4.66713	0.342068	0.471163
Monocytes	ICAM1	-0.20027	5.898773	-1.50923	0.134804	-5.37822	0.309454	0.426677
Monocytes	DIS3L	0.30939	2.828005	1.509202	0.134813	-4.66214	0.33734	0.46475
Monocytes	TOMM22	0.122102	6.905252	1.509173	0.13482	-5.48242	0.300881	0.414887
Monocytes	CISD2	0.126576	6.732173	1.508699	0.134941	-5.4669	0.302535	0.417012
Monocytes	SLC39A8	-0.58777	2.726982	-1.50849	0.134994	-4.47977	0.338578	0.466322
Monocytes	RPP21	0.173171	4.873001	1.508098	0.135095	-5.12288	0.318882	0.439547
Monocytes	GATA4	-0.64939	1.621315	-1.50799	0.135122	-4.41656	0.349497	0.48118
Monocytes	RANBP3	-0.18333	4.303701	-1.50756	0.135231	-4.96704	0.324025	0.446605
Monocytes	SLCO2A1	-0.82386	2.568296	-1.50746	0.135256	-4.48216	0.340264	0.468701
Monocytes	JADE2	-0.35279	3.270443	-1.50745	0.135262	-4.7397	0.333587	0.459635
Monocytes	SLC25A16	0.251621	3.724214	1.507295	0.1353	-4.81273	0.32935	0.453867
Monocytes	DPP6	0.769296	-0.15893	1.507242	0.135314	-4.40396	0.367616	0.505597
Monocytes	LRRC10B	-0.97406	0.595128	-1.50723	0.135317	-4.37526	0.359821	0.495124
Monocytes	CACNB4	0.567178	1.615307	1.506959	0.135386	-4.56312	0.349575	0.481324
Monocytes	FOXRED2	0.482103	1.168266	1.506906	0.135399	-4.47823	0.354032	0.487341
Monocytes	R3HDM4	0.165291	6.20323	1.506826	0.13542	-5.31602	0.307243	0.423667
Monocytes	AKT2	0.162327	5.234185	1.506232	0.135572	-5.14148	0.315966	0.435476
Monocytes	GFRA1	-0.52215	4.918403	-1.50592	0.135652	-4.70469	0.318797	0.439429
Monocytes	FAM241A	0.194027	5.59878	1.505849	0.13567	-5.21626	0.312777	0.431193
Monocytes	PIK3CA	0.156621	6.128996	1.50576	0.135693	-5.30345	0.308174	0.42493
Monocytes	PRDM1	0.302194	2.929068	1.505693	0.13571	-4.94729	0.33715	0.464557
Monocytes	MSRA	0.144559	6.629496	1.505216	0.135833	-5.38204	0.3041	0.41927
Monocytes	TMEM41A	0.577987	1.51992	1.504882	0.135919	-4.44209	0.351226	0.483551
Monocytes	SCML4	-0.34628	4.659948	-1.5047	0.135966	-4.76391	0.321493	0.443189
Monocytes	KLF16	-0.29184	3.164065	-1.50446	0.136028	-4.70959	0.335386	0.462111
Monocytes	ZFP212	-0.24882	3.640876	-1.50383	0.136188	-4.743	0.331097	0.45628
Monocytes	ZFP983	0.453904	2.38923	1.50382	0.136191	-4.5256	0.342998	0.472449
Monocytes	SMIM19	-0.17281	4.75168	-1.50378	0.136202	-5.09119	0.320917	0.442405
Monocytes	TBCC	0.290724	3.579816	1.50283	0.136446	-4.73575	0.332184	0.457331
Monocytes	CST7	0.485192	2.120473	1.502528	0.136524	-4.63389	0.346268	0.476298
Monocytes	KDM5D	2.141749	0.730109	1.502314	0.136579	-4.45364	0.360242	0.495134
Monocytes	1700123M08RI	0.650304	0.89752	1.5015	0.136789	-4.40888	0.358941	0.493271
Monocytes	ABR	0.205012	6.594873	1.501462	0.136799	-5.5571	0.305759	0.420961
Monocytes	TRIP4	0.261374	4.852518	1.501125	0.136886	-4.98777	0.321137	0.441884
Monocytes	LBP	0.333779	3.292366	1.501038	0.136909	-4.76753	0.335541	0.461496
Monocytes	MRPL48	0.133236	5.282039	1.500272	0.137107	-5.20345	0.31754	0.436919
Monocytes	DDX11	-0.44982	2.934033	-1.50024	0.137115	-4.55006	0.339214	0.466426
Monocytes	GM31243	-0.64879	2.779029	-1.50023	0.137116	-4.53497	0.340701	0.468442
Monocytes	GAS8	0.426719	1.529564	1.500123	0.137145	-4.49373	0.352955	0.485003
Monocytes	FBXL14	0.164487	5.203671	1.499833	0.13722	-5.12616	0.318284	0.438015
Monocytes	4930578M01RI	-0.58306	0.63262	-1.49968	0.13726	-4.45099	0.362101	0.497402
Monocytes	ATG2A	0.166809	5.710857	1.49967	0.137262	-5.34252	0.313795	0.431874
Monocytes	YIF1A	-0.2017	4.016242	-1.49953	0.1373	-4.89715	0.329085	0.452824
Monocytes	TPPP3	0.295479	1.071021	1.498304	0.137616	-5.02516	0.358376	0.492054
Monocytes	UBAP2L	-0.103	7.113568	-1.49814	0.137659	-5.51728	0.302403	0.415967
Monocytes	PNPLA1	-0.91335	0.231599	-1.49748	0.137829	-4.38426	0.367356	0.503999

Monocytes	IL1RL2	-0.72503	0.726101	-1.49734	0.137866	-4.42435	0.362233	0.497137
Monocytes	NAA50	-0.11656	6.371464	-1.49727	0.137884	-5.42572	0.308986	0.424896
Monocytes	WSB2	-0.19602	4.493162	-1.49719	0.137906	-5.06723	0.325674	0.447736
Monocytes	NXN	0.244897	5.325793	1.496942	0.13797	-5.08353	0.318166	0.437516
Monocytes	DDIT4	0.585737	3.744278	1.49692	0.137976	-4.62991	0.332616	0.457208
Monocytes	MGME1	0.406127	2.312135	1.496792	0.138009	-4.6208	0.346332	0.475843
Monocytes	RASGRP4	0.232394	1.734055	1.496052	0.138201	-5.06514	0.35245	0.483738
Monocytes	ZFP729A	0.322648	3.051831	1.495854	0.138253	-4.71564	0.339612	0.466372
Monocytes	CECR2	-0.45018	6.605628	-1.49566	0.138304	-4.99439	0.307424	0.42245
Monocytes	MAML1	0.172106	5.435368	1.495377	0.138377	-5.18824	0.317672	0.436487
Monocytes	EIF3J2	-0.55599	1.443832	-1.49526	0.138409	-4.44876	0.355484	0.487757
Monocytes	SLC9A3R2	-0.38372	2.701144	-1.49524	0.138413	-4.72959	0.343063	0.471
Monocytes	STK24	0.116298	7.291795	1.494881	0.138506	-5.56187	0.301748	0.414639
Monocytes	RNF157	-0.21436	5.375061	-1.49473	0.138545	-5.34836	0.318335	0.437462
Monocytes	SERPINE2	0.480207	1.29875	1.494651	0.138566	-4.56012	0.357091	0.490017
Monocytes	MOSPD2	-0.16316	4.445481	-1.49451	0.138603	-5.28313	0.326756	0.449
Monocytes	GM11464	-0.70328	-0.20123	-1.49418	0.138688	-4.40073	0.372783	0.511
Monocytes	STX8	0.103753	6.745581	1.493867	0.138771	-5.48753	0.306609	0.421327
Monocytes	GPCPD1	0.116835	7.329375	1.493684	0.138818	-5.67705	0.301697	0.414583
Monocytes	REM2	-0.89689	0.836601	-1.49346	0.138876	-4.39254	0.362185	0.496686
Monocytes	SEMA4A	-0.17699	3.364224	-1.49134	0.139433	-5.30213	0.338467	0.463764
Monocytes	ARC	-0.66981	0.905214	-1.49084	0.139563	-4.44507	0.363096	0.496766
Monocytes	ALDH16A1	-0.19332	4.358468	-1.49063	0.139618	-5.0597	0.32941	0.451274
Monocytes	2010310C07RIK	-0.63141	1.298313	-1.49045	0.139665	-4.53503	0.359166	0.49137
Monocytes	CDH2	-0.5236	1.947183	-1.49005	0.139769	-4.5145	0.352804	0.48267
Monocytes	SBDS	-0.13154	5.561152	-1.48938	0.139947	-5.26194	0.319009	0.436736
Monocytes	SRGAP3	-0.2297	4.261174	-1.48908	0.140025	-5.28388	0.330963	0.452981
Monocytes	UBE2I	-0.08851	7.624242	-1.48808	0.140288	-5.60343	0.301673	0.41286
Monocytes	GM37401	0.579238	0.875344	1.487996	0.14031	-4.45551	0.36467	0.498158
Monocytes	TMEM156	0.215265	3.855526	1.487986	0.140313	-5.01631	0.335212	0.458537
Monocytes	GM9949	-0.76624	0.483247	-1.48751	0.140438	-4.42165	0.368994	0.503741
Monocytes	MLLT6	-0.31836	3.270712	-1.48736	0.140477	-4.92956	0.341015	0.466186
Monocytes	GPC1	-0.62602	0.351919	-1.48724	0.140508	-4.47932	0.370385	0.505587
Monocytes	TOB2	-0.13224	6.656821	-1.48696	0.140583	-5.57358	0.310161	0.424307
Monocytes	UFD1	0.14994	5.129038	1.486559	0.140689	-5.16748	0.323698	0.44287
Monocytes	PIGP	-0.21658	4.420664	-1.48652	0.140699	-4.99086	0.330197	0.45169
Monocytes	CYP4A14	0.754038	1.833194	1.486506	0.140703	-4.47642	0.355194	0.485405
Monocytes	GM17066	-0.5287	1.747467	-1.48627	0.140764	-4.46816	0.356057	0.486563
Monocytes	REEP6	-0.60642	1.791912	-1.48626	0.140768	-4.48125	0.355609	0.485963
Monocytes	TMC6	-0.17631	4.632332	-1.48625	0.14077	-5.07587	0.32824	0.449037
Monocytes	SYVN1	-0.29704	4.228615	-1.48603	0.140829	-4.89204	0.331984	0.4542
Monocytes	UBAP1L	-0.51029	1.134289	-1.48598	0.140843	-4.45536	0.362297	0.495022
Monocytes	CRY2	0.321876	2.553643	1.485925	0.140856	-4.67203	0.348031	0.47587
Monocytes	GM33782	-0.57044	0.19699	-1.48521	0.141045	-4.4614	0.372478	0.508197
Monocytes	GAS5	-0.15142	6.391612	-1.48472	0.141175	-5.43413	0.312894	0.427946
Monocytes	CDK16	0.263669	3.39772	1.484706	0.141178	-4.73254	0.340306	0.465141
Monocytes	RSU1	0.123709	5.746299	1.484694	0.141182	-5.41261	0.318587	0.435705

Monocytes	LYVE1	0.812492	2.42534	1.483996	0.141366	-4.49424	0.350134	0.478111
Monocytes	DUBR	-0.33054	2.378269	-1.4839	0.141393	-4.72203	0.3506	0.478762
Monocytes	TAF2	0.195401	4.171185	1.483652	0.141457	-4.92542	0.333356	0.455498
Monocytes	PIP4K2A	0.132438	7.239298	1.483585	0.141475	-5.67537	0.305936	0.418207
Monocytes	ZFP85	0.811864	0.25725	1.483463	0.141508	-4.39871	0.372364	0.507873
Monocytes	GTF3C2	-0.13306	5.474786	-1.48307	0.141612	-5.22778	0.321544	0.439408
Monocytes	TMEM199	0.238927	3.379806	1.482602	0.141736	-4.82946	0.341256	0.465859
Monocytes	PLXDC2	0.353738	5.144109	1.482292	0.141818	-5.21589	0.324858	0.443577
Monocytes	GUSB	-0.12166	5.468903	-1.48194	0.141912	-5.47232	0.32205	0.439616
Monocytes	ADAMTS17	-0.86607	0.355692	-1.48182	0.141943	-4.40403	0.372033	0.506802
Monocytes	RAB3GAP2	0.169632	5.259956	1.480692	0.142244	-5.18984	0.324533	0.442583
Monocytes	ZSCAN2	-0.85109	1.067334	-1.48059	0.14227	-4.40556	0.365259	0.497342
Monocytes	EHMT2	0.171688	4.951712	1.48008	0.142407	-5.16509	0.327567	0.446622
Monocytes	GCNT7	0.302174	2.712006	1.479794	0.142483	-4.72853	0.34888	0.475394
Monocytes	GM29394	-0.70465	0.417159	-1.47976	0.142493	-4.41698	0.372311	0.506703
Monocytes	DTWD1	0.453442	1.928598	1.479616	0.14253	-4.51169	0.356692	0.485865
Monocytes	5033406O09RII	-0.69433	0.605012	-1.47962	0.14253	-4.44037	0.37033	0.504067
Monocytes	CNTRL	-0.14852	5.907012	-1.47943	0.142581	-5.30754	0.318935	0.434984
Monocytes	ELP1	0.176963	4.553493	1.479342	0.142603	-4.98018	0.331264	0.451695
Monocytes	BRD1	-0.15177	5.991255	-1.47833	0.142873	-5.2962	0.318649	0.434211
Monocytes	ATL2	0.159215	5.337211	1.478312	0.142878	-5.16566	0.324535	0.44219
Monocytes	WDR20	0.136427	5.843767	1.478017	0.142957	-5.31721	0.320048	0.436088
Monocytes	LAMTOR2	0.10459	6.577213	1.47777	0.143023	-5.52085	0.313558	0.427326
Monocytes	TMEM204	-0.59862	1.038857	-1.47777	0.143024	-4.42492	0.366443	0.498522
Monocytes	SLC4A1	0.88437	2.002694	1.477676	0.143048	-4.43944	0.356573	0.485364
Monocytes	GM10563	0.301588	3.607105	1.477545	0.143083	-4.80163	0.340788	0.464192
Monocytes	FMNL3	0.254563	4.258409	1.477289	0.143152	-4.91859	0.33462	0.455864
Monocytes	GM9725	0.454653	2.030059	1.477264	0.143158	-4.60204	0.356323	0.485021
Monocytes	TAF6L	-0.32562	2.800016	-1.47681	0.143279	-4.67215	0.34884	0.474902
Monocytes	CAAP1	0.188396	4.575271	1.476566	0.143345	-5.0403	0.331831	0.452076
Monocytes	APH1C	-0.1822	4.351444	-1.47652	0.143358	-5.40188	0.333924	0.454919
Monocytes	EXOSC7	-0.1861	4.629702	-1.47648	0.143368	-5.08709	0.331325	0.451424
Monocytes	NGDN	0.144045	5.424919	1.476175	0.14345	-5.2521	0.324128	0.441617
Monocytes	A1CF	-0.83328	0.569704	-1.47593	0.143515	-4.41706	0.371789	0.505562
Monocytes	2810402E24RIk	0.382725	2.412627	1.475231	0.143703	-4.57346	0.353255	0.480617
Monocytes	MEGF11	0.687484	0.651843	1.474623	0.143866	-4.44767	0.371567	0.504909
Monocytes	GTF3C4	-0.39422	2.57436	-1.47452	0.143893	-4.58704	0.351877	0.47864
Monocytes	MVD	0.367622	2.348846	1.474505	0.143898	-4.66588	0.354126	0.481651
Monocytes	HDAC10	-0.51485	1.608346	-1.47389	0.144062	-4.47432	0.361954	0.491918
Monocytes	COG8	0.189523	4.299473	1.47367	0.144122	-4.9655	0.33553	0.4565
Monocytes	SBF1	-0.16557	4.474225	-1.47339	0.144196	-5.1131	0.333887	0.454402
Monocytes	DAZAP2	0.110737	7.70346	1.473347	0.144209	-5.66476	0.30508	0.415341
Monocytes	IZUMO1R	-0.9226	0.001693	-1.47333	0.144215	-4.41178	0.378889	0.514582
Monocytes	CD300LG	0.356191	1.259921	1.472714	0.144379	-4.74837	0.365768	0.497129
Monocytes	PFDN4	0.179203	5.046958	1.472616	0.144406	-5.19817	0.328721	0.447418
Monocytes	1700056N10RII	-0.38164	2.033678	-1.47249	0.144439	-4.58738	0.357841	0.486621
Monocytes	JCHAIN	-2.03721	-0.4625	-1.47249	0.144441	-4.40296	0.38411	0.521554

Monocytes	F930017D23RIK	-0.60136	0.898224	-1.47245	0.144451	-4.60428	0.36954	0.502232
Monocytes	ADGRL2	-0.47927	4.208198	-1.47214	0.144533	-4.73411	0.336595	0.458215
Monocytes	LTBP4	-0.6593	2.297056	-1.47209	0.144547	-4.50976	0.355228	0.483241
Monocytes	ZFP395	-0.25763	4.289929	-1.47201	0.144569	-4.89757	0.335822	0.457184
Monocytes	GM13427	0.523556	2.062778	1.471779	0.144631	-4.47842	0.357661	0.48649
Monocytes	TIA1	0.13902	5.466514	1.471589	0.144683	-5.19186	0.325024	0.442548
Monocytes	ATG13	0.207335	4.765899	1.471015	0.144837	-5.06922	0.33174	0.451495
Monocytes	SEMA4F	-0.32138	-0.41218	-1.4709	0.144867	-4.74949	0.384044	0.521471
Monocytes	PDIA4	-0.154	5.653573	-1.47051	0.144973	-5.2338	0.323722	0.440693
Monocytes	PMVK	-0.1994	4.327226	-1.47041	0.145	-5.0826	0.33599	0.457292
Monocytes	LGALS8	0.18773	5.110017	1.470313	0.145027	-5.17218	0.328688	0.447482
Monocytes	LYSMD1	0.512254	1.591133	1.469945	0.145126	-4.45865	0.363085	0.493705
Monocytes	CBX4	-0.2032	5.468948	-1.46988	0.145145	-5.15763	0.325514	0.443233
Monocytes	NECAP2	0.145408	5.345804	1.469135	0.145346	-5.31407	0.327014	0.445078
Monocytes	PSMD13	-0.1253	6.203145	-1.46846	0.145529	-5.42241	0.319531	0.434671
Monocytes	THOC2	0.099173	7.106814	1.46844	0.145534	-5.56753	0.311579	0.423872
Monocytes	ESCO1	-0.14464	5.77786	-1.46812	0.145621	-5.33657	0.32343	0.439964
Monocytes	SIT1	-0.74687	2.062249	-1.46806	0.145636	-4.42896	0.359071	0.487915
Monocytes	TLN1	0.126192	7.109295	1.467438	0.145805	-5.64083	0.311923	0.424292
Monocytes	RMND1	0.235981	3.79439	1.467219	0.145865	-4.81758	0.342322	0.465466
Monocytes	ZFP811	0.982692	-1.02506	1.466697	0.146006	-4.40681	0.3927	0.532409
Monocytes	SPATA48	-0.48897	2.190427	-1.46624	0.146131	-4.55109	0.358659	0.486983
Monocytes	PLEKHG2	-0.23671	4.157105	-1.46558	0.146311	-5.0848	0.33958	0.461209
Monocytes	MMP14	0.519556	3.837765	1.465562	0.146315	-4.86503	0.342644	0.465327
Monocytes	NOTCH4	-0.53608	0.210596	-1.46523	0.146406	-4.5695	0.379818	0.514837
Monocytes	VPS26A	-0.1166	6.273847	-1.465	0.146468	-5.50649	0.320151	0.435001
Monocytes	SLC17A3	-0.75313	0.779648	-1.46497	0.146475	-4.44273	0.373732	0.506845
Monocytes	CCDC88A	-0.13985	5.372063	-1.46483	0.146513	-5.52448	0.328339	0.446068
Monocytes	BZW1	-0.09077	7.459579	-1.46438	0.146638	-5.58353	0.309947	0.420956
Monocytes	DHCR24	-0.38047	2.897631	-1.46398	0.146745	-4.68399	0.352392	0.478182
Monocytes	GM44702	0.787308	0.088947	1.463512	0.146873	-4.44477	0.381793	0.517132
Monocytes	NEDD9	0.162886	8.003683	1.463349	0.146918	-5.74274	0.305624	0.414931
Monocytes	D130062J10RIK	0.483439	1.642184	1.463307	0.14693	-4.54057	0.365332	0.495295
Monocytes	PHF19	0.419168	1.934693	1.463086	0.14699	-4.55132	0.362384	0.491322
Monocytes	GM36279	0.385584	2.959709	1.46254	0.147139	-4.6582	0.352315	0.477603
Monocytes	LONP2	-0.1282	5.87764	-1.46224	0.147222	-5.28849	0.324694	0.440433
Monocytes	LAMC1	0.330942	4.523339	1.462043	0.147275	-4.81915	0.337298	0.457373
Monocytes	ZFP36L2	0.140357	8.376464	1.461466	0.147433	-5.89006	0.303137	0.410983
Monocytes	MANBA	0.186657	4.766674	1.461396	0.147452	-5.13844	0.335251	0.454428
Monocytes	CYP2A12	-0.58082	2.85261	-1.46035	0.147738	-4.64332	0.354412	0.479704
Monocytes	NOL7	-0.10693	6.906907	-1.45908	0.148086	-5.51687	0.316941	0.42899
Monocytes	BRWD3	0.17963	5.109603	1.458938	0.148126	-5.23703	0.333269	0.451045
Monocytes	NOP14	-0.22615	4.004398	-1.45891	0.148133	-4.98634	0.343775	0.465137
Monocytes	BCAR3	-0.50804	4.496691	-1.45878	0.148168	-4.73034	0.339053	0.458867
Monocytes	OLR1	0.382805	1.333367	1.458237	0.148318	-5.03914	0.370926	0.501368
Monocytes	4833403J16RIK	-0.96777	0.509888	-1.45814	0.148346	-4.41879	0.379694	0.513
Monocytes	DDX3Y	3.233481	2.707432	1.458102	0.148356	-4.73504	0.356783	0.482604

Monocytes	SLC49A4	0.191274	6.30055	1.457866	0.14842	-5.56986	0.322604	0.436673
Monocytes	PAG1	0.184405	7.038518	1.457467	0.14853	-5.58875	0.316194	0.427871
Monocytes	CCSAP	0.433146	2.176115	1.456912	0.148683	-4.55548	0.362731	0.490061
Monocytes	SYN1	-0.61851	0.783746	-1.45633	0.148844	-4.51727	0.377648	0.509571
Monocytes	C920021L13RIK	0.407754	2.268205	1.456083	0.148912	-4.55969	0.362178	0.489003
Monocytes	LPAR1	0.808206	0.763125	1.45569	0.14902	-4.50729	0.378143	0.510153
Monocytes	CCNI	0.11702	6.783841	1.455501	0.149072	-5.5159	0.319242	0.431541
Monocytes	2310009B15RIK	-0.15766	4.473004	-1.45486	0.149248	-5.17627	0.340887	0.46044
Monocytes	AKAP9	-0.13831	6.105265	-1.45475	0.14928	-5.43516	0.32565	0.439985
Monocytes	LRRK1	-0.16763	5.612519	-1.45425	0.149418	-5.46496	0.330399	0.446276
Monocytes	ANXA1	0.196272	6.030034	1.45353	0.149617	-5.75961	0.326923	0.441407
Monocytes	RAPGEF3	-0.82195	1.358645	-1.45308	0.149742	-4.42549	0.3731	0.502811
Monocytes	RAB12	-0.14487	5.073594	-1.45263	0.149865	-5.24179	0.336167	0.453555
Monocytes	RHBDD1	0.210278	4.175534	1.45256	0.149886	-4.97564	0.344751	0.465064
Monocytes	GM14296	-0.48943	0.969349	-1.45204	0.150029	-4.48623	0.377699	0.508738
Monocytes	FAM53B	0.329115	4.652028	1.45185	0.150083	-4.8418	0.340409	0.45921
Monocytes	TEP1	0.171894	3.914158	1.451805	0.150095	-5.23377	0.347542	0.468739
Monocytes	KYNU	0.217658	3.853994	1.451469	0.150188	-5.17936	0.348268	0.469565
Monocytes	ASB8	0.252443	3.607063	1.45082	0.150369	-4.85457	0.350937	0.47307
Monocytes	CFAP20	-0.16185	5.191326	-1.45074	0.15039	-5.16819	0.335659	0.452662
Monocytes	OCEL1	0.236812	3.584489	1.45073	0.150394	-4.9063	0.35116	0.473368
Monocytes	IK	0.110255	6.288849	1.450351	0.150499	-5.48448	0.325664	0.439128
Monocytes	CD40LG	0.733576	-0.56972	1.4501	0.150569	-4.43201	0.395292	0.531533
Monocytes	BACH2IT1	-0.77343	-0.26225	-1.44951	0.150733	-4.41966	0.392186	0.527276
Monocytes	PA2G4	-0.17308	6.688076	-1.44876	0.150942	-5.48416	0.322783	0.434832
Monocytes	ARL6IP5	0.116176	7.048458	1.44806	0.151137	-5.65309	0.319898	0.430674
Monocytes	PDCD10	0.103477	6.713222	1.447837	0.1512	-5.54631	0.32296	0.434737
Monocytes	SH2B3	-0.14912	5.315208	-1.4476	0.151265	-5.3399	0.335857	0.452077
Monocytes	GM3235	0.734523	0.645113	1.447545	0.151281	-4.46233	0.383154	0.514789
Monocytes	CAPN5	0.647519	1.546779	1.447279	0.151355	-4.49886	0.373548	0.502103
Monocytes	GM40645	0.303222	0.751306	1.4472	0.151377	-4.94337	0.38207	0.513341
Monocytes	OAS3	0.558301	2.402295	1.446911	0.151458	-4.82037	0.364659	0.490453
Monocytes	NPC1	0.189093	4.971884	1.446887	0.151465	-5.23946	0.339208	0.456606
Monocytes	JMY	0.165586	6.282387	1.445904	0.15174	-5.47908	0.327458	0.440539
Monocytes	CAR13	0.561165	1.31675	1.445869	0.151749	-4.49409	0.376573	0.505819
Monocytes	PREPL	-0.46559	1.734988	-1.44555	0.15184	-4.53068	0.372216	0.500052
Monocytes	SLC4A1AP	0.195	4.278134	1.445508	0.151851	-5.00915	0.346446	0.465896
Monocytes	SCRIB	-0.24248	3.392621	-1.44523	0.151929	-4.84692	0.355293	0.4776
Monocytes	WSB1	-0.12132	6.461454	-1.44483	0.152041	-5.54317	0.326156	0.438702
Monocytes	EIF2AK3	-0.18958	6.399228	-1.44441	0.152158	-5.29734	0.326899	0.439556
Monocytes	ZMYND8	0.142887	6.108671	1.444191	0.15222	-5.35055	0.329622	0.443228
Monocytes	EIF5A2	0.57986	0.577892	1.443869	0.15231	-4.52285	0.385305	0.5171
Monocytes	MYO16	-0.8361	-0.30441	-1.44382	0.152324	-4.43807	0.39509	0.52992
Monocytes	PODXL	0.790324	0.550401	1.443235	0.152488	-4.44983	0.385934	0.517691
Monocytes	RTRAF	-0.11785	7.336334	-1.44301	0.152551	-5.70628	0.318926	0.428644
Monocytes	FAM126A	-0.1792	5.549539	-1.4429	0.152582	-5.3217	0.335233	0.450568
Monocytes	GALC	-0.18035	3.891293	-1.44239	0.152724	-5.27654	0.351453	0.472047

Monocytes	EIF1AD	-0.1886	4.923218	-1.44195	0.152851	-5.17926	0.341615	0.458833
Monocytes	LMO1	0.229274	1.523387	1.441564	0.152958	-5.19799	0.37616	0.504405
Monocytes	PALM	0.261642	4.231998	1.441278	0.153039	-4.99445	0.34851	0.467856
Monocytes	FOXN2	-0.10888	6.146142	-1.44126	0.153044	-5.55427	0.330307	0.443577
Monocytes	ATXN7L1OS2	-0.51079	1.337166	-1.44114	0.153077	-4.55821	0.378187	0.507123
Monocytes	ZZZ3	-0.15181	5.884403	-1.44056	0.153242	-5.39477	0.333017	0.446969
Monocytes	COP1	0.134537	7.053619	1.440359	0.153298	-5.60525	0.32234	0.432663
Monocytes	USP6NL	-0.14435	5.563085	-1.44029	0.153318	-5.40555	0.336039	0.450999
Monocytes	NKAPD1	0.203183	4.086425	1.44016	0.153354	-5.0199	0.350256	0.469984
Monocytes	MNDAL	0.217892	6.786829	1.43986	0.153438	-5.5731	0.324821	0.435947
Monocytes	5031425E22RIK	0.164855	5.390925	1.439686	0.153488	-5.29095	0.337741	0.453222
Monocytes	CDC42BPG	-0.33318	2.57838	-1.43967	0.153493	-4.84307	0.365549	0.490125
Monocytes	NEK11	-0.70394	0.082898	-1.43952	0.153533	-4.45517	0.392353	0.525324
Monocytes	MEIG1	0.773206	-0.47443	1.439213	0.153621	-4.44044	0.398761	0.533715
Monocytes	PEX11A	0.62408	0.072757	1.43899	0.153684	-4.49434	0.392674	0.525753
Monocytes	LAT2	0.166406	4.773693	1.438479	0.153829	-5.36683	0.344077	0.461513
Monocytes	CLK4	0.130651	6.012894	1.438334	0.15387	-5.40802	0.332354	0.445921
Monocytes	DOK3	0.218208	5.36627	1.438141	0.153924	-5.26935	0.338466	0.454038
Monocytes	IL10RB	0.141854	5.555085	1.437747	0.154036	-5.40735	0.336849	0.451795
Monocytes	SPPL3	-0.104	7.282604	-1.43726	0.154173	-5.62489	0.32119	0.430754
Monocytes	ST3GAL6	-0.30818	5.092875	-1.43711	0.154216	-4.87541	0.341449	0.457863
Monocytes	METTL25	0.191056	4.28478	1.437045	0.154235	-4.99423	0.349283	0.468304
Monocytes	COX6B2	0.390696	1.691068	1.436541	0.154378	-4.69299	0.376064	0.503545
Monocytes	MOCS2	-0.18799	5.094585	-1.4363	0.154445	-5.15083	0.341744	0.458126
Monocytes	ADCK5	-0.47165	1.510315	-1.43595	0.154545	-4.56226	0.378233	0.506381
Monocytes	IMP3	0.142749	5.264975	1.435503	0.154672	-5.26376	0.340462	0.456272
Monocytes	RBMS2	0.187208	4.872344	1.435374	0.154709	-5.04961	0.344235	0.461363
Monocytes	SGPL1	-0.13712	5.922758	-1.43507	0.154794	-5.53387	0.334365	0.44822
Monocytes	EAPP	0.121304	5.602282	1.434863	0.154854	-5.35444	0.33743	0.452332
Monocytes	JAGN1	0.228807	3.955321	1.434105	0.15507	-5.0154	0.353804	0.473729
Monocytes	H1FX	-0.52501	2.127581	-1.43388	0.155134	-4.61603	0.372578	0.498462
Monocytes	GULO	-0.58173	1.982596	-1.43378	0.155162	-4.54455	0.37411	0.500501
Monocytes	FZD1	-0.31391	-0.15494	-1.43358	0.155219	-4.8223	0.397557	0.531154
Monocytes	ZFP867	-0.65734	0.61031	-1.43331	0.155295	-4.4499	0.389035	0.520056
Monocytes	CCNK	-0.15576	4.857081	-1.43319	0.155332	-5.21691	0.345079	0.462119
Monocytes	EDIL3	0.293529	1.492552	1.43317	0.155336	-5.04681	0.379421	0.507464
Monocytes	OGDH	0.093467	6.625732	1.432763	0.155452	-5.55111	0.328592	0.439991
Monocytes	MACF1	0.116419	8.000182	1.43256	0.15551	-5.77852	0.316311	0.423555
Monocytes	CBX2	0.771763	-0.18673	1.43206	0.155653	-4.44143	0.398491	0.532071
Monocytes	TCF7L1	-0.50817	3.097343	-1.43167	0.155763	-4.60303	0.363262	0.485776
Monocytes	YAP1	-0.90852	1.070556	-1.43128	0.155876	-4.45868	0.384889	0.514045
Monocytes	RBM4	-0.16649	4.655948	-1.43113	0.155918	-5.11005	0.347864	0.465296
Monocytes	SNRPA1	-0.1958	5.077996	-1.4309	0.155985	-5.2406	0.343793	0.459958
Monocytes	MGAT2	-0.12383	5.873385	-1.43085	0.155999	-5.45926	0.336221	0.449892
Monocytes	SLC11A2	-0.27884	3.918763	-1.43057	0.156078	-4.97111	0.355273	0.475176
Monocytes	FHDC1	-0.70964	0.748905	-1.43006	0.156224	-4.47155	0.388732	0.519129
Monocytes	PAN2	0.269962	3.190864	1.430038	0.15623	-4.77841	0.362781	0.485055

Monocytes	RIOX1	-0.25894	3.521185	-1.42998	0.156247	-4.81278	0.359419	0.480617
Monocytes	GCC1	0.389509	2.459454	1.429571	0.156364	-4.68373	0.370543	0.495275
Monocytes	BAP1	-0.26591	3.565024	-1.42912	0.156493	-4.8378	0.359379	0.480468
Monocytes	KATNA1	-0.17832	4.864725	-1.42896	0.156539	-5.184	0.34651	0.463442
Monocytes	GM19605	0.630578	1.260305	1.42848	0.156677	-4.49824	0.383844	0.512512
Monocytes	NSUN6	-0.24128	3.821373	-1.42825	0.156744	-4.98864	0.357126	0.47732
Monocytes	ERCC5	0.282664	2.909402	1.42769	0.156904	-4.77605	0.366716	0.489847
Monocytes	MINDY3	0.135123	5.598106	1.427484	0.156963	-5.41898	0.340076	0.45456
Monocytes	SBK1	-0.35852	3.374185	-1.42722	0.157039	-4.69172	0.362092	0.48374
Monocytes	GM47889	-0.53953	1.560566	-1.427	0.157101	-4.56309	0.381195	0.50881
Monocytes	OAZ1	-0.1197	9.534615	-1.42682	0.157154	-5.98025	0.304995	0.407618
Monocytes	MAP4K1	-0.14191	4.801156	-1.42662	0.157211	-5.30963	0.347966	0.465112
Monocytes	SPNS1	0.264574	3.498213	1.426438	0.157264	-4.84427	0.360951	0.48232
Monocytes	SLC41A3	0.280198	1.73389	1.42636	0.157286	-4.85144	0.379394	0.506608
Monocytes	INPP4B	-0.25532	6.480863	-1.42624	0.157322	-5.4899	0.331989	0.443985
Monocytes	MECOM	1.164148	0.770813	1.426177	0.157339	-4.4611	0.389894	0.520446
Monocytes	ATXN2L	-0.13756	5.47603	-1.42592	0.157413	-5.34086	0.341531	0.456699
Monocytes	SLC27A4	0.285317	2.770367	1.425723	0.15747	-4.72555	0.368579	0.492448
Monocytes	CCND2	-0.22909	5.400515	-1.4254	0.157562	-5.25169	0.342357	0.457873
Monocytes	NUP155	0.163402	5.174039	1.425387	0.157566	-5.20748	0.344537	0.460769
Monocytes	BICDL1	-0.5169	1.878488	-1.42469	0.157768	-4.55462	0.378323	0.505309
Monocytes	TMED9	-0.10726	6.609324	-1.42426	0.157892	-5.54055	0.331221	0.443047
Monocytes	HSD17B4	-0.14232	5.027211	-1.42424	0.157897	-5.33807	0.346206	0.462998
Monocytes	PLA2G12A	0.276427	4.277535	1.424231	0.1579	-4.87448	0.353569	0.472762
Monocytes	ZFP677	0.605304	0.788621	1.424121	0.157931	-4.4811	0.390192	0.52098
Monocytes	DUSP10	-0.22935	4.392133	-1.42386	0.158006	-5.17329	0.352432	0.471385
Monocytes	FAH	-0.45605	3.892683	-1.42377	0.158032	-4.9015	0.357417	0.477984
Monocytes	GPR141B	-0.21127	-0.65516	-1.42343	0.158131	-5.03737	0.406541	0.542455
Monocytes	GPR89	0.243585	3.742407	1.423386	0.158143	-4.95344	0.358932	0.480055
Monocytes	MKLN1OS	0.747152	0.018205	1.423359	0.158151	-4.46201	0.398826	0.532416
Monocytes	GM20513	-0.29496	1.300029	-1.42322	0.158193	-5.18336	0.384573	0.513879
Monocytes	TTYH3	0.171293	5.141527	1.423046	0.158242	-5.3205	0.345098	0.46181
Monocytes	PIGQ	0.249224	3.519745	1.422996	0.158256	-4.86885	0.36119	0.483135
Monocytes	2010007H06RII	0.696496	0.459338	1.422826	0.158305	-4.45027	0.393857	0.526109
Monocytes	UTP15	-0.21411	3.757308	-1.42282	0.158307	-4.94162	0.358781	0.480013
Monocytes	CD55B	-1.00067	0.178504	-1.42271	0.158339	-4.44127	0.397012	0.53023
Monocytes	UVSSA	0.26576	3.709393	1.422683	0.158347	-4.86311	0.359266	0.480656
Monocytes	RNF146	0.160545	5.30614	1.422591	0.158373	-5.28704	0.34351	0.45976
Monocytes	ZFP959	-0.28863	3.241052	-1.42258	0.158376	-4.78242	0.364038	0.486962
Monocytes	FBXO46	0.410851	2.47185	1.422523	0.158393	-4.63791	0.372028	0.497494
Monocytes	CDC7	0.423474	2.873973	1.422307	0.158456	-4.63626	0.367827	0.491996
Monocytes	ANKRD54	0.259365	3.089479	1.422271	0.158466	-4.79851	0.365597	0.48907
Monocytes	LY6D	-0.41288	5.594266	-1.42219	0.158488	-4.90617	0.340749	0.456151
Monocytes	PBXIP1	0.197055	4.578633	1.421694	0.158633	-5.23328	0.350833	0.469529
Monocytes	ARRDC1	0.179748	4.471227	1.421264	0.158758	-5.2282	0.352092	0.47106
Monocytes	KLC2	-0.40522	2.681752	-1.42107	0.158814	-4.67836	0.370341	0.495113
Monocytes	GM16150	0.800353	0.174831	1.419957	0.159137	-4.44846	0.398324	0.53134

Monocytes	IGFALS	-0.47531	1.779759	-1.41969	0.159213	-4.60242	0.380694	0.508275
Monocytes	TNIP1	-0.20464	4.966974	-1.41937	0.159308	-5.32506	0.34808	0.465281
Monocytes	CALM3	0.117391	7.10732	1.419257	0.15934	-5.66247	0.32787	0.438389
Monocytes	APLP2	0.164474	5.805079	1.419189	0.15936	-5.46112	0.340004	0.454598
Monocytes	MICU3	0.23329	3.81398	1.418863	0.159455	-5.04128	0.359652	0.480662
Monocytes	GM31323	-0.56863	0.948728	-1.41878	0.15948	-4.50196	0.389987	0.520562
Monocytes	NES	-0.72838	0.578345	-1.41832	0.159611	-4.50753	0.394346	0.52603
Monocytes	BCCIP	0.124549	5.436859	1.418	0.159706	-5.33101	0.343967	0.459608
Monocytes	ZFP866	0.321265	2.993086	1.417786	0.159768	-4.75411	0.36849	0.491958
Monocytes	THOC7	0.111114	6.572603	1.417561	0.159834	-5.56898	0.333339	0.445378
Monocytes	AFF2	0.818471	0.04795	1.41632	0.160195	-4.48153	0.401373	0.534376
Monocytes	P2RX4	0.159347	5.534178	1.416295	0.160203	-5.48961	0.343794	0.458763
Monocytes	SLC37A4	0.288458	2.862467	1.416163	0.160241	-4.8235	0.370618	0.494213
Monocytes	DDRGK1	0.135551	5.272107	1.415868	0.160327	-5.35866	0.346372	0.462205
Monocytes	PITPNM1	-0.17987	3.730007	-1.41586	0.16033	-5.16005	0.361704	0.482478
Monocytes	TMA7	0.09807	6.564001	1.415711	0.160373	-5.58496	0.334101	0.445899
Monocytes	ANP32E	-0.14788	7.305672	-1.41538	0.16047	-5.64703	0.327393	0.436796
Monocytes	SUN1	0.196599	3.918531	1.415229	0.160514	-5.0241	0.359964	0.480036
Monocytes	NMRAL1	-0.26649	3.904291	-1.41446	0.160739	-4.98841	0.360534	0.480643
Monocytes	AP2S1	0.112205	6.932961	1.414291	0.160788	-5.63665	0.331245	0.441798
Monocytes	BMP2K	-0.11565	6.936172	-1.41397	0.160883	-5.6477	0.331337	0.441812
Monocytes	GM12089	-0.42042	0.306607	-1.41322	0.161102	-4.60366	0.399787	0.531564
Monocytes	TMEM191C	0.385774	1.703176	1.412884	0.161201	-4.57331	0.384409	0.51141
Monocytes	GM8113	-0.77771	-0.25687	-1.41263	0.161275	-4.45779	0.406499	0.540109
Monocytes	GM36756	-0.67363	0.926199	-1.4117	0.161548	-4.47549	0.39364	0.523008
Monocytes	ARHGAP12	0.191119	5.076663	1.411084	0.161729	-5.19306	0.350256	0.466185
Monocytes	ZMAT5	-0.16446	4.547775	-1.41106	0.161735	-5.14419	0.355493	0.4731
Monocytes	TRIB1	-0.15922	5.765307	-1.41104	0.161741	-5.61329	0.343566	0.457332
Monocytes	LDLR	0.186746	4.577033	1.411	0.161754	-5.52148	0.355201	0.472715
Monocytes	LFNG	-0.16226	4.413378	-1.41069	0.161845	-5.31271	0.356838	0.475004
Monocytes	ZFP366	-0.26502	1.840557	-1.41052	0.161894	-5.25912	0.383693	0.510285
Monocytes	FAM25C	-0.70515	1.148741	-1.41049	0.161903	-4.51243	0.391287	0.520198
Monocytes	MGARP	0.935587	-0.49619	1.410461	0.161912	-4.45321	0.410008	0.544503
Monocytes	RBM15	-0.14237	5.696792	-1.41039	0.161932	-5.34881	0.344225	0.458368
Monocytes	SLC35F6	0.215378	2.998461	1.410315	0.161955	-5.02918	0.37134	0.494144
Monocytes	POLE4	-0.12566	6.652817	-1.41002	0.162041	-5.5633	0.335187	0.446452
Monocytes	CLOCK	0.1474	5.071637	1.409911	0.162074	-5.38323	0.350339	0.466564
Monocytes	STFA3	0.365929	2.751543	1.409836	0.162096	-5.22849	0.373973	0.497686
Monocytes	CHST12	0.201675	5.763881	1.409753	0.162121	-5.35117	0.343613	0.457712
Monocytes	ARNT	0.163612	5.869604	1.409657	0.162149	-5.46319	0.342599	0.45637
Monocytes	AP5B1	0.497459	1.293299	1.40928	0.16226	-4.57727	0.389905	0.51852
Monocytes	CCT8	-0.11335	6.762623	-1.40903	0.162335	-5.63009	0.33436	0.445417
Monocytes	RASSF5	0.142384	5.485464	1.408968	0.162352	-5.43991	0.346506	0.461557
Monocytes	ZFP451	-0.16709	5.019065	-1.40859	0.162463	-5.24512	0.351228	0.467759
Monocytes	SLC7A8	-0.32393	2.86439	-1.4081	0.162609	-5.15246	0.373432	0.496904
Monocytes	ATP2A3	0.200317	5.210591	1.407616	0.162751	-5.14799	0.349744	0.46561
Monocytes	CD101	0.475773	0.741284	1.407602	0.162755	-4.62782	0.396744	0.527247

Monocytes	GM26802	-0.43229	1.069129	-1.40739	0.162817	-4.72503	0.393135	0.522526
Monocytes	TXNDC11	-0.16078	5.714713	-1.40726	0.162855	-5.31381	0.344901	0.4592
Monocytes	RAB11FIP5	0.516063	0.808074	1.40709	0.162906	-4.61067	0.3961	0.526375
Monocytes	NR1H2	0.192016	4.589379	1.40695	0.162948	-5.21612	0.356001	0.473893
Monocytes	TSPAN12	-0.78093	1.194761	-1.40683	0.162984	-4.48342	0.391793	0.520856
Monocytes	BIVM	0.48953	0.882383	1.406307	0.163138	-4.54349	0.395508	0.525435
Monocytes	PSMG3	0.288479	3.202758	1.406269	0.163149	-4.86259	0.37038	0.492607
Monocytes	TRIR	-0.12392	6.300182	-1.40612	0.163195	-5.50121	0.339565	0.45197
Monocytes	CAPZA1	0.082364	7.715586	1.405725	0.16331	-5.75314	0.326566	0.434625
Monocytes	COPA	-0.10743	6.661065	-1.40566	0.16333	-5.58624	0.336294	0.447596
Monocytes	UNKL	-0.29524	3.970057	-1.40529	0.163441	-4.86373	0.362759	0.482514
Monocytes	DNAJB9	-0.16833	5.168187	-1.40521	0.163462	-5.26212	0.350757	0.466692
Monocytes	TMEM141	-0.3317	2.239657	-1.40488	0.16356	-4.93934	0.381049	0.506451
Monocytes	GM13091	-0.69481	0.904415	-1.40471	0.163612	-4.48809	0.395776	0.525707
Monocytes	NFKBID	-0.17015	6.350031	-1.40458	0.16365	-5.53317	0.339515	0.451828
Monocytes	COX17	-0.11914	6.919214	-1.40446	0.163685	-5.64981	0.334168	0.444763
Monocytes	STK11	0.139532	5.696969	1.40368	0.163917	-5.36906	0.346138	0.460516
Monocytes	D130040H23RI	-0.44728	2.397794	-1.40363	0.163931	-4.61068	0.379793	0.504803
Monocytes	CCL3	0.3003	5.069599	1.403468	0.16398	-5.60064	0.352302	0.468722
Monocytes	1190007I07RIK	-0.26595	3.503332	-1.40316	0.164071	-4.86876	0.36828	0.489671
Monocytes	PSMC5	-0.1258	6.00886	-1.40277	0.164187	-5.46115	0.343444	0.456761
Monocytes	PPHLN1	-0.17454	4.932629	-1.40262	0.164233	-5.28322	0.353972	0.470686
Monocytes	KLHDC3	0.156813	4.338451	1.402255	0.164341	-5.13347	0.360029	0.47863
Monocytes	MIR22HG	0.194028	4.089128	1.402085	0.164391	-5.32832	0.362561	0.482013
Monocytes	ITGA2	-0.56225	0.926385	-1.40204	0.164405	-4.56013	0.396449	0.526343
Monocytes	GM15859	0.491234	1.208039	1.401976	0.164424	-4.51832	0.393294	0.522252
Monocytes	BC002059	0.353508	2.758564	1.401734	0.164496	-4.70043	0.376456	0.500245
Monocytes	CCT3	-0.12474	5.801241	-1.40163	0.164527	-5.43349	0.345601	0.459664
Monocytes	ATG2B	0.169604	4.861013	1.401326	0.164617	-5.21339	0.354825	0.471856
Monocytes	PIK3R3	-0.45422	3.43068	-1.40132	0.164618	-4.78903	0.369386	0.49102
Monocytes	RAB6B	-0.57591	1.697022	-1.40113	0.164676	-4.51712	0.387926	0.515277
Monocytes	PRKCG	-0.30601	4.448629	-1.40113	0.164677	-5.15897	0.358957	0.477304
Monocytes	GM31508	-0.63132	0.763062	-1.40107	0.164693	-4.51997	0.398335	0.528836
Monocytes	EPS15L1	-0.11763	6.525246	-1.40089	0.164747	-5.57476	0.338717	0.450569
Monocytes	RAB20	-0.23937	3.301078	-1.40057	0.164843	-5.2194	0.370913	0.492955
Monocytes	TRPC1	-0.71015	-0.17084	-1.40024	0.164941	-4.4764	0.409308	0.542974
Monocytes	CASTOR2	0.33243	3.643855	1.400237	0.164942	-5.02556	0.367408	0.48835
Monocytes	GM49189	0.575361	0.779988	1.399487	0.165166	-4.52298	0.398851	0.529023
Monocytes	CELSR2	0.890982	-0.09555	1.398281	0.165526	-4.46447	0.409701	0.542285
Monocytes	GM38190	-0.43806	1.823044	-1.398	0.165609	-4.60696	0.388103	0.514236
Monocytes	CD5	-0.36468	2.693985	-1.39776	0.165684	-4.72218	0.378706	0.502027
Monocytes	FBXL12	-0.25034	4.348122	-1.39765	0.165714	-4.93566	0.361464	0.479495
Monocytes	PRXL2A	-0.49482	4.184431	-1.39754	0.165748	-4.81184	0.363131	0.481749
Monocytes	APPBP2OS	0.456736	0.985935	1.397427	0.165782	-4.56456	0.397464	0.526632
Monocytes	RABEP1	-0.10979	6.897474	-1.39734	0.165808	-5.64263	0.336574	0.446757
Monocytes	ILKAP	0.137595	5.651644	1.396925	0.165933	-5.40262	0.348675	0.46266
Monocytes	PDRG1	-0.19914	4.496479	-1.3965	0.16606	-5.09315	0.36035	0.477921

Monocytes	ROCK2	-0.11897	7.468662	-1.39585	0.166254	-5.68605	0.33179	0.440174
Monocytes	CTSZ	0.148791	6.598627	1.395804	0.166269	-5.71694	0.339928	0.450965
Monocytes	MKLN1	0.114281	7.159858	1.395782	0.166275	-5.67448	0.334653	0.443974
Monocytes	CRYZL2	-0.29353	2.609637	-1.3957	0.1663	-4.77752	0.380212	0.503962
Monocytes	GM37233	0.739773	0.010375	1.395508	0.166358	-4.4786	0.40928	0.541746
Monocytes	SOX6	-0.75865	1.905309	-1.3955	0.16636	-4.57383	0.387859	0.513967
Monocytes	EIF5	-0.11361	8.418516	-1.39493	0.166533	-5.85621	0.32336	0.428795
Monocytes	PNKD	0.189802	4.867546	1.39491	0.166538	-5.22379	0.357021	0.473347
Monocytes	HYAL2	-0.52959	1.859326	-1.39433	0.166712	-4.56269	0.388919	0.514823
Monocytes	FBXO11	-0.12895	8.797772	-1.39423	0.166742	-6.00482	0.320236	0.424475
Monocytes	HIST2H4	-0.7565	0.951972	-1.39372	0.166895	-4.49921	0.399328	0.528214
Monocytes	TYK2	0.159101	3.992109	1.393563	0.166943	-5.23519	0.366483	0.485459
Monocytes	TANGO6	-0.22146	4.382985	-1.39341	0.166988	-5.05473	0.362498	0.480239
Monocytes	PTGR1	0.331329	3.377249	1.392213	0.16735	-4.8755	0.373631	0.494316
Monocytes	CHMP7	-0.35324	2.616172	-1.39201	0.16741	-4.72413	0.381794	0.504993
Monocytes	GM16014	0.808553	-0.09363	1.39189	0.167447	-4.4832	0.41228	0.544534
Monocytes	UACA	-0.66969	2.51718	-1.39105	0.167702	-4.52339	0.383359	0.506769
Monocytes	CCDC17	0.661697	0.893924	1.390915	0.167742	-4.5203	0.40139	0.53021
Monocytes	2610002M06RI	0.23682	4.208089	1.390611	0.167834	-5.01335	0.365518	0.483671
Monocytes	CTSO	0.264877	4.26707	1.39059	0.16784	-4.99257	0.364912	0.482878
Monocytes	LEMD3	-0.12644	5.687224	-1.39058	0.167844	-5.51742	0.350664	0.464159
Monocytes	TMEM56	-0.59792	2.147941	-1.39016	0.16797	-4.58139	0.387587	0.512333
Monocytes	TCRG-C4	-0.6226	0.327974	-1.38977	0.16809	-4.54628	0.408279	0.539078
Monocytes	HSPA13	-0.24697	3.082179	-1.38969	0.168112	-4.85097	0.37765	0.499348
Monocytes	BRAT1	0.400655	2.183649	1.389251	0.168246	-4.63918	0.387534	0.51216
Monocytes	ICE2	0.381982	2.160721	1.389197	0.168262	-4.63351	0.387785	0.512508
Monocytes	HIBCH	0.236721	3.478236	1.388946	0.168338	-4.88198	0.373717	0.494217
Monocytes	AGBL1	-0.67758	4.663698	-1.38875	0.168397	-4.72476	0.36151	0.478243
Monocytes	BDP1	0.139256	5.529011	1.38851	0.16847	-5.41989	0.352923	0.466947
Monocytes	RBM33	0.127661	5.898903	1.38791	0.168652	-5.42247	0.349592	0.462369
Monocytes	GM35769	0.468564	1.096204	1.387741	0.168703	-4.63015	0.400271	0.528596
Monocytes	ATP1A1	-0.12294	6.234757	-1.38741	0.168804	-5.64833	0.346488	0.458241
Monocytes	POLR2M	-0.12981	5.403452	-1.38724	0.168856	-5.34706	0.354672	0.468971
Monocytes	HAO1	-0.4303	3.070076	-1.38705	0.168915	-4.80009	0.378764	0.50044
Monocytes	1700010K24RIK	0.512484	0.709295	1.386729	0.169011	-4.53615	0.40506	0.534395
Monocytes	FFAR4	-0.30046	0.260828	-1.38657	0.169058	-4.9818	0.410253	0.541091
Monocytes	SIGMAR1	-0.19058	3.549056	-1.38647	0.169089	-5.1549	0.373801	0.493851
Monocytes	GM4566	0.45828	1.718905	1.386393	0.169113	-4.66042	0.39363	0.519657
Monocytes	GBP2	-0.47809	4.147241	-1.38592	0.169256	-5.30699	0.367721	0.485802
Monocytes	CD2	-0.42591	3.660392	-1.38587	0.169273	-4.71938	0.372794	0.492461
Monocytes	PLCD3	-0.66095	1.122441	-1.3857	0.169325	-4.49395	0.400515	0.528482
Monocytes	PARP4	-0.14144	5.335625	-1.38562	0.169348	-5.42245	0.355659	0.470031
Monocytes	CAR12	-0.74575	0.013745	-1.3855	0.169385	-4.48146	0.413325	0.545008
Monocytes	AXIN1	-0.11357	6.046226	-1.38538	0.169421	-5.4801	0.348657	0.460899
Monocytes	ENTPD7	0.186336	4.693758	1.385251	0.169461	-5.19384	0.362119	0.478665
Monocytes	ZCCHC24	0.256333	3.666755	1.385192	0.169479	-5.01028	0.372727	0.49255
Monocytes	SEC24C	0.155438	4.859773	1.384797	0.169599	-5.22705	0.360599	0.476585

Monocytes	CCDC171	0.231004	3.57202	1.384592	0.169662	-4.99291	0.373893	0.494025
Monocytes	CNRIP1	-0.70708	1.648634	-1.38454	0.169679	-4.50151	0.394766	0.521187
Monocytes	SUPT6	-0.13648	5.808893	-1.38446	0.169702	-5.50601	0.351138	0.464213
Monocytes	ITGA5	0.233739	3.54178	1.383841	0.169891	-5.22979	0.374547	0.494734
Monocytes	HPS3	0.154747	4.626547	1.38363	0.169955	-5.40633	0.36333	0.480091
Monocytes	TYMS	-0.28517	4.922548	-1.38355	0.169981	-5.27237	0.360324	0.47616
Monocytes	1600020E01RIK	-0.16805	6.223144	-1.3832	0.170088	-5.53237	0.347517	0.459342
Monocytes	ZFP142	-0.26535	3.207748	-1.38319	0.17009	-4.85029	0.378209	0.499563
Monocytes	ZFP422	-0.20499	4.395092	-1.38275	0.170223	-5.10904	0.36592	0.48346
Monocytes	ALDH4A1	-0.37862	2.912017	-1.38259	0.170274	-4.75339	0.381526	0.503936
Monocytes	DNAIC1	-0.73038	0.436784	-1.38256	0.170283	-4.49924	0.409226	0.539836
Monocytes	SNHG16	-0.41052	2.227913	-1.38249	0.170302	-4.68248	0.38897	0.513651
Monocytes	IRF5	-0.1298	5.351662	-1.38231	0.170358	-5.72402	0.356248	0.47099
Monocytes	TTPA	0.628441	1.860466	1.382198	0.170393	-4.59565	0.393054	0.519083
Monocytes	PAXIP1	-0.21691	4.269449	-1.38211	0.17042	-5.08701	0.367232	0.485482
Monocytes	BAD	0.215568	3.912661	1.381985	0.170458	-5.06791	0.370938	0.490353
Monocytes	TEPSIN	0.589893	0.762658	1.381766	0.170525	-4.54094	0.405496	0.535348
Monocytes	RNF20	-0.15563	5.755436	-1.3816	0.170576	-5.39413	0.352261	0.465939
Monocytes	GPRIN3	0.688864	0.638034	1.381565	0.170587	-4.51692	0.406933	0.537222
Monocytes	SEC22A	0.278345	3.176208	1.381485	0.170611	-4.82885	0.378729	0.500621
Monocytes	PLS3	-0.51351	2.162516	-1.38114	0.170718	-4.5762	0.389889	0.515081
Monocytes	LARS2	-0.32934	5.571824	-1.3809	0.17079	-5.30281	0.354258	0.468591
Monocytes	FBXW4	0.219913	4.272711	1.380621	0.170876	-5.15615	0.367405	0.485921
Monocytes	EEF1G	-0.12288	7.187877	-1.38061	0.170878	-5.69062	0.338632	0.448043
Monocytes	MFSD12	-0.24176	2.730413	-1.3806	0.170882	-4.92384	0.383721	0.50723
Monocytes	APOPT1	0.126778	4.910205	1.380409	0.170941	-5.29019	0.360935	0.477403
Monocytes	ARHGAP39	0.219636	4.38051	1.37989	0.1711	-5.27488	0.366422	0.484665
Monocytes	SLC45A1	-0.62488	0.608482	-1.37984	0.171116	-4.49152	0.407627	0.538269
Monocytes	PEMT	-0.39612	3.483706	-1.3798	0.171128	-4.86393	0.375786	0.496918
Monocytes	OGT	0.121289	6.649131	1.37971	0.171155	-5.5611	0.343874	0.455005
Monocytes	COG4	0.146473	5.336083	1.379691	0.171161	-5.34547	0.356729	0.471946
Monocytes	GM42477	-0.44573	1.273145	-1.37927	0.171291	-4.61697	0.400133	0.528526
Monocytes	4930595D18RIK	0.519631	1.248821	1.379246	0.171298	-4.59296	0.400409	0.528883
Monocytes	RHEBL1	-0.53666	1.532009	-1.37917	0.171322	-4.57949	0.397208	0.524737
Monocytes	LMAN2L	0.169614	4.447735	1.378908	0.171402	-5.18371	0.365844	0.483994
Monocytes	RP2	0.135613	5.226456	1.378888	0.171408	-5.47125	0.357937	0.473615
Monocytes	NDUFA11	0.131087	6.910675	1.378709	0.171463	-5.65153	0.341481	0.451982
Monocytes	RBM12B2	-0.38933	2.316248	-1.37866	0.171477	-4.65015	0.388493	0.513664
Monocytes	4632404H12RIK	0.583191	1.238383	1.378567	0.171506	-4.57371	0.40053	0.529328
Monocytes	KLHL9	0.169362	4.65586	1.378201	0.171619	-5.25014	0.363857	0.481503
Monocytes	SH3PXD2A	-0.21487	5.753145	-1.37776	0.171756	-5.29026	0.352839	0.467178
Monocytes	GOLPH3	-0.10246	5.902166	-1.37775	0.171759	-5.52743	0.351371	0.465244
Monocytes	LRRC45	0.349273	2.190744	1.377515	0.17183	-4.72641	0.39003	0.515982
Monocytes	TRAT1	-0.8427	-0.05747	-1.37746	0.171848	-4.52259	0.415709	0.549247
Monocytes	UBXN8	0.177072	4.404408	1.37741	0.171863	-5.17918	0.366436	0.485188
Monocytes	TMEM208	0.137819	4.89435	1.377286	0.171901	-5.26919	0.36143	0.478674
Monocytes	SH3D21	-0.39399	1.99564	-1.37708	0.171964	-4.75658	0.392189	0.518883

Monocytes	PARD3	-0.5984	2.300134	-1.37706	0.17197	-4.61115	0.388826	0.514508
Monocytes	ACTL6A	-0.14878	5.235041	-1.37703	0.17198	-5.31137	0.357994	0.474156
Monocytes	EXOG	0.5019	1.745846	1.376904	0.172018	-4.56851	0.394971	0.522499
Monocytes	CDK12	-0.11761	6.896517	-1.37688	0.172027	-5.66424	0.341753	0.452729
Monocytes	SNX24	-0.19801	4.299252	-1.3768	0.172051	-5.48791	0.36752	0.486699
Monocytes	SNRK	0.175498	4.865356	1.376458	0.172156	-5.28384	0.361868	0.479225
Monocytes	TMEM164	0.112318	6.945657	1.376229	0.172227	-5.73084	0.34146	0.452287
Monocytes	ZCCHC17	-0.1484	5.162664	-1.37616	0.172249	-5.28433	0.358905	0.475329
Monocytes	GM9844	-0.25393	2.309102	-1.37591	0.172325	-4.94592	0.389012	0.514758
Monocytes	UBE2J2	0.100088	6.638057	1.37548	0.172458	-5.64491	0.344476	0.456493
Monocytes	YWHAZ	-0.06722	8.482146	-1.37548	0.172458	-5.93475	0.327262	0.433654
Monocytes	RAD9A	-0.43743	2.338849	-1.37535	0.172499	-4.70547	0.388685	0.514543
Monocytes	EFCC1	-0.43133	0.001369	-1.37533	0.172503	-4.72188	0.415318	0.549069
Monocytes	ZC3H3	0.213465	3.468484	1.37521	0.172541	-4.98124	0.376489	0.498643
Monocytes	FBXO10	0.42334	1.355085	1.375203	0.172543	-4.62476	0.39966	0.528829
Monocytes	LACTB2	0.21641	3.94655	1.374682	0.172704	-5.0424	0.371723	0.492247
Monocytes	RANBP2	-0.14402	6.846596	-1.37453	0.17275	-5.67935	0.342745	0.454149
Monocytes	HIST1H1C	-0.32802	4.106663	-1.37436	0.172805	-5.00793	0.370112	0.490185
Monocytes	NPM3	0.184331	5.097579	1.374216	0.172848	-5.34577	0.35997	0.476849
Monocytes	ZBTB48	-0.58918	1.123594	-1.37406	0.172897	-4.56822	0.402689	0.532659
Monocytes	CCDC57	0.315862	2.546499	1.373752	0.172992	-4.77596	0.386848	0.512099
Monocytes	MYO15	-0.62323	1.234713	-1.37359	0.173041	-4.55783	0.40148	0.531149
Monocytes	SIDT1	-0.53328	3.376538	-1.37344	0.173087	-4.76902	0.377893	0.500522
Monocytes	TMEM120B	-0.28243	3.044848	-1.3734	0.173101	-5.08813	0.381444	0.505181
Monocytes	NT5M	0.233077	3.572881	1.373387	0.173105	-4.94376	0.375808	0.497813
Monocytes	COQ4	0.351589	2.66674	1.373084	0.173199	-4.71552	0.385664	0.510648
Monocytes	HJURP	0.198103	4.895427	1.37291	0.173252	-5.19176	0.362215	0.479977
Monocytes	PRPF4B	0.123759	6.312389	1.372564	0.17336	-5.55267	0.348132	0.461566
Monocytes	LDAH	0.176896	4.225642	1.372544	0.173366	-5.15402	0.369093	0.489174
Monocytes	RAB21	-0.10649	6.891793	-1.37237	0.17342	-5.64536	0.342551	0.454258
Monocytes	FANCB	-0.64977	0.677958	-1.37222	0.173468	-4.50769	0.408009	0.540021
Monocytes	DDR1	0.484874	0.137297	1.372136	0.173492	-4.58092	0.414324	0.548207
Monocytes	SLC31A2	0.25285	3.169586	1.37206	0.173516	-5.1143	0.380232	0.503897
Monocytes	FAF2	0.153256	5.155771	1.372029	0.173526	-5.30603	0.359581	0.476796
Monocytes	MTRF1L	0.35065	3.1332	1.372015	0.17353	-4.74881	0.380623	0.504407
Monocytes	PLEKHG1	0.186227	4.398533	1.371497	0.173691	-5.45958	0.367498	0.487174
Monocytes	MOB3A	-0.16556	4.775701	-1.37148	0.173695	-5.32106	0.363626	0.482086
Monocytes	TFRC	-0.18067	6.38061	-1.3713	0.173752	-5.35817	0.347682	0.461019
Monocytes	VPS50	0.156533	4.131766	1.3712	0.173783	-5.24492	0.370295	0.490805
Monocytes	MTMR2	0.142687	5.396075	1.371068	0.173824	-5.34939	0.357395	0.473897
Monocytes	PLCB2	-0.21213	3.320212	-1.37072	0.173933	-5.10209	0.379019	0.50222
Monocytes	SLFN3	0.551803	1.4477	1.370462	0.174012	-4.57014	0.399717	0.529172
Monocytes	NR1D2	-0.2568	3.478586	-1.37019	0.174097	-4.93975	0.377528	0.500279
Monocytes	GM30211	-0.78103	4.041214	-1.36981	0.174216	-4.60254	0.371771	0.492662
Monocytes	KDM5B	0.25463	5.362074	1.369536	0.1743	-5.21577	0.358338	0.474931
Monocytes	PDZD2	-0.48807	3.46937	-1.36942	0.174338	-4.74045	0.377909	0.500623
Monocytes	RDH5	-0.46181	2.368316	-1.36888	0.174504	-4.67692	0.390062	0.516374

Monocytes	THTPA	-0.46473	1.360701	-1.36846	0.174636	-4.59229	0.401346	0.531262
Monocytes	VAPA	0.076835	8.028548	1.36841	0.174651	-5.8564	0.332855	0.441288
Monocytes	UAP1L1	-0.21242	3.734573	-1.36838	0.174661	-5.07059	0.375314	0.497319
Monocytes	BCL11A	0.233718	5.196907	1.368336	0.174674	-5.32458	0.360209	0.477488
Monocytes	GPSM2	0.341984	2.440784	1.367991	0.174782	-4.83493	0.389264	0.515767
Monocytes	PEG3	-0.60098	1.53048	-1.36796	0.174793	-4.58537	0.39942	0.528989
Monocytes	FNDC7	-0.38673	-0.36573	-1.36795	0.174794	-4.78941	0.421515	0.55759
Monocytes	SUV39H2	0.324786	2.381659	1.36789	0.174813	-4.79328	0.389915	0.516659
Monocytes	LYAR	-0.19849	4.937131	-1.36778	0.174846	-5.2784	0.362843	0.481225
Monocytes	TRIM27	-0.14069	5.675636	-1.36769	0.174875	-5.40642	0.355413	0.471432
Monocytes	SLC22A4	0.305206	0.816422	1.367677	0.17488	-4.96273	0.407589	0.539669
Monocytes	TLL2	0.772542	0.725481	1.36721	0.175026	-4.55012	0.408815	0.541112
Monocytes	SNX20	0.137587	5.514815	1.367158	0.175042	-5.54874	0.357167	0.473637
Monocytes	AP4B1	0.383622	2.273778	1.367087	0.175064	-4.71462	0.39127	0.518344
Monocytes	ZBTB40	0.282429	3.163496	1.366801	0.175153	-4.85947	0.381677	0.505808
Monocytes	UQCC3	0.190621	4.016511	1.366068	0.175382	-5.10516	0.372996	0.494171
Monocytes	HSP90AB1	-0.0981	9.925498	-1.36599	0.175405	-6.14166	0.316395	0.419233
Monocytes	BYSL	-0.21702	3.67185	-1.36549	0.175564	-4.92818	0.376634	0.499067
Monocytes	PWWP2B	0.339399	2.082717	1.365453	0.175575	-4.80804	0.393911	0.521624
Monocytes	RAI1	-0.1646	6.004853	-1.36532	0.175617	-5.3916	0.35277	0.467715
Monocytes	TMEM218	0.442261	1.874833	1.365102	0.175685	-4.58511	0.396235	0.524756
Monocytes	NFKBIE	0.18266	4.879022	1.365056	0.175699	-5.42849	0.364069	0.482698
Monocytes	DCTN1	-0.13849	4.987161	-1.36497	0.175726	-5.35702	0.362967	0.481291
Monocytes	LZTS1	-0.64851	1.417164	-1.36482	0.175772	-4.53403	0.401404	0.531548
Monocytes	CITED4	-0.86808	-0.2568	-1.36451	0.17587	-4.51132	0.420944	0.557026
Monocytes	AFG1L	0.292485	3.691074	1.364462	0.175885	-4.9756	0.37643	0.499151
Monocytes	STEAP3	0.448032	1.980258	1.364235	0.175956	-4.62943	0.395055	0.523557
Monocytes	ENPP5	-0.58192	0.877362	-1.36421	0.175965	-4.61008	0.407595	0.539856
Monocytes	GM38843	-0.33885	0.985485	-1.36415	0.175983	-4.90078	0.406347	0.538237
Monocytes	CLEC10A	0.356066	0.930777	1.364123	0.175991	-4.94704	0.406978	0.539055
Monocytes	JAM3	-0.7291	0.244994	-1.36408	0.176004	-4.51347	0.414981	0.549423
Monocytes	EXOC3L4	-0.59082	0.594811	-1.36391	0.176057	-4.59999	0.410877	0.544144
Monocytes	SMPDL3B	-0.29568	1.9506	-1.36391	0.176059	-5.24844	0.395386	0.524025
Monocytes	2010320M18RI	0.388459	2.480423	1.363853	0.176076	-4.70233	0.389507	0.516361
Monocytes	SDAD1	-0.15096	4.473042	-1.36374	0.176113	-5.42025	0.368242	0.488544
Monocytes	NCLN	0.186636	3.839103	1.363657	0.176137	-5.15219	0.374865	0.49725
Monocytes	BSCL2	0.141718	4.542466	1.363639	0.176143	-5.32187	0.367525	0.487609
Monocytes	MYL6B	0.581477	0.862198	1.363329	0.17624	-4.53965	0.407911	0.540205
Monocytes	ACTG1	-0.12082	11.44474	-1.36307	0.176321	-6.4681	0.303644	0.402523
Monocytes	PLXNA4	-0.83553	2.166702	-1.36288	0.176381	-4.70836	0.393218	0.521167
Monocytes	BCDIN3D	0.389226	2.223563	1.362824	0.176399	-4.63222	0.392587	0.520364
Monocytes	VPS35L	0.12795	5.162292	1.36181	0.176718	-5.3676	0.361883	0.479909
Monocytes	B230307C23RIK	-0.22166	3.741811	-1.36179	0.176723	-5.02755	0.376615	0.499265
Monocytes	MAP2	-0.78526	0.187932	-1.36174	0.17674	-4.49893	0.416453	0.55111
Monocytes	INPP5J	0.516774	-1.12022	1.361559	0.176797	-4.56871	0.432301	0.571511
Monocytes	CLIP2	-0.22263	2.886513	-1.36132	0.176871	-4.98064	0.385931	0.511469
Monocytes	P4HTM	-0.2513	2.773581	-1.36109	0.176946	-5.22299	0.387245	0.513179

Monocytes	SERF2	0.073902	9.682679	1.360871	0.177013	-6.18966	0.31936	0.423433
Monocytes	BAG3	0.196724	2.967773	1.360162	0.177237	-5.23165	0.385601	0.51063
Monocytes	4930426D05RII	0.790209	-0.21569	1.359974	0.177296	-4.4942	0.422057	0.557945
Monocytes	NTMT1	0.190106	3.588232	1.359075	0.17758	-4.94787	0.379476	0.502269
Monocytes	GRB7	0.833703	0.183341	1.358934	0.177624	-4.49849	0.41786	0.552233
Monocytes	SRRT	-0.14634	5.528359	-1.35877	0.177675	-5.39199	0.359354	0.475996
Monocytes	TSEN2	0.493367	1.266976	1.358743	0.177685	-4.58813	0.405203	0.535923
Monocytes	EPHA4	-0.74927	0.083978	-1.35863	0.177721	-4.53928	0.419042	0.553867
Monocytes	COMMD8	-0.11632	5.188265	-1.35837	0.177802	-5.55828	0.362818	0.480619
Monocytes	GID8	-0.14601	5.195402	-1.35834	0.177813	-5.33159	0.362746	0.480524
Monocytes	A230059L01RIK	-0.86137	0.47539	-1.35824	0.177844	-4.50702	0.414435	0.54798
Monocytes	CHAC1	-0.53558	0.269965	-1.35803	0.177909	-4.62489	0.416925	0.551164
Monocytes	NR2C2	0.131488	6.925422	1.357548	0.178062	-5.61	0.345686	0.458115
Monocytes	ZFP433	0.636224	0.355661	1.357548	0.178062	-4.54589	0.41592	0.549984
Monocytes	RPN2	0.121441	6.097946	1.357522	0.17807	-5.58993	0.35376	0.468791
Monocytes	2310061I04RIK	0.232386	3.521012	1.357489	0.178081	-4.94748	0.380287	0.503655
Monocytes	STK11IP	0.338661	2.312647	1.357415	0.178104	-4.70153	0.393479	0.520874
Monocytes	PNRC2	0.144469	5.35165	1.357156	0.178186	-5.38972	0.361224	0.478722
Monocytes	1700094D03RII	0.446978	1.476408	1.35714	0.178191	-4.59194	0.402902	0.533224
Monocytes	MTFMT	-0.25942	2.527907	-1.35705	0.178219	-4.8002	0.391093	0.517867
Monocytes	PPM1J	-0.62543	-0.35731	-1.35697	0.178246	-4.53677	0.424438	0.561085
Monocytes	OVCA2	-0.7191	0.174482	-1.35683	0.17829	-4.52884	0.418083	0.552866
Monocytes	TIAM2	0.31214	2.628932	1.356369	0.178435	-5.08938	0.390189	0.516614
Monocytes	B3GNT8	0.425573	2.202747	1.35607	0.17853	-4.75976	0.394917	0.522837
Monocytes	REXO2	0.141514	6.270041	1.355991	0.178555	-5.58594	0.352254	0.466904
Monocytes	TMC4	0.441876	1.556285	1.355965	0.178563	-4.59501	0.402209	0.532351
Monocytes	WDR74	-0.18129	4.042303	-1.35588	0.17859	-5.07038	0.37495	0.496785
Monocytes	AKAP10	0.151126	5.860095	1.355844	0.178602	-5.54477	0.356313	0.47227
Monocytes	JAK1	0.098655	8.273816	1.355659	0.178661	-5.87331	0.333151	0.441647
Monocytes	SYNE3	0.3215	2.767053	1.355559	0.178692	-4.96051	0.388671	0.514868
Monocytes	NEK1	0.306067	3.697931	1.355492	0.178713	-4.91097	0.378603	0.501719
Monocytes	CNNM2	0.207139	5.427728	1.355044	0.178856	-5.62486	0.360864	0.478338
Monocytes	RAB14	0.083767	7.091867	1.354801	0.178933	-5.73532	0.344554	0.456697
Monocytes	RAP1GAP2	0.194113	4.692548	1.35455	0.179012	-5.57826	0.368549	0.48837
Monocytes	CLEC4N	0.259437	4.093598	1.354278	0.179099	-5.47685	0.374866	0.496598
Monocytes	PVR	0.227778	3.063552	1.354223	0.179116	-5.14338	0.385901	0.511039
Monocytes	SPINT1	0.310515	-1.0651	1.353621	0.179308	-4.78829	0.434216	0.573437
Monocytes	ZFP317	0.323955	2.898111	1.353268	0.17942	-4.7789	0.388203	0.513827
Monocytes	CLN3	-0.18017	4.768913	-1.35306	0.179486	-5.4471	0.368328	0.487839
Monocytes	PARD3B	-0.4272	4.55014	-1.3529	0.179536	-4.94649	0.370597	0.490838
Monocytes	POM121	-0.14717	4.883475	-1.35267	0.179611	-5.30538	0.367146	0.486398
Monocytes	TNRC18	0.110338	5.931838	1.352651	0.179616	-5.55673	0.356523	0.472407
Monocytes	PSD4	-0.18375	4.541706	-1.35263	0.179623	-5.23969	0.370685	0.491051
Monocytes	SCAI	-0.22383	4.434969	-1.35247	0.179674	-5.16745	0.371826	0.492574
Monocytes	RBM17	-0.133	5.789508	-1.35225	0.179745	-5.49501	0.358039	0.474406
Monocytes	MRPL28	0.130236	6.016074	1.352018	0.179818	-5.57349	0.355849	0.471455
Monocytes	NFKBIZ	0.170881	6.516715	1.351795	0.179889	-5.67264	0.350975	0.465028

Monocytes	GRIA3	0.347444	3.336555	1.351497	0.179984	-5.18204	0.383843	0.508129
Monocytes	PRRT1	-0.56355	0.466797	-1.35112	0.180103	-4.5816	0.416467	0.550427
Monocytes	CUX2	-0.63764	1.039079	-1.35106	0.180124	-4.53627	0.409757	0.541761
Monocytes	PLA2G12B	-0.72433	1.0508	-1.35075	0.180223	-4.53131	0.409759	0.541642
Monocytes	NDC1	-0.24833	3.748037	-1.35064	0.180259	-5.0005	0.379685	0.502537
Monocytes	GM11520	0.560164	1.043872	1.350409	0.180331	-4.5495	0.409919	0.541879
Monocytes	MAPKAP1	-0.12814	5.764174	-1.35005	0.180447	-5.51155	0.359025	0.475339
Monocytes	GM13710	-0.53093	1.36556	-1.34986	0.180507	-4.73161	0.406426	0.537313
Monocytes	TNNT1	-0.54015	1.520318	-1.34954	0.18061	-4.57576	0.404795	0.535012
Monocytes	AC149090.1	-0.32126	5.49949	-1.34778	0.181172	-5.34889	0.362923	0.479575
Monocytes	DECR2	-0.33972	2.741577	-1.34744	0.181282	-4.824	0.392325	0.517942
Monocytes	GM3448	-0.43089	1.9113	-1.34735	0.18131	-4.68344	0.401641	0.530069
Monocytes	EFCAB7	-0.65685	0.535523	-1.3472	0.181358	-4.51664	0.41764	0.550737
Monocytes	SESN2	-0.272	3.371941	-1.34702	0.181417	-4.96114	0.385473	0.509069
Monocytes	RTN4RL1	-0.40973	2.381813	-1.34691	0.181452	-4.87001	0.396397	0.523314
Monocytes	APMAP	0.199365	4.080552	1.346737	0.181507	-5.14296	0.377895	0.499233
Monocytes	ATP2B1	0.10376	8.327101	1.346358	0.181629	-6.10134	0.335788	0.443761
Monocytes	GPATCH3	0.261456	3.369567	1.345428	0.181927	-4.99432	0.38627	0.509685
Monocytes	AHDC1	-0.18773	4.689967	-1.34525	0.181983	-5.08145	0.372221	0.491352
Monocytes	TMX2	-0.27686	3.46498	-1.34503	0.182055	-4.90574	0.385268	0.50842
Monocytes	TUBA1B	-0.19455	7.795232	-1.34503	0.182057	-5.8496	0.341302	0.450684
Monocytes	KIF16B	-0.17222	4.843803	-1.34481	0.182126	-5.33036	0.370682	0.489304
Monocytes	N6AMT1	-0.33311	2.597729	-1.3445	0.182227	-4.79694	0.394979	0.52095
Monocytes	RASD1	-0.42405	3.88428	-1.34444	0.182247	-4.84344	0.380908	0.502631
Monocytes	ZBP1	0.377427	3.640152	1.34431	0.182287	-5.28495	0.383542	0.506058
Monocytes	AZGP1	-0.40904	3.926277	-1.34406	0.182368	-5.0025	0.380556	0.50216
Monocytes	TSTD3	0.324557	1.784277	1.343526	0.18254	-4.8082	0.40454	0.533229
Monocytes	PSMD7	-0.14623	5.828246	-1.34345	0.182564	-5.49659	0.36102	0.476489
Monocytes	ACVR2A	0.227444	4.7385	1.343245	0.18263	-5.28334	0.372273	0.491237
Monocytes	LZTS3	0.624638	0.323606	1.342959	0.182723	-4.54193	0.42183	0.555572
Monocytes	EPS8L2	-0.70906	0.893203	-1.34286	0.182754	-4.54696	0.415063	0.546872
Monocytes	HEATR5A	0.157049	5.656048	1.342669	0.182816	-5.54579	0.362967	0.479034
Monocytes	SNX3	-0.08717	7.730038	-1.34211	0.182995	-5.87246	0.342837	0.452357
Monocytes	INTS10	-0.25768	3.410048	-1.34136	0.183239	-4.93044	0.387335	0.510644
Monocytes	SNX6	-0.10207	6.568189	-1.34099	0.183359	-5.61689	0.354667	0.467813
Monocytes	ADAP2OS	0.404275	0.724917	1.340696	0.183454	-4.81614	0.418217	0.550527
Monocytes	GM43329	0.298862	3.105098	1.340135	0.183635	-4.93958	0.391265	0.515374
Monocytes	SMIM1	-0.59026	1.759237	-1.34005	0.183664	-4.6257	0.406432	0.53503
Monocytes	RPAIN	0.198981	3.771433	1.339383	0.183879	-5.07056	0.384338	0.506156
Monocytes	SMIM4	0.152112	5.255277	1.339193	0.183941	-5.33026	0.368649	0.485741
Monocytes	DCXR	0.20276	4.317514	1.339178	0.183945	-5.22683	0.378479	0.498589
Monocytes	PAPSS1	0.155935	4.181725	1.33824	0.18425	-5.26108	0.380412	0.500644
Monocytes	UBR4	-0.1063	6.056739	-1.33822	0.184256	-5.55759	0.360934	0.475182
Monocytes	TMED10	0.095605	7.769396	1.33777	0.184402	-5.88562	0.344318	0.453186
Monocytes	PSMD4	0.099388	6.691408	1.337265	0.184566	-5.68023	0.355015	0.467215
Monocytes	ELAVL1	-0.08014	7.604056	-1.33714	0.184606	-5.81306	0.346108	0.455553
Monocytes	TRIM34A	0.358489	3.308515	1.337025	0.184644	-4.92901	0.39034	0.513433

Monocytes	SLC28A2	0.445374	2.961181	1.33697	0.184662	-4.74265	0.394183	0.518444
Monocytes	RBM34	-0.1593	4.465573	-1.33683	0.184707	-5.19485	0.37785	0.497249
Monocytes	GM46430	0.404069	1.969969	1.336603	0.184782	-4.63355	0.405404	0.533128
Monocytes	IL31RA	-0.32885	3.109325	-1.33659	0.184787	-4.86371	0.392563	0.516498
Monocytes	UPB1	-0.3967	3.0781	-1.33642	0.184841	-4.83536	0.392944	0.517051
Monocytes	SMARCB1	-0.1237	5.410318	-1.3362	0.184913	-5.39406	0.368018	0.48465
Monocytes	AGBL3	-0.49175	1.72445	-1.33611	0.184943	-4.59949	0.408277	0.536996
Monocytes	EIF4G3	0.094559	7.91677	1.336059	0.184959	-5.8885	0.343173	0.451987
Monocytes	NCSTN	0.135617	5.576404	1.33548	0.185147	-5.51093	0.366609	0.482662
Monocytes	5031434O11RII	0.564842	0.524887	1.335363	0.185185	-4.56954	0.422748	0.555479
Monocytes	PROSCOS	-0.67127	0.287878	-1.33509	0.185274	-4.58562	0.42572	0.559269
Monocytes	TWSG1	-0.32985	3.230079	-1.33489	0.185338	-4.84941	0.391754	0.51542
Monocytes	TTC7	0.136999	5.873227	1.33461	0.185431	-5.63148	0.363837	0.478932
Monocytes	SENP7	0.159966	5.356774	1.334345	0.185517	-5.35108	0.369231	0.485944
Monocytes	OLA1	-0.1172	5.835409	-1.33417	0.185574	-5.51092	0.364355	0.479569
Monocytes	EEF1A1	-0.08345	10.55641	-1.3338	0.185695	-6.31503	0.319786	0.420665
Monocytes	HGS	-0.17884	4.094551	-1.33349	0.185796	-5.19418	0.382886	0.503671
Monocytes	GM11457	0.791772	0.10252	1.333354	0.185841	-4.54151	0.428666	0.562841
Monocytes	ZFP507	-0.63022	2.176566	-1.33316	0.185903	-4.56238	0.404237	0.531391
Monocytes	GM48302	0.420718	1.63727	1.332442	0.186139	-4.78373	0.41089	0.53962
Monocytes	DEF8	0.311963	2.04717	1.332202	0.186218	-4.81273	0.406239	0.53364
Monocytes	MYO1B	-0.48861	2.148687	-1.33182	0.186343	-4.73374	0.405228	0.53228
Monocytes	KIF3A	-0.3793	2.472633	-1.33169	0.186384	-4.69353	0.401534	0.527552
Monocytes	IRF4	-0.31196	4.497863	-1.33161	0.186411	-4.99	0.379264	0.498644
Monocytes	TASOR2	-0.14927	5.373883	-1.33152	0.18644	-5.44026	0.370059	0.48669
Monocytes	ARMCX6	-0.42454	0.977785	-1.33106	0.186591	-4.63707	0.419147	0.550175
Monocytes	ANKRD23	0.615861	0.33175	1.330811	0.186673	-4.54732	0.426926	0.560235
Monocytes	PCBP4	-0.76095	0.472145	-1.3308	0.186676	-4.51983	0.425227	0.558055
Monocytes	HSP90B1	-0.10996	8.976388	-1.33003	0.186928	-6.03774	0.335353	0.440804
Monocytes	GM49774	-0.30389	3.174932	-1.32984	0.186992	-5.05087	0.394416	0.518116
Monocytes	MAD1L1	0.139271	5.227133	1.329634	0.187059	-5.35574	0.372361	0.489369
Monocytes	TNFRSF23	0.38951	1.669914	1.32952	0.187097	-4.76729	0.411615	0.540353
Monocytes	PFDN6	-0.16043	4.874434	-1.32936	0.187149	-5.31181	0.376064	0.494326
Monocytes	TAF10	0.104989	6.856819	1.329284	0.187174	-5.76745	0.355785	0.4678
Monocytes	AKR1A1	-0.10189	7.188669	-1.32897	0.187277	-5.84558	0.352633	0.46358
Monocytes	SAA1	1.248092	-0.05604	1.328723	0.187358	-4.53013	0.432453	0.567243
Monocytes	CDK9	0.137288	5.578202	1.32871	0.187363	-5.45862	0.368867	0.484976
Monocytes	ETL4	-0.49895	2.396305	-1.32856	0.187412	-4.67902	0.403429	0.529918
Monocytes	GATAD2A	-0.10639	7.859185	-1.32832	0.187491	-5.86467	0.346234	0.455177
Monocytes	IL27	-0.65529	0.053926	-1.3282	0.187532	-4.62267	0.43123	0.56557
Monocytes	RNF144A	0.256771	3.619246	1.328006	0.187594	-4.99136	0.389889	0.512244
Monocytes	WDR44	0.178751	4.70447	1.327656	0.18771	-5.33323	0.378328	0.497047
Monocytes	ZSCAN20	-0.73383	1.073033	-1.32724	0.187847	-4.55056	0.419325	0.550043
Monocytes	GM17251	-0.45488	2.365949	-1.32693	0.187948	-4.6967	0.404253	0.530688
Monocytes	UXT	0.190141	4.452478	1.32685	0.187975	-5.17622	0.381168	0.500759
Monocytes	TAOK3	0.084499	7.012234	1.326728	0.188015	-5.81421	0.354828	0.466341
Monocytes	GPR132	-0.16101	6.00422	-1.32671	0.18802	-5.86787	0.364949	0.479614

Monocytes	MYO18A	-0.14591	4.439945	-1.32665	0.188041	-5.38699	0.381303	0.500985
Monocytes	TRIM23	0.290408	3.295163	1.326551	0.188073	-4.83082	0.393787	0.517221
Monocytes	AW209491	0.631833	1.448036	1.3265	0.18809	-4.58888	0.414891	0.544484
Monocytes	SWT1	0.150928	5.244266	1.325934	0.188277	-5.35919	0.372968	0.490119
Monocytes	UTP20	-0.27776	3.652111	-1.32593	0.188277	-5.05769	0.390028	0.512329
Monocytes	VAMP7	0.184039	4.132998	1.325887	0.188292	-5.17023	0.384786	0.505518
Monocytes	RAC1	0.07749	7.82414	1.325631	0.188377	-5.9458	0.347085	0.456222
Monocytes	GM26511	-0.46368	2.075738	-1.3256	0.188389	-4.68586	0.407795	0.535377
Monocytes	VSIG10L	0.5432	0.298869	1.32541	0.18845	-4.55676	0.428866	0.56248
Monocytes	CYB561A3	-0.17019	5.573777	-1.32539	0.188455	-5.40003	0.369562	0.485724
Monocytes	ZFP560	-0.22124	4.00302	-1.32499	0.18859	-5.02162	0.386339	0.507562
Monocytes	FKBPL	0.526601	1.019839	1.324937	0.188606	-4.60494	0.420311	0.551469
Monocytes	FASTKD5	0.502886	0.844458	1.324824	0.188643	-4.61248	0.422407	0.554162
Monocytes	DUSP7	0.290473	3.559127	1.324751	0.188668	-4.96968	0.391196	0.513878
Monocytes	SUGCT	-0.39265	2.835527	-1.32431	0.188812	-4.77664	0.399485	0.524509
Monocytes	KCNK13	-0.31926	2.912398	-1.32414	0.188871	-5.07267	0.398663	0.523466
Monocytes	TRIM2	-0.56916	2.325336	-1.32379	0.188985	-4.61328	0.405492	0.532243
Monocytes	ADIPOR1	0.134974	7.346972	1.32337	0.189124	-5.81385	0.352282	0.462865
Monocytes	MOSPD3	0.149457	4.656046	1.323357	0.189129	-5.35761	0.379799	0.498872
Monocytes	SPNS3	0.446309	3.650358	1.323327	0.189139	-4.78564	0.390692	0.513034
Monocytes	MRVI1	-0.54715	0.497468	-1.32319	0.189184	-4.61453	0.42715	0.560095
Monocytes	MID1	-0.47401	5.805162	-1.323	0.189247	-5.54223	0.367827	0.483249
Monocytes	TLR6	0.429096	1.070509	1.322823	0.189305	-4.78507	0.420361	0.551402
Monocytes	TRAPPC10	-0.14509	5.170629	-1.32267	0.189355	-5.3698	0.374482	0.491966
Monocytes	GM21762	-0.75668	-1.20491	-1.32234	0.189466	-4.50893	0.448661	0.587466
Monocytes	ATP6AP2	-0.08703	6.938045	-1.32155	0.189726	-5.79996	0.356973	0.468547
Monocytes	GM47096	-0.45525	1.734536	-1.32147	0.189756	-4.65409	0.413172	0.541557
Monocytes	MMAB	0.562626	1.326097	1.321047	0.189895	-4.60306	0.418151	0.547931
Monocytes	WDR45	0.2867	2.960786	1.321001	0.18991	-4.85077	0.39926	0.523598
Monocytes	GM16268	-0.59924	0.706579	-1.3207	0.190009	-4.61292	0.425701	0.557591
Monocytes	E030030I06RIK	0.334984	2.657085	1.320479	0.190083	-4.74256	0.402852	0.528274
Monocytes	LYRM1	0.277382	2.700522	1.320436	0.190097	-4.89941	0.402358	0.52766
Monocytes	NFAT5	-0.13365	7.501438	-1.32019	0.19018	-5.8649	0.351711	0.461731
Monocytes	SRP19	0.123968	6.048783	1.32004	0.190229	-5.58257	0.366246	0.480789
Monocytes	LCP2	0.169366	5.109024	1.320039	0.19023	-5.7027	0.376007	0.493534
Monocytes	PROX2	-0.68218	1.188752	-1.31995	0.190258	-4.54968	0.419959	0.550404
Monocytes	HMGA2	-1.15288	1.149583	-1.3191	0.190543	-4.53522	0.420971	0.551295
Monocytes	MPP6	-0.12101	6.194697	-1.31889	0.190613	-5.68336	0.36529	0.479161
Monocytes	RBMS3	-0.52856	3.072419	-1.31818	0.190849	-4.78174	0.399162	0.522864
Monocytes	DCN	-0.46852	3.138043	-1.31807	0.190884	-4.85539	0.398423	0.521956
Monocytes	2700038G22RII	-0.31377	2.577847	-1.31779	0.190978	-4.819	0.404852	0.530166
Monocytes	MIRT2	0.655889	-1.00119	1.317731	0.190998	-4.53574	0.448132	0.58561
Monocytes	UBN2	0.115953	7.076175	1.317247	0.19116	-5.78741	0.357082	0.467995
Monocytes	ZFP276	0.299557	2.479933	1.316964	0.191254	-4.8397	0.4063	0.531915
Monocytes	GM47350	-0.45328	1.355941	-1.31693	0.191267	-4.63225	0.419431	0.548799
Monocytes	DHRS1	-0.19559	4.762169	-1.31668	0.191348	-5.40808	0.38102	0.499212
Monocytes	ETAA1	-0.29325	3.334503	-1.31668	0.19135	-4.87327	0.396631	0.519448

Monocytes	MSL3	0.151144	4.998465	1.31623	0.191499	-5.35999	0.3787	0.49618
Monocytes	SERPINB1A	0.387385	3.600711	1.316146	0.191527	-5.04179	0.393873	0.515891
Monocytes	CPSF4	0.212039	4.317677	1.315759	0.191657	-5.16387	0.386191	0.505836
Monocytes	SRSF11	0.072227	7.871425	1.315368	0.191787	-5.89058	0.349817	0.458366
Monocytes	ADIPOR2	0.12049	6.368043	1.315318	0.191804	-5.64243	0.36477	0.477955
Monocytes	PITPNB	0.118939	5.507244	1.315176	0.191852	-5.47305	0.373672	0.489604
Monocytes	FMN1	-0.24223	3.350464	-1.3147	0.192011	-5.31111	0.397243	0.520121
Monocytes	GTPBP10	-0.32602	2.397542	-1.31461	0.19204	-4.77065	0.408073	0.534111
Monocytes	PLTP	-0.29406	5.244881	-1.31404	0.192232	-5.50007	0.376949	0.493585
Monocytes	ARMT1	0.250119	3.297769	1.31372	0.192339	-4.8804	0.398294	0.521074
Monocytes	CARD6	0.452519	3.06005	1.313562	0.192392	-4.72249	0.401003	0.524532
Monocytes	SPG7	0.142895	4.467655	1.313291	0.192483	-5.26679	0.385479	0.504436
Monocytes	EDARADD	-0.46161	3.159788	-1.31319	0.192518	-4.66278	0.399934	0.523157
Monocytes	AV099323	0.604739	1.209882	1.313135	0.192535	-4.60852	0.422605	0.552326
Monocytes	FOXJ2	0.204101	3.829076	1.312865	0.192626	-5.22191	0.39257	0.513683
Monocytes	GM45509	-0.52093	1.640462	-1.31264	0.1927	-4.65587	0.417671	0.545984
Monocytes	HEXIM1	-0.16809	5.48831	-1.31245	0.192766	-5.50307	0.374822	0.490627
Monocytes	ANLN	0.314578	3.747041	1.312337	0.192803	-5.09906	0.393602	0.514995
Monocytes	ANKS1	0.148652	5.759857	1.311903	0.192949	-5.57867	0.372191	0.487065
Monocytes	TSTA3	-0.18548	4.100613	-1.3117	0.193018	-5.22217	0.389985	0.510158
Monocytes	UHRF1	-0.31646	4.598357	-1.31135	0.193134	-5.15476	0.3847	0.503209
Monocytes	DGKH	-0.15464	4.909539	-1.31127	0.193162	-5.58909	0.381355	0.498869
Monocytes	1700047M11RI	0.712909	-0.15234	1.310978	0.19326	-4.55982	0.440135	0.574264
Monocytes	SHF	-0.46079	1.832889	-1.31069	0.193356	-4.63477	0.416149	0.543573
Monocytes	GM47371	0.580667	0.907518	1.309929	0.193613	-4.62952	0.427689	0.557991
Monocytes	ZFP512	0.243468	3.916117	1.309613	0.19372	-5.0333	0.392868	0.513213
Monocytes	RAB3IP	0.20845	4.587906	1.309329	0.193815	-5.23036	0.385519	0.503743
Monocytes	IGKC	-0.4963	7.88266	-1.30928	0.19383	-5.59757	0.35163	0.459595
Monocytes	FUT4	0.449643	-0.16835	1.309265	0.193837	-4.70788	0.441007	0.574962
Monocytes	ZFP213	0.385575	2.162406	1.309241	0.193845	-4.72367	0.412794	0.538914
Monocytes	DICER1	0.137848	4.846948	1.30907	0.193903	-5.34217	0.382726	0.500154
Monocytes	GORASP2	-0.10768	5.534187	-1.30897	0.193935	-5.5268	0.375425	0.490675
Monocytes	CD163L1	-0.65985	0.60908	-1.30895	0.193942	-4.61531	0.43137	0.56272
Monocytes	HIPK3	0.129001	5.445686	1.308683	0.194033	-5.53703	0.376459	0.491933
Monocytes	GAPT	0.290411	1.236779	1.308372	0.194138	-5.03627	0.423888	0.553128
Monocytes	TUT1	0.280437	2.638669	1.308355	0.194144	-4.79171	0.407402	0.531984
Monocytes	LRRC20	0.398867	2.057401	1.308316	0.194157	-4.73102	0.414151	0.540674
Monocytes	OASL2	0.427076	3.934807	1.307588	0.194403	-5.35231	0.393204	0.513437
Monocytes	1700061N14RII	-0.85351	-0.1438	-1.30727	0.19451	-4.55615	0.441462	0.575188
Monocytes	ARHGAP29	-0.48985	2.520124	-1.30712	0.19456	-4.70219	0.409371	0.534223
Monocytes	UBTD1	0.16654	4.402162	1.306748	0.194687	-5.40832	0.388337	0.507114
Monocytes	GM11290	0.354385	3.432889	1.306676	0.194711	-5.06852	0.399074	0.52103
Monocytes	GM28198	-0.27816	3.85447	-1.30662	0.194731	-5.05392	0.394364	0.51498
Monocytes	TRAF5	-0.16756	5.308971	-1.30647	0.19478	-5.42451	0.378602	0.494588
Monocytes	SETD7	0.158025	4.9704	1.306239	0.194859	-5.41136	0.382231	0.499399
Monocytes	OSBPL10	-0.67292	0.973543	-1.30622	0.194867	-4.56152	0.427858	0.558127
Monocytes	PPP1R16A	-0.31486	2.885752	-1.30563	0.195066	-4.83362	0.405623	0.529372

Monocytes	SLC9A1	0.134182	5.416377	1.305513	0.195105	-5.45042	0.377765	0.4934
Monocytes	PRPF18	0.168771	4.616004	1.305444	0.195129	-5.28834	0.386339	0.504533
Monocytes	WDTC1	0.254436	3.851337	1.30503	0.195269	-5.01191	0.394937	0.515538
Monocytes	FAM117B	0.120241	7.11596	1.304918	0.195307	-5.93458	0.36044	0.470768
Monocytes	TMEM79	-0.54047	0.778123	-1.30442	0.195475	-4.62745	0.431063	0.561771
Monocytes	ZFP992	0.20484	3.563813	1.304209	0.195547	-5.22559	0.398451	0.519985
Monocytes	CENPV	-0.26393	3.40694	-1.30414	0.195572	-5.19791	0.400215	0.522289
Monocytes	F830016B08RIK	-0.81198	0.444299	-1.30389	0.195654	-4.58094	0.435309	0.567238
Monocytes	PSMD5	-0.20918	3.835237	-1.30348	0.195797	-5.04996	0.395644	0.516257
Monocytes	RAB5C	0.107742	6.358629	1.303415	0.195817	-5.70132	0.368624	0.481235
Monocytes	HNRNPM	-0.08423	7.703885	-1.30335	0.19584	-5.87696	0.355068	0.463555
Monocytes	HIST1H2AN	-0.58491	3.254826	-1.30289	0.195996	-4.92262	0.402407	0.52495
Monocytes	CDC42EP4	-0.26694	2.155265	-1.30253	0.196118	-4.96718	0.415274	0.5414
Monocytes	GPR137C	0.49847	2.390141	1.302101	0.196264	-4.63704	0.412749	0.538033
Monocytes	TINAGL1	-0.59953	2.066409	-1.30185	0.19635	-4.66009	0.416572	0.543019
Monocytes	SLC38A6	-0.21636	4.233853	-1.30183	0.196355	-5.21427	0.391872	0.51124
Monocytes	HNRNPA2B1	-0.10135	9.287708	-1.30168	0.196406	-6.09586	0.340402	0.444175
Monocytes	DAPK1	-0.14682	4.932787	-1.30149	0.196473	-5.82101	0.384335	0.501458
Monocytes	GOLGA3	0.193422	4.032459	1.301298	0.196537	-5.13184	0.394229	0.514253
Monocytes	SFT2D1	0.098136	5.999884	1.301125	0.196596	-5.64986	0.373107	0.486903
Monocytes	GM13822	-0.60658	1.17949	-1.301	0.196639	-4.80059	0.427362	0.556793
Monocytes	TERF2	0.158665	4.800606	1.300717	0.196735	-5.26298	0.385861	0.503439
Monocytes	RUVBL1	-0.15326	4.77265	-1.30069	0.196745	-5.35157	0.386164	0.503831
Monocytes	COL4A1	0.414814	3.330895	1.300654	0.196757	-4.89911	0.402145	0.52446
Monocytes	TLCD2	-0.34574	2.659161	-1.30054	0.196797	-4.88451	0.409842	0.534398
Monocytes	DNTTIP2	-0.13101	5.107858	-1.29996	0.196994	-5.39651	0.382859	0.499388
Monocytes	HNRNPL	-0.08434	8.426349	-1.29897	0.19733	-5.96387	0.349549	0.455543
Monocytes	STRADB	0.290275	3.238382	1.298784	0.197395	-4.87677	0.40412	0.52634
Monocytes	SEMA5A	-0.79139	1.106724	-1.29857	0.197467	-4.56448	0.42923	0.558585
Monocytes	NARS	-0.11861	6.298026	-1.29853	0.197482	-5.6691	0.37087	0.483458
Monocytes	PCSK7	0.156808	5.282369	1.298521	0.197485	-5.42313	0.381557	0.497323
Monocytes	IDH1	0.129229	4.88393	1.298418	0.19752	-5.59215	0.385843	0.502878
Monocytes	BTBD10	-0.13333	5.583956	-1.29806	0.197644	-5.43647	0.37851	0.493351
Monocytes	USP50	0.229025	3.371827	1.297515	0.197829	-5.07392	0.403073	0.524878
Monocytes	GM17382	0.729607	0.002886	1.297203	0.197936	-4.55503	0.443566	0.576457
Monocytes	SLC36A3	-0.38583	0.101985	-1.29654	0.198162	-4.81538	0.442442	0.575084
Monocytes	DENND2D	-0.33002	2.202221	-1.29649	0.19818	-4.80104	0.416863	0.542521
Monocytes	PLXNB2	0.171777	3.959065	1.296312	0.198242	-5.38497	0.396709	0.516641
Monocytes	GM32250	0.650757	-0.2746	1.296235	0.198268	-4.56298	0.447206	0.581164
Monocytes	CDH23	0.262469	2.820164	1.29623	0.19827	-5.05996	0.409648	0.533279
Monocytes	SLC26A11	-0.28554	3.200265	-1.29618	0.198287	-5.06304	0.405279	0.527669
Monocytes	GM47601	0.512601	0.02249	1.296177	0.198288	-4.64749	0.443443	0.576393
Monocytes	AKAP17B	-0.44445	1.696468	-1.29613	0.198304	-4.71191	0.422872	0.550221
Monocytes	GM43773	0.474841	1.074002	1.295825	0.198408	-4.69019	0.430538	0.559916
Monocytes	ORAI2	-0.14365	5.87495	-1.29536	0.198569	-5.57047	0.376305	0.490096
Monocytes	NOL11	-0.20116	4.89374	-1.29495	0.19871	-5.29119	0.386967	0.503786
Monocytes	SLFN5	0.239268	4.230734	1.294865	0.198738	-5.56353	0.394239	0.51317

Monocytes	SLIT2	-0.77805	0.983319	-1.29401	0.199032	-4.58265	0.432497	0.561982
Monocytes	N4BP2	-0.16511	5.310122	-1.29394	0.199056	-5.43421	0.38282	0.498282
Monocytes	GM49067	-0.55592	0.406337	-1.29391	0.199065	-4.59249	0.43964	0.571096
Monocytes	PTDSS1	-0.12671	5.371518	-1.29389	0.199074	-5.52271	0.382163	0.497431
Monocytes	L1CAM	-0.27458	3.644556	-1.29369	0.199143	-5.1437	0.401216	0.521928
Monocytes	MAP3K10	-0.32008	2.731125	-1.29291	0.19941	-4.83812	0.412156	0.535673
Monocytes	GNL3L	-0.14382	4.467102	-1.29247	0.199563	-5.2604	0.392663	0.510539
Monocytes	ANKRD44	0.118846	8.18547	1.292426	0.199577	-6.01533	0.353946	0.460354
Monocytes	EXOSC2	0.291053	2.945711	1.2921	0.19969	-4.85563	0.409934	0.532719
Monocytes	SPRYD3	0.178444	3.699854	1.292078	0.199697	-5.16333	0.401312	0.521657
Monocytes	TMBIM1	0.221544	2.851295	1.291869	0.199769	-5.18175	0.411035	0.534169
Monocytes	GM50012	-0.3931	1.643382	-1.29184	0.199779	-4.70403	0.425315	0.552416
Monocytes	CHPF2	0.298062	2.64648	1.291637	0.199849	-4.93931	0.413454	0.537221
Monocytes	MOAP1	0.452353	0.547771	1.291509	0.199893	-4.62382	0.438775	0.569535
Monocytes	TTLL1	0.466753	1.626512	1.291441	0.199917	-4.65166	0.425555	0.552719
Monocytes	RHBDF2	-0.15921	4.650027	-1.29133	0.199954	-5.34416	0.390773	0.508127
Monocytes	HOOK3	-0.10937	6.076111	-1.29122	0.199993	-5.67031	0.375471	0.488349
Monocytes	DFFB	-0.29671	2.981393	-1.29096	0.200083	-4.91449	0.409606	0.532459
Monocytes	SENP3	-0.16791	4.324023	-1.29093	0.200092	-5.29099	0.394409	0.512931
Monocytes	ELOVL2	-0.52297	2.450205	-1.29062	0.200201	-4.78951	0.415942	0.540536
Monocytes	CRLF2	0.146976	5.010644	1.290257	0.200326	-5.52241	0.387179	0.503441
Monocytes	TMEM51OS1	-0.55112	-0.90367	-1.29005	0.200398	-4.59134	0.457711	0.593398
Monocytes	AK6	-0.1574	4.761906	-1.28998	0.200421	-5.37472	0.389923	0.506939
Monocytes	CISD1	0.185817	5.119405	1.289619	0.200546	-5.47453	0.386035	0.501926
Monocytes	FAM167B	-0.50781	2.127548	-1.28962	0.200548	-4.66474	0.419969	0.545502
Monocytes	GALNT4	0.390386	1.622606	1.289557	0.200568	-4.6831	0.426014	0.553214
Monocytes	MUS81	-0.42224	2.048507	-1.28952	0.20058	-4.71236	0.420909	0.546703
Monocytes	CD164L2	-0.51453	1.352514	-1.28937	0.200631	-4.68879	0.429312	0.557445
Monocytes	CCNDBP1	-0.17478	5.692836	-1.28916	0.200706	-5.56083	0.379908	0.494129
Monocytes	SMARCA4	-0.13356	7.5105	-1.28914	0.200713	-5.75635	0.361127	0.469747
Monocytes	DEPTOR	0.179068	3.469195	1.28893	0.200785	-5.41378	0.404386	0.525746
Monocytes	ZMIZ1OS1	0.70477	0.352543	1.28873	0.200854	-4.60058	0.44167	0.573419
Monocytes	TNFSF10	-0.47016	2.307037	-1.28872	0.200858	-4.69646	0.417869	0.543107
Monocytes	SH3YL1	0.512293	1.030881	1.288662	0.200877	-4.61429	0.433246	0.562723
Monocytes	PRR14	0.175141	4.524923	1.288577	0.200907	-5.30481	0.392553	0.510637
Monocytes	AMY1	-0.40711	2.627056	-1.28844	0.200956	-4.80071	0.414127	0.538361
Monocytes	5730522E02RIK	-0.19835	3.610742	-1.28831	0.201	-5.53694	0.402806	0.52384
Monocytes	1600022D10RII	-0.50343	-0.42787	-1.28815	0.201056	-4.59807	0.45165	0.58614
Monocytes	MTERF1A	0.468477	1.405346	1.287952	0.201123	-4.64071	0.428742	0.557087
Monocytes	CBLB	-0.12446	7.336459	-1.28786	0.201154	-5.93896	0.362939	0.472347
Monocytes	HUS1	0.417276	1.653586	1.2878	0.201176	-4.70846	0.425738	0.553304
Monocytes	VPREB3	-0.43056	6.013944	-1.28724	0.20137	-5.19355	0.376863	0.490292
Monocytes	NEK9	0.143536	5.27603	1.286675	0.201566	-5.55292	0.384937	0.50061
Monocytes	C8A	-0.63311	0.525618	-1.28663	0.201584	-4.59131	0.440155	0.571325
Monocytes	MARS	-0.29385	3.10364	-1.28658	0.201599	-4.94447	0.40918	0.531814
Monocytes	ACAT1	-0.19356	6.480379	-1.28618	0.201739	-5.69374	0.372374	0.484282
Monocytes	GM43305	0.269801	7.45195	1.286008	0.201798	-5.86073	0.36243	0.471359

Monocytes	MRGBP	0.25778	3.37422	1.285765	0.201882	-4.96089	0.406271	0.528102
Monocytes	PRR13	0.093268	6.510778	1.285741	0.201891	-5.76642	0.372058	0.48395
Monocytes	TMEM163	-0.31392	6.026941	-1.28573	0.201895	-5.16982	0.377119	0.490513
Monocytes	DLD	0.136559	4.599821	1.284991	0.202152	-5.36133	0.392924	0.510727
Monocytes	SERP2	-0.63237	0.429349	-1.28475	0.202236	-4.56058	0.442062	0.57367
Monocytes	STPG4	-0.36438	2.37884	-1.28468	0.202259	-4.80338	0.418304	0.543443
Monocytes	GYS1	0.309775	3.38284	1.284617	0.202282	-5.03397	0.406617	0.528498
Monocytes	GM12703	0.70887	-0.21463	1.28445	0.20234	-4.58408	0.450229	0.58407
Monocytes	NTAN1	-0.09585	6.261702	-1.28441	0.202353	-5.672	0.375064	0.487798
Monocytes	GM4258	0.351232	4.929368	1.284098	0.202462	-5.07995	0.389419	0.50632
Monocytes	FBXO42	0.143186	5.871653	1.283898	0.202532	-5.56521	0.379276	0.493269
Monocytes	SNAP29	-0.14265	5.5062	-1.28382	0.202559	-5.40567	0.383174	0.498345
Monocytes	HSP90AA1	-0.11682	7.657631	-1.28371	0.202596	-5.87609	0.360855	0.469397
Monocytes	UBXN6	0.169327	4.786672	1.283679	0.202608	-5.38209	0.390981	0.508454
Monocytes	USP10	-0.17991	4.567936	-1.28358	0.202644	-5.33092	0.393389	0.511563
Monocytes	FKBP1B	0.226512	0.793011	1.283219	0.202769	-5.1734	0.437734	0.568355
Monocytes	TFG	-0.11476	5.864765	-1.28302	0.202837	-5.59853	0.379432	0.493612
Monocytes	CDH24	-0.40149	2.179776	-1.28301	0.202843	-4.7233	0.420869	0.546921
Monocytes	KIF1A	0.410426	-0.59877	1.282919	0.202873	-4.73784	0.4554	0.590798
Monocytes	TRMT1	0.16997	4.050324	1.282884	0.202886	-5.18802	0.399241	0.519186
Monocytes	SERPINA3N	-0.38735	2.557516	-1.28243	0.203043	-4.79022	0.416641	0.541361
Monocytes	FHIT	-0.23977	6.167904	-1.28202	0.203189	-5.31952	0.376647	0.48972
Monocytes	BIRC2	-0.14394	5.673482	-1.28187	0.203239	-5.51941	0.38191	0.496539
Monocytes	RSPH9	-0.63409	1.514419	-1.28161	0.203332	-4.61546	0.42944	0.557458
Monocytes	PLCE1	-0.28388	1.279254	-1.28147	0.203379	-5.27275	0.432311	0.561114
Monocytes	MPV17L	0.421079	2.006778	1.281328	0.203429	-4.73203	0.423495	0.549885
Monocytes	H2-T23	-0.20515	6.358381	-1.28132	0.203432	-5.7288	0.374732	0.487164
Monocytes	SH3BP2	0.252728	3.288629	1.281125	0.2035	-5.1642	0.408496	0.530685
Monocytes	5330417C22RIK	-0.69497	0.541978	-1.28029	0.203792	-4.59492	0.441992	0.573133
Monocytes	GLIPR1	0.127913	5.059395	1.280176	0.203832	-5.704	0.389079	0.505462
Monocytes	UTY	3.006886	2.644423	1.280153	0.20384	-4.94688	0.416442	0.540602
Monocytes	CCZ1	0.109797	6.158644	1.279607	0.204031	-5.64642	0.377414	0.490272
Monocytes	GM15478	0.179301	4.735611	1.279604	0.204032	-5.37513	0.392757	0.510086
Monocytes	PEX16	0.218074	3.474463	1.279517	0.204063	-5.05321	0.406936	0.528341
Monocytes	GNG12	-0.11266	6.49518	-1.27923	0.204165	-5.82092	0.373885	0.485847
Monocytes	CHCHD6	0.222545	2.968098	1.279218	0.204168	-5.01802	0.412788	0.535968
Monocytes	HBP1	0.148867	5.788072	1.279187	0.204178	-5.50273	0.381344	0.495507
Monocytes	TIGAR	0.40442	1.851972	1.279172	0.204184	-4.74006	0.426016	0.552866
Monocytes	RNF185	0.151538	5.117108	1.278828	0.204304	-5.38293	0.388696	0.504922
Monocytes	TSHZ2	-0.51833	3.393266	-1.27877	0.204326	-4.84253	0.407994	0.52974
Monocytes	GM1604B	-0.6788	1.489369	-1.27863	0.204375	-4.61335	0.43055	0.558583
Monocytes	KNG2	-0.50438	2.382731	-1.27852	0.204414	-4.75572	0.419802	0.544927
Monocytes	ZFP429	0.294127	2.448075	1.278333	0.204478	-4.86722	0.419028	0.544021
Monocytes	OARD1	0.167161	4.902059	1.278309	0.204486	-5.36889	0.391052	0.508115
Monocytes	NAA40	0.145216	4.909944	1.278189	0.204528	-5.34296	0.39097	0.508069
Monocytes	ADGRL1	-0.38169	2.476047	-1.27807	0.20457	-4.75591	0.418707	0.543715
Monocytes	WBP1L	0.145793	5.329247	1.277907	0.204627	-5.51229	0.386438	0.50223

Monocytes	STRIP1	-0.1978	4.156745	-1.27751	0.204765	-5.1665	0.399473	0.518973
Monocytes	MRPL58	0.144934	5.199269	1.277361	0.204819	-5.52472	0.387952	0.504128
Monocytes	TRMT112	0.106116	7.277188	1.27727	0.204851	-5.85725	0.366079	0.475792
Monocytes	RNF38	0.110847	6.163439	1.277012	0.204942	-5.70158	0.377625	0.490822
Monocytes	USP31	-0.22915	3.715776	-1.27701	0.204943	-5.15568	0.40446	0.525431
Monocytes	SDF2L1	-0.15036	4.888426	-1.27684	0.205002	-5.59971	0.391348	0.508563
Monocytes	CLK3	-0.15086	4.807812	-1.27676	0.205029	-5.35991	0.392234	0.509734
Monocytes	PLEKHM2	0.161273	5.202185	1.276644	0.205071	-5.66061	0.38792	0.504172
Monocytes	PLA1A	-0.32796	1.841769	-1.27662	0.205081	-5.00816	0.426435	0.553589
Monocytes	HYPK	-0.11461	5.8151	-1.27654	0.205107	-5.59295	0.38132	0.495642
Monocytes	LYRM9	-0.35299	2.696887	-1.27621	0.205223	-4.82177	0.416249	0.540703
Monocytes	RIOK2	-0.16292	4.466641	-1.2761	0.205264	-5.26473	0.396009	0.514783
Monocytes	GM50071	0.5831	0.514178	1.276055	0.205278	-4.59181	0.442793	0.574601
Monocytes	PALB2	-0.30506	2.125531	-1.276	0.205296	-4.90202	0.423025	0.54944
Monocytes	ABCG2	-0.30502	3.149803	-1.27593	0.205322	-4.89854	0.410963	0.534033
Monocytes	FAS	0.221638	3.936884	1.275923	0.205325	-5.36898	0.401951	0.522463
Monocytes	UFL1	-0.1738	3.965196	-1.27558	0.205446	-5.20508	0.401631	0.522195
Monocytes	IL12A	-0.65855	2.215059	-1.27552	0.205466	-4.6203	0.421955	0.548251
Monocytes	HDAC3	-0.15351	4.707537	-1.27545	0.20549	-5.30818	0.393339	0.511515
Monocytes	GRCC10	0.101154	6.427807	1.275366	0.205521	-5.72883	0.374848	0.487594
Monocytes	ALOX15	1.19382	-0.82153	1.275191	0.205582	-4.60412	0.459939	0.5965
Monocytes	DMAP1	0.326186	2.496634	1.275186	0.205584	-4.80556	0.41861	0.543985
Monocytes	KDM1B	-0.29835	3.6758	-1.27503	0.205638	-4.94207	0.404916	0.526428
Monocytes	MRPL45	0.200813	4.340705	1.274876	0.205693	-5.23383	0.397413	0.516776
Monocytes	MFN1	-0.21658	3.297935	-1.27476	0.205733	-5.07651	0.40925	0.531993
Monocytes	MPG	-0.18984	3.63806	-1.27464	0.205776	-5.15057	0.405346	0.526981
Monocytes	SDCBP	0.097879	7.674968	1.274635	0.205778	-5.9626	0.362052	0.470966
Monocytes	SNHG6	0.256341	3.414627	1.274585	0.205796	-5.04593	0.407906	0.530268
Monocytes	PSIP1	-0.1289	5.994205	-1.27449	0.20583	-5.55256	0.379415	0.493574
Monocytes	APOL8	-0.27555	2.828251	-1.27432	0.205888	-4.9889	0.414744	0.539117
Monocytes	INTU	0.822015	0.880465	1.274002	0.206002	-4.58234	0.438408	0.569249
Monocytes	GM4869	-0.54115	1.447403	-1.27389	0.206041	-4.68521	0.431417	0.560412
Monocytes	ASXL2	0.117931	6.836454	1.273714	0.206103	-5.7554	0.370805	0.482469
Monocytes	SAMSN1	0.12032	7.050554	1.273504	0.206177	-5.97366	0.368602	0.479692
Monocytes	TRUB1	-0.39134	1.825881	-1.27349	0.206183	-4.72514	0.426865	0.554768
Monocytes	METTL15	-0.28204	3.231154	-1.27261	0.206493	-4.92301	0.410754	0.533928
Monocytes	GNG7	-0.66193	-0.40318	-1.27241	0.206562	-4.5866	0.455305	0.590667
Monocytes	MAPK13	0.282637	0.83712	1.272374	0.206577	-5.10508	0.439536	0.570677
Monocytes	USP54	-0.26392	2.915478	-1.27232	0.206595	-4.96132	0.414428	0.538638
Monocytes	LUC7L2	0.074329	8.527172	1.272176	0.206646	-6.0176	0.354244	0.460745
Monocytes	BAG1	0.094291	6.693589	1.271788	0.206784	-5.80082	0.372941	0.484994
Monocytes	GCA	0.430861	1.099128	1.271591	0.206853	-4.87943	0.436574	0.566696
Monocytes	5530601H04RII	0.234974	3.904215	1.271397	0.206922	-5.17541	0.403367	0.524187
Monocytes	ATP6V1G1	0.095921	7.177612	1.271265	0.206969	-5.8766	0.368062	0.478531
Monocytes	SEC61G	-0.12071	10.02398	-1.27114	0.207013	-6.30026	0.340167	0.442126
Monocytes	RNF24	0.161681	4.233052	1.271039	0.207049	-5.42464	0.399669	0.519492
Monocytes	USP21	-0.28448	3.140041	-1.27075	0.207151	-4.94233	0.412254	0.535726

Monocytes	ACTR1B	0.198669	4.215968	1.270681	0.207175	-5.21645	0.399952	0.519923
Monocytes	MLEC	-0.1385	5.064037	-1.27014	0.207368	-5.46924	0.390755	0.508095
Monocytes	1700126G02RII	0.51559	1.104608	1.270096	0.207383	-4.68504	0.436922	0.567259
Monocytes	GM16093	0.410067	2.640752	1.270025	0.207408	-4.78045	0.418334	0.543544
Monocytes	GM47802	0.688499	0.099495	1.269587	0.207563	-4.59081	0.449822	0.583449
Monocytes	REXO5	0.381793	1.950184	1.268892	0.20781	-4.76298	0.427244	0.554648
Monocytes	HGFAC	-0.54817	1.432763	-1.26846	0.207962	-4.67718	0.433784	0.562867
Monocytes	TPM3-RS7	0.536037	0.526188	1.267875	0.208171	-4.63236	0.445446	0.577362
Monocytes	ZFP518A	0.235052	3.684945	1.267529	0.208294	-5.07043	0.407461	0.528954
Monocytes	MCMBP	0.098494	7.089729	1.267453	0.208321	-5.82255	0.370403	0.481155
Monocytes	9930111J21RIK	0.251261	4.28297	1.26741	0.208337	-5.31124	0.400663	0.520268
Monocytes	EEA1	-0.11827	6.055281	-1.26721	0.208407	-5.68788	0.381247	0.495245
Monocytes	YIPF2	0.362695	1.926928	1.267162	0.208425	-4.78702	0.428193	0.555557
Monocytes	GNG4	-0.39976	1.462633	-1.26706	0.208463	-4.86419	0.433859	0.562843
Monocytes	EMC7	0.092158	6.059195	1.266861	0.208532	-5.70362	0.381206	0.495375
Monocytes	PPP2R3C	0.152967	4.333149	1.266386	0.208701	-5.37685	0.400099	0.519919
Monocytes	SELENOO	-0.25259	3.136064	-1.26634	0.208718	-5.00636	0.413813	0.537532
Monocytes	HMOX2	0.100317	6.471899	1.266328	0.208722	-5.68819	0.376837	0.489865
Monocytes	ACAD9	-0.30054	2.542008	-1.26631	0.208728	-4.87629	0.42081	0.546489
Monocytes	MYBL1	-0.46986	1.840587	-1.2663	0.208731	-4.70597	0.42924	0.557253
Monocytes	CBARP	-0.30572	1.923122	-1.26626	0.208746	-5.0149	0.428239	0.555975
Monocytes	ZMAT3	0.406626	1.902429	1.266078	0.208811	-4.85323	0.42849	0.556313
Monocytes	DUSP23	-0.33932	2.373334	-1.26607	0.208812	-4.80982	0.422821	0.549076
Monocytes	MTA1	0.157517	4.55479	1.265932	0.208863	-5.36412	0.397636	0.516761
Monocytes	RNASET2B	-0.19446	5.139167	-1.26573	0.208936	-5.44635	0.391231	0.508465
Monocytes	LMF2	-0.25398	3.103696	-1.26545	0.209035	-5.024	0.414279	0.538223
Monocytes	PRR7	0.263933	3.334702	1.265408	0.20905	-5.04404	0.41159	0.534775
Monocytes	GXYLT1	0.138623	5.093219	1.265311	0.209084	-5.49465	0.391737	0.50923
Monocytes	PDZK1	-0.69906	0.933701	-1.26528	0.209096	-4.61607	0.440509	0.571695
Monocytes	CBS	0.532339	1.874066	1.264514	0.209369	-4.71947	0.429402	0.55737
Monocytes	ARHGEF26	0.720217	0.379311	1.264273	0.209455	-4.59141	0.448094	0.581065
Monocytes	BCL9L	-0.29435	3.410778	-1.26349	0.209735	-4.90601	0.411697	0.534297
Monocytes	RSPRY1	-0.1143	5.833379	-1.26342	0.20976	-5.6015	0.384626	0.49947
Monocytes	ZMAT4	-0.80939	0.381713	-1.2628	0.209981	-4.64492	0.448895	0.581478
Monocytes	ARHGEF7	-0.17976	4.675352	-1.26274	0.210004	-5.35837	0.397621	0.516075
Monocytes	GM20033	0.58018	0.595708	1.262369	0.210136	-4.66106	0.44637	0.578245
Monocytes	MANEA	0.293392	3.728612	1.261548	0.21043	-4.92815	0.408975	0.53024
Monocytes	RPP25L	0.217851	3.672149	1.261485	0.210452	-5.10333	0.409626	0.531074
Monocytes	PLRG1	-0.16884	4.093452	-1.26139	0.210487	-5.21594	0.404798	0.524883
Monocytes	RTF1	0.08795	6.81408	1.261007	0.210624	-5.78774	0.375278	0.486828
Monocytes	NXPE3	0.324666	3.424156	1.26083	0.210687	-4.85387	0.412732	0.535045
Monocytes	SGCE	-0.78748	1.156412	-1.26062	0.210762	-4.58172	0.44013	0.570027
Monocytes	SLC25A3	-0.091	7.921066	-1.26048	0.210813	-6.00191	0.364016	0.472263
Monocytes	HHEX	-0.19079	4.675113	-1.26024	0.2109	-5.36382	0.398603	0.516955
Monocytes	XIAP	0.092247	6.725324	1.260176	0.210922	-5.78609	0.376382	0.48829
Monocytes	TM7SF3	0.191256	3.924878	1.260056	0.210965	-5.2052	0.407103	0.527934
Monocytes	PEX14	0.141921	5.405291	1.259704	0.211091	-5.50627	0.390691	0.506712

Monocytes	TRERF1	-0.16211	4.396216	-1.25929	0.211242	-5.76308	0.402006	0.521295
Monocytes	ATRIP	-0.3304	2.825397	-1.25923	0.211262	-4.91271	0.42019	0.544589
Monocytes	RAB11B	0.089159	7.147669	1.259225	0.211264	-5.85417	0.372228	0.482855
Monocytes	SNRPG	0.099595	8.018397	1.259095	0.21131	-6.00847	0.363338	0.471348
Monocytes	GM37240	0.189557	5.162317	1.258961	0.211358	-5.42531	0.393482	0.510406
Monocytes	4833419F23RIK	-0.43048	1.323387	-1.25869	0.211455	-4.80663	0.438459	0.568029
Monocytes	PHF11C	-0.3127	1.989779	-1.25869	0.211456	-4.91545	0.430263	0.557604
Monocytes	GM44777	0.595401	0.565153	1.258613	0.211483	-4.62054	0.447993	0.580155
Monocytes	RBM15B	-0.21072	4.047089	-1.25851	0.21152	-5.06465	0.406001	0.526693
Monocytes	USP28	0.195782	4.553264	1.258074	0.211677	-5.17519	0.400488	0.519474
Monocytes	EZH1	0.244456	3.600438	1.25787	0.211751	-5.09613	0.41143	0.533559
Monocytes	SERPINA1D	-0.38858	5.558333	-1.25755	0.211868	-5.50242	0.389497	0.505415
Monocytes	ROMO1	-0.10368	6.595619	-1.25753	0.211873	-5.75476	0.378369	0.491028
Monocytes	GOSR1	0.169098	4.182554	1.257158	0.212007	-5.25161	0.405005	0.525264
Monocytes	CORO2B	-0.59286	1.197161	-1.25694	0.212086	-4.60956	0.440707	0.570772
Monocytes	HNRNPA1	-0.11346	7.807246	-1.25647	0.212254	-5.94749	0.366272	0.47506
Monocytes	POLR2J	0.135108	4.922519	1.25554	0.212591	-5.43664	0.397541	0.515044
Monocytes	TM9SF2	-0.08103	6.683958	-1.25533	0.212665	-5.77787	0.378458	0.490449
Monocytes	AW549877	0.238792	3.352769	1.255275	0.212687	-5.10825	0.415511	0.538105
Monocytes	HOMER1	0.15235	6.109837	1.254971	0.212797	-5.70975	0.384662	0.498483
Monocytes	FAM50A	-0.12634	5.186332	-1.25481	0.212856	-5.53472	0.394733	0.511536
Monocytes	SNX9	-0.13591	6.331978	-1.25476	0.212873	-5.68174	0.382283	0.495502
Monocytes	OLFML2B	0.676992	0.874884	1.254619	0.212924	-4.62326	0.445781	0.576757
Monocytes	LEFTY1	0.595131	0.408138	1.254579	0.212938	-4.61828	0.451727	0.584282
Monocytes	INTS13	-0.182	4.149388	-1.25442	0.212997	-5.19547	0.406427	0.526579
Monocytes	TNNT3	-0.4005	1.772075	-1.25419	0.213079	-4.86438	0.434711	0.562665
Monocytes	TENM2	-0.81462	-0.35252	-1.25392	0.213178	-4.59749	0.461861	0.596945
Monocytes	PRKAA1	0.184622	4.45764	1.253732	0.213245	-5.3556	0.403145	0.522303
Monocytes	A730063M14RI	0.593344	1.218514	1.253644	0.213276	-4.65188	0.44174	0.571612
Monocytes	2010110K18RIK	0.776817	-0.03523	1.253049	0.213492	-4.59183	0.458115	0.592042
Monocytes	SMG7	-0.113	6.385921	-1.25295	0.213528	-5.69709	0.382258	0.495208
Monocytes	DPP4	-0.15566	5.100888	-1.25275	0.213601	-5.61005	0.396249	0.513251
Monocytes	P2RY10	-0.20694	3.930309	-1.25273	0.213608	-5.53868	0.409493	0.53025
Monocytes	D17H6S53E	0.17665	4.009289	1.25224	0.213785	-5.21267	0.408845	0.529186
Monocytes	DCLK2	-0.43611	2.72479	-1.25158	0.214023	-4.75109	0.42431	0.548641
Monocytes	TMEM170	-0.4696	1.517214	-1.25143	0.214078	-4.66488	0.439082	0.567397
Monocytes	ELAC1	0.282185	1.710286	1.251152	0.21418	-4.87869	0.436724	0.56447
Monocytes	CPSF6	-0.08651	6.68211	-1.25115	0.21418	-5.72458	0.379762	0.491531
Monocytes	CTPS	-0.25294	3.704287	-1.25101	0.214232	-5.05885	0.412814	0.534036
Monocytes	WDR25	0.373557	1.998808	1.250937	0.214257	-4.7482	0.433172	0.560003
Monocytes	TTC14	0.109072	5.803768	1.250693	0.214346	-5.58972	0.389281	0.503795
Monocytes	GM20492	-0.36185	1.798274	-1.25048	0.214423	-4.79205	0.4358	0.563282
Monocytes	CDKL2	-0.50412	1.163972	-1.25038	0.214459	-4.70636	0.443702	0.573329
Monocytes	CSNK1G3	0.106283	6.624241	1.249976	0.214607	-5.75354	0.38062	0.492515
Monocytes	ATP9B	0.107982	5.858499	1.249919	0.214628	-5.66664	0.388847	0.503128
Monocytes	RNF168	-0.22734	4.161361	-1.24962	0.214736	-5.15947	0.4078	0.527637
Monocytes	ZFP513	-0.29108	2.534011	-1.24959	0.214747	-4.92775	0.426941	0.552078

Monocytes	ZFP628	0.321046	2.635685	1.24941	0.214813	-4.93536	0.425716	0.550545
Monocytes	TMEM94	0.242805	2.610495	1.24929	0.214856	-4.95425	0.426019	0.550983
Monocytes	ASB13	0.291908	3.434394	1.249108	0.214923	-5.01404	0.416231	0.538554
Monocytes	NEFH	-0.77649	-0.09246	-1.2491	0.214925	-4.58057	0.459944	0.59402
Monocytes	E130215H24RII	0.669816	-0.13171	1.248967	0.214974	-4.59833	0.460457	0.594722
Monocytes	LILRA5	-0.38587	1.497471	-1.2488	0.215036	-4.98046	0.439646	0.568413
Monocytes	EIF4H	-0.09008	6.535904	-1.24878	0.215042	-5.7579	0.381559	0.494052
Monocytes	DYRK1A	-0.11656	7.419436	-1.24873	0.21506	-5.88481	0.372283	0.48205
Monocytes	9830166K06RIK	-0.62556	-1.08122	-1.24859	0.215109	-4.56805	0.473076	0.610679
Monocytes	CDC73	0.111071	6.174072	1.248437	0.215167	-5.68549	0.385433	0.499204
Monocytes	5830448L01RIK	0.444366	0.904406	1.24839	0.215184	-4.72035	0.447101	0.578055
Monocytes	TMEM86B	-0.26316	3.467162	-1.24832	0.215209	-4.98918	0.415847	0.538319
Monocytes	S100A16	-0.38835	2.552707	-1.24832	0.215209	-4.799	0.426715	0.55219
Monocytes	MYO1G	-0.12622	5.563064	-1.24822	0.215244	-5.81643	0.392075	0.50788
Monocytes	DISC1	-0.14286	3.43956	-1.24806	0.215303	-5.51086	0.416205	0.538846
Monocytes	BCR	-0.17913	5.853797	-1.24789	0.215365	-5.42532	0.38897	0.503883
Monocytes	SLC16A12	-0.69599	0.427781	-1.24739	0.215547	-4.60577	0.453574	0.586152
Monocytes	HIP1R	-0.18872	5.271328	-1.24668	0.215809	-5.31724	0.395878	0.512599
Monocytes	ATP5MD	-0.09732	8.035566	-1.24666	0.215815	-6.03603	0.366507	0.474634
Monocytes	GM14023	0.374019	1.123999	1.246617	0.215831	-4.88959	0.444984	0.575293
Monocytes	NDUFAF4	0.236272	3.867148	1.246571	0.215847	-5.08997	0.4118	0.533036
Monocytes	2810403D21RII	0.330909	2.774023	1.246228	0.215973	-4.85585	0.424853	0.549628
Monocytes	DNAJB13	-0.37996	2.103334	-1.2461	0.21602	-4.8045	0.432995	0.560055
Monocytes	CLCN3	0.131114	6.389845	1.245945	0.216076	-5.64953	0.383877	0.497072
Monocytes	TAF1	-0.12884	5.699019	-1.24582	0.21612	-5.61105	0.391366	0.506743
Monocytes	RO60	-0.32152	2.493379	-1.2455	0.216239	-4.82515	0.428441	0.554151
Monocytes	RNF150	0.202576	2.909798	1.245203	0.216347	-5.66322	0.423564	0.54786
Monocytes	PBX1	0.136915	5.465012	1.244347	0.216661	-5.90139	0.394481	0.51056
Monocytes	AFG3L1	0.106587	4.862083	1.244332	0.216666	-5.50525	0.401204	0.519202
Monocytes	MFAP4	-0.57957	1.50654	-1.24406	0.216767	-4.71272	0.441019	0.570175
Monocytes	4930532G15RII	-0.56235	1.429337	-1.24391	0.216819	-4.74908	0.441985	0.57144
Monocytes	EFCAB2	0.380244	2.668273	1.24391	0.216821	-4.85356	0.426762	0.55209
Monocytes	WWC2	-0.17604	5.107898	-1.24388	0.216832	-5.40552	0.398448	0.515849
Monocytes	LTC4S	0.66228	1.066227	1.243828	0.21685	-4.85599	0.446558	0.577234
Monocytes	MFSD4B4	0.627738	-0.0204	1.243704	0.216896	-4.62043	0.460553	0.594956
Monocytes	BOK	-0.66457	1.039523	-1.24369	0.2169	-4.62429	0.446896	0.577701
Monocytes	ARPC4	0.087875	7.957802	1.243679	0.216905	-6.04698	0.367995	0.476536
Monocytes	AW554918	0.132402	5.656196	1.24202	0.217514	-5.58852	0.393393	0.508632
Monocytes	TREM3	0.235231	2.200561	1.241924	0.217549	-5.53386	0.433561	0.559993
Monocytes	TMEM221	0.587002	-0.26558	1.241518	0.217698	-4.63263	0.465191	0.599843
Monocytes	CFAP126	-0.50678	1.069649	-1.24143	0.21773	-4.71135	0.447876	0.578023
Monocytes	FRMD6	-0.2929	3.47737	-1.24127	0.217791	-5.0727	0.418444	0.540649
Monocytes	CNOT7	0.108788	5.288607	1.241088	0.217856	-5.52008	0.397726	0.514135
Monocytes	HSF1	-0.15025	4.997353	-1.24075	0.217982	-5.4202	0.401143	0.518342
Monocytes	MYOF	0.270328	2.65687	1.239755	0.218347	-5.29947	0.429085	0.553707
Monocytes	INSIG1	-0.14843	5.457849	-1.23966	0.218383	-5.57422	0.39658	0.512181
Monocytes	SMDT1	0.08528	7.773748	1.239029	0.218614	-6.02122	0.372098	0.480354

Monocytes	TBC1D16	-0.28836	2.452428	-1.23872	0.218727	-5.12637	0.432067	0.557115
Monocytes	SCAMP2	-0.09843	6.357233	-1.2386	0.218773	-5.77003	0.387177	0.499804
Monocytes	AP1S1	0.155451	4.706161	1.238394	0.218848	-5.41321	0.405493	0.523447
Monocytes	ENO1B	0.437229	1.327345	1.238184	0.218925	-4.73706	0.446046	0.57514
Monocytes	NUDT14	-0.13982	4.342372	-1.23807	0.218969	-5.4192	0.409657	0.528922
Monocytes	MTMR4	-0.20676	2.648419	-1.23801	0.218989	-5.13971	0.429682	0.554454
Monocytes	PRIM1	-0.27913	4.178781	-1.23797	0.219005	-5.20927	0.411545	0.531339
Monocytes	MTERF3	0.175137	4.014483	1.237859	0.219045	-5.30563	0.413451	0.533821
Monocytes	DNTTIP1	-0.12622	4.812219	-1.2377	0.219104	-5.42742	0.404288	0.522113
Monocytes	PRKAR2B	0.213684	3.305356	1.237618	0.219134	-5.25683	0.42179	0.544487
Monocytes	PROX1OS	-0.64609	0.612207	-1.2374	0.219214	-4.64278	0.455188	0.586884
Monocytes	DOLPP1	0.326516	2.514642	1.237304	0.21925	-4.88429	0.431308	0.556658
Monocytes	GABPA	0.152598	4.662072	1.237304	0.21925	-5.35893	0.405995	0.524367
Monocytes	TRIO	-0.12252	6.069746	-1.23725	0.219269	-5.77933	0.390299	0.504215
Monocytes	KLHL42	0.406319	2.445253	1.237204	0.219287	-4.71786	0.432155	0.557735
Monocytes	ALG5	-0.17383	4.2129	-1.23682	0.219429	-5.31226	0.41134	0.531122
Monocytes	SOX12	0.498327	0.585357	1.236708	0.21947	-4.68119	0.455745	0.587564
Monocytes	4833407H14RIK	-0.25061	2.098821	-1.23598	0.219741	-4.98524	0.437066	0.563592
Monocytes	RPIA	-0.14343	5.458024	-1.23582	0.2198	-5.52753	0.397668	0.513317
Monocytes	ELK4	0.134505	5.882489	1.235565	0.219893	-5.55917	0.393065	0.507373
Monocytes	CPSF7	-0.11259	5.801172	-1.23535	0.219975	-5.60066	0.394032	0.508601
Monocytes	ERH	0.125215	7.372533	1.234773	0.220187	-5.91051	0.377427	0.487053
Monocytes	GM45606	0.593539	0.477564	1.234528	0.220277	-4.66482	0.458237	0.590146
Monocytes	GIMAP1OS	-0.53243	0.862202	-1.23451	0.220282	-4.67864	0.45326	0.583875
Monocytes	LCA5	-0.6334	1.313513	-1.2344	0.220324	-4.61	0.447497	0.576658
Monocytes	GM2449	-0.46054	1.279792	-1.23411	0.220432	-4.79403	0.448061	0.577326
Monocytes	GM30881	-0.31049	1.986079	-1.23393	0.220499	-4.9144	0.43924	0.566123
Monocytes	STAC2	-0.63639	1.824947	-1.23377	0.220558	-4.66433	0.441284	0.568685
Monocytes	NDFIP2	-0.11217	5.951714	-1.23365	0.220602	-5.66334	0.392911	0.506987
Monocytes	MRPL9	0.131465	4.787977	1.233518	0.220652	-5.40608	0.405946	0.523725
Monocytes	CD80	0.154471	3.929604	1.233345	0.220716	-5.83414	0.4159	0.53643
Monocytes	SGMS1	0.126241	7.102996	1.233139	0.220793	-6.02922	0.380608	0.491148
Monocytes	NIPA1	0.623687	0.13903	1.233011	0.22084	-4.63127	0.463062	0.596138
Monocytes	B230217C12RIK	-0.30556	1.478169	-1.23276	0.220934	-4.92883	0.445819	0.574372
Monocytes	TET1	0.590682	1.135429	1.232756	0.220935	-4.66766	0.450172	0.579868
Monocytes	LMBR1	0.416286	1.883516	1.232573	0.221003	-4.74815	0.440785	0.567936
Monocytes	RNF2	0.123821	5.723956	1.232024	0.221207	-5.55621	0.395918	0.510559
Monocytes	NANP	0.369897	2.821135	1.231752	0.221308	-4.84829	0.429577	0.55358
Monocytes	FLRT3	0.33361	-0.84839	1.231648	0.221346	-4.99017	0.476699	0.612993
Monocytes	MPPED2	-0.84271	-0.32287	-1.23158	0.221372	-4.58681	0.469619	0.604139
Monocytes	KLHL23	-0.61374	0.171341	-1.23158	0.221372	-4.63555	0.463065	0.595911
Monocytes	ORMDL3	-0.26641	3.803807	-1.23145	0.221421	-5.04544	0.417854	0.53869
Monocytes	1810026B05RIK	0.105441	6.570259	1.230907	0.221622	-5.70821	0.386962	0.498935
Monocytes	SLC4A4	-0.49412	2.065431	-1.23072	0.22169	-4.77111	0.439235	0.565673
Monocytes	GPKOW	0.137305	4.488998	1.229393	0.222186	-5.35771	0.411069	0.529208
Monocytes	ELL	0.144328	5.024631	1.228845	0.222391	-5.48816	0.405234	0.52157
Monocytes	TWISTNB	-0.11778	5.520145	-1.22835	0.222574	-5.61251	0.399902	0.51455

Monocytes	TMEM248	0.112307	5.858866	1.228209	0.222628	-5.66222	0.396153	0.509757
Monocytes	ZFP207	0.085129	6.934778	1.227877	0.222752	-5.84294	0.384575	0.494908
Monocytes	ACTR10	0.092555	6.186686	1.22761	0.222852	-5.71643	0.392731	0.505361
Monocytes	SETDB2	0.157188	4.580351	1.22751	0.222889	-5.42799	0.410803	0.528475
Monocytes	CHMP5	0.111375	5.632148	1.227478	0.222901	-5.63947	0.398867	0.513224
Monocytes	KIF3C	-0.33948	2.145051	-1.22734	0.222953	-4.84987	0.44001	0.565577
Monocytes	GM13481	-0.79022	0.231781	-1.22718	0.223011	-4.59329	0.464573	0.596506
Monocytes	POLR1C	-0.20795	3.945898	-1.22655	0.223246	-5.22586	0.418474	0.538079
Monocytes	HEXA	-0.11999	5.752202	-1.22652	0.223261	-5.81087	0.397795	0.511698
Monocytes	GM15337	-0.41043	2.140023	-1.2265	0.223268	-4.8778	0.440346	0.565812
Monocytes	ZFP800	0.1185	5.365723	1.226317	0.223336	-5.63455	0.402122	0.517274
Monocytes	RABEP2	-0.19608	4.414258	-1.22612	0.22341	-5.19627	0.413	0.531255
Monocytes	REV3L	-0.11025	6.713202	-1.22607	0.223429	-5.789	0.387261	0.498319
Monocytes	ATP8A2	-0.46079	2.725958	-1.22607	0.223429	-4.91228	0.433114	0.55681
Monocytes	ZFP652OS	0.539761	-0.171	1.225966	0.223467	-4.66745	0.47018	0.603548
Monocytes	FASTKD1	0.329116	2.189988	1.225867	0.223504	-4.86648	0.439724	0.565248
Monocytes	OGFOD3	-0.2571	3.187381	-1.22574	0.223553	-5.00903	0.427526	0.549828
Monocytes	EML5	-0.26001	3.697759	-1.22558	0.223613	-5.19277	0.421457	0.54216
Monocytes	ELP6	-0.36428	2.398694	-1.22533	0.223706	-4.83892	0.437291	0.56224
Monocytes	FAM91A1	0.111278	5.007003	1.22399	0.224208	-5.55297	0.407164	0.523325
Monocytes	ABCD1	0.131794	4.771292	1.223847	0.224262	-5.51225	0.409868	0.526796
Monocytes	UBQLN2	-0.17278	4.031371	-1.22377	0.224292	-5.23252	0.418478	0.537758
Monocytes	PANK4	0.253124	3.423163	1.223364	0.224443	-5.02953	0.425713	0.547016
Monocytes	RBIS	0.137653	5.209656	1.223344	0.22445	-5.52275	0.404868	0.52048
Monocytes	PAWR	-0.60037	0.662659	-1.22333	0.224454	-4.64069	0.46029	0.590653
Monocytes	RNF34	0.155741	4.455799	1.223314	0.224462	-5.36895	0.413523	0.531519
Monocytes	TECPR1	-0.24431	4.375591	-1.22274	0.224676	-5.19497	0.414776	0.532873
Monocytes	ATMIN	0.209776	2.969649	1.222428	0.224795	-5.2656	0.43167	0.554346
Monocytes	TM6SF1	-0.10585	6.252773	-1.22232	0.224834	-6.01616	0.393659	0.505942
Monocytes	GAMT	-0.31167	4.228598	-1.2219	0.224994	-5.34451	0.41685	0.535433
Monocytes	EIF4A1	-0.08721	7.526831	-1.22174	0.225054	-5.99078	0.38016	0.488479
Monocytes	SECISBP2	0.135592	4.786541	1.221114	0.225289	-5.48415	0.410757	0.527418
Monocytes	BCAP29	-0.13762	4.765797	-1.22047	0.225533	-5.54623	0.411276	0.527909
Monocytes	SLC20A1	-0.15626	5.219384	-1.22041	0.225556	-5.59437	0.406077	0.521296
Monocytes	PPP3CB	0.106582	5.980209	1.220377	0.225567	-5.64342	0.397522	0.510381
Monocytes	SFXN3	0.184077	3.370072	1.219771	0.225796	-5.33156	0.428096	0.549111
Monocytes	CHD7	0.115432	6.463817	1.219536	0.225884	-5.92776	0.392595	0.503877
Monocytes	ACOT9	0.135801	4.620859	1.218971	0.226098	-5.52127	0.413695	0.530602
Monocytes	TLK2	-0.09221	6.825344	-1.21886	0.226139	-5.83668	0.388953	0.499017
Monocytes	TVP23B	-0.16203	4.042523	-1.21772	0.226571	-5.30832	0.421199	0.539516
Monocytes	CTBP2	-0.1918	3.249836	-1.21755	0.226635	-5.34722	0.430711	0.55155
Monocytes	TUT4	-0.12853	7.475638	-1.21745	0.226671	-5.90798	0.382636	0.49034
Monocytes	ARMC2	0.632223	-0.1641	1.217301	0.226729	-4.61497	0.474441	0.606529
Monocytes	TMEM86A	-0.25611	2.885047	-1.21727	0.226739	-5.12615	0.435166	0.557257
Monocytes	GPR157	-0.30299	2.137362	-1.21694	0.226867	-4.92389	0.444517	0.569058
Monocytes	ZFP7	-0.63701	0.286794	-1.21691	0.226877	-4.64302	0.468461	0.599083
Monocytes	TIE1	-0.53706	1.482911	-1.21686	0.226895	-4.67797	0.452828	0.579506

Monocytes	GM13008	0.489999	1.087607	1.216627	0.226984	-4.70558	0.458027	0.585949
Monocytes	FUT8	-0.13163	5.850748	-1.21651	0.227027	-5.64655	0.400513	0.513232
Monocytes	CCNE2	-0.36088	3.75287	-1.21641	0.227068	-5.09043	0.424797	0.544145
Monocytes	IFI206	0.413195	3.940563	1.216086	0.227189	-5.29502	0.422707	0.541447
Monocytes	MIPEP	-0.2359	2.843457	-1.21545	0.227431	-4.9766	0.436364	0.55846
Monocytes	BIRC3	0.124596	7.111789	1.21534	0.227472	-5.93499	0.387139	0.495947
Monocytes	ZFP954	0.396791	2.050779	1.215101	0.227563	-4.8275	0.446316	0.571024
Monocytes	AHCY	0.283969	3.495963	1.215029	0.22759	-5.06761	0.428473	0.548547
Monocytes	LUC7L3	-0.09175	6.706417	-1.2145	0.22779	-5.801	0.391869	0.501823
Monocytes	ELL2	0.165385	6.734248	1.214004	0.227979	-5.92707	0.391818	0.501702
Monocytes	BTBD6	-0.46795	1.542046	-1.21388	0.228026	-4.74671	0.453409	0.579738
Monocytes	PLBD2	-0.14862	4.310294	-1.21353	0.22816	-5.43818	0.419495	0.536864
Monocytes	NEDD4L	0.102818	6.886893	1.213436	0.228195	-5.85786	0.390309	0.499713
Monocytes	FAM114A1	0.260706	2.398626	1.21294	0.228384	-5.17472	0.443002	0.566376
Monocytes	NVL	-0.14794	4.723925	-1.21273	0.228463	-5.37697	0.414984	0.530933
Monocytes	H2-K1	-0.23033	8.786019	-1.21253	0.228538	-6.16925	0.370611	0.474248
Monocytes	4833445I07RIK	0.42883	0.48418	1.212283	0.228634	-4.74936	0.467849	0.597596
Monocytes	ATP6V0C	0.093178	8.851286	1.212247	0.228647	-6.2103	0.369948	0.473487
Monocytes	ACOT2	-0.19736	4.416307	-1.21219	0.228667	-5.13296	0.418653	0.535701
Monocytes	GPR108	0.155109	4.177602	1.212071	0.228714	-5.41412	0.421481	0.539272
Monocytes	HSPB11	0.237782	2.925359	1.211955	0.228759	-5.13854	0.436618	0.558396
Monocytes	MDH2	-0.11269	6.490426	-1.21153	0.228922	-5.79494	0.395156	0.505797
Monocytes	PPIG	0.076368	6.974522	1.211504	0.228931	-5.91864	0.389859	0.499031
Monocytes	BUB1B	0.237631	4.016283	1.211455	0.228949	-5.28662	0.423522	0.541883
Monocytes	TIMM44	0.131968	4.84696	1.211331	0.228996	-5.46321	0.413752	0.529563
Monocytes	LRRC29	0.334695	1.690453	1.211236	0.229032	-4.76123	0.452257	0.578227
Monocytes	DUSP4	0.419785	0.909125	1.211116	0.229078	-4.73983	0.462393	0.590964
Monocytes	OMA1	0.205358	3.410326	1.211	0.229123	-5.22932	0.430821	0.551337
Monocytes	GTSE1	0.63814	1.404909	1.210777	0.229208	-4.74173	0.45597	0.583003
Monocytes	DNM2	0.090018	7.193868	1.210587	0.22928	-5.92129	0.387518	0.496338
Monocytes	COQ10A	0.198926	3.31768	1.210331	0.229378	-5.1314	0.431973	0.552957
Monocytes	RPP38	-0.53318	1.226405	-1.21026	0.229407	-4.70897	0.458283	0.586067
Monocytes	GM38604	0.381551	1.796583	1.2102	0.229428	-4.79566	0.45094	0.576853
Monocytes	PPFIA4	0.160128	2.735956	1.210197	0.229429	-5.54637	0.439121	0.561979
Monocytes	TBC1D30	-0.57366	1.06093	-1.21018	0.229434	-4.74332	0.460438	0.588768
Monocytes	TTC17	0.117274	5.324345	1.210087	0.229471	-5.63273	0.408287	0.522918
Monocytes	GM12227	-0.45511	1.113465	-1.20961	0.229653	-4.71138	0.460034	0.588106
Monocytes	RAB11FIP2	0.232238	3.576284	1.209242	0.229794	-5.17929	0.429284	0.549306
Monocytes	SNTA1	-0.31172	1.865421	-1.20873	0.22999	-4.95832	0.450806	0.576231
Monocytes	CHD1	0.089017	6.90203	1.208464	0.230091	-5.88061	0.391327	0.500865
Monocytes	CCDC18	-0.34054	2.722921	-1.20846	0.230094	-4.86964	0.440009	0.562653
Monocytes	USP53	0.170801	4.118641	1.208436	0.230102	-5.30733	0.423039	0.541219
Monocytes	LDHA	0.124164	8.792149	1.207533	0.230447	-6.17988	0.371806	0.475472
Monocytes	MTF2	0.133057	5.644163	1.2073	0.230537	-5.53434	0.405869	0.519085
Monocytes	MPP7	0.189375	7.383215	1.207289	0.230541	-6.10933	0.386645	0.494553
Monocytes	SELENOI	-0.21882	3.422495	-1.20689	0.230694	-5.18324	0.432126	0.552344
Monocytes	PUS7L	0.369208	1.839535	1.206731	0.230755	-4.84987	0.451884	0.577277

Monocytes	CDC26	0.100464	5.383294	1.206661	0.230781	-5.5719	0.408964	0.523069
Monocytes	GM47448	-0.82022	-0.89373	-1.20662	0.230795	-4.60258	0.488361	0.62279
Monocytes	ADGRG6	0.45864	0.969217	1.206557	0.230821	-4.95302	0.463167	0.59141
Monocytes	SFN	-0.50882	2.056108	-1.2064	0.230881	-4.74443	0.449123	0.573841
Monocytes	NDUFS4	0.102056	6.335519	1.206345	0.230903	-5.77522	0.398218	0.509409
Monocytes	EDA	-0.49935	1.445596	-1.20566	0.231167	-4.88074	0.45733	0.583874
Monocytes	ACTN2	-0.63342	-0.1007	-1.20553	0.231217	-4.6489	0.477853	0.609527
Monocytes	PRMT6	0.269088	2.45108	1.205521	0.231219	-4.92896	0.444501	0.567809
Monocytes	PPP3CA	0.093688	8.812084	1.204232	0.231714	-6.12371	0.372712	0.476082
Monocytes	TRAPPC12	0.152058	4.201517	1.204202	0.231725	-5.34843	0.423875	0.541276
Monocytes	HPX	-0.25302	5.670685	-1.20404	0.231786	-5.56639	0.406768	0.519593
Monocytes	HEYL	-0.77611	-0.20551	-1.20398	0.231813	-4.59376	0.480161	0.611831
Monocytes	MRPL32	0.121784	5.68103	1.203599	0.231957	-5.68992	0.406773	0.519609
Monocytes	CSTB	0.134284	6.672627	1.203417	0.232027	-5.92136	0.39566	0.505524
Monocytes	KIRREL3	-0.43607	1.272544	-1.20338	0.232042	-4.79525	0.460563	0.58749
Monocytes	2810013P06RIK	0.234843	4.123121	1.203313	0.232067	-5.26177	0.424944	0.542708
Monocytes	PRDX3	0.137681	5.400912	1.203247	0.232093	-5.61413	0.409975	0.523787
Monocytes	USP4	0.08763	5.660629	1.203036	0.232174	-5.68818	0.407074	0.520135
Monocytes	GTF3C6	-0.11353	5.226124	-1.20277	0.232276	-5.60087	0.412101	0.526475
Monocytes	LETM1	0.145936	4.54276	1.202753	0.232283	-5.38585	0.420077	0.536587
Monocytes	RAB2A	0.061693	8.094947	1.202402	0.232418	-6.08853	0.380512	0.486092
Monocytes	SUPT5	-0.11199	6.05341	-1.20235	0.232439	-5.73087	0.402771	0.51452
Monocytes	CDIPTOS	0.451658	-0.30875	1.202273	0.232468	-4.69994	0.481973	0.614145
Monocytes	HBS1L	-0.11643	5.694465	-1.20195	0.232594	-5.63184	0.406981	0.519807
Monocytes	FANCI	0.336902	2.266344	1.201555	0.232745	-4.82929	0.448393	0.572091
Monocytes	ICOSL	0.271376	1.327034	1.201176	0.232891	-5.07555	0.460655	0.58739
Monocytes	GCSH	0.154924	4.428205	1.201101	0.23292	-5.52373	0.422051	0.538866
Monocytes	FANCA	0.231095	3.05493	1.200744	0.233058	-5.18302	0.438868	0.560013
Monocytes	TLR8	0.340499	0.054908	1.200596	0.233115	-4.92973	0.477821	0.608706
Monocytes	TMEM256	0.115994	6.982152	1.200122	0.233298	-5.92473	0.393346	0.502132
Monocytes	ATAT1	-0.2713	2.722421	-1.20005	0.233325	-4.98916	0.443274	0.565347
Monocytes	MED19	-0.18601	3.778351	-1.19994	0.233368	-5.14267	0.430272	0.549022
Monocytes	GM27253	-0.57487	1.3478	-1.1992	0.233655	-4.65155	0.461336	0.587584
Monocytes	PLSCR1	0.180413	4.550033	1.19872	0.23384	-5.58612	0.421696	0.537626
Monocytes	MTM1	0.186507	4.433358	1.198593	0.233889	-5.48073	0.423081	0.539385
Monocytes	NIPSNAP1	-0.30428	3.134643	-1.19856	0.233902	-4.98062	0.438834	0.559237
Monocytes	ADPRM	0.240588	3.321315	1.198246	0.234023	-4.99548	0.436611	0.556347
Monocytes	VDAC2	-0.09338	7.488132	-1.19823	0.23403	-6.01626	0.388525	0.495418
Monocytes	NDUFB9	0.099995	7.069533	1.197887	0.234162	-5.94212	0.393228	0.501361
Monocytes	CMSS1	-0.21459	6.448827	-1.19754	0.234296	-5.69614	0.400248	0.510227
Monocytes	CTNS	0.211453	3.606042	1.197387	0.234356	-5.2379	0.433491	0.552306
Monocytes	IGF2BP2	-0.45088	1.457318	-1.19722	0.234421	-4.77071	0.460664	0.586397
Monocytes	NPRL2	-0.27027	2.790262	-1.1969	0.234544	-4.94478	0.443772	0.565175
Monocytes	ADCK1	0.232338	3.101425	1.19672	0.234615	-5.1409	0.439947	0.560414
Monocytes	MYO10	0.231041	5.227281	1.195923	0.234924	-5.40351	0.414786	0.528428
Monocytes	ETNK2	-0.52382	1.270533	-1.19589	0.234937	-4.71548	0.463732	0.589921
Monocytes	ABCB10	0.281577	2.977675	1.195716	0.235004	-5.00565	0.441873	0.562662

Monocytes	ASTN2	-0.63532	1.045238	-1.19569	0.235015	-4.65874	0.466704	0.59372
Monocytes	ANKZF1	0.222359	2.742515	1.195444	0.23511	-4.97221	0.444816	0.566448
Monocytes	4930402H24RII	0.161304	4.453202	1.195265	0.23518	-5.45111	0.423893	0.540148
Monocytes	OXNAD1	-0.36724	1.886539	-1.19521	0.235202	-4.78925	0.455711	0.580131
Monocytes	ACVRL1	-0.18344	2.619629	-1.1952	0.235207	-5.53051	0.446362	0.568424
Monocytes	MSTO1	-0.27662	2.681663	-1.19512	0.235235	-4.99822	0.445581	0.567463
Monocytes	MRPS26	-0.11301	5.167613	-1.19484	0.235347	-5.53619	0.41548	0.529638
Monocytes	ANKRD46	-0.29215	2.377526	-1.1948	0.235362	-4.8091	0.449426	0.572401
Monocytes	NOL12	-0.1448	4.012955	-1.19478	0.235369	-5.30372	0.429171	0.546938
Monocytes	MACROD1	0.353342	2.861084	1.194698	0.2354	-4.9337	0.44333	0.564753
Monocytes	WDR61	-0.12564	4.80713	-1.19459	0.235443	-5.47449	0.419701	0.53498
Monocytes	ISOC2A	-0.35672	2.013655	-1.19455	0.235457	-4.83127	0.454075	0.578237
Monocytes	NINJ2	-0.50799	0.701022	-1.19396	0.235688	-4.80254	0.471661	0.599892
Monocytes	BHMT	0.315317	5.13251	1.193848	0.235731	-5.4669	0.416223	0.530378
Monocytes	LRP8OS2	0.657572	0.163168	1.193696	0.23579	-4.69346	0.478958	0.609053
Monocytes	ZFP87	0.229112	3.396086	1.193426	0.235895	-5.14017	0.437129	0.556801
Monocytes	ANKRD10	-0.12778	5.371393	-1.19331	0.235942	-5.52772	0.413529	0.527034
Monocytes	ZFP112	0.529418	0.391108	1.193291	0.235948	-4.71197	0.475928	0.605337
Monocytes	DNAJA4	-0.59174	0.616749	-1.19309	0.236025	-4.66514	0.472957	0.601631
Monocytes	XRN1	0.120887	5.924264	1.192567	0.23623	-5.68308	0.40752	0.519278
Monocytes	MDM2	0.147275	6.41707	1.192278	0.236342	-5.8374	0.401976	0.512283
Monocytes	CD4	-0.6246	1.246276	-1.19208	0.236419	-4.69991	0.464936	0.591659
Monocytes	GM10603	-0.67884	0.220408	-1.19203	0.23644	-4.63948	0.478673	0.608781
Monocytes	MIS18A	0.160946	4.400206	1.191933	0.236477	-5.51587	0.425334	0.54205
Monocytes	NANOS1	0.468969	0.634448	1.19189	0.236494	-4.74556	0.473077	0.601923
Monocytes	DROSHA	-0.21739	3.121263	-1.19187	0.2365	-5.03545	0.440926	0.561717
Monocytes	PLCB3	0.264317	2.612	1.191732	0.236556	-5.01901	0.447334	0.569774
Monocytes	LHFP	-0.56759	1.237889	-1.19161	0.236604	-4.71535	0.465082	0.591985
Monocytes	CSMD3	0.429083	1.302147	1.191296	0.236726	-4.76222	0.464325	0.590966
Monocytes	DIP2B	-0.10819	7.880088	-1.19125	0.236745	-5.98543	0.386036	0.492111
Monocytes	INO80C	0.207289	3.401246	1.191166	0.236776	-5.15897	0.437577	0.557462
Monocytes	MAPK1	-0.07482	7.693924	-1.19092	0.236871	-6.00906	0.38806	0.494753
Monocytes	PPP2R5D	0.195179	3.682866	1.19081	0.236915	-5.21263	0.434145	0.55322
Monocytes	MRPL52	0.1017	7.102833	1.190804	0.236918	-5.99796	0.394493	0.50298
Monocytes	DOT1L	-0.13591	5.289755	-1.19067	0.236968	-5.68699	0.415	0.529119
Monocytes	SLC25A33	-0.20046	3.983563	-1.19029	0.237117	-5.30669	0.430661	0.548902
Monocytes	RNF217	-0.25183	2.071962	-1.19023	0.237141	-5.36063	0.454524	0.578897
Monocytes	NDUFA8	0.131669	5.798653	1.189891	0.237275	-5.71037	0.409305	0.521962
Monocytes	2900093K20RIK	0.32763	2.884005	1.189886	0.237277	-4.91424	0.444239	0.566072
Monocytes	SMLR1	-0.49848	1.822706	-1.18987	0.237282	-4.76309	0.457767	0.583028
Monocytes	MAP3K15	0.25723	3.326785	1.189495	0.237429	-5.22793	0.438918	0.559373
Monocytes	WDR78	-0.56794	0.561969	-1.18914	0.23757	-4.68253	0.47483	0.604204
Monocytes	NTPCR	0.181685	3.847172	1.188919	0.237655	-5.34715	0.432722	0.551521
Monocytes	UBE2T	-0.22309	4.06115	-1.1889	0.237661	-5.34688	0.430124	0.548246
Monocytes	SLC22A27	-0.62024	1.124082	-1.18854	0.237802	-4.69958	0.467428	0.595029
Monocytes	PTP4A1	0.446873	1.179279	1.188412	0.237854	-4.82674	0.466697	0.594195
Monocytes	PDE1A	-0.6759	0.441701	-1.1884	0.23786	-4.65651	0.476569	0.606494

Monocytes	ATG3	-0.08815	6.261249	-1.18833	0.237887	-5.83632	0.404489	0.515865
Monocytes	TPGS1	0.156496	4.393149	1.188231	0.237925	-5.48679	0.426219	0.543405
Monocytes	APH1A	0.127117	5.433388	1.187698	0.238133	-5.71262	0.414251	0.528018
Monocytes	PRUNE2	-0.4618	0.638929	-1.18747	0.238225	-4.76447	0.474329	0.603421
Monocytes	AQR	0.116612	4.812338	1.187353	0.238269	-5.48856	0.421607	0.537291
Monocytes	GM12064	0.467006	0.96957	1.187091	0.238372	-4.73712	0.469898	0.598033
Monocytes	MRM3	0.407164	2.005465	1.187059	0.238384	-4.83396	0.456308	0.581061
Monocytes	TECR	-0.11291	6.763011	-1.18696	0.238424	-5.84263	0.39922	0.508999
Monocytes	TGFBRAP1	0.174696	3.494101	1.186931	0.238434	-5.21739	0.437527	0.557494
Monocytes	POLR3GL	0.19748	3.878267	1.186674	0.238535	-5.16357	0.432925	0.551668
Monocytes	FILIP1	-0.63165	0.904589	-1.18647	0.238614	-4.66123	0.470953	0.599281
Monocytes	TNFSF9	0.181097	3.435421	1.186233	0.238708	-5.71806	0.43852	0.558605
Monocytes	TRAPPC5	0.161982	4.379372	1.186093	0.238763	-5.40943	0.427048	0.544174
Monocytes	LRR1	-0.46081	1.435876	-1.18576	0.238893	-4.74714	0.464114	0.590711
Monocytes	EYA1	-0.56643	2.426875	-1.18576	0.238893	-4.74083	0.45128	0.574658
Monocytes	NR2C2AP	0.181311	4.44571	1.185665	0.238932	-5.40581	0.426325	0.543324
Monocytes	KYAT1	-0.44519	1.503706	-1.18522	0.239106	-4.75666	0.463411	0.589828
Monocytes	PAIP2B	-0.20817	3.682629	-1.1852	0.239114	-5.14955	0.43575	0.555148
Monocytes	RAPGEF1	0.111622	6.775482	1.184974	0.239203	-5.82922	0.399614	0.509419
Monocytes	MCTS2	-0.54147	1.612847	-1.18476	0.239289	-4.73576	0.462025	0.588219
Monocytes	NUSAP1	-0.2518	5.649629	-1.18465	0.23933	-5.6565	0.41238	0.525722
Monocytes	FIRRE	-0.39668	2.608551	-1.18463	0.239339	-4.8766	0.449194	0.572157
Monocytes	UPF2	0.111915	6.075856	1.184587	0.239356	-5.69295	0.407494	0.519521
Monocytes	PLAU	-0.5356	0.814983	-1.18445	0.239411	-4.82034	0.472595	0.601452
Monocytes	POLR3D	-0.24277	3.27535	-1.18438	0.239435	-5.11259	0.440821	0.561685
Monocytes	ZFP623	0.606341	0.124749	1.184042	0.23957	-4.6677	0.482125	0.613248
Monocytes	KLRI2	-0.47465	0.447224	-1.18382	0.239657	-4.88698	0.477728	0.607894
Monocytes	ALAS1	0.245584	3.683631	1.183787	0.23967	-5.20952	0.435937	0.555588
Monocytes	PDZD4	-0.5829	1.698737	-1.18373	0.239692	-4.68233	0.461069	0.587135
Monocytes	EIF5A	-0.10062	9.061608	-1.1836	0.239742	-6.23292	0.375187	0.47842
Monocytes	SARS	0.10335	5.792679	1.183417	0.239816	-5.67702	0.410902	0.524092
Monocytes	ASAH2	-0.27074	2.945427	-1.18338	0.239832	-5.14776	0.445125	0.567324
Monocytes	SLC30A6	0.212702	3.417011	1.183095	0.239943	-5.14121	0.43937	0.560071
Monocytes	D330050I16RIK	-0.52199	1.118758	-1.18286	0.240035	-4.68931	0.468957	0.597152
Monocytes	GM15472	-0.37192	1.795978	-1.18273	0.240085	-4.97822	0.460045	0.586025
Monocytes	RPTOR	-0.10618	6.294365	-1.18265	0.240118	-5.80685	0.405376	0.517106
Monocytes	EBF1	-0.29552	8.683852	-1.18186	0.240432	-5.8848	0.379745	0.484071
Monocytes	COG1	-0.16413	3.677624	-1.18127	0.240663	-5.27035	0.437011	0.556619
Monocytes	TMLHE	-0.54957	2.31306	-1.18109	0.240734	-4.76039	0.454164	0.578201
Monocytes	CRAT	0.243889	3.198294	1.181041	0.240754	-5.14812	0.442954	0.564137
Monocytes	PDSS1	-0.1969	4.315269	-1.18104	0.240755	-5.36277	0.429242	0.54687
Monocytes	GRM8	-0.75904	1.381099	-1.18084	0.240835	-4.6824	0.466331	0.593419
Monocytes	FABP5	0.206395	7.278075	1.180683	0.240895	-5.97318	0.39513	0.50362
Monocytes	HCCS	0.133738	4.627385	1.180677	0.240898	-5.45882	0.42552	0.54219
Monocytes	CDCA5	-0.33247	3.028781	-1.18025	0.241066	-5.04437	0.445334	0.566984
Monocytes	TADA2A	0.28787	2.820969	1.180095	0.241128	-4.95433	0.447989	0.570271
Monocytes	GLCE	0.189346	3.79856	1.179859	0.241221	-5.30014	0.435904	0.555022

Monocytes	2610307P16RIK	-0.53899	4.550996	-1.17965	0.241304	-4.9652	0.4268	0.543571
Monocytes	GM44649	0.180156	3.949723	1.179554	0.241342	-5.42688	0.434075	0.552745
Monocytes	MAN2C1OS	0.187053	4.443794	1.179504	0.241362	-5.3181	0.428087	0.545196
Monocytes	HNF4A	-0.64545	0.691681	-1.17937	0.241413	-4.66744	0.475965	0.605161
Monocytes	RRS1	-0.19001	4.476059	-1.17924	0.241466	-5.29568	0.427733	0.544709
Monocytes	EDF1	-0.08942	7.047067	-1.17904	0.241547	-5.95082	0.398115	0.507196
Monocytes	A430072P03RIK	-0.8137	0.354986	-1.17869	0.241686	-4.62032	0.480774	0.611116
Monocytes	DPYD	-0.44114	3.567148	-1.17864	0.241703	-5.09023	0.439006	0.558909
Monocytes	MEN1	-0.30423	2.866329	-1.17846	0.241774	-4.95555	0.447821	0.57
Monocytes	CLSPN	-0.25938	4.411842	-1.17811	0.241913	-5.34368	0.428918	0.546043
Monocytes	HSPD1	-0.13821	7.46114	-1.17786	0.242013	-5.959	0.39389	0.501652
Monocytes	KLHDC2	-0.139	4.860142	-1.17779	0.24204	-5.47685	0.423575	0.539345
Monocytes	CFAP410	0.194188	2.119698	1.177603	0.242115	-5.15099	0.457573	0.582109
Monocytes	CDADC1	0.217224	4.609962	1.177583	0.242123	-5.3536	0.426559	0.543139
Monocytes	IKBKE	0.314988	3.116112	1.177536	0.242142	-5.31536	0.444874	0.566222
Monocytes	ARHGAP23	0.186568	3.694446	1.177434	0.242182	-5.41198	0.437683	0.557202
Monocytes	MXD4	-0.18394	5.790486	-1.17684	0.242418	-5.59294	0.412992	0.525817
Monocytes	NOX1	-0.57789	1.548335	-1.17675	0.242452	-4.71825	0.465387	0.591725
Monocytes	PKD1L3	0.463417	0.837305	1.176442	0.242576	-4.76503	0.475021	0.603629
Monocytes	TPMT	0.321323	2.097645	1.176336	0.242618	-4.89199	0.458358	0.582883
Monocytes	ZFP81	0.404764	1.873403	1.176125	0.242702	-4.78068	0.461353	0.586532
Monocytes	CAPN15	0.146072	4.710463	1.175918	0.242784	-5.4865	0.425946	0.542045
Monocytes	ARIH1	-0.09705	8.728541	-1.17583	0.242817	-6.23	0.380806	0.484686
Monocytes	IL1RL1	0.531403	-0.28284	1.175318	0.243023	-4.75209	0.490856	0.622969
Monocytes	NANS	-0.15237	4.873876	-1.17506	0.243127	-5.55388	0.424355	0.539847
Monocytes	SF3A2	-0.14129	5.260106	-1.175	0.243151	-5.60207	0.419784	0.534097
Monocytes	GNG5	0.093348	8.925654	1.1748	0.243229	-6.24318	0.379109	0.482358
Monocytes	BRCA2	-0.28085	3.555566	-1.17426	0.243443	-5.10995	0.440714	0.560313
Monocytes	TM2D3	-0.14709	4.227452	-1.17419	0.243473	-5.44653	0.43246	0.549958
Monocytes	5930430L01RIK	-0.7296	0.229599	-1.17409	0.24351	-4.61891	0.48421	0.614639
Monocytes	ALG10B	0.222013	2.952601	1.173926	0.243577	-5.11049	0.448271	0.569871
Monocytes	KLKB1	-0.40613	1.848942	-1.17388	0.243595	-4.8683	0.462473	0.587661
Monocytes	PTGER2	0.369183	1.047913	1.173117	0.2439	-4.90547	0.473392	0.60102
Monocytes	RPH3AL	-0.59704	1.299189	-1.1728	0.244027	-4.65576	0.470031	0.59692
Monocytes	TMUB2	0.221453	3.181282	1.172776	0.244036	-5.11666	0.445672	0.566482
Monocytes	1700094J05RIK	0.661178	0.092196	1.172704	0.244064	-4.67992	0.486415	0.617268
Monocytes	IDNK	0.122272	5.52475	1.172552	0.244125	-5.67144	0.41727	0.530718
Monocytes	SSBP1	0.114935	5.592236	1.172537	0.244131	-5.68043	0.416483	0.529722
Monocytes	ZFP952	0.35496	2.251647	1.172507	0.244143	-4.83763	0.457526	0.581331
Monocytes	2510009E07RIK	-0.2179	2.702942	-1.17244	0.244171	-5.2147	0.451729	0.57412
Monocytes	HDAC1	-0.10007	6.039987	-1.17219	0.244269	-5.72753	0.4113	0.523203
Monocytes	E4F1	0.273984	3.127007	1.172152	0.244285	-5.0638	0.446355	0.567395
Monocytes	TRIM62	-0.32633	1.410517	-1.17213	0.244293	-4.86217	0.46855	0.595135
Monocytes	DUT	-0.21215	5.882801	-1.17208	0.244316	-5.68587	0.413111	0.525498
Monocytes	TNFRSF12A	0.34915	1.479733	1.171913	0.24438	-4.91557	0.467632	0.593991
Monocytes	SEPHS1	-0.14548	4.119783	-1.17191	0.244381	-5.30068	0.434048	0.551933
Monocytes	SHLD3	0.363302	2.343634	1.171874	0.244396	-4.84314	0.456338	0.579895

Monocytes	AIP	0.146811	4.946962	1.171668	0.244478	-5.54913	0.424149	0.539481
Monocytes	SLC16A13	-0.60048	-0.01818	-1.17108	0.244715	-4.68742	0.488271	0.619642
Monocytes	ZSCAN22	-0.49618	1.316109	-1.17097	0.244758	-4.74479	0.470121	0.59711
Monocytes	TRMU	0.38286	1.548223	1.170873	0.244796	-4.75392	0.467039	0.593304
Monocytes	STAM2	0.14477	5.475543	1.170851	0.244805	-5.70186	0.418126	0.531887
Monocytes	CASC1	0.394518	1.810453	1.170607	0.244902	-4.93284	0.463584	0.589085
Monocytes	PTPN23	-0.21943	3.647447	-1.17033	0.245015	-5.1857	0.440151	0.559804
Monocytes	GM20219	-0.52912	0.646352	-1.17032	0.245016	-4.70261	0.479138	0.608521
Monocytes	4921516A02RIK	0.390012	0.76983	1.170143	0.245088	-4.83474	0.477462	0.606478
Monocytes	ZFP296	-0.32219	2.857765	-1.17007	0.245119	-5.04547	0.450061	0.572279
Monocytes	LAMTOR1	0.084245	6.503458	1.169879	0.245193	-5.94563	0.406283	0.517061
Monocytes	GPR137	0.22355	2.506946	1.169842	0.245208	-5.03971	0.454542	0.577898
Monocytes	MAP3K1	-0.09565	7.970553	-1.16982	0.245218	-6.10413	0.390028	0.49637
Monocytes	CYP4B1	-0.78866	0.266849	-1.1698	0.245225	-4.64473	0.484331	0.615016
Monocytes	TFEC	0.235216	2.368464	1.169754	0.245244	-5.3672	0.456324	0.580135
Monocytes	ZFP825	0.346083	2.050646	1.169496	0.245347	-4.85854	0.460443	0.585352
Monocytes	CARM1	-0.14626	4.727042	-1.16947	0.245358	-5.424	0.426992	0.543351
Monocytes	CHST3	-0.42053	4.312271	-1.16937	0.245397	-4.93187	0.431995	0.549681
Monocytes	FAM122A	-0.22132	3.563965	-1.16935	0.245404	-5.11035	0.441187	0.561249
Monocytes	HHAT	-0.42655	1.670909	-1.16925	0.245444	-4.81326	0.465419	0.59162
Monocytes	EXOC1	0.17111	4.095264	1.169206	0.245463	-5.35471	0.434639	0.553036
Monocytes	COL5A3	-0.70784	0.940726	-1.1686	0.245708	-4.65337	0.475542	0.603906
Monocytes	CDKN2AIP	0.172508	4.567595	1.168335	0.245812	-5.29522	0.429326	0.545964
Monocytes	GM38948	0.592237	0.463761	1.168287	0.245832	-4.63577	0.482098	0.611982
Monocytes	INTS6	-0.15566	6.588587	-1.16781	0.246021	-5.83019	0.405956	0.516199
Monocytes	MBL1	-0.49851	1.455107	-1.16769	0.246073	-4.75252	0.469024	0.595466
Monocytes	MMUT	0.260814	3.4125	1.167398	0.246188	-5.13939	0.443916	0.563959
Monocytes	GORASP1	0.500854	0.733344	1.166703	0.246468	-4.75706	0.479324	0.607868
Monocytes	FUNDC2	-0.12529	7.178207	-1.16608	0.24672	-5.90028	0.400194	0.508343
Monocytes	SERAC1	0.392967	2.177244	1.165754	0.246849	-4.85592	0.460661	0.584365
Monocytes	SDHC	0.129906	5.09505	1.16562	0.246903	-5.57711	0.424342	0.53889
Monocytes	GM29585	-0.51761	1.247477	-1.16538	0.247001	-4.73953	0.473074	0.599887
Monocytes	PRR5	-0.23917	3.479528	-1.16522	0.247065	-5.25393	0.44419	0.563868
Monocytes	IFI30	0.131426	6.746076	1.165108	0.247109	-6.07487	0.405325	0.514892
Monocytes	NIPBL	0.082481	8.615732	1.164944	0.247175	-6.15608	0.384841	0.488817
Monocytes	GM26881	0.527612	0.245715	1.16462	0.247305	-4.74913	0.48694	0.617173
Monocytes	NUDT7	0.421284	1.74453	1.164562	0.247329	-4.83445	0.466671	0.592017
Monocytes	KLRG1	-0.63711	-1.146	-1.16431	0.247431	-4.64101	0.506736	0.641586
Monocytes	A630052C17RIK	0.633133	0.189023	1.16411	0.247511	-4.66265	0.487914	0.618329
Monocytes	GM28981	0.418226	0.488246	1.163892	0.247599	-4.82413	0.483803	0.613352
Monocytes	LMO2	0.153223	4.903927	1.163709	0.247673	-5.63892	0.427093	0.542492
Monocytes	WHRN	-0.38939	3.066048	-1.16365	0.247698	-4.88978	0.449756	0.570964
Monocytes	BAZ2A	0.125759	6.97841	1.163638	0.247701	-5.84295	0.403029	0.512049
Monocytes	HPCAL1	0.097741	6.117607	1.163507	0.247754	-5.98813	0.412825	0.524476
Monocytes	SYTL1	-0.51951	1.59739	-1.16338	0.247808	-4.72274	0.46882	0.594776
Monocytes	ERCC4	0.271672	2.949475	1.163325	0.247828	-5.07052	0.451237	0.572837
Monocytes	BATF3	-0.15827	2.332688	-1.16313	0.247905	-5.69187	0.459167	0.582812

Monocytes	BTBD2	0.1915	3.477347	1.16307	0.247931	-5.27253	0.444571	0.564545
Monocytes	CBY1	0.252303	2.846037	1.163004	0.247957	-5.01594	0.452557	0.574595
Monocytes	CTDP1	-0.1257	4.660739	-1.16291	0.247997	-5.5492	0.430018	0.546296
Monocytes	MAGI1	-0.45777	4.373842	-1.16252	0.248152	-5.14158	0.433651	0.550737
Monocytes	METTL16	-0.15047	4.533537	-1.16241	0.248195	-5.39265	0.43171	0.548295
Monocytes	ADAMTS7	-0.71748	0.676356	-1.16236	0.248218	-4.63753	0.481397	0.610383
Monocytes	IFT22	0.231827	3.654091	1.162258	0.248258	-5.18153	0.44252	0.561883
Monocytes	PPP1R3F	0.468396	0.867721	1.162091	0.248326	-4.75397	0.478836	0.607191
Monocytes	SLU7	-0.12441	4.955244	-1.16147	0.248575	-5.53123	0.427026	0.54209
Monocytes	SLC25A15	-0.3298	2.488304	-1.16114	0.24871	-4.88675	0.457858	0.580793
Monocytes	NAIP6	0.184898	2.310523	1.161099	0.248727	-5.37696	0.460164	0.583685
Monocytes	ZSCAN12	0.386432	1.093811	1.160564	0.248943	-4.77552	0.476449	0.60399
Monocytes	GM30541	0.273849	0.952964	1.160494	0.248972	-5.06119	0.478356	0.606358
Monocytes	PRMT2	-0.38714	1.665423	-1.16048	0.248977	-4.81174	0.468793	0.594471
Monocytes	CSNK2A2	0.092491	5.948451	1.160471	0.248981	-5.76339	0.415557	0.527679
Monocytes	GM43062	-0.31878	1.638658	-1.16012	0.249124	-4.85331	0.469336	0.595011
Monocytes	RABL6	0.114051	5.136693	1.159726	0.249283	-5.57019	0.425475	0.539939
Monocytes	ERI3	-0.12968	5.177084	-1.15957	0.249347	-5.50408	0.425029	0.539332
Monocytes	DHRS9	-0.26774	0.196917	-1.15921	0.249493	-5.14813	0.489401	0.619595
Monocytes	UBE3A	0.088901	6.387077	1.158933	0.249604	-5.84507	0.411165	0.521625
Monocytes	DUSP8	-0.46886	1.274945	-1.15851	0.249776	-4.76509	0.475025	0.601567
Monocytes	KLF12	-0.38283	3.585919	-1.15831	0.249856	-5.05508	0.445061	0.564246
Monocytes	RFX3	0.113848	6.233922	1.15707	0.25036	-5.80475	0.413963	0.524428
Monocytes	GCC2	-0.1397	5.193926	-1.15677	0.250483	-5.574	0.42632	0.54001
Monocytes	GPRC5C	-0.44766	1.29284	-1.1566	0.25055	-4.93593	0.475924	0.601981
Monocytes	VCPKMT	-0.28046	2.877826	-1.15646	0.250609	-5.0598	0.455088	0.576062
Monocytes	PDCD1LG2	-0.32644	1.775707	-1.15618	0.250723	-5.06603	0.469526	0.594082
Monocytes	HAL	0.428375	2.215151	1.156084	0.250761	-4.99746	0.463724	0.586906
Monocytes	ACRBP	0.426636	1.566441	1.156065	0.250769	-4.78385	0.472317	0.59759
Monocytes	DRAM1	0.25677	2.581586	1.155981	0.250803	-5.35782	0.458947	0.580979
Monocytes	AMBRA1	0.091368	7.654516	1.155783	0.250884	-6.06537	0.398178	0.504547
Monocytes	TMEM186	-0.42028	2.111676	-1.1554	0.251041	-4.81577	0.465363	0.588724
Monocytes	M1AP	-0.38406	1.320039	-1.15527	0.251094	-4.86542	0.475929	0.601839
Monocytes	ITGB7	0.127001	4.620993	1.154031	0.251597	-5.94325	0.434399	0.549431
Monocytes	PIP5K1C	-0.12319	5.626433	-1.15389	0.251656	-5.74575	0.422352	0.534377
Monocytes	MCF2L	0.534891	0.46165	1.153388	0.25186	-4.78678	0.488896	0.617191
Monocytes	RCSD1	0.149798	7.091061	1.153176	0.251946	-5.78757	0.405758	0.51329
Monocytes	MAPK1IP1L	-0.08885	6.060276	-1.15287	0.25207	-5.7953	0.417728	0.52844
Monocytes	GM38134	0.535792	1.108316	1.152675	0.252151	-4.71968	0.48031	0.606505
Monocytes	PHAX	-0.1579	4.599165	-1.15239	0.252267	-5.46707	0.435369	0.550473
Monocytes	ZFP446	-0.48368	1.164128	-1.15218	0.252354	-4.72908	0.479716	0.605697
Monocytes	CHMP3	0.081432	5.884611	1.152141	0.252369	-5.80114	0.41999	0.531149
Monocytes	MCM4	-0.18475	5.68459	-1.15184	0.252492	-5.67148	0.422479	0.53417
Monocytes	RPP40	-0.45772	1.432828	-1.15169	0.252554	-4.75571	0.476259	0.601319
Monocytes	MAGOH	-0.11049	6.296682	-1.15158	0.252597	-5.81319	0.415342	0.525218
Monocytes	MMP13	0.946985	-1.01302	1.151263	0.252728	-4.63662	0.510718	0.643675
Monocytes	RPA2	-0.21307	4.645282	-1.15101	0.252832	-5.42581	0.435252	0.550096

Monocytes	GM42701	0.566562	0.844253	1.150828	0.252906	-4.71001	0.484615	0.611472
Monocytes	GM34084	0.217446	1.964286	1.150609	0.252996	-5.64451	0.469561	0.592823
Monocytes	RMDN1	0.169905	4.528231	1.150166	0.253177	-5.49162	0.43696	0.552126
Monocytes	RGS3	-0.24757	2.862627	-1.15015	0.253185	-5.30605	0.45794	0.578321
Monocytes	CASD1	-0.16656	4.363906	-1.15008	0.25321	-5.34379	0.438982	0.554657
Monocytes	2310008N11RII	0.699257	0.80481	1.149835	0.253313	-4.64719	0.485518	0.612369
Monocytes	GPAM	-0.40693	3.696477	-1.14946	0.253465	-4.92617	0.447596	0.565138
Monocytes	SMTNL2	-0.78282	0.4226	-1.14916	0.253588	-4.63735	0.491178	0.619029
Monocytes	CDK10	-0.30524	2.234353	-1.14879	0.253741	-4.9259	0.466794	0.588803
Monocytes	GM43696	-0.32502	2.459243	-1.14863	0.253805	-4.90079	0.463874	0.585181
Monocytes	HTR2B	0.487715	0.855091	1.148482	0.253867	-4.74202	0.485468	0.611898
Monocytes	4931414P19RIK	0.424993	2.099255	1.147983	0.254072	-4.82605	0.468817	0.591348
Monocytes	1110004F10RIK	-0.07807	6.204	-1.1478	0.254146	-5.80512	0.417719	0.527599
Monocytes	GABARAPL1	-0.17952	3.974288	-1.14773	0.254175	-5.38114	0.444663	0.561398
Monocytes	2410131K14RIK	0.326452	2.169111	1.147648	0.25421	-4.96977	0.467892	0.590338
Monocytes	ICK	-0.40343	2.147459	-1.14755	0.254251	-4.81137	0.468178	0.590696
Monocytes	REEP1	-0.59387	0.98372	-1.14754	0.254254	-4.69855	0.483868	0.610114
Monocytes	ZFP235	0.407143	1.919758	1.147518	0.254263	-4.8223	0.471204	0.594453
Monocytes	RASSF4	0.128357	4.553252	1.147227	0.254383	-5.829	0.437615	0.55256
Monocytes	C130046K22RIK	-0.46132	0.716514	-1.14677	0.254572	-4.7891	0.487974	0.614946
Monocytes	DOLK	0.322449	1.544978	1.146597	0.254642	-4.8306	0.476693	0.601027
Monocytes	LIAS	0.167321	4.310022	1.146132	0.254833	-5.34451	0.441126	0.556619
Monocytes	ZBTB41	0.265703	2.915286	1.145845	0.254951	-4.99776	0.458798	0.578756
Monocytes	POU2AF1	-0.43038	4.320007	-1.14574	0.254993	-4.96679	0.441002	0.556568
Monocytes	CYP39A1	0.404607	1.483874	1.145665	0.255025	-4.86483	0.477749	0.602258
Monocytes	REEP4	0.177838	4.356559	1.14548	0.255101	-5.45574	0.440549	0.556041
Monocytes	LY6E	0.122304	9.08245	1.145271	0.255188	-6.37455	0.386167	0.487575
Monocytes	CLMN	0.595432	0.334512	1.145264	0.25519	-4.70799	0.493586	0.621931
Monocytes	RANBP10	0.140014	5.698692	1.145081	0.255266	-5.6857	0.424278	0.535758
Monocytes	TOR4A	-0.40732	1.678835	-1.14506	0.255274	-4.84239	0.475119	0.599167
Monocytes	KDM3A	-0.12309	5.567098	-1.14502	0.255292	-5.67527	0.425843	0.537731
Monocytes	GM17103	-0.44907	1.028835	-1.14492	0.25533	-4.84832	0.483952	0.610158
Monocytes	RNF166	-0.10162	5.468282	-1.1449	0.255341	-5.74466	0.427023	0.53927
Monocytes	SCPEP1	0.120698	5.169388	1.144644	0.255446	-5.80474	0.430613	0.543787
Monocytes	NOP53	0.120974	5.740416	1.144636	0.255449	-5.76747	0.423783	0.53521
Monocytes	MVB12B	-0.19799	4.074675	-1.14459	0.255468	-5.33867	0.444054	0.560616
Monocytes	CDC25A	-0.23135	3.793744	-1.1445	0.255505	-5.21892	0.447578	0.565041
Monocytes	MNS1	-0.31232	2.821224	-1.14448	0.255512	-4.94947	0.460018	0.58054
Monocytes	GM26674	-0.5986	-1.15959	-1.14423	0.255618	-4.64583	0.515031	0.648486
Monocytes	PAM	0.172481	4.417759	1.144214	0.255623	-5.48239	0.439795	0.555381
Monocytes	PLK2	0.231718	5.006155	1.144161	0.255645	-5.55772	0.432591	0.54637
Monocytes	SLC12A6	0.134051	8.133974	1.14393	0.25574	-6.03879	0.396489	0.500882
Monocytes	E330020D12RIK	-0.22829	5.11024	-1.14388	0.25576	-5.49185	0.431376	0.544869
Monocytes	AXIN2	-0.6039	-0.02376	-1.14348	0.255925	-4.65979	0.498879	0.628501
Monocytes	IFI209	0.159077	5.809713	1.143434	0.255945	-5.81315	0.423167	0.534375
Monocytes	GM33524	-0.5309	0.491882	-1.14333	0.255987	-4.74348	0.491622	0.619593
Monocytes	CFL2	-0.17884	4.631141	-1.1422	0.256455	-5.35595	0.438101	0.552615

Monocytes	NR1D1	-0.53782	1.661874	-1.14197	0.25655	-4.80835	0.476459	0.600282
Monocytes	SLC25A36	0.113726	6.285868	1.14168	0.25667	-5.78222	0.418471	0.527903
Monocytes	DHX36	0.100385	5.392248	1.140971	0.256963	-5.66	0.429477	0.541399
Monocytes	PPIC	-0.36699	2.745716	-1.14075	0.257055	-4.88889	0.462688	0.582833
Monocytes	IFT46	0.129044	4.502973	1.140725	0.257065	-5.48497	0.440348	0.555023
Monocytes	CCNH	-0.12421	5.363256	-1.14025	0.257263	-5.59367	0.430107	0.542008
Monocytes	H13	-0.08972	6.42662	-1.1401	0.257324	-5.9097	0.417533	0.526227
Monocytes	LIPO3	0.24443	2.915424	1.139561	0.257547	-5.12533	0.461103	0.580449
Monocytes	CCDC126	0.447151	1.899321	1.139447	0.257595	-4.80677	0.474542	0.597107
Monocytes	BOD1L	0.101636	5.740505	1.139015	0.257774	-5.70871	0.426079	0.536736
Monocytes	ECT2	-0.29424	4.373594	-1.139	0.25778	-5.37791	0.442726	0.557555
Monocytes	CD82	-0.12788	5.476033	-1.13869	0.257908	-5.69143	0.429326	0.540852
Monocytes	TMCO3	-0.17391	3.656165	-1.13838	0.258038	-5.30185	0.451837	0.569069
Monocytes	PFKFB2	0.270221	2.465839	1.138365	0.258043	-4.96401	0.467266	0.588231
Monocytes	PAK2	0.070584	7.859842	1.138357	0.258047	-6.13204	0.401715	0.506225
Monocytes	PPM1N	0.55852	0.159456	1.138348	0.25805	-4.76744	0.498832	0.627164
Monocytes	APOLD1	-0.58999	1.979983	-1.13805	0.258176	-4.77023	0.473834	0.596381
Monocytes	MBD2	0.088227	7.044678	1.137997	0.258196	-5.96795	0.411015	0.517972
Monocytes	FZD6	0.693862	0.693274	1.137873	0.258248	-4.67209	0.491432	0.618091
Monocytes	ETFRF1	0.258621	3.163487	1.137748	0.2583	-5.13997	0.458254	0.577079
Monocytes	DBNL	-0.09503	6.127196	-1.13755	0.258382	-5.88373	0.42167	0.531484
Monocytes	TRIM30B	0.295589	1.730639	1.137494	0.258405	-5.12753	0.47719	0.600655
Monocytes	TTPAL	0.174113	4.234118	1.137467	0.258416	-5.28014	0.444646	0.560245
Monocytes	EPHX3	-0.45697	0.657793	-1.13714	0.258553	-4.77598	0.492104	0.618979
Monocytes	EPN2	-0.57944	1.664749	-1.13696	0.258628	-4.76062	0.47831	0.602017
Monocytes	PSMD12	-0.10642	5.784758	-1.13661	0.258772	-5.74956	0.426094	0.536919
Monocytes	1810059H22RII	-0.69734	1.799204	-1.13645	0.258838	-4.71774	0.476716	0.599943
Monocytes	PREX1	-0.12347	6.43388	-1.13626	0.25892	-5.8869	0.418514	0.527363
Monocytes	GMPR	-0.46519	1.923101	-1.13609	0.258989	-4.80602	0.475096	0.597941
Monocytes	IDH2	-0.1348	5.667278	-1.13607	0.258998	-5.68043	0.427577	0.538776
Monocytes	LRRC28	0.323062	3.874144	1.135528	0.259223	-5.11771	0.449914	0.566628
Monocytes	PSMC3IP	-0.35305	2.412129	-1.13531	0.259313	-4.93065	0.468853	0.590262
Monocytes	CCDC124	0.101007	5.595782	1.135275	0.259328	-5.74282	0.428691	0.540213
Monocytes	CCDC106	0.589837	0.51131	1.13527	0.25933	-4.69118	0.494795	0.622282
Monocytes	PUM1	-0.07587	7.56821	-1.13483	0.259514	-6.04661	0.405913	0.511368
Monocytes	SRSF7	-0.12683	6.098296	-1.13479	0.259529	-5.81934	0.422887	0.532744
Monocytes	GNAQ	0.097864	7.882192	1.134666	0.259582	-6.19297	0.402402	0.50698
Monocytes	POU5F2	0.371468	1.687999	1.134333	0.259721	-4.8554	0.47895	0.602522
Monocytes	IL7R	0.356605	4.966863	1.134093	0.259821	-5.21544	0.436765	0.550056
Monocytes	ARHGAP32	-0.239	3.193867	-1.13385	0.259924	-5.15771	0.459199	0.577996
Monocytes	DUSP2	-0.16457	5.991936	-1.1337	0.259985	-5.84011	0.42453	0.534734
Monocytes	PAICS	-0.11615	6.310596	-1.13347	0.260082	-5.83514	0.420853	0.530038
Monocytes	SNX5	0.097874	7.890887	1.133235	0.260179	-6.02734	0.402818	0.507288
Monocytes	RAMP2	-0.37883	3.013058	-1.13303	0.260265	-4.98172	0.461837	0.581181
Monocytes	SIPA1L3	0.120042	5.201146	1.132304	0.260568	-5.80391	0.434708	0.547113
Monocytes	ARID3B	0.202288	3.836648	1.131975	0.260706	-5.31264	0.451785	0.568294
Monocytes	SETD5	0.088617	6.628233	1.131961	0.260712	-5.89106	0.417792	0.525802

Monocytes	PKD1	0.217501	3.003757	1.131719	0.260813	-5.20858	0.462572	0.58165
Monocytes	CLK2	0.128511	4.63296	1.131618	0.260855	-5.47021	0.441839	0.555874
Monocytes	GM43581	0.444442	2.093907	1.131521	0.260896	-4.80089	0.474616	0.596601
Monocytes	KIFC5B	-0.41268	1.787655	-1.13137	0.260961	-4.82475	0.478747	0.601786
Monocytes	CRYBG1	0.134641	4.169377	1.131308	0.260985	-5.73322	0.447632	0.563228
Monocytes	ETFBKMT	-0.31829	2.261696	-1.13125	0.26101	-4.93127	0.472369	0.593975
Monocytes	JMJD7	0.359123	1.757778	1.131027	0.261102	-4.8493	0.479209	0.602466
Monocytes	TJAP1	-0.15115	4.452059	-1.13095	0.261136	-5.43139	0.444143	0.558958
Monocytes	BROX	0.114971	4.85097	1.130853	0.261175	-5.55855	0.439197	0.552785
Monocytes	CTNNB1	-0.0837	6.277504	-1.13062	0.261274	-5.84591	0.422072	0.531383
Monocytes	TRIM39	-0.23105	2.873416	-1.13054	0.261306	-5.04769	0.464406	0.584237
Monocytes	ME1	-0.63864	-0.00982	-1.12958	0.261708	-4.69874	0.504575	0.633305
Monocytes	UGT2B5	-0.54395	1.786068	-1.12951	0.261739	-4.83777	0.479508	0.602516
Monocytes	TMEM131L	-0.11794	7.26323	-1.12943	0.26177	-5.94093	0.411152	0.517352
Monocytes	BCAS3	0.107772	7.334935	1.129192	0.261871	-6.02489	0.410418	0.516409
Monocytes	SEC24A	-0.14742	6.616568	-1.12899	0.261956	-5.86569	0.418714	0.526853
Monocytes	FAM129B	0.15832	3.14002	1.128986	0.261958	-5.76522	0.461606	0.580388
Monocytes	MTREX	0.108686	5.477446	1.128759	0.262053	-5.66655	0.432341	0.543934
Monocytes	DDX51	-0.42507	1.37149	-1.12839	0.26221	-4.80599	0.485443	0.609911
Monocytes	GM48678	-0.26465	3.349644	-1.12835	0.262223	-5.17258	0.459046	0.577264
Monocytes	DIDO1	-0.12256	5.734022	-1.12834	0.262227	-5.71262	0.429314	0.540194
Monocytes	SYT11	0.420818	1.412759	1.127537	0.262566	-4.88055	0.485276	0.609592
Monocytes	TRF	-0.25782	9.658999	-1.12746	0.262598	-6.35706	0.385279	0.484606
Monocytes	NME1	0.143509	6.668403	1.12742	0.262615	-5.91041	0.4186	0.52666
Monocytes	ZFP664	-0.18099	4.050718	-1.1274	0.262622	-5.27634	0.450445	0.566474
Monocytes	MATN2	0.493021	0.980441	1.127111	0.262745	-4.77028	0.491314	0.617091
Monocytes	FXR2	-0.13225	5.267562	-1.12711	0.262747	-5.63947	0.435364	0.547714
Monocytes	LPIN2	0.123731	6.528551	1.127005	0.26279	-5.90264	0.420282	0.528866
Monocytes	VPREB2	-0.7498	-0.41404	-1.12691	0.262828	-4.64481	0.511173	0.641539
Monocytes	GTPBP6	-0.27196	2.84005	-1.12552	0.263415	-5.05244	0.467087	0.586851
Monocytes	AC160336.1	0.434443	1.371003	1.125355	0.263484	-4.81518	0.486946	0.611368
Monocytes	GIT2	-0.08723	7.020155	-1.1252	0.263549	-5.95909	0.415451	0.522455
Monocytes	FANCM	-0.24693	3.67592	-1.12506	0.263607	-5.16329	0.456248	0.573431
Monocytes	RPUSD4	0.260928	2.682051	1.125043	0.263616	-5.05655	0.469216	0.589509
Monocytes	GPALPP1	0.152961	3.737889	1.12488	0.263685	-5.37103	0.455494	0.572469
Monocytes	DPH5	0.189647	3.732552	1.124688	0.263765	-5.31435	0.45559	0.572627
Monocytes	METTL14	-0.25583	3.09472	-1.12455	0.263825	-5.07483	0.463853	0.58296
Monocytes	COX7A2L	0.083672	6.644119	1.124435	0.263872	-5.96516	0.419892	0.528136
Monocytes	CKS2	0.159614	5.959802	1.124355	0.263906	-5.79276	0.427992	0.538313
Monocytes	RUNX1	-0.10579	9.201324	-1.12431	0.263924	-6.38726	0.391117	0.491877
Monocytes	ARHGAP17	-0.10344	7.245574	-1.12404	0.26404	-6.02828	0.413027	0.519447
Monocytes	LONP1	0.196826	3.653778	1.123906	0.264095	-5.23789	0.456744	0.574084
Monocytes	HMGB1	-0.1077	9.807497	-1.12367	0.264193	-6.34473	0.384776	0.483757
Monocytes	EFCAB8	-0.38111	1.374064	-1.12365	0.264204	-4.85622	0.487165	0.61171
Monocytes	CSPRS	0.400462	0.292172	1.123417	0.264302	-4.81335	0.502455	0.630451
Monocytes	CD226	-0.30187	1.697205	-1.12264	0.26463	-5.28368	0.483341	0.606577
Monocytes	PLEKHA3	0.153438	3.98944	1.122194	0.264818	-5.39568	0.453305	0.569281

Monocytes	CPNE9	-0.44919	1.867904	-1.12172	0.26502	-4.944	0.481558	0.6039
Monocytes	LIN9	0.186123	4.133885	1.121327	0.265185	-5.34346	0.451884	0.567107
Monocytes	SEMA4B	-0.23706	5.763505	-1.12125	0.265217	-5.35932	0.431695	0.541954
Monocytes	ADGRA3	-0.53617	0.459311	-1.12119	0.265242	-4.69327	0.501346	0.628115
Monocytes	LCP1	-0.09323	8.858643	-1.121	0.265323	-6.38348	0.396129	0.497306
Monocytes	LMO7	0.88522	0.761873	1.120821	0.265399	-4.73568	0.497184	0.623016
Monocytes	NDUFB5	0.090859	7.037752	1.120573	0.265504	-6.02655	0.416811	0.523257
Monocytes	BPTF	-0.09702	7.356344	-1.12035	0.265598	-5.99009	0.413207	0.518722
Monocytes	CABP1	-0.74312	-0.13612	-1.12008	0.265713	-4.6472	0.510378	0.639036
Monocytes	ESRRA	0.161193	3.981496	1.119759	0.265849	-5.413	0.454403	0.569929
Monocytes	TXNL4A	-0.12524	4.968748	-1.1195	0.265959	-5.63004	0.442082	0.554618
Monocytes	SH2D3C	0.170945	4.693449	1.11924	0.266069	-5.55633	0.445621	0.558977
Monocytes	PAIP1	-0.11578	5.907952	-1.11911	0.266126	-5.75777	0.430733	0.540406
Monocytes	PIGC	0.218728	2.730331	1.11877	0.266269	-5.14388	0.471071	0.590436
Monocytes	GTF2F1	-0.11039	5.030746	-1.11875	0.266276	-5.61739	0.441543	0.55382
Monocytes	GABPB2	-0.09497	6.502447	-1.11851	0.266378	-5.86322	0.423802	0.53162
Monocytes	SLC23A1	-0.49289	1.528872	-1.11842	0.266416	-4.73647	0.487431	0.610533
Monocytes	ATRAID	0.133487	4.57765	1.118336	0.266453	-5.53369	0.447264	0.560921
Monocytes	CPM	-0.38891	4.160192	-1.11798	0.266604	-5.06045	0.452579	0.56766
Monocytes	AMT	-0.53019	1.565291	-1.11793	0.266626	-4.7808	0.486969	0.610157
Monocytes	ARHGEF11	0.129364	4.907611	1.117867	0.266652	-5.72889	0.443178	0.556005
Monocytes	SIDT2	0.140033	5.063847	1.117861	0.266655	-5.57783	0.441241	0.553592
Monocytes	OXLD1	0.339653	1.813163	1.117601	0.266765	-4.91975	0.483682	0.606039
Monocytes	NGRN	0.260741	3.173648	1.117082	0.266986	-5.09562	0.465749	0.583766
Monocytes	MEIS1	0.231936	3.866059	1.116654	0.267168	-5.29001	0.456918	0.572656
Monocytes	WDFY2	0.128768	5.19857	1.116575	0.267201	-5.78928	0.440135	0.551804
Monocytes	VPS72	-0.14785	4.357985	-1.11651	0.267229	-5.41362	0.45064	0.564868
Monocytes	PMM2	0.118956	4.561806	1.116432	0.267262	-5.53348	0.448068	0.561673
Monocytes	PGM2L1	-0.19707	5.192591	-1.11623	0.267349	-5.43959	0.440219	0.551912
Monocytes	TTC37	0.233013	3.798842	1.116109	0.2674	-5.27408	0.457794	0.573754
Monocytes	ASF1A	-0.16856	5.210724	-1.1161	0.267403	-5.48032	0.439995	0.551641
Monocytes	DONSON	0.156437	4.025423	1.115784	0.267538	-5.43132	0.454962	0.57023
Monocytes	LSM3	0.133079	5.624923	1.115781	0.267539	-5.76228	0.434995	0.545396
Monocytes	SH3BGRL3	0.086545	9.061448	1.115502	0.267658	-6.42862	0.395445	0.4957
Monocytes	CDC42EP1	-0.64631	0.752796	-1.11513	0.267815	-4.71751	0.499325	0.624688
Monocytes	CYP4A10	0.697853	1.117903	1.115116	0.267823	-4.78293	0.494179	0.618384
Monocytes	ALG2	0.239872	3.062397	1.114524	0.268075	-5.01138	0.467995	0.585986
Monocytes	FAM169B	-0.41713	3.62896	-1.11445	0.268105	-4.90539	0.460581	0.57682
Monocytes	CDYL2	0.199195	4.757934	1.114411	0.268123	-5.43009	0.446192	0.558976
Monocytes	ZNRD2	-0.1854	4.037669	-1.11434	0.268153	-5.3486	0.455313	0.570327
Monocytes	CATSPER2	-0.35392	1.800753	-1.11395	0.268321	-4.92923	0.485205	0.607119
Monocytes	PSMC2	-0.11975	5.362149	-1.11371	0.268423	-5.69026	0.438991	0.549914
Monocytes	NUMB	0.110693	6.956562	1.11352	0.268503	-5.93306	0.419922	0.526154
Monocytes	A930005H10RII	-0.22824	3.220496	-1.11299	0.26873	-5.07416	0.466603	0.583986
Monocytes	HDLBP	0.079243	6.655734	1.11285	0.268789	-5.96556	0.423767	0.530811
Monocytes	SLX4	-0.31478	1.820472	-1.1127	0.268855	-4.87083	0.485492	0.607287
Monocytes	GTF2B	-0.10668	6.738906	-1.11251	0.268936	-5.94653	0.422874	0.529617

Monocytes	DIRAS2	0.465726	0.718948	1.111995	0.269155	-4.80144	0.501125	0.626432
Monocytes	MCC	-0.38794	2.092188	-1.11193	0.269182	-4.93245	0.482	0.602991
Monocytes	ANO8	-0.5186	0.904446	-1.11187	0.26921	-4.75891	0.498494	0.62328
Monocytes	EIF4G1	-0.08925	6.604378	-1.1117	0.269282	-5.91667	0.424607	0.531905
Monocytes	MMRN1	1.047376	-0.22843	1.111641	0.269306	-4.68259	0.5148	0.643251
Monocytes	IRF9	0.202728	4.515749	1.111505	0.269364	-5.59697	0.450171	0.563842
Monocytes	RAB23	0.446957	1.279801	1.111428	0.269397	-4.79685	0.493216	0.616958
Monocytes	RHD	0.543921	1.667344	1.111339	0.269436	-4.78663	0.48783	0.610362
Monocytes	NDUFAF7	0.138698	4.007139	1.111148	0.269517	-5.49937	0.456653	0.571976
Monocytes	OTULIN	0.079585	6.57022	1.111132	0.269524	-5.96218	0.425011	0.532585
Monocytes	IGF2R	-0.15484	4.985937	-1.11081	0.269662	-5.61476	0.444269	0.556776
Monocytes	GPR182	-0.41858	2.557152	-1.11081	0.269663	-4.88444	0.475706	0.595711
Monocytes	TCEAL8	-0.20787	3.331023	-1.11078	0.269674	-5.18995	0.46543	0.583023
Monocytes	IKBKG	0.212424	3.339723	1.110751	0.269687	-5.16028	0.465316	0.582882
Monocytes	POLE2	-0.29717	3.014685	-1.11059	0.269756	-5.0872	0.469601	0.588201
Monocytes	TNFRSF14	0.522347	0.252225	1.110588	0.269757	-4.77858	0.507813	0.635142
Monocytes	NOP9	0.137286	4.555827	1.110488	0.2698	-5.51109	0.449664	0.563527
Monocytes	OSBPL5	-0.62818	0.3884	-1.1104	0.269839	-4.69514	0.505851	0.632784
Monocytes	AUTS2	-0.42422	4.397144	-1.11029	0.269883	-5.22649	0.451673	0.566067
Monocytes	RPA3	-0.15391	4.97161	-1.10989	0.270057	-5.6048	0.444659	0.557139
Monocytes	SDK1	-0.46827	3.195716	-1.10964	0.270163	-4.96704	0.467488	0.585381
Monocytes	UBR1	0.146009	5.323458	1.109567	0.270195	-5.62391	0.440349	0.551718
Monocytes	NINL	-0.52371	0.999292	-1.10949	0.270226	-4.74181	0.497451	0.622268
Monocytes	FBXL8	0.381709	1.99347	1.109371	0.270279	-4.87278	0.483636	0.605377
Monocytes	ZFP809	-0.21929	3.64912	-1.10929	0.270315	-5.25202	0.461553	0.57815
Monocytes	WDCP	0.234349	3.079235	1.109008	0.270434	-5.15378	0.469026	0.587406
Monocytes	PSPC1	-0.12958	5.702286	-1.10897	0.27045	-5.7271	0.435703	0.546047
Monocytes	ABITRAM	0.232922	2.584915	1.108947	0.27046	-5.08004	0.475616	0.59554
Monocytes	DMAC1	0.130589	4.611093	1.108853	0.270501	-5.58989	0.449235	0.562907
Monocytes	LBHD1	0.491372	0.16876	1.108645	0.27059	-4.71582	0.509322	0.63702
Monocytes	LMO4	0.139591	7.245458	1.108554	0.270629	-6.07716	0.417339	0.5232
Monocytes	B4GAT1	0.361779	1.65015	1.10855	0.270631	-4.89953	0.488359	0.611357
Monocytes	MLST8	0.391969	2.044303	1.107672	0.271008	-4.87593	0.48351	0.605021
Monocytes	DAAM1	-0.14187	4.764899	-1.10752	0.271074	-5.5837	0.447827	0.560925
Monocytes	OSBPL2	-0.1684	3.979026	-1.10732	0.271159	-5.29356	0.457826	0.573331
Monocytes	WWP1	-0.15843	5.227114	-1.10729	0.271174	-5.59774	0.442059	0.553753
Monocytes	LRRC47	0.143018	4.227098	1.107203	0.27121	-5.40163	0.454643	0.569386
Monocytes	TM2D2	0.102802	5.178235	1.107177	0.271221	-5.78383	0.442665	0.554507
Monocytes	SPRYD4	0.355979	1.803638	1.106863	0.271356	-4.83564	0.486975	0.609161
Monocytes	ZFAT	-0.20543	3.828322	-1.10608	0.271694	-5.32964	0.460367	0.576226
Monocytes	TESC	-0.4769	1.561476	-1.10604	0.271708	-4.87405	0.490799	0.613729
Monocytes	CCDC71	0.251933	2.73695	1.105757	0.271832	-5.08668	0.474777	0.593993
Monocytes	ERN1	0.118625	7.053542	1.105705	0.271854	-6.05469	0.420636	0.526744
Monocytes	DGKE	-0.14787	4.880229	-1.10569	0.271858	-5.64758	0.446982	0.559599
Monocytes	THADA	-0.12718	5.881845	-1.10527	0.272041	-5.74036	0.434837	0.544267
Monocytes	GM16536	0.362552	1.530823	1.105141	0.272097	-4.84	0.491519	0.614317
Monocytes	GTPBP3	-0.3535	2.225197	-1.10472	0.272277	-4.87031	0.482194	0.602782

Monocytes	GM12353	0.438789	1.278675	1.104396	0.272418	-4.83087	0.495463	0.618855
Monocytes	LTV1	0.164491	4.232009	1.104242	0.272484	-5.47409	0.455863	0.570049
Monocytes	LARP4B	0.081425	7.283403	1.104133	0.272531	-6.06692	0.41857	0.523648
Monocytes	FGL2	-0.21275	4.054695	-1.10392	0.272622	-5.85501	0.458221	0.573004
Monocytes	NDUFS1	0.096437	5.752161	1.103255	0.27291	-5.72997	0.437241	0.54688
Monocytes	MEIS2	-0.43459	2.894046	-1.10323	0.272922	-4.97877	0.473808	0.592174
Monocytes	ORC1	-0.26853	3.345968	-1.10312	0.272968	-5.1655	0.467807	0.584817
Monocytes	TAF1B	0.14901	4.199522	1.103029	0.273008	-5.41747	0.4567	0.571117
Monocytes	AP3S2	0.20095	3.43138	1.102818	0.273099	-5.21808	0.46676	0.583496
Monocytes	MGLL	-0.34551	3.495334	-1.10167	0.273594	-5.06466	0.466687	0.582749
Monocytes	COA4	0.26737	2.161432	1.101118	0.273834	-5.05758	0.484922	0.605004
Monocytes	CCL21A	1.628177	0.049265	1.101006	0.273883	-4.7038	0.514854	0.641587
Monocytes	KIT	0.202914	3.453389	1.10093	0.273915	-5.63355	0.467554	0.583704
Monocytes	DEDD2	0.172583	4.544003	1.100488	0.274107	-5.46722	0.45367	0.566336
Monocytes	LTBP1	0.871529	0.730726	1.100093	0.274278	-4.79299	0.505483	0.629905
Monocytes	ZYG11B	-0.12237	5.883084	-1.09997	0.27433	-5.74443	0.43717	0.545793
Monocytes	4930444A19RIK	0.206083	3.751804	1.09987	0.274374	-5.39923	0.46411	0.579178
Monocytes	PIAS3	0.218716	2.896492	1.098872	0.274807	-5.07768	0.476107	0.593581
Monocytes	RAP1A	0.056904	8.772339	1.098592	0.274929	-6.30709	0.404084	0.504088
Monocytes	NTN4	-0.59986	0.706687	-1.09838	0.27502	-4.74702	0.506692	0.630935
Monocytes	RBM22	-0.09121	5.603703	-1.09831	0.275049	-5.73446	0.441343	0.550583
Monocytes	SAMD8	0.11983	5.289431	1.098278	0.275065	-5.75422	0.445244	0.555422
Monocytes	VPS13A	-0.09664	6.447058	-1.09813	0.27513	-5.86232	0.431092	0.537816
Monocytes	SDC1	0.475475	2.216388	1.097665	0.275331	-4.91652	0.485784	0.605206
Monocytes	PRTN3	0.359615	3.6691	1.097366	0.275461	-5.89316	0.466291	0.581344
Monocytes	RASSF1	-0.09196	5.940086	-1.0973	0.27549	-5.92593	0.437504	0.545747
Monocytes	STFA1	0.247129	5.975982	1.097261	0.275507	-6.13327	0.437065	0.545202
Monocytes	MRPS11	-0.20018	3.950885	-1.09708	0.275587	-5.33241	0.462607	0.576843
Monocytes	ZFP994	0.228646	2.481603	1.09706	0.275594	-5.10555	0.482175	0.600914
Monocytes	GM17018	0.181163	3.620053	1.097011	0.275615	-5.3413	0.466935	0.582179
Monocytes	GM5544	0.415674	0.129876	1.096889	0.275668	-4.84803	0.515424	0.641573
Monocytes	IL18RAP	0.388895	1.983582	1.096379	0.27589	-5.07241	0.489178	0.609595
Monocytes	DNAJC25	0.151058	3.856746	1.096321	0.275916	-5.40482	0.463991	0.578642
Monocytes	IGF2	-0.45594	4.193244	-1.09627	0.275936	-5.34543	0.45962	0.573248
Monocytes	HARBI1	0.457297	1.346342	1.096272	0.275937	-4.82207	0.498086	0.620489
Monocytes	GM10138	-0.32802	2.471458	-1.09604	0.276039	-4.99324	0.482576	0.601433
Monocytes	SLC1A4	-0.44314	0.947937	-1.09592	0.276092	-4.82055	0.503861	0.62745
Monocytes	SLC25A14	0.27138	2.078186	1.095763	0.276158	-4.97879	0.488021	0.608122
Monocytes	4930484I04RIK	0.375185	1.660784	1.095388	0.276322	-4.87288	0.493957	0.615341
Monocytes	KIF18B	-0.36427	2.84215	-1.09524	0.276388	-5.01741	0.477731	0.59546
Monocytes	CDC37L1	0.125041	5.167302	1.095164	0.276419	-5.55624	0.447484	0.558146
Monocytes	5330438D12RIK	0.158978	4.842586	1.09513	0.276434	-5.49341	0.451577	0.563215
Monocytes	GNA11	-0.26982	3.954617	-1.09506	0.276463	-5.12188	0.462986	0.577321
Monocytes	NFYA	-0.15233	4.152707	-1.09487	0.276547	-5.50157	0.460476	0.574203
Monocytes	TSLP	-0.59068	-0.44209	-1.09461	0.276661	-4.72591	0.524512	0.652557
Monocytes	GOSR2	-0.10487	5.28486	-1.09455	0.276687	-5.83927	0.446152	0.556525
Monocytes	NFKBIA	-0.12748	8.130992	-1.09413	0.27687	-6.26159	0.412321	0.514266

Monocytes	TMEM150B	0.249429	1.12138	1.093529	0.277132	-5.32856	0.502364	0.625158
Monocytes	TMEM43	0.169065	3.43413	1.093162	0.277292	-5.39061	0.470656	0.586409
Monocytes	SAMD4B	0.113004	5.014196	1.093116	0.277312	-5.69933	0.450208	0.561198
Monocytes	MAP2K1	0.10235	6.311963	1.093027	0.277351	-5.97198	0.434153	0.541321
Monocytes	IDE	-0.13955	4.161112	-1.09299	0.277366	-5.40989	0.461123	0.574733
Monocytes	AA465934	-0.39099	1.66043	-1.09282	0.27744	-4.84068	0.49489	0.616216
Monocytes	AC142100.1	-0.44621	0.335219	-1.09231	0.277665	-4.88082	0.514107	0.639578
Monocytes	GM20324	-0.23098	2.098382	-1.09229	0.277672	-5.11538	0.489041	0.609
Monocytes	1810062O18RII	-0.37839	2.0989	-1.09204	0.277783	-4.88128	0.489121	0.60906
Monocytes	GLYAT	-0.44015	2.069154	-1.09188	0.27785	-4.89829	0.489533	0.609596
Monocytes	NFATC2IP	-0.24496	3.151572	-1.09186	0.277859	-5.11994	0.474793	0.591521
Monocytes	CD2BP2	-0.15766	4.011388	-1.09166	0.27795	-5.37748	0.463508	0.577626
Monocytes	PTDSS2	0.199799	3.06586	1.09083	0.278312	-5.20041	0.47656	0.593292
Monocytes	PCDH17	-0.42186	2.459624	-1.09037	0.278511	-4.97424	0.485055	0.603469
Monocytes	PCMT1	-0.09491	6.63523	-1.09011	0.278628	-5.90102	0.431476	0.537289
Monocytes	CLDN15	0.357693	0.423974	1.090037	0.278659	-4.88443	0.513958	0.638642
Monocytes	GM34680	0.565289	0.042973	1.089872	0.278731	-4.76508	0.519602	0.645511
Monocytes	TRIP10	-0.30268	1.355077	-1.08964	0.278834	-4.96363	0.500631	0.622502
Monocytes	ZFP646	0.213923	3.903418	1.089569	0.278864	-5.28792	0.465865	0.579915
Monocytes	OSTC	0.087634	6.387429	1.089525	0.278883	-5.93627	0.434533	0.541168
Monocytes	NDUFA10	0.096827	6.050526	1.08923	0.279012	-5.86067	0.438772	0.546408
Monocytes	ZFP287	-0.50702	0.566909	-1.08904	0.279095	-4.79128	0.512171	0.636597
Monocytes	ARHGAP1	-0.11218	4.473602	-1.08849	0.279339	-5.56529	0.458955	0.571188
Monocytes	CLIC5	0.592031	0.878367	1.088415	0.27937	-4.74974	0.507996	0.631258
Monocytes	YWHAG	-0.10202	6.203239	-1.08806	0.279528	-5.9455	0.437417	0.544372
Monocytes	PDCD11	0.146688	4.214015	1.087954	0.279572	-5.46928	0.462493	0.57542
Monocytes	HIST2H3B	-0.51232	1.047683	-1.08782	0.279631	-4.7843	0.505756	0.628415
Monocytes	EEF2	-0.09236	9.105144	-1.08753	0.279759	-6.36985	0.403528	0.502261
Monocytes	MYADM	0.12108	5.586342	1.08749	0.279776	-6.01011	0.445027	0.553942
Monocytes	E230014E18RIK	0.573443	-1.38289	1.087403	0.279814	-4.66839	0.54196	0.672341
Monocytes	CRISPLD2	0.56838	0.884481	1.08738	0.279825	-4.82819	0.508103	0.631352
Monocytes	GM17173	0.422184	0.150476	1.087333	0.279845	-4.84262	0.518806	0.644352
Monocytes	USF3	0.12094	4.977969	1.086761	0.280096	-5.75104	0.45301	0.563629
Monocytes	DCPS	-0.13184	5.004121	-1.08652	0.280202	-5.62282	0.452775	0.563311
Monocytes	GALT	-0.30444	2.389889	-1.08622	0.280336	-5.00199	0.487511	0.605895
Monocytes	TRIM69	-0.37049	2.05026	-1.08589	0.280481	-4.98832	0.492348	0.611803
Monocytes	CEPT1	0.137222	5.374134	1.085836	0.280503	-5.80569	0.448365	0.55776
Monocytes	TACO1OS	0.433699	1.705089	1.085711	0.280558	-4.82109	0.497197	0.617747
Monocytes	GM47469	0.570104	0.520329	1.085237	0.280767	-4.72805	0.514405	0.638617
Monocytes	D330041H03RII	0.330807	2.247493	1.085032	0.280857	-4.91037	0.489834	0.608788
Monocytes	CDC40	0.103213	6.047181	1.084906	0.280913	-5.85734	0.440203	0.547726
Monocytes	TAMM41	-0.26245	2.732365	-1.08486	0.280933	-5.11181	0.483169	0.600672
Monocytes	ZFP637	0.321293	2.315939	1.08485	0.280937	-4.96679	0.488887	0.607669
Monocytes	NRIP3	-0.56682	0.094317	-1.08472	0.280995	-4.81069	0.52067	0.646388
Monocytes	JAG1	-0.45602	1.3378	-1.08471	0.281	-4.94352	0.502612	0.624447
Monocytes	KIF18A	0.242756	3.977118	1.084414	0.281129	-5.3078	0.466554	0.580289
Monocytes	DBI	0.110609	7.067278	1.084341	0.281162	-6.11386	0.427891	0.532455

Monocytes	SEC23IP	0.114089	4.499732	1.084275	0.281191	-5.52	0.45975	0.571908
Monocytes	IMPDH2	-0.11871	5.196107	-1.08414	0.281251	-5.73682	0.450854	0.560928
Monocytes	GNAL	0.353395	1.371741	1.084079	0.281277	-4.88926	0.502174	0.623902
Monocytes	SHCBP1	-0.23205	4.69138	-1.08393	0.281342	-5.56913	0.457282	0.568938
Monocytes	CREB1	0.086399	6.332794	1.083918	0.281348	-5.94741	0.436744	0.543524
Monocytes	GANAB	0.131794	4.730059	1.083668	0.281458	-5.57026	0.456879	0.568424
Monocytes	PCBD1	-0.35741	3.53173	-1.08358	0.281497	-5.24167	0.472536	0.58772
Monocytes	ETV6	0.094693	8.156803	1.083125	0.281698	-6.38952	0.415381	0.516818
Monocytes	TMSB15B2	-0.29412	2.47439	-1.08304	0.281735	-5.03453	0.487043	0.605386
Monocytes	AADAT	-0.45491	1.2345	-1.08301	0.28175	-4.80831	0.504439	0.626617
Monocytes	AASDH	-0.29828	2.290623	-1.08269	0.281888	-5.09248	0.489627	0.608598
Monocytes	GM19967	-0.40668	1.377066	-1.08268	0.281893	-4.82064	0.502453	0.624253
Monocytes	GM4631	-0.46776	0.664941	-1.08263	0.281915	-4.75385	0.512706	0.63673
Monocytes	ZC2HC1A	-0.32831	1.741533	-1.08232	0.282052	-5.03748	0.497452	0.618038
Monocytes	FNIP1	-0.09899	7.760209	-1.0821	0.282149	-6.18873	0.420229	0.522789
Monocytes	PANK1	-0.25461	3.430383	-1.08175	0.282307	-5.24468	0.474477	0.589863
Monocytes	TTK	-0.37971	2.313922	-1.08174	0.282309	-4.95754	0.489667	0.608471
Monocytes	GM12216	-0.19373	4.554087	-1.08145	0.282439	-5.5463	0.459824	0.571747
Monocytes	FOXP4	-0.15196	4.962815	-1.08138	0.282469	-5.65081	0.454579	0.565278
Monocytes	1110032A03RIK	0.187455	3.592833	1.081052	0.282614	-5.25101	0.4725	0.587395
Monocytes	NT5C2	0.130793	5.867578	1.081007	0.282634	-5.86516	0.443276	0.551357
Monocytes	MYSM1	0.113446	5.388465	1.080966	0.282652	-5.71679	0.44926	0.558761
Monocytes	FKBP1A	-0.08334	7.336878	-1.08069	0.282776	-6.16269	0.425572	0.529351
Monocytes	CHTOP	0.131524	5.225925	1.080614	0.282808	-5.64057	0.451411	0.561403
Monocytes	MRPL41	-0.16742	4.003451	-1.08014	0.283018	-5.37738	0.467445	0.58107
Monocytes	SPAG1	0.48328	1.4147	1.079856	0.283143	-4.80123	0.503028	0.624527
Monocytes	SF3B1	0.063379	8.256825	1.079739	0.283195	-6.22894	0.415187	0.516274
Monocytes	HSCB	0.1876	4.143832	1.079657	0.283232	-5.41333	0.465728	0.578922
Monocytes	PML	0.236198	5.273859	1.079516	0.283294	-5.54906	0.451213	0.560999
Monocytes	DHRS3	-0.18737	4.170119	-1.07926	0.283405	-5.55351	0.465437	0.578596
Monocytes	ACVR2B	-0.41955	1.53111	-1.07921	0.283429	-4.86596	0.50143	0.62268
Monocytes	CD44	0.094781	8.587659	1.079166	0.283449	-6.46854	0.411445	0.511678
Monocytes	HIST1H2AP	0.26537	7.597755	1.078917	0.283559	-6.12839	0.422968	0.526026
Monocytes	RNF10	-0.107	6.51711	-1.07886	0.283586	-5.89505	0.435891	0.542119
Monocytes	OAS2	0.391205	1.571395	1.078642	0.283681	-5.07969	0.501023	0.622256
Monocytes	PET100	0.104854	5.786929	1.078346	0.283812	-5.80549	0.445008	0.553457
Monocytes	RBM6	0.084314	7.184868	1.078287	0.283839	-6.04235	0.427983	0.532343
Monocytes	OLFR1369-PS1	0.69643	-0.76388	1.078181	0.283886	-4.71971	0.535455	0.664039
Monocytes	4930557J02RIK	-0.55629	1.834427	-1.07814	0.283902	-4.82256	0.497369	0.617819
Monocytes	CD86	0.131646	6.31281	1.077909	0.284006	-6.30842	0.438543	0.545515
Monocytes	EIF1B	0.094715	5.828759	1.077897	0.284012	-5.83601	0.444515	0.552917
Monocytes	PHB	-0.17122	4.493205	-1.07765	0.284123	-5.47033	0.461576	0.573963
Monocytes	ZSCAN18	0.607333	0.363679	1.077427	0.284221	-4.75301	0.518786	0.643828
Monocytes	4931406C07RIK	-0.21546	3.540099	-1.07734	0.284261	-5.26779	0.474194	0.58942
Monocytes	RGS7BP	-0.58609	1.883105	-1.07706	0.284382	-4.84501	0.496946	0.61734
Monocytes	CCDC163	0.343219	2.446867	1.077048	0.284389	-5.02926	0.489086	0.607738
Monocytes	NEURL4	-0.35216	2.445552	-1.07697	0.284422	-5.01352	0.489104	0.607816

Monocytes	GM42937	-0.44782	0.198102	-1.07685	0.284475	-4.87168	0.521286	0.647044
Monocytes	PGRMC1	-0.15334	4.647619	-1.07657	0.284603	-5.49282	0.459733	0.571879
Monocytes	POLDIP2	-0.16847	4.278307	-1.07651	0.284627	-5.43196	0.464527	0.577823
Monocytes	GPR18	0.187803	4.514903	1.076495	0.284635	-5.52385	0.461449	0.574033
Monocytes	ZC3H7A	-0.08326	6.883008	-1.0761	0.284809	-6.04707	0.432061	0.537499
Monocytes	FBXL17	-0.08892	7.560342	-1.07578	0.28495	-6.14899	0.424134	0.527524
Monocytes	NOSIP	0.11672	5.158105	1.075543	0.285058	-5.63879	0.453649	0.564121
Monocytes	RSPH1	-0.70065	1.855808	-1.07536	0.285141	-4.75307	0.497889	0.618483
Monocytes	KCNMB4	0.487064	1.763375	1.075301	0.285166	-4.83643	0.499194	0.620087
Monocytes	GM10552	-0.31067	1.54086	-1.07513	0.285242	-5.05794	0.502349	0.623954
Monocytes	ELP2	0.139875	4.144774	1.074944	0.285325	-5.46384	0.466752	0.580372
Monocytes	PDLIM4	-0.2973	2.040162	-1.07484	0.285371	-5.27164	0.495299	0.615393
Monocytes	FARSA	-0.1123	4.90554	-1.07475	0.285413	-5.64296	0.456885	0.568234
Monocytes	VIL1	-0.54995	0.018277	-1.07472	0.285424	-4.79243	0.524533	0.650951
Monocytes	ZFYVE21	0.284944	2.542215	1.074662	0.28545	-5.04979	0.488319	0.606863
Monocytes	GM15411	0.607897	0.212426	1.07445	0.285545	-4.69377	0.521646	0.647551
Monocytes	CEP55	-0.30697	3.678611	-1.07438	0.285574	-5.33719	0.472915	0.588065
Monocytes	KCTD5	0.185282	3.640452	1.074383	0.285574	-5.31438	0.473424	0.58869
Monocytes	SLC10A1	-0.31109	3.248903	-1.07426	0.28563	-5.12231	0.478692	0.595181
Monocytes	TFEB	0.127023	5.158966	1.074091	0.285704	-5.67619	0.45371	0.564387
Monocytes	COMMD2	-0.11704	4.927522	-1.07397	0.285757	-5.63414	0.456674	0.568085
Monocytes	RANBP1	-0.12433	7.239938	-1.07386	0.285807	-6.08873	0.428104	0.532725
Monocytes	POLR1A	-0.1306	4.926327	-1.07336	0.286029	-5.73841	0.456921	0.568307
Monocytes	PARG	0.100717	5.560263	1.073249	0.28608	-5.76033	0.448876	0.55837
Monocytes	PEX13	-0.11308	5.524698	-1.07321	0.286096	-5.78104	0.449323	0.558923
Monocytes	GM49336	0.115437	4.881295	1.073129	0.286134	-5.65979	0.457499	0.569056
Monocytes	ABRACL	0.099435	7.550325	1.073017	0.286183	-6.17107	0.42464	0.52836
Monocytes	GM36551	0.476857	-0.80034	1.072722	0.286315	-4.68316	0.537419	0.666588
Monocytes	BC051537	-0.58069	0.044835	-1.07168	0.28678	-4.75044	0.525414	0.651433
Monocytes	PRORP	0.170165	3.853039	1.071569	0.28683	-5.32746	0.471751	0.585994
Monocytes	4930430E12RIK	0.429067	0.409202	1.071452	0.286882	-4.96995	0.520018	0.644965
Monocytes	SOCS1	-0.26033	5.152296	-1.0712	0.286993	-5.53105	0.454934	0.56531
Monocytes	CD207	-0.54524	-0.64958	-1.07113	0.287025	-4.7963	0.53601	0.664272
Monocytes	CCNB1IP1	-0.36431	1.863098	-1.07076	0.28719	-4.88407	0.499306	0.619575
Monocytes	UTP4	-0.12504	5.102474	-1.07066	0.287235	-5.63619	0.455756	0.566171
Monocytes	MED31	-0.19601	3.491551	-1.07047	0.287323	-5.23912	0.476933	0.592136
Monocytes	LIN54	0.1281	6.14118	1.069848	0.2876	-5.92153	0.443059	0.550287
Monocytes	CDKL4	0.380758	1.561956	1.069727	0.287654	-4.98006	0.503992	0.625071
Monocytes	CCDC114	0.39513	1.271818	1.069725	0.287655	-4.83396	0.508153	0.630132
Monocytes	PARP10	0.249432	3.565506	1.069251	0.287867	-5.26568	0.476535	0.591255
Monocytes	CTNNBIP1	-0.1256	4.359655	-1.06881	0.288065	-5.73003	0.46608	0.578387
Monocytes	WDR13	0.201981	2.570105	1.068776	0.28808	-5.14608	0.490183	0.607918
Monocytes	TIMM8A1	-0.20229	4.226992	-1.06876	0.288087	-5.3769	0.467821	0.580526
Monocytes	TFPI	0.263592	3.468648	1.068733	0.288099	-5.10032	0.477913	0.592909
Monocytes	ENTR1	-0.13072	4.880508	-1.06856	0.288179	-5.64126	0.459366	0.570106
Monocytes	PI4K2B	-0.16575	3.903982	-1.06837	0.288263	-5.44189	0.472207	0.585892
Monocytes	PHF23	-0.1329	5.240241	-1.06805	0.288404	-5.74066	0.454945	0.564557

Monocytes	GM43713	0.396114	2.135076	1.067978	0.288438	-4.92133	0.496511	0.61551
Monocytes	C2CD2L	-0.20406	2.991873	-1.06781	0.288514	-5.27212	0.484687	0.601113
Monocytes	PTK7	-0.66197	1.073787	-1.06749	0.288655	-4.765	0.511819	0.634185
Monocytes	IFT74	0.347841	2.322027	1.067424	0.288686	-4.94718	0.494045	0.612558
Monocytes	5830432E09RIK	0.309147	1.494203	1.067318	0.288734	-5.05443	0.505756	0.626864
Monocytes	CHMP1A	0.117482	5.305048	1.06717	0.2888	-5.7924	0.45426	0.563845
Monocytes	DET1	-0.33368	2.186379	-1.06716	0.288806	-4.9787	0.495944	0.614966
Monocytes	SLC16A4	-0.3726	1.428027	-1.06616	0.289256	-4.86515	0.507381	0.628402
Monocytes	6530402F18RIK	0.423083	-0.17553	1.066093	0.289284	-4.85042	0.531015	0.657018
Monocytes	CCDC28A	0.284553	2.307492	1.065475	0.289562	-5.00661	0.495303	0.613456
Monocytes	GM46652	0.515184	-0.73309	1.065232	0.289671	-4.77438	0.539978	0.667647
Monocytes	SOX5	-0.35531	4.684841	-1.06522	0.289675	-5.28834	0.463248	0.574305
Monocytes	WASHC5	-0.15164	4.472587	-1.06504	0.289756	-5.54735	0.466073	0.577777
Monocytes	PLXNA2	-0.48062	2.31233	-1.06424	0.29012	-4.87245	0.49587	0.614016
Monocytes	CIAPIN1	0.143328	4.643928	1.063571	0.290419	-5.58933	0.464747	0.575628
Monocytes	STRAP	-0.10425	6.174275	-1.06313	0.29062	-5.909	0.445486	0.551715
Monocytes	JAG2	-0.28204	1.093215	-1.06268	0.290821	-5.11527	0.514279	0.635586
Monocytes	1110019D14RIK	0.315039	1.751896	1.061806	0.291215	-4.98685	0.505202	0.624351
Monocytes	QTRT2	-0.40529	1.344368	-1.06166	0.29128	-4.93586	0.511068	0.631507
Monocytes	HNRNPDL	-0.0713	7.857159	-1.06159	0.291314	-6.15896	0.425677	0.526849
Monocytes	2700081O15RIK	-0.3045	3.137169	-1.06156	0.291325	-4.98766	0.485808	0.600833
Monocytes	AMPD1	-0.23619	2.911186	-1.0614	0.291399	-5.31343	0.488915	0.60468
Monocytes	RAPH1	0.121107	5.055385	1.061362	0.291416	-5.84966	0.460296	0.56965
Monocytes	FBRS	-0.12325	4.965395	-1.06132	0.291436	-5.67849	0.461459	0.571079
Monocytes	INPP5A	0.131705	6.256163	1.061226	0.291477	-5.86444	0.445086	0.550921
Monocytes	ANXA10	0.501516	0.643116	1.061193	0.291492	-4.78529	0.521336	0.644042
Monocytes	BRDT	-0.46558	1.567286	-1.06065	0.291738	-4.80348	0.508197	0.627917
Monocytes	ARID1A	0.083339	6.658177	1.060405	0.291848	-5.97611	0.440517	0.545037
Monocytes	MED25	0.130013	4.496149	1.06022	0.291932	-5.52884	0.468021	0.578866
Monocytes	2310058D17RIK	0.168302	2.785149	1.060167	0.291956	-5.28165	0.491124	0.607095
Monocytes	C2CD5	0.139012	4.918041	1.059638	0.292195	-5.59781	0.462815	0.572227
Monocytes	GM10501	0.306985	2.138944	1.059508	0.292255	-4.9762	0.500524	0.618255
Monocytes	ARPC3	0.066562	8.451234	1.05934	0.29233	-6.32583	0.419453	0.518733
Monocytes	NIPSNAP3B	0.110855	5.573981	1.058731	0.292606	-5.81784	0.454806	0.562311
Monocytes	SUOX	-0.31118	1.361958	-1.05852	0.292704	-4.94307	0.512197	0.632302
Monocytes	FRG2F1	0.410105	0.702787	1.0583	0.292802	-4.80079	0.521952	0.644063
Monocytes	UBE2L6	-0.27164	4.64931	-1.05812	0.292883	-5.54716	0.46691	0.57711
Monocytes	0610040F04RIK	-0.39004	0.269406	-1.05799	0.29294	-4.84146	0.528417	0.65186
Monocytes	CRIPT	0.087587	6.218829	1.057923	0.292973	-5.91707	0.446833	0.552455
Monocytes	KLHL12	0.206817	4.259168	1.057809	0.293024	-5.3273	0.472056	0.583482
Monocytes	ANKRD28	0.141076	5.835458	1.057784	0.293036	-5.71257	0.451647	0.558431
Monocytes	KLHL22	0.278651	3.001151	1.057483	0.293172	-5.03737	0.489224	0.604345
Monocytes	SLC7A2	0.484189	2.094729	1.057386	0.293216	-5.03394	0.501913	0.619783
Monocytes	PDZRN3	-0.52879	0.932125	-1.05695	0.293413	-4.79125	0.518984	0.640346
Monocytes	MUL1	0.228631	2.950066	1.056611	0.293568	-5.18569	0.490285	0.605464
Monocytes	PTPN18	-0.0761	7.11924	-1.05657	0.293586	-6.14642	0.436198	0.539122
Monocytes	1700020L24RIK	0.396965	0.390371	1.056477	0.293629	-4.86466	0.527141	0.65011

Monocytes	ELAC2	-0.19451	2.817476	-1.05632	0.2937	-5.25907	0.492123	0.60776
Monocytes	BCL10	0.085807	6.452929	1.056296	0.293711	-5.98655	0.444375	0.549269
Monocytes	CYFIP2	-0.11107	6.947339	-1.0561	0.293801	-6.02595	0.438329	0.541816
Monocytes	ERP27	0.405861	1.896721	1.055995	0.293848	-4.86601	0.505142	0.623588
Monocytes	5730455P16RIK	0.233206	2.914853	1.055931	0.293877	-5.08513	0.490814	0.606191
Monocytes	KXD1	0.097094	5.765423	1.055814	0.29393	-5.92115	0.453042	0.559952
Monocytes	SEC31A	-0.08412	5.620105	-1.0554	0.294119	-5.8298	0.454994	0.562408
Monocytes	RAB32	-0.10117	4.344985	-1.05531	0.294157	-5.97781	0.471559	0.582774
Monocytes	ARHGEF15	-0.60504	0.580885	-1.05516	0.294225	-4.75371	0.524474	0.647138
Monocytes	ABCA7	0.130115	3.89815	1.055079	0.294264	-5.52932	0.477522	0.590126
Monocytes	ARL2BP	-0.14227	5.086344	-1.05505	0.294276	-5.64579	0.461848	0.570927
Monocytes	GOLGA2	-0.15651	3.814242	-1.05483	0.294378	-5.38248	0.478651	0.591552
Monocytes	LRWD1	0.147283	3.781422	1.054777	0.294401	-5.40658	0.479093	0.592106
Monocytes	ABL1	0.12809	5.653787	1.054742	0.294417	-5.76301	0.454565	0.562039
Monocytes	GM19466	-0.52544	0.661272	-1.05466	0.294454	-4.77546	0.523279	0.645797
Monocytes	ANKRD6	-0.45595	0.95646	-1.05463	0.294469	-4.86204	0.518913	0.640526
Monocytes	IL10	0.275297	2.999738	1.054447	0.294551	-5.44221	0.489824	0.605213
Monocytes	5-Mar	-0.11617	6.018808	-1.05408	0.294717	-5.9014	0.450169	0.55649
Monocytes	RASGRP1	0.236524	4.119019	1.054002	0.294754	-5.3751	0.474797	0.586731
Monocytes	FBXW5	0.1826	3.39907	1.053766	0.294861	-5.28985	0.484588	0.598705
Monocytes	GM16066	-0.32079	2.012511	-1.05358	0.294945	-4.94018	0.503937	0.622379
Monocytes	GM29994	-0.29817	1.150146	-1.05357	0.294951	-4.98498	0.516398	0.637477
Monocytes	MYL6	-0.07774	9.689609	-1.0535	0.294984	-6.52997	0.406603	0.502676
Monocytes	MYO3B	-0.39418	1.289051	-1.05326	0.29509	-4.87711	0.51439	0.635082
Monocytes	HPS5	0.118395	4.448069	1.053261	0.295091	-5.66937	0.470514	0.581657
Monocytes	GM17494	0.23407	2.389013	1.052899	0.295256	-5.0416	0.498821	0.61607
Monocytes	SLC5A10	-0.5405	-0.1778	-1.05264	0.295376	-4.77021	0.536541	0.661663
Monocytes	ADPRHL2	-0.18414	3.241714	-1.05253	0.295427	-5.19397	0.487009	0.601692
Monocytes	HYOU1	-0.13281	4.380576	-1.05252	0.295429	-5.58449	0.471646	0.58292
Monocytes	GM17655	0.408617	1.183959	1.052318	0.295521	-4.85234	0.516265	0.637251
Monocytes	PSMD6	-0.12043	5.199485	-1.05213	0.295607	-5.74154	0.461018	0.569995
Monocytes	FAM3A	-0.23061	2.785704	-1.05209	0.295626	-5.14067	0.493409	0.609612
Monocytes	PHLDB1	-0.23001	1.355652	-1.05177	0.295769	-5.2717	0.513938	0.634469
Monocytes	ERF	-0.21213	3.477368	-1.05161	0.295843	-5.37383	0.484028	0.598114
Monocytes	ZMYND11	0.091913	6.466516	1.051542	0.295875	-5.92435	0.445107	0.550359
Monocytes	ARHGEF1	0.08847	7.10928	1.051442	0.295921	-6.05452	0.437203	0.540599
Monocytes	DPY19L3	-0.34927	2.757574	-1.05128	0.295996	-4.96112	0.493955	0.610253
Monocytes	SON	-0.06747	7.67203	-1.05121	0.296026	-6.16774	0.430413	0.532231
Monocytes	METAP1	-0.10428	4.718677	-1.05107	0.296089	-5.65693	0.467423	0.57784
Monocytes	GM16740	-0.2909	2.544974	-1.05106	0.296095	-5.02354	0.49693	0.613872
Monocytes	CDA	-0.55573	0.894078	-1.05084	0.296195	-4.78516	0.520802	0.642794
Monocytes	SCYL3	0.150826	3.642548	1.050636	0.296289	-5.39517	0.481943	0.59565
Monocytes	SCAF8	-0.08307	6.711338	-1.05048	0.296358	-6.00908	0.442252	0.546922
Monocytes	SLC16A10	0.142943	7.024829	1.050282	0.296451	-6.15667	0.438405	0.542194
Monocytes	MCPT8	-1.13004	-0.5048	-1.05021	0.296485	-4.75566	0.542043	0.6685
Monocytes	TMEM42	-0.21545	2.617603	-1.05018	0.296495	-5.12222	0.496107	0.612958
Monocytes	1190005I06RIK	-0.41181	1.185776	-1.05007	0.296547	-4.81233	0.516623	0.637878

Monocytes	SEC63	-0.08057	7.09953	-1.04996	0.296596	-6.03636	0.437494	0.541116
Monocytes	HOMER2	-0.34746	0.275337	-1.04987	0.296639	-5.01379	0.530148	0.654278
Monocytes	PRKRIP1	-0.14915	3.964552	-1.04977	0.296683	-5.39708	0.477623	0.590516
Monocytes	DBR1	-0.21712	3.262038	-1.04946	0.296826	-5.19903	0.487214	0.602307
Monocytes	USP11	0.353531	2.611991	1.049412	0.296849	-4.9036	0.496234	0.613324
Monocytes	PEG13	0.337717	2.114243	1.049273	0.296912	-4.93323	0.503265	0.621936
Monocytes	HIST1H3E	-0.23935	3.610481	-1.04915	0.296968	-5.29124	0.482453	0.596607
Monocytes	USP18	0.30414	2.737474	1.049112	0.296986	-5.24002	0.494478	0.611276
Monocytes	ERCC2	0.309191	1.983619	1.049093	0.296995	-4.9551	0.505128	0.624226
Monocytes	COL5A1	-0.55431	0.493409	-1.04894	0.297063	-4.77643	0.526962	0.650642
Monocytes	SLC43A1	0.648248	1.089863	1.048719	0.297166	-4.71822	0.518161	0.640023
Monocytes	CPSF4L	0.44615	0.499907	1.048673	0.297187	-4.77469	0.52691	0.650598
Monocytes	TAF3	0.104403	5.653075	1.048575	0.297232	-5.80422	0.455636	0.563776
Monocytes	PDPK1	-0.14657	6.462559	-1.04848	0.297277	-5.92049	0.445445	0.551257
Monocytes	BBS4	0.333765	2.313596	1.047511	0.297719	-4.92509	0.501173	0.619207
Monocytes	CYTH3	0.134267	4.88921	1.047417	0.297762	-5.74217	0.466106	0.576392
Monocytes	GM17259	-0.43378	1.365713	-1.04731	0.297812	-4.85531	0.514806	0.63581
Monocytes	BIN3	0.11134	5.271025	1.046414	0.298222	-5.78914	0.461637	0.570732
Monocytes	ICA1L	0.28131	1.590667	1.046412	0.298223	-5.17526	0.512079	0.632273
Monocytes	SPAST	-0.11868	5.207046	-1.0462	0.298319	-5.66126	0.462483	0.571806
Monocytes	TACC1	0.072062	7.703473	1.046182	0.298329	-6.15657	0.43136	0.533416
Monocytes	RBM27	-0.08441	6.399999	-1.04597	0.298428	-5.95267	0.447384	0.553126
Monocytes	AHRR	-0.40225	-0.36283	-1.04586	0.298477	-4.85498	0.541401	0.667611
Monocytes	ZBTB45	0.207953	2.916254	1.045293	0.298737	-5.20082	0.493599	0.609693
Monocytes	NDUFB11	-0.08097	7.55899	-1.04509	0.298829	-6.18678	0.433392	0.535832
Monocytes	GM8066	0.541225	0.498967	1.044958	0.298891	-4.73243	0.528565	0.652173
Monocytes	UBXN2B	0.211964	2.842475	1.044913	0.298912	-5.2223	0.494628	0.611045
Monocytes	GM4788	-0.50347	1.237901	-1.04485	0.298943	-4.8328	0.517597	0.638923
Monocytes	CRLF3	0.125248	6.899396	1.044785	0.298971	-6.00562	0.441422	0.545795
Monocytes	RBM14	-0.14864	3.712533	-1.04471	0.299005	-5.40509	0.482643	0.596464
Monocytes	3110001I22RIK	-0.21441	2.677347	-1.04467	0.299024	-5.15855	0.496939	0.613893
Monocytes	NOTCH3	0.523066	1.028947	1.044605	0.299053	-4.80701	0.520673	0.64268
Monocytes	LRRC61	-0.29202	2.011331	-1.04447	0.299116	-5.04198	0.506389	0.625394
Monocytes	RSPO3	-0.77285	0.723734	-1.04432	0.299186	-4.79081	0.525208	0.648238
Monocytes	VPS11	0.14186	4.072729	1.044289	0.299198	-5.46844	0.477781	0.590593
Monocytes	DHX29	0.196145	3.415036	1.044045	0.299311	-5.25085	0.486786	0.601503
Monocytes	TTLL4	0.19104	3.000367	1.043919	0.299369	-5.27502	0.492511	0.608483
Monocytes	UIMC1	0.092548	5.853468	1.043879	0.299387	-5.88848	0.454576	0.56203
Monocytes	TRIP12	-0.07393	7.435654	-1.04364	0.299499	-6.1592	0.434994	0.537812
Monocytes	ZFP715	-0.18731	3.662783	-1.04352	0.299553	-5.25378	0.483444	0.597371
Monocytes	PDE4B	0.106791	8.527906	1.043487	0.299568	-6.39286	0.422004	0.52171
Monocytes	MTMR10	-0.21243	2.897646	-1.04335	0.29963	-5.38108	0.493986	0.610226
Monocytes	DNAJB1	-0.13308	6.228259	-1.04317	0.299714	-5.91281	0.449881	0.556196
Monocytes	VTA1	-0.09291	5.707573	-1.04315	0.299721	-5.85371	0.456477	0.564318
Monocytes	RAVER2	-0.41213	1.423822	-1.0431	0.299745	-4.86205	0.51501	0.635753
Monocytes	GM13391	0.374395	0.765079	1.042903	0.299836	-4.92868	0.524784	0.647537
Monocytes	CENPL	0.21362	3.393274	1.042761	0.299902	-5.24795	0.487187	0.601956

Monocytes	KEAP1	-0.11013	5.115335	-1.04255	0.300001	-5.69698	0.464166	0.573854
Monocytes	NAP1L4	-0.08135	6.249563	-1.04254	0.300002	-5.90216	0.449666	0.556016
Monocytes	TMEM143	0.357499	1.491704	1.042442	0.300049	-4.87523	0.514081	0.634744
Monocytes	COASY	-0.21988	3.053875	-1.04241	0.300064	-5.24609	0.491871	0.607762
Monocytes	LARP1	-0.08842	6.798018	-1.04165	0.300415	-6.02906	0.443285	0.547909
Monocytes	HADHB	-0.11314	5.576702	-1.0415	0.300482	-5.76546	0.458699	0.566889
Monocytes	TFCP2	-0.3321	1.971586	-1.04132	0.300565	-4.93721	0.507703	0.626786
Monocytes	GM28375	-0.18681	3.206287	-1.04128	0.300585	-5.21621	0.490301	0.605627
Monocytes	MTA2	0.114824	5.782517	1.040926	0.300748	-5.81608	0.456253	0.563822
Monocytes	GM34225	-0.70731	-0.06364	-1.04056	0.300916	-4.6916	0.538243	0.663473
Monocytes	ANKRD11	-0.07925	9.391983	-1.04053	0.300933	-6.41956	0.412821	0.510027
Monocytes	DEPDC1A	-0.33491	3.001084	-1.04043	0.300978	-5.2539	0.493481	0.609369
Monocytes	SMPD2	0.324301	2.04317	1.040254	0.301058	-4.98146	0.507071	0.62587
Monocytes	TRIOBP	0.114639	4.877202	1.040116	0.301122	-5.64447	0.468156	0.578462
Monocytes	ATP2B4	0.179591	4.169834	1.040054	0.301151	-5.67033	0.47755	0.58997
Monocytes	D030028A08Rll	-0.19841	2.795422	-1.03963	0.301345	-5.40141	0.496647	0.613186
Monocytes	ARHGEF4	0.644064	0.759185	1.039473	0.301419	-4.81596	0.526163	0.648966
Monocytes	EP300	0.085257	6.696726	1.039274	0.301511	-6.0088	0.445249	0.550179
Monocytes	GM30239	-0.54276	-0.09316	-1.03893	0.301671	-4.7026	0.539343	0.664599
Monocytes	ZNRD1AS	0.506674	0.28937	1.038127	0.302042	-4.78053	0.53408	0.657862
Monocytes	2610021A01Rik	-0.33006	2.362444	-1.03771	0.302234	-4.93159	0.503634	0.621036
Monocytes	EIF1A	-0.10819	5.711772	-1.03765	0.302261	-5.91729	0.458356	0.565762
Monocytes	SERTAD2	-0.11882	6.12155	-1.03764	0.302267	-5.99608	0.453135	0.559344
Monocytes	GTF2H1	-0.10135	5.958143	-1.03756	0.302303	-5.88425	0.455209	0.561895
Monocytes	PPP1R15B	-0.10918	5.553021	-1.0375	0.302332	-5.8829	0.460397	0.568307
Monocytes	SATB1	-0.18024	7.143818	-1.03747	0.302344	-5.86909	0.440398	0.543687
Monocytes	KMT5A	0.088627	5.846575	1.037175	0.302483	-5.9342	0.456769	0.5638
Monocytes	MAP4K2	-0.16955	5.348887	-1.03687	0.302624	-5.55859	0.46325	0.571736
Monocytes	R3HCC1	-0.33394	2.353768	-1.03687	0.302626	-5.00887	0.50399	0.621434
Monocytes	DAGLB	0.122298	4.366228	1.036638	0.302731	-5.78023	0.47624	0.587608
Monocytes	CIT	-0.25306	4.461996	-1.0366	0.302747	-5.55657	0.47496	0.586044
Monocytes	DDB2	0.24571	3.43077	1.036191	0.302939	-5.25995	0.489176	0.603311
Monocytes	SLC15A2	-0.62712	4.597147	-1.03607	0.302995	-5.68591	0.473401	0.584065
Monocytes	ZHX3	-0.17614	4.441922	-1.03563	0.3032	-5.46525	0.475715	0.586789
Monocytes	PARP14	0.208905	5.480462	1.035444	0.303285	-5.84192	0.462116	0.570067
Monocytes	GM4356	-0.44979	0.868166	-1.03522	0.30339	-4.88129	0.526317	0.648111
Monocytes	HOGA1	-0.31264	2.370342	-1.03512	0.303434	-5.07248	0.504396	0.621607
Monocytes	NOL6	0.248901	2.582305	1.035104	0.303443	-5.10475	0.501384	0.617954
Monocytes	EZH2	-0.13858	6.663574	-1.03494	0.303519	-5.98581	0.447106	0.55163
Monocytes	NACA	-0.07177	9.080227	-1.03491	0.303535	-6.42942	0.418088	0.515757
Monocytes	OSGIN2	0.162952	3.18431	1.034468	0.303739	-5.53287	0.493049	0.607866
Monocytes	TRIM30A	0.171862	6.169574	1.034324	0.303805	-6.00011	0.453416	0.559433
Monocytes	GM49164	-0.45299	0.920748	-1.03422	0.303854	-4.83976	0.525652	0.647414
Monocytes	ZFP580	0.331446	2.166288	1.034196	0.303865	-4.96284	0.50743	0.625385
Monocytes	YAF2	-0.09354	6.056106	-1.03412	0.303903	-5.84165	0.454856	0.56124
Monocytes	GAB3	0.155503	4.746655	1.034096	0.303912	-5.68057	0.471847	0.582078
Monocytes	GTF2A1	-0.1111	5.65525	-1.03404	0.303937	-5.86747	0.459984	0.567538

Monocytes	INPP5B	-0.14782	4.216748	-1.03386	0.30402	-5.52939	0.478924	0.590807
Monocytes	ANKRD52	0.155729	3.881446	1.033834	0.304033	-5.4334	0.483462	0.596347
Monocytes	HIST4H4	-0.35472	1.887782	-1.03365	0.304118	-5.00699	0.511464	0.630461
Monocytes	ACP6	-0.21956	3.140192	-1.03361	0.304139	-5.21334	0.493682	0.608893
Monocytes	IGKV1-35	-0.62755	-0.53717	-1.03337	0.304251	-4.69594	0.547962	0.674513
Monocytes	TSKU	-0.53558	-0.3765	-1.03334	0.304262	-4.7823	0.545462	0.671514
Monocytes	ZFC3H1	-0.08161	6.914629	-1.03311	0.304368	-6.09294	0.444232	0.548301
Monocytes	SNX7	0.337887	1.212304	1.032653	0.304583	-4.9908	0.521519	0.642726
Monocytes	NKTR	-0.07701	7.193099	-1.03251	0.30465	-6.08288	0.440823	0.544186
Monocytes	MCM6	-0.16423	6.155584	-1.03247	0.304668	-5.88901	0.453763	0.560133
Monocytes	TCF20	0.103527	7.561094	1.032459	0.304673	-6.09376	0.436333	0.53864
Monocytes	CENPQ	-0.15895	5.314475	-1.03233	0.304733	-5.64074	0.464567	0.573435
Monocytes	TMEM39B	-0.17267	4.225197	-1.03225	0.304771	-5.4354	0.478988	0.591113
Monocytes	GPANK1	0.190129	3.300008	1.0322	0.304794	-5.26373	0.491627	0.606532
Monocytes	PIGO	-0.35186	1.804475	-1.03213	0.304827	-4.96065	0.512843	0.632294
Monocytes	TMEM70	-0.16138	3.930301	-1.03209	0.304844	-5.47963	0.482977	0.595986
Monocytes	ORAI3	0.15254	3.868765	1.031986	0.304893	-5.54918	0.483814	0.597032
Monocytes	SLC30A4	-0.29262	1.941057	-1.03196	0.304908	-5.01656	0.510864	0.629923
Monocytes	ERO1LB	0.091553	6.652932	1.031778	0.304991	-6.07431	0.44756	0.552544
Monocytes	CCR9	-0.27348	2.410766	-1.03138	0.305175	-5.21579	0.504408	0.621967
Monocytes	PNP2	-0.55481	0.616927	-1.03078	0.305454	-4.78793	0.531072	0.653998
Monocytes	PPP1CB	-0.09878	7.803763	-1.03072	0.305483	-6.15749	0.433948	0.535467
Monocytes	BRD4	0.05585	8.256086	1.030593	0.305543	-6.27155	0.428552	0.528793
Monocytes	ATPSCKMT	0.183642	3.937455	1.030474	0.305598	-5.44667	0.483521	0.596363
Monocytes	GM34095	-0.66669	-0.39051	-1.0296	0.306007	-4.70075	0.547136	0.672836
Monocytes	PIP5K1A	0.1153	5.595581	1.029553	0.306028	-5.78449	0.462042	0.569665
Monocytes	RIMS3	-0.41807	1.085548	-1.02923	0.30618	-4.94575	0.524812	0.645894
Monocytes	TWF1	0.095983	5.464598	1.02911	0.306235	-5.83193	0.463869	0.57181
Monocytes	SEC61B	0.076764	8.272683	1.028987	0.306292	-6.33826	0.428955	0.528848
Monocytes	GDF15	-0.46058	1.508239	-1.02896	0.306305	-5.04933	0.518561	0.638392
Monocytes	GM24362	0.43011	0.694716	1.028842	0.30636	-4.83927	0.530678	0.653028
Monocytes	FAM135A	0.257006	2.016455	1.027865	0.306817	-5.19726	0.511849	0.629925
Monocytes	F8A	0.254873	2.551871	1.027441	0.307016	-5.12242	0.504408	0.620879
Monocytes	NDUFAF5	0.244903	2.563951	1.026982	0.307231	-5.12533	0.50451	0.620843
Monocytes	CHD9	0.125066	6.065045	1.026285	0.307557	-5.86609	0.457651	0.563446
Monocytes	MFSD6	-0.14569	4.864614	-1.02592	0.307727	-5.85203	0.473484	0.58266
Monocytes	WDR60	-0.47498	0.883885	-1.02574	0.307811	-4.82346	0.529841	0.650988
Monocytes	TRIP6	0.339272	0.834471	1.025251	0.308041	-4.90079	0.530899	0.652001
Monocytes	LRCH4	0.137603	4.260162	1.024988	0.308165	-5.61108	0.481992	0.592841
Monocytes	DQX1	-0.78182	1.215444	-1.02495	0.308181	-4.83715	0.525258	0.645235
Monocytes	ZFP808	-0.36283	1.973033	-1.02486	0.308224	-4.87552	0.514108	0.631832
Monocytes	ITGB1BP2	-0.48891	0.43994	-1.02455	0.30837	-4.77317	0.537117	0.659519
Monocytes	MSH6	-0.17018	4.38755	-1.0244	0.308439	-5.52129	0.480456	0.591021
Monocytes	UBE2G2	0.115685	4.661055	1.024258	0.308508	-5.64673	0.47681	0.586599
Monocytes	GM49417	0.636336	1.078235	1.024084	0.30859	-4.79031	0.527604	0.648128
Monocytes	1500011B03RIK	0.149495	4.65787	1.023901	0.308675	-5.65855	0.476964	0.586791
Monocytes	CYP2A22	-0.48138	1.632221	-1.02357	0.308832	-4.87822	0.519625	0.638531

Monocytes	GFM2	0.194514	3.590998	1.023303	0.308956	-5.2811	0.491658	0.604747
Monocytes	GM16552	0.359214	1.517	1.023277	0.308969	-4.88412	0.521324	0.640657
Monocytes	MCOLN1	0.20926	2.728161	1.023268	0.308973	-5.17229	0.503768	0.619441
Monocytes	GM46224	0.162948	2.389468	1.022992	0.309102	-5.93175	0.508746	0.625334
Monocytes	ZFP949	0.307779	2.594224	1.022431	0.309366	-4.99219	0.506165	0.621951
Monocytes	UQCRB	-0.08694	8.047525	-1.02219	0.309482	-6.2995	0.434563	0.534361
Monocytes	KDM1A	-0.08917	5.813944	-1.02191	0.309612	-5.86248	0.462587	0.56867
Monocytes	NDUFAF3	0.144112	4.160988	1.021644	0.309737	-5.49721	0.48463	0.595519
Monocytes	COA7	-0.18404	3.404389	-1.02158	0.309769	-5.22126	0.495063	0.608238
Monocytes	TUBGCP3	-0.12747	3.88521	-1.02115	0.309969	-5.47724	0.488643	0.600314
Monocytes	CAPN11	-0.5935	1.046105	-1.01951	0.310743	-4.94983	0.530593	0.650296
Monocytes	ACAP1	-0.29231	3.923819	-1.01947	0.310759	-5.09082	0.48916	0.600283
Monocytes	INVS	0.265935	2.809082	1.019434	0.310778	-5.12873	0.504772	0.619195
Monocytes	NCKAP5L	-0.2244	4.041979	-1.01923	0.310875	-5.30429	0.487613	0.598426
Monocytes	TAF1C	0.29551	2.170342	1.018765	0.311094	-4.98499	0.514328	0.630526
Monocytes	ZFP58	0.429894	1.194809	1.018554	0.311194	-4.8426	0.528747	0.647858
Monocytes	SLC8A1	0.138542	6.330185	1.018345	0.311293	-6.39332	0.457588	0.56164
Monocytes	CCDC25	0.134409	4.712121	1.018251	0.311337	-5.56608	0.478792	0.587519
Monocytes	1700010I14RIK	0.424343	1.215796	1.018224	0.31135	-4.8117	0.528432	0.647533
Monocytes	DNAJC15	0.102522	5.723768	1.018206	0.311358	-5.92625	0.465409	0.571203
Monocytes	EGFL7	-0.31359	3.872106	-1.01808	0.311419	-5.17999	0.49023	0.60144
Monocytes	IQCK	-0.55449	0.158994	-1.01803	0.31144	-4.75198	0.544526	0.666847
Monocytes	CCDC85C	-0.39871	0.977037	-1.0175	0.311691	-4.85476	0.532366	0.652167
Monocytes	TMEM175	0.214841	3.375009	1.017403	0.311738	-5.27376	0.497463	0.610123
Monocytes	GM45267	0.250417	0.245242	1.017003	0.311927	-5.2489	0.54379	0.665798
Monocytes	ID3	-0.12507	6.599763	-1.01664	0.312097	-6.11813	0.454816	0.558058
Monocytes	PSMA7	0.080565	7.683799	1.016569	0.312132	-6.29135	0.441294	0.541469
Monocytes	ERMAP	0.592657	0.529243	1.016271	0.312273	-4.76956	0.539704	0.660731
Monocytes	MICU1	-0.09484	5.891562	-1.01611	0.312348	-5.86234	0.463983	0.56917
Monocytes	S1PR1	-0.30915	4.061514	-1.01603	0.312388	-5.16013	0.488418	0.598919
Monocytes	PPAT	-0.18233	4.107428	-1.01595	0.312425	-5.39538	0.487788	0.598154
Monocytes	BOLA3	-0.13355	5.736487	-1.01594	0.312429	-5.7802	0.466	0.571633
Monocytes	A730036I17RIK	0.550272	-0.20546	1.015826	0.312484	-4.82216	0.551105	0.67439
Monocytes	NEK8	-0.29074	2.113602	-1.01565	0.312569	-5.02901	0.516076	0.63235
Monocytes	EIF4ENIF1	-0.1032	5.556503	-1.01517	0.312795	-5.78338	0.468684	0.574758
Monocytes	GM42067	-0.43653	0.558955	-1.01454	0.313094	-4.79775	0.540062	0.660644
Monocytes	WRN	0.10783	5.831312	1.014247	0.313233	-5.84835	0.465601	0.570643
Monocytes	ELOVL5	-0.10759	6.195778	-1.01359	0.313543	-6.00387	0.461268	0.565157
Monocytes	SLC23A2	0.126085	5.113974	1.01334	0.313663	-5.76677	0.475542	0.582514
Monocytes	EFCAB14	-0.1449	4.713606	-1.01325	0.313704	-5.59853	0.480913	0.589078
Monocytes	MTG1	0.287954	2.186733	1.012912	0.313866	-5.1012	0.516588	0.632083
Monocytes	PDCD2L	-0.10322	4.660225	-1.01271	0.313964	-5.66475	0.481885	0.590099
Monocytes	RSRP1	-0.09871	6.653226	-1.01257	0.314029	-6.03346	0.455755	0.558264
Monocytes	USP7	-0.08032	6.215443	-1.01249	0.314068	-5.94152	0.461358	0.565118
Monocytes	CYP3A44	-0.46621	2.308322	-1.01238	0.314121	-5.02359	0.514915	0.630116
Monocytes	LSM1	-0.10308	5.251939	-1.01218	0.314214	-5.73647	0.474038	0.580639
Monocytes	PON2	0.105447	6.020273	1.011719	0.314433	-5.91365	0.464205	0.568443

Monocytes	CCL17	-0.38112	-0.70076	-1.01146	0.314556	-5.08393	0.561333	0.685308
Monocytes	NT5DC3	-0.11541	5.099601	-1.01116	0.314701	-5.77338	0.476569	0.583335
Monocytes	SNHG5	-0.29575	2.416156	-1.01107	0.31474	-5.05703	0.513963	0.628583
Monocytes	YTHDF3	0.076508	7.017555	1.010766	0.314886	-6.15133	0.451804	0.553075
Monocytes	GM41335	-0.70449	0.493808	-1.0106	0.314967	-4.74848	0.542859	0.663211
Monocytes	SMC5	0.104275	5.613071	1.010484	0.315021	-5.89048	0.469873	0.575211
Monocytes	VRK1	-0.09682	5.309256	-1.01034	0.315088	-5.90995	0.473887	0.58014
Monocytes	PRDX2	-0.11166	7.774205	-1.01033	0.315093	-6.26793	0.442394	0.541665
Monocytes	DHX34	0.261956	2.203437	1.010083	0.315212	-5.08607	0.517184	0.632656
Monocytes	RAB7	-0.06685	7.931545	-1.00999	0.315255	-6.29418	0.440465	0.539439
Monocytes	MTIF3	-0.22856	3.10156	-1.00987	0.315312	-5.21911	0.50423	0.617067
Monocytes	GM42595	-0.4134	0.917394	-1.00982	0.315337	-4.85865	0.536371	0.655701
Monocytes	SORCS2	-0.44934	3.3545	-1.00982	0.315337	-4.94441	0.500647	0.61274
Monocytes	SIRT2	-0.08922	5.688086	-1.0098	0.315347	-5.9223	0.468888	0.574204
Monocytes	ZER1	0.208477	3.262662	1.009099	0.315681	-5.16814	0.502398	0.61468
Monocytes	CHST10	0.4425	0.375693	1.008852	0.315799	-4.84972	0.545296	0.666172
Monocytes	WASL	-0.09397	5.483088	-1.00869	0.315874	-5.75349	0.472155	0.578014
Monocytes	FBXO31	0.223764	3.549697	1.008578	0.315929	-5.22767	0.498513	0.610054
Monocytes	SIK3	-0.09482	10.09847	-1.00844	0.315996	-6.6332	0.415384	0.508439
Monocytes	FNBP1L	-0.20558	3.079155	-1.00826	0.316079	-5.47129	0.505254	0.618158
Monocytes	AI837181	0.178971	3.127875	1.008074	0.31617	-5.2304	0.504627	0.61733
Monocytes	NLRC3	-0.37745	1.662225	-1.00765	0.316374	-4.88856	0.526227	0.64303
Monocytes	APBB2	-0.3974	4.02877	-1.00741	0.316488	-5.14318	0.492326	0.602104
Monocytes	NRXN1	-0.58943	2.142242	-1.00692	0.316721	-4.8843	0.519434	0.634715
Monocytes	RAB8B	-0.08918	7.724426	-1.00683	0.316765	-6.42739	0.444165	0.543392
Monocytes	6720489N17RII	-0.3863	0.964398	-1.00676	0.316796	-4.86514	0.537057	0.655871
Monocytes	UQCRC2	-0.07845	6.345631	-1.00675	0.316805	-6.00874	0.461553	0.56467
Monocytes	BORCS8	-0.12745	4.590375	-1.00656	0.316893	-5.63785	0.484864	0.592949
Monocytes	PRKAB2	-0.14706	3.392055	-1.0064	0.316968	-5.58465	0.50153	0.613059
Monocytes	NUDT4	-0.11288	5.949353	-1.00613	0.317098	-5.81942	0.466907	0.570938
Monocytes	ENY2	0.098835	5.972518	1.005753	0.317279	-5.90616	0.466661	0.570733
Monocytes	TEX14	-0.13866	5.64791	-1.0057	0.317305	-5.96106	0.470917	0.575927
Monocytes	LEF1OS1	-0.5603	-0.45613	-1.00558	0.317364	-4.71043	0.559506	0.682474
Monocytes	ZFP780B	0.227727	3.329247	1.00557	0.317366	-5.21786	0.502606	0.614332
Monocytes	GM13547	0.579498	0.514665	1.005545	0.317379	-4.82349	0.544274	0.664332
Monocytes	LIG1	-0.18971	5.051661	-1.00542	0.31744	-5.68864	0.478863	0.585587
Monocytes	OLFR164	0.615127	-0.16043	1.005251	0.317519	-4.72059	0.554835	0.676963
Monocytes	SMAD4	0.078355	6.23224	1.005146	0.31757	-5.99922	0.463301	0.566736
Monocytes	ORC4	0.102051	5.046376	1.005117	0.317583	-5.71733	0.478934	0.58576
Monocytes	BTBD1	-0.07817	6.497876	-1.00493	0.317671	-6.12097	0.459934	0.56259
Monocytes	EFHC1	-0.49408	-0.05796	-1.00477	0.317749	-4.77983	0.553339	0.675126
Monocytes	IL2RG	0.206644	6.294179	1.004356	0.317948	-5.91543	0.462791	0.565831
Monocytes	SPTBN5	0.463793	-0.00378	1.004293	0.317978	-4.84697	0.552717	0.674185
Monocytes	VEZT	-0.15002	4.178132	-1.00412	0.31806	-5.44672	0.491113	0.600211
Monocytes	DDX21	-0.10427	6.399043	-1.00399	0.318124	-6.01026	0.461509	0.564242
Monocytes	THA1	-0.52418	0.09883	-1.00373	0.318248	-4.79606	0.551321	0.672455
Monocytes	CINP	-0.243	3.163035	-1.00337	0.318419	-5.26782	0.505635	0.617679

Monocytes	ATP5G3	-0.09553	8.015505	-1.00335	0.318432	-6.28073	0.441429	0.539635
Monocytes	LTBR	0.13796	2.974023	1.003228	0.318489	-5.62366	0.50835	0.620943
Monocytes	GPC6	-0.569	1.763476	-1.00293	0.318633	-4.9501	0.526203	0.64232
Monocytes	HELLS	-0.21588	4.919254	-1.00255	0.318813	-5.62321	0.481555	0.588436
Monocytes	SRXN1	0.262977	1.094676	1.002539	0.318819	-5.14268	0.536419	0.654493
Monocytes	TXN2	-0.08647	6.315861	-1.00218	0.31899	-6.01074	0.463273	0.56612
Monocytes	RAD51C	0.246467	2.245463	1.001738	0.319204	-5.1444	0.519518	0.634201
Monocytes	A230072E10RIK	0.579808	0.278064	1.001696	0.319224	-4.7615	0.549315	0.669849
Monocytes	AHCTF1	-0.11427	5.405997	-1.0017	0.319224	-5.77352	0.475302	0.580813
Monocytes	CSRP2	0.139296	5.20682	1.00165	0.319246	-5.7236	0.477962	0.584058
Monocytes	IRAK1	-0.09302	5.224747	-1.00082	0.319645	-5.86011	0.478231	0.584016
Monocytes	MED15	0.095258	5.758733	1.000713	0.319697	-5.95192	0.471134	0.575406
Monocytes	TM7SF2	-0.33564	1.754706	-1.0006	0.319749	-4.95324	0.527343	0.643249
Monocytes	BTBD9	-0.08187	8.508871	-1.00054	0.319782	-6.35944	0.436408	0.533026
Monocytes	GNASAS1	0.513763	0.682179	0.999803	0.320134	-4.78477	0.54414	0.662958
Monocytes	RNF216	0.074851	6.677461	0.99944	0.320309	-6.10133	0.459815	0.561238
Monocytes	ZFP316	0.475785	0.380007	0.998911	0.320564	-4.84548	0.549366	0.668854
Monocytes	MTTP	-0.33666	2.566189	-0.99879	0.320622	-5.0141	0.516372	0.629427
Monocytes	PLAGL1	-0.32555	1.041672	-0.99875	0.320643	-5.1354	0.539144	0.656676
Monocytes	STX7	0.074763	6.176182	0.998332	0.320843	-6.06094	0.466785	0.569402
Monocytes	SLC14A1	-0.23819	3.335927	-0.99815	0.32093	-5.2367	0.505575	0.616292
Monocytes	GM5431	0.433707	0.742737	0.997614	0.321189	-4.98756	0.544413	0.662546
Monocytes	NME4	-0.29921	2.364464	-0.9967	0.321631	-5.16678	0.520606	0.633615
Monocytes	LIPE	-0.11881	3.783042	-0.99651	0.32172	-5.68036	0.50022	0.609149
Monocytes	COL1A1	0.459415	2.255466	0.996449	0.321752	-5.01036	0.522249	0.6356
Monocytes	SMCR8	-0.18759	3.28105	-0.99634	0.321802	-5.36716	0.507346	0.617743
Monocytes	NR3C1	-0.107	7.440275	-0.99588	0.322026	-6.14918	0.451656	0.550315
Monocytes	LRRC40	-0.17876	3.249731	-0.99582	0.322056	-5.35568	0.507904	0.618405
Monocytes	PRSS30	-0.30004	-0.33737	-0.99582	0.322056	-4.97236	0.562234	0.683225
Monocytes	GM10053	0.337205	1.424678	0.995794	0.322068	-5.04303	0.534793	0.650601
Monocytes	CSF3	0.642088	-0.096	0.995147	0.322381	-4.85781	0.558844	0.678757
Monocytes	REEP5	0.07109	7.566136	0.99473	0.322582	-6.25542	0.450573	0.54856
Monocytes	ABCC4	0.101666	4.55376	0.994682	0.322605	-5.84894	0.490144	0.596552
Monocytes	ALKBH7	-0.19818	3.373231	-0.99465	0.322619	-5.36013	0.506696	0.616491
Monocytes	CDKL1	-0.58538	1.100722	-0.99444	0.322724	-4.81761	0.54036	0.656804
Monocytes	DTD2	0.205899	3.491192	0.994402	0.322741	-5.34573	0.505051	0.614563
Monocytes	ZFP251	0.316733	2.407099	0.99386	0.323003	-4.9878	0.521061	0.633536
Monocytes	ERBB3	-0.489	1.113728	-0.99379	0.323038	-4.84557	0.540494	0.656745
Monocytes	DCP1B	0.226035	2.830948	0.9936	0.323129	-5.20247	0.514927	0.626247
Monocytes	CLK1	0.080315	7.55289	0.993068	0.323387	-6.17502	0.451338	0.54922
Monocytes	RCC1	-0.17643	3.989305	-0.99306	0.323392	-5.40974	0.498644	0.6065
Monocytes	RAD9B	-0.2054	3.275507	-0.99295	0.323446	-5.28633	0.508776	0.618718
Monocytes	IGBP1	0.114237	4.749526	0.992692	0.323569	-5.74295	0.488224	0.593892
Monocytes	TCF19	-0.22896	3.199871	-0.99238	0.323722	-5.38467	0.510141	0.620144
Monocytes	UBE2W	0.06233	6.081677	0.992041	0.323885	-5.99194	0.47066	0.572476
Monocytes	EXTL2	0.290757	2.165568	0.991865	0.32397	-5.10204	0.525505	0.638453
Monocytes	NDUFB10	0.082632	6.731387	0.991267	0.32426	-6.12017	0.462565	0.562517

Monocytes	GEN1	-0.37287	2.372171	-0.99121	0.324287	-5.03177	0.522796	0.635105
Monocytes	ZHX2	-0.15068	5.614006	-0.99067	0.324548	-5.79759	0.477542	0.580568
Monocytes	RRP12	0.217509	2.60063	0.990541	0.324613	-5.21975	0.519796	0.631416
Monocytes	AZIN1	-0.10035	6.940521	-0.99043	0.324668	-6.11393	0.460207	0.559594
Monocytes	CDIPT	0.113368	5.093616	0.990318	0.324721	-5.79096	0.484592	0.589178
Monocytes	PANX1	0.158895	4.208683	0.989987	0.324882	-5.40871	0.496952	0.603966
Monocytes	SPPL2A	0.107396	6.600738	0.989747	0.324999	-6.15448	0.464804	0.565149
Monocytes	U2AF1L4	0.227423	3.047054	0.989709	0.325017	-5.28177	0.513536	0.623922
Monocytes	MED23	-0.15157	3.692708	-0.98943	0.325154	-5.40924	0.504411	0.612861
Monocytes	COPS7A	0.140321	4.237724	0.989277	0.325228	-5.58283	0.496773	0.603663
Monocytes	CETN4	-0.32659	-0.08195	-0.98905	0.325338	-4.97856	0.561435	0.680781
Monocytes	THUMPD1	-0.13763	4.425949	-0.98891	0.325405	-5.53991	0.49427	0.600617
Monocytes	REST	0.106975	5.400614	0.988748	0.325485	-5.86022	0.480939	0.584578
Monocytes	RUBCNL	0.169395	3.191015	0.988711	0.325503	-5.52714	0.511753	0.62166
Monocytes	SCO1	0.323014	1.930574	0.988339	0.325684	-5.0055	0.530517	0.643956
Monocytes	GPR155	0.273344	3.05644	0.988033	0.325833	-5.16054	0.513906	0.624238
Monocytes	CDH17	-0.41443	0.13748	-0.98799	0.325852	-4.91148	0.558196	0.676992
Monocytes	KBTBD4	-0.23702	2.78551	-0.98799	0.325855	-5.14437	0.517851	0.628961
Monocytes	TENM4	0.453991	1.760128	0.987592	0.326047	-4.9475	0.533083	0.647154
Monocytes	AP2M1	-0.09216	6.941455	-0.98759	0.32605	-6.0967	0.460864	0.560252
Monocytes	ABHD17C	-0.1376	4.833665	-0.98756	0.326063	-5.64513	0.488842	0.594113
Monocytes	CBR3	-0.30727	0.624626	-0.98745	0.326116	-5.15851	0.550524	0.667897
Monocytes	TRAF3	-0.11218	6.856964	-0.98742	0.32613	-6.12205	0.46195	0.561572
Monocytes	HCLS1	0.090089	6.691521	0.987356	0.326162	-6.14702	0.464086	0.564185
Monocytes	MYH13	-0.50364	-1.61994	-0.98733	0.326178	-4.70725	0.586847	0.710839
Monocytes	GEMIN8	-0.30335	2.284507	-0.9867	0.326483	-4.98221	0.525515	0.637896
Monocytes	TNS4	0.377293	-0.27421	0.986687	0.326488	-4.96759	0.565074	0.684887
Monocytes	KHNYN	0.147747	4.263958	0.986661	0.326502	-5.48057	0.496993	0.603713
Monocytes	PRKAR1A	-0.06181	7.625008	-0.98636	0.32665	-6.19835	0.452467	0.549866
Monocytes	C530005A16RII	0.407134	0.742343	0.986336	0.32666	-4.82092	0.549035	0.665927
Monocytes	GM13610	0.470638	0.396658	0.986293	0.326681	-4.80008	0.554451	0.672371
Monocytes	C330011M18RI	-0.42195	0.592367	-0.98613	0.326763	-4.8191	0.551432	0.668847
Monocytes	PPARG	0.145746	2.579221	0.985866	0.326889	-5.87462	0.52135	0.633001
Monocytes	ZFP770	-0.32966	1.760027	-0.9858	0.326919	-4.98702	0.533569	0.647618
Monocytes	SP140	-0.11669	6.188606	-0.9856	0.327018	-6.07002	0.471112	0.572591
Monocytes	DPF1	-0.4855	0.292792	-0.98548	0.327079	-4.879	0.556289	0.674661
Monocytes	UBE2Z	-0.08934	5.437928	-0.98545	0.327094	-5.86612	0.481108	0.584706
Monocytes	AP2A1	0.147061	4.542647	0.985053	0.327286	-5.59483	0.493554	0.599636
Monocytes	GM45902	-0.30943	1.585805	-0.98492	0.327353	-4.99077	0.536514	0.651092
Monocytes	WWC1	-0.36779	1.216205	-0.98479	0.327413	-4.8911	0.542179	0.657847
Monocytes	PPM1H	0.112326	6.473739	0.984683	0.327467	-6.27139	0.467626	0.568371
Monocytes	ETV1	0.372242	1.377482	0.984261	0.327673	-4.9585	0.539926	0.655041
Monocytes	TUBB3	-0.40765	0.736309	-0.98422	0.327695	-4.8722	0.549835	0.66682
Monocytes	ZFP672	0.145252	4.004158	0.983949	0.327826	-5.457	0.501356	0.608994
Monocytes	A930001A20RII	-0.62286	-0.71301	-0.98394	0.327831	-4.7348	0.572998	0.694299
Monocytes	LRRC43	0.598192	-0.12202	0.983836	0.327881	-4.75241	0.56344	0.683071
Monocytes	MOCOS	0.284114	1.324581	0.983764	0.327916	-5.25523	0.540766	0.656188

Monocytes	MRPL11	0.137274	4.26095	0.983604	0.327994	-5.56794	0.497749	0.604735
Monocytes	PPDPF	0.098104	5.630234	0.983321	0.328133	-5.83397	0.478995	0.582183
Monocytes	GBP2B	-1.02677	0.300434	-0.98329	0.328147	-4.92999	0.556714	0.675214
Monocytes	RNF103	-0.14014	4.305165	-0.98327	0.328156	-5.55041	0.497131	0.60406
Monocytes	PIM3	0.137669	4.641176	0.983169	0.328207	-5.77731	0.49246	0.59848
Monocytes	GM43774	0.249027	2.238278	0.983081	0.32825	-5.20601	0.526958	0.639908
Monocytes	ARHGAP6	-0.18908	3.609197	-0.98307	0.328257	-5.6202	0.506961	0.615962
Monocytes	DEPDC7	0.213953	1.678465	0.982936	0.328321	-5.42936	0.535395	0.650014
Monocytes	PLPP2	-0.19628	1.875681	-0.98268	0.328448	-5.31652	0.532467	0.646489
Monocytes	CASC4	-0.37124	2.64714	-0.98267	0.328453	-4.90067	0.520979	0.632767
Monocytes	GLO1	-0.13824	5.029475	-0.98253	0.328521	-5.71451	0.487222	0.592235
Monocytes	PCDH7	0.637689	0.520175	0.98224	0.328662	-4.84376	0.553514	0.671487
Monocytes	RAB6A	0.07893	6.637549	0.982051	0.328754	-6.08158	0.465986	0.566432
Monocytes	CACTIN	0.172563	3.991663	0.981449	0.329049	-5.42101	0.502212	0.610043
Monocytes	PGP	0.12332	5.374059	0.980919	0.329309	-5.81329	0.483347	0.587233
Monocytes	AP4S1	-0.10502	4.920297	-0.9809	0.329319	-5.73331	0.489534	0.594698
Monocytes	SUPT3	-0.11245	5.480744	-0.98069	0.329423	-5.90289	0.481955	0.585491
Monocytes	PARK7	-0.08909	6.806683	-0.98063	0.329452	-6.11045	0.464429	0.564272
Monocytes	FLAD1	0.224016	2.957237	0.980316	0.329605	-5.2373	0.517548	0.628254
Monocytes	IQCE	0.303588	2.144332	0.979919	0.3298	-5.05125	0.529697	0.642768
Monocytes	CFH	0.139723	4.930751	0.979874	0.329822	-6.03143	0.489711	0.594815
Monocytes	PCX	-0.33339	2.70013	-0.97985	0.329832	-5.08461	0.521442	0.632917
Monocytes	BTD	0.297101	2.37707	0.979628	0.329943	-5.04776	0.52632	0.638763
Monocytes	CCR5	0.153468	4.693422	0.979498	0.330006	-5.85777	0.493095	0.59889
Monocytes	DESI2	0.072022	6.03278	0.979394	0.330058	-5.93314	0.474941	0.576987
Monocytes	SLFN9	0.311686	2.410472	0.979237	0.330135	-5.15389	0.525892	0.638244
Monocytes	GM28707	-0.44709	1.154193	-0.97905	0.330225	-4.86122	0.544998	0.660982
Monocytes	HEMGN	0.714	0.35427	0.978904	0.330298	-4.77038	0.557553	0.675873
Monocytes	WWTR1	-0.44202	2.126919	-0.97842	0.330535	-4.93057	0.530366	0.643476
Monocytes	SOCS4	0.117173	4.684801	0.978378	0.330557	-5.64348	0.493482	0.599269
Monocytes	EMC4	-0.12289	4.771124	-0.97834	0.330573	-5.73124	0.492288	0.597831
Monocytes	TRPM2	-0.17685	2.540831	-0.97832	0.330583	-5.68566	0.524197	0.636111
Monocytes	FIS1	0.080915	7.530663	0.977829	0.330827	-6.21952	0.455891	0.553741
Monocytes	PGM3	0.184513	2.89728	0.977822	0.33083	-5.21398	0.519092	0.629969
Monocytes	ELP4	0.124945	4.963596	0.977796	0.330843	-5.71438	0.489774	0.594765
Monocytes	ALDH3B1	0.130281	3.605843	0.977733	0.330874	-5.7165	0.508825	0.617683
Monocytes	POLR2I	0.10018	5.116859	0.977459	0.331009	-5.77424	0.487709	0.592314
Monocytes	TNFAIP2	0.151931	4.351498	0.977427	0.331025	-6.03777	0.498299	0.605069
Monocytes	AFTPH	-0.07897	6.658529	-0.97738	0.331048	-6.12829	0.467132	0.567449
Monocytes	RAD54L	0.259477	2.724058	0.977229	0.331122	-5.16526	0.521714	0.633204
Monocytes	STAB1	0.303733	3.176342	0.977027	0.331221	-5.2774	0.515172	0.625287
Monocytes	P2RX3	0.501768	2.219391	0.976701	0.331382	-4.88542	0.529283	0.642238
Monocytes	ZFP595	0.234065	1.84184	0.976689	0.331388	-5.03193	0.534967	0.649015
Monocytes	SF3B3	-0.08252	6.034328	-0.97665	0.331409	-5.94501	0.475448	0.577544
Monocytes	BCL2L11	0.1334	7.053365	0.976549	0.331457	-6.17657	0.462121	0.561397
Monocytes	SUFU	0.124806	5.025085	0.976515	0.331474	-5.63977	0.489073	0.594013
Monocytes	HCK	0.121325	5.693071	0.976426	0.331518	-6.11744	0.480007	0.58309

Monocytes	PSMD14	-0.07304	6.618882	-0.97629	0.331582	-6.09018	0.467772	0.568284
Monocytes	FBRSL1	-0.08426	5.766788	-0.97586	0.331794	-5.93971	0.479274	0.582116
Monocytes	NAE1	0.13651	3.958187	0.975638	0.331906	-5.53369	0.504289	0.612249
Monocytes	ACLY	0.078199	6.383195	0.975523	0.331963	-6.02401	0.471154	0.572284
Monocytes	UFSP2	0.107252	4.93604	0.975429	0.332009	-5.84309	0.490625	0.595812
Monocytes	PAM16	0.114011	4.987535	0.97527	0.332087	-5.70831	0.489917	0.594987
Monocytes	AGO4	-0.18571	3.167589	-0.97527	0.332088	-5.41613	0.515645	0.625902
Monocytes	HTRA2	-0.11264	3.681601	-0.97505	0.332196	-5.60569	0.50832	0.617055
Monocytes	FGFR1	0.281028	1.974297	0.974772	0.332333	-5.38625	0.533447	0.647157
Monocytes	PPIL6	-0.46576	0.219778	-0.97472	0.33236	-4.834	0.560661	0.679484
Monocytes	SNHG15	-0.16262	3.481407	-0.9744	0.332516	-5.51395	0.511223	0.620728
Monocytes	UBL4A	-0.1662	4.202976	-0.97435	0.332541	-5.53055	0.500946	0.608387
Monocytes	HNRNPH3	0.082612	5.447268	0.974253	0.332589	-5.81019	0.483758	0.587696
Monocytes	NAB1	0.084506	6.555305	0.974195	0.332618	-6.14792	0.469005	0.569856
Monocytes	FZD7	-0.28348	0.743156	-0.97416	0.332636	-5.07993	0.552388	0.669884
Monocytes	BVHT	0.55032	-0.0458	0.974073	0.332678	-4.80205	0.56491	0.684771
Monocytes	PROM1	-0.67542	0.403745	-0.97401	0.332709	-4.76108	0.557738	0.67628
Monocytes	GM9993	0.364367	1.16551	0.973945	0.332741	-4.95396	0.545809	0.662142
Monocytes	GBE1	0.127915	6.483019	0.973847	0.332789	-5.97427	0.469952	0.571119
Monocytes	GM26549	-0.1795	3.018851	-0.9738	0.332812	-5.35929	0.517934	0.628952
Monocytes	HIST2H2AA1	0.367641	2.225914	0.973488	0.332967	-5.01728	0.529743	0.643167
Monocytes	NDUFAB1	-0.11197	6.268706	-0.97347	0.332975	-5.98917	0.472842	0.574723
Monocytes	ARRDC4	0.183685	2.427989	0.973371	0.333024	-5.4772	0.526725	0.639606
Monocytes	NNT	0.303906	2.482711	0.97321	0.333104	-4.95672	0.525911	0.638718
Monocytes	ZCCHC7	0.124523	6.744533	0.973158	0.33313	-6.09487	0.466606	0.567288
Monocytes	EPB41L1	-0.36752	1.011227	-0.97305	0.333185	-5.04193	0.548284	0.665404
Monocytes	1700110K17RIK	-0.65545	-0.57203	-0.973	0.33321	-4.73596	0.573517	0.695312
Monocytes	GNS	0.097019	6.931874	0.972721	0.333346	-6.16193	0.464296	0.56442
Monocytes	ARID4A	-0.08518	7.062741	-0.97262	0.333396	-6.19827	0.462607	0.562373
Monocytes	TENT2	-0.07049	6.624422	-0.97247	0.33347	-6.14563	0.468301	0.569293
Monocytes	PRIMPOL	0.187745	3.486578	0.97239	0.333509	-5.35866	0.511366	0.621261
Monocytes	TEDC1	-0.33432	1.701406	-0.97231	0.333551	-4.9677	0.537812	0.652914
Monocytes	MFSD14A	-0.07369	6.156841	-0.97217	0.33362	-6.03821	0.474481	0.576816
Monocytes	NHEJ1	-0.27572	3.867688	-0.97171	0.333845	-5.43218	0.506128	0.614801
Monocytes	RNF167	-0.12005	5.334087	-0.97166	0.333872	-5.79869	0.485712	0.59021
Monocytes	ADGRE1	-0.18178	4.711233	-0.97148	0.333957	-6.00679	0.49427	0.600552
Monocytes	VTI1B	0.07389	5.690918	0.971414	0.333992	-5.93889	0.480884	0.584418
Monocytes	CLASP1	-0.08897	6.373608	-0.97114	0.334129	-6.01465	0.471796	0.573497
Monocytes	NIP7	-0.10733	4.635807	-0.97107	0.334161	-5.65783	0.495318	0.601926
Monocytes	PCK2	0.210026	3.244845	0.971026	0.334184	-5.34527	0.515087	0.625685
Monocytes	ASGR2	-0.45762	1.698173	-0.97098	0.334206	-4.9432	0.538096	0.65319
Monocytes	CHRAC1	0.091278	5.264928	0.970972	0.334211	-5.85724	0.486654	0.591478
Monocytes	CEP57	0.126156	5.139583	0.970826	0.334283	-5.65436	0.488367	0.593618
Monocytes	CRACR2A	0.321693	2.835916	0.970752	0.33432	-5.08591	0.521063	0.632947
Monocytes	SBNO1	-0.07878	7.359347	-0.97065	0.334371	-6.15774	0.459012	0.558098
Monocytes	RUFY1	0.093664	5.607628	0.970407	0.334491	-5.88757	0.482006	0.586098
Monocytes	RNF215	0.173706	2.627986	0.970394	0.334497	-5.28531	0.524131	0.636775

Monocytes	DTX3	-0.2663	2.572046	-0.97023	0.334579	-5.09362	0.52496	0.637791
Monocytes	GM47644	0.389871	1.060844	0.97021	0.334589	-5.01333	0.547903	0.665156
Monocytes	PQBP1	-0.13116	4.6495	-0.9702	0.334592	-5.61265	0.495127	0.601965
Monocytes	ELDR	-0.21786	3.256101	-0.96993	0.334729	-5.54836	0.515008	0.625775
Monocytes	GINS1	0.255239	3.197409	0.969806	0.334789	-5.29289	0.515861	0.626798
Monocytes	BBS5	0.293231	0.624034	0.969789	0.334797	-5.15976	0.554829	0.673283
Monocytes	CCDC189	0.547756	0.183787	0.96944	0.33497	-4.7979	0.562004	0.681743
Monocytes	GM20721	0.197968	3.72758	0.969357	0.335012	-5.31433	0.508387	0.617836
Monocytes	RIOK3	-0.08125	7.578172	-0.9692	0.335091	-6.25906	0.456464	0.555079
Monocytes	TRP53BP1	-0.1687	3.888271	-0.96915	0.335115	-5.42334	0.506098	0.615129
Monocytes	TOR2A	0.157509	3.670287	0.96887	0.335253	-5.40676	0.509347	0.618968
Monocytes	UBE2S	-0.11534	7.624597	-0.96871	0.335333	-6.22069	0.456036	0.554509
Monocytes	BC017158	-0.37412	1.449949	-0.96771	0.33583	-4.94803	0.543064	0.658909
Monocytes	MTPN	0.064313	6.898113	0.967639	0.335864	-6.16859	0.465949	0.566236
Monocytes	TRP53COR1	0.468461	1.008827	0.96745	0.335958	-4.92454	0.54997	0.667116
Monocytes	TRIP11	0.092468	6.069875	0.967122	0.336121	-5.95908	0.476985	0.579599
Monocytes	FDXACB1	0.23366	2.215747	0.96708	0.336142	-5.10155	0.531577	0.64524
Monocytes	ADGRF5	-0.42553	2.598167	-0.96704	0.336164	-5.0245	0.525863	0.638414
Monocytes	HIRA	-0.11841	5.652742	-0.96691	0.336225	-5.77726	0.482597	0.586382
Monocytes	GM26535	-0.33709	-0.60061	-0.9668	0.336283	-4.99845	0.575839	0.697737
Monocytes	INSL6	-0.34896	1.68935	-0.96625	0.336556	-5.028	0.539888	0.654857
Monocytes	BC005537	0.074039	7.116434	0.966182	0.336588	-6.23544	0.463549	0.563057
Monocytes	FCMR	0.426741	2.105124	0.966116	0.336622	-4.89196	0.533573	0.647351
Monocytes	VANGL2	-0.36212	2.225601	-0.96572	0.336817	-4.90875	0.531988	0.645317
Monocytes	ST3GAL2	0.204176	3.085043	0.965411	0.336972	-5.31879	0.519339	0.630215
Monocytes	CEP83	-0.09308	5.662564	-0.96538	0.336987	-5.92293	0.483057	0.586584
Monocytes	ZFAND3	0.068906	8.233036	0.965176	0.337089	-6.37972	0.449733	0.546156
Monocytes	MAF	-0.1783	5.654779	-0.96509	0.337132	-5.89317	0.483225	0.586794
Monocytes	SLC6A13	-0.28309	2.762131	-0.96499	0.337181	-5.15032	0.524161	0.636022
Monocytes	PNPLA8	0.097121	6.018439	0.964572	0.33739	-5.95704	0.478436	0.581001
Monocytes	RRAS	0.131983	4.338868	0.964561	0.337395	-5.87075	0.501496	0.608813
Monocytes	UMAD1	-0.11198	5.658219	-0.96441	0.337473	-5.85217	0.483281	0.586884
Monocytes	DIPK1A	0.119575	5.446127	0.964359	0.337496	-5.99391	0.486158	0.59036
Monocytes	DECR1	-0.22655	3.696206	-0.96435	0.337502	-5.39571	0.510645	0.619829
Monocytes	GCAT	0.252445	2.390839	0.964238	0.337557	-5.20094	0.529807	0.642754
Monocytes	ARCN1	0.088691	5.873425	0.963973	0.337689	-5.96295	0.480447	0.583473
Monocytes	RAB3A	-0.32042	2.619056	-0.96395	0.337699	-5.02601	0.526468	0.63879
Monocytes	GM43768	0.420196	0.70894	0.963652	0.337849	-4.90002	0.555862	0.673688
Monocytes	PCSK5	0.48737	0.558557	0.963589	0.33788	-4.86968	0.55824	0.676509
Monocytes	ILF2	0.103919	5.763945	0.963167	0.338091	-5.88076	0.482228	0.585368
Monocytes	MAT2A	-0.07866	6.522913	-0.96312	0.338115	-6.06731	0.472111	0.573129
Monocytes	BARD1	0.23302	3.42386	0.962988	0.33818	-5.33824	0.515001	0.624774
Monocytes	NCOR2	0.096622	4.956649	0.962277	0.338535	-5.86451	0.493646	0.598831
Monocytes	LRP10	0.091999	6.125795	0.962241	0.338553	-6.00805	0.477751	0.57966
Monocytes	ADSS	0.081335	6.271707	0.96212	0.338613	-6.03443	0.475808	0.577343
Monocytes	GM48236	-0.31403	0.1418	-0.96208	0.338632	-5.12726	0.56556	0.684674
Monocytes	CD59A	-0.28821	2.984691	-0.96184	0.338753	-5.10071	0.521876	0.632777

Monocytes	NUDT3	-0.09412	5.213023	-0.96179	0.338778	-5.7986	0.490177	0.594723
Monocytes	GM20300	-0.26467	1.343674	-0.96144	0.338954	-5.05481	0.546864	0.662493
Monocytes	ZFP710	-0.10547	6.297621	-0.96134	0.339001	-6.13291	0.4757	0.577226
Monocytes	RBBP9	0.422227	1.214794	0.961179	0.339083	-4.89933	0.548866	0.66495
Monocytes	GM9887	-0.31177	1.686181	-0.96114	0.339101	-5.04011	0.541583	0.656306
Monocytes	INTS8	-0.12216	4.693172	-0.96094	0.339202	-5.61078	0.497601	0.603651
Monocytes	NDUFV1	0.108191	5.20278	0.960757	0.339295	-5.81753	0.490539	0.595174
Monocytes	GM43388	-0.63061	0.386691	-0.9607	0.339321	-4.76831	0.561968	0.680503
Monocytes	MFSD13A	0.258501	2.008416	0.960683	0.339332	-5.16584	0.536714	0.650521
Monocytes	POLR1E	-0.28169	2.067812	-0.96003	0.339659	-5.09214	0.536056	0.649685
Monocytes	ZFP113	-0.48396	1.151185	-0.96	0.339672	-4.82148	0.550155	0.666451
Monocytes	INO80E	-0.10647	4.165534	-0.95999	0.339676	-5.57702	0.505261	0.612853
Monocytes	PLXNB3	-0.44896	0.164762	-0.95988	0.339733	-4.83813	0.56578	0.685019
Monocytes	LRMP	-0.10845	6.432878	-0.95979	0.339776	-5.99145	0.474165	0.57542
Monocytes	GM49463	-0.49385	-0.05016	-0.95977	0.339786	-4.84193	0.569247	0.689124
Monocytes	POSTN	-0.46167	1.260435	-0.95933	0.34001	-4.91475	0.548735	0.664673
Monocytes	ACOX1	-0.11932	5.892871	-0.95913	0.340107	-5.9333	0.481684	0.5843
Monocytes	AHNAK	0.132385	6.416793	0.958464	0.340443	-6.36275	0.475084	0.576074
Monocytes	GNAI3	0.072521	6.443605	0.958096	0.340627	-6.04963	0.474757	0.575772
Monocytes	SRCAP	-0.07525	6.652894	-0.95805	0.340649	-6.05507	0.471993	0.572427
Monocytes	DERL2	0.103082	4.925658	0.958037	0.340656	-5.80402	0.495355	0.600626
Monocytes	CBR4	-0.23644	2.335594	-0.95802	0.340664	-5.11801	0.532836	0.645515
Monocytes	YLPM1	-0.10175	5.660547	-0.95789	0.340732	-5.86714	0.485287	0.588488
Monocytes	SORT1	0.119876	4.067523	0.957741	0.340805	-5.89849	0.507501	0.615227
Monocytes	TCF25	0.06476	6.939299	0.957566	0.340893	-6.16185	0.468347	0.567999
Monocytes	GM45716	-0.20032	3.20355	-0.95732	0.341017	-5.31432	0.520124	0.630316
Monocytes	KCTD17	0.353834	0.958849	0.95728	0.341036	-5.05702	0.554219	0.670909
Monocytes	DIPK2A	-0.14712	4.112992	-0.9571	0.341127	-5.51729	0.507032	0.614601
Monocytes	RFC4	-0.18162	4.603054	-0.95661	0.34137	-5.63863	0.500225	0.606463
Monocytes	TLN2	-0.42249	1.298032	-0.95661	0.341371	-4.93553	0.549118	0.664864
Monocytes	TRIM68	-0.36141	0.946883	-0.95659	0.341385	-4.91088	0.554613	0.671382
Monocytes	GM29417	-0.47803	1.098886	-0.95653	0.341414	-4.77499	0.552227	0.668562
Monocytes	HASPIN	0.305087	2.299155	0.956239	0.341559	-5.05082	0.533914	0.646693
Monocytes	LNPK	0.155941	4.386002	0.956149	0.341604	-5.54163	0.503415	0.610233
Monocytes	STARD13	-0.50093	1.070842	-0.95596	0.341698	-4.90283	0.552843	0.669279
Monocytes	CHD4	-0.0769	7.633169	-0.95589	0.341733	-6.20815	0.459768	0.55758
Monocytes	VEGFC	-0.37319	1.200834	-0.95581	0.341775	-4.97609	0.550809	0.666908
Monocytes	STARD3NL	-0.07917	5.861943	-0.95565	0.341854	-6.14458	0.483054	0.585805
Monocytes	ZGRF1	-0.23641	3.956437	-0.95559	0.341884	-5.45332	0.509576	0.617744
Monocytes	NCOA3	-0.06903	6.960263	-0.95541	0.341973	-6.16384	0.468523	0.568246
Monocytes	TBC1D10A	-0.14066	3.56507	-0.9552	0.342079	-5.53238	0.515363	0.624717
Monocytes	RNASEL	0.208814	3.944163	0.954952	0.342206	-5.50848	0.510006	0.618208
Monocytes	FCF1	-0.08132	6.136138	-0.95478	0.342293	-6.00513	0.479656	0.581612
Monocytes	NLK	0.112734	5.966033	0.954422	0.342472	-5.92269	0.482124	0.584373
Monocytes	ELP3	-0.2393	2.716115	-0.9543	0.342534	-5.11932	0.528243	0.639758
Monocytes	AK3	-0.13628	4.328071	-0.9537	0.342837	-5.56714	0.505164	0.611854
Monocytes	GM13708	0.165554	3.088737	0.953365	0.343005	-5.51599	0.523275	0.633391

Monocytes	RALY	-0.08487	6.422986	-0.95327	0.343051	-6.06198	0.476534	0.57725
Monocytes	IST1	0.0862	5.536178	0.953121	0.343127	-5.94747	0.488531	0.591679
Monocytes	IL23A	0.545857	-0.31658	0.953	0.343188	-4.84249	0.576346	0.696253
Monocytes	TMEM242	0.119789	4.292008	0.952679	0.34335	-5.60064	0.506007	0.612603
Monocytes	LRRC58	-0.07927	5.945766	-0.95265	0.343363	-6.03169	0.483085	0.58504
Monocytes	CYTH4	0.082296	5.757599	0.95207	0.343657	-6.10803	0.485952	0.588175
Monocytes	H2-D1	-0.14041	8.452787	-0.95201	0.34369	-6.42808	0.45082	0.545685
Monocytes	AW112010	-0.2713	7.279978	-0.95188	0.343754	-6.22562	0.465761	0.56382
Monocytes	GM17387	-0.44384	0.498836	-0.95175	0.343819	-4.85596	0.563678	0.680922
Monocytes	GM50232	0.380092	1.372215	0.951591	0.343899	-4.97184	0.549929	0.664656
Monocytes	ARFGAP3	-0.15607	3.55174	-0.9514	0.343994	-5.48773	0.517125	0.625585
Monocytes	MST1	-0.50133	0.649079	-0.95121	0.344092	-4.88038	0.561371	0.67822
Monocytes	CSF2RA	0.081914	5.196056	0.951069	0.344162	-6.19995	0.493776	0.597662
Monocytes	DNAH17	-0.34052	3.275665	-0.95099	0.344201	-5.25405	0.521164	0.630485
Monocytes	PARN	0.157373	3.875182	0.950781	0.344307	-5.44127	0.512437	0.620143
Monocytes	LCORL	-0.08909	6.985276	-0.95068	0.344356	-6.16993	0.469697	0.568745
Monocytes	GM7072	0.150498	3.842908	0.950639	0.344379	-5.56619	0.512903	0.620739
Monocytes	5430416N02RII	-0.15691	3.547711	-0.95051	0.344446	-5.3904	0.517184	0.625921
Monocytes	SLC7A1	-0.12612	5.141375	-0.95046	0.344471	-5.95726	0.494534	0.598778
Monocytes	ITGB5	0.165629	3.058168	0.950316	0.344542	-5.71024	0.524371	0.634562
Monocytes	F2RL2	-0.35724	0.129948	-0.95028	0.344562	-5.03481	0.569712	0.688368
Monocytes	ARL6IP1	-0.07971	7.879749	-0.95023	0.344584	-6.29522	0.458157	0.554882
Monocytes	CAMTA2	0.126312	3.696678	0.950141	0.344631	-5.6507	0.515019	0.623457
Monocytes	CD6	-0.35004	0.929158	-0.95005	0.344675	-5.03059	0.556927	0.673353
Monocytes	GM17036	0.346811	2.038724	0.950038	0.344683	-5.00362	0.539692	0.652905
Monocytes	CUL2	0.112448	4.968394	0.949851	0.344777	-5.79056	0.496981	0.60187
Monocytes	2900060B14RIK	0.277332	2.614995	0.949669	0.344869	-5.04494	0.531017	0.642697
Monocytes	CTNNBL1	0.101251	5.139211	0.94956	0.344924	-5.7785	0.494606	0.599147
Monocytes	SLX4IP	0.120387	4.853222	0.949509	0.34495	-5.74826	0.498589	0.60396
Monocytes	YPEL2	0.202763	4.572346	0.94948	0.344965	-5.47612	0.502536	0.6087
Monocytes	UBE2V2	0.092296	5.614121	0.949299	0.345056	-5.91404	0.488129	0.591358
Monocytes	ZFP141	0.258265	3.367308	0.948821	0.345298	-5.17313	0.520058	0.629612
Monocytes	NR4A1	-0.12836	7.413157	-0.94881	0.345306	-6.44138	0.464349	0.562549
Monocytes	KLHL4	-0.62467	0.018315	-0.94877	0.345322	-4.76017	0.571785	0.691034
Monocytes	ACOX3	0.099008	4.372027	0.948728	0.345345	-5.88613	0.505559	0.61225
Monocytes	SGTB	-0.43642	0.3418	-0.94853	0.345444	-4.8514	0.566631	0.684987
Monocytes	TUBG1	-0.24661	3.393021	-0.94831	0.345558	-5.32467	0.51985	0.629344
Monocytes	GNGT2	0.13531	5.675602	0.948141	0.345642	-6.04134	0.487619	0.590635
Monocytes	G0S2	0.205658	3.133285	0.947643	0.345894	-5.462	0.523952	0.634099
Monocytes	GM15232	-0.29615	1.187425	-0.94746	0.345984	-5.0968	0.553593	0.669394
Monocytes	WDR11	0.173814	3.124121	0.947442	0.345996	-5.35406	0.524088	0.634299
Monocytes	INTS9	0.134522	4.394064	0.947441	0.345997	-5.57176	0.505682	0.612273
Monocytes	ADCY3	-0.43189	3.001893	-0.94725	0.346095	-5.06919	0.525971	0.636482
Monocytes	FERMT3	0.081213	7.235899	0.94671	0.346367	-6.23717	0.467367	0.565914
Monocytes	KCNK10	0.590711	0.557275	0.946603	0.346421	-4.77651	0.563966	0.681482
Monocytes	ZFP512B	-0.32296	2.377698	-0.94658	0.346435	-5.04804	0.535616	0.647862
Monocytes	PTBP3	0.060986	8.46802	0.946191	0.34663	-6.37325	0.451834	0.546958

Monocytes	PSAP	-0.07697	9.249596	-0.94551	0.346974	-6.79122	0.442489	0.535444
Monocytes	1810041H14RII	-0.32005	1.76748	-0.9455	0.346982	-5.08296	0.545563	0.659394
Monocytes	ZFP639	0.134318	4.128657	0.945308	0.347078	-5.58758	0.510465	0.617518
Monocytes	AGRN	-0.41378	1.254784	-0.94506	0.347205	-4.94512	0.553728	0.669042
Monocytes	TRAP1	0.109072	4.381095	0.944972	0.347248	-5.63075	0.506957	0.613345
Monocytes	NOP2	-0.15675	3.635097	-0.94441	0.347534	-5.40967	0.518059	0.626409
Monocytes	GNA12	-0.12761	4.652134	-0.94405	0.347715	-5.6964	0.503574	0.609031
Monocytes	SNHG17	0.337552	1.368538	0.943961	0.347762	-4.92964	0.552451	0.667333
Monocytes	ZFP945	-0.22154	3.17689	-0.94387	0.347807	-5.19561	0.524917	0.634606
Monocytes	SWI5	-0.09205	7.120072	-0.94385	0.34782	-6.17838	0.469996	0.568635
Monocytes	SMPD1	0.238726	2.643295	0.94364	0.347925	-5.18686	0.532966	0.644121
Monocytes	MSRB3	-0.29704	2.604277	-0.94342	0.348037	-5.15151	0.533648	0.64487
Monocytes	DNAJC4	0.158467	3.061383	0.943318	0.348089	-5.45153	0.526808	0.636733
Monocytes	CLU	-0.32554	5.440247	-0.94302	0.348238	-5.90012	0.492871	0.596077
Monocytes	CNOT8	-0.09143	5.284943	-0.94291	0.348295	-5.79068	0.49502	0.598662
Monocytes	GM16845	0.275931	2.527304	0.942796	0.348354	-5.10049	0.53496	0.646416
Monocytes	SPTLC1	0.120352	4.232218	0.942695	0.348406	-5.65263	0.509863	0.616478
Monocytes	STX5A	0.079254	5.871144	0.942629	0.348439	-5.95122	0.486964	0.588986
Monocytes	GBA	-0.12768	4.090254	-0.94239	0.34856	-5.64192	0.512005	0.61906
Monocytes	NAT10	-0.20398	3.270157	-0.94209	0.348715	-5.28516	0.524123	0.633402
Monocytes	ZMIZ2	-0.1096	4.532649	-0.94182	0.348849	-5.76865	0.505951	0.611533
Monocytes	ZBED4	-0.13926	4.650997	-0.94155	0.348987	-5.53935	0.504334	0.609618
Monocytes	DDAH1	-0.30354	2.481818	-0.94151	0.349009	-5.05257	0.536109	0.647557
Monocytes	XXYLT1	-0.17869	3.591278	-0.94142	0.349057	-5.36679	0.519589	0.627898
Monocytes	HAUS6	-0.1501	4.556972	-0.94121	0.349164	-5.60055	0.505667	0.611288
Monocytes	GTF2F2	-0.08256	5.881638	-0.94119	0.34917	-5.99929	0.48724	0.589155
Monocytes	DUS4L	-0.28608	1.929292	-0.94114	0.349196	-5.04473	0.544551	0.657654
Monocytes	LGALS2	0.437448	-0.89826	0.940847	0.349347	-4.82889	0.590181	0.711445
Monocytes	FANCL	-0.10709	4.144904	-0.94047	0.349537	-5.74017	0.511643	0.61849
Monocytes	MGAM	-0.57198	0.078138	-0.94042	0.349565	-4.8036	0.574006	0.692526
Monocytes	PKNOX2	-0.62281	-0.82371	-0.94032	0.349615	-4.75028	0.588929	0.710069
Monocytes	GALNT6	0.225221	3.115592	0.940318	0.349616	-5.38047	0.526692	0.636463
Monocytes	GSTT1	-0.38115	2.240293	-0.94026	0.349648	-5.05909	0.539873	0.652149
Monocytes	ELANE	0.343211	2.340078	0.940227	0.349663	-5.9093	0.538352	0.650342
Monocytes	COG6	-0.21965	3.137898	-0.94021	0.349674	-5.21193	0.52636	0.636068
Monocytes	NOL8	0.162253	4.078757	0.940132	0.349711	-5.49752	0.512596	0.619651
Monocytes	NLRP1A	0.280938	0.449959	0.939921	0.349819	-5.24486	0.568005	0.68549
Monocytes	HDDC3	0.410892	0.928077	0.939893	0.349833	-4.88124	0.560348	0.676448
Monocytes	SUPT7L	0.232312	2.767637	0.939611	0.349977	-5.18081	0.531989	0.642813
Monocytes	CEP85	0.14034	4.470348	0.939605	0.34998	-5.58175	0.50708	0.613065
Monocytes	ZFP831	-0.3564	3.063289	-0.93917	0.350204	-5.05213	0.52783	0.637645
Monocytes	PIKFYVE	0.121717	4.426045	0.939038	0.35027	-5.70655	0.507985	0.613946
Monocytes	KLHDC1	0.276977	2.152219	0.938863	0.350359	-5.06569	0.541671	0.654151
Monocytes	TMX1	-0.09949	5.362	-0.93869	0.35045	-5.81812	0.494883	0.598316
Monocytes	BRWD1	-0.08528	6.550877	-0.93864	0.350474	-6.07255	0.478706	0.578836
Monocytes	C1QTNF6	0.430622	1.904338	0.938476	0.350557	-4.9568	0.545493	0.658743
Monocytes	IPP	0.264919	2.465742	0.938453	0.350568	-5.06835	0.536901	0.648539

Monocytes	BHMT2	-0.40826	2.060069	-0.93829	0.350654	-5.00851	0.543149	0.656012
Monocytes	GM47428	-0.38868	0.207827	-0.93812	0.35074	-4.97589	0.57245	0.690688
Monocytes	RIC1	0.107903	6.710291	0.937923	0.350839	-6.16207	0.47664	0.576436
Monocytes	RINL	-0.15599	3.632551	-0.93778	0.350914	-5.56282	0.519578	0.628017
Monocytes	MDK	-0.37776	1.436872	-0.93774	0.350931	-4.97492	0.552828	0.667564
Monocytes	SLC46A1	-0.55093	0.761766	-0.93772	0.350942	-4.86472	0.563514	0.680203
Monocytes	EXOC6B	-0.10509	5.502152	-0.93768	0.350966	-5.9876	0.493004	0.596169
Monocytes	PPARGC1B	0.249872	2.799015	0.936532	0.351551	-5.20426	0.532734	0.643311
Monocytes	FOCAD	-0.25011	2.908895	-0.93638	0.351629	-5.18259	0.531084	0.641375
Monocytes	E2F8	0.183097	3.686572	0.936347	0.351645	-5.55285	0.519568	0.627646
Monocytes	ACSL4	0.095734	6.189637	0.936236	0.351702	-6.03284	0.484351	0.585424
Monocytes	IGFBP3	-0.50177	0.690335	-0.93606	0.351792	-4.87077	0.565526	0.682292
Monocytes	MYO19	-0.38111	1.357151	-0.93601	0.35182	-4.94059	0.55493	0.669781
Monocytes	CHST2	-0.60893	-0.02925	-0.93574	0.351957	-4.77783	0.577208	0.696112
Monocytes	ANKRD61	0.419557	0.799732	0.935728	0.351962	-4.86358	0.563773	0.680273
Monocytes	DENND5A	0.077623	6.395889	0.935667	0.351994	-6.16733	0.481578	0.582191
Monocytes	BBS7	-0.52125	0.558111	-0.93561	0.352022	-4.84144	0.567653	0.684855
Monocytes	YBEY	0.307465	1.366271	0.935528	0.352065	-4.95037	0.554787	0.669666
Monocytes	GINM1	0.096615	4.990227	0.935381	0.35214	-5.78652	0.500897	0.605484
Monocytes	UNC93B1	-0.07489	7.659349	-0.93533	0.352167	-6.34778	0.464928	0.562142
Monocytes	EIF4EBP3	0.231681	2.80817	0.934781	0.352447	-5.25113	0.532951	0.643713
Monocytes	GM15738	-0.43619	0.402397	-0.9347	0.35249	-4.84297	0.570529	0.688275
Monocytes	ANKRA2	-0.1355	3.792457	-0.93456	0.35256	-5.49849	0.51837	0.626402
Monocytes	PNN	-0.0788	6.310373	-0.93447	0.352609	-6.03142	0.483037	0.584042
Monocytes	CCDC186	0.113601	4.760025	0.934396	0.352645	-5.7364	0.504462	0.609836
Monocytes	NDUFA9	-0.10914	4.840567	-0.934	0.352848	-5.74564	0.503541	0.608596
Monocytes	PLIN2	0.113161	5.831377	0.933781	0.35296	-6.18264	0.489842	0.592126
Monocytes	FAM71F2	-0.31322	2.260769	-0.93313	0.353292	-5.19796	0.541938	0.654224
Monocytes	MMACHC	-0.46195	0.470101	-0.93296	0.35338	-4.86927	0.570149	0.687664
Monocytes	SNRPD1	-0.08673	7.17842	-0.93295	0.353387	-6.21741	0.472078	0.570587
Monocytes	BC003965	0.168296	3.701969	0.932915	0.353404	-5.43096	0.520343	0.628568
Monocytes	ZFP740	0.107903	4.76726	0.93284	0.353443	-5.68548	0.504991	0.610203
Monocytes	MOGS	-0.1493	4.095452	-0.9327	0.353516	-5.60424	0.514625	0.621734
Monocytes	GARNL3	-0.29453	2.487215	-0.93263	0.353553	-5.08051	0.538493	0.650222
Monocytes	FRS2	0.084356	5.84525	0.932401	0.353669	-5.93659	0.490013	0.592243
Monocytes	CWC25	0.122582	5.255579	0.932381	0.353679	-5.87799	0.498168	0.602046
Monocytes	SHLD2	0.14925	3.853936	0.932196	0.353774	-5.55381	0.518182	0.626016
Monocytes	LRRC8D	0.083177	7.366735	0.931977	0.353886	-6.34235	0.469665	0.567795
Monocytes	GLTP	-0.06653	6.759436	-0.93179	0.353983	-6.25472	0.477676	0.577509
Monocytes	TMEM259	0.126466	4.879001	0.931771	0.353992	-5.79369	0.50347	0.608548
Monocytes	KLHL26	0.247333	2.727028	0.931751	0.354003	-5.15196	0.534908	0.646108
Monocytes	GM14471	0.532048	0.24931	0.931484	0.35414	-4.77059	0.573804	0.692166
Monocytes	ABRAXAS1	0.27353	2.604808	0.931403	0.354182	-5.11683	0.536758	0.648308
Monocytes	MYLPF	0.383406	2.109786	0.931288	0.354241	-5.02728	0.544321	0.657318
Monocytes	GHDC	-0.34445	1.628326	-0.93115	0.354312	-4.92522	0.551789	0.666201
Monocytes	TSC22D1	-0.12296	4.872446	-0.93113	0.354322	-5.85564	0.503562	0.608704
Monocytes	NPM1	-0.08368	8.143673	-0.93112	0.354329	-6.35538	0.459637	0.555722

Monocytes	TNFRSF25	-0.46313	-1.26707	-0.93109	0.35434	-4.7588	0.599116	0.721948
Monocytes	WASF2	0.059422	8.064634	0.931063	0.354356	-6.33551	0.460646	0.55695
Monocytes	FAM102B	0.118076	4.23575	0.930763	0.354511	-5.8382	0.512647	0.61965
Monocytes	CRMP1	-0.56656	-1.07546	-0.93075	0.354517	-4.75996	0.595852	0.718196
Monocytes	FAM193A	-0.08273	6.704133	-0.93072	0.354532	-6.12172	0.478413	0.57851
Monocytes	CYP2C29	0.524112	1.011219	0.930649	0.35457	-4.8848	0.561525	0.677823
Monocytes	CPNE5	-0.50911	0.481176	-0.93057	0.354609	-4.81807	0.570037	0.68792
Monocytes	SPACA9	0.251045	1.758151	0.93048	0.354656	-5.11676	0.549764	0.663994
Monocytes	AGAP3	-0.14599	4.127556	-0.93017	0.354817	-5.61694	0.514367	0.621698
Monocytes	TNFRSF26	0.397529	2.617995	0.929965	0.354921	-4.97751	0.536804	0.648412
Monocytes	GM43259	-0.43109	1.452797	-0.92986	0.354975	-4.94537	0.554799	0.669768
Monocytes	KIZ	0.164212	3.359508	0.929624	0.355097	-5.35837	0.525726	0.635178
Monocytes	PARP9	0.169784	5.150171	0.929595	0.355112	-5.85457	0.499919	0.604295
Monocytes	SPATA32	-0.37456	0.244996	-0.92947	0.355179	-5.02137	0.574177	0.692684
Monocytes	EDNRB	0.641417	2.175551	0.929268	0.35528	-5.08454	0.543596	0.656555
Monocytes	NUP37	-0.19091	3.351508	-0.92927	0.355281	-5.38641	0.525844	0.635425
Monocytes	NT5DC1	0.138794	4.5014	0.929193	0.355319	-5.61253	0.509103	0.615436
Monocytes	ZFP65	0.333643	1.662117	0.929119	0.355357	-5.03263	0.551552	0.666066
Monocytes	APOE	-0.19544	9.241961	-0.92903	0.355404	-6.5844	0.446108	0.539434
Monocytes	ZFYVE1	0.134811	4.797159	0.928807	0.355518	-5.6902	0.504907	0.610588
Monocytes	TCERG1	-0.07353	6.296507	-0.92871	0.355566	-6.05937	0.484154	0.585644
Monocytes	TMEM260	-0.18212	3.273577	-0.92864	0.355605	-5.40386	0.527015	0.637058
Monocytes	PPP1R18	0.080444	6.994866	0.928614	0.355617	-6.1957	0.474815	0.574368
Monocytes	GM26737	-0.40932	0.478605	-0.92804	0.355913	-4.90732	0.570786	0.68878
Monocytes	4930513N10RII	-0.41916	0.475363	-0.92785	0.356011	-4.87433	0.570896	0.688912
Monocytes	MTG2	0.182034	3.031494	0.92771	0.356084	-5.30659	0.531042	0.641699
Monocytes	ZFP410	0.140934	4.138179	0.927676	0.356101	-5.49741	0.514745	0.622237
Monocytes	TET3	0.078814	6.827605	0.927336	0.356277	-6.16436	0.477533	0.577504
Monocytes	TRAF3IP2	-0.21587	3.229202	-0.9273	0.356296	-5.23183	0.528227	0.638357
Monocytes	SPSB2	0.220462	2.383882	0.927195	0.35635	-5.15021	0.540988	0.653556
Monocytes	KLRA3	-0.57426	-0.66191	-0.92693	0.356485	-4.85784	0.58996	0.711362
Monocytes	DDX18	-0.10479	4.987355	-0.92676	0.356576	-5.79334	0.502924	0.608038
Monocytes	MIR142HG	0.077454	8.019491	0.926549	0.356683	-6.33473	0.462199	0.55883
Monocytes	CD177	0.402913	0.17299	0.925992	0.356971	-4.98288	0.576649	0.695438
Monocytes	NLRP1B	-0.13691	1.79452	-0.92559	0.357181	-5.82607	0.550946	0.665048
Monocytes	PHF6	-0.11489	4.940138	-0.92544	0.357257	-5.77332	0.504206	0.60934
Monocytes	5830487J09RIK	0.500152	-0.01733	0.92538	0.357287	-4.768	0.58001	0.699404
Monocytes	GALK2	0.106869	4.897213	0.925164	0.357399	-5.75598	0.504814	0.610069
Monocytes	GSTK1	-0.25133	2.449061	-0.92515	0.357404	-5.15522	0.54084	0.65308
Monocytes	JHY	-0.48446	-0.37714	-0.9251	0.357432	-4.89828	0.585977	0.706421
Monocytes	RAB9	0.10172	4.887523	0.925023	0.357472	-5.74616	0.504951	0.610233
Monocytes	KMO	-0.1836	3.038738	-0.9249	0.357534	-5.46793	0.531927	0.642499
Monocytes	AQP11	-0.50325	0.397356	-0.92436	0.357817	-4.82005	0.573543	0.691533
Monocytes	MTF1	-0.12086	4.234341	-0.92433	0.35783	-5.62668	0.514594	0.621551
Monocytes	PMS1	0.239641	2.650773	0.924085	0.357957	-5.1627	0.538094	0.649675
Monocytes	CEP350	0.086417	6.505585	0.924033	0.357984	-6.14556	0.482894	0.583577
Monocytes	PJA2	0.12938	4.695864	0.923904	0.358051	-5.73322	0.507983	0.613735

Monocytes	LIPC	-0.25066	2.510562	-0.92389	0.358057	-5.25884	0.540229	0.652216
Monocytes	ACOT12	-0.45889	0.798615	-0.92378	0.358114	-4.85805	0.567064	0.684002
Monocytes	KMT2A	-0.10136	6.28827	-0.92368	0.358166	-5.99361	0.485832	0.587132
Monocytes	HECA	0.087585	6.23169	0.923571	0.358223	-6.04754	0.4866	0.588083
Monocytes	E2F3	-0.13836	5.460693	-0.92352	0.358251	-5.82027	0.497206	0.600843
Monocytes	PSMD2	-0.0774	6.149979	-0.92322	0.358404	-6.06907	0.487811	0.589474
Monocytes	ITPKA	-0.48584	0.529225	-0.92304	0.358497	-4.82483	0.571534	0.689262
Monocytes	9030025P20RIK	0.316219	1.737859	0.922994	0.358523	-5.02933	0.552278	0.666513
Monocytes	RNF169	0.110084	6.380124	0.922982	0.358529	-6.08587	0.484686	0.585737
Monocytes	CCDC180	0.272105	1.286108	0.922761	0.358643	-5.18517	0.55949	0.675057
Monocytes	TPST1	0.176453	3.472726	0.922647	0.358702	-5.53148	0.525972	0.63523
Monocytes	CHTF8	-0.33579	1.341638	-0.92218	0.358945	-4.97844	0.558921	0.674112
Monocytes	KCNC3	-0.40127	0.561324	-0.92195	0.359065	-4.89254	0.571541	0.688923
Monocytes	SRSF6	-0.08727	6.210068	-0.92163	0.359228	-6.04125	0.487595	0.588842
Monocytes	4930404I05RIK	-0.39201	0.086501	-0.9211	0.359504	-5.0049	0.579688	0.698492
Monocytes	TMEM101	0.220903	2.322989	0.921097	0.359506	-5.22915	0.544074	0.656408
Monocytes	RGS2	-0.09214	6.977171	-0.92109	0.359508	-6.28051	0.47744	0.576654
Monocytes	WRNIP1	-0.15051	3.743434	-0.92099	0.359559	-5.4046	0.522702	0.630989
Monocytes	GPR55	-0.24181	1.548876	-0.92076	0.359683	-5.19831	0.556202	0.670792
Monocytes	RNF5	-0.13358	4.122516	-0.92051	0.35981	-5.62313	0.51723	0.624475
Monocytes	HDAC7	0.185857	4.31747	0.920445	0.359845	-5.40445	0.514403	0.621096
Monocytes	HSPBP1	-0.13755	3.934559	-0.9204	0.359869	-5.52109	0.519972	0.627751
Monocytes	PSMB5	0.08885	6.258665	0.920348	0.359896	-6.09273	0.487174	0.588421
Monocytes	SPN	0.129321	3.586459	0.920276	0.359933	-5.69442	0.525092	0.633866
Monocytes	ENTPD6	-0.17563	2.776877	-0.92021	0.359969	-5.32811	0.537218	0.648329
Monocytes	EIF3B	-0.09073	5.890853	-0.92006	0.360047	-6.00674	0.492243	0.594552
Monocytes	VPS13D	0.095626	6.149191	0.919804	0.360178	-6.02354	0.488811	0.5903
Monocytes	NT5C3	-0.13806	4.85257	-0.91897	0.360611	-5.64867	0.507418	0.612216
Monocytes	PSTK	-0.17837	3.061289	-0.91839	0.360915	-5.28922	0.53402	0.643674
Monocytes	TNNI2	-0.11188	2.310806	-0.91821	0.361006	-5.83717	0.545518	0.657274
Monocytes	DYNLT1F	0.12463	5.068561	0.917451	0.361402	-5.88174	0.505245	0.608961
Monocytes	GM21887	-0.34017	2.133944	-0.9171	0.361584	-5.08448	0.548975	0.660849
Monocytes	ZC3H12D	0.187853	3.479104	0.916951	0.361663	-5.50729	0.528548	0.636578
Monocytes	MIER3	-0.14947	4.078409	-0.91687	0.361706	-5.50741	0.519706	0.626048
Monocytes	4931403E22RIK	-0.25278	1.501737	-0.91674	0.361775	-5.08656	0.55893	0.672586
Monocytes	RALGDS	-0.25303	3.822267	-0.91665	0.361817	-5.37153	0.523476	0.630556
Monocytes	SLC39A14	0.24631	3.169335	0.916343	0.36198	-5.237	0.533287	0.642202
Monocytes	LMLN	0.297039	1.750464	0.91611	0.362101	-5.03038	0.555102	0.668111
Monocytes	CD53	0.064847	7.920856	0.916023	0.362147	-6.38983	0.466877	0.562719
Monocytes	RGS14	0.147231	3.585563	0.915958	0.36218	-5.64276	0.527067	0.63493
Monocytes	GM26901	0.377899	0.57091	0.915753	0.362287	-4.87004	0.573982	0.690498
Monocytes	CPTP	-0.22984	2.248177	-0.91575	0.362291	-5.11685	0.54734	0.659052
Monocytes	FGL1	0.260143	3.523532	0.91566	0.362336	-5.34236	0.527989	0.636079
Monocytes	P2RY12	0.230929	2.616799	0.915602	0.362366	-5.37966	0.541668	0.652329
Monocytes	TRP53I13	0.192745	3.021819	0.915394	0.362475	-5.2345	0.53551	0.645086
Monocytes	ZFP24	-0.11976	4.243197	-0.91517	0.362591	-5.60456	0.517404	0.623562
Monocytes	MOB3B	0.086857	4.961752	0.915149	0.362603	-6.14373	0.507069	0.611213

Monocytes	IP6K2	0.17077	3.644965	0.915078	0.36264	-5.31929	0.526186	0.634066
Monocytes	PNRC1	-0.08015	8.470962	-0.91504	0.362658	-6.38685	0.459808	0.55436
Monocytes	CELF2	0.064503	8.597509	0.914966	0.362698	-6.46883	0.458199	0.552461
Monocytes	PROZ	0.292807	1.915226	0.914852	0.362758	-5.19792	0.552519	0.665396
Monocytes	MMGT1	-0.2118	2.857236	-0.91473	0.362824	-5.26543	0.538003	0.648231
Monocytes	SMYD3	0.100766	6.67093	0.914713	0.36283	-6.12639	0.483401	0.582944
Monocytes	TRMT61A	0.301295	1.593744	0.914616	0.362881	-5.01074	0.557571	0.671417
Monocytes	MFAP1A	0.103922	4.832089	0.914499	0.362942	-5.69326	0.508917	0.61357
Monocytes	CHMP6	0.15624	3.712475	0.914478	0.362953	-5.54368	0.525187	0.632992
Monocytes	CAV2	0.198048	2.771749	0.914364	0.363012	-5.34922	0.539303	0.64978
Monocytes	RNF31	-0.1898	3.162894	-0.91416	0.363117	-5.36009	0.533384	0.642819
Monocytes	C030034I22RIK	0.198661	2.630863	0.914062	0.36317	-5.26734	0.541453	0.652427
Monocytes	LRRFIP1	0.06673	7.249941	0.914057	0.363173	-6.37955	0.475666	0.573712
Monocytes	RASA1	-0.09174	6.030636	-0.91393	0.363238	-5.97733	0.492119	0.593604
Monocytes	CAB39	0.071202	6.974308	0.913859	0.363276	-6.20125	0.47933	0.578226
Monocytes	ASNA1	-0.10403	4.877847	-0.91381	0.3633	-5.81781	0.508264	0.61298
Monocytes	GM45370	-0.59943	-1.29914	-0.91378	0.36332	-4.76097	0.605355	0.727775
Monocytes	PSMF1	-0.12916	4.460205	-0.91341	0.36351	-5.62426	0.514386	0.620261
Monocytes	MYDGF	0.097707	4.485984	0.913317	0.36356	-5.75916	0.514013	0.619816
Monocytes	DHX8	0.101691	5.129659	0.913309	0.363564	-5.82705	0.504813	0.608809
Monocytes	ZKSCAN14	0.171077	3.081299	0.912982	0.363735	-5.31983	0.534891	0.64461
Monocytes	GM11696	-0.29831	1.871722	-0.91292	0.363767	-5.08916	0.553489	0.666669
Monocytes	4930522L14RIK	-0.19685	3.684756	-0.9127	0.363883	-5.31529	0.525958	0.633943
Monocytes	SERINC4	-0.46465	0.365548	-0.91261	0.36393	-4.85014	0.577736	0.695227
Monocytes	LYPLA1	0.080774	5.988433	0.912456	0.36401	-6.02842	0.493078	0.594604
Monocytes	UBAC2	0.076891	6.679645	0.9121	0.364196	-6.17306	0.483788	0.583438
Monocytes	OXSR1	-0.1547	3.496646	-0.91197	0.364263	-5.48961	0.528942	0.637545
Monocytes	APLF	-0.26158	2.05909	-0.91197	0.364266	-5.15111	0.55085	0.663557
Monocytes	DVL2	-0.20683	3.263703	-0.91183	0.364336	-5.33065	0.532453	0.641695
Monocytes	SLC43A3	0.218761	3.813488	0.91157	0.364474	-5.30331	0.524394	0.632004
Monocytes	DHRS11	0.146068	4.678666	0.911158	0.36469	-5.71548	0.511956	0.617054
Monocytes	GID4	-0.12195	4.230623	-0.91115	0.364692	-5.59934	0.518441	0.624798
Monocytes	BLOC1S3	0.244776	2.381624	0.9106	0.364982	-5.21903	0.54642	0.657863
Monocytes	SPATA21	-0.18564	3.397572	-0.91058	0.36499	-5.47983	0.530975	0.63953
Monocytes	HDAC4	0.121667	5.402881	0.910552	0.365007	-5.91179	0.501886	0.604811
Monocytes	MIER2	-0.23174	2.002333	-0.91037	0.365103	-5.20304	0.552379	0.664861
Monocytes	CRTC2	-0.14291	4.134514	-0.90982	0.365389	-5.64395	0.520361	0.626627
Monocytes	EFCAB9	-0.33493	0.443216	-0.90977	0.365416	-4.9186	0.577592	0.694361
Monocytes	LENG9	0.232742	2.352054	0.909678	0.365466	-5.20298	0.54718	0.658488
Monocytes	PPP1R10	-0.10062	6.277719	-0.90965	0.365481	-6.03893	0.490028	0.590336
Monocytes	RFWD3	0.098005	5.6362	0.909576	0.365519	-5.88154	0.498895	0.600982
Monocytes	PLD1	0.188372	3.080795	0.909381	0.365621	-5.57089	0.53604	0.645286
Monocytes	RBM47	-0.08972	6.404832	-0.90936	0.365634	-6.40924	0.488297	0.588258
Monocytes	MXD3	0.323652	1.735683	0.909275	0.365677	-5.08695	0.55681	0.669883
Monocytes	SCO2	-0.20041	2.851352	-0.90817	0.366257	-5.28273	0.540207	0.649869
Monocytes	SUMF1	-0.11557	4.240634	-0.9081	0.366293	-5.80892	0.519474	0.625266
Monocytes	TNFAIP3	-0.10521	6.763033	-0.9081	0.366296	-6.24143	0.484055	0.582864

Monocytes	MYG1	-0.12848	3.803891	-0.90772	0.366491	-5.5186	0.52606	0.633051
Monocytes	GAS2L1	0.223322	1.765951	0.907682	0.366514	-5.27424	0.557213	0.669981
Monocytes	POLD1	-0.17349	4.276679	-0.90752	0.366599	-5.48048	0.519158	0.624815
Monocytes	ALDH3A2	0.132358	3.596521	0.907209	0.366762	-5.54093	0.529232	0.636871
Monocytes	SASH3	0.103816	5.4972	0.907016	0.366864	-5.82738	0.501724	0.604091
Monocytes	KANK3	0.319293	2.000717	0.906982	0.366882	-5.13349	0.553619	0.665824
Monocytes	SNX22	0.209471	-0.37781	0.906979	0.366883	-5.39972	0.592278	0.711295
Monocytes	GM15892	0.249099	3.443855	0.906971	0.366887	-5.3433	0.531513	0.639625
Monocytes	E430018J23RIK	0.460111	0.653485	0.906762	0.366997	-4.82822	0.575188	0.691261
Monocytes	OPHN1	0.213534	2.997504	0.906749	0.367004	-5.49308	0.538264	0.647653
Monocytes	PSMG4	-0.09212	5.042688	-0.90649	0.367139	-5.89427	0.508288	0.611873
Monocytes	DDX19B	-0.15905	3.529612	-0.906	0.367397	-5.40545	0.530598	0.63827
Monocytes	ZFP777	0.210439	2.809648	0.905942	0.367429	-5.15758	0.541481	0.651185
Monocytes	CDK8	-0.15067	6.559883	-0.9059	0.367452	-6.06473	0.487379	0.586636
Monocytes	TGTP1	-0.63647	-0.20272	-0.90578	0.367514	-4.83997	0.58976	0.708057
Monocytes	SYDE2	-0.52749	-0.10373	-0.90539	0.367722	-4.8096	0.588351	0.706252
Monocytes	NUDCD1	-0.2055	3.226429	-0.90504	0.367904	-5.31559	0.535584	0.64389
Monocytes	MRPS33	-0.06885	6.714474	-0.90445	0.368213	-6.18066	0.485913	0.584548
Monocytes	NUDCD2	-0.13566	4.534449	-0.90442	0.368228	-5.69952	0.516481	0.621124
Monocytes	LRIG2	0.153921	4.256808	0.904402	0.36824	-5.49832	0.520526	0.625943
Monocytes	NAT9	0.184913	3.244632	0.90391	0.3685	-5.37286	0.535847	0.644045
Monocytes	YEATS4	0.082633	5.906755	0.903846	0.368533	-5.96284	0.497252	0.598045
Monocytes	1700109H08RII	0.185494	3.650709	0.903569	0.368679	-5.44651	0.529887	0.63691
Monocytes	SAPCD1	-0.43862	1.629002	-0.90327	0.368839	-4.8547	0.561105	0.67384
Monocytes	RMC1	0.12492	4.652621	0.903259	0.368843	-5.69091	0.515243	0.619475
Monocytes	TIMMDC1	0.116813	4.271519	0.903145	0.368903	-5.6505	0.520789	0.626091
Monocytes	6030458C11RIK	0.195671	2.74674	0.903059	0.368948	-5.22337	0.543647	0.653252
Monocytes	NHP2	-0.10339	5.748278	-0.90298	0.368993	-6.02687	0.499663	0.600904
Monocytes	ZFP60	0.325673	1.431044	0.902834	0.369067	-4.98984	0.564295	0.677627
Monocytes	ACACB	-0.56644	0.257652	-0.90249	0.369249	-4.78863	0.583512	0.700193
Monocytes	DOK2	-0.20135	2.811456	-0.90246	0.369267	-5.35779	0.542792	0.652219
Monocytes	3300002I08RIK	-0.28924	1.970632	-0.90241	0.369293	-5.08045	0.555845	0.66765
Monocytes	USP44	0.500046	-0.6309	0.90191	0.369555	-4.76229	0.598781	0.717767
Monocytes	CCDC191	0.262137	1.993228	0.901823	0.369601	-5.12177	0.555797	0.667339
Monocytes	SET	-0.07684	7.946372	-0.90144	0.369804	-6.3618	0.470488	0.565491
Monocytes	DPY30	-0.11372	5.414831	-0.90142	0.369812	-5.89442	0.504901	0.60678
Monocytes	CPEB1	-0.36117	0.696287	-0.90119	0.369934	-4.96426	0.576866	0.691985
Monocytes	SAV1	-0.10748	4.735258	-0.90107	0.369997	-5.75006	0.514694	0.618485
Monocytes	GM16316	0.344318	1.803894	0.901018	0.370026	-4.97736	0.559034	0.671061
Monocytes	RILPL2	-0.0825	7.158884	-0.90078	0.370151	-6.33456	0.481072	0.578224
Monocytes	SCRN2	-0.31196	1.43712	-0.90057	0.370261	-4.93691	0.565072	0.678098
Monocytes	LGR5	0.515336	1.073909	0.899908	0.370614	-4.80873	0.571383	0.685308
Monocytes	TAB1	0.20678	3.004453	0.899355	0.370907	-5.30099	0.541336	0.649673
Monocytes	GATD1	0.115375	4.394103	0.899296	0.370938	-5.71396	0.520564	0.625035
Monocytes	DOCK5	0.100272	4.189727	0.898965	0.371113	-6.14978	0.523661	0.628645
Monocytes	PHF20	0.079072	6.472356	0.898919	0.371137	-6.07739	0.491225	0.589939
Monocytes	S1PR5	-0.24892	-0.65106	-0.89887	0.371163	-5.22725	0.600561	0.719214

Monocytes	AGFG2	0.133204	4.605897	0.898336	0.371446	-5.63395	0.517896	0.621563
Monocytes	QDPR	-0.09918	5.245358	-0.89805	0.371597	-5.84947	0.508788	0.610643
Monocytes	SARDHOS	-0.67895	0.147254	-0.898	0.371622	-4.78095	0.587554	0.703659
Monocytes	UBTF	0.079999	5.879519	0.897433	0.371925	-5.97013	0.500173	0.600186
Monocytes	ZFP950	0.125305	4.197661	0.896975	0.372168	-5.66686	0.524595	0.629034
Monocytes	AIRN	-0.1607	5.663175	-0.89683	0.372246	-5.95605	0.503502	0.603922
Monocytes	MAJIN	-0.54365	0.644865	-0.89656	0.37239	-4.8702	0.580106	0.69453
Monocytes	MESD	-0.11991	4.085411	-0.89655	0.372396	-5.57336	0.526354	0.63116
Monocytes	PSRC1	0.383104	0.886949	0.896173	0.372594	-4.94415	0.57636	0.690013
Monocytes	LMNB2	0.276463	2.474409	0.895736	0.372826	-5.16588	0.551292	0.660385
Monocytes	CNOT3	0.092699	5.46868	0.895609	0.372893	-5.926	0.506766	0.607598
Monocytes	GM43378	-0.26602	1.243694	-0.89553	0.372937	-5.04302	0.570844	0.683401
Monocytes	GM10382	0.370993	0.439288	0.895208	0.373107	-4.97715	0.584208	0.698881
Monocytes	LYSMD3	0.104053	4.927993	0.894997	0.373219	-5.8456	0.514748	0.61686
Monocytes	GBA2	-0.21748	2.547922	-0.8947	0.373378	-5.14778	0.550447	0.659102
Monocytes	ZFP865	0.200193	3.265059	0.894675	0.37339	-5.31229	0.539419	0.646083
Monocytes	KCTD4	0.25929	2.200261	0.894668	0.373394	-5.20784	0.555883	0.665506
Monocytes	FDPS	-0.14133	4.623809	-0.89444	0.373513	-5.82136	0.519279	0.622182
Monocytes	MID2	-0.52162	0.27499	-0.89427	0.373604	-4.81463	0.58718	0.702242
Monocytes	XYLB	-0.40077	0.922746	-0.89424	0.373623	-4.90301	0.576479	0.689732
Monocytes	TRIM21	-0.25136	2.57157	-0.89412	0.373684	-5.26656	0.550199	0.65888
Monocytes	MOB3C	-0.1621	3.356469	-0.89373	0.37389	-5.43869	0.538276	0.644752
Monocytes	PHOSPHO2	0.125828	3.724911	0.893733	0.373891	-5.52552	0.532718	0.638175
Monocytes	MCRIP1	0.070628	5.919349	0.893661	0.373929	-6.04734	0.500906	0.600392
Monocytes	GRIK5	0.504906	0.167717	0.893441	0.374046	-4.85115	0.589228	0.704578
Monocytes	F3	-0.6214	0.113979	-0.89305	0.374253	-4.86516	0.590373	0.705766
Monocytes	HERC1	0.086507	7.056727	0.892843	0.374365	-6.18409	0.485597	0.581858
Monocytes	TCP11	-0.37676	0.648485	-0.89276	0.374411	-4.89242	0.58154	0.695448
Monocytes	DHX58OS	0.383926	0.831273	0.892652	0.374467	-5.00979	0.578531	0.691949
Monocytes	RMDN2	-0.30723	1.873328	-0.89215	0.374735	-5.05864	0.561695	0.672407
Monocytes	NOC3L	-0.22798	3.216026	-0.89211	0.374757	-5.3244	0.540788	0.647759
Monocytes	NUDT16L1	0.148578	4.234234	0.891977	0.374826	-5.57511	0.525506	0.629722
Monocytes	TXNDC12	0.134026	3.827411	0.891943	0.374844	-5.50792	0.531554	0.636894
Monocytes	GM16364.1	-0.29394	1.279847	-0.89181	0.374915	-5.21807	0.571217	0.683739
Monocytes	P2RY1	0.304235	0.13392	0.891792	0.374925	-5.0561	0.590104	0.70585
Monocytes	DCUN1D4	-0.26792	2.540378	-0.89175	0.374948	-5.19661	0.5512	0.660193
Monocytes	L3MBTL1	-0.42128	0.787342	-0.89174	0.374954	-4.91152	0.579253	0.693159
Monocytes	PPCS	-0.20663	2.732539	-0.89174	0.374954	-5.19146	0.548216	0.656673
Monocytes	GM47283	0.189106	7.990664	0.891624	0.375015	-6.38835	0.47315	0.567299
Monocytes	GM43065	-0.38597	0.153169	-0.89145	0.375107	-4.97201	0.589781	0.705562
Monocytes	SF3B4	-0.1005	5.494923	-0.8914	0.375135	-5.91931	0.507244	0.608155
Monocytes	BORCS5	0.126067	4.024358	0.891375	0.375147	-5.60499	0.528616	0.633569
Monocytes	PLCG1	-0.20678	3.409844	-0.89121	0.375234	-5.2482	0.537842	0.644514
Monocytes	PINK1	0.138887	5.115722	0.891211	0.375235	-5.73143	0.512661	0.614626
Monocytes	ROPN1L	0.267527	2.420763	0.891111	0.375289	-5.21351	0.553068	0.662509
Monocytes	GM12592	-0.23278	3.124779	-0.89081	0.37545	-5.17437	0.54229	0.649762
Monocytes	GM46620	-0.49008	0.683678	-0.89068	0.375518	-4.85965	0.581076	0.695449

Monocytes	FAM234A	0.131137	4.099085	0.890624	0.375548	-5.86734	0.527612	0.632436
Monocytes	ZDHHC13	-0.12328	3.693777	-0.89055	0.375587	-5.60197	0.533664	0.639641
Monocytes	COX7B	-0.07468	7.597144	-0.89048	0.375623	-6.3426	0.478446	0.57378
Monocytes	PCCB	-0.21655	3.081613	-0.88942	0.376192	-5.22999	0.543697	0.651055
Monocytes	MAP3K8	-0.10985	4.64612	-0.88897	0.376434	-5.88115	0.52054	0.623429
Monocytes	GPR27	0.346569	-1.60473	0.888825	0.376509	-4.77099	0.621373	0.741722
Monocytes	ETF1	-0.06633	7.536791	-0.88874	0.376553	-6.33898	0.480168	0.575274
Monocytes	DDX27	0.10419	4.974515	0.888546	0.376658	-5.80344	0.515858	0.61782
Monocytes	4930590J08RIK	-0.30955	1.547542	-0.88816	0.376864	-5.03944	0.568389	0.679696
Monocytes	COX4I1	0.058089	9.086822	0.888092	0.376901	-6.57175	0.460206	0.551088
Monocytes	USE1	-0.09357	5.084893	-0.88793	0.376989	-5.91023	0.514487	0.616101
Monocytes	KLC4	0.20003	2.884186	0.887834	0.377039	-5.27847	0.547345	0.655023
Monocytes	PCYOX1	0.122132	4.579765	0.887602	0.377163	-5.71624	0.52183	0.624919
Monocytes	C79798	0.22604	1.77973	0.887587	0.377171	-5.22664	0.564702	0.675548
Monocytes	COL9A3	0.467729	0.618437	0.887555	0.377188	-4.89946	0.583605	0.69771
Monocytes	FAM53C	0.131128	3.981286	0.887138	0.377412	-5.57326	0.53069	0.635552
Monocytes	DDX23	-0.10467	4.71777	-0.88701	0.377479	-5.76237	0.51982	0.622683
Monocytes	NASP	-0.0937	6.308139	-0.88694	0.377516	-6.04665	0.497183	0.595716
Monocytes	PTK6	-0.57104	-0.3416	-0.88685	0.377567	-4.85301	0.599759	0.716759
Monocytes	GM12185	0.185864	3.341306	0.886795	0.377595	-5.49174	0.540341	0.647044
Monocytes	PAQR5	0.474897	0.502008	0.88679	0.377598	-4.86543	0.585546	0.700187
Monocytes	DMTF1	0.104354	4.947065	0.886766	0.37761	-5.71457	0.516487	0.618761
Monocytes	IIGP1	-0.4003	4.709506	-0.88676	0.377613	-5.55274	0.519941	0.622867
Monocytes	CEACAM16	0.441189	1.137316	0.886615	0.377691	-4.87307	0.575112	0.687979
Monocytes	A2ML1	0.364132	2.471948	0.886532	0.377736	-5.10411	0.553789	0.662921
Monocytes	ZBTB6	0.254513	1.997204	0.886394	0.37781	-5.12199	0.561305	0.671797
Monocytes	RASAL1	-0.48767	0.712454	-0.88606	0.377988	-4.89127	0.582292	0.696302
Monocytes	TBC1D8	-0.09161	5.179428	-0.88599	0.378026	-6.24535	0.513339	0.614966
Monocytes	ZFP758	0.23548	2.74078	0.885832	0.378111	-5.13245	0.549841	0.658209
Monocytes	SERHL	0.143111	3.492794	0.885183	0.378459	-5.53357	0.538722	0.644786
Monocytes	VAMP5	-0.16458	4.717071	-0.88486	0.378633	-5.62594	0.520616	0.623269
Monocytes	TAX1BP3	0.1178	4.090035	0.884817	0.378655	-5.76913	0.529869	0.634243
Monocytes	FNDC3A	0.078138	7.526942	0.884656	0.378741	-6.31373	0.481318	0.576411
Monocytes	COX5B	0.075251	8.103976	0.88454	0.378804	-6.43093	0.473666	0.567228
Monocytes	HSPG2	-0.27588	2.741833	-0.88451	0.37882	-5.14743	0.550398	0.658547
Monocytes	LAS1L	-0.13161	4.172574	-0.88436	0.378899	-5.55981	0.528688	0.632915
Monocytes	GM4285	0.258276	1.640385	0.884172	0.379002	-5.10674	0.567926	0.679198
Monocytes	GM43112	-0.20685	0.075373	-0.88381	0.379198	-5.32255	0.593941	0.709525
Monocytes	PXDN	-0.3845	0.993778	-0.88359	0.379312	-4.91586	0.578744	0.691761
Monocytes	HK3	-0.21425	2.677613	-0.88324	0.379504	-5.64898	0.551852	0.660295
Monocytes	ATF3	-0.1355	6.1843	-0.88302	0.379618	-6.37742	0.500082	0.59896
Monocytes	TAB2	0.066631	6.780443	0.882993	0.379634	-6.21251	0.491832	0.589101
Monocytes	2610001J05RIK	0.131703	3.690163	0.882929	0.379669	-5.61301	0.536318	0.642026
Monocytes	BCORL1	-0.1877	3.355845	-0.88277	0.379753	-5.44819	0.541393	0.648042
Monocytes	TCF12	-0.08727	8.463403	-0.88267	0.379806	-6.31561	0.469357	0.562197
Monocytes	PTPRD	-0.36991	2.563535	-0.88266	0.379815	-5.1317	0.553633	0.662532
Monocytes	IGKV12-46	0.489802	-0.77013	0.882554	0.37987	-4.77844	0.608552	0.726825

Monocytes	WDR19	0.431578	0.508741	0.882485	0.379908	-4.88362	0.586814	0.701486
Monocytes	N4BP2L2	-0.06033	6.786437	-0.88246	0.37992	-6.14829	0.49175	0.589073
Monocytes	ARMC9	-0.23743	2.484131	-0.88246	0.37992	-5.09845	0.554877	0.664012
Monocytes	ZMYM3	-0.26735	2.31007	-0.88229	0.380011	-5.1481	0.557669	0.667265
Monocytes	PPM1A	0.068369	6.125745	0.88215	0.380088	-6.01683	0.500983	0.600067
Monocytes	ZDHHC24	-0.38674	1.089708	-0.88171	0.380323	-4.9459	0.577591	0.690463
Monocytes	CREBZF	0.117322	4.715073	0.881535	0.380418	-5.64432	0.52148	0.624229
Monocytes	HAVCR2	0.10887	2.301711	0.88114	0.380631	-6.24438	0.55825	0.667678
Monocytes	A630023P12RII	-0.58048	0.306091	-0.88111	0.380647	-4.80083	0.590737	0.705748
Monocytes	TNIP2	-0.10449	3.816648	-0.88109	0.380656	-5.75723	0.534896	0.640121
Monocytes	STK38L	-0.11971	4.003603	-0.88099	0.380713	-5.65209	0.532089	0.636834
Monocytes	SCFD1	0.116618	4.929173	0.880905	0.380758	-5.82414	0.518433	0.620666
Monocytes	MRPL34	0.083013	5.428125	0.880693	0.380872	-5.93352	0.511234	0.612177
Monocytes	GZMK	-0.50549	-1.47202	-0.88058	0.380934	-4.77907	0.621413	0.741614
Monocytes	PGM2	0.123271	4.115633	0.880437	0.381009	-5.6756	0.530415	0.634987
Monocytes	DNAAF2	-0.20526	2.845924	-0.88039	0.381033	-5.2218	0.549733	0.657824
Monocytes	MLYCD	0.167194	3.124459	0.880369	0.381046	-5.379	0.54543	0.652746
Monocytes	MND1	-0.31283	2.222735	-0.88029	0.381086	-5.02876	0.559498	0.669354
Monocytes	SIN3B	-0.06889	6.294475	-0.88026	0.381105	-6.09207	0.498997	0.59765
Monocytes	PISD	0.121945	4.8584	0.880081	0.381201	-5.78937	0.519524	0.622094
Monocytes	PILRA	0.132594	3.3927	0.879709	0.381402	-5.88394	0.541594	0.648064
Monocytes	EMCN	0.368914	1.650943	0.879111	0.381724	-4.97359	0.569313	0.680467
Monocytes	SLC9A8	0.104246	4.988676	0.878858	0.381861	-5.856	0.51831	0.620224
Monocytes	HNRNPC	-0.05251	7.730209	-0.87869	0.381949	-6.30791	0.480167	0.574726
Monocytes	SLC35E1	0.115677	4.084727	0.878554	0.382024	-5.66157	0.531696	0.636174
Monocytes	RAP2A	-0.08883	3.974658	-0.87849	0.382061	-6.01343	0.533344	0.638126
Monocytes	CSNK2B	-0.07371	7.247784	-0.87835	0.382137	-6.27878	0.48669	0.58255
Monocytes	CAPRIN1	0.058711	7.460311	0.878083	0.382279	-6.28183	0.483934	0.579114
Monocytes	COL5A2	0.465796	1.225753	0.877911	0.382371	-4.92733	0.576634	0.688868
Monocytes	MIS12	0.187767	3.322859	0.877217	0.382746	-5.4029	0.543753	0.650071
Monocytes	GM29093	0.346335	1.269702	0.877107	0.382806	-4.86601	0.576247	0.688267
Monocytes	GM47230	-0.38826	0.668652	-0.8771	0.38281	-4.95135	0.586157	0.699856
Monocytes	BRK1	0.064683	6.271039	0.877064	0.382829	-6.1942	0.500581	0.598852
Monocytes	HES6	0.151133	4.105884	0.876998	0.382864	-5.48168	0.531897	0.636074
Monocytes	AIMP2	-0.18652	3.206699	-0.87692	0.382907	-5.39691	0.545537	0.652236
Monocytes	ITGB3	0.269893	2.991713	0.876725	0.383012	-5.13687	0.54893	0.656238
Monocytes	MAST1	-0.50986	1.020113	-0.87646	0.383157	-4.93841	0.580556	0.693306
Monocytes	RBBP5	0.167752	3.457865	0.87634	0.38322	-5.40215	0.54189	0.647925
Monocytes	CCNL2	0.066401	6.219089	0.876183	0.383305	-6.08075	0.501495	0.600068
Monocytes	PLLP	-0.58928	-0.20457	-0.87617	0.383313	-4.77586	0.601109	0.717448
Monocytes	FEZ2	0.131903	3.693286	0.875827	0.383498	-5.51807	0.538399	0.643903
Monocytes	ICAM2	0.28058	4.282374	0.875777	0.383524	-5.4817	0.52955	0.633437
Monocytes	EEF1AKMT1	0.105762	4.588498	0.875757	0.383535	-5.95086	0.525015	0.628064
Monocytes	RAN	-0.10421	8.400943	-0.8755	0.383673	-6.45225	0.472071	0.564886
Monocytes	RBL2	0.121368	4.765126	0.875426	0.383714	-5.7258	0.522458	0.625076
Monocytes	TSPYL3	-0.45261	0.960124	-0.87534	0.383762	-4.90609	0.581686	0.694878
Monocytes	CASP8	0.083306	5.513709	0.875313	0.383775	-5.98345	0.511609	0.612202

Monocytes	NAPG	0.109575	4.542195	0.875058	0.383913	-5.79247	0.525773	0.629038
Monocytes	RNF170	0.153227	3.319753	0.874997	0.383946	-5.4046	0.544171	0.650828
Monocytes	SNX13	0.111177	5.413359	0.874829	0.384037	-5.91664	0.513083	0.614026
Monocytes	MBLAC1	0.391187	-0.19049	0.874823	0.38404	-4.87528	0.601054	0.717569
Monocytes	PEX3	-0.12785	3.770542	-0.87458	0.384173	-5.5392	0.537305	0.642811
Monocytes	GATAD1	0.078685	5.768843	0.874565	0.38418	-5.98791	0.508003	0.608029
Monocytes	THAP7	0.168067	3.607354	0.87454	0.384194	-5.47605	0.53978	0.645748
Monocytes	PRPS1	-0.16981	3.621673	-0.87449	0.384222	-5.41045	0.539562	0.645509
Monocytes	NRAP	-0.48895	-1.09404	-0.87427	0.384338	-4.78797	0.61679	0.7359
Monocytes	WRAP53	-0.20473	2.788872	-0.87413	0.384414	-5.20633	0.552445	0.6607
Monocytes	RCOR2	-0.5461	0.194015	-0.87393	0.384521	-4.8074	0.594586	0.710175
Monocytes	NCOA4	-0.13988	6.199192	-0.87393	0.384523	-5.93836	0.501986	0.600927
Monocytes	RBM25	-0.05301	8.296585	-0.87388	0.384554	-6.41868	0.473524	0.566848
Monocytes	MMS19	0.098197	4.947005	0.873822	0.384582	-5.79529	0.519892	0.622246
Monocytes	KCND1	-0.47845	-0.19065	-0.87346	0.38478	-4.85497	0.601191	0.717797
Monocytes	MIGA2	0.426498	0.892517	0.873429	0.384795	-4.89788	0.582972	0.696533
Monocytes	FBXO36	0.383413	1.228268	0.873291	0.38487	-4.98867	0.577447	0.690066
Monocytes	OCRL	0.196258	2.602911	0.873252	0.384892	-5.43209	0.555415	0.664191
Monocytes	DCP1A	0.100824	4.850131	0.873184	0.384928	-5.7946	0.521364	0.623926
Monocytes	GM50163	-0.43232	0.458993	-0.87316	0.384941	-4.86669	0.590191	0.70497
Monocytes	ATG14	0.149911	3.27883	0.872875	0.385096	-5.4068	0.545066	0.651873
Monocytes	TEX2	0.09557	6.723879	0.872492	0.385303	-6.17575	0.495074	0.592383
Monocytes	CPT2	-0.25138	2.829472	-0.87231	0.3854	-5.2386	0.552289	0.66024
Monocytes	NSMCE4A	-0.07644	6.083141	-0.87214	0.385496	-6.05506	0.504051	0.603122
Monocytes	CRIP1	-0.09236	8.825299	-0.87205	0.385546	-6.72782	0.467043	0.558804
Monocytes	GUCA1A	0.182809	1.4794	0.871972	0.385586	-5.4526	0.573784	0.685554
Monocytes	ZC3H10	0.15449	2.921486	0.871944	0.3856	-5.40811	0.550857	0.658606
Monocytes	HSPE1-RS1	0.496638	0.18545	0.871618	0.385778	-4.87807	0.595437	0.710794
Monocytes	SLC29A3	-0.12907	3.68183	-0.87151	0.385838	-5.64911	0.53936	0.64496
Monocytes	ZRANB3	-0.18298	3.148069	-0.87125	0.385976	-5.41885	0.547655	0.654623
Monocytes	ANGPT1	0.455941	0.973826	0.871106	0.386055	-4.90548	0.582435	0.695456
Monocytes	KANSL3	0.094908	4.995066	0.870667	0.386294	-5.85714	0.520205	0.621995
Monocytes	ADAM12	0.459201	0.815122	0.87044	0.386417	-4.90856	0.58533	0.698758
Monocytes	CTLA4	-0.3333	2.060232	-0.8704	0.386437	-5.18454	0.56504	0.67501
Monocytes	HRH4	-0.31738	0.27047	-0.87032	0.38648	-5.13347	0.594454	0.709425
Monocytes	STAT3	0.079248	7.950149	0.870297	0.386495	-6.42828	0.47903	0.57295
Monocytes	MRPL54	0.085145	5.906363	0.869752	0.386791	-6.08223	0.507418	0.606731
Monocytes	RPF1	-0.08642	5.149471	-0.86957	0.38689	-5.87196	0.518347	0.619691
Monocytes	TRAPPC9	-0.08412	6.178368	-0.86915	0.387117	-6.0864	0.503828	0.602278
Monocytes	CSNK1G1	0.08259	6.535289	0.869104	0.387144	-6.12104	0.498832	0.596336
Monocytes	KIF13B	0.09327	6.510852	0.868934	0.387236	-6.05127	0.499223	0.596794
Monocytes	MRGPRA2A	-0.45852	-1.27662	-0.86882	0.387298	-4.79084	0.621999	0.741093
Monocytes	GM13483	0.283145	1.85821	0.868696	0.387365	-5.13971	0.569014	0.679342
Monocytes	FLII	0.080337	5.969326	0.868332	0.387563	-6.08609	0.507052	0.605988
Monocytes	ADAMTS1	-0.36835	2.937185	-0.86801	0.387739	-5.1896	0.552306	0.659398
Monocytes	AP4E1	0.219524	3.305374	0.867798	0.387854	-5.27419	0.546688	0.652722
Monocytes	SPG21	0.068034	6.057126	0.86748	0.388027	-6.19363	0.506152	0.604626

Monocytes	1700037C18RIK	0.191496	3.065274	0.86746	0.388038	-5.32113	0.550513	0.657135
Monocytes	DKC1	-0.1386	4.56874	-0.86719	0.388187	-5.65298	0.527834	0.630346
Monocytes	CPED1	0.271266	2.524781	0.866912	0.388337	-5.29435	0.559196	0.66729
Monocytes	GM20274	0.191224	2.727126	0.866891	0.388349	-5.33173	0.556009	0.663545
Monocytes	AU020206	0.124672	5.320744	0.866571	0.388523	-5.7622	0.516994	0.617447
Monocytes	PRMT3	0.135327	4.041123	0.866554	0.388532	-5.63679	0.535902	0.639838
Monocytes	GFOD1	-0.09527	6.621215	-0.86634	0.388646	-6.31094	0.498616	0.595601
Monocytes	GSAP	-0.11017	5.642393	-0.86624	0.388701	-6.20877	0.512443	0.612055
Monocytes	TMTC4	-0.31582	1.357794	-0.86587	0.388906	-5.08867	0.578403	0.689675
Monocytes	TMEM161B	0.110825	4.545354	0.865775	0.388957	-5.68819	0.528651	0.631182
Monocytes	S100A11	0.074728	7.270251	0.86546	0.389129	-6.51432	0.490022	0.585151
Monocytes	CSDE1	0.049276	7.576149	0.865256	0.38924	-6.35783	0.485943	0.580308
Monocytes	RRM2	-0.1963	6.880861	-0.86483	0.389475	-6.1784	0.495613	0.591777
Monocytes	PTBP2	0.085104	6.156832	0.864728	0.389528	-6.08914	0.505727	0.603838
Monocytes	CRYL1	-0.12271	3.73595	-0.86471	0.389536	-5.70313	0.541258	0.645935
Monocytes	CCNG1	0.100467	4.873028	0.864589	0.389604	-5.83202	0.524254	0.625866
Monocytes	KCTD11	0.383765	0.546292	0.864223	0.389804	-4.93365	0.592576	0.706068
Monocytes	NR2C1	0.227899	2.276051	0.864151	0.389843	-5.26133	0.564231	0.672967
Monocytes	IPO4	-0.20112	2.384797	-0.86405	0.389898	-5.17982	0.562502	0.670979
Monocytes	ZXDC	0.141447	4.004625	0.863704	0.390088	-5.52423	0.537562	0.641437
Monocytes	CYBC1	0.124278	4.562008	0.863484	0.390208	-5.74928	0.529206	0.631692
Monocytes	NADSYN1	-0.35202	1.092531	-0.8633	0.39031	-4.94655	0.583653	0.695797
Monocytes	MRPL47	-0.21989	2.974642	-0.86314	0.390397	-5.27555	0.55339	0.660325
Monocytes	WDR66	-0.34883	1.900032	-0.86312	0.390408	-4.97583	0.570453	0.680358
Monocytes	ZFP946	-0.27065	1.39583	-0.86309	0.390422	-5.06028	0.578656	0.689961
Monocytes	CRYBB3	-0.45456	0.578324	-0.86307	0.390432	-4.87558	0.592233	0.705813
Monocytes	LGALS9	0.110944	7.157478	0.862878	0.390539	-6.14005	0.492158	0.587817
Monocytes	PRMT9	0.158065	4.074852	0.862797	0.390583	-5.52652	0.536501	0.640511
Monocytes	D830025C05RII	-0.25622	2.231904	-0.86276	0.390602	-5.17217	0.565122	0.674229
Monocytes	HADH	-0.13052	5.394105	-0.86269	0.390642	-5.89639	0.517	0.617468
Monocytes	SLC16A7	-0.15791	3.236151	-0.86263	0.390673	-5.5445	0.549323	0.655708
Monocytes	MAK16	-0.09252	5.072635	-0.86256	0.390715	-5.85724	0.521678	0.623033
Monocytes	EHD2	0.408082	1.82262	0.86243	0.390784	-4.91935	0.571727	0.682059
Monocytes	PLXNA1	-0.3643	0.899006	-0.86228	0.390865	-5.03106	0.586896	0.699817
Monocytes	ECD	-0.10094	4.899349	-0.86202	0.391008	-5.75343	0.524246	0.62624
Monocytes	GREB1L	-0.3717	1.947775	-0.86196	0.391042	-5.0267	0.569712	0.679877
Monocytes	TTC8	-0.31975	0.78875	-0.86195	0.391048	-5.04163	0.588735	0.702131
Monocytes	GM49692	-0.51324	0.104027	-0.86194	0.391054	-4.79642	0.600298	0.715608
Monocytes	LCN4	0.470132	-1.05402	0.861769	0.391146	-4.79766	0.620432	0.738999
Monocytes	TTLL12	-0.31014	2.217401	-0.86173	0.39117	-5.18641	0.565397	0.674835
Monocytes	PPP6R1	-0.07485	5.572544	-0.86132	0.39139	-5.9347	0.514625	0.614852
Monocytes	U2SURP	-0.06471	6.667671	-0.86107	0.391527	-6.1508	0.499117	0.5964
Monocytes	CD37	-0.09684	7.409059	-0.86106	0.391536	-6.16392	0.488917	0.584217
Monocytes	CTSL	0.103533	6.758061	0.861051	0.391539	-6.12631	0.497861	0.594901
Monocytes	HAT1	0.091108	6.105162	0.861015	0.391559	-6.0796	0.507016	0.605813
Monocytes	PDXDC1	-0.06447	6.616598	-0.86087	0.39164	-6.16276	0.499864	0.597256
Monocytes	SRA1	0.083543	5.239409	0.860492	0.391845	-5.97727	0.519624	0.620627

Monocytes	CCDC93	-0.13313	3.72019	-0.8604	0.391894	-5.66835	0.542278	0.647452
Monocytes	PLSCR2	-0.58115	-0.38835	-0.8603	0.391948	-4.83333	0.609178	0.725791
Monocytes	ABHD13	0.142408	3.557429	0.860289	0.391957	-5.44086	0.544769	0.650411
Monocytes	GM826	-0.48093	-0.25558	-0.85995	0.39214	-4.96034	0.606935	0.723158
Monocytes	ZFP263	0.112294	5.167548	0.85993	0.392153	-5.94709	0.520718	0.621934
Monocytes	RNASEH2B	-0.11796	4.594626	-0.85989	0.392174	-5.79379	0.529155	0.631934
Monocytes	ARL5C	0.12533	6.264356	0.859838	0.392204	-6.1108	0.504982	0.603236
Monocytes	TIPARP	0.085186	7.437182	0.859637	0.392314	-6.48832	0.488814	0.583885
Monocytes	B4GALT1	0.078455	7.137524	0.859332	0.392481	-6.15511	0.49305	0.588881
Monocytes	FTO	0.072081	6.592827	0.858918	0.392709	-6.16282	0.500814	0.597922
Monocytes	DPH3	0.080458	5.480129	0.85876	0.392795	-5.95683	0.516676	0.616745
Monocytes	4933408B17RIK	0.339108	1.941966	0.858642	0.39286	-5.00805	0.57081	0.680568
Monocytes	RAB13	-0.32454	1.635625	-0.85839	0.392997	-5.05743	0.575859	0.686394
Monocytes	EIF3I	-0.07356	6.822191	-0.85835	0.393019	-6.24897	0.497745	0.594139
Monocytes	ADRM1	-0.10324	5.477684	-0.85799	0.393217	-5.94167	0.516987	0.616912
Monocytes	BAHCC1	-0.3336	1.235974	-0.85754	0.393464	-5.02724	0.582918	0.69435
Monocytes	STRN3	0.072531	7.654054	0.857442	0.393519	-6.34292	0.486774	0.580816
Monocytes	GM31462	-0.36369	0.152102	-0.85735	0.393572	-4.95984	0.601135	0.715683
Monocytes	VDR	0.25696	0.938817	0.857157	0.393676	-5.27489	0.58793	0.700282
Monocytes	FAM189A1	-0.53211	2.327494	-0.85699	0.393767	-4.91649	0.565281	0.673818
Monocytes	AP1AR	-0.12928	5.002368	-0.85693	0.393799	-5.71101	0.524269	0.625515
Monocytes	ULK4	0.216749	2.667963	0.856607	0.393978	-5.22972	0.560042	0.667618
Monocytes	KRTCAP3	-0.28526	0.893962	-0.85652	0.394027	-5.06225	0.588886	0.701375
Monocytes	GDF11	-0.39477	1.639394	-0.85639	0.394096	-4.93721	0.576596	0.687071
Monocytes	SMARCAL1	-0.17312	2.628016	-0.85605	0.394287	-5.29026	0.560892	0.668508
Monocytes	DHDH	0.196924	2.654165	0.855866	0.394385	-5.33015	0.560542	0.668071
Monocytes	SLC35A4	-0.14671	3.561213	-0.85546	0.394609	-5.52697	0.54662	0.651519
Monocytes	GLS2	-0.3242	1.618045	-0.85518	0.394761	-5.11735	0.577511	0.687845
Monocytes	DNTT	0.672544	1.449877	0.855165	0.394771	-4.88049	0.580269	0.691068
Monocytes	B3GALT1	-0.42039	3.129188	-0.85495	0.394892	-5.20334	0.553365	0.659622
Monocytes	CALD1	-0.26185	3.777745	-0.8547	0.39503	-5.3971	0.543344	0.647948
Monocytes	CELF6	0.486081	-0.70622	0.854691	0.395032	-4.89661	0.616923	0.733931
Monocytes	ZFP597	0.217514	2.862663	0.854398	0.395194	-5.24149	0.557542	0.664684
Monocytes	GM16283	0.406987	0.252287	0.854384	0.395201	-4.85696	0.600328	0.714699
Monocytes	INTS1	-0.17956	3.347025	-0.85431	0.395244	-5.42102	0.549977	0.655787
Monocytes	GM11423	0.330041	0.892029	0.854294	0.395251	-4.96841	0.58952	0.702114
Monocytes	DHRS7	0.154555	4.016547	0.854234	0.395284	-5.75257	0.539705	0.643681
Monocytes	NOLC1	-0.11995	5.264292	-0.85421	0.3953	-5.85809	0.521126	0.621707
Monocytes	MRPL55	0.114543	4.367452	0.85414	0.395336	-5.717	0.534407	0.637424
Monocytes	RAB1A	0.059095	7.08553	0.854139	0.395337	-6.27745	0.495278	0.59097
Monocytes	ZC3H14	-0.07966	5.656337	-0.8539	0.395466	-5.94783	0.515479	0.615045
Monocytes	BSDC1	-0.1364	5.056629	-0.85389	0.395476	-5.75439	0.524211	0.6254
Monocytes	RHOJ	0.367921	1.992484	0.853611	0.395627	-5.02308	0.5716	0.681166
Monocytes	GIPC1	-0.11427	4.359749	-0.85353	0.395673	-5.72933	0.534689	0.637809
Monocytes	COPZ2	-0.24108	2.183281	-0.85338	0.395754	-5.18257	0.568563	0.677664
Monocytes	BACH1	0.094384	5.9952	0.853093	0.395913	-6.25363	0.510906	0.609563
Monocytes	TESK1	-0.12788	3.781202	-0.85288	0.396033	-5.62106	0.543735	0.648365

Monocytes	ZFP874B	-0.31976	2.178101	-0.8522	0.396408	-5.05743	0.569235	0.678158
Monocytes	GM31597	-0.24744	2.208627	-0.85219	0.396413	-5.10457	0.568744	0.677583
Monocytes	DOCK2	-0.06829	9.34967	-0.85196	0.396538	-6.62191	0.465795	0.555506
Monocytes	CARMIL1	-0.19532	4.075468	-0.85182	0.396617	-5.42576	0.539575	0.643346
Monocytes	PUS10	-0.11132	4.485499	-0.85163	0.396718	-5.82589	0.533392	0.636045
Monocytes	ASPH	0.113753	4.664011	0.851628	0.396721	-6.11966	0.530725	0.632892
Monocytes	DNAH10	-0.45737	0.024576	-0.85158	0.396747	-4.86851	0.605082	0.720036
Monocytes	VIM	0.107537	8.109275	0.851535	0.396773	-6.60151	0.482065	0.575011
Monocytes	GVIN1	0.259038	3.037272	0.851497	0.396794	-5.56168	0.555588	0.662204
Monocytes	PSMB7	-0.07464	5.624583	-0.85136	0.396871	-6.04681	0.516626	0.616254
Monocytes	SH2D4B	-0.18636	4.439997	-0.85131	0.3969	-5.68496	0.534075	0.63693
Monocytes	ZMYND19	-0.20603	3.409984	-0.85128	0.396911	-5.40487	0.549779	0.655454
Monocytes	GM32296	0.360252	-0.65404	0.851015	0.39706	-4.97223	0.617028	0.733895
Monocytes	SNED1	-0.25558	1.751149	-0.85089	0.397127	-5.30417	0.576313	0.6865
Monocytes	GM4129	0.360102	1.28611	0.850717	0.397225	-4.95021	0.583996	0.695431
Monocytes	4930557K07RIK	0.320057	1.483636	0.85065	0.397262	-5.03153	0.580736	0.691624
Monocytes	DTX2	0.122191	4.396914	0.850524	0.397331	-5.71168	0.534928	0.637784
Monocytes	DCAF11	-0.11017	4.559305	-0.85043	0.397385	-5.75958	0.532494	0.634907
Monocytes	NXPH4	-0.45016	-0.60095	-0.85023	0.397495	-4.88935	0.616274	0.732926
Monocytes	ZKSCAN17	-0.12035	3.498798	-0.84989	0.397681	-5.65393	0.548879	0.654108
Monocytes	MCM2	-0.18691	4.762793	-0.84953	0.397883	-5.72731	0.52979	0.631672
Monocytes	GM35867	-0.41864	0.456051	-0.84951	0.397892	-4.95339	0.598311	0.712048
Monocytes	MAOB	-0.41492	1.627366	-0.84932	0.397999	-5.00062	0.57876	0.68925
Monocytes	GM13963	-0.47193	-0.29578	-0.84927	0.398027	-4.9079	0.611237	0.727062
Monocytes	BEND5	0.44768	0.115495	0.849237	0.398043	-4.80747	0.604129	0.718811
Monocytes	PDXP	-0.34766	1.557173	-0.84898	0.398183	-5.04614	0.579912	0.690596
Monocytes	CARD10	-0.21091	1.098314	-0.84898	0.398187	-5.28753	0.587503	0.699458
Monocytes	CUL4B	0.123416	4.524951	0.848825	0.398271	-5.754	0.533339	0.635869
Monocytes	USP45	0.12426	4.074097	0.84878	0.398296	-5.70276	0.54014	0.643899
Monocytes	SNRPD2	-0.07341	6.391824	-0.84877	0.398304	-6.14209	0.506176	0.603664
Monocytes	PHGDH	-0.26073	4.379094	-0.84858	0.398405	-5.39823	0.535529	0.638456
Monocytes	PNP	0.099798	5.924728	0.848466	0.39847	-6.12238	0.512824	0.611601
Monocytes	EIF4G2	0.057362	7.71796	0.848198	0.398618	-6.33694	0.487823	0.581945
Monocytes	CALHM6	-0.24705	3.048969	-0.84815	0.398647	-5.68558	0.555965	0.662714
Monocytes	ADARB1	-0.29288	3.009129	-0.84791	0.398776	-5.1631	0.55659	0.663449
Monocytes	GALNT1	0.067774	6.628673	0.847903	0.398782	-6.16997	0.502842	0.599861
Monocytes	PPP4R2	-0.0724	6.318519	-0.84787	0.3988	-6.18392	0.507213	0.605063
Monocytes	ADRB2	0.153138	4.714467	0.847853	0.398809	-5.96103	0.530509	0.632696
Monocytes	RAB11FIP4	-0.21612	1.161785	-0.84776	0.398863	-5.26218	0.586447	0.698431
Monocytes	PIEZO1	-0.08829	5.046187	-0.84773	0.398875	-6.05605	0.525595	0.626893
Monocytes	TFAM	-0.13746	4.293459	-0.84767	0.398912	-5.54973	0.536819	0.640176
Monocytes	DDHD1	-0.10788	6.276227	-0.8476	0.398947	-6.22046	0.507812	0.605827
Monocytes	KMT2B	-0.13742	3.888591	-0.84752	0.398991	-5.54487	0.542967	0.64748
Monocytes	SERPINA11	-0.33646	1.813558	-0.84747	0.399022	-5.03862	0.575717	0.685958
Monocytes	UBLCP1	0.105739	4.773952	0.847298	0.399117	-5.74447	0.529679	0.631761
Monocytes	RGS5	0.499129	1.107497	0.846891	0.399342	-4.99569	0.587621	0.699719
Monocytes	SOX18	0.505238	0.720608	0.846849	0.399366	-4.8557	0.594107	0.707285

Monocytes	HTRA3	0.301308	0.502839	0.846604	0.399502	-5.07119	0.597837	0.711657
Monocytes	PDPR	0.165158	3.937711	0.846601	0.399503	-5.52056	0.542508	0.646833
Monocytes	HSD17B12	-0.08075	6.085483	-0.84634	0.399649	-6.16389	0.510917	0.609336
Monocytes	ARL6IP6	0.086407	5.278997	0.845988	0.399843	-5.96209	0.522687	0.623221
Monocytes	KDM4D	-0.42609	0.328103	-0.84597	0.399854	-4.89934	0.601077	0.71523
Monocytes	BOLA2	0.089889	5.686348	0.845898	0.399893	-6.06104	0.516759	0.616224
Monocytes	PRCC	0.089836	5.107381	0.84559	0.400064	-5.87175	0.525361	0.626343
Monocytes	ZBTB33	-0.1755	2.982117	-0.84549	0.400119	-5.31265	0.557724	0.664531
Monocytes	TMEM165	0.074546	5.311537	0.845119	0.400325	-6.02549	0.522491	0.622893
Monocytes	ARF4	-0.06701	8.479652	-0.84512	0.400328	-6.53748	0.478345	0.570313
Monocytes	STX6	-0.09361	5.2207	-0.84503	0.400375	-5.95646	0.523822	0.624487
Monocytes	FHOD1	-0.17545	2.907952	-0.84461	0.400609	-5.35932	0.559242	0.666126
Monocytes	POP5	0.09263	4.525116	0.844559	0.400637	-5.74615	0.534351	0.636827
Monocytes	NSRP1	0.097389	5.229533	0.844395	0.400728	-5.84174	0.523927	0.624482
Monocytes	KRT18	-0.2972	3.557982	-0.84427	0.400799	-5.42934	0.54912	0.654267
Monocytes	FAM126B	0.147694	3.772291	0.844171	0.400852	-5.45852	0.545817	0.650403
Monocytes	ZFP456	-0.39299	0.791994	-0.84413	0.400876	-4.93507	0.593795	0.706603
Monocytes	F13B	-0.37664	1.463355	-0.84377	0.401078	-4.99435	0.582813	0.693702
Monocytes	HIST1H3C	-0.47739	1.047316	-0.84324	0.401372	-4.95227	0.590082	0.701999
Monocytes	RFNG	0.206187	2.228203	0.843047	0.401477	-5.21275	0.57068	0.679407
Monocytes	FAM98A	-0.15786	3.607656	-0.84303	0.401484	-5.40035	0.548891	0.653828
Monocytes	SLC25A27	0.386457	0.260835	0.842105	0.402001	-4.90933	0.603847	0.717866
Monocytes	ABCC2	-0.29897	2.212583	-0.8421	0.402004	-5.1162	0.571344	0.679972
Monocytes	PDE7A	-0.10039	6.703412	-0.84191	0.402112	-6.0881	0.503636	0.600162
Monocytes	GM6787	-0.40428	0.188055	-0.84185	0.402141	-4.89213	0.605097	0.71944
Monocytes	ZFP41	0.39301	0.707145	0.841841	0.402148	-4.91626	0.59624	0.709145
Monocytes	ZFP850	0.32123	1.010206	0.84177	0.402188	-4.94915	0.591135	0.703231
Monocytes	SPIB	-0.32572	4.129765	-0.84171	0.402223	-5.15894	0.541275	0.644822
Monocytes	GM14085	0.472902	-1.35884	0.841603	0.402281	-4.80534	0.632343	0.751062
Monocytes	LY6G	0.543095	-0.81776	0.841519	0.402328	-4.82152	0.622665	0.739883
Monocytes	NEB	-0.38324	1.258562	-0.84147	0.402355	-4.93089	0.586986	0.698447
Monocytes	CACNB2	-0.22311	4.420818	-0.84142	0.40238	-5.74632	0.536865	0.639649
Monocytes	FGD3	0.115936	4.660813	0.841359	0.402417	-5.85145	0.533258	0.635399
Monocytes	EPOP	-0.29106	1.472765	-0.84123	0.402489	-5.08456	0.583461	0.694363
Monocytes	CCRL2	0.174141	4.741765	0.840958	0.40264	-6.24049	0.532202	0.634059
Monocytes	ASPDH	-0.40531	1.294554	-0.84082	0.402717	-4.9841	0.58659	0.697916
Monocytes	IER5	-0.1	6.692584	-0.84054	0.402872	-6.30702	0.504045	0.600682
Monocytes	PTPRG	0.38079	2.191113	0.840509	0.402891	-5.10262	0.571982	0.680856
Monocytes	DNAH2	0.276725	0.783062	0.840261	0.403029	-5.13373	0.595344	0.708081
Monocytes	PLEK	0.089377	7.520494	0.840215	0.403054	-6.55957	0.492627	0.58704
Monocytes	ZFP869	-0.13901	4.026546	-0.84	0.403174	-5.61414	0.543215	0.647079
Monocytes	PIK3CB	0.106612	4.688415	0.839756	0.40331	-6.10218	0.533205	0.63529
Monocytes	ZFPL1	-0.13373	3.466605	-0.83965	0.403372	-5.48982	0.551846	0.657304
Monocytes	GM16230	-0.49358	0.238127	-0.83959	0.403401	-4.88545	0.604645	0.71903
Monocytes	AOX3	-0.41904	0.998188	-0.83957	0.403412	-4.99299	0.591736	0.704013
Monocytes	PCBP1	-0.06138	8.199098	-0.83955	0.403428	-6.47956	0.483444	0.576192
Monocytes	RAB18	-0.07064	5.748093	-0.83952	0.40344	-6.04727	0.517604	0.616884

Monocytes	ACTR8	-0.14777	3.537225	-0.83929	0.40357	-5.48352	0.550851	0.656134
Monocytes	ACY3	-0.35592	2.04257	-0.8392	0.403622	-5.06811	0.574592	0.684004
Monocytes	TUSC2	-0.19782	3.017242	-0.83886	0.403809	-5.28834	0.559044	0.665858
Monocytes	1300002E11RIK	-0.24299	3.19234	-0.83881	0.403837	-5.15264	0.55629	0.662643
Monocytes	CEP162	-0.17515	3.080056	-0.83874	0.403875	-5.31959	0.558054	0.664731
Monocytes	GNG10	0.067232	6.940589	0.838736	0.403879	-6.44703	0.500801	0.597001
Monocytes	RILPL1	-0.29616	2.31814	-0.83841	0.404063	-5.06398	0.570279	0.679079
Monocytes	HIST1H4C	-0.44496	0.309171	-0.83832	0.404112	-4.8786	0.603693	0.718099
Monocytes	SEC23A	-0.12411	4.425257	-0.83828	0.404136	-5.63377	0.537399	0.64044
Monocytes	A930037H05RII	0.214932	3.242306	0.83824	0.404156	-5.51795	0.555592	0.661876
Monocytes	SLC33A1	0.117447	3.794664	0.838032	0.404272	-5.72323	0.547098	0.651805
Monocytes	LY96	-0.11232	4.287348	-0.83785	0.404376	-5.82446	0.539582	0.642949
Monocytes	TOMM40	-0.09674	5.225725	-0.83782	0.40439	-5.87378	0.525557	0.626371
Monocytes	USP38	0.079789	5.889969	0.837562	0.404535	-6.07254	0.515993	0.614917
Monocytes	ZSCAN29	-0.14066	3.485733	-0.83741	0.404622	-5.49457	0.552051	0.657543
Monocytes	VSIG10	-0.35813	0.47062	-0.83728	0.404691	-4.97865	0.601211	0.715079
Monocytes	THSD4	-0.53405	0.910314	-0.83723	0.404721	-4.97311	0.593754	0.706415
Monocytes	KCTD10	0.113346	4.043279	0.836991	0.404854	-5.7236	0.543503	0.647568
Monocytes	TIMP3	0.475245	2.197691	0.836972	0.404865	-5.03348	0.572543	0.681688
Monocytes	BPHL	0.137493	3.663858	0.836822	0.404949	-5.60835	0.549375	0.654493
Monocytes	TAB3	0.145861	3.680364	0.836629	0.405057	-5.4947	0.54912	0.654254
Monocytes	ZBTB14	-0.16348	3.004063	-0.83657	0.405089	-5.29402	0.559688	0.666683
Monocytes	ABCA8B	-0.41834	1.118002	-0.83654	0.405107	-4.98078	0.590359	0.702577
Monocytes	PAFAH2	-0.4318	0.619176	-0.83643	0.405167	-4.91922	0.598784	0.7124
Monocytes	HNRNPUL1	0.059764	7.28107	0.835624	0.405619	-6.24356	0.496899	0.592141
Monocytes	BCKDK	-0.10276	4.559456	-0.83552	0.405677	-5.74922	0.536183	0.638762
Monocytes	NID2	-0.27614	2.19946	-0.83552	0.405679	-5.23178	0.573057	0.682116
Monocytes	PRR3	-0.12005	3.987386	-0.83526	0.405822	-5.69034	0.544994	0.649077
Monocytes	BMF	0.25647	2.137288	0.835084	0.405921	-5.19923	0.574239	0.683479
Monocytes	GM10521	0.379108	0.308847	0.83501	0.405963	-4.89663	0.60479	0.71912
Monocytes	PHF2OS1	0.316102	1.058613	0.834835	0.406061	-4.95977	0.592118	0.704377
Monocytes	GM45051	-0.27163	1.845863	-0.83439	0.406312	-5.05805	0.579319	0.689325
Monocytes	VDAC1	0.079886	6.677223	0.834288	0.406367	-6.19875	0.505765	0.602624
Monocytes	FAM214B	0.173661	3.11159	0.834218	0.406406	-5.55567	0.558967	0.665506
Monocytes	UBE2G1	0.062548	7.307993	0.833868	0.406602	-6.28181	0.49713	0.592186
Monocytes	GBP5	-0.33789	2.014589	-0.83345	0.406836	-5.34272	0.577017	0.686357
Monocytes	SCAP	-0.15758	4.277309	-0.83328	0.406933	-5.56421	0.541403	0.644492
Monocytes	ATP5L	0.056545	8.67785	0.833056	0.407058	-6.55221	0.47893	0.570278
Monocytes	RNASEH1	0.156749	2.84733	0.832823	0.407189	-5.37442	0.563861	0.670847
Monocytes	HIST1H3A	0.361098	0.953909	0.832655	0.407283	-4.95147	0.59492	0.707163
Monocytes	MRTFB	-0.12521	4.475202	-0.8326	0.407311	-5.72251	0.538616	0.641219
Monocytes	TGFA	-0.47031	0.796415	-0.83233	0.407465	-4.88799	0.597731	0.710387
Monocytes	SLC39A13	-0.15741	2.796524	-0.83217	0.407557	-5.46118	0.564882	0.672033
Monocytes	DSTN	0.084115	6.886463	0.831883	0.407716	-6.27701	0.503777	0.599754
Monocytes	PFKFB3	-0.11151	6.273061	-0.83168	0.407833	-6.07675	0.512521	0.610161
Monocytes	ANAPC5	0.068559	6.399962	0.83162	0.407864	-6.12033	0.510709	0.60801
Monocytes	FAM199X	0.181357	2.776803	0.83141	0.407982	-5.37579	0.565486	0.672597

Monocytes	NRG2	0.162251	1.685011	0.831193	0.408104	-5.67596	0.583312	0.69337
Monocytes	YJU2	0.13439	3.454196	0.830724	0.408367	-5.46651	0.555162	0.660184
Monocytes	9930104L06RIK	0.31876	1.356879	0.830448	0.408522	-4.97829	0.589208	0.699867
Monocytes	COL4A3BP	0.075496	6.352484	0.830238	0.40864	-6.19092	0.512021	0.609039
Monocytes	GM4070	0.238708	2.887167	0.829852	0.408858	-5.56879	0.564534	0.670789
Monocytes	4930430F08RIK	0.269212	1.035161	0.829795	0.40889	-5.03599	0.594906	0.706232
Monocytes	TCEA2	-0.36126	0.335442	-0.8294	0.409109	-4.93591	0.607087	0.720196
Monocytes	CERKL	0.245463	1.852174	0.829078	0.409293	-5.26079	0.581629	0.69056
Monocytes	OSBPL7	0.158268	3.426262	0.829041	0.409314	-5.4132	0.556338	0.660978
Monocytes	RNF6	-0.07597	5.670645	-0.82898	0.409351	-6.02377	0.522356	0.620946
Monocytes	PTOV1	-0.11385	4.038474	-0.82889	0.409397	-5.69308	0.546831	0.649836
Monocytes	4933433G19RIK	0.312769	1.434512	0.82837	0.409692	-5.04719	0.588896	0.698864
Monocytes	SRGAP2	0.088513	6.60483	0.827952	0.409927	-6.22619	0.509364	0.605275
Monocytes	IKZF1	0.075164	8.378161	0.82794	0.409934	-6.40815	0.484858	0.576125
Monocytes	HMG20B	0.078795	5.119833	0.827526	0.410167	-5.93445	0.531192	0.630881
Monocytes	FSHR	-0.31276	0.419984	-0.82743	0.41022	-4.92481	0.606554	0.719003
Monocytes	GM26839	0.366568	0.656622	0.827137	0.410386	-4.97624	0.602583	0.714409
Monocytes	TRIM44	-0.08248	6.053928	-0.82703	0.410448	-6.0493	0.517563	0.614821
Monocytes	SMURF1	-0.11374	4.688664	-0.82702	0.410453	-5.77088	0.537738	0.63864
Monocytes	EIF2S3X	-0.14199	5.323448	-0.82694	0.410499	-5.94079	0.52825	0.627458
Monocytes	HAGHL	0.17896	2.911144	0.826346	0.410833	-5.46925	0.565712	0.671242
Monocytes	ASH1L	0.065393	7.367632	0.825915	0.411075	-6.34748	0.499517	0.59302
Monocytes	COX7A2	0.066307	8.039242	0.825726	0.411182	-6.43404	0.490345	0.582111
Monocytes	MCM5	-0.18494	4.910563	-0.82543	0.411347	-5.8541	0.535146	0.635125
Monocytes	PCDH15	-0.27516	1.323083	-0.82536	0.411387	-5.07112	0.592123	0.701793
Monocytes	LGALS3BP	-0.16293	4.826846	-0.82535	0.411397	-5.96267	0.536404	0.636615
Monocytes	KCNB1	0.461222	0.988195	0.825248	0.411452	-4.88223	0.597774	0.708392
Monocytes	GPD1	0.340446	1.749644	0.824978	0.411604	-5.04461	0.585092	0.693559
Monocytes	GM22146	0.294	2.073446	0.824948	0.411622	-5.0958	0.579755	0.687343
Monocytes	ZC3H12A	0.100131	5.128189	0.824861	0.41167	-5.99495	0.531963	0.631331
Monocytes	AFG3L2	0.081	4.593409	0.824513	0.411867	-5.84629	0.540158	0.640938
Monocytes	ADHFE1	-0.24892	2.690792	-0.82442	0.411918	-5.20602	0.569889	0.675789
Monocytes	DDX1	0.120498	4.964571	0.824345	0.411962	-5.77746	0.534561	0.634391
Monocytes	AMMECR1	0.126396	4.787165	0.82427	0.412004	-5.72188	0.537228	0.63756
Monocytes	FUNDC1	-0.08679	4.65588	-0.82385	0.412244	-5.937	0.539454	0.640074
Monocytes	KDM6B	-0.07593	8.152763	-0.82371	0.412322	-6.56155	0.489319	0.58074
Monocytes	TGFB3	-0.44884	0.567314	-0.82346	0.412462	-4.91091	0.605645	0.71722
Monocytes	HIST1H2AC	-0.31922	1.907677	-0.82312	0.412655	-5.17201	0.583104	0.691091
Monocytes	PPRC1	-0.11764	3.956149	-0.82294	0.412756	-5.68043	0.550353	0.652914
Monocytes	ZFP354C	-0.39103	1.777029	-0.82293	0.412761	-4.94584	0.585264	0.693717
Monocytes	GOLIM4	0.126835	5.140893	0.822847	0.412809	-5.9271	0.532343	0.631732
Monocytes	CES2A	-0.43494	0.898465	-0.82267	0.412909	-4.93558	0.60002	0.710947
Monocytes	RHBDD2	0.217449	2.284757	0.822602	0.412948	-5.19455	0.576918	0.684107
Monocytes	LCLAT1	0.170662	3.99918	0.822447	0.413035	-5.46665	0.549687	0.652299
Monocytes	ZFP763	0.456475	0.433803	0.822413	0.413054	-4.84576	0.607988	0.720283
Monocytes	D430001F17RIK	-0.56622	-0.87048	-0.82226	0.413142	-4.81668	0.630971	0.746845
Monocytes	MYL12A	-0.06573	7.929477	-0.82214	0.413211	-6.46436	0.492498	0.584714

Monocytes	PTPN21	0.369401	0.813665	0.822135	0.413212	-4.96138	0.601466	0.712744
Monocytes	OLFR543	0.462562	0.201065	0.822123	0.413219	-4.85449	0.612022	0.724972
Monocytes	SCYL2	0.105002	4.409687	0.822077	0.413244	-5.72257	0.54338	0.644909
Monocytes	THEMIS	-0.40691	1.160853	-0.82199	0.413294	-5.07561	0.595571	0.705951
Monocytes	GM31728	0.370302	-0.17439	0.821813	0.413394	-5.00666	0.618591	0.732637
Monocytes	ZFP319	-0.27898	2.544028	-0.82181	0.413396	-5.1787	0.572706	0.679341
Monocytes	POMT2	0.256301	1.813643	0.821766	0.413421	-5.09178	0.584658	0.693296
Monocytes	PHPT1	0.130295	4.330971	0.821682	0.413468	-5.63215	0.544583	0.646434
Monocytes	TRDV4	-0.47355	-1.36214	-0.8215	0.413572	-4.81719	0.639901	0.757287
Monocytes	TTC38	0.229609	2.505389	0.821385	0.413637	-5.24675	0.573353	0.680203
Monocytes	CD200R1	-0.19631	2.827999	-0.82133	0.413665	-5.48819	0.568153	0.674126
Monocytes	KDM5A	0.071553	7.014524	0.821271	0.413701	-6.22305	0.505208	0.599972
Monocytes	GM11714	-0.34361	1.498822	-0.82065	0.414052	-4.98918	0.59034	0.699725
Monocytes	FBXO6	-0.11072	3.653081	-0.8204	0.414194	-5.74741	0.555532	0.659125
Monocytes	PPP4C	-0.06381	6.80668	-0.8202	0.414307	-6.26167	0.508549	0.603784
Monocytes	TMEM87A	-0.09741	5.236916	-0.82016	0.414329	-5.88452	0.531357	0.630761
Monocytes	2510002D24RII	0.191773	3.199654	0.820137	0.414343	-5.31384	0.562676	0.667567
Monocytes	DNAL1	-0.20197	1.64713	-0.82011	0.414357	-5.36874	0.587913	0.697023
Monocytes	REPS2	-0.3434	0.905504	-0.81996	0.414442	-5.0424	0.600448	0.711607
Monocytes	HOXA7	0.469614	-0.09629	0.819749	0.414564	-4.85976	0.617816	0.731808
Monocytes	KCTD6	-0.16793	2.981269	-0.81973	0.414575	-5.42277	0.566227	0.671829
Monocytes	MRPS22	0.165126	3.085855	0.819388	0.414768	-5.37151	0.564729	0.669992
Monocytes	ZFP119A	0.193828	2.125414	0.819266	0.414838	-5.22463	0.580258	0.68816
Monocytes	HAP1	-0.37318	-0.45019	-0.81918	0.414887	-4.9337	0.624256	0.739216
Monocytes	FCRL1	0.221074	2.73778	0.819073	0.414947	-5.35372	0.570303	0.676561
Monocytes	ZMYM5	-0.08589	5.521459	-0.81903	0.414971	-5.93584	0.527366	0.626127
Monocytes	CCS	-0.1245	4.318203	-0.81883	0.415087	-5.73029	0.545551	0.647535
Monocytes	OIT3	-0.32058	2.345496	-0.8187	0.415157	-5.06471	0.576767	0.684079
Monocytes	GM9917	0.355628	0.773036	0.818491	0.415278	-5.0314	0.603133	0.7147
Monocytes	DIMT1	-0.15524	3.243055	-0.81801	0.415549	-5.43588	0.562635	0.667343
Monocytes	EEF1AKMT2	-0.16722	3.325831	-0.81791	0.415609	-5.51048	0.561323	0.665813
Monocytes	SCARB2	-0.07295	6.538743	-0.81777	0.415688	-6.31856	0.512952	0.608849
Monocytes	GLB1	0.09604	5.221188	0.81766	0.415749	-5.90662	0.532203	0.631624
Monocytes	EDEM2	0.112923	4.515035	0.817495	0.415843	-5.80051	0.542851	0.644243
Monocytes	MGAT1	0.11808	4.704416	0.817482	0.41585	-5.73722	0.539972	0.640856
Monocytes	SERPINA3G	-0.43202	3.597185	-0.81733	0.415935	-5.5876	0.557048	0.660914
Monocytes	RC3H2	0.073303	5.728871	0.817177	0.416024	-6.03109	0.524691	0.622836
Monocytes	ZFP993	0.229411	1.824761	0.817152	0.416038	-5.26169	0.585636	0.694314
Monocytes	CHSY3	-0.21645	1.978293	-0.81709	0.416072	-5.58497	0.583097	0.691357
Monocytes	ST8SIA1	0.366927	0.877835	0.817092	0.416072	-5.09071	0.601566	0.712827
Monocytes	KIF17	-0.33224	1.692096	-0.81692	0.416171	-5.10392	0.58784	0.69688
Monocytes	MRPL16	-0.11956	3.919331	-0.8169	0.416181	-5.6476	0.552019	0.655015
Monocytes	TRAPPC11	-0.13264	3.489165	-0.81688	0.416193	-5.50322	0.558745	0.662903
Monocytes	RGP1	-0.20437	2.34849	-0.81661	0.416347	-5.13185	0.577155	0.68433
Monocytes	MAD2L1	-0.15867	4.166093	-0.81645	0.416435	-5.65372	0.548325	0.650636
Monocytes	ASNS	0.349693	1.465562	0.816393	0.41647	-4.96773	0.591758	0.701394
Monocytes	G730013B05RII	-0.34511	-0.968	-0.81627	0.416538	-4.9123	0.634129	0.75045

Monocytes	GM5165	0.218143	2.638407	0.816223	0.416566	-5.19568	0.572447	0.678926
Monocytes	TMED3	0.08786	5.491352	0.816139	0.416614	-6.08437	0.52831	0.627102
Monocytes	SLC7A7	-0.14157	3.785978	-0.81559	0.416928	-5.70407	0.554566	0.657695
Monocytes	CCDC107	-0.09881	4.620705	-0.81534	0.417066	-5.8406	0.541812	0.64264
Monocytes	IFI35	-0.11677	5.333965	-0.81522	0.41714	-6.01912	0.531105	0.630039
Monocytes	1700016P03RIK	-0.12591	3.898827	-0.81488	0.417331	-6.16955	0.553109	0.655877
Monocytes	ZDHHC6	0.104006	4.609891	0.814712	0.417426	-5.7873	0.542164	0.643023
Monocytes	FSD2	-0.4697	-0.73763	-0.81469	0.417436	-4.82334	0.630719	0.74607
Monocytes	MRPL27	0.121111	4.210901	0.81459	0.417496	-5.72247	0.548276	0.650205
Monocytes	INTS14	-0.13474	4.691853	-0.81452	0.417537	-5.86421	0.540918	0.641558
Monocytes	GM49961	-0.41074	0.574285	-0.81417	0.417736	-4.92121	0.607819	0.719446
Monocytes	ZFP579	-0.35531	1.862923	-0.81408	0.417784	-4.93269	0.586014	0.69419
Monocytes	EIF2B1	-0.1406	3.62049	-0.81374	0.41798	-5.47509	0.55776	0.661222
Monocytes	SNHG4	-0.14808	3.683196	-0.81356	0.418084	-5.52814	0.556776	0.66007
Monocytes	COMMD3	-0.08149	5.85015	-0.81354	0.418095	-6.05139	0.523926	0.621429
Monocytes	GM47689	-0.14447	3.369026	-0.81341	0.418168	-5.42843	0.561726	0.665893
Monocytes	SIMC1	-0.10889	5.702765	-0.8133	0.418231	-5.93859	0.526091	0.62403
Monocytes	FADD	0.245496	2.421277	0.813294	0.418234	-5.24712	0.576953	0.683705
Monocytes	PMPCA	0.125916	4.024403	0.81319	0.418293	-5.63954	0.551455	0.653924
Monocytes	ABTB1	0.154201	4.303875	0.813167	0.418307	-5.62719	0.547139	0.648857
Monocytes	DOP1B	0.113935	4.289272	0.812958	0.418426	-5.83065	0.547405	0.649177
Monocytes	TMEM60	0.116478	4.112089	0.812907	0.418455	-5.65267	0.550138	0.652386
Monocytes	MRPL15	-0.08261	5.337746	-0.81282	0.418502	-5.99358	0.531535	0.630515
Monocytes	GLUL	-0.105	6.384395	-0.81261	0.418624	-6.11932	0.516226	0.612451
Monocytes	ASL	0.113575	4.484774	0.812601	0.41863	-5.85636	0.544431	0.645713
Monocytes	4732471J01RIK	-0.21216	1.996323	-0.81236	0.418767	-5.28832	0.5841	0.692132
Monocytes	10-Sep	0.138073	3.119761	0.812149	0.418887	-5.56733	0.565853	0.6709
Monocytes	SIK2	-0.0705	7.126745	-0.8121	0.418916	-6.34964	0.50574	0.600093
Monocytes	PCED1A	0.190334	2.508638	0.812092	0.41892	-5.31499	0.5757	0.682408
Monocytes	PPP4R3A	-0.05295	6.59174	-0.81183	0.419071	-6.19604	0.513419	0.609219
Monocytes	DNAH12	-0.26635	1.676699	-0.81178	0.419096	-5.36686	0.589502	0.698521
Monocytes	CHRM3	-0.37525	0.831294	-0.81157	0.419219	-5.08995	0.6039	0.715134
Monocytes	KIFC1	-0.26202	3.200672	-0.81135	0.419341	-5.43241	0.564837	0.669543
Monocytes	TAF6	0.144852	3.851886	0.810305	0.41994	-5.58344	0.555241	0.657835
Monocytes	LSM12	0.065124	6.360706	0.810274	0.419958	-6.19537	0.517534	0.613473
Monocytes	PMAIP1	0.10183	4.983149	0.810077	0.420071	-6.28177	0.537946	0.637493
Monocytes	IFIH1	0.14294	3.912874	0.80953	0.420384	-5.90348	0.554664	0.657014
Monocytes	HAUS5	0.183785	2.831524	0.809491	0.420406	-5.35619	0.571828	0.677088
Monocytes	IGKV9-124	-0.43553	-1.01492	-0.80884	0.420776	-4.7998	0.638171	0.753674
Monocytes	NAP1L1	-0.07769	7.439515	-0.80876	0.420825	-6.42018	0.502871	0.595773
Monocytes	GM32031	-0.20737	2.845461	-0.80873	0.420841	-5.2968	0.571972	0.677046
Monocytes	CCDC9	0.090785	4.238269	0.80849	0.420979	-5.76904	0.550074	0.651357
Monocytes	ARAF	-0.09898	4.478585	-0.80818	0.421159	-5.75329	0.546535	0.647144
Monocytes	CC2D1B	0.119698	3.659138	0.80742	0.421592	-5.5985	0.559472	0.662232
Monocytes	PSMA2	-0.06706	7.21693	-0.8074	0.421603	-6.32425	0.506423	0.599786
Monocytes	MAP1LC3A	0.120609	5.476214	0.807385	0.421611	-5.97971	0.531641	0.629568
Monocytes	RNPC3	-0.08703	4.741295	-0.80735	0.421631	-5.81097	0.542707	0.642582

Monocytes	AGA	-0.17021	3.27288	-0.80725	0.421691	-5.44462	0.565594	0.669388
Monocytes	PLPP6	-0.17745	2.754142	-0.80725	0.421691	-5.38886	0.573933	0.679119
Monocytes	IKBIP	0.224229	1.938543	0.807093	0.421779	-5.20554	0.587318	0.694725
Monocytes	SLC39A3	0.237244	1.91724	0.807084	0.421784	-5.193	0.587672	0.695137
Monocytes	CAGE1	0.306377	1.94977	0.807059	0.421798	-5.11533	0.587131	0.694508
Monocytes	GM26789	-0.41641	0.271556	-0.80683	0.42193	-4.92667	0.615862	0.727749
Monocytes	RBM8A	-0.07427	5.709751	-0.80662	0.42205	-6.07006	0.528355	0.625627
Monocytes	FUCA1	0.070015	5.963141	0.806453	0.422146	-6.21309	0.524675	0.621282
Monocytes	ANAPC1	0.102001	4.721072	0.806324	0.42222	-5.79442	0.543276	0.643182
Monocytes	SEC11C	0.072383	7.287381	0.805885	0.422472	-6.30389	0.505909	0.59892
Monocytes	ANKRD27	-0.15546	3.098779	-0.80574	0.422557	-5.43437	0.568925	0.672986
Monocytes	UBP1	0.079713	5.448392	0.805682	0.422588	-6.00729	0.532568	0.630382
Monocytes	MEMO1	-0.07769	6.363616	-0.80533	0.42279	-6.12013	0.519294	0.614615
Monocytes	ISG20L2	0.092227	5.083016	0.804978	0.422992	-5.92463	0.538422	0.637029
Monocytes	SHB	-0.14464	4.498582	-0.80484	0.423072	-5.71544	0.547359	0.647545
Monocytes	CCDC73	-0.16692	3.100981	-0.80464	0.423188	-5.35176	0.569394	0.67329
Monocytes	NOP56	-0.10971	4.591451	-0.80455	0.423236	-5.74859	0.546003	0.645933
Monocytes	PHACTR2	0.086868	6.036575	0.804228	0.423423	-6.2739	0.524503	0.620502
Monocytes	PTGES3	-0.05935	7.460041	-0.80405	0.423522	-6.38649	0.504098	0.596394
Monocytes	CTSE	0.13268	5.492809	0.803882	0.423621	-5.80036	0.532544	0.629978
Monocytes	RAP1GAP	-0.41594	0.58551	-0.80388	0.423624	-4.91009	0.611603	0.722117
Monocytes	NDUFS2	0.07987	6.235293	0.803768	0.423687	-6.15989	0.521601	0.61712
Monocytes	PPP3R1	0.072403	5.5618	0.803672	0.423742	-6.06841	0.531516	0.628827
Monocytes	9930022D16RIK	-0.33213	0.319301	-0.80365	0.423753	-5.07706	0.616245	0.727544
Monocytes	EGLN2	0.107769	4.887876	0.803553	0.42381	-5.88128	0.541653	0.640795
Monocytes	NR2F6	-0.13961	3.501892	-0.80236	0.424499	-5.63018	0.563978	0.666491
Monocytes	BICRAL	0.100608	5.023041	0.802312	0.424524	-5.86804	0.540372	0.63886
Monocytes	MIDN	-0.07463	6.064607	-0.80214	0.424625	-6.16992	0.524891	0.620658
Monocytes	BLOC1S6	-0.1292	3.751167	-0.80194	0.424738	-5.57075	0.560083	0.661997
Monocytes	GALNT16	-0.30471	0.933714	-0.80194	0.42474	-5.02297	0.606509	0.71595
Monocytes	ADGRE4	0.120213	3.108019	0.801836	0.424798	-6.12576	0.570326	0.673964
Monocytes	ODR4	-0.11604	4.042451	-0.80168	0.424888	-5.67995	0.555511	0.65671
Monocytes	LRRC18	-0.22514	1.588071	-0.80154	0.424966	-5.36405	0.595362	0.703147
Monocytes	EPN1	0.070012	6.109989	0.80152	0.42498	-6.21476	0.524226	0.620012
Monocytes	GM42670	0.37964	0.89839	0.801439	0.425026	-4.93213	0.607117	0.716796
Monocytes	LMF1	0.143215	3.239428	0.801386	0.425056	-5.54412	0.568216	0.67165
Monocytes	FIBP	0.093121	4.462532	0.801203	0.425162	-5.81041	0.549042	0.64923
Monocytes	4930509H03RIK	-0.23838	2.090644	-0.80106	0.425246	-5.09512	0.587009	0.693585
Monocytes	GM26670	-0.31822	0.402458	-0.80103	0.425262	-4.9329	0.615785	0.726895
Monocytes	TATDN2	0.085674	5.004884	0.800434	0.425605	-5.91382	0.541037	0.639706
Monocytes	PXN	0.076093	6.266447	0.800387	0.425632	-6.26521	0.522269	0.617642
Monocytes	1110012L19RIK	0.247791	1.829365	0.800349	0.425654	-5.13805	0.591681	0.698824
Monocytes	ULK2	-0.09881	5.457806	-0.80023	0.425723	-5.93334	0.534231	0.631761
Monocytes	PEX11B	-0.13778	3.446594	-0.79993	0.425895	-5.53877	0.565439	0.668242
Monocytes	TIPIN	-0.14332	5.238466	-0.79978	0.425983	-5.92763	0.537713	0.635746
Monocytes	FANCE	0.178313	2.916052	0.799521	0.426131	-5.35738	0.574131	0.678233
Monocytes	WDYHV1	-0.11899	4.174037	-0.79936	0.426225	-5.62198	0.554158	0.654984

Monocytes	SSU72	0.056206	6.693565	0.799238	0.426294	-6.26829	0.516407	0.610643
Monocytes	B020010K11RIK	0.348836	1.134374	0.799214	0.426308	-5.04068	0.603829	0.7128
Monocytes	GM19265	0.43528	0.22098	0.799054	0.4264	-4.85285	0.619745	0.731114
Monocytes	MICALL2	-0.27839	1.195719	-0.79892	0.426477	-5.14365	0.602863	0.711614
Monocytes	SLK	0.068884	6.477015	0.798627	0.426647	-6.3588	0.519748	0.614386
Monocytes	GINS4	-0.13302	3.753755	-0.79833	0.426819	-5.59082	0.561073	0.662775
Monocytes	EIF2B4	-0.09892	4.102435	-0.79828	0.426846	-5.68326	0.555595	0.656392
Monocytes	MORN3	-0.25312	1.323497	-0.79822	0.426884	-5.17299	0.600946	0.709163
Monocytes	1700007L15RIK	0.297879	1.663099	0.797885	0.427075	-5.04529	0.595382	0.70256
Monocytes	HIST2H2BE	0.367288	0.814865	0.797662	0.427204	-4.9428	0.609899	0.71936
Monocytes	SLC46A3	-0.12286	3.085031	-0.79754	0.427277	-5.73364	0.571953	0.675372
Monocytes	FUCA2	0.080263	4.624115	0.79748	0.427309	-5.99385	0.547708	0.647069
Monocytes	KATNB1	-0.23948	2.346793	-0.79747	0.427314	-5.20837	0.584001	0.689401
Monocytes	CENPN	-0.22184	2.920239	-0.79727	0.427431	-5.4002	0.574626	0.678551
Monocytes	ZMPSTE24	-0.1308	4.942134	-0.79721	0.427466	-5.75466	0.542849	0.641439
Monocytes	RAB2B	-0.15644	3.661593	-0.79711	0.42752	-5.44732	0.562741	0.664721
Monocytes	BMP8A	-0.30092	2.321943	-0.79708	0.427539	-5.16707	0.584419	0.689963
Monocytes	SLC30A5	-0.09383	5.799283	-0.79684	0.427681	-5.97816	0.530078	0.62638
Monocytes	GCN1	-0.09575	4.575371	-0.7967	0.427761	-5.75188	0.548609	0.6481
Monocytes	ATXN7L2	-0.23971	2.480154	-0.79648	0.427886	-5.21112	0.582041	0.687075
Monocytes	CEP104	0.159041	3.105243	0.796293	0.427995	-5.35723	0.57186	0.675266
Monocytes	DPH2	0.372699	0.879573	0.796214	0.42804	-4.95461	0.609028	0.718371
Monocytes	FICD	0.352711	0.917558	0.796142	0.428082	-4.9764	0.608372	0.717616
Monocytes	HIPK2	-0.08384	6.550097	-0.79604	0.428141	-6.2424	0.519176	0.613523
Monocytes	PAQR8	0.398871	0.223677	0.796027	0.428148	-4.95953	0.620478	0.731577
Monocytes	GART	0.113422	4.367941	0.795784	0.428289	-5.85276	0.551998	0.652022
Monocytes	VEZF1	-0.06914	6.00178	-0.79567	0.428356	-6.09667	0.527308	0.623061
Monocytes	GTF2I	-0.09617	5.762327	-0.79536	0.428532	-5.8766	0.530991	0.62733
Monocytes	ATIC	-0.11405	4.493549	-0.79528	0.42858	-5.84518	0.550215	0.649885
Monocytes	PRPF19	-0.0834	5.053951	-0.7945	0.429031	-5.95302	0.542101	0.640122
Monocytes	TMSB4X	-0.06055	12.24836	-0.79445	0.429063	-7.23462	0.444215	0.524031
Monocytes	APON	0.399978	1.166034	0.794204	0.429203	-5.01658	0.605043	0.71327
Monocytes	FAM193B	0.147745	4.050857	0.794077	0.429277	-5.46033	0.557724	0.658276
Monocytes	NDRG1	0.184486	2.72107	0.793829	0.42942	-5.55258	0.579041	0.683147
Monocytes	SRF	-0.12546	2.927184	-0.79383	0.429422	-5.4587	0.575681	0.679241
Monocytes	FANCC	-0.1171	4.74282	-0.79377	0.429456	-5.84718	0.547003	0.645772
Monocytes	GM47828	-0.44903	0.334612	-0.79339	0.429677	-4.83817	0.619777	0.730105
Monocytes	VAV1	0.071891	6.364742	0.792464	0.430211	-6.2016	0.523508	0.617569
Monocytes	BC147527	0.262583	1.109158	0.792233	0.430345	-5.1695	0.607086	0.714925
Monocytes	KAZN	-0.42445	0.348768	-0.79195	0.430512	-4.9344	0.620418	0.730255
Monocytes	LRFN1	-0.31419	1.097146	-0.79194	0.430515	-5.13598	0.607377	0.715233
Monocytes	MOCS3	-0.3097	1.446277	-0.79175	0.430623	-5.0635	0.60143	0.708398
Monocytes	TBCE	-0.08208	5.119789	-0.79171	0.43065	-5.92662	0.542267	0.639573
Monocytes	PCNX4	0.239875	2.141019	0.791307	0.430883	-5.14612	0.589768	0.694974
Monocytes	D930016D06RII	0.189715	2.876865	0.791303	0.430885	-5.27298	0.577633	0.680895
Monocytes	FPGT	-0.32939	1.345954	-0.79117	0.430961	-5.08081	0.603194	0.710532
Monocytes	CCNF	0.199942	3.438612	0.791166	0.430964	-5.53338	0.568554	0.670361

Monocytes	RBM7	0.079329	5.855126	0.790964	0.431082	-6.12586	0.531262	0.626786
Monocytes	PSMG1	-0.14493	3.325411	-0.79091	0.431113	-5.43095	0.570371	0.672514
Monocytes	KRAS	0.059422	7.278938	0.790886	0.431127	-6.35489	0.510575	0.602427
Monocytes	TUBB5	-0.10275	9.849372	-0.79087	0.431135	-6.74789	0.47549	0.560848
Monocytes	ZFP202	-0.37902	0.604939	-0.79072	0.431225	-5.00551	0.616008	0.725396
Monocytes	MRPL35	0.091092	5.21625	0.790632	0.431275	-5.95542	0.540849	0.638081
Monocytes	SNHG12	-0.16295	3.189103	-0.7906	0.431295	-5.45456	0.572567	0.675127
Monocytes	SENP6	-0.06055	6.681311	-0.78997	0.431658	-6.18386	0.519516	0.61278
Monocytes	RASSF8	0.210893	2.215433	0.789626	0.431859	-5.39649	0.589146	0.694025
Monocytes	ZFYVE28	-0.22482	0.624372	-0.78933	0.432033	-5.13643	0.616409	0.725438
Monocytes	CNOT9	0.094195	4.541441	0.789323	0.432035	-5.80142	0.551849	0.650563
Monocytes	CD200R4	-0.33186	1.662182	-0.78914	0.432142	-5.24024	0.598604	0.704897
Monocytes	FAM20B	0.13725	3.958304	0.789017	0.432213	-5.56246	0.561056	0.661314
Monocytes	RPRD1B	0.084483	5.480381	0.788859	0.432305	-6.02345	0.537637	0.633887
Monocytes	4921524J17RIK	-0.08335	5.474163	-0.7882	0.43269	-5.88796	0.538086	0.634196
Monocytes	ELK1	-0.23616	1.559348	-0.78816	0.432711	-5.06629	0.60082	0.707203
Monocytes	GM10785	0.237908	2.451276	0.788082	0.432756	-5.27074	0.585848	0.689884
Monocytes	ACADSB	0.140909	3.270603	0.787877	0.432876	-5.4887	0.57254	0.674338
Monocytes	BUD23	-0.13148	4.09892	-0.78759	0.433042	-5.68412	0.55948	0.659
Monocytes	TMX3	0.075204	5.505888	0.787502	0.433094	-6.04185	0.537822	0.633702
Monocytes	PPP1R35	0.147465	3.395179	0.787129	0.433311	-5.48752	0.570888	0.672264
Monocytes	ZFP46	-0.2154	1.77659	-0.78697	0.433404	-5.06016	0.597647	0.703282
Monocytes	CLN5	0.10368	4.008222	0.786584	0.433628	-5.74141	0.561379	0.661026
Monocytes	NELFE	-0.09012	4.393275	-0.78631	0.433788	-5.83529	0.555335	0.654041
Monocytes	ATG101	0.088368	5.222366	0.786197	0.433854	-5.93842	0.542564	0.639124
Monocytes	ATP23	-0.30869	1.824257	-0.78619	0.433861	-5.16814	0.597066	0.702506
Monocytes	E2F2	0.140207	4.99405	0.78606	0.433934	-5.8162	0.546048	0.643221
Monocytes	ADM	-0.40655	1.330377	-0.78599	0.433973	-5.01445	0.605477	0.712239
Monocytes	EPAS1	-0.22702	3.448567	-0.78586	0.434053	-5.35244	0.570294	0.671548
Monocytes	COA3	0.078611	5.812938	0.785744	0.434118	-6.11681	0.533667	0.628825
Monocytes	NPLOC4	-0.08224	5.537568	-0.78573	0.434124	-6.03848	0.537795	0.633664
Monocytes	GMFB	-0.07835	5.451593	-0.7857	0.434145	-6.01224	0.539091	0.635183
Monocytes	STON1	-0.39164	1.087662	-0.78558	0.434215	-4.97392	0.609658	0.717175
Monocytes	NDUFS5	0.07364	6.478554	0.785547	0.434233	-6.19802	0.523835	0.617279
Monocytes	ZFP955B	-0.25588	2.201747	-0.78545	0.43429	-5.12428	0.590726	0.695318
Monocytes	GM9967	-0.46657	0.569425	-0.78526	0.4344	-4.9366	0.618713	0.727651
Monocytes	SLC25A22	-0.24824	2.495595	-0.78523	0.434416	-5.27731	0.585859	0.689721
Monocytes	RCBTB1	-0.12538	3.826201	-0.78511	0.434485	-5.61092	0.564301	0.664702
Monocytes	PARP11	0.198812	3.46828	0.784493	0.434848	-5.44595	0.570418	0.671557
Monocytes	GM10101	0.353583	0.672014	0.784287	0.434968	-4.95565	0.617367	0.725873
Monocytes	BCAR1	-0.41478	0.776645	-0.78425	0.434992	-4.91643	0.615536	0.723767
Monocytes	ATF6	0.064265	6.979377	0.784211	0.435012	-6.38359	0.51697	0.609057
Monocytes	FMNL1	0.063158	6.443857	0.783997	0.435137	-6.36291	0.524753	0.618266
Monocytes	YY1	0.049773	7.144963	0.783973	0.435151	-6.30837	0.514598	0.606316
Monocytes	ISYNA1	-0.09275	4.852057	-0.78392	0.435184	-5.92505	0.548657	0.646319
Monocytes	CD46	0.276745	2.11028	0.783043	0.435694	-5.10355	0.593307	0.697775
Monocytes	RNF115	-0.05773	6.978362	-0.78299	0.435728	-6.29948	0.517505	0.609369

Monocytes	RRNAD1	0.128936	3.384404	0.782624	0.435939	-5.49789	0.572547	0.673572
Monocytes	GM21860	0.528891	-0.14186	0.782462	0.436034	-4.92284	0.632731	0.74301
Monocytes	D16ERTD472E	0.100737	4.914897	0.782268	0.436147	-5.97142	0.54854	0.645599
Monocytes	VILL	0.198914	1.228935	0.782104	0.436243	-5.37447	0.608639	0.715346
Monocytes	POLR2G	-0.0725	5.51542	-0.78201	0.436296	-6.05941	0.539386	0.634925
Monocytes	TBXAS1	-0.1464	3.468333	-0.78198	0.436318	-5.9788	0.571308	0.672161
Monocytes	IRF2	0.072669	6.689517	0.781896	0.436365	-6.25928	0.521978	0.614531
Monocytes	TXNDC9	-0.07163	5.512196	-0.78181	0.436415	-6.06103	0.539435	0.635009
Monocytes	PDHA1	0.087626	5.115043	0.781691	0.436485	-5.95242	0.545488	0.642087
Monocytes	TRMT13	0.13286	3.89491	0.781583	0.436548	-5.5916	0.564516	0.664326
Monocytes	ZFP951	0.247254	2.641469	0.781328	0.436697	-5.21137	0.584946	0.688031
Monocytes	NOM1	0.105885	4.141882	0.78112	0.436818	-5.73014	0.560731	0.659981
Monocytes	UTP23	0.121884	3.577367	0.781043	0.436864	-5.56855	0.56971	0.670432
Monocytes	CENPI	-0.19017	2.997583	-0.78104	0.436866	-5.46805	0.579099	0.681336
Monocytes	MLH1	0.148929	2.750298	0.780891	0.436952	-5.35599	0.583195	0.686117
Monocytes	ELOVL1	-0.08868	4.858115	-0.78056	0.437145	-6.01956	0.549773	0.647219
Monocytes	AI987944	-0.18201	2.764701	-0.78029	0.437304	-5.36565	0.583234	0.686106
Monocytes	GM29170	-0.2475	1.532706	-0.78009	0.437421	-5.06941	0.603913	0.710046
Monocytes	2700097O09RII	0.180701	3.090234	0.780073	0.437431	-5.37957	0.5779	0.679948
Monocytes	KLF10	-0.0842	5.248269	-0.78006	0.437437	-6.04366	0.543879	0.640309
Monocytes	GM46218	-0.35632	0.58606	-0.77984	0.437565	-4.96197	0.62045	0.728995
Monocytes	CSTF2	-0.12803	4.139659	-0.77952	0.437756	-5.63118	0.561204	0.660508
Monocytes	1700017B05RIK	0.12638	5.285252	0.779486	0.437775	-5.77384	0.543445	0.639776
Monocytes	EHD4	0.075588	6.076676	0.779435	0.437805	-6.242	0.531543	0.625833
Monocytes	SLC38A7	0.191338	2.59539	0.779411	0.437819	-5.3792	0.586169	0.689511
Monocytes	RAPGEF5	-0.27283	5.011787	-0.77931	0.437881	-5.51836	0.547634	0.644692
Monocytes	KAT7	-0.06965	5.68662	-0.77893	0.438101	-6.07014	0.537484	0.632782
Monocytes	HGH1	0.250288	1.856316	0.778892	0.438123	-5.11485	0.598672	0.703954
Monocytes	CAAA01147332	0.182952	2.131904	0.778875	0.438133	-5.32867	0.594022	0.698581
Monocytes	FUBP3	0.119279	4.08675	0.778661	0.438258	-5.68642	0.562215	0.661655
Monocytes	ACP2	-0.12697	3.54465	-0.7786	0.438294	-5.77676	0.570859	0.671721
Monocytes	TANK	-0.0847	6.451404	-0.77851	0.438345	-6.30866	0.526174	0.619537
Monocytes	VPS18	-0.09123	4.834066	-0.77814	0.438562	-5.92258	0.550737	0.648156
Monocytes	GTF2H3	-0.22186	2.299191	-0.77792	0.438691	-5.21668	0.591564	0.695623
Monocytes	SNX12	0.080235	4.718214	0.777702	0.438821	-5.87521	0.552593	0.65044
Monocytes	SELP	0.408693	1.452771	0.777691	0.438827	-5.11807	0.605906	0.712296
Monocytes	TXNRD1	-0.08047	5.871273	-0.77768	0.438834	-6.22053	0.535027	0.629891
Monocytes	WDR73	-0.16927	2.976485	-0.77728	0.439069	-5.3387	0.580493	0.682931
Monocytes	CKS1B	-0.13868	5.377173	-0.77725	0.439086	-6.05334	0.542606	0.63879
Monocytes	MLLT10	0.052167	7.464033	0.777217	0.439105	-6.37256	0.511905	0.602735
Monocytes	EME2	0.271627	0.924802	0.776816	0.439341	-5.00252	0.615374	0.723143
Monocytes	COX8A	0.052645	9.008145	0.776767	0.439369	-6.63361	0.490594	0.577513
Monocytes	EOMES	-0.50694	-0.77158	-0.77671	0.439403	-4.86771	0.645779	0.758059
Monocytes	PRKAG1	0.06849	5.515312	0.776248	0.439674	-6.01219	0.540891	0.636612
Monocytes	FAHD1	-0.28162	1.917334	-0.7762	0.439703	-5.12515	0.59855	0.703673
Monocytes	CTNND2	0.151745	1.726999	0.775924	0.439865	-5.77785	0.601784	0.707495
Monocytes	STARD4	-0.14548	3.526928	-0.7759	0.439878	-5.52841	0.571956	0.672947

Monocytes	RSAD2	0.271769	4.537164	0.775855	0.439905	-5.79689	0.555932	0.654303
Monocytes	ILK	-0.08255	5.628263	-0.77576	0.439962	-6.10208	0.539184	0.634749
Monocytes	TMEM183A	0.07166	5.16956	0.775647	0.440027	-5.95096	0.546155	0.642907
Monocytes	CD8B1	-0.43478	0.660348	-0.77565	0.440027	-4.98615	0.620261	0.728819
Monocytes	TUBGCP4	0.104815	4.42498	0.774955	0.440434	-5.78759	0.558066	0.656638
Monocytes	TOMM40L	0.213229	1.733558	0.774949	0.440438	-5.12171	0.602081	0.707693
Monocytes	MRPS18C	0.082428	5.696786	0.774592	0.440648	-6.05037	0.538624	0.633962
Monocytes	INO80B	0.114308	4.146114	0.774533	0.440682	-5.69511	0.562569	0.661935
Monocytes	TSIX	-1.88524	1.906429	-0.77451	0.440693	-4.98811	0.599262	0.704497
Monocytes	THRA	0.161765	3.771843	0.77438	0.440772	-5.49987	0.568554	0.668922
Monocytes	D830050J10RIK	0.244922	1.312085	0.774173	0.440894	-5.20181	0.609526	0.716364
Monocytes	HNRNPU	-0.05957	8.26781	-0.77401	0.440991	-6.48415	0.501474	0.590295
Monocytes	ZFP871	0.094445	5.249328	0.774007	0.440992	-5.94037	0.545497	0.642039
Monocytes	SPAG5	-0.21623	3.137614	-0.7739	0.441052	-5.41977	0.578862	0.680907
Monocytes	WDR27	-0.32929	0.499373	-0.77383	0.441094	-4.95389	0.623745	0.732721
Monocytes	PTAFR	0.122283	4.645313	0.773726	0.441156	-6.33268	0.554826	0.652969
Monocytes	SCCPDH	0.210445	2.465776	0.7734	0.441348	-5.19652	0.590039	0.693947
Monocytes	TNRC6C	0.066651	7.143837	0.773345	0.441381	-6.2757	0.517462	0.609224
Monocytes	MED8	0.080501	5.008062	0.773336	0.441386	-6.03233	0.549285	0.646547
Monocytes	RFXAP	0.099596	4.179927	0.773194	0.44147	-5.69789	0.56224	0.661675
Monocytes	NDUFB8	0.062092	7.487591	0.773043	0.441559	-6.42357	0.512566	0.603524
Monocytes	SARNP	-0.05081	7.797226	-0.77301	0.441579	-6.44472	0.508175	0.598345
Monocytes	ADGRG3	0.278492	2.366781	0.772758	0.441726	-5.13191	0.591776	0.695977
Monocytes	CBWD1	-0.14037	4.090993	-0.77268	0.441773	-5.6133	0.563695	0.663396
Monocytes	DUS2	0.201091	3.353616	0.772661	0.441784	-5.33224	0.57552	0.67715
Monocytes	MTFR2	0.142917	3.615592	0.772319	0.441985	-5.75782	0.571476	0.672376
Monocytes	JAK3	0.203585	2.778845	0.772126	0.442099	-5.35423	0.585197	0.688308
Monocytes	FKRP	-0.19125	2.332289	-0.77197	0.442191	-5.28002	0.592675	0.696936
Monocytes	WIPI2	0.086599	5.276718	0.771753	0.442319	-5.91802	0.545626	0.642227
Monocytes	RHOQ	-0.10309	5.771108	-0.77098	0.442772	-6.08318	0.538512	0.63364
Monocytes	MYH9	0.053498	7.80338	0.770905	0.442818	-6.48941	0.508853	0.598788
Monocytes	DTX4	0.193218	2.365512	0.7709	0.442821	-5.49005	0.592639	0.696637
Monocytes	GM5617	0.149429	3.238502	0.770784	0.44289	-5.5181	0.578212	0.679969
Monocytes	SMARCAD1	-0.08032	4.984115	-0.77074	0.442915	-5.90723	0.550514	0.647728
Monocytes	GM19696	0.454694	0.016291	0.770452	0.443086	-4.88631	0.63362	0.743822
Monocytes	ZFP790	0.229835	2.226902	0.770312	0.443168	-5.16987	0.595137	0.699513
Monocytes	YTHDF2	-0.06401	6.035232	-0.77024	0.44321	-6.14596	0.534704	0.629166
Monocytes	ARHGAP27OS2	-0.19129	2.032169	-0.76997	0.443371	-5.27077	0.59853	0.703422
Monocytes	SRL	0.346622	0.890591	0.769918	0.443401	-4.91706	0.618206	0.726103
Monocytes	SPSB3	-0.11944	3.587341	-0.76981	0.443466	-5.53535	0.572826	0.67372
Monocytes	GM37494	0.128765	3.825168	0.769724	0.443515	-5.60948	0.569003	0.669293
Monocytes	SLC36A1	0.145278	2.851189	0.769398	0.443708	-5.4084	0.58502	0.687877
Monocytes	RAB39	-0.18874	2.115142	-0.76926	0.443791	-5.50289	0.597352	0.702123
Monocytes	GTSF2	0.447269	0.091247	0.768766	0.444081	-4.90804	0.632972	0.742925
Monocytes	ULK3	0.22252	2.219052	0.768586	0.444187	-5.2133	0.59598	0.700373
Monocytes	CLUAP1	0.122355	3.885137	0.768141	0.44445	-5.55184	0.568854	0.668726
Monocytes	ESRRG	-0.36123	1.226258	-0.76808	0.444484	-4.99457	0.613225	0.720069

Monocytes	TGIF1	-0.09119	6.736671	-0.76758	0.444781	-6.3588	0.525393	0.61795
Monocytes	XRCC6	-0.17801	5.144906	-0.76755	0.444801	-5.61448	0.5493	0.645968
Monocytes	UCHL5	-0.07837	5.728606	-0.76726	0.444971	-6.0689	0.540395	0.635624
Monocytes	PLEKHB2	-0.08358	4.419141	-0.7672	0.445008	-5.93524	0.560603	0.659243
Monocytes	EPB41	-0.09655	8.026595	-0.76712	0.445055	-6.52226	0.506875	0.596285
Monocytes	SAFB	-0.07212	6.192591	-0.76706	0.445091	-6.13946	0.533431	0.627517
Monocytes	B230208H11RII	0.334773	0.861264	0.767011	0.445118	-5.12959	0.619857	0.727852
Monocytes	ALG3	-0.26273	1.77052	-0.76694	0.44516	-5.16091	0.604086	0.709692
Monocytes	ZKSCAN3	0.083595	4.82207	0.766928	0.445168	-5.82309	0.554296	0.651923
Monocytes	FTSJ3	-0.14172	4.016619	-0.76669	0.445308	-5.69178	0.566987	0.666804
Monocytes	SLC45A4	-0.14044	3.337998	-0.76665	0.445332	-5.47467	0.577926	0.679517
Monocytes	TRIM37	-0.12107	4.93302	-0.76664	0.445339	-5.73324	0.552578	0.650009
Monocytes	KDELR1	0.062228	6.091298	0.766347	0.445511	-6.19395	0.535086	0.629387
Monocytes	BHLHE41	-0.40723	1.739361	-0.76592	0.445762	-4.976	0.605046	0.710439
Monocytes	VSTM4	-0.48323	0.147061	-0.76553	0.445994	-4.89825	0.633221	0.742701
Monocytes	GRAMD1A	-0.13474	4.325204	-0.76548	0.446026	-5.67987	0.562673	0.661276
Monocytes	GM19710	0.219924	2.595654	0.76534	0.446107	-5.39158	0.590772	0.693895
Monocytes	DENND1A	0.060386	7.497877	0.765259	0.446155	-6.42839	0.514913	0.605398
Monocytes	E430024I08RIK	0.241303	1.898133	0.765018	0.446298	-5.11952	0.602538	0.707606
Monocytes	PIMREG	0.27933	2.104196	0.764947	0.44634	-5.28898	0.599036	0.703563
Monocytes	GM43466	0.293345	1.497294	0.764877	0.446381	-5.06881	0.609416	0.715534
Monocytes	ZSWIM6	-0.07118	8.155524	-0.76475	0.446455	-6.54471	0.505594	0.594508
Monocytes	MICAL1	-0.11283	3.487265	-0.76474	0.446462	-5.70705	0.576097	0.677008
Monocytes	COX11	0.11932	3.751078	0.764599	0.446546	-5.54959	0.571833	0.672128
Monocytes	GM29570	-0.35666	0.692445	-0.76455	0.446576	-4.97377	0.623486	0.731793
Monocytes	SNRNP48	0.090819	4.916287	0.764531	0.446586	-5.86299	0.553412	0.650682
Monocytes	UBA1	0.071687	6.00691	0.764419	0.446653	-6.13536	0.536779	0.631267
Monocytes	GM7160	0.243724	2.542262	0.764171	0.4468	-5.27257	0.591798	0.695265
Monocytes	GM16279	-0.35795	1.054801	-0.76399	0.446908	-5.00711	0.617321	0.724677
Monocytes	FAM192A	-0.08851	4.607769	-0.76387	0.44698	-5.78472	0.558436	0.656514
Monocytes	CCT2	-0.0637	6.286032	-0.7637	0.447081	-6.2088	0.532852	0.626618
Monocytes	CIITA	-0.13484	2.478401	-0.76336	0.447282	-5.94034	0.593154	0.696774
Monocytes	FCRLS	0.553041	-1.03209	0.763332	0.447297	-4.98071	0.6553	0.768121
Monocytes	LZTR1	-0.17064	2.612289	-0.76299	0.447497	-5.35869	0.591105	0.694354
Monocytes	NQO2	0.100318	3.942173	0.762864	0.447575	-5.76521	0.569381	0.669201
Monocytes	DHX16	-0.12523	4.087189	-0.76223	0.447951	-5.68115	0.567468	0.666689
Monocytes	PPP1CA	0.05475	8.233158	0.762035	0.448067	-6.53055	0.505481	0.594048
Monocytes	5830428M24RI	0.166057	2.548294	0.761873	0.448163	-5.47305	0.592741	0.695916
Monocytes	GM50340	0.317766	1.533078	0.761801	0.448206	-5.0486	0.610011	0.715869
Monocytes	SH3BP5	-0.08912	6.380773	-0.76127	0.448523	-6.20921	0.53245	0.625641
Monocytes	RAB11FIP4OS1	0.293062	-0.78929	0.761101	0.448622	-5.02603	0.651861	0.763721
Monocytes	SALL2	-0.37564	0.090711	-0.76107	0.448641	-4.94622	0.63574	0.745304
Monocytes	PTCD1	-0.18169	2.585716	-0.76095	0.44871	-5.29639	0.592339	0.695386
Monocytes	MRPL2	0.097742	4.862057	0.760857	0.448767	-5.92821	0.555568	0.652713
Monocytes	HIST1H2BM	-0.3145	2.139918	-0.76081	0.448797	-5.14328	0.59985	0.704086
Monocytes	HIC2	-0.17836	2.712677	-0.76078	0.448813	-5.42188	0.590218	0.692959
Monocytes	GLOD4	-0.07781	5.050654	-0.76076	0.448825	-5.97441	0.552637	0.649305

Monocytes	BUD13	0.128699	3.714639	0.760353	0.449067	-5.52257	0.573968	0.673974
Monocytes	RIF1	-0.09695	5.435862	-0.76028	0.44911	-5.94223	0.546886	0.642467
Monocytes	HDGF	0.065675	6.998077	0.760223	0.449144	-6.29816	0.523537	0.615145
Monocytes	FBP1	0.202481	5.143064	0.759957	0.449302	-5.88741	0.551425	0.647814
Monocytes	GM11131	0.37352	0.815809	0.759889	0.449343	-4.97161	0.623025	0.730697
Monocytes	BBS9	-0.13593	4.909527	-0.75987	0.449351	-5.81694	0.555048	0.652057
Monocytes	WARS2	0.147049	3.512477	0.75974	0.449431	-5.55034	0.577281	0.677965
Monocytes	ARL4C	0.076461	5.243589	0.759705	0.449452	-6.27515	0.549873	0.646075
Monocytes	MVP	-0.07858	5.108561	-0.75938	0.449647	-6.1045	0.552129	0.648595
Monocytes	RAI14	0.21643	2.228896	0.759205	0.449749	-5.3782	0.598804	0.702781
Monocytes	RING1	-0.1328	3.585545	-0.75899	0.449876	-5.59244	0.576311	0.676769
Monocytes	RANBP6	0.25071	1.620455	0.75896	0.449895	-5.08459	0.609204	0.714789
Monocytes	FAM221B	0.313104	0.343919	0.758947	0.449903	-5.04997	0.631669	0.740591
Monocytes	GMPPA	0.12816	3.453598	0.758636	0.450088	-5.64954	0.578579	0.679316
Monocytes	KCNH7	0.4185	-0.13979	0.758528	0.450152	-4.89885	0.640549	0.750665
Monocytes	4933423P22RIK	-0.20636	1.804113	-0.7585	0.450166	-5.2573	0.606171	0.71121
Monocytes	GOLGA1	0.134001	3.427325	0.758375	0.450243	-5.50214	0.579032	0.679813
Monocytes	UBQLN4	-0.14605	3.216072	-0.75824	0.450322	-5.40345	0.582491	0.683821
Monocytes	NENF	-0.08365	4.583677	-0.75819	0.450354	-5.91645	0.5605	0.658282
Monocytes	CNOT6	-0.05994	5.947416	-0.75779	0.450595	-6.09595	0.539708	0.633793
Monocytes	PSAT1	-0.14214	4.218142	-0.7576	0.450705	-5.7825	0.566583	0.665095
Monocytes	RWDD1	0.061329	6.105394	0.757509	0.450759	-6.21389	0.537392	0.631069
Monocytes	KRI1	0.09836	4.367396	0.75709	0.451009	-5.72503	0.564428	0.662492
Monocytes	2310057M21RI	0.153278	2.710086	0.75703	0.451045	-5.32001	0.591404	0.693776
Monocytes	LPCAT2	0.094904	4.726381	0.756739	0.451218	-6.16158	0.558848	0.655998
Monocytes	TRMT44	0.249559	1.793941	0.756731	0.451223	-5.15266	0.607011	0.711764
Monocytes	4833438C02RIK	0.16402	2.025244	0.75652	0.451348	-5.29039	0.603108	0.707232
Monocytes	ZDHHC5	0.07282	5.017726	0.756417	0.45141	-5.91941	0.554351	0.650746
Monocytes	CCDC115	0.090979	4.152653	0.756276	0.451494	-5.82898	0.567982	0.666612
Monocytes	GM44174	-0.37958	-0.56348	-0.75624	0.451515	-4.95456	0.649092	0.759985
Monocytes	B3GLCT	-0.15797	3.311117	-0.7561	0.451597	-5.50384	0.5816	0.682448
Monocytes	TRAPPC6A	0.072	5.057196	0.756054	0.451627	-5.97147	0.553738	0.650074
Monocytes	TRAFD1	-0.0994	5.468862	-0.75597	0.451677	-6.23062	0.547388	0.642666
Monocytes	MROH1	0.120411	4.199162	0.755903	0.451717	-5.72201	0.56724	0.665788
Monocytes	WIPF2	0.06914	5.343948	0.755542	0.451932	-6.00076	0.549491	0.644958
Monocytes	CFB	0.310946	4.663824	0.755459	0.451981	-5.96739	0.560072	0.657287
Monocytes	CASP3	-0.08688	4.814976	-0.75529	0.452083	-5.92489	0.557757	0.654599
Monocytes	MRPS30	0.082963	4.726011	0.754856	0.452341	-5.89847	0.559284	0.656353
Monocytes	DRC7	-0.49356	-0.15036	-0.75484	0.452349	-4.83319	0.641939	0.751657
Monocytes	SMC1B	-0.46585	-0.03473	-0.75483	0.45236	-4.90622	0.639831	0.74925
Monocytes	GM49173	-0.5022	-0.87955	-0.75455	0.452524	-4.89903	0.65556	0.7671
Monocytes	CSF1	0.373069	2.480595	0.75429	0.45268	-5.20697	0.596068	0.698888
Monocytes	TLR11	-0.11449	-0.67266	-0.75415	0.452766	-5.7433	0.651893	0.762872
Monocytes	TRAM1	-0.05667	7.121393	-0.75383	0.452954	-6.3239	0.52343	0.614227
Monocytes	G6PDX	0.089702	4.072997	0.753812	0.452965	-5.89495	0.570047	0.668629
Monocytes	SPTAN1	-0.07405	6.559592	-0.75369	0.453041	-6.23552	0.531713	0.623909
Monocytes	GRAMD2	-0.22626	0.533275	-0.75345	0.453181	-5.231	0.630179	0.737796

Monocytes	CSF2RB2	-0.13462	2.359571	-0.7531	0.453389	-5.79222	0.598419	0.701347
Monocytes	GNPNAT1	-0.10351	4.157449	-0.75293	0.453494	-5.77574	0.568843	0.667126
Monocytes	ADPGK	0.153051	4.903066	0.752925	0.453495	-5.74101	0.557055	0.653422
Monocytes	TIGIT	-0.32804	-0.09862	-0.7529	0.453511	-5.04633	0.64162	0.750919
Monocytes	P4HA2	0.334412	0.108055	0.752874	0.453526	-5.0086	0.63786	0.746624
Monocytes	PUM2	0.048418	7.324014	0.752678	0.453643	-6.37361	0.520622	0.610874
Monocytes	UBE2K	-0.04663	8.214477	-0.75265	0.453662	-6.55495	0.507902	0.595921
Monocytes	ACTR3	-0.04337	8.876057	-0.75259	0.453697	-6.71452	0.49868	0.585061
Monocytes	PCOLCE	0.360036	0.911461	0.752577	0.453704	-4.99769	0.623471	0.730214
Monocytes	NRP1	-0.1256	4.442319	-0.75209	0.453994	-6.04894	0.564598	0.661994
Monocytes	MPHOSPH9	0.121356	4.258614	0.75188	0.45412	-5.68489	0.567565	0.665388
Monocytes	UBXN1	-0.05503	6.759331	-0.75184	0.454143	-6.29724	0.529189	0.620648
Monocytes	FAU	0.035597	11.4506	0.751628	0.454271	-7.05753	0.464844	0.544773
Monocytes	GM49797	-0.09072	4.882769	-0.75162	0.454273	-5.8965	0.557709	0.653991
Monocytes	RFT1	0.192958	2.53189	0.751553	0.454316	-5.35908	0.595872	0.698226
Monocytes	FAM177A	-0.2029	1.731422	-0.7513	0.454465	-5.29097	0.60964	0.714055
Monocytes	EIF3G	-0.07886	4.982954	-0.75069	0.454835	-6.03251	0.55664	0.652545
Monocytes	WDR83	0.172726	2.732947	0.750501	0.454945	-5.35024	0.593098	0.694769
Monocytes	LMNB1	-0.08129	7.679763	-0.75	0.455244	-6.47992	0.516539	0.605533
Monocytes	GM49980	-0.18484	6.157222	-0.74997	0.455266	-6.01679	0.538923	0.63175
Monocytes	WDR33	0.051231	7.186641	0.749899	0.455306	-6.33302	0.523672	0.613927
Monocytes	SLC39A12	-0.45318	0.492802	-0.74982	0.455355	-5.05192	0.632199	0.739607
Monocytes	STAG1	-0.05032	8.353909	-0.74919	0.455733	-6.52183	0.507323	0.594488
Monocytes	IGHM	-0.18241	8.158942	-0.74892	0.455896	-6.3424	0.510106	0.5978
Monocytes	NECTIN4	0.351838	-0.02307	0.748805	0.455962	-4.97345	0.642031	0.75063
Monocytes	ACAD10	0.286323	1.501708	0.748789	0.455972	-5.06776	0.614837	0.719506
Monocytes	ZFP54	-0.36076	0.794625	-0.74872	0.456015	-4.96522	0.627288	0.733785
Monocytes	B3GNT3	0.398473	0.711737	0.748666	0.456046	-4.89771	0.628766	0.73549
Monocytes	CENPW	-0.1403	4.349764	-0.74842	0.456191	-5.78445	0.567467	0.664782
Monocytes	MATR3	-0.05273	6.50291	-0.74833	0.45625	-6.21044	0.534265	0.626103
Monocytes	RENBP	-0.09439	4.474184	-0.74803	0.456427	-5.96925	0.565561	0.662574
Monocytes	CCDC117	0.10139	4.699485	0.747979	0.456458	-5.75492	0.561994	0.658464
Monocytes	SIGIRR	-0.15456	2.175284	-0.74795	0.456477	-5.3833	0.603422	0.706382
Monocytes	SSRP1	-0.08323	6.322976	-0.74777	0.456583	-6.16822	0.537028	0.6294
Monocytes	GM14295	-0.26019	1.289141	-0.74775	0.456595	-5.04839	0.618759	0.724039
Monocytes	DYM	0.070059	5.781233	0.747375	0.456821	-6.12126	0.545411	0.639103
Monocytes	RETSAT	0.205205	1.405172	0.747201	0.456925	-5.14866	0.616944	0.72195
Monocytes	PPIF	-0.15044	3.577224	-0.7472	0.456925	-5.49345	0.580221	0.679629
Monocytes	DESI1	-0.11112	5.729904	-0.74688	0.457119	-5.89774	0.546294	0.640205
Monocytes	TBC1D31	-0.11293	4.166685	-0.74675	0.457198	-5.86972	0.570779	0.668706
Monocytes	YTHDC1	-0.05007	7.312924	-0.7467	0.457226	-6.35801	0.522691	0.612627
Monocytes	GM36486	0.197523	0.805963	0.746644	0.457259	-5.52903	0.627631	0.734219
Monocytes	LGMN	0.087497	7.188159	0.746274	0.457482	-6.46847	0.524509	0.61487
Monocytes	4930453N24RII	-0.10121	4.481233	-0.74625	0.457494	-5.76365	0.565756	0.662997
Monocytes	PIWIL2	0.307043	0.700548	0.746172	0.457543	-5.13259	0.629512	0.736506
Monocytes	GTF2H4	-0.18088	2.307317	-0.74614	0.457564	-5.23996	0.6015	0.704343
Monocytes	GM41556	-0.26935	1.259442	-0.746	0.457644	-5.10778	0.61961	0.725202

Monocytes	BRI3BP	0.069882	4.756436	0.745986	0.457655	-6.22441	0.561401	0.657976
Monocytes	MAGI2	0.345468	0.831391	0.745981	0.457658	-4.97714	0.627179	0.733877
Monocytes	BORCS6	0.1135	4.007961	0.745914	0.457698	-5.74668	0.573333	0.671836
Monocytes	RGMB	-0.17833	1.845211	-0.74581	0.457761	-5.4351	0.609414	0.713555
Monocytes	IFRD1	-0.07548	8.532379	-0.74574	0.4578	-6.71369	0.505291	0.592403
Monocytes	ZCCHC2	0.097231	5.422407	0.745642	0.457861	-5.97961	0.551022	0.646035
Monocytes	ZFP961	-0.19153	3.308556	-0.74545	0.457978	-5.32703	0.584819	0.685231
Monocytes	CEP70	-0.16472	3.195368	-0.74487	0.458323	-5.41902	0.586997	0.687495
Monocytes	2310040G24RII	-0.11975	1.291307	-0.74486	0.458332	-5.51099	0.619463	0.724877
Monocytes	TPGS2	-0.1228	3.805381	-0.74425	0.458696	-5.60714	0.577381	0.676123
Monocytes	CUL7	-0.25669	1.968845	-0.74415	0.458761	-5.138	0.608109	0.711576
Monocytes	LEPROT	-0.07293	5.065196	-0.74396	0.458871	-6.10539	0.557378	0.652876
Monocytes	STK25	-0.09753	4.440838	-0.74382	0.458957	-5.75616	0.567268	0.664372
Monocytes	GM9856	-0.28818	1.246414	-0.74366	0.459053	-5.03342	0.620844	0.726161
Monocytes	KAT14	-0.10765	3.838268	-0.74345	0.459178	-5.60192	0.577034	0.675763
Monocytes	SQLE	-0.22662	1.97366	-0.74344	0.459185	-5.25912	0.608211	0.711741
Monocytes	2410002F23RIK	-0.12917	3.766412	-0.7429	0.459512	-5.54329	0.578463	0.677269
Monocytes	PLEKHA7	0.293548	2.084938	0.742778	0.459585	-5.07029	0.606574	0.709721
Monocytes	NME7	-0.13885	3.562527	-0.74274	0.459607	-5.40618	0.581794	0.68114
Monocytes	SLAMF1	-0.35871	0.884468	-0.74272	0.459619	-5.03253	0.627558	0.7338
Monocytes	IDUA	-0.18597	2.137056	-0.74262	0.459677	-5.25769	0.605682	0.708712
Monocytes	GOLGA4	0.071951	5.30004	0.74239	0.459819	-6.00648	0.554146	0.649009
Monocytes	FAM92A	-0.16693	3.301539	-0.74234	0.459846	-5.43197	0.586161	0.686135
Monocytes	ANKRD39	0.111049	3.543857	0.741942	0.460089	-5.58187	0.582407	0.681543
Monocytes	JCAD	0.304029	1.404554	0.741664	0.460256	-4.99221	0.618847	0.723432
Monocytes	CHTF18	-0.2663	1.800347	-0.74119	0.460541	-5.16848	0.612251	0.715696
Monocytes	ZC3H6	0.161674	3.379852	0.740679	0.46085	-5.44756	0.585857	0.685165
Monocytes	B130034C11RIK	0.271336	1.038829	0.73987	0.461339	-4.98395	0.626465	0.731609
Monocytes	TRMT61B	-0.16132	4.178074	-0.73984	0.461355	-5.77616	0.573311	0.670442
Monocytes	GTPBP2	0.10393	4.751688	0.739644	0.461475	-5.81892	0.564147	0.659884
Monocytes	ZFP74	-0.22494	2.132709	-0.7396	0.461502	-5.1525	0.607353	0.709797
Monocytes	XAF1	0.200402	4.033526	0.739552	0.461531	-5.82175	0.575646	0.673236
Monocytes	CRELD1	-0.24325	1.843254	-0.73949	0.461571	-5.15718	0.612347	0.715533
Monocytes	POLR3H	-0.14807	3.509866	-0.73938	0.461634	-5.44883	0.584202	0.683141
Monocytes	PTPN11	-0.06614	5.063935	-0.73924	0.461717	-6.03615	0.559266	0.654274
Monocytes	YIPF4	0.081751	6.515782	0.739122	0.46179	-6.20832	0.537029	0.628372
Monocytes	MTHFR	0.095311	3.640415	0.73867	0.462064	-5.81539	0.582332	0.6809
Monocytes	CPEB2	0.070512	5.412476	0.738561	0.46213	-6.21526	0.554059	0.64816
Monocytes	ENTPD5	-0.12528	3.78751	-0.73855	0.462135	-5.47322	0.579925	0.678153
Monocytes	HILPDA	-0.18175	4.798635	-0.73794	0.462503	-5.85676	0.564055	0.659427
Monocytes	TTC39C	0.189427	2.230936	0.73761	0.462704	-5.48044	0.606466	0.708278
Monocytes	GM15489	-0.42255	0.208522	-0.73761	0.462707	-4.9236	0.642254	0.749218
Monocytes	ALG14	0.148767	3.562595	0.737536	0.462749	-5.51174	0.584096	0.682536
Monocytes	MRPL17	0.07161	5.426857	0.737334	0.462871	-6.09062	0.554377	0.648028
Monocytes	ENOPH1	0.13338	2.8863	0.736926	0.463118	-5.58094	0.595455	0.695662
Monocytes	TMEM219	0.081883	4.624458	0.736912	0.463127	-5.99591	0.56702	0.662813
Monocytes	DLG2	-0.35054	2.402232	-0.73681	0.46319	-5.0973	0.603652	0.705129

Monocytes	B230354K17RIK	0.265073	1.756956	0.736772	0.463212	-5.17117	0.614771	0.717894
Monocytes	USO1	0.069635	5.739311	0.736756	0.463221	-6.11422	0.549577	0.642591
Monocytes	GM47071	0.295558	1.555335	0.736723	0.463241	-5.18104	0.618291	0.721934
Monocytes	GM42997	-0.39735	0.465543	-0.73664	0.463293	-4.94853	0.637703	0.744142
Monocytes	C130036L24RIK	-0.26438	1.354595	-0.73654	0.463353	-5.05456	0.621821	0.726043
Monocytes	IWS1	0.067018	5.729671	0.736183	0.463568	-6.09488	0.549731	0.642913
Monocytes	ZDHHC9	0.104155	4.094804	0.736154	0.463586	-5.83592	0.575528	0.672856
Monocytes	PRDM2	0.073693	5.970241	0.736138	0.463596	-6.1802	0.546045	0.638627
Monocytes	NACC2	-0.19436	1.77434	-0.73609	0.463622	-5.39686	0.614475	0.717739
Monocytes	ZFP382	0.24289	2.008207	0.736064	0.46364	-5.21119	0.610422	0.713088
Monocytes	SRR	0.17833	2.599008	0.735775	0.463815	-5.28894	0.600343	0.701523
Monocytes	ZFP526	0.343478	0.37218	0.735769	0.463819	-4.96169	0.639437	0.74631
Monocytes	ZFP839	-0.2094	1.537463	-0.73571	0.463857	-5.19929	0.618644	0.722538
Monocytes	LSP1	-0.06439	8.230432	-0.73558	0.463931	-6.74995	0.512767	0.599746
Monocytes	LIG3	-0.15263	3.358188	-0.73552	0.46397	-5.51382	0.58762	0.686862
Monocytes	DOCK8	0.071292	7.656272	0.735459	0.464006	-6.45902	0.521006	0.609408
Monocytes	MAP3K2	0.067234	6.248682	0.735358	0.464068	-6.25028	0.541844	0.633778
Monocytes	FLI1	-0.06999	8.191063	-0.73512	0.464213	-6.44387	0.513329	0.600474
Monocytes	9330151L19RIK	0.265446	0.96571	0.735061	0.464248	-5.07613	0.628754	0.734195
Monocytes	ATP5A1	-0.05697	7.857813	-0.73488	0.464354	-6.4665	0.518098	0.606078
Monocytes	OCIAD2	-0.39863	0.485681	-0.73487	0.464362	-4.93041	0.63738	0.74406
Monocytes	ZNRD1	-0.07347	5.076686	-0.73481	0.464397	-6.06881	0.559908	0.654867
Monocytes	IFIT2	-0.33303	2.721315	-0.7348	0.464407	-5.39428	0.598275	0.699233
Monocytes	RNF113A1	0.415344	0.42846	0.734562	0.46455	-4.90846	0.63848	0.745278
Monocytes	PDSS2	0.095505	5.377267	0.734513	0.464579	-5.96838	0.555266	0.64944
Monocytes	PQLC1	-0.06759	4.395337	-0.73444	0.464623	-6.06301	0.570774	0.667441
Monocytes	GNG11	0.176124	3.831566	0.734075	0.464845	-5.61251	0.580102	0.678062
Monocytes	CSNK1G2	0.073696	5.468875	0.733853	0.46498	-6.01657	0.554133	0.647875
Monocytes	ACAT3	-0.33737	1.453412	-0.73369	0.465079	-5.05173	0.620561	0.724466
Monocytes	GM26827	0.283164	1.344192	0.733268	0.465335	-5.16862	0.622751	0.726754
Monocytes	DLG3	-0.39035	0.571754	-0.73314	0.465415	-4.90531	0.63658	0.742521
Monocytes	RASGRF2	-0.27779	0.116081	-0.73268	0.46569	-5.06354	0.64516	0.751999
Monocytes	9930111J21RIK	0.305502	1.279459	0.732525	0.465786	-5.11495	0.624201	0.728103
Monocytes	PIGK	0.090078	4.298401	0.732515	0.465792	-5.80891	0.573209	0.669457
Monocytes	SOX7	0.378852	0.442832	0.732399	0.465862	-4.93557	0.639204	0.745221
Monocytes	HIST1H4N	-0.3688	1.07648	-0.73232	0.465911	-4.99271	0.627811	0.732221
Monocytes	RCAN3	0.181451	2.117276	0.732066	0.466064	-5.32157	0.6097	0.711413
Monocytes	GH	-0.3893	-0.76502	-0.73189	0.466172	-4.84182	0.661727	0.77072
Monocytes	ORC6	-0.10221	5.216442	-0.73155	0.466375	-5.96933	0.558787	0.652729
Monocytes	UBE2B	-0.07133	8.25823	-0.73134	0.466508	-6.47874	0.513354	0.599769
Monocytes	GM44987	-0.38633	-0.34886	-0.7313	0.466529	-4.8724	0.653933	0.762012
Monocytes	ALDH8A1	-0.32459	2.197617	-0.73129	0.466538	-5.16096	0.608356	0.709975
Monocytes	GM42702	-0.24445	2.365724	-0.73114	0.466626	-5.17573	0.605472	0.706663
Monocytes	DLL4	-0.4506	0.717902	-0.73112	0.466638	-4.89031	0.634404	0.73978
Monocytes	ANKRD16	0.165247	3.026656	0.731003	0.46671	-5.37964	0.594276	0.69379
Monocytes	PCED1B	-0.16715	4.966857	-0.73094	0.466748	-5.66307	0.56271	0.657322
Monocytes	PDLIM2	-0.12693	3.759145	-0.73091	0.466766	-5.71561	0.582134	0.67981

Monocytes	AASDHPPT	0.123459	3.841771	0.730859	0.466798	-5.55473	0.580782	0.678272
Monocytes	ATG10	0.078968	5.64086	0.730794	0.466837	-6.13731	0.552185	0.645142
Monocytes	LYPLA2	-0.07938	4.945046	-0.73079	0.466842	-5.96733	0.563055	0.657761
Monocytes	TNKS2	0.053114	7.072389	0.730701	0.466894	-6.32268	0.530552	0.619936
Monocytes	PSMB4	-0.06428	6.172049	-0.73059	0.466959	-6.23676	0.544053	0.635739
Monocytes	PDLIM5	-0.06378	6.703681	-0.73038	0.467087	-6.46053	0.536101	0.626466
Monocytes	SNX29	0.095896	6.395771	0.73032	0.467125	-6.19469	0.540726	0.631883
Monocytes	TMEM243	-0.05629	6.427837	-0.73016	0.467222	-6.26599	0.540287	0.631381
Monocytes	SCOC	-0.12862	3.752146	-0.73002	0.467305	-5.63695	0.582405	0.680206
Monocytes	ECI2	-0.11154	4.557161	-0.72936	0.467712	-5.80768	0.569513	0.665257
Monocytes	POLA1	-0.11407	5.968201	-0.72935	0.467718	-6.14134	0.547436	0.63963
Monocytes	LARP4	-0.06526	6.410341	-0.72933	0.467729	-6.22791	0.540716	0.631804
Monocytes	NDUFS7	0.059848	6.320866	0.729316	0.467736	-6.22511	0.542068	0.63338
Monocytes	SGK1	-0.09054	5.355259	-0.72928	0.467756	-6.16194	0.556906	0.650656
Monocytes	CD79B	-0.14292	6.976941	-0.72927	0.467764	-6.01386	0.53224	0.621933
Monocytes	RER1	-0.06145	6.413933	-0.72911	0.46786	-6.29063	0.540706	0.631793
Monocytes	TSPAN18	-0.35922	1.764014	-0.72863	0.468152	-5.01427	0.616505	0.719291
Monocytes	ARPP21	0.561163	1.234602	0.728589	0.468178	-4.91257	0.625821	0.729953
Monocytes	ARPP19	-0.05388	6.840626	-0.72849	0.468239	-6.33503	0.534547	0.62452
Monocytes	GPC5	0.415901	0.598275	0.728258	0.46838	-4.98667	0.637342	0.74298
Monocytes	LEO1	-0.12476	4.138442	-0.728	0.468535	-5.64764	0.57674	0.673387
Monocytes	RHBDD3	-0.17391	2.194151	-0.7279	0.468598	-5.27754	0.609245	0.710842
Monocytes	ZDHHC1	-0.23808	1.415954	-0.72779	0.468664	-5.10274	0.622815	0.726409
Monocytes	CNOT11	-0.10475	4.112527	-0.72772	0.468707	-5.72903	0.577161	0.673916
Monocytes	MICOS10	-0.05886	7.131558	-0.72768	0.468731	-6.36292	0.530401	0.61959
Monocytes	PIGS	-0.09264	4.078714	-0.72732	0.46895	-5.7989	0.577864	0.674644
Monocytes	NSFL1C	-0.09437	4.168167	-0.72719	0.469033	-5.74178	0.576412	0.672975
Monocytes	4930469K13RIk	0.249899	1.311112	0.727174	0.469041	-5.30217	0.624835	0.728639
Monocytes	REEP3	-0.06297	6.727725	-0.72706	0.469111	-6.37667	0.536546	0.626689
Monocytes	CGAS	0.154697	2.747045	0.727008	0.469142	-5.67169	0.599961	0.700121
Monocytes	SCN1B	-0.23803	1.834445	-0.72671	0.469325	-5.27655	0.615755	0.718196
Monocytes	MPHOSPH6	0.103254	3.799303	0.726595	0.469393	-5.67892	0.582532	0.679995
Monocytes	BIRC5	-0.16369	5.35755	-0.72658	0.4694	-6.08019	0.557592	0.651129
Monocytes	ITGB1BP1	0.084159	4.649919	0.72599	0.469762	-5.88594	0.568917	0.664242
Monocytes	L2HGDH	-0.26527	1.767651	-0.72582	0.469864	-5.09801	0.617083	0.719755
Monocytes	CARS2	0.099525	3.502896	0.725742	0.469914	-5.56993	0.587569	0.685841
Monocytes	INCENP	-0.12117	5.454754	-0.72557	0.470016	-6.02156	0.556223	0.649572
Monocytes	TUBA4A	0.103107	4.513714	0.725515	0.470052	-5.90573	0.571098	0.666814
Monocytes	FAIM	0.10653	3.865848	0.725478	0.470075	-5.74135	0.581595	0.678948
Monocytes	MRS2	-0.11182	3.939507	-0.72547	0.470079	-5.62553	0.580391	0.677558
Monocytes	ZFP658	-0.39658	0.297611	-0.72543	0.470103	-4.91898	0.643356	0.749758
Monocytes	RASSF7	0.294294	0.86906	0.725385	0.470132	-5.04153	0.633001	0.737954
Monocytes	A430018G15RII	0.243585	1.329295	0.7253	0.470183	-5.07195	0.624793	0.728578
Monocytes	GM15614	-0.19679	2.981808	-0.72522	0.470234	-5.42011	0.596266	0.695858
Monocytes	IREB2	0.078126	5.493255	0.725172	0.470262	-6.03612	0.555624	0.648876
Monocytes	0610009B22RIk	-0.13263	3.780562	-0.72516	0.470267	-5.69199	0.582993	0.680562
Monocytes	FAM43A	-0.14344	4.098571	-0.72484	0.470466	-5.541	0.577974	0.674604

Monocytes	TTC23	-0.31129	1.101964	-0.72425	0.470823	-5.02385	0.62934	0.733409
Monocytes	BAK1	-0.08759	4.936771	-0.72416	0.470882	-6.05883	0.564811	0.659259
Monocytes	PRPF4	-0.1274	3.92555	-0.72408	0.470926	-5.60484	0.581087	0.67812
Monocytes	NDUFS6	0.076774	5.716521	0.724073	0.470932	-6.11954	0.552607	0.645139
Monocytes	TSGA10	-0.13686	2.968133	-0.72377	0.47112	-5.5182	0.597108	0.696439
Monocytes	GM16151	-0.4222	-0.28838	-0.72372	0.471151	-4.84668	0.654837	0.762397
Monocytes	GM14698	0.286118	0.738672	0.723465	0.471304	-5.0597	0.636058	0.741103
Monocytes	UNC50	0.089281	4.105649	0.723386	0.471352	-5.82389	0.57833	0.674879
Monocytes	SH3GL1	-0.09472	4.688824	-0.72336	0.471365	-5.80134	0.568932	0.664012
Monocytes	MON1A	0.117666	3.876268	0.723145	0.471499	-5.63045	0.582169	0.679272
Monocytes	AGPAT1	-0.11866	3.479015	-0.72272	0.471761	-5.71038	0.588954	0.686972
Monocytes	PAN3	-0.04903	8.767803	-0.72263	0.471813	-6.60156	0.508064	0.592967
Monocytes	PPP2R1B	-0.10344	4.339805	-0.72247	0.471914	-5.79572	0.57486	0.67079
Monocytes	ZFP61	0.32432	1.174198	0.722341	0.471991	-5.03338	0.628601	0.732559
Monocytes	GM17178	-0.26134	1.460682	-0.72233	0.471997	-5.06454	0.623518	0.72675
Monocytes	ALG9	0.12404	3.375018	0.722274	0.472032	-5.59673	0.590682	0.68911
Monocytes	RNF122	-0.24747	2.64878	-0.72202	0.472189	-5.2148	0.603025	0.703278
Monocytes	PIGH	0.232366	1.827535	0.721842	0.472297	-5.19347	0.617191	0.719535
Monocytes	CCDC137	-0.17712	2.673176	-0.72183	0.472304	-5.26286	0.60261	0.702812
Monocytes	UBE4A	0.087807	5.03345	0.721752	0.472351	-5.94667	0.563882	0.658154
Monocytes	AP3M2	0.173266	2.489505	0.721651	0.472413	-5.31169	0.60575	0.706472
Monocytes	GM50019	-0.36198	-0.06748	-0.72143	0.472549	-4.97499	0.651395	0.758537
Monocytes	ANKRD49	0.148166	3.032797	0.721342	0.472602	-5.41723	0.596623	0.695938
Monocytes	CYCS	-0.07561	7.712668	-0.72105	0.472781	-6.5406	0.52347	0.611095
Monocytes	HEATR6	-0.09982	4.812778	-0.72088	0.472887	-5.76197	0.567683	0.662535
Monocytes	SULT1A1	-0.1596	3.923228	-0.72039	0.473186	-5.73335	0.582271	0.679321
Monocytes	SORBS1	0.132803	4.747678	0.720343	0.473214	-5.72897	0.568935	0.663916
Monocytes	4833439L19RIK	0.09363	4.862518	0.720305	0.473238	-5.85665	0.567105	0.661797
Monocytes	UBE4B	0.083611	5.48847	0.720051	0.473393	-5.95919	0.557358	0.650422
Monocytes	TRIM47	-0.1871	2.41312	-0.71987	0.473505	-5.3144	0.607799	0.708574
Monocytes	LSM11	-0.23415	2.171179	-0.71959	0.473672	-5.24579	0.612049	0.713396
Monocytes	SIRT6	0.190278	2.652878	0.71958	0.473681	-5.31297	0.603774	0.703903
Monocytes	SMAP1	-0.0629	6.875842	-0.71932	0.473838	-6.31089	0.536377	0.625962
Monocytes	CNIH4	0.060615	5.763142	0.719286	0.473861	-6.27897	0.553302	0.645665
Monocytes	DHRS13	-0.30236	0.959658	-0.71834	0.474444	-5.1323	0.63419	0.738235
Monocytes	ACSS1	0.113127	3.946222	0.718177	0.474542	-5.84795	0.582867	0.679435
Monocytes	RHOB	0.139287	5.891039	0.71817	0.474546	-6.1707	0.551922	0.643628
Monocytes	MAN1A	-0.08082	8.000333	-0.71782	0.474763	-6.55352	0.520596	0.607023
Monocytes	DNAJC13	0.075262	6.16837	0.717687	0.474843	-6.15852	0.547842	0.638786
Monocytes	GM16153	0.214377	2.536009	0.717633	0.474875	-5.27025	0.606709	0.706745
Monocytes	GPATCH4	-0.16688	3.338867	-0.71719	0.475148	-5.40268	0.593329	0.691196
Monocytes	TATDN3	0.158319	2.490952	0.717068	0.475222	-5.43828	0.607699	0.707695
Monocytes	CLPTM1	0.066976	5.552635	0.716879	0.475339	-6.05804	0.557555	0.649935
Monocytes	RIOK1	-0.0676	6.007255	-0.71686	0.47535	-6.11704	0.55051	0.64176
Monocytes	MAP11	0.15577	2.866952	0.716696	0.475451	-5.47665	0.60128	0.700419
Monocytes	PMM1	-0.18013	3.07058	-0.71668	0.475463	-5.36788	0.597835	0.696469
Monocytes	4632428C04RIK	-0.34469	0.110114	-0.71658	0.475523	-4.96513	0.650122	0.756231

Monocytes	PPP6C	0.050183	6.737344	0.71652	0.475559	-6.30258	0.539404	0.628916
Monocytes	AFF1	-0.05978	8.730515	-0.71651	0.475562	-6.63318	0.510347	0.594978
Monocytes	HOTAIRM1	0.284596	0.698917	0.716325	0.475678	-5.17734	0.639371	0.744001
Monocytes	RAB40C	0.09379	4.838453	0.716289	0.475701	-5.8461	0.568856	0.663097
Monocytes	GM1123	-0.2642	0.265276	-0.71584	0.475976	-5.10668	0.647547	0.753286
Monocytes	GLRX3	0.064383	6.625536	0.715791	0.476007	-6.30649	0.541326	0.631132
Monocytes	DNAH1	-0.43042	-0.10849	-0.71562	0.476111	-4.87299	0.654464	0.761149
Monocytes	TRPV2	-0.07663	4.873594	-0.71561	0.476117	-6.09158	0.568517	0.662686
Monocytes	CIPC	0.181912	2.655218	0.715132	0.476412	-5.32364	0.605342	0.705087
Monocytes	TBC1D22B	0.118822	3.778303	0.715113	0.476423	-5.64445	0.586473	0.683394
Monocytes	WDR53	0.174675	2.715022	0.714951	0.476523	-5.37274	0.60432	0.70396
Monocytes	SLC20A2	-0.12733	4.036306	-0.71482	0.476605	-5.64681	0.58223	0.67857
Monocytes	COQ6	-0.18448	2.285167	-0.71478	0.476629	-5.20429	0.611704	0.712461
Monocytes	NOS2	0.582264	-1.12584	0.714667	0.476698	-4.87662	0.673912	0.783252
Monocytes	TOP1MT	-0.29827	1.70028	-0.71465	0.476707	-5.05826	0.621911	0.724155
Monocytes	PHF7	0.144113	3.28527	0.71462	0.476726	-5.50755	0.594675	0.692929
Monocytes	ABCF1	-0.05913	6.430861	-0.71419	0.476993	-6.26426	0.544685	0.634963
Monocytes	TMSB10	-0.06134	10.73818	-0.71365	0.477324	-7.11819	0.483692	0.563338
Monocytes	TMEM88	-0.16378	2.768173	-0.71358	0.477366	-5.63453	0.603993	0.703246
Monocytes	GRPEL1	-0.08063	5.877727	-0.71347	0.477434	-6.14275	0.553454	0.644963
Monocytes	EFTUD2	0.080931	5.105706	0.713354	0.477505	-5.95691	0.565548	0.658992
Monocytes	NEK4	-0.2648	1.202148	-0.71332	0.477529	-5.13782	0.631356	0.734587
Monocytes	VCL	0.073739	6.049702	0.713116	0.477652	-6.29008	0.550869	0.641972
Monocytes	NELFCD	0.103336	4.117826	0.712946	0.477757	-5.71906	0.581527	0.677511
Monocytes	GM26787	-0.32316	1.300668	-0.71294	0.477762	-5.08911	0.629674	0.732736
Monocytes	MIPOL1	-0.1019	4.257052	-0.71239	0.478099	-5.72332	0.579552	0.674962
Monocytes	AGFG1	0.06287	6.147106	0.712268	0.478174	-6.18616	0.549653	0.640365
Monocytes	METTL3	-0.12897	3.319562	-0.71226	0.478179	-5.51047	0.595049	0.692804
Monocytes	POLL	0.243924	1.158885	0.712092	0.478282	-5.09845	0.632563	0.735706
Monocytes	BCO2	0.186688	0.643324	0.712023	0.478325	-5.31459	0.641884	0.746328
Monocytes	SAP30BP	-0.08473	4.838744	-0.7118	0.478463	-5.88214	0.570187	0.66421
Monocytes	CIR1	0.086112	5.515333	0.71175	0.478493	-5.95429	0.559476	0.651816
Monocytes	KPNA4	-0.05823	8.246447	-0.71166	0.478548	-6.70606	0.518478	0.604101
Monocytes	JADE1	0.144213	4.165478	0.711605	0.478583	-5.60334	0.581072	0.676784
Monocytes	STOML3	-0.3289	0.359863	-0.71147	0.478668	-4.98786	0.647072	0.75234
Monocytes	CLEC4A4	0.222578	-1.00355	0.711465	0.478669	-5.09747	0.67267	0.78134
Monocytes	POP1	-0.17193	2.708556	-0.71138	0.47872	-5.33675	0.605423	0.704827
Monocytes	PXYLP1	-0.19927	2.352535	-0.7111	0.478896	-5.28222	0.611605	0.711916
Monocytes	EEF2KMT	0.115754	3.145957	0.711033	0.478935	-5.64017	0.598056	0.696375
Monocytes	SLC19A1	-0.31123	1.065999	-0.71102	0.478941	-5.01812	0.634295	0.737836
Monocytes	TMEM68	0.102176	3.807022	0.710737	0.479118	-5.70114	0.587101	0.683846
Monocytes	CDK4	-0.0786	5.844705	-0.71068	0.47915	-6.11438	0.554475	0.646167
Monocytes	NELFB	-0.09016	4.551192	-0.71064	0.479179	-5.79196	0.574944	0.669887
Monocytes	MYEF2	0.090213	5.328236	0.710273	0.479404	-5.98283	0.562615	0.655647
Monocytes	LRRC75A	-0.20479	2.6203	-0.71027	0.479407	-5.39182	0.60715	0.70697
Monocytes	CIAO1	0.115503	3.481337	0.710181	0.479461	-5.54602	0.59258	0.690258
Monocytes	PGPEP1L	0.415771	-0.29843	0.710013	0.479564	-4.85823	0.659532	0.76675

Monocytes	MAOA	0.317841	0.932781	0.710007	0.479568	-5.08852	0.636859	0.740985
Monocytes	SRBD1	-0.11061	4.393343	-0.70999	0.479582	-5.75369	0.577567	0.672991
Monocytes	CREB3L1	-0.20973	2.035248	-0.70971	0.47975	-5.27913	0.617309	0.718778
Monocytes	HTATIP2	-0.09136	4.480259	-0.7097	0.479761	-5.87823	0.576191	0.671507
Monocytes	MOB2	0.073989	5.416589	0.709662	0.479781	-5.99466	0.561256	0.654229
Monocytes	PARL	0.080074	4.391466	0.709387	0.479951	-5.92566	0.577764	0.67319
Monocytes	ERGIC3	0.06851	5.513051	0.709278	0.480018	-6.11178	0.559884	0.652527
Monocytes	DAZAP1	-0.05635	6.776612	-0.70906	0.480154	-6.27983	0.54051	0.63003
Monocytes	PYCRL	0.109351	3.541294	0.708896	0.480254	-5.65718	0.591812	0.689451
Monocytes	CHMP2B	-0.07329	4.813502	-0.70885	0.480283	-5.97217	0.571018	0.665464
Monocytes	FH1	-0.09201	5.484899	-0.70875	0.480346	-6.12567	0.560372	0.653167
Monocytes	4931406P16RIK	-0.07001	5.594349	-0.70871	0.48037	-6.0896	0.558658	0.651201
Monocytes	CEP95	0.106764	4.108354	0.708618	0.480426	-5.62258	0.582442	0.678711
Monocytes	CCL12	0.516816	0.036828	0.708497	0.4805	-5.02979	0.653529	0.760106
Monocytes	A330023F24RIK	0.148604	3.109027	0.708394	0.480564	-5.56681	0.599067	0.697932
Monocytes	PPP2R2A	-0.04831	7.180276	-0.70837	0.480578	-6.35591	0.534466	0.623153
Monocytes	PWP2	-0.249	1.891751	-0.70799	0.480812	-5.16634	0.620258	0.722085
Monocytes	CLN8	0.106537	3.202024	0.707836	0.480909	-5.93494	0.597761	0.696303
Monocytes	CCNA2	-0.1504	5.520186	-0.70775	0.480963	-6.12433	0.560066	0.652823
Monocytes	FXR1	-0.05679	6.109299	-0.70745	0.481149	-6.21495	0.551032	0.64234
Monocytes	ZFP523	0.218143	2.19531	0.707395	0.481182	-5.14996	0.615131	0.716272
Monocytes	ARL6	-0.14805	1.698649	-0.70678	0.481561	-5.43098	0.624214	0.72638
Monocytes	KCTD13	-0.15596	2.619439	-0.70674	0.481588	-5.39262	0.608169	0.708018
Monocytes	CPLX2	-0.30531	3.378009	-0.70647	0.481754	-5.17466	0.595376	0.693312
Monocytes	KLRA4	-0.49889	-1.21319	-0.70644	0.481772	-4.88896	0.678159	0.787617
Monocytes	TRMT11	0.115089	3.646744	0.706241	0.481895	-5.61625	0.590966	0.688146
Monocytes	IVD	-0.10836	4.435995	-0.70602	0.482035	-5.7448	0.578061	0.673194
Monocytes	ACOT4	-0.44779	0.046894	-0.70577	0.482185	-4.89835	0.654428	0.760643
Monocytes	FBH1	0.10181	3.425128	0.705764	0.48219	-5.69307	0.594739	0.69247
Monocytes	DDX3X	-0.06598	7.320412	-0.70564	0.482269	-6.46189	0.53327	0.621343
Monocytes	RIOX2	0.102567	3.769866	0.705556	0.482319	-5.59686	0.588992	0.685896
Monocytes	TMEM185B	0.095247	4.12483	0.705471	0.482372	-5.83424	0.583139	0.679178
Monocytes	RBAK	0.261778	1.336784	0.705466	0.482375	-5.10501	0.630898	0.733945
Monocytes	CNNM4	0.095997	4.077084	0.7054	0.482416	-5.80182	0.583922	0.680106
Monocytes	TOPBP1	-0.09555	5.680028	-0.70507	0.482621	-6.08748	0.558416	0.650511
Monocytes	AU040320	0.102897	4.589216	0.704873	0.482742	-5.79961	0.57576	0.670598
Monocytes	1810021B22RIK	0.317131	0.427969	0.704847	0.482758	-5.08867	0.647583	0.752869
Monocytes	2610044O15RII	-0.17229	2.24732	-0.70468	0.482859	-5.21372	0.615038	0.715753
Monocytes	NUFIP2	-0.059	7.215419	-0.70466	0.482877	-6.41704	0.534999	0.623318
Monocytes	PRKDC	0.088894	4.69925	0.704594	0.482915	-5.88767	0.573984	0.668547
Monocytes	TTI2	-0.16639	2.749363	-0.7045	0.482976	-5.29127	0.606379	0.705828
Monocytes	EIF2B2	0.082119	4.936837	0.704324	0.483082	-6.02027	0.570183	0.664171
Monocytes	FKBP2	0.078298	5.611404	0.704256	0.483124	-6.16628	0.559507	0.651814
Monocytes	PNPLA2	0.096208	5.509886	0.704166	0.48318	-6.01597	0.5611	0.653662
Monocytes	CAMSAP2	0.087859	4.496866	0.704108	0.483216	-5.92743	0.577268	0.672385
Monocytes	ATP5E	0.045928	8.783189	0.703818	0.483396	-6.68168	0.51223	0.596858
Monocytes	BIK	-0.36162	1.76969	-0.70355	0.483563	-5.04852	0.623419	0.725593

Monocytes	DBP	-0.30725	2.187718	-0.7035	0.483591	-5.09616	0.616088	0.717221
Monocytes	ITPK1	-0.07106	5.403224	-0.70342	0.483641	-6.08303	0.562778	0.65583
Monocytes	HELZ2	-0.14487	3.832468	-0.70341	0.48365	-5.66538	0.588153	0.685142
Monocytes	AASS	-0.35563	1.208483	-0.70339	0.483662	-5.02683	0.63341	0.737012
Monocytes	DELE1	-0.12447	3.426108	-0.70339	0.483663	-5.49638	0.594923	0.692934
Monocytes	E130307A14RIK	0.099579	4.490339	0.703319	0.483705	-5.70941	0.577374	0.67271
Monocytes	ACSL3	0.137611	3.828777	0.703272	0.483734	-5.63074	0.588214	0.685244
Monocytes	ZFP119B	0.25348	1.297642	0.702912	0.483957	-5.10274	0.632027	0.735347
Monocytes	WBP1	-0.12345	3.813733	-0.70258	0.484165	-5.7065	0.588847	0.685741
Monocytes	PBX3	-0.07663	5.496226	-0.70245	0.484245	-6.23426	0.561705	0.654368
Monocytes	CEP250	0.112948	4.458141	0.702122	0.484447	-5.75167	0.578471	0.673674
Monocytes	STT3A	-0.05564	6.169392	-0.7016	0.484769	-6.23913	0.551555	0.642448
Monocytes	MEST	-0.23877	3.792937	-0.70148	0.484846	-5.46344	0.589566	0.686388
Monocytes	MPC2	-0.06777	6.800876	-0.70146	0.484856	-6.36802	0.541921	0.631252
Monocytes	TRIB3	-0.39274	1.057747	-0.70134	0.484932	-4.99522	0.636943	0.74064
Monocytes	MAPK4	0.384617	0.274036	0.701268	0.484978	-4.92038	0.651273	0.756953
Monocytes	SLC22A5	-0.1792	3.042195	-0.70117	0.485037	-5.50581	0.602172	0.700912
Monocytes	GM17106	0.14709	3.395192	0.701098	0.485083	-5.61322	0.596208	0.694062
Monocytes	CHD1L	-0.11431	3.722264	-0.70109	0.485088	-5.58884	0.59074	0.687773
Monocytes	MAF1	0.092092	5.342723	0.700957	0.485171	-6.00551	0.564459	0.657438
Monocytes	NAIP5	0.115327	2.68586	0.700908	0.485201	-5.82802	0.608259	0.707895
Monocytes	SLC25A17	0.070979	5.308731	0.700814	0.48526	-6.06529	0.564996	0.658111
Monocytes	GM46440	0.306271	0.637929	0.700741	0.485304	-5.07893	0.644576	0.749438
Monocytes	TPP1	0.084988	5.393356	0.700682	0.485341	-6.06399	0.563659	0.656585
Monocytes	AATF	-0.08396	4.995367	-0.70053	0.485433	-5.9437	0.569981	0.663916
Monocytes	PON3	0.181148	3.489649	0.700496	0.485457	-5.44274	0.594623	0.692333
Monocytes	DMGDH	0.312845	1.667706	0.700187	0.485649	-5.09004	0.626099	0.728406
Monocytes	CNOT1	-0.05378	7.026328	-0.70006	0.485729	-6.34451	0.538589	0.627491
Monocytes	HAUS1	0.13231	3.65784	0.700028	0.485748	-5.63249	0.591881	0.68918
Monocytes	TBL3	-0.11059	3.91508	-0.69996	0.485787	-5.65513	0.587611	0.684263
Monocytes	SH3BP5L	0.197337	2.252113	0.699941	0.485802	-5.26037	0.61583	0.716667
Monocytes	ZFP956	0.269105	1.264702	0.699817	0.485879	-5.05324	0.633313	0.736629
Monocytes	CTIF	0.116033	2.596638	0.699323	0.486186	-5.54845	0.6102	0.710023
Monocytes	CETN3	-0.07176	5.807387	-0.69912	0.486311	-6.17737	0.557613	0.649391
Monocytes	ABHD4	0.143933	2.787772	0.698978	0.486401	-5.43037	0.607001	0.706372
Monocytes	NHLRC2	0.068495	4.994885	0.698934	0.486428	-6.08565	0.570448	0.664288
Monocytes	TRIM30D	0.129225	4.544173	0.698747	0.486544	-6.00956	0.577742	0.67273
Monocytes	PRRC1	0.087168	4.219989	0.698646	0.486607	-5.83985	0.583029	0.67884
Monocytes	PKD2L2	-0.23768	1.170955	-0.69861	0.486629	-5.0243	0.635449	0.738928
Monocytes	ADNP2	-0.16452	2.990665	-0.69815	0.486915	-5.46744	0.603663	0.702551
Monocytes	RFK	0.094267	5.042963	0.698006	0.487005	-5.89354	0.569801	0.663546
Monocytes	PWWP3A	0.105671	3.715755	0.697859	0.487096	-5.67865	0.591451	0.688519
Monocytes	HEBP1	-0.17211	4.67467	-0.69781	0.487126	-5.80916	0.575719	0.670384
Monocytes	INTS12	0.082788	4.380987	0.697803	0.487131	-5.89716	0.580488	0.675887
Monocytes	PPID	-0.0933	4.87562	-0.69777	0.487153	-5.88463	0.572482	0.666645
Monocytes	VCPIP1	0.061122	6.167501	0.697761	0.487157	-6.18978	0.552147	0.643094
Monocytes	TRMO	0.138873	3.013486	0.697743	0.487169	-5.50569	0.603275	0.702106

Monocytes	NUP43	-0.16486	2.795528	-0.69749	0.487329	-5.3697	0.607124	0.706431
Monocytes	GIN1	0.093832	3.954097	0.697372	0.4874	-5.62786	0.587636	0.68406
Monocytes	GM48099	0.356186	2.773722	0.697274	0.487461	-5.2913	0.607518	0.706922
Monocytes	OAS1G	-0.34122	-0.24881	-0.69711	0.487566	-5.03748	0.661926	0.768897
Monocytes	ZMAT2	0.066417	5.466404	0.6968	0.487756	-6.09059	0.563407	0.655987
Monocytes	DHX15	-0.05262	6.794688	-0.69672	0.487807	-6.32647	0.542882	0.632202
Monocytes	PLXNA4OS1	-0.23803	0.51532	-0.69664	0.487857	-5.23646	0.647859	0.752887
Monocytes	KNTC1	-0.21737	2.818917	-0.69649	0.487947	-5.34829	0.606966	0.706229
Monocytes	PEAK1	0.08963	6.584571	0.69632	0.488055	-6.27759	0.546089	0.635979
Monocytes	UBA7	-0.14851	3.784743	-0.69613	0.488175	-5.62405	0.590664	0.687607
Monocytes	FAM214A	0.122357	5.246692	0.696079	0.488205	-5.8805	0.566902	0.660186
Monocytes	PHF12	0.065983	6.186244	0.696031	0.488235	-6.19001	0.552195	0.64314
Monocytes	GM20275	-0.14761	3.071387	-0.69589	0.488325	-5.57459	0.602657	0.701392
Monocytes	IFT27	-0.14505	3.758551	-0.69586	0.488342	-5.56462	0.5911	0.688108
Monocytes	SLC39A10	-0.10139	3.903206	-0.69582	0.488369	-5.7703	0.588698	0.685354
Monocytes	GM16201	-0.13719	1.818935	-0.69578	0.488393	-5.6936	0.624369	0.726273
Monocytes	PRSS57	0.269379	-0.03051	0.695466	0.488587	-5.07104	0.65819	0.764592
Monocytes	NSUN2	-0.07416	5.0541	-0.69517	0.488775	-6.05435	0.570255	0.663845
Monocytes	RRBP1	0.050087	7.951353	0.695055	0.488844	-6.55678	0.525971	0.612415
Monocytes	IL11RA1	-0.16677	2.113905	-0.69501	0.488874	-5.32872	0.619488	0.720455
Monocytes	PUF60	-0.07085	5.949591	-0.69492	0.488927	-6.16989	0.556136	0.647523
Monocytes	COL11A2	-0.36417	0.72136	-0.69427	0.489336	-4.9919	0.644884	0.74919
Monocytes	CCT6A	-0.0691	5.802009	-0.69415	0.489409	-6.15567	0.558854	0.650488
Monocytes	PHLPP2	-0.10888	4.283952	-0.69383	0.48961	-5.77374	0.583224	0.678684
Monocytes	AA388235	-0.33573	0.991788	-0.69366	0.489711	-5.06462	0.64005	0.743833
Monocytes	KIF14	0.233975	2.55406	0.693576	0.489767	-5.41264	0.612359	0.712244
Monocytes	CD22	-0.23562	2.780412	-0.69353	0.489793	-5.18636	0.608457	0.707781
Monocytes	PARP6	0.172034	2.991541	0.693488	0.489821	-5.38226	0.604842	0.703636
Monocytes	SLX1B	-0.14625	3.074893	-0.69347	0.489831	-5.37365	0.603421	0.702006
Monocytes	CIC	0.083887	4.821881	0.693405	0.489873	-5.95842	0.574479	0.668689
Monocytes	ZFP362	-0.10653	3.9339	-0.6932	0.490001	-5.66323	0.589066	0.685505
Monocytes	TMEM63B	-0.10271	3.925117	-0.69312	0.490049	-5.87026	0.589211	0.685697
Monocytes	TOE1	0.170553	2.490022	0.692672	0.490331	-5.34986	0.613823	0.713834
Monocytes	KIF1BP	-0.10256	3.865166	-0.69238	0.490513	-5.73895	0.590625	0.687153
Monocytes	TXNL1	-0.06534	6.955558	-0.69206	0.490715	-6.33854	0.541817	0.630599
Monocytes	UQCR11	0.066171	7.224799	0.691253	0.491218	-6.40098	0.538201	0.626078
Monocytes	1110051M20RI	0.147703	3.263469	0.691128	0.491295	-5.45617	0.601377	0.699087
Monocytes	TMEM265	-0.12476	3.958769	-0.69109	0.491321	-5.63089	0.589715	0.68569
Monocytes	RAB4A	-0.26926	1.578469	-0.69095	0.491405	-5.11806	0.630706	0.732628
Monocytes	LENG1	-0.15336	2.925078	-0.69091	0.491429	-5.3457	0.607144	0.7057
Monocytes	TEFM	0.286818	1.160891	0.690831	0.491481	-5.10018	0.638214	0.741179
Monocytes	AP5S1	0.170247	2.440536	0.690779	0.491514	-5.38489	0.615509	0.715276
Monocytes	ING2	-0.08319	4.920364	-0.69044	0.491727	-5.90444	0.574178	0.667729
Monocytes	OXA1L	0.084224	4.658758	0.690124	0.491924	-5.8765	0.578485	0.672681
Monocytes	WDR38	-0.29159	0.617809	-0.69004	0.491979	-4.97899	0.648414	0.752717
Monocytes	PSMB2	0.051556	7.058131	0.690012	0.491994	-6.39798	0.540946	0.629238
Monocytes	SHMT2	-0.13539	4.430904	-0.6899	0.492063	-5.71156	0.582199	0.677039

Monocytes	COQ5	-0.12978	3.832511	-0.68984	0.492103	-5.65412	0.592078	0.688425
Monocytes	SLIRP	-0.07613	5.558884	-0.68978	0.492141	-6.17574	0.564071	0.656136
Monocytes	RC3H1	0.056996	6.887392	0.689436	0.492354	-6.31927	0.543633	0.632427
Monocytes	PIN1	-0.07485	5.245371	-0.68943	0.492356	-6.00502	0.569159	0.662009
Monocytes	FTX	-0.14655	4.311116	-0.68907	0.492581	-5.56535	0.584409	0.679584
Monocytes	UHRF2	-0.05544	6.43989	-0.68907	0.492582	-6.25905	0.550583	0.640479
Monocytes	ETOHD2	0.163808	2.615429	0.68845	0.492972	-5.3213	0.613421	0.712709
Monocytes	SGIP1	0.331874	0.175941	0.687859	0.493342	-4.99574	0.657417	0.762767
Monocytes	ABRAXAS2	-0.07394	4.960803	-0.68784	0.493357	-5.99821	0.574313	0.667734
Monocytes	EPB41L4AOS	0.164989	3.25392	0.687807	0.493375	-5.5422	0.602552	0.700223
Monocytes	DNAJB12	0.083744	4.757611	0.687793	0.493384	-5.90315	0.577596	0.671522
Monocytes	EIF3E	-0.04919	7.095265	-0.68776	0.493405	-6.41931	0.541055	0.629211
Monocytes	CDAN1	0.172539	2.943919	0.687753	0.493409	-5.38094	0.607844	0.706289
Monocytes	BOD1	0.158481	3.243589	0.687688	0.493449	-5.4234	0.602728	0.700424
Monocytes	INKA1	0.208905	2.703982	0.68751	0.493561	-5.21959	0.612041	0.711082
Monocytes	LRPPRC	-0.08699	5.200579	-0.68728	0.493706	-5.96882	0.570628	0.663383
Monocytes	EBNA1BP2	0.111779	4.017915	0.687159	0.493781	-5.73024	0.589914	0.685586
Monocytes	PLA2G6	-0.29612	1.28393	-0.68687	0.493964	-5.08598	0.637325	0.739796
Monocytes	DOHH	0.094062	4.347216	0.686866	0.493965	-5.83896	0.584532	0.679369
Monocytes	SLC3A1	0.301053	0.899213	0.686807	0.494002	-5.02778	0.644316	0.747765
Monocytes	MSH3	-0.10027	5.159864	-0.68653	0.494177	-5.82216	0.571355	0.664257
Monocytes	CCDC83	0.392383	0.420205	0.686522	0.494181	-5.07323	0.653143	0.757855
Monocytes	FYCO1	0.086392	4.137271	0.686466	0.494216	-5.92688	0.587996	0.683437
Monocytes	GNG2	0.063779	6.302982	0.68643	0.494238	-6.34789	0.553372	0.643449
Monocytes	DPF2	-0.07194	5.127706	-0.68621	0.494377	-6.01419	0.571963	0.664899
Monocytes	LAYN	0.228972	0.629576	0.686053	0.494475	-5.27933	0.649417	0.753555
Monocytes	TNS1	-0.22258	2.761945	-0.68597	0.494524	-5.3075	0.611367	0.710211
Monocytes	SKA1	-0.20238	2.918501	-0.68577	0.494649	-5.49684	0.608753	0.70714
Monocytes	NECAB3	-0.29459	1.186088	-0.68553	0.494806	-5.02186	0.639457	0.742094
Monocytes	EFCAB11	0.157585	3.410869	0.68536	0.49491	-5.66868	0.600492	0.697574
Monocytes	MBP	-0.07039	5.172136	-0.68527	0.494969	-6.29357	0.57149	0.6642
Monocytes	PIR	0.289158	1.198746	0.685213	0.495002	-5.07934	0.639241	0.741833
Monocytes	CMC4	-0.15015	3.064546	-0.68514	0.49505	-5.37906	0.606384	0.704328
Monocytes	TEX10	0.089666	5.266363	0.684992	0.495141	-6.01538	0.570021	0.662472
Monocytes	GPD1L	-0.11725	5.296236	-0.68462	0.495374	-5.76641	0.56955	0.662027
Monocytes	AP1S3	-0.08756	5.57812	-0.68425	0.495608	-6.2139	0.565072	0.656901
Monocytes	BABAM1	0.067804	5.569892	0.684202	0.495637	-6.11751	0.565202	0.657051
Monocytes	ETFB	-0.07595	6.979015	-0.68418	0.495652	-6.36452	0.543393	0.631783
Monocytes	TXNRD2	-0.14758	3.532111	-0.68409	0.495707	-5.56288	0.598489	0.69543
Monocytes	NR6A1OS	-0.26727	2.067272	-0.68407	0.49572	-5.15722	0.623757	0.724353
Monocytes	NUCB1	0.07764	5.383551	0.683929	0.495809	-6.08217	0.568158	0.66054
Monocytes	QARS	-0.0862	4.552856	-0.68391	0.495823	-5.94437	0.581551	0.676002
Monocytes	GM49482	-0.38839	-0.71551	-0.6838	0.495892	-4.91489	0.67503	0.782657
Monocytes	FBXL15	-0.15411	2.696607	-0.6836	0.496013	-5.42676	0.61276	0.711933
Monocytes	KHK	-0.09517	4.3658	-0.6836	0.496015	-5.92439	0.584614	0.679621
Monocytes	IRF7	-0.16407	6.139097	-0.68351	0.496071	-6.35964	0.556276	0.646884
Monocytes	TRIM17	-0.19769	2.197819	-0.68343	0.49612	-5.2662	0.621458	0.721882

Monocytes	DNAJC12	-0.16682	3.069108	-0.68342	0.496127	-5.48033	0.606352	0.704598
Monocytes	SLC25A45	-0.1006	3.260909	-0.68332	0.496193	-5.75009	0.603081	0.700848
Monocytes	TCF3	-0.08452	6.601305	-0.68323	0.49625	-6.09855	0.549145	0.638616
Monocytes	TMEM147	0.077506	4.948794	0.683224	0.496252	-6.02329	0.575124	0.668683
Monocytes	TOM1L2	0.084166	5.471299	0.682964	0.496416	-6.10997	0.566764	0.659027
Monocytes	GM44710	0.174261	2.000891	0.682943	0.496429	-5.47732	0.62493	0.725846
Monocytes	MBLAC2	0.164628	2.64789	0.682929	0.496437	-5.39075	0.613604	0.712903
Monocytes	TMEM198B	0.253603	0.786307	0.6829	0.496455	-5.12336	0.646813	0.750764
Monocytes	AHSA2	0.113402	3.646243	0.682855	0.496484	-5.59491	0.596568	0.693373
Monocytes	MALT1	0.10334	7.764534	0.682803	0.496516	-6.72263	0.531648	0.618269
Monocytes	SEC23B	-0.0683	5.223375	-0.68276	0.496546	-6.03127	0.570714	0.663591
Monocytes	SH3BP4	0.150649	1.369949	0.68259	0.496651	-5.59906	0.636255	0.738776
Monocytes	F11	-0.31716	0.954836	-0.68229	0.496842	-5.04962	0.643865	0.747417
Monocytes	IRF1	-0.14079	7.149071	-0.68218	0.496911	-6.35168	0.540941	0.629089
Monocytes	TOMM70A	0.061571	5.752051	0.682167	0.496917	-6.13499	0.562448	0.654038
Monocytes	GM31718	-0.11148	3.725877	-0.68213	0.496943	-5.77508	0.595359	0.691988
Monocytes	AIDA	0.083242	4.437691	0.68202	0.497009	-5.89367	0.583569	0.678452
Monocytes	GM44659	-0.29242	0.682551	-0.68183	0.497127	-5.01318	0.648947	0.753149
Monocytes	ASPSCR1	0.077345	4.685187	0.681491	0.497342	-5.93492	0.579736	0.673913
Monocytes	MAP7D3	0.300996	0.126284	0.681456	0.497364	-5.06941	0.659443	0.764995
Monocytes	EPM2A	0.271686	1.526051	0.681325	0.497446	-5.13602	0.633792	0.735852
Monocytes	RDH12	0.310428	2.275253	0.68104	0.497626	-5.13467	0.620608	0.72079
Monocytes	TRIM56	0.11848	3.810038	0.680996	0.497653	-5.59085	0.594311	0.69066
Monocytes	E130102H24RIK	0.233263	1.232168	0.680765	0.497799	-5.25617	0.639321	0.741999
Monocytes	SNX14	-0.09108	4.500746	-0.68064	0.497877	-5.92425	0.583005	0.677511
Monocytes	GT(ROSA)26SOR	-0.10655	4.594685	-0.6802	0.498153	-5.82767	0.581724	0.67582
Monocytes	BTG3	0.078444	5.347109	0.679845	0.498379	-5.95648	0.569708	0.661954
Monocytes	A430005L14RIK	0.099386	3.764054	0.679618	0.498522	-5.74593	0.595607	0.691836
Monocytes	OSBPL1A	-0.12947	3.288159	-0.6796	0.498534	-5.5903	0.603645	0.70106
Monocytes	MCCC1	-0.20734	2.50652	-0.67952	0.498586	-5.26913	0.61711	0.716481
Monocytes	IMPG2	-0.37477	0.6613	-0.67944	0.498634	-4.99153	0.650219	0.754199
Monocytes	REX1BD	0.05978	5.688469	0.679337	0.498699	-6.20514	0.56429	0.655775
Monocytes	CAMK2B	-0.27102	3.706454	-0.67928	0.498734	-5.28127	0.596573	0.692998
Monocytes	IMPACT	-0.07515	5.540939	-0.67919	0.498793	-6.20131	0.566624	0.658524
Monocytes	PDE3B	0.087524	6.955861	0.679098	0.49885	-6.45845	0.544671	0.633091
Monocytes	GM10642	0.381314	0.270003	0.679094	0.498853	-4.9359	0.657486	0.762505
Monocytes	GM9530	-0.1835	-0.31965	-0.67883	0.499021	-5.44138	0.668751	0.775214
Monocytes	RPP30	-0.10626	3.635635	-0.67841	0.499281	-5.63811	0.598137	0.694684
Monocytes	DLG1	-0.05594	7.219142	-0.67832	0.499341	-6.39345	0.541032	0.628778
Monocytes	C330018D20RIK	-0.17555	2.325358	-0.67814	0.499457	-5.29556	0.620738	0.720534
Monocytes	RBM39	-0.0343	9.586582	-0.67803	0.499522	-6.77531	0.506741	0.588695
Monocytes	BAG5	-0.13734	3.637068	-0.67768	0.499741	-5.62212	0.598384	0.694748
Monocytes	XK	-0.35252	1.158214	-0.6771	0.500113	-4.94858	0.64221	0.744587
Monocytes	ELOA	0.067262	5.789735	0.676953	0.500203	-6.186	0.563688	0.654625
Monocytes	CENPP	-0.12312	5.39603	-0.67665	0.500395	-6.0181	0.570086	0.661898
Monocytes	IPO5	-0.08799	5.437174	-0.67642	0.500541	-6.10623	0.569436	0.66115
Monocytes	SHPK	0.297766	0.252393	0.676274	0.500632	-5.03242	0.659161	0.763706

Monocytes	POU5F1	-0.23055	1.26222	-0.67627	0.500634	-5.16628	0.640536	0.742584
Monocytes	GLUD1	0.049089	7.653063	0.676231	0.500659	-6.5859	0.5353	0.621597
Monocytes	SHARPIN	-0.08024	4.530571	-0.67613	0.500723	-5.90936	0.584099	0.678049
Monocytes	CCDC91	0.177458	2.408137	0.676085	0.500751	-5.35411	0.620094	0.719301
Monocytes	A	-0.11164	4.78007	-0.6755	0.50112	-5.89756	0.580382	0.673419
Monocytes	ANXA7	-0.06136	5.409471	-0.67536	0.501209	-6.18954	0.570235	0.661722
Monocytes	FGD2	-0.09142	3.69567	-0.67531	0.501241	-6.07961	0.598351	0.694056
Monocytes	UPF3B	-0.09421	4.979585	-0.67512	0.501359	-5.8495	0.577215	0.669689
Monocytes	HOMEZ	-0.24914	1.333922	-0.67483	0.501548	-5.07891	0.639879	0.741231
Monocytes	CTSD	0.079584	7.133555	0.674567	0.501711	-6.44534	0.543751	0.630844
Monocytes	ATP5G1	-0.0641	7.643771	-0.67426	0.501907	-6.49966	0.536175	0.622025
Monocytes	CDC20	-0.18405	3.639962	-0.67425	0.501909	-5.63136	0.599739	0.695366
Monocytes	UQCRFS1	-0.05612	7.164384	-0.67358	0.502333	-6.46238	0.543625	0.630586
Monocytes	ASCC2	0.076902	4.605255	0.67354	0.502361	-5.87128	0.583949	0.677172
Monocytes	G2E3	-0.10961	3.938116	-0.67349	0.502395	-5.65306	0.595003	0.689878
Monocytes	GPLD1	-0.30677	0.934219	-0.67342	0.502436	-5.07797	0.647714	0.750012
Monocytes	PDP1	0.166859	2.180234	0.673325	0.502497	-5.29257	0.625252	0.724484
Monocytes	SMG9	-0.09223	4.52517	-0.67325	0.502546	-5.82261	0.585264	0.678722
Monocytes	MRPS31	-0.11392	3.653241	-0.67323	0.502555	-5.56638	0.599793	0.695396
Monocytes	DSCC1	-0.19092	2.435007	-0.67314	0.502612	-5.33133	0.620765	0.719383
Monocytes	GMPPB	-0.15204	2.629402	-0.67277	0.502848	-5.42757	0.617535	0.715565
Monocytes	VARS2	-0.25967	1.401859	-0.67274	0.502868	-5.15542	0.639358	0.740409
Monocytes	XRN2	0.042837	7.516217	0.672188	0.503217	-6.47876	0.538648	0.624776
Monocytes	BC024063	0.334826	0.183577	0.672125	0.503257	-4.94027	0.66206	0.766155
Monocytes	OPA1	0.074731	4.835691	0.67208	0.503285	-5.95546	0.580528	0.673183
Monocytes	GM17231	0.211526	2.734245	0.672064	0.503295	-5.33586	0.615904	0.71373
Monocytes	PDCD2	-0.11135	3.817161	-0.67202	0.50332	-5.65957	0.597385	0.692568
Monocytes	MED13	-0.05954	7.722854	-0.6718	0.503462	-6.47703	0.535572	0.621289
Monocytes	ZC3H4	-0.07314	4.818849	-0.67177	0.503484	-5.90748	0.580812	0.673604
Monocytes	ZFP667	0.142939	2.726732	0.671707	0.503522	-5.57966	0.616045	0.713995
Monocytes	HERC3	-0.10883	4.345366	-0.67156	0.503616	-5.77375	0.588586	0.68256
Monocytes	TAOK2	0.106495	4.127104	0.671434	0.503695	-5.68921	0.592208	0.68677
Monocytes	GDI2	-0.04061	8.891579	-0.67138	0.503726	-6.7365	0.518497	0.601471
Monocytes	CABLES2	0.116855	3.580958	0.671287	0.503788	-5.58797	0.601381	0.697306
Monocytes	SLC22A14	-0.17754	2.744823	-0.67116	0.503865	-5.42654	0.61573	0.713729
Monocytes	GM17745	0.268992	0.962497	0.671048	0.503939	-5.09636	0.647588	0.750043
Monocytes	CLINT1	0.050972	8.205941	0.67102	0.503957	-6.53536	0.528438	0.613129
Monocytes	WDR81	-0.11394	3.600126	-0.67088	0.504044	-5.63452	0.601056	0.697016
Monocytes	TMEFF1	-0.19925	-0.59624	-0.67064	0.504196	-5.4464	0.676923	0.783347
Monocytes	WDR5B	-0.3155	0.670974	-0.67057	0.50424	-5.02555	0.652968	0.756309
Monocytes	RUNX3	0.079513	5.712717	0.670454	0.504316	-6.39341	0.566446	0.657356
Monocytes	CERS5	0.064328	5.514163	0.67045	0.504318	-6.18074	0.569602	0.661
Monocytes	ZFP873	-0.2692	0.995152	-0.67044	0.504325	-5.01006	0.646988	0.749551
Monocytes	RFTN1	-0.0747	6.737622	-0.67044	0.504326	-6.32554	0.550461	0.638864
Monocytes	PUSL1	0.161048	2.317284	0.670343	0.504386	-5.34346	0.623214	0.722527
Monocytes	ZDHHC20	0.048648	6.740107	0.670252	0.504444	-6.33079	0.550424	0.638841
Monocytes	GM40841	-0.47101	0.40148	-0.66999	0.504608	-4.86927	0.658123	0.762066

Monocytes	RPRD1A	0.087648	4.057705	0.669873	0.504684	-5.72911	0.593501	0.688421
Monocytes	SHC1	0.080339	4.384809	0.669787	0.504739	-5.88334	0.588068	0.682192
Monocytes	AP3M1	0.066217	4.881049	0.669622	0.504844	-6.01207	0.579932	0.672888
Monocytes	POLR2L	-0.07037	5.414671	-0.66961	0.504852	-6.14505	0.571322	0.662966
Monocytes	PRPF38A	0.074808	5.176932	0.669494	0.504925	-6.04734	0.575157	0.667418
Monocytes	MMD	-0.10011	4.477186	-0.6694	0.504987	-5.96022	0.586564	0.680556
Monocytes	RBFOX3	-0.37614	-0.24977	-0.66923	0.505089	-4.96512	0.670456	0.776231
Monocytes	KLK1	-0.53561	-0.20745	-0.66921	0.505104	-4.85828	0.66965	0.77532
Monocytes	SLC9A5	0.317849	0.362304	0.668998	0.50524	-4.99783	0.658973	0.763246
Monocytes	SMIM13	0.123436	3.919727	0.668927	0.505285	-5.55136	0.595904	0.691385
Monocytes	NPTN	-0.04267	8.043161	-0.66865	0.505462	-6.57728	0.53108	0.616523
Monocytes	PDCD7	-0.08384	4.505243	-0.66853	0.505534	-5.86252	0.586224	0.680334
Monocytes	GM34086	-0.15905	2.658244	-0.66852	0.505541	-5.49829	0.617528	0.716217
Monocytes	LCOR	0.056243	7.538405	0.668464	0.505579	-6.46366	0.538578	0.625245
Monocytes	ARHGAP30	-0.04754	7.139253	-0.66839	0.505624	-6.43528	0.544593	0.63223
Monocytes	EMC9	-0.18772	1.600787	-0.66825	0.505717	-5.28279	0.636276	0.737602
Monocytes	INTS4	-0.10379	3.920928	-0.66821	0.505738	-5.71504	0.595933	0.691503
Monocytes	EHMT1	-0.06427	6.063885	-0.66806	0.505834	-6.20635	0.561187	0.651463
Monocytes	FAM20C	0.175136	1.641237	0.668013	0.505866	-5.81184	0.635561	0.736802
Monocytes	UBE2C	-0.1598	6.746366	-0.66782	0.505991	-6.33842	0.55067	0.639278
Monocytes	RCBTB2	0.069544	5.187796	0.667602	0.506127	-6.12914	0.575223	0.667684
Monocytes	C730034F03RIK	-0.19536	2.636781	-0.66759	0.506135	-5.34739	0.61803	0.71681
Monocytes	ZSWIM9	-0.38762	0.544388	-0.66745	0.506222	-4.94203	0.655799	0.759745
Monocytes	HIST1H2AI	-0.34235	2.588783	-0.66662	0.506749	-5.24851	0.619422	0.718016
Monocytes	GALK1	-0.11291	5.140655	-0.6666	0.506765	-5.80213	0.576499	0.668792
Monocytes	CCT6B	-0.3372	0.315333	-0.66647	0.506847	-5.01193	0.660638	0.764866
Monocytes	CFAP298	-0.10382	3.642504	-0.66641	0.506885	-5.73167	0.601288	0.697291
Monocytes	TSPAN9	-0.12317	3.290989	-0.66614	0.507057	-5.8395	0.607408	0.704206
Monocytes	WDSUB1	-0.13448	2.44185	-0.66592	0.507194	-5.4776	0.622178	0.721079
Monocytes	ISCA2	0.083239	4.968036	0.665907	0.507204	-5.99812	0.579463	0.67213
Monocytes	CFAP97	0.112591	3.29046	0.665571	0.507418	-5.51748	0.607638	0.704418
Monocytes	ACKR3	0.460363	0.835269	0.665476	0.507478	-5.01295	0.65134	0.754184
Monocytes	ABHD6	-0.1976	2.548845	-0.66529	0.507594	-5.2131	0.620547	0.719167
Monocytes	DCUN1D2	0.163763	2.743543	0.665219	0.507642	-5.44892	0.617145	0.715299
Monocytes	INIP	-0.08646	4.523666	-0.66472	0.507962	-5.82138	0.587208	0.680756
Monocytes	METTL2	-0.12474	3.27705	-0.66471	0.507967	-5.51657	0.608172	0.70479
Monocytes	A330040F15RIK	-0.29423	2.508264	-0.66447	0.508118	-5.37959	0.621574	0.720098
Monocytes	NABP2	0.070284	4.980112	0.664398	0.508165	-6.03959	0.579791	0.672235
Monocytes	MCM7	-0.12405	5.384269	-0.66431	0.508219	-6.03142	0.573261	0.664752
Monocytes	GRK3	0.079959	2.91909	0.664261	0.508252	-6.16898	0.614405	0.711979
Monocytes	UNC13A	-0.29715	1.183593	-0.66401	0.508411	-5.05214	0.645448	0.74726
Monocytes	PCLAF	-0.14969	7.982096	-0.66383	0.508527	-6.55896	0.53331	0.618467
Monocytes	BCLAF3	0.08479	4.966172	0.66372	0.508597	-5.91355	0.580134	0.67267
Monocytes	RECQL	0.13887	3.363727	0.663576	0.508688	-5.5639	0.60687	0.703403
Monocytes	WRAP73	-0.14033	2.923556	-0.66352	0.508722	-5.37856	0.614451	0.71207
Monocytes	ARHGAP25	-0.0763	6.214201	-0.66348	0.508752	-6.19293	0.560219	0.649747
Monocytes	TSPYL2	-0.17769	2.8065	-0.66345	0.508766	-5.36358	0.616484	0.714394

Monocytes	4933421O10RII	-0.15612	2.357702	-0.66333	0.508848	-5.47948	0.624378	0.723413
Monocytes	THEMIS2	0.090007	4.692319	0.662885	0.509129	-6.17261	0.584891	0.678006
Monocytes	SERBP1	-0.04789	8.529561	-0.66262	0.509299	-6.62148	0.525642	0.609419
Monocytes	THAP4	-0.10023	4.091332	-0.66237	0.509454	-5.70749	0.595053	0.689654
Monocytes	KBTBD3	-0.16199	2.727361	-0.66234	0.509479	-5.41227	0.618371	0.716344
Monocytes	RNGTT	-0.05389	6.852257	-0.66218	0.509578	-6.37092	0.550831	0.638687
Monocytes	PEF1	0.131758	3.347699	0.661841	0.509795	-5.53154	0.607879	0.70425
Monocytes	ZFP282	-0.11571	4.010784	-0.66137	0.510093	-5.6861	0.596915	0.691508
Monocytes	TFPT	0.132774	2.965912	0.661219	0.510192	-5.42536	0.614796	0.711994
Monocytes	FDX1	-0.10439	4.692703	-0.66099	0.510335	-5.89439	0.585707	0.678614
Monocytes	ASMT	0.247792	1.052723	0.660919	0.510383	-5.19634	0.649074	0.750923
Monocytes	SPECC1L	-0.07234	5.413581	-0.66084	0.510435	-6.26961	0.573987	0.665142
Monocytes	TARBP2	0.118388	3.242608	0.660644	0.510558	-5.60332	0.610116	0.706642
Monocytes	BIRC6	0.036355	7.8393	0.660626	0.51057	-6.53619	0.536457	0.621768
Monocytes	ATP2A1	0.36298	1.448126	0.660491	0.510656	-4.94016	0.641895	0.742839
Monocytes	LRRCC1	0.089329	3.748019	0.660143	0.510878	-5.70516	0.601689	0.696956
Monocytes	GM15964	0.174668	0.162356	0.660084	0.510916	-5.60173	0.665936	0.770054
Monocytes	PGAP3	0.311771	1.12543	0.659853	0.511064	-5.01796	0.647995	0.74982
Monocytes	GPIHBP1	0.168237	4.095185	0.659705	0.511158	-5.60585	0.59586	0.69042
Monocytes	KLC3	-0.43286	0.19256	-0.65963	0.511206	-4.87405	0.665386	0.76955
Monocytes	FUZ	0.261533	1.08456	0.659529	0.511271	-5.13411	0.648747	0.750721
Monocytes	NIPAL3	0.155467	3.198166	0.659505	0.511286	-5.46552	0.611104	0.707872
Monocytes	PCGF3	0.097257	3.998741	0.659504	0.511287	-5.6976	0.597479	0.692276
Monocytes	UPF3A	0.068839	4.634592	0.659403	0.511351	-5.93618	0.586903	0.680144
Monocytes	TPP2	0.049746	7.082191	0.659261	0.511442	-6.38976	0.548111	0.635355
Monocytes	GM32036	-0.08935	3.651119	-0.65901	0.511601	-5.76243	0.603453	0.699071
Monocytes	TMEM107	0.1949	1.616764	0.659002	0.511607	-5.23007	0.639141	0.739768
Monocytes	POLDIP3	0.055538	6.078814	0.658872	0.51169	-6.24212	0.563722	0.653394
Monocytes	GPN2	-0.13886	2.713988	-0.65882	0.511725	-5.45825	0.619613	0.717576
Monocytes	DPY19L1	0.070959	4.584338	0.658561	0.511889	-6.1719	0.587943	0.681208
Monocytes	ME2	0.07197	5.963937	0.65842	0.511979	-6.25301	0.565685	0.655566
Monocytes	FAM160B1	-0.10229	4.443367	-0.65812	0.512172	-5.72454	0.590357	0.683991
Monocytes	TMEM203	0.134888	3.168142	0.658117	0.512173	-5.56491	0.611933	0.708703
Monocytes	ZDHHC8	-0.12416	3.666743	-0.65802	0.512235	-5.57504	0.603395	0.698938
Monocytes	8430429K09RIK	0.212632	1.679457	0.657989	0.512255	-5.12356	0.638226	0.738678
Monocytes	TOR1AIP1	0.054865	7.42657	0.657643	0.512476	-6.49889	0.543197	0.629601
Monocytes	CANX	-0.04448	7.35016	-0.65761	0.512495	-6.46267	0.544353	0.630955
Monocytes	CTDSPL	0.150518	3.831098	0.657599	0.512504	-5.7495	0.600694	0.695898
Monocytes	MED6	0.089029	4.540282	0.656897	0.512954	-5.93012	0.589284	0.682454
Monocytes	GNB2	-0.04609	8.428837	-0.65677	0.513034	-6.65649	0.528715	0.61246
Monocytes	MORF4L1	0.031848	8.201561	0.656583	0.513155	-6.5736	0.532063	0.616376
Monocytes	PKMYT1	-0.13933	3.261877	-0.65658	0.513158	-5.62088	0.6109	0.707233
Monocytes	USP25	0.050931	7.051473	0.656434	0.51325	-6.4553	0.549379	0.636491
Monocytes	ARPC5L	-0.0573	6.300784	-0.65614	0.513435	-6.23664	0.561121	0.650027
Monocytes	TBC1D2	0.147446	1.45751	0.656078	0.513478	-5.59723	0.643043	0.743861
Monocytes	D330023K18RII	-0.21752	2.22594	-0.65569	0.513726	-5.24179	0.62944	0.728288
Monocytes	NR1H4	-0.30447	1.386536	-0.65555	0.513814	-5.07825	0.644612	0.745531

Monocytes	CCDC134	-0.11295	3.407824	-0.65544	0.513883	-5.71969	0.608832	0.704829
Monocytes	ERMARD	0.129428	3.076068	0.655181	0.514053	-5.52691	0.614657	0.711396
Monocytes	NSDHL	-0.16582	2.406095	-0.65509	0.514108	-5.39293	0.626395	0.724778
Monocytes	ARV1	0.234512	2.101251	0.655034	0.514147	-5.17916	0.631817	0.730988
Monocytes	IKZF5	0.109627	3.804869	0.654885	0.514242	-5.63305	0.602205	0.697205
Monocytes	TPM4	-0.05156	7.167543	-0.65451	0.514485	-6.49863	0.548293	0.634953
Monocytes	SPTSSA	0.060109	6.822761	0.654384	0.514563	-6.37195	0.553605	0.641077
Monocytes	PHETA1	-0.31507	0.450779	-0.65401	0.514804	-5.01746	0.66262	0.765515
Monocytes	USP5	0.096285	4.34395	0.653769	0.514957	-5.8102	0.593654	0.687109
Monocytes	CENPB	0.071289	5.450808	0.653539	0.515105	-6.13571	0.575505	0.666292
Monocytes	NEGR1	-0.26754	-0.13284	-0.65351	0.515121	-5.24372	0.67378	0.778214
Monocytes	DENND3	0.150245	3.548798	0.653495	0.515133	-5.53544	0.607085	0.70252
Monocytes	GM4890	0.299425	0.373848	0.653456	0.515159	-4.97895	0.664145	0.767358
Monocytes	1110002L01RIK	-0.1696	2.8153	-0.65332	0.515246	-5.35523	0.619808	0.717045
Monocytes	ACOT7	0.079662	4.076826	0.653153	0.515353	-5.97163	0.598217	0.692343
Monocytes	PTS	-0.0633	5.573329	-0.65284	0.51555	-6.13807	0.573683	0.664181
Monocytes	USP33	0.086921	4.710834	0.652795	0.515583	-5.89614	0.587721	0.680325
Monocytes	CAPZA2	0.034226	7.929486	0.652786	0.515588	-6.62875	0.537211	0.622017
Monocytes	CCDC173	-0.33456	0.767334	-0.65239	0.515842	-4.95007	0.657179	0.759269
Monocytes	RBX1	0.04919	7.700144	0.652169	0.515984	-6.51699	0.540844	0.626154
Monocytes	ZBTB22	0.138791	3.076444	0.652137	0.516005	-5.51648	0.61561	0.712167
Monocytes	LRIF1	0.073147	4.523869	0.65212	0.516015	-5.88749	0.591033	0.684049
Monocytes	MLH3	-0.17759	2.01272	-0.65196	0.516117	-5.3274	0.634443	0.733552
Monocytes	RNF11	-0.07163	6.207032	-0.65157	0.516367	-6.21148	0.563948	0.652847
Monocytes	TIAM1	0.073533	4.628891	0.65156	0.516375	-6.3076	0.589431	0.68217
Monocytes	AEN	0.130187	3.664015	0.651491	0.51642	-5.62572	0.605641	0.700756
Monocytes	WNT2	-0.38075	1.077377	-0.65146	0.516437	-5.01938	0.651576	0.753024
Monocytes	RBMX	-0.09161	4.578527	-0.65134	0.516518	-5.7686	0.590265	0.683173
Monocytes	XRCC1	-0.10885	4.144273	-0.65129	0.516551	-5.70823	0.597512	0.691483
Monocytes	GSKIP	0.067277	4.602025	0.651096	0.516673	-5.92702	0.589947	0.682728
Monocytes	RANGAP1	-0.07942	5.610379	-0.65094	0.516772	-6.09868	0.573548	0.663829
Monocytes	BOLA1	0.122079	3.767483	0.650366	0.517142	-5.65172	0.604363	0.698935
Monocytes	GM16062	-0.16682	1.874018	-0.6501	0.517314	-5.29211	0.637663	0.736897
Monocytes	GABARAP	0.042609	8.497974	0.650031	0.517358	-6.69203	0.52965	0.612834
Monocytes	HEY1	-0.38757	-0.2714	-0.64993	0.517423	-4.9214	0.677696	0.782144
Monocytes	HCAR2	-0.18181	2.377294	-0.64984	0.517478	-5.85185	0.628653	0.726674
Monocytes	RBL1	0.101141	4.466968	0.649458	0.517726	-5.88585	0.59277	0.685677
Monocytes	HPGD	-0.13006	5.673887	-0.64934	0.517801	-6.41535	0.573048	0.663022
Monocytes	MKI67	-0.14499	6.498445	-0.64924	0.517868	-6.33305	0.559995	0.647989
Monocytes	ZFP229	0.312732	-0.01049	0.649224	0.517877	-5.00398	0.672761	0.776597
Monocytes	SLTM	-0.04964	6.63584	-0.64918	0.517906	-6.33941	0.557853	0.645543
Monocytes	GM10802	-0.27516	-0.25172	-0.64901	0.518012	-5.091	0.677393	0.7819
Monocytes	SKAP2	0.04546	7.235487	0.64901	0.518014	-6.56338	0.548611	0.634901
Monocytes	GUK1	-0.0696	4.908743	-0.64895	0.518054	-6.06945	0.585465	0.6774
Monocytes	MUTYH	-0.30452	0.370874	-0.64892	0.51807	-4.95947	0.665508	0.768517
Monocytes	SAP30L	-0.07727	4.309459	-0.64837	0.518428	-5.84231	0.595743	0.688973
Monocytes	TMEM159	0.233251	1.553977	0.648139	0.518575	-5.27332	0.644022	0.743868

Monocytes	MAVS	-0.14827	2.932268	-0.64799	0.518671	-5.41952	0.619451	0.715931
Monocytes	ECSCR	-0.34193	1.207762	-0.64767	0.518875	-5.00525	0.650599	0.751116
Monocytes	PDCD6	0.062405	6.13963	0.647196	0.519182	-6.30451	0.566512	0.654939
Monocytes	ST18	0.317969	0.283819	0.647122	0.51923	-5.16038	0.668192	0.770825
Monocytes	D3ERTD751E	0.237693	1.61523	0.646665	0.519524	-5.14487	0.643672	0.743035
Monocytes	CDKN3	0.147618	4.160534	0.64644	0.519669	-5.87354	0.599057	0.692313
Monocytes	GSS	0.126029	3.399727	0.646432	0.519674	-5.63358	0.612025	0.707128
Monocytes	TUBGCP6	-0.18997	2.759296	-0.64642	0.519681	-5.27975	0.623181	0.719841
Monocytes	RWDD3	-0.2992	0.507573	-0.6462	0.519824	-5.00256	0.664214	0.766365
Monocytes	5430414B19RIK	0.31794	0.449396	0.646177	0.519839	-5.04402	0.665313	0.767604
Monocytes	HPRT	0.061325	6.310323	0.646137	0.519864	-6.28535	0.564035	0.652122
Monocytes	UBE2D2A	-0.0311	8.209532	-0.64607	0.519906	-6.56724	0.534999	0.618567
Monocytes	ELMOD2	0.13526	2.807903	0.645734	0.520124	-5.50038	0.622517	0.719068
Monocytes	FAM3C	0.090386	5.36557	0.645435	0.520317	-5.79679	0.579462	0.669778
Monocytes	ECM2	-0.39816	0.502848	-0.64528	0.520415	-4.95936	0.664726	0.766837
Monocytes	MAPK9	0.074887	4.751185	0.644956	0.520625	-5.97266	0.589634	0.681467
Monocytes	ELAVL3	-0.35799	1.03829	-0.64494	0.520633	-5.05293	0.654768	0.755611
Monocytes	SIAH1B	-0.20197	1.91846	-0.64485	0.520696	-5.21764	0.638642	0.737393
Monocytes	DTNB	-0.11256	5.293687	-0.64483	0.520706	-5.79864	0.580732	0.671291
Monocytes	THRAP3	-0.04696	8.006361	-0.64445	0.52095	-6.56304	0.538611	0.622559
Monocytes	SIL1	0.088942	5.149956	0.644265	0.521072	-5.96941	0.583323	0.674103
Monocytes	4930473A02RIK	-0.27485	0.38363	-0.64414	0.521153	-5.00816	0.667331	0.769717
Monocytes	1500009L16RIK	-0.34621	-0.02505	-0.64412	0.521164	-4.88913	0.675128	0.778498
Monocytes	CIRBP	0.06263	7.218243	0.643948	0.521276	-6.38767	0.550632	0.636479
Monocytes	POLR2B	0.072572	4.911643	0.643666	0.521458	-5.95783	0.58743	0.678799
Monocytes	MRPL14	0.073122	5.601025	0.643532	0.521545	-6.22732	0.576223	0.665927
Monocytes	SNAPC4	0.235727	1.856185	0.643351	0.521662	-5.18369	0.640357	0.7391
Monocytes	TMEM41B	-0.08942	4.300772	-0.64323	0.521741	-5.78878	0.597713	0.690531
Monocytes	MPI	-0.16224	2.225319	-0.64285	0.521987	-5.33898	0.633747	0.731707
Monocytes	PPP4R1	-0.07148	5.579566	-0.6428	0.522014	-6.11228	0.576671	0.666495
Monocytes	ZFP69	-0.20223	2.612118	-0.64276	0.522044	-5.29045	0.626858	0.723878
Monocytes	TTC33	0.117656	3.849347	0.642642	0.52212	-5.63491	0.605367	0.699388
Monocytes	VRK3	0.068455	4.98755	0.642605	0.522143	-6.0323	0.586315	0.677576
Monocytes	INO80DOS	-0.1438	4.442013	-0.64255	0.52218	-5.75355	0.595363	0.687961
Monocytes	SLC25A42	-0.24125	1.940911	-0.64246	0.522239	-5.18346	0.638867	0.737539
Monocytes	ADAP2	-0.12095	2.585365	-0.6424	0.522276	-5.8749	0.627332	0.724439
Monocytes	VPS28	-0.05856	6.686374	-0.64238	0.522292	-6.35979	0.559112	0.646291
Monocytes	MAFG	-0.06984	5.07367	-0.64204	0.522508	-6.09083	0.585064	0.676132
Monocytes	SHROOM3	-0.34619	0.981918	-0.64197	0.522555	-5.06612	0.656645	0.757675
Monocytes	FPGS	-0.16676	3.013479	-0.64183	0.522642	-5.33261	0.62	0.716066
Monocytes	MAP3K11	0.098097	3.876975	0.641696	0.522731	-5.79235	0.60513	0.699104
Monocytes	SOCS7	0.085581	4.207792	0.641199	0.523052	-5.85535	0.599579	0.692844
Monocytes	DIP2A	0.148621	2.079809	0.641191	0.523057	-5.42979	0.636663	0.735103
Monocytes	LONRF3	-0.14757	1.525709	-0.64116	0.523076	-5.53866	0.646728	0.746514
Monocytes	SMIM24	-0.10676	3.210803	-0.64111	0.523111	-5.64913	0.616648	0.712349
Monocytes	DTX3L	0.124002	4.876216	0.641023	0.523166	-6.02263	0.588428	0.680144
Monocytes	TAGAP	-0.11121	4.269177	-0.64093	0.523224	-6.04274	0.598545	0.691739

Monocytes	DCBLD2	-0.23648	1.32551	-0.6409	0.523248	-5.08747	0.650407	0.750763
Monocytes	EIF3K	-0.04158	7.871661	-0.64078	0.523323	-6.60627	0.541228	0.625743
Monocytes	ZFP28	-0.31874	0.2908	-0.64072	0.523365	-5.00413	0.669789	0.772667
Monocytes	KLC1	0.071503	4.702224	0.64066	0.523401	-5.93281	0.591308	0.683463
Monocytes	ALKBH2	0.168074	2.187068	0.640519	0.523492	-5.22936	0.634734	0.733025
Monocytes	8030453O22RII	-0.26547	-0.75227	-0.64052	0.523492	-5.02162	0.689963	0.79537
Monocytes	GM11973	-0.13999	3.179185	-0.64008	0.523778	-5.61802	0.617464	0.713233
Monocytes	PRDM11	-0.15333	3.444862	-0.63984	0.523933	-5.47969	0.612969	0.708042
Monocytes	CCT5	-0.06296	6.571584	-0.63964	0.524063	-6.33822	0.561531	0.649057
Monocytes	RRM1	-0.1022	5.449593	-0.63949	0.52416	-6.09141	0.57942	0.669651
Monocytes	DTYMK	-0.10212	5.126752	-0.63941	0.524212	-5.95635	0.584684	0.675707
Monocytes	MORC2A	-0.09178	4.295558	-0.63902	0.52446	-5.8662	0.598486	0.691615
Monocytes	STAT2	0.11941	5.210285	0.639004	0.524472	-6.11245	0.583317	0.674226
Monocytes	CBR1	0.125826	4.350162	0.638947	0.524509	-5.70163	0.597568	0.690565
Monocytes	HERC2	0.064151	5.98752	0.638943	0.524512	-6.22416	0.570765	0.659793
Monocytes	PCM1	0.060868	6.494399	0.63862	0.524721	-6.28173	0.562742	0.650619
Monocytes	NCKAP5	0.332188	1.217214	0.638561	0.524759	-5.05146	0.652826	0.753545
Monocytes	SMG8	0.139638	2.847118	0.638542	0.524772	-5.44574	0.623408	0.720158
Monocytes	ZFP3	-0.3076	0.562138	-0.63851	0.524795	-4.98905	0.665074	0.767397
Monocytes	PACRG	-0.34039	-0.43352	-0.63838	0.524877	-4.97511	0.684169	0.788955
Monocytes	MYLK	-0.25244	1.606214	-0.6383	0.524931	-5.15869	0.64567	0.745547
Monocytes	PPP1R14B	0.065152	6.546146	0.63824	0.524967	-6.26091	0.56193	0.649796
Monocytes	PPP1R7	-0.07571	4.409711	-0.63815	0.525024	-5.8235	0.596569	0.689653
Monocytes	FXYD1	-0.23828	2.943369	-0.638	0.525123	-5.34302	0.621717	0.718426
Monocytes	ANKLE1	-0.25031	0.64315	-0.63781	0.525246	-5.06804	0.663546	0.765907
Monocytes	DDX55	0.146672	2.934608	0.637794	0.525256	-5.44045	0.621871	0.718641
Monocytes	SLC52A2	0.174027	2.391257	0.637752	0.525283	-5.323	0.631487	0.729585
Monocytes	CRACR2B	0.300219	1.009115	0.637721	0.525304	-5.09131	0.65669	0.758161
Monocytes	GM20337	0.262576	1.078162	0.637451	0.525478	-5.09806	0.655405	0.756797
Monocytes	EIF4A2	-0.05817	5.530319	-0.6374	0.525513	-6.15304	0.578112	0.668642
Monocytes	OAZ2	0.080745	4.485342	0.63739	0.525518	-5.83296	0.595303	0.68838
Monocytes	NICN1	-0.30291	0.820121	-0.63732	0.525566	-5.01437	0.660221	0.762266
Monocytes	BAX	0.06105	6.141068	0.637235	0.525618	-6.33987	0.568321	0.657382
Monocytes	DAG1	0.097081	4.867236	0.637144	0.525677	-5.87872	0.588955	0.681114
Monocytes	CCL4	-0.13749	7.388292	-0.63712	0.525691	-6.70007	0.5489	0.634949
Monocytes	AHCYL2	-0.0599	6.28947	-0.6371	0.525706	-6.43744	0.56597	0.654672
Monocytes	MPV17	-0.08761	4.075124	-0.6371	0.525706	-5.7612	0.602207	0.696299
Monocytes	RNF44	0.074094	4.833037	0.636974	0.525788	-5.99315	0.589521	0.681763
Monocytes	GNAI2	0.034874	9.008515	0.636963	0.525795	-6.76845	0.524781	0.606957
Monocytes	F8	-0.25964	3.134673	-0.63668	0.525977	-5.245	0.618514	0.714873
Monocytes	HSPA9	-0.05756	6.483768	-0.63648	0.526108	-6.28855	0.563102	0.651295
Monocytes	CHURC1	0.056821	5.66012	0.636409	0.526154	-6.19273	0.576213	0.666418
Monocytes	LIMCH1	0.368992	0.57747	0.636214	0.52628	-4.97488	0.665076	0.767694
Monocytes	2210408F21RIK	-0.12066	2.856305	-0.63602	0.526405	-5.85238	0.623519	0.720643
Monocytes	CCDC15	-0.18737	2.810819	-0.63599	0.526425	-5.33027	0.62432	0.721556
Monocytes	GM41409	0.158601	3.031183	0.635938	0.52646	-5.68882	0.62045	0.717146
Monocytes	TMPRSS3	-0.39139	-0.48523	-0.63587	0.526502	-4.87299	0.685477	0.79075

Monocytes	ELF1	-0.04439	8.010369	-0.6354	0.526812	-6.50743	0.539987	0.624453
Monocytes	PHTF1	-0.09648	4.68503	-0.63517	0.526959	-5.89548	0.592604	0.68509
Monocytes	CD68	-0.08212	4.872198	-0.63509	0.52701	-6.30643	0.589499	0.681542
Monocytes	ATP2A2	-0.04724	6.456942	-0.63476	0.527221	-6.35087	0.564035	0.652183
Monocytes	DTWD2	-0.15021	2.777791	-0.63469	0.527268	-5.50961	0.62541	0.722511
Monocytes	GM15787	0.186905	2.641968	0.634663	0.527287	-5.36542	0.627813	0.725246
Monocytes	NPRL3	0.145434	2.892647	0.634457	0.527421	-5.42497	0.623473	0.720246
Monocytes	PRPF31	-0.09951	3.79385	-0.63434	0.527495	-5.68947	0.607841	0.70243
Monocytes	FGFR2	-0.07179	5.987704	-0.63427	0.527545	-6.17216	0.571561	0.66084
Monocytes	RDM1	-0.10478	5.028258	-0.63415	0.527621	-5.97486	0.587124	0.678762
Monocytes	DHX35	0.147262	2.187953	0.633992	0.527723	-5.45077	0.636018	0.734628
Monocytes	CACNA1B	0.36021	0.056619	0.633925	0.527767	-4.9544	0.675643	0.779442
Monocytes	ASTE1	0.144828	2.753642	0.63387	0.527802	-5.42066	0.625933	0.72316
Monocytes	4933411E06RIK	-0.31047	0.555657	-0.6338	0.527846	-5.01102	0.66613	0.768718
Monocytes	GM4876	-0.14671	1.628342	-0.63369	0.527917	-5.42551	0.646185	0.746149
Monocytes	WDR4	0.128782	2.999114	0.633484	0.528053	-5.4688	0.621714	0.718276
Monocytes	TLCD1	0.186135	1.624021	0.632791	0.528503	-5.24341	0.646788	0.746346
Monocytes	SP100	0.074378	7.43248	0.63276	0.528523	-6.50465	0.549452	0.634946
Monocytes	FBXO48	0.270251	0.24175	0.632661	0.528588	-5.0379	0.672662	0.775574
Monocytes	URI1	0.065936	5.89939	0.632351	0.52879	-6.18501	0.573567	0.662698
Monocytes	PDE6C	-0.33889	0.436862	-0.63232	0.528808	-4.99338	0.66907	0.771448
Monocytes	SH3D19	0.23726	2.244921	0.632153	0.528918	-5.18298	0.63571	0.733667
Monocytes	A430033K04RIK	0.334853	0.6406	0.632058	0.52898	-4.99369	0.665277	0.767119
Monocytes	GEMIN7	0.062957	5.084337	0.631636	0.529255	-6.02414	0.586978	0.678049
Monocytes	ATG16L2	-0.07498	5.680082	-0.63156	0.529301	-6.05723	0.577265	0.66691
Monocytes	OST4	-0.05669	8.049265	-0.6315	0.529341	-6.61418	0.540383	0.624366
Monocytes	SOCS5	-0.12115	4.029298	-0.63146	0.529371	-5.73311	0.604629	0.698298
Monocytes	CAPN3	0.243324	1.422788	0.631442	0.529381	-5.14844	0.650805	0.750806
Monocytes	1700012D14RIK	-0.21909	1.843803	-0.63098	0.529679	-5.18492	0.64329	0.742275
Monocytes	ACOT1	-0.19711	2.604682	-0.6309	0.529735	-5.57756	0.629598	0.726759
Monocytes	GM49602	0.208006	1.89388	0.630743	0.529836	-5.18867	0.642379	0.741275
Monocytes	MKKS	0.198224	1.995441	0.630726	0.529847	-5.19518	0.640536	0.739189
Monocytes	BCL7B	0.061923	5.531179	0.630597	0.52993	-6.13217	0.579855	0.66993
Monocytes	MAPK1IP1	-0.18249	2.304416	-0.63035	0.530092	-5.20958	0.634963	0.732994
Monocytes	DEAF1	-0.12008	3.281829	-0.63013	0.530232	-5.5172	0.617682	0.713345
Monocytes	MAN2B2	0.110437	3.799809	0.630114	0.530245	-5.76512	0.608734	0.703136
Monocytes	IFT80	0.179149	3.626112	0.629938	0.53036	-5.43911	0.611719	0.706605
Monocytes	ANKRD50	-0.12755	2.62264	-0.62973	0.530493	-5.39238	0.629279	0.726663
Monocytes	AKIP1	-0.13217	3.24519	-0.62943	0.530691	-5.57142	0.61832	0.714274
Monocytes	LIMK1	0.163429	2.1198	0.629297	0.530777	-5.40977	0.638286	0.736989
Monocytes	IDH3G	0.077347	5.124044	0.629292	0.530781	-6.10141	0.586506	0.677882
Monocytes	FBN1	-0.305	0.898383	-0.62924	0.530813	-5.0177	0.660759	0.762437
Monocytes	PICK1	0.22164	1.538716	0.629242	0.530814	-5.18392	0.648872	0.748991
Monocytes	BFAR	0.059983	4.85458	0.62915	0.530873	-6.05292	0.590955	0.682986
Monocytes	ZC3H11A	0.194802	2.01149	0.629116	0.530895	-5.27799	0.640245	0.739212
Monocytes	SHFL	-0.25582	1.832835	-0.62911	0.530901	-5.12399	0.64349	0.742893
Monocytes	GM45442	-0.41756	-0.57631	-0.62902	0.530958	-4.89952	0.689044	0.794332

Monocytes	PRPF40A	-0.03994	7.709067	-0.62897	0.530987	-6.53566	0.545681	0.630859
Monocytes	POC1A	-0.14172	3.229083	-0.62883	0.531085	-5.50407	0.618601	0.714671
Monocytes	AGTRAP	0.118766	4.534904	0.628815	0.531091	-5.88056	0.596282	0.689164
Monocytes	CCNJL	0.2729	1.734552	0.628786	0.53111	-5.06896	0.645283	0.745003
Monocytes	SCMH1	-0.08046	5.849321	-0.62876	0.531125	-6.15973	0.574716	0.664401
Monocytes	HS2ST1	-0.06515	5.689893	-0.62875	0.531136	-6.11856	0.577285	0.667356
Monocytes	1700097N02RII	-0.25	2.346622	-0.62855	0.531263	-5.18027	0.634206	0.73247
Monocytes	CYHR1	0.087577	4.48568	0.628495	0.5313	-5.82653	0.597107	0.690145
Monocytes	EFEMP2	0.194626	1.944258	0.628455	0.531326	-5.20259	0.641464	0.740719
Monocytes	SERPINE1	0.287559	0.805177	0.628428	0.531343	-5.09035	0.662509	0.764545
Monocytes	EEF1AKMT4	0.113185	2.582886	0.628352	0.531393	-5.56906	0.629986	0.727692
Monocytes	SCN4A	0.354726	0.410123	0.628346	0.531397	-4.93402	0.669982	0.772987
Monocytes	CNNM3	0.134298	3.157652	0.628174	0.53151	-5.47975	0.61991	0.716192
Monocytes	SENP5	-0.06422	5.992571	-0.62804	0.531598	-6.20307	0.572507	0.661899
Monocytes	LHPP	-0.15844	3.112672	-0.62734	0.532052	-5.35692	0.62117	0.717424
Monocytes	4732496C06RIK	-0.24777	0.689806	-0.62728	0.532094	-5.08612	0.665254	0.767413
Monocytes	ZFP593	-0.12502	3.359126	-0.62713	0.53219	-5.6488	0.61691	0.712607
Monocytes	4921511C10RIK	0.150272	2.59393	0.626982	0.532287	-5.46098	0.630409	0.728026
Monocytes	MMADHC	-0.07659	4.299266	-0.6269	0.532338	-5.85564	0.600833	0.694287
Monocytes	ARL8A	-0.05526	6.227073	-0.62672	0.53246	-6.3435	0.569266	0.658034
Monocytes	THOC3	-0.09662	4.102819	-0.62668	0.532482	-5.7618	0.604188	0.698137
Monocytes	FAM71D	0.363919	0.095273	0.626575	0.532552	-4.94761	0.676712	0.780421
Monocytes	RFC2	0.077059	5.227985	0.626475	0.532618	-6.08379	0.585419	0.676609
Monocytes	HAAO	-0.10701	3.64437	-0.62586	0.53302	-5.83392	0.612445	0.707319
Monocytes	PTPRA	0.045223	6.521638	0.625136	0.533492	-6.34665	0.565302	0.653009
Monocytes	MDGA1	0.270615	0.010829	0.625097	0.533517	-5.12229	0.679164	0.782655
Monocytes	DERA	0.07207	4.534652	0.625095	0.533519	-5.88696	0.597637	0.69015
Monocytes	TAGLN2	-0.07366	8.716101	-0.62503	0.53356	-6.7394	0.531855	0.614319
Monocytes	VWA5A	0.089147	3.406572	0.624592	0.533848	-5.9755	0.617128	0.71218
Monocytes	2610507B11RIK	-0.05466	5.431746	-0.62456	0.533869	-6.10415	0.582996	0.67315
Monocytes	PRDM16	0.311304	0.306557	0.624445	0.533944	-5.07602	0.673737	0.776301
Monocytes	FAM160B2	0.121644	3.264944	0.624232	0.534083	-5.49306	0.619639	0.715128
Monocytes	ARHGAP27	-0.10896	3.664658	-0.62423	0.534086	-5.67733	0.612699	0.707218
Monocytes	LY86	0.070036	6.704851	0.624048	0.534203	-6.56315	0.56272	0.64992
Monocytes	INPP5K	0.069948	5.371477	0.623852	0.534331	-6.04292	0.584159	0.674574
Monocytes	CRLS1	-0.09743	3.622878	-0.62366	0.534454	-5.66269	0.613637	0.708276
Monocytes	ZRANB2	0.062321	5.45391	0.623445	0.534597	-6.07655	0.582971	0.67308
Monocytes	RRAGB	-0.2735	0.125251	-0.62281	0.53501	-5.08471	0.677938	0.78081
Monocytes	THYN1	-0.11394	3.271604	-0.6228	0.535016	-5.58351	0.620156	0.715426
Monocytes	PUM3	-0.07061	4.640352	-0.6227	0.535083	-5.98131	0.596728	0.688684
Monocytes	GNPTAB	0.089178	4.589659	0.622659	0.535111	-5.93234	0.597578	0.689665
Monocytes	HIST1H1A	-0.22796	3.773403	-0.62242	0.535269	-5.65103	0.611563	0.705553
Monocytes	FZR1	0.090618	4.900739	0.622057	0.535505	-5.94383	0.592687	0.683842
Monocytes	MS4A7	0.18185	1.921581	0.621509	0.535864	-5.69667	0.644874	0.743076
Monocytes	GSTT2	-0.16968	3.554147	-0.62141	0.53593	-5.38133	0.615823	0.710162
Monocytes	ANKRD13A	-0.05603	6.215118	-0.62135	0.535969	-6.26114	0.571527	0.659466
Monocytes	GM37305	0.210325	1.078554	0.621263	0.536025	-5.19012	0.66046	0.760784

Monocytes	COLQ	-0.34901	0.244853	-0.6211	0.53613	-4.90363	0.676279	0.778684
Monocytes	PTPRS	-0.0908	4.390471	-0.62104	0.536172	-5.92294	0.601502	0.693927
Monocytes	ELMO2	0.078232	3.945024	0.620997	0.536199	-6.01138	0.609083	0.702594
Monocytes	CCDC59	0.05221	5.676842	0.620965	0.53622	-6.15844	0.580193	0.669536
Monocytes	CBX5	0.090816	5.103912	0.62084	0.536302	-5.978	0.589602	0.680305
Monocytes	MYOM1	0.207923	1.071853	0.620337	0.536631	-5.27523	0.660891	0.761087
Monocytes	FBXL6	-0.10526	3.399881	-0.62031	0.536648	-5.63852	0.618791	0.713378
Monocytes	MFAP1B	-0.07157	4.945536	-0.61966	0.537076	-5.92979	0.592768	0.683532
Monocytes	PAGR1A	0.341145	0.395669	0.619597	0.537117	-4.97036	0.674031	0.775754
Monocytes	SSBP2	0.07845	6.284595	0.619575	0.537131	-6.30837	0.570965	0.658535
Monocytes	SREK1IP1	-0.09605	4.172778	-0.61945	0.537213	-5.80153	0.605772	0.698468
Monocytes	TTC39B	-0.08344	4.653747	-0.61935	0.537277	-5.99354	0.597642	0.689189
Monocytes	LAPTM5	0.041934	8.326927	0.619351	0.537278	-6.70548	0.539424	0.622201
Monocytes	ZFP236	-0.07431	4.729945	-0.61924	0.537351	-5.96253	0.596365	0.687768
Monocytes	GM20743	-0.41052	0.031876	-0.61917	0.537394	-4.8907	0.681036	0.783766
Monocytes	GM11998	-0.33593	0.318253	-0.61886	0.537598	-4.98827	0.675674	0.777634
Monocytes	TRIAP1	0.090455	4.111675	0.618779	0.537653	-5.79788	0.606957	0.699818
Monocytes	RAB1B	-0.06051	5.58788	-0.61871	0.537701	-6.21896	0.582332	0.67167
Monocytes	EMC1	0.093193	3.622778	0.618301	0.537967	-5.69279	0.615491	0.709567
Monocytes	CCNJ	0.25484	2.015982	0.61827	0.537987	-5.14136	0.644051	0.742017
Monocytes	ASB4	-0.40622	1.68448	-0.61819	0.538036	-5.09182	0.650122	0.748904
Monocytes	ZBTB34	0.111247	3.592868	0.618172	0.538051	-5.6718	0.61601	0.710188
Monocytes	LY6G5B	-0.27703	1.345565	-0.61806	0.538124	-5.01988	0.65641	0.755998
Monocytes	1110006O24RII	-0.31149	0.319918	-0.61793	0.538209	-4.94203	0.675827	0.777886
Monocytes	ADGRG5	0.25033	0.105614	0.617442	0.53853	-5.22259	0.680219	0.782681
Monocytes	CASP6	0.081799	3.628567	0.617432	0.538537	-5.92319	0.615671	0.709666
Monocytes	TNFSF12	0.122964	2.295218	0.617223	0.538674	-5.62372	0.639367	0.736536
Monocytes	HACD1	-0.15027	3.002315	-0.61693	0.538864	-5.50138	0.626817	0.722236
Monocytes	DOCK11	-0.05555	7.111908	-0.6168	0.538949	-6.45965	0.558605	0.644232
Monocytes	FEM1C	0.053254	7.117684	0.616772	0.53897	-6.6226	0.558515	0.644128
Monocytes	UBE2H	0.059584	8.574917	0.616737	0.538993	-6.58078	0.536364	0.618527
Monocytes	ERP29	0.040794	7.289905	0.616537	0.539124	-6.57454	0.555869	0.641092
Monocytes	ITGA8	-0.14814	1.726804	-0.61637	0.539234	-5.76807	0.649857	0.748457
Monocytes	ZRANB1	-0.05273	6.142303	-0.61633	0.539258	-6.24497	0.573943	0.661916
Monocytes	DRAP1	-0.04647	6.508223	-0.61633	0.539258	-6.35157	0.56811	0.655207
Monocytes	GM14302	-0.31117	0.609708	-0.61618	0.53936	-4.94436	0.670771	0.7721
Monocytes	CEP112	-0.24325	1.570821	-0.61615	0.539379	-5.21564	0.652734	0.751756
Monocytes	AP5M1	0.09937	3.584616	0.615932	0.539522	-5.71935	0.616735	0.710853
Monocytes	P4HB	0.047684	7.267668	0.615723	0.539659	-6.51245	0.556348	0.641635
Monocytes	CWC27	-0.0655	5.662216	-0.61568	0.53969	-6.1265	0.58184	0.670963
Monocytes	CXCL13	-0.51837	-1.21101	-0.61549	0.53981	-4.87663	0.706682	0.812324
Monocytes	LARS	0.079549	4.738364	0.615339	0.539912	-5.99223	0.597165	0.688579
Monocytes	SPHK2	0.111238	3.388566	0.615315	0.539928	-5.65456	0.620271	0.71494
Monocytes	HIST1H2BJ	-0.2105	3.30978	-0.61519	0.540009	-5.45265	0.621656	0.716509
Monocytes	CTSK	0.285855	0.234472	0.614919	0.540188	-5.0656	0.6782	0.780514
Monocytes	SPATA7	0.228494	1.309288	0.614864	0.540224	-5.11797	0.657824	0.757588
Monocytes	SS18	0.060858	5.946104	0.614855	0.54023	-6.19966	0.577305	0.665888

Monocytes	FAM210A	-0.10366	3.636981	-0.61478	0.540278	-5.61744	0.615951	0.710119
Monocytes	CCPG1OS	-0.21537	1.576044	-0.61471	0.540322	-5.09799	0.65287	0.752026
Monocytes	TBCCD1	-0.15313	2.589728	-0.61465	0.540365	-5.40791	0.634416	0.731125
Monocytes	CARNMT1	-0.06652	5.353674	-0.61457	0.540414	-6.01974	0.586956	0.677012
Monocytes	IQGAP3	0.187081	2.400987	0.614347	0.540564	-5.47355	0.637884	0.735086
Monocytes	SNTB1	0.147249	4.264888	0.614151	0.540693	-5.91201	0.605236	0.697934
Monocytes	1700086O06RII	-0.22777	1.546442	-0.61405	0.540761	-5.08395	0.653495	0.75276
Monocytes	OLFR1259	0.420626	-1.34501	0.614021	0.540778	-4.89983	0.709477	0.815655
Monocytes	ST8SIA6	-0.26456	1.211567	-0.61371	0.540986	-5.34195	0.659726	0.75984
Monocytes	PWWP2A	0.076862	5.141602	0.613693	0.540994	-5.99194	0.590524	0.681144
Monocytes	TTC32	0.098656	4.101346	0.613621	0.541041	-5.72135	0.608026	0.701166
Monocytes	GM47863	-0.17827	0.552776	-0.61361	0.541052	-5.43437	0.672175	0.773883
Monocytes	EID2	0.35021	0.075956	0.61346	0.541148	-4.93592	0.681343	0.784195
Monocytes	NUP205	-0.09061	4.408914	-0.61339	0.541195	-5.82398	0.602792	0.695189
Monocytes	PLA2G4C	-0.32325	0.890136	-0.61337	0.541207	-5.01802	0.665768	0.766666
Monocytes	CISH	0.140273	2.887974	0.613304	0.54125	-5.70059	0.62917	0.725263
Monocytes	FAM129A	0.058798	5.667894	0.613288	0.541261	-6.47884	0.581884	0.671261
Monocytes	RTKN2	0.298762	1.228878	0.613076	0.5414	-5.02261	0.659499	0.759547
Monocytes	SMIM27	0.098654	3.590898	0.612646	0.541683	-5.67238	0.617133	0.711328
Monocytes	ARMCX5	-0.15155	2.433168	-0.6126	0.541713	-5.34105	0.637624	0.734613
Monocytes	SLC16A1	-0.13469	3.905544	-0.61222	0.541967	-5.56003	0.611909	0.705176
Monocytes	FGFR1OP2	0.057872	6.180589	0.612122	0.542028	-6.22383	0.574097	0.661935
Monocytes	CHCHD4	-0.0954	3.820424	-0.61203	0.54209	-5.80263	0.61338	0.706899
Monocytes	TMEM59	-0.04942	6.649746	-0.6119	0.542173	-6.35269	0.566631	0.653394
Monocytes	MED29	0.075709	3.806911	0.611756	0.542269	-5.85916	0.613614	0.707239
Monocytes	SLF1	0.104964	4.44199	0.611678	0.542321	-5.77287	0.602753	0.694859
Monocytes	PDE5A	-0.20192	2.569114	-0.61163	0.542352	-5.36147	0.63541	0.732062
Monocytes	NETO2	-0.1783	3.22493	-0.61157	0.542392	-5.46705	0.623758	0.718851
Monocytes	GM1604A	0.180604	2.804564	0.611223	0.54262	-5.4154	0.631325	0.727439
Monocytes	6530409C15RIK	-0.16235	1.661527	-0.61122	0.542623	-5.47187	0.652062	0.750933
Monocytes	MED10	0.075042	4.886924	0.61113	0.542682	-5.95102	0.595389	0.686491
Monocytes	CTCF	-0.04318	7.101448	-0.61089	0.542843	-6.37238	0.559654	0.645445
Monocytes	HIST2H2BB	-0.35653	-0.261	-0.61088	0.542849	-4.90365	0.688637	0.792129
Monocytes	PSMB10	-0.08292	5.706129	-0.61079	0.542906	-6.24497	0.58188	0.671012
Monocytes	HIBADH	0.081376	5.742005	0.610734	0.542942	-6.05876	0.581296	0.670342
Monocytes	FITM2	0.234688	0.918829	0.610675	0.542982	-5.04107	0.665935	0.766615
Monocytes	TTC1	-0.06063	4.740195	-0.61034	0.543204	-5.93494	0.597964	0.689346
Monocytes	CAPNS1	0.042754	7.25812	0.610329	0.54321	-6.53514	0.557328	0.642679
Monocytes	ZFP553	0.175029	2.414705	0.610198	0.543296	-5.22201	0.638472	0.735478
Monocytes	PRPF39	-0.06534	5.43876	-0.60976	0.543587	-6.12131	0.586585	0.676179
Monocytes	ERGIC1	0.084361	5.084699	0.60968	0.543638	-6.05549	0.592433	0.682886
Monocytes	ELF4	0.053461	6.785373	0.609657	0.543653	-6.37869	0.564925	0.651306
Monocytes	REPS1	0.058721	5.964436	0.609496	0.543759	-6.18289	0.578049	0.666366
Monocytes	ZBTB8OS	-0.06766	5.027468	-0.60943	0.5438	-6.08609	0.593414	0.683985
Monocytes	TMCC1	0.075343	7.946033	0.609012	0.544078	-6.54718	0.547125	0.630763
Monocytes	CEACAM10	0.315682	-1.04799	0.608893	0.544156	-4.92812	0.704837	0.810071
Monocytes	SREK1	-0.04384	6.429668	-0.60881	0.544211	-6.32295	0.570713	0.658022

Monocytes	FN3KRP	-0.2299	1.481688	-0.60864	0.544323	-5.17749	0.655944	0.755158
Monocytes	SLC35E2	0.086594	3.898481	0.608604	0.544347	-5.74926	0.612672	0.706062
Monocytes	BBOF1	-0.26888	1.302277	-0.60852	0.544401	-5.03792	0.659287	0.758932
Monocytes	UBIAD1	-0.16506	1.941616	-0.6085	0.544415	-5.20878	0.647457	0.745565
Monocytes	FBXO32	-0.19586	3.711448	-0.60825	0.544582	-5.57577	0.615906	0.709747
Monocytes	1810058I24RIK	-0.06985	7.005863	-0.6082	0.544612	-6.42821	0.561618	0.647551
Monocytes	NUP54	-0.06553	4.859149	-0.6082	0.544617	-6.06631	0.596355	0.687431
Monocytes	GM42982	0.245384	1.514483	0.608113	0.544672	-5.03765	0.655335	0.75447
Monocytes	PFAS	-0.1343	4.205445	-0.60801	0.544742	-5.73179	0.607405	0.700055
Monocytes	GM12743	0.151313	2.382516	0.608007	0.544742	-5.46579	0.639434	0.73648
Monocytes	B3GALT4	-0.2493	1.404829	-0.60794	0.544788	-5.17057	0.657374	0.756773
Monocytes	ZMAT1	-0.19841	2.090961	-0.60788	0.544826	-5.24923	0.644727	0.742475
Monocytes	NEPRO	-0.14218	2.709524	-0.60766	0.544971	-5.46133	0.63365	0.729811
Monocytes	ZFP958	0.164112	2.267977	0.607571	0.545029	-5.38361	0.641605	0.738884
Monocytes	NOA1	0.126331	3.137205	0.60729	0.545215	-5.54869	0.626192	0.721273
Monocytes	KEL	-0.42799	-0.44363	-0.60717	0.545294	-4.9219	0.693096	0.796767
Monocytes	MMAA	-0.20556	1.748976	-0.60707	0.545362	-5.23812	0.651277	0.749715
Monocytes	IBA57	-0.16638	1.934328	-0.60667	0.545626	-5.26567	0.648072	0.745939
Monocytes	MRM1	0.170417	2.067052	0.606627	0.545653	-5.26958	0.645643	0.743194
Monocytes	GM42941	-0.15629	1.267028	-0.60624	0.54591	-5.3965	0.660598	0.76001
Monocytes	DUSP11	-0.0461	6.33182	-0.60617	0.545954	-6.30942	0.57284	0.660107
Monocytes	RFC5	-0.13186	4.185223	-0.6061	0.546002	-5.77149	0.608351	0.700779
Monocytes	UBE4BOS1	0.340138	0.735049	0.606053	0.546033	-5.00131	0.670642	0.771369
Monocytes	YEATS2	-0.0805	4.376137	-0.60596	0.546095	-5.84271	0.605095	0.697109
Monocytes	WBP4	0.06127	5.270226	0.605876	0.546149	-6.10648	0.590104	0.679973
Monocytes	ZC3H7B	-0.14962	3.353116	-0.60575	0.546232	-5.40392	0.622795	0.717265
Monocytes	KIF23	-0.10208	5.173452	-0.60557	0.546355	-6.09633	0.591745	0.68186
Monocytes	CEP85L	0.082596	4.806918	0.605548	0.546366	-6.14733	0.597859	0.688854
Monocytes	GM14326	-0.10664	2.868013	-0.60533	0.54651	-5.71223	0.631453	0.727061
Monocytes	EPG5	-0.07834	4.420072	-0.60524	0.54657	-5.92446	0.604449	0.696353
Monocytes	PPARGC1A	-0.38167	1.419055	-0.60519	0.546602	-4.97946	0.657867	0.757009
Monocytes	COL14A1	-0.26375	1.863173	-0.6048	0.546859	-5.16769	0.649828	0.747812
Monocytes	HGD	-0.24521	2.465599	-0.60471	0.546917	-5.30296	0.638851	0.735398
Monocytes	TUBD1	-0.19681	2.095647	-0.60468	0.546938	-5.20642	0.645568	0.743012
Monocytes	DDX24	0.052586	6.717234	0.604394	0.54713	-6.34026	0.567103	0.653432
Monocytes	EIF4A3	-0.05719	5.877476	-0.60416	0.547287	-6.21612	0.580661	0.668997
Monocytes	CASP2	0.091068	4.01281	0.603837	0.547498	-5.71611	0.611918	0.704753
Monocytes	PHACTR4	0.05583	5.677431	0.603831	0.547502	-6.18185	0.583996	0.672839
Monocytes	LY6I	-0.18825	0.802083	-0.6037	0.547591	-5.9148	0.670036	0.770606
Monocytes	ATXN2	-0.05703	6.28335	-0.60368	0.547601	-6.28046	0.574189	0.661612
Monocytes	9230116N13RII	0.232795	0.920334	0.603473	0.547739	-5.14769	0.667875	0.768095
Monocytes	WDR83OS	0.060217	6.074614	0.603396	0.54779	-6.25684	0.577618	0.665517
Monocytes	INMT	-0.32181	0.639157	-0.60319	0.547928	-5.05329	0.673321	0.774259
Monocytes	SNRPD3	0.053769	6.518599	0.602825	0.548168	-6.33908	0.570705	0.657445
Monocytes	ZKSCAN6	0.127123	2.942031	0.602773	0.548203	-5.60424	0.630964	0.726275
Monocytes	TBC1D15	-0.05388	6.062519	-0.60274	0.548228	-6.27237	0.57802	0.665859
Monocytes	E2F4	-0.06341	5.334947	-0.60252	0.548371	-6.09664	0.589987	0.679583

Monocytes	PPP1R18OS	-0.21499	0.993134	-0.60244	0.548423	-5.05976	0.666825	0.766844
Monocytes	SART1	0.073955	4.872457	0.602062	0.548674	-5.97099	0.597879	0.688476
Monocytes	OTOA	-0.28693	0.757069	-0.60196	0.548743	-5.05472	0.671524	0.772021
Monocytes	COQ10B	0.049314	5.908947	0.601899	0.548782	-6.37783	0.580773	0.668936
Monocytes	GM11579	0.374472	-0.20636	0.601752	0.548879	-4.94313	0.690209	0.79296
Monocytes	IL27RA	0.302751	1.535873	0.601611	0.548973	-5.08753	0.656941	0.755525
Monocytes	AVEN	-0.08205	4.926701	-0.60131	0.549172	-6.03356	0.597191	0.687617
Monocytes	SORBS3	-0.29398	0.937867	-0.60123	0.549227	-5.01643	0.668334	0.768325
Monocytes	FOXM1	-0.14456	3.10354	-0.60075	0.549542	-5.58581	0.628849	0.72361
Monocytes	ATP6V1H	0.063165	6.803114	0.600738	0.549551	-6.41088	0.566874	0.652799
Monocytes	SLC35B4	0.132881	2.825471	0.600507	0.549705	-5.50329	0.63391	0.729269
Monocytes	ZFP764	0.236523	1.074799	0.600291	0.549848	-5.09966	0.666203	0.765665
Monocytes	ATXN7L3B	0.052485	5.941589	0.59998	0.550054	-6.2166	0.580973	0.668701
Monocytes	DDX50	-0.04521	6.369428	-0.59994	0.550078	-6.31165	0.574071	0.66079
Monocytes	TGFBR3	-0.1752	3.337964	-0.59976	0.550202	-5.47014	0.625037	0.71899
Monocytes	NT5C	0.064012	5.114948	0.599752	0.550205	-6.18418	0.594581	0.684293
Monocytes	RERE	0.05216	7.702335	0.599506	0.550369	-6.48292	0.553251	0.636804
Monocytes	FAM151B	0.204668	1.753415	0.599429	0.55042	-5.28869	0.653763	0.751492
Monocytes	GM21781	0.215513	1.561921	0.599299	0.550506	-5.16748	0.657348	0.755566
Monocytes	RAD54B	0.214121	2.207787	0.599053	0.55067	-5.29268	0.645514	0.742159
Monocytes	CMPK2	0.167992	3.672891	0.599024	0.550689	-5.69697	0.61937	0.712518
Monocytes	NSUN5	-0.17072	2.124735	-0.59875	0.550868	-5.27561	0.647142	0.744052
Monocytes	RCE1	0.0943	3.757774	0.598577	0.550985	-5.73938	0.617997	0.711039
Monocytes	PIP4K2B	-0.08831	4.178712	-0.59857	0.550991	-5.78281	0.610721	0.702757
Monocytes	UBALD1	0.084932	4.983613	0.598517	0.551025	-5.92001	0.597073	0.687198
Monocytes	0610010K14RIK	-0.06211	5.617735	-0.59828	0.551181	-6.16604	0.586565	0.675241
Monocytes	ATR	0.115205	3.862209	0.598213	0.551227	-5.76973	0.61619	0.70906
Monocytes	TMEM128	0.061082	5.718829	0.598088	0.55131	-6.24554	0.584908	0.673362
Monocytes	LY6C1	0.428092	-0.32417	0.598068	0.551324	-4.93169	0.693712	0.796579
Monocytes	CEP152	-0.10397	3.629164	-0.59789	0.551439	-5.66718	0.620246	0.713722
Monocytes	RNF219	0.158545	3.193241	0.597846	0.551471	-5.39312	0.627912	0.722439
Monocytes	GM17484	-0.27306	1.264186	-0.59783	0.551479	-5.12714	0.663112	0.76225
Monocytes	GM41611	-0.25025	0.284798	-0.59762	0.551621	-5.15625	0.681803	0.783347
Monocytes	UBL5	-0.04115	8.259007	-0.59761	0.551631	-6.62778	0.544953	0.627486
Monocytes	LIMK2	0.080194	4.779797	0.597572	0.551653	-5.92568	0.600503	0.691318
Monocytes	4930403P22RIK	-0.25203	0.612375	-0.59745	0.551732	-5.02374	0.675488	0.776295
Monocytes	CUL9	0.159103	2.579779	0.597269	0.551854	-5.39399	0.638878	0.734993
Monocytes	RELL1	0.060803	6.866963	0.597236	0.551876	-6.32841	0.566454	0.652317
Monocytes	TBPL1	0.064394	4.928003	0.597231	0.55188	-6.03573	0.598011	0.688497
Monocytes	ZMYM4	0.062605	5.733892	0.597109	0.551961	-6.16414	0.584676	0.673222
Monocytes	IFIT1BL1	0.346983	0.159661	0.597028	0.552014	-5.09994	0.684249	0.786123
Monocytes	ZFP408	-0.1072	3.716601	-0.59664	0.552274	-5.64073	0.618958	0.712168
Monocytes	A430073D23RII	-0.22178	1.815155	-0.59637	0.552455	-5.14995	0.653176	0.750938
Monocytes	TRAPPC8	-0.05482	6.225642	-0.59628	0.552514	-6.26435	0.576975	0.664213
Monocytes	KDELR2	0.053992	5.993878	0.59619	0.552572	-6.28031	0.580723	0.668544
Monocytes	ADGRV1	-0.2651	0.711845	-0.59617	0.552586	-5.16293	0.673928	0.774376
Monocytes	MTLN	0.098798	3.060484	0.596017	0.552687	-5.67601	0.630635	0.725502

Monocytes	RITA1	0.192817	1.367418	0.595906	0.55276	-5.16994	0.661576	0.760512
Monocytes	REPIN1	0.262529	1.082403	0.595696	0.5529	-5.06213	0.667024	0.766559
Monocytes	ATF6B	-0.07686	4.456866	-0.59557	0.552984	-5.97956	0.606414	0.697895
Monocytes	MAP2K5	0.051895	6.143251	0.595368	0.553119	-6.2533	0.57843	0.665941
Monocytes	ZCCHC3	0.234312	0.70709	0.595363	0.553122	-5.12868	0.674165	0.77467
Monocytes	CDK11B	0.057305	6.82853	0.595349	0.553131	-6.36848	0.567474	0.653356
Monocytes	PTPRF	-0.24926	1.276101	-0.59513	0.553274	-5.03294	0.663414	0.762556
Monocytes	NIM1K	-0.13726	2.88905	-0.59511	0.553288	-5.55608	0.633826	0.729112
Monocytes	COMMD4	0.060627	5.326168	0.594846	0.553466	-6.18091	0.591966	0.681295
Monocytes	DMD	-0.22923	2.419953	-0.59463	0.553609	-5.22858	0.642413	0.738692
Monocytes	CS	-0.04747	6.103857	-0.59457	0.553648	-6.29036	0.579226	0.666706
Monocytes	MRPS27	-0.11819	3.208477	-0.59445	0.55373	-5.59804	0.628271	0.722665
Monocytes	MCM8	-0.20946	1.54016	-0.59414	0.553938	-5.1859	0.658606	0.757031
Monocytes	CDK5RAP1	-0.27854	2.44604	-0.59386	0.55412	-5.25819	0.64194	0.738274
Monocytes	DSEL	-0.25225	0.375304	-0.59377	0.554182	-5.01461	0.680733	0.782004
Monocytes	PCGF2	-0.22859	0.79144	-0.59376	0.554188	-5.17798	0.672737	0.773019
Monocytes	KIF4	-0.13184	4.394266	-0.59372	0.554213	-5.91323	0.607648	0.699316
Monocytes	NUP160	-0.08353	5.017011	-0.59371	0.554219	-6.01341	0.597118	0.687297
Monocytes	SLC35E4	0.153754	0.657751	0.593679	0.554243	-5.52673	0.675295	0.775895
Monocytes	SUPT20	0.060567	5.30473	0.593666	0.554251	-6.11397	0.592322	0.681814
Monocytes	ABHD8	0.185312	2.172201	0.593572	0.554314	-5.23937	0.646928	0.743923
Monocytes	AKAP11	-0.08384	4.648059	-0.5935	0.554365	-5.92555	0.603332	0.694421
Monocytes	KLHL11	-0.14711	2.776613	-0.59347	0.554383	-5.4351	0.635974	0.731555
Monocytes	SRPK1	-0.04608	6.11152	-0.59334	0.554469	-6.26525	0.579102	0.666725
Monocytes	ADRB1	-0.26832	0.735849	-0.59333	0.554477	-5.27088	0.673799	0.774278
Monocytes	FAM136A	0.108324	4.090791	0.5933	0.554495	-5.69891	0.612853	0.705315
Monocytes	MRPL22	0.097246	3.858759	0.593014	0.554686	-5.77698	0.616893	0.70992
Monocytes	NIF3L1	0.129039	3.000203	0.592973	0.554713	-5.45912	0.631999	0.727085
Monocytes	FAF1	0.048805	6.739255	0.592758	0.554856	-6.37185	0.569067	0.655342
Monocytes	RBMX2	0.122563	3.368821	0.592637	0.554937	-5.57462	0.625464	0.719819
Monocytes	IL33	0.300175	0.25962	0.592478	0.555043	-5.00862	0.683003	0.784824
Monocytes	USP35	-0.29324	0.017078	-0.59246	0.555054	-5.1025	0.687728	0.790125
Monocytes	CNTD1	-0.27485	0.620711	-0.5924	0.555096	-5.05382	0.676033	0.777001
Monocytes	ECSIT	-0.10338	4.015765	-0.59221	0.555224	-5.65526	0.614174	0.707103
Monocytes	SLC27A1	0.157869	2.972091	0.592159	0.555256	-5.5189	0.632501	0.727951
Monocytes	A130010J15RIK	-0.20228	1.72998	-0.59206	0.555323	-5.22965	0.655102	0.753545
Monocytes	CNPPD1	0.070358	5.686802	0.591946	0.555398	-6.16418	0.586044	0.674981
Monocytes	KRCC1	0.043512	5.9705	0.591844	0.555465	-6.3079	0.581413	0.669686
Monocytes	EXOSC10	-0.07209	4.93082	-0.5918	0.555493	-6.01527	0.598588	0.689353
Monocytes	SDF4	0.044631	6.506152	0.591695	0.555565	-6.35034	0.572779	0.659773
Monocytes	PTCD2	-0.0641	4.58679	-0.59169	0.555568	-6.09183	0.604396	0.695987
Monocytes	COL6A1	0.307658	0.367808	0.591653	0.555593	-5.00475	0.680907	0.782627
Monocytes	PANK2	0.060621	5.327592	0.59153	0.555675	-6.1125	0.591967	0.681797
Monocytes	TMEM268	0.101354	3.100152	0.591449	0.555729	-5.69194	0.63022	0.725416
Monocytes	DDX59	0.171045	1.892836	0.59144	0.555735	-5.29719	0.652089	0.750189
Monocytes	GLT8D1	-0.10886	3.339159	-0.59139	0.555767	-5.58638	0.625987	0.720619
Monocytes	SMAD6	-0.12791	2.733914	-0.59135	0.555792	-5.85289	0.636767	0.732868

Monocytes	H2AFY	0.040638	7.697828	0.590871	0.556115	-6.60857	0.554346	0.638403
Monocytes	GM527	-0.29955	0.481195	-0.59059	0.556299	-5.07205	0.67907	0.780428
Monocytes	MPND	-0.07814	4.813143	-0.59059	0.556301	-6.01727	0.60088	0.691847
Monocytes	RPUSD3	-0.33802	0.640562	-0.59056	0.556324	-4.97378	0.676004	0.776982
Monocytes	PLEKHA6	-0.13328	1.808761	-0.59014	0.556599	-5.51765	0.654234	0.752224
Monocytes	2300009A05RIK	0.095159	4.080294	0.58971	0.556889	-5.81747	0.613865	0.706285
Monocytes	PHF5A	0.059923	6.072643	0.58962	0.556949	-6.27337	0.580517	0.6682
Monocytes	CDC45	0.142076	3.268129	0.589506	0.557025	-5.61425	0.628066	0.722501
Monocytes	MSANTD2	-0.06681	4.864755	-0.58944	0.557069	-5.96772	0.600486	0.691121
Monocytes	MCFD2	0.11724	3.702476	0.589079	0.55731	-5.67441	0.620628	0.713968
Monocytes	SPTBN1	0.068843	6.620496	0.588884	0.55744	-6.31567	0.571907	0.658327
Monocytes	TMEM168	-0.07283	4.583494	-0.58883	0.557475	-5.97982	0.605459	0.69674
Monocytes	ACAP3	-0.13275	2.238471	-0.58878	0.557509	-5.37296	0.646821	0.743746
Monocytes	GRB10	0.248157	3.090696	0.588669	0.557584	-5.36647	0.631455	0.726324
Monocytes	CHCHD1	-0.05787	5.813268	-0.58843	0.557745	-6.26358	0.585069	0.673388
Monocytes	2010013B24RIK	0.090014	3.545259	0.588271	0.55785	-5.95979	0.623578	0.717345
Monocytes	TERF1	-0.06637	5.254483	-0.58805	0.557996	-6.02273	0.594416	0.684047
Monocytes	NDE1	-0.09182	4.58489	-0.58769	0.558235	-5.88484	0.605686	0.697068
Monocytes	EPHB4	-0.36474	0.794939	-0.58766	0.558261	-5.02085	0.674098	0.774562
Monocytes	SUGP2	0.109883	4.059588	0.587572	0.558318	-5.74959	0.614694	0.707346
Monocytes	HES7	-0.22959	-0.49621	-0.58755	0.558329	-5.12519	0.699297	0.802831
Monocytes	RBM18	0.076792	4.236008	0.587524	0.55835	-5.79285	0.611652	0.703882
Monocytes	SRRM1	-0.03763	7.865839	-0.58749	0.558372	-6.57724	0.552654	0.636231
Monocytes	FTSJ1	-0.11057	3.078297	-0.58744	0.558408	-5.47256	0.631921	0.726929
Monocytes	SLC50A1	0.072871	5.247798	0.587201	0.558565	-6.04987	0.594585	0.684345
Monocytes	TSR2	-0.21293	2.118125	-0.58717	0.558584	-5.21298	0.649358	0.746631
Monocytes	CEP170	-0.05638	5.909007	-0.5867	0.558901	-6.32839	0.583787	0.672055
Monocytes	SLC26A10	-0.28189	0.914242	-0.58667	0.558921	-5.10044	0.672006	0.772244
Monocytes	SDE2	0.063845	6.013145	0.586668	0.558922	-6.26935	0.58209	0.670109
Monocytes	TMEM237	0.176373	2.157499	0.586605	0.558964	-5.35681	0.648751	0.746059
Monocytes	CHPF	0.193864	1.242066	0.586569	0.558988	-5.2231	0.665787	0.765289
Monocytes	CCDC157	0.229018	1.103128	0.585948	0.559404	-5.14499	0.668839	0.768415
Monocytes	ADAP1	0.070785	4.152492	0.585343	0.559808	-6.11048	0.614025	0.706076
Monocytes	GM10131	-0.28145	1.072727	-0.58504	0.560009	-5.12649	0.669938	0.769342
Monocytes	ALDH1A1	-0.37501	-5.25E-05	-0.58502	0.560023	-4.95642	0.690662	0.792605
Monocytes	TRMT6	0.080144	4.373913	0.584923	0.56009	-5.84878	0.610324	0.701908
Monocytes	OXR1	0.060091	6.336267	0.584336	0.560482	-6.27115	0.578018	0.664744
Monocytes	TPX2	-0.12574	5.047457	-0.58423	0.560555	-6.07126	0.599242	0.689033
Monocytes	GM13212	0.118453	3.205264	0.584162	0.560599	-5.62254	0.631102	0.725295
Monocytes	C430049B03RIK	0.227452	1.844436	0.583786	0.560851	-5.17274	0.656061	0.753346
Monocytes	FNDC9	0.153459	2.564671	0.583491	0.561048	-5.24264	0.642991	0.738451
Monocytes	5330439K02RIK	0.248305	0.538036	0.583339	0.561151	-5.14498	0.681033	0.781309
Monocytes	ZCWPW2	-0.26269	1.016841	-0.58311	0.561306	-5.1311	0.671899	0.771091
Monocytes	LBH	0.052325	6.308404	0.583085	0.561321	-6.28185	0.578899	0.665526
Monocytes	2810002D19RII	-0.18241	1.66458	-0.58283	0.561495	-5.15001	0.659809	0.757426
Monocytes	RALB	0.051105	4.247517	0.582604	0.561643	-6.20036	0.613527	0.704951
Monocytes	ACTA2	-0.27295	3.320545	-0.58244	0.561752	-5.63605	0.629801	0.723374

Monocytes	GM12367	-0.32242	0.051988	-0.58216	0.56194	-5.02724	0.690933	0.792257
Monocytes	MECR	0.106737	3.581314	0.582078	0.561995	-5.65111	0.625248	0.718223
Monocytes	TM2D1	0.045539	5.944576	0.581994	0.562052	-6.31698	0.585126	0.672507
Monocytes	ZFP689	-0.22345	1.407041	-0.58186	0.562144	-5.12438	0.664862	0.76305
Monocytes	GM6225	-0.12401	2.958455	-0.58176	0.562206	-5.66681	0.636324	0.730845
Monocytes	CSGALNACT1	-0.30061	2.818139	-0.58171	0.562242	-5.11758	0.638849	0.733726
Monocytes	STOML1	-0.18791	2.251865	-0.58164	0.56229	-5.25047	0.649152	0.745379
Monocytes	DCTN5	-0.0713	4.598222	-0.58164	0.562292	-5.97995	0.60762	0.698269
Monocytes	IGKV1-135	-0.36079	-0.02384	-0.58143	0.562427	-4.9244	0.692516	0.794127
Monocytes	2610306M01RI	0.264003	0.680103	0.580687	0.562928	-5.09547	0.679333	0.779024
Monocytes	GRIPAP1	0.056537	5.594976	0.580577	0.563003	-6.15898	0.59143	0.679505
Monocytes	PIK3C3	-0.07772	4.439233	-0.5803	0.56319	-5.89288	0.611033	0.701756
Monocytes	TMEM209	0.140142	3.362785	0.580229	0.563236	-5.52499	0.629824	0.723116
Monocytes	PTGR2	0.098412	3.734192	0.57984	0.563497	-5.68159	0.623436	0.715732
Monocytes	TALDO1	-0.03875	8.310001	-0.57979	0.563529	-6.77145	0.548602	0.630131
Monocytes	GM15265	-0.22456	1.797023	-0.57973	0.56357	-5.16433	0.658482	0.75537
Monocytes	PDE11A	-0.37177	0.072949	-0.57952	0.563715	-4.98159	0.69155	0.792436
Monocytes	GM48293	-0.24956	-0.15995	-0.57947	0.563748	-5.117	0.696145	0.797581
Monocytes	EVI5L	-0.17168	2.262161	-0.57929	0.563864	-5.26606	0.649929	0.745753
Monocytes	LLGL2	0.175072	2.291993	0.579245	0.563897	-5.29775	0.649381	0.745155
Monocytes	CIAO2B	-0.0728	4.566233	-0.57917	0.563948	-6.00811	0.609072	0.699504
Monocytes	MORRBID	0.08561	5.127394	0.579112	0.563986	-6.40419	0.599558	0.688699
Monocytes	TAF11	0.06601	4.933052	0.579023	0.564046	-5.99814	0.602834	0.692478
Monocytes	ALS2	0.078147	4.283789	0.578876	0.564145	-5.84911	0.613965	0.705191
Monocytes	CCDC50	-0.04658	5.994315	-0.57852	0.564384	-6.38537	0.585407	0.672511
Monocytes	ZBTB18	0.09065	3.894799	0.578302	0.56453	-5.74903	0.621008	0.713005
Monocytes	2510046G10RII	0.146229	2.620896	0.578161	0.564625	-5.37234	0.643721	0.738788
Monocytes	FPR1	0.188388	2.992339	0.578115	0.564656	-5.83984	0.637006	0.731193
Monocytes	SEC61A1	-0.05011	5.49084	-0.578	0.564736	-6.20444	0.593824	0.682108
Monocytes	SCD2	0.099541	4.726396	0.577577	0.565018	-5.96679	0.606871	0.69687
Monocytes	CLDN11	-0.25931	-0.29485	-0.57756	0.56503	-5.14032	0.699435	0.801272
Monocytes	ARL16	0.14152	2.250651	0.577363	0.565162	-5.31278	0.65074	0.7466
Monocytes	B3GNT2	0.068871	6.739389	0.577208	0.565266	-6.18324	0.573665	0.658971
Monocytes	TTLL3	-0.10087	4.027206	-0.57716	0.565297	-5.76295	0.618939	0.710654
Monocytes	MRPS25	0.076422	4.238863	0.577158	0.5653	-5.93628	0.615266	0.70648
Monocytes	CHMP4B	0.036719	7.889271	0.57704	0.565379	-6.63931	0.55562	0.638246
Monocytes	ZFP90	-0.17591	2.283533	-0.57643	0.565791	-5.34638	0.65056	0.746106
Monocytes	A530041M06RI	-0.18071	2.069945	-0.57627	0.565899	-5.20097	0.654555	0.750551
Monocytes	UPRT	0.217965	0.606501	0.576064	0.566035	-5.1438	0.682298	0.781776
Monocytes	LASP1	-0.0522	5.455474	-0.57598	0.56609	-6.26229	0.595076	0.683161
Monocytes	CNN3	-0.13974	4.531127	-0.57593	0.566127	-5.62156	0.610704	0.700985
Monocytes	GM50373	0.299578	0.358844	0.575779	0.566227	-5.04439	0.687114	0.78729
Monocytes	COX6A1	0.052264	6.768979	0.57571	0.566274	-6.43354	0.573633	0.658702
Monocytes	SKI	-0.06261	5.372605	-0.57569	0.566285	-6.19332	0.596459	0.684814
Monocytes	TRIM36	0.09911	3.146179	0.575098	0.566685	-5.80439	0.635304	0.728761
Monocytes	ENAH	0.217771	0.744942	0.575079	0.566698	-5.27012	0.67997	0.779072
Monocytes	ZSWIM3	0.240435	1.503758	0.574906	0.566815	-5.12849	0.665529	0.762919

Monocytes	UBE2D3	0.029424	8.966917	0.574855	0.56685	-6.75291	0.539975	0.619839
Monocytes	PLS1	-0.28092	0.910051	-0.57443	0.567133	-5.03976	0.677092	0.775686
Monocytes	LIMS2	-0.31123	0.424817	-0.57418	0.567304	-4.99666	0.68652	0.786297
Monocytes	PPP1R37	-0.08164	4.592802	-0.57418	0.567306	-5.8304	0.610263	0.700241
Monocytes	CLUH	-0.10786	3.417508	-0.57412	0.567347	-5.65978	0.630776	0.723531
Monocytes	GM11110	-0.14808	1.970897	-0.5739	0.567492	-5.35684	0.657133	0.753341
Monocytes	LPAR2	0.15127	2.187833	0.573862	0.567518	-5.26557	0.653113	0.74881
Monocytes	PAOX	0.137192	2.987177	0.573461	0.567789	-5.5158	0.638767	0.732414
Monocytes	MED11	0.089501	3.312748	0.573242	0.567936	-5.73512	0.632962	0.725942
Monocytes	TSNAX	-0.07443	4.49606	-0.57323	0.567941	-5.94755	0.612233	0.702423
Monocytes	SPOUT1	-0.15003	2.468742	-0.57271	0.568293	-5.35294	0.648538	0.743417
Monocytes	UBE2R2	0.050545	7.144409	0.572613	0.56836	-6.43606	0.568805	0.652698
Monocytes	NDUFC1	0.054712	7.367214	0.572394	0.568508	-6.51083	0.565289	0.648672
Monocytes	UPF1	-0.05459	5.35091	-0.57237	0.568522	-6.06494	0.59802	0.686125
Monocytes	SPCS1	0.042406	7.852067	0.572307	0.568566	-6.58442	0.557722	0.639977
Monocytes	TBC1D9	-0.06564	3.953614	-0.57227	0.568588	-6.49538	0.621945	0.71334
Monocytes	TRAF6	-0.06067	5.683882	-0.57213	0.568686	-6.1602	0.592471	0.679861
Monocytes	EIF3D	-0.05844	5.480464	-0.57189	0.568848	-6.16769	0.595854	0.68382
Monocytes	AIFM1	0.078402	4.370662	0.571833	0.568886	-5.84327	0.614695	0.705303
Monocytes	METTL6	0.081244	4.505421	0.571818	0.568896	-5.90745	0.612372	0.702662
Monocytes	APOBEC4	-0.33221	0.042895	-0.57174	0.568948	-4.93312	0.694704	0.795511
Monocytes	YOD1	0.121646	4.084266	0.57173	0.568955	-5.68206	0.619664	0.71097
Monocytes	FLT1	0.152619	4.243341	0.571515	0.569101	-6.05129	0.616989	0.707829
Monocytes	PIGU	0.090273	4.215968	0.571398	0.56918	-5.82101	0.617484	0.708403
Monocytes	AC125149.3	0.308379	-0.34803	0.570971	0.569468	-5.04807	0.702807	0.80445
Monocytes	ARFGEF1	0.052605	6.613928	0.570834	0.56956	-6.39158	0.577551	0.662866
Monocytes	SNAPC1	-0.07982	3.850567	-0.57077	0.569605	-5.89021	0.624049	0.715872
Monocytes	FNIP2	-0.08697	5.343736	-0.57074	0.569622	-6.60158	0.598425	0.686727
Monocytes	DGCR8	-0.09884	3.779114	-0.57066	0.569678	-5.68323	0.625305	0.717321
Monocytes	TMEM109	0.081622	3.841384	0.57054	0.569759	-5.99208	0.62421	0.716079
Monocytes	ZRSR1	0.189936	2.211301	0.57051	0.569779	-5.21696	0.653586	0.749306
Monocytes	SETD1A	-0.07234	4.19796	-0.57	0.570125	-5.84826	0.618289	0.709136
Monocytes	PYROXD2	-0.26215	0.182818	-0.5698	0.57026	-5.04518	0.692712	0.792922
Monocytes	IZUMO4	0.226161	1.453448	0.569423	0.570513	-5.25037	0.668231	0.765558
Monocytes	EPM2AIP1	-0.14836	2.609784	-0.56938	0.570541	-5.38058	0.646725	0.741345
Monocytes	GM43728	-0.1774	-0.48628	-0.56937	0.570546	-5.2755	0.706077	0.807929
Monocytes	GUCY2C	0.168638	-0.14698	0.569274	0.570614	-5.27382	0.699293	0.80039
Monocytes	PSENEN	-0.04226	6.713106	-0.56925	0.570632	-6.37972	0.576364	0.661354
Monocytes	DNAL4	-0.1597	1.951429	-0.56919	0.570674	-5.35362	0.658875	0.755092
Monocytes	E2F1	0.101698	4.409532	0.568972	0.570818	-5.85865	0.614783	0.705234
Monocytes	GM48226	-0.14839	2.396833	-0.56896	0.570825	-5.37296	0.65066	0.745858
Monocytes	PPP6R3	0.044634	7.283025	0.568392	0.57121	-6.45871	0.567636	0.651123
Monocytes	PHC1	-0.09236	3.5385	-0.56797	0.571497	-5.61708	0.630595	0.722684
Monocytes	4930539J05RIK	0.250851	0.706555	0.567873	0.571561	-5.08451	0.683179	0.781893
Monocytes	CACNA1F	0.274681	-0.45662	0.567821	0.571595	-5.07456	0.706145	0.807548
Monocytes	GM11713	-0.18022	2.37115	-0.5677	0.571676	-5.36759	0.651713	0.746564
Monocytes	NBDY	0.072372	3.659616	0.567664	0.571702	-5.9098	0.628447	0.720289

Monocytes	CHCHD5	0.115995	3.092559	0.567484	0.571823	-5.61669	0.638639	0.731795
Monocytes	SERGEF	0.081511	4.018452	0.567378	0.571896	-5.90228	0.622209	0.713227
Monocytes	4930556J24RIK	0.155237	2.185085	0.567054	0.572115	-5.3167	0.655271	0.750581
Monocytes	GBP4	-0.24579	3.363964	-0.56705	0.572118	-5.51397	0.633821	0.726366
Monocytes	CYP7B1	0.369949	0.01895	0.566912	0.57221	-5.00703	0.696785	0.797148
Monocytes	SEC61A2	0.101033	3.741527	0.566854	0.57225	-5.62419	0.627115	0.718783
Monocytes	2810405F17RIK	-0.25899	0.687122	-0.56679	0.572293	-5.07131	0.683682	0.782501
Monocytes	OPTN	-0.10272	4.651784	-0.56673	0.572332	-5.84134	0.611271	0.700804
Monocytes	SYT14	0.380281	0.008242	0.566427	0.572538	-4.97724	0.696997	0.797437
Monocytes	TACC3	-0.10999	5.072041	-0.56639	0.572566	-6.09032	0.604106	0.692694
Monocytes	POMGNT1	-0.1342	2.312495	-0.56637	0.57258	-5.38628	0.652915	0.747985
Monocytes	EPB42	-0.3374	-0.1647	-0.56633	0.572603	-4.94655	0.700433	0.801273
Monocytes	QK	-0.03622	8.142329	-0.56624	0.572663	-6.65268	0.554526	0.635949
Monocytes	AKT1S1	0.101327	3.589237	0.566236	0.572668	-5.61224	0.629811	0.721883
Monocytes	MEA1	0.059175	5.276389	0.566135	0.572736	-6.14683	0.600663	0.688767
Monocytes	RETNLA	-0.60467	-0.77811	-0.56593	0.572875	-5.37419	0.712875	0.81508
Monocytes	GM29114	-0.29567	-1.28439	-0.56579	0.57297	-4.9091	0.723277	0.82665
Monocytes	STAU1	0.05492	6.034578	0.5657	0.57303	-6.26865	0.588165	0.674482
Monocytes	MRPS14	-0.04412	6.892734	-0.56538	0.57325	-6.4607	0.574398	0.658612
Monocytes	SHKBP1	0.077438	4.068341	0.565297	0.573304	-5.88105	0.621664	0.712488
Monocytes	CSRNP2	0.171348	2.107884	0.565123	0.573422	-5.31686	0.657037	0.752461
Monocytes	FOXD2OS	0.275714	0.311339	0.565079	0.573451	-5.02928	0.691369	0.790975
Monocytes	HERC6	0.117661	4.719873	0.564877	0.573588	-5.9853	0.610493	0.699732
Monocytes	GM20139	0.279377	-0.63311	0.56473	0.573688	-4.95982	0.710335	0.812041
Monocytes	NRAS	0.047754	6.075126	0.564318	0.573967	-6.29685	0.587861	0.673924
Monocytes	PEX5	0.078547	4.071805	0.564292	0.573984	-5.80582	0.621823	0.712577
Monocytes	XLR4B	-0.19017	1.285179	-0.56426	0.574009	-5.18425	0.672731	0.770004
Monocytes	FAM173A	-0.06887	4.68182	-0.56423	0.574026	-5.9956	0.611255	0.700585
Monocytes	SDHD	0.060826	5.858408	0.564162	0.574072	-6.27555	0.591433	0.678008
Monocytes	2500004C02RIK	0.224071	1.043374	0.563961	0.574209	-5.08087	0.677359	0.775201
Monocytes	PLA2G15	0.097747	3.738633	0.563927	0.574232	-5.9053	0.62768	0.719221
Monocytes	1810024B03RIK	-0.14862	2.692562	-0.56386	0.574278	-5.39189	0.646469	0.740462
Monocytes	POLR2D	0.068721	5.184758	0.563818	0.574306	-6.06579	0.602693	0.690851
Monocytes	ATXN7L1	-0.04838	6.345264	-0.56353	0.574503	-6.46301	0.58358	0.66899
Monocytes	FCNB	0.122561	-0.23854	0.563135	0.574768	-5.58226	0.702896	0.803529
Monocytes	NFE2L2	0.05319	7.355477	0.562987	0.574869	-6.68375	0.567619	0.650559
Monocytes	SPRYD7	0.142979	2.133927	0.562683	0.575075	-5.41981	0.657354	0.752472
Monocytes	PDE4DIP	-0.08635	3.94998	-0.56242	0.575251	-5.80486	0.624628	0.715416
Monocytes	LMBRD1	-0.05058	6.356577	-0.56236	0.575292	-6.43117	0.583884	0.669055
Monocytes	UBE2Q1	-0.04367	6.334643	-0.56226	0.575359	-6.36966	0.584247	0.669464
Monocytes	JMJD4	0.197664	1.268434	0.561856	0.575636	-5.10402	0.674028	0.770845
Monocytes	CUTA	-0.04699	6.189562	-0.56174	0.575716	-6.33306	0.586836	0.672201
Monocytes	IRAK3	0.099563	4.187779	0.561314	0.576003	-6.17661	0.620699	0.710779
Monocytes	SURF1	0.07253	4.36834	0.561177	0.576097	-6.00058	0.617557	0.707215
Monocytes	KANSL1L	-0.09199	7.202302	-0.56108	0.576163	-6.40239	0.570496	0.653563
Monocytes	SNX10	0.062009	5.029489	0.56106	0.576176	-6.39095	0.606201	0.694318
Monocytes	GM50240	0.189507	1.572659	0.560987	0.576225	-5.23709	0.668243	0.764413

Monocytes	CBX8	0.200319	1.617418	0.560943	0.576256	-5.23449	0.667396	0.763478
Monocytes	ZFP386	0.088374	3.889207	0.560829	0.576333	-5.78952	0.625933	0.716776
Monocytes	CHM	0.060066	5.555869	0.560815	0.576342	-6.17759	0.597327	0.68428
Monocytes	GM41077	0.249899	0.721818	0.560811	0.576345	-5.10182	0.684561	0.782771
Monocytes	ANK3	-0.30559	1.681131	-0.56077	0.57637	-5.13032	0.666193	0.762185
Monocytes	MPEG1	0.069995	6.081136	0.560771	0.576372	-6.68831	0.588616	0.674349
Monocytes	GSTCD	0.105943	3.564196	0.560586	0.576497	-5.70583	0.631749	0.72331
Monocytes	GM550	0.250277	0.859238	0.560506	0.576552	-5.08309	0.681963	0.779812
Monocytes	AACS	-0.09021	3.861959	-0.56025	0.576729	-5.73496	0.626598	0.717391
Monocytes	SDHB	0.054174	7.274793	0.560157	0.576789	-6.51023	0.569514	0.652385
Monocytes	1700019L13RIK	-0.21791	0.774577	-0.55999	0.576905	-5.25174	0.683797	0.781771
Monocytes	POLR3A	0.119673	3.30507	0.559903	0.576962	-5.60636	0.636559	0.728666
Monocytes	GM3055	0.292407	0.319624	0.55979	0.577038	-4.98672	0.692698	0.791739
Monocytes	SRP9	0.038091	7.516452	0.55971	0.577092	-6.54357	0.565754	0.648079
Monocytes	PDZD11	0.08732	4.30203	0.559443	0.577274	-5.86086	0.619028	0.708747
Monocytes	EFR3A	-0.04598	5.780301	-0.55934	0.577341	-6.31386	0.593894	0.680171
Monocytes	RBM12B1	-0.25524	0.935508	-0.55933	0.577348	-5.09568	0.680773	0.778315
Monocytes	GPT	0.24233	1.203309	0.559112	0.577499	-5.12564	0.675665	0.772579
Monocytes	PBK	-0.13997	3.69023	-0.5591	0.577507	-5.72605	0.629814	0.72095
Monocytes	NEU1	0.079829	4.426111	0.558947	0.577611	-5.88893	0.616927	0.706334
Monocytes	SIPA1L1	-0.06992	7.327522	-0.5589	0.577645	-6.55531	0.568854	0.651524
Monocytes	YDJC	0.192618	1.21958	0.5587	0.577779	-5.28177	0.675455	0.772328
Monocytes	CCDC141	-0.20844	1.120838	-0.55858	0.577862	-5.21292	0.677374	0.774489
Monocytes	ZFP318	-0.12147	3.491008	-0.55845	0.577949	-5.72888	0.633504	0.72511
Monocytes	TTF1	0.109415	3.466533	0.558201	0.578118	-5.59786	0.634033	0.725627
Monocytes	APH1B	0.109615	2.578014	0.558144	0.578156	-5.72991	0.650128	0.74379
Monocytes	COL25A1	-0.3393	0.981282	-0.55781	0.578381	-5.01845	0.680326	0.777689
Monocytes	RIMS4	-0.34275	-0.99611	-0.55779	0.578398	-4.94708	0.719674	0.821569
Monocytes	TCEA1	-0.04117	7.486226	-0.5577	0.578458	-6.48937	0.566663	0.648969
Monocytes	RBM44	0.281498	0.135835	0.557412	0.578654	-5.08777	0.697019	0.796283
Monocytes	CCDC112	0.201476	1.151644	0.557218	0.578786	-5.27437	0.677287	0.77422
Monocytes	GGPS1	0.053032	5.393996	0.556978	0.578949	-6.07083	0.600983	0.688068
Monocytes	TRIM33	-0.05096	6.368235	-0.55698	0.578951	-6.30088	0.584832	0.669656
Monocytes	TARS	-0.07612	4.374393	-0.55682	0.579056	-5.91288	0.618422	0.707891
Monocytes	H2AFX	-0.09306	6.482628	-0.5568	0.57907	-6.38799	0.582968	0.667539
Monocytes	RSPH10B	-0.35734	0.413611	-0.55667	0.579157	-4.95402	0.691664	0.790345
Monocytes	CPNE1	0.048845	6.038278	0.556624	0.57919	-6.2679	0.590247	0.675874
Monocytes	SS18L2	0.106738	3.817204	0.556058	0.579575	-5.63249	0.628395	0.71918
Monocytes	GM39121	0.250665	-0.94202	0.556045	0.579585	-5.0576	0.719098	0.820861
Monocytes	COL4A2	0.196514	2.620499	0.555871	0.579703	-5.32481	0.64996	0.743545
Monocytes	IGLC2	0.398701	3.740399	0.55566	0.579847	-5.49649	0.629755	0.720763
Monocytes	PDS5A	0.042245	7.411745	0.555633	0.579865	-6.46722	0.568258	0.650738
Monocytes	LSAMP	-0.27954	0.929492	-0.55556	0.579913	-5.06569	0.681831	0.779364
Monocytes	PCGF5	0.061544	6.06196	0.555375	0.580041	-6.41952	0.59005	0.675718
Monocytes	LUZP1	-0.0603	5.787075	-0.55537	0.580042	-6.30435	0.594603	0.680909
Monocytes	ASB5	0.235295	0.515697	0.555341	0.580064	-5.1083	0.689887	0.788445
Monocytes	BLM	0.119852	3.897628	0.5553	0.580092	-5.82248	0.626974	0.717684

Monocytes	GM12764	-0.22689	1.453299	-0.55526	0.580119	-5.14544	0.67178	0.768176
Monocytes	CD3EAP	-0.1027	3.231663	-0.55519	0.580166	-5.69978	0.638846	0.73111
Monocytes	STYK1	-0.23423	0.538888	-0.55517	0.580183	-5.14757	0.689433	0.787952
Monocytes	AP1G1	0.053345	6.456316	0.554874	0.580382	-6.35999	0.583726	0.668483
Monocytes	APOBR	0.081595	2.373103	0.554776	0.580449	-6.01747	0.65468	0.74894
Monocytes	PLCL2	0.05735	6.985417	0.554091	0.580916	-6.37172	0.575531	0.658869
Monocytes	ATP5K	0.056256	7.486519	0.554079	0.580924	-6.55189	0.56756	0.649743
Monocytes	UNC45A	-0.0814	4.303611	-0.55387	0.581069	-5.85107	0.620386	0.709944
Monocytes	PPWD1	0.062155	4.68019	0.553709	0.581176	-5.9162	0.613859	0.70258
Monocytes	AI480526	-0.19261	2.064128	-0.5535	0.581316	-5.20478	0.660827	0.755652
Monocytes	DDX17	-0.05097	6.357267	-0.55349	0.581326	-6.3398	0.585704	0.670551
Monocytes	ARFRP1	-0.07387	4.120918	-0.55348	0.581333	-5.80393	0.623581	0.713625
Monocytes	COX7C	0.037616	8.585633	0.553439	0.58136	-6.73859	0.550512	0.630231
Monocytes	CHMP1B	0.06834	4.698125	0.553389	0.581394	-5.96926	0.61355	0.702259
Monocytes	BC049715	-0.26163	0.604594	-0.55323	0.581506	-5.17109	0.688736	0.786957
Monocytes	GZMM	-0.19081	2.051803	-0.55314	0.581563	-5.28308	0.661057	0.755936
Monocytes	CALML4	-0.25606	1.801954	-0.55311	0.581581	-5.15911	0.665748	0.761206
Monocytes	CD19	-0.20735	3.122136	-0.55305	0.581622	-5.24352	0.64137	0.733773
Monocytes	UBASH3A	0.257358	2.009253	0.552988	0.581667	-5.14574	0.661854	0.756876
Monocytes	DOK1	-0.08499	3.614618	-0.55279	0.581801	-5.78638	0.632608	0.723819
Monocytes	HMGXB4	0.079581	4.148587	0.552426	0.58205	-5.82484	0.623374	0.713257
Monocytes	MITD1	0.092249	4.433197	0.552251	0.58217	-5.85073	0.618468	0.707632
Monocytes	GM26759	0.145358	3.123372	0.551819	0.582464	-5.51481	0.641855	0.733914
Monocytes	ZHX1	0.080435	4.078556	0.551736	0.582521	-5.88506	0.624818	0.714662
Monocytes	SDHAF3	-0.11882	3.044533	-0.55165	0.58258	-5.46837	0.643284	0.735526
Monocytes	EDRF1	-0.0957	4.217889	-0.55163	0.582594	-5.75152	0.622375	0.711896
Monocytes	NONO	-0.04308	6.614635	-0.55159	0.582622	-6.37393	0.581972	0.665945
Monocytes	4930481A15RIK	0.19382	1.854963	0.551308	0.582813	-5.20106	0.665355	0.760322
Monocytes	C3AR1	-0.14274	2.290307	-0.5513	0.582815	-5.89545	0.657212	0.751174
Monocytes	GM20536	0.155256	2.340392	0.551147	0.582923	-5.33801	0.656334	0.750167
Monocytes	CCAR2	-0.11454	3.432111	-0.55101	0.583019	-5.5416	0.636456	0.727733
Monocytes	A530013C23RIK	0.102385	2.431674	0.550636	0.583272	-5.95658	0.654896	0.748334
Monocytes	ANP32B	0.05141	8.191649	0.550312	0.583493	-6.66946	0.557474	0.637506
Monocytes	NDC80	-0.11897	4.625978	-0.54998	0.58372	-5.95049	0.615982	0.704134
Monocytes	COQ8B	-0.12342	3.190292	-0.54963	0.583959	-5.57418	0.641441	0.732886
Monocytes	DYNC1I2	0.039501	6.441227	0.549618	0.583967	-6.3711	0.585521	0.669476
Monocytes	PRKCSH	0.057113	4.862373	0.549477	0.584064	-6.05051	0.61197	0.69956
Monocytes	GM6712	-0.16998	2.305447	-0.54946	0.584073	-5.31964	0.657668	0.751149
Monocytes	SMYD1	-0.27066	0.29641	-0.54942	0.584102	-5.06965	0.696205	0.794275
Monocytes	SRGN	0.04306	9.652655	0.54927	0.584205	-6.96664	0.535601	0.612242
Monocytes	HIST1H2AB	-0.30112	1.438759	-0.54926	0.584212	-5.18805	0.673999	0.769467
Monocytes	GM43260	-0.16069	2.252383	-0.54909	0.58433	-5.31455	0.658718	0.752294
Monocytes	EXOSC5	0.055339	5.376885	0.548115	0.584994	-6.20276	0.603844	0.690031
Monocytes	GM13184	0.115494	2.666577	0.548085	0.585015	-5.68686	0.651678	0.744095
Monocytes	EIF4E3	0.079188	4.420461	0.547737	0.585253	-5.99073	0.620451	0.708717
Monocytes	POC1B	-0.06756	4.642223	-0.54759	0.585356	-5.93598	0.616642	0.704381
Monocytes	KCNIP2	-0.22105	1.248736	-0.54723	0.585599	-5.18317	0.678797	0.774304

Monocytes	A530017D24RII	-0.12205	2.325165	-0.54716	0.585644	-5.49658	0.658424	0.751477
Monocytes	NEMP1	-0.15045	3.065406	-0.54697	0.585779	-5.51162	0.644884	0.736197
Monocytes	DNMBP	-0.10153	3.488772	-0.54667	0.58598	-5.68261	0.637244	0.727595
Monocytes	PHF1	-0.14767	2.695984	-0.54662	0.586018	-5.40875	0.651654	0.743822
Monocytes	TRAPPC1	0.066213	5.432947	0.546607	0.586026	-6.25917	0.603375	0.689265
Monocytes	CENPO	0.128212	2.773597	0.54659	0.586037	-5.43181	0.650228	0.742218
Monocytes	SGO2A	-0.18497	3.063462	-0.54636	0.586195	-5.51085	0.644982	0.736282
Monocytes	PSMC1	-0.04873	5.889053	-0.54634	0.586208	-6.21715	0.595771	0.680597
Monocytes	BC065397	0.200827	1.292469	0.546206	0.5863	-5.1458	0.678111	0.773489
Monocytes	ZFYVE16	0.099016	3.016629	0.546153	0.586337	-5.68361	0.64584	0.737307
Monocytes	ETFDH	0.06333	4.872939	0.545697	0.586648	-6.02082	0.613191	0.700308
Monocytes	ARL6IP4	0.052672	5.733017	0.545557	0.586744	-6.25935	0.598592	0.68376
Monocytes	PPP4R3B	0.039418	6.842276	0.545532	0.586762	-6.40699	0.580335	0.662967
Monocytes	STAMBP	-0.10437	3.374422	-0.54549	0.586792	-5.68264	0.639582	0.730159
Monocytes	EED	0.037952	6.114274	0.545088	0.587066	-6.35474	0.592459	0.676595
Monocytes	GABARAPL2	-0.06452	7.762001	-0.54473	0.587308	-6.5643	0.566039	0.646364
Monocytes	TTLL11	0.22209	1.608719	0.544661	0.587358	-5.11133	0.672735	0.767135
Monocytes	CD2AP	-0.05646	6.24029	-0.54437	0.587559	-6.44039	0.590656	0.674422
Monocytes	CSF2RB	0.064575	4.838003	0.544321	0.58759	-6.45705	0.614303	0.701272
Monocytes	ZBTB26	0.227365	0.850706	0.544097	0.587745	-5.11938	0.687582	0.783545
Monocytes	COIL	0.089763	3.741294	0.543975	0.587828	-5.731	0.633655	0.723054
Monocytes	B9D1	0.266902	0.371188	0.543754	0.587979	-5.04783	0.697068	0.79413
Monocytes	SELENOF	0.060205	5.422799	0.54375	0.587982	-6.19313	0.604462	0.690035
Monocytes	FKBP4	0.059059	5.691682	0.543534	0.58813	-6.18759	0.600017	0.68498
Monocytes	ACAT2	-0.12131	2.91084	-0.54344	0.588193	-5.46133	0.648791	0.740126
Monocytes	RAD52	-0.11259	3.121528	-0.54326	0.588318	-5.58364	0.645016	0.73588
Monocytes	ECI1	-0.08328	3.920606	-0.54313	0.588406	-5.80352	0.630686	0.719697
Monocytes	NABP1	0.070764	4.743721	0.543036	0.588472	-6.13846	0.616268	0.703413
Monocytes	ST5	-0.18009	2.235439	-0.54258	0.588783	-5.28393	0.661457	0.754462
Monocytes	EMC10	0.051923	5.502896	0.542411	0.5889	-6.19365	0.60335	0.688937
Monocytes	ARHGEF39	0.191005	1.960521	0.542408	0.588902	-5.43291	0.66662	0.760309
Monocytes	APBB3	0.171956	1.534528	0.542362	0.588933	-5.29341	0.674709	0.769366
Monocytes	CDC42BPA	0.151346	3.315927	0.542335	0.588952	-5.40838	0.641584	0.732185
Monocytes	LEAP2	0.197635	3.060255	0.542273	0.588995	-5.47484	0.646226	0.737429
Monocytes	TMEM35B	-0.1675	2.207613	-0.54212	0.5891	-5.39937	0.661977	0.755172
Monocytes	FDFT1	0.095173	3.796269	0.542096	0.589116	-5.72147	0.632961	0.722522
Monocytes	PPFIA1	-0.06262	5.566923	-0.54204	0.589153	-6.14177	0.60227	0.687791
Monocytes	TMEM185A	0.127155	2.540183	0.541952	0.589215	-5.47739	0.655784	0.748277
Monocytes	HDC	0.189636	3.642871	0.541915	0.58924	-5.90979	0.635701	0.725675
Monocytes	SNHG20	-0.13019	2.355617	-0.54187	0.589274	-5.34864	0.659213	0.752162
Monocytes	SGPP1	0.067968	5.017924	0.541557	0.589486	-6.12059	0.61172	0.698532
Monocytes	BICRA	0.071474	5.82128	0.541523	0.589509	-6.03714	0.598111	0.683084
Monocytes	ARSA	0.147656	1.69399	0.541323	0.589646	-5.35285	0.671817	0.766256
Monocytes	LETM2	0.119759	3.131534	0.541281	0.589675	-5.52172	0.645071	0.736225
Monocytes	4932438A13RIK	-0.06476	7.087671	-0.54122	0.589715	-6.43193	0.577361	0.659453
Monocytes	DPYSL2	0.049146	6.279623	0.540857	0.589966	-6.48069	0.590707	0.674477
Monocytes	BTG1	0.045387	9.314237	0.540746	0.590043	-6.90808	0.542974	0.619858

Monocytes	CIAO3	0.09346	3.316721	0.540487	0.59022	-5.67477	0.642058	0.732544
Monocytes	FKTN	-0.20289	1.595478	-0.54012	0.590474	-5.19169	0.674233	0.768585
Monocytes	GOPC	0.06751	4.545466	0.539995	0.590558	-5.87086	0.6204	0.708026
Monocytes	MED4	0.076123	4.020834	0.53995	0.590589	-5.748	0.629616	0.718442
Monocytes	SLC37A1	-0.1255	3.202303	-0.5399	0.590622	-5.56655	0.644299	0.734995
Monocytes	IRF3	-0.09533	3.786081	-0.53911	0.591163	-5.67659	0.634254	0.723367
Monocytes	GM16573	-0.16952	1.655105	-0.53909	0.591179	-5.27482	0.673588	0.767551
Monocytes	NUDT1	0.146549	3.00659	0.538903	0.591308	-5.47709	0.648414	0.739277
Monocytes	NPHS1	0.258242	0.439647	0.538757	0.591408	-5.08141	0.697338	0.793953
Monocytes	PINX1	-0.10293	3.022074	-0.53864	0.591486	-5.6639	0.648176	0.738959
Monocytes	TDRD7	0.087329	3.385152	0.538571	0.591536	-5.87787	0.641574	0.731531
Monocytes	MORF4L2	-0.05345	5.404884	-0.53744	0.592312	-6.1706	0.606912	0.691885
Monocytes	SNX17	0.049883	5.853791	0.537062	0.592573	-6.30373	0.599536	0.683421
Monocytes	SLC15A3	-0.06624	5.005968	-0.53657	0.592913	-6.33956	0.614193	0.69989
Monocytes	GLRA1	-0.17854	1.25067	-0.53634	0.593074	-5.51718	0.682807	0.777007
Monocytes	DYNLT1A	-0.06983	4.14434	-0.53633	0.593075	-6.03396	0.629235	0.716919
Monocytes	RNF126	0.066686	4.767243	0.536272	0.593118	-6.01238	0.61832	0.704606
Monocytes	GM47730	-0.31325	-0.65913	-0.53626	0.593129	-4.90633	0.720884	0.819325
Monocytes	OLFR920	0.218722	0.137933	0.535706	0.593506	-5.18433	0.705095	0.80166
Monocytes	BTNL9	0.319099	0.689973	0.535425	0.5937	-5.02334	0.694257	0.789531
Monocytes	DVL1	-0.14583	2.507773	-0.53528	0.593799	-5.39177	0.659418	0.750631
Monocytes	CYP3A25	0.238415	1.898523	0.535185	0.593866	-5.22674	0.670877	0.763457
Monocytes	TIMM10	0.11278	3.050855	0.535178	0.59387	-5.54322	0.649385	0.739376
Monocytes	DCTPP1	-0.07634	5.089369	-0.53504	0.593968	-6.14888	0.613238	0.69861
Monocytes	BAZ1B	0.053143	6.23949	0.534825	0.594114	-6.30619	0.5939	0.676642
Monocytes	LRIG1	-0.30372	0.178544	-0.5346	0.594267	-5.03329	0.704665	0.800923
Monocytes	PRR33	-0.24769	0.255516	-0.53432	0.594462	-5.07667	0.703282	0.799246
Monocytes	SRSF9	0.038413	6.428408	0.533816	0.594808	-6.43682	0.591082	0.673221
Monocytes	GM3435	-0.28435	0.205181	-0.5338	0.594818	-5.0459	0.704389	0.800458
Monocytes	FAAP20	0.090998	3.120662	0.533783	0.594831	-5.68991	0.648573	0.738151
Monocytes	ZBTB2	-0.05493	5.709287	-0.53377	0.594838	-6.17824	0.60308	0.686835
Monocytes	POLM	0.115713	3.245007	0.533743	0.594859	-5.45353	0.646303	0.735602
Monocytes	STMN1	-0.10851	7.897729	-0.53359	0.594962	-6.58542	0.567431	0.646241
Monocytes	HS3ST3B1	-0.14836	3.380647	-0.53328	0.595178	-5.48726	0.644037	0.732932
Monocytes	MTFR1	0.094585	4.079368	0.533202	0.595232	-5.72112	0.631491	0.718823
Monocytes	OSBPL3	-0.06773	3.353205	-0.53287	0.595463	-6.18654	0.644718	0.733655
Monocytes	AAMP	-0.03804	5.908148	-0.53204	0.596031	-6.29617	0.600572	0.68347
Monocytes	GM15543	-0.20541	0.901403	-0.53194	0.596103	-5.13055	0.691563	0.785622
Monocytes	FNTB	0.145421	2.971515	0.531894	0.596133	-5.32871	0.652217	0.741684
Monocytes	QSER1	-0.07179	4.323503	-0.53181	0.59619	-5.95573	0.627852	0.714297
Monocytes	LGALSL	-0.20925	1.281509	-0.53165	0.5963	-5.18803	0.684203	0.777377
Monocytes	PHKA2	0.085924	3.438409	0.531549	0.596371	-5.84963	0.643748	0.732118
Monocytes	RXYLT1	0.068711	3.864731	0.531174	0.59663	-5.87156	0.636109	0.723519
Monocytes	METRN	0.14833	2.538002	0.531054	0.596713	-5.41538	0.660357	0.750727
Monocytes	GLE1	-0.07267	4.4454	-0.53104	0.596722	-5.83576	0.625808	0.711919
Monocytes	9-Sep	-0.05439	5.640387	-0.53102	0.596735	-6.35644	0.605186	0.688625
Monocytes	EIF2B5	-0.06942	4.703803	-0.53101	0.596744	-5.97355	0.621283	0.706816

Monocytes	STRN4	0.061405	4.492619	0.530891	0.596825	-6.01128	0.624978	0.710984
Monocytes	GSTM7	-0.20424	1.02465	-0.53064	0.596999	-5.09145	0.689262	0.782983
Monocytes	PIM1	0.051495	9.488973	0.530604	0.597023	-6.95388	0.543788	0.618678
Monocytes	WIZ	-0.10219	3.836067	-0.53059	0.597034	-5.69836	0.636622	0.724096
Monocytes	C330013E15RIK	0.265866	0.677864	0.530583	0.597038	-5.05491	0.696079	0.790561
Monocytes	IFT122	-0.15065	1.594011	-0.53044	0.59714	-5.27703	0.678272	0.770699
Monocytes	TBC1D1	-0.05681	7.18844	-0.5303	0.597235	-6.45302	0.579632	0.659635
Monocytes	ARHGAP11A	0.079882	4.784218	0.530255	0.597265	-6.00681	0.619923	0.705302
Monocytes	CIP2A	-0.12118	3.506565	-0.52985	0.597543	-5.73939	0.642831	0.730943
Monocytes	RARRES1	-0.30652	0.889058	-0.52913	0.598044	-5.08532	0.69268	0.786169
Monocytes	FAM110A	0.087453	3.760358	0.528932	0.598178	-5.87497	0.638682	0.725837
Monocytes	GPAT4	-0.06203	4.264538	-0.52887	0.598224	-5.89929	0.629688	0.715723
Monocytes	BC050972	-0.23928	-0.27973	-0.52875	0.598304	-5.1449	0.71607	0.812095
Monocytes	AIMP1	0.043099	6.38044	0.528695	0.598342	-6.37803	0.593449	0.67478
Monocytes	PRDX4	-0.06522	5.118984	-0.52868	0.598349	-6.14234	0.614765	0.698899
Monocytes	FLT3	-0.06569	2.584668	-0.52851	0.598471	-6.27013	0.660213	0.750014
Monocytes	RCC2	-0.04988	6.222377	-0.52847	0.598499	-6.26399	0.596074	0.677793
Monocytes	GM30054	0.246388	1.953461	0.528301	0.598614	-5.12229	0.6721	0.763349
Monocytes	GATD3A	0.095462	3.544084	0.528269	0.598636	-5.68336	0.642584	0.73031
Monocytes	RYK	-0.20155	2.042505	-0.52824	0.598653	-5.17857	0.670409	0.761461
Monocytes	ZFP12	-0.17359	1.346551	-0.52818	0.598697	-5.19306	0.683752	0.776336
Monocytes	MCU	-0.06425	6.164566	-0.52802	0.598812	-6.2316	0.597077	0.678961
Monocytes	TRMT10B	0.167879	1.681609	0.527943	0.598862	-5.27771	0.677337	0.769172
Monocytes	DHRS7B	-0.10856	3.137991	-0.52764	0.599067	-5.56883	0.650144	0.73876
Monocytes	C1QTNF12	0.151531	2.21932	0.527602	0.599097	-5.39566	0.667232	0.757882
Monocytes	MSANTD4	-0.10473	2.89703	-0.52718	0.599386	-5.50621	0.654775	0.743764
Monocytes	NUP188	-0.07927	4.513741	-0.52708	0.599457	-5.86469	0.625639	0.711041
Monocytes	ZCCHC8	0.055207	5.108019	0.527009	0.599507	-6.08577	0.615292	0.699375
Monocytes	MAP2K7	0.066235	4.294187	0.526979	0.599528	-5.90316	0.62951	0.7154
Monocytes	HP1BP3	-0.05129	6.529858	-0.52681	0.599646	-6.34052	0.591323	0.672231
Monocytes	CCDC12	-0.03544	6.939585	-0.52677	0.599671	-6.53981	0.584606	0.664622
Monocytes	KIF5B	-0.03302	7.401742	-0.5265	0.599859	-6.5278	0.577253	0.656191
Monocytes	RHPN2	0.221697	0.915486	0.526292	0.600003	-5.09781	0.692801	0.786041
Monocytes	GM29019	-0.23459	0.361473	-0.52615	0.600099	-5.03567	0.703824	0.798259
Monocytes	ARHGAP35	-0.07503	5.237019	-0.5259	0.600278	-5.95621	0.613405	0.697141
Monocytes	NAA16	0.054775	5.024992	0.525873	0.600293	-6.04424	0.617062	0.701276
Monocytes	TMEM67	0.142264	1.688091	0.525736	0.600388	-5.44968	0.677945	0.769576
Monocytes	MIEN1	0.045029	5.845651	0.525549	0.600517	-6.23792	0.603142	0.685511
Monocytes	GM4013	-0.14368	1.984122	-0.52527	0.600712	-5.27391	0.672465	0.763363
Monocytes	CENPT	-0.12534	2.566821	-0.52524	0.600733	-5.37936	0.661477	0.751103
Monocytes	ZFP335	0.119214	2.842169	0.525053	0.600861	-5.44928	0.656372	0.745406
Monocytes	ST6GALNAC2	-0.23559	0.651842	-0.52503	0.600875	-5.09158	0.698362	0.792201
Monocytes	IFI211	-0.08402	2.960201	-0.52467	0.601124	-6.32922	0.654392	0.743051
Monocytes	4930594M22RI	-0.31	-0.02762	-0.52453	0.601226	-4.96762	0.712243	0.80744
Monocytes	GM32743	0.33473	-0.48509	0.524348	0.601348	-4.91395	0.721645	0.817796
Monocytes	RNASE6	-0.06304	4.491549	-0.52426	0.60141	-6.37709	0.626881	0.712144
Monocytes	CLDND1	0.047402	4.825073	0.524123	0.601505	-6.2115	0.621071	0.705631

Monocytes	GIGYF1	0.077015	4.441868	0.523743	0.601768	-5.82155	0.627953	0.713212
Monocytes	FILIP1L	-0.06195	5.411773	-0.52371	0.601787	-6.25326	0.6111	0.694214
Monocytes	NOD1	0.10367	3.387607	0.523541	0.601908	-5.89052	0.646922	0.734504
Monocytes	EXD2	0.108759	3.156926	0.523086	0.602223	-5.58136	0.651396	0.739289
Monocytes	ARHGDIA	-0.04407	7.188393	-0.52302	0.602267	-6.57907	0.581806	0.660809
Monocytes	POLR1D	0.037249	6.995878	0.522754	0.602453	-6.5049	0.585053	0.66441
Monocytes	COPS2	0.041459	6.020343	0.522621	0.602545	-6.30766	0.601226	0.682725
Monocytes	DDX47	0.047181	5.550552	0.52203	0.602955	-6.16348	0.609417	0.691886
Monocytes	PHKG1	0.240449	1.107052	0.522024	0.602959	-5.14776	0.690721	0.782932
Monocytes	GM15246	0.257028	1.046088	0.521893	0.60305	-5.11639	0.691917	0.784277
Monocytes	AS3MT	0.143225	2.69274	0.521889	0.603053	-5.41985	0.660411	0.749184
Monocytes	GCSAM	0.097108	-0.31734	0.521819	0.603101	-5.82248	0.719244	0.814539
Monocytes	PLOD1	-0.10694	3.410314	-0.52172	0.603172	-5.7748	0.647176	0.734381
Monocytes	GM11944	0.109968	4.498486	0.521646	0.603221	-5.7865	0.627663	0.712473
Monocytes	COX5A	-0.04626	8.546847	-0.52156	0.603284	-6.71431	0.560629	0.636574
Monocytes	AMD1	-0.07166	4.581194	-0.52139	0.603399	-5.99389	0.626261	0.710921
Monocytes	MCTP1	-0.07556	5.535347	-0.52083	0.603784	-6.65029	0.610032	0.692378
Monocytes	ADI1	-0.09286	3.749643	-0.52078	0.603824	-5.5981	0.641394	0.727665
Monocytes	PRKAB1	-0.10982	3.654114	-0.52043	0.604063	-5.61137	0.64322	0.729631
Monocytes	ING3	0.05679	4.830612	0.520413	0.604076	-6.04251	0.622296	0.706124
Monocytes	2310039H08RII	0.076717	4.246315	0.520371	0.604106	-5.87703	0.632592	0.717703
Monocytes	MYPOPOS	0.262727	1.654931	0.519944	0.604402	-5.08989	0.680799	0.771407
Monocytes	MS4A3	0.283918	-0.35863	0.519896	0.604435	-5.15948	0.720851	0.815771
Monocytes	GM45715	-0.24929	1.039938	-0.51982	0.604488	-4.99029	0.692769	0.784704
Monocytes	SLC39A7	-0.06404	4.500813	-0.5197	0.604569	-5.9426	0.628304	0.712733
Monocytes	MRPS9	0.056555	4.889322	0.519532	0.604688	-6.0241	0.621527	0.705138
Monocytes	TBKBP1	0.121419	1.449005	0.519398	0.604781	-5.65511	0.684844	0.776015
Monocytes	VWA8	-0.06893	4.975158	-0.51938	0.604794	-6.01554	0.620032	0.703501
Monocytes	RTP4	0.160442	4.174749	0.519279	0.604864	-5.93669	0.634133	0.719358
Monocytes	UGGT1	0.050705	5.389379	0.518879	0.605142	-6.17504	0.612977	0.69555
Monocytes	ADAMDEC1	-0.21081	0.746267	-0.51875	0.605232	-5.28927	0.698744	0.791446
Monocytes	SPC24	0.110942	4.768056	0.518658	0.605295	-6.09749	0.623748	0.707686
Monocytes	METTL8	0.144692	2.52734	0.518629	0.605315	-5.39518	0.664374	0.753219
Monocytes	ARMCX2	-0.23049	1.41393	-0.51854	0.605377	-5.09689	0.685638	0.776926
Monocytes	GAL3ST1	-0.25264	-0.29084	-0.51851	0.605398	-5.0595	0.719645	0.814602
Monocytes	GM17435	-0.19492	1.133905	-0.51845	0.60544	-5.19589	0.691102	0.783023
Monocytes	PPTC7	-0.05845	5.148669	-0.51836	0.605502	-6.1865	0.617125	0.700287
Monocytes	SART3	-0.05525	5.374984	-0.51833	0.605523	-6.125	0.613224	0.695888
Monocytes	IGFLR1	0.259592	0.807622	0.518017	0.60574	-5.1122	0.697706	0.790275
Monocytes	POLD4	-0.05192	5.76477	-0.51781	0.605887	-6.26398	0.606753	0.688508
Monocytes	GM41496	-0.24027	0.985043	-0.5178	0.605894	-5.14585	0.694235	0.786431
Monocytes	LIMD1	0.041784	6.016318	0.516979	0.606462	-6.35994	0.603002	0.683875
Monocytes	YBX3	0.065198	6.314911	0.516816	0.606575	-6.35167	0.598031	0.678284
Monocytes	NUDT16	-0.10959	2.966991	-0.51662	0.60671	-5.59234	0.656964	0.744537
Monocytes	SACS	-0.15207	2.596254	-0.51657	0.606746	-5.50041	0.663876	0.752256
Monocytes	GM26916	0.173763	0.934105	0.516561	0.606752	-5.2037	0.695862	0.787829
Monocytes	GM5914	0.144416	3.331997	0.516356	0.606895	-5.34527	0.650321	0.737037

Monocytes	CHAF1B	0.115382	3.382455	0.516211	0.606996	-5.63455	0.649415	0.736055
Monocytes	KRIT1	0.047701	5.937975	0.51615	0.607038	-6.26539	0.604458	0.685525
Monocytes	FHOD3	-0.3646	-0.11029	-0.51554	0.607465	-4.92325	0.717357	0.811297
Monocytes	PYGB	-0.06157	4.748164	-0.51545	0.607525	-6.00264	0.625315	0.708824
Monocytes	EID3	0.200515	0.925233	0.515178	0.607715	-5.30265	0.696641	0.788415
Monocytes	ARL1	-0.04764	5.538643	-0.51517	0.60772	-6.22159	0.611682	0.693451
Monocytes	GLMP	-0.05878	5.839868	-0.51491	0.607901	-6.32132	0.606666	0.687741
Monocytes	LRP8	0.141616	2.371033	0.514815	0.607967	-5.50769	0.668827	0.757447
Monocytes	PRKAG2	0.050491	5.050385	0.514683	0.608059	-6.32968	0.620235	0.703024
Monocytes	ZFP934	0.130756	2.990341	0.514535	0.608162	-5.47348	0.657237	0.744527
Monocytes	BRF1	-0.05725	4.828649	-0.51449	0.608193	-6.00683	0.624104	0.707393
Monocytes	GM42836	-0.32382	-0.40469	-0.51437	0.608275	-4.93983	0.723622	0.818179
Monocytes	PPP2R5E	0.038718	6.820761	0.514355	0.608287	-6.3953	0.590284	0.669228
Monocytes	NAA25	-0.08286	3.927641	-0.51422	0.608382	-5.90996	0.64014	0.725359
Monocytes	MOV10	-0.10028	3.628623	-0.51367	0.608764	-5.69638	0.645826	0.731618
Monocytes	MTUS2	-0.28913	0.654472	-0.51366	0.608768	-5.0683	0.702496	0.794691
Monocytes	GM42984	0.264063	0.916614	0.513378	0.608968	-5.05712	0.697414	0.78897
Monocytes	SHC4	0.222785	0.470109	0.513202	0.60909	-5.10901	0.70631	0.798861
Monocytes	RAP1GDS1	0.045649	6.711436	0.513151	0.609126	-6.47773	0.592476	0.671528
Monocytes	SMCHD1	-0.04798	7.994517	-0.51315	0.609129	-6.71309	0.5717	0.647968
Monocytes	GPATCH2	0.083335	4.253922	0.512982	0.609244	-5.81188	0.634706	0.71912
Monocytes	VPS33B	-0.11899	2.932531	-0.51291	0.609293	-5.43557	0.658773	0.746088
Monocytes	PPIA	-0.04623	10.92524	-0.51281	0.609361	-7.07597	0.5273	0.597315
Monocytes	SPEN	0.046023	5.922479	0.512743	0.60941	-6.30062	0.6057	0.686533
Monocytes	RAB34	0.278173	0.235405	0.512472	0.609598	-5.06603	0.711215	0.804268
Monocytes	NDUFB7	0.046331	6.480862	0.512083	0.60987	-6.41034	0.596555	0.676062
Monocytes	MEAK7	0.2292	0.666682	0.512069	0.60988	-5.10429	0.702681	0.794762
Monocytes	FRYL	-0.04343	7.564295	-0.51204	0.609896	-6.5228	0.578822	0.655977
Monocytes	GM17477	-0.22426	0.61424	-0.51184	0.610037	-5.07778	0.70374	0.795992
Monocytes	BBC3	0.109935	2.639565	0.511799	0.610067	-5.53859	0.664511	0.75242
Monocytes	RND1	-0.16195	1.145338	-0.51167	0.610154	-5.36849	0.693215	0.784366
Monocytes	ZBTB49	-0.1641	1.051258	-0.51167	0.61016	-5.15348	0.695067	0.786419
Monocytes	UHRF1BP1	0.166234	1.998967	0.511196	0.610488	-5.39824	0.676949	0.766106
Monocytes	ADGRA2	-0.15554	0.687625	-0.51105	0.610592	-5.54702	0.702628	0.794587
Monocytes	INTS3	0.078617	3.898156	0.51088	0.610708	-5.75256	0.641683	0.726748
Monocytes	TIMM9	-0.08805	3.882337	-0.51085	0.610732	-5.66698	0.641968	0.727068
Monocytes	TSPYL4	-0.20513	0.723815	-0.51054	0.610947	-5.0774	0.702096	0.793908
Monocytes	KMT5C	0.110949	2.877228	0.510308	0.611108	-5.59239	0.660582	0.747859
Monocytes	ACTB	-0.03556	13.9201	-0.51031	0.611108	-7.57726	0.486518	0.550133
Monocytes	GM37168	-0.13083	0.65738	-0.51012	0.611237	-5.50929	0.703427	0.79548
Monocytes	CENPM	-0.10473	3.916181	-0.51011	0.611246	-5.8426	0.641521	0.726566
Monocytes	SUGP1	-0.06519	4.615277	-0.50987	0.611414	-5.93588	0.629038	0.712663
Monocytes	OIP5	0.155813	2.207869	0.509773	0.61148	-5.33719	0.673193	0.762048
Monocytes	SYF2	0.039589	5.903221	0.509754	0.611494	-6.26342	0.606742	0.687577
Monocytes	GRHPR	0.108558	4.431109	0.509598	0.611603	-5.81775	0.6323	0.716357
Monocytes	FASTKD3	-0.15589	1.993657	-0.50958	0.611612	-5.3492	0.677284	0.766623
Monocytes	SUB1	-0.03753	8.911934	-0.50957	0.611624	-6.84834	0.558036	0.632356

Monocytes	EPC2	0.050784	6.14343	0.509535	0.611647	-6.27787	0.602683	0.683009
Monocytes	LYRM4	0.069238	4.142278	0.509313	0.611801	-5.84515	0.637549	0.722226
Monocytes	ACTR5	0.094214	3.477256	0.509045	0.611989	-5.66621	0.649728	0.735784
Monocytes	TMC7	-0.29284	-0.40026	-0.5089	0.612088	-4.91338	0.725195	0.819506
Monocytes	PLEKHM1	-0.06808	4.771883	-0.50866	0.612261	-5.92992	0.626602	0.709843
Monocytes	ZDHHC21	-0.06369	4.271545	-0.50856	0.612327	-6.02678	0.635469	0.719813
Monocytes	ADNP	-0.04642	6.50802	-0.50854	0.612342	-6.32306	0.596887	0.676364
Monocytes	PGAP1	0.084741	3.785443	0.508344	0.612478	-6.03967	0.644295	0.729652
Monocytes	NPHP1	-0.23707	0.652168	-0.50818	0.612594	-5.08472	0.704039	0.796119
Monocytes	NOC2L	-0.06359	5.059461	-0.50794	0.612762	-6.12717	0.621714	0.704353
Monocytes	APPBP2	0.042819	6.248663	0.507905	0.612785	-6.35072	0.601365	0.681418
Monocytes	HNRNPA3	0.038995	8.554364	0.507732	0.612906	-6.72869	0.56401	0.639105
Monocytes	CTNNA3	-0.33178	1.300067	-0.50767	0.612949	-5.11717	0.691238	0.782069
Monocytes	RARS2	0.092967	3.441715	0.507573	0.613017	-5.65167	0.650638	0.736867
Monocytes	ANGPTL4	0.221862	1.23193	0.507491	0.613074	-5.12906	0.692574	0.783572
Monocytes	1110038F14RIK	0.067919	4.590808	0.507322	0.613192	-5.98824	0.629944	0.713675
Monocytes	GPX3	0.164376	1.463214	0.507273	0.613226	-5.55338	0.688049	0.778573
Monocytes	PRIM2	-0.08496	5.536205	-0.50716	0.613306	-6.09839	0.613465	0.695167
Monocytes	ATP10A	0.098826	3.430551	0.507093	0.613352	-5.81228	0.650843	0.73714
Monocytes	SPARC	-0.12375	5.322477	-0.50709	0.613354	-6.01961	0.617148	0.699324
Monocytes	CEBPZOS	-0.06604	4.521965	-0.50694	0.613461	-5.99168	0.631163	0.715109
Monocytes	HIST1H2AF	-0.25842	0.617707	-0.50693	0.613469	-5.12729	0.704749	0.797128
Monocytes	GM2396	-0.16156	-0.85737	-0.50688	0.613503	-5.25604	0.734945	0.830445
Monocytes	UBE3C	0.052255	5.784905	0.506712	0.613619	-6.20798	0.609211	0.690445
Monocytes	TOLLIP	0.084431	3.832789	0.506636	0.613671	-5.80319	0.643512	0.729057
Monocytes	TPST2	0.048861	5.593782	0.506551	0.613731	-6.30754	0.612477	0.6942
Monocytes	PVRIG	0.248987	-0.53207	0.506497	0.613769	-4.9694	0.728168	0.823141
Monocytes	ADAMTS6	-0.07197	5.940983	-0.50634	0.613879	-6.28531	0.606558	0.687587
Monocytes	IPO11	0.081567	4.478076	0.506194	0.61398	-5.80669	0.631941	0.716197
Monocytes	MASTL	0.095551	3.782	0.506067	0.614069	-5.69953	0.644432	0.730236
Monocytes	NUMBL	-0.2028	1.04252	-0.50602	0.614103	-5.19211	0.696304	0.788057
Monocytes	ZADH2	-0.08982	3.381757	-0.50601	0.614109	-5.64539	0.651738	0.738428
Monocytes	CDH1	-0.23716	1.38751	-0.50592	0.614171	-5.14915	0.689527	0.780539
Monocytes	S1PR4	0.118799	4.183137	0.505818	0.614243	-5.6155	0.637201	0.72216
Monocytes	MOB4	0.039348	6.832732	0.505789	0.614263	-6.39024	0.591645	0.670814
Monocytes	FMR1	0.049423	5.971489	0.505709	0.61432	-6.26425	0.606041	0.687123
Monocytes	HMGCLL1	0.292378	1.17582	0.505586	0.614405	-4.98462	0.693702	0.785272
Monocytes	ICMT	-0.13678	2.429223	-0.50543	0.614515	-5.4129	0.669571	0.758432
Monocytes	TMOD1	-0.20736	1.823905	-0.505	0.614813	-5.21588	0.681322	0.771416
Monocytes	FURIN	0.061944	5.290193	0.504999	0.614816	-6.33789	0.617947	0.700487
Monocytes	GM10550	0.244251	0.590308	0.504915	0.614875	-5.05388	0.705572	0.798359
Monocytes	PSMC4	-0.05653	5.46131	-0.50477	0.614979	-6.16515	0.615002	0.697234
Monocytes	TMEM50B	-0.07575	4.107732	-0.50472	0.615011	-6.07558	0.638811	0.724007
Monocytes	GDPD1	-0.20622	1.600801	-0.50438	0.615248	-5.23665	0.685844	0.776365
Monocytes	MFNG	0.089684	3.090104	0.504265	0.615329	-5.66154	0.657581	0.744931
Monocytes	RAC2	0.048128	9.052768	0.50416	0.615403	-6.70066	0.556675	0.631038
Monocytes	ARRB1	0.056419	3.996665	0.504098	0.615447	-6.083	0.641	0.726404

Monocytes	AFF4	0.045698	7.842009	0.503726	0.615706	-6.62015	0.575851	0.65276
Monocytes	YWHAE	-0.02852	8.88218	-0.50349	0.615869	-6.77199	0.559576	0.634195
Monocytes	PDGFB	-0.15656	1.062654	-0.5032	0.616078	-5.56294	0.696894	0.788474
Monocytes	ITGA1	-0.08003	3.828562	-0.50295	0.616251	-6.19634	0.644617	0.730184
Monocytes	MTMR9	0.069698	3.972449	0.502767	0.616378	-5.7977	0.64207	0.727331
Monocytes	SEC22B	-0.05146	5.449712	-0.50269	0.616434	-6.17359	0.615991	0.698032
Monocytes	MTAP	-0.09227	3.671485	-0.50254	0.616536	-5.69475	0.647573	0.733485
Monocytes	IL6ST	0.084945	4.326252	0.502344	0.616675	-6.01278	0.635756	0.720304
Monocytes	CAND2	-0.23844	0.775357	-0.50228	0.616716	-5.02618	0.702833	0.795079
Monocytes	CCHCR1	0.190829	1.536464	0.502269	0.616727	-5.2217	0.687827	0.778444
Monocytes	PTMA	-0.04321	11.35991	-0.50183	0.617036	-7.14246	0.523179	0.592373
Monocytes	CHCHD2	-0.03354	8.94643	-0.50172	0.617109	-6.78315	0.559117	0.633484
Monocytes	PPP1R12C	0.050245	5.009564	0.501657	0.617156	-6.14877	0.623917	0.706849
Monocytes	GLYR1	0.039934	6.736527	0.501437	0.61731	-6.43672	0.594592	0.673695
Monocytes	NDUFA13	0.037121	7.128419	0.501199	0.617477	-6.53953	0.588182	0.666476
Monocytes	GM36839	-0.13726	2.346299	-0.5011	0.617547	-5.38348	0.672657	0.761391
Monocytes	TXN1	-0.05843	7.304482	-0.50101	0.617606	-6.56126	0.585307	0.663255
Monocytes	CBFA2T2	0.058063	5.563389	0.501005	0.617613	-6.12011	0.614451	0.696196
Monocytes	EBAG9	-0.07032	4.117531	-0.50055	0.617932	-5.86541	0.640012	0.724902
Monocytes	RAB35	-0.05579	4.720448	-0.50051	0.617961	-6.03987	0.629262	0.712843
Monocytes	HSH2D	0.106098	3.210107	0.500411	0.618029	-5.66351	0.656577	0.743456
Monocytes	GAS2L3	-0.07277	4.330262	-0.50022	0.618162	-6.10569	0.636196	0.72065
Monocytes	PSMB3	0.042112	7.724991	0.500059	0.618276	-6.60724	0.578614	0.655721
Monocytes	KCP	-0.24491	0.100139	-0.5	0.618317	-5.08064	0.71702	0.810675
Monocytes	RAD1	0.138517	2.477895	0.499988	0.618325	-5.37208	0.670288	0.758845
Monocytes	CCNB1	0.134341	4.032241	0.499915	0.618377	-5.90894	0.641549	0.726744
Monocytes	WDR35	-0.29387	0.509893	-0.49989	0.618391	-4.97746	0.70872	0.801526
Monocytes	PLEKHA4	0.169114	2.406918	0.499784	0.618469	-5.36151	0.671634	0.760388
Monocytes	TMEM33	0.04861	5.133185	0.499709	0.618522	-6.14389	0.622019	0.704843
Monocytes	ERLIN2	-0.0824	3.099067	-0.4996	0.6186	-5.76152	0.658636	0.745915
Monocytes	TMEM9	-0.18882	2.576914	-0.49959	0.618608	-5.23756	0.668415	0.756828
Monocytes	RSF1	0.04292	7.014648	0.499523	0.618652	-6.45932	0.590161	0.668897
Monocytes	HIST1H4M	0.232192	1.423329	0.49948	0.618682	-5.15322	0.690591	0.781509
Monocytes	COMTD1	-0.11251	2.92368	-0.49906	0.618977	-5.47798	0.662001	0.74965
Monocytes	TYW1	0.083689	3.875123	0.499043	0.618988	-5.773	0.644486	0.730063
Monocytes	IFI214	0.238401	1.667368	0.498865	0.619113	-5.16995	0.685935	0.77638
Monocytes	THUMPD3	-0.06953	4.262161	-0.49884	0.619131	-5.92035	0.637509	0.722312
Monocytes	ANKRD12	-0.05042	7.518738	-0.49881	0.619151	-6.49897	0.582026	0.659708
Monocytes	GM50020	-0.27778	-0.11132	-0.49881	0.619154	-4.93711	0.721451	0.815698
Monocytes	TRIB2	-0.2329	2.561151	-0.49844	0.619415	-5.13378	0.669025	0.757455
Monocytes	SIAE	-0.12673	2.612072	-0.49816	0.619605	-5.44232	0.668201	0.756496
Monocytes	PLEKHG5	-0.15842	1.864551	-0.49805	0.619686	-5.40928	0.682493	0.77245
Monocytes	RPGR	-0.17986	1.599467	-0.49775	0.619894	-5.16787	0.687795	0.778292
Monocytes	IRGM2	-0.18394	2.731293	-0.49736	0.620167	-5.41303	0.666216	0.754207
Monocytes	GM41555	-0.28422	0.571556	-0.49733	0.620189	-5.00999	0.708234	0.800876
Monocytes	IMPDH1	-0.11653	4.326873	-0.49732	0.6202	-5.65567	0.636934	0.721461
Monocytes	WDFY1	-0.06512	4.332443	-0.49724	0.620254	-6.04493	0.636834	0.721363

Monocytes	1810044D09RII	0.160916	1.915203	0.497185	0.620293	-5.32402	0.681764	0.771572
Monocytes	SERPINA3F	-0.35472	2.19929	-0.49674	0.620608	-5.22801	0.676582	0.765631
Monocytes	GM16152	0.22369	2.101073	0.496548	0.62074	-5.14844	0.678539	0.767727
Monocytes	ZFAS1	-0.07626	5.058651	-0.49635	0.620877	-6.07743	0.62438	0.707134
Monocytes	DNAJC19	0.043701	5.950115	0.496169	0.621006	-6.31604	0.609041	0.689802
Monocytes	ZFP747	0.162103	1.605909	0.496097	0.621057	-5.24491	0.688255	0.778441
Monocytes	PADI2	-0.0975	2.631853	-0.49596	0.621155	-5.86165	0.668608	0.756556
Monocytes	ZFP707	0.163439	1.958642	0.4958	0.621266	-5.23548	0.681506	0.770941
Monocytes	EFNB2	0.180491	2.469455	0.495427	0.621528	-5.3718	0.671925	0.760151
Monocytes	GM43647	0.292039	-0.94495	0.495367	0.62157	-4.98887	0.740296	0.835783
Monocytes	IFRD2	-0.11383	3.30652	-0.4952	0.621687	-5.52168	0.656278	0.742735
Monocytes	FRG1	-0.03915	6.660706	-0.49508	0.621773	-6.42003	0.597362	0.676572
Monocytes	GM16310	0.216974	1.197906	0.494969	0.62185	-5.16309	0.696592	0.787674
Monocytes	LRRC59	0.043273	5.556125	0.494951	0.621863	-6.28421	0.616086	0.697728
Monocytes	CARHSP1	0.077857	5.430457	0.494799	0.621969	-5.98069	0.6183	0.700228
Monocytes	COPS8	-0.04898	5.091019	-0.49451	0.62217	-6.09387	0.624346	0.706953
Monocytes	EXOC7	-0.06963	4.087686	-0.4944	0.622252	-5.83212	0.642206	0.726985
Monocytes	USP2	-0.11185	2.711696	-0.49419	0.622395	-5.62258	0.667682	0.755435
Monocytes	PSPH	-0.08778	3.906589	-0.49397	0.622554	-5.72448	0.645596	0.730761
Monocytes	MFAP3	0.053565	5.124697	0.493925	0.622584	-6.11223	0.623882	0.706397
Monocytes	NAPEPLD	-0.22118	1.056502	-0.49388	0.622613	-5.11287	0.699734	0.791041
Monocytes	SMPD3	0.31184	-0.20687	0.493753	0.622705	-4.96171	0.725335	0.819345
Monocytes	GTF2H5	-0.04103	6.547345	-0.49336	0.62298	-6.4058	0.599681	0.679082
Monocytes	CHN2	-0.08349	5.358074	-0.49331	0.623018	-6.42032	0.619953	0.701952
Monocytes	ITGA6	-0.1271	4.818835	-0.49329	0.62303	-5.82984	0.629397	0.71257
Monocytes	AMDHD2	-0.07551	3.693973	-0.49322	0.623082	-5.82934	0.649615	0.73526
Monocytes	EID1	-0.04949	5.155191	-0.49312	0.623154	-6.2176	0.623487	0.705954
Monocytes	4930526L06RIK	0.285038	-0.8055	0.492906	0.623301	-4.92861	0.737931	0.833224
Monocytes	RCCD1	-0.14748	3.170294	-0.49286	0.623333	-5.42908	0.659272	0.746119
Monocytes	CCM2	-0.04398	7.391127	-0.49266	0.623477	-6.47284	0.585749	0.66346
Monocytes	SPATA1	-0.07574	3.467697	-0.49265	0.623478	-5.68896	0.653769	0.740028
Monocytes	GPC3	0.181179	1.806193	0.492627	0.623497	-5.24774	0.685178	0.775031
Monocytes	HEATR5B	0.094909	3.730171	0.492526	0.623568	-5.70627	0.648953	0.734666
Monocytes	STARD7	0.054842	5.358518	0.492362	0.623684	-6.13829	0.619945	0.702188
Monocytes	4930503L19RIK	-0.10792	2.576564	-0.49235	0.623689	-5.5336	0.670413	0.758715
Monocytes	SERPING1	-0.15049	3.004813	-0.49196	0.623968	-5.46452	0.662357	0.749857
Monocytes	ZC4H2	-0.25558	0.054562	-0.49192	0.623999	-4.97395	0.720088	0.813957
Monocytes	IL16	0.100076	4.60831	0.491868	0.624032	-5.89109	0.633127	0.717166
Monocytes	DVL3	-0.08705	3.300094	-0.49185	0.624046	-5.6461	0.656864	0.74376
Monocytes	EMC6	0.046951	5.61708	0.491755	0.624111	-6.23691	0.615474	0.697305
Monocytes	UBQLN1	-0.04499	5.517917	-0.49174	0.624121	-6.19193	0.617184	0.699233
Monocytes	NCAPG2	-0.08366	5.068495	-0.49146	0.62432	-6.05416	0.625005	0.708054
Monocytes	DDX49	-0.06092	4.208396	-0.49142	0.62435	-5.89631	0.640281	0.725211
Monocytes	2810001G20RII	-0.13328	2.479465	-0.49141	0.624355	-5.26934	0.672255	0.760953
Monocytes	INO80D	-0.04329	5.995336	-0.49122	0.624486	-6.27365	0.608996	0.690056
Monocytes	SRP14	0.032828	7.234027	0.491091	0.624579	-6.50069	0.588315	0.666721
Monocytes	RBSN	0.092369	2.910659	0.491055	0.624604	-5.57159	0.664119	0.752005

Monocytes	RACK1	0.028622	8.637453	0.490958	0.624673	-6.79718	0.565835	0.641211
Monocytes	TNPO2	0.080571	4.122122	0.490882	0.624726	-5.81659	0.641836	0.727089
Monocytes	PPP1R13L	-0.26431	-0.0774	-0.49083	0.624765	-5.08038	0.722796	0.817128
Monocytes	GM20559	-0.08516	4.344461	-0.49067	0.624874	-5.92417	0.637837	0.722603
Monocytes	BCL7A	-0.13349	4.80999	-0.49063	0.624903	-5.59395	0.629553	0.713299
Monocytes	HELQ	0.142207	2.411841	0.490537	0.624969	-5.26503	0.673541	0.762526
Monocytes	PSMA6	-0.04519	6.941715	-0.49045	0.62503	-6.46013	0.593123	0.672198
Monocytes	H2AFZ	-0.05013	10.35837	-0.49036	0.625091	-7.10218	0.539589	0.611245
Monocytes	GM12359	0.154529	1.671224	0.490248	0.625173	-5.28257	0.687802	0.778401
Monocytes	SMG1	0.033993	7.499087	0.490197	0.625208	-6.56714	0.583993	0.661868
Monocytes	CHSY1	0.056859	5.079341	0.490036	0.625322	-6.20179	0.624815	0.707998
Monocytes	SEC16A	0.066815	4.57627	0.490034	0.625324	-6.00244	0.633697	0.717983
Monocytes	DDT	0.066802	5.541533	0.490015	0.625337	-6.17199	0.616776	0.698951
Monocytes	DUSP18	-0.1967	0.621447	-0.48999	0.625358	-5.27682	0.708583	0.801481
Monocytes	MMGT2	0.082589	3.166256	0.489944	0.625387	-5.67647	0.659347	0.746744
Monocytes	FBXO44	-0.26618	0.220079	-0.48981	0.625482	-5.04543	0.716708	0.810473
Monocytes	9130401M01RI	0.056041	4.02313	0.489755	0.625521	-6.0326	0.643625	0.729181
Monocytes	PBDC1	-0.0735	5.348225	-0.48965	0.625596	-6.18224	0.620124	0.702787
Monocytes	XNDC1	-0.1042	3.594505	-0.48943	0.625749	-5.57414	0.651437	0.737951
Monocytes	GM14636	-0.14152	2.274283	-0.48921	0.625902	-5.6842	0.676165	0.765587
Monocytes	1600002K03RIK	0.082392	3.214423	0.489147	0.625949	-5.69884	0.658452	0.745825
Monocytes	D6WSU163E	-0.10001	3.1191	-0.48912	0.625966	-5.52541	0.660225	0.747806
Monocytes	EIF2B3	0.117783	2.996786	0.48911	0.625975	-5.53025	0.662507	0.750355
Monocytes	NRBP1	0.044892	5.827937	0.489078	0.625998	-6.27782	0.611853	0.693493
Monocytes	RNASEH2A	0.068737	4.54522	0.489007	0.626048	-6.00714	0.634249	0.718706
Monocytes	SSBP4	-0.05229	4.90464	-0.48899	0.626057	-6.13204	0.627883	0.711551
Monocytes	MCMDC2	0.081654	3.465667	0.488523	0.626389	-6.04473	0.654086	0.74071
Monocytes	NCAPH2	-0.05002	5.344606	-0.48797	0.626782	-6.18309	0.620747	0.703148
Monocytes	ENKUR	-0.19903	0.339833	-0.48792	0.626813	-5.18591	0.714919	0.808164
Monocytes	ZFP35	0.119679	2.543067	0.487745	0.626938	-5.40732	0.671672	0.760166
Monocytes	MCM3	-0.08319	5.484871	-0.4876	0.62704	-6.2	0.61833	0.700439
Monocytes	SRSF1	-0.04895	6.067856	-0.48757	0.62706	-6.27827	0.608328	0.689162
Monocytes	EID2B	0.136036	1.703903	0.487538	0.627084	-5.28014	0.687804	0.778147
Monocytes	HRAS	0.052807	4.842094	0.48693	0.627513	-6.10235	0.629917	0.713225
Monocytes	EML2	-0.10677	2.655573	-0.48677	0.627628	-5.64755	0.669909	0.758005
Monocytes	NKAP	-0.05089	4.989943	-0.48675	0.627642	-6.0483	0.62731	0.710326
Monocytes	FAAP100	-0.08824	3.097229	-0.48658	0.627763	-5.6742	0.66161	0.748749
Monocytes	IGKV1-110	0.291116	0.050225	0.486544	0.627785	-4.93972	0.721243	0.814918
Monocytes	NCAPD2	-0.09646	4.81058	-0.48651	0.627811	-6.01405	0.630474	0.713894
Monocytes	TRIM16	-0.12144	2.341778	-0.4864	0.627889	-5.60259	0.675882	0.764664
Monocytes	PTPN2	0.035354	6.833794	0.486317	0.627945	-6.50738	0.595797	0.674804
Monocytes	SLC25A5	0.039001	7.608641	0.486211	0.62802	-6.61785	0.583096	0.66044
Monocytes	ZFP59	0.266293	0.230401	0.485964	0.628195	-5.00272	0.717618	0.81096
Monocytes	RELA	-0.05297	5.114793	-0.48589	0.628246	-6.18173	0.62517	0.707955
Monocytes	CLEC2I	0.127692	3.177972	0.485846	0.628279	-5.65582	0.66016	0.747167
Monocytes	GPATCH11	0.076753	3.800176	0.485802	0.628309	-5.79899	0.648688	0.734342
Monocytes	HELZ	-0.05056	6.25998	-0.48558	0.628467	-6.26121	0.605458	0.685842

Monocytes	CCT4	-0.03775	6.487167	-0.48553	0.628501	-6.42261	0.60163	0.681517
Monocytes	TK2	0.095373	3.825305	0.485528	0.628503	-5.71996	0.648232	0.733938
Monocytes	9430060I03RIK	0.208656	1.308677	0.485437	0.628567	-5.18003	0.695993	0.787189
Monocytes	BNIP1	0.099353	3.526643	0.485268	0.628686	-5.67063	0.653765	0.740118
Monocytes	YARS2	-0.11125	3.150094	-0.48501	0.62887	-5.53821	0.660756	0.747964
Monocytes	GMDS	-0.0483	6.245209	-0.485	0.628873	-6.30455	0.605776	0.686221
Monocytes	NMT1	-0.0354	6.941125	-0.48492	0.628936	-6.50485	0.59413	0.673091
Monocytes	VNN3	0.176347	1.614791	0.484832	0.628994	-5.35399	0.690059	0.780677
Monocytes	4732440D04RII	0.179572	2.03703	0.484706	0.629084	-5.26052	0.68186	0.771594
Monocytes	PTGIS	0.294211	-0.12832	0.484545	0.629198	-5.14481	0.725059	0.819459
Monocytes	RIIAD1	0.167063	0.993222	0.484483	0.629241	-5.24175	0.702324	0.79435
Monocytes	RPUSD1	-0.16893	1.47218	-0.48448	0.629243	-5.21439	0.692852	0.783848
Monocytes	DHODH	0.135651	2.109646	0.484231	0.629419	-5.34662	0.680461	0.770207
Monocytes	USP16	-0.05217	5.208005	-0.48423	0.629423	-6.18034	0.623611	0.706583
Monocytes	MGST2	0.149305	2.953494	0.484019	0.629569	-5.358	0.664431	0.752416
Monocytes	PIGM	0.078168	3.53133	0.483884	0.629665	-5.70258	0.653694	0.740416
Monocytes	OLFR77	-0.16716	1.376904	-0.48384	0.629693	-5.28779	0.694725	0.786136
Monocytes	SCG5	-0.23482	0.801405	-0.48383	0.629705	-5.12751	0.706157	0.798806
Monocytes	TMEM158	0.296448	0.271092	0.48381	0.629717	-5.04119	0.716872	0.810656
Monocytes	PFN2	-0.22246	1.339141	-0.48381	0.62972	-5.13872	0.695469	0.786961
Monocytes	MAP1S	-0.08261	3.781603	-0.48353	0.629912	-5.80125	0.649236	0.735336
Monocytes	XPOT	-0.06479	4.512589	-0.48318	0.63016	-5.98013	0.636165	0.72057
Monocytes	CCP110	0.143895	2.37539	0.483116	0.630208	-5.33603	0.675651	0.764722
Monocytes	SNAP47	-0.10942	2.473639	-0.48305	0.630251	-5.49171	0.673778	0.762636
Monocytes	PACSIN2	0.039019	5.573303	0.482981	0.630303	-6.33514	0.617523	0.699603
Monocytes	MZB1	-0.1509	5.956787	-0.48286	0.630389	-5.70438	0.610955	0.692194
Monocytes	ISG15	0.134298	6.408101	0.48272	0.630488	-6.43221	0.603335	0.683578
Monocytes	VWF	0.163695	1.565048	0.482534	0.630619	-5.65171	0.6914	0.782202
Monocytes	POLR3K	-0.0729	3.885777	-0.48251	0.630636	-5.76632	0.647547	0.733304
Monocytes	DNLZ	-0.08639	3.991855	-0.48239	0.630724	-5.75763	0.645617	0.731142
Monocytes	QRFP	0.298075	-0.61178	0.482341	0.630756	-4.94632	0.735495	0.830936
Monocytes	MOB1B	0.037493	6.203813	0.482162	0.630883	-6.36618	0.606854	0.68756
Monocytes	AGBL2	0.201976	0.26749	0.48208	0.630941	-5.10726	0.717392	0.811017
Monocytes	PHKB	-0.05509	5.588989	-0.48192	0.631055	-6.22182	0.617426	0.699474
Monocytes	SUZ12	0.041139	6.954117	0.481753	0.631172	-6.44069	0.594359	0.67342
Monocytes	ANKHD1	-0.03899	7.647791	-0.4817	0.631212	-6.59769	0.582997	0.660542
Monocytes	PHF11D	-0.1669	1.34229	-0.48141	0.631418	-5.52625	0.696085	0.78731
Monocytes	ZFP27	-0.18697	1.016419	-0.48124	0.631532	-5.15596	0.702603	0.794531
Monocytes	PIGA	0.155377	1.918295	0.481064	0.631659	-5.27416	0.684946	0.774907
Monocytes	RAB4B	0.04035	5.9254	0.480924	0.631759	-6.33096	0.611954	0.693201
Monocytes	THUMPD2	0.156069	1.928752	0.480524	0.632042	-5.18592	0.684964	0.774911
Monocytes	MAGED2	-0.21844	1.585547	-0.48049	0.632067	-5.1421	0.691652	0.78234
Monocytes	COQ2	-0.0619	4.467496	-0.48042	0.632118	-6.00691	0.637645	0.722064
Monocytes	SLF2	-0.05006	6.314028	-0.48015	0.632308	-6.24993	0.605627	0.685925
Monocytes	TTC7B	-0.05461	5.391283	-0.47981	0.632551	-6.18633	0.621625	0.703858
Monocytes	GM5535	0.312826	0.151841	0.479729	0.632605	-4.99346	0.720706	0.814285
Monocytes	SIRT5	-0.15363	1.958147	-0.47943	0.632815	-5.29308	0.684872	0.774572

Monocytes	SYNE1	-0.09674	4.964609	-0.47899	0.63313	-6.04843	0.629428	0.7125
Monocytes	CEP68	0.082664	4.299523	0.478898	0.633194	-5.81836	0.641292	0.72582
Monocytes	MRGPRA2B	0.297392	-0.43033	0.478819	0.633249	-5.01521	0.733118	0.827806
Monocytes	ANXA4	0.065403	3.74233	0.478817	0.633251	-6.16169	0.651422	0.737164
Monocytes	GNL2	0.057701	4.675227	0.478517	0.633463	-6.03743	0.634709	0.718358
Monocytes	BC029722	0.091438	3.061908	0.47829	0.633624	-5.67186	0.664291	0.751479
Monocytes	LRRC27	-0.24365	0.302993	-0.47811	0.633754	-5.01599	0.718341	0.811419
Monocytes	RCHY1	0.04467	5.661194	0.477803	0.63397	-6.25822	0.617715	0.699127
Monocytes	AK8	0.120591	2.179586	0.477582	0.634127	-5.49206	0.681319	0.77026
Monocytes	SF3A3	-0.05817	4.984502	-0.47758	0.634127	-6.08032	0.629566	0.712426
Monocytes	TPR	-0.03533	7.524789	-0.47745	0.634217	-6.5866	0.58647	0.663821
Monocytes	GALNT10	0.061992	4.614762	0.477374	0.634274	-6.12985	0.636151	0.719864
Monocytes	ZBTB44	0.052991	5.853417	0.477265	0.634351	-6.23384	0.614463	0.695496
Monocytes	HSPA12B	0.258796	0.480164	0.477069	0.63449	-4.97563	0.715022	0.807623
Monocytes	UQCRH	0.029097	8.592145	0.476814	0.634671	-6.76432	0.569404	0.644516
Monocytes	NOC4L	0.07559	3.728892	0.476748	0.634718	-5.75932	0.652252	0.737949
Monocytes	PRMT5	-0.09441	3.483009	-0.47669	0.634757	-5.6271	0.656784	0.743016
Monocytes	NFKBIL1	0.068006	3.861655	0.476676	0.634769	-5.82295	0.649819	0.735227
Monocytes	F730043M19RI	-0.20553	0.260543	-0.47667	0.634776	-5.22096	0.719498	0.812648
Monocytes	PPM1K	-0.10715	3.579795	-0.4763	0.635038	-5.64103	0.6552	0.741106
Monocytes	TGDS	0.091396	3.1411	0.476103	0.635175	-5.61645	0.663424	0.750296
Monocytes	UTP14B	0.112408	3.081232	0.476019	0.635235	-5.54143	0.664545	0.751562
Monocytes	VAC14	0.048632	4.624685	0.475543	0.635573	-6.06793	0.636522	0.720042
Monocytes	ATPAF2	0.107429	2.92038	0.475452	0.635637	-5.51792	0.667804	0.755046
Monocytes	ZSWIM7	0.078974	3.432711	0.475433	0.635651	-5.83329	0.658225	0.74436
Monocytes	APOBEC3	-0.05508	6.862598	-0.47529	0.635753	-6.4132	0.59793	0.676634
Monocytes	HAX1	0.068526	4.020737	0.47479	0.636107	-5.92158	0.647699	0.732479
Monocytes	TGS1	-0.04676	5.214453	-0.47468	0.636183	-6.16585	0.62635	0.708564
Monocytes	ANKRD55	-0.2018	0.009583	-0.47456	0.636272	-5.13619	0.725524	0.818917
Monocytes	GM10762	-0.12673	2.549426	-0.47448	0.636329	-5.38137	0.675128	0.763128
Monocytes	MBIP	0.08672	3.658726	0.474366	0.636408	-5.72613	0.654332	0.739935
Monocytes	GSDMD	0.065834	4.268046	0.474312	0.636447	-6.16329	0.64321	0.727492
Monocytes	B9D2	0.066871	4.930502	0.474108	0.636591	-6.08289	0.631356	0.714247
Monocytes	SLC4A9	-0.24648	0.122757	-0.4741	0.636594	-5.02255	0.723194	0.816417
Monocytes	CRY1	0.052926	5.502006	0.473986	0.636678	-6.24769	0.621325	0.702983
Monocytes	IFIT3	-0.17046	3.112471	-0.47398	0.636684	-5.80039	0.664483	0.751329
Monocytes	AKIRIN2	0.048685	5.756269	0.473963	0.636695	-6.20053	0.61692	0.698024
Monocytes	SIRT3	0.08601	3.344497	0.473719	0.636868	-5.63876	0.660229	0.746525
Monocytes	WDR18	-0.06162	4.82244	-0.47368	0.636899	-5.98314	0.633349	0.716439
Monocytes	AGPAT5	-0.04307	5.644905	-0.47327	0.637188	-6.2711	0.619088	0.70036
Monocytes	INSIG2	-0.06745	4.405764	-0.47325	0.637201	-5.94993	0.640977	0.724942
Monocytes	LAPTM4A	0.034429	7.210016	0.472868	0.637473	-6.58818	0.592802	0.670609
Monocytes	HMGXB3	-0.0623	4.408539	-0.47267	0.637612	-5.96051	0.641117	0.725054
Monocytes	GM13166	0.226026	-0.19353	0.47258	0.637677	-5.06575	0.73023	0.824071
Monocytes	SIRT4	-0.25512	0.56797	-0.47257	0.637688	-5.06754	0.714595	0.806838
Monocytes	MPHOSPH10	0.061247	4.827879	0.472465	0.637759	-5.99603	0.633613	0.716724
Monocytes	MRPL46	0.092893	3.264758	0.472444	0.637774	-5.62406	0.662091	0.748601

Monocytes	TUFT1	0.111774	2.2113	0.472096	0.638021	-5.42739	0.682277	0.77101
Monocytes	GM26887	0.102254	3.10483	0.471649	0.63834	-5.96848	0.665377	0.752157
Monocytes	TUBGCP2	-0.0673	3.994036	-0.47163	0.638351	-5.89075	0.648918	0.733773
Monocytes	STXBP4	-0.18647	2.23619	-0.47152	0.638433	-5.24862	0.681901	0.770578
Monocytes	PIGG	0.188411	0.963956	0.471453	0.638479	-5.17252	0.70692	0.798326
Monocytes	TRIM26	-0.0546	5.330265	-0.47136	0.638544	-6.14307	0.625026	0.707014
Monocytes	ZFP955A	0.210049	1.472841	0.471356	0.638548	-5.13791	0.696795	0.787117
Monocytes	DGLUCY	0.065472	2.944912	0.471328	0.638568	-5.97137	0.668386	0.755547
Monocytes	CNBD2	-0.09055	3.889219	-0.47086	0.638903	-5.79878	0.650943	0.735992
Monocytes	ZFP830	0.084889	3.541014	0.470809	0.638937	-5.66932	0.657356	0.743159
Monocytes	PAXX	-0.09076	3.270058	-0.4708	0.638946	-5.58094	0.662395	0.748783
Monocytes	RPA1	-0.06965	4.922792	-0.47065	0.639053	-5.98789	0.632312	0.715119
Monocytes	SMARCD2	-0.04235	5.611886	-0.47054	0.639129	-6.19498	0.620221	0.70153
Monocytes	GM26631	0.192685	1.219353	0.470511	0.639148	-5.14521	0.701936	0.792724
Monocytes	GM15728	-0.19672	0.500791	-0.4705	0.639155	-5.17999	0.716396	0.808705
Monocytes	TMEM115	-0.07238	3.447731	-0.47044	0.639198	-5.68099	0.659086	0.74511
Monocytes	B2M	-0.06777	9.94338	-0.47037	0.639251	-6.99441	0.549919	0.621913
Monocytes	KRT81	-0.26096	-1.53173	-0.46944	0.639908	-4.93355	0.759773	0.856036
Monocytes	HMCN1	0.28413	1.920186	0.468978	0.64024	-5.15896	0.689066	0.777953
Monocytes	NELFA	0.052977	4.630912	0.46872	0.640424	-5.99023	0.638454	0.721444
Monocytes	TTC9C	-0.04991	4.558959	-0.46853	0.640558	-6.01716	0.639746	0.7229
Monocytes	ARHGAP19	-0.10024	4.256309	-0.46851	0.640574	-5.87532	0.645209	0.729016
Monocytes	PDLIM7	0.088067	3.638312	0.468417	0.640639	-5.73746	0.656526	0.741681
Monocytes	TDP2	-0.07611	4.340054	-0.46832	0.640708	-5.79963	0.643692	0.727343
Monocytes	RETREG2	-0.05953	4.884018	-0.46832	0.640708	-6.0786	0.633935	0.716412
Monocytes	BRIP1OS	-0.05388	5.408086	-0.468	0.640938	-6.13178	0.624825	0.706125
Monocytes	ARNTL	0.082609	5.38742	0.467947	0.640974	-6.01844	0.625187	0.706532
Monocytes	OAF	0.129051	3.151249	0.467761	0.641107	-5.47681	0.665786	0.751953
Monocytes	ALG12	0.175157	1.395336	0.467707	0.641145	-5.24154	0.699671	0.789581
Monocytes	GM46367	-0.16563	2.175914	-0.46744	0.641337	-5.31823	0.684453	0.772722
Monocytes	RHOT2	-0.12163	2.679369	-0.46743	0.641341	-5.44944	0.674783	0.761977
Monocytes	ZFP467	0.097475	2.303773	0.466943	0.641689	-5.69715	0.682185	0.770043
Monocytes	POLRMT	0.157023	1.91437	0.466859	0.641749	-5.25755	0.689741	0.778426
Monocytes	SPOCK2	0.210271	0.349422	0.466716	0.641851	-5.08642	0.721038	0.813018
Monocytes	HBQ1A	-0.24237	-1.39639	-0.46657	0.641956	-4.95755	0.757779	0.853351
Monocytes	HDAC8	0.061711	6.530622	0.466514	0.641995	-6.28623	0.605807	0.684577
Monocytes	SHQ1	-0.11142	2.60612	-0.46646	0.642032	-5.5565	0.676382	0.763595
Monocytes	SLC18A2	-0.12503	2.251616	-0.46643	0.642052	-5.37485	0.683192	0.77116
Monocytes	POLR2E	0.063928	4.876402	0.466345	0.642115	-6.14063	0.634505	0.716843
Monocytes	METTL5	0.075687	3.739598	0.466314	0.642138	-5.75251	0.655104	0.739889
Monocytes	KLHL20	0.099565	3.27084	0.466107	0.642285	-5.69279	0.663813	0.749606
Monocytes	SLC25A13	0.060207	4.679744	0.466093	0.642295	-6.18787	0.638016	0.72078
Monocytes	TSC1	-0.06264	4.710552	-0.46608	0.642302	-6.00249	0.637464	0.720162
Monocytes	MRAS	0.160193	0.476226	0.465695	0.642579	-5.36269	0.718585	0.810328
Monocytes	BLVRA	0.048672	5.071733	0.465667	0.642599	-6.27583	0.631161	0.713103
Monocytes	GM43445	0.136008	1.896237	0.465599	0.642648	-5.3482	0.690228	0.779003
Monocytes	CD209D	0.203209	-0.32218	0.465441	0.64276	-5.30203	0.735082	0.828557

Monocytes	NOL9	-0.08078	3.728213	-0.46535	0.642824	-5.74028	0.655441	0.74037
Monocytes	CCDC97	0.087499	3.33761	0.46524	0.642903	-5.6046	0.662693	0.748461
Monocytes	MRM2	-0.19567	2.071278	-0.46522	0.642918	-5.27623	0.686818	0.775296
Monocytes	WARS	-0.08787	3.723103	-0.46519	0.642941	-5.76703	0.655535	0.740475
Monocytes	MAGI3	-0.06598	6.598022	-0.465	0.643071	-6.35947	0.604828	0.683539
Monocytes	MAD2L1BP	0.068642	4.194819	0.46481	0.64321	-5.8787	0.646937	0.730865
Monocytes	MARS2	0.110977	2.690445	0.464765	0.643242	-5.39356	0.67495	0.762116
Monocytes	CMTM7	0.037485	7.897065	0.46464	0.643331	-6.63483	0.583353	0.659308
Monocytes	HEXDC	-0.19251	1.86348	-0.46452	0.643417	-5.25545	0.690916	0.77986
Monocytes	GABPB1	-0.05365	5.380129	-0.46446	0.643461	-6.10049	0.625772	0.707161
Monocytes	PLSCR3	0.06761	4.301027	0.464438	0.643475	-5.9985	0.645008	0.728727
Monocytes	IGFBP1	0.201969	4.147259	0.464334	0.64355	-5.76731	0.647803	0.731853
Monocytes	ZFX	-0.03354	6.339182	-0.46422	0.643633	-6.40128	0.609213	0.688529
Monocytes	SESN1	0.055415	5.939971	0.464142	0.643687	-6.19847	0.616045	0.696224
Monocytes	ADAL	-0.13363	2.279066	-0.46398	0.6438	-5.29673	0.682841	0.7709
Monocytes	LMTK3	-0.22939	-0.70138	-0.46392	0.643845	-5.02678	0.743111	0.837427
Monocytes	LRP2BP	-0.2102	1.991491	-0.46369	0.644009	-5.23001	0.688418	0.777172
Monocytes	CCDC6	-0.06782	4.540905	-0.46367	0.644021	-5.96094	0.640676	0.723957
Monocytes	C030005K06RIK	-0.18308	0.674423	-0.46363	0.644051	-5.17991	0.714601	0.806153
Monocytes	DPP7	-0.15408	1.957266	-0.4636	0.644072	-5.38009	0.689085	0.777922
Monocytes	HERPUD2	-0.04176	5.474289	-0.4636	0.644076	-6.22317	0.624124	0.705394
Monocytes	CYB561D1	-0.16797	1.785161	-0.46301	0.644494	-5.25752	0.692762	0.781813
Monocytes	COX18	-0.08704	3.223872	-0.46301	0.644495	-5.67635	0.665166	0.751147
Monocytes	ANAPC10	0.054298	4.581596	0.462883	0.644586	-6.02008	0.640259	0.723332
Monocytes	QSOX1	-0.08228	4.311358	-0.46275	0.644678	-5.77529	0.645166	0.728803
Monocytes	ABCA9	0.148477	0.787541	0.462497	0.644861	-5.55372	0.712822	0.803942
Monocytes	MAP7	-0.09735	3.639692	-0.46215	0.645107	-5.6209	0.657779	0.742677
Monocytes	4732465J04RIK	0.232696	1.227323	0.461728	0.64541	-5.10382	0.704237	0.794328
Monocytes	FOSL1	0.102703	1.662098	0.46165	0.645466	-5.83783	0.695615	0.784788
Monocytes	ZFP771	0.064264	4.065583	0.461553	0.645535	-5.84719	0.649991	0.734049
Monocytes	SLC25A19	-0.0795	3.390435	-0.46149	0.645581	-5.77509	0.662465	0.747997
Monocytes	POFUT1	0.080713	3.458205	0.461224	0.64577	-5.68246	0.661201	0.746623
Monocytes	IRS2	-0.07048	5.572327	-0.46121	0.645778	-6.22818	0.623084	0.703953
Monocytes	KAT6B	0.04809	5.844837	0.461182	0.645801	-6.26954	0.618351	0.698632
Monocytes	CCDC84	0.112787	2.649641	0.461176	0.645805	-5.47121	0.676456	0.763609
Monocytes	SYNC	0.121786	1.973724	0.461133	0.645836	-5.33457	0.689505	0.778098
Monocytes	PLEKHA8	0.166842	0.886546	0.461128	0.645839	-5.17486	0.711076	0.801967
Monocytes	CLSTN1	-0.16578	1.774497	-0.46109	0.645863	-5.20287	0.693404	0.782433
Monocytes	TANGO2	-0.10173	3.93274	-0.4609	0.646003	-5.80793	0.652464	0.736849
Monocytes	HIST1H2BH	-0.24504	0.17295	-0.46082	0.646062	-5.03249	0.725678	0.818059
Monocytes	ZDHHC2	-0.1043	2.276539	-0.46077	0.646095	-5.69588	0.683667	0.771619
Monocytes	TCTN2	-0.20244	-0.37598	-0.46057	0.646238	-5.09428	0.737186	0.830694
Monocytes	RTN3	0.041776	8.060675	0.460301	0.64643	-6.65682	0.581556	0.656942
Monocytes	FXYD2	0.216238	-0.41192	0.460209	0.646496	-5.15835	0.738088	0.831528
Monocytes	TASOR	0.050608	5.742445	0.459899	0.646718	-6.26228	0.620481	0.700776
Monocytes	MED18	0.187898	1.412452	0.459766	0.646813	-5.14447	0.700953	0.790568
Monocytes	AREL1	0.069272	4.263334	0.459736	0.646834	-5.82399	0.646758	0.730307

Monocytes	CDCA7	0.134384	3.246784	0.459605	0.646928	-5.51707	0.665534	0.751291
Monocytes	DNAJC1	0.038074	6.915183	0.459587	0.646941	-6.50923	0.600494	0.678358
Monocytes	ENG	-0.1281	4.005662	-0.45918	0.647232	-5.68192	0.651689	0.735731
Monocytes	GM11755	0.307036	-0.115	0.459045	0.647329	-4.95136	0.732305	0.82505
Monocytes	GFI1	-0.2211	1.55053	-0.45896	0.647387	-5.06474	0.698489	0.787783
Monocytes	KCTD9	-0.08936	3.130956	-0.45884	0.647475	-5.6158	0.667997	0.753911
Monocytes	TMEM65	-0.06436	4.9205	-0.45869	0.64758	-6.11589	0.635213	0.71728
Monocytes	LRP5	0.071091	4.078112	0.458648	0.647612	-6.00779	0.650422	0.734298
Monocytes	GNPDA1	0.055587	4.395098	0.458311	0.647854	-6.11061	0.644828	0.727904
Monocytes	BBOX1	-0.22096	0.92351	-0.4581	0.648006	-5.12959	0.711289	0.801793
Monocytes	H19	0.149773	6.408472	0.458077	0.648021	-6.35083	0.609511	0.688312
Monocytes	GM10130	-0.1509	1.783227	-0.45794	0.648122	-5.27489	0.694206	0.782904
Monocytes	MTX2	0.053114	4.838218	0.457776	0.648236	-6.06461	0.636956	0.719166
Monocytes	USP32	0.045738	6.849617	0.457654	0.648323	-6.51897	0.602117	0.680011
Monocytes	9130230N09RII	-0.2789	0.640779	-0.45763	0.648343	-4.99602	0.717091	0.808237
Monocytes	MSMO1	0.080139	3.610529	0.457251	0.648612	-5.75889	0.659479	0.744268
Monocytes	GLDC	-0.20047	1.391092	-0.45723	0.648626	-5.20372	0.702157	0.791657
Monocytes	CDC16	0.073636	3.893321	0.457107	0.648716	-5.76415	0.654275	0.738456
Monocytes	NUP35	-0.10644	3.291207	-0.45679	0.648942	-5.53408	0.665632	0.751002
Monocytes	CEP83OS	0.157543	2.022979	0.456597	0.64908	-5.27311	0.689983	0.778037
Monocytes	CCDC28B	0.078839	3.504556	0.456349	0.649258	-5.82703	0.661833	0.746677
Monocytes	GGCT	0.105207	3.249782	0.456251	0.649328	-5.7005	0.666608	0.75199
Monocytes	CAMK2N1	0.22955	1.714702	0.456088	0.649445	-5.21136	0.696213	0.78485
Monocytes	ZFP267	0.152964	1.251689	0.455961	0.649536	-5.18635	0.705425	0.795057
Monocytes	ALKBH4	-0.12973	2.574916	-0.45588	0.649591	-5.33445	0.679498	0.76634
Monocytes	GM20508	0.188134	0.064606	0.455742	0.649693	-5.12132	0.729616	0.821728
Monocytes	TIMM50	-0.05825	4.827954	-0.45569	0.64973	-6.07624	0.637743	0.719788
Monocytes	RAB11FIP3	-0.12697	2.230172	-0.45557	0.649817	-5.37041	0.686186	0.773756
Monocytes	EHBP1L1	-0.04036	6.149657	-0.45508	0.650165	-6.4185	0.614685	0.693889
Monocytes	LGALS4	-0.17466	2.563012	-0.45507	0.650177	-5.23958	0.679861	0.766738
Monocytes	EBP	0.061414	4.791273	0.454824	0.65035	-6.08308	0.638515	0.720669
Monocytes	2010016I18RIK	0.183036	-0.03474	0.454813	0.650359	-5.17219	0.731812	0.824164
Monocytes	FGD5	-0.23534	1.663977	-0.45463	0.650493	-5.14892	0.69737	0.786186
Monocytes	FASTK	-0.08373	3.15048	-0.45455	0.650546	-5.65156	0.66868	0.754347
Monocytes	ITPA	-0.06238	4.105721	-0.45453	0.65056	-5.8275	0.650931	0.734558
Monocytes	SHROOM2	-0.24774	1.506317	-0.45453	0.65056	-5.10733	0.700491	0.789639
Monocytes	CLEC12A	-0.05873	4.937614	-0.45442	0.650644	-6.4373	0.635899	0.717761
Monocytes	PAPSS2	-0.09149	2.513577	-0.4544	0.650652	-5.89972	0.680811	0.767851
Monocytes	CWC22	-0.07682	3.900011	-0.45439	0.650661	-5.78223	0.654709	0.738795
Monocytes	SWSAP1	-0.22168	0.278314	-0.45435	0.650687	-5.06385	0.725329	0.81706
Monocytes	CALCOCO1	0.098677	3.863697	0.4542	0.650798	-5.60713	0.655378	0.739594
Monocytes	SAP18B	0.104682	2.771863	0.454177	0.650815	-5.54839	0.675863	0.762408
Monocytes	DDIT3	0.08852	4.008692	0.454068	0.650893	-5.71423	0.652724	0.736615
Monocytes	NQO1	0.200815	0.792254	0.453923	0.650996	-5.16277	0.714853	0.80559
Monocytes	EVI2A	0.04584	5.251447	0.453864	0.651039	-6.4298	0.630358	0.711641
Monocytes	DOCK10	-0.03822	8.80921	-0.45368	0.651168	-6.91005	0.570948	0.644603
Monocytes	FBXL12OS	-0.19701	0.679534	-0.45348	0.651315	-5.06516	0.71721	0.808287

Monocytes	SPTA1	-0.3199	0.279808	-0.45347	0.651324	-4.98412	0.725399	0.817308
Monocytes	SMC2	-0.08122	5.816825	-0.45332	0.651428	-6.27939	0.620518	0.700647
Monocytes	ASB6	-0.1151	2.575022	-0.45324	0.651486	-5.43953	0.679725	0.766812
Monocytes	DCAF6	0.060929	5.908083	0.453116	0.651575	-6.25417	0.618936	0.6989
Monocytes	FIZ1	-0.05404	4.553694	-0.45311	0.65158	-5.93229	0.642878	0.725756
Monocytes	SFXN1	-0.06182	5.121606	-0.45302	0.651648	-6.0647	0.632715	0.714374
Monocytes	EPO	0.231874	0.415776	0.452971	0.651679	-5.03177	0.722603	0.814274
Monocytes	GM5608	-0.16323	0.923456	-0.45287	0.651748	-5.29136	0.712267	0.802874
Monocytes	2310022B05RIK	0.094494	2.488564	0.452673	0.651893	-5.45888	0.681452	0.768722
Monocytes	CAMSAP1	0.093432	3.53399	0.452541	0.651988	-5.64442	0.661646	0.746716
Monocytes	AP1G2	0.107189	2.809774	0.452525	0.651999	-5.67415	0.675298	0.761919
Monocytes	DNAJC17	0.069003	3.568748	0.452085	0.652315	-5.6902	0.661253	0.746171
Monocytes	ACAA1A	0.047739	5.302507	0.451652	0.652626	-6.27859	0.630056	0.711015
Monocytes	B4GALNT1	0.043517	4.892601	0.451369	0.652829	-6.32372	0.637423	0.719279
Monocytes	NIPSNAP2	0.046604	4.810103	0.451273	0.652898	-6.0562	0.6389	0.720984
Monocytes	NSMCE3	0.054534	4.271532	0.451261	0.652906	-5.92766	0.648637	0.731874
Monocytes	PRKRA	0.053217	3.39958	0.45109	0.653029	-6.02353	0.664796	0.749935
Monocytes	EML4	-0.03917	7.138312	-0.45102	0.653078	-6.53326	0.598684	0.675811
Monocytes	GM19705	-0.20106	1.868138	-0.45074	0.653277	-5.20828	0.694324	0.782667
Monocytes	GM20045	0.14237	1.764585	0.450626	0.653363	-5.36358	0.696384	0.784923
Monocytes	ERCC6L	-0.10387	3.034059	-0.45017	0.653689	-5.70847	0.672112	0.757785
Monocytes	SUN2	-0.04627	6.122993	-0.45005	0.653776	-6.26523	0.616295	0.695352
Monocytes	CDC42EP2	0.097923	3.08028	0.449926	0.653865	-5.8402	0.671284	0.756826
Monocytes	ZFP944	-0.06834	4.397621	-0.4496	0.654099	-5.91011	0.646986	0.729693
Monocytes	GTDC1	0.045374	6.409058	0.449457	0.654202	-6.42393	0.611548	0.689954
Monocytes	GALNT18	-0.24784	2.025829	-0.44946	0.654202	-5.22606	0.691733	0.779469
Monocytes	SNUPN	0.097469	2.593487	0.449211	0.654379	-5.45087	0.680828	0.767266
Monocytes	FCRL6	-0.26232	-0.87747	-0.44905	0.654496	-4.92169	0.751291	0.844962
Monocytes	RFLNB	-0.10837	3.463749	-0.44904	0.654498	-5.73312	0.664316	0.748954
Monocytes	NFE2L3	0.143243	2.246069	0.448935	0.654577	-5.36855	0.68756	0.774771
Monocytes	AMER1	-0.11893	2.080672	-0.44882	0.654659	-5.30597	0.690802	0.778361
Monocytes	SCFD2	0.047355	5.78946	0.448541	0.65486	-6.27854	0.622484	0.702125
Monocytes	4930589L23RIK	0.166046	-0.68311	0.448394	0.654966	-5.18339	0.747357	0.840576
Monocytes	RBM5	-0.03357	6.623423	-0.44828	0.65505	-6.45077	0.60817	0.686054
Monocytes	LRRC51	-0.14854	1.76843	-0.44817	0.655126	-5.27542	0.697098	0.785302
Monocytes	API5	0.036274	6.03062	0.448155	0.655138	-6.39633	0.618319	0.69747
Monocytes	PLEKHO2	-0.03866	6.069061	-0.44804	0.655219	-6.52633	0.617671	0.696742
Monocytes	LAG3	-0.16828	1.429969	-0.44764	0.655507	-5.31263	0.70399	0.79283
Monocytes	ADAMTS14	-0.23559	0.543497	-0.44754	0.655583	-5.07157	0.721918	0.812597
Monocytes	LRRC63	-0.15883	0.544146	-0.44746	0.655637	-5.40153	0.721904	0.812583
Monocytes	TCHP	-0.16133	1.792962	-0.44742	0.655665	-5.19683	0.69679	0.784871
Monocytes	PARD6A	-0.10939	2.688858	-0.44733	0.655727	-5.59385	0.679361	0.765559
Monocytes	GM525	-0.12777	0.742971	-0.4473	0.655755	-5.32448	0.717841	0.808123
Monocytes	NFE2L1	-0.05697	4.98448	-0.44697	0.655987	-6.14136	0.637019	0.71824
Monocytes	XRCC5	-0.17241	2.083305	-0.44672	0.656166	-5.15595	0.691385	0.778703
Monocytes	IL5RA	-0.29742	0.975245	-0.4465	0.656326	-4.99245	0.713535	0.803112
Monocytes	CRYBA4	-0.20038	-0.18755	-0.44635	0.656435	-5.16209	0.737549	0.829503

Monocytes	MIS18BP1	-0.09783	3.964435	-0.44621	0.656536	-5.87151	0.655826	0.7391
Monocytes	ADCK2	0.099572	2.282482	0.445962	0.656715	-5.48624	0.6877	0.774525
Monocytes	POMK	0.181222	1.068205	0.445956	0.656719	-5.20165	0.711757	0.801114
Monocytes	NFS1	0.057684	4.533914	0.445923	0.656743	-5.98673	0.645424	0.727492
Monocytes	DENND4A	0.043484	10.03042	0.445811	0.656823	-7.05704	0.553912	0.6243
Monocytes	HABP4	-0.07648	3.054352	-0.44562	0.656958	-5.74328	0.672892	0.758073
Monocytes	BTAF1	-0.03323	7.190783	-0.44543	0.657095	-6.59687	0.59923	0.675681
Monocytes	TNKS1BP1	-0.14559	2.080541	-0.44539	0.657125	-5.29932	0.69166	0.778962
Monocytes	UBE2Q2	0.041329	5.51991	0.445319	0.657178	-6.29618	0.627843	0.707862
Monocytes	NDUFB6	0.050796	6.317819	0.445173	0.657282	-6.40195	0.613992	0.692331
Monocytes	AHCYL1	0.040867	5.179051	0.445089	0.657344	-6.14281	0.633866	0.714645
Monocytes	POLR3G	-0.14703	2.101279	-0.44504	0.657376	-5.33288	0.691254	0.778549
Monocytes	EOGT	-0.0999	2.532472	-0.44501	0.657403	-5.60227	0.682879	0.769269
Monocytes	TWF2	0.044874	5.240324	0.444996	0.65741	-6.29245	0.632779	0.713427
Monocytes	SUCO	-0.04752	6.354348	-0.4446	0.657693	-6.44286	0.613564	0.691713
Monocytes	FKBP8	-0.04574	6.067485	-0.44446	0.657797	-6.32383	0.6185	0.697283
Monocytes	HYLS1	-0.11227	3.183514	-0.44443	0.657816	-5.4338	0.670661	0.755568
Monocytes	ANKRD24	-0.17333	1.054272	-0.44401	0.658121	-5.16096	0.712532	0.801795
Monocytes	MIR17HG	-0.14595	2.192534	-0.44394	0.658169	-5.30755	0.689929	0.776856
Monocytes	SMO	-0.17442	1.198551	-0.44373	0.65832	-5.27901	0.709677	0.798662
Monocytes	DYNC1LI1	0.035907	6.412559	0.443643	0.658385	-6.4089	0.612822	0.690805
Monocytes	FBXO5	-0.12467	4.811103	-0.4436	0.658413	-5.89056	0.640914	0.722302
Monocytes	LPAR6	0.056709	4.523596	0.443395	0.658563	-6.27732	0.646184	0.728172
Monocytes	ATP13A1	0.061963	3.809105	0.443246	0.658671	-5.83741	0.659297	0.742838
Monocytes	DIO1	-0.19195	0.977554	-0.44316	0.658731	-5.12416	0.714224	0.803703
Monocytes	MADD	0.048087	5.091113	0.443135	0.65875	-6.2281	0.635975	0.716805
Monocytes	FCGR1	0.091424	2.432362	0.442877	0.658936	-6.00838	0.685516	0.771882
Monocytes	FCHSD1	0.173	1.15994	0.442688	0.659072	-5.15425	0.710658	0.799748
Monocytes	ANO6	-0.05139	6.75402	-0.44256	0.659164	-6.42999	0.607187	0.684498
Monocytes	TGM1	-0.20962	0.53017	-0.44246	0.659237	-5.16611	0.723472	0.813935
Monocytes	NUP153	-0.04062	5.848135	-0.44237	0.659301	-6.31464	0.622741	0.702037
Monocytes	DPM1	0.039639	5.979025	0.442313	0.659343	-6.31513	0.620466	0.699496
Monocytes	DHX58	0.102112	2.999964	0.44229	0.659359	-5.81913	0.674616	0.759977
Monocytes	MTERF4	0.142065	1.913823	0.442263	0.659379	-5.29405	0.695642	0.783288
Monocytes	GTF3C1	-0.05669	4.779184	-0.44208	0.659512	-6.00903	0.641694	0.723308
Monocytes	NAF1	0.068265	3.658508	0.441817	0.659701	-5.79483	0.662232	0.746327
Monocytes	SLC46A2	0.244148	-0.09426	0.441705	0.659781	-4.96609	0.736451	0.828383
Monocytes	1700112J16RIK	-0.18552	0.638697	-0.44162	0.659841	-5.23868	0.721271	0.811698
Monocytes	GM27241	-0.13023	2.603068	-0.4415	0.659927	-5.47114	0.682241	0.768568
Monocytes	CASP4	-0.08529	4.029936	-0.4415	0.659932	-6.13569	0.655345	0.738652
Monocytes	C2CD2	-0.0913	3.079676	-0.44118	0.660162	-5.64231	0.673124	0.758514
Monocytes	ACAD11	-0.12821	2.331168	-0.44094	0.660329	-5.32134	0.687503	0.774527
Monocytes	BAIAP2L1	0.097151	3.098445	0.440877	0.660378	-5.59227	0.672768	0.758203
Monocytes	NDUFA3	0.034172	8.000282	0.440748	0.660471	-6.70596	0.586518	0.661503
Monocytes	ZFP324	0.199026	0.798242	0.440627	0.660558	-5.14329	0.718012	0.808308
Monocytes	PPM1L	-0.08286	4.617569	-0.44049	0.660656	-5.90821	0.644612	0.726873
Monocytes	GM12462	-0.26209	-0.49881	-0.44049	0.66066	-4.93682	0.744978	0.837969

Monocytes	GM11508	-0.09992	3.437566	-0.44036	0.660752	-5.73791	0.666366	0.751164
Monocytes	ALDH2	0.046054	6.861181	0.44027	0.660816	-6.4764	0.605397	0.6829
Monocytes	XPO7	0.045374	6.710919	0.440195	0.66087	-6.39756	0.607938	0.685776
Monocytes	CNPY3	0.036083	5.300686	0.440169	0.660889	-6.29656	0.632379	0.713236
Monocytes	GM12689	-0.18775	-0.48996	-0.44004	0.660985	-5.14018	0.74479	0.837829
Monocytes	KAT2B	-0.04356	6.221283	-0.44003	0.660988	-6.46213	0.616302	0.695188
Monocytes	MAX	0.040571	6.499429	0.439968	0.661034	-6.36389	0.611535	0.689825
Monocytes	BTRC	-0.06298	5.211768	-0.43982	0.661137	-5.99398	0.633956	0.715004
Monocytes	HIST1H2AK	-0.22827	0.954719	-0.43981	0.66115	-5.11205	0.714831	0.804895
Monocytes	IRGC1	-0.20137	0.115465	-0.43979	0.661162	-5.09633	0.732073	0.823873
Monocytes	STARD5	-0.07272	4.526318	-0.43977	0.661177	-5.85188	0.646266	0.728779
Monocytes	HACD2	-0.03943	5.732831	-0.43976	0.661186	-6.32506	0.624774	0.704707
Monocytes	LRP4	0.086802	2.477171	0.439738	0.6612	-5.97669	0.684672	0.771543
Monocytes	TLE3	-0.04768	5.003886	-0.43967	0.661252	-6.18581	0.637662	0.719154
Monocytes	MRFAP1	0.033745	6.452488	0.439573	0.661319	-6.40979	0.612336	0.690762
Monocytes	NOXRED1	0.184742	0.546984	0.439496	0.661374	-5.16539	0.723152	0.814129
Monocytes	BLMH	0.041016	5.648784	0.439384	0.661455	-6.22204	0.626245	0.706432
Monocytes	ZYX	0.046835	6.244314	0.439347	0.661482	-6.56911	0.615905	0.694816
Monocytes	HLCS	-0.05871	4.15558	-0.43933	0.661491	-6.16825	0.653034	0.736417
Monocytes	ECHDC1	-0.08298	3.820316	-0.43927	0.661539	-5.76183	0.659222	0.743346
Monocytes	PTPA	0.040412	5.833043	0.438854	0.661838	-6.29633	0.623193	0.702995
Monocytes	CDCA8	-0.09631	5.109425	-0.43884	0.661846	-6.19937	0.635948	0.717301
Monocytes	9430091E24RIK	0.119388	2.439837	0.43845	0.662129	-5.39989	0.685805	0.77272
Monocytes	UGP2	0.05017	5.910415	0.438244	0.662278	-6.34229	0.62213	0.701655
Monocytes	ZFP428	-0.16291	2.058083	-0.43801	0.662449	-5.19652	0.69335	0.78111
Monocytes	CDK17	0.045081	6.780519	0.437921	0.662511	-6.39521	0.607213	0.684907
Monocytes	PRPS2	0.069913	4.382206	0.43789	0.662534	-5.80898	0.649372	0.732199
Monocytes	IPO9	-0.05455	4.771136	-0.43787	0.662551	-5.9401	0.642318	0.724314
Monocytes	SOAT2	-0.17626	0.810649	-0.43772	0.662654	-5.21309	0.718331	0.808688
Monocytes	ZCCHC10	0.070899	4.048466	0.437613	0.662734	-5.82227	0.655526	0.739077
Monocytes	SURF2	0.082317	3.331878	0.437513	0.662806	-5.65591	0.668887	0.754005
Monocytes	USP9X	-0.03787	7.387216	-0.43746	0.662844	-6.62716	0.59707	0.673537
Monocytes	BCLAF1	0.029233	7.155376	0.436858	0.663279	-6.54927	0.601241	0.678058
Monocytes	TEDC2	-0.1929	0.906107	-0.43681	0.663311	-5.09324	0.716753	0.806852
Monocytes	NUDT8	-0.10069	2.208161	-0.43664	0.663438	-5.56328	0.69083	0.778253
Monocytes	PRPF40B	-0.28078	0.534618	-0.43659	0.66347	-4.99542	0.724383	0.815313
Monocytes	ALKBH3	-0.07739	3.490416	-0.43633	0.663661	-5.76122	0.666363	0.751082
Monocytes	KIN	0.051416	4.642417	0.436292	0.663688	-6.01181	0.645118	0.727384
Monocytes	NRBF2	0.058053	4.314912	0.436106	0.663823	-5.96741	0.651099	0.734127
Monocytes	RAC3	0.245168	-0.22085	0.436087	0.663837	-5.04976	0.740226	0.832792
Monocytes	EEFSEC	-0.06225	4.399413	-0.43596	0.663932	-6.13514	0.649584	0.732409
Monocytes	GM12971	-0.15952	0.967071	-0.43586	0.664003	-5.18356	0.715701	0.80582
Monocytes	RBFOX2	-0.1759	2.758111	-0.43576	0.664075	-5.34644	0.680333	0.766708
Monocytes	PIAS1	-0.03119	7.42613	-0.43555	0.664224	-6.57745	0.596919	0.673286
Monocytes	ENSA	0.041423	5.388151	0.435534	0.664237	-6.26271	0.631858	0.71259
Monocytes	CD48	-0.04295	6.795574	-0.43543	0.664315	-6.57801	0.607504	0.685236
Monocytes	GM33370	-0.15152	0.508404	-0.43515	0.664514	-5.30224	0.725284	0.816324

Monocytes	MARK4	-0.06289	4.541737	-0.43501	0.664618	-5.97889	0.647198	0.72972
Monocytes	PCNX3	0.077402	3.30596	0.434945	0.664662	-5.70415	0.670098	0.755254
Monocytes	6330418K02RIK	-0.14996	1.506684	-0.43431	0.665124	-5.28292	0.705369	0.794138
Monocytes	TARDBP	-0.04168	5.771119	-0.4343	0.665126	-6.27209	0.625557	0.705282
Monocytes	GAK	-0.03283	6.280821	-0.43406	0.665304	-6.41735	0.616709	0.695366
Monocytes	PCBD2	-0.04882	5.24057	-0.43401	0.66534	-6.23996	0.634918	0.715799
Monocytes	MCUR1	-0.05315	4.462065	-0.43397	0.665367	-6.09333	0.648939	0.731483
Monocytes	VPS45	-0.07684	3.758422	-0.43391	0.665412	-5.75436	0.661908	0.745982
Monocytes	CYP27A1	-0.06658	2.194427	-0.43384	0.665458	-6.01838	0.691771	0.779191
Monocytes	STAT6	0.044349	5.031546	0.433816	0.665479	-6.22613	0.638649	0.720044
Monocytes	COL3A1	-0.15427	3.678331	-0.43339	0.665789	-5.71041	0.663612	0.747775
Monocytes	SNAPC2	0.099458	3.015281	0.433318	0.665839	-5.58345	0.676131	0.761708
Monocytes	DBF4	0.059605	5.205532	0.433241	0.665895	-6.17479	0.635743	0.71666
Monocytes	MAP4K4	-0.04009	7.459224	-0.43317	0.665949	-6.7695	0.596975	0.673077
Monocytes	ST6GALNAC4	0.075366	3.867281	0.433038	0.666042	-5.84058	0.660119	0.743905
Monocytes	1700048O20RII	0.320666	-0.24632	0.432892	0.666147	-4.96651	0.741665	0.834067
Monocytes	GM16675	0.188202	1.196561	0.432691	0.666293	-5.2314	0.711885	0.801326
Monocytes	HIST1H3D	-0.16747	1.963471	-0.43268	0.666303	-5.31068	0.696588	0.784426
Monocytes	GM15706	-0.1839	1.015787	-0.43263	0.666333	-5.12715	0.715544	0.805361
Monocytes	NLRC5	-0.10368	5.064856	-0.43225	0.66661	-6.17904	0.638442	0.719638
Monocytes	FAM49A	0.057691	5.390412	0.432184	0.66666	-6.42646	0.632644	0.713145
Monocytes	NGLY1	0.038943	4.849758	0.432179	0.666664	-6.06864	0.642305	0.72396
Monocytes	HRH2	0.178158	1.160498	0.431862	0.666893	-5.19185	0.712934	0.802259
Monocytes	1700102P08RIK	-0.18781	0.812589	-0.43129	0.667307	-5.18187	0.720382	0.810265
Monocytes	DLEU2	0.03766	8.40867	0.431055	0.667477	-6.8254	0.582155	0.655927
Monocytes	MTDH	-0.02427	7.470279	-0.43091	0.667586	-6.6091	0.597559	0.673289
Monocytes	DRG1	-0.03312	6.40915	-0.43075	0.6677	-6.39082	0.615528	0.693474
Monocytes	SLC35C2	-0.03713	4.846147	-0.43067	0.667759	-6.26417	0.643049	0.724317
Monocytes	STIM1	0.05528	7.712751	0.430387	0.667961	-6.53068	0.593705	0.66876
Monocytes	ORMDL1	-0.0718	3.609727	-0.43017	0.668121	-5.73538	0.666025	0.749727
Monocytes	F2RL1	-0.23629	-1.13295	-0.43003	0.668221	-4.97397	0.761894	0.855323
Monocytes	COLEC12	0.103112	2.890318	0.429526	0.668585	-5.92492	0.679961	0.765027
Monocytes	CPSF3	0.047728	4.770083	0.429505	0.668601	-6.0812	0.644922	0.726033
Monocytes	YIPF6	0.085947	3.457527	0.429099	0.668895	-5.68064	0.669274	0.753142
Monocytes	PRKACB	0.045816	5.548308	0.429038	0.668939	-6.13964	0.631104	0.710569
Monocytes	SLC2A8	0.112204	2.226604	0.429007	0.668962	-5.40425	0.692936	0.779387
Monocytes	PIP4K2C	0.068622	4.238405	0.428897	0.669041	-5.83289	0.654726	0.736959
Monocytes	HMGCR	-0.05574	4.712579	-0.4288	0.66911	-6.06194	0.646063	0.727295
Monocytes	ARHGAP4	0.047413	5.057347	0.428759	0.669141	-6.11564	0.639844	0.720348
Monocytes	POLR3C	0.041597	5.018329	0.428678	0.6692	-6.16396	0.640545	0.721131
Monocytes	IDS	0.063263	2.698638	0.428646	0.669224	-5.95705	0.683755	0.769221
Monocytes	PAK1IP1	-0.03274	5.901653	-0.4283	0.669477	-6.32653	0.625071	0.703783
Monocytes	RSF1OS1	0.098874	2.978349	0.428176	0.669564	-5.53907	0.678591	0.76345
Monocytes	MAP3K12	-0.09269	2.718206	-0.42797	0.669714	-5.54613	0.683614	0.769001
Monocytes	COX15	-0.08471	2.891641	-0.42797	0.669716	-5.52927	0.680275	0.765299
Monocytes	CAMK1	0.07444	2.966119	0.427752	0.669872	-5.88938	0.678939	0.763756
Monocytes	CHRNE	0.215853	-0.75209	0.4275	0.670055	-5.0009	0.754564	0.846864

Monocytes	SRPRB	-0.06016	4.104103	-0.42721	0.670265	-5.88176	0.657777	0.739995
Monocytes	1110038B12RIK	-0.0757	4.235292	-0.42677	0.670581	-5.82002	0.655449	0.737405
Monocytes	SLC26A2	0.061316	3.742645	0.426549	0.670745	-5.99182	0.664596	0.747613
Monocytes	GM15445	0.138428	0.994582	0.426493	0.670785	-5.28758	0.718272	0.806987
Monocytes	FTL1	-0.03864	11.26205	-0.42641	0.670846	-7.24014	0.539244	0.606474
Monocytes	AGRP	0.194167	1.935634	0.426408	0.670847	-5.21849	0.699374	0.786154
Monocytes	CCDC82	-0.06574	4.323551	-0.42629	0.67093	-5.86464	0.653825	0.735629
Monocytes	NAA20	-0.04138	5.164245	-0.42629	0.67093	-6.14448	0.638579	0.718613
Monocytes	PRKCI	-0.10205	3.113961	-0.42625	0.670964	-5.55626	0.676475	0.760814
Monocytes	REC114	-0.1063	3.19631	-0.42624	0.67097	-5.58031	0.674906	0.759073
Monocytes	4930579G24RII	0.110999	2.431609	0.42618	0.671012	-5.41211	0.689633	0.775393
Monocytes	PLATR25	0.096003	2.729731	0.42604	0.671114	-5.56426	0.683888	0.769031
Monocytes	GPRASP1	-0.06679	3.93667	-0.42531	0.671648	-5.78007	0.661474	0.743811
Monocytes	WDR82	-0.04371	4.82833	-0.42484	0.671985	-6.03026	0.645324	0.725671
Monocytes	PHF10	-0.04583	4.930175	-0.4247	0.672085	-6.21967	0.643483	0.723616
Monocytes	GM26542	0.063736	4.802534	0.424689	0.672095	-6.03129	0.645791	0.726192
Monocytes	FBXO9	0.06841	4.752411	0.424636	0.672133	-5.88207	0.6467	0.727212
Monocytes	WDR46	0.084613	3.429733	0.424368	0.672328	-5.72116	0.671338	0.754577
Monocytes	VPS13B	-0.03108	8.050399	-0.42371	0.672805	-6.68871	0.590304	0.663747
Monocytes	NEK3	-0.18913	1.761696	-0.42354	0.672931	-5.21424	0.70422	0.79073
Monocytes	HSPA2	-0.10206	3.291574	-0.42337	0.673055	-5.61363	0.67449	0.757806
Monocytes	ERCC8	0.10415	2.45611	0.422901	0.673394	-5.41229	0.690796	0.775682
Monocytes	GM14966	0.094188	2.847703	0.422843	0.673436	-5.49224	0.683199	0.767284
Monocytes	CDC25C	0.139887	2.030722	0.422813	0.673458	-5.40355	0.699155	0.784918
Monocytes	GM15446	-0.143	2.166881	-0.42272	0.673527	-5.31085	0.696471	0.781952
Monocytes	GM17491	0.132702	1.415352	0.422586	0.673623	-5.18036	0.71148	0.798505
Monocytes	SGTA	0.052624	4.959007	0.422232	0.673881	-6.08938	0.644009	0.723635
Monocytes	ZFP574	-0.09055	3.401172	-0.42195	0.674087	-5.583	0.672922	0.755787
Monocytes	CCL27A	-0.14432	1.684593	-0.42189	0.674128	-5.24617	0.706359	0.792746
Monocytes	TMEM240	-0.23728	0.240106	-0.42185	0.674156	-5.05659	0.735906	0.825203
Monocytes	KCNJ16	-0.32663	-0.02269	-0.42126	0.674586	-5.03168	0.741622	0.831342
Monocytes	IRGQ	-0.15455	2.194038	-0.42122	0.674613	-5.26451	0.696435	0.781688
Monocytes	GM26810	0.21774	0.423092	0.421216	0.67462	-5.11467	0.732285	0.821118
Monocytes	DPAGT1	-0.07968	3.285854	-0.42107	0.674727	-5.75395	0.675294	0.758305
Monocytes	LTB	-0.11199	3.446775	-0.42081	0.674915	-5.63991	0.672238	0.754944
Monocytes	GM15708	-0.09243	3.198583	-0.42075	0.674957	-5.66345	0.676957	0.760176
Monocytes	NDUFS8	-0.03606	6.232258	-0.42072	0.674979	-6.39714	0.621703	0.698618
Monocytes	TSPOAP1	-0.10504	2.650845	-0.42066	0.675027	-5.62034	0.687502	0.771848
Monocytes	BEND3	-0.12295	2.456588	-0.42051	0.675131	-5.429	0.691286	0.776038
Monocytes	NMT2	-0.04144	5.47269	-0.42045	0.675177	-6.22726	0.63505	0.713556
Monocytes	DCTN2	0.042491	5.367124	0.42033	0.675264	-6.24082	0.63693	0.715656
Monocytes	SLC39A6	0.058698	4.11285	0.420325	0.675268	-5.92389	0.659751	0.741086
Monocytes	ARAP1	-0.05365	5.049234	-0.42027	0.67531	-6.24131	0.642629	0.722027
Monocytes	JAML	-0.06574	2.748338	-0.42027	0.67531	-6.15531	0.685612	0.769775
Monocytes	ST6GALNAC6	-0.07457	3.214358	-0.42017	0.675378	-5.84973	0.676656	0.759883
Monocytes	PAFAH1B1	0.025073	8.064884	0.42005	0.675468	-6.68215	0.590779	0.663909
Monocytes	SRP54C	-0.13511	1.755516	-0.4198	0.675647	-5.32868	0.70513	0.791413

Monocytes	MED12L	-0.06959	3.141983	-0.41978	0.675662	-5.86604	0.678039	0.761477
Monocytes	FAM98B	-0.05714	4.474383	-0.41974	0.675691	-5.94885	0.653081	0.733757
Monocytes	HORMAD2	0.223427	1.357831	0.419611	0.675788	-5.20937	0.713119	0.800209
Monocytes	TMEM220	0.209686	0.299081	0.419451	0.675904	-5.0673	0.73487	0.824114
Monocytes	NAA10	0.05453	5.084078	0.419244	0.676055	-6.1598	0.642002	0.721483
Monocytes	GPR160	0.102879	2.407708	0.419242	0.676056	-5.71179	0.692241	0.777271
Monocytes	TMEM126B	0.09114	2.697386	0.419214	0.676076	-5.52713	0.686599	0.771034
Monocytes	ZNHIT3	-0.07432	3.532147	-0.41911	0.676155	-5.71554	0.670623	0.753338
Monocytes	TOX4	0.032909	5.903687	0.419025	0.676214	-6.36071	0.627437	0.705242
Monocytes	SPA17	0.162685	0.979282	0.418928	0.676285	-5.15624	0.720814	0.808809
Monocytes	APEX2	-0.06722	4.215093	-0.41885	0.676341	-5.80789	0.657857	0.739204
Monocytes	TUT7	0.031	7.206797	0.418829	0.676357	-6.54082	0.605037	0.68014
Monocytes	KLK1B27	-0.18576	-1.42677	-0.41868	0.676469	-4.9194	0.77188	0.864683
Monocytes	RARA	0.043099	5.561492	0.418596	0.676527	-6.3908	0.633473	0.712063
Monocytes	TMEM222	-0.0623	4.289191	-0.41852	0.676579	-5.93255	0.656488	0.737736
Monocytes	4933434E20RIK	0.048365	5.269667	0.418363	0.676696	-6.14314	0.638671	0.717872
Monocytes	DDX31	0.087898	3.220713	0.418361	0.676698	-5.58287	0.676535	0.760003
Monocytes	PECR	-0.17378	2.35098	-0.41833	0.676721	-5.31926	0.693352	0.778614
Monocytes	APBA3	0.113506	2.107298	0.418291	0.676748	-5.42633	0.698146	0.783909
Monocytes	GSK3A	-0.04082	5.557032	-0.41828	0.676754	-6.24717	0.633552	0.712151
Monocytes	CTR9	0.051145	4.518969	0.418099	0.676888	-5.96797	0.65233	0.733047
Monocytes	CAVIN2	-0.14094	2.454343	-0.41795	0.676999	-5.3573	0.691446	0.776409
Monocytes	ARHGEF10	-0.15933	2.266878	-0.41764	0.677225	-5.40519	0.695247	0.780517
Monocytes	RAB3IL1	0.124873	1.73222	0.417598	0.677253	-5.41966	0.705844	0.792223
Monocytes	POLK	0.075701	3.481233	0.417228	0.677523	-5.72252	0.672024	0.754718
Monocytes	GGT5	0.189635	0.649699	0.416486	0.678064	-5.19059	0.728508	0.816804
Monocytes	TOM1	0.053695	5.431971	0.416481	0.678067	-6.22374	0.636578	0.715064
Monocytes	YME1L1	0.028273	6.181942	0.416348	0.678164	-6.34057	0.623375	0.700291
Monocytes	VPS9D1	0.073009	3.05216	0.41619	0.67828	-5.78979	0.680629	0.764057
Monocytes	MRPS7	0.046405	5.010323	0.416118	0.678332	-6.1491	0.644155	0.723547
Monocytes	GSTZ1	-0.08306	4.205829	-0.4161	0.678344	-5.76582	0.658871	0.739935
Monocytes	TBC1D14	0.039912	5.680933	0.416009	0.678412	-6.33009	0.632168	0.710185
Monocytes	ICA1	-0.19507	2.463147	-0.41593	0.678471	-5.21116	0.692043	0.776726
Monocytes	WASHC3	-0.04666	4.517912	-0.41561	0.678701	-6.05298	0.65326	0.733672
Monocytes	TSTD2	0.06798	3.84075	0.415299	0.678929	-5.82103	0.665816	0.747745
Monocytes	TYW3	0.165564	0.995715	0.415267	0.678953	-5.18327	0.721557	0.809313
Monocytes	2510017J16RIK	0.166395	1.414805	0.415165	0.679027	-5.26429	0.713034	0.799957
Monocytes	MEF2A	-0.03291	7.727346	-0.41513	0.679055	-6.70344	0.597235	0.671079
Monocytes	DAB2	-0.11658	4.009728	-0.41503	0.679128	-6.05335	0.662658	0.744252
Monocytes	CFLAR	0.051402	5.971185	0.415011	0.679139	-6.39512	0.627192	0.704702
Monocytes	KRT83	-0.26318	-0.26141	-0.41501	0.679141	-4.97137	0.747793	0.838072
Monocytes	FAM222B	0.05434	5.370089	0.414907	0.679215	-6.21716	0.637838	0.716595
Monocytes	MIB2	0.097348	2.662247	0.41455	0.679475	-5.54451	0.688376	0.772751
Monocytes	GSTM2	-0.17902	1.633616	-0.41438	0.679598	-5.19838	0.708696	0.7952
Monocytes	LCMT1	-0.07562	3.124061	-0.41434	0.679631	-5.70574	0.679462	0.762913
Monocytes	BCL7C	-0.03922	5.355918	-0.41431	0.679653	-6.20712	0.638145	0.716961
Monocytes	5430401H09RII	-0.27894	-0.47304	-0.41428	0.67967	-4.99407	0.752382	0.843106

Monocytes	UCK1	0.097806	3.265642	0.414215	0.67972	-5.61658	0.676755	0.759914
Monocytes	SLC5A6	-0.18037	0.868812	-0.4142	0.67973	-5.1597	0.724229	0.812281
Monocytes	TCP1	-0.04109	6.67677	-0.41401	0.67987	-6.46709	0.615083	0.691107
Monocytes	PEX11G	-0.11162	2.394748	-0.41376	0.680052	-5.4	0.693788	0.778662
Monocytes	SLC11A1	0.062644	3.505984	0.413632	0.680145	-6.31692	0.672371	0.754969
Monocytes	GM20682	0.11139	1.711443	0.413586	0.680178	-5.38676	0.70733	0.793611
Monocytes	P2RX7	-0.07626	3.223954	-0.41343	0.680295	-5.96549	0.677784	0.760924
Monocytes	PPM1D	-0.04967	4.948971	-0.41331	0.680378	-6.01169	0.645687	0.725286
Monocytes	D11WSU47E	0.170793	1.299867	0.413147	0.680499	-5.1783	0.715675	0.802801
Monocytes	TFDP1	0.057721	5.887837	0.412952	0.680642	-6.2837	0.628932	0.706609
Monocytes	ARHGEF2	0.039958	5.586467	0.412939	0.680651	-6.2736	0.634257	0.712563
Monocytes	HDGFL3	-0.1142	1.511028	-0.41281	0.680742	-5.56736	0.711406	0.798132
Monocytes	CDCA2	-0.10418	3.79635	-0.4128	0.68075	-5.75112	0.666942	0.748977
Monocytes	ZFYVE19	-0.07725	3.132938	-0.41275	0.680791	-5.6559	0.679526	0.762941
Monocytes	CENPA	-0.06326	6.681539	-0.41265	0.680859	-6.48347	0.615146	0.691193
Monocytes	MAGT1	0.037052	5.872067	0.412625	0.68088	-6.40139	0.629209	0.706947
Monocytes	CHIL5	-0.17971	-0.02332	-0.41252	0.680954	-5.09401	0.743082	0.832936
Monocytes	COG2	-0.06515	3.567744	-0.41185	0.681445	-5.82392	0.671507	0.75381
Monocytes	WDR70	-0.03558	6.1341	-0.41178	0.681497	-6.36698	0.624858	0.701836
Monocytes	H2-T22	-0.09271	5.391347	-0.41167	0.681576	-6.2074	0.637976	0.7165
Monocytes	ZFP995	-0.09114	2.383748	-0.41162	0.681613	-5.57379	0.694319	0.779054
Monocytes	SLC25A1	0.073403	3.772843	0.411611	0.681621	-5.81299	0.66764	0.74952
Monocytes	SNHG14	0.143188	1.410261	0.411554	0.681662	-5.18015	0.713714	0.800426
Monocytes	ZBTB16	-0.0906	2.808614	-0.41155	0.681666	-5.95758	0.686036	0.769901
Monocytes	ZUP1	0.053398	5.168406	0.411401	0.681774	-6.19566	0.642013	0.720961
Monocytes	ASPM	-0.1252	3.870548	-0.41119	0.681925	-5.81905	0.665931	0.747497
Monocytes	CEP295	-0.06159	3.949897	-0.41091	0.682134	-5.86427	0.664585	0.745908
Monocytes	DCSTAMP	-0.07594	-1.33737	-0.41066	0.682318	-5.62872	0.772144	0.863991
Monocytes	CPS1	-0.13957	4.481272	-0.41047	0.682455	-5.89143	0.654914	0.735065
Monocytes	CDV3	-0.03675	7.090195	-0.41028	0.682593	-6.49632	0.608867	0.683603
Monocytes	GM20342	0.084433	3.499425	0.41015	0.682688	-5.68467	0.673301	0.755484
Monocytes	MED24	0.085252	3.187102	0.410062	0.682752	-5.55325	0.679254	0.762105
Monocytes	MRPS34	0.047444	4.826949	0.410047	0.682763	-6.07768	0.648633	0.728104
Monocytes	HEMK1	-0.1622	1.147753	-0.40993	0.682845	-5.19963	0.719597	0.806604
Monocytes	UTP25	0.098312	2.617836	0.409722	0.683001	-5.48477	0.690363	0.774372
Monocytes	ITPR1	-0.03798	6.992018	-0.40954	0.683136	-6.65514	0.610676	0.685642
Monocytes	CPLX1	-0.29361	-0.57566	-0.40947	0.683184	-4.98121	0.755871	0.846265
Monocytes	GSDMC4	0.13794	0.743558	0.409146	0.683422	-5.29271	0.728231	0.815886
Monocytes	ACTN4	0.030391	6.545001	0.408958	0.683559	-6.45154	0.61854	0.694274
Monocytes	GPHN	-0.03826	7.486182	-0.40889	0.683609	-6.56535	0.602536	0.676323
Monocytes	GINS2	-0.09792	3.493231	-0.40845	0.68393	-5.72224	0.674022	0.75589
Monocytes	WDR5	0.056894	4.681603	0.408253	0.684075	-5.97715	0.651868	0.731315
Monocytes	ILVBL	0.066714	3.757211	0.408172	0.684134	-5.81586	0.66903	0.750376
Monocytes	GBP7	0.119899	4.443583	0.408159	0.684144	-5.95224	0.65624	0.736177
Monocytes	1110065P20RIK	-0.06209	3.514697	-0.40813	0.684162	-5.798	0.673615	0.755457
Monocytes	WDR34	0.192667	1.224098	0.407974	0.684279	-5.19861	0.718713	0.805209
Monocytes	PFKFB1	0.172065	0.961216	0.407713	0.68447	-5.16949	0.724091	0.811159

Monocytes	SPRED3	0.178278	0.719893	0.407666	0.684505	-5.18834	0.729067	0.81662
Monocytes	1700084C06RIK	-0.09664	2.9115	-0.40767	0.684505	-5.59897	0.685213	0.768353
Monocytes	DNAJB14	-0.04212	4.692261	-0.40763	0.684534	-6.38941	0.651715	0.731224
Monocytes	LNCPPARA	0.197771	0.60974	0.407442	0.684668	-5.11532	0.731424	0.819229
Monocytes	TBC1D20	0.045686	5.789234	0.407231	0.684822	-6.25882	0.632123	0.709383
Monocytes	SELENBP2	-0.19668	1.116284	-0.40715	0.684879	-5.17455	0.721069	0.807881
Monocytes	ACSS2	-0.09941	3.033515	-0.40668	0.685226	-5.57585	0.683153	0.765984
Monocytes	EPOR	-0.28394	-0.66556	-0.4066	0.685282	-4.95106	0.758687	0.848922
Monocytes	RANBP17	0.207033	1.134101	0.406601	0.685284	-5.05972	0.72086	0.807555
Monocytes	ACO2	-0.03316	6.116578	-0.40659	0.685289	-6.35827	0.6265	0.703016
Monocytes	PTEN	0.028306	8.288084	0.40638	0.685445	-6.74722	0.589778	0.66179
Monocytes	SETD3	-0.0364	5.687067	-0.40638	0.685447	-6.31842	0.634094	0.711483
Monocytes	GDAP2	0.043576	4.778409	0.406004	0.685721	-6.19005	0.650644	0.729782
Monocytes	ISOC1	-0.04031	4.993923	-0.40583	0.685851	-6.31474	0.646751	0.725475
Monocytes	6330562C20RIK	0.154167	1.275201	0.405782	0.685883	-5.23106	0.71826	0.804557
Monocytes	KLHDC4	0.067969	3.945597	0.405472	0.68611	-5.80198	0.666202	0.747
Monocytes	SSR4	0.032692	7.573816	0.405398	0.686164	-6.71585	0.60191	0.675235
Monocytes	E430024P14RIK	-0.22129	0.53335	-0.40526	0.686266	-5.04167	0.733674	0.821371
Monocytes	HTATSF1	0.047354	4.697996	0.405196	0.686312	-6.03445	0.652264	0.73154
Monocytes	TYSND1	-0.08552	2.640407	-0.40511	0.686377	-5.49303	0.691171	0.774664
Monocytes	CSNK1A1	-0.02366	7.684452	-0.40507	0.686403	-6.64484	0.600062	0.673191
Monocytes	NAA80	0.105292	2.402845	0.404941	0.686499	-5.39807	0.695856	0.779799
Monocytes	5830418P13RIK	-0.24532	1.463039	-0.40462	0.686734	-5.06937	0.714763	0.800461
Monocytes	AKAP7	0.064585	3.374989	0.404473	0.686842	-5.81243	0.677168	0.75901
Monocytes	SEMA4G	-0.19667	0.909795	-0.40447	0.686843	-5.14254	0.726063	0.812861
Monocytes	EFL1	-0.05367	4.5955	-0.40437	0.686916	-5.94266	0.654319	0.733659
Monocytes	2810004N23RIK	-0.06144	4.315225	-0.40409	0.687121	-5.91282	0.659625	0.739484
Monocytes	2310015A10RIK	0.119677	2.091938	0.403996	0.687191	-5.37868	0.702301	0.786685
Monocytes	BLK	0.132509	4.074052	0.403911	0.687254	-5.39735	0.664113	0.744507
Monocytes	SLC35B1	-0.04322	5.751128	-0.40367	0.687433	-6.3081	0.6337	0.710618
Monocytes	2010001A14RIK	0.1515	1.389339	0.403519	0.687541	-5.18851	0.716574	0.802354
Monocytes	NR3C2	0.192917	1.939637	0.403345	0.687669	-5.2005	0.705557	0.790175
Monocytes	FBXO8	0.050972	4.606605	0.403215	0.687763	-5.962	0.654484	0.733722
Monocytes	TFR2	0.163588	1.213769	0.403021	0.687906	-5.16882	0.720324	0.806398
Monocytes	TMEM106B	0.060656	4.565283	0.402764	0.688094	-5.89991	0.655355	0.734661
Monocytes	ZFP414	0.071601	3.443642	0.402755	0.688101	-5.79222	0.676363	0.757958
Monocytes	KCTD7	0.167196	0.387211	0.402634	0.688189	-5.11766	0.737465	0.825176
Monocytes	NAGPA	0.055908	3.354165	0.402558	0.688245	-5.92292	0.678071	0.759848
Monocytes	SLC1A3	-0.26602	-0.29724	-0.40243	0.688335	-5.08077	0.751957	0.841
Monocytes	GMNN	0.063345	5.558334	0.402412	0.688352	-6.25828	0.63736	0.714629
Monocytes	ABCD4	0.088932	2.782602	0.402241	0.688478	-5.49382	0.689092	0.772027
Monocytes	PDE8B	0.175275	-0.24267	0.402111	0.688573	-5.1298	0.75079	0.839728
Monocytes	TMBIM6	-0.0332	9.142023	-0.40188	0.688745	-6.85473	0.576885	0.646788
Monocytes	GM16754	0.208134	0.04601	0.401791	0.688807	-5.06455	0.744651	0.83303
Monocytes	PCNP	0.03015	6.359657	0.401766	0.688826	-6.4062	0.623241	0.698863
Monocytes	BSN	-0.23569	0.764883	-0.40171	0.688864	-5.04856	0.729598	0.816583
Monocytes	PLK4	-0.08125	3.856529	-0.40158	0.688962	-5.88528	0.668544	0.749345

Monocytes	PAFAH1B2	-0.03944	5.489377	-0.40157	0.688972	-6.17164	0.638592	0.716047
Monocytes	FAM57B	-0.21082	0.285081	-0.4015	0.68902	-5.08719	0.739608	0.82757
Monocytes	VPS29	0.030762	6.521761	0.401443	0.689062	-6.48288	0.620428	0.695759
Monocytes	CNOT6L	0.03733	7.08541	0.401441	0.689064	-6.51188	0.610757	0.684927
Monocytes	PRRG1	-0.16973	1.915898	-0.40109	0.689323	-5.24764	0.706182	0.791003
Monocytes	RTN2	0.198606	-0.4318	0.401041	0.689357	-5.08752	0.754842	0.844296
Monocytes	CR1L	-0.03415	5.693128	-0.40087	0.689487	-6.27047	0.634961	0.712094
Monocytes	SPRED2	-0.04793	6.335393	-0.40082	0.689519	-6.27965	0.623664	0.699474
Monocytes	IL20RB	-0.0796	3.81612	-0.40082	0.689523	-5.7319	0.669305	0.750291
Monocytes	ZFP746	-0.06639	3.717554	-0.40076	0.689562	-5.74537	0.671165	0.752352
Monocytes	EI24	-0.04423	4.630195	-0.40073	0.689587	-6.07119	0.654162	0.733481
Monocytes	NCAPH	-0.0889	4.175802	-0.40054	0.689722	-5.91954	0.662567	0.742818
Monocytes	PDCD6IP	-0.02348	6.988379	-0.40044	0.689798	-6.56248	0.61241	0.686893
Monocytes	RBPMS2	-0.11581	1.246218	-0.40042	0.689813	-5.33244	0.719705	0.805904
Monocytes	GTF2IRD2	0.069455	3.944401	0.400371	0.689849	-5.78698	0.666893	0.747639
Monocytes	TCEA3	-0.1233	2.275566	-0.40037	0.689853	-5.53694	0.699034	0.783171
Monocytes	EME1	-0.11807	2.299791	-0.39974	0.690314	-5.4397	0.698901	0.782768
Monocytes	ACBD6	-0.03913	5.436733	-0.39972	0.690325	-6.19103	0.63985	0.71733
Monocytes	USP42	0.06924	3.500066	0.399545	0.690455	-5.66586	0.675647	0.757125
Monocytes	APOO	-0.0705	3.509146	-0.39945	0.690527	-5.70577	0.675474	0.75699
Monocytes	MOB1A	0.032354	5.970142	0.399372	0.690582	-6.44554	0.630394	0.706884
Monocytes	RAP1B	0.021724	8.543309	0.399304	0.690632	-6.87509	0.586832	0.658031
Monocytes	SNHG3	-0.06029	5.291632	-0.39899	0.690863	-6.1376	0.642479	0.720451
Monocytes	MRPL49	0.080432	3.778216	0.398952	0.690891	-5.69945	0.670375	0.751451
Monocytes	INPPL1	-0.09403	2.471527	-0.39886	0.690956	-5.4917	0.695542	0.779273
Monocytes	GM15492	-0.16833	0.742028	-0.39886	0.690957	-5.15809	0.730459	0.817649
Monocytes	OLFR56	0.176145	2.067818	0.398853	0.690963	-5.31478	0.703526	0.788071
Monocytes	STARD10	-0.10288	4.596544	-0.39869	0.691085	-5.94842	0.655128	0.734567
Monocytes	CHADL	-0.19578	0.647864	-0.39862	0.691131	-5.07947	0.732414	0.819875
Monocytes	RBM10	-0.04981	4.779494	-0.39861	0.691141	-5.99873	0.651772	0.730874
Monocytes	RBPMS	0.049759	5.842983	0.398523	0.691205	-6.44882	0.63264	0.709599
Monocytes	1700034P13RIK	0.191537	1.464772	0.398339	0.69134	-5.22647	0.71566	0.801533
Monocytes	FZD5	-0.09103	2.536377	-0.39832	0.691356	-5.55556	0.694288	0.778004
Monocytes	SUDS3	0.045775	5.515338	0.398106	0.691512	-6.21689	0.638567	0.716117
Monocytes	KCTD18	0.108162	3.044012	0.397963	0.691617	-5.57423	0.684537	0.767161
Monocytes	7-Mar	0.026343	6.926226	0.397816	0.691725	-6.54388	0.613965	0.688594
Monocytes	DNAJC14	-0.04196	4.252973	-0.39749	0.691966	-6.01717	0.661809	0.741867
Monocytes	ALDOC	0.149624	0.884667	0.39743	0.692008	-5.3094	0.727869	0.814723
Monocytes	ANP32A	-0.02187	7.495527	-0.39717	0.692199	-6.58892	0.604555	0.677873
Monocytes	SOD3	0.17555	1.286857	0.396973	0.692344	-5.22991	0.719826	0.805734
Monocytes	DOCK3	-0.15724	0.039329	-0.39675	0.692505	-5.27189	0.745879	0.834223
Monocytes	PDXK	0.047527	4.441778	0.396299	0.692839	-6.17538	0.658717	0.738211
Monocytes	ZFP39	-0.17916	1.023509	-0.39626	0.692869	-5.15193	0.725458	0.811842
Monocytes	TRIM30C	0.133972	1.320225	0.396239	0.692883	-5.42839	0.71938	0.805177
Monocytes	DPYS	-0.13531	2.829004	-0.39622	0.6929	-5.53168	0.689324	0.772097
Monocytes	MEX3C	-0.04376	5.133199	-0.39602	0.693043	-6.12888	0.646132	0.724152
Monocytes	GM13684	0.082695	3.516914	0.395875	0.693151	-5.73715	0.676197	0.757475

Monocytes	TESMIN	0.158148	0.971475	0.395635	0.693328	-5.33054	0.726678	0.813111
Monocytes	TICAM1	0.076737	2.819811	0.395634	0.693328	-5.73532	0.689644	0.772385
Monocytes	STK16	0.058772	4.37442	0.395525	0.693408	-5.97298	0.660099	0.739682
Monocytes	DEK	-0.03516	7.842	-0.39549	0.693436	-6.70058	0.599149	0.671617
Monocytes	RFC1	0.048773	5.999025	0.395278	0.69359	-6.28266	0.63072	0.707041
Monocytes	PLPP5	0.069972	3.394162	0.395263	0.693601	-5.7584	0.678564	0.760214
Monocytes	PIF1	0.170172	1.716548	0.394992	0.6938	-5.32278	0.711496	0.796574
Monocytes	CATSPERG1	-0.17235	0.160917	-0.39499	0.693804	-5.23458	0.7436	0.831751
Monocytes	GOLGB1	0.039481	5.4427	0.394893	0.693873	-6.24597	0.640614	0.718138
Monocytes	ZFP407	0.03132	7.039039	0.394724	0.693997	-6.52475	0.612681	0.686945
Monocytes	RECQL5	-0.0778	3.722047	-0.39471	0.694009	-5.65274	0.672325	0.753399
Monocytes	TIMM22	0.051303	4.4471	0.394669	0.694038	-5.96107	0.658755	0.738369
Monocytes	MGAT5	0.041854	6.916886	0.394623	0.694071	-6.43697	0.614769	0.68933
Monocytes	KLHL28	0.073779	3.44241	0.394588	0.694097	-5.65698	0.677642	0.759339
Monocytes	UFM1	-0.03963	5.448609	-0.39436	0.694268	-6.24265	0.640605	0.718097
Monocytes	LSM8	0.044248	5.264944	0.394126	0.694437	-6.17189	0.643957	0.721883
Monocytes	JUNOS	0.140885	1.820722	0.394106	0.694452	-5.34651	0.709562	0.79449
Monocytes	HEXIM2	-0.18801	0.998422	-0.394	0.694533	-5.11487	0.726306	0.812855
Monocytes	CHAMP1	0.091959	2.843874	0.393726	0.694732	-5.52508	0.689415	0.77224
Monocytes	PTGES2	0.098382	2.422157	0.393648	0.694789	-5.46525	0.697676	0.781383
Monocytes	CLTC	-0.02576	7.90636	-0.39364	0.694798	-6.68299	0.598287	0.670765
Monocytes	AKT3	0.043256	6.643383	0.393333	0.69502	-6.50072	0.619771	0.694806
Monocytes	GM16337	0.132446	1.967439	0.39333	0.695022	-5.35572	0.7068	0.791373
Monocytes	CHCHD3	0.026196	6.768694	0.393036	0.695239	-6.46726	0.61774	0.692471
Monocytes	ATG4A	0.070055	4.314693	0.392951	0.695302	-5.88035	0.661667	0.741433
Monocytes	YRDC	-0.04518	5.035051	-0.39279	0.69542	-6.18595	0.648446	0.726736
Monocytes	BBIP1	0.039096	5.888435	0.392729	0.695465	-6.32898	0.633135	0.709693
Monocytes	SUGT1	-0.03263	5.949959	-0.39259	0.69557	-6.35438	0.632047	0.708494
Monocytes	MINPP1	0.046169	4.734583	0.392458	0.695665	-6.04298	0.653935	0.732877
Monocytes	TELO2	0.119825	2.455163	0.392453	0.695668	-5.48167	0.697294	0.780871
Monocytes	PDE12	-0.05451	3.991564	-0.39218	0.695868	-5.84654	0.667732	0.748193
Monocytes	MFSD2B	0.162882	0.599514	0.392157	0.695886	-5.1525	0.734928	0.8222
Monocytes	RASSF2	-0.0531	4.259468	-0.39213	0.695904	-6.09594	0.662721	0.742634
Monocytes	TECPR2	-0.05697	3.905519	-0.39211	0.695922	-5.99184	0.66935	0.749987
Monocytes	SEL1L	-0.03857	5.161227	-0.39199	0.696008	-6.25205	0.646176	0.724253
Monocytes	KCNIP4	0.185806	0.608323	0.391793	0.696154	-5.33983	0.73485	0.822067
Monocytes	PLK1	0.116455	3.704872	0.391664	0.696249	-5.86593	0.673267	0.754305
Monocytes	SPATA24	-0.13827	2.193104	-0.39116	0.696622	-5.33771	0.70283	0.786814
Monocytes	UCP2	-0.03505	9.571709	-0.39114	0.696638	-6.97804	0.571842	0.640752
Monocytes	RCOR1	0.039082	6.85813	0.391037	0.696711	-6.4411	0.616535	0.691024
Monocytes	EIF1AX	0.038021	6.060349	0.390953	0.696772	-6.36983	0.630415	0.706562
Monocytes	RMND5A	0.038349	6.766957	0.390792	0.696891	-6.38897	0.618104	0.692818
Monocytes	PEX19	0.067032	3.955615	0.390758	0.696916	-5.75194	0.668743	0.74922
Monocytes	PRRG4	-0.22405	0.01648	-0.39073	0.696938	-4.99285	0.747579	0.835926
Monocytes	CCDC102A	-0.08025	2.048525	-0.39065	0.696996	-5.81677	0.70571	0.790044
Monocytes	CAVIN1	-0.20624	1.312054	-0.39053	0.697083	-5.14717	0.720604	0.806384
Monocytes	TMEM126A	0.037038	5.857631	0.390211	0.697319	-6.26221	0.634127	0.710656

Monocytes	GRIP1	-0.19944	2.360437	-0.39019	0.697336	-5.23233	0.699656	0.78331
Monocytes	FAM222A	-0.11841	2.331884	-0.3898	0.697623	-5.3167	0.700444	0.784005
Monocytes	GM19325	-0.15826	1.67424	-0.38948	0.697855	-5.29242	0.713674	0.798592
Monocytes	ADD1	-0.04104	5.795836	-0.38946	0.697873	-6.25541	0.635493	0.712058
Monocytes	BCOR	-0.05356	5.214585	-0.38943	0.697895	-6.08633	0.64592	0.723679
Monocytes	VRK2	0.038922	5.691956	0.389295	0.697994	-6.4209	0.637373	0.714143
Monocytes	TMCC2	-0.15743	1.686886	-0.38902	0.698196	-5.41752	0.713549	0.798488
Monocytes	4930403D09RII	-0.15265	1.041064	-0.38893	0.698265	-5.2248	0.72673	0.812966
Monocytes	IPMK	0.03969	5.867681	0.388897	0.698287	-6.3534	0.634334	0.710808
Monocytes	STX4A	-0.03753	4.972734	-0.38863	0.698481	-6.19127	0.650552	0.728832
Monocytes	PCMTD1	0.049726	5.924056	0.388351	0.69869	-6.18599	0.633581	0.709823
Monocytes	NIT1	0.067533	3.750254	0.388244	0.698768	-5.78061	0.673438	0.75414
Monocytes	LRRC49	0.12558	1.020554	0.387847	0.699062	-5.35318	0.727684	0.813771
Monocytes	MEF2B	-0.07322	3.605786	-0.38772	0.699152	-5.71818	0.676426	0.757357
Monocytes	PROK2	0.295663	-0.61756	0.387523	0.6993	-5.01987	0.762449	0.851671
Monocytes	MVB12A	0.036188	5.948563	0.387471	0.699339	-6.36961	0.633435	0.709544
Monocytes	USP30	0.108131	2.252852	0.387275	0.699483	-5.35634	0.702863	0.78641
Monocytes	ARL8B	-0.03023	5.97123	-0.38717	0.699561	-6.40517	0.633086	0.709105
Monocytes	SLC25A53	-0.07913	3.336328	-0.38711	0.699608	-5.58764	0.68169	0.763075
Monocytes	PAH	-0.14501	3.508909	-0.38704	0.69966	-5.66618	0.678383	0.759443
Monocytes	RNASEK	0.030063	6.735066	0.386924	0.699742	-6.50201	0.619743	0.694238
Monocytes	SLC37A2	-0.0942	3.315046	-0.38676	0.699865	-5.64755	0.682163	0.763597
Monocytes	PER2	-0.14754	2.222784	-0.38668	0.699924	-5.2997	0.703527	0.787178
Monocytes	CZIB	-0.06673	3.658795	-0.38621	0.70027	-5.76235	0.675858	0.756373
Monocytes	MRPS12	-0.04644	4.774775	-0.38602	0.700409	-6.11504	0.655041	0.733244
Monocytes	PRPSAP2	0.054065	4.045546	0.385948	0.700462	-5.91808	0.6686	0.74828
Monocytes	RND3	-0.06729	2.943599	-0.38579	0.700578	-6.01516	0.689719	0.771581
Monocytes	ALPK3	0.239933	-0.11417	0.385718	0.700632	-5.01535	0.752142	0.840036
Monocytes	YTHDC2	0.046853	4.693584	0.385469	0.700816	-6.12613	0.65668	0.735014
Monocytes	RABL2	-0.15292	1.106471	-0.38538	0.700881	-5.17089	0.726635	0.812139
Monocytes	FARSB	0.04383	5.008108	0.385135	0.701063	-6.12101	0.650982	0.728647
Monocytes	ZFP931	-0.10972	1.514653	-0.38505	0.701125	-5.35744	0.718356	0.803056
Monocytes	SPRTN	-0.07675	3.419835	-0.38501	0.701157	-5.68498	0.680707	0.761619
Monocytes	LRRK2	-0.04687	4.404591	-0.38466	0.701415	-6.46488	0.662236	0.741107
Monocytes	MBTPS1	0.046638	4.639311	0.384639	0.701429	-6.00485	0.657884	0.73628
Monocytes	NBAS	0.052843	4.535139	0.384353	0.70164	-5.9851	0.659948	0.738424
Monocytes	ART3	0.167353	0.944132	0.384172	0.701773	-5.16747	0.730436	0.815988
Monocytes	TMEM132B	-0.208	0.329919	-0.38388	0.701989	-5.05603	0.743396	0.830048
Monocytes	PRRG2	-0.08956	2.322172	-0.38385	0.70201	-5.51128	0.702583	0.785345
Monocytes	A630072M18RI	0.096036	3.151561	0.383743	0.702091	-5.55723	0.686327	0.767481
Monocytes	ITGB3BP	0.081524	3.52337	0.383664	0.702148	-5.50443	0.679172	0.759582
Monocytes	ACOT8	0.055097	4.129949	0.383448	0.702308	-5.92132	0.667764	0.746922
Monocytes	MAP1LC3B	0.041709	7.434486	0.383257	0.702449	-6.52779	0.608876	0.681277
Monocytes	IFI208	-0.15619	2.936618	-0.38287	0.702732	-5.45298	0.69083	0.772294
Monocytes	KIFAP3	0.063618	2.849059	0.382783	0.702799	-5.71578	0.69254	0.774191
Monocytes	EXOC6	0.036887	6.194009	0.382763	0.702814	-6.39472	0.630446	0.705363
Monocytes	USP34	0.028343	7.703229	0.382401	0.703082	-6.63339	0.604661	0.676506

Monocytes	AMIGO1	0.170418	0.613629	0.38201	0.70337	-5.08182	0.738107	0.824225
Monocytes	RAD51AP1	0.107895	3.61991	0.381858	0.703483	-5.78876	0.677944	0.758188
Monocytes	SMAD5	0.06333	3.702902	0.381714	0.703589	-5.68903	0.676362	0.7565
Monocytes	IAH1	-0.05213	4.521488	-0.38166	0.70363	-6.10404	0.66097	0.739469
Monocytes	GJA1	-0.21799	1.208053	-0.38158	0.703691	-5.11056	0.725763	0.810864
Monocytes	ZFP706	0.025679	7.871583	0.381512	0.703738	-6.66833	0.601928	0.673622
Monocytes	URB2	0.086245	2.571455	0.381429	0.7038	-5.57241	0.698292	0.780725
Monocytes	GM26801	-0.21794	0.557742	-0.38141	0.703814	-5.06399	0.739279	0.825674
Monocytes	IQGAP2	-0.03516	7.075634	-0.38119	0.70398	-6.61217	0.615399	0.688788
Monocytes	SNX16	-0.08413	2.847666	-0.38104	0.704086	-5.57995	0.692867	0.774875
Monocytes	ARFGEF2	-0.04218	6.285468	-0.38096	0.704144	-6.32797	0.62911	0.704173
Monocytes	GSTO1	0.034871	4.594828	0.380879	0.704206	-6.2587	0.65961	0.73811
Monocytes	FAAP24	0.161733	2.002599	0.380844	0.704233	-5.27549	0.709613	0.793313
Monocytes	ZW10	0.06468	4.014837	0.38081	0.704258	-5.81653	0.67045	0.750123
Monocytes	SENP8	-0.16986	0.568146	-0.38079	0.704276	-5.20419	0.739061	0.825573
Monocytes	AKAP8L	0.054366	4.8709	0.38073	0.704317	-6.04572	0.654519	0.732459
Monocytes	SPNS2	-0.16305	1.813184	-0.38069	0.704347	-5.15509	0.713427	0.797502
Monocytes	NUP133	-0.06357	3.691763	-0.38065	0.704377	-5.74048	0.676574	0.756898
Monocytes	MAFK	-0.06523	4.524193	-0.38044	0.704532	-6.05942	0.661003	0.739568
Monocytes	COA6	0.05438	4.110702	0.380305	0.704631	-5.93392	0.668739	0.748153
Monocytes	GSK3B	0.024246	8.105386	0.380178	0.704725	-6.67948	0.598119	0.66947
Monocytes	ANKRD22	-0.22519	-0.56253	-0.38015	0.704749	-4.9795	0.763316	0.851993
Monocytes	GM42659	0.049993	4.567867	0.380066	0.704808	-6.05055	0.660203	0.738754
Monocytes	PUS3	0.097685	2.37825	0.379857	0.704963	-5.38223	0.702303	0.785187
Monocytes	TXNL4B	-0.10708	1.740021	-0.37965	0.705117	-5.33984	0.715127	0.799245
Monocytes	HINT1	-0.02955	7.995864	-0.37963	0.705129	-6.74672	0.60004	0.671532
Monocytes	PRPF38B	0.023782	6.611272	0.379499	0.705227	-6.48028	0.623608	0.697919
Monocytes	6030443J06RIK	0.169179	0.848488	0.379291	0.705381	-5.12237	0.733429	0.819293
Monocytes	EXOC3	0.039754	5.06994	0.379278	0.705391	-6.20851	0.651076	0.72852
Monocytes	ALKBH6	-0.06806	2.755561	-0.37925	0.705415	-5.60809	0.694885	0.776989
Monocytes	GM16286	-0.03378	5.859685	-0.37919	0.705455	-6.34218	0.636836	0.712677
Monocytes	ZFP101	-0.08541	2.916235	-0.37858	0.70591	-5.54461	0.692049	0.773709
Monocytes	AK7	-0.1544	1.686834	-0.37856	0.705922	-5.30034	0.716524	0.800619
Monocytes	TOR1B	-0.05342	3.704769	-0.3785	0.705968	-5.92585	0.676836	0.756918
Monocytes	GALM	-0.10051	2.214948	-0.37834	0.706084	-5.58674	0.705945	0.788978
Monocytes	FLCN	0.054806	4.338996	0.377918	0.706397	-6.02022	0.664975	0.743749
Monocytes	MCPH1	0.04896	4.913551	0.377911	0.706403	-6.05415	0.654334	0.731948
Monocytes	SP4	-0.05466	5.254382	-0.3779	0.706412	-6.04643	0.648112	0.725035
Monocytes	ACYP1	0.066785	3.847714	0.377896	0.706414	-5.78904	0.674227	0.753989
Monocytes	NCKAP5LOS	-0.21389	0.041134	-0.37773	0.706538	-5.06648	0.750933	0.838174
Monocytes	2310016D23RII	0.223702	-0.3578	0.377636	0.706606	-5.01187	0.759503	0.847534
Monocytes	RCOR3	0.078713	2.937283	0.377546	0.706672	-5.64732	0.691782	0.773409
Monocytes	LEKR1	-0.1512	1.047991	-0.37749	0.706711	-5.31583	0.729766	0.815126
Monocytes	PLEKHJ1	-0.02893	5.969804	-0.37733	0.706831	-6.40735	0.635341	0.710836
Monocytes	CCDC61	-0.08965	2.934126	-0.37723	0.706908	-5.49036	0.691906	0.77355
Monocytes	TAF4	-0.05176	4.075399	-0.37689	0.707161	-5.88037	0.67019	0.749443
Monocytes	RPF2	0.053577	4.402771	0.37668	0.707314	-6.0669	0.664132	0.742702

Monocytes	TTL	0.1015	1.740811	0.376522	0.707431	-5.42105	0.715971	0.799802
Monocytes	PCF11	-0.03804	6.482932	-0.37635	0.707557	-6.45001	0.626606	0.700921
Monocytes	TOP2A	-0.07864	7.615909	-0.37633	0.707574	-6.6299	0.607144	0.67916
Monocytes	PSMB6	0.035689	6.304312	0.376007	0.707812	-6.43455	0.629891	0.704501
Monocytes	RETREG3	0.042566	5.527566	0.375867	0.707916	-6.17106	0.643723	0.719916
Monocytes	TNFRSF11A	-0.06653	2.190262	-0.37557	0.708136	-6.02401	0.707108	0.790065
Monocytes	9230111E07RIK	0.184712	-0.18455	0.375524	0.70817	-5.12559	0.75638	0.843944
Monocytes	2810408I11RIK	-0.13658	1.398496	-0.37541	0.708256	-5.31202	0.723135	0.807648
Monocytes	LUM	0.20808	0.551186	0.375342	0.708305	-5.09363	0.740728	0.826884
Monocytes	ALPL	-0.18413	1.490191	-0.37534	0.708307	-5.1185	0.721259	0.805592
Monocytes	XAB2	0.063622	3.922858	0.375326	0.708317	-5.84723	0.673379	0.752885
Monocytes	NUDCD3	-0.03164	5.998557	-0.37529	0.708345	-6.28326	0.635297	0.710613
Monocytes	SYMPK	0.050707	4.293778	0.374741	0.708751	-5.97016	0.66662	0.745161
Monocytes	RAD51D	-0.07451	2.984907	-0.37471	0.708776	-5.63504	0.69165	0.772803
Monocytes	D130020L05RIK	0.175769	0.782996	0.37469	0.708788	-5.13578	0.736122	0.821587
Monocytes	ARL14EP	0.074518	3.524764	0.3743	0.709077	-5.66298	0.6814	0.761317
Monocytes	GM553	-0.17071	0.241543	-0.37422	0.709134	-5.17515	0.747746	0.834083
Monocytes	GM5547	0.105752	1.560402	0.374144	0.709193	-5.42766	0.720282	0.804098
Monocytes	MRPL18	0.040214	6.633228	0.374038	0.709272	-6.45639	0.624545	0.69825
Monocytes	LSM4	0.032019	6.704001	0.373899	0.709375	-6.49871	0.623346	0.696893
Monocytes	ATP9A	-0.19274	0.837325	-0.37372	0.709506	-5.10196	0.735319	0.820539
Monocytes	EIPR1	-0.04023	4.924643	-0.37355	0.709633	-6.14453	0.655265	0.732428
Monocytes	MPST	0.06864	3.682692	0.373287	0.709829	-5.78514	0.678541	0.758268
Monocytes	ECHDC3	-0.19697	0.833138	-0.3731	0.70997	-5.13944	0.735467	0.820854
Monocytes	TBC1D5	-0.03308	7.293343	-0.373	0.710041	-6.64363	0.613303	0.685834
Monocytes	PRX	0.186109	1.104861	0.372961	0.71007	-5.08247	0.72982	0.814716
Monocytes	DHCR7	-0.10871	2.184949	-0.37294	0.710084	-5.30271	0.707834	0.790619
Monocytes	BEND6	-0.16424	0.170455	-0.37279	0.710199	-5.20023	0.74944	0.836133
Monocytes	PPP1R8	0.057543	4.114233	0.372705	0.71026	-5.85873	0.670351	0.7493
Monocytes	CASP12	0.18418	-0.47839	0.372702	0.710263	-5.07882	0.763401	0.851323
Monocytes	OGFOD2	0.059096	3.850672	0.372673	0.710284	-5.81673	0.67534	0.754817
Monocytes	SPACA6	-0.15772	1.094819	-0.37266	0.710295	-5.17007	0.730028	0.814945
Monocytes	RGL2	-0.07179	3.300422	-0.37236	0.710516	-5.72796	0.68602	0.766585
Monocytes	TBCK	0.041987	5.26974	0.372229	0.710613	-6.16185	0.649081	0.725707
Monocytes	PSMA4	-0.02994	6.877207	-0.37219	0.710645	-6.57026	0.620571	0.693945
Monocytes	MTHFD2L	-0.08232	3.033816	-0.37212	0.710697	-5.54713	0.691198	0.772311
Monocytes	SNRPC	-0.03145	6.245324	-0.372	0.710783	-6.39091	0.631625	0.706284
Monocytes	GM37612	-0.18294	0.985122	-0.37142	0.711211	-5.06164	0.732779	0.817672
Monocytes	DENND6B	-0.16002	0.94417	-0.37126	0.711331	-5.14098	0.73363	0.818662
Monocytes	MED12	0.064759	3.781462	0.371225	0.711358	-5.77098	0.677097	0.756556
Monocytes	PIGB	-0.09339	2.703863	-0.37119	0.711384	-5.48349	0.697984	0.779584
Monocytes	KCTD21	0.169268	0.182578	0.371082	0.711464	-5.21428	0.749669	0.836188
Monocytes	TUBE1	-0.15245	1.254723	-0.37097	0.711544	-5.19271	0.727198	0.811673
Monocytes	CCL2	0.087032	3.089272	0.370961	0.711554	-6.24189	0.690433	0.771306
Monocytes	6230400D17RIK	0.109985	2.163361	0.370842	0.711642	-5.36654	0.708749	0.791429
Monocytes	ZFHX3	-0.04246	5.163754	-0.37061	0.711817	-6.32724	0.651356	0.728104
Monocytes	DCTN3	0.03259	5.963361	0.370589	0.71183	-6.40055	0.636939	0.712074

Monocytes	ZFP991	0.07353	2.80432	0.370535	0.71187	-5.78981	0.696057	0.777521
Monocytes	MIEF1	0.061779	4.21125	0.370206	0.712114	-5.90473	0.669174	0.747668
Monocytes	DHDDS	0.050782	4.630539	0.369956	0.7123	-6.02875	0.661409	0.739021
Monocytes	CLPP	-0.04604	4.725598	-0.36992	0.712325	-6.07194	0.659647	0.737068
Monocytes	C9	0.257741	0.60037	0.369763	0.712443	-5.09289	0.741169	0.826687
Monocytes	LANCL2	-0.07773	2.807486	-0.36971	0.712483	-5.66049	0.696269	0.777494
Monocytes	TBP	-0.0782	3.21522	-0.36956	0.712595	-5.65765	0.68832	0.768724
Monocytes	SELENOH	-0.06127	5.20549	-0.36951	0.712632	-6.21414	0.650866	0.727313
Monocytes	GM28112	-0.20182	0.043725	-0.36931	0.712783	-5.09635	0.75309	0.839627
Monocytes	PSMC3	-0.03118	6.11628	-0.36865	0.713268	-6.40429	0.634935	0.709189
Monocytes	MZT1	0.038307	5.278837	0.368208	0.713598	-6.10528	0.650132	0.726072
Monocytes	GM32401	-0.08688	2.445198	-0.36818	0.71362	-5.67479	0.704098	0.785649
Monocytes	DDX54	-0.03742	5.820595	-0.36817	0.713626	-6.32495	0.640347	0.715203
Monocytes	BMYC	0.0654	3.380337	0.367494	0.714128	-6.06719	0.686179	0.765621
Monocytes	MAZ	0.050118	6.241133	0.367191	0.714354	-6.3728	0.633323	0.707087
Monocytes	ONECUT2	-0.16916	1.559667	-0.36714	0.714392	-5.21849	0.722484	0.80547
Monocytes	RFX1	-0.05771	3.263376	-0.36712	0.714409	-5.69832	0.688522	0.768195
Monocytes	PARVB	-0.07036	2.578711	-0.36682	0.714632	-5.88606	0.702046	0.783071
Monocytes	ILRUN	0.036282	5.988088	0.366745	0.714685	-6.42775	0.637899	0.712198
Monocytes	BTBD3	0.146615	1.337187	0.366717	0.714707	-5.29049	0.727147	0.810603
Monocytes	IL12RB1	-0.20837	0.500116	-0.36649	0.714874	-5.09546	0.744624	0.829708
Monocytes	CRBN	0.061056	4.060609	0.366464	0.714895	-5.74598	0.673325	0.751499
Monocytes	EIF3J1	-0.0266	7.268033	-0.3664	0.71494	-6.60987	0.615529	0.687305
Monocytes	PAPOLA	0.021942	7.055885	0.366365	0.714968	-6.55227	0.619175	0.691381
Monocytes	GM42829	0.0881	2.205641	0.366145	0.715132	-5.54138	0.709585	0.791325
Monocytes	MRE11A	0.045195	4.349103	0.36561	0.715529	-5.90897	0.66826	0.745634
Monocytes	1700113A16RII	0.113938	2.10081	0.365553	0.715572	-5.34848	0.711993	0.793778
Monocytes	ACAD12	-0.1705	0.714351	-0.36542	0.715669	-5.1278	0.740522	0.824967
Monocytes	RNF220	-0.04291	5.723291	-0.3653	0.715762	-6.25111	0.643	0.717668
Monocytes	PES1	-0.03783	4.691666	-0.36526	0.715792	-6.16663	0.661861	0.7386
Monocytes	WDR92	-0.083	3.083733	-0.3652	0.715834	-5.65112	0.692497	0.772412
Monocytes	ERAP1	-0.04088	5.228177	-0.3649	0.716058	-6.23937	0.652119	0.727684
Monocytes	CORO2A	0.042983	5.527432	0.364723	0.716189	-6.36988	0.646679	0.721646
Monocytes	GM43251	0.250163	-0.97845	0.364663	0.716234	-4.99654	0.777223	0.864709
Monocytes	GM13402	0.18368	0.307958	0.3646	0.71628	-5.10123	0.749286	0.834425
Monocytes	ACADL	0.037655	6.734458	0.364461	0.716384	-6.48374	0.625235	0.69789
Monocytes	SCRN3	0.122082	1.928035	0.364447	0.716394	-5.35189	0.715641	0.797778
Monocytes	MAIP1	0.058108	3.588251	0.364292	0.716509	-5.813	0.682914	0.761849
Monocytes	3300005D01RII	-0.1562	0.756323	-0.36404	0.7167	-5.2127	0.739852	0.824322
Monocytes	BTK	0.038013	6.033608	0.363965	0.716753	-6.38678	0.637627	0.711765
Monocytes	KDR	-0.12869	3.318647	-0.36396	0.716759	-5.53423	0.688121	0.767642
Monocytes	BICD1	0.194677	0.681795	0.363842	0.716845	-5.05259	0.741419	0.826051
Monocytes	CYYR1	-0.16898	2.282713	-0.36365	0.716988	-5.25879	0.708542	0.790128
Monocytes	AKAP6	-0.25499	0.299684	-0.36358	0.717037	-5.0884	0.749509	0.834894
Monocytes	CAD	-0.13693	1.795668	-0.36345	0.71714	-5.28747	0.718372	0.800929
Monocytes	EYA3	0.03758	5.473081	0.363229	0.717301	-6.3345	0.647704	0.723065
Monocytes	GTF3C3	-0.07652	3.102011	-0.36308	0.717414	-5.68094	0.692337	0.772395

Monocytes	FLT3L	-0.1061	2.201527	-0.36303	0.71745	-5.42018	0.71017	0.791993
Monocytes	PBRM1	-0.02482	7.416728	-0.36302	0.717457	-6.57634	0.613506	0.684985
Monocytes	EFHD2	-0.02823	6.92246	-0.36297	0.717491	-6.77436	0.622006	0.69449
Monocytes	DPF3	-0.15236	1.667384	-0.36286	0.717577	-5.25765	0.720986	0.803882
Monocytes	FAM220A.1	0.117146	2.200036	0.362594	0.717773	-5.38302	0.7102	0.792156
Monocytes	GM16638	0.11893	2.040296	0.362544	0.717811	-5.29162	0.713416	0.795682
Monocytes	RGS13	0.247756	-1.05629	0.362512	0.717835	-4.95696	0.778997	0.867117
Monocytes	SLAMF9	-0.09383	2.637226	-0.36242	0.7179	-5.74928	0.70148	0.782582
Monocytes	AKTIP	-0.07565	3.102695	-0.36242	0.717906	-5.56594	0.692324	0.772514
Monocytes	DLGAP1	0.148981	1.398192	0.362278	0.718009	-5.23474	0.726505	0.810012
Monocytes	ZBTB17	-0.05737	3.917458	-0.36227	0.718014	-5.9097	0.676614	0.755196
Monocytes	GM26930	0.228797	0.305585	0.362259	0.718023	-5.01288	0.749383	0.834972
Monocytes	GM13830	-0.20875	0.287554	-0.36222	0.718051	-5.06093	0.749767	0.83539
Monocytes	ZSCAN25	-0.11698	1.792602	-0.36221	0.71806	-5.34362	0.718435	0.801181
Monocytes	APIP	-0.04382	4.220389	-0.36214	0.718111	-6.01326	0.670873	0.748885
Monocytes	HIF1AN	-0.07754	3.373427	-0.36206	0.718172	-5.66505	0.68706	0.766779
Monocytes	ADAM32	-0.18819	0.784382	-0.36185	0.718331	-5.189	0.739358	0.824041
Monocytes	9530062K07RIK	-0.1681	0.793704	-0.36159	0.718522	-5.13812	0.739212	0.823889
Monocytes	PCP4L1	-0.20532	1.36121	-0.36152	0.718576	-5.15191	0.727409	0.811016
Monocytes	ZBTB25	0.073831	3.709262	0.361513	0.718579	-5.72719	0.680723	0.759744
Monocytes	SNHG8	-0.05423	4.013183	-0.36118	0.718823	-5.88661	0.675093	0.753458
Monocytes	THAP8	-0.22184	-0.29985	-0.36109	0.718896	-5.05794	0.762739	0.849449
Monocytes	TDG	0.042203	5.074746	0.36091	0.719028	-6.09415	0.655329	0.731594
Monocytes	ERCC1	0.066118	3.315868	0.360804	0.719106	-5.70087	0.68856	0.768331
Monocytes	NUDT19	-0.05962	4.544356	-0.3607	0.719185	-5.89411	0.665176	0.742556
Monocytes	2-Mar	0.037708	6.581903	0.360368	0.719432	-6.47564	0.628458	0.701682
Monocytes	MDM1	0.067779	3.41542	0.360204	0.719553	-5.86662	0.686861	0.766389
Monocytes	ERCC3	-0.05342	3.695125	-0.36012	0.719614	-5.8367	0.68147	0.760455
Monocytes	GYS2	0.152564	0.768164	0.359932	0.719756	-5.14782	0.740342	0.824949
Monocytes	GADD45GIP1	-0.043	5.128075	-0.35972	0.719916	-6.15224	0.654724	0.730772
Monocytes	CDKN2AIPNL	-0.05097	4.272414	-0.35946	0.720105	-5.90927	0.67064	0.748431
Monocytes	ZNRF3	-0.0585	5.517512	-0.35939	0.720161	-6.1194	0.64762	0.722956
Monocytes	CLPX	0.039985	5.328169	0.359263	0.720255	-6.14898	0.651064	0.72682
Monocytes	SAMD12	0.173204	0.266619	0.359226	0.720282	-5.24406	0.75105	0.836688
Monocytes	MED17	-0.03464	4.867425	-0.35921	0.720292	-6.09074	0.659528	0.736203
Monocytes	FAM172A	0.029045	7.557353	0.359145	0.720343	-6.60888	0.611794	0.683107
Monocytes	RNF25	0.068423	3.103588	0.359057	0.720409	-5.65875	0.693079	0.7733
Monocytes	HMGB3	-0.0694	4.658182	-0.35891	0.720522	-5.98515	0.663412	0.7406
Monocytes	FUOM	0.105995	2.61981	0.358809	0.720593	-5.60045	0.702608	0.783833
Monocytes	SDCCAG8	0.031838	5.914953	0.358777	0.720617	-6.44612	0.640459	0.715143
Monocytes	GM26632	0.151893	0.366747	0.358702	0.720673	-5.19441	0.748916	0.834516
Monocytes	TACO1	-0.08996	3.738306	-0.3586	0.720748	-5.76066	0.680793	0.759873
Monocytes	AMZ2	-0.05278	3.901939	-0.35857	0.720774	-5.84966	0.677664	0.756427
Monocytes	PEX26	0.145773	1.217016	0.358303	0.72097	-5.24978	0.73113	0.815096
Monocytes	SFT2D3	0.074552	2.864525	0.358265	0.720999	-5.53349	0.697836	0.778601
Monocytes	H2-M3	-0.06508	4.138363	-0.35819	0.721053	-6.05581	0.673237	0.751485
Monocytes	TMEM138	0.085968	2.910493	0.358015	0.721186	-5.4554	0.696931	0.777606

Monocytes	IQCC	-0.13472	1.695654	-0.35793	0.721246	-5.26473	0.721282	0.804325
Monocytes	FAM107B	-0.03417	7.858238	-0.3578	0.721346	-6.67404	0.606758	0.677558
Monocytes	SUMF2	0.087949	2.684259	0.357773	0.721366	-5.51135	0.701397	0.782515
Monocytes	SLC38A2	-0.02611	8.351985	-0.35748	0.721582	-6.74307	0.598503	0.668351
Monocytes	2810006K23RIK	0.110315	2.570884	0.357456	0.721603	-5.4675	0.703646	0.785033
Monocytes	MIER1	-0.02857	6.946828	-0.35745	0.721607	-6.53782	0.622337	0.695008
Monocytes	ARHGAP22	-0.06672	1.023886	-0.35745	0.721609	-6.09677	0.735145	0.81953
Monocytes	SLC24A5	-0.05515	4.423305	-0.35732	0.721701	-5.93524	0.667866	0.745609
Monocytes	PDCL3	0.041318	4.536557	0.357098	0.721869	-6.12443	0.665745	0.743307
Monocytes	GM36447	-0.20018	0.209364	-0.35696	0.721975	-5.03019	0.752345	0.838348
Monocytes	PTPN9	-0.03978	5.706865	-0.35693	0.721997	-6.28834	0.644259	0.719479
Monocytes	ZC3H18	0.039985	5.270525	0.356908	0.722011	-6.18532	0.652178	0.728273
Monocytes	B230118H07RIK	-0.09478	2.933488	-0.35691	0.722012	-5.50385	0.696479	0.777219
Monocytes	WDR47	-0.07317	3.140773	-0.35667	0.722188	-5.66101	0.692418	0.772749
Monocytes	PHKG2	-0.04657	4.528144	-0.35664	0.722208	-5.99579	0.665902	0.743481
Monocytes	CHST7	0.193076	0.725254	0.356644	0.722208	-5.10971	0.741401	0.826425
Monocytes	TMEM38A	-0.12599	1.868955	-0.35652	0.722298	-5.27496	0.717752	0.800573
Monocytes	WHAMM	-0.0499	4.147659	-0.35651	0.722311	-5.98815	0.673061	0.751397
Monocytes	TBC1D25	0.099513	2.839206	0.356072	0.722635	-5.46619	0.698537	0.779374
Monocytes	1700006J14RIK	-0.22065	-0.71534	-0.35605	0.722653	-5.0465	0.772624	0.860259
Monocytes	CNP	-0.05457	5.75023	-0.3558	0.722835	-6.16353	0.643766	0.718794
Monocytes	TRDMT1	0.081905	2.342131	0.355663	0.72294	-5.53998	0.708553	0.79031
Monocytes	NOP16	-0.05418	4.283714	-0.35558	0.723002	-5.96761	0.670817	0.748761
Monocytes	ENPEP	0.199557	0.401804	0.3555	0.723062	-5.04966	0.748605	0.834155
Monocytes	EXT2	0.051702	3.849599	0.355078	0.723377	-5.91298	0.679237	0.758004
Monocytes	BC005561	0.055952	4.273496	0.354819	0.72357	-5.8417	0.671187	0.749218
Monocytes	NPFF	0.139991	0.954229	0.35462	0.723719	-5.19242	0.737151	0.821683
Monocytes	GM48653	-0.1843	-0.16399	-0.35447	0.723831	-5.08399	0.760944	0.847603
Monocytes	KMT2E	0.025257	8.273581	0.354465	0.723835	-6.6826	0.600255	0.670281
Monocytes	CYP2R1	-0.15311	1.009263	-0.35435	0.723923	-5.18393	0.736001	0.820438
Monocytes	DDX20	-0.05918	3.596417	-0.35432	0.723942	-5.69782	0.684096	0.763485
Monocytes	PPP1R14A	-0.12772	0.601813	-0.35432	0.723943	-5.34131	0.744562	0.829775
Monocytes	CD302	0.051396	5.847821	0.354249	0.723996	-6.50641	0.642205	0.717104
Monocytes	AUNIP	0.118566	1.893508	0.354212	0.724023	-5.32135	0.717791	0.800521
Monocytes	GGCX	-0.11771	1.807957	-0.35417	0.724051	-5.31081	0.719531	0.802428
Monocytes	CDIP1	-0.03609	5.935336	-0.35417	0.724054	-6.2811	0.640635	0.715359
Monocytes	C77080	0.352006	0.694235	0.354032	0.724158	-5.05278	0.742628	0.827648
Monocytes	SLC35F5	0.069807	3.11698	0.353903	0.724255	-5.82004	0.693419	0.773739
Monocytes	APOL7E	-0.15356	-0.10248	-0.35388	0.724272	-5.15858	0.759632	0.846158
Monocytes	NEO1	-0.19892	0.660859	-0.35374	0.724377	-5.09913	0.743358	0.828465
Monocytes	ASCC1	0.047419	3.774861	0.353524	0.724537	-5.93763	0.680707	0.759817
Monocytes	CTDSPL2	0.038477	5.892856	0.353521	0.72454	-6.26099	0.641434	0.716313
Monocytes	ADCY10	-0.1644	0.912909	-0.35346	0.724587	-5.1692	0.73806	0.82276
Monocytes	EGF	0.154255	0.634522	0.353398	0.724631	-5.09955	0.743914	0.829146
Monocytes	PREP	-0.04481	5.298922	-0.35275	0.725114	-6.14547	0.652561	0.728451
Monocytes	SMARCA2	0.031981	6.123463	0.352487	0.725312	-6.44026	0.637795	0.712019
Monocytes	GM50334	-0.23741	-0.42372	-0.35216	0.725554	-4.96427	0.767398	0.854349

Monocytes	ATM	-0.06052	4.221236	-0.35205	0.725636	-5.78803	0.672891	0.750893
Monocytes	CENPF	-0.09281	5.050794	-0.35197	0.725696	-6.14858	0.6574	0.733751
Monocytes	CFAP53	-0.18902	0.830789	-0.35178	0.725842	-5.14063	0.740608	0.825241
Monocytes	ZFP239	0.198018	-0.06752	0.351516	0.726037	-4.99432	0.759843	0.846185
Monocytes	BC028528	-0.03328	4.105365	-0.35142	0.726109	-6.40056	0.675242	0.753557
Monocytes	TGFB2	-0.23508	0.46572	-0.35139	0.72613	-5.1504	0.748414	0.833813
Monocytes	FAM120B	0.054709	4.124917	0.351111	0.72634	-5.86948	0.675003	0.753215
Monocytes	UQCC1	0.056524	3.934429	0.350927	0.726478	-5.83165	0.678653	0.757303
Monocytes	HTR1F	0.115226	1.429334	0.350847	0.726538	-5.25967	0.7284	0.811972
Monocytes	WDR48	0.043396	4.298768	0.350787	0.726582	-5.98081	0.671735	0.74968
Monocytes	SYCE2	-0.06764	4.258668	-0.35068	0.72666	-6.02099	0.672492	0.750589
Monocytes	CNOT2	-0.02683	7.008824	-0.35057	0.726748	-6.49325	0.622679	0.6953
Monocytes	9230114K14Rik	-0.09368	2.574674	-0.35054	0.726766	-5.35328	0.705174	0.786636
Monocytes	SVBP	0.041022	5.267752	0.350354	0.726907	-6.19743	0.653726	0.729911
Monocytes	4833408A19Rik	0.177067	0.127774	0.350347	0.726911	-5.15979	0.755823	0.842073
Monocytes	PFKM	-0.13477	1.17862	-0.35019	0.727031	-5.21487	0.733631	0.817901
Monocytes	KRTCAP2	-0.02957	7.062528	-0.35014	0.727069	-6.57007	0.621779	0.694387
Monocytes	EPCAM	-0.13448	2.160039	-0.34974	0.727368	-5.3672	0.713751	0.796047
Monocytes	CBX1	0.035528	6.075378	0.349545	0.727512	-6.30183	0.639411	0.713863
Monocytes	KDM4A	-0.03984	4.161515	-0.34943	0.727598	-6.14594	0.674663	0.75293
Monocytes	TBK1	0.037616	5.964636	0.349348	0.727659	-6.38571	0.641404	0.716067
Monocytes	GPBP1	-0.03006	7.723603	-0.34892	0.727981	-6.62955	0.610935	0.68185
Monocytes	GM26944	0.141641	0.852005	0.348823	0.728051	-5.17724	0.74105	0.825546
Monocytes	FCER2A	0.232552	0.458562	0.348581	0.728233	-5.07964	0.749492	0.834653
Monocytes	MORN2	-0.11906	2.045949	-0.34843	0.728347	-5.29054	0.71656	0.798696
Monocytes	IL15	0.065492	3.93993	0.348115	0.728581	-6.19758	0.67943	0.757791
Monocytes	AURKA	-0.0706	3.318106	-0.34773	0.728869	-5.80475	0.691597	0.771087
Monocytes	DNAJC21	0.037384	5.890572	0.347705	0.728888	-6.29316	0.643406	0.717802
Monocytes	1600002D24Rik	-0.23497	0.257645	-0.34743	0.729091	-5.04768	0.754195	0.839651
Monocytes	TEAD1	-0.16571	1.756692	-0.34743	0.729091	-5.16358	0.722798	0.805402
Monocytes	COL1A2	0.134092	2.442649	0.347349	0.729155	-5.41203	0.708909	0.790234
Monocytes	KLHL36	0.10731	2.076404	0.347332	0.729167	-5.42747	0.716288	0.798323
Monocytes	FIGNL1	-0.0985	2.757262	-0.34715	0.729306	-5.55591	0.702707	0.783411
Monocytes	UBAP2	-0.03276	6.131148	-0.34674	0.729614	-6.34364	0.639261	0.713204
Monocytes	ZFP974	0.159771	0.877863	0.346721	0.729625	-5.20547	0.741211	0.825445
Monocytes	NID1	-0.1283	2.394724	-0.34657	0.72974	-5.30068	0.710045	0.791408
Monocytes	IER2	-0.04161	8.170209	-0.34649	0.729798	-6.78324	0.603984	0.673886
Monocytes	PCYOX1L	0.106449	1.613818	0.34646	0.729821	-5.36822	0.725907	0.808779
Monocytes	RRP7A	-0.05285	3.942345	-0.34643	0.729843	-5.95465	0.679723	0.758054
Monocytes	UFC1	0.026096	5.792898	0.346376	0.729883	-6.35013	0.645334	0.720005
Monocytes	METTL7A1	-0.06536	2.456994	-0.34566	0.730419	-5.70059	0.709252	0.790231
Monocytes	ITGB2	0.039116	6.734664	0.345356	0.730647	-6.69297	0.629027	0.701548
Monocytes	CATSPERE2	0.12437	1.174644	0.345306	0.730684	-5.19019	0.735516	0.818943
Monocytes	NBEAL1	-0.04194	4.937374	-0.34527	0.730708	-6.19215	0.661452	0.737554
Monocytes	GPR65	-0.04924	4.250918	-0.34506	0.730869	-6.37591	0.674325	0.751801
Monocytes	MRPL19	0.053351	3.923908	0.345056	0.730872	-5.86611	0.680555	0.758675
Monocytes	CCNC	0.048731	4.398502	0.345015	0.730902	-6.01219	0.671534	0.748734

Monocytes	EMG1	-0.03433	5.937655	-0.34494	0.73096	-6.37187	0.643181	0.717366
Monocytes	PPP1R15A	-0.04739	7.089434	-0.34488	0.731001	-6.6085	0.62284	0.694736
Monocytes	PELO	-0.06409	3.125716	-0.34458	0.731228	-5.7052	0.696183	0.775841
Monocytes	FAM131A	-0.13333	0.401139	-0.34441	0.731359	-5.36481	0.752049	0.836913
Monocytes	GM30948	0.267583	0.467484	0.344209	0.731506	-4.99298	0.750633	0.835423
Monocytes	2010315B03RIK	0.090799	2.024895	0.344167	0.731538	-5.39861	0.718213	0.800042
Monocytes	CREBL2	-0.0679	3.233581	-0.34417	0.731538	-5.73498	0.694109	0.773592
Monocytes	PRR5L	-0.07032	2.756439	-0.34402	0.73165	-6.01278	0.703534	0.783952
Monocytes	PCGF1	0.117841	1.17851	0.343923	0.731721	-5.2776	0.735657	0.81911
Monocytes	CLCN4	-0.0348	5.289066	-0.34382	0.731795	-6.27282	0.65516	0.730627
Monocytes	XPO1	0.039188	5.786847	0.343784	0.731825	-6.33026	0.646094	0.720571
Monocytes	THEM6	0.0842	3.448768	0.343404	0.73211	-5.59639	0.690047	0.769102
Monocytes	DSN1	-0.09004	2.622227	-0.34336	0.732142	-5.47564	0.706327	0.786993
Monocytes	SEC14L1	-0.04176	4.618739	-0.3433	0.732188	-6.16001	0.667708	0.744468
Monocytes	TRMT2B	0.063161	3.750232	0.343259	0.732219	-5.7642	0.684213	0.762698
Monocytes	ARRB2	-0.04051	5.195443	-0.34306	0.73237	-6.24906	0.657018	0.73268
Monocytes	SERTAD1	-0.04545	5.61417	-0.34302	0.732396	-6.26176	0.649358	0.724188
Monocytes	SMARCC2	0.027664	6.020787	0.342954	0.732447	-6.38163	0.642015	0.716045
Monocytes	1700001K19RIK	-0.12222	1.052633	-0.34249	0.732793	-5.38415	0.73852	0.822273
Monocytes	TIMM29	-0.07141	3.296538	-0.3424	0.732859	-5.63653	0.693114	0.772536
Monocytes	IGLL1	0.230917	3.916663	0.342343	0.732905	-5.45206	0.681113	0.759318
Monocytes	ATP5B	0.028161	8.182512	0.342333	0.732913	-6.73242	0.604576	0.674297
Monocytes	SMC6	-0.03035	7.579287	-0.34232	0.732922	-6.5837	0.614787	0.685713
Monocytes	PGGHG	-0.06879	2.820106	-0.34231	0.732927	-5.74286	0.702492	0.782844
Monocytes	TEX9	0.110994	1.820323	0.34213	0.733065	-5.38073	0.722669	0.80495
Monocytes	MMP11	-0.21368	0.128652	-0.34208	0.733103	-4.95882	0.758195	0.843694
Monocytes	TTBK2	-0.10739	1.314702	-0.34181	0.733303	-5.28863	0.733217	0.8165
Monocytes	POP4	-0.05615	4.085123	-0.34174	0.733359	-5.92339	0.678045	0.75596
Monocytes	LRSAM1	0.106018	1.610096	0.341417	0.7336	-5.35649	0.727246	0.809935
Monocytes	WDR12	-0.05515	4.283459	-0.34137	0.733635	-5.92367	0.674404	0.751886
Monocytes	KIF2C	-0.10824	2.663513	-0.34105	0.733877	-5.53437	0.705986	0.786631
Monocytes	P3H1	-0.16333	0.585168	-0.34102	0.733897	-5.1794	0.748787	0.833411
Monocytes	DLGAP5	0.095985	3.248568	0.340913	0.733978	-5.69933	0.694427	0.773931
Monocytes	FRMD4A	-0.06355	3.969412	-0.34089	0.733995	-6.03595	0.680471	0.75856
Monocytes	TMEM181A	0.043708	4.58903	0.340607	0.734207	-5.9819	0.668845	0.745623
Monocytes	FHAD1	0.096439	0.624492	0.340456	0.734321	-5.63252	0.748088	0.832538
Monocytes	DYRK1B	0.102617	1.826196	0.340346	0.734403	-5.30184	0.723031	0.805202
Monocytes	GM17529	0.187849	0.365187	0.340266	0.734463	-5.04156	0.753618	0.838577
Monocytes	UPP2	0.139134	1.652112	0.340158	0.734545	-5.33967	0.726604	0.809159
Monocytes	ABHD10	0.053848	3.649409	0.340111	0.734579	-5.73696	0.686753	0.765433
Monocytes	DAPK3	-0.0427	4.42231	-0.34005	0.734627	-5.99942	0.671984	0.749161
Monocytes	GM36660	0.162807	-0.14918	0.339875	0.734757	-5.07053	0.764718	0.850691
Monocytes	ASH2L	-0.0493	4.017187	-0.33978	0.734826	-5.84191	0.679681	0.757657
Monocytes	KIF22	-0.09182	4.043139	-0.33977	0.734832	-5.85232	0.679185	0.75711
Monocytes	GSDME	-0.05715	4.386903	-0.33964	0.734935	-6.01864	0.672653	0.749908
Monocytes	SSR1	0.026154	6.554701	0.339498	0.73504	-6.49036	0.633039	0.706023
Monocytes	PSMD10	0.060545	3.584601	0.339437	0.735085	-5.79956	0.688007	0.766886

Monocytes	KLRB1A	-0.16587	-0.08222	-0.33943	0.73509	-5.19357	0.763263	0.84917
Monocytes	4921531C22RIK	0.133817	1.642005	0.339277	0.735206	-5.23807	0.726812	0.809488
Monocytes	POLR2C	0.037558	5.080792	0.339227	0.735243	-6.18124	0.659679	0.735639
Monocytes	URM1	-0.04691	4.343407	-0.33919	0.73527	-5.90799	0.673475	0.750901
Monocytes	8030456M14RI	-0.11573	1.090368	-0.33896	0.735444	-5.32945	0.738361	0.822029
Monocytes	ABHD12	0.031077	5.584506	0.338883	0.735501	-6.4453	0.650522	0.725415
Monocytes	CHID1	-0.06566	2.453126	-0.33863	0.735694	-5.59426	0.710539	0.791547
Monocytes	MRTO4	0.050253	4.665156	0.33825	0.735977	-6.10681	0.667771	0.744392
Monocytes	PCDH9	-0.25146	0.472713	-0.33822	0.735997	-5.04239	0.751719	0.836445
Monocytes	EFNB1	-0.11624	1.912975	-0.33791	0.736234	-5.32045	0.721751	0.803735
Monocytes	VHL	-0.04312	3.486608	-0.33784	0.736286	-5.9335	0.690381	0.769313
Monocytes	GPR19	-0.1032	2.550401	-0.33781	0.73631	-5.37046	0.708859	0.789614
Monocytes	FCHO1	0.070286	3.772111	0.337449	0.736578	-5.74945	0.684987	0.763286
Monocytes	ALG1	-0.06511	3.312271	-0.33743	0.73659	-5.762	0.693918	0.77312
Monocytes	OGA	-0.03035	6.76343	-0.33722	0.73675	-6.47122	0.629998	0.702328
Monocytes	ANG	0.092569	4.211322	0.337108	0.736834	-5.86556	0.676677	0.754044
Monocytes	A930015D03RII	0.052928	4.760503	0.337004	0.736913	-5.95573	0.666329	0.742604
Monocytes	HSF2	-0.04342	4.185277	-0.3368	0.737066	-6.11853	0.677218	0.754634
Monocytes	DENND2C	-0.0807	2.008648	-0.33677	0.737085	-5.5428	0.720097	0.801745
Monocytes	HSD17B10	-0.04053	5.540491	-0.33615	0.737554	-6.26512	0.652154	0.72679
Monocytes	CD72	-0.10376	4.334961	-0.33615	0.737555	-5.53931	0.674583	0.751596
Monocytes	TTLL5	-0.04977	4.471737	-0.33613	0.73757	-5.91429	0.671996	0.74874
Monocytes	CANT1	0.050632	3.891787	0.336113	0.737582	-5.89785	0.683043	0.760924
Monocytes	TXNDC16	-0.05238	5.44957	-0.33569	0.7379	-6.22335	0.654039	0.728709
Monocytes	GM16023	0.099955	1.834877	0.335133	0.738319	-5.35121	0.724406	0.805975
Monocytes	SPDYA	0.176184	-0.02911	0.335035	0.738392	-5.05734	0.763748	0.848805
Monocytes	GRN	-0.03656	7.188808	-0.33503	0.738396	-6.67688	0.623286	0.6944
Monocytes	SNRPF	0.031121	6.76677	0.334896	0.738496	-6.52732	0.630665	0.702622
Monocytes	KCTD3	-0.06805	3.747291	-0.3346	0.73872	-5.61337	0.686342	0.764296
Monocytes	MYO1C	0.031272	5.436164	0.334572	0.73874	-6.27291	0.654554	0.729195
Monocytes	ANO10	0.079938	3.16746	0.33453	0.738772	-5.63144	0.697647	0.776728
Monocytes	OGG1	0.05906	3.189389	0.33452	0.738779	-5.67732	0.697216	0.776254
Monocytes	POGK	0.092721	2.454489	0.334282	0.738958	-5.49269	0.711831	0.792367
Monocytes	ZCWPW1	-0.1022	2.765843	-0.33424	0.738988	-5.35736	0.705598	0.785534
Monocytes	GUCD1	0.057458	3.805941	0.334169	0.739043	-5.89026	0.68521	0.763132
Monocytes	NATD1	0.094668	3.25628	0.334136	0.739068	-5.5186	0.695902	0.774895
Monocytes	BCAS3OS1	0.055455	3.793281	0.334043	0.739138	-5.83878	0.685454	0.763437
Monocytes	DDX10	-0.03385	5.436521	-0.33397	0.739194	-6.20687	0.654547	0.729303
Monocytes	CDPF1	-0.1012	2.220471	-0.33394	0.739213	-5.46837	0.716555	0.797586
Monocytes	1700008J07RIK	-0.12682	1.842472	-0.33366	0.73943	-5.28673	0.724406	0.806125
Monocytes	LMBR1L	-0.05098	4.517308	-0.33353	0.739522	-5.99937	0.671799	0.748375
Monocytes	ZMYM1	-0.07784	3.329996	-0.3332	0.739769	-5.52858	0.694791	0.773641
Monocytes	UXS1	-0.04276	4.429155	-0.333	0.739926	-6.10193	0.673646	0.750392
Monocytes	VPS4B	-0.02691	6.231035	-0.33298	0.739935	-6.42291	0.640487	0.713677
Monocytes	TENT4A	-0.05921	3.460072	-0.33291	0.73999	-5.82218	0.692266	0.770916
Monocytes	PSTPIP2	0.045858	4.482064	0.332664	0.740176	-6.46716	0.67271	0.749296
Monocytes	SASS6	0.050657	4.42508	0.332637	0.740196	-6.01849	0.673788	0.750486

Monocytes	TRA2A	0.026387	7.075692	0.332543	0.740266	-6.54523	0.625639	0.697133
Monocytes	ABCG3	0.053571	4.394356	0.332394	0.740379	-6.1978	0.674413	0.751167
Monocytes	PHLDA1	0.070995	4.035457	0.332279	0.740465	-5.99837	0.68127	0.758771
Monocytes	ARHGEF3	-0.06016	5.916073	-0.33218	0.740537	-6.33758	0.646274	0.720083
Monocytes	INAFM1	0.092296	1.866251	0.332003	0.740672	-5.45581	0.724344	0.805992
Monocytes	SAE1	-0.03424	6.342657	-0.33123	0.741251	-6.37792	0.639117	0.711743
Monocytes	TRAF2	-0.05412	3.917831	-0.331	0.741426	-5.83583	0.684081	0.761451
Monocytes	HIST2H2AC	0.155541	2.376405	0.330963	0.741455	-5.40121	0.714475	0.794823
Monocytes	ESR1	0.090937	3.611322	0.330874	0.741523	-5.69606	0.690009	0.768012
Monocytes	ERGIC2	-0.0253	6.017059	-0.33079	0.741585	-6.33734	0.644969	0.718297
Monocytes	ZFP605	0.129761	1.633403	0.330644	0.741696	-5.27196	0.729653	0.811517
Monocytes	MYO1E	-0.07126	6.198716	-0.33053	0.741778	-6.24175	0.641704	0.714725
Monocytes	GM26885	0.084936	2.317843	0.330415	0.741868	-5.68769	0.715658	0.796248
Monocytes	SLC2A12	0.113036	1.619007	0.330411	0.741871	-5.33217	0.72995	0.81187
Monocytes	PPIL2	-0.0332	5.124237	-0.33036	0.741909	-6.17526	0.661291	0.736429
Monocytes	SAA4	-0.18241	0.640047	-0.33032	0.74194	-5.11208	0.750498	0.834256
Monocytes	TMEM97	-0.06571	3.649444	-0.33016	0.742057	-5.72321	0.689315	0.76729
Monocytes	BLCAP	0.070463	3.009547	0.329875	0.742275	-5.59774	0.702003	0.781116
Monocytes	AGO1	0.066858	3.874896	0.32964	0.742452	-5.70362	0.685194	0.762594
Monocytes	D430042O09RI	-0.0521	3.792114	-0.32899	0.742938	-5.84532	0.686985	0.764477
Monocytes	GM45820	0.105791	1.425778	0.328989	0.742942	-5.28326	0.73447	0.816485
Monocytes	NAIF1	-0.10817	1.251393	-0.32897	0.742954	-5.31347	0.738109	0.820451
Monocytes	RNF138	-0.03115	5.857254	-0.32886	0.743038	-6.29709	0.64831	0.721783
Monocytes	TEX264	-0.04052	4.497588	-0.32886	0.74304	-5.99331	0.673492	0.749628
Monocytes	TAF7	0.035293	5.1473	0.328826	0.743065	-6.13959	0.661325	0.736199
Monocytes	ARHGEF6	0.033606	5.643537	0.328529	0.743288	-6.36321	0.652284	0.726139
Monocytes	PPM1E	0.115989	4.964118	0.328516	0.743298	-5.59979	0.664818	0.740004
Monocytes	CD47	0.022756	8.628324	0.328285	0.743473	-6.79632	0.600307	0.668262
Monocytes	GM48742	0.135085	0.618325	0.32828	0.743476	-5.18066	0.751628	0.8351
Monocytes	SERPINB8	-0.06502	-0.3114	-0.32819	0.743544	-5.78532	0.771757	0.856975
Monocytes	FBXO25	0.073088	2.127104	0.328088	0.743621	-5.45539	0.720178	0.800863
Monocytes	9530052E02RIK	-0.12282	1.000649	-0.32793	0.743742	-5.20406	0.743535	0.826338
Monocytes	TJP1	-0.12287	2.024999	-0.32787	0.743788	-5.27049	0.722269	0.803159
Monocytes	ACER1	-0.24239	-0.7216	-0.32781	0.743831	-5.00124	0.78084	0.866816
Monocytes	GM42047	0.095693	5.263713	0.327478	0.744081	-5.95879	0.659443	0.734018
Monocytes	KLHL32	0.179404	1.452777	0.327434	0.744113	-5.14064	0.734211	0.816143
Monocytes	LRP12	-0.09722	2.191669	-0.32731	0.74421	-5.59833	0.719032	0.799572
Monocytes	TBC1D22A	-0.03073	5.871527	-0.32723	0.744267	-6.37429	0.648334	0.721754
Monocytes	ZBTB12	0.110032	1.665552	0.326961	0.74447	-5.25111	0.729944	0.811416
Monocytes	GTPBP1	-0.04382	4.641527	-0.32688	0.744532	-6.06154	0.671185	0.74694
Monocytes	2510039O18RII	-0.03599	5.179646	-0.32649	0.744822	-6.17179	0.66117	0.735833
Monocytes	MFSD10	-0.04284	4.464143	-0.32647	0.744837	-6.12258	0.674578	0.750637
Monocytes	NFIC	0.04093	4.464453	0.326426	0.744874	-6.09808	0.674572	0.750631
Monocytes	IQGAP1	0.022773	9.247795	0.326421	0.744877	-6.97041	0.590393	0.657058
Monocytes	CCDC127	-0.04231	4.15296	-0.32632	0.744953	-5.91157	0.680503	0.757168
Monocytes	ASF1B	-0.06436	4.954931	-0.32628	0.744981	-6.16584	0.665348	0.740451
Monocytes	CEP170B	-0.14731	0.457754	-0.3261	0.745121	-5.09513	0.755442	0.839149

Monocytes	ALG6	-0.0993	2.044219	-0.32609	0.74513	-5.43667	0.72222	0.802961
Monocytes	TBCB	0.026896	6.070727	0.325924	0.745252	-6.4464	0.644915	0.717866
Monocytes	ABCF3	-0.05226	3.761251	-0.32588	0.745285	-5.78324	0.688076	0.765536
Monocytes	SGO1	0.101818	3.188246	0.325748	0.745385	-5.63148	0.699281	0.777876
Monocytes	MIIP	0.070725	3.057652	0.325692	0.745427	-5.57944	0.701861	0.780717
Monocytes	CD52	0.034363	9.30175	0.32546	0.745602	-7.05098	0.589632	0.656219
Monocytes	CSE1L	0.034279	5.5229	0.325254	0.745757	-6.28094	0.655024	0.729075
Monocytes	COPS3	-0.02859	5.92883	-0.3252	0.745795	-6.33427	0.647627	0.720882
Monocytes	4930455G09RII	-0.05327	1.816178	-0.32503	0.745924	-6.0999	0.727097	0.808316
Monocytes	GM39090	-0.14283	0.497553	-0.32501	0.745945	-5.11927	0.754798	0.83848
Monocytes	SPAG7	0.042904	4.55728	0.324855	0.746058	-6.08705	0.673018	0.748971
Monocytes	YWHAH	-0.02823	7.687722	-0.32482	0.746085	-6.69615	0.616658	0.68647
Monocytes	ANKIB1	0.03179	5.807453	0.324732	0.746151	-6.32362	0.649846	0.723378
Monocytes	CLPTM1L	-0.03673	4.883326	-0.32451	0.74632	-6.18892	0.666978	0.742246
Monocytes	ITFG2	-0.05801	3.893716	-0.32436	0.746432	-5.80621	0.685791	0.763029
Monocytes	SDHA	0.028198	5.691569	0.324254	0.746512	-6.3265	0.652051	0.725805
Monocytes	ATPAF1	0.068428	3.553171	0.324196	0.746555	-5.58903	0.692398	0.770339
Monocytes	PPP2R3D	-0.05985	3.751015	-0.32408	0.746642	-5.81517	0.688551	0.766153
Monocytes	NXT1	0.048571	4.399059	0.32405	0.746665	-5.99468	0.676115	0.752461
Monocytes	YTHDF1	-0.03117	5.78074	-0.32372	0.746913	-6.32912	0.650572	0.724087
Monocytes	KAT5	0.073536	2.921057	0.323615	0.746994	-5.66002	0.705006	0.784094
Monocytes	CAR1	0.237676	0.491473	0.323561	0.747035	-5.07515	0.755211	0.838875
Monocytes	PFDN2	-0.03264	5.031244	-0.32338	0.747169	-6.22027	0.664379	0.739399
Monocytes	2700062C07RIK	-0.06982	2.646093	-0.32331	0.747223	-5.5453	0.710507	0.790146
Monocytes	STK26	0.060843	4.048898	0.323283	0.747245	-5.78324	0.682963	0.759898
Monocytes	IGLV3	-0.2051	-0.70552	-0.32298	0.747471	-4.9595	0.781537	0.867278
Monocytes	RCL1	-0.0497	4.24025	-0.32283	0.747587	-5.88782	0.679442	0.755894
Monocytes	MED28	0.026966	6.023126	0.322805	0.747605	-6.41861	0.646321	0.719292
Monocytes	SIAH1A	-0.04339	5.079563	-0.32246	0.747866	-6.03483	0.663791	0.738476
Monocytes	TNFAIP8L1	-0.0844	2.396525	-0.32212	0.748122	-5.46499	0.716051	0.795814
Monocytes	MTRR	0.123212	1.505683	0.321725	0.748421	-5.26204	0.734457	0.815837
Monocytes	YBX1	0.026428	8.398771	0.321705	0.748435	-6.78504	0.60538	0.673429
Monocytes	TRDJ1	-0.15893	-1.29553	-0.32168	0.748453	-4.98863	0.795331	0.8818
Monocytes	PNPLA6	0.068288	2.760588	0.321377	0.748683	-5.56126	0.708893	0.787907
Monocytes	NUFIP1	-0.03675	4.171093	-0.32132	0.748729	-6.0819	0.681272	0.75759
Monocytes	DBNDD2	0.060857	2.836435	0.321262	0.74877	-5.83232	0.707376	0.78627
Monocytes	KCNG3	-0.11669	0.517714	-0.32124	0.748788	-5.37072	0.755379	0.838614
Monocytes	SLC4A2	-0.05266	3.559435	-0.32115	0.748858	-5.79195	0.693101	0.770615
Monocytes	ALPK2	-0.07532	1.260876	-0.32108	0.748907	-5.67034	0.739618	0.821492
Monocytes	ZFP189	0.132539	1.120206	0.320876	0.749062	-5.16222	0.74266	0.824725
Monocytes	CDC25B	-0.08687	4.400381	-0.3207	0.749192	-5.72361	0.677032	0.752809
Monocytes	NECTIN2	-0.09493	2.109704	-0.32047	0.749366	-5.38048	0.7223	0.802425
Monocytes	SRP54A	-0.05043	3.599318	-0.32011	0.749638	-5.78339	0.692748	0.770005
Monocytes	MYCN	0.204211	-0.18583	0.320012	0.749714	-4.96069	0.77112	0.855429
Monocytes	FDX2	-0.04237	4.192147	-0.31967	0.749975	-5.96662	0.681471	0.757471
Monocytes	RRM2B	-0.05324	4.437435	-0.31943	0.750153	-5.82821	0.676829	0.752376
Monocytes	GUF1	-0.06943	2.589786	-0.31943	0.750154	-5.48842	0.712992	0.792071

Monocytes	GM48027	0.080956	2.815176	0.319329	0.75023	-5.52449	0.708476	0.78712
Monocytes	CCNT2	-0.03095	5.37232	-0.31918	0.750344	-6.27731	0.65933	0.733048
Monocytes	PIBF1	0.039311	5.059147	0.319131	0.75038	-6.13371	0.665142	0.739467
Monocytes	OSGEP	-0.03432	4.907558	-0.31856	0.750811	-6.18834	0.668185	0.742743
Monocytes	STX11	-0.04174	4.829435	-0.31853	0.750833	-6.30371	0.669651	0.74436
Monocytes	CCDC14	0.159847	0.809905	0.318425	0.750913	-5.11951	0.750103	0.832441
Monocytes	JTB	-0.02443	5.842584	-0.3184	0.750929	-6.38118	0.650913	0.72369
Monocytes	SFXN2	0.046498	3.18033	0.318288	0.751017	-5.91351	0.701448	0.779378
Monocytes	2900089D17RIl	0.074848	2.58395	0.318076	0.751178	-5.66964	0.713355	0.792463
Monocytes	8-Mar	0.072329	3.141103	0.318058	0.751191	-5.67772	0.702224	0.780275
Monocytes	SERF1	0.066312	3.137334	0.318053	0.751195	-5.55134	0.702299	0.780357
Monocytes	TMEM170B	0.039377	4.646846	0.318014	0.751224	-6.13223	0.673092	0.748255
Monocytes	A530064D06RIl	0.080252	0.81889	0.317865	0.751337	-5.64065	0.749957	0.83234
Monocytes	TULP3	-0.10137	1.974251	-0.31778	0.751405	-5.35456	0.725801	0.806053
Monocytes	TOR1A	-0.03246	4.653633	-0.31731	0.751759	-6.23574	0.673202	0.748194
Monocytes	PDIA6	0.028919	6.97381	0.317304	0.751761	-6.61378	0.630905	0.701393
Monocytes	DNPH1	0.115463	0.708539	0.317171	0.751862	-5.2865	0.752551	0.834962
Monocytes	SLC5A11	0.158942	-0.01556	0.317098	0.751917	-5.1144	0.768194	0.851917
Monocytes	RBM41	0.051037	3.907296	0.316876	0.752084	-5.86103	0.687501	0.7639
Monocytes	GM14321	0.137094	-0.49943	0.316775	0.752161	-5.14169	0.778853	0.86345
Monocytes	ARMCX3	-0.05592	3.573363	-0.31676	0.752174	-5.79644	0.693995	0.77105
Monocytes	FIGNL2	-0.18788	-0.67701	-0.31673	0.752198	-5.03434	0.782801	0.867713
Monocytes	LRRC14	0.085335	2.339055	0.316487	0.752379	-5.47507	0.7187	0.798072
Monocytes	KCNIP3	-0.1161	0.193763	-0.31637	0.752468	-5.49876	0.763783	0.847104
Monocytes	H6PD	0.071132	2.776773	0.31611	0.752664	-5.55345	0.710009	0.788508
Monocytes	GM29243	-0.1631	-0.26495	-0.31537	0.753225	-5.05148	0.774451	0.858222
Monocytes	GM11707	0.129018	0.863582	0.315041	0.753473	-5.22172	0.75019	0.831842
Monocytes	ZDHHC23	-0.05268	2.221064	-0.31482	0.753638	-5.88299	0.72194	0.801107
Monocytes	FGGY	-0.09203	3.459854	-0.3147	0.75373	-5.57452	0.697132	0.774002
Monocytes	CCDC166	-0.1439	0.615517	-0.31466	0.753761	-5.11848	0.755543	0.837696
Monocytes	CCDC80	-0.12056	2.683481	-0.31438	0.753976	-5.39886	0.712568	0.790938
Monocytes	PHF8	-0.03255	5.563796	-0.31432	0.75402	-6.26818	0.657132	0.730007
Monocytes	DCAF7	-0.02964	5.327488	-0.31432	0.754022	-6.22386	0.661494	0.734825
Monocytes	NPL	0.107285	2.535175	0.314196	0.754112	-5.51337	0.715559	0.794221
Monocytes	ATP5H	0.0209	7.946365	0.314185	0.754121	-6.73896	0.614902	0.683171
Monocytes	ITGAD	-0.19479	0.789813	-0.31417	0.754135	-5.04328	0.751814	0.833702
Monocytes	ATF2	-0.0305	6.46092	-0.31406	0.754218	-6.416	0.640872	0.712016
Monocytes	YIPF3	0.033995	4.904587	0.313867	0.754362	-6.16879	0.669404	0.743611
Monocytes	SHOC2	0.027328	6.344774	0.313866	0.754362	-6.43514	0.64296	0.714385
Monocytes	2310009A05RIk	0.039885	4.692253	0.313712	0.754479	-6.0973	0.673448	0.748056
Monocytes	ZFP942	-0.05428	4.309464	-0.31342	0.754701	-5.84692	0.680812	0.75607
Monocytes	NRN1	-0.12095	3.109984	-0.31341	0.75471	-5.54394	0.704197	0.781735
Monocytes	DNAJC24	0.056077	3.89302	0.313149	0.754905	-5.86217	0.688832	0.764939
Monocytes	IGHD	-0.10428	3.245617	-0.31313	0.754917	-5.35994	0.701509	0.778846
Monocytes	COX6C	-0.02164	8.594079	-0.31307	0.754967	-6.8387	0.604091	0.671112
Monocytes	PADI6	-0.19686	0.058128	-0.31301	0.755011	-5.02284	0.767773	0.851003
Monocytes	JMJD1C	-0.02809	8.048596	-0.31296	0.755047	-6.77511	0.613295	0.681395

Monocytes	UBR3	-0.02907	6.575859	-0.31276	0.755198	-6.47844	0.639019	0.709971
Monocytes	THG1L	0.091282	2.021146	0.312576	0.755339	-5.42937	0.726297	0.805985
Monocytes	SF3B2	-0.02255	7.285583	-0.31255	0.755358	-6.5834	0.626526	0.696136
Monocytes	CARF	0.099885	2.216581	0.312411	0.755464	-5.35644	0.72233	0.801648
Monocytes	LPGAT1	-0.03892	6.245156	-0.31229	0.755553	-6.35549	0.645016	0.716622
Monocytes	SHPRH	0.045526	4.541567	0.312055	0.755733	-5.95868	0.676638	0.751429
Monocytes	SPEF2	0.166786	1.371663	0.311883	0.755864	-5.05585	0.740008	0.820753
Monocytes	NAPRT	-0.14863	0.897988	-0.31131	0.756298	-5.21297	0.750377	0.831652
Monocytes	GK5	-0.05387	4.404631	-0.3112	0.756384	-6.00896	0.679647	0.754418
Monocytes	LMAN2	0.024157	6.112382	0.310938	0.75658	-6.4414	0.648001	0.71943
Monocytes	SELENOW	-0.03097	7.046449	-0.31079	0.756689	-6.58177	0.631369	0.700964
Monocytes	EDC4	-0.07384	2.984777	-0.31065	0.756798	-5.62202	0.707565	0.784887
Monocytes	EMSY	0.031076	5.828738	0.310559	0.756867	-6.32899	0.653232	0.725169
Monocytes	SATB2	0.103547	1.643339	0.310181	0.757153	-5.4494	0.735087	0.814756
Monocytes	TMEM132A	-0.11247	0.400445	-0.31011	0.757208	-5.29011	0.761464	0.843393
Monocytes	TLE1	-0.03518	4.060562	-0.31005	0.75725	-6.15282	0.686607	0.761789
Monocytes	CLEC14A	-0.13417	2.196348	-0.30994	0.757336	-5.28645	0.72369	0.802369
Monocytes	ZFP341	-0.09821	1.984672	-0.3097	0.757516	-5.2803	0.728144	0.807146
Monocytes	SNX4	-0.02492	6.329814	-0.30926	0.757851	-6.42865	0.64464	0.715271
Monocytes	ATP5J2	0.027613	7.723844	0.309171	0.757919	-6.68134	0.62009	0.688045
Monocytes	PRKG1	-0.10694	3.504872	-0.30897	0.758071	-5.64492	0.69781	0.773812
Monocytes	CHUK	-0.03125	5.037427	-0.30891	0.758117	-6.26723	0.66839	0.741496
Monocytes	GMCL1	-0.03937	4.636774	-0.30888	0.758137	-5.96305	0.675946	0.749813
Monocytes	EML1	-0.14831	1.303195	-0.30877	0.758226	-5.16328	0.742618	0.82271
Monocytes	HDDC2	0.045124	3.817561	0.308692	0.758282	-5.92252	0.691702	0.76716
Monocytes	GM26532	-0.03798	5.294849	-0.30842	0.758485	-6.4084	0.663711	0.736311
Monocytes	PRDM15	-0.06344	3.462741	-0.30823	0.758632	-5.60933	0.698845	0.774902
Monocytes	RIPPLY3	-0.20505	0.048429	-0.30791	0.758876	-5.0019	0.769861	0.852069
Monocytes	N4BP3	-0.09215	2.955418	-0.3079	0.758879	-5.368	0.709003	0.785939
Monocytes	RSRC2	0.021685	6.479986	0.307836	0.758931	-6.43064	0.642225	0.712493
Monocytes	GM7854	0.136769	0.52802	0.307624	0.759092	-5.24424	0.759526	0.840891
Monocytes	SAMM50	0.032943	5.177169	0.307466	0.759212	-6.22701	0.666131	0.738898
Monocytes	9830107B12RIK	0.105125	-0.62676	0.307455	0.75922	-5.30213	0.784882	0.868282
Monocytes	MRRF	0.054549	3.454685	0.30729	0.759345	-5.76324	0.699222	0.775234
Monocytes	NUDT18	-0.06471	2.627052	-0.30717	0.75944	-5.54622	0.715765	0.793326
Monocytes	MON1B	0.089299	1.881481	0.306723	0.759775	-5.336	0.731237	0.809995
Monocytes	S100A13	0.02904	6.040644	0.30668	0.759808	-6.4355	0.650497	0.721464
Monocytes	FOXRED1	-0.08319	2.831724	-0.30643	0.759999	-5.53017	0.711916	0.788874
Monocytes	P2RY10B	-0.04687	3.709802	-0.30642	0.760006	-6.00551	0.694509	0.769834
Monocytes	NUTF2	0.110304	1.951713	0.306086	0.760259	-5.328	0.729934	0.808446
Monocytes	SLC13A3	-0.14481	1.11728	-0.30603	0.7603	-5.28034	0.747394	0.827414
Monocytes	HPS1	0.054436	3.445228	0.306027	0.760303	-5.83629	0.69979	0.775548
Monocytes	ATP5J	0.026244	7.873083	0.305749	0.760514	-6.70247	0.618314	0.685704
Monocytes	5730480H06RIK	0.072865	2.846707	0.305699	0.760553	-5.49065	0.711778	0.788683
Monocytes	SMIM8	0.045498	4.587829	0.305484	0.760716	-5.95771	0.677728	0.751381
Monocytes	DHFR	-0.0701	4.331798	-0.30547	0.760723	-5.8601	0.682619	0.756755
Monocytes	ESYT1	0.031896	5.654914	0.305473	0.760724	-6.37515	0.657755	0.729388

Monocytes	MIRT1	-0.04319	4.950639	-0.30496	0.761113	-6.24177	0.671146	0.743887
Monocytes	PEX1	-0.07557	3.071405	-0.30462	0.761375	-5.61444	0.707722	0.783909
Monocytes	EXTL3	0.048855	3.56619	0.304576	0.761405	-5.94719	0.69792	0.773211
Monocytes	4930562C15RIK	0.154139	0.146658	0.304049	0.761805	-5.07729	0.769175	0.850428
Monocytes	ZFP683	0.17381	-1.15907	0.303955	0.761876	-5.02238	0.798306	0.881798
Monocytes	USP36	-0.03808	4.970109	-0.30344	0.762269	-6.11805	0.671455	0.743774
Monocytes	GM34961	0.133802	0.869528	0.303411	0.762289	-5.21715	0.753821	0.833639
Monocytes	PPP1R42	0.163861	0.580161	0.303159	0.762481	-5.11969	0.760153	0.8404
Monocytes	CXCR5	0.135912	2.183855	0.303081	0.76254	-5.27407	0.72638	0.803782
Monocytes	DGKI	-0.19274	0.78	-0.30258	0.76292	-5.28089	0.755889	0.835862
Monocytes	TUFM	-0.04207	4.731912	-0.30244	0.763024	-6.0872	0.676091	0.748872
Monocytes	FLYWCH1	-0.05365	3.850858	-0.30228	0.763148	-5.75164	0.693042	0.767512
Monocytes	MTERF2	-0.10572	2.099595	-0.30226	0.763167	-5.30801	0.728148	0.805831
Monocytes	MB21D2	-0.15739	0.883088	-0.30221	0.763205	-5.12972	0.753681	0.833542
Monocytes	SHE	0.142914	0.946591	0.302165	0.763236	-5.21707	0.752324	0.832073
Monocytes	ASB3	-0.04057	4.711015	-0.3021	0.763286	-5.96835	0.676488	0.749354
Monocytes	KHDC4	0.026466	6.448578	0.302042	0.763329	-6.45857	0.644375	0.713971
Monocytes	GM15848	-0.17584	-0.86297	-0.30195	0.763398	-4.97592	0.792043	0.874937
Monocytes	ARGLU1	-0.01812	7.597485	-0.30182	0.7635	-6.62206	0.624082	0.691516
Monocytes	COG7	0.068316	2.604364	0.301815	0.763502	-5.54411	0.717833	0.794624
Monocytes	HADHA	-0.02942	5.770414	-0.30175	0.763551	-6.28752	0.656701	0.727632
Monocytes	PKN1	0.022953	6.766934	0.301609	0.763659	-6.51581	0.638678	0.70775
Monocytes	NAT8F1	0.134334	1.619362	0.301561	0.763695	-5.26619	0.738113	0.816758
Monocytes	CREB3L2	0.069574	3.55229	0.301558	0.763697	-5.70249	0.698893	0.774007
Monocytes	SH3BGR	0.19578	-0.59926	0.301531	0.763718	-4.94585	0.786116	0.868655
Monocytes	NEURL1B	-0.08413	0.43588	-0.30151	0.763735	-5.45091	0.763311	0.844063
Monocytes	PSMC6	-0.02618	6.141841	-0.30118	0.763981	-6.39086	0.650061	0.720301
Monocytes	6430548M08RI	-0.05195	2.407786	-0.30111	0.764039	-5.91062	0.721989	0.799208
Monocytes	USP24	-0.03284	5.459806	-0.30071	0.764342	-6.21533	0.662771	0.734257
Monocytes	USP39	-0.04692	4.614508	-0.30056	0.764455	-6.00625	0.678668	0.751774
Monocytes	GMIP	0.026475	5.964525	0.30053	0.764478	-6.40013	0.653478	0.724043
Monocytes	BCS1L	-0.11208	1.699946	-0.30046	0.764532	-5.33007	0.736804	0.815273
Monocytes	AFDN	-0.04659	3.267689	-0.30027	0.764673	-5.96734	0.704947	0.780557
Monocytes	GM35853	0.062869	0.184156	0.300158	0.764761	-5.75828	0.769274	0.850435
Monocytes	GMPR2	-0.04401	3.866899	-0.29994	0.764924	-5.97266	0.693244	0.767707
Monocytes	BID	0.046535	3.803317	0.299865	0.764984	-5.94335	0.694485	0.769067
Monocytes	1700028E10RIK	0.10451	1.36693	0.299418	0.765324	-5.22553	0.744192	0.823042
Monocytes	TNFSF11	-0.20487	0.706959	-0.29938	0.765356	-5.13278	0.758254	0.838272
Monocytes	GNAT3	0.137999	-0.04367	0.299054	0.7656	-5.15182	0.774772	0.856005
Monocytes	CHCHD7	0.03928	4.398636	0.298972	0.765663	-5.98964	0.683319	0.756551
Monocytes	HIVEP3	0.045474	5.144836	0.298652	0.765906	-6.16333	0.66931	0.741068
Monocytes	PFN1	0.021137	10.31807	0.298353	0.766134	-7.0927	0.579841	0.641707
Monocytes	SDC4	-0.05616	6.436865	-0.2982	0.766254	-6.23922	0.645732	0.714934
Monocytes	TMEM11	0.032946	5.070903	0.297981	0.766417	-6.21295	0.670924	0.742646
Monocytes	CAB39L	-0.04286	4.624571	-0.29787	0.766498	-5.98625	0.679379	0.751942
Monocytes	FOXK2	-0.03181	4.920069	-0.29787	0.766504	-6.16979	0.673768	0.745782
Monocytes	FMO1	-0.13838	2.23787	-0.29746	0.766813	-5.33283	0.726704	0.803636

Monocytes	WAC	0.020411	7.414892	0.297332	0.76691	-6.63648	0.628467	0.695834
Monocytes	A630001O12Ril	0.067793	2.282008	0.297294	0.766939	-5.63071	0.725798	0.802696
Monocytes	DGKD	0.028224	7.687633	0.297249	0.766974	-6.59042	0.62372	0.690571
Monocytes	NPC2	0.024488	7.639426	0.297246	0.766976	-6.82189	0.624556	0.691498
Monocytes	AAR2	-0.04075	4.021304	-0.29724	0.766982	-5.85893	0.691056	0.764777
Monocytes	JUNB	-0.02938	9.343714	-0.29703	0.767137	-7.05609	0.595794	0.659503
Monocytes	LYNX1	0.180251	0.898277	0.296944	0.767206	-5.03971	0.75486	0.834235
Monocytes	NCKAP1L	-0.02236	6.137906	-0.2969	0.76724	-6.47432	0.651287	0.721086
Monocytes	RALGAPA1	0.033372	6.84207	0.296544	0.76751	-6.54602	0.638735	0.707142
Monocytes	BLOC1S5	-0.09139	1.955013	-0.29653	0.76752	-5.3882	0.73273	0.81017
Monocytes	SDR39U1	-0.1175	1.131364	-0.2963	0.767697	-5.25222	0.75011	0.829033
Monocytes	DCAF4	0.077597	2.008291	0.296239	0.767742	-5.42001	0.731708	0.809071
Monocytes	TXLNA	0.035219	4.941372	0.295887	0.76801	-6.10627	0.673802	0.745751
Monocytes	SLC38A10	-0.03427	5.326136	-0.29587	0.768026	-6.15821	0.666574	0.737806
Monocytes	CLDN34C1	0.176838	0.809812	0.295573	0.768249	-5.02247	0.757169	0.836641
Monocytes	EPB41L2	0.025882	6.975502	0.295559	0.76826	-6.65718	0.636591	0.70475
Monocytes	LY9	-0.04311	4.262049	-0.29554	0.768272	-5.98939	0.686815	0.76005
Monocytes	PLEKHB1	0.116834	0.728002	0.295238	0.768504	-5.21438	0.759039	0.838566
Monocytes	UNG	-0.10235	3.369876	-0.29515	0.76857	-5.48731	0.704381	0.77921
Monocytes	ABT1	-0.05272	3.534654	-0.29507	0.768631	-5.69897	0.701119	0.77568
Monocytes	BCKDHB	-0.05946	3.929893	-0.29501	0.768675	-5.77485	0.69336	0.767202
Monocytes	DNAJC5	-0.0193	6.766699	-0.29487	0.768787	-6.5058	0.640398	0.708944
Monocytes	GM16196	0.108762	1.751417	0.29486	0.768792	-5.25842	0.737333	0.815133
Monocytes	MFF	0.027056	5.906654	0.29466	0.768944	-6.34059	0.656033	0.726181
Monocytes	ZFP933	-0.06173	3.490032	-0.2944	0.76914	-5.70509	0.702193	0.776742
Monocytes	SMYD4	-0.09005	2.820357	-0.29431	0.769213	-5.49124	0.715585	0.791369
Monocytes	ADH5	-0.03314	6.03865	-0.29416	0.769328	-6.38794	0.653768	0.723573
Monocytes	CDCA3	-0.06488	4.895561	-0.29338	0.769916	-6.18942	0.675319	0.747093
Monocytes	CCDC181	0.09119	1.514839	0.293359	0.769935	-5.36085	0.742859	0.820773
Monocytes	NHLRC3	-0.05767	3.16365	-0.29332	0.769968	-5.77631	0.709032	0.783988
Monocytes	FDXR	-0.06798	2.625449	-0.29328	0.769992	-5.55708	0.719884	0.795815
Monocytes	HAUS4	0.049035	4.204699	0.293278	0.769997	-5.90832	0.688549	0.7616
Monocytes	TTC25	0.153646	0.218704	0.293072	0.770154	-5.09357	0.770777	0.850901
Monocytes	DMXL1	-0.03463	6.604824	-0.29284	0.770334	-6.40514	0.643952	0.712434
Monocytes	TMCO1	0.022392	6.265444	0.292709	0.770431	-6.47023	0.650081	0.719223
Monocytes	VAT1	-0.05495	3.491225	-0.29263	0.770487	-5.82507	0.702694	0.77698
Monocytes	AKR1C13	0.098441	1.84784	0.292501	0.770589	-5.38341	0.736074	0.81335
Monocytes	ALS2CL	-0.1293	0.148166	-0.29235	0.770708	-5.21228	0.772432	0.852706
Monocytes	GBF1	-0.02217	6.562441	-0.29232	0.770727	-6.43115	0.644714	0.713363
Monocytes	RAB5A	0.021806	6.913368	0.292255	0.770776	-6.54408	0.638437	0.706428
Monocytes	HDHD5	0.081724	2.842941	0.292163	0.770847	-5.48393	0.715658	0.79119
Monocytes	HECTD1	0.021417	7.428886	0.292123	0.770877	-6.64337	0.62934	0.696373
Monocytes	5830411N06Ril	-0.16437	-1.25694	-0.29183	0.7711	-4.98121	0.804119	0.886637
Monocytes	GM16341	-0.14894	0.863635	-0.29168	0.771214	-5.07315	0.757091	0.836013
Monocytes	SMPD4	-0.06406	3.209019	-0.29139	0.771437	-5.66907	0.708605	0.783275
Monocytes	QRICH1	-0.02062	6.585338	-0.29128	0.771516	-6.48858	0.644575	0.713011
Monocytes	SULF2	0.051136	2.585463	0.291247	0.771545	-5.99825	0.721186	0.796981

Monocytes	GM29666	-0.16209	0.244861	-0.29107	0.771682	-5.10146	0.770698	0.850563
Monocytes	E130311K13RIK	0.143287	0.244935	0.290988	0.771742	-5.1524	0.770696	0.850567
Monocytes	PRAF2	0.056422	2.133854	0.290793	0.77189	-5.63035	0.730525	0.807108
Monocytes	CCAR1	-0.02107	6.820278	-0.29072	0.771949	-6.50047	0.640431	0.708433
Monocytes	HCFC1	0.035799	4.949877	0.290695	0.771965	-6.16497	0.674816	0.7463
Monocytes	TPPP	-0.19831	0.182012	-0.29056	0.772067	-5.05063	0.7721	0.852155
Monocytes	SSX2IP	0.085638	2.481205	0.290495	0.772118	-5.54646	0.723393	0.799414
Monocytes	SPOPL	0.038995	4.42179	0.290373	0.772211	-5.99694	0.6849	0.757413
Monocytes	NUF2	-0.07484	3.365247	-0.29032	0.772249	-5.72355	0.705569	0.780034
Monocytes	ZFP82	0.156263	0.121707	0.29019	0.77235	-5.0469	0.773425	0.853651
Monocytes	VAPB	-0.03427	5.031604	-0.29015	0.772382	-6.19675	0.673277	0.744701
Monocytes	IFT20	-0.03116	5.442542	-0.28989	0.772577	-6.23661	0.665677	0.736272
Monocytes	TMEM238	0.060513	2.938537	0.28966	0.772754	-5.8157	0.714256	0.789428
Monocytes	BMS1	0.038153	4.737717	0.289557	0.772833	-6.06753	0.678989	0.750873
Monocytes	NAV2	-0.05859	4.841571	-0.28944	0.772922	-5.95851	0.677013	0.748716
Monocytes	SLCO4A1	-0.12788	3.403213	-0.2893	0.773032	-5.30811	0.704958	0.779299
Monocytes	PPP1R2	0.017081	6.908394	0.28928	0.773044	-6.6276	0.638996	0.70685
Monocytes	GM12166	-0.11634	0.781167	-0.28925	0.77307	-5.23814	0.759227	0.838234
Monocytes	OAT	-0.03595	5.245936	-0.28922	0.773087	-6.25079	0.669381	0.740339
Monocytes	RBM38	-0.03728	6.898184	-0.28895	0.773293	-6.40894	0.639291	0.707118
Monocytes	CBFB	-0.01879	6.809866	-0.28853	0.773616	-6.55314	0.641001	0.708906
Monocytes	TIMELESS	-0.05313	3.491901	-0.28839	0.773721	-5.93399	0.70347	0.777503
Monocytes	4933433G15RII	-0.11526	0.742117	-0.2883	0.773789	-5.35333	0.760363	0.839279
Monocytes	LOCKD	0.069067	4.472202	0.288304	0.773789	-5.91924	0.684335	0.756576
Monocytes	PER1	-0.03733	4.926726	-0.28829	0.773797	-6.22622	0.675659	0.747063
Monocytes	TSPYL1	0.035274	4.888609	0.287893	0.774102	-6.13032	0.676507	0.747894
Monocytes	GM49521	0.126943	0.473447	0.287763	0.774202	-5.14625	0.766327	0.845614
Monocytes	CD151	-0.06882	2.758187	-0.28776	0.774207	-5.56868	0.718312	0.793585
Monocytes	NFU1	0.037914	4.549473	0.287747	0.774214	-6.12872	0.682977	0.754995
Monocytes	GM28417	0.113187	1.320479	0.287485	0.774414	-5.25981	0.748256	0.826008
Monocytes	1810055G02RII	-0.06946	2.268587	-0.28727	0.774575	-5.48611	0.72848	0.804604
Monocytes	RHNO1	0.044858	4.345115	0.28725	0.774593	-5.95638	0.687063	0.759433
Monocytes	FBXL19	-0.0923	2.333257	-0.28709	0.774712	-5.36697	0.727197	0.803246
Monocytes	ATAD3A	-0.0506	4.10261	-0.28691	0.774855	-5.90467	0.691875	0.764705
Monocytes	AU019990	0.08433	1.211418	0.286681	0.775027	-5.63153	0.750836	0.828758
Monocytes	AGMAT	-0.13549	1.438737	-0.28627	0.77534	-5.25512	0.746197	0.823622
Monocytes	CAAA01118383	0.028824	5.624186	0.286225	0.775375	-6.34478	0.663213	0.73308
Monocytes	LMAN1	-0.0406	4.627609	-0.28616	0.775423	-6.06951	0.682002	0.753704
Monocytes	ZKSCAN8	0.122102	0.632724	0.286002	0.775546	-5.14756	0.763506	0.842339
Monocytes	GM14410	-0.12011	0.850334	-0.28557	0.775872	-5.14921	0.758914	0.837332
Monocytes	4933439C10RIK	-0.13432	1.200712	-0.28555	0.775889	-5.12186	0.751409	0.829221
Monocytes	MTCH1	-0.02226	6.297903	-0.28555	0.77589	-6.47138	0.650979	0.719573
Monocytes	ETNK1	0.029483	5.999727	0.285496	0.775932	-6.33665	0.656424	0.725573
Monocytes	VMA21	0.029989	5.176423	0.285341	0.77605	-6.26373	0.67177	0.742437
Monocytes	P2RY6	-0.04128	2.305897	-0.28499	0.776316	-6.16398	0.728401	0.80423
Monocytes	HDAC2	-0.03404	5.300553	-0.28497	0.77633	-6.20822	0.669522	0.739923
Monocytes	AGAP2	-0.07138	2.925575	-0.28478	0.776475	-5.5064	0.715763	0.790486

Monocytes	GTPBP8	0.075308	2.317318	0.284759	0.776495	-5.42461	0.728166	0.803974
Monocytes	PPM1B	-0.02609	6.192574	-0.28472	0.776525	-6.37269	0.653021	0.721784
Monocytes	CUTC	-0.04464	3.519969	-0.28464	0.776585	-5.82853	0.703867	0.777542
Monocytes	FRAT2	0.045351	4.805593	0.284584	0.776628	-6.03725	0.678878	0.750222
Monocytes	TBRG4	-0.0483	4.075048	-0.28433	0.776824	-5.85938	0.693068	0.76567
Monocytes	CDO1	0.070019	3.756999	0.284155	0.776956	-5.77494	0.699356	0.772478
Monocytes	KTI12	0.05702	3.941057	0.283985	0.777086	-5.80493	0.695797	0.768531
Monocytes	E230032D23RII	-0.11381	1.879678	-0.28387	0.777177	-5.23963	0.737496	0.813891
Monocytes	RARS	-0.03155	5.121145	-0.28376	0.77726	-6.20589	0.673147	0.743714
Monocytes	SNRNP35	0.056051	3.056704	0.283341	0.777578	-5.69064	0.713514	0.787719
Monocytes	STK3	0.032894	5.985237	0.283221	0.77767	-6.37542	0.657179	0.726076
Monocytes	NOB1	-0.03865	4.287446	-0.28319	0.777693	-5.94575	0.68921	0.761225
Monocytes	GNB1L	0.069073	3.308245	0.283124	0.777743	-5.54389	0.708471	0.782262
Monocytes	TMEM119	-0.10212	0.305437	-0.28302	0.777826	-5.31	0.771322	0.850394
Monocytes	A930014D07RII	0.126261	-1.05861	0.282942	0.777883	-5.03769	0.801852	0.883209
Monocytes	ARID4B	0.022017	8.259309	0.28293	0.777892	-6.72497	0.61686	0.681594
Monocytes	KCTD1	-0.0633	1.719442	-0.28282	0.777973	-5.63367	0.740997	0.81767
Monocytes	GM12840	-0.09748	3.766389	-0.28276	0.778019	-5.78112	0.699386	0.772423
Monocytes	MCM3AP	0.041661	3.885875	0.282649	0.778106	-5.7956	0.697055	0.76986
Monocytes	GM49625	-0.09034	2.147374	-0.28234	0.778339	-5.35491	0.732251	0.808109
Monocytes	IGLV1	0.181008	-0.83837	0.281627	0.778888	-4.97025	0.797515	0.878166
Monocytes	DBT	-0.05792	3.826101	-0.28144	0.779031	-5.75155	0.698874	0.771483
Monocytes	ZFP91	0.021023	6.894237	0.281205	0.77921	-6.55342	0.641415	0.708351
Monocytes	SZT2	0.077363	2.318426	0.281048	0.779331	-5.35645	0.729389	0.804621
Monocytes	BTBD7	-0.01954	7.004982	-0.28084	0.779491	-6.56733	0.639539	0.706265
Monocytes	AKAP8	-0.02714	5.39229	-0.28078	0.779538	-6.22546	0.669006	0.73871
Monocytes	CALCRL	-0.05131	5.617508	-0.28029	0.779911	-6.21734	0.665041	0.734196
Monocytes	CLDN10	0.197389	0.159414	0.280229	0.779956	-4.99449	0.775774	0.854575
Monocytes	SLC25A47	0.060949	3.963	0.280112	0.780046	-5.805	0.696663	0.768856
Monocytes	ZBTB24	0.048951	3.32852	0.279829	0.780263	-5.66534	0.709358	0.782621
Monocytes	FHL3	0.104843	2.081558	0.279677	0.780378	-5.3403	0.734825	0.810316
Monocytes	ROBO1	-0.12511	2.137687	-0.27955	0.780478	-5.30719	0.733688	0.809052
Monocytes	TLK1	-0.02468	6.854126	-0.27919	0.780752	-6.48791	0.642806	0.709594
Monocytes	ZFP160	0.065305	2.950859	0.279147	0.780784	-5.54863	0.71718	0.791037
Monocytes	ARMC6	-0.09322	1.405639	-0.27868	0.781138	-5.27497	0.749379	0.825947
Monocytes	IGSF8	-0.03763	4.266842	-0.27867	0.781147	-6.0705	0.691239	0.762758
Monocytes	ETFA	-0.02827	6.599323	-0.27865	0.781163	-6.51262	0.647532	0.714802
Monocytes	NDNF	0.074285	-0.47189	0.278266	0.781458	-5.56234	0.790655	0.870193
Monocytes	SCAF11	0.023122	7.16109	0.278095	0.781589	-6.54105	0.63771	0.703778
Monocytes	EXOC4	-0.01916	8.03297	-0.27796	0.781691	-6.69791	0.622468	0.686928
Monocytes	PTAR1	-0.04416	3.690053	-0.27748	0.782059	-5.77974	0.703105	0.775301
Monocytes	TMEM150A	-0.1	2.327871	-0.27739	0.782132	-5.37398	0.730656	0.805262
Monocytes	EFCAB5	0.126733	0.504613	0.277112	0.782341	-5.18981	0.769478	0.847157
Monocytes	IFIT1	-0.12602	2.761439	-0.2771	0.782352	-5.68669	0.721837	0.795649
Monocytes	PIH1D1	0.031782	5.06576	0.27653	0.782786	-6.21208	0.676836	0.746284
Monocytes	TMEM91	-0.10278	2.459733	-0.27614	0.783086	-5.29391	0.728512	0.802438
Monocytes	AUP1	-0.02465	6.010505	-0.27613	0.783092	-6.4523	0.659312	0.726928

Monocytes	WDR55	-0.05397	2.979495	-0.27587	0.783288	-5.65025	0.717947	0.790988
Monocytes	SNX25	-0.03075	5.540177	-0.27568	0.783436	-6.20657	0.668089	0.736576
Monocytes	DEPDC1B	0.088114	2.954227	0.275565	0.783525	-5.62189	0.718459	0.791547
Monocytes	2310033P09RIK	0.041012	4.144026	0.275556	0.783532	-5.91521	0.694779	0.765768
Monocytes	RNF123	0.053855	3.804311	0.275476	0.783593	-5.80498	0.701451	0.773043
Monocytes	CKAP2	-0.06334	3.667324	-0.27544	0.78362	-5.86089	0.704161	0.775996
Monocytes	STAR	0.121111	1.465058	0.275309	0.783721	-5.25009	0.749358	0.825013
Monocytes	PITRM1	-0.05434	3.560314	-0.27528	0.783745	-5.71624	0.706286	0.77831
Monocytes	POLR1B	0.075555	2.442843	0.275251	0.783766	-5.48066	0.728909	0.802887
Monocytes	PLIN3	-0.0449	3.527331	-0.27489	0.784043	-5.85874	0.707088	0.779128
Monocytes	DDX39	-0.03364	6.372696	-0.27483	0.784091	-6.41369	0.652854	0.719814
Monocytes	RYR2	0.21295	0.059974	0.274698	0.784189	-5.01811	0.780006	0.857997
Monocytes	TBC1D12	-0.05742	3.768556	-0.27468	0.784202	-5.89331	0.702301	0.773949
Monocytes	UCKL1	0.039556	3.705129	0.274271	0.784516	-5.94242	0.703776	0.775338
Monocytes	PI4KA	0.027195	6.333121	0.274095	0.784651	-6.4254	0.653835	0.72064
Monocytes	HPD	0.084129	4.805638	0.27373	0.78493	-6.05661	0.682572	0.75203
Monocytes	DGKQ	-0.09458	1.470676	-0.27365	0.784991	-5.2877	0.749883	0.825165
Monocytes	LPIN1	0.055095	2.690777	0.273547	0.785071	-5.93314	0.724454	0.79765
Monocytes	UBE2O	-0.05807	4.874388	-0.27333	0.785234	-5.9739	0.681347	0.750701
Monocytes	ABCA1	0.041547	5.836161	0.273114	0.785402	-6.50977	0.663321	0.730941
Monocytes	GM42699	-0.13599	0.727533	-0.27292	0.785548	-5.17675	0.766129	0.842581
Monocytes	PIP4P2	0.050536	3.706325	0.272824	0.785625	-5.85856	0.704251	0.775635
Monocytes	FANCF	-0.09805	1.911025	-0.27273	0.785693	-5.34219	0.740862	0.81538
Monocytes	TESPA1	0.088115	2.504605	0.272409	0.785942	-5.44351	0.728587	0.802098
Monocytes	ZBTB8A	0.068395	1.978565	0.272358	0.785982	-5.48259	0.739505	0.813921
Monocytes	NLE1	-0.09149	2.257092	-0.27211	0.786173	-5.4144	0.733702	0.807674
Monocytes	POLR3B	0.038773	5.512738	0.272107	0.786174	-6.22502	0.669474	0.737713
Monocytes	XPO4	0.027976	5.623363	0.271977	0.786274	-6.30208	0.667405	0.735446
Monocytes	WBP2	0.031917	5.599979	0.271967	0.786282	-6.22167	0.667842	0.735925
Monocytes	BHLHB9	-0.09072	2.203905	-0.27195	0.786296	-5.26999	0.734806	0.808871
Monocytes	HCST	-0.03887	5.375869	-0.27194	0.786305	-6.30236	0.672045	0.740531
Monocytes	GM26936	-0.16068	0.478634	-0.27173	0.786464	-5.0113	0.77171	0.848599
Monocytes	PPP1R1C	0.07111	0.781145	0.271648	0.786526	-5.79943	0.76511	0.8415
Monocytes	MOSPD1	-0.04547	4.817896	-0.27127	0.786813	-6.019	0.682902	0.752154
Monocytes	PKP3	-0.06076	2.747004	-0.27106	0.786979	-5.79275	0.723933	0.796776
Monocytes	GM9828	-0.12475	1.076181	-0.27093	0.787077	-5.17691	0.758984	0.834661
Monocytes	EFNA1	0.1535	0.531324	0.270866	0.787125	-5.06629	0.770812	0.847388
Monocytes	MCM9	0.035737	4.75152	0.270768	0.787201	-6.09115	0.684212	0.753545
Monocytes	GLRX5	0.027661	6.17176	0.270724	0.787235	-6.37816	0.657536	0.724331
Monocytes	PTRH2	0.039925	4.181735	0.270678	0.78727	-5.94454	0.695251	0.765592
Monocytes	TCTN3	0.092259	1.366808	0.270388	0.787492	-5.39178	0.752879	0.828016
Monocytes	MTHFD1L	0.029117	5.661426	0.270331	0.787536	-6.39135	0.667094	0.734758
Monocytes	2900097C17RIK	-0.02877	4.909122	-0.26994	0.787832	-6.18909	0.681501	0.750405
Monocytes	MTMR12	0.028178	5.334196	0.269696	0.788022	-6.28113	0.673532	0.741592
Monocytes	9330020H09RII	0.075525	2.471892	0.26944	0.788219	-5.43248	0.730065	0.803084
Monocytes	TMEM263	-0.05016	3.98713	-0.26938	0.788265	-5.77519	0.699532	0.769929
Monocytes	PRCP	0.023887	5.424232	0.269364	0.788277	-6.48692	0.671876	0.739736

Monocytes	0610043K17RIK	0.082059	1.133363	0.269261	0.788356	-5.59934	0.758266	0.833529
Monocytes	NOL4L	0.065151	2.680623	0.269107	0.788474	-5.80812	0.72578	0.798435
Monocytes	PLEKHO1	0.023786	5.499121	0.26899	0.788564	-6.50441	0.670475	0.738195
Monocytes	PIAS2	-0.02933	6.02482	-0.26872	0.788769	-6.34605	0.660687	0.727469
Monocytes	KIF5A	-0.11221	0.429339	-0.26867	0.788812	-5.28299	0.77357	0.849989
Monocytes	ABHD14A	-0.11364	1.44344	-0.26863	0.788838	-5.1705	0.75163	0.826378
Monocytes	GNPDA2	-0.04854	3.100129	-0.26855	0.788899	-5.67936	0.717238	0.789171
Monocytes	LXN	0.044076	3.855864	0.268537	0.788912	-5.8448	0.702127	0.772747
Monocytes	STK4	-0.02218	7.52595	-0.26846	0.788972	-6.60421	0.633611	0.697695
Monocytes	VPS41	0.027138	5.101022	0.268335	0.789066	-6.25816	0.677995	0.746422
Monocytes	PSMA1	0.021653	6.9166	0.268329	0.789071	-6.60547	0.644448	0.709631
Monocytes	GM43623	-0.08184	-0.49767	-0.26827	0.789113	-5.48895	0.794233	0.872138
Monocytes	NRDE2	0.055302	3.371854	0.268144	0.789213	-5.77154	0.711792	0.783234
Monocytes	CAMKK1	-0.12858	0.590902	-0.26767	0.789578	-5.1162	0.770347	0.846265
Monocytes	SEH1L	-0.02396	5.334868	-0.26752	0.789688	-6.29259	0.67388	0.741703
Monocytes	TMEM176B	-0.03565	5.475623	-0.2674	0.789787	-6.4371	0.671254	0.738861
Monocytes	TPT1	-0.01587	10.10831	-0.26725	0.789899	-7.09795	0.590241	0.649509
Monocytes	1810037I17RIK	0.02552	6.482621	0.267169	0.789961	-6.53446	0.652653	0.718473
Monocytes	CPSF2	0.031158	5.649423	0.266947	0.790132	-6.24485	0.668113	0.735386
Monocytes	ACOT13	-0.03346	4.43558	-0.26681	0.79024	-5.97324	0.691267	0.760673
Monocytes	TAF8	0.055267	3.254648	0.266732	0.790296	-5.67148	0.714628	0.786099
Monocytes	TADA1	-0.03194	4.679062	-0.2663	0.790631	-6.08502	0.686749	0.755667
Monocytes	THOC5	0.043233	3.564433	0.26613	0.790758	-5.79932	0.708616	0.779496
Monocytes	DPP9	0.036204	4.420233	0.26612	0.790767	-6.04376	0.691759	0.761148
Monocytes	SMOC1	-0.14759	2.28252	-0.26608	0.790795	-5.20491	0.73472	0.807811
Monocytes	1-Mar	-0.11768	1.457886	-0.26598	0.790872	-5.2373	0.752076	0.826551
Monocytes	PIGZ	0.114582	0.017052	0.265386	0.79133	-5.25301	0.783857	0.860407
Monocytes	ST14	0.141119	1.167666	0.265287	0.791406	-5.1729	0.758667	0.833379
Monocytes	ULBP1	-0.05883	4.251838	-0.26482	0.791765	-5.81278	0.695537	0.764935
Monocytes	GREM2	0.143047	0.454759	0.264749	0.791819	-5.10885	0.774332	0.850119
Monocytes	CRP	-0.10723	2.076546	-0.26473	0.791832	-5.37464	0.73954	0.81266
Monocytes	RRP1	-0.01976	6.518197	-0.26462	0.791917	-6.47354	0.65276	0.718179
Monocytes	4930404N11RII	-0.16037	0.191025	-0.26458	0.791949	-4.99605	0.780156	0.856406
Monocytes	ATAD1	-0.02082	5.731262	-0.26435	0.792126	-6.30873	0.667271	0.734156
Monocytes	MED21	0.035916	5.21874	0.264339	0.792134	-6.22651	0.676916	0.744707
Monocytes	HIP1	-0.03601	5.505684	-0.26431	0.792153	-6.38751	0.671497	0.738791
Monocytes	DNAJC30	0.038898	3.959336	0.264265	0.79219	-5.93993	0.701282	0.771307
Monocytes	GM15559	0.049942	3.148018	0.263791	0.792555	-5.70441	0.717603	0.789046
Monocytes	OTUD6B	-0.03231	4.301102	-0.26374	0.792595	-5.95792	0.694681	0.764135
Monocytes	IGKV2-109	0.142125	-0.87987	0.26373	0.792602	-4.97009	0.80442	0.882475
Monocytes	GM43328	0.069929	2.238918	0.26343	0.792832	-5.53834	0.736264	0.809393
Monocytes	EMC8	-0.03407	4.721373	-0.26332	0.792914	-6.04135	0.686529	0.755377
Monocytes	ANKMY2	0.041894	3.602393	0.263305	0.792928	-5.78682	0.708472	0.779274
Monocytes	GM46560	-0.15665	-0.84515	-0.2633	0.792928	-4.95981	0.803624	0.881777
Monocytes	SH2B1	0.056234	3.699274	0.263267	0.792957	-5.70013	0.706542	0.777187
Monocytes	1700066M21RI	0.087432	1.288003	0.263167	0.793034	-5.26407	0.756354	0.831097
Monocytes	OLFML3	0.096819	1.284348	0.263148	0.793048	-5.28221	0.756432	0.831182

Monocytes	SCAF1	0.032963	4.690272	0.263118	0.793072	-6.04813	0.687129	0.75605
Monocytes	OXSM	-0.07626	1.788374	-0.26292	0.793223	-5.3644	0.745786	0.819636
Monocytes	A530072M11RI	-0.12536	0.764735	-0.26275	0.793357	-5.17288	0.767743	0.843318
Monocytes	SREBF1	-0.04441	3.760907	-0.26274	0.793361	-5.82626	0.70539	0.775905
Monocytes	TMEM216	0.053678	4.069162	0.262544	0.793513	-5.78338	0.69937	0.769281
Monocytes	CERK	0.031928	7.028222	0.262167	0.793802	-6.36696	0.643955	0.708516
Monocytes	KANK2	0.090233	2.38565	0.26202	0.793915	-5.35426	0.733612	0.806319
Monocytes	IRF2BP1	0.040842	4.152538	0.2618	0.794085	-5.86301	0.698017	0.767696
Monocytes	SLC12A4	0.081931	1.745624	0.261763	0.794113	-5.40787	0.747071	0.820864
Monocytes	BRIP1	-0.04615	4.898207	-0.26159	0.794242	-6.099	0.683602	0.75197
Monocytes	GBP9	-0.06659	3.036901	-0.26141	0.794381	-5.70253	0.720354	0.791952
Monocytes	MAPKBP1	0.06629	3.047991	0.261413	0.794382	-5.65344	0.720129	0.791708
Monocytes	KLHL7	0.031056	4.739495	0.260742	0.794898	-6.17874	0.687041	0.755407
Monocytes	GM10658	-0.09007	1.776179	-0.26043	0.795139	-5.33394	0.747037	0.82041
Monocytes	PRADC1	-0.04969	3.374688	-0.26038	0.79518	-5.72605	0.714055	0.784733
Monocytes	GM26782	0.047591	2.991571	0.260058	0.795424	-5.63151	0.72197	0.793216
Monocytes	ITPKB	0.025189	7.462345	0.25989	0.795553	-6.70519	0.637014	0.700401
Monocytes	WDR76	0.053559	4.504143	0.259655	0.795734	-5.90072	0.692019	0.760594
Monocytes	MRPL40	-0.03423	4.651456	-0.25957	0.7958	-6.12451	0.689162	0.757482
Monocytes	ATF1	-0.01855	6.564036	-0.25946	0.795885	-6.46865	0.653256	0.718203
Monocytes	SUPV3L1	-0.04119	3.910174	-0.25908	0.796173	-5.95878	0.703836	0.773287
Monocytes	CTBP1	-0.02409	6.500976	-0.25899	0.796242	-6.45417	0.654546	0.719498
Monocytes	CC2D1A	-0.05858	3.044755	-0.25897	0.796263	-5.60653	0.721208	0.792159
Monocytes	HNRNPF	-0.01635	9.158537	-0.25872	0.79645	-6.90106	0.608047	0.668207
Monocytes	PLAA	0.02198	6.069326	0.258669	0.796492	-6.4112	0.662558	0.728217
Monocytes	USP19	-0.03658	4.704682	-0.2585	0.796622	-6.0938	0.688425	0.756459
Monocytes	VPS8	0.035248	4.586793	0.258223	0.796835	-6.07905	0.690725	0.758986
Monocytes	BC004004	0.027227	5.147988	0.258188	0.796862	-6.18176	0.679935	0.747221
Monocytes	WNT4	-0.08077	1.526488	-0.25813	0.796906	-5.40669	0.753001	0.826454
Monocytes	MRPS35	-0.03327	4.266456	-0.25811	0.79692	-5.97808	0.696969	0.765802
Monocytes	PGPEP1	-0.05519	3.461593	-0.25781	0.797156	-5.60658	0.713056	0.783239
Monocytes	B930095G15RII	-0.14553	0.087994	-0.25776	0.797193	-5.09307	0.784509	0.860252
Monocytes	SNRNP200	-0.03184	5.038857	-0.25734	0.797512	-6.19383	0.682208	0.749642
Monocytes	TMC8	-0.05222	3.820781	-0.25731	0.797536	-5.71295	0.705957	0.775496
Monocytes	PTRHD1	-0.03117	4.56189	-0.25724	0.797591	-6.23301	0.6914	0.759686
Monocytes	BAMBI	0.0431	3.967331	0.257222	0.797605	-6.12463	0.703052	0.772368
Monocytes	WRB	-0.09043	1.437969	-0.25707	0.797719	-5.28857	0.755102	0.82867
Monocytes	NPHP3	-0.09797	1.097931	-0.25702	0.797759	-5.27146	0.762415	0.836537
Monocytes	GSG1L	-0.1558	-0.16326	-0.25696	0.797807	-5.06107	0.790222	0.866368
Monocytes	TRNAU1AP	0.037442	4.126118	0.256898	0.797854	-6.03678	0.699919	0.768983
Monocytes	PIP5K1B	0.052296	5.568352	0.256818	0.797916	-5.94683	0.672163	0.738751
Monocytes	ATAD5	0.053515	4.742851	0.256742	0.797974	-6.04402	0.687897	0.755945
Monocytes	DNMT3B	-0.06721	2.497904	-0.25626	0.798349	-5.4495	0.733072	0.804795
Monocytes	CADM4	-0.1034	1.741687	-0.25606	0.7985	-5.35143	0.748997	0.821964
Monocytes	GPAA1	0.03414	3.764843	0.255792	0.798706	-5.918	0.707533	0.777048
Monocytes	GM15972	0.144496	-0.2921	0.255628	0.798832	-5.06966	0.793669	0.869915
Monocytes	IFI47	-0.08308	5.277962	-0.25558	0.79887	-6.14316	0.678118	0.745099

Monocytes	KNSTRN	-0.06682	3.271497	-0.25528	0.799099	-5.83455	0.717579	0.787957
Monocytes	MAN2C1	0.040216	3.509599	0.255224	0.799143	-5.82306	0.71278	0.782762
Monocytes	PIDD1	-0.11174	0.824038	-0.2551	0.79924	-5.15748	0.769047	0.843452
Monocytes	CTNNAL1	0.110007	1.924908	0.254928	0.799371	-5.23462	0.745444	0.818102
Monocytes	FBXL3	-0.02658	5.215741	-0.25485	0.799434	-6.20606	0.679456	0.746516
Monocytes	IMPA1	-0.02733	5.285622	-0.2548	0.799469	-6.21602	0.678127	0.745065
Monocytes	KRT10	0.089979	2.100333	0.254551	0.799661	-5.26448	0.741781	0.814123
Monocytes	TAF1A	-0.05381	3.171877	-0.25452	0.799686	-5.68053	0.719668	0.790216
Monocytes	USP12	-0.02663	6.069178	-0.25444	0.799745	-6.44619	0.663445	0.728988
Monocytes	DGCR2	0.032862	4.849233	0.254257	0.799887	-6.12096	0.6865	0.754237
Monocytes	GM36445	0.092421	1.003387	0.254242	0.799899	-5.20323	0.765191	0.839398
Monocytes	ADCY7	0.023588	5.583087	0.254177	0.799949	-6.43071	0.672526	0.738994
Monocytes	LRIG3	0.149396	0.14201	0.254131	0.799984	-5.04508	0.784139	0.859734
Monocytes	NAV1	-0.03612	3.595293	-0.25381	0.800229	-6.31186	0.711287	0.781122
Monocytes	GM16576	0.102914	1.513376	0.253661	0.800346	-5.29819	0.754419	0.827691
Monocytes	KLHL21	-0.0633	2.689731	-0.25351	0.80046	-5.64113	0.72977	0.801065
Monocytes	APEH	0.046737	3.695339	0.253325	0.800606	-5.86812	0.709387	0.779033
Monocytes	TWNK	0.065723	2.886068	0.253264	0.800652	-5.56058	0.725757	0.796783
Monocytes	SSH3	0.108751	1.522336	0.253214	0.800691	-5.24301	0.75429	0.827579
Monocytes	DHX57	-0.04235	3.871504	-0.25296	0.80089	-5.80366	0.705992	0.775297
Monocytes	GM28791	-0.0459	3.918458	-0.25282	0.800991	-5.83881	0.705088	0.774319
Monocytes	PREX2	-0.10856	2.374714	-0.25264	0.801133	-5.3326	0.73653	0.80834
Monocytes	TRAPPC13	0.040061	3.808587	0.252333	0.80137	-5.81494	0.70734	0.776804
Monocytes	ATP6V1C1	0.024713	5.189889	0.252133	0.801523	-6.24484	0.680422	0.747507
Monocytes	ANAPC11	0.025541	5.923842	0.252102	0.801548	-6.43974	0.666586	0.732387
Monocytes	NFXL1	-0.04657	3.364621	-0.25207	0.801571	-5.83772	0.71624	0.786466
Monocytes	CYP4V3	-0.04799	2.78654	-0.25205	0.801589	-6.06468	0.728016	0.799217
Monocytes	FRA10AC1	-0.04979	3.408508	-0.25204	0.801597	-5.68424	0.715355	0.785506
Monocytes	DHX40	-0.03453	7.808142	-0.252	0.801624	-6.64193	0.632486	0.694968
Monocytes	DDHD2	0.032557	4.784447	0.251785	0.801792	-6.13335	0.688243	0.756034
Monocytes	SMG6	0.018014	8.045885	0.251704	0.801854	-6.6843	0.628362	0.690438
Monocytes	GM10353	0.063386	2.745067	0.251672	0.801879	-5.53443	0.728911	0.800218
Monocytes	RALGPS2	-0.03726	5.773254	-0.25143	0.802065	-6.16587	0.669455	0.735555
Monocytes	PLGRKT	-0.02791	5.796951	-0.25135	0.802125	-6.35824	0.669011	0.73507
Monocytes	MYO9B	0.024664	5.866728	0.251346	0.80213	-6.40349	0.667707	0.733644
Monocytes	CNIH1	0.029288	5.17624	0.25125	0.802204	-6.20025	0.68074	0.747884
Monocytes	2810454H06RII	0.071775	2.514759	0.25114	0.802289	-5.43931	0.733687	0.805382
Monocytes	VARS	-0.0287	5.713285	-0.25108	0.802332	-6.2799	0.670579	0.73679
Monocytes	AFAP1L1	0.127501	1.508026	0.251014	0.802386	-5.17697	0.754884	0.82825
Monocytes	VMP1	-0.02856	7.164309	-0.25078	0.802566	-6.61855	0.643974	0.707651
Monocytes	ZFP503	-0.09684	0.884395	-0.25075	0.802588	-5.25204	0.768361	0.842764
Monocytes	ESPN	0.126997	-0.17599	0.250729	0.802605	-5.13807	0.791861	0.867943
Monocytes	RALBP1	-0.02238	6.268527	-0.25029	0.802944	-6.39864	0.660395	0.725603
Monocytes	MKS1	0.09413	1.092934	0.250216	0.803001	-5.28582	0.763984	0.838004
Monocytes	DALRD3	-0.04128	3.729807	-0.25015	0.803048	-5.75781	0.709123	0.778751
Monocytes	GM43063	0.103234	0.779437	0.250101	0.80309	-5.19071	0.770809	0.845334
Monocytes	MAP3K13	0.133641	0.779749	0.250067	0.803116	-5.09537	0.770803	0.845327

Monocytes	11-Sep	0.035232	6.415184	0.249673	0.803419	-6.41543	0.657841	0.72278
Monocytes	TAT	-0.11396	1.826535	-0.24957	0.803497	-5.29792	0.748433	0.821263
Monocytes	ZFP703	0.036242	4.364442	0.249509	0.803546	-6.19644	0.696731	0.765276
Monocytes	PTER	-0.0817	2.433342	-0.24938	0.803644	-5.44358	0.735698	0.807522
Monocytes	GM4221	0.088824	1.846721	0.24928	0.803722	-5.29934	0.748006	0.820836
Monocytes	CHAC2	0.060531	2.893423	0.249248	0.803747	-5.45531	0.726201	0.797291
Monocytes	ITPKC	-0.08648	1.763821	-0.24921	0.803779	-5.37079	0.749763	0.822752
Monocytes	CENPX	0.023565	6.347296	0.248985	0.80395	-6.48951	0.659172	0.724293
Monocytes	NKIRAS1	-0.05451	3.432264	-0.24889	0.804024	-5.63922	0.715343	0.785536
Monocytes	CTSB	-0.02988	8.190873	-0.24851	0.804316	-6.86045	0.626312	0.688115
Monocytes	ARFIP1	0.038551	3.797972	0.248485	0.804336	-5.9386	0.708126	0.777624
Monocytes	TRAK1	0.02197	7.291138	0.248419	0.804386	-6.51564	0.642164	0.705565
Monocytes	ZFP629	0.108263	0.926	0.248287	0.804488	-5.22681	0.768003	0.842281
Monocytes	SPOP	-0.01606	7.366582	-0.24827	0.8045	-6.69374	0.640817	0.704087
Monocytes	PRKACA	-0.03549	4.156404	-0.24763	0.804992	-5.91455	0.70139	0.770078
Monocytes	EVI2	0.039528	3.35768	0.247494	0.8051	-6.11464	0.717373	0.787388
Monocytes	ISOC2B	0.064524	2.519105	0.247232	0.805302	-5.5674	0.734616	0.806017
Monocytes	TMEM229A	0.13151	-1.03017	0.246975	0.8055	-5.05182	0.812473	0.889602
Monocytes	RAE1	-0.03145	4.525674	-0.24689	0.805563	-6.03497	0.694243	0.762332
Monocytes	ABCG1	0.032916	6.591271	0.246812	0.805626	-6.50006	0.655241	0.719729
Monocytes	YWHAB	0.016372	7.88619	0.246749	0.805675	-6.73632	0.632047	0.694248
Monocytes	ZFP820	0.14613	-0.04486	0.246692	0.805719	-4.99391	0.789999	0.865632
Monocytes	NDUFC2	0.02285	6.741013	0.246611	0.805781	-6.56656	0.65251	0.716735
Monocytes	KDM4B	0.043322	4.441359	0.246569	0.805814	-5.92294	0.695889	0.764123
Monocytes	SH3PXD2B	0.040264	1.543779	0.246486	0.805878	-6.31877	0.755168	0.828269
Monocytes	CPSF1	-0.04708	3.349532	-0.24636	0.805977	-5.71406	0.717601	0.7877
Monocytes	PYM1	0.028836	4.692796	0.246285	0.806032	-6.0595	0.690993	0.758794
Monocytes	HSPA4	0.016102	7.667014	0.246261	0.806051	-6.69297	0.635907	0.698496
Monocytes	MDP1	-0.02897	4.373314	-0.24623	0.806075	-6.10153	0.697221	0.765572
Monocytes	RALYL	0.177854	0.59788	0.245638	0.806532	-5.05274	0.77597	0.85046
Monocytes	APC	0.023932	6.723147	0.245609	0.806554	-6.47429	0.653062	0.717211
Monocytes	CDCA4	-0.03631	4.453152	-0.2456	0.806563	-5.98317	0.6959	0.764
Monocytes	LIPT2	0.089996	1.430834	0.245452	0.806676	-5.29724	0.757861	0.831022
Monocytes	MAPK11	0.136733	0.260399	0.245404	0.806712	-5.02862	0.783455	0.858483
Monocytes	NFIA	-0.03959	5.904	-0.2453	0.806791	-6.26961	0.668186	0.733778
Monocytes	TBC1D9B	-0.03036	4.555284	-0.24514	0.806913	-6.04089	0.693968	0.761882
Monocytes	RIT1	0.033009	3.965996	0.244938	0.807072	-5.96479	0.705636	0.774527
Monocytes	ZFP768	-0.11011	0.794694	-0.24468	0.807274	-5.23244	0.771824	0.845975
Monocytes	TRP53RKB	-0.07532	2.444883	-0.24456	0.807365	-5.33522	0.736582	0.808049
Monocytes	FBXO7	0.038614	4.165071	0.244553	0.807369	-5.91753	0.701718	0.770283
Monocytes	GM43113	-0.08477	-0.69248	-0.24453	0.80739	-5.38261	0.805157	0.881622
Monocytes	INSYN2B	-0.03838	4.140533	-0.24437	0.807511	-5.97758	0.702202	0.770854
Monocytes	GM32916	-0.23329	-0.24915	-0.24431	0.807554	-5.0497	0.795061	0.870898
Monocytes	CASR	-0.09135	-0.61039	-0.2442	0.80764	-5.33866	0.803277	0.879678
Monocytes	ZFP692	-0.07016	2.030033	-0.24419	0.807653	-5.33277	0.745273	0.817482
Monocytes	PIFO	0.130806	1.246923	0.243944	0.807839	-5.15194	0.76201	0.835577
Monocytes	KBTBD11	0.102465	0.726015	0.243871	0.807895	-5.3668	0.773353	0.847761

Monocytes	NUPL2	0.06668	2.367649	0.243776	0.807969	-5.49862	0.738215	0.809949
Monocytes	RUVBL2	-0.04644	3.693884	-0.24372	0.808009	-5.81108	0.711104	0.780609
Monocytes	GM28529	-0.12747	0.724072	-0.2437	0.808025	-5.1109	0.773396	0.847806
Monocytes	PPP1R9B	0.030197	4.375184	0.243529	0.808159	-6.22437	0.697658	0.765946
Monocytes	CSAD	0.041334	3.965874	0.243445	0.808224	-5.95656	0.705734	0.774722
Monocytes	DAD1	0.019636	6.948328	0.243089	0.8085	-6.60052	0.649314	0.713071
Monocytes	PTPMT1	-0.02747	4.267152	-0.24299	0.808574	-6.134	0.699911	0.76836
Monocytes	FBXO17	0.121759	0.782245	0.242979	0.808585	-5.10252	0.772322	0.846556
Monocytes	SRPK2	0.026604	7.096447	0.242689	0.808808	-6.4667	0.646759	0.710221
Monocytes	AY036118	-0.08075	4.212426	-0.24243	0.809006	-5.90397	0.701117	0.769659
Monocytes	USP37	-0.02451	6.268931	-0.24239	0.80904	-6.43042	0.661857	0.726832
Monocytes	DIS3L2	-0.02324	5.906117	-0.24233	0.809084	-6.37359	0.668601	0.734211
Monocytes	IGLC3	-0.15354	4.04289	-0.24229	0.809114	-5.51541	0.704467	0.7733
Monocytes	CABYR	-0.09059	1.135391	-0.24223	0.809161	-5.39081	0.764763	0.838435
Monocytes	GM26771	0.090261	-0.2163	0.242153	0.809222	-5.37794	0.794696	0.870521
Monocytes	RFTN2	0.094445	2.237638	0.241991	0.809348	-5.29809	0.741262	0.813229
Monocytes	NDUFA5	-0.03177	5.432033	-0.24198	0.80936	-6.28786	0.67753	0.744055
Monocytes	ZC3H13	-0.02446	5.653384	-0.24186	0.809449	-6.32894	0.673361	0.739517
Monocytes	SPCS3	0.025409	4.704887	0.241673	0.809593	-6.18243	0.691502	0.759357
Monocytes	SERINC1	0.019562	6.230989	0.241486	0.809738	-6.46441	0.662576	0.727769
Monocytes	GTF3A	0.026499	4.569389	0.241474	0.809747	-6.10956	0.694138	0.762226
Monocytes	SLC9A3R1	-0.02469	6.690174	-0.24141	0.809795	-6.44291	0.654139	0.718525
Monocytes	RNF111	-0.0217	6.692893	-0.24141	0.809798	-6.51742	0.65409	0.71847
Monocytes	GM4707	0.051636	2.866085	0.240828	0.810246	-5.87672	0.728408	0.799375
Monocytes	EVL	-0.02579	6.485099	-0.24076	0.810295	-6.56033	0.658047	0.722787
Monocytes	CPEB4	-0.02721	6.51399	-0.24071	0.810339	-6.4299	0.657517	0.722209
Monocytes	KLHL15	0.031493	4.413728	0.240471	0.810522	-6.02646	0.697343	0.765707
Monocytes	A130014A01RII	0.067062	1.887317	0.240406	0.810572	-5.46598	0.74884	0.821465
Monocytes	FOXJ3	0.021178	5.947043	0.240385	0.810588	-6.343	0.668011	0.733709
Monocytes	CSF1R	0.036502	5.549681	0.240309	0.810647	-6.69119	0.675478	0.741871
Monocytes	AURKB	-0.05257	4.237652	-0.2403	0.810654	-6.07762	0.700802	0.76947
Monocytes	MPO	0.136465	1.827356	0.240214	0.810721	-5.85388	0.750111	0.822836
Monocytes	SELENOT	0.019585	6.182995	0.240106	0.810804	-6.44519	0.663621	0.728905
Monocytes	SCARF1	-0.09572	1.587665	-0.24003	0.810863	-5.24471	0.75522	0.828337
Monocytes	RIPOR1	0.044315	3.960811	0.240023	0.810868	-5.75702	0.70628	0.775422
Monocytes	1700030K09RIK	0.096565	1.730931	0.239909	0.810956	-5.27147	0.752162	0.825045
Monocytes	AARS2	-0.10004	0.821033	-0.23981	0.811029	-5.20418	0.771814	0.846205
Monocytes	TAF12	0.026923	5.021001	0.239772	0.811062	-6.17164	0.685558	0.752916
Monocytes	NME3	-0.13273	0.405363	-0.23962	0.811182	-5.10326	0.780978	0.856105
Monocytes	SLC9A7	-0.02886	5.115655	-0.23953	0.811247	-6.32109	0.683741	0.751016
Monocytes	SAAL1	-0.04792	3.298168	-0.23943	0.811328	-5.77164	0.719584	0.790037
Monocytes	ZDHHC3	0.025901	5.048938	0.239327	0.811406	-6.22647	0.685021	0.752485
Monocytes	SERINC5	-0.04659	5.287482	-0.23929	0.811431	-5.92368	0.680456	0.747508
Monocytes	ZFAND6	-0.01764	6.978135	-0.23916	0.811532	-6.54988	0.649063	0.713139
Monocytes	SLC35A5	0.029334	3.986264	0.239158	0.811537	-6.02083	0.705774	0.775081
Monocytes	TMEM8	0.048518	2.000346	0.239088	0.81159	-5.72311	0.746448	0.819109
Monocytes	CELSR1	-0.12783	1.626552	-0.23898	0.811675	-5.23275	0.754402	0.827665

Monocytes	ESF1	-0.02557	5.001622	-0.23885	0.811775	-6.136	0.685969	0.753491
Monocytes	ING4	0.039832	4.08954	0.238514	0.812034	-5.87856	0.703851	0.772892
Monocytes	LHFPL2	0.06125	2.227897	0.238414	0.812111	-5.62814	0.74179	0.81398
Monocytes	BCAM	0.181776	0.298097	0.238365	0.81215	-5.0705	0.783499	0.858819
Monocytes	TRIM12A	0.035763	5.048891	0.238363	0.812151	-6.07839	0.685142	0.752525
Monocytes	CLCF1	-0.06177	2.650892	-0.238	0.812433	-5.61035	0.733117	0.804541
Monocytes	ACTR1A	0.019587	6.140647	0.237886	0.81252	-6.41018	0.664651	0.730095
Monocytes	KRR1	0.037055	3.94276	0.237821	0.81257	-5.90766	0.706899	0.776162
Monocytes	PLP2	-0.02761	5.623922	-0.2378	0.812587	-6.53272	0.674324	0.740674
Monocytes	PRR11	-0.07111	2.847187	-0.2376	0.81274	-5.5895	0.72914	0.800207
Monocytes	OTUD4	0.027049	5.091742	0.237212	0.813041	-6.17285	0.684621	0.751798
Monocytes	GM31645	-0.10948	0.726126	-0.2371	0.813129	-5.19594	0.774373	0.848879
Monocytes	METTL22	0.083555	1.382795	0.237023	0.813187	-5.27282	0.760084	0.833533
Monocytes	CACYBP	0.02417	5.736095	0.236984	0.813217	-6.37605	0.672378	0.738444
Monocytes	APOL9B	-0.11606	0.574633	-0.23696	0.813238	-5.15393	0.777711	0.852458
Monocytes	TEAD2	-0.11246	1.154161	-0.23691	0.813276	-5.10614	0.765027	0.838845
Monocytes	NCDN	-0.05563	2.826122	-0.23663	0.813492	-5.60419	0.729728	0.800824
Monocytes	MCRS1	0.031619	4.318538	0.236501	0.813591	-6.00742	0.699689	0.768271
Monocytes	ZFP330	0.028802	4.432352	0.23649	0.8136	-6.05258	0.697455	0.765841
Monocytes	NEDD8	-0.01462	7.48397	-0.23648	0.813609	-6.69849	0.640427	0.703466
Monocytes	TTI1	0.05763	2.712153	0.236288	0.813756	-5.64521	0.73215	0.803459
Monocytes	GM10863	0.116205	0.229142	0.236182	0.813838	-5.19468	0.785521	0.860911
Monocytes	MKNK2	0.020743	6.23257	0.235612	0.814279	-6.45842	0.663531	0.728623
Monocytes	RAD54L2	0.036348	4.552757	0.234904	0.814826	-5.84569	0.695851	0.763592
Monocytes	IPPK	0.051826	3.400734	0.234871	0.814852	-5.6543	0.718773	0.788478
Monocytes	PABPN1	0.017789	6.640276	0.2348	0.814907	-6.48798	0.656361	0.720494
Monocytes	ADGRE5	0.020145	7.153842	0.23448	0.815155	-6.68329	0.647142	0.710325
Monocytes	GGA2	-0.04605	4.120751	-0.23421	0.815367	-5.82016	0.704468	0.772963
Monocytes	TSPAN13	0.02293	6.684078	0.234013	0.815516	-6.56286	0.655668	0.719754
Monocytes	SEC11A	0.017555	6.406384	0.233822	0.815663	-6.4593	0.660768	0.725372
Monocytes	SLC35B3	0.026878	4.482615	0.233818	0.815667	-6.00395	0.697339	0.76528
Monocytes	USP15	0.020452	7.650516	0.233631	0.815811	-6.64095	0.638264	0.700712
Monocytes	DHX9	-0.0291	6.492589	-0.23356	0.81587	-6.42391	0.65918	0.723701
Monocytes	MS4A1	-0.09753	3.936889	-0.23354	0.815883	-5.63014	0.70812	0.777067
Monocytes	ZBTB42	-0.05523	1.544195	-0.23348	0.815931	-5.49889	0.757611	0.830556
Monocytes	MNAT1	-0.03223	4.818214	-0.23337	0.816014	-6.05651	0.6908	0.758273
Monocytes	SLC25A51	-0.03003	5.697758	-0.23316	0.816176	-6.20529	0.673984	0.739987
Monocytes	MRI1	0.03336	3.963231	0.233129	0.8162	-5.90468	0.707596	0.776594
Monocytes	UBR2	0.020141	6.362518	0.23301	0.816293	-6.4325	0.661578	0.726438
Monocytes	BC005624	0.019492	5.761918	0.233002	0.816299	-6.3623	0.672775	0.738687
Monocytes	AKNA	-0.02434	4.948701	-0.23299	0.816311	-6.25436	0.688276	0.755602
Monocytes	LINS1	-0.07066	1.704648	-0.23284	0.816425	-5.297	0.754176	0.826983
Monocytes	METAP2	-0.01851	7.3726	-0.23283	0.816432	-6.61829	0.643215	0.706302
Monocytes	ZC3HAV1	0.018604	8.43854	0.232802	0.816453	-6.86663	0.624458	0.685657
Monocytes	EGFEM1	-0.17226	1.229611	-0.23276	0.816486	-5.10153	0.764395	0.837974
Monocytes	NLRP3	0.037934	4.527197	0.23261	0.816602	-6.57983	0.696467	0.764585
Monocytes	DHX30	0.031726	4.509922	0.232553	0.816646	-6.04588	0.696805	0.764971

Monocytes	SCYL1	0.035158	4.072604	0.232502	0.816686	-5.99776	0.705422	0.77434
Monocytes	WDR89	0.052446	2.643388	0.23249	0.816694	-5.58881	0.734418	0.805755
Monocytes	ZFP458	-0.11866	0.876726	-0.23231	0.816834	-5.17015	0.772084	0.846314
Monocytes	ZFP982	-0.14001	0.056297	-0.23194	0.817123	-5.07287	0.790287	0.865896
Monocytes	UBXN4	-0.01411	6.814787	-0.23191	0.817147	-6.55468	0.653284	0.717495
Monocytes	GM12905	-0.05269	2.138199	-0.23189	0.81716	-5.48117	0.744979	0.817234
Monocytes	RHEB	0.016244	7.039284	0.231851	0.81719	-6.65049	0.64921	0.713025
Monocytes	OIP5OS1	0.023626	6.02273	0.231804	0.817226	-6.33551	0.667887	0.733492
Monocytes	2610206C17RIK	0.114587	0.40853	0.231606	0.817379	-5.10206	0.782416	0.857517
Monocytes	HARS	-0.02454	5.114979	-0.23145	0.8175	-6.16933	0.685074	0.752306
Monocytes	PREB	0.024757	4.736405	0.231427	0.817518	-6.19724	0.692388	0.760277
Monocytes	PMS2	-0.04034	3.954235	-0.23141	0.817532	-5.76986	0.707775	0.777011
Monocytes	STXBP5	-0.03218	5.383452	-0.23133	0.817596	-6.24466	0.67994	0.746713
Monocytes	CACNB1	0.117009	0.846674	0.231257	0.81765	-5.13736	0.772743	0.847192
Monocytes	RPAP1	-0.06124	2.431442	-0.23117	0.817718	-5.4472	0.738829	0.810734
Monocytes	RABIF	0.028098	4.708703	0.231035	0.817821	-6.00255	0.692926	0.760988
Monocytes	ZFP773	0.148209	0.510873	0.230968	0.817874	-5.02437	0.780145	0.855224
Monocytes	SNX2	-0.01611	7.314048	-0.23089	0.817933	-6.58196	0.644263	0.707746
Monocytes	GM43111	-0.06643	-0.1905	-0.23079	0.818011	-5.51327	0.795854	0.872042
Monocytes	DDA1	-0.02707	5.082621	-0.23058	0.818174	-6.2061	0.685696	0.753153
Monocytes	ZFP180	0.040506	3.315586	0.230465	0.818263	-5.74447	0.72062	0.791134
Monocytes	PPP1R21	-0.02946	4.557871	-0.23043	0.818288	-6.18408	0.695867	0.764262
Monocytes	B3GNT6	-0.09619	-0.72192	-0.23031	0.818383	-5.1798	0.807983	0.885101
Monocytes	BATF2	0.115349	0.948583	0.230299	0.818391	-5.27366	0.770512	0.845001
Monocytes	TMEM144	0.10265	0.882973	0.230282	0.818405	-5.23946	0.771947	0.846543
Monocytes	ADAM10	0.018034	7.403441	0.230103	0.818543	-6.62097	0.642663	0.706132
Monocytes	GM17160	-0.0922	0.089084	-0.23002	0.818611	-5.21016	0.789551	0.865475
Monocytes	GSTM1	-0.04397	4.059268	-0.22986	0.818731	-6.07603	0.705687	0.775029
Monocytes	ODF2L	0.062493	2.682465	0.229852	0.818738	-5.43901	0.733608	0.805297
Monocytes	IARS	-0.02867	5.056202	-0.22972	0.81884	-6.19936	0.686204	0.75384
Monocytes	DNAJC16	0.062113	2.30093	0.229692	0.818861	-5.53883	0.741559	0.813919
Monocytes	FNTA	-0.02032	5.505108	-0.22955	0.818969	-6.28693	0.677628	0.744526
Monocytes	KARS	0.035514	4.273346	0.229528	0.818988	-6.00788	0.701452	0.7705
Monocytes	CASP7	-0.03778	4.140272	-0.22937	0.819113	-5.97401	0.704081	0.77336
Monocytes	UTRN	-0.02341	7.31422	-0.22924	0.819209	-6.67597	0.64426	0.707975
Monocytes	PRPF8	-0.01993	5.801492	-0.2292	0.819241	-6.35333	0.672031	0.738427
Monocytes	CLPB	-0.03314	4.010407	-0.22916	0.819273	-5.86165	0.706657	0.776179
Monocytes	TSEN15	0.05205	2.776415	0.229154	0.819278	-5.57332	0.731665	0.803294
Monocytes	0610040B10RIK	0.068759	2.051019	0.229138	0.819291	-5.41482	0.746819	0.819665
Monocytes	BTF3	-0.01442	8.999494	-0.22908	0.819339	-6.94119	0.614836	0.675539
Monocytes	SLC25A26	-0.04408	3.795963	-0.22904	0.819369	-5.80602	0.710934	0.780826
Monocytes	NDST2	0.036553	2.934923	0.228622	0.819691	-5.90019	0.728587	0.799833
Monocytes	PHEX	0.109377	1.372943	0.228482	0.819799	-5.26093	0.761493	0.835365
Monocytes	ATP5F1	-0.01655	8.084387	-0.22819	0.820028	-6.76876	0.630783	0.693095
Monocytes	FBXW8	-0.0351	3.868261	-0.22817	0.820041	-5.89868	0.709673	0.779408
Monocytes	LETMD1	-0.05092	2.91201	-0.22812	0.820081	-5.57582	0.729058	0.800425
Monocytes	FRMD8OS	-0.08552	0.736632	-0.22811	0.820085	-5.33447	0.775361	0.850323

Monocytes	2410004B18RIK	-0.02464	4.71052	-0.22795	0.820211	-6.10213	0.693071	0.761347
Monocytes	STOML2	-0.0291	4.805582	-0.22794	0.82022	-6.17115	0.691224	0.759335
Monocytes	HINFP	-0.04333	3.494398	-0.22783	0.820306	-5.71183	0.717183	0.787563
Monocytes	CORO1C	-0.02007	6.202322	-0.2277	0.820404	-6.46865	0.664716	0.730406
Monocytes	TSC2	0.042815	3.109391	0.227693	0.82041	-5.68993	0.72501	0.796076
Monocytes	ATF5	-0.06872	3.267979	-0.22768	0.820419	-5.57066	0.721775	0.792571
Monocytes	CYP20A1	-0.02805	4.104318	-0.22759	0.820494	-6.04191	0.70498	0.774346
Monocytes	SRP68	-0.02783	3.983827	-0.22727	0.820741	-5.86721	0.707527	0.776961
Monocytes	KIF3B	0.034254	3.495441	0.227122	0.820853	-5.88361	0.717359	0.787598
Monocytes	MICOS13	-0.02063	5.948201	-0.22697	0.820969	-6.40757	0.669673	0.735631
Monocytes	GM867	-0.14243	-0.30876	-0.2268	0.821105	-5.01145	0.799017	0.875507
Monocytes	PRKD2	-0.04351	4.348606	-0.22678	0.82112	-5.74595	0.700391	0.769186
Monocytes	UBALD2	0.023617	7.028455	0.226298	0.821492	-6.56502	0.650037	0.714002
Monocytes	POLR2K	-0.01772	6.77475	-0.2258	0.821879	-6.54662	0.654781	0.719179
Monocytes	PMEL	0.12212	0.339511	0.225798	0.82188	-5.08974	0.784872	0.860173
Monocytes	ZFP748	0.075538	1.938501	0.225649	0.821995	-5.38788	0.75008	0.822833
Monocytes	FSTL1	-0.10459	2.107543	-0.22561	0.822027	-5.25585	0.746501	0.818975
Monocytes	IGTP	-0.07782	3.443365	-0.22557	0.822057	-5.74787	0.718873	0.789113
Monocytes	SMARCA5	0.016425	7.448521	0.225462	0.82214	-6.63605	0.642612	0.705891
Monocytes	ZFP64	-0.03022	5.317866	-0.22542	0.822169	-6.3078	0.68199	0.749029
Monocytes	KLRB1B	-0.0523	1.981821	-0.22521	0.822336	-6.01746	0.749161	0.821903
Monocytes	S1PR2	0.071732	1.261742	0.225189	0.822352	-5.43977	0.764596	0.838523
Monocytes	ELMO1	-0.0169	8.719508	-0.22518	0.822357	-6.89601	0.620345	0.681372
Monocytes	NOTCH1	0.02604	5.292946	0.225076	0.82244	-6.16093	0.682477	0.74957
Monocytes	MGAT4B	-0.03279	3.847759	-0.22498	0.822512	-6.03149	0.710746	0.780343
Monocytes	UBE2F	0.019536	6.306137	0.224687	0.822742	-6.54627	0.663539	0.728791
Monocytes	GALNT3	-0.06647	1.767982	-0.22447	0.822911	-5.57867	0.753871	0.826938
Monocytes	TPM2	-0.11698	0.689833	-0.22442	0.822947	-5.19791	0.777269	0.852086
Monocytes	CDK2	-0.03988	4.350301	-0.22427	0.823069	-5.9246	0.700909	0.769639
Monocytes	CDC34	0.02164	6.050691	0.224226	0.823099	-6.42181	0.668292	0.734055
Monocytes	PRELID2	0.066074	2.621255	0.224153	0.823155	-5.67065	0.735899	0.807575
Monocytes	BUB1	-0.05544	3.743439	-0.22412	0.823181	-5.92529	0.712976	0.782755
Monocytes	ASXL1	0.020774	7.242343	0.224034	0.823247	-6.54748	0.646447	0.710128
Monocytes	GLMN	-0.06132	2.819036	-0.22396	0.823303	-5.45302	0.7318	0.803178
Monocytes	FTCD	-0.11165	1.403237	-0.22342	0.823728	-5.24975	0.762009	0.835501
Monocytes	CYLD	-0.01887	6.329731	-0.22335	0.823777	-6.5208	0.663373	0.728469
Monocytes	EIF3H	0.016465	7.353901	0.223158	0.823928	-6.67013	0.64473	0.70803
Monocytes	GLIS2	-0.08579	1.3513	-0.22298	0.824062	-5.27039	0.76316	0.836766
Monocytes	CDK13	-0.0178	7.414024	-0.22295	0.824087	-6.65166	0.643653	0.706857
Monocytes	GM37065	0.101615	2.706239	0.222839	0.824175	-5.29039	0.734461	0.805829
Monocytes	GM20712	0.12912	0.063822	0.222806	0.824201	-5.12389	0.791568	0.867227
Monocytes	EXOSC1	0.032306	4.175365	0.222767	0.824231	-5.90667	0.704677	0.77355
Monocytes	RBM42	0.017603	6.063078	0.22268	0.824298	-6.4176	0.668358	0.733957
Monocytes	RTCA	0.029247	4.282973	0.222508	0.824432	-6.06816	0.702603	0.771246
Monocytes	SLAMF6	0.060492	4.602908	0.22232	0.824578	-5.63855	0.696381	0.764469
Monocytes	TBC1D13	-0.03876	3.495587	-0.2221	0.82475	-5.86025	0.7185	0.788426
Monocytes	RHOT1	0.021076	5.400436	0.221863	0.824932	-6.2629	0.681132	0.747814

Monocytes	IGLC1	-0.14646	3.761408	-0.22175	0.825023	-5.60503	0.713214	0.782727
Monocytes	POLG	-0.03976	3.808912	-0.22172	0.825043	-5.81123	0.71226	0.781692
Monocytes	NTHL1	0.089637	0.544589	0.221461	0.825244	-5.13784	0.78117	0.855965
Monocytes	SMUG1	0.065001	1.976529	0.221446	0.825256	-5.32925	0.750096	0.822577
Monocytes	NBN	-0.03706	3.4476	-0.22135	0.825326	-5.8752	0.719576	0.789597
Monocytes	TTC19	0.026385	4.958514	0.221314	0.825359	-6.23966	0.689651	0.757084
Monocytes	RUSC2	0.080349	0.909936	0.220984	0.825615	-5.44629	0.773151	0.847457
Monocytes	BNIP2	0.016662	6.377564	0.220965	0.825629	-6.54874	0.662838	0.727865
Monocytes	TRIM32	0.096722	1.488698	0.220887	0.82569	-5.26337	0.760568	0.833938
Monocytes	GUCY1A1	-0.1304	-0.00341	-0.22082	0.825744	-5.07544	0.793471	0.869256
Monocytes	JPX	0.058064	3.810956	0.220704	0.825832	-5.71122	0.712287	0.781842
Monocytes	ADAMTS9	-0.12384	2.970839	-0.22069	0.825842	-5.41907	0.729353	0.800335
Monocytes	C530008M17RI	0.110049	0.74516	0.220416	0.826056	-5.18	0.776775	0.851487
Monocytes	ADO	0.036513	3.708631	0.220348	0.826108	-5.81267	0.714342	0.784148
Monocytes	PNPO	0.037413	3.744874	0.220343	0.826112	-5.96626	0.713614	0.783357
Monocytes	ARMCX1	-0.12613	0.550831	-0.22032	0.826133	-5.08718	0.781072	0.856109
Monocytes	IGF1OS	-0.13629	-1.03209	-0.22027	0.826171	-5.04273	0.817051	0.894542
Monocytes	MTRF1	0.085583	1.208712	0.219989	0.826387	-5.25931	0.766743	0.840635
Monocytes	GM4107	-0.07695	0.916265	-0.21993	0.826436	-5.53387	0.773129	0.847492
Monocytes	DHX37	0.055461	2.67509	0.219832	0.826508	-5.50358	0.735581	0.807052
Monocytes	HMGB2	-0.03165	9.132267	-0.21957	0.826712	-6.93761	0.614158	0.674304
Monocytes	PLEKHG3	0.028406	4.378559	0.219556	0.826723	-6.24519	0.701179	0.769699
Monocytes	GM10184	0.091928	1.11236	0.219441	0.826813	-5.276	0.76893	0.842937
Monocytes	TMEM250-PS	0.03178	4.629225	0.219065	0.827104	-6.06931	0.696331	0.764458
Monocytes	PEBP1	0.019392	6.440655	0.219052	0.827114	-6.48232	0.661901	0.726862
Monocytes	RABGGTA	-0.04504	2.703471	-0.21902	0.827137	-5.61143	0.735133	0.806545
Monocytes	4930505N22RII	0.065014	1.214516	0.21884	0.827279	-5.43925	0.766767	0.840634
Monocytes	CIART	0.063905	2.328452	0.218766	0.827336	-5.51016	0.742964	0.815001
Monocytes	DCLRE1A	-0.05533	1.621612	-0.21873	0.827366	-5.56862	0.757972	0.831178
Monocytes	9330159M07RI	0.075665	1.076034	0.218655	0.827422	-5.35004	0.769783	0.843881
Monocytes	HNRNPD	0.014545	7.908504	0.218636	0.827437	-6.73517	0.6354	0.697767
Monocytes	ALDH1B1	-0.06862	1.910302	-0.21817	0.8278	-5.34353	0.752013	0.82477
Monocytes	KLHL8	-0.06333	2.402928	-0.21805	0.827896	-5.5714	0.741609	0.813557
Monocytes	LRRC3	0.07637	0.375073	0.218029	0.827909	-5.32658	0.785473	0.86072
Monocytes	MAD2L2	0.038988	3.472942	0.217944	0.827975	-5.82263	0.719551	0.789709
Monocytes	A330032B11RII	-0.12911	-0.15579	-0.21789	0.828019	-5.09474	0.797417	0.873509
Monocytes	MEF2D	0.020341	7.288749	0.217399	0.828398	-6.57016	0.64686	0.710259
Monocytes	TCF4	-0.02871	7.694523	-0.21731	0.828465	-6.50925	0.639603	0.702291
Monocytes	KDM2A	-0.0139	7.465818	-0.21697	0.828728	-6.62279	0.643741	0.706791
Monocytes	EAF2	-0.04145	3.647314	-0.21677	0.828889	-5.72526	0.716355	0.786062
Monocytes	IER5L	-0.05133	2.89009	-0.21655	0.829062	-5.63199	0.731812	0.80286
Monocytes	FAM72A	0.091883	1.069392	0.216504	0.829094	-5.20873	0.770499	0.844547
Monocytes	PGAP2	-0.02476	5.75264	-0.2164	0.829175	-6.40529	0.675249	0.741377
Monocytes	SFR1	-0.01762	6.44096	-0.21631	0.829247	-6.49867	0.662386	0.727305
Monocytes	SYBU	0.140375	0.330557	0.216116	0.829395	-5.07766	0.78683	0.86206
Monocytes	COMMD1	0.026507	5.141948	0.21603	0.829462	-6.18073	0.686895	0.754089
Monocytes	DUSP12	0.041435	3.283921	0.216011	0.829476	-5.71006	0.723728	0.794115

Monocytes	APOL11B	-0.25547	-0.04187	-0.21599	0.829494	-5.06113	0.795204	0.871017
Monocytes	NCK1	0.024739	6.174664	0.215961	0.829516	-6.33203	0.667329	0.732717
Monocytes	PARP3	-0.05597	2.362796	-0.21585	0.829604	-5.56412	0.742793	0.814726
Monocytes	STT3B	-0.01725	6.666062	-0.21583	0.829614	-6.43237	0.658239	0.722761
Monocytes	ELOF1	0.026312	5.375272	0.215608	0.82979	-6.17674	0.682419	0.749285
Monocytes	FAM83F	0.062665	1.041009	0.215433	0.829926	-5.57521	0.77112	0.845338
Monocytes	ARL10	0.044143	2.295947	0.21543	0.829928	-5.68898	0.744198	0.816359
Monocytes	ZBTB1	-0.0256	5.505188	-0.21539	0.829959	-6.29961	0.679941	0.746608
Monocytes	KIF15	0.047049	4.90741	0.215077	0.830203	-6.19003	0.691428	0.759138
Monocytes	CROCC	-0.08874	1.025643	-0.21504	0.830228	-5.2089	0.771456	0.845699
Monocytes	VKORC1L1	0.023404	5.560377	0.215031	0.830238	-6.29	0.678892	0.745462
Monocytes	SOS1	0.019612	5.916592	0.215024	0.830244	-6.36519	0.67216	0.738106
Monocytes	EGFR	0.108316	2.469637	0.214979	0.830279	-5.4256	0.740553	0.812425
Monocytes	MTHFSD	-0.06068	2.433778	-0.2149	0.830342	-5.43908	0.741304	0.813237
Monocytes	SSPN	0.120187	-0.41075	0.214827	0.830397	-4.98918	0.803593	0.880135
Monocytes	A930006K02RII	-0.05865	2.074746	-0.21478	0.830433	-5.42726	0.748867	0.821435
Monocytes	VPREB1	0.14574	2.621584	0.214719	0.830481	-5.28035	0.737381	0.80905
Monocytes	ZFP788	0.059017	2.069958	0.214505	0.830647	-5.37438	0.748969	0.821587
Monocytes	GM19522	-0.10058	0.940675	-0.21439	0.830737	-5.11943	0.773318	0.847782
Monocytes	OVGP1	0.10237	0.492644	0.214386	0.83074	-5.07912	0.783215	0.858397
Monocytes	DMAC2L	-0.06108	2.257897	-0.21433	0.830781	-5.40528	0.744999	0.817304
Monocytes	CEP131	0.092485	1.3043	0.21433	0.830783	-5.30502	0.765384	0.83926
Monocytes	NXT2	-0.03947	2.785909	-0.21432	0.830792	-5.68497	0.733968	0.805389
Monocytes	PDK4	0.103158	1.417008	0.214143	0.830929	-5.1842	0.762943	0.836667
Monocytes	4930435F18RIK	0.079874	0.358699	0.214097	0.830964	-5.41374	0.786201	0.861628
Monocytes	RPGRIP1L	0.072843	1.613197	0.214013	0.83103	-5.28553	0.758715	0.832118
Monocytes	S100PBP	0.028024	4.405401	0.213897	0.83112	-5.97896	0.701241	0.769927
Monocytes	POLR3E	-0.03327	3.918387	-0.21388	0.831132	-5.85458	0.71091	0.780435
Monocytes	NOD2	0.0376	1.576786	0.213732	0.831248	-6.06751	0.75954	0.83298
Monocytes	COMMD10	-0.02591	4.694613	-0.21362	0.831334	-6.17527	0.695621	0.763774
Monocytes	ST3GAL4	0.019814	6.423085	0.213518	0.831415	-6.54962	0.662775	0.72789
Monocytes	EXOC3L2	-0.08294	2.269133	-0.21291	0.831884	-5.72214	0.745185	0.817145
Monocytes	ARMCX4	-0.11361	1.19399	-0.21259	0.832134	-5.09138	0.768316	0.841938
Monocytes	CAPN1	0.03309	4.246316	0.212525	0.832186	-6.06459	0.704873	0.773408
Monocytes	PPP6R2	0.030493	4.368985	0.212466	0.832233	-5.92504	0.702447	0.770771
Monocytes	1700030J22RIK	-0.09275	-0.14389	-0.21242	0.83227	-5.30091	0.798069	0.873814
Monocytes	ZFP940	-0.09225	0.734633	-0.21212	0.832501	-5.11318	0.778543	0.852869
Monocytes	ZBTB39	0.070029	1.975209	0.211967	0.832621	-5.39152	0.751691	0.823995
Monocytes	ZFP688	0.073894	1.695397	0.211632	0.832881	-5.3099	0.757806	0.830459
Monocytes	GM10851	-0.04093	4.366594	-0.21158	0.832922	-5.85016	0.702799	0.770982
Monocytes	TOMM5	0.02536	5.458336	0.211503	0.832982	-6.29027	0.681603	0.747912
Monocytes	ZFP335OS	-0.03441	4.456083	-0.21128	0.833152	-5.96351	0.701079	0.769137
Monocytes	MIEF2	0.083084	1.112018	0.211255	0.833175	-5.20939	0.770486	0.844126
Monocytes	MYCT1	-0.085	1.654516	-0.21089	0.833455	-5.23718	0.758854	0.831579
Monocytes	SIGLECE	0.05842	2.293576	0.21081	0.833521	-5.90621	0.745256	0.816951
Monocytes	BUD31	0.022865	5.474604	0.21076	0.833559	-6.2698	0.681446	0.747738
Monocytes	PACC1	-0.02875	5.421952	-0.21072	0.83359	-6.29373	0.682451	0.748834

Monocytes	MAP3K7	0.019617	5.219048	0.21052	0.833746	-6.28453	0.686368	0.753112
Monocytes	PGLS	-0.0182	7.788311	-0.21049	0.833767	-6.64317	0.638886	0.701126
Monocytes	PPIH	0.023261	5.305306	0.210409	0.833833	-6.28682	0.684711	0.751305
Monocytes	STIL	-0.04762	4.156047	-0.20994	0.834201	-5.97187	0.707363	0.775842
Monocytes	ZNRF2	-0.01854	5.763077	-0.20992	0.834215	-6.41573	0.676189	0.741898
Monocytes	CSK	-0.01888	6.929767	-0.20973	0.83436	-6.55429	0.654543	0.718217
Monocytes	ZFP644	0.018586	6.331418	0.209595	0.834466	-6.43823	0.665558	0.730319
Monocytes	MEX3D	0.055842	2.642534	0.209593	0.834467	-5.54393	0.738213	0.809291
Monocytes	GM15965	0.047025	2.027609	0.209415	0.834606	-5.76298	0.751157	0.823314
Monocytes	AMPD2	0.051904	2.339378	0.209347	0.834659	-5.55301	0.744564	0.81621
Monocytes	SEC22C	-0.06611	2.04121	-0.20903	0.834902	-5.36206	0.750868	0.823046
Monocytes	MNT	0.027216	4.606728	0.208966	0.834955	-6.17716	0.698487	0.766342
Monocytes	DCAF1	0.03766	5.222364	0.20891	0.834999	-5.86655	0.686528	0.75332
Monocytes	IKZF4	0.090323	-0.0706	0.208874	0.835027	-5.12487	0.797223	0.872774
Monocytes	GM36975	-0.04896	3.276788	-0.20875	0.83512	-5.76118	0.725119	0.79524
Monocytes	DIAPH3	-0.04249	6.026174	-0.20875	0.835125	-6.42577	0.671257	0.736649
Monocytes	PURG	-0.0366	3.260975	-0.20873	0.83514	-5.86138	0.725442	0.79559
Monocytes	AMFR	-0.01671	6.011988	-0.20871	0.835157	-6.40255	0.671523	0.73694
Monocytes	ZFP868	0.03725	3.676008	0.208594	0.835245	-5.83554	0.717014	0.786483
Monocytes	MZT2	-0.04691	2.206691	-0.20852	0.835304	-5.49757	0.747368	0.819313
Monocytes	TMEM106A	-0.03329	3.645492	-0.20819	0.83556	-6.0758	0.71779	0.787236
Monocytes	SERPINA1E	0.078612	5.030433	0.208006	0.835702	-6.13027	0.69045	0.757504
Monocytes	MMS22L	-0.03973	4.820836	-0.20753	0.836076	-6.09117	0.694617	0.762058
Monocytes	GM15545	-0.07979	1.102359	-0.20741	0.836166	-5.15997	0.771454	0.845113
Monocytes	RABEPK	0.041002	3.296184	0.207146	0.836372	-5.69063	0.725051	0.795123
Monocytes	GRPEL2	0.045047	3.176522	0.207141	0.836376	-5.56221	0.727502	0.797775
Monocytes	TIMP2	-0.04651	4.873204	-0.20711	0.836403	-6.09466	0.693597	0.760977
Monocytes	TRAF3IP1	0.07763	1.889467	0.207098	0.836409	-5.3506	0.754441	0.826851
Monocytes	CENPS	0.058145	3.529733	0.207044	0.836451	-5.6759	0.720295	0.789973
Monocytes	HIST1H2BC	0.036779	5.260139	0.206993	0.836491	-6.20365	0.686113	0.752824
Monocytes	MOK	0.105151	0.377087	0.206929	0.836541	-5.17165	0.7875	0.862335
Monocytes	TDO2	0.095608	3.218699	0.206923	0.836546	-5.57123	0.726637	0.796839
Monocytes	ZFP146	-0.04618	3.413704	-0.20676	0.836675	-5.60847	0.722654	0.792549
Monocytes	ERI1	-0.02342	5.459943	-0.20669	0.836728	-6.33426	0.682284	0.748668
Monocytes	TCTA	0.053518	2.098287	0.206638	0.836768	-5.44424	0.749997	0.822087
Monocytes	VPS35	-0.01656	6.232281	-0.2066	0.8368	-6.49824	0.667706	0.732742
Monocytes	NIFK	-0.02706	4.620947	-0.20628	0.837047	-6.09393	0.698655	0.766412
Monocytes	ARMC5	-0.03431	3.383082	-0.20622	0.837095	-5.74912	0.723412	0.793285
Monocytes	TCTN1	-0.10468	1.315572	-0.206	0.837267	-5.12924	0.767039	0.840252
Monocytes	CITED2	0.023721	6.149916	0.205807	0.837415	-6.44803	0.669511	0.734496
Monocytes	DNAJC7	0.020568	7.510277	0.20514	0.837934	-6.57431	0.644947	0.707411
Monocytes	TMEM258	-0.01824	6.825258	-0.2049	0.838117	-6.58653	0.657364	0.72108
Monocytes	IMMP1L	-0.02321	4.995599	-0.20488	0.83814	-6.15254	0.691867	0.758763
Monocytes	TXNDC5	0.024308	5.179694	0.204871	0.838144	-6.30809	0.688305	0.754884
Monocytes	PRDM10	0.025642	5.213796	0.204763	0.838228	-6.2192	0.687658	0.754219
Monocytes	PSKH1	0.032687	3.273493	0.204641	0.838323	-5.75288	0.726227	0.796125
Monocytes	9130019O22RII	0.109995	0.355077	0.204312	0.83858	-5.12041	0.788889	0.863417

Monocytes	FAM32A	0.01875	5.312501	0.204293	0.838594	-6.27515	0.685887	0.75221
Monocytes	LAX1	-0.07896	2.319766	-0.20406	0.838775	-5.3515	0.746173	0.817558
Monocytes	CXCL1	0.103642	2.815955	0.204026	0.838802	-5.79865	0.735787	0.806353
Monocytes	ATAD2	-0.02995	6.309192	-0.20388	0.838915	-6.41693	0.66704	0.731639
Monocytes	HMGN2	0.028678	7.53605	0.203835	0.838951	-6.61029	0.644624	0.707073
Monocytes	DDX52	0.02219	4.930462	0.203761	0.839009	-6.18071	0.693281	0.760269
Monocytes	KBTBD2	-0.0196	5.176548	-0.20374	0.839028	-6.18507	0.688514	0.755078
Monocytes	RIPK3	0.037381	3.285039	0.203595	0.839138	-5.86024	0.726149	0.795897
Monocytes	TBCD	0.025566	4.783335	0.20332	0.839352	-6.12149	0.696195	0.763389
Monocytes	AATK	-0.08998	0.48294	-0.20326	0.839397	-5.2615	0.786091	0.860374
Monocytes	RAD51B	-0.03611	6.152694	-0.20318	0.839461	-6.41256	0.670006	0.734834
Monocytes	FANCG	0.05691	2.222876	0.203171	0.839468	-5.43778	0.748269	0.819764
Monocytes	CTDSP1	0.021994	5.3236	0.202861	0.83971	-6.32522	0.685728	0.751993
Monocytes	OSTF1	-0.01249	7.480967	-0.20276	0.839786	-6.72109	0.645655	0.708158
Monocytes	LENG8	0.027714	4.564084	0.202745	0.8398	-6.05103	0.700493	0.768063
Monocytes	PYGO2	-0.04576	3.374512	-0.20263	0.83989	-5.80189	0.724333	0.793927
Monocytes	KIF20B	0.051882	4.170808	0.202605	0.839909	-5.97541	0.708277	0.776526
Monocytes	SYNRG	0.021127	5.053855	0.202552	0.83995	-6.25132	0.690932	0.75767
Monocytes	ADPRH	-0.02355	4.93883	-0.20252	0.839979	-6.24341	0.693164	0.7601
Monocytes	TMEM245	0.028529	4.753996	0.202493	0.839996	-6.07276	0.696768	0.764021
Monocytes	COPS7B	0.038313	3.34463	0.202261	0.840178	-5.72031	0.725039	0.7946
Monocytes	SUPT16	-0.0156	7.068147	-0.20205	0.840339	-6.55836	0.653233	0.716362
Monocytes	MRAP	-0.12164	1.214744	-0.20203	0.840359	-5.12025	0.770074	0.843085
Monocytes	LLGL1	0.042588	2.998446	0.20183	0.840513	-5.64013	0.732251	0.802291
Monocytes	ZFP566	-0.08481	1.254913	-0.20171	0.840608	-5.20759	0.769265	0.842144
Monocytes	CDT1	0.029891	4.370678	0.201663	0.840643	-5.95185	0.704498	0.77223
Monocytes	CKAP5	0.026547	6.118962	0.201464	0.840799	-6.37908	0.670886	0.735544
Monocytes	SH3GLB1	-0.01503	8.546317	-0.20137	0.840871	-6.84213	0.627077	0.687489
Monocytes	PIK3AP1	-0.02581	8.295612	-0.20099	0.84117	-6.7982	0.631621	0.692387
Monocytes	GM48089	-0.10114	1.135961	-0.20071	0.841387	-5.19387	0.772288	0.845056
Monocytes	2010300C02RIK	-0.10434	-0.0882	-0.20046	0.841578	-5.03488	0.799664	0.874314
Monocytes	TRPM4	-0.06651	1.109553	-0.20046	0.841585	-5.34207	0.77292	0.845714
Monocytes	DARS	0.022533	5.511648	0.200233	0.841758	-6.31016	0.682815	0.748313
Monocytes	DNAJA2	0.013393	7.208069	0.199777	0.842114	-6.62642	0.651295	0.713875
Monocytes	PCGF6	-0.04955	2.512206	-0.19974	0.842144	-5.44131	0.742996	0.813556
Monocytes	SCNM1	0.034983	3.621272	0.199736	0.842145	-5.77779	0.720111	0.788836
Monocytes	SLC36A3OS	-0.05154	1.010121	-0.1997	0.842178	-5.80456	0.775273	0.848245
Monocytes	GM38973	-0.07548	1.613034	-0.19968	0.842192	-5.34142	0.762137	0.834152
Monocytes	EIF3F	-0.01517	7.589953	-0.19945	0.842368	-6.7345	0.644495	0.706327
Monocytes	PAF1	-0.02068	4.600235	-0.19928	0.8425	-6.2165	0.700696	0.767631
Monocytes	ARF2	-0.02451	4.470722	-0.19911	0.842634	-6.31561	0.703263	0.770404
Monocytes	ZFP606	0.053369	2.487995	0.19908	0.842657	-5.39977	0.743666	0.814104
Monocytes	NCS1	0.0951	0.210797	0.198488	0.843119	-5.18175	0.793611	0.86741
Monocytes	RTF2	0.01589	5.957747	0.198175	0.843363	-6.42531	0.674944	0.739317
Monocytes	CHD6	-0.02248	5.997264	-0.19807	0.843447	-6.33406	0.674199	0.738534
Monocytes	TMEM134	0.018094	6.132006	0.198012	0.843491	-6.47637	0.671666	0.735768
Monocytes	1810046K07RIK	0.122071	-0.14929	0.197939	0.843547	-4.99686	0.801864	0.87622

Monocytes	DCUN1D5	0.012982	6.828224	0.197868	0.843602	-6.57135	0.658742	0.721649
Monocytes	GM49359	0.056375	2.260358	0.197831	0.843631	-5.43469	0.748894	0.819485
Monocytes	ANKRD26	0.065182	2.576265	0.197576	0.84383	-5.39538	0.742275	0.812393
Monocytes	RAMAC	-0.01831	5.913686	-0.19757	0.843838	-6.34247	0.67581	0.740329
Monocytes	ACOD1	-0.10847	2.318035	-0.19751	0.843884	-5.865	0.747711	0.818249
Monocytes	GM42031	0.060321	4.172175	0.197215	0.844112	-6.04169	0.709761	0.777225
Monocytes	REXO1	-0.02297	4.912691	-0.19672	0.8445	-6.1775	0.695414	0.76145
Monocytes	GPC4	0.094709	1.1587	0.196619	0.844577	-5.17275	0.773101	0.845292
Monocytes	LYSMD2	0.099115	1.265146	0.196471	0.844692	-5.14299	0.770813	0.842858
Monocytes	NUP50	0.020296	5.304963	0.196294	0.84483	-6.23568	0.687901	0.753268
Monocytes	SHCBP1L	0.078891	0.854389	0.196211	0.844895	-5.22526	0.779902	0.852546
Monocytes	RMND5B	0.026399	4.49308	0.195995	0.845064	-5.98881	0.703829	0.770488
Monocytes	CCDC34	0.034937	4.840796	0.195821	0.8452	-6.18873	0.697047	0.763065
Monocytes	RNPEPL1	-0.02708	4.75379	-0.19557	0.845393	-6.14851	0.698852	0.764928
Monocytes	FCRL5	-0.09574	-0.65204	-0.19521	0.845673	-5.10615	0.814452	0.888975
Monocytes	ADAM11	-0.08844	-1.14352	-0.19519	0.845689	-5.04467	0.825937	0.901169
Monocytes	GM43330	0.067444	1.526899	0.195031	0.845816	-5.31204	0.765624	0.836828
Monocytes	MEI4	-0.13506	1.201602	-0.19452	0.846213	-5.02697	0.772989	0.844526
Monocytes	PDIA5	-0.03082	2.273787	-0.19447	0.846257	-6.06036	0.749876	0.819726
Monocytes	CARMIL2	0.070222	2.704609	0.193304	0.847165	-5.26793	0.741263	0.810102
Monocytes	1700102H20RIK	-0.09023	0.529845	-0.19329	0.847175	-5.1327	0.788353	0.86058
Monocytes	D930030I03RIK	-0.06431	0.408475	-0.19327	0.847192	-5.46742	0.791076	0.863485
Monocytes	ARFGAP2	0.020314	4.901097	0.193266	0.847194	-6.14214	0.696823	0.762065
Monocytes	USP49	0.033694	4.534917	0.193238	0.847216	-6.03198	0.704022	0.769873
Monocytes	ITFG1	-0.01785	5.454025	-0.1932	0.847243	-6.27852	0.686107	0.750424
Monocytes	CBX3	0.018542	8.102288	0.193161	0.847276	-6.71147	0.637282	0.697091
Monocytes	LIN7C	0.02177	4.956273	0.192912	0.847471	-6.16474	0.695847	0.760934
Monocytes	HERC4	0.016912	7.008496	0.192651	0.847674	-6.62871	0.657089	0.71869
Monocytes	POLH	-0.03661	4.035992	-0.19259	0.847722	-5.78126	0.714106	0.780708
Monocytes	DUS3L	0.034039	3.300011	0.192582	0.847728	-5.73766	0.72906	0.796863
Monocytes	SDHAF4	0.025621	4.365947	0.192369	0.847895	-6.03949	0.707593	0.773611
Monocytes	GM49169	0.125467	-0.42801	0.192238	0.847997	-4.98052	0.810401	0.883958
Monocytes	PKD2	0.077922	0.719693	0.192033	0.848156	-5.1633	0.784473	0.856248
Monocytes	FAM133B	0.021743	4.911343	0.191937	0.848232	-6.11306	0.696944	0.76204
Monocytes	TOGARAM1	-0.01927	5.297825	-0.19184	0.848306	-6.2871	0.689437	0.753901
Monocytes	CEP128	-0.02385	6.336302	-0.19162	0.848482	-6.48554	0.669773	0.7325
Monocytes	GM38560	-0.06798	0.991541	-0.19135	0.848692	-5.42636	0.778536	0.850014
Monocytes	TRIP13	-0.05779	2.772205	-0.19123	0.848786	-5.60379	0.740274	0.808966
Monocytes	KPNA6	0.028657	3.896998	0.190953	0.849	-5.85983	0.717172	0.78407
Monocytes	EMD	-0.01584	6.102062	-0.19093	0.849015	-6.46112	0.674169	0.737389
Monocytes	PCBP3	-0.04931	2.616922	-0.19054	0.849323	-5.49575	0.743527	0.812676
Monocytes	FGFR1OP	-0.02381	4.601111	-0.19035	0.849467	-6.19589	0.703117	0.769024
Monocytes	CGRRF1	0.028608	3.907331	0.190242	0.849556	-5.8877	0.716964	0.784019
Monocytes	METTL21A	-0.04216	1.888583	-0.18992	0.849805	-5.5407	0.758997	0.829348
Monocytes	B4GALT4	-0.0433	1.774117	-0.18988	0.849836	-5.64275	0.76146	0.831999
Monocytes	MRPS2	0.048513	2.843371	0.189882	0.849837	-5.69842	0.738788	0.807623
Monocytes	CYB5D2	-0.06155	1.870839	-0.18986	0.849853	-5.41719	0.759378	0.829766

Monocytes	PIK3R2	0.047821	2.306399	0.189857	0.849857	-5.59027	0.75008	0.819777
Monocytes	CDC5L	-0.01417	5.768771	-0.1898	0.849902	-6.38868	0.68048	0.744471
Monocytes	PROS1	0.03732	2.741429	0.189774	0.849921	-5.88266	0.740917	0.809919
Monocytes	CYP2C69	0.089873	1.432054	0.189741	0.849947	-5.25862	0.768873	0.839949
Monocytes	HIST1H3B	0.099062	1.995292	0.189588	0.850067	-5.37708	0.756709	0.826915
Monocytes	GM33104	-0.07707	0.440079	-0.18958	0.85007	-5.35225	0.790819	0.863431
Monocytes	TBC1D32	-0.05019	2.141265	-0.18953	0.850112	-5.62102	0.75359	0.823565
Monocytes	NSD3	-0.01476	8.849458	-0.18953	0.850113	-6.8644	0.624584	0.683305
Monocytes	MAU2	-0.02273	6.192162	-0.18948	0.850149	-6.38449	0.672474	0.735775
Monocytes	ENGASE	0.06501	1.496503	0.189436	0.850185	-5.47961	0.767471	0.838479
Monocytes	GM42726	-0.02763	3.69069	-0.18944	0.850185	-5.93394	0.721349	0.788838
Monocytes	4930477G07RII	0.111504	-0.38448	0.189287	0.850301	-5.09013	0.809578	0.883483
Monocytes	MLX	0.030584	3.767938	0.189207	0.850364	-5.86278	0.719782	0.787209
Monocytes	IL1RAP	0.027292	4.731572	0.189178	0.850387	-6.26244	0.700547	0.76638
Monocytes	MDC1	0.039898	3.159139	0.189148	0.85041	-5.65167	0.732235	0.800666
Monocytes	BCAS2	0.014094	6.380106	0.188943	0.85057	-6.46251	0.668954	0.732066
Monocytes	NFYB	0.023615	5.103926	0.188864	0.850632	-6.16386	0.693268	0.758561
Monocytes	4930445E18RIK	0.073295	-0.41363	0.188809	0.850675	-5.23964	0.810251	0.884339
Monocytes	SURF4	0.019265	5.782118	0.188716	0.850748	-6.40181	0.680226	0.744381
Monocytes	ACADVL	0.025705	4.901912	0.188499	0.850918	-6.15765	0.697206	0.762847
Monocytes	PSD	-0.0663	1.633137	-0.18845	0.850957	-5.32678	0.764506	0.835478
Monocytes	IMP4	-0.02351	4.46298	-0.18841	0.850986	-6.05429	0.70585	0.772226
Monocytes	CLASRP	0.036819	3.334689	0.188358	0.851028	-5.71007	0.72862	0.79686
Monocytes	EXT1	0.019366	7.749502	0.188348	0.851035	-6.97192	0.643924	0.704708
Monocytes	SPP1	0.08579	2.353951	0.188346	0.851037	-5.66775	0.749072	0.8189
Monocytes	PEX12	-0.067	1.86574	-0.18811	0.851219	-5.30354	0.759488	0.8302
Monocytes	ZC3H8	0.055945	2.13952	0.187958	0.85134	-5.36536	0.753628	0.823946
Monocytes	IL18	-0.02449	3.422293	-0.18793	0.851363	-6.20601	0.726823	0.795063
Monocytes	TLE5	-0.01515	7.209326	-0.18791	0.851375	-6.65743	0.653669	0.715512
Monocytes	LIN52	0.025939	5.546829	0.18787	0.851409	-6.17975	0.684719	0.749415
Monocytes	MPV17L2	0.025961	4.459696	0.187797	0.851466	-6.12792	0.705915	0.772443
Monocytes	UBA2	-0.01583	6.457603	-0.18775	0.851504	-6.46626	0.667509	0.730657
Monocytes	SAYSD1	-0.03103	3.323883	-0.18724	0.851903	-5.77628	0.729019	0.79741
Monocytes	DCAF10	0.020242	5.193751	0.187192	0.851939	-6.20351	0.691693	0.756969
Monocytes	ACADM	0.027665	4.767805	0.187189	0.851941	-6.17321	0.700005	0.765999
Monocytes	GRK2	-0.01491	7.206149	-0.18712	0.851993	-6.62421	0.653886	0.715742
Monocytes	ACD	0.024031	4.940696	0.186798	0.852247	-6.08986	0.696722	0.762419
Monocytes	PPP2R3A	0.032732	5.003266	0.186779	0.852262	-6.14777	0.6955	0.761092
Monocytes	NUP88	-0.0242	4.421593	-0.18647	0.852504	-6.06291	0.70707	0.773573
Monocytes	GM43061	0.103156	0.306464	0.186412	0.852549	-5.06286	0.794274	0.867356
Monocytes	ELOVL6	-0.03692	4.451031	-0.18623	0.852687	-5.98105	0.706513	0.772965
Monocytes	4930599N23RII	-0.0483	1.733523	-0.18618	0.852729	-5.61029	0.762796	0.833671
Monocytes	DNAJC11	-0.02382	4.486406	-0.1861	0.852794	-6.068	0.705811	0.772206
Monocytes	SLC17A9	0.069403	2.888147	0.185901	0.852948	-5.3799	0.738372	0.807361
Monocytes	4833418N02RII	-0.06488	1.533706	-0.18543	0.853319	-5.38741	0.767469	0.838445
Monocytes	SNW1	0.013882	6.240739	0.185254	0.853454	-6.44198	0.67232	0.735524
Monocytes	2210408I21RIK	0.047752	3.277757	0.18506	0.853606	-5.59157	0.730639	0.798764

Monocytes	FCGR4	-0.0441	3.388468	-0.18499	0.853663	-6.25969	0.728363	0.796307
Monocytes	AI506816	0.029861	5.207229	0.184945	0.853696	-6.28032	0.692067	0.757012
Monocytes	ALDH6A1	-0.06738	2.786139	-0.18477	0.853831	-5.46352	0.74086	0.80979
Monocytes	SRSF3	-0.01341	7.532017	-0.18474	0.853857	-6.6647	0.648596	0.709578
Monocytes	IFT172	-0.03224	2.410455	-0.18391	0.854503	-5.83614	0.749245	0.818349
Monocytes	WDHD1	-0.03308	4.56722	-0.18386	0.854547	-6.0283	0.705074	0.77068
Monocytes	MEPCE	-0.03037	4.466254	-0.18372	0.854657	-5.97873	0.707088	0.772852
Monocytes	INF2	0.03328	3.252642	0.183635	0.854721	-5.94717	0.731655	0.799402
Monocytes	MYBL2	-0.0516	3.080165	-0.18357	0.854773	-5.60003	0.735223	0.803247
Monocytes	POT1A	-0.04391	3.012767	-0.1831	0.855135	-5.54086	0.736873	0.804847
Monocytes	NMRK1	0.033798	3.751312	0.18286	0.855326	-5.80631	0.721796	0.788513
Monocytes	ISCU	-0.01608	7.465509	-0.18263	0.855508	-6.71713	0.650589	0.711129
Monocytes	CCDC88B	0.023347	3.695402	0.182593	0.855535	-6.16535	0.72299	0.789821
Monocytes	SPR	0.029195	3.527522	0.182272	0.855787	-5.94282	0.726526	0.793565
Monocytes	SLC7A6	-0.02511	4.274758	-0.18225	0.855807	-6.01119	0.711409	0.777231
Monocytes	TFAP4	0.05259	2.748617	0.182153	0.85588	-5.51785	0.742665	0.810972
Monocytes	RNPS1	0.01566	6.167896	0.181749	0.856196	-6.40636	0.674847	0.737387
Monocytes	NR5A2	0.07904	1.145394	0.181593	0.856318	-5.16414	0.777401	0.847999
Monocytes	HIST3H2A	-0.06105	2.104979	-0.18133	0.856521	-5.42644	0.7566	0.825736
Monocytes	APOOL	0.02857	3.780654	0.181317	0.856534	-5.87724	0.721657	0.78813
Monocytes	RNF128	0.057022	2.499728	0.18119	0.856633	-5.47462	0.748204	0.816737
Monocytes	KLF2	-0.03126	8.937753	-0.18117	0.856648	-6.90839	0.624907	0.682752
Monocytes	CCDC43	0.034395	3.103767	0.180991	0.856789	-5.69462	0.735559	0.803178
Monocytes	9330136K24RIK	-0.04131	1.883233	-0.18098	0.856796	-5.73017	0.761368	0.830911
Monocytes	PPM1F	0.059544	2.206568	0.180705	0.857012	-5.3314	0.754564	0.823554
Monocytes	NIT2	0.038173	3.588667	0.180435	0.857224	-5.78721	0.725795	0.792576
Monocytes	KMT2C	-0.0146	7.717211	-0.18038	0.857269	-6.69717	0.646613	0.706554
Monocytes	9330111N05RII	0.080913	0.392898	0.180229	0.857385	-5.25092	0.794516	0.866253
Monocytes	MRPL4	-0.01805	5.176328	-0.1801	0.857483	-6.31875	0.694174	0.758344
Monocytes	TMEM167B	0.033272	3.755594	0.179975	0.857584	-5.83257	0.722438	0.788936
Monocytes	PKN2	-0.01698	6.774243	-0.17979	0.857726	-6.50459	0.66385	0.725347
Monocytes	A730081D07RII	0.040749	3.129627	0.179787	0.857731	-5.74692	0.735294	0.802799
Monocytes	PPIL1	0.035061	4.052063	0.179737	0.857771	-5.85655	0.716436	0.782453
Monocytes	ZFP472	-0.04005	3.448072	-0.17965	0.857836	-5.62819	0.728723	0.795717
Monocytes	SPATA13	-0.02588	5.147731	-0.17956	0.857912	-6.21732	0.694731	0.758948
Monocytes	GM42917	-0.05099	1.691044	-0.17935	0.858073	-5.4959	0.765804	0.835569
Monocytes	KAT2A	0.035533	3.199202	0.179295	0.858116	-5.73884	0.733853	0.801246
Monocytes	CDC14B	0.040443	3.454363	0.179238	0.858161	-5.76209	0.728593	0.795577
Monocytes	BAZ1A	-0.01398	7.694958	-0.17922	0.858174	-6.74992	0.647055	0.706993
Monocytes	GM16973	0.055588	1.894858	0.179058	0.858302	-5.40171	0.761448	0.830848
Monocytes	CPD	0.024138	4.333844	0.178369	0.858841	-6.1928	0.711058	0.776456
Monocytes	ABCB6	0.067846	1.101522	0.17833	0.858872	-5.17241	0.779006	0.849502
Monocytes	BC031181	0.015474	5.703047	0.178214	0.858963	-6.34188	0.684275	0.747444
Monocytes	TAF5	-0.02587	4.146651	-0.17816	0.859006	-5.96256	0.71481	0.780541
Monocytes	MIA2	0.012315	6.77012	0.178123	0.859034	-6.63718	0.664184	0.725566
Monocytes	PARP12	-0.02773	3.018486	-0.17794	0.85918	-6.14159	0.737888	0.805454
Monocytes	CLNK	-0.03709	1.404175	-0.17791	0.859201	-6.03113	0.772351	0.842446

Monocytes	ABI1	-0.01694	7.702393	-0.17781	0.859277	-6.7042	0.647172	0.707011
Monocytes	PSMD9	-0.01918	5.225742	-0.17773	0.859343	-6.17802	0.693482	0.757502
Monocytes	MOSMO	-0.02431	4.576833	-0.17772	0.859353	-6.0443	0.706221	0.771311
Monocytes	FAM78A	-0.05033	2.648934	-0.17754	0.85949	-5.48116	0.745626	0.813855
Monocytes	IFITM1	0.086411	3.489377	0.177404	0.859597	-5.9011	0.728157	0.795066
Monocytes	1810013L24RIK	0.017247	5.901803	0.177316	0.859666	-6.39899	0.680482	0.743444
Monocytes	MARVELD1	0.043832	1.253094	0.177195	0.85976	-5.59476	0.775665	0.846111
Monocytes	NLGN2	-0.09644	0.349554	-0.17698	0.859927	-5.18763	0.795813	0.867658
Monocytes	ORMDL2	0.018623	5.109617	0.176976	0.859932	-6.28441	0.695743	0.760071
Monocytes	HS1BP3	0.056303	1.087416	0.176931	0.859967	-5.34202	0.779318	0.850059
Monocytes	HIGD2A	-0.01221	6.065754	-0.17691	0.859985	-6.52713	0.67737	0.740105
Monocytes	SAPCD2	-0.06608	1.790194	-0.1769	0.859989	-5.32669	0.763953	0.833617
Monocytes	ZFP799	-0.07222	0.939029	-0.1769	0.859995	-5.20427	0.782605	0.85357
Monocytes	DCAF13	-0.01976	4.771338	-0.17656	0.860259	-6.15957	0.702531	0.767363
Monocytes	DENND1B	-0.01641	7.299486	-0.17647	0.860332	-6.63945	0.654608	0.715217
Monocytes	PSMD11	0.010025	7.032517	0.176381	0.860398	-6.59398	0.659491	0.720552
Monocytes	ABHD17A	0.013973	6.296745	0.176186	0.86055	-6.44695	0.673227	0.735464
Monocytes	DENND2A	0.065785	1.374468	0.175839	0.860822	-5.35697	0.773427	0.843479
Monocytes	ARF1	0.01123	8.02586	0.175661	0.860962	-6.76708	0.641789	0.700997
Monocytes	TICRR	-0.04968	3.009192	-0.17556	0.861043	-5.60837	0.738556	0.806051
Monocytes	BMT2	-0.01759	6.027835	-0.1752	0.86132	-6.42118	0.678686	0.741156
Monocytes	FAM71E1	0.092231	0.837279	0.174992	0.861486	-5.13302	0.785619	0.856381
Monocytes	SCLY	-0.03167	3.466279	-0.17494	0.861528	-5.75829	0.729329	0.796022
Monocytes	PARP1	-0.02307	5.30881	-0.1748	0.861639	-6.20432	0.692565	0.756257
Monocytes	CENPE	0.043473	4.9887	0.174665	0.861743	-6.21996	0.698833	0.76304
Monocytes	IQSEC1	-0.01926	6.277452	-0.1743	0.862025	-6.35506	0.674244	0.736203
Monocytes	TOP3B	0.028228	3.549805	0.173423	0.862715	-5.75284	0.728327	0.794486
Monocytes	EIF2A	0.015423	5.395584	0.173409	0.862726	-6.27276	0.691528	0.754697
Monocytes	ATP6V1G2	0.062282	1.237127	0.17331	0.862804	-5.30362	0.777526	0.847289
Monocytes	ANKS3	0.029251	4.082767	0.172758	0.863237	-5.84824	0.717789	0.782872
Monocytes	LYRM7	0.075449	1.247217	0.172497	0.863441	-5.17343	0.777673	0.847254
Monocytes	RSBN1L	0.015375	7.198782	0.172447	0.86348	-6.53562	0.657889	0.717957
Monocytes	RUFY2	0.035659	2.954919	0.17226	0.863627	-5.63409	0.741003	0.808014
Monocytes	CAMTA1	0.016111	5.835211	0.172259	0.863628	-6.35957	0.683401	0.745763
Monocytes	SCP2	-0.02375	7.526193	-0.17222	0.863658	-6.69151	0.651924	0.711472
Monocytes	SRM	-0.03568	4.842685	-0.17215	0.863711	-6.08678	0.702668	0.766675
Monocytes	H3F3B	0.0111	9.934295	0.171977	0.863849	-7.02317	0.609912	0.665393
Monocytes	PPP1R12B	0.018433	5.292478	0.171641	0.864112	-6.26916	0.69389	0.757148
Monocytes	MLXIP	0.018009	6.94185	0.171584	0.864156	-6.48474	0.662639	0.723167
Monocytes	ING5	0.030251	3.456639	0.171553	0.864181	-5.76264	0.730622	0.796845
Monocytes	UBAP1	0.019511	5.688984	0.171533	0.864197	-6.34603	0.686229	0.748836
Monocytes	MCTP2	-0.0313	6.80258	-0.17145	0.864262	-6.17516	0.665216	0.725976
Monocytes	CKLF	0.020179	4.804772	0.171394	0.864306	-6.2055	0.703443	0.767503
Monocytes	RBM28	0.01866	5.311789	0.171337	0.86435	-6.20724	0.693514	0.756759
Monocytes	SMC3	-0.01486	6.498954	-0.17129	0.864384	-6.46514	0.670872	0.732156
Monocytes	MRPL21	0.019422	5.233418	0.170995	0.864619	-6.25595	0.695145	0.758468
Monocytes	ZFP346	0.03572	3.658973	0.170777	0.864789	-5.77855	0.726581	0.792506

Monocytes	COMT	-0.01998	5.36738	-0.1707	0.86485	-6.24221	0.692541	0.755718
Monocytes	GM10790	-0.09323	-0.77429	-0.1706	0.864929	-4.98142	0.823815	0.896422
Monocytes	VMN2R19	0.081004	-0.79625	0.17059	0.864936	-5.20028	0.824331	0.896968
Monocytes	TIMM17A	-0.01715	5.135955	-0.17047	0.865033	-6.23043	0.697047	0.760612
Monocytes	GM11772	0.051057	0.302935	0.170406	0.86508	-5.43346	0.798951	0.870038
Monocytes	BC055324	0.046464	2.245694	0.1704	0.865085	-5.50042	0.756143	0.824316
Monocytes	MAP3K9	0.063331	0.308951	0.170273	0.865184	-5.38553	0.798839	0.869893
Monocytes	URAH	-0.05849	3.327083	-0.17018	0.865254	-5.66907	0.73343	0.799895
Monocytes	HGSNAT	0.02573	4.642285	0.169768	0.86558	-6.26129	0.706998	0.771252
Monocytes	ANKFY1	-0.01647	5.995175	-0.16939	0.86588	-6.4025	0.680786	0.742802
Monocytes	GCFC2	0.044	2.248325	0.169369	0.865893	-5.43581	0.756424	0.824443
Monocytes	GGACT	-0.03439	3.170013	-0.16936	0.865898	-5.58192	0.736991	0.803575
Monocytes	STX3	0.029113	2.314188	0.169138	0.866074	-6.08331	0.755064	0.822945
Monocytes	PUS1	-0.02741	3.723827	-0.16902	0.866165	-5.90836	0.725625	0.791296
Monocytes	UBE2M	-0.01356	7.20691	-0.16902	0.866165	-6.61675	0.658202	0.718172
Monocytes	TOMM7	0.011646	7.561253	0.168719	0.866403	-6.71958	0.651749	0.711178
Monocytes	FAM20A	0.065596	1.833508	0.168705	0.866414	-5.40974	0.765404	0.834083
Monocytes	BST2	-0.0268	6.606914	-0.16865	0.866454	-6.51353	0.669301	0.730324
Monocytes	9330175E14RIK	-0.08365	1.002962	-0.16861	0.866487	-5.25254	0.783631	0.853559
Monocytes	LDB1	-0.02484	4.482765	-0.16856	0.866524	-6.03195	0.710305	0.774816
Monocytes	GM15518	0.086485	0.80065	0.168228	0.866788	-5.15074	0.788227	0.85844
Monocytes	ADA	-0.06971	2.074866	-0.16811	0.866877	-5.27845	0.760276	0.828573
Monocytes	RNF7	-0.0163	6.742111	-0.1681	0.866891	-6.51705	0.666854	0.727641
Monocytes	2210016L21RIK	-0.02798	3.97848	-0.16808	0.866901	-5.92599	0.720523	0.785833
Monocytes	EXO5	-0.06133	2.006793	-0.16775	0.867162	-5.3682	0.761793	0.830213
Monocytes	PPOX	-0.03774	3.108757	-0.16774	0.867169	-5.59383	0.738444	0.805153
Monocytes	UROC1	0.076473	1.760508	0.167737	0.867174	-5.30406	0.767122	0.835917
Monocytes	TADA3	-0.03002	3.403164	-0.16739	0.867444	-5.78276	0.732429	0.798636
Monocytes	LTN1	0.018353	5.03701	0.167345	0.867481	-6.16563	0.699549	0.763146
Monocytes	3110009E18RIK	0.044784	2.175408	0.167215	0.867583	-5.4407	0.75826	0.826418
Monocytes	SLC35A2	0.035807	3.165869	0.167171	0.867617	-5.71123	0.737345	0.803961
Monocytes	RNF121	0.027923	4.41156	0.16715	0.867634	-5.95421	0.711938	0.776567
Monocytes	2610318N02RII	-0.07489	1.172871	-0.16688	0.867845	-5.2663	0.780138	0.849789
Monocytes	GM14455	-0.04756	1.315653	-0.16648	0.86816	-5.44254	0.776987	0.846483
Monocytes	4930549G23RII	-0.03783	2.714781	-0.16646	0.868175	-5.58206	0.746832	0.814191
Monocytes	GM17021	0.08638	0.125797	0.166232	0.868354	-5.1381	0.803678	0.874941
Monocytes	IFI27	-0.0465	4.998783	-0.16621	0.868369	-5.87489	0.700338	0.764059
Monocytes	SURF6	-0.03081	3.185009	-0.16621	0.868369	-5.61881	0.736988	0.803621
Monocytes	DGAT1	-0.02698	7.095267	-0.16616	0.868408	-6.65779	0.660486	0.72074
Monocytes	RIPOR2	-0.02521	7.617108	-0.16614	0.868423	-6.42692	0.650966	0.710346
Monocytes	CCDC69	-0.03112	3.297575	-0.1661	0.868455	-5.82214	0.734652	0.801108
Monocytes	RNF19A	0.02006	5.375931	0.166101	0.868457	-6.2142	0.692975	0.756078
Monocytes	PRR12	-0.03226	3.451612	-0.16569	0.868779	-5.67171	0.73147	0.797682
Monocytes	D030056L22RIK	-0.03215	3.888031	-0.16569	0.868779	-5.8431	0.722536	0.788052
Monocytes	TROAP	0.057792	1.774645	0.165661	0.868802	-5.39266	0.76695	0.83577
Monocytes	ATG16L1	-0.01858	5.446329	-0.16564	0.868819	-6.29523	0.69161	0.754598
Monocytes	GM4951	-0.06845	3.770894	-0.16549	0.868937	-5.72252	0.724922	0.790629

Monocytes	HIST1H3F	0.10105	0.34671	0.165385	0.869019	-5.08505	0.798648	0.869623
Monocytes	SRFBP1	0.035497	3.231687	0.165354	0.869043	-5.68019	0.736018	0.802606
Monocytes	PEX7	-0.01966	4.45855	-0.16529	0.869092	-6.06278	0.711038	0.775664
Monocytes	TRAJ18	0.075658	-1.3552	0.165214	0.869152	-5.00239	0.838293	0.911654
Monocytes	DACH2	0.101879	0.599965	0.164854	0.869435	-5.05967	0.792924	0.863644
Monocytes	GMPS	-0.01455	5.784964	-0.1647	0.869558	-6.37104	0.685086	0.747672
Monocytes	ARF5	0.015083	8.486216	0.16464	0.869603	-6.88263	0.635455	0.693515
Monocytes	ANAPC7	0.02256	4.359074	0.164621	0.869618	-5.94078	0.713028	0.777949
Monocytes	GM15133	0.050861	1.788244	0.164613	0.869624	-5.45587	0.766655	0.835628
Monocytes	PRRC2C	0.011543	7.744202	0.164601	0.869634	-6.70713	0.648671	0.707985
Monocytes	EIF3L	-0.01537	5.672102	-0.16455	0.869677	-6.38258	0.687253	0.750028
Monocytes	FAM234B	-0.05825	2.096184	-0.16448	0.869729	-5.35216	0.760003	0.828525
Monocytes	COPS9	-0.01397	6.68605	-0.16438	0.869809	-6.58678	0.66806	0.72917
Monocytes	BRIX1	0.013102	6.144863	0.164376	0.869811	-6.39431	0.678228	0.74024
Monocytes	DNMT3A	-0.01908	6.044028	-0.16437	0.869815	-6.35938	0.680142	0.742321
Monocytes	GM14798	0.025805	3.718232	0.164249	0.86991	-5.88092	0.726017	0.791978
Monocytes	WDR49	0.074694	0.34569	0.164049	0.870067	-5.19814	0.798772	0.869829
Monocytes	BRD8	0.013603	6.236165	0.163805	0.870259	-6.41317	0.676674	0.738418
Monocytes	PARP8	-0.01477	6.68237	-0.16358	0.870438	-6.71298	0.6683	0.729369
Monocytes	TJP2	-0.02552	3.845594	-0.16356	0.87045	-6.06477	0.723585	0.789305
Monocytes	EHD1	0.018194	6.1755	0.163542	0.870465	-6.48426	0.677821	0.739734
Monocytes	COPE	-0.01398	6.316581	-0.16341	0.870569	-6.5045	0.675181	0.736877
Monocytes	STEAP4	-0.08299	2.205553	-0.16329	0.87066	-5.25906	0.757897	0.826221
Monocytes	ADAT2	0.060111	1.285306	0.162986	0.870902	-5.31846	0.778054	0.84774
Monocytes	KNOP1	0.019031	4.90183	0.162682	0.87114	-6.14706	0.70274	0.766674
Monocytes	GM13012	-0.04283	2.775114	-0.16252	0.871263	-5.48575	0.746131	0.813441
Monocytes	SUCLG2	0.024061	4.835404	0.162289	0.871449	-6.08135	0.704185	0.768144
Monocytes	CCT7	0.013565	6.427225	0.161845	0.871797	-6.52456	0.673704	0.734882
Monocytes	XKR5	-0.0652	0.190356	-0.16174	0.871879	-5.18418	0.803162	0.87413
Monocytes	TMBIM4	-0.01405	6.749974	-0.16154	0.872033	-6.59759	0.667666	0.728334
Monocytes	TJP3	0.050886	1.707068	0.161429	0.872124	-5.42378	0.769336	0.838079
Monocytes	ZFP511	-0.0205	3.816412	-0.16131	0.87222	-5.97806	0.724857	0.79032
Monocytes	OGFR	0.020789	5.244677	0.161284	0.872238	-6.28118	0.696357	0.759515
Monocytes	MCAT	0.041098	2.553855	0.161242	0.87227	-5.52755	0.751129	0.818577
Monocytes	MRPL10	-0.02185	4.602348	-0.16123	0.872277	-6.10539	0.709017	0.773219
Monocytes	CEP63	0.023149	4.291786	0.161053	0.872419	-6.02343	0.715288	0.779992
Monocytes	CBX6	0.045824	2.859775	0.160844	0.872583	-5.41246	0.744808	0.811732
Monocytes	VPS37A	-0.01949	5.2037	-0.16073	0.872675	-6.24358	0.697305	0.760467
Monocytes	1700003F12RIK	-0.0579	2.027827	-0.16057	0.872801	-5.34751	0.762592	0.830758
Monocytes	MRPL53	0.025802	4.291701	0.160286	0.873022	-5.94349	0.715446	0.780052
Monocytes	GM48765	-0.06915	0.952243	-0.16026	0.87304	-5.18769	0.786207	0.855974
Monocytes	IL4RA	-0.02252	4.883706	-0.16015	0.873125	-6.24571	0.703651	0.76732
Monocytes	TOR3A	-0.03152	4.075925	-0.16	0.873248	-6.0828	0.719799	0.784799
Monocytes	KLF5	0.08084	0.36413	0.159949	0.873286	-5.21756	0.799444	0.870143
Monocytes	SAG	0.038389	3.5306	0.159909	0.873318	-5.81278	0.730933	0.796827
Monocytes	PRAMEF8	0.033777	3.188085	0.159904	0.873322	-5.74752	0.738024	0.804458
Monocytes	TENT4B	0.014935	6.368937	0.15957	0.873584	-6.44514	0.675074	0.736321

Monocytes	ADAM33	0.081248	-0.9471	0.159523	0.873621	-5.08413	0.829921	0.902429
Monocytes	ERG	-0.08666	3.921953	-0.15952	0.873627	-5.44326	0.723	0.788233
Monocytes	SP2	0.020068	4.742762	0.159297	0.873798	-6.1188	0.706568	0.770428
Monocytes	UPP1	-0.10895	0.646262	-0.15919	0.87388	-5.24789	0.793209	0.863446
Monocytes	RDH14	-0.02624	3.731822	-0.15883	0.874166	-5.7852	0.726935	0.792544
Monocytes	METTL18	0.06652	1.271701	0.158644	0.874311	-5.2053	0.779259	0.848674
Monocytes	GM37768	0.047074	2.087377	0.158478	0.874441	-5.43369	0.761466	0.829659
Monocytes	CHD2	0.014832	7.754934	0.158475	0.874444	-6.68743	0.649564	0.708587
Monocytes	CDK2AP1	-0.01465	5.884299	-0.15844	0.874471	-6.43677	0.68433	0.746464
Monocytes	ZWILCH	0.046553	3.028209	0.158414	0.874492	-5.63917	0.741494	0.80823
Monocytes	XPO5	0.02737	3.603677	0.158371	0.874525	-5.79393	0.729563	0.795391
Monocytes	CFAP45	-0.07394	0.728674	-0.15828	0.8746	-5.23562	0.791355	0.861585
Monocytes	NAA38	0.016589	5.467647	0.158264	0.87461	-6.36574	0.692355	0.755173
Monocytes	AP4M1	0.024378	3.581284	0.158253	0.874618	-5.78273	0.730023	0.795887
Monocytes	MN1	-0.10425	0.308399	-0.15825	0.87462	-4.98252	0.800857	0.871699
Monocytes	PWP1	-0.02613	3.903059	-0.15799	0.874821	-5.83415	0.723444	0.788843
Monocytes	COQ8A	-0.05156	2.303584	-0.15758	0.875148	-5.34286	0.756829	0.824789
Monocytes	ZNHIT2	0.026298	3.310699	0.157459	0.875242	-5.75436	0.735615	0.802002
Monocytes	RIPK1	0.014941	5.973193	0.15743	0.875265	-6.49435	0.682635	0.744712
Monocytes	CHST14	0.069133	0.872451	0.157404	0.875286	-5.23564	0.788137	0.858258
Monocytes	BORA	-0.03287	3.535011	-0.15736	0.875316	-5.71282	0.73098	0.797012
Monocytes	HIST1H3G	-0.07278	1.46486	-0.15731	0.875359	-5.24458	0.775009	0.844247
Monocytes	SH3TC1	-0.02562	2.768133	-0.15724	0.875411	-5.92617	0.746961	0.8142
Monocytes	CALM2	0.011189	8.565355	0.157231	0.875421	-6.78075	0.635129	0.692871
Monocytes	GEMIN6	0.041347	2.59595	0.15715	0.875485	-5.54013	0.750602	0.818109
Monocytes	CMAS	0.017714	5.862643	0.157082	0.875538	-6.41023	0.684748	0.747007
Monocytes	6720427I07RIK	-0.02032	4.894793	-0.15705	0.875561	-6.15203	0.703564	0.767409
Monocytes	GM28403	-0.05982	1.458517	-0.1569	0.875679	-5.39815	0.775148	0.844405
Monocytes	PDAP1	-0.01326	6.715812	-0.15689	0.875686	-6.53225	0.668628	0.729481
Monocytes	HVCN1	0.0604	4.117697	0.156797	0.875763	-5.50821	0.719089	0.784205
Monocytes	OGFOD1	0.027649	3.611469	0.156794	0.875765	-5.81744	0.729407	0.795332
Monocytes	ERBIN	-0.01333	7.975801	-0.15659	0.875925	-6.75234	0.645651	0.704401
Monocytes	GFER	-0.0156	4.406699	-0.15652	0.875981	-6.19875	0.713332	0.777984
Monocytes	UBA3	-0.02097	4.076756	-0.15642	0.876062	-5.9329	0.719986	0.785174
Monocytes	2700049A03RIK	0.029002	4.236674	0.156207	0.876226	-5.84498	0.716806	0.781702
Monocytes	SRRD	0.022652	3.465767	0.156106	0.876305	-5.94562	0.732528	0.798655
Monocytes	BCAT2	-0.0183	4.601375	-0.15607	0.876336	-6.17607	0.709499	0.773806
Monocytes	CD99L2	-0.03401	2.142314	-0.15581	0.876538	-5.60688	0.760458	0.828675
Monocytes	DNAJC8	-0.00939	6.901934	-0.15579	0.876552	-6.60406	0.665317	0.72586
Monocytes	EDEM3	-0.02384	5.974164	-0.15573	0.876602	-6.28863	0.682769	0.744852
Monocytes	ACBD3	-0.01382	5.707973	-0.15554	0.876747	-6.34399	0.687929	0.750402
Monocytes	QPCTL	0.029567	3.010203	0.155413	0.87685	-5.64563	0.74213	0.808942
Monocytes	NDUFB2	-0.01463	5.815553	-0.15531	0.87693	-6.41403	0.685894	0.748178
Monocytes	A530032D15RIK	-0.06522	0.594014	-0.15496	0.877204	-5.27539	0.794856	0.865232
Monocytes	NEAT1	0.016756	8.674976	0.15485	0.877293	-7.01419	0.633587	0.691016
Monocytes	CAML	-0.01981	4.427078	-0.15423	0.877781	-6.00256	0.713634	0.777906
Monocytes	TKTL1	0.045867	1.290296	0.154034	0.877934	-5.44575	0.779767	0.848867

Monocytes	ATP5MPL	0.011364	8.484004	0.153744	0.878162	-6.84006	0.637423	0.694885
Monocytes	HDHD2	-0.02334	3.552765	-0.15338	0.878448	-5.84345	0.731628	0.797207
Monocytes	STK35	0.022561	3.447827	0.153302	0.878509	-5.90349	0.733794	0.799542
Monocytes	HACE1	0.023115	4.490352	0.153288	0.878521	-6.02554	0.712585	0.776676
Monocytes	CCDC130	0.050627	2.056293	0.15317	0.878613	-5.41259	0.763198	0.831098
Monocytes	5430405H02RII	-0.02972	3.910079	-0.15315	0.878633	-5.80214	0.724305	0.789322
Monocytes	EEF1E1	-0.02105	4.824221	-0.15298	0.878759	-6.18223	0.705937	0.769533
Monocytes	E530011L22RIK	0.070741	1.095538	0.152983	0.87876	-5.23556	0.784252	0.853637
Monocytes	GFOD2	0.054404	2.309358	0.152894	0.87883	-5.31471	0.757756	0.825317
Monocytes	MORN1	-0.04725	1.227517	-0.15289	0.878835	-5.40475	0.781322	0.850514
Monocytes	GATC	0.040608	2.698485	0.152626	0.879041	-5.624	0.749537	0.816466
Monocytes	AHSA1	-0.01742	5.071608	-0.15259	0.879066	-6.24865	0.701118	0.764292
Monocytes	TOMT	-0.06121	1.736925	-0.15252	0.879126	-5.35469	0.770195	0.838647
Monocytes	GM16091	-0.04631	2.306149	-0.15228	0.879317	-5.44714	0.757931	0.825561
Monocytes	YIPF5	-0.01524	5.24625	-0.15216	0.879409	-6.23212	0.69773	0.760715
Monocytes	PHIP	-0.01376	7.276011	-0.15209	0.879459	-6.57611	0.659281	0.71892
Monocytes	GFM1	0.024064	3.89287	0.152091	0.879462	-5.88621	0.724758	0.789921
Monocytes	NOL10	0.017523	4.91748	0.152006	0.879529	-6.21508	0.704191	0.767729
Monocytes	ROBO3	0.086116	-0.8339	0.151828	0.879669	-4.96079	0.828618	0.900863
Monocytes	VAV3	-0.01397	7.363151	-0.15178	0.87971	-6.97795	0.657714	0.71723
Monocytes	GM12940	0.025101	4.51886	0.151585	0.87986	-5.96655	0.712188	0.776371
Monocytes	KIF20A	0.03871	3.768581	0.151485	0.879938	-5.97668	0.727373	0.792755
Monocytes	ATP8A1	0.013012	7.601268	0.151436	0.879977	-6.70147	0.653412	0.712529
Monocytes	TSN	-0.01118	6.360058	-0.15096	0.880348	-6.47116	0.676466	0.737677
Monocytes	ITPRIPL2	0.018802	3.897046	0.15091	0.88039	-6.2695	0.724819	0.790032
Monocytes	THNSL1	-0.05466	1.077135	-0.15076	0.880509	-5.37701	0.78493	0.854547
Monocytes	AMOT	-0.05877	0.863491	-0.15071	0.880548	-5.29436	0.789702	0.859654
Monocytes	AK5	-0.07498	0.348906	-0.15055	0.880671	-5.15272	0.801325	0.872043
Monocytes	JMJD8	-0.05388	1.564022	-0.15052	0.880701	-5.36659	0.774173	0.843098
Monocytes	HLTF	0.019885	4.64409	0.150458	0.880746	-6.16325	0.709758	0.773863
Monocytes	LRRC41	-0.01755	4.919545	-0.15027	0.88089	-6.21764	0.704293	0.767978
Monocytes	CCDC22	0.025323	3.49155	0.150249	0.880911	-5.84405	0.733142	0.799118
Monocytes	G3BP2	0.009971	6.759047	0.15024	0.880918	-6.56088	0.668978	0.729638
Monocytes	FAM114A2	-0.01516	5.166561	-0.15009	0.881036	-6.21589	0.699432	0.762758
Monocytes	BICD2	-0.02084	4.09822	-0.15006	0.881062	-6.04429	0.720729	0.785783
Monocytes	KCNAB1	-0.05764	1.324987	-0.15005	0.881065	-5.42543	0.779434	0.848795
Monocytes	RBM45	-0.03232	3.10947	-0.15004	0.881077	-5.60753	0.741081	0.807704
Monocytes	U2AF1	-0.01083	7.593832	-0.1499	0.881186	-6.68922	0.653612	0.712973
Monocytes	RNF8	-0.01482	4.800789	-0.14987	0.881205	-6.2896	0.706643	0.770623
Monocytes	TEX45	0.080685	0.222756	0.149541	0.881467	-5.11978	0.804341	0.87535
Monocytes	UGCG	-0.01794	6.436724	-0.1495	0.8815	-6.46601	0.675136	0.736397
Monocytes	GM10134	-0.05571	0.795093	-0.1494	0.881577	-5.435	0.791378	0.861581
Monocytes	LSR	0.030266	1.400914	0.149079	0.881831	-5.97228	0.777909	0.847298
Monocytes	CHEK2	0.029461	3.145946	0.149054	0.881851	-5.6795	0.740462	0.807163
Monocytes	CHML	-0.04002	1.690018	-0.14901	0.881884	-5.53239	0.771564	0.840517
Monocytes	OS9	0.014389	5.617189	0.149002	0.881892	-6.34126	0.690793	0.753507
Monocytes	ARID2	-0.01464	6.543762	-0.14893	0.881946	-6.47428	0.673137	0.734318

Monocytes	GM48960	0.050959	1.466163	0.148736	0.882101	-5.3468	0.776545	0.845817
Monocytes	SAP25	0.049024	2.354647	0.148473	0.882308	-5.46163	0.757379	0.82526
Monocytes	RWDD4A	-0.02284	3.983066	-0.14808	0.882617	-5.93086	0.723536	0.788788
Monocytes	KCNG2	0.067643	-0.23458	0.14783	0.882814	-5.20182	0.815258	0.886956
Monocytes	LAMTOR3	-0.01454	5.240813	-0.14773	0.882894	-6.30756	0.698431	0.761694
Monocytes	PELP1	0.0273	3.358177	0.147722	0.882898	-5.76527	0.73638	0.802673
Monocytes	ALG11	-0.033	2.610001	-0.14763	0.882973	-5.52685	0.752091	0.819556
Monocytes	TSEN54	-0.02888	2.874509	-0.14754	0.883044	-5.66612	0.746495	0.813548
Monocytes	KIF1B	0.016336	5.385583	0.147263	0.88326	-6.36257	0.695604	0.758664
Monocytes	THOC1	-0.01433	5.704802	-0.14724	0.883274	-6.3763	0.689416	0.751952
Monocytes	ZFP521	0.072888	2.100385	0.147207	0.883304	-5.29114	0.763003	0.831292
Monocytes	SUCLA2	-0.01399	5.593683	-0.14714	0.883353	-6.34695	0.691563	0.754282
Monocytes	ADAMTSL1	-0.104	0.587709	-0.14713	0.883363	-5.21019	0.796425	0.86698
Monocytes	GZF1	0.037378	2.752325	0.147103	0.883385	-5.58781	0.749074	0.816355
Monocytes	3-Sep	0.023619	-1.00287	0.14706	0.883419	-5.92827	0.833289	0.906096
Monocytes	KDM4C	0.01459	6.252217	0.146968	0.883492	-6.42993	0.678947	0.740591
Monocytes	FAM171B	0.089027	0.217464	0.146489	0.883869	-5.10887	0.804973	0.875963
Monocytes	COL20A1	-0.04474	1.340253	-0.14647	0.883884	-5.29059	0.779726	0.849067
Monocytes	GRK6	0.016182	5.764162	0.146363	0.883968	-6.27065	0.688381	0.750731
Monocytes	NUS1	-0.01416	4.837828	-0.14627	0.884038	-6.18974	0.70648	0.770358
Monocytes	ZCRB1	-0.0112	6.149583	-0.14625	0.884058	-6.44323	0.681004	0.742732
Monocytes	PRKCZ	-0.07152	0.606888	-0.14625	0.884059	-5.06346	0.796117	0.866559
Monocytes	NCBP1	-0.0141	5.256901	-0.14595	0.884296	-6.1802	0.698281	0.761499
Monocytes	P2RY14	0.019255	3.467146	0.145807	0.884405	-6.39008	0.734296	0.800422
Monocytes	EXOSC9	-0.02157	4.169312	-0.14579	0.884417	-5.96122	0.719926	0.784931
Monocytes	STN1	-0.01934	3.886297	-0.14574	0.884461	-6.03325	0.72568	0.791139
Monocytes	PFDN1	0.016041	5.109296	0.145707	0.884484	-6.14876	0.701175	0.764658
Monocytes	UQCRC1	0.012962	6.508803	0.1454	0.884726	-6.52784	0.674355	0.735483
Monocytes	MAST2	-0.02013	5.283184	-0.14502	0.885024	-6.19128	0.697864	0.761128
Monocytes	TMEM104	0.028271	3.273099	0.144962	0.885071	-5.83541	0.738426	0.804927
Monocytes	GRB14	0.056286	1.939569	0.144913	0.88511	-5.44252	0.76677	0.835344
Monocytes	UBE2L3	-0.00956	7.560477	-0.14491	0.885113	-6.70962	0.654889	0.714372
Monocytes	RFXANK	0.034368	2.879474	0.144841	0.885166	-5.54904	0.746671	0.813799
Monocytes	CAR9	0.050733	0.285801	0.144778	0.885215	-5.35623	0.803586	0.874633
Monocytes	GM45669	-0.07205	0.129811	-0.1447	0.885279	-5.19139	0.807157	0.87845
Monocytes	TM9SF4	0.017442	4.769644	0.144574	0.885376	-6.2191	0.707986	0.772137
Monocytes	RBCK1	0.014104	5.550838	0.144489	0.885443	-6.34861	0.692653	0.75553
Monocytes	CSNK2A1	-0.01003	6.59773	-0.14444	0.885484	-6.48953	0.672684	0.733829
Monocytes	THOC6	-0.02056	4.164901	-0.14441	0.885506	-6.03441	0.720116	0.785258
Monocytes	METTL17	-0.03655	2.488504	-0.14437	0.885537	-5.50548	0.75496	0.822765
Monocytes	HIST1H1B	-0.04609	5.277543	-0.1442	0.885674	-6.25489	0.698027	0.761381
Monocytes	LRRN3	0.089723	-0.11923	0.143837	0.885956	-5.0072	0.813123	0.884711
Monocytes	ZFP397	0.021742	3.944609	0.143555	0.886179	-5.83401	0.724797	0.79026
Monocytes	ZBTB5	-0.04458	2.463409	-0.14355	0.88618	-5.37849	0.75571	0.823509
Monocytes	BRF2	0.030342	2.326101	0.143496	0.886225	-5.61779	0.758648	0.82668
Monocytes	PRICKLE3	0.044236	2.034653	0.143366	0.886328	-5.38999	0.764927	0.833416
Monocytes	MAP2K6	-0.02628	3.533157	-0.14319	0.886466	-5.97396	0.733242	0.799424

Monocytes	TARBP1	-0.04429	2.687423	-0.14308	0.886553	-5.41431	0.750943	0.81848
Monocytes	FBXW7	-0.015	6.448567	-0.14308	0.886554	-6.47669	0.67568	0.737127
Monocytes	CEP89	-0.04788	2.805458	-0.14302	0.886596	-5.46818	0.748444	0.815797
Monocytes	ZNHIT6	-0.03352	3.231636	-0.14293	0.886673	-5.6467	0.739499	0.806181
Monocytes	USPL1	0.026517	3.533846	0.142887	0.886704	-5.72654	0.733227	0.79943
Monocytes	EIF4E2	-0.01283	5.989442	-0.14287	0.886717	-6.43768	0.684401	0.746609
Monocytes	MED26	0.022064	4.429093	0.142699	0.886852	-6.02688	0.715043	0.779798
Monocytes	PAPLN	-0.05851	0.894565	-0.1424	0.887084	-5.24768	0.790187	0.860466
Monocytes	SNRNP40	0.01243	5.982356	0.142386	0.887099	-6.41345	0.684664	0.746897
Monocytes	TMEM267	-0.0457	1.734822	-0.1423	0.887169	-5.4065	0.77159	0.840641
Monocytes	H2-KE6	-0.01875	5.147514	-0.14221	0.88724	-6.21564	0.700852	0.764496
Monocytes	ANKRD40	0.019135	4.238658	0.142069	0.887349	-5.99142	0.718993	0.784133
Monocytes	E230013L22RIK	0.065054	-0.72305	0.141976	0.887422	-5.12382	0.827409	0.900076
Monocytes	HS3ST1	0.0832	1.500377	0.141853	0.887519	-5.26655	0.776786	0.846221
Monocytes	NUDT21	-0.01287	6.148811	-0.14175	0.887601	-6.46893	0.681545	0.743556
Monocytes	VDAC3	-0.01083	7.191815	-0.14156	0.887752	-6.671	0.662046	0.722276
Monocytes	SNHG10	-0.07902	0.227889	-0.14141	0.887866	-5.09934	0.805404	0.876678
Monocytes	GM16272	0.065202	0.363126	0.141339	0.887923	-5.15584	0.802315	0.873393
Monocytes	COPS5	0.014381	5.288344	0.141319	0.887939	-6.26487	0.698192	0.761584
Monocytes	9930021J03RIK	-0.01343	6.407459	-0.14096	0.888219	-6.45905	0.676838	0.738271
Monocytes	TASP1	-0.03146	3.918389	-0.14049	0.888589	-5.77797	0.725986	0.791351
Monocytes	RBKS	0.021268	3.630745	0.140355	0.888698	-6.05285	0.73192	0.797737
Monocytes	BANF1	-0.01408	6.873409	-0.14012	0.888887	-6.58536	0.668441	0.72888
Monocytes	SPRY1	0.068364	1.345776	0.139815	0.889124	-5.19887	0.780963	0.850119
Monocytes	PBX4	-0.07155	0.976518	-0.13966	0.889248	-5.11336	0.789183	0.858886
Monocytes	GPBP1L1	-0.01446	5.913267	-0.13965	0.889253	-6.34676	0.686712	0.748662
Monocytes	DCP2	0.015904	5.103401	0.139565	0.889321	-6.28641	0.70246	0.765739
Monocytes	PCNT	-0.01621	5.233516	-0.1392	0.889612	-6.2576	0.700029	0.763056
Monocytes	ZFAND1	0.029263	2.727292	0.139181	0.889624	-5.58292	0.751165	0.818181
Monocytes	SMC4	-0.01495	7.287715	-0.13859	0.890089	-6.60088	0.661248	0.720763
Monocytes	1700027J07RIK	0.078728	2.481407	0.138579	0.890098	-5.20563	0.756682	0.823934
Monocytes	SH3GLB2	-0.02436	3.721025	-0.1384	0.890243	-5.81045	0.730683	0.796027
Monocytes	LAGE3	0.016832	4.771468	0.138348	0.89028	-6.14603	0.709423	0.773088
Monocytes	VAMP1	-0.02425	3.997211	-0.1383	0.890314	-5.82264	0.725026	0.789954
Monocytes	GGNBP1	-0.06647	1.044976	-0.13814	0.890443	-5.17656	0.788139	0.857575
Monocytes	USP22	0.016505	4.389395	0.137869	0.890658	-6.06052	0.717237	0.781399
Monocytes	GALE	-0.03447	2.330073	-0.13767	0.890815	-5.49413	0.76017	0.827482
Monocytes	AGK	0.033009	3.121713	0.137423	0.891009	-5.53922	0.743469	0.809479
Monocytes	NCOA5	-0.02222	4.146435	-0.13731	0.891094	-5.92912	0.722332	0.786754
Monocytes	MRPL39	0.020425	3.661118	0.136997	0.891344	-5.84664	0.73241	0.797575
Monocytes	APTX	-0.0249	3.052742	-0.13678	0.891513	-5.63875	0.745101	0.811232
Monocytes	MLLT1	-0.0297	2.735176	-0.13678	0.891516	-5.55582	0.751809	0.818431
Monocytes	CSNK1D	0.008735	6.6346	0.13665	0.891618	-6.54075	0.673889	0.734249
Monocytes	JOSD2	0.01832	4.09679	0.136401	0.891814	-5.99542	0.723625	0.788003
Monocytes	FHL1	-0.06304	0.58688	-0.13628	0.891913	-5.16985	0.799093	0.868792
Monocytes	FAM122B	0.03875	2.015817	0.136132	0.892027	-5.39277	0.767375	0.835013
Monocytes	DERL1	0.009086	6.22546	0.13608	0.892068	-6.48446	0.681714	0.742707

Monocytes	HECTD4	0.016333	5.548933	0.136049	0.892092	-6.234	0.694733	0.756833
Monocytes	PBLD1	0.0589	1.75083	0.1359	0.892209	-5.33915	0.773151	0.841274
Monocytes	RAG1	-0.0806	1.672599	-0.13587	0.892232	-5.15077	0.774866	0.843117
Monocytes	MAPT	0.048514	1.597756	0.135579	0.892462	-5.3051	0.776647	0.84491
Monocytes	ZFAND4	0.041268	3.4762	0.135372	0.892625	-5.75575	0.736583	0.801911
Monocytes	GM1976	0.040216	2.701657	0.135181	0.892776	-5.40015	0.752919	0.81938
Monocytes	NUDT22	-0.03036	2.557891	-0.13501	0.892909	-5.58608	0.756016	0.822685
Monocytes	FGFRL1	0.07675	0.299565	0.13495	0.892958	-5.10397	0.805973	0.87597
Monocytes	RBFA	-0.01846	4.826424	-0.13481	0.893069	-6.15528	0.709252	0.772373
Monocytes	APPL2	-0.03125	3.358029	-0.13474	0.893121	-5.77274	0.739154	0.804633
Monocytes	ZFP607A	-0.04133	1.840055	-0.13423	0.893523	-5.32433	0.771791	0.839444
Monocytes	ARL13B	-0.01864	3.775076	-0.13415	0.893593	-5.98028	0.730763	0.795471
Monocytes	ACER2	0.047088	2.516505	0.134028	0.893685	-5.4124	0.757167	0.823854
Monocytes	NAPA	-0.01088	5.92916	-0.134	0.893704	-6.42528	0.687908	0.749212
Monocytes	DYNLRB1	-0.0111	6.280147	-0.1337	0.893944	-6.48315	0.681324	0.742021
Monocytes	RBM24	-0.091	1.008251	-0.13337	0.894201	-5.05741	0.790511	0.859365
Monocytes	1700061G19RII	0.057059	1.070717	0.132979	0.894512	-5.21114	0.789323	0.857909
Monocytes	ZPBP	0.045734	1.648971	0.132828	0.894631	-5.32466	0.776534	0.844262
Monocytes	SNAI3	-0.07758	-0.34486	-0.13259	0.894822	-5.02546	0.821802	0.892398
Monocytes	H2AFV	-0.01495	8.095101	-0.13258	0.894824	-6.73765	0.648135	0.705668
Monocytes	PPP1CC	0.010558	7.584523	0.132293	0.895054	-6.60871	0.657488	0.715826
Monocytes	CRAMP1L	0.014686	4.984741	0.132234	0.895099	-6.2015	0.706992	0.769556
Monocytes	GM48512	-0.0522	1.068935	-0.13214	0.895175	-5.23401	0.789567	0.85814
Monocytes	MRPL37	0.017479	4.2912	0.131925	0.895344	-6.00359	0.720939	0.784601
Monocytes	ESS2	-0.0253	2.988114	-0.13159	0.895608	-5.63898	0.747886	0.813576
Monocytes	TARS2	0.023755	3.819829	0.131586	0.89561	-5.76546	0.730561	0.794963
Monocytes	NPAT	0.021139	4.485178	0.131544	0.895644	-5.97815	0.71702	0.780376
Monocytes	AVPI1	0.018372	2.712104	0.131528	0.895657	-6.1444	0.753736	0.819847
Monocytes	MKNK1	0.015944	4.508378	0.131526	0.895658	-6.09782	0.716553	0.779872
Monocytes	2310001H17RII	-0.0225	4.065364	-0.13138	0.895775	-6.04649	0.725568	0.789599
Monocytes	CFAP36	0.019081	4.102137	0.131011	0.896064	-5.91404	0.724863	0.788852
Monocytes	TTC4	0.022936	3.541793	0.130955	0.896109	-5.72722	0.736386	0.80126
Monocytes	CLIC1	0.011169	8.942892	0.130943	0.896118	-6.93971	0.633296	0.689417
Monocytes	MRPS5	0.016863	4.612666	0.130854	0.896188	-6.07877	0.714538	0.777755
Monocytes	ZFP608	-0.0201	6.820434	-0.13085	0.89619	-6.53053	0.671739	0.73139
Monocytes	RAD21	0.011702	7.022909	0.130475	0.896487	-6.55768	0.668128	0.727339
Monocytes	GM27003	-0.02105	4.204875	-0.12963	0.897155	-5.88385	0.723435	0.786846
Monocytes	GM49041	-0.03652	1.334735	-0.12952	0.897244	-5.59817	0.784508	0.852239
Monocytes	TTF2	-0.02922	3.091877	-0.12891	0.897723	-5.68474	0.746815	0.811627
Monocytes	ZC3HAV1L	0.023444	3.053639	0.128827	0.897787	-5.94086	0.747621	0.812491
Monocytes	TFB2M	-0.01918	3.953928	-0.12855	0.898005	-5.84677	0.729015	0.792471
Monocytes	FAN1	0.054912	1.159783	0.128432	0.898099	-5.22166	0.788907	0.856557
Monocytes	COX19	0.019731	4.496433	0.128251	0.898241	-6.04706	0.71803	0.78071
Monocytes	CYB5B	0.012121	5.866247	0.128201	0.898281	-6.34998	0.690986	0.751497
Monocytes	HECTD2	-0.04749	0.291184	-0.12799	0.898444	-5.31162	0.808717	0.877555
Monocytes	GM44699	0.052213	0.559613	0.127721	0.898659	-5.20196	0.802652	0.871095
Monocytes	NUBP2	0.018343	4.451117	0.1277	0.898676	-6.11747	0.719088	0.781745

Monocytes	COPS6	-0.01344	5.552293	-0.1275	0.898836	-6.33853	0.69729	0.758186
Monocytes	3010003L21RIK	0.044885	1.349271	0.127341	0.89896	-5.37288	0.784993	0.852296
Monocytes	PIK3R4	0.02797	3.189781	0.127229	0.899048	-5.67449	0.745205	0.80978
Monocytes	HIST1H2BE	-0.04496	2.079102	-0.127	0.899228	-5.36475	0.769045	0.835218
Monocytes	IGHMBP2	-0.02987	2.850148	-0.12681	0.899382	-5.52638	0.752477	0.817498
Monocytes	DCAF17	-0.01936	4.054876	-0.12663	0.89952	-5.90099	0.727363	0.790546
Monocytes	SCPEP1OS	0.046467	0.502575	0.126602	0.899543	-5.38974	0.804198	0.87262
Monocytes	CSTF3	0.01162	6.037918	0.126537	0.899594	-6.39349	0.688024	0.748086
Monocytes	NUMA1	-0.01289	5.705606	-0.1265	0.89962	-6.30122	0.694448	0.75504
Monocytes	FEM1A	-0.02535	2.924816	-0.12644	0.899668	-5.62061	0.750893	0.815808
Monocytes	ANAPC4	0.016467	4.541254	0.126191	0.899867	-5.99158	0.717589	0.779961
Monocytes	MKRN1	0.015429	7.149614	0.126056	0.899974	-6.65381	0.667136	0.725343
Monocytes	GAREM1	0.060692	0.757375	0.12591	0.900089	-5.15747	0.798548	0.866533
Monocytes	NME6	0.039168	2.576439	0.125842	0.900143	-5.41078	0.758456	0.823819
Monocytes	C4B	0.065967	2.480976	0.125696	0.900258	-5.46539	0.760504	0.826028
Monocytes	CPOX	0.020117	4.570271	0.125686	0.900266	-5.97377	0.717035	0.779373
Monocytes	ACAD8	-0.03001	2.476536	-0.12539	0.900496	-5.5287	0.760646	0.82615
Monocytes	TIAL1	0.01093	5.798885	0.125274	0.900591	-6.37276	0.692808	0.753184
Monocytes	LIX1L	0.052566	0.803407	0.12523	0.900626	-5.20199	0.797554	0.865478
Monocytes	MAPK6	0.015211	5.88266	0.125201	0.900649	-6.46774	0.691187	0.751429
Monocytes	MAPK8	0.012479	5.232129	0.125089	0.900737	-6.31319	0.703888	0.765163
Monocytes	EIF3C	-0.01016	7.172674	-0.12507	0.900753	-6.62153	0.666752	0.724922
Monocytes	ALDH18A1	-0.03347	3.070395	-0.12493	0.900859	-5.64317	0.748027	0.812628
Monocytes	CHDH	-0.06452	1.157054	-0.12473	0.901022	-5.16058	0.789635	0.857045
Monocytes	PROSER3	0.052159	1.105594	0.124674	0.901065	-5.19908	0.790788	0.858271
Monocytes	ITM2B	-0.01074	10.00633	-0.12443	0.901258	-7.09362	0.616463	0.670009
Monocytes	ERMP1	0.019445	3.633634	0.124428	0.901259	-5.83358	0.736257	0.800031
Monocytes	CALU	-0.01487	4.640586	-0.12439	0.901291	-6.08599	0.715703	0.777919
Monocytes	LDHB	-0.0222	3.00278	-0.12432	0.901346	-6.09654	0.749467	0.814198
Monocytes	HOOK2	0.023466	3.558904	0.124279	0.901376	-5.91946	0.737809	0.801696
Monocytes	LYZL4	-0.06393	-1.24765	-0.12404	0.901564	-5.04144	0.845611	0.916215
Monocytes	FAM204A	0.014441	4.894011	0.123908	0.901669	-6.10185	0.710707	0.772455
Monocytes	TAX1BP1	-0.00988	8.297296	-0.12388	0.901688	-6.78333	0.646345	0.702633
Monocytes	MLF1	-0.05879	0.512644	-0.1236	0.901912	-5.15045	0.804429	0.872632
Monocytes	NDRG3	0.015532	4.407312	0.123416	0.902057	-6.02388	0.720618	0.783073
Monocytes	RINT1	-0.02186	3.771156	-0.1234	0.902071	-5.7489	0.733626	0.797062
Monocytes	BOP1	0.018111	4.167475	0.123138	0.902278	-5.94875	0.7256	0.788362
Monocytes	CHKB	-0.01589	4.306938	-0.12285	0.902505	-6.00706	0.72281	0.7854
Monocytes	DUSP6	-0.02139	4.428378	-0.12273	0.902601	-6.02266	0.720348	0.782751
Monocytes	TTC13	-0.02004	3.938924	-0.12269	0.902634	-5.84397	0.730329	0.793488
Monocytes	RAPSN	0.041913	0.715904	0.122675	0.902643	-5.27154	0.799987	0.867889
Monocytes	PPIL4	0.010905	5.46202	0.122604	0.902699	-6.29261	0.699764	0.760557
Monocytes	PDCD5	0.010556	6.418317	0.122308	0.902933	-6.46852	0.681366	0.74066
Monocytes	SETD4	0.025674	2.964232	0.122213	0.903008	-5.61312	0.750734	0.815421
Monocytes	RNASE4	0.026019	5.17058	0.122138	0.903067	-6.19799	0.705565	0.766865
Monocytes	GM50386	0.062112	0.179027	0.122086	0.903108	-5.05728	0.812355	0.881068
Monocytes	A930001M01RI	-0.01852	3.967402	-0.12204	0.903142	-6.01972	0.729811	0.79299

Monocytes	SMIM26	0.018876	3.847983	0.121665	0.903441	-5.90879	0.732452	0.795699
Monocytes	ASPRV1	0.044077	1.33931	0.121405	0.903646	-5.59352	0.786352	0.853284
Monocytes	MCM10	-0.03159	3.112366	-0.12125	0.90377	-5.63393	0.747944	0.812243
Monocytes	TFB1M	0.037878	1.706255	0.121103	0.903884	-5.3778	0.778294	0.844649
Monocytes	CEP135	0.016257	3.853066	0.121022	0.903948	-5.91728	0.732525	0.795689
Monocytes	GM49864	-0.0593	0.452692	-0.12056	0.904313	-5.1347	0.806722	0.874702
Monocytes	GM43126	-0.06424	0.276018	-0.12033	0.904498	-5.09959	0.810883	0.879042
Monocytes	NACC1	-0.01476	4.380772	-0.12001	0.904745	-6.09032	0.722191	0.784378
Monocytes	TMEM176A	-0.01617	4.388779	-0.11984	0.904881	-6.30217	0.722028	0.784212
Monocytes	LIPH	0.065518	-0.05189	0.119511	0.905142	-5.09224	0.818627	0.887301
Monocytes	CAMK2A	-0.0444	0.846737	-0.11949	0.905154	-5.1656	0.797993	0.865437
Monocytes	PRR14L	0.011531	6.50182	0.119478	0.905168	-6.44892	0.680546	0.739441
Monocytes	AA386476	-0.04619	1.390731	-0.11935	0.905266	-5.29077	0.785778	0.852455
Monocytes	DDX42	0.010695	5.684077	0.119317	0.905295	-6.311	0.696275	0.756469
Monocytes	MRPL51	-0.01556	4.486929	-0.11929	0.905314	-6.07731	0.72004	0.782106
Monocytes	COL15A1	0.068025	0.332157	0.119129	0.905443	-5.14643	0.809739	0.877893
Monocytes	TMEM53	0.056511	0.637469	0.119012	0.905536	-5.17155	0.802748	0.870482
Monocytes	SARS2	0.023876	2.675724	0.118933	0.905598	-5.60147	0.757726	0.822536
Monocytes	CREBBP	0.008731	7.888027	0.118931	0.9056	-6.73503	0.654787	0.711452
Monocytes	POLR2A	0.009463	6.200996	0.11884	0.905672	-6.42894	0.686285	0.74566
Monocytes	ATL1	0.057604	0.498179	0.118839	0.905673	-5.20962	0.805929	0.873855
Monocytes	PP2D1	-0.04815	1.690903	-0.11856	0.90589	-5.36588	0.779231	0.84541
Monocytes	MIR99AHG	0.039457	3.430837	0.1185	0.90594	-5.74386	0.741857	0.805504
Monocytes	DNAH7A	0.073999	-0.11769	0.118225	0.906158	-5.03964	0.820312	0.889035
Monocytes	DYNC1LI2	0.015416	4.672528	0.118182	0.906191	-6.06202	0.71643	0.778174
Monocytes	TAZ	-0.01631	4.057371	-0.11817	0.906201	-5.92594	0.728921	0.791613
Monocytes	ABCB8	-0.02334	2.240197	-0.11791	0.906408	-5.54134	0.767314	0.832761
Monocytes	ATXN10	-0.01034	6.66392	-0.1177	0.906571	-6.55667	0.677657	0.736297
Monocytes	SGSH	-0.03332	1.453355	-0.11766	0.906601	-5.51224	0.784595	0.851187
Monocytes	GM20234	-0.03443	2.059362	-0.11764	0.906617	-5.41451	0.771248	0.836968
Monocytes	UNC13D	-0.02271	3.31115	-0.11758	0.90667	-5.76262	0.744461	0.808326
Monocytes	MELK	-0.03017	3.016502	-0.11753	0.906708	-5.77282	0.750673	0.814993
Monocytes	SNRPB	0.009277	7.300883	0.117393	0.906815	-6.67839	0.665756	0.723408
Monocytes	IMPAD1	0.014003	4.365894	0.117315	0.906876	-6.06436	0.722705	0.78501
Monocytes	MFGE8	-0.04002	2.292559	-0.1171	0.90705	-5.44279	0.766285	0.831696
Monocytes	GM48768	0.054538	0.621002	0.116947	0.907167	-5.14839	0.803489	0.871244
Monocytes	THSD1	-0.04427	1.60027	-0.11685	0.907243	-5.27141	0.781487	0.847859
Monocytes	IL3RA	0.017285	3.561284	0.116122	0.90782	-6.02415	0.739647	0.80294
Monocytes	MRPS18B	0.018215	3.873974	0.116111	0.907828	-5.91976	0.733164	0.795983
Monocytes	GM26724	0.038158	1.517501	0.115918	0.90798	-5.41544	0.78361	0.849955
Monocytes	PARPBP	-0.04947	2.056928	-0.11586	0.908023	-5.41576	0.771734	0.837304
Monocytes	NAGLU	-0.0234	2.6543	-0.11585	0.908037	-5.74462	0.758813	0.823509
Monocytes	IFT52	0.014103	4.249963	0.115797	0.908076	-6.03056	0.725452	0.78775
Monocytes	RPP14	0.025299	2.846249	0.115789	0.908083	-5.63517	0.754712	0.81913
Monocytes	BRCA1	-0.02772	4.143927	-0.11554	0.90828	-5.90713	0.72764	0.790117
Monocytes	SF3A1	-0.01247	4.851981	-0.11539	0.908398	-6.15588	0.713312	0.774697
Monocytes	PLXDC1	-0.02921	3.94302	-0.11518	0.908565	-5.88188	0.731763	0.794548

Monocytes	RACGAP1	-0.02218	5.205247	-0.11505	0.908663	-6.28044	0.706281	0.767115
Monocytes	METTL4	-0.02692	2.874732	-0.11499	0.908714	-5.52821	0.754128	0.818522
Monocytes	PLEKHG6	0.037108	-0.11425	0.11494	0.908753	-5.24449	0.820785	0.889411
Monocytes	LRCH3	0.010347	6.497112	0.114834	0.908837	-6.51337	0.681221	0.740034
Monocytes	IPO8	-0.01601	4.420007	-0.1148	0.908867	-5.9377	0.722015	0.784089
Monocytes	RTTN	-0.02268	3.609694	-0.1148	0.908868	-5.81377	0.738662	0.801974
Monocytes	DIS3	-0.02304	3.258023	-0.1147	0.908944	-5.69641	0.746018	0.809861
Monocytes	IPO13	0.031643	2.264795	0.114693	0.908948	-5.50127	0.767234	0.832549
Monocytes	PAQR4	-0.04137	1.484926	-0.1146	0.909025	-5.50684	0.784357	0.850809
Monocytes	SV2C	0.070035	-0.28271	0.114567	0.909048	-5.01749	0.824728	0.893617
Monocytes	AGPAT4	0.013139	5.023605	0.114419	0.909165	-6.63119	0.709921	0.771039
Monocytes	TREML2	0.026253	3.901235	0.114219	0.909323	-5.68936	0.732729	0.795521
Monocytes	PCBP2	0.00674	8.511828	0.114108	0.909411	-6.87124	0.644213	0.699726
Monocytes	ZFP518B	-0.04105	0.418092	-0.11367	0.909758	-5.2046	0.808711	0.876503
Monocytes	9530034E10RIK	-0.04506	0.330435	-0.11351	0.909886	-5.23611	0.810727	0.878675
Monocytes	PHYH	0.01933	5.40217	0.113444	0.909935	-6.34908	0.702614	0.763073
Monocytes	HNMT	-0.04724	-0.38409	-0.11317	0.910153	-5.15993	0.827369	0.896376
Monocytes	NAA30	0.016685	3.918894	0.113124	0.910189	-5.91404	0.732488	0.795333
Monocytes	EIF3M	0.007174	6.621301	0.113121	0.910191	-6.56621	0.679076	0.737693
Monocytes	ISY1	-0.01084	6.241546	-0.11287	0.910392	-6.54741	0.686312	0.745572
Monocytes	RSPH3B	0.021211	3.01938	0.112853	0.910403	-5.7266	0.75129	0.815533
Monocytes	CHMP2A	0.008499	6.544809	0.1128	0.910444	-6.59561	0.680526	0.739303
Monocytes	UBE2CBP	0.056757	1.761179	0.112774	0.910466	-5.29205	0.778487	0.844576
Monocytes	C230037L18RIK	0.048281	0.378737	0.112718	0.91051	-5.17387	0.809615	0.877648
Monocytes	HDAC6	0.030435	2.206503	0.112649	0.910564	-5.51506	0.768739	0.834189
Monocytes	DPH6	-0.01706	4.47347	-0.11264	0.910571	-6.05271	0.721156	0.783195
Monocytes	THEM4	0.037965	2.270582	0.112555	0.910638	-5.43546	0.767348	0.832704
Monocytes	ZFP810	-0.03034	2.914739	-0.11251	0.910676	-5.53097	0.753512	0.817921
Monocytes	TMOD3	-0.01005	7.218311	-0.11247	0.910702	-6.59244	0.667873	0.725587
Monocytes	ITGAL	0.01159	6.272091	0.112331	0.910816	-6.68672	0.68573	0.744984
Monocytes	MED20	-0.01658	4.104119	-0.11215	0.910961	-5.91791	0.728686	0.791332
Monocytes	RHAG	-0.08066	-0.3642	-0.11212	0.910986	-5.03116	0.826905	0.895983
Monocytes	MAP2K2	-0.00789	7.299123	-0.1121	0.910996	-6.6353	0.666377	0.723986
Monocytes	2010009K17RIK	-0.0772	0.112521	-0.11186	0.91119	-5.09345	0.815768	0.884271
Monocytes	HSDL1	-0.01519	4.167902	-0.11185	0.911195	-5.89196	0.72738	0.789984
Monocytes	KCMF1	-0.007	6.987433	-0.11172	0.911297	-6.62368	0.672185	0.730349
Monocytes	FCSK	-0.04883	0.928399	-0.11161	0.911385	-5.19767	0.797084	0.864466
Monocytes	PDCL	-0.01969	3.891934	-0.11153	0.911448	-5.87256	0.733048	0.796078
Monocytes	BUB3	-0.0121	5.956108	-0.11152	0.91146	-6.39301	0.691811	0.751622
Monocytes	8-Sep	-0.02993	1.954462	-0.11138	0.911566	-5.49811	0.774244	0.840167
Monocytes	SMIM5	0.044343	0.166279	0.111304	0.911628	-5.33022	0.814523	0.882959
Monocytes	ARPC1A	-0.0093	6.586291	-0.11128	0.911645	-6.54188	0.679743	0.738554
Monocytes	ELN	-0.04833	2.40021	-0.11088	0.911963	-5.45373	0.764749	0.829873
Monocytes	NDUFB4	-0.00954	6.598671	-0.11073	0.912079	-6.54731	0.679689	0.738348
Monocytes	TMED5	-0.0069	7.227678	-0.1106	0.912186	-6.67987	0.667881	0.725532
Monocytes	EIF6	-0.01051	5.835967	-0.11047	0.912285	-6.41897	0.694324	0.75423
Monocytes	C1GALT1	0.015852	6.353776	0.110449	0.912304	-6.21179	0.68435	0.743435

Monocytes	IFIT3B	-0.05785	1.43036	-0.11029	0.912427	-5.34206	0.786029	0.852612
Monocytes	HIST1H2BG	0.054401	1.128352	0.110171	0.912523	-5.19717	0.792787	0.859797
Monocytes	TTC27	0.023599	3.520631	0.110081	0.912595	-5.66759	0.740949	0.804459
Monocytes	GM13561	0.0305	1.307424	0.109956	0.912694	-5.47341	0.788772	0.855546
Monocytes	TRMT10A	-0.01883	3.81366	-0.10992	0.912725	-5.85428	0.73486	0.797959
Monocytes	SPINK10	-0.04685	0.791956	-0.10986	0.912772	-5.22837	0.800389	0.867909
Monocytes	AMMECR1L	-0.01414	4.488762	-0.10978	0.91283	-6.13998	0.721043	0.783117
Monocytes	PHYKPL	0.017634	3.470266	0.109721	0.912879	-5.77216	0.742001	0.805634
Monocytes	XIST	-0.40679	4.173151	-0.10964	0.912946	-5.65548	0.727466	0.790027
Monocytes	CDH22	0.082256	-0.85879	0.109606	0.91297	-4.96966	0.838856	0.908602
Monocytes	NEDD1	-0.02471	3.041246	-0.10951	0.913044	-5.66287	0.751032	0.815309
Monocytes	POGZ	-0.0141	4.431535	-0.10878	0.913625	-6.02829	0.722606	0.784495
Monocytes	N4BP2L1	-0.01305	4.595001	-0.10866	0.913722	-6.31971	0.719309	0.780939
Monocytes	ERRFI1	-0.01436	5.004787	-0.10857	0.913786	-6.36506	0.711085	0.772096
Monocytes	SMAD1	-0.01604	3.547772	-0.10807	0.914184	-5.98995	0.741008	0.804145
Monocytes	A530040E14RIK	0.045464	0.013043	0.107896	0.914323	-5.25976	0.818987	0.887166
Monocytes	GTF2E2	-0.00977	5.440806	-0.10782	0.914381	-6.38438	0.702637	0.762868
Monocytes	AIM2	-0.01255	5.579386	-0.10772	0.914461	-6.46076	0.699917	0.759945
Monocytes	BAG6	-0.01285	5.438014	-0.10768	0.914494	-6.29784	0.702692	0.762951
Monocytes	D10WSU102E	-0.02084	3.652731	-0.10763	0.914533	-5.81481	0.738821	0.801825
Monocytes	FRMD8	-0.01521	4.222806	-0.10758	0.914573	-6.0626	0.727065	0.789203
Monocytes	ITGB6	0.067919	-0.27325	0.107535	0.914608	-5.09008	0.825684	0.894271
Monocytes	WDR41	-0.01284	4.001344	-0.10721	0.914867	-6.10709	0.73172	0.794139
Monocytes	TENT5A	0.012651	5.169086	0.107174	0.914894	-6.43386	0.708116	0.768748
Monocytes	EMC3	0.010447	5.490353	0.107058	0.914986	-6.28665	0.701787	0.761942
Monocytes	GANC	0.02248	3.377098	0.106942	0.915077	-5.75801	0.744727	0.808138
Monocytes	STAG3	0.062353	0.607181	0.106761	0.915221	-5.13239	0.805499	0.872865
Monocytes	NCOA2	0.009452	7.684552	0.106543	0.915393	-6.71067	0.660252	0.716848
Monocytes	CYC1	0.011978	6.36195	0.106465	0.915455	-6.48585	0.685027	0.743733
Monocytes	STK19	-0.01446	4.61763	-0.10621	0.915658	-6.11744	0.719342	0.78078
Monocytes	2310011J03RIK	-0.01316	4.446593	-0.1061	0.915745	-6.14309	0.722806	0.78452
Monocytes	SKIV2L	0.016218	3.938774	0.105959	0.915855	-5.94872	0.7332	0.795712
Monocytes	PIGV	0.02044	2.921169	0.105895	0.915905	-5.60312	0.754528	0.818589
Monocytes	MED30	-0.01111	5.369422	-0.10578	0.916	-6.32006	0.704335	0.764683
Monocytes	GM48623	-0.06638	-0.24558	-0.10567	0.91608	-4.98556	0.825376	0.893917
Monocytes	GLA	0.014481	4.568639	0.105482	0.916232	-6.29346	0.720333	0.781956
Monocytes	HRH1	-0.03988	-0.56863	-0.10547	0.916245	-5.21278	0.833	0.901989
Monocytes	AGGF1	0.010288	5.384652	0.105454	0.916254	-6.27257	0.704035	0.764393
Monocytes	GM17056	-0.01914	2.866748	-0.10545	0.91626	-6.11784	0.755688	0.819883
Monocytes	ACVR1	0.017066	3.707524	0.105412	0.916288	-6.1224	0.737987	0.800935
Monocytes	STAT1	-0.02679	6.871703	-0.10489	0.9167	-6.62094	0.67558	0.73354
Monocytes	NUBP1	0.010576	5.473714	0.104881	0.916707	-6.3857	0.702485	0.762653
Monocytes	MED27	0.009488	5.275175	0.104748	0.916813	-6.27009	0.706402	0.76691
Monocytes	SKP2	0.025566	3.310393	0.104619	0.916915	-5.6003	0.746507	0.81002
Monocytes	CYP3A13	-0.05218	0.779768	-0.10455	0.916967	-5.16125	0.801907	0.869066
Monocytes	MED9	0.018153	3.526994	0.104437	0.917059	-5.87945	0.741965	0.805153
Monocytes	CD320	-0.03097	1.856288	-0.10439	0.917099	-5.33607	0.777808	0.843455

Monocytes	4931423N10RII	0.049792	0.653443	0.104203	0.917244	-5.19513	0.804787	0.872125
Monocytes	RYBP	0.011036	5.626574	0.104023	0.917387	-6.37264	0.699486	0.759538
Monocytes	LUC7L	0.00942	5.622585	0.103947	0.917447	-6.31872	0.699564	0.759622
Monocytes	HSPB6	0.050024	0.40248	0.103936	0.917455	-5.17371	0.810544	0.878327
Monocytes	CMC1	0.010761	4.601302	0.103909	0.917477	-6.21884	0.719881	0.78153
Monocytes	CGGBP1	-0.00845	6.845343	-0.10376	0.917592	-6.58015	0.676077	0.7342
Monocytes	CASS4	0.018768	3.04899	0.103695	0.917646	-6.18108	0.752031	0.816035
Monocytes	WNT5B	0.036679	1.562043	0.103626	0.917701	-5.31006	0.784316	0.850487
Monocytes	HSD17B7	0.038115	1.697013	0.103527	0.917779	-5.23317	0.781323	0.847302
Monocytes	GDPGP1	-0.04696	2.18615	-0.10344	0.917847	-5.28856	0.770583	0.835857
Monocytes	RMI2	-0.026	3.3602	-0.10327	0.917986	-5.62629	0.74546	0.809
Monocytes	MAEA	0.01059	5.40999	0.103259	0.917991	-6.23762	0.70374	0.764133
Monocytes	ZBTB9	-0.02816	2.511721	-0.10325	0.918	-5.44115	0.763525	0.828323
Monocytes	FAR1	-0.01079	6.64871	-0.10286	0.91831	-6.53361	0.679937	0.738329
Monocytes	CHCHD10	-0.02153	6.141333	-0.10282	0.918338	-6.17135	0.689635	0.748833
Monocytes	RNF227	0.032856	1.852006	0.102656	0.918469	-5.4329	0.778069	0.843786
Monocytes	DUS1L	0.016625	4.064535	0.102601	0.918512	-5.95269	0.730979	0.793416
Monocytes	AW146154	-0.03448	2.334177	-0.10254	0.918558	-5.40324	0.767529	0.832549
Monocytes	SPIN1	-0.01012	5.805516	-0.10229	0.918755	-6.30016	0.696236	0.755879
Monocytes	SCUBE2	0.049924	-0.26058	0.102054	0.918945	-5.24737	0.826351	0.894789
Monocytes	RTEL1	0.033131	2.682882	0.101979	0.919004	-5.43909	0.760194	0.824524
Monocytes	TMCO6	-0.02387	2.683081	-0.10167	0.919247	-5.5267	0.760272	0.82452
Monocytes	TRMT1L	0.011505	4.566129	0.101672	0.919247	-6.03683	0.721004	0.782426
Monocytes	TMEM30A	0.006983	6.581779	0.101404	0.919459	-6.5537	0.681506	0.739784
Monocytes	TSG101	-0.00991	5.622255	-0.10135	0.919505	-6.35719	0.700025	0.759813
Monocytes	SETD6	-0.03068	1.423905	-0.10131	0.919537	-5.32568	0.787903	0.853967
Monocytes	ELOB	-0.00762	8.913307	-0.10119	0.91963	-6.9024	0.63878	0.693318
Monocytes	ALOX5	-0.03774	0.791987	-0.10102	0.919766	-5.44319	0.802223	0.869145
Monocytes	TRADD	-0.01653	3.982911	-0.10076	0.919969	-5.92843	0.73315	0.795394
Monocytes	FBXO4	0.01164	3.431156	0.100642	0.920062	-6.17748	0.744646	0.80771
Monocytes	MAG	-0.05233	0.11897	-0.10038	0.920269	-5.15621	0.817938	0.885604
Monocytes	CYP4A31	0.052043	0.922941	0.100322	0.920316	-5.18413	0.799474	0.866079
Monocytes	CACUL1	-0.01051	6.117834	-0.09993	0.920628	-6.46449	0.690712	0.74957
Monocytes	AAMDC	-0.0164	3.299635	-0.09986	0.920685	-5.71451	0.747567	0.810749
Monocytes	USP46	-0.02511	3.281753	-0.09982	0.920713	-5.62515	0.747944	0.811154
Monocytes	RDH10	-0.02222	3.207433	-0.09966	0.920838	-5.72261	0.749513	0.812854
Monocytes	MATK	0.021136	2.36611	0.099523	0.920948	-5.81906	0.767528	0.832105
Monocytes	TRIM65	-0.02172	3.22435	-0.09949	0.920977	-5.67163	0.749155	0.812475
Monocytes	CD300LD	-0.01873	3.173108	-0.09943	0.921021	-6.17674	0.750239	0.813636
Monocytes	GM43727	-0.03297	-0.59426	-0.09943	0.921025	-5.37276	0.83478	0.90342
Monocytes	KIF11	0.02264	5.441855	0.099394	0.92105	-6.31279	0.703897	0.763857
Monocytes	NEK2	-0.02961	3.092142	-0.09897	0.921382	-5.65092	0.752166	0.81561
Monocytes	RIDA	-0.02931	4.316478	-0.09876	0.921556	-5.91798	0.726759	0.78833
Monocytes	PXMP4	-0.01665	3.831766	-0.09838	0.921853	-5.90904	0.736873	0.799076
Monocytes	ZFP68	0.016022	3.920899	0.098297	0.921919	-5.78709	0.735027	0.797096
Monocytes	GM50218	-0.0581	0.559001	-0.09826	0.921948	-5.07422	0.808318	0.875203
Monocytes	TGFBR1	0.01137	6.24383	0.098149	0.922036	-6.46331	0.688698	0.747193

Monocytes	ATP5O	-0.00904	7.142669	-0.09789	0.922243	-6.63081	0.671749	0.728807
Monocytes	RBM26	0.007718	6.821937	0.097594	0.922476	-6.52196	0.677895	0.735387
Monocytes	ENDOV	-0.03028	2.177707	-0.09719	0.922796	-5.44173	0.772501	0.83686
Monocytes	GGA3	0.015823	3.302395	0.09711	0.922859	-5.78643	0.748355	0.811084
Monocytes	KLHL3	-0.03555	2.005874	-0.09705	0.922908	-5.29559	0.776265	0.840868
Monocytes	ATXN7L3	0.012731	4.200833	0.096979	0.922962	-6.01368	0.729665	0.791055
Monocytes	PRDM4	0.018873	3.039382	0.096762	0.923135	-5.68285	0.753998	0.817114
Monocytes	SLC44A1	-0.01405	4.637679	-0.09669	0.923195	-6.12205	0.720832	0.781571
Monocytes	DPP3	-0.01249	4.728026	-0.09653	0.923321	-6.12542	0.719025	0.779609
Monocytes	INTS7	-0.0089	5.677826	-0.09647	0.923363	-6.27248	0.700139	0.759267
Monocytes	GATB	0.02219	3.11047	0.095725	0.923956	-5.69179	0.752873	0.815611
Monocytes	RAD50	0.01189	4.906644	0.095648	0.924017	-6.15278	0.715778	0.77584
Monocytes	ABCA3	0.010509	4.193838	0.095632	0.92403	-6.14594	0.730251	0.791388
Monocytes	PPP1R12A	0.006844	8.052992	0.095404	0.924209	-6.7849	0.655665	0.710803
Monocytes	MRPS10	-0.01378	4.162492	-0.09536	0.924247	-5.95687	0.73095	0.792115
Monocytes	GM16174	-0.03796	0.995634	-0.09527	0.924317	-5.20901	0.799347	0.865047
Monocytes	ALKBH8	-0.01861	3.835022	-0.09511	0.924444	-5.65424	0.737758	0.799368
Monocytes	PPNR	0.040891	1.051959	0.095012	0.92452	-5.2575	0.798119	0.863736
Monocytes	ABCB7	-0.00966	5.334608	-0.09483	0.924662	-6.2354	0.707337	0.766777
Monocytes	GOLT1B	-0.01318	4.631519	-0.09482	0.924674	-6.11796	0.721423	0.781935
Monocytes	STAM	0.011757	4.382468	0.094742	0.924734	-6.02767	0.726487	0.787388
Monocytes	MIB1	-0.00852	6.189415	-0.09453	0.924906	-6.50347	0.69067	0.748782
Monocytes	ZFP358	-0.02225	3.044691	-0.09448	0.92494	-5.62466	0.754422	0.817282
Monocytes	CDKN2C	0.02093	3.918847	0.094256	0.925119	-5.92811	0.736101	0.797685
Monocytes	TAOK1	-0.00707	7.033029	-0.09425	0.925126	-6.62225	0.674638	0.731442
Monocytes	ATG4B	-0.01209	4.800676	-0.094	0.925324	-6.10099	0.718155	0.778382
Monocytes	SLC6A19	0.05839	-0.35949	0.093794	0.925485	-4.99011	0.830947	0.898458
Monocytes	MEIS3	-0.04291	0.488054	-0.09375	0.925521	-5.16266	0.811164	0.877581
Monocytes	PLD4	-0.01074	6.186695	-0.09365	0.925596	-6.72294	0.69082	0.748951
Monocytes	PPM1G	0.007609	6.448565	0.093626	0.925618	-6.48735	0.685788	0.74351
Monocytes	GM27188	0.064691	0.619818	0.093547	0.92568	-5.02407	0.808134	0.874404
Monocytes	FAM210B	-0.01693	3.914295	-0.09339	0.925808	-5.88795	0.736267	0.797876
Monocytes	TMEM273	-0.02925	1.19098	-0.09327	0.925901	-5.4412	0.795143	0.860656
Monocytes	STIP1	0.011238	5.419076	0.093239	0.925925	-6.28398	0.705812	0.765146
Monocytes	NMNAT1	-0.039	1.326233	-0.09299	0.926121	-5.3103	0.792101	0.857461
Monocytes	BPGM	-0.02919	4.812429	-0.09285	0.926236	-5.81095	0.717918	0.778256
Monocytes	GM29340	-0.03833	-0.6731	-0.0928	0.926274	-5.20351	0.838399	0.906467
Monocytes	SNX11	0.024266	2.539196	0.092789	0.926281	-5.48451	0.765374	0.82909
Monocytes	ANXA3	-0.01502	3.542549	-0.09277	0.926294	-6.24857	0.744013	0.806266
Monocytes	VMAC	0.034819	1.5263	0.092682	0.926366	-5.25102	0.787623	0.852783
Monocytes	BLOC1S4	0.014348	3.695064	0.092669	0.926376	-5.86164	0.740824	0.802863
Monocytes	HARS2	-0.01884	2.848743	-0.09245	0.92655	-5.54641	0.758795	0.822073
Monocytes	TMPO	0.009723	7.067829	0.092019	0.926891	-6.70231	0.674277	0.73102
Monocytes	PLPPR1	-0.03817	1.149205	-0.09194	0.926951	-5.26739	0.796354	0.861901
Monocytes	AI467606	-0.01577	4.111903	-0.09191	0.926979	-6.0285	0.732432	0.793727
Monocytes	CAPN10	0.023958	2.598593	0.091649	0.927184	-5.50831	0.764371	0.827866
Monocytes	MRPS36	-0.00992	5.569029	-0.09157	0.927249	-6.36534	0.703114	0.762176

Monocytes	ISG20	-0.02606	4.233394	-0.09151	0.927295	-5.93111	0.729957	0.791049
Monocytes	TOP3A	-0.01746	3.860539	-0.09147	0.927323	-5.83056	0.737652	0.799301
Monocytes	FEN1	0.015512	4.525584	0.091368	0.927407	-6.05127	0.723988	0.784641
Monocytes	CABP4	-0.05502	-0.22452	-0.09124	0.927506	-5.06318	0.828066	0.895436
Monocytes	GM6710	-0.04352	0.761027	-0.09123	0.927515	-5.16451	0.805197	0.87127
Monocytes	CLP1	-0.01294	4.608479	-0.09098	0.927715	-5.99643	0.722405	0.782893
Monocytes	PTCH1	-0.02559	3.139748	-0.09085	0.927819	-5.60288	0.752914	0.815558
Monocytes	ZFP275	0.031581	1.581356	0.090698	0.927938	-5.2639	0.786841	0.851707
Monocytes	GM47507	0.038102	0.732528	0.090633	0.927989	-5.38632	0.806008	0.872043
Monocytes	1500015A07RIK	0.026997	1.76661	0.090489	0.928103	-5.3411	0.782727	0.847342
Monocytes	MIA3	-0.00768	5.849323	-0.09036	0.928209	-6.43763	0.697763	0.756341
Monocytes	MPDU1	0.010436	4.71579	0.090353	0.928211	-6.15023	0.720279	0.78059
Monocytes	ZFP975	-0.05243	0.810489	-0.08993	0.928544	-5.1357	0.804457	0.870232
Monocytes	SLC9A6	0.017047	2.81804	0.089702	0.928727	-5.70815	0.760098	0.823031
Monocytes	COQ3	-0.01333	3.500438	-0.0896	0.928807	-5.78974	0.74561	0.807562
Monocytes	ZFP932	0.018691	2.788655	0.089484	0.9289	-5.51792	0.760729	0.823726
Monocytes	PDS5B	0.00858	6.377064	0.089441	0.928934	-6.43655	0.687816	0.745425
Monocytes	AP3B1	0.006184	7.329007	0.089207	0.929119	-6.69395	0.669812	0.725942
Monocytes	OTUD3	-0.02432	2.558441	-0.08911	0.929196	-5.57169	0.765695	0.829031
Monocytes	SLC52A3	-0.03009	0.451484	-0.08911	0.929197	-5.38536	0.812789	0.879026
Monocytes	ZFP217	0.011078	4.011596	0.089069	0.929228	-6.00976	0.734961	0.796179
Monocytes	FAM219B	-0.02356	2.367885	-0.08878	0.929457	-5.56471	0.769958	0.833516
Monocytes	GM50431	0.040082	0.122176	0.088664	0.929549	-5.23632	0.820586	0.887201
Monocytes	2-Mar	-0.00898	5.868752	-0.08852	0.929667	-6.36309	0.697824	0.756189
Monocytes	CEP192	0.010517	5.27108	0.08823	0.929894	-6.19949	0.709684	0.768924
Monocytes	HPF1	-0.01243	5.467524	-0.08815	0.929953	-6.26276	0.70579	0.76473
Monocytes	POGLUT2	-0.03231	1.00166	-0.08796	0.930107	-5.3372	0.800483	0.865942
Monocytes	FAM78B	-0.019	2.115372	-0.08792	0.930138	-5.82494	0.775625	0.839548
Monocytes	RNF213	-0.01626	6.151249	-0.08791	0.930145	-6.53891	0.692421	0.750352
Monocytes	GM41442	0.036466	-0.19949	0.087771	0.930257	-5.29861	0.828293	0.895336
Monocytes	CDC6	-0.02489	3.269665	-0.08769	0.930318	-5.63574	0.750773	0.813064
Monocytes	TRABD	0.009813	5.363153	0.087491	0.930479	-6.25896	0.707913	0.767069
Monocytes	RRP8	-0.01586	3.730434	-0.08737	0.930576	-5.81724	0.741128	0.802766
Monocytes	NRXN3	0.044573	1.145579	0.087359	0.930584	-5.20421	0.797288	0.862598
Monocytes	EPHB2	0.048452	1.372664	0.086918	0.930933	-5.10439	0.792192	0.857186
Monocytes	RDH13	-0.03169	1.398416	-0.08682	0.93101	-5.27854	0.791614	0.856572
Monocytes	WDR45B	0.006511	6.330115	0.086803	0.931025	-6.487	0.689044	0.746733
Monocytes	CCNO	-0.04394	0.174468	-0.08678	0.931045	-5.16667	0.819596	0.886192
Monocytes	RASAL3	0.013877	4.10056	0.086677	0.931124	-5.86389	0.733468	0.794551
Monocytes	G6PC3	-0.01617	3.106653	-0.08661	0.93118	-5.86186	0.75429	0.816834
Monocytes	ERG28	-0.00827	5.118614	-0.08661	0.931181	-6.25436	0.712799	0.772349
Monocytes	SPINDOC	0.011677	4.427162	0.086341	0.93139	-6.02543	0.726766	0.787361
Monocytes	GM20707	-0.02353	1.898927	-0.08633	0.931402	-5.3745	0.780473	0.844739
Monocytes	TAF5L	0.011044	4.562119	0.086261	0.931454	-6.06426	0.724016	0.784434
Monocytes	CSRNP1	0.010294	6.641086	0.086083	0.931595	-6.62918	0.683091	0.740343
Monocytes	SAR1B	0.007464	5.553451	0.085896	0.931743	-6.34368	0.704169	0.763104
Monocytes	FAHD2A	-0.01822	2.845982	-0.08587	0.931761	-5.59771	0.759858	0.822832

Monocytes	RNF40	-0.01322	4.021973	-0.08587	0.931764	-5.84927	0.735091	0.796341
Monocytes	4933406I18RIK	0.017439	4.543564	0.085756	0.931854	-6.1177	0.724393	0.784884
Monocytes	SLC22A21	-0.01996	1.348839	-0.08569	0.931903	-5.76763	0.792727	0.857841
Monocytes	ZFP384	0.009764	4.90083	0.085686	0.93191	-6.17653	0.717165	0.777126
Monocytes	POP7	-0.0103	4.908142	-0.08555	0.932018	-6.18333	0.717018	0.777009
Monocytes	SCAF4	0.006954	6.317786	0.0855	0.932057	-6.55569	0.689282	0.747106
Monocytes	CENPK	0.021191	3.283005	0.085496	0.93206	-5.71514	0.750548	0.812963
Monocytes	STUB1	-0.00842	5.426852	-0.08537	0.932161	-6.29701	0.706688	0.765937
Monocytes	TEK	-0.03476	2.175307	-0.08528	0.932229	-5.34974	0.774414	0.838513
Monocytes	GM19684	0.034501	1.522121	0.084775	0.932632	-5.29636	0.789143	0.853964
Monocytes	SS18L1	-0.02673	2.325233	-0.08442	0.932913	-5.47748	0.771584	0.83511
Monocytes	TRIM3	-0.01856	2.274801	-0.08414	0.933136	-5.63182	0.772809	0.836282
Monocytes	FKBP7	-0.0308	1.867273	-0.08361	0.933552	-5.34666	0.782057	0.845952
Monocytes	USP14	-0.00711	5.793901	-0.0834	0.933722	-6.40084	0.700313	0.758406
Monocytes	EXOC5	0.006528	6.376842	0.083228	0.933859	-6.5062	0.689044	0.746216
Monocytes	MEGF8	0.031869	0.288303	0.082975	0.934059	-5.24761	0.818127	0.883969
Monocytes	ZFP1	0.016041	3.874226	0.082635	0.934329	-5.74681	0.739318	0.800093
Monocytes	ZFP619	-0.01981	2.21582	-0.08259	0.93436	-5.52691	0.774728	0.837886
Monocytes	SZRD1	0.006625	5.906997	0.082541	0.934403	-6.39789	0.698339	0.756098
Monocytes	CPN1	-0.03477	1.600653	-0.08234	0.934566	-5.26341	0.788409	0.852394
Monocytes	TNFRSF1A	0.00835	5.171275	0.08208	0.934769	-6.53971	0.713038	0.771828
Monocytes	CMYA5	0.05167	0.674059	0.08192	0.934896	-5.11329	0.809543	0.874716
Monocytes	METTL26	0.012445	4.639923	0.081801	0.93499	-6.09796	0.723774	0.783404
Monocytes	PLOD2	0.043982	1.261605	0.081624	0.93513	-5.14807	0.796161	0.860617
Monocytes	PCDHGC4	0.039097	0.256002	0.08158	0.935165	-5.17554	0.819213	0.884992
Monocytes	DTD1	-0.0183	3.881415	-0.08113	0.935519	-5.76523	0.739371	0.800201
Monocytes	MAN2A2	-0.01326	4.595669	-0.08112	0.935528	-5.99035	0.724674	0.784449
Monocytes	HLF	0.037502	1.023669	0.08112	0.93553	-5.23319	0.801551	0.866392
Monocytes	TLE4	0.007397	7.814076	0.081017	0.935611	-6.70674	0.662375	0.717221
Monocytes	POLA2	0.015599	4.050563	0.080977	0.935643	-5.85427	0.73586	0.796442
Monocytes	ALG13	0.008323	4.514642	0.080951	0.935664	-6.1164	0.726324	0.78622
Monocytes	FBXO33	0.007811	5.984659	0.080919	0.935689	-6.42819	0.697015	0.754693
Monocytes	BLOC1S2	-0.00925	4.833886	-0.08086	0.935738	-6.24744	0.719844	0.779264
Monocytes	SOWAHC	0.01221	2.917305	0.080842	0.935751	-6.25261	0.759735	0.821959
Monocytes	SAC3D1	0.016763	3.030557	0.080432	0.936075	-5.60275	0.757369	0.819373
Monocytes	B230369F24RIK	-0.01969	2.457789	-0.08042	0.936084	-5.5571	0.769717	0.832533
Monocytes	FAM207A	0.010928	4.223482	0.080322	0.936163	-5.98319	0.732347	0.792618
Monocytes	1700029H14RII	-0.02124	1.58753	-0.08028	0.936195	-5.53936	0.788903	0.852923
Monocytes	DDX46	0.007831	6.040748	0.080152	0.936298	-6.39442	0.695976	0.753516
Monocytes	GBP6	-0.0425	1.984096	-0.08004	0.936383	-5.36647	0.780096	0.843572
Monocytes	UBL7	0.010416	5.130384	0.07998	0.936434	-6.16906	0.713938	0.772858
Monocytes	TRAF3IP3	-0.00916	5.030914	-0.07996	0.93645	-6.23352	0.715932	0.775001
Monocytes	FAM13B	-0.0072	6.457749	-0.07993	0.936477	-6.48213	0.687918	0.744818
Monocytes	A930029G22RII	-0.02999	1.230907	-0.07981	0.93657	-5.31055	0.796933	0.861447
Monocytes	KIFC3	-0.02278	1.625707	-0.07946	0.936848	-5.53102	0.78824	0.852118
Monocytes	TSR3	0.015831	3.569257	0.07928	0.936989	-5.80771	0.746188	0.807318
Monocytes	BTLA	-0.01181	3.948652	-0.07917	0.937072	-6.42374	0.738269	0.798853

Monocytes	GM49101	-0.03414	-0.65342	-0.079	0.937209	-5.25784	0.841078	0.907872
Monocytes	L3MBTL2	-0.01472	3.445434	-0.07854	0.937576	-5.65128	0.748884	0.810243
Monocytes	PIGT	0.008303	5.640537	0.078504	0.937604	-6.38666	0.704113	0.762218
Monocytes	HDGFL2	-0.00941	4.979057	-0.07827	0.937786	-6.12129	0.717283	0.776385
Monocytes	NUCKS1	-0.00856	6.576295	-0.07826	0.937799	-6.52021	0.685942	0.742617
Monocytes	SIRT7	-0.00717	4.918849	-0.07818	0.93786	-6.23843	0.718495	0.777687
Monocytes	CMTM6	0.008091	4.866252	0.078029	0.937981	-6.23945	0.719556	0.778844
Monocytes	BAG4	0.010706	4.226706	0.077974	0.938025	-5.99512	0.732596	0.792839
Monocytes	GM14286	-0.03634	0.211974	-0.07792	0.938071	-5.12772	0.820655	0.886469
Monocytes	DARS2	-0.02342	2.849125	-0.0779	0.938082	-5.61192	0.761584	0.823822
Monocytes	NECTIN1	-0.01427	1.073651	-0.07789	0.938093	-6.02231	0.800822	0.865508
Monocytes	COPB2	0.006771	5.438302	0.077799	0.938164	-6.33553	0.70811	0.766543
Monocytes	LSM7	0.007123	6.346432	0.077768	0.938188	-6.49215	0.690356	0.747405
Monocytes	SNRNP25	0.013501	3.939991	0.077711	0.938234	-5.88989	0.738528	0.799191
Monocytes	POLQ	-0.01988	3.166551	-0.07755	0.938361	-5.60087	0.754794	0.816611
Monocytes	LSM2	-0.01075	5.695608	-0.07743	0.938455	-6.37318	0.703029	0.761104
Monocytes	ZFP281	-0.01126	4.496464	-0.07732	0.93854	-6.03369	0.727064	0.786963
Monocytes	LEPROTL1	0.009232	5.362087	0.077294	0.938564	-6.4006	0.709623	0.768225
Monocytes	ADORA2A	0.017352	3.148747	0.077163	0.938668	-5.85636	0.755173	0.817041
Monocytes	DUSP19	-0.02185	2.048701	-0.0771	0.938717	-5.4319	0.779007	0.842433
Monocytes	P2RX1	-0.0324	0.81074	-0.07684	0.938928	-5.32345	0.806817	0.871992
Monocytes	LMBRD2	0.012618	4.481892	0.076713	0.939025	-6.01399	0.727362	0.787359
Monocytes	BCAP31	-0.00647	5.901725	-0.07669	0.939041	-6.42424	0.698987	0.756843
Monocytes	KLF8	-0.03052	0.979838	-0.07664	0.939086	-5.40528	0.802956	0.867913
Monocytes	CCDC66	-0.02066	2.265203	-0.07661	0.93911	-5.43588	0.774251	0.837452
Monocytes	MON2	-0.00781	5.7408	-0.07658	0.939131	-6.33348	0.70214	0.760243
Monocytes	D2HGDH	-0.02425	2.08867	-0.07656	0.939143	-5.34819	0.778126	0.841574
Monocytes	VPS26C	-0.01221	4.084075	-0.07648	0.93921	-5.96146	0.73554	0.796147
Monocytes	PDE10A	-0.03577	2.555984	-0.07614	0.939482	-5.31343	0.768049	0.830801
Monocytes	ZFP40	0.031016	1.347612	0.07609	0.939519	-5.27354	0.794764	0.859203
Monocytes	1810034E14RIK	-0.02523	1.895787	-0.07594	0.939635	-5.36766	0.782557	0.846222
Monocytes	DPP8	-0.00717	5.667042	-0.07539	0.940071	-6.34778	0.703968	0.762027
Monocytes	DCAF5	-0.00858	5.52135	-0.07521	0.940214	-6.2953	0.706844	0.765146
Monocytes	TOM1L1	0.035234	0.714609	0.075083	0.940318	-5.18072	0.809455	0.874646
Monocytes	ASCC3	0.007212	6.882346	0.07505	0.940345	-6.65304	0.680478	0.736726
Monocytes	CHST15	-0.01484	3.54275	-0.07505	0.940347	-5.74949	0.747233	0.808503
Monocytes	CCDC152	0.022456	3.284464	0.075006	0.940379	-5.63878	0.752692	0.81434
Monocytes	ATP5C1	0.004703	8.358211	0.074898	0.940465	-6.87151	0.65313	0.707098
Monocytes	HSBP1	-0.00806	6.003464	-0.0748	0.940539	-6.39263	0.697375	0.755028
Monocytes	SIKE1	-0.00937	4.570739	-0.07474	0.940587	-6.0724	0.725938	0.785762
Monocytes	EAF1	0.011908	4.101569	0.074413	0.94085	-5.96117	0.735721	0.796182
Monocytes	KATNBL1	-0.00712	5.963648	-0.07423	0.940995	-6.37895	0.698298	0.755993
Monocytes	MRPL12	0.009765	5.090142	0.07422	0.941003	-6.19875	0.715585	0.774603
Monocytes	SLC35B2	0.008326	4.882082	0.074092	0.941104	-6.29488	0.719795	0.779116
Monocytes	PER3	0.029405	1.517982	0.073898	0.941258	-5.24852	0.791481	0.855558
Monocytes	AGBL5	-0.02113	2.244561	-0.07365	0.941452	-5.36753	0.775418	0.838486
Monocytes	DYNLL1	-0.00569	7.867224	-0.07364	0.941461	-6.71664	0.662344	0.717018

Monocytes	DNAJC10	-0.00765	4.533218	-0.07334	0.941702	-6.23579	0.727114	0.786812
Monocytes	ARFIP2	0.018486	2.138474	0.073231	0.941788	-5.4929	0.777898	0.841029
Monocytes	ATP6V1D	-0.00532	6.650485	-0.07306	0.941924	-6.52907	0.6853	0.741836
Monocytes	FAM71A	-0.02612	0.237403	-0.07298	0.941985	-5.40724	0.820992	0.886718
Monocytes	CHAF1A	0.015263	4.186029	0.072747	0.942172	-5.96579	0.734266	0.794543
Monocytes	RAB33B	-0.00989	4.405753	-0.07263	0.942263	-5.95046	0.729746	0.789711
Monocytes	TAF15	0.005455	6.905531	0.072572	0.942311	-6.54147	0.680445	0.736621
Monocytes	HIST1H1D	0.034049	1.531522	0.072567	0.942314	-5.28981	0.791392	0.855447
Monocytes	DEXI	0.013866	2.91114	0.072566	0.942316	-5.77878	0.761115	0.82325
Monocytes	ARL2	-0.01494	3.356926	-0.07237	0.942467	-5.73977	0.751668	0.813144
Monocytes	FBXL22	0.026632	2.248825	0.072223	0.942588	-5.36487	0.775593	0.838634
Monocytes	FAM118A	0.018456	2.069936	0.071909	0.942837	-5.51873	0.779661	0.842863
Monocytes	UNC5CL	-0.0484	0.465408	-0.07183	0.942896	-5.04703	0.815941	0.881318
Monocytes	ZFP322A	0.016036	3.041434	0.07143	0.943217	-5.67486	0.758691	0.820445
Monocytes	SPIRE1	-0.01322	2.681156	-0.07139	0.943248	-6.16007	0.766446	0.82871
Monocytes	ATL3	-0.00657	5.439187	-0.07128	0.943336	-6.35045	0.709241	0.767489
Monocytes	CKM	-0.04763	0.1057	-0.07125	0.943358	-5.13973	0.824473	0.890208
Monocytes	RUNDC1	-0.0152	2.994317	-0.07108	0.943496	-5.65155	0.759753	0.821587
Monocytes	GM15327	-0.02068	1.294764	-0.07075	0.94376	-5.38691	0.797317	0.861398
Monocytes	UQCR10	-0.00685	6.961957	-0.07066	0.943825	-6.59022	0.679886	0.735739
Monocytes	MAN1C1	0.009172	4.292733	0.070238	0.944163	-6.25212	0.732809	0.792504
Monocytes	CASTOR1	0.023483	0.626653	0.070128	0.94425	-5.43753	0.812787	0.877563
Monocytes	IARS2	-0.0101	4.475268	-0.07008	0.944292	-6.03413	0.72906	0.788487
Monocytes	DNMT1	-0.00957	5.753517	-0.06996	0.944384	-6.34291	0.703413	0.760921
Monocytes	MBNL1	-0.00676	8.978349	-0.06961	0.944663	-6.91861	0.643077	0.695617
Monocytes	NUP214	-0.00679	5.2678	-0.06954	0.944713	-6.33502	0.713061	0.771302
Monocytes	DZIP3	-0.0171	3.257554	-0.06945	0.944789	-5.65088	0.754512	0.81569
Monocytes	TRIM12C	-0.01076	4.726648	-0.06939	0.944835	-6.13165	0.723965	0.783036
Monocytes	CDC42SE1	0.005899	6.18973	0.069336	0.944879	-6.51305	0.694905	0.751784
Monocytes	ITGAV	-0.00828	6.550452	-0.06932	0.94489	-6.57068	0.687943	0.744272
Monocytes	GPR34	-0.0191	0.588061	-0.06927	0.944933	-5.78982	0.813715	0.878574
Monocytes	CTSG	0.040938	-0.2691	0.069171	0.94501	-5.45936	0.833784	0.899744
Monocytes	ANK1	0.023397	1.273318	0.069103	0.945064	-5.66156	0.798048	0.862039
Monocytes	INPP5D	0.00657	8.764735	0.068866	0.945252	-6.87989	0.646967	0.699882
Monocytes	GLT1D1	0.028676	0.611079	0.068699	0.945384	-5.27569	0.813287	0.878155
Monocytes	TG	-0.02053	1.91376	-0.06867	0.945408	-5.4546	0.783802	0.846937
Monocytes	GM15726	-0.02767	2.074088	-0.06857	0.945489	-5.59323	0.780254	0.84317
Monocytes	MTURN	-0.02815	2.270409	-0.06849	0.945549	-5.24823	0.775934	0.838584
Monocytes	NDUFAF1	0.015358	2.81597	0.068086	0.945871	-5.60391	0.764269	0.82604
Monocytes	FAM98C	-0.00965	3.737769	-0.06782	0.946082	-6.07682	0.74469	0.805203
Monocytes	STAMBPL1	-0.00945	6.55028	-0.0678	0.9461	-6.30595	0.68824	0.744564
Monocytes	MYO1D	-0.01866	3.025547	-0.06777	0.946124	-5.53178	0.759789	0.821323
Monocytes	CYP2AB1	0.02104	-0.30874	0.06759	0.946264	-5.55479	0.83514	0.90111
Monocytes	FAM216A	0.018925	2.582038	0.067349	0.946456	-5.43619	0.769513	0.831642
Monocytes	ENOX2	-0.0082	5.852601	-0.06671	0.946965	-6.43301	0.701991	0.759345
Monocytes	RAD18	-0.01223	4.103468	-0.06666	0.947001	-5.9454	0.737285	0.797239
Monocytes	NR1I3	0.033511	1.055611	0.066658	0.947004	-5.20335	0.803571	0.867767

Monocytes	PCTP	0.015791	2.252585	0.066606	0.947046	-5.6111	0.776789	0.839372
Monocytes	CRTAP	-0.0144	3.004923	-0.06659	0.947055	-5.6885	0.760457	0.82199
Monocytes	TMED1	-0.01871	2.452472	-0.06655	0.947093	-5.43348	0.772412	0.834719
Monocytes	PPA2	-0.00914	4.580694	-0.06654	0.947097	-6.10236	0.727464	0.786721
Monocytes	SERPINI1	0.016566	3.178888	0.066494	0.947135	-5.64664	0.756734	0.818021
Monocytes	FAM185A	-0.01895	2.579686	-0.06633	0.947267	-5.47311	0.769689	0.83179
Monocytes	TDRP	0.046672	-0.36439	0.066163	0.947398	-5.01589	0.836709	0.902695
Monocytes	COX14	-0.00613	5.656597	-0.06614	0.947418	-6.39724	0.705899	0.763534
Monocytes	MS4A4B	-0.01482	3.207847	-0.06598	0.947539	-5.93866	0.756206	0.817392
Monocytes	GM3604	0.027172	0.595301	0.065826	0.947665	-5.17942	0.814272	0.878968
Monocytes	CDNF	-0.03259	0.317972	-0.06574	0.94773	-5.14688	0.820711	0.885777
Monocytes	VAMP2	-0.00757	4.421644	-0.06534	0.948049	-6.0544	0.730856	0.790254
Monocytes	GM20457	-0.03497	-0.16272	-0.0653	0.948081	-5.06866	0.83202	0.897674
Monocytes	SLC39A1	-0.0058	6.161792	-0.0653	0.948083	-6.49623	0.696079	0.752882
Monocytes	COL27A1	0.025533	2.314283	0.065261	0.948114	-5.50166	0.775578	0.837979
Monocytes	TMEM202	0.02541	0.104736	0.064916	0.948387	-5.26179	0.825716	0.891036
Monocytes	DNPEP	-0.00954	4.077287	-0.06491	0.948395	-6.00231	0.737965	0.797865
Monocytes	SDR42E1	-0.02743	0.45745	-0.06485	0.948438	-5.15605	0.817481	0.882354
Monocytes	CTTNBP2NL	0.009383	2.52025	0.064849	0.948441	-6.09551	0.771077	0.833192
Monocytes	POLE3	0.010922	4.133596	0.064836	0.948451	-5.93416	0.736797	0.796616
Monocytes	TMEM184C	-0.01096	3.390595	-0.0647	0.948558	-5.79475	0.75237	0.81326
Monocytes	IFT140	-0.01255	3.507073	-0.06464	0.948606	-5.78942	0.749905	0.810628
Monocytes	DUSP16	0.007496	6.833204	0.064618	0.948624	-6.70922	0.68316	0.73894
Monocytes	SMARCC1	-0.00645	6.606315	-0.06437	0.948823	-6.50047	0.687566	0.74368
Monocytes	CFL1	0.005345	10.01399	0.064311	0.948868	-7.11149	0.625589	0.676406
Monocytes	DENND1C	-0.0101	3.966917	-0.06412	0.949019	-6.01144	0.740397	0.800407
Monocytes	GM50322	0.032878	0.358739	0.063674	0.949374	-5.14051	0.820093	0.884921
Monocytes	4921509O07RII	0.029182	0.064578	0.06363	0.949408	-5.27407	0.826978	0.89218
Monocytes	ZFP598	0.011822	3.44196	0.063539	0.949481	-5.80059	0.751572	0.812246
Monocytes	SCAMP3	-0.00684	4.819715	-0.06351	0.949501	-6.17911	0.723012	0.781689
Monocytes	LRRC25	0.008929	3.856093	0.063369	0.949616	-6.42876	0.742862	0.80299
Monocytes	CMTR2	-0.02557	1.0685	-0.06332	0.949653	-5.21802	0.80374	0.867728
Monocytes	GM34455	-0.01304	2.623429	-0.06292	0.949975	-5.94441	0.76919	0.831042
Monocytes	BMI1	0.012018	3.545427	0.062872	0.950011	-5.76223	0.749444	0.809996
Monocytes	CD59B	-0.03958	0.413	-0.06285	0.950029	-5.08558	0.818895	0.883697
Monocytes	NT5E	0.021596	2.431121	0.062764	0.950096	-5.53442	0.773381	0.835533
Monocytes	GM30025	-0.02383	1.597885	-0.06274	0.950112	-5.30716	0.791829	0.855116
Monocytes	ZDHHC12	0.02402	1.360226	0.062682	0.950161	-5.34101	0.797178	0.860786
Monocytes	SF3B6	0.004418	7.294759	0.062345	0.950429	-6.66293	0.674816	0.729782
Monocytes	PGS1	0.006637	4.740858	0.062292	0.950471	-6.26955	0.724743	0.783558
Monocytes	CRNKL1	-0.00638	5.293781	-0.0622	0.950542	-6.26357	0.713593	0.771589
Monocytes	GLIS1	-0.03338	0.394465	-0.06213	0.950599	-5.13802	0.819408	0.884232
Monocytes	ZFP760	0.0266	1.032725	0.062102	0.950621	-5.13726	0.804695	0.868695
Monocytes	MCCC2	-0.01989	2.436244	-0.06174	0.950908	-5.57916	0.773482	0.835456
Monocytes	SRPK3	-0.02978	1.292575	-0.06156	0.95105	-5.10928	0.798929	0.862446
Monocytes	1700052K11RIK	-0.02372	1.140287	-0.06139	0.951191	-5.20417	0.802386	0.866103
Monocytes	GM50013	0.018555	1.85467	0.061332	0.951233	-5.42065	0.786309	0.849077

Monocytes	RLIM	0.004274	6.654275	0.061261	0.951289	-6.54871	0.687085	0.74291
Monocytes	ALKBH5	-0.00526	7.148219	-0.06119	0.951343	-6.60248	0.677693	0.732765
Monocytes	CTDNEP1	-0.00651	5.516749	-0.06115	0.951381	-6.37443	0.709275	0.766814
Monocytes	CNOT4	-0.00459	7.523005	-0.06083	0.951631	-6.68467	0.670664	0.725206
Monocytes	SMTN	-0.01979	2.149503	-0.06068	0.951749	-5.38521	0.779778	0.842225
Monocytes	XRCC2	-0.02076	0.802718	-0.06065	0.951776	-5.3338	0.810106	0.874346
Monocytes	GZMC	0.042447	1.539474	0.060585	0.951826	-5.52405	0.793359	0.85663
Monocytes	TARSL2	0.01779	1.415252	0.060529	0.951871	-5.39261	0.796156	0.859592
Monocytes	PIK3IP1	0.021854	2.920799	0.060489	0.951902	-5.37575	0.76297	0.824349
Monocytes	SEC24B	0.005339	6.179882	0.060432	0.951948	-6.59359	0.696243	0.752858
Monocytes	GM17092	0.017852	2.1249	0.060397	0.951976	-5.4506	0.78032	0.842813
Monocytes	ARF4OS	-0.02348	0.845404	-0.06033	0.952032	-5.21986	0.809126	0.873346
Monocytes	ZBTB43	0.011778	3.163113	0.060313	0.952043	-5.70163	0.757772	0.818844
Monocytes	RFESD	-0.01629	2.541257	-0.06017	0.95216	-5.55801	0.77119	0.833151
Monocytes	GM9750	0.029284	0.476616	0.060144	0.952177	-5.20783	0.817642	0.882364
Monocytes	AGTR1A	-0.03972	0.270352	-0.05955	0.952647	-5.07214	0.822737	0.887528
Monocytes	RPGRIP1	0.006091	6.55945	0.059482	0.952703	-6.41909	0.689148	0.74508
Monocytes	SPCS2	-0.00429	7.595587	-0.05945	0.952731	-6.69857	0.669547	0.723904
Monocytes	REV1	0.006786	5.326262	0.059299	0.952848	-6.25443	0.713353	0.771156
Monocytes	SNX15	0.007591	4.880351	0.05899	0.953093	-6.11747	0.722344	0.780828
Monocytes	6330409D20RII	0.02941	0.333222	0.058958	0.953118	-5.12399	0.821325	0.886084
Monocytes	IQSEC2	-0.01776	2.267987	-0.05895	0.953124	-5.63985	0.777497	0.839704
Monocytes	GM27010	-0.01259	2.340877	-0.0589	0.953163	-5.71376	0.775897	0.838003
Monocytes	KLHL25	-0.01682	2.493825	-0.05876	0.953279	-5.3857	0.772585	0.834446
Monocytes	SMIM10L1	0.006843	4.931338	0.058248	0.953683	-6.19726	0.721562	0.779882
Monocytes	GPATCH2L	-0.00565	5.064269	-0.05821	0.953714	-6.25683	0.718877	0.777001
Monocytes	SNHG4.1	0.031779	0.471422	0.057956	0.953914	-5.17032	0.818501	0.88292
Monocytes	AHI1	-0.0143	3.003332	-0.05775	0.954079	-5.54452	0.761902	0.822917
Monocytes	KCNN4	0.009198	3.850661	0.057741	0.954085	-6.26732	0.743926	0.803741
Monocytes	ETAA1OS	-0.02463	0.543342	-0.05735	0.954396	-5.14722	0.816956	0.881216
Monocytes	GM50209	-0.02463	0.629068	-0.05734	0.954404	-5.19957	0.81497	0.87912
Monocytes	GM17227	0.013164	2.746669	0.05718	0.954531	-5.58128	0.76754	0.828914
Monocytes	ZMYM6	-0.01771	2.526746	-0.0571	0.954598	-5.49136	0.772322	0.834011
Monocytes	SCRG1	-0.03072	-0.98095	-0.05689	0.954763	-5.04558	0.853162	0.919403
Monocytes	3110040N11RII	0.009548	3.553549	0.056887	0.954763	-5.98729	0.750271	0.810574
Monocytes	ALDH9A1	-0.00559	5.005152	-0.05683	0.954806	-6.30688	0.720276	0.778476
Monocytes	IRGM1	-0.01384	4.93997	-0.05673	0.954886	-6.19706	0.721594	0.779906
Monocytes	TIMM23	0.004095	6.942518	0.056716	0.954899	-6.61732	0.682307	0.737615
Monocytes	MAPRE3	0.02019	1.502586	0.056585	0.955004	-5.33608	0.795028	0.858194
Monocytes	DYRK3	0.015319	2.520243	0.056494	0.955076	-5.6931	0.772464	0.834252
Monocytes	MPHOSPH8	0.00669	4.79616	0.056474	0.955092	-6.07417	0.724512	0.783053
Monocytes	PPME1	-0.0067	4.862972	-0.05639	0.955158	-6.17045	0.723155	0.781598
Monocytes	ARMC7	0.009084	3.924467	0.056245	0.955274	-5.9645	0.742502	0.802286
Monocytes	PNO1	0.007839	4.446222	0.05609	0.955396	-6.10152	0.73169	0.790718
Monocytes	FADS3	-0.02346	0.659123	-0.05607	0.95541	-5.1773	0.814302	0.878542
Monocytes	NTM	0.027164	0.913874	0.055909	0.95554	-5.19858	0.808484	0.872395
Monocytes	GM42722	0.022347	2.335621	0.055543	0.955831	-5.3372	0.77675	0.838655

Monocytes	GM12253	-0.03417	-0.86999	-0.0554	0.955944	-4.97495	0.850737	0.916785
Monocytes	C330007P06RIK	-0.0069	5.50907	-0.05527	0.956045	-6.20687	0.710402	0.767809
Monocytes	MANSC1	0.023968	0.242039	0.055055	0.956219	-5.27777	0.824239	0.888943
Monocytes	HMGCS1	0.008482	4.453186	0.05487	0.956365	-6.05105	0.731753	0.790714
Monocytes	GSTO2	0.024732	0.524993	0.054868	0.956367	-5.24732	0.81764	0.881987
Monocytes	ACCS	0.014015	2.079594	0.054816	0.956409	-5.54376	0.782394	0.844697
Monocytes	ACYP2	-0.01252	3.398712	-0.05481	0.95641	-5.68699	0.753789	0.814264
Monocytes	KANSL1	0.004617	8.964116	0.054813	0.956411	-6.85368	0.645276	0.697409
Monocytes	HYAL1	-0.01891	1.311575	-0.05446	0.956688	-5.27749	0.799766	0.862978
Monocytes	SP110	0.006507	5.924125	0.054185	0.95691	-6.45875	0.702427	0.75907
Monocytes	ZFP973	-0.02337	0.609416	-0.05409	0.956989	-5.17698	0.815945	0.880051
Monocytes	0610009L18RIK	-0.01628	2.030404	-0.05386	0.957166	-5.45509	0.783736	0.845998
Monocytes	MYBBP1A	-0.00786	4.85707	-0.05383	0.957195	-6.20818	0.723736	0.782008
Monocytes	COMMD9	0.012319	2.646144	0.053757	0.95725	-5.63152	0.770213	0.831635
Monocytes	CCL25	-0.0089	4.40296	-0.05372	0.95728	-6.05298	0.733023	0.791956
Monocytes	CCNL1	-0.00383	7.09056	-0.05342	0.957514	-6.69511	0.679933	0.734872
Monocytes	TYW5	0.012048	3.139549	0.053399	0.957534	-5.67821	0.759562	0.820308
Monocytes	PRM1	-0.03012	-0.44781	-0.05335	0.957573	-5.01646	0.840842	0.90629
Monocytes	GPR180	-0.01117	2.964205	-0.05334	0.957583	-5.65641	0.763328	0.82432
Monocytes	LRRC75AOS2	-0.02769	0.512247	-0.05328	0.957628	-5.10882	0.8182	0.882466
Monocytes	EXOSC3	-0.00602	5.047755	-0.05314	0.957744	-6.20016	0.719876	0.777899
Monocytes	CEP19	-0.01402	2.707827	-0.05311	0.957766	-5.63288	0.768873	0.830243
Monocytes	GM39326	-0.02326	1.289251	-0.05301	0.957847	-5.20147	0.800359	0.863681
Monocytes	SLC35A1	-0.00902	3.416075	-0.05295	0.957891	-5.73023	0.753664	0.814085
Monocytes	ACSL5	0.005698	6.00653	0.052726	0.958069	-6.46385	0.700888	0.757488
Monocytes	GM10135	0.025585	0.409376	0.052487	0.958259	-5.12862	0.820785	0.885156
Monocytes	DAP3	-0.00568	5.039126	-0.05229	0.958417	-6.23662	0.720281	0.778261
Monocytes	GM15943	0.027533	0.207344	0.052057	0.958601	-5.14561	0.825678	0.890265
Monocytes	FAM120C	-0.01384	3.050639	-0.05191	0.958718	-5.57066	0.761815	0.82265
Monocytes	EPHA2	-0.01361	2.408212	-0.05179	0.958811	-5.53362	0.775761	0.837483
Monocytes	GM6034	0.031704	0.50272	0.051757	0.958839	-5.09955	0.818793	0.883028
Monocytes	CASZ1	0.008951	3.474262	0.051546	0.959007	-5.9051	0.752846	0.813047
Monocytes	C130013H08RII	0.018752	0.498295	0.05083	0.959576	-5.19563	0.819268	0.883197
Monocytes	NCAPD3	-0.00763	5.127768	-0.05077	0.959621	-6.19111	0.718916	0.776492
Monocytes	CWC15	0.003959	6.384372	0.050715	0.959667	-6.50146	0.694083	0.749796
Monocytes	MRPL50	-0.00736	4.009842	-0.05057	0.959782	-5.90909	0.741842	0.801037
Monocytes	GM48383	0.018169	1.758143	0.050429	0.959894	-5.36932	0.790516	0.852817
Monocytes	WDR59	0.011969	3.072953	0.050418	0.959903	-5.61826	0.761682	0.822196
Monocytes	OTUD1	0.012899	3.007254	0.050398	0.959919	-5.52389	0.763095	0.8237
Monocytes	CD300C2	-0.00692	4.335062	-0.05032	0.959982	-6.49222	0.73509	0.793838
Monocytes	SNX19	0.009791	2.862011	0.050288	0.960007	-5.6233	0.766229	0.827056
Monocytes	PRC1	0.011746	5.285692	0.050124	0.960137	-6.25423	0.715783	0.773117
Monocytes	GM14858	-0.02275	2.296719	-0.05003	0.960211	-5.31468	0.778609	0.840175
Monocytes	SLC25A43	0.02139	-0.21871	0.049751	0.960433	-5.21111	0.836317	0.900986
Monocytes	DTX1	-0.02678	2.069415	-0.04931	0.960787	-5.15682	0.783913	0.845605
Monocytes	HIST1H4D	0.025619	1.04631	0.049239	0.96084	-5.19941	0.806963	0.869996
Monocytes	KIF1C	-0.00802	3.254466	-0.04922	0.960854	-5.88488	0.758111	0.818186

Monocytes	NEURL3	-0.00678	4.301516	-0.04904	0.960994	-6.23209	0.736093	0.794688
Monocytes	ANGPTL1	0.026121	0.376343	0.049042	0.960997	-5.09321	0.822457	0.886335
Monocytes	ZFP438	0.008159	3.204144	0.048898	0.961111	-5.89388	0.75922	0.819332
Monocytes	BCL2L12	0.007529	4.178233	0.048452	0.961466	-5.95016	0.738837	0.797435
Monocytes	LRRC57	-0.01394	2.732576	-0.0484	0.961506	-5.58273	0.769554	0.830168
Monocytes	ZCCHC14	0.019733	1.412865	0.048205	0.961662	-5.27094	0.798825	0.861223
Monocytes	NCBP3	-0.00516	5.506298	-0.04819	0.961677	-6.24702	0.711814	0.76852
Monocytes	SELENOP	0.007457	8.699951	0.048091	0.961752	-6.82206	0.651251	0.703174
Monocytes	AP1M1	0.005405	4.979621	0.048086	0.961756	-6.17939	0.722397	0.779898
Monocytes	ZGPAT	-0.00732	3.668613	-0.04775	0.96202	-5.79761	0.749581	0.808928
Monocytes	FOXR1	0.021677	-0.87975	0.047727	0.962041	-5.08998	0.852682	0.9179
Monocytes	VPS25	-0.01876	0.881521	-0.04767	0.962084	-5.22699	0.811031	0.874115
Monocytes	TMUB1	-0.00676	3.138278	-0.04755	0.962183	-5.87234	0.760868	0.820966
Monocytes	POLE	0.013127	3.301306	0.047507	0.962216	-5.71521	0.757379	0.817251
Monocytes	ITPRIPL1	0.006254	3.28736	0.047132	0.962515	-6.04545	0.75782	0.817642
Monocytes	E130308A19Rik	-0.00755	4.522919	-0.04697	0.962645	-5.92688	0.731925	0.790015
Monocytes	PDE8A	0.005725	5.847934	0.04693	0.962675	-6.54887	0.705243	0.761404
Monocytes	GMEB2	0.00443	5.764218	0.046845	0.962742	-6.34602	0.706897	0.763181
Monocytes	PLD3	-0.00872	4.413779	-0.04681	0.962767	-5.96863	0.734172	0.792419
Monocytes	TOP2B	-0.00505	6.593853	-0.04667	0.962885	-6.48453	0.690703	0.745755
Monocytes	MRPL38	-0.00735	3.863246	-0.04649	0.963028	-5.87487	0.745626	0.804697
Monocytes	DDX19A	0.00706	4.239703	0.046461	0.963047	-5.94259	0.737773	0.79631
Monocytes	FBXL4	0.010881	2.64655	0.046311	0.963167	-5.61316	0.771647	0.832438
Monocytes	CEBPZ	0.003791	6.141756	0.046158	0.963289	-6.47118	0.699475	0.755285
Monocytes	RBMXL1	0.004741	5.042632	0.046156	0.96329	-6.23254	0.721328	0.778755
Monocytes	SNX30	0.005792	5.755716	0.046099	0.963336	-6.33212	0.707065	0.763455
Monocytes	TRRAP	-0.00608	4.784536	-0.04607	0.963356	-6.11162	0.726569	0.784383
Monocytes	DEPP1	0.023119	1.043364	0.045951	0.963453	-5.20922	0.807468	0.87044
Monocytes	ARMH3	-0.00469	5.427651	-0.04589	0.963498	-6.35118	0.713587	0.770495
Monocytes	ABAT	-0.02406	1.196429	-0.04538	0.96391	-5.1818	0.804238	0.8668
Monocytes	KCTD12B	-0.01814	0.963206	-0.04531	0.963965	-5.32511	0.809576	0.872435
Monocytes	SLC25A24	0.007073	3.486183	0.045006	0.964204	-6.04686	0.753868	0.813363
Monocytes	MPPE1	-0.0059	4.363196	-0.04495	0.964251	-6.14855	0.735493	0.79375
Monocytes	TMPRSS5	-0.01987	0.722493	-0.04488	0.964301	-5.18795	0.815159	0.878328
Monocytes	MRPL24	-0.00376	5.658173	-0.04486	0.964322	-6.38947	0.709263	0.765644
Monocytes	F730311O21Rik	-0.02662	-0.31758	-0.04479	0.964373	-5.0536	0.839615	0.904055
Monocytes	GOLGA7	0.003694	5.94	0.044619	0.964511	-6.43857	0.70371	0.759676
Monocytes	CCDC77	-0.00944	3.291762	-0.04458	0.964541	-5.6064	0.758027	0.817805
Monocytes	FBXO45	-0.00691	3.654199	-0.04441	0.964679	-5.90933	0.750376	0.809635
Monocytes	PHF13	-0.00687	3.504231	-0.04418	0.964865	-5.85685	0.753608	0.813045
Monocytes	HSPE1	0.004945	7.179751	0.044133	0.964898	-6.61796	0.679884	0.733978
Monocytes	CYTIP	0.003648	8.087657	0.043956	0.965038	-6.98946	0.662978	0.715685
Monocytes	TPRN	0.01122	2.21301	0.043711	0.965233	-5.60594	0.78162	0.842887
Monocytes	CWF19L1	0.012675	2.200341	0.043562	0.965352	-5.46699	0.7819	0.843208
Monocytes	ALCAM	0.004982	6.646567	0.04354	0.965369	-6.90742	0.690095	0.74506
Monocytes	TMEM184B	-0.0055	4.707523	-0.04346	0.96543	-6.30212	0.728571	0.786387
Monocytes	TRPM7	0.003667	7.284405	0.043226	0.965619	-6.65376	0.677941	0.731996

Monocytes	ABCD3	0.007275	4.193442	0.043119	0.965704	-5.88069	0.739169	0.797774
Monocytes	LGR4	0.015031	2.632615	0.04297	0.965822	-5.4824	0.772407	0.833211
Monocytes	GM35188	0.014771	1.73277	0.042897	0.96588	-5.48321	0.792314	0.854323
Monocytes	DDOST	0.003717	5.788445	0.042861	0.965909	-6.45907	0.706835	0.763148
Monocytes	2010309G21RII	0.024062	1.223176	0.042808	0.965951	-5.10446	0.803836	0.866509
Monocytes	MGRN1	0.005186	5.682428	0.042798	0.965959	-6.33505	0.708935	0.765404
Monocytes	NDST1	-0.00791	4.570663	-0.0428	0.96596	-5.9549	0.731376	0.789464
Monocytes	NSUN4	0.00878	3.189091	0.042778	0.965975	-5.70894	0.760371	0.820411
Monocytes	ZBTB37	0.009729	2.901016	0.04245	0.966236	-5.61306	0.766725	0.827074
Monocytes	PLCL1	0.006778	5.328139	0.042278	0.966373	-6.43571	0.716188	0.773106
Monocytes	SSR2	-0.00496	5.189958	-0.04212	0.966501	-6.25662	0.719007	0.776104
Monocytes	ZFP131	-0.00419	5.815875	-0.04173	0.966809	-6.38119	0.706676	0.762786
Monocytes	SPI1	0.003394	6.411888	0.041657	0.966866	-6.74425	0.695005	0.750233
Monocytes	DGUOK	-0.00576	4.460054	-0.04149	0.966995	-6.12969	0.734092	0.792165
Monocytes	AVL9	-0.00446	6.010486	-0.04123	0.967208	-6.39343	0.702984	0.758741
Monocytes	FBXO34	0.004008	5.78838	0.040998	0.96739	-6.40621	0.707376	0.763479
Monocytes	ZFP420	0.021607	0.275776	0.040865	0.967496	-5.07916	0.826375	0.890014
Monocytes	SLC25A39	-0.00516	6.472882	-0.04083	0.967525	-6.5204	0.693976	0.749082
Monocytes	SAMD9L	-0.00562	5.184732	-0.0407	0.967626	-6.28329	0.719433	0.776439
Monocytes	WDR36	-0.00499	4.578146	-0.0407	0.967626	-6.10757	0.73178	0.789662
Monocytes	NEMP2	0.008247	2.854914	0.040458	0.967819	-5.62102	0.768159	0.828487
Monocytes	CCR4	-0.02145	-1.08113	-0.04044	0.967835	-5.05042	0.858912	0.924192
Monocytes	OLFR1033	-0.02408	0.583769	-0.04035	0.967901	-5.14277	0.819177	0.882476
Monocytes	RANBP9	-0.00344	6.962828	-0.04035	0.967904	-6.63598	0.68456	0.738968
Monocytes	PRDX1	0.003492	9.719927	0.040281	0.96796	-7.06527	0.634193	0.684396
Monocytes	INPP4A	0.004211	6.032154	0.040115	0.968092	-6.46893	0.702613	0.758364
Monocytes	PRKCD	-0.00316	6.897294	-0.03999	0.96819	-6.66946	0.685858	0.740325
Monocytes	MFN2	-0.00644	3.671067	-0.03978	0.968354	-5.80909	0.750737	0.809931
Monocytes	DSCAM	0.01824	0.029718	0.039415	0.968648	-5.31531	0.832232	0.89626
Monocytes	GTF2E1	-0.0102	2.399479	-0.03941	0.968655	-5.53243	0.778157	0.839148
Monocytes	ADGRG1	0.021626	1.720983	0.039378	0.968678	-5.20267	0.793237	0.855129
Monocytes	XPNPEP3	-0.00777	3.250052	-0.0393	0.96874	-5.68812	0.759696	0.819526
Monocytes	GM47664	-0.00601	3.774581	-0.03928	0.968759	-5.96804	0.748552	0.807656
Monocytes	PLAG1	0.00818	2.8948	0.039245	0.968784	-5.62034	0.767347	0.827678
Monocytes	PRELID3B	0.003793	5.574007	0.039223	0.968801	-6.36229	0.71168	0.768202
Monocytes	PYROXD1	0.008106	2.95488	0.038999	0.968979	-5.68001	0.766047	0.826352
Monocytes	SLC35F2	-0.02154	0.357488	-0.03897	0.969	-5.06851	0.824515	0.888225
Monocytes	ECH1	-0.00471	5.784916	-0.0388	0.969136	-6.32652	0.707492	0.763764
Monocytes	LTA4H	-0.00545	5.724021	-0.03866	0.969252	-6.32733	0.708699	0.765086
Monocytes	SIN3A	0.003995	5.871572	0.038607	0.969291	-6.33045	0.70578	0.761959
Monocytes	EVA1B	0.00728	3.639669	0.038581	0.969312	-5.86462	0.751401	0.810803
Monocytes	ERCC6	-0.00665	3.56729	-0.03852	0.969361	-5.90682	0.752935	0.812465
Monocytes	URB1	-0.0091	2.176346	-0.03848	0.969395	-5.4734	0.783082	0.84452
Monocytes	GM12655	0.020386	0.353725	0.038378	0.969473	-5.13035	0.824603	0.888425
Monocytes	MCAM	-0.02202	1.173297	-0.03835	0.969495	-5.11976	0.805643	0.868431
Monocytes	AIG1	0.004997	4.903663	0.037926	0.969832	-6.30472	0.725203	0.78289
Monocytes	THBS3	0.015557	1.01596	0.037726	0.969991	-5.37299	0.809277	0.872355

Monocytes	SHROOM4	0.017262	0.86592	0.037709	0.970005	-5.33472	0.812729	0.875999
Monocytes	PTPN7	0.005922	4.151708	0.037663	0.970042	-6.09605	0.740681	0.799498
Monocytes	NMNAT3	-0.00777	3.105259	-0.03762	0.970076	-5.8254	0.762834	0.823123
Monocytes	UBR5	-0.00324	6.888097	-0.03751	0.970163	-6.60085	0.686061	0.740876
Monocytes	LYRM2	0.007213	3.377583	0.037407	0.970245	-5.82549	0.756999	0.816933
Monocytes	COMMD6	0.004983	4.358964	0.037406	0.970245	-6.06747	0.736378	0.794922
Monocytes	SMARCD1	0.005653	4.031896	0.037391	0.970257	-5.81708	0.743181	0.802192
Monocytes	IL4	-0.02771	0.330173	-0.03723	0.970386	-5.15178	0.825187	0.889223
Monocytes	GTF3C5	0.010457	2.258469	0.037202	0.970407	-5.52001	0.781296	0.84283
Monocytes	GM49662	-0.00689	2.537113	-0.03715	0.970451	-6.27908	0.775167	0.836324
Monocytes	SEC62	0.00217	7.437896	0.037102	0.970487	-6.63817	0.67564	0.729679
Monocytes	PSME4	0.003544	7.130098	0.036824	0.970709	-6.58454	0.681556	0.735958
Monocytes	HMG20A	0.005075	4.897858	0.03659	0.970895	-6.09162	0.725516	0.783158
Monocytes	KBTBD7	-0.00943	2.137804	-0.03615	0.971242	-5.53057	0.784398	0.845767
Monocytes	PRPS1L3	0.01063	2.045409	0.035925	0.971423	-5.4164	0.786538	0.847947
Monocytes	WAPL	0.002949	7.165135	0.035799	0.971523	-6.70793	0.681256	0.735335
Monocytes	CSTF1	-0.00629	3.243385	-0.0356	0.971681	-5.75891	0.760458	0.820158
Monocytes	ACP1	-0.00359	5.923129	-0.03547	0.971788	-6.39589	0.705358	0.761205
Monocytes	4932422M17RI	-0.01459	0.972719	-0.03524	0.971969	-5.21493	0.810923	0.873608
Monocytes	RNF139	0.004374	5.218473	0.035214	0.971988	-6.2247	0.719409	0.776286
Monocytes	FBXW17	0.010097	1.479346	0.035048	0.972121	-5.51751	0.799359	0.861434
Monocytes	VAMP8	0.002728	7.209731	0.03493	0.972215	-6.69961	0.68049	0.734475
Monocytes	HIST1H1E	-0.01257	4.203103	-0.03491	0.972227	-5.97065	0.740207	0.798599
Monocytes	HAVCR1	-0.01432	-0.3843	-0.03486	0.972267	-5.27827	0.842802	0.907222
Monocytes	ATG5	0.003008	5.691584	0.034727	0.972376	-6.31675	0.709941	0.766218
Monocytes	DXO	0.007511	3.302702	0.034695	0.972401	-5.76033	0.75921	0.818901
Monocytes	ANKS1B	0.015045	1.030517	0.03464	0.972445	-5.24452	0.809594	0.872324
Monocytes	SAMD10	-0.0108	1.732501	-0.03432	0.972702	-5.42209	0.793798	0.855495
Monocytes	LSMEM1	0.005832	4.06136	0.034171	0.972818	-6.41133	0.743314	0.801833
Monocytes	NSMCE2	-0.00239	7.428191	-0.03385	0.973072	-6.6652	0.676504	0.730118
Monocytes	PPIB	0.002561	7.605608	0.033745	0.973157	-6.78726	0.673175	0.726541
Monocytes	CDC42EP3	0.004412	5.142958	0.033325	0.973491	-6.28924	0.721079	0.778179
Monocytes	GM36198	-0.00857	2.976425	-0.0333	0.973514	-5.60174	0.766383	0.826547
Monocytes	NSL1	-0.00892	2.25699	-0.03304	0.973718	-5.53654	0.782116	0.843354
Monocytes	4933407K13RIK	-0.01191	1.39879	-0.03295	0.973788	-5.21143	0.801348	0.863741
Monocytes	A230056P14RIK	0.015736	0.407448	0.032827	0.973886	-5.13598	0.824207	0.887857
Monocytes	GBP3	-0.00939	3.228626	-0.0328	0.97391	-5.81996	0.760951	0.820888
Monocytes	AFP	0.017703	4.371399	0.032769	0.973933	-5.92555	0.736863	0.795191
Monocytes	ZFP930	-0.0105	2.204594	-0.03275	0.973945	-5.37133	0.783275	0.844607
Monocytes	UBXN11	-0.01056	1.770334	-0.03274	0.973952	-5.31204	0.792959	0.854867
Monocytes	PARVA	-0.01423	1.329681	-0.03265	0.974024	-5.22925	0.802919	0.865444
Monocytes	ANAPC13	-0.00276	5.65737	-0.03259	0.974075	-6.41464	0.710765	0.767278
Monocytes	TNFAIP8	0.002873	7.424897	0.032544	0.974112	-6.69488	0.676565	0.730451
Monocytes	ZFP51	0.007778	2.86166	0.032526	0.974126	-5.48775	0.768869	0.829385
Monocytes	9130019P16RIK	0.013612	0.265967	0.032457	0.974181	-5.34955	0.827527	0.891447
Monocytes	FAM13A	0.017267	1.15315	0.032261	0.974337	-5.167	0.806947	0.869757
Monocytes	SLC24A1	-0.00805	1.860788	-0.03219	0.974397	-5.61669	0.790931	0.852818

Monocytes	GM42962	0.008018	-0.60259	0.032165	0.974413	-5.53181	0.848227	0.913198
Monocytes	ZFR	-0.00242	6.532381	-0.03215	0.974428	-6.52873	0.693598	0.748848
Monocytes	FUT11	0.005983	3.493603	0.032097	0.974467	-5.81259	0.755289	0.814951
Monocytes	DEDD	-0.00423	4.437275	-0.03209	0.974469	-6.01759	0.735501	0.793826
Monocytes	ZBTB21	0.005069	3.713914	0.032082	0.974479	-5.91408	0.750617	0.80997
Monocytes	CENPU	-0.00813	2.562499	-0.03192	0.974607	-5.55036	0.775427	0.836383
Monocytes	GM10974	-0.01114	0.824708	-0.03184	0.97467	-5.31786	0.814537	0.877791
Monocytes	NRM	-0.00456	5.099836	-0.03162	0.974844	-6.28398	0.722014	0.779372
Monocytes	ENC1	-0.00662	3.181392	-0.0316	0.974859	-5.72507	0.762032	0.82212
Monocytes	CPPED1	0.00444	3.886993	0.031263	0.97513	-5.87067	0.747131	0.806134
Monocytes	MRPS16	-0.00322	5.607422	-0.03124	0.97515	-6.36812	0.711914	0.768436
Monocytes	WDPCP	0.007794	3.269533	0.030831	0.975474	-5.60523	0.760239	0.820106
Monocytes	NUTF2-PS1	-0.00973	2.848853	-0.03074	0.975545	-5.67804	0.769315	0.829761
Monocytes	UMPS	-0.00478	4.099845	-0.03072	0.975562	-5.90615	0.742671	0.801373
Monocytes	SMIM11	0.003318	4.805042	0.03041	0.975809	-6.16433	0.728103	0.785795
Monocytes	POU3F1	0.017954	0.061626	0.0304	0.975817	-5.12408	0.832529	0.896587
Monocytes	COPRS	-0.01104	0.717795	-0.03039	0.975821	-5.32625	0.817153	0.880401
Monocytes	B4GALT7	0.00648	2.869796	0.03031	0.975888	-5.63323	0.76886	0.829278
Monocytes	CDK5RAP3	0.00393	4.379321	0.030304	0.975893	-6.098	0.736859	0.795163
Monocytes	PDK2	0.009882	2.10287	0.030098	0.976057	-5.38739	0.785703	0.847157
Monocytes	3830406C13Rik	0.003439	4.453206	0.030062	0.976086	-6.18183	0.735331	0.793529
Monocytes	CNEP1R1	-0.00393	4.587392	-0.0299	0.976214	-6.02668	0.732565	0.790571
Monocytes	APEX1	-0.00357	5.465947	-0.0299	0.976217	-6.32467	0.714739	0.771469
Monocytes	CTDSP2	0.00309	4.801724	0.029894	0.976219	-6.22222	0.728171	0.785868
Monocytes	PI4KB	0.003014	5.414205	0.029893	0.97622	-6.28169	0.715775	0.772581
Monocytes	INTS11	-0.00482	3.989645	-0.02987	0.976235	-5.96437	0.744977	0.803835
Monocytes	PTH1R	0.019164	0.264703	0.029689	0.976382	-5.08735	0.827761	0.891547
Monocytes	GGNBP2	-0.00218	7.268609	-0.02939	0.976623	-6.63669	0.679681	0.733707
Monocytes	CDK5	-0.00484	3.72259	-0.02937	0.976633	-5.90666	0.750618	0.809831
Monocytes	LSG1	-0.00321	4.928986	-0.02937	0.976635	-6.17199	0.725597	0.783088
Monocytes	STK40	-0.00284	5.69472	-0.02931	0.976683	-6.46348	0.710197	0.766571
Monocytes	SNAPC3	0.00409	4.825524	0.029284	0.976704	-6.10156	0.727706	0.785347
Monocytes	ABLIM2	0.014996	-0.47962	0.029164	0.9768	-5.1261	0.84549	0.910152
Monocytes	TMEM14A	-0.01358	0.419553	-0.0287	0.97717	-5.18393	0.824286	0.887797
Monocytes	FLVCR1	0.003304	4.670527	0.028483	0.977341	-6.24851	0.731019	0.788831
Monocytes	SMC1A	0.002841	6.748393	0.028467	0.977354	-6.54448	0.689734	0.744493
Monocytes	DCAF12	0.002823	6.638146	0.028373	0.977429	-6.52508	0.691858	0.746784
Monocytes	TIMM17B	0.002638	5.161183	0.028369	0.977432	-6.20124	0.721027	0.778132
Monocytes	USP47	0.00252	6.472249	0.028328	0.977464	-6.53378	0.695067	0.750241
Monocytes	ST7L	0.003753	4.451288	0.028307	0.977481	-6.126	0.735534	0.793663
Monocytes	JUN	0.006507	7.097589	0.028101	0.977645	-6.62488	0.683119	0.737294
Monocytes	FAM221A	0.012032	1.467642	0.027941	0.977772	-5.17951	0.800247	0.862349
Monocytes	GM8251	-0.00712	3.392286	-0.02778	0.977902	-5.78032	0.757886	0.817412
Monocytes	GALNT11	-0.00345	4.120023	-0.02777	0.97791	-6.10575	0.742517	0.801023
Monocytes	GPN3	-0.004	3.982026	-0.02759	0.97805	-5.91279	0.745455	0.804143
Monocytes	G530011O06Ri	-0.01133	1.80368	-0.02729	0.978291	-5.40662	0.792803	0.854498
Monocytes	AAAS	0.005251	3.794127	0.026918	0.978585	-5.82254	0.749484	0.808511

Monocytes	2610020C07RIK	0.003066	4.983149	0.026864	0.978629	-6.14947	0.724859	0.78219
Monocytes	PTCD3	0.003711	4.845166	0.026774	0.978701	-6.05974	0.72767	0.7852
Monocytes	PLPBP	0.003269	4.145683	0.026727	0.978738	-6.11594	0.742108	0.800639
Monocytes	FNBP4	0.002358	6.074991	0.026591	0.978846	-6.41701	0.703038	0.758779
Monocytes	KYAT3	0.007201	3.603362	0.02635	0.979038	-5.78535	0.75352	0.812825
Monocytes	GM34921	0.012757	0.841113	0.026273	0.979099	-5.14909	0.81473	0.877717
Monocytes	DNAJC27	-0.00636	2.442699	-0.02612	0.979222	-5.55426	0.778603	0.8395
Monocytes	LPCAT4	-0.00444	3.121289	-0.02611	0.979232	-6.14805	0.763828	0.823802
Monocytes	GM43462	0.005681	3.23183	0.02608	0.979252	-5.71714	0.761451	0.821272
Monocytes	ZFP369	0.006279	2.919936	0.026037	0.979286	-5.5853	0.76818	0.828429
Monocytes	ZXDB	-0.00517	3.1887	-0.02597	0.979342	-5.73634	0.762378	0.822258
Monocytes	PPP2R5C	-0.00155	7.456887	-0.02595	0.979352	-6.6898	0.676469	0.730143
Monocytes	INPP5E	0.006403	2.260693	0.025877	0.979414	-5.47918	0.782619	0.843777
Monocytes	CYB5RL	0.011065	0.380261	0.025725	0.979535	-5.15933	0.825461	0.889056
Monocytes	TTC41	0.013719	0.966475	0.025678	0.979572	-5.08033	0.811837	0.8747
Monocytes	DLAT	-0.00325	4.282042	-0.02554	0.979679	-6.01145	0.739268	0.797663
Monocytes	WASHC2	-0.00314	5.655214	-0.02546	0.979748	-6.33642	0.711339	0.767786
Monocytes	TM4SF1	0.01244	2.15291	0.025357	0.979828	-5.31078	0.785008	0.846416
Monocytes	RAG2	0.018422	0.168525	0.025316	0.97986	-5.01661	0.830443	0.894406
Monocytes	GEMIN2	-0.00508	3.260952	-0.02521	0.979943	-5.69359	0.760826	0.820759
Monocytes	STX12	0.002201	5.552332	0.025144	0.979996	-6.47712	0.71339	0.770051
Monocytes	TKFC	-0.00727	3.130684	-0.02458	0.980443	-5.55227	0.763626	0.823797
Monocytes	SAMD1	-0.00279	4.976188	-0.02453	0.980485	-6.27928	0.725	0.782551
Monocytes	HIST1H2AE	-0.00792	4.491316	-0.0245	0.980512	-6.0667	0.734934	0.793185
Monocytes	MGA	0.002686	5.917554	0.024366	0.980615	-6.38399	0.706138	0.762317
Monocytes	KSR1	0.004268	4.69672	0.024275	0.980688	-6.02062	0.730707	0.788673
Monocytes	LY6G2	0.014258	0.952461	0.02427	0.980692	-5.15553	0.81216	0.875241
Monocytes	PPP2R1A	-0.00236	6.00443	-0.02414	0.980794	-6.4336	0.704426	0.760511
Monocytes	GM16158	-0.01584	0.586451	-0.02405	0.980864	-5.01814	0.820641	0.884247
Monocytes	GM29707	-0.00882	-0.46354	-0.02391	0.980979	-5.23386	0.845508	0.910426
Monocytes	HAGH	0.003303	5.358255	0.023905	0.980982	-6.28423	0.717278	0.774363
Monocytes	CDC123	0.002696	5.404958	0.023674	0.981166	-6.27003	0.71634	0.773403
Monocytes	ABCB1B	-0.00465	3.934965	-0.02366	0.981178	-6.1613	0.746519	0.805708
Monocytes	6530413G14RIK	0.011198	-0.08741	0.023641	0.981192	-5.05425	0.836508	0.901033
Monocytes	TMEM69	-0.0081	1.585462	-0.02362	0.981213	-5.37003	0.797717	0.860127
Monocytes	RUBCN	-0.0048	3.888841	-0.02345	0.981344	-5.71581	0.747489	0.806747
Monocytes	KNL1	-0.00513	5.131736	-0.02342	0.981367	-6.28595	0.721845	0.779312
Monocytes	COQ7	-0.00391	4.79596	-0.02333	0.981436	-6.0175	0.728675	0.786666
Monocytes	GM21859	0.006704	1.780794	0.02329	0.981471	-5.84678	0.793317	0.855508
Monocytes	GM12992	-0.00815	2.041763	-0.02326	0.981495	-5.46532	0.787479	0.849324
Monocytes	XKR8	-0.01063	0.500884	-0.02318	0.981562	-5.1975	0.822638	0.886503
Monocytes	2410006H16RIK	-0.00301	7.535004	-0.02314	0.981593	-6.72896	0.675002	0.728931
Monocytes	RNF214	0.00279	4.961908	0.023101	0.981622	-6.16362	0.72529	0.783062
Monocytes	CDC42	0.001173	8.943013	0.023042	0.981668	-6.96633	0.649155	0.700923
Monocytes	TGM2	-0.00365	4.674438	-0.02303	0.981681	-6.50981	0.731165	0.789354
Monocytes	SDF2	-0.00228	5.410417	-0.02299	0.981709	-6.31869	0.716231	0.773345
Monocytes	RANGRF	-0.0096	1.262196	-0.02284	0.981829	-5.28371	0.805057	0.867966

Monocytes	KLF11	-0.0037	3.555665	-0.02284	0.981829	-5.75503	0.754533	0.814333
Monocytes	TFE3	-0.00285	4.149746	-0.02264	0.981985	-6.10956	0.742077	0.800997
Monocytes	5730409E04RIK	-0.01	0.23499	-0.02256	0.982048	-5.21774	0.828936	0.893108
Monocytes	ATP5G2	0.001636	8.844228	0.022415	0.982168	-6.93021	0.651008	0.702885
Monocytes	FBXO22	-0.00268	4.616588	-0.02232	0.982239	-6.10418	0.73244	0.790664
Monocytes	MR1	0.011616	0.68263	0.022068	0.982443	-5.10905	0.818562	0.882118
Monocytes	EEF2K	0.005642	4.644155	0.021968	0.982523	-5.61062	0.731929	0.790106
Monocytes	TBC1D7	-0.00513	2.220043	-0.02149	0.982903	-5.45323	0.783671	0.845252
Monocytes	GM45871	0.009066	0.985003	0.021484	0.982908	-5.21929	0.811568	0.874772
Monocytes	SLC19A2	-0.00596	1.834884	-0.02146	0.982929	-5.48027	0.792257	0.854353
Monocytes	IGHJ4	-0.01387	0.337645	-0.02143	0.982951	-5.00342	0.826622	0.890641
Monocytes	EXO1	-0.00863	2.019131	-0.02138	0.982994	-5.33984	0.788137	0.849988
Monocytes	ABCC5	-0.00351	4.301545	-0.02133	0.983031	-6.15411	0.739007	0.79769
Monocytes	ERAL1	-0.00593	1.918255	-0.02133	0.983032	-5.40558	0.79039	0.852375
Monocytes	CDKAL1	0.001968	6.364518	0.021224	0.983114	-6.46567	0.697511	0.753185
Monocytes	ALG8	0.003976	3.226775	0.021213	0.983123	-5.85389	0.761708	0.82193
Monocytes	ZFP787	-0.00219	5.128821	-0.02108	0.983227	-6.20569	0.722066	0.779573
Monocytes	MED22	0.006132	2.384243	0.020838	0.983422	-5.44683	0.780162	0.841513
Monocytes	SRPR	0.001683	5.794906	0.020513	0.98368	-6.37431	0.708896	0.765362
Monocytes	3110056K07RIK	-0.00236	4.723448	-0.02051	0.983687	-6.12108	0.730501	0.788537
Monocytes	HECW2	0.006302	3.162677	0.020408	0.983764	-5.65537	0.763297	0.823561
Monocytes	FBXO47	0.009963	1.069812	0.020264	0.983879	-5.2062	0.809858	0.872917
Monocytes	CORO7	-0.00196	6.215775	-0.0202	0.98393	-6.42784	0.700621	0.756487
Monocytes	SND1	-0.00145	7.195863	-0.01965	0.984368	-6.69918	0.681876	0.736213
Monocytes	GTF2H2	-0.00282	3.759481	-0.01964	0.984372	-5.87196	0.750739	0.810121
Monocytes	SPTB	-0.00876	1.120897	-0.01963	0.984384	-5.3063	0.808854	0.871804
Monocytes	ISCA1	0.003122	6.018689	0.019572	0.984429	-6.30431	0.704637	0.76075
Monocytes	CCDC174	0.002125	5.082834	0.019031	0.984859	-6.16666	0.723602	0.780958
Monocytes	VPS52	0.002378	3.923327	0.018652	0.985161	-5.94104	0.747679	0.806576
Monocytes	TRPV4	0.008203	-0.51202	0.018642	0.985169	-5.20177	0.847714	0.912391
Monocytes	CENPJ	0.004096	3.331867	0.018369	0.985386	-5.60553	0.760348	0.820037
Monocytes	F830208F22RIK	0.008723	-0.23725	0.018113	0.98559	-5.19164	0.84128	0.905588
Monocytes	SNRNP70	0.000984	7.511442	0.018074	0.985621	-6.66194	0.676408	0.730002
Monocytes	POLI	0.006473	1.527716	0.018022	0.985662	-5.2776	0.800164	0.862273
Monocytes	IDH3B	0.001953	5.850654	0.017886	0.98577	-6.41899	0.708495	0.764583
Monocytes	HAUS8	-0.002	4.722045	-0.01748	0.986096	-6.2455	0.731344	0.789098
Monocytes	HMMR	-0.00458	4.333361	-0.01744	0.986128	-6.0725	0.739371	0.797694
Monocytes	RAB10OS	-0.00232	4.429445	-0.01729	0.986247	-6.08548	0.737378	0.795566
Monocytes	ZBTB7A	-0.00127	7.04476	-0.01727	0.986256	-6.6116	0.685356	0.739699
Monocytes	GM17354	-0.00483	1.393239	-0.01727	0.986256	-5.46437	0.803342	0.865679
Monocytes	PATZ1	-0.00282	4.251704	-0.01669	0.986719	-5.86179	0.741109	0.79959
Monocytes	IGIP	-0.00722	0.675003	-0.01666	0.986742	-5.20966	0.819919	0.883212
Monocytes	RNF26	-0.00347	3.515898	-0.01656	0.986823	-5.76451	0.756614	0.81618
Monocytes	PRR16	-0.00793	1.720127	-0.01642	0.986936	-5.28144	0.79598	0.858001
Monocytes	PCK1	0.005708	4.182878	0.016346	0.986995	-5.89865	0.742544	0.801193
Monocytes	FECH	-0.00327	5.217188	-0.01633	0.987006	-6.19417	0.721296	0.778453
Monocytes	OSCP1	-0.00451	3.25254	-0.0162	0.987113	-5.73691	0.762251	0.822252

Monocytes	DCTN6	-0.00188	5.142412	-0.0161	0.987192	-6.16211	0.722809	0.780123
Monocytes	ASB7	0.003565	3.936004	0.016097	0.987194	-5.77671	0.747718	0.806766
Monocytes	CCNB2	0.003432	5.882225	0.015809	0.987422	-6.43311	0.707992	0.764269
Monocytes	SLC2A3	-0.00329	4.519005	-0.01573	0.987485	-5.91509	0.735563	0.793836
Monocytes	NFATC3	-0.00166	7.290076	-0.0156	0.98759	-6.50145	0.680727	0.734897
Monocytes	LATS1	0.001748	4.725566	0.015596	0.987592	-6.14699	0.73131	0.789284
Monocytes	TRIM14	0.002672	4.686216	0.015558	0.987622	-6.11261	0.732118	0.790149
Monocytes	CNPY2	0.002138	4.719728	0.015529	0.987645	-6.14006	0.73143	0.789412
Monocytes	PHKA1	-0.00333	3.411651	-0.0155	0.987668	-5.7573	0.75884	0.818683
Monocytes	ANGEL2	-0.00166	5.052327	-0.01538	0.987768	-6.14252	0.724637	0.782165
Monocytes	ACAA2	0.002492	5.01941	0.015197	0.987909	-6.21523	0.725306	0.782886
Monocytes	SNAI1	0.006459	0.389835	0.015124	0.987967	-5.1602	0.826586	0.890465
Monocytes	ABCA2	-0.00499	1.527476	-0.01509	0.987991	-5.44255	0.800335	0.862763
Monocytes	GM44686	0.00332	3.136727	0.015035	0.988038	-5.59846	0.764744	0.825004
Monocytes	DCUN1D1	-0.0015	5.787878	-0.01486	0.988178	-6.39734	0.709863	0.766314
Monocytes	GAA	-0.00292	3.191942	-0.01485	0.988182	-5.77485	0.763554	0.823737
Monocytes	BC024978	0.004827	1.865186	0.014829	0.988202	-5.43794	0.792718	0.854702
Monocytes	EIF2AK1	0.001564	5.326731	0.014791	0.988232	-6.27558	0.719085	0.776216
Monocytes	SLC10A3	0.00418	2.160549	0.014761	0.988256	-5.54958	0.786121	0.847712
Monocytes	GM40787	-0.00749	0.205726	-0.01465	0.988343	-5.14738	0.830921	0.895028
Monocytes	MRPS15	0.001305	5.910327	0.014616	0.988372	-6.46327	0.707436	0.763706
Monocytes	TRAPPC3	0.001662	4.917333	0.014325	0.988603	-6.22019	0.727481	0.785114
Monocytes	MTX1	-0.00196	4.556924	-0.01422	0.988689	-6.10774	0.734878	0.793031
Monocytes	ZFP280B	-0.00566	1.826966	-0.01417	0.988723	-5.24471	0.793681	0.855624
Monocytes	MED1	0.001086	5.883036	0.013844	0.988986	-6.38356	0.708205	0.764341
Monocytes	AP5Z1	0.002847	2.539751	0.013671	0.989123	-5.62714	0.778041	0.838903
Monocytes	2-Sep	-0.00365	2.22603	-0.01336	0.989367	-5.58393	0.785012	0.846301
Monocytes	ATN1	-0.00275	3.221914	-0.01332	0.989402	-5.76284	0.763245	0.823178
Monocytes	ZFP655	0.001712	4.453763	0.013249	0.989459	-6.08919	0.737237	0.795435
Monocytes	PASK	0.007451	1.295986	0.013236	0.98947	-5.16697	0.805956	0.868466
Monocytes	GRAP	-0.00258	4.536304	-0.01286	0.989766	-5.85155	0.735695	0.793643
Monocytes	TBX2	-0.00611	0.45083	-0.01262	0.989961	-5.18467	0.825805	0.88922
Monocytes	PRODH	0.004609	2.780405	0.012337	0.990184	-5.45511	0.773175	0.83352
Monocytes	PPP2R2D	-0.00119	5.459388	-0.01197	0.990479	-6.25892	0.71707	0.77363
Monocytes	BC049352	0.004113	1.083027	0.011967	0.990479	-5.45047	0.811218	0.873796
Monocytes	ABCF2	0.001546	4.411019	0.011954	0.990489	-6.04354	0.738469	0.796542
Monocytes	MAP2K3OS	-0.0063	-0.12812	-0.01187	0.990555	-5.21672	0.839609	0.903681
Monocytes	TPRKB	-0.00256	3.157978	-0.01187	0.990559	-5.693	0.764981	0.82481
Monocytes	RICTOR	0.001046	6.409431	0.011817	0.990598	-6.48173	0.698275	0.753436
Monocytes	MID1IP1	-0.00192	3.917342	-0.01167	0.990715	-6.01807	0.748824	0.807563
Monocytes	0610039K10RIK	-0.00708	0.022539	-0.01157	0.990792	-5.04203	0.836059	0.89991
Monocytes	POLD3	-0.00153	4.695421	-0.01126	0.99104	-6.10052	0.732731	0.790285
Monocytes	COX20	0.001265	5.583215	0.011169	0.991113	-6.33678	0.714721	0.770991
Monocytes	LMAN1L	0.004133	1.485183	0.011063	0.991198	-5.38397	0.802172	0.864123
Monocytes	CMBL	-0.00465	2.192141	-0.01096	0.991279	-5.38902	0.78628	0.847308
Monocytes	PCCA	-0.00157	4.599387	-0.01094	0.991298	-6.1417	0.734709	0.792415
Monocytes	ZFP687	-0.00229	2.919042	-0.01045	0.991685	-5.58739	0.770503	0.830483

Monocytes	NCAPG	0.002949	3.22832	0.010353	0.991763	-5.72251	0.76381	0.82339
Monocytes	UBAC1	0.001405	4.029628	0.010297	0.991807	-5.94178	0.746767	0.805236
Monocytes	MRC2	-0.00426	1.416971	-0.01015	0.991926	-5.30168	0.803938	0.865977
Monocytes	NDUFV2	-0.00101	6.136177	-0.01014	0.991931	-6.48384	0.703942	0.75941
Monocytes	2900076A07RIK	-0.00255	2.621325	-0.00996	0.992077	-5.45521	0.777044	0.837463
Monocytes	KCNRG	-0.00184	2.320944	-0.00971	0.992271	-5.82214	0.783668	0.844528
Monocytes	MTIF2	0.001189	4.285566	0.00955	0.992402	-6.07117	0.741448	0.799643
Monocytes	RBBP4	0.000698	7.676556	0.00946	0.992473	-6.67896	0.6744	0.727633
Monocytes	CAPSL	-0.00613	0.928873	-0.00939	0.99253	-5.09141	0.815181	0.877943
Monocytes	DAP	-0.00109	6.19848	-0.00926	0.992629	-6.4436	0.702752	0.758218
Monocytes	RASA4	-0.00144	4.228858	-0.00924	0.992651	-6.33182	0.742631	0.800955
Monocytes	LSM14A	0.000653	6.620649	0.009118	0.992745	-6.52606	0.694519	0.749361
Monocytes	ZFP444	-0.00176	3.63547	-0.0091	0.992763	-5.81471	0.755134	0.814295
Monocytes	COX10	0.001642	3.775159	0.009073	0.992782	-5.7934	0.75217	0.811143
Monocytes	XPA	-0.00139	4.138354	-0.00906	0.992791	-6.0138	0.744523	0.802985
Monocytes	ARHGEF5	-0.00544	0.327235	-0.00891	0.992908	-5.04852	0.829243	0.892786
Monocytes	NMI	-0.00117	5.064624	-0.00846	0.993271	-6.29191	0.725424	0.782698
Monocytes	MAT1A	-0.00249	4.65605	-0.0084	0.99332	-5.99564	0.733787	0.791661
Monocytes	PQLC2	0.001398	3.590318	0.008342	0.993363	-5.96371	0.756111	0.815498
Monocytes	MTSS1	-0.00139	6.472133	-0.00834	0.993368	-6.28472	0.697418	0.752631
Monocytes	RBM3	0.000647	9.070642	0.008336	0.993368	-6.96707	0.648853	0.700123
Monocytes	UBE2J1	-0.00068	6.282597	-0.00829	0.993402	-6.47179	0.701118	0.756613
Monocytes	H2-DMB2	0.001331	3.016521	0.008292	0.993403	-6.1318	0.768441	0.828623
Monocytes	TRIM10	-0.00552	0.349121	-0.00804	0.993604	-5.05867	0.828816	0.89242
Monocytes	ACACA	-0.00095	5.153719	-0.00778	0.993811	-6.29328	0.723692	0.780846
Monocytes	TPRGL	0.00068	6.927644	0.00775	0.993834	-6.57294	0.68869	0.743219
Monocytes	SOX5OS4	-0.00504	-0.23518	-0.00772	0.993856	-4.99611	0.8427	0.907072
Monocytes	RABGGTB	-0.0013	3.732854	-0.00761	0.993943	-5.83009	0.753163	0.812354
Monocytes	CYP51	0.0012	3.916341	0.007562	0.993983	-6.02333	0.749283	0.808227
Monocytes	AGO2	-0.00061	7.576219	-0.00743	0.99409	-6.71101	0.676371	0.729947
Monocytes	MTFP1	-0.00389	0.572509	-0.00732	0.994173	-5.10126	0.823574	0.88701
Monocytes	ING1	-0.00083	5.135774	-0.00728	0.99421	-6.22128	0.724056	0.781293
Monocytes	3300002A11RIK	-0.0029	0.451891	-0.00723	0.994247	-5.21982	0.8264	0.890002
Monocytes	FAM171A1	-0.00312	1.565475	-0.00703	0.994408	-5.22527	0.800774	0.862926
Monocytes	SNRPE	-0.00059	7.064225	-0.00676	0.994621	-6.62425	0.686231	0.740511
Monocytes	SNRNP27	0.000581	5.842837	0.006446	0.994871	-6.37421	0.710131	0.766203
Monocytes	RLN3	0.003435	-0.84713	0.006354	0.994945	-5.0264	0.857833	0.922857
Monocytes	B3GAT3	0.000937	4.307364	0.006295	0.994991	-6.03114	0.741369	0.799688
Monocytes	CETN2	-0.00057	5.798667	-0.00618	0.995081	-6.334	0.71102	0.767187
Monocytes	GM26982	0.001725	1.652146	0.005985	0.995238	-5.34318	0.79909	0.861048
Monocytes	WWP2	0.000507	6.537142	0.005847	0.995348	-6.49639	0.696533	0.751609
Monocytes	PJA1	-0.00098	3.699202	-0.00558	0.995556	-5.83463	0.754206	0.813438
Monocytes	KHSRP	0.000507	5.998752	0.005525	0.995604	-6.39681	0.707084	0.762998
Monocytes	FAM117A	0.000853	5.965212	0.005256	0.995818	-6.25763	0.707747	0.763711
Monocytes	NMB	-0.00185	1.642171	-0.00506	0.995974	-5.27994	0.799315	0.861409
Monocytes	FZD4	-0.00193	1.267832	-0.00496	0.996053	-5.28309	0.807838	0.870418
Monocytes	CEP57L1	0.001146	3.287731	0.004789	0.996189	-5.62572	0.763	0.82289

Monocytes	COL4A4	0.002007	1.009618	0.00478	0.996197	-5.22646	0.813775	0.876701
Monocytes	TNFAIP1	0.000824	3.775763	0.004778	0.996199	-5.96314	0.752582	0.811789
Monocytes	ACE	0.000904	0.341503	0.004729	0.996237	-6.25932	0.829357	0.89312
Monocytes	RNF14	-0.00048	5.242687	-0.00467	0.996282	-6.21953	0.722205	0.779308
Monocytes	ACTR3B	0.002121	-0.01555	0.004671	0.996283	-5.06949	0.837817	0.902015
Monocytes	ARMC10	-0.00085	3.275459	-0.0046	0.996344	-5.75435	0.763264	0.823171
Monocytes	ADORA3	-0.00104	-0.30137	-0.00441	0.996494	-5.7111	0.844657	0.909197
Monocytes	PLSCR4	-0.00226	0.494112	-0.00438	0.996515	-5.13764	0.82577	0.889344
Monocytes	LRRC4C	-0.00276	0.648779	-0.00437	0.996522	-5.08954	0.822151	0.885532
Monocytes	HMGN5	-0.00062	4.876393	-0.0043	0.996578	-6.07654	0.72966	0.787296
Monocytes	SERPINH1	0.001549	2.557349	0.004148	0.9967	-5.44799	0.77889	0.839781
Monocytes	GM13205	-0.00173	0.234435	-0.00409	0.996748	-5.20865	0.831885	0.895779
Monocytes	GM2A	-0.0003	7.22888	-0.00406	0.996769	-6.96177	0.683235	0.737386
Monocytes	MTCP1	0.001877	0.737147	0.004057	0.996772	-5.18105	0.820091	0.883362
Monocytes	ACADS	0.00055	4.490963	0.003959	0.99685	-6.03471	0.737598	0.795796
Monocytes	HNRNPH2	0.000296	6.435152	0.003901	0.996896	-6.52966	0.698518	0.753877
Monocytes	GCH1	0.000405	5.663529	0.00342	0.997279	-6.50154	0.713901	0.770327
Monocytes	RPAP3	-0.00064	3.841822	-0.00335	0.997331	-5.76612	0.751349	0.8104
Monocytes	RRAGA	-0.00035	5.020687	-0.00334	0.997344	-6.32422	0.726873	0.784238
Monocytes	TMC3	-0.00206	-1.08655	-0.00314	0.997505	-5.00528	0.863947	0.929378
Monocytes	PDCD4	-0.00031	7.282332	-0.00314	0.997506	-6.58982	0.682369	0.736424
Monocytes	POLB	0.000273	5.332268	0.003069	0.997558	-6.46025	0.720553	0.777519
Monocytes	GDI1	-0.00038	4.991215	-0.0028	0.997774	-6.12715	0.727578	0.784979
Monocytes	CCNY	-0.00023	6.67252	-0.00258	0.997948	-6.52129	0.694209	0.749161
Monocytes	PTPN3	0.000937	1.040176	0.002428	0.998068	-5.41474	0.813423	0.876309
Monocytes	ZNFX1	-0.00043	4.359029	-0.00237	0.998118	-6.1458	0.740658	0.799066
Monocytes	CYTH2	0.000383	3.933028	0.002336	0.998141	-5.93221	0.749584	0.808598
Monocytes	MIF4GD	-0.00025	4.964455	-0.0021	0.998328	-6.27719	0.72825	0.785765
Monocytes	BBS2	0.000792	0.404044	0.001943	0.998454	-5.24565	0.828329	0.892056
Monocytes	HCFC2	0.000359	3.758586	0.001869	0.998513	-5.72905	0.753349	0.812639
Monocytes	STX1A	-0.00091	1.792697	-0.00184	0.998538	-5.17603	0.796342	0.858315
Monocytes	ARFGAP1	0.000188	4.620741	0.001493	0.998812	-6.07523	0.735352	0.793423
Monocytes	NANOS3	-0.00075	0.427569	-0.00147	0.998834	-5.04887	0.827824	0.891555
Monocytes	RCN3	-0.0004	1.932131	-0.00146	0.998839	-5.50775	0.793253	0.855053
Monocytes	PLVAP	0.000635	1.394862	0.001392	0.998893	-5.20971	0.805414	0.867918
Monocytes	ORC5	0.000241	3.474755	0.001227	0.999024	-5.7779	0.759484	0.819163
Monocytes	DDX56	-0.00015	3.951871	-0.00099	0.999214	-5.92431	0.749387	0.808399
Monocytes	GPN1	0.00019	3.042468	0.000978	0.999222	-5.69886	0.768835	0.829118
Monocytes	TIMM21	0.0002	2.602996	0.000836	0.999335	-5.56269	0.778456	0.839323
Monocytes	RNF187	5.59E-05	6.12757	0.000566	0.99955	-6.41187	0.705059	0.760953
Monocytes	GRAMD1C	-0.00015	2.440736	-0.00053	0.999577	-5.53415	0.782033	0.843185
Monocytes	ERICH1	7.05E-05	3.61294	0.000423	0.999664	-5.91678	0.756594	0.81613
Monocytes	FABP7	0.000141	1.989157	0.000389	0.999691	-5.71504	0.792082	0.85384
Monocytes	STARD8	3.56E-05	3.359704	0.000201	0.99984	-5.93442	0.762011	0.821928
Monocytes	PPIP5K1	-6.60E-05	1.776843	-0.00019	0.999848	-5.42944	0.796856	0.858922
Monocytes	RAB8A	6.27E-06	5.454242	6.67E-05	0.999947	-6.36541	0.718464	0.775412
Monocytes	PTK2B	-3.34E-06	7.134222	-3.32E-05	0.999974	-6.64061	0.685539	0.739987

Monocytes	HIST1H2AG	5.01E-06	2.011327	1.22E-05	0.99999	-5.45795	0.791586	0.853386
