# Supplementary material for: Immune landscape in liver of neonatal mice with phlebotomy-induced anemia
Source: Pediatr Res. 2025 Sep 17;99(4):1602–12. doi: 10.1038/s41390-025-04361-x (PMC12659965; doi:10.1038/s41390-025-04361-x)
Supplement: Supplementary file 4 — Table S4 [file 41390_2025_4361_MOESM4_ESM.pdf]

immunecell	gene	logFC	AveExpr	t	P.Value	B	adj.P.Val. Within	adj.P.Val. Between
Neutrophils	CYP2E1	-2.43936	4.291137	-7.4532	5.84E-11	14.00059	6.05E-07	5.28E-08
Neutrophils	OSBPL9	0.525062	7.511715	6.981634	5.17E-10	12.54506	2.44E-06	3.33E-07
Neutrophils	7-Mar	0.479233	6.926226	6.686105	1.99E-09	11.26209	6.37E-06	1.06E-06
Neutrophils	FABP4	1.151377	6.966529	6.567909	3.40E-09	10.67999	8.14E-06	1.65E-06
Neutrophils	IL1RAP	0.744475	4.731572	6.449414	5.79E-09	10.24549	1.18E-05	2.65E-06
Neutrophils	GM2A	0.765078	7.22888	6.354664	8.85E-09	9.838323	1.40E-05	3.52E-06
Neutrophils	DUSP16	0.841837	6.833204	6.236373	1.50E-08	9.302839	2.06E-05	5.60E-06
Neutrophils	CASS4	1.008769	3.04899	6.045395	3.48E-08	8.569766	4.24E-05	1.30E-05
Neutrophils	GYPA	2.376869	3.611578	6.040052	3.56E-08	8.23378	4.17E-05	1.30E-05
Neutrophils	GK	0.750984	4.764608	5.898511	6.60E-08	7.934215	6.74E-05	2.14E-05
Neutrophils	POLR3B	-0.75127	5.512738	-5.82998	8.89E-08	7.66195	8.07E-05	2.71E-05
Neutrophils	HBB-BT	2.070137	8.105747	5.723668	1.41E-07	6.994299	0.000109	3.78E-05
Neutrophils	MT1	1.568576	6.314344	5.671101	1.76E-07	7.020884	0.000132	4.84E-05
Neutrophils	BNIP3	1.14359	5.086642	5.54454	3.02E-07	6.529913	0.000218	7.81E-05
Neutrophils	HBB-BS	1.973105	11.91128	5.512646	3.45E-07	5.669333	0.000186	7.07E-05
Neutrophils	SRGN	0.436453	9.652655	5.505133	3.57E-07	5.845149	0.000198	7.70E-05
Neutrophils	PICALM	0.46387	8.404518	5.464281	4.24E-07	5.926594	0.000226	9.11E-05
Neutrophils	MED12L	1.723154	3.141983	5.453973	4.42E-07	5.100361	0.000262	0.000109
Neutrophils	IL1B	0.936892	5.12575	5.441599	4.66E-07	5.851532	0.000248	0.000108
Neutrophils	SNCA	1.928967	4.248926	5.393823	5.70E-07	5.910765	0.000295	0.000133
Neutrophils	PLEKHM3	0.743861	6.068485	5.330838	7.41E-07	5.643986	0.000347	0.000158
Neutrophils	GM10076	0.979185	6.671496	5.290948	8.75E-07	5.528729	0.000384	0.00018
Neutrophils	CCR1	0.693961	2.32125	5.268742	9.59E-07	5.317718	0.000443	0.00022
Neutrophils	NDEL1	0.411326	6.927832	5.257906	1.00E-06	5.140623	0.000388	0.0002
Neutrophils	MIF	0.986239	7.650894	5.251287	1.03E-06	5.303994	0.00038	0.0002
Neutrophils	UBA52	0.637597	10.38927	5.246376	1.05E-06	4.968018	0.000352	0.000187
Neutrophils	FGF23	2.710828	0.594781	5.193691	1.31E-06	4.20725	0.000557	0.000289
Neutrophils	SLC2A1	0.758931	4.676925	5.174945	1.41E-06	5.086695	0.000516	0.000275
Neutrophils	BLNK	-1.28074	7.200732	-5.15503	1.53E-06	5.007117	0.000495	0.000274
Neutrophils	LYST	0.434353	6.729745	5.151014	1.56E-06	4.692125	0.000501	0.00028
Neutrophils	HBA-A2	1.910883	9.98555	5.123308	1.74E-06	4.33036	0.000495	0.000281
Neutrophils	HBA-A1	1.895514	10.46682	5.103082	1.90E-06	4.188197	0.000514	0.000296
Neutrophils	FAM162A	0.890575	5.640039	5.092288	1.98E-06	4.759202	0.000597	0.00035
Neutrophils	ST3GAL6	0.700455	5.092875	5.079009	2.09E-06	4.699957	0.000608	0.000374
Neutrophils	IL1R2	0.754777	3.320344	5.077453	2.10E-06	4.489094	0.000639	0.000395
Neutrophils	CHP1	0.409328	7.01438	5.058973	2.27E-06	4.421685	0.000603	0.000379
Neutrophils	MXD1	0.4304	6.036212	5.046163	2.39E-06	4.302389	0.000636	0.000407
Neutrophils	RABGEF1	0.555347	6.01057	5.014406	2.72E-06	4.271968	0.000704	0.000451
Neutrophils	SNAP23	0.440396	6.222753	4.998064	2.90E-06	4.241161	0.000729	0.000472
Neutrophils	HECTD1	0.388236	7.428886	4.991025	2.99E-06	4.160476	0.000707	0.000465
Neutrophils	BOP1	-1.00513	4.167475	-4.9732	3.21E-06	4.120247	0.000813	0.000535
Neutrophils	MIRT1	0.597019	4.950639	4.966983	3.29E-06	4.180079	0.000795	0.000533
Neutrophils	ZFP106	0.565484	6.288522	4.959139	3.40E-06	4.203259	0.000772	0.000527
Neutrophils	TBC1D23	0.451028	5.852371	4.942006	3.64E-06	4.0876	0.000818	0.000562

Neutrophils	IFITM2	0.538696	7.227901	4.91721	4.02E-06	3.763652	0.00085	0.000586
Neutrophils	BACH2	-0.91359	8.964274	-4.89642	4.37E-06	3.892837	0.00086	0.000596
Neutrophils	KLRC1	-2.63036	1.444197	-4.87596	4.74E-06	2.387405	0.001132	0.000785
Neutrophils	FABP2	-1.80917	3.257265	-4.86489	4.95E-06	3.558435	0.0011	0.000768
Neutrophils	KLHL2	0.45268	5.570335	4.83323	5.62E-06	3.620958	0.001135	0.000796
Neutrophils	GPI1	0.423145	7.844302	4.830072	5.69E-06	3.448322	0.001064	0.000754
Neutrophils	CD79B	-1.1604	6.976941	-4.81603	6.02E-06	3.742609	0.001131	0.000807
Neutrophils	SELPLG	0.593685	6.845492	4.777035	7.02E-06	3.484541	0.001299	0.000903
Neutrophils	ALCAM	0.576603	6.646567	4.766679	7.31E-06	3.129113	0.001335	0.000935
Neutrophils	A330023F2	0.832278	3.109027	4.697412	9.60E-06	3.313593	0.001893	0.001268
Neutrophils	VAMP4	0.438542	6.154005	4.692118	9.80E-06	3.153146	0.001736	0.001181
Neutrophils	SH2D2A	-2.4921	2.764079	-4.68927	9.91E-06	2.019156	0.001912	0.001309
Neutrophils	PPP1R3B	0.701973	2.491892	4.677932	1.04E-05	3.244324	0.001976	0.001367
Neutrophils	BSG	0.588819	7.716678	4.673825	1.05E-05	3.010467	0.001703	0.001189
Neutrophils	PLEK	0.55622	7.520494	4.648283	1.16E-05	2.730326	0.001844	0.001299
Neutrophils	ATP2B1	0.425245	8.327101	4.646195	1.17E-05	2.693575	0.001802	0.001276
Neutrophils	BTG2	0.554134	7.421641	4.636717	1.22E-05	2.801122	0.001887	0.00135
Neutrophils	FAM32A	0.441395	5.312501	4.625801	1.27E-05	2.996017	0.002057	0.001474
Neutrophils	EHD3	-1.013	3.517645	-4.60863	1.36E-05	2.988254	0.002277	0.001644
Neutrophils	CROT	-0.8051	5.259869	-4.59521	1.43E-05	2.944139	0.002247	0.00163
Neutrophils	KLRE1	-2.56494	1.626605	-4.58802	1.47E-05	1.878894	0.002523	0.001851
Neutrophils	FLII	0.559329	5.969326	4.570028	1.57E-05	2.822447	0.002353	0.001737
Neutrophils	CHMP4B	0.354657	7.889271	4.562019	1.62E-05	2.554313	0.002264	0.001679
Neutrophils	TPI1	0.674409	6.690385	4.552298	1.69E-05	2.675049	0.002396	0.001783
Neutrophils	AQP1	-1.1475	2.964821	-4.53413	1.81E-05	2.699205	0.002815	0.002065
Neutrophils	PIM1	0.503352	9.488973	4.499097	2.07E-05	2.044759	0.002637	0.001908
Neutrophils	DOP1B	0.644846	4.289272	4.487644	2.16E-05	2.548281	0.003115	0.002296
Neutrophils	STFA1	-0.57033	5.975982	-4.48673	2.17E-05	1.689547	0.002969	0.002195
Neutrophils	IL12RB2	-1.79484	3.672057	-4.46067	2.39E-05	2.039101	0.003453	0.002536
Neutrophils	GPCPD1	0.422722	7.329375	4.441771	2.57E-05	2.133996	0.003193	0.002446
Neutrophils	CSF1	1.190121	2.480595	4.437703	2.61E-05	2.400573	0.003666	0.002833
Neutrophils	MVP	0.723687	5.108561	4.43712	2.62E-05	2.400092	0.003401	0.002634
Neutrophils	CXXC5	-1.15107	5.11182	-4.43696	2.62E-05	2.145838	0.003401	0.002634
Neutrophils	PRELID1	0.421453	7.529242	4.433208	2.66E-05	2.107629	0.003175	0.002485
Neutrophils	ALAS2	1.660687	4.928547	4.43312	2.66E-05	2.364502	0.003419	0.002672
Neutrophils	LECT2	-1.52834	3.632928	-4.42956	2.69E-05	2.340347	0.003551	0.002793
Neutrophils	ASPRV1	0.713661	1.33931	4.421341	2.78E-05	2.211192	0.003862	0.003035
Neutrophils	SERPINA3K	2.961485	1.878609	4.414551	2.85E-05	1.685669	0.003855	0.003054
Neutrophils	TET2	0.440638	6.024646	4.402667	2.98E-05	2.107718	0.003539	0.002824
Neutrophils	CES1G	-2.54589	0.85155	-4.35667	3.55E-05	0.91314	0.00482	0.003741
Neutrophils	AKAP12	-1.24855	6.169014	-4.34787	3.67E-05	2.087008	0.004231	0.003298
Neutrophils	CYTH4	0.536654	5.757599	4.340493	3.77E-05	2.000516	0.004349	0.003409
Neutrophils	PPT1	0.533408	6.003515	4.328419	3.94E-05	1.888819	0.004467	0.00348
Neutrophils	ASPH	0.951459	4.664011	4.304938	4.31E-05	1.943809	0.005009	0.003821
Neutrophils	LRP6	-0.7992	5.378913	-4.30102	4.37E-05	1.936778	0.004924	0.003786
Neutrophils	SNX20	0.508947	5.514815	4.259869	5.09E-05	1.676497	0.005653	0.004263
Neutrophils	TNFRSF1B	0.59106	4.903674	4.24313	5.42E-05	1.612014	0.006053	0.004523

Neutrophils	ILRUN	0.603205	5.988088	4.228031	5.73E-05	1.640551	0.006077	0.004573
Neutrophils	UBXN4	0.306931	6.814787	4.227807	5.73E-05	1.496779	0.005936	0.004469
Neutrophils	PTPRCAP	-1.0588	5.807512	-4.22204	5.86E-05	1.643412	0.006174	0.004677
Neutrophils	PLPP1	-0.89958	4.327383	-4.21282	6.06E-05	1.626193	0.006592	0.005008
Neutrophils	MIR22HG	0.606115	4.089128	4.19084	6.57E-05	1.47071	0.007121	0.005292
Neutrophils	MAST1	-2.71905	1.020113	-4.17239	7.03E-05	0.432728	0.008231	0.006074
Neutrophils	KDR	-0.97874	3.318647	-4.15816	7.41E-05	1.456007	0.008038	0.005896
Neutrophils	ELL	0.505301	5.024631	4.149429	7.65E-05	1.356096	0.007825	0.005744
Neutrophils	CDKN1B	0.544329	6.470899	4.139496	7.93E-05	1.261317	0.007648	0.005664
Neutrophils	SMOX	0.543405	5.116761	4.138951	7.94E-05	1.055903	0.007948	0.005881
Neutrophils	CARD19	0.437998	5.613475	4.134977	8.06E-05	1.100701	0.007873	0.005863
Neutrophils	SLC16A3	0.471747	3.935418	4.12719	8.29E-05	1.037968	0.008413	0.006258
Neutrophils	PTGS2	0.895535	2.215449	4.123288	8.41E-05	1.159462	0.008877	0.006627
Neutrophils	HSF1	-0.53422	4.997353	-4.11954	8.53E-05	1.292294	0.008217	0.006196
Neutrophils	FUCA2	0.591545	4.624115	4.117468	8.59E-05	1.32077	0.008305	0.006276
Neutrophils	MYO16	-2.55033	-0.30441	-4.10989	8.83E-05	-0.09622	0.009735	0.007324
Neutrophils	DUSP1	0.508925	7.412842	4.105923	8.96E-05	0.780866	0.007853	0.005975
Neutrophils	NAPSA	0.61902	6.459379	4.100626	9.13E-05	1.100208	0.008149	0.006233
Neutrophils	CASC4	-2.03636	2.64714	-4.09272	9.40E-05	-0.02478	0.009236	0.007074
Neutrophils	TMEM248	0.49187	5.858866	4.090366	9.48E-05	1.178547	0.008428	0.006513
Neutrophils	TNFAIP2	0.61751	4.351498	4.088565	9.54E-05	0.928149	0.008797	0.006814
Neutrophils	KLF3	0.437373	6.281206	4.073997	0.000101	0.8905	0.0087	0.00672
Neutrophils	RAET1E	0.937822	4.113795	4.053872	0.000108	1.093587	0.009776	0.007521
Neutrophils	TRIO	-0.66642	6.069746	-4.05038	0.00011	1.080875	0.009247	0.007187
Neutrophils	GPR35	0.810723	2.516821	4.050306	0.00011	1.089016	0.010232	0.007934
Neutrophils	STFA3	-0.55367	2.751543	-4.04914	0.00011	0.576006	0.010163	0.007908
Neutrophils	DHRS9	0.591844	0.196917	4.04174	0.000113	0.994764	0.011132	0.00865
Neutrophils	SRA1	0.49894	5.239409	4.011633	0.000126	0.931715	0.010651	0.008117
Neutrophils	WDR47	-1.06605	3.140773	-4.00268	0.00013	0.79595	0.011353	0.008782
Neutrophils	GLCCI1	-0.64528	7.348661	-4.00219	0.00013	0.826042	0.010072	0.007816
Neutrophils	LITAF	0.300055	8.523937	4.002101	0.00013	0.369115	0.009742	0.007564
Neutrophils	ABCC1	0.811153	4.467713	4.00125	0.000131	0.955198	0.010932	0.008481
Neutrophils	FAU	0.23425	11.4506	3.990335	0.000136	0.087582	0.009251	0.007164
Neutrophils	ISG20	1.179152	4.233394	3.985549	0.000138	0.902405	0.011453	0.008859
Neutrophils	5430427O1	0.920694	3.846602	3.979299	0.000141	0.872193	0.011747	0.009106
Neutrophils	TNFSF14	1.06787	-0.05669	3.968065	0.000147	0.654597	0.01356	0.01048
Neutrophils	SNTB1	-0.97695	4.264888	-3.96042	0.000151	0.778353	0.012221	0.009487
Neutrophils	PGAM1	0.454738	6.892134	3.954734	0.000154	0.461438	0.011482	0.008965
Neutrophils	XCL1	-2.2753	2.335401	-3.9517	0.000156	0.440183	0.013113	0.010276
Neutrophils	BEND4	1.067227	3.557561	3.944051	0.00016	0.747402	0.012913	0.010159
Neutrophils	TRIM30B	0.732989	1.730639	3.928107	0.000169	0.693585	0.01421	0.011173
Neutrophils	ZFP263	0.753584	5.167548	3.927452	0.00017	0.710165	0.012884	0.010163
Neutrophils	RETREG1	0.37903	6.437217	3.923844	0.000172	0.2802	0.012443	0.009901
Neutrophils	KLRD1	-1.7543	3.767425	-3.92287	0.000173	0.582934	0.013425	0.010666
Neutrophils	GM19951	1.728533	3.23896	3.910143	0.000181	0.601203	0.01415	0.011173
Neutrophils	PFKP	0.669622	5.538024	3.907137	0.000182	0.577575	0.013297	0.01056
Neutrophils	CSRNP1	0.546734	6.641086	3.899286	0.000188	0.336716	0.013152	0.010485

Neutrophils	IRAK2	0.525561	6.796286	3.893487	0.000191	0.195449	0.013267	0.010596
Neutrophils	ZSWIM4	0.504095	4.579223	3.882605	0.000199	0.465551	0.014577	0.011583
Neutrophils	UHRF1BP1	-1.47346	1.998967	-3.86479	0.000212	0.262525	0.016583	0.012993
Neutrophils	RAPGEF1	0.475425	6.775482	3.852891	0.000221	0.334608	0.014947	0.011684
Neutrophils	UFSP2	0.646231	4.93604	3.851558	0.000222	0.478257	0.01575	0.012343
Neutrophils	LCOR	-0.31344	7.538405	-3.84562	0.000226	-0.03128	0.01483	0.011676
Neutrophils	BCL3	0.616371	5.236561	3.836013	0.000234	0.332139	0.016259	0.012782
Neutrophils	2410022M	1.630154	1.591485	3.832383	0.000237	-0.36115	0.018103	0.014279
Neutrophils	EBPL	-0.8574	4.469152	-3.82959	0.000239	0.405007	0.016676	0.013275
Neutrophils	MAPKAPK2	0.38213	7.207807	3.829124	0.00024	-0.00332	0.015427	0.012313
Neutrophils	IL13RA1	0.716712	3.558786	3.815895	0.000251	0.305742	0.0178	0.014081
Neutrophils	FGD3	0.552683	4.660813	3.811762	0.000255	0.265141	0.017383	0.013805
Neutrophils	GM26740	0.440721	7.068845	3.805483	0.00026	-0.07263	0.01648	0.01314
Neutrophils	CLEC4E	0.74061	2.622943	3.803059	0.000262	-0.00386	0.018716	0.01494
Neutrophils	SERPINA6	-2.55168	1.127419	-3.80149	0.000264	-0.41076	0.019533	0.015646
Neutrophils	CAAA0114	1.502354	2.131904	3.799401	0.000266	-0.30488	0.018994	0.015262
Neutrophils	PGK1	0.479253	7.765914	3.795766	0.000269	-0.16379	0.016279	0.013172
Neutrophils	KCNQ1	-1.68498	1.396097	-3.78973	0.000275	-0.03153	0.019801	0.01595
Neutrophils	GM4876	0.879147	1.628342	3.78591	0.000279	0.244911	0.019805	0.015988
Neutrophils	IKZF3	-1.15681	5.866917	-3.78047	0.000284	0.267	0.017771	0.014391
Neutrophils	ZFP619	-1.30386	2.21582	-3.77294	0.000291	-0.22753	0.020059	0.01626
Neutrophils	GM19585	-2.02993	2.331819	-3.76915	0.000295	-0.11155	0.019993	0.016371
Neutrophils	MAJIN	-1.94809	0.644865	-3.76873	0.000296	-0.22157	0.02098	0.017161
Neutrophils	TRAPPC4	0.498089	5.137813	3.76827	0.000296	0.183765	0.018456	0.01516
Neutrophils	MAF	-0.73052	5.654779	-3.76486	0.0003	0.024396	0.018289	0.015074
Neutrophils	GFRA1	-1.43868	4.918403	-3.75767	0.000307	0.175214	0.018986	0.015663
Neutrophils	GIMAP7	-2.19406	1.458503	-3.75457	0.00031	-0.58871	0.020955	0.017374
Neutrophils	CYSLTR2	-2.15402	1.496466	-3.75437	0.000311	-0.51847	0.020933	0.017357
Neutrophils	BPGM	1.215847	4.812429	3.753029	0.000312	0.188227	0.019044	0.015891
Neutrophils	ELL2	0.556842	6.734248	3.747157	0.000318	-0.22956	0.018289	0.015271
Neutrophils	POR	0.412752	5.6252	3.742107	0.000324	-0.1494	0.019091	0.015879
Neutrophils	ELANE	1.1838	2.340078	3.738853	0.000328	-0.18604	0.020992	0.017518
Neutrophils	PITPNA	0.286152	7.992513	3.738301	0.000328	-0.28227	0.017873	0.014978
Neutrophils	CD7	-1.73003	4.199176	-3.73352	0.000334	0.116366	0.02012	0.016863
Neutrophils	EHD1	0.44561	6.1755	3.727548	0.00034	-0.18337	0.0193	0.016187
Neutrophils	ZFP563	1.727548	0.800774	3.725513	0.000343	-0.9462	0.022523	0.018854
Neutrophils	GSTA3	-1.44978	4.0016	-3.71088	0.00036	0.061231	0.021287	0.017847
Neutrophils	TUBB2B	-1.90556	2.9596	-3.71002	0.000362	-0.70213	0.021929	0.018388
Neutrophils	CD44	0.35537	8.587659	3.709492	0.000362	-0.69157	0.018686	0.015727
Neutrophils	SULT2B1	-1.33181	2.700547	-3.70865	0.000363	-0.15995	0.022092	0.01857
Neutrophils	MKRN1	0.545768	7.149614	3.705882	0.000367	-0.29226	0.019539	0.016544
Neutrophils	CYP2C37	-1.46974	1.772648	-3.70097	0.000373	-0.30432	0.022955	0.019419
Neutrophils	PLA2G7	0.792787	4.021195	3.700272	0.000374	-0.35565	0.021529	0.018274
Neutrophils	FABP5	0.682711	7.278075	3.692935	0.000383	-0.43364	0.019929	0.016891
Neutrophils	ELP1	0.486546	4.553493	3.692529	0.000384	-0.01464	0.021534	0.018222
Neutrophils	CUL5	-0.50324	5.877947	-3.67975	0.000401	-0.06034	0.02154	0.018083
Neutrophils	SLC4A1	1.715755	2.002694	3.67419	0.000408	-0.23182	0.024382	0.020404

Neutrophils	GZMB	-1.86251	3.971899	-3.66898	0.000416	-0.06939	0.023335	0.019534
Neutrophils	SPRYD3	0.713373	3.699854	3.665344	0.000421	-0.09019	0.023627	0.019852
Neutrophils	ZFP971	1.098036	1.911606	3.663179	0.000424	-0.29267	0.024863	0.020995
Neutrophils	SUSD6	0.36532	7.690279	3.662875	0.000424	-0.56336	0.021092	0.017885
Neutrophils	CD244A	0.948755	3.429777	3.660088	0.000428	-0.0893	0.023909	0.020306
Neutrophils	E530011L2	1.92245	1.095538	3.656785	0.000433	-0.71477	0.025565	0.021772
Neutrophils	FKBPL	1.669414	1.019839	3.656579	0.000434	-0.74351	0.02562	0.021822
Neutrophils	CRYBG3	0.610183	4.474287	3.655355	0.000435	-0.15029	0.023216	0.019875
Neutrophils	RNF149	0.478614	5.786169	3.652889	0.000439	-0.54328	0.022436	0.01927
Neutrophils	TREM1	0.472798	1.639916	3.649625	0.000444	-0.43566	0.025285	0.021798
Neutrophils	LRG1	0.769561	2.460703	3.649454	0.000444	-0.2615	0.024699	0.021307
Neutrophils	MCL1	0.317113	8.364811	3.647367	0.000447	-0.80954	0.020922	0.018171
Neutrophils	ABLIM1	-0.8697	6.121137	-3.64438	0.000452	-0.13447	0.022317	0.019463
Neutrophils	F630028O1	0.577129	1.027185	3.644106	0.000452	-0.24759	0.025805	0.022436
Neutrophils	KLRB1C	-2.41239	1.954564	-3.64203	0.000455	-0.68191	0.025157	0.021973
Neutrophils	RAB11FIP1	0.480095	4.829945	3.640836	0.000457	-0.57957	0.023176	0.020312
Neutrophils	PTPRE	0.411221	5.539856	3.636513	0.000464	-0.49831	0.022932	0.020144
Neutrophils	GM26535	1.445132	-0.60061	3.632579	0.00047	-0.52266	0.027551	0.024064
Neutrophils	CD84	0.414381	5.366126	3.6303	0.000474	-0.54309	0.023303	0.020427
Neutrophils	APOC2	0.883319	4.203003	3.628749	0.000476	-0.23383	0.024096	0.021134
Neutrophils	EGFL7	-0.77669	3.872106	-3.62233	0.000487	-0.24223	0.024683	0.021662
Neutrophils	CALHM2	-1.31279	3.088827	-3.62101	0.000489	-0.70013	0.02524	0.022205
Neutrophils	CHMP3	0.30719	5.884611	3.620082	0.00049	-0.41293	0.023308	0.020575
Neutrophils	KLRA17	0.945001	0.224958	3.608421	0.00051	-0.2687	0.028323	0.024757
Neutrophils	PON1	-1.07964	4.304512	-3.60727	0.000512	-0.27488	0.02521	0.022165
Neutrophils	PRTN3	1.15067	3.6691	3.601817	0.000521	-0.6423	0.025703	0.022883
Neutrophils	DNM2	0.307174	7.193868	3.6017	0.000522	-0.64309	0.023251	0.020744
Neutrophils	NOP14	-1.20521	4.004398	-3.60113	0.000523	-0.52158	0.025459	0.022702
Neutrophils	DCLK2	-1.43269	2.72479	-3.60008	0.000524	-0.60422	0.026405	0.023568
Neutrophils	FAM133B	-0.5596	4.911343	-3.59987	0.000525	-0.27299	0.02481	0.022177
Neutrophils	CDCA7L	-0.93299	3.766654	-3.59404	0.000535	-0.28105	0.026016	0.023218
Neutrophils	NREP	-1.66554	1.234795	-3.58941	0.000543	-0.80974	0.028118	0.025166
Neutrophils	ARPC1B	0.272141	8.892807	3.588352	0.000545	-1.01727	0.022613	0.02037
Neutrophils	ESF1	-0.62917	5.001622	-3.58829	0.000545	-0.29837	0.025254	0.022701
Neutrophils	WFDC17	1.241282	5.906168	3.581888	0.000557	-0.65314	0.02503	0.022461
Neutrophils	CXCL3	1.769976	-0.07498	3.580307	0.00056	-0.45863	0.029707	0.026628
Neutrophils	SLC25A20	0.482243	5.597573	3.577307	0.000566	-0.56075	0.025409	0.022871
Neutrophils	ENTPD1	0.373753	6.9129	3.573978	0.000572	-0.99452	0.024639	0.022226
Neutrophils	HAVCR2	1.319084	2.301711	3.572475	0.000575	-0.36351	0.028112	0.02534
Neutrophils	CXCR6	-2.05643	1.255073	-3.56914	0.000582	-0.97738	0.029158	0.026254
Neutrophils	SPA17	-1.77729	0.979282	-3.56641	0.000587	-1.20771	0.029526	0.026647
Neutrophils	ITIH2	-1.45913	3.79432	-3.56384	0.000592	-0.36947	0.02736	0.024757
Neutrophils	FBXO34	-0.38524	5.78838	-3.56123	0.000597	-0.61803	0.025961	0.023547
Neutrophils	HSD17B6	-2.10302	0.767255	-3.5584	0.000603	-1.05286	0.030108	0.027281
Neutrophils	LYN	0.349943	10.03311	3.552509	0.000615	-1.1937	0.023489	0.021286
Neutrophils	EIF2AK1	0.553275	5.326731	3.550827	0.000618	-0.43596	0.026876	0.024378
Neutrophils	LCK	-1.15629	3.728178	-3.54466	0.000631	-0.52477	0.028454	0.025821

Neutrophils	DCLRE1C	0.437568	6.497189	3.543755	0.000633	-0.70521	0.026298	0.023955
Neutrophils	TOX	-1.41867	4.351569	-3.54346	0.000633	-0.42863	0.027953	0.025442
Neutrophils	CD80	0.511909	3.929604	3.539521	0.000642	-0.78232	0.028542	0.026033
Neutrophils	TIPARP	0.423236	7.437182	3.537006	0.000647	-0.97688	0.025938	0.023738
Neutrophils	FCMR	-1.70434	2.105124	-3.53484	0.000652	-0.95779	0.030231	0.027628
Neutrophils	TTYH2	-1.23839	3.075271	-3.53404	0.000653	-0.55494	0.029406	0.026936
Neutrophils	DMWD	-1.09432	2.228423	-3.53001	0.000662	-0.52593	0.030293	0.027773
Neutrophils	CAR3	-2.19159	3.476221	-3.52984	0.000663	-0.49909	0.029234	0.026826
Neutrophils	PSD3	-0.63842	5.794857	-3.52564	0.000672	-0.65758	0.027633	0.025408
Neutrophils	NAV1	-0.7678	3.595293	-3.52407	0.000675	-0.52489	0.02945	0.027107
Neutrophils	AKR1C6	-1.23124	4.841164	-3.52155	0.000681	-0.57717	0.02847	0.026279
Neutrophils	CCND3	0.457647	7.994145	3.520156	0.000684	-1.05881	0.02603	0.024149
Neutrophils	UNC119	0.35058	5.47027	3.519844	0.000685	-0.81611	0.027964	0.025922
Neutrophils	SRCAP	-0.35382	6.652894	-3.51849	0.000688	-0.71916	0.027051	0.02515
Neutrophils	FBXW2	0.361392	6.193608	3.513584	0.000699	-0.69401	0.027741	0.025715
Neutrophils	GAPDH	0.433325	10.59184	3.510491	0.000706	-1.43857	0.024643	0.022925
Neutrophils	CD300LG	-1.3249	1.259921	-3.50395	0.000722	-0.73312	0.032694	0.030194
Neutrophils	TRDC	-2.49498	1.265066	-3.4997	0.000732	-1.05172	0.032896	0.030463
Neutrophils	DDX6	0.286539	8.314732	3.499609	0.000732	-1.13156	0.026915	0.025036
Neutrophils	AKIRIN1	0.31811	6.287656	3.497754	0.000737	-0.79845	0.028569	0.026585
Neutrophils	GM26756	1.420052	0.354794	3.495367	0.000742	-0.68354	0.033966	0.031541
Neutrophils	DENND4A	0.365628	10.03042	3.492481	0.000749	-1.49054	0.025938	0.024275
Neutrophils	RARRES2	-1.17808	3.7462	-3.48763	0.000761	-0.58661	0.031379	0.029188
Neutrophils	PPP2R5C	0.245352	7.456887	3.484262	0.00077	-1.02149	0.028438	0.026477
Neutrophils	FBXL5	0.432037	6.173273	3.474961	0.000794	-1.0925	0.030288	0.028076
Neutrophils	SBNO2	0.39663	5.566821	3.469836	0.000807	-0.84083	0.031033	0.028896
Neutrophils	LDB2	-0.83079	3.160304	-3.46891	0.00081	-0.67914	0.033235	0.030963
Neutrophils	IL21R	-1.29109	5.150614	-3.46796	0.000812	-0.67886	0.031402	0.029339
Neutrophils	LPCAT2	0.398383	4.726381	3.467014	0.000815	-1.0726	0.031784	0.02971
Neutrophils	APOBR	0.551503	2.373103	3.466927	0.000815	-0.76513	0.03399	0.031721
Neutrophils	NRARP	-1.79369	1.780032	-3.46525	0.000819	-1.15745	0.034628	0.032343
Neutrophils	CUL9	-1.15665	2.579779	-3.46215	0.000828	-0.81298	0.033989	0.031819
Neutrophils	CUEDC1	-1.32271	2.113054	-3.46164	0.000829	-0.92939	0.034445	0.032274
Neutrophils	KRAS	0.305211	7.278938	3.459986	0.000834	-1.07685	0.029783	0.028034
Neutrophils	JAK2	0.463815	6.674709	3.458627	0.000837	-1.00141	0.030319	0.028599
Neutrophils	LDLR	0.80684	4.577033	3.449225	0.000863	-0.74776	0.032979	0.03099
Neutrophils	GM3550	1.324512	1.752212	3.448855	0.000864	-1.14077	0.035746	0.033536
Neutrophils	MRPL54	0.555911	5.906363	3.444572	0.000877	-0.77235	0.032082	0.030144
Neutrophils	MT2	1.728889	2.001416	3.442292	0.000883	-0.8699	0.035992	0.033757
Neutrophils	NRD1	0.339464	6.185357	3.433349	0.000909	-1.06911	0.032722	0.03068
Neutrophils	INTS6	-0.51092	6.588587	-3.43266	0.000911	-1.0679	0.032349	0.030378
Neutrophils	WBP1L	0.380274	5.329247	3.42965	0.00092	-1.03165	0.033735	0.031644
Neutrophils	TSPO2	1.728553	0.283153	3.424402	0.000936	-1.16606	0.039435	0.036783
Neutrophils	VAV1	0.384807	6.364742	3.423654	0.000938	-1.02893	0.033158	0.031083
Neutrophils	WNT2	-1.64238	1.077377	-3.42169	0.000944	-0.97654	0.038657	0.036166
Neutrophils	EGF	-1.87899	0.634522	-3.41884	0.000953	-1.68232	0.039371	0.036865
Neutrophils	ITPKB	0.395901	7.462345	3.41612	0.000962	-1.25465	0.032581	0.030657

Neutrophils	SHFL	-1.68076	1.832835	-3.41167	0.000975	-1.29793	0.03829	0.036152
Neutrophils	FAIM	-1.09838	3.865848	-3.41162	0.000976	-0.99639	0.036133	0.034164
Neutrophils	PAX5	-1.12792	5.141192	-3.41147	0.000976	-0.79715	0.034844	0.032973
Neutrophils	IGFBP6	1.86133	-0.02144	3.411366	0.000976	-1.22152	0.040372	0.038063
Neutrophils	CDKN1A	0.55349	5.267608	3.407758	0.000988	-1.04481	0.035002	0.033164
Neutrophils	GM39469	0.987295	2.328802	3.405239	0.000996	-0.9947	0.038184	0.036212
Neutrophils	FEM1C	0.332724	7.117684	3.404112	0.001	-1.39899	0.033317	0.031759
Neutrophils	PGRMC1	-0.62285	4.647619	-3.40351	0.001002	-0.83375	0.035741	0.034046
Neutrophils	STX18	0.540976	4.637177	3.399938	0.001013	-0.88208	0.036041	0.034342
Neutrophils	PRICKLE1	-0.97907	3.386121	-3.39778	0.00102	-0.84412	0.037481	0.035709
Neutrophils	BRMS1	0.800184	4.236998	3.392911	0.001036	-0.8625	0.037034	0.035201
Neutrophils	NFKBID	0.464191	6.350031	3.391788	0.00104	-1.17973	0.034879	0.033248
Neutrophils	CSTA2	-0.52511	1.96893	-3.38793	0.001053	-1.38581	0.039812	0.037823
Neutrophils	DBNL	0.361367	6.127196	3.386308	0.001059	-1.12409	0.035364	0.033803
Neutrophils	XYLT1	0.345703	7.355246	3.386285	0.001059	-1.60497	0.034152	0.032667
Neutrophils	POU6F1	-1.504	2.610644	-3.37496	0.001098	-1.32094	0.040403	0.038145
Neutrophils	GM156	-1.95581	-1.54566	-3.3722	0.001108	-1.81451	0.045692	0.043111
Neutrophils	VTI1B	0.306444	5.690918	3.371516	0.00111	-1.13069	0.037169	0.035307
Neutrophils	TJP3	1.619328	1.707068	3.364233	0.001137	-1.43299	0.042456	0.040074
Neutrophils	GNAI2	0.198394	9.008515	3.363364	0.00114	-1.70728	0.034494	0.032756
Neutrophils	PTPN22	-0.69842	5.324165	-3.3591	0.001155	-1.07485	0.038694	0.036616
Neutrophils	C130050O1	0.76151	1.379067	3.357617	0.001161	-0.95265	0.043309	0.041013
Neutrophils	PIAS4	0.625004	4.224377	3.35697	0.001163	-0.94653	0.039933	0.037928
Neutrophils	CYP3A11	-1.28808	3.352158	-3.35357	0.001176	-0.95515	0.04125	0.039182
Neutrophils	INPP5D	0.401659	8.764735	3.351222	0.001185	-1.5283	0.03543	0.0339
Neutrophils	ZFP36L1	0.436669	7.742233	3.350935	0.001186	-1.3674	0.036472	0.03489
Neutrophils	MYO1C	0.402087	5.436164	3.349938	0.00119	-1.10432	0.038941	0.037295
Neutrophils	EYA2	-1.95362	1.389743	-3.34621	0.001204	-1.61371	0.043875	0.042005
Neutrophils	SMAD4	0.317336	6.23224	3.346015	0.001205	-1.25948	0.038219	0.036717
Neutrophils	CEP78	-0.79657	3.001386	-3.34566	0.001206	-1.00601	0.041902	0.04019
Neutrophils	ALDOA	0.482515	8.900702	3.342036	0.00122	-1.77912	0.035482	0.034361
Neutrophils	KPNA4	0.328974	8.246447	3.341603	0.001222	-1.66204	0.036145	0.035015
Neutrophils	TBX21	-1.59291	2.437202	-3.34123	0.001223	-1.14425	0.042641	0.041167
Neutrophils	LILRA6	0.604128	1.213739	3.341205	0.001224	-1.11324	0.044156	0.042591
Neutrophils	AKR1B3	-0.64502	5.402163	-3.33751	0.001238	-1.00083	0.039525	0.03823
Neutrophils	GM48855	1.235262	1.463573	3.335434	0.001246	-1.33428	0.044374	0.042858
Neutrophils	ARHGEF7	-0.54715	4.675352	-3.3338	0.001253	-1.09536	0.040573	0.039307
Neutrophils	NUCB2	0.423325	4.304904	3.331949	0.00126	-1.12038	0.041116	0.039828
Neutrophils	STAMBPL1	-0.42674	6.55028	-3.32947	0.00127	-1.37182	0.038755	0.037588
Neutrophils	LRRC25	0.644713	3.856093	3.326802	0.001281	-1.19214	0.042068	0.040625
Neutrophils	NUP210L	-0.7032	6.407383	-3.32517	0.001288	-1.40673	0.039121	0.037968
Neutrophils	SMAP1	0.351563	6.875842	3.323691	0.001294	-1.3817	0.038604	0.037599
Neutrophils	MBNL2	0.326706	8.406165	3.323053	0.001296	-1.6855	0.036965	0.036055
Neutrophils	SLCO3A1	0.655156	4.38978	3.322894	0.001297	-1.22251	0.041433	0.04032
Neutrophils	SNX13	0.424637	5.413359	3.319342	0.001312	-1.32072	0.040575	0.039508
Neutrophils	CLEC4N	0.669531	4.093598	3.317352	0.00132	-1.3521	0.042266	0.041203
Neutrophils	IFITM1	1.090749	3.489377	3.303905	0.001378	-1.36732	0.044738	0.043094

Neutrophils	CDKN1C	-1.28917	3.302414	-3.30097	0.001391	-1.11172	0.045218	0.043527
Neutrophils	MED7	0.669293	3.583194	3.300289	0.001394	-1.09906	0.044857	0.04323
Neutrophils	UCHL1	-2.05984	0.862756	-3.29796	0.001404	-1.80866	0.048682	0.046798
Neutrophils	TSC22D4	0.318615	6.684783	3.296426	0.001411	-1.54313	0.04124	0.039938
Neutrophils	CD79A	-0.7764	6.200185	-3.29609	0.001412	-1.22194	0.041812	0.040495
Neutrophils	FXYD5	0.371969	6.974009	3.29247	0.001429	-1.83156	0.04125	0.03995
Neutrophils	GM13708	0.725697	3.088737	3.290218	0.001439	-1.13023	0.046263	0.044768
Neutrophils	ABCC9	1.270435	1.277502	3.28922	0.001443	-1.20763	0.048725	0.047148
Neutrophils	SUPV3L1	-0.75357	3.910174	-3.28706	0.001453	-1.13341	0.045373	0.044043
Neutrophils	HADH	-0.69983	5.394105	-3.28582	0.001459	-1.20628	0.043537	0.04239
Neutrophils	APBB1IP	0.284989	7.638967	3.284499	0.001465	-1.72945	0.040896	0.03994
Neutrophils	CAV2	-0.94338	2.771749	-3.27866	0.001492	-1.18916	0.047577	0.046349
Neutrophils	TUBB6	0.882294	3.749371	3.278325	0.001494	-1.16585	0.046269	0.045118
Neutrophils	PDCD6	0.339333	6.13963	3.277594	0.001497	-1.56371	0.043225	0.042254
Neutrophils	EBF1	-0.80741	8.683852	-3.2767	0.001502	-1.68646	0.040215	0.039436
Neutrophils	MERTK	-0.94589	3.774359	-3.27516	0.001509	-1.21855	0.046324	0.045409
Neutrophils	CYLD	0.314096	6.329731	3.271412	0.001527	-1.56455	0.043448	0.042637
Neutrophils	GRIPAP1	0.345027	5.594976	3.270368	0.001532	-1.43584	0.044365	0.043604
Neutrophils	PPP1R18OS	1.153486	0.993134	3.269653	0.001535	-1.48998	0.050585	0.049605
Neutrophils	RESF1	0.308156	6.500817	3.263722	0.001564	-1.75503	0.043925	0.043065
Neutrophils	NLRP3	0.518475	4.527197	3.261666	0.001574	-1.76925	0.046628	0.045722
Neutrophils	RPUSD4	-1.30997	2.682051	-3.26053	0.00158	-1.54303	0.04918	0.048264
Neutrophils	IRF4	-1.38418	4.497863	-3.25741	0.001596	-1.3786	0.047024	0.046213
Neutrophils	FOXO1	-0.54226	7.035015	-3.25595	0.001603	-1.35481	0.043827	0.043136
Neutrophils	OSTF1	0.200311	7.480967	3.251371	0.001626	-1.89597	0.043747	0.043041
Neutrophils	VTA1	0.370573	5.707573	3.2504	0.001631	-1.42502	0.046008	0.045342
Neutrophils	HJURP	0.436719	4.895427	3.24822	0.001642	-1.43557	0.047083	0.046527
Neutrophils	LRRC28	-0.54091	3.874144	-3.24786	0.001644	-1.46062	0.048473	0.047907
Neutrophils	JUNB	0.362509	9.343714	3.247564	0.001646	-2.12595	0.041499	0.041164
Neutrophils	IL2RB	-1.48923	3.130268	-3.24708	0.001648	-1.28134	0.049512	0.048948
Neutrophils	GRK5	0.641432	5.747033	3.241575	0.001677	-1.53889	0.046624	0.046056
Neutrophils	RNF41	0.531308	4.424549	3.239208	0.001689	-1.28173	0.048604	0.048044
Neutrophils	GM10552	0.940108	1.54086	3.23822	0.001695	-1.27071	0.052771	0.052149
Neutrophils	TPM4	0.305875	7.167543	3.23728	0.0017	-1.75519	0.044957	0.044654
Neutrophils	ENTHD1	2.085577	0.023444	3.236754	0.001702	-1.83863	0.055108	0.054518
Neutrophils	RAPH1	-0.52069	5.055385	-3.23431	0.001715	-1.42167	0.04775	0.04759
Neutrophils	PLPP3	-0.84507	4.693864	-3.23422	0.001716	-1.47907	0.048244	0.048071
Neutrophils	KLRA5	-1.95725	2.005641	-3.23403	0.001717	-1.43464	0.052088	0.051818
Neutrophils	PRF1	-2.06175	0.377462	-3.23092	0.001734	-1.86765	0.054949	0.054693
Neutrophils	NOCT	0.433989	5.04776	3.227649	0.001752	-1.65788	0.048256	0.048413
Neutrophils	THUMPD2	1.368286	1.928752	3.227499	0.001752	-1.78608	0.052744	0.05281
Neutrophils	SCAMP5	-1.31526	1.229049	-3.2267	0.001757	-1.56378	0.053808	0.05393
Neutrophils	SDCBP	0.266156	7.674968	3.226362	0.001759	-1.92712	0.044785	0.045092
Neutrophils	TLR13	0.570287	1.881588	3.220182	0.001793	-1.47329	0.053698	0.053699
Neutrophils	KNG1	-0.71901	6.039457	-3.2183	0.001803	-1.65678	0.047849	0.048051
Neutrophils	S100G	2.030389	0.605317	3.215433	0.00182	-1.71226	0.056101	0.056267
Neutrophils	PRDX4	-0.75286	5.118984	-3.21524	0.001821	-1.32496	0.049324	0.049643

Neutrophils	SGCZ	-1.50648	1.627249	-3.21224	0.001838	-1.55214	0.054852	0.055062
Neutrophils	MAML2	-0.56673	7.972039	-3.21045	0.001848	-1.66983	0.045921	0.046273
Neutrophils	BCL2L1	0.613402	6.15449	3.208666	0.001858	-1.58698	0.048403	0.048767
Neutrophils	ACAP2	0.328929	7.320314	3.208404	0.00186	-1.86265	0.046827	0.04721
Neutrophils	CNPY3	0.449316	5.300686	3.205407	0.001877	-1.40547	0.049925	0.0502
Neutrophils	HK1	0.396714	4.85354	3.2039	0.001886	-1.5835	0.050668	0.051003
Neutrophils	MYBPC2	-1.82591	2.760882	-3.20181	0.001898	-1.80855	0.053789	0.054242
Neutrophils	RIOK3	0.282951	7.578172	3.201707	0.001899	-1.97809	0.046899	0.047435
Neutrophils	VSIG4	-0.99395	4.823941	-3.20132	0.001901	-1.53816	0.050717	0.051261
Neutrophils	NMNAT2	-1.90324	2.111738	-3.19976	0.00191	-1.52154	0.054916	0.055439
Neutrophils	SYTL3	-1.56612	2.803793	-3.1976	0.001923	-1.45985	0.054064	0.054683
Neutrophils	GM11707	-1.64959	0.863582	-3.19191	0.001958	-1.83456	0.057955	0.058379
Neutrophils	GHITM	0.498268	7.4577	3.191379	0.001961	-1.76285	0.04803	0.048623
Neutrophils	RAB19	-1.44474	2.561986	-3.18874	0.001977	-1.68461	0.05535	0.056036
Neutrophils	GM33370	1.56768	0.508404	3.188142	0.001981	-1.78363	0.058692	0.059366
Neutrophils	ARL5B	0.52004	5.460574	3.187966	0.001982	-1.60567	0.050962	0.051739
Neutrophils	PAKAP.1	-0.79686	4.53559	-3.18685	0.001988	-1.4191	0.052321	0.053195
Neutrophils	REM2	-2.1571	0.836601	-3.18642	0.001991	-2.05991	0.058144	0.058996
Neutrophils	SKAP1	-1.02914	4.523681	-3.18342	0.00201	-1.44381	0.052692	0.05341
Neutrophils	M6PR	0.423321	6.293265	3.179785	0.002032	-1.62391	0.050543	0.05115
Neutrophils	TJP2	-0.99383	3.845594	-3.17811	0.002043	-1.44989	0.054255	0.054907
Neutrophils	BASP1	0.639287	5.646818	3.176893	0.002051	-1.81165	0.051542	0.052338
Neutrophils	CECR2	-0.97019	6.605628	-3.17652	0.002053	-1.56238	0.050156	0.050986
Neutrophils	HDAC4	0.409524	5.402881	3.176122	0.002056	-1.7807	0.051901	0.052752
Neutrophils	PRR13	0.231704	6.510778	3.173135	0.002075	-1.97186	0.05063	0.051423
Neutrophils	GM26532	0.453438	5.294849	3.172026	0.002082	-1.76155	0.05246	0.053344
Neutrophils	WASHC4	0.365583	5.792598	3.167485	0.002111	-1.63604	0.052301	0.053094
Neutrophils	PDK1	0.703062	4.164844	3.166798	0.002116	-1.44723	0.054782	0.05564
Neutrophils	DAND5	-1.13289	3.96611	-3.16483	0.002129	-1.45613	0.05529	0.056238
Neutrophils	SHLD2	0.891891	3.853936	3.161788	0.002149	-1.48759	0.055574	0.056814
Neutrophils	ENO1	0.34569	8.066335	3.161333	0.002152	-2.18986	0.049302	0.050578
Neutrophils	ZFP292	0.351508	7.046023	3.161051	0.002153	-1.8674	0.05075	0.052059
Neutrophils	TMEM205	-0.81996	3.71868	-3.16099	0.002154	-1.47288	0.055789	0.057111
Neutrophils	RHOG	0.319579	7.243088	3.159415	0.002164	-2.11654	0.050588	0.051947
Neutrophils	GM36551	0.81779	-0.80034	3.156082	0.002187	-1.54556	0.064064	0.065216
Neutrophils	STAP1	0.371456	4.891894	3.155422	0.002191	-1.8355	0.05446	0.055729
Neutrophils	SAMSN1	0.281206	7.050554	3.154783	0.002195	-2.25665	0.051218	0.05255
Neutrophils	BCL7A	-1.04185	4.80999	-3.15334	0.002205	-1.48813	0.054696	0.056114
Neutrophils	ITGA4	0.479458	7.657286	3.15032	0.002226	-1.91148	0.050791	0.052027
Neutrophils	HES1	-0.8015	5.161335	-3.14815	0.002241	-1.55343	0.054648	0.05601
Neutrophils	LRRFIP2	0.262494	5.900501	3.148004	0.002242	-1.97456	0.053511	0.054869
Neutrophils	RAB20	0.554338	3.301078	3.146153	0.002254	-1.70298	0.057791	0.059177
Neutrophils	CIB2	0.762144	2.152566	3.14549	0.002259	-1.50156	0.059716	0.06115
Neutrophils	WDR86	-1.56418	0.234245	-3.1438	0.002271	-2.13167	0.063254	0.064644
Neutrophils	SERPIND1	-1.17994	2.832849	-3.14176	0.002285	-1.52148	0.058958	0.060398
Neutrophils	HIST2H2AA	0.960623	2.225914	3.13399	0.00234	-1.53192	0.061288	0.062372
Neutrophils	RDH12	0.63375	2.275253	3.129762	0.002371	-1.67969	0.061821	0.062843

Neutrophils	P4HA1	0.633221	5.786072	3.129096	0.002375	-1.76209	0.055935	0.057043
Neutrophils	CSF3R	0.375739	2.933462	3.127878	0.002384	-2.06901	0.060671	0.061934
Neutrophils	GM26812	-2.055	0.020142	-3.1276	0.002386	-2.19739	0.065932	0.067195
Neutrophils	MYO1F	0.361459	5.259228	3.126544	0.002394	-2.10197	0.056829	0.058223
Neutrophils	ZNRF3	-0.95146	5.517512	-3.12308	0.00242	-1.55837	0.056808	0.05827
Neutrophils	CCRL2	0.658093	4.741765	3.122723	0.002422	-2.11433	0.058076	0.059555
Neutrophils	HSPA4L	0.42772	4.348791	3.120681	0.002437	-1.80275	0.058959	0.060414
Neutrophils	ACER1	-2.45506	-0.7216	-3.1196	0.002445	-2.22223	0.068193	0.069707
Neutrophils	ZFP1	-0.8679	3.874226	-3.11889	0.002451	-1.59949	0.059809	0.061379
Neutrophils	SCAMP1	0.685099	2.970331	3.118004	0.002457	-1.60994	0.061394	0.063075
Neutrophils	ANKRD37	0.721136	3.981977	3.115864	0.002473	-1.63962	0.059774	0.061508
Neutrophils	ALKBH5	0.324722	7.148219	3.115793	0.002474	-2.0016	0.054626	0.056319
Neutrophils	F10	0.882175	4.54109	3.114	0.002488	-1.834	0.059016	0.060728
Neutrophils	CD14	0.595377	4.260683	3.109891	0.002519	-2.16518	0.060082	0.061765
Neutrophils	UOX	0.909547	5.010934	3.109236	0.002524	-1.80262	0.058812	0.060488
Neutrophils	ZYX	0.350088	6.244314	3.107994	0.002534	-2.1886	0.056869	0.058608
Neutrophils	TEF	-1.0474	3.230001	-3.09973	0.002598	-1.75344	0.063361	0.064704
Neutrophils	CYP4F18	0.410948	3.551567	3.098625	0.002607	-1.92676	0.062783	0.064261
Neutrophils	MGMT	-1.0464	3.273792	-3.09842	0.002608	-1.66159	0.063282	0.064764
Neutrophils	TOP1MT	-1.64773	1.70028	-3.09297	0.002652	-2.06437	0.067109	0.068317
Neutrophils	GM44649	0.619145	3.949723	3.092375	0.002657	-1.68941	0.062938	0.064216
Neutrophils	DENND1B	0.304808	7.299486	3.089981	0.002676	-2.22641	0.057453	0.058699
Neutrophils	IKZF2	-1.19268	4.781138	-3.08954	0.00268	-1.69486	0.061717	0.063013
Neutrophils	CXCR5	0.943384	2.183855	3.088515	0.002688	-1.64636	0.066517	0.067833
Neutrophils	RAMP1	0.371134	4.507186	3.086999	0.0027	-1.83613	0.062399	0.063775
Neutrophils	APOA1	-0.8621	8.277086	-3.08573	0.002711	-2.36936	0.056152	0.057541
Neutrophils	HCLS1	0.32262	6.691521	3.081506	0.002746	-2.16155	0.059358	0.060737
Neutrophils	RNH1	0.47642	5.988026	3.079861	0.002759	-1.90797	0.060723	0.062092
Neutrophils	POLR2A	0.286635	6.200996	3.077697	0.002778	-2.00234	0.060618	0.061941
Neutrophils	DMXL2	0.554649	2.146865	3.074372	0.002806	-1.92357	0.068411	0.069805
Neutrophils	CYP1A2	-1.40019	1.633956	-3.07436	0.002806	-1.81549	0.06942	0.070808
Neutrophils	TRBC1	-1.27837	3.183542	-3.0737	0.002811	-1.68492	0.066417	0.06792
Neutrophils	TRIB1	0.342175	5.765307	3.06926	0.002849	-2.28856	0.062404	0.063771
Neutrophils	TMSB10	-0.45702	10.73818	-3.06824	0.002858	-2.49492	0.054251	0.055553
Neutrophils	NID2	-1.4268	2.19946	-3.06721	0.002867	-1.84514	0.069201	0.070557
Neutrophils	BRIX1	-0.40546	6.144863	-3.06538	0.002883	-1.80057	0.061875	0.063401
Neutrophils	MYCT1	-0.89026	1.654516	-3.06457	0.00289	-1.71011	0.070322	0.071932
Neutrophils	MALAT1	0.273505	14.72453	3.064504	0.002891	-3.2381	0.048572	0.050018
Neutrophils	IL1R1	1.094188	2.051839	3.064143	0.002894	-1.70622	0.069529	0.071152
Neutrophils	GM44686	0.849807	3.136727	3.062927	0.002904	-1.74559	0.067455	0.069229
Neutrophils	MIR142HG	0.263434	8.019491	3.062306	0.00291	-2.42426	0.058708	0.060496
Neutrophils	EFNA2	-1.6725	1.10216	-3.06176	0.002915	-1.87121	0.071487	0.073418
Neutrophils	CTNNBL1	-0.48157	5.139211	-3.0598	0.002932	-1.77921	0.063953	0.065764
Neutrophils	STEAP4	1.256719	2.205553	3.058593	0.002943	-1.74475	0.069635	0.071524
Neutrophils	SEMA4D	0.39614	6.446214	3.057069	0.002956	-2.31676	0.061862	0.063736
Neutrophils	CDH13	-1.3983	2.030566	-3.05502	0.002974	-1.73208	0.070437	0.072439
Neutrophils	ANXA11	0.25617	5.969579	3.05252	0.002997	-2.32852	0.063299	0.065254

Neutrophils	ST3GAL1	0.613055	5.796993	3.051754	0.003004	-1.99182	0.06362	0.065652
Neutrophils	RFX7	-0.37115	7.368214	-3.0482	0.003036	-2.22601	0.06132	0.063211
Neutrophils	EPB42	1.600574	-0.1647	3.047742	0.00304	-2.07365	0.076002	0.077946
Neutrophils	EYA3	0.435895	5.473081	3.044566	0.00307	-1.95142	0.065124	0.067111
Neutrophils	ERN1	-0.43105	7.053542	-3.04343	0.00308	-2.17714	0.062263	0.064308
Neutrophils	IK	0.284042	6.288849	3.043258	0.003082	-2.11861	0.063631	0.065702
Neutrophils	USP10	-0.94276	4.567936	-3.04239	0.00309	-1.79312	0.066824	0.068969
Neutrophils	APCS	0.640436	4.833138	3.042112	0.003092	-1.97091	0.066321	0.068501
Neutrophils	GM9844	-1.37869	2.309102	-3.03855	0.003125	-2.14113	0.071696	0.073907
Neutrophils	4930444A1	0.676395	3.751804	3.038486	0.003126	-1.8184	0.068806	0.070999
Neutrophils	SOAT2	1.366159	0.810649	3.038024	0.00313	-1.96675	0.07483	0.077117
Neutrophils	PRPS2	-0.94007	4.382206	-3.03611	0.003148	-1.85203	0.067692	0.07013
Neutrophils	CCL3	0.756371	5.069599	3.036053	0.003149	-2.22243	0.06638	0.068801
Neutrophils	SLC22A15	0.341977	3.077716	3.035391	0.003155	-2.13255	0.070256	0.072774
Neutrophils	LYRM1	0.747518	2.700522	3.033349	0.003175	-1.7833	0.071304	0.073785
Neutrophils	NUP155	-0.47115	5.174039	-3.03224	0.003185	-1.87213	0.066534	0.069017
Neutrophils	PPP4R3B	0.25359	6.842276	3.030745	0.003199	-2.23129	0.063605	0.066113
Neutrophils	NF2	-0.66715	4.781261	-3.02245	0.00328	-1.81381	0.068997	0.071085
Neutrophils	PIRA2	0.485971	3.589246	3.021328	0.003291	-2.16835	0.071474	0.073613
Neutrophils	ASNSD1	0.324926	5.360445	3.01888	0.003315	-2.10592	0.068199	0.070337
Neutrophils	COL13A1	-1.1806	0.62777	-3.01877	0.003316	-1.88127	0.078054	0.080233
Neutrophils	DDIAS	-1.32177	2.134705	-3.01568	0.003347	-2.0467	0.074872	0.077364
Neutrophils	PLEKHG1	-0.3833	4.398533	-3.01521	0.003352	-2.29568	0.070191	0.072699
Neutrophils	MGP	1.815169	0.463003	3.015075	0.003353	-2.0898	0.078532	0.081107
Neutrophils	RASGRP3	-0.74878	3.586569	-3.01419	0.003362	-1.90854	0.071834	0.074478
Neutrophils	BTG1	0.278607	9.314237	3.013557	0.003368	-2.76774	0.061048	0.063606
Neutrophils	SPI1	0.267384	6.411888	3.013437	0.003369	-2.49401	0.066283	0.068964
Neutrophils	TSPO	0.421182	7.399271	3.012823	0.003376	-2.46801	0.064451	0.067138
Neutrophils	FARP1	-0.61667	3.619271	-3.01071	0.003397	-1.90738	0.071767	0.0749
Neutrophils	MIER2	-1.40599	2.002333	-3.01063	0.003398	-2.18066	0.075155	0.078346
Neutrophils	CD3E	-1.4279	3.278256	-3.0104	0.0034	-1.84139	0.072469	0.075614
Neutrophils	ATP6V0E	0.244838	7.713213	3.009932	0.003405	-2.46038	0.063879	0.066871
Neutrophils	NCKIPSD	-1.00132	2.952621	-3.00935	0.003411	-1.97626	0.073145	0.076408
Neutrophils	SRPK3	-1.60149	1.292575	-3.0087	0.003417	-2.282	0.076694	0.080104
Neutrophils	COQ8A	-1.20216	2.303584	-3.00809	0.003424	-2.03003	0.074512	0.077958
Neutrophils	HMGN3	-1.59927	3.255903	-3.00593	0.003446	-2.10329	0.072515	0.076148
Neutrophils	CYFIP1	0.389979	5.64309	3.005668	0.003448	-2.19365	0.067748	0.071253
Neutrophils	AP3S1	0.233939	7.096627	3.005616	0.003449	-2.53667	0.065007	0.068427
Neutrophils	PRX	-1.31236	1.104861	-3.00561	0.003449	-2.01644	0.077106	0.080842
Neutrophils	SPTLC2	0.259797	6.245992	3.005495	0.00345	-2.28001	0.066596	0.070067
Neutrophils	MEF2C	-0.45395	7.538963	-3.00449	0.00346	-2.32319	0.064261	0.067691
Neutrophils	ARID5B	0.525583	7.244193	3.00153	0.003491	-2.35041	0.065247	0.068667
Neutrophils	METTL18	1.566673	1.271701	2.998686	0.003521	-2.2632	0.07785	0.081499
Neutrophils	FMNL2	-0.43216	7.100621	-2.99761	0.003532	-2.65275	0.066022	0.06946
Neutrophils	MFSD12	0.986369	2.730413	2.994682	0.003563	-1.9966	0.07519	0.078918
Neutrophils	NOTCH2	0.254168	7.16468	2.994405	0.003566	-2.60286	0.066274	0.069786
Neutrophils	SYK	0.328745	8.336181	2.993628	0.003574	-2.6053	0.064132	0.067643

Neutrophils	MORC3	0.335923	5.857789	2.992498	0.003586	-2.21483	0.068903	0.072597
Neutrophils	GM16272	-1.71132	0.363126	-2.9899	0.003614	-2.36887	0.081057	0.085116
Neutrophils	NMD3	-0.4804	4.999909	-2.98875	0.003626	-1.90974	0.071119	0.074977
Neutrophils	NOS2	2.301703	-1.12584	2.987281	0.003642	-2.30573	0.084909	0.089116
Neutrophils	HEY1	-1.94989	-0.2714	-2.98411	0.003677	-2.45436	0.083484	0.087512
Neutrophils	TTR	-0.85912	9.202897	-2.98079	0.003713	-2.79564	0.064068	0.067695
Neutrophils	GM26982	-1.13494	1.652146	-2.98076	0.003713	-2.16897	0.079415	0.083525
Neutrophils	RPF1	-0.47356	5.149471	-2.98001	0.003722	-1.96357	0.071878	0.075858
Neutrophils	KTN1	-0.48761	5.826158	-2.97925	0.00373	-1.96877	0.070508	0.074495
Neutrophils	MACROD2	0.747215	2.251559	2.979199	0.00373	-1.94539	0.078068	0.082287
Neutrophils	GDAP2	0.377494	4.778409	2.977921	0.003745	-2.14418	0.072778	0.076843
Neutrophils	APOF	-0.87953	3.847555	-2.97465	0.003781	-1.96914	0.075318	0.079324
Neutrophils	PSD4	0.46262	4.541706	2.971562	0.003816	-2.07811	0.074379	0.078239
Neutrophils	GM11808	-0.44037	7.262783	-2.96902	0.003845	-2.33328	0.069215	0.072905
Neutrophils	PLK3	0.415309	4.483011	2.96805	0.003856	-2.32835	0.074908	0.078844
Neutrophils	CSF2RA	0.301348	5.196056	2.967808	0.003858	-2.46414	0.073403	0.077315
Neutrophils	1600010M	0.438286	4.96425	2.966442	0.003874	-2.21738	0.07389	0.078001
Neutrophils	CHD3	-0.89972	4.533669	-2.96599	0.003879	-1.94781	0.074802	0.079028
Neutrophils	BRWD1	-0.30419	6.550877	-2.96589	0.00388	-2.38693	0.07063	0.074717
Neutrophils	OSBPL8	-0.296	7.62098	-2.96385	0.003904	-2.62785	0.068612	0.072747
Neutrophils	A630001O:	1.14894	2.282008	2.963719	0.003905	-2.08362	0.079872	0.084401
Neutrophils	SLC29A1	-0.59963	5.215066	-2.96352	0.003908	-2.04604	0.073466	0.077798
Neutrophils	SMIM3	0.342255	4.840554	2.961495	0.003931	-2.3576	0.074359	0.078806
Neutrophils	FCRLA	-1.18523	5.107141	-2.96116	0.003935	-1.9614	0.073797	0.078235
Neutrophils	PLEKHO2	0.344409	6.069061	2.961145	0.003935	-2.35602	0.071805	0.076168
Neutrophils	IGKC	-1.19313	7.88266	-2.96036	0.003944	-2.39061	0.068211	0.072496
Neutrophils	SIGLECG	-0.95947	4.076853	-2.95984	0.00395	-2.00428	0.076005	0.080665
Neutrophils	SKINT3	0.976508	0.115469	2.956012	0.003995	-1.98454	0.08591	0.09074
Neutrophils	HIST1H2BC	0.52071	5.260139	2.951873	0.004044	-2.30004	0.074574	0.079239
Neutrophils	OGFRL1	0.349598	5.082967	2.951101	0.004053	-2.44494	0.074951	0.079763
Neutrophils	FCGRT	-0.62522	4.823452	-2.95094	0.004055	-2.18875	0.075507	0.080341
Neutrophils	IFT122	-1.12669	1.594011	-2.95091	0.004055	-2.18742	0.082791	0.087894
Neutrophils	FAM45A	0.535597	3.979967	2.950698	0.004058	-1.99803	0.077343	0.082283
Neutrophils	PECAM1	-0.52848	7.734027	-2.95049	0.00406	-2.43993	0.069513	0.074128
Neutrophils	PDE4B	0.338476	8.527906	2.947373	0.004098	-2.81869	0.068333	0.072881
Neutrophils	SPINDOC	-0.66211	4.427162	-2.94696	0.004103	-1.99047	0.076776	0.081694
Neutrophils	PRXL2A	1.020934	4.184431	2.946808	0.004105	-2.0117	0.077309	0.082247
Neutrophils	GM15283	0.660747	4.261918	2.946089	0.004113	-2.05034	0.077161	0.082171
Neutrophils	CD3G	-1.26489	3.343766	-2.94462	0.004131	-1.99588	0.079406	0.084467
Neutrophils	BIRC3	0.34156	7.111789	2.942749	0.004154	-2.57932	0.07151	0.076198
Neutrophils	GFOD2	-1.17298	2.309358	-2.94254	0.004156	-2.12755	0.081988	0.087095
Neutrophils	ITGB6	-1.94631	-0.27325	-2.93991	0.004189	-2.37694	0.088789	0.094024
Neutrophils	PKP4	-0.61216	5.958306	-2.93702	0.004224	-2.20968	0.07483	0.079554
Neutrophils	HNRNPUL2	0.275404	6.468104	2.935274	0.004246	-2.39995	0.073995	0.07864
Neutrophils	RIN3	0.307232	5.640444	2.934687	0.004253	-2.47424	0.075757	0.080556
Neutrophils	CLPB	-0.69302	4.010407	-2.93247	0.004281	-2.03249	0.07973	0.084653
Neutrophils	CTSW	-1.55188	2.120663	-2.92927	0.004322	-2.14505	0.0844	0.089677

Neutrophils	RALGAPA2	-0.39375	6.409122	-2.92904	0.004324	-2.55061	0.074694	0.079588
Neutrophils	RLF	0.26045	6.699162	2.92903	0.004325	-2.63479	0.074081	0.078948
Neutrophils	CCL5	-1.64266	7.931718	-2.92901	0.004325	-2.64505	0.071535	0.076285
Neutrophils	SIRT1	-0.34955	5.043074	-2.92736	0.004346	-2.24095	0.077744	0.082876
Neutrophils	KLRA1	-2.42823	0.700337	-2.92679	0.004353	-2.45428	0.087996	0.093611
Neutrophils	FAM107B	0.286553	7.858238	2.926405	0.004358	-2.77567	0.071768	0.076756
Neutrophils	KCNQ5	-0.59614	7.024096	-2.9262	0.004361	-2.30799	0.073487	0.078559
Neutrophils	CES1C	-1.00544	4.023969	-2.92501	0.004376	-2.13353	0.080054	0.085593
Neutrophils	HPGD	-0.84883	5.673887	-2.92438	0.004384	-2.39633	0.076381	0.081801
Neutrophils	KCTD3	-0.84185	3.747291	-2.92413	0.004387	-2.07719	0.080688	0.08633
Neutrophils	NUP98	0.295116	7.461994	2.923474	0.004395	-2.67482	0.072598	0.077963
Neutrophils	UPP1	1.432518	0.646262	2.923114	0.0044	-2.04644	0.088154	0.094302
Neutrophils	ALG11	-0.93025	2.610001	-2.91602	0.004492	-2.16914	0.084946	0.090572
Neutrophils	IL10RA	0.5479	4.971561	2.908396	0.004593	-2.223	0.081061	0.085939
Neutrophils	CXCL2	0.751453	7.158496	2.906341	0.004621	-3.10429	0.0765	0.081117
Neutrophils	MEIS2	-0.71835	2.894046	-2.90428	0.004649	-2.2027	0.086741	0.091689
Neutrophils	GIMAP9	-0.96429	3.560572	-2.90335	0.004661	-2.10717	0.085191	0.09015
Neutrophils	WRN	0.378527	5.831312	2.899054	0.00472	-2.3787	0.080722	0.085227
Neutrophils	SASH1	-0.58544	5.303699	-2.89826	0.004731	-2.3982	0.081962	0.086613
Neutrophils	NSF	0.442381	6.770124	2.897785	0.004737	-2.4138	0.078616	0.083205
Neutrophils	VPREB3	-0.96845	6.013944	-2.89548	0.004769	-2.26605	0.080622	0.08529
Neutrophils	CD59B	-1.80504	0.413	-2.89532	0.004771	-2.406	0.094582	0.099674
Neutrophils	MRC1	-0.79539	5.240936	-2.89467	0.00478	-2.50593	0.08243	0.087281
Neutrophils	ZMPSTE24	-0.3443	4.942134	-2.89406	0.004789	-2.55855	0.083139	0.088123
Neutrophils	DIAPH1	0.225352	7.355352	2.892674	0.004808	-2.82726	0.077777	0.08264
Neutrophils	IGHD	-1.24561	3.245617	-2.8917	0.004822	-2.2643	0.087425	0.092818
Neutrophils	ACTR2	0.226624	8.066808	2.891363	0.004827	-2.83635	0.076224	0.081206
Neutrophils	TMEM14A	-1.76981	0.419553	-2.89105	0.004831	-2.54965	0.09477	0.1005
Neutrophils	GM40645	1.043014	0.751306	2.889461	0.004853	-2.13409	0.09415	0.099802
Neutrophils	URM1	-0.31059	4.343407	-2.88855	0.004866	-2.4421	0.085002	0.09051
Neutrophils	FECH	0.760015	5.217188	2.887998	0.004874	-2.2639	0.082913	0.088354
Neutrophils	STAT6	0.35877	5.031546	2.887621	0.004879	-2.39272	0.083352	0.088833
Neutrophils	MBL2	-0.94137	4.246085	-2.887	0.004888	-2.25142	0.085249	0.090807
Neutrophils	DLC1	-0.69575	4.570858	-2.88456	0.004923	-2.39225	0.084921	0.090396
Neutrophils	EMILIN2	0.500445	4.009199	2.883403	0.004939	-2.71203	0.086434	0.091951
Neutrophils	TNFRSF9	-1.43453	2.460851	-2.88163	0.004965	-2.20137	0.09052	0.096206
Neutrophils	ZFP281	0.499825	4.496464	2.881544	0.004966	-2.24639	0.085416	0.090911
Neutrophils	RNASEH2A	-0.73838	4.54522	-2.88081	0.004977	-2.14764	0.085336	0.090874
Neutrophils	RGL1	-0.60699	5.711711	-2.87885	0.005005	-2.36293	0.08283	0.088262
Neutrophils	PPRC1	-0.74853	3.956149	-2.87849	0.00501	-2.15366	0.087075	0.092715
Neutrophils	NT5C2	0.329182	5.867578	2.877133	0.00503	-2.70907	0.08265	0.088089
Neutrophils	CUX2	-1.36342	1.039079	-2.87517	0.005058	-2.37605	0.095187	0.10105
Neutrophils	MICOS13	0.370589	5.948201	2.874749	0.005065	-2.40119	0.082756	0.08822
Neutrophils	PTPN6	0.32918	6.758702	2.873371	0.005085	-2.72421	0.080956	0.08636
Neutrophils	HAUS8	0.442686	4.722045	2.873246	0.005087	-2.31843	0.085784	0.091412
Neutrophils	PTPRM	-0.66128	5.186994	-2.87133	0.005115	-2.54519	0.084986	0.090529
Neutrophils	VWF	1.372583	1.565048	2.870028	0.005134	-2.17377	0.094431	0.10019

Neutrophils	FAM220A	1.355815	2.03012	2.868792	0.005153	-2.28313	0.093289	0.099071
Neutrophils	LILRB4A	0.352962	4.163584	2.868507	0.005157	-2.82316	0.087782	0.093419
Neutrophils	IL4RA	0.630828	4.883706	2.864774	0.005213	-2.26871	0.086789	0.092154
Neutrophils	DLG4	-1.12744	3.571009	-2.86297	0.00524	-2.18878	0.090419	0.095895
Neutrophils	HSD17B2	-0.97912	2.446494	-2.85959	0.005291	-2.19994	0.094126	0.099443
Neutrophils	BORCS6	0.490343	4.007961	2.85797	0.005316	-2.31864	0.090301	0.095409
Neutrophils	DEGS1	-0.26994	6.525466	-2.85515	0.005359	-2.92153	0.084607	0.089365
Neutrophils	MYD88	0.449361	4.609439	2.852484	0.005401	-2.40982	0.089887	0.094658
Neutrophils	ARL2BP	0.422088	5.086344	2.851304	0.005419	-2.45828	0.088832	0.093593
Neutrophils	APOL8	-0.52374	2.828251	-2.84962	0.005445	-2.32015	0.095041	0.099923
Neutrophils	CSTDC6	-0.45197	-1.16323	-2.84808	0.005469	-2.30799	0.106708	0.111975
Neutrophils	SLC38A2	-0.23172	8.351985	-2.84787	0.005473	-2.97098	0.081371	0.085976
Neutrophils	UBE3A	0.289122	6.387077	2.846311	0.005497	-2.57406	0.086166	0.091083
Neutrophils	OAZ2	0.399238	4.485342	2.846225	0.005499	-2.36625	0.090957	0.096036
Neutrophils	EMID1	-1.24796	3.280299	-2.84553	0.00551	-2.42449	0.094173	0.099379
Neutrophils	BCL2A1B	0.488997	5.877283	2.843894	0.005536	-2.82092	0.087513	0.092807
Neutrophils	ZKSCAN14	0.722303	3.081299	2.84376	0.005538	-2.23705	0.094768	0.100318
Neutrophils	GM45353	-1.5109	-0.0712	-2.84336	0.005544	-2.68906	0.103692	0.10956
Neutrophils	SLFN2	0.391138	6.335056	2.843081	0.005548	-2.89591	0.086382	0.091684
Neutrophils	SLC2A6	0.755334	1.781297	2.841396	0.005575	-2.26021	0.098576	0.104381
Neutrophils	APOL7E	-1.6004	-0.10248	-2.84117	0.005579	-2.60529	0.104024	0.110008
Neutrophils	GCNT2	0.405439	4.580489	2.84051	0.00559	-2.7105	0.09104	0.09672
Neutrophils	PTK2B	0.362918	7.134222	2.839142	0.005612	-2.73361	0.084862	0.090299
Neutrophils	CSTDC5	-0.37099	5.46156	-2.83779	0.005633	-3.44509	0.089199	0.094858
Neutrophils	MIB2	-0.7975	2.662247	-2.83562	0.005668	-2.25056	0.097052	0.102851
Neutrophils	1810026B(	0.26923	6.570259	2.834065	0.005694	-2.6991	0.08703	0.092478
Neutrophils	ACOT11	-1.20612	1.943725	-2.83372	0.005699	-2.4777	0.09929	0.105268
Neutrophils	POFUT2	0.442173	4.257323	2.832054	0.005727	-2.41097	0.093247	0.09904
Neutrophils	UNKL	-0.77718	3.970057	-2.83081	0.005747	-2.25986	0.094013	0.100013
Neutrophils	GM13822	1.632198	1.17949	2.830807	0.005747	-2.29362	0.101804	0.108084
Neutrophils	SFXN5	0.40113	3.363367	2.830415	0.005754	-2.57699	0.095653	0.101766
Neutrophils	C2CD2	-0.85917	3.079676	-2.82792	0.005795	-2.27841	0.096968	0.102941
Neutrophils	SERPINC1	-0.69817	5.285455	-2.82665	0.005816	-2.57079	0.091251	0.097016
Neutrophils	NFATC3	-0.38732	7.290076	-2.82491	0.005845	-2.64709	0.086493	0.09198
Neutrophils	PNISR	0.293607	5.773836	2.822973	0.005877	-2.56715	0.09066	0.096271
Neutrophils	ATP6V0A1	0.410892	4.202735	2.821598	0.0059	-2.4987	0.095032	0.100795
Neutrophils	GM15336	0.876869	1.682133	2.820367	0.005921	-2.29654	0.102317	0.108349
Neutrophils	XBP1	0.340188	6.135195	2.818907	0.005946	-2.62397	0.090321	0.095981
Neutrophils	IGKV9-124	-1.55737	-1.01492	-2.81852	0.005953	-2.80161	0.110752	0.117115
Neutrophils	4933439C1	-1.57935	1.200712	-2.81708	0.005977	-2.63748	0.104121	0.110393
Neutrophils	CYP2D26	-1.13219	3.247258	-2.8169	0.00598	-2.30674	0.098213	0.104304
Neutrophils	CDA	-1.54572	0.894078	-2.81549	0.006004	-2.49164	0.105297	0.111535
Neutrophils	FKTN	-1.43364	1.595478	-2.81352	0.006038	-2.55616	0.103631	0.109805
Neutrophils	ORC3	0.493715	4.958044	2.810497	0.00609	-2.37145	0.094824	0.100628
Neutrophils	NMNAT1	-1.75677	1.326233	-2.80785	0.006136	-2.64835	0.105806	0.111692
Neutrophils	GCNT1	0.715958	2.451925	2.806116	0.006167	-2.31755	0.102687	0.108452
Neutrophils	SLFN1	0.609186	2.335521	2.805833	0.006172	-2.78782	0.103029	0.108804

Neutrophils	BATF	0.486761	4.183315	2.805459	0.006178	-2.45178	0.09774	0.103364
Neutrophils	DERL1	0.238909	6.22546	2.799019	0.006292	-2.71261	0.093783	0.098757
Neutrophils	EXOSC4	0.662174	3.784732	2.797643	0.006317	-2.34824	0.10058	0.105831
Neutrophils	RASAL2	-0.49276	5.468995	-2.79756	0.006318	-2.70389	0.095869	0.100983
Neutrophils	PIK3R3	-1.0358	3.43068	-2.79724	0.006324	-2.33724	0.1016	0.106935
Neutrophils	RAP1B	0.172173	8.543309	2.796468	0.006338	-3.22656	0.087917	0.092849
Neutrophils	HECA	0.286796	6.23169	2.794281	0.006378	-2.8349	0.09428	0.099484
Neutrophils	ADGRL2	-0.74137	4.208198	-2.79294	0.006402	-2.46882	0.099869	0.105403
Neutrophils	KDM7A	0.253216	7.129544	2.792867	0.006403	-3.08303	0.091906	0.09717
Neutrophils	LAG3	-1.64502	1.429969	-2.79283	0.006404	-2.61957	0.108106	0.113872
Neutrophils	VGLL4	-0.38079	6.477378	-2.79208	0.006418	-2.66396	0.093682	0.099016
Neutrophils	SGMS1	0.366301	7.102996	2.78978	0.00646	-2.97406	0.09237	0.097737
Neutrophils	ITIH4	-0.82186	3.988196	-2.78921	0.00647	-2.44739	0.100928	0.106634
Neutrophils	NEB	-1.49852	1.258562	-2.7892	0.00647	-2.63429	0.109102	0.115045
Neutrophils	CD320	-1.17915	1.856288	-2.78842	0.006485	-2.60149	0.107284	0.113256
Neutrophils	SIRPB1A	1.083216	-0.18195	2.787701	0.006498	-2.38009	0.113714	0.120033
Neutrophils	UGT2B34	-1.23555	1.748323	-2.78753	0.006501	-2.40936	0.107616	0.1138
Neutrophils	TMSB15B2	-1.11152	2.47439	-2.78538	0.006541	-2.45311	0.105895	0.111976
Neutrophils	CD247	-1.16689	3.01096	-2.7845	0.006557	-2.38934	0.10439	0.110466
Neutrophils	PIK3R5	0.495044	4.45137	2.782295	0.006598	-2.52481	0.10067	0.1066
Neutrophils	ITPKA	-1.69135	0.529225	-2.78122	0.006618	-2.75329	0.112765	0.119082
Neutrophils	GM14548	0.433862	1.47249	2.780644	0.006629	-2.67096	0.109785	0.116125
Neutrophils	DNAJC17	0.691048	3.568748	2.778718	0.006665	-2.39425	0.103822	0.109818
Neutrophils	XPO6	0.282036	5.572883	2.777252	0.006693	-2.77452	0.098321	0.104092
Neutrophils	ZSWIM7	0.75326	3.432711	2.776741	0.006702	-2.38144	0.104501	0.110503
Neutrophils	CLNK	-1.69648	1.404175	-2.77538	0.006728	-2.55387	0.110743	0.117118
Neutrophils	MARCKSL1	0.377885	7.792982	2.774789	0.006739	-3.23204	0.092325	0.098115
Neutrophils	CES1D	-1.15299	1.90006	-2.77468	0.006741	-2.39333	0.109186	0.115602
Neutrophils	TMCC3	-0.70019	5.475701	-2.77462	0.006743	-2.60615	0.098606	0.104653
Neutrophils	SULT2A8	-1.70838	0.571296	-2.76906	0.006849	-2.60408	0.114651	0.121119
Neutrophils	TCF21	-1.65871	0.477537	-2.76902	0.00685	-2.6849	0.114958	0.121435
Neutrophils	MANBAL	0.525272	4.531107	2.768609	0.006858	-2.41323	0.102403	0.108596
Neutrophils	ZFP955B	-1.35922	2.201747	-2.76824	0.006865	-2.65453	0.109436	0.115904
Neutrophils	MAFF	0.711639	3.876198	2.767912	0.006871	-2.47071	0.104332	0.11071
Neutrophils	SINHCAF	-0.67608	4.557551	-2.76766	0.006876	-2.40298	0.102326	0.10868
Neutrophils	PLAUR	0.332317	6.837467	2.766371	0.006901	-3.37895	0.096112	0.102168
Neutrophils	DCAF1	-0.59361	5.222364	-2.7656	0.006916	-2.45997	0.10067	0.10704
Neutrophils	GM20186	0.901011	3.559404	2.765198	0.006924	-2.40818	0.105554	0.112177
Neutrophils	CD46	0.911824	2.11028	2.763507	0.006957	-2.41902	0.110374	0.117177
Neutrophils	IER3IP1	0.248962	6.363638	2.761916	0.006989	-2.84149	0.098077	0.104373
Neutrophils	FAM111A	0.311192	5.742953	2.759205	0.007042	-2.91305	0.100443	0.106706
Neutrophils	4930526L0	-1.64108	-0.8055	-2.75851	0.007056	-2.84728	0.121135	0.128134
Neutrophils	ANP32A	0.168347	7.495527	2.756507	0.007096	-3.08404	0.096017	0.102053
Neutrophils	D130043K2	-1.42575	1.054045	-2.75397	0.007146	-2.63203	0.116011	0.122649
Neutrophils	FOXJ2	-0.60669	3.829076	-2.75262	0.007174	-2.48517	0.107231	0.113842
Neutrophils	GULO	-1.21603	1.982596	-2.7521	0.007184	-2.49044	0.11303	0.119936
Neutrophils	MFSD14A	0.267408	6.156841	2.751264	0.007201	-2.8374	0.100356	0.106809

Neutrophils	BCL6	0.439125	5.992679	2.751245	0.007201	-2.96936	0.100825	0.107299
Neutrophils	GOLPH3	0.242551	5.902166	2.751008	0.007206	-2.85994	0.101085	0.107601
Neutrophils	KCNJ10	-1.35176	-0.1202	-2.75032	0.00722	-2.72743	0.120025	0.127307
Neutrophils	CD226	-1.59066	1.697205	-2.75005	0.007226	-2.52529	0.113955	0.121108
Neutrophils	ETF1	0.231772	7.536791	2.749321	0.00724	-3.14295	0.096499	0.102987
Neutrophils	ASTE1	-0.94153	2.753642	-2.74912	0.007245	-2.49839	0.11057	0.1177
Neutrophils	ABI2	-0.76643	3.903545	-2.74838	0.007259	-2.448	0.107003	0.114146
Neutrophils	SLC35A4	0.726853	3.561213	2.748238	0.007262	-2.44422	0.108053	0.115245
Neutrophils	ZFP131	-0.29157	5.815875	-2.74625	0.007303	-2.9046	0.101756	0.108599
Neutrophils	SERPINF2	-0.9078	3.929119	-2.74395	0.00735	-2.50978	0.107914	0.114914
Neutrophils	NEAT1	0.306086	8.674976	2.742875	0.007372	-3.51805	0.094453	0.100908
Neutrophils	PLEKHF2	0.33298	5.470642	2.74217	0.007387	-2.73632	0.103506	0.110413
Neutrophils	LRP10	0.363371	6.125795	2.741127	0.007409	-2.77301	0.10175	0.108554
Neutrophils	TNFRSF13C	-1.03151	3.92598	-2.73805	0.007473	-2.49241	0.109001	0.116074
Neutrophils	CSF2RB2	0.931708	2.359571	2.737206	0.00749	-2.46846	0.113981	0.121444
Neutrophils	HSPG2	-0.59861	2.741833	-2.73713	0.007492	-2.55108	0.112745	0.120165
Neutrophils	SMDT1	0.246155	7.773748	2.736911	0.007497	-3.09083	0.097705	0.104505
Neutrophils	BLK	-0.91899	4.074052	-2.73338	0.007571	-2.47753	0.109466	0.116311
Neutrophils	CHD1	0.239209	6.90203	2.732038	0.007599	-3.02661	0.101225	0.10773
Neutrophils	NEK6	0.499904	3.873937	2.731575	0.007609	-2.59571	0.110332	0.117264
Neutrophils	TANC2	-0.67335	5.521032	-2.73023	0.007638	-2.62529	0.105277	0.112299
Neutrophils	PIRB	0.41753	4.941687	2.729836	0.007646	-3.10083	0.107027	0.114164
Neutrophils	DNAJC8	0.204594	6.901934	2.729747	0.007648	-3.00878	0.101225	0.108108
Neutrophils	FKBP5	0.483811	5.30259	2.729634	0.007651	-2.82331	0.105933	0.113035
Neutrophils	SIRPB1B	0.495745	1.445504	2.728015	0.007685	-2.8861	0.11862	0.126197
Neutrophils	RAB32	0.33754	4.344985	2.726462	0.007719	-2.91141	0.109395	0.116723
Neutrophils	GSR	0.239936	6.441975	2.726404	0.00772	-3.45721	0.103059	0.110103
Neutrophils	KLRC3	-1.36683	-1.20661	-2.72569	0.007735	-2.8933	0.128257	0.136343
Neutrophils	STT3A	-0.30916	6.169392	-2.7244	0.007763	-2.80537	0.1041	0.111372
Neutrophils	DMPK	-0.90658	2.786398	-2.72411	0.00777	-2.50594	0.114629	0.122407
Neutrophils	BAZ1A	0.258812	7.694958	2.722939	0.007795	-3.17367	0.099876	0.106988
Neutrophils	STFA2L1	-0.36544	2.448793	-2.72045	0.007849	-3.32463	0.116388	0.124434
Neutrophils	GNAQ	-0.24993	7.882192	-2.72015	0.007856	-3.36175	0.099715	0.10702
Neutrophils	GRAMD3	-0.74508	6.080829	-2.72015	0.007856	-2.58559	0.104947	0.112523
Neutrophils	MTTP	-0.99058	2.566189	-2.71871	0.007887	-2.52312	0.116305	0.124478
Neutrophils	SREBF2	0.387397	5.9605	2.716893	0.007927	-2.84578	0.105962	0.113596
Neutrophils	VPS25	-1.32336	0.881521	-2.71646	0.007937	-2.735	0.12247	0.130925
Neutrophils	SRXN1	0.924468	1.094676	2.715509	0.007958	-2.52841	0.121885	0.130335
Neutrophils	HIF1A	0.340703	6.608469	2.714106	0.007989	-3.02678	0.10443	0.112104
Neutrophils	PTEN	0.228904	8.288084	2.713096	0.008012	-3.26077	0.099716	0.107157
Neutrophils	4833407H1	0.438626	2.098821	2.711928	0.008038	-2.65337	0.119143	0.127481
Neutrophils	GM1043	-1.97304	1.915295	-2.71067	0.008066	-2.81144	0.120027	0.128367
Neutrophils	CMAS	0.461844	5.862643	2.708696	0.00811	-2.76405	0.107513	0.115413
Neutrophils	FOXJ3	-0.36258	5.947043	-2.70752	0.008137	-2.73023	0.107255	0.1154
Neutrophils	APOB	-0.83617	4.836413	-2.70715	0.008145	-2.78251	0.110699	0.119126
Neutrophils	TMEM208	-0.38966	4.89435	-2.7069	0.008151	-2.6502	0.110516	0.118957
Neutrophils	PFKL	0.455289	5.330071	2.706749	0.008154	-2.74822	0.109154	0.117522

Neutrophils	MPP7	0.417209	7.383215	2.706374	0.008163	-3.47102	0.102968	0.111041
Neutrophils	GPX1	0.368211	11.17498	2.706186	0.008167	-3.74085	0.092498	0.099945
Neutrophils	E2F5	-1.09094	2.69996	-2.70575	0.008177	-2.70619	0.117649	0.126612
Neutrophils	FARP2	-1.04917	2.6131	-2.70535	0.008186	-2.5752	0.117941	0.126957
Neutrophils	CCDC85C	-1.38985	0.977037	-2.70501	0.008194	-2.80561	0.12358	0.132918
Neutrophils	UBE2O	0.840979	4.874388	2.704493	0.008205	-2.57647	0.11059	0.119371
Neutrophils	FAM122A	0.475994	3.563965	2.702994	0.00824	-2.63476	0.115022	0.124057
Neutrophils	SRGAP3	-1.51391	4.261174	-2.70283	0.008243	-2.61931	0.112759	0.121673
Neutrophils	CEP170	0.419048	5.909007	2.701083	0.008283	-2.79042	0.107842	0.116462
Neutrophils	CMAH	0.683739	5.561409	2.70074	0.008291	-2.73081	0.108914	0.117673
Neutrophils	PRKAG1	0.24521	5.515312	2.700344	0.0083	-2.97674	0.109057	0.117854
Neutrophils	ZDHHC6	0.446982	4.609891	2.700097	0.008306	-2.68057	0.111904	0.120904
Neutrophils	SRGAP1	-0.73058	2.559229	-2.69868	0.008339	-2.60471	0.118815	0.12844
Neutrophils	HDC	0.504945	3.642871	2.698474	0.008344	-3.31131	0.115199	0.124625
Neutrophils	CD2	-1.1211	3.660392	-2.69815	0.008351	-2.58949	0.115142	0.124565
Neutrophils	GM4631	-1.49961	0.664941	-2.69735	0.00837	-2.91618	0.125531	0.135578
Neutrophils	CREBL2	0.529651	3.233581	2.696131	0.008398	-2.65275	0.116758	0.126563
Neutrophils	LY6D	-0.84923	5.594266	-2.69572	0.008407	-2.63594	0.109165	0.118588
Neutrophils	FOXP1	-0.2382	9.650428	-2.69562	0.00841	-3.39308	0.097303	0.105929
Neutrophils	AMN1	0.47353	3.42505	2.692842	0.008475	-2.67253	0.116868	0.126573
Neutrophils	DDX58	0.524641	4.759373	2.691913	0.008497	-2.78874	0.112598	0.122139
Neutrophils	SOCS4	0.42778	4.684801	2.69145	0.008507	-2.68666	0.112837	0.122436
Neutrophils	DUS3L	-0.81683	3.300011	-2.69106	0.008517	-2.59834	0.11738	0.127299
Neutrophils	CFI	-0.90474	3.743943	-2.69068	0.008526	-2.65781	0.115903	0.125816
Neutrophils	BFSP2	-1.39592	2.440103	-2.68795	0.00859	-2.68282	0.121048	0.131067
Neutrophils	ACAT1	-0.51726	6.480379	-2.68549	0.008649	-3.0018	0.108271	0.1175
Neutrophils	RUBCN	-0.82139	3.888841	-2.68537	0.008652	-2.58788	0.116558	0.12629
Neutrophils	TCRG-C2	-2.14327	0.685448	-2.68494	0.008662	-2.74902	0.127715	0.138143
Neutrophils	USP4	0.258177	5.660629	2.684685	0.008668	-2.89526	0.110824	0.120319
Neutrophils	TCRG-C1	-1.91039	0.880703	-2.68396	0.008685	-2.76322	0.127005	0.137616
Neutrophils	G0S2	0.292049	3.133285	2.683882	0.008687	-3.24217	0.119096	0.129261
Neutrophils	PKM	0.311187	8.60779	2.682486	0.008721	-3.55156	0.102193	0.111346
Neutrophils	TPBGL	-1.24	0.559425	-2.68151	0.008744	-2.64111	0.128687	0.139528
Neutrophils	POU2AF1	-0.97671	4.320007	-2.68013	0.008778	-2.60055	0.115887	0.125944
Neutrophils	GM36660	1.182588	-0.14918	2.678499	0.008817	-2.71353	0.132077	0.143061
Neutrophils	PIK3AP1	0.366988	8.295612	2.677162	0.00885	-3.45464	0.104093	0.113437
Neutrophils	NAB1	0.257442	6.555305	2.676542	0.008865	-3.23868	0.109409	0.119208
Neutrophils	WDR20	0.344312	5.843767	2.675266	0.008896	-2.86743	0.111855	0.121868
Neutrophils	ARIH2	0.241212	7.527213	2.674934	0.008904	-3.344	0.106633	0.11631
Neutrophils	MOB3A	0.410177	4.775701	2.671599	0.008986	-2.83341	0.116224	0.126316
Neutrophils	GM20234	0.811889	2.059362	2.669724	0.009033	-2.61798	0.126002	0.136597
Neutrophils	HIGD1A	0.357808	6.029655	2.669469	0.009039	-2.95265	0.112524	0.122369
Neutrophils	VEGFC	1.31098	1.200834	2.667914	0.009078	-2.73201	0.129385	0.140351
Neutrophils	MTMR6	0.330694	4.718718	2.66783	0.00908	-2.97236	0.117033	0.127267
Neutrophils	MAP2K1	0.298895	6.311963	2.666926	0.009103	-3.14786	0.111984	0.121909
Neutrophils	MYO9B	0.338703	5.866728	2.665793	0.009131	-2.98587	0.113621	0.123633
Neutrophils	SEMA4A	0.355918	3.364224	2.66491	0.009153	-3.05394	0.122157	0.132722

Neutrophils	LPXN	-0.71706	5.23201	-2.66442	0.009166	-2.64335	0.115838	0.126087
Neutrophils	CYTIP	0.320198	8.087657	2.663688	0.009184	-3.36843	0.106893	0.116628
Neutrophils	BDH1	-0.86969	3.282333	-2.66318	0.009197	-2.63071	0.122565	0.133458
Neutrophils	6030468B1	-1.85305	0.279697	-2.6611	0.00925	-2.91901	0.134128	0.145615
Neutrophils	FFAR4	1.52055	0.260828	2.660401	0.009267	-2.8237	0.134291	0.145841
Neutrophils	IQCB1	-0.63612	4.824078	-2.65939	0.009293	-2.64422	0.117995	0.128692
Neutrophils	PRKCA	-0.64187	7.401898	-2.65919	0.009298	-2.89252	0.109659	0.119825
Neutrophils	MET	1.095226	1.991608	2.657825	0.009333	-2.63909	0.128242	0.139483
Neutrophils	APTX	0.666571	3.052742	2.65655	0.009366	-2.64685	0.124667	0.13566
Neutrophils	LAIR1	0.731512	4.246709	2.655619	0.00939	-2.8642	0.120495	0.131438
Neutrophils	MICAL1	0.494659	3.487265	2.655595	0.00939	-2.73406	0.123132	0.134246
Neutrophils	CEP83	0.296009	5.662564	2.655283	0.009398	-3.05375	0.115735	0.126389
Neutrophils	POM121	-0.45748	4.883475	-2.65364	0.009441	-2.73086	0.118583	0.129559
Neutrophils	PTK2	-0.54013	4.164046	-2.6536	0.009442	-2.80094	0.121038	0.132179
Neutrophils	PER1	0.390585	4.926726	2.652863	0.009461	-2.98478	0.11853	0.129518
Neutrophils	NDUFS8	-0.33611	6.232258	-2.65203	0.009483	-2.95899	0.114331	0.125072
Neutrophils	PIP4K2A	0.350032	7.239298	2.650648	0.009519	-3.18024	0.11139	0.121859
Neutrophils	NUPR1	1.626718	2.41246	2.649444	0.00955	-2.66315	0.128062	0.139551
Neutrophils	BCLAF1	0.235604	7.155376	2.645472	0.009654	-3.18282	0.11297	0.123082
Neutrophils	TMEM219	0.43407	4.624458	2.643705	0.009701	-2.82956	0.121524	0.132492
Neutrophils	NDUFB9	0.273598	7.069533	2.643703	0.009701	-3.2518	0.113362	0.123773
Neutrophils	METTL7B	-1.28051	2.263028	-2.64358	0.009704	-2.66989	0.129988	0.141502
Neutrophils	SOCS3	0.654309	4.698376	2.643178	0.009715	-2.89935	0.121269	0.132301
Neutrophils	TRBC2	-1.41527	3.943953	-2.64275	0.009726	-2.70519	0.123903	0.135142
Neutrophils	NTM	1.379893	0.913874	2.642317	0.009738	-2.78929	0.135093	0.14715
Neutrophils	UGT2B36	-1.0687	2.198922	-2.64185	0.00975	-2.67732	0.130226	0.142077
Neutrophils	BMP6	-1.70461	0.450706	-2.64141	0.009762	-2.95969	0.136891	0.149282
Neutrophils	TGFBRAP1	0.600764	3.494101	2.641078	0.009771	-2.67638	0.125502	0.137203
Neutrophils	PCTP	-0.85198	2.252585	-2.63989	0.009803	-2.68455	0.130148	0.142271
Neutrophils	FADS2	-1.01493	3.530927	-2.63985	0.009804	-2.68143	0.125487	0.1373
Neutrophils	PLXNA2	-0.79552	2.31233	-2.63812	0.00985	-2.71654	0.130384	0.142313
Neutrophils	RBPJ	0.459006	6.055966	2.637619	0.009864	-3.18703	0.117212	0.12834
Neutrophils	AMY1	-0.93474	2.627056	-2.63696	0.009882	-2.68459	0.129316	0.141307
Neutrophils	TRIM8	0.335606	5.616506	2.63489	0.009937	-3.02453	0.119285	0.130329
Neutrophils	CPEB4	-0.27859	6.51399	-2.6341	0.009959	-3.34969	0.116389	0.12722
Neutrophils	RNF13	0.268184	6.065838	2.632294	0.010008	-3.24232	0.118143	0.129191
Neutrophils	A930001A2	-1.70342	-0.71301	-2.63203	0.010015	-3.00957	0.143341	0.156027
Neutrophils	FOXN2	0.25682	6.146142	2.631545	0.010028	-3.18085	0.117873	0.12899
Neutrophils	CD160	-1.66139	1.916487	-2.63052	0.010057	-2.77507	0.132971	0.145333
Neutrophils	LARS2	-0.70992	5.571824	-2.63047	0.010058	-3.00599	0.119814	0.131276
Neutrophils	UCP2	0.314879	9.571709	2.630376	0.01006	-3.58284	0.106965	0.117447
Neutrophils	CD3D	-0.78299	2.678526	-2.62922	0.010092	-2.7683	0.13011	0.142645
Neutrophils	TNRC18	0.224089	5.931838	2.628889	0.010101	-3.25936	0.118594	0.130352
Neutrophils	KPNA6	-0.66769	3.896998	-2.62856	0.01011	-2.70076	0.125667	0.137995
Neutrophils	TRIM30D	0.571401	4.544173	2.62854	0.010111	-3.00371	0.123371	0.135531
Neutrophils	EIF4EBP1	0.266576	5.454955	2.628462	0.010113	-3.2069	0.120213	0.132145
Neutrophils	PPP1R3E	-1.18479	1.031561	-2.6262	0.010176	-2.93917	0.137054	0.149839

Neutrophils	ALDH7A1	-0.88857	3.227154	-2.62476	0.010215	-2.70944	0.128957	0.141198
Neutrophils	NINJ1	0.370164	5.750418	2.624676	0.010218	-3.27849	0.120014	0.131621
Neutrophils	TMEM69	1.239943	1.585462	2.623514	0.01025	-2.83496	0.135409	0.14801
Neutrophils	TNFRSF19	-1.66381	0.886333	-2.62305	0.010263	-2.99006	0.138149	0.151027
Neutrophils	TPD52	0.272288	8.000639	2.622135	0.010289	-3.71514	0.112966	0.124141
Neutrophils	TARM1	0.580916	-0.18149	2.621231	0.010314	-2.88861	0.142798	0.156079
Neutrophils	KRT10	-1.28715	2.100333	-2.62008	0.010346	-2.92243	0.13405	0.146806
Neutrophils	LPCAT3	0.444484	5.162712	2.617548	0.010418	-2.91244	0.123469	0.135342
Neutrophils	PTPN1	0.300224	7.078745	2.617351	0.010423	-3.36887	0.116924	0.128321
Neutrophils	SBDS	0.328054	5.561152	2.61678	0.010439	-2.96495	0.122123	0.133963
Neutrophils	DISP1	-0.77641	3.443949	-2.61576	0.010468	-2.73018	0.129919	0.142335
Neutrophils	SLC44A1	0.368241	4.637679	2.614583	0.010502	-3.0858	0.12583	0.137978
Neutrophils	SLC5A3	-0.94917	3.816287	-2.61402	0.010518	-2.73358	0.128854	0.141254
Neutrophils	UBE2L6	0.874653	4.64931	2.61321	0.010541	-2.86593	0.125959	0.138258
Neutrophils	BCL10	0.221772	6.452929	2.612791	0.010553	-3.37349	0.11966	0.131551
Neutrophils	GNG12	0.248307	6.49518	2.611213	0.010598	-3.42986	0.119889	0.131703
Neutrophils	PZP	-0.8009	4.39586	-2.60907	0.01066	-2.93944	0.127861	0.14006
Neutrophils	PTP4A1	1.226293	1.179279	2.608031	0.01069	-2.79883	0.140379	0.153451
Neutrophils	ZFP410	-0.60592	4.138179	-2.60598	0.010749	-2.75608	0.129581	0.141734
Neutrophils	GATA3	-1.39405	1.116393	-2.60446	0.010794	-2.87407	0.141604	0.154545
Neutrophils	RGN	-0.79393	4.650255	-2.6042	0.010801	-3.02084	0.128027	0.140109
Neutrophils	RHOV	1.204106	-0.80702	2.603758	0.010814	-2.76395	0.149606	0.163136
Neutrophils	PLXND1	-0.44949	3.5086	-2.60288	0.01084	-2.89165	0.132425	0.144928
Neutrophils	GSK3A	0.306915	5.557032	2.602298	0.010857	-3.11041	0.124963	0.136967
Neutrophils	GM38394	-1.51846	0.848639	-2.6019	0.010869	-3.00858	0.142918	0.156209
Neutrophils	XPC	0.422589	3.385386	2.601132	0.010891	-2.91105	0.132953	0.145786
Neutrophils	SELENBP2	-1.43696	1.116284	-2.6008	0.010901	-2.87004	0.141847	0.155342
Neutrophils	4930523C0	0.26709	6.126408	2.600401	0.010913	-3.29901	0.122971	0.135148
Neutrophils	DNMT3B	-1.01547	2.497904	-2.60016	0.01092	-2.84483	0.136362	0.14951
Neutrophils	CD209F	-2.03644	2.767776	-2.59789	0.010987	-2.77837	0.135872	0.148816
Neutrophils	CAST	0.215547	6.266263	2.597791	0.01099	-3.40376	0.122987	0.135008
Neutrophils	4930486L2	-1.45198	0.484692	-2.59711	0.01101	-2.94618	0.145107	0.158705
Neutrophils	FERMT3	0.251982	7.235899	2.59666	0.011024	-3.47276	0.119716	0.13156
Neutrophils	PRODH2	-1.2114	2.255274	-2.59631	0.011034	-2.77245	0.137954	0.151141
Neutrophils	TENM3	-1.29474	1.688761	-2.59539	0.011062	-2.82718	0.140357	0.153587
Neutrophils	PDLIM1	-0.78964	4.944927	-2.59506	0.011072	-2.77882	0.127912	0.140341
Neutrophils	SESN3	-0.70369	4.83647	-2.59436	0.011092	-2.78417	0.128402	0.140944
Neutrophils	ISCU	0.294249	7.465509	2.591394	0.011181	-3.4172	0.119978	0.131717
Neutrophils	PDE4C	-1.14821	3.131277	-2.59015	0.011219	-2.81551	0.135925	0.148885
Neutrophils	TKTL1	-0.89457	1.290296	-2.58994	0.011225	-2.7885	0.143257	0.156771
Neutrophils	TIMD4	-0.83821	3.658253	-2.58959	0.011236	-2.90854	0.133898	0.146811
Neutrophils	NFE2L2	0.296205	7.355477	2.589165	0.011249	-3.57596	0.120531	0.132563
Neutrophils	GM44699	1.370069	0.559613	2.587956	0.011286	-3.00688	0.146591	0.160559
Neutrophils	ZFP607A	-1.14061	1.840055	-2.58677	0.011322	-2.95722	0.141621	0.155149
Neutrophils	2310016D2	-1.64938	-0.3578	-2.58509	0.011373	-3.05274	0.151308	0.165389
Neutrophils	CFH	-0.64116	4.930751	-2.58368	0.011416	-3.02168	0.130465	0.143107
Neutrophils	GLYCTK	-1.26179	0.979545	-2.58208	0.011466	-2.91301	0.146492	0.160091

Neutrophils	AGBL1	-1.20747	4.663698	-2.58059	0.011512	-2.82467	0.13226	0.144716
Neutrophils	ZC3H12A	0.367138	5.128189	2.579315	0.011551	-3.18264	0.13081	0.143076
Neutrophils	PKNOX2	-0.87502	-0.82371	-2.57893	0.011563	-2.80485	0.155022	0.168887
Neutrophils	GM9929	-1.17605	1.684976	-2.57846	0.011578	-2.95073	0.144325	0.157568
Neutrophils	TACC2	-0.80787	3.088667	-2.57729	0.011614	-2.80709	0.13894	0.151758
Neutrophils	TULP4	-0.46011	5.928036	-2.5756	0.011667	-2.99469	0.128462	0.14067
Neutrophils	IER5L	-0.98164	2.89009	-2.57554	0.011669	-2.83777	0.140073	0.153084
Neutrophils	GORASP2	0.32168	5.534187	2.575054	0.011684	-3.03402	0.129935	0.142347
Neutrophils	CTSG	1.672975	-0.2691	2.574424	0.011704	-2.81291	0.153415	0.167437
Neutrophils	GM11084	0.683181	3.368087	2.570894	0.011815	-2.82034	0.139305	0.15211
Neutrophils	TRIM25	0.429447	6.978283	2.570857	0.011816	-3.3147	0.125703	0.137566
Neutrophils	ZFPM1	-0.80465	4.248218	-2.56921	0.011869	-2.82455	0.136304	0.148893
Neutrophils	HCK	0.343259	5.693071	2.566308	0.011961	-3.50538	0.131586	0.143682
Neutrophils	SLA2	-1.0928	2.746588	-2.56618	0.011966	-2.87326	0.143109	0.155994
Neutrophils	ATP6V1G1	0.208914	7.177612	2.564562	0.012018	-3.58437	0.126448	0.138292
Neutrophils	PUM3	0.534104	4.640352	2.564442	0.012021	-2.86904	0.135907	0.148419
Neutrophils	LTBR	0.494148	2.974023	2.563851	0.01204	-2.95331	0.142518	0.155644
Neutrophils	ZAP70	-1.21098	1.982594	-2.5635	0.012052	-2.86301	0.146607	0.160059
Neutrophils	GPR137B	0.421003	5.316315	2.563242	0.01206	-3.19161	0.133317	0.145932
Neutrophils	IFITM10	-0.78502	3.144158	-2.5628	0.012074	-3.01116	0.141841	0.155123
Neutrophils	GM20536	0.676548	2.340392	2.561978	0.012101	-2.84881	0.14529	0.158887
Neutrophils	TRMT10B	1.098498	1.681609	2.558036	0.012229	-2.92453	0.149453	0.16277
Neutrophils	BCL11A	-0.61153	5.196907	-2.55546	0.012314	-3.04232	0.135986	0.14825
Neutrophils	PYGB	-0.60596	4.748164	-2.5542	0.012355	-2.86311	0.138046	0.150371
Neutrophils	TRAK1	-0.30476	7.291138	-2.55305	0.012393	-3.30994	0.128673	0.140276
Neutrophils	GCLM	0.374752	6.090034	2.552039	0.012427	-3.30843	0.133352	0.145213
Neutrophils	MARCKS	0.328698	7.242888	2.550134	0.01249	-3.56893	0.129541	0.140855
Neutrophils	TELO2	-1.46205	2.455163	-2.54982	0.012501	-3.02071	0.148456	0.161002
Neutrophils	DNAJC18	-0.79506	3.682567	-2.54849	0.012545	-2.86739	0.143702	0.1559
Neutrophils	GZMA	-1.67944	4.671597	-2.54696	0.012596	-3.04523	0.140043	0.152004
Neutrophils	INTS14	-0.41274	4.691853	-2.54678	0.012602	-3.28315	0.139962	0.151918
Neutrophils	ARF4	0.258522	8.479652	2.545867	0.012633	-3.67572	0.125775	0.13681
Neutrophils	GINS1	1.00692	3.197409	2.545694	0.012639	-2.87124	0.146156	0.15848
Neutrophils	FBXL15	0.84724	2.696607	2.544307	0.012685	-2.88284	0.14848	0.161018
Neutrophils	PARVB	-0.88061	2.578711	-2.54413	0.012691	-2.87375	0.148981	0.161547
Neutrophils	SCO1	1.008737	1.930574	2.543916	0.012699	-2.90337	0.151762	0.164485
Neutrophils	CWC27	-0.39045	5.662216	-2.54148	0.012781	-3.11126	0.137189	0.148743
Neutrophils	STYK1	-1.11191	0.538888	-2.54027	0.012822	-2.91629	0.158889	0.17183
Neutrophils	KLHL14	-1.30446	2.717642	-2.53955	0.012847	-2.91716	0.149307	0.161833
Neutrophils	ZFYVE28	-1.28803	0.624372	-2.53953	0.012848	-3.14942	0.158501	0.171528
Neutrophils	ZFP687	0.587791	2.919042	2.539216	0.012859	-2.90191	0.148452	0.160929
Neutrophils	SLC25A33	-0.34608	3.983563	-2.53912	0.012862	-3.27516	0.144013	0.156243
Neutrophils	IFNG	-1.6143	1.241502	-2.53848	0.012884	-2.94382	0.155732	0.168798
Neutrophils	CPSF4L	-1.29538	0.499907	-2.53778	0.012908	-3.10106	0.159066	0.172521
Neutrophils	TUSC2	0.793392	3.017242	2.537587	0.012914	-2.88802	0.148036	0.160862
Neutrophils	EHBP1L1	0.304433	6.149657	2.537567	0.012915	-3.32358	0.135402	0.14743
Neutrophils	PPP1R9A	-0.65419	3.383193	-2.53669	0.012945	-3.0076	0.146681	0.159338

Neutrophils	PADI6	-1.65923	0.058128	-2.53531	0.012993	-3.10307	0.161286	0.175229
Neutrophils	WIPF1	0.273978	7.425545	2.535144	0.012998	-3.66927	0.130746	0.142782
Neutrophils	APON	1.289583	1.166034	2.534962	0.013005	-2.92362	0.156262	0.169962
Neutrophils	ENDOU	-1.4155	0.913658	-2.53473	0.013013	-3.00591	0.157392	0.171217
Neutrophils	AFG1L	-0.84825	3.691074	-2.53434	0.013026	-2.89427	0.145399	0.158585
Neutrophils	IL1RN	0.545246	2.526967	2.534296	0.013028	-3.47921	0.150308	0.163806
Neutrophils	IFITM6	0.317257	3.518134	2.533676	0.013049	-3.83552	0.146183	0.159459
Neutrophils	WSB2	0.413677	4.493162	2.533286	0.013063	-3.1057	0.142177	0.155265
Neutrophils	GM15472	-1.24336	1.795978	-2.53293	0.013075	-2.9333	0.153545	0.16743
Neutrophils	ADIPOR2	0.273697	6.368043	2.531831	0.013113	-3.42126	0.134953	0.147695
Neutrophils	SH3BP1	0.350162	5.21446	2.531622	0.013121	-3.28476	0.139453	0.152579
Neutrophils	GM30211	-1.28653	4.041214	-2.53106	0.01314	-2.8993	0.144192	0.157781
Neutrophils	HPCAL1	0.259176	6.117607	2.529898	0.013181	-3.54297	0.135917	0.149215
Neutrophils	NEK11	-1.29993	0.082898	-2.52948	0.013195	-3.02291	0.161436	0.176535
Neutrophils	GOSR1	0.466394	4.182554	2.529106	0.013209	-2.98594	0.143612	0.157518
Neutrophils	NARF	0.360048	4.893244	2.528993	0.013212	-3.21516	0.140734	0.154432
Neutrophils	CD300LF	0.350955	3.385271	2.528592	0.013227	-3.51835	0.146913	0.161096
Neutrophils	NEIL1	-1.18838	3.000738	-2.52859	0.013227	-2.99699	0.148533	0.162828
Neutrophils	PYGL	0.271803	5.16283	2.52852	0.013229	-3.76435	0.139659	0.153319
Neutrophils	NXF7	-1.44113	-1.34285	-2.52733	0.013271	-3.21736	0.168502	0.183951
Neutrophils	CCNB1IP1	-0.97687	1.863098	-2.52541	0.013338	-2.94568	0.154308	0.16874
Neutrophils	SUPT5	-0.32678	6.05341	-2.5252	0.013346	-3.26607	0.13694	0.150233
Neutrophils	TRGV2	-1.69312	-0.51519	-2.52411	0.013384	-3.10927	0.165455	0.180559
Neutrophils	TM2D2	0.371387	5.178235	2.523396	0.01341	-3.13891	0.14077	0.154227
Neutrophils	KCTD9	0.607292	3.130956	2.519204	0.01356	-2.93967	0.150608	0.164121
Neutrophils	GM16364.1	-1.60753	1.279847	-2.51876	0.013576	-3.08155	0.158778	0.172814
Neutrophils	WWC2	-0.4463	5.107898	-2.51874	0.013576	-3.14364	0.142358	0.155356
Neutrophils	F12	-1.21413	1.691224	-2.51817	0.013597	-2.93608	0.156998	0.170945
Neutrophils	C1GALT1C1	0.51899	4.044817	2.517388	0.013625	-3.01622	0.146862	0.160316
Neutrophils	EIF4EBP3	0.838075	2.80817	2.517234	0.01363	-2.92667	0.152132	0.16597
Neutrophils	CSF2RB	0.40144	4.838003	2.516211	0.013667	-3.44645	0.14382	0.157088
Neutrophils	ATF4	-0.30894	6.443614	-2.51456	0.013727	-3.51282	0.137797	0.150638
Neutrophils	CAR2	0.780537	5.171918	2.514151	0.013742	-3.13792	0.142871	0.15619
Neutrophils	C1GALT1	-0.40125	6.353776	-2.51395	0.013749	-3.28849	0.138149	0.15118
Neutrophils	ANGPTL8	-1.10094	1.825356	-2.51335	0.013771	-2.93563	0.157261	0.17168
Neutrophils	AADAC	-1.01377	2.701819	-2.51237	0.013807	-2.95662	0.153615	0.167806
Neutrophils	RIPOR2	0.280885	7.617108	2.511815	0.013827	-3.84033	0.133622	0.14646
Neutrophils	TPM3	0.148984	8.661561	2.51077	0.013865	-3.8702	0.12995	0.142517
Neutrophils	COG4	0.395454	5.336083	2.508512	0.013948	-3.13672	0.143399	0.156908
Neutrophils	SLC11A1	0.429931	3.505984	2.508448	0.01395	-3.43564	0.151073	0.165109
Neutrophils	BLCAP	0.502611	3.009547	2.506279	0.01403	-3.03352	0.153949	0.168002
Neutrophils	NRP2	-0.6996	3.300945	-2.50482	0.014084	-3.13248	0.153106	0.16698
Neutrophils	E130308A1	-0.55026	4.522919	-2.50443	0.014099	-3.01139	0.147866	0.161471
Neutrophils	GM17056	0.612392	2.866748	2.503902	0.014119	-3.11799	0.155073	0.169239
Neutrophils	TM6SF1	0.30583	6.252773	2.502263	0.01418	-3.56774	0.141283	0.154458
Neutrophils	SLCO4A1	-0.88896	3.403213	-2.5016	0.014204	-2.9742	0.153323	0.167368
Neutrophils	SMYD4	1.213611	2.820357	2.501243	0.014218	-3.01987	0.155893	0.170164

Neutrophils	ZBTB37	-0.67664	2.901016	-2.5	0.014264	-2.96805	0.155884	0.17002
Neutrophils	SULT2A5	-1.16832	2.02078	-2.49935	0.014289	-2.96436	0.159901	0.174316
Neutrophils	NUP214	0.441876	5.2678	2.49911	0.014298	-3.08867	0.145765	0.159294
Neutrophils	ADORA3	1.215033	-0.30137	2.498096	0.014336	-3.05051	0.171011	0.186124
Neutrophils	TGFBI	0.425041	4.412001	2.498014	0.014339	-3.5055	0.149489	0.163266
Neutrophils	MPLKIP	0.370042	4.793719	2.4974	0.014362	-3.11461	0.147962	0.161668
Neutrophils	AU019990	0.571167	1.211418	2.495988	0.014416	-3.16882	0.164178	0.179085
Neutrophils	ZFP943	0.401382	4.280266	2.495187	0.014446	-3.08739	0.150416	0.164606
Neutrophils	PARP2	0.486177	4.841287	2.495128	0.014448	-3.02208	0.148032	0.162059
Neutrophils	CAMKK1	1.171262	0.590902	2.494987	0.014454	-3.04164	0.167113	0.182439
Neutrophils	PPME1	0.419193	4.862972	2.494692	0.014465	-3.13968	0.14794	0.162031
Neutrophils	XRCC6	-0.72915	5.144906	-2.49441	0.014476	-3.04819	0.146758	0.16083
Neutrophils	IFITM3	0.461571	7.709419	2.493874	0.014496	-3.97191	0.136499	0.1499
Neutrophils	GM35769	0.853483	1.096204	2.492892	0.014534	-2.97451	0.16488	0.180372
Neutrophils	RBM47	0.349308	6.404832	2.492891	0.014534	-3.64142	0.14173	0.155604
Neutrophils	GIMAP4	-0.7247	4.091849	-2.49219	0.01456	-3.0938	0.151392	0.166063
Neutrophils	DECR2	-0.86748	2.741577	-2.49176	0.014577	-2.97838	0.157334	0.172465
Neutrophils	KRT222	-1.56506	-0.05515	-2.4917	0.014579	-3.22855	0.170412	0.186403
Neutrophils	GIMAP1	-0.70251	4.06141	-2.49105	0.014604	-2.98807	0.151632	0.166394
Neutrophils	MZB1	-0.79064	5.956787	-2.4906	0.014621	-3.11919	0.143693	0.157924
Neutrophils	RP9	0.194489	6.834563	2.489245	0.014673	-3.648	0.140384	0.154241
Neutrophils	PNRC1	0.222742	8.470962	2.489217	0.014674	-3.93276	0.134018	0.147374
Neutrophils	ITGA9	-0.55007	5.58915	-2.48838	0.014707	-3.4112	0.145619	0.159899
Neutrophils	HCAR2	0.474554	2.377294	2.487757	0.014731	-3.60505	0.159677	0.174954
Neutrophils	WDR12	-0.62325	4.283459	-2.48632	0.014786	-3.02514	0.15165	0.166283
Neutrophils	GM5150	0.476463	2.574091	2.4859	0.014803	-3.42312	0.159242	0.17444
Neutrophils	DIS3L2	-0.42162	5.906117	-2.48413	0.014872	-3.17148	0.145351	0.15937
Neutrophils	VANGL2	-1.16476	2.225601	-2.48046	0.015015	-3.11217	0.162702	0.177375
Neutrophils	AI839979	0.612036	0.274557	2.480359	0.015019	-3.13042	0.172023	0.187256
Neutrophils	PSEN2	0.376991	5.03423	2.479565	0.01505	-3.20596	0.150347	0.164261
Neutrophils	GM3336	-1.16129	1.732452	-2.47908	0.01507	-3.00724	0.165236	0.180203
Neutrophils	ST3GAL4	0.264392	6.423085	2.478349	0.015098	-3.63937	0.1447	0.158364
Neutrophils	2310008N1	-1.5402	0.80481	-2.47488	0.015236	-3.20541	0.171105	0.186163
Neutrophils	GM20528	0.692197	-0.01428	2.474734	0.015242	-3.0489	0.175155	0.190481
Neutrophils	C5AR1	0.343004	4.179898	2.474318	0.015258	-3.95966	0.155413	0.16958
Neutrophils	2310058D1	0.760529	2.785149	2.473308	0.015299	-3.03419	0.161986	0.176599
Neutrophils	KLRC2	-1.5675	1.332802	-2.47225	0.015341	-3.09371	0.168998	0.184172
Neutrophils	GM20404	0.686402	1.661785	2.472205	0.015343	-3.03517	0.167418	0.182496
Neutrophils	USP25	0.225417	7.051473	2.471609	0.015367	-3.72189	0.143606	0.157261
Neutrophils	FAM110A	0.463262	3.760358	2.471341	0.015377	-3.2199	0.157703	0.172383
Neutrophils	DNA2	0.482636	3.583362	2.470686	0.015404	-3.1305	0.1585	0.173464
Neutrophils	SLAMF6	-0.97969	4.602908	-2.47052	0.015411	-3.01899	0.153962	0.168664
Neutrophils	CRK	0.265801	6.391053	2.470185	0.015424	-3.60016	0.146325	0.160521
Neutrophils	AI480526	1.186115	2.064128	2.469929	0.015434	-3.12943	0.165521	0.181073
Neutrophils	ABCC4	-0.34608	4.55376	-2.46827	0.015501	-3.32432	0.154697	0.1692
Neutrophils	GM44752	-1.04665	2.484975	-2.46748	0.015533	-3.10763	0.164274	0.179459
Neutrophils	DEFB1	-1.43542	0.86149	-2.46675	0.015563	-3.09286	0.172204	0.187935

Neutrophils	CLDN3	-1.08655	1.461002	-2.4663	0.015581	-3.0396	0.169281	0.184951
Neutrophils	LGR4	-0.60849	2.632615	-2.46606	0.015591	-3.16676	0.163714	0.179069
Neutrophils	WDR74	-0.63035	4.042303	-2.46534	0.01562	-3.02813	0.157374	0.172416
Neutrophils	ZFP579	-1.13555	1.862923	-2.46505	0.015632	-3.11622	0.167468	0.183287
Neutrophils	GGCX	-1.05569	1.807957	-2.46158	0.015774	-3.08791	0.169088	0.184324
Neutrophils	AOPEP	-0.31396	6.329542	-2.4607	0.01581	-3.4812	0.148697	0.162637
Neutrophils	CBFA2T3	-0.68273	5.507245	-2.46062	0.015814	-3.11446	0.152215	0.16642
Neutrophils	NFKBIL1	0.398549	3.861655	2.46035	0.015825	-3.19403	0.159517	0.174254
Neutrophils	AXL	-0.79707	3.708037	-2.45995	0.015841	-3.18178	0.160231	0.175117
Neutrophils	PRPF6	0.307548	5.300623	2.45943	0.015863	-3.34448	0.153184	0.167687
Neutrophils	IFIT1	1.161022	2.761439	2.458343	0.015907	-3.12902	0.164888	0.180115
Neutrophils	PLD2	-1.22762	1.042444	-2.45786	0.015928	-3.13908	0.17318	0.189004
Neutrophils	NADK	0.240093	6.266885	2.457838	0.015928	-3.79194	0.149221	0.163434
Neutrophils	SERPINF1	-0.89679	3.83888	-2.45698	0.015964	-3.08994	0.159915	0.175066
Neutrophils	PMS1	-0.95261	2.650773	-2.45659	0.01598	-3.07733	0.165426	0.181072
Neutrophils	PIGC	0.652699	2.730331	2.456377	0.015989	-3.05032	0.165051	0.180697
Neutrophils	ASPA	-1.39313	1.375432	-2.45624	0.015995	-3.06676	0.171559	0.187634
Neutrophils	CD55B	-1.5857	0.178504	-2.45555	0.016024	-3.19804	0.177525	0.19424
Neutrophils	FAM222A	-1.09726	2.331884	-2.45531	0.016033	-3.14985	0.166938	0.182991
Neutrophils	TALDO1	0.183663	8.310001	2.45523	0.016037	-4.05503	0.14083	0.154954
Neutrophils	ALOX5AP	0.367096	7.325815	2.453887	0.016093	-4.24584	0.144892	0.159576
Neutrophils	LIX1	-1.46496	0.723268	-2.4538	0.016097	-3.21568	0.174876	0.191776
Neutrophils	HIST1H4I	0.628904	4.409355	2.453654	0.016103	-3.22681	0.157419	0.17311
Neutrophils	ZFP623	-1.54221	0.124749	-2.45357	0.016106	-3.25926	0.177891	0.195012
Neutrophils	WDR91	-0.7241	4.750458	-2.45265	0.016145	-3.06246	0.156119	0.171709
Neutrophils	GM17529	-1.47011	0.365187	-2.45147	0.016194	-3.22974	0.177149	0.194207
Neutrophils	RNF141	0.302705	4.511433	2.451077	0.016211	-3.42197	0.157385	0.173115
Neutrophils	KLRA6	-1.59417	-0.71608	-2.45107	0.016211	-3.24196	0.182706	0.200188
Neutrophils	GM16023	0.990402	1.834877	2.450567	0.016232	-3.10798	0.169932	0.186675
Neutrophils	PTAFR	0.389222	4.645313	2.448781	0.016308	-3.87783	0.157425	0.172987
Neutrophils	ABCB1A	-1.39248	2.010725	-2.44829	0.016329	-3.08149	0.169765	0.186268
Neutrophils	SIN3B	0.205393	6.294475	2.447376	0.016367	-3.68255	0.150346	0.165464
Neutrophils	MBL1	-1.07194	1.455107	-2.44734	0.016369	-3.08494	0.172577	0.189316
Neutrophils	FANCL	-0.64444	4.144904	-2.44675	0.016394	-3.06592	0.159928	0.175799
Neutrophils	ZBTB2	-0.338	5.709287	-2.44574	0.016437	-3.37961	0.153216	0.168496
Neutrophils	PRRC2B	-0.33673	6.226887	-2.44518	0.016461	-3.38264	0.151056	0.166267
Neutrophils	SPATA5	-0.51997	5.905372	-2.44446	0.016492	-3.23982	0.152568	0.167952
Neutrophils	ANKFY1	0.319917	5.995175	2.443853	0.016517	-3.45732	0.152179	0.167651
Neutrophils	P2RX3	-1.51394	2.219391	-2.44303	0.016553	-3.16763	0.169463	0.186389
Neutrophils	PTPN4	-0.54458	5.463527	-2.44292	0.016557	-3.20564	0.154497	0.170299
Neutrophils	COPB1	0.237759	6.220287	2.442882	0.016559	-3.54221	0.151208	0.166748
Neutrophils	PCID2	0.414253	4.736836	2.442701	0.016567	-3.19396	0.157726	0.173779
Neutrophils	ANXA11OS	0.675186	0.812081	2.439653	0.016698	-3.13007	0.177595	0.194639
Neutrophils	AHSG	-0.65589	9.130873	-2.43894	0.016729	-4.13811	0.140163	0.15453
Neutrophils	FYB	0.386471	6.869674	2.438905	0.016731	-3.88161	0.14944	0.164566
Neutrophils	PTPN12	0.253828	6.049828	2.438681	0.01674	-3.81366	0.152962	0.168366
Neutrophils	GM12158	-1.54302	0.549233	-2.43735	0.016798	-3.25221	0.179386	0.196653

Neutrophils	DESI1	-0.39974	5.729904	-2.43681	0.016822	-3.42885	0.154784	0.170434
Neutrophils	LMO1	0.710073	1.523387	2.436545	0.016833	-3.14949	0.174502	0.191623
Neutrophils	ACBD6	-0.29088	5.436733	-2.43243	0.017013	-3.44147	0.157603	0.172819
Neutrophils	GM16599	0.369083	4.782001	2.431986	0.017033	-3.38062	0.160604	0.176053
Neutrophils	MOCS1	0.399511	3.367675	2.430563	0.017095	-3.30253	0.167668	0.183574
Neutrophils	PDE6C	-1.38307	0.436862	-2.43018	0.017112	-3.20149	0.182297	0.199231
Neutrophils	NDUFA4	-0.29257	8.596899	-2.42985	0.017127	-3.9506	0.144508	0.158844
Neutrophils	TRP53I11	-0.65452	4.565294	-2.42918	0.017156	-3.13717	0.162175	0.177897
Neutrophils	AKR1E1	-0.85499	2.622552	-2.42806	0.017206	-3.11325	0.171749	0.188175
Neutrophils	SERF2	0.153092	9.682679	2.426077	0.017294	-4.24958	0.141013	0.154925
Neutrophils	SPTAN1	-0.27703	6.559592	-2.42601	0.017297	-3.69924	0.154061	0.168991
Neutrophils	KIF5B	0.178961	7.401742	2.424274	0.017375	-3.82515	0.150959	0.165476
Neutrophils	TAF2	0.443164	4.171185	2.423879	0.017393	-3.18434	0.165501	0.181075
Neutrophils	RSF1OS1	0.733166	2.978349	2.42309	0.017428	-3.10988	0.171335	0.187361
Neutrophils	RAD54L2	-0.57556	4.552757	-2.42292	0.017436	-3.12706	0.163816	0.17934
Neutrophils	PSMA7	0.270085	7.683799	2.420792	0.017531	-3.79946	0.150385	0.164878
Neutrophils	HSPA2	-0.82421	3.291574	-2.42079	0.017532	-3.11494	0.170399	0.186321
Neutrophils	TCF4	-0.35107	7.694523	-2.42052	0.017544	-3.77577	0.15034	0.164859
Neutrophils	SFMBT1	-0.37078	6.08199	-2.41974	0.017579	-3.45419	0.157458	0.172594
Neutrophils	FCGR2B	-0.43382	4.881357	-2.41941	0.017594	-3.56373	0.162929	0.178461
Neutrophils	UCK2	0.421677	5.87449	2.419148	0.017606	-3.56933	0.158389	0.173609
Neutrophils	AFF3	-0.56774	7.930696	-2.41892	0.017616	-3.57978	0.149407	0.163982
Neutrophils	NDRG1	-0.58855	2.72107	-2.41855	0.017633	-3.27714	0.173285	0.189666
Neutrophils	COL4A3BP	0.258079	6.352484	2.41772	0.017671	-3.72254	0.15645	0.171559
Neutrophils	ASB8	0.623452	3.607063	2.417099	0.017699	-3.13298	0.169284	0.185371
Neutrophils	ANKRD50	-0.63949	2.62264	-2.41671	0.017717	-3.12065	0.174121	0.190664
Neutrophils	4930581F2	0.412857	3.634004	2.416229	0.017739	-3.3124	0.169226	0.185473
Neutrophils	HRG	-0.85749	3.61876	-2.41549	0.017773	-3.23311	0.16945	0.185628
Neutrophils	FBRS	-0.30201	4.965395	-2.41518	0.017786	-3.53872	0.163072	0.178861
Neutrophils	2010007H(	-1.2903	0.459338	-2.41482	0.017803	-3.30527	0.185446	0.202844
Neutrophils	TRAC	-1.35952	2.266621	-2.41233	0.017917	-3.15578	0.17709	0.193643
Neutrophils	MPO	1.142778	1.827356	2.411629	0.01795	-3.46626	0.179441	0.19611
Neutrophils	SLC39A8	-0.81388	2.726982	-2.41137	0.017961	-3.14943	0.174893	0.191265
Neutrophils	MKRN2	0.449795	4.074932	2.409396	0.018053	-3.18188	0.169002	0.184683
Neutrophils	CLYBL	0.628848	4.489599	2.408582	0.018091	-3.1807	0.167216	0.182817
Neutrophils	DNAH7A	-1.37667	-0.11769	-2.40769	0.018132	-3.22623	0.190845	0.208082
Neutrophils	ANXA2	0.292321	5.910919	2.407607	0.018136	-3.94803	0.1607	0.175969
Neutrophils	LRRC75A	-1.22842	2.6203	-2.40617	0.018203	-3.18596	0.176985	0.193234
Neutrophils	APP	0.269462	5.938302	2.405547	0.018232	-4.12558	0.161137	0.176405
Neutrophils	GDF11	-1.29368	1.639394	-2.40403	0.018303	-3.23783	0.182686	0.199223
Neutrophils	GSAP	0.349554	5.642393	2.403557	0.018325	-3.81996	0.163038	0.17836
Neutrophils	SARS2	0.852483	2.675724	2.402901	0.018356	-3.19288	0.177558	0.193791
Neutrophils	GAB2	0.305926	7.176286	2.402446	0.018377	-4.03435	0.15625	0.171089
Neutrophils	A530013C2	0.433275	2.431674	2.400541	0.018467	-3.5538	0.179562	0.19574
Neutrophils	GM29282	0.894009	1.712401	2.398673	0.018556	-3.1549	0.184001	0.200375
Neutrophils	GM867	1.510862	-0.30876	2.397919	0.018592	-3.31209	0.195074	0.212034
Neutrophils	ARPC3	0.167873	8.451234	2.397637	0.018605	-4.13773	0.152004	0.166198

Neutrophils	ARID3B	0.454885	3.836648	2.397351	0.018619	-3.35548	0.173306	0.189025
Neutrophils	UHRF1BP1l	0.245863	5.734451	2.39623	0.018672	-3.77352	0.164307	0.179368
Neutrophils	SPDL1	-0.99575	2.833783	-2.39618	0.018674	-3.17078	0.178461	0.19445
Neutrophils	HIST1H3A	-1.37082	0.953909	-2.39603	0.018682	-3.32136	0.188299	0.204915
Neutrophils	PADI2	-1.25049	2.631853	-2.39533	0.018715	-3.20496	0.179657	0.195741
Neutrophils	FAM71F2	0.780118	2.260769	2.393944	0.018782	-3.22185	0.181952	0.198206
Neutrophils	RBP1	-0.7908	3.578118	-2.39381	0.018788	-3.22129	0.175242	0.191108
Neutrophils	SEM1	0.163469	9.100384	2.393084	0.018823	-4.30659	0.149941	0.164024
Neutrophils	2610037D(	-0.29243	5.44268	-2.39047	0.018949	-3.66782	0.167305	0.182064
Neutrophils	IRAK4	0.350082	4.419459	2.390098	0.018967	-3.45124	0.172264	0.187416
Neutrophils	CFAP410	0.698709	2.119698	2.389434	0.018999	-3.17375	0.184008	0.199954
Neutrophils	AW112010	-0.97321	7.279978	-2.38926	0.019008	-3.74284	0.158866	0.173203
Neutrophils	CDK5RAP1	-1.40559	2.44604	-2.38876	0.019032	-3.18936	0.182344	0.198294
Neutrophils	SLC7A6	-0.73837	4.274758	-2.38848	0.019045	-3.1737	0.173079	0.188497
Neutrophils	FTL1	0.258005	11.26205	2.387529	0.019092	-4.52632	0.142195	0.155348
Neutrophils	CD34	1.624838	0.953495	2.3872	0.019108	-3.20657	0.190571	0.207038
Neutrophils	THAP3	0.708055	3.799217	2.386386	0.019148	-3.17803	0.175714	0.19153
Neutrophils	GID4	0.50756	4.230623	2.386331	0.01915	-3.22633	0.173567	0.18925
Neutrophils	NOP53	0.377505	5.740416	2.385981	0.019167	-3.49892	0.166266	0.18156
Neutrophils	IFT20	-0.36122	5.442542	-2.38581	0.019176	-3.44829	0.16768	0.183129
Neutrophils	AKAP1	-1.02629	1.799595	-2.38436	0.019247	-3.2284	0.186503	0.203213
Neutrophils	BC005624	0.27402	5.761918	2.384132	0.019258	-3.55206	0.166587	0.18206
Neutrophils	HSPA4	0.196381	7.667014	2.382875	0.01932	-3.90922	0.158181	0.172955
Neutrophils	SCAPER	0.258552	6.110801	2.381377	0.019394	-3.83076	0.165815	0.180811
Neutrophils	PDK3	0.420621	4.945028	2.380609	0.019432	-3.54243	0.171593	0.187014
Neutrophils	WAS	0.272536	5.349391	2.379534	0.019485	-3.59283	0.169946	0.185285
Neutrophils	ALKBH8	-0.65188	3.835022	-2.37609	0.019656	-3.19735	0.178844	0.194281
Neutrophils	PAPOLG	0.682142	3.005898	2.375422	0.01969	-3.20822	0.183165	0.198892
Neutrophils	SMARCA4	-0.33585	7.5105	-2.37524	0.019699	-3.72584	0.16113	0.175486
Neutrophils	HNRNPH1	-0.27537	6.424984	-2.37477	0.019722	-3.73854	0.166174	0.180958
Neutrophils	4931406P1	-0.31097	5.594349	-2.37463	0.019729	-3.55789	0.170144	0.185201
Neutrophils	ACSL4	0.281827	6.189637	2.373972	0.019762	-3.77035	0.167425	0.182367
Neutrophils	FAR1	-0.25282	6.64871	-2.37328	0.019797	-3.95564	0.165404	0.18022
Neutrophils	SLC45A4	-0.52175	3.337998	-2.37278	0.019822	-3.25635	0.181822	0.197692
Neutrophils	HOGA1	-0.84728	2.370342	-2.37238	0.019842	-3.20564	0.186939	0.203221
Neutrophils	TRP53RKB	-0.8204	2.444883	-2.37173	0.019875	-3.2094	0.18669	0.202972
Neutrophils	CRIP1	-0.52382	8.825299	-2.37134	0.019895	-3.83407	0.155734	0.169993
Neutrophils	RDH13	-1.16712	1.398416	-2.37086	0.019919	-3.30164	0.192447	0.209132
Neutrophils	TBC1D14	0.292768	5.680933	2.370306	0.019947	-3.65321	0.170422	0.185817
Neutrophils	PDGFC	-1.373	1.879122	-2.36968	0.019979	-3.20802	0.190029	0.206664
Neutrophils	FOLR2	-0.80573	3.655517	-2.36918	0.020005	-3.36766	0.180639	0.19685
Neutrophils	URB1	-0.98278	2.176346	-2.36908	0.02001	-3.31383	0.188424	0.205149
Neutrophils	TMEM150/	-0.89899	2.327871	-2.36844	0.020042	-3.21087	0.187615	0.204443
Neutrophils	COX14	0.310305	5.656597	2.367342	0.020098	-3.57463	0.170637	0.186721
Neutrophils	ZBTB12	1.10369	1.665552	2.367254	0.020102	-3.31383	0.191196	0.208653
Neutrophils	TDRD7	0.493069	3.385152	2.367081	0.020111	-3.37449	0.182041	0.198948
Neutrophils	ALAS1	0.427074	3.683631	2.367006	0.020115	-3.51336	0.180498	0.197303

Neutrophils	TCEA3	-0.89639	2.275566	-2.36691	0.02012	-3.22261	0.187895	0.205185
Neutrophils	ZFP654	0.307393	6.120389	2.366712	0.02013	-3.77813	0.168401	0.184415
Neutrophils	BCL2A1A	0.707836	2.399522	2.366372	0.020147	-3.45459	0.187234	0.2046
Neutrophils	BBC3	0.776312	2.639565	2.365612	0.020186	-3.21624	0.186016	0.203461
Neutrophils	CCNG2	-0.35757	5.189545	-2.36557	0.020188	-3.64879	0.172977	0.189522
Neutrophils	PKIG	-0.37819	7.198634	-2.36521	0.020207	-3.62611	0.163387	0.179216
Neutrophils	TSPOAP1	-0.75807	2.650845	-2.36357	0.020291	-3.24113	0.186586	0.203763
Neutrophils	GM12185	0.790726	3.341306	2.363108	0.020315	-3.23263	0.183006	0.200031
Neutrophils	SLC27A2	-0.75254	3.949769	-2.36238	0.020352	-3.41329	0.179904	0.196881
Neutrophils	GTF3C4	-0.84165	2.57436	-2.36234	0.020354	-3.22707	0.1871	0.204562
Neutrophils	DBT	-0.76463	3.826101	-2.36181	0.020382	-3.22659	0.180631	0.197654
Neutrophils	ADAM23	0.527902	3.184267	2.360895	0.020429	-3.55487	0.184214	0.20146
Neutrophils	RGS19	0.299099	5.393744	2.36061	0.020444	-3.7542	0.172976	0.189474
Neutrophils	TANC1	-0.5733	3.838336	-2.36013	0.020469	-3.41038	0.180879	0.197969
Neutrophils	ETFA	-0.32505	6.599323	-2.35979	0.020486	-3.74555	0.167211	0.183417
Neutrophils	ZFP709	0.995883	1.431477	2.359394	0.020507	-3.33426	0.193769	0.211893
Neutrophils	GPR155	-1.09276	3.05644	-2.35867	0.020544	-3.25797	0.185111	0.202668
Neutrophils	APOM	-0.69526	4.854579	-2.35844	0.020556	-3.60657	0.175864	0.192776
Neutrophils	EDEM2	0.501605	4.515035	2.358107	0.020574	-3.35562	0.177573	0.194606
Neutrophils	FAM91A1	-0.35204	5.007003	-2.3578	0.02059	-3.41054	0.175103	0.192086
Neutrophils	SLC17A3	-1.2769	0.779648	-2.35706	0.020628	-3.27815	0.197745	0.216316
Neutrophils	SLAIN1	-0.6909	3.6132	-2.35543	0.020713	-3.23507	0.182984	0.200357
Neutrophils	PHF3	0.216869	6.980017	2.354818	0.020745	-3.88681	0.166318	0.182556
Neutrophils	ABCB9	-0.77278	3.155352	-2.35418	0.020779	-3.24623	0.185445	0.2032
Neutrophils	ANKRD23	-1.30334	0.33175	-2.35404	0.020787	-3.38542	0.201011	0.21979
Neutrophils	OSGIN2	0.685962	3.18431	2.353701	0.020804	-3.26154	0.185292	0.203065
Neutrophils	FCRL1	0.82334	2.73778	2.35347	0.020816	-3.24769	0.187667	0.205669
Neutrophils	GAMT	-0.73825	4.228598	-2.35322	0.020829	-3.45221	0.179857	0.197367
Neutrophils	DEPP1	1.026468	1.043364	2.352828	0.02085	-3.23876	0.196967	0.215771
Neutrophils	CEBPB	0.341123	9.155958	2.35267	0.020858	-4.63636	0.156371	0.172181
Neutrophils	1110035H1	1.165368	1.216236	2.352203	0.020883	-3.29783	0.196065	0.214905
Neutrophils	RAB8B	0.234974	7.724426	2.351219	0.020935	-4.27572	0.163168	0.179546
Neutrophils	MMD	-0.50253	4.477186	-2.35075	0.02096	-3.44525	0.179007	0.196588
Neutrophils	WDR45B	0.20823	6.330115	2.349599	0.021021	-3.83267	0.17014	0.187005
Neutrophils	CBX7	-0.82345	2.651462	-2.34924	0.02104	-3.26914	0.188933	0.207244
Neutrophils	1700094D0	0.9174	1.476408	2.348835	0.021061	-3.28525	0.195377	0.214182
Neutrophils	LMNB1	0.225268	7.679763	2.348427	0.021083	-4.34662	0.163744	0.180285
Neutrophils	GM15345	-0.50925	2.98417	-2.34835	0.021087	-3.62329	0.187148	0.205459
Neutrophils	GM45267	0.781998	0.245242	2.347336	0.021141	-3.2643	0.20255	0.221964
Neutrophils	SERINC1	0.254298	6.230989	2.347115	0.021153	-3.73709	0.170773	0.187919
Neutrophils	ZNHIT6	-0.94155	3.231636	-2.34702	0.021158	-3.26256	0.185999	0.204282
Neutrophils	PRSS57	0.673652	-0.03051	2.346414	0.02119	-3.27408	0.204294	0.223806
Neutrophils	ADGRL1	-0.93783	2.476047	-2.34474	0.02128	-3.27548	0.190679	0.209195
Neutrophils	GM47802	1.424199	0.099495	2.344729	0.021281	-3.37607	0.204067	0.223476
Neutrophils	FERMT2	-0.51669	3.451103	-2.34424	0.021307	-3.4451	0.185487	0.203657
Neutrophils	PREB	0.311584	4.736405	2.343996	0.02132	-3.58608	0.178816	0.196555
Neutrophils	GM16552	-1.07387	1.517	-2.34232	0.02141	-3.33383	0.196681	0.215351

Neutrophils	EIF2AK3	-0.37667	6.399228	-2.34121	0.02147	-3.66609	0.171474	0.188263
Neutrophils	GK5	-0.41428	4.404631	-2.34005	0.021533	-3.60807	0.181867	0.199379
Neutrophils	ALDOC	1.37098	0.884667	2.339219	0.021578	-3.36181	0.201165	0.22016
Neutrophils	SEMA4C	-0.78262	2.202853	-2.33909	0.021585	-3.26366	0.193735	0.212247
Neutrophils	C330007PC	-0.35321	5.50907	-2.33891	0.021595	-3.60542	0.176313	0.19359
Neutrophils	CAPNS1	0.197925	7.25812	2.336824	0.021708	-4.04556	0.168509	0.18492
Neutrophils	GCNT7	0.636117	2.712006	2.334892	0.021814	-3.28815	0.192564	0.210352
Neutrophils	IVD	-0.65248	4.435995	-2.333	0.021918	-3.29032	0.184054	0.201136
Neutrophils	HDHD5	-1.07919	2.842941	-2.33178	0.021985	-3.32127	0.193038	0.21058
Neutrophils	SAMD4B	0.33424	5.014196	2.330957	0.022031	-3.5763	0.181681	0.198275
Neutrophils	SH3RF1	-0.56228	4.357213	-2.33014	0.022076	-3.39145	0.185341	0.202103
Neutrophils	TMEM108	-1.16147	5.137851	-2.32957	0.022108	-3.31581	0.181379	0.197897
Neutrophils	BRK1	0.236578	6.271039	2.328407	0.022172	-3.82099	0.175929	0.192083
Neutrophils	FGFR1OP2	0.273449	6.180589	2.328237	0.022182	-3.76671	0.176382	0.192593
Neutrophils	F2	-0.75124	4.237274	-2.32738	0.022229	-3.53056	0.186658	0.203542
Neutrophils	PDE8B	-1.3463	-0.24267	-2.32663	0.022271	-3.43075	0.212322	0.230755
Neutrophils	LUZP1	-0.41867	5.787075	-2.32622	0.022294	-3.58954	0.178777	0.195205
Neutrophils	MYO1E	-0.76112	6.198716	-2.32603	0.022305	-3.537	0.176697	0.193015
Neutrophils	PGM2L1	-0.64623	5.192591	-2.32543	0.022338	-3.36672	0.18195	0.198649
Neutrophils	ADAMTS9	-0.76566	2.970839	-2.32434	0.022399	-3.54629	0.194215	0.211591
Neutrophils	MAPRE2	0.265556	6.802434	2.323504	0.022446	-3.93646	0.174371	0.19026
Neutrophils	EBI3	0.279371	4.387447	2.32195	0.022534	-3.91286	0.187347	0.20388
Neutrophils	CIC	0.436724	4.821881	2.320916	0.022592	-3.46584	0.185225	0.201636
Neutrophils	INF2	1.110548	3.252642	2.320915	0.022592	-3.32964	0.193696	0.210638
Neutrophils	APOE	-0.58586	9.241961	-2.31891	0.022706	-4.36704	0.164076	0.178781
Neutrophils	MGST2	-0.3784	2.953494	-2.31839	0.022735	-3.64513	0.196277	0.213158
Neutrophils	ATG4A	0.593875	4.314693	2.317542	0.022784	-3.3864	0.188969	0.205452
Neutrophils	PCIF1	0.240334	6.021144	2.317341	0.022795	-3.80982	0.180011	0.19592
Neutrophils	CYP2C70	-0.75477	4.144913	-2.31709	0.02281	-3.56588	0.189885	0.206436
Neutrophils	GM19684	-1.34838	1.522121	-2.31671	0.022831	-3.39984	0.204663	0.222197
Neutrophils	SNX2	-0.22266	7.314048	-2.31616	0.022862	-3.87679	0.173623	0.189243
Neutrophils	CCL4	0.629713	7.388292	2.315638	0.022892	-4.22287	0.173257	0.18899
Neutrophils	MB21D2	-0.98172	0.883088	-2.31557	0.022896	-3.30542	0.208529	0.22649
Neutrophils	KIF17	-1.15298	1.692096	-2.31503	0.022927	-3.31829	0.20388	0.221653
Neutrophils	DNAJB4	0.508171	3.700143	2.314121	0.022979	-3.38047	0.192811	0.209863
Neutrophils	MSRB1	0.207388	5.958368	2.311449	0.023133	-4.25457	0.181869	0.19772
Neutrophils	NAPRT	-1.35766	0.897988	-2.31029	0.0232	-3.37258	0.210533	0.227961
Neutrophils	ASMT	1.278964	1.052723	2.309654	0.023237	-3.38284	0.209675	0.22716
Neutrophils	PARD6A	1.025696	2.688858	2.309412	0.023251	-3.33133	0.200107	0.2171
Neutrophils	RTN2	-1.4449	-0.4318	-2.3092	0.023263	-3.46465	0.218756	0.236838
Neutrophils	HSD3B3	-1.04936	2.014179	-2.30769	0.023351	-3.32055	0.204607	0.221596
Neutrophils	DSEL	1.195363	0.375304	2.307193	0.02338	-3.45525	0.214503	0.232128
Neutrophils	LDLRAP1	0.437779	4.099537	2.306589	0.023415	-3.5679	0.192883	0.209463
Neutrophils	TSC22D3	0.435346	5.71665	2.30648	0.023422	-3.80512	0.184204	0.200276
Neutrophils	NOB1	-0.62288	4.287446	-2.30588	0.023457	-3.32818	0.191853	0.208562
Neutrophils	RAB8A	0.262053	5.454242	2.30583	0.02346	-3.80166	0.185584	0.201896
Neutrophils	GM41409	0.777966	3.031183	2.305357	0.023487	-3.40141	0.198849	0.215983

Neutrophils	ACSS2	0.600825	3.033515	2.305274	0.023492	-3.39579	0.198835	0.215969
Neutrophils	SLX1B	-0.51149	3.074893	-2.3046	0.023532	-3.37374	0.198778	0.21587
Neutrophils	EEPD1	0.35773	4.881821	2.303714	0.023584	-3.80785	0.189037	0.205602
Neutrophils	FBXL18	-0.79091	2.985602	-2.30346	0.023598	-3.39287	0.199534	0.216799
Neutrophils	TIGIT	-1.40416	-0.09862	-2.30314	0.023618	-3.43824	0.217908	0.236336
Neutrophils	YWHAQ	-0.20776	7.746706	-2.30101	0.023743	-4.03897	0.174936	0.190483
Neutrophils	SPAG9	0.272348	9.266573	2.300598	0.023767	-4.48949	0.167566	0.182669
Neutrophils	DECR1	-0.71715	3.696206	-2.29997	0.023805	-3.39642	0.196293	0.21351
Neutrophils	MS4A1	-1.08814	3.936889	-2.29986	0.023811	-3.3859	0.194951	0.212119
Neutrophils	FYN	-0.50615	7.843798	-2.29968	0.023821	-3.95834	0.174455	0.190303
Neutrophils	VAV2	-0.73946	4.977829	-2.29904	0.023859	-3.3731	0.189255	0.206229
Neutrophils	EZR	0.201556	7.933064	2.298403	0.023897	-4.20385	0.174014	0.190137
Neutrophils	ATP1B1	-0.66697	5.798004	-2.29806	0.023918	-3.5253	0.18489	0.201839
Neutrophils	TRMT1	-0.61359	4.050324	-2.29771	0.023938	-3.33871	0.194321	0.212
Neutrophils	NTAN1	-0.23259	6.261702	-2.29735	0.02396	-3.82065	0.182468	0.199445
Neutrophils	GM19696	0.976548	0.016291	2.297234	0.023967	-3.33797	0.218032	0.237298
Neutrophils	TBC1D16	-1.04313	2.452428	-2.29698	0.023982	-3.34003	0.203381	0.221844
Neutrophils	CD93	-0.49939	3.89899	-2.29697	0.023983	-3.53448	0.195161	0.213093
Neutrophils	KRTCAP3	0.769842	0.893962	2.296925	0.023985	-3.3398	0.212634	0.231669
Neutrophils	DHDDS	0.374944	4.630539	2.296553	0.024008	-3.62151	0.191136	0.20887
Neutrophils	DNAJB9	-0.26481	5.168187	-2.2964	0.024016	-3.87625	0.188232	0.205791
Neutrophils	TIMD2	-1.12847	0.986598	-2.29571	0.024058	-3.37686	0.212072	0.231466
Neutrophils	LYPD6B	0.551154	0.004013	2.295382	0.024077	-3.56387	0.218108	0.237962
Neutrophils	NRP1	-0.46576	4.442319	-2.29534	0.02408	-3.78629	0.192163	0.210337
Neutrophils	GM29585	-1.18321	1.247477	-2.29531	0.024082	-3.38476	0.210498	0.229885
Neutrophils	CDKN2D	0.236408	6.09877	2.294506	0.02413	-4.05933	0.183539	0.201076
Neutrophils	CLEC4F	-0.90167	5.458864	-2.29358	0.024185	-3.95732	0.187193	0.204911
Neutrophils	4931428F0	-1.35137	0.65018	-2.29198	0.024282	-3.48942	0.215399	0.234706
Neutrophils	MEI4	-1.57975	1.201602	-2.29163	0.024303	-3.44662	0.212052	0.231262
Neutrophils	A3GALT2	-0.94936	2.675415	-2.29053	0.024369	-3.34994	0.203715	0.222257
Neutrophils	PKD1L2	0.685935	1.173969	2.28941	0.024437	-3.44886	0.213062	0.232157
Neutrophils	GDI1	0.324404	4.991215	2.288663	0.024482	-3.76367	0.191292	0.208944
Neutrophils	MTOR	-0.46296	4.766611	-2.2864	0.02462	-3.45124	0.193276	0.210755
Neutrophils	FYCO1	0.457823	4.137271	2.286164	0.024635	-3.50226	0.196772	0.214518
Neutrophils	GPR137	0.518341	2.506946	2.286138	0.024636	-3.39267	0.206136	0.224473
Neutrophils	ALB	-0.70257	9.441682	-2.28556	0.024672	-4.52436	0.16926	0.185172
Neutrophils	TTC7	0.358857	5.873227	2.28552	0.024674	-3.77019	0.187285	0.204506
Neutrophils	ATP6AP2	0.184389	6.938045	2.28522	0.024692	-4.09074	0.181703	0.198596
Neutrophils	TMSB15B1	-0.87252	2.422831	-2.28324	0.024814	-3.3689	0.207489	0.226056
Neutrophils	RANBP9	-0.19254	6.962828	-2.28085	0.024961	-4.27243	0.183165	0.199826
Neutrophils	CDIPT	0.337912	5.093616	2.280469	0.024984	-3.72277	0.193161	0.210623
Neutrophils	ABCC10	-1.15258	0.65899	-2.28046	0.024985	-3.47836	0.219206	0.238274
Neutrophils	RAB30	-1.27709	2.141089	-2.28015	0.025004	-3.40464	0.210126	0.228718
Neutrophils	TMEM204	-0.79842	1.038857	-2.27665	0.025221	-3.37522	0.21845	0.236906
Neutrophils	SLC45A1	-1.09189	0.608482	-2.27624	0.025247	-3.46494	0.221152	0.239887
Neutrophils	MAN1B1	0.338767	4.927787	2.275936	0.025266	-3.88722	0.195516	0.212814
Neutrophils	MAPK14	0.194356	6.656022	2.275925	0.025266	-4.15058	0.186139	0.202817

Neutrophils	GM20732	0.38946	4.58993	2.275409	0.025299	-3.57656	0.197433	0.214937
Neutrophils	GNA12	-0.38989	4.652134	-2.27511	0.025318	-3.64313	0.197084	0.21461
Neutrophils	IQGAP1	0.177738	9.247795	2.274946	0.025328	-4.53731	0.172978	0.188841
Neutrophils	LCP2	0.330936	5.109024	2.274407	0.025361	-4.08991	0.194648	0.212035
Neutrophils	PAK4	-0.82736	2.299468	-2.2735	0.025418	-3.38587	0.211181	0.229379
Neutrophils	GINS3	0.881482	2.330816	2.27321	0.025436	-3.38169	0.210992	0.229258
Neutrophils	POGLUT3	-1.12689	0.979034	-2.27172	0.02553	-3.43717	0.21988	0.238466
Neutrophils	RND3	-0.97808	2.943599	-2.27151	0.025543	-3.38261	0.207889	0.225863
Neutrophils	DUSP5	0.3301	6.834196	2.270594	0.025601	-4.07416	0.186376	0.20284
Neutrophils	MUC13	-1.26287	1.034673	-2.26994	0.025643	-3.41486	0.220052	0.238495
Neutrophils	GM32296	-1.28539	-0.65404	-2.26914	0.025693	-3.47772	0.231209	0.250285
Neutrophils	TSSC4	0.441538	3.902903	2.26877	0.025717	-3.55854	0.203038	0.220588
Neutrophils	CD101	0.481285	0.741284	2.268075	0.025761	-3.6117	0.222422	0.241
Neutrophils	PEMT	-0.70279	3.483706	-2.26739	0.025804	-3.4961	0.20587	0.223431
Neutrophils	GM10138	0.960757	2.471458	2.266513	0.02586	-3.39714	0.211962	0.230106
Neutrophils	GYG	0.254482	6.126163	2.266505	0.02586	-4.2375	0.191004	0.20785
Neutrophils	PKN2	0.258641	6.774243	2.266355	0.02587	-4.01825	0.187519	0.204134
Neutrophils	IGHM	-0.71715	8.158942	-2.2652	0.025943	-4.04547	0.18066	0.196723
Neutrophils	GM11290	-0.97481	3.432889	-2.26471	0.025975	-3.41846	0.206644	0.224582
Neutrophils	CUL7	-1.00657	1.968845	-2.26457	0.025984	-3.41415	0.215458	0.233917
Neutrophils	CABP4	-1.50822	-0.22452	-2.26432	0.026	-3.51617	0.229385	0.248669
Neutrophils	ESD	0.294208	7.081837	2.26227	0.026132	-4.09888	0.186964	0.203427
Neutrophils	MAN2A2	-0.53853	4.595669	-2.26218	0.026137	-3.50878	0.20066	0.21801
Neutrophils	0610009E0	-1.00894	0.843747	-2.26098	0.026214	-3.45113	0.223696	0.24215
Neutrophils	SGMS2	0.246481	3.643674	2.260905	0.026219	-4.48198	0.206518	0.224026
Neutrophils	ANKRD28	-0.29954	5.835458	-2.26053	0.026244	-3.9641	0.194057	0.210798
Neutrophils	IGFBP7	-0.3867	5.173401	-2.25992	0.026283	-4.00957	0.197895	0.214978
Neutrophils	UBAP2	-0.29144	6.131148	-2.25901	0.026342	-3.83348	0.192864	0.209607
Neutrophils	SH2D1A	-1.30115	0.051779	-2.25747	0.026442	-3.48985	0.230065	0.248724
Neutrophils	ERP27	-0.56666	1.896721	-2.25532	0.026582	-3.5121	0.219251	0.236656
Neutrophils	SLC31A1	0.286553	5.429507	2.253676	0.026689	-3.93123	0.198703	0.214934
Neutrophils	QRFP	-1.46293	-0.61178	-2.25349	0.026701	-3.5527	0.236075	0.254245
Neutrophils	GABARAP	0.167421	8.497974	2.253476	0.026702	-4.45626	0.182126	0.197338
Neutrophils	UGT2B5	-1.07615	1.786068	-2.253	0.026734	-3.41686	0.220546	0.237978
Neutrophils	EDEM1	0.280559	6.015715	2.252621	0.026758	-3.97123	0.195537	0.211646
Neutrophils	CHRAC1	0.328442	5.264928	2.25136	0.026841	-3.69033	0.200034	0.216433
Neutrophils	HACL1	-0.71945	3.523051	-2.25111	0.026858	-3.46252	0.21021	0.227239
Neutrophils	B4GALNT1	0.318572	4.892601	2.250376	0.026906	-3.81218	0.202165	0.219007
Neutrophils	ACER3	0.247645	6.311004	2.250111	0.026924	-4.1345	0.194171	0.210561
Neutrophils	FOS	0.282261	8.489188	2.249943	0.026935	-4.67185	0.182535	0.198174
Neutrophils	DIO1	-1.04989	0.977554	-2.24963	0.026955	-3.44757	0.226048	0.244276
Neutrophils	FAM122B	-0.89898	2.015817	-2.24956	0.02696	-3.44489	0.219446	0.237328
Neutrophils	SRSF10	-0.24486	6.4448	-2.24903	0.026995	-3.85981	0.193434	0.209999
Neutrophils	TEX30	-0.37738	5.223272	-2.24845	0.027034	-3.65966	0.200271	0.217357
Neutrophils	CD164	-0.25351	6.379209	-2.24818	0.027051	-4.05681	0.193795	0.210544
Neutrophils	CAMK4	-1.07802	3.194525	-2.24817	0.027052	-3.46329	0.212188	0.230061
Neutrophils	CSK	0.271578	6.929767	2.248126	0.027055	-4.01567	0.190788	0.207344

Neutrophils	MIF4GD	0.444007	4.964455	2.247798	0.027077	-3.61635	0.201752	0.219077
Neutrophils	GM49189	-1.25242	0.779988	-2.24708	0.027125	-3.52565	0.227327	0.246278
Neutrophils	INHBC	-1.29443	0.808252	-2.24686	0.027139	-3.46928	0.227144	0.246139
Neutrophils	SEMA6D	-0.70872	3.713919	-2.2467	0.02715	-3.63266	0.209069	0.227102
Neutrophils	GNG5	0.196981	8.925654	2.246471	0.027165	-4.52137	0.180293	0.196435
Neutrophils	2-Mar	-0.33956	5.868752	-2.24645	0.027166	-3.76605	0.196627	0.21388
Neutrophils	ZNHIT1	0.222844	5.422819	2.246347	0.027173	-4.0466	0.199137	0.216552
Neutrophils	BOLA2	0.370894	5.686348	2.245031	0.027261	-3.77127	0.198143	0.215315
Neutrophils	B230369F2	-0.78774	2.457789	-2.24445	0.027299	-3.42932	0.21735	0.235707
Neutrophils	IFNAR1	0.399785	5.246213	2.24422	0.027315	-3.65596	0.200745	0.218143
Neutrophils	C1QC	-0.59399	6.384084	-2.24379	0.027343	-4.16118	0.194414	0.211436
Neutrophils	GRB10	-0.69353	3.090696	-2.24339	0.02737	-3.62727	0.213583	0.231856
Neutrophils	SPIB	-0.96137	4.129765	-2.24277	0.027412	-3.43928	0.207509	0.225464
Neutrophils	RHOA	0.124537	9.258828	2.241756	0.02748	-4.53906	0.179424	0.195747
Neutrophils	CNRIP1	-1.12202	1.648634	-2.24165	0.027487	-3.46059	0.222779	0.241951
Neutrophils	FMNL1	0.195099	6.443857	2.241542	0.027494	-4.30429	0.194329	0.211726
Neutrophils	BNIP2	0.203222	6.377564	2.241433	0.027502	-4.12163	0.194696	0.212117
Neutrophils	DOCK3	-1.20205	0.039329	-2.24118	0.027518	-3.47131	0.233256	0.253074
Neutrophils	CD274	0.816425	5.805413	2.240635	0.027555	-4.02895	0.197888	0.215769
Neutrophils	HSH2D	0.464628	3.210107	2.240147	0.027588	-3.64218	0.21307	0.232114
Neutrophils	ISG15	0.75434	6.408101	2.239817	0.027611	-4.09642	0.194527	0.212371
Neutrophils	ABT1	-0.42654	3.534654	-2.23978	0.027613	-3.56983	0.211107	0.230056
Neutrophils	PIPOX	-0.96737	2.011866	-2.23971	0.027618	-3.43726	0.220481	0.240011
Neutrophils	RRAGC	0.275737	5.819399	2.239105	0.027659	-3.92377	0.197961	0.216037
Neutrophils	MATR3	0.211921	6.50291	2.237037	0.027799	-3.96027	0.194994	0.212659
Neutrophils	AKR1C20	-0.94263	2.839499	-2.23635	0.027845	-3.49041	0.216639	0.235639
Neutrophils	GAL3ST1	-1.40553	-0.29084	-2.23589	0.027876	-3.56629	0.236899	0.257123
Neutrophils	SEC61A2	-0.59539	3.741527	-2.23573	0.027887	-3.46433	0.211143	0.229865
Neutrophils	NFKBIB	0.363197	5.622292	2.235456	0.027906	-3.83308	0.200132	0.218238
Neutrophils	KLRA2	0.450809	2.524963	2.234695	0.027958	-4.01414	0.218847	0.238093
Neutrophils	RHEB	0.202989	7.039284	2.233649	0.028029	-4.16464	0.192805	0.210417
Neutrophils	SIAH1B	-0.9573	1.91846	-2.23321	0.02806	-3.46904	0.223156	0.242841
Neutrophils	FAM174A	0.348433	6.050716	2.232462	0.028111	-3.99489	0.198491	0.216675
Neutrophils	APIP	-0.45543	4.220389	-2.23211	0.028135	-3.53998	0.209106	0.228109
Neutrophils	S100PBP	-0.48265	4.405401	-2.23185	0.028153	-3.51658	0.208007	0.227
Neutrophils	REST	0.337786	5.400614	2.231775	0.028158	-3.81911	0.202196	0.220809
Neutrophils	RBM28	-0.35543	5.311789	-2.23123	0.028195	-3.70488	0.202832	0.221518
Neutrophils	SIRPB1C	0.48573	1.778958	2.230901	0.028218	-3.92562	0.224343	0.24452
Neutrophils	NLRC3	-0.96054	1.662225	-2.23046	0.028248	-3.46923	0.225172	0.245451
Neutrophils	CDH5	-0.5234	4.480665	-2.2298	0.028293	-3.86606	0.207965	0.22721
Neutrophils	ACTR1A	0.226111	6.140647	2.22934	0.028325	-4.00783	0.198452	0.216993
Neutrophils	TMEM167E	0.507305	3.755594	2.227524	0.028451	-3.58113	0.213107	0.232336
Neutrophils	GM13889	-0.95592	1.14195	-2.22738	0.028461	-3.45881	0.229608	0.249868
Neutrophils	KLF10	0.303908	5.248269	2.224899	0.028633	-3.91365	0.205248	0.2236
Neutrophils	USP54	-0.81179	2.915478	-2.22476	0.028643	-3.46614	0.219355	0.238628
Neutrophils	SIPA1L3	0.348326	5.201146	2.224462	0.028664	-3.86638	0.205528	0.223954
Neutrophils	PPARD	0.287903	5.613407	2.223513	0.02873	-4.02195	0.203456	0.221553

Neutrophils	GMPR2	0.515327	3.866899	2.221669	0.028859	-3.55996	0.214638	0.233229
Neutrophils	COX17	0.200509	6.919214	2.220612	0.028933	-4.31649	0.196991	0.214551
Neutrophils	TBL1X	-0.28025	7.155308	-2.22059	0.028935	-4.06457	0.195675	0.213145
Neutrophils	CLDN11	-1.33016	-0.29485	-2.22018	0.028964	-3.52865	0.241964	0.262414
Neutrophils	PPP2R3D	-0.54768	3.751015	-2.22007	0.028971	-3.56559	0.215574	0.23451
Neutrophils	TMEM221	-1.38857	-0.26558	-2.21946	0.029015	-3.6031	0.241951	0.262304
Neutrophils	HLX	0.334358	3.117664	2.218949	0.02905	-3.85312	0.219706	0.238905
Neutrophils	SREK1	-0.23482	6.429668	-2.21858	0.029077	-3.94223	0.199934	0.218001
Neutrophils	RIDA	-0.69531	4.316478	-2.21795	0.029121	-3.73789	0.212325	0.231368
Neutrophils	ORAI1	0.234457	6.491763	2.217815	0.029131	-4.21061	0.199582	0.217786
Neutrophils	MSN	0.206869	8.454473	2.217265	0.029169	-4.4778	0.188773	0.206303
Neutrophils	DHX58	0.820758	2.999964	2.217241	0.029171	-3.52024	0.220445	0.240129
Neutrophils	SYAP1	0.254272	5.053275	2.217093	0.029182	-3.91383	0.207916	0.22679
Neutrophils	PGD	0.196465	5.716274	2.217019	0.029187	-4.30778	0.20403	0.222643
Neutrophils	BORCS7	0.554663	3.716689	2.216783	0.029204	-3.51392	0.215985	0.235387
Neutrophils	CD300A	0.306148	4.623283	2.216067	0.029254	-4.20333	0.210551	0.229739
Neutrophils	LSS	-1.32682	0.604735	-2.216	0.029259	-3.59742	0.236127	0.256916
Neutrophils	GM12236	-1.08443	1.087964	-2.21577	0.029276	-3.47743	0.232891	0.25349
Neutrophils	FNDC3B	0.220364	6.549271	2.215363	0.029304	-4.34373	0.199383	0.217839
Neutrophils	AKR1D1	-0.91704	2.587501	-2.21438	0.029375	-3.51425	0.223573	0.243528
Neutrophils	PTH1R	1.507848	0.264703	2.213417	0.029443	-3.54761	0.239206	0.260212
Neutrophils	FGD6	-0.61595	4.002414	-2.21328	0.029453	-3.55819	0.215004	0.234538
Neutrophils	DUBR	0.526702	2.378269	2.212661	0.029497	-3.59924	0.225243	0.245412
Neutrophils	APOH	-0.63474	4.983199	-2.21262	0.0295	-3.91857	0.209123	0.228255
Neutrophils	LTBP1	1.961167	0.730726	2.209785	0.029703	-3.50616	0.237302	0.258018
Neutrophils	PLCG1	-0.67171	3.409844	-2.20936	0.029734	-3.49825	0.219833	0.239532
Neutrophils	1600014C1	0.456809	4.275444	2.209271	0.02974	-3.90894	0.214476	0.233853
Neutrophils	PAK1IP1	-0.22952	5.901653	-2.20901	0.029759	-3.95128	0.204774	0.223569
Neutrophils	NR4A1	0.381027	7.413157	2.208628	0.029787	-4.42803	0.196169	0.214414
Neutrophils	SLC36A3OS	0.975219	1.010121	2.208498	0.029796	-3.49836	0.235416	0.256231
Neutrophils	CRIPT	0.214733	6.218829	2.20849	0.029797	-4.11569	0.202936	0.221663
Neutrophils	TCF12	-0.28494	8.463403	-2.20796	0.029835	-4.23505	0.190441	0.208364
Neutrophils	FUCA1	0.257486	5.963141	2.207859	0.029842	-4.06177	0.204444	0.223382
Neutrophils	SERPINE1	1.128238	0.805177	2.206905	0.029911	-3.5085	0.237213	0.258147
Neutrophils	CFHR2	-0.85015	2.56401	-2.20621	0.029961	-3.52811	0.225786	0.246041
Neutrophils	NPLOC4	0.352731	5.537568	2.205984	0.029978	-3.80966	0.207443	0.226543
Neutrophils	ABCB11	-0.90802	1.681529	-2.20448	0.030087	-3.49822	0.232226	0.252846
Neutrophils	TTC28	-0.49407	4.798912	-2.20305	0.030191	-3.78302	0.213065	0.232312
Neutrophils	AHDC1	-0.42234	4.689967	-2.20205	0.030264	-3.61631	0.214094	0.233411
Neutrophils	SNHG4	-0.75562	3.683196	-2.20109	0.030334	-3.50275	0.220571	0.240269
Neutrophils	MLF1	1.307955	0.512644	2.200336	0.03039	-3.59337	0.241461	0.262591
Neutrophils	STK39	-0.62745	3.517793	-2.20022	0.030398	-3.72779	0.221614	0.241557
Neutrophils	OXR1	-0.2566	6.336267	-2.2002	0.0304	-4.13911	0.204527	0.223329
Neutrophils	SDAD1	-0.6837	4.473042	-2.20016	0.030403	-3.51827	0.215662	0.235224
Neutrophils	NMT1	0.217715	6.941125	2.199579	0.030445	-4.14699	0.201185	0.219733
Neutrophils	VMN2R19	-0.6737	-0.79625	-2.19871	0.030509	-3.58153	0.251189	0.27286
Neutrophils	NRXN3	1.065828	1.145579	2.19814	0.030551	-3.50737	0.237669	0.258704

Neutrophils	GM15247	1.504665	0.995367	2.198011	0.030561	-3.57312	0.238691	0.259789
Neutrophils	HIP1	0.392244	5.505684	2.197801	0.030576	-4.00826	0.209886	0.229244
Neutrophils	D16ERTD47	-0.28599	4.914897	-2.19723	0.030618	-4.09364	0.213592	0.233221
Neutrophils	RMI1	0.48612	3.629414	2.196237	0.030692	-3.56619	0.221891	0.242044
Neutrophils	BCKDHB	-0.63145	3.929893	-2.19586	0.03072	-3.56364	0.219999	0.240056
Neutrophils	IFRD1	0.264408	8.532379	2.195555	0.030742	-4.6171	0.193027	0.211189
Neutrophils	HTATIP2	0.373379	4.480259	2.195314	0.03076	-3.77349	0.216576	0.236429
Neutrophils	GPR171	-1.3429	4.027921	-2.195	0.030784	-3.52612	0.219385	0.239485
Neutrophils	CSTDC4	-0.34145	3.661016	-2.19466	0.030808	-4.66626	0.221691	0.241989
Neutrophils	SAMD9L	0.277532	5.184732	2.194623	0.030812	-4.19758	0.212275	0.23194
Neutrophils	B230217O1	-1.10102	1.757632	-2.19313	0.030922	-3.52915	0.234743	0.255609
Neutrophils	9330111N0	-1.24537	0.392898	-2.1925	0.03097	-3.55117	0.244155	0.265667
Neutrophils	NFAM1	0.212333	4.570884	2.192426	0.030975	-4.31355	0.21672	0.236538
Neutrophils	LAGE3	-0.4147	4.771468	-2.19113	0.031072	-3.70259	0.21576	0.23577
Neutrophils	MIEN1	0.265053	5.845651	2.19038	0.031128	-3.92428	0.209265	0.229044
Neutrophils	WSB1	-0.20957	6.461454	-2.19036	0.03113	-4.26913	0.205633	0.225151
Neutrophils	CHCHD4	0.609197	3.820424	2.190213	0.031141	-3.55924	0.221686	0.24237
Neutrophils	RARA	0.299548	5.561492	2.190078	0.031151	-4.05903	0.210963	0.230937
Neutrophils	AMPD1	-0.72223	2.911186	-2.18981	0.03117	-3.56795	0.227509	0.248715
Neutrophils	SGK3	0.286895	5.592861	2.189666	0.031182	-4.12554	0.210775	0.230827
Neutrophils	C530005A1	-1.03854	0.742343	-2.18947	0.031196	-3.57819	0.242039	0.264173
Neutrophils	GM15879	1.329118	0.515986	2.189357	0.031205	-3.60949	0.243609	0.265865
Neutrophils	CHKA	-0.25038	6.940391	-2.1877	0.031329	-4.32081	0.20331	0.22277
Neutrophils	RRP9	-0.801	2.59462	-2.18698	0.031384	-3.53017	0.230087	0.25151
Neutrophils	YPEL3	0.258547	7.069109	2.186667	0.031407	-4.40832	0.202568	0.222082
Neutrophils	MRPL33	0.202542	6.142277	2.186607	0.031412	-4.38042	0.207972	0.227887
Neutrophils	GSTM5	-1.27573	1.19189	-2.18592	0.031464	-3.55741	0.239487	0.2617
Neutrophils	FLOT1	0.251817	4.575358	2.185826	0.031471	-4.15424	0.217454	0.238223
Neutrophils	HYAL1	0.974773	1.311575	2.185327	0.031509	-3.55993	0.23867	0.261017
Neutrophils	GDA	0.314341	3.902395	2.185122	0.031524	-4.62078	0.221664	0.242892
Neutrophils	PLK2	0.458316	5.006155	2.18509	0.031527	-4.02784	0.214803	0.235542
Neutrophils	CXXC1	0.471885	4.307295	2.185079	0.031528	-3.63978	0.219121	0.24017
Neutrophils	SIVA1	0.368214	5.470666	2.184892	0.031542	-3.97852	0.211982	0.232568
Neutrophils	GIMAP6	-0.56157	5.672059	-2.18488	0.031543	-3.87112	0.210771	0.231268
Neutrophils	2700081O1	-0.79513	3.137169	-2.18476	0.031552	-3.52878	0.226553	0.248175
Neutrophils	6-Mar	0.178326	6.3538	2.184501	0.031571	-4.29026	0.206726	0.22698
Neutrophils	FXYD1	-0.73989	2.943369	-2.1841	0.031602	-3.63056	0.227875	0.249691
Neutrophils	SNHG15	-0.75978	3.481407	-2.18272	0.031707	-3.53649	0.224999	0.246297
Neutrophils	USP7	-0.20725	6.215443	-2.181	0.031838	-4.1359	0.208874	0.228588
Neutrophils	MTIF3	0.739735	3.10156	2.180501	0.031876	-3.54186	0.22836	0.24931
Neutrophils	ZFP36	0.272515	7.849088	2.179934	0.03192	-4.58449	0.199573	0.218496
Neutrophils	TIMM10	0.743619	3.050855	2.179755	0.031933	-3.53726	0.22876	0.249754
Neutrophils	RSAD2	0.776418	4.537164	2.179294	0.031969	-3.96823	0.219271	0.239702
Neutrophils	AU020206	-0.55468	5.320744	-2.17926	0.031971	-3.63997	0.214433	0.23453
Neutrophils	TACSTD2	0.622471	-0.35025	2.178413	0.032036	-3.64396	0.252301	0.274915
Neutrophils	KRT81	-0.5424	-1.53173	-2.17835	0.032041	-3.62901	0.260961	0.284067
Neutrophils	NR1D2	-0.64808	3.478586	-2.17774	0.032088	-3.55946	0.226358	0.247397

Neutrophils	ATCAYOS	-1.32184	1.220314	-2.17668	0.03217	-3.54832	0.241879	0.263622
Neutrophils	ZFP58	-1.22377	1.194809	-2.17632	0.032197	-3.61791	0.242101	0.263906
Neutrophils	GLRX2	0.324695	5.111058	2.175652	0.032249	-3.83565	0.216682	0.236832
Neutrophils	CLMN	1.318998	0.334512	2.175454	0.032264	-3.60336	0.24831	0.270512
Neutrophils	MRPL50	-0.46199	4.009842	-2.17515	0.032288	-3.633	0.223601	0.244343
Neutrophils	CCNC	0.421915	4.398502	2.174503	0.032338	-3.75494	0.221334	0.241907
Neutrophils	C8A	-1.15573	0.525618	-2.17382	0.032391	-3.5833	0.247433	0.269739
Neutrophils	GM30541	0.655223	0.952964	2.173073	0.032449	-3.58356	0.244708	0.266737
Neutrophils	ACTG1	0.227289	11.44474	2.172304	0.032509	-5.06437	0.18174	0.199352
Neutrophils	KDM2A	0.156802	7.465818	2.1722	0.032517	-4.39004	0.203378	0.222681
Neutrophils	0610043K1	-1.35962	1.133363	-2.17167	0.032558	-3.59052	0.243573	0.265768
Neutrophils	ITSN2	0.210932	6.989544	2.171595	0.032564	-4.34902	0.206145	0.225805
Neutrophils	TGM2	0.343828	4.674438	2.171264	0.03259	-4.37559	0.220173	0.240966
Neutrophils	KRT80	0.683263	0.161146	2.170887	0.032619	-3.55799	0.250431	0.273291
Neutrophils	HYAL3	-1.19106	0.035974	-2.17062	0.03264	-3.60409	0.251328	0.274327
Neutrophils	PRKAR2B	0.398632	3.305356	2.170282	0.032666	-3.86664	0.228933	0.25058
Neutrophils	DENND6A	-0.41434	5.102952	-2.17027	0.032667	-3.72139	0.217503	0.23835
Neutrophils	XLR4B	-1.16544	1.285179	-2.16963	0.032717	-3.64897	0.242519	0.265302
Neutrophils	PITPNM2	-0.65983	3.66119	-2.16949	0.032728	-3.58676	0.226622	0.248396
Neutrophils	SYNPO	-1.29827	0.219102	-2.16942	0.032734	-3.61217	0.250017	0.273342
Neutrophils	DNAH8	-0.51274	3.758702	-2.16928	0.032745	-3.75246	0.225993	0.247768
Neutrophils	SIAE	-0.72617	2.612072	-2.16862	0.032796	-3.5606	0.233717	0.255979
Neutrophils	EIF6	0.293604	5.835967	2.168039	0.032842	-4.004	0.213278	0.234177
Neutrophils	TRP53COR1	1.345814	1.008827	2.167807	0.03286	-3.58662	0.244744	0.267962
Neutrophils	AKAP17B	1.155129	1.696468	2.167663	0.032872	-3.60516	0.239985	0.262913
Neutrophils	ZFAND6	0.188868	6.978135	2.167173	0.03291	-4.29549	0.206574	0.227104
Neutrophils	R3HDM2	0.192876	6.193799	2.165419	0.033048	-4.19039	0.211977	0.232613
Neutrophils	WWP2	0.187807	6.537142	2.164231	0.033142	-4.37557	0.210377	0.230678
Neutrophils	LGALS1	-0.71644	6.036335	-2.1635	0.0332	-3.77847	0.213557	0.234088
Neutrophils	GM17021	1.354831	0.125797	2.163363	0.033211	-3.63218	0.252758	0.275982
Neutrophils	SLC10A3	0.85573	2.160549	2.162889	0.033249	-3.56531	0.238605	0.260903
Neutrophils	NRXN2	-1.33042	2.095803	-2.16132	0.033374	-3.57147	0.239789	0.26183
Neutrophils	GM50322	-1.25297	0.358739	-2.16042	0.033445	-3.61351	0.252355	0.275101
Neutrophils	MS4A4B	-0.91944	3.207847	-2.16	0.033479	-3.58688	0.232729	0.254316
Neutrophils	FCNB	0.316643	-0.23854	2.159412	0.033526	-4.02662	0.256982	0.280112
Neutrophils	GTDC1	0.210719	6.409058	2.157941	0.033643	-4.44498	0.213099	0.233169
Neutrophils	CD72	-0.85663	4.334961	-2.15791	0.033646	-3.58987	0.226053	0.247029
Neutrophils	BLOC1S3	0.659271	2.381624	2.154338	0.033933	-3.62475	0.240887	0.261835
Neutrophils	NIPSNAP2	0.284051	4.810103	2.153862	0.033972	-3.90317	0.224839	0.244796
Neutrophils	GPR141	0.3767	2.440611	2.153663	0.033988	-4.41823	0.240551	0.26152
Neutrophils	GM17036	1.040559	2.038724	2.153399	0.034009	-3.59052	0.243326	0.264467
Neutrophils	1300017J0	-0.65142	3.006144	-2.15283	0.034055	-3.6735	0.236865	0.257491
Neutrophils	TBCCD1	-0.87735	2.589728	-2.15164	0.034152	-3.58697	0.240224	0.260792
Neutrophils	FARSA	-0.43799	4.90554	-2.14985	0.034297	-3.68694	0.225605	0.244999
Neutrophils	DHCR7	-0.89963	2.184949	-2.14974	0.034307	-3.61335	0.2438	0.264318
Neutrophils	SERPINB9	-1.07787	3.483496	-2.1489	0.034375	-3.60828	0.235215	0.255236
Neutrophils	TPP1	-0.33188	5.393356	-2.14869	0.034392	-4.03179	0.222761	0.242037

Neutrophils	CFDP1	-0.2704	5.986865	-2.14835	0.03442	-4.06896	0.219066	0.238152
Neutrophils	GM26887	0.729006	3.10483	2.148081	0.034442	-3.73404	0.237806	0.258149
Neutrophils	TUBB3	-1.0696	0.736309	-2.14771	0.034472	-3.61836	0.254496	0.275832
Neutrophils	ATP9B	0.261802	5.858499	2.147141	0.034519	-4.05628	0.220075	0.239308
Neutrophils	DNAJC5	0.154919	6.766699	2.146116	0.034603	-4.37296	0.214569	0.233665
Neutrophils	ZKSCAN6	0.46673	2.942031	2.145897	0.034621	-3.7882	0.239251	0.259931
Neutrophils	ACAA1B	-1.03803	3.318751	-2.14586	0.034624	-3.71841	0.236695	0.257221
Neutrophils	MYDGF	-0.4351	4.485984	-2.14586	0.034625	-3.68863	0.22895	0.248999
Neutrophils	BC065397	-1.10812	1.292469	-2.14384	0.034791	-3.6577	0.251829	0.272838
Neutrophils	DEF6	0.208397	5.457612	2.142803	0.034876	-4.3133	0.224036	0.243236
Neutrophils	PRKAG2	0.354898	5.050385	2.141871	0.034954	-3.95306	0.227004	0.246243
Neutrophils	GM49482	-1.36947	-0.71551	-2.14116	0.035012	-3.69267	0.267727	0.289302
Neutrophils	POLDIP3	0.264939	6.078814	2.140936	0.035031	-4.05861	0.220567	0.239569
Neutrophils	SNX32	0.615173	2.812618	2.140854	0.035038	-3.61995	0.242071	0.262378
Neutrophils	SLC17A2	-1.02801	1.156237	-2.14051	0.035067	-3.60142	0.253836	0.274786
Neutrophils	CCS	-0.50822	4.318203	-2.13911	0.035183	-3.73767	0.23256	0.251962
Neutrophils	TFEC	-0.62578	2.368464	-2.13838	0.035244	-3.75617	0.246127	0.266257
Neutrophils	ACOT9	0.299213	4.620859	2.137834	0.03529	-4.01132	0.230969	0.250318
Neutrophils	BAZ2B	0.172234	8.430417	2.137464	0.035321	-4.68918	0.207328	0.225273
Neutrophils	H1FX	-1.0349	2.127581	-2.13704	0.035356	-3.60572	0.248134	0.268669
Neutrophils	KIF1BP	0.44112	3.865166	2.136636	0.03539	-3.75504	0.236214	0.25609
Neutrophils	TMEM26	-1.02752	2.190341	-2.13573	0.035466	-3.60819	0.248143	0.26851
Neutrophils	6430550D2	1.182423	1.142129	2.135138	0.035516	-3.65987	0.255879	0.276603
Neutrophils	SAMHD1	0.327888	7.45997	2.134199	0.035595	-4.65352	0.214064	0.232255
Neutrophils	SARNP	-0.17765	7.797226	-2.13396	0.035614	-4.44999	0.212026	0.230121
Neutrophils	PSMD8	0.273299	6.592353	2.133464	0.035657	-4.17924	0.219522	0.238115
Neutrophils	RASL11B	-0.78651	0.665224	-2.13138	0.035833	-3.6235	0.261041	0.281334
Neutrophils	SLC24A5	-0.66686	4.423305	-2.13064	0.035896	-3.6353	0.234718	0.25357
Neutrophils	MATK	0.946304	2.36611	2.130354	0.03592	-3.61704	0.248898	0.26856
Neutrophils	ISOC2B	0.833872	2.519105	2.13018	0.035935	-3.61756	0.247814	0.26742
Neutrophils	TNFRSF10B	1.241899	-0.0063	2.129004	0.036034	-3.68163	0.266915	0.287126
Neutrophils	BTD	-0.78455	2.37707	-2.1283	0.036095	-3.62247	0.249612	0.268928
Neutrophils	KLF13	0.206684	8.183848	2.126586	0.036241	-4.55805	0.212324	0.229213
Neutrophils	CYP2C68	-0.79374	2.791797	-2.12515	0.036364	-3.70014	0.248203	0.266891
Neutrophils	PDXDC1	0.209981	6.616598	2.124478	0.036421	-4.24011	0.222803	0.240152
Neutrophils	DRAM2	0.308801	4.847911	2.124087	0.036455	-4.06352	0.23433	0.252392
Neutrophils	IFI214	-1.46791	1.667368	-2.12388	0.036473	-3.69135	0.256576	0.275833
Neutrophils	ROMO1	0.221897	6.595619	2.123241	0.036528	-4.27965	0.223164	0.240612
Neutrophils	VPS33A	-0.43588	4.291318	-2.121	0.036721	-3.75992	0.239188	0.257181
Neutrophils	GTPBP2	0.378378	4.751688	2.120862	0.036733	-3.91678	0.236072	0.25397
Neutrophils	DSG2	-1.22734	0.740688	-2.12083	0.036736	-3.66687	0.264688	0.283944
Neutrophils	MFSD10	0.397337	4.464143	2.120567	0.036759	-3.90438	0.238013	0.25609
Neutrophils	NQO2	-0.32115	3.942173	-2.12023	0.036788	-3.95774	0.241617	0.259933
Neutrophils	DNAJC7	-0.2977	7.510277	-2.11925	0.036873	-4.25796	0.218428	0.235652
Neutrophils	NAIF1	0.875782	1.251393	2.119063	0.036889	-3.63656	0.261046	0.280509
Neutrophils	TRIB3	1.193542	1.057747	2.119047	0.036891	-3.64065	0.262494	0.282023
Neutrophils	MCRIP2	-0.87766	2.015335	-2.11878	0.036913	-3.64089	0.255414	0.274668

Neutrophils	PCOLCE2	-1.0615	1.678726	-2.11866	0.036924	-3.63989	0.25788	0.27725
Neutrophils	RBM15B	-0.44237	4.047089	-2.11742	0.037032	-3.74749	0.24153	0.259931
Neutrophils	CHMP2A	0.185903	6.544809	2.117252	0.037047	-4.4302	0.224959	0.242471
Neutrophils	GTF2E2	0.263418	5.440806	2.116684	0.037096	-4.10541	0.232185	0.250274
Neutrophils	TTLL12	-1.30667	2.217401	-2.11656	0.037107	-3.66135	0.254526	0.27381
Neutrophils	CTTNBP2NI	-0.67761	2.52025	-2.11636	0.037125	-3.66419	0.252336	0.271555
Neutrophils	ZFP788	0.839437	2.069958	2.115896	0.037165	-3.65308	0.255718	0.275145
Neutrophils	ELMOD3	-0.61682	3.437093	-2.1146	0.037279	-3.82299	0.246533	0.265344
Neutrophils	RHBDL3	-1.34932	0.747589	-2.11394	0.037337	-3.70078	0.266449	0.286256
Neutrophils	MAMLD1	1.185065	0.110917	2.113646	0.037362	-3.66672	0.271357	0.291431
Neutrophils	FADS1	-0.74935	3.059286	-2.11272	0.037444	-3.6517	0.249796	0.268871
Neutrophils	SIRT7	0.261771	4.918849	2.112279	0.037483	-4.0089	0.236903	0.255452
Neutrophils	DNAJA1	-0.15843	7.461326	-2.11202	0.037505	-4.55177	0.220388	0.238048
Neutrophils	OCIAD2	-1.2775	0.485681	-2.112	0.037507	-3.69595	0.268838	0.289036
Neutrophils	ATP6V1A	0.221156	6.534998	2.111682	0.037535	-4.44559	0.226289	0.244311
Neutrophils	WDPCP	-0.79142	3.269533	-2.11082	0.037611	-3.65043	0.24868	0.267832
Neutrophils	SERTAD1	0.303848	5.61417	2.110419	0.037647	-4.25107	0.232692	0.25111
Neutrophils	HNRNPH2	0.182624	6.435152	2.109691	0.037711	-4.39963	0.227572	0.245659
Neutrophils	TSPAN18	-0.69594	1.764014	-2.10936	0.03774	-3.72444	0.260005	0.279929
Neutrophils	THEMIS2	0.271133	4.692319	2.108614	0.037807	-4.43728	0.239315	0.258275
Neutrophils	ATXN7L3B	0.211151	5.941589	2.108585	0.037809	-4.2752	0.230957	0.249447
Neutrophils	TRDMT1	0.642401	2.342131	2.107921	0.037868	-3.67045	0.25614	0.275876
Neutrophils	FPR3	0.755837	-0.4789	2.107242	0.037929	-3.69528	0.277781	0.298612
Neutrophils	RIOK1	-0.22899	6.007255	-2.1068	0.037968	-4.25127	0.23087	0.249402
Neutrophils	LAX1	-1.13737	2.319766	-2.10646	0.037998	-3.66643	0.256445	0.276365
Neutrophils	DCLRE1A	-0.75573	1.621612	-2.10644	0.038	-3.66032	0.261607	0.281782
Neutrophils	ESPL1	-0.80309	2.563574	-2.10637	0.038006	-3.65695	0.254667	0.274497
Neutrophils	CHRNA9	1.006877	2.214161	2.105798	0.038057	-3.65889	0.257286	0.277379
Neutrophils	EPN1	0.260841	6.109989	2.105733	0.038063	-4.21952	0.230257	0.248895
Neutrophils	GADD45GII	-0.425	5.128075	-2.10536	0.038097	-3.85359	0.236842	0.255904
Neutrophils	C1QA	-0.59492	5.480946	-2.10495	0.038133	-4.2961	0.234499	0.253518
Neutrophils	PPP5C	-0.45083	4.346979	-2.10459	0.038165	-3.77538	0.242193	0.261787
Neutrophils	CEP135	0.394513	3.853066	2.104527	0.038171	-3.79068	0.245626	0.265424
Neutrophils	MAP3K1	-0.23878	7.970553	-2.10415	0.038204	-4.46372	0.21855	0.236798
Neutrophils	CARHSP1	0.285545	5.430457	2.103677	0.038247	-4.26471	0.235015	0.254229
Neutrophils	SEL1L	0.28442	5.161227	2.103271	0.038283	-4.08046	0.236903	0.256244
Neutrophils	WIZ	0.737619	3.836067	2.102957	0.038311	-3.66843	0.246046	0.265984
Neutrophils	HIST1H2BJ	-0.86333	3.30978	-2.10264	0.03834	-3.71398	0.249799	0.270025
Neutrophils	PGS1	0.278603	4.740858	2.101748	0.03842	-4.1161	0.240112	0.259838
Neutrophils	TLN1	0.210245	7.109295	2.10159	0.038434	-4.55434	0.224476	0.243279
Neutrophils	1500015A0	0.809671	1.76661	2.100949	0.038492	-3.66775	0.261519	0.282447
Neutrophils	VWA5A	0.630893	3.406572	2.100467	0.038535	-3.72848	0.249563	0.269985
Neutrophils	PADI4	0.465772	1.890862	2.100306	0.03855	-4.12678	0.260593	0.281638
Neutrophils	ARPC2	0.118638	9.318766	2.100297	0.038551	-4.91637	0.210977	0.229042
Neutrophils	CCDC62	-0.59775	2.858429	-2.09951	0.038622	-3.79886	0.253781	0.274534
Neutrophils	CAB39	0.184622	6.974308	2.099296	0.038641	-4.50615	0.225722	0.244836
Neutrophils	ATP2A2	-0.23144	6.456942	-2.09865	0.0387	-4.21237	0.229126	0.248596

Neutrophils	LDHA	0.237125	8.792149	2.098463	0.038717	-4.80699	0.214443	0.232981
Neutrophils	DOCK5	0.294354	4.189727	2.098399	0.038722	-4.35142	0.244395	0.264832
Neutrophils	SIDT2	0.301793	5.063847	2.097905	0.038767	-4.11061	0.238509	0.258653
Neutrophils	NR6A1	-0.53773	5.241175	-2.09766	0.03879	-3.85492	0.237308	0.257427
Neutrophils	HEXIM1	-0.29882	5.48831	-2.09583	0.038956	-4.27645	0.236513	0.256117
Neutrophils	APBA1	0.781634	4.384633	2.094897	0.039041	-3.73291	0.24414	0.264296
Neutrophils	DTD1	-0.40464	3.881415	-2.09488	0.039042	-4.00664	0.247666	0.268024
Neutrophils	ASTL	-0.96961	1.424213	-2.09466	0.039063	-3.67281	0.265651	0.287089
Neutrophils	ATP6V1D	0.178587	6.650485	2.0944	0.039086	-4.40756	0.228899	0.248244
Neutrophils	CTDSP2	-0.26429	4.801724	-2.09418	0.039106	-4.10573	0.241257	0.261424
Neutrophils	PDE5A	-1.13951	2.569114	-2.09392	0.03913	-3.68139	0.25711	0.278185
Neutrophils	ZFP87	0.521969	3.396086	2.09373	0.039147	-3.77386	0.251116	0.271858
Neutrophils	GM45370	-1.39796	-1.29914	-2.09362	0.039158	-3.75794	0.287139	0.309729
Neutrophils	WTAP	0.183856	6.931297	2.093038	0.039211	-4.43374	0.227127	0.246493
Neutrophils	COX4I2	0.972457	0.956141	2.092877	0.039225	-3.68395	0.26928	0.291125
Neutrophils	TSEN2	0.957927	1.266976	2.092756	0.039236	-3.69052	0.2669	0.288649
Neutrophils	FAM20C	1.236072	1.641237	2.091732	0.03933	-3.68631	0.264536	0.286109
Neutrophils	BOD1	-0.72954	3.243589	-2.09106	0.039392	-3.68319	0.252821	0.273748
Neutrophils	KDM6B	0.213169	8.152763	2.090887	0.039408	-4.81226	0.219879	0.238843
Neutrophils	RNF169	0.308191	6.380124	2.090628	0.039432	-4.34333	0.231223	0.250974
Neutrophils	AHI1	0.542047	3.003332	2.090246	0.039467	-3.75449	0.25456	0.275708
Neutrophils	BTBD1	0.254301	6.497876	2.090069	0.039483	-4.24334	0.23045	0.250164
Neutrophils	MBTPS1	-0.41403	4.639311	-2.09	0.03949	-3.81891	0.242962	0.263449
Neutrophils	EPC2	-0.23635	6.14343	-2.08956	0.03953	-4.33088	0.232885	0.252817
Neutrophils	PUS1	-0.67328	3.723827	-2.08925	0.039559	-3.69308	0.249519	0.270582
Neutrophils	CCNYL1	-0.27509	4.635175	-2.08894	0.039587	-4.18853	0.243153	0.263935
Neutrophils	ATXN7L1	0.216916	6.345264	2.088128	0.039662	-4.47115	0.231908	0.251987
Neutrophils	PIGA	0.69097	1.918295	2.086761	0.039789	-3.7012	0.26351	0.285496
Neutrophils	CAMK2A	-0.96487	0.846737	-2.08664	0.0398	-3.7321	0.271697	0.294133
Neutrophils	TBC1D10B	0.258113	5.147294	2.086178	0.039843	-4.22214	0.240336	0.261191
Neutrophils	N4BP1	0.235776	5.4056	2.086105	0.03985	-4.53257	0.238576	0.259323
Neutrophils	KDM8	-0.9094	1.814343	-2.08571	0.039887	-3.70743	0.264293	0.286733
Neutrophils	DUSP11	0.192837	6.33182	2.08533	0.039922	-4.35497	0.232374	0.252891
Neutrophils	YTHDC2	-0.44735	4.693584	-2.08523	0.039931	-3.85505	0.24346	0.264709
Neutrophils	ADGRL4	-0.50527	3.201942	-2.08523	0.039932	-3.97612	0.254033	0.27593
Neutrophils	LACTB	0.3528	5.139278	2.085122	0.039941	-4.00301	0.240391	0.261468
Neutrophils	IL10RB	0.229345	5.555085	2.084654	0.039985	-4.36252	0.237681	0.258621
Neutrophils	LIMD1	-0.36831	6.016318	-2.08417	0.04003	-3.90938	0.234708	0.255438
Neutrophils	MPRIP	-0.3647	5.481388	-2.08385	0.04006	-4.03599	0.238344	0.259382
Neutrophils	EXOSC5	0.358464	5.376885	2.083348	0.040107	-3.9784	0.239112	0.260301
Neutrophils	5033421B0	-1.4273	-0.4422	-2.08304	0.040136	-3.73828	0.282289	0.306138
Neutrophils	SUCO	0.20292	6.354348	2.082975	0.040141	-4.66661	0.232558	0.253426
Neutrophils	CIAPIN1	0.407945	4.643928	2.082172	0.040217	-3.93528	0.244273	0.265899
Neutrophils	MAP3K7	0.285703	5.219048	2.082003	0.040232	-4.05268	0.240306	0.261676
Neutrophils	GTF2H4	0.77977	2.307317	2.082	0.040233	-3.69778	0.261101	0.283762
Neutrophils	FAM83D	-0.69481	2.774945	-2.08131	0.040297	-3.71265	0.257836	0.280291
Neutrophils	NFKBIA	0.26949	8.130992	2.081166	0.040311	-4.78511	0.221399	0.241494

Neutrophils	FAM219A	-0.45664	4.171512	-2.0802	0.040401	-4.02353	0.248178	0.269885
Neutrophils	FBXO21	-0.75741	3.292952	-2.07986	0.040433	-3.6948	0.254489	0.276651
Neutrophils	KLRA3	-1.60095	-0.66191	-2.07967	0.040452	-3.75281	0.284908	0.308848
Neutrophils	TBK1	0.28529	5.964636	2.079111	0.040504	-4.28756	0.236011	0.257101
Neutrophils	MRGPRA2A	0.475789	-1.27662	2.078642	0.040548	-3.83249	0.290303	0.314556
Neutrophils	TRDV2-2	-0.8985	-1.43025	-2.07689	0.040714	-3.78244	0.292505	0.316606
Neutrophils	9130019P1	1.16713	0.265967	2.07677	0.040725	-3.70649	0.278672	0.302094
Neutrophils	AP2A1	0.435247	4.542647	2.075474	0.040848	-3.91976	0.247165	0.268732
Neutrophils	NCK2	-0.67726	4.870391	-2.07533	0.040861	-3.7845	0.244869	0.266349
Neutrophils	GGT5	0.776943	0.649699	2.074872	0.040905	-3.73874	0.276198	0.299641
Neutrophils	LSP1	0.236201	8.230432	2.074606	0.04093	-4.80628	0.222578	0.242748
Neutrophils	NFKBIE	0.463678	4.879022	2.073919	0.040995	-3.9968	0.244809	0.266754
Neutrophils	HIST1H2AP	0.606793	7.597755	2.073838	0.041003	-4.59251	0.226606	0.247336
Neutrophils	CNN3	-0.50859	4.531127	-2.0736	0.041026	-3.93789	0.247246	0.269404
Neutrophils	RER1	0.220262	6.413933	2.073009	0.041082	-4.40347	0.234351	0.255825
Neutrophils	ZFP945	-0.72616	3.17689	-2.07276	0.041106	-3.70799	0.256976	0.280023
Neutrophils	FGR	0.340052	4.732993	2.072373	0.041143	-4.56261	0.245829	0.268193
Neutrophils	CHCHD10	-0.45726	6.141333	-2.07227	0.041153	-4.22715	0.236173	0.257897
Neutrophils	MRPL43	0.309394	5.608686	2.072254	0.041154	-4.15168	0.239778	0.26175
Neutrophils	CCDC102A	-1.18786	2.048525	-2.07214	0.041165	-3.73369	0.265384	0.289035
Neutrophils	GM16867	0.825034	2.522419	2.071868	0.041191	-3.73631	0.261819	0.285313
Neutrophils	1700029H1	-0.67175	1.58753	-2.07169	0.041208	-3.73412	0.2689	0.292868
Neutrophils	RHOH	-0.46837	6.471792	-2.07136	0.04124	-4.12669	0.233966	0.255767
Neutrophils	ZFP169	0.867302	2.782441	2.071194	0.041255	-3.71712	0.259884	0.283463
Neutrophils	TAF7	0.244872	5.1473	2.071174	0.041257	-4.20637	0.242946	0.265407
Neutrophils	NES	-1.1434	0.578345	-2.07084	0.041289	-3.70992	0.276762	0.301406
Neutrophils	KEL	1.403041	-0.44363	2.070615	0.041311	-3.74222	0.28496	0.310122
Neutrophils	AMER1	-0.76855	2.080672	-2.0704	0.041332	-3.71535	0.265141	0.28914
Neutrophils	PROC	-0.71192	2.923674	-2.0701	0.04136	-3.80594	0.258839	0.282506
Neutrophils	GM48678	0.574828	3.349644	2.069588	0.041409	-3.83918	0.255713	0.279374
Neutrophils	IP6K2	0.497794	3.644965	2.06949	0.041419	-3.77607	0.253569	0.277088
Neutrophils	GNB1	0.130252	8.848488	2.069341	0.041433	-4.78873	0.218716	0.239725
Neutrophils	UAP1	0.323792	5.112787	2.069333	0.041434	-4.12906	0.243185	0.265994
Neutrophils	RSPH1	-1.28553	1.855808	-2.06852	0.041512	-3.71405	0.267197	0.291572
Neutrophils	PXDN	-0.74588	0.993778	-2.06809	0.041554	-3.72673	0.273861	0.298785
Neutrophils	SOCS5	-0.72327	4.029298	-2.06796	0.041566	-3.74703	0.25114	0.274622
Neutrophils	MAGOH	-0.2186	6.296682	-2.06762	0.041599	-4.45114	0.235445	0.257901
Neutrophils	MACO1	-0.27355	6.27208	-2.06751	0.041609	-4.2031	0.23561	0.258086
Neutrophils	5830418P1	-1.38106	1.463039	-2.06725	0.041635	-3.74273	0.270223	0.295153
Neutrophils	SYTL2	-1.28754	1.257049	-2.06644	0.041712	-3.72659	0.272	0.297109
Neutrophils	CRIP2	-0.45613	4.565121	-2.06636	0.04172	-4.03355	0.247508	0.270999
Neutrophils	PIEZO1	-0.41683	5.046187	-2.06575	0.041779	-3.96581	0.244141	0.267611
Neutrophils	EGLN2	0.411973	4.887876	2.065596	0.041794	-3.99388	0.245244	0.268833
Neutrophils	UBE2J2	0.204814	6.638057	2.065116	0.04184	-4.36854	0.233336	0.256201
Neutrophils	GM16153	-0.65262	2.536009	-2.06503	0.041848	-3.76848	0.262249	0.287188
Neutrophils	TXK	-0.80369	3.287256	-2.06497	0.041855	-3.78802	0.256689	0.281252
Neutrophils	DRAM1	-0.40612	2.581586	-2.06492	0.04186	-4.22007	0.261908	0.286831

Neutrophils	OSGEP	-0.4451	4.907558	-2.06471	0.04188	-3.84736	0.245107	0.268902
Neutrophils	ACOD1	0.66281	2.318035	2.063091	0.042037	-4.4833	0.26471	0.289604
Neutrophils	PYGM	-0.50638	4.300481	-2.06287	0.042059	-4.00636	0.250161	0.274143
Neutrophils	ACOXL	0.975442	1.285897	2.06215	0.042129	-3.72521	0.27292	0.298302
Neutrophils	BAG1	0.204211	6.693589	2.06174	0.042169	-4.41306	0.234037	0.256757
Neutrophils	PWP2	-1.0795	1.891751	-2.06126	0.042216	-3.74299	0.268349	0.29355
Neutrophils	GM6712	-0.95712	2.305447	-2.06123	0.042218	-3.72823	0.265199	0.290192
Neutrophils	CORO1B	0.5109	4.252422	2.060857	0.042255	-3.86907	0.250948	0.274928
Neutrophils	RENBP	-0.44037	4.474184	-2.06058	0.042282	-3.95082	0.249386	0.273328
Neutrophils	RAB7	0.133641	7.931545	2.058699	0.042466	-4.89506	0.226854	0.248745
Neutrophils	ARMC1	-0.35311	4.827556	-2.05854	0.042482	-3.95155	0.24777	0.271223
Neutrophils	PDGFA	-1.13862	2.114128	-2.05829	0.042506	-3.72993	0.267701	0.292566
Neutrophils	VWA8	-0.34755	4.975158	-2.05749	0.042584	-4.08338	0.247046	0.270513
Neutrophils	ROBO3	-1.20721	-0.8339	-2.05644	0.042688	-3.82717	0.292124	0.318355
Neutrophils	4933411E0	1.167847	0.555657	2.055912	0.04274	-3.76187	0.280822	0.30637
Neutrophils	ZFP422	-0.50296	4.395092	-2.05585	0.042746	-3.83982	0.251685	0.275344
Neutrophils	MSANTD4	-0.52713	2.89703	-2.05517	0.042814	-3.77491	0.262919	0.287206
Neutrophils	GM49625	-0.9276	2.147374	-2.05484	0.042846	-3.73835	0.268604	0.293283
Neutrophils	SCARB2	0.255752	6.538743	2.054688	0.042861	-4.43639	0.237022	0.259542
Neutrophils	GGA2	-0.64386	4.120751	-2.05319	0.04301	-3.78794	0.254639	0.278005
Neutrophils	SHC4	-1.10547	0.470109	-2.05265	0.043062	-3.76249	0.282783	0.307951
Neutrophils	MBP	0.260144	5.172136	2.052083	0.043119	-4.4378	0.247345	0.270343
Neutrophils	PARVG	0.278387	5.071901	2.052066	0.043121	-4.24365	0.248051	0.271099
Neutrophils	CD209G	-2.39268	1.089148	-2.05157	0.04317	-3.73758	0.278046	0.30305
Neutrophils	PAQR5	1.124994	0.502008	2.051207	0.043206	-3.74928	0.282825	0.3082
Neutrophils	POC1B	-0.26267	4.642223	-2.05015	0.043312	-4.19941	0.251665	0.275088
Neutrophils	HACD3	-0.40162	4.907611	-2.05006	0.04332	-3.90156	0.249771	0.273063
Neutrophils	CROCC	-1.05835	1.025643	-2.04987	0.04334	-3.76065	0.279014	0.304263
Neutrophils	WDSUB1	-0.5955	2.44185	-2.04907	0.043419	-3.76965	0.268296	0.292879
Neutrophils	ATP6V0D1	0.134302	7.228201	2.048376	0.043489	-4.71276	0.234363	0.256555
Neutrophils	APOA2	-0.51706	8.419445	-2.04623	0.043705	-4.88485	0.227471	0.248809
Neutrophils	GLDC	0.967574	1.391092	2.046179	0.043709	-3.74623	0.277841	0.302559
Neutrophils	MMRN1	1.961716	-0.22843	2.04563	0.043764	-3.77897	0.291068	0.316794
Neutrophils	DMD	0.637375	2.419953	2.045357	0.043792	-3.85137	0.269869	0.294399
Neutrophils	ETV6	0.242714	8.156803	2.044788	0.043849	-4.77973	0.229229	0.250952
Neutrophils	CAR8	-0.67782	2.375063	-2.04468	0.04386	-3.85082	0.270215	0.294767
Neutrophils	ABCD1	0.326058	4.771292	2.044552	0.043873	-4.06555	0.252372	0.275753
Neutrophils	IL5RA	-1.44563	0.975245	-2.04443	0.043885	-3.7935	0.281232	0.306463
Neutrophils	KIFC5B	-0.87335	1.787655	-2.04435	0.043894	-3.74813	0.274783	0.299636
Neutrophils	KNOP1	-0.38873	4.90183	-2.04404	0.043925	-3.95428	0.251472	0.274877
Neutrophils	ESRRG	-0.85413	1.226258	-2.04341	0.043988	-3.75832	0.279509	0.304736
Neutrophils	GM14305	-0.81435	2.260659	-2.04287	0.044043	-3.76067	0.27156	0.296203
Neutrophils	5830428M	0.516746	2.548294	2.042276	0.044103	-3.89092	0.269558	0.293929
Neutrophils	NT5DC2	-0.80645	2.432435	-2.04144	0.044188	-3.75794	0.270752	0.295247
Neutrophils	SLC23A3	-1.13015	1.461516	-2.0413	0.044202	-3.75435	0.278361	0.303327
Neutrophils	SPINT1	1.020675	-1.0651	2.040209	0.044313	-3.80447	0.299355	0.3258
Neutrophils	DENND3	0.318327	3.548798	2.039937	0.044341	-4.25537	0.26241	0.286678

Neutrophils	RAB31	0.247889	4.770711	2.039846	0.04435	-4.4629	0.253431	0.277092
Neutrophils	ZFP236	0.272748	4.729945	2.039842	0.04435	-4.21942	0.253725	0.277407
Neutrophils	OCEL1	0.460731	3.584489	2.039651	0.04437	-3.88462	0.262143	0.286394
Neutrophils	TRAF1	0.908181	3.343827	2.039599	0.044375	-3.81868	0.263948	0.288331
Neutrophils	LCP1	0.176294	8.858643	2.039093	0.044427	-5.07998	0.225793	0.247392
Neutrophils	EEF1AKMT	-0.70684	2.582886	-2.03866	0.044471	-3.75684	0.270018	0.294734
Neutrophils	COLGALT1	-0.31156	5.889717	-2.03818	0.04452	-4.16987	0.245844	0.268899
Neutrophils	I830077J02	0.373414	2.758256	2.037995	0.044539	-4.22389	0.268782	0.293439
Neutrophils	MFSD2B	0.981725	0.599514	2.037297	0.04461	-3.76859	0.286166	0.311835
Neutrophils	ENTPD6	-0.74125	2.776877	-2.03697	0.044644	-3.75913	0.268971	0.293552
Neutrophils	PLEKHA5	-0.56401	4.693302	-2.03591	0.044753	-3.8688	0.255152	0.278784
Neutrophils	RNF227	-1.11012	1.852006	-2.0356	0.044784	-3.78702	0.276722	0.301772
Neutrophils	SLC2A2	-0.90033	1.627926	-2.03375	0.044974	-3.77475	0.279527	0.304287
Neutrophils	SVBP	0.378391	5.267752	2.033348	0.045016	-4.05339	0.252068	0.275066
Neutrophils	ITGAD	-1.3185	0.789813	-2.03294	0.045058	-3.79408	0.286514	0.311714
Neutrophils	CADM4	-0.75128	1.741687	-2.03173	0.045183	-3.82833	0.279094	0.304038
Neutrophils	MNDAL	-0.52181	6.786829	-2.03167	0.045189	-4.26313	0.241734	0.264211
Neutrophils	ARID1B	-0.18617	8.198201	-2.03165	0.045191	-4.67736	0.232247	0.254033
Neutrophils	CLDN5	-0.84971	1.006603	-2.03156	0.045201	-3.78465	0.285014	0.310339
Neutrophils	MPV17	-0.41816	4.075124	-2.03085	0.045274	-3.90173	0.261389	0.285206
Neutrophils	TXNRD2	0.739247	3.532111	2.030625	0.045297	-3.7888	0.265466	0.289567
Neutrophils	GM28981	0.610881	0.488246	2.029925	0.04537	-3.85424	0.289864	0.315479
Neutrophils	ELOVL1	0.335861	4.858115	2.029243	0.045441	-4.16801	0.256149	0.27942
Neutrophils	CAP2	-1.39133	0.071585	-2.02855	0.045513	-3.82843	0.293934	0.319637
Neutrophils	SLC2A12	0.852946	1.619007	2.027944	0.045576	-3.77324	0.281235	0.306373
Neutrophils	MXRA7	-0.76976	1.654737	-2.02779	0.045592	-3.79868	0.280948	0.306118
Neutrophils	0610040B1	0.747687	2.051019	2.027349	0.045638	-3.78083	0.277788	0.30293
Neutrophils	ICE1	0.418856	4.081492	2.027012	0.045673	-4.01562	0.262157	0.286389
Neutrophils	GM16124	-0.93787	2.669782	-2.02684	0.045691	-3.77494	0.272926	0.297917
Neutrophils	PTK7	-1.1841	1.073787	-2.02667	0.045709	-3.77423	0.285647	0.311479
Neutrophils	ZFAND2A	0.376189	3.630572	2.026647	0.045711	-3.99439	0.265548	0.290102
Neutrophils	MLH3	-0.84905	2.01272	-2.0266	0.045716	-3.77458	0.278092	0.303464
Neutrophils	HILPDA	0.520519	4.798635	2.025969	0.045782	-4.27385	0.257053	0.280882
Neutrophils	SGCE	-0.95806	1.156412	-2.02567	0.045814	-3.77991	0.285193	0.310927
Neutrophils	SLC35A1	-0.52033	3.416075	-2.02555	0.045827	-3.81239	0.267383	0.292025
Neutrophils	CDH2	-0.77558	1.947183	-2.02471	0.045914	-3.79758	0.279009	0.304438
Neutrophils	CYP2B9	-1.07406	1.9577	-2.02459	0.045927	-3.78907	0.278925	0.304401
Neutrophils	ZFP598	0.562785	3.44196	2.024471	0.045939	-3.84252	0.26736	0.292089
Neutrophils	LAMTOR2	0.171898	6.577213	2.024089	0.045979	-4.57651	0.244534	0.267776
Neutrophils	RDH16F2	-1.12067	1.257357	-2.02406	0.045983	-3.77846	0.284558	0.310499
Neutrophils	AP1S3	-0.42517	5.57812	-2.02311	0.046082	-4.18796	0.251931	0.275675
Neutrophils	METRNL	0.569436	3.205494	2.022761	0.046119	-4.03864	0.269546	0.294603
Neutrophils	COX15	-0.56613	2.891641	-2.02239	0.046158	-3.79953	0.27197	0.297228
Neutrophils	GM30198	-1.12295	1.865066	-2.02217	0.046181	-3.78078	0.280055	0.305895
Neutrophils	ABHD18	0.613023	3.182254	2.022129	0.046186	-3.85653	0.269725	0.294891
Neutrophils	MAP3K14	0.386277	5.189544	2.022004	0.046199	-4.17917	0.254732	0.27888
Neutrophils	PTGS2OS2	0.460381	-0.48663	2.021576	0.046244	-4.0267	0.299639	0.326693

Neutrophils	DHX33	-0.69804	3.32737	-2.02075	0.046332	-3.78592	0.268762	0.294088
Neutrophils	GM26759	0.758817	3.123372	2.02068	0.046339	-3.81082	0.27033	0.295762
Neutrophils	S1PR5	1.175357	-0.65106	2.020636	0.046343	-3.81704	0.301089	0.328462
Neutrophils	ZFP68	0.429863	3.920899	2.020511	0.046356	-3.90142	0.264252	0.28927
Neutrophils	IRGQ	-0.87133	2.194038	-2.02019	0.04639	-3.7844	0.277594	0.303507
Neutrophils	CRLF2	0.282923	5.010644	2.020084	0.046402	-4.33089	0.256176	0.280627
Neutrophils	PSME2B	0.769596	2.815452	2.019538	0.046459	-3.80325	0.272782	0.298376
Neutrophils	DTD2	0.632115	3.491192	2.019277	0.046487	-3.84514	0.267575	0.292871
Neutrophils	CCL6	0.261615	5.306299	2.01926	0.046489	-4.88669	0.254092	0.278441
Neutrophils	XNDC1	-0.62269	3.594505	-2.01902	0.046514	-3.81851	0.266788	0.292125
Neutrophils	GM43305	-0.84983	7.45195	-2.01864	0.046555	-4.22435	0.239141	0.262489
Neutrophils	COL9A3	-1.29396	0.618437	-2.01828	0.046593	-3.81367	0.290613	0.317461
Neutrophils	CCL21A	2.404048	0.049265	2.018023	0.04662	-3.78854	0.29539	0.322544
Neutrophils	GM12596	0.604826	2.627238	2.017612	0.046664	-3.86426	0.274523	0.30031
Neutrophils	NCKAP1	-0.52259	2.425386	-2.0174	0.046687	-3.88634	0.276109	0.302076
Neutrophils	IFNGR2	0.350548	5.782195	2.016428	0.04679	-4.34694	0.251174	0.275311
Neutrophils	MYLPF	0.960937	2.109786	2.016069	0.046828	-3.79545	0.278884	0.304992
Neutrophils	BACH1	0.309374	5.9952	2.015555	0.046883	-4.48402	0.249657	0.273918
Neutrophils	TNFRSF14	1.173539	0.252225	2.015271	0.046913	-3.80628	0.294076	0.32148
Neutrophils	9130230L2	0.440633	3.687563	2.015155	0.046926	-4.34339	0.266611	0.292221
Neutrophils	PPM1G	0.237315	6.448565	2.01503	0.046939	-4.3417	0.246461	0.270602
Neutrophils	ADAM8	0.253694	2.602795	2.015019	0.04694	-4.62777	0.274988	0.301176
Neutrophils	TRIM47	-0.66973	2.41312	-2.01494	0.046948	-3.81446	0.27648	0.302799
Neutrophils	NCF2	0.181285	6.576577	2.014823	0.046961	-4.9556	0.245566	0.269703
Neutrophils	CRY1	0.250669	5.502006	2.014445	0.047002	-4.44891	0.253265	0.278029
Neutrophils	CDKL5	-1.12465	2.066071	-2.0139	0.04706	-3.7978	0.279518	0.306182
Neutrophils	HAGHL	0.658408	2.911144	2.013182	0.047137	-3.84917	0.273065	0.299281
Neutrophils	RNFT1	0.369239	4.495375	2.013095	0.047146	-4.01028	0.261008	0.286372
Neutrophils	STXBP5	-0.21217	5.383452	-2.01261	0.047199	-4.71753	0.254602	0.279512
Neutrophils	PARP10	0.401192	3.565506	2.012431	0.047218	-4.17756	0.268133	0.294055
Neutrophils	TRMT2B	-0.54112	3.750232	-2.01201	0.047263	-3.87884	0.266835	0.292761
Neutrophils	AW011738	0.633342	2.812185	2.011666	0.0473	-3.94535	0.274138	0.300503
Neutrophils	CISD3	-0.77708	3.000972	-2.01092	0.04738	-3.80595	0.272825	0.299151
Neutrophils	ETS1	-0.22805	7.418769	-2.01072	0.047402	-4.73385	0.240596	0.264535
Neutrophils	COPG2	0.289195	5.004811	2.010702	0.047404	-4.2622	0.257682	0.282924
Neutrophils	IYD	-1.03212	0.499692	-2.01003	0.047476	-3.80522	0.29331	0.32096
Neutrophils	TNIK	-0.64112	4.035038	-2.00902	0.047585	-3.96657	0.265528	0.291154
Neutrophils	TRAFD1	0.398771	5.468862	2.008951	0.047593	-4.33677	0.254905	0.279778
Neutrophils	RAG2	-1.53236	0.168525	-2.00859	0.047632	-3.85272	0.29651	0.324355
Neutrophils	RIN2	-0.29551	3.687666	-2.00847	0.047645	-4.38942	0.268175	0.294152
Neutrophils	STAB1	-0.55674	3.176342	-2.00802	0.047694	-4.09027	0.272248	0.298532
Neutrophils	CD9	0.227093	6.033746	2.007645	0.047734	-4.97873	0.25105	0.275844
Neutrophils	ASH2L	0.432951	4.017187	2.007186	0.047784	-3.91653	0.266019	0.291943
Neutrophils	MRPS6	-0.33091	6.008603	-2.00661	0.047846	-4.22006	0.251429	0.276331
Neutrophils	MRPL55	-0.43094	4.367452	-2.00659	0.047848	-3.97185	0.263451	0.289249
Neutrophils	2210408I2:	-0.58292	3.277757	-2.00606	0.047907	-3.89861	0.271866	0.298305
Neutrophils	IRF8	-0.63607	6.49623	-2.00575	0.04794	-4.07306	0.248064	0.2728

Neutrophils	RASAL3	-0.46316	4.10056	-2.00549	0.047968	-3.91907	0.265564	0.291709
Neutrophils	VMP1	-0.2503	7.164309	-2.00548	0.047969	-4.74952	0.243402	0.26786
Neutrophils	FAM214A	-0.40627	5.246692	-2.00467	0.048058	-4.23026	0.25724	0.282786
Neutrophils	GM37982	0.772417	2.996043	2.004666	0.048058	-3.86689	0.274277	0.301067
Neutrophils	GSN	0.224495	5.844895	2.003949	0.048136	-4.78578	0.253128	0.278446
Neutrophils	WDR41	-0.31111	4.001344	-2.0038	0.048153	-4.14424	0.266768	0.293189
Neutrophils	PLSCR1	0.24802	4.550033	2.003151	0.048224	-4.67093	0.262877	0.288869
Neutrophils	FRMD5	-1.22453	2.390592	-2.00264	0.04828	-3.84212	0.279749	0.306989
Neutrophils	TAF1	0.2634	5.699019	2.002095	0.048339	-4.36727	0.254608	0.280125
Neutrophils	PRDX2	0.349816	7.774205	2.00184	0.048367	-4.53871	0.240036	0.264401
Neutrophils	CD300LB	0.316524	1.973752	2.001802	0.048371	-4.53621	0.283127	0.310724
Neutrophils	CCSAP	-0.76472	2.176115	-2.00166	0.048387	-3.81178	0.281496	0.309061
Neutrophils	PIK3C2B	-0.64191	2.667746	-2.0007	0.048493	-3.89145	0.27794	0.305147
Neutrophils	ZFP950	0.381533	4.197661	2.000608	0.048502	-4.06918	0.266076	0.292446
Neutrophils	COMTD1	-0.41958	2.92368	-2.00023	0.048544	-3.99285	0.276009	0.303113
Neutrophils	TMEM88	-0.52764	2.768173	-1.99993	0.048577	-4.05809	0.277254	0.30451
Neutrophils	GM5086	1.103515	-0.01663	1.999734	0.048599	-3.85442	0.300198	0.329075
Neutrophils	VEGFA	0.337124	4.223513	1.999448	0.04863	-4.36759	0.266017	0.292608
Neutrophils	RGS12	-0.82555	2.240588	-1.99829	0.048758	-3.81569	0.281884	0.30965
Neutrophils	AFF4	0.214784	7.842009	1.998283	0.048759	-4.78198	0.240348	0.264949
Neutrophils	ERC1	-0.33629	4.872109	-1.99795	0.048795	-4.27896	0.261511	0.287917
Neutrophils	TNNI2	0.664928	2.310806	1.997904	0.0488	-3.84506	0.28132	0.309187
Neutrophils	SERPINA11	-0.80769	1.813558	-1.99656	0.048949	-3.8336	0.286061	0.31387
Neutrophils	BHLHE40	0.361996	6.323106	1.996025	0.049008	-4.55111	0.251599	0.276966
Neutrophils	GTF3C1	-0.39283	4.779184	-1.9959	0.049023	-4.03891	0.262896	0.289131
Neutrophils	GRIK4	-1.16781	0.238216	-1.99581	0.049032	-3.8191	0.299259	0.328047
Neutrophils	SDHAF4	-0.4449	4.365947	-1.99552	0.049065	-3.96299	0.266045	0.292548
Neutrophils	NOD2	0.531947	1.576786	1.995177	0.049103	-4.03924	0.288145	0.316287
Neutrophils	FBXO25	-0.65329	2.127104	-1.99466	0.04916	-3.82127	0.283834	0.311651
Neutrophils	LONP2	0.185601	5.87764	1.994216	0.049209	-4.49773	0.255107	0.280959
Neutrophils	ITK	-0.69195	4.224474	-1.99413	0.049219	-4.18916	0.267399	0.294222
Neutrophils	PDCD2L	0.338429	4.660225	1.993715	0.049265	-4.02801	0.264166	0.290818
Neutrophils	FUS	-0.14747	7.820328	-1.99355	0.049283	-4.81614	0.241476	0.266344
Neutrophils	MTA1	-0.48239	4.55479	-1.9932	0.049322	-3.93083	0.265031	0.291837
Neutrophils	TRIM10	1.184529	0.349121	1.992508	0.0494	-3.82433	0.298892	0.328401
Neutrophils	RAB3GAP2	0.295397	5.259956	1.992403	0.049412	-4.26923	0.259827	0.286505
Neutrophils	DDI2	0.301437	5.943537	1.992402	0.049412	-4.28593	0.254823	0.281104
Neutrophils	KDM4A	0.432472	4.161515	1.991983	0.049458	-3.98297	0.268196	0.295478
Neutrophils	ZFP322A	0.66436	3.041434	1.990604	0.049613	-3.85596	0.277618	0.305261
Neutrophils	CMIP	0.197462	9.635584	1.989611	0.049724	-5.13969	0.230588	0.254421
Neutrophils	PRKAA1	0.338267	4.45764	1.988458	0.049854	-4.25167	0.267556	0.294257
Neutrophils	4930503L1	-0.68473	2.576564	-1.98838	0.049862	-3.83995	0.282297	0.31007
Neutrophils	PLEKHG2	-0.55127	4.157105	-1.98799	0.049906	-3.98915	0.269954	0.296805
Neutrophils	GM44987	-1.15917	-0.34886	-1.9874	0.049973	-3.88772	0.307252	0.336684
Neutrophils	DST	-0.55515	4.335211	-1.98701	0.050016	-4.05775	0.268839	0.295713
Neutrophils	CLK4	-0.2025	6.012894	-1.98688	0.050031	-4.54582	0.256304	0.282219
Neutrophils	ICAM1	0.354258	5.898773	1.986222	0.050106	-4.45687	0.257388	0.283288

Neutrophils	PAK2	0.136888	7.859842	1.984387	0.050314	-4.89354	0.244256	0.268818
Neutrophils	CAMK2G	0.313702	4.893224	1.983854	0.050374	-4.28321	0.265738	0.292134
Neutrophils	PVT1	-0.74395	5.282726	-1.98367	0.050395	-4.04406	0.262808	0.289004
Neutrophils	DLD	0.29429	4.599821	1.983481	0.050417	-4.15724	0.267968	0.294604
Neutrophils	MIER1	0.203426	6.946828	1.983023	0.050469	-4.603	0.250666	0.275989
Neutrophils	SBNO1	0.160725	7.359347	1.983001	0.050471	-4.85381	0.247748	0.272838
Neutrophils	AP2B1	0.188039	6.179239	1.982907	0.050482	-4.53746	0.256192	0.281951
Neutrophils	PCCA	-0.51766	4.599387	-1.98286	0.050487	-3.98668	0.267971	0.294628
Neutrophils	SOCS6	0.42238	3.829946	1.982681	0.050508	-3.96759	0.273911	0.30103
Neutrophils	ATOX1	0.198149	8.244709	1.981651	0.050625	-4.90491	0.242042	0.266583
Neutrophils	TNF	0.502077	4.405834	1.980958	0.050704	-4.48775	0.270038	0.296931
Neutrophils	NPRL3	0.722088	2.892647	1.980803	0.050722	-3.84418	0.281942	0.309768
Neutrophils	STXBP3	0.295117	5.278015	1.98047	0.05076	-4.32258	0.263415	0.289916
Neutrophils	LGALS4	-0.76564	2.563012	-1.98034	0.050774	-3.8624	0.284606	0.312671
Neutrophils	GGACT	-0.56082	3.170013	-1.98034	0.050774	-3.8647	0.27972	0.307436
Neutrophils	MTMR10	0.785809	2.897646	1.979728	0.050844	-3.86361	0.282094	0.309962
Neutrophils	RAB2A	0.111961	8.094947	1.97958	0.050861	-4.94074	0.243327	0.26822
Neutrophils	CYP39A1	0.940992	1.483874	1.979008	0.050927	-3.84276	0.293935	0.322599
Neutrophils	EPS15	0.205246	6.183956	1.97829	0.051009	-4.53213	0.25733	0.283242
Neutrophils	NMI	0.404904	5.064624	1.978117	0.051029	-4.22776	0.265655	0.292237
Neutrophils	NXF1	-0.27387	5.180351	-1.9779	0.051054	-4.28659	0.264781	0.291336
Neutrophils	SRSF5	-0.15588	6.955445	-1.97762	0.051086	-4.78512	0.25178	0.27735
Neutrophils	TOX4	0.195554	5.903687	1.976552	0.051209	-4.49344	0.259716	0.285995
Neutrophils	SSR4	0.233141	7.573816	1.976446	0.051221	-4.67739	0.247685	0.273023
Neutrophils	TMEM156	0.290862	3.855526	1.976429	0.051223	-4.33893	0.275311	0.302798
Neutrophils	TMEM132E	-1.18435	0.984501	-1.97618	0.051252	-3.84889	0.29882	0.328039
Neutrophils	LRRFIP1	0.169279	7.249941	1.975964	0.051277	-4.87903	0.249975	0.275632
Neutrophils	CD52	0.242766	9.30175	1.975541	0.051325	-5.18567	0.235887	0.260515
Neutrophils	MORRBID	0.308345	5.127394	1.975394	0.051343	-4.76597	0.265551	0.29267
Neutrophils	SNX3	0.160791	7.730038	1.975228	0.051362	-4.78821	0.246623	0.272242
Neutrophils	JMJD1C	0.211189	8.048596	1.97459	0.051435	-4.89506	0.244631	0.270027
Neutrophils	TLR1	0.973503	1.219121	1.973825	0.051524	-3.85167	0.297352	0.326901
Neutrophils	ERO1L	0.248208	5.171152	1.973808	0.051526	-4.68662	0.265661	0.292864
Neutrophils	MPEG1	0.39452	6.081136	1.973358	0.051578	-4.64456	0.259002	0.285729
Neutrophils	RTP4	0.578838	4.174749	1.972779	0.051645	-4.40433	0.273659	0.301494
Neutrophils	BRI3BP	0.377771	4.756436	1.972533	0.051673	-4.16096	0.269174	0.296656
Neutrophils	CHD8	0.244545	5.733086	1.971994	0.051736	-4.33678	0.261979	0.288893
Neutrophils	OXLD1	-0.88982	1.813163	-1.9714	0.051805	-3.85464	0.293161	0.32235
Neutrophils	ISYNA1	0.338604	4.852057	1.971211	0.051827	-4.2044	0.268828	0.296273
Neutrophils	BICC1	1.211929	0.332453	1.970675	0.051889	-3.86202	0.306035	0.336151
Neutrophils	CD19	-0.83519	3.122136	-1.97012	0.051954	-3.86721	0.282819	0.3112
Neutrophils	ADH1	-0.75703	3.503888	-1.96922	0.052059	-4.0565	0.280176	0.308395
Neutrophils	FAM135A	-0.73676	2.016455	-1.96858	0.052134	-3.88546	0.292593	0.321735
Neutrophils	LMAN2	0.218011	6.112382	1.968001	0.052202	-4.44042	0.26057	0.287174
Neutrophils	PAFAH1B3	-0.54465	5.814072	-1.96739	0.052273	-4.06801	0.263018	0.289679
Neutrophils	DMAC2	-0.75987	3.093682	-1.96682	0.05234	-3.86338	0.284351	0.312661
Neutrophils	FBLIM1	-1.05672	0.928973	-1.96655	0.052372	-3.8616	0.302474	0.332169

Neutrophils	XCR1	-1.40879	0.412393	-1.96648	0.052379	-3.88344	0.30697	0.336991
Neutrophils	GLIPR2	0.296648	4.6029	1.966222	0.05241	-4.66095	0.2724	0.299981
Neutrophils	PARP4	0.248044	5.335625	1.965511	0.052494	-4.44836	0.267068	0.29414
Neutrophils	NPM1	-0.25309	8.143673	-1.96501	0.052552	-4.70662	0.246677	0.272089
Neutrophils	FCHO2	0.184727	6.902577	1.964913	0.052564	-4.80758	0.255516	0.281678
Neutrophils	2010110K1	-1.34631	-0.03523	-1.96395	0.052677	-3.89246	0.311897	0.341868
Neutrophils	PPP1R16A	0.703992	2.885752	1.963043	0.052784	-3.87301	0.287204	0.315463
Neutrophils	RBP4	-0.50484	8.031076	-1.96298	0.052792	-4.9651	0.2481	0.273373
Neutrophils	MTMR3	-0.15444	7.886466	-1.96266	0.052829	-4.97554	0.249119	0.27453
Neutrophils	WDR66	-1.05228	1.900032	-1.9625	0.052848	-3.87935	0.295397	0.324364
Neutrophils	TRIM2	-0.82855	2.325336	-1.96242	0.052859	-3.87804	0.291833	0.320551
Neutrophils	RAPGEFL1	-0.89401	2.784487	-1.96207	0.0529	-3.86881	0.288113	0.316541
Neutrophils	PPP1R16B	-0.44667	6.504104	-1.96143	0.052975	-4.28945	0.259308	0.285532
Neutrophils	C130046K2	-1.14651	0.716514	-1.96136	0.052984	-3.89003	0.305812	0.335428
Neutrophils	GM47889	-1.13951	1.560566	-1.96098	0.053029	-3.88195	0.298631	0.327777
Neutrophils	PDCD11	0.50239	4.214015	1.960027	0.053142	-3.95049	0.277313	0.304801
Neutrophils	MTM1	0.390243	4.433358	1.959697	0.053181	-4.27811	0.275601	0.30313
Neutrophils	KLK8	-0.80606	1.605933	-1.95923	0.053237	-3.8714	0.298747	0.328006
Neutrophils	DENND5A	0.176068	6.395889	1.959199	0.05324	-4.80058	0.260634	0.287073
Neutrophils	YPEL5	0.245318	6.583092	1.959102	0.053252	-4.65843	0.259251	0.285617
Neutrophils	GM11476	0.303365	2.536389	1.958447	0.05333	-4.40002	0.291197	0.319895
Neutrophils	OAS3	0.61286	2.402295	1.957913	0.053393	-4.29781	0.292516	0.321307
Neutrophils	HIST1H3G	-1.07019	1.46486	-1.95759	0.053433	-3.87332	0.300518	0.329867
Neutrophils	CYTH3	-0.47631	4.88921	-1.95724	0.053474	-4.08572	0.272632	0.300051
Neutrophils	TTC13	-0.40383	3.938924	-1.9566	0.05355	-4.06281	0.280373	0.308405
Neutrophils	DELE1	-0.50332	3.426108	-1.95623	0.053594	-3.93108	0.284593	0.313069
Neutrophils	GM46620	1.125626	0.683678	1.955409	0.053693	-3.88174	0.308177	0.338235
Neutrophils	VPS39	-0.58748	3.331542	-1.95496	0.053746	-3.90811	0.285841	0.314397
Neutrophils	RNASE4	-0.45067	5.17058	-1.95481	0.053765	-4.4503	0.27125	0.298708
Neutrophils	PPP1R15A	0.321162	7.089434	1.954314	0.053824	-4.78205	0.256863	0.283299
Neutrophils	CAPZB	0.134778	8.722615	1.953915	0.053872	-5.02992	0.245242	0.270825
Neutrophils	SEC22A	-0.57732	3.176208	-1.9539	0.053874	-3.91544	0.287123	0.316037
Neutrophils	VCAN	1.459752	0.76602	1.953894	0.053875	-3.88476	0.30757	0.337939
Neutrophils	PGLYRP2	-0.84129	2.088786	-1.95203	0.054099	-3.89648	0.297163	0.326317
Neutrophils	NOL11	-0.51082	4.89374	-1.95176	0.054132	-4.05188	0.274324	0.301871
Neutrophils	SSBP4	0.310599	4.90464	1.951549	0.054157	-4.24074	0.274239	0.301796
Neutrophils	HSPA5	-0.19532	8.355671	-1.9515	0.054163	-4.90264	0.248633	0.274152
Neutrophils	CNP	-0.57116	5.75023	-1.95127	0.054191	-4.06293	0.267724	0.294778
Neutrophils	GBE1	-0.22535	6.483019	-1.95091	0.054234	-4.85725	0.262282	0.288825
Neutrophils	MAST4	-0.61257	6.39259	-1.95041	0.054295	-4.27126	0.263122	0.289725
Neutrophils	ZNRD1AS	-1.16605	0.28937	-1.94998	0.054347	-3.91058	0.313269	0.343529
Neutrophils	ACVR1B	0.548435	2.543644	1.948762	0.054495	-3.95295	0.29436	0.32306
Neutrophils	DTX4	0.587614	2.365512	1.94859	0.054516	-4.04813	0.29586	0.324726
Neutrophils	RFTN2	-0.69172	2.237638	-1.94814	0.05457	-3.94135	0.297091	0.326029
Neutrophils	RAC1	0.137883	7.82414	1.947619	0.054634	-4.92191	0.253589	0.279248
Neutrophils	CASP8AP2	0.292834	5.182109	1.946786	0.054735	-4.30269	0.273453	0.300944
Neutrophils	TCERG1	0.241299	6.296507	1.946718	0.054743	-4.39732	0.264922	0.291775

Neutrophils	ZFP53	-0.37511	4.791451	-1.94667	0.054749	-4.19782	0.276511	0.304275
Neutrophils	PLEKHA2	-0.28238	7.455833	-1.94657	0.054762	-4.57586	0.256343	0.282527
Neutrophils	COL18A1	-0.74798	2.743841	-1.94624	0.054802	-3.98883	0.29313	0.322231
Neutrophils	LTB	0.427617	3.446775	1.946123	0.054816	-4.26792	0.287312	0.316002
Neutrophils	6030458C1	-0.67172	2.74674	-1.94552	0.05489	-3.89266	0.293356	0.322414
Neutrophils	UMAD1	-0.27366	5.658219	-1.94418	0.055054	-4.46103	0.270269	0.297778
Neutrophils	PGLYRP1	0.188363	4.959034	1.944136	0.055059	-5.22746	0.2757	0.303631
Neutrophils	PTPRG	-0.70109	2.191113	-1.94409	0.055065	-4.04152	0.298337	0.327942
Neutrophils	NLRX1	0.553383	1.80919	1.944083	0.055065	-4.04303	0.301607	0.331443
Neutrophils	COX16	-0.20385	6.153691	-1.94397	0.055079	-4.45929	0.266488	0.293699
Neutrophils	TRIM44	-0.35917	6.053928	-1.94306	0.05519	-4.19958	0.267537	0.294706
Neutrophils	MARK2	0.165498	6.879023	1.943025	0.055195	-4.77781	0.261338	0.288014
Neutrophils	GRAMD1C	0.742555	2.440736	1.942811	0.055221	-3.91438	0.296543	0.325991
Neutrophils	MYL4	-0.77884	4.638008	-1.94207	0.055312	-4.07825	0.278706	0.306988
Neutrophils	POGLUT1	-0.67394	2.752407	-1.94183	0.055342	-3.90997	0.294097	0.323553
Neutrophils	RAPGEF2	-0.28078	7.429198	-1.9416	0.05537	-4.8505	0.257446	0.284119
Neutrophils	ZDHHC5	0.249442	5.017726	1.941441	0.05539	-4.29935	0.275709	0.303925
Neutrophils	PPARA	-0.79131	2.784821	-1.94122	0.055416	-3.977	0.293825	0.323477
Neutrophils	DYNC1LI1	0.196112	6.412559	1.941208	0.055418	-4.61925	0.264986	0.292408
Neutrophils	PRKCD	0.188392	6.897294	1.940566	0.055497	-4.78445	0.261607	0.288489
Neutrophils	NDUFS1	-0.22057	5.752161	-1.93988	0.055582	-4.42523	0.27054	0.298004
Neutrophils	BLOC1S2	0.412787	4.833886	1.939262	0.055658	-4.11384	0.277821	0.305893
Neutrophils	ISCA2	0.327926	4.968036	1.939247	0.05566	-4.30638	0.276762	0.304753
Neutrophils	PEX11A	1.136399	0.072757	1.938837	0.055711	-3.91314	0.318375	0.349278
Neutrophils	SWT1	0.301268	5.244266	1.938253	0.055783	-4.29393	0.274857	0.302596
Neutrophils	GIMAP5	-0.67759	3.319876	-1.93815	0.055795	-3.97707	0.290347	0.319255
Neutrophils	BC005537	-0.17837	7.116434	-1.93756	0.055868	-4.8374	0.26079	0.287423
Neutrophils	CCDC77	-0.50693	3.291762	-1.93739	0.05589	-3.97392	0.290776	0.319781
Neutrophils	FRMD8	0.36625	4.222806	1.936853	0.055957	-4.20879	0.283161	0.311925
Neutrophils	GM29394	-1.11875	0.417159	-1.9368	0.055963	-3.92382	0.315641	0.346743
Neutrophils	TMED7	0.170232	6.308978	1.936748	0.05597	-4.63239	0.26684	0.294325
Neutrophils	CD164L2	-1.04568	1.352514	-1.93613	0.056047	-3.90384	0.307454	0.338109
Neutrophils	GPR132	0.379194	6.00422	1.936113	0.056048	-4.60922	0.269278	0.29706
Neutrophils	COPB2	0.248573	5.438302	1.935137	0.05617	-4.34667	0.274037	0.301986
Neutrophils	AMBRA1	0.200373	7.654516	1.935031	0.056183	-4.78888	0.257316	0.283921
Neutrophils	EPAS1	-0.46567	3.448567	-1.93451	0.056249	-4.20409	0.290213	0.319426
Neutrophils	GLE1	-0.38154	4.4454	-1.93364	0.056356	-4.09902	0.282488	0.310984
Neutrophils	SIRT4	1.233524	0.56797	1.933327	0.056396	-3.92204	0.315599	0.346545
Neutrophils	SESN2	0.399524	3.371941	1.932733	0.05647	-4.18991	0.291454	0.320724
Neutrophils	GM47828	-1.1312	0.334612	-1.93265	0.056481	-3.93665	0.31785	0.348979
Neutrophils	RRAD	-0.97126	2.552475	-1.93218	0.056539	-3.90767	0.298347	0.328286
Neutrophils	INKA1	-0.62971	2.703982	-1.93211	0.056547	-3.95072	0.297061	0.326906
Neutrophils	4931423N1	-0.99984	0.653443	-1.93203	0.056558	-3.9078	0.314969	0.34608
Neutrophils	ARHGEF3	0.394858	5.916073	1.931812	0.056585	-4.62187	0.271085	0.298948
Neutrophils	NSRP1	0.250589	5.229533	1.931368	0.056641	-4.444	0.276569	0.304842
Neutrophils	TRIM12A	0.293666	5.048891	1.931066	0.056679	-4.46127	0.278046	0.306473
Neutrophils	GM47754	1.048868	0.667894	1.930463	0.056755	-3.91026	0.315322	0.346357

Neutrophils	LZTR1	0.690102	2.612289	1.930089	0.056802	-3.91933	0.298398	0.328228
Neutrophils	E130311K1	-1.24814	0.244935	-1.9293	0.056901	-3.93645	0.31967	0.350829
Neutrophils	TMEM176/	-0.54346	4.388779	-1.92884	0.056959	-4.09932	0.284173	0.312816
Neutrophils	PBLD2	-0.87917	0.872415	-1.92841	0.057012	-3.91314	0.314301	0.345123
Neutrophils	FGA	-0.41243	5.727993	-1.92756	0.05712	-4.63259	0.2739	0.301619
Neutrophils	OPRM1	0.371139	2.803916	1.927543	0.057122	-4.40269	0.297694	0.327193
Neutrophils	NRROS	0.214335	7.067702	1.927375	0.057144	-4.76955	0.263669	0.290602
Neutrophils	B9D2	0.382883	4.930502	1.926696	0.057229	-4.28171	0.280423	0.308646
Neutrophils	TSTD1	-0.78381	2.589608	-1.92656	0.057247	-3.97032	0.299772	0.329482
Neutrophils	GM41077	-0.93621	0.721818	-1.9261	0.057305	-3.91592	0.316357	0.347125
Neutrophils	CDK13	0.18453	7.414024	1.925722	0.057353	-4.83994	0.261473	0.288246
Neutrophils	2010309G2	-1.17315	1.223176	-1.92562	0.057366	-3.92679	0.31189	0.342492
Neutrophils	KLRB1B	-1.11326	1.981821	-1.92493	0.057454	-3.93082	0.30538	0.335577
Neutrophils	SERPINA1C	-0.559	7.348457	-1.92482	0.057467	-4.93179	0.262109	0.289017
Neutrophils	TMEM87B	-0.28786	5.127611	-1.92471	0.057481	-4.3263	0.279186	0.307482
Neutrophils	GM16158	1.077715	0.586451	1.923909	0.057583	-3.91964	0.318205	0.349096
Neutrophils	FAM114A1	0.389176	2.398626	1.923581	0.057625	-4.31897	0.302214	0.33207
Neutrophils	LHFPL2	-0.38361	2.227897	-1.92339	0.057649	-4.29816	0.30369	0.33367
Neutrophils	FXN	-0.48747	4.085717	-1.9226	0.057749	-4.01052	0.288322	0.317054
Neutrophils	EIF1AD	0.412744	4.923218	1.922264	0.057793	-4.17121	0.281526	0.309868
Neutrophils	ZFP595	0.528016	1.84184	1.922262	0.057793	-3.95029	0.30738	0.337607
Neutrophils	SETD4	0.667836	2.964232	1.921407	0.057902	-3.92833	0.297989	0.327639
Neutrophils	ABCB4	-0.77216	3.391454	-1.92137	0.057906	-3.96257	0.29438	0.323768
Neutrophils	KLRI2	-1.31811	0.447224	-1.92115	0.057935	-3.92834	0.320195	0.351452
Neutrophils	1810059H2	-1.22987	1.799204	-1.9209	0.057967	-3.92322	0.308084	0.338554
Neutrophils	CPNE9	-1.04203	1.867904	-1.92044	0.058026	-3.93171	0.307481	0.337998
Neutrophils	MYLIP	-0.25021	5.991118	-1.92012	0.058067	-4.69003	0.273394	0.301423
Neutrophils	UBE2N	0.168052	7.48412	1.920104	0.058069	-4.78416	0.262044	0.289151
Neutrophils	CD5L	-0.55347	6.068559	-1.91996	0.058087	-4.76675	0.272793	0.300773
Neutrophils	SAT1	0.195299	8.474503	1.919825	0.058104	-5.2284	0.25479	0.28129
Neutrophils	KLHL4	-1.00509	0.018315	-1.91929	0.058173	-3.92543	0.324383	0.356019
Neutrophils	SLC25A25	-0.38862	4.951297	-1.91892	0.05822	-4.18745	0.281894	0.310486
Neutrophils	ADGRE5	0.19599	7.153842	1.918318	0.058298	-4.8942	0.265012	0.292134
Neutrophils	SMARCC1	-0.29188	6.606315	-1.9178	0.058364	-4.49042	0.269343	0.296792
Neutrophils	RAB40C	0.390078	4.838453	1.917525	0.0584	-4.1598	0.283269	0.311859
Neutrophils	GM45606	1.191353	0.477564	1.916103	0.058583	-3.94411	0.321618	0.352512
Neutrophils	CNPPD1	0.255221	5.686802	1.915928	0.058606	-4.60625	0.277223	0.305022
Neutrophils	SLCO1B2	-0.56633	4.25024	-1.91563	0.058644	-4.37851	0.288847	0.317525
Neutrophils	EVI2A	0.252573	5.251447	1.915318	0.058684	-4.64978	0.280788	0.308873
Neutrophils	PDCD1LG2	0.746335	1.775707	1.914884	0.058741	-3.9893	0.310046	0.340395
Neutrophils	GM26737	-1.06889	0.478605	-1.91487	0.058742	-3.93908	0.321745	0.352886
Neutrophils	ADARB1	-0.57707	3.009129	-1.91451	0.058789	-4.09178	0.299421	0.328999
Neutrophils	CHST11	0.258163	6.242747	1.914063	0.058847	-5.07758	0.273218	0.300859
Neutrophils	H6PD	-0.4326	2.776773	-1.91328	0.058949	-4.10484	0.301876	0.331533
Neutrophils	CYP4V3	0.816231	2.78654	1.913163	0.058964	-3.9896	0.301792	0.331465
Neutrophils	BRF1	-0.32819	4.828649	-1.9129	0.058998	-4.20108	0.284759	0.313181
Neutrophils	GPRIN3	1.130284	0.638034	1.912597	0.059037	-3.94312	0.320977	0.35206

Neutrophils	AGPAT4	0.267519	5.023605	1.911896	0.059128	-4.8151	0.283413	0.311866
Neutrophils	AGBL3	-0.7409	1.72445	-1.91188	0.05913	-3.9412	0.311367	0.341849
Neutrophils	AVIL	-0.69637	2.092681	-1.91138	0.059195	-3.98124	0.308306	0.338584
Neutrophils	RAB11FIP5	0.984454	0.808074	1.911101	0.059232	-3.93613	0.319869	0.35099
Neutrophils	9930021J0	0.216168	6.407459	1.910445	0.059317	-4.68583	0.27295	0.300566
Neutrophils	WIPF2	0.216603	5.343948	1.909655	0.05942	-4.48232	0.281686	0.309849
Neutrophils	DNAH2	-0.91744	0.783062	-1.90918	0.059482	-3.93852	0.320816	0.351914
Neutrophils	ARG1	0.79246	3.498182	1.909022	0.059503	-4.13747	0.296895	0.326356
Neutrophils	TNFRSF23	0.373172	1.669914	1.908893	0.05952	-4.3111	0.312793	0.343457
Neutrophils	GM1123	-0.89534	0.265276	-1.90879	0.059533	-3.93903	0.325595	0.357135
Neutrophils	CGGBP1	0.184029	6.845343	1.908234	0.059606	-4.79939	0.270071	0.297581
Neutrophils	AMOTL1	-0.64539	1.696223	-1.90811	0.059623	-4.02424	0.312735	0.343501
Neutrophils	NAA15	-0.20643	6.743192	-1.9066	0.059821	-4.55807	0.271629	0.299001
Neutrophils	TRAF3IP2	-0.67345	3.229202	-1.90499	0.060032	-3.96314	0.300723	0.330433
Neutrophils	GM15559	-0.55211	3.148018	-1.90498	0.060034	-4.00005	0.30142	0.33118
Neutrophils	SLPI	0.270318	4.206306	1.904651	0.060077	-5.09602	0.292462	0.32166
Neutrophils	MRPS34	0.387084	4.826949	1.904478	0.060099	-4.14989	0.287338	0.316231
Neutrophils	TMEM35B	0.831856	2.207613	1.904251	0.060129	-3.95957	0.309617	0.340211
Neutrophils	MED15	0.281445	5.758733	1.904244	0.06013	-4.44636	0.279819	0.308197
Neutrophils	USP40	0.643558	3.64171	1.904179	0.060139	-4.0291	0.297207	0.32691
Neutrophils	RXRA	-0.48376	3.1994	-1.90381	0.060188	-4.1053	0.300979	0.330959
Neutrophils	MSH3	-0.3672	5.159864	-1.90376	0.060194	-4.25911	0.284627	0.313383
Neutrophils	SMAGP	-0.45583	3.61538	-1.90367	0.060206	-4.1803	0.29743	0.327166
Neutrophils	ADGRV1	0.772122	0.711845	1.903011	0.060293	-4.00075	0.323381	0.355009
Neutrophils	PFKFB1	-1.00997	0.961216	-1.90288	0.06031	-3.95099	0.321086	0.352606
Neutrophils	NCKAP5L	-0.60683	4.041979	-1.90242	0.060372	-4.04243	0.294085	0.323823
Neutrophils	TTC41	-1.18715	0.966475	-1.90216	0.060405	-3.97601	0.321057	0.352867
Neutrophils	NEU1	0.270951	4.426111	1.902142	0.060408	-4.46737	0.290884	0.320495
Neutrophils	CDC42EP2	0.42069	3.08028	1.901631	0.060476	-4.36724	0.30226	0.332861
Neutrophils	OLFML3	-0.59357	1.284348	-1.90162	0.060477	-4.03493	0.318156	0.349906
Neutrophils	PTCH1	-0.90017	3.139748	-1.90158	0.060483	-3.95297	0.301748	0.332323
Neutrophils	ARRDC4	0.403871	2.427989	1.90113	0.060542	-4.28065	0.308095	0.339155
Neutrophils	SEH1L	0.253535	5.334868	1.900613	0.060611	-4.39203	0.283788	0.312901
Neutrophils	LPL	-0.43652	5.132487	-1.90018	0.060669	-4.52285	0.285567	0.314821
Neutrophils	GPATCH2	-0.45902	4.253922	-1.89955	0.060753	-4.11821	0.29307	0.322899
Neutrophils	MTHFD1L	-0.30616	5.661426	-1.89898	0.060828	-4.47239	0.281651	0.310787
Neutrophils	KLRK1	-1.11503	3.149025	-1.89897	0.060829	-3.97421	0.302546	0.333299
Neutrophils	RAD17	0.225937	5.023722	1.89868	0.060868	-4.51864	0.286842	0.316421
Neutrophils	NR2F2	-0.46857	3.13223	-1.89849	0.060893	-4.34411	0.302727	0.333613
Neutrophils	CD200R2	-1.26544	0.789771	-1.89761	0.061011	-3.95392	0.324137	0.35637
Neutrophils	RELT	0.387577	3.728351	1.897101	0.061079	-4.25572	0.298256	0.328532
Neutrophils	PHF11C	-0.79786	1.989779	-1.89653	0.061155	-3.95885	0.313531	0.345036
Neutrophils	PRKRA	0.429873	3.39958	1.896517	0.061157	-4.09238	0.30117	0.331767
Neutrophils	CD22	-0.90316	2.780412	-1.89608	0.061216	-3.95583	0.30669	0.337684
Neutrophils	GAR1	-0.54933	4.430651	-1.8958	0.061253	-4.08109	0.292637	0.322527
Neutrophils	ARL5C	-0.63321	6.264356	-1.89556	0.061285	-4.20174	0.277777	0.306509
Neutrophils	ADAM3	0.660128	-0.17326	1.895153	0.06134	-4.00379	0.333885	0.366752

Neutrophils	OGFOD2	0.419761	3.850672	1.893648	0.061542	-4.12997	0.298243	0.328533
Neutrophils	PEX10	-1.02638	0.82136	-1.89324	0.061597	-3.96956	0.325171	0.357424
Neutrophils	ITGA6	-0.496	4.818835	-1.89286	0.061648	-4.38951	0.290129	0.319959
Neutrophils	PDCD4	-0.2356	7.282332	-1.89272	0.061667	-4.78303	0.270506	0.298753
Neutrophils	SIRT2	0.22832	5.688086	1.892146	0.061744	-4.55543	0.28304	0.312413
Neutrophils	MOSPD2	-0.26181	4.445481	-1.89214	0.061745	-4.57008	0.29323	0.32341
Neutrophils	FASL	-0.85899	1.037842	-1.89207	0.061755	-3.98512	0.323167	0.355563
Neutrophils	GNG11	-0.37533	3.831566	-1.89203	0.06176	-4.48501	0.298405	0.328984
Neutrophils	CMTR1	0.28069	5.166737	1.891954	0.06177	-4.67387	0.28727	0.316981
Neutrophils	OIT3	-0.46851	2.345496	-1.89195	0.06177	-4.21791	0.311325	0.342871
Neutrophils	CMTM8	-0.43149	4.192364	-1.89193	0.061773	-4.34449	0.295353	0.325697
Neutrophils	SAT2	-1.36756	-0.24549	-1.89145	0.061838	-3.9861	0.335428	0.368534
Neutrophils	PLAU	-1.02463	0.814983	-1.89042	0.061977	-3.97645	0.326003	0.358431
Neutrophils	PLG	-0.61673	4.004656	-1.88973	0.062071	-4.31691	0.297766	0.328165
Neutrophils	POLR2H	-0.4568	4.512761	-1.8896	0.062088	-4.07238	0.293486	0.323562
Neutrophils	TINAGL1	-0.45678	2.066409	-1.88919	0.062143	-4.32986	0.314691	0.34652
Neutrophils	CLUH	-0.64971	3.417508	-1.88914	0.062151	-3.99591	0.302792	0.33374
Neutrophils	IL2RA	-1.29445	2.557963	-1.88887	0.062187	-3.96556	0.310307	0.341856
Neutrophils	DTX1	-1.05367	2.069415	-1.88882	0.062195	-3.96554	0.314664	0.346533
Neutrophils	INMT	-1.13056	0.639157	-1.88876	0.062203	-3.9661	0.32778	0.360602
Neutrophils	TMEM71	0.308068	3.858441	1.888547	0.062231	-4.53673	0.299009	0.32977
Neutrophils	JPT1	-0.20753	7.832843	-1.88818	0.062281	-4.90522	0.267151	0.295366
Neutrophils	ACP6	-0.67659	3.140192	-1.88787	0.062324	-3.98575	0.305369	0.336692
Neutrophils	GM29170	0.757007	1.532706	1.88754	0.062368	-3.9671	0.319785	0.352161
Neutrophils	CD53	0.141305	7.920856	1.886931	0.062451	-5.15391	0.266792	0.295019
Neutrophils	ANKS1B	-0.95459	1.030517	-1.88681	0.062467	-3.96816	0.324622	0.357421
Neutrophils	SBF2	-0.33421	5.368073	-1.8865	0.062509	-4.50835	0.286915	0.316942
Neutrophils	MRS2	0.420919	3.939507	1.886161	0.062556	-4.1168	0.298916	0.329952
Neutrophils	GM50240	0.736369	1.572659	1.885806	0.062604	-3.98632	0.319807	0.35253
Neutrophils	PSMG2	-0.53871	3.919974	-1.88573	0.062615	-4.0787	0.299092	0.330265
Neutrophils	KRCC1	0.170607	5.9705	1.885462	0.062651	-4.70947	0.282166	0.31201
Neutrophils	CHST3	-0.72641	4.312271	-1.8847	0.062755	-4.06689	0.296156	0.326998
Neutrophils	KIF13B	-0.18874	6.510852	-1.88416	0.06283	-4.90756	0.278404	0.307696
Neutrophils	SSNA1	0.225452	5.922585	1.883784	0.062881	-4.63154	0.283102	0.312871
Neutrophils	TUFT1	-0.73491	2.2113	-1.88373	0.062888	-3.97233	0.314679	0.346914
Neutrophils	KNTC1	0.617984	2.818917	1.88332	0.062944	-4.10554	0.309373	0.341283
Neutrophils	CASD1	-0.28626	4.363906	-1.88316	0.062966	-4.39858	0.296042	0.327015
Neutrophils	ANKRD13A	0.191927	6.215118	1.882417	0.063068	-4.76358	0.281054	0.310886
Neutrophils	DPP4	-0.40823	5.100888	-1.88225	0.063091	-4.34022	0.290104	0.320739
Neutrophils	H2-Q6	-1.4989	3.115257	-1.8822	0.063098	-3.98219	0.306991	0.338982
Neutrophils	CITED4	-1.36286	-0.2568	-1.88081	0.063289	-3.9884	0.338882	0.37296
Neutrophils	6720489N1	-1.00551	0.964398	-1.88003	0.063397	-3.99172	0.327544	0.360852
Neutrophils	STARD7	0.244397	5.358518	1.879932	0.06341	-4.56572	0.28897	0.319331
Neutrophils	KIF22	0.592453	4.043139	1.879797	0.063429	-4.2823	0.299999	0.331284
Neutrophils	PANX1	-0.5536	4.208683	-1.87884	0.063562	-4.02588	0.299075	0.33008
Neutrophils	TRIM12C	0.306724	4.726648	1.877768	0.06371	-4.54883	0.295247	0.325618
Neutrophils	ETHE1	0.240262	4.997365	1.875789	0.063984	-4.618	0.294109	0.323849

Neutrophils	ODF2	0.241042	5.483537	1.875044	0.064088	-4.4831	0.29031	0.319879
Neutrophils	ZC3H3	0.433244	3.468484	1.874862	0.064113	-4.10992	0.30746	0.33844
Neutrophils	RERG	0.939888	0.522461	1.874779	0.064125	-3.99243	0.334431	0.367358
Neutrophils	E330020D1	-0.85791	5.11024	-1.87406	0.064225	-4.04937	0.293737	0.323625
Neutrophils	ITGB1BP1	0.300178	4.649919	1.873695	0.064276	-4.3476	0.297616	0.328005
Neutrophils	POPDC3	-1.3794	0.894439	-1.87356	0.064294	-3.99573	0.33127	0.364132
Neutrophils	RNF44	-0.24518	4.833037	-1.87343	0.064313	-4.5497	0.296068	0.326404
Neutrophils	CD200	1.088177	1.910379	1.872391	0.064458	-3.98674	0.32229	0.354454
Neutrophils	SLU7	0.245919	4.955244	1.872308	0.06447	-4.47327	0.295491	0.3257
Neutrophils	SPP2	-0.65512	3.372982	-1.8718	0.064541	-4.25642	0.309146	0.340469
Neutrophils	HAO2	-1.06967	1.393032	-1.87166	0.064561	-4.00734	0.327116	0.35979
Neutrophils	RALGPS2	-0.37227	5.773254	-1.87164	0.064563	-4.41753	0.288719	0.318507
Neutrophils	SCAP	-0.59251	4.277309	-1.87125	0.064618	-4.08097	0.301279	0.332115
Neutrophils	CCL24	-0.92233	3.596362	-1.87123	0.064621	-4.31664	0.307183	0.33847
Neutrophils	TNFAIP1	-0.4671	3.775763	-1.87035	0.064744	-4.19811	0.305995	0.337129
Neutrophils	NCF1	0.203861	5.301832	1.870219	0.064762	-4.99271	0.29298	0.323167
Neutrophils	9330136K2	0.863971	1.883233	1.869494	0.064864	-3.99413	0.32297	0.355607
Neutrophils	STAT5B	0.242566	6.043742	1.86943	0.064873	-4.67231	0.286862	0.316734
Neutrophils	IQCK	-1.05208	0.158994	-1.86933	0.064887	-3.99618	0.33927	0.373101
Neutrophils	RAB4B	0.187729	5.9254	1.869174	0.064909	-4.73059	0.287829	0.317899
Neutrophils	SORD	-0.47277	4.176195	-1.869	0.064934	-4.31586	0.302523	0.333809
Neutrophils	FAM3A	-0.57507	2.785704	-1.86899	0.064934	-4.04213	0.314758	0.346979
Neutrophils	IMPACT	-0.27731	5.540939	-1.86853	0.064999	-4.61793	0.291154	0.321407
Neutrophils	STK11	0.21253	5.696969	1.867929	0.065084	-4.6623	0.289903	0.32007
Neutrophils	ANKRD33B	0.303785	4.882196	1.86788	0.065091	-5.01887	0.296703	0.327413
Neutrophils	GALNT12	-0.96057	2.991393	-1.86782	0.0651	-3.99688	0.31313	0.345117
Neutrophils	DIP2C	-0.40382	6.172429	-1.86716	0.065193	-4.67492	0.28601	0.31611
Neutrophils	PFKFB4	0.301549	3.15878	1.867161	0.065193	-4.46073	0.311639	0.343772
Neutrophils	CYP3A44	-0.86635	2.308322	-1.86703	0.065211	-4.0886	0.319293	0.352048
Neutrophils	PGM1	0.330175	4.592397	1.866933	0.065225	-4.41945	0.299162	0.330394
Neutrophils	HK1OS	0.758036	0.741866	1.866818	0.065241	-4.01989	0.333897	0.367765
Neutrophils	PTPN11	0.243796	5.063935	1.865987	0.065359	-4.44723	0.295406	0.326419
Neutrophils	TMEM119	0.92457	0.305437	1.865913	0.065369	-3.99496	0.338352	0.372606
Neutrophils	PSMA4	-0.21149	6.877207	-1.86552	0.065425	-4.76821	0.280562	0.310525
Neutrophils	IGKV1-35	-1.17571	-0.53717	-1.86547	0.065432	-4.02632	0.346594	0.381635
Neutrophils	CREBBP	-0.15471	7.888027	-1.86536	0.065447	-5.06796	0.27263	0.30194
Neutrophils	LRIF1	-0.23773	4.523869	-1.865	0.065498	-4.47278	0.299983	0.331669
Neutrophils	TXNDC5	-0.51495	5.179694	-1.86476	0.065533	-4.15573	0.294434	0.325695
Neutrophils	FRMD4B	-0.42807	5.267906	-1.86472	0.065539	-4.61615	0.293696	0.324896
Neutrophils	BTK	0.226409	6.033608	1.864598	0.065555	-4.77119	0.287368	0.318069
Neutrophils	LIPC	-0.70803	2.510562	-1.8641	0.065626	-4.05354	0.31778	0.351031
Neutrophils	ZBTB24	0.439894	3.32852	1.863933	0.06565	-4.09669	0.310451	0.343134
Neutrophils	UGDH	-0.25433	4.250685	-1.86387	0.065658	-4.59625	0.302397	0.334439
Neutrophils	BACH2OS	-0.93064	1.526179	-1.86325	0.065747	-3.99865	0.326896	0.361
Neutrophils	DHX40	-0.26861	7.808142	-1.86316	0.06576	-5.07346	0.273362	0.303111
Neutrophils	LMTK2	-0.2024	5.616268	-1.86309	0.06577	-4.77779	0.29092	0.322194
Neutrophils	HSPA8	-0.18144	10.08692	-1.86293	0.065792	-5.32268	0.256291	0.284566

Neutrophils	NUDT18	0.513486	2.627052	1.862762	0.065816	-4.06009	0.316784	0.350301
Neutrophils	DNAJC19	0.24168	5.950115	1.862211	0.065895	-4.55799	0.288387	0.319615
Neutrophils	LMBR1L	0.388256	4.517308	1.861782	0.065956	-4.31187	0.300446	0.332697
Neutrophils	KLHL15	-0.38667	4.413728	-1.86147	0.066001	-4.21607	0.301334	0.33373
Neutrophils	PMAIP1	0.410668	4.983149	1.86139	0.066012	-4.55776	0.296487	0.328483
Neutrophils	ARHGAP25	0.253068	6.214201	1.861175	0.066043	-4.72317	0.286284	0.317483
Neutrophils	TRAPPC6A	0.253481	5.057196	1.861098	0.066054	-4.4288	0.295863	0.327892
Neutrophils	GOLGA5	0.280457	4.913418	1.860282	0.06617	-4.40887	0.297469	0.329538
Neutrophils	TRAPPC5	0.353695	4.379372	1.859133	0.066335	-4.321	0.302645	0.334777
Neutrophils	GOLGA2	0.361331	3.814242	1.858172	0.066472	-4.23766	0.30806	0.340552
Neutrophils	GM9733	0.378203	0.76406	1.857101	0.066626	-4.64776	0.336705	0.371295
Neutrophils	TAF13	-0.2644	4.566883	-1.85668	0.066686	-4.55672	0.302228	0.334207
Neutrophils	TGFA	-0.93021	0.796415	-1.85627	0.066746	-4.01478	0.336658	0.371281
Neutrophils	MAMDC2	-1.11096	0.366618	-1.85612	0.066767	-4.01059	0.340816	0.375792
Neutrophils	PDSS2	-0.29964	5.377267	-1.85552	0.066854	-4.53436	0.295542	0.327137
Neutrophils	MARF1	0.220858	6.147381	1.855343	0.066879	-4.65707	0.289139	0.320216
Neutrophils	EFCC1	-1.15556	0.001369	-1.85496	0.066935	-4.01912	0.344519	0.379994
Neutrophils	SEMA4F	0.741512	-0.41218	1.854798	0.066958	-4.02251	0.348613	0.384422
Neutrophils	LANCL2	-0.64587	2.807486	-1.85473	0.066968	-4.04648	0.317995	0.351547
Neutrophils	SELENOH	-0.5249	5.20549	-1.85469	0.066974	-4.33401	0.29699	0.328861
Neutrophils	CCDC138	-0.40474	4.628632	-1.85452	0.066998	-4.45983	0.301908	0.334266
Neutrophils	WDR73	-0.55896	2.976485	-1.85429	0.067032	-4.05988	0.316487	0.350091
Neutrophils	MASP2	-0.87591	1.894509	-1.85346	0.067152	-4.05207	0.326852	0.361159
Neutrophils	RNF114	0.295328	5.645299	1.852799	0.067247	-4.58429	0.294	0.325617
Neutrophils	ZZEF1	0.210883	5.867775	1.852266	0.067325	-4.68975	0.292352	0.323696
Neutrophils	AU022252	0.664944	2.452615	1.85187	0.067382	-4.03562	0.322362	0.356092
Neutrophils	SLC16A1	-0.54329	3.905544	-1.85141	0.067448	-4.13598	0.309358	0.342141
Neutrophils	TLR4	0.284152	3.122783	1.851342	0.067459	-4.70277	0.31634	0.349684
Neutrophils	GM4869	-1.02671	1.447403	-1.85054	0.067575	-4.01449	0.332154	0.366658
Neutrophils	D8ERTD73	0.150906	7.358725	1.850158	0.067631	-5.00021	0.28069	0.311146
Neutrophils	TAGLN2	0.267922	8.716101	1.849673	0.067702	-5.20085	0.270097	0.29977
Neutrophils	PAIP2B	-0.47069	3.682629	-1.84955	0.067719	-4.13102	0.311632	0.344856
Neutrophils	FBXL14	0.212801	5.203671	1.84943	0.067737	-4.66326	0.298418	0.330559
Neutrophils	EML6	-1.09027	2.764743	-1.84912	0.067783	-4.02746	0.319897	0.353846
Neutrophils	SF3B2	0.150629	7.285583	1.848863	0.06782	-4.99291	0.281273	0.312112
Neutrophils	MCMBP	0.175122	7.089729	1.848861	0.06782	-4.90504	0.282841	0.313818
Neutrophils	BYSL	-0.33941	3.67185	-1.84867	0.067848	-4.24442	0.311727	0.345194
Neutrophils	NOL12	-0.39747	4.012955	-1.84854	0.067867	-4.13159	0.308711	0.341962
Neutrophils	GM43260	-0.42953	2.252383	-1.84851	0.067871	-4.23821	0.324608	0.359129
Neutrophils	TRIM62	-0.94322	1.410517	-1.84841	0.067886	-4.03274	0.332504	0.367634
Neutrophils	ZBTB4	-0.52471	3.655496	-1.8482	0.067917	-4.11593	0.311873	0.345404
Neutrophils	RPP38	-1.07775	1.226405	-1.84787	0.067965	-4.02147	0.334256	0.369654
Neutrophils	TCF25	0.154758	6.939299	1.847847	0.067968	-4.89395	0.284052	0.315322
Neutrophils	C1QB	-0.46772	6.51237	-1.84639	0.068181	-4.94123	0.288221	0.319707
Neutrophils	UBXN2B	-0.65244	2.842475	-1.84625	0.068202	-4.0434	0.31997	0.354124
Neutrophils	ACOT4	-1.22018	0.046894	-1.8461	0.068224	-4.02892	0.346555	0.382759
Neutrophils	H2AFJ	0.157374	7.521162	1.845497	0.068312	-5.0722	0.280327	0.311094

Neutrophils	NFIB	-0.46467	3.880029	-1.84523	0.068351	-4.56944	0.310953	0.344331
Neutrophils	MINPP1	-0.40124	4.734583	-1.84429	0.06849	-4.19365	0.303695	0.336558
Neutrophils	CPLX2	-0.87966	3.378009	-1.84399	0.068534	-4.06023	0.315665	0.349593
Neutrophils	PTGER2	0.546322	1.047913	1.843909	0.068546	-4.17571	0.33737	0.372994
Neutrophils	TNFSF10	-0.83284	2.307037	-1.84389	0.068549	-4.02959	0.325458	0.360165
Neutrophils	PLVAP	-0.74225	1.394862	-1.84388	0.06855	-4.06217	0.334044	0.369415
Neutrophils	OGFR	0.360029	5.244677	1.842354	0.068775	-4.45164	0.300033	0.332555
Neutrophils	PRKAR1B	1.26438	-0.37005	1.842342	0.068777	-4.03947	0.352154	0.388744
Neutrophils	GRINA	0.221174	6.205395	1.842166	0.068803	-5.18981	0.291946	0.323864
Neutrophils	H2-M3	-0.76846	4.138363	-1.84177	0.068862	-4.09767	0.309767	0.343261
Neutrophils	PDLIM5	0.174812	6.703681	1.840994	0.068976	-5.13179	0.288317	0.31984
Neutrophils	CSPRS	0.840853	0.292172	1.840108	0.069108	-4.02772	0.346374	0.382433
Neutrophils	TM9SF4	0.318551	4.769644	1.840101	0.069109	-4.44627	0.304838	0.33766
Neutrophils	AIF1	-0.54886	4.896333	-1.84005	0.069115	-4.47164	0.303741	0.336478
Neutrophils	CLEC4D	0.291715	2.949351	1.838878	0.06929	-5.17902	0.321608	0.355655
Neutrophils	KBTBD3	0.608564	2.727361	1.838874	0.069291	-4.09069	0.323651	0.357858
Neutrophils	WBP1	0.43863	3.813733	1.838641	0.069325	-4.27307	0.313799	0.347212
Neutrophils	DNAAF2	0.622922	2.845924	1.837553	0.069487	-4.05575	0.323174	0.356979
Neutrophils	ARAP1	0.283396	5.049234	1.83738	0.069513	-4.67327	0.303505	0.335814
Neutrophils	SARAF	-0.25129	5.365255	-1.83644	0.069654	-4.62297	0.301267	0.333129
Neutrophils	JAK3	0.660936	2.778845	1.835617	0.069776	-4.09689	0.324739	0.358258
Neutrophils	RORA	-0.38682	5.020685	-1.83515	0.069846	-4.60068	0.304811	0.33673
Neutrophils	DNTT	-1.61263	1.449877	-1.83481	0.069896	-4.03743	0.337583	0.372087
Neutrophils	ARID4A	0.205951	7.062741	1.834285	0.069975	-4.93403	0.287906	0.318369
Neutrophils	GIT2	0.178003	7.020155	1.834046	0.070011	-4.87844	0.288278	0.318781
Neutrophils	BUB3	-0.25511	5.956108	-1.83378	0.070052	-4.69173	0.297171	0.328397
Neutrophils	BRD1	0.24643	5.991255	1.832897	0.070184	-4.6366	0.297052	0.328313
Neutrophils	BORA	-0.50411	3.535011	-1.83275	0.070206	-4.16822	0.318571	0.351568
Neutrophils	TACO1OS	0.772599	1.705089	1.832702	0.070213	-4.05131	0.335645	0.369925
Neutrophils	BMYC	-1.02587	3.380337	-1.83263	0.070224	-4.04086	0.319979	0.353107
Neutrophils	FAM117A	-0.34199	5.965212	-1.83249	0.070244	-4.66192	0.297272	0.328642
Neutrophils	GEM	-0.62296	5.207345	-1.83122	0.070437	-4.24377	0.30445	0.336095
Neutrophils	GM16090	-1.32522	-0.96132	-1.831	0.070469	-4.05439	0.363048	0.399041
Neutrophils	CHRNB1	-1.0783	0.775917	-1.83052	0.070541	-4.04896	0.345544	0.380426
Neutrophils	BCL2A1D	0.491186	4.122114	1.830382	0.070562	-4.58861	0.314079	0.346655
Neutrophils	EGR2	0.765846	2.237621	1.830274	0.070578	-4.1604	0.33142	0.365329
Neutrophils	PHLPP2	-0.42123	4.283952	-1.83011	0.070604	-4.281	0.312634	0.345168
Neutrophils	GARNL3	-0.66999	2.487215	-1.82907	0.07076	-4.09029	0.329184	0.363174
Neutrophils	GM12802	0.801162	0.658153	1.82902	0.070768	-4.04151	0.346831	0.382114
Neutrophils	CNOT8	0.264826	5.284943	1.828939	0.07078	-4.42829	0.303956	0.335984
Neutrophils	BC024386	-0.84349	1.614053	-1.8289	0.070787	-4.07122	0.337491	0.372115
Neutrophils	AMBP	-0.4855	4.730423	-1.8288	0.070802	-4.59326	0.308792	0.341264
Neutrophils	GM42067	-1.03655	0.558955	-1.82874	0.07081	-4.06202	0.347815	0.383268
Neutrophils	CXCR2	0.344143	-0.00657	1.828353	0.070869	-4.58562	0.35362	0.38954
Neutrophils	SLCO5A1	-0.93368	-0.20706	-1.82786	0.070944	-4.06288	0.355875	0.391911
Neutrophils	TMEM199	0.45932	3.379806	1.827422	0.07101	-4.15395	0.321402	0.354896
Neutrophils	SHANK3	-0.63218	1.430051	-1.82679	0.071106	-4.21051	0.340105	0.374903

Neutrophils	SORBS3	-0.77106	0.937867	-1.82588	0.071244	-4.06687	0.345442	0.380327
Neutrophils	TNFAIP8L2	0.376561	3.852483	1.825047	0.071371	-4.34283	0.318305	0.350996
Neutrophils	CFAP43	0.302571	3.09234	1.824419	0.071466	-4.4588	0.325576	0.358801
Neutrophils	ASB6	0.70996	2.575022	1.824092	0.071516	-4.05845	0.330425	0.364104
Neutrophils	TCIM	-0.73842	1.634355	-1.82391	0.071544	-4.15559	0.339416	0.373798
Neutrophils	SSU72	0.142971	6.693565	1.823811	0.071559	-5.00393	0.293859	0.324743
Neutrophils	PROX1	-0.7969	2.15604	-1.82339	0.071624	-4.13861	0.334482	0.368686
Neutrophils	ARL8B	0.198527	5.97123	1.823296	0.071638	-4.70805	0.300028	0.331582
Neutrophils	BZW2	-0.42211	5.869307	-1.82232	0.071787	-4.3089	0.301397	0.332761
Neutrophils	ORAI3	0.446854	3.868765	1.820842	0.072014	-4.25514	0.319862	0.352454
Neutrophils	JAK1	0.139866	8.273816	1.820626	0.072047	-5.24945	0.282218	0.311806
Neutrophils	RUBCNL	0.530558	3.191015	1.820549	0.072059	-4.20292	0.326103	0.359195
Neutrophils	DHX36	0.241914	5.392248	1.820188	0.072114	-4.48966	0.306386	0.337996
Neutrophils	GSTA4	-0.78973	1.583658	-1.81994	0.072152	-4.07311	0.341561	0.375781
Neutrophils	RCN3	0.632441	1.932131	1.819734	0.072184	-4.10478	0.338186	0.372192
Neutrophils	ELF4	0.199544	6.785373	1.819487	0.072222	-4.88603	0.294541	0.325247
Neutrophils	8030456M	0.84212	1.090368	1.819311	0.072249	-4.0573	0.346431	0.381127
Neutrophils	ELAVL1	0.136248	7.604056	1.819061	0.072287	-5.01042	0.287808	0.318057
Neutrophils	ZFP189	-0.88018	1.120206	-1.81843	0.072385	-4.05438	0.346495	0.381044
Neutrophils	PLAC8	0.544807	8.2646	1.818047	0.072444	-5.12612	0.28284	0.31246
Neutrophils	IFNGR1	0.219872	7.03854	1.816193	0.07273	-4.97547	0.293783	0.323839
Neutrophils	PCMTD1	0.267883	5.924056	1.815964	0.072765	-4.69034	0.303234	0.334049
Neutrophils	NOTCH3	-0.94628	1.028947	-1.81595	0.072767	-4.06268	0.348643	0.382787
Neutrophils	F2R	-0.35847	3.599158	-1.81551	0.072836	-4.48105	0.324024	0.356524
Neutrophils	PREP	-0.36038	5.298922	-1.81528	0.072871	-4.40656	0.308709	0.340097
Neutrophils	STRBP	-0.24093	7.195368	-1.81494	0.072923	-4.86897	0.292511	0.322767
Neutrophils	SNX10	0.23886	5.029489	1.814891	0.072932	-4.9237	0.311086	0.342848
Neutrophils	GM46652	1.091899	-0.73309	1.814572	0.072981	-4.06512	0.36668	0.402466
Neutrophils	VTN	-0.53088	4.267725	-1.81455	0.072984	-4.51536	0.317908	0.350242
Neutrophils	ZFP207	-0.15145	6.934778	-1.81433	0.073018	-4.94996	0.294683	0.325245
Neutrophils	SYDE1	-0.95641	0.308678	-1.81432	0.073021	-4.06437	0.35593	0.391081
Neutrophils	RCSD1	0.22798	7.091061	1.814115	0.073052	-5.01388	0.29338	0.323934
Neutrophils	ALG1	-0.60959	3.312271	-1.81385	0.073094	-4.1216	0.326736	0.359985
Neutrophils	ACTB	0.145536	13.9201	1.813461	0.073154	-6.08912	0.242017	0.268072
Neutrophils	SLC35B3	-0.26667	4.482615	-1.81343	0.073159	-4.42084	0.316034	0.348498
Neutrophils	MOK	-1.25944	0.377087	-1.81282	0.073254	-4.07398	0.355512	0.39102
Neutrophils	HMGCS2	-0.59458	4.456909	-1.81276	0.073262	-4.54992	0.316446	0.349104
Neutrophils	SHTN1	-0.60593	3.187667	-1.8125	0.073304	-4.19424	0.328119	0.361745
Neutrophils	PDRG1	-0.47942	4.496479	-1.81192	0.073394	-4.18486	0.316104	0.349116
Neutrophils	RTN1	-0.86219	2.561189	-1.8118	0.073413	-4.13486	0.334036	0.368475
Neutrophils	RMND5B	0.296981	4.49308	1.811712	0.073426	-4.44227	0.316135	0.34922
Neutrophils	ZFP866	0.558325	2.993086	1.811665	0.073433	-4.14027	0.329945	0.364107
Neutrophils	5031425E2	0.228387	5.390925	1.811558	0.07345	-4.76581	0.308156	0.340639
Neutrophils	NLE1	-1.00338	2.257092	-1.81095	0.073545	-4.06355	0.337191	0.371979
Neutrophils	TROAP	-0.7827	1.774645	-1.81057	0.073605	-4.08822	0.341867	0.377071
Neutrophils	GM29417	-0.92343	1.098886	-1.81054	0.073608	-4.06738	0.348527	0.38422
Neutrophils	RAD21	-0.18484	7.022909	-1.81043	0.073626	-4.9909	0.294403	0.325829

Neutrophils	TMEM234	0.153135	7.040364	1.808284	0.073962	-4.93802	0.295362	0.326352
Neutrophils	CYB561D1	0.733897	1.785161	1.808269	0.073965	-4.0967	0.343048	0.37775
Neutrophils	UNC119B	-0.63428	3.551645	-1.80804	0.074	-4.10385	0.326209	0.3597
Neutrophils	8030462N1	0.163673	6.073398	1.807709	0.074052	-4.80281	0.303695	0.335363
Neutrophils	MRPL18	0.252908	6.633228	1.807344	0.07411	-4.83735	0.299009	0.330232
Neutrophils	ZFP871	-0.2801	5.249328	-1.80675	0.074203	-4.59957	0.311272	0.34343
Neutrophils	BLM	0.548893	3.897628	1.805987	0.074323	-4.32267	0.323729	0.35688
Neutrophils	ATP6V0B	0.134136	8.046898	1.805956	0.074328	-5.25326	0.287709	0.31795
Neutrophils	LAMC1	-0.53064	4.523339	-1.80578	0.074356	-4.25457	0.318009	0.350757
Neutrophils	IDH2	-0.32934	5.667278	-1.80561	0.074382	-4.51302	0.307821	0.33979
Neutrophils	SHANK2	-0.9304	1.410698	-1.80513	0.074458	-4.07158	0.347615	0.382691
Neutrophils	RGS1	-0.55636	5.563816	-1.80504	0.074473	-4.57765	0.308801	0.340979
Neutrophils	DYRK1B	0.749147	1.826196	1.804913	0.074493	-4.07205	0.343516	0.378384
Neutrophils	FILIP1	-0.75231	0.904589	-1.80446	0.074564	-4.1136	0.352825	0.388388
Neutrophils	FLT4	-0.46911	2.321562	-1.80429	0.074592	-4.3299	0.338837	0.373451
Neutrophils	SLC11A2	-0.65352	3.918763	-1.80375	0.074677	-4.15504	0.323748	0.357219
Neutrophils	KAT14	-0.42372	3.838268	-1.80362	0.074696	-4.18387	0.324491	0.358036
Neutrophils	BPNT1	-0.56662	3.339718	-1.8034	0.074732	-4.11263	0.329137	0.363166
Neutrophils	GM48960	0.776401	1.466163	1.803369	0.074737	-4.08412	0.347212	0.382592
Neutrophils	GATC	0.746481	2.698485	1.803232	0.074758	-4.10964	0.335212	0.369772
Neutrophils	MCC	-0.58369	2.092188	-1.80307	0.074784	-4.22186	0.341062	0.376088
Neutrophils	INPP5K	0.254559	5.371477	1.80295	0.074803	-4.61411	0.310627	0.343335
Neutrophils	CYTH1	0.186162	8.072227	1.802572	0.074863	-5.23656	0.287757	0.318504
Neutrophils	LIPE	0.360962	3.783042	1.802434	0.074885	-4.3437	0.325076	0.358897
Neutrophils	TAOK3	0.150797	7.012234	1.802077	0.074941	-4.98174	0.296541	0.328168
Neutrophils	SOAT1	0.24194	5.176779	1.802051	0.074945	-4.91572	0.312423	0.345374
Neutrophils	HPGDS	-0.65203	3.647133	-1.80187	0.074975	-4.19965	0.326338	0.360453
Neutrophils	GM16573	0.676464	1.655105	1.801513	0.075031	-4.10568	0.345496	0.381109
Neutrophils	UTP15	-0.50476	3.757308	-1.80138	0.075053	-4.13914	0.325384	0.35947
Neutrophils	MYH10	0.390617	2.573809	1.80101	0.075111	-4.38056	0.336593	0.371619
Neutrophils	ACOT8	0.392397	4.129949	1.800841	0.075138	-4.3	0.321984	0.355911
Neutrophils	RHD	0.885064	1.667344	1.800736	0.075154	-4.09856	0.345415	0.381212
Neutrophils	SLAMF7	-0.76399	5.151332	-1.80033	0.075218	-4.1799	0.31289	0.346192
Neutrophils	NT5DC3	-0.26541	5.099601	-1.7998	0.075304	-4.6302	0.313578	0.346902
Neutrophils	LZIC	0.506884	3.63991	1.799514	0.075349	-4.17173	0.326953	0.36142
Neutrophils	ICOS	-0.88933	2.283057	-1.7991	0.075415	-4.11281	0.340015	0.375562
Neutrophils	TSPAN33	0.836143	2.55857	1.798643	0.075488	-4.11702	0.337538	0.372888
Neutrophils	GM15232	-0.63129	1.187425	-1.79774	0.075632	-4.16099	0.351197	0.387619
Neutrophils	ERP29	0.186473	7.289905	1.797734	0.075633	-4.9042	0.295164	0.327081
Neutrophils	SPTA1	1.092694	0.279808	1.797434	0.075681	-4.08358	0.360419	0.397671
Neutrophils	SGK1	-0.27052	5.355259	-1.79718	0.075721	-4.82455	0.311845	0.345356
Neutrophils	PLIN2	0.237038	5.831377	1.797149	0.075726	-5.10042	0.30765	0.340809
Neutrophils	FGL1	0.507011	3.523532	1.796994	0.075751	-4.38866	0.328548	0.363497
Neutrophils	ZFP512	-0.4845	3.916117	-1.79684	0.075775	-4.22626	0.324892	0.359573
Neutrophils	ABR	0.321692	6.594873	1.796838	0.075776	-4.97656	0.301046	0.333734
Neutrophils	TSHZ3	-0.7696	2.187789	-1.79579	0.075944	-4.19052	0.341802	0.377731
Neutrophils	MFSD7A	1.065208	-0.44923	1.795767	0.075948	-4.08342	0.368535	0.406436

Neutrophils	SARDHOS	-1.39282	0.147254	-1.79556	0.07598	-4.0919	0.362315	0.399784
Neutrophils	ZDHHC14	-0.47334	7.022709	-1.79519	0.07604	-4.73571	0.297895	0.330394
Neutrophils	TMEM184I	0.374732	4.707523	1.795097	0.076055	-4.47325	0.318166	0.352422
Neutrophils	TRUB1	0.851964	1.825881	1.794728	0.076115	-4.08372	0.345443	0.381925
Neutrophils	FBXL22	-0.70471	2.248825	-1.79467	0.076124	-4.13902	0.341298	0.37746
Neutrophils	GMEB2	0.22013	5.764218	1.79442	0.076164	-4.70303	0.308803	0.3424
Neutrophils	TCP11L2	-0.22153	6.196085	-1.7938	0.076264	-5.11744	0.305168	0.338582
Neutrophils	GADD45G	-0.33066	4.465474	-1.79374	0.076274	-4.78051	0.320572	0.355294
Neutrophils	PPIL6	-1.0188	0.219778	-1.79363	0.076291	-4.08581	0.361857	0.399828
Neutrophils	CHD2	0.183439	7.754934	1.793031	0.076388	-5.18595	0.292209	0.324436
Neutrophils	OS9	0.239483	5.617189	1.792473	0.076478	-4.65672	0.310615	0.344496
Neutrophils	SLC25A46	0.267641	4.587825	1.792469	0.076478	-4.42316	0.31985	0.354511
Neutrophils	FBXO10	-0.40756	1.355085	-1.79168	0.076606	-4.31854	0.351052	0.388059
Neutrophils	CIZ1	0.392075	3.940619	1.791634	0.076613	-4.29335	0.326089	0.361132
Neutrophils	PHLDA1	-0.33356	4.035457	-1.7914	0.076651	-4.78469	0.325209	0.360218
Neutrophils	PXK	0.17999	6.270189	1.79128	0.07667	-5.03631	0.305169	0.338501
Neutrophils	PDE3B	-0.31825	6.955861	-1.7897	0.076926	-4.9635	0.300159	0.33269
Neutrophils	TNIP1	0.362567	4.966974	1.788772	0.077077	-4.60456	0.318034	0.351884
Neutrophils	ALG8	-0.62474	3.226775	-1.7887	0.077089	-4.14933	0.334202	0.369347
Neutrophils	PKP3	1.01917	2.747004	1.788427	0.077133	-4.09058	0.338862	0.374338
Neutrophils	GM15445	0.830103	0.994582	1.788234	0.077164	-4.09158	0.356244	0.393044
Neutrophils	KLRA9	-1.25415	-0.30711	-1.78665	0.077422	-4.099	0.370691	0.408068
Neutrophils	GRAMD1B	-0.25675	5.536445	-1.78641	0.077461	-4.97144	0.313779	0.346958
Neutrophils	RAB5C	0.172194	6.358629	1.786357	0.077469	-4.98052	0.306529	0.339106
Neutrophils	HIC1	-0.94738	2.158311	-1.78624	0.077488	-4.14574	0.345491	0.381199
Neutrophils	IP6K1	0.21603	6.598351	1.785419	0.077622	-4.91778	0.304851	0.337076
Neutrophils	1700056N1	0.640325	2.033678	1.785205	0.077657	-4.12058	0.347196	0.382788
Neutrophils	ZCCHC24	-0.52775	3.666755	-1.78424	0.077815	-4.24107	0.331837	0.366148
Neutrophils	LENG8	0.304778	4.564084	1.784047	0.077847	-4.51821	0.323459	0.357196
Neutrophils	ATP11C	-0.167	6.869185	-1.78394	0.077864	-5.00873	0.302934	0.334993
Neutrophils	SRGAP2	-0.22952	6.60483	-1.78361	0.077918	-4.98446	0.305217	0.337484
Neutrophils	CCDC136	-1.23119	0.119291	-1.78361	0.077919	-4.09697	0.367196	0.404206
Neutrophils	NMNAT3	-0.77198	3.105259	-1.78334	0.077962	-4.11709	0.337222	0.372077
Neutrophils	MIPOL1	-0.41587	4.257052	-1.78302	0.078015	-4.26842	0.32633	0.360459
Neutrophils	GM43813	-0.40763	4.535806	-1.78295	0.078027	-4.44921	0.323749	0.357673
Neutrophils	KIT	-0.63402	3.453389	-1.7828	0.078052	-4.32725	0.333891	0.36863
Neutrophils	RBM22	0.212755	5.603703	1.782393	0.078118	-4.61596	0.31409	0.347302
Neutrophils	UBE2J1	0.20522	6.282597	1.782265	0.078139	-4.77032	0.308085	0.340848
Neutrophils	CD55	-0.40123	4.863884	-1.78208	0.078169	-4.74463	0.320773	0.354627
Neutrophils	LSM14A	0.157396	6.620649	1.781691	0.078233	-4.92984	0.30514	0.33773
Neutrophils	CEP83OS	-0.71105	2.022979	-1.78169	0.078234	-4.12927	0.347836	0.383832
Neutrophils	YIPF2	-0.75639	1.926928	-1.78136	0.078288	-4.10514	0.348791	0.384937
Neutrophils	ST6GALNA(	-0.40747	5.127632	-1.781	0.078346	-4.6969	0.318374	0.3523
Neutrophils	ARHGEF26	-0.71987	0.379311	-1.78094	0.078357	-4.16834	0.364551	0.402038
Neutrophils	ZFP12	0.844698	1.346551	1.78076	0.078386	-4.10028	0.354619	0.391423
Neutrophils	LRRC10B	-1.21992	0.595128	-1.78064	0.078406	-4.10534	0.362311	0.39968
Neutrophils	ALDH3B1	0.23172	3.605843	1.780634	0.078407	-4.81874	0.332479	0.367585

Neutrophils	FILIP1L	0.429223	5.411773	1.780412	0.078444	-4.40912	0.31583	0.34963
Neutrophils	BC052040	-0.43279	4.165796	-1.78016	0.078485	-4.39168	0.32725	0.362082
Neutrophils	PDE2A	-0.28103	6.050057	-1.77994	0.078522	-4.97765	0.310162	0.343685
Neutrophils	MTFMT	-0.56071	2.527907	-1.77917	0.078648	-4.11922	0.342897	0.379372
Neutrophils	ALDH9A1	-0.32952	5.005152	-1.77912	0.078657	-4.40946	0.31952	0.354102
Neutrophils	DVL3	0.440422	3.300094	1.778969	0.078682	-4.23789	0.335427	0.371355
Neutrophils	RABL6	-0.28053	5.136693	-1.77893	0.078688	-4.45689	0.318326	0.352848
Neutrophils	FPR1	0.358448	2.992339	1.778898	0.078693	-5.06457	0.338384	0.374548
Neutrophils	ARHGAP45	0.190355	6.710119	1.778863	0.078699	-5.0796	0.304399	0.337725
Neutrophils	CPTP	-0.57373	2.248177	-1.77783	0.07887	-4.14224	0.346258	0.382869
Neutrophils	SLC2A3	0.216355	4.519005	1.777403	0.078941	-5.04225	0.324707	0.359541
Neutrophils	GSDMD	0.309218	4.268046	1.776972	0.079012	-4.69644	0.327202	0.36225
Neutrophils	BACH2IT1	-0.99362	-0.26225	-1.77602	0.07917	-4.12091	0.372955	0.411188
Neutrophils	IRF9	0.443037	4.515749	1.775757	0.079214	-4.55984	0.32547	0.360159
Neutrophils	CXCL12	-0.54229	2.838235	-1.77538	0.079277	-4.30203	0.341547	0.377545
Neutrophils	SSH1	-0.4334	3.775324	-1.77497	0.079345	-4.27504	0.332568	0.367963
Neutrophils	AMIGO1	-0.98669	0.613629	-1.77488	0.07936	-4.11362	0.363971	0.4018
Neutrophils	CWC22	0.478074	3.900011	1.774767	0.079378	-4.24596	0.331388	0.36671
Neutrophils	CCDC148	-1.14938	1.762244	-1.77452	0.079419	-4.16752	0.352267	0.389232
Neutrophils	GM31763	0.476997	3.257819	1.774321	0.079452	-4.38337	0.337558	0.373393
Neutrophils	SNRPC	-0.18853	6.245324	-1.77382	0.079536	-4.86463	0.310233	0.343849
Neutrophils	RNF181	0.365993	4.477136	1.773311	0.079621	-4.46151	0.326459	0.361376
Neutrophils	PAFAH1B1	0.120622	8.064884	1.773061	0.079662	-5.24808	0.294852	0.327038
Neutrophils	GM26771	-0.71597	-0.2163	-1.77284	0.0797	-4.17904	0.37332	0.411851
Neutrophils	SH3PXD2A	-0.42688	5.753145	-1.77163	0.079902	-4.48239	0.315517	0.349251
Neutrophils	1700123M	-1.01184	0.89752	-1.77149	0.079926	-4.11461	0.362365	0.399789
Neutrophils	TNFRSF18	0.525926	2.322619	1.771102	0.07999	-4.2232	0.348059	0.384287
Neutrophils	CARF	0.666567	2.216581	1.770815	0.080038	-4.1528	0.349185	0.385554
Neutrophils	TCTEX1D2	0.255519	3.301638	1.770388	0.080109	-4.71709	0.338709	0.374229
Neutrophils	C8B	-0.84817	0.571894	-1.7698	0.080209	-4.11696	0.366462	0.403916
Neutrophils	CHST14	0.9697	0.872451	1.769028	0.080338	-4.11412	0.363696	0.400672
Neutrophils	FAM118B	0.429815	3.464677	1.768936	0.080353	-4.27239	0.337761	0.372808
Neutrophils	ZFYVE26	0.264512	5.03643	1.768695	0.080394	-4.69063	0.322973	0.356974
Neutrophils	CYP4A14	0.851038	1.833194	1.768318	0.080457	-4.1947	0.353859	0.390376
Neutrophils	GPR182	-0.43353	2.557152	-1.7682	0.080476	-4.44352	0.346622	0.382646
Neutrophils	ACADL	0.226859	6.734458	1.767858	0.080535	-4.96846	0.30775	0.340702
Neutrophils	GM43647	-1.16022	-0.94495	-1.76779	0.080546	-4.12032	0.383078	0.421805
Neutrophils	ESR1	-0.64174	3.611322	-1.76772	0.080558	-4.27809	0.336355	0.371676
Neutrophils	TEC	-0.23086	5.751113	-1.7676	0.080577	-4.91752	0.316472	0.350246
Neutrophils	SULT2A1	-0.65342	3.901678	-1.76722	0.080643	-4.52872	0.333721	0.368915
Neutrophils	ZFP119B	0.746665	1.297642	1.766914	0.080693	-4.12891	0.359545	0.396736
Neutrophils	SET	0.20614	7.946372	1.766704	0.080729	-5.08262	0.297553	0.329721
Neutrophils	ZFP296	-0.74281	2.857765	-1.76603	0.080843	-4.17133	0.344116	0.380198
Neutrophils	MGST1	-0.23609	7.132587	-1.76603	0.080843	-5.42577	0.304692	0.337545
Neutrophils	RFX2	0.548199	3.630115	1.765643	0.080908	-4.25545	0.336756	0.372277
Neutrophils	CHD7	0.193923	6.463817	1.765414	0.080947	-5.14025	0.310686	0.344155
Neutrophils	GLCE	-0.47708	3.79856	-1.76483	0.081046	-4.27267	0.335449	0.370917

Neutrophils	SUPT3	-0.26503	5.480744	-1.76416	0.081159	-4.76762	0.319901	0.35415
Neutrophils	ELMO2	0.483234	3.945024	1.763529	0.081266	-4.27676	0.334201	0.369933
Neutrophils	ZFP719	0.525697	2.119349	1.763249	0.081313	-4.19032	0.352065	0.389208
Neutrophils	PPIL4	-0.20968	5.46202	-1.76321	0.08132	-4.68893	0.320071	0.354641
Neutrophils	EFTUD2	-0.34158	5.105706	-1.76317	0.081327	-4.42673	0.323333	0.358176
Neutrophils	THRB	-0.63109	3.815098	-1.76296	0.081363	-4.38609	0.335441	0.371353
Neutrophils	EVL	-0.37858	6.485099	-1.76285	0.081382	-4.60181	0.310895	0.344771
Neutrophils	C9ORF72	0.547399	3.893192	1.762649	0.081415	-4.36279	0.334695	0.370596
Neutrophils	ARHGAP26	0.21013	6.685583	1.762614	0.081421	-5.19785	0.309129	0.342878
Neutrophils	RFC5	0.547664	4.185223	1.762538	0.081434	-4.36597	0.331921	0.367613
Neutrophils	SLC16A13	-1.05124	-0.01818	-1.76204	0.081519	-4.12165	0.374223	0.413311
Neutrophils	RNF34	0.229466	4.455799	1.761832	0.081554	-4.72353	0.329372	0.364979
Neutrophils	PMPCA	0.405295	4.024403	1.761599	0.081594	-4.34714	0.333445	0.369407
Neutrophils	IFIT3B	0.933108	1.43036	1.761578	0.081598	-4.19524	0.359058	0.397047
Neutrophils	MAP3K9	0.728387	0.308951	1.761443	0.081621	-4.18403	0.370743	0.409652
Neutrophils	TOMM20	-0.21968	6.725117	-1.76143	0.081623	-4.8364	0.308782	0.342687
Neutrophils	ABCC3	-0.68249	2.689429	-1.76133	0.081639	-4.31442	0.346384	0.383454
Neutrophils	ECI1	-0.41848	3.920606	-1.76122	0.081658	-4.3179	0.334433	0.37054
Neutrophils	CMTM4	-0.57459	3.141567	-1.7606	0.081765	-4.21395	0.342047	0.3789
Neutrophils	TMED5	0.128341	7.227678	1.760501	0.081781	-5.16588	0.304499	0.338163
Neutrophils	HOPX	0.305358	4.034194	1.760417	0.081795	-4.79232	0.333452	0.369612
Neutrophils	BHMT	0.472611	5.13251	1.76033	0.08181	-4.79321	0.323183	0.358526
Neutrophils	TYROBP	0.181633	9.095091	1.760078	0.081853	-5.69298	0.288844	0.321087
Neutrophils	4930404N1	1.042247	0.191025	1.759444	0.081961	-4.1258	0.372345	0.411557
Neutrophils	FMN2	-1.2942	2.359589	-1.75935	0.081978	-4.12484	0.349989	0.387504
Neutrophils	GM2000	-0.49369	4.799183	-1.75927	0.081991	-4.42128	0.326474	0.36207
Neutrophils	1700010K2	-0.82405	0.709295	-1.75873	0.082083	-4.1252	0.367098	0.405903
Neutrophils	NMRK1	0.359252	3.751312	1.758602	0.082105	-4.45398	0.336574	0.373029
Neutrophils	REXO5	0.664738	1.950184	1.758034	0.082202	-4.15779	0.354464	0.392496
Neutrophils	CENPB	0.312591	5.450808	1.758019	0.082205	-4.57563	0.320802	0.356065
Neutrophils	0610010F0	-0.37765	4.458879	-1.75784	0.082235	-4.48073	0.32999	0.366111
Neutrophils	GM28192	0.353534	0.064757	1.757488	0.082296	-4.48414	0.37413	0.413846
Neutrophils	NOP58	-0.34622	5.838554	-1.7574	0.082312	-4.61865	0.317333	0.352467
Neutrophils	3110009E1	-0.70903	2.175408	-1.75715	0.082353	-4.13499	0.352281	0.390366
Neutrophils	EEF1B2	0.188619	8.556525	1.756977	0.082384	-5.18976	0.293803	0.326852
Neutrophils	SORL1	0.202687	5.66068	1.756256	0.082507	-5.33876	0.319125	0.354543
Neutrophils	CDC16	-0.44211	3.893321	-1.75616	0.082523	-4.27395	0.335596	0.372434
Neutrophils	GMIP	0.165885	5.964525	1.756032	0.082546	-5.02451	0.31638	0.351567
Neutrophils	SON	0.12897	7.67203	1.755871	0.082574	-5.22058	0.301405	0.335281
Neutrophils	IQSEC1	0.165538	6.277452	1.755813	0.082584	-5.16536	0.313578	0.348559
Neutrophils	ZFP825	0.658464	2.050646	1.755304	0.082671	-4.15887	0.353944	0.392187
Neutrophils	PPP2R3C	0.333981	4.333149	1.754949	0.082732	-4.45245	0.331755	0.368173
Neutrophils	SAA1	1.66245	-0.05604	1.754177	0.082865	-4.13046	0.37642	0.41636
Neutrophils	NDNF	-1.07842	-0.47189	-1.75407	0.082884	-4.13682	0.380918	0.421211
Neutrophils	GPATCH2L	0.202311	5.064269	1.753849	0.082922	-4.76791	0.325285	0.361134
Neutrophils	DIPK2A	-0.38916	4.112992	-1.7535	0.082981	-4.37503	0.334328	0.370946
Neutrophils	EPHX1	-0.70863	3.053436	-1.75168	0.083297	-4.19787	0.345614	0.382767

Neutrophils	ZFP318	-0.73056	3.491008	-1.75153	0.083322	-4.19904	0.341328	0.378213
Neutrophils	HEPACAM2	-1.22702	0.779743	-1.75151	0.083327	-4.13349	0.368788	0.407828
Neutrophils	SLC16A2	-0.53497	2.626868	-1.75048	0.083504	-4.30452	0.350356	0.387746
Neutrophils	GADD45A	-0.20574	4.876654	-1.75043	0.083513	-5.17161	0.328594	0.364214
Neutrophils	TRIT1	-0.55538	4.076961	-1.74988	0.083609	-4.19369	0.33642	0.372725
Neutrophils	GIMAP8	-0.82053	2.465426	-1.74913	0.08374	-4.17198	0.352658	0.390153
Neutrophils	CAMK2B	-0.88527	3.706454	-1.74825	0.083892	-4.18969	0.340786	0.377305
Neutrophils	SENP7	0.2241	5.356774	1.748197	0.083901	-4.82088	0.325138	0.360386
Neutrophils	ZFP235	0.828898	1.919758	1.747199	0.084075	-4.14352	0.359081	0.396901
Neutrophils	REEP6	-0.75902	1.791912	-1.74719	0.084077	-4.18851	0.360394	0.398314
Neutrophils	ZEB2	0.260034	9.36649	1.746732	0.084157	-5.5557	0.290627	0.322745
Neutrophils	VIM	0.292654	8.109275	1.746509	0.084196	-5.33445	0.301157	0.334278
Neutrophils	DMAP1	0.586551	2.496634	1.746337	0.084226	-4.18454	0.353297	0.390853
Neutrophils	RTL8B	-0.67658	3.297356	-1.74633	0.084226	-4.20415	0.345319	0.382243
Neutrophils	MASP1	-0.66888	1.673868	-1.74548	0.084376	-4.23615	0.362061	0.400348
Neutrophils	NECAP1	0.238682	4.774239	1.745473	0.084377	-4.67447	0.331424	0.367261
Neutrophils	ZFP949	0.491265	2.594224	1.744434	0.084559	-4.29161	0.353188	0.390651
Neutrophils	MNT	0.371446	4.606728	1.744399	0.084565	-4.48289	0.333494	0.369374
Neutrophils	GM32250	0.752449	-0.2746	1.743455	0.08473	-4.1626	0.38394	0.423417
Neutrophils	MANF	-0.22015	6.808848	-1.74288	0.084832	-4.85115	0.313997	0.347765
Neutrophils	USP21	-0.5664	3.140041	-1.7424	0.084916	-4.22392	0.348562	0.385404
Neutrophils	ANKRD52	-0.45738	3.881446	-1.74237	0.084921	-4.27883	0.341272	0.377534
Neutrophils	MRC2	-0.83471	1.416971	-1.74231	0.084931	-4.16822	0.366131	0.404349
Neutrophils	PLEKHM2	0.377742	5.202185	1.742134	0.084963	-4.65183	0.328672	0.363938
Neutrophils	JKAMP	-0.32047	3.680975	-1.74145	0.085083	-4.40785	0.343508	0.379891
Neutrophils	GCHFR	-0.58332	3.389908	-1.74123	0.085122	-4.44467	0.346371	0.383056
Neutrophils	RAP2B	0.32795	4.614993	1.741183	0.08513	-4.51768	0.334486	0.370215
Neutrophils	CYB5D2	0.710105	1.870839	1.740874	0.085184	-4.18712	0.361806	0.399702
Neutrophils	COL5A1	0.935122	0.493409	1.74051	0.085249	-4.1533	0.37646	0.415479
Neutrophils	TRAF3	0.215103	6.856964	1.73989	0.085358	-5.18821	0.314305	0.348305
Neutrophils	GDE1	0.283604	4.883129	1.739609	0.085408	-4.88122	0.3325	0.368045
Neutrophils	IFT52	0.322106	4.249963	1.738446	0.085614	-4.42587	0.33855	0.375095
Neutrophils	TMA7	0.157896	6.564001	1.738273	0.085644	-4.98545	0.316978	0.351745
Neutrophils	ATRN	0.164081	5.965865	1.738228	0.085652	-5.29133	0.322413	0.357651
Neutrophils	CNEP1R1	0.314071	4.587392	1.738191	0.085659	-4.48762	0.335312	0.37165
Neutrophils	PRUNE1	0.265083	3.600837	1.73777	0.085733	-4.6692	0.344872	0.382056
Neutrophils	NRBP1	0.206208	5.827937	1.737659	0.085753	-4.84282	0.32368	0.359124
Neutrophils	RUNDC3B	-0.8087	3.848947	-1.73759	0.085766	-4.22328	0.342441	0.379475
Neutrophils	USP8	0.210611	5.372996	1.737523	0.085777	-4.79197	0.327896	0.363712
Neutrophils	FAM199X	0.563293	2.776803	1.737174	0.085839	-4.23581	0.353073	0.39109
Neutrophils	ZFAND3	0.135111	8.233036	1.736674	0.085928	-5.42308	0.302318	0.336105
Neutrophils	GPR27	-0.34277	-1.60473	-1.73658	0.085945	-4.29678	0.40013	0.441841
Neutrophils	GNB2	0.138991	8.428837	1.736537	0.085952	-5.40737	0.300646	0.334279
Neutrophils	EXT1	-0.34022	7.749502	-1.73615	0.086021	-5.03789	0.30649	0.340768
Neutrophils	MTDH	0.128818	7.470279	1.736096	0.08603	-5.10955	0.308927	0.343427
Neutrophils	ZSCAN26	-0.46589	4.652438	-1.73608	0.086033	-4.32042	0.334691	0.371453
Neutrophils	SPNS1	-0.48858	3.498213	-1.73604	0.08604	-4.26528	0.345882	0.38358

Neutrophils	CCM2	-0.18419	7.391127	-1.73594	0.086058	-5.10302	0.309622	0.3442
Neutrophils	HSP90B1	-0.18894	8.976388	-1.73581	0.086081	-5.31301	0.296022	0.329349
Neutrophils	ZFP239	1.075219	-0.06752	1.735777	0.086087	-4.15837	0.382942	0.423567
Neutrophils	ENPEP	-0.97661	0.401804	-1.73575	0.086091	-4.15299	0.377842	0.418085
Neutrophils	LPIN2	0.243082	6.528551	1.735514	0.086134	-4.97736	0.317298	0.35262
Neutrophils	RHOBTB2	0.6326	2.943311	1.735387	0.086156	-4.19811	0.3514	0.389636
Neutrophils	GM11342	0.798882	2.120734	1.735291	0.086173	-4.20379	0.359745	0.3987
Neutrophils	NACC2	-0.81873	1.77434	-1.73505	0.086217	-4.16952	0.36332	0.402637
Neutrophils	CYB5R3	0.301048	4.85016	1.735026	0.086221	-4.58019	0.332812	0.369613
Neutrophils	TMEM127	0.249317	4.290896	1.734477	0.086319	-4.62187	0.338411	0.375731
Neutrophils	PLA2G12B	-0.81106	1.0508	-1.73406	0.086393	-4.18427	0.371363	0.411289
Neutrophils	LAMTOR3	0.232487	5.240813	1.733738	0.08645	-4.73689	0.329634	0.366118
Neutrophils	RBMS1	0.135001	7.558573	1.733209	0.086545	-5.3709	0.308828	0.343454
Neutrophils	PLS3	-0.59037	2.162516	-1.73305	0.086573	-4.24495	0.360101	0.399127
Neutrophils	SERINC5	-0.36756	5.287482	-1.73267	0.086641	-4.60841	0.329547	0.365945
Neutrophils	BEX3	-0.46864	4.990762	-1.73191	0.086777	-4.36165	0.332737	0.369375
Neutrophils	TIRAP	0.290773	3.005516	1.731642	0.086825	-4.61332	0.352133	0.39041
Neutrophils	LYZ1	1.085078	-0.68152	1.731506	0.086849	-4.1561	0.391224	0.432563
Neutrophils	PPP1R18	0.160308	6.994866	1.730831	0.08697	-5.2377	0.314654	0.349602
Neutrophils	NDUFAF3	0.321408	4.160988	1.73049	0.087032	-4.50219	0.34118	0.378405
Neutrophils	GM15987	0.934642	2.537923	1.729606	0.08719	-4.17592	0.357858	0.396402
Neutrophils	PRPF4B	0.215105	6.312389	1.728941	0.08731	-4.86981	0.321643	0.357074
Neutrophils	STXBP2	0.194867	5.31968	1.728726	0.087349	-4.9652	0.330852	0.367208
Neutrophils	CPLX1	-1.18574	-0.57566	-1.72782	0.087513	-4.16028	0.391449	0.433056
Neutrophils	LRMP	-0.25144	6.432878	-1.7277	0.087534	-4.90555	0.320544	0.356378
Neutrophils	ZFP60	-0.86312	1.431044	-1.72709	0.087643	-4.16195	0.369645	0.409868
Neutrophils	HBP1	-0.21763	5.788072	-1.72691	0.087677	-4.92513	0.326473	0.363118
Neutrophils	DAPP1	0.187303	6.482475	1.726747	0.087706	-5.06414	0.320092	0.35618
Neutrophils	SLC41A2	-0.9114	2.900939	-1.72668	0.087717	-4.17989	0.354459	0.39353
Neutrophils	WFDC18	0.856629	-0.88323	1.726539	0.087743	-4.16237	0.394903	0.437153
Neutrophils	AGO2	0.153982	7.576219	1.726434	0.087762	-5.31724	0.310306	0.345522
Neutrophils	PTPRC	0.163122	8.915439	1.726343	0.087779	-5.57891	0.298753	0.332917
Neutrophils	RHOT2	-0.60322	2.679369	-1.72622	0.087802	-4.2283	0.356707	0.39606
Neutrophils	MFAP1A	0.220482	4.832089	1.726029	0.087835	-4.71461	0.335476	0.373058
Neutrophils	NR1H3	-0.58654	3.325046	-1.72602	0.087837	-4.4469	0.350197	0.389039
Neutrophils	MED24	-0.41285	3.187102	-1.72568	0.087898	-4.34298	0.351577	0.390595
Neutrophils	CENPC1	-0.23941	5.265605	-1.72563	0.087908	-4.64731	0.331362	0.368639
Neutrophils	PDXP	-0.94383	1.557173	-1.72561	0.087911	-4.16882	0.368317	0.408708
Neutrophils	CAPN3	-0.85147	1.422788	-1.72559	0.087915	-4.17003	0.369732	0.410237
Neutrophils	GALNT11	0.360521	4.120023	1.725582	0.087916	-4.4277	0.34235	0.380585
Neutrophils	AVPI1	0.651472	2.712104	1.725561	0.08792	-4.21875	0.356374	0.395791
Neutrophils	NOL10	-0.30153	4.91748	-1.72335	0.088321	-4.59181	0.336063	0.372819
Neutrophils	RAB3GAP1	0.21975	6.044858	1.723026	0.08838	-4.70694	0.325477	0.361418
Neutrophils	2010109A1	-0.55002	2.521995	-1.72295	0.088393	-4.2841	0.359836	0.398651
Neutrophils	UBAP1	0.206196	5.688984	1.722307	0.088511	-5.01874	0.328918	0.365297
Neutrophils	FBXO4	0.417604	3.431156	1.721949	0.088576	-4.34928	0.350764	0.38913
Neutrophils	DENND1A	-0.18801	7.497877	-1.72195	0.088576	-5.10426	0.312444	0.34748

Neutrophils	DTNBP1	-0.14373	6.683213	-1.72187	0.08859	-5.07144	0.319754	0.35545
Neutrophils	FBRSL1	-0.2008	5.766788	-1.72167	0.088626	-4.84037	0.328191	0.36466
Neutrophils	PPP1R10	-0.20601	6.277719	-1.72157	0.088645	-5.07408	0.323459	0.35955
Neutrophils	RASGRF2	-1.01082	0.116081	-1.72149	0.08866	-4.17212	0.385577	0.426796
Neutrophils	CYP2C23	-0.86574	0.860959	-1.7208	0.088784	-4.18374	0.377848	0.418301
Neutrophils	MBNL1	-0.19111	8.978349	-1.72039	0.088861	-5.48019	0.300042	0.333781
Neutrophils	ERCC2	0.771918	1.983619	1.720239	0.088887	-4.17161	0.366081	0.405519
Neutrophils	MAVS	-0.62179	2.932268	-1.7198	0.088967	-4.20821	0.356366	0.395085
Neutrophils	DOLPP1	-0.68522	2.514642	-1.71979	0.088969	-4.19922	0.360638	0.399702
Neutrophils	CAMKMT	0.398597	4.501622	1.719493	0.089024	-4.49298	0.340857	0.378223
Neutrophils	TMEM97	-0.50939	3.649444	-1.71921	0.089076	-4.30398	0.349246	0.387358
Neutrophils	PTPRB	-0.43626	3.708559	-1.71911	0.089094	-4.80514	0.348658	0.386746
Neutrophils	PRPF4	-0.43149	3.92555	-1.71893	0.089126	-4.36995	0.346509	0.384461
Neutrophils	SPATA21	-0.543	3.397572	-1.71873	0.089163	-4.33466	0.351778	0.390255
Neutrophils	KANK2	-0.63886	2.38565	-1.7184	0.089225	-4.24686	0.362097	0.401597
Neutrophils	YOD1	0.518411	4.084266	1.718211	0.089258	-4.36558	0.344974	0.383179
Neutrophils	TNPO3	0.200885	6.365771	1.718169	0.089266	-4.8997	0.323287	0.359598
Neutrophils	FABP1	-0.47293	7.428716	-1.71739	0.089408	-5.33166	0.313947	0.349383
Neutrophils	HS3ST3B1	-0.56652	3.380647	-1.71739	0.08941	-4.3297	0.352268	0.391058
Neutrophils	KDM3A	0.235225	5.567098	1.716896	0.0895	-4.81507	0.331162	0.368157
Neutrophils	EEF2K	-0.47087	4.644155	-1.71668	0.089539	-4.38797	0.339977	0.377812
Neutrophils	NAA38	0.298337	5.467647	1.716604	0.089553	-4.65545	0.3321	0.36929
Neutrophils	CDC27	0.189025	6.009717	1.71607	0.089651	-5.01274	0.327256	0.363899
Neutrophils	DGKE	-0.53988	4.880229	-1.71586	0.089689	-4.32793	0.337961	0.375579
Neutrophils	CAPN11	-1.41167	1.046105	-1.7153	0.089793	-4.17969	0.377311	0.418109
Neutrophils	PITRM1	-0.55395	3.560314	-1.71509	0.089831	-4.25789	0.351209	0.389952
Neutrophils	RDH7	-0.64983	2.864625	-1.71461	0.089921	-4.39681	0.358261	0.39773
Neutrophils	UTP4	0.282856	5.102474	1.714471	0.089946	-4.63032	0.336126	0.373732
Neutrophils	MOB4	0.154111	6.832732	1.714191	0.089997	-5.09293	0.319991	0.356195
Neutrophils	KLHL5	0.196508	4.645979	1.71418	0.089999	-4.86398	0.340523	0.378554
Neutrophils	DYSF	-0.72156	2.592245	-1.71417	0.090001	-4.41519	0.361055	0.400819
Neutrophils	ATP2B4	-0.55144	4.169834	-1.71396	0.09004	-4.43714	0.345174	0.383646
Neutrophils	CHMP1A	0.267211	5.305048	1.713811	0.090068	-4.76191	0.334194	0.371711
Neutrophils	GM48696	0.582342	2.476417	1.713389	0.090145	-4.27881	0.36229	0.402359
Neutrophils	RNF138	0.214162	5.857254	1.713288	0.090164	-4.82485	0.329021	0.366243
Neutrophils	TRIP11	0.198695	6.069875	1.713218	0.090177	-4.95857	0.327038	0.364087
Neutrophils	LRPAP1	0.388677	4.499631	1.712548	0.090301	-4.45565	0.342174	0.380666
Neutrophils	TPGS2	0.438627	3.805381	1.712487	0.090312	-4.34295	0.34901	0.388093
Neutrophils	PXN	0.184415	6.266447	1.712405	0.090327	-5.17582	0.325398	0.362415
Neutrophils	MEF2D	-0.19431	7.288749	-1.7122	0.090366	-5.12396	0.316104	0.35228
Neutrophils	KANSL1L	-0.2675	7.202302	-1.71159	0.090478	-5.31657	0.317101	0.353402
Neutrophils	SLC25A34	-1.03305	-0.47643	-1.71141	0.090511	-4.17986	0.394679	0.437595
Neutrophils	GM20219	-0.90878	0.646352	-1.71132	0.090528	-4.17938	0.382222	0.42418
Neutrophils	TMEM154	0.269003	2.583653	1.710881	0.09061	-4.86189	0.361848	0.402206
Neutrophils	SIRT3	0.476012	3.344497	1.710359	0.090706	-4.2866	0.354269	0.394068
Neutrophils	PELI1	-0.16679	7.676753	-1.71025	0.090727	-5.2548	0.313194	0.349327
Neutrophils	TDP2	-0.35921	4.340054	-1.71008	0.090758	-4.48196	0.344357	0.383367

Neutrophils	PDCD10	0.144845	6.713222	1.70967	0.090835	-5.13047	0.32203	0.359026
Neutrophils	ACLY	-0.16913	6.383195	-1.70943	0.09088	-5.01773	0.325072	0.362409
Neutrophils	RHOF	0.382485	3.558194	1.709298	0.090904	-4.58482	0.352294	0.39211
Neutrophils	CPLANE2	-0.95164	0.331465	-1.70907	0.090947	-4.18191	0.386321	0.429044
Neutrophils	P2RY12	0.58981	2.616799	1.708837	0.090989	-4.35473	0.361957	0.402733
Neutrophils	RBM43	0.50531	2.961078	1.708429	0.091065	-4.31344	0.358481	0.398971
Neutrophils	EOGT	-0.67528	2.532472	-1.70828	0.091093	-4.22099	0.362891	0.403756
Neutrophils	BIK	-0.73595	1.76969	-1.70819	0.091111	-4.28025	0.370878	0.412412
Neutrophils	PTTG1	0.219695	5.78	1.707945	0.091156	-4.95715	0.33082	0.368909
Neutrophils	LMO2	0.311157	4.903927	1.707721	0.091197	-4.74674	0.339171	0.37808
Neutrophils	H3F3B	0.124257	9.934295	1.707679	0.091205	-5.62892	0.294067	0.328689
Neutrophils	MAP2K5	-0.21049	6.143251	-1.70727	0.091281	-4.84326	0.327575	0.36539
Neutrophils	MITD1	0.366389	4.433197	1.706909	0.091349	-4.56072	0.344038	0.383331
Neutrophils	PRR3	-0.54326	3.987386	-1.70629	0.091465	-4.26612	0.348575	0.388423
Neutrophils	MAX	0.162162	6.499429	1.706206	0.09148	-5.15742	0.324527	0.362211
Neutrophils	GM43707	-1.05802	0.407926	-1.70603	0.091513	-4.1856	0.386064	0.429101
Neutrophils	CCDC57	-0.33128	2.546499	-1.706	0.091519	-4.54201	0.363197	0.404341
Neutrophils	ZFP213	0.715095	2.162406	1.705594	0.091595	-4.19974	0.367319	0.408835
Neutrophils	GANC	-0.48815	3.377098	-1.70548	0.091616	-4.36404	0.354808	0.395303
Neutrophils	C1S1	-0.70217	1.787052	-1.70526	0.091657	-4.23842	0.37131	0.413303
Neutrophils	C9	-1.20544	0.60037	-1.70411	0.091873	-4.19638	0.384811	0.42767
Neutrophils	GM12979	-0.93187	0.049111	-1.70401	0.091892	-4.18733	0.390917	0.434312
Neutrophils	IFI209	-0.60095	5.809713	-1.70351	0.091986	-4.42914	0.331912	0.370244
Neutrophils	SDF4	0.169331	6.506152	1.702573	0.092162	-4.9984	0.325792	0.363539
Neutrophils	PRPF8	-0.21782	5.801492	-1.70257	0.092162	-4.77547	0.332383	0.370742
Neutrophils	MID2	-0.83954	0.27499	-1.70205	0.092261	-4.20741	0.38917	0.432575
Neutrophils	A730063M	0.923297	1.218514	1.702017	0.092266	-4.19592	0.378824	0.421391
Neutrophils	NR1I3	-0.82797	1.055611	-1.70167	0.092331	-4.22854	0.38059	0.423437
Neutrophils	CTSH	-0.26579	5.779307	-1.70151	0.092361	-5.02697	0.332638	0.371339
Neutrophils	PRORSD1	0.28501	4.586531	1.701344	0.092393	-4.62025	0.344124	0.383916
Neutrophils	CHIL5	0.551342	-0.02332	1.701115	0.092436	-4.2943	0.3925	0.436552
Neutrophils	PEPD	-0.40684	5.308275	-1.70107	0.092444	-4.44357	0.337126	0.376396
Neutrophils	APEX1	-0.40493	5.465947	-1.70104	0.09245	-4.50262	0.335617	0.374754
Neutrophils	KEAP1	0.265473	5.115335	1.700902	0.092476	-4.64417	0.338982	0.378463
Neutrophils	GM32036	0.298947	3.651119	1.700489	0.092554	-4.54267	0.35359	0.394425
Neutrophils	2610307P1	-0.89021	4.550996	-1.69979	0.092687	-4.34242	0.344812	0.384877
Neutrophils	AB124611	0.194587	5.452214	1.69975	0.092694	-5.12705	0.336078	0.375341
Neutrophils	CERS6	0.195243	7.330325	1.699674	0.092708	-5.52251	0.318613	0.356241
Neutrophils	LARP4	-0.22472	6.410341	-1.69953	0.092735	-4.87304	0.327046	0.365503
Neutrophils	OAS1G	0.988206	-0.24881	1.699185	0.0928	-4.19621	0.395532	0.440051
Neutrophils	GM41555	-1.08954	0.571556	-1.69874	0.092886	-4.19215	0.386372	0.430372
Neutrophils	ZFP110	0.3782	4.039873	1.69861	0.092909	-4.4629	0.349964	0.390823
Neutrophils	SUGT1	0.169252	5.949959	1.69851	0.092928	-5.00496	0.331443	0.370584
Neutrophils	ZDHHC18	0.213977	5.57552	1.698474	0.092935	-5.00613	0.334991	0.374467
Neutrophils	ZFP27	-0.90384	1.016419	-1.69833	0.092961	-4.1924	0.381494	0.425139
Neutrophils	RABGAP1L	-0.23915	7.908574	-1.69802	0.09302	-5.16402	0.3136	0.351006
Neutrophils	COQ5	0.553757	3.832511	1.697548	0.093111	-4.31552	0.352348	0.39343

Neutrophils	BPHL	-0.42082	3.663858	-1.6973	0.093158	-4.42578	0.3541	0.395371
Neutrophils	LY6G5B	-0.64643	1.345565	-1.69699	0.093216	-4.24871	0.378412	0.421881
Neutrophils	ZBTB11OS1	-0.57585	2.065369	-1.69655	0.093301	-4.24343	0.370841	0.413722
Neutrophils	NUS1	0.261775	4.837828	1.696313	0.093345	-4.57684	0.342656	0.383048
Neutrophils	GFI1	-0.63354	1.55053	-1.6963	0.093348	-4.27415	0.376332	0.419733
Neutrophils	ZC3HAV1L	-0.40719	3.053639	-1.69603	0.093399	-4.52334	0.360594	0.402639
Neutrophils	LCLAT1	-0.43928	3.99918	-1.69539	0.09352	-4.43678	0.35122	0.392195
Neutrophils	SELENOI	0.501851	3.422495	1.695374	0.093524	-4.33632	0.357041	0.39854
Neutrophils	HAVCR1	-1.11089	-0.3843	-1.69481	0.093631	-4.19969	0.398205	0.443275
Neutrophils	ADPRM	0.326797	3.321315	1.694803	0.093632	-4.49636	0.358232	0.3999
Neutrophils	GPAT3	0.23485	5.178486	1.694625	0.093666	-4.96846	0.33977	0.37977
Neutrophils	TGIF2	0.479784	3.122183	1.693386	0.093903	-4.33381	0.361052	0.402473
Neutrophils	PIFO	0.955006	1.246923	1.69309	0.093959	-4.21161	0.380994	0.42421
Neutrophils	NUP210	-0.37202	5.041054	-1.69271	0.094031	-4.49244	0.342061	0.381952
Neutrophils	DNAJB14	-0.24577	4.692261	-1.69243	0.094085	-4.91141	0.345548	0.38582
Neutrophils	SERPINA3N	-0.51274	3.688152	-1.69126	0.094309	-4.59243	0.356269	0.397254
Neutrophils	SLC38A6	-0.34422	4.233853	-1.69078	0.094402	-4.63516	0.350771	0.391462
Neutrophils	DSTYK	-0.2425	3.925134	-1.69077	0.094403	-4.71591	0.353871	0.39484
Neutrophils	CALM1	0.130511	9.782329	1.690771	0.094403	-5.62128	0.299659	0.335446
Neutrophils	ABRAXAS2	0.237857	4.960803	1.690344	0.094485	-4.76194	0.343678	0.383754
Neutrophils	VEGFB	-0.52497	3.802219	-1.69028	0.094497	-4.31466	0.355211	0.396332
Neutrophils	AFM	-0.65119	2.294567	-1.6899	0.09457	-4.31758	0.370935	0.413391
Neutrophils	CCL12	-1.6771	0.036828	-1.68978	0.094594	-4.20181	0.395635	0.440153
Neutrophils	PDE4D	-0.22846	6.428002	-1.68879	0.094784	-5.36769	0.330126	0.368859
Neutrophils	ZFP707	0.698899	1.958642	1.688524	0.094835	-4.22423	0.374952	0.417838
Neutrophils	WBP11	0.176113	6.346343	1.688362	0.094866	-5.01702	0.330893	0.369894
Neutrophils	0610030E2	0.251491	4.591291	1.688196	0.094898	-4.74856	0.347834	0.388493
Neutrophils	CYP2J5	-0.85562	1.06057	-1.68813	0.09491	-4.23319	0.384691	0.428617
Neutrophils	SERPINB6B	-0.90676	2.936635	-1.68796	0.094944	-4.2825	0.364633	0.40684
Neutrophils	NICN1	-0.9416	0.820121	-1.68789	0.094958	-4.20427	0.387341	0.431496
Neutrophils	RLN3	-0.92928	-0.84713	-1.68776	0.094981	-4.2038	0.406233	0.45193
Neutrophils	USP37	0.228401	6.268931	1.687607	0.095012	-4.95107	0.331622	0.370855
Neutrophils	PDHB	-0.23577	5.703174	-1.68665	0.095196	-4.76646	0.337412	0.377151
Neutrophils	PNPO	0.481441	3.744874	1.686541	0.095217	-4.41073	0.356762	0.398333
Neutrophils	STIM1	-0.17848	7.712751	-1.68648	0.09523	-5.56036	0.318694	0.356689
Neutrophils	BLOC1S1	0.208268	6.24963	1.68603	0.095316	-5.10371	0.332339	0.371649
Neutrophils	TXNDC15	0.338586	4.911319	1.685812	0.095358	-4.58501	0.345235	0.385779
Neutrophils	FAM129C	-0.60182	3.433215	-1.68576	0.095369	-4.28194	0.360085	0.402003
Neutrophils	SPEF2	0.899483	1.371663	1.684915	0.095532	-4.21144	0.382118	0.426079
Neutrophils	CXCR4	0.236458	6.146916	1.684885	0.095538	-5.22522	0.333498	0.373064
Neutrophils	KBTBD2	0.211948	5.176548	1.684881	0.095538	-4.75227	0.342831	0.38328
Neutrophils	INSYN2B	0.352671	4.140533	1.684779	0.095558	-4.57633	0.353096	0.394533
Neutrophils	GM11579	-1.15208	-0.20636	-1.68452	0.095608	-4.20796	0.399799	0.445317
Neutrophils	SLC22A23	-0.8867	3.017213	-1.6842	0.09567	-4.29005	0.364702	0.407251
Neutrophils	SOX7	-0.67433	0.442832	-1.6841	0.09569	-4.2765	0.392513	0.4375
Neutrophils	AK3	-0.39643	4.328071	-1.68373	0.095762	-4.43134	0.351463	0.392832
Neutrophils	LRP8	0.631594	2.371033	1.68275	0.095952	-4.31825	0.372235	0.415196

Neutrophils	GM10184	-0.86082	1.11236	-1.68258	0.095985	-4.22756	0.385853	0.430056
Neutrophils	EPO	-0.89825	0.415776	-1.68227	0.096044	-4.21227	0.393708	0.438487
Neutrophils	AI182371	-0.66031	2.439191	-1.6821	0.096078	-4.36584	0.371607	0.41462
Neutrophils	I730030J21	-0.98813	0.707146	-1.68181	0.096134	-4.21013	0.390465	0.435196
Neutrophils	CDYL	0.181164	5.659184	1.681728	0.096151	-4.97974	0.339046	0.379216
Neutrophils	CRYZ	0.793943	1.288432	1.681364	0.096222	-4.22047	0.384184	0.428356
Neutrophils	PDE7B	0.330723	5.37371	1.681006	0.096291	-5.35315	0.342069	0.382462
Neutrophils	STX8	-0.16442	6.745581	-1.67962	0.096562	-5.02807	0.329792	0.368451
Neutrophils	ASS1	0.440179	6.535258	1.679355	0.096614	-5.06395	0.331829	0.370691
Neutrophils	KHDC4	0.153046	6.448578	1.678614	0.096759	-5.23569	0.333028	0.371811
Neutrophils	FAM149A	-0.98887	-0.42534	-1.67762	0.096954	-4.21438	0.405709	0.450398
Neutrophils	CUX1	0.148312	7.953864	1.677562	0.096965	-5.40242	0.319554	0.35676
Neutrophils	CDC42SE1	0.170464	6.18973	1.6771	0.097055	-5.12163	0.336159	0.374913
Neutrophils	PPARGC1A	1.080187	1.419055	1.676808	0.097112	-4.21993	0.38513	0.428283
Neutrophils	SOCS7	-0.40213	4.207792	-1.67673	0.097128	-4.4246	0.355679	0.396316
Neutrophils	PPP3CC	-0.67655	3.993482	-1.67507	0.097454	-4.2988	0.358771	0.399001
Neutrophils	RARB	-0.72037	1.586463	-1.67495	0.097478	-4.38577	0.384272	0.426679
Neutrophils	WDR92	0.647329	3.083733	1.674944	0.097479	-4.28153	0.368199	0.409277
Neutrophils	WDR38	0.854017	0.617809	1.674536	0.097559	-4.21761	0.395208	0.438476
Neutrophils	SRRD	0.358916	3.465767	1.674008	0.097663	-4.48671	0.364355	0.405286
Neutrophils	GM43388	-1.11846	0.386691	-1.67388	0.097689	-4.21851	0.397825	0.441506
Neutrophils	POU2F2	0.448348	5.772063	1.673816	0.097701	-4.77438	0.341193	0.380086
Neutrophils	NAIP1	1.054379	-0.47909	1.673668	0.09773	-4.21873	0.407786	0.452315
Neutrophils	XRN2	0.153959	7.516217	1.673598	0.097744	-5.12907	0.324702	0.362142
Neutrophils	GM4285	0.595999	1.640385	1.673525	0.097759	-4.28272	0.383835	0.426506
Neutrophils	MBTD1	-0.1943	7.420536	-1.67261	0.097939	-5.2387	0.325948	0.363459
Neutrophils	CTSZ	0.285612	6.598627	1.672569	0.097947	-5.01167	0.333643	0.371871
Neutrophils	MAPK7	-0.31398	3.195275	-1.67245	0.097971	-4.67845	0.367586	0.408885
Neutrophils	GMPS	0.237704	5.784964	1.671339	0.098191	-4.7549	0.341914	0.380874
Neutrophils	DDX51	-0.95767	1.37149	-1.67122	0.098214	-4.22119	0.387752	0.4307
Neutrophils	IGF2BP1	-1.08013	-0.96106	-1.67108	0.098243	-4.22327	0.414464	0.459579
Neutrophils	NOC4L	-0.45933	3.728892	-1.67064	0.098329	-4.34744	0.362529	0.403596
Neutrophils	DHCR24	-0.62236	2.897631	-1.67029	0.098398	-4.3095	0.371225	0.413206
Neutrophils	PKN1	0.153386	6.766934	1.669447	0.098566	-5.16675	0.332504	0.371227
Neutrophils	GM28417	0.880571	1.320479	1.669425	0.09857	-4.22914	0.388317	0.431977
Neutrophils	VAMP3	0.174623	5.320751	1.669379	0.09858	-4.94067	0.346459	0.386491
Neutrophils	EMC1	-0.37186	3.622778	-1.66929	0.098597	-4.43635	0.363627	0.405235
Neutrophils	DONSON	0.339826	4.025423	1.669022	0.098651	-4.55018	0.359478	0.400817
Neutrophils	TIMM21	-0.64911	2.602996	-1.66891	0.098673	-4.25335	0.374359	0.417021
Neutrophils	UBA7	0.451899	3.784743	1.668718	0.098711	-4.51702	0.361952	0.403558
Neutrophils	TRIM72	-0.83868	0.692552	-1.66866	0.098722	-4.24295	0.395343	0.439792
Neutrophils	GATAD2A	0.165406	7.859185	1.668601	0.098734	-5.31057	0.322356	0.360285
Neutrophils	ATAD1	0.193634	5.731262	1.668488	0.098757	-4.79716	0.342436	0.382293
Neutrophils	UVRAG	-0.1727	8.569242	-1.66825	0.098805	-5.56149	0.315936	0.353319
Neutrophils	GAB1	0.566776	4.473058	1.668207	0.098813	-4.60937	0.354922	0.395992
Neutrophils	ABCG3	0.405865	4.394356	1.668146	0.098825	-4.70205	0.355719	0.396891
Neutrophils	NCF4	0.17963	5.316123	1.667905	0.098873	-5.30602	0.346504	0.386864

Neutrophils	EPB41L5	-0.64438	3.379051	-1.66775	0.098903	-4.312	0.366163	0.408296
Neutrophils	GM31645	-1.04875	0.726126	-1.66769	0.098916	-4.22473	0.394964	0.439551
Neutrophils	AMOTL2	-0.67123	0.684568	-1.66763	0.098928	-4.35709	0.395433	0.440058
Neutrophils	GRAMD2	-0.59846	0.533275	-1.66749	0.098955	-4.31677	0.397146	0.44197
Neutrophils	ATP2A3	-0.22714	5.210591	-1.66733	0.098987	-4.93981	0.347547	0.388142
Neutrophils	MSR1	0.394843	3.816756	1.667293	0.098995	-4.70155	0.361622	0.4035
Neutrophils	ACSF2	-0.53874	4.286847	-1.66726	0.099001	-4.36345	0.35681	0.398255
Neutrophils	SNX30	-0.4141	5.755716	-1.66683	0.099087	-4.56632	0.342378	0.382437
Neutrophils	MFHAS1	-0.66447	3.729832	-1.66644	0.099165	-4.31113	0.362865	0.404751
Neutrophils	ITPA	0.336015	4.105721	1.666223	0.099208	-4.47952	0.359031	0.400636
Neutrophils	STK16	0.282186	4.37442	1.665735	0.099306	-4.75924	0.356519	0.397856
Neutrophils	NELFE	0.278712	4.393275	1.665338	0.099385	-4.63678	0.356405	0.397676
Neutrophils	NCBP2	0.354927	4.599992	1.665281	0.099396	-4.50861	0.354313	0.395418
Neutrophils	ZFP354C	-0.92248	1.777029	-1.66476	0.0995	-4.23298	0.384285	0.427948
Neutrophils	MMRN2	-0.53575	1.228883	-1.66424	0.099605	-4.38122	0.390531	0.434682
Neutrophils	GIT1	-0.36298	3.282244	-1.66401	0.099651	-4.40854	0.368304	0.410557
Neutrophils	MTSS1	-0.32722	6.472133	-1.66381	0.099691	-4.94214	0.336331	0.375673
Neutrophils	CXCR3	-1.16165	1.731497	-1.66371	0.099711	-4.23127	0.384967	0.428652
Neutrophils	HPSE	0.672155	3.426146	1.663618	0.09973	-4.29815	0.366795	0.408916
Neutrophils	NTHL1	-0.84262	0.544589	-1.66349	0.099755	-4.22906	0.398238	0.443048
Neutrophils	SERAC1	0.782033	2.177244	1.663096	0.099834	-4.25165	0.38027	0.423528
Neutrophils	UBC	-0.16416	8.132317	-1.66287	0.09988	-5.56911	0.321033	0.35887
Neutrophils	HSPBP1	-0.49127	3.934559	-1.66269	0.099916	-4.32553	0.361725	0.403389
Neutrophils	AHRR	0.870203	-0.36283	1.662379	0.099978	-4.2321	0.409042	0.454704
Neutrophils	TMEM38B	0.180346	4.174266	1.66207	0.10004	-5.13079	0.359405	0.400996
Neutrophils	HPN	-0.61846	2.642028	-1.66186	0.100083	-4.3562	0.375456	0.418463
Neutrophils	DGAT1	0.21815	7.095267	1.661802	0.100094	-5.66693	0.330752	0.369678
Neutrophils	MAN2C1OS	0.353023	4.443794	1.661466	0.100162	-4.58139	0.35673	0.398096
Neutrophils	ASXL1	-0.16093	7.242343	-1.66135	0.100184	-5.3255	0.329443	0.368257
Neutrophils	IKBKB	0.236496	5.778617	1.661171	0.100221	-4.8383	0.343439	0.383614
Neutrophils	ZFYVE1	-0.29567	4.797159	-1.66098	0.100259	-4.75469	0.353178	0.394265
Neutrophils	GC	-0.48572	5.287073	-1.6607	0.100316	-5.01848	0.348364	0.389021
Neutrophils	AK7	-0.81666	1.686834	-1.66018	0.10042	-4.2766	0.386158	0.430182
Neutrophils	GM17178	-0.74459	1.460682	-1.65983	0.100491	-4.24391	0.38866	0.432894
Neutrophils	NGLY1	0.182459	4.849758	1.659706	0.100517	-4.80866	0.352854	0.393943
Neutrophils	ABCC2	-0.62288	2.212583	-1.65968	0.100523	-4.34516	0.380407	0.423939
Neutrophils	E2F4	0.289918	5.334947	1.659663	0.100525	-4.65947	0.348015	0.388658
Neutrophils	CLEC4G	-0.37198	3.468481	-1.65944	0.100571	-4.73047	0.367058	0.409502
Neutrophils	CTSL	0.191824	6.758061	1.659216	0.100616	-5.32896	0.334259	0.373731
Neutrophils	USP42	-0.43903	3.500066	-1.65908	0.100642	-4.35762	0.366735	0.409301
Neutrophils	RFC3	-0.36694	4.31335	-1.65855	0.10075	-4.52056	0.358594	0.400369
Neutrophils	GNG10	0.212901	6.940589	1.65834	0.100793	-5.14523	0.332799	0.372232
Neutrophils	CCDC174	0.224774	5.082834	1.65786	0.10089	-4.82242	0.351066	0.392102
Neutrophils	DLG2	-0.88179	2.402232	-1.65752	0.100958	-4.28209	0.378946	0.422569
Neutrophils	GTF3C3	-0.61758	3.102011	-1.65751	0.10096	-4.30203	0.371455	0.414423
Neutrophils	MEFV	0.836398	-0.03339	1.657135	0.101037	-4.27747	0.406302	0.452283
Neutrophils	GM49463	1.112375	-0.05016	1.656918	0.101081	-4.23625	0.406497	0.452541

Neutrophils	STK40	0.185828	5.69472	1.656762	0.101112	-5.13571	0.345065	0.385765
Neutrophils	ATXN7L1OS	0.631416	1.337166	1.656562	0.101153	-4.32889	0.390706	0.435595
Neutrophils	GYS2	-0.76087	0.768164	-1.65612	0.101242	-4.2554	0.397106	0.442793
Neutrophils	YDJC	-0.94031	1.21958	-1.65591	0.101285	-4.23934	0.39202	0.43736
Neutrophils	SS18	-0.21295	5.946104	-1.65558	0.101352	-4.96079	0.342607	0.383594
Neutrophils	AP4S1	0.260989	4.920297	1.655484	0.101372	-4.69698	0.352754	0.394733
Neutrophils	ELP5	-0.35266	4.633093	-1.6554	0.101388	-4.54233	0.355651	0.397902
Neutrophils	RPUSD3	0.987385	0.640562	1.655364	0.101396	-4.24034	0.398556	0.444631
Neutrophils	4930599N2	0.635267	1.733523	1.655331	0.101403	-4.32238	0.38631	0.431331
Neutrophils	ELK3	-0.27257	5.288608	-1.65523	0.101423	-4.8579	0.349075	0.390706
Neutrophils	CORO1A	0.164263	8.516071	1.655208	0.101428	-5.63973	0.31851	0.357135
Neutrophils	SETD5	0.169846	6.628233	1.654617	0.101548	-5.07761	0.336313	0.376643
Neutrophils	CCDC130	0.644358	2.056293	1.654303	0.101612	-4.31838	0.383187	0.427879
Neutrophils	LMAN2L	0.285319	4.447735	1.653995	0.101675	-4.6479	0.357926	0.400513
Neutrophils	YARS	-0.43814	5.391202	-1.65381	0.101712	-4.61292	0.34844	0.390139
Neutrophils	TONSL	0.793919	1.456718	1.653807	0.101713	-4.25311	0.389803	0.435273
Neutrophils	LIMK1	0.749383	2.1198	1.652519	0.101976	-4.26274	0.383351	0.428042
Neutrophils	WARS	0.481291	3.723103	1.651707	0.102142	-4.43509	0.366684	0.409672
Neutrophils	IIGP1	0.611444	4.709506	1.65127	0.102231	-4.94414	0.356662	0.398767
Neutrophils	LRRC61	0.647951	2.011331	1.65117	0.102252	-4.29515	0.385185	0.429919
Neutrophils	RILPL2	0.181569	7.158884	1.650989	0.102289	-5.36885	0.332675	0.372595
Neutrophils	PLCB1	0.340823	4.622522	1.650081	0.102475	-5.09362	0.358082	0.400072
Neutrophils	TMOD3	0.165918	7.218311	1.649596	0.102574	-5.29916	0.332717	0.372313
Neutrophils	ATF3	0.34719	6.1843	1.649573	0.102579	-5.27238	0.342633	0.383186
Neutrophils	CDAN1	0.584322	2.943919	1.649093	0.102678	-4.33099	0.375878	0.419519
Neutrophils	GFRA2	-0.67625	2.545769	-1.64907	0.102682	-4.33637	0.380172	0.424191
Neutrophils	GM33104	-0.98051	0.440079	-1.64876	0.102747	-4.25353	0.403826	0.449846
Neutrophils	SRL	-0.89635	0.890591	-1.64859	0.102782	-4.24437	0.398664	0.444282
Neutrophils	ANGPTL4	0.778144	1.23193	1.648439	0.102812	-4.27342	0.394797	0.440104
Neutrophils	GM44751	-0.80747	1.310612	-1.64807	0.102888	-4.26043	0.394067	0.439247
Neutrophils	SPTB	0.915594	1.120897	1.647728	0.102958	-4.2684	0.396269	0.441686
Neutrophils	GM46218	-0.8896	0.58606	-1.64765	0.102974	-4.24938	0.402368	0.448323
Neutrophils	WDR35	-1.08897	0.509893	-1.64735	0.103037	-4.24594	0.403354	0.449405
Neutrophils	USB1	0.441005	3.245957	1.646909	0.103127	-4.47557	0.373086	0.416509
Neutrophils	INSL6	-0.86121	1.68935	-1.64689	0.103131	-4.26442	0.390032	0.434937
Neutrophils	RGS7BP	-0.81168	1.883105	-1.64665	0.103179	-4.3603	0.38788	0.432672
Neutrophils	DHX9	-0.30087	6.492589	-1.64662	0.103186	-4.92838	0.340148	0.380587
Neutrophils	DNASE2A	-0.38748	4.427932	-1.64619	0.103276	-4.53119	0.360918	0.403305
Neutrophils	B3GALT4	0.867628	1.404829	1.646	0.103314	-4.26583	0.393434	0.438718
Neutrophils	SLC25A17	0.262474	5.308731	1.645434	0.103431	-4.74674	0.35227	0.393712
Neutrophils	NOD1	0.365641	3.387607	1.645044	0.103512	-4.78065	0.372126	0.415357
Neutrophils	CD33	0.243213	2.518088	1.645041	0.103512	-5.20014	0.381471	0.425527
Neutrophils	NEDD9	0.169305	8.003683	1.644297	0.103666	-5.6904	0.326627	0.365683
Neutrophils	M1AP	-0.80894	1.320039	-1.64407	0.103714	-4.26328	0.395082	0.440511
Neutrophils	YWHAZ	0.091567	8.482146	1.643934	0.103741	-5.55939	0.32223	0.36093
Neutrophils	SRSF1	0.2169	6.067856	1.643932	0.103742	-4.97812	0.345079	0.386019
Neutrophils	BC028528	-0.32931	4.105365	-1.64358	0.103815	-4.64708	0.364954	0.407855

Neutrophils	GPN2	-0.53562	2.713988	-1.64333	0.103866	-4.33191	0.379724	0.424088
Neutrophils	GCLC	-0.29276	6.841762	-1.64299	0.103936	-5.07083	0.337621	0.378175
Neutrophils	PPDPF	-0.27692	5.630234	-1.64288	0.10396	-4.69552	0.349449	0.391148
Neutrophils	CYP4B1	-0.94143	0.266849	-1.64286	0.103963	-4.27812	0.407202	0.454075
Neutrophils	DPAGT1	-0.46914	3.285854	-1.64273	0.103992	-4.44064	0.37358	0.417525
Neutrophils	GM32051	1.095804	-0.14913	1.642612	0.104015	-4.25195	0.41207	0.459361
Neutrophils	MYCL	-0.95776	1.305739	-1.64251	0.104037	-4.2574	0.395298	0.44123
Neutrophils	MFSD6	0.285074	4.864614	1.641258	0.104297	-5.01888	0.357544	0.399995
Neutrophils	PALD1	-0.73674	1.292537	-1.64125	0.104298	-4.31349	0.395888	0.441786
Neutrophils	PDLIM2	0.368957	3.759145	1.641214	0.104306	-4.63329	0.368984	0.412505
Neutrophils	IST1	0.194789	5.536178	1.641175	0.104314	-4.98077	0.350775	0.392607
Neutrophils	1700030KC	-0.8011	1.730931	-1.64093	0.104365	-4.27818	0.390964	0.436585
Neutrophils	GRAMD1A	0.26021	4.325204	1.64086	0.104379	-4.91202	0.363079	0.406186
Neutrophils	ADAM11	0.878415	-1.14352	1.640522	0.10445	-4.25428	0.424414	0.473025
Neutrophils	SHROOM4	-0.79463	0.86592	-1.64049	0.104456	-4.33541	0.400741	0.447374
Neutrophils	BEX4	-0.84061	0.397887	-1.64038	0.10448	-4.26138	0.406133	0.453236
Neutrophils	STX7	0.158689	6.176182	1.640261	0.104504	-5.14276	0.344449	0.385979
Neutrophils	TNFRSF1A	0.178866	5.171275	1.639838	0.104592	-5.30726	0.354617	0.397141
Neutrophils	BCKDK	0.267264	4.559456	1.639584	0.104645	-4.7095	0.360909	0.404075
Neutrophils	2810006K2	-0.69666	2.570884	-1.63941	0.104681	-4.32483	0.381963	0.427098
Neutrophils	ARRB2	0.171922	5.195443	1.638217	0.104931	-5.25829	0.35508	0.397441
Neutrophils	CP	-0.42614	4.588111	-1.63816	0.104942	-4.8194	0.361273	0.404218
Neutrophils	PRMT2	-0.70178	1.665423	-1.63772	0.105035	-4.2805	0.392892	0.438544
Neutrophils	TCRG-C4	-0.86806	0.327974	-1.63714	0.105157	-4.27562	0.408526	0.455409
Neutrophils	SH3BGRL3	0.149239	9.061448	1.636936	0.105199	-5.71425	0.318639	0.357184
Neutrophils	SPHK2	0.341978	3.388566	1.636724	0.105243	-4.61418	0.374381	0.418383
Neutrophils	GM3055	-1.01877	0.319624	-1.63659	0.105271	-4.25659	0.408656	0.455725
Neutrophils	SMIM5	0.627596	0.166279	1.636247	0.105343	-4.38024	0.410592	0.457821
Neutrophils	PHKG2	-0.22256	4.528144	-1.63606	0.105382	-4.88717	0.362558	0.405622
Neutrophils	GM27188	-1.25761	0.619818	-1.63583	0.10543	-4.25821	0.40537	0.452257
Neutrophils	ABHD10	-0.30594	3.649409	-1.63544	0.105512	-4.5709	0.371958	0.415865
Neutrophils	TXNDC11	-0.28698	5.714713	-1.63503	0.105598	-4.75279	0.350747	0.39277
Neutrophils	EFHC1	-0.97475	-0.05796	-1.63487	0.105633	-4.25811	0.413517	0.46121
Neutrophils	GM20139	-0.93053	-0.63311	-1.63473	0.105661	-4.25868	0.420367	0.468627
Neutrophils	A730081D(	0.408804	3.129627	1.634567	0.105696	-4.59756	0.377549	0.422137
Neutrophils	NAT9	0.381942	3.244632	1.634554	0.105698	-4.55958	0.376312	0.420788
Neutrophils	MAT2A	-0.17101	6.522913	-1.63428	0.105757	-5.13362	0.342856	0.384113
Neutrophils	FGF13	-1.02956	3.379693	-1.63376	0.105866	-4.30245	0.375155	0.419368
Neutrophils	TLCD2	0.44205	2.659161	1.633663	0.105886	-4.54735	0.382945	0.427869
Neutrophils	GM43328	0.624307	2.238918	1.63327	0.105969	-4.33616	0.387738	0.433041
Neutrophils	SPPL3	-0.14585	7.282604	-1.63283	0.106061	-5.27885	0.336055	0.376383
Neutrophils	MMP14	-0.63369	3.837765	-1.6324	0.106151	-4.67615	0.370844	0.414508
Neutrophils	SVIP	0.320711	1.34626	1.632001	0.106236	-4.73038	0.398246	0.444528
Neutrophils	B3GNT3	-0.68484	0.711737	-1.63193	0.106252	-4.31692	0.405528	0.45244
Neutrophils	TSPAN7	-0.32116	3.257326	-1.63175	0.106289	-4.78796	0.377109	0.421588
Neutrophils	BACE2	-0.65354	1.120623	-1.63163	0.106314	-4.36425	0.400821	0.447395
Neutrophils	ANGPT1	1.018622	0.973826	1.630382	0.106578	-4.2663	0.402808	0.449678

Neutrophils	TMEM241	-0.41144	4.310016	-1.63034	0.106588	-4.59534	0.366238	0.40983
Neutrophils	LNCPINT	-0.34843	8.328661	-1.6302	0.106616	-5.2801	0.326716	0.366454
Neutrophils	QRSL1	0.614446	3.18623	1.630125	0.106633	-4.33418	0.37816	0.42285
Neutrophils	UBR7	0.387024	4.241439	1.630107	0.106637	-4.54165	0.366954	0.410613
Neutrophils	RNF217	0.726158	2.071962	1.630027	0.106654	-4.39461	0.390375	0.436161
Neutrophils	TRABD	0.246098	5.363153	1.629959	0.106668	-4.86237	0.355419	0.397989
Neutrophils	TMEM64	-0.42938	5.051385	-1.62991	0.106678	-4.60616	0.358587	0.401478
Neutrophils	TRIM26	0.308355	5.330265	1.629507	0.106764	-4.69936	0.355833	0.398434
Neutrophils	PFDN1	-0.26501	5.109296	-1.62946	0.106773	-4.70284	0.358078	0.400893
Neutrophils	USP44	-0.93678	-0.6309	-1.62906	0.106857	-4.26502	0.421902	0.470495
Neutrophils	FIGNL2	-1.09867	-0.67701	-1.62867	0.106942	-4.26416	0.422458	0.471228
Neutrophils	SH3D19	-0.58618	2.244921	-1.62854	0.106969	-4.39461	0.388639	0.434498
Neutrophils	TAPBP	0.287983	6.597076	1.628526	0.106972	-5.15859	0.34333	0.38493
Neutrophils	IRF5	0.396429	5.351662	1.628456	0.106987	-4.67241	0.355705	0.398511
Neutrophils	DPH7	-0.89064	1.586984	-1.62821	0.107039	-4.2721	0.396007	0.442577
Neutrophils	BCAS3OS1	0.362987	3.793281	1.628168	0.107048	-4.5693	0.371848	0.416251
Neutrophils	IER5	0.251186	6.692584	1.627742	0.107138	-5.25703	0.342457	0.384138
Neutrophils	SERTAD3	0.391936	4.131362	1.627599	0.107169	-4.64945	0.368343	0.412599
Neutrophils	GM17387	-0.9497	0.498836	-1.62727	0.107238	-4.2667	0.408572	0.456523
Neutrophils	SHQ1	-0.7486	2.60612	-1.62721	0.107252	-4.28907	0.384717	0.430553
Neutrophils	SLC22A27	-0.87352	1.124082	-1.62701	0.107293	-4.30044	0.401342	0.448663
Neutrophils	ICOSL	0.600779	1.327034	1.627014	0.107293	-4.36316	0.399022	0.44614
Neutrophils	CTNNB1	0.138151	6.277504	1.626917	0.107314	-5.19811	0.346519	0.388729
Neutrophils	TIMM50	-0.35183	4.827954	-1.62639	0.107426	-4.62127	0.361107	0.404931
Neutrophils	RBMX	-0.2317	4.578527	-1.62632	0.107441	-4.84771	0.363681	0.407772
Neutrophils	NR1H2	0.280548	4.589379	1.626226	0.107461	-4.83388	0.363568	0.407683
Neutrophils	CPPED1	-0.36537	3.886993	-1.62614	0.107479	-4.45859	0.370917	0.415732
Neutrophils	FUBP1	-0.16446	6.678668	-1.62564	0.107587	-5.05697	0.342592	0.384801
Neutrophils	TJAP1	-0.35983	4.452059	-1.62524	0.107672	-4.5387	0.364993	0.40955
Neutrophils	CASP2	-0.36847	4.01281	-1.6252	0.107679	-4.49873	0.36959	0.414588
Neutrophils	ZADH2	0.435004	3.381757	1.625105	0.1077	-4.42224	0.376299	0.42195
Neutrophils	SAP18	0.149221	7.043708	1.624987	0.107725	-5.28432	0.339059	0.381089
Neutrophils	ELAC2	-0.61047	2.817476	-1.62497	0.107728	-4.30741	0.382404	0.428664
Neutrophils	ATP8B1	-0.76225	0.813793	-1.62497	0.107728	-4.31164	0.404914	0.453218
Neutrophils	ADGRL3	-0.64824	3.47827	-1.6249	0.107744	-4.77024	0.375264	0.420875
Neutrophils	CXADR	-0.44844	1.645302	-1.62473	0.10778	-4.55092	0.395413	0.442923
Neutrophils	4930594M	-1.08874	-0.02762	-1.62459	0.10781	-4.26835	0.414762	0.464015
Neutrophils	FBXL7	-0.41282	3.621991	-1.62429	0.107874	-4.99246	0.373831	0.419355
Neutrophils	IPMK	-0.18089	5.867681	-1.62405	0.107926	-5.20616	0.350727	0.394044
Neutrophils	KLHL18	0.308961	4.310869	1.623804	0.107978	-4.69778	0.366659	0.411587
Neutrophils	UFD1	0.226327	5.129038	1.623659	0.108009	-4.82117	0.358215	0.402373
Neutrophils	FANCA	0.314591	3.05493	1.623368	0.108071	-4.75088	0.380118	0.426407
Neutrophils	MDM1	0.419435	3.41542	1.622846	0.108183	-4.5884	0.376442	0.422347
Neutrophils	MS4A4A	0.995978	0.617812	1.622754	0.108203	-4.27114	0.407732	0.456515
Neutrophils	ZFP36L2	0.210528	8.376464	1.621994	0.108366	-5.50447	0.327274	0.368054
Neutrophils	1810046K0	-1.04553	-0.14929	-1.62178	0.108412	-4.27096	0.417213	0.466624
Neutrophils	LLPH	0.192389	6.447819	1.621713	0.108426	-5.00895	0.34568	0.388416

Neutrophils	TRIM30A	0.344105	6.169574	1.621048	0.108568	-5.15915	0.348769	0.391637
Neutrophils	BATF3	-0.77624	2.332688	-1.62045	0.108698	-4.33491	0.389388	0.436006
Neutrophils	EDARADD	-0.5846	3.159788	-1.62014	0.108765	-4.36752	0.380417	0.426171
Neutrophils	PIP5K1B	0.197137	5.568352	1.619262	0.108953	-5.33663	0.355369	0.398717
Neutrophils	CREB3L3	-0.71746	1.62088	-1.6189	0.10903	-4.33761	0.397694	0.445046
Neutrophils	FCGR3	0.25885	4.189291	1.618902	0.10903	-5.20565	0.369599	0.414357
Neutrophils	ARPC4	0.133299	7.957802	1.618889	0.109033	-5.5212	0.332054	0.37309
Neutrophils	ANXA4	0.503383	3.74233	1.618807	0.109051	-4.50398	0.374337	0.419594
Neutrophils	ALS2	0.372812	4.283789	1.618739	0.109065	-4.51035	0.368605	0.413325
Neutrophils	AGO3	-0.2363	5.940327	-1.61873	0.109068	-4.95423	0.351629	0.394697
Neutrophils	SERINC3	-0.12929	9.707046	-1.6184	0.109138	-5.66475	0.316088	0.355485
Neutrophils	G730013B(	-0.88523	-0.968	-1.61802	0.10922	-4.27467	0.428312	0.478416
Neutrophils	PGGHG	0.413625	2.820106	1.617836	0.10926	-4.55221	0.384401	0.430637
Neutrophils	USP38	0.183422	5.889969	1.617731	0.109283	-5.12648	0.352214	0.395368
Neutrophils	SLC25A21	-0.86505	1.867089	-1.61757	0.109317	-4.41448	0.394999	0.442203
Neutrophils	ETNK1	0.231241	5.999727	1.617521	0.109328	-4.95734	0.351117	0.394162
Neutrophils	RGS14	0.285278	3.585563	1.617453	0.109343	-4.85883	0.3761	0.421562
Neutrophils	SMURF1	-0.33175	4.688664	-1.61634	0.109584	-4.66715	0.364903	0.409211
Neutrophils	MMP11	-1.04511	0.128652	-1.61628	0.109597	-4.27653	0.415605	0.464532
Neutrophils	GM46224	0.469389	2.389468	1.616272	0.109598	-4.78877	0.389624	0.436248
Neutrophils	TSKU	-0.95274	-0.3765	-1.61618	0.109618	-4.28184	0.421645	0.471105
Neutrophils	RMC1	0.25937	4.652621	1.615891	0.109681	-4.84883	0.365369	0.409635
Neutrophils	DYRK2	-0.36362	4.884934	-1.61503	0.109868	-4.55659	0.363431	0.407165
Neutrophils	JUND	0.210547	10.62781	1.614906	0.109894	-5.79695	0.308825	0.347077
Neutrophils	AI413582	0.279759	4.68032	1.614035	0.110083	-4.85609	0.366016	0.409692
Neutrophils	RNF145	0.257339	5.159727	1.613864	0.11012	-4.91161	0.361054	0.404332
Neutrophils	KLF12	-0.58452	3.585919	-1.61373	0.11015	-4.54525	0.37761	0.422498
Neutrophils	LYPLA1	0.181131	5.988433	1.613419	0.110217	-5.09481	0.352642	0.395293
Neutrophils	SLC9A9	-0.30501	7.120003	-1.61331	0.11024	-5.3638	0.341486	0.383065
Neutrophils	UBA3	0.303657	4.076756	1.613238	0.110257	-4.5997	0.372363	0.416934
Neutrophils	SFXN3	0.531641	3.370072	1.613114	0.110284	-4.40236	0.379941	0.425243
Neutrophils	RNF11	0.162201	6.207032	1.612939	0.110322	-5.3803	0.350465	0.392997
Neutrophils	ERCC3	0.3688	3.695125	1.61238	0.110443	-4.50862	0.376627	0.4216
Neutrophils	HSD3B7	-0.44558	3.537133	-1.61236	0.110447	-4.59664	0.378327	0.423458
Neutrophils	GM16337	0.477258	1.967439	1.611213	0.110698	-4.51877	0.396424	0.442783
Neutrophils	NIPAL3	0.527782	3.198166	1.610639	0.110823	-4.42453	0.383054	0.427988
Neutrophils	JAG2	-0.95146	1.093215	-1.61011	0.110938	-4.28355	0.407061	0.453891
Neutrophils	KIF3C	-0.80008	2.145051	-1.60984	0.110998	-4.28779	0.395107	0.440869
Neutrophils	TMEM189	0.193659	6.387922	1.609508	0.11107	-5.39296	0.350239	0.391938
Neutrophils	MYH9	0.115528	7.80338	1.609109	0.111158	-5.62373	0.336479	0.376909
Neutrophils	RAD23B	-0.16888	6.360558	-1.60891	0.111201	-5.01629	0.350545	0.392355
Neutrophils	C1RA	-0.6423	1.359965	-1.60874	0.111238	-4.34763	0.404232	0.450936
Neutrophils	RRP12	-0.61751	2.60063	-1.60834	0.111325	-4.33529	0.390168	0.435772
Neutrophils	PLEKHA7	-0.84196	2.084938	-1.6082	0.111357	-4.2959	0.395952	0.442079
Neutrophils	NCBP1	0.179231	5.256901	1.608187	0.11136	-4.96252	0.361722	0.40473
Neutrophils	ETAA1OS	-0.87286	0.543342	-1.60817	0.111363	-4.28463	0.41377	0.461428
Neutrophils	SERPINA1A	-0.44296	8.012506	-1.60815	0.111368	-5.57623	0.33449	0.374844

Neutrophils	SULT1D1	-0.65429	2.290136	-1.60797	0.111407	-4.44461	0.39365	0.439592
Neutrophils	SNX24	-0.37207	4.299252	-1.60758	0.111493	-4.87496	0.371802	0.415783
Neutrophils	MRPL42	-0.26878	6.521235	-1.60754	0.111501	-5.04961	0.349024	0.390848
Neutrophils	CD59A	-0.38025	2.984691	-1.60709	0.111601	-4.65137	0.386086	0.431468
Neutrophils	BAG2	0.853175	1.435408	1.607062	0.111607	-4.29005	0.40354	0.450464
Neutrophils	ZFP608	0.235793	6.820434	1.60694	0.111633	-5.46376	0.346148	0.387834
Neutrophils	GPC3	0.561215	1.806193	1.606565	0.111716	-4.4306	0.399457	0.445994
Neutrophils	STOX2	-0.42562	3.196694	-1.60575	0.111894	-4.90915	0.384406	0.429354
Neutrophils	LHFP	0.824804	1.237889	1.605554	0.111938	-4.32502	0.406537	0.453411
Neutrophils	GJB2	-0.74529	1.638244	-1.60494	0.112074	-4.35445	0.402276	0.448639
Neutrophils	PRKX	0.213136	5.379346	1.604667	0.112133	-4.97789	0.361656	0.404387
Neutrophils	EML1	-0.4781	1.303195	-1.60375	0.112336	-4.56373	0.40683	0.453136
Neutrophils	SHPK	-0.90844	0.252393	-1.60307	0.112487	-4.29213	0.419527	0.466915
Neutrophils	JMJD7	-0.82637	1.757778	-1.60305	0.11249	-4.29076	0.401875	0.447791
Neutrophils	PDPR	-0.48781	3.937711	-1.60181	0.112764	-4.48782	0.378446	0.422123
Neutrophils	MRPL40	0.335242	4.651456	1.601203	0.112899	-4.68889	0.371152	0.413935
Neutrophils	GM10874	-0.98039	-0.06869	-1.60087	0.112974	-4.29154	0.424806	0.472145
Neutrophils	SWSAP1	-0.98016	0.278314	-1.60047	0.113061	-4.29173	0.420806	0.467669
Neutrophils	GDF15	-0.96498	1.508239	-1.60001	0.113163	-4.36943	0.406513	0.452149
Neutrophils	ITIH1	-0.6409	2.749811	-1.59986	0.113198	-4.52876	0.39236	0.436876
Neutrophils	CHTF18	0.766248	1.800347	1.599376	0.113305	-4.33359	0.403388	0.448786
Neutrophils	L3MBTL3	0.347928	5.067059	1.598111	0.113586	-4.73436	0.368037	0.410272
Neutrophils	STAT5A	0.386221	3.992851	1.597934	0.113626	-4.57884	0.379471	0.422726
Neutrophils	HSD17B4	0.284005	5.027211	1.597869	0.11364	-4.73502	0.368454	0.410728
Neutrophils	VAC14	0.296941	4.624685	1.597818	0.113652	-4.61343	0.372701	0.415378
Neutrophils	GRIP1	-0.8253	2.360437	-1.59762	0.113697	-4.38461	0.397555	0.442401
Neutrophils	BROX	-0.2247	4.85097	-1.59759	0.113702	-4.81257	0.370308	0.412778
Neutrophils	H2-OB	-0.79704	4.007971	-1.59749	0.113725	-4.42201	0.379308	0.422581
Neutrophils	TASOR2	0.325436	5.373883	1.597346	0.113757	-4.70311	0.364837	0.406835
Neutrophils	PJA1	-0.45266	3.699202	-1.59684	0.113869	-4.46731	0.38277	0.426344
Neutrophils	ZFP511	-0.3368	3.816412	-1.59674	0.113892	-4.54615	0.381493	0.424988
Neutrophils	CZIB	-0.32184	3.658795	-1.59665	0.113913	-4.66721	0.383211	0.426873
Neutrophils	TCEAL9	-0.27488	5.693961	-1.59655	0.113935	-4.93961	0.361633	0.403362
Neutrophils	SPOP	0.122843	7.366582	1.596008	0.114055	-5.45157	0.345045	0.385089
Neutrophils	PLXNB3	-0.80901	0.164762	-1.59583	0.114096	-4.31197	0.423636	0.47055
Neutrophils	A930006K(	-0.76819	2.074746	-1.59577	0.114108	-4.30104	0.40115	0.446232
Neutrophils	B230219D2	0.148728	6.252623	1.595225	0.114231	-5.13385	0.356323	0.39741
Neutrophils	MIDN	0.175126	6.064607	1.595177	0.114241	-5.23441	0.358232	0.399496
Neutrophils	FKBP7	-0.75024	1.867273	-1.5948	0.114325	-4.33722	0.403804	0.449172
Neutrophils	2310039H(	-0.35912	4.246315	-1.5947	0.114348	-4.613	0.377313	0.42047
Neutrophils	MSRB3	-0.45446	2.604277	-1.59457	0.114377	-4.60956	0.395399	0.440136
Neutrophils	OPHN1	-0.46764	2.997504	-1.59445	0.114403	-4.6574	0.390988	0.43541
Neutrophils	A230056P1	0.91099	0.407448	1.594085	0.114486	-4.30073	0.421028	0.468083
Neutrophils	TMEM94	-0.41202	2.610495	-1.59407	0.11449	-4.45707	0.395362	0.440284
Neutrophils	UBL3	0.123619	7.408809	1.593633	0.114587	-5.45832	0.344893	0.385357
Neutrophils	BRPF3	-0.24993	4.451036	-1.59321	0.114682	-4.76267	0.375151	0.418595
Neutrophils	PGM2	0.242432	4.115633	1.593043	0.11472	-4.90569	0.378753	0.422521

Neutrophils	STFA2	-0.19053	2.469209	-1.59299	0.114731	-5.5392	0.396959	0.442328
Neutrophils	TXNDC12	-0.29309	3.827411	-1.59292	0.114747	-4.67377	0.381877	0.425925
Neutrophils	ZFP69	-0.52669	2.612118	-1.59288	0.114757	-4.47293	0.395344	0.440573
Neutrophils	SMLR1	-0.62151	1.822706	-1.59249	0.114844	-4.43049	0.404352	0.450493
Neutrophils	CDH17	-1.02699	0.13748	-1.59232	0.114881	-4.29967	0.424287	0.472097
Neutrophils	LRRC32	-0.70832	1.241998	-1.59224	0.1149	-4.35656	0.411112	0.457865
Neutrophils	QPCT	-0.62479	2.264701	-1.59222	0.114903	-4.40558	0.399283	0.445032
Neutrophils	GM21859	-0.34513	1.780794	-1.59206	0.114941	-5.04005	0.404836	0.451085
Neutrophils	FTX	-0.48696	4.311116	-1.59193	0.114969	-4.50426	0.376649	0.420422
Neutrophils	DBNDD2	-0.53798	2.836435	-1.59191	0.114974	-4.44711	0.392822	0.438035
Neutrophils	POMGNT1	-0.60331	2.312495	-1.59181	0.114996	-4.33764	0.398739	0.444465
Neutrophils	URI1	0.284887	5.89939	1.591468	0.115074	-4.82133	0.360089	0.402283
Neutrophils	RCC2	-0.21574	6.222377	-1.59137	0.115097	-4.98689	0.356798	0.39873
Neutrophils	PPP2R2D	0.197105	5.459388	1.590703	0.115246	-4.98688	0.364947	0.407407
Neutrophils	ITSN1	-0.2886	5.300914	-1.59059	0.115272	-5.05314	0.366597	0.409245
Neutrophils	GM26674	-0.93392	-1.15959	-1.58974	0.115463	-4.30176	0.441391	0.490129
Neutrophils	PCCB	-0.52245	3.081613	-1.58923	0.115579	-4.41941	0.391319	0.435862
Neutrophils	GM12462	-0.98602	-0.49881	-1.58842	0.115761	-4.30276	0.433902	0.481844
Neutrophils	IL17RA	0.210626	5.907729	1.588353	0.115776	-5.26101	0.361443	0.403237
Neutrophils	LETMD1	-0.58284	2.91201	-1.58809	0.115836	-4.35903	0.393688	0.438418
Neutrophils	TAF1A	-0.62983	3.171877	-1.58797	0.115864	-4.3575	0.390781	0.435335
Neutrophils	CUEDC2	-0.23216	5.245055	-1.58749	0.115972	-4.94222	0.368485	0.411019
Neutrophils	LAMP1	0.150228	7.757865	1.587452	0.11598	-5.524	0.343097	0.383249
Neutrophils	ZCCHC2	0.219138	5.422407	1.587317	0.116011	-5.1233	0.366631	0.409018
Neutrophils	PGPEP1L	0.777744	-0.29843	1.58712	0.116056	-4.3242	0.431648	0.479628
Neutrophils	PTS	0.192208	5.573329	1.586854	0.116116	-5.06619	0.365159	0.407477
Neutrophils	APOC4	-0.42698	5.632488	-1.58632	0.116238	-5.22017	0.364721	0.406881
Neutrophils	PIK3R2	0.618923	2.306399	1.586023	0.116304	-4.38427	0.400981	0.446468
Neutrophils	RASGRP2	0.173986	6.609517	1.585994	0.116311	-5.31451	0.354731	0.396079
Neutrophils	TARS2	0.349141	3.819829	1.585679	0.116382	-4.60812	0.384041	0.428151
Neutrophils	IFI203	-0.40639	6.302394	-1.58565	0.11639	-4.94108	0.35784	0.399568
Neutrophils	IQCC	0.662889	1.695654	1.58552	0.116419	-4.37647	0.408033	0.454224
Neutrophils	CIRBP	0.179788	7.218243	1.585232	0.116484	-5.36124	0.348652	0.389595
Neutrophils	PKMYT1	0.464328	3.261877	1.584527	0.116644	-4.56263	0.390199	0.435192
Neutrophils	CRYZL2	-0.49675	2.609637	-1.58438	0.116678	-4.41057	0.397526	0.443191
Neutrophils	AI837181	0.345179	3.127875	1.584171	0.116725	-4.54261	0.391693	0.436901
Neutrophils	TAF10	0.163668	6.856819	1.584152	0.11673	-5.29767	0.352248	0.393824
Neutrophils	LIG1	0.366336	5.051661	1.584063	0.11675	-4.9337	0.370799	0.414122
Neutrophils	F630040K0	0.843054	0.611295	1.583827	0.116804	-4.3506	0.420865	0.468601
Neutrophils	PSMD13	0.182521	6.203145	1.583799	0.11681	-5.11794	0.35885	0.401091
Neutrophils	EEA1	0.15327	6.055281	1.583409	0.116899	-5.35958	0.360362	0.402884
Neutrophils	EIF2S3X	-0.41477	5.323448	-1.58309	0.116971	-4.8388	0.367941	0.411265
Neutrophils	ADAMTS6	-0.48105	5.940983	-1.58307	0.116977	-4.69008	0.361535	0.404255
Neutrophils	DIMT1	-0.54182	3.243055	-1.58294	0.117007	-4.38731	0.390408	0.435802
Neutrophils	TUG1	0.169822	6.098897	1.582908	0.117013	-5.22914	0.359915	0.402499
Neutrophils	GM9887	-0.8324	1.686181	-1.58281	0.117036	-4.31949	0.408143	0.455087
Neutrophils	SULT2A2	-0.52345	4.037802	-1.58277	0.117045	-4.86652	0.381662	0.426269

Neutrophils	ZFP143	0.324139	3.815014	1.582653	0.117071	-4.62675	0.384093	0.428946
Neutrophils	GM49602	-0.63346	1.89388	-1.58239	0.117131	-4.36422	0.40573	0.452504
Neutrophils	ATXN3	0.252059	5.054807	1.582337	0.117144	-4.80302	0.370765	0.414405
Neutrophils	GM15448	0.51914	-0.00747	1.582184	0.117178	-4.42079	0.428369	0.477046
Neutrophils	CDC26	-0.18313	5.383294	-1.58206	0.117207	-4.91945	0.367315	0.410632
Neutrophils	KANSL2	-0.28371	5.119718	-1.58198	0.117224	-4.75037	0.370081	0.413657
Neutrophils	BIVM	-0.71197	0.882383	-1.58195	0.117233	-4.32842	0.417619	0.465405
Neutrophils	1500004A1	0.64987	2.078931	1.581942	0.117234	-4.33529	0.403593	0.450182
Neutrophils	GOSR2	0.234801	5.28486	1.581732	0.117282	-4.95781	0.368381	0.411802
Neutrophils	HSPA13	0.375546	3.082179	1.581156	0.117413	-4.5164	0.392513	0.437966
Neutrophils	INTS2	-0.40568	4.120259	-1.58066	0.117528	-4.64467	0.381067	0.425693
Neutrophils	VAMP8	0.140273	7.209731	1.580608	0.117539	-5.4956	0.349012	0.39061
Neutrophils	NAA30	0.324042	3.918894	1.580588	0.117543	-4.63474	0.38326	0.428085
Neutrophils	COL4A1	-0.36601	3.330895	-1.58057	0.117546	-4.89314	0.389739	0.435148
Neutrophils	LZTS2	-0.94041	1.249256	-1.58026	0.117619	-4.31989	0.413719	0.46127
Neutrophils	SELENOS	-0.21153	6.113957	-1.58005	0.117668	-5.02621	0.360159	0.402922
Neutrophils	SGCB	-0.7664	2.212559	-1.57993	0.117693	-4.35193	0.402501	0.449198
Neutrophils	ERCC8	-0.65392	2.45611	-1.57911	0.117881	-4.33185	0.400225	0.446469
Neutrophils	PFN2	0.885646	1.339141	1.578829	0.117946	-4.32884	0.413293	0.460633
Neutrophils	WDR59	-0.50438	3.072953	-1.57831	0.118065	-4.4605	0.393497	0.439126
Neutrophils	RNF135	0.6238	2.08421	1.578305	0.118067	-4.37591	0.404756	0.45137
Neutrophils	TCEANC2	-0.28651	4.247598	-1.57789	0.118162	-4.70003	0.380725	0.425135
Neutrophils	DHX15	-0.14946	6.794688	-1.5777	0.118205	-5.27991	0.354134	0.396047
Neutrophils	ZFP691	0.533546	3.075803	1.577421	0.11827	-4.45507	0.393709	0.439303
Neutrophils	GM4356	0.741721	0.868166	1.577249	0.11831	-4.38137	0.419316	0.467204
Neutrophils	BRD4	0.099029	8.256086	1.577134	0.118336	-5.57772	0.339779	0.380405
Neutrophils	NFE2	0.218611	1.59738	1.577021	0.118362	-5.138	0.410675	0.457908
Neutrophils	HSD17B13	-0.58322	2.671988	-1.57687	0.118398	-4.55653	0.398271	0.444506
Neutrophils	METTL17	-0.66281	2.488504	-1.57656	0.118468	-4.34367	0.400387	0.446936
Neutrophils	MRVI1	0.827999	0.497468	1.576515	0.118479	-4.33204	0.423807	0.472367
Neutrophils	RNF144A	0.192741	3.619246	1.576317	0.118524	-5.16666	0.387709	0.433156
Neutrophils	GM29019	-0.62105	0.361473	-1.57595	0.118608	-4.37644	0.425547	0.474413
Neutrophils	COBLL1	-0.46605	5.069977	-1.57574	0.118657	-4.58655	0.372064	0.416272
Neutrophils	RAD52	0.495742	3.121528	1.57566	0.118676	-4.4591	0.393306	0.43952
Neutrophils	YWHAH	0.156836	7.687722	1.575032	0.118821	-5.54317	0.345394	0.387323
Neutrophils	CDK5	-0.3363	3.72259	-1.57496	0.118839	-4.68232	0.386623	0.432525
Neutrophils	CHSY1	-0.26457	5.079341	-1.57495	0.118841	-4.93285	0.371965	0.416495
Neutrophils	DCAF11	-0.26998	4.559305	-1.57493	0.118845	-4.82525	0.377514	0.422568
Neutrophils	CKAP4	0.17688	4.030233	1.574762	0.118883	-5.42362	0.383247	0.428837
Neutrophils	RAB27B	-0.97242	0.615378	-1.57472	0.118893	-4.33088	0.422472	0.471555
Neutrophils	SORBS1	-0.28377	4.747678	-1.57459	0.118922	-4.95934	0.375494	0.420371
Neutrophils	NDUFAF7	0.220566	4.007139	1.574492	0.118945	-4.94487	0.3835	0.429158
Neutrophils	XKR6	0.726851	0.862903	1.573597	0.119152	-4.35864	0.420097	0.468782
Neutrophils	HSPB6	-0.89239	0.40248	-1.57335	0.119209	-4.32989	0.425729	0.474848
Neutrophils	DCAKD	0.350554	4.076447	1.572966	0.119299	-4.70192	0.383435	0.428832
Neutrophils	THYN1	-0.44453	3.271604	-1.57293	0.119308	-4.47788	0.392335	0.438546
Neutrophils	SF3B1	0.105441	8.256825	1.572746	0.11935	-5.60661	0.340495	0.381752

Neutrophils	OLFR543	-0.87944	0.201065	-1.57256	0.119393	-4.32923	0.428317	0.477713
Neutrophils	DLAT	0.305384	4.282042	1.572098	0.1195	-4.64978	0.381455	0.426642
Neutrophils	PIGN	0.331474	4.40092	1.571736	0.119584	-4.77756	0.380316	0.425364
Neutrophils	TRDJ1	-0.83193	-1.29553	-1.5707	0.119825	-4.31945	0.448226	0.498711
Neutrophils	TMEM131	0.17546	6.606387	1.570384	0.119898	-5.29625	0.357846	0.400351
Neutrophils	TMEM230	-0.21019	4.742854	-1.57026	0.119928	-4.90847	0.377325	0.42167
Neutrophils	MRM2	-0.8624	2.071278	-1.57015	0.119952	-4.36385	0.407191	0.454211
Neutrophils	CES2A	-0.83227	0.898465	-1.56954	0.120095	-4.36449	0.421318	0.469466
Neutrophils	11-Sep	-0.33439	6.415184	-1.5695	0.120103	-5.01617	0.36002	0.402676
Neutrophils	SDR42E1	-0.82684	0.45745	-1.56935	0.120138	-4.32446	0.426659	0.475278
Neutrophils	ZFP429	0.475325	2.448075	1.569182	0.120178	-4.45448	0.403096	0.449727
Neutrophils	ACBD5	0.177492	6.231959	1.568975	0.120226	-5.18768	0.361939	0.404815
Neutrophils	MTFR1L	0.255967	4.250609	1.568628	0.120307	-4.77171	0.382933	0.427915
Neutrophils	DMTF1	-0.23701	4.947065	-1.5685	0.120337	-4.8455	0.375413	0.419714
Neutrophils	TAF4	0.268715	4.075399	1.568249	0.120395	-4.75041	0.38485	0.430107
Neutrophils	FAM110B	-0.97591	0.653098	-1.56803	0.120446	-4.33169	0.424328	0.47313
Neutrophils	RABGGTB	-0.39524	3.732854	-1.56802	0.120448	-4.55211	0.388626	0.434301
Neutrophils	RAB24	0.177921	5.118704	1.567987	0.120456	-5.10652	0.373583	0.417866
Neutrophils	PIP5K1A	0.2128	5.595581	1.567724	0.120518	-5.07865	0.368548	0.412451
Neutrophils	CCL27A	-0.74842	1.684593	-1.56771	0.120521	-4.33889	0.412012	0.459871
Neutrophils	ZDHHC8	-0.54148	3.666743	-1.56744	0.120584	-4.41476	0.389442	0.435257
Neutrophils	POLR2B	-0.25489	4.911643	-1.56711	0.120661	-4.8112	0.375998	0.420539
Neutrophils	3-Mar	-0.23944	6.392222	-1.56635	0.120839	-5.53211	0.360914	0.403962
Neutrophils	SF3B5	0.183423	6.277364	1.566071	0.120904	-5.19659	0.362126	0.405256
Neutrophils	MAP3K8	0.306906	4.64612	1.565796	0.120969	-4.82619	0.379328	0.424085
Neutrophils	NOTCH4	-0.8927	0.210596	-1.56576	0.120977	-4.35086	0.430502	0.479754
Neutrophils	MARVELD1	0.542139	1.253094	1.565671	0.120998	-4.45225	0.417873	0.466066
Neutrophils	EDNRB	-0.98381	2.175551	-1.56528	0.121089	-4.57518	0.407193	0.454499
Neutrophils	REPS1	-0.18096	5.964436	-1.56432	0.121316	-5.15188	0.366042	0.409401
Neutrophils	STPG4	-0.41937	2.37884	-1.56423	0.121335	-4.59351	0.405412	0.45241
Neutrophils	PDE6H	0.364439	2.093346	1.563907	0.121412	-4.64978	0.408863	0.456138
Neutrophils	SPG21	0.169355	6.057126	1.563167	0.121586	-5.21213	0.365608	0.408687
Neutrophils	FGFR1OP	-0.42138	4.601111	-1.56298	0.121629	-4.57582	0.381074	0.425687
Neutrophils	CSRP1	-0.26941	5.881401	-1.56279	0.121675	-5.04075	0.367439	0.410806
Neutrophils	POLR1B	-0.67567	2.442843	-1.56268	0.121701	-4.37181	0.405259	0.452063
Neutrophils	MLH1	-0.46098	2.750298	-1.56254	0.121734	-4.42119	0.40172	0.448214
Neutrophils	ZFP85	-0.98994	0.25725	-1.562	0.12186	-4.32766	0.431572	0.480608
Neutrophils	GM43126	-0.949	0.276018	-1.56196	0.12187	-4.33257	0.431341	0.480376
Neutrophils	KDM6A	-0.41001	6.914035	-1.56103	0.122089	-5.30565	0.357528	0.399706
Neutrophils	CAR1	1.062069	0.491473	1.55999	0.122335	-4.37273	0.430069	0.478362
Neutrophils	TSPAN9	-0.42204	3.290989	-1.55952	0.122446	-4.72641	0.397109	0.442643
Neutrophils	TERF1	0.244829	5.254483	1.559412	0.122471	-4.8782	0.375505	0.4191
Neutrophils	4833418N(	0.635425	1.533706	1.559227	0.122515	-4.43256	0.41753	0.464867
Neutrophils	PSMG4	-0.31439	5.042688	-1.55886	0.122601	-4.69099	0.377775	0.421799
Neutrophils	TDP1	-0.52011	3.397629	-1.55879	0.122619	-4.48132	0.395903	0.441562
Neutrophils	ARF3	0.164742	6.29303	1.558663	0.122649	-5.1805	0.364576	0.407381
Neutrophils	CRYBA4	0.89157	-0.18755	1.558571	0.12267	-4.34576	0.438567	0.48785

Neutrophils	COPZ1	0.156996	6.366584	1.558521	0.122682	-5.2528	0.363815	0.406578
Neutrophils	ABCA5	-1.05925	0.444818	-1.55825	0.122747	-4.33083	0.430717	0.479503
Neutrophils	MTA2	-0.24159	5.782517	-1.55816	0.122767	-5.00421	0.369906	0.413361
Neutrophils	GM13610	0.901689	0.396658	1.558064	0.122791	-4.33291	0.43131	0.480172
Neutrophils	SLC7A11	0.218584	4.278698	1.558009	0.122804	-5.87541	0.386084	0.43104
Neutrophils	SNX8	-0.42681	5.468943	-1.55785	0.122841	-4.86313	0.37322	0.417032
Neutrophils	HAUS2	0.399529	3.755191	1.557465	0.122933	-4.59576	0.392061	0.437481
Neutrophils	CLEC4A1	0.59383	3.158732	1.557185	0.122999	-4.70658	0.398824	0.444829
Neutrophils	CDH24	-0.59762	2.179776	-1.55694	0.123058	-4.40234	0.410121	0.457176
Neutrophils	RND1	0.385043	1.145338	1.55693	0.12306	-4.72181	0.422413	0.470512
Neutrophils	RIT1	0.255389	3.965996	1.556786	0.123094	-4.8232	0.38975	0.435097
Neutrophils	PHACTR4	0.214323	5.677431	1.556128	0.12325	-4.97223	0.371572	0.415125
Neutrophils	TOP3B	0.327729	3.549805	1.555725	0.123346	-4.60592	0.394787	0.440603
Neutrophils	IL18RAP	0.275933	1.983582	1.555699	0.123352	-5.20665	0.412827	0.460223
Neutrophils	PAIP2	-0.12963	7.620191	-1.5556	0.123375	-5.45506	0.351627	0.393417
Neutrophils	SNRPE	-0.1914	7.064225	-1.55549	0.123401	-5.25305	0.357218	0.399579
Neutrophils	GM16794	0.87461	0.41499	1.555204	0.12347	-4.33533	0.431849	0.480804
Neutrophils	MRPL35	-0.30481	5.21625	-1.55496	0.123527	-4.78279	0.376641	0.420797
Neutrophils	TADA1	-0.27402	4.679062	-1.55452	0.123633	-4.78378	0.382554	0.42732
Neutrophils	NTN4	0.724565	0.706687	1.554413	0.123658	-4.42898	0.428457	0.477238
Neutrophils	PLA2G4C	-0.87389	0.890136	-1.55435	0.123675	-4.35874	0.426218	0.474847
Neutrophils	ZXDC	-0.24145	4.004625	-1.55385	0.123792	-4.92157	0.390167	0.435671
Neutrophils	MND1	-0.68693	2.222735	-1.55378	0.12381	-4.38399	0.410508	0.457843
Neutrophils	TMEM185/	-0.56817	2.540183	-1.55356	0.123863	-4.42116	0.406818	0.45384
Neutrophils	RASGRP4	0.240146	1.734055	1.553322	0.123919	-5.11839	0.416287	0.464158
Neutrophils	GM42556	0.60637	0.793712	1.553101	0.123971	-4.47507	0.427616	0.476546
Neutrophils	SMUG1	0.593256	1.976529	1.55307	0.123979	-4.37947	0.413415	0.461136
Neutrophils	TRAP1	-0.33111	4.381095	-1.55294	0.124011	-4.58243	0.386016	0.431295
Neutrophils	TPMT	-0.58489	2.097645	-1.55277	0.124051	-4.40561	0.411989	0.459586
Neutrophils	KARS	-0.41519	4.273346	-1.55256	0.124101	-4.60933	0.387202	0.43263
Neutrophils	2310011J0	0.283387	4.446593	1.552471	0.124122	-4.80918	0.385296	0.430564
Neutrophils	GAS5	-0.24235	6.391612	-1.55229	0.124165	-5.08723	0.364553	0.407869
Neutrophils	PSMB5	0.207058	6.258665	1.552196	0.124188	-5.17712	0.365933	0.409408
Neutrophils	PRAG1	0.77763	1.923732	1.55195	0.124247	-4.36921	0.414078	0.462046
Neutrophils	REEP3	0.14492	6.727725	1.551833	0.124274	-5.52966	0.361124	0.404265
Neutrophils	ZFP933	-0.49134	3.490032	-1.55157	0.124337	-4.50804	0.396061	0.442474
Neutrophils	HDDC2	-0.47718	3.817561	-1.55122	0.12442	-4.46519	0.39238	0.438499
Neutrophils	NFIL3	0.287024	4.515288	1.551218	0.124422	-5.21075	0.384656	0.430065
Neutrophils	QSER1	-0.37893	4.323503	-1.55086	0.124507	-4.62148	0.386764	0.432432
Neutrophils	PIP4K2B	-0.26972	4.178712	-1.55082	0.124517	-4.82205	0.388362	0.434179
Neutrophils	CYBC1	0.312462	4.562008	1.550733	0.124538	-4.81611	0.384145	0.429575
Neutrophils	IGFBP1	0.625389	4.147259	1.550562	0.124579	-4.94284	0.388711	0.434604
Neutrophils	MKNK2	0.17018	6.23257	1.55048	0.124599	-5.2507	0.366313	0.410139
Neutrophils	SP140	0.172743	6.188606	1.549961	0.124723	-5.57452	0.366939	0.410876
Neutrophils	FURIN	0.236759	5.290193	1.549859	0.124748	-5.23184	0.376435	0.421288
Neutrophils	KHK	0.305278	4.3658	1.54962	0.124805	-4.84468	0.386475	0.432326
Neutrophils	HMGCS1	0.314593	4.453186	1.549422	0.124853	-4.82341	0.385514	0.431414

Neutrophils	CTNND1	-0.30019	4.294756	-1.54919	0.124909	-4.9332	0.387258	0.433429
Neutrophils	MTIF2	0.365146	4.285566	1.549156	0.124917	-4.58253	0.387359	0.43355
Neutrophils	ZFP113	-0.88876	1.151185	-1.54908	0.124936	-4.34858	0.423592	0.473095
Neutrophils	RTL4	-0.9913	0.402884	-1.54899	0.124955	-4.36293	0.432741	0.483031
Neutrophils	CPN1	-0.65586	1.600653	-1.54839	0.1251	-4.43513	0.418385	0.467436
Neutrophils	SENP8	-0.95156	0.568146	-1.54829	0.125125	-4.34971	0.430904	0.481039
Neutrophils	SPATA13	0.253129	5.147731	1.54753	0.125308	-5.10573	0.378141	0.423796
Neutrophils	ARHGAP35	-0.34417	5.237019	-1.54725	0.125376	-4.72651	0.377181	0.42277
Neutrophils	TMEM245	-0.37421	4.753996	-1.54706	0.12542	-4.66222	0.382403	0.428594
Neutrophils	TRPS1	0.190215	7.683996	1.546978	0.125441	-5.71975	0.35185	0.395038
Neutrophils	MIPEP	0.388003	2.843457	1.546835	0.125475	-4.52317	0.403805	0.452017
Neutrophils	CYB5R1	0.275699	3.659051	1.546826	0.125477	-4.84711	0.394521	0.441875
Neutrophils	ELP6	-0.74775	2.398694	-1.54676	0.125494	-4.36574	0.408962	0.457644
Neutrophils	GSG1L	-1.00616	-0.16326	-1.54652	0.125552	-4.35036	0.440001	0.491491
Neutrophils	KLRB1A	-0.98166	-0.08222	-1.5465	0.125557	-4.35544	0.438984	0.490387
Neutrophils	ALOX5	0.29909	0.791987	1.546116	0.125649	-5.03637	0.428158	0.478794
Neutrophils	GM45715	0.554496	1.039938	1.546098	0.125653	-4.44258	0.425137	0.475507
Neutrophils	GM16066	0.574503	2.012511	1.545905	0.1257	-4.42109	0.413495	0.462915
Neutrophils	SCARF1	-0.59649	1.587665	-1.54578	0.125729	-4.45315	0.41854	0.468415
Neutrophils	MPG	0.446234	3.63806	1.545759	0.125735	-4.47884	0.394758	0.442449
Neutrophils	MYL12B	0.113654	8.183707	1.54567	0.125756	-5.76174	0.346901	0.389887
Neutrophils	STARD3	0.331009	4.116736	1.54551	0.125795	-4.75154	0.389408	0.436663
Neutrophils	FLYWCH1	-0.34137	3.850858	-1.54542	0.125816	-4.66502	0.39237	0.43992
Neutrophils	SLC36A4	-0.51832	3.21113	-1.54542	0.125816	-4.54797	0.399593	0.447822
Neutrophils	LDB1	0.335501	4.482765	1.545395	0.125823	-4.737	0.385369	0.43225
Neutrophils	ITCH	0.17277	7.100446	1.545335	0.125837	-5.41438	0.357724	0.401881
Neutrophils	WDHD1	0.358082	4.56722	1.544673	0.125997	-4.83853	0.384753	0.431628
Neutrophils	EFCAB14	-0.22219	4.713606	-1.5446	0.126014	-5.08321	0.383153	0.429873
Neutrophils	BOD1L	0.176308	5.740505	1.544374	0.12607	-5.10223	0.372138	0.417842
Neutrophils	PLP2	0.183372	5.623922	1.544266	0.126096	-5.51585	0.373374	0.419238
Neutrophils	RASA2	0.157065	6.158678	1.543804	0.126208	-5.61979	0.367816	0.413129
Neutrophils	NUAK2	0.241771	4.289152	1.543677	0.126238	-4.9966	0.387915	0.435281
Neutrophils	S100A1	0.266173	4.086611	1.543542	0.126271	-4.96684	0.39016	0.437773
Neutrophils	PPNR	0.84827	1.051959	1.543538	0.126272	-4.36744	0.425447	0.476325
Neutrophils	NDUFA7	0.122936	7.909207	1.542734	0.126467	-5.57193	0.350421	0.393855
Neutrophils	KMT2C	-0.14489	7.717211	-1.54218	0.126601	-5.54634	0.352465	0.396189
Neutrophils	GLT8D1	0.400916	3.339159	1.542053	0.126633	-4.55718	0.399208	0.447539
Neutrophils	GM15133	0.699591	1.788244	1.542019	0.126641	-4.39954	0.41727	0.467267
Neutrophils	FNBP1L	-0.27299	3.079155	-1.54192	0.126664	-5.08536	0.402179	0.450805
Neutrophils	ENAH	0.583386	0.744942	1.54143	0.126784	-4.52867	0.430167	0.481269
Neutrophils	BCAS3	0.175457	7.334935	1.541064	0.126873	-5.46539	0.356584	0.400692
Neutrophils	SPATA2	-0.34875	4.217056	-1.54105	0.126875	-4.7611	0.389644	0.437026
Neutrophils	MZT1	-0.2114	5.278837	-1.54087	0.126919	-4.93201	0.378058	0.424334
Neutrophils	NMRAL1	-0.54838	3.904291	-1.54031	0.127056	-4.5042	0.39346	0.441091
Neutrophils	EVA1B	-0.37573	3.639669	-1.53995	0.127143	-4.70001	0.396532	0.444532
Neutrophils	9430038I0	-0.30234	4.248537	-1.53988	0.127161	-4.65719	0.389711	0.437071
Neutrophils	ARFRP1	-0.2863	4.120918	-1.53943	0.12727	-4.71097	0.391219	0.43871

Neutrophils	LENG9	0.552839	2.352054	1.539378	0.127284	-4.45802	0.41146	0.460853
Neutrophils	ZC3H18	0.252779	5.270525	1.539024	0.12737	-4.91314	0.378619	0.425038
Neutrophils	DOK3	0.212654	5.36627	1.539018	0.127371	-5.34856	0.377588	0.423907
Neutrophils	GM11110	0.596593	1.970897	1.53886	0.12741	-4.4065	0.41596	0.465949
Neutrophils	AP3M1	0.182952	4.881049	1.538812	0.127422	-5.06489	0.382839	0.429711
Neutrophils	G530011O(	-1.02389	1.80368	-1.53863	0.127467	-4.3657	0.417951	0.468125
Neutrophils	SMPD4	0.542384	3.209019	1.538528	0.127491	-4.47522	0.401522	0.450196
Neutrophils	DGUOK	-0.37741	4.460054	-1.538	0.12762	-4.68209	0.387698	0.435007
Neutrophils	BET1L	0.363627	3.860623	1.537894	0.127646	-4.61802	0.394377	0.442328
Neutrophils	LAS1L	-0.35245	4.172574	-1.53715	0.127827	-4.63036	0.39128	0.438731
Neutrophils	BAK1	0.297228	4.936771	1.536623	0.127957	-4.90263	0.382855	0.429608
Neutrophils	ARL4C	0.24121	5.243589	1.536536	0.127978	-5.1382	0.379526	0.426008
Neutrophils	SLC35B2	0.264127	4.882082	1.536509	0.127985	-4.92452	0.383452	0.430322
Neutrophils	SAFB2	0.161313	5.993263	1.536421	0.128006	-5.17108	0.371519	0.417234
Neutrophils	DPYD	-0.58499	3.567148	-1.53622	0.128055	-4.75584	0.39809	0.446484
Neutrophils	DHRS7	0.204705	4.016547	1.536069	0.128093	-5.39083	0.393023	0.440979
Neutrophils	CLEC2D	0.462247	4.7558	1.53592	0.128129	-4.86564	0.384833	0.432069
Neutrophils	PSTPIP1	0.192507	4.472927	1.535858	0.128144	-5.30572	0.387946	0.435518
Neutrophils	NCK1	-0.17237	6.174664	-1.5357	0.128184	-5.37067	0.369608	0.415448
Neutrophils	RAD50	0.318545	4.906644	1.535676	0.128189	-4.74536	0.383184	0.430376
Neutrophils	GM30054	-0.80487	1.953461	-1.53506	0.128341	-4.39368	0.417139	0.467425
Neutrophils	GM33677	-1.12616	-1.06476	-1.53485	0.128391	-4.35248	0.45469	0.508344
Neutrophils	CPED1	0.447379	2.524781	1.534848	0.128392	-4.74956	0.410392	0.460115
Neutrophils	C130036L2	0.684948	1.354595	1.534511	0.128475	-4.3807	0.424481	0.475462
Neutrophils	LRRC43	-0.95587	-0.12202	-1.53433	0.128519	-4.35925	0.442788	0.495458
Neutrophils	ISG20L2	-0.3179	5.083016	-1.53405	0.128588	-4.75085	0.381778	0.428789
Neutrophils	RAB3IL1	-0.58342	1.73222	-1.53384	0.12864	-4.47376	0.420098	0.470843
Neutrophils	SEMA7A	0.555476	2.017527	1.533324	0.128767	-4.51869	0.416739	0.467381
Neutrophils	IRF2	0.165345	6.689517	1.533171	0.128804	-5.41394	0.364814	0.410419
Neutrophils	WDR33	0.135229	7.186641	1.533091	0.128824	-5.36219	0.359701	0.404778
Neutrophils	PAN3	-0.11851	8.767803	-1.53304	0.128837	-5.72206	0.343935	0.387358
Neutrophils	ASXL2	-0.20308	6.836454	-1.53295	0.128858	-5.21291	0.363294	0.408753
Neutrophils	AQP9	0.297741	2.330322	1.532859	0.128881	-5.00918	0.413035	0.463409
Neutrophils	JAML	0.251725	2.748338	1.532536	0.128961	-5.20439	0.40827	0.458078
Neutrophils	SURF6	-0.42597	3.185009	-1.53224	0.129033	-4.47422	0.403323	0.452607
Neutrophils	SERPINB2	1.471959	0.991964	1.53196	0.129103	-4.57363	0.429465	0.481205
Neutrophils	GM43560	-0.74522	0.578606	-1.53175	0.129155	-4.35727	0.434565	0.486844
Neutrophils	VAPA	0.097605	8.028548	1.531674	0.129173	-5.64935	0.351494	0.395726
Neutrophils	AGPAT3	-0.36673	5.164345	-1.53118	0.129294	-4.71663	0.381357	0.428838
Neutrophils	PHLDB3	-0.53974	2.97973	-1.53104	0.129329	-4.46165	0.405851	0.455778
Neutrophils	DNAJC14	0.209651	4.252973	1.530993	0.129342	-4.89316	0.391384	0.439915
Neutrophils	AADAT	-0.66052	1.2345	-1.53093	0.129358	-4.42954	0.426579	0.478468
Neutrophils	IER2	0.193409	8.170209	1.530813	0.129386	-5.78417	0.35015	0.394509
Neutrophils	KLKB1	-0.68565	1.848942	-1.53037	0.129495	-4.43882	0.419279	0.470478
Neutrophils	PLAG1	0.441278	2.8948	1.53036	0.129498	-4.50874	0.40695	0.456991
Neutrophils	NINL	0.87281	0.999292	1.529779	0.129642	-4.36686	0.429926	0.48183
Neutrophils	EPHX2	-0.62829	2.477792	-1.52941	0.129733	-4.54986	0.412238	0.46259

Neutrophils	NME3	-1.01883	0.405363	-1.52937	0.129743	-4.36159	0.437366	0.490014
Neutrophils	PPIC	-0.37248	2.745716	-1.52909	0.129812	-4.72709	0.409198	0.459294
Neutrophils	CERK	-0.26495	7.028222	-1.52882	0.129879	-5.08658	0.362314	0.407739
Neutrophils	MEX3A	-0.83915	1.577184	-1.52844	0.129974	-4.37124	0.423356	0.474566
Neutrophils	CHD4	0.142403	7.633169	1.528032	0.130074	-5.51812	0.356477	0.401135
Neutrophils	EFCAB11	-0.41802	3.410869	-1.5273	0.130257	-4.79498	0.402048	0.451515
Neutrophils	TM7SF3	-0.25536	3.924878	-1.52725	0.130268	-4.9054	0.396199	0.445102
Neutrophils	RIOX2	-0.32747	3.769866	-1.52677	0.130388	-4.60398	0.397954	0.447069
Neutrophils	1600012H(	0.383514	2.771016	1.526703	0.130404	-4.55559	0.409452	0.459668
Neutrophils	AUTS2	-0.47706	4.397144	-1.52666	0.130415	-5.00972	0.390904	0.439331
Neutrophils	ZC3H7A	0.15468	6.883008	1.526564	0.130439	-5.36542	0.364219	0.409955
Neutrophils	RALY	-0.16399	6.422986	-1.52652	0.130451	-5.33795	0.369009	0.415239
Neutrophils	ERMARD	0.452604	3.076068	1.526492	0.130457	-4.52925	0.405905	0.455784
Neutrophils	SPRY1	-0.92553	1.345776	-1.52645	0.130467	-4.37655	0.426452	0.47825
Neutrophils	AP1S2	0.162702	5.081901	1.526072	0.130561	-5.35424	0.383355	0.431237
Neutrophils	IKZF4	-0.83922	-0.0706	-1.52602	0.130575	-4.36225	0.444056	0.497678
Neutrophils	SSR1	0.153427	6.554701	1.525745	0.130643	-5.30271	0.367631	0.413922
Neutrophils	ZCCHC18	-0.91509	1.04036	-1.5256	0.13068	-4.36387	0.430188	0.482563
Neutrophils	ATP6V0C	0.126881	8.851286	1.525447	0.130717	-5.86016	0.344452	0.388324
Neutrophils	RGS9	-1.05257	0.03543	-1.52542	0.130723	-4.36205	0.442714	0.496258
Neutrophils	TMCC2	0.805807	1.686886	1.52518	0.130784	-4.46683	0.422319	0.474117
Neutrophils	SNU13	-0.17713	6.96176	-1.52515	0.13079	-5.26013	0.363405	0.409385
Neutrophils	EGLN1	0.230686	5.631732	1.525092	0.130805	-5.04706	0.377404	0.424845
Neutrophils	RAP1GAP2	0.216544	4.692548	1.525033	0.13082	-5.49145	0.387628	0.436125
Neutrophils	GM7030	-1.00571	2.084686	-1.52476	0.130888	-4.38367	0.417646	0.468991
Neutrophils	IFT46	-0.26864	4.502973	-1.5244	0.130978	-4.77195	0.38987	0.438591
Neutrophils	NR6A1OS	-0.7686	2.067272	-1.52403	0.13107	-4.41084	0.417912	0.469395
Neutrophils	GM44067	0.873752	1.717425	1.523994	0.131079	-4.36888	0.422107	0.473982
Neutrophils	1110020A2	-0.89807	0.468534	-1.52377	0.131136	-4.3674	0.437432	0.490763
Neutrophils	FADD	0.60783	2.421277	1.523583	0.131182	-4.48798	0.413711	0.464865
Neutrophils	SFPQ	-0.12826	8.189863	-1.52358	0.131183	-5.60308	0.351094	0.395909
Neutrophils	GM17231	0.500533	2.734245	1.523539	0.131193	-4.61335	0.410033	0.460837
Neutrophils	LIMS1	-0.12123	7.288787	-1.52347	0.13121	-5.75145	0.36018	0.405985
Neutrophils	5730455P1	-0.43819	2.914853	-1.52301	0.131326	-4.51513	0.40817	0.45874
Neutrophils	TPR	0.141542	7.524789	1.522573	0.131434	-5.4876	0.358183	0.403671
Neutrophils	ASGR2	-0.74389	1.698173	-1.52233	0.131495	-4.47215	0.422891	0.474844
Neutrophils	ENDOD1	-0.62434	2.901638	-1.52184	0.131617	-4.41202	0.408875	0.459378
Neutrophils	CIDEB	-0.54871	1.942054	-1.52153	0.131697	-4.51222	0.420238	0.471884
Neutrophils	LONP1	0.412019	3.653778	1.521524	0.131697	-4.57698	0.40021	0.449941
Neutrophils	GM42658	-0.59008	1.544714	-1.5209	0.131853	-4.46599	0.425412	0.477425
Neutrophils	ARF6	0.195128	6.770128	1.520153	0.132041	-5.32363	0.36699	0.41315
Neutrophils	HNMT	0.513517	-0.38409	1.51997	0.132087	-4.50005	0.450042	0.504203
Neutrophils	FAM76A	0.195727	5.119697	1.519422	0.132224	-5.10426	0.384849	0.432877
Neutrophils	SAPCD1	-0.77847	1.629002	-1.51939	0.132233	-4.3907	0.425124	0.477029
Neutrophils	ASB2	-0.77358	2.906427	-1.51902	0.132326	-4.48109	0.409983	0.460414
Neutrophils	EMCN	-0.44644	1.650943	-1.51898	0.132335	-4.65708	0.424939	0.476785
Neutrophils	GSDME	0.213999	4.386903	1.518825	0.132374	-5.27069	0.393044	0.44194

Neutrophils	CYP4F16	0.740992	1.441368	1.518552	0.132443	-4.42861	0.42757	0.479716
Neutrophils	RPAP1	-0.59095	2.431442	-1.51825	0.13252	-4.42685	0.415656	0.466784
Neutrophils	RAI1	-0.28477	6.004853	-1.51819	0.132533	-4.93264	0.375423	0.422594
Neutrophils	HAO1	-0.45239	3.070076	-1.51813	0.13255	-4.76574	0.408151	0.458586
Neutrophils	STN1	-0.28308	3.886297	-1.5173	0.132757	-4.80869	0.39927	0.448562
Neutrophils	GM34471	-0.45948	0.232245	-1.51691	0.132858	-4.62652	0.443364	0.496748
Neutrophils	AA388235	-0.9711	0.991788	-1.51668	0.132916	-4.38366	0.433914	0.486491
Neutrophils	PCM1	0.1936	6.494399	1.515716	0.133158	-5.23329	0.371507	0.417777
Neutrophils	FOXO3	0.199102	6.397005	1.515461	0.133223	-5.36773	0.37261	0.418957
Neutrophils	RNF166	0.175837	5.468282	1.515243	0.133278	-5.16549	0.382582	0.429969
Neutrophils	BC003965	0.414954	3.701969	1.515066	0.133323	-4.60555	0.40232	0.451653
Neutrophils	DPM2	0.338167	3.936241	1.515004	0.133338	-4.70537	0.399643	0.448718
Neutrophils	TRP53	-0.23739	5.681235	-1.51464	0.13343	-4.97606	0.380425	0.427587
Neutrophils	HPS3	0.289906	4.626547	1.514268	0.133525	-4.89567	0.392183	0.440403
Neutrophils	SUCLA2	0.212014	5.593683	1.513986	0.133596	-5.04021	0.3816	0.428806
Neutrophils	ALDOB	-0.46572	4.949636	-1.51387	0.133624	-5.12372	0.388658	0.436575
Neutrophils	GM47730	-0.92486	-0.65913	-1.5135	0.13372	-4.37004	0.456151	0.510343
Neutrophils	RALA	0.178707	5.871704	1.513475	0.133726	-5.24469	0.378628	0.425597
Neutrophils	PPP1R14B	-0.25237	6.546146	-1.51333	0.133763	-5.04359	0.371439	0.417716
Neutrophils	PLOD2	0.819045	1.261605	1.513225	0.133789	-4.41957	0.431801	0.483944
Neutrophils	BC035044	-0.72631	5.146379	-1.513	0.133847	-4.53702	0.386578	0.434388
Neutrophils	NR2C1	-0.57864	2.276051	-1.51215	0.134063	-4.48975	0.420094	0.470891
Neutrophils	PCSK7	0.223883	5.282369	1.511857	0.134136	-5.09206	0.385598	0.433149
Neutrophils	CD300LD	0.238264	3.173108	1.511841	0.134141	-5.38527	0.409481	0.45934
Neutrophils	CEP350	0.182129	6.505585	1.511525	0.134221	-5.35372	0.372534	0.418759
Neutrophils	LDLRAD3	0.442244	4.591753	1.511296	0.134279	-4.73545	0.393436	0.44176
Neutrophils	5830432E0	0.429771	1.494203	1.510945	0.134369	-4.67712	0.429939	0.481689
Neutrophils	IL27	0.899653	0.053926	1.510638	0.134447	-4.42196	0.447995	0.501544
Neutrophils	HACE1	-0.29895	4.490352	-1.51051	0.13448	-4.86552	0.394727	0.4434
Neutrophils	EPS8	0.623075	4.963297	1.510406	0.134506	-4.72238	0.389446	0.43762
Neutrophils	RNF130	0.128061	7.282781	1.510339	0.134523	-5.67433	0.364598	0.410257
Neutrophils	DDR1	0.81616	0.137297	1.510168	0.134566	-4.3786	0.446947	0.500543
Neutrophils	RDX	0.12357	6.872179	1.509853	0.134647	-5.44284	0.369001	0.415134
Neutrophils	DDX10	-0.28434	5.436521	-1.50951	0.134734	-4.78165	0.38437	0.432153
Neutrophils	B4GALT3	0.227499	3.874826	1.509176	0.134819	-4.9054	0.40185	0.451407
Neutrophils	APOPT1	-0.21706	4.910205	-1.50914	0.134828	-4.86738	0.390171	0.438583
Neutrophils	PIGT	0.248802	5.640537	1.509037	0.134855	-5.02687	0.382146	0.429769
Neutrophils	ANAPC2	-0.31211	4.497598	-1.50898	0.134868	-4.78744	0.394783	0.443676
Neutrophils	GM43660	0.597887	0.127169	1.508827	0.134908	-4.54925	0.447215	0.501076
Neutrophils	PDS5B	-0.20433	6.377064	-1.5088	0.134915	-5.15812	0.374227	0.421122
Neutrophils	RBM4B	0.23582	5.747385	1.507865	0.135154	-5.03758	0.381352	0.428988
Neutrophils	PDAP1	0.16934	6.715812	1.507632	0.135214	-5.30974	0.370999	0.417599
Neutrophils	TET1	-0.96561	1.135429	-1.50758	0.135228	-4.38348	0.434938	0.487748
Neutrophils	GM5914	0.509054	3.331997	1.507507	0.135246	-4.5087	0.40851	0.458848
Neutrophils	FBXO32	-0.88535	3.711448	-1.50732	0.135293	-4.47497	0.404114	0.454028
Neutrophils	DTX3	-0.54029	2.572046	-1.5073	0.135298	-4.49102	0.417462	0.468652
Neutrophils	CLIC5	-1.01346	0.878367	-1.50713	0.135343	-4.38358	0.438142	0.491244

Neutrophils	ZFP715	-0.34854	3.662783	-1.50699	0.135379	-4.65883	0.404675	0.454679
Neutrophils	SRP14	0.123423	7.234027	1.506941	0.13539	-5.45788	0.365581	0.41165
Neutrophils	COQ10B	0.133841	5.908947	1.506256	0.135566	-5.51291	0.379676	0.427372
Neutrophils	4-Sep	-0.69862	1.735848	-1.50624	0.135569	-4.46196	0.427626	0.479998
Neutrophils	IFI35	0.348242	5.333965	1.506173	0.135587	-4.96245	0.385937	0.434284
Neutrophils	ZBTB42	0.452066	1.544195	1.506134	0.135597	-4.52447	0.429973	0.482579
Neutrophils	NXT2	-0.31289	2.785909	-1.50609	0.135608	-4.71838	0.415001	0.466202
Neutrophils	KCNC3	-0.82386	0.561324	-1.50585	0.135669	-4.3911	0.442248	0.496052
Neutrophils	PARP6	-0.43266	2.991541	-1.50563	0.135726	-4.61834	0.412609	0.463755
Neutrophils	NIM1K	-0.37368	2.88905	-1.5056	0.135735	-4.76004	0.413817	0.465079
Neutrophils	COQ3	-0.32884	3.500438	-1.50437	0.136051	-4.6136	0.407473	0.457838
Neutrophils	CLEC10A	1.005132	0.930777	1.504246	0.136082	-4.39683	0.438477	0.491843
Neutrophils	LRRK1	-0.35461	5.612519	-1.50355	0.136262	-4.90248	0.383892	0.432001
Neutrophils	MPHOSPH1	0.284964	4.827879	1.503532	0.136266	-4.84592	0.39256	0.441542
Neutrophils	SPPL2A	0.217098	6.600738	1.503511	0.136271	-5.40326	0.373258	0.420278
Neutrophils	CD28	-0.69354	2.935145	-1.50306	0.136388	-4.51837	0.414549	0.465595
Neutrophils	GM39556	0.309274	3.384757	1.50279	0.136457	-5.0023	0.409267	0.45982
Neutrophils	ZC3HAV1	0.140722	8.43854	1.502767	0.136463	-5.77074	0.354495	0.399477
Neutrophils	TBRG1	0.218417	5.618457	1.502589	0.136509	-5.23327	0.384061	0.432101
Neutrophils	DNAIC1	-0.85722	0.436784	-1.50149	0.136792	-4.40227	0.445605	0.499624
Neutrophils	TYW5	-0.51093	3.139549	-1.50145	0.136803	-4.53448	0.412519	0.463462
Neutrophils	PANK1	-0.56348	3.430383	-1.50133	0.136833	-4.56153	0.409112	0.459727
Neutrophils	VEZT	-0.34919	4.178132	-1.50122	0.136862	-4.6652	0.400484	0.450258
Neutrophils	GTPBP1	-0.23618	4.641527	-1.50111	0.136891	-5.03509	0.395232	0.444487
Neutrophils	UQCRQ	0.169079	7.858453	1.501069	0.136901	-5.60808	0.360702	0.406412
Neutrophils	HHEX	-0.24463	4.675113	-1.50095	0.136932	-5.11748	0.394854	0.444072
Neutrophils	PI4K2B	0.336138	3.903982	1.50082	0.136965	-4.76165	0.403625	0.453751
Neutrophils	JRKL	0.682777	1.537523	1.500785	0.136974	-4.4524	0.431816	0.484638
Neutrophils	PIANP	-0.84677	-0.06426	-1.5006	0.137023	-4.38756	0.452062	0.506826
Neutrophils	HPS1	0.4862	3.445228	1.500103	0.13715	-4.55232	0.409141	0.459839
Neutrophils	GOLPH3L	0.357357	4.39604	1.500075	0.137158	-4.75103	0.398202	0.44783
Neutrophils	GNL1	0.319987	4.299889	1.499017	0.137432	-4.73545	0.399769	0.449528
Neutrophils	SLFN4	0.316204	0.48225	1.498899	0.137462	-5.22367	0.445776	0.499956
Neutrophils	ZFP984	0.272672	3.878402	1.498855	0.137473	-4.82418	0.4046	0.454906
Neutrophils	USP36	-0.2221	4.970109	-1.49885	0.137475	-5.0775	0.392211	0.44129
Neutrophils	CBX1	-0.21522	6.075378	-1.49861	0.137538	-5.10112	0.380138	0.428005
Neutrophils	TAF12	0.201807	5.021001	1.498402	0.137591	-5.09777	0.391752	0.440829
Neutrophils	TMEM131L	-0.17048	7.26323	-1.49818	0.137649	-5.53161	0.367616	0.414238
Neutrophils	KYAT3	-0.51365	3.603362	-1.49784	0.137737	-4.71724	0.408054	0.458751
Neutrophils	TMBIM6	0.136223	9.142023	1.497617	0.137794	-5.87955	0.348642	0.393298
Neutrophils	NCAM1	0.298816	1.275146	1.497603	0.137798	-5.30382	0.436084	0.489549
Neutrophils	NAIP2	0.270742	3.006951	1.497401	0.137851	-5.03667	0.415095	0.466583
Neutrophils	RBM26	-0.16421	6.821937	-1.49693	0.137972	-5.26972	0.372526	0.419666
Neutrophils	XPA	0.337185	4.138354	1.496891	0.137983	-4.78066	0.40208	0.45222
Neutrophils	LPGAT1	-0.18896	6.245156	-1.49638	0.138116	-5.4434	0.378733	0.426615
Neutrophils	RAMP2	-0.36037	3.013058	-1.49636	0.138122	-4.91082	0.415246	0.466778
Neutrophils	NFATC1	0.208459	5.77824	1.496238	0.138153	-5.18948	0.383794	0.432251

Neutrophils	ZFP580	-0.64236	2.166288	-1.49622	0.138157	-4.44951	0.425401	0.47796
Neutrophils	NCOA1	0.174196	7.07231	1.496059	0.1382	-5.53575	0.369948	0.416956
Neutrophils	MRPS11	-0.4437	3.950885	-1.49523	0.138416	-4.62849	0.404721	0.454903
Neutrophils	TMEM53	-0.69229	0.637469	-1.49521	0.138421	-4.46072	0.444855	0.498807
Neutrophils	ZDHHC15	-0.8456	1.125862	-1.49497	0.138483	-4.40943	0.438768	0.492228
Neutrophils	ZFP26	0.34936	3.995742	1.494709	0.138551	-4.65122	0.404358	0.454588
Neutrophils	SLC25A36	-0.191	6.285868	-1.49433	0.138649	-5.24463	0.378988	0.426639
Neutrophils	MRI1	-0.421	3.963231	-1.49421	0.138682	-4.54769	0.404888	0.455141
Neutrophils	2310009B1	0.260272	4.473004	1.493832	0.13878	-4.85586	0.399113	0.448857
Neutrophils	GM42722	0.597206	2.335621	1.49379	0.138791	-4.54804	0.424193	0.476365
Neutrophils	EIF4H	0.146957	6.535904	1.493516	0.138862	-5.28634	0.376365	0.423853
Neutrophils	4930403P2	-0.6357	0.612375	-1.4934	0.138893	-4.43677	0.445586	0.499788
Neutrophils	TADA2A	-0.55137	2.820969	-1.49325	0.138932	-4.47714	0.418359	0.470029
Neutrophils	OAF	0.493832	3.151249	1.493233	0.138936	-4.60057	0.414436	0.46573
Neutrophils	ARHGAP28	-1.03142	0.598833	-1.49264	0.139092	-4.4109	0.446133	0.500201
Neutrophils	ZBTB25	0.476858	3.709262	1.492286	0.139184	-4.58904	0.408303	0.458969
Neutrophils	OGFOD1	-0.41092	3.611469	-1.49226	0.139192	-4.61609	0.409443	0.460219
Neutrophils	STX12	0.181829	5.552332	1.492089	0.139236	-5.228	0.387438	0.43607
Neutrophils	TRAF3IP1	-0.84403	1.889467	-1.49194	0.139275	-4.41506	0.430076	0.482907
Neutrophils	DDX21	-0.26105	6.399043	-1.49151	0.139386	-5.08665	0.378421	0.426133
Neutrophils	TEDC2	-0.74703	0.906107	-1.49123	0.139462	-4.41583	0.442669	0.49665
Neutrophils	DENND1C	0.266315	3.966917	1.490845	0.139561	-4.99015	0.405826	0.456219
Neutrophils	STARD8	-0.27274	3.359704	-1.48979	0.139837	-4.99686	0.413583	0.464196
Neutrophils	CAMSAP2	-0.33899	4.496866	-1.48968	0.139868	-4.75111	0.400394	0.449798
Neutrophils	TRP53BP1	-0.39602	3.888271	-1.48945	0.139927	-4.65178	0.407397	0.457535
Neutrophils	SUSD3	0.374454	3.170727	1.489316	0.139963	-4.70476	0.415818	0.466802
Neutrophils	PRR5L	0.478529	2.756439	1.489239	0.139983	-4.7848	0.420761	0.472263
Neutrophils	MSL2	0.147263	6.010868	1.488852	0.140085	-5.41341	0.383579	0.431512
Neutrophils	PHTF2	-0.24948	5.998759	-1.48868	0.140131	-5.10641	0.383711	0.431708
Neutrophils	GRHPR	0.371232	4.431109	1.488558	0.140162	-4.90441	0.401218	0.45099
Neutrophils	WDR27	0.705374	0.499373	1.48855	0.140164	-4.42219	0.448847	0.503097
Neutrophils	GM26511	0.668128	2.075738	1.487836	0.140352	-4.46945	0.429548	0.481894
Neutrophils	DNAAF5	0.538261	2.988111	1.487337	0.140484	-4.47776	0.418677	0.469866
Neutrophils	GALC	0.218998	3.891293	1.487228	0.140512	-5.20311	0.408033	0.458204
Neutrophils	ABHD17B	0.156192	6.542955	1.487179	0.140525	-5.32547	0.378375	0.425621
Neutrophils	RBMS2	0.282994	4.872344	1.486884	0.140603	-4.80325	0.396815	0.445966
Neutrophils	ZFP644	-0.19889	6.331418	-1.48685	0.140613	-5.1485	0.380683	0.428221
Neutrophils	AKR1B8	0.54817	1.592252	1.486597	0.140679	-4.60484	0.43578	0.488667
Neutrophils	CPSF7	-0.16262	5.801172	-1.48648	0.140709	-5.24447	0.386515	0.43466
Neutrophils	ITPRIPL2	-0.2079	3.897046	-1.48627	0.140766	-5.24756	0.408063	0.458322
Neutrophils	TRAPPC6B	0.131025	6.134079	1.48615	0.140797	-5.37975	0.382889	0.430701
Neutrophils	ZFP777	0.49126	2.809648	1.485881	0.140868	-4.48848	0.420915	0.472493
Neutrophils	DENND2A	-0.52753	1.374468	-1.48579	0.140892	-4.60318	0.438514	0.49173
Neutrophils	HERC6	0.508912	4.719873	1.485722	0.14091	-4.80989	0.398609	0.448031
Neutrophils	SP3OS	0.218306	4.205373	1.4854	0.140995	-5.08625	0.404526	0.454593
Neutrophils	C030006K1	-0.80325	1.498775	-1.48532	0.141016	-4.41735	0.436996	0.490141
Neutrophils	SOS2	0.171062	5.600384	1.485236	0.141038	-5.37295	0.388775	0.437281

Neutrophils	FLT1	-0.35744	4.243341	-1.48501	0.141099	-5.38234	0.404151	0.45425
Neutrophils	GTF3C2	-0.19376	5.474786	-1.48468	0.141185	-5.03163	0.390265	0.439011
Neutrophils	LRRC8B	-0.43349	3.358799	-1.48466	0.141191	-4.53472	0.414511	0.465645
Neutrophils	SLC7A7	-0.39872	3.785978	-1.48426	0.141295	-4.76493	0.409683	0.460245
Neutrophils	PLEC	0.190941	4.805597	1.484027	0.141358	-5.28002	0.398021	0.447468
Neutrophils	ST3GAL3	-0.28798	5.293649	-1.48358	0.141477	-4.90696	0.39275	0.441485
Neutrophils	CNTLN	-0.30485	4.34763	-1.48319	0.14158	-4.99809	0.40357	0.453347
Neutrophils	FRMD8OS	0.700283	0.736632	1.482863	0.141667	-4.4697	0.447364	0.50136
Neutrophils	KLF6	0.208866	7.581942	1.482628	0.141729	-5.68503	0.368116	0.414431
Neutrophils	LCT	-0.79899	0.170332	-1.48238	0.141795	-4.42909	0.454659	0.509379
Neutrophils	TBX2	-0.73725	0.45083	-1.48233	0.141809	-4.46166	0.451031	0.505428
Neutrophils	APBB2	-0.39438	4.02877	-1.4823	0.141816	-5.03868	0.407253	0.457565
Neutrophils	PSMD6	0.252198	5.199485	1.482223	0.141837	-5.00811	0.393898	0.442902
Neutrophils	LTBP4	-0.56289	2.297056	-1.48191	0.141919	-4.66108	0.427871	0.480186
Neutrophils	ZDHHC17	-0.32507	4.015064	-1.48174	0.141965	-4.74546	0.407412	0.457773
Neutrophils	GM14286	0.578872	0.211974	1.481674	0.141982	-4.50743	0.454118	0.508828
Neutrophils	9430015G1	0.663029	1.911053	1.481534	0.14202	-4.43634	0.432611	0.485396
Neutrophils	SEC23IP	0.211234	4.499732	1.48149	0.142031	-4.91616	0.401825	0.451667
Neutrophils	FAAP100	0.396881	3.097229	1.481265	0.142091	-4.63261	0.418214	0.469769
Neutrophils	USP30	-0.60804	2.252852	-1.48126	0.142093	-4.44771	0.428411	0.480933
Neutrophils	PRR7	0.293736	3.334702	1.481192	0.142111	-4.93209	0.415391	0.466675
Neutrophils	ANKHD1	0.146003	7.647791	1.481098	0.142136	-5.59006	0.367429	0.413919
Neutrophils	CDKN2C	-0.47903	3.918847	-1.48068	0.142247	-4.74666	0.408704	0.459418
Neutrophils	GM50431	-0.63848	0.122176	-1.48058	0.142274	-4.52078	0.455477	0.510568
Neutrophils	SUFU	-0.30822	5.025085	-1.48027	0.142357	-4.7906	0.396147	0.44562
Neutrophils	GM21887	-0.86988	2.133944	-1.47993	0.142445	-4.43285	0.430201	0.48308
Neutrophils	DDX60	0.375304	2.866859	1.479685	0.142512	-5.06513	0.421298	0.473351
Neutrophils	AK2	0.172862	5.995761	1.479674	0.142515	-5.38102	0.385375	0.43387
Neutrophils	MTPN	0.123429	6.898113	1.479655	0.14252	-5.48533	0.375621	0.423106
Neutrophils	NIT1	0.330829	3.750254	1.479304	0.142613	-4.77411	0.41094	0.461908
Neutrophils	CAPRIN2	-0.56825	3.181523	-1.4792	0.14264	-4.61085	0.417658	0.469306
Neutrophils	UBLCP1	0.186835	4.773952	1.479053	0.14268	-5.17081	0.399129	0.449022
Neutrophils	LYSMD4	0.372673	3.330154	1.478875	0.142728	-4.75044	0.415918	0.467472
Neutrophils	ELAVL3	0.975728	1.03829	1.478613	0.142798	-4.43143	0.444128	0.498309
Neutrophils	LEF1	-0.57372	5.638758	-1.47819	0.14291	-4.91435	0.389732	0.43852
Neutrophils	ENG	-0.35105	4.005662	-1.47795	0.142974	-5.03525	0.408354	0.458993
Neutrophils	DCTN3	0.168086	5.963361	1.477636	0.143059	-5.30644	0.386342	0.434708
Neutrophils	POLD4	0.204604	5.76477	1.477366	0.143131	-5.18124	0.388619	0.43714
Neutrophils	PEAK1	-0.22058	6.584571	-1.47684	0.143271	-5.45341	0.379862	0.427415
Neutrophils	MON1A	0.342221	3.876268	1.476574	0.143343	-4.72429	0.410296	0.460958
Neutrophils	RIMS4	0.985869	-0.99611	1.476318	0.143411	-4.40735	0.471521	0.527916
Neutrophils	D130040H2	-0.6788	2.397794	-1.47612	0.143464	-4.45059	0.427967	0.480434
Neutrophils	CCZ1	0.182393	6.158644	1.476068	0.143478	-5.19227	0.384487	0.432688
Neutrophils	ARFIP2	0.553256	2.138474	1.476053	0.143482	-4.48021	0.431146	0.483911
Neutrophils	GM4316	-0.89744	0.707324	-1.47588	0.143529	-4.41818	0.449129	0.503635
Neutrophils	ITM2A	-0.64973	2.544803	-1.47585	0.143536	-4.52217	0.426176	0.478564
Neutrophils	BAZ2A	0.180214	6.97841	1.475776	0.143557	-5.47084	0.375637	0.423029

Neutrophils	CDH23	0.260711	2.820164	1.47494	0.143781	-5.0658	0.423382	0.475309
Neutrophils	INAFM1	0.511578	1.866251	1.474799	0.143819	-4.55872	0.435065	0.488136
Neutrophils	PHF7	0.460905	3.28527	1.474638	0.143862	-4.58689	0.417813	0.469264
Neutrophils	RBM15	-0.21133	5.696792	-1.47409	0.144009	-5.086	0.390371	0.438996
Neutrophils	IFNAR2	0.153994	7.128736	1.473779	0.144093	-5.57569	0.374922	0.421899
Neutrophils	MYSM1	0.219748	5.388465	1.473612	0.144138	-5.06218	0.393947	0.442884
Neutrophils	MTBP	-0.44853	3.256156	-1.47337	0.144201	-4.63145	0.41869	0.470071
Neutrophils	OSM	0.253682	2.305551	1.473177	0.144255	-5.43291	0.430242	0.482704
Neutrophils	ERBIN	0.142466	7.975801	1.472886	0.144333	-5.71803	0.366233	0.412334
Neutrophils	IRGC1	0.620887	0.115465	1.471592	0.144682	-4.50027	0.459112	0.513561
Neutrophils	SCAF8	0.16089	6.711338	1.471341	0.144749	-5.33292	0.380499	0.427525
Neutrophils	FBXL8	-0.62705	1.99347	-1.47091	0.144866	-4.48851	0.435456	0.487768
Neutrophils	SLC44A2	0.245732	6.242042	1.469324	0.145294	-5.21243	0.386813	0.434073
Neutrophils	SUPT20	0.156695	5.30473	1.469181	0.145333	-5.34123	0.397263	0.445581
Neutrophils	TSHZ1	-0.35114	5.060403	-1.46908	0.145362	-4.89791	0.400035	0.44865
Neutrophils	RNF19B	0.211454	5.758355	1.468811	0.145433	-5.34212	0.392169	0.440087
Neutrophils	CYBA	0.131785	8.55691	1.468694	0.145465	-5.95368	0.362225	0.407106
Neutrophils	EFL1	-0.32353	4.5955	-1.46861	0.145488	-4.73759	0.405365	0.454568
Neutrophils	2200002D(	0.814097	1.337585	1.468496	0.145519	-4.47535	0.444839	0.49769
Neutrophils	ARHGAP5	0.354882	4.809305	1.468231	0.14559	-4.99993	0.402993	0.451947
Neutrophils	CHCHD5	-0.50035	3.092559	-1.46797	0.145662	-4.57134	0.423293	0.474192
Neutrophils	VCAM1	-0.45226	4.334733	-1.46711	0.145895	-5.18743	0.40911	0.458563
Neutrophils	ZFP180	-0.36066	3.315586	-1.46673	0.145997	-4.67445	0.421302	0.47193
Neutrophils	GM16754	0.862949	0.04601	1.466207	0.14614	-4.42812	0.462517	0.516878
Neutrophils	TNFRSF25	-0.772	-1.26707	-1.4662	0.146141	-4.41224	0.480194	0.536046
Neutrophils	ELOVL6	-0.56957	4.451031	-1.46614	0.146159	-4.62253	0.407886	0.457299
Neutrophils	HP	0.199568	7.365035	1.46608	0.146174	-6.01987	0.375454	0.421666
Neutrophils	ZKSCAN1	0.329835	4.068204	1.46598	0.146201	-4.71568	0.41236	0.462198
Neutrophils	ARHGEF1	0.138008	7.10928	1.465917	0.146218	-5.58291	0.378189	0.424679
Neutrophils	ATMIN	0.543142	2.969649	1.465124	0.146434	-4.5559	0.425982	0.476868
Neutrophils	TTC39A	0.616869	2.027911	1.464898	0.146496	-4.57561	0.437584	0.489571
Neutrophils	ATP11B	0.131965	7.035822	1.464846	0.14651	-5.77909	0.379426	0.425877
Neutrophils	SMIM12	0.283727	4.0506	1.464375	0.146638	-4.8371	0.41313	0.462863
Neutrophils	AA465934	-0.66149	1.66043	-1.4643	0.146659	-4.46235	0.44228	0.494699
Neutrophils	DHX29	-0.35073	3.415036	-1.46385	0.146782	-4.71905	0.420683	0.471397
Neutrophils	ZBTB20	-0.17187	8.246328	-1.46377	0.146803	-5.66792	0.366688	0.412079
Neutrophils	RPP30	-0.35294	3.635635	-1.46353	0.14687	-4.67529	0.418045	0.46858
Neutrophils	FETUB	-0.5029	3.175824	-1.46346	0.146887	-4.80807	0.423562	0.474614
Neutrophils	CERCAM	-0.82901	0.652443	-1.46344	0.146893	-4.42137	0.455195	0.509105
Neutrophils	PRDM11	-0.5603	3.444862	-1.46341	0.146901	-4.5428	0.420325	0.471074
Neutrophils	AKT2	0.188545	5.234185	1.463338	0.146921	-5.17788	0.399436	0.448187
Neutrophils	CCDC73	0.483694	3.100981	1.463292	0.146934	-4.53186	0.424467	0.475603
Neutrophils	DOCK9	-0.42237	4.197961	-1.46303	0.147006	-4.81952	0.411406	0.461339
Neutrophils	YIPF4	0.194306	6.515782	1.462992	0.147016	-5.40371	0.385149	0.432514
Neutrophils	NPHP4	-0.96027	0.297045	-1.4626	0.147122	-4.41611	0.460051	0.514284
Neutrophils	GNPDA2	0.379098	3.100129	1.462356	0.147189	-4.62772	0.424673	0.47579
Neutrophils	CEP70	0.425714	3.195368	1.462317	0.1472	-4.64717	0.423521	0.474539

Neutrophils	FCNA	-0.53958	4.555419	-1.46202	0.14728	-5.2308	0.407418	0.456965
Neutrophils	ARHGAP4	0.174021	5.057347	1.461731	0.14736	-5.24907	0.401636	0.450755
Neutrophils	B3GAT3	0.283924	4.307364	1.461651	0.147382	-4.93171	0.410307	0.460275
Neutrophils	UBE2E1	-0.15697	6.229498	-1.46161	0.147394	-5.22294	0.388466	0.436293
Neutrophils	GPD1L	-0.26721	5.296236	-1.46159	0.147398	-5.05852	0.398915	0.447777
Neutrophils	ZFP652OS	-0.72561	-0.171	-1.46144	0.147441	-4.45848	0.466253	0.5213
Neutrophils	GM49980	-0.60505	6.157222	-1.46094	0.147576	-4.94767	0.389505	0.437251
Neutrophils	GYS1	0.276527	3.38284	1.460827	0.147608	-5.13252	0.421522	0.472441
Neutrophils	MEIS3	-0.81322	0.488054	-1.46051	0.147693	-4.43427	0.457905	0.512202
Neutrophils	SNHG4.1	-1.0814	0.471422	-1.46032	0.147748	-4.42046	0.458123	0.512528
Neutrophils	CASP1	0.327962	3.51676	1.460303	0.147751	-4.91357	0.419985	0.470921
Neutrophils	NAPA	0.158368	5.92916	1.459489	0.147975	-5.27068	0.392574	0.44072
Neutrophils	H2-Q7	-1.11903	3.889878	-1.45937	0.148008	-4.55469	0.41604	0.466519
Neutrophils	ARL6IP5	0.177151	7.048458	1.458927	0.148129	-5.39103	0.380418	0.427344
Neutrophils	SPRTN	0.448253	3.419835	1.458637	0.148208	-4.61725	0.421798	0.472923
Neutrophils	MLLT6	0.664932	3.270712	1.458251	0.148315	-4.56827	0.423596	0.475071
Neutrophils	IL7R	-0.70185	4.966863	-1.45816	0.148341	-4.65361	0.40361	0.453192
Neutrophils	SLC22A1	0.804177	0.740269	1.457968	0.148392	-4.46358	0.455321	0.509728
Neutrophils	CR1L	0.154608	5.693128	1.457835	0.148429	-5.25841	0.395354	0.444122
Neutrophils	ATF6	0.179305	6.979377	1.457647	0.148481	-5.40416	0.381164	0.428541
Neutrophils	RFTN1	-0.34209	6.737622	-1.45754	0.14851	-4.98262	0.38379	0.431442
Neutrophils	ARID2	-0.1615	6.543762	-1.45716	0.148616	-5.47764	0.385909	0.433915
Neutrophils	BC049352	-0.73463	1.083027	-1.45711	0.148629	-4.48631	0.450886	0.50513
Neutrophils	DUSP12	-0.46451	3.283921	-1.45709	0.148635	-4.56815	0.423436	0.475143
Neutrophils	1700047M	-0.32179	-0.15234	-1.45697	0.148667	-4.98571	0.467079	0.522808
Neutrophils	UBA5	-0.24846	4.489487	-1.45695	0.148672	-4.84764	0.409134	0.45951
Neutrophils	CCHCR1	0.679296	1.536464	1.456683	0.148746	-4.49099	0.445086	0.498854
Neutrophils	VPS26C	-0.34477	4.084075	-1.45668	0.148747	-4.78945	0.413887	0.464725
Neutrophils	COX8A	0.118864	9.008145	1.456678	0.148748	-5.85125	0.359865	0.405201
Neutrophils	MSANTD2	-0.24993	4.864755	-1.45635	0.148837	-4.90824	0.404785	0.45489
Neutrophils	C87436	-0.35677	3.905756	-1.45622	0.148875	-4.72852	0.415995	0.46722
Neutrophils	SMIM11	0.217268	4.805042	1.456177	0.148886	-5.01491	0.405474	0.455669
Neutrophils	RBBP7	-0.19931	6.2357	-1.45615	0.148893	-5.25741	0.389302	0.437877
Neutrophils	GM43672	0.493065	1.700182	1.456146	0.148894	-4.65536	0.443011	0.496783
Neutrophils	2700049A(	-0.33729	4.236674	-1.45594	0.148951	-4.83077	0.412139	0.463014
Neutrophils	CYP7A1	-0.74967	0.739983	-1.4548	0.149265	-4.4991	0.456125	0.510814
Neutrophils	ERCC1	-0.40223	3.315868	-1.4547	0.149294	-4.6388	0.423794	0.47551
Neutrophils	UQCRFS1	0.169352	7.164384	1.454515	0.149345	-5.47392	0.379834	0.427297
Neutrophils	GM28809	0.487903	-0.12461	1.454477	0.149355	-4.64591	0.467529	0.523345
Neutrophils	ARHGAP10	-0.37666	5.105594	-1.4542	0.149432	-5.10589	0.402826	0.452646
Neutrophils	HNRNPF	0.111155	9.158537	1.453622	0.149592	-5.8506	0.359263	0.404598
Neutrophils	CDK2	-0.26064	4.350301	-1.45356	0.149609	-5.08162	0.411821	0.462538
Neutrophils	ZFP276	0.413114	2.479933	1.453454	0.149638	-4.63668	0.43438	0.487272
Neutrophils	LZTS1	-0.82083	1.417164	-1.45314	0.149724	-4.44615	0.447896	0.501975
Neutrophils	IGF2	-0.61704	4.193244	-1.45217	0.149995	-5.03337	0.414433	0.46504
Neutrophils	KANSL1	-0.12873	8.964116	-1.45147	0.150189	-5.91076	0.362246	0.407306
Neutrophils	GM16310	-0.81562	1.197906	-1.45138	0.150213	-4.45833	0.45182	0.505566

Neutrophils	HDDC3	-0.8076	0.928077	-1.45062	0.150426	-4.44169	0.455669	0.509674
Neutrophils	MTHFR	-0.40018	3.640415	-1.45058	0.150435	-4.63923	0.421729	0.472647
Neutrophils	GCDH	-0.52293	3.116618	-1.45053	0.150449	-4.70317	0.428076	0.479586
Neutrophils	SCAND1	0.123072	7.018464	1.45038	0.150491	-5.60669	0.383085	0.43029
Neutrophils	MIEF1	0.371677	4.21125	1.449805	0.150651	-4.77746	0.415183	0.465481
Neutrophils	D930030I0	0.593172	0.408475	1.44976	0.150664	-4.58393	0.46277	0.517422
Neutrophils	GIMAP1OS	-0.84179	0.862202	-1.44909	0.15085	-4.44178	0.457232	0.511306
Neutrophils	MTA3	0.255646	6.109959	1.448838	0.15092	-5.17445	0.393704	0.441887
Neutrophils	MINDY1	0.152156	3.987424	1.448831	0.150922	-5.4097	0.418223	0.468785
Neutrophils	GREM2	0.862355	0.454759	1.448519	0.151009	-4.45573	0.462706	0.517245
Neutrophils	NAP1L4	0.138223	6.249563	1.448378	0.151049	-5.367	0.392248	0.440265
Neutrophils	STX5A	-0.14892	5.871144	-1.44807	0.151135	-5.32175	0.396491	0.445024
Neutrophils	CENPP	0.320306	5.39603	1.447871	0.15119	-5.19329	0.401885	0.450974
Neutrophils	SIRT5	-0.55885	1.958147	-1.44786	0.151192	-4.54019	0.443263	0.496249
Neutrophils	B630019A1	-0.62326	1.596371	-1.4475	0.151294	-4.55274	0.447864	0.501361
Neutrophils	METTL2	-0.46996	3.27705	-1.44743	0.151313	-4.55256	0.426892	0.478497
Neutrophils	APOL11B	1.654434	-0.04187	1.447392	0.151324	-4.46719	0.469316	0.52475
Neutrophils	AACS	-0.29546	3.861959	-1.44728	0.151355	-4.85019	0.419832	0.470788
Neutrophils	GRAMD4	-0.46434	5.165751	-1.44725	0.151363	-4.73501	0.404528	0.454012
Neutrophils	MYCBP	0.295525	4.22905	1.446767	0.151498	-4.88208	0.415544	0.466193
Neutrophils	PDF	-0.8413	0.794298	-1.44672	0.151513	-4.43458	0.458329	0.512933
Neutrophils	ARNT	0.224774	5.869604	1.446574	0.151552	-5.28455	0.396585	0.445411
Neutrophils	GM29340	-0.8505	-0.6731	-1.44636	0.151612	-4.43241	0.477948	0.534376
Neutrophils	FBXO11	0.171188	8.797772	1.4463	0.151629	-5.8398	0.364968	0.410652
Neutrophils	KLHDC2	-0.23406	4.860142	-1.44629	0.151633	-5.00548	0.408142	0.458221
Neutrophils	PPAN	-0.4931	4.072585	-1.4458	0.151769	-4.61802	0.417595	0.468495
Neutrophils	VKORC1L1	0.193048	5.560377	1.445747	0.151784	-5.25446	0.400275	0.449488
Neutrophils	MRPL53	-0.29229	4.291701	-1.44553	0.151846	-4.92016	0.41503	0.46572
Neutrophils	PRRG1	-0.57076	1.915898	-1.4454	0.151879	-4.61891	0.444127	0.497603
Neutrophils	INIP	-0.31524	4.523666	-1.44527	0.151917	-4.76845	0.412297	0.4628
Neutrophils	FEM1A	-0.44967	2.924816	-1.44494	0.152009	-4.55941	0.431671	0.484042
Neutrophils	ISY1	0.179615	6.241546	1.444788	0.152052	-5.43164	0.392772	0.441402
Neutrophils	CRIM1	-0.32784	5.191827	-1.44464	0.152094	-5.23032	0.40468	0.454569
Neutrophils	KCTD1	0.542492	1.719442	1.444459	0.152144	-4.54652	0.446823	0.500693
Neutrophils	IZUMO1R	-1.06201	0.001693	-1.4432	0.152498	-4.43743	0.470254	0.525637
Neutrophils	HEATR9	-0.83938	-0.41964	-1.443	0.152555	-4.4431	0.476001	0.531844
Neutrophils	PIK3CB	0.204127	4.688415	1.442823	0.152604	-5.47394	0.411463	0.461535
Neutrophils	VIPR1	0.703233	0.964939	1.442224	0.152772	-4.5123	0.457898	0.512139
Neutrophils	ORC4	-0.22858	5.046376	-1.44198	0.152841	-4.96052	0.407582	0.457262
Neutrophils	IFIT3	0.726582	3.112471	1.441898	0.152864	-4.80391	0.430674	0.482575
Neutrophils	ZFP931	0.59012	1.514653	1.441687	0.152924	-4.49623	0.450766	0.504523
Neutrophils	GRAP	-0.33846	4.536304	-1.44164	0.152937	-4.8927	0.413545	0.463846
Neutrophils	CCDC61	-0.43436	2.934126	-1.44158	0.152955	-4.61954	0.43287	0.485
Neutrophils	RAB27A	0.160265	4.50985	1.441395	0.153006	-5.48045	0.413886	0.464238
Neutrophils	POU5F1	0.595821	1.26222	1.441024	0.15311	-4.52444	0.45425	0.508244
Neutrophils	GM3235	-0.97604	0.645113	-1.44052	0.153254	-4.44201	0.462411	0.517101
Neutrophils	DESI2	0.152738	6.03278	1.440424	0.153279	-5.20076	0.396572	0.445136

Neutrophils	PDPN	0.659959	-0.21403	1.440377	0.153293	-4.65223	0.473899	0.529593
Neutrophils	ZFR2	-0.76791	0.817601	-1.44008	0.153377	-4.44843	0.460139	0.51471
Neutrophils	GM11714	0.363227	1.498822	1.439942	0.153415	-4.83958	0.451274	0.505059
Neutrophils	ADGRA2	-0.71402	0.687625	-1.43981	0.153453	-4.56157	0.46185	0.516571
Neutrophils	CNBP	-0.12122	8.132464	-1.4398	0.153455	-5.62301	0.373627	0.41992
Neutrophils	MTX1	-0.34701	4.556924	-1.43978	0.153461	-4.80348	0.413582	0.463868
Neutrophils	EFHD2	0.133607	6.92246	1.43898	0.153687	-5.7919	0.387142	0.434542
Neutrophils	MYO1G	0.243073	5.563064	1.43826	0.153891	-5.23603	0.402659	0.451555
Neutrophils	ZCWPW1	-0.50984	2.765843	-1.43822	0.153901	-4.57786	0.436064	0.488115
Neutrophils	2310022B(	-0.47707	2.488564	-1.43819	0.15391	-4.51877	0.439527	0.491897
Neutrophils	ZFP446	-0.70219	1.164128	-1.43777	0.15403	-4.47467	0.456698	0.510501
Neutrophils	TUBA4A	-0.18796	4.513714	-1.43744	0.154123	-5.38208	0.415128	0.465201
Neutrophils	SFT2D1	0.130377	5.999884	1.437299	0.154163	-5.4388	0.397937	0.446414
Neutrophils	CAPZA1	0.0896	7.715586	1.437279	0.154168	-5.79524	0.379016	0.425593
Neutrophils	ECE1	-0.40153	5.613294	-1.4363	0.154445	-4.94811	0.402953	0.451557
Neutrophils	A1CF	-0.83568	0.569704	-1.43614	0.154492	-4.48213	0.4653	0.519683
Neutrophils	RALYL	-1.09645	0.59788	-1.43584	0.154576	-4.4569	0.464981	0.51945
Neutrophils	TXNRD1	0.1695	5.871273	1.43565	0.15463	-5.52902	0.400071	0.448576
Neutrophils	CSMD3	-0.60121	1.302147	-1.43565	0.154631	-4.5232	0.455723	0.509388
Neutrophils	SSBP2	0.197379	6.284595	1.435504	0.154672	-5.55544	0.395398	0.443501
Neutrophils	NUP188	-0.314	4.513741	-1.43512	0.15478	-4.85565	0.416012	0.466125
Neutrophils	SLC15A4	0.350659	4.804844	1.434474	0.154965	-4.83326	0.412872	0.462533
Neutrophils	RUVBL2	0.377428	3.693884	1.434451	0.154971	-4.77308	0.426149	0.47706
Neutrophils	SRRM2	0.115463	8.62357	1.433797	0.155157	-5.80766	0.370515	0.41604
Neutrophils	INO80B	0.331585	4.146114	1.433572	0.155222	-4.79074	0.420778	0.471296
Neutrophils	TDG	0.211445	5.074746	1.43353	0.155233	-5.1061	0.409796	0.459283
Neutrophils	FAM168B	0.149165	6.280422	1.433368	0.15528	-5.28767	0.395981	0.444164
Neutrophils	CCR9	0.6945	2.410766	1.433281	0.155304	-4.5335	0.442125	0.494673
Neutrophils	BC050972	-0.63766	-0.27973	-1.43327	0.155306	-4.56637	0.47743	0.533064
Neutrophils	CLIC1	0.149213	8.942892	1.433227	0.15532	-5.88433	0.367181	0.41244
Neutrophils	SELENOF	-0.21015	5.422799	-1.43316	0.155338	-5.22689	0.405757	0.454897
Neutrophils	GM44777	-0.74055	0.565153	-1.43306	0.155368	-4.48045	0.466047	0.520709
Neutrophils	RAI14	-0.57988	2.228896	-1.43247	0.155537	-4.61956	0.444779	0.497412
Neutrophils	EXO1	0.67547	2.019131	1.432342	0.155572	-4.54599	0.44745	0.500365
Neutrophils	HLCS	-0.30273	4.15558	-1.43213	0.155632	-5.01101	0.421017	0.471569
Neutrophils	EXOSC9	-0.35924	4.169312	-1.43203	0.15566	-4.73488	0.420852	0.471389
Neutrophils	KHNYN	0.20258	4.263958	1.431591	0.155786	-5.156	0.419951	0.470282
Neutrophils	GM16853	-0.59971	2.351019	-1.43127	0.155878	-4.53633	0.44364	0.49607
Neutrophils	EGR3	0.43794	3.533307	1.430731	0.156032	-5.14916	0.429084	0.480244
Neutrophils	CTSF	-0.66962	1.860603	-1.43063	0.156062	-4.57881	0.450057	0.503162
Neutrophils	SLC15A3	0.189927	5.005968	1.430552	0.156083	-5.55583	0.411453	0.461011
Neutrophils	STAT4	0.261074	5.177658	1.430373	0.156135	-5.44484	0.409447	0.458883
Neutrophils	DPY19L3	-0.60571	2.757574	-1.43027	0.156163	-4.54695	0.438683	0.490888
Neutrophils	SAMD4	-0.63347	3.187151	-1.43022	0.156178	-4.80613	0.43334	0.485056
Neutrophils	YPEL2	-0.47509	4.572346	-1.43001	0.156238	-4.74079	0.416617	0.466759
Neutrophils	SIK1	0.145091	6.868808	1.429682	0.156332	-5.64239	0.390318	0.438015
Neutrophils	TNFRSF13E	0.42263	4.518479	1.429669	0.156336	-4.66773	0.417302	0.467634

Neutrophils	GM41556	-0.56368	1.259442	-1.4292	0.15647	-4.56858	0.458227	0.512245
Neutrophils	TAF15	0.137759	6.905531	1.429009	0.156525	-5.45335	0.390181	0.437845
Neutrophils	MAPK6	0.249027	5.88266	1.428172	0.156765	-5.28796	0.4022	0.450733
Neutrophils	IL23R	0.785854	-0.9447	1.427781	0.156878	-4.48534	0.48882	0.545167
Neutrophils	GM47071	-0.36058	1.555335	-1.42772	0.156895	-4.9889	0.455134	0.508643
Neutrophils	COX18	-0.4091	3.223872	-1.42756	0.156942	-4.6745	0.43399	0.485616
Neutrophils	HIST2H4	0.877963	0.951972	1.427108	0.157071	-4.47419	0.463125	0.517488
Neutrophils	ASAH1	0.164387	6.301867	1.427106	0.157072	-5.51189	0.397627	0.445918
Neutrophils	MATN2	0.803102	0.980441	1.427058	0.157085	-4.47233	0.462749	0.517079
Neutrophils	IQCE	0.61833	2.144332	1.426418	0.157269	-4.5335	0.448033	0.500832
Neutrophils	SSH3	-0.76863	1.522336	-1.42579	0.157451	-4.49403	0.456467	0.509829
Neutrophils	SIGLECE	0.308792	2.293576	1.425563	0.157516	-5.32293	0.446579	0.49915
Neutrophils	OFCC1	0.44149	-0.63548	1.425349	0.157577	-4.73897	0.485539	0.541542
Neutrophils	CREBRF	-0.1826	6.98351	-1.4253	0.157591	-5.58424	0.390753	0.438141
Neutrophils	BMP2	-0.48719	1.613957	-1.42463	0.157784	-4.71946	0.455542	0.509039
Neutrophils	GRTP1	0.677801	0.35364	1.42462	0.157787	-4.49418	0.472235	0.527177
Neutrophils	VAMP7	-0.30846	4.132998	-1.42453	0.157813	-4.81892	0.423956	0.474633
Neutrophils	SPATA2L	0.830171	0.521707	1.424477	0.157829	-4.47288	0.469974	0.524806
Neutrophils	FZD4	-0.52319	1.267832	-1.42415	0.157924	-4.61858	0.460163	0.51409
Neutrophils	COLGALT2	-0.86639	-0.12576	-1.42396	0.157978	-4.49103	0.478848	0.534437
Neutrophils	AW146154	-0.61401	2.334177	-1.42383	0.158016	-4.55208	0.446367	0.499166
Neutrophils	TUBD1	0.549796	2.095647	1.423516	0.158106	-4.55447	0.449416	0.502536
Neutrophils	CARD11	-0.6634	4.618562	-1.42352	0.158106	-4.62824	0.418221	0.468468
Neutrophils	MRPS10	0.333264	4.162492	1.423513	0.158107	-4.7681	0.42369	0.474454
Neutrophils	LXN	0.338527	3.855864	1.423017	0.15825	-4.78394	0.42749	0.478636
Neutrophils	MTCH2	-0.19073	5.950226	-1.42297	0.158263	-5.23465	0.402746	0.451528
Neutrophils	ID3	-0.2484	6.599763	-1.42275	0.158328	-5.44805	0.39538	0.443496
Neutrophils	GLG1	0.172447	6.951102	1.422668	0.158351	-5.44885	0.391454	0.43918
Neutrophils	CAB39L	0.234735	4.624571	1.422615	0.158366	-5.08694	0.418229	0.468567
Neutrophils	4632404H1	-0.76487	1.238383	-1.42256	0.158382	-4.4994	0.460639	0.51485
Neutrophils	NKG7	-0.35891	4.013022	-1.42203	0.158536	-5.22674	0.425885	0.476807
Neutrophils	ZSWIM6	-0.14389	8.155524	-1.42135	0.158734	-5.90289	0.378682	0.425141
Neutrophils	ESYT2	0.121329	6.802347	1.421232	0.158767	-5.75095	0.393501	0.44146
Neutrophils	FOXD2OS	0.815987	0.311339	1.421038	0.158823	-4.46879	0.473466	0.528829
Neutrophils	WIPI2	0.20169	5.276718	1.421001	0.158834	-5.16434	0.410943	0.460616
Neutrophils	INO80	-0.1898	6.666636	-1.42099	0.158838	-5.39634	0.39502	0.443132
Neutrophils	LARP1	-0.20423	6.798018	-1.42094	0.158852	-5.23097	0.393549	0.441538
Neutrophils	PHF23	0.244394	5.240241	1.420574	0.158958	-5.16729	0.41137	0.461273
Neutrophils	MPHOSPH	0.347414	4.258614	1.420533	0.15897	-4.81011	0.423031	0.47407
Neutrophils	TATDN3	-0.49593	2.490952	-1.42039	0.159011	-4.59925	0.4449	0.497989
Neutrophils	1700086O(	-0.56403	1.546442	-1.42017	0.159074	-4.53014	0.457057	0.511399
Neutrophils	TIA1	-0.18976	5.466514	-1.42009	0.159099	-5.09355	0.40873	0.458593
Neutrophils	KDSR	-0.24642	3.963038	-1.42004	0.159113	-5.00688	0.426609	0.478202
Neutrophils	RPTOR	-0.21338	6.294365	-1.41974	0.159199	-5.17923	0.399221	0.448263
Neutrophils	TMEM117	-0.9502	-0.04691	-1.41973	0.159204	-4.48468	0.478335	0.534726
Neutrophils	SNX5	-0.17259	7.890887	-1.41958	0.159247	-5.4909	0.381533	0.428797
Neutrophils	TSR2	-0.80834	2.118125	-1.41944	0.159287	-4.47145	0.449659	0.503627

Neutrophils	PRDX6	-0.10726	7.110346	-1.41931	0.159324	-5.88017	0.390075	0.438332
Neutrophils	GM50209	0.749274	0.629068	1.419068	0.159395	-4.4819	0.469189	0.525082
Neutrophils	PIGQ	-0.35614	3.519745	-1.41875	0.159488	-4.73096	0.432034	0.484647
Neutrophils	ADIPOR1	0.142343	7.346972	1.418464	0.159571	-5.84771	0.387464	0.435716
Neutrophils	STRIP1	0.281337	4.156745	1.41792	0.159729	-4.91687	0.42426	0.476282
Neutrophils	SLC25A19	0.486087	3.390435	1.417854	0.159748	-4.60178	0.43363	0.486549
Neutrophils	OTUD5	0.156593	5.568497	1.417811	0.159761	-5.33089	0.407546	0.457927
Neutrophils	FDX1	-0.32529	4.692703	-1.41775	0.159778	-4.96572	0.417832	0.469241
Neutrophils	GM43259	-0.54453	1.452797	-1.41765	0.159808	-4.68635	0.458281	0.513553
Neutrophils	NAP1L1	0.1804	7.439515	1.41763	0.159814	-5.63031	0.386448	0.43474
Neutrophils	SHARPIN	0.253974	4.530571	1.41742	0.159875	-4.95671	0.419766	0.471521
Neutrophils	EIF5	0.126107	8.418516	1.41742	0.159875	-5.8862	0.375871	0.423153
Neutrophils	CBY1	0.389222	2.846037	1.417316	0.159905	-4.68296	0.440415	0.494212
Neutrophils	SUGP2	-0.43258	4.059588	-1.41727	0.159918	-4.73627	0.425437	0.477806
Neutrophils	BCL7B	-0.1972	5.531179	-1.41715	0.159955	-5.17912	0.407978	0.458629
Neutrophils	AGMO	-0.45138	3.650343	-1.41712	0.159961	-4.85159	0.430428	0.48328
Neutrophils	DCAF17	0.344849	4.054876	1.417045	0.159984	-4.75068	0.425494	0.47787
Neutrophils	DTWD1	0.526819	1.928598	1.416997	0.159998	-4.5637	0.452098	0.50699
Neutrophils	ZFP119A	0.454576	2.125414	1.416957	0.16001	-4.58862	0.449565	0.504223
Neutrophils	FRAT2	-0.22553	4.805593	-1.41647	0.160152	-5.25744	0.416732	0.468131
Neutrophils	RBM44	-0.89585	0.135835	-1.41636	0.160184	-4.4791	0.47612	0.533075
Neutrophils	GM6034	-0.96167	0.50272	-1.4162	0.160231	-4.47429	0.471174	0.527715
Neutrophils	DCAF5	0.207421	5.52135	1.415937	0.160307	-5.24065	0.408344	0.459009
Neutrophils	BCR	-0.29723	5.853797	-1.41588	0.160323	-5.02006	0.404501	0.45478
Neutrophils	EEF1AKMT[illegible]	-0.48381	3.325831	-1.4155	0.160435	-4.66354	0.434697	0.48797
Neutrophils	SERPINH1	-0.45201	2.557349	-1.41546	0.160447	-4.81879	0.444331	0.498515
Neutrophils	ZFP433	-0.91731	0.355661	-1.41542	0.160458	-4.45781	0.473158	0.52997
Neutrophils	UHMK1	0.2441	4.630018	1.415359	0.160476	-4.95736	0.418836	0.470575
Neutrophils	EIF2AK2	0.266785	4.927436	1.415014	0.160577	-5.22205	0.41546	0.466886
Neutrophils	TMEM185[illegible]	-0.25104	4.12483	-1.41439	0.160759	-5.02307	0.425367	0.477701
Neutrophils	TET3	-0.15308	6.827605	-1.41435	0.160772	-5.5226	0.393886	0.443081
Neutrophils	SMAD5	-0.33468	3.702902	-1.41419	0.160819	-4.71776	0.430522	0.483366
Neutrophils	CPD	0.173728	4.333844	1.414013	0.16087	-5.47344	0.42285	0.474998
Neutrophils	SLC12A9	0.382272	4.242634	1.413915	0.160898	-4.82254	0.423951	0.476216
Neutrophils	GM14455	0.458266	1.315653	1.413475	0.161027	-4.64913	0.460958	0.516767
Neutrophils	SNF8	0.172216	5.731869	1.41325	0.161093	-5.28375	0.406431	0.457109
Neutrophils	1600022D[illegible]	0.489114	-0.42787	1.412928	0.161188	-4.57833	0.484493	0.542587
Neutrophils	CENPH	0.486811	3.059161	1.412882	0.161201	-4.74477	0.438582	0.492483
Neutrophils	WRAP73	-0.41101	2.923556	-1.41287	0.161205	-4.62086	0.440282	0.494345
Neutrophils	PRMT9	-0.3118	4.074852	-1.41285	0.16121	-4.92556	0.426064	0.478758
Neutrophils	MRNIP	0.513705	2.538227	1.412832	0.161216	-4.6674	0.445149	0.499674
Neutrophils	SLC39A10	-0.40187	3.903206	-1.41239	0.161347	-4.69039	0.428328	0.481181
Neutrophils	NCSTN	0.187493	5.576404	1.412115	0.161426	-5.30385	0.408399	0.459409
Neutrophils	ETFRF1	0.340619	3.163487	1.412046	0.161447	-4.89673	0.437458	0.491325
Neutrophils	GM10101	0.649836	0.672014	1.41184	0.161507	-4.52868	0.4697	0.526634
Neutrophils	YARS2	-0.45298	3.150094	-1.41174	0.161537	-4.65795	0.437625	0.491633
Neutrophils	HECW2	-0.32924	3.162677	-1.4115	0.161607	-5.10993	0.437468	0.491568

Neutrophils	PTPN14	-0.72796	0.524362	-1.41149	0.16161	-4.52697	0.471685	0.528975
Neutrophils	SLC23A1	-0.67354	1.528872	-1.41127	0.161675	-4.50786	0.458347	0.514499
Neutrophils	HPF1	0.262627	5.467524	1.41122	0.161689	-5.1951	0.409666	0.461095
Neutrophils	SH3BP4	0.815735	1.369949	1.411213	0.161691	-4.4863	0.460432	0.516777
Neutrophils	ST8SIA4	0.2197	7.324778	1.410597	0.161872	-5.5678	0.388945	0.438052
Neutrophils	BIN3	0.166126	5.271025	1.4102	0.161989	-5.40441	0.412428	0.463886
Neutrophils	COMMD10	-0.29221	4.694613	-1.41007	0.162029	-4.92584	0.419251	0.471451
Neutrophils	SGIP1	-0.80008	0.175941	-1.40995	0.162062	-4.4858	0.47694	0.534591
Neutrophils	URAH	-0.5051	3.327083	-1.40984	0.162095	-4.88036	0.435912	0.48978
Neutrophils	LMNB2	-0.54717	2.474409	-1.40967	0.162144	-4.63108	0.446645	0.501598
Neutrophils	LAPTM5	0.127345	8.326927	1.409613	0.162162	-5.78117	0.378147	0.426216
Neutrophils	BAP1	-0.36956	3.565024	-1.4094	0.162226	-4.73281	0.432965	0.486739
Neutrophils	IMPA2	0.181275	4.492173	1.40934	0.162242	-5.36229	0.421675	0.474346
Neutrophils	ENC1	-0.36509	3.181392	-1.40912	0.162308	-4.79477	0.437795	0.492062
Neutrophils	DNAJC16	0.620591	2.30093	1.408878	0.162379	-4.54683	0.449012	0.504367
Neutrophils	TRMO	0.483908	3.013486	1.408354	0.162533	-4.60218	0.440241	0.494716
Neutrophils	TEPSIN	-0.70707	0.762658	-1.40827	0.162558	-4.51171	0.469454	0.526725
Neutrophils	ARFGAP3	-0.35191	3.55174	-1.40748	0.162792	-4.79225	0.433842	0.487641
Neutrophils	UTP6	-0.34053	4.42275	-1.40718	0.162879	-4.79973	0.423206	0.476025
Neutrophils	GM47350	-0.62425	1.355941	-1.40718	0.162879	-4.50861	0.461895	0.518417
Neutrophils	GM14325	-0.49657	2.324076	-1.40716	0.162887	-4.54196	0.449304	0.504656
Neutrophils	GM47863	0.621438	0.552776	1.406783	0.162997	-4.59605	0.472611	0.530228
Neutrophils	SYF2	0.129292	5.903221	1.406736	0.163011	-5.41329	0.405744	0.456902
Neutrophils	TNNT1	-0.37779	1.520318	-1.4066	0.163051	-4.80077	0.459732	0.516195
Neutrophils	SPINT2	0.224198	4.563442	1.406587	0.163055	-5.15218	0.421513	0.474293
Neutrophils	SLC25A13	0.309121	4.679744	1.406298	0.163141	-5.01223	0.420119	0.472768
Neutrophils	PPA1	-0.35577	4.797054	-1.40624	0.163157	-4.9093	0.418718	0.471226
Neutrophils	RWDD2B	-0.75945	1.849393	-1.40623	0.16316	-4.49081	0.455433	0.511503
Neutrophils	POP4	-0.37495	4.085123	-1.40621	0.163166	-4.80117	0.427296	0.480658
Neutrophils	ATP5E	0.112768	8.783189	1.405812	0.163285	-5.89673	0.373967	0.421897
Neutrophils	RAMAC	0.15596	5.913686	1.405661	0.163329	-5.37681	0.405688	0.457007
Neutrophils	ZFP397	0.280032	3.944609	1.405536	0.163366	-4.8468	0.429079	0.482785
Neutrophils	MEN1	-0.47206	2.866329	-1.40553	0.163369	-4.64533	0.442477	0.49749
Neutrophils	YAP1	-0.7121	1.070556	-1.40529	0.163438	-4.6123	0.465748	0.523036
Neutrophils	ZFP740	-0.23156	4.76726	-1.40529	0.163439	-4.99548	0.41914	0.471925
Neutrophils	FMN1	-0.41185	3.350464	-1.40512	0.163489	-4.93809	0.436434	0.490919
Neutrophils	AGAP2	-0.40168	2.925575	-1.40456	0.163654	-4.71644	0.441991	0.497017
Neutrophils	SNX22	-0.9095	-0.37781	-1.40455	0.163659	-4.47063	0.485707	0.544794
Neutrophils	TLNRD1	-0.33452	4.581796	-1.40382	0.163875	-4.96983	0.422018	0.475009
Neutrophils	D430020J0	0.821514	0.440007	1.403738	0.1639	-4.49445	0.474952	0.533007
Neutrophils	GM15417	0.279377	2.477041	1.403262	0.164041	-5.01797	0.448162	0.503822
Neutrophils	GM13166	-0.85955	-0.19353	-1.40319	0.164062	-4.47096	0.483673	0.542659
Neutrophils	SEC23A	-0.24998	4.425257	-1.40314	0.164077	-5.01706	0.423946	0.477266
Neutrophils	CYP2A22	-0.65912	1.632221	-1.40313	0.16408	-4.60434	0.4591	0.515829
Neutrophils	ARMC9	-0.51375	2.484131	-1.40287	0.164158	-4.55386	0.448092	0.503815
Neutrophils	FGD2	-0.45874	3.69567	-1.40283	0.16417	-4.76612	0.432872	0.487135
Neutrophils	DNAJA4	0.746284	0.616749	1.402344	0.164314	-4.5083	0.472925	0.530782

Neutrophils	PGM3	-0.40826	2.89728	-1.40191	0.164444	-4.61368	0.443357	0.49829
Neutrophils	MCM2	0.436891	4.762793	1.401533	0.164555	-4.97964	0.4205	0.473147
Neutrophils	CYP7B1	0.773264	0.01895	1.401404	0.164594	-4.58031	0.481445	0.539863
Neutrophils	S100A10	-0.324	6.601647	-1.40136	0.164607	-5.23553	0.399072	0.449534
Neutrophils	TRMT44	-0.69453	1.793941	-1.4011	0.164684	-4.50928	0.457748	0.51389
Neutrophils	BHLHE41	0.887935	1.739361	1.400403	0.164892	-4.49943	0.458528	0.514917
Neutrophils	GAA	-0.43912	3.191942	-1.40036	0.164904	-4.67991	0.439911	0.49453
Neutrophils	SPCS3	0.234254	4.704887	1.400202	0.164952	-5.00475	0.421346	0.474138
Neutrophils	GM34466	-0.80068	-1.10689	-1.40018	0.164958	-4.46795	0.497358	0.557252
Neutrophils	VSIG10L	0.500004	0.298869	1.400127	0.164975	-4.58533	0.477783	0.535941
Neutrophils	MOB3B	0.28752	4.961752	1.400103	0.164982	-5.04431	0.418276	0.47076
Neutrophils	NUDT2	-0.39393	3.238769	-1.39989	0.165047	-4.67727	0.439324	0.493991
Neutrophils	ISCA1	0.229267	6.018689	1.399808	0.16507	-5.47122	0.405885	0.457239
Neutrophils	KCNB1	-0.71001	0.988195	-1.39978	0.165079	-4.54691	0.468469	0.525929
Neutrophils	KLHDC3	0.235325	4.338451	1.399282	0.165227	-4.93026	0.425973	0.479175
Neutrophils	HERC3	-0.30418	4.345366	-1.39908	0.165288	-4.95537	0.425889	0.479083
Neutrophils	ZFP84	-0.44443	3.7569	-1.39899	0.165313	-4.68998	0.433091	0.487019
Neutrophils	SLC35C1	0.440725	2.749333	1.398959	0.165324	-4.67983	0.445716	0.500881
Neutrophils	TOGARAM1	-0.21956	5.297825	-1.39869	0.165403	-5.10141	0.41459	0.466655
Neutrophils	ALDH6A1	-0.52695	2.786139	-1.39786	0.165652	-4.73858	0.445911	0.500726
Neutrophils	D1ERTD622	0.152417	5.295192	1.397318	0.165815	-5.49449	0.415446	0.467137
Neutrophils	H2AFX	-0.28836	6.482628	-1.39705	0.165896	-5.52928	0.401715	0.45201
Neutrophils	CEP68	0.297712	4.299523	1.396922	0.165934	-4.97185	0.427459	0.480408
Neutrophils	L3MBTL2	-0.40294	3.445434	-1.39682	0.165964	-4.63168	0.437992	0.491982
Neutrophils	RAB29	0.376457	3.635461	1.39625	0.166135	-4.81236	0.435817	0.489622
Neutrophils	NIPSNAP3B	0.185907	5.573981	1.396177	0.166157	-5.33745	0.412409	0.463917
Neutrophils	PGP	0.190461	5.374059	1.396162	0.166161	-5.40256	0.414761	0.466506
Neutrophils	ST7	0.316742	5.144851	1.395604	0.166329	-4.99618	0.417697	0.469657
Neutrophils	EVI5	-0.22235	5.166502	-1.39559	0.166332	-5.33333	0.41744	0.469374
Neutrophils	AHR	-0.56468	4.719836	-1.39523	0.16644	-4.83085	0.422884	0.475297
Neutrophils	FASTKD3	-0.65298	1.993657	-1.395	0.166511	-4.53867	0.457068	0.512753
Neutrophils	GM5617	0.344125	3.238502	1.394957	0.166524	-4.84185	0.441118	0.495303
Neutrophils	SRSF11	0.097674	7.871425	1.394909	0.166538	-5.69752	0.386657	0.435374
Neutrophils	TXNL4A	-0.2445	4.968748	-1.39443	0.166682	-5.04505	0.420157	0.47211
Neutrophils	CEPT1	0.22052	5.374134	1.394119	0.166775	-5.36995	0.415406	0.466951
Neutrophils	MRPL48	-0.16173	5.282039	-1.39406	0.166793	-5.24684	0.416496	0.46816
Neutrophils	TMEM67	-0.67939	1.688091	-1.39388	0.166848	-4.53017	0.461471	0.517486
Neutrophils	GOLM1	0.367399	3.2957	1.393696	0.166903	-4.78821	0.440817	0.49489
Neutrophils	AURKA	0.367535	3.318106	1.393393	0.166994	-4.88456	0.440667	0.494685
Neutrophils	MYO1B	-0.60652	2.148687	-1.39315	0.167068	-4.65519	0.455649	0.511066
Neutrophils	AOAH	-0.24861	4.784961	-1.39308	0.167089	-5.73366	0.422656	0.474943
Neutrophils	SEC24B	0.159734	6.179882	1.392576	0.16724	-5.5402	0.40648	0.457132
Neutrophils	B4GALT7	-0.44696	2.869796	-1.39219	0.167356	-4.63746	0.446861	0.501345
Neutrophils	CLDND1	0.242016	4.825073	1.39193	0.167435	-4.97662	0.422735	0.47491
Neutrophils	G430095P1	0.688074	0.465518	1.391602	0.167534	-4.52314	0.478768	0.536267
Neutrophils	L2HGDH	-0.62364	1.767651	-1.3916	0.167536	-4.54927	0.461293	0.517213
Neutrophils	DPYS	-0.52594	2.829004	-1.39102	0.167709	-4.79573	0.447882	0.502392

Neutrophils	GDI2	0.111634	8.891579	1.390166	0.167969	-5.8756	0.377287	0.424659
Neutrophils	PIGO	-0.64679	1.804475	-1.39013	0.167978	-4.55256	0.461553	0.517249
Neutrophils	SLC7A6OS	0.25619	4.789888	1.3901	0.167988	-4.97118	0.423887	0.476029
Neutrophils	SP100	-0.16019	7.43248	-1.38972	0.168104	-5.91661	0.393213	0.442396
Neutrophils	CYB5A	-0.26904	7.336341	-1.38961	0.168138	-5.72923	0.394287	0.443582
Neutrophils	GTF2H3	-0.57483	2.299191	-1.3895	0.168171	-4.56881	0.455082	0.51041
Neutrophils	HCFC2	0.263763	3.758586	1.389472	0.168179	-4.98567	0.436527	0.490106
Neutrophils	NCOA4	-0.18548	6.199192	-1.38934	0.168218	-5.68614	0.407228	0.457959
Neutrophils	NDUFA6	0.15225	6.56293	1.38926	0.168243	-5.57877	0.403041	0.45339
Neutrophils	SMC5	-0.24231	5.613071	-1.38919	0.168264	-5.12098	0.414072	0.465559
Neutrophils	CSTF3	0.14597	6.037918	1.388807	0.16838	-5.47797	0.409281	0.460291
Neutrophils	ZFP954	-0.61707	2.050779	-1.38861	0.168438	-4.57482	0.458568	0.514372
Neutrophils	SERTAD2	-0.24605	6.12155	-1.38813	0.168584	-5.32009	0.408511	0.459411
Neutrophils	ABCB10	0.45394	2.977675	1.388121	0.168588	-4.68252	0.446776	0.501426
Neutrophils	GBP6	-0.8456	1.984096	-1.3877	0.168715	-4.61876	0.45984	0.515723
Neutrophils	MBLAC2	0.467829	2.64789	1.387369	0.168816	-4.65582	0.451211	0.506352
Neutrophils	H2AFV	-0.18624	8.095101	-1.3872	0.168869	-5.77473	0.386438	0.435115
Neutrophils	PRKCE	-0.19475	7.233184	-1.38709	0.168902	-5.60504	0.396001	0.44568
Neutrophils	FBXL20	0.292354	5.495646	1.387074	0.168906	-5.22763	0.416043	0.467769
Neutrophils	A930007I1	0.476288	4.385631	1.387038	0.168917	-4.91595	0.4294	0.482449
Neutrophils	GM1673	-0.29031	2.42109	-1.38683	0.168982	-5.01947	0.454202	0.509556
Neutrophils	CD36	-0.33752	5.712063	-1.38663	0.169042	-5.42645	0.413592	0.464967
Neutrophils	SLC35A5	0.155172	3.986264	1.386385	0.169116	-5.35231	0.434504	0.487893
Neutrophils	MRPL10	0.249616	4.602348	1.385835	0.169283	-5.09061	0.427261	0.479717
Neutrophils	RINL	0.244281	3.632551	1.38564	0.169342	-5.15596	0.439243	0.492849
Neutrophils	FHIT	-0.35182	6.167904	-1.38555	0.16937	-5.05993	0.408655	0.459266
Neutrophils	TRIM13	0.776459	0.46615	1.385029	0.169528	-4.51671	0.481109	0.538472
Neutrophils	HMG20A	-0.24267	4.897858	-1.3849	0.169567	-5.11156	0.423975	0.476048
Neutrophils	WDR49	-0.82462	0.34569	-1.38474	0.169618	-4.50152	0.482792	0.540359
Neutrophils	SLIT1	-0.64391	1.464859	-1.38445	0.169705	-4.59765	0.467732	0.523856
Neutrophils	ACVR2B	-0.74352	1.53111	-1.38389	0.169877	-4.52364	0.467207	0.523165
Neutrophils	2610021A0	-0.63145	2.362444	-1.38374	0.169922	-4.52913	0.456258	0.511278
Neutrophils	HIPK1	-0.14173	6.269668	-1.38359	0.169969	-5.69214	0.408212	0.458633
Neutrophils	ADHFE1	-0.50061	2.690792	-1.38329	0.170058	-4.67824	0.452143	0.506751
Neutrophils	GOLIM4	-0.16739	5.140893	-1.38277	0.170219	-5.69853	0.421945	0.473526
Neutrophils	VPS18	0.203122	4.834066	1.38224	0.17038	-5.23213	0.425944	0.477843
Neutrophils	TBC1D2B	0.220543	4.091379	1.382014	0.170449	-5.26519	0.435121	0.487911
Neutrophils	CCNO	-0.36606	0.174468	-1.38171	0.170542	-4.95035	0.486625	0.544181
Neutrophils	5330417C2	-0.71758	0.541978	-1.38164	0.170564	-4.56643	0.481544	0.538657
Neutrophils	LSM7	-0.21159	6.346432	-1.38155	0.17059	-5.23285	0.408111	0.458335
Neutrophils	P4HB	0.132965	7.267668	1.381396	0.170638	-5.69748	0.397587	0.446775
Neutrophils	GIMAP3	-0.9282	2.932139	-1.38115	0.170713	-4.65006	0.449888	0.504172
Neutrophils	4933408B1	0.391676	1.941966	1.380843	0.170808	-4.83884	0.462865	0.518403
Neutrophils	HEMK1	0.641744	1.147753	1.380777	0.170828	-4.55808	0.47348	0.530002
Neutrophils	LRRC40	-0.37891	3.249731	-1.38048	0.170919	-4.75204	0.446041	0.499985
Neutrophils	RSRC1	0.180683	6.605429	1.380226	0.170997	-5.37847	0.405415	0.455395
Neutrophils	CD63	0.204573	4.105165	1.380191	0.171008	-5.57404	0.435307	0.488222

Neutrophils	ADAP1	0.243063	4.152492	1.379655	0.171172	-5.16108	0.434835	0.487771
Neutrophils	TMBIM4	0.134469	6.749974	1.379598	0.17119	-5.71124	0.403861	0.453745
Neutrophils	MLLT1	-0.42704	2.735176	-1.37954	0.171206	-4.63713	0.452768	0.507393
Neutrophils	DCBLD1	-0.34934	3.33755	-1.37946	0.171233	-4.99424	0.445055	0.499007
Neutrophils	TTLL11	0.527757	1.608719	1.379362	0.171262	-4.62785	0.467561	0.523619
Neutrophils	GM17259	0.719677	1.365713	1.378817	0.17143	-4.53579	0.471127	0.527343
Neutrophils	SMYD1	0.881918	0.29641	1.378721	0.171459	-4.49824	0.485736	0.543292
Neutrophils	KIF5A	-0.89759	0.429339	-1.37781	0.171741	-4.50007	0.484572	0.541747
Neutrophils	HES6	-0.33953	4.105884	-1.37766	0.171785	-4.81738	0.436315	0.489159
Neutrophils	NSD1	0.133776	7.273059	1.377116	0.171953	-5.58369	0.398859	0.448002
Neutrophils	ADAMTS10	-0.414	3.015513	-1.37705	0.171972	-4.70808	0.450236	0.504371
Neutrophils	PNRC2	0.191322	5.35165	1.376934	0.172009	-5.26141	0.421243	0.472619
Neutrophils	NAA80	0.447383	2.402845	1.376931	0.17201	-4.64781	0.458175	0.51304
Neutrophils	AMZ2	-0.30573	3.901939	-1.37673	0.172071	-4.83642	0.438999	0.492111
Neutrophils	HCFC1	-0.26495	4.949877	-1.37667	0.172092	-5.04521	0.426088	0.477967
Neutrophils	PRDM9	0.689228	1.146615	1.37644	0.172161	-4.53656	0.474934	0.531455
Neutrophils	TMF1	0.148028	6.243916	1.37627	0.172214	-5.4487	0.410715	0.46121
Neutrophils	SLC6A6	0.125246	7.287991	1.376223	0.172228	-5.8639	0.398716	0.447997
Neutrophils	ADCY10	-0.5211	0.912909	-1.37579	0.172363	-4.70301	0.478363	0.535179
Neutrophils	CCDC146	0.33629	2.867299	1.37566	0.172402	-5.05083	0.452407	0.506956
Neutrophils	SLC19A2	-0.53477	1.834884	-1.37525	0.172527	-4.60461	0.466159	0.521824
Neutrophils	GM16083	-0.50279	1.977218	-1.37504	0.172592	-4.72667	0.464332	0.519778
Neutrophils	SRSF6	-0.1652	6.210068	-1.37448	0.172767	-5.45555	0.411894	0.46229
Neutrophils	PTBP3	0.096583	8.46802	1.374219	0.172847	-5.94742	0.386374	0.434144
Neutrophils	NDUFV3	0.149314	7.098589	1.374147	0.17287	-5.71055	0.401672	0.451017
Neutrophils	UBE2W	0.106889	6.081677	1.37364	0.173027	-5.49526	0.413719	0.464137
Neutrophils	1810009A1	0.425798	2.243882	1.373317	0.173126	-4.65021	0.461655	0.516593
Neutrophils	SEPHS2	0.18554	6.39868	1.373205	0.173161	-5.73088	0.410128	0.460228
Neutrophils	PLCL2	-0.14311	6.985417	-1.37303	0.173215	-5.73705	0.403377	0.452809
Neutrophils	SLC12A7	-0.51079	3.37429	-1.37288	0.173263	-4.66915	0.44705	0.500735
Neutrophils	RRAS2	-0.38444	5.198697	-1.3727	0.173319	-4.86686	0.424442	0.47604
Neutrophils	GM47230	0.595102	0.668652	1.372397	0.173411	-4.62264	0.482981	0.540128
Neutrophils	TREML4	-0.20444	2.758058	-1.37233	0.173431	-5.63081	0.455015	0.509652
Neutrophils	UBE2D1	0.175711	5.377113	1.372287	0.173446	-5.22473	0.422294	0.473837
Neutrophils	GCH1	0.212567	5.663529	1.371874	0.173574	-5.47157	0.419074	0.4702
Neutrophils	FAM49B	0.095467	9.086243	1.371601	0.173658	-6.05669	0.380297	0.427567
Neutrophils	TARBP1	-0.64234	2.687423	-1.3716	0.17366	-4.55141	0.456164	0.510918
Neutrophils	HEATR1	-0.26615	4.84445	-1.37131	0.173749	-5.1419	0.429079	0.481164
Neutrophils	PRDX1	-0.16345	9.719927	-1.37099	0.173848	-5.96931	0.373755	0.420173
Neutrophils	GTPBP3	-0.61961	2.225197	-1.37085	0.173891	-4.54317	0.462482	0.517663
Neutrophils	SEC23B	0.221326	5.223375	1.370632	0.173959	-5.07031	0.4246	0.47632
Neutrophils	CSNK1G3	-0.16161	6.624241	-1.3706	0.173969	-5.43726	0.40802	0.45811
Neutrophils	SIPA1L1	-0.18359	7.327522	-1.37038	0.174039	-5.86455	0.400018	0.449322
Neutrophils	CSNK1A1	0.098128	7.684452	1.369627	0.174272	-5.75048	0.396335	0.445197
Neutrophils	CCT2	-0.16909	6.286032	-1.36947	0.174322	-5.35756	0.412386	0.46287
Neutrophils	RHAG	0.936681	-0.3642	1.369426	0.174335	-4.52802	0.498491	0.556903
Neutrophils	LGALS8	-0.22553	5.110017	-1.36934	0.17436	-5.21765	0.426413	0.478276

Neutrophils	GNPDA1	0.267199	4.395098	1.369189	0.174408	-5.03672	0.435194	0.487993
Neutrophils	ATXN2	-0.17617	6.28335	-1.36906	0.174449	-5.39978	0.412428	0.463005
Neutrophils	CKB	-0.37909	4.578345	-1.36859	0.174594	-5.06686	0.433177	0.485752
Neutrophils	FBF1	-0.72941	1.539498	-1.36838	0.174662	-4.61692	0.472395	0.528628
Neutrophils	CCDC32	-0.40052	3.31271	-1.36833	0.174675	-4.72986	0.449087	0.503199
Neutrophils	ALG6	0.611465	2.044219	1.367642	0.174891	-4.5926	0.46583	0.521565
Neutrophils	MFSD14B	-0.10674	5.841678	-1.36757	0.174914	-5.81944	0.418052	0.469258
Neutrophils	SERPINB6A	-0.22924	5.570318	-1.36755	0.17492	-5.41739	0.421291	0.472817
Neutrophils	EMP1	0.793807	1.978152	1.367498	0.174935	-4.61481	0.466709	0.522538
Neutrophils	INPPL1	-0.47343	2.471527	-1.36742	0.174959	-4.63388	0.460184	0.515422
Neutrophils	GM16268	-0.71323	0.706579	-1.36708	0.175066	-4.57238	0.483989	0.54142
Neutrophils	GM26885	0.429232	2.317843	1.367042	0.175078	-4.86348	0.462229	0.517709
Neutrophils	RAB5IF	0.113227	7.307335	1.366477	0.175255	-5.86012	0.401023	0.450831
Neutrophils	NPC1	0.276595	4.971884	1.366433	0.175268	-5.1001	0.428548	0.481124
Neutrophils	GM22146	0.475004	2.073446	1.366383	0.175284	-4.72569	0.465465	0.521536
Neutrophils	EP400	-0.1291	6.870294	-1.36626	0.175321	-5.60247	0.406029	0.456376
Neutrophils	MRPL2	-0.28697	4.862057	-1.36617	0.17535	-5.02423	0.42989	0.482651
Neutrophils	GRPEL2	-0.39672	3.176522	-1.36604	0.17539	-4.69633	0.451043	0.505859
Neutrophils	SRRM1	0.111082	7.865839	1.365905	0.175433	-5.7444	0.394721	0.443958
Neutrophils	BRI3	0.134882	7.328714	1.365866	0.175446	-5.76749	0.40078	0.450645
Neutrophils	FRG2F1	-0.69525	0.702787	-1.36579	0.175468	-4.51244	0.484042	0.541879
Neutrophils	EHHADH	-0.64497	1.66442	-1.36577	0.175474	-4.59794	0.470931	0.527593
Neutrophils	GATA4	-0.43943	1.621315	-1.36545	0.175575	-4.7803	0.471584	0.528358
Neutrophils	MICAL2	-0.55207	1.966497	-1.36542	0.175586	-4.71203	0.466959	0.523327
Neutrophils	GM20682	0.55928	1.711443	1.365219	0.175648	-4.57058	0.470426	0.527103
Neutrophils	RIOX1	-0.28597	3.521185	-1.36479	0.175781	-4.89727	0.446908	0.501336
Neutrophils	GM16740	0.403352	2.544974	1.364753	0.175794	-4.76208	0.459527	0.515155
Neutrophils	CALML4	-0.60218	1.801954	-1.36443	0.175896	-4.67447	0.469535	0.526082
Neutrophils	NKIRAS2	0.243161	4.042562	1.363858	0.176075	-5.03518	0.440773	0.494398
Neutrophils	GPANK1	0.295167	3.300008	1.363606	0.176154	-4.90163	0.450203	0.504766
Neutrophils	ZFP869	0.268068	4.026546	1.363515	0.176182	-5.06128	0.440974	0.494663
Neutrophils	TAF1D	-0.16925	6.027224	-1.3635	0.176188	-5.35427	0.416563	0.467865
Neutrophils	ITPK1	0.211877	5.403224	1.363218	0.176275	-5.17739	0.424129	0.476162
Neutrophils	PHF20	-0.15629	6.472356	-1.36284	0.176394	-5.4616	0.411608	0.462329
Neutrophils	NDUFAF8	0.211861	5.052226	1.362337	0.176552	-5.1165	0.428853	0.481173
Neutrophils	GM14221	-0.62663	1.910062	-1.36215	0.176611	-4.71527	0.469045	0.525248
Neutrophils	EIF2D	-0.34529	3.5699	-1.36178	0.176727	-4.79084	0.447351	0.501659
Neutrophils	MUS81	0.543883	2.048507	1.361718	0.176747	-4.61655	0.467195	0.523346
Neutrophils	GM32569	0.944769	1.845691	1.361687	0.176757	-4.54544	0.469907	0.526305
Neutrophils	BRCC3	0.237629	4.874732	1.361664	0.176764	-5.08251	0.431025	0.483766
Neutrophils	MTG1	0.64296	2.186733	1.361191	0.176913	-4.57753	0.465605	0.521593
Neutrophils	NDUFAF6	-0.46123	2.973661	-1.36109	0.176943	-4.64771	0.455267	0.51033
Neutrophils	SNX6	0.121479	6.568189	1.360853	0.177019	-5.58335	0.411056	0.461768
Neutrophils	BPTF	-0.14251	7.356344	-1.36036	0.177173	-5.66259	0.401961	0.451908
Neutrophils	MSI2	-0.20776	7.509903	-1.36034	0.177181	-5.36698	0.400214	0.44998
Neutrophils	FBXO45	-0.29821	3.654199	-1.36015	0.177242	-4.90453	0.446602	0.50097
Neutrophils	TCF3	-0.22117	6.601305	-1.36012	0.17725	-5.32012	0.41067	0.461515

Neutrophils	CRNKL1	-0.18338	5.293781	-1.35981	0.177348	-5.26495	0.426225	0.478751
Neutrophils	GM34095	-0.92474	-0.39051	-1.3598	0.177351	-4.49962	0.50126	0.560726
Neutrophils	CTH	0.495204	3.512521	1.359756	0.177365	-5.01923	0.44841	0.503087
Neutrophils	ACOX1	-0.19223	5.892871	-1.35973	0.177374	-5.50563	0.419023	0.470842
Neutrophils	GM26810	0.917119	0.423092	1.359592	0.177416	-4.51425	0.489746	0.548322
Neutrophils	OASL2	0.497356	3.934807	1.359504	0.177444	-5.32023	0.443044	0.497327
Neutrophils	GM31814	0.276625	-0.06084	1.35936	0.17749	-5.03217	0.496562	0.555806
Neutrophils	ARL5A	0.230096	5.831093	1.359175	0.177548	-5.18058	0.41976	0.471828
Neutrophils	QPRT	-0.50368	2.660001	-1.3591	0.177571	-4.78729	0.459447	0.51541
Neutrophils	MRPL34	0.185975	5.428125	1.358794	0.177668	-5.22078	0.424731	0.477322
Neutrophils	HECTD4	0.232272	5.548933	1.358509	0.177758	-5.17713	0.423387	0.47578
Neutrophils	SDC3	-0.34948	4.594842	-1.35774	0.178001	-5.28442	0.435496	0.488824
Neutrophils	A430018G1	-0.59875	1.329295	-1.35762	0.17804	-4.54654	0.478008	0.535309
Neutrophils	EDEM3	-0.28219	5.974164	-1.35752	0.178071	-5.27092	0.418733	0.470409
Neutrophils	TYW1	-0.35954	3.875123	-1.35686	0.17828	-4.8137	0.44491	0.498815
Neutrophils	UBE2Z	0.166897	5.437928	1.356756	0.178313	-5.30815	0.425543	0.477594
Neutrophils	MYB	-0.29879	5.137938	-1.35648	0.178401	-5.19121	0.429302	0.481722
Neutrophils	SCNN1A	0.314072	0.542515	1.356243	0.178475	-5.14072	0.489513	0.547511
Neutrophils	LEAP2	0.468277	3.060255	1.356082	0.178526	-4.95138	0.455588	0.510514
Neutrophils	ZFP467	-0.40248	2.303773	-1.35593	0.178573	-4.81824	0.46554	0.521402
Neutrophils	EMG1	-0.21905	5.937655	-1.35516	0.178818	-5.23476	0.420226	0.471533
Neutrophils	SHISA5	0.24	6.572889	1.354865	0.178912	-5.53022	0.412827	0.463335
Neutrophils	SLC30A9	0.187937	5.265307	1.354266	0.179103	-5.23237	0.428752	0.480662
Neutrophils	SMNDC1	0.129051	6.059801	1.354179	0.17913	-5.49162	0.419172	0.470146
Neutrophils	AP2S1	0.12466	6.932961	1.354084	0.179161	-5.72013	0.408902	0.458883
Neutrophils	TRAF5	-0.32781	5.308971	-1.35374	0.179269	-4.95981	0.428356	0.480289
Neutrophils	DCTN1	0.16999	4.987161	1.353636	0.179303	-5.31638	0.432298	0.484651
Neutrophils	TMEM181A	0.279086	4.58903	1.353416	0.179373	-4.91615	0.437241	0.490141
Neutrophils	LILR4B	0.202748	3.079256	1.353349	0.179394	-5.77738	0.456468	0.511203
Neutrophils	SNTB2	0.161603	5.607555	1.35306	0.179486	-5.73225	0.424783	0.47658
Neutrophils	EFCAB5	-0.56615	0.504613	-1.35292	0.179531	-4.6682	0.49132	0.549286
Neutrophils	TBP	-0.42206	3.21522	-1.35276	0.179583	-4.74476	0.454739	0.509485
Neutrophils	FBXW11	0.175347	7.259166	1.352691	0.179603	-5.65834	0.40531	0.455269
Neutrophils	SPATS2	-0.45407	2.720159	-1.35252	0.179659	-4.81234	0.461206	0.516649
Neutrophils	MRPL21	-0.24559	5.233418	-1.35241	0.179692	-5.11163	0.429329	0.481777
Neutrophils	GM49521	0.725394	0.473447	1.352365	0.179708	-4.53653	0.491758	0.549989
Neutrophils	NCR1	-0.80166	1.170957	-1.35161	0.179949	-4.62899	0.48239	0.539665
Neutrophils	NR1D1	-0.75506	1.661874	-1.35155	0.179966	-4.60451	0.475675	0.532356
Neutrophils	B020010K1	0.680351	1.134374	1.351552	0.179966	-4.6037	0.482894	0.540217
Neutrophils	TRIM69	-0.52745	2.05026	-1.35144	0.180002	-4.73192	0.470431	0.526639
Neutrophils	TAF9B	0.742524	0.956005	1.351171	0.180088	-4.53966	0.485478	0.542999
Neutrophils	PCYT1A	0.210179	5.255992	1.350749	0.180223	-5.37259	0.429638	0.481843
Neutrophils	MOB1A	-0.13533	5.970142	-1.35066	0.180251	-5.57348	0.420998	0.472392
Neutrophils	NEK1	-0.45404	3.697931	-1.35035	0.180349	-4.75244	0.449175	0.503324
Neutrophils	9230114K1	-0.35812	2.574674	-1.35034	0.180353	-4.7623	0.463798	0.519303
Neutrophils	APBB3	0.516835	1.534528	1.34992	0.180487	-4.6612	0.478017	0.534772
Neutrophils	WHRN	-0.68359	3.066048	-1.34976	0.180538	-4.58142	0.457594	0.512502

Neutrophils	PACS2	0.260553	4.036916	1.349559	0.180603	-4.98181	0.445157	0.49884
Neutrophils	DPH2	-0.85742	0.879573	-1.34926	0.180698	-4.51432	0.487264	0.544688
Neutrophils	ADAR	0.286632	4.311216	1.349123	0.180742	-5.08561	0.441823	0.495143
Neutrophils	PTPN13	-0.88867	0.415506	-1.34856	0.180924	-4.5266	0.494148	0.552057
Neutrophils	CAP1	0.102857	6.878742	1.348299	0.181006	-5.86664	0.411111	0.461333
Neutrophils	AV099323	0.729817	1.209882	1.348044	0.181088	-4.57214	0.483273	0.54025
Neutrophils	VKORC1	-0.31356	4.701765	-1.34725	0.181341	-5.08931	0.437843	0.490477
Neutrophils	GPR65	0.341318	4.250918	1.347016	0.181417	-5.1723	0.443502	0.496767
Neutrophils	STX6	0.220434	5.2207	1.347002	0.181422	-5.21468	0.431422	0.483535
Neutrophils	CXCL16	-0.44605	3.468405	-1.3469	0.181455	-4.9552	0.453505	0.507732
Neutrophils	OTC	-0.47241	3.438444	-1.34688	0.181461	-5.01745	0.453893	0.508155
Neutrophils	NUDT14	-0.3052	4.342372	-1.34632	0.181642	-4.85361	0.442686	0.495823
Neutrophils	ECSCR	0.536587	1.207762	1.345856	0.18179	-4.72362	0.484375	0.541102
Neutrophils	PHRF1	0.189789	5.804424	1.345675	0.181848	-5.26031	0.424923	0.47619
Neutrophils	COPS2	0.162111	6.020343	1.345277	0.181976	-5.31633	0.422349	0.473486
Neutrophils	RAE1	-0.28336	4.525674	-1.34505	0.182049	-4.92604	0.440701	0.49363
Neutrophils	ITM2C	0.265356	5.637577	1.344944	0.182083	-5.19707	0.426971	0.478584
Neutrophils	KLHL22	-0.51594	3.001151	-1.34494	0.182084	-4.6331	0.460274	0.515024
Neutrophils	RNPS1	-0.16133	6.167896	-1.34492	0.18209	-5.37674	0.420581	0.471569
Neutrophils	PDHX	-0.35183	3.395129	-1.3448	0.182129	-4.73005	0.455132	0.509437
Neutrophils	XRCC3	-0.76342	0.159666	-1.34471	0.18216	-4.51828	0.499167	0.557385
Neutrophils	CLUAP1	0.284577	3.885137	1.344146	0.18234	-4.89845	0.44908	0.502725
Neutrophils	GM27017	0.36199	3.311249	1.344108	0.182352	-4.91358	0.456488	0.510829
Neutrophils	CYP2D10	-0.58387	1.738911	-1.34322	0.182639	-4.66966	0.478072	0.533993
Neutrophils	IGHMBP2	-0.46788	2.850148	-1.34284	0.182762	-4.64134	0.463296	0.517915
Neutrophils	PDLIM7	0.193612	3.638312	1.342755	0.182788	-5.3637	0.452999	0.506694
Neutrophils	PLXNA4OS1	0.717838	0.51532	1.34264	0.182826	-4.57301	0.495229	0.552674
Neutrophils	UGT1A7C	0.508932	-0.45047	1.342325	0.182927	-4.7105	0.509112	0.567914
Neutrophils	GPSM2	-0.49233	2.440784	-1.34225	0.182953	-4.71987	0.468768	0.524093
Neutrophils	ZFP385A	-0.37419	3.938147	-1.34221	0.182966	-4.84178	0.449171	0.502711
Neutrophils	TMEM63B	0.349167	3.925117	1.340788	0.183424	-4.91479	0.450339	0.503642
Neutrophils	2610318N(	-0.91415	1.172871	-1.34068	0.183459	-4.52768	0.487128	0.543718
Neutrophils	TTC27	-0.46522	3.520631	-1.34044	0.183538	-4.67806	0.455648	0.509442
Neutrophils	TRIM7	0.814508	0.591087	1.340256	0.183596	-4.53089	0.495425	0.552681
Neutrophils	OSGIN1	-0.23218	3.869004	-1.34002	0.183672	-5.3973	0.451264	0.504534
Neutrophils	FLI1	-0.13949	8.191063	-1.3396	0.183807	-5.88658	0.399293	0.447503
Neutrophils	CDC25A	0.270351	3.793744	1.339213	0.183934	-5.1216	0.452666	0.505911
Neutrophils	VPS8	0.253182	4.586793	1.33904	0.18399	-5.10974	0.442583	0.494865
Neutrophils	PI4KB	0.174282	5.414205	1.338859	0.184049	-5.32241	0.432299	0.483614
Neutrophils	NQO1	0.522968	0.792254	1.338719	0.184094	-4.71748	0.493203	0.549991
Neutrophils	WBP2	0.184793	5.599979	1.338498	0.184166	-5.38339	0.43002	0.481225
Neutrophils	GM41790	-0.86159	-0.09939	-1.33848	0.184171	-4.55178	0.505924	0.563876
Neutrophils	HMGB1	-0.14821	9.807497	-1.33792	0.184353	-6.09128	0.381947	0.428194
Neutrophils	EIF4G3	0.114449	7.91677	1.33758	0.184464	-5.84952	0.403091	0.451409
Neutrophils	MED10	-0.2432	4.886924	-1.33734	0.184542	-5.07421	0.439412	0.491286
Neutrophils	ZMYM1	-0.43282	3.329996	-1.33711	0.184618	-4.70526	0.459427	0.513109
Neutrophils	HS2ST1	-0.23631	5.689893	-1.33665	0.184767	-5.11291	0.429808	0.480695

Neutrophils	GNGT2	0.266103	5.675602	1.336122	0.184938	-5.44199	0.43028	0.481085
Neutrophils	ABHD17C	-0.2281	4.833665	-1.33541	0.185169	-5.21728	0.441161	0.492746
Neutrophils	ABAT	0.645608	1.196429	1.335164	0.18525	-4.66397	0.489483	0.545326
Neutrophils	SMAD2	-0.13058	6.426685	-1.33482	0.185362	-5.56482	0.421834	0.471551
Neutrophils	IGFBP4	-0.38786	5.679023	-1.33472	0.185394	-5.56902	0.430896	0.481494
Neutrophils	OAS1C	0.600916	1.903745	1.333633	0.18575	-4.66096	0.480484	0.535254
Neutrophils	EGLN3	0.371409	3.294296	1.333503	0.185792	-5.0877	0.461793	0.515014
Neutrophils	PRPS1	-0.41166	3.621673	-1.33326	0.185873	-4.74713	0.457503	0.510412
Neutrophils	DHPS	-0.2643	4.486343	-1.33313	0.185913	-4.96533	0.446367	0.498313
Neutrophils	F13B	-0.60727	1.463355	-1.333	0.185955	-4.6625	0.486563	0.542067
Neutrophils	GM32401	0.280064	2.445198	1.33295	0.185973	-5.14861	0.473115	0.527482
Neutrophils	GJB1	-0.59586	1.59761	-1.33289	0.185992	-4.69573	0.484701	0.54009
Neutrophils	LAMTOR1	0.119252	6.503458	1.332853	0.186005	-5.68421	0.421474	0.471168
Neutrophils	P4HA3	-0.82168	0.308298	-1.33276	0.186036	-4.53099	0.50288	0.559804
Neutrophils	ZFP458	0.796854	0.876726	1.332583	0.186093	-4.57098	0.494782	0.551103
Neutrophils	CFHR1	-0.8085	0.449486	-1.33251	0.186118	-4.592	0.500856	0.557721
Neutrophils	BMI1	0.395317	3.545427	1.33203	0.186274	-4.76672	0.458776	0.511914
Neutrophils	CCDC85B	-0.25919	3.779227	-1.33167	0.186392	-4.97982	0.45591	0.508718
Neutrophils	SCAI	-0.30248	4.434969	-1.33097	0.186619	-5.05798	0.447913	0.499735
Neutrophils	MYO1D	0.204813	3.025547	1.330467	0.186786	-5.35359	0.466438	0.52006
Neutrophils	ZFP637	-0.54153	2.315939	-1.33039	0.186811	-4.64244	0.475978	0.530449
Neutrophils	DNASE1L3	-0.27931	6.30437	-1.33017	0.186884	-5.66259	0.424859	0.474769
Neutrophils	S100A16	-0.32797	2.552707	-1.32992	0.186966	-4.96441	0.472773	0.527144
Neutrophils	ZFP438	0.381853	3.204144	1.329785	0.18701	-4.79376	0.464068	0.517695
Neutrophils	DNAH10	-0.82905	0.024576	-1.32976	0.187019	-4.53991	0.508161	0.565527
Neutrophils	GM34084	0.253484	1.964286	1.329679	0.187044	-5.56247	0.480779	0.535881
Neutrophils	TMEM192	-0.29488	4.694631	-1.32966	0.187051	-4.93294	0.444768	0.496678
Neutrophils	CLDN34C1	0.746139	0.809812	1.329426	0.187128	-4.57531	0.496891	0.553479
Neutrophils	RAD23A	0.242424	5.807004	1.329299	0.187169	-5.2462	0.430909	0.481674
Neutrophils	FKBP4	0.238549	5.691682	1.329198	0.187203	-5.16664	0.432325	0.483258
Neutrophils	PPP1CC	0.116383	7.584523	1.329118	0.187229	-5.80448	0.409695	0.458453
Neutrophils	TUBGCP4	0.277859	4.42498	1.329053	0.18725	-5.00404	0.448197	0.500609
Neutrophils	TMEM163	-0.373	6.026941	-1.3284	0.187464	-5.1278	0.428608	0.478988
Neutrophils	STMN1	-0.32217	7.897729	-1.32827	0.187506	-5.83241	0.406438	0.454689
Neutrophils	SPACA6	0.536897	1.094819	1.327896	0.187631	-4.67491	0.493435	0.549509
Neutrophils	KDM4B	-0.39626	4.441359	-1.32778	0.187668	-4.84378	0.448508	0.500667
Neutrophils	HES7	-0.77544	-0.49621	-1.32773	0.187685	-4.53493	0.516375	0.574333
Neutrophils	NEK10	-0.79551	1.324395	-1.32705	0.187909	-4.58265	0.49042	0.546279
Neutrophils	GM13391	0.64676	0.765079	1.326816	0.187986	-4.60236	0.498316	0.554855
Neutrophils	CSNK1E	0.187372	4.953409	1.326735	0.188013	-5.41055	0.442204	0.493852
Neutrophils	NDUFC2	-0.1743	6.741013	-1.32662	0.188052	-5.54597	0.420287	0.46991
Neutrophils	GM17435	-0.52703	1.133905	-1.32657	0.188067	-4.67903	0.493095	0.549258
Neutrophils	PYCRL	-0.39201	3.541294	-1.32654	0.188076	-4.73168	0.460357	0.51369
Neutrophils	PSPC1	-0.20462	5.702286	-1.3265	0.188089	-5.35791	0.43288	0.4837
Neutrophils	CNTROB	-0.59734	2.044211	-1.32643	0.188113	-4.61737	0.480445	0.535547
Neutrophils	SPTSSA	-0.15211	6.822761	-1.32615	0.188204	-5.65312	0.419418	0.468975
Neutrophils	TLE4	-0.1358	7.814076	-1.32552	0.188415	-5.8779	0.408147	0.456356

Neutrophils	P2RY14	-0.62031	3.467146	-1.32534	0.188474	-4.68976	0.461896	0.51508
Neutrophils	A430035B1	-0.67622	3.071834	-1.32509	0.188555	-4.64511	0.467224	0.520839
Neutrophils	TTYH3	0.207978	5.141527	1.324918	0.188612	-5.34678	0.440499	0.491728
Neutrophils	ZFP507	-0.5635	2.176566	-1.32462	0.188711	-4.66992	0.479487	0.534046
Neutrophils	QTRT1	-0.37807	4.980734	-1.32385	0.188964	-5.0438	0.442861	0.494309
Neutrophils	TEX2	-0.2054	6.723879	-1.32379	0.188984	-5.50784	0.421445	0.470914
Neutrophils	MBNL3	-0.19761	4.597519	-1.32367	0.189025	-5.47957	0.44772	0.499686
Neutrophils	FBXO22	0.231136	4.616588	1.323654	0.18903	-5.08179	0.447477	0.49942
Neutrophils	ITGA1	-0.36712	3.828562	-1.32342	0.189107	-5.11871	0.457637	0.510496
Neutrophils	MFF	0.140229	5.906654	1.323355	0.189129	-5.5386	0.431347	0.481802
Neutrophils	DLG1	-0.12821	7.219142	-1.32333	0.189139	-5.75078	0.41556	0.464515
Neutrophils	GM8369	-0.70905	3.324129	-1.32316	0.189192	-4.66285	0.464266	0.517767
Neutrophils	PLBD2	-0.32133	4.310294	-1.32305	0.189229	-4.88156	0.451398	0.503776
Neutrophils	ZFP710	-0.20117	6.297621	-1.32303	0.189238	-5.56466	0.426579	0.47666
Neutrophils	CCND1	-0.39167	4.140959	-1.32257	0.189389	-5.02946	0.453702	0.506294
Neutrophils	MOAP1	0.536074	0.547771	1.322507	0.18941	-4.59723	0.502689	0.559498
Neutrophils	PIK3R6	0.327321	2.596377	1.322183	0.189518	-5.21778	0.474132	0.528628
Neutrophils	SNHG12	-0.43898	3.189103	-1.32215	0.189528	-4.73877	0.466182	0.519983
Neutrophils	LRRC20	0.556871	2.057401	1.322124	0.189537	-4.63399	0.481481	0.536611
Neutrophils	C6	-0.55713	2.802964	-1.32208	0.189551	-4.954	0.471345	0.525599
Neutrophils	GM3435	-0.8234	0.205181	-1.32185	0.18963	-4.56044	0.507639	0.564988
Neutrophils	PLTP	-0.38868	5.244881	-1.3217	0.189677	-5.37992	0.439668	0.491121
Neutrophils	RUFY2	-0.42855	2.954919	-1.32168	0.189684	-4.69969	0.469313	0.523443
Neutrophils	RNF125	0.232392	3.852901	1.32147	0.189754	-5.38983	0.457512	0.510567
Neutrophils	ASNA1	0.243095	4.877847	1.320961	0.189923	-5.10025	0.44464	0.496393
Neutrophils	PRRT1	-0.80292	0.466797	-1.32062	0.190036	-4.54349	0.504331	0.561332
Neutrophils	GM15478	0.242593	4.735611	1.32062	0.190036	-5.22085	0.446505	0.498495
Neutrophils	CD3EAP	0.415772	3.231663	1.319901	0.190276	-4.7763	0.466417	0.520009
Neutrophils	FNIP1	0.134361	7.760209	1.319832	0.190298	-5.95134	0.410043	0.458433
Neutrophils	UBIAD1	-0.46971	1.941616	-1.31979	0.190314	-4.62199	0.483905	0.539023
Neutrophils	MEA1	0.177556	5.276389	1.319338	0.190463	-5.36908	0.440262	0.491428
Neutrophils	SLC30A6	0.339824	3.417011	1.319211	0.190505	-4.84049	0.464211	0.517594
Neutrophils	KLHL9	0.222634	4.65586	1.319016	0.19057	-5.18249	0.448158	0.500083
Neutrophils	LIN54	0.229584	6.14118	1.31878	0.190648	-5.42695	0.429637	0.479906
Neutrophils	ATG3	0.109453	6.261249	1.31862	0.190702	-5.71426	0.428173	0.478349
Neutrophils	CFAP298	0.366479	3.642504	1.31859	0.190712	-4.81441	0.461313	0.514523
Neutrophils	USP48	-0.20068	5.391239	-1.3184	0.190775	-5.20982	0.438944	0.490103
Neutrophils	PHYHD1	-0.4525	2.730001	-1.31789	0.190946	-4.75937	0.473677	0.527927
Neutrophils	AP4E1	-0.45488	3.305374	-1.31787	0.19095	-4.75105	0.465967	0.519543
Neutrophils	KDM5C	-0.20901	6.827805	-1.31779	0.190979	-5.64027	0.421516	0.471017
Neutrophils	MAB21L3	-0.78935	-0.30498	-1.31758	0.191049	-4.61176	0.516555	0.574497
Neutrophils	UPF1	0.170511	5.35091	1.317542	0.191061	-5.25291	0.439589	0.490893
Neutrophils	C2CD3	-0.25636	4.657214	-1.31731	0.191138	-4.96045	0.448356	0.500597
Neutrophils	MXD3	-0.64243	1.735683	-1.3173	0.191143	-4.6422	0.487311	0.542986
Neutrophils	ZFP112	0.799615	0.391108	1.316996	0.191243	-4.55368	0.506531	0.563706
Neutrophils	OSBPL3	-0.40677	3.353205	-1.31677	0.191319	-4.87925	0.465465	0.519178
Neutrophils	ART2B	-1.08119	0.097769	-1.31674	0.191329	-4.54667	0.510793	0.568348

Neutrophils	ACSS1	-0.49226	3.946222	-1.31604	0.191563	-4.74409	0.458116	0.510958
Neutrophils	ELOVL5	-0.19624	6.195778	-1.31583	0.191633	-5.48579	0.42976	0.480038
Neutrophils	TMEM237	-0.59043	2.157499	-1.31458	0.192052	-4.64886	0.483103	0.537689
Neutrophils	TIMM44	-0.2295	4.84696	-1.31394	0.192266	-5.0696	0.447674	0.499083
Neutrophils	ENY2	-0.18518	5.972518	-1.31387	0.19229	-5.36948	0.433565	0.483687
Neutrophils	SLC26A10	-0.62527	0.914242	-1.31386	0.192291	-4.67494	0.500816	0.556777
Neutrophils	ALG13	-0.21404	4.514642	-1.31359	0.192383	-5.03991	0.451931	0.503722
Neutrophils	CCAR2	-0.33115	3.432111	-1.3133	0.192482	-4.88441	0.466091	0.519186
Neutrophils	UBE2F	0.160154	6.306137	1.313265	0.192492	-5.5491	0.429472	0.479267
Neutrophils	ELF2	0.131186	7.39491	1.313254	0.192496	-5.76611	0.416398	0.464958
Neutrophils	MBOAT1	-0.75733	0.682144	-1.31323	0.192502	-4.61783	0.504147	0.560439
Neutrophils	2810405F1	-0.75502	0.687122	-1.31279	0.192652	-4.56505	0.504352	0.560617
Neutrophils	PMS2	-0.34514	3.954235	-1.31266	0.192696	-4.82696	0.459458	0.51195
Neutrophils	CCR7	-0.7591	4.535564	-1.3123	0.192814	-4.75317	0.452083	0.503817
Neutrophils	RNASEH1	0.39696	2.84733	1.311973	0.192925	-4.71805	0.474372	0.528194
Neutrophils	C330018D2	0.445705	2.325358	1.311925	0.192941	-4.70548	0.481489	0.535919
Neutrophils	GM50020	-0.78814	-0.11132	-1.31191	0.192945	-4.53755	0.516183	0.573455
Neutrophils	TCF7L2	-0.23928	7.34951	-1.31165	0.193033	-5.74593	0.417339	0.466042
Neutrophils	HSDL2	-0.24787	4.684002	-1.31163	0.193041	-5.12139	0.450192	0.501941
Neutrophils	GM49797	-0.20934	4.882769	-1.31097	0.193263	-5.23357	0.448006	0.499241
Neutrophils	UCHL3	-0.2032	6.235223	-1.31091	0.193282	-5.37824	0.431097	0.480817
Neutrophils	GM2245	0.491628	1.558462	1.31076	0.193333	-4.77214	0.492563	0.547721
Neutrophils	GM48302	0.406512	1.63727	1.310642	0.193373	-4.92113	0.491456	0.54656
Neutrophils	BNIP3L	0.162354	7.263429	1.310463	0.193433	-5.85624	0.418733	0.467397
Neutrophils	4930522L1	-0.39494	3.684756	-1.30987	0.193632	-4.8027	0.463648	0.51652
Neutrophils	OPA3	0.150438	5.128547	1.309767	0.193668	-5.53639	0.444966	0.496173
Neutrophils	MAP4K1	-0.33961	4.801156	-1.30975	0.193673	-4.81851	0.449132	0.500716
Neutrophils	COX10	-0.37842	3.775159	-1.30972	0.193682	-4.79393	0.462454	0.515222
Neutrophils	CACNA1D	-0.32622	3.396539	-1.30972	0.193683	-5.08031	0.467473	0.52068
Neutrophils	DAPK2	-0.27817	2.818088	-1.30963	0.193714	-5.1935	0.47525	0.529136
Neutrophils	TRAF6	0.206761	5.683882	1.309345	0.19381	-5.26891	0.438066	0.488605
Neutrophils	GM15848	-0.8168	-0.86297	-1.30927	0.193836	-4.53887	0.528013	0.586145
Neutrophils	PRMT7	-0.57127	3.247895	-1.30904	0.193912	-4.70051	0.469618	0.522916
Neutrophils	MMUT	-0.39448	3.4125	-1.30889	0.193964	-4.87673	0.467438	0.520527
Neutrophils	SDC4	-0.34919	6.436865	-1.30854	0.194081	-5.27933	0.428927	0.478567
Neutrophils	E4F1	0.35759	3.127007	1.308533	0.194084	-4.91198	0.471314	0.524755
Neutrophils	PTPRK	-0.36729	3.178658	-1.30843	0.194119	-5.19058	0.47062	0.524052
Neutrophils	SARDH	-0.57364	2.900243	-1.30799	0.194266	-4.83498	0.474624	0.528299
Neutrophils	CCDC163	-0.56404	2.446867	-1.30758	0.194405	-4.71325	0.480822	0.535139
Neutrophils	PRR16	-0.61938	1.720127	-1.30748	0.194439	-4.72553	0.490899	0.546075
Neutrophils	INTS13	-0.24345	4.149388	-1.30746	0.194446	-5.05401	0.458035	0.510391
Neutrophils	RALGDS	0.422227	3.822267	1.307451	0.194449	-4.97745	0.462325	0.515059
Neutrophils	GM12216	-0.27941	4.554087	-1.30716	0.194548	-5.26938	0.452911	0.504811
Neutrophils	ZFP638	0.14819	6.22747	1.306233	0.194861	-5.52704	0.432388	0.482223
Neutrophils	PEX7	-0.20162	4.45855	-1.30605	0.194922	-5.17322	0.454709	0.506596
Neutrophils	KAT6A	0.149049	6.479855	1.305896	0.194976	-5.60463	0.429298	0.478914
Neutrophils	HERPUD1	-0.21232	7.853564	-1.30586	0.194988	-5.72967	0.412882	0.460951

Neutrophils	KCTD6	0.411197	2.981269	1.305794	0.19501	-4.77602	0.474266	0.527918
Neutrophils	9930111J2	0.354765	4.28297	1.305583	0.195082	-5.12721	0.457044	0.509193
Neutrophils	RRP1	0.142398	6.518197	1.305464	0.195122	-5.46232	0.428883	0.478507
Neutrophils	SMARCE1	0.145608	6.302182	1.30528	0.195184	-5.43521	0.431534	0.481446
Neutrophils	CC2D1A	-0.43373	3.044755	-1.30519	0.195215	-4.74454	0.473477	0.527158
Neutrophils	MPZL1	-0.35926	2.78686	-1.30496	0.195292	-5.09571	0.477052	0.531036
Neutrophils	NCDN	-0.50932	2.826122	-1.30469	0.195383	-4.67787	0.476607	0.530505
Neutrophils	PHLDB1	-0.58403	1.355652	-1.3046	0.195416	-4.65727	0.497035	0.552698
Neutrophils	IDH3G	-0.23477	5.124044	-1.30425	0.195534	-5.26668	0.446563	0.497866
Neutrophils	SERPINA10	-0.59359	0.966902	-1.30404	0.195605	-4.6557	0.50279	0.558989
Neutrophils	CMPK2	0.310121	3.672891	1.303969	0.195629	-5.34687	0.465426	0.518516
Neutrophils	PRELID3B	0.182758	5.574007	1.303799	0.195687	-5.33798	0.440928	0.491811
Neutrophils	MDH2	0.14259	6.490426	1.303415	0.195817	-5.6724	0.429684	0.479473
Neutrophils	ARMCX4	0.537189	1.19399	1.303309	0.195853	-4.72353	0.499686	0.555626
Neutrophils	PHLDB2	-0.38051	2.509693	-1.30321	0.195888	-5.02015	0.481267	0.535733
Neutrophils	SUN1	-0.27771	3.918531	-1.30315	0.195907	-4.9488	0.462313	0.515159
Neutrophils	PIN4	0.184756	4.949389	1.302874	0.196001	-5.22913	0.449049	0.500766
Neutrophils	GNG7	0.704653	-0.40318	1.302368	0.196173	-4.59391	0.523381	0.581338
Neutrophils	ST6GALNA(	0.360131	3.867281	1.3023	0.196196	-4.90139	0.46332	0.516275
Neutrophils	SPRED3	0.738088	0.719893	1.302218	0.196224	-4.59285	0.506859	0.563551
Neutrophils	KDM2B	-0.31609	7.133876	-1.302	0.1963	-5.53332	0.422273	0.471549
Neutrophils	1700097N(	-0.53881	2.346622	-1.30172	0.196393	-4.73704	0.484055	0.53883
Neutrophils	FRMD4A	-0.39637	3.969412	-1.30113	0.196594	-5.00845	0.462532	0.515218
Neutrophils	CD207	0.892485	-0.64958	1.300624	0.196767	-4.56376	0.528061	0.585801
Neutrophils	AKT1	0.162229	6.230392	1.300435	0.196831	-5.59005	0.434032	0.48386
Neutrophils	POLR3D	-0.51221	3.27535	-1.30016	0.196926	-4.67534	0.472251	0.525444
Neutrophils	CATSPER2	-0.57661	1.800753	-1.29989	0.197016	-4.65754	0.492661	0.547557
Neutrophils	ASCC3	0.154363	6.882346	1.29953	0.19714	-5.76596	0.426448	0.47547
Neutrophils	4930473A(	0.669422	0.38363	1.299175	0.197262	-4.57373	0.513415	0.56975
Neutrophils	CAMTA2	-0.30595	3.696678	-1.29889	0.197358	-4.9432	0.467217	0.519653
Neutrophils	TK1	-0.35559	4.882981	-1.29846	0.197506	-5.20159	0.451808	0.502891
Neutrophils	UQCC3	0.280591	4.016511	1.298418	0.19752	-4.96321	0.463098	0.515168
Neutrophils	AKR7A5	-0.30413	4.13514	-1.29807	0.19764	-4.97188	0.461535	0.513656
Neutrophils	THEM4	-0.6311	2.270582	-1.29799	0.197666	-4.64836	0.486741	0.541016
Neutrophils	GM37305	0.511086	1.078554	1.297974	0.197672	-4.68848	0.50359	0.559243
Neutrophils	SYNGR2	0.165555	6.111593	1.297962	0.197676	-5.56237	0.436288	0.486165
Neutrophils	VDR	0.846193	0.938817	1.297501	0.197834	-4.57141	0.505656	0.561615
Neutrophils	NUDT15	0.580738	1.647281	1.297466	0.197846	-4.61713	0.49553	0.550667
Neutrophils	PCDHGC4	-0.74382	0.256002	-1.29744	0.197855	-4.59048	0.515614	0.572372
Neutrophils	PON2	0.194983	6.020273	1.297357	0.197883	-5.39774	0.437468	0.487579
Neutrophils	CTR9	0.210727	4.518969	1.297253	0.197919	-5.14363	0.456563	0.50842
Neutrophils	FASTKD1	0.599994	2.189988	1.296983	0.198011	-4.60632	0.487994	0.542502
Neutrophils	RMI2	-0.30296	3.3602	-1.2969	0.198041	-5.06282	0.471971	0.525157
Neutrophils	CYP2A12	-0.51008	2.85261	-1.29672	0.198101	-4.94328	0.478893	0.53268
Neutrophils	MYO10	-0.27196	5.227281	-1.29593	0.198374	-5.34611	0.448074	0.498798
Neutrophils	ARFIP1	0.281354	3.797972	1.295633	0.198474	-5.03282	0.466756	0.519177
Neutrophils	POLR3GL	0.279204	3.878267	1.295524	0.198512	-4.96068	0.465689	0.518069

Neutrophils	GNMT	0.432471	4.232803	1.295457	0.198535	-5.24509	0.461008	0.512987
Neutrophils	MAPRE3	-0.61456	1.502586	-1.29459	0.198831	-4.63154	0.49898	0.553725
Neutrophils	RCC1L	-0.52523	3.072184	-1.29426	0.198947	-4.67205	0.477292	0.530212
Neutrophils	RBM25	0.099814	8.296585	1.293612	0.199169	-5.88448	0.411764	0.458565
Neutrophils	DUSP19	0.545413	2.048701	1.293337	0.199264	-4.6366	0.491937	0.545696
Neutrophils	GM35154	-0.66601	1.526848	-1.29328	0.199284	-4.68314	0.499321	0.553688
Neutrophils	NAXE	-0.21373	5.283838	-1.29283	0.19944	-5.2799	0.448698	0.498971
Neutrophils	NAA16	-0.19101	5.024992	-1.29276	0.199464	-5.20458	0.452016	0.502606
Neutrophils	DNMT3A	-0.19997	6.044028	-1.2927	0.199485	-5.43577	0.4391	0.488547
Neutrophils	YME1L1	0.102551	6.181942	1.292634	0.199506	-5.60668	0.437382	0.486681
Neutrophils	SART1	0.223442	4.872457	1.292366	0.199598	-5.18328	0.45403	0.504837
Neutrophils	CELF2	0.103738	8.597509	1.292316	0.199615	-6.07876	0.408453	0.455112
Neutrophils	CNST	-0.227	3.943862	-1.29215	0.199673	-5.07876	0.466232	0.518126
Neutrophils	PRXL2C	0.202516	4.932963	1.291828	0.199784	-5.27174	0.453283	0.504101
Neutrophils	INPP5E	-0.44076	2.260693	-1.29169	0.19983	-4.69571	0.489164	0.543016
Neutrophils	PITPNC1	-0.13771	8.799744	-1.29168	0.199833	-6.1974	0.406153	0.45266
Neutrophils	CPLANE1	-0.28877	4.78519	-1.29162	0.199854	-5.13796	0.455194	0.506179
Neutrophils	CDIP1	-0.18804	5.935336	-1.29142	0.199923	-5.34718	0.440577	0.490266
Neutrophils	ARL4D	-0.63233	1.705958	-1.29131	0.19996	-4.66931	0.497015	0.5515
Neutrophils	GM16576	0.60431	1.513376	1.29057	0.200217	-4.68846	0.500085	0.554812
Neutrophils	ABCE1	0.28192	5.007871	1.29054	0.200228	-5.05448	0.452655	0.503422
Neutrophils	ASH1L	0.124931	7.367632	1.290521	0.200234	-5.81681	0.423292	0.471414
Neutrophils	TLR3	-0.64394	1.534056	-1.29042	0.200271	-4.71981	0.499789	0.554516
Neutrophils	PPIL2	0.186559	5.124237	1.290143	0.200365	-5.24001	0.451191	0.501851
Neutrophils	SLC39A9	-0.22978	4.172579	-1.29012	0.200375	-5.00399	0.463586	0.515324
Neutrophils	TRMU	0.549538	1.548223	1.289949	0.200432	-4.6057	0.499656	0.554352
Neutrophils	ANKRD16	-0.40265	3.026656	-1.2898	0.200482	-4.78141	0.479026	0.532023
Neutrophils	CD151	-0.38919	2.758187	-1.28964	0.200539	-4.83021	0.482736	0.536018
Neutrophils	VPS53	0.173457	4.462382	1.289506	0.200585	-5.20922	0.459848	0.511191
Neutrophils	TLR6	0.395261	1.070509	1.288985	0.200766	-4.96259	0.506803	0.56201
Neutrophils	RAPGEF3	-0.57341	1.358645	-1.28898	0.200769	-4.6967	0.50265	0.557528
Neutrophils	PPP6R1	0.154307	5.572544	1.288766	0.200841	-5.34	0.445814	0.495926
Neutrophils	COL25A1	-0.83248	0.981282	-1.28818	0.201043	-4.60909	0.508294	0.563647
Neutrophils	GBP9	0.459734	3.036901	1.288087	0.201076	-4.85816	0.479329	0.532365
Neutrophils	MEGF9	-0.16196	4.103779	-1.28799	0.20111	-5.73997	0.464969	0.516803
Neutrophils	KBTBD11	0.940762	0.726015	1.287965	0.201119	-4.57394	0.512013	0.567701
Neutrophils	SEMA4G	-0.66044	0.909795	-1.28791	0.201137	-4.6568	0.509333	0.564832
Neutrophils	SLC37A1	0.385402	3.202303	1.287673	0.20122	-4.888	0.477074	0.530037
Neutrophils	GRIA3	0.68012	3.336555	1.287619	0.201239	-4.82982	0.47525	0.528073
Neutrophils	SAA2	1.205725	-1.02347	1.287589	0.201249	-4.56982	0.538249	0.596082
Neutrophils	GM43063	-0.59573	0.779437	-1.28723	0.201373	-4.63979	0.511434	0.567151
Neutrophils	GCC2	-0.169	5.193926	-1.28709	0.201422	-5.48312	0.450942	0.501659
Neutrophils	MFNG	-0.45081	3.090104	-1.28695	0.201472	-4.69376	0.478812	0.531911
Neutrophils	ZSCAN12	-0.61968	1.093811	-1.28677	0.201534	-4.5849	0.506901	0.562366
Neutrophils	GHR	-0.45624	4.215001	-1.28667	0.201567	-5.27708	0.463715	0.515618
Neutrophils	GM20743	-0.89152	0.031876	-1.2861	0.201767	-4.56	0.522911	0.579424
Neutrophils	TULP3	-0.51141	1.974251	-1.28581	0.201867	-4.70027	0.494833	0.549069

Neutrophils	7-Sep	0.090347	8.277792	1.285673	0.201915	-5.97483	0.413588	0.460726
Neutrophils	DCP1A	-0.20384	4.850131	-1.28545	0.201994	-5.22632	0.455903	0.506963
Neutrophils	CYP20A1	-0.29199	4.104318	-1.28523	0.202069	-4.91949	0.465692	0.517715
Neutrophils	SORCS2	-0.62445	3.3545	-1.28516	0.202092	-4.73332	0.475753	0.528635
Neutrophils	ADPRH	0.171425	4.93883	1.285132	0.202102	-5.47868	0.454753	0.505836
Neutrophils	RABEP2	-0.31953	4.414258	-1.28455	0.202304	-4.90973	0.461908	0.513543
Neutrophils	BMP1	0.687147	0.911089	1.284485	0.202328	-4.65466	0.510456	0.566098
Neutrophils	NR2C2	-0.14074	6.925422	-1.28435	0.202376	-5.73015	0.430074	0.478877
Neutrophils	NLRP12	0.328004	-0.12085	1.284194	0.202429	-5.07935	0.525756	0.582603
Neutrophils	1810041H1	-0.69758	1.76748	-1.28397	0.202506	-4.62452	0.498156	0.552948
Neutrophils	CALM3	0.124453	7.10732	1.283935	0.202519	-5.83371	0.427879	0.476589
Neutrophils	RNF123	-0.44232	3.804311	-1.2838	0.202567	-4.79306	0.470041	0.522528
Neutrophils	GANAB	-0.24626	4.730059	-1.28312	0.202803	-5.09008	0.458238	0.509445
Neutrophils	F13A1	1.418293	2.512117	1.282887	0.202885	-4.62972	0.488236	0.54189
Neutrophils	DNAJC21	-0.19978	5.890572	-1.28242	0.203047	-5.35489	0.443689	0.493399
Neutrophils	PTGES3	0.127419	7.460041	1.281932	0.203218	-5.75662	0.424609	0.47243
Neutrophils	TMEM201	-0.47084	2.102835	-1.28147	0.20338	-4.72152	0.494849	0.548414
Neutrophils	RTN4IP1	0.593995	1.927586	1.281132	0.203497	-4.67173	0.497509	0.55116
Neutrophils	RAG1	-0.87404	1.672599	-1.28096	0.203558	-4.60742	0.501181	0.555173
Neutrophils	IGFBP2	-0.48562	4.701389	-1.28081	0.203611	-5.44767	0.459729	0.510415
Neutrophils	2610206C1	-0.76721	0.40853	-1.28057	0.203694	-4.57709	0.519721	0.575199
Neutrophils	ACVRL1	0.257473	2.619629	1.280222	0.203816	-5.32447	0.488105	0.540974
Neutrophils	LSG1	0.230025	4.928986	1.280101	0.203858	-5.09508	0.45701	0.5073
Neutrophils	CEP104	-0.36013	3.105243	-1.27953	0.204057	-4.7958	0.481753	0.533797
Neutrophils	STX11	0.125812	4.829435	1.278833	0.204302	-5.97368	0.459104	0.508982
Neutrophils	OGG1	-0.26757	3.189389	-1.27867	0.204359	-4.98075	0.481097	0.532778
Neutrophils	DCAF13	-0.22384	4.771338	-1.27837	0.204466	-5.13769	0.46003	0.509948
Neutrophils	ROR1	-0.70387	1.374979	-1.27823	0.204513	-4.65317	0.506823	0.56056
Neutrophils	ZFP428	-0.53669	2.058083	-1.27794	0.204616	-4.67726	0.497061	0.550083
Neutrophils	TRERF1	-0.36095	4.396216	-1.27779	0.204667	-5.1712	0.464998	0.515446
Neutrophils	FZD7	-0.58043	0.743156	-1.27776	0.204679	-4.65157	0.516077	0.570566
Neutrophils	GM39302	0.6834	0.880898	1.277689	0.204704	-4.62834	0.514051	0.568434
Neutrophils	AFG3L2	0.2196	4.593409	1.277482	0.204777	-5.04966	0.462403	0.512703
Neutrophils	SCYL2	0.173192	4.409687	1.277311	0.204836	-5.35186	0.464829	0.515332
Neutrophils	PHC3	0.276471	4.499127	1.277292	0.204843	-5.03135	0.463647	0.51405
Neutrophils	SALL2	-0.74148	0.090711	-1.27716	0.204889	-4.59587	0.525796	0.581127
Neutrophils	EHMT1	-0.18082	6.063885	-1.27683	0.205005	-5.41461	0.443608	0.492189
Neutrophils	ITGB3BP	-0.33493	3.52337	-1.27666	0.205067	-4.83104	0.476925	0.528291
Neutrophils	UBR5	-0.13876	6.888097	-1.27639	0.205159	-5.72414	0.433476	0.481085
Neutrophils	TM9SF1	-0.23365	5.042685	-1.27571	0.2054	-5.19934	0.457223	0.506738
Neutrophils	BC049715	0.791981	0.604594	1.27548	0.205481	-4.62904	0.518925	0.573345
Neutrophils	EXOC1	0.245226	4.095264	1.275393	0.205511	-5.13279	0.469728	0.520308
Neutrophils	GM46430	-0.43789	1.969969	-1.27538	0.205517	-4.70518	0.499083	0.552
Neutrophils	NEMP2	0.390198	2.854914	1.27515	0.205597	-4.75461	0.486719	0.538591
Neutrophils	METAP1D	-0.39086	3.768583	-1.27488	0.205692	-4.85087	0.47425	0.525071
Neutrophils	ANKRD22	-0.25018	-0.56253	-1.27483	0.205708	-5.26124	0.53666	0.592232
Neutrophils	ACBD3	0.149563	5.707973	1.274397	0.205862	-5.45055	0.448938	0.49756

Neutrophils	1700037C1	0.340869	3.065274	1.274359	0.205876	-4.91086	0.484038	0.535559
Neutrophils	AZI2	0.138472	5.839646	1.274161	0.205946	-5.52022	0.447314	0.495825
Neutrophils	UBTD2	-0.41268	3.37685	-1.2738	0.206072	-4.84188	0.479954	0.53118
Neutrophils	GM15543	0.592838	0.901403	1.273646	0.206127	-4.6354	0.515089	0.569137
Neutrophils	CD300C	0.651043	0.72621	1.273606	0.206141	-4.62582	0.517672	0.571932
Neutrophils	NDFIP2	0.137317	5.951714	1.273125	0.206311	-5.59745	0.44629	0.494742
Neutrophils	CHD1L	0.292772	3.722264	1.27299	0.206359	-4.93126	0.475543	0.526481
Neutrophils	ABL2	-0.23122	6.21523	-1.2727	0.20646	-5.38615	0.44305	0.491369
Neutrophils	APOA5	-0.61036	1.793969	-1.27262	0.206489	-4.77093	0.502533	0.555703
Neutrophils	CLASP1	-0.11003	6.373608	-1.27212	0.206666	-5.88454	0.441343	0.489226
Neutrophils	DAP3	-0.19897	5.039126	-1.27198	0.206715	-5.26978	0.458424	0.507791
Neutrophils	TTPAL	-0.24619	4.234118	-1.27169	0.20682	-5.06555	0.46919	0.519375
Neutrophils	SH2B2	0.162695	4.550186	1.27148	0.206893	-5.64957	0.464985	0.514846
Neutrophils	ENHO	-0.46699	2.481211	-1.27143	0.206911	-4.86361	0.493239	0.545358
Neutrophils	ABL1	-0.23372	5.653787	-1.27067	0.207181	-5.28636	0.450913	0.499522
Neutrophils	POLR2E	-0.32956	4.876402	-1.27064	0.207189	-5.0553	0.460999	0.510451
Neutrophils	MMP25	0.235284	0.499533	1.270633	0.207192	-5.38674	0.522305	0.576528
Neutrophils	BTG3	-0.22353	5.347109	-1.27052	0.207233	-5.17242	0.454864	0.503852
Neutrophils	GM17276	0.835904	0.785789	1.27003	0.207406	-4.59791	0.518373	0.572305
Neutrophils	DNMT1	0.223314	5.753517	1.269705	0.207521	-5.47085	0.450065	0.498535
Neutrophils	UPF3B	-0.21025	4.979585	-1.26946	0.207608	-5.2853	0.460178	0.50948
Neutrophils	GM43623	-0.8255	-0.49767	-1.26907	0.207747	-4.57749	0.538119	0.593585
Neutrophils	CRAT	0.305449	3.198294	1.268927	0.207798	-5.04968	0.484231	0.535723
Neutrophils	SRPK1	0.137541	6.11152	1.268858	0.207822	-5.48414	0.445681	0.493995
Neutrophils	ETS2	0.130295	5.865691	1.268783	0.207849	-6.0111	0.448807	0.497421
Neutrophils	NKIRAS1	-0.39337	3.432264	-1.26871	0.207875	-4.82498	0.481011	0.532303
Neutrophils	PEX16	0.302974	3.474463	1.26851	0.207946	-4.92847	0.480468	0.531783
Neutrophils	GM37233	-0.79655	0.010375	-1.2684	0.207985	-4.5781	0.530405	0.585594
Neutrophils	UPF2	0.131774	6.075856	1.268284	0.208026	-5.64618	0.446166	0.494718
Neutrophils	MCF2L	0.671955	0.46165	1.267377	0.208348	-4.6923	0.52431	0.578613
Neutrophils	LRP5	-0.28501	4.078112	-1.26695	0.208499	-5.15618	0.473138	0.52341
Neutrophils	GPAM	-0.54665	3.696477	-1.26652	0.208653	-4.78275	0.478523	0.529207
Neutrophils	POLG2	-0.18821	5.080463	-1.26644	0.208682	-5.40323	0.460025	0.509214
Neutrophils	CEBPZOS	-0.23449	4.521965	-1.2661	0.208805	-5.17957	0.467572	0.517209
Neutrophils	TOMM34	-0.15135	5.601463	-1.2659	0.208876	-5.49373	0.453479	0.501921
Neutrophils	SEC63	0.126508	7.09953	1.265687	0.20895	-5.68554	0.43464	0.481464
Neutrophils	TNKS	0.182992	6.066905	1.265284	0.209094	-5.53757	0.447789	0.495611
Neutrophils	CAPZA2	0.08195	7.929486	1.264741	0.209288	-5.99019	0.424959	0.47085
Neutrophils	SLC28A2	0.442113	2.961181	1.264708	0.2093	-4.88538	0.48947	0.540693
Neutrophils	UBP1	0.148814	5.448392	1.264523	0.209366	-5.52779	0.45603	0.5046
Neutrophils	CATSPERG:	-0.71972	0.160917	-1.26417	0.209491	-4.64481	0.530304	0.584599
Neutrophils	DFFA	0.443063	2.939835	1.264095	0.209519	-4.7564	0.489858	0.541148
Neutrophils	ADGRF5	-0.3776	2.598167	-1.26409	0.20952	-5.13109	0.494656	0.546316
Neutrophils	PDSS1	-0.25928	4.315269	-1.26385	0.209606	-5.22047	0.471113	0.520924
Neutrophils	1110004F1	0.117126	6.204	1.263577	0.209704	-5.52313	0.44657	0.494317
Neutrophils	STK38L	0.278031	4.003603	1.263289	0.209807	-5.00161	0.475435	0.525623
Neutrophils	TAF6L	-0.41332	2.800016	-1.26319	0.20984	-4.77175	0.492035	0.543539

Neutrophils	SNX18	0.13458	6.77742	1.263192	0.209841	-5.98629	0.439356	0.486574
Neutrophils	SNAP29	0.217105	5.5062	1.262961	0.209924	-5.18464	0.455592	0.504193
Neutrophils	ARHGAP17	-0.1747	7.245574	-1.26285	0.209965	-5.6239	0.433622	0.480391
Neutrophils	GGPS1	0.16933	5.393996	1.262514	0.210084	-5.28508	0.457182	0.50589
Neutrophils	RAP1A	0.080756	8.772339	1.262322	0.210152	-6.05841	0.415379	0.460606
Neutrophils	CORO1C	0.147917	6.202322	1.262293	0.210163	-5.58895	0.446792	0.494772
Neutrophils	JDP2	0.15334	5.304027	1.262058	0.210247	-5.9495	0.458438	0.507331
Neutrophils	ARHGAP9	-0.15404	5.14536	-1.26142	0.210474	-5.63689	0.46091	0.509736
Neutrophils	LDHB	-0.3668	3.00278	-1.2604	0.210841	-5.0673	0.490495	0.541572
Neutrophils	PTMS	-0.19328	6.015415	-1.26023	0.210904	-5.55316	0.450163	0.49802
Neutrophils	SECISBP2L	0.221805	4.964176	1.260051	0.210967	-5.28118	0.46383	0.512879
Neutrophils	IRF7	0.411582	6.139097	1.259675	0.211102	-5.66216	0.448583	0.496465
Neutrophils	NOP10	-0.15829	7.15867	-1.2595	0.211164	-5.64807	0.435779	0.482655
Neutrophils	SRSF2	-0.12996	7.941182	-1.2594	0.211201	-5.91705	0.426213	0.472287
Neutrophils	PES1	0.25928	4.691666	1.259383	0.211207	-5.0186	0.467443	0.517003
Neutrophils	SRSF7	-0.18591	6.098296	-1.25923	0.21126	-5.55675	0.449104	0.49717
Neutrophils	PLA2G15	-0.42506	3.738633	-1.25922	0.211265	-4.9034	0.48031	0.530924
Neutrophils	PSMA6	-0.14582	6.941715	-1.25918	0.211279	-5.72656	0.438471	0.485639
Neutrophils	TOMM7	0.108611	7.561253	1.258989	0.211348	-5.8816	0.430829	0.477432
Neutrophils	BCL6B	-0.55084	0.649778	-1.25894	0.211364	-4.70509	0.524571	0.578665
Neutrophils	IL6RA	0.19649	5.504339	1.258828	0.211406	-5.62043	0.456754	0.505612
Neutrophils	FKBP15	0.17931	5.239594	1.25876	0.211431	-5.38486	0.460208	0.509391
Neutrophils	RALBP1	0.125012	6.268527	1.258744	0.211436	-5.6993	0.446936	0.495006
Neutrophils	ANKRD54	0.37572	3.089479	1.258709	0.211449	-4.77063	0.489283	0.540807
Neutrophils	THEM6	-0.564	3.448768	-1.25826	0.21161	-4.68892	0.484558	0.535596
Neutrophils	EPB41L3	-0.62048	2.334313	-1.2581	0.211667	-4.85552	0.500239	0.55255
Neutrophils	GM43661	0.217842	2.981343	1.257784	0.211782	-5.54023	0.491249	0.542894
Neutrophils	HAUS6	0.314305	4.556972	1.25708	0.212035	-5.03314	0.470134	0.519818
Neutrophils	COPS3	-0.15293	5.92883	-1.25655	0.212225	-5.45791	0.452438	0.500523
Neutrophils	ASF1A	-0.24643	5.210724	-1.25643	0.21227	-5.22929	0.461776	0.51068
Neutrophils	CDC34	0.147929	6.050691	1.256173	0.212362	-5.63084	0.450885	0.49895
Neutrophils	CLCC1	0.226983	4.329275	1.256157	0.212368	-5.0465	0.473524	0.523444
Neutrophils	GM4707	0.541924	2.866085	1.255936	0.212448	-4.785	0.493695	0.54529
Neutrophils	GALNT16	-0.69368	0.933714	-1.25591	0.212459	-4.60231	0.521691	0.575385
Neutrophils	FTO	-0.14153	6.592827	-1.25562	0.212562	-5.61827	0.444111	0.491642
Neutrophils	GID8	0.154753	5.195402	1.255442	0.212626	-5.47273	0.462145	0.511199
Neutrophils	MEGF8	-0.58262	0.288303	-1.25533	0.212668	-4.65945	0.531577	0.585953
Neutrophils	KLF4	-0.2445	6.447469	-1.25497	0.212797	-5.52057	0.446155	0.493819
Neutrophils	FCF1	-0.16086	6.136138	-1.25469	0.212897	-5.44591	0.450215	0.498222
Neutrophils	NASP	-0.16722	6.308139	-1.25459	0.212934	-5.58845	0.448019	0.495841
Neutrophils	ELMO1	-0.09882	8.719508	-1.25416	0.213091	-6.24102	0.418618	0.463745
Neutrophils	CD27	-0.38774	3.318035	-1.25345	0.213347	-4.93825	0.488407	0.539195
Neutrophils	MTUS2	-0.80852	0.654472	-1.25341	0.213359	-4.63272	0.526985	0.580639
Neutrophils	GSTT1	-0.5137	2.240293	-1.25339	0.213369	-4.86784	0.503657	0.555614
Neutrophils	MOB3C	-0.29954	3.356469	-1.25323	0.213428	-4.99379	0.487901	0.538715
Neutrophils	GPR141B	0.468083	-0.65516	1.253026	0.2135	-4.71758	0.547168	0.602312
Neutrophils	TOMM70A	-0.15629	5.752051	-1.2529	0.213545	-5.44198	0.455776	0.504025

Neutrophils	LRP4	0.251188	2.477171	1.25235	0.213745	-5.44435	0.500714	0.552211
Neutrophils	ZBTB49	0.549484	1.051258	1.252164	0.213813	-4.61852	0.521539	0.574569
Neutrophils	OFD1	-0.29632	3.31561	-1.25207	0.213846	-4.90273	0.488902	0.53953
Neutrophils	SLC19A1	0.720551	1.065999	1.251144	0.214183	-4.60869	0.522028	0.574668
Neutrophils	CEMIP2	-0.15328	5.494454	-1.25081	0.214303	-5.80077	0.460115	0.508151
Neutrophils	OLR1	0.263928	1.333367	1.250804	0.214306	-5.6346	0.518069	0.570523
Neutrophils	EFNB1	-0.45453	1.912975	-1.25075	0.214325	-4.80386	0.509567	0.561406
Neutrophils	DDX59	-0.60409	1.892836	-1.25037	0.214465	-4.63535	0.510082	0.561832
Neutrophils	MTRF1L	-0.41364	3.1332	-1.24993	0.214624	-4.81691	0.492441	0.54278
Neutrophils	LACTB2	0.263279	3.94655	1.249836	0.214658	-5.08093	0.481153	0.530692
Neutrophils	ABCB1B	-0.69915	3.934965	-1.24981	0.214666	-4.75102	0.481312	0.53087
Neutrophils	S100A6	0.210428	7.580063	1.249744	0.214691	-6.40412	0.43392	0.479685
Neutrophils	TMEM242	-0.26804	4.292008	-1.24953	0.214769	-4.98746	0.476484	0.525721
Neutrophils	SLC5A6	-0.69892	0.868812	-1.24944	0.214803	-4.62835	0.525367	0.578261
Neutrophils	GM37240	0.244789	5.162317	1.249308	0.21485	-5.33775	0.464822	0.51321
Neutrophils	RGS16	-0.81784	1.351563	-1.24892	0.214991	-4.62265	0.518406	0.570723
Neutrophils	FLOT2	0.134132	4.443449	1.247959	0.215341	-5.66049	0.475066	0.524032
Neutrophils	PPP3R1	0.144063	5.5618	1.247766	0.215411	-5.53701	0.46018	0.508005
Neutrophils	PRKRIP1	-0.24707	3.964552	-1.24776	0.215415	-5.04399	0.481593	0.531103
Neutrophils	LAMP2	0.097533	7.069003	1.24775	0.215417	-6.05359	0.440886	0.48713
Neutrophils	MED8	0.173736	5.008062	1.247728	0.215425	-5.44175	0.467489	0.515898
Neutrophils	GM50232	-0.71619	1.372215	-1.24754	0.215494	-4.63687	0.518561	0.570924
Neutrophils	TMEM267	-0.58078	1.734822	-1.24743	0.215534	-4.67941	0.513221	0.56524
Neutrophils	KLHL20	0.375332	3.27084	1.247383	0.215551	-4.94288	0.491212	0.541609
Neutrophils	SDHAF3	-0.40486	3.044533	-1.2469	0.215729	-4.76787	0.494694	0.545279
Neutrophils	PSMD7	-0.21742	5.828246	-1.24639	0.215915	-5.32908	0.457251	0.504799
Neutrophils	SEC14L1	0.169049	4.618739	1.246299	0.215947	-5.50777	0.473264	0.522116
Neutrophils	SMAD1	0.299429	3.547772	1.245936	0.216079	-5.00157	0.488125	0.537997
Neutrophils	PDE4A	-0.47846	3.81993	-1.24562	0.216193	-4.90653	0.484504	0.534055
Neutrophils	SELP	0.458635	1.452771	1.245295	0.216314	-5.08287	0.518404	0.570476
Neutrophils	RSPH10B	0.824559	0.413611	1.24523	0.216337	-4.61724	0.534019	0.587222
Neutrophils	TOM1	0.193026	5.431971	1.245191	0.216352	-5.52658	0.462807	0.510719
Neutrophils	TXNDC16	-0.23435	5.44957	-1.24506	0.216399	-5.49532	0.462578	0.510493
Neutrophils	HGSNAT	0.215883	4.642285	1.244424	0.216632	-5.54113	0.47374	0.522381
Neutrophils	RPP40	-0.69224	1.432828	-1.24417	0.216724	-4.61225	0.519171	0.571294
Neutrophils	GUK1	0.203542	4.908743	1.243708	0.216894	-5.28582	0.470177	0.518751
Neutrophils	ARHGAP27	0.692988	0.465307	1.243485	0.216976	-4.60157	0.533716	0.587082
Neutrophils	EXO5	-0.65419	2.006793	-1.24341	0.217003	-4.66798	0.510733	0.562497
Neutrophils	CNTRL	0.145354	5.907012	1.243386	0.217013	-5.61562	0.45701	0.50464
Neutrophils	NDUFB3	-0.1544	5.72376	-1.24331	0.217041	-5.54484	0.459398	0.507249
Neutrophils	GM17160	0.734051	0.089084	1.243304	0.217042	-4.59717	0.539482	0.593309
Neutrophils	CDKL4	0.30522	1.561956	1.243282	0.217051	-5.19236	0.51726	0.569532
Neutrophils	GM29966	-0.69246	1.411825	-1.24326	0.217057	-4.73948	0.519482	0.571915
Neutrophils	ERC2	-0.62606	0.862583	-1.24289	0.217194	-4.89364	0.527802	0.580815
Neutrophils	PCBD2	0.178476	5.24057	1.242892	0.217194	-5.49699	0.465852	0.514218
Neutrophils	TSFM	-0.28215	4.258618	-1.24253	0.217326	-5.00192	0.47925	0.528603
Neutrophils	SLC8A2	-0.54783	1.394752	-1.24206	0.2175	-4.92742	0.52021	0.572662

Neutrophils	IGF1	-0.3436	5.292109	-1.24198	0.217528	-5.50603	0.465499	0.513821
Neutrophils	H2-T24	0.528423	1.828462	1.241967	0.217533	-4.80132	0.513808	0.565808
Neutrophils	CPM	-0.52094	4.160192	-1.24164	0.217652	-4.91031	0.480766	0.530373
Neutrophils	ZNRD2	-0.35981	4.037669	-1.2416	0.217668	-4.90513	0.482447	0.532185
Neutrophils	PAXBP1	0.23264	5.312121	1.241534	0.217692	-5.30751	0.465252	0.513666
Neutrophils	RFLNB	0.169831	3.463749	1.241425	0.217732	-5.75592	0.490405	0.540795
Neutrophils	MTCP1	-0.70661	0.737147	-1.24131	0.217773	-4.63036	0.530091	0.583447
Neutrophils	BIN2	0.16286	5.165118	1.240761	0.217976	-5.58401	0.467539	0.515927
Neutrophils	VPS13B	-0.10465	8.050399	-1.24027	0.218158	-6.02784	0.431018	0.476332
Neutrophils	SMG7	-0.14139	6.385921	-1.23998	0.218264	-5.635	0.451989	0.49905
Neutrophils	RETREG2	0.199756	4.884018	1.239816	0.218324	-5.37046	0.471747	0.520416
Neutrophils	NAT8F1	-0.61936	1.619362	-1.23897	0.218637	-4.73761	0.518338	0.570094
Neutrophils	SMIM7	0.184595	4.932589	1.238916	0.218655	-5.31865	0.471609	0.519906
Neutrophils	DHRS13	0.695712	0.959658	1.238762	0.218712	-4.67849	0.52822	0.580718
Neutrophils	USO1	0.159854	5.739311	1.238373	0.218856	-5.48603	0.461133	0.508605
Neutrophils	NKAP	0.177877	4.989943	1.238232	0.218908	-5.28244	0.471083	0.519401
Neutrophils	OLFM1	-0.45619	2.469141	-1.23751	0.219173	-4.88947	0.506494	0.557363
Neutrophils	ATP6V1E1	0.100433	7.59223	1.237388	0.219219	-6.1089	0.437776	0.483291
Neutrophils	C8G	-0.42235	3.782028	-1.23738	0.219224	-5.23204	0.487879	0.537371
Neutrophils	SPN	0.204919	3.586459	1.237359	0.21923	-5.3588	0.490607	0.540304
Neutrophils	PCBP2	-0.08944	8.511828	-1.23715	0.219307	-6.06907	0.426572	0.471091
Neutrophils	RANBP6	-0.49748	1.620455	-1.237	0.219364	-4.70333	0.51901	0.570761
Neutrophils	MMGT2	-0.38739	3.166256	-1.23682	0.219428	-4.77729	0.496613	0.546819
Neutrophils	SCMH1	-0.24088	5.849321	-1.23669	0.219477	-5.36672	0.460077	0.507528
Neutrophils	MICU2	0.205384	4.939969	1.236582	0.219517	-5.32739	0.472136	0.520542
Neutrophils	HECTD3	0.349894	2.789878	1.236419	0.219577	-4.8548	0.501973	0.552683
Neutrophils	HAAO	0.319193	3.64437	1.236255	0.219638	-5.08488	0.489889	0.53971
Neutrophils	DCUN1D2	-0.45368	2.743543	-1.23624	0.219643	-4.8124	0.502637	0.553408
Neutrophils	ARHGEF10I	0.432619	2.695098	1.235592	0.219883	-4.91753	0.503732	0.554442
Neutrophils	MED16	-0.27642	4.12299	-1.23552	0.21991	-4.98925	0.483634	0.532844
Neutrophils	HOOK2	-0.38807	3.558904	-1.23525	0.220009	-4.94158	0.491591	0.541405
Neutrophils	SERP1	0.115463	7.981742	1.235033	0.22009	-5.97984	0.433514	0.478765
Neutrophils	OLA1	-0.1728	5.835409	-1.23498	0.220112	-5.34996	0.460753	0.508273
Neutrophils	FBXO9	-0.22638	4.752411	-1.23468	0.220221	-5.27116	0.475311	0.523908
Neutrophils	DPP9	0.240314	4.420233	1.234309	0.220358	-5.17835	0.480026	0.528957
Neutrophils	SUPT6	-0.16729	5.808893	-1.23373	0.220575	-5.5416	0.461708	0.509007
Neutrophils	SELENBP1	0.362931	3.795416	1.233691	0.220588	-5.10069	0.488951	0.538352
Neutrophils	OTOA	-0.37016	0.757069	-1.23316	0.220786	-4.99788	0.533398	0.585995
Neutrophils	CXCL10	0.398841	4.788598	1.233114	0.220802	-5.56892	0.475453	0.523836
Neutrophils	RAB21	-0.10336	6.891793	-1.23288	0.22089	-5.89277	0.447851	0.494044
Neutrophils	TMEM51OS	-0.70056	-0.90367	-1.2328	0.220918	-4.60078	0.559309	0.613632
Neutrophils	GM15860	-0.72618	0.373729	-1.23279	0.22092	-4.60678	0.53927	0.592266
Neutrophils	MSH6	0.33004	4.38755	1.232569	0.221004	-5.03753	0.480915	0.529717
Neutrophils	ITFG2	-0.41591	3.893716	-1.23254	0.221017	-4.81502	0.48773	0.537048
Neutrophils	POLR2I	-0.1969	5.116859	-1.23251	0.221027	-5.24738	0.47103	0.519071
Neutrophils	THSD4	-1.00873	0.910314	-1.23223	0.22113	-4.64489	0.531205	0.583622
Neutrophils	OSBPL2	0.196811	3.979026	1.231587	0.221369	-5.24446	0.487093	0.536051

Neutrophils	TRIM28	-0.24237	5.460856	-1.23128	0.221483	-5.24299	0.467048	0.514445
Neutrophils	BECN1	0.142033	5.896307	1.231193	0.221516	-5.60516	0.461299	0.508292
Neutrophils	FAM13B	0.124857	6.457749	1.23103	0.221577	-5.77421	0.453995	0.500465
Neutrophils	GM15494	-0.64051	0.759331	-1.23098	0.221594	-4.74716	0.534056	0.586474
Neutrophils	MMADHC	-0.198	4.299266	-1.23062	0.221727	-5.22072	0.48294	0.531645
Neutrophils	GABARAPL	-0.33238	3.974288	-1.23024	0.22187	-4.9774	0.487626	0.536628
Neutrophils	BCL2L13	-0.187	5.133362	-1.23002	0.221952	-5.45854	0.471791	0.51968
Neutrophils	NAMPT	0.197017	6.301782	1.229938	0.221983	-5.86735	0.456371	0.503047
Neutrophils	SLTM	-0.12723	6.63584	-1.22978	0.222044	-5.66275	0.45206	0.498436
Neutrophils	GM20342	0.262818	3.499425	1.229744	0.222055	-5.16732	0.494272	0.543911
Neutrophils	GM26936	-0.8046	0.478634	-1.22953	0.222136	-4.60385	0.538778	0.591633
Neutrophils	UTY	3.854081	2.644423	1.229447	0.222166	-4.90721	0.506474	0.557071
Neutrophils	ZFP266	-0.27632	4.029092	-1.22926	0.222236	-4.94428	0.486865	0.536049
Neutrophils	NPAS2	-0.70497	0.089778	-1.2292	0.222257	-4.66409	0.544795	0.598109
Neutrophils	EGR1	0.247701	6.545199	1.229136	0.222282	-5.9597	0.453225	0.499828
Neutrophils	CYB561A3	0.206361	5.573777	1.228842	0.222392	-5.41277	0.466048	0.513656
Neutrophils	ZFP317	0.404609	2.898111	1.2285	0.222519	-4.81107	0.503044	0.553456
Neutrophils	TMEM238	-0.45446	2.938537	-1.22841	0.222553	-4.8569	0.502464	0.552874
Neutrophils	RPAP3	-0.33691	3.841822	-1.22837	0.222567	-4.92509	0.489687	0.53916
Neutrophils	TMEM60	0.254067	4.112089	1.227559	0.222871	-5.08262	0.48649	0.535408
Neutrophils	CCNT1	0.102611	6.841664	1.227368	0.222942	-5.85396	0.450192	0.496274
Neutrophils	NOA1	0.399712	3.137205	1.227219	0.222998	-4.83924	0.500276	0.550228
Neutrophils	PAPSS2	-0.51568	2.513577	-1.22663	0.22322	-4.85193	0.509554	0.559941
Neutrophils	MYBPC3	0.380811	1.123244	1.22662	0.223222	-4.95414	0.530184	0.582007
Neutrophils	PTAR1	-0.29484	3.690053	-1.22635	0.223325	-4.93434	0.492795	0.541965
Neutrophils	ARRDC1	0.224812	4.471227	1.226303	0.223341	-5.2807	0.481946	0.530317
Neutrophils	SURF1	0.258913	4.36834	1.225823	0.22352	-5.14411	0.48355	0.532006
Neutrophils	SLX4	-0.48442	1.820472	-1.22582	0.223522	-4.68333	0.519996	0.571085
Neutrophils	RCBTB2	-0.20537	5.187796	-1.22564	0.22359	-5.32599	0.472442	0.520106
Neutrophils	ARHGDIA	0.150799	7.188393	1.225111	0.223787	-5.75621	0.446545	0.492133
Neutrophils	MAP4K3	-0.29578	4.283134	-1.2251	0.223792	-5.12833	0.485006	0.533591
Neutrophils	TMEM70	0.313438	3.930301	1.224841	0.223888	-5.00588	0.489936	0.538941
Neutrophils	GPBP1L1	0.150189	5.913267	1.224778	0.223912	-5.61017	0.463044	0.510036
Neutrophils	ADGRA3	-0.56955	0.459311	-1.22469	0.223946	-4.67765	0.540951	0.593647
Neutrophils	ZFP553	0.488267	2.414705	1.224494	0.224018	-4.69374	0.511636	0.562341
Neutrophils	PRAM1	0.147256	3.279629	1.224073	0.224176	-5.61381	0.499414	0.5491
Neutrophils	ARMCX1	-0.61855	0.550831	-1.22373	0.224303	-4.73069	0.539967	0.592406
Neutrophils	CTNNA3	-0.45209	1.300067	-1.22365	0.224335	-5.12085	0.528536	0.580262
Neutrophils	DUSP28	-0.47949	1.99306	-1.22348	0.224399	-4.74048	0.518182	0.569215
Neutrophils	HIGD2A	0.111726	6.065754	1.223464	0.224405	-5.68635	0.461407	0.508221
Neutrophils	HMGXB4	0.26777	4.148587	1.223	0.22458	-5.05293	0.487413	0.536172
Neutrophils	IRF2BPL	-0.48639	4.091669	-1.22299	0.224585	-4.85941	0.488204	0.537022
Neutrophils	KDM5A	-0.12507	7.014524	-1.22293	0.224605	-5.81261	0.449255	0.495055
Neutrophils	OCIAD1	0.151908	5.885315	1.22208	0.224926	-5.45398	0.464383	0.511254
Neutrophils	ERRFI1	-0.24104	5.004787	-1.22206	0.224932	-5.41779	0.476164	0.523943
Neutrophils	UFM1	0.190368	5.448609	1.221807	0.225028	-5.33676	0.470284	0.517556
Neutrophils	BUD13	0.254087	3.714639	1.221691	0.225072	-5.06599	0.494092	0.54317

Neutrophils	EARS2	-0.57208	1.644083	-1.22144	0.225166	-4.6556	0.524233	0.575471
Neutrophils	MYO6	-0.31462	3.1857	-1.22136	0.225198	-5.07266	0.501671	0.551321
Neutrophils	BAZ1B	0.15485	6.23949	1.220929	0.225359	-5.63157	0.459914	0.506574
Neutrophils	FGFR1	0.702194	1.974297	1.220876	0.225379	-4.77598	0.51934	0.570439
Neutrophils	IRAK1BP1	-0.87765	0.288762	-1.2208	0.225409	-4.60809	0.544948	0.597834
Neutrophils	SLC12A8	-0.69049	0.657727	-1.22077	0.22542	-4.6564	0.539235	0.591741
Neutrophils	2310009A(	-0.1798	4.692253	-1.2207	0.225444	-5.43528	0.480611	0.528935
Neutrophils	BID	0.240819	3.803317	1.220416	0.225552	-5.21124	0.493072	0.542256
Neutrophils	PCDH17	-0.30587	2.459624	-1.22014	0.225658	-5.28717	0.512401	0.563031
Neutrophils	KCNQ1OT1	-0.23861	6.399833	-1.21994	0.22573	-5.48018	0.458006	0.504638
Neutrophils	GABBR1	-0.31368	2.766527	-1.21976	0.2258	-5.41381	0.507934	0.55841
Neutrophils	CSF3	0.603094	-0.096	1.219564	0.225874	-4.90479	0.55119	0.604721
Neutrophils	UCHL5	0.160795	5.728606	1.21914	0.226033	-5.53775	0.46683	0.514488
Neutrophils	ENDOV	-0.4611	2.177707	-1.21903	0.226076	-4.81257	0.516541	0.567928
Neutrophils	N4BP3	-0.44092	2.955418	-1.21899	0.22609	-4.78828	0.505204	0.555774
Neutrophils	NUAK1	-0.54741	3.219187	-1.21898	0.226095	-4.82024	0.501418	0.551711
Neutrophils	GM11423	-0.64574	0.892029	-1.21897	0.226099	-4.63723	0.535853	0.588589
Neutrophils	LARGE1	-0.29573	5.482959	-1.21884	0.226145	-5.35741	0.470103	0.518019
Neutrophils	EMC9	-0.53971	1.600787	-1.21878	0.226171	-4.69732	0.525118	0.57711
Neutrophils	GSE1	-0.17478	5.664856	-1.21877	0.226172	-5.47488	0.467677	0.515402
Neutrophils	GPSM3	0.106519	6.022347	1.21872	0.226192	-5.97928	0.462946	0.510316
Neutrophils	A630072M	0.358535	3.151561	1.218536	0.226262	-4.96628	0.502436	0.552802
Neutrophils	SLC27A5	-0.5972	1.457834	-1.21809	0.22643	-4.77166	0.527599	0.579673
Neutrophils	IL12RB1	-0.78143	0.500116	-1.21763	0.226604	-4.65852	0.542471	0.595364
Neutrophils	COPS7B	0.322813	3.34463	1.217517	0.226647	-4.93808	0.500167	0.550097
Neutrophils	SLC18A1	0.718059	0.497721	1.217451	0.226672	-4.68064	0.542508	0.595404
Neutrophils	NDUFS4	-0.13174	6.335519	-1.21678	0.226927	-5.67344	0.459668	0.506306
Neutrophils	ENO3	0.570647	2.077848	1.216773	0.226929	-4.78443	0.518946	0.57
Neutrophils	PLEKHO1	-0.27286	5.499121	-1.21648	0.227038	-5.12311	0.470861	0.518283
Neutrophils	ECHDC2	-0.57446	1.612612	-1.21601	0.227218	-4.78308	0.526255	0.577722
Neutrophils	HIVEP2	-0.24753	8.386816	-1.21569	0.227338	-5.86564	0.433985	0.478579
Neutrophils	DOLK	-0.48205	1.544978	-1.21557	0.227385	-4.68079	0.527272	0.578989
Neutrophils	SP1	0.135767	5.924217	1.215441	0.227434	-5.58938	0.465403	0.512568
Neutrophils	H2-KE6	-0.18559	5.147514	-1.21542	0.227443	-5.51727	0.475801	0.523773
Neutrophils	FGD5	-0.44514	1.663977	-1.21527	0.227497	-4.97706	0.525484	0.57714
Neutrophils	ZFP790	0.491076	2.226902	1.215227	0.227515	-4.73622	0.517108	0.568198
Neutrophils	NHLRC2	0.200397	4.994885	1.215084	0.227569	-5.27906	0.477872	0.526109
Neutrophils	ATP6AP1	0.133408	5.990589	1.214969	0.227613	-5.70734	0.464525	0.511746
Neutrophils	DDX3Y	3.164202	2.707432	1.214961	0.227616	-5.01228	0.510066	0.560714
Neutrophils	2310040G2	0.390475	1.291307	1.214859	0.227654	-4.74723	0.531105	0.583299
Neutrophils	MTRF1	-0.63906	1.208712	-1.21465	0.227734	-4.66576	0.532435	0.584763
Neutrophils	SLC13A3	-0.71354	1.11728	-1.21448	0.227797	-4.72181	0.533866	0.586263
Neutrophils	SNHG17	-0.62654	1.368538	-1.21425	0.227885	-4.63603	0.530142	0.582305
Neutrophils	VPS45	-0.32632	3.758422	-1.21396	0.227995	-4.96374	0.49532	0.545034
Neutrophils	GM20470	0.536209	1.403571	1.213856	0.228035	-4.74544	0.529743	0.58198
Neutrophils	SOX4	-0.47908	5.508952	-1.21348	0.228176	-5.1824	0.471427	0.519255
Neutrophils	OSBPL11	-0.14184	5.368512	-1.21303	0.228351	-5.76286	0.473514	0.521415

Neutrophils	ATG14	-0.33028	3.27883	-1.21289	0.228402	-4.87138	0.502559	0.552689
Neutrophils	GM34455	0.303659	2.623429	1.212804	0.228435	-5.2271	0.512043	0.562885
Neutrophils	GGCT	0.345739	3.249782	1.212735	0.228462	-5.07864	0.502975	0.553169
Neutrophils	9930111J2	0.69526	1.279459	1.212197	0.228666	-4.68372	0.532352	0.584544
Neutrophils	CAGE1	-0.46415	1.94977	-1.21217	0.228678	-4.87707	0.522261	0.573754
Neutrophils	ARMC3	0.309119	1.900651	1.211933	0.228767	-5.09619	0.523089	0.574538
Neutrophils	TNR	-0.63234	0.036927	-1.21179	0.228822	-4.65662	0.551702	0.605133
Neutrophils	METTL1	0.475868	3.431367	1.211404	0.228969	-4.81786	0.50095	0.550803
Neutrophils	PLCXD2	-0.39073	3.46805	-1.2113	0.229007	-4.93179	0.500426	0.550293
Neutrophils	SAYSD1	0.347629	3.323883	1.210616	0.229269	-4.84655	0.502847	0.552795
Neutrophils	ZFP358	-0.44092	3.044691	-1.21057	0.229288	-4.80602	0.506867	0.557106
Neutrophils	BCAP29	0.277928	4.765797	1.210503	0.229312	-5.08184	0.482604	0.531076
Neutrophils	AIDA	-0.24465	4.437691	-1.20994	0.229525	-5.12144	0.487393	0.536019
Neutrophils	POLA1	-0.281	5.968201	-1.20994	0.229528	-5.46437	0.466619	0.513659
Neutrophils	GM14029	-0.77478	0.297438	-1.20978	0.229588	-4.62812	0.548538	0.601462
Neutrophils	VNN3	0.325688	1.614791	1.209411	0.229729	-5.17336	0.528424	0.580069
Neutrophils	2610507B1	-0.17121	5.431746	-1.2093	0.229773	-5.29291	0.473942	0.521655
Neutrophils	CHDH	-0.55058	1.157054	-1.20923	0.2298	-4.79441	0.535376	0.587534
Neutrophils	BEND3	-0.56073	2.456588	-1.20913	0.229837	-4.7214	0.515879	0.566689
Neutrophils	TXLNG	-0.22467	5.107247	-1.20869	0.230003	-5.23388	0.478465	0.526528
Neutrophils	RET	-0.63282	0.727544	-1.20862	0.230033	-4.68731	0.542126	0.594742
Neutrophils	OLFR56	0.629253	2.067818	1.208496	0.230079	-4.79255	0.521772	0.572998
Neutrophils	ZFP830	0.271112	3.541014	1.20843	0.230104	-5.05711	0.500297	0.54999
Neutrophils	GSTT2	-0.44239	3.554147	-1.20825	0.230174	-4.83587	0.50011	0.549828
Neutrophils	AURKB	0.366702	4.237652	1.208211	0.230188	-5.2575	0.490462	0.539467
Neutrophils	PLPPR1	0.569172	1.149205	1.208094	0.230233	-4.74094	0.535637	0.587909
Neutrophils	TRAK2	0.148456	5.153104	1.20798	0.230276	-5.64627	0.477841	0.525995
Neutrophils	SNX33	0.613993	0.759185	1.207738	0.230369	-4.6635	0.541743	0.594456
Neutrophils	GTF2H1	-0.1555	5.958143	-1.20742	0.230491	-5.58328	0.467126	0.514553
Neutrophils	SERINC4	0.674791	0.365548	1.207366	0.230511	-4.66732	0.547881	0.601179
Neutrophils	CNOT11	0.214661	4.112527	1.207344	0.23052	-5.24391	0.492323	0.541697
Neutrophils	LMAN1L	-0.5631	1.485183	-1.20723	0.230564	-4.7674	0.53064	0.582796
Neutrophils	POLB	0.206098	5.332268	1.206861	0.230705	-5.29427	0.475708	0.523758
Neutrophils	ACTN2	-0.79743	-0.1007	-1.20634	0.230903	-4.62523	0.555701	0.609272
Neutrophils	GM42836	-0.77258	-0.40469	-1.20634	0.230904	-4.63576	0.560548	0.614433
Neutrophils	SMYD2	-0.48424	3.028651	-1.20608	0.231003	-4.80961	0.508323	0.558561
Neutrophils	OTULIN	0.129976	6.57022	1.205704	0.231149	-5.61335	0.459651	0.506276
Neutrophils	ZFP182	0.276629	4.211679	1.20561	0.231185	-5.17008	0.491555	0.540634
Neutrophils	RAB12	-0.19545	5.073594	-1.20548	0.231234	-5.26113	0.479636	0.527845
Neutrophils	PSMB7	-0.16169	5.624583	-1.20546	0.231241	-5.44254	0.472175	0.51981
Neutrophils	GM31522	0.568853	-1.00368	1.205355	0.231282	-4.6289	0.570454	0.625045
Neutrophils	COL4A2	-0.30381	2.620499	-1.20515	0.231362	-5.21004	0.514383	0.565195
Neutrophils	MEPCE	-0.22746	4.466254	-1.20476	0.231509	-5.32729	0.488015	0.537064
Neutrophils	HAUS4	0.300745	4.204699	1.204642	0.231556	-5.06985	0.491665	0.541038
Neutrophils	NCLN	0.22815	3.839103	1.204532	0.231599	-5.1973	0.496814	0.546615
Neutrophils	CS	0.132932	6.103857	1.204495	0.231613	-5.59366	0.465795	0.513234
Neutrophils	CPT2	-0.40459	2.829472	-1.20436	0.231663	-4.94273	0.511325	0.562268

Neutrophils	FANCD2	-0.40065	2.80256	-1.20415	0.231744	-4.88154	0.511718	0.562763
Neutrophils	GTF2A2	0.146726	5.923594	1.20412	0.231757	-5.56864	0.468188	0.515939
Neutrophils	SYMPK	0.25905	4.293778	1.204077	0.231773	-5.09425	0.490418	0.539896
Neutrophils	YKT6	-0.19319	4.483546	-1.20401	0.231801	-5.28484	0.487774	0.537052
Neutrophils	CTNNBIP1	-0.15983	4.359655	-1.20398	0.23181	-5.6093	0.489499	0.538907
Neutrophils	GM11973	-0.46722	3.179185	-1.20384	0.231866	-4.84798	0.50627	0.556996
Neutrophils	HIST1H1D	0.625326	1.531522	1.203612	0.231952	-4.77118	0.530732	0.583204
Neutrophils	LRCH4	0.202778	4.260162	1.203403	0.232033	-5.35591	0.490992	0.540642
Neutrophils	TSG101	-0.15026	5.622255	-1.20332	0.232064	-5.57153	0.472317	0.520525
Neutrophils	FAM216A	-0.39246	2.582038	-1.2032	0.232112	-4.84872	0.515056	0.566565
Neutrophils	NACC1	0.237747	4.380772	1.203014	0.232182	-5.13684	0.489307	0.539002
Neutrophils	SMU1	-0.14725	5.608875	-1.20299	0.23219	-5.49813	0.472497	0.520885
Neutrophils	UBXN11	0.462977	1.770334	1.202711	0.232299	-4.74842	0.52721	0.579614
Neutrophils	GPR84	0.379687	0.260814	1.202654	0.232321	-5.04781	0.550433	0.604453
Neutrophils	TLK2	-0.11523	6.825344	-1.20215	0.232516	-5.77545	0.4568	0.503621
Neutrophils	MRPL11	0.296063	4.26095	1.201497	0.232767	-5.0105	0.491734	0.541228
Neutrophils	PBXIP1	0.16579	4.578633	1.201281	0.23285	-5.65092	0.487304	0.536584
Neutrophils	MAPK3	0.133634	4.874121	1.200882	0.233005	-5.74115	0.483221	0.532327
Neutrophils	LRRCC1	-0.28968	3.748019	-1.20062	0.233107	-4.91812	0.498975	0.549316
Neutrophils	YBX3	0.247452	6.314911	1.200566	0.233126	-5.4641	0.463817	0.511432
Neutrophils	NANOS3	-0.68827	0.427569	-1.20055	0.233133	-4.62983	0.548573	0.602442
Neutrophils	GM16174	0.366923	0.995634	1.200532	0.233139	-4.92619	0.539745	0.593012
Neutrophils	WDFY1	0.191527	4.332443	1.200436	0.233177	-5.43208	0.490734	0.540495
Neutrophils	HSP90AA1	-0.13424	7.657631	-1.20034	0.233212	-5.92474	0.446469	0.492731
Neutrophils	UBE2Q2	0.160858	5.51991	1.200284	0.233235	-5.49254	0.474421	0.522949
Neutrophils	OXA1L	0.240293	4.658758	1.200194	0.23327	-5.12971	0.486194	0.535685
Neutrophils	ZDHHC16	0.302442	2.888345	1.200171	0.233279	-4.89726	0.511359	0.56274
Neutrophils	AXIN2	-0.66778	-0.02376	-1.19995	0.233365	-4.64852	0.55569	0.610226
Neutrophils	PNPLA2	0.185776	5.509886	1.199679	0.233469	-5.58518	0.474556	0.523268
Neutrophils	MRPS31	-0.28815	3.653241	-1.19953	0.233525	-4.97315	0.500325	0.55106
Neutrophils	STK25	-0.23235	4.440838	-1.19953	0.233526	-5.14137	0.489221	0.539112
Neutrophils	PPP1R12C	0.150471	5.009564	1.199333	0.233603	-5.50811	0.481362	0.530651
Neutrophils	B3GALT5	-0.80003	0.779445	-1.19932	0.233607	-4.68921	0.543088	0.596911
Neutrophils	ZFP974	-0.8081	0.877863	-1.19917	0.233665	-4.63992	0.541563	0.595306
Neutrophils	HSCB	0.394402	4.143832	1.199039	0.233716	-4.91666	0.493378	0.543617
Neutrophils	IMP3	-0.20972	5.264975	-1.199	0.233733	-5.20167	0.477875	0.526913
Neutrophils	PCBD1	-0.42832	3.53173	-1.19888	0.233779	-5.17986	0.502061	0.553052
Neutrophils	E230016K2	0.307753	0.143576	1.198778	0.233818	-5.13934	0.553041	0.607704
Neutrophils	CDS2	0.155963	4.708784	1.198695	0.23385	-5.52869	0.485502	0.535255
Neutrophils	LNPK	0.180216	4.386002	1.198471	0.233936	-5.50197	0.489986	0.540096
Neutrophils	B130055M	-0.58454	2.013314	-1.1984	0.233964	-4.69034	0.524288	0.576954
Neutrophils	CD40	0.791729	2.17854	1.198381	0.233971	-4.70467	0.521821	0.574309
Neutrophils	GM826	-0.89813	-0.25558	-1.19817	0.234052	-4.6582	0.559461	0.614534
Neutrophils	HTRA2	0.238768	3.681601	1.197897	0.234158	-5.0557	0.50007	0.550948
Neutrophils	REPS2	-0.50791	0.905504	-1.1978	0.234195	-4.82944	0.541297	0.595206
Neutrophils	STK35	-0.27903	3.447827	-1.19763	0.23426	-5.00693	0.503414	0.554653
Neutrophils	CEACAM1	0.152355	3.675486	1.197588	0.234278	-5.63627	0.500157	0.551153

Neutrophils	CDCA2	0.337769	3.79635	1.196939	0.23453	-5.20467	0.49887	0.549562
Neutrophils	GM29093	-0.55319	1.269702	-1.19646	0.234714	-4.65077	0.536323	0.589759
Neutrophils	FCER1G	0.17578	8.628675	1.19643	0.234727	-6.44133	0.434989	0.480562
Neutrophils	ABCD3	-0.23914	4.193442	-1.19639	0.234743	-5.2039	0.493404	0.543732
Neutrophils	FANCC	-0.31891	4.74282	-1.1961	0.234854	-5.09602	0.485874	0.535589
Neutrophils	CFAP53	-0.75659	0.830789	-1.19593	0.234922	-4.68414	0.543282	0.597171
Neutrophils	RPH3AL	-0.49334	1.299189	-1.19545	0.235108	-4.78606	0.536379	0.589642
Neutrophils	PCP4L1	-0.75381	1.36121	-1.19462	0.235432	-4.7169	0.536059	0.588804
Neutrophils	ABHD12	-0.19595	5.584506	-1.19417	0.235606	-5.40761	0.475507	0.523662
Neutrophils	ASL	-0.25303	4.484774	-1.19381	0.235744	-5.25998	0.490763	0.540044
Neutrophils	CST7	0.397822	2.120473	1.193562	0.235842	-5.0792	0.524991	0.576868
Neutrophils	ETOHD2	0.241753	2.615429	1.193458	0.235883	-5.14795	0.517628	0.569019
Neutrophils	ITGB5	-0.38728	3.058168	-1.19331	0.235941	-5.07111	0.511132	0.562084
Neutrophils	ATF1	0.099783	6.564036	1.193303	0.235943	-5.79873	0.462576	0.509822
Neutrophils	PSME2	0.230768	7.278608	1.193209	0.23598	-5.8337	0.453286	0.499813
Neutrophils	4930469K1	0.511122	1.311112	1.193145	0.236005	-4.88057	0.537263	0.590122
Neutrophils	USP45	0.28934	4.074097	1.192888	0.236105	-5.08155	0.496649	0.546566
Neutrophils	9330151L1	-0.62896	0.96571	-1.19276	0.236156	-4.65749	0.542717	0.596032
Neutrophils	ACVR1	-0.36675	3.707524	-1.19238	0.236302	-5.04445	0.502082	0.552359
Neutrophils	TRIM32	0.669547	1.488698	1.192143	0.236395	-4.70715	0.534998	0.587675
Neutrophils	C330011M	-0.59954	0.592367	-1.19168	0.236574	-4.66718	0.549173	0.602689
Neutrophils	GPNMB	0.881056	0.898381	1.191337	0.236709	-4.66447	0.544511	0.597632
Neutrophils	USP50	0.302412	3.371827	1.19131	0.23672	-5.03067	0.507398	0.557912
Neutrophils	CDK20	0.591168	0.593617	1.19083	0.236908	-4.72912	0.549595	0.60284
Neutrophils	PLPP6	0.377332	2.754142	1.190288	0.237119	-4.90218	0.517078	0.568005
Neutrophils	SKIL	0.138808	7.304614	1.190112	0.237188	-6.0097	0.4543	0.500473
Neutrophils	ZCCHC14	-0.44743	1.412865	-1.19	0.237233	-4.87417	0.537299	0.5897
Neutrophils	MIA3	0.128481	5.849323	1.189844	0.237293	-5.6676	0.473498	0.521226
Neutrophils	TMED3	0.148569	5.491352	1.189548	0.237409	-5.7227	0.478454	0.526633
Neutrophils	1810034E1	0.582569	1.895787	1.189386	0.237472	-4.6996	0.530091	0.582152
Neutrophils	MRPL1	-0.19632	4.619868	-1.18934	0.237491	-5.20184	0.490472	0.53966
Neutrophils	CC2D2B	-0.3005	2.70829	-1.18873	0.237729	-5.68959	0.518308	0.569363
Neutrophils	1110012L1	-0.51735	1.829365	-1.18858	0.237786	-4.73402	0.531473	0.583474
Neutrophils	CCNDBP1	0.19457	5.692836	1.18854	0.237803	-5.64658	0.476057	0.524003
Neutrophils	LMBRD1	0.14968	6.356577	1.188409	0.237855	-5.71725	0.467165	0.514477
Neutrophils	MRPL52	0.179056	7.102833	1.188213	0.237932	-5.60741	0.457423	0.50392
Neutrophils	CHST10	-0.74578	0.375693	-1.18809	0.237979	-4.63908	0.554074	0.607687
Neutrophils	9230116N1	-0.68341	0.920334	-1.18783	0.238083	-4.6571	0.545544	0.598596
Neutrophils	ABHD5	0.139492	3.453436	1.187822	0.238085	-5.72	0.507497	0.557885
Neutrophils	GPAT4	-0.20676	4.264538	-1.18766	0.23815	-5.16199	0.495936	0.545487
Neutrophils	COX7B	-0.13393	7.597144	-1.18673	0.238514	-5.89962	0.451637	0.497395
Neutrophils	SLC16A7	-0.33539	3.236151	-1.18669	0.238528	-5.01803	0.511293	0.561613
Neutrophils	MRPL28	0.176174	6.016074	1.186571	0.238575	-5.51679	0.472377	0.51984
Neutrophils	PRMT5	-0.41849	3.483009	-1.18585	0.238858	-4.81438	0.508135	0.558009
Neutrophils	MSH5	-0.50316	2.9838	-1.18581	0.238873	-4.8659	0.515421	0.565814
Neutrophils	P3H2	-0.83635	1.190669	-1.18531	0.239073	-4.77116	0.542831	0.594915
Neutrophils	IGFALS	-0.51011	1.779759	-1.18435	0.239449	-4.79057	0.534511	0.585542

Neutrophils	GALE	0.361696	2.330073	1.183963	0.239601	-4.88415	0.526409	0.576851
Neutrophils	ZFP668	0.293977	3.829078	1.183381	0.23983	-4.98669	0.504575	0.553544
Neutrophils	MGL2	1.067703	-0.18973	1.183281	0.23987	-4.69654	0.565903	0.618996
Neutrophils	PPP2R5A	0.08596	7.861178	1.183249	0.239882	-6.13042	0.44992	0.494796
Neutrophils	ANK	0.473405	3.446389	1.183235	0.239888	-4.89594	0.51011	0.559499
Neutrophils	GNA11	-0.35939	3.954617	-1.18289	0.240023	-4.99278	0.502955	0.551864
Neutrophils	POU5F2	-0.50996	1.687999	-1.18262	0.240129	-4.74915	0.536664	0.58784
Neutrophils	PXMP2	-0.38608	4.067436	-1.18238	0.240224	-5.30431	0.501447	0.550294
Neutrophils	ANKLE2	-0.21862	5.177792	-1.18219	0.240299	-5.28849	0.485838	0.533612
Neutrophils	PRNP	0.404376	2.012971	1.182115	0.24033	-4.9008	0.531709	0.582748
Neutrophils	RASSF4	-0.38359	4.553252	-1.18187	0.240428	-5.00831	0.494555	0.543056
Neutrophils	UTP3	0.185961	5.450096	1.181821	0.240446	-5.43486	0.482088	0.52967
Neutrophils	RBM24	-0.86266	1.008251	-1.18179	0.240457	-4.6635	0.547182	0.599338
Neutrophils	WASHC5	0.238499	4.472587	1.181787	0.240459	-5.25381	0.495692	0.544282
Neutrophils	FBXO7	-0.29975	4.165071	-1.18161	0.240528	-5.03712	0.500097	0.549025
Neutrophils	PLEKHB1	0.45001	0.728002	1.181433	0.240599	-4.83429	0.551679	0.604138
Neutrophils	ZFP341	-0.46313	1.984672	-1.1812	0.24069	-4.75354	0.53233	0.58346
Neutrophils	ZFP445	0.174766	4.93802	1.180806	0.240847	-5.38079	0.489502	0.537517
Neutrophils	WDR83	-0.40143	2.732947	-1.18076	0.240866	-4.83882	0.521254	0.571536
Neutrophils	MYO5A	0.179824	5.016572	1.180587	0.240933	-5.6793	0.488448	0.536349
Neutrophils	MS4A3	0.351104	-0.35863	1.180264	0.241061	-5.25545	0.569455	0.622943
Neutrophils	CACNA2D1	-0.78533	0.864423	-1.18021	0.241083	-4.70381	0.549906	0.602148
Neutrophils	GM17477	-0.61966	0.61424	-1.18017	0.241099	-4.68166	0.553849	0.606347
Neutrophils	MESD	-0.25042	4.085411	-1.18002	0.241159	-5.05206	0.501645	0.550555
Neutrophils	ADSS	0.147852	6.271707	1.179288	0.241447	-5.58146	0.4717	0.518145
Neutrophils	CEP85	0.303174	4.470348	1.17928	0.241451	-5.0308	0.496507	0.544793
Neutrophils	KLHL21	-0.50779	2.689731	-1.17924	0.241466	-4.80155	0.522363	0.57247
Neutrophils	SHOC2	0.112392	6.344774	1.178719	0.241673	-5.86016	0.470815	0.517321
Neutrophils	DRC7	-0.65539	-0.15036	-1.17865	0.241699	-4.70295	0.566599	0.619748
Neutrophils	ANKRD10	-0.22756	5.371393	-1.17852	0.241751	-5.16822	0.484029	0.53159
Neutrophils	SLC25A12	0.169482	5.092687	1.178201	0.241878	-5.42311	0.487883	0.535791
Neutrophils	ABCA3	0.205636	4.193838	1.178183	0.241885	-5.21759	0.500533	0.549366
Neutrophils	VAMP2	0.170263	4.421644	1.178118	0.241911	-5.33775	0.497295	0.545894
Neutrophils	CEP162	-0.39759	3.080056	-1.1781	0.241916	-4.81247	0.516681	0.566661
Neutrophils	SPSB3	0.276213	3.587341	1.178058	0.241935	-4.97785	0.509261	0.558743
Neutrophils	DENND2C	-0.39897	2.008648	-1.17798	0.241967	-4.86502	0.532721	0.583827
Neutrophils	WDR6	0.458925	3.277321	1.177901	0.241997	-4.79949	0.513783	0.563612
Neutrophils	VSTM4	-0.62384	0.147061	-1.17772	0.242068	-4.78065	0.56186	0.614874
Neutrophils	D330041H(	-0.41603	2.247493	-1.17748	0.242164	-4.83036	0.529255	0.580089
Neutrophils	USP19	-0.22924	4.704682	-1.17713	0.242304	-5.31705	0.493452	0.541927
Neutrophils	MDGA1	-0.75807	0.010829	-1.17697	0.242364	-4.65874	0.564166	0.617499
Neutrophils	POT1B	0.333389	4.421725	1.176955	0.242372	-5.09125	0.497444	0.54623
Neutrophils	ELOVL7	0.399967	-0.19718	1.176876	0.242404	-4.96943	0.567528	0.621086
Neutrophils	RAB3A	-0.58316	2.619056	-1.17682	0.242427	-4.72058	0.523679	0.574344
Neutrophils	GM48796	-0.58066	0.350478	-1.1767	0.242473	-4.70412	0.558719	0.611776
Neutrophils	TIMM8B	0.188359	5.242586	1.176623	0.242504	-5.54004	0.485953	0.533971
Neutrophils	LY6I	0.454113	0.802083	1.175828	0.24282	-5.37612	0.552168	0.604292

Neutrophils	UPB1	-0.45353	3.0781	-1.17541	0.242985	-4.93372	0.517577	0.567402
Neutrophils	VPS11	-0.21704	4.072729	-1.17539	0.242995	-5.2027	0.503106	0.551913
Neutrophils	SNUPN	-0.38652	2.593487	-1.17525	0.243051	-4.79263	0.524782	0.575159
Neutrophils	1810006J0	0.462768	-0.01116	1.175158	0.243086	-4.84497	0.565297	0.618367
Neutrophils	ADAM32	-0.77328	0.784382	-1.17505	0.243128	-4.70203	0.552597	0.604882
Neutrophils	ZBTB6	0.437453	1.997204	1.174855	0.243207	-4.81704	0.533857	0.584905
Neutrophils	ARHGAP30	0.094341	7.139253	1.174458	0.243365	-6.00477	0.461276	0.507112
Neutrophils	FTSJ3	0.458887	4.016619	1.174435	0.243374	-4.87693	0.504122	0.553169
Neutrophils	TBC1D20	0.146452	5.789234	1.174288	0.243433	-5.71175	0.479333	0.526535
Neutrophils	TBCK	0.164331	5.26974	1.173976	0.243557	-5.50577	0.486565	0.534279
Neutrophils	TUSC3	-0.25219	4.978446	-1.17392	0.243578	-5.19494	0.490616	0.538629
Neutrophils	COL5A2	0.497249	1.225753	1.173657	0.243685	-4.91453	0.546162	0.598002
Neutrophils	TAF1B	-0.26243	4.199522	-1.17335	0.243806	-5.05894	0.501738	0.55058
Neutrophils	TNK2	-0.35279	3.209858	-1.17334	0.243812	-4.95271	0.516095	0.565955
Neutrophils	ADCY9	-0.25864	3.136828	-1.1733	0.243828	-5.29692	0.517171	0.567106
Neutrophils	JMJD6	-0.18908	4.999369	-1.17312	0.243897	-5.43858	0.49048	0.538456
Neutrophils	REL	0.15699	8.12408	1.172862	0.244001	-6.05403	0.448836	0.493665
Neutrophils	ZDHHC3	0.140968	5.048938	1.172783	0.244033	-5.63205	0.489805	0.537787
Neutrophils	MTERF4	0.446052	1.913823	1.172633	0.244093	-4.81901	0.535602	0.586809
Neutrophils	SHF	-0.47152	1.832889	-1.17262	0.244099	-4.76866	0.536841	0.58813
Neutrophils	RRP1B	0.230312	4.132089	1.172175	0.244276	-5.3886	0.503028	0.551744
Neutrophils	1700066M	-0.48942	1.288003	-1.17185	0.244404	-4.73762	0.545718	0.597165
Neutrophils	FGL2	0.28307	4.054695	1.171737	0.24445	-5.70998	0.504299	0.553015
Neutrophils	IPO11	-0.24857	4.478076	-1.1714	0.244584	-5.18502	0.498427	0.546708
Neutrophils	GM45669	-0.77875	0.129811	-1.17126	0.244642	-4.6668	0.564289	0.617051
Neutrophils	CCNJ	-0.46118	2.015982	-1.17109	0.244708	-4.87962	0.534738	0.585598
Neutrophils	SCRN2	-0.47064	1.43712	-1.17091	0.244783	-4.7376	0.543658	0.595128
Neutrophils	APOBEC3	-0.24829	6.862598	-1.17084	0.244809	-5.43448	0.465802	0.511739
Neutrophils	GABARAPL	0.162771	7.762001	1.169952	0.245165	-6.0259	0.454635	0.49926
Neutrophils	TRIM5	0.291728	3.589282	1.169513	0.24534	-5.14617	0.512184	0.560825
Neutrophils	9130019O2	-0.78267	0.355077	-1.16919	0.245471	-4.66094	0.561799	0.613647
Neutrophils	HARS	0.18693	5.114979	1.169136	0.245491	-5.30978	0.490473	0.537573
Neutrophils	NDUFV2	0.156245	6.136177	1.169057	0.245523	-5.67546	0.476431	0.522519
Neutrophils	JARID2	-0.12442	8.144583	-1.16889	0.245589	-6.10543	0.450064	0.49415
Neutrophils	TSHZ2	-0.30511	3.393266	-1.16865	0.245684	-5.42225	0.515268	0.564096
Neutrophils	DNAJC3	0.149862	6.725717	1.168272	0.245838	-5.74181	0.46879	0.51414
Neutrophils	CLDN1	0.340531	1.493829	1.167971	0.245958	-5.27449	0.54414	0.594826
Neutrophils	SWAP70	-0.30588	5.61218	-1.16792	0.24598	-5.28628	0.483865	0.530424
Neutrophils	GM10563	0.215819	3.607105	1.167838	0.246012	-5.37894	0.512299	0.560872
Neutrophils	FKBP3	-0.20886	6.130579	-1.16767	0.246078	-5.51497	0.476784	0.522824
Neutrophils	TRAPPC13	-0.26381	3.808587	-1.16766	0.246083	-5.06022	0.509365	0.557735
Neutrophils	TOPORS	-0.19835	5.749708	-1.1675	0.246147	-5.54569	0.481976	0.528407
Neutrophils	PATL2	-0.52611	2.023827	-1.16745	0.246169	-4.81322	0.535969	0.58617
Neutrophils	UGGT1	0.18311	5.389379	1.167369	0.2462	-5.3569	0.486941	0.533764
Neutrophils	ADTRP	-0.30264	1.835711	-1.16697	0.246362	-5.20124	0.539103	0.589422
Neutrophils	UBE3B	-0.19911	4.424846	-1.16662	0.2465	-5.24945	0.50091	0.548579
Neutrophils	SDR39U1	0.648426	1.131364	1.1662	0.24667	-4.69511	0.550362	0.601422

Neutrophils	LCORL	0.14113	6.985276	1.166031	0.246738	-5.84665	0.465822	0.51108
Neutrophils	GM32089	0.659652	-1.09921	1.165895	0.246793	-4.65954	0.586566	0.639981
Neutrophils	ZFP217	0.197767	4.011596	1.165749	0.246851	-5.23752	0.506943	0.55529
Neutrophils	GLIS3	0.471622	3.068689	1.165747	0.246852	-5.14358	0.520756	0.570053
Neutrophils	AI987944	-0.3995	2.764701	-1.16553	0.24694	-4.88216	0.525292	0.575055
Neutrophils	EPM2A	-0.37886	1.526051	-1.16546	0.246969	-5.01509	0.544194	0.59523
Neutrophils	FASTKD2	-0.3769	2.952537	-1.16541	0.246989	-4.80671	0.522484	0.572099
Neutrophils	IBTK	0.2144	4.642613	1.165316	0.247026	-5.38536	0.497911	0.545854
Neutrophils	FKBP1A	-0.13056	7.336878	-1.16519	0.247076	-5.85613	0.461197	0.506457
Neutrophils	PIGG	0.633514	0.963956	1.165056	0.24713	-4.69181	0.552999	0.604777
Neutrophils	PHF21A	0.124867	7.069098	1.165005	0.24715	-6.03309	0.464715	0.510295
Neutrophils	ZFP839	-0.52588	1.537463	-1.16493	0.24718	-4.71503	0.544017	0.595245
Neutrophils	MAP3K11	0.2164	3.876975	1.164441	0.247378	-5.31164	0.509198	0.557957
Neutrophils	BRAF	0.118225	7.588384	1.163991	0.247559	-6.2378	0.458368	0.503363
Neutrophils	CHM	0.136161	5.555869	1.163815	0.24763	-5.73168	0.485612	0.532735
Neutrophils	TSTD2	0.257159	3.84075	1.163806	0.247634	-5.15178	0.509918	0.558787
Neutrophils	GM43768	0.559917	0.70894	1.163724	0.247667	-4.77162	0.557589	0.609631
Neutrophils	KCTD4	0.5466	2.200261	1.163249	0.247858	-4.77732	0.534434	0.584957
Neutrophils	PSTPIP2	0.172809	4.482064	1.163164	0.247893	-5.93041	0.500768	0.548977
Neutrophils	TRAIP	-0.48358	2.119414	-1.16314	0.247903	-4.82418	0.535669	0.586279
Neutrophils	SIAH2	-0.24409	4.793136	-1.16314	0.247904	-5.1426	0.496352	0.544244
Neutrophils	ZFP617	0.479707	2.165547	1.162941	0.247983	-4.81416	0.534964	0.585573
Neutrophils	HMGN2	0.142136	7.53605	1.162899	0.248	-6.24712	0.459125	0.504272
Neutrophils	SMIM10L1	-0.20176	4.931338	-1.16244	0.248184	-5.3376	0.494435	0.542256
Neutrophils	DYM	0.120849	5.781233	1.162355	0.248219	-5.78062	0.482621	0.529576
Neutrophils	ZFP955A	-0.69645	1.472841	-1.16226	0.248257	-4.68009	0.545684	0.597027
Neutrophils	RUNX2	0.262375	4.240789	1.162185	0.248288	-5.64445	0.504256	0.552783
Neutrophils	LNPEP	0.104822	7.335044	1.162073	0.248333	-6.06211	0.461782	0.507194
Neutrophils	TMLHE	-0.53304	2.31306	-1.16202	0.248356	-4.86647	0.532752	0.583292
Neutrophils	SNX9	-0.1891	6.331978	-1.16181	0.248438	-5.55354	0.475124	0.521629
Neutrophils	PEX12	0.458295	1.86574	1.161734	0.24847	-4.82687	0.539598	0.590721
Neutrophils	GM26881	-0.65139	0.245715	-1.16168	0.248492	-4.72462	0.565146	0.617914
Neutrophils	FRS2	0.151027	5.84525	1.161591	0.248528	-5.51038	0.481744	0.528823
Neutrophils	NFX1	0.184469	4.900546	1.161489	0.248569	-5.33853	0.494869	0.542916
Neutrophils	GM12166	0.557229	0.781167	1.161412	0.2486	-4.75746	0.556569	0.608856
Neutrophils	AKAP9	0.15461	6.105265	1.160895	0.248809	-5.63374	0.478504	0.525287
Neutrophils	HIBADH	-0.19328	5.742005	-1.16003	0.249159	-5.52444	0.484056	0.530849
Neutrophils	TEX14	-0.19253	5.64791	-1.15981	0.249247	-5.76628	0.48543	0.532308
Neutrophils	RNF170	-0.26716	3.319753	-1.15917	0.249509	-5.05138	0.519126	0.56808
Neutrophils	SERPINB1A	0.203506	3.600711	1.159082	0.249544	-5.81396	0.514983	0.563661
Neutrophils	COMMD9	-0.45569	2.646144	-1.15892	0.249608	-4.80028	0.52923	0.578868
Neutrophils	U2AF1L4	0.395543	3.047054	1.158688	0.249703	-4.945	0.523309	0.572565
Neutrophils	GLO1	0.240868	5.029475	1.157979	0.249991	-5.32454	0.494598	0.542071
Neutrophils	TRIM41	-0.19299	4.185669	-1.15792	0.250013	-5.26912	0.506627	0.554955
Neutrophils	TBC1D19	-0.45438	1.749793	-1.15791	0.250021	-4.75405	0.54308	0.593865
Neutrophils	TMEM106I	-0.23311	4.565283	-1.15787	0.250034	-5.23023	0.501178	0.549121
Neutrophils	ARF5	0.133118	8.486216	1.157801	0.250063	-6.15639	0.448353	0.492341

Neutrophils	HSPE1	-0.19045	7.179751	-1.15766	0.25012	-5.73735	0.465272	0.510611
Neutrophils	KMT2B	0.25675	3.888591	1.157609	0.250141	-5.12857	0.510935	0.559604
Neutrophils	EXOC8	0.426595	1.951234	1.157545	0.250167	-4.83691	0.539966	0.590611
Neutrophils	OPTN	0.153743	4.651784	1.157453	0.250205	-5.7816	0.499945	0.547906
Neutrophils	EZH1	0.296626	3.600438	1.157446	0.250208	-5.10788	0.515149	0.564176
Neutrophils	IER3	0.193035	6.13903	1.157246	0.250289	-6.11704	0.479292	0.52578
Neutrophils	RBM39	-0.06771	9.586582	-1.1566	0.250552	-6.34464	0.434968	0.477917
Neutrophils	HCCS	0.187217	4.627385	1.156454	0.250611	-5.3067	0.500705	0.548686
Neutrophils	OLFR920	-0.59141	0.137933	-1.15635	0.250655	-4.7539	0.569131	0.621701
Neutrophils	GM49173	0.890721	-0.87955	1.156288	0.250678	-4.66428	0.585914	0.639526
Neutrophils	PHF2OS1	0.525527	1.058613	1.156197	0.250715	-4.72163	0.554361	0.606041
Neutrophils	CDT1	0.23907	4.370678	1.155991	0.250799	-5.19277	0.50438	0.552763
Neutrophils	S1PR1	-0.32442	4.061514	-1.15584	0.25086	-5.20582	0.508842	0.557605
Neutrophils	NET1	-0.37148	4.035875	-1.15572	0.250908	-5.01768	0.509214	0.558022
Neutrophils	CNN2	0.135502	6.538205	1.15563	0.250946	-6.08209	0.474216	0.520472
Neutrophils	DNAJB2	0.38336	2.766414	1.15548	0.251007	-4.90272	0.527984	0.5781
Neutrophils	GPD2	0.1877	6.625661	1.155426	0.251029	-5.95519	0.473039	0.519218
Neutrophils	CPNE8	-0.36599	2.649239	-1.15525	0.251101	-5.12612	0.529752	0.580003
Neutrophils	ARHGAP33	-0.72327	0.549492	-1.15518	0.25113	-4.67817	0.56248	0.614873
Neutrophils	A930024E(	-0.74446	1.108621	-1.15514	0.251147	-4.6741	0.553569	0.605414
Neutrophils	GM15956	0.458297	0.27605	1.154831	0.251271	-4.84369	0.56706	0.619743
Neutrophils	SLC46A2	-0.70526	-0.09426	-1.15455	0.251387	-4.65289	0.573163	0.626198
Neutrophils	ZFP942	0.270441	4.309464	1.154513	0.251401	-5.15242	0.505476	0.55404
Neutrophils	GM43113	0.736548	-0.69248	1.154094	0.251572	-4.6651	0.583323	0.636875
Neutrophils	CLASP2	-0.14625	6.927855	-1.15338	0.251863	-5.73193	0.469723	0.515575
Neutrophils	TTC4	0.295208	3.541793	1.153304	0.251894	-4.97048	0.517236	0.566557
Neutrophils	RDH9	-0.59657	0.805989	-1.15328	0.251903	-4.78716	0.559239	0.611337
Neutrophils	TIMM29	0.32693	3.296538	1.153222	0.251927	-4.98486	0.520866	0.570463
Neutrophils	PARD3	-0.4755	2.300134	-1.15284	0.252085	-4.95251	0.535989	0.586741
Neutrophils	TICAM2	0.460369	1.274799	1.1528	0.2521	-4.82512	0.551909	0.603706
Neutrophils	GPR183	-0.57533	3.488292	-1.15274	0.252123	-4.8609	0.518127	0.567664
Neutrophils	CD180	-0.37556	5.032672	-1.15245	0.252243	-5.06972	0.495962	0.543831
Neutrophils	SPP1	0.464967	2.353951	1.152322	0.252295	-5.31166	0.535323	0.585973
Neutrophils	TMC7	0.635093	-0.40026	1.152068	0.252399	-4.67679	0.579243	0.632598
Neutrophils	GM10634	0.500676	0.651485	1.151956	0.252445	-4.83855	0.562101	0.614402
Neutrophils	TTLL4	0.389545	3.000367	1.151633	0.252577	-4.85014	0.525738	0.575685
Neutrophils	THOP1	0.540629	2.236401	1.151611	0.252586	-4.77206	0.537324	0.588053
Neutrophils	WRAP53	-0.3827	2.788872	-1.15119	0.252759	-4.84621	0.529179	0.579186
Neutrophils	MRPL20	0.13914	6.1388	1.151048	0.252816	-5.71215	0.481033	0.52768
Neutrophils	STAB2	-0.27595	5.148934	-1.15094	0.252862	-5.85772	0.494769	0.542453
Neutrophils	BMF	0.550875	2.137288	1.150455	0.253059	-4.77321	0.539436	0.590063
Neutrophils	HS6ST1	-0.27162	4.156867	-1.1502	0.253164	-5.15061	0.509258	0.557863
Neutrophils	ATAD3A	-0.37562	4.10261	-1.15018	0.253172	-4.96734	0.510046	0.558706
Neutrophils	FOPNL	0.188399	4.468642	1.150078	0.253213	-5.38369	0.504755	0.553042
Neutrophils	NRN1	0.414384	3.109984	1.149783	0.253334	-5.18018	0.524733	0.574355
Neutrophils	STRN4	0.197524	4.492619	1.14966	0.253384	-5.30088	0.504455	0.552673
Neutrophils	LDLRAD4	-0.23342	6.109073	-1.14959	0.253411	-5.60204	0.481777	0.528387

Neutrophils	GM4952	-0.41992	2.667628	-1.14941	0.253487	-5.09629	0.531397	0.58155
Neutrophils	MORF4L2	0.147803	5.404884	1.149368	0.253504	-5.57314	0.491523	0.538892
Neutrophils	CSNK2A2	0.133033	5.948451	1.149305	0.25353	-5.57054	0.483982	0.530812
Neutrophils	TSC22D1	-0.26861	4.872446	-1.14916	0.253589	-5.26768	0.499049	0.546972
Neutrophils	ALAD	-0.32503	4.057214	-1.14871	0.253776	-5.14441	0.510969	0.559716
Neutrophils	FTSJ1	-0.28897	3.078297	-1.14867	0.253791	-4.95129	0.525431	0.575173
Neutrophils	UFC1	-0.13434	5.792898	-1.14849	0.253863	-5.49971	0.486334	0.533359
Neutrophils	2410131K1	0.409629	2.169111	1.148444	0.253883	-4.92016	0.539239	0.589954
Neutrophils	GM17484	-0.56889	1.264186	-1.14819	0.253987	-4.809	0.553472	0.605125
Neutrophils	APPBP2	-0.10455	6.248663	-1.14769	0.254192	-5.90242	0.480472	0.526955
Neutrophils	GTSF2	0.644615	0.091247	1.147558	0.254247	-4.75078	0.57269	0.625423
Neutrophils	ICE2	-0.44165	2.160721	-1.1473	0.254352	-4.77767	0.539853	0.590575
Neutrophils	PLCB2	0.213355	3.320212	1.147291	0.254357	-5.33611	0.522287	0.571831
Neutrophils	GM45894	-0.27365	2.290972	-1.14697	0.254488	-5.02674	0.538013	0.588579
Neutrophils	SH3BGRL	0.138144	6.824052	1.146864	0.254532	-5.80257	0.472857	0.518819
Neutrophils	SCRIB	-0.26905	3.392621	-1.14646	0.254697	-5.0546	0.521606	0.570974
Neutrophils	BCL11B	-0.82657	1.080616	-1.14627	0.254775	-4.73837	0.557245	0.608931
Neutrophils	CACTIN	0.301255	3.991663	1.146149	0.254826	-5.06393	0.512836	0.561574
Neutrophils	IFT43	0.410404	1.54627	1.145594	0.255055	-4.86213	0.550266	0.601222
Neutrophils	ARPC1A	0.120255	6.586291	1.145486	0.255099	-5.83023	0.476662	0.522574
Neutrophils	FBXO30	-0.15825	5.032199	-1.14535	0.255154	-5.70562	0.498211	0.545727
Neutrophils	PIGB	0.232156	2.703863	1.144821	0.255373	-5.26295	0.532597	0.582399
Neutrophils	SLC16A10	-0.22168	7.024829	-1.14481	0.255377	-5.86182	0.470952	0.5164
Neutrophils	CSAD	-0.29736	3.965874	-1.14476	0.255398	-5.14319	0.513771	0.562303
Neutrophils	IL11RA1	-0.37863	2.113905	-1.14437	0.255557	-4.87124	0.541657	0.592095
Neutrophils	TOR1AIP1	0.115826	7.42657	1.144315	0.255582	-6.02566	0.465629	0.51072
Neutrophils	CDO1	0.306544	3.756999	1.14415	0.25565	-5.26838	0.516856	0.565702
Neutrophils	TMEM170	-0.38731	1.517214	-1.14392	0.255746	-4.8906	0.550963	0.602154
Neutrophils	UBA1	0.133539	6.00691	1.143827	0.255783	-5.71918	0.484787	0.531435
Neutrophils	TOR2A	-0.26703	3.670287	-1.14357	0.25589	-5.07376	0.518135	0.567168
Neutrophils	BTBD19	0.253631	2.725168	1.143468	0.255931	-5.18661	0.532291	0.58228
Neutrophils	UBN2	0.141686	7.076175	1.143434	0.255945	-5.81467	0.470282	0.515863
Neutrophils	AP1AR	-0.31901	5.002368	-1.14338	0.255966	-5.06264	0.498841	0.546526
Neutrophils	TMEM198I	0.580084	0.786307	1.143333	0.255987	-4.7313	0.562583	0.614548
Neutrophils	FBXL17	-0.1188	7.560342	-1.14316	0.256058	-6.00069	0.463865	0.509
Neutrophils	PDE12	-0.228	3.991564	-1.14314	0.256067	-5.13978	0.513412	0.562166
Neutrophils	LPAR6	-0.27555	4.523596	-1.14305	0.256103	-5.30533	0.505688	0.553948
Neutrophils	DDX28	0.468501	1.558733	1.143011	0.25612	-4.76278	0.55031	0.601584
Neutrophils	MOB1B	0.113091	6.203813	1.142839	0.256191	-5.75595	0.482081	0.528727
Neutrophils	ZFP3	-0.65379	0.562138	-1.14283	0.256194	-4.68951	0.566196	0.618575
Neutrophils	VASP	0.114765	6.541669	1.14229	0.256418	-6.08633	0.477799	0.523971
Neutrophils	ZWINT	-0.19241	5.039001	-1.1419	0.25658	-5.34729	0.498857	0.546491
Neutrophils	PSMD1	-0.13508	6.546401	-1.14169	0.256666	-5.72233	0.477924	0.524088
Neutrophils	DSTN	-0.09588	6.886463	-1.14169	0.256667	-6.30385	0.47333	0.51915
Neutrophils	ARL11	0.269023	1.439587	1.141558	0.25672	-5.16255	0.552788	0.604154
Neutrophils	HDAC8	-0.21709	6.530622	-1.14099	0.256955	-5.56188	0.478393	0.524509
Neutrophils	EAPP	0.118233	5.602282	1.140887	0.256998	-5.67025	0.491186	0.538253

Neutrophils	5330438D1	-0.24175	4.842586	-1.14088	0.257001	-5.25165	0.501922	0.549758
Neutrophils	CCDC71L	0.245482	4.455272	1.140585	0.257123	-5.30665	0.507632	0.555802
Neutrophils	NCALD	-0.42518	2.377149	-1.14036	0.257217	-4.94062	0.538714	0.588928
Neutrophils	ADAM17	0.194505	6.061586	1.140157	0.2573	-5.67965	0.485052	0.531602
Neutrophils	SLC48A1	0.175934	5.074284	1.139994	0.257368	-5.44016	0.498868	0.546417
Neutrophils	WFS1	-0.51974	1.279726	-1.13998	0.257374	-4.70667	0.555879	0.607255
Neutrophils	SHE	-0.5079	0.946591	-1.13944	0.257598	-4.88883	0.561572	0.613128
Neutrophils	PTPN7	0.198363	4.151708	1.139196	0.257699	-5.52194	0.512599	0.560945
Neutrophils	INTS12	-0.11885	4.380987	-1.13907	0.257751	-5.76748	0.509268	0.557435
Neutrophils	DAB2	-0.36496	4.009728	-1.13891	0.257816	-5.53295	0.514714	0.563282
Neutrophils	WDR3	0.251901	4.225869	1.138458	0.258005	-5.10891	0.511789	0.560117
Neutrophils	TMEM229A	-0.68241	-1.03017	-1.13838	0.258037	-4.68296	0.594625	0.648203
Neutrophils	PAXX	-0.24561	3.270058	-1.13823	0.258099	-5.13383	0.525924	0.575254
Neutrophils	NT5M	-0.30784	3.572881	-1.13796	0.25821	-4.97689	0.521403	0.570532
Neutrophils	PNKD	-0.23348	4.867546	-1.13783	0.258266	-5.3062	0.50252	0.550339
Neutrophils	SLC6A19	-0.74653	-0.35949	-1.13783	0.258267	-4.66249	0.583343	0.636419
Neutrophils	PELP1	-0.3456	3.358177	-1.1378	0.25828	-4.93736	0.524604	0.573951
Neutrophils	NSMAF	0.261984	4.903953	1.137476	0.258413	-5.24634	0.502152	0.549896
Neutrophils	KLHL42	-0.42743	2.445253	-1.13737	0.258457	-4.81491	0.538609	0.588879
Neutrophils	EIF2B1	-0.28806	3.62049	-1.13638	0.258866	-5.01641	0.521579	0.570326
Neutrophils	SYNJ1	0.114943	6.731012	1.136088	0.25899	-6.01041	0.477528	0.523054
Neutrophils	ZFP865	0.332259	3.265059	1.135881	0.259076	-5.01214	0.527117	0.576091
Neutrophils	FAM53A	-0.29893	3.797306	-1.13561	0.259189	-5.00858	0.519304	0.567779
Neutrophils	CCSER2	-0.24278	4.778998	-1.13543	0.259262	-5.27127	0.505028	0.552576
Neutrophils	ZFP251	-0.55536	2.407099	-1.13521	0.259357	-4.73205	0.540366	0.590345
Neutrophils	IGFBP3	-0.65836	0.690335	-1.13519	0.259365	-4.76131	0.567511	0.619196
Neutrophils	MYEF2	-0.30235	5.328236	-1.13485	0.259504	-5.10714	0.497372	0.544267
Neutrophils	ERAP1	-0.23358	5.228177	-1.13466	0.259583	-5.27555	0.498848	0.545856
Neutrophils	E2F7	-0.38589	2.983824	-1.13439	0.259697	-5.09799	0.531839	0.58118
Neutrophils	GATAD2B	-0.11844	7.433227	-1.13416	0.259795	-5.99006	0.46859	0.513555
Neutrophils	AURKAIP1	0.131003	5.837172	1.134119	0.25981	-5.74625	0.490323	0.536888
Neutrophils	IQCG	-0.77836	0.050135	-1.13403	0.259848	-4.70185	0.5783	0.630661
Neutrophils	SPDEF	-0.73244	-0.4296	-1.13402	0.259853	-4.69335	0.586279	0.639112
Neutrophils	A130010J1	0.456273	1.72998	1.133723	0.259976	-4.85464	0.551368	0.602068
Neutrophils	GSTM4	0.580116	1.121715	1.133418	0.260103	-4.77186	0.561175	0.612364
Neutrophils	MMS19	0.208767	4.947005	1.133321	0.260143	-5.24997	0.503174	0.550577
Neutrophils	NOP16	-0.33502	4.283714	-1.1328	0.260361	-5.0134	0.512996	0.561058
Neutrophils	PRG3	-0.88803	1.171663	-1.1327	0.260402	-4.71116	0.560622	0.611803
Neutrophils	ISOC1	-0.25008	4.993923	-1.13269	0.260407	-5.19942	0.502724	0.550088
Neutrophils	CHKB	0.19785	4.306938	1.132527	0.260475	-5.2813	0.512693	0.560823
Neutrophils	GNB1L	-0.41019	3.308245	-1.13227	0.260582	-4.8688	0.527581	0.576712
Neutrophils	GM9993	-0.62394	1.16551	-1.13209	0.260658	-4.71891	0.560849	0.612119
Neutrophils	RAPGEF5	-0.27093	5.011787	-1.13207	0.260665	-5.67595	0.502584	0.550005
Neutrophils	TOMM6	0.102507	7.550466	1.131849	0.260759	-6.06317	0.46763	0.512475
Neutrophils	WIPI1	0.258808	2.277802	1.131798	0.26078	-5.33664	0.543353	0.593493
Neutrophils	MAOA	-0.71121	0.932781	-1.13161	0.260857	-4.7171	0.564667	0.616132
Neutrophils	RNF144B	-0.31019	2.562148	-1.13151	0.260899	-5.10957	0.539004	0.588883

Neutrophils	KRT8	-0.48342	1.975849	-1.13107	0.261084	-4.94944	0.548151	0.598825
Neutrophils	ELK1	0.380355	1.559348	1.131024	0.261104	-4.83671	0.554707	0.605798
Neutrophils	4930430E1	0.592736	0.409202	1.131017	0.261106	-4.84754	0.573229	0.625467
Neutrophils	RERE	-0.12485	7.702335	-1.13095	0.261133	-5.94634	0.465701	0.510602
Neutrophils	MAP2K3OS	0.784713	-0.12812	1.130861	0.261172	-4.7127	0.582094	0.634865
Neutrophils	SMARCA5	0.099886	7.448521	1.130698	0.26124	-5.98998	0.469099	0.514307
Neutrophils	TAPT1	0.19352	5.802801	1.130431	0.261352	-5.5576	0.491665	0.538502
Neutrophils	SYNRG	-0.17333	5.053855	-1.12986	0.26159	-5.44169	0.502526	0.550054
Neutrophils	UBE2M	0.109395	7.20691	1.129717	0.261651	-5.9658	0.472693	0.518083
Neutrophils	EIF2S3Y	2.786617	1.945318	1.129623	0.26169	-4.94706	0.549094	0.59982
Neutrophils	TBXA2R	-0.68185	2.36173	-1.12962	0.261692	-4.76144	0.542607	0.592914
Neutrophils	FAP	0.662265	0.744801	1.129227	0.261857	-4.85119	0.568246	0.620329
Neutrophils	4921531C2	-0.45465	1.642005	-1.12919	0.261873	-4.87543	0.553872	0.605063
Neutrophils	THBS3	-0.67825	1.01596	-1.12908	0.26192	-4.75507	0.563862	0.615697
Neutrophils	ATM	-0.3493	4.221236	-1.12898	0.26196	-4.93677	0.514587	0.563184
Neutrophils	HRH4	-0.57914	0.27047	-1.12896	0.261968	-4.81417	0.575996	0.628577
Neutrophils	GMEB1	0.188002	5.025748	1.12887	0.262006	-5.44778	0.50293	0.55076
Neutrophils	B930036N1	-0.1981	5.605823	-1.12879	0.26204	-5.74915	0.494697	0.541993
Neutrophils	PPM1E	-0.38068	4.964118	-1.12835	0.262223	-5.24012	0.50407	0.551898
Neutrophils	9230111E0	0.495957	-0.18455	1.128214	0.262282	-4.86689	0.583848	0.636881
Neutrophils	SNRNP25	-0.34259	3.939991	-1.12805	0.262351	-4.99054	0.519047	0.567986
Neutrophils	SPRYD4	0.456704	1.803638	1.127906	0.262411	-4.77891	0.551685	0.602843
Neutrophils	ALKBH6	-0.31839	2.755561	-1.12757	0.262554	-4.90227	0.53709	0.587251
Neutrophils	POLR2K	-0.11218	6.77475	-1.12717	0.262719	-5.86431	0.479195	0.525206
Neutrophils	GM15337	-0.4629	2.140023	-1.12709	0.262752	-4.94039	0.546812	0.597519
Neutrophils	3830406C1	-0.22927	4.453206	-1.12693	0.262822	-5.20683	0.511946	0.560353
Neutrophils	WDR24	-0.48528	1.796393	-1.12644	0.263025	-4.79031	0.552567	0.603535
Neutrophils	TGS1	-0.17797	5.214453	-1.12599	0.263216	-5.37405	0.501442	0.549012
Neutrophils	NUDT5	-0.21142	5.148841	-1.12598	0.26322	-5.37612	0.50238	0.550016
Neutrophils	ERG28	-0.18182	5.118614	-1.12562	0.263371	-5.31445	0.502884	0.550714
Neutrophils	UBE2V1	-0.11463	6.983076	-1.12548	0.263433	-5.84903	0.476924	0.52293
Neutrophils	FHL1	-0.58442	0.58688	-1.12526	0.263524	-4.75548	0.572273	0.624858
Neutrophils	IQCH	-0.88661	0.250926	-1.12525	0.263529	-4.70204	0.57779	0.630714
Neutrophils	MRPS16	0.166927	5.607422	1.125189	0.263554	-5.55537	0.495938	0.543413
Neutrophils	SELENON	0.143595	3.694822	1.125185	0.263556	-5.82945	0.5237	0.573129
Neutrophils	SLC25A37	0.208437	5.267981	1.124953	0.263654	-5.69488	0.500842	0.548573
Neutrophils	NBDY	-0.25157	3.659616	-1.12445	0.263865	-5.12281	0.524643	0.573889
Neutrophils	SHKBP1	0.19595	4.068341	1.123995	0.264057	-5.35728	0.518742	0.567498
Neutrophils	TMEM106A	-0.28427	3.645492	-1.12393	0.264086	-5.23201	0.525032	0.574216
Neutrophils	COPS6	0.175066	5.552293	1.123885	0.264104	-5.51265	0.49728	0.544532
Neutrophils	HERC1	0.12422	7.056727	1.123615	0.264218	-5.97455	0.476584	0.522237
Neutrophils	GAS6	-0.46985	1.545619	-1.12298	0.264485	-4.8975	0.557786	0.609063
Neutrophils	IGLC3	-0.8535	4.04289	-1.12287	0.264534	-4.98296	0.519432	0.568194
Neutrophils	CASP12	-0.60486	-0.47839	-1.12275	0.264583	-4.73736	0.590979	0.644287
Neutrophils	SSB	-0.10942	6.803522	-1.12236	0.264749	-5.79959	0.480195	0.526364
Neutrophils	TCF7L1	-0.29593	3.097343	-1.12233	0.264759	-5.24679	0.533625	0.583567
Neutrophils	ZCCHC8	-0.12922	5.108019	-1.12227	0.264785	-5.66501	0.503911	0.551806

Neutrophils	ACADM	-0.21868	4.767805	-1.12223	0.264801	-5.4718	0.508815	0.557076
Neutrophils	ITFG1	0.125474	5.454025	1.121817	0.264978	-5.64928	0.498974	0.546546
Neutrophils	ACTR10	0.102665	6.186686	1.121781	0.264993	-5.7971	0.488685	0.535511
Neutrophils	GM37168	0.299802	0.65738	1.121735	0.265013	-5.15628	0.572115	0.624559
Neutrophils	GM12743	-0.45548	2.382516	-1.12173	0.265015	-4.85728	0.544619	0.59532
Neutrophils	TMEM43	0.204876	3.43413	1.121692	0.26503	-5.34842	0.528524	0.578162
Neutrophils	UNC45B	-0.56731	0.423376	-1.12159	0.265075	-4.84519	0.575951	0.62869
Neutrophils	2500004C0	0.492984	1.043374	1.121537	0.265096	-4.7757	0.565843	0.617957
Neutrophils	SOD2	0.252319	6.929524	1.121509	0.265108	-6.03773	0.47848	0.524599
Neutrophils	RBM12B2	0.363475	2.316248	1.121502	0.265111	-4.91501	0.54565	0.596475
Neutrophils	ZBTB26	-0.66395	0.850706	-1.12118	0.265246	-4.69624	0.569063	0.621325
Neutrophils	A230059L0	-0.7409	0.47539	-1.12116	0.265256	-4.72419	0.575195	0.627835
Neutrophils	KBTBD8	0.661779	0.251382	1.120926	0.265355	-4.68749	0.578993	0.631876
Neutrophils	KLRI1	-0.69797	-0.19768	-1.12068	0.265461	-4.7087	0.586575	0.639883
Neutrophils	GBP2	0.606345	4.147241	1.120419	0.265569	-5.35948	0.518168	0.567176
Neutrophils	ZFP651	-0.34016	1.578642	-1.12029	0.265624	-4.99641	0.55756	0.609223
Neutrophils	DNAJA3	-0.33682	3.76841	-1.12027	0.265631	-4.98784	0.523793	0.573224
Neutrophils	CDK4	-0.19742	5.844705	-1.12022	0.265653	-5.46351	0.493725	0.541058
Neutrophils	PSMB1	-0.12067	7.299863	-1.11987	0.265804	-5.88699	0.473782	0.519636
Neutrophils	GTF3C5	0.481011	2.258469	1.119825	0.265821	-4.8262	0.546903	0.597915
Neutrophils	MAP11	0.291532	2.866952	1.119803	0.26583	-5.12414	0.537489	0.58788
Neutrophils	THAP11	-0.23535	4.457867	-1.1194	0.266	-5.24769	0.513794	0.562584
Neutrophils	GM14321	0.542509	-0.49943	1.119401	0.266001	-4.76135	0.591873	0.645704
Neutrophils	SNHG9	-0.40358	4.309776	-1.11906	0.266146	-5.26681	0.515994	0.564999
Neutrophils	RYR2	-0.86411	0.059974	-1.11905	0.266148	-4.72244	0.582522	0.635863
Neutrophils	KDM1A	0.14568	5.813944	1.118934	0.266199	-5.53324	0.494367	0.541865
Neutrophils	BTBD10	-0.11807	5.583956	-1.11872	0.266292	-5.75925	0.497612	0.545361
Neutrophils	TMEM86A	-0.34697	2.885047	-1.11868	0.266309	-5.04963	0.537384	0.587916
Neutrophils	ACACA	0.230671	5.153719	1.118596	0.266343	-5.35573	0.503741	0.551958
Neutrophils	TMEM164	0.096588	6.945657	1.118522	0.266374	-6.13683	0.478722	0.525101
Neutrophils	CRELD1	-0.49553	1.843254	-1.11831	0.266464	-4.80086	0.5536	0.60527
Neutrophils	HIPK2	-0.13519	6.550097	-1.11824	0.266493	-5.95287	0.484131	0.530993
Neutrophils	CCPG1	0.104579	6.037912	1.118191	0.266514	-6.0824	0.491229	0.538633
Neutrophils	6330562C2	-0.37017	1.275201	-1.11796	0.266611	-4.99581	0.562676	0.61509
Neutrophils	TMEM68	-0.25708	3.807022	-1.11793	0.266626	-5.0986	0.523462	0.573273
Neutrophils	CTLA2B	-0.52937	3.353706	-1.11761	0.266761	-5.08303	0.530439	0.580619
Neutrophils	SCFD2	-0.1923	5.78946	-1.11736	0.266867	-5.46069	0.49489	0.542668
Neutrophils	9830107B1	-0.18041	-0.62676	-1.11716	0.266952	-5.53836	0.594275	0.648761
Neutrophils	ARHGAP29	-0.33146	2.520124	-1.11701	0.267016	-5.14513	0.543203	0.594488
Neutrophils	EDIL3	0.742617	1.492552	1.116949	0.267042	-4.73346	0.559372	0.611738
Neutrophils	KLRA4	-0.88267	-1.21319	-1.11689	0.267066	-4.68159	0.604313	0.659446
Neutrophils	SCN1B	-0.44723	1.834445	-1.11683	0.267091	-4.94377	0.553939	0.605952
Neutrophils	MCEE	-0.17421	4.194477	-1.11663	0.267178	-5.55658	0.517878	0.567445
Neutrophils	STRIP2	-0.71403	1.016356	-1.11656	0.267207	-4.71806	0.56703	0.619886
Neutrophils	PPM1B	-0.126	6.192574	-1.11636	0.267292	-5.74977	0.48925	0.536742
Neutrophils	IL1RL1	-0.5426	-0.28284	-1.11626	0.267335	-4.84229	0.588466	0.642662
Neutrophils	ERMP1	-0.22978	3.633634	-1.11622	0.267353	-5.16774	0.526222	0.576389

Neutrophils	PTRH2	0.210146	4.181735	1.116186	0.267367	-5.31427	0.518066	0.567672
Neutrophils	ZMYND8	0.142922	6.108671	1.116087	0.267409	-5.65612	0.490419	0.538022
Neutrophils	GM5165	-0.44086	2.638407	-1.11582	0.267523	-4.8173	0.541503	0.592667
Neutrophils	HGH1	-0.54414	1.856316	-1.11552	0.267652	-4.73738	0.553864	0.605766
Neutrophils	ZMAT5	0.180297	4.547775	1.115274	0.267755	-5.3433	0.512943	0.56208
Neutrophils	GLUL	-0.17433	6.384395	-1.11509	0.267835	-5.79798	0.486829	0.534095
Neutrophils	GLYAT	-0.43584	2.069154	-1.11505	0.267852	-4.99259	0.55051	0.602255
Neutrophils	GM3448	0.468923	1.9113	1.114988	0.267877	-4.84139	0.552996	0.604918
Neutrophils	PI16	0.174671	1.135522	1.114816	0.267951	-5.67144	0.565381	0.618178
Neutrophils	SNRNP40	0.149222	5.982356	1.114708	0.267997	-5.56042	0.492424	0.540191
Neutrophils	FNTA	0.139107	5.505108	1.114703	0.267999	-5.53342	0.499154	0.547416
Neutrophils	MRPL19	0.246555	3.923908	1.114607	0.26804	-5.17361	0.522142	0.572075
Neutrophils	HDAC10	-0.49576	1.608346	-1.11388	0.268351	-4.77404	0.558284	0.610381
Neutrophils	IPO7	0.209972	5.656347	1.113827	0.268372	-5.49579	0.497443	0.545365
Neutrophils	NIP7	-0.22427	4.635807	-1.11332	0.268589	-5.14129	0.512325	0.561316
Neutrophils	SLC41A3	0.350627	1.73389	1.113279	0.268606	-4.973	0.556527	0.608528
Neutrophils	POU3F1	-0.82235	0.061626	-1.1132	0.268639	-4.6938	0.583749	0.637489
Neutrophils	WWTR1	-0.34872	2.126919	-1.11301	0.268722	-5.16594	0.550387	0.602023
Neutrophils	MARK4	0.209193	4.541737	1.112793	0.268814	-5.38327	0.513843	0.563005
Neutrophils	PNN	0.137203	6.310373	1.112109	0.269106	-5.64036	0.489067	0.536131
Neutrophils	EXOSC7	0.229362	4.629702	1.111819	0.26923	-5.24021	0.513074	0.561959
Neutrophils	PIDD1	0.570933	0.824038	1.111781	0.269246	-4.74978	0.571911	0.624712
Neutrophils	DDX47	-0.14587	5.550552	-1.11169	0.269285	-5.49543	0.499801	0.547742
Neutrophils	IGKV12-46	-0.64749	-0.77013	-1.11149	0.269371	-4.68164	0.598633	0.653076
Neutrophils	CHFR	0.135981	5.641975	1.111296	0.269454	-5.63476	0.498571	0.546465
Neutrophils	GRK6	0.131106	5.764162	1.111253	0.269472	-5.7628	0.496841	0.54462
Neutrophils	COQ7	-0.27532	4.79596	-1.11109	0.269542	-5.23107	0.510758	0.559563
Neutrophils	6330409D2	0.33526	0.333222	1.110695	0.269711	-5.09185	0.580218	0.633702
Neutrophils	ZFP212	-0.24568	3.640876	-1.11069	0.269712	-5.09562	0.527949	0.578028
Neutrophils	DHRS11	0.167001	4.678666	1.110618	0.269744	-5.72046	0.512566	0.561582
Neutrophils	GM13710	-0.55066	1.36556	-1.11028	0.26989	-4.92348	0.563434	0.615874
Neutrophils	STAG1	-0.08988	8.353909	-1.11023	0.269912	-6.15548	0.461809	0.507038
Neutrophils	MICALL2	-0.46177	1.195719	-1.10999	0.270015	-4.89096	0.566173	0.618838
Neutrophils	PROSER3	0.601061	1.105594	1.10989	0.270056	-4.72568	0.567632	0.620398
Neutrophils	SNRPA	-0.19145	5.131803	-1.10983	0.270084	-5.30538	0.50606	0.55467
Neutrophils	1110002J0	0.602647	-0.51304	1.109596	0.270182	-4.8098	0.59449	0.648923
Neutrophils	ZEB2OS	0.325723	4.678362	1.109446	0.270247	-5.12804	0.512636	0.561761
Neutrophils	6230400D1	-0.49216	2.163361	-1.10942	0.270259	-4.78655	0.550747	0.602495
Neutrophils	DBI	-0.15777	7.067278	-1.10933	0.270295	-5.91316	0.47897	0.525657
Neutrophils	NAXD	0.22721	4.128354	1.109221	0.270343	-5.21229	0.520731	0.570505
Neutrophils	VPS13C	0.214621	4.373385	1.109189	0.270357	-5.38302	0.517108	0.566627
Neutrophils	MFN1	0.305449	3.297935	1.108995	0.27044	-5.00586	0.533205	0.583843
Neutrophils	RRS1	0.281887	4.476059	1.108947	0.27046	-5.12406	0.515598	0.565009
Neutrophils	NSMCE2	-0.09924	7.428191	-1.10872	0.270558	-5.97699	0.474089	0.520506
Neutrophils	BMPR1A	-0.25809	2.310682	-1.10846	0.270668	-5.44239	0.548436	0.600242
Neutrophils	ZFP229	-0.68663	-0.01049	-1.10841	0.270691	-4.69862	0.586017	0.640227
Neutrophils	SMIM13	-0.2644	3.919727	-1.10839	0.270701	-5.11752	0.523836	0.573963

Neutrophils	STOM	0.257829	3.274711	1.108377	0.270705	-5.294	0.533558	0.584359
Neutrophils	PPID	-0.21417	4.87562	-1.10826	0.270756	-5.34183	0.509764	0.558892
Neutrophils	GM48383	-0.47428	1.758143	-1.1082	0.270781	-4.87321	0.557154	0.609535
Neutrophils	ZFP770	-0.57161	1.760027	-1.10759	0.271041	-4.74626	0.557391	0.609826
Neutrophils	TAF11	-0.16345	4.933052	-1.10744	0.271106	-5.47557	0.509175	0.558305
Neutrophils	BLOC1S6	0.220369	3.751167	1.107379	0.271134	-5.25268	0.526611	0.576978
Neutrophils	2900026A(	-0.49228	3.595436	-1.10724	0.271195	-4.99664	0.528954	0.579484
Neutrophils	CREB5	0.384715	2.17534	1.107041	0.271279	-5.26085	0.550822	0.602849
Neutrophils	SWI5	-0.14326	7.120072	-1.10701	0.271292	-5.88838	0.478482	0.525341
Neutrophils	ANKRD12	-0.1093	7.518738	-1.10697	0.271311	-6.18684	0.473099	0.519544
Neutrophils	IMP4	-0.21944	4.46298	-1.10685	0.271362	-5.22419	0.516037	0.565718
Neutrophils	TRP53BP2	0.218028	3.957204	1.106784	0.271389	-5.31831	0.523527	0.573754
Neutrophils	WASF2	0.08417	8.064634	1.106329	0.271585	-6.13578	0.465833	0.512027
Neutrophils	TEX10	-0.22535	5.266363	-1.10615	0.271661	-5.392	0.504367	0.553594
Neutrophils	PPP4R1	0.162136	5.579566	1.105979	0.271736	-5.60459	0.499893	0.548809
Neutrophils	PODNL1	-0.62151	-1.02387	-1.10589	0.271775	-4.68875	0.603517	0.659392
Neutrophils	NOM1	-0.26128	4.141882	-1.10587	0.271784	-5.11103	0.520779	0.571219
Neutrophils	CATSPERD	-0.50525	1.329786	-1.10573	0.271844	-4.89062	0.56428	0.617694
Neutrophils	SCFD1	0.216875	4.929173	1.105674	0.271867	-5.39516	0.509231	0.558837
Neutrophils	SEMA6A	-0.35159	2.821738	-1.10563	0.271885	-5.44685	0.540755	0.592594
Neutrophils	UBXN2A	0.146894	5.233503	1.105447	0.271965	-5.58449	0.504839	0.554177
Neutrophils	SEMA4B	-0.23816	5.763505	-1.10543	0.271971	-5.49124	0.497284	0.54606
Neutrophils	OMA1	0.296436	3.410326	1.105376	0.271995	-5.09777	0.531753	0.58303
Neutrophils	TRAPPC9	0.122158	6.178368	1.10516	0.272089	-5.87656	0.491453	0.539792
Neutrophils	ZBTB34	0.232579	3.592868	1.105153	0.272092	-5.28153	0.528993	0.580076
Neutrophils	3300002A1	0.628018	0.451891	1.105082	0.272122	-4.70443	0.578605	0.633029
Neutrophils	AAMP	0.113515	5.908148	1.105066	0.272129	-5.64115	0.495243	0.543886
Neutrophils	NHP2	0.228711	5.748278	1.104938	0.272184	-5.42749	0.4975	0.546378
Neutrophils	EPCAM	0.658556	2.160039	1.10487	0.272213	-4.77059	0.551062	0.603792
Neutrophils	MCCC1	0.450666	2.50652	1.104514	0.272367	-4.87278	0.54567	0.598086
Neutrophils	KALRN	-0.393	2.750236	-1.10451	0.27237	-5.08363	0.541889	0.594045
Neutrophils	TGOLN1	0.121267	5.839155	1.104407	0.272413	-5.90619	0.496242	0.545106
Neutrophils	FCOR	0.269996	0.865885	1.104371	0.272429	-5.23978	0.571835	0.625993
Neutrophils	MON2	0.132199	5.7408	1.104128	0.272534	-5.73494	0.497732	0.546626
Neutrophils	SENP5	-0.16309	5.992571	-1.1032	0.272932	-5.60107	0.494814	0.543248
Neutrophils	MECP2	0.135732	5.847525	1.10307	0.27299	-5.6568	0.496867	0.545506
Neutrophils	2610020C(	-0.18675	4.983149	-1.10296	0.273038	-5.35296	0.509238	0.558827
Neutrophils	ZFP763	0.654849	0.433803	1.102837	0.273091	-4.71283	0.57981	0.634205
Neutrophils	ATP5D	0.103055	7.898894	1.10221	0.273362	-6.06646	0.469135	0.515484
Neutrophils	KANSL3	0.190956	4.995066	1.101997	0.273454	-5.36109	0.509481	0.558901
Neutrophils	VSIR	0.128536	4.468275	1.10198	0.273461	-5.93542	0.517181	0.567157
Neutrophils	PLAGL2	0.188931	4.367209	1.101626	0.273614	-5.39666	0.518867	0.568778
Neutrophils	PIM3	0.27141	4.641176	1.101505	0.273667	-5.29484	0.514838	0.564522
Neutrophils	MYH13	-0.58333	-1.61994	-1.10133	0.273743	-4.67381	0.615589	0.671874
Neutrophils	ZC3H10	0.303583	2.921486	1.101166	0.273813	-4.99103	0.540721	0.592343
Neutrophils	SMCHD1	0.14688	7.994517	1.101088	0.273847	-6.04458	0.46806	0.514349
Neutrophils	RBM7	0.162715	5.855126	1.10102	0.273877	-5.62007	0.497372	0.545917

Neutrophils	SUV39H1	-0.2533	3.622039	-1.10086	0.273946	-5.14608	0.530063	0.580985
Neutrophils	FAM20A	-0.45407	1.833508	-1.10063	0.274045	-4.98944	0.55791	0.610668
Neutrophils	XKR5	0.463753	0.190356	1.100452	0.274123	-4.82054	0.584771	0.639224
Neutrophils	LRRC29	0.372746	1.690453	1.100281	0.274196	-4.83095	0.560298	0.613232
Neutrophils	TRA2B	-0.09755	7.832072	-1.09987	0.274373	-6.05488	0.47063	0.51699
Neutrophils	ZC2HC1A	0.565183	1.741533	1.099772	0.274417	-4.7998	0.559726	0.612511
Neutrophils	DEPTOR	-0.3682	3.469195	-1.09894	0.274776	-5.02974	0.533054	0.584195
Neutrophils	SMAD7	-0.21311	5.164939	-1.09878	0.274846	-5.46819	0.507915	0.557314
Neutrophils	SLC1A2	0.259134	2.197177	1.098769	0.274852	-5.27204	0.552753	0.605294
Neutrophils	ELAC1	0.309493	1.710286	1.098637	0.274909	-4.98189	0.560488	0.613566
Neutrophils	AGPAT2	0.148577	4.355109	1.098549	0.274947	-5.9099	0.519763	0.570041
Neutrophils	1190007I0	-0.33005	3.503332	-1.09839	0.275016	-4.98322	0.532536	0.583734
Neutrophils	THAP1	0.326182	2.879636	1.098309	0.275051	-4.99394	0.542093	0.593952
Neutrophils	SLC9A3R1	0.110354	6.690174	1.098195	0.275101	-6.04166	0.486359	0.534189
Neutrophils	PLPP2	0.503937	1.875681	1.098154	0.275119	-4.77536	0.557848	0.610781
Neutrophils	FRMPD4	-0.6154	0.351798	-1.09811	0.275138	-4.80288	0.582659	0.637196
Neutrophils	AGXT	-0.42473	3.279368	-1.09811	0.275138	-5.19855	0.535947	0.587394
Neutrophils	E2F8	0.232771	3.686572	1.097939	0.275212	-5.46065	0.529761	0.580855
Neutrophils	UTP20	-0.44379	3.652111	-1.09782	0.275263	-4.93667	0.530281	0.581472
Neutrophils	MAT2B	0.13415	5.679223	1.097751	0.275294	-5.68594	0.500537	0.549577
Neutrophils	MOCS3	-0.61977	1.446277	-1.09772	0.275308	-4.74419	0.564729	0.618288
Neutrophils	ZBTB44	-0.16087	5.853417	-1.09762	0.275351	-5.64182	0.498063	0.546953
Neutrophils	MRPL45	-0.1757	4.340705	-1.09728	0.275499	-5.5501	0.52016	0.570596
Neutrophils	HIRIP3	-0.36588	3.169999	-1.09715	0.275554	-4.97523	0.537821	0.589516
Neutrophils	NAIP5	0.297257	2.68586	1.09697	0.275633	-5.19808	0.545338	0.597532
Neutrophils	TUBE1	-0.52191	1.254723	-1.0968	0.275707	-4.80742	0.568076	0.621886
Neutrophils	CMKLR1	0.600393	2.151939	1.096603	0.275793	-4.91313	0.55371	0.606634
Neutrophils	BRWD3	0.170869	5.109603	1.096499	0.275838	-5.60285	0.508939	0.558757
Neutrophils	SHMT2	-0.3293	4.430904	-1.0964	0.275881	-5.1502	0.51887	0.569468
Neutrophils	PEX1	-0.42697	3.071405	-1.09606	0.27603	-4.92007	0.539373	0.591479
Neutrophils	NFAT5	0.11996	7.501438	1.096056	0.276031	-6.20165	0.475496	0.522857
Neutrophils	SLCO4C1	0.207623	-1.1708	1.095996	0.276057	-5.39388	0.608826	0.665443
Neutrophils	PLD1	-0.21371	3.080795	-1.09599	0.27606	-5.58348	0.539228	0.591324
Neutrophils	DDX3X	-0.10827	7.320412	-1.09582	0.276133	-6.20844	0.477944	0.52555
Neutrophils	PACSIN2	0.105834	5.573303	1.095742	0.276167	-5.83611	0.502268	0.551759
Neutrophils	EID3	-0.52629	0.925233	-1.09567	0.276198	-4.89091	0.573446	0.627924
Neutrophils	GM36199	0.64842	-0.13106	1.095604	0.276228	-4.75029	0.59101	0.646631
Neutrophils	FAM8A1	-0.27689	3.231881	-1.0952	0.276402	-5.09375	0.537	0.589039
Neutrophils	B230217C1	-0.36606	1.478169	-1.09503	0.27648	-4.96522	0.564558	0.61849
Neutrophils	HDAC5	0.261286	4.378269	1.095002	0.27649	-5.24227	0.519736	0.570553
Neutrophils	GARS	0.165859	5.511566	1.094957	0.276509	-5.53814	0.503235	0.552853
Neutrophils	ERG	-0.25198	3.921953	-1.09492	0.276526	-5.79441	0.526538	0.577873
Neutrophils	NFS1	-0.23174	4.533914	-1.09464	0.276647	-5.22168	0.517531	0.568256
Neutrophils	GM43258	-0.45957	0.724157	-1.09457	0.276677	-4.86231	0.576951	0.631793
Neutrophils	SLC4A4	0.475866	2.065431	1.09429	0.2768	-4.93813	0.555385	0.608804
Neutrophils	ACSL5	0.160265	6.00653	1.094214	0.276833	-5.73482	0.496391	0.545586
Neutrophils	ANP32E	-0.15213	7.305672	-1.0938	0.277012	-5.91183	0.478544	0.526233

Neutrophils	PTPN23	0.231661	3.647447	1.093682	0.277065	-5.28859	0.531029	0.582677
Neutrophils	CREB3L1	-0.32135	2.035248	-1.09359	0.277104	-5.10104	0.556022	0.609445
Neutrophils	FGG	-0.22124	6.166651	-1.09357	0.277114	-5.95408	0.494278	0.543257
Neutrophils	PEX2	0.246471	3.914925	1.093449	0.277167	-5.27465	0.527001	0.578484
Neutrophils	VPS37A	-0.15142	5.2037	-1.09282	0.277443	-5.69486	0.508422	0.558363
Neutrophils	9130401M	0.26926	4.02313	1.092527	0.27757	-5.06789	0.52595	0.577171
Neutrophils	GDPD5	-0.49254	1.750804	-1.09227	0.277684	-4.86181	0.561173	0.614914
Neutrophils	ABCD4	-0.35888	2.782602	-1.09225	0.27769	-4.8931	0.544889	0.597521
Neutrophils	MKS1	0.607898	1.092934	1.092179	0.277722	-4.74231	0.571813	0.62626
Neutrophils	LST1	0.130135	5.23223	1.091983	0.277807	-6.13875	0.508213	0.558204
Neutrophils	LTC4S	0.725223	1.066227	1.091599	0.277975	-4.95797	0.572424	0.626937
Neutrophils	MRPL41	0.224002	4.003451	1.091596	0.277976	-5.25827	0.526407	0.577759
Neutrophils	R3HDM4	0.104147	6.20323	1.091508	0.278015	-6.11527	0.494459	0.543465
Neutrophils	POLR3G	0.531965	2.101279	1.091343	0.278087	-4.80179	0.555756	0.60927
Neutrophils	DEF8	0.319397	2.04717	1.091249	0.278128	-5.01164	0.556615	0.610187
Neutrophils	MRPL36	0.176746	5.835469	1.091177	0.27816	-5.5803	0.499656	0.549168
Neutrophils	ZFP683	-0.70773	-1.15907	-1.09092	0.27827	-4.70198	0.61012	0.667117
Neutrophils	GNG2	0.13843	6.302982	1.090732	0.278354	-5.84202	0.493225	0.542194
Neutrophils	PAQR9	-0.29851	4.029832	-1.09052	0.278447	-5.34809	0.526226	0.577671
Neutrophils	B430010I2	0.385375	-0.41456	1.090395	0.278502	-4.96371	0.597396	0.653627
Neutrophils	CAR5A	0.645283	0.542195	1.090342	0.278525	-4.76046	0.581292	0.6365
Neutrophils	RPP25L	-0.29405	3.672149	-1.09006	0.278647	-5.05964	0.531754	0.583487
Neutrophils	NMT2	-0.17994	5.47269	-1.08974	0.278787	-5.40822	0.505257	0.555099
Neutrophils	MBOAT2	-0.74103	0.666194	-1.08973	0.278794	-4.78433	0.579481	0.634507
Neutrophils	HPS6	-0.55235	1.22115	-1.08947	0.278909	-4.76048	0.570502	0.624857
Neutrophils	ZNRD1	-0.19624	5.076686	-1.08898	0.279124	-5.3631	0.511402	0.561559
Neutrophils	OTULINL	0.242447	5.347398	1.088587	0.279295	-5.47642	0.507609	0.557461
Neutrophils	TAF6	0.297303	3.851886	1.088579	0.279298	-5.12167	0.529697	0.581159
Neutrophils	TMEM30A	0.086301	6.581779	1.087853	0.279616	-5.98559	0.490574	0.538732
Neutrophils	FDPS	0.256784	4.623809	1.087696	0.279685	-5.41448	0.5187	0.568935
Neutrophils	IL16	0.238381	4.60831	1.087534	0.279757	-5.51526	0.518929	0.569232
Neutrophils	ASAP1	0.101952	7.200045	1.087354	0.279836	-6.20628	0.482064	0.529607
Neutrophils	SFI1	0.228638	5.320089	1.087291	0.279863	-5.36465	0.50852	0.558064
Neutrophils	TASP1	-0.42807	3.918389	-1.08724	0.279886	-4.94864	0.52923	0.580303
Neutrophils	GM36862	-0.53121	0.340355	-1.08688	0.280045	-4.83002	0.586126	0.641182
Neutrophils	VIL1	-0.65527	0.018277	-1.08686	0.280051	-4.77764	0.591543	0.64694
Neutrophils	CASK	0.112083	5.500078	1.08674	0.280106	-6.11495	0.505922	0.555495
Neutrophils	TMCO6	0.257667	2.683081	1.086686	0.28013	-5.13202	0.548206	0.60082
Neutrophils	UROD	0.22816	4.467162	1.086576	0.280178	-5.32886	0.521019	0.571777
Neutrophils	9330160F1	0.287565	2.295032	1.086402	0.280254	-5.13575	0.55431	0.60745
Neutrophils	GM16341	-0.64059	0.863635	-1.08637	0.280269	-4.72705	0.577431	0.632105
Neutrophils	PPP4C	0.098509	6.80668	1.086321	0.28029	-6.01072	0.487477	0.535766
Neutrophils	RAP1GDS1	0.118009	6.711436	1.086191	0.280347	-5.97972	0.488809	0.537228
Neutrophils	EYA1	0.688513	2.426875	1.085995	0.280433	-4.7626	0.552261	0.605372
Neutrophils	AZIN1	0.129663	6.940521	1.085937	0.280459	-6.00437	0.485657	0.533928
Neutrophils	AMDHD1	-0.45018	2.26974	-1.08576	0.280537	-4.97899	0.554798	0.608119
Neutrophils	PSPH	0.29781	3.906589	1.085513	0.280646	-5.07548	0.529602	0.581161

Neutrophils	ZFP672	0.206316	4.004158	1.085377	0.280705	-5.27523	0.528148	0.579633
Neutrophils	PAQR4	0.581135	1.484926	1.085165	0.280799	-4.86581	0.56759	0.621731
Neutrophils	MAP7D1	0.128428	5.36793	1.084982	0.280879	-5.7012	0.508162	0.558169
Neutrophils	LAMTOR5	0.154888	5.527925	1.08478	0.280968	-5.58517	0.505922	0.555731
Neutrophils	GM28529	0.601157	0.724072	1.083841	0.281382	-4.78252	0.580856	0.635357
Neutrophils	SKAP2	0.082841	7.235487	1.083826	0.281389	-6.25306	0.482508	0.530074
Neutrophils	CAPSL	-0.80607	0.928873	-1.08318	0.281674	-4.72907	0.57795	0.631947
Neutrophils	MTHFD2	0.205005	5.486209	1.082772	0.281854	-5.62453	0.507754	0.556804
Neutrophils	GM4924	-0.66048	0.278075	-1.08253	0.281958	-4.71641	0.589065	0.643606
Neutrophils	ATP2C1	0.11153	5.85373	1.082476	0.281984	-5.87629	0.502476	0.551172
Neutrophils	KIF2C	0.379985	2.663513	1.082424	0.282008	-5.11519	0.550283	0.602362
Neutrophils	SLC12A2	0.364883	3.544469	1.082309	0.282058	-5.11164	0.536629	0.587809
Neutrophils	DCTD	-0.54571	1.018621	-1.08207	0.282166	-4.75635	0.576776	0.630672
Neutrophils	COLEC10	-0.55223	0.557157	-1.08201	0.282192	-4.78411	0.584427	0.63883
Neutrophils	USP32	0.104152	6.849617	1.081922	0.282229	-6.22805	0.488488	0.536259
Neutrophils	CMC4	-0.28829	3.064546	-1.08142	0.282453	-5.04569	0.544392	0.595947
Neutrophils	FADS3	-0.5758	0.659123	-1.08118	0.282555	-4.7382	0.583191	0.637272
Neutrophils	MCPT8	1.279644	-0.5048	1.080733	0.282755	-4.75666	0.603223	0.658359
Neutrophils	CKS1B	-0.30576	5.377173	-1.08061	0.282808	-5.49703	0.510044	0.559044
Neutrophils	KAT7	-0.13872	5.68662	-1.07966	0.28323	-5.60339	0.506102	0.554584
Neutrophils	TMEM129	-0.4629	2.084833	-1.07963	0.283242	-4.88076	0.560812	0.613058
Neutrophils	PHF13	0.283602	3.504231	1.079545	0.283281	-5.03349	0.538557	0.589325
Neutrophils	HNRNPU	0.100417	8.26781	1.079487	0.283307	-6.16723	0.470342	0.516138
Neutrophils	CRYBG2	-0.65436	0.051796	-1.07924	0.283418	-4.72394	0.594459	0.648797
Neutrophils	CSGALNAC	-0.35505	2.818139	-1.07892	0.283557	-5.21238	0.549399	0.600879
Neutrophils	ZFP414	-0.21911	3.443642	-1.07876	0.283631	-5.34448	0.539685	0.590592
Neutrophils	GM48099	0.708014	2.773722	1.078614	0.283694	-4.94403	0.550096	0.601808
Neutrophils	ZFP97	0.547099	1.263623	1.078526	0.283733	-4.77183	0.574325	0.627644
Neutrophils	TRMT61A	-0.53612	1.593744	-1.07848	0.283752	-4.75772	0.568937	0.621922
Neutrophils	9530068E0	-0.14022	4.851397	-1.07839	0.283792	-5.7509	0.518465	0.568038
Neutrophils	MCU	-0.09695	6.164566	-1.07827	0.283848	-6.2365	0.499454	0.54768
Neutrophils	GM48512	-0.62082	1.068935	-1.07822	0.283869	-4.7227	0.577527	0.631103
Neutrophils	NECTIN3	-0.4473	1.220532	-1.07801	0.28396	-4.87933	0.575114	0.628514
Neutrophils	GM27008	-0.62328	1.012976	-1.07761	0.284141	-4.71386	0.578632	0.632309
Neutrophils	SAR1B	0.132738	5.553451	1.077551	0.284165	-5.59943	0.508368	0.557288
Neutrophils	GPR157	0.319012	2.137362	1.077476	0.284199	-5.04435	0.560352	0.612867
Neutrophils	MAN1C1	0.174127	4.292733	1.077418	0.284225	-5.63577	0.526946	0.577194
Neutrophils	HOMER2	-0.67363	0.275337	-1.07733	0.284263	-4.73674	0.590951	0.645418
Neutrophils	PLCG2	0.098952	7.02313	1.077192	0.284325	-6.0285	0.487591	0.534972
Neutrophils	PRDM4	-0.2789	3.039382	-1.07672	0.284533	-5.07215	0.546436	0.597967
Neutrophils	RNF31	-0.29673	3.162894	-1.07644	0.28466	-5.1139	0.544615	0.596079
Neutrophils	SLC25A45	0.220011	3.260909	1.076321	0.284712	-5.2769	0.543095	0.594455
Neutrophils	GM6787	0.616045	0.188055	1.076261	0.284739	-4.73638	0.592885	0.647466
Neutrophils	GCAT	0.456112	2.390839	1.075861	0.284916	-4.88603	0.556992	0.609145
Neutrophils	RP2	0.131384	5.226456	1.075115	0.285249	-5.76978	0.514175	0.563141
Neutrophils	ACADS	-0.22387	4.490963	-1.07496	0.285318	-5.27107	0.525055	0.574806
Neutrophils	CMTM6	0.11721	4.866252	1.074933	0.285329	-5.77737	0.519474	0.568832

Neutrophils	PPP3CA	-0.08226	8.812084	-1.07474	0.285417	-6.43339	0.464416	0.509685
Neutrophils	NFYB	-0.15388	5.103926	-1.07471	0.28543	-5.63977	0.515971	0.565105
Neutrophils	CCDC71	0.364633	2.73695	1.07464	0.28546	-4.9471	0.551978	0.603585
Neutrophils	AMMECR1	-0.18692	4.787165	-1.07433	0.285599	-5.5197	0.520725	0.570197
Neutrophils	GM17354	-0.46898	1.393239	-1.07417	0.285668	-4.81799	0.573648	0.626704
Neutrophils	CALCRL	0.157669	5.617508	1.074165	0.285671	-6.0488	0.508564	0.557208
Neutrophils	ZFP346	0.383626	3.658973	1.074083	0.285708	-4.98239	0.537736	0.588452
Neutrophils	PPM1J	0.634018	-0.35731	1.073447	0.285992	-4.72888	0.603547	0.658199
Neutrophils	MCFD2	-0.25804	3.702476	-1.07279	0.286286	-5.2741	0.537514	0.588192
Neutrophils	FBXO38	0.124962	5.443447	1.072745	0.286305	-5.68613	0.511512	0.560366
Neutrophils	TEDC1	-0.48021	1.701406	-1.07268	0.286333	-4.82854	0.569094	0.62187
Neutrophils	RBIS	0.193436	5.209656	1.072598	0.28637	-5.40259	0.514926	0.564095
Neutrophils	MYNN	0.210081	4.303762	1.072303	0.286502	-5.23156	0.528382	0.578602
Neutrophils	WDR75	-0.36437	3.575364	-1.07216	0.286565	-4.92459	0.539465	0.590495
Neutrophils	PDE7A	-0.20781	6.703412	-1.0721	0.286592	-5.56383	0.493515	0.541251
Neutrophils	VPS51	0.265942	3.730024	1.072064	0.286609	-5.15732	0.537092	0.587959
Neutrophils	GLTP	0.125318	6.759436	1.07205	0.286615	-5.79062	0.49273	0.540407
Neutrophils	RSRP1	-0.11118	6.653226	-1.07197	0.286649	-6.05873	0.494219	0.542007
Neutrophils	ZCCHC9	0.151034	5.404138	1.071959	0.286656	-5.52305	0.512084	0.561187
Neutrophils	TSPAN14	0.135931	6.835826	1.071944	0.286662	-5.85517	0.491663	0.539259
Neutrophils	SYNCRIP	-0.11728	7.375906	-1.07178	0.286734	-5.92196	0.484183	0.531224
Neutrophils	COMMD2	0.16996	4.927522	1.071672	0.286784	-5.43679	0.519078	0.568727
Neutrophils	PIAS2	-0.16821	6.02482	-1.07163	0.286801	-5.62459	0.503124	0.551615
Neutrophils	EAF2	-0.29003	3.647314	-1.07161	0.286813	-5.07898	0.53836	0.589359
Neutrophils	SLC25A39	0.191402	6.472882	1.071375	0.286917	-5.77226	0.496789	0.544891
Neutrophils	SORBS2	-0.58958	1.878636	-1.0712	0.286996	-4.90539	0.566258	0.619224
Neutrophils	DNAJC13	0.142764	6.16837	1.070964	0.287101	-5.76727	0.501107	0.549605
Neutrophils	NFXL1	-0.33422	3.364621	-1.07095	0.287109	-5.0482	0.542751	0.594219
Neutrophils	VPS36	0.132671	5.315956	1.070812	0.287169	-5.64533	0.513404	0.562816
Neutrophils	SNAPC1	0.260163	3.850567	1.070799	0.287174	-5.16771	0.535284	0.586249
Neutrophils	AKAP10	-0.15266	5.860095	-1.07059	0.287267	-5.79082	0.505518	0.554354
Neutrophils	MRPS18B	0.304557	3.873974	1.070283	0.287405	-5.04334	0.534927	0.585981
Neutrophils	PINX1	-0.43234	3.022074	-1.06991	0.287574	-4.86183	0.54808	0.600199
Neutrophils	GM15832	0.251021	1.685015	1.069718	0.287658	-5.29941	0.569396	0.622937
Neutrophils	PQLC3	0.180587	3.812077	1.069663	0.287683	-5.55632	0.535872	0.587149
Neutrophils	PLCD3	-0.63015	1.122441	-1.06954	0.287736	-4.71826	0.578616	0.632759
Neutrophils	TRAM2	-0.25212	4.061754	-1.06945	0.28778	-5.14357	0.532072	0.58309
Neutrophils	GGH	0.177239	4.555539	1.069301	0.287845	-5.74135	0.524639	0.575166
Neutrophils	GM27010	0.259113	2.340877	1.069185	0.287897	-5.22273	0.558836	0.61177
Neutrophils	KMT2D	0.108021	5.945956	1.069166	0.287905	-5.85691	0.504285	0.553367
Neutrophils	RFT1	-0.42528	2.53189	-1.06909	0.287941	-4.91696	0.555798	0.608565
Neutrophils	HMBS	0.261811	4.451319	1.068872	0.288037	-5.33828	0.526199	0.576913
Neutrophils	APOLD1	-0.41541	1.979983	-1.06884	0.288052	-5.097	0.564622	0.617977
Neutrophils	PDZRN3	-0.50729	0.932125	-1.06883	0.288054	-4.88499	0.58177	0.636238
Neutrophils	NOC3L	-0.49919	3.216026	-1.06881	0.288067	-4.87048	0.545056	0.597092
Neutrophils	CLEC2G	0.715648	0.015976	1.068759	0.288088	-4.71944	0.597193	0.65263
Neutrophils	NADSYN1	0.497052	1.092531	1.068714	0.288108	-4.81567	0.579111	0.633409

Neutrophils	TRADD	0.289195	3.982911	1.068677	0.288124	-5.11847	0.533269	0.584487
Neutrophils	IRF2BP2	0.109531	7.59206	1.068407	0.288246	-6.06914	0.481254	0.528703
Neutrophils	PGGT1B	-0.19725	4.818529	-1.06833	0.288278	-5.3319	0.520725	0.571165
Neutrophils	NEPRO	-0.44494	2.709524	-1.06831	0.288287	-4.85132	0.552988	0.605691
Neutrophils	BBOX1	-0.5507	0.92351	-1.0683	0.288293	-4.84281	0.581913	0.636523
Neutrophils	USF2	0.121506	6.021725	1.068106	0.288381	-5.82561	0.5032	0.552406
Neutrophils	AP5S1	0.317001	2.440536	1.068103	0.288382	-5.07305	0.557249	0.610303
Neutrophils	WDR4	0.352091	2.999114	1.068028	0.288416	-4.91834	0.548439	0.600894
Neutrophils	DGKA	-0.23028	5.023185	-1.06763	0.288595	-5.46864	0.517809	0.568132
Neutrophils	LAMB3	0.160706	4.067884	1.067328	0.288729	-5.81571	0.532091	0.583555
Neutrophils	CTNNAL1	0.487371	1.924908	1.067142	0.288813	-4.89006	0.56563	0.619453
Neutrophils	ITGB2	0.129536	6.734664	1.067132	0.288817	-6.2679	0.493213	0.541844
Neutrophils	CEP72	0.381541	2.208429	1.067083	0.28884	-4.93633	0.561071	0.614608
Neutrophils	BBS4	-0.23915	2.313596	-1.06686	0.288938	-5.26528	0.559389	0.612912
Neutrophils	PTPN18	-0.10532	7.11924	-1.06684	0.288947	-5.97788	0.487857	0.536178
Neutrophils	IGHJ4	0.69458	0.337645	1.066755	0.288987	-4.73161	0.591856	0.647537
Neutrophils	CORO2A	-0.21742	5.527432	-1.06666	0.289028	-5.51332	0.510432	0.560544
Neutrophils	CCR5	-0.33853	4.693422	-1.06665	0.289032	-5.28132	0.522694	0.573711
Neutrophils	RNASET2B	-0.24027	5.139167	-1.06661	0.28905	-5.46081	0.516102	0.566645
Neutrophils	NPR1	-0.39437	1.263007	-1.06653	0.28909	-5.03489	0.57642	0.63117
Neutrophils	PSMA2	-0.11125	7.21693	-1.06639	0.289151	-6.01156	0.486518	0.534827
Neutrophils	EBAG9	-0.23319	4.117531	-1.06606	0.289298	-5.20657	0.531352	0.583106
Neutrophils	GSTZ1	0.254927	4.205829	1.066061	0.289298	-5.29792	0.530017	0.581675
Neutrophils	PIN1	0.160025	5.245371	1.066055	0.289301	-5.5298	0.514557	0.565083
Neutrophils	RSPRY1	-0.11508	5.833379	-1.06574	0.289441	-5.80009	0.506177	0.555963
Neutrophils	NCOR2	0.219407	4.956649	1.065529	0.289538	-5.2642	0.51902	0.569837
Neutrophils	UNG	-0.53968	3.369876	-1.06545	0.289575	-4.89877	0.543024	0.595599
Neutrophils	ESRRA	0.220064	3.981496	1.065326	0.289629	-5.29052	0.533643	0.585621
Neutrophils	DCAF10	-0.15722	5.193751	-1.06488	0.289831	-5.52816	0.515746	0.566347
Neutrophils	TCF20	-0.11073	7.561094	-1.06464	0.28994	-6.17157	0.482195	0.530344
Neutrophils	GM8797	-0.34898	2.19543	-1.06463	0.289943	-4.98696	0.561764	0.615767
Neutrophils	TNRC6A	-0.12176	6.648204	-1.06461	0.289953	-5.8591	0.494853	0.544001
Neutrophils	CDC37L1	0.150869	5.167302	1.064267	0.290106	-5.52963	0.516264	0.56712
Neutrophils	DCXR	-0.25532	4.317514	-1.06422	0.290128	-5.27382	0.528909	0.580699
Neutrophils	AI597479	0.40655	1.688645	1.063867	0.290286	-4.87821	0.570164	0.624851
Neutrophils	FRY	0.111415	6.378637	1.063855	0.290291	-6.23249	0.498844	0.548396
Neutrophils	NIN	0.111655	5.920519	1.063772	0.290329	-6.05932	0.505383	0.555468
Neutrophils	H2-D1	0.188145	8.452787	1.06357	0.29042	-6.28705	0.470337	0.51772
Neutrophils	ZFP444	-0.33382	3.63547	-1.06357	0.29042	-5.04384	0.539358	0.592015
Neutrophils	RRN3	-0.2366	4.400393	-1.06312	0.290622	-5.17207	0.528	0.579645
Neutrophils	HYOU1	-0.20786	4.380576	-1.06294	0.290706	-5.32495	0.528327	0.580021
Neutrophils	PAFAH2	-0.48275	0.619176	-1.06286	0.290741	-4.91676	0.58818	0.643987
Neutrophils	ATAD2	0.191086	6.309192	1.062747	0.290791	-5.83633	0.500115	0.549717
Neutrophils	IMPG2	0.575657	0.6613	1.062329	0.29098	-4.84794	0.587648	0.643301
Neutrophils	IFT80	-0.32335	3.626112	-1.06228	0.291001	-5.18368	0.539971	0.592397
Neutrophils	RICTOR	-0.11434	6.409431	-1.06221	0.291031	-5.88566	0.498841	0.548237
Neutrophils	BBIP1	0.113589	5.888435	1.061904	0.291171	-5.9288	0.506438	0.556429

Neutrophils	GM16973	-0.44979	1.894858	-1.0614	0.2914	-4.8658	0.567664	0.622016
Neutrophils	GM12227	-0.39629	1.113465	-1.06129	0.29145	-4.91986	0.580471	0.635672
Neutrophils	NUDT3	-0.15219	5.213023	-1.06124	0.291473	-5.49321	0.516428	0.567156
Neutrophils	GM47662	-0.63944	-0.79475	-1.06115	0.291514	-4.7194	0.612986	0.670282
Neutrophils	BFAR	0.163574	4.85458	1.061073	0.291547	-5.40642	0.521724	0.572877
Neutrophils	ALKBH7	-0.35194	3.373231	-1.06102	0.291572	-5.02693	0.544218	0.596991
Neutrophils	MTERF2	-0.40032	2.099595	-1.06078	0.291681	-4.9508	0.564441	0.618665
Neutrophils	STX16	0.174324	5.756821	1.060694	0.291718	-5.58345	0.508576	0.558822
Neutrophils	XPNPEP1	-0.19999	4.365592	-1.06049	0.291809	-5.31912	0.529165	0.580935
Neutrophils	PARP11	0.416463	3.46828	1.060291	0.2919	-5.00836	0.542873	0.595665
Neutrophils	DNPEP	0.243265	4.077287	1.060234	0.291926	-5.22677	0.53353	0.585654
Neutrophils	MSRB2	0.427387	1.023579	1.060053	0.292008	-4.96661	0.5821	0.63762
Neutrophils	SNX29	-0.20737	6.395771	-1.05998	0.292041	-5.67496	0.499464	0.549105
Neutrophils	MOSPD1	-0.16861	4.817896	-1.05988	0.292088	-5.70502	0.522392	0.573818
Neutrophils	GM26782	0.335353	2.991571	1.059845	0.292102	-4.89776	0.550304	0.603739
Neutrophils	SYCE2	-0.34651	4.258668	-1.0596	0.292212	-5.22574	0.530887	0.582827
Neutrophils	TWNK	0.378242	2.886068	1.058853	0.292551	-4.98671	0.552437	0.60593
Neutrophils	METTL3	0.289883	3.319562	1.058852	0.292551	-5.03909	0.545648	0.598666
Neutrophils	SMURF2	0.111424	6.82379	1.05874	0.292602	-6.01904	0.493852	0.543082
Neutrophils	TRAPPC2L	0.130652	5.811456	1.058724	0.292609	-5.78346	0.508266	0.558606
Neutrophils	TM4SF5	0.760894	0.261552	1.058164	0.292863	-4.74239	0.595768	0.652002
Neutrophils	MGARP	0.730003	-0.49619	1.058099	0.292893	-4.73573	0.608803	0.665856
Neutrophils	GM20707	0.350167	1.898927	1.058006	0.292935	-4.90789	0.568553	0.623007
Neutrophils	MDN1	-0.22428	5.788045	-1.05764	0.293102	-5.59131	0.509096	0.559206
Neutrophils	FNTB	-0.35756	2.971515	-1.05753	0.293149	-4.94792	0.551624	0.604843
Neutrophils	PCX	-0.40795	2.70013	-1.05717	0.293314	-5.0366	0.556128	0.609499
Neutrophils	ZSCAN21	0.349426	3.394527	1.056935	0.293421	-5.01713	0.545324	0.597972
Neutrophils	GM16286	0.134862	5.859685	1.05673	0.293514	-5.67279	0.508425	0.558362
Neutrophils	LSMEM1	-0.20619	4.06136	-1.05654	0.2936	-5.88218	0.535161	0.587027
Neutrophils	RRP15	0.302911	3.823389	1.056467	0.293633	-5.04036	0.538803	0.590928
Neutrophils	1700112J1	-0.50606	0.638697	-1.05628	0.293716	-4.89818	0.59013	0.645706
Neutrophils	YLPM1	-0.17366	5.660547	-1.056	0.293846	-5.51129	0.511534	0.561578
Neutrophils	PFKFB3	0.145702	6.273061	1.05523	0.294195	-5.98719	0.503166	0.552353
Neutrophils	RASA3	0.119712	6.702935	1.055085	0.294261	-5.94096	0.497058	0.5458
Neutrophils	STK11IP	-0.35939	2.312647	-1.05506	0.294271	-4.90034	0.563258	0.61674
Neutrophils	ING5	0.231629	3.456639	1.054247	0.294642	-5.2158	0.545765	0.597598
Neutrophils	TIE1	-0.34039	1.482911	-1.05411	0.294703	-5.08264	0.577402	0.631354
Neutrophils	POP1	-0.39993	2.708556	-1.05398	0.294766	-4.88717	0.557574	0.610217
Neutrophils	RCN2	0.198044	5.075901	1.053821	0.294836	-5.503	0.521229	0.57138
Neutrophils	ZC3H15	-0.0977	7.266033	-1.05333	0.295061	-5.996	0.490059	0.537769
Neutrophils	GRK3	-0.34878	2.91909	-1.05313	0.295149	-5.10489	0.554638	0.606985
Neutrophils	GM10974	-0.59235	0.824708	-1.05293	0.295241	-4.74164	0.588808	0.643448
Neutrophils	COX7C	0.090497	8.585633	1.052898	0.295257	-6.25876	0.472102	0.518474
Neutrophils	NIPA2	0.140946	6.042829	1.052765	0.295318	-5.73632	0.507423	0.556531
Neutrophils	MAP2K3	0.141141	6.01457	1.052713	0.295341	-5.97289	0.507831	0.556984
Neutrophils	TRIM35	-0.21214	5.607952	-1.05229	0.295534	-5.52283	0.513984	0.563462
Neutrophils	RNASEH2B	-0.21098	4.594626	-1.05203	0.295653	-5.41611	0.529145	0.579703

Neutrophils	PTGIS	0.885468	-0.12832	1.051828	0.295745	-4.75362	0.605565	0.661161
Neutrophils	2900060B1	0.244237	2.614995	1.05154	0.295876	-5.23595	0.560093	0.612638
Neutrophils	AGT	-0.37433	4.084746	-1.05118	0.29604	-5.47155	0.537255	0.588127
Neutrophils	DCSTAMP	0.507011	-1.33737	1.051132	0.296062	-4.73543	0.627188	0.683745
Neutrophils	PIWIL2	0.436789	0.700548	1.050883	0.296176	-5.00768	0.591791	0.646239
Neutrophils	ADSSL1	0.137194	4.426295	1.050832	0.2962	-5.72408	0.532114	0.582648
Neutrophils	GM29243	0.49667	-0.26495	1.050562	0.296323	-4.84294	0.608463	0.664023
Neutrophils	RASSF8	-0.46653	2.215433	-1.05042	0.296386	-4.93679	0.566857	0.619841
Neutrophils	GM26632	0.568488	0.366747	1.050359	0.296416	-4.79526	0.597583	0.652514
Neutrophils	MRM3	0.525683	2.005465	1.049871	0.296639	-4.82943	0.570594	0.623571
Neutrophils	ARIH1	0.100871	8.728541	1.049679	0.296727	-6.36787	0.471332	0.517218
Neutrophils	INO80E	-0.18336	4.165534	-1.04932	0.29689	-5.2376	0.536769	0.587404
Neutrophils	MUL1	-0.33356	2.950066	-1.04914	0.296972	-5.02307	0.555698	0.607671
Neutrophils	TANK	0.130862	6.451404	1.049003	0.297036	-6.07948	0.502968	0.551245
Neutrophils	ZC3H14	-0.12563	5.656337	-1.04898	0.297046	-5.67713	0.514466	0.56359
Neutrophils	ZFP956	-0.51386	1.264702	-1.0483	0.297359	-4.79129	0.583532	0.637111
Neutrophils	FAM217B	0.290996	0.616789	1.048226	0.297391	-5.22627	0.594429	0.648716
Neutrophils	GPT	0.527415	1.203309	1.048134	0.297434	-4.8416	0.584556	0.638303
Neutrophils	OGA	0.10046	6.76343	1.048028	0.297482	-6.07177	0.498914	0.546826
Neutrophils	AZGP1	-0.31544	3.926277	-1.04775	0.297608	-5.46286	0.540996	0.591925
Neutrophils	OIP5OS1	0.121627	6.02273	1.047591	0.297683	-5.84641	0.509693	0.558431
Neutrophils	PEG3	-0.46407	1.53048	-1.04719	0.297867	-4.95489	0.579567	0.63295
Neutrophils	TGFBR3	-0.28457	3.337964	-1.04684	0.298027	-5.32489	0.550529	0.602082
Neutrophils	MRPS28	-0.16094	6.885185	-1.04679	0.298049	-6.27746	0.497663	0.545482
Neutrophils	ACKR3	-0.7747	0.835269	-1.04674	0.298075	-4.83005	0.591291	0.64551
Neutrophils	AKR1A1	-0.11623	7.188669	-1.04638	0.298238	-5.96353	0.493578	0.541063
Neutrophils	GOLGB1	0.154451	5.4427	1.0462	0.29832	-5.57099	0.518739	0.568066
Neutrophils	SART3	0.161263	5.374984	1.04606	0.298385	-5.52828	0.519745	0.569138
Neutrophils	GM17491	0.401831	1.415352	1.045968	0.298427	-4.82157	0.581861	0.635424
Neutrophils	NMB	-0.40367	1.642171	-1.0453	0.298732	-4.92524	0.578596	0.631682
Neutrophils	EIF3K	0.093628	7.871661	1.044898	0.298918	-6.10254	0.484606	0.531222
Neutrophils	ARL14EP	-0.24267	3.524764	-1.04425	0.299217	-5.2331	0.548373	0.599669
Neutrophils	SOX18	-0.42685	0.720608	-1.04421	0.299235	-5.01483	0.594063	0.648295
Neutrophils	RNF103	-0.2117	4.305165	-1.0442	0.299242	-5.32052	0.53631	0.586784
Neutrophils	CENPK	0.307788	3.283005	1.044129	0.299272	-5.21149	0.552166	0.603717
Neutrophils	RBMXL1	-0.17769	5.042632	-1.04411	0.299279	-5.39184	0.525165	0.574861
Neutrophils	CYSLTR1	-0.4911	2.70023	-1.04393	0.299362	-4.91921	0.561422	0.613585
Neutrophils	ZFP810	0.427045	2.914739	1.043847	0.299402	-4.9026	0.557996	0.609935
Neutrophils	GM29488	-0.57561	0.172443	-1.04381	0.299418	-4.8103	0.603437	0.658239
Neutrophils	CCDC167	-0.1955	4.065969	-1.04357	0.299529	-5.46043	0.539978	0.590705
Neutrophils	IMPDH2	-0.20334	5.196107	-1.04353	0.299548	-5.3971	0.522875	0.572411
Neutrophils	RHBDF2	0.146104	4.650027	1.04337	0.299621	-5.64095	0.531068	0.581179
Neutrophils	THG1L	0.502898	2.021146	1.043351	0.29963	-4.81778	0.572408	0.625284
Neutrophils	PECR	-0.4427	2.35098	-1.0433	0.299655	-5.03952	0.567045	0.619575
Neutrophils	PRKCSH	0.178238	4.862373	1.043251	0.299676	-5.36684	0.527867	0.577754
Neutrophils	RIPK2	0.28397	4.205503	1.04323	0.299686	-5.24186	0.537835	0.588416
Neutrophils	ALPK2	-0.60685	1.260876	-1.04317	0.299712	-4.76503	0.584968	0.638639

Neutrophils	PDCL	-0.22039	3.891934	-1.04305	0.299769	-5.3499	0.542663	0.593574
Neutrophils	COP1	0.10119	7.053619	1.043044	0.299771	-6.19657	0.495984	0.543562
Neutrophils	TSR1	-0.41272	3.584651	-1.04301	0.299789	-4.91454	0.547437	0.598672
Neutrophils	ANGPTL6	-0.54259	0.97577	-1.04278	0.299893	-4.81146	0.589787	0.643808
Neutrophils	ALG2	0.260157	3.062397	1.042687	0.299936	-5.07942	0.555687	0.607552
Neutrophils	TWSG1	-0.33423	3.230079	-1.04263	0.299964	-5.0713	0.553035	0.604746
Neutrophils	ELDR	-0.23778	3.256101	-1.04152	0.300474	-5.60102	0.553413	0.604715
Neutrophils	RBM10	-0.17075	4.779494	-1.04147	0.300495	-5.45209	0.529902	0.579613
Neutrophils	DGCR2	0.207679	4.849233	1.041306	0.300573	-5.38279	0.528875	0.578556
Neutrophils	GM13547	-0.63647	0.514665	-1.04122	0.300613	-4.84255	0.598485	0.652699
Neutrophils	KIF3A	0.379496	2.472633	1.041023	0.300703	-4.91032	0.566019	0.618196
Neutrophils	TMEM140	0.34031	3.514222	1.040734	0.300836	-5.24248	0.549535	0.600633
Neutrophils	RNF126	0.20523	4.767243	1.040659	0.300871	-5.35933	0.530261	0.580046
Neutrophils	APH1C	-0.14753	4.351444	-1.04049	0.30095	-5.94541	0.536578	0.586867
Neutrophils	GM10658	-0.45587	1.776179	-1.04041	0.300986	-4.88601	0.577473	0.630488
Neutrophils	POLG	-0.21851	3.808912	-1.04033	0.301023	-5.33027	0.544938	0.59586
Neutrophils	ARPC5	0.068507	8.144041	1.040247	0.301062	-6.38308	0.481754	0.52813
Neutrophils	DOT1L	0.194297	5.289755	1.040038	0.301158	-5.53641	0.52251	0.57186
Neutrophils	AP5Z1	0.313344	2.539751	1.039542	0.301387	-4.99946	0.565416	0.617592
Neutrophils	SAMD3	-0.64861	0.184815	-1.03928	0.301508	-4.8109	0.60474	0.659439
Neutrophils	TSPAN32	0.251915	2.972272	1.039177	0.301556	-5.34746	0.558482	0.61028
Neutrophils	IGKV1-135	-0.68611	-0.02384	-1.03917	0.301559	-4.74122	0.608355	0.663269
Neutrophils	NCS1	-0.68324	0.210797	-1.03908	0.301599	-4.74752	0.604291	0.658963
Neutrophils	DARS2	0.287995	2.849125	1.039008	0.301634	-5.32674	0.560447	0.612418
Neutrophils	XPO7	0.147855	6.710919	1.038414	0.301909	-5.80123	0.502265	0.550233
Neutrophils	CD8A	-0.75643	1.360615	-1.03835	0.301941	-4.81237	0.584971	0.638566
Neutrophils	POLN	-0.4425	3.191009	-1.03829	0.301966	-5.02434	0.555198	0.606885
Neutrophils	MYLK	-0.38392	1.606214	-1.03818	0.302018	-5.03941	0.580883	0.634232
Neutrophils	ADAP2	-0.41907	2.585365	-1.03812	0.302044	-5.06374	0.564874	0.617197
Neutrophils	S100A11	0.083243	7.270251	1.038064	0.302071	-6.573	0.494352	0.54174
Neutrophils	2810001G2	-0.28249	2.479465	-1.038	0.302102	-5.03515	0.566583	0.619018
Neutrophils	TMEM8	-0.25011	2.000346	-1.03743	0.302365	-5.24553	0.574687	0.627372
Neutrophils	C77080	-1.2315	0.694235	-1.03735	0.302402	-4.74293	0.596523	0.650561
Neutrophils	PDE1C	-0.42572	3.989093	-1.03711	0.302515	-5.25643	0.542994	0.593758
Neutrophils	RAD54B	0.481788	2.207787	1.037059	0.302536	-4.93088	0.571294	0.623942
Neutrophils	HINT2	-0.21857	4.356845	-1.03698	0.302573	-5.31365	0.537334	0.587727
Neutrophils	DIAPH2	0.097591	7.666379	1.036794	0.302659	-6.37358	0.489085	0.536028
Neutrophils	AHNAK	0.299487	6.416793	1.036726	0.302691	-5.72562	0.506746	0.555002
Neutrophils	ALG9	-0.30952	3.375018	-1.03666	0.302723	-5.03638	0.552583	0.604044
Neutrophils	TTL	0.452672	1.740811	1.036603	0.302748	-4.83782	0.57896	0.632133
Neutrophils	4930426D0	-0.65564	-0.21569	-1.03653	0.302781	-4.7383	0.61223	0.667484
Neutrophils	APOC1	-0.27199	7.949726	-1.03635	0.302864	-6.27708	0.485221	0.531937
Neutrophils	ATP1A3	0.216159	1.709416	1.035879	0.303084	-5.55877	0.579859	0.633067
Neutrophils	GM43062	-0.35078	1.638658	-1.03573	0.303155	-4.92656	0.581067	0.634373
Neutrophils	CMYA5	-0.71349	0.674059	-1.0355	0.303259	-4.79402	0.597302	0.651615
Neutrophils	TIFA	-0.29076	6.262161	-1.03534	0.303334	-5.49725	0.509349	0.557794
Neutrophils	ADRB2	0.1847	4.714467	1.035188	0.303404	-5.94449	0.532277	0.582354

Neutrophils	A930014D(	0.446506	-1.05861	1.035078	0.303455	-4.81113	0.627607	0.683704
Neutrophils	TPST1	-0.41808	3.472726	-1.035	0.303489	-4.97565	0.551448	0.602847
Neutrophils	PPP1R2	0.062738	6.908394	1.034982	0.3035	-6.26083	0.500083	0.547866
Neutrophils	GM14698	0.553389	0.738672	1.034933	0.303523	-4.78808	0.596201	0.650487
Neutrophils	CHRM3	0.318395	0.831294	1.034682	0.303639	-5.33372	0.594696	0.648895
Neutrophils	MTERF3	-0.22579	4.014483	-1.03463	0.303663	-5.28259	0.543061	0.593947
Neutrophils	PPFIBP2	-0.37825	4.285147	-1.03446	0.303742	-5.27911	0.538919	0.589526
Neutrophils	CCDC22	-0.26453	3.49155	-1.03432	0.303809	-5.14872	0.551247	0.602732
Neutrophils	DCTPP1	0.264288	5.089369	1.03423	0.303849	-5.38353	0.526717	0.576529
Neutrophils	POLD3	0.197709	4.695421	1.034075	0.303921	-5.42152	0.532657	0.582908
Neutrophils	SIT1	-0.57524	2.062249	-1.03404	0.30394	-4.81314	0.57419	0.62722
Neutrophils	GM35867	0.633535	0.456051	1.033657	0.304115	-4.79982	0.601381	0.656046
Neutrophils	GM36723	0.206209	3.324777	1.033404	0.304233	-5.9303	0.554195	0.605879
Neutrophils	ECPAS	0.113299	6.743157	1.033322	0.304272	-5.92781	0.502813	0.550882
Neutrophils	CHD6	-0.17871	5.997264	-1.03302	0.304414	-5.58443	0.513621	0.562491
Neutrophils	TRIM14	-0.2896	4.686216	-1.03297	0.304433	-5.3093	0.533144	0.583402
Neutrophils	IDH1	-0.1421	4.88393	-1.03289	0.304472	-5.73073	0.530151	0.580203
Neutrophils	NFIC	0.162531	4.464453	1.032825	0.304503	-5.569	0.536522	0.58705
Neutrophils	UBASH3B	0.200915	5.794781	1.032645	0.304586	-5.868	0.516637	0.565788
Neutrophils	RBM41	0.248117	3.907296	1.032519	0.304645	-5.20754	0.54516	0.596312
Neutrophils	PBDC1	-0.25062	5.348225	-1.03243	0.304688	-5.48279	0.523241	0.572876
Neutrophils	PRMT6	0.368237	2.45108	1.032313	0.304741	-4.89081	0.568279	0.621003
Neutrophils	RBM42	0.099321	6.063078	1.032094	0.304843	-5.87711	0.512796	0.56163
Neutrophils	C530008M	0.516527	0.74516	1.031919	0.304924	-4.90036	0.596767	0.651215
Neutrophils	ATG4D	0.215185	3.954415	1.031804	0.304978	-5.31728	0.544545	0.595652
Neutrophils	C2	-0.5068	2.413631	-1.03173	0.305014	-4.98417	0.569007	0.621749
Neutrophils	AMT	-0.47739	1.565291	-1.0314	0.305165	-4.94489	0.583091	0.636761
Neutrophils	RAF1	0.100238	5.986456	1.031269	0.305227	-6.08186	0.51406	0.563091
Neutrophils	MCOLN2	0.467766	3.161941	1.031237	0.305242	-4.82197	0.55712	0.609152
Neutrophils	TMEM168	0.196085	4.583494	1.030986	0.305359	-5.39093	0.535076	0.585668
Neutrophils	FBH1	-0.19539	3.425128	-1.03091	0.305396	-5.32725	0.553035	0.60488
Neutrophils	GM50373	0.607697	0.358844	1.030797	0.305448	-4.80925	0.603618	0.658701
Neutrophils	NEK2	-0.45132	3.092142	-1.03061	0.305533	-5.02068	0.558311	0.610537
Neutrophils	ZC3H8	0.444549	2.13952	1.030583	0.305548	-4.83578	0.573695	0.626932
Neutrophils	A330032B1	-0.76501	-0.15579	-1.03028	0.305688	-4.73993	0.612702	0.668291
Neutrophils	HMGN1	-0.19877	7.26929	-1.0302	0.305724	-5.72336	0.495855	0.543613
Neutrophils	RWDD2A	0.573852	0.48641	1.029713	0.305954	-4.79049	0.601772	0.656658
Neutrophils	MYO3B	-0.48435	1.289051	-1.02963	0.30599	-4.85431	0.588135	0.64218
Neutrophils	BC031181	0.106082	5.703047	1.02962	0.305997	-5.82187	0.518596	0.567963
Neutrophils	ANO6	-0.14307	6.75402	-1.02953	0.306038	-5.97829	0.503334	0.551585
Neutrophils	DAGLA	-0.67152	-0.78197	-1.02918	0.306202	-4.73468	0.624147	0.680256
Neutrophils	FGFR2	-0.12637	5.987704	-1.02913	0.306228	-5.91829	0.514558	0.563543
Neutrophils	PBK	-0.30609	3.69023	-1.02869	0.306431	-5.36324	0.549506	0.600886
Neutrophils	STAU1	-0.13652	6.034578	-1.0286	0.306474	-5.75652	0.514027	0.562967
Neutrophils	KLHL28	0.228747	3.44241	1.028595	0.306476	-5.23367	0.553403	0.605091
Neutrophils	RRP8	0.311679	3.730434	1.028417	0.306559	-5.05859	0.548897	0.600343
Neutrophils	HORMAD2	-0.54401	1.357831	-1.02835	0.306592	-4.97314	0.587342	0.641336

Neutrophils	PALLD	-0.17953	3.882236	-1.02818	0.306669	-5.75894	0.546573	0.597918
Neutrophils	IAH1	0.198281	4.521488	1.027132	0.30716	-5.49243	0.537475	0.587866
Neutrophils	ZFYVE21	-0.36955	2.542215	-1.0267	0.307361	-4.98794	0.568856	0.621363
Neutrophils	CEP76	-0.32627	2.990307	-1.02668	0.307371	-5.05189	0.56163	0.613667
Neutrophils	ZEB1	-0.15563	6.848689	-1.02652	0.307448	-6.11092	0.50321	0.551239
Neutrophils	TTC36	-0.29938	5.028124	-1.02641	0.307499	-5.73177	0.529944	0.57993
Neutrophils	LMAN1	-0.224	4.627609	-1.02637	0.307519	-5.36139	0.536022	0.586444
Neutrophils	TMEM240	-0.73613	0.240106	-1.0261	0.307642	-4.73875	0.607588	0.6626
Neutrophils	ABCB8	-0.32632	2.240197	-1.02605	0.307667	-4.8917	0.573862	0.626781
Neutrophils	IL27RA	-0.65095	1.535873	-1.02565	0.307852	-4.80367	0.585734	0.639315
Neutrophils	LRRC4C	-0.7024	0.648779	-1.02558	0.307886	-4.78675	0.600762	0.655279
Neutrophils	CLEC1B	-0.28146	3.741016	-1.02541	0.307967	-5.49942	0.55003	0.60134
Neutrophils	GBA	0.223777	4.090254	1.025345	0.307998	-5.28617	0.544583	0.595524
Neutrophils	ZFP706	-0.09796	7.871583	-1.02512	0.308102	-6.02756	0.489161	0.536095
Neutrophils	MTPAP	0.229262	4.284578	1.024839	0.308235	-5.30184	0.541809	0.592477
Neutrophils	BCAT1	-0.67771	0.852062	-1.02446	0.308412	-4.80892	0.597775	0.65207
Neutrophils	C1QBP	0.233306	6.055545	1.024364	0.308458	-5.48819	0.515368	0.564207
Neutrophils	USP9X	0.114309	7.387216	1.024261	0.308506	-6.10329	0.49624	0.543727
Neutrophils	THA1	0.659937	0.09883	1.024068	0.308597	-4.75913	0.61085	0.665998
Neutrophils	NOS1AP	0.165805	3.163666	1.023757	0.308743	-5.87322	0.559756	0.611788
Neutrophils	DHRS3	-0.22373	4.170119	-1.02375	0.308745	-5.53837	0.543924	0.59489
Neutrophils	MTFP1	-0.60197	0.572509	-1.02339	0.308916	-4.78666	0.602758	0.657687
Neutrophils	FAM53C	0.178058	3.981286	1.023346	0.308936	-5.44174	0.546888	0.598214
Neutrophils	DDA1	0.162343	5.082621	1.023321	0.308948	-5.58903	0.529999	0.580152
Neutrophils	GM4107	-0.24555	0.916265	-1.02304	0.309082	-5.53644	0.59687	0.651464
Neutrophils	SGO2A	-0.34767	3.063462	-1.02303	0.309086	-5.29844	0.561388	0.613711
Neutrophils	TCEA1	0.094175	7.486226	1.022938	0.309128	-6.05959	0.495005	0.542634
Neutrophils	GM29264	0.363086	0.505466	1.022601	0.309287	-5.00567	0.603914	0.659075
Neutrophils	CSPP1	-0.12272	6.098729	-1.0226	0.309287	-5.94274	0.514899	0.564106
Neutrophils	SERPINA1E	0.3899	5.030433	1.022579	0.309297	-5.78035	0.530787	0.581142
Neutrophils	THAP8	-0.7683	-0.29985	-1.02252	0.309325	-4.74765	0.617966	0.67401
Neutrophils	NUDT21	-0.14283	6.148811	-1.0224	0.30938	-5.70362	0.514166	0.563364
Neutrophils	IKBKE	0.374596	3.116112	1.02238	0.30939	-5.33553	0.560545	0.612987
Neutrophils	MLF2	0.140019	5.85253	1.022082	0.309531	-5.69478	0.518578	0.56803
Neutrophils	TMEM144	-0.66539	0.882973	-1.02196	0.309588	-4.76863	0.597509	0.652285
Neutrophils	PPM1A	-0.08388	6.125745	-1.02195	0.309595	-5.97656	0.514565	0.563754
Neutrophils	FBXW5	-0.26212	3.39907	-1.02186	0.309637	-5.13163	0.556107	0.608212
Neutrophils	GM19710	0.49936	2.595654	1.021354	0.309873	-4.93084	0.569336	0.622135
Neutrophils	CORO2B	-0.38597	1.197161	-1.02111	0.309987	-4.97982	0.592546	0.646938
Neutrophils	CLIP1	0.114014	5.440971	1.021004	0.310038	-5.96068	0.525015	0.57494
Neutrophils	9030622O2	-0.61989	1.05914	-1.021	0.310042	-4.85108	0.594886	0.649478
Neutrophils	SPOCK2	-0.5954	0.349422	-1.02081	0.310128	-4.76222	0.607132	0.662473
Neutrophils	RTN3	-0.09614	8.060675	-1.02028	0.310382	-6.35707	0.487742	0.534655
Neutrophils	GM9856	0.525198	1.246414	1.02011	0.31046	-4.79495	0.592209	0.646382
Neutrophils	A530040E1	0.636451	0.013043	1.019651	0.310676	-4.76723	0.613489	0.669165
Neutrophils	RNF219	0.36955	3.193241	1.019593	0.310704	-4.99271	0.560247	0.612585
Neutrophils	ST7L	-0.14664	4.451288	-1.01952	0.310736	-5.69224	0.540513	0.591531

Neutrophils	KAZN	-0.55796	0.348768	-1.01945	0.31077	-4.85884	0.607635	0.663017
Neutrophils	PRLR	-0.45358	3.152323	-1.01943	0.310779	-5.15724	0.560901	0.61331
Neutrophils	PTBP1	-0.14471	6.221744	-1.01927	0.310856	-5.69373	0.513958	0.563192
Neutrophils	SLC3A1	-0.53207	0.899213	-1.01922	0.310881	-4.81884	0.598157	0.653083
Neutrophils	ZBED3	-0.33875	3.559356	-1.01896	0.311003	-4.99129	0.554428	0.606596
Neutrophils	PSMC2	0.168152	5.362149	1.018957	0.311004	-5.53525	0.526676	0.576907
Neutrophils	GPRASP1	-0.20724	3.93667	-1.01895	0.311008	-5.29482	0.548497	0.600259
Neutrophils	SETBP1	-0.48332	4.621756	-1.0187	0.311127	-5.16561	0.538009	0.588951
Neutrophils	PSMB8	0.21434	7.077604	1.018222	0.311351	-5.87527	0.501933	0.550074
Neutrophils	NRBF2	0.184643	4.314912	1.018195	0.311364	-5.42694	0.542962	0.594051
Neutrophils	HSD17B11	-0.12275	4.796644	-1.018	0.311458	-5.85072	0.535611	0.586184
Neutrophils	HPX	-0.21407	5.670685	-1.01791	0.311497	-5.90652	0.522453	0.572114
Neutrophils	TST	-0.25452	3.470042	-1.0177	0.311595	-5.6218	0.556327	0.608381
Neutrophils	EFNB2	-0.39298	2.469455	-1.01759	0.311652	-5.10908	0.572441	0.625654
Neutrophils	GBF1	-0.09224	6.562441	-1.01744	0.311722	-5.93936	0.509486	0.558314
Neutrophils	GALNT18	-0.34444	2.025829	-1.01694	0.311958	-5.3688	0.580109	0.633696
Neutrophils	PPM1L	-0.25814	4.617569	-1.01678	0.312032	-5.38379	0.538814	0.589693
Neutrophils	NFYC	-0.09728	6.202043	-1.01668	0.312081	-5.79821	0.515063	0.564271
Neutrophils	TBC1D7	-0.35428	2.220043	-1.01641	0.312208	-4.88985	0.576999	0.630404
Neutrophils	ANAPC5	0.115211	6.399962	1.016383	0.31222	-5.79593	0.51223	0.561171
Neutrophils	ZHX3	-0.22486	4.441922	-1.01593	0.312435	-5.38777	0.541755	0.59261
Neutrophils	ZFPL1	0.237178	3.466605	1.015861	0.312467	-5.15767	0.557025	0.608953
Neutrophils	NEDD8	0.081082	7.48397	1.015749	0.312521	-6.11442	0.496869	0.544537
Neutrophils	PTGES2	0.295365	2.422157	1.015656	0.312564	-5.09287	0.57387	0.626947
Neutrophils	NSUN5	0.410829	2.124735	1.01561	0.312586	-4.88503	0.578762	0.632154
Neutrophils	YTHDC1	0.079532	7.312924	1.014866	0.312939	-6.12095	0.499693	0.547402
Neutrophils	ARHGAP27	0.255232	3.664658	1.01485	0.312946	-5.20863	0.55434	0.605919
Neutrophils	GM19466	0.559863	0.661272	1.014362	0.313178	-4.82551	0.604201	0.658855
Neutrophils	WDTC1	0.183758	3.851337	1.014353	0.313182	-5.50755	0.551628	0.602929
Neutrophils	PIGH	0.508471	1.827535	1.014004	0.313348	-4.82765	0.584623	0.63804
Neutrophils	FAM102B	-0.21456	4.23575	-1.01364	0.31352	-5.43682	0.546023	0.596852
Neutrophils	MRPS26	-0.15619	5.167613	-1.01347	0.313601	-5.42722	0.531736	0.581593
Neutrophils	RPGRIP1L	0.407813	1.613197	1.013404	0.313632	-4.90355	0.588448	0.642074
Neutrophils	SEPSECS	-0.38503	3.143326	-1.01323	0.313713	-4.96501	0.563357	0.615394
Neutrophils	TOLLIP	0.208118	3.832789	1.013039	0.313806	-5.39464	0.552451	0.603749
Neutrophils	FKBP11	-0.37087	1.995525	-1.01283	0.313905	-4.92536	0.582176	0.635489
Neutrophils	SLC35E3	0.395447	1.909396	1.012793	0.313923	-4.98433	0.58361	0.637025
Neutrophils	NSDHL	-0.3701	2.406095	-1.0127	0.313967	-4.98763	0.575394	0.628299
Neutrophils	GM36839	0.366728	2.346299	1.012607	0.314011	-4.97614	0.576377	0.62936
Neutrophils	ADNP2	0.276505	2.990665	1.01246	0.314081	-5.23159	0.565908	0.618203
Neutrophils	GM26631	-0.53502	1.219353	-1.01224	0.314187	-4.80742	0.595308	0.649533
Neutrophils	GM15246	0.55462	1.046088	1.012059	0.314271	-4.87173	0.59826	0.652778
Neutrophils	PRDM1	0.458489	2.929068	1.012009	0.314295	-5.00795	0.566955	0.6195
Neutrophils	HIKESHI	-0.16756	5.228907	-1.01196	0.31432	-5.50861	0.530991	0.581088
Neutrophils	FARSB	0.201513	5.008108	1.01175	0.314419	-5.34069	0.534343	0.584689
Neutrophils	MRPL39	-0.24508	3.661118	-1.0116	0.314489	-5.1189	0.555246	0.607036
Neutrophils	COLEC12	-0.40289	2.890318	-1.0115	0.314536	-5.23093	0.567586	0.620233

Neutrophils	HNRNPL	-0.08043	8.426349	-1.01142	0.314574	-6.24059	0.484909	0.531664
Neutrophils	SLC7A8	-0.40423	2.86439	-1.01136	0.314605	-5.26266	0.568006	0.62073
Neutrophils	HIST1H3I	0.580012	1.296054	1.011186	0.314687	-4.84309	0.59401	0.648446
Neutrophils	TEFM	-0.5747	1.160891	-1.01093	0.314809	-4.81508	0.596307	0.650923
Neutrophils	MECR	-0.33047	3.581314	-1.01082	0.314861	-5.0187	0.556511	0.608584
Neutrophils	CTIF	-0.25002	2.596638	-1.01077	0.314885	-5.11554	0.572362	0.625498
Neutrophils	TMEM62	0.360633	2.375873	1.010766	0.314887	-5.00144	0.575979	0.62935
Neutrophils	NPRL2	-0.33745	2.790262	-1.01073	0.314902	-4.959	0.569209	0.622153
Neutrophils	AHCYL2	0.150545	6.28947	1.010486	0.31502	-5.83944	0.515325	0.56448
Neutrophils	CASP6	-0.28692	3.628567	-1.01013	0.31519	-5.11337	0.556082	0.608062
Neutrophils	RASIP1	-0.28202	1.808725	-1.00959	0.315447	-5.2873	0.586097	0.639751
Neutrophils	PTPN21	0.558524	0.813665	1.009242	0.315613	-4.81448	0.60312	0.657947
Neutrophils	CHST7	-0.56141	0.725254	-1.00918	0.315641	-4.86928	0.604645	0.659595
Neutrophils	ZKSCAN5	-0.4088	2.402977	-1.00911	0.315675	-4.91396	0.576366	0.629558
Neutrophils	TMEM173	-0.29605	4.250581	-1.00871	0.315867	-5.12995	0.547027	0.598189
Neutrophils	EIF5B	-0.10094	7.158051	-1.0084	0.316013	-5.97942	0.503664	0.551782
Neutrophils	SATB2	-0.54279	1.643339	-1.00838	0.316025	-4.86655	0.589318	0.643309
Neutrophils	IMMP2L	-0.2656	6.534254	-1.00831	0.316057	-5.64161	0.512665	0.561447
Neutrophils	ZFP780B	0.331348	3.329247	1.008021	0.316195	-5.05385	0.561791	0.61388
Neutrophils	BGN	-0.3087	2.420901	-1.00762	0.316389	-5.18005	0.576653	0.629608
Neutrophils	TMEM141	-0.4397	2.239657	-1.00756	0.316417	-5.02462	0.579643	0.63279
Neutrophils	NUCKS1	-0.14899	6.576295	-1.00747	0.316459	-5.88081	0.512292	0.560868
Neutrophils	PIGM	0.234079	3.53133	1.007367	0.316508	-5.17663	0.558674	0.610498
Neutrophils	ZSCAN25	-0.52363	1.792602	-1.00734	0.316522	-4.81291	0.587087	0.640763
Neutrophils	MAST3	-0.19187	4.169613	-1.00721	0.316581	-5.53107	0.548613	0.599762
Neutrophils	ETV5	-0.30087	3.199847	-1.00697	0.316697	-5.24494	0.564082	0.616285
Neutrophils	CFP	-0.1592	5.518079	-1.00674	0.316807	-6.04252	0.528035	0.577831
Neutrophils	SMAD3	0.143784	6.315623	1.006699	0.316827	-6.02982	0.516195	0.565142
Neutrophils	FIS1	0.091527	7.530663	1.006601	0.316874	-6.24688	0.498691	0.546346
Neutrophils	ARMH2	-0.39601	0.301427	-1.00655	0.316896	-5.04688	0.612738	0.668059
Neutrophils	CDC42	0.058538	8.943013	1.006288	0.317023	-6.47973	0.479183	0.52529
Neutrophils	TRAF4	-0.45357	4.196822	-1.00626	0.317037	-5.05693	0.548338	0.599481
Neutrophils	2410018L1	0.566675	0.727626	1.00614	0.317094	-4.78449	0.605399	0.660203
Neutrophils	RFC1	0.146653	5.999025	1.00596	0.31718	-5.85098	0.52098	0.57022
Neutrophils	RALGPS1	0.17548	4.92479	1.005817	0.317248	-5.6226	0.537175	0.587608
Neutrophils	AASDH	0.489614	2.290623	1.005556	0.317373	-4.89001	0.579198	0.632341
Neutrophils	A530017D:	-0.31046	2.325165	-1.00539	0.317453	-5.059	0.578677	0.631746
Neutrophils	DCAF15	0.284007	3.456621	1.004654	0.317805	-5.13829	0.560745	0.612294
Neutrophils	PRAMEF8	0.240156	3.188085	1.004424	0.317915	-5.32405	0.565055	0.616931
Neutrophils	STX17	0.193438	4.034327	1.004373	0.31794	-5.51018	0.551586	0.602572
Neutrophils	NEMF	0.130842	5.95092	1.004307	0.317972	-5.65155	0.522299	0.571285
Neutrophils	TAX1BP1	0.087647	8.297296	1.004272	0.317988	-6.34953	0.488648	0.535166
Neutrophils	F2RL2	0.597414	0.129948	1.004196	0.318025	-4.82327	0.616602	0.671715
Neutrophils	SIDT1	-0.56677	3.376538	-1.00403	0.318102	-4.99082	0.562035	0.613771
Neutrophils	ZFP827	-0.45577	2.85843	-1.00397	0.318135	-4.95389	0.570402	0.622685
Neutrophils	ERI3	-0.16792	5.177084	-1.00366	0.31828	-5.4415	0.53405	0.583909
Neutrophils	FAS	0.154071	3.936884	1.003597	0.318312	-5.92015	0.553252	0.604434

Neutrophils	GUCY2C	-0.32638	-0.14698	-1.00323	0.31849	-5.03732	0.621861	0.677201
Neutrophils	NUDT22	-0.29014	2.557891	-1.00306	0.318571	-5.08442	0.575641	0.628214
Neutrophils	KCNIP4	0.767668	0.608323	1.002762	0.318712	-4.8177	0.608589	0.663377
Neutrophils	MDH1	-0.11635	8.120076	-1.00268	0.31875	-6.13254	0.491394	0.538258
Neutrophils	JUP	-0.21366	3.562597	-1.00266	0.318763	-5.4371	0.559379	0.611066
Neutrophils	TFPI	-0.21081	3.468648	-1.00262	0.318782	-5.40875	0.560879	0.612677
Neutrophils	INPP5F	0.273115	4.366787	1.002492	0.318842	-5.26168	0.546704	0.597579
Neutrophils	A430073D2	0.513346	1.815155	1.002485	0.318845	-4.82109	0.587974	0.641542
Neutrophils	MRPL37	-0.24615	4.2912	-1.00193	0.319109	-5.15379	0.548245	0.599042
Neutrophils	GADD45B	0.172968	5.322838	1.001318	0.319406	-5.92391	0.532569	0.582289
Neutrophils	LTO1	0.25788	3.526077	1.001259	0.319434	-5.16475	0.560536	0.61218
Neutrophils	CYB5R4	0.084475	5.869945	1.001226	0.31945	-6.17342	0.524344	0.573521
Neutrophils	CHMP2B	0.134949	4.813502	1.001147	0.319488	-5.62736	0.540346	0.590645
Neutrophils	WDYHV1	0.200548	4.174037	1.001142	0.31949	-5.33002	0.550278	0.601253
Neutrophils	MTERF1A	-0.37879	1.405346	-1.0009	0.319607	-4.93958	0.595622	0.649471
Neutrophils	CCDC59	0.127171	5.676842	1.000609	0.319746	-5.61729	0.527468	0.57674
Neutrophils	EPHX3	-0.51279	0.657793	-1.0005	0.319799	-4.83568	0.608625	0.663183
Neutrophils	FHL3	-0.29185	2.081558	-1.0004	0.319846	-5.2624	0.58438	0.637467
Neutrophils	TWISTNB	0.143637	5.520145	1.000174	0.319956	-5.62504	0.529919	0.579386
Neutrophils	1110019D1	-0.40696	1.751896	-0.9997	0.320182	-4.93558	0.590255	0.643583
Neutrophils	PRORP	-0.29494	3.853039	-0.99962	0.320222	-5.03743	0.555914	0.607107
Neutrophils	CCDC112	-0.54178	1.151644	-0.99947	0.320294	-4.85387	0.600458	0.654521
Neutrophils	ZFP524	0.246012	3.672126	0.999457	0.320301	-5.31309	0.558788	0.610214
Neutrophils	MRPS15	0.115052	5.910327	0.998847	0.320595	-5.87568	0.524625	0.573486
Neutrophils	TECPR1	0.278914	4.375591	0.998705	0.320663	-5.27218	0.548049	0.59852
Neutrophils	MED18	0.504829	1.412452	0.998699	0.320666	-4.8332	0.596386	0.649945
Neutrophils	ESCO2	0.326042	3.734708	0.998338	0.32084	-5.34304	0.558362	0.609447
Neutrophils	SRPK2	-0.0941	7.096447	-0.99784	0.321081	-6.29811	0.507672	0.555148
Neutrophils	GM42962	0.505104	-0.60259	0.997696	0.32115	-4.78996	0.632252	0.687718
Neutrophils	KAT5	-0.30566	2.921057	-0.99769	0.321151	-5.1479	0.571738	0.623585
Neutrophils	EPS8L1	-0.81536	0.408966	-0.99747	0.32126	-4.80544	0.61432	0.668777
Neutrophils	PIGU	-0.18621	4.215968	-0.99726	0.321362	-5.49329	0.551082	0.601624
Neutrophils	NAALADL2	-0.36025	1.964286	-0.9972	0.321391	-5.02895	0.587634	0.640536
Neutrophils	CFL1	0.09898	10.01399	0.997172	0.321403	-6.59104	0.467474	0.511992
Neutrophils	DSCC1	0.371615	2.435007	0.996777	0.321593	-5.01936	0.580039	0.632373
Neutrophils	FNDC5	0.607735	0.474524	0.996439	0.321756	-4.81039	0.613554	0.667848
Neutrophils	DISC1	-0.25826	3.43956	-0.99642	0.321763	-5.20719	0.563766	0.615004
Neutrophils	GM44284	-0.54725	-0.05378	-0.99603	0.321954	-4.84149	0.623149	0.677871
Neutrophils	GEMIN5	0.311728	3.385628	0.995507	0.322207	-5.04668	0.564975	0.616092
Neutrophils	SAP130	0.144075	5.619105	0.995309	0.322302	-5.72195	0.530154	0.579013
Neutrophils	PDP1	0.375857	2.180234	0.995286	0.322313	-4.90138	0.584742	0.637165
Neutrophils	FAM104A	0.112448	6.153765	0.995257	0.322327	-5.90627	0.522155	0.570458
Neutrophils	NACA	0.093748	9.080227	0.995237	0.322337	-6.31753	0.480568	0.525837
Neutrophils	CCDC90B	-0.31922	3.165156	-0.9949	0.322498	-5.03328	0.568539	0.619998
Neutrophils	ACSBG1	-0.61678	-0.5064	-0.99487	0.322514	-4.76897	0.63137	0.686587
Neutrophils	ME2	0.132655	5.963937	0.994832	0.322533	-5.93558	0.52498	0.57353
Neutrophils	TMEM14C	0.113143	6.950542	0.994826	0.322536	-6.04745	0.510469	0.557991

Neutrophils	CHPT1	0.251799	3.607446	0.994811	0.322543	-5.18339	0.561413	0.612414
Neutrophils	RNF4	-0.11871	5.964365	-0.99469	0.322599	-5.70608	0.524979	0.573578
Neutrophils	LGALS2	-0.59619	-0.89826	-0.99442	0.322731	-4.75524	0.638637	0.694273
Neutrophils	PSMD4	0.099372	6.691408	0.994086	0.322894	-5.93205	0.514534	0.562274
Neutrophils	ZFP277	-0.17466	5.309649	-0.99399	0.322941	-5.41693	0.535149	0.584349
Neutrophils	SPR	-0.27028	3.527522	-0.99341	0.323221	-5.18426	0.563412	0.614262
Neutrophils	PURA	0.137172	5.343404	0.993103	0.32337	-5.5456	0.535165	0.584048
Neutrophils	RNF6	-0.12068	5.670645	-0.993	0.323421	-5.75612	0.530206	0.578788
Neutrophils	KIFAP3	-0.30133	2.849059	-0.99289	0.323475	-5.00054	0.574587	0.62611
Neutrophils	GM13919	-0.4413	3.111975	-0.99268	0.323573	-4.92682	0.570373	0.621586
Neutrophils	DNAJC15	-0.16652	5.723768	-0.99233	0.323745	-5.61787	0.529588	0.578072
Neutrophils	A430072P(	0.557364	0.354986	0.992327	0.323746	-4.88762	0.617202	0.671177
Neutrophils	SGF29	0.181956	4.671195	0.992108	0.323852	-5.37346	0.545782	0.595381
Neutrophils	SMPDL3B	0.363321	1.9506	0.991765	0.324019	-5.3389	0.590015	0.642332
Neutrophils	SFSWAP	0.105852	5.786795	0.99124	0.324273	-5.75658	0.529222	0.577516
Neutrophils	NUBP1	-0.20188	5.473714	-0.99115	0.324316	-5.41143	0.533956	0.582623
Neutrophils	SKI	-0.11996	5.372605	-0.99075	0.32451	-5.91827	0.535641	0.584472
Neutrophils	MCM10	0.242056	3.112366	0.990747	0.324513	-5.38188	0.571268	0.622423
Neutrophils	ITGB2L	0.157015	-0.61932	0.990594	0.324587	-5.62975	0.635492	0.690473
Neutrophils	PFKFB2	0.299474	2.465839	0.99053	0.324618	-5.03361	0.581903	0.633787
Neutrophils	TMEM176I	-0.22837	5.475623	-0.99035	0.324704	-5.62149	0.534127	0.58293
Neutrophils	SFMBT2	-0.34699	0.60235	-0.99006	0.324848	-5.1136	0.61382	0.667663
Neutrophils	RCN1	-0.289	3.060814	-0.99001	0.324871	-5.13534	0.57222	0.623568
Neutrophils	HMGA1	0.269408	4.670399	0.98996	0.324895	-5.30306	0.546562	0.596264
Neutrophils	E230014E1	0.266766	-1.38289	0.989794	0.324976	-5.16533	0.649652	0.705572
Neutrophils	ATG12	0.152164	4.873947	0.989424	0.325156	-5.63757	0.543421	0.593037
Neutrophils	2810454H(	-0.3176	2.514759	-0.98934	0.325197	-5.08431	0.581223	0.633279
Neutrophils	MR1	0.56407	0.68263	0.989336	0.325199	-4.82485	0.612433	0.666363
Neutrophils	GM9725	0.35503	2.030059	0.989336	0.325199	-5.10258	0.589319	0.641873
Neutrophils	ZFP384	-0.14295	4.90083	-0.98921	0.32526	-5.63022	0.543005	0.592623
Neutrophils	ZFHX2	0.394317	2.765643	0.989052	0.325337	-5.02645	0.577077	0.628989
Neutrophils	H13	0.105292	6.42662	0.988994	0.325365	-5.92306	0.519944	0.568065
Neutrophils	TPCN1	0.236554	4.187411	0.98895	0.325386	-5.46658	0.554152	0.604609
Neutrophils	GM17655	0.52692	1.183959	0.988793	0.325463	-4.81781	0.603767	0.657321
Neutrophils	SGTA	-0.17364	4.959007	-0.98869	0.325514	-5.53232	0.542142	0.59181
Neutrophils	RAB9	-0.14399	4.887523	-0.9885	0.325604	-5.57078	0.543307	0.593026
Neutrophils	1700017B(	0.22137	5.285252	0.987895	0.3259	-5.44759	0.537492	0.58669
Neutrophils	CYB5D1	0.537902	0.775244	0.987745	0.325973	-4.86355	0.611266	0.665109
Neutrophils	MEIG1	0.499799	-0.47443	0.98772	0.325985	-4.8728	0.633479	0.688584
Neutrophils	THSD1	-0.36422	1.60027	-0.98769	0.326	-5.01999	0.597034	0.650036
Neutrophils	RELB	0.158988	6.088989	0.98739	0.326146	-5.79304	0.525492	0.573712
Neutrophils	ZBTB39	0.369043	1.975209	0.987101	0.326287	-5.03442	0.591003	0.643389
Neutrophils	CEP97	-0.3955	2.922474	-0.98697	0.326352	-4.93224	0.575262	0.626676
Neutrophils	IDE	-0.21453	4.161112	-0.98674	0.326465	-5.19861	0.555403	0.605443
Neutrophils	PLEKHB2	0.191271	4.419141	0.986551	0.326555	-5.37352	0.551396	0.60112
Neutrophils	CUL2	0.206401	4.968394	0.986328	0.326664	-5.38523	0.542931	0.592075
Neutrophils	FES	0.126837	4.284767	0.985714	0.326963	-5.93332	0.554022	0.603708

Neutrophils	CXCL9	0.793896	2.080321	0.985466	0.327085	-5.03464	0.59007	0.641972
Neutrophils	CCDC117	-0.22624	4.699485	-0.98533	0.327149	-5.24446	0.547608	0.596851
Neutrophils	SNRNP200	-0.17146	5.038857	-0.98518	0.327224	-5.58384	0.542343	0.591337
Neutrophils	RNF24	0.144792	4.233052	0.985162	0.327233	-5.73932	0.554932	0.604754
Neutrophils	SZT2	0.288682	2.318426	0.984958	0.327332	-5.05893	0.586084	0.637964
Neutrophils	PHOSPHO2	-0.19621	3.724911	-0.98486	0.327381	-5.28328	0.563037	0.613501
Neutrophils	SREK1IP1	0.203424	4.172778	0.984705	0.327456	-5.40874	0.555896	0.605964
Neutrophils	PID1	0.206227	5.484883	0.984627	0.327494	-5.79217	0.535512	0.584259
Neutrophils	KDM5B	0.154753	5.362074	0.98451	0.327551	-5.94137	0.537386	0.586302
Neutrophils	ZFP773	0.436409	0.510873	0.984492	0.32756	-5.01611	0.617125	0.671001
Neutrophils	RAD51B	0.237654	6.152694	0.984254	0.327676	-5.81189	0.525455	0.573538
Neutrophils	TRIP12	0.083117	7.435654	0.984252	0.327678	-6.19484	0.506655	0.553415
Neutrophils	TRRAP	0.167796	4.784536	0.983982	0.327809	-5.55511	0.546385	0.595906
Neutrophils	ZFP260	-0.25839	4.245881	-0.98387	0.327863	-5.07884	0.554833	0.604944
Neutrophils	MCAM	-0.36361	1.173297	-0.98377	0.327913	-5.15552	0.605662	0.658953
Neutrophils	STUB1	0.129555	5.426852	0.983723	0.327936	-5.70198	0.536487	0.58541
Neutrophils	4930402H2	-0.15997	4.453202	-0.98336	0.328116	-5.63844	0.551778	0.601576
Neutrophils	SLC4A9	-0.62235	0.122757	-0.9832	0.328193	-4.77002	0.624394	0.6786
Neutrophils	TAOK1	0.084345	7.033029	0.9829	0.328339	-6.1651	0.512925	0.560023
Neutrophils	4833417C1	-0.5579	0.707506	-0.98274	0.328418	-4.80099	0.614273	0.667805
Neutrophils	DENND2D	-0.26624	2.202221	-0.9826	0.328487	-5.16351	0.588637	0.640686
Neutrophils	DYNLL2	-0.23125	5.082989	-0.98232	0.328624	-5.45711	0.542299	0.591426
Neutrophils	KLRB1F	-0.60063	0.424065	-0.98229	0.328636	-4.87929	0.619374	0.673257
Neutrophils	C3AR1	-0.48851	2.290307	-0.98216	0.328701	-5.16687	0.587253	0.639269
Neutrophils	YTHDF3	0.084845	7.017555	0.982022	0.328769	-6.19796	0.51332	0.560471
Neutrophils	MAP1LC3B	0.106577	7.434486	0.981558	0.328996	-6.27386	0.50755	0.554234
Neutrophils	SERPINA1B	-0.27141	7.994808	-0.98131	0.32912	-6.37052	0.499655	0.545794
Neutrophils	NDUFAF4	0.304674	3.867148	0.981173	0.329185	-5.09398	0.561887	0.612291
Neutrophils	HIST1H1E	0.545733	4.203103	0.980834	0.329351	-5.27961	0.556575	0.606691
Neutrophils	ADAM15	0.231005	2.475369	0.980708	0.329413	-5.48078	0.584683	0.636569
Neutrophils	HMGXB3	-0.21373	4.408539	-0.98064	0.329447	-5.30729	0.553327	0.603231
Neutrophils	ZNHIT3	0.261452	3.532147	0.980622	0.329455	-5.15483	0.567321	0.618126
Neutrophils	BTBD2	-0.26535	3.477347	-0.98058	0.329478	-5.17367	0.568208	0.619092
Neutrophils	COPG1	0.174748	5.141092	0.979987	0.329767	-5.52805	0.542195	0.591306
Neutrophils	KIF7	0.627653	-0.46987	0.979894	0.329812	-4.77622	0.636322	0.691177
Neutrophils	RBBP9	0.477348	1.214794	0.979888	0.329815	-4.91216	0.60643	0.659595
Neutrophils	PDGFB	-0.69412	1.062654	-0.97975	0.329884	-4.82391	0.609098	0.662457
Neutrophils	MOB2	0.125096	5.416589	0.979523	0.329995	-5.72701	0.538054	0.586954
Neutrophils	GM34983	-0.41451	0.754004	-0.97943	0.330038	-4.99942	0.614569	0.66826
Neutrophils	ZC3H12B	-0.5909	1.084598	-0.97913	0.330186	-4.80942	0.608794	0.662252
Neutrophils	TAP1	0.397844	5.446269	0.979088	0.330208	-5.50764	0.5376	0.586579
Neutrophils	LIN9	-0.2144	4.133885	-0.97888	0.330308	-5.37503	0.558069	0.608448
Neutrophils	LIG4	-0.51045	1.902715	-0.97885	0.330323	-4.89531	0.594738	0.647413
Neutrophils	USE1	0.139724	5.084893	0.978828	0.330336	-5.68741	0.543157	0.592552
Neutrophils	TRP53RKA	0.361629	2.472703	0.978665	0.330416	-4.93716	0.585141	0.637278
Neutrophils	HIST2H3B	-0.56465	1.047683	-0.97847	0.330514	-4.83813	0.609436	0.663071
Neutrophils	ZC3H6	0.302783	3.379852	0.978362	0.330565	-5.11615	0.570194	0.621435

Neutrophils	DGCR8	-0.26449	3.779114	-0.97836	0.330565	-5.15952	0.56374	0.614568
Neutrophils	KMT2E	0.077991	8.273581	0.978315	0.330588	-6.30852	0.496128	0.542293
Neutrophils	A	-0.1873	4.78007	-0.97824	0.330624	-5.66225	0.547891	0.597705
Neutrophils	IFI27L2A	0.326062	7.373467	0.978031	0.330728	-6.10494	0.509009	0.556165
Neutrophils	VCP	-0.09171	7.431716	-0.9779	0.330792	-6.08905	0.508169	0.555268
Neutrophils	HSF5	-0.53356	0.277217	-0.97768	0.330901	-4.85491	0.623064	0.677569
Neutrophils	UBL4A	0.223165	4.202976	0.977649	0.330916	-5.40661	0.557033	0.607503
Neutrophils	SAMM50	0.162974	5.177169	0.977621	0.330929	-5.5446	0.541792	0.591249
Neutrophils	MTHFD2L	-0.27487	3.033816	-0.97748	0.330998	-5.12552	0.575939	0.62767
Neutrophils	EGFL6	-0.64375	0.548891	-0.97682	0.331322	-4.78249	0.61878	0.672755
Neutrophils	FBXW17	0.433424	1.479346	0.976715	0.331375	-4.9329	0.602556	0.655588
Neutrophils	TCEAL8	-0.24104	3.331023	-0.97647	0.331497	-5.21634	0.571654	0.622717
Neutrophils	D030056L2	0.260999	3.888031	0.976327	0.331566	-5.27579	0.562648	0.613167
Neutrophils	PRDX3	0.202967	5.400912	0.976257	0.331601	-5.45011	0.538922	0.587885
Neutrophils	SLC38A4	-0.3462	3.153421	-0.97578	0.331838	-5.33927	0.574803	0.626056
Neutrophils	ZSCAN22	-0.51381	1.316109	-0.97575	0.33185	-4.86214	0.605747	0.658885
Neutrophils	ANAPC10	0.189507	4.581596	0.975632	0.331909	-5.3588	0.551882	0.601717
Neutrophils	GM11655	-0.61268	0.316941	-0.97515	0.332149	-4.76907	0.623441	0.677531
Neutrophils	FBXO3	0.12444	5.012921	0.975136	0.332153	-5.68586	0.545278	0.594581
Neutrophils	PGPEP1	-0.29271	3.461593	-0.97514	0.332154	-5.08087	0.56992	0.620817
Neutrophils	GSTO2	0.495218	0.524993	0.974862	0.332288	-4.91782	0.619747	0.673751
Neutrophils	GM15564	0.376666	2.474453	0.974833	0.332303	-5.08529	0.586196	0.638219
Neutrophils	IPO4	-0.35926	2.384797	-0.97471	0.332362	-4.90237	0.587697	0.639812
Neutrophils	ZC3H12D	0.329097	3.479104	0.974705	0.332366	-5.17487	0.569635	0.620627
Neutrophils	FNBP4	0.118916	6.074991	0.974285	0.332573	-5.79418	0.529274	0.5775
Neutrophils	4921511C1	0.293726	2.59393	0.974203	0.332614	-5.15811	0.584447	0.636285
Neutrophils	PIGP	-0.22057	4.420664	-0.97394	0.332742	-5.33259	0.554912	0.604823
Neutrophils	GM26542	0.180496	4.802534	0.97372	0.332852	-5.60989	0.549001	0.598536
Neutrophils	B230118H(	0.291271	2.933488	0.973384	0.333018	-5.1757	0.579165	0.630676
Neutrophils	CHN2	0.229218	5.358074	0.973327	0.333046	-5.90343	0.540505	0.589546
Neutrophils	EFEMP2	0.358171	1.944258	0.973248	0.333085	-4.97297	0.595747	0.648332
Neutrophils	MTCH1	0.103788	6.297903	0.973122	0.333148	-5.89142	0.526264	0.574386
Neutrophils	VPS72	0.180606	4.357985	0.97264	0.333386	-5.39321	0.556358	0.606354
Neutrophils	ANKS1	0.113709	5.759857	0.97245	0.33348	-6.06922	0.534594	0.583162
Neutrophils	HACD2	0.093092	5.732831	0.972423	0.333493	-6.03897	0.535005	0.583601
Neutrophils	CAMK2N1	0.505541	1.714702	0.972246	0.333581	-5.00548	0.599924	0.652673
Neutrophils	EAR2	-0.20844	3.55304	-0.97216	0.333624	-5.78658	0.569269	0.620178
Neutrophils	DDX50	0.090736	6.369428	0.972064	0.333671	-5.96502	0.52541	0.57342
Neutrophils	COX6A2	0.630153	2.070766	0.97202	0.333693	-4.88119	0.593857	0.646299
Neutrophils	H2-AB1	-0.58908	6.764416	-0.97191	0.333749	-5.61408	0.519547	0.567169
Neutrophils	GM16556	0.207692	1.676001	0.971747	0.333827	-5.59145	0.600587	0.653449
Neutrophils	SNX17	0.13451	5.853791	0.97173	0.333836	-5.75004	0.533168	0.581732
Neutrophils	GM15441	0.338107	1.344167	0.971648	0.333876	-5.03612	0.606304	0.659503
Neutrophils	TMEM186	-0.50261	2.111676	-0.97128	0.334056	-4.85602	0.593226	0.645714
Neutrophils	2410006H1	0.181651	7.535004	0.971263	0.334067	-6.05761	0.508361	0.555246
Neutrophils	CAVIN2	-0.2407	2.454343	-0.97125	0.334073	-5.39408	0.587453	0.63959
Neutrophils	EHD4	-0.17976	6.076676	-0.9711	0.334149	-5.61468	0.52989	0.578258

Neutrophils	MLKL	-0.33269	2.335281	-0.97028	0.334555	-5.18031	0.589939	0.642005
Neutrophils	MDC1	-0.28436	3.159139	-0.97019	0.334598	-5.11657	0.576235	0.627459
Neutrophils	ZFP456	0.529177	0.791994	0.970118	0.334634	-4.85268	0.616514	0.670142
Neutrophils	HTR1F	0.368861	1.429334	0.970081	0.334652	-4.95085	0.605395	0.65838
Neutrophils	TRIM16	-0.33168	2.341778	-0.97004	0.334673	-5.16129	0.58983	0.641889
Neutrophils	SDCBP2	-0.5732	1.097863	-0.96963	0.334876	-4.84174	0.611426	0.664574
Neutrophils	TTK	-0.37971	2.313922	-0.96926	0.33506	-5.07936	0.590793	0.64266
Neutrophils	ZCCHC10	-0.26053	4.048466	-0.96899	0.335192	-5.18493	0.562408	0.612436
Neutrophils	TRNAU1AP	0.223351	4.126118	0.968577	0.335399	-5.35497	0.561421	0.611275
Neutrophils	ZKSCAN17	0.264628	3.498798	0.96824	0.335566	-5.13993	0.571738	0.622156
Neutrophils	POLR2M	-0.1472	5.403452	-0.96814	0.335617	-5.51766	0.541546	0.590083
Neutrophils	USP15	-0.0891	7.650516	-0.96802	0.335677	-6.30263	0.508065	0.554365
Neutrophils	PRPF39	0.148129	5.43876	0.967538	0.335915	-5.65786	0.541308	0.589742
Neutrophils	GM33524	0.528414	0.491882	0.967054	0.336155	-4.86811	0.623509	0.676902
Neutrophils	TIMM10B	0.102947	6.181583	0.966869	0.336247	-6.02509	0.53015	0.577811
Neutrophils	ABI1	0.10575	7.702393	0.96682	0.336271	-6.26924	0.507746	0.55386
Neutrophils	GAS7	-0.16983	6.417925	-0.9668	0.336283	-6.12005	0.526601	0.574022
Neutrophils	SRSF3	-0.08836	7.532017	-0.96679	0.336284	-6.14248	0.510205	0.556493
Neutrophils	SMIM4	-0.10535	5.255277	-0.96663	0.336365	-5.92023	0.544346	0.592917
Neutrophils	COL15A1	0.658704	0.332157	0.966421	0.33647	-4.82477	0.626504	0.680001
Neutrophils	ZFP946	-0.49031	1.39583	-0.96623	0.336566	-4.81271	0.607836	0.660222
Neutrophils	TMEM175	-0.29589	3.375009	-0.96597	0.336695	-5.14868	0.574582	0.624928
Neutrophils	LUC7L2	0.066651	8.527172	0.965863	0.336747	-6.34052	0.4963	0.541476
Neutrophils	GM15675	-0.30302	2.317552	-0.96575	0.336804	-5.25278	0.592184	0.643667
Neutrophils	SCP2	-0.14383	7.526193	-0.9655	0.336929	-6.31639	0.510693	0.556903
Neutrophils	PET100	0.100383	5.786929	0.965284	0.337035	-5.99753	0.536639	0.584584
Neutrophils	PPIP5K2	-0.18382	4.496011	-0.96511	0.33712	-5.40485	0.556725	0.606033
Neutrophils	PTMA	-0.1088	11.35991	-0.96506	0.337145	-6.68596	0.458309	0.500679
Neutrophils	ZBTB8A	-0.38117	1.978565	-0.96488	0.337234	-4.92687	0.598206	0.650034
Neutrophils	SNRPD2	-0.12302	6.391824	-0.9648	0.337278	-5.8151	0.527531	0.574903
Neutrophils	MAPK1	0.07834	7.693924	0.96442	0.337466	-6.14685	0.508456	0.554549
Neutrophils	CD47	0.074073	8.628324	0.964417	0.337468	-6.43526	0.49517	0.540315
Neutrophils	POGLUT2	-0.51601	1.00166	-0.96421	0.337573	-4.85485	0.615209	0.668059
Neutrophils	1190005I0(	-0.44557	1.185776	-0.96418	0.337587	-4.86643	0.611983	0.66465
Neutrophils	RTF1	0.09024	6.81408	0.964163	0.337594	-5.97394	0.521311	0.568299
Neutrophils	TUT7	0.068003	7.206797	0.963751	0.337799	-6.36245	0.515697	0.562266
Neutrophils	AGPAT5	0.10653	5.644905	0.963727	0.337811	-5.88646	0.539099	0.587254
Neutrophils	WASL	0.123982	5.483088	0.963569	0.33789	-5.639	0.541597	0.589907
Neutrophils	OAS1A	0.379679	2.355225	0.963496	0.337927	-5.2805	0.592092	0.64357
Neutrophils	QRICH1	0.089228	6.585338	0.963203	0.338073	-5.96113	0.5249	0.572134
Neutrophils	SLC25A15	-0.34251	2.488304	-0.96318	0.338085	-5.00925	0.589861	0.641243
Neutrophils	SAMD12	0.588471	0.266619	0.963087	0.33813	-4.8951	0.628489	0.682082
Neutrophils	PTPMT1	-0.20322	4.267152	-0.96294	0.338202	-5.3055	0.560687	0.610281
Neutrophils	APOBEC4	-0.61154	0.042895	-0.96288	0.338236	-4.78195	0.632518	0.686335
Neutrophils	SNRNP48	-0.17811	4.916287	-0.96276	0.338296	-5.44049	0.550417	0.59937
Neutrophils	F3	0.435323	0.113979	0.962714	0.338316	-5.17476	0.631235	0.685002
Neutrophils	TGFB2	-0.8675	0.46572	-0.96261	0.338367	-4.80183	0.624925	0.678387

Neutrophils	CLEC5A	0.162651	0.512385	0.962346	0.3385	-5.82023	0.624239	0.67762
Neutrophils	PYCR2	0.276039	3.687049	0.962181	0.338583	-5.187	0.570215	0.620409
Neutrophils	MRGPRA2E	0.204793	-0.43033	0.961652	0.338847	-5.6588	0.641728	0.695819
Neutrophils	PCMT1	-0.08334	6.63523	-0.96151	0.338918	-6.17969	0.524675	0.571754
Neutrophils	NDUFA2	0.093553	7.238115	0.961203	0.339071	-6.14125	0.515921	0.562336
Neutrophils	S100A4	-0.50214	2.87429	-0.96089	0.339227	-5.08217	0.584322	0.634997
Neutrophils	AMD2	-0.53592	0.044662	-0.96068	0.339333	-4.79353	0.633561	0.686995
Neutrophils	MTO1	-0.24332	3.553315	-0.96059	0.339376	-5.18397	0.573184	0.623171
Neutrophils	KATNB1	0.367755	2.346793	0.960424	0.339461	-5.02811	0.593308	0.644498
Neutrophils	EVI5L	0.371993	2.262161	0.960104	0.339621	-4.95813	0.594928	0.64616
Neutrophils	HAX1	0.23421	4.020737	0.959932	0.339707	-5.27492	0.565875	0.61536
Neutrophils	SLC9A1	0.117734	5.416377	0.959728	0.339809	-5.80307	0.543827	0.591961
Neutrophils	5830411N(	0.567854	-1.25694	0.959719	0.339814	-4.78326	0.657881	0.712603
Neutrophils	FN1	0.432886	5.580946	0.959378	0.339985	-5.77864	0.541432	0.58938
Neutrophils	ZFP395	0.189998	4.289929	0.959276	0.340036	-5.4947	0.561702	0.610965
Neutrophils	SIL1	-0.22808	5.149956	-0.95921	0.340068	-5.38109	0.548112	0.596511
Neutrophils	MRPL14	0.148258	5.601025	0.959064	0.340142	-5.83239	0.541154	0.589067
Neutrophils	SLC37A4	0.28029	2.862467	0.958888	0.34023	-5.14447	0.585125	0.635796
Neutrophils	GM45509	0.464501	1.640462	0.958486	0.340431	-4.93174	0.606153	0.657958
Neutrophils	DYNLL1	-0.08619	7.867224	-0.95825	0.340552	-6.26403	0.50772	0.553386
Neutrophils	GART	0.252334	4.367941	0.958232	0.340559	-5.31388	0.560816	0.610007
Neutrophils	CLCN5	-0.22689	5.160349	-0.95771	0.340822	-5.45853	0.548592	0.596793
Neutrophils	FAM207A	0.195829	4.223482	0.957659	0.340846	-5.33507	0.563425	0.612589
Neutrophils	TMEM183/	-0.1224	5.16956	-0.95755	0.340901	-5.6398	0.548448	0.596687
Neutrophils	ASNS	0.253978	1.465562	0.957325	0.341014	-5.30333	0.609646	0.661529
Neutrophils	ENPP2	-0.362	2.411456	-0.95683	0.341262	-5.13858	0.593667	0.644533
Neutrophils	TSPYL2	0.399696	2.8065	0.956785	0.341285	-4.96419	0.587013	0.637513
Neutrophils	ERI2	0.469213	1.310882	0.9567	0.341327	-4.89733	0.612615	0.664618
Neutrophils	GLT1D1	0.381538	0.611079	0.956579	0.341388	-5.05745	0.624992	0.677673
Neutrophils	SYN1	-0.45871	0.783746	-0.95647	0.341445	-4.99609	0.621922	0.674449
Neutrophils	KPNA1	0.109815	6.778024	0.955997	0.34168	-6.02683	0.524519	0.570988
Neutrophils	ARMT1	0.216941	3.297769	0.955923	0.341718	-5.21518	0.579138	0.629101
Neutrophils	SGSH	-0.42793	1.453355	-0.95579	0.341783	-4.93315	0.610455	0.662245
Neutrophils	GM36279	-0.31224	2.959709	-0.95516	0.342099	-5.13394	0.585151	0.635337
Neutrophils	ECHDC3	0.520677	0.833138	0.955073	0.342145	-4.93364	0.621771	0.674074
Neutrophils	ZC3H4	-0.13404	4.818849	-0.95502	0.34217	-5.59458	0.554952	0.603338
Neutrophils	UROS	-0.30261	2.669254	-0.95478	0.342293	-4.98993	0.590059	0.640532
Neutrophils	AA386476	0.463826	1.390731	0.954665	0.34235	-4.9081	0.61199	0.663712
Neutrophils	FAM3C	0.153853	5.36557	0.954652	0.342357	-5.57334	0.54642	0.594224
Neutrophils	CYCS	-0.12577	7.712668	-0.95431	0.342529	-6.2716	0.511318	0.556691
Neutrophils	FBXO6	0.264161	3.653081	0.95401	0.34268	-5.16355	0.573894	0.623397
Neutrophils	FANCF	-0.43508	1.911025	-0.95389	0.342742	-4.95822	0.603136	0.654384
Neutrophils	GM36447	-0.60123	0.209364	-0.95387	0.342749	-4.80365	0.633167	0.686062
Neutrophils	ZFP729B	0.199471	3.639068	0.953852	0.342759	-5.34648	0.574123	0.623678
Neutrophils	CPB2	0.257733	3.209826	0.95375	0.34281	-5.36098	0.581194	0.631195
Neutrophils	MYO15	-0.58529	1.234713	-0.95361	0.342882	-4.83367	0.614895	0.666838
Neutrophils	GPLD1	0.488083	0.934219	0.953595	0.342889	-4.93135	0.620194	0.672429

Neutrophils	SFXN1	0.20133	5.121606	0.953445	0.342964	-5.47182	0.550415	0.598533
Neutrophils	SLC20A1	-0.12929	5.219384	-0.95302	0.343176	-5.96326	0.548997	0.596921
Neutrophils	ACYP1	-0.19622	3.847714	-0.95296	0.34321	-5.43421	0.57087	0.620154
Neutrophils	DCK	0.14025	5.832657	0.95252	0.34343	-5.90128	0.539502	0.586932
Neutrophils	SLC25A47	0.239517	3.963	0.952518	0.343431	-5.42853	0.568997	0.61829
Neutrophils	CASR	0.58875	-0.61039	0.952514	0.343433	-4.8175	0.64834	0.702089
Neutrophils	IGSF5	-0.2205	1.922281	-0.95249	0.343444	-5.63113	0.603102	0.654414
Neutrophils	SLC30A4	-0.4034	1.941057	-0.95243	0.343475	-4.92526	0.602779	0.654076
Neutrophils	SQLE	-0.45225	1.97366	-0.95242	0.343478	-4.92582	0.602218	0.653483
Neutrophils	FAM171A1	-0.31998	1.565475	-0.9521	0.343643	-5.17577	0.609277	0.660947
Neutrophils	PRXL2B	-0.32755	2.256934	-0.95197	0.343705	-5.02296	0.597369	0.648389
Neutrophils	TNPO1	-0.10501	7.082293	-0.95191	0.343737	-6.04028	0.520683	0.566922
Neutrophils	LETM1	-0.18541	4.54276	-0.95184	0.343771	-5.36184	0.559676	0.608465
Neutrophils	ZFP330	-0.18386	4.432352	-0.95158	0.343903	-5.37715	0.561439	0.610418
Neutrophils	PIGL	0.240428	2.746364	0.951508	0.343941	-5.14945	0.589084	0.639761
Neutrophils	ESPN	-0.63109	-0.17599	-0.95145	0.343969	-4.79955	0.640345	0.693877
Neutrophils	ACAT3	-0.45348	1.453412	-0.95141	0.343992	-4.98485	0.611229	0.66318
Neutrophils	GM21188	0.22559	1.578983	0.951395	0.343997	-5.73417	0.609042	0.66087
Neutrophils	ATF5	0.306146	3.267979	0.951295	0.344048	-5.26757	0.580384	0.630594
Neutrophils	ZDHHC20	0.089933	6.740107	0.95125	0.344071	-5.99853	0.525767	0.572498
Neutrophils	CRLF3	0.130224	6.899396	0.951145	0.344124	-6.11493	0.523394	0.569965
Neutrophils	VPS33B	-0.29139	2.932531	-0.95103	0.344182	-5.07165	0.58597	0.636533
Neutrophils	SCD1	-0.47733	3.037555	-0.95087	0.34426	-5.03709	0.584228	0.63472
Neutrophils	ZFP629	-0.54843	0.926	-0.9508	0.344297	-4.85208	0.620522	0.673145
Neutrophils	XRCC4	0.132454	5.185209	0.950091	0.344656	-5.76985	0.550033	0.59816
Neutrophils	DPH3	0.104724	5.480129	0.949762	0.344822	-5.86622	0.545605	0.593421
Neutrophils	ALOX15	1.127476	-0.82153	0.949664	0.344872	-4.80622	0.653059	0.707066
Neutrophils	FNIP2	0.229182	5.343736	0.949464	0.344973	-6.0181	0.547797	0.595768
Neutrophils	TENT5A	-0.14571	5.169086	-0.94936	0.345025	-5.89215	0.550526	0.598705
Neutrophils	NDUFS7	0.098519	6.320866	0.949052	0.345181	-5.96452	0.532835	0.579917
Neutrophils	GM13427	-0.43898	2.062778	-0.94902	0.345196	-4.88669	0.601556	0.652879
Neutrophils	PTPRD	-0.38184	2.563535	-0.94883	0.345292	-5.22172	0.59302	0.64385
Neutrophils	UBR3	0.117825	6.575859	0.948744	0.345337	-5.95152	0.528989	0.575815
Neutrophils	RNF14	0.129446	5.242687	0.948736	0.345341	-5.65466	0.549426	0.597589
Neutrophils	ASGR1	-0.29226	3.372794	-0.94865	0.345385	-5.40798	0.579488	0.629549
Neutrophils	F8A	-0.40099	2.551871	-0.94784	0.345796	-4.93602	0.593729	0.644364
Neutrophils	SLC5A11	-0.53052	-0.01556	-0.94781	0.345809	-4.86611	0.638888	0.692016
Neutrophils	PPP1R12B	-0.15211	5.292478	-0.94774	0.345846	-5.61723	0.549121	0.597087
Neutrophils	IFT74	-0.41102	2.322027	-0.94755	0.345942	-4.95974	0.597658	0.648611
Neutrophils	HEMGN	-0.59927	0.35427	-0.9475	0.345968	-4.92857	0.632198	0.685065
Neutrophils	SPECC1L	-0.23393	5.413581	-0.94706	0.346192	-5.43834	0.547459	0.595222
Neutrophils	MAP4	0.094877	6.676158	0.947022	0.346209	-6.12582	0.528158	0.574668
Neutrophils	FZD1	0.489568	-0.15494	0.946168	0.346642	-4.86504	0.642405	0.695233
Neutrophils	LRPPRC	-0.22663	5.200579	-0.94599	0.346733	-5.33832	0.551448	0.599097
Neutrophils	ZFP202	-0.62042	0.604939	-0.94585	0.346803	-4.82854	0.628707	0.680858
Neutrophils	ACAT2	0.287701	2.91084	0.945541	0.34696	-5.0873	0.588788	0.638757
Neutrophils	GPS1	0.177077	4.905295	0.945462	0.347	-5.45458	0.556253	0.604274

Neutrophils	CAND1	-0.15326	5.132169	-0.94537	0.347046	-5.49993	0.552672	0.600486
Neutrophils	TMED10	0.078281	7.769396	0.945067	0.3472	-6.32997	0.5128	0.558111
Neutrophils	TBC1D12	-0.35199	3.768556	-0.94487	0.347302	-5.16505	0.574589	0.623927
Neutrophils	LYSMD3	0.127792	4.927993	0.944827	0.347322	-5.80145	0.55592	0.604112
Neutrophils	SLC2A8	-0.37403	2.226604	-0.94479	0.347338	-4.94075	0.600424	0.651275
Neutrophils	PAK1	-0.1654	5.334473	-0.94471	0.347382	-6.17985	0.549525	0.597352
Neutrophils	NFKB2	-0.16121	5.096214	-0.94459	0.347443	-5.82912	0.553264	0.60135
Neutrophils	LCN2	0.151042	3.398879	0.944585	0.347445	-6.52839	0.580676	0.630443
Neutrophils	SUN2	0.089934	6.122993	0.94446	0.347508	-6.14687	0.537352	0.584474
Neutrophils	INVS	0.343983	2.809082	0.944167	0.347657	-5.06203	0.590646	0.641056
Neutrophils	LRCH3	0.111878	6.497112	0.9441	0.347691	-5.96394	0.531763	0.578525
Neutrophils	LZTFL1	0.295677	5.075487	0.943902	0.347792	-5.24437	0.553702	0.601942
Neutrophils	STX1A	-0.54679	1.792697	-0.94373	0.347877	-4.86621	0.608029	0.659539
Neutrophils	TMED8	0.248847	2.954093	0.94366	0.347915	-5.18535	0.588207	0.638576
Neutrophils	COL27A1	-0.47297	2.314283	-0.94349	0.347999	-5.06178	0.599044	0.650084
Neutrophils	CDC14B	-0.36334	3.454363	-0.94348	0.348004	-5.07933	0.579875	0.629789
Neutrophils	CALCA	-0.73137	-0.96013	-0.9434	0.348045	-4.79469	0.657761	0.711977
Neutrophils	6530413G1	-0.52362	-0.08741	-0.94328	0.348107	-4.78313	0.641567	0.694975
Neutrophils	SMARCAL1	-0.27786	2.628016	-0.94322	0.348141	-5.06021	0.593705	0.644516
Neutrophils	ZFP74	-0.38846	2.132709	-0.94307	0.348213	-4.92669	0.602156	0.653523
Neutrophils	BRD9	0.125716	5.185663	0.942983	0.348259	-5.7447	0.551968	0.600319
Neutrophils	NEK7	-0.1222	6.476805	-0.94294	0.348281	-5.88729	0.532069	0.579123
Neutrophils	FAM193A	0.107195	6.704133	0.942181	0.348667	-6.01965	0.528992	0.575658
Neutrophils	DDIT4	0.382003	3.744278	0.941995	0.348762	-5.30051	0.57548	0.6251
Neutrophils	SEC14L2	-0.40439	1.70477	-0.94199	0.348765	-5.04567	0.609958	0.661588
Neutrophils	STON2	0.177209	3.518101	0.941884	0.348818	-5.70391	0.579203	0.629047
Neutrophils	KCTD13	-0.2927	2.619439	-0.94188	0.348823	-5.08472	0.594241	0.644973
Neutrophils	BAD	0.19649	3.912661	0.941789	0.348867	-5.42293	0.572725	0.622178
Neutrophils	5430401H(	0.623099	-0.47304	0.941689	0.348918	-4.85312	0.6491	0.702883
Neutrophils	ANKRD39	-0.23161	3.543857	-0.94153	0.348998	-5.15353	0.578778	0.628696
Neutrophils	ARL15	-0.09071	7.591924	-0.94142	0.349056	-6.40278	0.515829	0.561722
Neutrophils	CLU	0.354786	5.440247	0.941415	0.349058	-5.96174	0.548344	0.596398
Neutrophils	POLR3A	0.355615	3.30507	0.941004	0.349267	-5.03696	0.58277	0.633019
Neutrophils	BIN1	-0.20258	5.230633	-0.94099	0.349276	-5.38091	0.551661	0.599984
Neutrophils	CNOT6	-0.09799	5.947416	-0.94095	0.349294	-5.82813	0.540527	0.588135
Neutrophils	AFF2	-0.19246	0.04795	-0.9408	0.349371	-5.74193	0.639554	0.693033
Neutrophils	PEX11G	-0.30437	2.394748	-0.94069	0.349426	-5.08264	0.598103	0.649346
Neutrophils	YWHAB	0.071008	7.88619	0.940674	0.349435	-6.34912	0.511577	0.557308
Neutrophils	ACAA2	-0.20352	5.01941	-0.94055	0.349498	-5.67207	0.554988	0.603615
Neutrophils	NABP1	0.126916	4.743721	0.94051	0.349519	-5.96222	0.559361	0.608266
Neutrophils	SFT2D3	0.266362	2.864525	0.940294	0.349629	-5.09184	0.590146	0.640924
Neutrophils	SLC9A7	-0.27864	5.115655	-0.94029	0.349632	-5.25922	0.553476	0.602
Neutrophils	ECHS1	-0.17646	5.549982	-0.93977	0.349894	-5.75127	0.547	0.594865
Neutrophils	NSMCE1	0.182808	4.829662	0.939562	0.350002	-5.51556	0.558333	0.606963
Neutrophils	KIF1C	-0.23895	3.254466	-0.93951	0.350028	-5.23416	0.583966	0.634166
Neutrophils	WDR37	0.108313	5.478401	0.939336	0.350117	-5.92003	0.548118	0.596167
Neutrophils	ARHGAP39	-0.28329	4.38051	-0.93914	0.350215	-5.33482	0.565521	0.614693

Neutrophils	DHX34	0.340839	2.203437	0.939143	0.350216	-5.01836	0.601742	0.653079
Neutrophils	ARL2	0.267479	3.356926	0.939024	0.350277	-5.20854	0.582262	0.632489
Neutrophils	L3MBTL1	-0.49927	0.787342	-0.93886	0.350362	-4.89859	0.626569	0.679441
Neutrophils	STAG3	0.556003	0.607181	0.938788	0.350397	-4.91242	0.629802	0.682896
Neutrophils	CYP2C29	0.530035	1.011219	0.93875	0.350416	-4.96088	0.622576	0.675278
Neutrophils	GM7160	0.240432	2.542262	0.938669	0.350458	-5.44122	0.595952	0.647161
Neutrophils	PIGF	-0.33766	3.388301	-0.93859	0.350498	-5.05728	0.581742	0.632106
Neutrophils	USP16	0.15486	5.208005	0.938276	0.350659	-5.61386	0.5524	0.600991
Neutrophils	HS3ST1	0.67887	1.500377	0.938186	0.350705	-4.91286	0.613995	0.666324
Neutrophils	BEND5	-0.52622	0.115495	-0.93817	0.350713	-4.79588	0.638765	0.692454
Neutrophils	RAB2B	-0.2618	3.661593	-0.93805	0.350775	-5.18047	0.577277	0.627481
Neutrophils	IGLL1	-0.59821	3.916663	-0.93793	0.350833	-5.28855	0.573095	0.623055
Neutrophils	SCARB1	0.16555	4.995715	0.937692	0.350957	-5.57425	0.555748	0.604634
Neutrophils	NFKBIZ	0.116133	6.516715	0.937631	0.350988	-6.30211	0.532221	0.579565
Neutrophils	CEBPE	0.167125	0.2028	0.937564	0.351023	-5.74588	0.637174	0.690893
Neutrophils	STARD13	-0.55826	1.070842	-0.93727	0.351171	-4.92835	0.621572	0.674613
Neutrophils	ZBTB18	-0.23154	3.894799	-0.93716	0.351227	-5.24533	0.573453	0.6237
Neutrophils	ACTR3	0.060471	8.876057	0.937159	0.35123	-6.59699	0.497778	0.542947
Neutrophils	XYLB	0.451042	0.922746	0.937142	0.351238	-4.9178	0.624206	0.677442
Neutrophils	FBN1	0.433456	0.898383	0.937098	0.351261	-4.96101	0.624641	0.677917
Neutrophils	FAM167B	-0.27475	2.127548	-0.93703	0.351296	-5.26592	0.6031	0.655194
Neutrophils	4930481A1	0.315864	1.854963	0.936888	0.351368	-5.10739	0.607841	0.660242
Neutrophils	WASHC3	-0.15727	4.517912	-0.93648	0.351579	-5.49089	0.563526	0.613289
Neutrophils	AP1G1	-0.09647	6.456316	-0.9364	0.351617	-6.15952	0.533291	0.58107
Neutrophils	NR4A3	0.17904	6.012984	0.936365	0.351636	-6.09386	0.540053	0.588286
Neutrophils	ROPN1L	0.294129	2.420763	0.936293	0.351673	-5.25915	0.598249	0.650143
Neutrophils	CD4	-0.60064	1.246276	-0.93575	0.351953	-4.86406	0.619043	0.671886
Neutrophils	MTLN	0.28173	3.060484	0.935482	0.352088	-5.09047	0.587939	0.638861
Neutrophils	AP1G2	-0.39987	2.809774	-0.93502	0.352324	-5.0414	0.592442	0.643565
Neutrophils	CCDC192	0.598492	-0.69514	0.934939	0.352366	-4.8325	0.6548	0.709411
Neutrophils	GNAT3	-0.6071	-0.04367	-0.93422	0.352734	-4.7941	0.64308	0.697064
Neutrophils	DOCK1	-0.13736	4.079995	-0.93415	0.352771	-5.88216	0.571683	0.621583
Neutrophils	MREG	0.335828	3.199109	0.934145	0.352773	-5.39595	0.586221	0.637009
Neutrophils	CDC42BPB	-0.26579	3.262023	-0.93404	0.352825	-5.32446	0.58517	0.635931
Neutrophils	GM15492	-0.46629	0.742028	-0.93403	0.352834	-4.89494	0.62881	0.682083
Neutrophils	NUDT6	-0.3936	2.183509	-0.93374	0.352981	-4.95092	0.603475	0.655422
Neutrophils	TMED9	-0.10497	6.609324	-0.93371	0.352996	-5.86687	0.532002	0.579475
Neutrophils	PPP6R3	-0.08447	7.283025	-0.93368	0.353011	-6.19741	0.521923	0.568713
Neutrophils	INPP5J	0.297349	-1.12022	0.933256	0.353229	-5.12809	0.663492	0.718567
Neutrophils	OLFM4	-0.172	-0.01496	-0.933	0.353361	-5.90151	0.643011	0.696986
Neutrophils	IFT22	-0.34183	3.654091	-0.93276	0.353485	-5.05348	0.579109	0.629406
Neutrophils	GM11457	-0.63433	0.10252	-0.93275	0.35349	-4.83931	0.640892	0.694717
Neutrophils	LRCH1	0.114517	7.432191	0.932561	0.353586	-6.095	0.520161	0.566617
Neutrophils	GM48086	-0.41989	1.511775	-0.93235	0.353696	-4.93147	0.615778	0.668141
Neutrophils	ILF2	-0.13638	5.763945	-0.93207	0.353839	-5.76623	0.545624	0.593651
Neutrophils	ZFP963	0.412077	1.15161	0.931902	0.353925	-4.90589	0.622352	0.675034
Neutrophils	FAM98C	0.236412	3.737769	0.931796	0.353979	-5.29801	0.57808	0.62819

Neutrophils	TLE3	0.122033	5.003886	0.931595	0.354083	-5.82331	0.557655	0.606436
Neutrophils	N4BP2	0.165417	5.310122	0.931427	0.354169	-5.65659	0.552816	0.601288
Neutrophils	SEMA5A	0.593032	1.106724	0.931408	0.354179	-4.87204	0.623211	0.675878
Neutrophils	MRPS5	-0.20062	4.612666	-0.93092	0.354431	-5.35777	0.56413	0.613165
Neutrophils	E030030I0(	-0.1808	2.657085	-0.93085	0.354466	-5.42619	0.596473	0.647459
Neutrophils	INSIG1	0.154736	5.457849	0.930809	0.354487	-5.7334	0.55072	0.598906
Neutrophils	KIF3B	0.171416	3.495441	0.930683	0.354552	-5.46177	0.582395	0.632563
Neutrophils	GM15489	0.574544	0.208522	0.930482	0.354655	-4.85622	0.639726	0.693202
Neutrophils	FAM124A	-0.26802	1.64482	-0.93042	0.354687	-5.23677	0.614018	0.666099
Neutrophils	RDH11	0.182337	3.215453	0.930227	0.354786	-5.46199	0.58717	0.637662
Neutrophils	BC029722	0.297547	3.061908	0.930131	0.354836	-5.10299	0.589747	0.640411
Neutrophils	LRRC75AOS	0.579024	0.512247	0.929812	0.355	-4.81785	0.634383	0.687499
Neutrophils	CASP4	0.194797	4.029936	0.929794	0.35501	-5.87756	0.573795	0.623436
Neutrophils	DMC1	-0.42114	0.152078	-0.92968	0.355069	-4.92707	0.64095	0.694408
Neutrophils	PRADC1	0.249838	3.374688	0.929399	0.355213	-5.21332	0.584768	0.635011
Neutrophils	BICD1	-0.58024	0.681795	-0.92866	0.355592	-4.81951	0.632061	0.684657
Neutrophils	SLC49A4	0.133548	6.30055	0.928382	0.355737	-6.24432	0.538623	0.585686
Neutrophils	EIF4G1	0.107144	6.604378	0.928317	0.35577	-5.89824	0.533993	0.580753
Neutrophils	LILRA5	-0.42125	1.497471	-0.92808	0.355892	-5.11279	0.617675	0.669558
Neutrophils	SUDS3	0.13269	5.515338	0.92805	0.355908	-5.81324	0.550827	0.598737
Neutrophils	MPC2	-0.09865	6.800876	-0.92792	0.355976	-6.24007	0.531082	0.577716
Neutrophils	INSR	0.127448	5.770425	0.927711	0.356083	-6.0314	0.54689	0.594596
Neutrophils	RAB37	0.192785	2.340912	0.927672	0.356103	-5.48181	0.603031	0.654145
Neutrophils	FAM76B	0.133323	5.401156	0.927368	0.35626	-5.63861	0.552752	0.6009
Neutrophils	GPR34	0.661284	0.588061	0.92735	0.356269	-4.85817	0.634078	0.686983
Neutrophils	KLRA7	-0.82985	0.646446	-0.92722	0.356336	-4.89717	0.633041	0.68589
Neutrophils	JUNOS	0.267859	1.820722	0.926838	0.356534	-5.33358	0.6122	0.664024
Neutrophils	GM26510	0.267882	4.122817	0.926827	0.356539	-5.24756	0.573292	0.62283
Neutrophils	ZFP426	0.280585	2.769149	0.926745	0.356582	-5.11916	0.595852	0.646758
Neutrophils	BBS7	-0.5653	0.558111	-0.92669	0.35661	-4.87596	0.634674	0.687755
Neutrophils	ZFP959	-0.25131	3.241052	-0.92663	0.356641	-5.18734	0.587885	0.638333
Neutrophils	POLD2	-0.35827	3.401936	-0.92654	0.356686	-5.08603	0.585194	0.635482
Neutrophils	TTC39C	0.311126	2.230936	0.926192	0.356867	-5.24632	0.605266	0.656709
Neutrophils	LTV1	0.201223	4.232009	0.925988	0.356973	-5.44636	0.571693	0.62122
Neutrophils	BATF2	-0.54857	0.948583	-0.92593	0.357001	-4.95971	0.627836	0.68066
Neutrophils	GM43848	-0.2485	2.767038	-0.9259	0.35702	-5.21738	0.596077	0.647115
Neutrophils	REX1BD	0.122631	5.688469	0.925674	0.357135	-5.7696	0.548566	0.596711
Neutrophils	GSDMC4	-0.34841	0.743558	-0.92525	0.357352	-5.04833	0.631915	0.684784
Neutrophils	2700038G2	-0.34917	2.577847	-0.92469	0.357645	-4.98344	0.599969	0.650846
Neutrophils	JAGN1	-0.26243	3.955321	-0.92458	0.357703	-5.22219	0.576858	0.626395
Neutrophils	ZBP1	0.513229	3.640152	0.92443	0.357779	-5.2802	0.582063	0.631913
Neutrophils	ZFP369	0.346033	2.919936	0.924421	0.357783	-5.01403	0.594142	0.644705
Neutrophils	VTI1A	0.087986	7.233705	0.924099	0.35795	-6.24614	0.525498	0.571867
Neutrophils	ITGAL	0.107007	6.272091	0.924017	0.357992	-6.30476	0.540047	0.587385
Neutrophils	PEA15A	-0.21802	3.90143	-0.92387	0.358066	-5.37554	0.577744	0.627458
Neutrophils	CACNA1A	-0.4766	1.424509	-0.9238	0.358107	-4.91005	0.620049	0.672211
Neutrophils	CDK11B	0.085695	6.82853	0.923788	0.358111	-6.30538	0.531577	0.578356

Neutrophils	MRPL17	0.133711	5.426857	0.923658	0.358179	-5.70933	0.553184	0.601383
Neutrophils	TIAL1	-0.11748	5.798885	-0.92365	0.358185	-5.75235	0.547361	0.595186
Neutrophils	BRMS1L	-0.19641	4.269409	-0.92364	0.35819	-5.33624	0.571718	0.621076
Neutrophils	JPT2	0.274974	3.513173	0.923295	0.358367	-5.24145	0.584373	0.63437
Neutrophils	PAFAH1B2	0.113404	5.489377	0.922962	0.358539	-5.76984	0.552392	0.600449
Neutrophils	2610002M	0.198762	4.208089	0.922938	0.358551	-5.44085	0.572916	0.622263
Neutrophils	CLEC2I	0.500274	3.177972	0.922722	0.358663	-4.96465	0.589989	0.640462
Neutrophils	ADCK1	0.32156	3.101425	0.922651	0.3587	-5.07326	0.591279	0.641827
Neutrophils	RBPMS2	-0.3935	1.246218	-0.92252	0.358766	-4.91303	0.623429	0.675834
Neutrophils	GM4221	0.264163	1.846721	0.922444	0.358808	-5.25461	0.612832	0.664698
Neutrophils	VPS4B	0.078495	6.231035	0.922339	0.358862	-6.15509	0.540864	0.588364
Neutrophils	UXT	-0.19544	4.452478	-0.92233	0.358869	-5.39472	0.568941	0.61823
Neutrophils	TBCE	0.129839	5.119789	0.922236	0.358915	-5.67653	0.558232	0.606851
Neutrophils	DNAJB13	-0.38873	2.103334	-0.92215	0.35896	-4.97831	0.608359	0.659989
Neutrophils	GM14023	0.358974	1.123999	0.921991	0.359042	-5.1096	0.625609	0.67824
Neutrophils	ANXA5	0.118478	6.137389	0.921917	0.359081	-6.07473	0.542306	0.589933
Neutrophils	CNNM3	-0.24587	3.157652	-0.92128	0.359411	-5.22456	0.590331	0.641273
Neutrophils	SNAPC4	-0.48051	1.856185	-0.9211	0.359507	-4.89821	0.612666	0.664984
Neutrophils	ZRSR2	0.10878	5.063714	0.921041	0.359536	-5.72086	0.559124	0.608215
Neutrophils	RAD51D	0.266289	2.984907	0.921036	0.359538	-5.1478	0.593247	0.644443
Neutrophils	SCO2	-0.34139	2.851352	-0.92103	0.359542	-5.0112	0.595512	0.646842
Neutrophils	CLTA	-0.07526	8.442682	-0.9209	0.359606	-6.34559	0.507966	0.553667
Neutrophils	GM614	-0.56424	-0.15476	-0.92079	0.359664	-4.80286	0.64888	0.703292
Neutrophils	MZT2	-0.28354	2.206691	-0.92075	0.359685	-5.04491	0.606567	0.658636
Neutrophils	RAB11A	0.073305	7.145444	0.920658	0.359734	-6.23615	0.526998	0.574055
Neutrophils	CDK18	-0.54919	-0.22726	-0.92042	0.359856	-4.86334	0.650225	0.70474
Neutrophils	GM13431	-0.28402	0.492651	-0.92031	0.359917	-5.25314	0.636991	0.690797
Neutrophils	LRRC8C	-0.1812	6.412612	-0.9202	0.35997	-5.77078	0.538081	0.585928
Neutrophils	RTF2	0.092256	5.957747	0.920069	0.36004	-5.96054	0.545083	0.593446
Neutrophils	ZFP746	-0.2191	3.717554	-0.92005	0.36005	-5.2581	0.580982	0.631633
Neutrophils	4930414N(	0.34314	3.331114	0.919964	0.360095	-5.10611	0.587418	0.638471
Neutrophils	USP47	0.101106	6.472249	0.919955	0.3601	-6.05944	0.53717	0.585014
Neutrophils	FZD5	-0.25347	2.536377	-0.91986	0.36015	-5.22654	0.600887	0.652745
Neutrophils	TRNT1	0.159432	4.772449	0.919838	0.36016	-5.522	0.56378	0.613365
Neutrophils	ABRAXAS1	-0.37279	2.604808	-0.91983	0.360162	-5.01161	0.599715	0.651504
Neutrophils	LYRM2	0.186479	3.377583	0.919797	0.360182	-5.52392	0.58664	0.637646
Neutrophils	DPH5	-0.28265	3.732552	-0.91974	0.360213	-5.14799	0.580733	0.631378
Neutrophils	GM10501	0.38583	2.138944	0.919671	0.360247	-4.95077	0.607741	0.660007
Neutrophils	USP39	0.159806	4.614508	0.919234	0.360474	-5.68696	0.566476	0.616261
Neutrophils	POLI	0.372728	1.527716	0.919088	0.36055	-4.97925	0.618607	0.671545
Neutrophils	CACNA1E	-0.54529	4.907691	-0.91909	0.360552	-5.24483	0.561766	0.611274
Neutrophils	TARSL2	0.391573	1.415252	0.919057	0.360566	-4.92254	0.620597	0.673648
Neutrophils	SUPT7L	-0.34402	2.767637	-0.91852	0.360848	-5.01503	0.597435	0.648954
Neutrophils	GM47507	0.321702	0.732528	0.918385	0.360916	-5.29615	0.633172	0.686725
Neutrophils	GRASP	0.329693	3.781204	0.918288	0.360967	-5.14728	0.580414	0.630928
Neutrophils	PCF11	-0.10127	6.482932	-0.91817	0.361027	-6.16671	0.537456	0.585215
Neutrophils	EIF3J1	0.089743	7.268033	0.918135	0.361046	-6.11786	0.525607	0.572563

Neutrophils	EPB41L4AC	-0.37767	3.25392	-0.91788	0.361179	-5.08068	0.589332	0.640342
Neutrophils	GM21781	-0.3215	1.561921	-0.91756	0.361348	-5.09873	0.618679	0.671301
Neutrophils	COA5	0.207735	4.468388	0.917422	0.361418	-5.49925	0.569483	0.619229
Neutrophils	GM16014	0.615598	-0.09363	0.916999	0.361638	-4.82654	0.648904	0.703036
Neutrophils	FAM83F	0.561436	1.041009	0.916947	0.361665	-4.85718	0.628212	0.681233
Neutrophils	PEAR1	0.330397	2.352213	0.916804	0.36174	-5.08307	0.605164	0.656856
Neutrophils	KLK1B27	-0.42303	-1.42677	-0.91647	0.361915	-4.78321	0.674214	0.729613
Neutrophils	GRN	-0.12563	7.188808	-0.91645	0.361922	-6.24124	0.527387	0.574202
Neutrophils	LRRC27	0.399267	0.302993	0.916394	0.361953	-4.98279	0.641713	0.695452
Neutrophils	RPN2	-0.12482	6.097946	-0.91615	0.362081	-5.82359	0.544034	0.591979
Neutrophils	PHKA2	0.228981	3.438409	0.916117	0.362097	-5.34435	0.586832	0.637469
Neutrophils	ZDHHC2	0.37231	2.276539	0.915866	0.362228	-5.07798	0.606735	0.658495
Neutrophils	DBR1	-0.27793	3.262038	-0.91561	0.36236	-5.13899	0.589963	0.640724
Neutrophils	EMC6	0.137966	5.61708	0.915529	0.362404	-5.66332	0.551685	0.600077
Neutrophils	PDK2	0.436754	2.10287	0.915403	0.36247	-4.91927	0.6098	0.661786
Neutrophils	NR1H4	-0.44208	1.386536	-0.91533	0.362506	-4.99604	0.622399	0.675121
Neutrophils	FPR2	0.190268	1.833327	0.915278	0.362535	-6.09811	0.61451	0.666797
Neutrophils	PIAS1	0.074716	7.42613	0.915164	0.362595	-6.27646	0.524054	0.570686
Neutrophils	TMEM40	0.199908	0.497552	0.914827	0.362771	-5.5965	0.63855	0.692129
Neutrophils	PPP1R35	-0.19234	3.395179	-0.9146	0.362888	-5.41912	0.587863	0.638606
Neutrophils	KCNJ16	-0.76075	-0.02269	-0.91453	0.362924	-4.86116	0.648109	0.702248
Neutrophils	SCLY	-0.23685	3.466279	-0.91447	0.362958	-5.22631	0.586673	0.637344
Neutrophils	P2RY6	0.278984	2.305897	0.91424	0.363077	-5.28345	0.606418	0.658313
Neutrophils	ACOT1	-0.21092	2.604682	-0.91422	0.363087	-5.78028	0.601269	0.652865
Neutrophils	FUT4	0.42862	-0.16835	0.914183	0.363107	-4.95646	0.650812	0.705167
Neutrophils	CGNL1	-0.44646	1.622928	-0.9141	0.363153	-5.0427	0.618356	0.670971
Neutrophils	SLC25A23	-0.35098	2.25448	-0.91406	0.363173	-5.03303	0.607308	0.659292
Neutrophils	DRG1	0.09555	6.40915	0.913783	0.363316	-5.92344	0.539577	0.587396
Neutrophils	AKAP13	0.083345	8.500476	0.913779	0.363318	-6.4162	0.508496	0.55418
Neutrophils	ECSIT	-0.27982	4.015765	-0.91356	0.363435	-5.11338	0.57764	0.627964
Neutrophils	ZFP592	-0.11212	5.89966	-0.91353	0.363448	-5.77152	0.547477	0.595879
Neutrophils	UNC50	0.150595	4.105649	0.913354	0.36354	-5.55245	0.576221	0.626413
Neutrophils	4930590J0	-0.38244	1.547542	-0.91295	0.363752	-5.00171	0.619992	0.672704
Neutrophils	HOMER1	-0.11085	6.109837	-0.91287	0.363794	-6.21666	0.544402	0.5925
Neutrophils	YTHDF1	-0.11958	5.78074	-0.91287	0.363794	-5.82586	0.54952	0.597954
Neutrophils	EXOSC2	-0.31574	2.945711	-0.91264	0.363915	-5.01959	0.595806	0.647114
Neutrophils	NENF	-0.17621	4.583677	-0.91258	0.363946	-5.42898	0.568627	0.618291
Neutrophils	EXOC5	0.086797	6.376842	0.912376	0.364052	-6.05992	0.54035	0.588247
Neutrophils	SESN1	-0.1535	5.939971	-0.91223	0.364128	-5.72065	0.547102	0.595511
Neutrophils	EIF2AK4	0.234849	4.653928	0.911951	0.364274	-5.35579	0.567495	0.617336
Neutrophils	UTP14A	0.141445	5.238791	0.91195	0.364275	-5.6812	0.558124	0.607366
Neutrophils	CEP57L1	-0.30972	3.287731	-0.9119	0.364299	-5.11364	0.590026	0.641264
Neutrophils	AKAP11	-0.14642	4.648059	-0.91187	0.364319	-5.71811	0.56759	0.617439
Neutrophils	PRRC2A	-0.10947	5.517966	-0.91178	0.364366	-5.83816	0.553708	0.602679
Neutrophils	MTRR	-0.49936	1.505683	-0.91169	0.364413	-4.87275	0.620807	0.673893
Neutrophils	RYBP	0.097905	5.626574	0.911563	0.364478	-6.10446	0.552	0.600897
Neutrophils	FAM185A	-0.40451	2.579686	-0.91147	0.364528	-4.94449	0.602064	0.654085

Neutrophils	IMMT	0.115043	6.049157	0.911409	0.364558	-5.77487	0.545406	0.593883
Neutrophils	CERS4	-0.29947	3.651416	-0.91112	0.364711	-5.15655	0.583948	0.634952
Neutrophils	RNF187	0.153377	6.12757	0.911108	0.364716	-5.65027	0.5442	0.592659
Neutrophils	RFC2	0.155241	5.227985	0.911078	0.364732	-5.71018	0.558304	0.607698
Neutrophils	PYGO2	0.279422	3.374512	0.910956	0.364795	-5.31155	0.588577	0.639891
Neutrophils	B130034C1	-0.38065	1.038829	-0.91087	0.364841	-4.89775	0.629147	0.68283
Neutrophils	ADAMTS1	-0.25201	2.937185	-0.91068	0.364941	-5.63435	0.596037	0.647775
Neutrophils	UBALD2	0.111137	7.028455	0.910049	0.365271	-6.17574	0.530801	0.578118
Neutrophils	MSL1	0.110085	5.419802	0.910043	0.365274	-5.96663	0.555633	0.604598
Neutrophils	MED25	0.180932	4.496149	0.909798	0.365403	-5.38023	0.570437	0.620351
Neutrophils	GM50386	-0.51558	0.179027	-0.90972	0.365443	-4.82779	0.645215	0.699473
Neutrophils	ZFAND2B	0.133256	4.313639	0.909681	0.365464	-5.72942	0.573411	0.62351
Neutrophils	STK24	0.08439	7.291795	0.909602	0.365506	-6.22592	0.526849	0.573911
Neutrophils	B3GNT2	-0.09282	6.739389	-0.90956	0.365526	-6.23753	0.535176	0.582804
Neutrophils	CNKSR3	-0.22557	4.754523	-0.9092	0.365718	-5.61094	0.566366	0.615956
Neutrophils	PER3	-0.34917	1.517982	-0.90913	0.365756	-5.062	0.621131	0.673995
Neutrophils	PRG4	1.159016	1.084056	0.908855	0.365897	-4.93893	0.628875	0.682252
Neutrophils	EPC1	0.106652	6.811099	0.908644	0.366008	-6.0761	0.534192	0.581833
Neutrophils	9-Sep	-0.11775	5.640387	-0.90859	0.366036	-6.00916	0.552265	0.601132
Neutrophils	ZDHHC4	0.166878	4.474275	0.90859	0.366036	-5.5263	0.570905	0.620967
Neutrophils	GM16151	-0.55667	-0.28838	-0.90844	0.366115	-4.80189	0.654015	0.708959
Neutrophils	PNPLA7	0.104251	6.039516	0.908363	0.366156	-6.20137	0.546033	0.59459
Neutrophils	NEGR1	-0.70061	-0.13284	-0.90831	0.366185	-4.81695	0.651116	0.705983
Neutrophils	BRPF1	0.136417	4.929241	0.908297	0.36619	-5.77584	0.563556	0.61329
Neutrophils	SPSB4	-0.57371	0.417547	-0.90826	0.366208	-4.84563	0.64096	0.695286
Neutrophils	TMEM170B	-0.10134	4.646846	-0.90804	0.366325	-6.02367	0.568164	0.618248
Neutrophils	CDON	0.473597	1.952332	0.907931	0.366383	-4.9287	0.613539	0.666413
Neutrophils	CTSK	0.493264	0.234472	0.907876	0.366412	-4.90636	0.644386	0.699025
Neutrophils	CDC6	0.292795	3.269665	0.907535	0.366591	-5.29448	0.590956	0.642573
Neutrophils	CD74	-0.3536	11.27972	-0.90749	0.366616	-6.60538	0.470841	0.514196
Neutrophils	4931406G(	0.650737	0.347467	0.907447	0.366637	-4.87068	0.642364	0.696963
Neutrophils	LBH	-0.129	6.308404	-0.9073	0.366712	-5.75495	0.541976	0.59053
Neutrophils	ZSCAN2	0.511863	1.067334	0.90729	0.36672	-4.91416	0.629292	0.683241
Neutrophils	TTF2	0.315372	3.091877	0.907088	0.366826	-5.11569	0.594015	0.64592
Neutrophils	ERGIC2	-0.08723	6.017059	-0.90693	0.366908	-5.91502	0.546534	0.595422
Neutrophils	CEP170B	0.50427	0.457754	0.906908	0.36692	-4.83318	0.640403	0.695014
Neutrophils	ABCA9	-0.33817	0.787541	-0.90669	0.367033	-5.28643	0.634498	0.688699
Neutrophils	DDAH2	-0.24977	3.503269	-0.90657	0.367096	-5.32103	0.587187	0.638627
Neutrophils	GM15563	0.427647	1.923249	0.906473	0.367149	-5.00002	0.614262	0.667357
Neutrophils	FAM171B	-0.60588	0.217464	-0.90626	0.36726	-4.87537	0.644931	0.699821
Neutrophils	ZBTB45	-0.31706	2.916254	-0.90608	0.367357	-5.02191	0.597107	0.649256
Neutrophils	APLP2	-0.13808	5.805079	-0.90606	0.367367	-5.9237	0.549938	0.599094
Neutrophils	4930539J0	0.471383	0.706555	0.905974	0.367412	-4.90291	0.635984	0.690388
Neutrophils	HADHB	-0.13189	5.576702	-0.9059	0.367451	-5.76142	0.553522	0.602916
Neutrophils	IRS1	-0.50987	0.821216	-0.90583	0.367487	-4.8857	0.633905	0.688193
Neutrophils	SRMS	-0.53222	-0.72874	-0.90561	0.367605	-4.86397	0.662694	0.7185
Neutrophils	LRRK2	-0.10604	4.404591	-0.90552	0.36765	-6.31894	0.572385	0.623005

Neutrophils	PPP6C	0.078738	6.737344	0.905184	0.367828	-6.08922	0.535813	0.583929
Neutrophils	GM4951	0.305402	3.770894	0.904619	0.368126	-5.67273	0.583392	0.634401
Neutrophils	TSC2	0.243444	3.109391	0.904412	0.368235	-5.18436	0.594587	0.646253
Neutrophils	FAM160A2	0.161506	2.604929	0.904149	0.368373	-5.56834	0.603304	0.655542
Neutrophils	RASAL1	-0.66639	0.712454	-0.90409	0.368406	-4.81633	0.636795	0.690977
Neutrophils	SGPP1	0.148145	5.017924	0.903829	0.368542	-5.75951	0.563241	0.613029
Neutrophils	ATG2A	0.101249	5.710857	0.903687	0.368617	-6.17466	0.552244	0.601369
Neutrophils	A330040F1	0.533968	2.508264	0.903399	0.368769	-5.11815	0.605008	0.657562
Neutrophils	KYAT1	-0.42744	1.503706	-0.90334	0.368798	-4.93021	0.622607	0.676191
Neutrophils	LRRC59	0.126541	5.556125	0.90334	0.3688	-5.70616	0.55468	0.604087
Neutrophils	ASCC2	-0.16687	4.605255	-0.9033	0.368819	-5.40945	0.569898	0.620292
Neutrophils	GM11099	-0.64692	-0.70994	-0.90328	0.368832	-4.80598	0.663241	0.719051
Neutrophils	ADA	-0.38759	2.074866	-0.90301	0.368972	-5.05468	0.612538	0.665584
Neutrophils	TOPBP1	0.152727	5.680028	0.902957	0.369002	-5.86668	0.552729	0.602047
Neutrophils	CSNK1G1	0.095411	6.535289	0.902823	0.369073	-6.13512	0.539453	0.587902
Neutrophils	ARHGAP12	-0.20295	5.076663	-0.90268	0.369148	-5.43672	0.5623	0.612329
Neutrophils	ECHDC1	-0.24139	3.820316	-0.90244	0.369275	-5.31164	0.582787	0.634165
Neutrophils	B9D1	-0.56969	0.371188	-0.90235	0.369321	-4.82791	0.643073	0.69799
Neutrophils	CDC42EP1	-0.46503	0.752796	-0.90221	0.369395	-5.00268	0.636102	0.690697
Neutrophils	GM31323	-0.44817	0.948728	-0.90211	0.369452	-4.89066	0.632552	0.686977
Neutrophils	CAPRIN1	0.078215	7.460311	0.902064	0.369474	-6.15572	0.525471	0.57313
Neutrophils	MLLT3	-0.26263	5.728512	-0.90206	0.369477	-5.467	0.551967	0.601438
Neutrophils	TUBA1B	-0.16353	7.795232	-0.90205	0.369483	-6.26622	0.520503	0.567812
Neutrophils	LRRC8D	-0.09189	7.366735	-0.90181	0.369607	-6.38805	0.526868	0.57464
Neutrophils	CCNG1	0.146072	4.873028	0.901735	0.369648	-5.63538	0.565569	0.615951
Neutrophils	9030404E1	0.618803	-0.16276	0.901727	0.369652	-4.81795	0.652955	0.708526
Neutrophils	GM42567	0.533733	1.12927	0.901592	0.369723	-4.95573	0.6293	0.683615
Neutrophils	EME1	0.347761	2.299791	0.901565	0.369738	-5.08331	0.608618	0.66173
Neutrophils	TBPL1	0.133218	4.928003	0.901516	0.369763	-5.66556	0.564685	0.61506
Neutrophils	FBXO47	0.567977	1.069812	0.901146	0.369959	-4.86908	0.630575	0.684842
Neutrophils	4930404I0!	-0.60628	0.086501	-0.90083	0.370125	-4.85175	0.648535	0.703911
Neutrophils	GAB3	-0.14258	4.746655	-0.90066	0.370217	-5.89779	0.567793	0.618426
Neutrophils	DUS1L	0.232777	4.064535	0.900638	0.370228	-5.30958	0.578934	0.630287
Neutrophils	STIMATE	0.16675	4.159047	0.900615	0.37024	-5.55596	0.577377	0.62864
Neutrophils	GALM	-0.22061	2.214948	-0.90058	0.370259	-5.48412	0.610293	0.663593
Neutrophils	ABHD15	-0.29239	2.977881	-0.90052	0.370288	-5.26325	0.597151	0.649694
Neutrophils	IRS2	-0.17281	5.572327	-0.90038	0.370362	-5.86494	0.554606	0.60452
Neutrophils	1700110K1	0.60468	-0.57203	0.900257	0.370429	-4.83458	0.660849	0.71717
Neutrophils	ATP6V0A2	-0.16911	4.619482	-0.90024	0.37044	-5.47148	0.569853	0.6208
Neutrophils	PYCARD	0.139993	5.876191	0.89985	0.370644	-5.87569	0.549942	0.599608
Neutrophils	RIPOR1	-0.22126	3.960811	-0.89984	0.370649	-5.30328	0.580763	0.632447
Neutrophils	ADRM1	0.149238	5.477684	0.899436	0.370864	-5.74826	0.556211	0.606443
Neutrophils	GM20274	-0.30896	2.727126	-0.8994	0.370883	-5.08995	0.601558	0.654692
Neutrophils	MTMR9	0.174853	3.972449	0.899136	0.371023	-5.37905	0.580571	0.632435
Neutrophils	TECR	-0.08375	6.763011	-0.89909	0.371048	-6.30317	0.536256	0.585183
Neutrophils	4933412E1	-0.4954	1.908564	-0.89908	0.371053	-4.90553	0.615776	0.669833
Neutrophils	RIPK3	-0.29914	3.285039	-0.89907	0.371057	-5.14981	0.59206	0.644673

Neutrophils	SYNE3	0.367571	2.767053	0.899056	0.371065	-5.12067	0.600873	0.654032
Neutrophils	GM8251	-0.32964	3.392286	-0.89894	0.371129	-5.24514	0.590252	0.642753
Neutrophils	CFAP45	-0.54241	0.728674	-0.89893	0.371131	-4.89326	0.636874	0.692161
Neutrophils	RUFY3	0.15329	5.26954	0.898813	0.371193	-5.66007	0.559515	0.610069
Neutrophils	STAR	-0.50876	1.465058	-0.89873	0.371236	-4.91925	0.623622	0.678188
Neutrophils	TMEM18	-0.39358	1.593876	-0.89819	0.371522	-4.95615	0.621717	0.675893
Neutrophils	MAFG	0.113975	5.07367	0.898009	0.37162	-5.89688	0.563027	0.613569
Neutrophils	DGKD	0.094793	7.687633	0.89786	0.371699	-6.27939	0.522724	0.570567
Neutrophils	LY6C1	0.599038	-0.32417	0.897832	0.371713	-4.90007	0.656765	0.713002
Neutrophils	GM42418	-0.22083	11.10924	-0.89773	0.371768	-6.79104	0.474524	0.518823
Neutrophils	FABP7	-0.45946	1.989157	-0.89732	0.371983	-5.20363	0.614895	0.668842
Neutrophils	GM13212	0.252254	3.205264	0.897258	0.372018	-5.26937	0.593925	0.646645
Neutrophils	HMGCL	0.133026	5.187114	0.896989	0.372161	-5.82549	0.561317	0.611944
Neutrophils	AAMDC	0.22812	3.299635	0.896983	0.372164	-5.1376	0.592329	0.644953
Neutrophils	HCFC1R1	0.108149	5.33224	0.896969	0.372171	-5.95514	0.559004	0.609477
Neutrophils	GPR174	-0.46816	2.267506	-0.89697	0.372172	-4.928	0.610029	0.663739
Neutrophils	CPNE2	0.133869	3.37335	0.896594	0.37237	-5.97886	0.591227	0.643777
Neutrophils	TMEM42	0.270727	2.617603	0.89659	0.372372	-5.08112	0.604111	0.657456
Neutrophils	ABHD16A	0.160638	4.808556	0.896361	0.372494	-5.55364	0.56758	0.61864
Neutrophils	SYNJ2	0.339579	2.324633	0.896326	0.372513	-5.00998	0.60923	0.662914
Neutrophils	TMEM41A	-0.44309	1.51992	-0.89604	0.372664	-4.92622	0.623546	0.67801
Neutrophils	5330439K0	-0.54338	0.538036	-0.89571	0.372841	-4.86521	0.641428	0.696899
Neutrophils	ARSA	-0.35078	1.69399	-0.89567	0.372863	-5.00156	0.620601	0.674872
Neutrophils	CSNK1G2	0.115235	5.468875	0.895541	0.37293	-5.7989	0.557302	0.607607
Neutrophils	BANK1	-0.20967	6.09331	-0.8949	0.373271	-6.00762	0.547767	0.597168
Neutrophils	SURF4	0.115323	5.782118	0.894893	0.373274	-5.94923	0.552636	0.602365
Neutrophils	SNN	-0.37482	3.649853	-0.89487	0.373284	-5.06225	0.58723	0.639201
Neutrophils	DDX18	-0.17435	4.987355	-0.89463	0.373412	-5.46916	0.565332	0.615842
Neutrophils	TPM3-RS7	0.439942	0.526188	0.894493	0.373487	-4.91205	0.64205	0.697264
Neutrophils	GABPB2	-0.11184	6.502447	-0.89444	0.373515	-5.86012	0.54149	0.590477
Neutrophils	4833403J1	0.574395	0.509888	0.894385	0.373544	-4.89609	0.642349	0.697607
Neutrophils	HSPH1	-0.43413	4.050366	-0.89406	0.373718	-5.11721	0.580807	0.632324
Neutrophils	EVI2	0.156612	3.35768	0.89343	0.374053	-5.81445	0.592831	0.644793
Neutrophils	MGME1	-0.35625	2.312135	-0.89316	0.374198	-5.06934	0.610925	0.663914
Neutrophils	TRAPPC10	-0.11704	5.170629	-0.89289	0.374341	-5.80054	0.563214	0.613228
Neutrophils	1700028E1	0.372799	1.36693	0.892833	0.37437	-4.9484	0.627736	0.681681
Neutrophils	DUSP2	-0.16598	5.991936	-0.89255	0.374521	-5.98662	0.550248	0.59941
Neutrophils	KCTD11	0.437839	0.546292	0.892524	0.374535	-4.94446	0.642667	0.697452
Neutrophils	GM13091	0.512981	0.904415	0.892468	0.374565	-4.87613	0.636128	0.690568
Neutrophils	GM1604A	-0.17957	2.804564	-0.89197	0.37483	-5.68495	0.602879	0.65528
Neutrophils	GM5089	0.564801	0.395428	0.891649	0.375001	-4.83564	0.645807	0.700787
Neutrophils	BCL2	-0.27246	5.016128	-0.89161	0.375021	-5.61804	0.566057	0.616275
Neutrophils	HPS5	-0.17807	4.448069	-0.89157	0.375041	-5.44237	0.575288	0.62612
Neutrophils	MEG3	-0.67328	1.040515	-0.89139	0.375137	-4.95316	0.634018	0.688389
Neutrophils	EPHA4	-0.61355	0.083978	-0.89125	0.375216	-4.85997	0.651578	0.706938
Neutrophils	PROCR	0.790427	0.279761	0.89118	0.375252	-4.85404	0.647945	0.703106
Neutrophils	RABAC1	0.087203	5.988734	0.891087	0.375301	-6.13914	0.550609	0.599885

Neutrophils	ZFP120	0.255772	2.536502	0.89091	0.375396	-5.15124	0.607512	0.660465
Neutrophils	ANAPC4	-0.16641	4.541254	-0.89064	0.375539	-5.44306	0.573763	0.6247
Neutrophils	PCDH7	-0.52541	0.520175	-0.89056	0.375583	-5.04663	0.643511	0.698647
Neutrophils	NT5C3	0.17679	4.85257	0.890434	0.37565	-5.55935	0.568699	0.619358
Neutrophils	SMC1B	0.590556	-0.03473	0.890319	0.375711	-4.87297	0.653791	0.70957
Neutrophils	TMEM191(	0.343234	1.703176	0.890206	0.375771	-4.92375	0.622135	0.676131
Neutrophils	TIGD2	0.220615	3.85213	0.890198	0.375775	-5.26059	0.58514	0.636899
Neutrophils	CCNJL	0.30332	1.734552	0.890128	0.375813	-5.1811	0.621578	0.675542
Neutrophils	SH2D1B1	-0.42905	1.695197	-0.89011	0.375821	-5.05497	0.622277	0.676281
Neutrophils	MLXIP	-0.1115	6.94185	-0.89009	0.375832	-6.08483	0.535899	0.584406
Neutrophils	0610009B2	-0.23219	3.780562	-0.89001	0.375874	-5.39289	0.586335	0.638177
Neutrophils	MAPK1IP1I	0.093418	6.060276	0.889954	0.375906	-5.9074	0.54949	0.598965
Neutrophils	EXOC3L2	-0.48992	2.269133	-0.88979	0.375992	-5.22967	0.612165	0.665729
Neutrophils	MLLT11	-0.26783	3.193389	-0.88967	0.376056	-5.09309	0.596234	0.648892
Neutrophils	SAP18B	0.288508	2.771863	0.889373	0.376216	-5.14743	0.603446	0.656715
Neutrophils	TICAM1	0.288895	2.819811	0.889197	0.37631	-5.15717	0.602622	0.655928
Neutrophils	ILKAP	0.121802	5.651644	0.889153	0.376333	-5.79586	0.555913	0.606219
Neutrophils	ZDHHC12	0.447959	1.360226	0.889041	0.376394	-4.96009	0.628257	0.683103
Neutrophils	ATP5O	-0.10757	7.142669	-0.88901	0.376412	-6.12966	0.532852	0.58157
Neutrophils	TEK	-0.25555	2.175307	-0.88896	0.376436	-5.41795	0.613807	0.667799
Neutrophils	4833420G1	0.168058	5.494494	0.888902	0.376468	-5.70963	0.558403	0.608877
Neutrophils	SP110	0.133389	5.924125	0.888891	0.376474	-6.00375	0.551621	0.601638
Neutrophils	DTNB	0.183909	5.293687	0.888729	0.37656	-5.60554	0.561603	0.612343
Neutrophils	PGLS	-0.08931	7.788311	-0.88872	0.376564	-6.27503	0.523181	0.57126
Neutrophils	TTI2	-0.26387	2.749363	-0.8885	0.376685	-5.11311	0.603916	0.6573
Neutrophils	FLNB	-0.22739	5.398208	-0.88842	0.376727	-5.99213	0.560011	0.610597
Neutrophils	LIPH	-0.58204	-0.05189	-0.88789	0.377009	-4.83376	0.654505	0.710701
Neutrophils	MCAT	-0.3498	2.553855	-0.88788	0.377014	-5.00798	0.607577	0.661063
Neutrophils	ACADVL	-0.16316	4.901912	-0.88759	0.37717	-5.66054	0.568372	0.61934
Neutrophils	MIR99AHG	-0.28256	3.430837	-0.88741	0.377267	-5.56112	0.592702	0.645328
Neutrophils	GM47601	-0.40455	0.02249	-0.88738	0.377281	-4.98115	0.653265	0.709466
Neutrophils	NECTIN2	-0.23993	2.109704	-0.88724	0.377358	-5.28101	0.615467	0.669504
Neutrophils	GM15886	0.235485	1.881502	0.887096	0.377434	-5.18707	0.619489	0.673783
Neutrophils	TLE5	0.081055	7.209326	0.887093	0.377435	-6.31067	0.532286	0.580929
Neutrophils	ZFP868	0.22797	3.676008	0.886989	0.377491	-5.3147	0.588573	0.641003
Neutrophils	THEMIS	0.55833	1.160853	0.886535	0.377734	-4.97812	0.632485	0.687529
Neutrophils	FGF1	0.425231	0.63081	0.886505	0.37775	-4.94316	0.642132	0.697727
Neutrophils	EIF4EBP2	0.072375	6.850744	0.886437	0.377787	-6.16094	0.537833	0.586889
Neutrophils	PI4K2A	-0.16623	5.639245	-0.88636	0.377827	-5.71485	0.556674	0.607045
Neutrophils	PIP5K1C	0.144083	5.626433	0.886227	0.377899	-5.75888	0.556877	0.60727
Neutrophils	IGFLR1	-0.60089	0.807622	-0.88603	0.378004	-4.85343	0.638898	0.694468
Neutrophils	TLR11	-0.4846	-0.67266	-0.88597	0.378035	-4.83984	0.66649	0.723615
Neutrophils	KLC4	-0.24709	2.884186	-0.88594	0.378052	-5.23198	0.602127	0.655533
Neutrophils	RILPL1	-0.31954	2.31814	-0.88583	0.378114	-5.11922	0.611931	0.665992
Neutrophils	ARHGAP18	0.151927	6.528672	0.885776	0.378141	-6.11933	0.542775	0.592345
Neutrophils	TMEM179I	0.089648	5.864754	0.88571	0.378177	-5.98113	0.553115	0.603412
Neutrophils	IGLV3	0.577237	-0.70552	0.885522	0.378277	-4.82157	0.667195	0.724418

Neutrophils	PNKP	0.089556	4.959191	0.885071	0.378519	-6.2511	0.567898	0.619061
Neutrophils	SDHC	-0.14562	5.09505	-0.88483	0.37865	-5.63534	0.565818	0.616745
Neutrophils	GATAD1	0.094848	5.768843	0.884454	0.37885	-5.95649	0.555285	0.605364
Neutrophils	TEAD1	-0.3153	1.756692	-0.88391	0.379142	-5.21838	0.622864	0.677114
Neutrophils	CHMP5	-0.09218	5.632148	-0.88388	0.379159	-6.00265	0.557724	0.607908
Neutrophils	PTGS1	-0.14907	3.986721	-0.88379	0.379205	-5.89657	0.584475	0.636418
Neutrophils	CALD1	-0.24578	3.777745	-0.88342	0.379406	-5.58667	0.588191	0.640315
Neutrophils	SLC22A5	0.374542	3.042195	0.883017	0.379622	-5.10626	0.600892	0.653706
Neutrophils	LASP1	0.081627	5.455474	0.882536	0.37988	-6.16921	0.560967	0.611311
Neutrophils	2700097O(	-0.31528	3.090234	-0.88245	0.379925	-5.09583	0.60007	0.65291
Neutrophils	TUBB2A	-0.22552	5.517234	-0.88244	0.379932	-5.60151	0.559982	0.610261
Neutrophils	4930589L2	-0.48987	-0.68311	-0.88241	0.379948	-4.84071	0.668327	0.725073
Neutrophils	LSM6	-0.12014	6.681324	-0.88241	0.379949	-6.0321	0.541755	0.5908
Neutrophils	ANGPTL1	-0.5525	0.376343	-0.88235	0.379978	-4.84228	0.648406	0.704095
Neutrophils	PARP1	-0.22672	5.30881	-0.88229	0.38001	-5.38632	0.563313	0.61384
Neutrophils	BOK	-0.42043	1.039523	-0.88217	0.380079	-5.02431	0.63624	0.691287
Neutrophils	CD86	0.189756	6.31281	0.8821	0.380115	-6.0953	0.547457	0.596963
Neutrophils	MMP13	0.780607	-1.01302	0.881774	0.38029	-4.82112	0.674833	0.731953
Neutrophils	NFKB1	0.121746	8.743793	0.881678	0.380341	-6.52403	0.511114	0.558008
Neutrophils	BAG4	-0.19531	4.226706	-0.8816	0.380384	-5.35009	0.581094	0.632773
Neutrophils	PTPRF	-0.37047	1.276101	-0.88069	0.380874	-4.97035	0.632763	0.687142
Neutrophils	GM49417	0.633147	1.078235	0.880578	0.380934	-4.88336	0.636348	0.690956
Neutrophils	SLC38A7	-0.35643	2.59539	-0.88042	0.381019	-5.05208	0.609377	0.662424
Neutrophils	SLC46A3	-0.2629	3.085031	-0.88038	0.381043	-5.23816	0.600924	0.653462
Neutrophils	ZFP658	-0.55001	0.297611	-0.88023	0.381121	-4.84922	0.650695	0.706162
Neutrophils	8-Mar	-0.17793	3.141103	-0.88009	0.381196	-5.60526	0.599964	0.65255
Neutrophils	UVSSA	0.184783	3.709393	0.880051	0.381217	-5.45512	0.59032	0.642312
Neutrophils	GSKIP	0.124448	4.602025	0.879948	0.381273	-5.61208	0.575495	0.62657
Neutrophils	SAR1A	0.118672	5.508767	0.879948	0.381273	-5.82575	0.560831	0.610951
Neutrophils	RPP14	-0.30367	2.846249	-0.87984	0.381333	-5.07567	0.605037	0.657963
Neutrophils	STK38	0.082865	6.531625	0.87971	0.381401	-6.27757	0.54478	0.59384
Neutrophils	STK26	-0.11494	4.048898	-0.87947	0.381529	-5.94269	0.584694	0.636425
Neutrophils	5031439G(	0.142593	5.696635	0.879461	0.381535	-5.79412	0.557897	0.607891
Neutrophils	NCKAP5	-0.47795	1.217214	-0.87921	0.38167	-4.98404	0.634021	0.688675
Neutrophils	KLF2	0.173604	8.937753	0.878866	0.381856	-6.55537	0.509158	0.55566
Neutrophils	ARNTL	-0.27771	5.38742	-0.87852	0.382044	-5.37602	0.563329	0.613517
Neutrophils	TMEM33	0.115031	5.133185	0.87841	0.382102	-5.75533	0.567422	0.617875
Neutrophils	PLGRKT	0.138538	5.796951	0.878302	0.38216	-5.82397	0.55681	0.606561
Neutrophils	CYP3A16	-0.4275	0.777697	-0.87793	0.382362	-5.00254	0.642704	0.697493
Neutrophils	ATRAID	-0.16531	4.57765	-0.87757	0.382556	-5.4907	0.576676	0.62764
Neutrophils	4732440D(	0.322335	2.03703	0.877378	0.382659	-5.1603	0.620005	0.673719
Neutrophils	6330418K(	0.309662	1.506684	0.877295	0.382704	-5.1426	0.629463	0.683741
Neutrophils	NUDCD1	0.342484	3.226429	0.877197	0.382757	-5.05123	0.599318	0.651818
Neutrophils	0610040J0	0.322155	2.378015	0.877077	0.382822	-5.1926	0.614001	0.667385
Neutrophils	PTOV1	-0.1845	4.038474	-0.87705	0.382837	-5.41506	0.585603	0.637253
Neutrophils	TSPAN5	0.107057	5.373256	0.877044	0.38284	-6.11291	0.563762	0.614009
Neutrophils	GM15614	-0.26243	2.981808	-0.87704	0.38284	-5.35596	0.603514	0.656269

Neutrophils	TBC1D31	0.281232	4.166685	0.87701	0.382858	-5.25416	0.583467	0.634998
Neutrophils	DHODH	-0.37837	2.109646	-0.8769	0.382916	-4.94986	0.618723	0.672446
Neutrophils	SUOX	0.363976	1.361958	0.876451	0.38316	-4.96232	0.632372	0.686752
Neutrophils	A630052C1	-0.56338	0.189023	-0.87633	0.383224	-4.83861	0.653913	0.709557
Neutrophils	SLK	0.094778	6.477015	0.876255	0.383266	-6.22181	0.546605	0.595737
Neutrophils	1600020E0	0.144051	6.223144	0.876115	0.383342	-5.94539	0.55059	0.600024
Neutrophils	SRSF4	-0.10162	6.225555	-0.8757	0.383564	-5.89162	0.550773	0.600084
Neutrophils	WEE1	-0.25993	3.947727	-0.87562	0.383607	-5.29987	0.587664	0.639395
Neutrophils	HEXA	-0.11641	5.752202	-0.87547	0.38369	-6.02265	0.558274	0.608125
Neutrophils	ZBTB8OS	-0.15464	5.027468	-0.87516	0.383857	-5.60784	0.570022	0.620589
Neutrophils	BORCS5	0.180129	4.024358	0.875117	0.383882	-5.42437	0.586543	0.63817
Neutrophils	EEF2KMT	0.298302	3.145957	0.874942	0.383976	-5.06443	0.601477	0.653982
Neutrophils	TMEM37	-0.2509	3.751452	-0.87479	0.384057	-5.47186	0.591221	0.6431
Neutrophils	SGPL1	0.100005	5.922758	0.874656	0.384131	-6.13764	0.555803	0.605389
Neutrophils	CFAP36	0.193822	4.102137	0.874419	0.384259	-5.33725	0.585415	0.636937
Neutrophils	MINDY2	0.093226	6.610817	0.874273	0.384338	-6.08528	0.545085	0.593994
Neutrophils	MAPK1IP1	0.266641	2.304416	0.87426	0.384345	-5.1323	0.616212	0.669632
Neutrophils	4930556J2	-0.34706	2.185085	-0.87418	0.384387	-4.99093	0.618314	0.671868
Neutrophils	GM47469	0.557244	0.520329	0.87383	0.384578	-4.8409	0.648627	0.703771
Neutrophils	RAC2	0.077599	9.052768	0.873623	0.38469	-6.66743	0.508791	0.555047
Neutrophils	COPS7A	0.208277	4.237724	0.873604	0.3847	-5.36619	0.58335	0.634654
Neutrophils	ALG14	-0.23115	3.562595	-0.87348	0.384766	-5.31447	0.594684	0.646692
Neutrophils	DDX24	0.105333	6.717234	0.873293	0.384869	-5.97738	0.543618	0.592322
Neutrophils	FAM71A	-0.44541	0.237403	-0.87324	0.3849	-4.97269	0.65389	0.70932
Neutrophils	MIEF2	-0.37595	1.112018	-0.87313	0.384956	-4.98587	0.637759	0.692299
Neutrophils	KIF19A	-0.48203	1.071139	-0.8731	0.384974	-4.85204	0.638504	0.693094
Neutrophils	H2-T23	0.194249	6.358381	0.873014	0.385021	-5.98836	0.549189	0.598293
Neutrophils	NADK2	-0.13873	4.994117	-0.87263	0.385227	-5.76473	0.57106	0.621564
Neutrophils	ABHD14A	-0.46538	1.44344	-0.87263	0.385229	-4.88679	0.631909	0.686101
Neutrophils	CD302	-0.17625	5.847821	-0.87241	0.38535	-6.02038	0.55745	0.607018
Neutrophils	PRRG2	0.204239	2.322172	0.87205	0.385543	-5.36336	0.616566	0.669678
Neutrophils	IMPAD1	-0.13575	4.365894	-0.87196	0.385592	-5.6054	0.581662	0.632658
Neutrophils	RMND5A	0.101853	6.766957	0.871436	0.385876	-6.06857	0.543267	0.591879
Neutrophils	FICD	-0.4356	0.917558	-0.87143	0.385879	-4.94914	0.641801	0.696492
Neutrophils	FAAP24	0.459234	2.002599	0.871414	0.385888	-4.99754	0.622223	0.675803
Neutrophils	MRPL9	-0.16042	4.787977	-0.87138	0.385904	-5.4199	0.574718	0.625408
Neutrophils	DSCAM	-0.59849	0.029718	-0.87134	0.385929	-4.8878	0.658284	0.713879
Neutrophils	TXNL4B	0.247052	1.740021	0.871258	0.385973	-5.19631	0.626905	0.680767
Neutrophils	HDLBP	0.077577	6.655734	0.871161	0.386026	-6.13102	0.544986	0.593759
Neutrophils	NEDD4	-0.12203	5.933587	-0.87112	0.386048	-6.08227	0.556286	0.605817
Neutrophils	PARVA	-0.33093	1.329681	-0.8709	0.386168	-5.13945	0.634397	0.688654
Neutrophils	LNX2	-0.23527	3.592505	-0.87016	0.386571	-5.29461	0.595227	0.646888
Neutrophils	MRTO4	0.22774	4.665156	0.870097	0.386604	-5.42952	0.577309	0.627889
Neutrophils	SCG5	0.4862	0.801405	0.869649	0.386847	-4.94729	0.644804	0.699347
Neutrophils	AI467606	0.159007	4.111903	0.869641	0.386851	-5.7535	0.586685	0.637837
Neutrophils	B3GALT2	0.530242	0.771125	0.869313	0.38703	-4.91389	0.645392	0.700046
Neutrophils	A530032D1	0.50659	0.594014	0.869231	0.387074	-4.90304	0.648665	0.703502

Neutrophils	1-Sep	-0.11906	6.037433	-0.86897	0.387218	-5.97207	0.555422	0.604707
Neutrophils	MCOLN1	0.26721	2.728161	0.86881	0.387303	-5.10969	0.610325	0.663094
Neutrophils	MKLN1	-0.0881	7.159858	-0.86876	0.38733	-6.19795	0.537992	0.58614
Neutrophils	EDF1	0.083239	7.047067	0.868747	0.387337	-6.18302	0.539717	0.587983
Neutrophils	AMZ1	-0.31823	3.157688	-0.86864	0.387398	-5.29796	0.602892	0.655262
Neutrophils	OSTC	-0.09794	6.387429	-0.86846	0.387495	-5.96986	0.549924	0.598975
Neutrophils	PSMC5	-0.11616	6.00886	-0.86846	0.387496	-5.81559	0.555873	0.605321
Neutrophils	YWHAG	0.121369	6.203239	0.868408	0.387522	-5.91974	0.55281	0.602054
Neutrophils	KLHL26	0.359658	2.727028	0.868351	0.387553	-5.00153	0.610344	0.663219
Neutrophils	VPS4A	-0.16547	4.241466	-0.86822	0.387627	-5.45914	0.584551	0.635876
Neutrophils	GM38832	0.340464	0.763984	0.867982	0.387754	-5.09506	0.645524	0.700468
Neutrophils	PREX2	-0.23138	2.374714	-0.86797	0.387761	-5.55058	0.616511	0.669801
Neutrophils	1600002D2	0.413377	0.257645	0.867671	0.387923	-5.14146	0.654927	0.710462
Neutrophils	NOLC1	-0.20652	5.264292	-0.86761	0.387959	-5.50776	0.567771	0.61812
Neutrophils	CEP290	-0.29516	2.468766	-0.86759	0.387967	-5.08618	0.614859	0.66813
Neutrophils	ITPR3	-0.31084	4.288295	-0.86748	0.388027	-5.17224	0.583771	0.635145
Neutrophils	SEC31A	-0.10283	5.620105	-0.86746	0.388039	-5.77543	0.562052	0.612027
Neutrophils	KDM1B	-0.31298	3.6758	-0.86745	0.388043	-5.11437	0.594051	0.646066
Neutrophils	ATG7	0.091198	5.786606	0.86743	0.388055	-6.26481	0.559397	0.609197
Neutrophils	SIK2	0.084247	7.126745	0.867168	0.388197	-6.34171	0.538618	0.587017
Neutrophils	ACE	0.499926	0.341503	0.867054	0.388259	-5.0975	0.653515	0.709014
Neutrophils	MIRT2	0.188963	-1.00119	0.866917	0.388334	-5.48434	0.679098	0.735938
Neutrophils	GM15965	0.316692	2.027609	0.866572	0.388522	-5.17526	0.622947	0.676703
Neutrophils	4833445I0	-0.47009	0.48418	-0.86632	0.388659	-4.86986	0.651014	0.706486
Neutrophils	TREML2	-0.13359	3.901235	-0.86627	0.388688	-5.84447	0.590528	0.642452
Neutrophils	PFN1	0.065862	10.31807	0.866087	0.388787	-6.84177	0.492218	0.537431
Neutrophils	ZC3H13	0.124791	5.653384	0.866026	0.38882	-5.80103	0.561789	0.611945
Neutrophils	GEMIN7	0.114832	5.084337	0.866023	0.388821	-5.73089	0.57096	0.621717
Neutrophils	ACSL3	-0.20614	3.828777	-0.86597	0.38885	-5.47443	0.591749	0.643854
Neutrophils	MED26	0.198889	4.429093	0.865897	0.38889	-5.46538	0.581713	0.63319
Neutrophils	SLC27A4	0.098432	2.770367	0.865847	0.388918	-5.90529	0.609882	0.663113
Neutrophils	WDR11	-0.23964	3.124121	-0.86562	0.389039	-5.21424	0.603824	0.656713
Neutrophils	DROSHA	-0.24393	3.121263	-0.86547	0.389124	-5.11783	0.603873	0.65679
Neutrophils	HMGA2	-0.60102	1.149583	-0.86546	0.389129	-5.03628	0.638829	0.693803
Neutrophils	MRPS18C	-0.1103	5.696786	-0.86497	0.389399	-5.95033	0.561464	0.611568
Neutrophils	MS4A6C	0.36858	5.165825	0.864789	0.389495	-5.54693	0.570069	0.620728
Neutrophils	GUCD1	0.216547	3.805941	0.864262	0.389783	-5.39894	0.592923	0.644872
Neutrophils	ZFP329	0.273292	2.511051	0.863991	0.389931	-5.07241	0.61523	0.668629
Neutrophils	THOC7	0.092027	6.572603	0.863915	0.389972	-6.03606	0.548029	0.597158
Neutrophils	FCHSD2	-0.16443	6.823892	-0.86391	0.389977	-6.08624	0.544132	0.592995
Neutrophils	DGKG	0.112771	2.506906	0.863785	0.390043	-6.13861	0.615303	0.668713
Neutrophils	TBCD	0.193701	4.783335	0.863724	0.390077	-5.40618	0.576641	0.627683
Neutrophils	PRKCB	0.073735	8.511819	0.863446	0.390229	-6.53578	0.518699	0.565928
Neutrophils	AHSA2	0.197828	3.646243	0.86342	0.390243	-5.32612	0.595629	0.648022
Neutrophils	GM47200	-0.49223	0.11613	-0.86331	0.390301	-4.90031	0.658771	0.714917
Neutrophils	IL20RB	-0.16604	3.81612	-0.86314	0.390395	-5.62398	0.592751	0.645043
Neutrophils	NAPG	0.164663	4.542195	0.863099	0.390418	-5.55898	0.580614	0.632138

Neutrophils	CADM1	-0.32096	5.440386	-0.86289	0.390533	-5.76476	0.565957	0.616599
Neutrophils	SPRY2	-0.17853	5.158508	-0.86284	0.390561	-5.8522	0.570515	0.621457
Neutrophils	MAGOHB	-0.22656	4.560419	-0.86272	0.390628	-5.48887	0.580313	0.63196
Neutrophils	MAP7	-0.22006	3.639692	-0.86268	0.390647	-5.34969	0.59574	0.648372
Neutrophils	DCUN1D3	0.143852	4.424811	0.862568	0.390709	-5.72239	0.582559	0.634371
Neutrophils	ASB3	-0.15486	4.711015	-0.86235	0.390828	-5.53608	0.57783	0.629399
Neutrophils	CCDC180	0.197989	1.286108	0.862193	0.390914	-5.56155	0.637123	0.692367
Neutrophils	NME2	0.129917	8.557653	0.861871	0.39109	-6.28356	0.518026	0.565654
Neutrophils	4933434E2	0.138075	5.269667	0.861842	0.391106	-5.70715	0.568713	0.619869
Neutrophils	ORC5	-0.25616	3.474755	-0.86183	0.39111	-5.2275	0.598548	0.651625
Neutrophils	SH3BP5L	-0.27213	2.252113	-0.86175	0.391158	-5.18339	0.619793	0.674179
Neutrophils	GM44127	-0.49649	-0.52381	-0.86155	0.391263	-4.94325	0.670923	0.728327
Neutrophils	MCM3	0.212195	5.484871	0.861498	0.391295	-5.75335	0.565241	0.616299
Neutrophils	UTP23	0.216571	3.577367	0.861426	0.391334	-5.24193	0.5968	0.649921
Neutrophils	VCL	-0.08624	6.049702	-0.86114	0.391488	-6.35343	0.556234	0.606749
Neutrophils	ARHGEF11	0.124843	4.907611	0.860927	0.391607	-5.90521	0.574604	0.626355
Neutrophils	GM13684	0.313587	3.516914	0.860912	0.391615	-5.15042	0.597829	0.651077
Neutrophils	RANGAP1	-0.16272	5.610379	-0.86087	0.391637	-5.67951	0.563226	0.614224
Neutrophils	ZFP951	-0.43708	2.641469	-0.86084	0.391655	-4.95167	0.612945	0.667141
Neutrophils	KRT18	-0.30475	3.557982	-0.86078	0.391686	-5.54096	0.59713	0.650344
Neutrophils	PUS10	0.198626	4.485499	0.86078	0.391688	-5.447	0.581553	0.63377
Neutrophils	ITPRIP	0.121068	3.468077	0.860632	0.391769	-5.8933	0.598662	0.651995
Neutrophils	STOML3	-0.45263	0.359863	-0.86061	0.39178	-4.91993	0.654201	0.710838
Neutrophils	2010016I1	0.453653	-0.03474	0.860495	0.391843	-4.91152	0.661616	0.718666
Neutrophils	STXBP1	-0.28513	3.786972	-0.86049	0.391849	-5.24432	0.593244	0.646234
Neutrophils	5031425F1	-0.55507	-0.17235	-0.86047	0.391855	-4.86261	0.664222	0.721416
Neutrophils	COPS9	0.088197	6.68605	0.86043	0.391879	-6.199	0.546266	0.596133
Neutrophils	MRTFB	-0.20505	4.475202	-0.86035	0.391923	-5.42946	0.581723	0.634004
Neutrophils	SGO1	-0.30498	3.188246	-0.86001	0.392111	-5.36973	0.603592	0.657159
Neutrophils	GFOD1	0.103864	6.621215	0.859886	0.392178	-6.37251	0.547394	0.597266
Neutrophils	SLC39A11	0.115764	4.419343	0.859868	0.392187	-5.99637	0.582779	0.635025
Neutrophils	SMIM14	0.063382	7.74729	0.859746	0.392254	-6.48017	0.530177	0.578841
Neutrophils	DUT	0.20319	5.882801	0.859676	0.392292	-5.88466	0.559003	0.609706
Neutrophils	MELK	-0.3233	3.016502	-0.85931	0.392494	-5.25582	0.606742	0.660461
Neutrophils	GM45820	0.305212	1.425778	0.859154	0.392579	-5.07363	0.634922	0.690382
Neutrophils	TNS1	0.258501	2.761945	0.85884	0.392751	-5.37177	0.611164	0.66521
Neutrophils	NDUFB4	-0.09438	6.598671	-0.85879	0.39278	-6.10071	0.547913	0.597835
Neutrophils	SOCS1	-0.21641	5.152296	-0.85877	0.39279	-5.83147	0.570917	0.6224
Neutrophils	DKC1	-0.21116	4.56874	-0.85875	0.392799	-5.41693	0.580482	0.632594
Neutrophils	NT5C3B	-0.2897	3.396774	-0.85874	0.392809	-5.07001	0.600198	0.653569
Neutrophils	D230025D1	0.156778	4.57771	0.858387	0.393	-5.61493	0.580412	0.63245
Neutrophils	E130307A1	-0.14442	4.490339	-0.85836	0.393017	-5.57738	0.581858	0.63399
Neutrophils	CRYBB3	0.482519	0.578324	0.85833	0.393031	-4.92162	0.650562	0.706856
Neutrophils	NECTIN1	-0.49008	1.073651	-0.85823	0.393089	-4.93335	0.641427	0.697249
Neutrophils	1200007C1	0.452284	-0.38465	0.858092	0.393162	-4.9935	0.668735	0.726109
Neutrophils	LOXL2	0.329689	1.406795	0.857919	0.393257	-5.14373	0.635443	0.6909
Neutrophils	EEF1E1	-0.22444	4.824221	-0.85769	0.393383	-5.40276	0.576535	0.628341

Neutrophils	GM42997	-0.55043	0.465543	-0.85711	0.393702	-4.87442	0.653306	0.709575
Neutrophils	LSM5	-0.1294	5.652829	-0.85677	0.393888	-5.86406	0.56366	0.614341
Neutrophils	CNNM2	-0.12703	5.427728	-0.85606	0.394277	-6.33848	0.56776	0.618395
Neutrophils	TNFSF8	-0.64799	-0.51498	-0.8558	0.394423	-4.84501	0.672687	0.729571
Neutrophils	PCSK5	0.492709	0.558557	0.855762	0.394443	-4.95016	0.652373	0.708155
Neutrophils	MOV10	0.178142	3.628623	0.855696	0.394479	-5.55677	0.597645	0.650229
Neutrophils	SEC62	-0.05684	7.437896	-0.85561	0.394526	-6.29974	0.536272	0.584845
Neutrophils	GM5244	-0.59496	-0.39473	-0.8555	0.394586	-4.84621	0.670387	0.727221
Neutrophils	CTSO	-0.23572	4.26707	-0.85537	0.394658	-5.3613	0.586897	0.638852
Neutrophils	ATXN7L3	0.132839	4.200833	0.855215	0.394744	-5.65611	0.588048	0.640145
Neutrophils	SPSB2	0.241555	2.383882	0.854665	0.395047	-5.18874	0.619707	0.673569
Neutrophils	TNPO2	-0.20448	4.122122	-0.85437	0.395212	-5.39443	0.589861	0.641792
Neutrophils	HYAL2	-0.31921	1.859326	-0.85431	0.395243	-5.12562	0.629187	0.683492
Neutrophils	IPCEF1	0.11104	4.679593	0.853822	0.395511	-6.31204	0.580877	0.631999
Neutrophils	CDK16	0.20104	3.39772	0.853681	0.395589	-5.28217	0.602521	0.655027
Neutrophils	LGALS3BP	-0.26261	4.826846	-0.85346	0.395709	-5.66959	0.578569	0.629575
Neutrophils	PRR5	-0.44083	3.479528	-0.8533	0.395799	-4.97043	0.601264	0.653714
Neutrophils	COPS8	-0.11114	5.091019	-0.853	0.395966	-5.74699	0.574385	0.625127
Neutrophils	TRIM30C	-0.43202	1.320225	-0.85297	0.395983	-5.018	0.639591	0.694271
Neutrophils	GM38843	-0.19121	0.985485	-0.85252	0.396229	-5.62631	0.646036	0.700899
Neutrophils	1700052K1	0.376277	1.140287	0.852425	0.396282	-4.95797	0.643186	0.697944
Neutrophils	BE692007	0.486197	2.816357	0.852271	0.396367	-4.99778	0.613183	0.666213
Neutrophils	SRP72	0.089156	6.657086	0.851969	0.396533	-6.02974	0.549768	0.59873
Neutrophils	SCYL3	0.189482	3.642548	0.851847	0.3966	-5.32925	0.599018	0.651144
Neutrophils	PIGZ	-0.53684	0.017052	-0.85165	0.39671	-4.88347	0.664327	0.720186
Neutrophils	AC160336.	-0.3697	1.371003	-0.85163	0.396722	-5.03936	0.639129	0.693609
Neutrophils	ULK2	-0.129	5.457806	-0.85162	0.396727	-5.84287	0.568835	0.619059
Neutrophils	MLST8	0.25609	2.044303	0.851497	0.396794	-5.29983	0.626961	0.680748
Neutrophils	RNF213	-0.18244	6.151249	-0.85133	0.396888	-6.18929	0.557727	0.607295
Neutrophils	ATF7IP	0.097129	6.540023	0.851295	0.396905	-6.19094	0.5516	0.60076
Neutrophils	DXO	-0.2846	3.302702	-0.85115	0.396987	-5.20676	0.60485	0.65747
Neutrophils	TNKS1BP1	-0.31295	2.080541	-0.85102	0.397057	-5.11301	0.626313	0.680225
Neutrophils	RCHY1	0.087435	5.661194	0.850906	0.39712	-6.0703	0.565553	0.615726
Neutrophils	MAGI3	-0.11249	6.598022	-0.8508	0.397177	-6.32486	0.550692	0.599874
Neutrophils	ARPP21	-0.70776	1.234602	-0.85073	0.397217	-4.88748	0.641623	0.696427
Neutrophils	G3BP1	-0.10287	6.728766	-0.85066	0.397256	-6.05975	0.54865	0.597695
Neutrophils	KCTD10	0.131085	4.043279	0.85056	0.397311	-5.73279	0.592215	0.644093
Neutrophils	DHFR	-0.26215	4.331798	-0.85045	0.397373	-5.44261	0.587366	0.638941
Neutrophils	SPTLC1	0.152916	4.232218	0.850289	0.397461	-5.56008	0.589035	0.640786
Neutrophils	FAM43A	-0.21249	4.098571	-0.85025	0.397483	-5.38277	0.591282	0.643183
Neutrophils	GM15972	-0.52339	-0.2921	-0.85018	0.397522	-4.8934	0.670219	0.726694
Neutrophils	GLRX3	0.13026	6.625536	0.849729	0.397771	-5.84049	0.550528	0.599661
Neutrophils	EEFSEC	-0.27056	4.399413	-0.84957	0.39786	-5.3083	0.586567	0.638084
Neutrophils	GTF2H2	-0.20559	3.759481	-0.84934	0.397988	-5.23497	0.597388	0.649562
Neutrophils	LIPA	-0.12848	5.64326	-0.84926	0.398032	-5.99194	0.566187	0.616377
Neutrophils	TOR1A	0.122137	4.653633	0.849194	0.398067	-5.75788	0.582361	0.633595
Neutrophils	PLEKHA6	-0.34314	1.808761	-0.84902	0.398163	-5.03898	0.631576	0.685783

Neutrophils	SUPT16	-0.08874	7.068147	-0.84886	0.398251	-6.07648	0.54372	0.592404
Neutrophils	MEF2B	0.204278	3.605786	0.848807	0.398281	-5.3818	0.600011	0.652346
Neutrophils	SMG5	-0.12715	5.55464	-0.84869	0.398345	-5.71263	0.567616	0.6179
Neutrophils	MFAP3	-0.15229	5.124697	-0.84864	0.398372	-5.56173	0.574603	0.62534
Neutrophils	CLEC4A3	0.298196	3.297848	0.848611	0.398389	-5.55002	0.605303	0.65796
Neutrophils	GM20508	-0.43814	0.064606	-0.84801	0.398724	-4.90349	0.664265	0.720025
Neutrophils	MYG1	-0.17944	3.803891	-0.84793	0.398765	-5.35507	0.597024	0.648937
Neutrophils	TARDBP	0.114613	5.771119	0.84768	0.398905	-5.83319	0.564531	0.614376
Neutrophils	RASGEF1B	-0.21271	5.547786	-0.84754	0.39898	-5.87053	0.568129	0.618254
Neutrophils	FBXL3	0.096703	5.215741	0.847521	0.398993	-5.94729	0.573522	0.623996
Neutrophils	DSE	-0.24022	3.834005	-0.84744	0.39904	-5.50626	0.596543	0.648468
Neutrophils	LMNA	-0.2849	4.617164	-0.84735	0.399087	-5.41936	0.583379	0.634512
Neutrophils	MEX3B	-0.32974	2.233947	-0.84728	0.399127	-5.09187	0.624399	0.678034
Neutrophils	GM48653	0.488975	-0.16399	0.847047	0.399256	-4.91308	0.668737	0.724815
Neutrophils	TBC1D22B	-0.22221	3.778303	-0.84698	0.399292	-5.29478	0.597567	0.649595
Neutrophils	EIF4A2	0.095575	5.530319	0.846845	0.399368	-5.94371	0.568511	0.618711
Neutrophils	RSAD1	-0.55206	1.298807	-0.84663	0.399489	-4.90802	0.641508	0.696094
Neutrophils	4732496CC	0.317669	0.689806	0.846506	0.399556	-5.0961	0.652779	0.707994
Neutrophils	CIP2A	0.2379	3.506565	0.84616	0.399748	-5.49011	0.602559	0.654714
Neutrophils	TM7SF2	-0.3467	1.754706	-0.84591	0.399885	-5.03271	0.633569	0.687468
Neutrophils	AK6	-0.15827	4.761906	-0.84581	0.399942	-5.5774	0.581502	0.632322
Neutrophils	FEN1	0.212868	4.525584	0.845287	0.400232	-5.49972	0.585563	0.636673
Neutrophils	RSF1	-0.08429	7.014648	-0.84513	0.400322	-6.23216	0.545547	0.594048
Neutrophils	SECTM1A	-0.55907	-1.13086	-0.84505	0.400361	-4.83774	0.68815	0.745066
Neutrophils	CAPG	0.13589	5.264343	0.845042	0.400369	-6.1596	0.573375	0.623729
Neutrophils	PHC1	-0.2446	3.5385	-0.84495	0.400421	-5.11227	0.602268	0.654444
Neutrophils	RTTN	-0.27486	3.609694	-0.84491	0.400442	-5.21178	0.601047	0.653154
Neutrophils	CD82	0.113781	5.476033	0.844812	0.400496	-5.94488	0.569932	0.620142
Neutrophils	BSCL2	-0.16477	4.542466	-0.84465	0.400584	-5.44292	0.585281	0.636501
Neutrophils	CRP	-0.36538	2.076546	-0.84464	0.400592	-5.17571	0.627918	0.681706
Neutrophils	MID1	-0.49729	5.805162	-0.84455	0.400641	-5.72238	0.564621	0.614532
Neutrophils	RGL2	-0.17233	3.300422	-0.84452	0.40066	-5.54677	0.606371	0.658914
Neutrophils	GM34680	0.319263	0.042973	0.844362	0.400746	-5.23036	0.665509	0.721378
Neutrophils	MPST	-0.1919	3.682692	-0.84421	0.40083	-5.46813	0.599882	0.65202
Neutrophils	HVCN1	-0.24056	4.117697	-0.84389	0.401009	-5.58851	0.592628	0.644275
Neutrophils	STAM2	0.147259	5.475543	0.843838	0.401037	-5.83974	0.570152	0.620376
Neutrophils	FAM120A	0.088289	6.352078	0.843708	0.401109	-5.9862	0.556139	0.605484
Neutrophils	AMDHD2	-0.16382	3.693973	-0.8434	0.401282	-5.53373	0.599926	0.65216
Neutrophils	SBK1	-0.30898	3.374185	-0.84337	0.401296	-5.09215	0.605421	0.658
Neutrophils	HDAC7	-0.19976	4.31747	-0.84326	0.401357	-5.45928	0.58936	0.640966
Neutrophils	CLEC9A	0.261845	2.519945	0.843149	0.40142	-5.50616	0.620355	0.673906
Neutrophils	PPP2R5B	-0.47581	1.645269	-0.84309	0.401452	-4.951	0.636037	0.690498
Neutrophils	FPGS	0.24366	3.013479	0.843003	0.401502	-5.24048	0.611681	0.66473
Neutrophils	RANBP1	-0.13725	7.239938	-0.84284	0.401593	-6.12329	0.542377	0.590979
Neutrophils	SAA3	1.336267	0.738679	0.842775	0.401629	-4.98671	0.652738	0.708182
Neutrophils	PRUNE2	-0.41955	0.638929	-0.8425	0.401779	-4.97039	0.654753	0.710283
Neutrophils	RDH5	0.306782	2.368316	0.84235	0.401865	-5.20293	0.623255	0.677008

Neutrophils	DGCR6	0.219538	3.650268	0.842114	0.401996	-5.29395	0.600956	0.653313
Neutrophils	VPS26B	0.139441	3.835261	0.841965	0.40208	-5.69735	0.597795	0.650041
Neutrophils	RPRD1A	-0.15043	4.057705	-0.84195	0.402089	-5.46815	0.594017	0.646033
Neutrophils	EBP	0.157848	4.791273	0.841738	0.402206	-5.67899	0.581797	0.633041
Neutrophils	RASL11A	0.519627	0.28073	0.841494	0.402342	-4.88949	0.661694	0.717676
Neutrophils	SEC24D	-0.19194	4.470665	-0.84133	0.402435	-5.45759	0.587134	0.638787
Neutrophils	MIB1	-0.09033	6.189415	-0.84118	0.402517	-6.13656	0.55911	0.608985
Neutrophils	LRP2BP	-0.46824	1.991491	-0.84104	0.402596	-5.00804	0.630147	0.684485
Neutrophils	IL6ST	0.195784	4.326252	0.841022	0.402604	-5.64152	0.589554	0.641436
Neutrophils	BAIAP2	-0.17736	5.269985	-0.841	0.402618	-5.75916	0.573923	0.624803
Neutrophils	EML5	-0.26626	3.697759	-0.84097	0.402635	-5.34949	0.600209	0.652755
Neutrophils	CDK5RAP3	0.189318	4.379321	0.840867	0.402691	-5.40869	0.588664	0.640525
Neutrophils	DPP7	-0.39705	1.957266	-0.84084	0.402704	-5.04016	0.630763	0.685178
Neutrophils	SEC61G	0.108994	10.02398	0.840712	0.402777	-6.59309	0.501554	0.547466
Neutrophils	TNIP3	0.696374	1.037658	0.840486	0.402903	-5.00262	0.647559	0.703039
Neutrophils	SBF1	0.151812	4.474225	0.840455	0.40292	-5.51866	0.587089	0.638953
Neutrophils	NUP43	-0.29426	2.795528	-0.84025	0.403037	-5.09907	0.615868	0.669556
Neutrophils	PDK4	-0.35015	1.417008	-0.84023	0.403048	-5.1375	0.640583	0.695718
Neutrophils	GM43466	-0.38996	1.497294	-0.84014	0.403095	-4.99936	0.639116	0.694191
Neutrophils	1110032A(	0.208429	3.592833	0.840049	0.403147	-5.29196	0.602022	0.654937
Neutrophils	SAG	0.200803	3.5306	0.839956	0.403199	-5.63611	0.603091	0.656072
Neutrophils	YES1	-0.17449	4.740029	-0.83976	0.403307	-5.94204	0.582662	0.634378
Neutrophils	EAF1	-0.19164	4.101569	-0.83963	0.403378	-5.45456	0.593355	0.645791
Neutrophils	GRIK5	-0.59081	0.167717	-0.83957	0.403412	-4.84986	0.66385	0.720437
Neutrophils	MED13	-0.08662	7.722854	-0.83949	0.403457	-6.35097	0.535304	0.5839
Neutrophils	MAPK9	-0.16791	4.751185	-0.83932	0.403556	-5.48245	0.582476	0.634322
Neutrophils	B3GALNT2	0.230298	3.643954	0.839257	0.403589	-5.31322	0.601145	0.654193
Neutrophils	LY96	-0.15176	4.287348	-0.83918	0.403631	-5.68785	0.590223	0.642591
Neutrophils	UQCR10	0.099332	6.961957	0.83909	0.403682	-6.19804	0.546987	0.596521
Neutrophils	PCNX	-0.11654	5.518161	-0.83901	0.403724	-5.96874	0.569899	0.621015
Neutrophils	LEMD2	0.158787	4.232797	0.838984	0.403741	-5.50542	0.591141	0.643648
Neutrophils	RTN4	0.07116	7.592315	0.838186	0.404186	-6.3323	0.537563	0.586391
Neutrophils	GM15787	0.316345	2.641968	0.838147	0.404208	-5.16642	0.618887	0.673023
Neutrophils	ELOVL2	-0.36433	2.450205	-0.83814	0.404215	-5.24802	0.622282	0.676622
Neutrophils	THOC3	0.213744	4.102819	0.838093	0.404239	-5.31905	0.593636	0.64622
Neutrophils	CERS5	0.119931	5.514163	0.838073	0.404249	-5.82324	0.570253	0.621328
Neutrophils	0610039K1	0.549852	0.022539	0.837976	0.404304	-4.86953	0.666947	0.723889
Neutrophils	CCDC125	-0.12449	4.497843	-0.83782	0.404389	-5.98749	0.586993	0.639193
Neutrophils	WBP4	0.112135	5.270226	0.837734	0.404439	-5.82335	0.574225	0.625619
Neutrophils	SHB	0.273258	4.498582	0.837697	0.40446	-5.3102	0.58698	0.639204
Neutrophils	P2RY13	0.373951	1.100765	0.837556	0.404538	-5.18074	0.646756	0.702564
Neutrophils	ZFP367	-0.16698	5.436634	-0.83678	0.404974	-5.83048	0.57193	0.622987
Neutrophils	GPHN	0.094048	7.486182	0.836626	0.405058	-6.27052	0.539577	0.588484
Neutrophils	CD48	-0.1885	6.795574	-0.83658	0.405086	-5.68746	0.550258	0.599906
Neutrophils	NCOA6	0.090519	5.600455	0.836484	0.405138	-5.97973	0.569271	0.620214
Neutrophils	G6PC	0.412485	1.911843	0.836463	0.405149	-5.18586	0.63238	0.687258
Neutrophils	FAM129A	0.095317	5.667894	0.836453	0.405155	-6.298	0.56818	0.61905

Neutrophils	ANAPC11	-0.12075	5.923842	-0.83614	0.405331	-5.93406	0.564105	0.614742
Neutrophils	BC030867	0.410242	2.099983	0.836095	0.405355	-5.08434	0.629043	0.683797
Neutrophils	1700010I1	-0.32494	1.215796	-0.83599	0.405413	-5.05603	0.645124	0.700859
Neutrophils	CEP192	-0.15627	5.27108	-0.83599	0.405416	-5.59378	0.574677	0.626084
Neutrophils	CENPQ	0.145858	5.314475	0.835567	0.405651	-5.84047	0.574112	0.625476
Neutrophils	MFN2	0.158136	3.671067	0.835385	0.405753	-5.48449	0.601625	0.65479
Neutrophils	SLC14A1	-0.3923	3.335927	-0.83538	0.405753	-4.99436	0.607401	0.660926
Neutrophils	WDR60	0.471163	0.883885	0.835333	0.405782	-4.92207	0.651431	0.707567
Neutrophils	CCNB1	-0.28096	4.032241	-0.83529	0.405804	-5.63087	0.595463	0.64825
Neutrophils	GM7854	0.282762	0.52802	0.834976	0.405982	-5.31876	0.658282	0.714766
Neutrophils	RBBP4	-0.07408	7.676556	-0.83485	0.406051	-6.30126	0.537025	0.585906
Neutrophils	LRBA	-0.17291	6.139874	-0.83419	0.406422	-5.78756	0.56122	0.611707
Neutrophils	CLINT1	0.070682	8.205941	0.834183	0.406426	-6.45479	0.529258	0.577519
Neutrophils	UBE2L3	0.07097	7.560477	0.834157	0.406441	-6.27469	0.539032	0.587987
Neutrophils	MS4A4C	0.767614	3.224383	0.833959	0.406551	-4.98682	0.609802	0.663459
Neutrophils	GM26801	-0.57319	0.557742	-0.83394	0.40656	-4.87027	0.65803	0.714513
Neutrophils	PSMB9	0.215184	6.228284	0.833924	0.406571	-5.93014	0.559812	0.610244
Neutrophils	POLR3C	0.133089	5.018329	0.833655	0.406722	-5.59924	0.579463	0.631244
Neutrophils	MAGI1	-0.22788	4.373842	-0.83355	0.406779	-5.89889	0.590196	0.642722
Neutrophils	TIPRL	-0.11695	5.525539	-0.83352	0.406798	-5.79378	0.571159	0.622442
Neutrophils	CD40LG	-0.47985	-0.56972	-0.83337	0.406881	-4.87871	0.679623	0.737441
Neutrophils	GM10130	-0.38545	1.783227	-0.83335	0.406891	-4.96856	0.635455	0.690798
Neutrophils	ABHD14B	-0.31702	2.125702	-0.83301	0.407086	-5.1043	0.629431	0.68436
Neutrophils	1500011B0	0.236731	4.65787	0.832917	0.407136	-5.3756	0.585587	0.637867
Neutrophils	PRMT3	-0.27466	4.041123	-0.83287	0.407165	-5.17385	0.595967	0.648921
Neutrophils	GM4129	-0.27813	1.28611	-0.83254	0.407346	-5.21608	0.644885	0.70073
Neutrophils	TMEM39A	0.168715	4.395391	0.832452	0.407397	-5.47797	0.590151	0.64271
Neutrophils	MYOM1	-0.3822	1.071853	-0.83235	0.407454	-5.04022	0.648842	0.704965
Neutrophils	C030005K0	0.402016	0.674423	0.83222	0.407527	-4.97404	0.656248	0.712855
Neutrophils	LEF1OS1	-0.48422	-0.45613	-0.83215	0.407568	-4.83935	0.677786	0.735595
Neutrophils	CARMIL2	-0.3018	2.704609	-0.83203	0.407634	-5.12159	0.61931	0.673758
Neutrophils	SMARCC2	0.095207	6.020787	0.831704	0.407817	-5.91137	0.56366	0.614462
Neutrophils	GCSH	-0.18884	4.428205	-0.8316	0.407877	-5.51048	0.589799	0.642319
Neutrophils	CMTR2	0.466778	1.0685	0.831392	0.407992	-4.87831	0.649215	0.705312
Neutrophils	GM26520	-0.57536	2.362216	-0.83091	0.408265	-4.92789	0.625965	0.680513
Neutrophils	BEND6	-0.53546	0.170455	-0.83085	0.408296	-4.87566	0.666391	0.723245
Neutrophils	2-Mar	0.112798	6.581903	0.830602	0.408436	-6.09438	0.555147	0.605161
Neutrophils	TFIP11	0.194125	3.322157	0.830512	0.408486	-5.39426	0.609127	0.662643
Neutrophils	PLK1	0.310099	3.704872	0.830461	0.408515	-5.54728	0.602517	0.655624
Neutrophils	NFU1	-0.13084	4.549473	-0.83034	0.408584	-5.81089	0.588196	0.64039
Neutrophils	NDRG2	-0.29515	2.964877	-0.83025	0.408634	-5.35898	0.615373	0.669263
Neutrophils	PQLC2	-0.25197	3.590318	-0.82994	0.408808	-5.2683	0.60457	0.657792
Neutrophils	GM29114	-0.45469	-1.28439	-0.8299	0.408833	-4.84942	0.694842	0.753179
Neutrophils	SAV1	-0.12002	4.735258	-0.82985	0.408857	-5.77096	0.585165	0.63716
Neutrophils	CYP27A1	-0.39088	2.194427	-0.82956	0.409019	-5.00963	0.629149	0.683853
Neutrophils	DNAJA2	-0.07185	7.208069	-0.8295	0.409057	-6.20384	0.545456	0.594815
Neutrophils	SF3A3	-0.15831	4.984502	-0.82943	0.409094	-5.58793	0.581045	0.632847

Neutrophils	KDELR2	0.090414	5.993878	0.82938	0.409123	-6.08461	0.564597	0.61532
Neutrophils	PER2	-0.1808	2.222784	-0.82925	0.409195	-5.65527	0.62864	0.683468
Neutrophils	APPL2	-0.26189	3.358029	-0.82918	0.409234	-5.33716	0.608607	0.662234
Neutrophils	CEP85L	0.143624	4.806918	0.829117	0.409271	-5.90852	0.583991	0.636059
Neutrophils	ACSF3	-0.3813	1.59243	-0.82883	0.409431	-4.95732	0.640137	0.695604
Neutrophils	HNRNPM	-0.06483	7.703885	-0.82882	0.409439	-6.3358	0.537907	0.586809
Neutrophils	MOCS2	-0.14117	5.094585	-0.82865	0.409536	-5.7014	0.579359	0.631109
Neutrophils	QPCTL	0.210872	3.010203	0.828343	0.409707	-5.24271	0.614836	0.668904
Neutrophils	LRRN3	-0.5596	-0.11923	-0.8282	0.409788	-4.84742	0.672296	0.729743
Neutrophils	PREX1	-0.11737	6.43388	-0.8282	0.409789	-6.07341	0.557727	0.608153
Neutrophils	4933433G1	-0.43994	1.434512	-0.82812	0.409834	-4.93599	0.643116	0.698925
Neutrophils	CCNL2	0.083796	6.219089	0.828108	0.409839	-5.98946	0.561142	0.611801
Neutrophils	SPTBN4	0.486077	-0.18469	0.827922	0.409944	-4.86584	0.673554	0.731081
Neutrophils	PDIA4	-0.14173	5.653573	-0.82783	0.409997	-5.63036	0.570238	0.621522
Neutrophils	AVL9	0.11155	6.010486	0.827713	0.410062	-5.99384	0.564479	0.615384
Neutrophils	MCM4	0.167149	5.68459	0.827612	0.410119	-5.90741	0.569735	0.620993
Neutrophils	TMEM120A	0.140212	3.799392	0.827587	0.410133	-5.68107	0.601155	0.654459
Neutrophils	TBC1D15	0.086158	6.062519	0.827428	0.410222	-6.11358	0.563645	0.614559
Neutrophils	KIF14	0.377349	2.55406	0.827283	0.410304	-5.20811	0.622889	0.677668
Neutrophils	4632428CC	-0.4931	0.110114	-0.82714	0.410383	-4.87766	0.667906	0.725331
Neutrophils	ASPSCR1	0.131437	4.685187	0.827037	0.410442	-5.66025	0.586173	0.638702
Neutrophils	PATL1	-0.11788	5.222719	-0.82695	0.410494	-5.82552	0.577271	0.62922
Neutrophils	CAR9	0.452645	0.285801	0.826887	0.410527	-4.9189	0.664563	0.721843
Neutrophils	ATP13A2	0.187866	5.610722	0.826824	0.410563	-5.64391	0.570933	0.622477
Neutrophils	AI847159	-0.52088	-0.25702	-0.82679	0.410582	-4.86718	0.674947	0.732826
Neutrophils	BC051537	-0.53891	0.044835	-0.8265	0.410744	-4.88598	0.669194	0.726853
Neutrophils	FHAD1	-0.50348	0.624492	-0.82648	0.410757	-4.95168	0.658206	0.71525
Neutrophils	STYX	-0.14757	4.526153	-0.82644	0.410781	-5.52681	0.58887	0.64172
Neutrophils	NDE1	-0.20479	4.58489	-0.82634	0.410837	-5.45194	0.587886	0.640692
Neutrophils	PHAX	-0.18215	4.599165	-0.82621	0.410908	-5.4855	0.587666	0.640438
Neutrophils	ANGPTL7	-0.5859	-0.05517	-0.82608	0.410983	-4.8545	0.67116	0.728975
Neutrophils	1700094J0	0.566671	0.092196	0.825734	0.411178	-4.88533	0.6684	0.726143
Neutrophils	USP2	0.260575	2.711696	0.825727	0.411182	-5.27929	0.620235	0.675162
Neutrophils	SNHG16	-0.40879	2.227913	-0.82566	0.411221	-4.95569	0.628857	0.684308
Neutrophils	GM12764	0.415869	1.453299	0.825599	0.411254	-4.97366	0.642917	0.699204
Neutrophils	IGLC1	0.601451	3.761408	0.825506	0.411306	-5.37006	0.601943	0.655728
Neutrophils	CXCL1	-0.52029	2.815955	-0.8254	0.411363	-5.44971	0.618393	0.673252
Neutrophils	CDC42BPG	0.224048	2.57838	0.825036	0.411572	-5.46334	0.622757	0.677759
Neutrophils	PROK2	-0.22047	-0.61756	-0.82502	0.411582	-5.7237	0.682263	0.740679
Neutrophils	CUL3	-0.05898	7.198802	-0.82486	0.411673	-6.36016	0.54605	0.595966
Neutrophils	WNT5B	-0.34957	1.562043	-0.82462	0.411809	-5.0349	0.641214	0.697303
Neutrophils	ITIH3	-0.27024	3.636116	-0.82452	0.411865	-5.53785	0.604369	0.658246
Neutrophils	FBXO33	-0.09029	5.984659	-0.82447	0.411893	-6.1219	0.565278	0.616588
Neutrophils	GM26787	-0.45147	1.300668	-0.82428	0.411996	-5.00213	0.646088	0.702496
Neutrophils	GM550	-0.44	0.859238	-0.82384	0.41225	-4.94022	0.654596	0.711364
Neutrophils	TPST2	0.098096	5.593782	0.823181	0.41262	-6.04375	0.572366	0.623698
Neutrophils	PHKG1	-0.47126	1.107052	-0.82291	0.412774	-4.96129	0.650489	0.706755

Neutrophils	RBM19	-0.32912	2.925866	-0.82289	0.412785	-5.02622	0.617581	0.671903
Neutrophils	SLC36A1	-0.21319	2.851189	-0.82286	0.412802	-5.24096	0.618898	0.673301
Neutrophils	IFI204	0.171327	4.390085	0.822584	0.412958	-6.08311	0.592354	0.645187
Neutrophils	SRI	0.080648	6.74315	0.822542	0.412982	-6.32321	0.554002	0.60429
Neutrophils	FAM25C	-0.42646	1.148741	-0.82239	0.413069	-5.04629	0.649738	0.706123
Neutrophils	NAA10	-0.17349	5.084078	-0.82236	0.413085	-5.61313	0.580763	0.632911
Neutrophils	UBXN6	0.143826	4.786672	0.822256	0.413143	-5.72573	0.585701	0.638176
Neutrophils	PABPC1	0.098591	10.47512	0.822224	0.413161	-6.71723	0.498451	0.544745
Neutrophils	RTL8A	-0.26381	3.632481	-0.82178	0.413411	-5.27534	0.605511	0.659089
Neutrophils	TTBK2	-0.32295	1.314702	-0.82158	0.413526	-5.02583	0.646911	0.703049
Neutrophils	LRMDA	0.130784	7.760594	0.821432	0.41361	-6.62548	0.538439	0.587629
Neutrophils	UQCRB	-0.08666	8.047525	-0.82131	0.413681	-6.41991	0.534077	0.582966
Neutrophils	INTS7	0.111723	5.677826	0.821303	0.413683	-5.72912	0.57125	0.62272
Neutrophils	TFDP2	-0.20903	5.591554	-0.82124	0.413719	-5.62546	0.572653	0.624217
Neutrophils	TTC7B	-0.09976	5.391283	-0.82124	0.41372	-6.05214	0.575925	0.627707
Neutrophils	MAN2B1	0.107878	6.851978	0.821063	0.413819	-6.30125	0.552557	0.602703
Neutrophils	CCT4	-0.09287	6.487167	-0.82051	0.414131	-5.99243	0.558656	0.609065
Neutrophils	ATP5J2	-0.09126	7.723844	-0.82041	0.414191	-6.32825	0.53939	0.588509
Neutrophils	MVB12B	-0.11895	4.074675	-0.82016	0.414333	-5.93705	0.598481	0.651502
Neutrophils	ARAP2	-0.19733	5.924266	-0.81985	0.414509	-5.71148	0.567948	0.618923
Neutrophils	DCUN1D1	0.094842	5.787878	0.819516	0.414696	-6.08523	0.570211	0.621399
Neutrophils	CTDP1	-0.15972	4.660739	-0.81946	0.414728	-5.48249	0.588799	0.641224
Neutrophils	TBC1D10C	0.142989	4.736281	0.819257	0.414843	-5.90603	0.587534	0.639939
Neutrophils	TMEM126/	-0.11715	5.857631	-0.81918	0.414889	-5.76757	0.569081	0.620289
Neutrophils	SHLD1	0.200683	3.33904	0.819098	0.414933	-5.39357	0.6114	0.665338
Neutrophils	HIST2H2BE	-0.48445	0.814865	-0.81909	0.414937	-4.88142	0.657072	0.71371
Neutrophils	ZFP273	-0.47244	0.440289	-0.81901	0.414983	-4.86476	0.664139	0.721187
Neutrophils	AKT1S1	-0.19175	3.589237	-0.81883	0.415086	-5.35645	0.607053	0.660807
Neutrophils	NEK3	0.435035	1.761696	0.818784	0.415111	-5.01617	0.639545	0.695267
Neutrophils	AHCY	0.293771	3.495963	0.818754	0.415128	-5.20935	0.60867	0.662524
Neutrophils	PLCE1	0.48657	1.279254	0.818658	0.415183	-5.06209	0.648416	0.70466
Neutrophils	LRFN1	-0.42018	1.097146	-0.81819	0.415451	-5.04875	0.652054	0.708341
Neutrophils	VPS41	0.132737	5.101022	0.818085	0.415508	-5.67063	0.581694	0.633703
Neutrophils	GALNS	-0.14168	3.757313	-0.81806	0.415523	-5.68321	0.60439	0.657861
Neutrophils	TMPRSS6	-0.41175	1.467899	-0.81795	0.415584	-5.06787	0.645187	0.70112
Neutrophils	4933406I1	-0.24483	4.543564	-0.81782	0.415661	-5.58781	0.591	0.643625
Neutrophils	DPF2	0.121767	5.127706	0.817761	0.415692	-5.73375	0.581252	0.633243
Neutrophils	STAT3	0.086637	7.950149	0.817322	0.415942	-6.47199	0.5366	0.585489
Neutrophils	DYNLT3	-0.12513	4.662522	-0.8173	0.415957	-5.69022	0.589147	0.641603
Neutrophils	GM30025	0.370844	1.597885	0.817199	0.416011	-5.03639	0.642956	0.698707
Neutrophils	2510046G1	0.289799	2.620896	0.817135	0.416048	-5.0986	0.624454	0.679112
Neutrophils	CACFD1	0.229448	3.174111	0.817074	0.416082	-5.20341	0.614676	0.66874
Neutrophils	TESK2	0.141369	4.072329	0.81681	0.416233	-5.90675	0.599139	0.652231
Neutrophils	KLRG1	-0.49831	-1.146	-0.81678	0.416251	-4.86195	0.695372	0.754001
Neutrophils	FBXL12	0.188048	4.348122	0.816767	0.416257	-5.43675	0.594449	0.647243
Neutrophils	ING1	0.111777	5.135774	0.816633	0.416333	-5.86662	0.581291	0.633255
Neutrophils	SH2B3	-0.11168	5.315208	-0.81615	0.41661	-5.90504	0.578482	0.630185

Neutrophils	SLC39A4	-0.2761	1.149584	-0.81614	0.416612	-5.24272	0.651442	0.707617
Neutrophils	ICMT	-0.31124	2.429223	-0.81608	0.416649	-5.10184	0.628075	0.682907
Neutrophils	RPGR	-0.35693	1.599467	-0.81592	0.416739	-4.9898	0.643128	0.698887
Neutrophils	MED28	0.095778	6.023126	0.815895	0.416753	-5.9626	0.566951	0.617941
Neutrophils	C2CD2L	0.229408	2.991873	0.815833	0.416788	-5.29538	0.618073	0.672343
Neutrophils	VEZF1	-0.08885	6.00178	-0.81542	0.417022	-6.00976	0.567474	0.618389
Neutrophils	LYNX1	-0.52715	0.898277	-0.81539	0.417041	-4.90466	0.656341	0.712731
Neutrophils	XPO4	0.137692	5.623363	0.815299	0.417092	-5.6938	0.573614	0.624957
Neutrophils	RAB1B	-0.106	5.58788	-0.81461	0.417483	-5.95413	0.574473	0.625752
Neutrophils	1110059G1	0.179814	3.928256	0.8146	0.41749	-5.37533	0.602273	0.65534
Neutrophils	ASB5	-0.44084	0.515697	-0.81447	0.417567	-4.92968	0.663875	0.72057
Neutrophils	DOCK6	-0.28476	1.914885	-0.81444	0.417582	-5.20114	0.637875	0.693094
Neutrophils	POLR1D	0.081589	6.995878	0.814436	0.417584	-6.12903	0.55194	0.601698
Neutrophils	DDX39	0.142482	6.372696	0.814269	0.417679	-5.92926	0.561847	0.612265
Neutrophils	SPG20	-0.27548	2.615271	-0.81403	0.417815	-5.11789	0.625425	0.679838
Neutrophils	B4GALT5	0.116187	5.975954	0.813663	0.418024	-6.16855	0.568531	0.619257
Neutrophils	GM28050	-0.46523	0.410517	-0.81347	0.418133	-4.90113	0.666382	0.72295
Neutrophils	FAM57A	0.422247	1.118395	0.813165	0.418308	-4.9861	0.653062	0.708971
Neutrophils	CUL4A	-0.11606	5.002429	-0.81301	0.418396	-5.74843	0.584586	0.636416
Neutrophils	DAPK3	-0.15976	4.42231	-0.81294	0.418436	-5.47791	0.594323	0.646777
Neutrophils	CERS2	0.10367	5.262709	0.812933	0.41844	-5.9384	0.580271	0.631821
Neutrophils	ZBTB46	-0.23679	3.021217	-0.81293	0.418442	-5.38567	0.61854	0.672494
Neutrophils	ARID1A	-0.07865	6.658177	-0.8127	0.418571	-6.14873	0.557699	0.607801
Neutrophils	HNRNPA0	-0.07727	7.214278	-0.81267	0.418589	-6.16869	0.548963	0.598464
Neutrophils	PPP4R3A	0.072801	6.59174	0.812643	0.418606	-6.03618	0.558753	0.608926
Neutrophils	KCNG3	0.510637	0.517714	0.812456	0.418712	-4.90552	0.664439	0.721109
Neutrophils	H2AFY	0.078489	7.697828	0.812284	0.41881	-6.27045	0.541599	0.590572
Neutrophils	GM45871	-0.40222	0.985003	-0.81216	0.418883	-4.97772	0.655719	0.711927
Neutrophils	ZDHHC24	0.411919	1.089708	0.812054	0.418942	-4.9917	0.653762	0.709875
Neutrophils	GSTK1	0.255103	2.449061	0.811889	0.419036	-5.24257	0.628921	0.683656
Neutrophils	TRP53INP1	0.134659	5.187354	0.811802	0.419086	-5.94485	0.581702	0.633511
Neutrophils	SLC8A1	-0.18957	6.330185	-0.81155	0.41923	-6.20124	0.563148	0.613684
Neutrophils	FUOM	-0.33436	2.61981	-0.81154	0.419237	-5.22432	0.625917	0.68045
Neutrophils	UTP25	-0.29851	2.617836	-0.81138	0.419326	-5.0767	0.626	0.680488
Neutrophils	COL11A2	0.48566	0.72136	0.811085	0.419495	-4.94259	0.660869	0.717404
Neutrophils	DDX17	-0.09081	6.357267	-0.81103	0.419528	-6.12471	0.562793	0.613303
Neutrophils	GRPEL1	0.125856	5.877727	0.810957	0.419568	-5.91313	0.570518	0.621555
Neutrophils	TRIP4	0.130766	4.852518	0.810875	0.419615	-5.94941	0.587406	0.639583
Neutrophils	FAM214B	0.21854	3.11159	0.810829	0.419641	-5.4811	0.617284	0.671337
Neutrophils	CCND2	-0.18011	5.400515	-0.8102	0.420003	-5.74998	0.57872	0.630094
Neutrophils	NSD3	0.071122	8.849458	0.810046	0.420088	-6.58606	0.524755	0.572459
Neutrophils	ILK	0.106789	5.628263	0.809868	0.42019	-6.01556	0.574982	0.626214
Neutrophils	GM31728	0.454279	-0.17439	0.809836	0.420209	-4.97413	0.67847	0.73589
Neutrophils	INSIG2	-0.15887	4.405764	-0.80981	0.420224	-5.59346	0.595344	0.647908
Neutrophils	ABCD2	-0.13022	2.633823	-0.80957	0.420358	-5.93897	0.626307	0.680813
Neutrophils	PTPN9	-0.11513	5.706865	-0.80917	0.420589	-5.94292	0.574039	0.625144
Neutrophils	CLCN7	0.251604	3.608444	0.808683	0.420868	-5.32498	0.609635	0.662824

Neutrophils	ALPK3	-0.52362	-0.11417	-0.80866	0.420882	-4.91478	0.677981	0.735081
Neutrophils	PRDX5	0.107372	7.635313	0.808474	0.420988	-6.73217	0.543663	0.592581
Neutrophils	PEX6	0.191449	4.039128	0.808469	0.420991	-5.49223	0.602197	0.654977
Neutrophils	SNIP1	0.190504	3.633076	0.808238	0.421123	-5.2845	0.609315	0.662523
Neutrophils	PLXNA1	0.495299	0.899006	0.807898	0.421318	-4.93567	0.658828	0.714945
Neutrophils	CPNE1	0.081443	6.038278	0.807855	0.421342	-6.11207	0.569045	0.619717
Neutrophils	SPAG1	0.383859	1.4147	0.807803	0.421372	-5.03859	0.649197	0.704805
Neutrophils	ADSL	0.206333	4.033205	0.807607	0.421484	-5.31592	0.602468	0.655373
Neutrophils	PTPN2	-0.07241	6.833794	-0.80757	0.421508	-6.24522	0.556327	0.606217
Neutrophils	AASDHPPT	0.189867	3.841771	0.807518	0.421535	-5.35174	0.605764	0.658875
Neutrophils	AGFG2	-0.16624	4.605897	-0.80745	0.421572	-5.54995	0.592718	0.645009
Neutrophils	BCAR1	0.353328	0.776645	0.807018	0.421822	-5.10469	0.661239	0.71753
Neutrophils	SLC35C2	0.116521	4.846147	0.80701	0.421827	-5.69778	0.58877	0.64074
Neutrophils	SMAP2	-0.06776	6.821529	-0.80696	0.421855	-6.34035	0.556609	0.606468
Neutrophils	ANO10	0.14062	3.16746	0.806858	0.421914	-5.80053	0.617621	0.671454
Neutrophils	RRAGA	0.13559	5.020687	0.806508	0.422114	-5.71157	0.585852	0.637791
Neutrophils	GM47819	0.50588	-0.22199	0.806501	0.422118	-4.976	0.680371	0.737882
Neutrophils	SYVN1	-0.22627	4.228615	-0.80637	0.422192	-5.44899	0.599217	0.65206
Neutrophils	TNFRSF26	0.240074	2.617995	0.806338	0.422211	-5.4428	0.627378	0.681949
Neutrophils	ZFP606	0.286645	2.487995	0.806314	0.422225	-5.06377	0.629709	0.684419
Neutrophils	E2F3	-0.14229	5.460693	-0.80628	0.422244	-5.9064	0.578561	0.630076
Neutrophils	SERPINA7	-0.45203	0.531403	-0.80619	0.422299	-4.98615	0.665887	0.722669
Neutrophils	TRAT1	-0.55673	-0.05747	-0.80613	0.422332	-4.9611	0.677181	0.734593
Neutrophils	PLD3	-0.21106	4.413779	-0.80573	0.422561	-5.49702	0.596307	0.648802
Neutrophils	WNT4	-0.28686	1.526488	-0.80561	0.422632	-5.18427	0.647513	0.703065
Neutrophils	CASC3	-0.13925	5.292913	-0.80519	0.422869	-5.68081	0.581813	0.633273
Neutrophils	ERMAP	0.503606	0.529243	0.805021	0.422967	-4.93237	0.666481	0.723032
Neutrophils	ANXA1	-0.08435	6.030034	-0.80501	0.422972	-6.87936	0.569741	0.620441
Neutrophils	SLC46A1	-0.56445	0.761766	-0.80484	0.423071	-4.92981	0.662102	0.718466
Neutrophils	SRR	0.270083	2.599008	0.804754	0.42312	-5.11869	0.62827	0.682724
Neutrophils	GM12840	0.27654	3.766389	0.804644	0.423183	-5.65593	0.607698	0.660938
Neutrophils	NAAA	0.242374	3.389339	0.804539	0.423244	-5.38283	0.614265	0.66791
Neutrophils	METTL26	-0.16872	4.639923	-0.80441	0.423315	-5.6374	0.592759	0.645085
Neutrophils	LATS2	0.089967	5.889771	0.804371	0.42334	-6.08349	0.572047	0.623029
Neutrophils	USP22	0.128532	4.389395	0.80422	0.423427	-5.63657	0.597044	0.649619
Neutrophils	RREB1	0.126776	6.814971	0.803951	0.423582	-6.20418	0.557329	0.6072
Neutrophils	TGFBR1	0.113019	6.24383	0.803902	0.42361	-6.1096	0.566445	0.616942
Neutrophils	SSBP1	-0.11804	5.592236	-0.80373	0.423707	-5.79674	0.577092	0.628279
Neutrophils	PDLIM4	-0.37694	2.040162	-0.80312	0.424057	-5.20202	0.638982	0.693869
Neutrophils	GDPD1	0.449225	1.600801	0.803061	0.424093	-4.98477	0.647046	0.70241
Neutrophils	XLR4A	0.452091	0.802973	0.802891	0.424191	-4.90141	0.662003	0.718189
Neutrophils	JAKMIP1	-0.2998	3.981458	-0.80281	0.424238	-5.21397	0.604604	0.657471
Neutrophils	CIAO2A	0.099807	6.679979	0.802399	0.424474	-6.11401	0.560152	0.610059
Neutrophils	GM10642	-0.46672	0.270003	-0.80209	0.424654	-4.937	0.672631	0.729226
Neutrophils	RGCC	0.105208	5.613883	0.801835	0.424799	-6.33841	0.57762	0.628557
Neutrophils	CAPN1	0.114337	4.246316	0.801696	0.424878	-6.00223	0.600552	0.652994
Neutrophils	CENPV	-0.34503	3.40694	-0.80164	0.424912	-5.19448	0.615094	0.66843

Neutrophils	POLR2L	0.139781	5.414671	0.801596	0.424936	-5.7192	0.580903	0.632111
Neutrophils	KIF21A	-0.42194	0.515306	-0.80122	0.42515	-4.99069	0.66817	0.724554
Neutrophils	TBL1XR1	-0.07601	6.669065	-0.80122	0.425151	-6.25413	0.560684	0.610548
Neutrophils	TNFAIP3	-0.12177	6.763033	-0.80095	0.425309	-6.21504	0.55926	0.609009
Neutrophils	SERPINA3G	0.588974	3.597185	0.800927	0.425321	-5.44589	0.611987	0.665118
Neutrophils	FAM78B	0.379347	2.115372	0.800812	0.425387	-5.05018	0.638424	0.693153
Neutrophils	E230013L2	-0.43787	-0.72305	-0.8007	0.425452	-4.9056	0.692339	0.750018
Neutrophils	STARD9	-0.18557	3.529605	-0.80037	0.425642	-5.55251	0.61335	0.666541
Neutrophils	TCN2	0.109157	4.791081	0.800094	0.425801	-6.18397	0.591695	0.643665
Neutrophils	GM10851	0.13488	4.366594	0.800087	0.425805	-5.83465	0.598892	0.651316
Neutrophils	SETD1A	-0.15643	4.19796	-0.80003	0.425838	-5.45364	0.601776	0.654396
Neutrophils	B4GALT1	-0.07992	7.137524	-0.8	0.425854	-6.26279	0.55351	0.602977
Neutrophils	RBM48	-0.23779	3.037834	-0.79987	0.425928	-5.17514	0.622015	0.675886
Neutrophils	PSAP	0.110361	9.249596	0.799702	0.426027	-6.54626	0.52136	0.568588
Neutrophils	KLHL8	-0.34846	2.402928	-0.7997	0.42603	-5.126	0.633385	0.687946
Neutrophils	H2-Q10	-0.15115	4.038149	-0.7994	0.426203	-5.95769	0.60469	0.657459
Neutrophils	CLCN6	-0.2385	3.649755	-0.79918	0.426327	-5.30913	0.611516	0.664642
Neutrophils	GM50012	-0.36919	1.643382	-0.79904	0.42641	-4.94357	0.64757	0.702789
Neutrophils	PHF11D	-0.48156	1.34229	-0.79873	0.426588	-5.05649	0.653161	0.708751
Neutrophils	SLCO2A1	-0.29537	2.568296	-0.79872	0.426591	-5.54906	0.630698	0.685
Neutrophils	TMEM79	0.422488	0.778123	0.798611	0.426656	-4.95898	0.663769	0.719947
Neutrophils	SKA3	-0.26287	2.738324	-0.7986	0.426664	-5.23443	0.627646	0.681768
Neutrophils	R74862	-0.40422	1.054776	-0.79847	0.426735	-5.05949	0.658545	0.714436
Neutrophils	ARFGAP2	-0.13196	4.901097	-0.79837	0.426795	-5.58031	0.590124	0.641942
Neutrophils	CYB5RL	0.454879	0.380261	0.798345	0.42681	-4.87751	0.671354	0.727946
Neutrophils	GM28375	-0.24184	3.206287	-0.79799	0.427016	-5.13084	0.619539	0.67301
Neutrophils	GM36161	0.229374	0.563057	0.797845	0.427098	-5.5779	0.668099	0.724377
Neutrophils	ST13	0.080802	6.987135	0.797721	0.42717	-6.12696	0.556341	0.605846
Neutrophils	SCAMP3	0.131798	4.819715	0.797676	0.427196	-5.62125	0.591706	0.643529
Neutrophils	AFDN	-0.18142	3.267689	-0.7972	0.427468	-5.50463	0.618773	0.672041
Neutrophils	ITGA2B	-0.38916	0.938407	-0.79705	0.427559	-4.97559	0.661358	0.717031
Neutrophils	LRWD1	-0.15389	3.781422	-0.79675	0.427729	-5.47849	0.60983	0.662573
Neutrophils	PCK1	-0.27937	4.182878	-0.79674	0.427736	-5.7346	0.602893	0.655211
Neutrophils	CGRRF1	0.155043	3.907331	0.796737	0.427738	-5.5154	0.607645	0.660255
Neutrophils	TRIM17	0.32311	2.197819	0.796576	0.427831	-5.06853	0.638047	0.692453
Neutrophils	ATPIF1	0.096041	7.745006	0.79649	0.427881	-6.36002	0.544861	0.593419
Neutrophils	C1D	0.084179	5.843961	0.796383	0.427943	-6.00482	0.575089	0.62567
Neutrophils	CRYL1	-0.24303	3.73595	-0.79608	0.428115	-5.23804	0.610711	0.663521
Neutrophils	STRAP	0.108675	6.174275	0.79608	0.428118	-5.98039	0.569757	0.619976
Neutrophils	CLIP2	-0.1897	2.886513	-0.79602	0.42815	-5.34841	0.625685	0.67939
Neutrophils	GBP7	0.30176	4.443583	0.795867	0.428241	-5.6371	0.598565	0.650627
Neutrophils	NARS	0.103046	6.298026	0.795709	0.428333	-5.99378	0.567839	0.617927
Neutrophils	2410002F2	0.223771	3.766412	0.795581	0.428406	-5.23987	0.610269	0.663076
Neutrophils	SPOPL	0.157051	4.42179	0.795516	0.428444	-5.52837	0.598977	0.651124
Neutrophils	MILR1	0.115003	4.330073	0.795318	0.428558	-5.84173	0.600623	0.652849
Neutrophils	SLC4A1AP	0.147305	4.278134	0.795003	0.428741	-5.54265	0.601571	0.653915
Neutrophils	RNF146	0.105224	5.30614	0.794969	0.42876	-5.9477	0.584217	0.635466

Neutrophils	HBQ1B	0.539845	-0.16343	0.794945	0.428774	-4.91851	0.682864	0.739854
Neutrophils	ECH1	-0.11118	5.784916	-0.79482	0.428845	-6.02804	0.576332	0.627123
Neutrophils	ARHGEF15	-0.36799	0.580885	-0.79466	0.42894	-5.12652	0.668577	0.724891
Neutrophils	TNFRSF12A	0.351838	1.479733	0.794363	0.429111	-5.05461	0.651647	0.70705
Neutrophils	FOXO4	-0.28444	2.699869	-0.79435	0.429117	-5.15633	0.629344	0.683465
Neutrophils	AASS	-0.37435	1.208483	-0.79424	0.429181	-5.11754	0.656714	0.71243
Neutrophils	PSENEN	-0.06597	6.713106	-0.79411	0.42926	-6.26645	0.561385	0.611252
Neutrophils	TSPAN2	0.162097	3.159011	0.794071	0.42928	-5.55201	0.621155	0.674818
Neutrophils	LONRF1	0.152093	3.091293	0.794042	0.429297	-5.71055	0.622356	0.676091
Neutrophils	NIFK	0.178645	4.620947	0.793728	0.429479	-5.46735	0.595865	0.647966
Neutrophils	POLR2G	-0.10108	5.51542	-0.79368	0.429504	-5.89357	0.580886	0.63203
Neutrophils	RWDD4A	-0.20628	3.983066	-0.7936	0.429553	-5.33143	0.606793	0.659607
Neutrophils	SDE2	0.089458	6.013145	0.793542	0.429586	-6.24124	0.572721	0.623363
Neutrophils	POC1A	-0.23261	3.229083	-0.79347	0.429631	-5.31013	0.619977	0.673614
Neutrophils	ITGB1	0.085152	6.871704	0.793299	0.429727	-6.19754	0.558922	0.608753
Neutrophils	BRAP	-0.11314	4.924367	-0.79311	0.429837	-5.80021	0.590743	0.642733
Neutrophils	FOSB	0.127321	6.636378	0.793014	0.429893	-6.3306	0.56267	0.612828
Neutrophils	ZFP518B	0.399797	0.418092	0.792934	0.429939	-4.88923	0.671778	0.72859
Neutrophils	SFR1	-0.08672	6.44096	-0.79277	0.430033	-6.07414	0.565803	0.616238
Neutrophils	EXTL3	-0.2705	3.56619	-0.79271	0.430068	-5.19506	0.61405	0.667582
Neutrophils	SDHA	-0.09308	5.691569	-0.79263	0.430117	-5.92943	0.577986	0.629246
Neutrophils	HEXDC	0.435335	1.86348	0.792506	0.430187	-5.0125	0.644616	0.699983
Neutrophils	COQ2	0.188382	4.467496	0.792475	0.430205	-5.41063	0.598479	0.651066
Neutrophils	ATP5L	0.064798	8.67785	0.792292	0.430311	-6.55066	0.531019	0.579126
Neutrophils	IRF3	-0.22083	3.786081	-0.79228	0.43032	-5.2736	0.610213	0.663607
Neutrophils	E430024P1	0.381882	0.53335	0.792211	0.430358	-5.04857	0.66957	0.726456
Neutrophils	GGTA1	-0.14642	5.365487	-0.79206	0.430447	-5.84681	0.583372	0.635138
Neutrophils	DAG1	-0.1809	4.867236	-0.79206	0.430447	-5.54672	0.591705	0.64401
Neutrophils	GPIHBP1	-0.14222	4.095185	-0.79168	0.430669	-5.97372	0.604964	0.658078
Neutrophils	2310061I0	-0.18399	3.521012	-0.79154	0.430748	-5.36831	0.614946	0.668713
Neutrophils	9130230N(	0.391527	0.640779	0.791371	0.430845	-5.06742	0.667633	0.724519
Neutrophils	LFNG	0.136931	4.413378	0.791213	0.430937	-5.72973	0.599504	0.652419
Neutrophils	TCP1	-0.1191	6.67677	-0.79119	0.430948	-5.98878	0.562121	0.612577
Neutrophils	USF3	-0.11774	4.977969	-0.79113	0.430984	-5.88885	0.589943	0.642245
Neutrophils	SLC25A22	-0.38939	2.495595	-0.79112	0.430993	-5.06692	0.633199	0.688181
Neutrophils	RNPEPL1	0.152649	4.75379	0.791106	0.430999	-5.69974	0.59372	0.646266
Neutrophils	PSMB4	0.096662	6.172049	0.790931	0.431101	-6.02195	0.57024	0.62128
Neutrophils	CCDC88B	0.103903	3.695402	0.790928	0.431102	-5.99636	0.611897	0.665625
Neutrophils	DDX49	-0.17784	4.208396	-0.79053	0.431331	-5.33388	0.60318	0.65626
Neutrophils	SLC25A53	0.24986	3.336328	0.790525	0.431336	-5.16035	0.618362	0.672382
Neutrophils	OTUD6B	0.132612	4.301102	0.790331	0.431449	-5.55083	0.601666	0.654658
Neutrophils	RC3H1	0.078343	6.887392	0.78985	0.431729	-6.21633	0.559228	0.609342
Neutrophils	MICALL1	-0.2104	2.97359	-0.78982	0.431748	-5.27207	0.625136	0.679484
Neutrophils	CHST8	-0.59223	-0.80735	-0.78964	0.431848	-4.88346	0.696467	0.7548
Neutrophils	SMIM8	0.164149	4.587829	0.789466	0.431952	-5.54523	0.597102	0.649723
Neutrophils	ZBTB16	-0.30133	2.808614	-0.78942	0.43198	-5.39481	0.628168	0.682704
Neutrophils	6530402F1	0.278446	-0.17553	0.789024	0.432209	-5.22523	0.684305	0.741844

Neutrophils	HIST1H2BN	-0.38369	2.139918	-0.78892	0.432268	-5.11839	0.640524	0.69564
Neutrophils	4933421O1	0.30958	2.357702	0.788676	0.432411	-5.12589	0.636607	0.691502
Neutrophils	APLF	0.34454	2.05909	0.788662	0.432419	-5.05218	0.642055	0.697268
Neutrophils	GH	-0.43324	-0.76502	-0.78844	0.43255	-4.86838	0.696039	0.754249
Neutrophils	NEDD4L	0.085172	6.886893	0.788401	0.432571	-6.18646	0.559623	0.609679
Neutrophils	SC5D	0.131239	4.248539	0.788101	0.432745	-5.81426	0.603377	0.656232
Neutrophils	CNTD1	0.387608	0.620711	0.788031	0.432786	-5.01427	0.66918	0.725892
Neutrophils	EHBP1	0.269608	3.52414	0.787916	0.432853	-5.33921	0.61597	0.669597
Neutrophils	MORN3	0.291777	1.323497	0.787749	0.432951	-5.17689	0.65589	0.711858
Neutrophils	MBD6	-0.13901	3.632499	-0.78772	0.432965	-5.65719	0.61407	0.667581
Neutrophils	ZFP91	-0.08132	6.894237	-0.7875	0.433098	-6.12035	0.559736	0.609675
Neutrophils	MON1B	-0.23952	1.881481	-0.7868	0.433501	-5.20314	0.64615	0.701263
Neutrophils	OARD1	0.127445	4.902059	0.786599	0.43362	-5.79479	0.592893	0.644782
Neutrophils	EIF3E	-0.08317	7.095265	-0.78644	0.433712	-6.11577	0.557054	0.606637
Neutrophils	KCNK10	-0.53783	0.557275	-0.78639	0.433739	-4.8858	0.671122	0.727663
Neutrophils	PFDN5	0.060408	7.413663	0.786322	0.433781	-6.35632	0.552043	0.601294
Neutrophils	MYBL1	-0.30285	1.840587	-0.78615	0.433882	-5.15875	0.647038	0.702201
Neutrophils	TMX3	0.084375	5.505888	0.786032	0.43395	-6.07576	0.582862	0.63412
Neutrophils	LDHC	0.311295	0.610466	0.785866	0.434047	-5.32692	0.670237	0.726684
Neutrophils	AU040320	-0.1452	4.589216	-0.78569	0.434148	-5.66614	0.598318	0.650617
Neutrophils	ZFP760	0.354961	1.032725	0.785674	0.434159	-4.98653	0.662203	0.718271
Neutrophils	SERGEF	-0.26542	4.018452	-0.78514	0.434469	-5.17678	0.608367	0.661141
Neutrophils	SCAF1	0.146253	4.690272	0.784977	0.434565	-5.56649	0.596834	0.648991
Neutrophils	SNRK	-0.09077	4.865356	-0.78492	0.434597	-6.13083	0.593866	0.645836
Neutrophils	DUSP10	-0.26925	4.392133	-0.78491	0.434606	-5.31887	0.601924	0.654401
Neutrophils	SLC15A2	-0.61382	4.597147	-0.78488	0.434622	-5.80829	0.598419	0.650677
Neutrophils	CENPI	0.273778	2.997583	0.784257	0.434985	-5.29605	0.626773	0.680431
Neutrophils	AGPS	-0.06936	6.813953	-0.78416	0.435044	-6.34409	0.562248	0.611872
Neutrophils	TMEM218	-0.31569	1.874833	-0.784	0.435134	-5.00687	0.647226	0.702099
Neutrophils	BCL7C	-0.11954	5.355918	-0.78356	0.43539	-5.6904	0.586352	0.637356
Neutrophils	NECAP2	0.126655	5.345804	0.783402	0.435485	-5.74322	0.586569	0.637571
Neutrophils	CYP2D22	-0.36046	0.864397	-0.78263	0.435937	-5.06504	0.667046	0.722461
Neutrophils	STOML1	-0.3483	2.251865	-0.78262	0.435939	-5.03053	0.641139	0.695131
Neutrophils	SKP2	-0.26748	3.310393	-0.78243	0.436052	-5.19702	0.622067	0.675042
Neutrophils	METTL14	-0.26891	3.09472	-0.78238	0.436085	-5.16151	0.625905	0.679135
Neutrophils	RCBTB1	0.218032	3.826201	0.782325	0.436114	-5.27574	0.612986	0.665451
Neutrophils	BBS2	0.454087	0.404044	0.782189	0.436193	-4.90981	0.675888	0.731891
Neutrophils	NUMB	-0.06915	6.956562	-0.7821	0.436248	-6.50272	0.560764	0.609955
Neutrophils	2310015A1	-0.24605	2.091938	-0.78201	0.436299	-5.26203	0.644086	0.69839
Neutrophils	SLC25A32	-0.21832	3.120627	-0.78185	0.436394	-5.26366	0.625509	0.678719
Neutrophils	MRPL4	0.138766	5.176328	0.781731	0.436461	-5.64746	0.589934	0.640992
Neutrophils	GM50218	-0.47316	0.559001	-0.78153	0.43658	-4.95193	0.673031	0.728924
Neutrophils	SH3TC1	0.219535	2.768133	0.781436	0.436633	-5.31861	0.631897	0.685571
Neutrophils	GM50163	-0.44094	0.458993	-0.78133	0.436694	-4.92626	0.674956	0.731057
Neutrophils	MRPL15	0.124039	5.337746	0.781267	0.436732	-5.74352	0.587281	0.638325
Neutrophils	DGAT2	-0.08873	3.862389	-0.78111	0.436824	-6.25578	0.61253	0.665135
Neutrophils	GM38115	-0.18953	3.861728	-0.78066	0.437086	-5.42304	0.612827	0.665345

Neutrophils	GM28875	0.133096	4.612323	0.780545	0.437155	-5.89438	0.599875	0.651667
Neutrophils	GMFB	0.102556	5.451593	0.780238	0.437335	-5.89875	0.585831	0.636686
Neutrophils	GPC4	-0.35877	1.1587	-0.78012	0.437404	-5.09508	0.66211	0.717465
Neutrophils	ADCY7	-0.09321	5.583087	-0.78005	0.437443	-6.06615	0.583643	0.634368
Neutrophils	QK	-0.05749	8.142329	-0.77994	0.43751	-6.51914	0.542735	0.590758
Neutrophils	RRBP1	0.073126	7.951353	0.779895	0.437535	-6.38522	0.545681	0.593906
Neutrophils	RHOJ	0.236528	1.992484	0.779781	0.437602	-5.44483	0.646547	0.701078
Neutrophils	GPKOW	0.124762	4.488998	0.779648	0.43768	-5.59806	0.602144	0.654081
Neutrophils	RFXAP	-0.12656	4.179927	-0.77937	0.437844	-5.62163	0.607477	0.659785
Neutrophils	TMEM143	0.333221	1.491704	0.779329	0.437867	-5.01167	0.655894	0.711015
Neutrophils	BCAS2	-0.08243	6.380106	-0.77912	0.437989	-5.99203	0.57061	0.620656
Neutrophils	IGKV1-110	-0.48888	0.050225	-0.77912	0.437992	-4.88084	0.683459	0.740143
Neutrophils	PDP2	-0.25065	3.724878	-0.77911	0.437996	-5.1525	0.615407	0.668272
Neutrophils	GM42701	-0.3091	0.844253	-0.77904	0.438037	-5.1843	0.668133	0.724029
Neutrophils	C130013H(	-0.39522	0.498295	-0.77883	0.438158	-4.8947	0.674864	0.731024
Neutrophils	LY75	0.149344	3.332572	0.778571	0.438311	-6.1062	0.622553	0.675712
Neutrophils	GM42937	0.486156	0.198102	0.778205	0.438526	-4.93243	0.681064	0.737319
Neutrophils	COMT	-0.13893	5.36738	-0.77752	0.438925	-5.71142	0.588123	0.638888
Neutrophils	GM5547	-0.41927	1.560402	-0.7773	0.439058	-4.91975	0.655548	0.710361
Neutrophils	AHCYL1	-0.09597	5.179051	-0.77725	0.439084	-5.81111	0.591284	0.642323
Neutrophils	GBP3	0.403904	3.228626	0.77725	0.439086	-5.18647	0.625072	0.678157
Neutrophils	TAP2	0.160855	5.110651	0.777168	0.439134	-5.84984	0.592436	0.643575
Neutrophils	SNAPC5	0.107601	4.755447	0.777056	0.4392	-5.89472	0.598466	0.649992
Neutrophils	NBR1	0.104084	5.455137	0.776955	0.439259	-5.85453	0.586667	0.637473
Neutrophils	AMIGO2	-0.47332	0.934211	-0.77646	0.439548	-5.00877	0.667428	0.723092
Neutrophils	GTF2H5	-0.08654	6.547345	-0.7764	0.439585	-6.09266	0.568764	0.61854
Neutrophils	SF3A1	-0.14606	4.851981	-0.77627	0.439663	-5.50979	0.596862	0.648477
Neutrophils	A530064D(	-0.12044	0.81889	-0.77626	0.439667	-5.98847	0.669629	0.725461
Neutrophils	GM16201	-0.24979	1.818935	-0.77613	0.439741	-5.35094	0.650779	0.70563
Neutrophils	BABAM1	-0.10885	5.569892	-0.77606	0.439784	-5.8675	0.58479	0.635716
Neutrophils	KCNAB2	-0.13528	4.287693	-0.77601	0.439812	-5.79096	0.606532	0.658826
Neutrophils	CUL1	0.078379	6.562305	0.775924	0.439865	-6.09638	0.568523	0.61842
Neutrophils	NAA25	0.277876	3.927641	0.775914	0.43987	-5.20867	0.612787	0.665484
Neutrophils	GUF1	0.231738	2.589786	0.775792	0.439942	-5.15307	0.636618	0.690761
Neutrophils	1700048O(	0.634034	-0.24632	0.775681	0.440007	-4.87042	0.690316	0.747394
Neutrophils	TMEM87A	-0.12955	5.236916	-0.77565	0.440025	-5.74347	0.590357	0.641703
Neutrophils	ADGRE4	-0.24352	3.108019	-0.77561	0.440048	-5.60097	0.627275	0.680885
Neutrophils	TMEM216	0.129076	4.069162	0.775426	0.440157	-5.85994	0.610344	0.663031
Neutrophils	SLC25A27	0.388099	0.260835	0.775249	0.440261	-4.98108	0.680415	0.737163
Neutrophils	NR3C2	-0.37616	1.939637	-0.7752	0.440292	-5.12747	0.648566	0.703594
Neutrophils	MMP19	0.457102	1.213067	0.77506	0.440372	-5.02852	0.66216	0.717991
Neutrophils	RASSF2	0.129197	4.259468	0.775042	0.440383	-5.8281	0.607044	0.659658
Neutrophils	CIR1	-0.11217	5.515333	-0.77495	0.440434	-5.88289	0.585722	0.637005
Neutrophils	GM10053	-0.34956	1.424678	-0.77488	0.440476	-5.10849	0.658171	0.713795
Neutrophils	CHPF	-0.40661	1.242066	-0.77471	0.440581	-4.93448	0.661644	0.717555
Neutrophils	GM43713	-0.39397	2.135076	-0.77465	0.440615	-5.02767	0.644989	0.69998
Neutrophils	CCDC47	0.110122	5.110399	0.774461	0.440724	-5.79427	0.592608	0.64442

Neutrophils	KATNAL1	0.464719	0.973387	0.773882	0.441065	-4.90994	0.666819	0.723138
Neutrophils	SNAI1	0.249072	0.389835	0.773834	0.441093	-5.23934	0.678026	0.734953
Neutrophils	TTC5	-0.15112	4.64029	-0.77374	0.44115	-5.4819	0.600595	0.653061
Neutrophils	ATRNL1	0.087632	6.820202	0.77348	0.441302	-6.4677	0.56449	0.614706
Neutrophils	PEX3	-0.14821	3.770542	-0.77334	0.441384	-5.51259	0.615664	0.669207
Neutrophils	GSTP2	-0.37504	0.861498	-0.77321	0.441458	-4.99598	0.668953	0.725647
Neutrophils	IQGAP2	0.081458	7.075634	0.773039	0.441561	-6.40567	0.560411	0.610484
Neutrophils	IARS	-0.19767	5.056202	-0.77297	0.4416	-5.43013	0.593524	0.64579
Neutrophils	SLC20A2	-0.21629	4.036306	-0.77295	0.441614	-5.35303	0.611018	0.664387
Neutrophils	KIFC1	-0.30139	3.200672	-0.77292	0.441632	-5.42874	0.625749	0.680019
Neutrophils	NRGN	0.435862	3.663682	0.772905	0.44164	-5.18106	0.617542	0.671313
Neutrophils	GM42699	-0.3568	0.727533	-0.77288	0.441653	-5.13618	0.671518	0.72842
Neutrophils	TSIX	-1.99405	1.906429	-0.77283	0.441684	-5.04481	0.64929	0.704944
Neutrophils	LYRM7	-0.38881	1.247217	-0.77282	0.441693	-4.98223	0.661625	0.717979
Neutrophils	DDHD1	-0.16005	6.276227	-0.77281	0.441695	-5.95898	0.573281	0.624223
Neutrophils	4833419F2	0.368468	1.323387	0.772762	0.441724	-5.08534	0.660188	0.716461
Neutrophils	HEXB	-0.09476	5.741415	-0.77275	0.441733	-6.11856	0.582065	0.633587
Neutrophils	PALM	0.12983	4.231998	0.772666	0.441781	-5.89332	0.60762	0.660797
Neutrophils	TCIRG1	0.124347	5.455039	0.77236	0.441961	-5.90327	0.586987	0.638791
Neutrophils	CCDC127	0.147514	4.15296	0.772211	0.442049	-5.47473	0.609198	0.662445
Neutrophils	TRAF3IP3	-0.08609	5.030914	-0.77204	0.44215	-6.11722	0.594179	0.646513
Neutrophils	GM12353	0.305541	1.278675	0.771904	0.44223	-5.16937	0.661284	0.717689
Neutrophils	BHLHB9	0.32129	2.203905	0.771881	0.442243	-5.02789	0.644046	0.699474
Neutrophils	LCAT	-0.29193	2.143834	-0.77146	0.442492	-5.2411	0.645216	0.700826
Neutrophils	TMEM132/	-0.32634	0.400445	-0.77141	0.442518	-5.09535	0.678147	0.735613
Neutrophils	ARRB1	0.093407	3.996665	0.771273	0.442601	-5.98659	0.612004	0.66566
Neutrophils	GM44899	0.339068	0.774696	0.771224	0.442631	-5.05035	0.670937	0.728092
Neutrophils	ZFP280C	-0.20197	4.164903	-0.77116	0.442669	-5.32593	0.609077	0.662595
Neutrophils	GNE	-0.18044	4.467546	-0.77106	0.44273	-5.40509	0.603848	0.657058
Neutrophils	TAGAP	0.207118	4.269177	0.77103	0.442745	-5.69027	0.60727	0.660696
Neutrophils	BRAT1	0.278305	2.183649	0.770972	0.442779	-5.12368	0.644483	0.700168
Neutrophils	SNX7	-0.33858	1.212304	-0.77091	0.442815	-5.08388	0.662605	0.719332
Neutrophils	AIMP2	0.279035	3.206699	0.770736	0.442918	-5.17715	0.626006	0.680577
Neutrophils	GM14326	-0.26734	2.868013	-0.77024	0.443208	-5.18094	0.632413	0.68715
Neutrophils	PTPN3	0.466955	1.040176	0.769098	0.443885	-4.98096	0.667005	0.723508
Neutrophils	NAT10	-0.25405	3.270157	-0.76887	0.444019	-5.20496	0.625877	0.680082
Neutrophils	ID2	-0.11048	6.76154	-0.76882	0.444047	-6.31069	0.566669	0.617095
Neutrophils	ARPP19	0.066056	6.840626	0.768809	0.444056	-6.30666	0.565398	0.615739
Neutrophils	TMEM107	-0.3415	1.616764	-0.76854	0.444213	-5.00121	0.656114	0.7122
Neutrophils	ZFAND5	0.062285	7.203792	0.768523	0.444224	-6.40151	0.559598	0.609643
Neutrophils	PEBP1	0.101038	6.440655	0.768509	0.444233	-5.98157	0.571858	0.622733
Neutrophils	ABHD13	0.148267	3.557429	0.768495	0.444241	-5.50727	0.620771	0.674777
Neutrophils	NUFIP1	0.185218	4.171093	0.768394	0.4443	-5.33109	0.610008	0.663386
Neutrophils	NDUFB11	0.07195	7.55899	0.768334	0.444336	-6.39735	0.553987	0.603678
Neutrophils	GM24362	0.386264	0.694716	0.768293	0.44436	-4.98656	0.673618	0.730727
Neutrophils	TNFSF13OS	0.552599	0.055873	0.768238	0.444393	-4.91645	0.686022	0.743807
Neutrophils	EPRS	-0.15847	6.581496	-0.76776	0.444677	-5.93784	0.569831	0.620523

Neutrophils	ZBTB9	-0.26596	2.511721	-0.7677	0.444712	-5.14042	0.639854	0.694991
Neutrophils	CASP7	0.15186	4.140272	0.767447	0.44486	-5.6912	0.610819	0.66433
Neutrophils	1110038F1	-0.16317	4.590808	-0.76744	0.444867	-5.54991	0.60303	0.656051
Neutrophils	RASA4	-0.32917	4.228858	-0.7674	0.444887	-5.28719	0.609279	0.6627
Neutrophils	TIGAR	0.311435	1.851972	0.767176	0.445021	-5.08901	0.652126	0.708059
Neutrophils	TENM4	-0.48392	1.760128	-0.76697	0.445141	-4.99342	0.653928	0.709897
Neutrophils	AY036118	0.272091	4.212426	0.76667	0.44532	-5.73508	0.609823	0.66318
Neutrophils	SLC22A17	0.469555	-0.51359	0.766633	0.445342	-4.90165	0.697882	0.756277
Neutrophils	REXO4	0.171038	4.291897	0.766586	0.44537	-5.41624	0.608444	0.661719
Neutrophils	MCUB	-0.45763	1.557619	-0.76632	0.445528	-5.02158	0.657837	0.714015
Neutrophils	COG3	0.167973	4.292574	0.766115	0.445648	-5.50252	0.608468	0.661706
Neutrophils	ZFP280D	-0.13808	5.625842	-0.7661	0.445658	-5.72549	0.585806	0.637596
Neutrophils	PITHD1	-0.15006	4.744392	-0.76602	0.445704	-5.61865	0.600688	0.653436
Neutrophils	GM49439	0.497586	-0.46286	0.765998	0.445718	-4.88428	0.696913	0.755219
Neutrophils	PAM	0.097168	4.417759	0.765937	0.445754	-6.13156	0.606302	0.659424
Neutrophils	GM43111	0.454494	-0.1905	0.765748	0.445866	-4.91617	0.691513	0.749582
Neutrophils	TERF2IP	-0.17741	3.58313	-0.76575	0.445867	-5.36785	0.620896	0.674945
Neutrophils	FXR1	0.086066	6.109299	0.76547	0.44603	-5.99594	0.577944	0.629233
Neutrophils	PPIE	0.171122	4.586932	0.765301	0.44613	-5.42012	0.603585	0.656498
Neutrophils	3110082I1	0.176945	4.199896	0.764906	0.446364	-5.62093	0.610516	0.663713
Neutrophils	ARL4A	-0.18102	4.453783	-0.76475	0.446457	-5.45071	0.606162	0.659039
Neutrophils	RPA2	0.199134	4.645282	0.764519	0.446594	-5.60494	0.60297	0.65561
Neutrophils	RBM38	-0.09322	6.898184	-0.76422	0.446774	-6.36394	0.565703	0.615874
Neutrophils	GCC1	0.214285	2.459454	0.7641	0.446842	-5.40076	0.641929	0.69688
Neutrophils	BORCS8	0.124317	4.590375	0.76388	0.446972	-5.75131	0.604142	0.656832
Neutrophils	FAM49A	-0.2281	5.390412	-0.76384	0.446998	-5.62087	0.590539	0.642372
Neutrophils	TMEM80	-0.17422	3.276451	-0.76356	0.447165	-5.35237	0.627346	0.68139
Neutrophils	HIF1AN	0.242912	3.373427	0.76332	0.447305	-5.26502	0.625726	0.679639
Neutrophils	ABCA7	0.106017	3.89815	0.762825	0.447598	-5.81028	0.61665	0.670026
Neutrophils	BMP2K	0.089753	6.936172	0.76279	0.447619	-6.13664	0.565589	0.615692
Neutrophils	SLC52A2	0.261011	2.391257	0.762759	0.447637	-5.19422	0.64373	0.698735
Neutrophils	GM49336	-0.10539	4.881295	-0.76232	0.4479	-5.84126	0.599892	0.652105
Neutrophils	GM2788	0.34306	0.569462	0.762062	0.448051	-5.09149	0.678438	0.735236
Neutrophils	USHBP1	-0.28698	1.085061	-0.76203	0.448071	-5.20433	0.668521	0.724782
Neutrophils	GM14410	0.427772	0.850334	0.761984	0.448097	-4.90416	0.673018	0.729549
Neutrophils	MRPL57	0.094444	5.995401	0.761762	0.448229	-6.05355	0.581296	0.632284
Neutrophils	H2-DMB1	-0.41746	4.478206	-0.76144	0.448423	-5.37078	0.607128	0.659652
Neutrophils	MED17	0.101664	4.867425	0.761321	0.448491	-5.71395	0.600447	0.65255
Neutrophils	CLCN3	-0.10406	6.389845	-0.76102	0.448673	-6.01724	0.575158	0.625517
Neutrophils	CAD	-0.43508	1.795668	-0.76045	0.449011	-4.95196	0.655932	0.710927
Neutrophils	EXOSC8	-0.16126	5.033192	-0.76042	0.449025	-5.66577	0.598095	0.649684
Neutrophils	FMO5	0.118756	3.247756	0.75998	0.449288	-5.94589	0.629596	0.682899
Neutrophils	ADD3	0.102663	6.047415	0.759478	0.449587	-6.32148	0.5815	0.631843
Neutrophils	PSKH1	-0.18214	3.273493	-0.7594	0.449632	-5.30026	0.629297	0.682584
Neutrophils	CYP2R1	-0.45129	1.009263	-0.75923	0.449737	-4.92741	0.671306	0.727013
Neutrophils	IFI213	-0.54351	3.327221	-0.75903	0.449855	-5.19978	0.628333	0.681651
Neutrophils	8-Sep	-0.27767	1.954462	-0.75899	0.449879	-5.14614	0.653432	0.708182

Neutrophils	TMEM98	-0.39385	0.620631	-0.75897	0.44989	-5.00362	0.678798	0.734923
Neutrophils	CEP120	-0.10597	5.494784	-0.75895	0.449898	-5.88389	0.590711	0.64174
Neutrophils	TRIM56	0.155779	3.810038	0.758889	0.449938	-5.52741	0.619744	0.672563
Neutrophils	CREB1	-0.07374	6.332794	-0.75885	0.44996	-6.19589	0.576802	0.626949
Neutrophils	CD8B1	0.568645	0.660348	0.758792	0.449995	-4.91982	0.678028	0.734162
Neutrophils	PHF14	-0.08375	6.331947	-0.75858	0.450124	-6.11248	0.576906	0.627009
Neutrophils	NCOR1	0.050947	8.098707	0.758406	0.450225	-6.53333	0.548744	0.596919
Neutrophils	GFPT1	0.136579	5.095805	0.758054	0.450434	-5.91192	0.597804	0.648964
Neutrophils	FAAP20	0.177821	3.120662	0.757493	0.450769	-5.41435	0.6328	0.685739
Neutrophils	1-Mar	0.147071	4.477654	0.757266	0.450904	-6.29308	0.608827	0.660433
Neutrophils	SNAPC2	-0.26114	3.015281	-0.75724	0.450919	-5.21259	0.634742	0.687866
Neutrophils	ACY3	-0.3293	2.04257	-0.75714	0.450978	-5.20779	0.652607	0.70676
Neutrophils	HNRNPD	-0.06384	7.908504	-0.75696	0.451087	-6.39006	0.552264	0.60032
Neutrophils	MAPKAPK5	0.222967	2.128701	0.756952	0.451091	-5.21912	0.651004	0.705094
Neutrophils	PKD1L3	-0.40899	0.837305	-0.75675	0.451211	-4.96899	0.675546	0.730939
Neutrophils	CDCA4	0.157733	4.453152	0.756634	0.45128	-5.56893	0.609348	0.661029
Neutrophils	COG7	-0.24569	2.604364	-0.75616	0.451562	-5.17714	0.642645	0.695999
Neutrophils	METTL7A1	0.254218	2.456994	0.755876	0.451733	-5.16286	0.645385	0.698858
Neutrophils	CCNI	0.072386	6.783841	0.755858	0.451743	-6.24813	0.570571	0.619563
Neutrophils	TRMT1L	0.113496	4.566129	0.75566	0.451862	-5.65094	0.60772	0.659077
Neutrophils	MPPE1	-0.09553	4.363196	-0.75564	0.451875	-6.05231	0.611243	0.66281
Neutrophils	F2RL1	-0.46014	-1.13295	-0.75549	0.451964	-4.88349	0.715057	0.77218
Neutrophils	LNCPPARA	-0.40749	0.60974	-0.75543	0.451996	-5.00216	0.680335	0.735736
Neutrophils	TOR4A	0.34806	1.678835	0.75543	0.451999	-5.06065	0.65988	0.714203
Neutrophils	UMPS	-0.18667	4.099845	-0.75495	0.452284	-5.3748	0.616155	0.667894
Neutrophils	WASHC1	-0.12961	4.182087	-0.75482	0.452366	-5.58924	0.614743	0.666367
Neutrophils	FAM89A	-0.35992	0.879902	-0.75465	0.452465	-4.96879	0.675537	0.730545
Neutrophils	ARHGEF17	-0.47443	-0.37609	-0.75384	0.452946	-4.92504	0.700825	0.756647
Neutrophils	PHACTR2	0.095354	6.036575	0.753603	0.45309	-6.30609	0.583699	0.63306
Neutrophils	CLSPN	0.203207	4.411842	0.753513	0.453144	-5.68232	0.611321	0.662353
Neutrophils	ABHD17A	-0.08959	6.296745	-0.75339	0.453218	-5.94149	0.579398	0.628497
Neutrophils	PRIM2	0.15763	5.536205	0.753364	0.453232	-5.88176	0.592064	0.641947
Neutrophils	MPV17L	0.299504	2.006778	0.752974	0.453466	-5.12942	0.654722	0.708261
Neutrophils	SH3BGR	0.4897	-0.59926	0.752965	0.453471	-4.88305	0.705307	0.76142
Neutrophils	GPATCH1	-0.25191	2.73622	-0.75293	0.453494	-5.14117	0.641234	0.694038
Neutrophils	GM47371	0.485319	0.907518	0.752851	0.453539	-4.92343	0.675597	0.730233
Neutrophils	SKA2	0.221198	3.293834	0.75283	0.453552	-5.36983	0.631115	0.683354
Neutrophils	TNFSF13B	0.173205	0.855901	0.752804	0.453567	-5.68075	0.676593	0.731291
Neutrophils	PSRC1	-0.4145	0.886949	-0.75279	0.453573	-4.97047	0.675994	0.73066
Neutrophils	CAAA0111[illegible]	0.097684	5.624186	0.752506	0.453746	-6.00995	0.590621	0.640524
Neutrophils	TTC3	-0.14073	5.355192	-0.75238	0.453819	-5.69538	0.595158	0.645356
Neutrophils	FKBP2	-0.13049	5.611404	-0.75228	0.453882	-5.85484	0.590835	0.640793
Neutrophils	FXR2	0.124386	5.267562	0.752196	0.453931	-5.80042	0.596644	0.646959
Neutrophils	PARP9	0.207317	5.150171	0.752157	0.453955	-5.75696	0.598641	0.649077
Neutrophils	NDUFC1	-0.09211	7.367214	-0.75215	0.453957	-6.29911	0.562086	0.610226
Neutrophils	POLR1A	-0.12524	4.926327	-0.75193	0.454093	-5.86948	0.602538	0.653134
Neutrophils	NSMCE3	-0.13317	4.271532	-0.75187	0.454129	-5.55043	0.61388	0.665149

Neutrophils	ZCCHC3	-0.42804	0.70709	-0.75166	0.454249	-4.9268	0.679688	0.73453
Neutrophils	TMEM202	0.275089	0.104736	0.751433	0.454388	-5.20168	0.691524	0.74701
Neutrophils	TMEM104	0.18253	3.273099	0.751343	0.454442	-5.52479	0.631725	0.684085
Neutrophils	TRIM36	-0.34021	3.146179	-0.75132	0.454454	-5.05445	0.634015	0.686505
Neutrophils	WARS2	0.264274	3.512477	0.751087	0.454595	-5.19811	0.627539	0.679637
Neutrophils	USP14	0.097603	5.793901	0.750939	0.454683	-5.8717	0.588076	0.637911
Neutrophils	ENTPD7	-0.13339	4.693758	-0.75087	0.454722	-5.7202	0.606779	0.657762
Neutrophils	CBFB	0.063984	6.809866	0.750585	0.454896	-6.20176	0.571482	0.620207
Neutrophils	CASZ1	-0.11439	3.474262	-0.75044	0.454982	-5.90038	0.628392	0.680608
Neutrophils	FCHSD1	-0.33854	1.15994	-0.75035	0.455038	-5.02664	0.671294	0.725851
Neutrophils	GM14085	0.443703	-1.35884	0.750289	0.455073	-4.89065	0.721367	0.778415
Neutrophils	CHCHD6	0.235439	2.968098	0.750026	0.45523	-5.15786	0.637612	0.690395
Neutrophils	CHIL1	0.119605	-0.07877	0.749991	0.455251	-5.96913	0.695559	0.751397
Neutrophils	PINK1	0.143307	5.115722	0.749807	0.455361	-5.79359	0.599771	0.650369
Neutrophils	TM9SF3	0.054282	7.288714	0.749774	0.455381	-6.39743	0.563849	0.612191
Neutrophils	PWWP3A	-0.17957	3.715755	-0.74968	0.455437	-5.38162	0.624172	0.676242
Neutrophils	WAC	0.059721	7.414892	0.749305	0.455663	-6.40384	0.56204	0.610198
Neutrophils	KDM5D	1.329765	0.730109	0.748898	0.455907	-5.04818	0.680142	0.735067
Neutrophils	TMEM256	0.094424	6.982152	0.748845	0.455938	-6.24579	0.569159	0.61773
Neutrophils	MYBBP1A	0.228139	4.85707	0.748704	0.456023	-5.41763	0.604609	0.655383
Neutrophils	COA4	0.236652	2.161432	0.748656	0.456051	-5.25126	0.652907	0.706447
Neutrophils	FDXR	-0.27527	2.625449	-0.74853	0.456126	-5.12092	0.644319	0.697435
Neutrophils	ZRANB1	0.071008	6.142303	0.748478	0.456158	-6.20275	0.582901	0.632401
Neutrophils	GM27003	-0.13765	4.204875	-0.7483	0.456262	-5.65417	0.615986	0.667475
Neutrophils	SLC25A10	-0.24031	3.077126	-0.74823	0.456308	-5.26914	0.636112	0.688778
Neutrophils	GM43149	0.299529	0.774872	0.747777	0.456579	-5.14529	0.679634	0.734451
Neutrophils	ZFP24	-0.15672	4.243197	-0.74758	0.456696	-5.45581	0.615637	0.667003
Neutrophils	E130215H2	-0.49324	-0.13171	-0.74751	0.456737	-4.88274	0.697504	0.753297
Neutrophils	GM20045	-0.30285	1.764585	-0.74739	0.456814	-5.14763	0.660736	0.714683
Neutrophils	MFSD4B4	0.471217	-0.0204	0.747335	0.456844	-4.90611	0.69529	0.751025
Neutrophils	NAGA	0.193943	4.159018	0.747151	0.456955	-5.50777	0.61715	0.668746
Neutrophils	DNAJB12	0.129513	4.757611	0.746991	0.457051	-5.67782	0.606717	0.657756
Neutrophils	APC	0.092545	6.723147	0.74691	0.4571	-6.14348	0.573732	0.622733
Neutrophils	D3ERTD75:	-0.34873	1.61523	-0.74681	0.457158	-5.03599	0.663597	0.717876
Neutrophils	CHAC1	-0.31093	0.269965	-0.7467	0.457226	-5.18715	0.689586	0.745229
Neutrophils	SAA4	-0.44504	0.640047	-0.7467	0.457226	-5.00336	0.682335	0.73761
Neutrophils	A730036I1	-0.36937	-0.20546	-0.74646	0.45737	-5.13764	0.699119	0.755213
Neutrophils	UBE4B	-0.11507	5.48847	-0.74623	0.457508	-5.8175	0.594315	0.644664
Neutrophils	CD81	-0.09898	7.604661	-0.7462	0.457526	-6.46363	0.559639	0.607782
Neutrophils	HYPK	-0.10297	5.8151	-0.74609	0.457595	-5.851	0.588819	0.638853
Neutrophils	LSM12	0.087556	6.360706	0.746005	0.457643	-6.02667	0.579757	0.629235
Neutrophils	DUSP23	-0.25941	2.373334	-0.74599	0.457652	-5.16235	0.649489	0.703089
Neutrophils	SELENOM	0.300175	2.384919	0.745429	0.457989	-5.25067	0.649575	0.703065
Neutrophils	HGD	-0.31051	2.465599	-0.74523	0.458112	-5.31932	0.648081	0.701534
Neutrophils	NIF3L1	-0.20483	3.000203	-0.74517	0.458142	-5.30494	0.638272	0.691178
Neutrophils	1110065P2	0.165552	3.514697	0.745119	0.458176	-5.42281	0.628975	0.681371
Neutrophils	ALPL	-0.4291	1.490191	-0.74501	0.458242	-4.97145	0.666379	0.720869

Neutrophils	CCNQ	0.254898	3.060749	0.744912	0.458301	-5.14872	0.63717	0.690075
Neutrophils	EPN2	-0.34914	1.664749	-0.74488	0.458321	-5.1789	0.663066	0.717393
Neutrophils	HIC2	-0.28001	2.712677	-0.7448	0.458366	-5.18371	0.643529	0.696789
Neutrophils	LGMN	-0.11442	7.188159	-0.74475	0.458399	-6.35342	0.566555	0.615175
Neutrophils	1810058I24	0.09117	7.005863	0.744175	0.458744	-6.4219	0.569814	0.618498
Neutrophils	IPO9	-0.13862	4.771136	-0.74412	0.458775	-5.54465	0.607196	0.658201
Neutrophils	EXOC7	0.153569	4.087686	0.743753	0.458998	-5.48503	0.619222	0.670969
Neutrophils	ACVR2A	-0.2002	4.7385	-0.74372	0.459019	-5.59615	0.607848	0.658935
Neutrophils	ATXN7	-0.09876	6.23066	-0.74365	0.459062	-6.08523	0.582584	0.632124
Neutrophils	ETL4	-0.2128	2.396305	-0.74362	0.459079	-5.48508	0.649822	0.703322
Neutrophils	TXNDC17	-0.09083	6.116505	-0.74323	0.459316	-6.17133	0.584551	0.634283
Neutrophils	MTHFSL	0.09125	5.211119	0.743123	0.459377	-6.06879	0.5998	0.650495
Neutrophils	PPARGC1B	0.310707	2.799015	0.743119	0.45938	-5.12433	0.642479	0.695664
Neutrophils	FAM57B	-0.47625	0.285081	-0.74307	0.459412	-4.93119	0.690285	0.746021
Neutrophils	GM10143	0.306552	1.245831	0.742672	0.459649	-5.1054	0.671602	0.726489
Neutrophils	CCSER1	-0.47114	1.697174	-0.74265	0.45966	-5.10036	0.663003	0.717433
Neutrophils	CPNE3	-0.07295	5.367342	-0.74244	0.459789	-6.38881	0.597139	0.647794
Neutrophils	SPRED2	-0.09906	6.335393	-0.74239	0.459817	-6.12876	0.580926	0.630568
Neutrophils	MCRS1	0.14947	4.318538	0.742379	0.459825	-5.54215	0.61524	0.666986
Neutrophils	SIGMAR1	0.210191	3.549056	0.742336	0.459851	-5.31099	0.628881	0.681425
Neutrophils	TRPV2	0.124262	4.873594	0.742273	0.459889	-5.79566	0.60559	0.656778
Neutrophils	HSF2	-0.20877	4.185277	-0.7421	0.459994	-5.3554	0.61758	0.669484
Neutrophils	ZFP867	-0.39012	0.61031	-0.74202	0.460039	-4.93121	0.683902	0.739461
Neutrophils	ROGDI	0.101185	4.014028	0.741932	0.460095	-6.014	0.620601	0.672683
Neutrophils	FOXRED2	-0.30361	1.168266	-0.7419	0.460112	-5.08589	0.673091	0.728091
Neutrophils	TMOD1	0.322376	1.823905	0.741855	0.460142	-5.16737	0.660609	0.714965
Neutrophils	PWP1	-0.11007	3.903059	-0.74184	0.460149	-5.82537	0.622567	0.674786
Neutrophils	CTSB	-0.11578	8.190873	-0.74174	0.460209	-6.50911	0.55113	0.598902
Neutrophils	KTI12	-0.25187	3.941057	-0.74144	0.460391	-5.27914	0.62204	0.67419
Neutrophils	FAM114A2	0.105516	5.166561	0.741366	0.460436	-5.78241	0.600703	0.651616
Neutrophils	MAN1A2	0.079412	6.392544	0.741053	0.460625	-6.08516	0.580236	0.629908
Neutrophils	A530076I1	0.433179	-0.88246	0.74094	0.460693	-4.91829	0.714004	0.77119
Neutrophils	GM36486	0.528283	0.805963	0.740918	0.460707	-4.93537	0.680388	0.735891
Neutrophils	ACOX2	-0.38675	1.030725	-0.7407	0.460839	-5.09026	0.676113	0.731415
Neutrophils	SCAF4	-0.07412	6.317786	-0.74051	0.46095	-6.23393	0.581536	0.631422
Neutrophils	BTBD11	-0.27407	4.309098	-0.74039	0.461027	-5.54742	0.615744	0.667794
Neutrophils	CEP131	-0.36467	1.3043	-0.74035	0.461051	-5.12391	0.670851	0.726024
Neutrophils	AK4	0.385278	1.048143	0.740264	0.461101	-5.07732	0.675776	0.731243
Neutrophils	ATN1	-0.18359	3.221914	-0.74024	0.461117	-5.48599	0.635125	0.688362
Neutrophils	TMEM19	0.137967	3.958743	0.740098	0.461202	-5.58161	0.621956	0.674474
Neutrophils	AMD1	-0.13638	4.581194	-0.73969	0.461449	-5.73113	0.611078	0.663007
Neutrophils	SCLT1	0.16693	4.474106	0.7395	0.461562	-5.64216	0.612945	0.66503
Neutrophils	LEPROT	0.131162	5.065196	0.7394	0.461623	-5.6971	0.602715	0.654197
Neutrophils	CTDSPL2	-0.10912	5.892856	-0.73935	0.461653	-5.91075	0.588688	0.639297
Neutrophils	UTRN	-0.08289	7.31422	-0.73934	0.461657	-6.48742	0.565393	0.614494
Neutrophils	MYADM	0.091166	5.586342	0.739307	0.461679	-6.37057	0.593843	0.644785
Neutrophils	OASL1	0.382141	2.957835	0.739176	0.461758	-5.44145	0.640019	0.693784

Neutrophils	PSAT1	-0.29625	4.218142	-0.73917	0.461762	-5.27759	0.617431	0.669873
Neutrophils	BCL9	0.228342	3.399574	0.739142	0.461779	-5.29141	0.632006	0.685309
Neutrophils	MLEC	0.112229	5.064037	0.738497	0.462168	-5.81441	0.603126	0.654497
Neutrophils	KCNN4	-0.35009	3.850661	-0.73839	0.462234	-5.1969	0.624336	0.676997
Neutrophils	NBEAL1	0.118554	4.937374	0.738349	0.462258	-5.86575	0.605305	0.656824
Neutrophils	5730480H(	-0.24559	2.846707	-0.73822	0.462333	-5.1637	0.642475	0.696176
Neutrophils	GLB1L	0.215413	2.192734	0.738021	0.462456	-5.25915	0.654577	0.70902
Neutrophils	COX7A2	-0.07077	8.039242	-0.73776	0.462616	-6.47624	0.554253	0.602552
Neutrophils	MED19	-0.16824	3.778351	-0.73775	0.462618	-5.35419	0.625631	0.678433
Neutrophils	CSDE1	0.050621	7.576149	0.737719	0.462639	-6.42474	0.561578	0.610368
Neutrophils	RBMS3	-0.25096	3.072419	-0.73754	0.462747	-5.53922	0.638352	0.691909
Neutrophils	SMCR8	-0.19224	3.28105	-0.73752	0.462761	-5.4249	0.634565	0.687911
Neutrophils	SLC7A1	-0.20026	5.141375	-0.73735	0.462864	-5.60979	0.601806	0.653199
Neutrophils	UBE2V2	-0.10928	5.614121	-0.7373	0.462894	-5.83377	0.593765	0.644659
Neutrophils	PUSL1	0.281274	2.317284	0.737284	0.462902	-5.08111	0.652254	0.706606
Neutrophils	P4HA2	0.406684	0.108055	0.737257	0.462918	-4.97914	0.694725	0.751347
Neutrophils	FUT11	-0.21209	3.493603	-0.73696	0.463096	-5.32826	0.630891	0.684006
Neutrophils	0610005C1	-0.27952	1.911141	-0.73667	0.463271	-5.20641	0.660192	0.714843
Neutrophils	GSS	-0.19852	3.399727	-0.73652	0.463364	-5.4295	0.632786	0.685907
Neutrophils	COA6	-0.15701	4.110702	-0.73632	0.463485	-5.52587	0.620136	0.672518
Neutrophils	SMYD5	-0.42244	2.056268	-0.73625	0.463526	-4.97888	0.657559	0.712077
Neutrophils	STAT1	0.247698	6.871703	0.735953	0.463707	-6.23727	0.573291	0.622842
Neutrophils	PIK3R1	-0.10497	7.694648	-0.73594	0.463716	-6.36665	0.560058	0.608737
Neutrophils	ATAT1	-0.2399	2.722421	-0.73591	0.463736	-5.21411	0.645177	0.699096
Neutrophils	ST8SIA1	0.46659	0.877835	0.735873	0.463756	-5.01592	0.68006	0.7359
Neutrophils	NRG4	0.220877	2.017195	0.735771	0.463818	-5.57548	0.658295	0.712966
Neutrophils	SDC1	0.386061	2.216388	0.735528	0.463965	-5.1533	0.654594	0.709028
Neutrophils	SH3BP5	-0.08481	6.380773	-0.73539	0.464046	-6.38971	0.581371	0.631436
Neutrophils	TIFAB	-0.37541	2.73524	-0.73531	0.464097	-5.12345	0.644974	0.698904
Neutrophils	DDB1	-0.12277	5.611857	-0.73511	0.464216	-5.82284	0.594221	0.645172
Neutrophils	TRIOBP	0.069893	4.877202	0.735066	0.464245	-6.18794	0.606775	0.658504
Neutrophils	GM43462	-0.2342	3.23183	-0.73505	0.464253	-5.28909	0.635902	0.689362
Neutrophils	MYL6B	0.382741	0.862198	0.734998	0.464286	-4.96333	0.680398	0.736313
Neutrophils	PRCC	-0.12895	5.107381	-0.73495	0.464316	-5.63514	0.602812	0.654308
Neutrophils	WDR25	0.247577	1.998808	0.73478	0.464418	-5.17032	0.658678	0.713426
Neutrophils	EPOP	-0.41714	1.472765	-0.73474	0.464442	-4.94057	0.668644	0.723954
Neutrophils	ZRSR1	0.246816	2.211301	0.734577	0.464541	-5.23471	0.654695	0.709255
Neutrophils	PHEX	-0.50551	1.372943	-0.73455	0.464559	-4.95294	0.670553	0.725979
Neutrophils	MAP4K2	-0.10045	5.348887	-0.73434	0.464687	-6.12324	0.598697	0.649966
Neutrophils	MBD5	0.070086	6.903175	0.73418	0.464782	-6.37977	0.572821	0.622458
Neutrophils	UBXN1	0.067955	6.759331	0.734144	0.464803	-6.22934	0.575166	0.624964
Neutrophils	RBBP6	-0.07152	7.269431	-0.73401	0.464885	-6.26902	0.566897	0.616177
Neutrophils	CEP41	0.310268	1.886422	0.733958	0.464916	-5.06973	0.660804	0.715789
Neutrophils	CTSC	-0.15866	7.322442	-0.73378	0.465025	-6.29614	0.566045	0.615291
Neutrophils	GPT2	-0.35027	1.738624	-0.73371	0.46507	-5.08056	0.663597	0.718745
Neutrophils	ZCCHC4	0.259694	3.455439	0.733488	0.465201	-5.22096	0.631875	0.685255
Neutrophils	ZSCAN20	-0.4649	1.073033	-0.73329	0.465322	-4.95564	0.676329	0.73225

Neutrophils	A430090L1	-0.30485	0.628221	-0.73325	0.465345	-5.07245	0.684976	0.741354
Neutrophils	SYNE1	-0.08571	4.964609	-0.73321	0.465371	-6.57256	0.605281	0.65712
Neutrophils	ELOB	0.06491	8.913307	0.733196	0.465378	-6.62943	0.541093	0.588721
Neutrophils	ULK1	-0.13824	3.637915	-0.73312	0.465426	-5.67571	0.628596	0.681844
Neutrophils	RFNG	-0.2549	2.228203	-0.73308	0.465448	-5.13979	0.654388	0.709115
Neutrophils	CEP295	0.170624	3.949897	0.733032	0.465478	-5.44079	0.623031	0.675949
Neutrophils	TMED4	0.188556	3.553347	0.732963	0.46552	-5.37157	0.630113	0.68345
Neutrophils	TTC25	-0.45923	0.218704	-0.73277	0.465636	-4.93159	0.693038	0.74986
Neutrophils	GAS2L3	-0.1797	4.330262	-0.73275	0.465647	-5.62717	0.616318	0.668853
Neutrophils	CHURC1	-0.11346	5.66012	-0.73259	0.465747	-5.754	0.593422	0.644554
Neutrophils	TRMT11	-0.22047	3.646744	-0.7325	0.465804	-5.2227	0.628441	0.68171
Neutrophils	EID2	0.421828	0.075956	0.732363	0.465884	-4.95982	0.695869	0.75285
Neutrophils	LIG3	0.189747	3.358188	0.732154	0.466011	-5.45609	0.633633	0.687263
Neutrophils	CELA1	0.285293	2.24977	0.732142	0.466018	-5.26461	0.653989	0.708784
Neutrophils	E030042O2	-0.50517	0.180529	-0.73199	0.466113	-4.92483	0.693794	0.750743
Neutrophils	ZFP874A	0.282934	2.146516	0.731984	0.466114	-5.12428	0.655919	0.710835
Neutrophils	D17H6S53E	0.115376	4.009289	0.731603	0.466346	-5.72973	0.62198	0.675036
Neutrophils	GM32031	-0.19209	2.845461	-0.73159	0.466356	-5.45224	0.642967	0.697255
Neutrophils	1700001K1	-0.40684	1.052633	-0.73144	0.466444	-5.01869	0.676727	0.732921
Neutrophils	GM37612	0.420851	0.985122	0.73133	0.466511	-4.93164	0.678033	0.734309
Neutrophils	CSTB	0.103408	6.672627	0.731272	0.466546	-6.28722	0.576587	0.626833
Neutrophils	CLEC4B1	0.432086	-0.61033	0.731224	0.466576	-4.91789	0.709645	0.767559
Neutrophils	AP4B1	-0.23728	2.273778	-0.73121	0.466584	-5.27317	0.653541	0.708472
Neutrophils	4930532G1	-0.46703	1.429337	-0.73121	0.466586	-5.01827	0.669486	0.725301
Neutrophils	1700027J0	-0.40739	2.481407	-0.73119	0.466595	-5.13082	0.64968	0.704393
Neutrophils	MARK3	0.076196	6.304211	0.731086	0.46666	-6.08497	0.58266	0.633295
Neutrophils	GM47644	-0.33214	1.060844	-0.73069	0.4669	-5.19572	0.676836	0.732814
Neutrophils	MACROD1	-0.29108	2.861084	-0.73048	0.467029	-5.20213	0.64303	0.697164
Neutrophils	DIDO1	-0.1078	5.734022	-0.73037	0.467093	-5.92458	0.592504	0.643609
Neutrophils	PLXDC1	-0.39922	3.94302	-0.72998	0.46733	-5.14539	0.623738	0.676603
Neutrophils	CLCN4	0.115484	5.289066	0.729473	0.46764	-5.79346	0.600602	0.651839
Neutrophils	ANAPC13	0.083365	5.65737	0.72914	0.467843	-6.01327	0.594523	0.645238
Neutrophils	IFRD2	-0.26079	3.30652	-0.72858	0.468187	-5.17348	0.635999	0.689085
Neutrophils	ZFP932	-0.22327	2.788655	-0.72848	0.468246	-5.12723	0.645463	0.699085
Neutrophils	ERCC6L	-0.27053	3.034059	-0.72848	0.468246	-5.29136	0.64096	0.694328
Neutrophils	ATXN7L2	-0.32668	2.480154	-0.72826	0.468378	-5.0809	0.651269	0.705118
Neutrophils	BTNL9	-0.29038	0.689973	-0.72809	0.46848	-5.30932	0.685483	0.741117
Neutrophils	BAHCC1	-0.37823	1.235974	-0.72793	0.468582	-5.01865	0.674885	0.730028
Neutrophils	ZMIZ1	0.075793	6.978265	0.727888	0.468605	-6.26538	0.573041	0.622221
Neutrophils	AVEN	-0.20404	4.926701	-0.7277	0.468717	-5.4438	0.607528	0.658814
Neutrophils	SUMO3	0.110061	5.724941	0.727529	0.468824	-5.87937	0.593916	0.644357
Neutrophils	ACSM1	-0.43319	1.026687	-0.72746	0.468868	-5.09394	0.679043	0.734371
Neutrophils	RAB3IP	-0.19894	4.587906	-0.72737	0.468922	-5.44642	0.61345	0.665149
Neutrophils	HOTAIRM1	-0.36127	0.698917	-0.72726	0.468987	-5.12139	0.685436	0.741137
Neutrophils	TUBA1A	-0.15194	5.430541	-0.72706	0.469107	-5.91787	0.598976	0.649735
Neutrophils	TXLNA	-0.12622	4.941372	-0.72699	0.469152	-5.6869	0.607373	0.658644
Neutrophils	OXNAD1	0.27014	1.886539	0.726661	0.469353	-5.11174	0.662846	0.717157

Neutrophils	CCDC66	0.265442	2.265203	0.726516	0.469441	-5.11472	0.655759	0.709681
Neutrophils	YIF1B	0.138095	4.886916	0.726336	0.469551	-5.65583	0.608534	0.659796
Neutrophils	ARMCX5	0.235892	2.433168	0.726321	0.46956	-5.18768	0.652624	0.706424
Neutrophils	USP18	0.327596	2.737474	0.72613	0.469677	-5.26339	0.64706	0.700522
Neutrophils	SPINK10	-0.39611	0.791956	-0.72603	0.46974	-4.98546	0.684018	0.739472
Neutrophils	ATP5H	-0.06001	7.946365	-0.72553	0.470043	-6.46091	0.55823	0.606044
Neutrophils	GM48027	-0.22372	2.815176	-0.72511	0.470301	-5.30618	0.646029	0.699091
Neutrophils	FCER2A	-0.55805	0.458562	-0.7251	0.470307	-4.95198	0.690987	0.746415
Neutrophils	FAM83G	0.373947	0.263823	0.724907	0.470423	-4.94781	0.694841	0.750503
Neutrophils	GM17023	-0.4876	-0.64235	-0.72488	0.470437	-4.98091	0.713061	0.769613
Neutrophils	COPS4	0.113034	5.581159	0.724867	0.470448	-5.73431	0.597073	0.647327
Neutrophils	HIST1H1A	-0.4437	3.773403	-0.72472	0.470537	-5.28834	0.628615	0.680784
Neutrophils	METRN	-0.26964	2.538002	-0.7247	0.470552	-5.20959	0.651159	0.704593
Neutrophils	ARFGAP1	0.131111	4.620741	0.724669	0.470568	-5.6326	0.61362	0.664926
Neutrophils	ZW10	0.161862	4.014837	0.723875	0.471054	-5.53217	0.624869	0.67644
Neutrophils	SLFN3	-0.20249	1.4477	-0.72353	0.471265	-5.4466	0.672436	0.726603
Neutrophils	ABHD2	-0.07965	5.471153	-0.72342	0.471334	-6.12327	0.599563	0.649699
Neutrophils	GM16104	0.435234	-0.60575	0.723369	0.471363	-5.0084	0.713052	0.769298
Neutrophils	GM11520	-0.31418	1.043872	-0.7231	0.471529	-5.05507	0.680234	0.734945
Neutrophils	GM28112	-0.45121	0.043725	-0.72299	0.471592	-4.97732	0.699945	0.755677
Neutrophils	GRB7	-0.45059	0.183341	-0.72298	0.471597	-4.95514	0.69716	0.752755
Neutrophils	SNAPC3	-0.16301	4.825524	-0.72296	0.47161	-5.5569	0.610682	0.661599
Neutrophils	MBIP	-0.20493	3.658726	-0.72292	0.471639	-5.34837	0.631325	0.683453
Neutrophils	CRISPLD2	0.193033	0.884481	0.722826	0.471695	-5.72409	0.683338	0.738302
Neutrophils	CTC1	-0.15949	3.25694	-0.72272	0.47176	-5.44967	0.638599	0.691231
Neutrophils	H2-DMB2	0.295289	3.016521	0.722697	0.471773	-5.21603	0.642993	0.695876
Neutrophils	PSME1	0.122456	8.384368	0.722551	0.471862	-6.42089	0.55197	0.599362
Neutrophils	FBXO28	0.114492	4.523471	0.72243	0.471937	-5.85685	0.61596	0.667373
Neutrophils	MTHFS	0.124806	5.861718	0.722401	0.471954	-6.38378	0.592941	0.64297
Neutrophils	GM42659	-0.08814	4.567867	-0.72213	0.472123	-6.08273	0.615323	0.666674
Neutrophils	GM26802	-0.30337	1.069129	-0.72194	0.472234	-5.2247	0.679925	0.734862
Neutrophils	SENP3	-0.14057	4.324023	-0.7218	0.472323	-5.60585	0.619632	0.671263
Neutrophils	MAP3K10	-0.26526	2.731125	-0.72178	0.472334	-5.14669	0.648422	0.701692
Neutrophils	DEPDC5	-0.14402	4.978079	-0.72165	0.472413	-5.59171	0.608197	0.659201
Neutrophils	CDK2AP2	0.061435	6.467925	0.72158	0.472457	-6.46178	0.582965	0.632418
Neutrophils	2010013B2	0.126532	3.545259	0.721465	0.472527	-5.89001	0.633539	0.686061
Neutrophils	1810044D(	0.344538	1.915203	0.721414	0.472558	-5.05182	0.663697	0.71789
Neutrophils	ESCO1	0.088457	5.77786	0.721125	0.472735	-6.06327	0.594628	0.644882
Neutrophils	LYPLA2	-0.10044	4.945046	-0.72106	0.472776	-5.88295	0.608888	0.66004
Neutrophils	OGDH	0.065866	6.625732	0.72097	0.47283	-6.12111	0.58047	0.629902
Neutrophils	UGCG	0.104353	6.436724	0.72085	0.472903	-6.18612	0.583612	0.633251
Neutrophils	SRBD1	-0.16359	4.393343	-0.72049	0.473125	-5.54666	0.618744	0.670468
Neutrophils	SLC22A14	-0.22058	2.744823	-0.72026	0.473264	-5.40054	0.648519	0.701976
Neutrophils	GPATCH3	0.226805	3.369567	0.72023	0.473283	-5.30818	0.637065	0.689882
Neutrophils	EPB41L4B	-0.3462	1.976143	-0.7202	0.473303	-5.09828	0.662902	0.717146
Neutrophils	ZFP503	-0.24391	0.884395	-0.72002	0.473411	-5.28103	0.683891	0.739299
Neutrophils	NBEA	-0.1977	2.602645	-0.72002	0.473414	-5.51412	0.651155	0.704818

Neutrophils	PRODH	-0.22681	2.780405	-0.71968	0.473617	-5.49807	0.647976	0.70141
Neutrophils	HLTF	0.152687	4.64409	0.719564	0.473691	-5.6303	0.614451	0.665959
Neutrophils	FKBP1B	0.330414	0.793011	0.719266	0.473874	-5.06556	0.685801	0.741405
Neutrophils	HINT3	0.14767	4.643869	0.719256	0.47388	-5.62575	0.614454	0.666101
Neutrophils	MTSS2	-0.34177	0.842863	-0.71918	0.473926	-5.09377	0.684825	0.740394
Neutrophils	ZFP472	0.255006	3.448072	0.719152	0.473944	-5.1742	0.635754	0.688658
Neutrophils	FOXA3	-0.30677	1.425712	-0.71909	0.473985	-5.15819	0.673521	0.728533
Neutrophils	WDCP	0.235125	3.079235	0.719056	0.474003	-5.24446	0.642476	0.695798
Neutrophils	TRIM27	0.097719	5.675636	0.718913	0.47409	-5.9394	0.596673	0.647322
Neutrophils	TBC1D22A	-0.08968	5.871527	-0.7189	0.474098	-6.04628	0.593358	0.643801
Neutrophils	GM46440	-0.44716	0.637929	-0.71843	0.474385	-4.95157	0.68916	0.744861
Neutrophils	SCYL1	0.177989	4.072604	0.718328	0.474449	-5.4765	0.624822	0.67701
Neutrophils	ADAMTS7	0.421412	0.676356	0.718253	0.474495	-4.98205	0.688404	0.744105
Neutrophils	10-Sep	-0.15662	3.119761	-0.71805	0.474621	-5.54846	0.642117	0.695289
Neutrophils	MRTFA	0.105234	7.084199	0.71794	0.474687	-6.21565	0.573607	0.622692
Neutrophils	CACNA1B	-0.49188	0.056619	-0.71779	0.47478	-4.90148	0.700798	0.757149
Neutrophils	AHSA1	0.119179	5.071608	0.717705	0.474831	-5.80119	0.607381	0.658592
Neutrophils	CRMP1	-0.45319	-1.07546	-0.71765	0.474863	-4.91041	0.723829	0.781302
Neutrophils	ENPP5	0.379429	0.877362	0.717555	0.474923	-5.0814	0.684561	0.740132
Neutrophils	1700019L1	0.290396	0.774577	0.717204	0.475139	-5.25315	0.686799	0.742275
Neutrophils	GLIS2	-0.33459	1.3513	-0.71637	0.475653	-5.06781	0.676226	0.730638
Neutrophils	PKD2L2	0.264328	1.170955	0.71621	0.475749	-5.05811	0.679769	0.734351
Neutrophils	TRAF7	0.107255	4.585124	0.715774	0.476017	-5.7584	0.616853	0.668022
Neutrophils	RASSF5	0.079272	5.485464	0.715673	0.476079	-6.21181	0.601245	0.651486
Neutrophils	PRR12	-0.19217	3.451612	-0.71567	0.47608	-5.31752	0.637104	0.689433
Neutrophils	NSMF	-0.20256	2.696993	-0.71561	0.476115	-5.35171	0.650966	0.704069
Neutrophils	NECAB2	0.306139	0.442881	0.715222	0.476356	-5.21597	0.694466	0.749795
Neutrophils	ALG12	0.368577	1.395336	0.71516	0.476394	-5.02145	0.675831	0.730224
Neutrophils	RAD51C	-0.21037	2.245463	-0.71504	0.476466	-5.33459	0.659643	0.713168
Neutrophils	GZF1	-0.18803	2.752325	-0.71487	0.476574	-5.47053	0.650231	0.703253
Neutrophils	GALK1	-0.15906	5.140655	-0.71477	0.476631	-5.6609	0.607446	0.658037
Neutrophils	RASA1	-0.09923	6.030636	-0.71468	0.47669	-5.97111	0.592261	0.641944
Neutrophils	UBE2E3	-0.06921	7.066891	-0.71458	0.476751	-6.22314	0.57508	0.623705
Neutrophils	LYRM9	-0.27105	2.696887	-0.71446	0.476825	-5.16778	0.65128	0.704414
Neutrophils	PI4KA	-0.10503	6.333121	-0.71428	0.476936	-5.98038	0.587271	0.636648
Neutrophils	COLQ	-0.43013	0.244853	-0.71417	0.477003	-4.90616	0.698592	0.75419
Neutrophils	PRPSAP2	0.161182	4.045546	0.713841	0.477206	-5.4621	0.626972	0.678593
Neutrophils	PHF2	0.108396	5.076145	0.713641	0.477329	-5.72617	0.608836	0.659465
Neutrophils	ITPR2	-0.0726	6.912603	-0.71356	0.477376	-6.38485	0.577863	0.626627
Neutrophils	HTR7	0.511975	0.754108	0.713437	0.477454	-5.10963	0.688709	0.743769
Neutrophils	ARHGAP1	0.099734	4.473602	0.71343	0.477459	-5.74669	0.619372	0.670674
Neutrophils	1810021B2	0.360431	0.427969	0.713344	0.477512	-5.10726	0.695154	0.750554
Neutrophils	CMTM3	-0.17274	3.593966	-0.71322	0.477589	-5.52837	0.635094	0.687341
Neutrophils	CAMK2D	-0.14533	7.214604	-0.71306	0.477684	-6.07303	0.572931	0.621501
Neutrophils	RNF215	0.203478	2.627986	0.71306	0.477686	-5.2291	0.652835	0.706135
Neutrophils	AATF	-0.15119	4.995367	-0.71274	0.477885	-5.55551	0.610376	0.661213
Neutrophils	SLC30A1	-0.23493	3.386735	-0.71258	0.477983	-5.35406	0.639003	0.69151

Neutrophils	ZFP287	-0.43576	0.566909	-0.71256	0.477995	-4.96207	0.692559	0.747903
Neutrophils	A230072E1	-0.47605	0.278064	-0.7124	0.478091	-4.91731	0.698296	0.753925
Neutrophils	EFCAB8	-0.26287	1.374064	-0.71239	0.478102	-5.19718	0.676778	0.73132
Neutrophils	SPIN1	-0.07833	5.805516	-0.7123	0.478156	-6.10168	0.596467	0.646484
Neutrophils	CDK1	-0.17049	5.506863	-0.71187	0.478423	-6.11807	0.601815	0.651936
Neutrophils	UPRT	-0.41809	0.606501	-0.71157	0.478602	-4.91484	0.692247	0.747191
Neutrophils	MRPS21	0.060237	7.034242	0.711332	0.478751	-6.34401	0.576494	0.624882
Neutrophils	PIP4P2	-0.16494	3.706325	-0.71124	0.478806	-5.5926	0.633748	0.685552
Neutrophils	GM13986	-0.13285	1.376917	-0.7111	0.478897	-6.27498	0.677345	0.731489
Neutrophils	ANKRD42	-0.42412	0.346625	-0.71084	0.479052	-5.02467	0.697709	0.752852
Neutrophils	NONO	-0.07637	6.614635	-0.71054	0.479241	-6.11341	0.583715	0.632492
Neutrophils	SLC39A7	0.118339	4.500813	0.710345	0.47936	-5.69388	0.619905	0.67092
Neutrophils	JHY	-0.51426	-0.37714	-0.71028	0.479398	-4.92666	0.71249	0.768363
Neutrophils	MPZL3	0.171669	1.727945	0.710226	0.479433	-5.63986	0.670919	0.724732
Neutrophils	SMIM24	0.209396	3.210803	0.709774	0.479712	-5.26081	0.643416	0.69563
Neutrophils	DGLUCY	0.148471	2.944912	0.709535	0.479859	-5.60395	0.648353	0.700843
Neutrophils	DIAPH3	-0.19778	6.026174	-0.70951	0.479874	-6.04358	0.593879	0.643239
Neutrophils	MAPKBP1	-0.27522	3.047991	-0.70943	0.479922	-5.24217	0.64645	0.698836
Neutrophils	S1PR2	0.315728	1.261742	0.709091	0.480134	-5.12177	0.680443	0.734525
Neutrophils	TBC1D8	0.072125	5.179428	0.708967	0.48021	-6.54863	0.60852	0.658719
Neutrophils	SEC11C	-0.07688	7.287381	-0.70893	0.480232	-6.34023	0.573135	0.621174
Neutrophils	PLA2G4A	0.141557	3.294743	0.70862	0.480424	-5.9011	0.642265	0.694254
Neutrophils	NIPA1	0.41756	0.13903	0.708478	0.480512	-4.96396	0.702847	0.757956
Neutrophils	B230208H1	0.142186	0.861264	0.708181	0.480696	-5.94693	0.688607	0.743026
Neutrophils	TIMM13	-0.08806	6.732513	-0.70809	0.48075	-6.12293	0.58252	0.631072
Neutrophils	RNASET2A	-0.13682	5.439463	-0.70805	0.480774	-5.94044	0.60433	0.654205
Neutrophils	LYSMD1	0.288669	1.591133	0.707964	0.48083	-5.04977	0.674404	0.728158
Neutrophils	RAB44	0.109165	1.487929	0.707839	0.480907	-6.23333	0.676418	0.730299
Neutrophils	SNX1	-0.08806	5.483558	-0.7073	0.48124	-5.84397	0.603937	0.653666
Neutrophils	TMEM29	0.119331	4.706351	0.706862	0.481511	-5.75666	0.617673	0.668063
Neutrophils	IREB2	0.098904	5.493255	0.706818	0.481538	-5.9449	0.603992	0.65359
Neutrophils	YIPF3	0.104118	4.904587	0.706498	0.481736	-5.82876	0.614372	0.66447
Neutrophils	SLC31A2	0.13968	3.169586	0.706294	0.481862	-5.83657	0.645536	0.697344
Neutrophils	IPO13	-0.29352	2.264795	-0.70626	0.481883	-5.11031	0.662417	0.715118
Neutrophils	TENT5C	0.185832	5.86321	0.706147	0.481954	-6.11394	0.597864	0.64697
Neutrophils	GM12905	-0.19509	2.138199	-0.70607	0.482	-5.25986	0.664815	0.71764
Neutrophils	FAM83E	-0.33622	1.10583	-0.70588	0.48212	-5.05044	0.684701	0.738585
Neutrophils	RHOQ	-0.18574	5.771108	-0.70586	0.482131	-5.6426	0.599432	0.648679
Neutrophils	CIT	0.191909	4.461996	0.705784	0.482178	-5.87047	0.622189	0.672772
Neutrophils	APBA3	0.246934	2.107298	0.705651	0.482261	-5.21331	0.665432	0.718365
Neutrophils	GM31462	0.426041	0.152102	0.705403	0.482414	-4.93145	0.703702	0.758523
Neutrophils	SNX12	-0.10774	4.718214	-0.70537	0.482432	-5.70521	0.617748	0.668088
Neutrophils	CX3CR1	0.180422	3.268892	0.705202	0.482538	-5.81004	0.643797	0.695661
Neutrophils	PPP1R21	0.14928	4.557871	0.705138	0.482578	-5.64539	0.620575	0.671146
Neutrophils	D2HGDH	0.255037	2.08867	0.705093	0.482606	-5.16825	0.665845	0.718878
Neutrophils	EXOC4	0.061192	8.03297	0.704807	0.482783	-6.41164	0.562358	0.609295
Neutrophils	PFDN6	-0.12132	4.874434	-0.70467	0.482865	-5.73016	0.615159	0.665295

Neutrophils	NUP93	0.167907	4.586111	0.704542	0.482947	-5.46217	0.62023	0.670656
Neutrophils	KIZ	0.131362	3.359508	0.704509	0.482968	-5.60887	0.642295	0.693947
Neutrophils	ATP5G3	-0.09016	8.015505	-0.70441	0.483028	-6.39209	0.56264	0.609615
Neutrophils	GM49961	0.445974	0.574285	0.704161	0.483183	-4.93626	0.695577	0.749855
Neutrophils	HACD4	0.10423	3.532004	0.703983	0.483293	-6.23621	0.639336	0.690772
Neutrophils	CIAO3	-0.17437	3.316721	-0.70359	0.483536	-5.40537	0.643408	0.695034
Neutrophils	STK17B	0.061167	8.129238	0.703558	0.483557	-6.65851	0.561116	0.607892
Neutrophils	RAB5A	0.062932	6.913368	0.703383	0.483666	-6.31223	0.580806	0.628828
Neutrophils	F9	-0.34366	0.896192	-0.70335	0.483688	-5.09492	0.689428	0.743435
Neutrophils	ANKRD17	-0.06257	7.985576	-0.70333	0.483698	-6.50591	0.563405	0.610338
Neutrophils	KIN	0.105419	4.642417	0.703085	0.48385	-5.78379	0.619607	0.669975
Neutrophils	LRRC63	-0.28623	0.544146	-0.70307	0.483861	-5.28931	0.696456	0.750859
Neutrophils	DGKZ	0.09468	6.2774	0.702875	0.48398	-6.21625	0.59149	0.640208
Neutrophils	CDC42BPA	-0.18481	3.315927	-0.70281	0.484018	-5.49526	0.643527	0.695255
Neutrophils	MTX3	-0.43414	0.591443	-0.70271	0.484081	-4.9533	0.69557	0.749986
Neutrophils	SLC33A1	0.196658	3.794664	0.702472	0.48423	-5.35388	0.634898	0.686105
Neutrophils	GNA15	-0.21391	3.584316	-0.7024	0.484272	-5.42195	0.638716	0.690159
Neutrophils	UBE2B	0.073325	8.25823	0.701928	0.484568	-6.58084	0.559514	0.606078
Neutrophils	PCBP1	-0.06503	8.199098	-0.70177	0.484668	-6.50081	0.560481	0.607098
Neutrophils	MRAS	0.432098	0.476226	0.701693	0.484713	-4.96836	0.698339	0.752657
Neutrophils	ZFP704	-0.22687	3.792086	-0.70132	0.484948	-5.5355	0.635513	0.686408
Neutrophils	KIDINS220	0.083378	5.419693	0.700955	0.485172	-6.07606	0.606931	0.656031
Neutrophils	DCTN5	0.136006	4.598222	0.700622	0.485379	-5.62609	0.621453	0.671281
Neutrophils	TCTN1	-0.38062	1.315572	-0.70048	0.485468	-5.0276	0.682451	0.735484
Neutrophils	CC2D2A	0.174366	0.81427	0.700248	0.485611	-5.66254	0.692291	0.745875
Neutrophils	A630001G2	-0.13021	5.126842	-0.70024	0.485618	-5.7307	0.61217	0.661557
Neutrophils	DNAJC1	0.07466	6.915183	0.700033	0.485745	-6.27294	0.581825	0.629435
Neutrophils	CHAF1A	0.188658	4.186029	0.699927	0.485811	-5.64817	0.628794	0.679124
Neutrophils	SNX25	-0.11958	5.540177	-0.69971	0.485943	-5.75017	0.605012	0.654062
Neutrophils	DPP3	0.141736	4.728026	0.699652	0.485982	-5.62486	0.61916	0.669052
Neutrophils	H2-T22	0.250681	5.391347	0.699569	0.486033	-5.75984	0.607579	0.656842
Neutrophils	DPP6	-0.45444	-0.15893	-0.69949	0.48608	-4.96738	0.711804	0.766487
Neutrophils	SPIC	-0.37416	1.863637	-0.69947	0.486097	-5.31239	0.671855	0.724615
Neutrophils	ZFP62	0.128597	4.580193	0.69944	0.486113	-5.67515	0.621773	0.671865
Neutrophils	UBXN7	0.08768	5.704222	0.699395	0.486141	-6.06206	0.602196	0.651166
Neutrophils	HECTD2	-0.39859	0.291184	-0.69939	0.486147	-4.96051	0.702711	0.756972
Neutrophils	GM13561	-0.35375	1.307424	-0.69926	0.486226	-4.97659	0.682623	0.73596
Neutrophils	MPC1	0.066359	7.569491	0.699119	0.486313	-6.54294	0.571132	0.618304
Neutrophils	VAT1	0.1933	3.491225	0.699079	0.486338	-5.43582	0.641383	0.692652
Neutrophils	ZDHHC9	0.126431	4.094804	0.698494	0.486701	-5.77434	0.630824	0.681224
Neutrophils	GLRA1	-0.37066	1.25067	-0.69841	0.486753	-5.18586	0.684143	0.73733
Neutrophils	CARS2	-0.15259	3.502896	-0.69825	0.486854	-5.3263	0.641611	0.692596
Neutrophils	SLC10A7	-0.09685	5.908741	-0.69788	0.487081	-5.96802	0.599249	0.647743
Neutrophils	GM28403	-0.34084	1.458517	-0.69785	0.487101	-5.143	0.680291	0.733131
Neutrophils	RBM6	0.071162	7.184868	0.697753	0.487163	-6.26394	0.577914	0.625135
Neutrophils	KRR1	-0.12085	3.94276	-0.69766	0.487221	-5.74678	0.633745	0.684224
Neutrophils	A430093F1	-0.28198	2.363356	-0.69759	0.487267	-5.16362	0.662945	0.714974

Neutrophils	KDELR1	-0.07766	6.091298	-0.6975	0.487318	-6.08568	0.596147	0.64451
Neutrophils	CCDC189	-0.26161	0.183787	-0.69728	0.487454	-5.30925	0.705563	0.75968
Neutrophils	HIST1H2AI	-0.45572	2.588783	-0.6972	0.487509	-5.16696	0.658741	0.710592
Neutrophils	ADM	0.364972	1.330377	0.69709	0.487576	-5.15418	0.682833	0.735912
Neutrophils	ACAA1A	0.101816	5.302507	0.697057	0.487596	-5.977	0.609716	0.658923
Neutrophils	CRCP	0.118189	4.449755	0.696913	0.487686	-5.62506	0.624701	0.67477
Neutrophils	NUMA1	-0.09051	5.705606	-0.69686	0.487717	-5.98562	0.602764	0.651606
Neutrophils	TUBGCP3	-0.14593	3.88521	-0.69609	0.488199	-5.4204	0.635315	0.685687
Neutrophils	CD5	-0.29399	2.693985	-0.69606	0.488218	-5.10543	0.657267	0.708795
Neutrophils	ZFP768	-0.40922	0.794694	-0.69597	0.48827	-5.00518	0.693888	0.747222
Neutrophils	MFGE8	0.287137	2.292559	0.695821	0.488366	-5.22132	0.664853	0.716765
Neutrophils	RAC3	0.47334	-0.22085	0.695763	0.488402	-4.934	0.714324	0.768608
Neutrophils	IFI207	0.211061	4.721848	0.695568	0.488524	-5.86281	0.620442	0.669949
Neutrophils	SP2	0.124062	4.742762	0.69537	0.488647	-5.71802	0.620152	0.669559
Neutrophils	ARG2	0.090211	3.174154	0.695087	0.488824	-6.42561	0.648657	0.699518
Neutrophils	FAM160B1	0.112448	4.443367	0.694976	0.488893	-5.80215	0.625621	0.67524
Neutrophils	SEC24C	-0.10633	4.859773	-0.69485	0.48897	-5.7637	0.618267	0.667494
Neutrophils	CHTOP	-0.11349	5.225925	-0.69429	0.489322	-5.83629	0.611909	0.66096
Neutrophils	CCT6B	-0.36164	0.315333	-0.69427	0.489335	-5.07288	0.703909	0.757684
Neutrophils	BRD8	0.070879	6.236165	0.69425	0.489346	-6.13659	0.594578	0.642631
Neutrophils	PPARG	0.267527	2.579221	0.694207	0.489372	-5.38851	0.659851	0.71151
Neutrophils	EMC3	-0.10102	5.490353	-0.6942	0.489377	-5.82481	0.607322	0.656136
Neutrophils	UBR4	-0.06829	6.056739	-0.69412	0.489426	-6.1342	0.597618	0.645898
Neutrophils	ZMIZ1OS1	0.498348	0.352543	0.694103	0.489438	-4.93387	0.703162	0.756963
Neutrophils	EPB41L1	-0.42741	1.011227	-0.6939	0.489563	-5.00743	0.690082	0.743359
Neutrophils	FBXO42	0.094863	5.871653	0.69388	0.489577	-6.08124	0.600793	0.649337
Neutrophils	MAN1A	0.117901	8.000333	0.693673	0.489706	-6.31597	0.565637	0.612038
Neutrophils	HSPA14	0.128492	5.034968	0.693375	0.489892	-5.76004	0.615509	0.664738
Neutrophils	SYNC	0.235012	1.973724	0.692464	0.49046	-5.1551	0.672108	0.724265
Neutrophils	WRB	-0.33431	1.437969	-0.69245	0.490467	-5.02465	0.682467	0.735132
Neutrophils	KIF2A	0.078716	6.338703	0.692397	0.490503	-6.12763	0.593515	0.641428
Neutrophils	ZMAT3	0.415529	1.902429	0.69235	0.490532	-5.00239	0.673477	0.725729
Neutrophils	AGRN	-0.2907	1.254784	-0.69215	0.490654	-5.23424	0.686046	0.738965
Neutrophils	POLR3E	-0.2049	3.918387	-0.69195	0.490784	-5.24714	0.635841	0.686304
Neutrophils	PPIG	0.057475	6.974522	0.691872	0.49083	-6.2838	0.582893	0.630357
Neutrophils	OTUB1	0.073626	5.728978	0.691823	0.490861	-5.98603	0.603893	0.652599
Neutrophils	APH1A	-0.11136	5.433388	-0.69179	0.490885	-5.92259	0.608992	0.657999
Neutrophils	IPPK	-0.1917	3.400734	-0.69172	0.490928	-5.40218	0.645294	0.696302
Neutrophils	MICAL3	0.222271	3.436889	0.6916	0.491	-5.3248	0.644629	0.695608
Neutrophils	SMARCA2	-0.06835	6.123463	-0.69157	0.491017	-6.32963	0.597157	0.645484
Neutrophils	ZFP90	-0.32049	2.283533	-0.69146	0.491087	-5.09614	0.666191	0.718289
Neutrophils	PLXNC1	-0.0915	5.427157	-0.69136	0.49115	-6.38358	0.6091	0.658157
Neutrophils	SV2C	0.448604	-0.28271	0.691259	0.491214	-4.92716	0.716843	0.771468
Neutrophils	BARD1	0.212737	3.42386	0.691233	0.49123	-5.46595	0.644869	0.695955
Neutrophils	NEURL1B	-0.34864	0.43588	-0.69118	0.491266	-4.99256	0.702279	0.756251
Neutrophils	CPSF2	-0.10826	5.649423	-0.69101	0.491369	-5.91244	0.605261	0.654166
Neutrophils	ANKRA2	0.139607	3.792457	0.690982	0.491387	-5.52459	0.638127	0.688872

Neutrophils	GLS	-0.06771	7.7257	-0.69049	0.491693	-6.5571	0.570847	0.617558
Neutrophils	GM26901	0.308693	0.57091	0.690448	0.491721	-5.03358	0.699878	0.753594
Neutrophils	AGTPBP1	0.093332	5.128653	0.690104	0.491936	-6.12675	0.614755	0.663939
Neutrophils	PHETA1	-0.41387	0.450779	-0.68987	0.492085	-4.96311	0.702573	0.756288
Neutrophils	HERPUD2	0.065585	5.474289	0.689699	0.49219	-6.189	0.608797	0.657683
Neutrophils	RAB11B	0.058263	7.147669	0.689593	0.492256	-6.37486	0.580524	0.627791
Neutrophils	FMO1	-0.2813	2.23787	-0.68959	0.492257	-5.39472	0.667623	0.71972
Neutrophils	TIMM17B	0.078861	5.161183	0.689541	0.492289	-5.91927	0.614246	0.663517
Neutrophils	COG8	-0.1385	4.299473	-0.6893	0.492441	-5.49761	0.629622	0.679696
Neutrophils	NLK	-0.10901	5.966033	-0.68843	0.492984	-6.00072	0.600609	0.649248
Neutrophils	SLC12A3	0.37443	1.874484	0.688351	0.493034	-5.0774	0.674884	0.727594
Neutrophils	SETDB1	0.133052	4.832864	0.688335	0.493044	-5.6725	0.620289	0.670104
Neutrophils	GTF3C6	-0.10367	5.226124	-0.6883	0.493066	-5.77624	0.613384	0.66282
Neutrophils	GOLGA1	-0.16954	3.427325	-0.68804	0.493228	-5.39648	0.645636	0.696852
Neutrophils	CAMK1D	0.083231	8.628354	0.68801	0.493248	-6.62892	0.556908	0.602929
Neutrophils	RHOBTB1	-0.24688	3.180961	-0.68797	0.493274	-5.41971	0.650188	0.701646
Neutrophils	PHGDH	0.27614	4.379094	0.6879	0.493316	-5.41237	0.628358	0.678635
Neutrophils	INTS6L	0.085369	5.229737	0.687846	0.49335	-6.17771	0.613321	0.662754
Neutrophils	KCNIP3	0.423233	0.193763	0.687642	0.493478	-5.07703	0.708064	0.762416
Neutrophils	ATP13A3	0.059361	7.315105	0.687575	0.49352	-6.35258	0.578029	0.625397
Neutrophils	NOMO1	-0.17924	3.5373	-0.68729	0.493699	-5.39339	0.643615	0.694818
Neutrophils	GM27253	-0.38942	1.3478	-0.68726	0.49372	-4.95524	0.685109	0.738442
Neutrophils	ATP5MD	-0.06711	8.035566	-0.6872	0.493756	-6.49916	0.566339	0.613041
Neutrophils	SPIDR	0.076849	5.228567	0.687165	0.493777	-6.18563	0.613342	0.662866
Neutrophils	SEPHS1	-0.13579	4.119783	-0.68702	0.493866	-5.44827	0.633017	0.683645
Neutrophils	LIN7C	-0.09516	4.956273	-0.68702	0.493867	-5.90456	0.618114	0.66791
Neutrophils	PLEKHG3	0.116098	4.378559	0.686926	0.493927	-5.9299	0.628367	0.678771
Neutrophils	ACAP3	0.197905	2.238471	0.68682	0.493994	-5.24832	0.667909	0.720442
Neutrophils	GNA13	-0.0847	7.201206	-0.68681	0.493998	-6.3257	0.579901	0.627496
Neutrophils	TBC1D32	-0.321	2.141265	-0.68676	0.494029	-5.11602	0.669764	0.722395
Neutrophils	KITL	-0.34607	1.956944	-0.68662	0.49412	-5.39377	0.673297	0.726169
Neutrophils	GAN	0.176276	4.031697	0.686574	0.494148	-5.52596	0.634608	0.685442
Neutrophils	GM26789	0.377212	0.271556	0.686419	0.494245	-5.03856	0.706493	0.760989
Neutrophils	CHAC2	-0.18436	2.893423	-0.68641	0.494251	-5.32334	0.655543	0.707508
Neutrophils	SHPRH	0.157363	4.541567	0.686339	0.494295	-5.51536	0.625456	0.675812
Neutrophils	RCL1	-0.17581	4.24025	-0.68631	0.494313	-5.40711	0.630848	0.681509
Neutrophils	PSMC4	-0.10936	5.46131	-0.6863	0.494323	-5.87079	0.609293	0.658732
Neutrophils	CTBP2	0.157963	3.249836	0.686273	0.494337	-5.61968	0.648912	0.700557
Neutrophils	VRK1	0.081204	5.309256	0.68618	0.494395	-6.12113	0.611935	0.661561
Neutrophils	NME6	0.214908	2.576439	0.685645	0.494731	-5.3358	0.661731	0.713917
Neutrophils	MAU2	0.113657	6.192162	0.685598	0.49476	-6.00755	0.596971	0.645585
Neutrophils	EIF5A	0.071178	9.061608	0.685532	0.494802	-6.64637	0.550315	0.596049
Neutrophils	LYPLAL1	-0.37875	1.634017	-0.68545	0.494854	-5.08319	0.679772	0.732914
Neutrophils	RRM2B	0.139409	4.437435	0.685417	0.494874	-5.61502	0.627536	0.677925
Neutrophils	ZFP282	-0.20941	4.010784	-0.68511	0.495066	-5.32853	0.635293	0.686024
Neutrophils	CLPP	0.149838	4.725598	0.685107	0.495069	-5.54055	0.622487	0.672509
Neutrophils	AMFR	0.060911	6.011988	0.685023	0.495121	-6.23806	0.600114	0.64886

Neutrophils	NFIX	-0.17105	3.676603	-0.68471	0.495321	-5.59947	0.641537	0.69254
Neutrophils	AK5	-0.43799	0.348906	-0.68444	0.49549	-4.94616	0.705454	0.759706
Neutrophils	GM13205	-0.37198	0.234435	-0.68441	0.495507	-4.98454	0.707765	0.762125
Neutrophils	ZFP597	0.1748	2.862663	0.684402	0.495512	-5.47456	0.656602	0.70844
Neutrophils	TRA2A	-0.05663	7.075692	-0.68434	0.495552	-6.47486	0.5824	0.630062
Neutrophils	THY1	-0.36363	1.573983	-0.68412	0.49569	-5.09979	0.681307	0.734351
Neutrophils	PROX1OS	-0.3563	0.612207	-0.68395	0.495798	-5.09622	0.700298	0.754286
Neutrophils	TPM1	-0.16306	5.177563	-0.68383	0.49587	-5.69946	0.614799	0.66438
Neutrophils	5930403N2	0.437334	-0.48768	0.683759	0.495915	-4.94412	0.722648	0.777746
Neutrophils	TIAM2	-0.15235	2.628932	-0.68366	0.495977	-5.89657	0.661117	0.713262
Neutrophils	MBD1	-0.12871	4.580853	-0.68361	0.496009	-5.69111	0.625333	0.675564
Neutrophils	FZR1	0.146216	4.900739	0.682742	0.496555	-5.69157	0.620131	0.669579
Neutrophils	GPX7	-0.39829	0.610424	-0.68271	0.496575	-5.02401	0.700862	0.75444
Neutrophils	GM26549	0.143196	3.018851	0.682575	0.49666	-5.59822	0.654297	0.705668
Neutrophils	CELSR2	0.483983	-0.09555	0.682562	0.496668	-4.91471	0.715138	0.769463
Neutrophils	TTPA	0.307249	1.860466	0.682273	0.49685	-5.26496	0.676287	0.728841
Neutrophils	CLPTM1	-0.09457	5.552635	-0.68226	0.496857	-5.85307	0.608732	0.657676
Neutrophils	PPCS	-0.22895	2.732539	-0.68226	0.49686	-5.16856	0.659663	0.711378
Neutrophils	B230354K1	-0.35462	1.756956	-0.68222	0.496881	-5.05672	0.678288	0.730944
Neutrophils	AP3S2	0.201173	3.43138	0.682061	0.496983	-5.28796	0.646645	0.69772
Neutrophils	ADCY3	-0.40671	3.001893	-0.68201	0.497014	-5.16124	0.654614	0.70611
Neutrophils	NXPE2	0.331902	1.56591	0.681948	0.497054	-5.12804	0.681998	0.734878
Neutrophils	PIKFYVE	0.114614	4.426045	0.681821	0.497134	-5.80546	0.628595	0.678682
Neutrophils	SOGA1	0.194238	3.807296	0.681681	0.497222	-5.503	0.639811	0.690538
Neutrophils	LIMA1	-0.17904	4.507655	-0.68141	0.49739	-5.84813	0.627304	0.677252
Neutrophils	TADA3	0.152121	3.403164	0.68125	0.497494	-5.52426	0.647416	0.698467
Neutrophils	FAM120C	-0.27255	3.050639	-0.68107	0.497604	-5.17854	0.653958	0.70541
Neutrophils	MMAA	-0.31203	1.748976	-0.68101	0.497644	-5.0966	0.678706	0.731415
Neutrophils	TBC1D9	-0.24921	3.953614	-0.68087	0.497733	-5.49101	0.637337	0.687908
Neutrophils	GM50019	-0.36483	-0.06748	-0.68078	0.497789	-5.04457	0.714842	0.769267
Neutrophils	PPWD1	0.096501	4.68019	0.680646	0.497874	-5.76616	0.62428	0.674135
Neutrophils	B4GAT1	-0.27595	1.65015	-0.68064	0.497881	-5.15784	0.680624	0.733429
Neutrophils	SUGCT	0.175385	2.835527	0.680576	0.497919	-5.55767	0.657983	0.709647
Neutrophils	WAPL	0.062978	7.165135	0.680326	0.498076	-6.51264	0.581744	0.629138
Neutrophils	ZFP64	-0.18751	5.317866	-0.68031	0.498083	-5.58302	0.613101	0.66234
Neutrophils	NBAS	-0.14961	4.535139	-0.68016	0.498183	-5.54995	0.626967	0.676951
Neutrophils	IGIP	-0.37257	0.675003	-0.67993	0.498323	-4.99547	0.700067	0.753821
Neutrophils	VPS28	0.068322	6.686374	0.679709	0.498464	-6.38772	0.58994	0.637746
Neutrophils	ABCA13	-0.11811	0.73813	-0.67953	0.498578	-6.25599	0.698992	0.752578
Neutrophils	2310057M	-0.19279	2.710086	-0.67909	0.498855	-5.22503	0.661017	0.712512
Neutrophils	NUDT19	0.183071	4.544356	0.678915	0.498965	-5.44411	0.627402	0.677135
Neutrophils	TANGO6	-0.12625	4.382985	-0.67814	0.499454	-5.73911	0.630596	0.680439
Neutrophils	ACTR3B	-0.36611	-0.01555	-0.67805	0.499513	-4.91673	0.714927	0.768954
Neutrophils	CWC25	0.094487	5.255579	0.678014	0.499534	-6.16575	0.615121	0.664109
Neutrophils	TRIM59	0.147892	4.368452	0.677962	0.499566	-5.75147	0.630857	0.680715
Neutrophils	INTS4	-0.18222	3.920928	-0.6779	0.499605	-5.36948	0.638953	0.689247
Neutrophils	MPHOSPH8	-0.11945	4.79616	-0.67789	0.49961	-5.63343	0.623219	0.672658

Neutrophils	D5ERTD579	0.122706	5.221315	0.677839	0.499644	-5.75145	0.615721	0.664767
Neutrophils	CYSTM1	0.261709	3.36709	0.677441	0.499895	-5.54367	0.649121	0.700065
Neutrophils	TMED2	0.057138	8.136615	0.677373	0.499938	-6.54265	0.566794	0.613058
Neutrophils	ORMDL2	-0.11348	5.109617	-0.67707	0.500126	-5.78264	0.617682	0.667076
Neutrophils	ENGASE	-0.33843	1.496503	-0.67701	0.500168	-5.13858	0.684711	0.737655
Neutrophils	DAZAP1	-0.07076	6.776612	-0.67691	0.500228	-6.18313	0.589089	0.63689
Neutrophils	CCNL1	0.054881	7.09056	0.676854	0.500266	-6.49582	0.583861	0.63137
Neutrophils	GM12971	-0.35319	0.967071	-0.67683	0.500282	-4.96451	0.695141	0.748657
Neutrophils	CD300C2	0.116611	4.335062	0.676813	0.500292	-6.19651	0.631457	0.681711
Neutrophils	SNHG20	-0.24727	2.355617	-0.67662	0.500416	-5.09124	0.668124	0.720342
Neutrophils	ANAPC15	0.11552	4.974484	0.6765	0.500489	-5.82669	0.620062	0.669722
Neutrophils	AP5M1	0.152389	3.584616	0.676249	0.500648	-5.52308	0.645108	0.696139
Neutrophils	DAZAP2	0.057247	7.70346	0.676238	0.500655	-6.53824	0.573796	0.620726
Neutrophils	COMMD3	0.098177	5.85015	0.676121	0.500729	-5.94266	0.604805	0.653592
Neutrophils	RXRB	0.136526	4.393184	0.676097	0.500744	-5.63131	0.630412	0.680647
Neutrophils	FBXL2	0.169551	3.231347	0.675996	0.500808	-5.49678	0.651639	0.703035
Neutrophils	EIF4E3	-0.11401	4.420461	-0.67595	0.500834	-5.91275	0.629923	0.680158
Neutrophils	KNG2	-0.26679	2.382731	-0.67594	0.500844	-5.34387	0.667607	0.719838
Neutrophils	JCAD	-0.24802	1.404554	-0.6759	0.50087	-5.1966	0.686511	0.739683
Neutrophils	ZFP267	-0.32955	1.251689	-0.67584	0.500904	-4.96189	0.689514	0.742836
Neutrophils	ZFP639	0.159144	4.128657	0.675831	0.500912	-5.48185	0.635182	0.68571
Neutrophils	NDUFA8	0.090332	5.798653	0.675749	0.500964	-6.14111	0.605691	0.654576
Neutrophils	ARFGEF2	-0.07108	6.285468	-0.67565	0.501029	-6.37129	0.597366	0.645779
Neutrophils	SMARCAD1	0.124094	4.984115	0.675621	0.501045	-5.59501	0.619892	0.669605
Neutrophils	FGD4	-0.07723	4.134435	-0.67537	0.501206	-6.54883	0.635077	0.685632
Neutrophils	ZFP157	-0.28612	2.446699	-0.67531	0.50124	-5.13264	0.66639	0.718597
Neutrophils	ASAH2	0.196097	2.945427	0.67529	0.501254	-5.50139	0.656975	0.708697
Neutrophils	AAGAB	0.119804	4.862045	0.675244	0.501283	-5.71841	0.622051	0.671884
Neutrophils	SCD2	0.169782	4.726396	0.675092	0.501379	-5.70052	0.624467	0.674426
Neutrophils	GM44710	-0.27206	2.000891	-0.67504	0.501413	-5.23954	0.674933	0.727561
Neutrophils	ZMIZ2	0.171848	4.532649	0.674932	0.50148	-5.41436	0.627931	0.678074
Neutrophils	TSNAX	-0.16828	4.49606	-0.67435	0.501848	-5.44272	0.628969	0.678981
Neutrophils	ATP6V1G2	0.285309	1.237127	0.674052	0.502037	-5.13208	0.690269	0.743451
Neutrophils	ATP11A	0.106911	2.676915	0.674034	0.502048	-5.93386	0.662476	0.714279
Neutrophils	GM43445	-0.23088	1.896237	-0.67399	0.502076	-5.24493	0.677401	0.72997
Neutrophils	TRMT112	0.07669	7.277188	0.673933	0.502112	-6.27728	0.581171	0.628465
Neutrophils	TTC23	-0.2886	1.101964	-0.67383	0.502179	-5.14965	0.692946	0.746315
Neutrophils	CACNA1F	-0.43865	-0.45662	-0.67358	0.502334	-4.94705	0.724625	0.779468
Neutrophils	MVD	0.205328	2.348846	0.673471	0.502404	-5.36816	0.668851	0.721067
Neutrophils	SNED1	-0.16699	1.751149	-0.67311	0.502635	-5.71885	0.680552	0.733199
Neutrophils	USP34	-0.05861	7.703229	-0.67305	0.502669	-6.47143	0.574476	0.621272
Neutrophils	PRM1	-0.41031	-0.44781	-0.67285	0.5028	-4.92272	0.724728	0.779396
Neutrophils	STK32C	-0.37742	0.553606	-0.67255	0.502985	-4.98094	0.704292	0.758134
Neutrophils	NT5DC1	0.107649	4.5014	0.672553	0.502985	-5.88282	0.629271	0.679306
Neutrophils	HMMR	-0.19361	4.333361	-0.67245	0.503049	-5.91116	0.63229	0.682546
Neutrophils	UBE2G2	-0.11181	4.661055	-0.67242	0.503069	-5.7161	0.626416	0.676363
Neutrophils	ATRX	0.062161	7.666179	0.6724	0.503083	-6.45139	0.575133	0.622088

Neutrophils	GTPBP8	-0.22461	2.317318	-0.67213	0.503256	-5.20999	0.669808	0.722052
Neutrophils	HSDL1	0.112611	4.167902	0.672084	0.503283	-5.63486	0.635376	0.685814
Neutrophils	8430429K0	-0.22995	1.679457	-0.67194	0.503375	-5.18096	0.682157	0.735013
Neutrophils	FBP1	0.176268	5.143064	0.671745	0.503498	-6.10588	0.618087	0.667551
Neutrophils	SPIRE1	-0.31114	2.681156	-0.6716	0.503588	-5.28198	0.663051	0.714913
Neutrophils	GM12992	-0.31274	2.041763	-0.67125	0.503808	-5.18011	0.675393	0.727815
Neutrophils	FITM2	-0.31555	0.918829	-0.67117	0.503865	-4.98572	0.697398	0.750894
Neutrophils	TEAD2	-0.29569	1.154161	-0.67104	0.503947	-5.11347	0.692727	0.74606
Neutrophils	SEC13	-0.09746	5.361757	-0.6709	0.504031	-5.88119	0.614409	0.663661
Neutrophils	ATL2	0.098317	5.337211	0.67087	0.504052	-5.84947	0.614839	0.664115
Neutrophils	SEC22B	0.086585	5.449712	0.670866	0.504055	-6.00129	0.612874	0.662038
Neutrophils	DDRGK1	0.108463	5.272107	0.670649	0.504192	-5.76104	0.616072	0.665423
Neutrophils	ATP1B3	0.114588	7.281647	0.670215	0.504467	-6.33841	0.582058	0.629293
Neutrophils	KIF20B	0.225096	4.170808	0.670137	0.504517	-5.67387	0.635899	0.686234
Neutrophils	YEATS2	0.138776	4.376137	0.670084	0.504551	-5.54333	0.63219	0.682326
Neutrophils	PA2G4	-0.12321	6.688076	-0.67001	0.504596	-6.02026	0.591949	0.639792
Neutrophils	P2RX7	-0.2989	3.223954	-0.66986	0.504693	-5.21786	0.65332	0.704581
Neutrophils	VIPAS39	-0.11089	4.131233	-0.66979	0.504738	-5.65325	0.63664	0.687024
Neutrophils	SLAMF8	-0.52676	0.718707	-0.66955	0.504891	-5.01351	0.701748	0.755502
Neutrophils	SHISA8	0.466879	-0.21439	0.66945	0.504953	-4.94207	0.720702	0.775372
Neutrophils	FCSK	-0.35477	0.928399	-0.66944	0.504962	-5.01917	0.697558	0.751158
Neutrophils	TSPYL3	-0.39337	0.960124	-0.6694	0.504982	-5.02874	0.696926	0.750496
Neutrophils	HERC4	-0.06277	7.008496	-0.66884	0.50534	-6.50791	0.586954	0.634477
Neutrophils	ITPKC	0.305428	1.763821	0.668646	0.505463	-5.12952	0.681599	0.734254
Neutrophils	TNFAIP8	-0.06153	7.424897	-0.66838	0.505632	-6.58067	0.580193	0.627223
Neutrophils	RNASEH2C	-0.08005	4.998911	-0.66822	0.505733	-6.12612	0.621608	0.671061
Neutrophils	1810037I1	0.067492	6.482621	0.668205	0.505744	-6.44209	0.595924	0.643906
Neutrophils	ARHGAP22	-0.46551	1.023886	-0.66808	0.505823	-5.01086	0.696228	0.749531
Neutrophils	KCTD18	0.226874	3.044012	0.668077	0.505825	-5.38857	0.65722	0.708592
Neutrophils	CYBB	-0.09942	7.155496	-0.66779	0.506007	-6.7888	0.584701	0.632039
Neutrophils	MAP2	0.441891	0.187932	0.667638	0.506104	-4.9256	0.713119	0.767281
Neutrophils	VCPIP1	-0.06724	6.167501	-0.66763	0.506106	-6.22128	0.601342	0.649711
Neutrophils	ATG13	-0.12706	4.765899	-0.66755	0.506161	-5.74542	0.625806	0.67558
Neutrophils	LOCKD	-0.17443	4.472202	-0.66748	0.506205	-5.80725	0.631062	0.681126
Neutrophils	LEO1	0.15915	4.138442	0.667262	0.506343	-5.53004	0.637091	0.687541
Neutrophils	RIC8A	0.126001	3.980741	0.667251	0.506349	-5.64896	0.639961	0.690565
Neutrophils	CRBN	0.147055	4.060609	0.667229	0.506364	-5.49271	0.638506	0.689038
Neutrophils	LAP3	-0.18078	4.901491	-0.66694	0.506545	-5.65755	0.623456	0.673182
Neutrophils	MNAT1	-0.15208	4.818214	-0.66666	0.506722	-5.55512	0.624936	0.674822
Neutrophils	GM16675	-0.29588	1.196561	-0.66666	0.506724	-5.22559	0.692939	0.746352
Neutrophils	RBAK	-0.34777	1.336784	-0.66657	0.50678	-5.00696	0.69017	0.74345
Neutrophils	GM37529	0.351937	1.962925	0.666559	0.506789	-5.10186	0.677942	0.730622
Neutrophils	SEMA6B	-0.24819	1.154578	-0.66653	0.506808	-5.2692	0.69377	0.747223
Neutrophils	FMNL3	0.097575	4.258409	0.666475	0.506843	-6.01522	0.63498	0.68544
Neutrophils	GM8113	0.434496	-0.25687	0.666294	0.506958	-4.91245	0.722385	0.777132
Neutrophils	PGAP1	-0.24804	3.785443	-0.66604	0.50712	-5.37546	0.643792	0.694599
Neutrophils	USP46	-0.12143	3.281753	-0.66577	0.507293	-5.85056	0.653145	0.70451

Neutrophils	TSEN54	-0.2498	2.874509	-0.66569	0.50734	-5.10161	0.660776	0.712581
Neutrophils	MCCC2	-0.32676	2.436244	-0.66565	0.507368	-5.18354	0.669091	0.721348
Neutrophils	SLC25A26	-0.21927	3.795963	-0.6656	0.507397	-5.30495	0.64364	0.694579
Neutrophils	NLRC4	0.161687	3.885651	0.665452	0.507494	-5.60736	0.642041	0.692847
Neutrophils	GLIPR1	-0.07002	5.059395	-0.66533	0.507571	-6.38517	0.62095	0.670626
Neutrophils	DEK	0.075591	7.842	0.665176	0.507669	-6.46613	0.573764	0.620671
Neutrophils	GM10790	0.388578	-0.77429	0.665119	0.507706	-4.90648	0.733424	0.788757
Neutrophils	RTEL1	0.252192	2.682882	0.664729	0.507954	-5.25863	0.664726	0.716537
Neutrophils	CRYZL1	0.099003	4.570122	0.664475	0.508116	-5.8915	0.629991	0.679944
Neutrophils	HDGFL3	-0.27862	1.511028	-0.66443	0.508144	-5.23965	0.687426	0.740343
Neutrophils	UGP2	-0.07509	5.910415	-0.66366	0.508637	-6.37835	0.606753	0.655247
Neutrophils	LRRC47	-0.14268	4.227098	-0.66356	0.508698	-5.45668	0.636533	0.686702
Neutrophils	CAML	0.130223	4.427078	0.663513	0.508729	-5.56159	0.632917	0.68289
Neutrophils	GNPAT	-0.1321	4.15461	-0.66347	0.508757	-5.61581	0.637849	0.688091
Neutrophils	H2AFZ	-0.08246	10.35837	-0.6632	0.508931	-6.95822	0.534905	0.579027
Neutrophils	HBQ1A	0.361543	-1.39639	0.663172	0.508946	-4.94417	0.747363	0.80289
Neutrophils	UBAP2L	-0.05938	7.113568	-0.66316	0.508953	-6.29059	0.586364	0.633728
Neutrophils	PAQR3	-0.27721	1.469925	-0.66316	0.508954	-5.09381	0.688618	0.7415
Neutrophils	ZER1	-0.1804	3.262662	-0.66301	0.509052	-5.32107	0.654275	0.705471
Neutrophils	PLOD3	0.136663	3.277379	0.662964	0.509078	-5.71701	0.654	0.705185
Neutrophils	TERF2	-0.13041	4.800606	-0.66287	0.509138	-5.56226	0.626219	0.675965
Neutrophils	MALSU1	-0.10003	4.843801	-0.66283	0.509165	-5.94789	0.625449	0.675163
Neutrophils	CENPW	0.159409	4.349764	0.662701	0.509246	-5.75222	0.634337	0.684558
Neutrophils	CTNS	-0.16316	3.606042	-0.66255	0.509345	-5.53985	0.647957	0.698957
Neutrophils	NECAB3	0.312166	1.186088	0.662469	0.509394	-5.06901	0.694283	0.747647
Neutrophils	FNDC3A	0.07485	7.526942	0.66236	0.509464	-6.39102	0.579587	0.626751
Neutrophils	ARHGAP15	0.056914	9.872031	0.662219	0.509553	-6.965	0.542383	0.587204
Neutrophils	MRPS17	-0.10043	5.224333	-0.66213	0.509613	-5.85532	0.618804	0.668325
Neutrophils	TNFSF9	0.340049	3.435421	0.662034	0.509671	-5.25341	0.651157	0.702494
Neutrophils	RAP1GAP	-0.36323	0.58551	-0.66179	0.509826	-5.03115	0.706446	0.760473
Neutrophils	DICER1	0.109253	4.846948	0.661718	0.509873	-5.67779	0.625585	0.675508
Neutrophils	CARMIL1	-0.22029	4.075468	-0.66147	0.510033	-5.37137	0.639607	0.690264
Neutrophils	AFMID	-0.17693	4.371552	-0.66125	0.51017	-5.4978	0.634329	0.684672
Neutrophils	GLRX	0.065893	6.214402	0.661132	0.510247	-6.59156	0.601939	0.650463
Neutrophils	CNBD2	-0.13367	3.889219	-0.66084	0.510435	-5.7898	0.643286	0.693975
Neutrophils	D10WSU1(	0.200036	3.652731	0.66073	0.510503	-5.36993	0.647646	0.698599
Neutrophils	PDCD1	-0.57174	0.743001	-0.6605	0.510648	-4.96752	0.703745	0.757535
Neutrophils	SULF2	-0.27294	2.585463	-0.66048	0.51066	-5.23649	0.667685	0.719716
Neutrophils	ZFP930	0.308418	2.204594	0.660327	0.510761	-5.04902	0.674982	0.727434
Neutrophils	ZFP566	-0.36991	1.254913	-0.6603	0.51078	-4.98503	0.693532	0.74691
Neutrophils	DOCK8	0.07048	7.656272	0.660222	0.510828	-6.5649	0.577965	0.625064
Neutrophils	NAA50	-0.07821	6.371464	-0.65989	0.511042	-6.04793	0.599614	0.647966
Neutrophils	HGS	0.132674	4.094551	0.659645	0.511196	-5.59854	0.63987	0.690432
Neutrophils	MGAM	0.194173	0.078138	0.659525	0.511273	-5.56899	0.717602	0.771998
Neutrophils	CYTH2	-0.14155	3.933028	-0.65927	0.511433	-5.62183	0.642896	0.693571
Neutrophils	FAM167A	0.302452	1.837572	0.659119	0.511532	-5.25557	0.682496	0.735242
Neutrophils	KCTD21	-0.37803	0.182578	-0.65911	0.511539	-5.05459	0.715526	0.769834

Neutrophils	DHX57	-0.16619	3.871504	-0.65903	0.51159	-5.41286	0.644024	0.694797
Neutrophils	OSGEPL1	0.195152	2.472736	0.658976	0.511624	-5.26534	0.670235	0.72237
Neutrophils	ZFHX3	-0.0794	5.163754	-0.65877	0.511755	-6.27887	0.620783	0.670299
Neutrophils	DFFB	-0.22887	2.981393	-0.65874	0.511774	-5.24789	0.660614	0.712276
Neutrophils	GM12940	-0.12292	4.51886	-0.65857	0.511881	-5.80143	0.632335	0.682451
Neutrophils	ATP5K	0.082251	7.486519	0.658488	0.511935	-6.43445	0.581175	0.628335
Neutrophils	SLC25A44	0.161869	3.239164	0.658153	0.51215	-5.48204	0.655962	0.707304
Neutrophils	4933432I0:	0.143125	0.408312	0.658079	0.512198	-5.79527	0.711168	0.765212
Neutrophils	MCEMP1	0.101108	1.650063	0.658032	0.512228	-6.39907	0.686392	0.739264
Neutrophils	CLK2	0.111366	4.63296	0.657807	0.512371	-5.6477	0.630517	0.680469
Neutrophils	CKLF	0.085368	4.804772	0.657692	0.512445	-6.06104	0.627454	0.677256
Neutrophils	CYP2D9	-0.30768	0.530177	-0.65757	0.512524	-5.09922	0.708851	0.762809
Neutrophils	ZCCHC17	-0.10043	5.162664	-0.65713	0.512802	-5.80019	0.621139	0.67071
Neutrophils	STXBP4	-0.33788	2.23619	-0.65703	0.512871	-5.09254	0.675177	0.727618
Neutrophils	RAPGEF4	0.268911	2.563789	0.656832	0.512995	-5.27082	0.668894	0.721041
Neutrophils	GM17103	-0.28039	1.028835	-0.65683	0.512997	-5.24171	0.698855	0.752474
Neutrophils	ZFP973	-0.37416	0.609416	-0.65672	0.513065	-4.95784	0.707276	0.761325
Neutrophils	NSA2	-0.05522	7.622863	-0.65665	0.513115	-6.35363	0.579202	0.626384
Neutrophils	MTHFD1	-0.19123	3.79072	-0.65662	0.51313	-5.37038	0.645893	0.696901
Neutrophils	DYNLT1A	0.153769	4.14434	0.656589	0.51315	-5.56946	0.639416	0.690076
Neutrophils	AK8	-0.26872	2.179586	-0.65651	0.513202	-5.15385	0.676268	0.728899
Neutrophils	EGFEM1	-0.39302	1.229611	-0.6565	0.513209	-5.21052	0.69486	0.748403
Neutrophils	NTNG2	0.096628	3.05713	0.656382	0.513283	-6.28549	0.659546	0.711381
Neutrophils	COMMD8	0.092922	5.188265	0.656343	0.513308	-5.91296	0.620687	0.670428
Neutrophils	CBX4	0.102938	5.468948	0.656287	0.513344	-6.00752	0.615749	0.665224
Neutrophils	ANKDD1A	-0.23205	2.346094	-0.65595	0.513558	-5.73905	0.673262	0.725756
Neutrophils	CTPS2	-0.09781	5.066744	-0.65518	0.514053	-5.90901	0.623513	0.673052
Neutrophils	PLOD1	-0.15198	3.410314	-0.65513	0.514086	-5.72633	0.653644	0.70481
Neutrophils	DENND4C	-0.10936	5.068456	-0.65495	0.514197	-5.85895	0.623519	0.673091
Neutrophils	PROZ	0.264794	1.915226	0.654807	0.514292	-5.30789	0.682169	0.734872
Neutrophils	ITGAX	-0.16026	3.098724	-0.65479	0.5143	-5.73248	0.659517	0.711077
Neutrophils	SLC39A1	0.082818	6.161792	0.654595	0.514428	-6.0917	0.604497	0.652982
Neutrophils	CACNB1	-0.44242	0.846674	-0.65443	0.514531	-4.93484	0.703385	0.757047
Neutrophils	MYZAP	-0.21886	3.284402	-0.65441	0.514545	-5.34933	0.65611	0.707436
Neutrophils	FCGR4	0.156796	3.388468	0.654116	0.514735	-6.2077	0.654241	0.705457
Neutrophils	PKD1	-0.2042	3.003757	-0.65404	0.514781	-5.31979	0.661459	0.713066
Neutrophils	IFI30	-0.12983	6.746076	-0.65403	0.514789	-6.13237	0.594612	0.642523
Neutrophils	WDR89	0.215618	2.643388	0.653655	0.51503	-5.24635	0.668456	0.720441
Neutrophils	RAB6B	-0.30175	1.697022	-0.65364	0.515037	-5.07893	0.686757	0.739651
Neutrophils	1700037H(	-0.13095	3.714085	-0.65308	0.515399	-5.67175	0.64859	0.699405
Neutrophils	NUP54	0.108631	4.859149	0.653058	0.515413	-5.77548	0.627773	0.677464
Neutrophils	1700012D1	-0.27319	1.843803	-0.65296	0.515478	-5.14964	0.684136	0.736782
Neutrophils	ZFP316	0.437812	0.380007	0.652925	0.515499	-4.93856	0.713338	0.76737
Neutrophils	RGMB	0.273242	1.845211	0.65287	0.515534	-5.17542	0.684109	0.736786
Neutrophils	TMEM41B	0.106473	4.300772	0.652691	0.515649	-5.77515	0.637902	0.68816
Neutrophils	CHERP	-0.09612	5.016827	-0.6525	0.515769	-5.82469	0.625096	0.67461
Neutrophils	ZBTB33	-0.16547	2.982117	-0.65221	0.515957	-5.39915	0.662512	0.714022

Neutrophils	PFKM	-0.31544	1.17862	-0.65198	0.516103	-5.05369	0.697507	0.750757
Neutrophils	EXOG	0.295618	1.745846	0.651965	0.516115	-5.06684	0.686302	0.739015
Neutrophils	SCIMP	-0.29923	3.702449	-0.65194	0.516133	-5.27677	0.649042	0.699869
Neutrophils	LMBR1	0.236722	1.883516	0.651836	0.516198	-5.22614	0.68361	0.7362
Neutrophils	EIF3C	-0.07304	7.172674	-0.65181	0.516216	-6.25329	0.588026	0.635428
Neutrophils	GM45435	-0.40894	0.920103	-0.651	0.516734	-5.04605	0.703241	0.756352
Neutrophils	XLR	-0.3366	2.01867	-0.65095	0.516769	-5.23007	0.681525	0.733609
Neutrophils	VPS29	0.064815	6.521761	0.650874	0.516816	-6.24594	0.599478	0.647197
Neutrophils	OSBPL5	-0.40864	0.3884	-0.65067	0.516946	-4.97003	0.714039	0.767723
Neutrophils	UQCC1	-0.17306	3.934429	-0.65056	0.517019	-5.3922	0.645317	0.695642
Neutrophils	SLC9A8	0.103694	4.988676	0.650543	0.517029	-5.8894	0.626226	0.675524
Neutrophils	PPP1R8	-0.163	4.114233	-0.65005	0.517348	-5.43678	0.642251	0.692373
Neutrophils	GM12703	0.448827	-0.21463	0.650005	0.517374	-4.94193	0.726707	0.78091
Neutrophils	DNTTIP2	-0.09373	5.107858	-0.64994	0.517419	-5.81159	0.624331	0.673487
Neutrophils	SNRPG	-0.0684	8.018397	-0.64983	0.517487	-6.45106	0.574789	0.621083
Neutrophils	HIST3H2BA	-0.35255	0.997637	-0.64963	0.517619	-4.94375	0.701977	0.75514
Neutrophils	PRPF18	0.104746	4.616004	0.649591	0.517641	-5.83256	0.633136	0.682838
Neutrophils	ATP5J	-0.0661	7.873083	-0.64958	0.517648	-6.52063	0.577162	0.623647
Neutrophils	PKIB	0.288749	5.453591	0.649438	0.517739	-5.60356	0.618254	0.6671
Neutrophils	PPP1CA	0.055343	8.233158	0.64927	0.517847	-6.59038	0.571378	0.617439
Neutrophils	4921516A(	-0.29587	0.76983	-0.64907	0.517978	-5.09831	0.706653	0.760009
Neutrophils	IL31RA	0.207433	3.109325	0.64899	0.518027	-5.3716	0.661006	0.712185
Neutrophils	RAB10	0.072	7.670572	0.648969	0.518041	-6.41988	0.580563	0.627268
Neutrophils	SLC35B1	-0.1125	5.751128	-0.6489	0.518088	-5.86898	0.613091	0.661711
Neutrophils	ECM2	0.380542	0.502848	0.648748	0.518183	-5.07541	0.712107	0.765784
Neutrophils	PPP1R42	-0.16527	0.580161	-0.64838	0.518417	-5.69681	0.710699	0.764262
Neutrophils	DCAF7	-0.08702	5.327488	-0.64824	0.518507	-5.87767	0.620706	0.669723
Neutrophils	DET1	-0.28367	2.186379	-0.64813	0.518579	-5.14019	0.678842	0.730904
Neutrophils	PPP2R5E	-0.06003	6.820761	-0.64805	0.518634	-6.26436	0.594908	0.642486
Neutrophils	LRR1	-0.27602	1.435876	-0.64783	0.518775	-5.15107	0.693543	0.74637
Neutrophils	SNTA1	0.237151	1.865421	0.647779	0.518806	-5.2523	0.68509	0.737511
Neutrophils	REEP1	-0.24773	0.98372	-0.64771	0.518853	-5.29801	0.702555	0.755806
Neutrophils	ATG16L1	-0.09159	5.446329	-0.6477	0.518856	-6.02758	0.618611	0.667565
Neutrophils	DQX1	-0.65592	1.215444	-0.6477	0.518858	-4.98302	0.697922	0.750956
Neutrophils	GPSM1	-0.22835	2.672071	-0.64742	0.519041	-5.22588	0.669607	0.72125
Neutrophils	2010310C(	0.228481	1.298313	0.647327	0.519098	-5.53309	0.696386	0.749365
Neutrophils	MEGF11	-0.32285	0.651843	-0.64728	0.519128	-5.12333	0.709361	0.762944
Neutrophils	1110017D1	-0.38967	0.201816	-0.64716	0.519207	-5.06305	0.718562	0.772582
Neutrophils	SMG9	0.101873	4.52517	0.646705	0.519498	-5.85003	0.635452	0.685252
Neutrophils	TOE1	-0.24478	2.490022	-0.64606	0.519916	-5.16678	0.673876	0.725312
Neutrophils	DDHD2	0.11048	4.784447	0.645952	0.519983	-5.84316	0.631216	0.680526
Neutrophils	AFG3L1	0.081079	4.862083	0.645758	0.520108	-5.83403	0.62985	0.679113
Neutrophils	GNA14	-0.48843	-0.0617	-0.64572	0.52013	-4.97359	0.724847	0.778755
Neutrophils	TMEM268	-0.18769	3.100152	-0.64529	0.520411	-5.34307	0.662512	0.713423
Neutrophils	4930513N1	0.394187	0.475363	0.645255	0.520433	-4.94311	0.714058	0.767423
Neutrophils	NDUFB6	-0.09886	6.317819	-0.64474	0.520765	-6.15582	0.604801	0.652421
Neutrophils	AMMECR1	0.136401	4.488762	0.644673	0.520808	-5.631	0.637109	0.686503

Neutrophils	CDS1	-0.33771	2.788548	-0.64444	0.520958	-5.15416	0.668757	0.719875
Neutrophils	NDUFA9	-0.12311	4.840567	-0.64435	0.52102	-5.65358	0.630764	0.679916
Neutrophils	GM48765	0.349641	0.952243	0.644329	0.52103	-5.00421	0.704746	0.757579
Neutrophils	CENPT	0.186821	2.566821	0.644281	0.521061	-5.2874	0.673001	0.724348
Neutrophils	H2-K1	-0.1655	8.786019	-0.64386	0.521334	-6.54353	0.564093	0.60932
Neutrophils	PRCP	-0.11081	5.424232	-0.64365	0.52147	-5.83225	0.620569	0.669181
Neutrophils	SRP54A	-0.14986	3.599318	-0.64363	0.52148	-5.43496	0.653678	0.704052
Neutrophils	IGF2R	0.101688	4.985937	0.643506	0.521562	-6.0336	0.628358	0.677429
Neutrophils	GZMM	0.281299	2.051803	0.643388	0.521637	-5.17784	0.683182	0.73509
Neutrophils	A930005H1	-0.16982	3.220496	-0.64331	0.521687	-5.35161	0.660777	0.711579
Neutrophils	SCCPDH	-0.22124	2.465776	-0.64313	0.521804	-5.21089	0.675158	0.726746
Neutrophils	GM10382	-0.35155	0.439288	-0.64304	0.521862	-5.04205	0.715374	0.768874
Neutrophils	ANKS3	0.134421	4.082767	0.642999	0.521889	-5.61756	0.644732	0.694812
Neutrophils	TRMT10A	0.183335	3.81366	0.64297	0.521908	-5.36484	0.649696	0.70004
Neutrophils	ECD	-0.11774	4.899349	-0.64291	0.521947	-5.64178	0.629909	0.67922
Neutrophils	SCPEP1	-0.17607	5.169388	-0.64276	0.522043	-5.50461	0.625086	0.67415
Neutrophils	ANKRD9	-0.18354	2.821177	-0.64274	0.522056	-5.50875	0.668346	0.719681
Neutrophils	TMUB1	0.137105	3.138278	0.642682	0.522094	-5.46112	0.662328	0.713365
Neutrophils	ZC3H7B	-0.22511	3.353116	-0.64251	0.522206	-5.23163	0.658346	0.70913
Neutrophils	NEURL4	-0.29159	2.445552	-0.64237	0.522295	-5.19447	0.675648	0.727262
Neutrophils	MPP6	0.088405	6.194697	0.641733	0.522707	-6.08728	0.607617	0.65542
Neutrophils	RPA3	-0.12028	4.97161	-0.64158	0.522803	-5.85863	0.629153	0.678149
Neutrophils	SGSM3	-0.17382	4.063557	-0.64145	0.522888	-5.52597	0.645637	0.69558
Neutrophils	KCNG2	-0.40255	-0.23458	-0.64141	0.522913	-4.96736	0.7299	0.783869
Neutrophils	CHMP7	-0.20777	2.616172	-0.64126	0.523014	-5.29848	0.672842	0.724219
Neutrophils	PANK2	0.088041	5.327592	0.641161	0.523077	-5.94927	0.62281	0.671579
Neutrophils	NRF1	-0.06213	6.474245	-0.64095	0.523213	-6.22578	0.602831	0.650551
Neutrophils	PJA2	0.118878	4.695864	0.6409	0.523246	-5.82497	0.634112	0.683571
Neutrophils	TMEM258	-0.07517	6.825258	-0.64087	0.523267	-6.28306	0.596851	0.644232
Neutrophils	TXNL1	-0.06725	6.955558	-0.64084	0.523284	-6.4168	0.594647	0.641904
Neutrophils	PABPC1L	-0.11221	2.906471	-0.64068	0.52339	-5.97176	0.667348	0.718488
Neutrophils	GM19705	0.167053	1.868138	0.640378	0.523583	-5.67806	0.687524	0.739608
Neutrophils	BTBD3	0.312841	1.337187	0.64036	0.523594	-5.14872	0.698025	0.750604
Neutrophils	E2F1	-0.16436	4.409532	-0.6398	0.523959	-5.62773	0.639825	0.689284
Neutrophils	SMPD5	0.249871	-0.18019	0.639668	0.524042	-5.15841	0.729391	0.78314
Neutrophils	IL15	-0.09825	3.93993	-0.63945	0.524185	-6.39868	0.64857	0.698393
Neutrophils	BST2	-0.13922	6.606914	-0.63898	0.524489	-6.1675	0.601447	0.648513
Neutrophils	SPATA6	0.152126	4.52297	0.638738	0.524644	-5.61893	0.638293	0.687303
Neutrophils	PPP1R1C	0.320428	0.781145	0.63847	0.524818	-5.23682	0.710307	0.762744
Neutrophils	SUMF1	-0.1711	4.240634	-0.6384	0.524864	-5.47633	0.643545	0.692822
Neutrophils	EWSR1	-0.05532	7.421625	-0.63823	0.524972	-6.37061	0.58789	0.634151
Neutrophils	ILF3	-0.0884	6.541502	-0.63823	0.524973	-6.07658	0.602765	0.64988
Neutrophils	BLVRB	-0.14504	7.092023	-0.63801	0.525115	-6.24222	0.593461	0.640071
Neutrophils	SLC1A4	-0.33432	0.947937	-0.63797	0.525139	-5.04521	0.706986	0.759409
Neutrophils	WDFY3	0.081149	5.666809	0.637881	0.5252	-6.46989	0.617988	0.666003
Neutrophils	CUTA	0.076127	6.189562	0.63758	0.525395	-6.09179	0.608949	0.656448
Neutrophils	H19	0.212063	6.408472	0.63753	0.525428	-6.38429	0.605172	0.652482

Neutrophils	SLC7A5	-0.14576	6.118367	-0.63726	0.525601	-5.94061	0.610182	0.657798
Neutrophils	ARHGAP21	-0.11465	5.511128	-0.63715	0.525673	-5.87465	0.620811	0.669013
Neutrophils	BBX	-0.09775	6.494425	-0.63698	0.525785	-6.13968	0.603696	0.650947
Neutrophils	NSMCE4A	0.077917	6.083141	0.636974	0.525788	-6.05456	0.610794	0.658443
Neutrophils	2700062C0	0.215433	2.646093	0.636827	0.525883	-5.18708	0.673618	0.724624
Neutrophils	OAZ1	0.064988	9.534615	0.636788	0.525908	-6.74246	0.553856	0.598208
Neutrophils	ARF1	0.046544	8.02586	0.636762	0.525925	-6.59758	0.578022	0.623856
Neutrophils	AKR1C14	-0.28536	0.911524	-0.63676	0.525927	-5.13846	0.707813	0.760429
Neutrophils	GRK2	-0.05789	7.206149	-0.6367	0.525963	-6.45957	0.591618	0.638274
Neutrophils	WLS	-0.10142	4.263574	-0.63663	0.526013	-6.08711	0.643257	0.692774
Neutrophils	5730409E0	-0.39321	0.23499	-0.63639	0.526165	-4.96699	0.721633	0.774959
Neutrophils	SLC35D2	0.125758	4.232	0.636296	0.526228	-5.83396	0.643845	0.693503
Neutrophils	NUF2	-0.1763	3.365247	-0.63624	0.526266	-5.64691	0.65995	0.710437
Neutrophils	SAP30BP	0.127185	4.838744	0.636232	0.526269	-5.59813	0.632814	0.68189
Neutrophils	ALKBH1	0.151631	4.99535	0.635958	0.526447	-5.47501	0.630119	0.678994
Neutrophils	4930435F1	-0.33685	0.358699	-0.6358	0.526547	-5.12537	0.719224	0.772447
Neutrophils	DMGDH	-0.28029	1.667706	-0.63579	0.526556	-5.23756	0.692836	0.744861
Neutrophils	AP3M2	0.217091	2.489505	0.635519	0.526732	-5.20892	0.67692	0.728113
Neutrophils	NDFIP1	0.060405	7.521506	0.635348	0.526842	-6.4565	0.586606	0.633008
Neutrophils	TFPT	-0.16756	2.965912	-0.63531	0.526866	-5.3423	0.667794	0.718601
Neutrophils	SERPINB8	0.367645	-0.3114	0.635147	0.526973	-4.96923	0.7333	0.787181
Neutrophils	NDUFB1-PS	0.047515	8.902498	0.635115	0.526994	-6.73279	0.5641	0.609206
Neutrophils	TPPP3	0.377053	1.071021	0.634927	0.527116	-5.11427	0.704964	0.757602
Neutrophils	GM9530	-0.39358	-0.31965	-0.63486	0.527157	-4.9861	0.733528	0.787424
Neutrophils	NUPL2	-0.2727	2.367649	-0.63453	0.527374	-5.13214	0.679545	0.730838
Neutrophils	AIG1	-0.2004	4.903663	-0.63441	0.527449	-5.45532	0.632167	0.681042
Neutrophils	ZFP335OS	0.129625	4.456083	0.634176	0.527603	-5.72558	0.640303	0.68966
Neutrophils	NCOA7	0.153262	5.299685	0.634118	0.527641	-5.63331	0.625107	0.673652
Neutrophils	NATD1	-0.24588	3.25628	-0.63396	0.527746	-5.26647	0.662578	0.713118
Neutrophils	LIAS	-0.12157	4.310022	-0.63395	0.527748	-5.66192	0.642973	0.692508
Neutrophils	WDFY2	-0.07061	5.19857	-0.6339	0.527782	-6.38117	0.626908	0.675589
Neutrophils	NFE2L3	0.233584	2.246069	0.633753	0.527878	-5.29135	0.681996	0.733441
Neutrophils	TMEM50B	0.12669	4.107732	0.633532	0.528022	-5.87777	0.646756	0.696451
Neutrophils	UCK1	-0.22076	3.265642	-0.63344	0.528079	-5.31995	0.662469	0.712988
Neutrophils	CLNS1A	0.100608	5.340846	0.633303	0.528171	-5.74533	0.624439	0.672987
Neutrophils	IFI47	0.364699	5.277962	0.633237	0.528213	-5.59806	0.625558	0.674167
Neutrophils	EPS8L2	0.405788	0.893203	0.633107	0.528298	-4.99044	0.70888	0.761624
Neutrophils	EPHB4	-0.2928	0.794939	-0.63311	0.528299	-5.30296	0.710872	0.763706
Neutrophils	SLC25A14	-0.21642	2.078186	-0.63305	0.528334	-5.18423	0.685298	0.73695
Neutrophils	FBXW9	-0.20592	2.418265	-0.6328	0.528497	-5.22489	0.678713	0.730045
Neutrophils	D130020L0	0.330275	0.782996	0.632775	0.528514	-5.07262	0.711151	0.763993
Neutrophils	RIMS3	-0.35098	1.085548	-0.63262	0.528613	-5.09534	0.705033	0.757613
Neutrophils	NPEPPS	-0.05138	7.184287	-0.63258	0.52864	-6.72506	0.592597	0.639371
Neutrophils	PTRHD1	0.132514	4.56189	0.632467	0.528714	-5.70342	0.638474	0.687812
Neutrophils	KPNA2	0.121727	4.937072	0.632437	0.528734	-5.97225	0.631689	0.680681
Neutrophils	MBD3	-0.10502	5.696238	-0.63182	0.529137	-5.81469	0.618515	0.66651
Neutrophils	MDK	-0.30149	1.436872	-0.63178	0.529164	-5.14579	0.698363	0.750399

Neutrophils	STAT2	0.210865	5.210285	0.631721	0.529199	-5.71782	0.627126	0.675637
Neutrophils	HIST1H2BE	0.218871	2.079102	0.631559	0.529305	-5.34294	0.685714	0.737206
Neutrophils	PMM2	0.105072	4.561806	0.631485	0.529352	-5.66906	0.638847	0.688022
Neutrophils	KLHL12	-0.13292	4.259168	-0.63132	0.529458	-5.72761	0.644432	0.693854
Neutrophils	DNAJC10	0.10178	4.533218	0.631082	0.529615	-5.77976	0.639534	0.688722
Neutrophils	STX2	-0.09895	3.698442	-0.63084	0.52977	-5.77895	0.654994	0.704983
Neutrophils	APEX2	-0.15508	4.215093	-0.63079	0.529805	-5.46556	0.64542	0.694918
Neutrophils	CPSF1	-0.17143	3.349532	-0.63071	0.529858	-5.39326	0.661542	0.71187
Neutrophils	WDR34	0.400172	1.224098	0.630619	0.529916	-5.02312	0.70291	0.755218
Neutrophils	9330020H(	0.190717	2.471892	0.630268	0.530145	-5.35596	0.678527	0.729588
Neutrophils	RNF180	-0.29159	2.247716	-0.62992	0.530371	-5.24977	0.683026	0.734227
Neutrophils	MGAT4A	0.085928	5.666978	0.62975	0.530482	-6.16064	0.619613	0.667628
Neutrophils	WDR13	-0.18183	2.570105	-0.62974	0.530491	-5.25806	0.676771	0.727726
Neutrophils	CCL25	-0.16563	4.40296	-0.62963	0.530559	-5.5856	0.642313	0.691557
Neutrophils	CEBPA	-0.21075	2.906324	-0.62961	0.530574	-5.48372	0.670311	0.720969
Neutrophils	TWF1	0.084345	5.464598	0.629521	0.530631	-5.9542	0.62319	0.671449
Neutrophils	FRMD6	-0.21572	3.47737	-0.62938	0.530723	-5.40327	0.659519	0.709676
Neutrophils	PDZD2	-0.2548	3.46937	-0.62918	0.530851	-5.38373	0.659751	0.709834
Neutrophils	TAGLN	-0.28834	2.304796	-0.62902	0.530955	-5.44869	0.68209	0.733244
Neutrophils	IL1F9	0.110325	-0.11555	0.628832	0.53108	-6.04719	0.730929	0.784287
Neutrophils	ZHX1	-0.14449	4.078556	-0.6288	0.531102	-5.56985	0.648472	0.697974
Neutrophils	SURF2	0.190347	3.331878	0.628478	0.531311	-5.29814	0.662555	0.712667
Neutrophils	TEX261	-0.0933	5.229001	-0.62844	0.531333	-5.8406	0.627696	0.676017
Neutrophils	HEATR3	-0.18523	4.273336	-0.62812	0.531546	-5.3805	0.645195	0.694313
Neutrophils	EIF2A	-0.09573	5.395584	-0.62748	0.531961	-5.73415	0.625287	0.673209
Neutrophils	NEK4	0.333012	1.202148	0.627427	0.531996	-5.0472	0.704704	0.756542
Neutrophils	IFI205	0.327785	2.758001	0.626935	0.532317	-5.41559	0.674294	0.724712
Neutrophils	ANGPTL2	0.380932	0.84131	0.626765	0.532429	-5.04336	0.712215	0.764375
Neutrophils	TBCC	-0.16424	3.579816	-0.62676	0.532431	-5.44306	0.658673	0.708331
Neutrophils	PYM1	0.116378	4.692796	0.626675	0.532487	-5.61956	0.638112	0.686765
Neutrophils	PRKD2	0.106776	4.348606	0.626607	0.532531	-5.83248	0.644399	0.693377
Neutrophils	SF3B4	-0.10404	5.494923	-0.62654	0.532578	-5.89219	0.623708	0.671599
Neutrophils	MANSC1	-0.36579	0.242039	-0.62652	0.532591	-5.04089	0.72451	0.777248
Neutrophils	RRM2	0.140946	6.880861	0.625824	0.533043	-6.50911	0.599872	0.646386
Neutrophils	LCN4	-0.3562	-1.05402	-0.62574	0.533099	-4.94997	0.752155	0.805956
Neutrophils	CIAO2B	-0.15613	4.566233	-0.62569	0.533131	-5.50042	0.64069	0.689415
Neutrophils	IFIT2	0.350736	2.721315	0.62568	0.533136	-5.4092	0.675288	0.725735
Neutrophils	XPNPEP3	-0.15857	3.250052	-0.6256	0.533191	-5.43223	0.66518	0.715137
Neutrophils	RNF168	0.147438	4.161361	0.62557	0.533209	-5.60447	0.648122	0.697228
Neutrophils	CDC42SE2	-0.07732	7.179375	-0.6255	0.533255	-6.15388	0.59481	0.641071
Neutrophils	PIGW	-0.34286	0.765108	-0.62533	0.533364	-4.96195	0.714134	0.766426
Neutrophils	TMEM165	0.064666	5.311537	0.625223	0.533435	-6.17578	0.627304	0.675384
Neutrophils	OIP5	0.22541	2.207869	0.625007	0.533576	-5.27273	0.685396	0.736311
Neutrophils	KATNA1	-0.0863	4.864725	-0.62495	0.533615	-6.0184	0.635398	0.683838
Neutrophils	THADA	-0.09944	5.881845	-0.62481	0.533707	-5.97962	0.617306	0.664745
Neutrophils	TNFAIP8L1	-0.20147	2.396525	-0.62471	0.533771	-5.30002	0.681756	0.732475
Neutrophils	GM31718	0.152525	3.725877	0.624595	0.533845	-5.58673	0.656404	0.705879

Neutrophils	CCNY	-0.08005	6.67252	-0.62441	0.533969	-6.14054	0.603605	0.65031
Neutrophils	TLR7	0.194805	2.963787	0.624404	0.53397	-5.56062	0.670825	0.721033
Neutrophils	GM16536	-0.22205	1.530823	-0.62374	0.534404	-5.20408	0.699314	0.750685
Neutrophils	ITGAV	-0.10853	6.550452	-0.62353	0.534544	-6.16239	0.606131	0.652842
Neutrophils	CCDC51	-0.23518	1.810129	-0.6235	0.534564	-5.15129	0.693769	0.744906
Neutrophils	TMX1	0.08265	5.362	0.623443	0.534599	-5.98768	0.626962	0.674805
Neutrophils	GM36445	-0.28812	1.003387	-0.62312	0.534809	-5.03126	0.710116	0.761919
Neutrophils	SF3B3	-0.08329	6.034328	-0.62302	0.534875	-5.92923	0.615244	0.662448
Neutrophils	CBX6	0.229834	2.859775	0.622948	0.534922	-5.18675	0.673467	0.72367
Neutrophils	ZFP362	-0.17285	3.9339	-0.62264	0.535123	-5.33442	0.653322	0.702374
Neutrophils	CSPG5	-0.45399	0.28413	-0.62244	0.535253	-4.97852	0.725054	0.777426
Neutrophils	MORN1	0.258156	1.227517	0.622436	0.535257	-5.15461	0.70578	0.757321
Neutrophils	LYSMD2	-0.37863	1.265146	-0.62222	0.535397	-5.00355	0.705124	0.756586
Neutrophils	JADE3	-0.15704	3.471551	-0.62188	0.535621	-5.4316	0.662119	0.711643
Neutrophils	E230032D2	0.192307	1.879678	0.621848	0.535642	-5.43316	0.692884	0.743862
Neutrophils	FEZ2	-0.12606	3.693286	-0.62183	0.535652	-5.56597	0.657947	0.707265
Neutrophils	CDH22	-0.48485	-0.85879	-0.62181	0.535665	-4.9218	0.749248	0.802627
Neutrophils	5031434O1	0.321416	0.524887	0.621657	0.535767	-5.02234	0.720266	0.772467
Neutrophils	SLC17A9	0.1317	2.888147	0.6212	0.536067	-5.84371	0.673575	0.723478
Neutrophils	HSD17B7	-0.223	1.697013	-0.6208	0.536331	-5.21706	0.696947	0.747949
Neutrophils	CASP9	-0.18105	2.447605	-0.62078	0.536339	-5.27759	0.682174	0.732501
Neutrophils	RNF167	0.081587	5.334087	0.620754	0.536359	-6.15436	0.628302	0.67596
Neutrophils	MED21	-0.09431	5.21874	-0.62068	0.536407	-6.11076	0.630368	0.678148
Neutrophils	QSOX1	0.080358	4.311358	0.620627	0.536442	-6.13136	0.646867	0.695496
Neutrophils	FAM98A	-0.17113	3.607656	-0.6204	0.536592	-5.35188	0.660079	0.709265
Neutrophils	FASN	-0.23346	3.14635	-0.62022	0.53671	-5.26339	0.668824	0.718445
Neutrophils	GNAS	-0.05503	8.931692	-0.6202	0.536721	-6.62044	0.567412	0.611587
Neutrophils	GM15327	-0.29074	1.294764	-0.62004	0.536824	-5.02186	0.70517	0.756436
Neutrophils	RABL3	0.235969	2.218563	0.619675	0.537066	-5.17968	0.686971	0.737419
Neutrophils	ZBTB40	-0.20666	3.163496	-0.61967	0.53707	-5.25458	0.668698	0.718289
Neutrophils	CENPL	-0.14197	3.393274	-0.61955	0.537149	-5.5987	0.664351	0.713742
Neutrophils	6030443J0	0.32015	0.848488	0.619387	0.537254	-5.04206	0.714454	0.766122
Neutrophils	GALNT7	0.120982	6.060844	0.619228	0.537358	-5.87744	0.615853	0.662751
Neutrophils	GUCY1A1	-0.39819	-0.00341	-0.61912	0.537426	-5.00603	0.732115	0.784541
Neutrophils	ZPBP	-0.28069	1.648971	-0.61895	0.537538	-5.09887	0.698407	0.74939
Neutrophils	ADGRG5	-0.42802	0.105614	-0.61888	0.537589	-4.97728	0.729878	0.782237
Neutrophils	RITA1	-0.27727	1.367418	-0.61876	0.537664	-5.04645	0.704044	0.755329
Neutrophils	JADE1	0.133663	4.165478	0.618699	0.537706	-5.73859	0.650031	0.6988
Neutrophils	SF1	-0.05076	7.215593	-0.61856	0.537796	-6.40153	0.59605	0.641967
Neutrophils	VPREB2	-0.42335	-0.41404	-0.61831	0.537961	-4.94799	0.740972	0.793811
Neutrophils	PITPNM1	0.107414	3.730007	0.617741	0.538334	-5.78043	0.658688	0.707567
Neutrophils	PARP12	0.179303	3.018486	0.61762	0.538413	-5.54732	0.672209	0.721761
Neutrophils	NDOR1	0.221143	3.086341	0.617466	0.538514	-5.24158	0.670957	0.720454
Neutrophils	TTC17	-0.08686	5.324345	-0.61726	0.538651	-5.9482	0.629596	0.676992
Neutrophils	GM14966	0.140831	2.847703	0.616959	0.538847	-5.51024	0.675785	0.725349
Neutrophils	GNPTAB	-0.17522	4.589659	-0.61688	0.538898	-5.46096	0.643048	0.691045
Neutrophils	VMA21	-0.10271	5.176423	-0.61674	0.538988	-5.82791	0.632427	0.679898

Neutrophils	PILRB2	-0.09456	2.237136	-0.61651	0.539143	-6.18488	0.687789	0.737903
Neutrophils	TXNDC9	-0.07832	5.512196	-0.61643	0.539194	-6.0067	0.626497	0.673631
Neutrophils	SDC2	-0.26278	2.267759	-0.61631	0.539275	-5.27038	0.687188	0.737284
Neutrophils	PTER	0.226451	2.433342	0.616253	0.539311	-5.33975	0.683949	0.733908
Neutrophils	4930549G2	0.204823	2.714781	0.615884	0.539553	-5.24945	0.678638	0.728281
Neutrophils	ZFP35	0.211585	2.543067	0.615829	0.539589	-5.20802	0.681972	0.731784
Neutrophils	CCDC97	0.163905	3.33761	0.615773	0.539627	-5.36966	0.666689	0.715815
Neutrophils	TGFB1	0.061991	8.800362	0.615013	0.540126	-6.5756	0.571095	0.61501
Neutrophils	SELENOO	-0.15284	3.136064	-0.61498	0.54015	-5.49618	0.670865	0.720037
Neutrophils	MRPL16	-0.16799	3.919331	-0.61483	0.540248	-5.37594	0.656049	0.704542
Neutrophils	CSGALNACT	0.072019	4.10884	0.614762	0.540291	-6.08452	0.652515	0.700861
Neutrophils	YAE1D1	-0.22612	3.240113	-0.61471	0.540324	-5.31315	0.668877	0.718016
Neutrophils	ZKSCAN3	0.08753	4.82207	0.61468	0.540345	-5.81636	0.639393	0.687087
Neutrophils	PHF11B	0.297702	4.313119	0.614539	0.540437	-5.34288	0.648728	0.69692
Neutrophils	RECK	-0.20418	1.480341	-0.61416	0.540685	-5.55384	0.703324	0.754144
Neutrophils	SOS1	-0.07366	5.916592	-0.61413	0.540708	-6.08566	0.619786	0.666568
Neutrophils	GM48089	0.309664	1.135961	0.614104	0.540724	-5.17373	0.710274	0.761396
Neutrophils	BCAT2	0.114321	4.601375	0.614037	0.540768	-5.71828	0.643424	0.691427
Neutrophils	GPR55	-0.28524	1.548876	-0.614	0.540791	-5.09399	0.701949	0.752709
Neutrophils	GLMP	0.105485	5.839868	0.613948	0.540826	-6.02007	0.62114	0.667993
Neutrophils	DHDH	-0.18881	2.654165	-0.61381	0.540917	-5.37339	0.680151	0.729938
Neutrophils	TPX2	-0.15758	5.047457	-0.61379	0.54093	-6.03038	0.635303	0.682904
Neutrophils	CD37	0.071467	7.409059	0.613788	0.540932	-6.45698	0.594062	0.639449
Neutrophils	GM33782	0.276695	0.19699	0.613743	0.540961	-5.13338	0.729579	0.781542
Neutrophils	TLL2	-0.44367	0.725481	-0.61357	0.541072	-5.01791	0.718684	0.77018
Neutrophils	P2RY10	-0.25329	3.930309	-0.61351	0.541113	-5.42694	0.655876	0.704512
Neutrophils	TRIM11	-0.11762	5.06784	-0.61339	0.541194	-5.71647	0.634987	0.682553
Neutrophils	PLEKHN1	-0.26462	1.876111	-0.61328	0.541263	-5.11823	0.695488	0.745962
Neutrophils	TAGAP1	-0.30622	2.214287	-0.61308	0.541398	-5.1122	0.688899	0.739024
Neutrophils	GM19325	-0.31783	1.67424	-0.61274	0.54162	-5.14	0.699807	0.750253
Neutrophils	PIGK	0.120464	4.298401	0.612455	0.541809	-5.59855	0.649448	0.697458
Neutrophils	THOC1	-0.11051	5.704802	-0.61241	0.541836	-5.76524	0.62396	0.670678
Neutrophils	HIST1H2AB	0.410978	1.438759	0.612228	0.541958	-5.12557	0.704722	0.755259
Neutrophils	GNL2	-0.12477	4.675227	-0.61208	0.542055	-5.6265	0.642625	0.690278
Neutrophils	SLC35F2	-0.39771	0.357488	-0.61189	0.542183	-4.94783	0.726956	0.778391
Neutrophils	LHPP	-0.21352	3.112672	-0.61152	0.542428	-5.23346	0.672131	0.721115
Neutrophils	BRF2	-0.17615	2.326101	-0.6115	0.542437	-5.33482	0.687384	0.737067
Neutrophils	GM11131	0.375685	0.815809	0.611341	0.542543	-4.99949	0.717682	0.768737
Neutrophils	ZMYM3	0.255854	2.31007	0.611212	0.542627	-5.18895	0.687709	0.737513
Neutrophils	RUNX3	-0.15637	5.712717	-0.61118	0.542649	-5.84206	0.624159	0.670877
Neutrophils	A930037H0	-0.18708	3.242306	-0.61101	0.542759	-5.64663	0.66966	0.718646
Neutrophils	VPS35	0.060876	6.232281	0.610975	0.542784	-6.27092	0.615007	0.661254
Neutrophils	SYNJ2BP	-0.08588	5.520643	-0.61075	0.54293	-5.91173	0.627579	0.674486
Neutrophils	CNIH4	0.067365	5.763142	0.610681	0.542977	-6.23596	0.623265	0.669956
Neutrophils	DEDD2	0.084268	4.544003	0.610616	0.54302	-6.12668	0.645271	0.693101
Neutrophils	ZFYVE27	0.082423	4.063949	0.610594	0.543035	-5.95987	0.654156	0.702424
Neutrophils	DIPK1A	0.148603	5.446127	0.610513	0.543088	-5.77364	0.62891	0.675912

Neutrophils	CHCHD1	0.095171	5.813268	0.610417	0.543152	-5.95573	0.622377	0.669056
Neutrophils	MAP7D3	0.396663	0.126284	0.610346	0.543199	-4.96387	0.731956	0.783713
Neutrophils	ARHGEF39	-0.25071	1.960521	-0.61011	0.543352	-5.3969	0.694639	0.744834
Neutrophils	GM27216	0.334126	0.912751	0.610078	0.543375	-5.12444	0.715734	0.766842
Neutrophils	SIMC1	-0.1008	5.702765	-0.61002	0.543414	-6.02281	0.624367	0.67118
Neutrophils	CRTC1	0.238549	3.126748	0.609783	0.54357	-5.22738	0.672008	0.721124
Neutrophils	CRTAP	0.191632	3.004923	0.609701	0.543624	-5.34135	0.674347	0.723581
Neutrophils	MBOAT7	0.080856	4.278521	0.609453	0.543787	-6.05209	0.650411	0.698395
Neutrophils	LEPR	-0.26624	1.823369	-0.60937	0.54384	-5.35923	0.697588	0.74778
Neutrophils	PLA2G12A	-0.17525	4.277535	-0.60913	0.544002	-5.41981	0.650546	0.698533
Neutrophils	TMEM9	-0.28461	2.576914	-0.60903	0.544064	-5.14818	0.682868	0.732434
Neutrophils	RPRD1B	0.076365	5.480381	0.608294	0.544552	-6.13907	0.629092	0.675644
Neutrophils	TATDN1	-0.12972	3.630234	-0.60825	0.544582	-5.57636	0.66313	0.711353
Neutrophils	ASPM	-0.23682	3.870548	-0.60816	0.54464	-5.64089	0.658603	0.706611
Neutrophils	DENND6B	0.355265	0.94417	0.608006	0.544742	-4.97134	0.715964	0.766521
Neutrophils	TXN1	0.07655	7.304482	0.607965	0.544769	-6.57574	0.59732	0.64219
Neutrophils	ZFPM2	-0.19866	2.777013	-0.60773	0.544922	-5.73498	0.679544	0.728443
Neutrophils	LYAR	-0.13641	4.937131	-0.60768	0.544955	-5.73274	0.638969	0.685941
Neutrophils	NAF1	-0.18528	3.658508	-0.60704	0.54538	-5.27272	0.662977	0.710935
Neutrophils	PPIL1	-0.15628	4.052063	-0.60702	0.545396	-5.58883	0.655582	0.703191
Neutrophils	IRF2BP1	-0.13857	4.152538	-0.60696	0.54543	-5.52189	0.653707	0.701232
Neutrophils	LRRC49	0.258467	1.020554	0.60678	0.545552	-5.1759	0.71481	0.765115
Neutrophils	SLC38A3	-0.27581	1.479322	-0.60669	0.545609	-5.219	0.705508	0.755434
Neutrophils	ZFP526	0.324825	0.37218	0.606653	0.545636	-5.02799	0.72817	0.779028
Neutrophils	ERF	-0.1985	3.477368	-0.60664	0.545645	-5.42916	0.66641	0.714602
Neutrophils	TACC1	-0.05063	7.703473	-0.60638	0.545818	-6.47544	0.591049	0.635359
Neutrophils	PCDH15	-0.26873	1.323083	-0.60619	0.545941	-5.10219	0.708801	0.758827
Neutrophils	GM15265	0.258282	1.797023	0.605914	0.546124	-5.19121	0.699275	0.748967
Neutrophils	HIST1H2AF	0.418696	0.617707	0.605899	0.546134	-4.99598	0.723224	0.773916
Neutrophils	RHBDD2	0.21774	2.284757	0.605873	0.546151	-5.20703	0.689607	0.73888
Neutrophils	NCAPG	-0.19711	3.22832	-0.60584	0.546174	-5.57271	0.671291	0.71974
Neutrophils	NRIP3	0.472641	0.094317	0.6058	0.5462	-4.95675	0.734116	0.785257
Neutrophils	GM5608	0.262979	0.923456	0.605714	0.546256	-5.202	0.716936	0.767402
Neutrophils	ORMDL3	-0.19849	3.803807	-0.60538	0.54648	-5.34211	0.660561	0.708373
Neutrophils	ASCC1	-0.15797	3.774861	-0.60524	0.546572	-5.37775	0.661142	0.708972
Neutrophils	GPN3	-0.11037	3.982026	-0.60499	0.546738	-5.67138	0.657372	0.704963
Neutrophils	CHMP1B	-0.09106	4.698125	-0.60473	0.546908	-5.8969	0.644223	0.691101
Neutrophils	DALRD3	0.138712	3.729807	0.604455	0.547089	-5.46654	0.662284	0.710089
Neutrophils	MFSD5	0.134648	4.282812	0.604448	0.547094	-5.64385	0.651928	0.699243
Neutrophils	ZFP820	-0.38176	-0.04486	-0.6044	0.547129	-4.95052	0.737623	0.788655
Neutrophils	MED1	0.066456	5.883036	0.604272	0.54721	-6.02679	0.622921	0.668839
Neutrophils	RHOT1	-0.08952	5.400436	-0.60403	0.547369	-5.83369	0.631564	0.677998
Neutrophils	BCL2L2	-0.23398	1.276497	-0.60403	0.547372	-5.15006	0.710367	0.760428
Neutrophils	GM10125	-0.20734	2.003063	-0.60394	0.547428	-5.30324	0.695784	0.745225
Neutrophils	PAIP1	0.080894	5.907952	0.603629	0.547636	-6.08702	0.622677	0.668563
Neutrophils	MAP3K2	0.074018	6.248682	0.6033	0.547854	-6.19936	0.616842	0.66235
Neutrophils	LRRC1	0.249548	3.264954	0.603214	0.547911	-5.27432	0.671538	0.71975

Neutrophils	VRK2	0.099125	5.691956	0.602922	0.548104	-5.98534	0.626749	0.672751
Neutrophils	ZFP605	0.318352	1.633403	0.602908	0.548114	-5.0778	0.703614	0.753183
Neutrophils	METTL15	0.18084	3.231154	0.602843	0.548156	-5.34856	0.672255	0.720448
Neutrophils	1700003F1	-0.26595	2.027827	-0.60239	0.548453	-5.17834	0.695937	0.745105
Neutrophils	PAH	-0.22927	3.508909	-0.60234	0.548493	-5.68893	0.667144	0.715034
Neutrophils	BC051226	0.241403	2.179737	0.602319	0.548503	-5.10138	0.692926	0.741967
Neutrophils	ATL1	-0.38281	0.498179	-0.60207	0.548667	-5.01212	0.727026	0.777542
Neutrophils	SHLD3	-0.25158	2.343634	-0.60206	0.548674	-5.14145	0.68971	0.73867
Neutrophils	CLEC4A4	0.293621	-1.00355	0.601999	0.548715	-4.97234	0.75889	0.81067
Neutrophils	RNF38	0.059324	6.163439	0.601917	0.54877	-6.34525	0.618597	0.664213
Neutrophils	POLR1E	0.244114	2.067812	0.601671	0.548933	-5.19607	0.695237	0.744433
Neutrophils	SSH2	-0.06787	8.396317	-0.60164	0.548956	-6.75409	0.580681	0.624211
Neutrophils	MGLL	-0.12357	3.495334	-0.60146	0.549072	-5.94196	0.667556	0.715497
Neutrophils	ALDH1B1	0.264057	1.910302	0.601029	0.549359	-5.09454	0.698722	0.747931
Neutrophils	TMTC3	-0.17621	2.514058	-0.60068	0.549591	-5.27302	0.686888	0.735613
Neutrophils	ANKRD24	0.282605	1.054272	0.60065	0.54961	-5.079	0.716117	0.766079
Neutrophils	WDR26	-0.04878	7.53429	-0.60054	0.549686	-6.56739	0.595434	0.639681
Neutrophils	TAF4B	-0.1439	5.130162	-0.60052	0.549694	-5.89091	0.637534	0.683959
Neutrophils	LAT	-0.25348	2.414617	-0.60023	0.549887	-5.14984	0.688862	0.737673
Neutrophils	AQP11	0.35078	0.397356	0.60018	0.549922	-5.02083	0.729702	0.780207
Neutrophils	EFCAB2	0.238754	2.668273	0.600099	0.549975	-5.26363	0.683894	0.732487
Neutrophils	CRACR2B	-0.25386	1.009115	-0.60007	0.549995	-5.29115	0.717064	0.767063
Neutrophils	HEATR6	-0.07402	4.812778	-0.59986	0.550136	-6.08753	0.64334	0.690063
Neutrophils	MRPS2	-0.26927	2.843371	-0.5998	0.550173	-5.22369	0.680486	0.728941
Neutrophils	EPHA2	-0.25392	2.408212	-0.59971	0.550232	-5.14015	0.688987	0.737842
Neutrophils	RAB5B	0.084287	4.382557	0.599668	0.550261	-6.00521	0.651272	0.698408
Neutrophils	GM20457	-0.37395	-0.16272	-0.59957	0.550326	-4.95822	0.741469	0.792527
Neutrophils	1110046J0	-0.35989	0.031359	-0.59955	0.550342	-4.98914	0.73737	0.788282
Neutrophils	DOCK7	-0.14063	3.830588	-0.59943	0.55042	-5.65532	0.661614	0.709301
Neutrophils	GM42477	0.180329	1.273145	0.599328	0.550487	-5.38978	0.711701	0.761638
Neutrophils	GTF2I	-0.10194	5.762327	-0.59903	0.550684	-5.83094	0.626363	0.672257
Neutrophils	SLC25A43	0.362084	-0.21871	0.598646	0.55094	-4.97226	0.743049	0.79412
Neutrophils	HAP1	-0.35819	-0.45019	-0.59859	0.550979	-4.97109	0.747978	0.799252
Neutrophils	CETN3	-0.0852	5.807387	-0.59854	0.551012	-6.07308	0.625718	0.671617
Neutrophils	UBB	0.061471	10.85501	0.598331	0.551149	-7.05942	0.542431	0.583666
Neutrophils	DIRAS2	0.344255	0.718948	0.597878	0.55145	-5.01966	0.723766	0.773877
Neutrophils	D11WSU47	-0.31241	1.299867	-0.59769	0.551577	-5.05741	0.711858	0.761541
Neutrophils	DNAH17	-0.21443	3.275665	-0.59761	0.551626	-5.62395	0.67283	0.720856
Neutrophils	1700102H2	-0.32913	0.529845	-0.59742	0.551752	-5.01686	0.727685	0.778084
Neutrophils	ARMC10	-0.18781	3.275459	-0.59741	0.551762	-5.28133	0.672834	0.720905
Neutrophils	AKR1C13	-0.26401	1.84784	-0.59738	0.551781	-5.18494	0.700808	0.750106
Neutrophils	RSBN1L	0.056912	7.198782	0.597371	0.551786	-6.45681	0.60176	0.646319
Neutrophils	SMARCD2	0.07637	5.611886	0.597151	0.551933	-5.93989	0.629609	0.675513
Neutrophils	FBXW7	-0.07822	6.448567	-0.59691	0.552092	-6.2418	0.614846	0.659959
Neutrophils	ADAM10	-0.05874	7.403441	-0.59682	0.552152	-6.39589	0.598401	0.642652
Neutrophils	5530601H0	-0.15214	3.904215	-0.59682	0.552155	-5.60964	0.66102	0.708405
Neutrophils	6720427I0	-0.11267	4.894793	-0.59654	0.552342	-5.78191	0.642772	0.689229

Neutrophils	TRPM4	-0.28941	1.109553	-0.59573	0.552876	-5.06348	0.716342	0.765981
Neutrophils	CRTC3	0.074373	5.658838	0.595621	0.55295	-6.21533	0.629205	0.674916
Neutrophils	TCTA	0.191614	2.098287	0.595463	0.553055	-5.2648	0.696404	0.745273
Neutrophils	HIST1H1B	0.264713	5.277543	0.595437	0.553073	-5.924	0.636068	0.68217
Neutrophils	STK10	0.057441	7.315855	0.595099	0.553297	-6.53833	0.600271	0.644583
Neutrophils	4930453N2	0.12182	4.481233	0.595059	0.553324	-5.62482	0.650654	0.697511
Neutrophils	CCDC84	0.188604	2.649641	0.595032	0.553342	-5.32453	0.685532	0.733984
Neutrophils	SH3BP2	0.167272	3.288629	0.594964	0.553387	-5.58245	0.67315	0.721051
Neutrophils	PACSIN1	-0.27489	2.953403	-0.59485	0.553465	-5.14425	0.679617	0.727808
Neutrophils	GM43330	-0.25417	1.526899	-0.59481	0.553488	-5.14796	0.707856	0.757259
Neutrophils	DYNLT1C	-0.28083	1.100031	-0.59481	0.553492	-5.08405	0.716537	0.766295
Neutrophils	POFUT1	-0.16126	3.458205	-0.59471	0.553555	-5.3774	0.669903	0.717661
Neutrophils	NOL4L	0.250627	2.680623	0.59465	0.553596	-5.33896	0.684927	0.733356
Neutrophils	COL23A1	-0.33788	1.376373	-0.59454	0.553666	-5.08533	0.710905	0.760438
Neutrophils	NXN	0.083742	5.325793	0.594529	0.553677	-6.26914	0.635195	0.681306
Neutrophils	MAP3K13	-0.33878	0.779749	-0.5945	0.553697	-5.05168	0.723121	0.773148
Neutrophils	SNX27	-0.05512	5.803763	-0.59445	0.553729	-6.3265	0.626617	0.6723
Neutrophils	INPP1	0.082751	4.03687	0.594397	0.553765	-6.13299	0.658943	0.706196
Neutrophils	BBS5	-0.36743	0.624034	-0.59439	0.55377	-5.01473	0.726344	0.776498
Neutrophils	CDC123	0.094248	5.404958	0.594158	0.553924	-5.92851	0.633869	0.679849
Neutrophils	MKLN1OS	0.365751	0.018205	0.593992	0.554034	-4.99274	0.739204	0.789737
Neutrophils	EMC8	-0.127	4.721373	-0.5939	0.554096	-5.58902	0.646378	0.692977
Neutrophils	MVB12A	-0.09376	5.948563	-0.5937	0.554229	-5.91444	0.62424	0.669701
Neutrophils	HIRA	0.095185	5.652742	0.593612	0.554287	-5.94703	0.629513	0.675241
Neutrophils	NUCB1	0.089315	5.383551	0.593438	0.554403	-6.01425	0.634353	0.680355
Neutrophils	LSR	-0.30728	1.400914	-0.5934	0.55443	-5.16967	0.710632	0.760093
Neutrophils	ALKBH3	-0.16681	3.490416	-0.59327	0.554518	-5.42133	0.6695	0.717202
Neutrophils	NPFF	-0.29088	0.954229	-0.59308	0.554643	-5.06811	0.719754	0.769642
Neutrophils	SNHG14	0.252161	1.410261	0.592973	0.554713	-5.08173	0.710442	0.759952
Neutrophils	GM10131	-0.39133	1.072727	-0.59275	0.554865	-5.02027	0.717322	0.767263
Neutrophils	ETFDH	-0.09246	4.872939	-0.5927	0.554892	-5.86023	0.643639	0.690306
Neutrophils	ZBTB48	-0.32747	1.123594	-0.59264	0.554933	-5.0657	0.716281	0.766193
Neutrophils	TESK1	0.183661	3.781202	0.592594	0.554965	-5.30879	0.663972	0.711615
Neutrophils	DDAH1	-0.20193	2.481818	-0.59258	0.554972	-5.34201	0.689041	0.737812
Neutrophils	STAC2	-0.40484	1.824947	-0.59251	0.555021	-4.99618	0.70208	0.751426
Neutrophils	ENOX2	-0.10408	5.852601	-0.59242	0.555082	-6.0652	0.625945	0.671754
Neutrophils	GM19967	-0.28351	1.377066	-0.59242	0.555084	-5.06243	0.711116	0.760839
Neutrophils	CREBZF	0.109595	4.715073	0.592079	0.555309	-5.68398	0.646728	0.693487
Neutrophils	FAM126B	-0.11065	3.772291	-0.59185	0.555459	-5.69899	0.664438	0.711946
Neutrophils	GM28379	0.377505	0.264238	0.591633	0.555606	-4.99024	0.734479	0.78488
Neutrophils	MRPS33	-0.06374	6.714474	-0.59154	0.555668	-6.22132	0.611133	0.655973
Neutrophils	ZSWIM1	-0.36826	1.4089	-0.59149	0.555704	-5.01935	0.710858	0.760339
Neutrophils	GM7072	0.164864	3.842908	0.591159	0.555922	-5.46262	0.663353	0.710651
Neutrophils	MED4	0.113795	4.020834	0.590903	0.556093	-5.57938	0.660126	0.707213
Neutrophils	RUNDC1	-0.1934	2.994317	-0.59071	0.556224	-5.2791	0.679771	0.72777
Neutrophils	GM21762	-0.35004	-1.20491	-0.59066	0.556252	-4.92419	0.766364	0.817796
Neutrophils	PAN2	-0.13635	3.190864	-0.59055	0.556325	-5.48533	0.675982	0.723801

Neutrophils	GM19557	0.289519	-0.95554	0.590457	0.556391	-4.96776	0.760937	0.812187
Neutrophils	GM28198	0.173489	3.85447	0.590366	0.556451	-5.52684	0.663312	0.710612
Neutrophils	TCTN2	0.352133	-0.37598	0.590258	0.556523	-4.96015	0.748456	0.799278
Neutrophils	LTF	0.075269	2.942911	0.590116	0.556618	-6.6995	0.680826	0.728868
Neutrophils	XAF1	0.267459	4.033526	0.589886	0.556772	-5.58845	0.659981	0.707158
Neutrophils	ELMOD2	-0.12985	2.807903	-0.58985	0.556796	-5.61909	0.683453	0.731692
Neutrophils	SCOC	-0.16852	3.752146	-0.58977	0.55685	-5.43616	0.665295	0.712759
Neutrophils	OSBPL7	0.157275	3.426262	0.58974	0.556869	-5.41255	0.671505	0.719252
Neutrophils	ALDH16A1	0.122163	4.358468	0.589219	0.557217	-5.65865	0.654233	0.701003
Neutrophils	RUSC2	0.299894	0.909936	0.588905	0.557426	-5.18515	0.721981	0.771695
Neutrophils	HDAC9	-0.1194	7.563843	-0.5889	0.557428	-6.40704	0.59734	0.641264
Neutrophils	NUP35	-0.20695	3.291207	-0.58866	0.557587	-5.26731	0.67459	0.722283
Neutrophils	POSTN	0.303763	1.260435	0.588655	0.557594	-5.18071	0.714841	0.764246
Neutrophils	TLK1	0.067394	6.854126	0.588554	0.557661	-6.25132	0.60954	0.654113
Neutrophils	CPQ	-0.11283	5.056734	-0.58843	0.557742	-5.94237	0.641519	0.687722
Neutrophils	NOXRED1	0.250231	0.546984	0.588215	0.557887	-5.20505	0.729659	0.779711
Neutrophils	PRIM1	0.178051	4.178781	0.588156	0.557927	-5.63283	0.65783	0.704832
Neutrophils	FAM192A	-0.08766	4.607769	-0.58803	0.558015	-5.80518	0.649868	0.696485
Neutrophils	BICRA	0.090839	5.82128	0.587796	0.558167	-6.00586	0.627852	0.67344
Neutrophils	ZNRF1	0.058478	6.496008	0.587777	0.558181	-6.49915	0.615925	0.660908
Neutrophils	OSBPL10	-0.247	0.973543	-0.58734	0.558473	-5.22478	0.721153	0.770738
Neutrophils	ATP10A	-0.19467	3.430551	-0.58729	0.558508	-5.44081	0.672318	0.719852
Neutrophils	ARHGAP11	-0.10821	4.784218	-0.58709	0.558638	-5.92909	0.646878	0.693269
Neutrophils	TMX2	0.168799	3.46498	0.587084	0.558643	-5.40557	0.671658	0.719199
Neutrophils	ANKRD13D	-0.18385	2.738056	-0.58687	0.558784	-5.35841	0.685729	0.734014
Neutrophils	2610008E1	0.273436	2.466966	0.586828	0.558815	-5.11572	0.691054	0.739578
Neutrophils	2010009K1	-0.46889	0.112521	-0.5868	0.558833	-4.99007	0.739107	0.789583
Neutrophils	BRD2	0.064041	7.085393	0.586731	0.558879	-6.28939	0.60591	0.65039
Neutrophils	GM31597	-0.23128	2.208627	-0.58659	0.558977	-5.14784	0.69621	0.744999
Neutrophils	BTLA	0.312046	3.948652	0.585976	0.559385	-5.26456	0.66281	0.709948
Neutrophils	IWS1	0.072552	5.729671	0.585959	0.559396	-6.04279	0.630038	0.675606
Neutrophils	NEK8	-0.2382	2.113602	-0.58591	0.559428	-5.1662	0.698426	0.747134
Neutrophils	PPP2R3A	-0.1276	5.003266	-0.58541	0.559765	-5.9365	0.643511	0.689545
Neutrophils	XRN1	-0.08689	5.924264	-0.58488	0.560119	-5.98848	0.627157	0.672273
Neutrophils	SLC39A12	-0.38009	0.492802	-0.58476	0.560197	-5.18721	0.732204	0.781967
Neutrophils	ZZZ3	-0.08982	5.884403	-0.58474	0.560214	-6.02057	0.627868	0.673059
Neutrophils	ZBTB14	0.14053	3.004063	0.584627	0.560287	-5.4042	0.681564	0.729271
Neutrophils	UNC45A	-0.10455	4.303611	-0.58449	0.56038	-5.79319	0.656787	0.70341
Neutrophils	STARD4	-0.13181	3.526928	-0.58437	0.560459	-5.61827	0.671488	0.718815
Neutrophils	CEP250	-0.1378	4.458141	-0.58433	0.560487	-5.61561	0.653902	0.700426
Neutrophils	SETX	0.081974	5.905752	0.584069	0.560661	-6.11464	0.627599	0.672773
Neutrophils	FGB	-0.11987	5.797972	-0.58394	0.56075	-6.28313	0.629525	0.674821
Neutrophils	LUC7L	0.083163	5.622585	0.583924	0.560758	-5.87364	0.632673	0.678124
Neutrophils	BCAM	-0.42226	0.298097	-0.58377	0.560864	-5.10274	0.736474	0.786416
Neutrophils	SLC25A42	0.27408	1.940911	0.583246	0.561212	-5.16062	0.70308	0.751472
Neutrophils	HSPA12A	-0.32722	0.150275	-0.58305	0.561341	-5.04214	0.740051	0.789886
Neutrophils	MARS	-0.18101	3.10364	-0.58295	0.561412	-5.39689	0.680224	0.727671

Neutrophils	ABTB1	0.080856	4.303875	0.582756	0.561541	-6.21363	0.657358	0.703805
Neutrophils	PARG	-0.07101	5.560263	-0.58274	0.561551	-6.04986	0.634261	0.679616
Neutrophils	GM14798	0.132826	3.718232	0.582618	0.561634	-5.54064	0.668422	0.715413
Neutrophils	DENND4B	0.133978	3.747946	0.582544	0.561683	-5.55586	0.667856	0.714821
Neutrophils	GM2629	0.246649	0.511245	0.582456	0.561742	-5.15656	0.732486	0.782134
Neutrophils	CHST15	-0.10194	3.54275	-0.58224	0.56189	-5.84284	0.671874	0.718983
Neutrophils	RSPH3A	-0.20099	2.558451	-0.58198	0.562058	-5.32202	0.691134	0.73897
Neutrophils	ZFP513	-0.18812	2.534011	-0.58134	0.562491	-5.31599	0.69199	0.739676
Neutrophils	ATL3	-0.07811	5.439187	-0.58132	0.562506	-5.97376	0.637009	0.682232
Neutrophils	GNAI3	0.045979	6.443605	0.581218	0.562573	-6.43878	0.619074	0.663469
Neutrophils	DDC	-0.28336	1.455602	-0.58113	0.562632	-5.14662	0.713622	0.762293
Neutrophils	CELF4	-0.3188	0.6776	-0.58104	0.562692	-5.03024	0.729654	0.778967
Neutrophils	MRPS18A	-0.10578	5.09387	-0.58092	0.56277	-5.76623	0.643299	0.688923
Neutrophils	SPTBN1	-0.07485	6.620496	-0.58062	0.562971	-6.37249	0.615971	0.660372
Neutrophils	RSPH9	-0.33643	1.514419	-0.58056	0.563017	-5.06099	0.712425	0.761207
Neutrophils	SLC24A1	0.133643	1.860788	0.580455	0.563085	-5.6713	0.705415	0.753943
Neutrophils	CRLS1	-0.12904	3.622878	-0.5804	0.563121	-5.5114	0.67083	0.717883
Neutrophils	CYFIP2	-0.06113	6.947339	-0.58031	0.563179	-6.55539	0.61028	0.654427
Neutrophils	FOXN3	-0.0757	8.899803	-0.58024	0.563227	-6.55792	0.57742	0.619831
Neutrophils	EIF3B	-0.09496	5.890853	-0.58007	0.563344	-5.91838	0.628877	0.673996
Neutrophils	ADCK5	0.320724	1.510315	0.57996	0.563417	-4.99909	0.712508	0.761401
Neutrophils	PDE1A	-0.22863	0.441701	-0.57996	0.563419	-5.35698	0.734587	0.784343
Neutrophils	IGKV1-117	-0.31782	-0.39916	-0.57992	0.56344	-4.95715	0.752443	0.802861
Neutrophils	PPM1N	0.331323	0.159456	0.579793	0.563529	-5.11338	0.740532	0.790513
Neutrophils	ZCRB1	0.053374	6.149583	0.579686	0.563601	-6.25621	0.624269	0.669208
Neutrophils	AKAP7	-0.12638	3.374989	-0.57967	0.563613	-5.5903	0.675588	0.722943
Neutrophils	ZHX2	-0.0946	5.614006	-0.57964	0.563635	-6.1876	0.633848	0.679261
Neutrophils	OTUD3	0.194929	2.558441	0.579444	0.563763	-5.38278	0.691587	0.739564
Neutrophils	GM29666	-0.3231	0.244861	-0.5793	0.563859	-5.11083	0.738856	0.788748
Neutrophils	LRRC18	-0.29315	1.588071	-0.57902	0.564045	-5.16218	0.711115	0.760018
Neutrophils	OST4	0.062104	8.049265	0.578783	0.564207	-6.61926	0.591653	0.635062
Neutrophils	GMDS	-0.07432	6.245209	-0.57873	0.564241	-6.15665	0.622737	0.667768
Neutrophils	TFB2M	-0.12327	3.953928	-0.57869	0.564269	-5.52052	0.664703	0.71176
Neutrophils	POLR2D	-0.1131	5.184758	-0.57867	0.564286	-5.7593	0.641805	0.687795
Neutrophils	AGMAT	-0.27768	1.438737	-0.57845	0.564431	-5.25773	0.714153	0.763412
Neutrophils	GM20337	-0.23896	1.078162	-0.57841	0.564455	-5.22743	0.721544	0.771099
Neutrophils	NLRP1B	-0.21711	1.79452	-0.57839	0.564472	-5.38396	0.706936	0.7559
Neutrophils	CALM2	-0.0456	8.565355	-0.57832	0.564518	-6.67439	0.583066	0.626075
Neutrophils	RACK1	0.048013	8.637453	0.578279	0.564546	-6.55149	0.581877	0.624832
Neutrophils	PHF11A	-0.37349	1.004104	-0.57798	0.564745	-5.03045	0.723245	0.772792
Neutrophils	SMPD2	-0.20941	2.04317	-0.57789	0.564808	-5.28494	0.702104	0.750828
Neutrophils	MAP4K4	0.070685	7.459224	0.577559	0.56503	-6.57084	0.601885	0.645818
Neutrophils	ID1	0.09363	4.07039	0.577552	0.565035	-6.26964	0.662775	0.709712
Neutrophils	LONRF3	-0.13143	1.525709	-0.57738	0.565148	-5.71692	0.71274	0.761792
Neutrophils	TIPIN	0.132884	5.238466	0.57726	0.565231	-5.98227	0.64117	0.687081
Neutrophils	LDAH	0.107903	4.225642	0.576997	0.565408	-5.68426	0.660062	0.706782
Neutrophils	PARN	0.16537	3.875182	0.576736	0.565583	-5.36703	0.666727	0.713716

Neutrophils	GM15964	0.34636	0.162356	0.576734	0.565585	-5.12348	0.741255	0.791322
Neutrophils	TMEM134	-0.06489	6.132006	-0.57666	0.565632	-6.2767	0.625242	0.670259
Neutrophils	DLG3	-0.3535	0.571754	-0.57631	0.565867	-4.97395	0.732638	0.782457
Neutrophils	DNAJB6	-0.04905	8.310874	-0.57595	0.566111	-6.61741	0.587752	0.630976
Neutrophils	UBE4BOS1	-0.39799	0.735049	-0.57577	0.566236	-4.98105	0.72923	0.779109
Neutrophils	POGZ	-0.11814	4.431535	-0.57571	0.566271	-5.59893	0.656242	0.702992
Neutrophils	ZFAS1	-0.13973	5.058651	-0.57571	0.566275	-5.75257	0.644627	0.690826
Neutrophils	SPC24	-0.1707	4.768056	-0.57562	0.566337	-5.90244	0.649982	0.696475
Neutrophils	UBE2Q1	-0.05528	6.334643	-0.57554	0.566387	-6.30307	0.621652	0.66679
Neutrophils	ESYT1	0.101103	5.654914	0.575463	0.56644	-5.90773	0.633782	0.679536
Neutrophils	POGK	0.24999	2.454489	0.575308	0.566545	-5.17467	0.694297	0.742857
Neutrophils	SDF2	0.079489	5.410417	0.575301	0.566549	-5.98393	0.638206	0.684192
Neutrophils	IGLV1	-0.38671	-0.83837	-0.57529	0.566555	-4.9532	0.76275	0.813997
Neutrophils	PIGYL	0.101972	4.270837	0.575117	0.566673	-5.81404	0.659253	0.706305
Neutrophils	IGSF3	-0.30916	0.826018	-0.57509	0.566688	-5.09793	0.727337	0.77732
Neutrophils	DOCK4	-0.08907	6.427831	-0.57509	0.566692	-6.3541	0.620008	0.665139
Neutrophils	LACC1	0.190364	3.279516	0.574989	0.566759	-5.45157	0.678147	0.726111
Neutrophils	GINS4	0.135953	3.753755	0.574888	0.566827	-5.55816	0.669039	0.716637
Neutrophils	SECISBP2	0.099915	4.786541	0.574744	0.566924	-5.76572	0.64964	0.696353
Neutrophils	LRATD2	0.224781	1.700967	0.5747	0.566953	-5.18651	0.709391	0.758775
Neutrophils	PSMD11	0.0438	7.032517	0.574693	0.566958	-6.30754	0.609451	0.654145
Neutrophils	GM11713	0.166872	2.37115	0.574651	0.566987	-5.56346	0.69595	0.744767
Neutrophils	LRRC45	-0.22549	2.190744	-0.57457	0.567043	-5.17244	0.699543	0.748513
Neutrophils	LMO7	-0.4979	0.761873	-0.57449	0.567092	-5.08606	0.728671	0.778865
Neutrophils	SERPINI1	-0.16997	3.178888	-0.57449	0.567097	-5.48506	0.680096	0.728247
Neutrophils	THRA	-0.12694	3.771843	-0.57423	0.567271	-5.70672	0.668774	0.716427
Neutrophils	THBS1	-0.09329	4.967156	-0.57415	0.567321	-6.95176	0.646385	0.692982
Neutrophils	ACCS	-0.25013	2.079594	-0.57385	0.567524	-5.12156	0.701849	0.751067
Neutrophils	ATP6V1H	0.080615	6.803114	0.573798	0.567562	-6.29196	0.613507	0.658566
Neutrophils	RRP36	-0.12603	3.775241	-0.5737	0.567626	-5.48056	0.66871	0.716494
Neutrophils	THAP2	-0.17966	3.329281	-0.57368	0.56764	-5.37977	0.677266	0.725441
Neutrophils	RABGAP1	0.078913	5.920481	0.573648	0.567662	-6.0785	0.629089	0.674959
Neutrophils	WDR70	0.075749	6.1341	0.573625	0.567678	-6.02734	0.62528	0.670957
Neutrophils	HDAC6	-0.24807	2.206503	-0.57354	0.567738	-5.14429	0.699312	0.748464
Neutrophils	FKBP8	0.075577	6.067485	0.573368	0.567851	-6.16679	0.626489	0.672297
Neutrophils	TRMT12	0.238586	1.30746	0.573314	0.567887	-5.16087	0.717519	0.767529
Neutrophils	P4HTM	-0.18699	2.773581	-0.57321	0.56796	-5.56919	0.688123	0.736927
Neutrophils	GUCA1A	0.225672	1.4794	0.572933	0.568144	-5.25725	0.714087	0.7641
Neutrophils	5930430L0	0.338808	0.229599	0.572858	0.568195	-5.01745	0.740034	0.791097
Neutrophils	KCMF1	0.044463	6.987433	0.572783	0.568245	-6.40529	0.610397	0.655536
Neutrophils	ARID4B	0.049842	8.259309	0.572669	0.568322	-6.64026	0.588771	0.632755
Neutrophils	ARRDC2	-0.252	1.79539	-0.57264	0.568339	-5.15053	0.707673	0.757468
Neutrophils	2510009E0	-0.16226	2.702942	-0.57245	0.568471	-5.46206	0.689579	0.738601
Neutrophils	MBD2	0.046967	7.044678	0.572347	0.568539	-6.53642	0.609406	0.654522
Neutrophils	RNF185	0.072694	5.117108	0.572332	0.56855	-6.08611	0.64373	0.690606
Neutrophils	INTS8	0.091244	4.693172	0.572274	0.568589	-5.82417	0.651546	0.698805
Neutrophils	PRKN	-0.17934	3.428362	-0.57195	0.568807	-5.64152	0.675641	0.723976

Neutrophils	MYO19	-0.32102	1.357151	-0.57164	0.569016	-5.03587	0.716935	0.766999
Neutrophils	TDRP	-0.39495	-0.36439	-0.57149	0.569115	-5.01488	0.753066	0.804552
Neutrophils	MYO18A	0.095987	4.439945	0.571482	0.569123	-5.82164	0.656585	0.703965
Neutrophils	NPM3	-0.13471	5.097579	-0.57106	0.569407	-5.71406	0.644653	0.691392
Neutrophils	DPF1	-0.36317	0.292792	-0.5709	0.569515	-5.0459	0.739405	0.790296
Neutrophils	ZFP961	0.159457	3.308556	0.570717	0.569639	-5.48192	0.678417	0.726752
Neutrophils	NINJ2	-0.35873	0.701022	-0.57071	0.569642	-5.07177	0.730834	0.781395
Neutrophils	SLC25A28	0.091828	4.806244	0.570618	0.569706	-5.91223	0.650072	0.697103
Neutrophils	PCGF5	0.094044	6.06196	0.570522	0.569771	-6.17333	0.627258	0.673157
Neutrophils	GM10550	0.243944	0.590308	0.570147	0.570024	-5.20986	0.733372	0.783937
Neutrophils	AKAP8	-0.08453	5.39229	-0.57008	0.570071	-5.84606	0.639513	0.685942
Neutrophils	SH3GLB2	0.150279	3.721025	0.569978	0.570138	-5.45267	0.670693	0.718612
Neutrophils	SKIV2L	-0.1247	3.938774	-0.56982	0.570245	-5.61639	0.666557	0.714299
Neutrophils	CRACR2A	0.160802	2.835916	0.569772	0.570277	-5.60163	0.687852	0.73656
Neutrophils	DLGAP4	-0.09218	5.015503	-0.56935	0.570563	-6.02189	0.646539	0.693338
Neutrophils	TMSB4X	0.052461	12.24836	0.569326	0.570578	-7.35037	0.526838	0.566995
Neutrophils	GOT2	0.070691	6.235083	0.569273	0.570615	-6.18197	0.624495	0.670214
Neutrophils	SPEN	-0.05789	5.922479	-0.56925	0.570633	-6.29623	0.630069	0.676073
Neutrophils	CKAP5	0.091851	6.118962	0.568979	0.570813	-6.19037	0.62662	0.672458
Neutrophils	METTL27	-0.40325	0.539053	-0.56897	0.570816	-4.98152	0.734667	0.785378
Neutrophils	SIKE1	-0.08788	4.570739	-0.56872	0.570987	-5.86628	0.654873	0.702193
Neutrophils	ILDR1	-0.34916	1.791756	-0.56866	0.571029	-5.1046	0.708884	0.758638
Neutrophils	TADA2B	-0.15067	3.468615	-0.56865	0.571034	-5.4826	0.675768	0.724067
Neutrophils	CIPC	-0.2186	2.655218	-0.56847	0.571158	-5.20443	0.691637	0.740639
Neutrophils	PHF6	-0.11553	4.940138	-0.56843	0.571185	-5.71451	0.648028	0.695009
Neutrophils	BCKDHA	0.125588	4.820285	0.568359	0.571232	-5.71397	0.650243	0.697332
Neutrophils	NUP205	-0.14581	4.408914	-0.56823	0.571319	-5.49445	0.657933	0.705386
Neutrophils	IRAK3	0.087616	4.187779	0.567984	0.571486	-6.45482	0.662182	0.709742
Neutrophils	MITF	-0.09719	4.369202	-0.56793	0.571525	-6.18188	0.658768	0.706171
Neutrophils	MRPS35	0.108839	4.266456	0.56783	0.571589	-5.63502	0.660699	0.708193
Neutrophils	4930509H(	0.189012	2.090644	0.567508	0.571807	-5.24815	0.70312	0.752465
Neutrophils	GATB	-0.21703	3.11047	-0.56733	0.571927	-5.2695	0.682955	0.731483
Neutrophils	CCDC50	-0.09159	5.994315	-0.56732	0.571938	-5.87282	0.629104	0.675059
Neutrophils	ZFP451	0.108679	5.019065	0.567184	0.572027	-5.73676	0.646803	0.693663
Neutrophils	DAPK1	-0.13701	4.932787	-0.56686	0.572249	-5.91032	0.648394	0.695339
Neutrophils	SYBU	0.365116	0.330557	0.566827	0.572268	-5.08529	0.73936	0.790286
Neutrophils	MTF2	-0.07838	5.644163	-0.56678	0.572299	-6.01013	0.6354	0.681707
Neutrophils	MAK16	-0.11263	5.072635	-0.56669	0.572358	-5.6339	0.645817	0.692645
Neutrophils	DOP1A	-0.12689	4.09244	-0.56661	0.572416	-5.66283	0.664101	0.711821
Neutrophils	FRAT1	0.087681	3.463579	0.566481	0.572502	-5.872	0.676112	0.724412
Neutrophils	NUP37	-0.17868	3.351508	-0.56646	0.572519	-5.39895	0.678276	0.726675
Neutrophils	3300002I0	-0.2862	1.970632	-0.56631	0.572615	-5.06989	0.705532	0.755129
Neutrophils	ZBTB11	-0.05745	7.088839	-0.56624	0.572667	-6.45538	0.609844	0.654873
Neutrophils	SCN4A	-0.3477	0.410123	-0.56622	0.572681	-4.99269	0.737682	0.788591
Neutrophils	GIPC1	-0.11286	4.359749	-0.56612	0.57275	-5.69802	0.659062	0.706569
Neutrophils	LARP4B	0.044546	7.283403	0.566087	0.572769	-6.59501	0.606486	0.651338
Neutrophils	PPP1R13B	0.108718	5.30235	0.566077	0.572775	-5.82517	0.641609	0.688269

Neutrophils	SIGLEC1	-0.36189	0.449661	-0.56576	0.572989	-5.12251	0.736902	0.787837
Neutrophils	CENPN	-0.22634	2.920239	-0.56574	0.573005	-5.36615	0.68672	0.735552
Neutrophils	FBXO31	0.107307	3.549697	0.565575	0.573116	-5.81037	0.674502	0.722802
Neutrophils	RNF150	0.261617	2.909798	0.565546	0.573135	-5.44799	0.686925	0.735789
Neutrophils	PARD3B	-0.14053	4.55014	-0.56551	0.573159	-5.98587	0.655544	0.70296
Neutrophils	SLC1A3	-0.4231	-0.29724	-0.56545	0.573199	-5.04456	0.752791	0.804405
Neutrophils	ZFP408	-0.13592	3.716601	-0.56524	0.573344	-5.53568	0.671368	0.719498
Neutrophils	BICRAL	0.093685	5.023041	0.565177	0.573384	-5.90447	0.646841	0.693815
Neutrophils	BDP1	0.076417	5.529011	0.565071	0.573457	-6.05812	0.637604	0.684127
Neutrophils	PELO	0.178057	3.125716	0.564916	0.573562	-5.30407	0.682811	0.731472
Neutrophils	GAS8	-0.23605	1.529564	-0.56483	0.57362	-5.09344	0.714632	0.764687
Neutrophils	SLC5A10	0.373422	-0.1778	0.564757	0.573669	-4.97439	0.750342	0.801842
Neutrophils	ERBB4	-0.31394	0.909465	-0.56456	0.573805	-5.18044	0.727416	0.778014
Neutrophils	GM49774	0.190564	3.174932	0.564548	0.57381	-5.50933	0.681869	0.73052
Neutrophils	KCTD2	-0.12919	3.355478	-0.56434	0.573953	-5.57512	0.67846	0.726925
Neutrophils	PPIH	-0.10436	5.305306	-0.56386	0.574275	-5.81154	0.64209	0.688529
Neutrophils	SERPING1	-0.17891	3.004813	-0.56357	0.57447	-5.52719	0.685653	0.734175
Neutrophils	GABPA	0.093551	4.662072	0.563569	0.574474	-5.8004	0.654018	0.701082
Neutrophils	RIF1	-0.09905	5.435862	-0.56341	0.574585	-5.9086	0.63977	0.686156
Neutrophils	PSMD3	-0.11211	4.642778	-0.56337	0.574609	-5.66933	0.654377	0.701474
Neutrophils	GM6225	0.197065	2.958455	0.563311	0.574649	-5.38469	0.68656	0.73514
Neutrophils	PMEPA1	-0.24829	4.361383	-0.56304	0.574833	-5.40928	0.659718	0.707109
Neutrophils	MBD4	0.170367	3.212172	0.563027	0.574841	-5.38736	0.681686	0.730091
Neutrophils	TMPRSS3	0.35155	-0.48523	0.562634	0.575108	-4.96428	0.757761	0.809263
Neutrophils	RBM18	-0.08898	4.236008	-0.56255	0.575162	-5.77043	0.662238	0.709743
Neutrophils	GM35853	0.156431	0.184156	0.56254	0.575171	-5.53956	0.74341	0.794379
Neutrophils	GALNT3	0.172493	1.767982	0.562182	0.575415	-5.56037	0.710758	0.760206
Neutrophils	HELZ	-0.07461	6.25998	-0.56183	0.575651	-6.13393	0.625428	0.670884
Neutrophils	TMEM158	0.374255	0.271092	0.561744	0.575712	-5.05466	0.741871	0.792496
Neutrophils	ROBO1	-0.20237	2.137687	-0.5617	0.575738	-5.50355	0.703365	0.752442
Neutrophils	ATP13A1	0.1219	3.809105	0.561649	0.575776	-5.527	0.670618	0.718255
Neutrophils	GM43256	-0.25229	0.309245	-0.56153	0.575858	-5.18192	0.741063	0.791684
Neutrophils	TCEA2	-0.33188	0.335442	-0.56151	0.575867	-4.97502	0.740508	0.791108
Neutrophils	DDX42	0.071223	5.684077	0.561439	0.575919	-5.97791	0.635753	0.681757
Neutrophils	NRG1	0.210937	2.541088	0.561263	0.576038	-5.70966	0.695336	0.744104
Neutrophils	TEN1	0.075853	5.852604	0.561224	0.576064	-5.99357	0.632733	0.678588
Neutrophils	TOMM40L	-0.19778	1.733558	-0.56057	0.576505	-5.16537	0.711933	0.761094
Neutrophils	MCM8	0.247743	1.54016	0.560571	0.576508	-5.14905	0.715875	0.765196
Neutrophils	DNAL4	0.214648	1.951429	0.560482	0.576568	-5.23631	0.707519	0.756498
Neutrophils	3010003L2	0.308302	1.349271	0.560272	0.576711	-5.05499	0.719852	0.76932
Neutrophils	GM45716	-0.17239	3.20355	-0.56022	0.576748	-5.38944	0.682749	0.730658
Neutrophils	KHDRBS1	-0.0469	7.151511	-0.56006	0.576853	-6.35183	0.610206	0.65467
Neutrophils	S100A9	0.082525	6.814997	0.559947	0.576932	-7.40109	0.616063	0.660838
Neutrophils	TOR3A	-0.20633	4.075925	-0.55992	0.57695	-5.519	0.665991	0.7132
Neutrophils	AS3MT	0.221343	2.69274	0.559729	0.57708	-5.23758	0.692814	0.741208
Neutrophils	BNIP1	0.181044	3.526643	0.559693	0.577104	-5.37772	0.67653	0.724209
Neutrophils	DIP2B	-0.06335	7.880088	-0.55902	0.57756	-6.46259	0.598091	0.64158

Neutrophils	DIABLO	-0.13495	4.259159	-0.55902	0.577561	-5.50933	0.66293	0.709624
Neutrophils	HSD11B1	0.067904	4.593839	0.55836	0.57801	-6.44535	0.657066	0.703195
Neutrophils	MAIP1	0.15059	3.588251	0.55828	0.578064	-5.40852	0.676167	0.723155
Neutrophils	LPCAT4	-0.11948	3.121289	-0.55809	0.578194	-5.92367	0.685308	0.732643
Neutrophils	AIRN	-0.13986	5.663175	-0.55789	0.578332	-6.02053	0.637523	0.682683
Neutrophils	TMUB2	0.13418	3.181282	0.557372	0.578682	-5.5165	0.684524	0.731654
Neutrophils	TIMM17A	-0.09682	5.135955	-0.55733	0.578713	-5.72688	0.647442	0.692911
Neutrophils	B3GLCT	-0.19086	3.311117	-0.55712	0.578856	-5.33717	0.682086	0.729109
Neutrophils	C920021L1	-0.17835	2.268205	-0.55694	0.578979	-5.29641	0.702698	0.750605
Neutrophils	EPS15L1	-0.06581	6.525246	-0.55691	0.578994	-6.19836	0.622457	0.666737
Neutrophils	HHAT	-0.28443	1.670909	-0.55675	0.579105	-5.09723	0.714837	0.763202
Neutrophils	ADI1	-0.15494	3.749643	-0.5566	0.579204	-5.33798	0.673676	0.720336
Neutrophils	CD209D	0.441768	-0.32218	0.556516	0.579264	-4.97572	0.756706	0.806676
Neutrophils	G3BP2	0.049546	6.759047	0.556469	0.579296	-6.28036	0.618386	0.662486
Neutrophils	HPRT	-0.0711	6.310323	-0.55627	0.579429	-6.19718	0.626355	0.670873
Neutrophils	MLX	0.105762	3.767938	0.556081	0.57956	-5.72522	0.673363	0.720158
Neutrophils	ZMAT1	0.17743	2.090961	0.555971	0.579635	-5.40919	0.706357	0.754576
Neutrophils	CRKL	-0.07742	5.071994	-0.55591	0.579676	-5.95378	0.648808	0.694507
Neutrophils	KIF18A	0.137976	3.977118	0.555899	0.579684	-5.75023	0.66936	0.716
Neutrophils	MRPL46	-0.178	3.264758	-0.55586	0.579713	-5.30942	0.683093	0.730336
Neutrophils	RARS2	-0.16091	3.441715	-0.55574	0.579795	-5.37115	0.679675	0.726774
Neutrophils	TMEM106(	-0.17479	3.205819	-0.55561	0.579882	-5.29343	0.68429	0.731566
Neutrophils	HNRNPUL1	0.047148	7.28107	0.555489	0.579963	-6.41645	0.609384	0.653109
Neutrophils	KCTD20	-0.06469	4.885027	-0.55533	0.580074	-6.14356	0.652388	0.698226
Neutrophils	AGL	-0.08494	3.963587	-0.55476	0.580458	-6.16213	0.670057	0.716521
Neutrophils	FRG1	-0.05137	6.660706	-0.55473	0.580478	-6.38274	0.620556	0.66469
Neutrophils	BTBD6	0.257917	1.542046	0.554535	0.580613	-5.16335	0.718069	0.766506
Neutrophils	MLLT10	0.044695	7.464033	0.554293	0.580778	-6.48812	0.606672	0.650135
Neutrophils	PPP4R2	0.06173	6.318519	0.554161	0.580868	-6.27562	0.626728	0.671212
Neutrophils	PCDH9	-0.40325	0.472713	-0.55404	0.58095	-5.08398	0.740375	0.789749
Neutrophils	FAM189B	-0.24502	2.569109	-0.55402	0.580964	-5.21324	0.697362	0.74505
Neutrophils	PITPNB	0.069147	5.507244	0.553983	0.580989	-5.99869	0.641354	0.686549
Neutrophils	HMOX1	-0.13826	4.807778	-0.55389	0.58105	-6.1886	0.65425	0.70005
Neutrophils	ENSA	0.073835	5.388151	0.55383	0.581093	-6.02769	0.64353	0.68883
Neutrophils	GM26724	0.259435	1.517501	0.553562	0.581276	-5.15047	0.718673	0.767223
Neutrophils	NCL	-0.07995	7.783001	-0.55336	0.581413	-6.42331	0.60126	0.644466
Neutrophils	SNRPB2	-0.06335	6.282619	-0.5532	0.58152	-6.14082	0.627422	0.671968
Neutrophils	LAPTM4B	0.185334	3.501356	0.553061	0.581617	-5.57457	0.679122	0.726071
Neutrophils	ZFP873	0.27231	0.995152	0.552913	0.581719	-5.0275	0.729473	0.778512
Neutrophils	SLAIN2	-0.04757	5.670672	-0.55287	0.581748	-6.4004	0.638434	0.683555
Neutrophils	HKDC1	0.278094	0.37121	0.552808	0.58179	-5.27485	0.742588	0.792119
Neutrophils	LMTK3	-0.33389	-0.70138	-0.55279	0.581802	-4.97126	0.765691	0.816047
Neutrophils	DAP	0.081889	6.19848	0.552739	0.581837	-6.22111	0.628925	0.673584
Neutrophils	MNS1	-0.18824	2.821224	-0.55245	0.582033	-5.31434	0.692424	0.740096
Neutrophils	A630023P1	-0.39436	0.306091	-0.55238	0.582081	-4.97429	0.743971	0.793681
Neutrophils	KLHL13	-0.32757	0.360663	-0.55233	0.582116	-5.11826	0.742812	0.79248
Neutrophils	ORC2	0.130951	4.279461	0.552179	0.582219	-5.51804	0.664226	0.710677

Neutrophils	2010315B(	-0.19838	2.024895	-0.55209	0.582278	-5.20468	0.708338	0.756684
Neutrophils	TOR1B	0.136837	3.704769	0.552036	0.582317	-5.48557	0.675195	0.722136
Neutrophils	SIK3	0.057871	10.09847	0.552011	0.582333	-7.03066	0.563127	0.604421
Neutrophils	H2-DMA	0.209644	5.184297	0.551944	0.582379	-5.6388	0.64733	0.692999
Neutrophils	ARL6IP6	0.064992	5.278997	0.551925	0.582392	-6.17072	0.645588	0.691175
Neutrophils	ARVCF	0.343309	1.228411	0.551678	0.582561	-5.01778	0.72463	0.773622
Neutrophils	RBFA	-0.07344	4.826424	-0.55166	0.582572	-6.18805	0.653959	0.699938
Neutrophils	EIF2B4	-0.11917	4.102435	-0.55164	0.582586	-5.48349	0.667585	0.714187
Neutrophils	ANK3	-0.29227	1.681131	-0.55162	0.582603	-5.26068	0.715324	0.76395
Neutrophils	TFR2	-0.24054	1.213769	-0.55151	0.582678	-5.17108	0.724933	0.773954
Neutrophils	TECPR2	-0.1317	3.905519	-0.5515	0.582683	-5.60743	0.671342	0.718128
Neutrophils	XPO5	0.130682	3.603677	0.551233	0.582864	-5.52272	0.677279	0.724237
Neutrophils	KCNAB1	-0.29254	1.324987	-0.55112	0.58294	-5.18375	0.722794	0.771647
Neutrophils	SLF2	0.077061	6.314028	0.550874	0.583109	-6.07782	0.627114	0.671642
Neutrophils	COPZ2	0.18199	2.183281	0.550723	0.583212	-5.35589	0.705473	0.753489
Neutrophils	NHLRC3	0.161347	3.16365	0.550595	0.5833	-5.46312	0.686041	0.733251
Neutrophils	ABCF2	-0.10787	4.411019	-0.5504	0.583434	-5.65917	0.662156	0.708258
Neutrophils	ZFP994	-0.1858	2.481603	-0.55029	0.583506	-5.23878	0.699614	0.747322
Neutrophils	PURG	0.13166	3.260975	0.550034	0.583683	-5.59891	0.684363	0.731392
Neutrophils	BC005561	-0.12957	4.273496	-0.54992	0.583763	-5.53872	0.664911	0.711078
Neutrophils	ALDH1L1	-0.18202	3.098676	-0.54955	0.584015	-5.52588	0.687777	0.734756
Neutrophils	PLATR25	-0.20645	2.729731	-0.54944	0.584085	-5.22715	0.695061	0.742337
Neutrophils	EIF3J2	0.260544	1.443832	0.549076	0.584337	-5.1024	0.721277	0.769396
Neutrophils	SNAPIN	0.099432	4.083623	0.548943	0.584428	-5.71297	0.668985	0.714977
Neutrophils	CKM	-0.45153	0.1057	-0.54867	0.584613	-5.00751	0.749564	0.798729
Neutrophils	TRIAP1	-0.12733	4.111675	-0.54832	0.584851	-5.55902	0.668625	0.714711
Neutrophils	CCAR1	-0.05121	6.820278	-0.54814	0.584978	-6.29424	0.619038	0.662852
Neutrophils	BHMT2	-0.23693	2.060069	-0.54808	0.585021	-5.35543	0.708917	0.756753
Neutrophils	RFESD	0.202661	2.541257	0.548044	0.585043	-5.30336	0.699248	0.746694
Neutrophils	ERCC5	0.17481	2.909402	0.547974	0.58509	-5.27989	0.691942	0.739086
Neutrophils	NOL8	-0.17377	4.078757	-0.54781	0.585202	-5.37704	0.669253	0.715485
Neutrophils	CDC7	0.16411	2.873973	0.547725	0.585261	-5.46216	0.692642	0.739885
Neutrophils	IQGAP3	0.201149	2.400987	0.547712	0.58527	-5.47755	0.702053	0.749683
Neutrophils	GFM2	-0.1421	3.590998	-0.54769	0.585287	-5.48226	0.678622	0.72527
Neutrophils	NOTCH1	-0.0605	5.292946	-0.54769	0.585288	-6.22905	0.646508	0.691711
Neutrophils	GM11998	-0.261	0.318253	-0.5475	0.585413	-5.196	0.745144	0.794427
Neutrophils	UBA6	0.087398	5.172539	0.547158	0.585649	-5.90743	0.648836	0.694228
Neutrophils	COX6B1	-0.05433	7.821955	-0.54714	0.585662	-6.55706	0.601792	0.644867
Neutrophils	PNP	-0.08242	5.924728	-0.54698	0.585772	-6.23578	0.6351	0.679874
Neutrophils	RAB6A	0.0425	6.637549	0.546892	0.585831	-6.59001	0.622363	0.666533
Neutrophils	BMX	0.101209	0.02253	0.546846	0.585862	-6.09577	0.751514	0.801185
Neutrophils	PGAP3	-0.32984	1.12543	-0.54681	0.585885	-5.00959	0.728211	0.77702
Neutrophils	5430405H(	0.112054	3.910079	0.546751	0.585927	-5.7621	0.672591	0.719114
Neutrophils	URB2	-0.2055	2.571455	-0.5466	0.586029	-5.22385	0.698764	0.746403
Neutrophils	CREM	-0.1114	6.0952	-0.5466	0.58603	-6.16255	0.632029	0.676673
Neutrophils	TOMM5	-0.11221	5.458336	-0.54649	0.586108	-5.80343	0.643596	0.688778
Neutrophils	DNAJC27	-0.23763	2.442699	-0.54628	0.586246	-5.10895	0.701375	0.74913

Neutrophils	1700061G1	0.290667	1.070717	0.546271	0.586256	-5.06975	0.729391	0.778254
Neutrophils	RC3H2	0.058158	5.728871	0.545909	0.586503	-6.19171	0.638852	0.683754
Neutrophils	EVA1A	-0.24657	1.060527	-0.54578	0.586592	-5.17201	0.729796	0.778622
Neutrophils	RGP1	0.14482	2.34849	0.545762	0.586604	-5.35156	0.703449	0.751239
Neutrophils	ARAF	0.091705	4.478585	0.545601	0.586714	-5.7705	0.662048	0.708061
Neutrophils	HIST2H2AC	0.35242	2.376405	0.545382	0.586864	-5.1998	0.702949	0.750729
Neutrophils	CALCOCO1	0.147445	3.863697	0.545362	0.586877	-5.50883	0.673754	0.720297
Neutrophils	KIF16B	-0.0964	4.843803	-0.54531	0.586911	-5.89411	0.655203	0.700912
Neutrophils	CCDC12	0.048879	6.939585	0.545139	0.58703	-6.39718	0.617299	0.661226
Neutrophils	NFE2L1	-0.0972	4.98448	-0.54511	0.587053	-5.92022	0.652584	0.698215
Neutrophils	ACYP2	0.170241	3.398712	0.544864	0.587219	-5.4197	0.682745	0.729823
Neutrophils	FAM98B	0.12497	4.474383	0.544782	0.587275	-5.55932	0.662132	0.708313
Neutrophils	DYNLT1F	0.092044	5.068561	0.544779	0.587277	-6.08256	0.651024	0.696693
Neutrophils	ST3GAL2	-0.11759	3.085043	-0.54475	0.587297	-5.72117	0.68888	0.736237
Neutrophils	ASRGL1	0.198823	3.315534	0.544652	0.587364	-5.35398	0.68437	0.73157
Neutrophils	BACE1	0.142996	2.654059	0.544431	0.587515	-5.46216	0.697508	0.745215
Neutrophils	GM4117	0.271582	0.660809	0.544238	0.587647	-5.08893	0.738363	0.78767
Neutrophils	AXIN1	0.063898	6.046226	0.544148	0.587709	-6.06987	0.633275	0.678092
Neutrophils	DDX54	-0.1014	5.820595	-0.54406	0.58777	-5.81693	0.637351	0.682372
Neutrophils	PRAF2	-0.21633	2.133854	-0.54395	0.587847	-5.10593	0.707953	0.756121
Neutrophils	SPATA1	0.110796	3.467697	0.543946	0.587848	-5.55127	0.68152	0.72858
Neutrophils	TCP11	-0.23756	0.648485	-0.5433	0.588287	-5.13805	0.738849	0.788234
Neutrophils	RPRD2	0.065076	5.916624	0.543286	0.5883	-6.22491	0.635807	0.680786
Neutrophils	RDM1	-0.11225	5.028258	-0.5432	0.588359	-5.95843	0.652082	0.697845
Neutrophils	TEX9	-0.30371	1.820323	-0.54316	0.58839	-5.03884	0.714536	0.762999
Neutrophils	LY86	-0.13586	6.704851	-0.54313	0.58841	-5.98232	0.621723	0.66602
Neutrophils	GNASAS1	0.319315	0.682179	0.54312	0.588414	-5.0201	0.738139	0.787515
Neutrophils	CENPM	-0.18186	3.916181	-0.54291	0.588561	-5.52656	0.673163	0.719863
Neutrophils	CHPF2	0.188206	2.64648	0.542727	0.588683	-5.3372	0.698019	0.745821
Neutrophils	PCK2	-0.1979	3.244845	-0.5426	0.588768	-5.33096	0.686206	0.733563
Neutrophils	ST18	0.384018	0.283819	0.542466	0.588863	-5.06595	0.746728	0.796566
Neutrophils	TMEM222	0.115777	4.289191	0.542375	0.588925	-5.68591	0.66608	0.712612
Neutrophils	TRIM33	-0.05772	6.368235	-0.54233	0.588957	-6.30901	0.627818	0.672543
Neutrophils	TKT	0.05041	6.932084	0.542298	0.588978	-6.66878	0.617843	0.662082
Neutrophils	QDPR	0.102546	5.245358	0.542104	0.589111	-5.73788	0.648199	0.693957
Neutrophils	ACOX3	0.087218	4.372027	0.541984	0.589193	-5.91439	0.66452	0.711084
Neutrophils	ATP5F1	-0.04808	8.084387	-0.54195	0.589215	-6.59894	0.597984	0.641272
Neutrophils	MAPK13	0.076338	0.83712	0.541909	0.589244	-6.28269	0.735032	0.784558
Neutrophils	AIFM1	0.107456	4.370662	0.541648	0.589424	-5.66177	0.664676	0.711225
Neutrophils	9830166K0	-0.15019	-1.08122	-0.5411	0.589798	-5.40646	0.776991	0.827646
Neutrophils	YWHAE	-0.03824	8.88218	-0.54092	0.589923	-6.68322	0.585106	0.627348
Neutrophils	ADCY6	0.279917	0.433361	0.540747	0.590042	-5.10147	0.74421	0.793599
Neutrophils	CSNK1D	-0.03888	6.6346	-0.54063	0.590125	-6.43813	0.623642	0.667887
Neutrophils	CDK5R1	-0.16065	2.111423	-0.54059	0.590148	-5.47772	0.709393	0.757502
Neutrophils	SLC25A1	-0.1638	3.772843	-0.54049	0.590221	-5.4281	0.676567	0.723305
Neutrophils	MAPK8	-0.08292	5.232129	-0.54028	0.590365	-5.9138	0.649089	0.694594
Neutrophils	USP11	-0.2226	2.611991	-0.54018	0.590434	-5.18008	0.699413	0.747165

Neutrophils	ALKBH2	-0.21147	2.187068	-0.54009	0.59049	-5.10296	0.707946	0.756047
Neutrophils	SLC25A24	-0.07741	3.486183	-0.54002	0.59054	-6.13838	0.682189	0.729212
Neutrophils	CAPN5	-0.32952	1.546779	-0.53973	0.590741	-5.06113	0.721167	0.769674
Neutrophils	BAIAP2L1	0.159624	3.098445	0.539581	0.590843	-5.42925	0.68993	0.737177
Neutrophils	PPM1F	-0.19509	2.206568	-0.53955	0.590866	-5.27324	0.707712	0.7557
Neutrophils	CCT5	0.079302	6.571584	0.539247	0.591072	-6.15487	0.624975	0.669392
Neutrophils	GTF2IRD1	-0.15197	3.292146	-0.53914	0.591143	-5.60167	0.68613	0.733378
Neutrophils	RBM5	0.049293	6.623423	0.538998	0.591243	-6.35928	0.624055	0.668472
Neutrophils	DCTN4	0.052781	6.423452	0.538942	0.591281	-6.31901	0.62761	0.672204
Neutrophils	SAP30	0.085426	5.143228	0.538907	0.591305	-6.04009	0.650882	0.696595
Neutrophils	DAD1	-0.06743	6.948328	-0.53887	0.591332	-6.20784	0.618323	0.662453
Neutrophils	2310001H1	0.069597	4.065364	0.538808	0.591374	-6.32896	0.671172	0.717813
Neutrophils	EIF3A	-0.05045	7.379667	-0.53868	0.591462	-6.34802	0.6108	0.654546
Neutrophils	CDK14	0.162483	5.455475	0.538554	0.591548	-6.10121	0.645124	0.690565
Neutrophils	TWF2	0.064643	5.240324	0.538523	0.59157	-6.22527	0.649085	0.694714
Neutrophils	SNRPA1	0.127099	5.077996	0.538347	0.59169	-5.71831	0.652091	0.697941
Neutrophils	GM16093	-0.14667	2.640752	-0.53831	0.591715	-5.58228	0.698998	0.746924
Neutrophils	GRHL1	-0.28633	1.025537	-0.53827	0.591743	-5.11833	0.731982	0.781228
Neutrophils	ATP2A1	-0.27664	1.448126	-0.53823	0.59177	-5.11719	0.723203	0.772108
Neutrophils	UHRF1	-0.16547	4.598357	-0.53798	0.59194	-5.73967	0.661176	0.707387
Neutrophils	IRF1	0.133231	7.149071	0.537771	0.592086	-6.40815	0.615004	0.658992
Neutrophils	GM17745	-0.303	0.962497	-0.53768	0.592151	-5.03551	0.733534	0.782797
Neutrophils	ZFP800	-0.07641	5.365723	-0.53744	0.592313	-6.00121	0.647085	0.69264
Neutrophils	TEX45	-0.28279	0.222756	-0.53727	0.592434	-5.14299	0.749391	0.799192
Neutrophils	CCDC82	0.086497	4.323551	0.536925	0.592668	-5.91395	0.6668	0.713165
Neutrophils	CPNE5	0.303176	0.481176	0.536859	0.592714	-5.07121	0.744062	0.793601
Neutrophils	GM12359	-0.23783	1.671224	-0.53677	0.592772	-5.13027	0.719201	0.767853
Neutrophils	N6AMT1	0.209754	2.597729	0.536448	0.592996	-5.2124	0.700551	0.748433
Neutrophils	GM36371	-0.22557	0.884673	-0.53644	0.593002	-5.10481	0.735662	0.784934
Neutrophils	C3	0.098442	6.327835	0.536236	0.593142	-6.60933	0.630022	0.674648
Neutrophils	APPBP2OS	0.209767	0.985935	0.535742	0.593482	-5.1715	0.733803	0.782991
Neutrophils	ZNHIT2	0.136154	3.310699	0.535738	0.593485	-5.40776	0.686696	0.733978
Neutrophils	FASTK	-0.14771	3.15048	-0.53569	0.593518	-5.40283	0.689841	0.737269
Neutrophils	PDE3A	-0.35667	0.411945	-0.53558	0.593596	-5.10902	0.745931	0.795634
Neutrophils	MRPS27	0.198315	3.208477	0.535565	0.593604	-5.24598	0.688701	0.736125
Neutrophils	MS4A6D	-0.2558	2.866447	-0.53531	0.593782	-5.46057	0.695455	0.743179
Neutrophils	TFG	-0.07189	5.864765	-0.53529	0.593792	-6.01629	0.638523	0.683674
Neutrophils	SMPD3	-0.3797	-0.20687	-0.53525	0.593824	-4.96752	0.759236	0.809434
Neutrophils	MIGA2	-0.30199	0.892517	-0.53518	0.593869	-5.0633	0.735766	0.785102
Neutrophils	PROCA1	-0.15698	2.988273	-0.53509	0.593929	-5.48245	0.693042	0.740682
Neutrophils	ZFP808	0.204165	1.973033	0.534801	0.59413	-5.211	0.713577	0.761948
Neutrophils	FAM126A	-0.08165	5.549539	-0.53448	0.59435	-6.13635	0.64459	0.689816
Neutrophils	CGAS	0.174705	2.747045	0.534307	0.59447	-5.53947	0.698221	0.745776
Neutrophils	EIF1B	0.060053	5.828759	0.534232	0.594522	-6.17234	0.639537	0.684492
Neutrophils	FASTKD5	0.284453	0.844458	0.534076	0.594629	-5.03058	0.737245	0.786354
Neutrophils	H2-Q4	-0.20441	4.933329	-0.53375	0.594857	-5.57216	0.656207	0.701834
Neutrophils	TAB1	0.169166	3.004453	0.533578	0.594972	-5.39227	0.693289	0.740542

Neutrophils	PODXL	-0.2373	0.550401	-0.53353	0.595005	-5.28192	0.743597	0.792822
Neutrophils	POP5	0.099183	4.525116	0.533407	0.595091	-5.60922	0.663879	0.709901
Neutrophils	DDX52	0.080715	4.930462	0.533389	0.595103	-5.89624	0.656261	0.701938
Neutrophils	CNNM4	-0.08307	4.077084	-0.53308	0.595317	-5.91805	0.672408	0.718924
Neutrophils	INTS1	-0.17212	3.347025	-0.53294	0.595412	-5.38902	0.686548	0.733748
Neutrophils	PQBP1	-0.09595	4.6495	-0.53284	0.59548	-5.80017	0.661532	0.707657
Neutrophils	ATP9A	0.25921	0.837325	0.532744	0.595548	-5.19064	0.737529	0.786824
Neutrophils	OPLAH	0.174151	1.500611	0.532742	0.595549	-5.39962	0.723691	0.772456
Neutrophils	DYNC2H1	0.151365	3.842041	0.532719	0.595564	-5.48008	0.676927	0.723752
Neutrophils	SPATA32	-0.33377	0.244996	-0.53269	0.595582	-5.05424	0.750112	0.799873
Neutrophils	RELCH	0.063756	6.238997	0.532331	0.595832	-6.11872	0.632282	0.677178
Neutrophils	JOSD1	-0.0982	3.99961	-0.53213	0.595969	-5.7247	0.673894	0.720766
Neutrophils	DHX32	-0.15529	3.380004	-0.5321	0.595992	-5.33653	0.685903	0.733302
Neutrophils	SIPA1L2	0.072838	5.103799	0.532041	0.596032	-6.26883	0.65303	0.698948
Neutrophils	TSLP	-0.31946	-0.44209	-0.53202	0.596047	-5.04743	0.764979	0.815474
Neutrophils	2010001A1	0.244879	1.389339	0.531881	0.596142	-5.10958	0.725994	0.775043
Neutrophils	FUT8	-0.08539	5.850748	-0.53181	0.596194	-5.99642	0.639299	0.684563
Neutrophils	THRAP3	-0.0495	8.006361	-0.53177	0.596218	-6.53726	0.601346	0.644693
Neutrophils	PROSER1	-0.13375	4.213066	-0.53175	0.596232	-5.54735	0.669807	0.716496
Neutrophils	LEFTY1	0.304927	0.408138	0.531742	0.596238	-5.02324	0.746625	0.796458
Neutrophils	GM5535	0.354822	0.151841	0.531712	0.596259	-5.05079	0.75211	0.802145
Neutrophils	EIF3H	0.054277	7.353901	0.531489	0.596413	-6.38471	0.612577	0.656516
Neutrophils	DYNC1I2	0.043884	6.441227	0.531461	0.596432	-6.39297	0.628659	0.67341
Neutrophils	SELENOT	0.057045	6.182995	0.531257	0.596573	-6.21509	0.633289	0.6783
Neutrophils	3110040N1	-0.17631	3.553549	-0.53121	0.596607	-5.39368	0.682517	0.729812
Neutrophils	NVL	-0.09542	4.723925	-0.53114	0.596654	-5.72467	0.660131	0.706421
Neutrophils	SETD3	0.06633	5.687067	0.53108	0.596695	-6.08757	0.642282	0.68773
Neutrophils	2610203C2	-0.26854	0.354453	-0.53083	0.596867	-5.14476	0.74777	0.797693
Neutrophils	B3GALT1	-0.19122	3.129188	-0.53076	0.596918	-5.79194	0.690827	0.738481
Neutrophils	ZC3H12C	-0.22779	4.431325	-0.53066	0.596985	-5.55404	0.665655	0.712198
Neutrophils	GM41496	0.312453	0.985043	0.530642	0.596998	-5.08441	0.734424	0.783845
Neutrophils	UBE2K	0.041447	8.214477	0.530583	0.597038	-6.61747	0.59781	0.641008
Neutrophils	FTL1-PS1	-0.13881	3.902493	-0.53053	0.597076	-5.81744	0.675762	0.722759
Neutrophils	CSTF1	0.15293	3.243385	0.530491	0.597102	-5.34926	0.68858	0.736138
Neutrophils	SYPL	-0.05264	5.860238	-0.53045	0.597127	-6.41425	0.639127	0.684426
Neutrophils	RHOU	0.11108	1.27725	0.530291	0.597239	-6.0457	0.728379	0.777575
Neutrophils	GM11755	-0.38776	-0.115	-0.53015	0.597335	-4.96301	0.757965	0.808238
Neutrophils	PLAA	0.067394	6.069326	0.530053	0.597404	-6.06446	0.635426	0.680555
Neutrophils	MAOB	-0.25695	1.627366	-0.52994	0.597481	-5.27857	0.721193	0.770139
Neutrophils	RWDD1	0.061701	6.105394	0.529828	0.597559	-6.15338	0.634803	0.679931
Neutrophils	SCAMP2	0.049023	6.357233	0.529623	0.597701	-6.38298	0.63033	0.6752
Neutrophils	NEBL	-0.34221	1.182115	-0.52945	0.597822	-5.15233	0.730498	0.779794
Neutrophils	TSPAN15	-0.13994	2.388192	-0.52939	0.597863	-5.54957	0.705776	0.754083
Neutrophils	KANTR	0.200454	2.529607	0.529379	0.597869	-5.27352	0.702933	0.751123
Neutrophils	ARMC6	-0.26649	1.405639	-0.52906	0.598087	-5.03031	0.725886	0.774965
Neutrophils	RETNLG	0.080721	3.313557	0.529042	0.598102	-6.73016	0.687421	0.734918
Neutrophils	TSEN34	0.103681	4.195681	0.528976	0.598147	-5.78705	0.670351	0.717096

Neutrophils	MYCBP2	-0.06043	7.768752	-0.52894	0.598175	-6.57537	0.605602	0.649199
Neutrophils	EXOC6B	-0.07804	5.502152	-0.52873	0.598316	-6.16164	0.645874	0.691483
Neutrophils	SENP1	-0.08119	5.013417	-0.52865	0.598373	-5.83308	0.65492	0.700964
Neutrophils	KCND1	-0.31853	-0.19065	-0.52863	0.59839	-5.04147	0.759745	0.810101
Neutrophils	APH1B	0.09582	2.578014	0.528589	0.598415	-5.9579	0.701997	0.750132
Neutrophils	RNF139	-0.08931	5.218473	-0.52826	0.59864	-5.9419	0.651283	0.697053
Neutrophils	GM1604B	-0.26364	1.489369	-0.52814	0.598727	-5.2198	0.724371	0.77326
Neutrophils	FRA10AC1	-0.157	3.408508	-0.52805	0.598789	-5.37301	0.685769	0.733095
Neutrophils	AKTIP	-0.1217	3.102695	-0.52776	0.598987	-5.54508	0.691863	0.739428
Neutrophils	RNASEK	0.045787	6.735066	0.527751	0.598994	-6.47834	0.623897	0.668318
Neutrophils	D130062J1	0.180317	1.642184	0.527634	0.599075	-5.369	0.721308	0.770141
Neutrophils	GM26930	-0.38123	0.305585	-0.52746	0.599194	-4.97731	0.749372	0.79931
Neutrophils	FBXL6	-0.12954	3.399881	-0.52739	0.59924	-5.49821	0.686024	0.73343
Neutrophils	EML4	0.060356	7.138312	0.527378	0.599252	-6.37459	0.616795	0.66094
Neutrophils	CREB3L2	0.144417	3.55229	0.527227	0.599356	-5.59992	0.683094	0.730326
Neutrophils	FAM71E1	0.321563	0.837279	0.527117	0.599433	-5.05307	0.738141	0.787595
Neutrophils	CLOCK	-0.10124	5.071637	-0.52699	0.599522	-5.78183	0.654198	0.700084
Neutrophils	GLUD1	-0.04874	7.653063	-0.52689	0.599591	-6.58583	0.607933	0.651537
Neutrophils	4933433G1	0.314447	0.742117	0.526646	0.599758	-5.10122	0.740312	0.789707
Neutrophils	ICAM2	-0.17199	4.282374	-0.52648	0.59987	-5.82675	0.669239	0.715723
Neutrophils	ALG5	0.119371	4.2129	0.526375	0.599946	-5.60844	0.670565	0.717145
Neutrophils	DYNLT1B	-0.2622	1.188553	-0.5263	0.599998	-5.15685	0.73099	0.7801
Neutrophils	CLN6	0.134156	3.482895	0.526194	0.600071	-5.58449	0.684673	0.73191
Neutrophils	COMMD4	0.103783	5.326168	0.526057	0.600166	-5.74181	0.649683	0.695331
Neutrophils	ZFP574	0.141144	3.401172	0.525922	0.600259	-5.47829	0.686337	0.733679
Neutrophils	SPARCL1	0.343822	0.3267	0.525636	0.600457	-5.2429	0.749423	0.799247
Neutrophils	SMC1A	-0.06353	6.748393	-0.52558	0.600495	-6.36911	0.624079	0.668492
Neutrophils	DHRS7B	-0.1756	3.137991	-0.52522	0.600745	-5.29791	0.691795	0.739263
Neutrophils	NCKAP5LO:	-0.36982	0.041134	-0.52519	0.600767	-4.98556	0.75574	0.805715
Neutrophils	HEATR5B	-0.13186	3.730171	-0.52508	0.600842	-5.59123	0.680223	0.727214
Neutrophils	OTUD7B	0.079714	5.17042	0.524973	0.600916	-6.0037	0.652886	0.698658
Neutrophils	DPP10	0.357009	0.411958	0.524888	0.600975	-5.04209	0.747794	0.797529
Neutrophils	PRKACB	-0.07838	5.548308	-0.52456	0.601201	-5.9216	0.645915	0.691454
Neutrophils	NEMP1	-0.1707	3.065406	-0.5245	0.601241	-5.50798	0.693257	0.740933
Neutrophils	CCL9	0.170952	2.831707	0.524466	0.601267	-5.59662	0.697894	0.745766
Neutrophils	POLR2J	0.082909	4.922519	0.524271	0.601402	-5.84263	0.657521	0.703651
Neutrophils	MED29	-0.11704	3.806911	-0.52409	0.601528	-5.51489	0.678754	0.725915
Neutrophils	KLHL7	-0.09365	4.739495	-0.52404	0.601559	-5.84051	0.660957	0.707314
Neutrophils	GM26670	0.250319	0.402458	0.523896	0.601662	-5.03644	0.748011	0.797998
Neutrophils	ABCA6	-0.36061	0.705764	-0.52388	0.601669	-5.05172	0.741559	0.791306
Neutrophils	NOSTRIN	-0.12033	3.426931	-0.52375	0.601763	-5.84284	0.686146	0.733646
Neutrophils	PKD2	-0.215	0.719693	-0.52367	0.601815	-5.17312	0.741265	0.791005
Neutrophils	CAPN7	-0.06656	5.340998	-0.52357	0.601887	-6.00601	0.649736	0.695631
Neutrophils	MXD4	-0.10402	5.790486	-0.52338	0.60202	-6.04494	0.641481	0.687009
Neutrophils	PCNP	0.046118	6.359657	0.523353	0.602038	-6.34171	0.631185	0.676211
Neutrophils	TMEM120I	-0.23309	3.044848	-0.52324	0.602115	-5.31072	0.693664	0.741598
Neutrophils	MLYCD	-0.13896	3.124459	-0.52323	0.602121	-5.44873	0.692091	0.739957

Neutrophils	GM15545	-0.24251	1.102359	-0.52307	0.602236	-5.05833	0.733208	0.782753
Neutrophils	POLK	-0.14319	3.481233	-0.5229	0.60235	-5.45346	0.685085	0.732702
Neutrophils	2300009A(	-0.12944	4.080294	-0.52287	0.602376	-5.60421	0.673486	0.720588
Neutrophils	VDAC1	0.063602	6.677223	0.522848	0.602388	-6.32146	0.625516	0.67033
Neutrophils	ARHGEF12	-0.11181	3.963016	-0.52278	0.602436	-5.86665	0.67574	0.722944
Neutrophils	GNL3	-0.13215	5.420028	-0.52273	0.602469	-5.62918	0.648277	0.694208
Neutrophils	ALDH1A1	-0.33084	-5.25E-05	-0.52273	0.60247	-5.07283	0.756661	0.807145
Neutrophils	PDE11A	-0.36598	0.072949	-0.52261	0.602552	-5.03817	0.755084	0.805539
Neutrophils	PRKCQ	-0.16138	3.12892	-0.52247	0.60265	-5.61913	0.692002	0.739993
Neutrophils	MPND	-0.09854	4.813143	-0.5224	0.602696	-5.86366	0.659572	0.706132
Neutrophils	CD163L1	0.24442	0.60908	0.522348	0.602734	-5.2931	0.74361	0.793736
Neutrophils	HRH2	0.201329	1.160498	0.522323	0.602752	-5.3194	0.731992	0.781671
Neutrophils	FAF2	-0.08634	5.155771	-0.5221	0.602906	-5.85237	0.65317	0.699519
Neutrophils	ARSB	0.110329	4.938464	0.522078	0.602921	-6.16683	0.657223	0.703769
Neutrophils	PLEKHG5	0.240808	1.864551	0.52207	0.602927	-5.23012	0.717425	0.766612
Neutrophils	GM2682	-0.24184	2.755703	-0.52196	0.603002	-5.49229	0.69942	0.747873
Neutrophils	LY9	-0.17828	4.262049	-0.52181	0.603105	-5.3004	0.67006	0.717178
Neutrophils	ATRIP	0.216113	2.825397	0.521646	0.603221	-5.24123	0.698135	0.746431
Neutrophils	A530072M	-0.32547	0.764735	-0.52112	0.603583	-5.02888	0.740599	0.790615
Neutrophils	GM16283	-0.29982	0.252287	-0.52107	0.603619	-4.9802	0.751518	0.801947
Neutrophils	GM13830	0.333267	0.287554	0.52105	0.603635	-5.04343	0.750762	0.801162
Neutrophils	SND1	0.059185	7.195863	0.520824	0.603791	-6.28975	0.616612	0.661099
Neutrophils	MAN2A1	0.06323	7.183028	0.520786	0.603818	-6.5154	0.616837	0.661335
Neutrophils	TTI1	0.182119	2.712153	0.520759	0.603837	-5.3764	0.70055	0.748977
Neutrophils	MPV17L2	-0.12112	4.459696	-0.52068	0.603888	-5.67537	0.666503	0.713425
Neutrophils	EIPR1	0.098986	4.924643	0.520632	0.603925	-5.69521	0.657737	0.704268
Neutrophils	GM20406	0.115844	-0.53168	0.520521	0.604001	-5.70411	0.768537	0.819638
Neutrophils	TRAJ18	-0.25441	-1.3552	-0.52052	0.604003	-4.97777	0.786828	0.838563
Neutrophils	KCTD17	0.256086	0.958849	0.520269	0.604176	-5.21942	0.736567	0.786418
Neutrophils	NDUFV1	0.090229	5.20278	0.520215	0.604214	-5.86954	0.652605	0.698846
Neutrophils	2-Sep	0.206452	2.22603	0.520147	0.604261	-5.2906	0.710396	0.759211
Neutrophils	APPL1	0.080509	5.974209	0.520072	0.604313	-6.10136	0.63844	0.684015
Neutrophils	ZFP655	-0.10439	4.453763	-0.51988	0.604447	-5.6946	0.666749	0.713621
Neutrophils	CYP3A25	0.222273	1.898523	0.519618	0.604628	-5.37947	0.717289	0.766295
Neutrophils	RCAN3	-0.18867	2.117276	-0.51947	0.604728	-5.25568	0.712865	0.761698
Neutrophils	ATP7A	0.054524	5.8288	0.519194	0.604923	-6.36386	0.641458	0.686985
Neutrophils	BMT2	0.059745	6.027835	0.51863	0.605315	-6.31745	0.638183	0.683295
Neutrophils	BRIP1OS	-0.07408	5.408086	-0.51848	0.605417	-6.04627	0.649571	0.695228
Neutrophils	RFXANK	0.151662	2.879474	0.518216	0.605602	-5.39489	0.698225	0.746006
Neutrophils	CRAMP1L	-0.08266	4.984741	-0.51813	0.605663	-5.88186	0.657564	0.703566
Neutrophils	CSNK2A1	0.04307	6.59773	0.518045	0.605721	-6.33901	0.628082	0.67269
Neutrophils	TMEM65	-0.11028	4.9205	-0.51774	0.605936	-5.83879	0.658814	0.704884
Neutrophils	C230037L1	-0.28697	0.378737	-0.5176	0.606033	-5.02068	0.749947	0.799842
Neutrophils	MAST2	-0.10063	5.283184	-0.51758	0.606045	-5.89744	0.652048	0.697851
Neutrophils	BANF1	-0.07508	6.873409	-0.51757	0.60605	-6.25268	0.623228	0.667629
Neutrophils	PCGF3	-0.11939	3.998741	-0.51752	0.606088	-5.51525	0.676341	0.723265
Neutrophils	ZBTB17	0.152626	3.917458	0.51728	0.606252	-5.43343	0.677958	0.72496

Neutrophils	DDX1	-0.11437	4.964571	-0.51721	0.606301	-5.73271	0.658035	0.704152
Neutrophils	TMEM273	0.219291	1.19098	0.517137	0.606352	-5.22176	0.732803	0.782092
Neutrophils	ZFP65	-0.27679	1.662117	-0.51705	0.606415	-5.09681	0.723011	0.771918
Neutrophils	PPP1R13L	-0.33964	-0.0774	-0.51695	0.606484	-5.04244	0.759836	0.810198
Neutrophils	SMG6	-0.04676	8.045885	-0.51671	0.606648	-6.50132	0.602882	0.64638
Neutrophils	DCUN1D5	-0.04614	6.828224	-0.51669	0.606662	-6.30587	0.624073	0.668664
Neutrophils	GM49164	0.232833	0.920748	0.516655	0.606687	-5.21192	0.73848	0.788121
Neutrophils	CASC1	0.229684	1.810453	0.516616	0.606714	-5.2979	0.719956	0.768874
Neutrophils	ANXA3	-0.07068	3.542549	-0.5164	0.606862	-6.43121	0.685335	0.732731
Neutrophils	TRP53I13	-0.14964	3.021819	-0.51611	0.607067	-5.34657	0.69575	0.743494
Neutrophils	PLCB3	0.155412	2.612	0.515953	0.607175	-5.39752	0.703982	0.752071
Neutrophils	SLC39A3	-0.1969	1.91724	-0.51579	0.607286	-5.2684	0.718102	0.766789
Neutrophils	4930430F0	-0.23674	1.035161	-0.51568	0.607362	-5.06663	0.736417	0.785869
Neutrophils	GM17066	-0.25874	1.747467	-0.51565	0.607389	-5.06978	0.721591	0.770475
Neutrophils	CAT	0.101493	6.72379	0.515413	0.607551	-6.23168	0.626321	0.67089
Neutrophils	FIZ1	0.083764	4.553694	0.515054	0.607801	-5.75814	0.666402	0.71278
Neutrophils	SRC	0.299605	0.980292	0.514932	0.607885	-5.03877	0.737938	0.787302
Neutrophils	GVIN1	0.307964	3.037272	0.514667	0.60807	-5.32982	0.695949	0.743539
Neutrophils	FAM172A	0.0523	7.557353	0.514606	0.608112	-6.46594	0.611961	0.655643
Neutrophils	TYW3	0.293729	0.995715	0.51454	0.608158	-5.01917	0.737708	0.786982
Neutrophils	ERICH1	-0.09905	3.61294	-0.51426	0.608353	-5.79946	0.684764	0.73174
Neutrophils	PHB	0.133624	4.493205	0.513896	0.608607	-5.58721	0.668008	0.714152
Neutrophils	FSTL1	-0.22595	2.107543	-0.51363	0.608794	-5.32555	0.715144	0.76325
Neutrophils	ATG4B	-0.06889	4.800676	-0.51351	0.608877	-6.08176	0.662287	0.708146
Neutrophils	CTSD	-0.06399	7.133555	-0.51348	0.608896	-6.63502	0.619785	0.663615
Neutrophils	MYPOPOS	0.311871	1.654931	0.513234	0.609068	-5.07152	0.724571	0.772993
Neutrophils	CCDC92	0.297496	-0.17821	0.512898	0.609302	-5.00982	0.763696	0.81345
Neutrophils	TMEM147	-0.10511	4.948794	-0.51272	0.609425	-5.71324	0.659773	0.705402
Neutrophils	DDX43	-0.2969	0.153087	-0.51266	0.609466	-5.0598	0.756503	0.806047
Neutrophils	LRAT	0.30594	-0.03713	0.512652	0.609473	-5.04783	0.760624	0.810314
Neutrophils	SDK1	-0.22225	3.195716	-0.51218	0.609799	-5.46962	0.693822	0.740802
Neutrophils	ARID5A	0.100415	4.681741	0.512111	0.60985	-5.83518	0.665051	0.710788
Neutrophils	ATG4C	0.112743	3.11799	0.51203	0.609907	-5.67344	0.695362	0.742405
Neutrophils	MTFR2	0.150183	3.615592	0.51196	0.609955	-5.63617	0.685565	0.732245
Neutrophils	FGGY	0.180768	3.459854	0.511516	0.610265	-5.47128	0.688892	0.735497
Neutrophils	EHD2	-0.24402	1.82262	-0.51085	0.61073	-5.18001	0.722172	0.769943
Neutrophils	CCDC191	-0.22695	1.993228	-0.51079	0.610772	-5.14996	0.718664	0.766312
Neutrophils	GPN1	-0.16572	3.042468	-0.51067	0.610854	-5.28576	0.697466	0.744323
Neutrophils	GALNT2	-0.08865	5.565067	-0.51066	0.610864	-5.9979	0.649102	0.693854
Neutrophils	ERGIC1	0.110188	5.084699	0.510641	0.610875	-5.83472	0.658036	0.703195
Neutrophils	PPP2R1A	0.062484	6.00443	0.510286	0.611123	-6.23484	0.641152	0.685515
Neutrophils	ADRB1	-0.34688	0.735849	-0.51014	0.611223	-5.12508	0.745061	0.793749
Neutrophils	EFCAB7	0.293896	0.535523	0.510129	0.611232	-4.98535	0.749336	0.798177
Neutrophils	IFI27	0.113775	4.998783	0.510117	0.611241	-6.11878	0.65976	0.704997
Neutrophils	SELL	0.06769	5.472373	0.509991	0.611329	-6.49971	0.650927	0.695809
Neutrophils	KCNH7	-0.17234	-0.13979	-0.50993	0.61137	-5.44675	0.76393	0.813374
Neutrophils	2210408F2	0.214036	2.856305	0.509622	0.611586	-5.3609	0.701447	0.74845

Neutrophils	PRKAR2A	0.092437	5.222556	0.509534	0.611648	-6.34174	0.655709	0.700746
Neutrophils	ZSWIM8	0.077324	4.703109	0.509375	0.611759	-5.88251	0.665477	0.711035
Neutrophils	CTNND2	-0.3208	1.726999	-0.50919	0.611886	-5.10599	0.724422	0.772452
Neutrophils	CDPF1	-0.2567	2.220471	-0.50918	0.611894	-5.1404	0.71429	0.761926
Neutrophils	GM34961	-0.20587	0.869528	-0.50911	0.611945	-5.32892	0.742378	0.791118
Neutrophils	ZFP319	-0.21126	2.544028	-0.50909	0.611954	-5.31226	0.707725	0.755138
Neutrophils	NDRG3	-0.10829	4.407312	-0.50896	0.61205	-5.57178	0.671141	0.716982
Neutrophils	LSM1	0.071645	5.251939	0.50882	0.612146	-5.9432	0.655227	0.700326
Neutrophils	PPM1K	-0.16581	3.579795	-0.50835	0.612476	-5.43983	0.687347	0.73384
Neutrophils	RARG	-0.17616	1.622679	-0.50827	0.612529	-5.44148	0.726821	0.774909
Neutrophils	ARCN1	-0.06211	5.873425	-0.50822	0.612566	-6.16352	0.64389	0.688477
Neutrophils	GM43727	-0.3058	-0.59426	-0.50787	0.61281	-4.98398	0.774327	0.824252
Neutrophils	FAM120B	0.100992	4.124917	0.507686	0.612938	-5.72222	0.67675	0.722972
Neutrophils	ACOT12	-0.23788	0.798615	-0.50756	0.613029	-5.19473	0.744126	0.793062
Neutrophils	UBE2A	0.044238	6.786705	0.50744	0.61311	-6.37714	0.627394	0.671352
Neutrophils	BLVRA	-0.09419	5.071733	-0.50741	0.613127	-5.8558	0.658745	0.704184
Neutrophils	2810402E2	-0.16663	2.412627	-0.50737	0.613158	-5.29684	0.710616	0.75827
Neutrophils	IGSF9	-0.29767	0.790152	-0.50726	0.613234	-4.98731	0.744306	0.793269
Neutrophils	SOX5	-0.18577	4.684841	-0.50724	0.61325	-5.7412	0.666041	0.711828
Neutrophils	FAM221A	-0.19755	1.467642	-0.50724	0.613251	-5.26396	0.730045	0.778477
Neutrophils	PPP1R7	0.093809	4.409711	0.507216	0.613266	-5.63008	0.671281	0.717309
Neutrophils	SYDE2	0.337794	-0.10373	0.507196	0.61328	-4.98956	0.763554	0.813213
Neutrophils	CASP8	-0.0695	5.513709	-0.50713	0.613323	-6.02821	0.650512	0.695603
Neutrophils	GM19605	-0.24958	1.260305	-0.50713	0.613326	-5.16419	0.73438	0.782989
Neutrophils	FAM177A	0.190973	1.731422	0.506588	0.613705	-5.27945	0.724938	0.772949
Neutrophils	SMG1	0.041373	7.499087	0.506317	0.613895	-6.54664	0.615242	0.6584
Neutrophils	RHOC	-0.09941	4.059106	-0.50627	0.613926	-5.93216	0.678468	0.724582
Neutrophils	UBE2S	-0.06934	7.624597	-0.50611	0.614037	-6.56879	0.613101	0.656138
Neutrophils	CARNS1	-0.15294	2.476067	-0.5059	0.614188	-5.61488	0.709895	0.75727
Neutrophils	ONECUT2	0.233514	1.559667	0.505873	0.614205	-5.28113	0.728708	0.776821
Neutrophils	UBTF	-0.06165	5.879519	-0.50544	0.614505	-6.08322	0.644537	0.688989
Neutrophils	MPI	-0.16991	2.225319	-0.50532	0.61459	-5.29959	0.715283	0.76277
Neutrophils	TXNIP	0.072811	5.791358	0.505067	0.614768	-6.38075	0.64617	0.690781
Neutrophils	GM29707	-0.22077	-0.46354	-0.50506	0.614776	-5.12864	0.772368	0.822009
Neutrophils	TRIP6	0.225279	0.834471	0.50505	0.61478	-5.10263	0.744258	0.79292
Neutrophils	PLK4	-0.12423	3.856529	-0.50489	0.614895	-5.80043	0.682821	0.729062
Neutrophils	IL18R1	0.169471	0.978465	0.50456	0.615123	-5.5179	0.741387	0.789891
Neutrophils	TAT	0.228391	1.826535	0.504553	0.615128	-5.34378	0.72365	0.771492
Neutrophils	PCNT	0.083454	5.233516	0.504176	0.615392	-5.95396	0.656714	0.701814
Neutrophils	MYC	0.247445	3.533741	0.504106	0.615441	-5.39763	0.689294	0.735824
Neutrophils	GM11508	-0.21848	3.437566	-0.5041	0.615443	-5.29648	0.691187	0.737796
Neutrophils	PTGER4	-0.12937	4.681638	-0.50399	0.615522	-5.76824	0.667113	0.712729
Neutrophils	MYL9	0.281333	1.95475	0.503932	0.615562	-5.32141	0.721055	0.76891
Neutrophils	ZSCAN18	-0.35858	0.363679	-0.50392	0.615571	-5.00595	0.75457	0.803664
Neutrophils	HC	-0.20106	2.358321	-0.50373	0.615701	-5.45989	0.712798	0.760397
Neutrophils	UBR2	-0.05123	6.362518	-0.50365	0.615759	-6.32315	0.635963	0.680228
Neutrophils	INPP4A	-0.05213	6.032154	-0.5036	0.615797	-6.50568	0.641963	0.686528

Neutrophils	GM16091	0.170222	2.306149	0.503562	0.615822	-5.38772	0.71386	0.761551
Neutrophils	MIS12	-0.15349	3.322859	-0.50332	0.615989	-5.46355	0.693565	0.740407
Neutrophils	VMAC	0.235924	1.5263	0.503233	0.616052	-5.11384	0.730048	0.778379
Neutrophils	AFP	0.269359	4.371399	0.503009	0.616209	-5.95542	0.673243	0.719226
Neutrophils	STK3	0.08463	5.985237	0.50275	0.61639	-6.07983	0.643141	0.687697
Neutrophils	KMO	0.209987	3.038738	0.501623	0.617179	-5.2534	0.700148	0.746705
Neutrophils	TOP1	-0.04969	8.354587	-0.5016	0.617194	-6.73073	0.601921	0.644001
Neutrophils	GBP2B	0.737876	0.300434	0.501583	0.617207	-5.07314	0.757075	0.805757
Neutrophils	PPM1H	0.077817	6.473739	0.501307	0.617401	-6.47361	0.634981	0.678706
Neutrophils	GM42982	-0.21185	1.514483	-0.50128	0.617421	-5.14638	0.731355	0.779152
Neutrophils	OVCA2	0.336411	0.174482	0.501209	0.61747	-5.00204	0.759885	0.808721
Neutrophils	MIGA1	0.170319	2.677211	0.50108	0.61756	-5.20687	0.707511	0.754416
Neutrophils	SLX4IP	-0.09221	4.853222	-0.50051	0.617958	-5.8536	0.66526	0.710213
Neutrophils	PPBP	-0.45007	0.842206	-0.50037	0.618056	-5.0838	0.745896	0.794049
Neutrophils	HIVEP1	0.065107	6.40877	0.500346	0.618075	-6.33136	0.636466	0.68012
Neutrophils	MDM2	0.068828	6.41707	0.500311	0.618099	-6.45445	0.636316	0.679963
Neutrophils	AIM2	0.099527	5.579386	0.500063	0.618273	-5.97549	0.651767	0.696081
Neutrophils	RBBP8	-0.05662	6.609426	-0.49995	0.618354	-6.3535	0.632975	0.676425
Neutrophils	SERTAD4	-0.35329	-0.0023	-0.49964	0.618573	-4.98584	0.76445	0.813046
Neutrophils	HTRA3	-0.27117	0.502839	-0.49952	0.618657	-5.07021	0.753502	0.80175
Neutrophils	SLC35D1	-0.12292	4.114625	-0.49945	0.618706	-5.53868	0.679712	0.72514
Neutrophils	WDR48	-0.08475	4.298768	-0.49911	0.618939	-5.76878	0.676339	0.721505
Neutrophils	ITGB1BP2	0.276898	0.43994	0.498837	0.619133	-5.03182	0.755082	0.803312
Neutrophils	DCUN1D4	-0.21864	2.540378	-0.49877	0.619178	-5.25321	0.711132	0.757766
Neutrophils	TLN2	0.283834	1.298032	0.498643	0.61927	-5.11541	0.736803	0.78447
Neutrophils	BIRC6	-0.03457	7.8393	-0.49856	0.619324	-6.55128	0.6116	0.653928
Neutrophils	GM33280	0.343787	-0.66423	0.498538	0.619343	-4.99537	0.779276	0.828412
Neutrophils	CNOT7	-0.04805	5.288607	-0.49853	0.619348	-6.19765	0.65756	0.702055
Neutrophils	ERBB3	0.317063	1.113728	0.498096	0.619653	-5.03795	0.740962	0.788721
Neutrophils	RPA1	0.110375	4.922792	0.497733	0.619908	-5.74222	0.664685	0.709562
Neutrophils	JCHAIN	-0.74146	-0.4625	-0.4977	0.619928	-4.98706	0.775081	0.824173
Neutrophils	LAMTOR4	0.051524	6.205335	0.497697	0.619933	-6.52244	0.640872	0.684682
Neutrophils	GM3604	-0.25379	0.595301	-0.49752	0.620055	-5.05717	0.752014	0.800341
Neutrophils	RCCD1	-0.19575	3.170294	-0.4975	0.62007	-5.32982	0.698722	0.745052
Neutrophils	NSFL1C	0.108322	4.168167	0.497385	0.620152	-5.5477	0.679126	0.724646
Neutrophils	CPT1A	-0.06372	5.446894	-0.49738	0.620156	-6.07756	0.654844	0.699304
Neutrophils	ATG101	-0.06256	5.222366	-0.49737	0.620163	-6.15505	0.659041	0.703689
Neutrophils	DUSP4	0.250493	0.909125	0.497132	0.62033	-5.05181	0.745416	0.793464
Neutrophils	CD209A	0.331414	0.102824	0.496941	0.620464	-5.11412	0.762782	0.811467
Neutrophils	BC002059	0.166599	2.758564	0.496906	0.620489	-5.31918	0.707084	0.753737
Neutrophils	EIF3F	0.05382	7.589953	0.496863	0.620519	-6.43333	0.616258	0.658902
Neutrophils	GM1976	0.175004	2.701657	0.496498	0.620775	-5.29378	0.708321	0.755032
Neutrophils	DIS3L	-0.17774	2.828005	-0.49643	0.620824	-5.23068	0.705772	0.752401
Neutrophils	RTN4RL1	-0.25517	2.381813	-0.49641	0.620834	-5.26162	0.714815	0.7618
Neutrophils	GM15952	0.244782	1.028007	0.496381	0.620857	-5.15121	0.742982	0.791029
Neutrophils	PRPF38B	0.0429	6.611272	0.49612	0.621041	-6.26418	0.633725	0.677221
Neutrophils	RNF216	0.045727	6.677461	0.496048	0.621092	-6.41734	0.632534	0.675984

Neutrophils	CAPN15	-0.11215	4.710463	-0.49602	0.621114	-5.6214	0.66893	0.714034
Neutrophils	SLC50A1	0.092021	5.247798	0.495726	0.621318	-5.86206	0.658775	0.703513
Neutrophils	KLF16	-0.15517	3.164065	-0.49572	0.621323	-5.33727	0.69907	0.745522
Neutrophils	MBLAC1	0.261206	-0.19049	0.495704	0.621333	-5.03844	0.769329	0.818365
Neutrophils	PRELID2	-0.2227	2.621255	-0.49569	0.621343	-5.35936	0.709979	0.756866
Neutrophils	NUDT13	-0.19563	2.358115	-0.49492	0.621885	-5.21636	0.715849	0.762681
Neutrophils	4933423P2	0.185014	1.804113	0.494862	0.621925	-5.2674	0.727259	0.77454
Neutrophils	MTMR14	-0.08239	5.427711	-0.49468	0.622052	-5.97859	0.655952	0.700319
Neutrophils	GLP2R	0.20868	0.940346	0.494488	0.622188	-5.24729	0.745501	0.793477
Neutrophils	ENPP4	0.158634	2.924155	0.494451	0.622214	-5.4275	0.70446	0.7509
Neutrophils	PML	-0.16399	5.273859	-0.49436	0.622276	-5.7442	0.658838	0.70339
Neutrophils	CDK19	0.065943	6.390727	0.494298	0.622322	-6.28507	0.638242	0.681886
Neutrophils	TNFRSF11A	-0.20978	2.190262	-0.49406	0.622487	-5.34348	0.719483	0.766488
Neutrophils	TRIM3	0.181255	2.274801	0.493882	0.622614	-5.24348	0.717804	0.764701
Neutrophils	GM17494	-0.14794	2.389013	-0.49355	0.622851	-5.29013	0.715468	0.762388
Neutrophils	CDH1	0.255378	1.38751	0.493434	0.622929	-5.18976	0.736217	0.783933
Neutrophils	CELF6	-0.32453	-0.70622	-0.49338	0.622965	-5.06256	0.781585	0.830849
Neutrophils	EIF1AX	-0.06608	6.060349	-0.49337	0.622977	-6.16259	0.644416	0.688352
Neutrophils	GSK3B	-0.03444	8.105386	-0.49326	0.623054	-6.69018	0.608074	0.650286
Neutrophils	FAM241A	0.069604	5.59878	0.493195	0.623098	-6.25057	0.65293	0.697289
Neutrophils	RYR1	-0.24465	1.831874	-0.49318	0.623112	-5.21787	0.726936	0.774349
Neutrophils	HELZ2	-0.11907	3.832468	-0.49316	0.623124	-5.77571	0.686611	0.732432
Neutrophils	GM42047	-0.16442	5.263713	-0.49298	0.623248	-5.91205	0.65919	0.703838
Neutrophils	SMN1	-0.08421	4.851728	-0.49283	0.623358	-5.92931	0.666966	0.712015
Neutrophils	ERGIC3	-0.07337	5.513051	-0.49264	0.623485	-5.97095	0.65453	0.699072
Neutrophils	SNHG6	0.131317	3.414627	0.492626	0.623498	-5.55886	0.694844	0.741121
Neutrophils	NGP	0.060325	5.022684	0.492461	0.623614	-7.08237	0.663727	0.708737
Neutrophils	ARL3	0.108017	4.04145	0.492179	0.623813	-5.66373	0.682539	0.728481
Neutrophils	DNTTIP1	-0.06587	4.812219	-0.4918	0.624077	-5.93086	0.667716	0.713106
Neutrophils	CDC14A	-0.07536	5.803629	-0.4918	0.624079	-6.29964	0.649142	0.693693
Neutrophils	CAPN2	0.110771	4.14309	0.4918	0.62408	-5.60975	0.680565	0.726513
Neutrophils	COQ8B	-0.1334	3.190292	-0.49175	0.624117	-5.56866	0.699304	0.746035
Neutrophils	LY6E	-0.08883	9.08245	-0.49169	0.624156	-6.53751	0.591486	0.63319
Neutrophils	IL12A	-0.26754	2.215059	-0.49169	0.624158	-5.16278	0.719034	0.76655
Neutrophils	CFAP97	0.103552	3.29046	0.491644	0.624189	-5.54953	0.697309	0.743974
Neutrophils	TNKS2	-0.04453	7.072389	-0.4916	0.624218	-6.40001	0.626158	0.669634
Neutrophils	ANXA6	0.073766	6.174347	0.491497	0.624293	-6.19506	0.642336	0.686586
Neutrophils	CD1D1	-0.19415	3.932282	-0.49144	0.624331	-5.42242	0.684666	0.730806
Neutrophils	CTU1	-0.28779	0.850388	-0.49141	0.624352	-5.05339	0.747602	0.796202
Neutrophils	MS4A7	-0.28797	1.921581	-0.49135	0.6244	-5.30599	0.725083	0.772883
Neutrophils	SLC35G1	-0.33073	1.026313	-0.49133	0.624411	-5.02865	0.743856	0.792355
Neutrophils	GM45902	0.211756	1.585805	0.491289	0.624439	-5.16133	0.732066	0.780131
Neutrophils	SVIL	0.041036	6.860609	0.491106	0.624569	-6.70764	0.629998	0.673685
Neutrophils	GM47689	-0.13945	3.369026	-0.49089	0.624723	-5.35315	0.695822	0.742489
Neutrophils	GM16845	0.197574	2.527304	0.490845	0.624752	-5.24171	0.712731	0.760081
Neutrophils	RFX3	-0.05769	6.233922	-0.49081	0.624775	-6.31611	0.641317	0.685575
Neutrophils	GM46367	0.145377	2.175914	0.490731	0.624832	-5.57916	0.719914	0.767545

Neutrophils	ZFP366	-0.22885	1.840557	-0.49062	0.62491	-5.41937	0.726839	0.774736
Neutrophils	CTTN	0.185399	1.717261	0.490364	0.625091	-5.31861	0.729401	0.777396
Neutrophils	HFE	-0.12157	3.452689	-0.4903	0.625133	-5.68274	0.694164	0.740762
Neutrophils	FBXO46	0.172008	2.47185	0.490293	0.625141	-5.33897	0.71386	0.761254
Neutrophils	TESC	0.155612	1.561476	0.490246	0.625174	-5.63056	0.732653	0.780779
Neutrophils	9930104L0	-0.24071	1.356879	-0.48965	0.625597	-5.07831	0.736945	0.785329
Neutrophils	TMEM126I	-0.15943	2.697386	-0.48964	0.625602	-5.29686	0.709281	0.756597
Neutrophils	SS18L1	0.205666	2.325233	0.489634	0.625605	-5.27868	0.716853	0.764469
Neutrophils	NOX1	0.259297	1.548335	0.489621	0.625615	-5.17354	0.732928	0.781161
Neutrophils	CDK9	0.063184	5.578202	0.48952	0.625686	-6.12861	0.653387	0.698301
Neutrophils	HPD	-0.15542	4.805638	-0.48948	0.625711	-6.08987	0.667912	0.713483
Neutrophils	RING1	0.130253	3.585545	0.489463	0.625726	-5.51372	0.691539	0.738135
Neutrophils	ACOT7	-0.10205	4.076826	-0.4894	0.62577	-5.7062	0.681923	0.728117
Neutrophils	HLF	0.174684	1.023669	0.489342	0.625811	-5.45764	0.74399	0.792655
Neutrophils	PAF1	0.08066	4.600235	0.489248	0.625877	-5.82746	0.67183	0.717606
Neutrophils	GSTM7	-0.19075	1.02465	-0.48924	0.625881	-5.19986	0.74397	0.792647
Neutrophils	PSMB2	0.048596	7.058131	0.489012	0.626044	-6.38466	0.626575	0.670144
Neutrophils	GLOD4	0.070706	5.050654	0.488869	0.626145	-5.95463	0.663394	0.708709
Neutrophils	OAS2	0.177979	1.571395	0.488694	0.626268	-5.60413	0.732582	0.780781
Neutrophils	MAN2B2	-0.13327	3.799809	-0.48868	0.626278	-5.57986	0.687457	0.733854
Neutrophils	MTMR12	-0.07267	5.334196	-0.48852	0.626391	-6.00774	0.658062	0.703169
Neutrophils	FAHD2A	0.145044	2.845982	0.48852	0.626391	-5.347	0.706412	0.753592
Neutrophils	KIF1B	0.062796	5.385583	0.488295	0.62655	-6.25965	0.657113	0.702323
Neutrophils	GM45051	-0.19051	1.845863	-0.48827	0.626564	-5.21347	0.726878	0.775028
Neutrophils	EPHB2	-0.3254	1.372664	-0.48813	0.626664	-5.01388	0.736765	0.785309
Neutrophils	LGR5	-0.27518	1.073909	-0.48806	0.626712	-5.07792	0.743077	0.791855
Neutrophils	KIF23	0.09857	5.173452	0.487955	0.62679	-6.13059	0.661092	0.706506
Neutrophils	ESAM	0.135611	2.065322	0.487945	0.626796	-5.61592	0.722339	0.770333
Neutrophils	TOP3A	-0.14811	3.860539	-0.48768	0.62698	-5.45405	0.686283	0.732834
Neutrophils	TAX1BP3	-0.10564	4.090035	-0.48735	0.627214	-5.72069	0.681809	0.728211
Neutrophils	SLC25A51	0.092389	5.697758	0.487271	0.627273	-5.89494	0.651305	0.696337
Neutrophils	PHF20L1	0.041362	7.046788	0.487255	0.627284	-6.65593	0.62681	0.67067
Neutrophils	MAP3K3	0.0541	6.795781	0.487129	0.627373	-6.48809	0.631294	0.675423
Neutrophils	TMCO3	0.078638	3.656165	0.48708	0.627407	-5.88183	0.690293	0.737124
Neutrophils	FAM234B	-0.27437	2.096184	-0.487	0.627461	-5.05668	0.721705	0.769857
Neutrophils	ACAD11	-0.13618	2.331168	-0.48697	0.627482	-5.45524	0.716881	0.764842
Neutrophils	DPH6	-0.13621	4.47347	-0.48685	0.627572	-5.53095	0.674402	0.720592
Neutrophils	GM9750	0.325961	0.476616	0.486744	0.627645	-5.02107	0.755864	0.805329
Neutrophils	RNF115	-0.04159	6.978362	-0.48672	0.627659	-6.48897	0.628029	0.672072
Neutrophils	COMMD6	-0.09872	4.358964	-0.48645	0.627854	-5.70777	0.676605	0.722927
Neutrophils	PRRG4	-0.31944	0.01648	-0.48627	0.627976	-4.97625	0.765863	0.815715
Neutrophils	CCDC18	-0.14037	2.722921	-0.48617	0.628049	-5.47977	0.708912	0.756665
Neutrophils	KCP	0.264374	0.100139	0.485976	0.628187	-5.11511	0.764035	0.813897
Neutrophils	DCAF6	-0.06547	5.908083	-0.48597	0.628191	-6.38375	0.647421	0.69249
Neutrophils	DNAJC4	0.128484	3.061383	0.485965	0.628194	-5.49024	0.702101	0.749595
Neutrophils	EML2	0.111591	2.655573	0.485933	0.628217	-5.74189	0.710276	0.758105
Neutrophils	ACY1	-0.25245	1.799123	-0.48586	0.628266	-5.12017	0.727851	0.776377

Neutrophils	ZFP52	0.143183	3.859767	0.485863	0.628266	-5.46802	0.686298	0.733125
Neutrophils	BMP8A	-0.19047	2.321943	-0.48586	0.628269	-5.41983	0.71707	0.765172
Neutrophils	MAPK8IP3	0.096075	4.521476	0.485858	0.62827	-5.66213	0.67348	0.719745
Neutrophils	DLST	-0.0448	5.852364	-0.48581	0.628305	-6.31356	0.648447	0.693565
Neutrophils	RSPH3B	-0.1329	3.01938	-0.48527	0.628683	-5.43492	0.703292	0.750638
Neutrophils	INTS3	-0.1002	3.898156	-0.48467	0.629106	-5.63632	0.686278	0.73266
Neutrophils	PLXNB2	-0.12563	3.959065	-0.48455	0.629196	-5.60238	0.685094	0.731445
Neutrophils	LPP	-0.0675	7.6885	-0.48448	0.629243	-6.5432	0.616163	0.659327
Neutrophils	FAAH	-0.20894	1.451163	-0.48414	0.629486	-5.22391	0.736073	0.784494
Neutrophils	DOK2	0.130748	2.811456	0.484014	0.629573	-5.58413	0.708043	0.755395
Neutrophils	ZFYVE19	-0.14765	3.132938	-0.484	0.629581	-5.34651	0.70158	0.748669
Neutrophils	FOXK1	0.090115	4.627409	0.483819	0.62971	-5.7446	0.672392	0.718168
Neutrophils	ZFP141	0.124003	3.367308	0.483338	0.630051	-5.63882	0.697179	0.743909
Neutrophils	NDUFA13	0.044496	7.128419	0.483203	0.630146	-6.48327	0.626417	0.66996
Neutrophils	AMPD2	-0.13483	2.339378	-0.48317	0.63017	-5.49845	0.717926	0.765489
Neutrophils	BTF3L4	0.086924	4.269235	0.483114	0.630209	-5.70232	0.679484	0.725468
Neutrophils	SRSF9	0.04955	6.428408	0.483097	0.630221	-6.26507	0.638995	0.683144
Neutrophils	TIMM8A1	0.145158	4.226992	0.482618	0.63056	-5.52491	0.680597	0.726372
Neutrophils	PRDM10	-0.08272	5.213796	-0.48242	0.630698	-5.97224	0.661809	0.70669
Neutrophils	RBL1	0.104721	4.466968	0.482337	0.630759	-5.80868	0.676032	0.721536
Neutrophils	OSER1	-0.05907	6.59749	-0.48204	0.630966	-6.30513	0.636388	0.680092
Neutrophils	ARC	-0.30646	0.905214	-0.48199	0.631001	-5.04472	0.748466	0.796814
Neutrophils	RANBP2	-0.06376	6.846596	-0.48185	0.631106	-6.41237	0.631941	0.675409
Neutrophils	CLTC	-0.03344	7.90636	-0.48163	0.631256	-6.73555	0.613305	0.655805
Neutrophils	ATF7	-0.06593	6.314357	-0.48139	0.631431	-6.19629	0.641668	0.685588
Neutrophils	ST6GALNA(	-0.27759	0.651842	-0.48134	0.631463	-5.04506	0.754066	0.802577
Neutrophils	MN1	-0.29212	0.308399	-0.48134	0.631467	-5.05309	0.761499	0.810267
Neutrophils	MSMO1	0.089999	3.610529	0.481066	0.631658	-5.82684	0.693073	0.739275
Neutrophils	PARPBP	-0.23924	2.056928	-0.48107	0.631658	-5.32588	0.724481	0.771925
Neutrophils	GM49041	0.167776	1.334735	0.480727	0.631898	-5.45279	0.739637	0.787722
Neutrophils	1810030O(	0.085873	4.824335	0.480726	0.631899	-5.82394	0.669571	0.714845
Neutrophils	GM4013	-0.15432	1.984122	-0.48071	0.631909	-5.28527	0.72605	0.773631
Neutrophils	ATP5C1	0.039396	8.358211	0.48029	0.632207	-6.64486	0.605789	0.647966
Neutrophils	ZFAND4	0.106835	3.4762	0.480192	0.632277	-6.08853	0.695981	0.742301
Neutrophils	REXO2	0.09219	6.270041	0.479893	0.632488	-5.93311	0.642767	0.686847
Neutrophils	PAM16	0.089971	4.987535	0.479862	0.63251	-5.75686	0.666649	0.711815
Neutrophils	ARHGEF40	0.283547	0.550928	0.479832	0.632532	-5.07914	0.756585	0.805299
Neutrophils	ACP5	-0.14775	5.117131	-0.47982	0.632537	-5.7959	0.664194	0.709251
Neutrophils	1110059E2	-0.10689	4.393524	-0.47977	0.632575	-5.63287	0.678022	0.723683
Neutrophils	GDPGP1	-0.28208	2.18615	-0.47968	0.632642	-5.13164	0.722072	0.769548
Neutrophils	HUS1	0.191522	1.653586	0.479604	0.632693	-5.22394	0.733133	0.781059
Neutrophils	BCOR	0.08863	5.214585	0.47954	0.632738	-5.9132	0.662354	0.707407
Neutrophils	SARS	0.062304	5.792679	0.47934	0.63288	-6.01124	0.65156	0.696215
Neutrophils	MICU1	-0.04128	5.891562	-0.47929	0.632914	-6.47883	0.64973	0.694301
Neutrophils	FBXW8	-0.11497	3.868261	-0.47925	0.632945	-5.55525	0.688256	0.734532
Neutrophils	CFAP126	0.279883	1.069649	0.478986	0.633131	-5.0661	0.745542	0.794037
Neutrophils	SASS6	0.102294	4.42508	0.478977	0.633137	-5.78909	0.677487	0.723283

Neutrophils	RHEBL1	0.238219	1.532009	0.478428	0.633526	-5.14138	0.736139	0.784061
Neutrophils	RBCK1	0.0742	5.550838	0.478164	0.633714	-5.94991	0.65652	0.701215
Neutrophils	1700120C1	-0.2034	1.482076	-0.47803	0.63381	-5.14186	0.737263	0.785289
Neutrophils	PPM1D	0.068085	4.948971	0.477994	0.633834	-5.96665	0.667862	0.713082
Neutrophils	GM2449	0.261746	1.279792	0.477921	0.633886	-5.14576	0.741533	0.789715
Neutrophils	AP2M1	-0.05484	6.941455	-0.47788	0.633918	-6.41047	0.631079	0.674625
Neutrophils	WDR19	-0.26074	0.508741	-0.47776	0.634	-5.0939	0.758062	0.806864
Neutrophils	LMLN	-0.17907	1.750464	-0.47755	0.63415	-5.27532	0.731726	0.779505
Neutrophils	WWP1	0.080688	5.227114	0.477348	0.634292	-6.06294	0.662678	0.707701
Neutrophils	TMEM250-	0.095377	4.629225	0.477329	0.634306	-5.82635	0.674054	0.719579
Neutrophils	GM26916	-0.18785	0.934105	-0.47717	0.63442	-5.21441	0.748983	0.797505
Neutrophils	SEC61B	0.045411	8.272683	0.477152	0.634431	-6.66017	0.607772	0.650202
Neutrophils	USP28	-0.1094	4.553264	-0.47711	0.634458	-5.59368	0.675514	0.721133
Neutrophils	MRPS24	0.058486	6.031221	0.476944	0.634579	-6.14034	0.647702	0.692114
Neutrophils	GM48768	-0.18957	0.621002	-0.47692	0.634598	-5.29081	0.755722	0.80454
Neutrophils	ZFP995	0.213782	2.383748	0.476734	0.634728	-5.12401	0.718701	0.766089
Neutrophils	TBC1D8B	0.119401	3.257326	0.476535	0.634869	-5.52795	0.70108	0.747722
Neutrophils	NSUN4	-0.15446	3.189091	-0.47641	0.634959	-5.35361	0.702446	0.749164
Neutrophils	MED31	-0.08613	3.491551	-0.47637	0.634987	-5.80215	0.696413	0.74289
Neutrophils	MEX3D	-0.19944	2.642534	-0.47592	0.635308	-5.23041	0.71377	0.76079
Neutrophils	CSRP2	0.07871	5.20682	0.475722	0.635446	-6.07688	0.66348	0.708406
Neutrophils	MED20	-0.07968	4.104119	-0.47571	0.635451	-5.84172	0.684646	0.730489
Neutrophils	FANCI	-0.16356	2.266344	-0.47539	0.635679	-5.27862	0.721626	0.768926
Neutrophils	UQCRH	-0.03776	8.592145	-0.47536	0.635705	-6.65431	0.602794	0.644812
Neutrophils	2610001J0	0.110548	3.690163	0.475091	0.635894	-5.61011	0.692974	0.739174
Neutrophils	ARHGAP6	0.173392	3.609197	0.475081	0.6359	-5.53312	0.694576	0.740842
Neutrophils	GRK4	-0.11754	3.108893	-0.47498	0.635975	-5.60849	0.704566	0.751274
Neutrophils	CRPPA	0.201254	2.552625	0.474742	0.636142	-5.26208	0.715843	0.763031
Neutrophils	CD2AP	-0.09711	6.24029	-0.47463	0.636221	-5.96845	0.644467	0.688614
Neutrophils	NUDT8	0.17714	2.208161	0.474454	0.636346	-5.28174	0.722915	0.770406
Neutrophils	SLC24A3	0.224885	1.280307	0.474427	0.636365	-5.39154	0.742319	0.790535
Neutrophils	HSPA12B	-0.22551	0.480164	-0.47435	0.636423	-5.14825	0.759477	0.808302
Neutrophils	SEC24A	0.082838	6.616568	0.474343	0.636425	-6.24504	0.637613	0.681449
Neutrophils	FDX2	-0.11992	4.192147	-0.47432	0.636443	-5.47038	0.683147	0.729031
Neutrophils	GOPC	-0.07623	4.545466	-0.47422	0.636513	-5.81038	0.676311	0.721905
Neutrophils	FAM222B	0.067053	5.370089	0.474071	0.636618	-6.26314	0.660659	0.705569
Neutrophils	WDR62	0.150925	3.526858	0.473962	0.636695	-5.47108	0.696285	0.742715
Neutrophils	SUSD1	0.079214	5.183728	0.473814	0.636801	-6.10374	0.664225	0.709269
Neutrophils	FAR1OS	0.152037	2.788806	0.473565	0.636977	-5.61002	0.711265	0.758167
Neutrophils	LSM8	0.06398	5.264944	0.472967	0.637402	-6.10522	0.663054	0.707925
Neutrophils	TMEM263	0.110398	3.98713	0.47288	0.637464	-5.63939	0.687628	0.733559
Neutrophils	ARHGEF2	0.052707	5.586467	0.472868	0.637473	-6.25718	0.657016	0.701616
Neutrophils	EEF1G	-0.06568	7.187877	-0.47279	0.63753	-6.23116	0.627789	0.671038
Neutrophils	1700113A1	-0.17967	2.10081	-0.47277	0.637544	-5.27668	0.725637	0.773109
Neutrophils	POLR2F	0.067348	5.381223	0.472665	0.637617	-5.9631	0.660871	0.705684
Neutrophils	OLFML2B	-0.12088	0.874884	-0.4725	0.637734	-5.78436	0.751539	0.800015
Neutrophils	LPIN1	-0.23105	2.690777	-0.47235	0.637839	-5.24332	0.713574	0.760667

Neutrophils	CDC73	-0.04999	6.174072	-0.47233	0.637854	-6.31588	0.646176	0.690379
Neutrophils	GNS	-0.0643	6.931874	-0.47223	0.637928	-6.36291	0.632422	0.676019
Neutrophils	DNAJB1	-0.07177	6.228259	-0.47172	0.638287	-6.31256	0.645486	0.689524
Neutrophils	KIF9	0.179269	1.680578	0.471294	0.638592	-5.30475	0.735069	0.782594
Neutrophils	NCKAP1L	0.040757	6.137906	0.470876	0.638889	-6.42005	0.647524	0.691331
Neutrophils	RNF8	0.075728	4.800789	0.470789	0.638951	-5.87582	0.672632	0.717552
Neutrophils	TRIM24	-0.08713	5.087563	-0.47073	0.63899	-5.84882	0.667162	0.711846
Neutrophils	DNLZ	-0.11797	3.991855	-0.47065	0.639052	-5.5988	0.688312	0.733893
Neutrophils	HADHA	-0.06032	5.770414	-0.47064	0.639055	-6.1068	0.654326	0.698441
Neutrophils	RBPMS	-0.08131	5.842983	-0.47052	0.639143	-6.24954	0.652999	0.697031
Neutrophils	STRADA	0.095327	4.58715	0.469791	0.639661	-5.78349	0.677098	0.72209
Neutrophils	D830025C(	0.14245	2.231904	0.46979	0.639662	-5.49771	0.724131	0.770998
Neutrophils	RFX1	-0.10803	3.263376	-0.46966	0.639755	-5.46291	0.703132	0.749251
Neutrophils	TTC14	-0.05875	5.803768	-0.46955	0.639835	-6.0418	0.654055	0.698144
Neutrophils	A530041M	-0.1605	2.069945	-0.46952	0.639851	-5.29264	0.727486	0.774596
Neutrophils	BCL9L	0.131272	3.410778	0.469512	0.639859	-5.47514	0.700183	0.746237
Neutrophils	GM17092	0.180086	2.1249	0.469302	0.640009	-5.27675	0.726405	0.773431
Neutrophils	CEP152	-0.09912	3.629164	-0.46916	0.640112	-5.69174	0.695894	0.741785
Neutrophils	SMARCD1	-0.11358	4.031896	-0.46911	0.640149	-5.44131	0.687951	0.733521
Neutrophils	BLOC1S5	0.211275	1.955013	0.469067	0.640176	-5.16733	0.729936	0.777164
Neutrophils	GM37768	0.187853	2.087377	0.46887	0.640317	-5.24143	0.727267	0.774347
Neutrophils	SH2B1	0.103296	3.699274	0.468661	0.640465	-5.73868	0.694584	0.740398
Neutrophils	NEO1	-0.25963	0.660859	-0.4686	0.640509	-5.17609	0.757501	0.805677
Neutrophils	SLC43A1	0.277739	1.089863	0.468541	0.640551	-5.09343	0.748277	0.796133
Neutrophils	1810024B(	-0.16932	2.692562	-0.4685	0.64058	-5.3041	0.714815	0.761439
Neutrophils	PPP1CB	-0.04633	7.803763	-0.46828	0.640738	-6.69182	0.618107	0.660484
Neutrophils	MTHFSD	0.186551	2.433778	0.46821	0.640787	-5.22158	0.720161	0.767004
Neutrophils	DOK1	0.135995	3.614618	0.468162	0.640821	-5.40621	0.696309	0.742242
Neutrophils	PSMD2	0.056097	6.149979	0.468018	0.640923	-6.18102	0.647847	0.69169
Neutrophils	SUGP1	-0.09434	4.615277	-0.46792	0.640995	-5.67767	0.676764	0.721884
Neutrophils	AKR1B10	0.164689	3.494629	0.467845	0.641046	-5.33964	0.698725	0.744775
Neutrophils	PPFIA4	0.202936	2.735956	0.467493	0.641297	-5.2447	0.714148	0.760779
Neutrophils	BRCA1	0.133064	4.143927	0.467486	0.641303	-5.79324	0.686045	0.731549
Neutrophils	MAP2K4	-0.04429	6.321239	-0.46732	0.641422	-6.68684	0.644867	0.688551
Neutrophils	PROM1	-0.14593	0.403745	-0.4671	0.641575	-5.6782	0.763364	0.811793
Neutrophils	THTPA	0.216316	1.360701	0.466814	0.641781	-5.10618	0.742785	0.790498
Neutrophils	PAXIP1	-0.1041	4.269449	-0.46679	0.641797	-5.69746	0.683639	0.729057
Neutrophils	BTAF1	0.04876	7.190783	0.466697	0.641865	-6.40597	0.629137	0.672111
Neutrophils	COX19	-0.1093	4.496433	-0.46663	0.641913	-5.70456	0.679233	0.724465
Neutrophils	NAA40	-0.08322	4.909944	-0.46657	0.641956	-5.74108	0.671282	0.716174
Neutrophils	C230066G2	-0.3155	0.025289	-0.46646	0.642035	-4.9947	0.77166	0.820366
Neutrophils	UBAP1L	-0.20856	1.134289	-0.46639	0.642087	-5.11187	0.747602	0.795487
Neutrophils	INPP4B	-0.10869	6.480863	-0.46632	0.64213	-6.26696	0.641949	0.685526
Neutrophils	PLEKHJ1	0.049328	5.969804	0.466296	0.64215	-6.20561	0.651342	0.69535
Neutrophils	PPP1R3F	-0.23176	0.867721	-0.46626	0.642177	-5.12736	0.753315	0.801403
Neutrophils	BTBD7	0.038328	7.004982	0.466113	0.642281	-6.48324	0.632464	0.675599
Neutrophils	UBQLN4	-0.13131	3.216072	-0.46608	0.642306	-5.37275	0.704478	0.750749

Neutrophils	XIST	-2.59102	4.173151	-0.46604	0.642334	-5.35576	0.685518	0.731016
Neutrophils	ITPR1	-0.07978	6.992018	-0.46572	0.642559	-6.18098	0.632829	0.675978
Neutrophils	UNC5CL	0.323843	0.465408	0.465586	0.642656	-5.05184	0.76218	0.810597
Neutrophils	CD200R4	-0.23449	1.662182	-0.46557	0.642666	-5.38789	0.736572	0.784089
Neutrophils	GPX3	0.197315	1.463214	0.465306	0.642856	-5.47208	0.740785	0.788512
Neutrophils	MDP1	-0.11302	4.373314	-0.46528	0.642872	-5.50992	0.681777	0.727195
Neutrophils	TSEN15	0.157995	2.776415	0.46514	0.642975	-5.30057	0.713534	0.760243
Neutrophils	MFSD8	0.112216	2.979793	0.465098	0.643005	-5.45592	0.709406	0.755953
Neutrophils	ING2	-0.06839	4.920364	-0.46509	0.643011	-5.9844	0.671238	0.716208
Neutrophils	FUBP3	-0.13126	4.08675	-0.46475	0.64325	-5.46476	0.687518	0.733073
Neutrophils	0610009L1	-0.14954	2.030404	-0.46457	0.643382	-5.43769	0.729048	0.776249
Neutrophils	6430548M	0.064434	2.407786	0.464499	0.643432	-6.1999	0.721237	0.768145
Neutrophils	SERPINA3F	0.317931	2.19929	0.464491	0.643438	-5.38805	0.725542	0.772612
Neutrophils	TMEM220	0.234363	0.299081	0.464351	0.643538	-5.14465	0.765995	0.814502
Neutrophils	ACSL1	0.064856	5.758511	0.464335	0.643549	-6.51743	0.655563	0.69975
Neutrophils	BCAR3	-0.15795	4.496691	-0.46385	0.643897	-5.72494	0.679833	0.724794
Neutrophils	GNL3L	0.081549	4.467102	0.463614	0.644064	-5.6691	0.680492	0.725452
Neutrophils	CREG1	0.059589	7.514034	0.463549	0.64411	-6.60804	0.624027	0.666479
Neutrophils	HMOX2	0.045073	6.471899	0.463292	0.644293	-6.31445	0.642882	0.686158
Neutrophils	WDR5	-0.07297	4.681603	-0.46287	0.644597	-5.98071	0.67669	0.721321
Neutrophils	SMIM1	0.218363	1.759237	0.462699	0.644717	-5.28467	0.7355	0.782452
Neutrophils	GM48293	-0.18389	-0.15995	-0.46265	0.644753	-5.33024	0.776936	0.825306
Neutrophils	TMEM150I	-0.22434	1.12138	-0.46252	0.644848	-5.27731	0.749018	0.79652
Neutrophils	GTF2B	0.049937	6.738906	0.462515	0.644848	-6.4817	0.638232	0.681259
Neutrophils	PIGS	0.104237	4.078714	0.462443	0.6449	-5.57082	0.688412	0.733639
Neutrophils	D730003I1	0.144896	2.591958	0.462323	0.644985	-5.39858	0.718226	0.764631
Neutrophils	ITGA2	-0.22531	0.926385	-0.46221	0.645069	-5.18951	0.7532	0.80088
Neutrophils	C330013E1	-0.24469	0.677864	-0.46217	0.645098	-5.13997	0.758565	0.806428
Neutrophils	PCOLCE	-0.21448	0.911461	-0.46169	0.64544	-5.24754	0.753568	0.801329
Neutrophils	NAA20	-0.06517	5.164245	-0.46167	0.64545	-5.91867	0.667497	0.711923
Neutrophils	MAP2K2	0.040476	7.299123	0.461639	0.645474	-6.4686	0.628201	0.670843
Neutrophils	CEP55	-0.19991	3.678611	-0.46155	0.645537	-5.53935	0.696351	0.741997
Neutrophils	SPON1	0.286938	1.404705	0.461276	0.645734	-5.16777	0.743028	0.790504
Neutrophils	SLC36A3	-0.22578	0.101985	-0.4612	0.64579	-5.17272	0.771193	0.819629
Neutrophils	BC004004	0.064196	5.147988	0.461174	0.645806	-5.99138	0.667806	0.712318
Neutrophils	CCDC80	-0.12313	2.683481	-0.46108	0.645875	-5.79044	0.716397	0.762903
Neutrophils	KCNIP2	0.253627	1.248736	0.46107	0.645881	-5.10899	0.746345	0.79395
Neutrophils	JADE2	0.169622	3.270443	0.461007	0.645926	-5.41081	0.704502	0.750546
Neutrophils	HIST1H2AG	0.254039	2.011327	0.460921	0.645987	-5.2606	0.730271	0.777308
Neutrophils	CORO7	-0.06045	6.215775	-0.4608	0.646075	-6.17922	0.647829	0.691491
Neutrophils	AEBP2	0.072295	5.787827	0.460777	0.64609	-6.05643	0.655759	0.699782
Neutrophils	TNIP2	0.112412	3.816648	0.460731	0.646123	-5.51382	0.693616	0.739276
Neutrophils	CAMSAP1	0.131539	3.53399	0.460721	0.64613	-5.4927	0.699228	0.745114
Neutrophils	CDV3	0.052018	7.090195	0.460468	0.646311	-6.39595	0.632049	0.674927
Neutrophils	TUBB5	-0.07452	9.849372	-0.46026	0.646463	-6.88637	0.584617	0.625053
Neutrophils	UROC1	0.217625	1.760508	0.460038	0.646618	-5.3188	0.735848	0.782863
Neutrophils	U2SURP	0.047373	6.667671	0.459907	0.646712	-6.25263	0.639878	0.682956

Neutrophils	RTRAF	-0.06056	7.336334	-0.45959	0.646937	-6.30787	0.627967	0.670452
Neutrophils	ZFP934	0.155453	2.990341	0.459547	0.646969	-5.38659	0.710639	0.756685
Neutrophils	HARS2	-0.1297	2.848743	-0.45937	0.647098	-5.31977	0.713584	0.759735
Neutrophils	NXPH4	-0.29724	-0.60095	-0.45904	0.647333	-5.0281	0.787591	0.836247
Neutrophils	MEAF6	0.076914	4.941914	0.459017	0.647348	-5.76719	0.672381	0.716826
Neutrophils	MAPK11	-0.26977	0.260399	-0.45872	0.647563	-5.03881	0.768595	0.816529
Neutrophils	BRCA2	0.12508	3.555566	0.458663	0.647602	-5.65223	0.699602	0.745056
Neutrophils	RNPC3	0.066465	4.741295	0.458397	0.647792	-5.88709	0.676487	0.720923
Neutrophils	PUM2	0.033502	7.324014	0.458307	0.647856	-6.57318	0.628598	0.670907
Neutrophils	TOB1	-0.05424	5.050855	-0.45792	0.648132	-6.33515	0.670698	0.714862
Neutrophils	ACD	-0.0825	4.940696	-0.45767	0.648313	-5.83295	0.672805	0.717128
Neutrophils	POMT1	0.21905	2.21731	0.457575	0.648381	-5.21397	0.727122	0.77359
Neutrophils	BRD3	-0.05307	5.712362	-0.45753	0.648414	-6.31176	0.658192	0.701906
Neutrophils	PFDN2	-0.0736	5.031244	-0.45744	0.648478	-5.87243	0.671073	0.71539
Neutrophils	UBE2D3	0.025376	8.966917	0.457434	0.648481	-6.88354	0.600136	0.641159
Neutrophils	GM42702	-0.10635	2.365724	-0.45742	0.648491	-5.73773	0.724049	0.770475
Neutrophils	GAPVD1	-0.04466	6.832806	-0.45733	0.648557	-6.40239	0.637564	0.680402
Neutrophils	APAF1	-0.05582	5.726771	-0.45727	0.6486	-6.31733	0.657923	0.70169
Neutrophils	CARS	-0.12795	4.118134	-0.4571	0.648719	-5.62157	0.688754	0.73385
Neutrophils	PATJ	-0.25566	3.348038	-0.45707	0.648745	-5.26716	0.704041	0.749748
Neutrophils	GM12592	-0.12565	3.124779	-0.457	0.648793	-5.50386	0.708538	0.75442
Neutrophils	DAXX	-0.10202	4.352332	-0.45656	0.649105	-5.79858	0.684432	0.729221
Neutrophils	AIFM2	0.140863	1.347203	0.456301	0.649292	-5.54129	0.745764	0.792848
Neutrophils	CDK5RAP2	-0.08743	4.446752	-0.45628	0.64931	-5.75013	0.68266	0.727351
Neutrophils	NME4	0.244243	2.364464	0.456197	0.649367	-5.178	0.72442	0.770761
Neutrophils	SENP2	-0.04923	6.656089	-0.45594	0.649548	-6.39654	0.641145	0.684049
Neutrophils	PDZD4	-0.26723	1.698737	-0.45585	0.649614	-5.06944	0.738396	0.785246
Neutrophils	PRPF31	0.123381	3.79385	0.455736	0.649697	-5.4381	0.695554	0.740807
Neutrophils	GSPT1	-0.0519	6.707096	-0.45573	0.649702	-6.18187	0.640217	0.683098
Neutrophils	TANGO2	0.145352	3.93274	0.455521	0.649851	-5.61604	0.692895	0.737959
Neutrophils	SSPN	0.2576	-0.41075	0.455281	0.650023	-5.0196	0.78436	0.832722
Neutrophils	KLHL3	0.14793	2.005874	0.455271	0.65003	-5.42839	0.732055	0.778665
Neutrophils	NAA35	0.06291	4.851078	0.455229	0.65006	-6.0175	0.675014	0.719411
Neutrophils	SSBP3	-0.07127	5.600863	-0.45496	0.650251	-6.02571	0.660885	0.704584
Neutrophils	DNAJC9	0.091896	5.617283	0.454876	0.650313	-6.03802	0.660576	0.704262
Neutrophils	CBX8	-0.20124	1.617418	-0.45464	0.650479	-5.23049	0.740363	0.787204
Neutrophils	ZFP799	-0.23886	0.939029	-0.4546	0.65051	-5.07625	0.754844	0.802184
Neutrophils	EID1	0.060688	5.155191	0.454589	0.650519	-6.10711	0.669327	0.713408
Neutrophils	FTH1	0.074394	12.5537	0.454331	0.650704	-7.44125	0.542947	0.580748
Neutrophils	NSUN2	-0.09994	5.0541	-0.45418	0.650811	-5.65812	0.671314	0.715488
Neutrophils	NSL1	-0.16691	2.25699	-0.4541	0.650867	-5.32335	0.727033	0.773404
Neutrophils	COPS5	0.067432	5.288344	0.454099	0.65087	-5.95935	0.666853	0.710837
Neutrophils	GM11613	-0.23125	2.020755	-0.45405	0.650908	-5.28463	0.731952	0.778502
Neutrophils	RAB3D	0.066724	3.11921	0.45368	0.651171	-6.17295	0.709466	0.755224
Neutrophils	DOCK2	-0.03958	9.34967	-0.45352	0.651288	-6.94698	0.594356	0.635035
Neutrophils	5430416N(	0.122073	3.547711	0.453472	0.65132	-5.42071	0.700849	0.746332
Neutrophils	PMVK	-0.0795	4.327226	-0.45342	0.651358	-5.91225	0.685451	0.730314

Neutrophils	SH3KBP1	-0.03963	7.863843	-0.45338	0.651383	-6.66292	0.619895	0.661837
Neutrophils	EIF5A2	-0.29429	0.577892	-0.45331	0.651435	-5.02186	0.762843	0.810572
Neutrophils	AC142100.	0.19792	0.335219	0.453081	0.651601	-5.32914	0.768149	0.816087
Neutrophils	KREMEN1	-0.13716	3.039966	-0.45301	0.651654	-5.3614	0.711071	0.756986
Neutrophils	MSTO1	0.173577	2.681663	0.452911	0.651722	-5.2512	0.718376	0.764576
Neutrophils	ZFP46	-0.13532	1.77659	-0.45281	0.651795	-5.26326	0.737174	0.784064
Neutrophils	NARS2	-0.14769	4.271035	-0.45262	0.651929	-5.40079	0.686549	0.731531
Neutrophils	SRP54C	-0.20486	1.755516	-0.45258	0.651958	-5.17859	0.737618	0.784559
Neutrophils	KMT2A	0.060725	6.28827	0.452445	0.652056	-6.26249	0.648248	0.691626
Neutrophils	TAOK2	0.085176	4.127104	0.452427	0.652069	-5.78577	0.68937	0.734516
Neutrophils	SMPD1	-0.15702	2.643295	-0.45234	0.652135	-5.34899	0.719163	0.765516
Neutrophils	RAPGEF6	-0.03925	8.049527	-0.45219	0.652237	-6.72675	0.61664	0.658629
Neutrophils	NDUFAF5	0.170875	2.563951	0.452031	0.652354	-5.24892	0.720793	0.767325
Neutrophils	KCNMB4	-0.2724	1.763375	-0.45202	0.652361	-5.09846	0.737452	0.784598
Neutrophils	1110051M	-0.15069	3.263469	-0.45178	0.652535	-5.33643	0.706553	0.752537
Neutrophils	DDX19B	0.119886	3.529612	0.451729	0.65257	-5.46128	0.701211	0.746984
Neutrophils	GM43065	-0.28116	0.153169	-0.45168	0.652602	-5.05315	0.772154	0.82049
Neutrophils	STON1	-0.22111	1.087662	-0.45165	0.652628	-5.23525	0.751818	0.799471
Neutrophils	GM14295	0.207509	1.289141	0.45162	0.652649	-5.09512	0.747505	0.795008
Neutrophils	CCDC14	-0.262	0.809905	-0.45161	0.652654	-5.08888	0.757805	0.805664
Neutrophils	SLC30A5	-0.08609	5.799283	-0.45161	0.652657	-5.87599	0.657323	0.701249
Neutrophils	METAP2	0.046556	7.3726	0.451357	0.652838	-6.43767	0.628644	0.671294
Neutrophils	GATD3A	-0.13291	3.544084	-0.45135	0.652844	-5.42885	0.70098	0.746791
Neutrophils	PRR33	0.23372	0.255516	0.451144	0.65299	-5.13123	0.770058	0.8184
Neutrophils	PLEKHA4	-0.1625	2.406918	-0.45105	0.653057	-5.45248	0.72418	0.770937
Neutrophils	TRAPPC1	0.085438	5.432947	0.450805	0.653234	-6.02259	0.664458	0.708735
Neutrophils	PMF1	0.078474	5.697613	0.450496	0.653456	-6.12277	0.659627	0.703584
Neutrophils	GM4890	0.2631	0.373848	0.450248	0.653634	-4.99896	0.767769	0.815942
Neutrophils	CDKAL1	-0.06409	6.364518	-0.4502	0.653671	-6.09921	0.647238	0.690692
Neutrophils	POLDIP2	-0.1101	4.278307	-0.4501	0.653743	-5.61539	0.686824	0.731995
Neutrophils	GM43774	-0.12079	2.238278	-0.45009	0.65375	-5.62182	0.727965	0.774746
Neutrophils	LRP8OS2	0.328346	0.163168	0.450039	0.653784	-5.03749	0.772403	0.820728
Neutrophils	KLHL25	0.12996	2.493825	0.449894	0.653888	-5.41774	0.722718	0.769274
Neutrophils	ZFP386	0.136526	3.889207	0.449739	0.653999	-5.40419	0.69457	0.740042
Neutrophils	GPBP1	-0.04583	7.723603	-0.4494	0.654246	-6.57261	0.622834	0.665167
Neutrophils	ENOPH1	0.152938	2.8863	0.449367	0.654267	-5.33298	0.714733	0.761041
Neutrophils	AOX3	-0.26798	0.998188	-0.4493	0.654313	-5.15945	0.75431	0.802058
Neutrophils	2610301B2	0.217135	1.613471	0.449278	0.654331	-5.08058	0.741173	0.788461
Neutrophils	CKAP2	-0.14421	3.667324	-0.44926	0.654343	-5.63878	0.69899	0.744681
Neutrophils	SDF2L1	-0.11877	4.888426	-0.44893	0.654583	-5.67982	0.675209	0.719847
Neutrophils	SNX11	-0.13497	2.539196	-0.44892	0.654591	-5.41298	0.721973	0.768481
Neutrophils	FBXL19	-0.20629	2.333257	-0.44872	0.654734	-5.16878	0.726267	0.772954
Neutrophils	OTUD1	-0.16084	3.007254	-0.44862	0.654802	-5.29293	0.712434	0.758627
Neutrophils	ATIC	-0.15076	4.493549	-0.4485	0.65489	-5.42766	0.682881	0.727926
Neutrophils	CENPF	-0.13867	5.050794	-0.4485	0.654891	-6.10366	0.672131	0.716721
Neutrophils	GM41335	0.341592	0.493808	0.44825	0.655069	-5.03193	0.765559	0.813611
Neutrophils	MTUS1	-0.07358	3.721745	-0.4481	0.655174	-6.32541	0.698225	0.743784

Neutrophils	FBXO17	0.236852	0.782245	0.447896	0.655324	-5.11728	0.75942	0.807144
Neutrophils	FAM50A	0.066347	5.186332	0.447663	0.655491	-5.97611	0.669858	0.714154
Neutrophils	2900093K2	-0.12724	2.884005	-0.44761	0.655531	-5.52386	0.715279	0.761414
Neutrophils	MTX2	0.080987	4.838218	0.446991	0.655975	-5.79525	0.676918	0.721171
Neutrophils	CHML	0.201809	1.690018	0.446542	0.656297	-5.17545	0.740787	0.78731
Neutrophils	PRKACA	-0.09169	4.156404	-0.44628	0.656486	-5.66125	0.690588	0.735159
Neutrophils	CNPY2	0.093033	4.719728	0.446191	0.65655	-5.79892	0.679595	0.72373
Neutrophils	SOX6	-0.22578	1.905309	-0.44601	0.656679	-5.4096	0.736452	0.782691
Neutrophils	GM10785	0.158742	2.451276	0.445751	0.656867	-5.43943	0.72513	0.770898
Neutrophils	ZFYVE9	0.165892	2.603777	0.445621	0.65696	-5.50643	0.721982	0.767635
Neutrophils	SMO	-0.22804	1.198551	-0.44541	0.657114	-5.19647	0.751531	0.798295
Neutrophils	ACBD4	-0.22191	1.764413	-0.44535	0.657152	-5.09918	0.739486	0.785839
Neutrophils	GON4L	0.072531	5.15234	0.445223	0.657247	-5.8609	0.6714	0.715153
Neutrophils	CRY2	0.1413	2.553643	0.445079	0.657351	-5.35853	0.723015	0.768816
Neutrophils	MCM5	0.135599	4.910563	0.444983	0.65742	-5.93993	0.676036	0.720007
Neutrophils	YIPF6	-0.12941	3.457527	-0.44498	0.657421	-5.48353	0.704612	0.749724
Neutrophils	AGAP1	0.057736	4.225875	0.444889	0.657487	-6.4291	0.689349	0.733879
Neutrophils	RTL5	0.243328	0.491888	0.44486	0.657508	-5.03108	0.766851	0.814176
Neutrophils	U2AF2	0.055608	6.245648	0.44486	0.657508	-6.18679	0.650843	0.693752
Neutrophils	NDUFA10	0.056593	6.050526	0.444822	0.657535	-6.14463	0.654463	0.697534
Neutrophils	RNF214	0.069986	4.961908	0.444606	0.657691	-5.96143	0.67514	0.719055
Neutrophils	HIST1H3B	0.299089	1.995292	0.444383	0.657852	-5.23624	0.734833	0.780956
Neutrophils	GPR146	0.129956	3.278936	0.443756	0.658303	-5.43821	0.708797	0.75377
Neutrophils	KIF15	0.116867	4.90741	0.44366	0.658372	-6.08423	0.676658	0.720368
Neutrophils	SPCS2	0.042046	7.595587	0.443603	0.658414	-6.483	0.626889	0.668425
Neutrophils	AFF1	-0.04878	8.730515	-0.44341	0.658552	-6.66765	0.607102	0.647703
Neutrophils	NUDT4	0.039185	5.949353	0.443338	0.658604	-6.59515	0.65695	0.699842
Neutrophils	EGFR	-0.24138	2.469637	-0.44299	0.658852	-5.42208	0.725486	0.771064
Neutrophils	CACNB2	0.115353	4.420818	0.442992	0.658854	-6.15135	0.686227	0.730316
Neutrophils	CENPJ	0.103803	3.331867	0.44295	0.658884	-5.6069	0.707859	0.752796
Neutrophils	SLF1	-0.11238	4.44199	-0.44288	0.658935	-5.64053	0.685813	0.729903
Neutrophils	ANKRD44	0.043864	8.18547	0.442711	0.659056	-6.82052	0.61661	0.657729
Neutrophils	SLC35A3	0.064703	4.775291	0.442689	0.659072	-5.90151	0.679338	0.723229
Neutrophils	XRCC5	-0.18492	2.083305	-0.44227	0.659376	-5.20679	0.733798	0.779606
Neutrophils	ABHD8	-0.18646	2.172201	-0.44203	0.659545	-5.19268	0.731992	0.777747
Neutrophils	CREB3	-0.08422	3.923009	-0.44201	0.659558	-5.70115	0.696336	0.740777
Neutrophils	LSM11	0.189345	2.171179	0.441797	0.659715	-5.28351	0.732029	0.777855
Neutrophils	CHADL	-0.25301	0.647864	-0.44174	0.659757	-5.06597	0.76457	0.811492
Neutrophils	MARS2	0.130769	2.690445	0.441668	0.659808	-5.35179	0.721262	0.766702
Neutrophils	NDUFAF2	-0.09845	4.440034	-0.44159	0.659865	-5.65191	0.686168	0.730268
Neutrophils	GM26827	0.242408	1.344192	0.441445	0.659969	-5.16058	0.749517	0.795945
Neutrophils	CCDC152	-0.1479	3.284464	-0.44144	0.659973	-5.58837	0.709144	0.754136
Neutrophils	KAT2B	0.05903	6.221283	0.441272	0.660094	-6.31585	0.652256	0.694968
Neutrophils	NCAPH	0.128328	4.175802	0.441256	0.660105	-5.79393	0.691352	0.735692
Neutrophils	CERKL	0.22855	1.852174	0.441108	0.660212	-5.16187	0.73877	0.784858
Neutrophils	ABRACL	0.048337	7.550325	0.440844	0.660402	-6.62813	0.628228	0.669834
Neutrophils	POLE	0.171334	3.301306	0.44079	0.660441	-5.46666	0.708947	0.753944

Neutrophils	ANKIB1	-0.0646	5.807453	-0.4407	0.660506	-6.03878	0.660109	0.703152
Neutrophils	ATG10	-0.08334	5.64086	-0.44058	0.660593	-5.91522	0.663265	0.706451
Neutrophils	CHIC2	0.032693	7.043743	0.440315	0.660783	-6.56625	0.637427	0.679438
Neutrophils	SERHL	-0.10921	3.492794	-0.44027	0.660813	-5.56267	0.705202	0.750033
Neutrophils	SPG11	0.093789	4.094018	0.440064	0.660964	-5.75122	0.693302	0.737602
Neutrophils	PCGF2	0.230076	0.79144	0.439894	0.661087	-5.13136	0.761811	0.808554
Neutrophils	CWC15	0.046636	6.384372	0.439726	0.661208	-6.25674	0.649554	0.692039
Neutrophils	RALGAPA1	0.043149	6.84207	0.439683	0.66124	-6.77719	0.641164	0.683277
Neutrophils	SMIM41	-0.23144	0.89661	-0.43963	0.661276	-5.12384	0.759526	0.806215
Neutrophils	CCT6A	-0.07281	5.802009	-0.4396	0.661302	-5.95605	0.660397	0.703386
Neutrophils	MTMR4	-0.13825	2.648419	-0.43945	0.661408	-5.34613	0.722478	0.767958
Neutrophils	OLFR1033	0.258484	0.583769	0.439336	0.66149	-5.18772	0.766343	0.813317
Neutrophils	ZSWIM9	-0.2767	0.544388	-0.43933	0.661491	-5.06272	0.767205	0.814207
Neutrophils	GOLT1B	0.094352	4.631519	0.439108	0.661654	-5.76615	0.682866	0.726825
Neutrophils	TRMT13	-0.11626	3.89491	-0.43856	0.662048	-5.52796	0.697654	0.742043
Neutrophils	LAMA4	0.190085	1.088771	0.438521	0.662078	-5.42633	0.75581	0.802279
Neutrophils	CNIH1	0.078684	5.17624	0.438325	0.66222	-5.87384	0.672652	0.716091
Neutrophils	TMEM265	-0.10386	3.958769	-0.43822	0.662295	-5.65509	0.696385	0.740841
Neutrophils	PPA2	0.087801	4.580694	0.438129	0.662361	-5.80239	0.684155	0.728139
Neutrophils	GM12253	0.280282	-0.86999	0.438056	0.662414	-4.98296	0.7993	0.847272
Neutrophils	DYNC1H1	0.045621	6.255557	0.437901	0.662526	-6.24626	0.652317	0.69499
Neutrophils	FAM210B	-0.11523	3.914295	-0.43785	0.662561	-5.60481	0.697268	0.741797
Neutrophils	UBQLN2	-0.09163	4.031371	-0.43779	0.662606	-5.62351	0.694946	0.739405
Neutrophils	AI506816	0.127504	5.207229	0.437773	0.662619	-5.79184	0.672059	0.715594
Neutrophils	GMPR	0.184294	1.923101	0.437504	0.662812	-5.31078	0.738019	0.784137
Neutrophils	HNRNPA2E	-0.04096	9.287708	-0.43737	0.662912	-6.808	0.598579	0.638844
Neutrophils	SEC16A	-0.07598	4.57627	-0.43734	0.662928	-5.94053	0.684241	0.728363
Neutrophils	ALS2CL	0.272778	0.148166	0.437322	0.662944	-5.04941	0.776389	0.823809
Neutrophils	PIK3CG	-0.05687	4.936727	-0.43716	0.663061	-6.21832	0.677254	0.721142
Neutrophils	SRM	0.160851	4.842685	0.437147	0.66307	-5.62383	0.679069	0.723033
Neutrophils	GM39326	-0.23637	1.289251	-0.43714	0.663077	-5.09725	0.751496	0.798159
Neutrophils	TMEM161E	0.081161	4.545354	0.436939	0.663221	-5.75044	0.684878	0.729127
Neutrophils	GM34086	-0.11494	2.658244	-0.43674	0.663365	-5.65734	0.722733	0.768527
Neutrophils	CRTAM	-0.28038	1.176627	-0.43656	0.663493	-5.19239	0.753954	0.800903
Neutrophils	BCLAF3	-0.07669	4.966172	-0.43654	0.663506	-5.87726	0.67672	0.720769
Neutrophils	TRF	-0.10601	9.658999	-0.43641	0.6636	-6.98461	0.592359	0.63255
Neutrophils	SERBP1	0.041105	8.529561	0.436288	0.663691	-6.63334	0.611591	0.652786
Neutrophils	GALT	-0.16851	2.389889	-0.43628	0.663698	-5.28393	0.728288	0.774437
Neutrophils	SPSB1	-0.273	1.685361	-0.43619	0.663759	-5.07637	0.743082	0.789799
Neutrophils	EI24	-0.07389	4.630195	-0.43597	0.663918	-5.79869	0.683225	0.727718
Neutrophils	H3F3A	0.033583	10.71708	0.435917	0.66396	-7.1208	0.574925	0.614345
Neutrophils	KLF11	0.087692	3.555665	0.4359	0.663972	-5.61184	0.704469	0.74983
Neutrophils	ADAMDEC1	0.241189	0.746267	0.435813	0.664034	-5.20484	0.763278	0.810797
Neutrophils	UST	-0.20463	6.028853	-0.43572	0.6641	-5.75614	0.656567	0.699991
Neutrophils	CBLB	-0.07171	7.336459	-0.43572	0.664103	-6.29201	0.63263	0.674968
Neutrophils	SIAH1A	0.069912	5.079563	0.435708	0.664111	-5.97044	0.674539	0.718738
Neutrophils	4732471J0	-0.15899	1.996323	-0.4353	0.664403	-5.34277	0.736609	0.783216

Neutrophils	SNHG8	-0.09938	4.013183	-0.435	0.664626	-5.61771	0.69543	0.740535
Neutrophils	RARS	0.075817	5.121145	0.434948	0.66466	-5.8723	0.673827	0.718044
Neutrophils	PCBP3	0.156285	2.616922	0.434915	0.664684	-5.29197	0.723678	0.769872
Neutrophils	EML3	0.10173	4.139794	0.434875	0.664712	-5.63173	0.692925	0.73794
Neutrophils	COL5A3	-0.26066	0.940726	-0.43481	0.664758	-5.11475	0.759147	0.80664
Neutrophils	POP7	-0.08189	4.908142	-0.43477	0.664789	-5.81862	0.677925	0.722368
Neutrophils	RABEPK	0.132561	3.296184	0.434689	0.664847	-5.38931	0.709791	0.755519
Neutrophils	HIST1H2BN	-0.19552	1.240034	-0.43468	0.664853	-5.22226	0.752687	0.799977
Neutrophils	KIF1A	-0.24906	-0.59877	-0.43459	0.664922	-5.02559	0.793273	0.841873
Neutrophils	UBE3C	0.064512	5.784905	0.434523	0.664968	-6.03776	0.661222	0.704952
Neutrophils	GM39121	-0.12235	-0.94202	-0.43445	0.665023	-5.57183	0.801089	0.849941
Neutrophils	NUP50	0.062683	5.304963	0.43432	0.665115	-5.98983	0.670311	0.714495
Neutrophils	NANS	0.094478	4.873876	0.434295	0.665132	-5.78616	0.678587	0.723122
Neutrophils	COX7A1	0.222026	0.986976	0.434201	0.6652	-5.07752	0.758145	0.805714
Neutrophils	XIAP	0.037826	6.725324	0.434115	0.665262	-6.4507	0.643783	0.686813
Neutrophils	GM43112	0.251703	0.075373	0.434017	0.665333	-5.02678	0.778149	0.82637
Neutrophils	PRC1	-0.11687	5.285692	-0.43362	0.665622	-6.17492	0.670907	0.71497
Neutrophils	FAM78A	-0.1934	2.648934	-0.43303	0.666047	-5.18646	0.72353	0.769556
Neutrophils	2900097C1	0.063099	4.909122	0.433023	0.666052	-5.98052	0.678387	0.722641
Neutrophils	RNF128	-0.13243	2.499728	-0.43289	0.666146	-5.55918	0.726617	0.772802
Neutrophils	CHST12	0.05909	5.763881	0.432875	0.66616	-6.41295	0.662086	0.705689
Neutrophils	CHAF1B	0.123448	3.382455	0.432817	0.666201	-5.58965	0.708549	0.75406
Neutrophils	MRPL51	0.078252	4.486929	0.432554	0.666392	-5.83098	0.68672	0.731273
Neutrophils	LSM4	-0.05458	6.704001	-0.43231	0.666565	-6.24546	0.644826	0.687534
Neutrophils	MAP3K12	0.136716	2.718206	0.432213	0.666639	-5.35799	0.722321	0.768206
Neutrophils	SGSM2	0.125535	3.786605	0.432168	0.666671	-5.48389	0.700645	0.745705
Neutrophils	STARD3NL	-0.06816	5.861943	-0.43198	0.666808	-6.02805	0.660513	0.703882
Neutrophils	GBA2	0.111658	2.547922	0.431692	0.667016	-5.47692	0.726035	0.771949
Neutrophils	WDR43	0.085077	6.033494	0.431601	0.667082	-5.8607	0.657408	0.700593
Neutrophils	TM9SF2	0.035043	6.683958	0.43139	0.667235	-6.38306	0.645368	0.688121
Neutrophils	CABYR	0.210165	1.135391	0.431352	0.667263	-5.25347	0.755909	0.803009
Neutrophils	USP53	-0.07872	4.118641	-0.43124	0.667341	-5.81042	0.694233	0.739104
Neutrophils	BUD31	-0.06078	5.474604	-0.4312	0.667375	-6.0951	0.667941	0.711728
Neutrophils	CUL4B	-0.08384	4.524951	-0.43118	0.667386	-5.87952	0.686244	0.730795
Neutrophils	ERO1LB	-0.06432	6.652932	-0.43104	0.667488	-6.15069	0.645971	0.68878
Neutrophils	NXPE4	0.167535	2.181788	0.43093	0.667568	-5.44724	0.733715	0.780058
Neutrophils	ARHGEF18	0.056153	5.591269	0.430724	0.667718	-6.37009	0.665809	0.709446
Neutrophils	CINP	0.150193	3.163035	0.430642	0.667776	-5.455	0.713494	0.759055
Neutrophils	CHRNE	-0.25337	-0.75209	-0.4306	0.667807	-4.99916	0.797876	0.846276
Neutrophils	E130309D0	-0.09844	3.983364	-0.43033	0.668005	-5.6078	0.697	0.741923
Neutrophils	E2F6	0.169658	2.266229	0.430244	0.668065	-5.2122	0.731985	0.778261
Neutrophils	FKRP	0.147832	2.332289	0.430026	0.668223	-5.31504	0.730606	0.77686
Neutrophils	TINF2	-0.08622	4.054948	-0.43002	0.668227	-5.8323	0.69558	0.740503
Neutrophils	ATP6V1B2	0.040177	5.98524	0.429807	0.668382	-6.41006	0.658391	0.701837
Neutrophils	SIN3A	-0.05653	5.871572	-0.42964	0.668506	-6.15558	0.660522	0.704106
Neutrophils	9530077C0	0.152296	1.213471	0.429624	0.668514	-5.45658	0.754318	0.801539
Neutrophils	ADAT2	-0.21101	1.285306	-0.42955	0.66857	-5.1612	0.752772	0.799941

Neutrophils	UBE2T	-0.1121	4.06115	-0.42953	0.668586	-5.76237	0.695457	0.740503
Neutrophils	SATB1	-0.07644	7.143818	-0.42947	0.668625	-6.42805	0.637074	0.679604
Neutrophils	FAM102A	-0.08242	4.182226	-0.42933	0.668727	-5.93029	0.693062	0.738018
Neutrophils	NUP107	-0.05988	5.353646	-0.42922	0.668804	-6.17318	0.670325	0.714371
Neutrophils	D930016D(	-0.14108	2.876865	-0.42922	0.668804	-5.33043	0.719341	0.765354
Neutrophils	TMTC4	0.240068	1.357794	0.428828	0.669091	-5.11797	0.751215	0.79844
Neutrophils	LARS	-0.09248	4.738364	-0.42876	0.669142	-5.7514	0.682169	0.726773
Neutrophils	ATXN10	-0.03451	6.66392	-0.42869	0.66919	-6.70647	0.645814	0.688838
Neutrophils	TMC8	0.089663	3.820781	0.428664	0.669211	-5.76793	0.700238	0.745575
Neutrophils	LSAMP	-0.22415	0.929492	-0.42854	0.669304	-5.18423	0.76046	0.808057
Neutrophils	NUSAP1	-0.10698	5.649629	-0.42852	0.669316	-6.23974	0.664705	0.708619
Neutrophils	HASPIN	-0.16244	2.299155	-0.42844	0.66937	-5.33104	0.731297	0.777925
Neutrophils	NDUFB10	-0.04812	6.731387	-0.42829	0.66948	-6.37077	0.644578	0.687712
Neutrophils	PSTK	-0.12894	3.061289	-0.42828	0.66949	-5.34531	0.715567	0.761686
Neutrophils	GAS2L1	-0.14595	1.765951	-0.42821	0.669539	-5.41079	0.742512	0.78963
Neutrophils	TNRC6B	0.037471	8.234075	0.428121	0.669604	-6.84424	0.617678	0.659546
Neutrophils	PPP2CA	0.026979	7.762516	0.428046	0.669659	-6.63656	0.62599	0.668263
Neutrophils	PIP4K2C	0.094083	4.238405	0.427714	0.669899	-5.68256	0.691953	0.737187
Neutrophils	HRAS	0.067239	4.842094	0.427698	0.669911	-5.92116	0.680157	0.724903
Neutrophils	DUSP8	0.233102	1.274945	0.427646	0.669949	-5.09366	0.752995	0.800529
Neutrophils	ING3	0.049581	4.830612	0.427613	0.669973	-6.1715	0.68038	0.725134
Neutrophils	MAGT1	0.063718	5.872067	0.427612	0.669973	-6.05378	0.660513	0.704411
Neutrophils	VARS2	-0.25227	1.401859	-0.42753	0.67003	-5.07428	0.750271	0.79771
Neutrophils	DDB2	0.133008	3.43077	0.427525	0.670037	-5.55758	0.708067	0.753945
Neutrophils	HIST1H4H	-0.22567	0.513275	-0.42751	0.670047	-5.10189	0.769553	0.817646
Neutrophils	SUPT4A	0.052432	6.836136	0.427471	0.670076	-6.36376	0.642663	0.685756
Neutrophils	GSTCD	-0.0992	3.564196	-0.42727	0.670219	-5.71782	0.705378	0.751182
Neutrophils	ENTPD5	-0.06805	3.78751	-0.42726	0.670231	-5.82896	0.700902	0.746528
Neutrophils	HNRNPH3	-0.05166	5.447268	-0.42709	0.670353	-5.98822	0.668542	0.712885
Neutrophils	TM4SF4	0.171749	2.168937	0.427068	0.670368	-5.44736	0.73402	0.780982
Neutrophils	FNDC9	0.100966	2.564671	0.427016	0.670406	-5.51287	0.725777	0.77244
Neutrophils	GTF2E1	-0.16021	2.399479	-0.42682	0.670551	-5.28143	0.729252	0.776044
Neutrophils	F730043M	0.284724	0.260543	0.426774	0.670581	-5.04873	0.775176	0.823575
Neutrophils	PSMB10	0.11683	5.706129	0.42668	0.670649	-5.81792	0.66368	0.707821
Neutrophils	PPP2R1B	-0.09233	4.339805	-0.42654	0.670754	-5.71731	0.690001	0.73529
Neutrophils	CCR3	0.427705	-0.02075	0.426451	0.670815	-5.11873	0.781431	0.830061
Neutrophils	TRAPPC2	-0.07269	4.171105	-0.4264	0.670849	-5.93925	0.693326	0.738751
Neutrophils	ARMCX6	0.177453	0.977785	0.426259	0.670955	-5.15952	0.759497	0.80737
Neutrophils	PRKAB2	0.114667	3.392055	0.42614	0.671041	-5.56537	0.708928	0.754953
Neutrophils	INTS5	0.131932	2.704825	0.425903	0.671214	-5.32067	0.722962	0.769638
Neutrophils	NDUFS5	-0.0481	6.478554	-0.42589	0.671226	-6.37841	0.649297	0.6929
Neutrophils	GM28960	-0.24191	-0.82667	-0.42577	0.671307	-5.03738	0.799667	0.849002
Neutrophils	THUMPD3	0.097507	4.262161	0.42575	0.671325	-5.63874	0.691563	0.737034
Neutrophils	RGS5	0.269697	1.107497	0.425701	0.67136	-5.25062	0.756689	0.804626
Neutrophils	PWWP2A	0.073784	5.141602	0.425624	0.671416	-5.92282	0.674458	0.719209
Neutrophils	P2RY1	-0.16492	0.13392	-0.42551	0.671499	-5.3174	0.778042	0.826683
Neutrophils	3830403N1	-0.29512	0.704685	-0.42535	0.671615	-5.15932	0.765518	0.813738

Neutrophils	POLR3K	-0.09731	3.885777	-0.4252	0.671726	-5.55713	0.699134	0.744882
Neutrophils	CDCA3	-0.11541	4.895561	-0.42453	0.672209	-6.09107	0.679731	0.724356
Neutrophils	GM43696	0.142247	2.459243	0.423841	0.672711	-5.35092	0.728973	0.775474
Neutrophils	FAM219B	0.166477	2.367885	0.423755	0.672773	-5.29806	0.730876	0.777479
Neutrophils	9530062K(	0.194346	0.793704	0.423586	0.672896	-5.23454	0.764472	0.812287
Neutrophils	MICU3	0.127219	3.81398	0.423507	0.672954	-5.49903	0.701344	0.746853
Neutrophils	ERAL1	-0.16715	1.918255	-0.42346	0.672991	-5.18653	0.740315	0.787296
Neutrophils	CYP2C69	-0.18171	1.432054	-0.42345	0.672999	-5.39385	0.750663	0.798008
Neutrophils	MFAP2	-0.24974	0.586159	-0.42333	0.673084	-5.14288	0.769017	0.817001
Neutrophils	ZFP626	-0.15585	2.562798	-0.42327	0.673123	-5.2292	0.726822	0.773327
Neutrophils	ASB4	0.186085	1.68448	0.423161	0.673206	-5.59932	0.745273	0.792446
Neutrophils	GLIS1	0.24936	0.394465	0.423159	0.673207	-5.11683	0.773239	0.821361
Neutrophils	DEPDC7	-0.21241	1.678465	-0.42272	0.673525	-5.22634	0.745582	0.792642
Neutrophils	SZRD1	0.045134	5.906997	0.422708	0.673535	-6.18421	0.660932	0.704652
Neutrophils	D430042O(	0.100566	3.792114	0.422657	0.673572	-5.5848	0.701952	0.747405
Neutrophils	NECTIN4	-0.27294	-0.02307	-0.42226	0.673864	-5.00352	0.782867	0.831157
Neutrophils	ZFP516	0.05239	4.66128	0.422063	0.674004	-6.33079	0.684923	0.729732
Neutrophils	HIST1H1C	0.102063	4.106663	0.421983	0.674062	-5.92402	0.69583	0.741092
Neutrophils	AREL1	0.076571	4.263334	0.421981	0.674063	-5.80799	0.692731	0.737868
Neutrophils	ZFP944	0.083439	4.397621	0.421748	0.674233	-5.81808	0.690086	0.735115
Neutrophils	GM10847	0.252132	0.165978	0.421714	0.674257	-5.03852	0.778651	0.826903
Neutrophils	CD177	0.069299	0.17299	0.421676	0.674285	-6.22872	0.778495	0.826742
Neutrophils	TNFRSF21	-0.06265	4.10783	-0.42164	0.674308	-6.26041	0.695807	0.741068
Neutrophils	RHBDD1	0.070265	4.175534	0.421415	0.674475	-5.88595	0.694466	0.739759
Neutrophils	4833438C(	-0.11681	2.025244	-0.4214	0.674487	-5.3823	0.738389	0.785366
Neutrophils	SLC25A38	0.110856	3.517886	0.421253	0.674593	-5.52919	0.707606	0.753424
Neutrophils	TMEM56	-0.17393	2.147941	-0.42121	0.674624	-5.39789	0.735808	0.782691
Neutrophils	EIF2B5	-0.09745	4.703803	-0.42119	0.674635	-5.64014	0.684094	0.728961
Neutrophils	FAM168A	-0.05647	5.93211	-0.42114	0.674674	-6.15875	0.660596	0.704454
Neutrophils	ACTL6A	-0.07613	5.235041	-0.42107	0.674723	-5.79711	0.673826	0.718259
Neutrophils	ZFP738	-0.11643	2.843416	-0.421	0.67478	-5.42242	0.721349	0.7677
Neutrophils	SLIT2	-0.30639	0.983319	-0.42085	0.674882	-5.05102	0.760724	0.808465
Neutrophils	PSMA3	-0.03732	7.755055	-0.42057	0.67509	-6.5077	0.62742	0.669622
Neutrophils	C730034F0	0.145339	2.636781	0.420484	0.675152	-5.44978	0.725787	0.772158
Neutrophils	HIST1H2AC	0.191591	1.907677	0.420321	0.675271	-5.37032	0.741048	0.787974
Neutrophils	GM5544	-0.22923	0.129876	-0.42031	0.675276	-5.09318	0.77964	0.827868
Neutrophils	KSR2	-0.176	2.695294	-0.42013	0.675409	-5.5234	0.724648	0.770936
Neutrophils	COG6	0.119209	3.137898	0.419889	0.675586	-5.4828	0.715581	0.761563
Neutrophils	AARSD1	0.114876	3.977143	0.419834	0.675625	-5.54314	0.698663	0.743983
Neutrophils	TBL3	-0.12102	3.91508	-0.41982	0.675633	-5.41196	0.6999	0.74527
Neutrophils	HIST1H4M	-0.22745	1.423329	-0.4193	0.676014	-5.17814	0.751815	0.798822
Neutrophils	GM12089	-0.22685	0.306607	-0.41909	0.676168	-5.02574	0.776218	0.824011
Neutrophils	C1QTNF6	-0.15667	1.904338	-0.41892	0.676294	-5.50052	0.7416	0.788281
Neutrophils	KCNA2	-0.2564	0.96787	-0.41889	0.676313	-5.24897	0.761696	0.80907
Neutrophils	HELLS	-0.11782	4.919254	-0.41888	0.676317	-5.91102	0.680513	0.724854
Neutrophils	ST8SIA6	-0.35468	1.211567	-0.41822	0.676797	-5.05157	0.756728	0.803672
Neutrophils	SLC25A11	-0.05932	5.162778	-0.41814	0.67686	-6.15007	0.676094	0.720026

Neutrophils	ANGPT2	0.209145	0.571482	0.418068	0.676911	-5.16171	0.770688	0.818105
Neutrophils	SEC11A	-0.04494	6.406384	-0.41797	0.676985	-6.19915	0.652601	0.695536
Neutrophils	C1RL	-0.12719	1.632594	-0.41785	0.677072	-5.62289	0.747686	0.794353
Neutrophils	ARL1	-0.06275	5.538643	-0.4178	0.677109	-5.94869	0.668901	0.712543
Neutrophils	BAG6	-0.06914	5.438014	-0.41778	0.677121	-6.05057	0.670819	0.714543
Neutrophils	KRIT1	0.051804	5.937975	0.417762	0.677134	-6.17306	0.661347	0.704665
Neutrophils	RRAGD	-0.15765	2.299081	-0.41749	0.677332	-5.34401	0.733738	0.779895
Neutrophils	CCT7	0.051656	6.427225	0.417228	0.677523	-6.21995	0.65246	0.695294
Neutrophils	TFE3	-0.06248	4.149746	-0.41689	0.677766	-6.00465	0.696277	0.740928
Neutrophils	PTCD2	-0.09174	4.58679	-0.41687	0.677786	-5.66118	0.687662	0.731971
Neutrophils	NABP2	0.059387	4.980112	0.416721	0.677892	-6.03012	0.680014	0.724055
Neutrophils	SLC35A2	-0.12108	3.165869	-0.41667	0.67793	-5.51297	0.716094	0.761565
Neutrophils	ZFP831	0.09665	3.063289	0.416329	0.678178	-5.89234	0.718346	0.7638
Neutrophils	SLC26A11	0.110198	3.200265	0.416286	0.678209	-5.72397	0.715545	0.76091
Neutrophils	PTPRO	0.198875	2.363394	0.41597	0.67844	-5.31755	0.73301	0.778881
Neutrophils	ULBP1	-0.09693	4.251838	-0.41569	0.678646	-5.85909	0.694723	0.739097
Neutrophils	ZFP787	0.051454	5.128821	0.415425	0.678837	-6.09696	0.677712	0.721393
Neutrophils	HSPD1	-0.0663	7.46114	-0.41511	0.679065	-6.40439	0.634292	0.67607
Neutrophils	VWCE	-0.23539	0.865529	-0.41511	0.679067	-5.08658	0.765384	0.812259
Neutrophils	SIGLECH	0.278227	0.771994	0.415086	0.679084	-5.14192	0.767432	0.814372
Neutrophils	RNF2	0.054295	5.723956	0.414989	0.679155	-6.09986	0.666381	0.709569
Neutrophils	CCL2	0.199797	3.089272	0.414632	0.679416	-5.69537	0.71842	0.763625
Neutrophils	F11R	-0.10404	3.432174	-0.41459	0.679449	-5.80065	0.711429	0.756374
Neutrophils	GPATCH11	-0.08297	3.800176	-0.41458	0.679454	-5.75159	0.704004	0.748667
Neutrophils	SAC3D1	0.12891	3.030557	0.414373	0.679605	-5.32815	0.719671	0.7649
Neutrophils	SIPA1	-0.0469	5.681522	-0.41432	0.679641	-6.26216	0.667325	0.710491
Neutrophils	FER	-0.07876	3.489418	-0.41417	0.679754	-5.98818	0.710315	0.755197
Neutrophils	CCDC157	0.218929	1.103128	0.414151	0.679766	-5.10731	0.760371	0.807011
Neutrophils	AMPD3	0.080304	2.578161	0.413866	0.679974	-6.00347	0.729171	0.774672
Neutrophils	MRPS9	-0.06973	4.889322	-0.41369	0.680105	-5.80526	0.682695	0.726452
Neutrophils	CYP51	-0.11157	3.916341	-0.41365	0.680133	-5.59931	0.701881	0.746396
Neutrophils	RAD1	0.163125	2.477895	0.413526	0.680223	-5.24965	0.731274	0.776876
Neutrophils	LYZ2	-0.08374	7.170673	-0.41341	0.680309	-7.14496	0.639823	0.681754
Neutrophils	PILRB1	-0.07836	2.049283	-0.41316	0.680489	-6.10731	0.740274	0.78625
Neutrophils	PRSS30	-0.21982	-0.33737	-0.41307	0.680558	-5.01666	0.792486	0.840159
Neutrophils	CLDN10	-0.31711	0.159414	-0.41297	0.680631	-5.00339	0.78132	0.828659
Neutrophils	ZMYND11	0.039979	6.466516	0.412805	0.680749	-6.43022	0.652746	0.695322
Neutrophils	PRMT1	-0.06733	5.803475	-0.41276	0.680783	-5.95352	0.665165	0.708284
Neutrophils	PIP4P1	0.052232	5.856707	0.412599	0.6809	-6.00007	0.664159	0.707249
Neutrophils	EXOSC10	-0.07915	4.93082	-0.41241	0.681036	-5.81891	0.681889	0.725746
Neutrophils	PSMA5	0.07151	5.884469	0.4124	0.681045	-6.05001	0.663635	0.70673
Neutrophils	BMS1	0.091281	4.737717	0.412376	0.681062	-5.68845	0.685648	0.729657
Neutrophils	SLC1A5	-0.08304	6.46392	-0.41234	0.68109	-5.98842	0.652794	0.695421
Neutrophils	YIF1A	0.088919	4.016242	0.412325	0.681099	-5.57655	0.699886	0.744459
Neutrophils	CACNA1I	0.204628	-0.17458	0.412251	0.681153	-5.00632	0.78881	0.83644
Neutrophils	MAF1	0.068971	5.342723	0.412235	0.681165	-6.07526	0.673941	0.717471
Neutrophils	GTF3A	0.081244	4.569389	0.412193	0.681195	-5.65705	0.688943	0.733085

Neutrophils	ANKRD26	-0.18065	2.576265	-0.41209	0.681274	-5.24214	0.729236	0.774893
Neutrophils	CMC1	0.084299	4.601302	0.411915	0.681399	-5.65242	0.688387	0.732434
Neutrophils	ARHGEF37	0.313042	0.616444	0.411737	0.681529	-5.1529	0.771337	0.818274
Neutrophils	YIPF5	0.057239	5.24625	0.411481	0.681715	-5.9928	0.676041	0.7194
Neutrophils	TMC6	0.061125	4.632332	0.411393	0.68178	-5.97594	0.68796	0.731801
Neutrophils	RCAN1	0.091238	3.708543	0.410878	0.682156	-5.69916	0.706629	0.750935
Neutrophils	BAG3	0.118346	2.967773	0.410711	0.682278	-5.54481	0.721753	0.766606
Neutrophils	PDE4DIP	0.117242	3.94998	0.410645	0.682327	-5.46168	0.70182	0.74596
Neutrophils	PABPN1	-0.04	6.640276	-0.41038	0.682519	-6.33422	0.650217	0.692197
Neutrophils	COLEC11	0.171256	1.589372	0.40977	0.682966	-5.2783	0.751206	0.796828
Neutrophils	MAGED1	-0.15123	2.409871	-0.4097	0.683014	-5.3867	0.733817	0.778869
Neutrophils	WDR36	0.104801	4.578146	0.409646	0.683057	-5.50271	0.689826	0.733292
Neutrophils	LBHD1	0.212969	0.16876	0.409569	0.683113	-5.05324	0.782308	0.828956
Neutrophils	MAG	0.269601	0.11897	0.409377	0.683253	-5.05127	0.783432	0.830112
Neutrophils	CHMP6	0.111163	3.712475	0.409334	0.683285	-5.57794	0.707063	0.751211
Neutrophils	MFSD3	0.248043	0.642198	0.409281	0.683323	-5.03154	0.771811	0.81817
Neutrophils	LPAR1	0.331248	0.763125	0.408784	0.683686	-5.09184	0.769483	0.815457
Neutrophils	CCDC106	-0.23922	0.51131	-0.40866	0.683776	-5.06831	0.775063	0.821196
Neutrophils	PEX5	0.073309	4.071805	0.408551	0.683857	-5.78319	0.700198	0.743811
Neutrophils	ARL6IP1	-0.04099	7.879749	-0.40837	0.683986	-6.63259	0.628412	0.669007
Neutrophils	PSMD10	0.097044	3.584601	0.408251	0.684076	-5.6542	0.710052	0.753989
Neutrophils	TIMMDC1	0.07124	4.271519	0.408144	0.684155	-5.82124	0.696286	0.739711
Neutrophils	CABIN1	0.074552	5.4487	0.408101	0.684186	-5.86791	0.673336	0.715872
Neutrophils	POLR3H	-0.1431	3.509866	-0.40797	0.684281	-5.28492	0.711595	0.755617
Neutrophils	ZFP729A	0.12862	3.051831	0.40757	0.684574	-5.44245	0.721186	0.765399
Neutrophils	PCNA	-0.06276	6.710032	-0.40748	0.684639	-6.39676	0.649858	0.691334
Neutrophils	9430060I0:	-0.1797	1.308677	-0.40693	0.685042	-5.29723	0.758345	0.803609
Neutrophils	RNGTT	0.045788	6.852257	0.406777	0.685155	-6.33745	0.647587	0.688731
Neutrophils	ACTR8	0.102952	3.537225	0.406539	0.685329	-5.5448	0.711655	0.755351
Neutrophils	PHC2	0.077529	5.798035	0.406498	0.685359	-5.99765	0.667281	0.709288
Neutrophils	TARBP2	0.120125	3.242608	0.406463	0.685384	-5.42821	0.717659	0.761579
Neutrophils	CDK17	-0.04151	6.780519	-0.40625	0.685542	-6.56363	0.648908	0.690158
Neutrophils	WDR55	0.120171	2.979495	0.406232	0.685554	-5.39117	0.723064	0.767188
Neutrophils	2810013PC	0.097859	4.123121	0.406098	0.685652	-5.80596	0.699869	0.743198
Neutrophils	JMJD4	-0.18684	1.268434	-0.40602	0.685709	-5.08178	0.759252	0.804632
Neutrophils	HSD17B12	-0.06348	6.085483	-0.40589	0.685804	-6.10472	0.66185	0.703745
Neutrophils	REEP4	-0.10089	4.356559	-0.40588	0.685815	-5.70794	0.695229	0.73845
Neutrophils	MRPL32	0.065352	5.68103	0.405717	0.685931	-6.01567	0.669505	0.71174
Neutrophils	ARMC2	0.247699	-0.1641	0.405694	0.685948	-5.01119	0.79096	0.837368
Neutrophils	SOX5OS4	-0.26308	-0.23518	-0.40555	0.686053	-5.03697	0.792567	0.839043
Neutrophils	PVRIG	-0.22461	-0.53207	-0.40543	0.686138	-5.0149	0.799317	0.845994
Neutrophils	EIF3L	0.062666	5.672102	0.405352	0.686198	-5.97865	0.669675	0.711955
Neutrophils	GM15706	0.201351	1.015787	0.405283	0.686248	-5.13213	0.76475	0.810438
Neutrophils	MGAT5	0.051029	6.916886	0.405255	0.686269	-6.41302	0.6464	0.687716
Neutrophils	LRP1	0.086747	4.178873	0.405232	0.686286	-5.93839	0.698758	0.742187
Neutrophils	INTU	0.270029	0.880465	0.404895	0.686533	-5.11527	0.767773	0.813505
Neutrophils	TPPP	-0.24031	0.182012	-0.40483	0.686578	-5.21425	0.783242	0.829437

Neutrophils	SLC35F5	0.117807	3.11698	0.404723	0.686659	-5.59055	0.720293	0.764473
Neutrophils	ULK3	-0.13895	2.219052	-0.40469	0.686685	-5.37461	0.738984	0.783813
Neutrophils	H2-EB1	-0.23027	6.754688	-0.40466	0.686708	-6.10928	0.649436	0.690856
Neutrophils	ZBTB7A	0.037275	7.04476	0.404604	0.686746	-6.45996	0.644109	0.6853
Neutrophils	CNOT9	-0.07685	4.541441	-0.40447	0.686847	-5.73182	0.691668	0.734764
Neutrophils	PTCD1	0.132749	2.585716	0.404217	0.687029	-5.33967	0.731437	0.775916
Neutrophils	GM10802	0.182403	-0.25172	0.404143	0.687083	-5.24883	0.793163	0.839584
Neutrophils	SCRG1	0.077873	-0.98095	0.403779	0.68735	-5.92121	0.810018	0.856823
Neutrophils	WDR90	-0.17436	1.959845	-0.40364	0.687449	-5.21019	0.744767	0.789699
Neutrophils	CCNF	0.130911	3.438612	0.403599	0.687482	-5.65874	0.713999	0.757894
Neutrophils	PLXDC2	0.200223	5.144109	0.403431	0.687605	-5.65689	0.680137	0.722766
Neutrophils	DNPH1	-0.21328	0.708539	-0.40341	0.687621	-5.10875	0.771858	0.817674
Neutrophils	RPF2	-0.12047	4.402771	-0.40341	0.687624	-5.5232	0.694646	0.737838
Neutrophils	ZFP618	0.211628	0.531603	0.403122	0.687832	-5.15293	0.775926	0.821802
Neutrophils	GM5577	0.159609	0.20371	0.402995	0.687925	-5.32088	0.783232	0.82934
Neutrophils	D6WSU163	-0.10692	3.1191	-0.40289	0.688001	-5.46718	0.720686	0.764829
Neutrophils	AAAS	-0.1052	3.794127	-0.40284	0.688038	-5.62332	0.706948	0.750601
Neutrophils	MRPL30	-0.04463	6.172662	-0.40274	0.688113	-6.21815	0.660673	0.70251
Neutrophils	LEPROTL1	-0.06299	5.362087	-0.40245	0.688327	-6.20911	0.676174	0.718544
Neutrophils	PRIMPOL	0.095355	3.486578	0.402423	0.688344	-5.66864	0.713281	0.757053
Neutrophils	EMC10	0.062506	5.502896	0.402245	0.688475	-5.9418	0.673533	0.715733
Neutrophils	CCPG1OS	-0.14079	1.576044	-0.402	0.688657	-5.28603	0.753384	0.798369
Neutrophils	RHNO1	0.085447	4.345115	0.401972	0.688674	-5.77464	0.696169	0.739178
Neutrophils	IFI206	-0.2271	3.940563	-0.4018	0.688798	-5.59233	0.704298	0.747549
Neutrophils	WDR44	-0.08094	4.70447	-0.40139	0.689103	-5.86345	0.689268	0.731866
Neutrophils	SYT14	0.29031	0.008242	0.40135	0.689131	-5.07304	0.788098	0.833954
Neutrophils	PIM2	-0.13582	3.150665	-0.40135	0.689132	-5.37828	0.720477	0.76421
Neutrophils	NPEPL1	-0.08242	5.049543	-0.40125	0.689201	-5.74875	0.682531	0.724878
Neutrophils	TSPYL1	0.063189	4.888609	0.401041	0.689358	-5.99793	0.685754	0.728208
Neutrophils	GM36738	-0.09853	3.095803	-0.40095	0.689425	-5.66808	0.7217	0.765482
Neutrophils	FAH	-0.13166	3.892683	-0.40074	0.689579	-5.86868	0.705502	0.748694
Neutrophils	ACPP	-0.05339	2.739914	-0.40068	0.689622	-6.515	0.729079	0.773099
Neutrophils	GM15943	0.262641	0.207344	0.400623	0.689664	-5.05326	0.783748	0.829472
Neutrophils	COMMD7	-0.05131	5.29863	-0.40047	0.689774	-5.94816	0.677811	0.719976
Neutrophils	IKBKG	0.08148	3.339723	0.40042	0.689813	-5.72006	0.716712	0.760326
Neutrophils	AP5B1	0.151882	1.293299	0.400334	0.689877	-5.34591	0.759814	0.804859
Neutrophils	GM43378	0.153756	1.243694	0.400287	0.689911	-5.21772	0.760891	0.805979
Neutrophils	FKBP9	-0.18095	1.265181	-0.40007	0.69007	-5.33554	0.760448	0.805579
Neutrophils	AGO1	0.125262	3.874896	0.399916	0.690183	-5.4384	0.705882	0.749179
Neutrophils	TMEM51	0.110766	3.130512	0.399913	0.690185	-5.65914	0.721023	0.764859
Neutrophils	ACAD10	0.185451	1.501708	0.39989	0.690202	-5.1886	0.755329	0.800311
Neutrophils	F830208F2	0.195277	-0.23725	0.39979	0.690275	-5.23049	0.793795	0.839927
Neutrophils	XRCC2	0.194108	0.802718	0.399665	0.690367	-5.13321	0.77059	0.816049
Neutrophils	FIG4	0.073486	4.603641	0.39956	0.690444	-5.69243	0.691416	0.734219
Neutrophils	ADORA2A	0.144089	3.148747	0.399222	0.690692	-5.50307	0.720825	0.764672
Neutrophils	IL18	-0.10327	3.422293	-0.3992	0.690708	-5.65233	0.715224	0.758874
Neutrophils	SOWAHC	-0.10996	2.917305	-0.39883	0.690983	-5.75744	0.725817	0.76967

Neutrophils	D430001F1	0.291976	-0.87048	0.398683	0.691088	-5.01151	0.808761	0.855111
Neutrophils	DOCK11	0.038779	7.111908	0.39836	0.691325	-6.64182	0.644231	0.684969
Neutrophils	PHKA1	0.149781	3.411651	0.398347	0.691335	-5.34207	0.715763	0.759287
Neutrophils	HAGH	-0.07327	5.358255	-0.39832	0.691358	-6.07824	0.677153	0.719241
Neutrophils	TOMM22	0.039835	6.905252	0.398182	0.691456	-6.46034	0.648022	0.688936
Neutrophils	ATXN2L	0.049135	5.47603	0.398133	0.691492	-6.15629	0.674888	0.716901
Neutrophils	RELA	0.061953	5.114793	0.397846	0.691703	-6.02414	0.681897	0.724211
Neutrophils	2010320M	-0.14322	2.480423	-0.39783	0.691713	-5.33844	0.735069	0.7793
Neutrophils	AC149090.	-0.13225	5.49949	-0.39778	0.691754	-5.97404	0.674473	0.716508
Neutrophils	PPP1R12A	-0.02974	8.052992	-0.39759	0.691892	-6.81872	0.627295	0.667397
Neutrophils	NCAPD3	0.073217	5.127768	0.397543	0.691926	-6.06614	0.681645	0.724019
Neutrophils	DPYSL2	-0.07794	6.279623	-0.39753	0.691936	-5.95344	0.659676	0.701169
Neutrophils	TCP11L1	-0.13284	2.689841	-0.39727	0.692123	-5.41466	0.73069	0.774892
Neutrophils	HMG20B	-0.05788	5.119833	-0.3972	0.692174	-5.93081	0.681799	0.72422
Neutrophils	MCMDC2	-0.08633	3.465667	-0.39719	0.692181	-5.97867	0.714699	0.758345
Neutrophils	MDM4	0.049956	5.749077	0.397016	0.692312	-6.09136	0.669701	0.711659
Neutrophils	PLPBP	0.08426	4.145683	0.396993	0.69233	-5.67329	0.700979	0.744145
Neutrophils	CXCL13	0.347671	-1.21101	0.396902	0.692396	-4.9951	0.816787	0.863573
Neutrophils	PWWP2B	0.145984	2.082717	0.396882	0.692411	-5.35322	0.74346	0.788116
Neutrophils	GM47096	-0.12888	1.734536	-0.39665	0.692582	-5.42504	0.750886	0.79589
Neutrophils	IFI211	0.184812	2.960201	0.396592	0.692624	-5.53802	0.725076	0.769237
Neutrophils	PPFIBP1	-0.09304	3.366596	-0.39648	0.692707	-5.65708	0.716721	0.760635
Neutrophils	HDAC11	-0.25553	0.5008	-0.39628	0.692851	-5.05252	0.777811	0.823715
Neutrophils	PIK3C3	-0.0751	4.439233	-0.39614	0.692955	-5.79717	0.695141	0.738263
Neutrophils	KLF9	0.107339	3.693914	0.396139	0.692957	-5.74897	0.710063	0.753738
Neutrophils	SLC35E4	-0.21625	0.657751	-0.39608	0.692998	-5.14908	0.774332	0.820147
Neutrophils	KLHL36	0.138961	2.076404	0.396064	0.693012	-5.43189	0.743594	0.788443
Neutrophils	GOLGA7	0.046339	5.94	0.396064	0.693012	-6.17446	0.666075	0.708066
Neutrophils	CBX2	-0.25021	-0.18673	-0.39591	0.693122	-5.00351	0.793285	0.839602
Neutrophils	RECQL4	0.229921	-0.06717	0.395727	0.69326	-5.02253	0.79066	0.83686
Neutrophils	CTPS	-0.13019	3.704287	-0.39555	0.693387	-5.41004	0.710003	0.753608
Neutrophils	IPO8	-0.08431	4.420007	-0.39547	0.693448	-5.62463	0.695669	0.738744
Neutrophils	GCSAM	0.244305	-0.31734	0.395328	0.693553	-5.0463	0.79637	0.842809
Neutrophils	SENP6	0.03309	6.681311	0.395313	0.693564	-6.4758	0.652328	0.693735
Neutrophils	PTPRJ	0.04067	8.855581	0.395177	0.693664	-6.81224	0.613355	0.653021
Neutrophils	GM12064	-0.21513	0.96957	-0.39478	0.693958	-5.08022	0.767845	0.813321
Neutrophils	COG1	-0.08561	3.677624	-0.39472	0.694001	-5.58491	0.710742	0.754331
Neutrophils	NAE1	-0.07714	3.958187	-0.39456	0.694121	-5.74111	0.705081	0.748517
Neutrophils	POU2F1	0.077692	5.39401	0.394519	0.694148	-6.04129	0.676833	0.719189
Neutrophils	MMP12	-0.33263	-0.05819	-0.3945	0.694163	-5.07958	0.790718	0.836934
Neutrophils	H2-AA	-0.19276	8.331767	-0.39402	0.694513	-6.44536	0.622804	0.662812
Neutrophils	UBE4A	0.058905	5.03345	0.394014	0.694519	-6.07133	0.683966	0.72655
Neutrophils	NCEH1	-0.08016	4.906119	-0.39401	0.69452	-6.02237	0.686449	0.72913
Neutrophils	POT1A	-0.11322	3.012767	-0.3938	0.69468	-5.46352	0.724601	0.768609
Neutrophils	ZFP747	-0.16463	1.605909	-0.39363	0.694801	-5.21091	0.754285	0.799319
Neutrophils	PLD4	-0.11685	6.186695	-0.39362	0.694813	-5.88102	0.661979	0.703658
Neutrophils	CPEB2	-0.03613	5.412476	-0.39333	0.695022	-6.59322	0.676853	0.71903

Neutrophils	TMPRSS5	0.193074	0.722493	0.393203	0.695116	-5.16631	0.773742	0.81928
Neutrophils	MRPL13	0.073345	4.834872	0.393111	0.695184	-5.8268	0.6881	0.730756
Neutrophils	IL18BP	-0.12923	3.740937	-0.3927	0.695485	-5.96318	0.709941	0.753417
Neutrophils	GM40787	-0.1521	0.205726	-0.39266	0.695513	-5.40633	0.785312	0.831238
Neutrophils	PSMC1	0.04713	5.889053	0.392534	0.695608	-6.14755	0.667821	0.709696
Neutrophils	PMEL	-0.21047	0.339511	-0.39252	0.695616	-5.15908	0.782317	0.828157
Neutrophils	FAM131A	0.267301	0.401139	0.392468	0.695657	-5.06125	0.780941	0.826741
Neutrophils	PUM1	0.033764	7.56821	0.392363	0.695734	-6.50738	0.636721	0.677285
Neutrophils	BC024063	0.233581	0.183577	0.392336	0.695754	-5.01796	0.785809	0.831749
Neutrophils	RXYLT1	-0.08947	3.864731	-0.39225	0.695818	-5.55898	0.707441	0.750839
Neutrophils	NEFH	-0.26855	-0.09246	-0.39217	0.695876	-5.02437	0.792029	0.838146
Neutrophils	ATR	0.13347	3.862209	0.392115	0.695917	-5.49603	0.707491	0.750891
Neutrophils	CCL7	-0.3168	1.659903	-0.3919	0.696076	-5.34391	0.753467	0.798375
Neutrophils	THUMPD1	-0.1018	4.425949	-0.39161	0.696286	-5.50733	0.696376	0.739317
Neutrophils	ABI3	0.141059	4.401261	0.391587	0.696305	-5.62152	0.696866	0.739825
Neutrophils	GM6710	-0.24876	0.761027	-0.39153	0.696346	-5.03119	0.773131	0.81865
Neutrophils	SLC22A18	0.184344	1.143718	0.39144	0.696414	-5.2249	0.764729	0.810025
Neutrophils	PSMF1	-0.08888	4.460205	-0.39118	0.696605	-5.65323	0.695809	0.738714
Neutrophils	ZGRF1	0.127555	3.956437	0.391105	0.696661	-5.69319	0.705868	0.749145
Neutrophils	CETN4	0.224746	-0.08195	0.390911	0.696803	-5.03136	0.792128	0.838199
Neutrophils	IGSF6	0.057289	3.689623	0.390887	0.696821	-6.54591	0.711283	0.754775
Neutrophils	TMEM251	0.063894	4.619848	0.39051	0.697099	-5.9545	0.692787	0.735494
Neutrophils	RNF121	-0.09698	4.41156	-0.39043	0.697159	-5.67283	0.696909	0.739771
Neutrophils	CEP164	0.083072	4.052145	0.390338	0.697226	-5.70212	0.704083	0.74724
Neutrophils	DCP2	-0.06445	5.103401	-0.39004	0.697446	-6.01186	0.683314	0.725788
Neutrophils	2810004N2	0.09058	4.315225	0.390029	0.697453	-5.67071	0.698825	0.741889
Neutrophils	GM14471	-0.22169	0.24931	-0.38995	0.697508	-5.06555	0.784792	0.830711
Neutrophils	ITGB7	0.112376	4.620993	0.389905	0.697544	-5.75341	0.692764	0.735622
Neutrophils	CCDC186	-0.05619	4.760025	-0.38989	0.697556	-6.06517	0.690027	0.73278
Neutrophils	MCM6	0.08161	6.155584	0.389827	0.697602	-6.21669	0.663167	0.704855
Neutrophils	GTF2F2	-0.05341	5.881638	-0.38979	0.697627	-6.07566	0.668352	0.710253
Neutrophils	GM49662	-0.19431	2.537113	-0.38973	0.697675	-5.44689	0.735168	0.779547
Neutrophils	GM16230	0.245128	0.238127	0.389602	0.697768	-5.11762	0.785044	0.831044
Neutrophils	CPT1B	-0.14955	1.536488	-0.38948	0.69786	-5.23119	0.756469	0.801656
Neutrophils	USP3	0.042333	6.873279	0.389451	0.697879	-6.46726	0.649783	0.691016
Neutrophils	MEIS1	0.124334	3.866059	0.38885	0.698322	-5.54873	0.708209	0.751558
Neutrophils	VPS37B	-0.05638	7.797142	-0.38873	0.698408	-6.70765	0.633327	0.673694
Neutrophils	KCTD14	-0.2327	1.055743	-0.38862	0.698488	-5.03362	0.767372	0.812735
Neutrophils	FOXP4	0.116269	4.962815	0.388306	0.698723	-5.58703	0.68652	0.729083
Neutrophils	RIC1	0.058856	6.710291	0.388236	0.698775	-6.4002	0.653239	0.694488
Neutrophils	N4BP2L2	0.033284	6.786437	0.388209	0.698794	-6.38441	0.651828	0.693019
Neutrophils	NPL	-0.13951	2.535175	-0.38771	0.699163	-5.575	0.735708	0.780255
Neutrophils	ITGAM	0.049854	4.619395	0.387655	0.699203	-6.77984	0.693266	0.736292
Neutrophils	2010300C0	-0.22767	-0.0882	-0.38753	0.699298	-5.01895	0.792932	0.839256
Neutrophils	HELB	-0.10334	3.704754	-0.38751	0.699311	-5.58267	0.711571	0.755278
Neutrophils	PDE6D	-0.06821	3.812865	-0.38751	0.699313	-5.74688	0.709382	0.753009
Neutrophils	NXT1	0.075721	4.399059	0.387447	0.699357	-5.86714	0.697631	0.740822

Neutrophils	IGLC2	-0.35624	3.740399	-0.38744	0.69936	-5.48473	0.710849	0.754529
Neutrophils	GHDC	0.138668	1.628326	0.387391	0.699398	-5.32118	0.754999	0.800184
Neutrophils	PIF1	-0.21512	1.716548	-0.38733	0.699442	-5.24818	0.7531	0.798224
Neutrophils	THOC2	-0.03225	7.106814	-0.38731	0.699455	-6.49475	0.645926	0.68703
Neutrophils	MCTS2	-0.19789	1.612847	-0.38725	0.699501	-5.22092	0.755333	0.800537
Neutrophils	USP24	-0.06882	5.459806	-0.38696	0.699718	-5.86954	0.677022	0.719329
Neutrophils	FUNDC1	0.07471	4.65588	0.386794	0.699838	-5.76814	0.692714	0.735625
Neutrophils	FBXO48	0.184591	0.24175	0.386741	0.699877	-5.1515	0.785685	0.831697
Neutrophils	NOL7	-0.0381	6.906907	-0.38664	0.699953	-6.34452	0.64976	0.690972
Neutrophils	ANKRD49	0.110706	3.032797	0.386562	0.700009	-5.42945	0.725513	0.769663
Neutrophils	GT(ROSA)2	-0.07815	4.594685	-0.38621	0.700268	-5.87955	0.693971	0.736924
Neutrophils	ZFP664	-0.10062	4.050718	-0.38619	0.700283	-5.50224	0.704809	0.748167
Neutrophils	ZFP523	-0.07942	2.19531	-0.3861	0.700351	-5.75245	0.74311	0.787803
Neutrophils	FAM169B	0.091503	3.62896	0.386043	0.700392	-5.86788	0.713333	0.757006
Neutrophils	SNX19	0.111553	2.862011	0.385972	0.700445	-5.35851	0.729107	0.773334
Neutrophils	1810055G(	-0.06815	2.268587	-0.38593	0.700476	-5.86676	0.741558	0.786205
Neutrophils	RBBP5	-0.10848	3.457865	-0.38582	0.700559	-5.48181	0.716821	0.760619
Neutrophils	PDCD6IP	0.027672	6.988379	0.385763	0.700599	-6.51911	0.648303	0.689427
Neutrophils	HARBI1	-0.19595	1.346342	-0.38567	0.700667	-5.17992	0.76134	0.806675
Neutrophils	RNF26	-0.09251	3.515898	-0.38546	0.700823	-5.71125	0.715679	0.759506
Neutrophils	RTL6	0.264098	0.292529	0.385371	0.700888	-5.01186	0.784648	0.830706
Neutrophils	USP49	-0.07439	4.534917	-0.38527	0.700963	-6.02534	0.695195	0.738267
Neutrophils	SFT2D2	0.060749	4.677531	0.385179	0.70103	-6.06944	0.692377	0.735376
Neutrophils	FAM173A	-0.0638	4.68182	-0.38516	0.701047	-5.93258	0.692293	0.735288
Neutrophils	NCAPD2	-0.1005	4.81058	-0.38469	0.701394	-5.93967	0.690029	0.732778
Neutrophils	NGRN	0.122809	3.173648	0.384436	0.701578	-5.44292	0.722981	0.766941
Neutrophils	GM20324	-0.12277	2.098382	-0.38428	0.701693	-5.35928	0.745505	0.790226
Neutrophils	NEURL3	0.062834	4.301516	0.384212	0.701744	-6.14574	0.700106	0.743242
Neutrophils	CHD9	0.073269	6.065045	0.384209	0.701746	-6.02447	0.665835	0.707633
Neutrophils	CCNB2	-0.10089	5.882225	-0.38415	0.701787	-6.3228	0.669305	0.711244
Neutrophils	JAZF1	0.112301	3.086872	0.384067	0.701851	-5.59009	0.724773	0.768795
Neutrophils	UQCC2	-0.06522	6.141014	-0.38403	0.701881	-6.10772	0.664399	0.706138
Neutrophils	MCTS1	0.042461	6.207891	0.383959	0.701931	-6.27893	0.663137	0.704838
Neutrophils	PAQR8	0.24444	0.223677	0.383796	0.702051	-5.07166	0.786559	0.832528
Neutrophils	GNG3	0.205785	1.449153	0.383672	0.702143	-5.13158	0.759508	0.80469
Neutrophils	ANK2	-0.16194	2.998552	-0.38361	0.702186	-5.75816	0.726655	0.770756
Neutrophils	CD99L2	0.135551	2.142314	0.383305	0.702414	-5.2861	0.744754	0.789393
Neutrophils	GM527	-0.23593	0.481195	-0.38326	0.702445	-5.11529	0.780929	0.826685
Neutrophils	GP9	-0.25347	0.659096	-0.38315	0.702528	-5.20854	0.776971	0.82266
Neutrophils	GM13963	0.257795	-0.29578	0.383011	0.702631	-5.0763	0.798454	0.844803
Neutrophils	MAFB	0.101426	4.60884	0.382962	0.702667	-6.04322	0.694175	0.737143
Neutrophils	CCDC107	0.093213	4.620705	0.3829	0.702713	-5.59089	0.693941	0.736933
Neutrophils	CHEK1	-0.13781	2.722134	-0.3825	0.703008	-5.39986	0.73277	0.777068
Neutrophils	2410004B1	0.060018	4.71052	0.38225	0.703193	-5.84965	0.692409	0.735358
Neutrophils	TBC1D9B	-0.06197	4.555284	-0.38212	0.703288	-5.88952	0.695477	0.738616
Neutrophils	NUDT9	-0.09886	4.362001	-0.38192	0.703435	-5.68028	0.699317	0.742662
Neutrophils	HOMER3	-0.10446	3.103095	-0.3819	0.703452	-5.43784	0.724868	0.769145

Neutrophils	ALDH8A1	-0.16577	2.197617	-0.3817	0.703603	-5.45296	0.743839	0.788853
Neutrophils	TASOR	0.058972	5.742445	0.381652	0.703635	-6.11395	0.67237	0.714756
Neutrophils	ATF2	0.042442	6.46092	0.381591	0.70368	-6.40797	0.658777	0.7006
Neutrophils	GAPT	-0.09086	1.236779	-0.38158	0.70369	-5.82521	0.764525	0.810217
Neutrophils	4932438H2	0.254637	-0.27879	0.381575	0.703692	-5.07655	0.798344	0.845039
Neutrophils	COG2	-0.09179	3.567744	-0.38146	0.70378	-5.59579	0.715327	0.759381
Neutrophils	MGRN1	0.062426	5.682428	0.381441	0.703791	-6.12157	0.673519	0.71597
Neutrophils	1700008J0	-0.17249	1.842472	-0.38138	0.703838	-5.25676	0.751417	0.796727
Neutrophils	PF4	-0.23701	4.278945	-0.38127	0.70392	-5.87606	0.700988	0.744543
Neutrophils	MYL6	-0.03113	9.689609	-0.38074	0.704311	-7.0595	0.601463	0.640528
Neutrophils	CABP1	0.257739	-0.13612	0.380599	0.704414	-5.0212	0.795518	0.841871
Neutrophils	UBA2	0.042779	6.457603	0.379937	0.704904	-6.27894	0.659583	0.700911
Neutrophils	G2E3	0.076942	3.938116	0.379534	0.705201	-5.73488	0.708821	0.751838
Neutrophils	PQLC1	0.043544	4.395337	0.379351	0.705337	-6.18902	0.699646	0.742331
Neutrophils	CSTA3	-0.07087	0.791872	-0.37932	0.705362	-6.42443	0.775401	0.820582
Neutrophils	OSCP1	-0.05993	3.25254	-0.37929	0.705383	-6.34337	0.722811	0.76632
Neutrophils	PBX4	0.220085	0.976518	0.379199	0.705449	-5.08912	0.771323	0.816384
Neutrophils	MED13L	0.035514	7.196724	0.379058	0.705554	-6.60447	0.6461	0.686654
Neutrophils	MROH1	0.081552	4.199162	0.378975	0.705615	-5.78674	0.703597	0.746429
Neutrophils	TSPAN17	-0.15681	1.115501	-0.37882	0.70573	-5.23521	0.7683	0.813334
Neutrophils	NUP160	-0.08055	5.017011	-0.37879	0.70575	-5.86864	0.687398	0.729687
Neutrophils	CDK6	0.074523	6.322112	0.378473	0.705986	-6.30099	0.662507	0.703728
Neutrophils	OLFR164	-0.24294	-0.16043	-0.37816	0.706222	-5.01939	0.797194	0.842847
Neutrophils	HABP4	-0.11923	3.054352	-0.37776	0.706511	-5.37774	0.727513	0.770841
Neutrophils	METTL23	0.047257	5.675126	0.377475	0.706725	-6.0925	0.675214	0.716671
Neutrophils	XDH	-0.05766	4.807401	-0.37736	0.706809	-6.67725	0.692095	0.734216
Neutrophils	SERP2	-0.20835	0.429349	-0.37733	0.706831	-5.03571	0.784165	0.829282
Neutrophils	ALYREF2	-0.11866	3.243584	-0.37732	0.706844	-5.38694	0.723636	0.766883
Neutrophils	ZFP354B	-0.19098	-0.81695	-0.37722	0.70691	-4.99801	0.812582	0.858503
Neutrophils	ARHGAP23	0.065464	3.694446	0.377163	0.706956	-6.05966	0.714393	0.757368
Neutrophils	AGRP	-0.21234	1.935634	-0.37699	0.707083	-5.19	0.751214	0.795391
Neutrophils	AP3B1	-0.03278	7.329007	-0.37687	0.707172	-6.55293	0.644294	0.684544
Neutrophils	COL1A2	-0.15081	2.442649	-0.37665	0.707335	-5.4974	0.740465	0.784336
Neutrophils	MRGBP	0.107458	3.37422	0.376511	0.707439	-5.46144	0.721046	0.76428
Neutrophils	METTL8	-0.16782	2.52734	-0.37629	0.7076	-5.20555	0.738677	0.782516
Neutrophils	PPIA	-0.04352	10.92524	-0.37613	0.70772	-7.03918	0.581991	0.619512
Neutrophils	VPREB1	-0.26458	2.621584	-0.3761	0.707742	-5.33074	0.736693	0.780547
Neutrophils	ST14	-0.25244	1.167666	-0.3759	0.707892	-5.09453	0.767911	0.812758
Neutrophils	NDUFAF1	-0.12131	2.81597	-0.37584	0.707935	-5.37384	0.732619	0.776363
Neutrophils	PIMREG	-0.17471	2.104196	-0.37579	0.707971	-5.4049	0.747651	0.791894
Neutrophils	SETDB2	0.07706	4.580351	0.375731	0.708017	-5.78388	0.696683	0.739184
Neutrophils	FAM129B	-0.1743	3.14002	-0.37572	0.708024	-5.37405	0.725878	0.769417
Neutrophils	VAMP5	0.053173	4.717071	0.375568	0.708138	-6.33329	0.693975	0.736423
Neutrophils	ATG16L2	0.040699	5.680082	0.375439	0.708233	-6.41675	0.675214	0.716942
Neutrophils	KLF5	0.090331	0.36413	0.375269	0.708359	-5.91908	0.785737	0.83119
Neutrophils	POLQ	0.116769	3.166551	0.37526	0.708366	-5.50596	0.725329	0.768898
Neutrophils	MEX3C	-0.05232	5.133199	-0.37514	0.708457	-6.05936	0.685802	0.727941

Neutrophils	INTS10	0.092306	3.410048	0.375119	0.708471	-5.59072	0.72031	0.763706
Neutrophils	FFAR1	-0.21169	-0.22509	-0.37508	0.708498	-5.00799	0.799073	0.844894
Neutrophils	MSRA	-0.02674	6.629496	-0.375	0.708556	-6.85911	0.657236	0.698239
Neutrophils	CCL22	-0.36294	-0.37257	-0.37499	0.708567	-5.04345	0.802446	0.848358
Neutrophils	GM47167	0.10107	2.927116	0.374913	0.708623	-5.54567	0.7303	0.774038
Neutrophils	SPATA24	-0.1923	2.193104	-0.37491	0.708627	-5.1802	0.745757	0.790003
Neutrophils	TAF5L	-0.08244	4.562119	-0.37479	0.708716	-5.64475	0.697045	0.739615
Neutrophils	NAPEPLD	-0.20659	1.056502	-0.37474	0.708753	-5.11222	0.770353	0.815369
Neutrophils	FHOD3	-0.2342	-0.11029	-0.37451	0.708925	-5.11879	0.796477	0.842224
Neutrophils	ALDH3A2	0.058137	3.596521	0.374479	0.708945	-5.94294	0.716508	0.759769
Neutrophils	WDR82	0.052975	4.82833	0.374193	0.709156	-5.89244	0.691797	0.734204
Neutrophils	PAPSS1	-0.07268	4.181725	-0.37402	0.709288	-5.71472	0.704657	0.747569
Neutrophils	GNPNAT1	0.078455	4.157449	0.374016	0.709288	-5.7297	0.705144	0.748074
Neutrophils	FIBP	0.064023	4.462532	0.373958	0.709331	-5.83115	0.699042	0.741748
Neutrophils	CARD10	-0.09616	1.098314	-0.37386	0.709403	-5.64147	0.769452	0.814533
Neutrophils	RAMP3	0.225885	0.947281	0.373831	0.709425	-5.15395	0.772778	0.817974
Neutrophils	PPP1R11	0.065114	4.640461	0.373702	0.709521	-5.97023	0.695508	0.738176
Neutrophils	CENPA	-0.07519	6.681539	-0.3736	0.709599	-6.38077	0.656281	0.697439
Neutrophils	KLC1	-0.06658	4.702224	-0.37353	0.709649	-5.7689	0.694286	0.736981
Neutrophils	4930403D(	-0.19209	1.041064	-0.3735	0.709674	-5.14652	0.770711	0.815993
Neutrophils	EID2B	0.140323	1.703903	0.373477	0.709687	-5.21125	0.756262	0.801106
Neutrophils	LAD1	0.304008	-0.99444	0.373432	0.709721	-5.02041	0.816847	0.863439
Neutrophils	ARL6IP4	0.054774	5.733017	0.373185	0.709904	-6.08022	0.674324	0.716237
Neutrophils	UBR1	0.063142	5.323458	0.372913	0.710105	-6.05454	0.682353	0.724496
Neutrophils	ZFP653	-0.12282	3.131364	-0.37282	0.710172	-5.36037	0.726328	0.770078
Neutrophils	CBX3	-0.04143	8.102288	-0.37245	0.71045	-6.66472	0.630759	0.670656
Neutrophils	PDE8A	0.056385	5.847934	0.372232	0.710611	-6.4177	0.672523	0.714118
Neutrophils	RPAIN	-0.08678	3.771433	-0.37208	0.710727	-5.57056	0.713484	0.756632
Neutrophils	GM21860	-0.31684	-0.14186	-0.37207	0.710734	-5.03039	0.797798	0.843578
Neutrophils	TUSC1	0.088148	3.124075	0.371754	0.710965	-5.64345	0.726852	0.770416
Neutrophils	DOHH	0.093578	4.347216	0.371746	0.710971	-5.58369	0.701946	0.744632
Neutrophils	CCDC141	-0.17937	1.120838	-0.37165	0.711044	-5.20365	0.76962	0.814546
Neutrophils	9430091E2	0.135437	2.439837	0.371599	0.71108	-5.31218	0.74118	0.78522
Neutrophils	KCTD12	-0.03725	6.383969	-0.37132	0.711288	-6.64597	0.662519	0.703668
Neutrophils	SLC25A30	0.082341	3.550303	0.371277	0.711319	-5.67028	0.718176	0.761433
Neutrophils	OLFR1259	-0.26544	-1.34501	-0.37111	0.711442	-5.02725	0.825962	0.872361
Neutrophils	SLC38A1	0.052893	7.937408	0.370453	0.711931	-6.50134	0.634096	0.673999
Neutrophils	HBEGF	-0.13268	2.789221	-0.37038	0.711984	-5.70407	0.734111	0.777848
Neutrophils	EBNA1BP2	-0.08946	4.017915	-0.37032	0.712033	-5.66675	0.708837	0.751702
Neutrophils	ALMS1	-0.10676	3.824261	-0.37032	0.712033	-5.56231	0.71276	0.755765
Neutrophils	ZFP758	0.112155	2.74078	0.370263	0.712072	-5.41653	0.735127	0.778896
Neutrophils	GMCL1	-0.06619	4.636774	-0.37026	0.712076	-5.7713	0.696449	0.738864
Neutrophils	ARID3A	0.043966	5.513727	0.370193	0.712124	-6.37808	0.67928	0.721044
Neutrophils	MAN2C1	-0.09591	3.509599	-0.37018	0.712131	-5.48725	0.719182	0.762412
Neutrophils	CALHM6	-0.28053	3.048969	-0.3701	0.712194	-5.27181	0.728692	0.772247
Neutrophils	TES	-0.03812	5.486918	-0.36989	0.712346	-6.60682	0.679798	0.72161
Neutrophils	IGTP	0.170858	3.443365	0.369845	0.712382	-5.58512	0.720542	0.763854

Neutrophils	RBKS	-0.05563	3.630745	-0.36975	0.712452	-6.14709	0.716703	0.759887
Neutrophils	DVL1	-0.12152	2.507773	-0.3696	0.712561	-5.42536	0.74003	0.784003
Neutrophils	NKAIN2	0.205188	1.350359	0.369593	0.712569	-5.25921	0.764888	0.809641
Neutrophils	NUDC	0.057454	5.698731	0.36957	0.712586	-5.98674	0.675714	0.717378
Neutrophils	GAK	-0.0331	6.280821	-0.36944	0.712682	-6.42516	0.664624	0.705883
Neutrophils	PISD	-0.08655	4.8584	-0.3694	0.712714	-5.74903	0.692068	0.734398
Neutrophils	ANKMY2	-0.10604	3.602393	-0.36896	0.713039	-5.38046	0.717393	0.760596
Neutrophils	SYNE2	0.055386	5.373755	0.368732	0.713209	-6.32673	0.682095	0.724002
Neutrophils	IZUMO4	0.192513	1.453448	0.368691	0.713239	-5.23428	0.762757	0.807441
Neutrophils	HNRNPC	-0.02593	7.730209	-0.36869	0.713241	-6.6118	0.63793	0.678031
Neutrophils	TVP23B	0.079523	4.042523	0.368662	0.713261	-5.64167	0.708449	0.751336
Neutrophils	ANKRD13C	-0.04236	5.868682	-0.36859	0.713313	-6.3877	0.67256	0.714118
Neutrophils	JMJD8	0.192194	1.564022	0.368568	0.713331	-5.15864	0.760353	0.804991
Neutrophils	MRPS25	0.087597	4.238863	0.368519	0.713367	-5.63444	0.704496	0.747278
Neutrophils	MIS18A	0.078375	4.400206	0.368351	0.713492	-5.84909	0.701321	0.744014
Neutrophils	SLFN8	0.174244	3.326481	0.367937	0.7138	-5.43492	0.723163	0.766683
Neutrophils	PAPLN	-0.15979	0.894565	-0.3678	0.713901	-5.27447	0.775139	0.820356
Neutrophils	GM20300	-0.1648	1.343674	-0.36773	0.713951	-5.13181	0.765262	0.810225
Neutrophils	HSPB11	-0.14079	2.925359	-0.36764	0.71402	-5.31282	0.731484	0.775372
Neutrophils	PRRC2C	-0.03178	7.744202	-0.36758	0.714068	-6.59627	0.637768	0.678039
Neutrophils	MCM7	0.091652	5.384269	0.367536	0.714098	-6.04871	0.681989	0.724083
Neutrophils	PLLP	0.243567	-0.20457	0.367503	0.714122	-5.03875	0.799859	0.84583
Neutrophils	MFSD11	-0.0906	3.854162	-0.36739	0.714209	-5.67012	0.712365	0.755606
Neutrophils	PFDN4	0.069962	5.046958	0.367377	0.714216	-5.89532	0.688568	0.730929
Neutrophils	GRB2	0.035196	7.898248	0.367332	0.714249	-6.65993	0.634989	0.675169
Neutrophils	KNL1	0.102238	5.131736	0.367282	0.714286	-6.13563	0.686908	0.72924
Neutrophils	TRIM34A	0.131254	3.308515	0.367031	0.714473	-5.55093	0.723654	0.767268
Neutrophils	4930505N2	-0.17996	1.214516	-0.36679	0.714655	-5.14436	0.768339	0.813346
Neutrophils	SLC25A4	0.068565	7.14787	0.366294	0.715021	-6.16282	0.648876	0.689624
Neutrophils	CC2D1B	-0.08412	3.659138	-0.36629	0.715027	-5.58448	0.716586	0.759961
Neutrophils	SNRPD1	-0.04721	7.17842	-0.36628	0.71503	-6.40989	0.648314	0.689038
Neutrophils	TTC1	0.049996	4.740195	0.366269	0.715039	-5.89354	0.69485	0.737434
Neutrophils	HIST1H2BH	0.214101	0.17295	0.366262	0.715044	-5.09261	0.791557	0.837298
Neutrophils	PDS5A	0.031572	7.411745	0.366034	0.715214	-6.57851	0.644036	0.684599
Neutrophils	SQSTM1	0.045226	7.466775	0.366011	0.715231	-6.59357	0.643031	0.683552
Neutrophils	TMEM63A	0.082307	3.49863	0.365985	0.71525	-5.85032	0.719873	0.763388
Neutrophils	TIMP2	0.059626	4.873204	0.365852	0.715349	-6.50611	0.692224	0.734752
Neutrophils	EFCAB9	-0.17444	0.443216	-0.36582	0.715371	-5.09528	0.78547	0.831089
Neutrophils	4931413K1	0.129111	1.943113	0.365757	0.71542	-5.3469	0.752539	0.797183
Neutrophils	HSBP1	0.049292	6.003464	0.365495	0.715615	-6.25942	0.670437	0.712082
Neutrophils	ADGRE1	0.090465	4.711233	0.365362	0.715714	-6.27954	0.695577	0.738177
Neutrophils	E230016M	0.095177	2.465953	0.365153	0.715869	-5.71253	0.741576	0.785816
Neutrophils	RUSC1	0.108842	3.288288	0.3651	0.715908	-5.52571	0.724382	0.768043
Neutrophils	VPS13A	-0.04719	6.447058	-0.36506	0.715937	-6.16242	0.662083	0.703403
Neutrophils	XYLT2	0.145911	1.972195	0.364951	0.71602	-5.21731	0.752116	0.796693
Neutrophils	A230072C0	0.163785	1.545767	0.36443	0.716407	-5.19008	0.761586	0.806305
Neutrophils	SLIRP	-0.0638	5.558884	-0.36438	0.716444	-6.04649	0.679258	0.721143

Neutrophils	MED22	0.138189	2.384243	0.364351	0.716466	-5.31221	0.743574	0.787767
Neutrophils	FAM71D	-0.22021	0.095273	-0.3642	0.716578	-5.09522	0.793797	0.839543
Neutrophils	CHUK	0.05712	5.037427	0.364142	0.716621	-5.97563	0.689414	0.731747
Neutrophils	NETO2	-0.08994	3.22493	-0.36398	0.716739	-5.85616	0.725955	0.769644
Neutrophils	CTDNEP1	-0.05921	5.516749	-0.36398	0.71674	-6.05074	0.680074	0.72208
Neutrophils	GM10603	-0.23403	0.220408	-0.36387	0.716826	-5.05757	0.790984	0.836689
Neutrophils	RGS3	0.092017	2.862627	0.363337	0.71722	-5.85066	0.733848	0.777523
Neutrophils	GTF2A1	-0.05029	5.65525	-0.36314	0.717371	-6.22628	0.677803	0.719407
Neutrophils	MRPL22	-0.09693	3.858759	-0.36304	0.717439	-5.57941	0.713378	0.756306
Neutrophils	GSTM2	-0.14292	1.633616	-0.36282	0.717602	-5.39788	0.760232	0.804638
Neutrophils	1300002E1	-0.13549	3.19234	-0.36237	0.717943	-5.30662	0.72733	0.770589
Neutrophils	ADAT1	0.172253	2.003267	0.362323	0.717975	-5.18669	0.75243	0.796502
Neutrophils	SHCBP1	-0.09386	4.69138	-0.36225	0.71803	-6.03117	0.696912	0.739101
Neutrophils	MIER3	-0.06418	4.078409	-0.36207	0.71816	-5.90015	0.709188	0.751881
Neutrophils	ATP8A1	-0.03559	7.601268	-0.36195	0.718256	-6.66397	0.64159	0.681654
Neutrophils	TRPM7	0.038273	7.284405	0.361935	0.718264	-6.52198	0.647384	0.687693
Neutrophils	ACOT2	0.077578	4.416307	0.361894	0.718295	-5.63349	0.702393	0.74486
Neutrophils	2610507I0	0.161572	0.583661	0.361864	0.718317	-5.33187	0.783556	0.828642
Neutrophils	B3GNTL1	0.130498	2.673331	0.361709	0.718433	-5.36856	0.7382	0.781916
Neutrophils	ADD1	-0.04294	5.795836	-0.36165	0.718474	-6.29652	0.675367	0.716843
Neutrophils	MUTYH	0.180415	0.370874	0.361476	0.718606	-5.10745	0.78836	0.833637
Neutrophils	SAMD8	0.043001	5.289431	0.361465	0.718614	-6.34024	0.685171	0.727052
Neutrophils	PTK6	0.23539	-0.3416	0.361351	0.718699	-5.15356	0.804587	0.850324
Neutrophils	UAP1L1	-0.09107	3.734573	-0.36091	0.719027	-5.58264	0.716472	0.759402
Neutrophils	RSL1	-0.22349	1.039109	-0.36076	0.719143	-5.08207	0.773806	0.818567
Neutrophils	CLTB	-0.0974	4.396264	-0.3605	0.719335	-5.55894	0.703256	0.745683
Neutrophils	EIF3D	-0.05781	5.480464	-0.36033	0.719462	-5.9786	0.68189	0.723539
Neutrophils	ETV1	-0.16164	1.377482	-0.3603	0.71948	-5.26897	0.766506	0.811044
Neutrophils	DIP2A	0.11876	2.079809	0.35991	0.719773	-5.38634	0.751403	0.79542
Neutrophils	PPP2R2A	-0.0296	7.180276	-0.35991	0.719773	-6.50488	0.649831	0.690153
Neutrophils	SELENOK	-0.03174	7.584969	-0.35985	0.719817	-6.71307	0.642413	0.682438
Neutrophils	SLC16A9	-0.23062	0.530927	-0.35976	0.719882	-5.20551	0.78538	0.830428
Neutrophils	C430049B0	0.123968	1.844436	0.359702	0.719928	-5.46774	0.756468	0.80069
Neutrophils	METTL9	0.033191	5.718204	0.359549	0.720042	-6.60307	0.677437	0.718894
Neutrophils	ODR4	-0.0743	4.042451	-0.35933	0.720205	-5.73445	0.710635	0.753288
Neutrophils	ZBTB10	-0.1206	4.077959	-0.3592	0.720299	-5.49862	0.709941	0.752561
Neutrophils	C1QTNF12	0.09316	2.21932	0.358964	0.720478	-5.67543	0.748658	0.792591
Neutrophils	CCT8	0.043215	6.762623	0.358933	0.720501	-6.29561	0.657795	0.698445
Neutrophils	ELOA	0.046541	5.789735	0.358121	0.721106	-6.23469	0.676544	0.717719
Neutrophils	CMPK1	-0.02878	6.881961	-0.35806	0.721152	-6.54537	0.655868	0.696223
Neutrophils	DCN	-0.12775	3.138043	-0.35782	0.721333	-5.68189	0.729621	0.772699
Neutrophils	MIR155HG	-0.22402	4.086438	-0.35779	0.721357	-5.33341	0.710158	0.752572
Neutrophils	CCDC137	0.126869	2.673176	0.357755	0.721379	-5.25889	0.739361	0.782756
Neutrophils	THAP4	0.052095	4.091332	0.357746	0.721386	-6.02972	0.710059	0.752469
Neutrophils	MAP2K6	-0.12904	3.533157	-0.35773	0.7214	-5.45967	0.721447	0.76425
Neutrophils	SLC12A4	0.172624	1.745624	0.357704	0.721417	-5.17413	0.759195	0.803216
Neutrophils	MID1IP1	-0.05341	3.917342	-0.35741	0.721637	-6.19044	0.713589	0.756169

Neutrophils	CENPU	-0.09039	2.562499	-0.35732	0.721704	-5.63167	0.7417	0.785234
Neutrophils	GM37494	-0.07991	3.825168	-0.35725	0.721752	-5.70007	0.715466	0.758151
Neutrophils	AGO4	-0.09327	3.167589	-0.35722	0.721779	-5.6494	0.729007	0.772162
Neutrophils	GM28694	-0.22567	-0.23311	-0.35716	0.721823	-5.03385	0.803332	0.848706
Neutrophils	CABLES2	0.066498	3.580958	0.357095	0.721871	-5.81199	0.720464	0.763342
Neutrophils	BAHD1	-0.11795	2.543391	-0.35705	0.721906	-5.3585	0.742104	0.785699
Neutrophils	SLC7A2	-0.21013	2.094729	-0.35691	0.722009	-5.36967	0.751667	0.795563
Neutrophils	GABPB1	-0.04289	5.380129	-0.35686	0.722047	-6.25062	0.684473	0.726054
Neutrophils	POLE2	-0.14062	3.014685	-0.35675	0.72213	-5.37624	0.732193	0.775465
Neutrophils	MALT1	0.067524	7.764534	0.356716	0.722155	-6.82034	0.639647	0.679424
Neutrophils	HRH1	-0.20213	-0.56863	-0.35666	0.722194	-5.01213	0.811068	0.856641
Neutrophils	GM13012	0.12862	2.775114	0.356359	0.72242	-5.31417	0.737375	0.780699
Neutrophils	CCNK	0.056273	4.857081	0.35623	0.722517	-5.93024	0.694918	0.736784
Neutrophils	1500009L1	-0.20194	-0.02505	-0.35582	0.722822	-5.02414	0.79904	0.844035
Neutrophils	SNX15	0.073463	4.880351	0.355725	0.722894	-5.76001	0.694689	0.736414
Neutrophils	EIF4E2	-0.04422	5.989442	-0.35541	0.723128	-6.21669	0.673265	0.714104
Neutrophils	RACGAP1	-0.09559	5.205247	-0.3551	0.72336	-6.05103	0.688505	0.729933
Neutrophils	AARS	0.076813	5.020707	0.355084	0.723372	-5.77245	0.692131	0.733692
Neutrophils	NUP88	-0.07176	4.421593	-0.35506	0.723388	-5.74994	0.70404	0.746032
Neutrophils	MED14	0.052021	6.321429	0.354167	0.724057	-6.35369	0.667512	0.707711
Neutrophils	PXDC1	-0.17345	2.191629	-0.35412	0.724089	-5.36421	0.750848	0.793913
Neutrophils	SNRPD3	0.042757	6.518599	0.353898	0.724258	-6.30874	0.663842	0.703836
Neutrophils	GM12689	-0.16645	-0.48996	-0.35366	0.724439	-5.20151	0.810677	0.855379
Neutrophils	TAF8	-0.10526	3.254648	-0.35348	0.72457	-5.46688	0.728484	0.770896
Neutrophils	PIGX	-0.04172	5.308726	-0.35339	0.724639	-6.28101	0.687077	0.728057
Neutrophils	DDX20	-0.08651	3.596417	-0.35339	0.72464	-5.49827	0.721419	0.7636
Neutrophils	FHDC1	0.180187	0.748905	0.353368	0.724654	-5.20436	0.782493	0.826516
Neutrophils	KLHL24	-0.04302	6.228518	-0.35329	0.724709	-6.37937	0.669337	0.709682
Neutrophils	ARHGAP32	0.0925	3.193867	0.353212	0.72477	-5.62247	0.729747	0.772257
Neutrophils	GM11772	0.189506	0.302935	0.353116	0.724842	-5.08439	0.792523	0.836927
Neutrophils	SUV39H2	-0.13419	2.381659	-0.35307	0.724878	-5.29212	0.746854	0.789973
Neutrophils	TMCC1	0.037211	7.946033	0.353031	0.724905	-6.97747	0.637488	0.676646
Neutrophils	RRNAD1	0.093066	3.384404	0.352834	0.725053	-5.44589	0.725803	0.768324
Neutrophils	BZW1	0.0273	7.459579	0.352791	0.725085	-6.50571	0.646352	0.685925
Neutrophils	CNOT4	-0.03567	7.523005	-0.35274	0.72512	-6.48677	0.64519	0.684715
Neutrophils	QTRT2	-0.23199	1.344368	-0.35248	0.72532	-5.06175	0.769383	0.813287
Neutrophils	MANBA	-0.06669	4.766674	-0.35247	0.725326	-5.8715	0.697837	0.739429
Neutrophils	GINS2	-0.13276	3.493231	-0.35214	0.72557	-5.48036	0.723774	0.766233
Neutrophils	MMGT1	-0.0987	2.857236	-0.35201	0.72567	-5.54447	0.737022	0.779942
Neutrophils	GM20033	0.181536	0.595708	0.351991	0.725682	-5.21728	0.786173	0.830546
Neutrophils	NRM	0.080031	5.099836	0.35182	0.72581	-5.93593	0.691449	0.732821
Neutrophils	PRKCI	-0.09664	3.113961	-0.35173	0.725879	-5.59366	0.731704	0.774485
Neutrophils	OSBPL1A	0.104431	3.288159	0.351167	0.726299	-5.48997	0.728378	0.770961
Neutrophils	STXBP6	0.180168	2.953526	0.351145	0.726315	-5.43801	0.735363	0.778173
Neutrophils	KIF20A	-0.11736	3.768581	-0.35102	0.726406	-5.82075	0.718492	0.760758
Neutrophils	LMBRD2	-0.08902	4.481892	-0.3508	0.726573	-5.70006	0.704131	0.745868
Neutrophils	VAMP1	-0.07518	3.997211	-0.35056	0.726754	-5.72964	0.713982	0.756108

Neutrophils	IL1RL2	-0.19729	0.726101	-0.35054	0.72677	-5.17687	0.783834	0.828111
Neutrophils	RGS2	-0.04066	6.977171	-0.35036	0.726899	-6.64263	0.656003	0.695987
Neutrophils	DNAJC11	-0.08543	4.486406	-0.3502	0.727024	-5.57795	0.704212	0.746004
Neutrophils	RPGRIP1	0.048698	6.55945	0.349808	0.727314	-6.21011	0.664064	0.704303
Neutrophils	RAB11FIP4	0.114851	1.161785	0.349748	0.727359	-5.43521	0.774478	0.818445
Neutrophils	THAP7	-0.1053	3.607354	-0.34939	0.727628	-5.51041	0.722275	0.764632
Neutrophils	TUT4	0.048782	7.475638	0.349377	0.727637	-6.45251	0.647017	0.686571
Neutrophils	SAPCD2	-0.16864	1.790194	-0.34937	0.727646	-5.21434	0.760708	0.804275
Neutrophils	RMDN1	0.05258	4.528231	0.349167	0.727794	-6.19147	0.703568	0.745284
Neutrophils	ELP3	-0.11622	2.716115	-0.34916	0.727802	-5.3455	0.74087	0.783831
Neutrophils	KIF21B	-0.05224	4.865707	-0.34908	0.727857	-6.12525	0.696839	0.738315
Neutrophils	TXNRD3	0.163697	1.950944	0.349005	0.727915	-5.1487	0.757226	0.800689
Neutrophils	SCML4	0.09708	4.659948	0.348908	0.727988	-5.69915	0.700934	0.742556
Neutrophils	SACS	0.16357	2.596254	0.348783	0.728081	-5.30893	0.743408	0.786473
Neutrophils	2210016F1	0.062666	4.954824	0.348773	0.728089	-5.80383	0.695073	0.736508
Neutrophils	WASHC2	0.04466	5.655214	0.348738	0.728115	-6.38645	0.681357	0.722286
Neutrophils	ACO2	0.041508	6.116578	0.348608	0.728213	-6.16074	0.672504	0.713096
Neutrophils	TUBGCP6	-0.12074	2.759296	-0.34849	0.728303	-5.39162	0.74001	0.782987
Neutrophils	RAB23	-0.15414	1.279801	-0.34797	0.728692	-5.24287	0.772269	0.816001
Neutrophils	CARD9	-0.09714	0.997223	-0.34779	0.728828	-5.69581	0.778572	0.822489
Neutrophils	HIST1H4C	-0.21509	0.309171	-0.34773	0.728872	-5.06435	0.794023	0.838356
Neutrophils	SLC4A8	-0.24545	0.467759	-0.34753	0.729022	-5.04382	0.790522	0.834718
Neutrophils	TUFM	-0.08257	4.731912	-0.34733	0.72917	-5.7177	0.700048	0.741462
Neutrophils	RCOR2	-0.24159	0.194015	-0.34724	0.729238	-5.01988	0.79681	0.841225
Neutrophils	TFB1M	0.165578	1.706255	0.347002	0.729415	-5.13992	0.763133	0.806655
Neutrophils	MRPL58	0.064175	5.199269	0.346971	0.729438	-5.9366	0.690796	0.731944
Neutrophils	PHYH	0.069912	5.40217	0.346888	0.7295	-6.28041	0.686819	0.727821
Neutrophils	ZFP398	-0.1045	4.517006	-0.34688	0.729509	-5.57117	0.704346	0.745979
Neutrophils	MTURN	-0.1354	2.270409	-0.34658	0.729727	-5.35631	0.751096	0.794149
Neutrophils	APOL7C	-0.28776	-0.99321	-0.34648	0.729807	-5.01584	0.824477	0.869481
Neutrophils	CCDC166	0.199434	0.615517	0.346333	0.729915	-5.06182	0.787487	0.831559
Neutrophils	4930557K0	0.156248	1.483636	0.346247	0.72998	-5.23982	0.768205	0.811761
Neutrophils	TTC9C	-0.06109	4.558959	-0.34585	0.730276	-5.7372	0.70387	0.745309
Neutrophils	HAUS7	0.106385	2.992661	0.345736	0.730363	-5.38734	0.736007	0.778516
Neutrophils	HAUS1	0.097602	3.65784	0.345729	0.730368	-5.58232	0.722178	0.764239
Neutrophils	LRP12	-0.18055	2.191669	-0.34543	0.730592	-5.25945	0.753182	0.796091
Neutrophils	PSEN1	0.032674	6.297037	0.345191	0.730771	-6.42942	0.670112	0.710172
Neutrophils	EMB	0.052747	5.68619	0.345077	0.730856	-6.65057	0.681852	0.722354
Neutrophils	FTCD	0.170451	1.403237	0.345068	0.730863	-5.34586	0.770396	0.813776
Neutrophils	TMEM50A	0.030248	7.711684	0.344846	0.731029	-6.68349	0.643761	0.682806
Neutrophils	ZFP622	0.041995	5.377893	0.344843	0.731031	-6.26315	0.687888	0.728641
Neutrophils	DDX19A	-0.07851	4.239703	-0.34448	0.731301	-5.65792	0.710713	0.752174
Neutrophils	RNF19A	0.037974	5.375931	0.344414	0.731353	-6.40412	0.688088	0.728766
Neutrophils	GM44702	0.227251	0.088947	0.344298	0.73144	-5.10388	0.800085	0.844235
Neutrophils	NUP62	0.097344	4.181003	0.34407	0.73161	-5.71138	0.711903	0.753471
Neutrophils	RBM4	-0.06191	4.655948	-0.34407	0.731611	-5.76073	0.702336	0.743575
Neutrophils	PRPF40B	-0.22031	0.534618	-0.34398	0.731676	-5.11192	0.789965	0.833892

Neutrophils	RECQL5	0.067043	3.722047	0.343972	0.731684	-5.8159	0.721276	0.76317
Neutrophils	GM525	-0.1821	0.742971	-0.34374	0.731859	-5.03552	0.78535	0.829143
Neutrophils	GM36975	0.156334	3.276788	0.3437	0.731888	-5.30044	0.730558	0.77275
Neutrophils	1700030J2	-0.21945	-0.14389	-0.34351	0.732029	-5.1132	0.805527	0.849863
Neutrophils	SLC27A1	-0.15728	2.972091	-0.34343	0.732092	-5.30306	0.736962	0.77937
Neutrophils	KBTBD7	-0.07459	2.137804	-0.34339	0.732121	-5.77193	0.754716	0.797667
Neutrophils	CLN3	-0.04618	4.768913	-0.34287	0.732507	-6.41305	0.700474	0.741455
Neutrophils	ALDH2	0.032709	6.861181	0.342714	0.732627	-6.75312	0.660028	0.699531
Neutrophils	KCNK5	-0.19321	1.47886	-0.34245	0.732823	-5.10708	0.769397	0.812592
Neutrophils	HIST3H2A	0.164168	2.104979	0.342392	0.732868	-5.23756	0.755768	0.79857
Neutrophils	TPRKB	0.116023	3.157978	0.342377	0.73288	-5.38828	0.733399	0.77552
Neutrophils	POLH	0.082562	4.035992	0.342157	0.733045	-5.66663	0.715268	0.75683
Neutrophils	MAD2L1BP	-0.07296	4.194819	-0.34204	0.733131	-5.72763	0.712039	0.753518
Neutrophils	EFNA1	0.169543	0.531324	0.341802	0.733311	-5.24189	0.790501	0.834463
Neutrophils	4930477G(	-0.18919	-0.38448	-0.34174	0.733356	-5.20468	0.811451	0.855961
Neutrophils	LRRC42	-0.09989	3.272197	-0.34159	0.733471	-5.41878	0.731014	0.773291
Neutrophils	ZFP952	-0.13554	2.251647	-0.3415	0.733537	-5.24759	0.75261	0.795593
Neutrophils	GM26839	0.220649	0.656622	0.341383	0.733625	-5.03559	0.787677	0.831678
Neutrophils	SNAI3	0.216792	-0.34486	0.341332	0.733663	-5.03594	0.810534	0.855142
Neutrophils	NPHP3	-0.1901	1.097931	-0.34113	0.733819	-5.09594	0.777812	0.821625
Neutrophils	LZTS3	-0.18686	0.323606	-0.34101	0.733906	-5.0908	0.795204	0.839485
Neutrophils	FCGR1	0.132755	2.432362	0.340948	0.733951	-5.60607	0.748739	0.791705
Neutrophils	PCNX4	-0.14121	2.141019	-0.34092	0.733975	-5.23036	0.754991	0.798145
Neutrophils	CMBL	0.161945	2.192141	0.340893	0.733993	-5.37161	0.75389	0.797011
Neutrophils	CABCOCO1	-0.19285	0.315412	-0.34086	0.734021	-5.05653	0.79539	0.839676
Neutrophils	AGK	-0.10774	3.121713	-0.34084	0.734031	-5.39654	0.734158	0.776667
Neutrophils	DENR	-0.03695	6.279481	-0.34084	0.734034	-6.331	0.671025	0.711313
Neutrophils	IDH3A	-0.08045	4.865883	-0.34071	0.734128	-5.69132	0.698558	0.739898
Neutrophils	ACAP1	-0.05997	3.923819	-0.3405	0.734285	-6.05722	0.717559	0.759557
Neutrophils	OPA1	-0.0609	4.835691	-0.34049	0.734295	-5.81462	0.699159	0.74052
Neutrophils	MPP1	0.040032	6.203718	0.340484	0.7343	-6.51118	0.672472	0.712846
Neutrophils	RHPN2	0.174455	0.915486	0.340484	0.7343	-5.125	0.781875	0.825836
Neutrophils	R3HCC1L	-0.04619	5.540599	-0.34047	0.734307	-6.18451	0.685272	0.726129
Neutrophils	B230307C2	0.087969	3.741811	0.340236	0.734486	-5.55409	0.721316	0.763451
Neutrophils	CCDC69	-0.12976	3.297575	-0.34016	0.734547	-5.30915	0.73051	0.772964
Neutrophils	0610040F0	-0.15958	0.269406	-0.34014	0.734558	-5.14744	0.796464	0.840844
Neutrophils	HNRNPA1	0.041348	7.807246	0.339891	0.734745	-6.52595	0.642568	0.68179
Neutrophils	ZFP521	-0.17946	2.100385	-0.33985	0.734772	-5.32061	0.755893	0.799158
Neutrophils	ZBTB7B	0.075004	2.473173	0.339831	0.73479	-5.89787	0.747894	0.790916
Neutrophils	5033406O(	-0.22184	0.605012	-0.33976	0.734846	-5.05696	0.788866	0.833073
Neutrophils	ADPGK	-0.04174	4.903066	-0.33971	0.734882	-6.58517	0.697843	0.73921
Neutrophils	WDR46	-0.12184	3.429733	-0.3393	0.735185	-5.37859	0.727994	0.770217
Neutrophils	NPHP1	0.205065	0.652168	0.339174	0.735283	-5.0656	0.788087	0.832084
Neutrophils	GM47448	-0.25547	-0.89373	-0.33907	0.735359	-5.01144	0.823674	0.868565
Neutrophils	ATP23	-0.21631	1.824257	-0.33891	0.735479	-5.12949	0.762162	0.805433
Neutrophils	CLN5	0.051108	4.008222	0.338881	0.735503	-5.99649	0.71613	0.757955
Neutrophils	MMS22L	0.089398	4.820836	0.338625	0.735695	-5.89752	0.699814	0.741074

Neutrophils	MYO9A	-0.05897	6.336763	-0.3386	0.735715	-6.33481	0.670278	0.710448
Neutrophils	RCOR1	0.037595	6.85813	0.338366	0.73589	-6.5045	0.660519	0.700293
Neutrophils	CEP128	-0.04746	6.336302	-0.33811	0.736083	-6.47771	0.670497	0.710552
Neutrophils	UQCR11	0.039887	7.224799	0.337744	0.736357	-6.52369	0.653962	0.693296
Neutrophils	NELFCD	0.066945	4.117826	0.337668	0.736414	-5.75647	0.714381	0.755951
Neutrophils	UFL1	0.074089	3.965196	0.337176	0.736783	-5.61897	0.717788	0.759227
Neutrophils	TTC38	-0.14	2.505389	-0.33689	0.736997	-5.29119	0.748313	0.790712
Neutrophils	TBC1D5	-0.03795	7.293343	-0.33689	0.736997	-6.57026	0.652969	0.692054
Neutrophils	SAE1	-0.04823	6.342657	-0.33689	0.737001	-6.20748	0.670837	0.710614
Neutrophils	MCTP2	0.042723	6.80258	0.336705	0.737137	-6.63462	0.662189	0.701604
Neutrophils	TYK2	0.075698	3.992109	0.336246	0.737482	-5.62483	0.717518	0.758803
Neutrophils	MIS18BP1	0.104039	3.964435	0.336244	0.737484	-5.73556	0.718084	0.759387
Neutrophils	GM20513	0.248521	1.300029	0.336101	0.737591	-5.14923	0.774809	0.817802
Neutrophils	ADNP	-0.03735	6.50802	-0.33602	0.737652	-6.35037	0.667952	0.707509
Neutrophils	2610044O1	0.104143	2.24732	0.335956	0.7377	-5.30299	0.754137	0.796586
Neutrophils	ILVBL	-0.11456	3.757211	-0.33584	0.737786	-5.34055	0.722365	0.763839
Neutrophils	PDPK1	-0.05451	6.462559	-0.33552	0.738024	-6.40511	0.668966	0.708445
Neutrophils	CDNF	-0.21481	0.317972	-0.33546	0.738073	-5.04545	0.797027	0.84055
Neutrophils	FAM20B	0.076641	3.958304	0.335211	0.73826	-5.68866	0.718384	0.759744
Neutrophils	AIP	0.047948	4.946962	0.335187	0.738278	-6.17407	0.698436	0.739121
Neutrophils	ARHGAP31	-0.0447	6.152696	-0.3351	0.738344	-6.57944	0.674885	0.714721
Neutrophils	ANKRD61	0.17671	0.799732	0.334748	0.738608	-5.11571	0.786139	0.829579
Neutrophils	ROCK1	0.026848	8.134703	0.33473	0.738622	-6.79756	0.637968	0.676425
Neutrophils	C4BP	-0.14489	1.968581	-0.33466	0.738672	-5.42223	0.760336	0.803065
Neutrophils	CLPX	-0.05233	5.328169	-0.33465	0.738685	-5.9784	0.690898	0.731391
Neutrophils	ST3GAL5	0.035498	6.23691	0.334609	0.738712	-6.80536	0.673272	0.713119
Neutrophils	SLC35B4	0.112014	2.825471	0.334584	0.738732	-5.3941	0.741969	0.784153
Neutrophils	CCT3	-0.05382	5.801241	-0.33453	0.73877	-5.91401	0.681663	0.721821
Neutrophils	UXS1	0.068937	4.429155	0.334449	0.738833	-5.80116	0.708811	0.749964
Neutrophils	AP1M1	0.04163	4.979621	0.334202	0.739019	-6.17287	0.697849	0.738597
Neutrophils	9330159M	-0.10752	1.076034	-0.33418	0.739037	-5.50508	0.78003	0.823325
Neutrophils	LTA4H	0.037161	5.724021	0.33389	0.739253	-6.65238	0.68336	0.723493
Neutrophils	ARMC5	-0.08513	3.383082	-0.33359	0.739479	-5.47888	0.730629	0.772341
Neutrophils	MYCN	0.208086	-0.18583	0.333462	0.739575	-5.0556	0.809018	0.852917
Neutrophils	ADK	-0.05332	6.968234	-0.33321	0.739768	-6.4972	0.659896	0.699124
Neutrophils	PHF1	0.082484	2.695984	0.332756	0.740106	-5.69406	0.74541	0.78748
Neutrophils	ERCC6L2	-0.10648	3.372895	-0.33272	0.740135	-5.43111	0.731156	0.772788
Neutrophils	GM15859	-0.11948	1.208039	-0.33271	0.740143	-5.26469	0.777752	0.82074
Neutrophils	SCAF11	-0.03355	7.16109	-0.33256	0.740256	-6.48339	0.656481	0.695477
Neutrophils	EPB41L2	-0.04167	6.975502	-0.33208	0.740612	-6.44156	0.659955	0.69916
Neutrophils	TRMT10C	-0.06148	4.996252	-0.33199	0.740682	-5.83413	0.698156	0.738767
Neutrophils	TFAP4	0.128166	2.748617	0.331785	0.740837	-5.37155	0.744344	0.786521
Neutrophils	GPALPP1	0.069307	3.737889	0.331613	0.740966	-5.66046	0.723637	0.765208
Neutrophils	CENPE	-0.10693	4.9887	-0.3315	0.74105	-6.07844	0.698306	0.739032
Neutrophils	FCRL5	0.207005	-0.65204	0.331429	0.741104	-5.02817	0.820245	0.864541
Neutrophils	GPR180	0.097479	2.964205	0.331406	0.741122	-5.44952	0.73978	0.78191
Neutrophils	MCM9	-0.06965	4.75152	-0.33138	0.741143	-5.78169	0.703038	0.743969

Neutrophils	A430033K0	0.211519	0.6406	0.331303	0.7412	-5.06724	0.790512	0.834097
Neutrophils	MCRIP1	-0.03724	5.919349	-0.33124	0.741246	-6.19081	0.680059	0.72018
Neutrophils	AKNA	0.039343	4.948701	0.331042	0.741396	-6.24881	0.699102	0.739908
Neutrophils	GREB1L	-0.1214	1.947775	-0.33092	0.74149	-5.51791	0.761551	0.804361
Neutrophils	CPOX	0.077413	4.570271	0.330712	0.741645	-5.72031	0.706676	0.747848
Neutrophils	SLC16A4	-0.1497	1.428027	-0.33071	0.741647	-5.17666	0.772935	0.816162
Neutrophils	1810062O1	0.152305	2.0989	0.330663	0.741681	-5.20559	0.758273	0.801082
Neutrophils	SMC4	-0.04011	7.287715	-0.33037	0.741899	-6.58902	0.654133	0.693382
Neutrophils	GM43251	-0.25574	-0.97845	-0.33034	0.741926	-5.02083	0.827928	0.872572
Neutrophils	FH1	-0.07892	5.484899	-0.33033	0.741935	-5.88277	0.688515	0.729071
Neutrophils	NPHS1	0.192359	0.439647	0.330303	0.741953	-5.116	0.795062	0.838911
Neutrophils	PPP3CB	-0.038	5.980209	-0.33009	0.742109	-6.19466	0.678883	0.719088
Neutrophils	CDKN2AIP	0.053538	4.567595	0.329988	0.74219	-5.9344	0.70673	0.747941
Neutrophils	MTR	-0.05826	4.54186	-0.32998	0.742192	-5.86964	0.707248	0.748477
Neutrophils	RFK	0.062425	5.042963	0.329906	0.742252	-5.90086	0.697228	0.738137
Neutrophils	ZFP61	-0.20453	1.174198	-0.32989	0.742263	-5.06981	0.778557	0.822013
Neutrophils	ASB7	0.063317	3.936004	0.329803	0.742329	-5.99782	0.719562	0.761258
Neutrophils	TIMM9	0.094783	3.882337	0.329784	0.742343	-5.42756	0.720664	0.762395
Neutrophils	TUBGCP5	-0.09467	3.976604	-0.32966	0.742437	-5.45897	0.71873	0.760432
Neutrophils	SLC12A6	0.039206	8.133974	0.329583	0.742495	-6.76577	0.638622	0.677344
Neutrophils	MGA	0.044425	5.917554	0.32958	0.742497	-6.28285	0.680094	0.720451
Neutrophils	IRAK1	-0.04455	5.224747	-0.32958	0.742498	-6.08129	0.69363	0.734483
Neutrophils	CHCHD2	-0.02617	8.94643	-0.32937	0.742655	-6.83338	0.624097	0.662234
Neutrophils	MAGI2	-0.16416	0.831391	-0.32931	0.742698	-5.18342	0.786217	0.829998
Neutrophils	AKT3	-0.06282	6.643383	-0.32922	0.742769	-6.13666	0.666209	0.706094
Neutrophils	ZBTB38	0.060275	5.068147	0.329218	0.74277	-5.94849	0.696729	0.737749
Neutrophils	DTX2	-0.08341	4.396914	-0.32918	0.7428	-5.62553	0.710174	0.751665
Neutrophils	BC048403	-0.11944	1.255612	-0.32898	0.742952	-5.22888	0.77675	0.820355
Neutrophils	GM20721	0.087583	3.72758	0.328931	0.742986	-5.58977	0.72385	0.765857
Neutrophils	GM43581	-0.15029	2.093907	-0.32886	0.74304	-5.23833	0.758381	0.801478
Neutrophils	XKR8	-0.15863	0.500884	-0.32884	0.743055	-5.22975	0.793673	0.83776
Neutrophils	WDR83OS	-0.0428	6.074614	-0.32881	0.743076	-6.27181	0.677064	0.717438
Neutrophils	EIF3G	0.067848	4.982954	0.328666	0.743186	-5.76261	0.698421	0.739592
Neutrophils	DDX56	-0.09311	3.951871	-0.32859	0.743243	-5.4543	0.719237	0.761144
Neutrophils	DUSP3	-0.04641	5.553941	-0.32854	0.743281	-6.16655	0.687164	0.727952
Neutrophils	ZDBF2	0.238659	-0.23932	0.328524	0.743292	-5.08404	0.810632	0.8552
Neutrophils	HBS1L	0.045138	5.694465	0.328399	0.743387	-6.09075	0.684446	0.725126
Neutrophils	IL3RA	0.088313	3.561284	0.328071	0.743633	-5.58056	0.727489	0.76954
Neutrophils	ERI1	0.052851	5.459943	0.327812	0.743828	-6.10936	0.689309	0.729944
Neutrophils	INPP5A	-0.03929	6.256163	-0.32769	0.743921	-6.54392	0.673891	0.713964
Neutrophils	CYP4A31	-0.17477	0.922941	-0.32761	0.743981	-5.2361	0.784527	0.828187
Neutrophils	GM50340	-0.1525	1.533078	-0.3274	0.74414	-5.25395	0.77103	0.814333
Neutrophils	CCNH	-0.05211	5.363256	-0.32736	0.744169	-5.95299	0.691269	0.732017
Neutrophils	ANK1	0.203356	1.273318	0.327042	0.744409	-5.24772	0.776948	0.820309
Neutrophils	PDCD7	-0.06296	4.505243	-0.32682	0.744576	-5.76793	0.708618	0.749779
Neutrophils	CD300E	0.231814	0.628868	0.326592	0.744748	-5.29029	0.791484	0.835127
Neutrophils	PTGR2	0.074124	3.734192	0.326503	0.744815	-5.66273	0.72436	0.766044

Neutrophils	GXYLT1	-0.0503	5.093219	-0.3265	0.744817	-6.03585	0.696854	0.737606
Neutrophils	D830036C2	-0.15418	0.420218	-0.32645	0.744852	-5.26856	0.796215	0.83998
Neutrophils	GALNT6	-0.13345	3.115592	-0.32613	0.745097	-5.40657	0.737424	0.779383
Neutrophils	PHYKPL	-0.077	3.470266	-0.3259	0.745267	-5.52496	0.730102	0.771759
Neutrophils	GIGYF2	0.043658	5.464029	0.325755	0.74538	-6.18753	0.689836	0.730129
Neutrophils	GM42670	0.150062	0.89839	0.325663	0.745449	-5.23523	0.785753	0.82902
Neutrophils	NTMT1	-0.07957	3.588232	-0.32547	0.745592	-5.39047	0.727738	0.769334
Neutrophils	LYRM4	0.071868	4.142278	0.325421	0.745632	-5.61624	0.716336	0.757572
Neutrophils	POMT2	-0.1479	1.813643	-0.32528	0.745738	-5.15338	0.76557	0.808349
Neutrophils	PGRMC2	-0.07411	3.628957	-0.32498	0.745968	-5.51914	0.727086	0.768567
Neutrophils	DDX46	0.044864	6.040748	0.324726	0.746155	-6.15773	0.678837	0.718695
Neutrophils	MIIP	0.088055	3.057652	0.32452	0.746311	-5.51891	0.739031	0.780936
Neutrophils	SNW1	-0.03485	6.240739	-0.32429	0.746486	-6.20474	0.674989	0.714787
Neutrophils	SPRYD7	0.125225	2.133927	0.324249	0.746516	-5.29702	0.758768	0.801343
Neutrophils	GM10353	0.108272	2.745067	0.324245	0.746518	-5.40871	0.745651	0.787838
Neutrophils	MRPS23	0.074819	4.411962	0.324175	0.746572	-5.73421	0.711044	0.752146
Neutrophils	PYROXD1	0.096153	2.95488	0.32393	0.746756	-5.45941	0.741201	0.783338
Neutrophils	TRMT5	0.168435	1.213824	0.323779	0.74687	-5.15057	0.778964	0.822192
Neutrophils	SMS	-0.05018	6.477089	-0.32371	0.746921	-6.04397	0.670471	0.710185
Neutrophils	PALB2	-0.12911	2.125531	-0.3236	0.747004	-5.2737	0.75895	0.801636
Neutrophils	COQ9	-0.09181	3.205352	-0.32356	0.747035	-5.50544	0.735924	0.777916
Neutrophils	EMSY	-0.04255	5.828738	-0.32353	0.747058	-6.20736	0.682942	0.72314
Neutrophils	RBL2	-0.06076	4.765126	-0.32349	0.747091	-5.89941	0.703928	0.744875
Neutrophils	SSRP1	0.048903	6.322976	0.323435	0.747129	-6.24152	0.673414	0.713276
Neutrophils	POLD1	-0.09103	4.276679	-0.3233	0.747231	-5.60301	0.71379	0.755095
Neutrophils	ZFX	-0.03087	6.339182	-0.32327	0.747256	-6.33207	0.673103	0.712959
Neutrophils	HOOK3	0.029795	6.076111	0.323198	0.747309	-6.49982	0.678155	0.7182
Neutrophils	PUS3	0.12286	2.37825	0.323118	0.747369	-5.21691	0.753496	0.796071
Neutrophils	HIBCH	0.055992	3.478236	0.323107	0.747377	-5.82447	0.73022	0.772079
Neutrophils	COA7	0.090824	3.404389	0.322979	0.747474	-5.39279	0.731759	0.773685
Neutrophils	AI662270	-0.06406	6.090129	-0.32295	0.747499	-6.0131	0.677885	0.717951
Neutrophils	SORT1	0.039135	4.067523	0.322911	0.747525	-6.47327	0.718057	0.759537
Neutrophils	GDPD3	-0.0687	1.793559	-0.32273	0.747661	-5.97815	0.766176	0.809137
Neutrophils	MOCOS	0.069944	1.324581	0.322545	0.747801	-6.04079	0.776504	0.819782
Neutrophils	TRAF2	0.084268	3.917831	0.322435	0.747885	-5.55621	0.721126	0.762747
Neutrophils	ZFP7	-0.20584	0.286794	-0.32234	0.747953	-5.06495	0.799862	0.843788
Neutrophils	UBE2R2	0.035811	7.144409	0.322332	0.747962	-6.47004	0.657885	0.697236
Neutrophils	EME2	0.146375	0.924802	0.322217	0.748049	-5.10807	0.785419	0.829044
Neutrophils	ME1	-0.20171	-0.00982	-0.32221	0.748051	-5.10657	0.806668	0.85084
Neutrophils	GGA1	0.046605	4.730426	0.322208	0.748056	-5.98685	0.704624	0.745771
Neutrophils	MRPL24	-0.0392	5.658173	-0.32214	0.74811	-6.1252	0.686263	0.726756
Neutrophils	MAP3K5	0.033902	7.255072	0.322048	0.748177	-6.71465	0.655822	0.695162
Neutrophils	RCC1	-0.08901	3.989305	-0.32167	0.748464	-5.5596	0.719705	0.761387
Neutrophils	GPR68	0.163808	1.433054	0.321615	0.748503	-5.3093	0.774152	0.817496
Neutrophils	TUBA1C	0.039779	7.576145	0.321575	0.748533	-6.77297	0.649917	0.689034
Neutrophils	PANK4	0.102021	3.423163	0.321364	0.748693	-5.41602	0.731415	0.773516
Neutrophils	HSP90AB1	0.031742	9.925498	0.321308	0.748735	-6.85087	0.608101	0.645476

Neutrophils	HDAC2	0.056798	5.300553	0.321143	0.74886	-5.96881	0.693325	0.734157
Neutrophils	TRIB2	-0.14691	2.561151	-0.32105	0.748933	-5.31178	0.749622	0.792336
Neutrophils	CCR2	0.198182	3.092746	0.320914	0.749033	-5.32549	0.738339	0.78071
Neutrophils	BTRC	-0.06131	5.211768	-0.3209	0.749044	-5.909	0.695079	0.735995
Neutrophils	MED30	-0.04307	5.369422	-0.32083	0.749095	-6.18482	0.691968	0.732772
Neutrophils	LIMK2	-0.04421	4.779797	-0.32071	0.749187	-6.16602	0.703679	0.7449
Neutrophils	MIA2	0.028264	6.77012	0.320703	0.749192	-6.51223	0.664956	0.704747
Neutrophils	CDK8	0.074764	6.559883	0.320701	0.749193	-6.23673	0.668939	0.708883
Neutrophils	FCRLS	-0.33225	-1.03209	-0.3207	0.749194	-5.01939	0.830623	0.875487
Neutrophils	GM42031	-0.22722	4.172175	-0.32061	0.749259	-5.40241	0.715965	0.757622
Neutrophils	ZFP991	-0.11793	2.80432	-0.32008	0.749665	-5.40263	0.744566	0.787228
Neutrophils	PSMB6	-0.04368	6.304312	-0.32002	0.749705	-6.24814	0.673929	0.714152
Neutrophils	CYP8B1	0.170617	1.074088	0.319997	0.749726	-5.16138	0.782262	0.826022
Neutrophils	GM49169	0.218192	-0.42801	0.319961	0.749753	-5.02133	0.816553	0.861195
Neutrophils	REV1	0.04906	5.326262	0.319885	0.74981	-6.07131	0.692937	0.733895
Neutrophils	SETD7	-0.05279	4.9704	-0.31987	0.749822	-6.03208	0.699991	0.741202
Neutrophils	TMEM209	-0.10434	3.362785	-0.31967	0.749975	-5.50131	0.7328	0.775158
Neutrophils	ZFP628	-0.11817	2.635685	-0.31966	0.749983	-5.39421	0.748157	0.790998
Neutrophils	TRMT61B	-0.134	4.178074	-0.31962	0.750009	-5.53603	0.715967	0.757767
Neutrophils	RDH10	0.094056	3.207433	0.319367	0.750202	-5.56995	0.736109	0.778577
Neutrophils	OAT	-0.05713	5.245936	-0.31926	0.750281	-6.03869	0.694575	0.735632
Neutrophils	DVL2	0.103973	3.263703	0.319194	0.750332	-5.47714	0.734929	0.77736
Neutrophils	LBP	-0.04796	3.292366	-0.31918	0.750341	-6.24987	0.734328	0.77674
Neutrophils	ASB1	-0.13776	1.712368	-0.31905	0.750441	-5.20528	0.768224	0.811662
Neutrophils	SP3	-0.02741	6.891028	-0.3189	0.750558	-6.58551	0.66291	0.702768
Neutrophils	ATP5MPL	-0.02992	8.484004	-0.3188	0.750627	-6.71473	0.63363	0.672314
Neutrophils	PUS7L	-0.14475	1.839535	-0.31852	0.750842	-5.21863	0.76557	0.808932
Neutrophils	TLR2	-0.04197	3.875493	-0.31847	0.750881	-6.52781	0.722374	0.764402
Neutrophils	GNB4	-0.10677	2.895468	-0.31836	0.750962	-5.44567	0.742845	0.785577
Neutrophils	TMC4	0.094128	1.556285	0.318263	0.751036	-5.43253	0.771786	0.815385
Neutrophils	SLC41A1	-0.14025	2.387756	-0.31824	0.751051	-5.19247	0.753685	0.796752
Neutrophils	HDAC3	-0.05192	4.707537	-0.3176	0.751535	-5.88815	0.705797	0.747067
Neutrophils	1700025G(	-0.07463	4.957756	-0.31757	0.751557	-6.03083	0.700786	0.741885
Neutrophils	CHIL3	-0.05997	2.447415	-0.31735	0.751723	-6.93492	0.752868	0.795567
Neutrophils	ATXN1L	0.078417	2.959513	0.317092	0.751921	-5.61741	0.74196	0.784315
Neutrophils	ARMC7	0.045978	3.924467	0.316981	0.752005	-6.16283	0.721824	0.763535
Neutrophils	FDFT1	0.074814	3.796269	0.31691	0.752059	-5.68094	0.724466	0.766264
Neutrophils	LIMD2	0.032743	7.333227	0.316897	0.752069	-6.72164	0.655126	0.69441
Neutrophils	FOXRED1	0.101529	2.831724	0.316874	0.752086	-5.51523	0.744669	0.787108
Neutrophils	DEPDC1A	0.105666	3.001084	0.316791	0.752149	-5.80153	0.74108	0.783425
Neutrophils	B930095G1	0.175917	0.087994	0.316278	0.752537	-5.19206	0.805689	0.849641
Neutrophils	GLB1	-0.06089	5.221188	-0.31595	0.752783	-5.85842	0.69611	0.7367
Neutrophils	CASP3	-0.04046	4.814976	-0.3158	0.7529	-6.19411	0.704252	0.745154
Neutrophils	CISH	0.083524	2.887974	0.315642	0.753018	-5.83433	0.744061	0.786223
Neutrophils	ENTR1	-0.04756	4.880508	-0.31549	0.753135	-6.08823	0.703028	0.743813
Neutrophils	H2-OA	0.188515	3.19042	0.315398	0.753203	-5.2046	0.737716	0.779638
Neutrophils	COL14A1	-0.13285	1.863173	-0.31521	0.753343	-5.41339	0.766202	0.809007

Neutrophils	SPTY2D1	-0.03443	5.765398	-0.31515	0.753393	-6.36056	0.685557	0.725767
Neutrophils	SLC43A2	0.065479	6.179865	0.31503	0.753481	-6.20356	0.677526	0.717472
Neutrophils	LRRC8A	-0.04513	5.427019	-0.31485	0.753614	-6.11724	0.692188	0.732728
Neutrophils	LPAR2	0.087502	2.187833	0.314679	0.753747	-5.43666	0.759134	0.801881
Neutrophils	N4BP2L1	0.05104	4.595001	0.314656	0.753764	-6.13383	0.708778	0.74993
Neutrophils	LINS1	-0.0939	1.704648	-0.31461	0.753796	-5.41015	0.769677	0.812726
Neutrophils	ARFGEF1	-0.0271	6.613928	-0.3145	0.753885	-6.71851	0.669221	0.708964
Neutrophils	D330023K1	0.085793	2.22594	0.314447	0.753922	-5.66795	0.758309	0.801067
Neutrophils	RPAP2	-0.10204	3.02458	-0.31434	0.754003	-5.35645	0.741225	0.783468
Neutrophils	EXOSC3	-0.05886	5.047755	-0.31432	0.754021	-5.82458	0.699699	0.74057
Neutrophils	POLM	-0.08897	3.245007	-0.31429	0.754044	-5.4382	0.73658	0.778688
Neutrophils	TNFAIP6	0.059672	1.129558	0.313789	0.754421	-6.27068	0.782601	0.825983
Neutrophils	GM34921	-0.1474	0.841113	-0.31366	0.75452	-5.24269	0.789074	0.832634
Neutrophils	1700061N1	-0.22154	-0.1438	-0.31365	0.754524	-5.10964	0.811586	0.855716
Neutrophils	PIAS3	-0.08085	2.896492	-0.31359	0.754569	-5.46943	0.74411	0.786392
Neutrophils	TMEM128	-0.05074	5.718829	-0.31358	0.754582	-6.07614	0.686624	0.726979
Neutrophils	RNF20	0.039253	5.755436	0.313509	0.754633	-6.31437	0.68591	0.726239
Neutrophils	SLC39A6	0.078533	4.11285	0.313331	0.754767	-5.57048	0.718792	0.760219
Neutrophils	EPG5	-0.06499	4.420072	-0.31326	0.754819	-5.75627	0.712528	0.753763
Neutrophils	GM50333	-0.14208	1.191453	-0.31303	0.754995	-5.18152	0.781378	0.824629
Neutrophils	TMEM229E	0.105314	4.03146	0.312781	0.755184	-5.39344	0.720573	0.762083
Neutrophils	YPEL1	-0.11119	2.4444	-0.31274	0.755212	-5.3163	0.753935	0.796492
Neutrophils	AC166172.	-0.14603	0.939575	-0.31274	0.755212	-5.2271	0.78703	0.830515
Neutrophils	AA467197	-0.07795	0.801131	-0.31265	0.755284	-6.14373	0.79015	0.833766
Neutrophils	UACA	0.104027	2.51718	0.312401	0.755472	-5.57112	0.752491	0.794974
Neutrophils	SLC25A3	0.028248	7.921066	0.312215	0.755612	-6.66624	0.645255	0.683958
Neutrophils	1700006J1	-0.21271	-0.71534	-0.3122	0.755622	-5.09062	0.82527	0.869653
Neutrophils	YIPF1	0.041955	5.234712	0.311793	0.755932	-6.04446	0.696643	0.737122
Neutrophils	ZBTB41	-0.09723	2.915286	-0.31169	0.756008	-5.39924	0.744249	0.786295
Neutrophils	CEACAM10	-0.05761	-1.04799	-0.31157	0.756103	-5.9404	0.833442	0.877794
Neutrophils	THBD	-0.08622	3.145231	-0.31149	0.756165	-5.91435	0.739401	0.78128
Neutrophils	CAPN10	0.089481	2.598593	0.311071	0.756479	-5.51727	0.751105	0.793323
Neutrophils	HEBP1	-0.09043	4.67467	-0.31106	0.75649	-5.98264	0.707941	0.74879
Neutrophils	ALKBH4	-0.10493	2.574916	-0.31101	0.756524	-5.36484	0.751612	0.793853
Neutrophils	ATPAF2	0.113801	2.92038	0.310937	0.75658	-5.30574	0.744241	0.786277
Neutrophils	ZFP809	-0.09855	3.64912	-0.31074	0.756731	-5.49098	0.728934	0.770505
Neutrophils	TRPV4	0.195242	-0.51202	0.31058	0.756851	-5.03703	0.820871	0.864992
Neutrophils	TEP1	-0.09579	3.914158	-0.3105	0.756912	-5.46947	0.723448	0.764873
Neutrophils	ANGEL2	0.043126	5.052327	0.310369	0.757011	-5.99844	0.70037	0.741028
Neutrophils	ANO8	-0.19622	0.904446	-0.31034	0.757036	-5.05807	0.788323	0.831657
Neutrophils	F5	-0.04999	4.094976	-0.31023	0.757117	-6.6388	0.719729	0.761042
Neutrophils	THAP12	0.065051	4.362104	0.310056	0.757248	-5.63095	0.714272	0.755422
Neutrophils	PNPLA8	0.030635	6.018439	0.310034	0.757264	-6.53131	0.681383	0.721384
Neutrophils	SCHIP1	-0.09101	1.591487	-0.30995	0.75733	-5.64829	0.773008	0.815976
Neutrophils	SMIM40	0.193051	0.301755	0.309816	0.75743	-5.06446	0.80201	0.845768
Neutrophils	TREM2	0.213261	0.639882	0.309695	0.757522	-5.24892	0.794302	0.837879
Neutrophils	CDCA7	0.092187	3.246784	0.309529	0.757647	-5.68367	0.737344	0.779324

Neutrophils	ATPAF1	0.086384	3.553171	0.309413	0.757735	-5.48425	0.73093	0.772747
Neutrophils	SERPINE2	-0.06932	1.29875	-0.30933	0.757798	-5.89534	0.779497	0.822747
Neutrophils	9530082P2	-0.16747	0.699259	-0.30932	0.75781	-5.06111	0.792956	0.836566
Neutrophils	ARF4OS	-0.16013	0.845404	-0.30925	0.757857	-5.0976	0.789653	0.833179
Neutrophils	MED9	-0.09647	3.526994	-0.30913	0.757947	-5.4601	0.731476	0.773314
Neutrophils	TAF5	-0.06006	4.146651	-0.30913	0.757953	-5.80358	0.71867	0.760092
Neutrophils	CYB561D2	0.096203	3.329402	0.309124	0.757954	-5.39136	0.735609	0.777578
Neutrophils	SLC26A2	0.063547	3.742645	0.309085	0.757984	-5.84083	0.726993	0.768687
Neutrophils	ALYREF	-0.03802	8.216984	-0.30908	0.757987	-6.73643	0.640163	0.678667
Neutrophils	BCL2L11	0.049431	7.053365	0.308898	0.758126	-6.59407	0.661641	0.701027
Neutrophils	E330009J0	0.064741	2.552592	0.30887	0.758147	-5.98596	0.752091	0.794587
Neutrophils	EDA	-0.21181	1.445596	-0.30882	0.758185	-5.17868	0.776235	0.819443
Neutrophils	HIST1H4N	0.174442	1.07648	0.308559	0.758383	-5.16975	0.78455	0.827991
Neutrophils	PIR	-0.14987	1.198746	-0.30852	0.758411	-5.22483	0.781816	0.825183
Neutrophils	LIX1L	-0.1324	0.803407	-0.30816	0.758683	-5.26499	0.790777	0.834374
Neutrophils	DR1	-0.04491	4.953577	-0.30813	0.758705	-5.96843	0.702498	0.743406
Neutrophils	GYPC	-0.12753	3.080733	-0.30803	0.758784	-5.41003	0.74101	0.783185
Neutrophils	GM48623	0.202067	-0.24558	0.307887	0.758893	-5.02143	0.814829	0.859049
Neutrophils	ZFP992	0.087724	3.563813	0.307875	0.758902	-5.58171	0.730872	0.772746
Neutrophils	DNAJC24	0.083869	3.89302	0.307818	0.758945	-5.6226	0.724045	0.765704
Neutrophils	NF1	0.035164	6.550388	0.307775	0.758977	-6.3952	0.671309	0.71112
Neutrophils	PMM1	-0.08516	3.07058	-0.30767	0.759055	-5.59728	0.741232	0.783498
Neutrophils	RLIM	0.024054	6.654275	0.307453	0.759221	-6.5398	0.669381	0.709128
Neutrophils	TM2D1	0.035595	5.944576	0.307331	0.759315	-6.20422	0.683018	0.723316
Neutrophils	RFFL	0.034674	5.915131	0.307323	0.75932	-6.6076	0.68359	0.723909
Neutrophils	GATM	-0.04916	4.530012	-0.30713	0.759464	-6.33035	0.711075	0.752415
Neutrophils	YEATS4	-0.0317	5.906755	-0.30708	0.759507	-6.34474	0.683753	0.724138
Neutrophils	KLHDC10	-0.0337	5.434437	-0.30703	0.759543	-6.41499	0.693001	0.733741
Neutrophils	0610012G(	-0.04356	5.039442	-0.30697	0.759587	-6.02119	0.700835	0.741857
Neutrophils	CTLA4	-0.21127	2.060232	-0.30686	0.759674	-5.13765	0.762962	0.805979
Neutrophils	HGFAC	-0.13745	1.432763	-0.30674	0.759761	-5.32583	0.776752	0.820161
Neutrophils	5-Mar	0.055463	6.018808	0.306696	0.759796	-6.06875	0.68158	0.721903
Neutrophils	GM43728	0.170351	-0.48628	0.306465	0.759972	-5.06735	0.820577	0.865052
Neutrophils	MOSPD3	0.047749	4.656046	0.306422	0.760003	-5.99147	0.708584	0.749823
Neutrophils	SULT1A1	-0.08965	3.923228	-0.30627	0.760122	-5.81669	0.723533	0.765296
Neutrophils	HMGCLL1	-0.18668	1.17582	-0.30618	0.760191	-5.09416	0.782533	0.826069
Neutrophils	SLC9A6	0.085502	2.81804	0.306063	0.760276	-5.46909	0.746698	0.7892
Neutrophils	GALNT1	-0.03408	6.628673	-0.30605	0.760284	-6.30292	0.669921	0.709776
Neutrophils	PLIN3	-0.05472	3.527331	-0.30598	0.76034	-5.90622	0.731745	0.773783
Neutrophils	MAP1S	-0.08758	3.781603	-0.30568	0.76057	-5.54184	0.726567	0.768433
Neutrophils	VPS35L	0.042312	5.162292	0.305543	0.760671	-5.9918	0.698548	0.73946
Neutrophils	ATP6V1F	-0.02773	6.902317	-0.30531	0.760844	-6.48816	0.664834	0.704492
Neutrophils	AP1S1	-0.05639	4.706161	-0.30518	0.76095	-5.88152	0.707678	0.74895
Neutrophils	FGFRL1	-0.21058	0.299565	-0.3051	0.761009	-5.05384	0.802481	0.846588
Neutrophils	SNRNP27	-0.03462	5.842837	-0.30508	0.761023	-6.2519	0.685153	0.72562
Neutrophils	PRR14	-0.05141	4.524923	-0.30499	0.761088	-5.99	0.71134	0.752755
Neutrophils	GM15708	-0.0955	3.198583	-0.30497	0.761106	-5.52504	0.738746	0.781062

Neutrophils	NUB1	0.036772	5.534309	0.304964	0.761111	-6.18479	0.691192	0.731897
Neutrophils	AP2A2	-0.03294	5.931782	-0.3049	0.761156	-6.35719	0.683423	0.723848
Neutrophils	GM42726	0.061713	3.69069	0.304828	0.761213	-5.74562	0.728453	0.770467
Neutrophils	PPP1R15B	-0.03645	5.553021	-0.30419	0.761694	-6.38487	0.691181	0.731701
Neutrophils	PTDSS1	0.04532	5.371518	0.304068	0.76179	-6.03315	0.694759	0.735411
Neutrophils	ASB13	0.125401	3.434394	0.303956	0.761875	-5.32337	0.734174	0.776161
Neutrophils	DAAM1	-0.05233	4.764899	-0.30379	0.762	-5.99143	0.70686	0.747977
Neutrophils	ARL13B	0.063432	3.775076	0.303224	0.762432	-5.70942	0.727078	0.768955
Neutrophils	TTC32	-0.05448	4.101346	-0.30314	0.762496	-5.89115	0.720348	0.762012
Neutrophils	TNNI1	-0.2179	-0.907	-0.30296	0.762635	-5.04256	0.831048	0.875816
Neutrophils	9030025P2	0.134514	1.737859	0.302926	0.762658	-5.24842	0.770587	0.813815
Neutrophils	KPTN	-0.06793	4.098478	-0.30284	0.762721	-5.93653	0.720407	0.762125
Neutrophils	SEC61A1	0.042287	5.49084	0.302842	0.762722	-6.01205	0.692405	0.73315
Neutrophils	IKZF5	-0.06799	3.804869	-0.30283	0.762729	-5.66125	0.72646	0.768379
Neutrophils	H2-EB2	-0.20398	0.547733	-0.30275	0.762793	-5.05162	0.797225	0.841238
Neutrophils	MECOM	0.199915	0.770813	0.302727	0.762809	-5.34426	0.792162	0.836042
Neutrophils	IL10	0.089171	2.999738	0.30269	0.762837	-5.94978	0.743331	0.785809
Neutrophils	UBL7	0.05155	5.130384	0.302554	0.762941	-6.01661	0.699543	0.740599
Neutrophils	AKAP6	-0.2238	0.299684	-0.30255	0.762943	-5.16663	0.802893	0.847091
Neutrophils	PTPRA	-0.0305	6.521638	-0.30251	0.762977	-6.31871	0.672408	0.712462
Neutrophils	NAT2	-0.11456	2.532818	-0.30247	0.763002	-5.327	0.753299	0.796126
Neutrophils	ABCF3	0.068971	3.761251	0.302123	0.763268	-5.61056	0.727364	0.769428
Neutrophils	NSG2	-0.18112	0.587521	-0.30212	0.763271	-5.04638	0.796319	0.840413
Neutrophils	CDKN2AIPN	0.051811	4.272414	0.302112	0.763276	-5.89109	0.716845	0.758559
Neutrophils	ZFP692	-0.1098	2.030033	-0.30209	0.76329	-5.25743	0.764186	0.807391
Neutrophils	CMTM7	0.030512	7.897065	0.301906	0.763432	-6.62415	0.646666	0.685761
Neutrophils	CBL	-0.02717	7.22396	-0.30188	0.763456	-6.67348	0.659131	0.698737
Neutrophils	IDNK	0.043628	5.52475	0.301634	0.76364	-6.12664	0.691737	0.732655
Neutrophils	BAIAP3	-0.18579	-0.32152	-0.3015	0.763742	-5.09318	0.817266	0.861993
Neutrophils	USP6NL	0.057468	5.563085	0.301176	0.763988	-5.90112	0.690983	0.732001
Neutrophils	TSPAN12	0.131506	1.194761	0.301038	0.764092	-5.38518	0.782629	0.826647
Neutrophils	1810020O(	-0.20138	-0.23349	-0.30101	0.764114	-5.12315	0.815214	0.860088
Neutrophils	NXPE3	0.085584	3.424156	0.301008	0.764115	-5.53096	0.734388	0.776954
Neutrophils	PPT2	0.05935	4.199673	0.300882	0.764211	-5.89419	0.718333	0.76042
Neutrophils	DYRK1A	0.035321	7.419436	0.300868	0.764222	-6.53216	0.655485	0.695225
Neutrophils	KPNA3	-0.04137	6.307968	-0.30078	0.764288	-6.20199	0.676502	0.717097
Neutrophils	NRXN1	-0.17961	2.142242	-0.30075	0.76431	-5.32209	0.761743	0.80526
Neutrophils	FOXM1	-0.09685	3.10354	-0.3007	0.764349	-5.58561	0.741134	0.784027
Neutrophils	BIRC5	-0.08584	5.35755	-0.30067	0.764374	-6.21151	0.695035	0.736365
Neutrophils	AREG	0.416071	1.634087	0.300635	0.764399	-5.07911	0.772873	0.816756
Neutrophils	SDCCAG8	0.039046	5.914953	0.300556	0.764459	-6.24214	0.684103	0.725043
Neutrophils	CD6	-0.11723	0.929158	-0.3005	0.764504	-5.43083	0.788587	0.832924
Neutrophils	HIVEP3	0.060094	5.144836	0.300494	0.764506	-6.04742	0.699256	0.740756
Neutrophils	PGAM5	-0.07281	3.934831	-0.30045	0.764536	-5.57882	0.723774	0.766132
Neutrophils	MORF4L1	-0.01739	8.201561	-0.30025	0.764688	-6.71785	0.641109	0.680391
Neutrophils	HDHD2	0.075338	3.552765	0.30021	0.764722	-5.50597	0.7317	0.774408
Neutrophils	AC154200.	0.145433	0.660722	0.299858	0.764989	-5.13264	0.794656	0.839294

Neutrophils	DCP1B	-0.11587	2.830948	-0.29984	0.765006	-5.28934	0.746919	0.790159
Neutrophils	MAGED2	0.130671	1.585547	0.299703	0.765107	-5.33542	0.773945	0.818042
Neutrophils	CSTF2	0.075216	4.139659	0.299686	0.76512	-5.62791	0.719562	0.761937
Neutrophils	STK4	-0.02886	7.52595	-0.29958	0.765203	-6.59316	0.653507	0.693403
Neutrophils	4930445E1	0.134244	-0.41363	0.299571	0.765208	-5.22522	0.819419	0.864754
Neutrophils	FBL	0.059241	6.091074	0.299412	0.765328	-5.90969	0.680686	0.721661
Neutrophils	PHLPP1	-0.05846	7.671226	-0.29938	0.765352	-6.42847	0.65082	0.690605
Neutrophils	ZPR1	0.059065	4.397865	0.299372	0.765359	-5.73694	0.714288	0.756494
Neutrophils	CYP3A13	0.13391	0.779768	0.299338	0.765385	-5.32076	0.791959	0.836578
Neutrophils	DIS3	-0.09317	3.258023	-0.29934	0.765386	-5.41905	0.737876	0.780877
Neutrophils	RELL1	0.036391	6.866963	0.299298	0.765415	-6.43459	0.665845	0.70624
Neutrophils	TARS	0.074988	4.374393	0.298864	0.765745	-5.60364	0.715009	0.75709
Neutrophils	CDK7	0.065476	4.595584	0.298641	0.765915	-5.77007	0.710566	0.752487
Neutrophils	RIIAD1	0.130007	0.993222	0.298487	0.766032	-5.23758	0.787466	0.831793
Neutrophils	MTREX	-0.04204	5.477446	-0.29848	0.766037	-6.02238	0.69295	0.734237
Neutrophils	MSS51	-0.10735	2.436698	-0.29843	0.766073	-5.38543	0.755676	0.799078
Neutrophils	UNC93B1	0.033534	7.659349	0.298269	0.766198	-6.55307	0.651352	0.690995
Neutrophils	GORAB	0.126241	1.673364	0.297831	0.766531	-5.23114	0.772613	0.816287
Neutrophils	DHRS1	0.05421	4.762169	0.297778	0.766571	-6.15084	0.707471	0.749076
Neutrophils	SLFN9	0.114139	2.410472	0.297227	0.76699	-5.49793	0.756837	0.799833
Neutrophils	FIRRE	-0.12903	2.608551	-0.29718	0.767022	-5.33111	0.752571	0.795439
Neutrophils	5830487J0	0.171639	-0.01733	0.296963	0.767191	-5.03307	0.811266	0.855715
Neutrophils	IFIT1BL1	0.21679	0.159661	0.296875	0.767258	-5.14447	0.807175	0.851538
Neutrophils	6530409C1	-0.15428	1.661527	-0.29663	0.767443	-5.1873	0.773405	0.816795
Neutrophils	ZFP59	-0.1902	0.230401	-0.29639	0.767625	-5.04504	0.805785	0.850005
Neutrophils	CRYBG1	-0.07878	4.169377	-0.29604	0.767897	-5.70172	0.720194	0.761939
Neutrophils	CANT1	-0.06083	3.891787	-0.29601	0.76792	-5.76392	0.725914	0.767849
Neutrophils	TPP2	-0.02909	7.082191	-0.296	0.767925	-6.45102	0.662931	0.702591
Neutrophils	PCGF1	-0.12896	1.17851	-0.29592	0.767985	-5.22403	0.784344	0.828048
Neutrophils	VPS50	0.075158	4.131766	0.295586	0.768239	-5.51255	0.72114	0.762811
Neutrophils	NOP56	-0.06388	4.591451	-0.29543	0.768358	-5.73004	0.711792	0.753113
Neutrophils	MOSMO	0.059303	4.576833	0.295347	0.768421	-5.80205	0.712089	0.753419
Neutrophils	SNAP47	-0.09672	2.473639	-0.29526	0.768485	-5.3762	0.756094	0.798848
Neutrophils	GM42984	0.182696	0.916614	0.295141	0.768578	-5.09486	0.790485	0.834217
Neutrophils	DDX27	-0.06339	4.974515	-0.29498	0.768704	-5.72003	0.704113	0.745214
Neutrophils	GOLGA4	0.042156	5.30004	0.29493	0.768739	-6.00633	0.697619	0.738492
Neutrophils	CTU2	-0.08916	3.206943	-0.29451	0.769056	-5.43556	0.740594	0.782885
Neutrophils	9530052E0	-0.12947	1.000649	-0.29442	0.769126	-5.2052	0.788729	0.832415
Neutrophils	AARS2	-0.16918	0.821033	-0.2944	0.76914	-5.08188	0.792785	0.836579
Neutrophils	MAP3K4	0.071417	4.074187	0.294237	0.769266	-5.61539	0.722508	0.764234
Neutrophils	UQCRC1	-0.03644	6.508803	-0.29423	0.76927	-6.32266	0.674148	0.714152
Neutrophils	SPRED1	-0.07938	4.710293	-0.29421	0.769285	-5.77947	0.709534	0.750826
Neutrophils	ACTN4	0.02733	6.545001	0.293908	0.769517	-6.42046	0.673455	0.713474
Neutrophils	SAP25	-0.10088	2.354647	-0.29382	0.769586	-5.52799	0.758823	0.801766
Neutrophils	RPP21	-0.04597	4.873001	-0.29376	0.769626	-6.02093	0.706254	0.747505
Neutrophils	A130014A0	0.085648	1.887317	0.293723	0.769658	-5.54346	0.769012	0.812256
Neutrophils	PROS1	-0.07446	2.741429	-0.29352	0.769809	-5.76502	0.750494	0.793247

Neutrophils	RAB34	-0.18329	0.235405	-0.29347	0.769847	-5.13327	0.806157	0.850467
Neutrophils	MAP3K20	-0.0628	3.798494	-0.29334	0.76995	-5.91537	0.728208	0.770259
Neutrophils	TPCN2	0.066715	2.597655	0.293337	0.769952	-5.78063	0.753579	0.79643
Neutrophils	KNSTRN	0.104164	3.271497	0.293329	0.769958	-5.6862	0.739232	0.781639
Neutrophils	TBC1D1	-0.03871	7.18844	-0.29332	0.769962	-6.49367	0.661263	0.700893
Neutrophils	COASY	-0.10024	3.053875	-0.29307	0.770154	-5.4145	0.743947	0.786423
Neutrophils	NUDCD2	-0.06837	4.534449	-0.2929	0.770283	-5.77945	0.713204	0.754676
Neutrophils	CCDC17	-0.16162	0.893924	-0.2929	0.770287	-5.16503	0.791256	0.835095
Neutrophils	WDR61	0.043849	4.80713	0.292655	0.770472	-5.92647	0.707793	0.749037
Neutrophils	TMEM167	-0.0273	6.804318	-0.29252	0.770576	-6.46319	0.668745	0.70855
Neutrophils	REEP5	0.027055	7.566136	0.292217	0.770805	-6.59786	0.654576	0.693707
Neutrophils	CCDC28A	-0.09284	2.307492	-0.29213	0.770874	-5.425	0.760264	0.803062
Neutrophils	NDUFS6	0.04095	5.716521	0.291915	0.771035	-6.21041	0.689912	0.730398
Neutrophils	CTSS	0.07055	7.501211	0.291904	0.771044	-6.46581	0.655809	0.694996
Neutrophils	PCED1B	-0.07094	4.966857	-0.29167	0.771221	-5.95638	0.704886	0.745838
Neutrophils	NRIP1	-0.063	6.353871	-0.29129	0.771514	-6.03413	0.677691	0.717621
Neutrophils	GM16287	-0.15128	-0.44109	-0.29127	0.771527	-5.10524	0.822576	0.866888
Neutrophils	RAD9A	0.123896	2.338849	0.291226	0.77156	-5.34605	0.759802	0.80247
Neutrophils	GM47428	-0.19208	0.207827	-0.2912	0.771581	-5.06656	0.807469	0.851426
Neutrophils	TAMM41	-0.11555	2.732365	-0.29095	0.771774	-5.30927	0.751439	0.793766
Neutrophils	ATG9A	0.043431	3.85849	0.290807	0.77188	-6.06735	0.727724	0.769297
Neutrophils	ETFBKMT	0.11535	2.261696	0.290589	0.772046	-5.27383	0.761639	0.804309
Neutrophils	DDX11	-0.10064	2.934033	-0.29052	0.7721	-5.45093	0.747167	0.789408
Neutrophils	GTPBP4	-0.03935	5.956432	-0.29041	0.772179	-6.11526	0.685536	0.725735
Neutrophils	ANKRD6	-0.1789	0.95646	-0.29031	0.772261	-5.10651	0.790556	0.834068
Neutrophils	RB1CC1	-0.03082	6.500368	-0.29024	0.772316	-6.53092	0.675019	0.714863
Neutrophils	VAV3	-0.03051	7.363151	-0.29004	0.772462	-6.96526	0.658685	0.697932
Neutrophils	GM17251	0.107599	2.365949	0.289871	0.772594	-5.42429	0.759376	0.80209
Neutrophils	CSRNP2	-0.11303	2.107884	-0.28987	0.772595	-5.3349	0.76499	0.807867
Neutrophils	DTYMK	0.061062	5.126752	0.289839	0.772618	-5.99402	0.701909	0.742773
Neutrophils	GGNBP2	0.024143	7.268609	0.289802	0.772646	-6.62968	0.660454	0.699781
Neutrophils	ARMC8	-0.03407	5.019279	-0.2898	0.772648	-6.14517	0.704059	0.745003
Neutrophils	EIF4A1	0.02555	7.526831	0.289548	0.77284	-6.62716	0.655737	0.694775
Neutrophils	DEGS2	-0.18602	0.674225	-0.28929	0.77304	-5.04959	0.797118	0.840728
Neutrophils	ATG5	0.030414	5.691584	0.289272	0.773051	-6.23556	0.690861	0.731206
Neutrophils	ZFP811	-0.21218	-1.02506	-0.28915	0.773142	-5.01283	0.836762	0.881336
Neutrophils	ABHD4	0.090068	2.787772	0.289017	0.773245	-5.38997	0.750446	0.792817
Neutrophils	PON3	-0.09916	3.489649	-0.289	0.773255	-5.51094	0.735573	0.777484
Neutrophils	LRP1B	0.119624	1.219155	0.288858	0.773366	-5.28208	0.78481	0.828207
Neutrophils	EIF4G2	-0.02117	7.71796	-0.28876	0.773441	-6.72176	0.652224	0.691204
Neutrophils	EIF2B2	-0.05869	4.936837	-0.28867	0.773508	-5.83963	0.70586	0.746853
Neutrophils	PELI2	0.046319	2.831902	0.288636	0.773536	-6.2682	0.749502	0.791913
Neutrophils	SMCO4	-0.08017	4.10439	-0.2885	0.77364	-5.62861	0.722796	0.764362
Neutrophils	4732465J0	0.068617	1.227323	0.288497	0.773642	-5.89014	0.784627	0.828043
Neutrophils	WWC1	-0.12226	1.216205	-0.28831	0.773788	-5.23226	0.784953	0.828298
Neutrophils	TMEM203	-0.07676	3.168142	-0.28817	0.773891	-5.62634	0.742453	0.784539
Neutrophils	CCDC58	-0.06517	4.247902	-0.28766	0.774282	-5.68805	0.720189	0.761375

Neutrophils	ADAM33	-0.17672	-0.9471	-0.28755	0.774368	-5.04395	0.835299	0.879667
Neutrophils	4930579G2	-0.10354	2.431609	-0.28751	0.774392	-5.31667	0.758472	0.800882
Neutrophils	XK	0.110867	1.158214	0.287434	0.774453	-5.38846	0.786551	0.829751
Neutrophils	VPS13D	-0.03272	6.149191	-0.28738	0.774497	-6.45667	0.682256	0.722184
Neutrophils	TMC3	0.20238	-1.08655	0.28709	0.774715	-5.02606	0.83864	0.883261
Neutrophils	4930562C1	0.168945	0.146658	0.287082	0.774721	-5.0819	0.809613	0.853561
Neutrophils	IDH3B	-0.04966	5.850654	-0.28683	0.77491	-6.08098	0.688077	0.728382
Neutrophils	AGA	0.084061	3.27288	0.286804	0.774934	-5.54696	0.740493	0.782568
Neutrophils	GNAL	0.105036	1.371741	0.286781	0.774951	-5.34244	0.781777	0.825055
Neutrophils	B230206L0	0.133813	1.02404	0.286764	0.774964	-5.48171	0.789578	0.833065
Neutrophils	RPE	-0.04028	4.554255	-0.28665	0.775053	-6.15782	0.713938	0.755173
Neutrophils	ICK	-0.12476	2.147459	-0.28664	0.775061	-5.23727	0.764655	0.807477
Neutrophils	MAPT	0.110592	1.597756	0.286507	0.77516	-5.33805	0.776778	0.81998
Neutrophils	DDIT3	0.052486	4.008692	0.286268	0.775343	-5.9674	0.725226	0.766801
Neutrophils	ZDHHC1	-0.10677	1.415954	-0.28616	0.775424	-5.28849	0.780903	0.824144
Neutrophils	APOBEC1	0.059929	6.087599	0.286128	0.77545	-6.26832	0.683555	0.723682
Neutrophils	RAB18	-0.03265	5.748093	-0.28587	0.775646	-6.20337	0.690195	0.730649
Neutrophils	PASK	-0.16914	1.295986	-0.28584	0.77567	-5.20846	0.783592	0.827008
Neutrophils	KRT83	-0.06543	-0.26141	-0.2856	0.775854	-5.909	0.819233	0.863577
Neutrophils	AU041133	-0.14283	1.264215	-0.28558	0.77587	-5.09775	0.784303	0.827759
Neutrophils	PNPLA6	0.082494	2.760588	0.285571	0.775875	-5.44028	0.75151	0.794027
Neutrophils	BRDT	0.153579	1.567286	0.285568	0.775877	-5.13537	0.777546	0.820816
Neutrophils	SMG8	0.078988	2.847118	0.285194	0.776163	-5.51687	0.74976	0.792163
Neutrophils	DPY19L4	0.054257	4.417697	0.28483	0.776441	-5.82217	0.716931	0.758334
Neutrophils	SH3YL1	0.109343	1.030881	0.28475	0.776502	-5.298	0.789655	0.833243
Neutrophils	PSMC3	-0.03436	6.11628	-0.28468	0.776556	-6.23346	0.6831	0.723326
Neutrophils	PDIK1L	0.077673	3.065249	0.284609	0.776609	-5.54116	0.745109	0.787455
Neutrophils	BLOC1S4	0.061767	3.695064	0.284304	0.776842	-5.65949	0.731847	0.773811
Neutrophils	CSNK2B	-0.0285	7.247784	-0.2843	0.776845	-6.59473	0.661496	0.700942
Neutrophils	WDR53	0.095491	2.715022	0.284226	0.776902	-5.42097	0.752591	0.795202
Neutrophils	PDXK	0.070331	4.441778	0.284218	0.776908	-5.67994	0.716439	0.757894
Neutrophils	GM5431	-0.18305	0.742737	-0.28405	0.777033	-5.17384	0.79618	0.840015
Neutrophils	PARP8	0.039164	6.68237	0.284022	0.777058	-6.41937	0.6722	0.712062
Neutrophils	AGXT2	-0.13162	1.612637	-0.28401	0.777068	-5.36363	0.776646	0.819955
Neutrophils	EMC4	-0.05681	4.771124	-0.28396	0.777106	-5.85867	0.70975	0.750977
Neutrophils	1110002L0	0.102884	2.8153	0.283941	0.777119	-5.35554	0.750441	0.792987
Neutrophils	COIL	-0.09784	3.741294	-0.28391	0.777141	-5.33182	0.730883	0.772816
Neutrophils	TBRG4	0.065237	4.075048	0.283874	0.777171	-5.72255	0.723964	0.76567
Neutrophils	CETN2	-0.0353	5.798667	-0.28382	0.777213	-6.16003	0.689297	0.729816
Neutrophils	HNRNPK	-0.01919	8.860645	-0.28379	0.777235	-6.81693	0.631939	0.670193
Neutrophils	GCFC2	-0.10575	2.248325	-0.28363	0.777357	-5.25262	0.762707	0.805611
Neutrophils	AGFG1	0.029217	6.147106	0.283544	0.777423	-6.39253	0.682526	0.722771
Neutrophils	TIAM1	0.036523	4.628891	0.283451	0.777494	-6.4958	0.712656	0.753995
Neutrophils	ZFP335	-0.08255	2.842169	-0.28335	0.777574	-5.4521	0.749892	0.792434
Neutrophils	EPSTI1	0.059688	7.192656	0.283166	0.777711	-6.47044	0.662555	0.702081
Neutrophils	TMPO	0.038302	7.067829	0.283161	0.777716	-6.57262	0.664906	0.704525
Neutrophils	MAD2L1	0.064493	4.166093	0.283081	0.777776	-5.92289	0.722113	0.763821

Neutrophils	UBAC1	-0.06908	4.029628	-0.28303	0.777818	-5.52129	0.724927	0.766744
Neutrophils	RAB43	-0.03616	6.948549	-0.28288	0.777933	-6.56903	0.667165	0.706923
Neutrophils	MAP2K7	0.056174	4.294187	0.282771	0.778013	-5.71582	0.719486	0.761159
Neutrophils	FEM1B	-0.06758	4.728435	-0.28268	0.778085	-5.71539	0.710641	0.752015
Neutrophils	PEX11B	-0.0807	3.446594	-0.28262	0.778126	-5.48731	0.737079	0.77933
Neutrophils	SKA1	0.09516	2.918501	0.282567	0.778169	-5.68946	0.748264	0.790874
Neutrophils	PPP1R9B	0.054623	4.375184	0.282378	0.778314	-5.93642	0.717896	0.759538
Neutrophils	HP1BP3	-0.03315	6.529858	-0.28213	0.778503	-6.40236	0.675226	0.715383
Neutrophils	DCPS	0.054639	5.004121	0.282129	0.778504	-5.85564	0.705169	0.746426
Neutrophils	LGALS9	0.038957	7.157478	0.282014	0.778592	-6.54527	0.663299	0.703012
Neutrophils	ATG2B	-0.05041	4.861013	-0.28199	0.77861	-5.88877	0.708048	0.749427
Neutrophils	CUTC	0.072056	3.519969	0.281853	0.778715	-5.53113	0.735659	0.777969
Neutrophils	ACRBP	-0.10669	1.566441	-0.28155	0.778944	-5.34932	0.777989	0.821493
Neutrophils	UBXN8	0.045338	4.404408	0.281344	0.779104	-5.97095	0.717578	0.759181
Neutrophils	ZFP748	0.124318	1.938501	0.281133	0.779265	-5.27882	0.769949	0.813138
Neutrophils	SRPR	0.028154	5.794906	0.280993	0.779372	-6.28852	0.689847	0.73043
Neutrophils	ACP1	0.03995	5.923129	0.280894	0.779448	-6.17668	0.687341	0.727842
Neutrophils	CLP1	0.050661	4.608479	0.280763	0.779548	-5.88272	0.713569	0.755007
Neutrophils	ZFP982	-0.19358	0.056297	-0.28067	0.779616	-5.07604	0.81254	0.856946
Neutrophils	STAM	0.048026	4.382468	0.280524	0.779731	-5.83616	0.71822	0.759865
Neutrophils	TOMM40	-0.05525	5.225725	-0.28041	0.779815	-5.8159	0.701194	0.742235
Neutrophils	CENPX	0.035776	6.347296	0.280124	0.780037	-6.33696	0.679314	0.719479
Neutrophils	UTP14B	0.073558	3.081232	0.279954	0.780166	-5.64592	0.745546	0.787992
Neutrophils	GM16618	0.150114	0.733543	0.279849	0.780247	-5.09411	0.797219	0.841172
Neutrophils	CBR4	-0.09363	2.335594	-0.27971	0.780351	-5.34813	0.761576	0.804608
Neutrophils	SIRT6	-0.09676	2.652878	-0.27965	0.780398	-5.40414	0.754712	0.797542
Neutrophils	PHB2	-0.03569	6.570723	-0.27958	0.780454	-6.2446	0.675039	0.715144
Neutrophils	LGALSL	-0.14023	1.281509	-0.27954	0.780482	-5.2	0.784841	0.828538
Neutrophils	MS4A6B	-0.14175	4.588359	-0.27933	0.780643	-5.57863	0.714282	0.755797
Neutrophils	MBTPS2	-0.0691	3.917498	-0.2792	0.780743	-5.60424	0.728086	0.770043
Neutrophils	YBX1	0.029148	8.398771	0.27912	0.780805	-6.71038	0.641019	0.679739
Neutrophils	AGBL2	0.140149	0.26749	0.278737	0.781097	-5.14064	0.808251	0.852454
Neutrophils	FLNA	0.030676	6.097597	0.278526	0.781259	-6.72775	0.684552	0.724856
Neutrophils	IGSF8	0.076515	4.266842	0.278016	0.781649	-5.56123	0.721342	0.76284
Neutrophils	TACC3	0.066968	5.072041	0.277906	0.781733	-6.14695	0.704989	0.745949
Neutrophils	TTC39B	0.04405	4.653747	0.27786	0.781769	-6.13063	0.713436	0.754686
Neutrophils	FAM53B	-0.07264	4.652028	-0.27785	0.781776	-5.7718	0.713471	0.754722
Neutrophils	RETREG3	0.046653	5.527566	0.27782	0.781799	-5.97197	0.69591	0.736548
Neutrophils	RSRC2	0.022444	6.479986	0.277538	0.782015	-6.45242	0.67733	0.717363
Neutrophils	SGTB	-0.15893	0.3418	-0.2774	0.782119	-5.08631	0.806842	0.850948
Neutrophils	UPP2	-0.13248	1.652112	-0.27719	0.782278	-5.35187	0.77721	0.820576
Neutrophils	ZC4H2	-0.16457	0.054562	-0.27719	0.78228	-5.0339	0.813489	0.857808
Neutrophils	CBR3	-0.16494	0.624626	-0.27719	0.782285	-5.16266	0.800351	0.844339
Neutrophils	AKIRIN2	-0.03897	5.756269	-0.27715	0.782309	-6.11671	0.691408	0.732016
Neutrophils	BSPRY	-0.1682	0.341542	-0.27713	0.782331	-5.08218	0.806848	0.851002
Neutrophils	PCBP4	-0.17417	0.472145	-0.27704	0.7824	-5.05079	0.803844	0.847921
Neutrophils	RAB39	-0.14489	2.115142	-0.27692	0.782491	-5.23915	0.767004	0.810112

Neutrophils	CNDP2	0.057849	5.051865	0.276894	0.782508	-5.87525	0.705405	0.746543
Neutrophils	GM49359	-0.09901	2.260358	-0.27673	0.782635	-5.3585	0.763888	0.806874
Neutrophils	RAD51AP1	0.102473	3.61991	0.276617	0.78272	-5.7129	0.734844	0.776931
Neutrophils	PTGS2OS	0.134281	-1.09526	0.276374	0.782906	-5.31837	0.840762	0.885695
Neutrophils	ZFP821	-0.05706	3.905173	-0.27627	0.782982	-5.80277	0.728924	0.770807
Neutrophils	LRRC57	0.094382	2.732576	0.276169	0.783063	-5.53346	0.75371	0.796371
Neutrophils	GM9949	0.106766	0.483247	0.276114	0.783105	-5.4886	0.803697	0.847748
Neutrophils	NCOA5	-0.07776	4.146435	-0.2761	0.783114	-5.54272	0.723929	0.765648
Neutrophils	ERLIN1	0.05888	4.765624	0.275916	0.783256	-5.79922	0.711273	0.752607
Neutrophils	SH2D4B	-0.07424	4.439997	-0.27583	0.783325	-5.94108	0.7179	0.759483
Neutrophils	GM50399	-0.20085	0.166375	-0.2758	0.783347	-5.07028	0.811004	0.855313
Neutrophils	EXOC6	0.028957	6.194009	0.275655	0.783456	-6.49305	0.682948	0.723289
Neutrophils	ZFP532	-0.11891	1.223667	-0.27564	0.783466	-5.40985	0.786881	0.830566
Neutrophils	LRRC4	0.093936	3.774431	0.275548	0.783538	-5.47516	0.731645	0.773686
Neutrophils	FCRL6	-0.16783	-0.87747	-0.27546	0.783606	-5.01466	0.835549	0.880444
Neutrophils	PACS1	-0.0339	5.972963	-0.27538	0.783665	-6.50019	0.687253	0.727754
Neutrophils	MRPL27	-0.06595	4.210901	-0.27521	0.783795	-5.70267	0.722601	0.764344
Neutrophils	PIK3IP1	-0.11033	2.920799	-0.27521	0.783797	-5.38412	0.749673	0.792283
Neutrophils	GM31812	-0.16459	0.191942	-0.2748	0.784107	-5.10133	0.810647	0.854845
Neutrophils	ADORA2B	-0.15472	0.37813	-0.27468	0.784203	-5.2044	0.806348	0.850453
Neutrophils	ZFP677	0.138318	0.788621	0.274605	0.784261	-5.13554	0.79695	0.840815
Neutrophils	SAMD1	-0.04946	4.976188	-0.27455	0.784304	-5.95084	0.707227	0.748369
Neutrophils	CCDC34	0.058134	4.840796	0.274218	0.784557	-6.15064	0.710092	0.751211
Neutrophils	KLF7	-0.03062	6.159891	-0.27417	0.784591	-6.7226	0.683938	0.724124
Neutrophils	GZMC	0.290907	1.539474	0.274029	0.784701	-5.28389	0.780231	0.823476
Neutrophils	PUS7	0.115926	2.95762	0.273762	0.784906	-5.22325	0.749413	0.791709
Neutrophils	CYP4F13	-0.07104	2.829707	-0.27363	0.785008	-5.63454	0.752183	0.794572
Neutrophils	PSMG3	-0.09227	3.202758	-0.27313	0.785392	-5.42174	0.744354	0.786532
Neutrophils	MRPL3	0.067089	4.701467	0.273016	0.785478	-5.70006	0.71323	0.754414
Neutrophils	WDR78	0.152732	0.561969	0.272936	0.785539	-5.13246	0.802631	0.846464
Neutrophils	RSL1D1	-0.0502	6.043414	-0.27291	0.785558	-5.97084	0.68651	0.726749
Neutrophils	BTBD8	-0.1355	1.572677	-0.2729	0.785566	-5.21088	0.779797	0.823026
Neutrophils	DNHD1	0.12328	1.01891	0.272608	0.78579	-5.26572	0.792225	0.835849
Neutrophils	MTAP	0.095802	3.671485	0.27246	0.785904	-5.35287	0.734472	0.776454
Neutrophils	CEP95	0.048865	4.108354	0.272386	0.785961	-5.79972	0.725383	0.767092
Neutrophils	CPEB3	-0.07202	4.234811	-0.27231	0.786016	-5.88036	0.722774	0.764408
Neutrophils	NDUFAB1	-0.04384	6.268706	-0.27224	0.786076	-6.23836	0.682128	0.722331
Neutrophils	4833408A1	-0.17574	0.127774	-0.2722	0.786103	-5.11464	0.812647	0.856892
Neutrophils	KAT6B	-0.03603	5.844837	-0.27217	0.786124	-6.25311	0.690397	0.730921
Neutrophils	HEYL	0.179874	-0.20551	0.272137	0.786151	-5.03785	0.82042	0.864862
Neutrophils	SETD6	-0.12494	1.423905	-0.27207	0.786202	-5.11449	0.783116	0.826602
Neutrophils	HNF4A	-0.15147	0.691681	-0.27191	0.786329	-5.18392	0.799663	0.843594
Neutrophils	LIN52	-0.05251	5.546829	-0.27179	0.786418	-5.98596	0.696274	0.737018
Neutrophils	SPAG7	0.050156	4.55728	0.271748	0.78645	-5.92971	0.716165	0.757604
Neutrophils	TSPAN6	0.156747	1.103606	0.271622	0.786546	-5.19766	0.790311	0.833996
Neutrophils	ZRANB2	0.037455	5.45391	0.271537	0.786611	-6.06674	0.698117	0.738953
Neutrophils	B430306N(	-0.04661	2.692804	-0.27151	0.786633	-6.12588	0.755261	0.797979

Neutrophils	ECM1	-0.10998	3.680244	-0.27146	0.786671	-5.76785	0.734289	0.776353
Neutrophils	NAT8L	-0.09606	0.332665	-0.2714	0.786716	-5.45936	0.807905	0.852075
Neutrophils	TATDN2	0.038326	5.004884	0.271353	0.786752	-6.06828	0.707095	0.748249
Neutrophils	VSIG10	-0.12439	0.47062	-0.27114	0.786918	-5.26477	0.804779	0.848818
Neutrophils	XAB2	0.062597	3.922858	0.271052	0.786983	-5.73494	0.729275	0.771129
Neutrophils	CCDC173	-0.13039	0.767334	-0.27101	0.787011	-5.20181	0.797988	0.841852
Neutrophils	SPACA9	0.091422	1.758151	0.270825	0.787157	-5.3881	0.775803	0.818991
Neutrophils	GM49864	0.148722	0.452692	0.270413	0.787473	-5.14107	0.805453	0.84931
Neutrophils	RELN	0.108263	3.647841	0.270408	0.787476	-5.66367	0.735253	0.777117
Neutrophils	ZFP219	-0.10819	1.771622	-0.27023	0.787611	-5.22117	0.775746	0.818765
Neutrophils	RAD9B	0.081081	3.275507	0.27002	0.787774	-5.50605	0.74325	0.785291
Neutrophils	UNC13B	0.123882	0.758993	0.269784	0.787955	-5.2976	0.798659	0.842243
Neutrophils	UBL5	0.021681	8.259007	0.269736	0.787992	-6.76621	0.645099	0.683605
Neutrophils	GPX4	0.022591	7.153816	0.269668	0.788044	-6.64586	0.665637	0.70496
Neutrophils	IDI1	0.058025	3.90584	0.269557	0.788129	-5.76352	0.730071	0.771673
Neutrophils	CDR2	0.089903	2.488604	0.26942	0.788234	-5.54568	0.760184	0.80271
Neutrophils	STARD10	-0.07473	4.596544	-0.26922	0.788386	-6.06348	0.715845	0.756979
Neutrophils	HELQ	-0.10358	2.411841	-0.26921	0.788394	-5.23317	0.761851	0.804426
Neutrophils	LTB4R1	0.03659	2.689349	0.269143	0.788447	-6.58758	0.755842	0.798241
Neutrophils	RYK	0.095344	2.042505	0.269117	0.788466	-5.43121	0.769924	0.81273
Neutrophils	NSUN3	0.07184	2.658689	0.268832	0.788686	-5.50278	0.756539	0.798922
Neutrophils	GAS2	0.094354	2.456745	0.268757	0.788743	-5.35794	0.760911	0.803422
Neutrophils	GM10863	-0.19074	0.229142	-0.26875	0.788749	-5.04956	0.810879	0.854726
Neutrophils	ADAM19	-0.06804	5.568234	-0.26845	0.788975	-6.11343	0.696349	0.736776
Neutrophils	ETNK2	-0.13847	1.270533	-0.2684	0.789015	-5.19547	0.787118	0.830359
Neutrophils	GALK2	0.058251	4.897213	0.268337	0.789065	-5.68944	0.709775	0.750669
Neutrophils	WFDC21	0.060344	2.947804	0.268254	0.789129	-6.77641	0.750325	0.792522
Neutrophils	CLN8	0.08817	3.202024	0.268242	0.789138	-5.56633	0.744904	0.786936
Neutrophils	CELF1	-0.02205	7.40812	-0.26815	0.789206	-6.53231	0.660885	0.699994
Neutrophils	FARS2	0.029606	6.899126	0.268	0.789324	-6.2855	0.670502	0.710018
Neutrophils	PHPT1	0.066877	4.330971	0.267989	0.789332	-5.6422	0.721314	0.762641
Neutrophils	LAYN	-0.12498	0.629576	-0.26798	0.789341	-5.30126	0.801658	0.845326
Neutrophils	HDGFL2	0.042917	4.979057	0.267144	0.78998	-5.95928	0.708591	0.749188
Neutrophils	TMA16	-0.08234	3.964843	-0.26712	0.790002	-5.64365	0.72936	0.770653
Neutrophils	1110038B1	-0.07568	4.235292	-0.267	0.79009	-5.58782	0.723778	0.764914
Neutrophils	CALU	0.03959	4.640586	0.266821	0.790228	-5.9982	0.715504	0.756344
Neutrophils	STAP2	-0.12079	0.504981	-0.26677	0.790268	-5.31693	0.805105	0.848544
Neutrophils	GM8066	0.157994	0.498967	0.266545	0.79044	-5.05461	0.80526	0.84871
Neutrophils	PTDSS2	-0.08955	3.06586	-0.26629	0.790634	-5.32335	0.748366	0.790368
Neutrophils	POLRMT	0.133872	1.91437	0.266059	0.790813	-5.1356	0.773363	0.816122
Neutrophils	ATE1	-0.04748	3.945361	-0.26603	0.790832	-5.79287	0.729833	0.771292
Neutrophils	ZMAT4	-0.20585	0.381713	-0.26603	0.790836	-5.15746	0.807961	0.851629
Neutrophils	PIK3CD	0.043393	6.624599	0.265899	0.790936	-6.29263	0.676259	0.715864
Neutrophils	IFT27	0.0845	3.758551	0.265821	0.790996	-5.51724	0.733729	0.775321
Neutrophils	TSTD3	0.107759	1.784277	0.265753	0.791048	-5.26226	0.776241	0.819094
Neutrophils	ZFP407	0.02268	7.039039	0.265561	0.791196	-6.64345	0.668346	0.707705
Neutrophils	METTL4	-0.08671	2.874732	-0.26551	0.791235	-5.35636	0.752457	0.794692

Neutrophils	SHMT1	0.074718	4.012421	0.265444	0.791285	-5.68954	0.728439	0.769951
Neutrophils	NDUFA11	-0.03295	6.910675	-0.26531	0.791386	-6.43652	0.670787	0.710292
Neutrophils	ZNFX1	0.068069	4.359029	0.265251	0.791434	-5.93417	0.721281	0.762597
Neutrophils	BC017158	-0.13451	1.449949	-0.26523	0.791453	-5.19107	0.783685	0.826877
Neutrophils	USP31	-0.08283	3.715776	-0.2652	0.791472	-5.52312	0.734624	0.776376
Neutrophils	AATK	0.09618	0.48294	0.265147	0.791513	-5.52824	0.805628	0.849419
Neutrophils	ICA1	-0.05684	2.463147	-0.26511	0.791539	-6.05456	0.761345	0.803937
Neutrophils	SSX2IP	-0.10238	2.481205	-0.26511	0.791542	-5.45329	0.760953	0.803533
Neutrophils	NAGPA	-0.07473	3.354165	-0.26481	0.791771	-5.47078	0.742281	0.784286
Neutrophils	CAMK1	-0.0513	2.966119	-0.26479	0.791785	-5.98404	0.750542	0.7928
Neutrophils	LLGL1	-0.08099	2.998446	-0.26478	0.791798	-5.44098	0.74985	0.792087
Neutrophils	LSM2	0.053003	5.695608	0.264421	0.792071	-6.13376	0.694478	0.734876
Neutrophils	IL33	0.13851	0.25962	0.264408	0.792081	-5.17939	0.810929	0.854828
Neutrophils	RBM34	-0.05132	4.465573	-0.26413	0.792294	-5.69624	0.719223	0.760557
Neutrophils	TFEB	0.041721	5.158966	0.264085	0.792329	-6.06798	0.705163	0.746016
Neutrophils	ERLIN2	0.069518	3.099067	0.264048	0.792357	-5.56392	0.747791	0.790038
Neutrophils	ACAD8	-0.07026	2.476536	-0.26401	0.792383	-5.54361	0.76119	0.803839
Neutrophils	BCO2	0.121887	0.643324	0.263876	0.792489	-5.18175	0.80209	0.845902
Neutrophils	COX4I1	0.02104	9.086822	0.263622	0.792685	-6.88329	0.630776	0.668861
Neutrophils	2900089D1	-0.09453	2.58395	-0.26361	0.792697	-5.4529	0.758861	0.80157
Neutrophils	FNDC7	-0.16549	-0.36573	-0.26353	0.792758	-5.08171	0.825544	0.870078
Neutrophils	GBP4	0.167916	3.363964	0.263205	0.793005	-5.4276	0.742163	0.784476
Neutrophils	GATD1	0.054573	4.394103	0.26319	0.793016	-5.76273	0.720689	0.762304
Neutrophils	MAPRE1	0.0208	7.148653	0.26318	0.793024	-6.57486	0.666389	0.706031
Neutrophils	NELFB	-0.04581	4.551192	-0.26318	0.793027	-5.84585	0.717472	0.758978
Neutrophils	TXN2	0.033492	6.315861	0.263154	0.793044	-6.24002	0.682339	0.722591
Neutrophils	2510039O1	-0.03886	5.179646	-0.26315	0.793049	-6.05557	0.704748	0.745815
Neutrophils	2610027K0	0.128116	0.681173	0.263107	0.793081	-5.17573	0.801223	0.845232
Neutrophils	PHKB	0.040056	5.588989	0.263011	0.793154	-6.17111	0.696588	0.737378
Neutrophils	CDC40	-0.03275	6.047181	-0.26277	0.793342	-6.28449	0.68757	0.72806
Neutrophils	RAB11FIP3	0.098972	2.230172	0.262712	0.793384	-5.30984	0.766562	0.809661
Neutrophils	FOSL1	-0.04309	1.662098	-0.26257	0.793494	-6.33896	0.779093	0.822555
Neutrophils	DNAL1	-0.10387	1.64713	-0.26251	0.79354	-5.36755	0.779426	0.822897
Neutrophils	RBFOX3	0.160077	-0.24977	0.262337	0.793672	-5.13236	0.822815	0.867421
Neutrophils	STX3	0.114179	2.314188	0.262335	0.793674	-5.37756	0.764726	0.807786
Neutrophils	ORC1	-0.08364	3.345968	-0.26231	0.793693	-5.57597	0.742545	0.784939
Neutrophils	E230029C0	0.072013	3.508508	0.262287	0.79371	-5.79762	0.739111	0.781397
Neutrophils	PSME4	0.030268	7.130098	0.261969	0.793955	-6.52027	0.666888	0.706588
Neutrophils	ASPDH	-0.12653	1.294554	-0.26154	0.794284	-5.32667	0.787743	0.831265
Neutrophils	SLC30A7	-0.03332	5.62501	-0.26137	0.794416	-6.33131	0.696309	0.736908
Neutrophils	GM16541	-0.07691	2.915994	-0.26124	0.794518	-5.51866	0.752207	0.794623
Neutrophils	ABITRAM	0.090137	2.584915	0.261113	0.794613	-5.30571	0.759364	0.802024
Neutrophils	BLZF1	-0.06872	3.200504	-0.26103	0.794677	-5.52864	0.746145	0.788443
Neutrophils	NR4A2	-0.08175	6.380914	-0.26086	0.794808	-6.19673	0.681563	0.721649
Neutrophils	ELP4	-0.05566	4.963596	-0.26079	0.794858	-5.80367	0.7096	0.750709
Neutrophils	AKR1C12	0.122507	0.756271	0.260734	0.794904	-5.19563	0.800076	0.843909
Neutrophils	CTBS	-0.05316	2.934998	-0.26064	0.79498	-5.76656	0.751839	0.794345

Neutrophils	PTPRS	-0.10104	4.390471	-0.26038	0.795179	-5.34901	0.721376	0.762858
Neutrophils	PARP3	0.09637	2.362796	0.260319	0.795223	-5.38591	0.764314	0.807139
Neutrophils	GM28501	-0.15414	0.476037	-0.26003	0.795442	-5.04717	0.806758	0.850666
Neutrophils	CCDC115	-0.0479	4.152653	-0.25996	0.795502	-5.81429	0.726408	0.768001
Neutrophils	BMPR2	0.036997	5.519027	0.259854	0.79558	-6.39062	0.698698	0.739337
Neutrophils	MED27	-0.03934	5.275175	-0.25945	0.795893	-5.89816	0.703747	0.744443
Neutrophils	1700034P1	0.143873	1.464772	0.259305	0.796003	-5.2671	0.784511	0.827705
Neutrophils	CLIC4	-0.05493	7.449524	-0.2592	0.796085	-6.40951	0.661582	0.700719
Neutrophils	ZDHHC23	0.131799	2.221064	0.259191	0.796091	-5.11643	0.767755	0.810484
Neutrophils	PBRM1	0.021964	7.416728	0.259128	0.796139	-6.54622	0.662198	0.701368
Neutrophils	SLC6A13	0.048927	2.762131	0.258942	0.796282	-6.05366	0.756	0.798412
Neutrophils	RETNLA	0.582585	-0.77811	0.258797	0.796393	-5.05109	0.836417	0.880921
Neutrophils	MAML3	0.042631	6.974808	0.258792	0.796397	-6.5138	0.670562	0.710073
Neutrophils	PIBF1	-0.04323	5.059147	-0.25875	0.796426	-6.00513	0.708094	0.748971
Neutrophils	UTP18	0.046899	5.338906	0.258429	0.796677	-5.89005	0.702527	0.743244
Neutrophils	INO80DOS	0.058067	4.442013	0.258412	0.796689	-6.05451	0.720697	0.762036
Neutrophils	RETSAT	0.088111	1.405172	0.258389	0.796708	-5.29382	0.785909	0.829209
Neutrophils	IL4	-0.20549	0.330173	-0.25832	0.796761	-5.18687	0.81041	0.85436
Neutrophils	HIST1H2AE	0.119483	4.491316	0.258077	0.796947	-5.8421	0.719791	0.761029
Neutrophils	ZBTB21	-0.0683	3.713914	-0.25795	0.797048	-5.55754	0.735942	0.77772
Neutrophils	RABEP1	-0.03236	6.897474	-0.25782	0.797147	-6.25965	0.672231	0.711795
Neutrophils	IFT140	0.080756	3.507073	0.257598	0.797316	-5.47617	0.740413	0.782296
Neutrophils	REC114	0.087371	3.19631	0.257399	0.797469	-5.49734	0.74706	0.78909
Neutrophils	B3GALT6	-0.12124	1.806345	-0.25734	0.797515	-5.2823	0.777285	0.820193
Neutrophils	SRP19	0.032832	6.048783	0.257139	0.797669	-6.24927	0.688846	0.72889
Neutrophils	PSMD5	-0.05619	3.835237	-0.25704	0.797745	-5.65621	0.733656	0.775218
Neutrophils	GM14634	0.104664	1.834434	0.256847	0.797894	-5.36184	0.776826	0.819626
Neutrophils	B2M	0.045005	9.94338	0.256367	0.798263	-7.0266	0.617053	0.654155
Neutrophils	SF3B6	-0.02247	7.294759	-0.25636	0.798272	-6.57196	0.665106	0.70418
Neutrophils	LYVE1	-0.08914	2.42534	-0.25626	0.798343	-5.8373	0.763997	0.806421
Neutrophils	5730522E0	-0.12156	3.610742	-0.25622	0.798378	-5.42547	0.738596	0.780265
Neutrophils	FSCN1	0.114009	3.378992	0.256171	0.798414	-5.74443	0.743493	0.785323
Neutrophils	PDCD2	-0.07878	3.817161	-0.25589	0.798632	-5.4313	0.734351	0.775898
Neutrophils	ADO	-0.07125	3.708631	-0.25587	0.798649	-5.4831	0.736626	0.778253
Neutrophils	THOC5	-0.06926	3.564433	-0.2555	0.798929	-5.48634	0.739751	0.781502
Neutrophils	TCHP	0.121623	1.792962	0.255405	0.799004	-5.15823	0.7781	0.821016
Neutrophils	KAT8	-0.05994	3.624929	-0.2554	0.799006	-5.66173	0.738477	0.780227
Neutrophils	ADAL	0.103809	2.279066	0.255295	0.799088	-5.21222	0.767378	0.810013
Neutrophils	SSR3	-0.02989	6.071068	-0.25513	0.799213	-6.30567	0.688796	0.728891
Neutrophils	JAM3	-0.13843	0.244994	-0.2551	0.799236	-5.1782	0.813267	0.857123
Neutrophils	DMTN	-0.13312	0.637339	-0.2551	0.799237	-5.19731	0.804204	0.847836
Neutrophils	2810002D1	-0.08968	1.66458	-0.25478	0.799482	-5.27821	0.781092	0.824046
Neutrophils	FAM210A	0.063045	3.636981	0.25474	0.799515	-5.55634	0.73835	0.780055
Neutrophils	FSHR	-0.11033	0.419984	-0.25463	0.799598	-5.14508	0.809365	0.853037
Neutrophils	HSPA1B	0.185236	4.531616	0.254516	0.799688	-5.7982	0.719794	0.76087
Neutrophils	ARF2	0.058119	4.470722	0.254209	0.799924	-5.86053	0.721131	0.762233
Neutrophils	TMEM11	-0.04607	5.070903	-0.25414	0.799981	-5.90014	0.708911	0.74961

Neutrophils	GM11837	0.163777	0.292319	0.25407	0.800031	-5.1331	0.812439	0.856186
Neutrophils	TGM1	-0.12712	0.53017	-0.25388	0.800178	-5.28413	0.806939	0.850608
Neutrophils	ACIN1	-0.02042	7.577859	-0.25383	0.800215	-6.5916	0.660166	0.69915
Neutrophils	ADAM22	0.152046	0.796933	0.253828	0.800217	-5.15935	0.800814	0.844329
Neutrophils	C2CD5	-0.04639	4.918041	-0.25364	0.800365	-5.93447	0.712003	0.752888
Neutrophils	UTP11	-0.03955	4.845019	-0.2536	0.80039	-5.98805	0.713485	0.75442
Neutrophils	DTX3L	0.074981	4.876216	0.253572	0.800415	-5.93233	0.712851	0.753765
Neutrophils	MAP1LC3A	0.040747	5.476214	0.253102	0.800777	-6.33985	0.701038	0.741385
Neutrophils	EDC4	-0.08705	2.984777	-0.25261	0.801154	-5.44974	0.752871	0.79475
Neutrophils	RGS10	-0.06135	5.509055	-0.25256	0.801198	-5.72614	0.700628	0.740838
Neutrophils	ADAM12	-0.13118	0.815122	-0.25237	0.801341	-5.26349	0.801044	0.844234
Neutrophils	EXOSC1	0.060156	4.175365	0.252274	0.801415	-5.58948	0.727814	0.768919
Neutrophils	AI504432	-0.11748	2.840905	-0.25211	0.801538	-5.15512	0.75609	0.798009
Neutrophils	CNPY4	-0.08695	2.433697	-0.2516	0.801934	-5.35156	0.765159	0.807196
Neutrophils	COPE	-0.02996	6.316581	-0.25138	0.802105	-6.31115	0.685044	0.724513
Neutrophils	HAT1	0.039704	6.105162	0.251276	0.802184	-6.17133	0.689173	0.72879
Neutrophils	GPRC5C	-0.13068	1.29284	-0.25125	0.802206	-5.35318	0.790487	0.833212
Neutrophils	IGF1R	-0.02508	6.357541	-0.25124	0.802211	-6.84184	0.684247	0.723687
Neutrophils	ANXA10	0.128298	0.643116	0.251186	0.802253	-5.20055	0.805291	0.848387
Neutrophils	APMAP	0.036417	4.080552	0.25116	0.802273	-6.12772	0.730054	0.771046
Neutrophils	CPSF4	0.056667	4.317677	0.250813	0.80254	-5.77408	0.725192	0.766013
Neutrophils	CTNNA1	-0.03536	6.643473	-0.25072	0.802614	-6.3997	0.678762	0.717986
Neutrophils	KLF8	0.170711	0.979838	0.250216	0.803001	-5.10345	0.797644	0.840641
Neutrophils	SACM1L	-0.03431	5.504292	-0.2501	0.803088	-6.19437	0.701103	0.741218
Neutrophils	POLR2C	0.03998	5.080792	0.250035	0.80314	-6.0012	0.709603	0.750009
Neutrophils	XPO1	-0.04072	5.786847	-0.24997	0.803194	-6.15945	0.69549	0.735424
Neutrophils	SNRNP35	0.07452	3.056704	0.249956	0.803201	-5.47579	0.751735	0.79349
Neutrophils	XXYLT1	-0.08379	3.591278	-0.2499	0.803245	-5.38565	0.740362	0.781791
Neutrophils	NAV2	-0.07029	4.841571	-0.24985	0.803279	-5.80437	0.714452	0.755068
Neutrophils	GM16638	-0.07922	2.040296	-0.24982	0.803304	-5.46619	0.773856	0.816282
Neutrophils	CCP110	0.08945	2.37539	0.249814	0.803311	-5.37998	0.76649	0.808711
Neutrophils	HIST1H3C	0.161018	1.047316	0.249618	0.803461	-5.19646	0.796108	0.839242
Neutrophils	BST1	0.04405	3.315218	0.249596	0.803478	-6.29011	0.746213	0.787952
Neutrophils	GALNT10	0.061943	4.614762	0.249545	0.803518	-5.74629	0.719081	0.759962
Neutrophils	NFATC2IP	-0.07909	3.151572	-0.24949	0.803564	-5.45274	0.749703	0.791548
Neutrophils	FOSL2	-0.0288	5.561041	-0.24947	0.803578	-6.69784	0.699972	0.740202
Neutrophils	GEMIN2	0.0716	3.260952	0.24944	0.803599	-5.49144	0.747368	0.789143
Neutrophils	PBLD1	0.116406	1.75083	0.249439	0.8036	-5.38234	0.780276	0.822993
Neutrophils	POLE3	-0.06411	4.133596	-0.24935	0.803666	-5.66088	0.729006	0.770212
Neutrophils	EPHA1	0.10786	0.461905	0.249186	0.803795	-5.36331	0.80959	0.853065
Neutrophils	HIST1H2BG	-0.14405	1.128352	-0.24891	0.804009	-5.17438	0.794365	0.83751
Neutrophils	NOL6	0.09989	2.582305	0.248703	0.804167	-5.25157	0.762071	0.804342
Neutrophils	LRRC41	0.041545	4.919545	0.248693	0.804175	-6.00224	0.712955	0.75369
Neutrophils	NME7	-0.07352	3.562527	-0.24866	0.804198	-5.36741	0.74106	0.782708
Neutrophils	SDHB	0.031686	7.274793	0.248601	0.804246	-6.51303	0.666786	0.705885
Neutrophils	AEN	0.090858	3.664015	0.248532	0.804299	-5.40304	0.738919	0.780528
Neutrophils	MRAP	-0.16038	1.214744	-0.24846	0.804355	-5.16754	0.792408	0.835547

Neutrophils	NKRF	0.092298	2.983842	0.248362	0.804431	-5.34177	0.753391	0.795445
Neutrophils	SNX16	-0.09159	2.847666	-0.24832	0.804466	-5.31949	0.756323	0.798464
Neutrophils	PPCDC	-0.05985	3.567947	-0.24815	0.804598	-5.65542	0.741002	0.782669
Neutrophils	GPD1	-0.11426	1.749644	-0.24776	0.804896	-5.31096	0.780564	0.823386
Neutrophils	HIST1H2AK	0.139627	0.954719	0.247714	0.80493	-5.20551	0.798484	0.841795
Neutrophils	PEG13	0.066194	2.114243	0.247678	0.804958	-5.62277	0.772483	0.815099
Neutrophils	GM16152	-0.14596	2.101073	-0.24761	0.805011	-5.13537	0.772773	0.815397
Neutrophils	ACAD9	0.080443	2.542008	0.247549	0.805058	-5.40206	0.76311	0.80546
Neutrophils	ADGRG1	0.153217	1.720983	0.247406	0.805167	-5.20321	0.781241	0.824059
Neutrophils	SMC2	-0.05187	5.816825	-0.24713	0.80538	-6.34358	0.695288	0.735381
Neutrophils	6-Sep	-0.04963	5.624977	-0.24685	0.805598	-6.05177	0.69922	0.739355
Neutrophils	GM17106	0.083309	3.395192	0.246707	0.805707	-5.52614	0.745103	0.786699
Neutrophils	1810013L2	-0.02809	5.901803	-0.24654	0.805832	-6.37785	0.693782	0.733678
Neutrophils	RNF25	0.061316	3.103588	0.246508	0.805861	-5.58873	0.751338	0.793108
Neutrophils	NDUFS2	0.034161	6.235293	0.246392	0.80595	-6.27145	0.687251	0.726898
Neutrophils	NDUFA1	-0.02596	6.729501	-0.24625	0.806058	-6.51033	0.677696	0.716965
Neutrophils	CPA6	0.04415	-0.07229	0.246168	0.806122	-6.12271	0.822694	0.866313
Neutrophils	AW549877	0.07351	3.352769	0.246038	0.806223	-5.53804	0.746092	0.787652
Neutrophils	EXT2	-0.05734	3.849599	-0.24573	0.806463	-5.65306	0.735754	0.776922
Neutrophils	D330050I1	-0.12082	1.118758	-0.24518	0.806882	-5.17301	0.795696	0.838305
Neutrophils	NCBP3	0.036096	5.506298	0.245013	0.807014	-6.0595	0.70213	0.741982
Neutrophils	E130317F2	0.129579	0.77534	0.244989	0.807033	-5.06051	0.803538	0.846359
Neutrophils	JPX	0.100171	3.810956	0.244885	0.807113	-5.45692	0.736861	0.777883
Neutrophils	ROBO2	0.155251	0.744725	0.244811	0.80717	-5.20904	0.804241	0.84714
Neutrophils	LMF2	-0.07413	3.103696	-0.24474	0.807222	-5.49277	0.751871	0.793372
Neutrophils	TLR8	0.091823	0.054908	0.244671	0.807279	-5.41164	0.820243	0.863531
Neutrophils	HSPE1-RS1	0.178645	0.18545	0.244413	0.807477	-5.04829	0.81726	0.860452
Neutrophils	4921524J1	0.034355	5.474163	0.2444	0.807487	-6.03242	0.702832	0.742756
Neutrophils	TSACC	0.139174	1.881856	0.244239	0.807611	-5.21971	0.778621	0.820856
Neutrophils	TGTP2	0.181813	0.773598	0.244191	0.807649	-5.18906	0.803654	0.84653
Neutrophils	4930484I0	-0.09125	1.660784	-0.24388	0.807886	-5.3641	0.783551	0.826025
Neutrophils	4930417O1	-0.15478	0.963159	-0.24385	0.807909	-5.12091	0.799315	0.842195
Neutrophils	AAK1	-0.0344	6.392459	-0.24381	0.807941	-6.31327	0.684726	0.724115
Neutrophils	IFT57	0.062441	2.891644	0.243783	0.807964	-5.63468	0.756504	0.79823
Neutrophils	BOLA3	-0.04956	5.736487	-0.24362	0.808087	-5.98074	0.697614	0.737461
Neutrophils	RTCB	0.048383	5.290693	0.243559	0.808136	-5.82458	0.706518	0.746671
Neutrophils	ARHGEF5	-0.14226	0.327235	-0.24347	0.808202	-5.15326	0.813965	0.8572
Neutrophils	RAB1A	0.019053	7.08553	0.243447	0.808223	-6.63679	0.671379	0.710278
Neutrophils	GPC5	0.145475	0.598275	0.243388	0.808268	-5.22185	0.807688	0.850773
Neutrophils	SNX4	0.022485	6.329814	0.242906	0.808641	-6.45926	0.686112	0.725496
Neutrophils	ZBTB5	-0.09769	2.463409	-0.24283	0.808703	-5.28068	0.765991	0.807951
Neutrophils	GM553	-0.13419	0.241543	-0.24279	0.808731	-5.17624	0.816158	0.859408
Neutrophils	MAPK4	0.079457	0.274036	0.242768	0.808747	-5.54816	0.815401	0.858633
Neutrophils	HNRNPR	0.027725	6.29021	0.242679	0.808817	-6.20914	0.686885	0.726342
Neutrophils	EMC7	0.025025	6.059195	0.242479	0.808971	-6.26789	0.691414	0.731089
Neutrophils	COQ4	-0.03962	2.66674	-0.24232	0.809092	-6.01614	0.761563	0.803486
Neutrophils	ABCB7	-0.03693	5.334608	-0.24232	0.809096	-5.97235	0.705811	0.745987

Neutrophils	RRM1	-0.04697	5.449593	-0.24231	0.809102	-6.22199	0.703506	0.743603
Neutrophils	EEF1AKNM	-0.07786	2.318289	-0.24214	0.809231	-5.42883	0.769229	0.811334
Neutrophils	COL20A1	-0.0873	1.340253	-0.24201	0.809335	-5.26528	0.791041	0.833772
Neutrophils	STRN3	0.02482	7.654054	0.241559	0.809682	-6.6362	0.661102	0.699502
Neutrophils	PLAAT3	-0.08785	5.19136	-0.24139	0.809814	-5.7212	0.709072	0.749142
Neutrophils	DEXI	-0.08757	2.91114	-0.24113	0.810015	-5.33968	0.756796	0.798336
Neutrophils	ZFC3H1	-0.02296	6.914629	-0.24086	0.810221	-6.55547	0.67535	0.714181
Neutrophils	PAWR	0.112704	0.662659	0.240803	0.810265	-5.25243	0.807047	0.849868
Neutrophils	NDUFB2	-0.03157	5.815553	-0.24075	0.81031	-6.22374	0.696775	0.736387
Neutrophils	CANX	-0.02243	7.35016	-0.24066	0.810379	-6.48234	0.667052	0.705597
Neutrophils	MRPL44	0.07418	2.945204	0.240451	0.810537	-5.46345	0.756174	0.797649
Neutrophils	NUBPL	-0.08042	2.913085	-0.24043	0.810557	-5.37362	0.756867	0.798362
Neutrophils	RTCA	0.056819	4.282973	0.240227	0.810711	-5.66361	0.727911	0.768499
Neutrophils	GM20492	0.075717	1.798274	0.240121	0.810792	-5.45548	0.781368	0.823501
Neutrophils	OXCT1	-0.03825	5.719852	-0.23995	0.810926	-6.00489	0.698738	0.738365
Neutrophils	GPR137C	0.049367	2.390141	0.239929	0.810941	-5.8886	0.768279	0.81006
Neutrophils	SSR2	0.046715	5.189958	0.239908	0.810957	-5.89756	0.709352	0.749339
Neutrophils	ZFP148	0.023458	6.830997	0.239832	0.811016	-6.42959	0.677017	0.715902
Neutrophils	NLRC5	-0.11699	5.064856	-0.23905	0.811624	-5.7175	0.711964	0.752156
Neutrophils	GM47283	-0.07859	7.990664	-0.23898	0.811672	-6.47282	0.65518	0.693321
Neutrophils	PTP4A2	-0.01616	8.699413	-0.2389	0.811732	-6.78019	0.642157	0.679782
Neutrophils	TBC1D24	0.06794	1.799284	0.238886	0.811747	-5.61269	0.781435	0.8237
Neutrophils	ATF6B	-0.05149	4.456866	-0.23873	0.811867	-5.78527	0.724397	0.765024
Neutrophils	ATP8A2	-0.06637	2.725958	-0.23869	0.811895	-5.93453	0.76104	0.802781
Neutrophils	INO80C	-0.05389	3.401246	-0.23868	0.811903	-5.68319	0.746522	0.787839
Neutrophils	IKZF1	0.022433	8.378161	0.23866	0.811922	-6.83546	0.648026	0.685917
Neutrophils	GM44174	-0.15246	-0.56348	-0.2386	0.811965	-5.07059	0.835984	0.879597
Neutrophils	YBEY	0.093804	1.366271	0.238525	0.812026	-5.23603	0.791156	0.833712
Neutrophils	FLVCR1	-0.03026	4.670527	-0.23849	0.812056	-6.28044	0.720002	0.760493
Neutrophils	GM10371	-0.12821	-0.68835	-0.23845	0.812088	-5.10463	0.838972	0.882648
Neutrophils	CYP2AB1	0.138532	-0.30874	0.238439	0.812092	-5.16763	0.829924	0.873404
Neutrophils	GM34225	0.169326	-0.06364	0.238427	0.812102	-5.02994	0.824134	0.867485
Neutrophils	HMGN5	-0.0447	4.876393	-0.2382	0.812278	-5.94428	0.715794	0.756147
Neutrophils	DTL	0.060517	4.961318	0.238165	0.812304	-6.04163	0.714065	0.754362
Neutrophils	UCP1	-0.1333	-1.00774	-0.23816	0.812305	-5.04058	0.84666	0.890498
Neutrophils	ATAD2B	0.024964	6.740991	0.237764	0.812614	-6.49754	0.678967	0.718003
Neutrophils	KMT5B	-0.02681	5.91383	-0.23773	0.812642	-6.34072	0.695115	0.734737
Neutrophils	DHX16	-0.05588	4.087189	-0.23753	0.812795	-5.73784	0.732216	0.773066
Neutrophils	GM36198	0.08301	2.976425	0.237506	0.812814	-5.4474	0.755776	0.797335
Neutrophils	ITM2B	-0.02256	10.00633	-0.23749	0.812823	-7.1284	0.618985	0.655651
Neutrophils	ARMH3	0.036852	5.427651	0.237359	0.812928	-6.06785	0.704804	0.744753
Neutrophils	SESTD1	-0.14377	2.300083	-0.2373	0.812974	-5.23857	0.770512	0.812492
Neutrophils	PSMC6	-0.02738	6.141841	-0.23679	0.813369	-6.27784	0.690909	0.730149
Neutrophils	SPDYA	-0.14993	-0.02911	-0.23653	0.813567	-5.05367	0.823958	0.866981
Neutrophils	DCBLD2	0.0847	1.32551	0.236379	0.813686	-5.35174	0.792737	0.835017
Neutrophils	ABCA8B	-0.12625	1.118002	-0.23628	0.813765	-5.27646	0.797455	0.839869
Neutrophils	DANCR	-0.1132	1.545559	-0.23619	0.813834	-5.19346	0.787779	0.829999

Neutrophils	ENKD1	0.106018	1.3576	0.235937	0.814027	-5.22169	0.792138	0.834432
Neutrophils	TRAPPC8	0.027437	6.225642	0.235751	0.814171	-6.32981	0.689539	0.728748
Neutrophils	GM15503	0.121488	0.81285	0.235734	0.814185	-5.06227	0.804573	0.847191
Neutrophils	ALPK1	-0.04854	4.802266	-0.23559	0.814293	-6.13033	0.718066	0.758222
Neutrophils	RNF157	0.075923	5.375061	0.235326	0.8145	-5.784	0.706556	0.746229
Neutrophils	TSPAN4	-0.05606	3.356981	-0.23526	0.814551	-5.68422	0.748365	0.789371
Neutrophils	PRR11	0.082916	2.847187	0.235118	0.814661	-5.57904	0.759334	0.800696
Neutrophils	GM48236	-0.16383	0.1418	-0.23507	0.814701	-5.05336	0.820307	0.863218
Neutrophils	PHACTR1	-0.09211	1.519212	-0.23485	0.814868	-5.40154	0.788749	0.830841
Neutrophils	FBXL4	-0.07832	2.64655	-0.23477	0.814927	-5.42308	0.763773	0.805219
Neutrophils	ZDHHC7	0.050297	3.834677	0.234642	0.815029	-5.74318	0.738328	0.779066
Neutrophils	LIFR	-0.06148	3.842745	-0.23446	0.815169	-5.93781	0.738158	0.778913
Neutrophils	AHCTF1	-0.03923	5.405997	-0.23439	0.815226	-6.01214	0.706016	0.745768
Neutrophils	WNK1	0.023795	9.07644	0.234371	0.815239	-6.89715	0.636181	0.673341
Neutrophils	MRPS30	-0.04413	4.726011	-0.23424	0.815337	-5.78485	0.719814	0.760038
Neutrophils	DACH2	-0.16146	0.599965	-0.23406	0.815479	-5.07633	0.809728	0.852503
Neutrophils	SNHG1	-0.04683	5.230493	-0.23401	0.815518	-5.8641	0.709551	0.749479
Neutrophils	ADAM9	-0.0461	5.178783	-0.23387	0.815626	-5.92043	0.710596	0.750588
Neutrophils	ZFP646	0.065137	3.903418	0.233821	0.815665	-5.63106	0.736882	0.777718
Neutrophils	TGTP1	-0.2002	-0.20272	-0.23375	0.815721	-5.05669	0.828506	0.871773
Neutrophils	MEST	-0.06268	3.792937	-0.2337	0.815759	-6.01521	0.739206	0.780147
Neutrophils	PLBD1	-0.02854	5.389651	-0.23368	0.815772	-6.84085	0.706344	0.74623
Neutrophils	RUNX1	0.028582	9.201324	0.233524	0.815895	-6.87898	0.633977	0.671151
Neutrophils	SNX14	0.037203	4.500746	0.233402	0.815989	-6.09859	0.724514	0.764993
Neutrophils	KIF13A	0.040454	4.605824	0.233297	0.81607	-6.07917	0.722359	0.762804
Neutrophils	GM47917	0.132997	0.467794	0.23313	0.8162	-5.06235	0.81292	0.855862
Neutrophils	AP3D1	0.031672	4.873786	0.233058	0.816255	-5.93872	0.716906	0.75718
Neutrophils	SPNS3	0.094514	3.650358	0.232734	0.816506	-5.473	0.742424	0.783416
Neutrophils	USP1	-0.03532	5.737466	-0.23244	0.816734	-6.21585	0.699585	0.73927
Neutrophils	DNAJC25	0.050753	3.856746	0.232388	0.816774	-5.66279	0.738069	0.779006
Neutrophils	TMEM109	-0.07623	3.841384	-0.23233	0.816815	-5.50789	0.738392	0.77934
Neutrophils	RB1	0.02828	7.240705	0.232277	0.81686	-6.51576	0.670336	0.708971
Neutrophils	PURB	0.021798	6.824936	0.232261	0.816872	-6.48097	0.678295	0.717224
Neutrophils	GKAP1	0.069182	3.541031	0.232238	0.81689	-5.5793	0.744742	0.785881
Neutrophils	SNRPN	-0.17216	-0.6475	-0.2322	0.816916	-5.04554	0.839334	0.882885
Neutrophils	HK2	0.039241	5.335441	0.231976	0.817093	-6.38785	0.707721	0.747656
Neutrophils	AP4M1	-0.05677	3.581284	-0.23189	0.817159	-5.49729	0.743981	0.785076
Neutrophils	PPTC7	0.041554	5.148669	0.231568	0.817409	-5.99277	0.711625	0.751601
Neutrophils	USP33	-0.0256	4.710834	-0.23151	0.817452	-6.39896	0.720551	0.760818
Neutrophils	LTN1	0.037837	5.03701	0.23089	0.817934	-5.91731	0.714165	0.754064
Neutrophils	BCORL1	0.055345	3.355845	0.230866	0.817952	-5.80951	0.749207	0.790188
Neutrophils	CRELD2	0.057254	4.759514	0.230529	0.818213	-5.78409	0.71983	0.759966
Neutrophils	ZFR	0.022982	6.532381	0.230507	0.81823	-6.3784	0.684431	0.723369
Neutrophils	PAPOLA	0.017662	7.055885	0.230471	0.818258	-6.49931	0.674332	0.712904
Neutrophils	WDR45	-0.06403	2.960786	-0.23026	0.818424	-5.50307	0.757696	0.799027
Neutrophils	COL6A1	0.118489	0.367808	0.230123	0.818528	-5.2106	0.81591	0.858805
Neutrophils	LMF1	-0.08059	3.239428	-0.23002	0.818611	-5.36856	0.751698	0.792915

Neutrophils	AUNIP	0.08657	1.893508	0.229946	0.818665	-5.37678	0.781127	0.823161
Neutrophils	PCGF6	-0.08564	2.512206	-0.2299	0.818701	-5.23909	0.767455	0.80912
Neutrophils	OSBP	-0.0353	5.016429	-0.22983	0.818753	-6.04979	0.714584	0.754652
Neutrophils	OXSM	-0.07891	1.788374	-0.22981	0.818768	-5.3573	0.783475	0.825571
Neutrophils	DUS4L	0.10099	1.929292	0.229801	0.818777	-5.2161	0.78033	0.822343
Neutrophils	RABGGTA	-0.0636	2.703471	-0.2298	0.818778	-5.48522	0.763278	0.804827
Neutrophils	TESMIN	0.124019	0.971475	0.229733	0.81883	-5.25859	0.801964	0.844553
Neutrophils	ARL10	-0.09975	2.295947	-0.22961	0.818927	-5.20218	0.772206	0.814061
Neutrophils	NIPBL	-0.01863	8.615732	-0.22946	0.819042	-6.81716	0.64516	0.682773
Neutrophils	KCTD12B	-0.10249	0.963206	-0.22941	0.819083	-5.33739	0.802153	0.844804
Neutrophils	NISCH	-0.02241	6.194239	-0.22929	0.819176	-6.49822	0.69104	0.730379
Neutrophils	PICK1	-0.10666	1.538716	-0.22928	0.819178	-5.22398	0.789079	0.831402
Neutrophils	CCR4	-0.13994	-1.08113	-0.22887	0.819499	-5.04061	0.850549	0.894182
Neutrophils	CMC2	0.064509	4.535117	0.228796	0.819556	-5.86182	0.724584	0.764969
Neutrophils	RECQL	0.067772	3.363727	0.22875	0.819591	-5.56807	0.749182	0.790322
Neutrophils	PAOX	-0.07314	2.987177	-0.22863	0.819682	-5.51905	0.757271	0.798671
Neutrophils	TM2D3	0.049902	4.227452	0.22855	0.819746	-5.70583	0.730962	0.771589
Neutrophils	GM4788	0.111197	1.237901	0.228535	0.819758	-5.31017	0.796038	0.838493
Neutrophils	GM20069	-0.14986	-0.52038	-0.22836	0.819892	-5.06476	0.837088	0.880477
Neutrophils	UBE2E2	-0.06488	3.465951	-0.22829	0.819951	-5.87775	0.747049	0.788179
Neutrophils	RASSF7	0.125388	0.86906	0.227905	0.820246	-5.10983	0.80466	0.847205
Neutrophils	FAM118A	0.086505	2.069936	0.227666	0.820431	-5.3062	0.777539	0.819428
Neutrophils	EIF3M	-0.02147	6.621301	-0.22767	0.820432	-6.30778	0.682999	0.721935
Neutrophils	RAD51	-0.07175	3.891445	-0.22765	0.820446	-5.7807	0.738172	0.778943
Neutrophils	DYRK3	0.131255	2.520243	0.227593	0.820488	-5.18825	0.76761	0.80924
Neutrophils	ADAMTSL1	0.178585	0.587709	0.227554	0.820519	-5.23257	0.811152	0.853899
Neutrophils	DPP8	-0.03016	5.667042	-0.22728	0.820731	-6.14987	0.701803	0.741394
Neutrophils	EIF2B3	0.085523	2.996786	0.227249	0.820755	-5.36787	0.75727	0.798597
Neutrophils	UHRF2	-0.02441	6.43989	-0.22724	0.820762	-6.33366	0.686552	0.725612
Neutrophils	GM48742	0.107912	0.618325	0.227154	0.820829	-5.2063	0.81047	0.853183
Neutrophils	RBSN	-0.06479	2.910659	-0.2268	0.821104	-5.4303	0.759322	0.80059
Neutrophils	SP4	-0.03765	5.254382	-0.22645	0.821372	-6.1019	0.710313	0.750131
Neutrophils	TAZ	-0.04098	4.057371	-0.22645	0.821378	-5.79844	0.734944	0.775548
Neutrophils	5430414B1	0.13407	0.449396	0.226344	0.821456	-5.14934	0.814645	0.8574
Neutrophils	TAB2	-0.02026	6.780443	-0.22614	0.821617	-6.5523	0.680155	0.719002
Neutrophils	COQ6	0.074069	2.285167	0.226074	0.821665	-5.32246	0.773045	0.814843
Neutrophils	GM41611	0.130859	0.284798	0.226049	0.821685	-5.15023	0.818483	0.861423
Neutrophils	TPRN	0.090305	2.21301	0.226003	0.821721	-5.34501	0.774638	0.816493
Neutrophils	IMPA1	-0.03023	5.285622	-0.22596	0.821755	-6.15603	0.709682	0.749595
Neutrophils	HOOK1	-0.08409	2.667358	-0.22591	0.821791	-5.3379	0.76466	0.806257
Neutrophils	DUSP22	0.067289	4.486105	0.22577	0.821901	-5.59483	0.72604	0.766503
Neutrophils	ZFP39	-0.13299	1.023509	-0.22564	0.821999	-5.11346	0.801417	0.844026
Neutrophils	RBM45	-0.06447	3.10947	-0.22552	0.822094	-5.48341	0.755096	0.796483
Neutrophils	NUP133	-0.05554	3.691763	-0.22552	0.822095	-5.57404	0.742662	0.783681
Neutrophils	SELENOP	-0.03685	8.699951	-0.22542	0.822172	-6.8951	0.644146	0.681731
Neutrophils	GM42869	0.084249	1.342309	0.225246	0.822308	-5.32019	0.794202	0.836633
Neutrophils	PHF19	-0.08465	1.934693	-0.2248	0.82265	-5.29359	0.780883	0.823061

Neutrophils	4930557J0	-0.13961	1.834427	-0.22468	0.822743	-5.20414	0.783121	0.82537
Neutrophils	RO60	0.067113	2.493379	0.22466	0.822763	-5.44867	0.768531	0.810384
Neutrophils	ZFP846	-0.09006	2.431015	-0.22466	0.822764	-5.27808	0.7699	0.811791
Neutrophils	HAUS3	-0.04552	4.199727	-0.22463	0.822788	-5.70834	0.732031	0.772808
Neutrophils	0610010K1	-0.03396	5.617735	-0.22444	0.822931	-6.10018	0.703068	0.742932
Neutrophils	ADAMTS17	-0.12999	0.355692	-0.22437	0.822989	-5.17865	0.816898	0.860029
Neutrophils	5830408C2	-0.07789	2.989299	-0.22437	0.822991	-5.33267	0.757734	0.799322
Neutrophils	GNB5	0.100713	0.819995	0.224361	0.822994	-5.20061	0.806136	0.849011
Neutrophils	STEAP3	-0.10163	1.980258	-0.22436	0.822994	-5.20953	0.779868	0.822072
Neutrophils	DGKQ	-0.09206	1.470676	-0.22427	0.823061	-5.2817	0.791296	0.833828
Neutrophils	D030028A(	0.066731	2.795422	0.224155	0.823154	-5.61755	0.761957	0.803672
Neutrophils	TGDS	0.045371	3.1411	0.224035	0.823247	-5.78405	0.754501	0.796009
Neutrophils	ATAD5	0.054996	4.742851	0.223863	0.82338	-6.0393	0.720888	0.761316
Neutrophils	4932422M	0.132702	0.972719	0.223671	0.823529	-5.06103	0.802773	0.845537
Neutrophils	VILL	0.063822	1.228935	0.223629	0.823562	-5.65428	0.796921	0.83954
Neutrophils	ACTR6	0.046645	3.522286	0.223489	0.823671	-5.77951	0.746448	0.787684
Neutrophils	DMXL1	-0.03509	6.604824	-0.22325	0.823855	-6.29352	0.683747	0.722947
Neutrophils	KIF4	-0.06146	4.394266	-0.22309	0.823979	-5.99753	0.728127	0.76884
Neutrophils	COMMD1	-0.03316	5.141948	-0.22307	0.823996	-6.14061	0.712789	0.753003
Neutrophils	EMD	0.020656	6.102062	0.223051	0.824011	-6.49493	0.693586	0.733141
Neutrophils	ZFP54	0.126991	0.794625	0.222965	0.824077	-5.12803	0.806877	0.849808
Neutrophils	TMCO4	0.067192	4.054604	0.222895	0.824132	-5.57383	0.735207	0.776155
Neutrophils	CD200R1	0.06208	2.827999	0.222879	0.824144	-5.77282	0.761376	0.803104
Neutrophils	ZDHHC21	0.061848	4.271545	0.222711	0.824274	-5.54042	0.730677	0.771482
Neutrophils	GFER	0.045026	4.406699	0.222704	0.824279	-5.67098	0.727869	0.768585
Neutrophils	QARS	-0.04687	4.552856	-0.22251	0.824432	-5.80739	0.724861	0.765474
Neutrophils	COTL1	0.021586	6.629821	0.222444	0.824481	-6.67066	0.683276	0.722487
Neutrophils	BICD2	-0.04899	4.09822	-0.2224	0.824517	-5.74508	0.73431	0.775269
Neutrophils	PARL	-0.0435	4.391466	-0.22232	0.82458	-5.77259	0.728201	0.768982
Neutrophils	EEF1AKMT	0.054711	4.588498	0.222031	0.824802	-5.77197	0.724256	0.764819
Neutrophils	USPL1	-0.06219	3.533846	-0.22176	0.82501	-5.48736	0.746353	0.787699
Neutrophils	LRRC51	0.082895	1.76843	0.221687	0.825069	-5.35913	0.78491	0.827345
Neutrophils	RPUSD1	-0.10903	1.47218	-0.22168	0.825073	-5.14853	0.791576	0.834186
Neutrophils	NAGLU	-0.07764	2.6543	-0.22158	0.825155	-5.38845	0.765314	0.807213
Neutrophils	RAB13	-0.08608	1.635625	-0.22155	0.825174	-5.4241	0.787891	0.830405
Neutrophils	EXOC2	-0.02656	5.578749	-0.22149	0.825223	-6.2635	0.704127	0.744116
Neutrophils	ANKRD46	0.047073	2.377526	0.221323	0.825352	-5.59359	0.771436	0.813461
Neutrophils	QSOX2	0.112702	0.948725	0.220984	0.825614	-5.15447	0.803693	0.846545
Neutrophils	CASTOR1	0.105061	0.626653	0.220675	0.825854	-5.26421	0.81112	0.854225
Neutrophils	PDIA3	0.022622	8.546649	0.220659	0.825866	-6.75051	0.647396	0.68528
Neutrophils	MPPED2	-0.16486	-0.32287	-0.22065	0.825873	-5.04411	0.833419	0.877037
Neutrophils	TSTA3	0.051894	4.100613	0.220587	0.825923	-5.68386	0.734572	0.775575
Neutrophils	ZBTB43	0.059938	3.163113	0.220297	0.826148	-5.52521	0.75447	0.796142
Neutrophils	RGS18	0.033409	3.108266	0.220175	0.826242	-6.29023	0.755651	0.797364
Neutrophils	NPAT	-0.05156	4.485178	-0.22008	0.826316	-5.75594	0.726568	0.767407
Neutrophils	AGTRAP	0.038014	4.534904	0.219956	0.826413	-6.27598	0.72554	0.766345
Neutrophils	ZBED4	-0.04693	4.650997	-0.21989	0.826466	-5.73582	0.723145	0.763873

Neutrophils	INO80D	0.023069	5.995336	0.219851	0.826494	-6.34581	0.696004	0.735812
Neutrophils	CDYL2	-0.04859	4.757934	-0.21985	0.826496	-5.93716	0.720946	0.761602
Neutrophils	ATP5B	0.022962	8.182512	0.219819	0.826519	-6.68049	0.654108	0.69235
Neutrophils	ABCC5	-0.04388	4.301545	-0.2198	0.826533	-6.0922	0.730379	0.77134
Neutrophils	INCENP	0.043394	5.454754	0.219622	0.826672	-6.23807	0.706788	0.746975
Neutrophils	UNK	0.063049	4.287183	0.219524	0.826748	-5.59395	0.730678	0.771666
Neutrophils	HIST1H3E	-0.06993	3.610481	-0.21942	0.826829	-5.60921	0.744906	0.786373
Neutrophils	CBLL1	-0.03917	4.917555	-0.21912	0.827059	-5.86186	0.717677	0.758347
Neutrophils	SMIM27	0.049845	3.590898	0.219107	0.827072	-5.64966	0.745322	0.786874
Neutrophils	INPP5B	0.042366	4.216748	0.219088	0.827086	-5.87306	0.732146	0.773286
Neutrophils	VIRMA	0.032433	5.71037	0.219036	0.827127	-6.15982	0.701667	0.741792
Neutrophils	PHF10	0.03286	4.930175	0.218995	0.827159	-6.11324	0.717419	0.75808
Neutrophils	CALR	-0.02713	7.35156	-0.21896	0.827188	-6.51521	0.669703	0.708662
Neutrophils	LSM3	0.03607	5.624923	0.218955	0.82719	-6.18027	0.703374	0.743558
Neutrophils	ITPRIPL1	-0.06463	3.28736	-0.21875	0.827351	-5.44549	0.751883	0.793549
Neutrophils	KANK1	-0.09149	1.172928	-0.21838	0.827635	-5.33981	0.798857	0.841759
Neutrophils	NAB2	0.078143	3.217989	0.218149	0.827816	-5.4597	0.753584	0.795311
Neutrophils	ZSCAN29	0.034904	3.485733	0.218033	0.827906	-5.97935	0.747852	0.789415
Neutrophils	PPIP5K1	0.106362	1.776843	0.217904	0.828006	-5.25833	0.785219	0.827857
Neutrophils	TBC1D30	-0.1194	1.06093	-0.2179	0.828011	-5.28247	0.801435	0.844493
Neutrophils	NLGN2	0.143151	0.349554	0.217829	0.828065	-5.15734	0.817884	0.861372
Neutrophils	PMP22	0.097448	1.847479	0.21761	0.828235	-5.45946	0.783637	0.826335
Neutrophils	DSCAML1	-0.14466	-0.596	-0.21757	0.828267	-5.04528	0.840274	0.884364
Neutrophils	ZDHHC13	-0.05818	3.693777	-0.21741	0.828388	-5.50072	0.74343	0.785017
Neutrophils	ARSG	0.040064	1.867658	0.217351	0.828436	-5.84797	0.783186	0.825936
Neutrophils	NR2F6	-0.05862	3.501892	-0.21707	0.828658	-5.6489	0.747508	0.789249
Neutrophils	PGAP2	0.028513	5.75264	0.217055	0.828665	-6.41812	0.701098	0.741317
Neutrophils	PPM1M	0.027858	4.583696	0.217051	0.828669	-6.2667	0.724815	0.765839
Neutrophils	ABCA1	-0.03677	5.836161	-0.21698	0.828726	-6.56588	0.699434	0.739595
Neutrophils	MAML1	-0.02627	5.435368	-0.21696	0.828737	-6.33911	0.707454	0.747894
Neutrophils	EXD2	0.073766	3.156926	0.216904	0.828783	-5.39506	0.754898	0.796862
Neutrophils	NNT	0.077544	2.482711	0.21676	0.828895	-5.31465	0.769558	0.811948
Neutrophils	PTTG1IP	-0.02756	4.790183	-0.21673	0.828916	-6.12268	0.720565	0.761449
Neutrophils	ITGA8	-0.07731	1.726804	-0.21672	0.828926	-5.70265	0.786342	0.829195
Neutrophils	TSPAN3	-0.06414	4.739337	-0.21631	0.829244	-5.63268	0.721774	0.762589
Neutrophils	ZFP518A	0.041023	3.684945	0.216296	0.829256	-5.89965	0.743786	0.785302
Neutrophils	SPATS2L	-0.09758	0.9742	-0.21614	0.829375	-5.40208	0.803606	0.846788
Neutrophils	GM32743	-0.14417	-0.48509	-0.21611	0.829398	-5.03693	0.837809	0.881794
Neutrophils	CAVIN1	0.09007	1.312054	0.215933	0.829537	-5.48142	0.795935	0.838876
Neutrophils	3300005D(	0.13333	0.756323	0.215836	0.829612	-5.09923	0.808667	0.851928
Neutrophils	SPAST	0.034067	5.207046	0.215602	0.829794	-5.99514	0.712268	0.752802
Neutrophils	TTC37	-0.07811	3.798842	-0.21539	0.829959	-5.47563	0.741418	0.782928
Neutrophils	HMCES	-0.04319	4.456587	-0.21538	0.829969	-5.91679	0.727651	0.768725
Neutrophils	KIRREL3	-0.08832	1.272544	-0.21535	0.82999	-5.36793	0.796834	0.839917
Neutrophils	WDR77	-0.04897	3.895337	-0.21522	0.830095	-5.73315	0.739382	0.780828
Neutrophils	STIL	-0.05249	4.156047	-0.21521	0.830101	-6.04489	0.733909	0.775183
Neutrophils	HDGF	0.024975	6.998077	0.215127	0.830164	-6.42007	0.676913	0.716214

Neutrophils	GM42941	0.093087	1.267028	0.214997	0.830265	-5.24415	0.796959	0.840046
Neutrophils	HMGB2	0.031587	9.132267	0.214983	0.830275	-7.06964	0.637184	0.674915
Neutrophils	GRB14	-0.09917	1.939569	-0.21486	0.830374	-5.41494	0.781803	0.824514
Neutrophils	OTUD4	-0.0408	5.091742	-0.21479	0.830426	-5.84037	0.71461	0.755289
Neutrophils	BOLA1	0.062523	3.767483	0.214631	0.830549	-5.57024	0.742081	0.783689
Neutrophils	FANCE	0.0693	2.916052	0.214626	0.830553	-5.43452	0.760319	0.802478
Neutrophils	POLR1C	-0.06524	3.945898	-0.2146	0.830575	-5.52065	0.738317	0.779807
Neutrophils	AGAP3	-0.05652	4.127556	-0.2143	0.830806	-5.66911	0.734567	0.775875
Neutrophils	GM16150	-0.12289	0.174831	-0.21428	0.830823	-5.10629	0.82228	0.866004
Neutrophils	CARM1	-0.03451	4.727042	-0.21421	0.830878	-5.95656	0.72213	0.763064
Neutrophils	RRAS	0.04953	4.338868	0.214126	0.830942	-5.94577	0.730158	0.771354
Neutrophils	NIPAL1	0.113271	1.060638	0.214052	0.831	-5.20632	0.801739	0.844994
Neutrophils	ACTR5	0.063852	3.477256	0.213806	0.831191	-5.47951	0.748321	0.790082
Neutrophils	SUMO1	-0.01246	7.992553	-0.21379	0.831203	-6.70069	0.658149	0.696764
Neutrophils	FAN1	0.11771	1.159783	0.213764	0.831223	-5.14123	0.799483	0.84269
Neutrophils	MCTP1	0.030544	5.535347	0.213399	0.831507	-6.92581	0.705824	0.746105
Neutrophils	C030034I2:	-0.07063	2.630863	-0.21332	0.831566	-5.38973	0.766724	0.808951
Neutrophils	GM9967	-0.13704	0.569425	-0.21331	0.831578	-5.17714	0.813201	0.856654
Neutrophils	OGFOD3	0.069514	3.187381	0.213236	0.831634	-5.40984	0.754648	0.796523
Neutrophils	WDR76	0.052569	4.504143	0.212904	0.831892	-5.88472	0.727014	0.767874
Neutrophils	FAM189A1	-0.12337	2.327494	-0.21281	0.831963	-5.31088	0.773565	0.815822
Neutrophils	MRE11A	0.040456	4.349103	0.212652	0.832088	-5.69532	0.730279	0.771198
Neutrophils	DLL4	0.11571	0.717902	0.212563	0.832157	-5.2172	0.809995	0.853147
Neutrophils	TACO1	-0.07966	3.738306	-0.21236	0.832318	-5.57665	0.743181	0.784434
Neutrophils	ZMYND15	-0.17226	0.109664	-0.21212	0.832505	-5.09401	0.824393	0.867797
Neutrophils	CCDC126	-0.06363	1.899321	-0.21198	0.832609	-5.62226	0.783327	0.825736
Neutrophils	B3GALNT1	0.13666	0.351359	0.211653	0.832865	-5.13296	0.818729	0.862053
Neutrophils	CHCHD7	-0.04095	4.398636	-0.21165	0.832869	-5.79267	0.729436	0.770295
Neutrophils	NANOS1	-0.0841	0.634448	-0.21164	0.832873	-5.36442	0.812136	0.855304
Neutrophils	LIMS2	0.110194	0.424817	0.211616	0.832894	-5.2166	0.817013	0.860297
Neutrophils	SMTN	-0.06125	2.149503	-0.21156	0.832936	-5.59236	0.777753	0.820046
Neutrophils	KDM3B	0.022277	6.131762	0.211311	0.833131	-6.34955	0.694436	0.734075
Neutrophils	GCKR	-0.0969	1.581851	-0.21097	0.833394	-5.34868	0.790759	0.833261
Neutrophils	MEF2A	-0.01935	7.727346	-0.21083	0.833501	-6.77183	0.663834	0.702284
Neutrophils	UIMC1	0.02595	5.853468	0.210788	0.833538	-6.22871	0.700116	0.739892
Neutrophils	ANKRD55	-0.1311	0.009583	-0.21053	0.83374	-5.05121	0.827206	0.870513
Neutrophils	CNOT1	0.020171	7.026328	0.210264	0.833946	-6.4957	0.677386	0.716201
Neutrophils	CFAP77	-0.12652	0.931954	-0.20988	0.834248	-5.09539	0.805991	0.848624
Neutrophils	G6PDX	0.023203	4.072997	0.209867	0.834254	-6.36443	0.7369	0.777638
Neutrophils	PBX2	-0.02764	4.930622	-0.20977	0.834331	-6.33434	0.719122	0.759319
Neutrophils	SMIM26	0.053023	3.847983	0.209399	0.834618	-5.59133	0.741738	0.782657
Neutrophils	GM12248	-0.08979	2.619411	-0.20936	0.834652	-5.31418	0.768187	0.809877
Neutrophils	EPM2AIP1	0.068335	2.609784	0.209328	0.834674	-5.43853	0.768398	0.8101
Neutrophils	HIST1H3F	0.140108	0.34671	0.209271	0.834718	-5.11782	0.819685	0.862704
Neutrophils	GM28791	0.051815	3.918458	0.208965	0.834956	-5.70294	0.740398	0.781165
Neutrophils	MICOS10	-0.02204	7.131558	-0.20878	0.835098	-6.49124	0.675783	0.714325
Neutrophils	SASH3	0.031588	5.4972	0.208671	0.835185	-6.08192	0.707923	0.747578

Neutrophils	ZFP142	-0.05414	3.207748	-0.20844	0.835367	-5.55016	0.75569	0.796814
Neutrophils	PDE1B	-0.03977	3.114849	-0.20798	0.835724	-5.91595	0.757695	0.799
Neutrophils	OTUB2	-0.11136	1.885457	-0.20779	0.835871	-5.15312	0.784747	0.826885
Neutrophils	FOXR1	-0.1187	-0.87975	-0.20769	0.835946	-5.02992	0.849232	0.89287
Neutrophils	HDAC1	-0.02134	6.039987	-0.20769	0.835951	-6.3439	0.697133	0.736603
Neutrophils	PRKAB1	0.041562	3.654114	0.207678	0.835958	-5.90065	0.746133	0.787187
Neutrophils	NUMBL	-0.10144	1.04252	-0.20766	0.83597	-5.23856	0.803863	0.846485
Neutrophils	GMFG	0.020416	7.424355	0.207601	0.836018	-6.83659	0.670255	0.708767
Neutrophils	CCL8	0.119082	-1.46778	0.207576	0.836037	-5.02481	0.863615	0.907554
Neutrophils	EIF4B	0.027424	6.136378	0.207494	0.836101	-6.17157	0.695225	0.734655
Neutrophils	GM12367	-0.14821	0.051988	-0.20743	0.836149	-5.06114	0.826929	0.870126
Neutrophils	TNNT3	-0.08793	1.772075	-0.20734	0.836223	-5.40333	0.787291	0.829528
Neutrophils	SMIM19	0.030049	4.75168	0.207283	0.836265	-6.07357	0.723161	0.763545
Neutrophils	KCNJ2	-0.05579	-0.01521	-0.20721	0.836323	-5.54266	0.828518	0.87177
Neutrophils	TRP53INP2	0.025684	4.134264	0.207083	0.836421	-6.30593	0.735992	0.776792
Neutrophils	RBFOX2	-0.06096	2.758111	-0.20698	0.836498	-5.80942	0.765445	0.807116
Neutrophils	TNRC6C	0.020955	7.143837	0.20669	0.836728	-6.53927	0.675612	0.714381
Neutrophils	GM14858	0.118642	2.296719	0.206652	0.836756	-5.23856	0.775589	0.817563
Neutrophils	PNLDC1	-0.12236	0.924032	-0.20658	0.836815	-5.12178	0.806587	0.849359
Neutrophils	MYL12A	-0.01918	7.929477	-0.20653	0.836854	-6.78065	0.660722	0.698929
Neutrophils	GOT1	-0.03933	6.081478	-0.20651	0.836868	-6.19794	0.696311	0.735825
Neutrophils	TFRC	0.027417	6.38061	0.206492	0.836882	-6.38357	0.690416	0.729722
Neutrophils	S100A8	-0.03049	6.633628	-0.20627	0.837055	-7.59844	0.685471	0.724648
Neutrophils	GM31508	0.116004	0.763062	0.206234	0.837083	-5.15205	0.810304	0.853226
Neutrophils	4632427E1	-0.03382	4.404143	-0.20614	0.837158	-6.02952	0.730354	0.771075
Neutrophils	ARL8A	-0.02347	6.227073	-0.20608	0.837202	-6.41475	0.693435	0.73292
Neutrophils	WDR5B	-0.12476	0.670974	-0.20602	0.837251	-5.10065	0.812438	0.855434
Neutrophils	HSPA1A	-0.14753	3.985226	-0.20599	0.837269	-5.66863	0.739124	0.780119
Neutrophils	ROCK2	-0.01847	7.468662	-0.20592	0.837324	-6.72187	0.669413	0.70802
Neutrophils	CBS	0.088161	1.874066	0.20586	0.837374	-5.4276	0.785002	0.827309
Neutrophils	PYROXD2	0.100972	0.182818	0.205699	0.837499	-5.2003	0.823898	0.867145
Neutrophils	A2ML1	-0.07813	2.471948	-0.20534	0.83778	-5.59721	0.771965	0.813856
Neutrophils	SLC8B1	0.034493	4.721857	0.205212	0.837878	-6.14293	0.724028	0.764487
Neutrophils	RALGAPB	0.029969	4.920242	0.205095	0.837969	-6.07516	0.719967	0.760319
Neutrophils	1700109H(	0.055904	3.650709	0.204909	0.838115	-5.68293	0.74655	0.787706
Neutrophils	ANP32B	0.023608	8.191649	0.20461	0.838347	-6.74059	0.656178	0.694264
Neutrophils	GM17749	-0.04063	4.634129	-0.20461	0.838351	-6.46142	0.725969	0.766539
Neutrophils	GM49692	0.129152	0.104027	0.204564	0.838383	-5.04676	0.826137	0.869452
Neutrophils	AFAP1L1	-0.08875	1.508026	-0.20428	0.838606	-5.41601	0.793785	0.836202
Neutrophils	PDZD11	-0.04482	4.30203	-0.20421	0.838663	-5.81406	0.732978	0.773674
Neutrophils	PNO1	-0.05572	4.446222	-0.20403	0.838796	-5.60884	0.729973	0.770587
Neutrophils	PDIA6	0.029171	6.97381	0.203886	0.838911	-6.3503	0.67934	0.718226
Neutrophils	BCDIN3D	-0.06853	2.223563	-0.20369	0.839066	-5.33177	0.777736	0.819749
Neutrophils	PFAS	0.062936	4.205445	0.203623	0.839116	-5.7173	0.734997	0.775769
Neutrophils	ANXA7	-0.02377	5.409471	-0.20362	0.839117	-6.29676	0.710227	0.750198
Neutrophils	KRI1	0.030625	4.367396	0.20356	0.839165	-5.98102	0.731614	0.77228
Neutrophils	CAR13	-0.10494	1.31675	-0.2035	0.83921	-5.15188	0.798132	0.840676

Neutrophils	ZFP28	-0.13022	0.2908	-0.20349	0.839222	-5.06427	0.821863	0.864976
Neutrophils	IQSEC2	0.095844	2.267987	0.203394	0.839294	-5.37608	0.77675	0.818736
Neutrophils	ABCB6	-0.08467	1.101522	-0.2031	0.839527	-5.21074	0.803052	0.845718
Neutrophils	PILRA	0.025017	3.3927	0.203063	0.839552	-6.58291	0.752224	0.793517
Neutrophils	TMEM59	-0.01986	6.649746	-0.20295	0.839637	-6.47789	0.685621	0.724735
Neutrophils	TICRR	-0.0594	3.009192	-0.20288	0.839693	-5.70706	0.760496	0.802029
Neutrophils	GM43936	-0.13272	-1.34803	-0.20284	0.839729	-5.0257	0.861249	0.905196
Neutrophils	PACRG	-0.1367	-0.43352	-0.20283	0.839733	-5.04267	0.839044	0.882538
Neutrophils	2500002B1	0.079655	1.915535	0.202768	0.839783	-5.29544	0.784604	0.8268
Neutrophils	MTF1	-0.03078	4.234341	-0.20258	0.839927	-6.01392	0.734407	0.775154
Neutrophils	CARD6	0.068384	3.06005	0.202565	0.83994	-5.544	0.759408	0.800904
Neutrophils	FANCB	0.10356	0.677958	0.202447	0.840032	-5.1484	0.812861	0.855741
Neutrophils	CNOT6L	-0.02448	7.08541	-0.20228	0.840164	-6.46189	0.677245	0.716018
Neutrophils	SEC22C	-0.04343	2.04121	-0.20195	0.840423	-5.82401	0.781856	0.823938
Neutrophils	TRAPPC11	-0.04065	3.489165	-0.2019	0.840459	-5.73322	0.750217	0.791411
Neutrophils	SH3GLB1	0.017245	8.546317	0.201814	0.840525	-6.85813	0.649766	0.687489
Neutrophils	MAP3K15	-0.04423	3.326785	-0.20178	0.840552	-5.9926	0.753699	0.794995
Neutrophils	GM2396	0.079157	-0.85737	0.201466	0.840797	-5.29716	0.849331	0.892996
Neutrophils	ZFP993	-0.1014	1.824761	-0.20136	0.840879	-5.1936	0.786702	0.828911
Neutrophils	PEF1	0.039788	3.347699	0.201314	0.840915	-5.85501	0.753249	0.79455
Neutrophils	HABP2	0.118256	0.622763	0.20128	0.840942	-5.16803	0.814172	0.857091
Neutrophils	TOB2	-0.03099	6.656821	-0.20125	0.840962	-6.25226	0.685537	0.724635
Neutrophils	ABHD11	-0.0554	3.541149	-0.20123	0.840981	-5.5969	0.749106	0.790292
Neutrophils	GINM1	0.027565	4.990227	0.200915	0.841226	-6.06329	0.718809	0.75913
Neutrophils	TDRKH	0.103639	1.697145	0.200887	0.841249	-5.17482	0.789573	0.831971
Neutrophils	DCAF8	-0.02363	5.699638	-0.20088	0.841251	-6.10356	0.704444	0.744284
Neutrophils	SPG7	0.032687	4.467655	0.200843	0.841282	-5.8326	0.729585	0.770261
Neutrophils	CHAMP1	-0.07507	2.843874	-0.20081	0.841311	-5.31529	0.764152	0.805866
Neutrophils	TAF1C	-0.07123	2.170342	-0.20078	0.841331	-5.34684	0.77898	0.821105
Neutrophils	RAB14	-0.01509	7.091867	-0.20062	0.841457	-6.57477	0.677121	0.715994
Neutrophils	HOMEZ	-0.07855	1.333922	-0.20051	0.84154	-5.29916	0.797804	0.840449
Neutrophils	CDC42EP3	0.026563	5.142958	0.200408	0.841622	-6.41581	0.715691	0.755967
Neutrophils	NHSL2	-0.03218	2.487556	-0.20039	0.841632	-6.40064	0.77196	0.81395
Neutrophils	TDRD3	0.049637	4.059195	0.200371	0.841651	-5.63596	0.738125	0.779124
Neutrophils	KXD1	-0.03096	5.765423	-0.2001	0.841858	-6.09259	0.703128	0.743098
Neutrophils	RBX1	0.018432	7.700144	0.199996	0.841943	-6.66026	0.665535	0.70414
Neutrophils	DEAF1	-0.05308	3.281829	-0.19994	0.841989	-5.52144	0.754665	0.796311
Neutrophils	MAZ	0.038523	6.241133	0.199765	0.842123	-6.23141	0.693682	0.733352
Neutrophils	KMT5C	0.057562	2.877228	0.199724	0.842155	-5.57754	0.763425	0.805337
Neutrophils	ANKRD40	0.041151	4.238658	0.199724	0.842155	-5.7305	0.734361	0.775397
Neutrophils	FZD6	0.119102	0.693274	0.199718	0.842159	-5.16832	0.812534	0.855744
Neutrophils	CEBPG	-0.0279	5.586056	-0.19953	0.842306	-6.09029	0.706724	0.74685
Neutrophils	CCDC25	-0.03696	4.712121	-0.19949	0.84234	-5.86507	0.724523	0.765244
Neutrophils	ELP2	0.044275	4.144774	0.199442	0.842375	-5.64216	0.736328	0.777425
Neutrophils	CLEC1A	0.045423	0.972503	0.199303	0.842484	-5.99944	0.80608	0.849132
Neutrophils	DOCK10	0.024794	8.80921	0.199215	0.842552	-6.75689	0.644948	0.682764
Neutrophils	PDIA5	0.070201	2.273787	0.199182	0.842578	-5.47794	0.776684	0.818968

Neutrophils	TTC33	0.050704	3.849347	0.199136	0.842613	-5.64723	0.742553	0.783844
Neutrophils	3110056K0	-0.02893	4.723448	-0.19886	0.842831	-6.02451	0.724311	0.765061
Neutrophils	TBKBP1	-0.06275	1.449005	-0.19875	0.842917	-5.62782	0.79521	0.838046
Neutrophils	GORASP1	0.115903	0.733344	0.198696	0.842957	-5.13506	0.811629	0.854875
Neutrophils	CEP19	-0.03397	2.707827	-0.19853	0.843088	-6.15482	0.767146	0.809258
Neutrophils	PARD6G	-0.11515	1.981117	-0.19843	0.843167	-5.22234	0.783222	0.825782
Neutrophils	MAD2L2	0.065003	3.472942	0.198397	0.84319	-5.4319	0.750586	0.792215
Neutrophils	GBP8	-0.06913	3.65151	-0.1983	0.843263	-5.62691	0.746774	0.788298
Neutrophils	CMSS1	0.049837	6.448827	0.198291	0.843272	-6.19579	0.68962	0.729241
Neutrophils	XRCC1	-0.04994	4.144273	-0.19829	0.843276	-5.6544	0.73636	0.777561
Neutrophils	FIGNL1	-0.07352	2.757262	-0.19819	0.843351	-5.49249	0.766068	0.808199
Neutrophils	METAP1	-0.03296	4.718677	-0.198	0.843497	-5.82656	0.724425	0.765296
Neutrophils	ZMAT2	0.027641	5.466404	0.197991	0.843507	-6.08767	0.70917	0.74953
Neutrophils	UPF3A	0.028438	4.634592	0.197842	0.843623	-5.9675	0.726186	0.767095
Neutrophils	RSBN1	0.031223	5.326631	0.197773	0.843676	-6.07839	0.712019	0.752479
Neutrophils	ZC3H11A	-0.07861	2.01149	-0.19747	0.843911	-5.34545	0.782738	0.825271
Neutrophils	COL1A1	0.081636	2.255466	0.197295	0.844049	-5.61281	0.777368	0.819756
Neutrophils	CLPTM1L	0.030673	4.883326	0.19708	0.844217	-6.06159	0.721341	0.762053
Neutrophils	TOP2B	-0.02889	6.593853	-0.19688	0.844371	-6.31696	0.687156	0.72663
Neutrophils	ADGRG6	-0.12933	0.969217	-0.1967	0.844511	-5.24767	0.806617	0.849731
Neutrophils	RNF113A1	-0.10303	0.42846	-0.1967	0.844514	-5.2149	0.819171	0.862589
Neutrophils	DBP	0.088101	2.187718	0.196605	0.844588	-5.36373	0.779042	0.821456
Neutrophils	SERPINA3N	0.058613	2.557516	0.196118	0.844968	-5.59023	0.771116	0.813125
Neutrophils	GEMIN8	-0.08237	2.284507	-0.1959	0.845139	-5.23014	0.777147	0.819323
Neutrophils	FAM204A	0.034296	4.894011	0.195892	0.845144	-5.85164	0.721432	0.761936
Neutrophils	ICA1L	-0.08356	1.590667	-0.19589	0.845148	-5.38262	0.792693	0.835281
Neutrophils	REV3L	0.022921	6.713202	0.195784	0.845229	-6.39804	0.685067	0.724307
Neutrophils	4930578M	-0.11711	0.63262	-0.19555	0.845409	-5.08605	0.81472	0.857827
Neutrophils	CAND2	0.10964	0.775357	0.195504	0.845447	-5.0858	0.811406	0.854433
Neutrophils	OSTM1	-0.03153	4.762239	-0.19547	0.845476	-5.95255	0.724181	0.764748
Neutrophils	1110006O	-0.10257	0.319918	-0.1948	0.845995	-5.11356	0.82208	0.865403
Neutrophils	5430427M	-0.0851	1.237849	-0.19479	0.846006	-5.39566	0.800809	0.843618
Neutrophils	SLC45A3	-0.09797	1.111286	-0.19466	0.846104	-5.29975	0.803709	0.846631
Neutrophils	HOXA7	-0.13288	-0.09629	-0.19464	0.846119	-5.03995	0.831911	0.8755
Neutrophils	GM15518	-0.1122	0.80065	-0.19462	0.846139	-5.17236	0.81087	0.853968
Neutrophils	P2RX4	0.044592	5.534178	0.19451	0.846223	-5.9142	0.708487	0.748622
Neutrophils	SQOR	-0.02675	3.496234	-0.19449	0.846237	-6.39932	0.750828	0.792318
Neutrophils	GPAA1	-0.03391	3.764843	-0.19444	0.846274	-5.82384	0.745101	0.786417
Neutrophils	GPR108	-0.03964	4.177602	-0.19425	0.846425	-5.8033	0.736387	0.777468
Neutrophils	PSMD12	0.027171	5.784758	0.194153	0.846501	-6.12002	0.703456	0.743473
Neutrophils	GPR89	0.063808	3.742407	0.193913	0.846689	-5.4326	0.745577	0.787004
Neutrophils	DPY19L1	-0.05391	4.584338	-0.19385	0.846737	-5.62302	0.727904	0.768775
Neutrophils	IFI208	-0.09733	2.936618	-0.19384	0.846747	-5.45261	0.762908	0.804849
Neutrophils	TGFB3	0.121272	0.567314	0.193834	0.846751	-5.13278	0.816292	0.859641
Neutrophils	NRAS	0.019601	6.075126	0.193833	0.846751	-6.40906	0.697672	0.737521
Neutrophils	ATP8B4	0.024684	4.781456	0.193826	0.846757	-6.79895	0.723829	0.764568
Neutrophils	PSIP1	0.023854	5.994205	0.193759	0.846809	-6.27933	0.699278	0.739188

Neutrophils	CFB	-0.12155	4.663824	-0.19366	0.846886	-5.96911	0.726258	0.767086
Neutrophils	TMEM86B	-0.05328	3.467162	-0.19363	0.846907	-5.56375	0.751451	0.793077
Neutrophils	CBR2	-0.1607	-1.3198	-0.19358	0.846946	-5.03089	0.861498	0.905867
Neutrophils	NIPSNAP1	-0.06044	3.134643	-0.19309	0.847334	-5.53877	0.758894	0.800468
Neutrophils	MAPKAPK3	0.024085	5.306805	0.192813	0.847547	-6.42706	0.713415	0.753544
Neutrophils	COX6B2	-0.07186	1.691068	-0.19281	0.84755	-5.42614	0.790878	0.833301
Neutrophils	VDAC2	0.017675	7.488132	0.192528	0.84777	-6.69538	0.670608	0.709172
Neutrophils	SPICE1	0.084923	1.890422	0.192472	0.847814	-5.23879	0.78647	0.828734
Neutrophils	NOL9	-0.06283	3.728213	-0.19221	0.848016	-5.40691	0.7463	0.787527
Neutrophils	TM4SF1	-0.06429	2.15291	-0.19213	0.848078	-5.74292	0.7806	0.822808
Neutrophils	TUBG1	0.07233	3.393021	0.192118	0.84809	-5.52323	0.753466	0.794908
Neutrophils	UBAC2	0.025957	6.679645	0.191901	0.84826	-6.25282	0.686176	0.725444
Neutrophils	IGF2BP3	0.02528	6.5516	0.191842	0.848306	-6.43387	0.688676	0.728039
Neutrophils	PDCD5	-0.02222	6.418317	-0.19181	0.848329	-6.31879	0.691289	0.730751
Neutrophils	CBARP	0.078531	1.923122	0.191719	0.848402	-5.39788	0.785737	0.828171
Neutrophils	GM44659	-0.08145	0.682551	-0.19163	0.848474	-5.27515	0.814068	0.857272
Neutrophils	BSN	-0.11987	0.764883	-0.19152	0.848555	-5.14475	0.812156	0.855337
Neutrophils	ECI2	0.041655	4.557161	0.191487	0.848583	-5.85638	0.728878	0.769723
Neutrophils	ZFP689	-0.09545	1.407041	-0.19138	0.84867	-5.15849	0.797399	0.840257
Neutrophils	S1PR4	0.038369	4.183137	0.191336	0.848701	-5.99895	0.736686	0.777814
Neutrophils	BTBD9	-0.0172	8.508871	-0.1912	0.848807	-6.89487	0.651484	0.689578
Neutrophils	GM38560	0.084326	0.991541	0.191062	0.848915	-5.37388	0.806916	0.850051
Neutrophils	RUVBL1	0.041408	4.77265	0.191023	0.848946	-5.71852	0.724419	0.76519
Neutrophils	GIN1	0.034384	3.954097	0.190982	0.848978	-5.70972	0.74151	0.782825
Neutrophils	ZC3HC1	0.040265	4.34425	0.190972	0.848985	-5.73422	0.733312	0.77437
Neutrophils	ETV3	0.053507	4.950898	0.190758	0.849152	-5.87005	0.720833	0.761475
Neutrophils	GM17382	-0.12757	0.002886	-0.19026	0.849542	-5.04413	0.83043	0.873907
Neutrophils	IKBIP	0.066703	1.938543	0.189644	0.850022	-5.39373	0.786102	0.82822
Neutrophils	GLA	0.029147	4.568639	0.189545	0.8501	-6.31803	0.729299	0.769778
Neutrophils	SERPINA1D	-0.05827	5.558333	-0.18946	0.850169	-6.40033	0.709041	0.748874
Neutrophils	PLEKHA1	0.062545	4.707996	0.189443	0.85018	-5.65383	0.726411	0.766814
Neutrophils	CIITA	-0.12029	2.478401	-0.18929	0.850301	-5.22337	0.7741	0.81594
Neutrophils	GM40841	-0.13887	0.40148	-0.18916	0.850402	-5.04478	0.821391	0.864456
Neutrophils	STIM2	-0.01662	6.210089	-0.1891	0.850447	-6.78672	0.696037	0.735476
Neutrophils	IDUA	0.062296	2.137056	0.189054	0.850484	-5.37437	0.781678	0.823769
Neutrophils	BC055324	-0.07011	2.245694	-0.1889	0.850603	-5.37003	0.779259	0.82136
Neutrophils	PEX13	0.027689	5.524698	0.188852	0.850641	-6.15223	0.709736	0.749727
Neutrophils	CYYR1	-0.05203	2.282713	-0.1887	0.850764	-5.86855	0.778436	0.820524
Neutrophils	PHTF1	0.037515	4.68503	0.188669	0.850785	-5.96059	0.726903	0.767467
Neutrophils	HACD1	0.061122	3.002315	0.18851	0.850909	-5.53872	0.762616	0.804264
Neutrophils	VRK3	0.028774	4.98755	0.188507	0.850911	-6.01711	0.720669	0.761031
Neutrophils	SMARCB1	0.028226	5.410318	0.188318	0.851059	-5.92467	0.712093	0.752137
Neutrophils	MYBL2	0.068005	3.080165	0.188079	0.851246	-5.51355	0.76097	0.802627
Neutrophils	JOSD2	-0.03718	4.09679	-0.18791	0.851379	-5.76957	0.739231	0.780277
Neutrophils	TENM2	-0.15126	-0.35252	-0.1879	0.851386	-5.04298	0.839324	0.882999
Neutrophils	LAT2	-0.05131	4.773693	-0.18782	0.851449	-5.69006	0.725114	0.765721
Neutrophils	KIF18B	-0.06541	2.84215	-0.18775	0.851501	-5.60011	0.766154	0.808015

Neutrophils	NTPCR	-0.04951	3.847172	-0.18773	0.851522	-5.63841	0.744509	0.785734
Neutrophils	PIH1D1	-0.02787	5.06576	-0.18771	0.85153	-6.13187	0.71911	0.759528
Neutrophils	TGFBR2	0.021243	7.292661	0.18695	0.852128	-6.46856	0.675422	0.714072
Neutrophils	APRT	0.0232	6.416788	0.186774	0.852266	-6.63583	0.69248	0.731722
Neutrophils	GFM1	-0.04545	3.89287	-0.18649	0.852486	-5.62815	0.744052	0.785042
Neutrophils	TRIP10	-0.08417	1.355077	-0.18646	0.85251	-5.18457	0.799923	0.842457
Neutrophils	MKKS	0.066315	1.995441	0.186459	0.852512	-5.35944	0.785432	0.827594
Neutrophils	KLHDC4	-0.02662	3.945597	-0.18636	0.852593	-6.15519	0.742935	0.783891
Neutrophils	SLC29A3	0.055395	3.68183	0.186328	0.852615	-5.50382	0.748541	0.789667
Neutrophils	PP2D1	-0.09092	1.690903	-0.18617	0.852735	-5.33197	0.792327	0.834659
Neutrophils	STAMBP	-0.05254	3.374422	-0.1861	0.852796	-5.59249	0.755166	0.796478
Neutrophils	OXSR1	-0.04333	3.496646	-0.18597	0.852896	-5.70897	0.752564	0.793798
Neutrophils	HIP1R	0.025326	5.271328	0.185807	0.853021	-6.28222	0.715486	0.755562
Neutrophils	TOM1L2	-0.03336	5.471299	-0.18575	0.853065	-6.10476	0.711427	0.751368
Neutrophils	MCPH1	-0.03154	4.913551	-0.18558	0.853198	-5.99294	0.722862	0.763164
Neutrophils	SOD1	-0.02699	5.993562	-0.1854	0.853342	-6.27382	0.701001	0.74059
Neutrophils	TGFB1I1	-0.08664	0.90381	-0.18535	0.853378	-5.30937	0.810461	0.85326
Neutrophils	CLDN15	-0.04018	0.423974	-0.1852	0.8535	-5.81619	0.821645	0.864711
Neutrophils	ACTA2	-0.07234	3.320545	-0.18514	0.853541	-6.08559	0.756444	0.797814
Neutrophils	1700096K1	-0.0504	2.613489	-0.18511	0.853569	-5.62521	0.771856	0.813663
Neutrophils	DUSP18	-0.12047	0.621447	-0.18487	0.853753	-5.11913	0.817131	0.86
Neutrophils	PRRC1	0.034392	4.219989	0.184775	0.853829	-5.82516	0.737399	0.778115
Neutrophils	ZFP160	-0.064	2.950859	-0.1845	0.854041	-5.36789	0.764674	0.806088
Neutrophils	RASSF3	-0.01904	6.834864	-0.18435	0.85416	-6.81506	0.684632	0.723483
Neutrophils	3-Sep	0.098751	-1.00287	0.184351	0.85416	-5.07445	0.856059	0.899656
Neutrophils	ADGRG3	0.032498	2.366781	0.184254	0.854236	-6.1218	0.777528	0.819291
Neutrophils	BLMH	-0.02675	5.648784	-0.18415	0.85432	-6.02179	0.708118	0.747799
Neutrophils	PBX1	0.017734	5.465012	0.183953	0.854472	-6.92821	0.711897	0.751656
Neutrophils	TSR3	-0.06012	3.569257	-0.18361	0.854739	-5.49469	0.751541	0.79239
Neutrophils	LIN37	0.046002	3.646925	0.183556	0.854782	-5.64586	0.749878	0.790679
Neutrophils	CLCF1	0.09337	2.650892	0.18331	0.854975	-5.20412	0.771595	0.81295
Neutrophils	VPS9D1	0.04858	3.05216	0.18302	0.855202	-5.61909	0.762952	0.803986
Neutrophils	ATP8B2	0.062725	2.774077	0.182768	0.855398	-5.30177	0.769035	0.810296
Neutrophils	ZFP775	-0.10748	0.81684	-0.18275	0.855415	-5.08207	0.813225	0.855604
Neutrophils	MIOS	0.044554	3.665658	0.182724	0.855433	-5.57757	0.749727	0.79045
Neutrophils	METTL21A	-0.06959	1.888583	-0.18255	0.855572	-5.23413	0.788716	0.830533
Neutrophils	6430590A(	0.069427	1.647577	0.182381	0.855701	-5.24174	0.794161	0.836147
Neutrophils	EIF1	0.017532	9.721973	0.182373	0.855707	-7.05205	0.63127	0.667881
Neutrophils	PDHA1	-0.02719	5.115043	-0.18236	0.855714	-6.10472	0.719403	0.759247
Neutrophils	GPATCH8	0.019769	7.002079	0.182192	0.855849	-6.36721	0.681828	0.720422
Neutrophils	TRAPPC12	-0.0372	4.201517	-0.18209	0.85593	-5.71535	0.738364	0.778857
Neutrophils	TPM2	-0.11972	0.689833	-0.18195	0.856039	-5.14808	0.81618	0.858769
Neutrophils	USP20	0.055582	2.877475	0.181941	0.856045	-5.39693	0.76677	0.808101
Neutrophils	TMEM39B	-0.05069	4.225197	-0.18164	0.856281	-5.59398	0.737866	0.778366
Neutrophils	GM49085	0.065962	2.025817	0.18137	0.856492	-5.31751	0.785632	0.827547
Neutrophils	PPP1R37	0.038437	4.592802	0.1812	0.856625	-5.7423	0.73018	0.770525
Neutrophils	4931414P1	0.087027	2.099255	0.181074	0.856724	-5.24179	0.783987	0.825925

Neutrophils	LRRC58	-0.02056	5.945766	-0.18104	0.856752	-6.30631	0.702601	0.742079
Neutrophils	ZFP512B	0.089225	2.377698	0.180701	0.857016	-5.23081	0.777782	0.819644
Neutrophils	PPFIA1	0.031746	5.566923	0.180542	0.85714	-6.01271	0.710211	0.750095
Neutrophils	2900076A(	0.053873	2.621325	0.180492	0.85718	-5.44152	0.772394	0.814186
Neutrophils	NCOA2	0.019246	7.684552	0.180485	0.857185	-6.6679	0.668751	0.707158
Neutrophils	OGT	-0.01728	6.649131	-0.18046	0.857208	-6.53987	0.688696	0.727842
Neutrophils	DSN1	-0.05683	2.622227	-0.18032	0.857315	-5.48487	0.772374	0.814211
Neutrophils	ZFP593	0.070616	3.359126	0.180213	0.857398	-5.36576	0.756308	0.797689
Neutrophils	C79798	0.074295	1.77973	0.180137	0.857457	-5.28608	0.791171	0.833512
Neutrophils	RAB11FIP4	-0.10474	-0.78929	-0.18006	0.857516	-5.05277	0.851401	0.895137
Neutrophils	TPRGL	0.016945	6.927644	0.180042	0.857531	-6.63633	0.68327	0.722256
Neutrophils	2810403D2	0.058145	2.774023	0.179881	0.857658	-5.45677	0.769036	0.81078
Neutrophils	LBR	0.013258	7.219631	0.179851	0.857681	-6.75945	0.67763	0.716409
Neutrophils	TENT4A	0.052771	3.460072	0.179752	0.857758	-5.54778	0.754134	0.795451
Neutrophils	NKAPD1	-0.0372	4.086425	-0.1797	0.857796	-5.76345	0.740789	0.781705
Neutrophils	NDUFA5	-0.03478	5.432033	-0.1797	0.857803	-6.07377	0.712942	0.752965
Neutrophils	WDR1	0.017771	7.335798	0.179675	0.857819	-6.73355	0.6754	0.714096
Neutrophils	GNPTG	0.04666	3.368723	0.179609	0.857871	-5.53145	0.756101	0.797476
Neutrophils	CCL17	-0.14534	-0.70076	-0.17959	0.857884	-5.05495	0.84925	0.892942
Neutrophils	TMEM223	-0.03365	4.652686	-0.1795	0.857957	-5.85316	0.728935	0.76948
Neutrophils	SMC3	-0.01973	6.498954	-0.17943	0.858007	-6.38035	0.69164	0.730927
Neutrophils	UBE2I	0.013546	7.624242	0.179398	0.858035	-6.59062	0.669896	0.708386
Neutrophils	EXOC3	0.025427	5.06994	0.179317	0.858099	-6.11834	0.720327	0.760593
Neutrophils	ENO1B	0.063417	1.327345	0.179303	0.85811	-5.43198	0.801455	0.844058
Neutrophils	COPA	0.017551	6.661065	0.179272	0.858135	-6.44442	0.688462	0.727636
Neutrophils	AMACR	-0.07579	2.326832	-0.1792	0.85819	-5.42808	0.778912	0.820928
Neutrophils	ARL16	-0.05511	2.250651	-0.17915	0.858232	-5.37642	0.780607	0.822669
Neutrophils	DPF3	-0.08599	1.667384	-0.17895	0.858384	-5.30839	0.793712	0.836156
Neutrophils	ZFP326	0.029632	5.21559	0.17884	0.858473	-5.92492	0.717347	0.757599
Neutrophils	SPPL2B	-0.07574	2.17345	-0.17864	0.858631	-5.30712	0.782329	0.824554
Neutrophils	AR	0.136518	0.218656	0.178597	0.858663	-5.07742	0.827238	0.870578
Neutrophils	TAF3	-0.02665	5.653075	-0.17849	0.858747	-6.08069	0.708473	0.748452
Neutrophils	SNX21	0.059762	2.718125	0.178474	0.858759	-5.50404	0.770263	0.812158
Neutrophils	TPD52L2	0.021082	5.433659	0.17842	0.858801	-6.17298	0.712909	0.753038
Neutrophils	EMC2	-0.01909	5.762132	-0.1784	0.858818	-6.32947	0.706279	0.746183
Neutrophils	MARCO	-0.06591	5.795483	-0.17837	0.858839	-6.49103	0.705609	0.745496
Neutrophils	CEBPD	0.019287	3.956744	0.17815	0.859013	-6.56734	0.743576	0.784678
Neutrophils	ENKUR	-0.10834	0.339833	-0.17813	0.859032	-5.09321	0.824428	0.867692
Neutrophils	SDHAF1	0.051932	4.132974	0.177668	0.85939	-5.58344	0.739978	0.780915
Neutrophils	SLC25A18	-0.05164	4.491338	-0.17766	0.859397	-5.9558	0.732462	0.773164
Neutrophils	ZFP703	-0.03702	4.364442	-0.17762	0.859429	-6.01169	0.735114	0.775915
Neutrophils	AQR	0.030122	4.812338	0.177422	0.859583	-5.77997	0.725796	0.766322
Neutrophils	TNFRSF4	-0.12806	0.30724	-0.17726	0.859708	-5.07078	0.825338	0.868625
Neutrophils	FDXACB1	0.060888	2.215747	0.177224	0.859738	-5.32006	0.781565	0.82377
Neutrophils	EPOR	-0.12538	-0.66556	-0.17722	0.859744	-5.06842	0.848593	0.892398
Neutrophils	MPDU1	-0.02903	4.71579	-0.17709	0.859839	-5.95116	0.727794	0.768411
Neutrophils	PLXNA4	-0.09477	2.166702	-0.17709	0.859843	-5.71139	0.78266	0.824894

Neutrophils	LSM10	-0.03919	3.07773	-0.17696	0.859946	-5.77963	0.762578	0.804295
Neutrophils	GM17173	-0.05251	0.150476	-0.17693	0.859966	-5.61632	0.829041	0.872467
Neutrophils	GM16316	-0.08105	1.803894	-0.17685	0.860034	-5.24805	0.790807	0.833312
Neutrophils	PSMD9	0.028862	5.225742	0.176659	0.86018	-5.92936	0.717367	0.757693
Neutrophils	MGAT2	0.023941	5.873385	0.176409	0.860376	-6.15388	0.704374	0.744184
Neutrophils	IFI44	-0.12281	0.579228	-0.17627	0.860486	-5.22009	0.819178	0.862264
Neutrophils	LUM	-0.09856	0.551186	-0.17601	0.860688	-5.22776	0.819909	0.862966
Neutrophils	MMP8	-0.0235	1.517071	-0.17599	0.860707	-6.79159	0.797603	0.840117
Neutrophils	DDX23	0.035579	4.71777	0.175687	0.860942	-5.76356	0.728151	0.768615
Neutrophils	TOR1AIP2	-0.01823	6.580878	-0.17561	0.861003	-6.38798	0.690569	0.729767
Neutrophils	TCEANC	-0.06886	1.730178	-0.1755	0.861091	-5.40204	0.792922	0.835238
Neutrophils	DPM3	0.020955	6.309203	0.175354	0.861202	-6.32349	0.695966	0.735325
Neutrophils	ADAP2OS	-0.08824	0.724917	-0.17501	0.861476	-5.28569	0.816224	0.859058
Neutrophils	ATP5G1	0.022398	7.643771	0.174931	0.861534	-6.56652	0.670233	0.708631
Neutrophils	5830448L0	-0.07557	0.904406	-0.17466	0.861744	-5.27062	0.812051	0.854847
Neutrophils	TMEFF1	-0.12681	-0.59624	-0.17453	0.861848	-5.06772	0.847614	0.891205
Neutrophils	TRMT2A	-0.05088	3.456488	-0.17452	0.861858	-5.45668	0.755009	0.796287
Neutrophils	CDC25B	-0.05926	4.400381	-0.1745	0.861872	-5.68966	0.734969	0.77564
Neutrophils	DRAP1	0.016857	6.508223	0.174483	0.861885	-6.42994	0.692189	0.731433
Neutrophils	GM38973	-0.09364	1.613034	-0.17431	0.862016	-5.19712	0.795791	0.838242
Neutrophils	CBX5	0.034579	5.103912	0.174291	0.862036	-6.00386	0.720396	0.760656
Neutrophils	RTKN2	-0.06215	1.228878	-0.1742	0.862105	-5.48887	0.804567	0.847256
Neutrophils	HTATSF1	0.03165	4.697996	0.17394	0.86231	-5.82659	0.728884	0.769361
Neutrophils	SPOUT1	-0.06564	2.468742	-0.17376	0.862449	-5.30733	0.776756	0.818627
Neutrophils	GM36756	0.066939	0.926199	0.173657	0.862532	-5.3754	0.811726	0.854532
Neutrophils	MOGS	-0.05307	4.095452	-0.17339	0.862741	-5.5538	0.741546	0.782512
Neutrophils	DYNLRB1	-0.01865	6.280147	-0.17334	0.862778	-6.37205	0.696844	0.736354
Neutrophils	SFN	0.066237	2.056108	0.173213	0.86288	-5.47765	0.785957	0.828219
Neutrophils	KLHDC1	0.07672	2.152219	0.173106	0.862964	-5.2177	0.783804	0.826018
Neutrophils	MAD1L1	0.024941	5.227133	0.173089	0.862977	-6.03115	0.718028	0.758276
Neutrophils	SLC39A14	-0.068	3.169335	-0.17307	0.862992	-5.39973	0.761385	0.802975
Neutrophils	TMEM159	0.066292	1.553977	0.173057	0.863002	-5.50765	0.797304	0.839871
Neutrophils	SYTL1	-0.05585	1.59739	-0.1729	0.863122	-5.5577	0.796355	0.838882
Neutrophils	CCDC171	0.053419	3.57202	0.172823	0.863186	-5.45552	0.752728	0.794066
Neutrophils	DPY30	0.030274	5.414831	0.172401	0.863517	-6.10309	0.714369	0.754528
Neutrophils	RABL2	-0.09081	1.106471	-0.17235	0.863553	-5.12372	0.807745	0.85062
Neutrophils	LRIG3	0.107794	0.14201	0.172196	0.863677	-5.11097	0.830303	0.873726
Neutrophils	WDR81	-0.049	3.600126	-0.17212	0.863738	-5.50737	0.752264	0.793661
Neutrophils	CLEC16A	0.033788	4.763553	0.172056	0.863787	-5.89822	0.727735	0.76838
Neutrophils	FANCG	-0.06017	2.222876	-0.172	0.863833	-5.38545	0.782406	0.824669
Neutrophils	KYNU	-0.05552	3.853994	-0.17194	0.863875	-5.60726	0.746839	0.788082
Neutrophils	FAM160B2	0.042427	3.264944	0.171795	0.863992	-5.57893	0.759488	0.801107
Neutrophils	ZMYM4	-0.02851	5.733892	-0.17172	0.864052	-6.02894	0.707914	0.7479
Neutrophils	SLC10A1	0.048254	3.248903	0.171665	0.864093	-5.77618	0.759835	0.801465
Neutrophils	MXI1	0.01892	7.063981	0.171588	0.864153	-6.84453	0.681659	0.720712
Neutrophils	MRPS36	-0.03181	5.569029	-0.17149	0.864231	-5.98522	0.711242	0.751341
Neutrophils	PCLAF	0.044637	7.982096	0.171437	0.864272	-6.77114	0.664135	0.702528

Neutrophils	MDFIC	-0.05348	4.328783	-0.17131	0.864374	-5.77838	0.736811	0.777754
Neutrophils	TRIM68	-0.09241	0.946883	-0.17124	0.864424	-5.09544	0.811443	0.854482
Neutrophils	SNHG3	-0.03955	5.291632	-0.17106	0.864564	-5.94128	0.716943	0.757231
Neutrophils	ART3	0.069878	0.944132	0.170973	0.864636	-5.37136	0.811572	0.854594
Neutrophils	ABCG1	-0.03007	6.591271	-0.17064	0.864899	-6.39167	0.691032	0.730401
Neutrophils	KLK1	-0.13777	-0.20745	-0.17063	0.864902	-5.05178	0.838829	0.882439
Neutrophils	PRKG1	0.046497	3.504872	0.170439	0.865055	-6.03185	0.754539	0.795941
Neutrophils	PRDM15	-0.04795	3.462741	-0.17035	0.865128	-5.50014	0.755446	0.796875
Neutrophils	DHX35	0.069793	2.187953	0.170284	0.865176	-5.27656	0.783424	0.825641
Neutrophils	BCL2L14	-0.12707	0.459361	-0.17003	0.865378	-5.15341	0.82309	0.8663
Neutrophils	2510017J1	0.080223	1.414805	0.170005	0.865394	-5.311	0.800936	0.84361
Neutrophils	NHSL1	-0.06004	1.728678	-0.16997	0.865421	-5.4711	0.793791	0.83629
Neutrophils	CSF1R	0.03737	5.549681	0.169626	0.865692	-6.48918	0.711996	0.752053
Neutrophils	LEKR1	-0.08538	1.047991	-0.1696	0.865711	-5.30159	0.809507	0.852392
Neutrophils	SLFN5	0.048603	4.230734	0.169511	0.865782	-6.08855	0.739241	0.780197
Neutrophils	PLS1	-0.09572	0.910051	-0.16941	0.865863	-5.14636	0.812711	0.855687
Neutrophils	KHDRBS3	0.064212	1.943216	0.16871	0.86641	-5.51987	0.789521	0.831543
Neutrophils	ABCA8A	-0.10615	1.028779	-0.16861	0.866491	-5.26038	0.810414	0.852947
Neutrophils	GM42917	-0.06581	1.691044	-0.16842	0.866634	-5.36238	0.795299	0.837429
Neutrophils	ATPSCKMT	-0.04603	3.937455	-0.16833	0.866707	-5.67899	0.745933	0.78671
Neutrophils	SIGIRR	-0.05811	2.175284	-0.16822	0.866797	-5.31912	0.7844	0.826263
Neutrophils	MRPL38	-0.04641	3.863246	-0.16811	0.866882	-5.50439	0.747541	0.788358
Neutrophils	RASSF1	0.024184	5.940086	0.16787	0.867069	-6.12616	0.704716	0.744106
Neutrophils	PROX2	-0.07041	1.188752	-0.16773	0.867181	-5.35247	0.806938	0.849306
Neutrophils	MGAT1	-0.03075	4.704416	-0.16751	0.867348	-5.95909	0.729947	0.770191
Neutrophils	ADCK2	-0.05238	2.282482	-0.16745	0.867402	-5.40599	0.782132	0.823912
Neutrophils	VPS54	0.018266	6.506686	0.1674	0.867438	-6.56546	0.693469	0.732535
Neutrophils	CISD1	0.041103	5.119405	0.167398	0.867439	-5.90319	0.721374	0.761381
Neutrophils	UQCRC2	0.018034	6.345631	0.167248	0.867557	-6.32454	0.696687	0.735854
Neutrophils	CRADD	-0.02462	5.224096	-0.16694	0.867801	-6.07546	0.719271	0.759213
Neutrophils	USP35	-0.12116	0.017078	-0.16693	0.867806	-5.06619	0.834449	0.877506
Neutrophils	TMEM101	-0.06217	2.322989	-0.16692	0.867816	-5.34861	0.781276	0.82305
Neutrophils	COQ10A	-0.04433	3.31768	-0.16688	0.867849	-5.4991	0.759418	0.800588
Neutrophils	LENG1	-0.04401	2.925078	-0.16674	0.867954	-5.5203	0.768	0.809394
Neutrophils	ANKZF1	0.035782	2.742515	0.166522	0.868127	-5.58442	0.772082	0.813556
Neutrophils	GSTO1	0.025391	4.594828	0.166375	0.868242	-5.97308	0.732371	0.7727
Neutrophils	PARK7	0.020921	6.806683	0.166292	0.868307	-6.40099	0.687719	0.726553
Neutrophils	EMP3	-0.01756	7.421524	-0.16627	0.868321	-6.77562	0.675821	0.714224
Neutrophils	GRAP2	-0.06145	4.975054	-0.16608	0.868477	-5.71275	0.724505	0.764564
Neutrophils	MAP4K5	0.034563	4.52083	0.16606	0.868489	-5.95079	0.733937	0.774293
Neutrophils	GEMIN6	-0.06454	2.59595	-0.16592	0.868603	-5.34376	0.775378	0.816899
Neutrophils	ENPP1	0.086809	2.31349	0.165749	0.868733	-5.28388	0.781705	0.823342
Neutrophils	NAIP6	0.047298	2.310523	0.165528	0.868907	-5.60706	0.781774	0.823413
Neutrophils	TUBB4B	-0.03044	6.422022	-0.1655	0.86893	-6.45188	0.695377	0.734379
Neutrophils	ZFP146	-0.0624	3.413704	-0.16547	0.868949	-5.31001	0.757554	0.798527
Neutrophils	DNM1L	0.026539	5.639142	0.165296	0.869089	-6.03526	0.711068	0.750584
Neutrophils	COX6C	-0.01432	8.594079	-0.16523	0.869142	-6.80016	0.653864	0.691318

Neutrophils	ZMYM6	-0.06906	2.526746	-0.16499	0.869325	-5.36143	0.777056	0.818608
Neutrophils	ZFP652	0.019574	5.654413	0.164979	0.869337	-6.51782	0.710802	0.750362
Neutrophils	ZFP688	0.077301	1.695397	0.164501	0.869712	-5.20953	0.795993	0.837801
Neutrophils	GM37401	0.0791	0.875344	0.164181	0.869964	-5.21951	0.814995	0.857128
Neutrophils	GMPPB	0.055314	2.629402	0.164075	0.870046	-5.40653	0.77519	0.816338
Neutrophils	GM49067	0.085252	0.406337	0.164033	0.87008	-5.12166	0.825985	0.868365
Neutrophils	HNRNPDL	-0.01337	7.857159	-0.16388	0.870196	-6.66147	0.668078	0.705838
Neutrophils	COX20	0.024373	5.583215	0.163736	0.870312	-6.20637	0.712647	0.751982
Neutrophils	CD68	0.034615	4.872198	0.163714	0.87033	-6.16261	0.727214	0.767021
Neutrophils	TNS4	-0.09425	-0.27421	-0.16362	0.870408	-5.15049	0.842239	0.885055
Neutrophils	CTSA	-0.02864	6.259221	-0.16352	0.870479	-6.27817	0.699084	0.738012
Neutrophils	MED6	-0.03559	4.540282	-0.16291	0.870957	-5.82005	0.734365	0.774379
Neutrophils	FAM72A	-0.07591	1.069392	-0.16286	0.871004	-5.25775	0.810795	0.852897
Neutrophils	ADPRHL2	0.043884	3.241714	0.162819	0.871032	-5.43661	0.762053	0.802905
Neutrophils	UBE2H	-0.0174	8.574917	-0.1628	0.871049	-6.8068	0.654846	0.692137
Neutrophils	TPGS1	0.036849	4.393149	0.162663	0.871155	-5.78602	0.737458	0.77761
Neutrophils	HYLS1	-0.04194	3.183514	-0.16253	0.871263	-5.61783	0.763329	0.804251
Neutrophils	ZYG11B	-0.02214	5.883084	-0.16243	0.871336	-6.2703	0.706839	0.746058
Neutrophils	PLSCR4	-0.08455	0.494112	-0.16225	0.871478	-5.2256	0.824236	0.86672
Neutrophils	RAB28	0.019674	4.828065	0.162154	0.871554	-6.33968	0.72838	0.768304
Neutrophils	TMBIM1	0.056785	2.851295	0.162029	0.871653	-5.4522	0.770597	0.811783
Neutrophils	RARRES1	-0.10395	0.889058	-0.16191	0.871747	-5.20763	0.814991	0.857305
Neutrophils	RAPSN	-0.06263	0.715904	-0.16191	0.87175	-5.28758	0.819031	0.861438
Neutrophils	PPP6R2	0.032124	4.368985	0.161683	0.871924	-5.7742	0.737966	0.778245
Neutrophils	TENT4B	-0.02055	6.368937	-0.16164	0.871959	-6.29449	0.697144	0.736098
Neutrophils	NR5A2	0.059079	1.145394	0.161629	0.871967	-5.40962	0.809047	0.85125
Neutrophils	CNOT2	-0.01594	7.008824	-0.16145	0.87211	-6.44041	0.684588	0.723113
Neutrophils	LY6C2	0.031824	5.53324	0.161437	0.872118	-6.85983	0.713907	0.753437
Neutrophils	2610306M	0.097798	0.680103	0.161411	0.872138	-5.11515	0.819869	0.862338
Neutrophils	E430018J2	0.090377	0.653485	0.161273	0.872246	-5.11147	0.820493	0.862976
Neutrophils	RNF17	0.126244	-0.02892	0.161257	0.872259	-5.05474	0.836642	0.87948
Neutrophils	CCNT2	0.024727	5.37232	0.161106	0.872377	-6.02008	0.717183	0.756874
Neutrophils	SOD3	-0.08691	1.286857	-0.16106	0.872412	-5.24473	0.805786	0.84799
Neutrophils	RIPPLY3	0.091293	0.048429	0.161018	0.872446	-5.22419	0.834795	0.877678
Neutrophils	CHST2	0.094304	-0.02925	0.160828	0.872595	-5.19076	0.83665	0.879588
Neutrophils	MED11	-0.03951	3.312748	-0.16082	0.872604	-5.60529	0.760521	0.801566
Neutrophils	H1F0	-0.02901	6.001224	-0.16078	0.872633	-6.33901	0.704469	0.743768
Neutrophils	STARD5	0.032929	4.526318	0.160667	0.872723	-5.87215	0.734682	0.774962
Neutrophils	MRFAP1	-0.01562	6.452488	-0.16057	0.872799	-6.40896	0.695509	0.734505
Neutrophils	VOPP1	0.047546	3.965239	0.16045	0.872892	-5.64968	0.746543	0.787172
Neutrophils	PRKCZ	-0.08718	0.606888	-0.16016	0.873122	-5.08947	0.821776	0.864244
Neutrophils	B3GNT6	-0.0903	-0.72192	-0.16007	0.873193	-5.09056	0.853566	0.896726
Neutrophils	PRDM16	0.095308	0.306557	0.159924	0.873306	-5.1876	0.828895	0.871543
Neutrophils	GM46560	0.099133	-0.84515	0.15973	0.873458	-5.03233	0.856653	0.899876
Neutrophils	GM15411	0.098942	0.212426	0.159694	0.873486	-5.05775	0.831162	0.873859
Neutrophils	EP300	0.013987	6.696726	0.159301	0.873795	-6.60974	0.691093	0.729637
Neutrophils	ARHGDIB	-0.0153	8.995183	-0.15883	0.874168	-7.13976	0.64752	0.684537

Neutrophils	CBFA2T2	0.027052	5.563389	0.158803	0.874186	-6.00724	0.713747	0.753149
Neutrophils	PLEKHA8	-0.06539	0.886546	-0.15876	0.874218	-5.26005	0.815566	0.857799
Neutrophils	DTWD2	0.062751	2.777791	0.158759	0.874221	-5.37449	0.772703	0.813864
Neutrophils	HK3	0.033054	2.677613	0.158753	0.874225	-6.29723	0.774915	0.816136
Neutrophils	GZMK	-0.09544	-1.47202	-0.15871	0.874258	-5.0454	0.8724	0.915802
Neutrophils	SPATA7	0.071839	1.309288	0.158594	0.87435	-5.21507	0.805799	0.847799
Neutrophils	MINDY3	0.017078	5.598106	0.158356	0.874537	-6.4184	0.713122	0.752435
Neutrophils	STX4A	0.023688	4.972734	0.158252	0.874619	-5.97968	0.725927	0.765651
Neutrophils	PROSCOS	-0.0979	0.287878	-0.15822	0.874642	-5.1727	0.829723	0.8722
Neutrophils	NID1	-0.04035	2.394724	-0.15795	0.874856	-5.78105	0.78141	0.822653
Neutrophils	PPP2CB	0.015434	5.60171	0.157857	0.874929	-6.38746	0.713166	0.752432
Neutrophils	RIPK1	-0.01899	5.973193	-0.15774	0.87502	-6.40968	0.705688	0.744712
Neutrophils	WDR18	-0.03495	4.82244	-0.15755	0.875168	-5.72524	0.729239	0.768957
Neutrophils	PIGV	-0.03445	2.921169	-0.15718	0.875462	-5.61816	0.770015	0.810741
Neutrophils	HIST1H3D	-0.06945	1.963471	-0.15713	0.875503	-5.3906	0.791349	0.832626
Neutrophils	PANK3	-0.02649	4.591077	-0.15699	0.875608	-5.9058	0.734225	0.773934
Neutrophils	TRUB2	-0.01866	4.526877	-0.15694	0.875652	-6.32817	0.735569	0.775318
Neutrophils	RNASE6	0.054446	4.491549	0.15676	0.875791	-5.64547	0.736354	0.776087
Neutrophils	MRPS7	-0.03149	5.010323	-0.15659	0.875925	-5.80942	0.725556	0.76502
Neutrophils	ALG10B	-0.04242	2.952601	-0.15644	0.876046	-5.53652	0.769376	0.810125
Neutrophils	GM11696	0.078474	1.871722	0.156336	0.876124	-5.20808	0.793476	0.834863
Neutrophils	MRPL49	0.045833	3.778216	0.15629	0.876161	-5.56428	0.751474	0.79174
Neutrophils	BCAP31	-0.01635	5.901725	-0.15621	0.876223	-6.35221	0.707386	0.746294
Neutrophils	NAA60	0.02436	4.697726	0.156158	0.876264	-6.03857	0.732042	0.771733
Neutrophils	GM20712	-0.10543	0.063822	-0.15609	0.876314	-5.13117	0.835517	0.877862
Neutrophils	RAD18	-0.04166	4.103468	-0.15605	0.876348	-5.73157	0.74454	0.784605
Neutrophils	A230083N1	-0.08905	0.546139	-0.15534	0.876911	-5.17657	0.824411	0.866293
Neutrophils	GOLGA3	-0.03119	4.032459	-0.15533	0.876918	-5.77098	0.746343	0.786254
Neutrophils	CLMP	-0.05618	1.140521	-0.15527	0.87696	-5.53993	0.810536	0.852108
Neutrophils	HEATR5A	0.023513	5.656048	0.155247	0.876981	-6.30894	0.712627	0.751518
Neutrophils	RASD1	0.054245	3.88428	0.155036	0.877146	-5.66503	0.749581	0.789574
Neutrophils	GNG4	-0.04234	1.462633	-0.15486	0.877283	-5.79738	0.80326	0.844611
Neutrophils	USP12	0.026417	6.069178	0.154598	0.877491	-6.13877	0.704521	0.743073
Neutrophils	GM32916	-0.17775	-0.24915	-0.15452	0.877549	-5.03994	0.843612	0.88585
Neutrophils	PSMC3IP	0.058732	2.412129	0.154379	0.877662	-5.41217	0.78188	0.822757
Neutrophils	UBE2CBP	0.109629	1.761179	0.154358	0.87768	-5.15047	0.796543	0.837784
Neutrophils	RNMT	-0.02701	4.970618	-0.15391	0.878034	-5.91786	0.727119	0.766265
Neutrophils	TRPC4AP	-0.01697	5.651799	-0.15374	0.878162	-6.263	0.71316	0.751885
Neutrophils	1700020L2	0.039743	0.390371	0.153727	0.878175	-5.70808	0.828606	0.870381
Neutrophils	ASAP2	-0.04822	3.068411	-0.15333	0.878487	-5.62671	0.767804	0.808028
Neutrophils	TMEM135	-0.02468	5.7512	-0.15321	0.878581	-6.16555	0.711316	0.749886
Neutrophils	LMO4	0.019249	7.245458	0.153136	0.87864	-6.82415	0.681751	0.719331
Neutrophils	RCE1	0.042481	3.757774	0.153055	0.878704	-5.52684	0.752859	0.7927
Neutrophils	ZFP948	-0.06221	2.965976	-0.15301	0.878742	-5.45344	0.770051	0.810378
Neutrophils	GTF2F1	0.021191	5.030746	0.152644	0.879027	-6.02906	0.726224	0.765154
Neutrophils	KIF24	0.031445	3.667581	0.15242	0.879203	-5.7928	0.75507	0.794845
Neutrophils	DLGAP1	-0.06864	1.398192	-0.15231	0.879288	-5.32072	0.805592	0.846649

Neutrophils	HCST	0.018699	5.375869	0.151634	0.879821	-6.61923	0.719594	0.758056
Neutrophils	GM11523	0.116051	-0.95948	0.151252	0.880122	-5.0541	0.862153	0.904113
Neutrophils	NPTN	0.011505	8.043161	0.151142	0.880208	-6.7641	0.667096	0.703875
Neutrophils	SLC37A2	-0.05561	3.315046	-0.15112	0.880228	-5.47935	0.763104	0.802898
Neutrophils	IL6	-0.11795	1.106723	-0.15108	0.880255	-5.34553	0.812746	0.853764
Neutrophils	NHEJ1	-0.04409	3.867688	-0.15105	0.880283	-5.9734	0.751175	0.790662
Neutrophils	SYS1	0.015211	6.417243	0.150983	0.880333	-6.41587	0.698599	0.736503
Neutrophils	EOMES	0.106613	-0.77158	0.150928	0.880376	-5.08585	0.857539	0.899474
Neutrophils	IVNS1ABP	0.022237	6.027762	0.150912	0.880389	-6.17659	0.706375	0.744533
Neutrophils	GM43331	0.05226	2.057137	0.150609	0.880627	-5.33506	0.791067	0.83162
Neutrophils	SRF	-0.03378	2.927184	-0.15058	0.880652	-5.55939	0.77167	0.811737
Neutrophils	SH3D21	-0.06331	1.99564	-0.15044	0.880759	-5.36613	0.792457	0.833043
Neutrophils	LRRC14	-0.06392	2.339055	-0.15044	0.880763	-5.26765	0.784728	0.825126
Neutrophils	CLEC4A2	-0.01852	3.416946	-0.15022	0.88093	-6.64251	0.761048	0.80078
Neutrophils	TRIM65	-0.05509	3.22435	-0.14998	0.881118	-5.35741	0.765332	0.805156
Neutrophils	CYC1	0.022939	6.36195	0.149906	0.88118	-6.32834	0.699927	0.737847
Neutrophils	RAB7B	0.068112	2.804284	0.14969	0.88135	-5.50829	0.774609	0.814727
Neutrophils	TEX264	0.029028	4.497588	0.149648	0.881383	-5.75455	0.738106	0.777239
Neutrophils	SUMF2	0.057356	2.684259	0.149414	0.881567	-5.31808	0.777365	0.817503
Neutrophils	PTGR1	0.039263	3.377249	0.14931	0.881649	-5.78366	0.762159	0.801918
Neutrophils	EMILIN1	-0.03151	2.608435	-0.14915	0.881777	-5.74298	0.779107	0.819315
Neutrophils	SMC6	0.017296	7.579287	0.148718	0.882115	-6.51308	0.676371	0.713498
Neutrophils	BCCIP	-0.02227	5.436859	-0.14871	0.882119	-5.92875	0.718818	0.757348
Neutrophils	SF3A2	0.026139	5.260106	0.148685	0.882141	-6.04136	0.722442	0.761085
Neutrophils	NIT2	0.039978	3.588667	0.148654	0.882165	-5.7122	0.757671	0.797346
Neutrophils	CCDC9	0.025175	4.238269	0.148627	0.882187	-5.82858	0.743772	0.78307
Neutrophils	GM9828	0.081833	1.076181	0.148444	0.88233	-5.1753	0.814055	0.855115
Neutrophils	NLN	0.029213	4.446951	0.148298	0.882445	-5.9385	0.73946	0.778604
Neutrophils	BET1	0.029814	4.246255	0.148023	0.882661	-5.81708	0.743764	0.783046
Neutrophils	TENT2	0.015897	6.624422	0.147941	0.882726	-6.36525	0.695105	0.732888
Neutrophils	HNRNPA3	0.014307	8.554364	0.147826	0.882817	-6.7676	0.658079	0.69458
Neutrophils	CTDSP1	0.019209	5.3236	0.147517	0.88306	-6.3039	0.721294	0.75994
Neutrophils	C130026I2	0.042771	2.944263	0.147486	0.883084	-5.72753	0.77189	0.811971
Neutrophils	AGTR1A	-0.09618	0.270352	-0.14749	0.883085	-5.17966	0.833119	0.874614
Neutrophils	SAMD10	-0.05636	1.732501	-0.14742	0.883135	-5.37929	0.79905	0.839801
Neutrophils	ZGPAT	-0.03155	3.668613	-0.1474	0.883148	-5.6258	0.756109	0.79577
Neutrophils	HERC2	0.017777	5.98752	0.14738	0.883168	-6.38282	0.707801	0.746031
Neutrophils	TESPA1	0.077547	2.504605	0.147064	0.883417	-5.20621	0.781747	0.821999
Neutrophils	FXYD2	0.103385	-0.41192	0.147034	0.88344	-5.06067	0.849639	0.89137
Neutrophils	EIF2S2	-0.01403	7.925804	-0.14691	0.883537	-6.65668	0.670002	0.706888
Neutrophils	A630089N(	0.054655	2.672384	0.14685	0.883585	-5.40403	0.778015	0.818195
Neutrophils	TRIM21	0.063762	2.57157	0.146686	0.883714	-5.37089	0.780288	0.820493
Neutrophils	JAM2	-0.05886	1.449339	-0.14652	0.883841	-5.57657	0.805687	0.846497
Neutrophils	ZFP850	0.076567	1.010206	0.146481	0.883875	-5.08359	0.815853	0.85689
Neutrophils	SLC43A3	0.031177	3.813488	0.146423	0.883921	-5.90893	0.753134	0.792625
Neutrophils	RSF1OS2	-0.06633	1.663513	-0.14635	0.883979	-5.29252	0.800775	0.841473
Neutrophils	PNP2	0.075459	0.616927	0.146218	0.884082	-5.27121	0.825097	0.866331

Neutrophils	TRMT6	-0.03187	4.373913	-0.14595	0.884296	-5.71197	0.741276	0.780449
Neutrophils	FAM151B	0.060231	1.753415	0.145917	0.884319	-5.39658	0.798801	0.839473
Neutrophils	SLC9A3R2	0.040417	2.701144	0.145873	0.884354	-5.78685	0.777485	0.817653
Neutrophils	DARS	0.019014	5.511648	0.145513	0.884637	-6.32128	0.717822	0.756141
Neutrophils	TK2	-0.03141	3.825305	-0.1453	0.884807	-5.87008	0.753139	0.792571
Neutrophils	CDK2AP1	-0.01788	5.884299	-0.14528	0.884823	-6.30803	0.710257	0.748419
Neutrophils	FHL2	-0.10238	0.017257	-0.14516	0.884916	-5.1404	0.839609	0.881142
Neutrophils	NUBP2	0.035715	4.451117	0.145143	0.884929	-5.75538	0.739828	0.778945
Neutrophils	RRP7A	-0.04261	3.942345	-0.14493	0.885097	-5.54683	0.750665	0.790075
Neutrophils	GM43061	0.079546	0.306464	0.144883	0.885133	-5.17029	0.832739	0.874127
Neutrophils	GPR160	-0.03742	2.407708	-0.14474	0.885245	-5.87358	0.784249	0.824564
Neutrophils	ELOF1	0.026294	5.375272	0.144727	0.885256	-5.9555	0.720649	0.759202
Neutrophils	CCDC122	0.066507	1.315452	0.144532	0.885409	-5.24682	0.809118	0.850021
Neutrophils	SRP9	-0.01188	7.516452	-0.1445	0.885434	-6.6517	0.678141	0.715297
Neutrophils	TMEM184(	0.044755	3.390595	0.144316	0.885579	-5.39728	0.762658	0.802387
Neutrophils	PDZK1	-0.07488	0.933701	-0.14414	0.885719	-5.29976	0.818078	0.859175
Neutrophils	GPS2	0.014112	5.859455	0.144101	0.885749	-6.301	0.710899	0.749147
Neutrophils	B4GALT4	-0.05501	1.774117	-0.14398	0.885841	-5.34605	0.798699	0.839372
Neutrophils	MS4A8A	-0.09341	0.670009	-0.14384	0.885954	-5.21099	0.824319	0.865538
Neutrophils	THOC6	-0.03259	4.164901	-0.14368	0.886082	-5.74394	0.74612	0.785401
Neutrophils	SDHD	0.020769	5.858408	0.143403	0.886298	-6.2825	0.711004	0.749286
Neutrophils	ARHGEF4	0.089646	0.759185	0.143336	0.886351	-5.29	0.822263	0.863487
Neutrophils	SPATC1	-0.03558	-1.06407	-0.14333	0.886357	-5.60841	0.866219	0.908293
Neutrophils	WDR7	-0.02164	5.188411	-0.14329	0.886386	-6.28407	0.724686	0.763397
Neutrophils	GM19522	-0.06855	0.940675	-0.14324	0.886429	-5.21792	0.818012	0.859181
Neutrophils	RAP2A	0.02253	3.974658	0.143018	0.886601	-6.10018	0.750176	0.789683
Neutrophils	APOO	-0.03963	3.509146	-0.143	0.886617	-5.50724	0.760197	0.79998
Neutrophils	NME1	0.029873	6.668403	0.142963	0.886644	-6.19117	0.694824	0.732629
Neutrophils	TPT1	0.010925	10.10831	0.14279	0.886781	-7.02194	0.630365	0.665767
Neutrophils	ITGA5	-0.03952	3.54178	-0.14232	0.887153	-5.81097	0.759572	0.799434
Neutrophils	DAPL1	0.080634	-1.31502	0.14227	0.887191	-5.05529	0.872544	0.9149
Neutrophils	AUH	-0.0235	5.641378	-0.14226	0.887195	-6.07514	0.715484	0.75405
Neutrophils	KCTD7	-0.06121	0.387211	-0.14222	0.887228	-5.24834	0.831135	0.872719
Neutrophils	1600002K(	0.042214	3.214423	0.142217	0.887232	-5.43947	0.766696	0.806757
Neutrophils	RANGRF	0.081206	1.262196	0.141895	0.887486	-5.16427	0.810624	0.851834
Neutrophils	ERLEC1	0.033455	4.341715	0.141792	0.887567	-5.71532	0.742451	0.781921
Neutrophils	HMCN1	0.05859	1.920186	0.141684	0.887652	-5.66396	0.795538	0.836415
Neutrophils	REPIN1	-0.08578	1.082403	-0.14162	0.887704	-5.07051	0.814797	0.856119
Neutrophils	CAR12	0.088633	0.013745	0.141603	0.887716	-5.05535	0.840049	0.881904
Neutrophils	UNC13A	0.073938	1.183593	0.141547	0.88776	-5.19982	0.812446	0.853715
Neutrophils	2310033P(	-0.03042	4.144026	-0.14143	0.887852	-5.74288	0.746645	0.786235
Neutrophils	ZFYVE16	-0.03818	3.016629	-0.14141	0.887866	-5.59224	0.771033	0.811294
Neutrophils	TRDV4	-0.08612	-1.36214	-0.14138	0.887893	-5.05812	0.873719	0.9162
Neutrophils	FAM92A	-0.0449	3.301539	-0.14119	0.88804	-5.50081	0.764793	0.804933
Neutrophils	TBC1D17	-0.04007	3.823308	-0.14113	0.888086	-5.56182	0.753501	0.79333
Neutrophils	CCDC15	-0.04497	2.810819	-0.14112	0.888096	-5.55402	0.775573	0.815998
Neutrophils	CCNE1	0.039027	3.530373	0.141006	0.888186	-5.73777	0.759819	0.799833

Neutrophils	LRIG1	-0.08887	0.178544	-0.14096	0.888226	-5.15241	0.836104	0.877939
Neutrophils	CAAP1	-0.02493	4.575271	-0.14092	0.88825	-5.89792	0.737528	0.776906
Neutrophils	ZFP771	0.031189	4.065583	0.140622	0.888488	-5.65294	0.748456	0.788079
Neutrophils	DDX5	-0.00829	9.318404	-0.14046	0.888619	-7.0037	0.644745	0.680924
Neutrophils	GTPBP6	-0.05289	2.84005	-0.14043	0.888642	-5.35325	0.775081	0.815472
Neutrophils	PLSCR2	-0.05654	-0.38835	-0.14022	0.888803	-5.54415	0.849928	0.89201
Neutrophils	TPRA1	0.046472	2.802051	0.140204	0.888818	-5.42841	0.775928	0.816345
Neutrophils	SMOC1	-0.07811	2.28252	-0.14014	0.888865	-5.33251	0.787516	0.828227
Neutrophils	FAM89B	-0.02251	5.277421	-0.14002	0.888966	-6.11168	0.723105	0.762004
Neutrophils	GM4070	-0.07661	2.887167	-0.13973	0.88919	-5.44731	0.774177	0.814453
Neutrophils	BCS1L	-0.06909	1.699946	-0.13968	0.889232	-5.25891	0.800857	0.841791
Neutrophils	MAT1A	-0.04079	4.65605	-0.13932	0.889515	-6.14085	0.736286	0.77539
Neutrophils	RABIF	0.01978	4.708703	0.139158	0.889642	-6.01444	0.735228	0.774287
Neutrophils	PSD	-0.04779	1.633137	-0.13883	0.889898	-5.47164	0.802756	0.843544
Neutrophils	CD163	0.095936	1.13156	0.138765	0.889951	-5.32609	0.814336	0.855394
Neutrophils	SIRPA	0.017753	5.832373	0.138701	0.890002	-6.6716	0.712205	0.750543
Neutrophils	TMEM9B	-0.01485	5.558526	-0.13855	0.890121	-6.32412	0.717782	0.75632
Neutrophils	PAICS	-0.02135	6.310596	-0.13851	0.890155	-6.20611	0.702596	0.740659
Neutrophils	AW209491	0.060395	1.448036	0.13815	0.890436	-5.38212	0.807208	0.848024
Neutrophils	RIOK2	-0.02651	4.466641	-0.13775	0.890753	-5.78546	0.74082	0.779721
Neutrophils	TG	0.045885	1.91376	0.137345	0.891071	-5.48191	0.796933	0.837172
Neutrophils	DHX58OS	0.079611	0.831273	0.137327	0.891084	-5.20067	0.82195	0.862739
Neutrophils	RNPEP	-0.01849	5.72187	-0.13666	0.891613	-6.47916	0.715217	0.753146
Neutrophils	HMGCR	0.019465	4.712579	0.136568	0.891683	-6.19841	0.736058	0.77461
Neutrophils	RAP2C	0.015508	4.944479	0.136477	0.891754	-6.34127	0.731214	0.769626
Neutrophils	TYMS	-0.03621	4.922548	-0.13638	0.89183	-6.08405	0.731671	0.770096
Neutrophils	CTDSPL	-0.02917	3.831098	-0.13629	0.891899	-6.15069	0.754778	0.793852
Neutrophils	R3HCC1	0.068125	2.353768	0.136278	0.891911	-5.22203	0.78726	0.827161
Neutrophils	NGDN	-0.02078	5.424919	-0.13614	0.892023	-6.01252	0.721284	0.759439
Neutrophils	CCDC91	-0.04944	2.408137	-0.13612	0.892036	-5.39929	0.78604	0.825953
Neutrophils	SOCS2	-0.04641	3.962092	-0.13605	0.892095	-5.67122	0.751965	0.791015
Neutrophils	TBCB	-0.01558	6.070727	-0.13591	0.892205	-6.31747	0.708157	0.745983
Neutrophils	HTT	-0.0229	5.362069	-0.13576	0.892322	-5.98468	0.722575	0.760882
Neutrophils	ARHGAP27	0.059366	2.032169	0.135722	0.892349	-5.19375	0.79452	0.834766
Neutrophils	DNAJC2	-0.02	5.81945	-0.13566	0.892396	-6.07558	0.713235	0.751263
Neutrophils	TMTC1	0.076195	2.714836	0.135642	0.892412	-5.38291	0.779191	0.819069
Neutrophils	CRTC2	0.031792	4.134514	0.135565	0.892473	-5.79701	0.748279	0.787342
Neutrophils	GM11464	-0.07397	-0.20123	-0.13547	0.89255	-5.12562	0.846848	0.888207
Neutrophils	FUNDC2	-0.0197	7.178207	-0.13531	0.892673	-6.41118	0.68627	0.723385
Neutrophils	CHORDC1	0.024229	5.082056	0.135087	0.89285	-5.85004	0.728487	0.766856
Neutrophils	GM43914	-0.06561	0.384486	-0.13489	0.893002	-5.22858	0.832975	0.873918
Neutrophils	ULK4	0.041994	2.667963	0.134862	0.893028	-5.48859	0.780404	0.820183
Neutrophils	TBL2	0.041993	2.392625	0.134457	0.893347	-5.32234	0.786777	0.826624
Neutrophils	ETFB	-0.0177	6.979015	-0.13424	0.893521	-6.56713	0.690514	0.727576
Neutrophils	GM43329	0.044071	3.105098	0.134184	0.893562	-5.53385	0.771007	0.810422
Neutrophils	STRN	0.014784	5.530644	0.133539	0.894071	-6.48822	0.719882	0.757713
Neutrophils	TUBGCP2	-0.02899	3.994036	-0.13331	0.894252	-5.73936	0.752171	0.790899

Neutrophils	ZRANB3	0.046995	3.148069	0.133092	0.894423	-5.43841	0.77058	0.809759
Neutrophils	GM15726	-0.08766	2.074088	-0.13306	0.89445	-5.31134	0.794558	0.834314
Neutrophils	PKDCC	-0.05563	1.303453	-0.13276	0.894686	-5.34903	0.812381	0.852434
Neutrophils	RFWD3	0.016774	5.6362	0.132553	0.894848	-6.26592	0.717991	0.755662
Neutrophils	SCNM1	0.031527	3.621272	0.13245	0.89493	-5.64912	0.760396	0.799296
Neutrophils	YY1	-0.00963	7.144963	-0.13238	0.894981	-6.61891	0.68786	0.72463
Neutrophils	SMIM20	0.024203	4.914811	0.132239	0.895096	-5.88778	0.732883	0.771056
Neutrophils	ARHGEF6	-0.01586	5.643537	-0.13211	0.895199	-6.39918	0.717841	0.755601
Neutrophils	CYHR1	0.023763	4.48568	0.132051	0.895244	-5.90827	0.741894	0.780361
Neutrophils	AGBL5	0.046529	2.244561	0.13204	0.895253	-5.32048	0.790851	0.830574
Neutrophils	EEF1D	0.016272	7.27018	0.13202	0.895268	-6.40533	0.68542	0.722157
Neutrophils	LRIG2	-0.02729	4.256808	-0.13186	0.895398	-5.82928	0.746789	0.785352
Neutrophils	ELF1	0.010813	8.010369	0.131664	0.895549	-6.68922	0.671219	0.707431
Neutrophils	ANKRD27	0.034999	3.098779	0.131621	0.895583	-5.57091	0.771853	0.811075
Neutrophils	PLEKHA3	0.031593	3.98944	0.13159	0.895607	-5.6074	0.7525	0.791219
Neutrophils	CCDC86	0.022171	5.146452	0.13133	0.895812	-6.02447	0.7282	0.766199
Neutrophils	PCNX3	-0.03176	3.30596	-0.13126	0.895865	-5.64395	0.767404	0.80648
Neutrophils	EIF2S1	-0.01897	6.052768	-0.13081	0.896219	-6.13123	0.709762	0.747205
Neutrophils	CEP63	0.033688	4.291786	0.13061	0.89638	-5.63211	0.746241	0.784793
Neutrophils	ARSK	0.049628	2.705302	0.130523	0.896449	-5.42887	0.780771	0.82022
Neutrophils	GRSF1	-0.01628	5.185543	-0.13045	0.896508	-6.11168	0.727489	0.765513
Neutrophils	PXYLP1	0.024174	2.352535	0.130373	0.896568	-5.99186	0.78867	0.828319
Neutrophils	SMTNL2	0.083976	0.4226	0.130314	0.896614	-5.13086	0.833348	0.873967
Neutrophils	SLC38A10	-0.0203	5.326136	-0.13029	0.896635	-6.05421	0.724584	0.762552
Neutrophils	LAPTM4A	0.012586	7.210016	0.1302	0.896704	-6.56118	0.686811	0.723629
Neutrophils	NRG2	-0.06146	1.685011	-0.12997	0.896887	-5.35392	0.803841	0.843959
Neutrophils	CITED2	-0.01505	6.149916	-0.1298	0.897018	-6.60319	0.707805	0.745372
Neutrophils	RAB10OS	0.026207	4.429445	0.129782	0.897033	-5.83685	0.743321	0.781942
Neutrophils	RNF111	0.013027	6.692893	0.129517	0.897243	-6.57601	0.696969	0.734189
Neutrophils	LOXL3	-0.08254	0.451305	-0.12944	0.897303	-5.15168	0.832665	0.873412
Neutrophils	TKFC	0.042887	3.130684	0.129414	0.897324	-5.57795	0.771354	0.810723
Neutrophils	GM38190	-0.05456	1.823044	-0.12926	0.897447	-5.26024	0.80068	0.84075
Neutrophils	GUSB	0.018419	5.468903	0.129231	0.897468	-6.15072	0.721646	0.759639
Neutrophils	FBXL12OS	0.066547	0.679534	0.129217	0.897479	-5.11582	0.827255	0.867894
Neutrophils	GM11944	-0.03192	4.498486	-0.12915	0.897535	-5.89917	0.74186	0.780441
Neutrophils	DMAC2L	-0.04574	2.257897	-0.12914	0.897543	-5.36471	0.790803	0.830646
Neutrophils	FMC1	-0.03031	4.072806	-0.1291	0.897573	-5.79833	0.750912	0.789743
Neutrophils	SYT11	-0.06639	1.412759	-0.12909	0.897582	-5.2514	0.810114	0.850393
Neutrophils	LCMT2	-0.04172	2.687418	-0.12904	0.897623	-5.39867	0.78117	0.820803
Neutrophils	TMED1	0.043293	2.452472	0.129006	0.897646	-5.40761	0.786424	0.826185
Neutrophils	RHBDD3	0.04401	2.194151	0.128649	0.897928	-5.3272	0.792383	0.832188
Neutrophils	IQCN	-0.05918	1.791223	-0.12822	0.898264	-5.38659	0.801549	0.841736
Neutrophils	BANP	0.022368	4.601582	0.128149	0.898322	-5.87673	0.739816	0.778428
Neutrophils	MORN2	0.053426	2.045949	0.128122	0.898343	-5.29515	0.795742	0.835796
Neutrophils	MARVELD2	0.067262	1.369704	0.128049	0.898401	-5.21733	0.811254	0.851656
Neutrophils	KATNBL1	0.017736	5.963648	0.128039	0.898409	-6.16512	0.711689	0.749462
Neutrophils	PAQR7	-0.03714	1.552734	-0.12804	0.898409	-5.68645	0.807025	0.847335

Neutrophils	DYNC1LI2	-0.02447	4.672528	-0.12804	0.89841	-5.84557	0.738323	0.776892
Neutrophils	LETM2	0.02759	3.131534	0.12792	0.898503	-5.80267	0.771472	0.810958
Neutrophils	ERH	0.018634	7.372533	0.127844	0.898563	-6.44725	0.683772	0.720677
Neutrophils	LPCAT1	-0.02572	4.107714	-0.12771	0.898666	-5.86565	0.750298	0.789253
Neutrophils	GM14302	0.068538	0.609708	0.127707	0.898671	-5.11165	0.829053	0.869884
Neutrophils	CNOT3	-0.01693	5.46868	-0.12737	0.898936	-6.21344	0.721886	0.759992
Neutrophils	GM4673	-0.03751	2.578427	-0.12717	0.899092	-5.47644	0.783857	0.82367
Neutrophils	SUCLG1	-0.02062	5.422184	-0.12711	0.899139	-6.12594	0.722841	0.761008
Neutrophils	MTFR1	0.02395	4.079368	0.126996	0.899232	-5.92887	0.751016	0.790007
Neutrophils	FBXO44	0.088477	0.220079	0.126928	0.899286	-5.05801	0.838455	0.879491
Neutrophils	IL2RG	0.043728	6.294179	0.126902	0.899306	-6.05105	0.705138	0.742768
Neutrophils	RSL24D1	0.018191	5.674542	0.126899	0.899309	-6.11867	0.71767	0.755693
Neutrophils	FPGT	-0.07355	1.345954	-0.1268	0.899385	-5.1847	0.811925	0.852414
Neutrophils	DGKH	-0.01444	4.909539	-0.12671	0.899454	-6.60197	0.733466	0.77196
Neutrophils	1700021F0	-0.03635	3.209032	-0.12636	0.899733	-5.50943	0.770019	0.809394
Neutrophils	ANKRD11	0.011551	9.391983	0.126262	0.899811	-6.91924	0.645967	0.681455
Neutrophils	PREPL	-0.05857	1.734988	-0.12622	0.899844	-5.17305	0.803099	0.843296
Neutrophils	KCNK6	-0.0405	2.306704	-0.12604	0.899989	-5.48528	0.790099	0.830011
Neutrophils	D830050J1	0.053244	1.312085	0.126013	0.900008	-5.33273	0.812854	0.853282
Neutrophils	PRICKLE3	-0.03879	2.034653	-0.12596	0.900046	-5.51755	0.796258	0.836314
Neutrophils	DLGAP5	0.042725	3.248568	0.125657	0.900288	-5.71161	0.769297	0.808582
Neutrophils	WDFY4	0.019236	7.251372	0.125531	0.900388	-6.60691	0.686501	0.723335
Neutrophils	NUDT1	-0.04896	3.00659	-0.12539	0.900496	-5.42096	0.774679	0.814058
Neutrophils	EEF1A1	0.010683	10.55641	0.125132	0.900703	-7.03448	0.625304	0.659798
Neutrophils	MRPS14	-0.01332	6.892734	-0.12493	0.900864	-6.45423	0.693635	0.730605
Neutrophils	GM17268	-0.07497	0.383225	-0.12493	0.900865	-5.27788	0.835021	0.875651
Neutrophils	FCHO1	0.042438	3.772111	0.124758	0.900998	-5.49774	0.758043	0.796927
Neutrophils	MYO7A	-0.03085	3.25231	-0.12474	0.901013	-5.67341	0.769361	0.808538
Neutrophils	FUT10	0.068063	-0.01434	0.124674	0.901065	-5.14277	0.844557	0.885381
Neutrophils	GLS2	-0.059	1.618045	-0.12462	0.901109	-5.35131	0.806089	0.846164
Neutrophils	GJA1	-0.04695	1.208053	-0.12441	0.90127	-5.5961	0.815616	0.85586
Neutrophils	IGBP1	-0.0243	4.749526	-0.12428	0.901377	-5.8549	0.737256	0.775564
Neutrophils	ANAPC16	-0.01656	5.656735	-0.12425	0.901401	-6.10575	0.718464	0.756221
Neutrophils	TRIM39	-0.03074	2.873416	-0.1242	0.901436	-5.5622	0.777754	0.817134
Neutrophils	ZMYM2	-0.01565	5.970893	-0.12398	0.901609	-6.35143	0.712107	0.749637
Neutrophils	NDC80	-0.03301	4.625978	-0.12396	0.901625	-6.03475	0.739888	0.77824
Neutrophils	FMR1	-0.01463	5.971489	-0.12388	0.901694	-6.34511	0.712094	0.749627
Neutrophils	MORC2A	0.027939	4.295558	0.123395	0.902075	-5.77217	0.747141	0.785512
Neutrophils	AMOT	-0.06421	0.863491	-0.12326	0.902185	-5.19668	0.824005	0.864212
Neutrophils	ACP2	-0.03123	3.54465	-0.12315	0.90227	-5.78926	0.763307	0.802147
Neutrophils	NUTF2	0.065783	1.951713	0.123058	0.902341	-5.17983	0.798796	0.83849
Neutrophils	DHX37	0.050833	2.67509	0.123023	0.902368	-5.24782	0.782475	0.821791
Neutrophils	9530034E1	-0.07364	0.330435	-0.12276	0.902574	-5.0612	0.836657	0.877228
Neutrophils	TTLL5	-0.02563	4.471737	-0.12263	0.902676	-5.78238	0.743414	0.781812
Neutrophils	F7	-0.06873	0.719823	-0.12246	0.90281	-5.21646	0.827404	0.867872
Neutrophils	ZFP958	-0.05403	2.267977	-0.12244	0.902829	-5.26495	0.791629	0.831323
Neutrophils	DERA	0.023244	4.534652	0.12241	0.902852	-5.74486	0.742083	0.780509

Neutrophils	GALNT4	0.032778	1.622606	0.122394	0.902865	-5.53411	0.806347	0.846374
Neutrophils	UEVLD	0.039421	2.944551	0.122089	0.903106	-5.5306	0.776493	0.815886
Neutrophils	WWOX	-0.01444	6.961551	-0.12204	0.903145	-6.53565	0.692593	0.729571
Neutrophils	1-Mar	0.058104	1.457886	0.122003	0.903174	-5.31634	0.810148	0.850335
Neutrophils	INTS9	-0.02521	4.394064	-0.12199	0.903187	-5.78176	0.745061	0.783641
Neutrophils	METTL6	0.026026	4.505421	0.121945	0.90322	-5.81866	0.742701	0.781231
Neutrophils	4931406C0	-0.03371	3.540099	-0.12182	0.90332	-5.63628	0.763416	0.802519
Neutrophils	FFAR2	-0.03453	-0.53401	-0.12178	0.903346	-5.79259	0.857573	0.898734
Neutrophils	4930595D1	0.052893	1.248821	0.121726	0.903392	-5.32007	0.814999	0.855338
Neutrophils	MASTL	-0.02881	3.782	-0.12148	0.90359	-5.74131	0.758276	0.797213
Neutrophils	POLA2	-0.03503	4.050563	-0.12108	0.903903	-5.62143	0.752637	0.791323
Neutrophils	ADAMTS14	-0.06835	0.543497	-0.12107	0.903907	-5.18881	0.831856	0.872381
Neutrophils	SLAMF9	-0.0756	2.637226	-0.12083	0.904101	-5.20164	0.783695	0.823084
Neutrophils	SRRT	-0.01885	5.528359	-0.12065	0.90424	-6.06606	0.721775	0.759498
Neutrophils	GM13402	-0.0779	0.307958	-0.12044	0.90441	-5.07791	0.837736	0.878243
Neutrophils	GM15788	-0.07477	0.240256	-0.1201	0.904676	-5.12516	0.839388	0.88003
Neutrophils	MIR17HG	0.045163	2.192534	0.119943	0.9048	-5.39327	0.793877	0.833628
Neutrophils	R3HDM1	-0.01438	6.683532	-0.11991	0.904827	-6.25488	0.69856	0.735678
Neutrophils	RHOB	-0.02807	5.891039	-0.11986	0.904864	-6.38071	0.714471	0.752091
Neutrophils	CHTF8	0.057546	1.341638	0.119758	0.904947	-5.20446	0.813397	0.853611
Neutrophils	HIST2H2BB	0.074038	-0.261	0.119709	0.904985	-5.05999	0.851493	0.892456
Neutrophils	FN3KRP	0.064137	1.481688	0.119699	0.904993	-5.17128	0.810151	0.850295
Neutrophils	CACYBP	-0.01922	5.736095	-0.11966	0.905021	-6.10487	0.717626	0.755374
Neutrophils	GSTP1	0.019374	6.928594	0.119459	0.905183	-6.40307	0.69372	0.730708
Neutrophils	CPEB1	0.061255	0.696287	0.119385	0.905241	-5.19189	0.828532	0.869061
Neutrophils	HIST1H2AN	-0.07981	3.254826	-0.11938	0.905244	-5.45648	0.770183	0.80939
Neutrophils	FNBP1	0.01715	8.248905	0.119199	0.905388	-6.61799	0.668274	0.704343
Neutrophils	MCM3AP	0.020327	3.885875	0.119045	0.90551	-5.82992	0.75651	0.795301
Neutrophils	ADCY4	-0.05191	1.413611	-0.11902	0.905532	-5.512	0.811797	0.851913
Neutrophils	TMEM115	0.028063	3.447731	0.118764	0.905732	-5.53205	0.766031	0.805057
Neutrophils	RDH14	0.02542	3.731822	0.118744	0.905748	-5.69333	0.759851	0.798718
Neutrophils	RMDN3	-0.03275	3.398108	-0.11872	0.905764	-5.4547	0.767115	0.80617
Neutrophils	KCNRG	-0.02513	2.320944	-0.11858	0.905875	-5.85233	0.791088	0.83073
Neutrophils	EFNA5	0.078219	2.313548	0.118356	0.906054	-5.29792	0.791331	0.830964
Neutrophils	TBCA	-0.01244	7.625569	-0.11829	0.906107	-6.60613	0.680293	0.716759
Neutrophils	PRDM2	-0.01424	5.970241	-0.11768	0.906589	-6.40238	0.713314	0.750655
Neutrophils	TCF7	-0.06826	2.524319	-0.11739	0.906819	-5.29872	0.786892	0.826274
Neutrophils	BICDL1	-0.04864	1.878488	-0.11732	0.90687	-5.34583	0.801531	0.841259
Neutrophils	P2RX1	0.046666	0.81074	0.117315	0.906877	-5.48817	0.826344	0.866595
Neutrophils	SUMO2	-0.00943	8.656817	-0.11729	0.906894	-6.79482	0.660959	0.696635
Neutrophils	ARL6	0.054286	1.698649	0.11719	0.906975	-5.14251	0.805657	0.845518
Neutrophils	DDT	0.021728	5.541533	0.116953	0.907162	-6.13062	0.722065	0.759794
Neutrophils	ST6GAL1	-0.04138	5.862113	-0.11694	0.907177	-5.89387	0.715511	0.753043
Neutrophils	DHX38	0.01552	4.560036	0.116905	0.9072	-6.25462	0.742522	0.780842
Neutrophils	CDC23	-0.0318	2.927739	-0.11672	0.907343	-5.51149	0.777887	0.817174
Neutrophils	HYKK	-0.0553	0.618386	-0.11656	0.907474	-5.24177	0.830896	0.871353
Neutrophils	PMPCB	0.0222	4.974651	0.11655	0.907481	-5.91782	0.733808	0.771922

Neutrophils	TBC1D13	-0.03315	3.495587	-0.11653	0.907497	-5.57538	0.765391	0.804365
Neutrophils	ST6GALNA(	-0.0369	3.214358	-0.11643	0.907578	-5.5431	0.771554	0.810706
Neutrophils	FBXW4	-0.02558	4.272711	-0.11642	0.907583	-5.89213	0.748625	0.787175
Neutrophils	CCNA2	0.030373	5.520186	0.116373	0.907621	-6.31043	0.722503	0.760307
Neutrophils	NLRP1A	0.053989	0.449959	0.11623	0.907734	-5.38533	0.834903	0.875481
Neutrophils	CCDC93	-0.02762	3.72019	-0.11619	0.907762	-5.75635	0.760506	0.799392
Neutrophils	SLC35E1	0.016027	4.084727	0.116098	0.907838	-6.12014	0.752648	0.791328
Neutrophils	GM44148	0.056275	1.90428	0.116003	0.907913	-5.29635	0.800947	0.840865
Neutrophils	RUFY1	-0.01717	5.607628	-0.1159	0.907998	-6.07455	0.720723	0.758525
Neutrophils	UBTD1	0.015397	4.402162	0.115527	0.90829	-6.3272	0.746041	0.784458
Neutrophils	GM20275	0.044308	3.071387	0.115471	0.908334	-5.4101	0.774886	0.814056
Neutrophils	CIB1	0.016311	5.732242	0.11536	0.908422	-6.22752	0.718337	0.755942
Neutrophils	SUB1	-0.01102	8.911934	-0.11495	0.908743	-6.86029	0.656546	0.691914
Neutrophils	PRKAR1A	0.007873	7.625008	0.114802	0.908863	-6.73371	0.680936	0.71716
Neutrophils	ZXDB	-0.03683	3.1887	-0.11475	0.908902	-5.4501	0.772534	0.811424
Neutrophils	TSGA10	0.03588	2.968133	0.11464	0.90899	-5.44621	0.777423	0.816426
Neutrophils	ZWILCH	-0.04211	3.028209	-0.11432	0.909243	-5.57981	0.776246	0.815071
Neutrophils	GM48226	-0.04655	2.396833	-0.11414	0.909384	-5.2704	0.790385	0.829539
Neutrophils	TTLL3	0.023646	4.027206	0.114096	0.909421	-5.89533	0.754476	0.792765
Neutrophils	ABLIM2	0.074903	-0.47962	0.113992	0.909503	-5.06297	0.858055	0.898586
Neutrophils	NOC2L	0.028405	5.059461	0.113871	0.909598	-5.74266	0.732643	0.770334
Neutrophils	TBCEL	0.020095	4.594507	0.11369	0.909741	-5.98464	0.742465	0.780393
Neutrophils	C5AR2	0.023475	1.231827	0.113555	0.909848	-6.05869	0.817236	0.856949
Neutrophils	ZFP560	-0.02673	4.00302	-0.11301	0.910279	-5.64233	0.755409	0.793509
Neutrophils	KLHL11	0.039807	2.776613	0.112763	0.910474	-5.41405	0.78233	0.82106
Neutrophils	MSH2	0.030271	4.128132	0.112747	0.910487	-5.56624	0.752756	0.790754
Neutrophils	PEX26	0.065115	1.217016	0.112679	0.91054	-5.15155	0.817943	0.85745
Neutrophils	NBEAL2	0.019724	2.635242	0.112415	0.910749	-5.90008	0.785609	0.824331
Neutrophils	BUB1	0.037228	3.743439	0.112325	0.910821	-5.81276	0.761168	0.799316
Neutrophils	TOMT	-0.04797	1.736925	-0.11203	0.911053	-5.42794	0.80611	0.845209
Neutrophils	ETAA1	-0.03472	3.334503	-0.1119	0.911154	-5.47229	0.770189	0.808521
Neutrophils	SLA	0.016713	5.280678	0.111849	0.911196	-6.35277	0.728649	0.765887
Neutrophils	CKAP2L	0.025469	4.357364	0.111817	0.911222	-5.97402	0.748059	0.785829
Neutrophils	CFLAR	0.013791	5.971185	0.111448	0.911514	-6.62317	0.714497	0.75131
Neutrophils	TBXAS1	-0.03294	3.468333	-0.11144	0.911522	-5.97944	0.767277	0.805522
Neutrophils	VDAC3	0.011316	7.191815	0.111345	0.911595	-6.54436	0.690147	0.726208
Neutrophils	LY6G	-0.02265	-0.81776	-0.11118	0.911728	-6.07913	0.867147	0.907414
Neutrophils	DNAAF3	-0.07008	-0.6587	-0.11116	0.911745	-5.06181	0.863217	0.903421
Neutrophils	TMEM161/	0.036676	2.853132	0.111153	0.911747	-5.46385	0.780858	0.819447
Neutrophils	F830016B0	0.082134	0.444299	0.111121	0.911772	-5.13249	0.836445	0.8762
Neutrophils	BAX	0.016935	6.141068	0.110785	0.912038	-6.22756	0.711055	0.747815
Neutrophils	1700016P0	0.048408	3.898827	0.110673	0.912127	-5.65398	0.757918	0.795982
Neutrophils	AFTPH	-0.01225	6.658529	-0.11066	0.91214	-6.4338	0.700676	0.737119
Neutrophils	TBC1D2	-0.01774	1.45751	-0.1105	0.912263	-6.24749	0.812589	0.851951
Neutrophils	FYTTD1	-0.01599	5.267108	-0.11032	0.912404	-5.96557	0.728951	0.766286
Neutrophils	PRKDC	0.02432	4.69925	0.110298	0.912423	-5.74235	0.740831	0.778497
Neutrophils	TSPAN31	-0.02698	4.424978	-0.11011	0.912571	-5.74688	0.74664	0.784463

Neutrophils	1110008P1	0.012491	5.226277	0.11011	0.912572	-6.40572	0.729798	0.767158
Neutrophils	GPC1	0.030483	0.351919	0.110099	0.912581	-5.80355	0.838655	0.87854
Neutrophils	IL1A	-0.03443	2.787687	-0.1101	0.912583	-5.91481	0.782318	0.821033
Neutrophils	SPECC1	-0.02265	4.276803	-0.11006	0.912609	-6.30633	0.749799	0.787706
Neutrophils	EED	-0.01027	6.114274	-0.11004	0.912624	-6.35767	0.711596	0.748424
Neutrophils	ALDH4A1	-0.03343	2.912017	-0.1098	0.91282	-5.60721	0.779653	0.818245
Neutrophils	ARAP3	0.015709	3.537048	0.109672	0.912918	-6.36904	0.765894	0.804138
Neutrophils	PRKD3	-0.013	5.198034	-0.10955	0.913017	-6.39365	0.730499	0.767806
Neutrophils	SS18L2	0.019615	3.817204	0.109498	0.913056	-5.97138	0.759802	0.797893
Neutrophils	F730311O2	-0.07084	-0.31758	-0.10922	0.913274	-5.08969	0.855069	0.895108
Neutrophils	GM16196	0.048979	1.751417	0.109194	0.913296	-5.25412	0.806009	0.845125
Neutrophils	NFATC2	-0.05302	2.489047	-0.10865	0.913729	-5.23209	0.789448	0.828072
Neutrophils	RNF10	0.012634	6.51711	0.108583	0.913779	-6.45177	0.703885	0.740263
Neutrophils	SLBP	0.012365	7.147492	0.10849	0.913853	-6.60098	0.691397	0.727401
Neutrophils	FLCN	-0.02278	4.338996	-0.10837	0.913945	-5.87728	0.748885	0.786599
Neutrophils	LARP1B	0.013091	5.599705	0.108353	0.913961	-6.4416	0.722484	0.759474
Neutrophils	UBN1	0.008803	6.944041	0.108307	0.913998	-6.5777	0.695402	0.731574
Neutrophils	CELSR1	-0.07352	1.626552	-0.10811	0.914157	-5.18632	0.80916	0.848284
Neutrophils	CLSTN1	0.047165	1.774497	0.107933	0.914293	-5.24648	0.805749	0.844803
Neutrophils	NRAP	-0.06539	-1.09404	-0.1079	0.914323	-5.039	0.874539	0.914827
Neutrophils	TMX4	-0.01372	4.420261	-0.10774	0.914443	-6.41463	0.747186	0.784868
Neutrophils	GPR19	0.037437	2.550401	0.107588	0.914566	-5.42934	0.788102	0.826797
Neutrophils	SMPDL3A	-0.01883	5.614644	-0.10754	0.914601	-6.44643	0.722209	0.75921
Neutrophils	BC024978	0.055457	1.865186	0.10753	0.914613	-5.19854	0.803666	0.842704
Neutrophils	FAF1	0.010825	6.739255	0.107309	0.914787	-6.48925	0.699489	0.735824
Neutrophils	UBFD1	0.025535	4.520398	0.106922	0.915093	-5.66882	0.745058	0.782828
Neutrophils	CD24A	-0.01612	7.730753	-0.1069	0.915113	-6.91486	0.680081	0.715899
Neutrophils	USP5	0.021637	4.34395	0.106858	0.915144	-5.85229	0.748812	0.786689
Neutrophils	CARNMT1	-0.01548	5.353674	-0.10671	0.915261	-6.06219	0.727591	0.764906
Neutrophils	ARHGAP24	0.01409	6.825889	0.106634	0.915321	-6.70525	0.69777	0.734189
Neutrophils	KRTCAP2	-0.0138	7.062528	-0.10662	0.915331	-6.35603	0.693097	0.729368
Neutrophils	TFCP2	0.043596	1.971586	0.106621	0.915331	-5.28572	0.801229	0.840391
Neutrophils	MAFK	-0.0228	4.524193	-0.10654	0.915396	-6.05678	0.744978	0.782775
Neutrophils	NT5E	-0.01802	2.431121	-0.10653	0.915401	-6.32494	0.790789	0.82972
Neutrophils	CLEC12A	-0.01445	4.937614	-0.1065	0.91543	-6.63442	0.736259	0.773818
Neutrophils	SLC16A12	0.054579	0.427781	0.106454	0.915463	-5.258	0.837339	0.877224
Neutrophils	HGF	0.029399	2.743326	0.106422	0.915488	-5.96335	0.783776	0.82255
Neutrophils	TIMM22	0.020799	4.4471	0.106298	0.915586	-5.79112	0.746636	0.784495
Neutrophils	NFRKB	-0.0214	4.478568	-0.10601	0.915813	-5.68732	0.746093	0.78384
Neutrophils	TRPC1	0.066779	-0.17084	0.10578	0.915997	-5.04528	0.85205	0.892052
Neutrophils	NUDT16	-0.03587	2.966991	-0.10529	0.91638	-5.43204	0.779252	0.817636
Neutrophils	PRPS1L3	0.044951	2.045409	0.105121	0.916518	-5.24585	0.800016	0.838924
Neutrophils	GLYR1	-0.0092	6.736527	-0.10503	0.916587	-6.61129	0.699957	0.736278
Neutrophils	MGST3	-0.03622	3.846236	-0.10476	0.9168	-5.60747	0.759958	0.798031
Neutrophils	LIPO3	0.038736	2.915424	0.104628	0.916908	-5.36842	0.780399	0.818971
Neutrophils	1700123O2	0.015058	4.989927	0.104624	0.916911	-6.16375	0.735599	0.773025
Neutrophils	YJU2	0.024215	3.454196	0.104597	0.916933	-5.59311	0.768499	0.806785

Neutrophils	DEPDC1B	0.027069	2.954227	0.104473	0.91703	-5.98032	0.779536	0.818087
Neutrophils	GMNN	0.020048	5.558334	0.104472	0.917031	-6.287	0.723796	0.760888
Neutrophils	CATSPERE2	-0.04429	1.174644	-0.10447	0.917035	-5.22123	0.820154	0.859584
Neutrophils	EIF4A3	0.014128	5.877476	0.104453	0.917046	-6.19133	0.717256	0.754157
Neutrophils	ZCCHC7	0.017281	6.744533	0.104251	0.917206	-6.4513	0.699829	0.736175
Neutrophils	CHP2	-0.08293	-0.09865	-0.10422	0.917232	-5.1764	0.850566	0.890524
Neutrophils	ADGB	0.032188	2.335955	0.103901	0.917483	-5.88939	0.793599	0.832381
Neutrophils	IPP	-0.04325	2.465742	-0.10362	0.917704	-5.23866	0.790788	0.829383
Neutrophils	HIST1H2BB	-0.04581	1.116611	-0.10354	0.917771	-5.28133	0.821838	0.86108
Neutrophils	EPB41	-0.01573	8.026595	-0.10332	0.91794	-6.77134	0.675098	0.710452
Neutrophils	CYB5B	0.014792	5.866247	0.1033	0.917959	-6.0993	0.717801	0.754488
Neutrophils	PPIF	-0.03531	3.577224	-0.1032	0.918035	-5.41707	0.76615	0.804149
Neutrophils	LUC7L3	-0.01033	6.706417	-0.10278	0.918372	-6.38887	0.70107	0.737144
Neutrophils	B3GNT8	0.049441	2.202747	0.102395	0.918675	-5.3098	0.797216	0.835597
Neutrophils	RNF43	0.049679	1.563577	0.102291	0.918757	-5.35209	0.811903	0.850578
Neutrophils	F930017D2	0.043882	0.898224	0.102197	0.918832	-5.59462	0.827478	0.866462
Neutrophils	GM30239	-0.05551	-0.09316	-0.1021	0.918912	-5.06769	0.85125	0.890642
Neutrophils	BSDC1	0.019489	5.056629	0.101856	0.919102	-6.07084	0.735035	0.771814
Neutrophils	SPARC	-0.02018	5.322477	-0.10156	0.919335	-6.48379	0.729622	0.766147
Neutrophils	PNPT1	0.024397	4.198204	0.101147	0.919663	-5.69984	0.753516	0.790566
Neutrophils	EDRF1	0.027727	4.217889	0.101134	0.919673	-5.61336	0.753093	0.790134
Neutrophils	SLC4A2	-0.02061	3.559435	-0.10093	0.919837	-5.77277	0.767394	0.804781
Neutrophils	VPS52	0.018186	3.923327	0.100899	0.919859	-5.75749	0.759475	0.796675
Neutrophils	ASTN2	0.051892	1.045238	0.100442	0.920221	-5.24786	0.824599	0.863116
Neutrophils	DBN1	0.042162	1.388658	0.100429	0.920231	-5.47694	0.816553	0.854919
Neutrophils	ATP5A1	-0.01049	7.857813	-0.10042	0.920235	-6.58592	0.679198	0.714067
Neutrophils	DUSP7	-0.03025	3.559127	-0.10036	0.920287	-5.61021	0.767516	0.804846
Neutrophils	CCDC88C	-0.01288	5.294187	-0.10021	0.920401	-6.37793	0.730537	0.766922
Neutrophils	GCA	-0.01887	1.099128	-0.10002	0.920552	-6.24919	0.823438	0.861899
Neutrophils	PRPF40A	-0.00762	7.709067	-0.09973	0.920784	-6.69468	0.682197	0.717111
Neutrophils	SRPRB	-0.02731	4.104103	-0.09971	0.920803	-5.50444	0.755828	0.792821
Neutrophils	LY6G2	-0.05608	0.952461	-0.0996	0.920887	-5.2904	0.826943	0.865453
Neutrophils	TGIF1	-0.01476	6.736671	-0.09952	0.92095	-6.57701	0.701285	0.736797
Neutrophils	DZIP3	0.030142	3.257554	0.099496	0.92097	-5.59985	0.77429	0.811724
Neutrophils	2810408I1	0.040792	1.398496	0.099278	0.921142	-5.3876	0.816566	0.854868
Neutrophils	FAM136A	0.02895	4.090791	0.09916	0.921236	-5.57417	0.756214	0.793191
Neutrophils	2210016L2	0.02656	3.97848	0.099041	0.92133	-5.63977	0.758656	0.795717
Neutrophils	BRIP1	-0.02274	4.898207	-0.09866	0.921632	-6.01726	0.739099	0.775667
Neutrophils	AUP1	-0.01226	6.010505	-0.0985	0.92176	-6.30961	0.716076	0.752015
Neutrophils	CKS2	0.013478	5.959802	0.098448	0.921799	-6.58915	0.717109	0.753095
Neutrophils	RBM33	-0.01051	5.898903	-0.09835	0.921876	-6.35389	0.718352	0.754391
Neutrophils	NUDT16L1	0.024265	4.234234	0.098285	0.921928	-5.72264	0.753209	0.790175
Neutrophils	METTL25	0.018208	4.28478	0.098275	0.921936	-5.78722	0.752125	0.789064
Neutrophils	POLR3F	-0.02436	3.882542	-0.09821	0.921987	-5.60869	0.760796	0.797952
Neutrophils	GM15446	0.044311	2.166881	0.098067	0.922101	-5.27259	0.798952	0.837001
Neutrophils	TREM3	0.013908	2.200561	0.098046	0.922118	-6.70573	0.798185	0.836218
Neutrophils	RRAGB	0.064168	0.125251	0.098027	0.922133	-5.05764	0.846914	0.885851

Neutrophils	UGT3A2	-0.04424	1.174889	-0.09791	0.922222	-5.33457	0.821905	0.860459
Neutrophils	CENPO	0.030539	2.773597	0.097616	0.922458	-5.44245	0.785242	0.823105
Neutrophils	HINFP	-0.02392	3.494398	-0.09751	0.922545	-5.63961	0.769264	0.806762
Neutrophils	METTL16	-0.02391	4.533537	-0.09743	0.922609	-5.58448	0.746817	0.783763
Neutrophils	ORAI2	0.01018	5.87495	0.097418	0.922615	-6.58867	0.718844	0.755033
Neutrophils	PSMD14	0.009894	6.618882	0.097385	0.922641	-6.40428	0.703805	0.739556
Neutrophils	AW554918	-0.01534	5.656196	-0.09738	0.922645	-6.10362	0.72333	0.759646
Neutrophils	SLC22A4	0.031736	0.816422	0.097177	0.922806	-5.60767	0.830402	0.869128
Neutrophils	ZBTB22	0.024714	3.076444	0.097115	0.922855	-5.67917	0.778525	0.816199
Neutrophils	BAG5	-0.03165	3.637068	-0.09699	0.922952	-5.54218	0.766178	0.803565
Neutrophils	RBM12B1	-0.05716	0.935508	-0.09696	0.92298	-5.13232	0.827582	0.866258
Neutrophils	GLMN	-0.0384	2.819036	-0.0967	0.923181	-5.29554	0.784371	0.822147
Neutrophils	SLC17A5	0.026587	3.572742	0.096563	0.923292	-5.65156	0.767722	0.805086
Neutrophils	GM38604	0.041605	1.796583	0.096326	0.92348	-5.24692	0.80768	0.845888
Neutrophils	CCDC134	0.036616	3.407824	0.096282	0.923515	-5.36127	0.771389	0.808818
Neutrophils	MRPL12	-0.02105	5.090142	-0.09621	0.923568	-5.86041	0.735289	0.771817
Neutrophils	CCDC83	0.063025	0.420205	0.095921	0.9238	-5.25146	0.840139	0.878913
Neutrophils	ADAM30	0.031209	0.62147	0.09581	0.923888	-5.57732	0.835323	0.874044
Neutrophils	MPP5	-0.01234	5.379551	-0.09572	0.923958	-6.31638	0.729329	0.765692
Neutrophils	TMEM214	0.0206	4.338367	0.095672	0.923997	-5.6622	0.751273	0.788211
Neutrophils	CD69	-0.02965	5.620451	-0.09558	0.924068	-6.04723	0.724348	0.760582
Neutrophils	DACH1	-0.01554	1.877933	-0.09555	0.924095	-6.46135	0.805886	0.844065
Neutrophils	5430431A1	-0.04259	1.423829	-0.0952	0.924369	-5.19506	0.816534	0.854816
Neutrophils	GPATCH4	-0.03746	3.338867	-0.09516	0.924403	-5.31716	0.77311	0.810481
Neutrophils	DCAF4	-0.04179	2.008291	-0.09497	0.924552	-5.17442	0.803023	0.841081
Neutrophils	MANEA	0.019708	3.728612	0.094912	0.924599	-5.85627	0.764566	0.801794
Neutrophils	UBQLN1	-0.01221	5.517917	-0.09471	0.924759	-6.1218	0.726582	0.762892
Neutrophils	SH2D3C	-0.01043	4.693449	-0.09462	0.924831	-6.61704	0.743835	0.78062
Neutrophils	ARHGEF10	-0.04775	2.266878	-0.09449	0.924936	-5.36961	0.797118	0.835157
Neutrophils	ZFP41	0.057899	0.707145	0.094452	0.924964	-5.07564	0.833418	0.872155
Neutrophils	NCAPH2	-0.01345	5.344606	-0.09442	0.924988	-6.1245	0.730174	0.766603
Neutrophils	ARGLU1	-0.00672	7.597485	-0.09432	0.925068	-6.6431	0.684902	0.720037
Neutrophils	POMP	-0.0103	7.268227	-0.09429	0.925094	-6.55641	0.69133	0.726669
Neutrophils	SLC23A2	0.018011	5.113974	0.094257	0.925118	-5.99568	0.734982	0.771584
Neutrophils	ASF1B	-0.02243	4.954931	-0.09398	0.92534	-6.17566	0.738399	0.775046
Neutrophils	BBOF1	0.03546	1.302277	0.093911	0.925393	-5.39015	0.819464	0.857945
Neutrophils	CFL2	0.019049	4.631141	0.093852	0.925439	-5.86396	0.745238	0.782064
Neutrophils	SETD1B	0.012999	5.026478	0.093509	0.925711	-6.21447	0.736992	0.773538
Neutrophils	PLEKHM1	-0.0139	4.771883	-0.0935	0.925715	-6.09331	0.742353	0.77904
Neutrophils	SLC2A9	0.021402	2.879571	0.093399	0.925798	-5.90711	0.783492	0.821183
Neutrophils	L1CAM	-0.01963	3.644556	-0.0932	0.925959	-6.06798	0.766585	0.803905
Neutrophils	COX11	0.02278	3.751078	0.093099	0.926036	-5.55061	0.76426	0.801539
Neutrophils	TJP1	0.024828	2.024999	0.093066	0.926061	-5.71839	0.802833	0.840967
Neutrophils	TTLL1	0.035876	1.626512	0.092971	0.926137	-5.32975	0.812018	0.850335
Neutrophils	PRKCH	-0.01787	6.999653	-0.09297	0.926141	-6.62343	0.696787	0.732233
Neutrophils	GM38134	-0.04873	1.108316	-0.09274	0.92632	-5.19426	0.824167	0.862734
Neutrophils	ABHD6	-0.02972	2.548845	-0.09249	0.926515	-5.44604	0.790963	0.828959

Neutrophils	PUF60	-0.01301	5.949591	-0.09237	0.926614	-6.23631	0.717928	0.754106
Neutrophils	CASTOR2	-0.04899	3.643855	-0.09227	0.926694	-5.36598	0.766642	0.804126
Neutrophils	CEP89	0.042509	2.805458	0.092263	0.926698	-5.33854	0.785193	0.823105
Neutrophils	TBC1D4	-0.03219	4.706501	-0.09224	0.926718	-5.78789	0.743777	0.78069
Neutrophils	MPHOSPH(	-0.02091	3.799303	-0.09211	0.926819	-5.63574	0.763252	0.800657
Neutrophils	BRD7	0.008293	6.307707	0.092048	0.926868	-6.45279	0.710657	0.746654
Neutrophils	SLC35F6	-0.02652	2.998461	-0.09203	0.926883	-5.52147	0.780882	0.8187
Neutrophils	CDCA8	0.024908	5.109425	0.091924	0.926966	-6.23194	0.735294	0.77199
Neutrophils	CACNB3	0.074071	-0.407	0.091796	0.927068	-5.07629	0.860619	0.899959
Neutrophils	NDUFB5	0.010681	7.037752	0.091691	0.927151	-6.43572	0.696072	0.73164
Neutrophils	XPR1	-0.01065	6.866995	-0.09161	0.927218	-6.59032	0.699455	0.735126
Neutrophils	METTL22	-0.04601	1.382795	-0.09156	0.927251	-5.14665	0.817733	0.856328
Neutrophils	MADD	-0.01607	5.091113	-0.09153	0.927282	-6.005	0.735677	0.772385
Neutrophils	ZBTB1	-0.01747	5.505188	-0.09104	0.927667	-6.00483	0.727204	0.76359
Neutrophils	B4GALT6	0.017077	2.991751	0.090836	0.927828	-6.12509	0.781187	0.818924
Neutrophils	SPNS2	-0.03848	1.813184	-0.09079	0.927866	-5.32296	0.807907	0.846218
Neutrophils	FBXO8	-0.01631	4.606605	-0.09032	0.928236	-5.84444	0.746045	0.782967
Neutrophils	4833439L1	-0.0166	4.862518	-0.0903	0.928257	-5.91779	0.740628	0.777408
Neutrophils	SKP1A	0.008896	6.639256	0.090259	0.928285	-6.46027	0.704134	0.739889
Neutrophils	AKAP8L	-0.017	4.8709	-0.09026	0.928287	-5.99265	0.740451	0.777227
Neutrophils	RBM14	0.021435	3.712533	0.090212	0.928323	-5.58569	0.765295	0.802696
Neutrophils	ARMCX2	0.042552	1.41393	0.090154	0.928369	-5.25371	0.817168	0.85569
Neutrophils	A930029G:	0.042324	1.230907	0.090095	0.928416	-5.25625	0.82145	0.860071
Neutrophils	GM50334	0.063404	-0.42372	0.090019	0.928476	-5.05891	0.861202	0.900543
Neutrophils	ACTN1	0.009476	5.757216	0.089915	0.928558	-6.69571	0.722009	0.758349
Neutrophils	EIF1A	-0.0152	5.711772	-0.0899	0.928567	-6.12079	0.722943	0.759309
Neutrophils	IL15RA	0.039709	2.38833	0.08987	0.928594	-5.35935	0.794752	0.8329
Neutrophils	FBXO5	-0.02475	4.811103	-0.08977	0.928676	-6.14138	0.741713	0.778622
Neutrophils	GM13008	-0.04418	1.087607	-0.08974	0.928698	-5.20537	0.824818	0.863595
Neutrophils	GRM8	0.057628	1.381099	0.089683	0.928742	-5.25391	0.817935	0.856581
Neutrophils	SH3BGRL2	0.038289	2.700678	0.089639	0.928777	-5.50708	0.787701	0.825728
Neutrophils	CDKL2	0.042542	1.163972	0.08936	0.928998	-5.28155	0.823066	0.861787
Neutrophils	TCF19	0.031835	3.199871	0.089282	0.92906	-5.58186	0.776606	0.814356
Neutrophils	ZFP51	-0.02399	2.86166	-0.08915	0.929168	-5.51257	0.784134	0.822068
Neutrophils	ELK4	0.011293	5.882489	0.089086	0.929215	-6.29421	0.719481	0.755773
Neutrophils	GM4258	0.036309	4.929368	0.08907	0.929228	-5.61138	0.739259	0.776096
Neutrophils	DERL2	-0.01534	4.925658	-0.08897	0.929305	-5.94129	0.739337	0.776186
Neutrophils	UCKL1	-0.01637	3.705129	-0.08893	0.929336	-5.88836	0.765498	0.803021
Neutrophils	D430040D:	-0.05316	-0.34621	-0.08866	0.929554	-5.13302	0.859478	0.898807
Neutrophils	MACF1	0.008494	8.000182	0.088252	0.929876	-6.77003	0.677784	0.71267
Neutrophils	ODC1	0.017794	5.188947	0.088162	0.929947	-5.94797	0.734127	0.770684
Neutrophils	ZMYM5	-0.01151	5.521459	-0.08784	0.9302	-6.18952	0.727355	0.763637
Neutrophils	IFIT1BL2	0.033974	-0.46837	0.08743	0.930528	-5.45744	0.863089	0.902127
Neutrophils	FAHD1	0.035968	1.917334	0.08734	0.930599	-5.37967	0.806245	0.844293
Neutrophils	NDUFA3	0.007823	8.000282	0.087281	0.930645	-6.80532	0.678078	0.7128
Neutrophils	PLPP5	-0.02074	3.394162	-0.08712	0.930773	-5.69616	0.773024	0.810326
Neutrophils	PCYT2	0.021789	4.635801	0.087021	0.930852	-5.73947	0.746155	0.7828

Neutrophils	PSMA1	0.009063	6.9166	0.086849	0.930988	-6.53376	0.699297	0.734639
Neutrophils	UBE2G1	0.007846	7.307993	0.086778	0.931044	-6.5874	0.691571	0.726679
Neutrophils	IGF2BP2	0.035121	1.457318	0.086654	0.931143	-5.3776	0.81696	0.855171
Neutrophils	GM26590	-0.03608	1.314817	-0.08659	0.931191	-5.17139	0.82029	0.858565
Neutrophils	KIFC3	-0.03535	1.625707	-0.08651	0.931259	-5.36461	0.813041	0.851178
Neutrophils	RMDN2	0.042365	1.873328	0.086399	0.931344	-5.21983	0.807314	0.84534
Neutrophils	SLC40A1	-0.01543	5.835325	-0.08635	0.93138	-6.69414	0.721116	0.757091
Neutrophils	INAFM2	0.023456	3.225336	0.086033	0.931635	-5.59478	0.776763	0.814193
Neutrophils	LGALS3	0.011476	6.142988	0.086016	0.931648	-6.83575	0.714836	0.750681
Neutrophils	CEP44	0.018576	3.531339	0.086013	0.931651	-5.60815	0.770015	0.807289
Neutrophils	FAM234A	0.021192	4.099085	0.085927	0.931719	-5.90327	0.757654	0.794634
Neutrophils	HS1BP3	-0.03649	1.087416	-0.08582	0.931802	-5.24705	0.825634	0.864072
Neutrophils	STAG2	0.008458	7.464582	0.085822	0.931802	-6.72855	0.688505	0.723573
Neutrophils	CST3	0.016857	9.157123	0.085517	0.932043	-6.9586	0.65639	0.69034
Neutrophils	BCL2L12	-0.02005	4.178233	-0.08523	0.932274	-5.70726	0.756096	0.793063
Neutrophils	GM38948	-0.04391	0.463761	-0.08518	0.932314	-5.11623	0.840637	0.879358
Neutrophils	GAREM1	-0.04277	0.757375	-0.08516	0.93233	-5.23771	0.833617	0.872217
Neutrophils	STRADB	-0.02032	3.238382	-0.08513	0.93235	-5.68143	0.776627	0.814089
Neutrophils	TAB3	-0.01698	3.680364	-0.08502	0.93244	-5.82706	0.766915	0.804146
Neutrophils	CDKN3	-0.02236	4.160534	-0.08472	0.932678	-6.07624	0.756626	0.793535
Neutrophils	GRWD1	-0.03399	2.674334	-0.08437	0.932955	-5.30204	0.789451	0.82704
Neutrophils	GM9917	-0.04712	0.773036	-0.0843	0.933006	-5.2077	0.833484	0.871926
Neutrophils	TFDP1	-0.01085	5.887837	-0.08423	0.933065	-6.59179	0.720389	0.75628
Neutrophils	GLRX5	0.012535	6.17176	0.08417	0.933112	-6.19284	0.714597	0.750325
Neutrophils	AAR2	-0.01858	4.021304	-0.08415	0.933128	-5.60463	0.759703	0.796617
Neutrophils	CDKL3	0.027692	3.044103	0.083551	0.933603	-5.59668	0.781394	0.818712
Neutrophils	GSTP3	-0.0278	3.39573	-0.08353	0.933616	-5.75774	0.773597	0.810741
Neutrophils	ERCC6	-0.0211	3.56729	-0.08324	0.933848	-5.69197	0.769823	0.806921
Neutrophils	RCOR3	-0.02389	2.937283	-0.08306	0.933989	-5.55956	0.783779	0.821224
Neutrophils	SLC25A5	-0.00903	7.608641	-0.08288	0.934133	-6.57961	0.686229	0.721093
Neutrophils	PEX14	-0.01534	5.405291	-0.08267	0.934299	-5.9399	0.730557	0.766709
Neutrophils	DENND5B	0.020872	4.198991	0.082647	0.934319	-5.7623	0.756086	0.792893
Neutrophils	SLC38A9	0.01709	5.16902	0.082401	0.934514	-5.91837	0.735486	0.771825
Neutrophils	CHSY3	-0.0387	1.978293	-0.08231	0.93459	-5.51785	0.805523	0.843499
Neutrophils	1700084CC	0.029229	2.9115	0.082201	0.934673	-5.4532	0.784355	0.821913
Neutrophils	CDC20	-0.02636	3.639962	-0.08216	0.934709	-5.82895	0.768229	0.805421
Neutrophils	ECT2	0.025531	4.373594	0.081966	0.934859	-5.95209	0.752334	0.789188
Neutrophils	DMAC1	-0.01814	4.611093	-0.08187	0.934939	-5.73043	0.747262	0.784018
Neutrophils	VPS37C	-0.01365	3.950923	-0.0817	0.935072	-5.89756	0.76145	0.798592
Neutrophils	RAB35	0.01244	4.720448	0.081671	0.935093	-6.00499	0.744939	0.781668
Neutrophils	TOP2A	-0.01913	7.615909	-0.08163	0.935122	-6.73327	0.686088	0.721132
Neutrophils	BABAM2	0.006945	7.216423	0.081605	0.935145	-6.74114	0.693908	0.729195
Neutrophils	GTSE1	0.047752	1.404909	0.081581	0.935164	-5.32694	0.818818	0.857196
Neutrophils	ANG	0.023602	4.211322	0.081435	0.93528	-6.00682	0.755821	0.792872
Neutrophils	EZH2	0.015025	6.663574	0.081341	0.935354	-6.34324	0.704886	0.740563
Neutrophils	GM13184	-0.02436	2.666577	-0.08131	0.935378	-5.64287	0.789855	0.827712
Neutrophils	PACC1	-0.01636	5.421952	-0.08121	0.935457	-6.08393	0.730211	0.76661

Neutrophils	GM10135	0.033483	0.409376	0.08113	0.935522	-5.35665	0.842431	0.881301
Neutrophils	TRIM37	0.016637	4.93302	0.080918	0.93569	-5.90377	0.740443	0.777116
Neutrophils	DDX31	0.027419	3.220713	0.080853	0.935741	-5.38281	0.777468	0.815049
Neutrophils	NDST1	-0.01445	4.570663	-0.08085	0.935742	-6.12789	0.748123	0.784995
Neutrophils	TMCO1	-0.00853	6.265444	-0.08071	0.935854	-6.3422	0.712905	0.748817
Neutrophils	NUTF2-PS1	0.040478	2.848853	0.080704	0.93586	-5.41335	0.785758	0.823526
Neutrophils	GM29994	0.029365	1.150146	0.080689	0.935872	-5.3565	0.824796	0.863353
Neutrophils	UBASH3A	0.034922	2.009253	0.080564	0.935971	-5.42888	0.804812	0.842982
Neutrophils	SLC39A13	-0.0232	2.796524	-0.08048	0.936035	-5.53682	0.786932	0.824725
Neutrophils	TBC1D10A	-0.02283	3.56507	-0.08045	0.936062	-5.50766	0.769871	0.807277
Neutrophils	MFSD4A	-0.03332	2.041521	-0.0804	0.936099	-5.34716	0.804071	0.842226
Neutrophils	JMY	0.01284	6.282387	0.080361	0.936132	-6.32053	0.712561	0.748464
Neutrophils	LANCL1	-0.02061	3.448045	-0.08031	0.936168	-5.4759	0.772444	0.80991
Neutrophils	PEX19	-0.01796	3.955615	-0.0802	0.93626	-5.71526	0.761348	0.79855
Neutrophils	ZFP568	0.022741	4.08691	0.080191	0.936266	-5.59694	0.758505	0.795637
Neutrophils	PARP16	0.039051	1.117003	0.080185	0.936271	-5.25903	0.825577	0.864149
Neutrophils	SLAMF1	0.052434	0.884468	0.080178	0.936277	-5.12608	0.831077	0.869749
Neutrophils	PLEKHG6	0.023824	-0.11425	0.08012	0.936323	-5.42716	0.855125	0.894203
Neutrophils	PIK3C2A	0.008486	6.854141	0.079943	0.936463	-6.65741	0.701081	0.736644
Neutrophils	GM42595	-0.0414	0.917394	-0.07992	0.936483	-5.16442	0.830296	0.868954
Neutrophils	SLC25A16	-0.01806	3.724214	-0.07985	0.936534	-5.71346	0.766386	0.803713
Neutrophils	CLASRP	0.020721	3.334689	0.0796	0.936735	-5.60413	0.77504	0.812537
Neutrophils	BC147527	-0.04901	1.109158	-0.07953	0.936794	-5.08394	0.825863	0.864437
Neutrophils	CWF19L1	-0.03487	2.200341	-0.07943	0.936871	-5.26164	0.800536	0.838625
Neutrophils	P3H1	-0.04514	0.585168	-0.07928	0.936991	-5.20376	0.83836	0.877157
Neutrophils	CPSF6	0.007319	6.68211	0.078886	0.937302	-6.34928	0.704768	0.740341
Neutrophils	COL3A1	-0.02903	3.678331	-0.07888	0.937307	-5.90212	0.767665	0.80491
Neutrophils	PAGR1A	0.049661	0.395669	0.078609	0.937521	-5.09909	0.843074	0.881923
Neutrophils	SMAD6	0.029411	2.733914	0.078591	0.937536	-5.65346	0.788633	0.826432
Neutrophils	RNF40	0.011474	4.021973	0.078587	0.937539	-6.04056	0.760192	0.797336
Neutrophils	TMEM132I	-0.02771	0.329919	-0.07833	0.937741	-5.53615	0.844664	0.883547
Neutrophils	GMPPA	0.022711	3.453598	0.078292	0.937773	-5.56711	0.772614	0.81006
Neutrophils	JAG1	-0.03767	1.3378	-0.07826	0.937801	-5.43093	0.820699	0.859155
Neutrophils	TTC8	-0.03955	0.78875	-0.07799	0.938014	-5.20785	0.833668	0.872385
Neutrophils	ACO1	-0.01996	3.712371	-0.07794	0.938054	-5.56751	0.766935	0.80427
Neutrophils	IL12B	0.081513	-0.29714	0.077892	0.93809	-5.12065	0.859929	0.899082
Neutrophils	1700102PC	-0.04091	0.812589	-0.07769	0.938249	-5.22041	0.8331	0.871808
Neutrophils	SELENOW	0.013362	7.046449	0.077554	0.938358	-6.24089	0.697528	0.733009
Neutrophils	NUDT7	-0.02741	1.74453	-0.07751	0.938393	-5.50867	0.811223	0.849558
Neutrophils	DHX8	-0.01237	5.129659	-0.07745	0.938441	-6.028	0.736589	0.773191
Neutrophils	COPRS	-0.0393	0.717795	-0.07739	0.938491	-5.18968	0.835359	0.874159
Neutrophils	COA3	-0.0099	5.812938	-0.07711	0.93871	-6.3217	0.722408	0.758648
Neutrophils	PHF8	-0.00994	5.563796	-0.07702	0.93878	-6.24772	0.727546	0.763946
Neutrophils	PXMP4	-0.01626	3.831766	-0.07696	0.938829	-5.84599	0.76433	0.801705
Neutrophils	ADH5	-0.0124	6.03865	-0.07687	0.9389	-6.22674	0.717787	0.753952
Neutrophils	UBE2C	-0.02111	6.746366	-0.07686	0.938905	-6.56927	0.703497	0.739242
Neutrophils	TNFSF12	-0.02738	2.295218	-0.07683	0.938931	-5.42386	0.798572	0.836747

Neutrophils	ELMSAN1	0.01008	6.924696	0.07671	0.939028	-6.66663	0.699943	0.735592
Neutrophils	4932438A1	0.00814	7.087671	0.07663	0.939091	-6.84042	0.696712	0.732263
Neutrophils	PCED1A	0.0257	2.508638	0.07644	0.939241	-5.42446	0.793723	0.831835
Neutrophils	TRPM2	-0.01366	2.540831	-0.07643	0.939253	-6.29864	0.792994	0.831093
Neutrophils	TLE1	-0.01147	4.060562	-0.0763	0.939355	-6.07237	0.759362	0.796729
Neutrophils	SLC9A5	0.045956	0.362304	0.076264	0.939381	-5.09086	0.843883	0.883022
Neutrophils	RFX5	0.03329	1.943489	0.076009	0.939583	-5.24131	0.806629	0.845099
Neutrophils	CES2E	-0.03417	1.228112	-0.07586	0.939705	-5.35304	0.823273	0.862115
Neutrophils	RANBP3	-0.01585	4.303701	-0.07579	0.939758	-5.67607	0.754119	0.791437
Neutrophils	USF1	0.015465	4.934416	0.075717	0.939815	-5.91787	0.740694	0.777663
Neutrophils	MMACHC	-0.04817	0.470101	-0.07565	0.939872	-5.09945	0.841289	0.880469
Neutrophils	HSPA9	0.009971	6.483768	0.075623	0.93989	-6.26622	0.708764	0.744831
Neutrophils	NAGK	-0.01651	3.956557	-0.07561	0.939899	-5.79143	0.761616	0.799121
Neutrophils	SCIN	0.051825	0.090871	0.07551	0.93998	-5.17272	0.850451	0.889829
Neutrophils	ACTR1B	-0.01758	4.215968	-0.0754	0.94007	-5.74276	0.756007	0.793441
Neutrophils	TNFSF11	-0.06839	0.706959	-0.07537	0.94009	-5.07681	0.835617	0.874772
Neutrophils	GM13481	0.049139	0.231781	0.074885	0.940476	-5.11056	0.847035	0.886479
Neutrophils	E2F2	-0.01438	4.99405	-0.07481	0.940531	-6.18284	0.739438	0.776547
Neutrophils	VCPKMT	-0.03076	2.877826	-0.07478	0.94056	-5.33387	0.785406	0.82366
Neutrophils	MTMR1	-0.0134	4.622377	-0.07476	0.940575	-5.93317	0.747305	0.784631
Neutrophils	SFXN2	0.01793	3.18033	0.07467	0.940646	-5.64468	0.778659	0.816784
Neutrophils	UBE2D2A	-0.00421	8.209532	-0.07457	0.940724	-6.76411	0.674894	0.710114
Neutrophils	CWF19L2	0.011626	5.038452	0.07452	0.940765	-6.04879	0.738504	0.775652
Neutrophils	NEURL2	0.047599	0.801683	0.074494	0.940785	-5.11007	0.83336	0.87267
Neutrophils	POLE4	-0.00943	6.652817	-0.07434	0.940911	-6.33756	0.705368	0.741615
Neutrophils	KSR1	-0.0123	4.69672	-0.07433	0.940912	-6.22011	0.745724	0.783122
Neutrophils	FGFBP3	-0.05564	0.028033	-0.07429	0.940944	-5.06703	0.851979	0.891691
Neutrophils	JUN	-0.02169	7.097589	-0.07426	0.940973	-6.54306	0.696516	0.7325
Neutrophils	FLT3L	-0.02832	2.201527	-0.07421	0.941008	-5.37966	0.80071	0.839439
Neutrophils	HSPBAP1	0.013583	3.764205	0.074183	0.941032	-6.11068	0.765803	0.803727
Neutrophils	RSU1	0.006779	5.746299	0.074038	0.941147	-6.54149	0.723779	0.760594
Neutrophils	GM4566	0.034908	1.718905	0.073998	0.941179	-5.28227	0.811817	0.850793
Neutrophils	ARHGAP42	-0.03698	1.752056	-0.07397	0.941201	-5.28399	0.811049	0.85001
Neutrophils	SHC1	-0.01072	4.384809	-0.07386	0.941289	-6.0588	0.752379	0.789976
Neutrophils	MST1	0.04617	0.649079	0.0738	0.941336	-5.18314	0.837	0.876473
Neutrophils	PHIP	-0.00792	7.276011	-0.07351	0.941565	-6.56239	0.693084	0.728934
Neutrophils	GM42829	-0.01867	2.205641	-0.07347	0.941599	-5.68236	0.800715	0.839438
Neutrophils	DGKI	0.063631	0.78	0.07308	0.941908	-5.19618	0.834076	0.873458
Neutrophils	9930022D1	-0.04741	0.319301	-0.07305	0.941934	-5.11221	0.845123	0.884704
Neutrophils	GM37065	-0.04045	2.706239	-0.07297	0.941993	-5.27448	0.78945	0.827919
Neutrophils	PLA2G6	-0.0396	1.28393	-0.07296	0.942	-5.20218	0.822159	0.861315
Neutrophils	CTCF	0.006527	7.101448	0.072816	0.942117	-6.46989	0.696626	0.732602
Neutrophils	SAAL1	0.024488	3.298168	0.072747	0.942172	-5.47402	0.776254	0.81442
Neutrophils	NCAPG2	0.014618	5.068495	0.072527	0.942346	-6.15161	0.738131	0.775277
Neutrophils	CEP57	0.011824	5.139583	0.072405	0.942443	-6.02961	0.736639	0.773781
Neutrophils	TOM1L1	0.037847	0.714609	0.072331	0.942502	-5.21054	0.835727	0.87513
Neutrophils	KDM4D	0.033693	0.328103	0.072218	0.942591	-5.29757	0.845004	0.884609

Neutrophils	SNRPF	-0.01024	6.76677	-0.07219	0.942614	-6.31536	0.703335	0.73954
Neutrophils	EHMT2	-0.01609	4.951712	-0.07208	0.942701	-5.74344	0.740592	0.777898
Neutrophils	ANAPC1	-0.01392	4.721072	-0.07197	0.942789	-5.84781	0.745472	0.782925
Neutrophils	BAMBI	0.019621	3.967331	0.071855	0.942879	-5.83793	0.761652	0.799544
Neutrophils	ITPRID2	-0.011	5.08881	-0.0717	0.943003	-6.17967	0.737708	0.774995
Neutrophils	SNRPB	0.007717	7.300883	0.071639	0.943051	-6.5271	0.692755	0.728712
Neutrophils	FAM221B	0.037813	0.343919	0.071633	0.943056	-5.19371	0.844626	0.884327
Neutrophils	PLCL1	-0.02737	5.328139	-0.07145	0.943199	-5.75357	0.732724	0.769853
Neutrophils	B3GNT5	0.03082	2.546338	0.07141	0.943233	-5.37542	0.793177	0.831823
Neutrophils	GM16279	-0.04079	1.054801	-0.0713	0.943319	-5.18051	0.82769	0.867052
Neutrophils	TRAM1	-0.00693	7.121393	-0.07101	0.943548	-6.49522	0.696392	0.732448
Neutrophils	ANLN	0.019107	3.747041	0.070972	0.94358	-5.9275	0.766559	0.804595
Neutrophils	PHF12	0.009031	6.186244	0.070838	0.943686	-6.28501	0.715138	0.751778
Neutrophils	RGS13	0.050991	-1.05629	0.070819	0.943702	-5.0488	0.879217	0.919471
Neutrophils	MRRF	0.019421	3.454685	0.07038	0.944051	-5.54843	0.773201	0.811206
Neutrophils	NUDCD3	-0.00853	5.998557	-0.06999	0.944357	-6.14144	0.71926	0.755748
Neutrophils	HNRNPLL	-0.00751	4.231786	-0.06996	0.944384	-6.50917	0.756354	0.793849
Neutrophils	IARS2	-0.01512	4.475268	-0.06994	0.944403	-5.79833	0.751126	0.788487
Neutrophils	TSPAN13	0.009576	6.684078	0.069802	0.944509	-6.40655	0.705384	0.741482
Neutrophils	RPIA	0.009133	5.458024	0.069753	0.944547	-6.29316	0.730403	0.767228
Neutrophils	RANBP17	0.038455	1.134101	0.069612	0.944659	-5.15814	0.826238	0.865304
Neutrophils	REXO1	-0.0118	4.912691	-0.06953	0.944728	-5.98217	0.741828	0.778966
Neutrophils	IFFO2	-0.02018	3.364648	-0.0695	0.944748	-5.61606	0.775282	0.813263
Neutrophils	4930455G(	-0.03044	1.816178	-0.06936	0.944858	-5.40287	0.810335	0.849086
Neutrophils	ISOC2A	-0.02761	2.013655	-0.06923	0.944967	-5.29481	0.80581	0.844451
Neutrophils	STAU2	-0.02931	2.900653	-0.06912	0.945051	-5.32476	0.78568	0.8239
Neutrophils	NELFA	-0.01165	4.630912	-0.06882	0.945288	-5.85724	0.748	0.785248
Neutrophils	RAVER2	-0.03193	1.423822	-0.06858	0.945477	-5.25392	0.819727	0.85855
Neutrophils	VARS	0.01412	5.713285	0.068407	0.945616	-5.8911	0.725381	0.761987
Neutrophils	KDM4C	0.007915	6.252217	0.068393	0.945627	-6.43637	0.714351	0.750639
Neutrophils	GM6377	0.025941	3.091942	0.068247	0.945743	-5.52524	0.781618	0.81969
Neutrophils	MFSD1	0.010014	5.149534	0.068243	0.945747	-6.13218	0.737109	0.774065
Neutrophils	GM29570	-0.03708	0.692445	-0.06815	0.945824	-5.17265	0.837031	0.876244
Neutrophils	ZFAT	-0.01498	3.828322	-0.0679	0.946018	-5.87725	0.765479	0.803174
Neutrophils	TFAM	0.017338	4.293459	0.067794	0.946103	-5.60855	0.755409	0.792845
Neutrophils	DNAJB11	-0.00843	6.073023	-0.06765	0.94622	-6.24188	0.718136	0.754557
Neutrophils	KMT5A	-0.00658	5.846575	-0.06747	0.946362	-6.4784	0.722828	0.759386
Neutrophils	TMEM177	0.042691	0.336385	0.067264	0.946523	-5.08146	0.845815	0.885198
Neutrophils	4930438A(	0.012226	-0.84008	0.067219	0.946559	-5.91163	0.874721	0.914566
Neutrophils	RAB33B	0.010575	4.405753	0.06718	0.94659	-5.95633	0.753093	0.790491
Neutrophils	PERP	-0.03105	1.228461	-0.06708	0.946671	-5.35144	0.824545	0.863563
Neutrophils	HOXB4	0.029195	1.361865	0.06684	0.94686	-5.27069	0.821437	0.860387
Neutrophils	OCRL	0.021035	2.602911	0.066811	0.946883	-5.61287	0.792848	0.831209
Neutrophils	GM10134	0.03407	0.795093	0.066752	0.94693	-5.31393	0.83484	0.87405
Neutrophils	STK19	-0.01563	4.61763	-0.06616	0.947402	-5.74934	0.748592	0.785942
Neutrophils	RNF7	-0.00805	6.742111	-0.06605	0.94749	-6.45291	0.704699	0.740804
Neutrophils	LRRC3	0.032076	0.375073	0.065967	0.947553	-5.21219	0.844915	0.88436

Neutrophils	VBP1	-0.00913	4.92592	-0.06593	0.94758	-6.06363	0.74205	0.779225
Neutrophils	KIF11	0.017543	5.441855	0.065877	0.947625	-6.31071	0.731233	0.768113
Neutrophils	MRM1	0.018612	2.067052	0.065842	0.947653	-5.53795	0.805065	0.843746
Neutrophils	KBTBD4	0.023802	2.78551	0.065809	0.947679	-5.35001	0.788728	0.827053
Neutrophils	DNAH1	0.042049	-0.10849	0.065642	0.947811	-5.07198	0.856666	0.896324
Neutrophils	ELOC	0.006908	7.093954	0.065631	0.94782	-6.57115	0.697694	0.733596
Neutrophils	EIF4E	0.009281	6.950147	0.065519	0.947909	-6.45454	0.700548	0.736538
Neutrophils	RBM17	0.009499	5.789508	0.06551	0.947916	-6.15389	0.724038	0.760726
Neutrophils	PIK3CA	-0.00854	6.128996	-0.06544	0.947968	-6.3297	0.717082	0.75357
Neutrophils	PIK3R4	0.020804	3.189781	0.065311	0.948074	-5.49084	0.779685	0.817817
Neutrophils	ANAPC7	0.013025	4.359074	0.065292	0.948089	-5.74581	0.754126	0.791632
Neutrophils	TPK1	0.014071	4.865838	0.065283	0.948096	-5.86651	0.74332	0.780543
Neutrophils	TIMELESS	0.020908	3.491901	0.065131	0.948217	-5.60546	0.772996	0.810971
Neutrophils	ITGAE	-0.03369	2.127038	-0.06509	0.948246	-5.32668	0.803688	0.842353
Neutrophils	GM17018	-0.01629	3.620053	-0.06501	0.948312	-5.65841	0.770177	0.808084
Neutrophils	OLFR77	0.034426	1.376904	0.064867	0.948426	-5.17381	0.821084	0.860103
Neutrophils	IFIH1	0.01566	3.912874	0.06486	0.948432	-6.06453	0.763775	0.801525
Neutrophils	ABCG2	0.017287	3.149803	0.064832	0.948454	-5.64006	0.780574	0.818727
Neutrophils	ZFP938	0.032552	1.450658	0.064664	0.948587	-5.14123	0.819399	0.858342
Neutrophils	SCUBE2	0.039536	-0.26058	0.064577	0.948657	-5.19745	0.86044	0.900114
Neutrophils	TMEM160	0.009279	5.666014	0.064495	0.948722	-6.12058	0.726622	0.76335
Neutrophils	RASGRP1	0.025706	4.119019	0.064297	0.948879	-5.51136	0.759408	0.797001
Neutrophils	PLCB4	0.012497	3.876337	0.064198	0.948958	-6.16232	0.764683	0.802415
Neutrophils	RINT1	0.016103	3.771156	0.063982	0.949129	-5.57771	0.767058	0.80478
Neutrophils	DEDD	-0.01137	4.437275	-0.06378	0.949291	-5.87539	0.752706	0.790033
Neutrophils	IFT172	-0.02568	2.410455	-0.06353	0.949491	-5.2726	0.797525	0.835892
Neutrophils	NDUFS3	-0.0095	6.050235	-0.0635	0.949511	-6.16168	0.71897	0.755363
Neutrophils	SMYD3	-0.00976	6.67093	-0.06345	0.949555	-6.35852	0.7064	0.742435
Neutrophils	YTHDF2	0.008147	6.035232	0.063257	0.949704	-6.15831	0.71928	0.755734
Neutrophils	LEMD3	-0.00834	5.687224	-0.06306	0.949862	-6.24304	0.726433	0.763114
Neutrophils	FRYL	0.006364	7.564295	0.062992	0.949915	-6.62792	0.688716	0.724261
Neutrophils	CEACAM16	0.028837	1.137316	0.062927	0.949967	-5.29779	0.827047	0.866096
Neutrophils	NOP9	0.014073	4.555827	0.062919	0.949973	-5.69011	0.750207	0.787534
Neutrophils	RAD54L	0.021777	2.724058	0.062878	0.950006	-5.47963	0.790423	0.82872
Neutrophils	PBX3	-0.01156	5.496226	-0.06267	0.950169	-6.11444	0.730391	0.767217
Neutrophils	GTF2IRD2	0.012016	3.944401	0.062632	0.950201	-5.90653	0.76339	0.801104
Neutrophils	HAL	-0.01952	2.215151	-0.06261	0.95022	-5.85976	0.801985	0.840589
Neutrophils	HIST1H4D	-0.03663	1.04631	-0.06247	0.950333	-5.22129	0.829199	0.868368
Neutrophils	API5	0.006638	6.03062	0.062429	0.950362	-6.37511	0.719374	0.755922
Neutrophils	RANBP10	-0.01224	5.698692	-0.06233	0.950437	-6.01304	0.726197	0.76294
Neutrophils	RIC8B	0.011932	4.502862	0.062095	0.950628	-5.84834	0.751434	0.788767
Neutrophils	NDUFA12	0.010035	5.362792	0.061911	0.950773	-6.03532	0.733264	0.770108
Neutrophils	CDKL1	0.031576	1.100722	0.061805	0.950857	-5.36311	0.828019	0.867056
Neutrophils	SNHG10	-0.04299	0.227889	-0.06173	0.95092	-5.0616	0.848919	0.88833
Neutrophils	SCRN3	-0.02318	1.928035	-0.06172	0.950924	-5.43399	0.80869	0.847347
Neutrophils	SLMAP	0.006984	6.393027	0.061575	0.95104	-6.43766	0.712127	0.748333
Neutrophils	PPOX	0.013597	3.108757	0.061305	0.951255	-5.75107	0.781956	0.819972

Neutrophils	RBM3	-0.0062	9.070642	-0.06123	0.951316	-6.85096	0.660079	0.694594
Neutrophils	FLRT3	0.040099	-0.84839	0.061182	0.951352	-5.09276	0.875487	0.915253
Neutrophils	MINK1	0.017233	4.171023	0.061173	0.951359	-5.54823	0.75863	0.796087
Neutrophils	HSD17B10	-0.00844	5.540491	-0.06071	0.951731	-6.32738	0.729662	0.76638
Neutrophils	LSM14B	-0.013	3.604671	-0.06068	0.951752	-5.67308	0.77102	0.808811
Neutrophils	EEF2	0.007261	9.105144	0.060552	0.951852	-6.76527	0.659473	0.693993
Neutrophils	TDO2	0.025647	3.218699	0.060535	0.951866	-5.79968	0.779552	0.817545
Neutrophils	IMPDH1	-0.01674	4.326873	-0.06048	0.951907	-5.75309	0.755313	0.792716
Neutrophils	GM47664	-0.0111	3.774581	-0.06044	0.951942	-5.93829	0.767294	0.804996
Neutrophils	AGPAT1	0.013298	3.479015	0.060334	0.952026	-5.82237	0.773787	0.811677
Neutrophils	MFSD13A	0.016833	2.008416	0.060287	0.952063	-5.66055	0.806942	0.845569
Neutrophils	ODF2L	0.021545	2.682465	0.060168	0.952158	-5.35983	0.791568	0.829885
Neutrophils	2310010J1	-0.01164	3.692212	-0.06013	0.95219	-6.03021	0.769098	0.806901
Neutrophils	ZKSCAN16	-0.03527	-0.59074	-0.06004	0.95226	-5.05083	0.869117	0.908916
Neutrophils	CLK1	-0.00518	7.55289	-0.06	0.952291	-6.74544	0.689118	0.724702
Neutrophils	ZFP764	0.032168	1.074799	0.059835	0.952422	-5.1199	0.828791	0.867859
Neutrophils	ATP6V1C1	0.007122	5.189889	0.059447	0.95273	-6.2266	0.737115	0.774046
Neutrophils	A430005L1	0.01425	3.764054	0.059182	0.95294	-5.64637	0.767668	0.805461
Neutrophils	1700056E2	-0.03294	1.466557	-0.05913	0.952981	-5.14269	0.819675	0.858612
Neutrophils	PSMG1	-0.01371	3.325411	-0.05906	0.953037	-5.63573	0.777329	0.815363
Neutrophils	FRRS1	0.007181	4.851486	0.058954	0.953122	-6.40017	0.74425	0.781455
Neutrophils	ANPEP	-0.03801	1.184937	-0.05892	0.95315	-5.20541	0.826293	0.865369
Neutrophils	PSMB3	-0.00598	7.724991	-0.05887	0.953185	-6.69466	0.685891	0.721377
Neutrophils	TRIM23	-0.01825	3.295163	-0.05883	0.953219	-5.42367	0.778	0.81605
Neutrophils	GM13483	-0.02259	1.85821	-0.05877	0.953271	-5.43811	0.810561	0.849326
Neutrophils	PDCL3	0.008767	4.536557	0.058763	0.953273	-6.08638	0.750955	0.788337
Neutrophils	CCDC88A	0.011866	5.372063	0.058284	0.953654	-5.99035	0.733433	0.770371
Neutrophils	ACADSB	0.011598	3.270603	0.058284	0.953654	-5.81065	0.778682	0.816777
Neutrophils	SRD5A3	0.009686	4.511422	0.05825	0.953681	-6.13334	0.751626	0.789052
Neutrophils	CDCA5	0.020671	3.028781	0.058183	0.953734	-5.58765	0.784071	0.822291
Neutrophils	F11	-0.02948	0.954836	-0.05793	0.953939	-5.25927	0.831957	0.871145
Neutrophils	SREBF1	0.015204	3.760907	0.057876	0.953978	-5.59774	0.767937	0.805751
Neutrophils	TUT1	0.017426	2.638669	0.057772	0.954061	-5.35466	0.792913	0.831308
Neutrophils	FAM117B	-0.00667	7.11596	-0.05771	0.954106	-6.7	0.698027	0.733902
Neutrophils	NFIA	-0.00877	5.904	-0.05759	0.954204	-6.50103	0.722499	0.759087
Neutrophils	GM45442	0.035063	-0.57631	0.057424	0.954337	-5.15736	0.869225	0.909001
Neutrophils	TTC21B	-0.027	1.614885	-0.0573	0.954438	-5.14479	0.816512	0.855385
Neutrophils	ITGA3	-0.03206	-0.28405	-0.05722	0.954501	-5.07755	0.862015	0.901727
Neutrophils	CNR2	-0.00891	3.101889	-0.05711	0.954589	-6.13994	0.7826	0.820773
Neutrophils	IGF1OS	-0.04037	-1.03209	-0.05699	0.95468	-5.06627	0.880662	0.920682
Neutrophils	A930015D(	0.01073	4.760503	0.056626	0.954971	-5.97402	0.746586	0.783831
Neutrophils	TTF1	0.015168	3.466533	0.05655	0.955031	-5.59456	0.774627	0.812576
Neutrophils	CPS1	0.019312	4.481272	0.056547	0.955034	-6.10836	0.752547	0.789947
Neutrophils	CDC37	-0.00579	6.605127	-0.05636	0.955179	-6.36126	0.708445	0.744593
Neutrophils	HMGB3	0.011801	4.658182	0.05633	0.955206	-6.11278	0.748785	0.786066
Neutrophils	CENPS	-0.01982	3.529733	-0.05604	0.955437	-5.62395	0.773289	0.811148
Neutrophils	MMP9	-0.01001	1.381978	-0.05592	0.955528	-6.41324	0.822163	0.861119

Neutrophils	LIMCH1	0.025455	0.57747	0.055874	0.955568	-5.38776	0.84127	0.880589
Neutrophils	DCAF12	0.006205	6.638146	0.055756	0.955662	-6.56269	0.707813	0.74399
Neutrophils	GM16062	0.017391	1.874018	0.055706	0.955702	-5.43011	0.810694	0.849475
Neutrophils	SPTBN5	-0.03603	-0.00378	-0.05568	0.955719	-5.07896	0.855353	0.894961
Neutrophils	FLAD1	-0.01792	2.957237	-0.05563	0.955761	-5.49701	0.786018	0.824265
Neutrophils	HUWE1	0.00679	7.155414	0.055293	0.95603	-6.46981	0.697543	0.733412
Neutrophils	PTP4A3	0.009834	5.77385	0.055211	0.956095	-5.99318	0.725468	0.76217
Neutrophils	HIPK3	-0.00709	5.445686	-0.05516	0.956138	-6.25744	0.732272	0.769165
Neutrophils	DUS2	0.021392	3.353616	0.055147	0.956146	-5.38491	0.777237	0.815287
Neutrophils	PPP2R5D	-0.01225	3.682866	-0.05509	0.956195	-5.72639	0.769975	0.807851
Neutrophils	FOXK2	0.009215	4.920069	0.054843	0.956387	-5.92566	0.743347	0.780504
Neutrophils	SHCBP1L	-0.03159	0.854389	-0.05484	0.95639	-5.09665	0.834745	0.873955
Neutrophils	ZFP975	0.036075	0.810489	0.054598	0.956582	-5.1549	0.835861	0.87507
Neutrophils	TMEM260	-0.016	3.273577	-0.05437	0.956763	-5.57167	0.779118	0.817154
Neutrophils	CDK10	0.019751	2.234353	0.054368	0.956765	-5.34244	0.802563	0.841122
Neutrophils	SLC25A40	-0.0174	3.227708	-0.05427	0.956839	-5.48618	0.780137	0.818237
Neutrophils	NOSIP	0.008373	5.158105	0.054226	0.956877	-6.00342	0.738388	0.775433
Neutrophils	GM31243	-0.02605	2.779029	-0.05419	0.956908	-5.49219	0.790186	0.828515
Neutrophils	SLC18A2	0.014367	2.251616	0.05373	0.957272	-5.59425	0.80238	0.840811
Neutrophils	DCTN2	0.007329	5.367124	0.053567	0.957401	-6.18006	0.734202	0.770991
Neutrophils	IRGM2	0.02217	2.731293	0.053505	0.95745	-5.54888	0.791472	0.829678
Neutrophils	SH3PXD2B	0.037691	1.543779	0.053412	0.957524	-5.19795	0.818757	0.857532
Neutrophils	ESS2	-0.01464	2.988114	-0.05341	0.957527	-5.47221	0.785694	0.823772
Neutrophils	VPS16	0.00949	4.436967	0.0533	0.957613	-5.79887	0.753915	0.791217
Neutrophils	PHF5A	0.008259	6.072643	0.053144	0.957737	-6.17863	0.719637	0.756017
Neutrophils	NKTR	-0.00421	7.193099	-0.0531	0.957773	-6.69654	0.697094	0.732798
Neutrophils	RNASEL	0.011412	3.944163	0.052987	0.957862	-6.09107	0.7646	0.802205
Neutrophils	STIP1	0.009013	5.419076	0.052858	0.957965	-6.09929	0.733169	0.76995
Neutrophils	RNF122	0.025183	2.64878	0.052772	0.958032	-5.28641	0.793392	0.831686
Neutrophils	SOX12	0.028065	0.585357	0.05228	0.958424	-5.13749	0.841776	0.880895
Neutrophils	SNRNP70	-0.00375	7.511442	-0.05224	0.958458	-6.53787	0.691054	0.726512
Neutrophils	AGGF1	0.006017	5.384652	0.05205	0.958607	-6.25907	0.734099	0.770877
Neutrophils	GM26917	-0.01244	5.74366	-0.05199	0.958656	-6.0215	0.72664	0.763235
Neutrophils	SERF1	0.013407	3.137334	0.051964	0.958674	-5.54147	0.782637	0.820664
Neutrophils	NSUN6	-0.01148	3.821373	-0.05177	0.958832	-5.82496	0.767521	0.805194
Neutrophils	SETD2	0.00535	6.922415	0.051592	0.95897	-6.55385	0.702703	0.738617
Neutrophils	ZMYND19	-0.01969	3.409984	-0.05155	0.959006	-5.46769	0.776576	0.814502
Neutrophils	COX5A	0.005618	8.546847	0.05145	0.959083	-6.79304	0.671069	0.705997
Neutrophils	LATS1	0.007706	4.725566	0.051281	0.959218	-6.01123	0.748001	0.785235
Neutrophils	ACER2	0.013412	2.516505	0.051269	0.959227	-5.80453	0.796622	0.835022
Neutrophils	APEH	-0.01523	3.695339	-0.05123	0.959256	-5.60434	0.770283	0.808079
Neutrophils	TLCD1	-0.02026	1.624021	-0.05123	0.95926	-5.29198	0.817176	0.856003
Neutrophils	FAM193B	0.012067	4.050857	0.051093	0.959366	-5.66723	0.762544	0.800123
Neutrophils	TRIR	0.005462	6.300182	0.050961	0.959472	-6.42167	0.715266	0.751556
Neutrophils	TIMM23	0.005351	6.942518	0.050896	0.959523	-6.39823	0.702334	0.738236
Neutrophils	ABCF1	0.006368	6.430861	0.050537	0.959809	-6.25361	0.712657	0.748827
Neutrophils	TSC22D2	0.005194	6.616128	0.050514	0.959827	-6.4954	0.708916	0.744974

Neutrophils	METTL5	-0.01176	3.739598	-0.05046	0.959867	-5.66234	0.769393	0.807084
Neutrophils	INHBA	-0.01411	1.050066	-0.05046	0.959873	-6.10036	0.830765	0.869765
Neutrophils	NR3C1	0.006051	7.440275	0.050258	0.96003	-6.67512	0.692582	0.7281
Neutrophils	CISD2	-0.0053	6.732173	-0.05017	0.960101	-6.50316	0.706644	0.742601
Neutrophils	WHAMM	0.010323	4.147659	0.050057	0.96019	-5.82768	0.760574	0.798001
Neutrophils	ABTB2	-0.00746	7.412469	-0.04983	0.960371	-6.88368	0.693157	0.72867
Neutrophils	ZFP324	0.029408	0.798242	0.049698	0.960475	-5.1308	0.836867	0.875927
Neutrophils	VAPB	-0.00799	5.031604	-0.04966	0.960505	-6.08578	0.741683	0.778633
Neutrophils	CSTF2T	0.012867	3.017029	0.049602	0.960551	-5.56274	0.785509	0.823555
Neutrophils	HPS4	0.011405	4.16681	0.049566	0.96058	-5.86881	0.760178	0.797637
Neutrophils	PPHLN1	-0.00918	4.932629	-0.04909	0.960958	-5.9846	0.744002	0.780848
Neutrophils	ZFP874B	0.022611	2.178101	0.048984	0.961042	-5.29691	0.804783	0.843053
Neutrophils	COX6A1	-0.00558	6.768979	-0.04892	0.961092	-6.52186	0.706149	0.74193
Neutrophils	2310022A1	0.015975	2.998253	0.048458	0.961461	-5.40838	0.786354	0.824055
Neutrophils	GM12655	-0.03201	0.353725	-0.04845	0.961465	-5.08714	0.848017	0.88691
Neutrophils	NEIL3	0.010596	4.375558	0.048323	0.961568	-6.17619	0.756083	0.793078
Neutrophils	TMEM38A	0.014632	1.868955	0.04826	0.961618	-5.57172	0.812113	0.850374
Neutrophils	ZFP101	0.013647	2.916235	0.047986	0.961836	-5.5376	0.78832	0.826026
Neutrophils	POMK	0.021872	1.068205	0.047821	0.961966	-5.30033	0.831024	0.869593
Neutrophils	CHEK2	0.013083	3.145946	0.047794	0.961988	-5.53699	0.783176	0.820779
Neutrophils	TCOF1	-0.01155	5.717375	-0.04754	0.962187	-5.85298	0.727917	0.764095
Neutrophils	GEN1	-0.02164	2.372171	-0.04752	0.962207	-5.40001	0.800717	0.838649
Neutrophils	CCDC162	0.020747	2.876554	0.047235	0.962433	-5.53521	0.789382	0.827037
Neutrophils	CD2BP2	-0.01002	4.011388	-0.04717	0.962482	-5.71944	0.764248	0.801328
Neutrophils	GM39090	0.027555	0.497553	0.046811	0.96277	-5.0641	0.844981	0.883593
Neutrophils	CIAO1	0.010933	3.481337	0.046807	0.962772	-5.58881	0.775999	0.813253
Neutrophils	NDC1	0.011367	3.748037	0.046613	0.962927	-5.71688	0.770133	0.807255
Neutrophils	PRPF19	0.008619	5.053951	0.046605	0.962933	-5.86627	0.74201	0.778437
Neutrophils	NRDE2	-0.01529	3.371854	-0.04639	0.963106	-5.52418	0.778458	0.815808
Neutrophils	CBR1	0.011662	4.350162	0.046351	0.963135	-5.80517	0.757051	0.793895
Neutrophils	CDADC1	0.005714	4.609962	0.046147	0.963297	-6.5365	0.751469	0.788191
Neutrophils	HNRNPAB	-0.00509	8.05587	-0.04602	0.963396	-6.6487	0.681376	0.716121
Neutrophils	CACNB4	-0.02445	1.615307	-0.046	0.963412	-5.35644	0.81847	0.856709
Neutrophils	CTBP1	-0.00523	6.500976	-0.04592	0.963474	-6.43665	0.712113	0.747807
Neutrophils	VPS26A	0.004375	6.273847	0.045772	0.963595	-6.52523	0.716724	0.752558
Neutrophils	NEDD1	0.013629	3.041246	0.045642	0.963699	-5.55554	0.785832	0.823432
Neutrophils	NUP85	-0.01216	4.345133	-0.04548	0.963826	-5.73179	0.757159	0.794113
Neutrophils	CAMP	-0.00614	4.463959	-0.04539	0.9639	-7.11219	0.754601	0.791491
Neutrophils	PTGES	0.013807	0.477516	0.045308	0.963964	-5.8726	0.845499	0.884283
Neutrophils	GM27201	-0.01447	2.321008	-0.04527	0.963996	-5.38341	0.802147	0.840134
Neutrophils	ZFP983	0.019005	2.38923	0.045218	0.964035	-5.30454	0.800587	0.838546
Neutrophils	CTLA2A	0.012654	4.28991	0.045207	0.964045	-6.00741	0.758352	0.795351
Neutrophils	KCNK13	-0.01878	2.912398	-0.04519	0.964062	-5.53247	0.788726	0.826432
Neutrophils	TSPYL4	-0.02234	0.723815	-0.04504	0.964174	-5.1267	0.839573	0.878295
Neutrophils	KAT2A	-0.0161	3.199202	-0.04487	0.964312	-5.37621	0.7823	0.819884
Neutrophils	DNAJB5	-0.02104	1.175316	-0.04475	0.964406	-5.21831	0.828817	0.867348
Neutrophils	CYREN	-0.01069	3.374354	-0.04473	0.964422	-5.76548	0.778402	0.815898

Neutrophils	MRPS22	0.014549	3.085855	0.044729	0.964425	-5.39312	0.784833	0.822474
Neutrophils	MRPL47	-0.01664	2.974642	-0.04461	0.964516	-5.40714	0.787342	0.825041
Neutrophils	FSD2	0.028204	-0.73763	0.044518	0.964592	-5.04469	0.875379	0.914696
Neutrophils	PCMTD2	0.01059	3.680281	0.044312	0.964756	-5.75319	0.771673	0.809072
Neutrophils	LCMT1	-0.01135	3.124061	-0.04424	0.96481	-5.61529	0.784009	0.821692
Neutrophils	FBXO36	0.024458	1.228268	0.044219	0.96483	-5.21488	0.827598	0.866169
Neutrophils	DDX39B	-0.00506	7.251694	-0.04396	0.965033	-6.52277	0.697169	0.732532
Neutrophils	GM14963	0.021708	0.898614	0.043934	0.965056	-5.35857	0.835481	0.874176
Neutrophils	TMEM138	0.01154	2.910493	0.04377	0.965187	-5.55793	0.788853	0.826665
Neutrophils	ZFP280B	0.019923	1.826966	0.043769	0.965188	-5.25942	0.813626	0.851955
Neutrophils	BUD23	-0.01221	4.09892	-0.04328	0.965575	-5.63671	0.76282	0.799875
Neutrophils	XPOT	-0.00914	4.512589	-0.0429	0.965879	-5.80049	0.753922	0.790742
Neutrophils	ZFP82	0.027676	0.121707	0.042851	0.965917	-5.054	0.854551	0.893424
Neutrophils	COX5B	-0.00458	8.103976	-0.04274	0.966004	-6.74128	0.680778	0.715509
Neutrophils	DCTN6	-0.00629	5.142412	-0.04266	0.966069	-6.08272	0.740523	0.777017
Neutrophils	PRPSAP1	0.009268	4.34081	0.042571	0.96614	-5.67399	0.757621	0.79457
Neutrophils	PKNOX1	0.007917	4.569323	0.042551	0.966156	-5.99909	0.752705	0.789531
Neutrophils	RAB11FIP2	0.012689	3.576284	0.042502	0.966194	-5.59312	0.774309	0.811659
Neutrophils	GM15892	-0.01448	3.443855	-0.04248	0.966213	-5.65462	0.777239	0.814665
Neutrophils	MEAK7	-0.02117	0.666682	-0.04216	0.966466	-5.22194	0.841509	0.88016
Neutrophils	SLC4A7	0.007419	5.767425	0.04171	0.966824	-6.13543	0.727617	0.763816
Neutrophils	OLFR1369-	0.032699	-0.76388	0.041678	0.96685	-5.05551	0.876619	0.91591
Neutrophils	SAFB	-0.00528	6.192591	-0.04163	0.96689	-6.28387	0.718875	0.754828
Neutrophils	ZUP1	-0.00958	5.168406	-0.04163	0.966891	-5.87839	0.740124	0.776661
Neutrophils	S100A13	0.004771	6.040644	0.041596	0.966915	-6.4417	0.721986	0.758028
Neutrophils	BIRC2	0.006586	5.673482	0.041588	0.966921	-6.1302	0.729564	0.765816
Neutrophils	KPNB1	-0.00544	5.739818	-0.0414	0.967073	-6.19136	0.72822	0.764457
Neutrophils	TMEM116	0.018878	2.038927	0.041351	0.96711	-5.32108	0.809222	0.847393
Neutrophils	GM49101	0.025861	-0.65342	0.041068	0.967335	-5.1044	0.874032	0.91323
Neutrophils	DPM1	0.005146	5.979025	0.040868	0.967494	-6.22774	0.723401	0.759452
Neutrophils	SLC52A3	-0.02484	0.451484	-0.04086	0.967503	-5.07676	0.846884	0.885683
Neutrophils	JTB	0.004843	5.842584	0.040671	0.96765	-6.15193	0.726213	0.762359
Neutrophils	TNFSF13	0.015004	1.863945	0.040636	0.967678	-5.59119	0.813406	0.851624
Neutrophils	GM26944	0.018679	0.852005	0.040599	0.967707	-5.23953	0.837252	0.875918
Neutrophils	ARRDC3	0.009097	3.403751	0.040291	0.967952	-5.93588	0.778582	0.815932
Neutrophils	GGNBP1	-0.01937	1.044976	-0.04013	0.96808	-5.28967	0.832845	0.871243
Neutrophils	CCDC6	-0.01008	4.540905	-0.04004	0.968153	-5.70161	0.753797	0.790521
Neutrophils	CDK12	-0.00412	6.896517	-0.03992	0.968247	-6.55715	0.704967	0.74035
Neutrophils	IL23A	0.021331	-0.31658	0.039409	0.968653	-5.29505	0.866187	0.904913
Neutrophils	PPIB	0.004115	7.605608	0.03923	0.968795	-6.61737	0.691209	0.72596
Neutrophils	GM30948	0.029461	0.467484	0.03908	0.968915	-5.14042	0.847104	0.885474
Neutrophils	RBM8A	0.00497	5.709751	0.038851	0.969097	-6.21639	0.729505	0.765364
Neutrophils	PATZ1	0.007678	4.251704	0.038761	0.969168	-5.85273	0.760422	0.797076
Neutrophils	RHOD	0.017396	1.328394	0.038665	0.969244	-5.28562	0.826554	0.864622
Neutrophils	GPM6B	-0.02129	2.439081	-0.03863	0.969272	-5.21912	0.80076	0.838341
Neutrophils	PLRG1	0.007149	4.093452	0.03849	0.969384	-5.79761	0.763858	0.800654
Neutrophils	TBC1D25	0.012407	2.839206	0.038194	0.969619	-5.52036	0.79167	0.829135

Neutrophils	MKNK1	-0.00534	4.508378	-0.03816	0.969644	-6.11301	0.754882	0.791501
Neutrophils	PLSCR3	0.007268	4.301027	0.038123	0.969676	-5.9895	0.759354	0.796087
Neutrophils	4931403E2	-0.0111	1.501737	-0.03786	0.969884	-5.50286	0.822474	0.860612
Neutrophils	NEK9	0.004695	5.27603	0.03786	0.969885	-6.40117	0.738563	0.77482
Neutrophils	KCTD5	-0.01003	3.640452	-0.03783	0.969906	-5.59675	0.773785	0.810913
Neutrophils	DRG2	-0.01044	3.507943	-0.03781	0.969928	-5.47069	0.776714	0.81392
Neutrophils	ZFP667	0.016891	2.726732	0.037578	0.970109	-5.2854	0.794214	0.83184
Neutrophils	PARP14	-0.01077	5.480462	-0.03741	0.970239	-6.16798	0.734279	0.770487
Neutrophils	DNASE1L1	-0.00984	3.048154	-0.03728	0.970342	-5.6639	0.786965	0.82451
Neutrophils	PTBP2	-0.00438	6.156832	-0.03715	0.970446	-6.42784	0.72029	0.756171
Neutrophils	GGA3	-0.01009	3.302395	-0.03713	0.970468	-5.47593	0.78128	0.818707
Neutrophils	AXDND1	-0.01157	1.557778	-0.03705	0.970529	-5.73872	0.821159	0.85941
Neutrophils	GM17227	-0.01084	2.746669	-0.03695	0.970612	-5.50496	0.793763	0.831463
Neutrophils	YAF2	-0.00345	6.056106	-0.03685	0.97069	-6.47774	0.722356	0.758295
Neutrophils	CDC42EP4	0.009135	2.155265	0.036821	0.970711	-5.69194	0.807272	0.845253
Neutrophils	DCLRE1B	-0.00785	2.770832	-0.03679	0.970734	-5.71185	0.793216	0.830905
Neutrophils	MED23	-0.00938	3.692708	-0.03675	0.970764	-5.54231	0.772633	0.809863
Neutrophils	RAN	0.005609	8.400943	0.036616	0.970874	-6.73344	0.675852	0.710393
Neutrophils	EIF3I	0.004891	6.822191	0.036568	0.970912	-6.32598	0.706802	0.742305
Neutrophils	COX7A2L	0.003769	6.644119	0.036531	0.970941	-6.4159	0.710386	0.745997
Neutrophils	GPC6	0.020776	1.763476	0.036465	0.970994	-5.4588	0.816351	0.854547
Neutrophils	CCDC43	-0.01245	3.103767	-0.03639	0.971054	-5.34223	0.785718	0.823305
Neutrophils	ZFP40	-0.02018	1.347612	-0.03631	0.97112	-5.16224	0.826101	0.864541
Neutrophils	CHCHD3	0.003542	6.768694	0.036246	0.971168	-6.31784	0.707877	0.743482
Neutrophils	MRPS12	-0.00666	4.774775	-0.03618	0.971218	-5.9275	0.749177	0.785925
Neutrophils	SLC37A3	-0.00703	3.658041	-0.03579	0.971531	-5.81796	0.773588	0.810748
Neutrophils	CFAP20	-0.00593	5.191326	-0.03565	0.971638	-5.93993	0.740554	0.776873
Neutrophils	TSC1	0.008088	4.710552	0.035469	0.971785	-5.75564	0.750762	0.787341
Neutrophils	TRAPPC3	-0.00608	4.917333	-0.03544	0.971811	-5.99147	0.746355	0.782822
Neutrophils	1700007L1	0.015803	1.663099	0.035372	0.971863	-5.26371	0.818927	0.857008
Neutrophils	DNAH12	-0.01805	1.676699	-0.03527	0.971946	-5.41087	0.818618	0.856684
Neutrophils	GM27241	-0.01482	2.603068	-0.03514	0.972044	-5.38469	0.797277	0.834903
Neutrophils	8030453O2	0.01844	-0.75227	0.03499	0.972167	-5.10295	0.877452	0.916502
Neutrophils	ACAD12	0.018804	0.714351	0.034912	0.972229	-5.17898	0.841454	0.879963
Neutrophils	ZFP420	0.020907	0.275776	0.034747	0.97236	-5.0931	0.852061	0.890785
Neutrophils	2610035D1	-0.00982	4.872984	-0.03453	0.972531	-5.94486	0.747332	0.783917
Neutrophils	MLXIPL	0.023042	0.355448	0.034355	0.972671	-5.19834	0.850124	0.888858
Neutrophils	MEMO1	-0.00461	6.363616	-0.03427	0.972739	-6.26269	0.716307	0.75205
Neutrophils	BUB1B	-0.00973	4.016283	-0.03425	0.972754	-5.77366	0.765793	0.802839
Neutrophils	STOML2	0.006764	4.805582	0.034222	0.972777	-5.92552	0.748767	0.785392
Neutrophils	WRNIP1	0.008584	3.743434	0.034155	0.97283	-5.54575	0.771772	0.808959
Neutrophils	LYL1	-0.0086	3.172684	-0.03412	0.972855	-5.81758	0.784435	0.82191
Neutrophils	F8	0.008326	3.134673	0.033987	0.972964	-5.99201	0.785285	0.822786
Neutrophils	CDC45	-0.00973	3.268129	-0.03398	0.972971	-5.75374	0.782302	0.819737
Neutrophils	HINT1	0.003723	7.995864	0.033923	0.973015	-6.62876	0.683881	0.718658
Neutrophils	ITIH5	-0.00871	1.943297	-0.03384	0.973082	-5.79521	0.81244	0.850531
Neutrophils	ABCA2	0.017283	1.527476	0.03373	0.973168	-5.22139	0.822153	0.860442

Neutrophils	PRPF3	0.006064	4.472577	0.033522	0.973334	-5.78072	0.755931	0.792818
Neutrophils	APOOL	0.008625	3.780654	0.033515	0.973339	-5.58789	0.770983	0.808235
Neutrophils	CIART	-0.01202	2.328452	-0.0334	0.973429	-5.48232	0.803592	0.841573
Neutrophils	CCNE2	-0.00891	3.75287	-0.03332	0.973491	-5.9947	0.771598	0.80888
Neutrophils	TTC19	0.006234	4.958514	0.033119	0.973654	-5.97243	0.745561	0.782243
Neutrophils	IFT88	-0.01439	1.778503	-0.03312	0.973657	-5.20733	0.816322	0.854611
Neutrophils	NUFIP2	-0.00317	7.215419	-0.03219	0.974389	-6.65241	0.699302	0.734743
Neutrophils	GM15728	-0.01497	0.500791	-0.03218	0.974401	-5.29523	0.846741	0.885661
Neutrophils	HTR2B	-0.01802	0.855091	-0.03217	0.974405	-5.12346	0.838216	0.876989
Neutrophils	DBF4	0.006032	5.205532	0.032167	0.974412	-6.11395	0.740412	0.777028
Neutrophils	NR2C2AP	-0.01003	4.44571	-0.03214	0.974431	-5.56299	0.756605	0.793639
Neutrophils	SPCS1	-0.00291	7.852067	-0.03212	0.974452	-6.69834	0.686786	0.721838
Neutrophils	EFR3A	-0.00466	5.780301	-0.0321	0.974466	-6.06591	0.728403	0.764693
Neutrophils	SLC35E2	-0.00736	3.898481	-0.03209	0.974472	-5.62814	0.768495	0.80582
Neutrophils	PDE10A	-0.01176	2.555984	-0.03207	0.974485	-5.64464	0.798488	0.83649
Neutrophils	IPO5	-0.00697	5.437174	-0.03207	0.97449	-5.98348	0.735547	0.772033
Neutrophils	GPR18	-0.01152	4.514903	-0.03189	0.974634	-5.55183	0.755128	0.792167
Neutrophils	TCAIM	-0.01394	1.789758	-0.03179	0.974712	-5.28325	0.816156	0.854564
Neutrophils	URGCP	0.00743	4.434418	0.031663	0.974812	-5.79412	0.756861	0.793944
Neutrophils	MAEA	-0.00328	5.40999	-0.03162	0.974848	-6.37899	0.736129	0.772672
Neutrophils	GM15738	0.017642	0.402397	0.031586	0.974873	-5.113	0.849138	0.888146
Neutrophils	GM10762	-0.01072	2.549426	-0.0314	0.97502	-5.39843	0.798702	0.836701
Neutrophils	CTSE	-0.00477	5.492809	-0.03131	0.975092	-6.33125	0.734443	0.770892
Neutrophils	PPIL3	-0.0058	4.605985	-0.03123	0.975156	-5.84884	0.753219	0.790161
Neutrophils	WTIP	0.012395	1.051984	0.03092	0.975403	-5.32846	0.833609	0.872266
Neutrophils	NPC2	-0.00404	7.639426	-0.03082	0.975481	-6.55696	0.691017	0.726173
Neutrophils	DNAJC12	0.009266	3.069108	0.03067	0.975602	-5.63441	0.786972	0.824692
Neutrophils	ZCWPW2	-0.01811	1.016841	-0.0306	0.97566	-5.17058	0.834446	0.873118
Neutrophils	CDIPTOS	-0.01423	-0.30875	-0.03046	0.975769	-5.16615	0.866645	0.905849
Neutrophils	ARMCX3	-0.00791	3.573363	-0.03007	0.976081	-5.62031	0.775736	0.813202
Neutrophils	GM35188	-0.016	1.73277	-0.02994	0.976181	-5.25732	0.817562	0.855919
Neutrophils	CHID1	-0.00915	2.453126	-0.0299	0.976218	-5.38824	0.800924	0.838945
Neutrophils	TMEM123	0.004326	6.484305	0.029831	0.976269	-6.33832	0.714051	0.749903
Neutrophils	LYZL4	0.017444	-1.24765	0.029825	0.976274	-5.06068	0.890201	0.929738
Neutrophils	PTCD3	-0.00696	4.845166	-0.02982	0.976279	-5.71776	0.748129	0.784918
Neutrophils	SH3GL1	0.005389	4.688824	0.029752	0.976332	-5.88834	0.751467	0.788341
Neutrophils	PRR14L	-0.00355	6.50182	-0.02961	0.976445	-6.39063	0.713696	0.749538
Neutrophils	RBMX2	0.007985	3.368821	0.029396	0.976615	-5.62581	0.780273	0.817843
Neutrophils	POC5	0.010654	2.510705	0.029273	0.976713	-5.3294	0.799609	0.837603
Neutrophils	CCDC28B	-0.00766	3.504556	-0.02925	0.976732	-5.68383	0.777259	0.81476
Neutrophils	CYP4A10	-0.01971	1.117903	-0.02923	0.976751	-5.2876	0.832041	0.87067
Neutrophils	KLC2	0.011784	2.681752	0.029147	0.976813	-5.36912	0.795716	0.833627
Neutrophils	ZNRF2	-0.00328	5.763077	-0.02913	0.976827	-6.34921	0.728841	0.765113
Neutrophils	IDS	0.009385	2.698638	0.029127	0.976829	-5.47995	0.795333	0.833236
Neutrophils	RWDD3	0.015978	0.507573	0.029044	0.976895	-5.12843	0.846671	0.885573
Neutrophils	PCYOX1L	0.013143	1.613818	0.028813	0.977078	-5.23034	0.820343	0.858822
Neutrophils	KLHL23	0.014717	0.171341	0.028799	0.97709	-5.22431	0.854841	0.893932

Neutrophils	A930001M	0.006413	3.967402	0.028784	0.977102	-5.81012	0.767072	0.804397
Neutrophils	CCDC124	-0.0036	5.595782	-0.02874	0.977133	-6.18579	0.732317	0.768747
Neutrophils	SLC16A6	-0.00526	4.554893	-0.02871	0.977157	-6.19745	0.754339	0.791349
Neutrophils	CD83	0.011285	5.198788	0.028603	0.977246	-5.95161	0.740636	0.777311
Neutrophils	RNF5	0.005101	4.122516	0.028444	0.977372	-5.96785	0.763689	0.800957
Neutrophils	NBN	-0.00775	3.4476	-0.02832	0.97747	-5.59223	0.778523	0.816146
Neutrophils	FANCM	-0.008	3.67592	-0.02826	0.97752	-5.64171	0.773471	0.810977
Neutrophils	MED12	0.00671	3.781462	0.028242	0.977532	-5.69209	0.771148	0.808599
Neutrophils	KLC3	-0.01828	0.19256	-0.02824	0.977536	-5.09179	0.854323	0.893437
Neutrophils	CLEC14A	0.007671	2.196348	0.027561	0.978074	-5.82814	0.807162	0.845103
Neutrophils	INTS11	-0.0065	3.989645	-0.02714	0.978413	-5.7407	0.766916	0.804099
Neutrophils	NEURL1A	0.019149	-0.39095	0.027125	0.978421	-5.05736	0.869056	0.908216
Neutrophils	PABPC4	-0.00441	5.897724	-0.02693	0.978575	-6.08802	0.726368	0.762525
Neutrophils	ZSWIM3	-0.01081	1.503758	-0.02686	0.978636	-5.3948	0.823279	0.861694
Neutrophils	ZFP81	-0.01275	1.873403	-0.02682	0.978662	-5.18137	0.814637	0.852886
Neutrophils	SHROOM2	0.009478	1.506317	0.02679	0.978687	-5.64189	0.823219	0.861633
Neutrophils	YRDC	0.005148	5.035051	0.026751	0.978718	-5.91632	0.744417	0.78106
Neutrophils	BOLL	-0.01551	1.308178	-0.02668	0.978774	-5.19345	0.827889	0.866391
Neutrophils	GM28707	0.016964	1.154193	0.026558	0.978872	-5.07667	0.831538	0.870116
Neutrophils	GTPBP10	-0.00933	2.397542	-0.02652	0.978902	-5.33133	0.802541	0.840555
Neutrophils	NUP153	-0.00364	5.848135	-0.02651	0.97891	-6.16713	0.727393	0.763587
Neutrophils	2510002D2	-0.00817	3.199654	-0.02633	0.979051	-5.49552	0.784385	0.822007
Neutrophils	LCA5	0.014608	1.313513	0.026093	0.979242	-5.12734	0.827763	0.866272
Neutrophils	RNF220	0.004887	5.723291	0.026091	0.979243	-6.01478	0.72998	0.766246
Neutrophils	ORC6	0.004868	5.216442	0.026041	0.979283	-6.09472	0.740583	0.777135
Neutrophils	ATXN1	-0.00332	6.919224	-0.02602	0.979301	-6.73364	0.705588	0.741153
Neutrophils	TCTN3	0.007929	1.366808	0.025963	0.979346	-5.6429	0.826505	0.86499
Neutrophils	SPATA48	-0.01064	2.190427	-0.02595	0.979354	-5.3705	0.807298	0.845411
Neutrophils	KLHL32	0.01396	1.452777	0.025775	0.979495	-5.29525	0.824524	0.86296
Neutrophils	MTG2	0.007513	3.031494	0.025699	0.979555	-5.47793	0.7882	0.825894
Neutrophils	ZFAND1	-0.00798	2.727292	-0.02526	0.979903	-5.37857	0.795118	0.833027
Neutrophils	PAG1	0.002975	7.038518	0.025227	0.97993	-6.85037	0.703285	0.738833
Neutrophils	MGAT4B	-0.00529	3.847759	-0.02518	0.979965	-5.85553	0.770114	0.807469
Neutrophils	TLR12	-0.01424	2.045397	-0.02504	0.980081	-5.279	0.810742	0.848994
Neutrophils	CSE1L	0.00426	5.5229	0.025038	0.980081	-6.01888	0.734239	0.770684
Neutrophils	IMMP1L	-0.00406	4.995599	-0.02503	0.980085	-5.97587	0.745341	0.782081
Neutrophils	SCPEP1OS	0.016557	0.502575	0.024976	0.98013	-5.08119	0.847256	0.886185
Neutrophils	IRGM1	-0.00898	4.93997	-0.02478	0.98029	-5.96691	0.746588	0.78334
Neutrophils	MCUR1	0.004238	4.462065	0.024649	0.980391	-6.00576	0.756839	0.793831
Neutrophils	ZKSCAN8	0.01346	0.632724	0.024525	0.980489	-5.1094	0.844215	0.883037
Neutrophils	ARHGAP19	-0.00495	4.256309	-0.0244	0.980585	-6.17349	0.761292	0.7984
Neutrophils	EIF4ENIF1	-0.00321	5.556503	-0.02436	0.980617	-6.18045	0.733627	0.770019
Neutrophils	DAB2IP	-0.00701	2.225574	-0.02417	0.98077	-5.64087	0.806722	0.844838
Neutrophils	GM26749	-0.01009	2.222722	-0.02403	0.980881	-5.28988	0.806787	0.844939
Neutrophils	PPAT	-0.00649	4.107428	-0.02399	0.980916	-5.63951	0.764566	0.801793
Neutrophils	TMEM91	0.010156	2.459733	0.023934	0.980959	-5.33881	0.801349	0.839418
Neutrophils	NSD2	-0.00315	6.699324	-0.02382	0.981049	-6.43658	0.710224	0.746017

Neutrophils	NCOA3	0.002222	6.960263	0.023428	0.981362	-6.49642	0.705153	0.740632
Neutrophils	CAMTA1	0.002852	5.835211	0.022984	0.981715	-6.24676	0.728267	0.764253
Neutrophils	OVGP1	0.008246	0.492644	0.022598	0.982022	-5.39622	0.848305	0.886738
Neutrophils	3110001I2:	0.00626	2.677347	0.022067	0.982445	-5.49094	0.797214	0.834539
Neutrophils	GM19265	-0.01316	0.22098	-0.02194	0.982542	-5.07607	0.855129	0.893517
Neutrophils	PVR	-0.00875	3.063552	-0.02183	0.982633	-5.37579	0.788479	0.825641
Neutrophils	SUCLG2	-0.00474	4.835404	-0.02175	0.9827	-5.87466	0.749652	0.785923
Neutrophils	ATP5G2	0.0022	8.844228	0.021655	0.982772	-6.75297	0.669	0.702939
Neutrophils	MMAB	-0.01096	1.326097	-0.02164	0.982785	-5.21339	0.828562	0.866521
Neutrophils	ARV1	0.011299	2.101251	0.021581	0.982831	-5.17912	0.810428	0.848049
Neutrophils	ING4	0.004255	4.08954	0.021545	0.982859	-5.88987	0.765749	0.802411
Neutrophils	CNOT10	-0.00334	5.238486	-0.02106	0.983244	-6.00378	0.741224	0.777196
Neutrophils	IGKV2-109	-0.01188	-0.87987	-0.02102	0.983281	-5.04843	0.882594	0.921289
Neutrophils	SLC22A21	-0.01197	1.348839	-0.02097	0.983321	-5.16701	0.828164	0.866039
Neutrophils	MFAP4	0.009892	1.50654	0.020828	0.98343	-5.39904	0.824443	0.8623
Neutrophils	CBWD1	-0.00526	4.090993	-0.0208	0.983449	-5.7287	0.765846	0.802486
Neutrophils	SPAG5	-0.00678	3.137614	-0.02073	0.983508	-5.68158	0.786947	0.82408
Neutrophils	UBALD1	-0.00378	4.983613	-0.02061	0.983605	-5.99261	0.746621	0.782846
Neutrophils	GET4	0.00307	5.110437	0.020594	0.983616	-6.11573	0.74393	0.780087
Neutrophils	AFAP1	0.00711	1.955044	0.020505	0.983687	-5.39289	0.813954	0.851677
Neutrophils	VHL	-0.00406	3.486608	-0.02033	0.983827	-5.71529	0.779207	0.81618
Neutrophils	HAUS5	0.006311	2.831524	0.019966	0.984115	-5.49184	0.793929	0.831283
Neutrophils	CPSF3	0.002994	4.770083	0.019947	0.984131	-6.02838	0.75125	0.787622
Neutrophils	HMBOX1	0.002959	5.578159	0.019924	0.984149	-6.2276	0.734169	0.770101
Neutrophils	POLL	-0.0084	1.158885	-0.01992	0.984154	-5.24836	0.832752	0.870854
Neutrophils	MSL3	0.002989	4.998465	0.019306	0.984641	-6.05084	0.746584	0.782727
Neutrophils	AKIP1	0.006245	3.24519	0.019262	0.984675	-5.50463	0.784829	0.821885
Neutrophils	HEG1	-0.00367	5.971346	-0.01906	0.98484	-6.15136	0.726203	0.761876
Neutrophils	PPP1R14A	0.009003	0.601813	0.0189	0.984963	-5.293	0.846336	0.884619
Neutrophils	CACUL1	-0.00254	6.117834	-0.01889	0.984972	-6.37572	0.723185	0.758775
Neutrophils	RPN1	-0.00245	5.957169	-0.01881	0.985034	-6.31551	0.726495	0.762177
Neutrophils	AIMP1	-0.0022	6.38044	-0.01874	0.985091	-6.30774	0.717807	0.753248
Neutrophils	EDC3	-0.00453	3.628178	-0.01859	0.985208	-5.60292	0.776305	0.81324
Neutrophils	DDOST	-0.00268	5.788445	-0.01855	0.985244	-6.11289	0.729989	0.765797
Neutrophils	SMIM15	-0.00295	4.858356	-0.01852	0.985264	-5.96934	0.749567	0.785888
Neutrophils	SLC3A2	-0.00271	6.772545	-0.01838	0.985378	-6.43707	0.709856	0.745138
Neutrophils	RAB4A	-0.00818	1.578469	-0.01836	0.985394	-5.31297	0.823059	0.861017
Neutrophils	AP1B1	0.002977	5.190975	0.018353	0.985398	-6.04134	0.742504	0.778681
Neutrophils	DNAJC30	-0.00393	3.959336	-0.01823	0.985497	-5.76194	0.769012	0.805872
Neutrophils	KHSRP	0.002405	5.998752	0.018107	0.985595	-6.18498	0.725637	0.7614
Neutrophils	ELN	0.008105	2.40021	0.018097	0.985602	-5.5629	0.803978	0.841607
Neutrophils	CLK3	0.002758	4.807812	0.017893	0.985764	-6.03028	0.750647	0.787107
Neutrophils	ENDOG	0.005511	2.943039	0.017836	0.98581	-5.43947	0.791622	0.829049
Neutrophils	MKI67	-0.00462	6.498445	-0.01782	0.985821	-6.54267	0.715405	0.750929
Neutrophils	TMEM259	0.003673	4.879001	0.017592	0.986004	-5.94839	0.749177	0.785636
Neutrophils	MVK	-0.00604	2.634356	-0.01755	0.986034	-5.44792	0.798678	0.836288
Neutrophils	ATP1A1	0.00246	6.234757	0.01725	0.986276	-6.27711	0.720908	0.756569

Neutrophils	IFFO1	-0.00728	2.880878	-0.01721	0.98631	-5.42071	0.793162	0.830603
Neutrophils	SPEF1	-0.01007	0.343281	-0.01715	0.986357	-5.11287	0.852754	0.891301
Neutrophils	ZFP382	0.008089	2.008207	0.017028	0.986453	-5.26735	0.81318	0.851009
Neutrophils	CEP112	-0.00787	1.570821	-0.0169	0.986552	-5.35502	0.823418	0.861431
Neutrophils	GM50013	-0.00704	1.85467	-0.01643	0.98693	-5.26925	0.816919	0.854798
Neutrophils	RMND1	-0.00353	3.79439	-0.0164	0.986949	-5.6686	0.772943	0.809896
Neutrophils	ERP44	0.001587	6.074446	0.016403	0.98695	-6.26563	0.724366	0.760087
Neutrophils	U2AF1	-0.00146	7.593832	-0.01604	0.987237	-6.62469	0.693928	0.728681
Neutrophils	GM30881	0.005299	1.986079	0.015878	0.987368	-5.45757	0.814072	0.851795
Neutrophils	GM14296	-0.00716	0.969349	-0.01576	0.987458	-5.15317	0.83805	0.876212
Neutrophils	G6PC3	0.005269	3.106653	0.015658	0.987543	-5.47985	0.788455	0.82566
Neutrophils	CAMKK2	-0.00203	5.188179	-0.01562	0.987575	-6.18711	0.743051	0.779184
Neutrophils	DUSP6	-0.00292	4.428378	-0.01548	0.987686	-6.09916	0.759331	0.795848
Neutrophils	DLEU2	-0.00147	8.40867	-0.01502	0.988047	-6.95692	0.678153	0.7124
Neutrophils	GM43773	-0.00605	1.074002	-0.01492	0.988132	-5.37929	0.835597	0.873704
Neutrophils	GM41442	-0.00822	-0.19949	-0.0149	0.988147	-5.20468	0.866549	0.905149
Neutrophils	IL4I1	0.009394	2.841673	0.014855	0.988181	-5.24574	0.794482	0.831804
Neutrophils	LRSAM1	-0.00497	1.610096	-0.01477	0.98825	-5.46191	0.822904	0.860784
Neutrophils	GRCC10	-0.00152	6.427807	-0.01473	0.98828	-6.42035	0.717354	0.752782
Neutrophils	NDUFB8	-0.00153	7.487591	-0.01471	0.988293	-6.61625	0.696092	0.730898
Neutrophils	PSME3	0.002977	5.282258	0.014713	0.988294	-5.96818	0.741107	0.777178
Neutrophils	TAPBPL	-0.00418	4.125165	-0.01448	0.98848	-5.62538	0.766016	0.802619
Neutrophils	GCN1	0.002483	4.575371	0.014395	0.988548	-5.8582	0.756254	0.792624
Neutrophils	C4B	0.00813	2.480976	0.014295	0.988627	-5.53129	0.802791	0.840191
Neutrophils	TRIP13	0.005274	2.772205	0.0141	0.988782	-5.56624	0.7962	0.833425
Neutrophils	SRP68	0.002761	3.983827	0.014028	0.988839	-5.60085	0.769162	0.805795
Neutrophils	NT5C	-0.00274	5.114948	-0.01363	0.989153	-5.9034	0.744945	0.780885
Neutrophils	EXOC3L4	0.006608	0.594811	0.013488	0.989269	-5.32474	0.847452	0.885544
Neutrophils	GM14636	0.00683	2.274283	0.013471	0.989282	-5.43885	0.807775	0.845162
Neutrophils	DHRS4	-0.00311	4.374568	-0.01324	0.989468	-5.82906	0.760866	0.79719
Neutrophils	PLAGL1	0.007338	1.041672	0.013151	0.989537	-5.25565	0.83676	0.874625
Neutrophils	UNC13D	-0.00247	3.31115	-0.01303	0.989636	-5.92319	0.784281	0.821137
Neutrophils	ARPC5L	-0.00178	6.300784	-0.01302	0.989641	-6.1628	0.720285	0.755566
Neutrophils	PRPF38A	0.00188	5.176932	0.012741	0.989863	-6.14389	0.743775	0.779669
Neutrophils	RNF113A2	-0.00284	4.271264	-0.01256	0.990009	-5.7321	0.763206	0.799598
Neutrophils	HEXIM2	-0.00716	0.998422	-0.01235	0.990174	-5.13962	0.837901	0.875853
Neutrophils	FXYD4	0.003901	3.001862	0.012295	0.990218	-5.58345	0.791331	0.8284
Neutrophils	SPC25	-0.00257	4.372308	-0.01212	0.990357	-6.19141	0.761012	0.797401
Neutrophils	MTMR2	0.002011	5.396075	0.012093	0.990379	-5.95408	0.739151	0.774995
Neutrophils	GM20559	0.002525	4.344461	0.011896	0.990536	-5.99188	0.761616	0.798019
Neutrophils	THNSL1	0.006379	1.077135	0.01184	0.99058	-5.2023	0.83602	0.873939
Neutrophils	GIGYF1	-0.00219	4.441868	-0.01177	0.990633	-5.8727	0.759506	0.795858
Neutrophils	SHROOM3	-0.00667	0.981918	-0.01169	0.990696	-5.28275	0.838296	0.876254
Neutrophils	ZFP609	0.001358	6.067152	0.01159	0.990779	-6.38367	0.725177	0.76065
Neutrophils	FAM220A.:	0.005392	2.200036	0.011554	0.990807	-5.2685	0.809644	0.847083
Neutrophils	PLA1A	-0.00256	1.841769	-0.01153	0.990823	-6.01502	0.817966	0.855564
Neutrophils	GBP5	0.008237	2.014589	0.011423	0.990912	-5.30058	0.813941	0.851463

Neutrophils	ANTXR2	-0.00163	7.654982	-0.01137	0.990951	-7.23719	0.693208	0.727766
Neutrophils	FAM13A	-0.00716	1.15315	-0.01117	0.991115	-5.14779	0.834207	0.872122
Neutrophils	9330175E1	0.007618	1.002962	0.011143	0.991135	-5.14409	0.837793	0.875769
Neutrophils	ACACB	0.007552	0.257652	0.011092	0.991175	-5.06585	0.855817	0.89409
Neutrophils	SRFBP1	-0.00381	3.231687	-0.01103	0.991225	-5.41569	0.786161	0.823157
Neutrophils	ALDH18A1	0.004325	3.070395	0.011008	0.991242	-5.45286	0.789786	0.82686
Neutrophils	IBA57	-0.00451	1.934328	-0.01061	0.991559	-5.19803	0.815925	0.853537
Neutrophils	GM10521	-0.00411	0.308847	-0.01049	0.991651	-5.3408	0.85469	0.892968
Neutrophils	RFC4	-0.00284	4.603054	-0.01048	0.991664	-5.88181	0.756136	0.792456
Neutrophils	RSPO3	0.007243	0.723734	0.010381	0.991741	-5.31326	0.844622	0.882761
Neutrophils	TYSND1	-0.00297	2.640407	-0.01031	0.9918	-5.43138	0.799649	0.836972
Neutrophils	SUZ12	0.001103	6.954117	0.010272	0.991828	-6.47979	0.707234	0.742275
Neutrophils	CABLES1	0.003011	4.513404	0.010056	0.991999	-6.10039	0.758069	0.794506
Neutrophils	PCYOX1	-0.00185	4.579765	-0.00997	0.992071	-5.95238	0.756638	0.793043
Neutrophils	AC125149.	-0.00717	-0.34803	-0.00992	0.992109	-5.06864	0.870877	0.909488
Neutrophils	ZFP275	0.003566	1.581356	0.009901	0.992122	-5.35631	0.824189	0.862042
Neutrophils	MFAP1B	-0.0013	4.945536	-0.00977	0.992228	-6.12568	0.748822	0.785045
Neutrophils	TMEM243	0.000926	6.427837	0.009579	0.992379	-6.45627	0.717935	0.753346
Neutrophils	NOP2	0.002441	3.635097	0.009535	0.992414	-5.56904	0.777337	0.814251
Neutrophils	DPYSL3	-0.00397	0.753878	-0.00931	0.992592	-5.38821	0.843998	0.882223
Neutrophils	1700126G(	-0.005	1.104608	-0.00916	0.992714	-5.18561	0.835586	0.873672
Neutrophils	PDZD8	0.001042	5.970732	0.009132	0.992735	-6.52225	0.727361	0.763043
Neutrophils	PRKCG	-0.00246	4.448629	-0.0091	0.992759	-6.0337	0.759561	0.796072
Neutrophils	LARP7	0.001062	5.919166	0.008771	0.993021	-6.23865	0.728481	0.764193
Neutrophils	TIMP3	-0.003	2.197691	-0.00877	0.993022	-5.85933	0.809972	0.847585
Neutrophils	CDC25C	0.003725	2.030722	0.008733	0.993052	-5.3947	0.813841	0.851536
Neutrophils	MRPL23	0.001036	6.069484	0.008335	0.993368	-6.14033	0.725551	0.761115
Neutrophils	DDX55	0.002954	2.934608	0.008094	0.99356	-5.36125	0.793407	0.830535
Neutrophils	KANK3	0.003003	2.000717	0.00794	0.993683	-5.54298	0.814852	0.852393
Neutrophils	MMP27	0.002573	-0.20195	0.007674	0.993895	-5.53869	0.867753	0.90624
Neutrophils	BBS9	-0.00154	4.909527	-0.00764	0.993923	-6.12245	0.749999	0.786177
Neutrophils	STT3B	0.000744	6.666062	0.007608	0.993947	-6.40637	0.713455	0.748656
Neutrophils	4933407K1	0.003249	1.39879	0.007604	0.99395	-5.20053	0.828975	0.866839
Neutrophils	DNMBP	-0.00156	3.488772	-0.00748	0.994052	-5.84944	0.781005	0.817902
Neutrophils	A530088E(	0.003258	1.760356	0.00724	0.99424	-5.29793	0.82051	0.858228
Neutrophils	BVHT	-0.00407	-0.0458	-0.00717	0.994292	-5.20584	0.863942	0.902398
Neutrophils	PTPA	-0.00108	5.833043	-0.00714	0.994316	-6.05717	0.730591	0.766289
Neutrophils	E430024I0	0.002692	1.898133	0.006994	0.994435	-5.31211	0.817289	0.854987
Neutrophils	NDUFB7	0.00072	6.480862	0.00691	0.994502	-6.55061	0.717261	0.752618
Neutrophils	PNPLA1	-0.00248	0.231599	-0.00686	0.994543	-5.54538	0.857124	0.895529
Neutrophils	CCDC114	-0.00251	1.271818	-0.00671	0.99466	-5.36083	0.832035	0.87003
Neutrophils	DDX41	-0.00141	4.356626	-0.00654	0.994795	-5.71925	0.761947	0.798546
Neutrophils	ITGB3	0.002068	2.991713	0.006527	0.994807	-5.55679	0.792179	0.829462
Neutrophils	KLHL6	-0.00117	6.109159	-0.00633	0.99496	-6.22813	0.724878	0.760564
Neutrophils	ANKLE1	0.002903	0.64315	0.00619	0.995075	-5.19469	0.847108	0.885497
Neutrophils	CD38	-0.00164	5.882237	-0.00615	0.995107	-6.2374	0.729569	0.765404
Neutrophils	DAGLB	-0.00109	4.366228	-0.00611	0.995142	-6.0499	0.761739	0.798402

Neutrophils	GM50071	-0.00301	0.514178	-0.00605	0.995186	-5.16521	0.850234	0.888676
Neutrophils	MYOF	0.003351	2.65687	0.005952	0.995264	-5.3296	0.799782	0.837327
Neutrophils	ACOT13	0.000987	4.43558	0.005891	0.995313	-5.87803	0.760236	0.79689
Neutrophils	CEBPZ	-0.0005	6.141756	-0.00586	0.995341	-6.5913	0.724206	0.759928
Neutrophils	FAM149B	-0.00166	2.992688	-0.00577	0.995411	-5.52523	0.792157	0.829568
Neutrophils	RBM27	0.000598	6.399999	0.005628	0.995522	-6.36987	0.718938	0.754522
Neutrophils	APOL9B	0.002467	0.574633	0.005359	0.995736	-5.32889	0.848872	0.887267
Neutrophils	HIST4H4	0.002453	1.887782	0.005316	0.99577	-5.33047	0.817632	0.855477
Neutrophils	TMTC2	-0.00151	4.574445	-0.00511	0.995935	-6.19099	0.757328	0.793921
Neutrophils	SDHAF2	-0.0009	4.655098	-0.00506	0.995971	-5.94286	0.75559	0.792147
Neutrophils	FOCAD	-0.002	2.908895	-0.005	0.996025	-5.35112	0.79415	0.831594
Neutrophils	LIPT2	-0.00234	1.430834	-0.00496	0.99605	-5.24552	0.828368	0.866484
Neutrophils	LLGL2	-0.00181	2.291993	-0.00454	0.996389	-5.33662	0.808429	0.846002
Neutrophils	MAPKAP1	-0.00057	5.764174	-0.00451	0.996415	-6.26642	0.732273	0.768075
Neutrophils	P2RY10B	0.001539	3.709802	0.004302	0.996577	-5.42875	0.776405	0.813281
Neutrophils	SNHG5	0.00143	2.416156	0.004293	0.996585	-5.50145	0.805587	0.843085
Neutrophils	ORMDL1	-0.00091	3.609727	-0.00414	0.996704	-5.70848	0.77865	0.815549
Neutrophils	FIP1L1	-0.00036	6.639254	-0.00406	0.996767	-6.39147	0.714325	0.749589
Neutrophils	TSN	0.000432	6.360058	0.003947	0.99686	-6.24958	0.720027	0.755445
Neutrophils	BTF3	0.0003	8.999494	0.003588	0.997145	-6.83557	0.668182	0.702021
Neutrophils	ALG3	0.00177	1.77052	0.003445	0.997259	-5.20614	0.820713	0.858465
Neutrophils	CDC5L	0.000346	5.768771	0.003439	0.997264	-6.26333	0.732321	0.768074
Neutrophils	EXTL2	-0.00105	2.165568	-0.00342	0.997282	-5.60577	0.81151	0.849088
Neutrophils	GSTM1	-0.00085	4.059268	-0.00333	0.99735	-5.97758	0.768843	0.80551
Neutrophils	4921509O(	-0.00115	0.064578	-0.00324	0.997422	-5.60784	0.861685	0.900148
Neutrophils	LY6A	0.002764	4.339309	0.003102	0.997532	-5.55955	0.76276	0.799302
Neutrophils	NANP	-0.00108	2.821135	-0.00279	0.997778	-5.39529	0.796637	0.83385
Neutrophils	FLT3	0.001438	2.584668	0.002693	0.997858	-5.24599	0.802034	0.839399
Neutrophils	FUZ	0.001249	1.08456	0.002438	0.99806	-5.22279	0.837233	0.875229
Neutrophils	UBOX5	-0.00098	1.390449	-0.00202	0.998395	-5.18361	0.830159	0.867913
Neutrophils	ST5	-0.00066	2.235439	-0.00188	0.998506	-5.48412	0.810374	0.847814
Neutrophils	E130102H2	0.000945	1.232168	0.001835	0.99854	-5.24857	0.833919	0.871792
Neutrophils	CD96	-0.00117	0.686804	-0.00175	0.998604	-5.21194	0.847009	0.8851
Neutrophils	NDST2	0.000496	2.934923	0.001531	0.998782	-5.43571	0.794431	0.831535
Neutrophils	COG5	0.000182	6.582266	0.001421	0.998869	-6.39561	0.71607	0.751257
Neutrophils	GPR107	0.000227	5.057833	0.001363	0.998915	-5.95753	0.747795	0.783827
Neutrophils	NFYA	0.000195	4.152707	0.001082	0.99914	-5.97131	0.767388	0.803893
Neutrophils	COL4A4	0.000493	1.009618	0.000991	0.999212	-5.21378	0.839383	0.877329
Neutrophils	DHX30	-0.00023	4.509922	-0.00097	0.999231	-5.70342	0.759618	0.795939
Neutrophils	ZFP940	-0.00048	0.734633	-0.00087	0.999312	-5.07324	0.846001	0.884061
Neutrophils	FHOD1	-0.00019	2.907952	-0.0007	0.999446	-5.57808	0.795111	0.832255
Neutrophils	SAP30L	8.50E-05	4.309459	0.000536	0.999574	-5.91638	0.763968	0.800461
Neutrophils	CCDC181	0.000239	1.514839	0.000534	0.999575	-5.23223	0.827361	0.865173
Neutrophils	RAB22A	-6.52E-05	5.60028	-0.00048	0.999621	-6.14548	0.736404	0.772207
Neutrophils	RALB	-3.48E-05	4.247517	-0.00036	0.999713	-6.42107	0.765318	0.801842
Neutrophils	TNS3	-4.34E-05	5.235143	-0.00024	0.99981	-6.27967	0.744094	0.780117
Neutrophils	ERCC4	9.22E-05	2.949475	0.000234	0.999814	-5.35984	0.79417	0.831358

Neutrophils	CCN1	-4.87E-05	1.098666	-8.64E-05	0.999931	-5.34054	0.837288	0.875297
