# Supplementary material for: Immune landscape in liver of neonatal mice with phlebotomy-induced anemia
Source: Pediatr Res. 2025 Sep 17;99(4):1602–12. doi: 10.1038/s41390-025-04361-x (PMC12659965; doi:10.1038/s41390-025-04361-x)
Supplement: Supplementary file 5 — Table S5 [file 41390_2025_4361_MOESM5_ESM.pdf]

immunecell	gene	logFC	AveExpr	t	P.Value	B	adj.P.Val. Within	adj.P.Val. Between
Dendritic.cells	FABP4	1.269077	6.966529	7.081526	3.27E-10	12.85587	3.37E-06	2.25E-07
Dendritic.cells	SGK1	-1.91147	5.355259	-6.2061	1.71E-08	8.87732	9.10E-05	6.49E-06
Dendritic.cells	ABHD17B	0.377557	6.542955	5.986607	4.50E-08	7.976389	0.000156	1.44E-05
Dendritic.cells	MAJIN	-3.24066	0.644865	-5.88027	7.15E-08	6.445974	0.000178	2.55E-05
Dendritic.cells	DNASE1L3	-0.98221	6.30437	-5.86423	7.66E-08	7.567136	0.00016	2.31E-05
Dendritic.cells	PLPP3	-1.59101	4.693864	-5.60433	2.34E-07	6.75498	0.00042	6.42E-05
Dendritic.cells	CYP2E1	-2.49248	4.291137	-5.53185	3.19E-07	6.203453	0.000493	8.34E-05
Dendritic.cells	COL13A1	-2.39791	0.62777	-5.48159	3.94E-07	5.356925	0.000573	0.000107
Dendritic.cells	AQP1	-1.46327	2.964821	-5.3508	6.82E-07	5.714218	0.000843	0.00016
Dendritic.cells	GP9	3.126251	0.659096	5.303517	8.30E-07	4.68331	0.000966	0.000203
Dendritic.cells	FOSB	-0.81152	6.636378	-5.24184	1.07E-06	5.062432	0.001011	0.000211
Dendritic.cells	RUBCNL	0.950381	3.191015	5.03515	2.50E-06	4.555595	0.002306	0.000456
Dendritic.cells	PLPP1	-1.26845	4.327383	-4.97098	3.24E-06	4.316808	0.002699	0.000535
Dendritic.cells	BCL11A	0.512918	5.196907	4.862996	4.99E-06	3.446626	0.003438	0.000731
Dendritic.cells	PPBP	3.590172	0.842206	4.842953	5.41E-06	3.083349	0.003739	0.000882
Dendritic.cells	FGF23	2.710535	0.594781	4.832638	5.63E-06	3.369004	0.003757	0.000914
Dendritic.cells	TCIM	-2.32778	1.634355	-4.82871	5.72E-06	3.326243	0.003682	0.000899
Dendritic.cells	JMJD7	-1.9879	1.757778	-4.81954	5.93E-06	2.863722	0.003673	0.000925
Dendritic.cells	IKZF1	0.424762	8.378161	4.804044	6.31E-06	3.152867	0.003237	0.000803
Dendritic.cells	UBA52	0.532004	10.38927	4.798367	6.45E-06	2.943062	0.003119	0.000771
Dendritic.cells	MIF	0.948617	7.650894	4.778924	6.97E-06	3.385678	0.003375	0.000879
Dendritic.cells	TUBB2A	-0.64793	5.517234	-4.74751	7.89E-06	3.22013	0.003797	0.001018
Dendritic.cells	HPGDS	-0.99175	3.647133	-4.69851	9.56E-06	3.300005	0.004562	0.001248
Dendritic.cells	BHLHE40	-0.64268	6.323106	-4.68607	1.00E-05	2.852685	0.004362	0.001197
Dendritic.cells	LRCH1	0.391028	7.432191	4.572918	1.56E-05	2.320808	0.0061	0.001654
Dendritic.cells	RGCC	-0.6477	5.613883	-4.57124	1.57E-05	2.556538	0.006313	0.001749
Dendritic.cells	GM10076	0.764635	6.671496	4.563934	1.61E-05	2.63648	0.006188	0.001728
Dendritic.cells	CEP192	0.50872	5.27108	4.554011	1.67E-05	2.612018	0.006366	0.001854
Dendritic.cells	RAI14	-2.42578	2.228896	-4.53867	1.78E-05	1.978053	0.006913	0.002092
Dendritic.cells	CTLA2A	1.247463	4.28991	4.528566	1.85E-05	2.702272	0.006677	0.002015
Dendritic.cells	UBC	-0.48583	8.132317	-4.49822	2.07E-05	2.07606	0.006748	0.001986
Dendritic.cells	PLEKHG1	-0.89865	4.398533	-4.4886	2.15E-05	2.520465	0.007279	0.002283
Dendritic.cells	STOX2	-1.04579	3.196694	-4.4787	2.24E-05	2.432442	0.007502	0.002431
Dendritic.cells	MYL9	2.580271	1.95475	4.424596	2.75E-05	1.992664	0.009161	0.002958
Dendritic.cells	TMEM192	0.563477	4.694631	4.406154	2.94E-05	2.182599	0.008891	0.002902
Dendritic.cells	DUSP18	-1.44152	0.621447	-4.40344	2.97E-05	2.236388	0.00962	0.003276
Dendritic.cells	LECT2	-2.45404	3.632928	-4.3859	3.18E-05	2.013899	0.009433	0.003175
Dendritic.cells	PTPRG	-2.17626	2.191113	-4.35861	3.52E-05	1.854861	0.010466	0.003586
Dendritic.cells	FUT4	2.189457	-0.16835	4.323114	4.02E-05	1.262968	0.012141	0.004188
Dendritic.cells	KLF4	-0.64934	6.447469	-4.31756	4.11E-05	1.674422	0.010688	0.003531
Dendritic.cells	TRIT1	-0.4338	4.076961	-4.3107	4.21E-05	1.696387	0.011192	0.003844
Dendritic.cells	UFL1	0.687707	3.965196	4.291048	4.53E-05	1.890949	0.011564	0.004057
Dendritic.cells	TTR	-1.47922	9.202897	-4.28515	4.64E-05	1.288109	0.010473	0.003567
Dendritic.cells	DNAJC7	0.369048	7.510277	4.282304	4.68E-05	1.252216	0.01081	0.003775

Dendritic.cells	TPI1	0.864072	6.690385	4.272428	4.86E-05	1.727916	0.010978	0.003982
Dendritic.cells	KMO	0.503715	3.038738	4.266955	4.96E-05	1.517665	0.011772	0.004467
Dendritic.cells	REEP5	0.323592	7.566136	4.265825	4.98E-05	1.243559	0.010799	0.00395
Dendritic.cells	YARS	-0.59386	5.391202	-4.25789	5.13E-05	1.505558	0.011347	0.004301
Dendritic.cells	EMC6	0.544012	5.61708	4.251098	5.26E-05	1.650413	0.01135	0.004357
Dendritic.cells	CDYL	0.474915	5.659184	4.243739	5.41E-05	1.572859	0.011421	0.004424
Dendritic.cells	NAV1	-1.12295	3.595293	-4.20239	6.30E-05	1.603765	0.013569	0.005227
Dendritic.cells	H2AFJ	0.350597	7.521162	4.17739	6.90E-05	1.003585	0.013537	0.005005
Dendritic.cells	ARHGAP23	-1.08515	3.694446	-4.16286	7.28E-05	1.473036	0.015066	0.005765
Dendritic.cells	TRIM14	0.940491	4.686216	4.152856	7.55E-05	1.414404	0.014911	0.005755
Dendritic.cells	ZMAT5	0.733775	4.547775	4.148366	7.68E-05	1.429414	0.01495	0.005838
Dendritic.cells	PGK1	0.706353	7.765914	4.145429	7.76E-05	1.071706	0.014065	0.005387
Dendritic.cells	SYN1	-2.43224	0.783746	-4.1227	8.43E-05	0.69511	0.016855	0.006899
Dendritic.cells	ITGB6	-2.60663	-0.27325	-4.11882	8.55E-05	0.66127	0.017206	0.007187
Dendritic.cells	GATM	-1.0946	4.530012	-4.11817	8.57E-05	1.323946	0.015678	0.006292
Dendritic.cells	RUNX2	0.443621	4.240789	4.107262	8.91E-05	0.519741	0.016129	0.006508
Dendritic.cells	SGK3	0.53766	5.592861	4.078864	9.88E-05	0.930799	0.017135	0.006773
Dendritic.cells	RASGRP3	-1.21788	3.586569	-4.05665	0.000107	1.130768	0.018982	0.007574
Dendritic.cells	ABCC9	1.587532	1.277502	4.040606	0.000113	0.981714	0.020696	0.008415
Dendritic.cells	MTAP	1.068505	3.671485	4.035032	0.000116	1.030665	0.0198	0.007977
Dendritic.cells	CORO1B	0.851596	4.252422	4.031334	0.000117	1.038054	0.01958	0.00791
Dendritic.cells	HSPG2	-1.00188	2.741833	-4.01132	0.000126	0.987631	0.021191	0.008701
Dendritic.cells	MSRB3	-1.26002	2.604277	-4.00895	0.000127	0.964818	0.021247	0.008763
Dendritic.cells	ITGA9	-1.17995	5.58915	-3.98826	0.000137	0.842358	0.020886	0.008477
Dendritic.cells	CYP2C37	-2.03732	1.772648	-3.98661	0.000138	0.386516	0.022476	0.009461
Dendritic.cells	CAR8	-1.61443	2.375063	-3.98546	0.000138	0.806824	0.022215	0.009329
Dendritic.cells	ADAMTS9	-1.43881	2.970839	-3.97471	0.000144	0.838537	0.022266	0.009457
Dendritic.cells	PLCG2	0.306647	7.02313	3.968314	0.000147	0.271144	0.020608	0.008606
Dendritic.cells	RND3	0.498975	2.943599	3.963335	0.00015	0.44136	0.022277	0.009783
Dendritic.cells	CYP2C68	-2.04324	2.791797	-3.9623	0.00015	0.616641	0.022343	0.009847
Dendritic.cells	FERMT2	-0.99602	3.451103	-3.96075	0.000151	0.822845	0.02206	0.0097
Dendritic.cells	SOX7	-1.71391	0.442832	-3.95849	0.000152	0.55092	0.023386	0.010593
Dendritic.cells	DLC1	-1.18403	4.570858	-3.93562	0.000165	0.675137	0.023111	0.010121
Dendritic.cells	AARS	-0.67791	5.020707	-3.93196	0.000167	0.668184	0.022914	0.010086
Dendritic.cells	ARHGEF7	-0.62415	4.675352	-3.90843	0.000182	0.534809	0.024747	0.01079
Dendritic.cells	MTHFD1L	-0.66286	5.661426	-3.89861	0.000188	0.514649	0.024827	0.010785
Dendritic.cells	RAPH1	-0.69262	5.055385	-3.89182	0.000193	0.50079	0.025396	0.011165
Dendritic.cells	PSD3	-0.70787	5.794857	-3.88843	0.000195	0.345637	0.025041	0.011046
Dendritic.cells	FLT4	-1.05305	2.321562	-3.88308	0.000199	0.585143	0.026946	0.012331
Dendritic.cells	LDAH	0.695274	4.225642	3.874973	0.000204	0.543745	0.026406	0.011908
Dendritic.cells	ZBTB14	0.925879	3.004063	3.868619	0.000209	0.496288	0.027318	0.012508
Dendritic.cells	AHSG	-1.22126	9.130873	-3.86334	0.000213	-0.1638	0.024486	0.010701
Dendritic.cells	CLEC4G	-0.83146	3.468481	-3.8581	0.000217	0.422461	0.027275	0.01271
Dendritic.cells	EMCN	-1.29626	1.650943	-3.8566	0.000218	0.462	0.028251	0.013381
Dendritic.cells	EPCAM	-1.11635	2.160039	-3.8528	0.000221	0.489125	0.028028	0.013286
Dendritic.cells	JUND	-0.42711	10.62781	-3.82544	0.000243	-0.63927	0.025951	0.011299
Dendritic.cells	LY6D	0.44353	5.594266	3.822544	0.000245	-0.29434	0.028517	0.013088

Dendritic.cells	PEA15A	-0.67281	3.90143	-3.8178	0.000249	0.305935	0.02962	0.013879
Dendritic.cells	GM5914	0.893014	3.331997	3.804606	0.000261	0.344263	0.030819	0.014609
Dendritic.cells	PRR13	0.353912	6.510778	3.801372	0.000264	-0.04104	0.029003	0.01347
Dendritic.cells	CPEB4	-0.41813	6.51399	-3.79987	0.000265	-0.16936	0.029001	0.01351
Dendritic.cells	FAM167B	-1.20269	2.127548	-3.79726	0.000268	0.298964	0.031544	0.01534
Dendritic.cells	WNT2	-1.71223	1.077377	-3.79192	0.000273	0.224706	0.032457	0.016009
Dendritic.cells	TENM4	-0.60997	1.760128	-3.7852	0.000279	-0.03227	0.032076	0.01595
Dendritic.cells	ANAPC16	0.427973	5.656735	3.784498	0.00028	0.061026	0.029761	0.014328
Dendritic.cells	APOA1	-1.28974	8.277086	-3.78268	0.000282	-0.2797	0.028325	0.013379
Dendritic.cells	CYP1A2	-1.9445	1.633956	-3.76693	0.000297	-0.05069	0.033506	0.01677
Dendritic.cells	C1RA	-1.85787	1.359965	-3.76501	0.000299	-0.24552	0.033685	0.016988
Dendritic.cells	ELK3	-0.66605	5.288608	-3.75044	0.000315	0.064306	0.031833	0.01579
Dendritic.cells	PRKCG	-0.69974	4.448629	-3.75035	0.000315	-0.13265	0.032349	0.016163
Dendritic.cells	DGKG	1.885494	2.506906	3.749442	0.000316	-0.09877	0.033581	0.017094
Dendritic.cells	MASP1	-1.6242	1.673868	-3.74448	0.000321	0.024463	0.034127	0.01769
Dendritic.cells	TPBGL	-1.90061	0.559425	-3.74351	0.000322	-0.10578	0.034876	0.018246
Dendritic.cells	EFCAB8	-1.81829	1.374064	-3.74092	0.000325	-0.29843	0.034327	0.017909
Dendritic.cells	DNTT	1.80165	1.449877	3.738404	0.000328	0.133573	0.034276	0.01797
Dendritic.cells	ATF4	-0.51909	6.443614	-3.73748	0.000329	-0.19446	0.031142	0.015672
Dendritic.cells	PRKDC	0.541451	4.69925	3.732128	0.000335	-0.0203	0.032233	0.01669
Dendritic.cells	PPP2CA	-0.22056	7.762516	-3.73187	0.000335	-0.52761	0.030414	0.01533
Dendritic.cells	DEPP1	1.858503	1.043364	3.722266	0.000347	-0.11628	0.035378	0.018902
Dendritic.cells	FGD5	-1.36616	1.663977	-3.71914	0.00035	0.078162	0.034954	0.018736
Dendritic.cells	GPI1	0.392456	7.844302	3.717566	0.000352	-0.51118	0.03106	0.015842
Dendritic.cells	GM2000	-0.93263	4.799183	-3.7055	0.000367	0.000452	0.033748	0.017665
Dendritic.cells	TANC1	-1.02515	3.838336	-3.7051	0.000368	0.02747	0.034376	0.018159
Dendritic.cells	PON1	-1.78708	4.304512	-3.69307	0.000383	0.003852	0.034945	0.018348
Dendritic.cells	HBA-A2	1.559029	9.98555	3.692683	0.000384	-0.93734	0.031397	0.015665
Dendritic.cells	GM15503	1.172437	0.81285	3.687438	0.00039	-0.10437	0.037507	0.020476
Dendritic.cells	HBA-A1	1.54929	10.46682	3.686829	0.000391	-1.02395	0.03122	0.015673
Dendritic.cells	ZSWIM6	-0.44715	8.155524	-3.67675	0.000405	-0.64282	0.03345	0.017099
Dendritic.cells	SSH1	-0.89839	3.775324	-3.67172	0.000412	-0.05662	0.036657	0.019564
Dendritic.cells	SCUBE2	-2.3059	-0.26058	-3.66948	0.000415	-0.50382	0.039644	0.021967
Dendritic.cells	INPP5K	0.455217	5.371477	3.658572	0.000431	-0.34254	0.03617	0.019269
Dendritic.cells	SPP2	-1.9975	3.372982	-3.65836	0.000431	-0.16166	0.037582	0.020371
Dendritic.cells	PITPNC1	-0.57548	8.799744	-3.65736	0.000432	-0.63529	0.033905	0.017554
Dendritic.cells	FABP2	-1.80106	3.257265	-3.64985	0.000444	-0.20812	0.038332	0.020834
Dendritic.cells	SNCA	1.788259	4.248926	3.647157	0.000448	-0.14756	0.037658	0.020377
Dendritic.cells	CLDN5	-1.52828	1.006603	-3.63976	0.000459	-0.22676	0.040631	0.02265
Dendritic.cells	MIR142HG	0.338903	8.019491	3.638254	0.000461	-0.83828	0.035529	0.01872
Dendritic.cells	DGKD	0.362015	7.687633	3.634621	0.000467	-0.78968	0.035751	0.019008
Dendritic.cells	RFX2	-0.60319	3.630115	-3.63303	0.000469	-0.34588	0.038619	0.021373
Dendritic.cells	HBB-BT	1.616282	8.105747	3.631934	0.000471	-0.80388	0.035471	0.018891
Dendritic.cells	0610040F0	-1.69738	0.269406	-3.62496	0.000482	-0.59315	0.041886	0.02377
Dendritic.cells	MGLL	-1.08493	3.495334	-3.61883	0.000492	-0.21601	0.03987	0.02206
Dendritic.cells	NEAT1	-0.44142	8.674976	-3.60521	0.000515	-1.1736	0.037199	0.019736
Dendritic.cells	CLPTM1	-0.40929	5.552635	-3.60517	0.000516	-0.50123	0.039455	0.021528

Dendritic.cells	RAB30	1.139288	2.141089	3.603719	0.000518	-0.25365	0.042127	0.023763
Dendritic.cells	TNFAIP8	0.411086	7.424897	3.599668	0.000525	-0.77359	0.038317	0.02068
Dendritic.cells	STAB2	-0.81833	5.148934	-3.59761	0.000529	-0.75341	0.040006	0.022141
Dendritic.cells	EFNA2	-2.22686	1.10216	-3.59087	0.000541	-0.54854	0.043928	0.025171
Dendritic.cells	PKM	0.536517	8.60779	3.585552	0.000551	-0.97367	0.038473	0.020641
Dendritic.cells	HBB-BS	1.466483	11.91128	3.576646	0.000567	-1.5533	0.037031	0.01922
Dendritic.cells	RTKN2	1.879436	1.228878	3.571382	0.000577	-0.70008	0.045785	0.026165
Dendritic.cells	RALGAPA2	-0.49447	6.409122	-3.56129	0.000597	-0.88639	0.041448	0.023143
Dendritic.cells	LDB2	-0.80718	3.160304	-3.56045	0.000599	-0.53625	0.044104	0.025387
Dendritic.cells	SNX5	0.251257	7.890887	3.558109	0.000603	-1.28092	0.040306	0.022382
Dendritic.cells	SH3BP5	-0.80011	6.380773	-3.55788	0.000604	-0.48728	0.04147	0.023343
Dendritic.cells	PROX1	-1.92077	2.15604	-3.55699	0.000606	-0.51708	0.044968	0.026289
Dendritic.cells	MTCH1	0.322423	6.297903	3.556935	0.000606	-0.84036	0.041535	0.023427
Dendritic.cells	KCTD1	1.684697	1.719442	3.556081	0.000607	-0.75666	0.045351	0.026634
Dendritic.cells	RNF19B	-0.49311	5.758355	-3.55348	0.000613	-0.78087	0.041963	0.023943
Dendritic.cells	PRICKLE1	-0.58359	3.386121	-3.55265	0.000614	-0.81043	0.043912	0.025612
Dendritic.cells	GATAD1	0.327801	5.768843	3.549912	0.00062	-0.83888	0.041955	0.024131
Dendritic.cells	SLC44A2	0.314003	6.242042	3.549493	0.000621	-1.18213	0.041579	0.023831
Dendritic.cells	PTPRM	-1.01819	5.186994	-3.53804	0.000645	-0.66633	0.043662	0.025227
Dendritic.cells	SIKE1	0.522627	4.570739	3.536968	0.000647	-0.57765	0.044179	0.02571
Dendritic.cells	FERMT3	0.381992	7.235899	3.531103	0.00066	-0.99608	0.042552	0.02413
Dendritic.cells	ALDOA	0.621538	8.900702	3.52689	0.000669	-1.23792	0.041563	0.023229
Dendritic.cells	DLG2	-0.57702	2.402232	-3.50933	0.000709	-0.89118	0.049286	0.02889
Dendritic.cells	SLC39A4	-1.86102	1.149584	-3.50893	0.00071	-0.89077	0.050499	0.029927
Dendritic.cells	RAMP1	0.293606	4.507186	3.502431	0.000725	-1.06564	0.047788	0.027688
Dendritic.cells	WDR47	-0.91528	3.140773	-3.50228	0.000726	-0.5433	0.049059	0.028761
Dendritic.cells	ADGRL4	-0.8867	3.201942	-3.49584	0.000741	-0.63837	0.049747	0.02911
Dendritic.cells	IGFBP7	-0.61888	5.173401	-3.49338	0.000747	-0.964	0.048	0.027719
Dendritic.cells	SERPINA1A	-1.15523	8.012506	-3.4905	0.000754	-1.17996	0.045637	0.025788
Dendritic.cells	TSPAN18	-1.19925	1.764014	-3.48812	0.00076	-0.58455	0.051421	0.030819
Dendritic.cells	MAF	-0.84642	5.654779	-3.48645	0.000764	-0.83779	0.047715	0.027736
Dendritic.cells	GM46430	1.181963	1.969969	3.485147	0.000768	-0.71898	0.051216	0.030769
Dendritic.cells	KYNU	0.338971	3.853994	3.48087	0.000779	-1.28706	0.049754	0.029532
Dendritic.cells	RBP4	-1.0607	8.031076	-3.47933	0.000782	-1.22601	0.04596	0.02638
Dendritic.cells	TIMM10B	0.37454	6.181583	3.474116	0.000796	-1.06172	0.047943	0.028113
Dendritic.cells	FILIP1	-1.48977	0.904589	-3.47355	0.000797	-0.71213	0.053066	0.032559
Dendritic.cells	GTF3C6	0.412596	5.226124	3.463144	0.000825	-0.90355	0.050225	0.029514
Dendritic.cells	VSIG10	1.846732	0.47062	3.45937	0.000835	-0.99746	0.05542	0.033933
Dendritic.cells	SRGAP1	-0.93207	2.559229	-3.45568	0.000845	-0.71326	0.053557	0.032303
Dendritic.cells	RPAP2	0.857694	3.02458	3.453592	0.000851	-0.68006	0.053133	0.032034
Dendritic.cells	PFKFB4	0.74884	3.15878	3.451917	0.000856	-0.71944	0.052996	0.032063
Dendritic.cells	ADAMTS6	-0.3209	5.940983	-3.44532	0.000874	-1.59764	0.050698	0.030067
Dendritic.cells	GM26532	-0.49265	5.294849	-3.44509	0.000875	-1.13713	0.051325	0.030622
Dendritic.cells	TUBGCP5	0.276537	3.976604	3.443987	0.000878	-1.34745	0.052637	0.031842
Dendritic.cells	PML	0.648408	5.273859	3.442395	0.000883	-1.09561	0.051346	0.030819
Dendritic.cells	ADGRE5	-0.35905	7.153842	-3.43294	0.00091	-1.35218	0.05071	0.029892
Dendritic.cells	NUP210L	-1.01686	6.407383	-3.43193	0.000913	-1.00723	0.051431	0.030582

Dendritic.cells	SLC38A3	-1.91049	1.479322	-3.42497	0.000934	-1.02976	0.05709	0.035563
Dendritic.cells	GM43126	-1.8578	0.276018	-3.42399	0.000937	-1.05971	0.058444	0.036812
Dendritic.cells	HIST3H2BA	1.121107	0.997637	3.423965	0.000937	-0.80604	0.057627	0.036082
Dendritic.cells	MIOS	0.639333	3.665658	3.420392	0.000948	-0.81044	0.055071	0.033755
Dendritic.cells	SKI	-0.46117	5.372605	-3.41644	0.000961	-1.09358	0.053704	0.032487
Dendritic.cells	EFNB1	-1.25978	1.912975	-3.41336	0.00097	-0.8391	0.057587	0.035983
Dendritic.cells	TRAPPC2L	0.416426	5.811456	3.412197	0.000974	-1.14202	0.053432	0.032363
Dendritic.cells	PHKA2	0.964673	3.438409	3.40626	0.000993	-0.81103	0.056703	0.035024
Dendritic.cells	DMPK	-1.70262	2.786398	-3.40458	0.000998	-0.91323	0.057435	0.035787
Dendritic.cells	SERPINA3K	2.866245	1.878609	3.400693	0.001011	-1.01502	0.058761	0.03702
Dendritic.cells	CFI	-1.67807	3.743943	-3.39979	0.001014	-0.83129	0.056682	0.03521
Dendritic.cells	PTPRA	0.278838	6.521638	3.395346	0.001028	-1.3763	0.053834	0.032853
Dendritic.cells	BRD3	0.4552	5.712362	3.395035	0.001029	-1.14306	0.054667	0.033622
Dendritic.cells	GEMIN2	0.919328	3.260952	3.394631	0.001031	-0.84587	0.057294	0.036029
Dendritic.cells	SP3	0.285463	6.891028	3.392269	0.001039	-1.46657	0.053599	0.032713
Dendritic.cells	SIK2	-0.32041	7.126745	-3.38916	0.001049	-1.55183	0.053446	0.032724
Dendritic.cells	KIZ	0.725649	3.359508	3.388689	0.001051	-0.8684	0.057427	0.036356
Dendritic.cells	KSR1	-1.33317	4.69672	-3.37972	0.001081	-0.9234	0.056514	0.03572
Dendritic.cells	PRAM1	1.176775	3.279629	3.379064	0.001084	-0.95806	0.058073	0.037221
Dendritic.cells	CIPC	1.117221	2.655218	3.376645	0.001092	-0.92568	0.058777	0.038022
Dendritic.cells	PATJ	-1.91811	3.348038	-3.37608	0.001094	-0.99875	0.057996	0.037296
Dendritic.cells	AKAP7	0.74037	3.374989	3.376059	0.001094	-0.90885	0.057966	0.037268
Dendritic.cells	CXCL12	-1.51042	2.838235	-3.37564	0.001096	-0.97501	0.058569	0.037852
Dendritic.cells	PIK3C2B	-1.38999	2.667746	-3.37511	0.001098	-0.95973	0.058763	0.038081
Dendritic.cells	SARAF	-0.37121	5.365255	-3.36913	0.001119	-1.3519	0.056541	0.035794
Dendritic.cells	MUC13	-1.86252	1.034673	-3.36802	0.001123	-1.10331	0.061463	0.040474
Dendritic.cells	DDI2	0.321501	5.943537	3.359117	0.001155	-1.52169	0.057272	0.03599
Dendritic.cells	ORAI1	-0.32275	6.491763	-3.35496	0.001171	-1.59624	0.05717	0.035792
Dendritic.cells	GSTA3	-2.04649	4.0016	-3.35331	0.001177	-0.96098	0.059985	0.038488
Dendritic.cells	FLT3	0.418895	2.584668	3.34872	0.001195	-1.50236	0.061989	0.040443
Dendritic.cells	CD47	0.216472	8.628324	3.34867	0.001195	-1.97762	0.055257	0.034181
Dendritic.cells	DYNLT1F	0.706578	5.068561	3.34721	0.0012	-1.1086	0.059103	0.037853
Dendritic.cells	CHMP3	0.314742	5.884611	3.34029	0.001227	-1.33809	0.05922	0.037482
Dendritic.cells	SH3BGR	0.527484	-0.59926	3.336516	0.001242	-1.2849	0.067626	0.045282
Dendritic.cells	LBP	-1.36902	3.292366	-3.33252	0.001258	-1.06929	0.062742	0.040931
Dendritic.cells	WASF2	0.239438	8.064634	3.331437	0.001262	-1.77543	0.057299	0.035877
Dendritic.cells	CTDSPL	-1.31999	3.831098	-3.33078	0.001265	-1.01643	0.062095	0.040419
Dendritic.cells	NRGN	1.59015	3.663682	3.330623	0.001266	-1.01837	0.062295	0.040613
Dendritic.cells	ABHD14A	-1.57493	1.44344	-3.32843	0.001274	-1.23798	0.065193	0.043362
Dendritic.cells	UQCR11	0.370147	7.224799	3.326471	0.001282	-1.6465	0.058461	0.037001
Dendritic.cells	PLXNA2	-0.88914	2.31233	-3.32017	0.001308	-1.11449	0.06509	0.043003
Dendritic.cells	PROC	-1.49125	2.923674	-3.31945	0.001311	-1.07658	0.064325	0.042343
Dendritic.cells	ASAP1	-0.49646	7.200045	-3.31248	0.001341	-1.43081	0.060346	0.0382
Dendritic.cells	RGS12	1.164835	2.240588	3.304914	0.001373	-1.13897	0.067664	0.044541
Dendritic.cells	IER3IP1	0.294726	6.363638	3.302681	0.001383	-1.55912	0.062546	0.039867
Dendritic.cells	MEIS2	-0.78413	2.894046	-3.30206	0.001386	-1.27726	0.066839	0.043975
Dendritic.cells	PPM1M	0.390367	4.583696	3.298295	0.001403	-1.49991	0.065146	0.042167

Dendritic.cells	LAG3	0.567391	1.429969	3.297135	0.001408	-1.39804	0.069234	0.046155
Dendritic.cells	SERPINA1C	-1.20878	7.348457	-3.29377	0.001423	-1.63192	0.061814	0.039455
Dendritic.cells	SASH1	-0.82639	5.303699	-3.29331	0.001425	-1.32502	0.064256	0.041787
Dendritic.cells	MTX3	1.769873	0.591443	3.293135	0.001426	-1.34075	0.070373	0.047645
Dendritic.cells	BIN2	0.577747	5.165118	3.28486	0.001463	-1.30895	0.065164	0.042754
Dendritic.cells	GM11808	-0.44166	7.262783	-3.28345	0.00147	-1.71696	0.062623	0.040453
Dendritic.cells	TCEA2	1.61224	0.335442	3.283062	0.001472	-1.38282	0.071536	0.049077
Dendritic.cells	RMND5A	0.345009	6.766957	3.282923	0.001472	-1.69259	0.063212	0.041041
Dendritic.cells	GM10847	1.59887	0.165978	3.281975	0.001477	-1.33239	0.071773	0.049444
Dendritic.cells	PTPRK	-1.11678	3.178658	-3.28159	0.001479	-1.19678	0.067695	0.045496
Dendritic.cells	SLC38A2	-0.29802	8.351985	-3.27693	0.001501	-1.88265	0.062008	0.039786
Dendritic.cells	FGFR1OP2	0.276575	6.180589	3.272598	0.001521	-1.8288	0.064914	0.0427
Dendritic.cells	COX7A1	1.590617	0.986976	3.270907	0.001529	-1.44042	0.071733	0.049501
Dendritic.cells	GM36839	1.084845	2.346299	3.270248	0.001532	-1.1977	0.069865	0.047732
Dendritic.cells	FIBP	0.518169	4.462532	3.268279	0.001542	-1.30649	0.067075	0.045175
Dendritic.cells	FITM2	-1.36691	0.918829	-3.26774	0.001545	-1.3883	0.071829	0.04987
Dendritic.cells	CARD19	0.687318	5.613475	3.26764	0.001545	-1.32835	0.065617	0.043764
Dendritic.cells	ATF7IP	0.400369	6.540023	3.260673	0.001579	-1.69859	0.065442	0.043345
Dendritic.cells	SIK3	-0.33404	10.09847	-3.26036	0.001581	-2.3705	0.061227	0.039271
Dendritic.cells	STX16	0.39733	5.756821	3.252061	0.001623	-1.66589	0.067885	0.04509
Dendritic.cells	DHDH	-1.12524	2.654165	-3.24878	0.001639	-1.25911	0.072047	0.049483
Dendritic.cells	ALAS2	1.619326	4.928547	3.248418	0.001641	-1.36275	0.068966	0.046476
Dendritic.cells	RBIS	0.495125	5.209656	3.248378	0.001642	-1.44384	0.068597	0.046114
Dendritic.cells	HSD17B2	-1.43442	2.446494	-3.23592	0.001707	-1.32961	0.074923	0.050986
Dendritic.cells	PDE4C	-1.80154	3.131277	-3.23202	0.001728	-1.27907	0.074553	0.050505
Dendritic.cells	CFHR2	-1.72699	2.56401	-3.23024	0.001737	-1.3704	0.075501	0.051545
Dendritic.cells	SHMT1	-1.06998	4.012421	-3.22528	0.001765	-1.29608	0.074281	0.050052
Dendritic.cells	SORBS1	-0.75968	4.747678	-3.22242	0.00178	-1.39081	0.073343	0.049381
Dendritic.cells	CSK	0.32617	6.929767	3.221713	0.001784	-1.92622	0.070364	0.046532
Dendritic.cells	GIMAP4	-1.25842	4.091849	-3.22027	0.001792	-1.31312	0.074271	0.050497
Dendritic.cells	MAFB	-1.46784	4.60884	-3.21993	0.001794	-1.31234	0.073538	0.049799
Dendritic.cells	CORO2B	-1.13811	1.197161	-3.21768	0.001807	-1.33208	0.078656	0.055021
Dendritic.cells	PUS7L	-1.3493	1.839535	-3.21652	0.001813	-1.40506	0.07768	0.054222
Dendritic.cells	ENO1	0.506454	8.066335	3.215834	0.001817	-1.90869	0.068973	0.045677
Dendritic.cells	TIMD2	-1.48771	0.986598	-3.20971	0.001852	-1.44347	0.079612	0.056282
Dendritic.cells	CEMIP2	-0.54076	5.494454	-3.20915	0.001856	-1.67047	0.072988	0.049672
Dendritic.cells	SPNS3	0.553525	3.650358	3.208768	0.001858	-1.68672	0.07561	0.052288
Dendritic.cells	CDH13	-1.54382	2.030566	-3.20847	0.001859	-1.3416	0.078013	0.054698
Dendritic.cells	ADCY7	0.392943	5.583087	3.206999	0.001868	-1.73925	0.07293	0.049655
Dendritic.cells	ELP3	0.984868	2.716115	3.205406	0.001877	-1.36089	0.077152	0.053944
Dendritic.cells	PLEKHJ1	0.329977	5.969804	3.197314	0.001925	-1.76378	0.074061	0.050099
Dendritic.cells	TMEM204	-1.16209	1.038857	-3.19329	0.001949	-1.43745	0.082154	0.057914
Dendritic.cells	ALB	-1.12391	9.441682	-3.18386	0.002007	-2.33323	0.071524	0.046557
Dendritic.cells	GM39469	1.009477	2.328802	3.183605	0.002009	-1.44819	0.081867	0.056774
Dendritic.cells	FLI1	-0.28365	8.191063	-3.18206	0.002018	-2.33093	0.073212	0.048384
Dendritic.cells	C2	-1.87108	2.413631	-3.18166	0.002021	-1.52102	0.081732	0.056825
Dendritic.cells	HPGD	-1.44173	5.673887	-3.17771	0.002046	-1.56626	0.07744	0.052199

Dendritic.cells	CMAH	0.370328	5.561409	3.174465	0.002066	-2.27511	0.078107	0.052664
Dendritic.cells	ANKRA2	0.651406	3.792457	3.16958	0.002098	-1.46864	0.081523	0.055874
Dendritic.cells	SSNA1	0.430131	5.922585	3.169259	0.0021	-1.74817	0.078271	0.052677
Dendritic.cells	PEX7	0.512234	4.45855	3.160609	0.002156	-1.58187	0.082371	0.055969
Dendritic.cells	ETL4	-1.04482	2.396305	-3.15749	0.002177	-1.47439	0.08594	0.059642
Dendritic.cells	DUSP3	-0.43303	5.553941	-3.15674	0.002182	-1.80828	0.080885	0.054636
Dendritic.cells	ZEB2	0.466169	9.36649	3.156058	0.002187	-2.54032	0.075286	0.049153
Dendritic.cells	ELOVL2	-1.76506	2.450205	-3.15517	0.002193	-1.53273	0.085851	0.059676
Dendritic.cells	APOE	-0.89014	9.241961	-3.15288	0.002208	-2.36435	0.07573	0.049655
Dendritic.cells	LPCAT2	1.030259	4.726381	3.150551	0.002224	-1.50695	0.082546	0.05645
Dendritic.cells	ADGRF5	-0.95051	2.598167	-3.1497	0.00223	-1.57593	0.085995	0.059973
Dendritic.cells	BMT2	0.360781	6.027835	3.149193	0.002233	-1.99863	0.080524	0.05455
Dendritic.cells	PELI2	0.403849	2.831902	3.147109	0.002248	-2.06589	0.085781	0.059849
Dendritic.cells	ELAC1	1.012701	1.710286	3.1463	0.002253	-1.53611	0.087665	0.061844
Dendritic.cells	EML1	-1.39869	1.303195	-3.14333	0.002274	-1.54829	0.088793	0.062806
Dendritic.cells	ZFP229	1.64497	-0.01049	3.139857	0.002298	-1.70476	0.091096	0.065607
Dendritic.cells	KNG1	-0.9586	6.039457	-3.1394	0.002302	-1.81219	0.081064	0.055507
Dendritic.cells	STAT2	0.507226	5.210285	3.139162	0.002303	-2.154	0.082353	0.056804
Dendritic.cells	NRP2	-1.12528	3.300945	-3.13855	0.002308	-1.57054	0.085424	0.059973
Dendritic.cells	SLC12A6	0.410818	8.133974	3.138106	0.002311	-2.26907	0.077924	0.05248
Dendritic.cells	SYNE1	-0.84843	4.964609	-3.1359	0.002326	-1.70804	0.083023	0.057572
Dendritic.cells	IARS	-0.45259	5.056202	-3.13144	0.002358	-1.86509	0.08374	0.057916
Dendritic.cells	GM12253	0.545789	-0.86999	3.126279	0.002396	-1.78429	0.095105	0.069054
Dendritic.cells	APOBEC3	0.344651	6.862598	3.123311	0.002418	-2.30741	0.082405	0.056104
Dendritic.cells	CNIH4	0.406448	5.763142	3.121934	0.002428	-1.91389	0.084158	0.057986
Dendritic.cells	GPCPD1	0.279355	7.329375	3.12109	0.002434	-2.37941	0.0817	0.05557
Dendritic.cells	TIMD4	-1.81597	3.658253	-3.11976	0.002444	-1.57956	0.087684	0.061719
Dendritic.cells	APOA2	-0.95966	8.419445	-3.11732	0.002462	-2.33966	0.080445	0.054249
Dendritic.cells	PALD1	-1.59285	1.292537	-3.10775	0.002535	-1.70185	0.094598	0.067266
Dendritic.cells	PDLIM4	-2.09402	2.040162	-3.10502	0.002557	-1.77924	0.093414	0.066196
Dendritic.cells	CREB3L3	-1.69724	1.62088	-3.10488	0.002558	-1.77285	0.094177	0.066976
Dendritic.cells	EIF2S2	-0.26592	7.925804	-3.10154	0.002584	-2.41633	0.083486	0.05658
Dendritic.cells	AKR1C6	-1.68802	4.841164	-3.10068	0.002591	-1.66735	0.088509	0.061763
Dendritic.cells	TTYH2	-1.50496	3.075271	-3.09961	0.002599	-1.68883	0.091562	0.064983
Dendritic.cells	CYP2A22	-1.66792	1.632221	-3.09848	0.002608	-1.7088	0.094156	0.067789
Dendritic.cells	FDPS	0.379411	4.623809	3.098065	0.002611	-2.27079	0.088878	0.062408
Dendritic.cells	GAPDH	0.43614	10.59184	3.096138	0.002627	-2.74892	0.079449	0.053071
Dendritic.cells	RHPN2	-1.57568	0.915486	-3.09611	0.002627	-1.82775	0.095478	0.069467
Dendritic.cells	OGFOD2	0.577088	3.850672	3.09494	0.002636	-1.72902	0.090205	0.064184
Dendritic.cells	TUT7	-0.2609	7.206797	-3.09282	0.002653	-2.32594	0.084622	0.058611
Dendritic.cells	CD101	1.721726	0.741284	3.092779	0.002654	-1.83473	0.095802	0.070162
Dendritic.cells	RALGPS1	0.381842	4.92479	3.092107	0.002659	-2.1447	0.088368	0.062497
Dendritic.cells	CIRBP	0.293061	7.218243	3.090795	0.00267	-2.44025	0.084604	0.058787
Dendritic.cells	HIST2H2AA	1.543666	2.225914	3.090774	0.00267	-1.71221	0.093078	0.067551
Dendritic.cells	KCNQ1	-1.46355	1.396097	-3.09037	0.002673	-1.66518	0.094589	0.06913
Dendritic.cells	COL14A1	1.603461	1.863173	3.089444	0.00268	-1.72057	0.093735	0.068342
Dendritic.cells	PF4	2.274076	4.278945	3.085969	0.002709	-1.71284	0.08998	0.064312

Dendritic.cells	BICRA	0.447012	5.82128	3.085535	0.002712	-1.99565	0.087369	0.061622
Dendritic.cells	ANKRD26	1.11672	2.576265	3.081105	0.002749	-1.66916	0.093414	0.068154
Dendritic.cells	DCBLD1	-1.27235	3.33755	-3.0805	0.002754	-1.67341	0.092052	0.066794
Dendritic.cells	TAZ	0.513226	4.057371	3.080358	0.002755	-1.78199	0.090786	0.065479
Dendritic.cells	CLYBL	0.436811	4.489599	3.079982	0.002758	-2.04131	0.090037	0.064734
Dendritic.cells	AVIL	-1.53463	2.092681	-3.07642	0.002788	-1.80102	0.094849	0.069626
Dendritic.cells	MEF2C	0.259724	7.538963	3.076051	0.002792	-2.85329	0.085474	0.059875
Dendritic.cells	LRP8	-0.28444	2.371033	-3.07064	0.002838	-2.52884	0.095608	0.069941
Dendritic.cells	FBXL18	-1.31175	2.985602	-3.06784	0.002862	-1.69652	0.09457	0.068973
Dendritic.cells	CUEDC1	-1.12255	2.113054	-3.06776	0.002862	-1.70876	0.096179	0.070667
Dendritic.cells	LUZP1	-0.57442	5.787075	-3.06725	0.002867	-1.92899	0.089628	0.063853
Dendritic.cells	ADAM12	-1.74024	0.815122	-3.06557	0.002881	-1.83175	0.098636	0.073513
Dendritic.cells	ADORA2B	1.87177	0.37813	3.064437	0.002891	-1.88793	0.09948	0.074528
Dendritic.cells	SERP1	0.227865	7.981742	3.064425	0.002891	-2.69488	0.085989	0.060318
Dendritic.cells	ITGB5	-1.48534	3.058168	-3.06234	0.00291	-1.7396	0.094589	0.069455
Dendritic.cells	FAM220A.:	-0.58321	2.200036	-3.06195	0.002913	-1.92002	0.096171	0.07119
Dendritic.cells	ZCCHC14	-1.2637	1.412865	-3.05805	0.002947	-1.74489	0.098385	0.073184
Dendritic.cells	UGT2B34	-1.59424	1.748323	-3.05754	0.002952	-1.80028	0.097746	0.072554
Dendritic.cells	CCT3	-0.35083	5.801241	-3.05634	0.002963	-2.11788	0.090483	0.065015
Dendritic.cells	NDUFV3	0.347325	7.098589	3.052981	0.002993	-2.38797	0.088906	0.063165
Dendritic.cells	AKR1C20	-1.87344	2.839499	-3.05178	0.003004	-1.78462	0.096439	0.071285
Dendritic.cells	TRMT10B	1.35401	1.681609	3.049952	0.00302	-1.841	0.098626	0.0739
Dendritic.cells	ZFR2	1.121649	0.817601	3.049328	0.003026	-1.76313	0.100297	0.075758
Dendritic.cells	SLF2	-0.23042	6.314028	-3.04858	0.003033	-2.61331	0.090234	0.065093
Dendritic.cells	IRF4	-0.52905	4.497863	-3.0483	0.003035	-2.08608	0.093411	0.068469
Dendritic.cells	PPIP5K2	0.447125	4.496011	3.043028	0.003084	-1.99371	0.0946	0.069065
Dendritic.cells	UGT2B36	-1.55539	2.198922	-3.04136	0.003099	-1.81656	0.09888	0.073821
Dendritic.cells	DHX40	-0.36728	7.808142	-3.0402	0.00311	-2.75002	0.08884	0.063266
Dendritic.cells	EPS15	0.259359	6.183956	3.039641	0.003115	-2.43709	0.091605	0.066241
Dendritic.cells	1700112J1(	-1.71995	0.638697	-3.03939	0.003118	-1.90573	0.101925	0.077308
Dendritic.cells	RDH7	-1.66079	2.864625	-3.03696	0.00314	-1.79316	0.098054	0.073028
Dendritic.cells	SRSF4	0.285423	6.225555	3.03252	0.003183	-2.33236	0.092917	0.066987
Dendritic.cells	PPP1R9A	-1.19651	3.383193	-3.0286	0.00322	-1.79388	0.098988	0.073105
Dendritic.cells	GATA4	-0.9963	1.621315	-3.02727	0.003233	-1.79936	0.102543	0.076976
Dendritic.cells	ELMOD3	-1.13229	3.437093	-3.02626	0.003243	-1.81107	0.099028	0.073257
Dendritic.cells	GM15614	-1.37002	2.981808	-3.02519	0.003253	-1.83293	0.099945	0.074372
Dendritic.cells	SH2D1A	-1.68386	0.051779	-3.02288	0.003276	-1.98427	0.1061	0.081014
Dendritic.cells	VCL	0.438203	6.049702	3.021192	0.003292	-2.27102	0.094511	0.068739
Dendritic.cells	UCK2	-0.36457	5.87449	-3.02022	0.003302	-2.5785	0.094826	0.069197
Dendritic.cells	CORO2A	0.322388	5.527432	3.019055	0.003313	-2.55909	0.095455	0.070011
Dendritic.cells	STFA2L1	2.226163	2.448793	3.018915	0.003315	-1.86907	0.101267	0.076274
Dendritic.cells	2210016F1	0.435269	4.954824	3.018744	0.003316	-2.05165	0.096503	0.071136
Dendritic.cells	SLC8A2	-2.04106	1.394752	-3.01732	0.003331	-1.91177	0.103518	0.078778
Dendritic.cells	WDFY3	-0.75172	5.666809	-3.01606	0.003343	-2.02819	0.095433	0.070075
Dendritic.cells	LEF1	-1.63787	5.638758	-3.01442	0.00336	-1.84199	0.095484	0.070334
Dendritic.cells	OXR1	-0.4415	6.336267	-3.01431	0.003361	-2.19556	0.094228	0.068982
Dendritic.cells	YWHAH	0.346351	7.687722	3.0128	0.003376	-2.52355	0.092023	0.066601

Dendritic.cells	SULT2A5	-1.76538	2.02078	-3.01018	0.003402	-1.90456	0.103075	0.078318
Dendritic.cells	DCTN3	0.360539	5.963361	3.007598	0.003429	-2.27094	0.096042	0.070493
Dendritic.cells	HGFAC	-1.67886	1.432763	-3.00592	0.003446	-1.98432	0.105026	0.080109
Dendritic.cells	PCBP3	-0.96314	2.616922	-3.00224	0.003484	-1.85949	0.103245	0.077979
Dendritic.cells	TGFBR1	-0.28663	6.24383	-3.00194	0.003487	-2.72269	0.096317	0.070539
Dendritic.cells	GK5	-1.02543	4.404631	-3.00075	0.003499	-1.86643	0.099753	0.074398
Dendritic.cells	WSB1	-0.43243	6.461454	-3.0004	0.003503	-2.19029	0.09592	0.070279
Dendritic.cells	WNK1	-0.26116	9.07644	-2.99689	0.00354	-2.99214	0.091814	0.06583
Dendritic.cells	TMEM88	-1.33178	2.768173	-2.99684	0.00354	-1.87688	0.103496	0.078465
Dendritic.cells	CTU2	0.778635	3.206943	2.993963	0.003571	-1.8877	0.103235	0.077969
Dendritic.cells	SYNPO	-1.52533	0.219102	-2.99302	0.003581	-1.96097	0.10942	0.084855
Dendritic.cells	EHD3	-0.694	3.517645	-2.99092	0.003603	-2.06099	0.103012	0.077771
Dendritic.cells	TTC23	-1.37562	1.101964	-2.98632	0.003653	-1.96597	0.109146	0.08389
Dendritic.cells	KLHL7	-0.40109	4.739495	-2.98483	0.003669	-2.27317	0.101749	0.076023
Dendritic.cells	GM29585	-1.40372	1.247477	-2.98395	0.003678	-1.95456	0.108841	0.083917
Dendritic.cells	KDM1B	0.458269	3.6758	2.981926	0.0037	-2.23944	0.103846	0.078757
Dendritic.cells	CREM	-0.49948	6.0952	-2.9799	0.003723	-2.50249	0.099155	0.073893
Dendritic.cells	VSIG4	-1.6117	4.823941	-2.97958	0.003726	-1.96508	0.101585	0.076589
Dendritic.cells	THBS1	2.520234	4.967156	2.979324	0.003729	-1.91518	0.101307	0.076298
Dendritic.cells	DAAM1	-0.3643	4.764899	-2.97909	0.003732	-2.3889	0.101699	0.076735
Dendritic.cells	USP8	0.39344	5.372996	2.97879	0.003735	-2.22119	0.100526	0.075474
Dendritic.cells	MBL1	-1.51821	1.455107	-2.97853	0.003738	-2.01149	0.108403	0.084175
Dendritic.cells	TMED4	0.717211	3.553347	2.977628	0.003748	-1.94468	0.104106	0.079559
Dendritic.cells	ZFP273	1.444984	0.440289	2.976089	0.003765	-2.06178	0.110808	0.086959
Dendritic.cells	GM11714	1.608655	1.498822	2.971227	0.00382	-2.10018	0.109484	0.085214
Dendritic.cells	GM32051	1.902194	-0.14913	2.968893	0.003846	-2.10449	0.113059	0.089609
Dendritic.cells	AAMP	-0.28291	5.908148	-2.96854	0.00385	-2.36672	0.100586	0.075777
Dendritic.cells	LY6A	1.016657	4.339309	2.965408	0.003886	-2.49765	0.103642	0.079532
Dendritic.cells	PTPRB	-0.70945	3.708559	-2.96526	0.003887	-2.35117	0.104905	0.08094
Dendritic.cells	ITGB1BP2	-1.43418	0.43994	-2.96521	0.003888	-2.06375	0.111765	0.08864
Dendritic.cells	FBXL7	-0.7296	3.621991	-2.96502	0.00389	-2.36553	0.10508	0.081153
Dendritic.cells	GULO	-1.35368	1.982596	-2.9641	0.003901	-1.96934	0.108461	0.085107
Dendritic.cells	SOAT2	-1.50472	0.810649	-2.96365	0.003906	-2.04863	0.11096	0.087925
Dendritic.cells	SCAND1	0.247712	7.018464	2.962921	0.003914	-2.68815	0.098493	0.074038
Dendritic.cells	IRF8	0.308696	6.49623	2.962895	0.003915	-3.0106	0.09947	0.075122
Dendritic.cells	SMARCD2	0.350131	5.611886	2.96265	0.003918	-2.28927	0.101154	0.077014
Dendritic.cells	DNAJB9	-0.40303	5.168187	-2.96112	0.003935	-2.32956	0.102013	0.078103
Dendritic.cells	YPEL3	0.481482	7.069109	2.960818	0.003939	-2.48252	0.098399	0.074098
Dendritic.cells	RBM12B2	0.957419	2.316248	2.960627	0.003941	-1.96177	0.107762	0.084599
Dendritic.cells	FOXO1	-0.28936	7.035015	-2.95702	0.003983	-2.76529	0.098883	0.074725
Dendritic.cells	HOMER1	-0.44259	6.109837	-2.95651	0.003989	-2.50032	0.100629	0.076727
Dendritic.cells	GM20234	1.197771	2.059362	2.956194	0.003993	-2.02313	0.108762	0.085944
Dendritic.cells	YWHAQ	-0.21207	7.746706	-2.95563	0.003999	-2.85699	0.097567	0.073432
Dendritic.cells	ARPC5L	0.287945	6.300784	2.955088	0.004006	-2.56089	0.100266	0.076517
Dendritic.cells	TSPAN14	-0.26179	6.835826	-2.95345	0.004025	-2.8187	0.099497	0.075671
Dendritic.cells	ATG3	0.256694	6.261249	2.950516	0.00406	-2.60192	0.101215	0.077231
Dendritic.cells	SLC16A3	1.300822	3.935418	2.94914	0.004077	-1.98569	0.105887	0.08263

Dendritic.cells	REXO5	1.216486	1.950184	2.94832	0.004086	-2.04137	0.110024	0.087411
Dendritic.cells	CBS	-1.48899	1.874066	-2.94745	0.004097	-2.02652	0.110186	0.087707
Dendritic.cells	DLST	0.293297	5.852364	2.946975	0.004103	-2.46757	0.102079	0.078516
Dendritic.cells	CTNNBL1	-0.29524	5.139211	-2.94623	0.004112	-2.52503	0.103475	0.080185
Dendritic.cells	FBXO8	0.407789	4.606605	2.943046	0.00415	-2.27276	0.105076	0.081702
Dendritic.cells	MYD88	0.65213	4.609439	2.94287	0.004152	-2.11346	0.10507	0.081696
Dendritic.cells	TOMM40L	1.014421	1.733558	2.940959	0.004176	-2.04764	0.111424	0.088753
Dendritic.cells	KDR	-0.63277	3.318647	-2.93869	0.004204	-2.35302	0.108527	0.08528
Dendritic.cells	SLCO2A1	-1.05207	2.568296	-2.9377	0.004216	-2.14041	0.110175	0.087293
Dendritic.cells	AKAP13	-0.22374	8.500476	-2.93588	0.004239	-3.09376	0.098555	0.074251
Dendritic.cells	SERPINC1	-0.98861	5.285455	-2.93529	0.004246	-2.23299	0.104723	0.081273
Dendritic.cells	SRRM1	0.211169	7.865839	2.934831	0.004252	-2.88265	0.099734	0.075703
Dendritic.cells	DDR1	0.456334	0.137297	2.932153	0.004285	-2.32831	0.116048	0.094278
Dendritic.cells	EGFL7	-0.68397	3.872106	-2.93215	0.004285	-2.25257	0.107944	0.08498
Dendritic.cells	ZFP277	0.349833	5.309649	2.929724	0.004316	-2.41835	0.105485	0.081997
Dendritic.cells	GM45606	1.623455	0.477564	2.928817	0.004327	-2.1402	0.115797	0.093887
Dendritic.cells	ZFP954	-1.24742	2.050779	-2.92827	0.004334	-2.06004	0.112307	0.089954
Dendritic.cells	RETNLG	2.267716	3.313557	2.926294	0.004359	-2.05573	0.109984	0.087108
Dendritic.cells	BCAR1	-1.46053	0.776645	-2.92397	0.004389	-2.10766	0.11605	0.093786
Dendritic.cells	PCDH17	-0.94179	2.459624	-2.92078	0.00443	-2.12219	0.113115	0.090159
Dendritic.cells	MARVELD1	-1.29376	1.253094	-2.91994	0.004441	-2.11732	0.115814	0.093416
Dendritic.cells	DHDDS	0.509766	4.630539	2.918695	0.004457	-2.27504	0.108528	0.085213
Dendritic.cells	GM1976	0.730778	2.701657	2.918322	0.004462	-2.09811	0.11263	0.089978
Dendritic.cells	3830406C1	0.483243	4.453206	2.916368	0.004488	-2.25734	0.109274	0.085978
Dendritic.cells	G0S2	1.300123	3.133285	2.914062	0.004518	-2.07715	0.112585	0.08951
Dendritic.cells	DUSP19	-0.99155	2.048701	-2.91102	0.004558	-2.07972	0.115463	0.092816
Dendritic.cells	LAMTOR2	0.283468	6.577213	2.909802	0.004574	-2.62675	0.105865	0.081918
Dendritic.cells	LAYN	-1.56519	0.629576	-2.90958	0.004577	-2.1658	0.118693	0.096701
Dendritic.cells	INPP5F	0.520675	4.366787	2.908651	0.00459	-2.36613	0.110417	0.08737
Dendritic.cells	MCC	-1.21034	2.092188	-2.90822	0.004596	-2.08476	0.115365	0.093119
Dendritic.cells	GM43063	-1.45093	0.779437	-2.90625	0.004622	-2.20511	0.118347	0.096874
Dendritic.cells	GM21762	-0.42091	-1.20491	-2.90559	0.004631	-2.4349	0.123031	0.102531
Dendritic.cells	AKAP12	-0.96407	6.169014	-2.90537	0.004634	-2.31675	0.106687	0.083551
Dendritic.cells	5430427O1	0.419361	3.846602	2.90399	0.004653	-2.54048	0.111524	0.089347
Dendritic.cells	LTBP4	-1.28946	2.297056	-2.90383	0.004655	-2.09152	0.114908	0.093302
Dendritic.cells	MAPK1IP1	0.859375	2.304416	2.903756	0.004656	-2.09159	0.114892	0.093283
Dendritic.cells	AADAC	-1.65857	2.701819	-2.9031	0.004665	-2.10438	0.114012	0.092375
Dendritic.cells	4930486L2	-1.63917	0.484692	-2.90272	0.00467	-2.23101	0.119029	0.098256
Dendritic.cells	SPOP	0.23749	7.366582	2.900429	0.004701	-2.84292	0.104615	0.081494
Dendritic.cells	ISG20	1.303538	4.233394	2.899494	0.004714	-2.13587	0.111034	0.089043
Dendritic.cells	SLC25A53	0.473834	3.336328	2.899436	0.004715	-2.31557	0.112966	0.091294
Dendritic.cells	ADAM32	-1.91118	0.784382	-2.89717	0.004746	-2.23336	0.119066	0.098357
Dendritic.cells	SHB	-0.30909	4.498582	-2.89631	0.004758	-2.88338	0.110818	0.088866
Dendritic.cells	MCMBP	0.243891	7.089729	2.895902	0.004763	-2.89297	0.105492	0.082726
Dendritic.cells	RBL2	0.533025	4.765126	2.895319	0.004771	-2.30666	0.110254	0.088311
Dendritic.cells	PLS1	1.503833	0.910051	2.894649	0.004781	-2.20404	0.118774	0.098456
Dendritic.cells	SMLR1	-1.52683	1.822706	-2.89274	0.004807	-2.18922	0.117083	0.096397

Dendritic.cells	IGLV3	1.409289	-0.70552	2.891251	0.004828	-2.18167	0.123116	0.103657
Dendritic.cells	GM44127	-1.67193	-0.52381	-2.89044	0.00484	-2.22122	0.122678	0.10329
Dendritic.cells	H2AFY	0.225299	7.697828	2.889883	0.004847	-3.02546	0.104744	0.082256
Dendritic.cells	CLPP	0.475963	4.725598	2.889481	0.004853	-2.35408	0.110817	0.089358
Dendritic.cells	ZFP771	0.546018	4.065583	2.886613	0.004894	-2.23171	0.112925	0.091272
Dendritic.cells	CROT	-0.65487	5.259869	-2.8843	0.004927	-2.33316	0.110518	0.088707
Dendritic.cells	BRMS1	0.535836	4.236998	2.884143	0.004929	-2.30197	0.112701	0.09129
Dendritic.cells	SMAGP	-0.94522	3.61538	-2.88398	0.004931	-2.14367	0.114055	0.092889
Dendritic.cells	KRT80	1.379996	0.161146	2.882743	0.004949	-2.29983	0.122137	0.102357
Dendritic.cells	BC051537	-1.65989	0.044835	-2.88113	0.004972	-2.25393	0.12273	0.102947
Dendritic.cells	NUDT13	-0.92426	2.358115	-2.8795	0.004995	-2.14819	0.117638	0.096811
Dendritic.cells	SLC4A1AP	0.511914	4.278134	2.877048	0.005031	-2.29296	0.113927	0.092074
Dendritic.cells	SCARB1	0.3968	4.995715	2.876351	0.005041	-2.54445	0.112374	0.090343
Dendritic.cells	MAP2K6	0.924062	3.533157	2.875411	0.005055	-2.18024	0.115643	0.094254
Dendritic.cells	ZDHHC13	0.39638	3.693777	2.874087	0.005074	-2.47114	0.115489	0.093996
Dendritic.cells	HRG	-1.44593	3.61876	-2.87303	0.00509	-2.16989	0.11577	0.094283
Dendritic.cells	NOL10	-0.37234	4.91748	-2.87021	0.005131	-2.58824	0.1132	0.091268
Dendritic.cells	AMPD3	1.266586	2.578161	2.870171	0.005132	-2.18193	0.118407	0.097407
Dendritic.cells	PRODH2	-1.65023	2.255274	-2.87004	0.005134	-2.19823	0.119149	0.098286
Dendritic.cells	KIFC3	-1.4201	1.625707	-2.86908	0.005148	-2.25008	0.120698	0.100181
Dendritic.cells	RMI2	-1.24966	3.3602	-2.86779	0.005168	-2.20585	0.116737	0.095635
Dendritic.cells	LONRF1	-0.92708	3.091293	-2.8676	0.00517	-2.182	0.117343	0.096353
Dendritic.cells	ADGRL2	-0.65031	4.208198	-2.86553	0.005201	-2.53049	0.115302	0.093797
Dendritic.cells	MRC2	-1.56806	1.416971	-2.86311	0.005238	-2.26739	0.122046	0.101813
Dendritic.cells	SLC25A17	0.386041	5.308731	2.863099	0.005238	-2.53991	0.113233	0.091365
Dendritic.cells	AKR1E1	-0.98964	2.622552	-2.86	0.005285	-2.19992	0.119756	0.0989
Dendritic.cells	ITPKB	-0.28881	7.462345	-2.85927	0.005296	-3.14324	0.109189	0.086485
Dendritic.cells	RFTN2	-1.41836	2.237638	-2.85907	0.005299	-2.25039	0.120651	0.100033
Dendritic.cells	HDC	2.387935	3.642871	2.858352	0.00531	-2.19911	0.117423	0.096346
Dendritic.cells	PHKG1	-1.63076	1.107052	-2.85805	0.005315	-2.28853	0.123329	0.103422
Dendritic.cells	ST6GALNA(	-1.0417	3.214358	-2.85554	0.005353	-2.20387	0.118962	0.097944
Dendritic.cells	PARD3B	-0.76938	4.55014	-2.85452	0.005369	-2.47674	0.115948	0.094498
Dendritic.cells	FKBP7	-1.11719	1.867273	-2.85431	0.005372	-2.20736	0.122102	0.10185
Dendritic.cells	SHANK3	-0.94916	1.430051	-2.85353	0.005384	-2.26663	0.123171	0.103226
Dendritic.cells	ZFP1	0.447401	3.874226	2.850207	0.005436	-2.50586	0.118305	0.097006
Dendritic.cells	METTL23	0.287221	5.675126	2.849314	0.00545	-2.73602	0.114301	0.092355
Dendritic.cells	CDKN1C	-1.31327	3.302414	-2.84905	0.005454	-2.22373	0.119614	0.098677
Dendritic.cells	TNNT1	1.455655	1.520318	2.845098	0.005516	-2.35044	0.124976	0.104471
Dendritic.cells	GINS1	1.36645	3.197409	2.841516	0.005573	-2.25164	0.121993	0.100336
Dendritic.cells	TIE1	-0.95126	1.482911	-2.83656	0.005653	-2.25308	0.127664	0.106144
Dendritic.cells	DTNB	-0.36998	5.293687	-2.83439	0.005688	-2.81708	0.119134	0.095774
Dendritic.cells	XLR4A	1.379814	0.802973	2.827381	0.005804	-2.34834	0.132143	0.109773
Dendritic.cells	SIN3B	0.279091	6.294475	2.826476	0.005819	-2.81525	0.118895	0.094343
Dendritic.cells	PIK3CD	0.341433	6.624599	2.825804	0.00583	-3.01932	0.118154	0.093569
Dendritic.cells	AKT3	0.32637	6.643383	2.825704	0.005832	-3.07222	0.118112	0.09352
Dendritic.cells	CTSF	-1.61401	1.860603	-2.82479	0.005847	-2.33054	0.129478	0.106984
Dendritic.cells	CHCHD2	0.224884	8.94643	2.823501	0.005868	-3.27262	0.113136	0.088046

Dendritic.cells	GLCCI1	-0.22948	7.348661	-2.82347	0.005869	-3.45267	0.116573	0.09205
Dendritic.cells	SLC26A10	-1.25696	0.914242	-2.82291	0.005878	-2.28022	0.131882	0.110208
Dendritic.cells	ADCK5	1.159907	1.510315	2.822045	0.005893	-2.30005	0.130433	0.108539
Dendritic.cells	EPHX2	-1.58088	2.477792	-2.82108	0.005909	-2.31293	0.128115	0.105867
Dendritic.cells	FNIP1	-0.28292	7.760209	-2.81931	0.005939	-3.2285	0.115923	0.091674
Dendritic.cells	PREX2	-0.80147	2.374714	-2.8188	0.005948	-2.42083	0.12847	0.106571
Dendritic.cells	DNTTIP2	-0.39328	5.107858	-2.81879	0.005948	-2.55303	0.121893	0.098766
Dendritic.cells	AGO2	-0.22967	7.576219	-2.81725	0.005974	-3.27359	0.116612	0.092447
Dendritic.cells	MKRN1	0.467516	7.149614	2.815869	0.005998	-2.94568	0.11779	0.093688
Dendritic.cells	EFCAB9	-1.3153	0.443216	-2.80999	0.006099	-2.4295	0.135614	0.11419
Dendritic.cells	TUBGCP2	0.61332	3.994036	2.809526	0.006107	-2.39072	0.126602	0.10353
Dendritic.cells	9930111J2	1.325342	1.279459	2.809322	0.006111	-2.31974	0.133422	0.111671
Dendritic.cells	1110059E2	0.391799	4.393524	2.808279	0.006129	-2.67837	0.125635	0.102528
Dendritic.cells	ELAC2	0.817301	2.817476	2.807294	0.006146	-2.31653	0.129503	0.107262
Dendritic.cells	TRIM30D	0.579832	4.544173	2.806795	0.006155	-2.77371	0.125273	0.10231
Dendritic.cells	ENDOV	-1.04164	2.177707	-2.80632	0.006163	-2.31523	0.131116	0.109279
Dendritic.cells	TAPT1	0.340209	5.802801	2.806029	0.006168	-2.96624	0.122301	0.098795
Dendritic.cells	TAOK3	0.196601	7.012234	2.805637	0.006175	-3.17883	0.119531	0.095524
Dendritic.cells	ADGRE4	-1.71917	3.108019	-2.8044	0.006197	-2.34066	0.128992	0.106658
Dendritic.cells	OTULINL	0.554887	5.347398	2.79998	0.006275	-2.68022	0.124346	0.101079
Dendritic.cells	HSD17B6	-1.72546	0.767255	-2.7992	0.006289	-2.42311	0.13583	0.114932
Dendritic.cells	PINX1	0.823712	3.022074	2.798774	0.006297	-2.34517	0.130018	0.107979
Dendritic.cells	RNF123	0.615237	3.804311	2.7987	0.006298	-2.48749	0.128074	0.105654
Dendritic.cells	CDIP1	0.238202	5.935336	2.798302	0.006305	-3.13224	0.122963	0.099616
Dendritic.cells	MGAT4A	0.3495	5.666978	2.79776	0.006315	-2.92961	0.123592	0.100428
Dendritic.cells	PRELID1	0.291147	7.529242	2.797602	0.006318	-3.10725	0.119313	0.095354
Dendritic.cells	SRSF2	-0.26669	7.941182	-2.79433	0.006377	-3.24565	0.119279	0.09486
Dendritic.cells	CEACAM16	-0.80426	1.137316	-2.79085	0.00644	-2.40776	0.136755	0.115207
Dendritic.cells	SFT2D3	0.665609	2.864525	2.790717	0.006442	-2.37817	0.132251	0.109826
Dendritic.cells	VAMP2	0.371973	4.421644	2.789776	0.00646	-2.64317	0.128455	0.105312
Dendritic.cells	C77080	-2.35817	0.694235	-2.78799	0.006492	-2.37486	0.138501	0.117075
Dendritic.cells	PTPN12	-0.52723	6.049828	-2.78674	0.006515	-2.67351	0.125019	0.101073
Dendritic.cells	TFAM	0.641912	4.293459	2.786152	0.006526	-2.42738	0.12928	0.106296
Dendritic.cells	MRPS35	0.521657	4.266456	2.785799	0.006533	-2.47939	0.129347	0.106452
Dendritic.cells	POT1B	0.753227	4.421725	2.782215	0.006599	-2.46037	0.129862	0.106688
Dendritic.cells	SRGAP3	0.271816	4.261174	2.782056	0.006602	-3.44701	0.130262	0.10717
Dendritic.cells	HES1	-0.9782	5.161335	-2.77865	0.006666	-2.44813	0.12884	0.105056
Dendritic.cells	GM33370	1.142396	0.508404	2.778298	0.006673	-2.38261	0.140951	0.119625
Dendritic.cells	KIF17	-1.55996	1.692096	-2.77711	0.006695	-2.42961	0.13774	0.115962
Dendritic.cells	TNFAIP3	-0.52903	6.763033	-2.77675	0.006702	-2.87155	0.12498	0.100719
Dendritic.cells	ORAI3	0.459017	3.868765	2.776161	0.006713	-2.69636	0.132069	0.109268
Dendritic.cells	PDE7A	0.266266	6.703412	2.776009	0.006716	-3.31282	0.125121	0.100983
Dendritic.cells	GALNT18	-0.99314	2.025829	-2.77462	0.006743	-2.50177	0.136926	0.115198
Dendritic.cells	PPP1R35	0.841427	3.395179	2.774115	0.006752	-2.39016	0.13335	0.110989
Dendritic.cells	BLOC1S1	0.380003	6.24963	2.773911	0.006756	-2.94149	0.126269	0.102513
Dendritic.cells	TOB1	-0.59267	5.050855	-2.77277	0.006778	-2.55346	0.129367	0.106177
Dendritic.cells	STAG3	1.583713	0.607181	2.772076	0.006791	-2.43005	0.140983	0.120319

Dendritic.cells	ETS2	-0.80904	5.865691	-2.7707	0.006818	-2.51587	0.127665	0.104211
Dendritic.cells	FBXL20	0.483845	5.495646	2.767324	0.006883	-2.90761	0.129443	0.105888
Dendritic.cells	SERPINB9	-1.56529	3.483496	-2.76703	0.006888	-2.40284	0.134528	0.112004
Dendritic.cells	MICAL2	-1.03207	1.966497	-2.76495	0.006929	-2.42435	0.139101	0.117299
Dendritic.cells	AGRP	1.42831	1.935634	2.763188	0.006964	-2.42911	0.13956	0.117772
Dendritic.cells	SRC	1.192331	0.980292	2.762733	0.006972	-2.42088	0.142176	0.121025
Dendritic.cells	AMBP	-1.09118	4.730423	-2.76155	0.006996	-2.54129	0.132451	0.109319
Dendritic.cells	GLB1L	0.929777	2.192734	2.760607	0.007014	-2.42021	0.139209	0.117524
Dendritic.cells	RAD1	0.994749	2.477895	2.759323	0.00704	-2.42144	0.138593	0.116847
Dendritic.cells	NOP10	0.317595	7.15867	2.758975	0.007047	-3.08819	0.126733	0.102599
Dendritic.cells	NUCB2	0.217331	4.304904	2.75811	0.007064	-3.20293	0.133892	0.111176
Dendritic.cells	FGD2	0.530435	3.69567	2.755997	0.007106	-2.75938	0.136037	0.113561
Dendritic.cells	OPLAH	1.317533	1.500611	2.75429	0.00714	-2.4853	0.142309	0.121051
Dendritic.cells	PEMT	-1.18136	3.483706	-2.75382	0.007149	-2.43798	0.136953	0.114664
Dendritic.cells	DIABLO	0.549039	4.259159	2.75254	0.007175	-2.56834	0.135101	0.112487
Dendritic.cells	TSPAN9	-0.9072	3.290989	-2.75168	0.007193	-2.49041	0.137639	0.115679
Dendritic.cells	PRX	-1.39969	1.104861	-2.75151	0.007196	-2.49274	0.143592	0.12293
Dendritic.cells	IGF1	-1.25623	5.292109	-2.74968	0.007233	-2.55309	0.132912	0.109597
Dendritic.cells	BTBD9	-0.23417	8.508871	-2.74547	0.007319	-3.55029	0.126347	0.100928
Dendritic.cells	CCDC17	1.390679	0.893924	2.744542	0.007338	-2.48693	0.146271	0.124918
Dendritic.cells	CYB561A3	0.199384	5.573777	2.743712	0.007355	-3.54263	0.133739	0.109826
Dendritic.cells	RNF122	0.389555	2.64878	2.742481	0.007381	-3.00745	0.141705	0.119403
Dendritic.cells	CD300LD	-1.57075	3.173108	-2.74111	0.007409	-2.46929	0.140536	0.117854
Dendritic.cells	TREM1	1.728439	1.639916	2.740601	0.00742	-2.54245	0.144768	0.123076
Dendritic.cells	ZFP940	1.249046	0.734633	2.738731	0.007459	-2.53123	0.147861	0.126654
Dendritic.cells	MSMO1	0.396351	3.610529	2.737805	0.007478	-2.85854	0.139974	0.117157
Dendritic.cells	SLC4A1	1.611	2.002694	2.733871	0.007561	-2.48472	0.145738	0.123188
Dendritic.cells	ARSG	-1.13357	1.867658	-2.73198	0.007601	-2.54584	0.146413	0.123933
Dendritic.cells	CCND3	0.441094	7.994145	2.731942	0.007601	-3.2774	0.130248	0.104505
Dendritic.cells	KLK1B27	0.381985	-1.42677	2.73107	0.00762	-2.72483	0.156257	0.135988
Dendritic.cells	HMGA1	0.542186	4.670399	2.730009	0.007643	-2.76883	0.138995	0.114994
Dendritic.cells	MLXIP	0.355683	6.94185	2.727228	0.007702	-3.1028	0.133933	0.108416
Dendritic.cells	NIPSNAP2	0.329946	4.810103	2.724538	0.00776	-2.86635	0.140283	0.115661
Dendritic.cells	MS4A6C	0.853636	5.165825	2.722453	0.007806	-2.90206	0.13991	0.114869
Dendritic.cells	DCAF8	0.235884	5.699638	2.716776	0.00793	-3.11191	0.139808	0.114441
Dendritic.cells	MBL2	-1.26307	4.246085	-2.71605	0.007946	-2.60256	0.143744	0.119319
Dendritic.cells	HC	-1.46029	2.358321	-2.71601	0.007947	-2.52277	0.149068	0.125754
Dendritic.cells	ACD	0.411868	4.940696	2.715833	0.007951	-2.84634	0.141845	0.11705
Dendritic.cells	TASOR	0.354791	5.742445	2.715542	0.007957	-3.05429	0.139694	0.114525
Dendritic.cells	BRWD3	0.409013	5.109603	2.715463	0.007959	-2.94276	0.141388	0.116561
Dendritic.cells	FLT1	-0.74372	4.243341	-2.71386	0.007995	-3.04499	0.143819	0.119794
Dendritic.cells	ADAM19	0.474142	5.568234	2.713677	0.007999	-3.20223	0.140224	0.115476
Dendritic.cells	2310015A1	0.984228	2.091938	2.713027	0.008013	-2.52096	0.14991	0.127325
Dendritic.cells	ZNHIT3	0.796118	3.532147	2.71215	0.008033	-2.52979	0.145797	0.122495
Dendritic.cells	SLC25A12	0.214574	5.092687	2.712095	0.008034	-3.34843	0.1415	0.117288
Dendritic.cells	ERGIC1	0.359054	5.084699	2.711723	0.008042	-3.13286	0.141522	0.117317
Dendritic.cells	DDX60	-1.76844	2.866859	-2.70979	0.008086	-2.54819	0.147961	0.125221

Dendritic.cells	BAG3	-0.92913	2.967773	-2.70928	0.008097	-2.5336	0.147673	0.125003
Dendritic.cells	KANSL3	0.384086	4.995066	2.709264	0.008097	-2.88054	0.142035	0.118145
Dendritic.cells	ZFP873	1.159166	0.995152	2.708061	0.008125	-2.57592	0.153692	0.132271
Dendritic.cells	BDH1	-1.12825	3.282333	-2.70669	0.008156	-2.53508	0.147351	0.124416
Dendritic.cells	FCGR2B	-0.6238	4.881357	-2.70438	0.008208	-2.87693	0.143199	0.119364
Dendritic.cells	ABCB8	-0.64599	2.240197	-2.7032	0.008235	-2.55523	0.150665	0.128711
Dendritic.cells	APOA5	-1.6534	1.793969	-2.70317	0.008236	-2.57784	0.151974	0.130318
Dendritic.cells	ZMYM6	0.872305	2.526746	2.703051	0.008238	-2.57568	0.149832	0.127689
Dendritic.cells	4833445I0	1.368609	0.48418	2.702901	0.008242	-2.62936	0.155897	0.135147
Dendritic.cells	E2F6	0.879126	2.266229	2.701995	0.008262	-2.54566	0.150589	0.128745
Dendritic.cells	ADPRM	0.683391	3.321315	2.701831	0.008266	-2.56802	0.147551	0.125035
Dendritic.cells	SERPINF2	-1.17913	3.929119	-2.69937	0.008323	-2.59342	0.146597	0.123445
Dendritic.cells	ATP6V1D	0.199493	6.650485	2.696934	0.008379	-3.38783	0.139903	0.114882
Dendritic.cells	GATD1	0.48387	4.394103	2.695677	0.008408	-2.77353	0.146313	0.122617
Dendritic.cells	PLCB1	-0.79673	4.622522	-2.69501	0.008424	-2.87736	0.145674	0.121958
Dendritic.cells	MAP3K9	-1.4599	0.308951	-2.69453	0.008435	-2.60195	0.158349	0.13759
Dendritic.cells	CARS	-0.62498	4.118134	-2.69206	0.008493	-2.75463	0.147861	0.124319
Dendritic.cells	GM17092	-1.07659	2.1249	-2.69133	0.00851	-2.5734	0.153719	0.131496
Dendritic.cells	CCDC130	1.049775	2.056293	2.690528	0.008529	-2.56953	0.153978	0.131864
Dendritic.cells	CEP95	0.500628	4.108354	2.690053	0.00854	-2.70629	0.148002	0.124668
Dendritic.cells	MGST1	-0.7921	7.132587	-2.68886	0.008569	-2.98716	0.139964	0.11482
Dendritic.cells	MRPL54	0.351466	5.906363	2.686114	0.008634	-3.10336	0.144113	0.1193
Dendritic.cells	KLF16	-0.6471	3.164065	-2.68391	0.008687	-2.64788	0.152566	0.129151
Dendritic.cells	ETNK1	0.282911	5.999727	2.680845	0.00876	-3.279	0.14529	0.120094
Dendritic.cells	CCDC148	-2.28639	1.762244	-2.68053	0.008768	-2.62954	0.157611	0.135152
Dendritic.cells	SMIM14	0.19749	7.74729	2.680233	0.008775	-3.60225	0.140573	0.114471
Dendritic.cells	LMF2	-0.72661	3.103696	-2.67708	0.008852	-2.64357	0.154668	0.131077
Dendritic.cells	NUDT3	-0.33279	5.213023	-2.67504	0.008902	-2.99909	0.14914	0.124036
Dendritic.cells	PRORSD1	0.540377	4.586531	2.671627	0.008986	-2.74469	0.151976	0.126983
Dendritic.cells	C9	-1.88458	0.60037	-2.6714	0.008991	-2.64342	0.16415	0.141872
Dendritic.cells	ASGR2	-1.62427	1.698173	-2.66867	0.009059	-2.65378	0.161639	0.138217
Dendritic.cells	TSHZ2	-0.71837	3.393266	-2.6676	0.009086	-2.92359	0.156642	0.132079
Dendritic.cells	ZFP825	1.098123	2.050646	2.666454	0.009115	-2.63947	0.161016	0.13737
Dendritic.cells	JAML	0.464534	2.748338	2.662652	0.00921	-3.1291	0.160274	0.135573
Dendritic.cells	4931406C0	0.525008	3.540099	2.660498	0.009265	-2.87305	0.158504	0.133126
Dendritic.cells	RWDD4A	0.55262	3.983066	2.660003	0.009278	-2.78011	0.15716	0.131583
Dendritic.cells	AFM	-1.27272	2.294567	-2.65812	0.009326	-2.64247	0.162895	0.138312
Dendritic.cells	NSMAF	0.373253	4.903953	2.657677	0.009337	-3.16505	0.154919	0.12863
Dendritic.cells	NFKB2	-0.65208	5.096214	-2.65601	0.00938	-2.86086	0.154819	0.128307
Dendritic.cells	2610035D1	-0.36937	4.872984	-2.65537	0.009396	-3.46237	0.155515	0.129189
Dendritic.cells	TFG	-0.29277	5.864765	-2.65309	0.009455	-3.20552	0.153086	0.126149
Dendritic.cells	2610020C0	-0.34659	4.983149	-2.65309	0.009455	-3.05517	0.155679	0.129283
Dendritic.cells	PHKB	0.257761	5.588989	2.652042	0.009482	-3.47712	0.154096	0.127333
Dendritic.cells	PDK3	0.542542	4.945028	2.650161	0.009531	-2.98301	0.156134	0.129966
Dendritic.cells	TMEM189	-0.31835	6.387922	-2.64979	0.009541	-3.50386	0.151906	0.124934
Dendritic.cells	TIMM17A	0.34723	5.135955	2.649516	0.009548	-3.0403	0.155566	0.129365
Dendritic.cells	GTF2B	-0.31195	6.738906	-2.6492	0.009556	-3.3564	0.150901	0.123764

Dendritic.cells	CELF2	0.216389	8.597509	2.648632	0.009571	-3.71954	0.145722	0.117611
Dendritic.cells	NOP16	0.617882	4.283714	2.648137	0.009584	-2.78836	0.158122	0.132697
Dendritic.cells	CCDC138	-0.8197	4.628632	-2.64756	0.009599	-2.77751	0.157081	0.131524
Dendritic.cells	IMPACT	-0.35911	5.540939	-2.64727	0.009607	-3.24881	0.154369	0.128289
Dendritic.cells	GM48226	0.7264	2.396833	2.645447	0.009655	-2.70836	0.164516	0.140513
Dendritic.cells	HSD3B3	-1.41776	2.014179	-2.64496	0.009668	-2.66898	0.165739	0.14212
Dendritic.cells	RAB5IF	0.275275	7.307335	2.642925	0.009722	-3.42163	0.150124	0.123042
Dendritic.cells	SLC9A3R2	-1.04197	2.701144	-2.64286	0.009723	-2.68213	0.163924	0.139876
Dendritic.cells	CLDN34C1	-1.49078	0.809812	-2.64247	0.009734	-2.72622	0.170053	0.147539
Dendritic.cells	H2-Q10	-1.06623	4.038149	-2.64105	0.009772	-2.70862	0.160118	0.135141
Dendritic.cells	SRP68	0.358362	3.983827	2.640538	0.009785	-2.91073	0.160285	0.135423
Dendritic.cells	GMFG	0.429064	7.424355	2.639649	0.009809	-3.25668	0.150272	0.123197
Dendritic.cells	CALHM2	0.466752	3.088827	2.637171	0.009876	-2.90653	0.163615	0.139502
Dendritic.cells	EIF1B	0.334867	5.828759	2.637015	0.00988	-3.11753	0.155251	0.129259
Dendritic.cells	GM19605	1.165772	1.260305	2.636881	0.009884	-2.68502	0.169515	0.146779
Dendritic.cells	MTLN	-0.69597	3.060484	-2.63658	0.009892	-2.71605	0.163704	0.139685
Dendritic.cells	INSIG2	0.521838	4.405764	2.635031	0.009934	-2.89234	0.159959	0.134797
Dendritic.cells	SMDT1	0.205267	7.773748	2.633428	0.009977	-3.67867	0.150273	0.12299
Dendritic.cells	IRGM2	1.33202	2.731293	2.633413	0.009977	-2.69033	0.165436	0.141523
Dendritic.cells	AMOTL1	-0.91045	1.696223	-2.63176	0.010023	-2.7213	0.169298	0.145994
Dendritic.cells	CD244A	0.749489	3.429777	2.631214	0.010037	-2.82698	0.163717	0.13917
Dendritic.cells	CES1C	-1.39681	4.023969	-2.62802	0.010125	-2.7533	0.16242	0.137545
Dendritic.cells	PLPPR1	-1.37062	1.149205	-2.62801	0.010125	-2.73739	0.171703	0.148994
Dendritic.cells	USP20	0.405832	2.877475	2.627955	0.010127	-2.99665	0.166045	0.142004
Dendritic.cells	PDE2A	-0.77481	6.050057	-2.62738	0.010143	-2.92924	0.156257	0.130126
Dendritic.cells	EPHB4	-1.37592	0.794939	-2.62717	0.010148	-2.7034	0.172892	0.150613
Dendritic.cells	G6PDX	0.693134	4.072997	2.626462	0.010168	-2.74402	0.162267	0.137649
Dendritic.cells	CASP1	0.986784	3.51676	2.626128	0.010177	-2.7108	0.164011	0.139837
Dendritic.cells	SLC25A30	-0.75468	3.550303	-2.62482	0.010214	-2.72573	0.163905	0.139934
Dendritic.cells	RNF180	-0.47654	2.247716	-2.62472	0.010216	-3.19884	0.16808	0.145098
Dendritic.cells	ARRB1	-0.73414	3.996665	-2.62442	0.010225	-2.72708	0.162505	0.13824
Dendritic.cells	MPI	0.965066	2.225319	2.624009	0.010236	-2.71228	0.168153	0.145272
Dendritic.cells	DPH2	1.379416	0.879573	2.621443	0.010308	-2.75887	0.173563	0.151514
Dendritic.cells	VKORC1L1	0.270514	5.560377	2.61985	0.010353	-3.4355	0.159038	0.133365
Dendritic.cells	ZFP451	-0.40678	5.019065	-2.61934	0.010367	-3.07074	0.160688	0.135453
Dendritic.cells	GDA	2.098111	3.902395	2.618278	0.010397	-2.7224	0.164398	0.140047
Dendritic.cells	ADAMTS1	-0.86878	2.937185	-2.61704	0.010432	-2.91266	0.167804	0.144035
Dendritic.cells	DGKH	-0.67878	4.909539	-2.61477	0.010496	-2.89483	0.161822	0.136932
Dendritic.cells	GM17103	-1.33874	1.028835	-2.61464	0.0105	-2.74136	0.174406	0.152548
Dendritic.cells	STAB1	-0.82769	3.176342	-2.61459	0.010501	-2.89724	0.167297	0.143705
Dendritic.cells	ATP6V1H	-0.27426	6.803114	-2.61433	0.010509	-3.66194	0.156103	0.129924
Dendritic.cells	KEAP1	0.37084	5.115335	2.613007	0.010547	-3.08624	0.161312	0.136495
Dendritic.cells	GRN	-0.23869	7.188808	-2.61299	0.010547	-3.96973	0.155091	0.128839
Dendritic.cells	FOS	-0.41442	8.489188	-2.6119	0.010579	-3.8313	0.151522	0.124461
Dendritic.cells	ASPRV1	1.80201	1.33931	2.611515	0.010589	-2.77123	0.173685	0.151943
Dendritic.cells	LAGE3	0.453895	4.771468	2.609418	0.01065	-3.00084	0.163252	0.138514
Dendritic.cells	IRF2BP1	0.448954	4.152538	2.607489	0.010706	-2.99366	0.165794	0.141423

Dendritic.cells	MTA3	0.396504	6.109959	2.607037	0.010719	-3.29772	0.159716	0.134025
Dendritic.cells	MRC1	-0.88112	5.240936	-2.60473	0.010786	-3.24042	0.163056	0.137771
Dendritic.cells	SLA2	0.259006	2.746588	2.604449	0.010794	-3.62235	0.171055	0.147697
Dendritic.cells	NARS	-0.28252	6.298026	-2.60267	0.010846	-3.44303	0.160218	0.134129
Dendritic.cells	BMYC	0.377185	3.380337	2.602462	0.010852	-3.44638	0.169407	0.145523
Dendritic.cells	0610012G(	0.392815	5.039442	2.599071	0.010952	-3.05924	0.165281	0.139542
Dendritic.cells	RGS18	0.443032	3.108266	2.598767	0.010961	-3.215	0.171522	0.147292
Dendritic.cells	IDNK	0.374461	5.52475	2.597859	0.010988	-3.19415	0.163813	0.137824
Dendritic.cells	ATAD1	-0.22489	5.731262	-2.59658	0.011026	-3.38953	0.163171	0.137188
Dendritic.cells	BACH2OS	-1.35747	1.526179	-2.59637	0.011032	-2.82181	0.176916	0.154233
Dendritic.cells	GM38948	-1.23877	0.463761	-2.59596	0.011044	-2.82184	0.180614	0.158894
Dendritic.cells	GM42556	1.4877	0.793712	2.595776	0.01105	-2.82038	0.179455	0.157455
Dendritic.cells	ZFYVE28	0.85648	0.624372	2.595523	0.011058	-2.76936	0.180049	0.158197
Dendritic.cells	PGPEP1L	1.327742	-0.29843	2.590987	0.011194	-2.82759	0.185141	0.163599
Dendritic.cells	APOF	-1.03854	3.847555	-2.59052	0.011208	-2.84962	0.170831	0.145867
Dendritic.cells	GNPTAB	0.381529	4.589659	2.590274	0.011215	-3.26459	0.168417	0.142937
Dendritic.cells	PSMD10	0.726682	3.584601	2.589798	0.01123	-2.82716	0.171697	0.147097
Dendritic.cells	MYO19	-1.23845	1.357151	-2.58751	0.011299	-2.79952	0.180117	0.157091
Dendritic.cells	CPLX2	-1.50333	3.378009	-2.58676	0.011322	-2.79976	0.173317	0.148652
Dendritic.cells	SLC25A38	0.637396	3.517886	2.584987	0.011376	-2.8732	0.173437	0.14852
Dendritic.cells	MRPL48	0.335923	5.282039	2.583314	0.011428	-3.1437	0.167965	0.141729
Dendritic.cells	HOXA7	0.667474	-0.09629	2.583311	0.011428	-2.96064	0.186365	0.164593
Dendritic.cells	SLC49A4	0.308096	6.30055	2.581931	0.01147	-3.84951	0.165129	0.13807
Dendritic.cells	PPDPF	0.257335	5.630234	2.579731	0.011538	-3.47774	0.167653	0.141056
Dendritic.cells	JUNB	-0.38184	9.343714	-2.57953	0.011544	-3.85063	0.156342	0.127214
Dendritic.cells	IQCN	-1.44852	1.791223	-2.57949	0.011546	-2.81943	0.180495	0.15697
Dendritic.cells	CTNND1	-0.72717	4.294756	-2.57722	0.011616	-2.91957	0.172576	0.146749
Dendritic.cells	MRPL23	0.25685	6.069484	2.57719	0.011617	-3.39587	0.166831	0.139674
Dendritic.cells	D8ERTD73(	0.221358	7.358725	2.574582	0.011699	-3.64405	0.163725	0.13539
Dendritic.cells	PLOD1	-0.87411	3.410314	-2.57409	0.011714	-2.86196	0.176516	0.151194
Dendritic.cells	PPM1K	0.728198	3.579795	2.572936	0.011751	-2.90517	0.176248	0.150725
Dendritic.cells	BAHCC1	-1.18575	1.235974	-2.5724	0.011768	-2.82107	0.18444	0.161032
Dendritic.cells	GPT	1.325314	1.203309	2.571056	0.01181	-2.84235	0.184971	0.161509
Dendritic.cells	C1QB	-1.00366	6.51237	-2.57023	0.011836	-3.26518	0.167181	0.139546
Dendritic.cells	SLC12A7	-1.05994	3.37429	-2.56943	0.011862	-2.82209	0.177509	0.152513
Dendritic.cells	GM43773	-1.30904	1.074002	-2.56915	0.011871	-2.85228	0.185596	0.162639
Dendritic.cells	GINS3	1.044959	2.330816	2.568714	0.011885	-2.82413	0.181122	0.157147
Dendritic.cells	CD55B	-1.61576	0.178504	-2.56711	0.011936	-2.87873	0.189425	0.167215
Dendritic.cells	SMIM5	0.446299	0.166279	2.566033	0.01197	-3.29619	0.189759	0.167632
Dendritic.cells	MYO5A	0.303816	5.016572	2.564498	0.01202	-3.65317	0.173255	0.146872
Dendritic.cells	SHKBP1	0.585715	4.068341	2.563837	0.012041	-2.93988	0.176503	0.150976
Dendritic.cells	GALNT2	0.386944	5.565067	2.562911	0.012071	-3.34663	0.17172	0.145015
Dendritic.cells	EML3	0.484431	4.139794	2.562397	0.012087	-3.0747	0.176467	0.151007
Dendritic.cells	APOH	-1.04825	4.983199	-2.56087	0.012137	-3.06919	0.1741	0.147933
Dendritic.cells	TMEM138	0.597797	2.910493	2.559956	0.012167	-2.92882	0.18117	0.156885
Dendritic.cells	ITIH1	-1.39878	2.749811	-2.55991	0.012168	-2.84253	0.181733	0.157588
Dendritic.cells	SNRK	-0.40386	4.865356	-2.55833	0.012219	-3.25346	0.174784	0.148914

Dendritic.cells	LRRK1	0.35433	5.612519	2.558286	0.012221	-3.54229	0.172311	0.145849
Dendritic.cells	PROZ	-1.39242	1.915226	-2.55774	0.012239	-2.85814	0.185025	0.16182
Dendritic.cells	PLCE1	-1.78701	1.279254	-2.55678	0.01227	-2.87847	0.187552	0.165035
Dendritic.cells	ERBB4	-1.72989	0.909465	-2.55601	0.012296	-2.9004	0.188907	0.16694
Dendritic.cells	S100A16	-0.68327	2.552707	-2.55511	0.012325	-2.95338	0.182976	0.159596
Dendritic.cells	ATP5D	0.218437	7.898894	2.554497	0.012345	-3.81929	0.165245	0.137697
Dendritic.cells	RNASE6	0.253447	4.491549	2.554144	0.012357	-3.91173	0.176274	0.151449
Dendritic.cells	ZFP280D	0.25618	5.625842	2.553784	0.012369	-3.64635	0.172498	0.146783
Dendritic.cells	ZFP532	-1.24946	1.223667	-2.55298	0.012396	-2.85545	0.187755	0.166077
Dendritic.cells	CEP63	-0.51317	4.291786	-2.55166	0.012439	-3.06618	0.17695	0.152667
Dendritic.cells	CD8A	0.735801	1.360615	2.551606	0.012441	-3.13875	0.187255	0.165637
Dendritic.cells	GJA1	-1.07125	1.208053	-2.55158	0.012442	-2.88453	0.187812	0.16634
Dendritic.cells	NET1	0.364931	4.035875	2.551327	0.01245	-3.45415	0.177821	0.153759
Dendritic.cells	RPUSD3	-1.49443	0.640562	-2.55022	0.012487	-2.90758	0.189899	0.169245
Dendritic.cells	ZFP692	0.859616	2.030033	2.549719	0.012504	-2.86186	0.184837	0.162921
Dendritic.cells	CDK11B	-0.29268	6.82853	-2.54888	0.012532	-3.61259	0.168609	0.142824
Dendritic.cells	SLC8B1	0.522461	4.721857	2.548467	0.012546	-3.15293	0.175499	0.151454
Dendritic.cells	S100G	1.69486	0.605317	2.548316	0.012551	-2.89343	0.19003	0.169826
Dendritic.cells	ZFP217	0.510313	4.011596	2.54808	0.012559	-2.98182	0.177904	0.154545
Dendritic.cells	DOCK1	-0.57121	4.079995	-2.54797	0.012562	-3.10402	0.17767	0.154273
Dendritic.cells	ARL6IP5	0.223917	7.048458	2.547534	0.012577	-3.82558	0.16791	0.142163
Dendritic.cells	MMGT2	0.543367	3.166256	2.546794	0.012602	-2.98243	0.180821	0.158507
Dendritic.cells	ATP11A	0.826236	2.676915	2.546791	0.012602	-2.89209	0.182537	0.16068
Dendritic.cells	MED8	0.477925	5.008062	2.546468	0.012613	-3.10849	0.174541	0.150634
Dendritic.cells	SLC39A3	-0.98068	1.91724	-2.54565	0.01264	-2.87042	0.185313	0.164224
Dendritic.cells	APOC1	-0.8868	7.949726	-2.54535	0.01265	-3.63244	0.165151	0.138896
Dendritic.cells	SKAP1	-1.12393	4.523681	-2.54464	0.012674	-2.97142	0.176339	0.152955
Dendritic.cells	ARAP3	-0.7703	3.537048	-2.54368	0.012707	-2.96934	0.17994	0.157327
Dendritic.cells	LPL	-1.01816	5.132487	-2.54255	0.012745	-3.00023	0.174822	0.150726
Dendritic.cells	OIP5	1.241577	2.207869	2.539996	0.012832	-2.90952	0.185607	0.164072
Dendritic.cells	CCNI	0.236366	6.783841	2.539333	0.012855	-3.67086	0.170039	0.144515
Dendritic.cells	SNRPD3	0.314929	6.518599	2.539322	0.012855	-3.47816	0.170894	0.145586
Dendritic.cells	IFRD1	-0.3629	8.532379	-2.53927	0.012857	-3.89838	0.164542	0.13765
Dendritic.cells	SNX24	-0.88084	4.299252	-2.53818	0.012894	-3.00863	0.178563	0.155094
Dendritic.cells	USP2	-0.79301	2.711696	-2.53707	0.012932	-2.94111	0.18413	0.162284
Dendritic.cells	BC147527	0.493541	1.109158	2.536752	0.012943	-3.22398	0.189943	0.169739
Dendritic.cells	ABHD10	-0.54559	3.649409	-2.53669	0.012945	-2.97353	0.180831	0.158162
Dendritic.cells	MT1	1.104083	6.314344	2.533793	0.013045	-3.22496	0.172959	0.147512
Dendritic.cells	PLXND1	-0.48871	3.5086	-2.53321	0.013065	-3.11508	0.182483	0.159589
Dendritic.cells	NABP2	0.373593	4.980112	2.532847	0.013078	-3.27054	0.177406	0.153234
Dendritic.cells	SERPINA1B	-0.83084	7.994808	-2.53141	0.013128	-3.8039	0.167976	0.141254
Dendritic.cells	GM16151	1.20301	-0.28838	2.530971	0.013143	-2.92081	0.196927	0.17796
Dendritic.cells	SEC24D	-0.25197	4.470665	-2.52987	0.013182	-3.70452	0.179878	0.156216
Dendritic.cells	MLLT10	0.183893	7.464033	2.529223	0.013204	-3.8676	0.170018	0.143766
Dendritic.cells	NEK7	0.231313	6.476805	2.52837	0.013234	-3.79232	0.17339	0.147813
Dendritic.cells	GM17529	-1.32365	0.365187	-2.52785	0.013253	-2.92738	0.195057	0.175304
Dendritic.cells	METTL4	0.501148	2.874732	2.527025	0.013282	-3.08865	0.185953	0.163658

Dendritic.cells	MRPL28	0.317089	6.016074	2.525944	0.01332	-3.51936	0.175377	0.150186
Dendritic.cells	FOPNL	0.477164	4.468642	2.524623	0.013366	-3.13482	0.180644	0.157081
Dendritic.cells	GM2682	-1.51711	2.755703	-2.52451	0.013371	-2.91541	0.186697	0.164775
Dendritic.cells	GM15848	-1.34008	-0.86297	-2.52367	0.0134	-2.96415	0.200326	0.182403
Dendritic.cells	RUNX1	0.297269	9.201324	2.522959	0.013425	-4.07523	0.165199	0.13798
Dendritic.cells	BABAM1	0.339261	5.569892	2.522628	0.013437	-3.39018	0.176886	0.152705
Dendritic.cells	BIVM	1.008575	0.882383	2.522494	0.013442	-2.91964	0.193608	0.174013
Dendritic.cells	MAN1A	-0.314	8.000333	-2.52225	0.013451	-3.99661	0.168946	0.14282
Dendritic.cells	ANTXR2	-0.41083	7.654982	-2.52214	0.013455	-4.23865	0.170045	0.14422
Dendritic.cells	CHIL1	1.405297	-0.07877	2.520434	0.013515	-2.96267	0.197635	0.179212
Dendritic.cells	RTL4	-1.64387	0.402884	-2.52038	0.013518	-2.95262	0.195784	0.176832
Dendritic.cells	ABTB1	0.80821	4.303875	2.519251	0.013558	-2.98692	0.181547	0.15885
Dendritic.cells	GM42067	1.216683	0.558955	2.519077	0.013564	-2.96493	0.195189	0.176285
Dendritic.cells	TIMM17B	0.318555	5.161183	2.518378	0.013589	-3.28117	0.178595	0.155203
Dendritic.cells	NUDT1	-1.14581	3.00659	-2.51826	0.013594	-2.92582	0.186134	0.164805
Dendritic.cells	RNF227	1.067386	1.852006	2.51818	0.013596	-2.92479	0.19034	0.170183
Dendritic.cells	CYP2C23	-1.43458	0.860959	-2.51738	0.013625	-2.96022	0.194216	0.175159
Dendritic.cells	DYSF	-1.20761	2.592245	-2.51641	0.01366	-2.99546	0.187907	0.167153
Dendritic.cells	GM4952	-1.4043	2.667628	-2.51604	0.013673	-2.92906	0.187633	0.16692
Dendritic.cells	PLEKHO2	-0.30811	6.069061	-2.51511	0.013707	-3.61179	0.175795	0.152073
Dendritic.cells	CLEC4F	-1.91688	5.458864	-2.5149	0.013715	-3.13889	0.177846	0.154745
Dendritic.cells	GRTP1	-1.28609	0.35364	-2.51465	0.013724	-2.97572	0.196261	0.178447
Dendritic.cells	ENAH	1.464566	0.744942	2.514375	0.013734	-2.95993	0.194769	0.176577
Dendritic.cells	AGAP1	-0.81645	4.225875	-2.51379	0.013755	-3.02878	0.182146	0.160445
Dendritic.cells	ATP2B4	-0.32632	4.169834	-2.50972	0.013904	-3.79302	0.184089	0.161804
Dendritic.cells	GAS6	-1.15663	1.545619	-2.509	0.01393	-2.94296	0.193789	0.174271
Dendritic.cells	LY96	-0.65274	4.287348	-2.50854	0.013947	-3.05409	0.1838	0.161557
Dendritic.cells	ITGA2B	0.989907	0.938407	2.50648	0.014023	-2.94847	0.19692	0.177942
Dendritic.cells	TTL	-1.06419	1.740811	-2.50575	0.01405	-2.95977	0.194011	0.174145
Dendritic.cells	2700062CC	0.733745	2.646093	2.504395	0.0141	-2.96904	0.191087	0.170128
Dendritic.cells	TARSL2	1.042129	1.415252	2.503817	0.014122	-2.96331	0.195762	0.176226
Dendritic.cells	GM48960	1.063018	1.466163	2.502738	0.014162	-2.95606	0.195889	0.17636
Dendritic.cells	URI1	0.280161	5.89939	2.501345	0.014214	-3.66894	0.180327	0.156188
Dendritic.cells	SGF29	0.422006	4.671195	2.500865	0.014232	-3.20795	0.18461	0.161728
Dendritic.cells	MTM1	0.518166	4.433358	2.500004	0.014264	-3.31437	0.18565	0.162923
Dendritic.cells	TALDO1	0.216904	8.310001	2.499146	0.014297	-4.02234	0.172618	0.146359
Dendritic.cells	ARL15	-0.29996	7.591924	-2.49884	0.014308	-3.83005	0.174958	0.149357
Dendritic.cells	ZFP65	1.148583	1.662117	2.496397	0.0144	-2.96873	0.197001	0.176708
Dendritic.cells	PEAK1	-0.43333	6.584571	-2.49583	0.014422	-3.60406	0.179305	0.154241
Dendritic.cells	SHC1	-0.44795	4.384809	-2.49466	0.014466	-3.19332	0.187324	0.164202
Dendritic.cells	SFXN5	1.090348	3.363367	2.493302	0.014518	-2.97382	0.191492	0.169299
Dendritic.cells	ZCCHC18	0.819494	1.04036	2.492007	0.014567	-3.0212	0.200747	0.180808
Dendritic.cells	LRRC42	0.680503	3.272197	2.491188	0.014599	-3.01595	0.192437	0.17005
Dendritic.cells	SCD2	0.574426	4.726396	2.490269	0.014634	-3.29996	0.187349	0.163514
Dendritic.cells	RXRA	-0.87031	3.1994	-2.48985	0.01465	-2.99136	0.192925	0.170665
Dendritic.cells	BLOC1S2	0.271317	4.833886	2.486909	0.014764	-3.68824	0.188189	0.163605
Dendritic.cells	PBX3	-0.5573	5.496226	-2.48593	0.014802	-3.26292	0.186085	0.160815

Dendritic.cells	ZFP251	0.625768	2.407099	2.484254	0.014867	-3.11811	0.197981	0.175655
Dendritic.cells	BCAS2	-0.22774	6.380106	-2.48276	0.014925	-3.68224	0.183472	0.157595
Dendritic.cells	CFP	-0.57327	5.518079	-2.4823	0.014943	-3.46293	0.1865	0.161493
Dendritic.cells	MED17	0.288879	4.867425	2.482296	0.014943	-3.41617	0.188829	0.164445
Dendritic.cells	ZFPL1	0.560196	3.466605	2.482244	0.014945	-3.08411	0.193973	0.170984
Dendritic.cells	B4GALT7	0.658483	2.869796	2.481717	0.014966	-3.0571	0.196218	0.173935
Dendritic.cells	CTSE	-0.24799	5.492809	-2.48132	0.014982	-3.98243	0.18659	0.161749
Dendritic.cells	TMEM179E	0.261753	5.864754	2.481312	0.014982	-3.59471	0.185274	0.160082
Dendritic.cells	ENPEP	-1.39521	0.401804	-2.47791	0.015116	-3.02846	0.207315	0.18737
Dendritic.cells	EBAG9	0.480498	4.117531	2.477707	0.015124	-3.20585	0.192924	0.169024
Dendritic.cells	FRMD8	-0.58086	4.222806	-2.477	0.015152	-3.15273	0.192663	0.168774
Dendritic.cells	TCF4	0.166461	7.694523	2.475071	0.015228	-4.58505	0.181073	0.153659
Dendritic.cells	TMSB4X	0.273879	12.24836	2.472793	0.015319	-4.57019	0.167244	0.135838
Dendritic.cells	GM10642	1.405407	0.270003	2.472244	0.015341	-3.04563	0.209854	0.18969
Dendritic.cells	S100A6	0.711893	7.580063	2.469488	0.015452	-3.92004	0.18302	0.155389
Dendritic.cells	NR2F2	-0.67147	3.13223	-2.46935	0.015458	-3.30232	0.199207	0.175912
Dendritic.cells	GM43813	0.718663	4.535806	2.469169	0.015465	-3.14253	0.193906	0.169203
Dendritic.cells	MAP4K4	0.288889	7.459224	2.469123	0.015467	-4.04504	0.183437	0.155977
Dendritic.cells	PIGYL	0.53769	4.270837	2.468033	0.015511	-3.19739	0.195121	0.170588
Dendritic.cells	HDDC2	0.545745	3.817561	2.467785	0.015521	-3.16465	0.196826	0.172811
Dendritic.cells	TOP3B	0.52742	3.549805	2.466653	0.015567	-3.12829	0.198198	0.174407
Dendritic.cells	TEX45	-1.50287	0.222756	-2.46614	0.015588	-3.06801	0.211448	0.191457
Dendritic.cells	GM42982	0.848118	1.514483	2.465603	0.015609	-3.03378	0.206243	0.184897
Dendritic.cells	LMAN2	0.237686	6.112382	2.464808	0.015642	-3.72343	0.188871	0.162926
Dendritic.cells	PEX12	-1.01473	1.86574	-2.46417	0.015668	-3.03024	0.204914	0.183475
Dendritic.cells	GM2449	-1.33342	1.279792	-2.46414	0.015669	-3.03877	0.20726	0.186488
Dendritic.cells	TFEC	-1.25224	2.368464	-2.46276	0.015726	-3.0333	0.203427	0.181125
Dendritic.cells	D130043K2	-1.32183	1.054045	-2.46219	0.015749	-3.05608	0.208753	0.188107
Dendritic.cells	FGR	0.439848	4.732993	2.461651	0.015771	-3.85717	0.19449	0.169908
Dendritic.cells	MRPS27	-0.73006	3.208477	-2.46051	0.015818	-3.08598	0.200608	0.177427
Dendritic.cells	NRARP	-1.31809	1.780032	-2.46012	0.015834	-3.04805	0.206229	0.184719
Dendritic.cells	SLC45A3	-1.52733	1.111286	-2.45923	0.015871	-3.07236	0.209173	0.188443
Dendritic.cells	VAV3	0.417412	7.363151	2.458405	0.015905	-3.83802	0.185738	0.158455
Dendritic.cells	NDUFA13	0.225598	7.128419	2.453037	0.016128	-3.91108	0.188404	0.16059
Dendritic.cells	TCF20	0.257704	7.561094	2.452269	0.016161	-4.02843	0.186875	0.15884
Dendritic.cells	HIVEP1	0.267876	6.40877	2.452238	0.016162	-3.93706	0.190983	0.164018
Dendritic.cells	TMEM256	0.307904	6.982152	2.452065	0.016169	-3.84469	0.188924	0.161452
Dendritic.cells	GM12064	0.702143	0.96957	2.451845	0.016178	-3.14677	0.212034	0.190892
Dendritic.cells	RAB10OS	-0.41391	4.429445	-2.45168	0.016185	-3.39923	0.198322	0.17342
Dendritic.cells	ECSCR	-0.96552	1.207762	-2.45156	0.01619	-3.08332	0.211054	0.189701
Dendritic.cells	BIK	-1.21533	1.76969	-2.44999	0.016257	-3.06081	0.209257	0.187089
Dendritic.cells	DCUN1D3	-0.55618	4.424811	-2.44978	0.016265	-3.24381	0.19881	0.173794
Dendritic.cells	PPM1E	0.373038	4.964118	2.448134	0.016335	-3.90693	0.19739	0.1716
Dendritic.cells	MT2	1.631333	2.001416	2.446674	0.016397	-3.06792	0.209313	0.186613
Dendritic.cells	L3MBTL3	0.260603	5.067059	2.446405	0.016409	-3.92824	0.197322	0.17142
Dendritic.cells	MRPL18	0.331631	6.633228	2.445916	0.016429	-3.78552	0.191535	0.164186
Dendritic.cells	ECH1	0.292174	5.784916	2.445773	0.016435	-3.82556	0.194641	0.168142

Dendritic.cells	L3MBTL1	-1.32425	0.787342	-2.44458	0.016486	-3.08425	0.214735	0.193624
Dendritic.cells	DYRK1A	-0.23894	7.419436	-2.444	0.016511	-4.07989	0.18916	0.161096
Dendritic.cells	ARAF	0.323012	4.478585	2.443008	0.016554	-3.51957	0.200203	0.175033
Dendritic.cells	THEM6	0.386647	3.448768	2.44279	0.016563	-3.56881	0.204202	0.180122
Dendritic.cells	ESAM	-0.75042	2.065322	-2.44187	0.016603	-3.16844	0.210001	0.18732
Dendritic.cells	FZD4	-0.92774	1.267832	-2.44142	0.016622	-3.08635	0.213286	0.191651
Dendritic.cells	LIMS1	0.199808	7.288787	2.440988	0.016641	-4.19516	0.190045	0.16215
Dendritic.cells	RAD51B	0.466021	6.152694	2.439631	0.016699	-3.85798	0.194638	0.167765
Dendritic.cells	PLBD2	0.379493	4.310294	2.438758	0.016737	-3.46615	0.201829	0.17672
Dendritic.cells	LIFR	0.322241	3.842745	2.436585	0.016831	-3.97044	0.204056	0.179651
Dendritic.cells	POLI	0.898737	1.527716	2.436578	0.016832	-3.0877	0.213393	0.191599
Dendritic.cells	FXYD1	-1.12827	2.943369	-2.43631	0.016843	-3.10817	0.207622	0.184213
Dendritic.cells	CHD9	0.283982	6.065045	2.436269	0.016845	-3.92277	0.195576	0.168882
Dendritic.cells	EPAS1	-0.73802	3.448567	-2.43354	0.016964	-3.23091	0.20671	0.182327
Dendritic.cells	GPR157	-0.91657	2.137362	-2.43335	0.016973	-3.09056	0.212012	0.18915
Dendritic.cells	S100A11	0.339902	7.270251	2.43215	0.017025	-3.9232	0.192329	0.164233
Dendritic.cells	DCAF12	0.288572	6.638146	2.431859	0.017038	-3.86749	0.194638	0.167221
Dendritic.cells	SPA17	0.950815	0.979282	2.431792	0.017041	-3.09253	0.216991	0.195746
Dendritic.cells	ME2	0.277216	5.963937	2.431284	0.017063	-3.93555	0.197183	0.17054
Dendritic.cells	SGCZ	-1.30156	1.627249	-2.43039	0.017103	-3.09858	0.214497	0.192741
Dendritic.cells	FAM32A	0.36701	5.312501	2.430112	0.017115	-3.4114	0.199807	0.17397
Dendritic.cells	POLR1B	-0.9326	2.442843	-2.42737	0.017237	-3.10413	0.212398	0.189242
Dendritic.cells	CCNC	-0.5602	4.398502	-2.42561	0.017315	-3.27208	0.205035	0.179555
Dendritic.cells	RELN	-1.44501	3.647841	-2.4256	0.017316	-3.10752	0.208011	0.183346
Dendritic.cells	CLEC2D	0.665624	4.7558	2.424764	0.017353	-3.49184	0.203771	0.177969
Dendritic.cells	ARHGAP31	-0.51418	6.152696	-2.42408	0.017383	-3.60252	0.198421	0.171337
Dendritic.cells	GM21188	1.450629	1.578983	2.423897	0.017392	-3.10918	0.216642	0.194615
Dendritic.cells	NFX1	0.330697	4.900546	2.423651	0.017403	-3.54579	0.203208	0.177485
Dendritic.cells	ENKUR	-1.12153	0.339833	-2.42298	0.017433	-3.10883	0.222076	0.20163
Dendritic.cells	PLD3	-0.71628	4.413779	-2.42032	0.017553	-3.24143	0.206086	0.180676
Dendritic.cells	UTP11	-0.38702	4.845019	-2.42001	0.017567	-3.45668	0.204392	0.178589
Dendritic.cells	SLC9A7	0.335456	5.115655	2.419754	0.017578	-3.76602	0.203338	0.177301
Dendritic.cells	LGALS3	0.551882	6.142988	2.419295	0.017599	-3.72598	0.199402	0.172301
Dendritic.cells	GM26885	1.2876	2.317843	2.419278	0.0176	-3.11782	0.214576	0.191657
Dendritic.cells	NIN	0.410381	5.920519	2.418528	0.017634	-3.5872	0.200308	0.173506
Dendritic.cells	AUH	-0.28913	5.641378	-2.41832	0.017644	-3.80181	0.201374	0.174883
Dendritic.cells	SLC50A1	0.337257	5.247798	2.416624	0.017721	-3.6757	0.203555	0.177249
Dendritic.cells	PTGS2	2.235584	2.215449	2.415813	0.017758	-3.12908	0.215963	0.192939
Dendritic.cells	PABPC1L	-0.94187	2.906471	-2.41538	0.017777	-3.14571	0.213096	0.189343
Dendritic.cells	EPB41L4B	-1.16804	1.976143	-2.41477	0.017805	-3.12429	0.216967	0.194474
Dendritic.cells	CES1D	-1.45119	1.90006	-2.41461	0.017813	-3.12916	0.217287	0.194898
Dendritic.cells	SMPD4	0.705916	3.209019	2.414033	0.017839	-3.20405	0.211882	0.188073
Dendritic.cells	ZFP62	0.397374	4.580193	2.413727	0.017853	-3.4798	0.206374	0.181152
Dendritic.cells	EXOC6	0.267672	6.194009	2.411906	0.017937	-3.89015	0.200597	0.173657
Dendritic.cells	VMP1	-0.35603	7.164309	-2.41125	0.017967	-3.95318	0.19695	0.169041
Dendritic.cells	DENND2C	-0.9065	2.008648	-2.4112	0.017969	-3.13338	0.21737	0.195124
Dendritic.cells	NAT10	-0.7139	3.270157	-2.41114	0.017972	-3.18747	0.212133	0.188396

Dendritic.cells	CEBPA	-0.88341	2.906324	-2.4094	0.018053	-3.2092	0.214264	0.190788
Dendritic.cells	GM13427	-0.91729	2.062778	-2.40915	0.018064	-3.13777	0.217789	0.195392
Dendritic.cells	ABHD15	0.344843	2.977881	2.40678	0.018174	-3.81485	0.214876	0.191198
Dendritic.cells	PASK	1.52199	1.295986	2.406418	0.018191	-3.15463	0.221994	0.200444
Dendritic.cells	4833418N(	1.044335	1.533706	2.406162	0.018203	-3.14352	0.22097	0.199162
Dendritic.cells	ZBTB44	0.356932	5.853417	2.405847	0.018218	-3.66673	0.20336	0.176726
Dendritic.cells	CTDSPL2	-0.30047	5.892856	-2.40516	0.01825	-3.77674	0.203348	0.176699
Dendritic.cells	RBM26	0.1803	6.821937	2.403228	0.018341	-4.14419	0.200267	0.172598
Dendritic.cells	AKR1C13	-1.31682	1.84784	-2.40309	0.018347	-3.16842	0.220292	0.19825
Dendritic.cells	NAT2	0.788451	2.532818	2.402892	0.018356	-3.18056	0.217389	0.194563
Dendritic.cells	FCNA	-1.76216	4.555419	-2.4026	0.01837	-3.24475	0.209089	0.183987
Dendritic.cells	CAR2	1.102154	5.171918	2.402185	0.01839	-3.28416	0.206644	0.181008
Dendritic.cells	EED	0.245541	6.114274	2.401724	0.018411	-3.73114	0.202998	0.176467
Dendritic.cells	DAB2	-0.97516	4.009728	-2.40074	0.018458	-3.44973	0.211632	0.187332
Dendritic.cells	SNTA1	-1.15905	1.865421	-2.39982	0.018502	-3.16081	0.220731	0.199127
Dendritic.cells	NDUFB7	0.307333	6.480862	2.399639	0.01851	-3.80118	0.202033	0.175142
Dendritic.cells	SCAMP2	0.21103	6.357233	2.398552	0.018562	-4.00769	0.202757	0.176083
Dendritic.cells	UBTD2	-0.71901	3.37685	-2.39834	0.018572	-3.23871	0.214639	0.191342
Dendritic.cells	PDCD10	0.198014	6.713222	2.397792	0.018598	-4.01786	0.201466	0.174419
Dendritic.cells	CD209F	-2.95859	2.767776	-2.3973	0.018621	-3.15686	0.2173	0.194731
Dendritic.cells	LRRC49	-1.1851	1.020554	-2.39685	0.018642	-3.16707	0.224811	0.20446
Dendritic.cells	CAAA0114:	0.922652	2.131904	2.395792	0.018693	-3.16004	0.220302	0.198367
Dendritic.cells	NECTIN2	-0.75717	2.109704	-2.39552	0.018706	-3.20044	0.220396	0.198589
Dendritic.cells	SURF6	-0.55724	3.185009	-2.39506	0.018728	-3.23145	0.215891	0.192823
Dendritic.cells	LY6C2	0.476356	5.53324	2.393636	0.018796	-4.29647	0.206929	0.180991
Dendritic.cells	ACOX3	0.436483	4.372027	2.39129	0.018909	-3.47405	0.212347	0.187536
Dendritic.cells	TANC2	-0.41559	5.521032	-2.39122	0.018913	-3.83628	0.207737	0.181632
Dendritic.cells	NFIX	-0.78649	3.676603	-2.39098	0.018924	-3.24247	0.215201	0.191201
Dendritic.cells	NDUFS5	0.282021	6.478554	2.389905	0.018976	-3.89202	0.204346	0.177021
Dendritic.cells	ZFP46	-0.72266	1.77659	-2.38936	0.019003	-3.17371	0.223713	0.20187
Dendritic.cells	IGF1R	0.262647	6.357541	2.388694	0.019035	-4.22553	0.204949	0.177843
Dendritic.cells	DENND2A	-1.08905	1.374468	-2.38843	0.019048	-3.17533	0.225535	0.204336
Dendritic.cells	ERI2	-1.22841	1.310882	-2.38779	0.019079	-3.18667	0.22595	0.204894
Dendritic.cells	UACA	-0.83363	2.51718	-2.38342	0.019293	-3.25811	0.221929	0.199495
Dendritic.cells	SLC24A1	-0.98978	1.860788	-2.38299	0.019314	-3.18483	0.224769	0.203254
Dendritic.cells	TRF	-0.67549	9.658999	-2.38271	0.019328	-4.38238	0.193817	0.163667
Dendritic.cells	LHFPL2	-0.80631	2.227897	-2.38245	0.019341	-3.24818	0.223175	0.201282
Dendritic.cells	STARD13	-1.30712	1.070842	-2.38208	0.019359	-3.18565	0.228246	0.207994
Dendritic.cells	LIN37	0.552039	3.646925	2.381942	0.019366	-3.34989	0.217145	0.193615
Dendritic.cells	ANKLE2	0.292068	5.177792	2.381923	0.019367	-3.82688	0.210869	0.185538
Dendritic.cells	ANGPTL4	1.278927	1.23193	2.381888	0.019368	-3.19961	0.227532	0.207065
Dendritic.cells	FAM189A1	-1.65522	2.327494	-2.38164	0.019381	-3.19698	0.222745	0.200888
Dendritic.cells	ANKRD27	0.511281	3.098779	2.381634	0.019381	-3.37444	0.21945	0.196624
Dendritic.cells	MICU1	-0.32255	5.891562	-2.38105	0.01941	-3.67137	0.208121	0.18203
Dendritic.cells	FGA	-0.70293	5.727993	-2.38031	0.019446	-3.63996	0.20895	0.183081
Dendritic.cells	AGO3	-0.26588	5.940327	-2.37831	0.019545	-3.95505	0.208401	0.182618
Dendritic.cells	MRPS10	0.477735	4.162492	2.378163	0.019553	-3.40124	0.215596	0.191936

Dendritic.cells	RGN	-1.14678	4.650255	-2.37781	0.019571	-3.37328	0.21359	0.189435
Dendritic.cells	SPG21	0.330057	6.057126	2.377697	0.019576	-3.68297	0.20794	0.182159
Dendritic.cells	PLEKHM3	0.211541	6.068485	2.377568	0.019583	-4.4069	0.207895	0.182101
Dendritic.cells	SLC36A4	0.69289	3.21113	2.377003	0.019611	-3.33506	0.219577	0.197401
Dendritic.cells	PLXNC1	-0.2936	5.427157	-2.37681	0.01962	-4.16664	0.210445	0.185641
Dendritic.cells	IFT57	0.433956	2.891644	2.37633	0.019644	-3.53182	0.220934	0.199348
Dendritic.cells	HDGFL3	-1.17439	1.511028	-2.37575	0.019673	-3.19667	0.226923	0.207314
Dendritic.cells	WDR11	0.605581	3.124121	2.375607	0.01968	-3.29032	0.219945	0.198228
Dendritic.cells	BCL2L11	-0.30986	7.053365	-2.37516	0.019703	-4.31824	0.204056	0.177734
Dendritic.cells	ARCN1	-0.24763	5.873425	-2.37431	0.019745	-3.94344	0.208666	0.183898
Dendritic.cells	TOMM7	0.192367	7.561253	2.374253	0.019748	-4.20726	0.202113	0.175463
Dendritic.cells	FAF2	0.33045	5.155771	2.37419	0.019751	-3.67912	0.211537	0.187624
Dendritic.cells	CRCP	0.442499	4.449755	2.372915	0.019816	-3.39018	0.214711	0.19166
Dendritic.cells	SSU72	0.223657	6.693565	2.372843	0.019819	-3.94866	0.205735	0.180054
Dendritic.cells	GM49164	1.345593	0.920748	2.372371	0.019843	-3.22754	0.229864	0.211577
Dendritic.cells	FYB	0.301192	6.869674	2.372063	0.019858	-4.44948	0.205051	0.179408
Dendritic.cells	FER	-0.7494	3.489418	-2.37156	0.019884	-3.29971	0.218768	0.197191
Dendritic.cells	TTLL3	-0.62645	4.027206	-2.37062	0.019932	-3.34315	0.216821	0.19446
Dendritic.cells	ZKSCAN3	0.259585	4.82207	2.369628	0.019982	-3.77576	0.213868	0.190415
Dendritic.cells	FBXL8	0.961149	1.99347	2.368689	0.020029	-3.21385	0.226081	0.206283
Dendritic.cells	CDC42SE2	-0.17159	7.179375	-2.36842	0.020043	-4.35898	0.204725	0.178619
Dendritic.cells	GM44752	0.550368	2.484975	2.3671	0.02011	-3.37736	0.22433	0.203994
Dendritic.cells	FBXO34	-0.32387	5.78838	-2.36695	0.020118	-3.7667	0.210555	0.186075
Dendritic.cells	MSRA	0.38622	6.629496	2.365076	0.020214	-3.6787	0.207934	0.182078
Dendritic.cells	RARRES2	-1.22204	3.7462	-2.36482	0.020227	-3.26983	0.219691	0.197328
Dendritic.cells	TMEM273	-1.23625	1.19098	-2.36419	0.020259	-3.23336	0.230965	0.212036
Dendritic.cells	SENP1	0.306701	5.013417	2.363793	0.02028	-3.68473	0.214553	0.190739
Dendritic.cells	EGLN1	0.283197	5.631732	2.362607	0.020341	-3.91197	0.21245	0.187832
Dendritic.cells	ADGRL3	-1.85884	3.47827	-2.36208	0.020368	-3.2387	0.221391	0.199549
Dendritic.cells	RFC3	0.58817	4.31335	2.361103	0.020418	-3.34358	0.217867	0.195228
Dendritic.cells	CD300C	0.275806	0.72621	2.360975	0.020425	-4.02065	0.233518	0.215709
Dendritic.cells	GM28375	0.535247	3.206287	2.360878	0.02043	-3.33315	0.222554	0.201337
Dendritic.cells	JTB	0.195754	5.842584	2.360693	0.020439	-4.02848	0.2116	0.187121
Dendritic.cells	IFI27L2A	0.670474	7.373467	2.359953	0.020478	-4.24792	0.205693	0.179453
Dendritic.cells	SOS1	0.233738	5.916592	2.359144	0.02052	-3.98736	0.211439	0.187079
Dendritic.cells	SAMHD1	0.493778	7.45997	2.359052	0.020525	-4.09502	0.205358	0.179233
Dendritic.cells	PHLDA1	-1.29175	4.035457	-2.35884	0.020535	-3.23423	0.219174	0.197179
Dendritic.cells	CREBZF	0.331285	4.715073	2.3585	0.020553	-3.67974	0.216338	0.193526
Dendritic.cells	CFAP77	-1.24616	0.931954	-2.35723	0.020619	-3.23887	0.233016	0.215333
Dendritic.cells	RER1	0.259805	6.413933	2.35659	0.020653	-3.97826	0.209707	0.185033
Dendritic.cells	ATG4C	0.689069	3.11799	2.356543	0.020655	-3.29932	0.223347	0.202812
Dendritic.cells	FUCA2	0.33508	4.624115	2.356477	0.020659	-3.69464	0.216977	0.194495
Dendritic.cells	EYA1	0.513903	2.426875	2.355567	0.020706	-3.65349	0.226651	0.207049
Dendritic.cells	SNTB1	-0.47046	4.264888	-2.35482	0.020745	-3.95974	0.218966	0.196889
Dendritic.cells	CYHR1	0.412406	4.48568	2.354306	0.020772	-3.5575	0.218112	0.195814
Dendritic.cells	PCNX4	0.713348	2.141019	2.353761	0.020801	-3.29556	0.228284	0.209044
Dendritic.cells	2610037D(	0.343323	5.44268	2.352682	0.020858	-3.78843	0.214625	0.190928

Dendritic.cells	GFRA2	-1.28589	2.545769	-2.35199	0.020894	-3.24475	0.227078	0.207157
Dendritic.cells	TMEM160	0.264485	5.666014	2.351418	0.020924	-3.91758	0.213983	0.190118
Dendritic.cells	BLK	0.482165	4.074052	2.350525	0.020972	-3.62709	0.220877	0.198878
Dendritic.cells	DPYSL2	0.306235	6.279623	2.350002	0.020999	-3.97662	0.211851	0.187159
Dendritic.cells	FAM241A	0.20404	5.59878	2.349504	0.021026	-4.37881	0.214671	0.190877
Dendritic.cells	PSMA7	0.237271	7.683799	2.348961	0.021055	-4.25356	0.206454	0.180202
Dendritic.cells	TRIM30A	0.395515	6.169574	2.348144	0.021098	-4.20511	0.212675	0.188064
Dendritic.cells	NR4A3	-0.39446	6.012984	-2.3469	0.021165	-4.19895	0.213765	0.189063
Dendritic.cells	INF2	0.388819	3.252642	2.346486	0.021187	-3.77964	0.225359	0.204273
Dendritic.cells	ASCC1	0.425277	3.774861	2.346151	0.021204	-3.51192	0.223105	0.201409
Dendritic.cells	FHL3	-1.05668	2.081558	-2.34496	0.021268	-3.25858	0.230987	0.211502
Dendritic.cells	RAB8A	0.36935	5.454242	2.343586	0.021342	-3.63896	0.216729	0.19277
Dendritic.cells	SULT1D1	-1.21603	2.290136	-2.34337	0.021353	-3.26011	0.230311	0.210528
Dendritic.cells	GALE	0.630798	2.330073	2.342836	0.021382	-3.33869	0.230133	0.210452
Dendritic.cells	B3GNTL1	0.747139	2.673331	2.342581	0.021396	-3.31827	0.22861	0.208537
Dendritic.cells	PPTC7	0.316908	5.148669	2.34254	0.021398	-3.87296	0.217995	0.194654
Dendritic.cells	PLEKHA8	-1.08779	0.886546	-2.34224	0.021414	-3.26804	0.236677	0.219157
Dendritic.cells	RBFOX2	-0.79943	2.758111	-2.34158	0.02145	-3.41216	0.2284	0.20824
Dendritic.cells	MDK	-1.30867	1.436872	-2.34062	0.021502	-3.27091	0.234573	0.216321
Dendritic.cells	CAR3	-1.77199	3.476221	-2.34022	0.021524	-3.31688	0.225498	0.204558
Dendritic.cells	KANSL1L	-0.39532	7.202302	-2.33977	0.021548	-4.23847	0.210041	0.1845
Dendritic.cells	CDK10	0.759324	2.234353	2.339646	0.021555	-3.28956	0.230971	0.211874
Dendritic.cells	TIFAB	0.535358	2.73524	2.33827	0.021629	-3.66579	0.228935	0.209311
Dendritic.cells	RBM10	-0.36785	4.779494	-2.33705	0.021696	-3.64004	0.220113	0.198131
Dendritic.cells	ZFP397	0.362183	3.944609	2.336997	0.021699	-3.61066	0.223663	0.20279
Dendritic.cells	TFIP11	-0.64582	3.322157	-2.3368	0.021709	-3.34521	0.226357	0.206333
Dendritic.cells	D17H6S53I	-0.40057	4.009289	-2.33674	0.021713	-3.56685	0.223385	0.202425
Dendritic.cells	CASP6	0.536803	3.628567	2.336732	0.021713	-3.44757	0.225026	0.204581
Dendritic.cells	LAX1	-1.08497	2.319766	-2.33668	0.021716	-3.27371	0.230783	0.212167
Dendritic.cells	SCYL1	0.517783	4.072604	2.33575	0.021767	-3.49897	0.223425	0.202338
Dendritic.cells	CDC5L	0.250203	5.768771	2.334337	0.021844	-3.816	0.216866	0.193362
Dendritic.cells	GRCC10	0.214987	6.427807	2.333568	0.021887	-4.11159	0.21436	0.190113
Dendritic.cells	ZFP606	0.740865	2.487995	2.333238	0.021905	-3.29257	0.231158	0.212255
Dendritic.cells	C1QC	-0.988	6.384084	-2.33226	0.021959	-3.76122	0.214862	0.190707
Dendritic.cells	EGR1	-0.69148	6.545199	-2.33169	0.02199	-3.93233	0.214313	0.18996
Dendritic.cells	PFN1	0.201186	10.31807	2.33109	0.022023	-4.65794	0.199718	0.171068
Dendritic.cells	ERG28	0.245461	5.118614	2.330895	0.022034	-3.91797	0.220203	0.1977
Dendritic.cells	ANAPC13	0.292406	5.65737	2.330572	0.022052	-3.80634	0.217954	0.194819
Dendritic.cells	NUP155	-0.36843	5.174039	-2.32998	0.022085	-3.66998	0.219989	0.197619
Dendritic.cells	FKBP11	-0.79208	1.995525	-2.32979	0.022095	-3.29305	0.233862	0.215929
Dendritic.cells	GM26982	-0.69102	1.652146	-2.32801	0.022194	-3.31417	0.236212	0.218515
Dendritic.cells	ASB3	0.388838	4.711015	2.327371	0.02223	-3.62044	0.222685	0.200875
Dendritic.cells	ABT1	-0.54554	3.534654	-2.32735	0.022231	-3.40332	0.227769	0.207562
Dendritic.cells	4930455G(	-1.32972	1.816178	-2.32685	0.022259	-3.29007	0.235535	0.217901
Dendritic.cells	SNX8	-0.91569	5.468943	-2.3257	0.022323	-3.42793	0.219838	0.197034
Dendritic.cells	ACSL4	0.254193	6.189637	2.325387	0.022341	-4.14625	0.216847	0.193211
Dendritic.cells	HEXIM1	-0.40416	5.48831	-2.32523	0.022349	-3.82476	0.219757	0.197044

Dendritic.cells	PSME2B	0.814895	2.815452	2.324229	0.022406	-3.34864	0.231395	0.212527
Dendritic.cells	ATP6V1F	0.216881	6.902317	2.323882	0.022425	-4.11366	0.214012	0.18973
Dendritic.cells	RAB1A	-0.19735	7.08553	-2.32356	0.022443	-4.18758	0.213274	0.188767
Dendritic.cells	MS4A4B	-1.13191	3.207847	-2.32319	0.022464	-3.30995	0.229649	0.210294
Dendritic.cells	SLC7A11	1.48401	4.278698	2.322263	0.022516	-3.4103	0.224969	0.204472
Dendritic.cells	GM34086	-1.00865	2.658244	-2.32225	0.022517	-3.29732	0.232099	0.213903
Dendritic.cells	NHP2	0.355446	5.748278	2.322173	0.022521	-3.92045	0.218744	0.196275
Dendritic.cells	DUS4L	0.858698	1.929292	2.322146	0.022523	-3.30487	0.235398	0.218283
Dendritic.cells	GM10552	0.810073	1.54086	2.321564	0.022556	-3.35843	0.237288	0.220803
Dendritic.cells	KCTD13	0.583581	2.619439	2.321208	0.022576	-3.41446	0.232379	0.214363
Dendritic.cells	VPREB3	-1.02483	6.013944	-2.3208	0.022599	-3.5616	0.217742	0.195076
Dendritic.cells	AGBL1	-0.44454	4.663698	-2.32013	0.022637	-4.16897	0.223419	0.202704
Dendritic.cells	PRPS1L3	-0.86792	2.045409	-2.32006	0.022641	-3.3058	0.234976	0.218023
Dendritic.cells	GSTM1	-1.08726	4.059268	-2.31976	0.022658	-3.32818	0.226022	0.206267
Dendritic.cells	SCN1B	-1.14055	1.834445	-2.31836	0.022737	-3.30161	0.236402	0.219896
Dendritic.cells	XCL1	-1.58	2.335401	-2.31758	0.022781	-3.30681	0.234118	0.217085
Dendritic.cells	WBP1L	0.348081	5.329247	2.317452	0.022789	-3.83612	0.22103	0.199731
Dendritic.cells	DNAJB13	1.076022	2.103334	2.317216	0.022802	-3.30377	0.235173	0.21849
Dendritic.cells	CCR9	-0.20069	2.410766	-2.31715	0.022806	-4.50801	0.233777	0.21663
Dendritic.cells	DOCK6	-0.83981	1.914885	-2.31653	0.022841	-3.33897	0.236161	0.219862
Dendritic.cells	CNOT10	-0.30957	5.238486	-2.31621	0.02286	-3.82411	0.221532	0.200498
Dendritic.cells	AMN1	0.576746	3.42505	2.314803	0.02294	-3.43321	0.229965	0.211255
Dendritic.cells	CRYBG3	0.369986	4.474287	2.313612	0.023009	-3.83225	0.225843	0.205545
Dendritic.cells	NCF1	0.248027	5.301832	2.312811	0.023055	-4.33554	0.222543	0.201057
Dendritic.cells	SH2D4B	-0.81267	4.439997	-2.31122	0.023146	-3.49721	0.226607	0.206333
Dendritic.cells	9430038I0:	0.411105	4.248537	2.311163	0.02315	-3.54464	0.22744	0.207443
Dendritic.cells	HTR7	1.715632	0.754108	2.31107	0.023155	-3.31373	0.243343	0.228644
Dendritic.cells	CEP162	-0.57793	3.080056	-2.31007	0.023213	-3.4222	0.23295	0.214592
Dendritic.cells	UBIAD1	-0.74918	1.941616	-2.30976	0.023231	-3.32175	0.238136	0.221581
Dendritic.cells	PHETA1	1.268566	0.450779	2.30907	0.023271	-3.3257	0.245345	0.231135
Dendritic.cells	KIF18B	-1.16757	2.84215	-2.30756	0.023359	-3.32252	0.234831	0.216576
Dendritic.cells	IL17RA	0.390181	5.907729	2.307003	0.023391	-3.91934	0.221434	0.199076
Dendritic.cells	TNIK	-0.88141	4.035038	-2.30693	0.023395	-3.43048	0.229498	0.209747
Dendritic.cells	MAGI1	-0.69937	4.373842	-2.30556	0.023476	-3.81999	0.228453	0.208061
Dendritic.cells	WDR6	0.656015	3.277321	2.305433	0.023483	-3.42688	0.233318	0.214509
Dendritic.cells	ANKRD17	-0.18475	7.985576	-2.30395	0.02357	-4.48695	0.213929	0.188512
Dendritic.cells	ZFP948	-0.93436	2.965976	-2.301	0.023743	-3.3747	0.236901	0.217596
Dendritic.cells	ATXN2L	0.302714	5.47603	2.298383	0.023899	-3.86526	0.22704	0.203597
Dendritic.cells	CBL	0.199262	7.22396	2.297372	0.023959	-4.40567	0.219999	0.194173
Dendritic.cells	RELL1	0.160148	6.866963	2.297014	0.02398	-4.66972	0.221486	0.196194
Dendritic.cells	GPR182	-0.5562	2.557152	-2.29536	0.024079	-3.63921	0.24129	0.221665
Dendritic.cells	NSD3	-0.18795	8.849458	-2.29344	0.024194	-4.63324	0.214687	0.186486
Dendritic.cells	SRP19	0.257351	6.048783	2.293171	0.02421	-4.08694	0.226304	0.201682
Dendritic.cells	ADAT2	1.170428	1.285306	2.292808	0.024232	-3.34904	0.24801	0.230306
Dendritic.cells	HSPA12B	1.203695	0.480164	2.292473	0.024252	-3.35748	0.251931	0.235639
Dendritic.cells	RSPH9	-1.2762	1.514419	-2.29243	0.024255	-3.34901	0.246908	0.228965
Dendritic.cells	DHRS3	-0.77015	4.170119	-2.29197	0.024283	-3.48034	0.234628	0.212821

Dendritic.cells	FRG1	0.270831	6.660706	2.291233	0.024327	-3.99184	0.223964	0.198818
Dendritic.cells	VTN	-0.98241	4.267725	-2.29074	0.024357	-3.5239	0.234487	0.212624
Dendritic.cells	GRASP	-0.23779	3.781204	-2.28921	0.024449	-4.3339	0.237377	0.215944
Dendritic.cells	CKAP2L	-0.81191	4.357364	-2.28766	0.024543	-3.45365	0.2348	0.212956
Dendritic.cells	EXOSC5	0.297512	5.376885	2.287596	0.024547	-3.9587	0.23027	0.206997
Dendritic.cells	CLTB	-0.3335	4.396264	-2.28758	0.024548	-3.90026	0.234625	0.212726
Dendritic.cells	HARS2	0.565777	2.848743	2.287207	0.024571	-3.42222	0.241715	0.222094
Dendritic.cells	IPP	0.707374	2.465742	2.286935	0.024587	-3.40645	0.243512	0.224601
Dendritic.cells	DIP2A	-0.96885	2.079809	-2.28673	0.0246	-3.35619	0.245338	0.227072
Dendritic.cells	PILRA	-0.88007	3.3927	-2.28665	0.024605	-3.4344	0.239193	0.218943
Dendritic.cells	NFU1	0.49843	4.549473	2.285314	0.024686	-3.62402	0.234509	0.212228
Dendritic.cells	SRGN	0.245827	9.652655	2.281179	0.02494	-4.63126	0.215	0.18536
Dendritic.cells	RNF213	-0.58236	6.151249	-2.28044	0.024986	-4.08595	0.229781	0.204512
Dendritic.cells	TRAPPC5	0.308683	4.379372	2.280144	0.025004	-3.95704	0.237677	0.214909
Dendritic.cells	ZFAND2B	0.486988	4.313639	2.279693	0.025032	-3.59059	0.238033	0.215478
Dendritic.cells	SPATA1	-0.45337	3.467697	-2.27865	0.025097	-3.54559	0.242348	0.22103
Dendritic.cells	SIVA1	0.474015	5.470666	2.27827	0.02512	-3.79571	0.233239	0.209113
Dendritic.cells	VRK1	0.318533	5.309256	2.27764	0.02516	-3.92614	0.234031	0.210195
Dendritic.cells	LMF1	0.479986	3.239428	2.277436	0.025172	-3.63337	0.243504	0.222706
Dendritic.cells	C2CD5	0.309966	4.918041	2.276869	0.025208	-3.97634	0.235909	0.212649
Dendritic.cells	ARID4A	0.24111	7.062741	2.275676	0.025282	-4.33123	0.226826	0.200628
Dendritic.cells	GM3235	1.306425	0.645113	2.275573	0.025288	-3.3778	0.256577	0.239849
Dendritic.cells	COX4I1	0.173294	9.086822	2.274909	0.02533	-4.65639	0.218551	0.189689
Dendritic.cells	GSTT1	-1.21744	2.240293	-2.27377	0.025401	-3.38462	0.249401	0.229701
Dendritic.cells	UNC13B	-1.0916	0.758993	-2.27345	0.025422	-3.38175	0.256683	0.239413
Dendritic.cells	GM3336	-1.07509	1.732452	-2.27234	0.025491	-3.41298	0.252161	0.233396
Dendritic.cells	MARS2	-0.71546	2.690445	-2.27227	0.025495	-3.40153	0.247525	0.22726
Dendritic.cells	PPP1R3F	-1.08407	0.867721	-2.27042	0.025612	-3.38466	0.257211	0.239469
Dendritic.cells	GM11772	0.933028	0.302935	2.27035	0.025617	-3.39032	0.26006	0.243255
Dendritic.cells	NID2	-1.04604	2.19946	-2.26889	0.025709	-3.40711	0.251129	0.231294
Dendritic.cells	CLEC4D	1.895343	2.949351	2.268644	0.025725	-3.39231	0.247513	0.22652
Dendritic.cells	D330041H(	0.942709	2.247493	2.268509	0.025733	-3.388	0.250895	0.230985
Dendritic.cells	DTNBP1	0.180295	6.683213	2.26752	0.025796	-4.28571	0.230828	0.204391
Dendritic.cells	SNHG16	-0.83213	2.227913	-2.2668	0.025842	-3.42394	0.251501	0.231574
Dendritic.cells	PFDN1	-0.39291	5.109296	-2.26659	0.025855	-3.68968	0.237943	0.213819
Dendritic.cells	0610005C1	-1.00654	1.911141	-2.26599	0.025894	-3.39653	0.25305	0.233848
Dendritic.cells	SELENOP	-0.38357	8.699951	-2.26579	0.025906	-4.60001	0.222348	0.193616
Dendritic.cells	DDX41	0.4465	4.356626	2.265584	0.025919	-3.63599	0.241393	0.21859
Dendritic.cells	FANCI	0.907551	2.266344	2.26499	0.025957	-3.394	0.251466	0.231862
Dendritic.cells	TRMT1	0.402017	4.050324	2.264596	0.025982	-3.65892	0.242989	0.220756
Dendritic.cells	VWF	1.273485	1.565048	2.26405	0.026017	-3.43998	0.255062	0.236698
Dendritic.cells	TBC1D14	-0.26378	5.680933	-2.26315	0.026075	-4.12031	0.235654	0.211284
Dendritic.cells	TTC39C	-1.26345	2.230936	-2.26311	0.026077	-3.39791	0.251795	0.232576
Dendritic.cells	EDEM2	0.264928	4.515035	2.262905	0.026091	-4.17007	0.240957	0.218282
Dendritic.cells	PHLPP2	0.317741	4.283952	2.262372	0.026125	-4.01254	0.242026	0.219872
Dendritic.cells	TMEM37	-0.98328	3.751452	-2.26221	0.026135	-3.44719	0.244513	0.223194
Dendritic.cells	COL4A2	-0.6787	2.620499	-2.26172	0.026167	-3.59231	0.249904	0.230348

Dendritic.cells	DUSP10	-0.65912	4.392133	-2.2611	0.026207	-3.59739	0.241525	0.219387
Dendritic.cells	KLRA9	-1.46545	-0.30711	-2.26097	0.026215	-3.41195	0.264552	0.250011
Dendritic.cells	ICA1	-0.946	2.463147	-2.2608	0.026226	-3.46031	0.250665	0.231523
Dendritic.cells	GM13919	0.276181	3.111975	2.260569	0.026241	-4.21054	0.247543	0.227399
Dendritic.cells	PPP4R3A	0.168043	6.59174	2.259324	0.026321	-4.28362	0.23213	0.206859
Dendritic.cells	ZFP524	0.555727	3.672126	2.258142	0.026398	-3.62887	0.245934	0.224676
Dendritic.cells	ESR1	-1.1738	3.611322	-2.25775	0.026424	-3.41672	0.246253	0.225191
Dendritic.cells	HUS1	0.926251	1.653586	2.25648	0.026506	-3.41172	0.256048	0.238004
Dendritic.cells	F2	-1.05908	4.237274	-2.25633	0.026516	-3.58177	0.243592	0.221498
Dendritic.cells	CLDN3	-1.18483	1.461002	-2.25605	0.026534	-3.40943	0.257007	0.239331
Dendritic.cells	PARP4	0.310848	5.335625	2.255613	0.026563	-3.9901	0.23853	0.215015
Dendritic.cells	RIPK1	-0.2604	5.973193	-2.2553	0.026583	-4.21967	0.235654	0.211276
Dendritic.cells	MRPS9	0.314982	4.889322	2.254848	0.026613	-3.86329	0.24057	0.217908
Dendritic.cells	OXCT1	0.270293	5.719852	2.254845	0.026613	-4.07599	0.236791	0.212927
Dendritic.cells	MOB3A	0.333664	4.775701	2.254519	0.026634	-3.96181	0.241093	0.218659
Dendritic.cells	ATP7A	0.264278	5.8288	2.254061	0.026664	-4.15458	0.236368	0.212467
Dendritic.cells	CLEC4E	2.090996	2.622943	2.253645	0.026691	-3.41349	0.2514	0.232396
Dendritic.cells	ZFP595	-0.67008	1.84184	-2.2525	0.026767	-3.43363	0.255737	0.237718
Dendritic.cells	CD209A	-0.38669	0.102824	-2.25116	0.026854	-4.16634	0.265083	0.249903
Dendritic.cells	CYP3A11	-1.19536	3.352158	-2.25079	0.026879	-3.48715	0.248881	0.228456
Dendritic.cells	1110006O2	1.095276	0.319918	2.25039	0.026905	-3.42319	0.263961	0.248705
Dendritic.cells	KLHL42	0.357718	2.445253	2.2503	0.026911	-3.86961	0.253278	0.234442
Dendritic.cells	LUC7L3	0.1998	6.706417	2.248676	0.027018	-4.28169	0.234179	0.208555
Dendritic.cells	ACAA1B	-1.47661	3.318751	-2.24772	0.027082	-3.49017	0.250204	0.229347
Dendritic.cells	RAB7B	-0.51792	2.804284	-2.24643	0.027168	-3.94384	0.253289	0.232923
Dendritic.cells	CALM3	0.243911	7.10732	2.245143	0.027253	-4.30751	0.233845	0.206951
Dendritic.cells	DIPK1A	0.325333	5.446127	2.243824	0.027341	-4.22759	0.241548	0.21703
Dendritic.cells	TEK	-0.73601	2.175307	-2.24371	0.027349	-3.58697	0.257218	0.237736
Dendritic.cells	SCAF1	0.332749	4.690272	2.24357	0.027358	-3.88981	0.245058	0.221725
Dendritic.cells	BC031181	0.238276	5.703047	2.243088	0.027391	-4.10415	0.240369	0.215733
Dendritic.cells	FLCN	-0.45492	4.338996	-2.24273	0.027415	-3.77032	0.246712	0.224155
Dendritic.cells	AMER1	0.834216	2.080672	2.242725	0.027415	-3.43212	0.25769	0.238691
Dendritic.cells	SLC2A3	0.379898	4.519005	2.241856	0.027473	-4.06267	0.246182	0.223353
Dendritic.cells	ACAT1	-0.42543	6.480379	-2.24069	0.027552	-4.21546	0.23748	0.211752
Dendritic.cells	MTFMT	0.537992	2.527907	2.24062	0.027556	-3.51979	0.256148	0.236377
Dendritic.cells	ATP9A	-1.04612	0.837325	-2.23862	0.027692	-3.44965	0.265773	0.248334
Dendritic.cells	FETUB	-1.07786	3.175824	-2.23611	0.027862	-3.50504	0.255341	0.233466
Dendritic.cells	CEP170B	0.898844	0.457754	2.235047	0.027934	-3.45434	0.269168	0.252137
Dendritic.cells	DCUN1D1	0.33107	5.787878	2.235021	0.027936	-4.00303	0.242883	0.217393
Dendritic.cells	KDM2B	-0.34076	7.133876	-2.23493	0.027942	-4.59559	0.236771	0.209422
Dendritic.cells	LAIR1	0.273527	4.246709	2.234758	0.027954	-4.70129	0.250137	0.226955
Dendritic.cells	ETAA1	-0.76278	3.334503	-2.23427	0.027987	-3.47658	0.254654	0.232988
Dendritic.cells	PAK1	-0.86233	5.334473	-2.23199	0.028143	-3.75086	0.24624	0.221185
Dendritic.cells	SOX18	-0.92564	0.720608	-2.23144	0.028181	-3.50215	0.269242	0.251602
Dendritic.cells	SMIM20	0.347941	4.914811	2.231195	0.028198	-3.89942	0.248295	0.22398
Dendritic.cells	GM12236	-1.36213	1.087964	-2.23046	0.028248	-3.4528	0.267578	0.249399
Dendritic.cells	DAGLB	-0.86538	4.366228	-2.22973	0.028299	-3.47467	0.251396	0.227938

Dendritic.cells	MXI1	0.576765	7.063981	2.229398	0.028321	-3.89009	0.238824	0.211486
Dendritic.cells	SLCO3A1	0.300767	4.38978	2.228131	0.028409	-4.47168	0.25184	0.228113
Dendritic.cells	TOMM6	0.216769	7.550466	2.2274	0.02846	-4.43189	0.237171	0.209056
Dendritic.cells	VPS54	0.278845	6.506686	2.227278	0.028468	-4.26206	0.241888	0.215225
Dendritic.cells	IL1A	-1.11179	2.787687	-2.22714	0.028477	-3.53263	0.259724	0.23873
Dendritic.cells	GM33104	-1.3976	0.440079	-2.226	0.028556	-3.46632	0.272148	0.255215
Dendritic.cells	ZFP810	0.317165	2.914739	2.225761	0.028573	-4.0975	0.259382	0.238369
Dendritic.cells	TNIP2	0.413561	3.816648	2.225673	0.028579	-3.75493	0.254914	0.232489
Dendritic.cells	TPCN1	0.63415	4.187411	2.22496	0.028629	-3.69238	0.253218	0.230301
Dendritic.cells	CD200R1	0.516434	2.827999	2.224489	0.028662	-3.84571	0.259932	0.239246
Dendritic.cells	AGO1	0.464383	3.874896	2.224445	0.028665	-3.76808	0.254742	0.232377
Dendritic.cells	ASGR1	-1.02665	3.372794	-2.22415	0.028685	-3.52158	0.257215	0.235706
Dendritic.cells	TCF7L1	-0.61731	3.097343	-2.22364	0.028721	-3.70559	0.258698	0.237597
Dendritic.cells	GM12248	-0.7974	2.619411	-2.22293	0.028771	-3.5265	0.261336	0.240993
Dendritic.cells	SEMA6A	-0.68037	2.821738	-2.22163	0.028862	-3.93175	0.260771	0.240111
Dendritic.cells	UFC1	0.199281	5.792898	2.221542	0.028868	-4.22023	0.246335	0.221079
Dendritic.cells	NRXN2	-0.59519	2.095803	-2.2205	0.028941	-3.8999	0.264915	0.245409
Dendritic.cells	TIA1	0.221514	5.466514	2.219339	0.029023	-4.16877	0.248772	0.223681
Dendritic.cells	KPNA1	-0.20859	6.778024	-2.2189	0.029054	-4.49413	0.242659	0.215767
Dendritic.cells	TRIM36	-0.38069	3.146179	-2.21797	0.029119	-3.97843	0.260087	0.239028
Dendritic.cells	RAB35	-0.35437	4.720448	-2.21792	0.029123	-3.85871	0.25234	0.228781
Dendritic.cells	ATP1A3	0.510406	1.709416	2.217868	0.029127	-3.87024	0.267419	0.248781
Dendritic.cells	RGS19	0.380813	5.393744	2.217719	0.029137	-3.98952	0.249118	0.22456
Dendritic.cells	ZFYVE26	0.345877	5.03643	2.21682	0.029201	-4.00749	0.251167	0.226897
Dendritic.cells	SLC27A2	-0.99911	3.949769	-2.21626	0.029241	-3.63634	0.25659	0.234042
Dendritic.cells	SMC3	0.241205	6.498954	2.215681	0.029282	-4.25039	0.244414	0.218059
Dendritic.cells	GYPA	1.146528	3.611578	2.215599	0.029288	-3.64686	0.258263	0.23631
Dendritic.cells	SELENOO	0.545482	3.136064	2.214934	0.029335	-3.65111	0.26064	0.239713
Dendritic.cells	RUBCN	0.445283	3.888841	2.214288	0.029381	-3.79189	0.25689	0.234898
Dendritic.cells	GM32250	1.215327	-0.2746	2.213707	0.029422	-3.49227	0.278501	0.26404
Dendritic.cells	HOGA1	-0.94313	2.370342	-2.21338	0.029445	-3.50491	0.264525	0.245424
Dendritic.cells	GMFB	-0.27009	5.451593	-2.21337	0.029447	-4.12616	0.249324	0.225255
Dendritic.cells	CADM1	-0.49703	5.440386	-2.21324	0.029456	-4.44781	0.249377	0.225335
Dendritic.cells	DOCK11	0.260432	7.111908	2.212685	0.029495	-4.37616	0.2416	0.215117
Dendritic.cells	TST	-1.19354	3.470042	-2.2123	0.029522	-3.52732	0.258968	0.238141
Dendritic.cells	PIK3R5	0.513811	4.45137	2.212274	0.029525	-3.76335	0.254133	0.231725
Dendritic.cells	ANKRD12	-0.23413	7.518738	-2.21203	0.029542	-4.55989	0.239755	0.212828
Dendritic.cells	ESRRG	-1.04943	1.226258	-2.21169	0.029566	-3.49283	0.270463	0.253601
Dendritic.cells	NEK1	-0.2821	3.697931	-2.21165	0.02957	-4.26936	0.257835	0.23676
Dendritic.cells	CUTC	0.561377	3.519969	2.211033	0.029614	-3.59775	0.258898	0.238246
Dendritic.cells	SLC23A3	-1.51519	1.461516	-2.2102	0.029674	-3.48769	0.269582	0.25268
Dendritic.cells	RHOA	0.12269	9.258828	2.209704	0.029709	-4.82058	0.232383	0.203515
Dendritic.cells	NDUFAF8	0.292173	5.052226	2.209542	0.029721	-4.02493	0.251559	0.228812
Dendritic.cells	PPARGC1A	1.080236	1.419055	2.209248	0.029742	-3.5182	0.269804	0.253192
Dendritic.cells	TMEM229I	0.177911	4.03146	2.208553	0.029792	-4.60656	0.256523	0.23558
Dendritic.cells	EMP3	0.225024	7.421524	2.208513	0.029795	-4.66267	0.240509	0.214364
Dendritic.cells	GM11084	0.343183	3.368087	2.207999	0.029832	-3.96144	0.259817	0.240116

Dendritic.cells	CARMIL1	0.207369	4.075468	2.207716	0.029852	-4.59576	0.256307	0.235435
Dendritic.cells	TMEM42	0.537612	2.617603	2.207616	0.02986	-3.61175	0.263607	0.245219
Dendritic.cells	SLC25A44	-0.62573	3.239164	-2.20737	0.029877	-3.59399	0.260463	0.241079
Dendritic.cells	ZFP512B	0.677412	2.377698	2.207184	0.029891	-3.63716	0.264834	0.247007
Dendritic.cells	AC166172.	-1.05609	0.939575	-2.20618	0.029964	-3.49427	0.272783	0.257406
Dendritic.cells	OGT	0.201356	6.649131	2.205342	0.030024	-4.4564	0.244744	0.219922
Dendritic.cells	TMEM141	-1.1643	2.239657	-2.20501	0.030048	-3.50143	0.266312	0.248791
Dendritic.cells	1700010K2	-1.0533	0.709295	-2.20418	0.030108	-3.50322	0.274685	0.259933
Dendritic.cells	MYL6	0.208246	9.689609	2.203406	0.030165	-4.83231	0.231735	0.20273
Dendritic.cells	HFE	-0.75509	3.452689	-2.20176	0.030286	-3.5822	0.261584	0.241716
Dendritic.cells	NSMCE2	-0.16305	7.428191	-2.20127	0.030322	-4.62374	0.242607	0.216482
Dendritic.cells	ATP5J	0.239885	7.873083	2.200809	0.030355	-4.52286	0.240665	0.213818
Dendritic.cells	ATP2A2	-0.19799	6.456942	-2.19954	0.030448	-4.3826	0.247739	0.222714
Dendritic.cells	GM47644	1.083604	1.060844	2.198434	0.03053	-3.52544	0.275329	0.259232
Dendritic.cells	SPACA6	-1.05993	1.094819	-2.19677	0.030653	-3.50948	0.275316	0.259521
Dendritic.cells	PAQR5	0.369491	0.502008	2.196235	0.030692	-4.1377	0.278515	0.264032
Dendritic.cells	SERINC5	0.322222	5.287482	2.196086	0.030703	-4.25342	0.253942	0.231142
Dendritic.cells	PSKH1	0.427085	3.273493	2.195884	0.030718	-3.72733	0.263938	0.244481
Dendritic.cells	ASNA1	-0.35007	4.877847	-2.19578	0.030726	-3.97092	0.255935	0.233804
Dendritic.cells	CLEC5A	1.324102	0.512385	2.195668	0.030734	-3.51334	0.278459	0.263986
Dendritic.cells	NEK3	1.107196	1.761696	2.195449	0.03075	-3.52035	0.27177	0.255004
Dendritic.cells	HSPA1B	1.877597	4.531616	2.195413	0.030753	-3.69566	0.257636	0.236091
Dendritic.cells	EFCAB2	-0.72107	2.668273	-2.19461	0.030813	-3.62749	0.26704	0.248767
Dendritic.cells	GID8	0.310075	5.195402	2.19455	0.030817	-4.02194	0.254388	0.231871
Dendritic.cells	MERTK	-0.92855	3.774359	-2.19435	0.030832	-3.69779	0.261406	0.241278
Dendritic.cells	DNAJC30	0.453185	3.959336	2.193866	0.030868	-3.78035	0.260478	0.240214
Dendritic.cells	ANK2	-1.5078	2.998552	-2.19371	0.03088	-3.55194	0.265342	0.246764
Dendritic.cells	FBRSL1	-0.20705	5.766788	-2.19367	0.030882	-4.3523	0.251635	0.228465
Dendritic.cells	GM14455	-0.91192	1.315653	-2.19267	0.030957	-3.52065	0.274583	0.258916
Dendritic.cells	RAPGEF1	-0.16516	6.775482	-2.19144	0.031049	-4.75877	0.247572	0.222887
Dendritic.cells	TICRR	1.042604	3.009192	2.191384	0.031053	-3.53055	0.266042	0.247535
Dendritic.cells	VCAN	1.655812	0.76602	2.191208	0.031066	-3.51991	0.277873	0.263551
Dendritic.cells	KNG2	-1.12244	2.382731	-2.19008	0.03115	-3.5348	0.269737	0.252299
Dendritic.cells	NFAT5	-0.29967	7.501438	-2.18987	0.031166	-4.46461	0.244618	0.218882
Dendritic.cells	FCRL1	-1.17255	2.73778	-2.18931	0.031208	-3.52139	0.268045	0.249946
Dendritic.cells	ATP6AP2	-0.15602	6.938045	-2.18827	0.031287	-4.61446	0.247752	0.222649
Dendritic.cells	DCLK2	-0.94079	2.72479	-2.18803	0.031305	-3.53702	0.268526	0.250395
Dendritic.cells	MRPL52	0.203949	7.102833	2.187289	0.03136	-4.64549	0.247229	0.221849
Dendritic.cells	TNFAIP8L2	0.495124	3.852483	2.18698	0.031384	-3.77814	0.263015	0.242859
Dendritic.cells	LDHC	-1.49257	0.610466	-2.1862	0.031443	-3.52854	0.28019	0.265911
Dendritic.cells	MAPK9	0.330768	4.751185	2.186145	0.031447	-4.00787	0.258645	0.236986
Dendritic.cells	ZFP426	0.62149	2.769149	2.185129	0.031524	-3.62381	0.269142	0.250673
Dendritic.cells	MAN2C1	0.418299	3.509599	2.184593	0.031564	-3.80879	0.265465	0.24567
Dendritic.cells	TBC1D32	1.020285	2.141265	2.182741	0.031705	-3.54049	0.273387	0.255652
Dendritic.cells	NHSL2	-1.01303	2.487556	-2.18262	0.031715	-3.5887	0.27156	0.253202
Dendritic.cells	HTT	-0.2772	5.362069	-2.18219	0.031747	-4.18955	0.256974	0.23384
Dendritic.cells	CCNYL1	-0.43109	4.635175	-2.18205	0.031758	-3.86468	0.260566	0.23862

Dendritic.cells	DHX37	-0.4173	2.67509	-2.18177	0.031779	-3.88507	0.270577	0.252047
Dendritic.cells	USF3	0.359899	4.977969	2.181327	0.031813	-4.04087	0.25894	0.236555
Dendritic.cells	DTWD1	0.787098	1.928598	2.180901	0.031846	-3.56042	0.274669	0.257552
Dendritic.cells	GCNT2	0.279158	4.580489	2.180311	0.031891	-4.54023	0.261155	0.239278
Dendritic.cells	PAKAP.1	-0.3197	4.53559	-2.17973	0.031935	-4.34304	0.261396	0.239644
Dendritic.cells	CAV2	-0.6438	2.771749	-2.17965	0.031941	-3.66573	0.270416	0.251702
Dendritic.cells	SERPIND1	-1.15922	2.832849	-2.17869	0.032015	-3.56095	0.270515	0.251555
Dendritic.cells	REPIN1	-1.10822	1.082403	-2.17766	0.032094	-3.54067	0.280127	0.264446
Dendritic.cells	CNN2	0.292682	6.538205	2.177584	0.0321	-4.51955	0.252251	0.227201
Dendritic.cells	VAMP4	0.276813	6.154005	2.17691	0.032152	-4.21192	0.254094	0.229779
Dendritic.cells	UHRF1BP1l	0.211502	5.734451	2.176766	0.032163	-4.49287	0.256127	0.232478
Dendritic.cells	MCCC2	-1.01418	2.436244	-2.17623	0.032204	-3.58145	0.272862	0.254954
Dendritic.cells	ITPKA	1.066166	0.529225	2.176202	0.032207	-3.54476	0.283163	0.26883
Dendritic.cells	PTGS1	-0.75509	3.986721	-2.17608	0.032216	-3.68536	0.264827	0.244204
Dendritic.cells	SERPINA11	-1.04586	1.813558	-2.17491	0.032306	-3.55182	0.276743	0.259759
Dendritic.cells	SUPT16	0.182942	7.068147	2.174233	0.032359	-4.55872	0.250291	0.224667
Dendritic.cells	A	-0.45977	4.78007	-2.1736	0.032408	-3.99993	0.261408	0.23968
Dendritic.cells	SLC38A4	-1.08699	3.153421	-2.17357	0.03241	-3.61761	0.269703	0.250781
Dendritic.cells	CEP97	0.681695	2.922474	2.17342	0.032422	-3.62959	0.270908	0.25245
Dendritic.cells	NDUFB1-PS	0.170498	8.902498	2.172993	0.032455	-4.79713	0.241828	0.213804
Dendritic.cells	9430015G1	0.844232	1.911053	2.172965	0.032457	-3.56083	0.276262	0.259729
Dendritic.cells	GM16573	-0.87208	1.655105	-2.17222	0.032515	-3.56375	0.277924	0.261766
Dendritic.cells	PTK2	-0.36135	4.164046	-2.17162	0.032562	-4.16677	0.264968	0.244284
Dendritic.cells	PNPT1	0.492126	4.198204	2.170264	0.032668	-3.79759	0.265444	0.24443
Dendritic.cells	RANBP9	0.2314	6.962828	2.169964	0.032691	-4.49493	0.251839	0.226435
Dendritic.cells	NRN1	-1.25895	3.109984	-2.16966	0.032715	-3.58428	0.271059	0.252176
Dendritic.cells	AGPS	0.200084	6.813953	2.168994	0.032767	-4.53617	0.252759	0.227641
Dendritic.cells	FGFBP3	1.261395	0.028033	2.167769	0.032863	-3.55789	0.288396	0.275361
Dendritic.cells	KIF21B	0.46049	4.865707	2.167493	0.032885	-3.87358	0.262635	0.2408
Dendritic.cells	PARP8	0.224413	6.68237	2.166476	0.032965	-4.66808	0.253719	0.229206
Dendritic.cells	GRAMD1B	0.241776	5.536445	2.166285	0.03298	-4.69856	0.259296	0.236703
Dendritic.cells	FBXL4	0.549778	2.64655	2.16625	0.032983	-3.74953	0.274082	0.256528
Dendritic.cells	DBR1	-0.6313	3.262038	-2.16569	0.033027	-3.65719	0.270845	0.252341
Dendritic.cells	CYYR1	-0.6598	2.282713	-2.16569	0.033027	-3.83125	0.276018	0.25931
Dendritic.cells	SIAH2	-0.2945	4.793136	-2.16565	0.03303	-4.12976	0.262999	0.241813
Dendritic.cells	CSGALNAC	0.418325	4.10884	2.165477	0.033044	-3.83454	0.266469	0.246515
Dendritic.cells	GALNT6	0.848833	3.115592	2.165232	0.033063	-3.63861	0.27161	0.253429
Dendritic.cells	TNRC18	0.25223	5.931838	2.165028	0.033079	-4.21305	0.257353	0.234374
Dendritic.cells	ERN1	-0.21666	7.053542	-2.16313	0.03323	-4.80645	0.252483	0.227677
Dendritic.cells	ZFP688	0.915337	1.695397	2.162824	0.033254	-3.57472	0.279774	0.264299
Dendritic.cells	COX17	0.240296	6.919214	2.162432	0.033285	-4.45026	0.253124	0.228691
Dendritic.cells	RFWD3	0.364723	5.6362	2.162336	0.033293	-4.00093	0.259355	0.237007
Dendritic.cells	NKAP	0.297644	4.989943	2.162291	0.033296	-4.03846	0.26257	0.241313
Dendritic.cells	CMPK1	0.195482	6.881961	2.162072	0.033313	-4.44257	0.253302	0.229011
Dendritic.cells	TSPAN15	-0.87152	2.388192	-2.16196	0.033322	-3.58266	0.276041	0.259532
Dendritic.cells	MTBP	0.694219	3.256156	2.161664	0.033346	-3.65723	0.271452	0.253385
Dendritic.cells	AMPD1	-0.95954	2.911186	-2.15929	0.033535	-3.60011	0.27422	0.256691

Dendritic.cells	ATAD2B	0.185931	6.740991	2.159044	0.033555	-4.66439	0.254866	0.230807
Dendritic.cells	EBF1	-0.64849	8.683852	-2.15885	0.033571	-4.49344	0.245732	0.218727
Dendritic.cells	ANKDD1A	-1.77456	2.346094	-2.15874	0.033579	-3.57206	0.277232	0.260965
Dendritic.cells	JAK2	0.302179	6.674709	2.158611	0.03359	-4.46734	0.255185	0.231359
Dendritic.cells	COX5B	0.228931	8.103976	2.158243	0.033619	-4.69641	0.248411	0.222391
Dendritic.cells	RYK	-0.88499	2.042505	-2.15805	0.033635	-3.59508	0.278868	0.263299
Dendritic.cells	MRPL22	0.593527	3.858759	2.156973	0.033721	-3.72359	0.269753	0.25066
Dendritic.cells	ZGPAT	-0.44236	3.668613	-2.15613	0.033789	-3.76439	0.271022	0.252206
Dendritic.cells	TOP1	-0.20563	8.354587	-2.15592	0.033805	-4.83884	0.247957	0.221363
Dendritic.cells	ZKSCAN8	0.936334	0.632724	2.15543	0.033845	-3.58268	0.287537	0.274533
Dendritic.cells	2510046G1	0.547445	2.620896	2.154277	0.033938	-3.74673	0.276647	0.260114
Dendritic.cells	COL27A1	-1.29042	2.314283	-2.15422	0.033943	-3.5871	0.278293	0.262356
Dendritic.cells	RASAL2	-0.34597	5.468995	-2.15405	0.033956	-4.44223	0.26193	0.24031
Dendritic.cells	CYSTM1	-0.58457	3.36709	-2.15389	0.03397	-4.08466	0.272692	0.254829
Dendritic.cells	GM15965	0.93851	2.027609	2.153659	0.033988	-3.60384	0.279843	0.264541
Dendritic.cells	IYD	-1.18308	0.499692	-2.15355	0.033997	-3.58064	0.288285	0.276021
Dendritic.cells	CRLF3	0.330908	6.899396	2.153279	0.034019	-4.41795	0.25492	0.231006
Dendritic.cells	JARID2	0.217549	8.144583	2.153029	0.034039	-4.78637	0.249019	0.223144
Dendritic.cells	ZC3HAV1L	-0.91627	3.053639	-2.15263	0.034071	-3.60401	0.274368	0.257167
Dendritic.cells	5330438D1	0.363476	4.842586	2.152059	0.034118	-4.0746	0.265102	0.244842
Dendritic.cells	PTPRC	0.197934	8.915439	2.151688	0.034148	-5.02611	0.245479	0.218691
Dendritic.cells	PGRMC2	0.380202	3.628957	2.151642	0.034152	-3.85597	0.271344	0.25336
Dendritic.cells	HOOK3	-0.23435	6.076111	-2.15085	0.034216	-4.36733	0.258946	0.236937
Dendritic.cells	TIMELESS	0.650564	3.491901	2.150759	0.034224	-3.76584	0.272061	0.254646
Dendritic.cells	RPGRIP1	0.168174	6.55945	2.150587	0.034238	-4.79195	0.256585	0.233875
Dendritic.cells	CRTAP	-0.57418	3.004923	-2.15056	0.03424	-3.75224	0.274626	0.258199
Dendritic.cells	MFSD10	0.461646	4.464143	2.150009	0.034285	-3.90697	0.267056	0.248027
Dendritic.cells	SFSWAP	0.203605	5.786795	2.149909	0.034293	-4.3319	0.2604	0.239061
Dendritic.cells	CSTDC4	1.736939	3.661016	2.148861	0.034378	-3.65769	0.271686	0.253978
Dendritic.cells	DYNLT1C	0.912059	1.100031	2.148338	0.034421	-3.59756	0.285542	0.272837
Dendritic.cells	TICAM2	1.04607	1.274799	2.148051	0.034445	-3.58818	0.284572	0.271625
Dendritic.cells	KDM3B	0.213008	6.131762	2.147878	0.034459	-4.41292	0.259204	0.237345
Dendritic.cells	PURA	-0.23937	5.343404	-2.14689	0.03454	-4.21842	0.263548	0.242846
Dendritic.cells	UNC93B1	0.169409	7.659349	2.146381	0.034582	-4.90954	0.252369	0.227806
Dendritic.cells	GM15232	1.056879	1.187425	2.145626	0.034644	-3.59398	0.28583	0.272999
Dendritic.cells	DUSP4	1.034251	0.909125	2.145529	0.034652	-3.59336	0.287383	0.275138
Dendritic.cells	SAMD4	-0.99262	3.187151	-2.14497	0.034698	-3.79103	0.275141	0.258487
Dendritic.cells	ARL4D	-1.20551	1.705958	-2.14372	0.034801	-3.59513	0.283592	0.269745
Dendritic.cells	CCR4	-1.14214	-1.08113	-2.14341	0.034826	-3.59872	0.299449	0.291443
Dendritic.cells	4930556J2	0.683493	2.185085	2.14339	0.034828	-3.66051	0.280969	0.266175
Dendritic.cells	S100A4	1.174735	2.87429	2.142753	0.034881	-3.65149	0.277405	0.261379
Dendritic.cells	DTX3L	0.708047	4.876216	2.14255	0.034897	-3.92624	0.266938	0.247269
Dendritic.cells	FAM120A	0.195535	6.352078	2.141943	0.034948	-4.45807	0.259627	0.237466
Dendritic.cells	ZSCAN22	1.00916	1.316109	2.141625	0.034974	-3.6067	0.285999	0.273278
Dendritic.cells	DTD2	0.586448	3.491192	2.141422	0.034991	-3.79906	0.274211	0.257294
Dendritic.cells	HIST1H4I	0.920334	4.409355	2.141216	0.035008	-3.69944	0.269418	0.250883
Dendritic.cells	RASGRF2	-1.08893	0.116081	-2.14072	0.035049	-3.60102	0.292901	0.282828

Dendritic.cells	JAK3	0.788393	2.778845	2.140296	0.035084	-3.67156	0.278167	0.262731
Dendritic.cells	SPCS1	0.174252	7.852067	2.139662	0.035137	-4.78454	0.252533	0.228247
Dendritic.cells	GM48512	0.873485	1.068935	2.139491	0.035151	-3.62264	0.287546	0.275675
Dendritic.cells	TSPAN3	0.355879	4.739337	2.139472	0.035153	-4.20875	0.267878	0.248918
Dendritic.cells	CD247	-1.12145	3.01096	-2.13862	0.035224	-3.61432	0.277285	0.261516
Dendritic.cells	SNIP1	0.433771	3.633076	2.137141	0.035348	-3.81693	0.274748	0.257611
Dendritic.cells	STAMBPL1	0.291168	6.55028	2.136596	0.035393	-4.47942	0.259902	0.237648
Dendritic.cells	RBM47	-0.22353	6.404832	-2.13649	0.035402	-4.89655	0.260619	0.238632
Dendritic.cells	MRPL40	-0.45836	4.651456	-2.13614	0.035432	-3.92513	0.269502	0.250677
Dendritic.cells	KDM6B	-0.25448	8.152763	-2.13545	0.035489	-4.7854	0.252442	0.227541
Dendritic.cells	EIF3D	0.287342	5.480464	2.134842	0.03554	-4.23524	0.26565	0.245245
Dendritic.cells	ITIH2	-1.12187	3.79432	-2.13464	0.035558	-3.76037	0.274361	0.257091
Dendritic.cells	FGG	-0.61028	6.166651	-2.13416	0.035598	-4.2353	0.262319	0.240769
Dendritic.cells	ARHGEF12	-0.55524	3.963016	-2.13305	0.035691	-3.90976	0.273992	0.256282
Dendritic.cells	FBXW17	-0.94892	1.479346	-2.13294	0.0357	-3.62425	0.287461	0.274613
Dendritic.cells	CLTC	-0.16615	7.90636	-2.13198	0.035782	-4.73607	0.254599	0.229827
Dendritic.cells	PPP1R14A	0.964823	0.601813	2.131725	0.035804	-3.6368	0.292847	0.2817
Dendritic.cells	TAGAP	0.341264	4.269177	2.130483	0.035909	-4.48148	0.273204	0.254709
Dendritic.cells	DGLUCY	0.520551	2.944912	2.130353	0.03592	-3.82945	0.280254	0.264271
Dendritic.cells	CCZ1	0.203297	6.158644	2.130192	0.035933	-4.50425	0.263533	0.241657
Dendritic.cells	ANAPC7	0.363806	4.359074	2.129579	0.035986	-3.97489	0.272938	0.25419
Dendritic.cells	KLRE1	-1.37616	1.626605	-2.12853	0.036075	-3.62181	0.288188	0.274588
Dendritic.cells	SYDE2	1.200705	-0.10373	2.128291	0.036095	-3.61968	0.298072	0.288117
Dendritic.cells	GOLGB1	0.230079	5.4427	2.127498	0.036163	-4.41869	0.267888	0.247159
Dendritic.cells	INO80	-0.2243	6.666636	-2.12731	0.036179	-4.62167	0.261736	0.238945
Dendritic.cells	ACTR10	0.206978	6.186686	2.126723	0.036229	-4.38274	0.264126	0.242313
Dendritic.cells	EBI3	-0.41453	4.387447	-2.1267	0.036231	-4.09959	0.273344	0.254759
Dendritic.cells	ATCAYOS	-1.18177	1.220314	-2.12665	0.036235	-3.6555	0.290586	0.278226
Dendritic.cells	TJP2	-0.62731	3.845594	-2.12558	0.036327	-3.79562	0.276708	0.258981
Dendritic.cells	SPI1	0.37918	6.411888	2.124981	0.036378	-4.18541	0.26367	0.241383
Dendritic.cells	DHRS9	1.239665	0.196917	2.124404	0.036428	-3.62885	0.297394	0.287106
Dendritic.cells	NSUN4	-0.48258	3.189091	-2.12346	0.036509	-3.88345	0.280967	0.264474
Dendritic.cells	SSR4	0.189884	7.573816	2.123144	0.036536	-4.8584	0.258451	0.234156
Dendritic.cells	SLC40A1	-0.83093	5.835325	-2.12276	0.036569	-4.03008	0.267086	0.245821
Dendritic.cells	MSH3	0.342082	5.159864	2.122689	0.036575	-4.16162	0.270544	0.250488
Dendritic.cells	GM26881	-1.27379	0.245715	-2.12077	0.036741	-3.63277	0.298568	0.287888
Dendritic.cells	KYAT3	-0.87283	3.603362	-2.12056	0.036759	-3.75897	0.279747	0.262299
Dendritic.cells	LRRC14	-0.67235	2.339055	-2.12005	0.036804	-3.73229	0.286807	0.271844
Dendritic.cells	CCDC124	0.292538	5.595782	2.118889	0.036904	-4.16664	0.269779	0.248607
Dendritic.cells	KLHL36	0.873824	2.076404	2.118666	0.036924	-3.6706	0.288653	0.274202
Dendritic.cells	SNX30	0.267705	5.755716	2.118467	0.036941	-4.5133	0.26896	0.247607
Dendritic.cells	PPARGC1B	0.734647	2.799015	2.118308	0.036955	-3.72582	0.284645	0.268878
Dendritic.cells	GSE1	-0.41185	5.664856	-2.11768	0.03701	-4.00736	0.269623	0.248436
Dendritic.cells	TRMT13	-0.3958	3.89491	-2.11741	0.037033	-4.02943	0.278903	0.261035
Dendritic.cells	LILRA5	-1.41626	1.497471	-2.11651	0.037112	-3.63814	0.29247	0.279345
Dendritic.cells	CACNB2	-0.29475	4.420818	-2.11605	0.037152	-4.88636	0.276425	0.257709
Dendritic.cells	AACS	-0.33268	3.861959	-2.11604	0.037153	-4.12996	0.279405	0.261749

Dendritic.cells	ATR	0.526954	3.862209	2.114581	0.03728	-3.96477	0.280169	0.262224
Dendritic.cells	TBX21	-1.1362	2.437202	-2.11392	0.037338	-3.67407	0.288224	0.273123
Dendritic.cells	BUB3	0.333853	5.956108	2.112531	0.03746	-4.25666	0.270014	0.248098
Dendritic.cells	HINFP	0.526636	3.494398	2.112364	0.037475	-3.81837	0.28303	0.265774
Dendritic.cells	MAN1A2	-0.1579	6.392544	-2.11149	0.037552	-4.74722	0.267788	0.24531
Dendritic.cells	TAF15	0.180138	6.905531	2.111465	0.037554	-4.61576	0.265203	0.241832
Dendritic.cells	AGPAT5	0.226745	5.644905	2.111386	0.037561	-4.4272	0.271616	0.250485
Dendritic.cells	9330159M	1.045591	1.076034	2.111226	0.037575	-3.64776	0.296598	0.284453
Dendritic.cells	TMPRSS5	-1.13229	0.722493	-2.10918	0.037756	-3.64996	0.299821	0.288184
Dendritic.cells	RAPSN	1.005523	0.715904	2.108761	0.037794	-3.65133	0.29986	0.288428
Dendritic.cells	MIPEP	0.562688	2.843457	2.108683	0.0378	-3.73752	0.287731	0.271893
Dendritic.cells	DNAH2	-1.16834	0.783062	-2.1082	0.037843	-3.65231	0.299574	0.287995
Dendritic.cells	LMNA	-0.60756	4.617164	-2.10795	0.037865	-4.01329	0.278183	0.258953
Dendritic.cells	UBE2R2	0.214407	7.144409	2.107616	0.037895	-4.66104	0.265169	0.241493
Dendritic.cells	SMTN	-0.7198	2.149503	-2.10698	0.037952	-3.73655	0.291989	0.277659
Dendritic.cells	METTL7A1	-0.6521	2.456994	-2.10641	0.038002	-3.73146	0.290446	0.275312
Dendritic.cells	SLC37A1	0.701112	3.202303	2.105947	0.038044	-3.76246	0.286412	0.269786
Dendritic.cells	C87436	0.462008	3.905756	2.105299	0.038102	-3.92177	0.282786	0.264769
Dendritic.cells	ARHGEF6	0.192876	5.643537	2.105029	0.038126	-4.72808	0.273544	0.252373
Dendritic.cells	ANKRD55	1.089239	0.009583	2.1045	0.038173	-3.65689	0.305146	0.295353
Dendritic.cells	EPB41L2	-0.26984	6.975502	-2.1038	0.038236	-4.54252	0.267131	0.243474
Dendritic.cells	NCOA7	0.427941	5.299685	2.10328	0.038282	-4.09819	0.275852	0.255258
Dendritic.cells	COL18A1	-1.02714	2.743841	-2.10303	0.038305	-3.71707	0.289725	0.274168
Dendritic.cells	AI182371	-1.07158	2.439191	-2.10279	0.038326	-3.69435	0.291436	0.276589
Dendritic.cells	GREB1L	-0.92191	1.947775	-2.1023	0.03837	-3.71894	0.294364	0.280585
Dendritic.cells	SLC6A6	-0.19165	7.287991	-2.10184	0.038412	-4.85559	0.265886	0.242019
Dendritic.cells	TGFA	-1.11755	0.796415	-2.10121	0.038469	-3.67006	0.30139	0.290105
Dendritic.cells	GM14221	-1.2849	1.910062	-2.10025	0.038555	-3.67215	0.295257	0.281497
Dendritic.cells	TOM1L1	-1.20868	0.714609	-2.10016	0.038563	-3.66452	0.302202	0.291017
Dendritic.cells	ZNHIT1	0.297029	5.422819	2.099248	0.038646	-4.23193	0.276228	0.255643
Dendritic.cells	1810026B0	0.173342	6.570259	2.099083	0.03866	-4.70237	0.270275	0.247659
Dendritic.cells	GSTP1	0.300552	6.928594	2.098843	0.038682	-4.60617	0.268451	0.24528
Dendritic.cells	PLEKHG2	-0.56647	4.157105	-2.09861	0.038703	-3.94075	0.282994	0.265034
Dendritic.cells	C8A	-1.10896	0.525618	-2.09699	0.03885	-3.66894	0.304551	0.293678
Dendritic.cells	TOMM70A	0.218852	5.752051	2.095822	0.038956	-4.45894	0.27593	0.254243
Dendritic.cells	NRROS	0.196311	7.067702	2.094487	0.039078	-4.81607	0.269484	0.245366
Dendritic.cells	2610203C2	-1.03765	0.354453	-2.0943	0.039095	-3.6822	0.306592	0.295785
Dendritic.cells	HAO2	-1.4242	1.393032	-2.0939	0.039132	-3.67836	0.300451	0.287432
Dendritic.cells	PET100	0.24645	5.786929	2.093579	0.039161	-4.43197	0.2761	0.254354
Dendritic.cells	FSD2	1.148623	-0.73763	2.093547	0.039164	-3.67545	0.313212	0.304946
Dendritic.cells	SNED1	-1.207	1.751149	-2.09345	0.039173	-3.67675	0.298368	0.284603
Dendritic.cells	PTPRD	-0.98765	2.563535	-2.09327	0.03919	-3.7653	0.293709	0.278324
Dendritic.cells	CMBL	-1.26456	2.192141	-2.09235	0.039274	-3.68028	0.296267	0.281534
Dendritic.cells	SP100	0.284781	7.43248	2.091272	0.039373	-4.78964	0.268528	0.243574
Dendritic.cells	REEP4	0.489792	4.356559	2.09093	0.039404	-4.00831	0.284724	0.26546
Dendritic.cells	RBM27	-0.19429	6.399999	-2.09058	0.039436	-4.59259	0.273891	0.250835
Dendritic.cells	RAB4A	-1.11726	1.578469	-2.09027	0.039465	-3.67889	0.300466	0.286852

Dendritic.cells	D030056L2	0.550609	3.888031	2.088797	0.039601	-3.92062	0.288152	0.269552
Dendritic.cells	IGFBP4	-0.75876	5.679023	-2.08804	0.039671	-4.3403	0.278758	0.256735
Dendritic.cells	KCTD2	0.518704	3.355478	2.087465	0.039724	-3.8753	0.291609	0.274108
Dendritic.cells	RAPGEF5	-0.5387	5.011787	-2.08722	0.039747	-4.34484	0.28249	0.26184
Dendritic.cells	C1D	0.228292	5.843961	2.085569	0.0399	-4.39824	0.278903	0.25635
Dendritic.cells	PPRC1	-0.42671	3.956149	-2.08482	0.039969	-3.99749	0.289147	0.270368
Dendritic.cells	TRAF3IP3	0.466092	5.030914	2.084715	0.039979	-3.95541	0.283256	0.262409
Dendritic.cells	NOL11	-0.39859	4.89374	-2.08457	0.039993	-4.18261	0.283999	0.263437
Dendritic.cells	MNDAL	0.360537	6.786829	2.084477	0.040001	-4.70673	0.273964	0.249928
Dendritic.cells	RNF220	0.16884	5.723291	2.083997	0.040046	-4.92121	0.279666	0.257608
Dendritic.cells	TMEM219	0.293029	4.624458	2.082876	0.040151	-4.31156	0.28559	0.265929
Dendritic.cells	PTPN11	0.246374	5.063935	2.082731	0.040164	-4.31266	0.283201	0.262696
Dendritic.cells	ATG5	0.203017	5.691584	2.082707	0.040167	-4.4676	0.279834	0.258145
Dendritic.cells	IFI203	0.398923	6.302394	2.082613	0.040175	-4.53082	0.276608	0.253792
Dendritic.cells	SKA1	-1.24084	2.918501	-2.08259	0.040178	-3.69952	0.295111	0.278859
Dendritic.cells	NCKAP1	-0.5511	2.425386	-2.08163	0.040268	-3.87514	0.298407	0.28295
Dendritic.cells	ANGPTL7	0.927684	-0.05517	2.080514	0.040372	-3.7459	0.313766	0.303435
Dendritic.cells	PSMD1	-0.20945	6.546401	-2.07989	0.040431	-4.62737	0.276519	0.252697
Dendritic.cells	MED19	0.417481	3.778351	2.07956	0.040462	-3.92903	0.291545	0.273034
Dendritic.cells	A230072E1	-1.11808	0.278064	-2.0788	0.040534	-3.6998	0.312359	0.301254
Dendritic.cells	GM12802	0.906662	0.658153	2.078487	0.040563	-3.69748	0.31007	0.29818
Dendritic.cells	GM16093	0.729543	2.640752	2.077848	0.040623	-3.79959	0.298457	0.282436
Dendritic.cells	CORO7	0.232004	6.215775	2.077783	0.040629	-4.5608	0.278704	0.255688
Dendritic.cells	TINF2	-0.58951	4.054948	-2.07661	0.04074	-3.86855	0.291038	0.271928
Dendritic.cells	TNFSF13B	-1.2646	0.855901	-2.07627	0.040773	-3.70078	0.309663	0.297346
Dendritic.cells	ARHGEF5	-1.1188	0.327235	-2.07516	0.040877	-3.70703	0.313415	0.302288
Dendritic.cells	LIMA1	-0.82645	4.507655	-2.07495	0.040897	-3.92624	0.289065	0.269145
Dendritic.cells	TCF7	-1.3045	2.524319	-2.074	0.040988	-3.71146	0.300783	0.284788
Dendritic.cells	CPEB2	-0.30612	5.412476	-2.07367	0.041019	-4.35606	0.284566	0.262874
Dendritic.cells	UBE2L6	0.90758	4.64931	2.073076	0.041076	-3.95832	0.288743	0.268707
Dendritic.cells	FNDC3B	-0.1759	6.549271	-2.07302	0.041081	-4.99373	0.278489	0.254863
Dendritic.cells	GM16853	-0.78714	2.351019	-2.07263	0.041119	-3.75979	0.301804	0.286553
Dendritic.cells	SLFN2	0.769445	6.335056	2.072578	0.041123	-4.01389	0.279621	0.256485
Dendritic.cells	GM34680	0.466103	0.042973	2.072038	0.041175	-4.17835	0.315786	0.305602
Dendritic.cells	TNFRSF13B	0.209294	4.518479	2.071849	0.041193	-4.65936	0.289579	0.269897
Dendritic.cells	RNF166	0.211292	5.468282	2.071025	0.041272	-4.53342	0.284559	0.263048
Dendritic.cells	JADE2	-0.88905	3.270443	-2.07101	0.041273	-3.75519	0.296799	0.279643
Dendritic.cells	POMP	0.192955	7.268227	2.070583	0.041314	-4.8395	0.275111	0.250253
Dendritic.cells	MYL6B	0.87814	0.862198	2.070077	0.041362	-3.72801	0.311247	0.29917
Dendritic.cells	ABCC3	-1.29182	2.689429	-2.06955	0.041413	-3.72704	0.300575	0.284553
Dendritic.cells	MAML1	-0.31141	5.435368	-2.06904	0.041462	-4.31526	0.285294	0.263749
Dendritic.cells	IFFO2	-0.31306	3.364648	-2.06813	0.04155	-4.37771	0.297278	0.279723
Dendritic.cells	SCRIB	-0.63541	3.392621	-2.06779	0.041582	-3.77298	0.297155	0.279573
Dendritic.cells	GM16023	0.649042	1.834877	2.067492	0.041611	-3.80957	0.306257	0.292007
Dendritic.cells	TOMM22	0.212645	6.905252	2.066873	0.041671	-4.65024	0.278128	0.253773
Dendritic.cells	NOTCH1	-0.26132	5.292946	-2.06632	0.041724	-4.40403	0.286949	0.265567
Dendritic.cells	MLH3	-0.78611	2.01272	-2.06533	0.04182	-3.75823	0.306138	0.29133

Dendritic.cells	PRIMPOL	0.556871	3.486578	2.065008	0.041851	-3.90387	0.297574	0.279693
Dendritic.cells	MED11	0.525711	3.312748	2.064388	0.041911	-3.87786	0.298609	0.281216
Dendritic.cells	CPEB1	-1.03157	0.696287	-2.0641	0.041939	-3.72236	0.314146	0.302548
Dendritic.cells	6330562C2	0.775267	1.275201	2.064035	0.041945	-3.787	0.310625	0.297741
Dendritic.cells	FAM174A	0.215512	6.050716	2.06384	0.041964	-4.90307	0.283372	0.260733
Dendritic.cells	ATXN10	0.242153	6.66392	2.062047	0.042139	-4.58057	0.280879	0.256866
Dendritic.cells	GM13008	0.994895	1.087607	2.061515	0.04219	-3.72738	0.312629	0.300184
Dendritic.cells	ORMDL2	0.30761	5.109617	2.061251	0.042216	-4.2924	0.289298	0.268408
Dendritic.cells	WWC2	-0.30055	5.107898	-2.06114	0.042227	-4.42971	0.289307	0.268437
Dendritic.cells	FECH	0.690043	5.217188	2.060949	0.042246	-4.07609	0.288704	0.267642
Dendritic.cells	GM50020	1.091275	-0.11132	2.060846	0.042256	-3.72444	0.320029	0.310388
Dendritic.cells	CCDC107	0.286351	4.620705	2.060692	0.042271	-4.35117	0.292013	0.2722
Dendritic.cells	PMAIP1	0.440893	4.983149	2.058874	0.042449	-4.40925	0.290875	0.269993
Dendritic.cells	GRB2	-0.15667	7.898248	-2.05883	0.042453	-5.0942	0.275254	0.248948
Dendritic.cells	CCDC58	0.450502	4.247902	2.058461	0.042489	-4.00733	0.295059	0.275634
Dendritic.cells	GRB10	-0.7297	3.090696	-2.05729	0.042604	-4.04088	0.302324	0.285129
Dendritic.cells	CHD3	-0.67298	4.533669	-2.0559	0.042742	-3.87683	0.294556	0.274284
Dendritic.cells	TBCCD1	0.716159	2.589728	2.055738	0.042757	-3.79995	0.305784	0.28953
Dendritic.cells	SOCS7	0.350519	4.207792	2.05572	0.042759	-4.17722	0.296402	0.276784
Dendritic.cells	DCP2	0.324302	5.103401	2.054742	0.042855	-4.32148	0.291838	0.270124
Dendritic.cells	4930473A0	0.95167	0.38363	2.054218	0.042907	-3.73539	0.319752	0.308103
Dendritic.cells	ZC3H10	-0.5176	2.921486	-2.05398	0.042931	-3.89035	0.304378	0.287212
Dendritic.cells	PDE6C	-1.2104	0.436862	-2.05383	0.042945	-3.73407	0.31942	0.307806
Dendritic.cells	GM8369	1.252463	3.324129	2.053208	0.043007	-3.74406	0.302267	0.284237
Dendritic.cells	TSLP	-1.2394	-0.44209	-2.05282	0.043046	-3.73583	0.325303	0.315818
Dendritic.cells	LANCL1	0.352903	3.448045	2.052448	0.043083	-4.07454	0.301692	0.28351
Dendritic.cells	BMP8A	-0.98736	2.321943	-2.04972	0.043355	-3.78217	0.310072	0.293551
Dendritic.cells	GEMIN8	0.557924	2.284507	2.049246	0.043402	-3.9094	0.310438	0.294125
Dendritic.cells	LOXL2	-0.79491	1.406795	-2.04828	0.043499	-3.82262	0.316273	0.301706
Dendritic.cells	KDM5C	-0.27339	6.827805	-2.04793	0.043534	-4.82833	0.285086	0.259521
Dendritic.cells	TRP53BP1	-0.37807	3.888271	-2.04679	0.043648	-4.17701	0.302114	0.28206
Dendritic.cells	GM34961	1.080503	0.869528	2.045439	0.043784	-3.75378	0.32081	0.307364
Dendritic.cells	CYSLTR1	0.91325	2.70023	2.045424	0.043785	-3.80244	0.309613	0.292113
Dendritic.cells	BC048403	0.69928	1.255612	2.045041	0.043824	-3.77615	0.318408	0.304085
Dendritic.cells	PRUNE2	1.095473	0.638929	2.044962	0.043832	-3.74794	0.322255	0.309339
Dendritic.cells	GGACT	0.435598	3.170013	2.044451	0.043883	-3.98068	0.306818	0.288321
Dendritic.cells	APOB	-0.89543	4.836413	-2.04415	0.043914	-4.11532	0.297156	0.27533
Dendritic.cells	KLHL21	-0.74247	2.689731	-2.04394	0.043935	-3.85062	0.309676	0.292377
Dendritic.cells	GTF3C2	-0.2182	5.474786	-2.04346	0.043984	-4.46782	0.293559	0.270763
Dendritic.cells	COQ9	0.52923	3.205352	2.043392	0.04399	-3.94409	0.306609	0.288418
Dendritic.cells	DRG1	0.17433	6.40915	2.043335	0.043996	-4.71275	0.288395	0.26381
Dendritic.cells	SMCR8	-0.47607	3.28105	-2.04238	0.044093	-4.04465	0.306522	0.287987
Dendritic.cells	GM26670	-0.79779	0.402458	-2.04228	0.044103	-3.76697	0.324127	0.311991
Dendritic.cells	CYB5A	-0.45766	7.336341	-2.04196	0.044135	-4.72258	0.283749	0.257396
Dendritic.cells	APOC4	-0.83424	5.632488	-2.04082	0.044251	-4.27184	0.293412	0.270206
Dendritic.cells	MAP4K5	0.31286	4.52083	2.040141	0.04432	-4.46272	0.299705	0.278975
Dendritic.cells	NAA30	0.372236	3.918894	2.039455	0.04439	-4.12522	0.303184	0.283803

Dendritic.cells	SRP14	0.152379	7.234027	2.03932	0.044404	-4.84576	0.284647	0.258791
Dendritic.cells	CD99L2	-0.77703	2.142314	-2.03928	0.044408	-3.77692	0.313757	0.298197
Dendritic.cells	ATXN7L3B	0.205403	5.941589	2.039175	0.044418	-4.60244	0.291693	0.268303
Dendritic.cells	FANCA	0.875549	3.05493	2.038893	0.044447	-3.7798	0.308269	0.290854
Dendritic.cells	PGGHG	-0.6905	2.820106	-2.03869	0.044468	-3.8572	0.30967	0.292795
Dendritic.cells	PRPF4B	0.219417	6.312389	2.038627	0.044474	-4.64542	0.289648	0.265673
Dendritic.cells	PHB	0.453431	4.493205	2.038618	0.044475	-4.11443	0.299863	0.279475
Dendritic.cells	GGPS1	0.225214	5.393996	2.037965	0.044542	-4.43903	0.294747	0.272684
Dendritic.cells	LXN	-0.51367	3.855864	-2.0379	0.044549	-3.95348	0.303552	0.284613
Dendritic.cells	FRMD4A	0.374679	3.969412	2.037874	0.044551	-4.46301	0.30289	0.283715
Dendritic.cells	ATF1	-0.17675	6.564036	-2.03761	0.044578	-4.65984	0.288271	0.264025
Dendritic.cells	POU3F1	-1.34022	0.061626	-2.03706	0.044634	-3.75895	0.326923	0.316545
Dendritic.cells	SERPINA3M	-0.90146	3.688152	-2.0368	0.044661	-3.91901	0.304718	0.286251
Dendritic.cells	KAT14	0.398493	3.838268	2.035663	0.044778	-4.02342	0.304446	0.28555
Dendritic.cells	LSM7	0.22778	6.346432	2.034897	0.044856	-4.65181	0.29041	0.266493
Dendritic.cells	ABHD18	0.530623	3.182254	2.034704	0.044876	-4.03586	0.308522	0.291102
Dendritic.cells	GCDH	-0.93601	3.116618	-2.03456	0.044891	-3.83324	0.308912	0.291681
Dendritic.cells	GM43560	-0.68557	0.578606	-2.03425	0.044923	-3.80433	0.324533	0.313025
Dendritic.cells	1700066M	0.882852	1.288003	2.033962	0.044953	-3.76774	0.32009	0.307072
Dendritic.cells	ZFHX2	-0.7611	2.765643	-2.03338	0.045012	-3.86771	0.311131	0.294904
Dendritic.cells	PABPC4	-0.29996	5.897724	-2.03331	0.04502	-4.41549	0.293005	0.270284
Dendritic.cells	PTPN18	0.181273	7.11924	2.032514	0.045102	-4.83778	0.286652	0.261486
Dendritic.cells	CD44	-0.20873	8.587659	-2.03167	0.045189	-5.23469	0.279227	0.251295
Dendritic.cells	GGTA1	0.359639	5.365487	2.031306	0.045227	-4.42139	0.296797	0.274956
Dendritic.cells	SERF2	0.131114	9.682679	2.029843	0.045379	-5.28201	0.274029	0.244272
Dendritic.cells	4930599N2	-1.07023	1.733523	-2.02973	0.04539	-3.7716	0.318717	0.30475
Dendritic.cells	KHK	-0.27141	4.3658	-2.02971	0.045392	-4.55611	0.302936	0.283231
Dendritic.cells	GM13561	0.88082	1.307424	2.029368	0.045428	-3.78237	0.321366	0.30843
Dendritic.cells	MYCL	-0.31991	1.305739	-2.02936	0.045429	-4.53966	0.321377	0.308445
Dendritic.cells	PHYHD1	-0.75048	2.730001	-2.02715	0.045659	-3.84844	0.313384	0.297336
Dendritic.cells	COLGALT2	-1.24237	-0.12576	-2.0269	0.045685	-3.77708	0.331279	0.321959
Dendritic.cells	RBBP7	-0.27356	6.2357	-2.02672	0.045704	-4.52451	0.293038	0.269808
Dendritic.cells	H2-AA	-0.55747	8.331767	-2.02643	0.045734	-5.29078	0.281693	0.254551
Dendritic.cells	DDX5	-0.12353	9.318404	-2.02605	0.045774	-5.18453	0.276558	0.247679
Dendritic.cells	SLC39A12	-1.38938	0.492802	-2.02605	0.045774	-3.79578	0.3273	0.316615
Dendritic.cells	PAM16	0.288836	4.987535	2.025512	0.04583	-4.37037	0.300082	0.279527
Dendritic.cells	GM16283	0.970796	0.252287	2.025295	0.045853	-3.77815	0.32884	0.318943
Dendritic.cells	C430049B0	0.891633	1.844436	2.024977	0.045886	-3.81252	0.318805	0.305277
Dendritic.cells	3-Mar	-0.58042	6.392222	-2.02486	0.045899	-4.37727	0.29217	0.269001
Dendritic.cells	CRIP1	0.327401	8.825299	2.024738	0.045911	-5.0475	0.279109	0.251399
Dendritic.cells	GM15708	-0.61558	3.198583	-2.02456	0.04593	-3.95207	0.310563	0.294074
Dendritic.cells	GM49336	-0.29497	4.881295	-2.02449	0.045937	-4.32798	0.300691	0.280618
Dendritic.cells	ZDHHC3	0.329314	5.048938	2.02445	0.045942	-4.27586	0.29973	0.279312
Dendritic.cells	LEFTY1	0.300846	0.408138	2.024147	0.045973	-4.50403	0.327841	0.31791
Dendritic.cells	R3HDM2	0.167189	6.193799	2.023974	0.045991	-4.73748	0.293271	0.270649
Dendritic.cells	FABP5	0.426732	7.278075	2.023743	0.046016	-4.84333	0.287321	0.262703
Dendritic.cells	FYCO1	0.338039	4.137271	2.022864	0.046108	-4.29455	0.305104	0.287058

Dendritic.cells	1700061G1	-0.95108	1.070717	-2.02279	0.046116	-3.79609	0.323738	0.312619
Dendritic.cells	JADE1	0.346109	4.165478	2.02262	0.046134	-4.39096	0.304939	0.286836
Dendritic.cells	GM15545	-0.76289	1.102359	-2.02254	0.046143	-3.80735	0.323539	0.312345
Dendritic.cells	ADAM11	0.32898	-1.14352	2.022019	0.046197	-4.27927	0.338252	0.332555
Dendritic.cells	DGKQ	0.803565	1.470676	2.021116	0.046292	-3.81888	0.32157	0.309621
Dendritic.cells	CD5L	-0.9298	6.068559	-2.02092	0.046313	-4.3639	0.294374	0.272482
Dendritic.cells	OTUB1	0.187288	5.728978	2.020507	0.046357	-4.58991	0.29628	0.275071
Dendritic.cells	STK16	0.473056	4.37442	2.020478	0.04636	-4.08472	0.304041	0.285642
Dendritic.cells	GNG10	0.168992	6.940589	2.020255	0.046383	-5.12036	0.289555	0.265947
Dendritic.cells	FAM222B	0.30149	5.370089	2.019901	0.046421	-4.56309	0.298311	0.277833
Dendritic.cells	RNF24	0.339591	4.233052	2.019892	0.046422	-4.34466	0.304867	0.286768
Dendritic.cells	CLNK	1.137088	1.404175	2.019681	0.046444	-3.81648	0.321986	0.31026
Dendritic.cells	CMTM8	-0.55338	4.192364	-2.01937	0.046478	-4.14553	0.305139	0.287203
Dendritic.cells	TTC3	-0.24467	5.355192	-2.01837	0.046584	-4.6176	0.29893	0.278272
Dendritic.cells	WDFY1	0.406849	4.332443	2.017927	0.04663	-4.1956	0.304952	0.286333
Dendritic.cells	MXRA7	-0.79493	1.654737	-2.01752	0.046673	-3.87732	0.321225	0.308572
Dendritic.cells	ELAVL3	-1.36318	1.03829	-2.01692	0.046738	-3.78952	0.325102	0.31403
Dendritic.cells	EP400	-0.17674	6.870294	-2.01679	0.046751	-4.82265	0.290669	0.267001
Dendritic.cells	WLS	-0.67962	4.263574	-2.01673	0.046758	-3.96637	0.305455	0.287098
Dendritic.cells	KIF1B	-0.41215	5.385583	-2.01548	0.046891	-4.22811	0.299506	0.278637
Dendritic.cells	WRN	0.208093	5.831312	2.015416	0.046898	-4.76409	0.296976	0.275219
Dendritic.cells	ERRFI1	-0.56521	5.004787	-2.0149	0.046954	-4.12271	0.301866	0.281741
Dendritic.cells	XPO6	0.277469	5.572883	2.01379	0.047072	-4.38885	0.299054	0.277714
Dendritic.cells	ZDHHC20	0.159104	6.740107	2.013383	0.047115	-4.85584	0.292505	0.269018
Dendritic.cells	NF1	-0.20824	6.550388	-2.01331	0.047123	-4.78064	0.293557	0.270448
Dendritic.cells	SPINK10	1.048227	0.791956	2.012946	0.047162	-3.79872	0.327922	0.31749
Dendritic.cells	NTHL1	0.954639	0.544589	2.01293	0.047164	-3.79443	0.329507	0.319679
Dendritic.cells	USP18	0.764641	2.737474	2.012503	0.04721	-4.00031	0.315888	0.300902
Dendritic.cells	ARF5	0.229704	8.486216	2.011741	0.047292	-5.06228	0.283434	0.256602
Dendritic.cells	PARVA	-0.91419	1.329681	-2.01156	0.047311	-3.8291	0.324935	0.313166
Dendritic.cells	A930006K0	0.536652	2.074746	2.011021	0.047369	-3.97761	0.320318	0.306958
Dendritic.cells	ERH	0.252379	7.372533	2.010967	0.047375	-4.845	0.289463	0.264876
Dendritic.cells	GM4258	0.338328	4.929368	2.010329	0.047444	-4.69471	0.303356	0.28364
Dendritic.cells	ABCB9	-0.76149	3.155352	-2.01007	0.047472	-3.88665	0.313864	0.298119
Dendritic.cells	BGN	-0.68316	2.420901	-2.00994	0.047485	-3.943	0.318349	0.304286
Dendritic.cells	PRPF31	-0.43788	3.79385	-2.00964	0.047518	-4.06604	0.310058	0.292999
Dendritic.cells	5430431A1	-0.73652	1.423829	-2.00922	0.047564	-3.84606	0.324715	0.313058
Dendritic.cells	GM15518	0.921234	0.80065	2.008181	0.047676	-3.85132	0.329255	0.318842
Dendritic.cells	ATF6B	0.277706	4.456866	2.0074	0.047761	-4.50214	0.307152	0.288324
Dendritic.cells	CDC42EP3	0.591042	5.142958	2.006611	0.047846	-4.04856	0.303351	0.283071
Dendritic.cells	MTDH	0.11348	7.470279	2.006492	0.047859	-5.09079	0.290262	0.265354
Dendritic.cells	TRIM35	-0.34143	5.607952	-2.00632	0.047878	-4.48706	0.300675	0.279513
Dendritic.cells	TM4SF4	-1.07506	2.168937	-2.00531	0.047988	-3.83371	0.321486	0.307899
Dendritic.cells	NXT1	0.466159	4.399059	2.005206	0.047999	-4.12135	0.307963	0.2894
Dendritic.cells	USF1	0.401686	4.934416	2.005157	0.048005	-4.25105	0.304824	0.285131
Dendritic.cells	NECTIN3	-0.88422	1.220532	-2.00463	0.048062	-3.83048	0.327491	0.316303
Dendritic.cells	GIMAP8	-0.95035	2.465426	-2.00459	0.048066	-3.85488	0.319676	0.305545

Dendritic.cells	SPSB1	-0.3946	1.685361	-2.0037	0.048163	-4.40728	0.325011	0.312725
Dendritic.cells	FOLR2	-1.09575	3.655517	-2.00338	0.048198	-3.9311	0.312889	0.296057
Dendritic.cells	UGCG	0.281562	6.436724	2.00315	0.048224	-4.73473	0.29671	0.274087
Dendritic.cells	PIP4K2A	0.181155	7.239298	2.002826	0.048259	-5.14636	0.292287	0.268191
Dendritic.cells	RNF34	0.368878	4.455799	2.002403	0.048305	-4.20449	0.308277	0.289928
Dendritic.cells	ACSS1	0.565509	3.946222	2.001276	0.048429	-4.06591	0.311918	0.294341
Dendritic.cells	TMEM40	1.167694	0.497552	2.000477	0.048517	-3.81388	0.333869	0.32419
Dendritic.cells	NAT8F1	-1.14171	1.619362	-1.99972	0.048601	-3.8245	0.327027	0.314457
Dendritic.cells	EVA1A	-1.03607	1.060527	-1.99943	0.048632	-3.81727	0.330616	0.319516
Dendritic.cells	LCK	-0.71737	3.728178	-1.99904	0.048675	-3.93393	0.314005	0.296851
Dendritic.cells	TINAGL1	-0.78787	2.066409	-1.99888	0.048693	-3.96967	0.324243	0.310924
Dendritic.cells	MTMR9	0.384468	3.972449	1.997773	0.048815	-4.1165	0.313136	0.295239
Dendritic.cells	SLC27A5	-1.10038	1.457834	-1.99738	0.048859	-3.8362	0.328827	0.316775
Dendritic.cells	MAST1	-1.17907	1.020113	-1.99664	0.04894	-3.85369	0.331703	0.320868
Dendritic.cells	THBD	-0.91008	3.145231	-1.99659	0.048945	-3.95295	0.31832	0.302458
Dendritic.cells	SULF2	-0.8905	2.585463	-1.99652	0.048954	-3.86979	0.321779	0.307205
Dendritic.cells	C5AR1	1.350112	4.179898	1.996021	0.049009	-3.91018	0.312213	0.293995
Dendritic.cells	ACTR8	0.484602	3.537225	1.994588	0.049168	-4.04804	0.316774	0.299694
Dendritic.cells	SUMO1	0.109579	7.992553	1.994551	0.049172	-5.04991	0.291051	0.264741
Dendritic.cells	GAA	0.395648	3.191942	1.993946	0.04924	-4.24264	0.319012	0.30286
Dendritic.cells	RNF185	-0.23174	5.117108	-1.99384	0.049252	-4.62176	0.307444	0.28709
Dendritic.cells	1810034E1	0.490206	1.895787	1.993604	0.049278	-4.1243	0.32711	0.31409
Dendritic.cells	GRK4	-0.5868	3.108893	-1.99323	0.049319	-3.98606	0.319534	0.30382
Dendritic.cells	D930030I0	1.188183	0.408475	1.992962	0.049349	-3.82388	0.336726	0.327615
Dendritic.cells	LPAR6	-0.47934	4.523596	-1.99236	0.049417	-4.25554	0.310963	0.292439
Dendritic.cells	IL12A	0.699283	2.215059	1.992037	0.049452	-4.01878	0.325102	0.311921
Dendritic.cells	GM11755	-1.26782	-0.115	-1.99201	0.049456	-3.82437	0.340188	0.33278
Dendritic.cells	KDM4D	1.144444	0.328103	1.991996	0.049457	-3.82498	0.337255	0.328712
Dendritic.cells	PSMA3	-0.15081	7.755055	-1.99147	0.049516	-5.02667	0.292474	0.267545
Dendritic.cells	PDZRN3	-1.14587	0.932125	-1.99142	0.049521	-3.82901	0.333306	0.323481
Dendritic.cells	GM50333	-0.87103	1.191453	-1.99127	0.049538	-3.84072	0.331628	0.321223
Dendritic.cells	SDF2L1	0.371519	4.888426	1.99066	0.049607	-4.41433	0.308914	0.289965
Dendritic.cells	HSF1	-0.29665	4.997353	-1.99056	0.049618	-4.42135	0.308272	0.289088
Dendritic.cells	TMEM50A	0.172988	7.711684	1.990199	0.049658	-5.02943	0.292821	0.268164
Dendritic.cells	CYP8B1	-1.06943	1.074088	-1.99008	0.049672	-3.83215	0.332508	0.322621
Dendritic.cells	PTPRF	0.292226	1.276101	1.989366	0.049752	-4.48733	0.331543	0.321121
Dendritic.cells	CKM	1.543931	0.1057	1.989086	0.049783	-3.8288	0.339213	0.331849
Dendritic.cells	TRIM12C	0.41018	4.726648	1.988808	0.049814	-4.33976	0.31022	0.291957
Dendritic.cells	2700097O(	0.559802	3.090234	1.988347	0.049866	-4.02909	0.320206	0.305671
Dendritic.cells	AC142100.	1.187672	0.335219	1.988186	0.049884	-3.83301	0.337796	0.330028
Dendritic.cells	TRIM37	-0.27438	4.93302	-1.98714	0.050003	-4.59141	0.309471	0.290785
Dendritic.cells	CYP3A44	-1.33508	2.308322	-1.98678	0.050043	-3.85431	0.325497	0.312869
Dendritic.cells	HCST	0.491564	5.375869	1.986735	0.050048	-4.30265	0.306867	0.287269
Dendritic.cells	FIS1	0.201434	7.530663	1.986595	0.050064	-4.9303	0.294599	0.27059
Dendritic.cells	ST18	1.330177	0.283819	1.986054	0.050125	-3.84042	0.338786	0.33121
Dendritic.cells	PZP	-0.84956	4.39586	-1.98506	0.050237	-4.15144	0.312989	0.295889
Dendritic.cells	GPR146	0.289314	3.278936	1.985024	0.050241	-4.47908	0.319779	0.305231

Dendritic.cells	STK4	0.179569	7.52595	1.984683	0.05028	-4.98937	0.294927	0.271295
Dendritic.cells	GM17655	0.858434	1.183959	1.984568	0.050293	-3.86616	0.333021	0.323661
Dendritic.cells	ADGRE1	-1.05032	4.711233	-1.98398	0.05036	-4.05442	0.311105	0.293574
Dendritic.cells	GM37065	0.807836	2.706239	1.983794	0.050381	-3.97965	0.323333	0.310487
Dendritic.cells	ACAD9	0.621111	2.542008	1.98363	0.0504	-3.94219	0.324362	0.311918
Dendritic.cells	GCKR	-1.01991	1.581851	-1.9835	0.050414	-3.86325	0.330455	0.320407
Dendritic.cells	MED28	0.218264	6.023126	1.983455	0.050419	-4.6581	0.303423	0.283158
Dendritic.cells	DIAPH1	0.166595	7.355352	1.983333	0.050433	-5.0372	0.295876	0.272869
Dendritic.cells	MDGA1	-1.12524	0.010829	-1.98286	0.050487	-3.84149	0.340735	0.334722
Dendritic.cells	GJB2	-1.09345	1.638244	-1.98282	0.050492	-3.84918	0.3301	0.319928
Dendritic.cells	TSEN15	-0.7171	2.776415	-1.98213	0.05057	-3.88674	0.323216	0.310225
Dendritic.cells	ACACB	-1.02787	0.257652	-1.98184	0.050603	-3.85661	0.339451	0.332888
Dendritic.cells	PXDN	-0.70354	0.993778	-1.98154	0.050638	-3.94664	0.334652	0.326221
Dendritic.cells	IQCB1	0.267845	4.824078	1.980746	0.050728	-4.5239	0.311178	0.293575
Dendritic.cells	ETHE1	0.332761	4.997365	1.980369	0.050771	-4.38782	0.310231	0.29219
Dendritic.cells	TGOLN1	-0.26809	5.839155	-1.98012	0.050799	-4.62005	0.305295	0.285487
Dendritic.cells	BDP1	-0.25041	5.529011	-1.97969	0.050849	-4.59861	0.307228	0.288072
Dendritic.cells	HECW2	-0.544	3.162677	-1.97892	0.050937	-4.26502	0.321709	0.307901
Dendritic.cells	PRR3	0.417275	3.987386	1.978659	0.050967	-4.19116	0.316643	0.30099
Dendritic.cells	FAM102A	-0.62504	4.182226	-1.97843	0.050993	-4.01735	0.315462	0.299455
Dendritic.cells	LY6G5B	0.915324	1.345565	1.97817	0.051023	-3.85796	0.333231	0.324077
Dendritic.cells	AKAP10	0.257731	5.860095	1.977973	0.051046	-4.75091	0.305515	0.285852
Dendritic.cells	SULT2A8	-1.3338	0.571296	-1.97763	0.051085	-3.84602	0.338294	0.331258
Dendritic.cells	CABCOCO1	-0.95213	0.315412	-1.9776	0.051088	-3.85229	0.339987	0.33362
Dendritic.cells	GM2A	0.201438	7.22888	1.977299	0.051123	-5.10469	0.297735	0.275303
Dendritic.cells	BTBD3	0.594526	1.337187	1.97643	0.051223	-4.12053	0.333783	0.324767
Dendritic.cells	NUP35	-0.51504	3.291207	-1.97576	0.0513	-4.08591	0.321692	0.307833
Dendritic.cells	GM30881	-0.74009	1.986079	-1.97508	0.051379	-3.90957	0.330232	0.319501
Dendritic.cells	TPPP3	1.098618	1.071021	1.974718	0.05142	-3.88875	0.336237	0.327878
Dendritic.cells	MTMR14	0.272089	5.427711	1.973593	0.051551	-4.60987	0.309769	0.290901
Dendritic.cells	ASS1	0.530771	6.535258	1.972614	0.051664	-4.73181	0.303693	0.282323
Dendritic.cells	CHERP	-0.28362	5.016827	-1.97255	0.051672	-4.40693	0.312588	0.294514
Dendritic.cells	PCOLCE2	-1.25532	1.678726	-1.97182	0.051756	-3.86867	0.333516	0.323395
Dendritic.cells	SPDL1	-0.86622	2.833783	-1.97182	0.051757	-3.89598	0.326139	0.313172
Dendritic.cells	PWP2	-0.89457	1.891751	-1.97133	0.051813	-3.88406	0.33222	0.321661
Dendritic.cells	VMAC	0.695942	1.5263	1.971206	0.051828	-3.94219	0.334585	0.324995
Dendritic.cells	PXMP2	-0.96736	4.067436	-1.97045	0.051916	-4.04711	0.318879	0.303049
Dendritic.cells	CTNS	-0.43932	3.606042	-1.97026	0.051938	-4.22441	0.321719	0.307013
Dendritic.cells	CPEB3	-0.82599	4.234811	-1.96886	0.052101	-3.99468	0.318619	0.30237
Dendritic.cells	RAET1E	0.638577	4.113795	1.968686	0.052121	-4.01033	0.319359	0.303448
Dendritic.cells	DOCK8	0.219715	7.656272	1.968063	0.052194	-5.05797	0.298712	0.275093
Dendritic.cells	COPS4	0.194408	5.581159	1.967979	0.052204	-4.7538	0.31067	0.291453
Dendritic.cells	DISP1	0.399722	3.443949	1.967666	0.052241	-4.23414	0.323692	0.309433
Dendritic.cells	TSPO	0.307873	7.399271	1.967144	0.052302	-4.99194	0.300383	0.277252
Dendritic.cells	DEK	0.218882	7.842	1.966895	0.052331	-5.02987	0.297894	0.273939
Dendritic.cells	CBFB	-0.16039	6.809866	-1.96588	0.05245	-4.85442	0.304262	0.282208
Dendritic.cells	UBAC2	0.194096	6.679645	1.963795	0.052696	-4.91019	0.306267	0.28369

Dendritic.cells	PRKD2	-0.44042	4.348606	-1.96281	0.052812	-4.18697	0.320612	0.302925
Dendritic.cells	KITL	-1.10666	1.956944	-1.96214	0.052892	-4.00514	0.335744	0.323911
Dendritic.cells	LAMC1	-0.42984	4.523339	-1.96209	0.052897	-4.34592	0.319541	0.301588
Dendritic.cells	MRPS34	0.279261	4.826949	1.961997	0.052908	-4.47554	0.31769	0.29905
Dendritic.cells	4933434E2	0.260906	5.269667	1.961855	0.052925	-4.59655	0.315016	0.29541
Dendritic.cells	GNA15	0.282183	3.584316	1.961671	0.052947	-4.68981	0.325353	0.309629
Dendritic.cells	MAGI3	-0.31107	6.598022	-1.96131	0.052989	-4.8469	0.307173	0.284796
Dendritic.cells	MAPK1	0.141009	7.693924	1.961164	0.053007	-5.05985	0.300891	0.276266
Dendritic.cells	HDAC3	0.27667	4.707537	1.960579	0.053076	-4.44987	0.31866	0.30049
Dendritic.cells	CD81	-0.39591	7.604661	-1.9594	0.053217	-4.68396	0.302252	0.277574
Dendritic.cells	TMEM109	0.462091	3.841384	1.958695	0.0533	-4.2398	0.324941	0.308489
Dendritic.cells	TUBGCP4	0.345035	4.42498	1.958529	0.05332	-4.36146	0.321322	0.303518
Dendritic.cells	STAP2	-1.06975	0.504981	-1.95809	0.053372	-3.88215	0.346728	0.338622
Dendritic.cells	PAQR9	-0.8303	4.029832	-1.95782	0.053405	-4.01772	0.323854	0.307081
Dendritic.cells	PARP6	0.613335	2.991541	1.957661	0.053424	-4.02365	0.330392	0.316145
Dendritic.cells	SBK1	-0.56294	3.374185	-1.95685	0.053521	-4.05926	0.328378	0.313201
Dendritic.cells	AFF1	0.1935	8.730515	1.955671	0.053662	-5.21043	0.297258	0.270261
Dendritic.cells	SPRY1	-0.56477	1.345776	-1.9551	0.05373	-4.19699	0.342335	0.332247
Dendritic.cells	VPS37B	-0.31986	7.797142	-1.95504	0.053737	-5.01129	0.302586	0.277646
Dendritic.cells	AQR	-0.22846	4.812338	-1.95426	0.053831	-4.52333	0.320576	0.30184
Dendritic.cells	SPTAN1	-0.22451	6.559592	-1.95323	0.053955	-4.86686	0.310544	0.287816
Dendritic.cells	B4GAT1	-0.7525	1.65015	-1.95312	0.053968	-3.95145	0.341213	0.329952
Dendritic.cells	UCHL3	0.209701	6.235223	1.952365	0.054059	-4.91636	0.312809	0.290677
Dendritic.cells	ETV6	0.186118	8.156803	1.951508	0.054162	-5.38227	0.302056	0.275674
Dendritic.cells	SLC16A10	-0.45762	7.024829	-1.95131	0.054186	-4.68977	0.308553	0.284501
Dendritic.cells	2810006K2	-0.81259	2.570884	-1.94967	0.054385	-3.96733	0.336904	0.322563
Dendritic.cells	RAC1	-0.15279	7.82414	-1.94957	0.054396	-5.03218	0.30479	0.278688
Dendritic.cells	FASTKD3	0.756753	1.993657	1.949006	0.054465	-3.96199	0.34091	0.328006
Dendritic.cells	EMC1	0.376449	3.622778	1.948789	0.054491	-4.22542	0.330349	0.313505
Dendritic.cells	ZFP993	-0.77542	1.824761	-1.94829	0.054552	-3.96242	0.34222	0.329737
Dendritic.cells	GC	-0.85982	5.287073	-1.94651	0.054768	-4.37334	0.321255	0.300111
Dendritic.cells	ALKBH3	0.479239	3.490416	1.946045	0.054825	-4.15929	0.332671	0.315626
Dendritic.cells	ZCCHC3	0.810685	0.70709	1.945482	0.054894	-3.94213	0.351352	0.341227
Dendritic.cells	DEAF1	0.535571	3.281829	1.944089	0.055064	-4.03761	0.334448	0.318142
Dendritic.cells	MEA1	0.29334	5.276389	1.943832	0.055096	-4.50031	0.321901	0.300961
Dendritic.cells	CFAP36	-0.31655	4.102137	-1.94357	0.055127	-4.38071	0.329214	0.311006
Dendritic.cells	ATG2B	-0.30034	4.861013	-1.94352	0.055134	-4.47127	0.324463	0.304505
Dendritic.cells	TBXA2R	0.42483	2.36173	1.943512	0.055135	-4.49305	0.340445	0.326437
Dendritic.cells	SLC9A1	0.204399	5.416377	1.943476	0.05514	-4.77897	0.321043	0.299833
Dendritic.cells	ZFP467	0.560727	2.303773	1.943355	0.055154	-4.12998	0.340827	0.327009
Dendritic.cells	BHMT2	-1.11892	2.060069	-1.94314	0.055181	-3.91794	0.34244	0.329347
Dendritic.cells	2410022M	0.662462	1.591485	1.942985	0.0552	-3.9849	0.345568	0.333673
Dendritic.cells	SBF2	-0.38638	5.368073	-1.94188	0.055335	-4.49766	0.321952	0.300836
Dendritic.cells	1810009A1	0.596704	2.243882	1.940576	0.055496	-3.98885	0.342169	0.328381
Dendritic.cells	1700123O2	0.321634	4.989927	1.940396	0.055518	-4.4522	0.324562	0.304222
Dendritic.cells	PTPN13	-1.2242	0.415506	-1.94037	0.055522	-3.89775	0.354552	0.345496
Dendritic.cells	MLH1	-0.50486	2.750298	-1.94034	0.055526	-4.03206	0.338833	0.323788

Dendritic.cells	TRMO	-0.515	3.013486	-1.93992	0.055577	-4.10884	0.337115	0.321609
Dendritic.cells	PIK3CB	0.406443	4.688415	1.939635	0.055612	-4.52132	0.326438	0.30701
Dendritic.cells	1810037I1	0.262248	6.482621	1.939125	0.055675	-4.76787	0.315479	0.292169
Dendritic.cells	TMEM65	-0.42553	4.9205	-1.93908	0.05568	-4.37048	0.324993	0.305156
Dendritic.cells	ZDHHC12	0.910464	1.360226	1.938811	0.055714	-3.94095	0.348087	0.33701
Dendritic.cells	TRPC1	0.84316	-0.17084	1.938787	0.055717	-3.94076	0.358637	0.351647
Dendritic.cells	RRNAD1	0.401068	3.384404	1.938595	0.055741	-4.18655	0.334713	0.318634
Dendritic.cells	ZFP74	0.551283	2.132709	1.938391	0.055766	-4.07357	0.342906	0.329965
Dendritic.cells	TFPI	-0.44936	3.468648	-1.9383	0.055777	-4.24664	0.334171	0.317928
Dendritic.cells	NEDD8	0.155066	7.48397	1.937815	0.055837	-5.00235	0.309737	0.284408
Dendritic.cells	TEX9	0.869563	1.820323	1.936368	0.056017	-3.94087	0.345597	0.333653
Dendritic.cells	THEMIS2	0.521833	4.692319	1.936177	0.056041	-4.32697	0.326988	0.308111
Dendritic.cells	AHSA1	0.367887	5.071608	1.935948	0.056069	-4.36138	0.324627	0.304967
Dendritic.cells	LRRC8D	-0.19028	7.366735	-1.93571	0.056099	-5.2063	0.310803	0.286134
Dendritic.cells	FAM234B	-0.33386	2.096184	-1.93543	0.056134	-4.5511	0.343753	0.331367
Dendritic.cells	GM5535	-1.0883	0.151841	-1.93537	0.056141	-3.93308	0.357009	0.34976
Dendritic.cells	GTF2A2	0.22709	5.923594	1.935337	0.056145	-4.70797	0.319402	0.297886
Dendritic.cells	OTC	-0.96293	3.438444	-1.93531	0.056149	-4.07361	0.334953	0.319223
Dendritic.cells	MCUB	1.054268	1.557619	1.934692	0.056225	-3.94735	0.347649	0.336602
Dendritic.cells	GM20045	0.892952	1.764585	1.934226	0.056284	-3.93579	0.346425	0.334845
Dendritic.cells	SNAP47	-0.69678	2.473639	-1.93358	0.056364	-3.9631	0.342004	0.328528
Dendritic.cells	PGM1	0.428467	4.592397	1.933271	0.056403	-4.30119	0.328384	0.30985
Dendritic.cells	CYB5D1	-1.01823	0.775244	-1.93299	0.056438	-3.92653	0.353553	0.344639
Dendritic.cells	UBL7	0.270855	5.130384	1.93251	0.056498	-4.65791	0.325222	0.305441
Dendritic.cells	TBC1D22B	0.379552	3.778303	1.931796	0.056587	-4.32476	0.334102	0.317278
Dendritic.cells	GM4566	1.064269	1.718905	1.931118	0.056672	-3.91529	0.347975	0.336211
Dendritic.cells	TUFM	0.413242	4.731912	1.930218	0.056785	-4.32831	0.328342	0.309411
Dendritic.cells	MIR17HG	0.742936	2.192534	1.930146	0.056794	-3.98978	0.344793	0.332065
Dendritic.cells	ZDHHC1	-0.70711	1.415954	-1.93	0.056813	-3.96772	0.350028	0.339331
Dendritic.cells	PEX3	0.423912	3.770542	1.929609	0.056862	-4.16859	0.334451	0.317945
Dendritic.cells	DNAJA1	-0.15963	7.461326	-1.92959	0.056864	-5.01959	0.31177	0.2869
Dendritic.cells	PBX2	0.346627	4.930622	1.929203	0.056913	-4.49329	0.327097	0.308011
Dendritic.cells	ARL6	0.645181	1.698649	1.929054	0.056932	-3.99397	0.348111	0.337063
Dendritic.cells	9430060I0	1.023429	1.308677	1.928974	0.056942	-3.92996	0.350759	0.340736
Dendritic.cells	OAF	0.687374	3.151249	1.928964	0.056943	-4.04802	0.338463	0.323718
Dendritic.cells	S100A9	1.268971	6.814997	1.928625	0.056986	-4.52008	0.31566	0.292457
Dendritic.cells	GM48796	-0.97667	0.350478	-1.92787	0.057081	-3.91874	0.357843	0.350283
Dendritic.cells	ITPR1	0.16475	6.992018	1.927556	0.057121	-5.35984	0.314956	0.291189
Dendritic.cells	PALB2	0.828	2.125531	1.927397	0.057141	-3.94249	0.345698	0.333454
Dendritic.cells	CNPY3	0.171094	5.300686	1.926922	0.057201	-4.86906	0.325369	0.305421
Dendritic.cells	TAF3	0.214515	5.653075	1.926713	0.057227	-4.79084	0.323193	0.302499
Dendritic.cells	NUMB	-0.24355	6.956562	-1.92646	0.057259	-4.86466	0.315314	0.291777
Dendritic.cells	HGS	-0.34902	4.094551	-1.92531	0.057405	-4.3579	0.3335	0.316242
Dendritic.cells	PON2	0.286576	6.020273	1.924693	0.057483	-4.6436	0.321463	0.299934
Dendritic.cells	SLC25A33	-0.58199	3.983563	-1.9246	0.057496	-4.11768	0.334212	0.317439
Dendritic.cells	MED31	-0.47853	3.491551	-1.92444	0.057516	-4.14649	0.337388	0.321869
Dendritic.cells	4930523C0	0.319858	6.126408	1.924367	0.057525	-4.5006	0.320816	0.299122

Dendritic.cells	MLKL	-0.98034	2.335281	-1.92384	0.057592	-3.95141	0.345004	0.332535
Dendritic.cells	SLC30A6	0.460394	3.417011	1.923578	0.057625	-4.17354	0.337873	0.322794
Dendritic.cells	WASHC4	0.174757	5.792598	1.923562	0.057627	-4.9052	0.322856	0.302139
Dendritic.cells	GM15283	0.396739	4.261918	1.923531	0.057631	-4.46803	0.332431	0.315293
Dendritic.cells	POLR3GL	0.260606	3.878267	1.923179	0.057676	-4.57776	0.334888	0.318725
Dendritic.cells	GM7160	-0.98883	2.542262	-1.92309	0.057687	-3.95003	0.343625	0.330799
Dendritic.cells	CYP2A12	-1.106	2.85261	-1.92223	0.057797	-3.99674	0.341901	0.328249
Dendritic.cells	RARB	-0.9508	1.586463	-1.92217	0.057804	-4.05109	0.35039	0.340012
Dendritic.cells	4933412E1	0.895956	1.908564	1.921322	0.057913	-3.97052	0.348541	0.337401
Dendritic.cells	KIF3B	0.460483	3.495441	1.92109	0.057943	-4.15592	0.338015	0.322896
Dendritic.cells	COL4A1	-0.47793	3.330895	-1.92101	0.057952	-4.46141	0.339088	0.324412
Dendritic.cells	CYTH3	-0.36844	4.88921	-1.92002	0.05808	-4.48769	0.329488	0.310813
Dendritic.cells	MYL12B	0.168688	8.183707	1.919997	0.058082	-5.1758	0.309574	0.283576
Dendritic.cells	GM47889	0.939483	1.560566	1.919404	0.058158	-3.96058	0.351594	0.341091
Dendritic.cells	TMEM8	0.583602	2.000346	1.918448	0.058281	-4.093	0.348975	0.337174
Dendritic.cells	TMEM9	0.783825	2.576914	1.918437	0.058282	-4.01399	0.345101	0.331811
Dendritic.cells	ZCCHC4	0.549156	3.455439	1.918204	0.058312	-4.15767	0.3393	0.323854
Dendritic.cells	APPBP2	-0.19984	6.248663	-1.91773	0.058374	-4.8456	0.321831	0.299792
Dendritic.cells	SERPINB6B	-0.74163	2.936635	-1.91691	0.058479	-4.22567	0.343011	0.328999
Dendritic.cells	HSPA5	-0.15982	8.355671	-1.91689	0.058481	-5.35444	0.309418	0.282934
Dendritic.cells	GM28707	0.713185	1.154193	1.916849	0.058487	-4.06114	0.355069	0.345722
Dendritic.cells	EPB42	1.094506	-0.1647	1.916258	0.058563	-3.93521	0.364607	0.358797
Dendritic.cells	ARL4A	-0.27562	4.453783	-1.91573	0.058631	-4.6242	0.33359	0.315731
Dendritic.cells	HIC1	-1.1195	2.158311	-1.91551	0.05866	-3.99649	0.348676	0.336555
Dendritic.cells	TUSC3	-0.35136	4.978446	-1.91496	0.05873	-4.44422	0.330259	0.311369
Dendritic.cells	CFAP97	0.407598	3.29046	1.914829	0.058748	-4.18205	0.341131	0.326354
Dendritic.cells	PHLDB2	-0.66917	2.509693	-1.91481	0.05875	-4.17082	0.346312	0.33352
Dendritic.cells	RNF130	0.181199	7.282781	1.913663	0.058899	-5.11323	0.316429	0.292385
Dendritic.cells	DCAF5	-0.23872	5.52135	-1.91331	0.058945	-4.79572	0.327164	0.307151
Dendritic.cells	SNRPN	1.277168	-0.6475	1.913299	0.058946	-3.9346	0.368617	0.364619
Dendritic.cells	GM26740	0.28055	7.068845	1.913133	0.058967	-4.95513	0.317707	0.294219
Dendritic.cells	KLHL6	0.305661	6.109159	1.913042	0.058979	-4.80341	0.323529	0.302208
Dendritic.cells	TMEM120/	-0.52543	3.799392	-1.91296	0.05899	-4.1294	0.338122	0.322284
Dendritic.cells	CEACAM1	0.43418	3.675486	1.911371	0.059196	-4.29434	0.339884	0.323999
Dendritic.cells	SRR	0.506915	2.599008	1.911204	0.059218	-4.13382	0.347013	0.33391
Dendritic.cells	SLX1B	0.557899	3.074893	1.910855	0.059264	-4.0501	0.343923	0.329598
Dendritic.cells	LCN2	1.858309	3.398879	1.909697	0.059415	-3.9856	0.342474	0.327087
Dendritic.cells	TNFSF13	-1.14219	1.863945	-1.90732	0.059726	-3.95734	0.354363	0.342181
Dendritic.cells	TENT2	0.171469	6.624422	1.906982	0.05977	-4.99015	0.323456	0.299848
Dendritic.cells	4930402H2	-0.33955	4.453202	-1.90696	0.059773	-4.49504	0.337106	0.318532
Dendritic.cells	STFA2	1.067462	2.469209	1.906526	0.05983	-4.01664	0.350233	0.336804
Dendritic.cells	PEF1	0.58332	3.347699	1.906481	0.059836	-4.08793	0.344342	0.328685
Dendritic.cells	KATNAL1	-0.98717	0.973387	-1.90482	0.060055	-3.95895	0.36168	0.351839
Dendritic.cells	ITGA4	0.210734	7.657286	1.90416	0.060141	-5.32096	0.318506	0.292335
Dendritic.cells	2-Mar	0.242785	5.868752	1.903897	0.060176	-4.75206	0.329474	0.307259
Dendritic.cells	D030028A(	-0.84954	2.795422	-1.90365	0.060208	-3.99663	0.349468	0.334716
Dendritic.cells	PIK3R3	-0.72301	3.43068	-1.90285	0.060315	-4.20006	0.345524	0.329213

Dendritic.cells	GM47601	-1.03593	0.02249	-1.90277	0.060325	-3.95172	0.369152	0.361963
Dendritic.cells	TMEM59	0.170016	6.649746	1.902209	0.060399	-5.0343	0.32516	0.301249
Dendritic.cells	GM50240	0.780974	1.572659	1.901391	0.060508	-4.00906	0.358505	0.347222
Dendritic.cells	UBASH3A	-1.14541	2.009253	-1.90125	0.060526	-3.95991	0.35548	0.343055
Dendritic.cells	DUSP1	-0.45486	7.412842	-1.90092	0.06057	-4.76296	0.320586	0.29528
Dendritic.cells	VTI1A	0.178372	7.233705	1.900806	0.060585	-5.13016	0.32167	0.296789
Dendritic.cells	GM39326	0.852562	1.289251	1.900718	0.060597	-3.99698	0.360485	0.350178
Dendritic.cells	GM36862	-0.97374	0.340355	-1.90068	0.060602	-3.97425	0.367213	0.35953
Dendritic.cells	LRRC47	-0.28614	4.227098	-1.90038	0.060642	-4.47284	0.340645	0.322765
Dendritic.cells	CAPRIN2	-0.74842	3.181523	-1.89985	0.060713	-4.11509	0.34779	0.332584
Dendritic.cells	FCGRT	-0.50392	4.823452	-1.89949	0.06076	-4.49762	0.337085	0.317839
Dendritic.cells	CP	-0.70565	4.588111	-1.89888	0.060841	-4.29235	0.338678	0.320259
Dendritic.cells	PRRG4	0.747448	0.01648	1.898373	0.060909	-4.05468	0.370013	0.363851
Dendritic.cells	HDAC1	-0.19377	6.039987	-1.89819	0.060934	-4.83642	0.329433	0.307764
Dendritic.cells	FGB	-0.54457	5.797972	-1.89815	0.060939	-4.61113	0.330951	0.309846
Dendritic.cells	EEF1AKMT	0.329374	4.588498	1.898108	0.060944	-4.60584	0.338675	0.320464
Dendritic.cells	SUN1	0.361868	3.918531	1.897982	0.060961	-4.33416	0.343054	0.326527
Dendritic.cells	SLC38A1	-0.17716	7.937408	-1.89774	0.060993	-5.43409	0.317848	0.29206
Dendritic.cells	LY86	0.191097	6.704851	1.896718	0.06113	-5.46045	0.325714	0.302537
Dendritic.cells	TADA2A	0.574166	2.820969	1.896709	0.061131	-4.10751	0.35082	0.337077
Dendritic.cells	TMEM91	0.714288	2.459733	1.896406	0.061172	-4.07165	0.35333	0.340603
Dendritic.cells	C3AR1	-1.34313	2.290307	-1.89575	0.06126	-4.00472	0.354647	0.342318
Dendritic.cells	KLHL20	0.539978	3.27084	1.895711	0.061265	-4.23114	0.347992	0.333107
Dendritic.cells	POP7	-0.29664	4.908142	-1.8955	0.061293	-4.57385	0.337228	0.318314
Dendritic.cells	DEF6	0.223326	5.457612	1.894402	0.061441	-4.81432	0.334106	0.313889
Dendritic.cells	FAU	0.106782	11.4506	1.894322	0.061451	-5.76608	0.298707	0.265687
Dendritic.cells	ZFPM2	-0.65979	2.777013	-1.89412	0.061479	-4.37563	0.351739	0.338315
Dendritic.cells	GM17251	0.786733	2.365949	1.894022	0.061492	-4.04025	0.354547	0.342233
Dendritic.cells	HPS3	0.358312	4.626547	1.893519	0.061559	-4.48503	0.339649	0.321529
Dendritic.cells	TRIM11	0.197985	5.06784	1.893206	0.061601	-4.9402	0.336854	0.317606
Dendritic.cells	PPARA	-0.93865	2.784821	-1.89283	0.061652	-4.0653	0.352031	0.338595
Dendritic.cells	DBNL	0.197143	6.127196	1.892614	0.061681	-4.92236	0.330203	0.308536
Dendritic.cells	MARCKS	-0.28634	7.242888	-1.89197	0.061768	-5.00293	0.32331	0.299179
Dendritic.cells	TMEM263	-0.35078	3.98713	-1.89175	0.061797	-4.46706	0.343972	0.327607
Dendritic.cells	SLFN3	0.972167	1.4477	1.891729	0.0618	-3.97566	0.361272	0.351584
Dendritic.cells	TARS2	-0.48425	3.819829	-1.89161	0.061817	-4.1854	0.34508	0.329157
Dendritic.cells	MRPS14	0.184175	6.892734	1.891469	0.061836	-5.019	0.325453	0.30217
Dendritic.cells	DENND4C	-0.32713	5.068456	-1.89025	0.062001	-4.53766	0.337432	0.318483
Dendritic.cells	PFDN4	0.352619	5.046958	1.890013	0.062033	-4.49269	0.33757	0.318698
Dendritic.cells	CDC42EP2	-0.74614	3.08028	-1.88968	0.062077	-4.14735	0.350556	0.336722
Dendritic.cells	TERF1	0.274773	5.254483	1.889563	0.062094	-4.59476	0.336236	0.31695
Dendritic.cells	BMPR1A	-1.12434	2.310682	-1.88952	0.062099	-3.98734	0.355809	0.344013
Dendritic.cells	HNRNPH3	0.210049	5.447268	1.88938	0.062118	-4.65235	0.335003	0.315315
Dendritic.cells	ESCO1	0.21355	5.77786	1.88907	0.06216	-4.87634	0.332958	0.312525
Dendritic.cells	GNB5	0.864012	0.819995	1.888342	0.062259	-3.98381	0.366572	0.358924
Dendritic.cells	FTO	-0.17151	6.592827	-1.88811	0.06229	-5.06999	0.328073	0.305761
Dendritic.cells	KDM7A	0.270351	7.129544	1.888063	0.062297	-4.85157	0.324764	0.301226

Dendritic.cells	STAU2	-0.68134	2.900653	-1.88719	0.062416	-4.09863	0.352396	0.339196
Dendritic.cells	PDCD5	0.201481	6.418317	1.886967	0.062446	-4.8987	0.329463	0.30762
Dendritic.cells	TXNDC5	0.201472	5.179694	1.886941	0.06245	-5.05473	0.337312	0.318413
Dendritic.cells	CPNE8	-0.68071	2.649239	-1.88598	0.062581	-4.20375	0.354677	0.342029
Dendritic.cells	PPIF	0.309067	3.577224	1.88543	0.062656	-4.58842	0.348625	0.333506
Dendritic.cells	PALM	0.328869	4.231998	1.885037	0.062709	-4.65064	0.344388	0.327606
Dendritic.cells	SDHAF2	0.363661	4.655098	1.884735	0.062751	-4.40587	0.341609	0.323892
Dendritic.cells	TASOR2	0.21667	5.373883	1.884341	0.062805	-4.90773	0.336952	0.317547
Dendritic.cells	RDH13	-0.79275	1.398416	-1.88434	0.062805	-4.01819	0.363761	0.354687
Dendritic.cells	RNF150	1.073388	2.909798	1.883596	0.062907	-4.08025	0.353658	0.340258
Dendritic.cells	SUPT4A	0.220516	6.836136	1.883015	0.062986	-4.98496	0.328347	0.305343
Dendritic.cells	SLC7A1	-0.33404	5.141375	-1.88245	0.063064	-4.76449	0.339332	0.320317
Dendritic.cells	LIPA	-0.3677	5.64326	-1.88205	0.063118	-4.61322	0.336222	0.31603
Dendritic.cells	SERPINA6	-1.23507	1.127419	-1.88152	0.063191	-4.01389	0.366932	0.35856
Dendritic.cells	GM19967	-0.68412	1.377066	-1.8813	0.063222	-4.07128	0.365154	0.356226
Dendritic.cells	LNCPINT	-0.2566	8.328661	-1.88115	0.063242	-5.49732	0.319751	0.293614
Dendritic.cells	BTG2	0.250755	7.421641	1.88052	0.063329	-5.17255	0.325525	0.301374
Dendritic.cells	EVI5	0.22975	5.166502	1.880143	0.063381	-5.02096	0.339768	0.32103
Dendritic.cells	ITFG1	0.186196	5.454025	1.879984	0.063403	-4.87351	0.33791	0.318483
Dendritic.cells	PAQR8	-1.01595	0.223677	-1.87979	0.06343	-4.00439	0.373813	0.3684
Dendritic.cells	HORMAD2	-1.35425	1.357831	-1.87902	0.063536	-3.98793	0.365835	0.357296
Dendritic.cells	CDKN2AIPN	-0.46267	4.272414	-1.87887	0.063557	-4.21338	0.34581	0.329495
Dendritic.cells	AP1S3	0.281636	5.57812	1.878654	0.063587	-4.93399	0.337291	0.317756
Dendritic.cells	FABP1	-0.6925	7.428716	-1.87862	0.063591	-4.98728	0.32568	0.301801
Dendritic.cells	MANBA	0.248786	4.766674	1.878363	0.063627	-4.77575	0.342576	0.325113
Dendritic.cells	ELMSAN1	-0.21304	6.924696	-1.87728	0.063778	-5.30027	0.329341	0.306451
Dendritic.cells	DAD1	0.167305	6.948328	1.876879	0.063833	-5.17218	0.329194	0.30644
Dendritic.cells	RABL6	0.210686	5.136693	1.876786	0.063846	-4.74919	0.340711	0.322332
Dendritic.cells	HIST1H2BH	-1.09316	0.17295	-1.8767	0.063857	-3.98611	0.375009	0.370054
Dendritic.cells	FKBP2	0.269494	5.611404	1.876419	0.063897	-4.81603	0.337681	0.318189
Dendritic.cells	FGFR2	-0.27588	5.987704	-1.87608	0.063943	-4.77399	0.335351	0.314934
Dendritic.cells	CELSR1	-0.40291	1.626552	-1.87582	0.06398	-4.70127	0.364692	0.355689
Dendritic.cells	CTC1	0.539254	3.25694	1.875388	0.06404	-4.11861	0.353521	0.340158
Dendritic.cells	NAPSA	0.217401	6.459379	1.87456	0.064155	-5.24474	0.332936	0.31151
Dendritic.cells	NCKIPSD	0.27079	2.952621	1.874374	0.064181	-4.63203	0.356031	0.343516
Dendritic.cells	GOLM1	0.405155	3.2957	1.873222	0.064342	-4.38353	0.354006	0.340765
Dendritic.cells	AMDHD1	-0.88733	2.26974	-1.87312	0.064356	-4.06814	0.361092	0.350675
Dendritic.cells	DHX57	0.343506	3.871504	1.873011	0.064371	-4.44539	0.350105	0.335402
Dendritic.cells	RBM42	-0.18742	6.063078	-1.87295	0.06438	-4.87093	0.335753	0.315573
Dendritic.cells	2900026A0	-0.95202	3.595436	-1.87255	0.064436	-4.09228	0.351969	0.338188
Dendritic.cells	SLC38A6	-0.47854	4.233853	-1.87245	0.064449	-4.3365	0.347679	0.332261
Dendritic.cells	GM14966	-0.54045	2.847703	-1.87222	0.064482	-4.13733	0.357079	0.345421
Dendritic.cells	SLU7	0.283151	4.955244	1.871901	0.064526	-4.5396	0.342911	0.3258
Dendritic.cells	GLMP	0.247988	5.839868	1.871784	0.064543	-4.96246	0.33718	0.317874
Dendritic.cells	SETD2	0.171929	6.922415	1.871662	0.06456	-5.17423	0.330335	0.308478
Dendritic.cells	GPC1	-1.11717	0.351919	-1.87143	0.064592	-4.00142	0.374813	0.370418
Dendritic.cells	RNF146	-0.24456	5.30614	-1.87117	0.064629	-4.78681	0.34065	0.322738

Dendritic.cells	SREK1IP1	0.411489	4.172778	1.870426	0.064733	-4.37561	0.348502	0.33341
Dendritic.cells	GSTA4	-1.01137	1.583658	-1.86902	0.064931	-4.00543	0.367065	0.358773
Dendritic.cells	TTF1	0.476154	3.466533	1.868532	0.064999	-4.25468	0.353938	0.340512
Dendritic.cells	SELL	0.199601	5.472373	1.868365	0.065023	-5.34439	0.340599	0.322043
Dendritic.cells	SERPINE1	1.116663	0.805177	1.86805	0.065067	-4.00024	0.372667	0.36673
Dendritic.cells	4933411E0	0.996636	0.555657	1.86803	0.06507	-4.00896	0.374484	0.36928
Dendritic.cells	SENP6	0.137854	6.681311	1.867991	0.065076	-5.18341	0.332873	0.311417
Dendritic.cells	HSBP1	0.260663	6.003464	1.867951	0.065081	-4.81586	0.337176	0.31735
Dendritic.cells	ICOS	-1.21625	2.283057	-1.86765	0.065124	-4.00616	0.362178	0.352064
Dendritic.cells	AMD2	-0.95587	0.044662	-1.8672	0.065188	-3.9986	0.378373	0.374851
Dendritic.cells	STXBP3	0.282497	5.278015	1.866917	0.065227	-4.7006	0.341984	0.324148
Dendritic.cells	CDCA7L	-0.54109	3.766654	-1.86657	0.065276	-4.34895	0.352025	0.338239
Dendritic.cells	OSBPL11	-0.30127	5.368512	-1.86646	0.065292	-4.70732	0.341394	0.323528
Dendritic.cells	NDUFC1	0.213874	7.367214	1.866422	0.065297	-5.15441	0.32871	0.306024
Dendritic.cells	ACSF2	0.354368	4.286847	1.865984	0.065359	-4.56325	0.348686	0.333557
Dendritic.cells	PFKM	0.842375	1.17862	1.865517	0.065425	-4.03272	0.370273	0.363884
Dendritic.cells	ZFP444	0.447954	3.63547	1.865215	0.065468	-4.3501	0.353081	0.339915
Dendritic.cells	ZC3H12B	-0.29206	1.084598	-1.8651	0.065484	-4.8066	0.370951	0.364948
Dendritic.cells	SH3PXD2A	0.168248	5.753145	1.865068	0.065489	-5.39137	0.339064	0.3205
Dendritic.cells	CD209G	-2.5008	1.089148	-1.8648	0.065528	-4.01527	0.370955	0.364949
Dendritic.cells	BCL11B	-1.37728	1.080616	-1.86399	0.065641	-4.01255	0.371298	0.365266
Dendritic.cells	ZFP329	0.495498	2.511051	1.863917	0.065652	-4.17038	0.361133	0.351026
Dendritic.cells	SMIM3	0.245907	4.840554	1.863237	0.065749	-4.88408	0.345311	0.329201
Dendritic.cells	MSL2	0.223007	6.010868	1.863188	0.065756	-4.84125	0.337698	0.318662
Dendritic.cells	GM26812	-1.02056	0.020142	-1.86295	0.06579	-4.02237	0.379059	0.376535
Dendritic.cells	PGGT1B	0.263949	4.818529	1.862877	0.0658	-4.65506	0.345457	0.329524
Dendritic.cells	UBE2N	-0.14909	7.48412	-1.86275	0.065818	-5.17922	0.328424	0.306004
Dendritic.cells	CCDC171	-0.38227	3.57202	-1.86263	0.065836	-4.41081	0.353816	0.341264
Dendritic.cells	DNAJC15	0.329309	5.723768	1.86225	0.065889	-4.59415	0.339545	0.321627
Dendritic.cells	GM49359	-0.59408	2.260358	-1.86218	0.065899	-4.15588	0.36289	0.354183
Dendritic.cells	KLHL18	0.287349	4.310869	1.860483	0.066141	-4.6645	0.349569	0.335026
Dendritic.cells	GAMT	-0.87165	4.228598	-1.86045	0.066146	-4.26356	0.350121	0.335795
Dendritic.cells	NUDT19	0.282101	4.544356	1.860159	0.066188	-4.72634	0.348009	0.332947
Dendritic.cells	PTPN7	-0.70916	4.151708	-1.86016	0.066188	-4.13232	0.350638	0.336606
Dendritic.cells	BCR	-0.14184	5.853797	-1.85981	0.066237	-5.45407	0.339424	0.321096
Dendritic.cells	NELFE	0.32381	4.393275	1.85979	0.066241	-4.46213	0.349017	0.334421
Dendritic.cells	DDIT3	-0.46284	4.008692	-1.85956	0.066274	-4.33435	0.351601	0.338097
Dendritic.cells	TMCO6	0.4816	2.683081	1.859399	0.066297	-4.22338	0.360696	0.350869
Dendritic.cells	GM13547	-1.15485	0.514665	-1.85826	0.066459	-4.03238	0.37695	0.373221
Dendritic.cells	POLR2K	0.179801	6.77475	1.857916	0.066509	-5.04521	0.334296	0.313693
Dendritic.cells	HNF4A	-1.10481	0.691681	-1.85679	0.06667	-4.01808	0.376479	0.372203
Dendritic.cells	OSTF1	0.155181	7.480967	1.856416	0.066725	-5.1203	0.330622	0.308246
Dendritic.cells	MMAA	0.72158	1.748976	1.853902	0.067088	-4.11671	0.370583	0.362464
Dendritic.cells	ORC6	0.394485	5.216442	1.853753	0.067109	-4.5511	0.346656	0.329214
Dendritic.cells	NAF1	0.34866	3.658508	1.853677	0.06712	-4.44108	0.357157	0.343835
Dendritic.cells	FBXL5	0.351226	6.173273	1.852895	0.067233	-4.86725	0.340813	0.320835
Dendritic.cells	SLFN8	0.578363	3.326481	1.852617	0.067274	-4.4555	0.35992	0.347375

Dendritic.cells	MTMR2	-0.22415	5.396075	-1.85217	0.067339	-4.81046	0.346093	0.327979
Dendritic.cells	VDAC2	0.183844	7.488132	1.85155	0.067428	-5.17384	0.332929	0.30964
Dendritic.cells	ZFP148	0.150297	6.830997	1.851131	0.067489	-5.17823	0.337079	0.315389
Dendritic.cells	LZTFL1	-0.18732	5.075487	-1.85111	0.067493	-5.24323	0.348507	0.331186
Dendritic.cells	HNRNPUL1	0.132438	7.28107	1.850872	0.067527	-5.27264	0.334236	0.311548
Dendritic.cells	NPC2	0.181656	7.639426	1.84991	0.067667	-5.37685	0.332518	0.308819
Dendritic.cells	AFAP1L1	-0.75815	1.508026	-1.84923	0.067766	-4.17527	0.374272	0.366421
Dendritic.cells	6430590A(	0.538132	1.647577	1.848453	0.06788	-4.1655	0.373438	0.365219
Dendritic.cells	SLC39A11	0.255259	4.419343	1.848416	0.067885	-4.88909	0.353993	0.338116
Dendritic.cells	NAT8L	-1.00962	0.332665	-1.84828	0.067905	-4.02875	0.383123	0.378807
Dendritic.cells	TRNAU1AP	0.512646	4.126118	1.84811	0.06793	-4.27199	0.355989	0.340936
Dendritic.cells	RDH9	-1.08856	0.805989	-1.84734	0.068042	-4.03814	0.379856	0.374277
Dendritic.cells	LAMTOR4	0.225443	6.205335	1.847141	0.068071	-5.03473	0.342372	0.322163
Dendritic.cells	RNF170	0.379958	3.319753	1.847128	0.068073	-4.36945	0.361793	0.349109
Dendritic.cells	HERC4	0.215439	7.008496	1.846468	0.06817	-5.06704	0.337526	0.31531
Dendritic.cells	RTRAF	0.176041	7.336334	1.845909	0.068252	-5.27324	0.335658	0.312583
Dendritic.cells	MAP7D3	-1.03438	0.126284	-1.84568	0.068285	-4.03654	0.385531	0.382094
Dendritic.cells	MAST4	-0.3201	6.39259	-1.84543	0.068322	-5.17271	0.341695	0.32103
Dendritic.cells	ABLIM1	-0.18931	6.121137	-1.84522	0.068354	-5.26269	0.343458	0.323505
Dendritic.cells	AP3S2	0.465859	3.43138	1.84502	0.068382	-4.27456	0.361582	0.348744
Dendritic.cells	POLG2	0.353902	5.080463	1.844835	0.06841	-4.53017	0.350328	0.333153
Dendritic.cells	CRTC2	-0.40789	4.134514	-1.84386	0.068553	-4.47014	0.357306	0.342309
Dendritic.cells	PARN	0.310515	3.875182	1.843582	0.068594	-4.57852	0.359131	0.344904
Dendritic.cells	GM42477	0.666693	1.273145	1.843243	0.068644	-4.13128	0.377756	0.370987
Dendritic.cells	KLF6	-0.28564	7.581942	-1.8428	0.068709	-5.22596	0.33493	0.31144
Dendritic.cells	DCBLD2	-0.74416	1.32551	-1.84247	0.068758	-4.07758	0.377636	0.370849
Dendritic.cells	PSMD9	-0.23917	5.225742	-1.84158	0.068889	-4.74113	0.350796	0.333082
Dendritic.cells	CEP112	-1.02121	1.570821	-1.84134	0.068925	-4.05679	0.376395	0.368805
Dendritic.cells	FMO5	0.650605	3.247756	1.840401	0.069064	-4.19049	0.364935	0.352264
Dendritic.cells	2010110K1	-1.19894	-0.03523	-1.83977	0.069158	-4.03647	0.389189	0.386023
Dendritic.cells	CPTP	0.594634	2.248177	1.83968	0.069171	-4.13128	0.372273	0.362295
Dendritic.cells	TENT5C	-0.36246	5.86321	-1.83855	0.069339	-5.20044	0.347668	0.327957
Dendritic.cells	SIPA1L3	0.327856	5.201146	1.838139	0.0694	-4.66969	0.352078	0.334175
Dendritic.cells	TNFAIP1	-0.43257	3.775763	-1.83776	0.069457	-4.44407	0.361821	0.347823
Dendritic.cells	A3GALT2	-0.7551	2.675415	-1.83774	0.069459	-4.15015	0.369577	0.358636
Dendritic.cells	WDR73	-0.44925	2.976485	-1.8377	0.069465	-4.30025	0.367435	0.355645
Dendritic.cells	EIF4EBP2	0.158542	6.850744	1.837668	0.06947	-5.06156	0.341223	0.319287
Dendritic.cells	SLC1A4	-0.89443	0.947937	-1.83766	0.069472	-4.06768	0.382176	0.376279
Dendritic.cells	CCNJ	0.658054	2.015982	1.837109	0.069553	-4.21933	0.374413	0.365458
Dendritic.cells	RIOK1	-0.19291	6.007255	-1.83711	0.069554	-4.98301	0.346799	0.327038
Dendritic.cells	TKTL1	-0.83466	1.290296	-1.83679	0.0696	-4.07073	0.379756	0.373026
Dendritic.cells	FAM3C	0.175266	5.36557	1.836472	0.069648	-5.07701	0.351085	0.333126
Dendritic.cells	A630001G2	0.356965	5.126842	1.836405	0.069658	-4.53537	0.352687	0.335351
Dendritic.cells	ZEB1	-0.29753	6.848689	-1.83581	0.069747	-5.13355	0.341616	0.319887
Dendritic.cells	GM45715	0.372904	1.039938	1.835056	0.06986	-4.50475	0.382092	0.376219
Dendritic.cells	EXTL2	-0.88118	2.165568	-1.83472	0.06991	-4.07492	0.373829	0.364769
Dendritic.cells	NDUFA11	0.213583	6.910675	1.834702	0.069913	-5.10506	0.341374	0.319641

Dendritic.cells	PRKX	0.286376	5.379346	1.834495	0.069944	-4.65791	0.351436	0.333604
Dendritic.cells	TUBB4B	-0.4531	6.422022	-1.83449	0.069945	-4.77094	0.344543	0.324059
Dendritic.cells	TMEM64	-0.40556	5.051385	-1.8341	0.070003	-4.58594	0.353767	0.336742
Dendritic.cells	DCTPP1	0.348848	5.089369	1.833142	0.070147	-4.75227	0.353864	0.336601
Dendritic.cells	SLC2A6	0.530403	1.781297	1.83293	0.070179	-4.38193	0.377136	0.36912
Dendritic.cells	SRA1	0.264247	5.239409	1.832895	0.070184	-4.70433	0.352852	0.335257
Dendritic.cells	NUP98	-0.18228	7.461994	-1.83275	0.070205	-5.32301	0.338302	0.315161
Dendritic.cells	LRP2BP	1.108368	1.991491	1.832481	0.070246	-4.06507	0.375642	0.367071
Dendritic.cells	NDUFB3	0.271987	5.72376	1.83222	0.070285	-4.72196	0.349681	0.330918
Dendritic.cells	CD164L2	-1.03605	1.352514	-1.83199	0.070319	-4.05702	0.38037	0.373785
Dendritic.cells	GM20406	0.97662	-0.53168	1.830906	0.070483	-4.04501	0.395184	0.394353
Dendritic.cells	DAPK2	-0.78976	2.818088	-1.83079	0.0705	-4.14243	0.370249	0.359334
Dendritic.cells	KLRA17	-0.26212	0.224958	-1.83066	0.07052	-4.87011	0.38938	0.386231
Dendritic.cells	SCN4A	-0.92847	0.410123	-1.83017	0.070595	-4.07239	0.388045	0.384419
Dendritic.cells	TKT	0.228036	6.932084	1.830117	0.070602	-5.10614	0.342323	0.320636
Dendritic.cells	PRMT2	-0.7668	1.665423	-1.82981	0.070648	-4.09246	0.378676	0.371295
Dendritic.cells	JAG1	-1.21799	1.3378	-1.8295	0.070696	-4.06173	0.381095	0.374791
Dendritic.cells	EDA	-1.0653	1.445596	-1.82948	0.070699	-4.12126	0.380297	0.37367
Dendritic.cells	GM13391	-1.01677	0.765079	-1.82831	0.070876	-4.05285	0.386057	0.381283
Dendritic.cells	ZFP446	-0.8831	1.164128	-1.828	0.070923	-4.06755	0.383068	0.377193
Dendritic.cells	INTS1	-0.51198	3.347025	-1.82798	0.070925	-4.31458	0.367213	0.354978
Dendritic.cells	LY6C1	1.300163	-0.32417	1.827314	0.071026	-4.05189	0.394735	0.393374
Dendritic.cells	ZFP668	0.374501	3.829078	1.826766	0.071109	-4.41948	0.364349	0.350703
Dendritic.cells	TMEM51	-0.68607	3.130512	-1.82657	0.071139	-4.20304	0.369283	0.357663
Dendritic.cells	NOTCH2	0.201261	7.16468	1.826059	0.071217	-5.19917	0.341929	0.319796
Dendritic.cells	STT3B	-0.12636	6.666062	-1.82591	0.07124	-5.32511	0.345163	0.324294
Dendritic.cells	CD33	-0.44571	2.518088	-1.82573	0.071267	-4.65467	0.373677	0.36403
Dendritic.cells	UQCC2	0.26176	6.141014	1.825646	0.07128	-4.93775	0.348613	0.329151
Dendritic.cells	BCDIN3D	0.5168	2.223563	1.82515	0.071355	-4.2173	0.375813	0.367258
Dendritic.cells	GM4129	-0.90449	1.28611	-1.82512	0.07136	-4.07235	0.382714	0.376955
Dendritic.cells	NDUFB9	0.184058	7.069533	1.825113	0.071361	-5.19763	0.342543	0.320919
Dendritic.cells	SNX32	0.53441	2.812618	1.824559	0.071445	-4.22055	0.371822	0.361535
Dendritic.cells	ANGPTL8	-1.07666	1.825356	-1.82408	0.071519	-4.08988	0.379155	0.371774
Dendritic.cells	POMGNT1	-0.51578	2.312495	-1.82393	0.071542	-4.22872	0.375591	0.366816
Dendritic.cells	GTF2E2	0.2417	5.440806	1.823425	0.071618	-4.77964	0.353774	0.33647
Dendritic.cells	RO60	-0.45323	2.493379	-1.82317	0.071657	-4.31826	0.374374	0.365283
Dendritic.cells	DNAAF2	0.536727	2.845924	1.823153	0.07166	-4.21942	0.371831	0.361718
Dendritic.cells	ST3GAL5	-0.54873	6.23691	-1.82255	0.071752	-4.50262	0.348691	0.329324
Dendritic.cells	UBR2	-0.16919	6.362518	-1.82241	0.071774	-5.12953	0.347861	0.328198
Dendritic.cells	GFM2	0.36187	3.590998	1.822084	0.071823	-4.50225	0.366853	0.354669
Dendritic.cells	WDR49	-1.03991	0.34569	-1.8214	0.071927	-4.06419	0.390838	0.388437
Dendritic.cells	COMMD3	0.223495	5.85015	1.821001	0.071989	-4.9296	0.351484	0.333522
Dendritic.cells	ALDH8A1	-1.02585	2.197617	-1.82085	0.072012	-4.12019	0.377011	0.369228
Dendritic.cells	RASAL1	1.120642	0.712454	1.820565	0.072056	-4.0731	0.388051	0.384819
Dendritic.cells	CD55	-0.75429	4.863884	-1.82055	0.072058	-4.37633	0.358153	0.342854
Dendritic.cells	MRGPRA2/	0.915494	-1.27662	1.820327	0.072093	-4.05869	0.40345	0.40677
Dendritic.cells	HTATIP2	0.403465	4.480259	1.820124	0.072124	-4.42433	0.360791	0.346688

Dendritic.cells	DNAJC17	0.280602	3.568748	1.820123	0.072124	-4.55704	0.367161	0.355596
Dendritic.cells	PPIL2	0.245009	5.124237	1.819933	0.072153	-4.71686	0.356377	0.340528
Dendritic.cells	THY1	-0.80522	1.573983	-1.81941	0.072234	-4.18244	0.381735	0.376009
Dendritic.cells	CASZ1	-0.59892	3.474262	-1.81912	0.072279	-4.21871	0.367959	0.356784
Dendritic.cells	NDOR1	0.492149	3.086341	1.818899	0.072312	-4.32826	0.37072	0.360703
Dendritic.cells	GCLM	0.360521	6.090034	1.818736	0.072337	-4.73168	0.35001	0.331815
Dendritic.cells	CRTC3	0.218383	5.658838	1.818518	0.072371	-5.0309	0.352889	0.335842
Dendritic.cells	TREML4	-1.14005	2.758058	-1.81847	0.072379	-4.1032	0.373077	0.364082
Dendritic.cells	NR2C2	0.172499	6.925422	1.817461	0.072534	-5.27677	0.344949	0.324522
Dendritic.cells	LIPT2	-0.85907	1.430834	-1.81732	0.072556	-4.09807	0.383276	0.378169
Dendritic.cells	DNAJB4	-0.55036	3.700143	-1.81723	0.07257	-4.2085	0.36682	0.355048
Dendritic.cells	SERTAD2	-0.28418	6.12155	-1.81671	0.072651	-4.97873	0.350466	0.332034
Dendritic.cells	DEDD	0.288389	4.437275	1.816026	0.072756	-4.62605	0.361958	0.348166
Dendritic.cells	SMAD7	-0.25583	5.164939	-1.81594	0.072769	-4.95917	0.356957	0.341184
Dendritic.cells	1700010I1	-0.80756	1.215796	-1.81578	0.072794	-4.09347	0.385191	0.380805
Dendritic.cells	SMIM1	-0.8246	1.759237	-1.81577	0.072796	-4.17797	0.381145	0.375097
Dendritic.cells	RNF187	0.141289	6.12757	1.815043	0.072908	-5.39169	0.350733	0.332455
Dendritic.cells	CMTM7	0.166619	7.897065	1.814686	0.072963	-5.36293	0.339216	0.316584
Dendritic.cells	CCM2	-0.2017	7.391127	-1.81459	0.072978	-5.13277	0.342457	0.321074
Dendritic.cells	SLC48A1	0.30685	5.074284	1.814564	0.072982	-4.61667	0.357835	0.342466
Dendritic.cells	LYRM4	0.295189	4.142278	1.81425	0.073031	-4.59093	0.364307	0.351569
Dendritic.cells	TIMP3	-0.68634	2.197691	-1.81409	0.073056	-4.44413	0.378226	0.371198
Dendritic.cells	SNX6	0.16985	6.568189	1.813451	0.073155	-5.05365	0.348162	0.328839
Dendritic.cells	GM5608	-0.92045	0.923456	-1.81282	0.073253	-4.09855	0.38835	0.385132
Dendritic.cells	WDR86	0.817986	0.234245	1.812662	0.073278	-4.07984	0.393606	0.39264
Dendritic.cells	HSPB11	0.548554	2.925359	1.811785	0.073415	-4.28808	0.374086	0.364761
Dendritic.cells	DLGAP4	-0.36963	5.015503	-1.81022	0.073659	-4.61313	0.360339	0.344592
Dendritic.cells	TTC36	-0.77883	5.028124	-1.80993	0.073704	-4.54017	0.360252	0.34455
Dendritic.cells	MRPS21	0.161963	7.034242	1.809869	0.073714	-5.1542	0.346791	0.325834
Dendritic.cells	LILRB4A	0.95891	4.163584	1.809505	0.073771	-4.18241	0.366378	0.353026
Dendritic.cells	PRR14L	-0.17208	6.50182	-1.80776	0.074044	-5.23264	0.351548	0.331386
Dendritic.cells	ANKRD23	0.817758	0.33175	1.806896	0.07418	-4.11298	0.396424	0.393765
Dendritic.cells	IGKV1-117	0.89701	-0.39916	1.806674	0.074215	-4.07632	0.402134	0.401833
Dendritic.cells	CCR2	0.551018	3.092746	1.80633	0.074269	-4.6415	0.375845	0.364805
Dendritic.cells	ITGAX	-0.28804	3.098724	-1.80594	0.074331	-4.97157	0.375944	0.364903
Dendritic.cells	VPS13D	-0.19405	6.149191	-1.80547	0.074405	-5.17511	0.354662	0.335277
Dendritic.cells	COX5A	0.19777	8.546847	1.805461	0.074406	-5.40557	0.339007	0.313632
Dendritic.cells	TAF4B	-0.47985	5.130162	-1.8049	0.074494	-4.62792	0.361768	0.345149
Dendritic.cells	MREG	-1.0691	3.199109	-1.80482	0.074507	-4.17595	0.37542	0.364207
Dendritic.cells	IL1F9	1.14621	-0.11555	1.804114	0.074619	-4.08318	0.400627	0.399612
Dendritic.cells	ALG13	0.272663	4.514642	1.803896	0.074653	-4.57206	0.366269	0.351343
Dendritic.cells	BOLA3	0.285443	5.736487	1.803329	0.074743	-4.8878	0.357828	0.339739
Dendritic.cells	ARHGAP10	-0.78314	5.105594	-1.80314	0.074772	-4.35815	0.362154	0.345823
Dendritic.cells	CTNNB1	-0.16726	6.277504	-1.80286	0.074818	-5.03207	0.354171	0.334791
Dendritic.cells	PIM2	-0.54399	3.150665	-1.80274	0.074836	-4.28868	0.375997	0.365269
Dendritic.cells	SHANK2	-0.95962	1.410698	-1.80267	0.074848	-4.1011	0.38888	0.383377
Dendritic.cells	CELA1	-0.95114	2.24977	-1.80262	0.074854	-4.12203	0.3826	0.374538

Dendritic.cells	SFI1	0.225481	5.320089	1.802446	0.074883	-5.04383	0.360676	0.343865
Dendritic.cells	DEFB1	-1.21629	0.86149	-1.80237	0.074894	-4.0888	0.393059	0.38928
Dendritic.cells	SLC39A8	-0.61742	2.726982	-1.80218	0.074925	-4.3379	0.379084	0.369737
Dendritic.cells	SNHG15	-0.45829	3.481407	-1.80164	0.07501	-4.42046	0.373696	0.362204
Dendritic.cells	POLN	-0.85691	3.191009	-1.80142	0.075046	-4.18946	0.375792	0.36518
Dendritic.cells	RAB18	-0.18255	5.748093	-1.80137	0.075053	-4.99544	0.357832	0.34009
Dendritic.cells	GPR141	1.375055	2.440611	1.801214	0.075078	-4.1554	0.381279	0.372957
Dendritic.cells	MTIF2	0.269298	4.285566	1.800987	0.075114	-4.70194	0.367977	0.354333
Dendritic.cells	PSMB3	0.181517	7.724991	1.799603	0.075335	-5.32977	0.345573	0.322527
Dendritic.cells	ST3GAL2	0.595155	3.085043	1.79859	0.075496	-4.25595	0.378042	0.367481
Dendritic.cells	LGR4	-0.94171	2.632615	-1.79831	0.07554	-4.14228	0.38136	0.372201
Dendritic.cells	WDR24	-0.69089	1.796393	-1.79829	0.075544	-4.17221	0.387587	0.380957
Dendritic.cells	ZFP867	-0.88516	0.61031	-1.79782	0.075619	-4.09476	0.396763	0.393883
Dendritic.cells	ABHD6	-0.27543	2.548845	-1.79771	0.075636	-4.7742	0.382101	0.373222
Dendritic.cells	GATD3A	0.419289	3.544084	1.797162	0.075724	-4.43164	0.375101	0.363214
Dendritic.cells	POM121	-0.25528	4.883475	-1.79651	0.075828	-4.73754	0.365924	0.350184
Dendritic.cells	NRG2	0.338931	1.685011	1.796006	0.075909	-4.83603	0.389428	0.38303
Dendritic.cells	LNPEP	0.17579	7.335044	1.795578	0.075978	-5.28474	0.349541	0.327329
Dendritic.cells	SELENOT	0.161089	6.182995	1.795539	0.075984	-5.16771	0.357235	0.337998
Dendritic.cells	CYP2D26	-0.99881	3.247258	-1.79487	0.076092	-4.25889	0.378082	0.367083
Dendritic.cells	POLDIP3	0.182318	6.078814	1.794843	0.076096	-5.06625	0.358146	0.339261
Dendritic.cells	SF1	-0.13483	7.215593	-1.79431	0.076182	-5.31416	0.350629	0.328881
Dendritic.cells	PHRF1	0.184839	5.804424	1.794282	0.076186	-5.07225	0.360121	0.342061
Dendritic.cells	MCEMP1	1.414147	1.650063	1.793411	0.076326	-4.10345	0.390602	0.384313
Dendritic.cells	CCDC32	0.424097	3.31271	1.792719	0.076438	-4.42625	0.378476	0.367267
Dendritic.cells	ZWILCH	0.757698	3.028209	1.792689	0.076443	-4.256	0.380558	0.370187
Dendritic.cells	RALGDS	-0.56089	3.822267	-1.79186	0.076576	-4.48724	0.375072	0.362324
Dendritic.cells	POLR2F	0.249089	5.381223	1.791692	0.076604	-4.76489	0.364046	0.346936
Dendritic.cells	PSMB2	0.162296	7.058131	1.791636	0.076613	-5.23719	0.352653	0.331112
Dendritic.cells	IDH1	-0.41406	4.88393	-1.79141	0.076649	-4.4758	0.367517	0.35181
Dendritic.cells	C030034I2	0.44391	2.630863	1.791045	0.076708	-4.39024	0.383785	0.374683
Dendritic.cells	MRPL35	0.310635	5.21625	1.791028	0.076711	-4.69375	0.365193	0.348671
Dendritic.cells	PTPA	0.210738	5.833043	1.790839	0.076742	-4.95785	0.360929	0.342799
Dendritic.cells	TRIAP1	0.391392	4.111675	1.790267	0.076834	-4.46713	0.373276	0.359911
Dendritic.cells	BC004004	0.220989	5.147988	1.789987	0.076879	-4.87337	0.365976	0.349729
Dendritic.cells	SMIM4	0.253391	5.255277	1.789805	0.076909	-4.79686	0.365228	0.348761
Dendritic.cells	TMEM132E	-1.07703	0.984501	-1.78925	0.076998	-4.13084	0.396861	0.39292
Dendritic.cells	STON1	-0.91195	1.087662	-1.78891	0.077054	-4.15552	0.396092	0.391972
Dendritic.cells	RIPPLY3	-1.00393	0.048429	-1.78856	0.077111	-4.13732	0.404203	0.40347
Dendritic.cells	D930016D0	-0.49146	2.876865	-1.78801	0.0772	-4.32096	0.382586	0.373096
Dendritic.cells	PITPNM1	-0.50353	3.730007	-1.78791	0.077217	-4.30358	0.376348	0.364364
Dendritic.cells	AP3M2	0.389348	2.489505	1.787559	0.077274	-4.4748	0.385462	0.377293
Dendritic.cells	PP2D1	-0.94259	1.690903	-1.78729	0.077318	-4.15958	0.391474	0.385823
Dendritic.cells	CPLANE2	-1.00682	0.331465	-1.78726	0.077323	-4.11003	0.401974	0.400678
Dendritic.cells	ZFP746	0.309591	3.717554	1.787165	0.077338	-4.61577	0.376438	0.364695
Dendritic.cells	MBD2	0.164476	7.044678	1.786979	0.077368	-5.23837	0.353316	0.332511
Dendritic.cells	SGCB	0.505731	2.212559	1.786937	0.077375	-4.44751	0.387534	0.380377

Dendritic.cells	PPP1R16A	-0.52178	2.885752	-1.7869	0.077381	-4.30568	0.382521	0.373321
Dendritic.cells	GSTZ1	-0.43563	4.205829	-1.78576	0.077567	-4.51843	0.373654	0.360209
Dendritic.cells	ANGPT2	0.942987	0.571482	1.785503	0.077609	-4.12132	0.400916	0.398609
Dendritic.cells	APBA3	-0.62095	2.107298	-1.78492	0.077704	-4.23665	0.389279	0.382141
Dendritic.cells	AA467197	1.471846	0.801131	1.784693	0.077741	-4.11594	0.399287	0.396343
Dendritic.cells	LYST	0.202163	6.729745	1.784678	0.077743	-5.27374	0.356297	0.336073
Dendritic.cells	AASDHPPT	-0.36223	3.841771	-1.78381	0.077886	-4.49583	0.37679	0.364463
Dendritic.cells	PKIB	0.273051	5.453591	1.783681	0.077907	-5.47336	0.365347	0.348476
Dendritic.cells	CSTF2	0.32498	4.139659	1.783635	0.077914	-4.65855	0.374641	0.361459
Dendritic.cells	CALM2	-0.14074	8.565355	-1.78342	0.07795	-5.49214	0.344519	0.319615
Dendritic.cells	FBXO10	-0.75273	1.355085	-1.78304	0.078011	-4.14578	0.395489	0.390762
Dendritic.cells	QTRT1	-0.39027	4.980734	-1.78214	0.07816	-4.78496	0.368994	0.353453
Dendritic.cells	2310033PC	0.377632	4.144026	1.782055	0.078173	-4.49479	0.374952	0.361805
Dendritic.cells	DCTN6	0.175052	5.142412	1.782001	0.078182	-5.13956	0.367857	0.351891
Dendritic.cells	H2-AB1	-0.57017	6.764416	-1.78194	0.078192	-5.341	0.356698	0.336361
Dendritic.cells	IMMP1L	0.267465	4.995599	1.7814	0.078281	-4.74184	0.369145	0.353433
Dendritic.cells	IGLL1	-1.63335	3.916663	-1.78091	0.078362	-4.21528	0.376881	0.364379
Dendritic.cells	GMPR2	-0.38546	3.866899	-1.78074	0.07839	-4.52186	0.377241	0.364924
Dendritic.cells	ORAI2	0.23371	5.87495	1.780641	0.078406	-5.04229	0.363039	0.345084
Dendritic.cells	SPATS2L	-0.93339	0.9742	-1.78053	0.078425	-4.17761	0.398958	0.395516
Dendritic.cells	RANBP6	0.595742	1.620455	1.779862	0.078534	-4.23941	0.394352	0.388771
Dendritic.cells	PTK2B	0.202413	7.134222	1.779554	0.078585	-5.33018	0.354901	0.33356
Dendritic.cells	DUS2	0.596043	3.353616	1.778971	0.078681	-4.30167	0.38162	0.370802
Dendritic.cells	USHBP1	-0.70251	1.085061	-1.77888	0.078697	-4.23612	0.398762	0.394946
Dendritic.cells	ITPRIP	-0.45589	3.468077	-1.77869	0.078728	-4.44184	0.38078	0.369701
Dendritic.cells	GPR35	0.597302	2.516821	1.778363	0.078782	-4.42989	0.387928	0.379733
Dendritic.cells	POLR2C	0.28263	5.080792	1.777687	0.078894	-4.73682	0.369637	0.353905
Dendritic.cells	IMPA2	0.421462	4.492173	1.777386	0.078944	-4.48866	0.373893	0.35981
Dendritic.cells	GM28501	0.746074	0.476037	1.776285	0.079126	-4.18423	0.40485	0.402759
Dendritic.cells	DESI2	-0.13657	6.03278	-1.7752	0.079307	-5.1811	0.363924	0.345476
Dendritic.cells	LSM4	0.189915	6.704001	1.775145	0.079315	-5.17355	0.359326	0.339098
Dendritic.cells	SERTAD1	-0.36933	5.61417	-1.77499	0.079341	-4.73762	0.366832	0.349601
Dendritic.cells	RAB3GAP2	-0.25145	5.259956	-1.77482	0.079369	-4.87788	0.369315	0.353087
Dendritic.cells	PTP4A2	-0.10582	8.699413	-1.77466	0.079396	-5.59885	0.346106	0.320885
Dendritic.cells	ZC3H12D	0.590777	3.479104	1.774433	0.079434	-4.37563	0.382136	0.371092
Dendritic.cells	CDK5	0.404463	3.72259	1.773863	0.079529	-4.49486	0.38035	0.368852
Dendritic.cells	ALDOC	0.638053	0.884667	1.773703	0.079555	-4.3292	0.401831	0.399197
Dendritic.cells	PSME2	0.291244	7.278608	1.773703	0.079555	-5.3654	0.35545	0.334135
Dendritic.cells	MSH2	0.394773	4.128132	1.773685	0.079558	-4.48482	0.377398	0.364764
Dendritic.cells	FOXK1	0.298442	4.627409	1.773669	0.079561	-4.66412	0.373805	0.35973
Dendritic.cells	OTULIN	0.153938	6.57022	1.773552	0.07958	-5.20706	0.360236	0.340837
Dendritic.cells	DYM	0.204425	5.781233	1.77323	0.079634	-5.02682	0.365707	0.348502
Dendritic.cells	APLP2	-0.30428	5.805079	-1.77308	0.079659	-4.90309	0.365542	0.348272
Dendritic.cells	TROAP	-0.82761	1.774645	-1.77218	0.07981	-4.18483	0.395559	0.389925
Dendritic.cells	NUCB1	-0.2393	5.383551	-1.77188	0.079859	-4.97964	0.369092	0.352776
Dendritic.cells	GRAP	0.375541	4.536304	1.771429	0.079935	-4.66107	0.375123	0.361308
Dendritic.cells	GLTP	0.122801	6.759436	1.77118	0.079977	-5.53559	0.359588	0.339644

Dendritic.cells	KANK1	0.974874	1.172928	1.770723	0.080053	-4.13224	0.400293	0.396918
Dendritic.cells	ZFP622	0.287809	5.377893	1.770567	0.080079	-4.75624	0.369141	0.353134
Dendritic.cells	B3GNT8	0.633595	2.202747	1.770547	0.080083	-4.35078	0.392369	0.385757
Dendritic.cells	FRMD4B	-0.55623	5.267906	-1.77028	0.080128	-4.69972	0.369916	0.354323
Dendritic.cells	SMYD2	0.685503	3.028651	1.770064	0.080164	-4.25745	0.386153	0.3772
Dendritic.cells	FNBP4	0.178847	6.074991	1.769976	0.080179	-5.124	0.364279	0.346543
Dendritic.cells	REXO4	0.324114	4.291897	1.76983	0.080203	-4.59165	0.376884	0.36422
Dendritic.cells	GPAT3	-0.57281	5.178486	-1.76963	0.080237	-4.36052	0.370547	0.355375
Dendritic.cells	MARCKSL1	-0.39484	7.792982	-1.76946	0.080265	-5.12977	0.352654	0.330457
Dendritic.cells	PTPN22	-0.68317	5.324165	-1.76925	0.080301	-4.42502	0.369519	0.354003
Dendritic.cells	KSR2	-1.02962	2.695294	-1.76898	0.080346	-4.20797	0.388647	0.380875
Dendritic.cells	COPB1	0.154442	6.220287	1.76897	0.080348	-5.16923	0.363277	0.34528
Dendritic.cells	PLXNA4OS1	1.03426	0.51532	1.768118	0.080491	-4.13238	0.406002	0.40502
Dendritic.cells	AIG1	-0.32289	4.903663	-1.76788	0.08053	-4.84496	0.373021	0.358518
Dendritic.cells	GM20536	-0.67411	2.340392	-1.76738	0.080615	-4.21706	0.392118	0.385248
Dendritic.cells	RETSAT	0.665403	1.405172	1.766687	0.080732	-4.16959	0.399704	0.395552
Dendritic.cells	AMZ1	-0.48646	3.157688	-1.76579	0.080884	-4.62389	0.386735	0.377168
Dendritic.cells	PARP12	-0.79373	3.018486	-1.76571	0.080897	-4.22402	0.387775	0.378644
Dendritic.cells	ARHGEF15	-0.83735	0.580885	-1.76558	0.080919	-4.22889	0.406557	0.40525
Dendritic.cells	ZSCAN25	-0.63226	1.792602	-1.76541	0.080948	-4.27531	0.397084	0.391933
Dendritic.cells	CHRNE	0.932649	-0.75209	1.765191	0.080985	-4.12992	0.4173	0.420663
Dendritic.cells	L2HGDH	0.769406	1.767651	1.76442	0.081115	-4.18839	0.397752	0.39256
Dendritic.cells	NFIB	-0.50798	3.880029	-1.76333	0.0813	-4.78157	0.382559	0.370603
Dendritic.cells	GM35188	0.527174	1.73277	1.762948	0.081365	-4.50279	0.398806	0.393498
Dendritic.cells	4933433G1	1.055016	0.742117	1.762858	0.08138	-4.15562	0.406566	0.404503
Dendritic.cells	3110001I2	-0.52454	2.677347	-1.76235	0.081466	-4.29042	0.391702	0.383386
Dendritic.cells	FARSB	0.272516	5.008108	1.761911	0.081541	-4.77067	0.374546	0.359411
Dendritic.cells	0610030E2	0.323278	4.591291	1.76177	0.081565	-4.64175	0.377543	0.363621
Dendritic.cells	TMEM86B	-0.40656	3.467162	-1.76171	0.081576	-4.50253	0.385779	0.375177
Dendritic.cells	KCNQ1OT1	-0.2752	6.399833	-1.76158	0.081598	-5.11506	0.364762	0.345769
Dendritic.cells	SEC14L1	-0.39535	4.618739	-1.76147	0.081616	-4.54428	0.377344	0.363384
Dendritic.cells	RAB32	-0.57003	4.344985	-1.76027	0.08182	-4.34904	0.379998	0.366475
Dendritic.cells	AU041133	0.637567	1.264215	1.760147	0.081841	-4.23564	0.403295	0.399265
Dendritic.cells	2010001A1	0.690377	1.389339	1.760005	0.081866	-4.22016	0.402315	0.397879
Dendritic.cells	SELENOW	0.186115	7.046449	1.759323	0.081982	-5.41551	0.361325	0.340112
Dendritic.cells	MRPL43	0.257715	5.608686	1.758865	0.08206	-4.91746	0.3715	0.354135
Dendritic.cells	SMAD3	-0.23566	6.315623	-1.7576	0.082277	-5.29261	0.367357	0.347756
Dendritic.cells	9330020H0	0.564112	2.471892	1.756985	0.082382	-4.31453	0.395764	0.38717
Dendritic.cells	WEE1	-0.53158	3.947727	-1.75652	0.082462	-4.42048	0.384715	0.371816
Dendritic.cells	HTATSF1	0.268033	4.697996	1.75634	0.082493	-4.74317	0.379222	0.364166
Dendritic.cells	FTSJ1	0.424274	3.078297	1.756304	0.082499	-4.36055	0.391207	0.380966
Dendritic.cells	ANGPTL1	0.936355	0.376343	1.75605	0.082543	-4.16331	0.412279	0.410692
Dendritic.cells	GRAMD2	-0.86007	0.533275	-1.75584	0.082579	-4.16855	0.411018	0.408952
Dendritic.cells	LAMTOR1	0.172318	6.503458	1.755681	0.082606	-5.17492	0.366419	0.34642
Dendritic.cells	DHPS	-0.2883	4.486343	-1.75535	0.082664	-4.68981	0.380806	0.366489
Dendritic.cells	NME3	0.906669	0.405363	1.75514	0.082699	-4.20011	0.412087	0.410586
Dendritic.cells	CARD6	0.401722	3.06005	1.755018	0.08272	-4.66157	0.391391	0.381451

Dendritic.cells	IMMT	0.192814	6.049157	1.754607	0.082791	-5.02735	0.369784	0.351121
Dendritic.cells	PLVAP	-0.7134	1.394862	-1.7538	0.08293	-4.2777	0.404858	0.399949
Dendritic.cells	ERC2	-1.29914	0.862583	-1.75359	0.082967	-4.18809	0.409074	0.406029
Dendritic.cells	GNL3	-0.23806	5.420028	-1.75344	0.082992	-5.05803	0.374665	0.357692
Dendritic.cells	SMAP1	0.191831	6.875842	1.753226	0.083029	-5.20807	0.364468	0.343538
Dendritic.cells	GM9828	0.976432	1.076181	1.753	0.083068	-4.16096	0.407392	0.40387
Dendritic.cells	SMPDL3B	1.268111	1.9506	1.752478	0.083158	-4.18052	0.400529	0.394443
Dendritic.cells	DUSP23	-0.60797	2.373334	-1.75245	0.083164	-4.26425	0.397262	0.389834
Dendritic.cells	CATSPER2	-0.58907	1.800753	-1.75236	0.083179	-4.34827	0.401695	0.396105
Dendritic.cells	REXO2	0.159356	6.270041	1.752196	0.083207	-5.47545	0.368676	0.34983
Dendritic.cells	TSPAN33	0.864876	2.55857	1.751147	0.083389	-4.2422	0.396372	0.388268
Dendritic.cells	TEX14	-0.44827	5.64791	-1.75114	0.08339	-4.75535	0.373559	0.356279
Dendritic.cells	EMILIN2	0.92841	4.009199	1.748737	0.083807	-4.42235	0.386968	0.373934
Dendritic.cells	GM16675	1.065117	1.196561	1.748678	0.083817	-4.16441	0.408593	0.40439
Dendritic.cells	PHYKPL	0.371506	3.470266	1.748386	0.083868	-4.46623	0.390999	0.379765
Dendritic.cells	SDC3	-0.62984	4.594842	-1.74831	0.083882	-4.6215	0.382648	0.36809
Dendritic.cells	MFSD13A	-0.73903	2.008416	-1.74771	0.083985	-4.23185	0.4022	0.39578
Dendritic.cells	GM10138	0.457426	2.471458	1.747645	0.083997	-4.53317	0.398609	0.390727
Dendritic.cells	CPSF4L	-0.71456	0.499907	-1.74759	0.084007	-4.21016	0.414176	0.412739
Dendritic.cells	1110008P1	0.237259	5.226277	1.747544	0.084015	-4.97247	0.378059	0.361912
Dendritic.cells	ARHGAP39	0.31572	4.38051	1.747409	0.084039	-4.94792	0.384222	0.370562
Dendritic.cells	SGSM2	-0.3546	3.786605	-1.74681	0.084143	-4.65323	0.388786	0.37694
Dendritic.cells	HACD3	-0.28088	4.907611	-1.74668	0.084166	-4.72056	0.380522	0.365401
Dendritic.cells	WDR19	0.989823	0.508741	1.746601	0.08418	-4.16016	0.414275	0.412966
Dendritic.cells	CBR3	-1.12701	0.624626	-1.74581	0.084317	-4.15749	0.413842	0.411991
Dendritic.cells	SCARB2	0.177307	6.538743	1.745411	0.084388	-5.39137	0.369482	0.349662
Dendritic.cells	PDE8B	-0.95634	-0.24267	-1.74479	0.084497	-4.15862	0.421066	0.422517
Dendritic.cells	TMEM106/	-0.5743	3.645492	-1.74473	0.084507	-4.40498	0.390458	0.379246
Dendritic.cells	SAP18	0.160181	7.043708	1.744235	0.084593	-5.29958	0.365974	0.345238
Dendritic.cells	GM43259	-0.86732	1.452797	-1.74422	0.084596	-4.24193	0.407371	0.403356
Dendritic.cells	CISD3	0.639396	3.000972	1.744178	0.084603	-4.30578	0.395337	0.386359
Dendritic.cells	ABCD3	-0.39189	4.193442	-1.74405	0.084627	-4.5499	0.38637	0.373748
Dendritic.cells	RASAL3	-0.2365	4.10056	-1.74389	0.084654	-4.91152	0.387059	0.374727
Dendritic.cells	DENND4A	-0.17927	10.03042	-1.74381	0.084668	-5.96935	0.346112	0.317732
Dendritic.cells	EEF2K	-0.33854	4.644155	-1.74304	0.084804	-4.76332	0.383451	0.369314
Dendritic.cells	CPNE1	-0.18039	6.038278	-1.74279	0.084848	-5.14061	0.373396	0.355343
Dendritic.cells	COX7C	0.143446	8.585633	1.742694	0.084864	-5.57299	0.355905	0.33106
Dendritic.cells	UBE2A	0.157819	6.786705	1.742253	0.084942	-5.19907	0.368322	0.348226
Dendritic.cells	LONRF3	-0.95495	1.525709	-1.74188	0.085008	-4.16756	0.407445	0.403273
Dendritic.cells	KCTD17	0.853017	0.958849	1.741823	0.085017	-4.24558	0.411962	0.409675
Dendritic.cells	HACD2	0.240943	5.732831	1.741272	0.085114	-4.89883	0.376046	0.358871
Dendritic.cells	MAP7D1	-0.16473	5.36793	-1.74089	0.085182	-5.22288	0.378811	0.362639
Dendritic.cells	GSR	0.294061	6.441975	1.740044	0.085331	-5.13427	0.371561	0.352325
Dendritic.cells	NMB	-0.76651	1.642171	-1.73996	0.085346	-4.21003	0.407412	0.402687
Dendritic.cells	RCSD1	0.206709	7.091061	1.739632	0.085404	-5.40386	0.367127	0.346103
Dendritic.cells	DMAC2L	0.59158	2.257897	1.738278	0.085643	-4.29498	0.403405	0.396531
Dendritic.cells	PTK7	-0.92416	1.073787	-1.73797	0.085697	-4.275	0.412788	0.409813

Dendritic.cells	ATP2B1	-0.23246	8.327101	-1.73794	0.085704	-5.3409	0.359346	0.334915
Dendritic.cells	TBCD	0.255683	4.783335	1.737845	0.08572	-4.89383	0.384266	0.369672
Dendritic.cells	RRAGC	-0.20156	5.819399	-1.73775	0.085736	-5.10927	0.376751	0.359175
Dendritic.cells	ZFP764	0.733191	1.074799	1.737541	0.085774	-4.23191	0.412785	0.40991
Dendritic.cells	NIPAL3	0.470918	3.198166	1.737344	0.085809	-4.48808	0.396147	0.386475
Dendritic.cells	PLK3	-0.62097	4.483011	-1.73667	0.085929	-4.42995	0.386778	0.373089
Dendritic.cells	CD209D	-0.29846	-0.32218	-1.73657	0.085945	-5.11857	0.424517	0.42641
Dendritic.cells	POLR3F	-0.34618	3.882542	-1.73607	0.086035	-4.604	0.391507	0.379499
Dendritic.cells	OLFR164	0.292904	-0.16043	1.735084	0.08621	-4.93242	0.423971	0.424866
Dendritic.cells	SARS	0.186863	5.792679	1.735061	0.086214	-5.05412	0.377941	0.36004
Dendritic.cells	FTL1	-0.19732	11.26205	-1.7344	0.086333	-5.98589	0.341562	0.309514
Dendritic.cells	ALG2	0.304728	3.062397	1.734124	0.086382	-4.64756	0.398674	0.388917
Dendritic.cells	COLQ	-0.94609	0.244853	-1.7337	0.086456	-4.17001	0.421279	0.420692
Dendritic.cells	RNF216	0.160634	6.677461	1.733449	0.086502	-5.27844	0.372231	0.351882
Dendritic.cells	UBE2Z	0.2178	5.437928	1.733226	0.086542	-4.93365	0.381092	0.364341
Dendritic.cells	TBC1D8	0.15763	5.179428	1.733044	0.086574	-5.64612	0.382975	0.366987
Dendritic.cells	KMT2D	-0.1794	5.945956	-1.7329	0.0866	-5.14329	0.377427	0.359277
Dendritic.cells	LARP1B	-0.29802	5.599705	-1.732	0.08676	-4.87607	0.380136	0.363151
Dendritic.cells	SP110	0.238194	5.924125	1.731869	0.086784	-5.22469	0.377799	0.359913
Dendritic.cells	GM43062	-0.60845	1.638658	-1.73184	0.086789	-4.25328	0.410231	0.405497
Dendritic.cells	PROCA1	-0.56606	2.988273	-1.73182	0.086793	-4.38027	0.39965	0.39056
Dendritic.cells	VAV1	0.162469	6.364742	1.731157	0.086912	-5.37183	0.375014	0.355746
Dendritic.cells	XLR4B	0.719859	1.285179	1.730832	0.08697	-4.22656	0.413561	0.409805
Dendritic.cells	ALKBH1	0.197202	4.99535	1.730621	0.087008	-5.10112	0.385018	0.369717
Dendritic.cells	CD7	0.28813	4.199176	1.730407	0.087046	-5.5262	0.39094	0.378003
Dendritic.cells	SRSF10	0.157862	6.4448	1.729658	0.087181	-5.18038	0.374984	0.355537
Dendritic.cells	TSC22D4	0.208863	6.684783	1.729313	0.087243	-5.17001	0.373396	0.353311
Dendritic.cells	GM31597	0.506405	2.208627	1.729007	0.087298	-4.38301	0.406904	0.40025
Dendritic.cells	EIF1A	-0.24079	5.711772	-1.72856	0.087378	-5.0726	0.380623	0.363264
Dendritic.cells	AW146154	-0.63176	2.334177	-1.72795	0.087489	-4.34916	0.406465	0.399433
Dendritic.cells	QPCTL	-0.34721	3.010203	-1.72677	0.087701	-4.56375	0.401996	0.392325
Dendritic.cells	ZFP865	0.429173	3.265059	1.726507	0.087749	-4.53709	0.40008	0.389567
Dendritic.cells	CSTA3	1.405783	0.791872	1.72598	0.087844	-4.20245	0.419835	0.417437
Dendritic.cells	GM44067	0.690881	1.717425	1.725938	0.087852	-4.29445	0.412345	0.406838
Dendritic.cells	3110040N1	0.468293	3.553549	1.725781	0.08788	-4.52106	0.397965	0.38663
Dendritic.cells	MAPK7	0.544121	3.195275	1.725222	0.087981	-4.39187	0.400951	0.390663
Dendritic.cells	DIP2B	-0.16183	7.880088	-1.72489	0.088041	-5.55553	0.366747	0.343019
Dendritic.cells	CLEC2G	1.012754	0.015976	1.724844	0.08805	-4.1906	0.426486	0.426903
Dendritic.cells	NXPE3	0.358942	3.424156	1.724699	0.088076	-4.65699	0.399187	0.388318
Dendritic.cells	PRIM1	-0.67821	4.178781	-1.7245	0.088112	-4.42395	0.393439	0.3803
Dendritic.cells	NDUFB6	0.275218	6.317819	1.724067	0.088191	-5.08932	0.377884	0.358458
Dendritic.cells	PRDM10	0.249567	5.213796	1.723885	0.088224	-5.01914	0.385899	0.369675
Dendritic.cells	PRKAA1	0.338653	4.45764	1.723528	0.088288	-4.77076	0.391558	0.377622
Dendritic.cells	CLIC4	-0.2117	7.449524	-1.72344	0.088305	-5.73124	0.369932	0.347443
Dendritic.cells	MECP2	-0.18041	5.847525	-1.72294	0.088395	-5.1685	0.381442	0.363409
Dendritic.cells	IFNG	-1.3005	1.241502	-1.72287	0.088408	-4.20026	0.416789	0.4131
Dendritic.cells	FAM168B	0.136253	6.280422	1.722478	0.088479	-5.30825	0.37847	0.359283

Dendritic.cells	1500011BC	0.365509	4.65787	1.722229	0.088525	-4.752	0.390388	0.37596
Dendritic.cells	PCK2	-0.60827	3.244845	-1.72203	0.088561	-4.36933	0.401128	0.391153
Dendritic.cells	BPGM	0.605975	4.812429	1.7217	0.088621	-4.66358	0.389341	0.374479
Dendritic.cells	MRPL53	0.382387	4.291701	1.720963	0.088755	-4.61993	0.393356	0.380234
Dendritic.cells	BBS5	-0.85096	0.624034	-1.72078	0.088789	-4.25425	0.422278	0.421063
Dendritic.cells	ADNP	0.157904	6.50802	1.720593	0.088823	-5.35742	0.377092	0.357482
Dendritic.cells	SAR1B	0.213738	5.553451	1.720571	0.088827	-4.93408	0.383987	0.36711
Dendritic.cells	OSTM1	0.208647	4.762239	1.720564	0.088828	-5.02087	0.389828	0.375286
Dendritic.cells	RARS2	0.423292	3.441715	1.719955	0.088939	-4.49281	0.40017	0.389503
Dendritic.cells	ZBTB11OS1	0.526993	2.065369	1.719496	0.089023	-4.35034	0.410985	0.40475
Dendritic.cells	LNCPPARA	0.932484	0.60974	1.719355	0.089049	-4.21454	0.422784	0.421467
Dendritic.cells	CDKL4	-0.98399	1.561956	-1.71923	0.089071	-4.21563	0.41502	0.410514
Dendritic.cells	CHST10	0.962736	0.375693	1.719124	0.089091	-4.19532	0.424719	0.424294
Dendritic.cells	ATP8B2	0.298047	2.774077	1.718906	0.089131	-4.71164	0.405403	0.396982
Dendritic.cells	GLUD1	-0.17426	7.653063	-1.71735	0.089417	-5.40127	0.369566	0.34722
Dendritic.cells	SNRNP25	0.4164	3.939991	1.716937	0.089492	-4.5666	0.39659	0.385216
Dendritic.cells	TOR2A	0.336602	3.670287	1.716908	0.089497	-4.63383	0.398651	0.388119
Dendritic.cells	TMEM108	-0.28256	5.137851	-1.71682	0.089514	-5.46051	0.387598	0.372625
Dendritic.cells	C1QA	-0.83545	5.480946	-1.71654	0.089565	-4.72027	0.385071	0.369163
Dendritic.cells	DDX19B	-0.41872	3.529612	-1.71652	0.089569	-4.483	0.399731	0.38977
Dendritic.cells	PFDN5	0.132871	7.413663	1.716218	0.089624	-5.42494	0.371234	0.349949
Dendritic.cells	MED7	0.347924	3.583194	1.716146	0.089637	-4.59465	0.399319	0.389328
Dendritic.cells	SLC25A21	-1.27842	1.867089	-1.71608	0.08965	-4.23777	0.412783	0.408364
Dendritic.cells	GNA12	-0.27631	4.652134	-1.71603	0.089659	-4.89735	0.391212	0.377915
Dendritic.cells	ITIH3	-0.78398	3.636116	-1.71573	0.089714	-4.43482	0.398913	0.388863
Dendritic.cells	PSPC1	-0.22183	5.702286	-1.71536	0.089782	-5.15439	0.383452	0.367308
Dendritic.cells	1810041H1	0.951341	1.76748	1.715332	0.089787	-4.21566	0.413582	0.40981
Dendritic.cells	GM42829	-0.59236	2.205641	-1.71526	0.0898	-4.34185	0.410083	0.404861
Dendritic.cells	PRPF39	0.225889	5.43876	1.714992	0.08985	-5.03751	0.385381	0.370102
Dendritic.cells	DHRS7	0.497004	4.016547	1.714711	0.089902	-4.63461	0.396007	0.385183
Dendritic.cells	ATF3	-0.4763	6.1843	-1.71464	0.089915	-5.11587	0.379958	0.362626
Dendritic.cells	TARBP2	-0.46897	3.242608	-1.71461	0.08992	-4.44133	0.401947	0.393569
Dendritic.cells	IFI44	0.812351	0.579228	1.714536	0.089934	-4.42066	0.423255	0.423837
Dendritic.cells	BORCS5	0.287978	4.024358	1.714493	0.089942	-4.77213	0.395948	0.385118
Dendritic.cells	P3H2	1.229377	1.190669	1.714375	0.089963	-4.26444	0.418244	0.416718
Dendritic.cells	GPALPP1	0.364201	3.737889	1.714279	0.089981	-4.54522	0.398133	0.388218
Dendritic.cells	MORC3	0.215388	5.857789	1.713792	0.090071	-5.08503	0.38232	0.366035
Dendritic.cells	1700056N1	0.625781	2.033678	1.713783	0.090073	-4.28782	0.411452	0.407137
Dendritic.cells	TAF6	0.446422	3.851886	1.713631	0.090101	-4.53771	0.397262	0.387139
Dendritic.cells	CIC	0.264932	4.821881	1.713418	0.09014	-4.90156	0.389944	0.376935
Dendritic.cells	GNAQ	0.157963	7.882192	1.713263	0.090169	-5.68505	0.367979	0.346169
Dendritic.cells	SERPINA1D	-0.83168	5.558333	-1.71306	0.090206	-4.7925	0.384504	0.369364
Dendritic.cells	CD4	-0.27651	1.246276	-1.71295	0.090227	-5.14896	0.417792	0.416514
Dendritic.cells	HIST1H2BN	0.910719	1.240034	1.711582	0.09048	-4.21383	0.418843	0.417181
Dendritic.cells	TCF21	-1.02798	0.477537	-1.71036	0.090706	-4.20804	0.425861	0.426761
Dendritic.cells	VSTM4	-0.98332	0.147061	-1.71033	0.090712	-4.24734	0.428618	0.430702
Dendritic.cells	ARRDC1	0.323405	4.471227	1.709445	0.090876	-4.77831	0.394757	0.382223

Dendritic.cells	FCER1G	0.295606	8.628675	1.708167	0.091114	-5.76934	0.365633	0.340744
Dendritic.cells	DDX51	0.637268	1.37149	1.708065	0.091133	-4.34174	0.419946	0.417001
Dendritic.cells	SFPQ	-0.13096	8.189863	-1.70766	0.091209	-5.61795	0.368804	0.345032
Dendritic.cells	PMEPA1	-0.19875	4.361383	-1.7073	0.091276	-5.58362	0.396626	0.383973
Dendritic.cells	STIMATE	0.387229	4.159047	1.707011	0.09133	-4.60143	0.398168	0.386267
Dendritic.cells	GM36975	0.204371	3.276788	1.706988	0.091334	-5.35315	0.40498	0.395869
Dendritic.cells	SERGEF	0.350694	4.018452	1.706239	0.091474	-4.69904	0.399695	0.388106
Dendritic.cells	EIF2A	0.188601	5.395584	1.705901	0.091537	-5.02775	0.389416	0.373563
Dendritic.cells	NPLOC4	-0.18753	5.537568	-1.70521	0.091667	-5.23386	0.388731	0.372344
Dendritic.cells	PHC1	-0.16616	3.5385	-1.70479	0.091745	-5.18803	0.403897	0.393857
Dendritic.cells	RAB2A	0.095965	8.094947	1.704668	0.091768	-5.64735	0.370397	0.346986
Dendritic.cells	CMTR1	0.321446	5.166737	1.704656	0.09177	-4.97739	0.391488	0.376456
Dendritic.cells	BLCAP	-0.35604	3.009547	-1.70442	0.091814	-4.64896	0.408056	0.399845
Dendritic.cells	SLC11A1	-0.95634	3.505984	-1.70424	0.091849	-4.31302	0.404171	0.394431
Dendritic.cells	ITGB3	0.290774	2.991713	1.703544	0.091979	-4.93559	0.408613	0.400451
Dendritic.cells	PEX11A	0.992224	0.072757	1.702803	0.092118	-4.21472	0.432798	0.434779
Dendritic.cells	SPPL2B	0.625951	2.17345	1.702761	0.092126	-4.36548	0.415462	0.41013
Dendritic.cells	ARHGAP24	-0.18725	6.825889	-1.70251	0.092174	-5.67988	0.380103	0.360344
Dendritic.cells	USP54	-0.4183	2.915478	-1.70216	0.092239	-4.53753	0.409719	0.401912
Dendritic.cells	A530040E1	0.654483	0.013043	1.701904	0.092287	-4.38128	0.433543	0.435794
Dendritic.cells	FBXO3	0.219708	5.012921	1.701409	0.092381	-4.94866	0.393702	0.379379
Dendritic.cells	DAB2IP	-0.60563	2.225574	-1.70138	0.092387	-4.3906	0.415388	0.409977
Dendritic.cells	GLIS2	-0.70148	1.3513	-1.70078	0.092498	-4.31721	0.42283	0.420307
Dendritic.cells	MRPS18A	0.271205	5.09387	1.700517	0.092549	-4.87453	0.393404	0.378895
Dendritic.cells	TDRKH	-0.59605	1.697145	-1.70027	0.092595	-4.40602	0.419998	0.416571
Dendritic.cells	CDK19	0.250206	6.390727	1.700208	0.092607	-5.11857	0.383821	0.365564
Dendritic.cells	CRIM1	-0.49073	5.191827	-1.69939	0.092763	-4.83559	0.393172	0.37817
Dendritic.cells	NUBP1	0.233538	5.473714	1.698342	0.09296	-5.053	0.391621	0.375558
Dendritic.cells	COX14	0.209841	5.656597	1.698157	0.092995	-5.08141	0.39026	0.373678
Dendritic.cells	MPP5	0.333106	5.379551	1.698104	0.093005	-4.7638	0.392324	0.376571
Dendritic.cells	GMPR	0.74546	1.923101	1.697858	0.093052	-4.3232	0.419334	0.414731
Dendritic.cells	BORCS8	0.27449	4.590375	1.697491	0.093121	-4.86635	0.398463	0.385226
Dendritic.cells	MRPS16	0.221708	5.607422	1.697053	0.093204	-5.1005	0.390996	0.374697
Dendritic.cells	POLR3B	-0.3356	5.512738	-1.69625	0.093357	-4.92065	0.391887	0.375918
Dendritic.cells	HDAC4	-0.24155	5.402881	-1.69625	0.093357	-5.21167	0.392708	0.377069
Dendritic.cells	GM14634	0.822309	1.834434	1.696237	0.09336	-4.27015	0.420611	0.416433
Dendritic.cells	PGAM1	0.275077	6.892134	1.695716	0.093459	-5.18042	0.381928	0.361847
Dendritic.cells	TRMT44	0.671083	1.793941	1.69553	0.093494	-4.32752	0.421117	0.417008
Dendritic.cells	LIPC	-0.77655	2.510562	-1.69545	0.093509	-4.30989	0.41531	0.408781
Dendritic.cells	SLC38A9	0.218776	5.16902	1.694828	0.093628	-5.18811	0.394847	0.379853
Dendritic.cells	HP1BP3	0.180806	6.529858	1.694624	0.093666	-5.3445	0.384767	0.365747
Dendritic.cells	PHF12	0.177495	6.186244	1.694592	0.093673	-5.28907	0.38728	0.369263
Dendritic.cells	HMGCS2	-0.8126	4.456909	-1.6943	0.093728	-4.61857	0.400318	0.387476
Dendritic.cells	B3GNT5	0.79583	2.546338	1.694131	0.09376	-4.303	0.415315	0.408684
Dendritic.cells	SPINT2	0.203343	4.563442	1.693466	0.093887	-5.18079	0.399885	0.386603
Dendritic.cells	HPD	-0.86998	4.805638	-1.69299	0.093979	-4.6282	0.398267	0.384288
Dendritic.cells	APOL8	-0.69981	2.828251	-1.69231	0.094108	-4.2998	0.413839	0.406413

Dendritic.cells	SYNE2	0.220174	5.373755	1.691838	0.094199	-5.34687	0.394111	0.378816
Dendritic.cells	PRKAR1A	-0.11728	7.625008	-1.69159	0.094245	-5.5382	0.377669	0.355912
Dendritic.cells	RFX1	-0.3481	3.263376	-1.69157	0.094251	-4.5471	0.41038	0.401861
Dendritic.cells	SCML4	-0.22986	4.659948	-1.69153	0.094258	-5.2497	0.399519	0.386553
Dendritic.cells	PSTPIP1	0.320897	4.472927	1.691235	0.094314	-4.89466	0.400952	0.388672
Dendritic.cells	TBC1D2	0.893257	1.45751	1.690613	0.094433	-4.25808	0.424978	0.42295
Dendritic.cells	CATSPERD	-1.00863	1.329786	-1.69032	0.09449	-4.24253	0.426035	0.424545
Dendritic.cells	LRR1	0.912261	1.435876	1.690183	0.094516	-4.2301	0.425157	0.423295
Dendritic.cells	9230116N1	0.766731	0.920334	1.690124	0.094527	-4.28952	0.429443	0.429403
Dendritic.cells	ZFP934	0.4868	2.990341	1.690111	0.09453	-4.51506	0.412546	0.405389
Dendritic.cells	CPD	-0.51535	4.333844	-1.68994	0.094563	-4.5056	0.402022	0.390588
Dendritic.cells	ETS1	-0.19389	7.418769	-1.68992	0.094566	-5.5035	0.379137	0.358443
Dendritic.cells	GAS8	0.529242	1.529564	1.689809	0.094588	-4.38034	0.424383	0.422278
Dendritic.cells	NUBPL	0.451623	2.913085	1.68965	0.094618	-4.49404	0.413162	0.40637
Dendritic.cells	GATC	0.606221	2.698485	1.689535	0.09464	-4.43748	0.414878	0.408836
Dendritic.cells	EIF2B4	-0.31545	4.102435	-1.68952	0.094643	-4.62552	0.40381	0.393172
Dendritic.cells	TUBB2B	-0.3411	2.9596	-1.6881	0.094917	-4.86298	0.413576	0.40658
Dendritic.cells	ZFP438	-0.4123	3.204144	-1.68775	0.094984	-4.57856	0.41163	0.403823
Dendritic.cells	GM5150	-1.1162	2.574091	-1.68768	0.094997	-4.2802	0.416668	0.410988
Dendritic.cells	PIK3R6	-1.00309	2.596377	-1.68751	0.09503	-4.29732	0.416489	0.410827
Dendritic.cells	ISG15	0.733519	6.408101	1.687482	0.095036	-5.09253	0.387176	0.369479
Dendritic.cells	TRAM1	0.123839	7.121393	1.68743	0.095046	-5.59245	0.381992	0.362236
Dendritic.cells	ARID1A	0.153389	6.658177	1.687025	0.095124	-5.35586	0.385515	0.367121
Dendritic.cells	TRIM2	-0.76313	2.325336	-1.68643	0.095238	-4.32389	0.419159	0.414393
Dendritic.cells	LPGAT1	-0.14441	6.245156	-1.68629	0.095266	-5.7351	0.38882	0.371619
Dendritic.cells	SENP8	-1.03186	0.568146	-1.68595	0.095331	-4.23512	0.433845	0.435307
Dendritic.cells	MAGI2	-0.86749	0.831391	-1.68534	0.095449	-4.25843	0.431991	0.432317
Dendritic.cells	SLC7A8	-1.06335	2.86439	-1.68478	0.095558	-4.33155	0.415596	0.408795
Dendritic.cells	GM12592	-0.48411	3.124779	-1.68424	0.095662	-4.48183	0.413681	0.406034
Dendritic.cells	GM20219	-0.8331	0.646352	-1.68409	0.095692	-4.26664	0.434054	0.435035
Dendritic.cells	FAM78A	0.637454	2.648934	1.684001	0.095709	-4.35963	0.4175	0.411497
Dendritic.cells	SNX4	-0.15991	6.329814	-1.68355	0.095797	-5.29245	0.38917	0.371573
Dendritic.cells	NSL1	-0.77245	2.25699	-1.68351	0.095804	-4.27809	0.420767	0.416172
Dendritic.cells	COA3	0.223962	5.812938	1.683149	0.095874	-5.12726	0.393142	0.377085
Dendritic.cells	GLOD4	-0.22937	5.050654	-1.68275	0.095953	-4.97332	0.398963	0.385361
Dendritic.cells	IQGAP3	-0.87973	2.400987	-1.68268	0.095966	-4.32304	0.419814	0.414867
Dendritic.cells	SLC16A6	-0.5052	4.554893	-1.68149	0.096197	-4.62915	0.403576	0.39115
Dendritic.cells	UBE2I	0.119361	7.624242	1.680429	0.096404	-5.495	0.38109	0.359493
Dendritic.cells	TRIM47	-0.57056	2.41312	-1.68035	0.09642	-4.40257	0.420947	0.415608
Dendritic.cells	OXSM	0.6717	1.788374	1.680348	0.09642	-4.29478	0.426074	0.42289
Dendritic.cells	KLKB1	-0.843	1.848942	-1.68015	0.096458	-4.2927	0.425574	0.422186
Dendritic.cells	TIMM50	0.299774	4.827954	1.680001	0.096487	-4.86601	0.401839	0.388603
Dendritic.cells	M1AP	-0.89864	1.320039	-1.67989	0.096509	-4.24909	0.429969	0.428438
Dendritic.cells	RESF1	0.261403	6.500817	1.679412	0.096602	-5.12615	0.389477	0.371038
Dendritic.cells	CPM	-0.88886	4.160192	-1.67878	0.096727	-4.36591	0.407612	0.396226
Dendritic.cells	TMEM150/	-0.60725	2.327871	-1.67816	0.096848	-4.46827	0.422269	0.41708
Dendritic.cells	NDFIP2	-0.18671	5.951714	-1.67805	0.096869	-5.19898	0.393911	0.377066

Dendritic.cells	TMEM230	-0.26398	4.742854	-1.67802	0.096874	-4.79539	0.403092	0.389972
Dendritic.cells	ALG12	-0.65242	1.395336	-1.67797	0.096886	-4.39026	0.429978	0.428046
Dendritic.cells	TMED2	0.119287	8.136615	1.677814	0.096915	-5.76778	0.378005	0.354902
Dendritic.cells	TNFSF12	-0.69313	2.295218	-1.67756	0.096965	-4.32474	0.422588	0.417616
Dendritic.cells	YTHDC1	-0.12633	7.312924	-1.67713	0.097048	-5.50237	0.384129	0.363371
Dendritic.cells	SUFU	0.259619	5.025085	1.675764	0.097318	-4.92083	0.401977	0.387637
Dendritic.cells	ZFAS1	-0.41894	5.058651	-1.67575	0.09732	-4.76219	0.40172	0.387275
Dendritic.cells	SEMA6D	-0.7788	3.713919	-1.67546	0.097378	-4.5228	0.412292	0.402059
Dendritic.cells	WARS2	0.272602	3.512477	1.675194	0.09743	-4.97989	0.413902	0.404338
Dendritic.cells	RGS16	-1.07013	1.351563	-1.67492	0.097483	-4.25395	0.431575	0.429473
Dendritic.cells	UPF2	0.184203	6.075856	1.674479	0.097571	-5.22328	0.394107	0.376733
Dendritic.cells	RHOBTB2	0.297461	2.943311	1.674132	0.097639	-4.88241	0.418468	0.411209
Dendritic.cells	POMK	-0.91403	1.068205	-1.67406	0.097652	-4.26162	0.43396	0.433227
Dendritic.cells	GRIP1	-1.06326	2.360437	-1.67395	0.097674	-4.29266	0.423209	0.417942
Dendritic.cells	MYO6	-0.5004	3.1857	-1.67394	0.097677	-4.54082	0.416515	0.408456
Dendritic.cells	MTFR1	0.403868	4.079368	1.673918	0.097681	-4.62029	0.409416	0.398423
Dendritic.cells	CD93	-0.47553	3.89899	-1.6735	0.097764	-4.61413	0.410922	0.400538
Dendritic.cells	RPAP3	-0.42382	3.841822	-1.67343	0.097778	-4.56537	0.411374	0.401196
Dendritic.cells	ASB1	0.638291	1.712368	1.673243	0.097814	-4.34561	0.428649	0.425757
Dendritic.cells	TMSB10	0.21239	10.73818	1.673008	0.097861	-5.96983	0.361316	0.331296
Dendritic.cells	TRAK2	0.283251	5.153104	1.672678	0.097926	-4.91875	0.401262	0.387111
Dendritic.cells	GM9967	-1.09976	0.569425	-1.67251	0.097959	-4.2651	0.438388	0.439817
Dendritic.cells	4930469K1	1.090182	1.311112	1.672371	0.097987	-4.25572	0.4321	0.430927
Dendritic.cells	LGALSL	0.778015	1.281509	1.671376	0.098183	-4.318	0.433052	0.431705
Dendritic.cells	A630072M	0.512021	3.151561	1.670819	0.098294	-4.51765	0.417956	0.410061
Dendritic.cells	CNNM3	-0.47763	3.157652	-1.67012	0.098432	-4.48559	0.418256	0.410228
Dendritic.cells	RAB27A	0.234507	4.50985	1.670024	0.098451	-5.12805	0.407527	0.39513
Dendritic.cells	PDLIM1	-0.2354	4.944927	-1.66973	0.098509	-5.22192	0.404233	0.390422
Dendritic.cells	PSME1	0.21382	8.384368	1.668698	0.098715	-5.7986	0.379374	0.355058
Dendritic.cells	CYB561D2	0.405176	3.329402	1.668577	0.098739	-4.57042	0.417611	0.408704
Dendritic.cells	STX1A	-0.54678	1.792697	-1.66823	0.098809	-4.55604	0.430348	0.426651
Dendritic.cells	CLCN6	0.329114	3.649755	1.667943	0.098865	-4.84529	0.415257	0.405232
Dendritic.cells	MTERF4	-0.68296	1.913823	-1.66736	0.098981	-4.33341	0.429756	0.425427
Dendritic.cells	SNAPC4	-0.64531	1.856185	-1.66704	0.099046	-4.40996	0.430329	0.42621
Dendritic.cells	NRXN3	-0.89192	1.145579	-1.66688	0.099078	-4.32356	0.436312	0.434772
Dendritic.cells	CD164	-0.14716	6.379209	-1.66662	0.09913	-5.57328	0.39462	0.375934
Dendritic.cells	RHD	0.923651	1.667344	1.6664	0.099173	-4.30808	0.431993	0.428651
Dendritic.cells	DHX34	-0.54533	2.203437	-1.66557	0.099339	-4.43084	0.427721	0.422629
Dendritic.cells	NUB1	0.186407	5.534309	1.665529	0.099347	-5.16689	0.401208	0.385214
Dendritic.cells	CYP4F16	0.743575	1.441368	1.665524	0.099348	-4.37039	0.434092	0.431678
Dendritic.cells	PROX1OS	-1.0269	0.612207	-1.66541	0.09937	-4.26391	0.441157	0.441743
Dendritic.cells	CDH1	0.350249	1.38751	1.664784	0.099496	-4.94406	0.434743	0.432607
Dendritic.cells	GALK1	0.213116	5.140655	1.664618	0.099529	-5.28815	0.404412	0.389713
Dendritic.cells	ITPR3	0.442542	4.288295	1.664509	0.099551	-4.66396	0.411059	0.3991
Dendritic.cells	SPIC	-1.25098	1.863637	-1.66443	0.099566	-4.31023	0.430741	0.426975
Dendritic.cells	NBN	0.467317	3.4476	1.664191	0.099615	-4.52192	0.417798	0.408672
Dendritic.cells	SEMA6B	-0.73432	1.154578	-1.66364	0.099725	-4.34827	0.436939	0.43558

Dendritic.cells	PCED1A	-0.61619	2.508638	-1.66361	0.099731	-4.36761	0.425609	0.419489
Dendritic.cells	FXYD5	0.25422	6.974009	1.661816	0.100092	-5.51251	0.392019	0.370928
Dendritic.cells	COMMD7	0.173222	5.29863	1.661661	0.100123	-5.09646	0.404679	0.388714
Dendritic.cells	RBM4	0.221069	4.655948	1.661112	0.100233	-4.98122	0.409879	0.395813
Dendritic.cells	ADPRHL2	-0.33248	3.241714	-1.66103	0.10025	-4.63246	0.421164	0.411709
Dendritic.cells	RHOD	-0.73752	1.328394	-1.66076	0.100304	-4.33939	0.43706	0.434298
Dendritic.cells	PGAM5	0.346616	3.934831	1.66066	0.100324	-4.67358	0.415584	0.403944
Dendritic.cells	PAFAH1B3	0.210487	5.814072	1.659766	0.100505	-5.42177	0.401448	0.383508
Dendritic.cells	NIFK	-0.33868	4.620947	-1.65952	0.100553	-4.75405	0.410688	0.396525
Dendritic.cells	TRIM16	-0.81037	2.341778	-1.65926	0.100607	-4.33207	0.429109	0.422598
Dendritic.cells	WDCP	0.548093	3.079235	1.659256	0.100608	-4.44031	0.423037	0.414018
Dendritic.cells	RPP25L	0.388606	3.672149	1.658987	0.100662	-4.62896	0.4183	0.407351
Dendritic.cells	ING4	0.404383	4.08954	1.658233	0.100814	-4.63327	0.415349	0.402997
Dendritic.cells	IGF2BP2	-0.80822	1.457318	-1.65814	0.100833	-4.31283	0.437015	0.433649
Dendritic.cells	TMEM267	0.698704	1.734822	1.657494	0.100964	-4.35776	0.435067	0.430484
Dendritic.cells	GM36738	0.447785	3.095803	1.657232	0.101017	-4.60043	0.42376	0.414612
Dendritic.cells	DDAH1	-0.62886	2.481818	-1.65709	0.101045	-4.40995	0.428816	0.421755
Dendritic.cells	SGMS2	0.912287	3.643674	1.656837	0.101097	-4.4426	0.419311	0.408415
Dendritic.cells	4930403P2	-0.73807	0.612375	-1.65674	0.101117	-4.29977	0.444675	0.444347
Dendritic.cells	GADD45GII	0.27785	5.128075	1.655392	0.101391	-4.93393	0.408447	0.392456
Dendritic.cells	LHFP	-0.88369	1.237889	-1.65527	0.101416	-4.322	0.440255	0.437314
Dendritic.cells	CBX8	0.850711	1.617418	1.654967	0.101477	-4.29748	0.437118	0.432783
Dendritic.cells	SUGCT	-0.585	2.835527	-1.65456	0.10156	-4.47764	0.427004	0.418594
Dendritic.cells	STT3A	-0.13654	6.169392	-1.6545	0.101572	-5.46294	0.400599	0.381506
Dendritic.cells	1110059G1	0.331107	3.928256	1.653749	0.101725	-4.68981	0.41831	0.406373
Dendritic.cells	A430035B1	-0.21357	3.071834	-1.65371	0.101733	-5.43645	0.425263	0.416174
Dendritic.cells	USP1	0.273529	5.737466	1.653697	0.101736	-5.05234	0.404091	0.386413
Dendritic.cells	SLC41A2	0.169311	2.900939	1.651934	0.102095	-5.65648	0.428016	0.419153
Dendritic.cells	GM43696	-0.52735	2.459243	-1.6511	0.102266	-4.46214	0.432197	0.424636
Dendritic.cells	SLC16A4	-0.6829	1.428027	-1.65097	0.102293	-4.34435	0.440927	0.437018
Dendritic.cells	SPAG9	-0.20296	9.266573	-1.6504	0.10241	-5.90297	0.380019	0.351506
Dendritic.cells	ZMYND8	0.162301	6.108671	1.650149	0.102461	-5.39671	0.403246	0.383856
Dendritic.cells	PLG	-0.75159	4.004656	-1.65009	0.102472	-4.62056	0.419772	0.407012
Dendritic.cells	PTPN23	-0.37038	3.647447	-1.64937	0.10262	-4.7205	0.422994	0.4113
Dendritic.cells	IFITM6	1.1432	3.518134	1.649123	0.102671	-4.38664	0.424048	0.41287
Dendritic.cells	SPARCL1	1.29839	0.3267	1.649029	0.102691	-4.31112	0.451121	0.451181
Dendritic.cells	KLC3	0.805626	0.19256	1.648953	0.102706	-4.34985	0.452304	0.452902
Dendritic.cells	CHCHD4	0.396694	3.820424	1.648648	0.102769	-4.66582	0.421666	0.40952
Dendritic.cells	CCDC50	0.166909	5.994315	1.648487	0.102802	-5.36002	0.404511	0.385485
Dendritic.cells	CAPRIN1	0.11933	7.460311	1.64812	0.102877	-5.61349	0.393597	0.370154
Dendritic.cells	CAMK1	-0.65176	2.966119	-1.64793	0.102916	-4.41381	0.428817	0.419512
Dendritic.cells	GM43328	0.368356	2.238918	1.647302	0.103046	-4.7848	0.434967	0.428322
Dendritic.cells	ZCCHC7	0.196345	6.744533	1.647211	0.103064	-5.66018	0.399019	0.377841
Dendritic.cells	GM553	-0.93842	0.241543	-1.64698	0.103112	-4.29658	0.452199	0.452786
Dendritic.cells	DPP7	-0.691	1.957266	-1.64691	0.103127	-4.42526	0.437348	0.431709
Dendritic.cells	TUBA1B	-0.3274	7.795232	-1.6469	0.103128	-5.46329	0.391198	0.366967
Dendritic.cells	CARMIL2	0.511137	2.704609	1.646168	0.103279	-4.51597	0.431293	0.423118

Dendritic.cells	ZBTB25	0.441931	3.709262	1.646076	0.103299	-4.63416	0.42302	0.411481
Dendritic.cells	CCDC77	-0.43543	3.291762	-1.64605	0.103304	-4.52968	0.426433	0.416289
Dendritic.cells	MND1	-0.78813	2.222735	-1.6458	0.103355	-4.31827	0.435333	0.428893
Dendritic.cells	OFD1	-0.36435	3.31561	-1.64571	0.103375	-4.6003	0.426237	0.41605
Dendritic.cells	RP2	0.231524	5.226456	1.645132	0.103493	-5.09918	0.411223	0.394632
Dendritic.cells	IFT172	0.516476	2.410455	1.644165	0.103693	-4.47758	0.434654	0.427344
Dendritic.cells	DDIT4	0.741452	3.744278	1.644108	0.103705	-4.57767	0.423613	0.411789
Dendritic.cells	D430020J0	-0.90503	0.440007	-1.64359	0.103812	-4.31139	0.451715	0.451616
Dendritic.cells	ZFP64	-0.33456	5.317866	-1.64345	0.103842	-4.99069	0.411124	0.394368
Dendritic.cells	D1ERTD62	0.234297	5.295192	1.643041	0.103926	-5.09417	0.411302	0.394814
Dendritic.cells	UROS	0.442622	2.669254	1.64299	0.103937	-4.48211	0.432572	0.424749
Dendritic.cells	TCRG-C1	-1.20476	0.880703	-1.64293	0.103949	-4.30642	0.44785	0.446401
Dendritic.cells	TSTD2	-0.3739	3.84075	-1.64275	0.103988	-4.6904	0.422914	0.411127
Dendritic.cells	MEIS3	-0.89036	0.488054	-1.6427	0.103997	-4.29252	0.451292	0.451295
Dendritic.cells	AKR1D1	-0.87556	2.587501	-1.64233	0.104074	-4.41579	0.433306	0.425842
Dendritic.cells	NPR1	-0.66233	1.263007	-1.64227	0.104086	-4.45923	0.444582	0.441815
Dendritic.cells	HMGCL	0.269063	5.187114	1.641572	0.104231	-5.03227	0.412339	0.396369
Dendritic.cells	APOM	-0.7416	4.854579	-1.64156	0.104234	-4.76626	0.414966	0.400055
Dendritic.cells	P4HA1	0.297279	5.786072	1.641436	0.10426	-5.29822	0.407663	0.389824
Dendritic.cells	4930414N(	-0.32073	3.331114	-1.64135	0.104277	-4.95272	0.427277	0.4174
Dendritic.cells	SAPCD2	-0.82801	1.790194	-1.64085	0.104381	-4.32849	0.440333	0.435866
Dendritic.cells	DIO1	-0.80374	0.977554	-1.64072	0.104408	-4.32161	0.447344	0.445877
Dendritic.cells	IGFALS	0.675955	1.779759	1.640628	0.104428	-4.40976	0.440422	0.436109
Dendritic.cells	ARPC3	0.122403	8.451234	1.640461	0.104463	-5.75265	0.387798	0.362247
Dendritic.cells	SPATA5	-0.18325	5.905372	-1.64001	0.104557	-5.4867	0.407077	0.389027
Dendritic.cells	LEPROTL1	0.249276	5.362087	1.63934	0.104696	-5.22192	0.411428	0.395183
Dendritic.cells	SCO1	0.632663	1.930574	1.639154	0.104735	-4.42421	0.439497	0.434875
Dendritic.cells	TG	-0.55171	1.91376	-1.63896	0.104776	-4.49292	0.439641	0.435154
Dendritic.cells	HNRNPLL	-0.52689	4.231786	-1.63894	0.10478	-4.50082	0.420415	0.407977
Dendritic.cells	UROC1	-0.91247	1.760508	-1.6388	0.104808	-4.35845	0.44095	0.437089
Dendritic.cells	ZFP748	0.648069	1.938501	1.638747	0.10482	-4.43187	0.43943	0.434939
Dendritic.cells	SLC9A5	-0.93796	0.362304	-1.63844	0.104883	-4.28693	0.453224	0.454585
Dendritic.cells	FAM110B	1.000021	0.653098	1.638254	0.104923	-4.30191	0.450663	0.450966
Dendritic.cells	NCOA1	0.162757	7.07231	1.637816	0.105014	-5.68488	0.398484	0.377264
Dendritic.cells	CLTA	-0.12618	8.442682	-1.63777	0.105023	-5.71909	0.388359	0.363154
Dendritic.cells	XKR5	0.848052	0.190356	1.63714	0.105156	-4.28866	0.455257	0.457152
Dendritic.cells	TTC14	0.142675	5.803768	1.636926	0.105201	-5.40088	0.40856	0.391084
Dendritic.cells	DRAM2	0.335285	4.847911	1.636464	0.105298	-4.81573	0.416302	0.401865
Dendritic.cells	CUL5	-0.16236	5.877947	-1.63534	0.105534	-5.30026	0.408787	0.390861
Dendritic.cells	GGH	-0.53709	4.555539	-1.63533	0.105535	-4.63499	0.419229	0.405519
Dendritic.cells	SNX3	0.133345	7.730038	1.635198	0.105563	-5.5877	0.39473	0.371269
Dendritic.cells	RHOJ	-0.53909	1.992484	-1.63468	0.105672	-4.62406	0.44075	0.435674
Dendritic.cells	ZBTB5	0.518969	2.463409	1.633903	0.105835	-4.50603	0.43726	0.430144
Dendritic.cells	F13A1	1.621024	2.512117	1.633495	0.105921	-4.37335	0.437042	0.429635
Dendritic.cells	TMEM33	-0.20896	5.133185	-1.63308	0.106008	-5.0724	0.415761	0.399554
Dendritic.cells	C330007P(	-0.2576	5.50907	-1.63286	0.106054	-5.05582	0.41282	0.395433
Dendritic.cells	GBP4	0.683761	3.363964	1.631697	0.1063	-4.77954	0.43027	0.420338

Dendritic.cells	COL9A3	-0.92128	0.618437	-1.63159	0.106323	-4.34047	0.453793	0.453694
Dendritic.cells	BNIP3	0.540802	5.086642	1.631345	0.106375	-4.80708	0.416288	0.400744
Dendritic.cells	RASA4	0.417135	4.228858	1.631255	0.106394	-4.94296	0.423177	0.410463
Dendritic.cells	PPP1R12A	0.133595	8.052992	1.631166	0.106413	-5.71279	0.393568	0.369031
Dendritic.cells	NFIC	-0.25926	4.464453	-1.631	0.106449	-5.00131	0.42127	0.407879
Dendritic.cells	CCDC12	0.144963	6.939585	1.630948	0.106459	-5.46335	0.401899	0.38072
Dendritic.cells	DOHH	0.31317	4.347216	1.630912	0.106466	-4.83925	0.422217	0.409213
Dendritic.cells	RAB28	-0.2428	4.828065	-1.63091	0.106466	-5.0831	0.418349	0.403772
Dendritic.cells	BLZF1	0.386133	3.200504	1.630862	0.106477	-4.63403	0.431628	0.422486
Dendritic.cells	A930015D(	0.249605	4.760503	1.63061	0.10653	-5.1046	0.418947	0.404635
Dendritic.cells	HK2	0.521586	5.335441	1.630156	0.106626	-4.77022	0.414541	0.398296
Dendritic.cells	TSPAN32	0.925088	2.972272	1.630037	0.106651	-4.30695	0.433767	0.425375
Dendritic.cells	EIF2AK4	0.334978	4.653928	1.62909	0.106852	-4.88472	0.420506	0.406211
Dendritic.cells	YPEL2	0.28805	4.572346	1.628878	0.106897	-5.1444	0.421163	0.407179
Dendritic.cells	PEX11B	0.365219	3.446594	1.628812	0.106911	-4.70541	0.430366	0.420139
Dendritic.cells	WDR34	-0.90099	1.224098	-1.62857	0.106961	-4.35898	0.449285	0.447005
Dendritic.cells	PKD2	-0.69605	0.719693	-1.62825	0.10703	-4.35451	0.45372	0.453372
Dendritic.cells	NDFIP1	-0.16242	7.521506	-1.62823	0.107034	-5.52304	0.398237	0.3752
Dendritic.cells	DCAF17	0.317972	4.054876	1.628109	0.10706	-4.7843	0.425362	0.413243
Dendritic.cells	WDFY2	-0.37751	5.19857	-1.6266	0.107381	-4.83433	0.417247	0.400677
Dendritic.cells	TNKS	0.172392	6.066905	1.626197	0.107467	-5.53343	0.410589	0.391253
Dendritic.cells	PRXL2C	0.168217	4.932963	1.625624	0.107589	-5.38485	0.419789	0.403965
Dendritic.cells	UHRF1	0.659249	4.598357	1.625395	0.107638	-4.62218	0.422484	0.407833
Dendritic.cells	GM9733	1.268888	0.76406	1.624881	0.107748	-4.30996	0.454976	0.453866
Dendritic.cells	TGIF1	-0.39516	6.736671	-1.62473	0.10778	-5.04331	0.405643	0.384409
Dendritic.cells	FHDC1	0.887952	0.748905	1.624625	0.107802	-4.33575	0.45511	0.454108
Dendritic.cells	ANKRD9	-0.37089	2.821177	-1.62453	0.107822	-4.85593	0.437171	0.42873
Dendritic.cells	IL18	-0.6248	3.422293	-1.62446	0.107837	-4.44544	0.432132	0.421656
Dendritic.cells	TRP53INP2	-0.41183	4.134264	-1.62433	0.107865	-4.65128	0.426258	0.413416
Dendritic.cells	A630052C1	-0.87092	0.189023	-1.62426	0.107881	-4.33459	0.46011	0.461354
Dendritic.cells	CNOT7	0.168049	5.288607	1.623986	0.107939	-5.18357	0.417024	0.400462
Dendritic.cells	ZFP956	0.653132	1.264702	1.623482	0.108047	-4.39684	0.450831	0.448109
Dendritic.cells	LIPE	0.380468	3.783042	1.623408	0.108062	-4.66328	0.429399	0.41784
Dendritic.cells	3010003L2	0.706217	1.349271	1.622882	0.108175	-4.41217	0.450396	0.447322
Dendritic.cells	TIMM8B	0.283207	5.242586	1.622538	0.108249	-5.06668	0.417981	0.40149
Dendritic.cells	NSD1	0.119554	7.273059	1.6218	0.108407	-5.70311	0.402638	0.379579
Dendritic.cells	ZFP82	-0.874	0.121707	-1.62162	0.108446	-4.32558	0.461954	0.463171
Dendritic.cells	PAPOLA	0.105034	7.055885	1.621412	0.10849	-5.613	0.40431	0.381944
Dendritic.cells	TPRGL	0.168092	6.927644	1.620884	0.108604	-5.48355	0.405566	0.383504
Dendritic.cells	BNIP2	0.182662	6.377564	1.619893	0.108817	-5.26389	0.410385	0.389725
Dendritic.cells	5830428M	0.424978	2.548294	1.61974	0.10885	-4.69606	0.44161	0.433617
Dendritic.cells	TMEM199	0.380508	3.379806	1.619547	0.108891	-4.6358	0.434579	0.423762
Dendritic.cells	RNF40	0.2826	4.021973	1.619275	0.10895	-4.8456	0.429245	0.416256
Dendritic.cells	PHTF2	-0.24679	5.998759	-1.61918	0.108971	-5.19606	0.413344	0.393972
Dendritic.cells	SNRPD1	0.188083	7.17842	1.619074	0.108993	-5.50652	0.404221	0.381272
Dendritic.cells	FKBP3	0.254496	6.130579	1.618391	0.10914	-5.23315	0.412719	0.392686
Dendritic.cells	ACACA	0.234029	5.153719	1.617955	0.109235	-5.21934	0.42053	0.403556

Dendritic.cells	NDUFB10	0.169919	6.731387	1.61784	0.109259	-5.45917	0.408116	0.386214
Dendritic.cells	SATB2	-0.95549	1.643339	-1.61775	0.109279	-4.3298	0.44994	0.444962
Dendritic.cells	DAPK3	0.255987	4.42231	1.617447	0.109344	-4.94085	0.426452	0.411857
Dendritic.cells	EFTUD2	0.218722	5.105706	1.617396	0.109355	-5.12699	0.420916	0.404107
Dendritic.cells	PTCD2	-0.2987	4.58679	-1.61672	0.109501	-4.87001	0.425114	0.41023
Dendritic.cells	TBC1D30	-0.94952	1.06093	-1.61667	0.109512	-4.37082	0.455068	0.45251
Dendritic.cells	CC2D1B	-0.34525	3.659138	-1.61661	0.109524	-4.70333	0.432749	0.420995
Dendritic.cells	HSPBAP1	0.440403	3.764205	1.616496	0.10955	-4.73407	0.431876	0.419795
Dendritic.cells	PSMD4	0.151333	6.691408	1.61596	0.109666	-5.41585	0.408428	0.387032
Dendritic.cells	ANKRD42	-0.99307	0.346625	-1.61596	0.109667	-4.33816	0.461449	0.461745
Dendritic.cells	STFA3	1.482404	2.751543	1.615943	0.10967	-4.35495	0.440388	0.431891
Dendritic.cells	RELCH	0.135509	6.238997	1.615823	0.109696	-5.53681	0.411941	0.391937
Dendritic.cells	PSMD5	-0.33984	3.835237	-1.61575	0.109711	-4.71468	0.431286	0.419069
Dendritic.cells	MSRB2	-0.92897	1.023579	-1.61529	0.109811	-4.32749	0.455442	0.453304
Dendritic.cells	TMTC2	-0.44418	4.574445	-1.61525	0.10982	-5.20499	0.425255	0.410672
Dendritic.cells	2-Mar	0.225453	6.581903	1.61479	0.10992	-5.34672	0.409313	0.388431
Dendritic.cells	SIRT5	0.638411	1.958147	1.614767	0.109924	-4.4269	0.447246	0.441774
Dendritic.cells	UBR4	-0.15665	6.056739	-1.61462	0.109956	-5.32544	0.413407	0.39418
Dendritic.cells	CSNK2A1	0.123568	6.59773	1.614528	0.109976	-5.48877	0.40919	0.388296
Dendritic.cells	KLF11	0.294346	3.555665	1.614432	0.109997	-4.77277	0.433652	0.422611
Dendritic.cells	ATAT1	0.5129	2.722421	1.613832	0.110128	-4.5088	0.441042	0.43272
Dendritic.cells	SLAMF8	1.212875	0.718707	1.61336	0.11023	-4.34888	0.458796	0.457733
Dendritic.cells	MRPS26	0.193532	5.167613	1.612854	0.11034	-5.09798	0.42119	0.404535
Dendritic.cells	AGRN	-0.83606	1.254784	-1.61283	0.110346	-4.38562	0.454183	0.451059
Dendritic.cells	PDE10A	0.348684	2.555984	1.612563	0.110403	-5.08916	0.442936	0.435135
Dendritic.cells	LSS	0.636934	0.604735	1.611687	0.110594	-4.45224	0.460677	0.459703
Dendritic.cells	EMC8	0.241218	4.721373	1.611327	0.110673	-5.04275	0.425598	0.410109
Dendritic.cells	PQLC1	-0.32848	4.395337	-1.61101	0.110743	-4.72303	0.428267	0.41389
Dendritic.cells	1-Mar	0.563435	4.477654	1.61081	0.110786	-4.97817	0.427592	0.412979
Dendritic.cells	ROBO2	-0.70351	0.744725	-1.61078	0.110793	-4.5631	0.459588	0.45816
Dendritic.cells	GM48383	0.636417	1.758143	1.610455	0.110863	-4.48514	0.4507	0.445517
Dendritic.cells	HPN	-0.74558	2.642028	-1.61024	0.110911	-4.42711	0.443049	0.434737
Dendritic.cells	GM41442	0.967594	-0.19949	1.61014	0.110932	-4.32869	0.468227	0.470478
Dendritic.cells	CIB1	0.177789	5.732242	1.60991	0.110982	-5.4197	0.417594	0.398982
Dendritic.cells	LYSMD1	-0.5862	1.591133	-1.60792	0.111418	-4.4205	0.45362	0.448232
Dendritic.cells	AGBL2	-0.84136	0.26749	-1.60781	0.111443	-4.3291	0.465467	0.465054
Dendritic.cells	TMEM26	-1.00731	2.190341	-1.60771	0.111464	-4.34586	0.448378	0.440906
Dendritic.cells	ZFP384	0.265091	4.90083	1.607602	0.111488	-4.9502	0.42559	0.408878
Dendritic.cells	ANKHD1	-0.14605	7.647791	-1.60742	0.111528	-5.69176	0.403998	0.378796
Dendritic.cells	SLCO1B2	-0.79081	4.25024	-1.60703	0.111614	-4.65904	0.431022	0.416547
Dendritic.cells	FAM83G	-0.80081	0.263823	-1.6068	0.111664	-4.33109	0.465607	0.465503
Dendritic.cells	FAM219A	-0.44023	4.171512	-1.60676	0.111672	-4.8242	0.431674	0.417577
Dendritic.cells	EIF4ENIF1	0.168074	5.556503	1.604919	0.112078	-5.35774	0.421576	0.402297
Dendritic.cells	NDUFS3	0.205006	6.050235	1.604648	0.112138	-5.27581	0.417638	0.396904
Dendritic.cells	RAMP2	-0.4365	3.013058	-1.60439	0.112194	-4.83649	0.442641	0.431999
Dendritic.cells	SPR	-0.42942	3.527522	-1.60435	0.112204	-4.65623	0.438274	0.425859
Dendritic.cells	TCP11L2	-0.3545	6.196085	-1.6043	0.112215	-5.09608	0.416484	0.39537

Dendritic.cells	ZC3HC1	0.314035	4.34425	1.604255	0.112224	-4.81629	0.431452	0.416287
Dendritic.cells	1110004F1	0.133434	6.204	1.603946	0.112292	-5.35899	0.416452	0.395372
Dendritic.cells	RIOK2	-0.24195	4.466641	-1.60365	0.112357	-4.9987	0.430474	0.415
Dendritic.cells	4930581F2	0.252928	3.634004	1.603511	0.112388	-5.0414	0.437409	0.424808
Dendritic.cells	CD74	-0.3806	11.27972	-1.6035	0.112392	-6.45225	0.378832	0.343402
Dendritic.cells	ZFP433	-0.85003	0.355661	-1.60261	0.112588	-4.36205	0.466758	0.465831
Dendritic.cells	B130034C1	0.612623	1.038829	1.60192	0.11274	-4.39022	0.460747	0.457505
Dendritic.cells	FAM171A1	-0.55101	1.565475	-1.6019	0.112745	-4.59281	0.456052	0.450859
Dendritic.cells	DHRS1	-0.52075	4.762169	-1.60176	0.112776	-4.68197	0.428793	0.412553
Dendritic.cells	ZBTB21	-0.27636	3.713914	-1.60142	0.112851	-4.95965	0.4375	0.424879
Dendritic.cells	MACROD1	-0.57298	2.861084	-1.60078	0.112993	-4.56459	0.444749	0.435252
Dendritic.cells	SLC35A4	0.390547	3.561213	1.600655	0.113021	-4.70648	0.438787	0.426877
Dendritic.cells	GM13483	-0.59233	1.85821	-1.60064	0.113024	-4.53723	0.453467	0.447586
Dendritic.cells	SLC22A21	0.942603	1.348839	1.600638	0.113024	-4.34009	0.457976	0.453968
Dendritic.cells	ABRACL	0.183401	7.550325	1.600512	0.113052	-5.63677	0.406706	0.382011
Dendritic.cells	SLC9A9	-0.21772	7.120003	-1.60045	0.113066	-5.91494	0.410015	0.386614
Dendritic.cells	SLC35E2	0.253461	3.898481	1.600447	0.113067	-4.9528	0.43595	0.422889
Dendritic.cells	CMTR2	-0.66966	1.0685	-1.60019	0.113123	-4.42431	0.460546	0.457604
Dendritic.cells	PTGR1	-0.65717	3.377249	-1.59959	0.113256	-4.49428	0.440762	0.429455
Dendritic.cells	MEGF11	-0.87825	0.651843	-1.59942	0.113294	-4.37221	0.464675	0.463326
Dendritic.cells	4833403J1	-0.98927	0.509888	-1.59912	0.113361	-4.36545	0.466059	0.46525
Dendritic.cells	CHD8	0.163116	5.733086	1.598574	0.113483	-5.33373	0.421413	0.402623
Dendritic.cells	ARL6IP4	0.174986	5.733017	1.598521	0.113495	-5.39568	0.421414	0.402641
Dendritic.cells	TNFAIP8L1	0.537544	2.396525	1.598509	0.113498	-4.50891	0.449281	0.44182
Dendritic.cells	SUDS3	0.244191	5.515338	1.598421	0.113517	-5.14353	0.423163	0.405106
Dendritic.cells	GPR137B	-0.15705	5.316315	-1.598	0.113611	-5.74249	0.424837	0.407435
Dendritic.cells	DDT	0.324331	5.541533	1.597983	0.113615	-5.03825	0.423018	0.404888
Dendritic.cells	USE1	0.232995	5.084893	1.597738	0.113669	-5.10759	0.426718	0.41009
Dendritic.cells	FRMPD4	0.666659	0.351798	1.597543	0.113713	-4.54161	0.467574	0.467796
Dendritic.cells	CREBBP	-0.12815	7.888027	-1.59744	0.113735	-5.76179	0.404666	0.379327
Dendritic.cells	MFAP1A	0.248154	4.832089	1.596711	0.113898	-4.93702	0.428892	0.413147
Dendritic.cells	NAA20	-0.20864	5.164245	-1.59649	0.113947	-5.07762	0.42618	0.409354
Dendritic.cells	TM7SF2	0.526522	1.754706	1.596233	0.114005	-4.54901	0.455092	0.450092
Dendritic.cells	TRIP4	0.412068	4.852518	1.596074	0.114041	-4.8345	0.428724	0.412989
Dendritic.cells	OVCA2	0.895454	0.174482	1.596035	0.114049	-4.3705	0.469314	0.470382
Dendritic.cells	IBTK	0.329151	4.642613	1.595984	0.114061	-4.92003	0.430449	0.41543
Dendritic.cells	ZFP949	0.572526	2.594224	1.595905	0.114078	-4.48999	0.44775	0.439831
Dendritic.cells	RETREG3	0.186813	5.527566	1.595883	0.114083	-5.32594	0.423239	0.40535
Dendritic.cells	HSD17B13	-0.82285	2.671988	-1.59558	0.114151	-4.46892	0.44716	0.439006
Dendritic.cells	ESD	0.201492	7.081837	1.595411	0.114189	-5.55817	0.41103	0.388341
Dendritic.cells	ZFP458	-0.88072	0.876726	-1.59513	0.114253	-4.39378	0.463015	0.461534
Dendritic.cells	FAM117A	0.281654	5.965212	1.595074	0.114264	-5.29669	0.419809	0.400634
Dendritic.cells	IL10RA	0.35849	4.971561	1.594814	0.114323	-4.92949	0.427898	0.412002
Dendritic.cells	STARD8	-0.40007	3.359704	-1.5946	0.11437	-4.77283	0.441347	0.430982
Dendritic.cells	S100A10	0.301863	6.601647	1.5944	0.114415	-5.342	0.414852	0.393866
Dendritic.cells	ZFP113	0.794537	1.151185	1.594265	0.114445	-4.39157	0.460632	0.458412
Dendritic.cells	CD3G	-0.83384	3.343766	-1.594	0.114504	-4.58853	0.441556	0.431428

Dendritic.cells	WDR83	0.507574	2.732947	1.59377	0.114556	-4.53093	0.446838	0.438873
Dendritic.cells	LRIG2	0.316045	4.256808	1.593283	0.114666	-4.88432	0.434174	0.420864
Dendritic.cells	METAP2	0.149382	7.3726	1.593054	0.114717	-5.59991	0.40921	0.385896
Dendritic.cells	LDHB	0.312072	3.00278	1.592921	0.114747	-5.14566	0.444775	0.435811
Dendritic.cells	ID1	-0.62705	4.07039	-1.59276	0.114783	-4.65235	0.435729	0.42307
Dendritic.cells	LYRM7	0.638745	1.247217	1.592501	0.114841	-4.46992	0.460181	0.457756
Dendritic.cells	SMC6	-0.11468	7.579287	-1.59239	0.114867	-5.96003	0.407635	0.383814
Dendritic.cells	ITIH4	-0.69502	3.988196	-1.59209	0.114934	-4.65573	0.436535	0.424218
Dendritic.cells	HIST1H2BC	0.546564	5.260139	1.591873	0.114983	-4.77497	0.426068	0.409461
Dendritic.cells	SERPINB6A	-0.33734	5.570318	-1.59097	0.115185	-5.09033	0.424102	0.406141
Dendritic.cells	HINT2	0.293638	4.356845	1.59078	0.115229	-4.93439	0.434047	0.420105
Dendritic.cells	SMC4	0.22088	7.287715	1.590682	0.115251	-5.48616	0.410531	0.387188
Dendritic.cells	TRPV4	-0.86016	-0.51202	-1.5905	0.115292	-4.35995	0.477023	0.481061
Dendritic.cells	ZYX	0.2014	6.244314	1.590106	0.11538	-5.61657	0.418887	0.398785
Dendritic.cells	SEC13	0.224582	5.361757	1.589818	0.115446	-5.11621	0.426066	0.408843
Dendritic.cells	CCDC137	-0.39626	2.673176	-1.58953	0.115512	-4.64948	0.448735	0.440752
Dendritic.cells	ARRDC2	0.574446	1.79539	1.589241	0.115576	-4.52038	0.456524	0.451736
Dendritic.cells	ACTR5	0.330419	3.477256	1.588721	0.115693	-4.83773	0.442003	0.43139
Dendritic.cells	IPO4	-0.45625	2.384797	-1.58864	0.115712	-4.53438	0.451423	0.444702
Dendritic.cells	FAM71F2	-0.91601	2.260769	-1.58861	0.115718	-4.41335	0.452509	0.446239
Dendritic.cells	2610044O1	-0.4675	2.24732	-1.58791	0.115876	-4.4951	0.453043	0.446676
Dendritic.cells	SLC12A3	-0.74964	1.874484	-1.58762	0.115943	-4.47599	0.456329	0.451418
Dendritic.cells	ACTR3	0.124839	8.876057	1.587609	0.115945	-5.81226	0.399364	0.371495
Dendritic.cells	GM19710	0.620049	2.595654	1.5865	0.116196	-4.59123	0.450819	0.442718
Dendritic.cells	PYCRL	0.409947	3.541294	1.586226	0.116258	-4.62999	0.44269	0.431292
Dendritic.cells	DNAJC1	-0.13039	6.915183	-1.58612	0.116282	-5.7467	0.415105	0.392674
Dendritic.cells	ATP5H	0.12633	7.946365	1.585272	0.116475	-5.7368	0.407661	0.381766
Dendritic.cells	9330151L1	-0.691	0.96571	-1.58482	0.116578	-4.4125	0.466191	0.463753
Dendritic.cells	CDC14A	0.238466	5.803629	1.584511	0.116648	-5.42254	0.424832	0.405465
Dendritic.cells	SIL1	0.180144	5.149956	1.584143	0.116732	-5.48914	0.430238	0.412991
Dendritic.cells	AATK	-0.97504	0.48294	-1.58402	0.116759	-4.36159	0.47081	0.470234
Dendritic.cells	DACH1	1.064108	1.877933	1.583879	0.116792	-4.39025	0.458204	0.452386
Dendritic.cells	GPC4	0.789917	1.1587	1.58328	0.116928	-4.42498	0.465034	0.461732
Dendritic.cells	ZFP672	0.302696	4.004158	1.583048	0.116981	-4.84007	0.440188	0.42665
Dendritic.cells	RCOR3	0.369034	2.937283	1.582254	0.117162	-4.78632	0.449754	0.439562
Dendritic.cells	KLHL4	-0.83174	0.018315	-1.5822	0.117174	-4.4241	0.476	0.476705
Dendritic.cells	TTC33	0.39088	3.849347	1.581999	0.117221	-4.73953	0.441945	0.428547
Dendritic.cells	GRB7	-0.98765	0.183341	-1.58182	0.117262	-4.35365	0.474497	0.474569
Dendritic.cells	TET2	0.167563	6.024646	1.581552	0.117323	-5.49642	0.423961	0.403481
Dendritic.cells	APPBP2OS	-0.67722	0.985935	-1.58143	0.117351	-4.39605	0.467127	0.464226
Dendritic.cells	TRP53BP2	0.38139	3.957204	1.581251	0.117392	-4.75013	0.441035	0.42743
Dendritic.cells	PNLDC1	-0.8383	0.924032	-1.58111	0.117424	-4.39521	0.467691	0.465072
Dendritic.cells	GM20743	0.841323	0.031876	1.580109	0.117653	-4.40891	0.476461	0.477188
Dendritic.cells	CEP164	0.253732	4.052145	1.580037	0.117669	-5.0461	0.440748	0.426726
Dendritic.cells	GM15987	0.670876	2.537923	1.579769	0.117731	-4.56768	0.453798	0.445201
Dendritic.cells	SMU1	0.200121	5.608875	1.579685	0.11775	-5.16689	0.427826	0.40873
Dendritic.cells	KCTD12B	-0.7971	0.963206	-1.57963	0.117764	-4.42717	0.467882	0.465137

Dendritic.cells	ATP6V0B	0.11489	8.046898	1.578987	0.11791	-5.8606	0.408936	0.382176
Dendritic.cells	KLRB1C	-1.08255	1.954564	-1.57869	0.117978	-4.42736	0.459472	0.452849
Dendritic.cells	ELOB	0.137976	8.913307	1.577533	0.118244	-5.89699	0.403025	0.373417
Dendritic.cells	USP11	0.399749	2.611991	1.577452	0.118263	-4.7472	0.454297	0.445008
Dendritic.cells	DCTN4	-0.1605	6.423452	-1.57744	0.118265	-5.42247	0.422328	0.400216
Dendritic.cells	KDELR1	0.145693	6.091298	1.576687	0.118439	-5.50102	0.425349	0.404244
Dendritic.cells	NOP9	-0.29325	4.555827	-1.57666	0.118444	-4.89297	0.437982	0.4219
Dendritic.cells	CD163	-1.21255	1.13156	-1.57639	0.118508	-4.42408	0.468011	0.464177
Dendritic.cells	SLC35F5	-0.56877	3.11698	-1.57595	0.118609	-4.57739	0.450578	0.439433
Dendritic.cells	GM20508	-0.84004	0.064606	-1.57561	0.118688	-4.35975	0.478249	0.478494
Dendritic.cells	HLF	-0.95885	1.023669	-1.57541	0.118733	-4.37112	0.469405	0.466013
Dendritic.cells	MSL3	0.242213	4.998465	1.575113	0.118802	-5.07669	0.434857	0.417433
Dendritic.cells	MTUS2	-0.92017	0.654472	-1.57465	0.118909	-4.41744	0.473167	0.471043
Dendritic.cells	CUTA	0.171007	6.189562	1.573821	0.119101	-5.3859	0.425872	0.404106
Dendritic.cells	YES1	-0.50427	4.740029	-1.57358	0.119157	-4.91177	0.437793	0.420858
Dendritic.cells	TCEAL8	-0.36402	3.331023	-1.57349	0.119178	-4.75296	0.449796	0.437699
Dendritic.cells	PPIC	-0.44612	2.745716	-1.57326	0.119231	-4.71619	0.454952	0.444931
Dendritic.cells	RNASEH2A	-0.37033	4.54522	-1.57297	0.119297	-4.83081	0.439488	0.423273
Dendritic.cells	CENPS	-0.78077	3.529733	-1.57289	0.119316	-4.48553	0.448139	0.435409
Dendritic.cells	GM43581	-0.83988	2.093907	-1.57239	0.119433	-4.38322	0.461036	0.453315
Dendritic.cells	GPD1	-0.81947	1.749644	-1.5717	0.119592	-4.42389	0.464583	0.457925
Dendritic.cells	TYROBP	0.150477	9.095091	1.57133	0.119678	-6.29236	0.404106	0.373354
Dendritic.cells	HNRNPA0	-0.13009	7.214278	-1.57097	0.119761	-5.63138	0.418745	0.393489
Dendritic.cells	NEK2	0.663556	3.092142	1.570504	0.11987	-4.59215	0.453221	0.441495
Dendritic.cells	ZFP651	-0.71877	1.578642	-1.57037	0.119901	-4.40615	0.466697	0.460474
Dendritic.cells	CACNA1B	0.944998	0.056619	1.570047	0.119976	-4.37331	0.480838	0.480358
Dendritic.cells	CYP2C70	-0.84691	4.144913	-1.56992	0.120006	-4.6772	0.44422	0.428748
Dendritic.cells	AMT	-0.88908	1.565291	-1.56927	0.120156	-4.41014	0.467	0.460873
Dendritic.cells	RANGRF	0.876698	1.262196	1.569262	0.120159	-4.39697	0.46976	0.464771
Dendritic.cells	HYAL2	-0.6165	1.859326	-1.56926	0.12016	-4.51612	0.464342	0.457121
Dendritic.cells	ZFP58	0.674088	1.194809	1.569116	0.120193	-4.46766	0.470376	0.465674
Dendritic.cells	SLC33A1	-0.24648	3.794664	-1.56884	0.120259	-5.1192	0.447398	0.433267
Dendritic.cells	ADTRP	-0.74174	1.835711	-1.56845	0.120349	-4.47228	0.464793	0.45769
Dendritic.cells	FUBP3	0.285333	4.08675	1.56827	0.12039	-4.99054	0.445031	0.429971
Dendritic.cells	DHRS4	0.378049	4.374568	1.568151	0.120418	-4.85877	0.442582	0.426564
Dendritic.cells	MEMO1	-0.18414	6.363616	-1.56741	0.120592	-5.40803	0.42659	0.403777
Dendritic.cells	CANT1	-0.32593	3.891787	-1.56708	0.120668	-4.84888	0.447235	0.432649
Dendritic.cells	STARD5	0.211243	4.526318	1.567009	0.120685	-5.29998	0.441825	0.425098
Dendritic.cells	SLC29A1	-0.32645	5.215066	-1.56617	0.120881	-5.03639	0.436091	0.417421
Dendritic.cells	DERA	0.272452	4.534652	1.566055	0.120908	-4.88386	0.441798	0.425403
Dendritic.cells	ALDH1L1	-0.67741	3.098676	-1.56602	0.120916	-4.5568	0.454161	0.442748
Dendritic.cells	BRF2	0.525879	2.326101	1.56585	0.120956	-4.51027	0.460992	0.452366
Dendritic.cells	TNS3	-0.21194	5.235143	-1.56582	0.120964	-5.57944	0.435924	0.417187
Dendritic.cells	PSMA4	0.157862	6.877207	1.565736	0.120983	-5.57735	0.422547	0.398545
Dendritic.cells	RSF1OS2	0.695495	1.663513	1.565734	0.120983	-4.47599	0.466953	0.460777
Dendritic.cells	FDX2	0.290243	4.192147	1.565102	0.121131	-4.8962	0.445101	0.429779
Dendritic.cells	RIOX2	0.257524	3.769866	1.564836	0.121194	-4.90369	0.448767	0.435013

Dendritic.cells	ZC3H8	0.507341	2.13952	1.564539	0.121263	-4.55782	0.463114	0.455341
Dendritic.cells	NSUN6	-0.44846	3.821373	-1.56444	0.121286	-4.68646	0.448323	0.434536
Dendritic.cells	ATP5G2	0.132439	8.844228	1.564355	0.121307	-5.94083	0.407632	0.377862
Dendritic.cells	IRF9	0.375517	4.515749	1.563931	0.121406	-5.10853	0.442604	0.426407
Dendritic.cells	GM45871	0.775899	0.985003	1.563652	0.121472	-4.4164	0.473943	0.470544
Dendritic.cells	MPRIP	-0.24777	5.481388	-1.56335	0.121543	-5.18798	0.434715	0.415253
Dendritic.cells	CCNQ	0.40955	3.060749	1.563159	0.121588	-4.68169	0.455376	0.444236
Dendritic.cells	MIR99AHG	-0.60658	3.430837	-1.5621	0.121836	-4.81655	0.452912	0.439957
Dendritic.cells	LRPPRC	-0.23682	5.200579	-1.56184	0.121897	-5.20135	0.43784	0.418917
Dendritic.cells	SRPK2	0.167995	7.096447	1.561714	0.121928	-5.67819	0.422373	0.397415
Dendritic.cells	GM16576	0.856186	1.513376	1.561509	0.121976	-4.41932	0.470092	0.464306
Dendritic.cells	HS6ST1	-0.32935	4.156867	-1.56113	0.122066	-4.90828	0.446716	0.431592
Dendritic.cells	SRSF9	0.134876	6.428408	1.561118	0.122068	-5.56593	0.427784	0.405144
Dendritic.cells	BCKDHB	-0.37031	3.929893	-1.56015	0.122298	-4.86827	0.448667	0.434722
Dendritic.cells	ACP1	0.224947	5.923129	1.560047	0.122321	-5.23483	0.431904	0.411257
Dendritic.cells	ANKZF1	0.369951	2.742515	1.560038	0.122323	-4.62455	0.459049	0.449325
Dendritic.cells	CLIC1	0.165693	8.942892	1.559964	0.122341	-5.89555	0.408045	0.378128
Dendritic.cells	GUCY2C	0.78273	-0.14698	1.559621	0.122422	-4.38033	0.48558	0.487015
Dendritic.cells	KRI1	0.231481	4.367396	1.559589	0.12243	-5.05883	0.444917	0.429572
Dendritic.cells	DPY19L3	0.478925	2.757574	1.559484	0.122454	-4.69402	0.458916	0.449261
Dendritic.cells	SNAPC3	0.230628	4.825524	1.559452	0.122462	-5.22621	0.441033	0.424135
Dendritic.cells	NXF1	-0.21651	5.180351	-1.55934	0.122489	-5.16612	0.438054	0.419966
Dendritic.cells	SLC25A16	0.339011	3.724214	1.559311	0.122495	-4.82382	0.450444	0.437339
Dendritic.cells	SMNDC1	-0.15499	6.059801	-1.55877	0.122624	-5.37101	0.430784	0.410022
Dendritic.cells	LAMB3	-0.64193	4.067884	-1.55876	0.122625	-4.5791	0.44748	0.433402
Dendritic.cells	E330009J0	-0.80265	2.552592	-1.55862	0.122659	-4.44882	0.460738	0.452079
Dendritic.cells	CREBL2	0.493864	3.233581	1.558602	0.122663	-4.62209	0.454719	0.443595
Dendritic.cells	HAL	-1.03855	2.215151	-1.55809	0.122784	-4.43138	0.463757	0.456511
Dendritic.cells	GTPBP6	-0.46053	2.84005	-1.55805	0.122793	-4.66486	0.458185	0.448645
Dendritic.cells	ZFP420	0.839426	0.275776	1.557894	0.122831	-4.39943	0.481585	0.481834
Dendritic.cells	WAS	0.227396	5.349391	1.557794	0.122854	-5.08181	0.436644	0.418422
Dendritic.cells	HSDL1	0.246228	4.167902	1.557755	0.122864	-4.95552	0.446622	0.432413
Dendritic.cells	CEP57	0.220008	5.139583	1.557606	0.122899	-5.20834	0.438395	0.420924
Dendritic.cells	TNFRSF21	0.655769	4.10783	1.55743	0.122941	-4.54527	0.447137	0.433208
Dendritic.cells	CIITA	-0.73773	2.478401	-1.55731	0.12297	-4.59151	0.461399	0.453301
Dendritic.cells	NCALD	0.162693	2.377149	1.557144	0.123009	-5.69126	0.462304	0.454606
Dendritic.cells	AKAP11	0.235513	4.648059	1.556665	0.123123	-5.21293	0.442793	0.426962
Dendritic.cells	ANKS1B	-0.76519	1.030517	-1.55638	0.12319	-4.4559	0.474929	0.472266
Dendritic.cells	COX8A	0.120086	9.008145	1.555028	0.123512	-5.97665	0.408604	0.378643
Dendritic.cells	CKAP5	0.264763	6.118962	1.554998	0.123519	-5.33347	0.431415	0.410353
Dendritic.cells	MCF2L	-1.04652	0.46165	-1.55494	0.123533	-4.38601	0.481083	0.480292
Dendritic.cells	TRIP13	0.834234	2.772205	1.554671	0.123597	-4.4648	0.460057	0.450522
Dendritic.cells	GHR	-0.73181	4.215001	-1.55426	0.123695	-4.87174	0.447662	0.432913
Dendritic.cells	PTPN2	-0.14437	6.833794	-1.5538	0.123805	-5.56282	0.425937	0.402698
Dendritic.cells	DOCK7	-0.43394	3.830588	-1.55369	0.123831	-4.75677	0.451028	0.437835
Dendritic.cells	SLAMF6	0.321222	4.602908	1.553507	0.123874	-5.10284	0.444397	0.428523
Dendritic.cells	FUNDC1	0.244064	4.65588	1.553363	0.123909	-5.08295	0.443946	0.427919

Dendritic.cells	SLTM	-0.13313	6.63584	-1.55334	0.123915	-5.61402	0.427534	0.404969
Dendritic.cells	VEZF1	-0.18041	6.00178	-1.55298	0.124002	-5.31268	0.432846	0.412268
Dendritic.cells	THOC5	-0.35399	3.564433	-1.55282	0.124039	-4.75726	0.453493	0.441211
Dendritic.cells	PISD	-0.21317	4.8584	-1.55232	0.124157	-5.35601	0.442526	0.425656
Dendritic.cells	PAG1	0.266469	7.038518	1.5523	0.124163	-5.51515	0.424577	0.400589
Dendritic.cells	ADORA2A	0.625823	3.148747	1.551902	0.124258	-4.60944	0.457422	0.446591
Dendritic.cells	BAG2	0.66798	1.435408	1.551581	0.124335	-4.47761	0.472852	0.468495
Dendritic.cells	GM11707	-0.81236	0.863582	-1.55135	0.124389	-4.41993	0.478143	0.476041
Dendritic.cells	TENT5A	-0.45924	5.169086	-1.55119	0.124427	-4.80349	0.440033	0.422309
Dendritic.cells	ROGDI	0.27369	4.014028	1.551147	0.124439	-5.09199	0.449869	0.436128
Dendritic.cells	CCL3	0.459672	5.069599	1.550972	0.124481	-5.43453	0.440869	0.423543
Dendritic.cells	CCDC88C	0.273107	5.294187	1.550946	0.124487	-5.13344	0.438984	0.420903
Dendritic.cells	KLRD1	0.26835	3.767425	1.55056	0.12458	-5.57085	0.452192	0.439223
Dendritic.cells	GM14963	-0.98742	0.898614	-1.54993	0.12473	-4.41195	0.478157	0.476025
Dendritic.cells	4930426D(	0.889445	-0.21569	1.54988	0.124743	-4.39145	0.488672	0.490987
Dendritic.cells	DPM3	0.154214	6.309203	1.549838	0.124753	-5.55367	0.430902	0.409504
Dendritic.cells	MRPL45	0.389683	4.340705	1.549715	0.124782	-4.79973	0.447378	0.432616
Dendritic.cells	NUP54	-0.27992	4.859149	-1.54927	0.124888	-4.96997	0.443106	0.42662
Dendritic.cells	CDK5RAP1	-0.86819	2.44604	-1.54905	0.124943	-4.54029	0.464157	0.456369
Dendritic.cells	COLEC11	-0.7233	1.589372	-1.54856	0.125059	-4.46257	0.47193	0.467555
Dendritic.cells	SMPDL3A	-0.62121	5.614644	-1.54851	0.125072	-4.77075	0.436767	0.418013
Dendritic.cells	SPON1	0.960915	1.404705	1.54846	0.125084	-4.45531	0.473627	0.469964
Dendritic.cells	DCAF4	0.477924	2.008291	1.548197	0.125147	-4.58972	0.468109	0.462169
Dendritic.cells	COQ8B	-0.45873	3.190292	-1.54802	0.125189	-4.71063	0.457532	0.447291
Dendritic.cells	GET4	0.266534	5.110437	1.547959	0.125204	-5.0448	0.440985	0.424014
Dendritic.cells	WWOX	0.159209	6.961551	1.547859	0.125228	-5.71666	0.425758	0.402752
Dendritic.cells	LRPAP1	0.324534	4.499631	1.547827	0.125236	-4.92422	0.446164	0.431335
Dendritic.cells	CBLB	0.205884	7.336459	1.547397	0.12534	-5.63154	0.422969	0.398766
Dendritic.cells	HAAO	0.253459	3.64437	1.546943	0.125449	-5.25706	0.453911	0.442056
Dendritic.cells	LPCAT4	-0.87372	3.121289	-1.5469	0.12546	-4.45134	0.458508	0.448537
Dendritic.cells	ST6GALNA(	-0.41573	5.127632	-1.54586	0.12571	-5.11768	0.441925	0.424478
Dendritic.cells	DYNC1LI1	0.14278	6.412559	1.54548	0.125802	-5.56777	0.431366	0.409634
Dendritic.cells	CCDC51	0.53395	1.810129	1.54539	0.125824	-4.53647	0.471185	0.465616
Dendritic.cells	CD2AP	0.165408	6.24029	1.544608	0.126013	-5.74514	0.433084	0.411985
Dendritic.cells	H2-EB1	-0.53707	6.754688	-1.54445	0.126051	-5.70939	0.428887	0.406189
Dendritic.cells	FRYL	0.136385	7.564295	1.544443	0.126053	-5.80222	0.422391	0.397137
Dendritic.cells	GDI2	0.105964	8.891579	1.544125	0.12613	-6.02416	0.412024	0.382838
Dendritic.cells	BAG4	-0.27489	4.226706	-1.54408	0.126142	-4.97466	0.450038	0.43593
Dendritic.cells	PWP1	0.369994	3.903059	1.543993	0.126162	-4.75691	0.452842	0.439875
Dendritic.cells	CSMD3	-0.73168	1.302147	-1.5435	0.126281	-4.43884	0.47649	0.47303
Dendritic.cells	ZFP652	0.271223	5.654413	1.543003	0.126402	-5.21156	0.438237	0.419333
Dendritic.cells	TRAM2	0.327698	4.061754	1.542851	0.126439	-4.83736	0.451781	0.438372
Dendritic.cells	TRMT61A	0.592842	1.593744	1.542657	0.126486	-4.51702	0.473837	0.46958
Dendritic.cells	CDCA5	0.744272	3.028781	1.542164	0.126605	-4.52037	0.460853	0.451421
Dendritic.cells	NFE2	0.81999	1.59738	1.542157	0.126607	-4.41999	0.473803	0.469753
Dendritic.cells	COPZ2	-0.61708	2.183281	-1.54203	0.126638	-4.51518	0.468449	0.462163
Dendritic.cells	DPYS	-0.78127	2.829004	-1.54202	0.12664	-4.5624	0.462634	0.453937

Dendritic.cells	CD6	0.904897	0.929158	1.541919	0.126665	-4.41491	0.480002	0.478574
Dendritic.cells	TTC8	-0.73057	0.78875	-1.54192	0.126665	-4.47014	0.481317	0.480444
Dendritic.cells	GM14029	0.755314	0.297438	1.541485	0.126771	-4.47651	0.486196	0.48727
Dendritic.cells	CCNG1	0.240388	4.873028	1.541229	0.126833	-5.12597	0.445112	0.42911
Dendritic.cells	NDUFAF3	0.301794	4.160988	1.540492	0.127012	-4.91538	0.451427	0.437863
Dendritic.cells	USP36	-0.2437	4.970109	-1.54048	0.127016	-5.1547	0.444489	0.428111
Dendritic.cells	RBM41	0.288573	3.907296	1.540367	0.127042	-4.99039	0.453631	0.441024
Dendritic.cells	DDX18	-0.25551	4.987355	-1.54009	0.127111	-5.06369	0.444342	0.428107
Dendritic.cells	OCRL	0.662695	2.602911	1.539962	0.127141	-4.53568	0.46518	0.457542
Dendritic.cells	FAM207A	0.280433	4.223482	1.539862	0.127166	-4.94378	0.450886	0.437376
Dendritic.cells	TXNDC16	0.309647	5.44957	1.539547	0.127242	-5.24428	0.440442	0.422756
Dendritic.cells	MAPK8IP3	0.206465	4.521476	1.539382	0.127283	-5.19914	0.448319	0.433856
Dendritic.cells	CCR1	0.836154	2.32125	1.539342	0.127292	-4.54231	0.467722	0.461254
Dendritic.cells	PIRA2	0.546875	3.589246	1.539288	0.127305	-4.80812	0.456413	0.445266
Dendritic.cells	ATP5MPL	0.134175	8.484004	1.538951	0.127388	-5.88522	0.415958	0.388668
Dendritic.cells	AXL	-0.87624	3.708037	-1.53879	0.127426	-4.59317	0.455405	0.443973
Dendritic.cells	UBLCP1	0.321434	4.773952	1.53869	0.127451	-4.85168	0.446191	0.430994
Dendritic.cells	TMEM115	0.28956	3.447731	1.538367	0.12753	-4.84066	0.457692	0.447285
Dendritic.cells	HGD	-0.87211	2.465599	-1.53829	0.127548	-4.50614	0.466452	0.459719
Dendritic.cells	LZTS3	-0.83803	0.323606	-1.53803	0.127613	-4.40657	0.486283	0.488027
Dendritic.cells	PAICS	0.199816	6.310596	1.537874	0.127651	-5.43945	0.433327	0.413165
Dendritic.cells	RNF217	0.955504	2.071962	1.537859	0.127654	-4.46225	0.470021	0.464895
Dendritic.cells	FGGY	0.316328	3.459854	1.537686	0.127697	-5.15144	0.457585	0.447383
Dendritic.cells	NDUFB4	0.168432	6.598671	1.537411	0.127764	-5.52297	0.43097	0.410037
Dendritic.cells	COMMD2	0.230114	4.927522	1.537311	0.127788	-5.10828	0.444883	0.429565
Dendritic.cells	ANKIB1	0.154158	5.807453	1.537149	0.127828	-5.55554	0.437485	0.419211
Dendritic.cells	2310039H(	0.261882	4.246315	1.53704	0.127855	-5.09036	0.450722	0.437889
Dendritic.cells	TWF1	-0.18025	5.464598	-1.5367	0.127938	-5.33729	0.440492	0.423341
Dendritic.cells	CABYR	-0.94716	1.135391	-1.53614	0.128076	-4.4195	0.479089	0.477758
Dendritic.cells	IGFBP2	-0.96789	4.701389	-1.53563	0.1282	-4.86657	0.447222	0.432842
Dendritic.cells	AK6	-0.28082	4.761906	-1.5355	0.128232	-5.01994	0.446704	0.432136
Dendritic.cells	TARS	-0.3053	4.374393	-1.5353	0.128282	-4.9471	0.45003	0.43693
Dendritic.cells	CSF2RA	0.335356	5.196056	1.535293	0.128283	-4.98852	0.443016	0.427049
Dendritic.cells	RBM7	0.195668	5.855126	1.535211	0.128303	-5.41097	0.437491	0.41929
Dendritic.cells	CTLA4	0.926488	2.060232	1.535204	0.128305	-4.48131	0.47056	0.465994
Dendritic.cells	HSPA9	-0.16278	6.483768	-1.53484	0.128394	-5.54054	0.432394	0.412107
Dendritic.cells	ST3GAL4	-0.15105	6.423085	-1.53476	0.128414	-5.71916	0.432891	0.412836
Dendritic.cells	GM15133	0.657524	1.788244	1.534578	0.128458	-4.52288	0.473156	0.469755
Dendritic.cells	CDPF1	0.586657	2.220471	1.534408	0.1285	-4.60996	0.469208	0.464209
Dendritic.cells	DNAJB2	0.424331	2.766414	1.534045	0.128589	-4.73423	0.464451	0.457345
Dendritic.cells	CNPY2	0.240398	4.719728	1.533551	0.128711	-5.23507	0.447522	0.433426
Dendritic.cells	7-Sep	0.100007	8.277792	1.533087	0.128825	-5.92158	0.418376	0.39267
Dendritic.cells	RSRC1	0.143012	6.605429	1.532981	0.128851	-5.68959	0.431752	0.41139
Dendritic.cells	RWDD3	-0.82274	0.507573	-1.53295	0.128858	-4.41802	0.485482	0.487449
Dendritic.cells	TLR4	0.711726	3.122783	1.532798	0.128896	-4.54305	0.461462	0.453306
Dendritic.cells	NBEA	-0.4803	2.602645	-1.53267	0.128928	-4.75898	0.46612	0.459938
Dendritic.cells	PERP	-0.88555	1.228461	-1.53252	0.128966	-4.44116	0.478711	0.47785

Dendritic.cells	SPOUT1	0.461282	2.468742	1.532472	0.128977	-4.65302	0.467329	0.461654
Dendritic.cells	HBEGF	-0.73984	2.789221	-1.5321	0.129067	-4.65718	0.464619	0.457662
Dendritic.cells	DEXI	0.47083	2.91114	1.531884	0.129121	-4.65135	0.463572	0.456233
Dendritic.cells	TMEM53	-0.91086	0.637469	-1.53123	0.129282	-4.41131	0.484793	0.486355
Dendritic.cells	THUMPD2	0.454146	1.928752	1.531162	0.1293	-4.62477	0.472769	0.46924
Dendritic.cells	2310011J0	0.313828	4.446593	1.531024	0.129334	-4.92398	0.450369	0.437547
Dendritic.cells	ARL6IP1	-0.16445	7.879749	-1.53053	0.129456	-5.7111	0.421978	0.397756
Dendritic.cells	N4BP3	0.306556	2.955418	1.530518	0.129459	-4.98666	0.46347	0.456191
Dendritic.cells	SERINC3	0.10519	9.707046	1.530507	0.129462	-6.16436	0.407839	0.378041
Dendritic.cells	PNKP	0.306404	4.959191	1.530272	0.12952	-5.07088	0.445974	0.431483
Dendritic.cells	INTS3	-0.31784	3.898156	-1.53013	0.129554	-4.85372	0.455132	0.444418
Dendritic.cells	EXOC8	0.519595	1.951234	1.529888	0.129615	-4.60147	0.472563	0.469217
Dendritic.cells	HCFC2	0.460383	3.758586	1.529599	0.129686	-4.63301	0.456354	0.446303
Dendritic.cells	PPA2	0.260274	4.580694	1.529388	0.129738	-5.13235	0.449214	0.436297
Dendritic.cells	H2-T24	0.600389	1.828462	1.529299	0.12976	-4.65103	0.47369	0.471056
Dendritic.cells	SEC23A	0.268505	4.425257	1.529167	0.129793	-5.04168	0.450553	0.438291
Dendritic.cells	ORC2	0.292627	4.279461	1.528995	0.129836	-4.95068	0.451813	0.440132
Dendritic.cells	MMP8	1.252783	1.517071	1.528787	0.129887	-4.43787	0.476563	0.475323
Dendritic.cells	CYFIP1	0.171233	5.64309	1.528692	0.129911	-5.63667	0.440197	0.423769
Dendritic.cells	RNF43	-0.92956	1.563577	-1.52869	0.129912	-4.44603	0.476132	0.474709
Dendritic.cells	ITGB3BP	0.360119	3.52337	1.528469	0.129966	-4.74601	0.458469	0.449561
Dendritic.cells	KLRC3	0.706133	-1.20661	1.527891	0.130109	-4.41254	0.503046	0.512928
Dendritic.cells	SERBP1	0.12437	8.529561	1.526858	0.130366	-5.93654	0.41793	0.391591
Dendritic.cells	METTL1	0.390431	3.431367	1.52648	0.13046	-4.89245	0.460584	0.4513
Dendritic.cells	RHOU	-0.98821	1.27725	-1.52626	0.130515	-4.45801	0.480243	0.479282
Dendritic.cells	PES1	-0.26455	4.691666	-1.52538	0.130734	-4.99264	0.450231	0.435993
Dendritic.cells	ARL6IP6	0.237817	5.278997	1.524826	0.130872	-5.144	0.445467	0.429089
Dendritic.cells	TSGA10	-0.44526	2.968133	-1.52446	0.130964	-4.66985	0.465663	0.457723
Dendritic.cells	ST8SIA6	1.080226	1.211567	1.524453	0.130965	-4.47426	0.481789	0.480634
Dendritic.cells	REPS2	-0.91377	0.905504	-1.5243	0.131002	-4.43018	0.484668	0.484755
Dendritic.cells	GM43330	-0.36876	1.526899	-1.52424	0.131019	-4.87665	0.478844	0.476464
Dendritic.cells	TUBA1C	-0.30765	7.576145	-1.52397	0.131085	-5.42484	0.426559	0.40267
Dendritic.cells	NOXRED1	-0.8429	0.546984	-1.52383	0.13112	-4.43081	0.48813	0.489684
Dendritic.cells	PRAG1	0.564801	1.923732	1.52359	0.13118	-4.63551	0.475299	0.471341
Dendritic.cells	TAF10	0.15476	6.856819	1.523212	0.131275	-5.61433	0.432501	0.410993
Dendritic.cells	CHMP4B	0.111012	7.889271	1.523184	0.131282	-5.89158	0.424176	0.39935
Dendritic.cells	INPP5E	-0.45365	2.260693	-1.52227	0.131511	-4.62771	0.472906	0.467513
Dendritic.cells	ACAD10	0.611346	1.501708	1.522062	0.131562	-4.55867	0.47992	0.477614
Dendritic.cells	KLHL25	-0.53883	2.493825	-1.52179	0.13163	-4.54716	0.470777	0.464619
Dendritic.cells	ASPSCR1	0.244229	4.685187	1.521685	0.131657	-5.07754	0.45134	0.437158
Dendritic.cells	NME2	0.17941	8.557653	1.521638	0.131668	-5.96609	0.419466	0.392502
Dendritic.cells	PCYT1A	0.172715	5.255992	1.52134	0.131743	-5.63709	0.446556	0.430418
Dendritic.cells	XRN1	0.170482	5.924264	1.520637	0.131919	-5.50804	0.441362	0.422766
Dendritic.cells	IQGAP1	-0.11611	9.247795	-1.52044	0.131968	-6.15648	0.414651	0.385464
Dendritic.cells	MORN1	0.710703	1.227517	1.519775	0.132136	-4.47031	0.483581	0.482243
Dendritic.cells	CCN1	-0.97538	1.098666	-1.5194	0.132231	-4.48709	0.484858	0.484112
Dendritic.cells	CCDC83	-1.13087	0.420205	-1.51922	0.132276	-4.44457	0.491312	0.493321

Dendritic.cells	ENOX2	0.246626	5.852601	1.519206	0.132279	-5.40054	0.442464	0.424134
Dendritic.cells	PDZD4	0.192288	1.698737	1.518761	0.132391	-5.56185	0.479249	0.476258
Dendritic.cells	ACVR1	-0.36768	3.707524	-1.51863	0.132424	-5.00639	0.461001	0.450413
Dendritic.cells	DOCK9	-0.41978	4.197961	-1.51858	0.132436	-4.95024	0.456679	0.444307
Dendritic.cells	ANKRD44	0.169404	8.18547	1.518401	0.132481	-5.87932	0.423423	0.397664
Dendritic.cells	CTSK	-0.89094	0.234472	-1.51835	0.132493	-4.43378	0.493111	0.496118
Dendritic.cells	TANK	0.307417	6.451404	1.518181	0.132537	-5.24965	0.437484	0.417372
Dendritic.cells	SLC27A1	0.270858	2.972091	1.517801	0.132632	-5.28406	0.467725	0.459942
Dendritic.cells	PICK1	0.80465	1.538716	1.517567	0.132691	-4.43877	0.48089	0.47878
Dendritic.cells	I730030J21	-0.88421	0.707146	-1.51752	0.132702	-4.42978	0.488736	0.48997
Dendritic.cells	PACS1	0.249198	5.972963	1.516988	0.132837	-5.35116	0.441913	0.423398
Dendritic.cells	CTNNAL1	0.639736	1.924908	1.516444	0.132975	-4.59157	0.477975	0.474138
Dendritic.cells	PCYOX1L	-0.59935	1.613818	-1.51604	0.133076	-4.55332	0.481027	0.478472
Dendritic.cells	GM11523	-1.04121	-0.95948	-1.51594	0.133102	-4.43042	0.505807	0.513928
Dendritic.cells	GLO1	0.337856	5.029475	1.515385	0.133242	-5.00781	0.450636	0.435216
Dendritic.cells	GM17066	-0.5147	1.747467	-1.51532	0.133258	-4.63977	0.480038	0.476838
Dendritic.cells	PICALM	0.131686	8.404518	1.514908	0.133363	-6.00231	0.422983	0.396262
Dendritic.cells	CD9	0.427356	6.033746	1.514189	0.133545	-5.17023	0.442772	0.4235
Dendritic.cells	GM16638	0.646913	2.040296	1.513862	0.133628	-4.50344	0.478075	0.473388
Dendritic.cells	RNF138	0.17214	5.857254	1.513711	0.133666	-5.41612	0.444288	0.425708
Dendritic.cells	MLLT1	-0.41736	2.735176	-1.51357	0.133701	-4.66929	0.471688	0.464422
Dendritic.cells	DAND5	-0.62093	3.96611	-1.51349	0.133722	-4.8334	0.460634	0.448784
Dendritic.cells	KLF10	-0.23086	5.248269	-1.51317	0.133803	-5.25785	0.449507	0.433141
Dendritic.cells	C330011M	0.686891	0.592367	1.51311	0.133818	-4.47438	0.491769	0.493033
Dendritic.cells	ZFHX3	0.23515	5.163754	1.512358	0.134009	-5.3469	0.450732	0.434455
Dendritic.cells	GM10125	0.579128	2.003063	1.511991	0.134102	-4.57856	0.479175	0.474534
Dendritic.cells	1110017D1	-0.97561	0.201816	-1.51164	0.134191	-4.45215	0.496443	0.498979
Dendritic.cells	HACE1	0.319089	4.490352	1.51125	0.134291	-4.99163	0.457099	0.443013
Dendritic.cells	ZFP27	-0.6946	1.016419	-1.51103	0.134347	-4.48898	0.488876	0.488084
Dendritic.cells	SUSD6	0.138689	7.690279	1.509853	0.134647	-5.96785	0.430852	0.405787
Dendritic.cells	LAS1L	-0.3042	4.172574	-1.50954	0.134727	-4.92445	0.460619	0.447629
Dendritic.cells	GM17477	0.62067	0.61424	1.509489	0.134739	-4.55217	0.493453	0.494189
Dendritic.cells	TSTD1	-0.7115	2.589608	-1.50946	0.134747	-4.59627	0.47488	0.467787
Dendritic.cells	SMC1A	0.19842	6.748393	1.509418	0.134757	-5.5228	0.438568	0.416654
Dendritic.cells	BRAF	-0.18923	7.588384	-1.50909	0.13484	-5.7963	0.431807	0.407072
Dendritic.cells	LTA4H	0.328631	5.724021	1.508711	0.134938	-5.17309	0.447412	0.428882
Dendritic.cells	MED15	-0.18191	5.758733	-1.50863	0.134958	-5.46244	0.447117	0.428506
Dendritic.cells	PCNT	0.254939	5.233516	1.508235	0.13506	-5.17294	0.451688	0.435062
Dendritic.cells	ANXA6	0.212028	6.174347	1.507998	0.13512	-5.47917	0.443678	0.423918
Dendritic.cells	SPNS2	-0.56186	1.813184	-1.508	0.13512	-4.62895	0.482421	0.47862
Dendritic.cells	LCLAT1	0.286574	3.99918	1.507879	0.135151	-5.11768	0.462485	0.450382
Dendritic.cells	NCOA6	0.162761	5.600455	1.507319	0.135294	-5.42861	0.448858	0.43077
Dendritic.cells	TAF7	-0.20007	5.1473	-1.50717	0.135331	-5.24762	0.452751	0.436239
Dendritic.cells	KLRC1	-0.89871	1.444197	-1.50697	0.135382	-4.47836	0.486266	0.483619
Dendritic.cells	PCYT2	0.263609	4.635801	1.506631	0.13547	-5.19317	0.457381	0.442676
Dendritic.cells	NUDT14	0.191496	4.342372	1.506244	0.135569	-5.29769	0.460122	0.446423
Dendritic.cells	CNNM4	-0.35072	4.077084	-1.50611	0.135603	-4.83986	0.462469	0.449748

Dendritic.cells	GLYAT	-0.81656	2.069154	-1.50548	0.135766	-4.51389	0.481065	0.475887
Dendritic.cells	ATP8B4	0.65255	4.781456	1.505283	0.135816	-4.81274	0.456585	0.441405
Dendritic.cells	MSTO1	0.444117	2.681663	1.505251	0.135824	-4.72303	0.475395	0.467969
Dendritic.cells	FGD4	-0.6572	4.134435	-1.50438	0.136048	-4.70173	0.462896	0.449831
Dendritic.cells	KRT18	-0.81209	3.557982	-1.50418	0.136098	-4.69878	0.468079	0.457229
Dendritic.cells	GM48855	0.524357	1.463573	1.503584	0.136252	-4.5838	0.487483	0.484865
Dendritic.cells	2410018L1	0.8239	0.727626	1.503	0.136403	-4.44385	0.494516	0.495069
Dendritic.cells	XRCC2	0.699772	0.802718	1.502866	0.136437	-4.49287	0.493792	0.494045
Dendritic.cells	SIRT4	0.913814	0.56797	1.502857	0.13644	-4.46199	0.496058	0.497276
Dendritic.cells	GM43672	0.471461	1.700182	1.50269	0.136483	-4.79406	0.485247	0.481877
Dendritic.cells	GLUL	-0.29891	6.384395	-1.50266	0.13649	-5.29151	0.44353	0.422984
Dendritic.cells	RTL5	0.721434	0.491888	1.502631	0.136498	-4.47095	0.496794	0.498328
Dendritic.cells	PCED1B	-0.51764	4.966857	-1.50261	0.136503	-4.83948	0.455652	0.440011
Dendritic.cells	ZFP760	-0.65758	1.032725	-1.50258	0.136511	-4.49878	0.491585	0.490899
Dendritic.cells	RNASEK	0.131778	6.735066	1.502204	0.136608	-5.71096	0.440596	0.418982
Dendritic.cells	CASP4	-0.68655	4.029936	-1.5016	0.136764	-4.75494	0.463901	0.452114
Dendritic.cells	ACOT13	0.265029	4.43558	1.501466	0.136798	-4.97494	0.460307	0.447043
Dendritic.cells	PKD2L2	0.532493	1.170955	1.501387	0.136819	-4.52985	0.490264	0.489539
Dendritic.cells	YWHAE	0.08859	8.88218	1.501332	0.136833	-6.10917	0.42319	0.394998
Dendritic.cells	KCTD5	-0.3615	3.640452	-1.50129	0.136843	-4.84427	0.467387	0.457047
Dendritic.cells	ATXN7L1	0.156437	6.345264	1.501261	0.136851	-5.68608	0.443858	0.423896
Dendritic.cells	GMCL1	-0.23559	4.636774	-1.501	0.136919	-5.08968	0.458537	0.444546
Dendritic.cells	TMEM41A	-0.72548	1.51992	-1.50099	0.136921	-4.50091	0.486949	0.484814
Dendritic.cells	SLC3A2	-0.16646	6.772545	-1.50096	0.136929	-5.82156	0.440284	0.418884
Dendritic.cells	GM46218	0.704456	0.58606	1.500824	0.136964	-4.52041	0.495883	0.497605
Dendritic.cells	MVB12B	0.344456	4.074675	1.500507	0.137046	-4.97671	0.463521	0.451767
Dendritic.cells	UBE2E2	-0.50381	3.465951	-1.50043	0.137067	-4.90467	0.468977	0.459518
Dendritic.cells	HSPA12A	-0.86854	0.150275	-1.50031	0.137097	-4.45078	0.500138	0.503945
Dendritic.cells	AASS	-0.7455	1.208483	-1.49967	0.137262	-4.56571	0.490242	0.489509
Dendritic.cells	KLF5	-1.01147	0.36413	-1.49953	0.137297	-4.44918	0.498375	0.501222
Dendritic.cells	KIF3C	0.412467	2.145051	1.499474	0.137313	-4.78324	0.481407	0.477052
Dendritic.cells	NXPE2	0.558858	1.56591	1.499072	0.137417	-4.71219	0.486967	0.48505
Dendritic.cells	ZFP369	0.385197	2.919936	1.499014	0.137432	-4.83057	0.474363	0.467126
Dendritic.cells	SPTB	0.955826	1.120897	1.498804	0.137487	-4.46364	0.491241	0.491187
Dendritic.cells	HNRNPD	0.11253	7.908504	1.498599	0.13754	-5.89643	0.431437	0.406712
Dendritic.cells	DDX52	-0.23325	4.930462	-1.49809	0.137672	-5.13985	0.456765	0.442059
Dendritic.cells	CHDH	0.412263	1.157054	1.497872	0.137728	-4.95537	0.491302	0.491046
Dendritic.cells	4833420G1	0.238147	5.494494	1.497149	0.137916	-5.37552	0.452398	0.435427
Dendritic.cells	BCL6B	-0.63077	0.649778	-1.49691	0.137977	-4.57578	0.49677	0.49829
Dendritic.cells	GM9844	-0.53461	2.309102	-1.49643	0.138103	-4.60232	0.481106	0.475994
Dendritic.cells	EIF3A	-0.11285	7.379667	-1.49638	0.138115	-5.81594	0.436682	0.413351
Dendritic.cells	ADARB1	-0.52182	3.009129	-1.49618	0.138169	-4.76524	0.47464	0.466885
Dendritic.cells	RAB33B	0.144567	4.405753	1.49596	0.138225	-5.54429	0.462062	0.449104
Dendritic.cells	ZFP97	-0.60576	1.263623	-1.49591	0.138239	-4.57647	0.490967	0.490145
Dendritic.cells	AU040320	0.201807	4.589216	1.49556	0.138329	-5.38082	0.460442	0.446844
Dendritic.cells	PLLP	-0.92683	-0.20457	-1.49529	0.1384	-4.44966	0.505232	0.510604
Dendritic.cells	BCAR3	-0.37459	4.496691	-1.49518	0.138428	-5.24372	0.461258	0.448044

Dendritic.cells	GM16196	0.645703	1.751417	1.495171	0.138431	-4.54465	0.486336	0.483605
Dendritic.cells	RCE1	0.31143	3.757774	1.495163	0.138433	-4.94733	0.467845	0.457354
Dendritic.cells	ACY3	-0.80914	2.04257	-1.49452	0.138601	-4.53621	0.483872	0.480045
Dendritic.cells	GPT2	0.760017	1.738624	1.494461	0.138616	-4.51353	0.486734	0.484126
Dendritic.cells	4930594M	0.808578	-0.02762	1.494083	0.138714	-4.496	0.503774	0.508564
Dendritic.cells	CLPX	-0.18911	5.328169	-1.49362	0.138834	-5.36209	0.454247	0.438345
Dendritic.cells	BBOX1	-0.89884	0.92351	-1.49362	0.138837	-4.46795	0.49451	0.495486
Dendritic.cells	2500002B1	0.584042	1.915535	1.493479	0.138872	-4.56558	0.485066	0.482014
Dendritic.cells	SHISA5	0.253786	6.572889	1.493382	0.138897	-5.58692	0.443634	0.423417
Dendritic.cells	SNHG5	-0.66069	2.416156	-1.49337	0.138899	-4.54007	0.480384	0.475351
Dendritic.cells	EMC7	0.13503	6.059195	1.493236	0.138936	-5.54764	0.447974	0.429517
Dendritic.cells	GM10501	0.490716	2.138944	1.493069	0.138979	-4.6689	0.482969	0.479088
Dendritic.cells	CEP83	-0.18793	5.662564	-1.4929	0.139022	-5.39534	0.451363	0.434371
Dendritic.cells	LACTB	-0.21511	5.139278	-1.4929	0.139024	-5.30804	0.455886	0.440746
Dendritic.cells	GM13184	-0.28484	2.666577	-1.49227	0.139187	-5.19842	0.478476	0.472479
Dendritic.cells	WFDC21	1.643344	2.947804	1.492102	0.139232	-4.58795	0.475891	0.468856
Dendritic.cells	SFN	0.758454	2.056108	1.491885	0.139289	-4.53493	0.48422	0.480688
Dendritic.cells	EIF2AK2	0.315581	4.927436	1.491548	0.139377	-5.2109	0.458214	0.443954
Dendritic.cells	TRMT6	-0.25879	4.373913	-1.49152	0.139384	-5.08152	0.463093	0.450848
Dendritic.cells	ARHGDIA	0.150879	7.188393	1.490889	0.13955	-5.80424	0.439263	0.417085
Dendritic.cells	GM15952	-0.75267	1.028007	-1.49084	0.139563	-4.51025	0.494355	0.495074
Dendritic.cells	TGFB2	1.15247	0.46572	1.490464	0.139661	-4.49522	0.499804	0.502866
Dendritic.cells	DHRS13	-0.86706	0.959658	-1.49034	0.139693	-4.49943	0.495014	0.496015
Dendritic.cells	SCYL2	-0.3005	4.409687	-1.49018	0.139735	-4.92924	0.463088	0.45065
Dendritic.cells	PCX	-0.67373	2.70013	-1.49016	0.139741	-4.61443	0.478577	0.472607
Dendritic.cells	CHST7	-0.86834	0.725254	-1.49002	0.139778	-4.50035	0.49728	0.499353
Dendritic.cells	WDR45B	0.139468	6.330115	1.489604	0.139887	-5.55012	0.44666	0.427417
Dendritic.cells	BAIAP2L1	-0.38019	3.098445	-1.48944	0.13993	-4.86149	0.475138	0.467702
Dendritic.cells	SLC41A3	0.610484	1.73389	1.487771	0.140369	-4.5501	0.488225	0.486499
Dendritic.cells	MGAM	-0.96281	0.078138	-1.48769	0.140391	-4.46416	0.504227	0.509401
Dendritic.cells	SYMPK	0.275621	4.293778	1.48761	0.140412	-5.04385	0.464695	0.453068
Dendritic.cells	FRY	0.272261	6.378637	1.48754	0.14043	-5.38532	0.446592	0.427544
Dendritic.cells	RAD51AP1	0.674037	3.61991	1.487526	0.140434	-4.76863	0.470748	0.461672
Dendritic.cells	HOXB4	0.523983	1.361865	1.487313	0.14049	-4.66467	0.491766	0.491601
Dendritic.cells	ABCA1	-0.50502	5.836161	-1.48725	0.140507	-5.07785	0.451212	0.434055
Dendritic.cells	SIDT2	0.235685	5.063847	1.487087	0.140549	-5.2434	0.457899	0.443548
Dendritic.cells	CXCL16	-0.65435	3.468405	-1.48698	0.140578	-4.73423	0.472123	0.46369
Dendritic.cells	GM47664	-0.40801	3.774581	-1.48677	0.140632	-4.77658	0.46935	0.459812
Dendritic.cells	PHPT1	0.304679	4.330971	1.486656	0.140663	-5.01532	0.464364	0.452814
Dendritic.cells	RNF103	0.2638	4.305165	1.48658	0.140683	-5.09966	0.464594	0.453145
Dendritic.cells	PKD1L2	0.904388	1.173969	1.486425	0.140724	-4.49741	0.493567	0.494381
Dendritic.cells	APBB2	-0.33904	4.02877	-1.48641	0.140728	-5.3657	0.467064	0.456644
Dendritic.cells	ULK4	-0.48089	2.667963	-1.48608	0.140815	-4.68506	0.47947	0.474344
Dendritic.cells	SETX	0.206225	5.905752	1.485889	0.140866	-5.42937	0.450616	0.43348
Dendritic.cells	CEP78	0.360795	3.001386	1.485766	0.140898	-4.81355	0.476392	0.46998
Dendritic.cells	GM33524	-0.77573	0.491882	-1.48576	0.1409	-4.51896	0.50017	0.503934
Dendritic.cells	SLC25A13	-0.34541	4.679744	-1.48571	0.140914	-5.04236	0.461273	0.448532

Dendritic.cells	CRYZL2	0.413808	2.609637	1.485632	0.140933	-4.72643	0.480011	0.475129
Dendritic.cells	CEPT1	0.306688	5.374134	1.485066	0.141083	-5.20506	0.455197	0.440151
Dendritic.cells	NECAP2	0.182009	5.345804	1.484941	0.141116	-5.4386	0.455443	0.44052
Dendritic.cells	BC030867	0.900536	2.099983	1.48469	0.141183	-4.50392	0.48477	0.482239
Dendritic.cells	MYL12A	0.153652	7.929477	1.484639	0.141196	-5.82459	0.433724	0.410013
Dendritic.cells	LRRC28	0.421562	3.874144	1.484354	0.141272	-4.9991	0.468453	0.459107
Dendritic.cells	CALCA	-1.04456	-0.96013	-1.48429	0.14129	-4.46444	0.514581	0.525122
Dendritic.cells	SETDB1	0.233507	4.832864	1.483746	0.141433	-5.23664	0.459924	0.447185
Dendritic.cells	RELB	-0.24343	6.088989	-1.48373	0.141436	-5.39026	0.449051	0.431817
Dendritic.cells	SIN3A	0.172495	5.871572	1.483637	0.141462	-5.50347	0.450909	0.434438
Dendritic.cells	MCM10	-0.65213	3.112366	-1.48351	0.141496	-4.59016	0.475373	0.469127
Dendritic.cells	GM49439	0.873081	-0.46286	1.483462	0.141508	-4.46378	0.509591	0.518124
Dendritic.cells	ACAP2	0.150363	7.320314	1.48346	0.141508	-5.85266	0.438718	0.417272
Dendritic.cells	1-Mar	-0.73027	1.457886	-1.48341	0.141521	-4.56569	0.490849	0.491215
Dendritic.cells	NOD1	-0.70803	3.387607	-1.48314	0.141593	-4.62429	0.472858	0.465649
Dendritic.cells	CSNK1A1	-0.10087	7.684452	-1.48298	0.141636	-5.8567	0.435724	0.413207
Dendritic.cells	GIN1	-0.26388	3.954097	-1.48295	0.141643	-4.91723	0.467734	0.458425
Dendritic.cells	HIST1H3B	1.134607	1.995292	1.482768	0.141692	-4.51541	0.485755	0.484127
Dendritic.cells	KRT222	-0.86091	-0.05515	-1.48274	0.141698	-4.46341	0.505542	0.512503
Dendritic.cells	SORL1	0.286417	5.66068	1.482706	0.141708	-5.48969	0.452721	0.437184
Dendritic.cells	ALS2CL	-0.82902	0.148166	-1.48245	0.141777	-4.49632	0.5036	0.509692
Dendritic.cells	UCP2	0.161974	9.571709	1.482317	0.141812	-6.137	0.420689	0.392149
Dendritic.cells	PHF21A	0.187428	7.069098	1.481907	0.141921	-5.67758	0.440909	0.420461
Dendritic.cells	SPAG1	-0.75125	1.4147	-1.48188	0.141927	-4.52821	0.491382	0.49211
Dendritic.cells	NAPA	-0.14437	5.92916	-1.48171	0.141972	-5.54379	0.450527	0.434018
Dendritic.cells	1500009L1	-0.6645	-0.02505	-1.48161	0.142	-4.51746	0.50537	0.512186
Dendritic.cells	GM42869	0.476701	1.342309	1.481407	0.142053	-4.68792	0.49211	0.493173
Dendritic.cells	SORCS2	0.65643	3.3545	1.481094	0.142137	-4.69252	0.473315	0.466465
Dendritic.cells	UBE2S	-0.23452	7.624597	-1.48108	0.142141	-5.6911	0.436357	0.414186
Dendritic.cells	ADAP1	0.486806	4.152492	1.480636	0.142258	-4.72053	0.466354	0.456441
Dendritic.cells	ARSB	0.418452	4.938464	1.480374	0.142328	-5.18073	0.459476	0.446639
Dendritic.cells	CDC25C	0.770778	2.030722	1.479905	0.142453	-4.53413	0.486162	0.484469
Dendritic.cells	LIMD2	0.23012	7.333227	1.479695	0.142509	-5.51478	0.439281	0.418012
Dendritic.cells	CALU	-0.25884	4.640586	-1.47961	0.142531	-5.049	0.462323	0.450552
Dendritic.cells	MORN3	-0.54192	1.323497	-1.47947	0.142569	-4.67435	0.492884	0.494148
Dendritic.cells	ACY1	-0.58603	1.799123	-1.47931	0.142611	-4.65133	0.488351	0.48767
Dendritic.cells	SEMA4B	0.16807	5.763505	1.478899	0.142722	-5.83089	0.452739	0.436856
Dendritic.cells	GM12940	-0.34657	4.51886	-1.47862	0.142797	-4.97605	0.463672	0.452328
Dendritic.cells	TOGARAM:	-0.16472	5.297825	-1.47852	0.142823	-5.49758	0.456822	0.442675
Dendritic.cells	KLF7	-0.24904	6.159891	-1.4783	0.142882	-5.50364	0.449444	0.432177
Dendritic.cells	TMEM203	0.399904	3.168142	1.477806	0.143013	-4.84818	0.476211	0.469864
Dendritic.cells	DNAJB5	-0.59361	1.175316	-1.47739	0.143124	-4.59997	0.49519	0.49668
Dendritic.cells	KLRA3	1.0958	-0.66191	1.477058	0.143213	-4.47106	0.513444	0.522775
Dendritic.cells	PGLS	0.111217	7.788311	1.476616	0.143332	-6.08887	0.436562	0.413394
Dendritic.cells	TMEM131L	-0.21932	7.26323	-1.47661	0.143334	-5.52088	0.440894	0.419481
Dendritic.cells	ACKR3	-1.05156	0.835269	-1.47607	0.143477	-4.54396	0.498995	0.501759
Dendritic.cells	MAPK1IP1L	0.129368	6.060276	1.475743	0.143565	-5.61454	0.451252	0.433982

Dendritic.cells	PGLYRP2	-0.77559	2.088786	-1.47538	0.143664	-4.54118	0.486986	0.484913
Dendritic.cells	COQ2	0.218824	4.467496	1.475364	0.143667	-5.28786	0.465167	0.453858
Dendritic.cells	SHF	0.555819	1.832889	1.475265	0.143694	-4.60048	0.489409	0.488427
Dendritic.cells	CHST11	-0.22076	6.242747	-1.47519	0.143714	-5.91245	0.449692	0.432018
Dendritic.cells	WFS1	0.205406	1.279726	1.475071	0.143746	-5.30006	0.494696	0.496038
Dendritic.cells	ZFP768	0.858875	0.794694	1.474853	0.143804	-4.50162	0.49939	0.502889
Dendritic.cells	ZFP637	0.542143	2.315939	1.474809	0.143816	-4.64865	0.484848	0.48207
Dendritic.cells	COG7	-0.42731	2.604364	-1.47475	0.143833	-4.72996	0.48215	0.478218
Dendritic.cells	CYBB	0.219694	7.155496	1.474591	0.143875	-6.15134	0.441999	0.421335
Dendritic.cells	DEPDC5	0.213436	4.978079	1.474246	0.143967	-5.29384	0.460806	0.447755
Dendritic.cells	UEVLD	0.469769	2.944551	1.473177	0.144255	-4.76865	0.479773	0.474201
Dendritic.cells	GM20707	0.423542	1.898927	1.472905	0.144328	-4.69567	0.48958	0.488316
Dendritic.cells	HSPA1A	1.190177	3.985226	1.472841	0.144345	-4.79771	0.470256	0.460799
Dendritic.cells	LRRC8C	-0.19997	6.412612	-1.47266	0.144393	-5.68891	0.448979	0.430726
Dendritic.cells	DOCK10	0.162723	8.80921	1.472602	0.144409	-6.03838	0.429202	0.402933
Dendritic.cells	GM28379	0.858435	0.264238	1.472062	0.144555	-4.49522	0.505408	0.511323
Dendritic.cells	MDC1	0.39739	3.159139	1.471992	0.144574	-4.80074	0.477791	0.471801
Dendritic.cells	LAP3	-0.38861	4.901491	-1.47195	0.144586	-5.06883	0.462074	0.449475
Dendritic.cells	THRB	-0.83839	3.815098	-1.47171	0.144651	-4.64438	0.471795	0.463367
Dendritic.cells	NRIP3	-1.03166	0.094317	-1.47168	0.144658	-4.4829	0.507088	0.513863
Dendritic.cells	GATAD2A	-0.12983	7.859185	-1.47153	0.144697	-5.9365	0.436899	0.414031
Dendritic.cells	GM2788	0.820812	0.569462	1.471524	0.1447	-4.49467	0.502406	0.507129
Dendritic.cells	WDR12	-0.39138	4.283459	-1.47148	0.144713	-4.86764	0.467572	0.457366
Dendritic.cells	KLHDC1	0.41966	2.152219	1.470707	0.14492	-4.79774	0.487481	0.485727
Dendritic.cells	HIST1H2BE	0.779276	2.079102	1.470551	0.144963	-4.50949	0.488172	0.486769
Dendritic.cells	APBA1	0.135133	4.384633	1.470409	0.145001	-6.087	0.466953	0.456561
Dendritic.cells	EXOC2	0.173678	5.578749	1.47028	0.145036	-5.46148	0.456424	0.441692
Dendritic.cells	DQX1	-1.16689	1.215444	-1.47015	0.145072	-4.57809	0.496428	0.49879
Dendritic.cells	MICU3	0.312754	3.81398	1.470055	0.145097	-5.06885	0.472095	0.464048
Dendritic.cells	EPOP	0.649635	1.472765	1.470029	0.145104	-4.57867	0.493951	0.495272
Dendritic.cells	UQCC1	0.279213	3.934429	1.469693	0.145195	-5.06101	0.47109	0.462675
Dendritic.cells	MATN2	0.343749	0.980441	1.469612	0.145217	-5.06255	0.498795	0.50232
Dendritic.cells	CYP51	0.215361	3.916341	1.469228	0.14532	-5.40652	0.47145	0.463126
Dendritic.cells	MAPT	-0.69574	1.597756	-1.46858	0.145497	-4.53132	0.493375	0.494104
Dendritic.cells	CCSAP	-0.6184	2.176115	-1.46847	0.145527	-4.55393	0.487871	0.486223
Dendritic.cells	FCHSD1	-0.5865	1.15994	-1.46828	0.145578	-4.60066	0.497593	0.500237
Dendritic.cells	OAS1G	-0.92984	-0.24881	-1.46738	0.14582	-4.48708	0.511461	0.520689
Dendritic.cells	YME1L1	-0.12294	6.181942	-1.46715	0.145884	-5.53047	0.451793	0.435577
Dendritic.cells	4930557J0	-0.8515	1.834427	-1.46705	0.145911	-4.56848	0.491114	0.491634
Dendritic.cells	FBLIM1	0.931927	0.928973	1.466833	0.145969	-4.51523	0.499835	0.504276
Dendritic.cells	HNMT	-0.75921	-0.38409	-1.46674	0.145995	-4.47954	0.512817	0.523031
Dendritic.cells	CYP4F13	0.400655	2.829707	1.46669	0.146008	-4.84068	0.481742	0.478353
Dendritic.cells	BTLA	0.261339	3.948652	1.466562	0.146043	-5.56281	0.47147	0.463724
Dendritic.cells	MTTP	-0.59891	2.566189	-1.46653	0.146051	-4.64995	0.484201	0.481909
Dendritic.cells	STAT6	-0.24308	5.031546	-1.46652	0.146054	-5.18055	0.461793	0.449954
Dendritic.cells	GM11342	0.36093	2.120734	1.46632	0.146109	-5.12608	0.488395	0.487928
Dendritic.cells	GNPAT	0.270417	4.15461	1.466093	0.146171	-5.07727	0.46961	0.461088

Dendritic.cells	CAND2	-0.67402	0.775357	-1.46589	0.146227	-4.54434	0.501333	0.506521
Dendritic.cells	SNAPIN	0.295664	4.083623	1.465876	0.146229	-4.95641	0.47025	0.462
Dendritic.cells	PPP2R1B	-0.29592	4.339805	-1.46573	0.14627	-5.05575	0.467945	0.458795
Dendritic.cells	SGMS1	0.177412	7.102996	1.465529	0.146324	-5.92164	0.443991	0.424897
Dendritic.cells	SIGIRR	-0.43561	2.175284	-1.46533	0.146378	-4.69997	0.487879	0.48744
Dendritic.cells	CLN8	0.41979	3.202024	1.465273	0.146394	-4.9253	0.478293	0.473749
Dendritic.cells	TREML2	0.362103	3.901235	1.465222	0.146408	-4.96717	0.4719	0.464631
Dendritic.cells	VPS37A	0.162638	5.2037	1.465093	0.146443	-5.65058	0.460279	0.448121
Dendritic.cells	ZFP710	0.262093	6.297621	1.465083	0.146445	-5.42889	0.450803	0.434679
Dendritic.cells	MEAF6	0.208962	4.941914	1.464729	0.146542	-5.19623	0.462585	0.451441
Dendritic.cells	GPATCH11	0.338546	3.800176	1.464701	0.146549	-4.91043	0.472817	0.466019
Dendritic.cells	PTTG1	-0.26542	5.78	-1.46464	0.146566	-5.21685	0.455255	0.441047
Dendritic.cells	DFFB	-0.45396	2.981393	-1.46458	0.146583	-4.75671	0.480333	0.476781
Dendritic.cells	SYNJ2BP	0.183395	5.520643	1.464009	0.146738	-5.37318	0.457857	0.444515
Dendritic.cells	SELENBP2	-0.8375	1.116284	-1.46337	0.146913	-4.54042	0.498552	0.502587
Dendritic.cells	SEMA5A	-0.604	1.106724	-1.46325	0.146946	-4.74407	0.498645	0.502721
Dendritic.cells	TRBC1	-0.78657	3.183542	-1.46305	0.147	-4.69988	0.478979	0.474548
Dendritic.cells	GM47730	0.79086	-0.65913	1.462915	0.147036	-4.48344	0.516142	0.528059
Dendritic.cells	PIP4K2B	0.283715	4.178712	1.462907	0.147039	-5.03575	0.469899	0.461623
Dendritic.cells	CDKN2D	0.168419	6.09877	1.462843	0.147056	-5.6526	0.452994	0.437599
Dendritic.cells	GTPBP1	0.291448	4.641527	1.462549	0.147137	-5.0766	0.46575	0.455763
Dendritic.cells	CATSPERG1	-0.88398	0.160917	-1.46238	0.147183	-4.50954	0.507927	0.51629
Dendritic.cells	LRRC3	0.782596	0.375073	1.462346	0.147192	-4.49795	0.505807	0.51323
Dendritic.cells	TMEM123	0.15565	6.484305	1.462241	0.147221	-5.80341	0.449696	0.433057
Dendritic.cells	LGMN	-0.23628	7.188159	-1.4621	0.14726	-5.74239	0.443757	0.42467
Dendritic.cells	ARL13B	-0.27824	3.775076	-1.46139	0.147454	-5.03624	0.473829	0.467145
Dendritic.cells	LEMD3	-0.16292	5.687224	-1.46127	0.147486	-5.57901	0.456814	0.442964
Dendritic.cells	ZFP182	0.242058	4.211679	1.461098	0.147533	-5.32236	0.469873	0.461539
Dendritic.cells	ADH1	-0.75103	3.503888	-1.46101	0.147557	-4.78089	0.476307	0.470769
Dendritic.cells	BC052040	-0.33928	4.165796	-1.461	0.147561	-5.06977	0.470287	0.462176
Dendritic.cells	CCNO	-0.89798	0.174468	-1.45996	0.147847	-4.49655	0.50892	0.516956
Dendritic.cells	SLC6A13	-0.65198	2.762131	-1.45965	0.14793	-4.61742	0.484094	0.481278
Dendritic.cells	B020010K1	0.867898	1.134374	1.459169	0.148062	-4.51258	0.499727	0.503699
Dendritic.cells	CSTF2T	0.349907	3.017029	1.45898	0.148114	-4.85794	0.481821	0.478087
Dendritic.cells	NUDT16	0.344208	2.966991	1.458837	0.148153	-4.96419	0.482287	0.478811
Dendritic.cells	TANGO6	-0.30855	4.382985	-1.45875	0.148179	-5.0405	0.469329	0.460339
Dendritic.cells	TM2D3	0.298795	4.227452	1.458575	0.148226	-4.99504	0.470731	0.462432
Dendritic.cells	RBKS	-0.45783	3.630745	-1.45838	0.14828	-4.78145	0.476157	0.470316
Dendritic.cells	TRIM17	0.423537	2.197819	1.458293	0.148303	-4.81839	0.489514	0.489463
Dendritic.cells	APOL11B	1.731238	-0.04187	1.458273	0.148309	-4.51604	0.511325	0.520886
Dendritic.cells	CPT1A	-0.24891	5.446894	-1.45805	0.148369	-5.12232	0.459912	0.447175
Dendritic.cells	MRPL13	0.260644	4.834872	1.457926	0.148404	-5.13452	0.465315	0.45486
Dendritic.cells	AATF	-0.2073	4.995367	-1.45775	0.148453	-5.34483	0.463908	0.452878
Dendritic.cells	PHF11B	0.426018	4.313119	1.457447	0.148536	-5.1482	0.470085	0.4616
Dendritic.cells	OCIAD1	0.140939	5.885315	1.457339	0.148565	-5.58747	0.456189	0.441904
Dendritic.cells	SPECC1	0.719614	4.276803	1.456897	0.148687	-4.75745	0.470498	0.462239
Dendritic.cells	MGRN1	-0.22985	5.682428	-1.45679	0.148716	-5.40862	0.458035	0.4445

Dendritic.cells	YBEY	0.568062	1.366271	1.456769	0.148723	-4.57478	0.497702	0.501219
Dendritic.cells	BBS4	0.400211	2.313596	1.456352	0.148837	-4.83683	0.488872	0.488358
Dendritic.cells	ADGRG6	0.974856	0.969217	1.456043	0.148923	-4.56598	0.501944	0.507001
Dendritic.cells	AFAP1	-0.60305	1.955044	-1.45541	0.149097	-4.57184	0.492851	0.493663
Dendritic.cells	ACTR2	0.11531	8.066808	1.454811	0.149263	-5.9863	0.438796	0.416605
Dendritic.cells	PGLYRP1	0.807091	4.959034	1.454718	0.149289	-4.7054	0.465379	0.454252
Dendritic.cells	CHST3	0.429417	4.312271	1.454682	0.149299	-5.02358	0.471174	0.462505
Dendritic.cells	ATP11C	-0.12757	6.869185	-1.45384	0.149533	-5.79619	0.449368	0.431124
Dendritic.cells	HNRNPU	0.121925	8.26781	1.453269	0.149689	-6.01894	0.437993	0.4148
Dendritic.cells	SLC16A2	-0.4999	2.626868	-1.45302	0.149758	-4.78203	0.487657	0.48539
Dendritic.cells	VWCE	-0.6419	0.865529	-1.45269	0.14985	-4.62135	0.504617	0.509878
Dendritic.cells	PACC1	-0.28625	5.421952	-1.45268	0.149852	-5.30043	0.462181	0.449177
Dendritic.cells	RUNX3	-0.15528	5.712717	-1.45263	0.149866	-6.04084	0.45963	0.445556
Dendritic.cells	XPC	0.390321	3.385386	1.452398	0.149931	-4.79728	0.480571	0.475496
Dendritic.cells	RBM17	0.198731	5.789508	1.452379	0.149936	-5.39979	0.458959	0.444716
Dendritic.cells	PHACTR1	-0.63175	1.519212	-1.45179	0.150099	-4.63526	0.498376	0.501029
Dendritic.cells	COPG2	0.235166	5.004811	1.451765	0.150106	-5.2575	0.466	0.454722
Dendritic.cells	ARGLU1	0.092375	7.597485	1.45176	0.150108	-5.93459	0.443657	0.423058
Dendritic.cells	ERGIC2	-0.13108	6.017059	-1.45123	0.150256	-5.56081	0.457244	0.442236
Dendritic.cells	GM19466	0.787152	0.661272	1.451145	0.150278	-4.53727	0.506923	0.513294
Dendritic.cells	USP45	0.324154	4.074097	1.451124	0.150284	-5.01114	0.474523	0.46682
Dendritic.cells	CIAO2B	0.258834	4.566233	1.450938	0.150336	-5.18407	0.470073	0.460578
Dendritic.cells	ERI1	0.241	5.459943	1.450805	0.150373	-5.32669	0.462119	0.449289
Dendritic.cells	PTAFR	-0.24943	4.645313	-1.45054	0.150446	-5.87332	0.469393	0.45961
Dendritic.cells	ZHX1	0.289225	4.078556	1.450327	0.150506	-5.08957	0.474519	0.467002
Dendritic.cells	CRBN	0.274638	4.060609	1.450185	0.150545	-5.04433	0.474683	0.467341
Dendritic.cells	GLE1	0.222466	4.4454	1.450141	0.150557	-5.20574	0.471193	0.462366
Dendritic.cells	CTDNEP1	0.201135	5.516749	1.449968	0.150606	-5.45666	0.461664	0.448843
Dendritic.cells	NOD2	-0.88741	1.576786	-1.44932	0.150787	-4.52017	0.498479	0.501165
Dendritic.cells	TREM3	1.094787	2.200561	1.448852	0.150916	-4.54654	0.492667	0.492679
Dendritic.cells	APC	0.123291	6.723147	1.448493	0.151016	-5.96571	0.451797	0.434476
Dendritic.cells	TNFSF9	0.506366	3.435421	1.448454	0.151027	-4.98875	0.481055	0.476134
Dendritic.cells	PIGT	0.188577	5.640537	1.448369	0.151051	-5.5242	0.46117	0.447794
Dendritic.cells	ATP13A3	-0.10208	7.315105	-1.44831	0.151069	-5.96643	0.446779	0.427432
Dendritic.cells	ARHGAP42	-0.70776	1.752056	-1.44818	0.151105	-4.61198	0.496971	0.499036
Dendritic.cells	TTC9C	0.23018	4.558959	1.447858	0.151194	-5.10208	0.47093	0.461607
Dendritic.cells	GNA13	-0.15153	7.201206	-1.44728	0.151356	-5.90233	0.448225	0.428996
Dendritic.cells	LRRC63	0.954246	0.544146	1.447021	0.151427	-4.5068	0.509423	0.516401
Dendritic.cells	PKIG	0.138545	7.198634	1.446878	0.151467	-5.95346	0.448317	0.42916
Dendritic.cells	HSD17B7	0.421971	1.697013	1.446455	0.151586	-4.79752	0.498366	0.500308
Dendritic.cells	RCOR1	0.150532	6.85813	1.446181	0.151662	-5.81079	0.451464	0.433482
Dendritic.cells	TMEM258	0.152048	6.825258	1.4461	0.151685	-5.77889	0.451745	0.433891
Dendritic.cells	NDRG2	-0.66257	2.964877	-1.44566	0.151809	-4.72394	0.486577	0.483207
Dendritic.cells	PIK3IP1	0.37547	2.920799	1.445284	0.151913	-5.01478	0.487167	0.483911
Dendritic.cells	CASD1	0.225671	4.363906	1.445066	0.151974	-5.24286	0.473829	0.464966
Dendritic.cells	IPO13	-0.55021	2.264795	-1.44488	0.152026	-4.66279	0.493386	0.493049
Dendritic.cells	WWTR1	-0.4421	2.126919	-1.44484	0.152038	-4.97452	0.494705	0.494951

Dendritic.cells	GM15336	-0.70562	1.682133	-1.44456	0.152117	-4.5316	0.499104	0.501208
Dendritic.cells	DACH2	0.788876	0.599965	1.443956	0.152286	-4.58275	0.510147	0.516757
Dendritic.cells	PRRC1	-0.28511	4.219989	-1.4435	0.152414	-5.0151	0.4759	0.467422
Dendritic.cells	TRIM44	-0.13156	6.053928	-1.44308	0.152531	-5.8099	0.459746	0.444223
Dendritic.cells	RBM15	-0.17674	5.696792	-1.44287	0.15259	-5.46951	0.462876	0.448733
Dendritic.cells	MID2	-0.67562	0.27499	-1.44278	0.152615	-4.62416	0.513912	0.521804
Dendritic.cells	ACTR6	0.399442	3.522286	1.442532	0.152686	-4.81244	0.482553	0.476849
Dendritic.cells	ZRSR2	-0.18004	5.063714	-1.44246	0.152706	-5.28365	0.468502	0.456866
Dendritic.cells	GM17749	-0.62815	4.634129	-1.44201	0.152833	-4.9739	0.472624	0.46254
Dendritic.cells	CLEC2I	0.305836	3.177972	1.441811	0.152889	-5.31127	0.486067	0.481717
Dendritic.cells	CENPL	0.468822	3.393274	1.441652	0.152933	-4.74575	0.484058	0.47884
Dendritic.cells	HIVEP2	-0.2961	8.386816	-1.44095	0.15313	-5.77766	0.440377	0.416857
Dendritic.cells	USP31	-0.3929	3.715776	-1.44076	0.153183	-4.91616	0.481194	0.474783
Dendritic.cells	TRIM69	-0.674	2.05026	-1.44073	0.153194	-4.65225	0.49692	0.497299
Dendritic.cells	AKAP9	0.148896	6.105265	1.440597	0.15323	-5.72882	0.459721	0.444229
Dendritic.cells	MIER1	0.133469	6.946828	1.44057	0.153238	-5.7985	0.452456	0.433942
Dendritic.cells	CHML	0.51119	1.690018	1.440569	0.153238	-4.72029	0.500405	0.502305
Dendritic.cells	ADSS	0.14402	6.271707	1.439281	0.153602	-5.67094	0.459177	0.4424
Dendritic.cells	D10WSU1C	-0.398	3.652731	-1.43918	0.153629	-4.8878	0.48273	0.475879
Dendritic.cells	CCDC134	0.232457	3.407824	1.438925	0.153703	-5.33524	0.48506	0.479225
Dendritic.cells	PANK1	-0.35966	3.430383	-1.43881	0.153736	-5.08803	0.484849	0.478956
Dendritic.cells	CERKL	0.647298	1.852174	1.438444	0.153839	-4.64861	0.499869	0.500595
Dendritic.cells	RNF7	0.163198	6.742111	1.438312	0.153876	-5.72386	0.455153	0.436962
Dendritic.cells	SIRPB1C	1.136218	1.778958	1.438239	0.153897	-4.53833	0.50058	0.501697
Dendritic.cells	RBP1	-0.62116	3.578118	-1.43806	0.153947	-4.77317	0.483472	0.47721
Dendritic.cells	ARNT	-0.20322	5.869604	-1.43788	0.153997	-5.58943	0.462742	0.447803
Dendritic.cells	PRR5	0.187231	3.479528	1.437778	0.154027	-5.66262	0.484391	0.478666
Dendritic.cells	RNASET2A	-0.22527	5.439463	-1.43773	0.15404	-5.58784	0.466544	0.453251
Dendritic.cells	VCAM1	-0.83343	4.334733	-1.43715	0.154204	-4.88987	0.476697	0.46763
Dendritic.cells	TMEM240	0.780613	0.240106	1.437001	0.154247	-4.58378	0.516021	0.52407
Dendritic.cells	HAGH	-0.29325	5.358255	-1.43687	0.154284	-5.27314	0.467462	0.454628
Dendritic.cells	LPCAT3	0.222352	5.162712	1.436702	0.154332	-5.36798	0.469209	0.457173
Dendritic.cells	CD300LG	-0.52193	1.259921	-1.43655	0.154374	-4.77166	0.505865	0.509615
Dendritic.cells	IFITM1	1.410969	3.489377	1.436464	0.1544	-4.72268	0.484501	0.479004
Dendritic.cells	TSPYL4	0.433451	0.723815	1.436417	0.154413	-4.77827	0.511174	0.51729
Dendritic.cells	MVD	0.403335	2.348846	1.435236	0.154748	-4.89133	0.496132	0.495035
Dendritic.cells	STON2	-0.3765	3.518101	-1.43518	0.154764	-5.05487	0.485059	0.479197
Dendritic.cells	IGFBP6	0.958975	-0.02144	1.434509	0.154955	-4.52231	0.520041	0.528963
Dendritic.cells	GM38973	-0.67046	1.613034	-1.43345	0.155255	-4.63397	0.504571	0.505765
Dendritic.cells	ALG11	0.371141	2.610001	1.433145	0.155343	-4.81372	0.494977	0.49194
Dendritic.cells	SUGP1	0.203253	4.615277	1.433076	0.155363	-5.32501	0.476245	0.46524
Dendritic.cells	CNTROB	0.649684	2.044211	1.432796	0.155443	-4.6013	0.500437	0.499828
Dendritic.cells	RBM33	0.149622	5.898903	1.432757	0.155454	-5.59086	0.464737	0.448985
Dendritic.cells	SEC61B	0.111592	8.272683	1.432597	0.155499	-6.17226	0.444394	0.420341
Dendritic.cells	BLOC1S4	0.290067	3.695064	1.431657	0.155768	-5.00527	0.485438	0.477764
Dendritic.cells	MBLAC2	-0.3819	2.64789	-1.43141	0.155839	-4.89885	0.495425	0.491964
Dendritic.cells	CAB39L	0.266347	4.624571	1.431203	0.155897	-5.16211	0.476985	0.465684

Dendritic.cells	CTSH	0.153621	5.779307	1.429538	0.156374	-6.00393	0.467566	0.451566
Dendritic.cells	SNX19	0.317937	2.862011	1.429067	0.156509	-4.90427	0.494454	0.489992
Dendritic.cells	SETD5	-0.11657	6.628233	-1.42896	0.15654	-5.83831	0.460099	0.441238
Dendritic.cells	DNA2	0.457857	3.583362	1.428861	0.156568	-4.74208	0.487626	0.480332
Dendritic.cells	APP	-0.2974	5.938302	-1.42885	0.156572	-5.54122	0.466155	0.449853
Dendritic.cells	TSPAN5	0.254191	5.373256	1.428766	0.156595	-5.3518	0.471194	0.457022
Dendritic.cells	SUMF2	0.433608	2.684259	1.428563	0.156653	-4.79992	0.496155	0.492618
Dendritic.cells	WDR92	0.463459	3.083733	1.428549	0.156657	-4.8139	0.492342	0.487174
Dendritic.cells	STXBP1	-0.51847	3.786972	-1.42847	0.156681	-4.77555	0.48572	0.477733
Dendritic.cells	EML4	0.123931	7.138312	1.4284	0.1567	-5.99248	0.455688	0.435188
Dendritic.cells	PLAU	-0.95316	0.814983	-1.42835	0.156714	-4.56468	0.514486	0.518913
Dendritic.cells	STX2	-0.31406	3.698442	-1.4281	0.156785	-4.92196	0.486547	0.479018
Dendritic.cells	OTUD1	0.471448	3.007254	1.428055	0.156799	-4.73834	0.493069	0.488345
Dendritic.cells	PTPMT1	0.283343	4.267152	1.427001	0.157102	-5.02885	0.481988	0.471907
Dendritic.cells	RRAGB	-0.67795	0.125251	-1.42692	0.157124	-4.61919	0.522243	0.529561
Dendritic.cells	PFKFB3	0.330878	6.273061	1.426608	0.157215	-5.26722	0.464018	0.446437
Dendritic.cells	MAPKAPK2	0.140139	7.207807	1.426477	0.157252	-5.99328	0.455892	0.434998
Dendritic.cells	B3GAT3	0.294005	4.307364	1.425398	0.157563	-5.11309	0.482466	0.472021
Dendritic.cells	HS2ST1	-0.12563	5.689893	-1.42534	0.157579	-5.83201	0.469895	0.454204
Dendritic.cells	ROBO1	-0.54731	2.137687	-1.42503	0.157668	-4.86424	0.503213	0.501526
Dendritic.cells	BCL2A1A	1.125775	2.399522	1.42465	0.157779	-4.57985	0.500685	0.498041
Dendritic.cells	RAB10	0.132954	7.670572	1.424625	0.157786	-6.01243	0.452764	0.430079
Dendritic.cells	ADGRG1	0.823667	1.720983	1.424328	0.157872	-4.62048	0.507313	0.507611
Dendritic.cells	OPRM1	-0.89401	2.803916	-1.4243	0.157879	-4.54302	0.496785	0.492552
Dendritic.cells	RAB3IP	0.211083	4.587906	1.424245	0.157896	-5.47928	0.480031	0.468695
Dendritic.cells	FDFT1	0.234598	3.796269	1.423588	0.158085	-5.29352	0.487823	0.4793
Dendritic.cells	GPSM2	0.755652	2.440784	1.422968	0.158264	-4.55916	0.501157	0.497857
Dendritic.cells	B3GALNT2	0.315018	3.643954	1.422829	0.158305	-5.06731	0.489657	0.48153
Dendritic.cells	HECTD3	0.377125	2.789878	1.422623	0.158364	-4.83398	0.497833	0.493113
Dendritic.cells	IFNAR2	0.105624	7.128736	1.422219	0.158481	-6.1946	0.45834	0.437075
Dendritic.cells	IRF2BPL	-0.36464	4.091669	-1.4222	0.158486	-5.11716	0.485608	0.475637
Dendritic.cells	COX6B2	0.724656	1.691068	1.4216	0.15866	-4.57517	0.509062	0.508841
Dendritic.cells	KCNG2	-0.83816	-0.23458	-1.42132	0.158742	-4.53736	0.528631	0.5369
Dendritic.cells	COPS6	0.196983	5.552293	1.421072	0.158813	-5.45894	0.472713	0.457096
Dendritic.cells	MBD3	0.157735	5.696238	1.421035	0.158824	-5.61671	0.47142	0.455268
Dendritic.cells	BANK1	-0.55181	6.09331	-1.42058	0.158955	-5.16528	0.467885	0.450446
Dendritic.cells	STIP1	0.225004	5.419076	1.420573	0.158958	-5.40486	0.473922	0.458982
Dendritic.cells	TSTA3	-0.28389	4.100613	-1.42047	0.158987	-5.1065	0.486022	0.476164
Dendritic.cells	ABHD4	-0.38343	2.787772	-1.42041	0.159005	-4.83277	0.498465	0.493895
Dendritic.cells	SH2D1B1	-0.35747	1.695197	-1.41992	0.159148	-5.18165	0.509232	0.509368
Dendritic.cells	FAM71D	-0.82715	0.095273	-1.41972	0.159207	-4.55376	0.525352	0.532621
Dendritic.cells	PPP1R3E	-0.38525	1.031561	-1.41936	0.15931	-4.82102	0.515845	0.51911
Dendritic.cells	UOX	0.614949	5.010934	1.4193	0.159327	-5.14946	0.477726	0.464715
Dendritic.cells	KIFAP3	-0.3532	2.849059	-1.41922	0.159351	-4.84835	0.49798	0.493573
Dendritic.cells	GM44686	0.314278	3.136727	1.419104	0.159384	-4.99587	0.495223	0.489708
Dendritic.cells	PIGX	0.209755	5.308726	1.418995	0.159416	-5.36399	0.475019	0.461021
Dendritic.cells	CCDC84	0.446404	2.649641	1.418651	0.159516	-4.78118	0.499903	0.496544

Dendritic.cells	GM45509	0.662701	1.640462	1.418561	0.159543	-4.64605	0.509774	0.510695
Dendritic.cells	SLC41A1	-0.40402	2.387756	-1.41853	0.159553	-4.86293	0.502441	0.500193
Dendritic.cells	RAMP3	-0.75456	0.947281	-1.4185	0.159561	-4.64212	0.516693	0.520629
Dendritic.cells	TYK2	0.222583	3.992109	1.41821	0.159644	-5.25094	0.487138	0.478444
Dendritic.cells	NOSTRIN	-0.4249	3.426931	-1.41807	0.159686	-4.99661	0.492461	0.486055
Dendritic.cells	SS18	0.194144	5.946104	1.418041	0.159694	-5.45316	0.469293	0.453138
Dendritic.cells	ANK1	1.059565	1.273318	1.417844	0.159751	-4.55128	0.513424	0.516055
Dendritic.cells	OPA1	0.196511	4.835691	1.417836	0.159753	-5.34813	0.479328	0.467365
Dendritic.cells	SMIM13	0.255018	3.919727	1.416562	0.160125	-5.16942	0.48866	0.479818
Dendritic.cells	ST7	-0.26771	5.144851	-1.41644	0.16016	-5.3134	0.477331	0.46373
Dendritic.cells	KANK3	-0.49765	2.000717	-1.41643	0.160164	-4.89125	0.507099	0.50613
Dendritic.cells	RASSF5	0.208625	5.485464	1.416218	0.160225	-5.42691	0.47429	0.459418
Dendritic.cells	KDSR	0.258013	3.963038	1.416057	0.160272	-5.15658	0.48831	0.479324
Dendritic.cells	GM45894	0.415085	2.290972	1.41567	0.160385	-4.74186	0.504383	0.502212
Dendritic.cells	RETREG2	-0.28498	4.884018	-1.41563	0.160398	-5.15006	0.479836	0.46725
Dendritic.cells	ACTR1A	0.138912	6.140647	1.415522	0.160429	-5.64726	0.468491	0.451195
Dendritic.cells	SH3BGRL2	0.727722	2.700678	1.414624	0.160691	-4.69275	0.50108	0.496936
Dendritic.cells	RALBP1	0.150897	6.268527	1.414261	0.160797	-5.63954	0.468169	0.450046
Dendritic.cells	FAM120B	0.245437	4.124917	1.413906	0.160901	-5.22131	0.487717	0.477779
Dendritic.cells	POLR1D	0.125297	6.995878	1.413817	0.160927	-5.80661	0.46178	0.441134
Dendritic.cells	HDAC5	0.264399	4.378269	1.413542	0.161008	-5.2654	0.485353	0.474566
Dendritic.cells	AK8	-0.28097	2.179586	-1.4135	0.161021	-5.19926	0.50636	0.504504
Dendritic.cells	NFKBID	0.250792	6.350031	1.413486	0.161024	-5.51338	0.467454	0.449217
Dendritic.cells	NSDHL	-0.40227	2.406095	-1.4131	0.161137	-4.89728	0.504358	0.501447
Dendritic.cells	TSPOAP1	0.677409	2.650845	1.412942	0.161183	-4.64065	0.50198	0.498108
Dendritic.cells	BC024386	-0.73966	1.614053	-1.41234	0.161361	-4.64063	0.512304	0.512846
Dendritic.cells	C1QTNF12	0.56348	2.21932	1.412247	0.161387	-4.70298	0.506325	0.504336
Dendritic.cells	MGAT4B	-0.35987	3.847759	-1.41222	0.161396	-4.9619	0.490663	0.482006
Dendritic.cells	LIN9	0.381491	4.133885	1.412046	0.161446	-4.91479	0.487975	0.478234
Dendritic.cells	F11R	-0.42401	3.432174	-1.41192	0.161483	-4.99454	0.494602	0.48771
Dendritic.cells	ADAP2OS	-0.71777	0.724917	-1.41188	0.161496	-4.64587	0.521244	0.525843
Dendritic.cells	MGA	-0.18156	5.917554	-1.41158	0.161584	-5.62054	0.471763	0.455239
Dendritic.cells	U2AF2	-0.15829	6.245648	-1.41139	0.161639	-5.62751	0.468863	0.45119
Dendritic.cells	ALDH2	-0.19769	6.861181	-1.41103	0.161745	-5.67311	0.463543	0.44359
Dendritic.cells	ZFP609	0.159746	6.067152	1.410955	0.161767	-5.65597	0.470565	0.45352
Dendritic.cells	CSTDC5	0.872017	5.46156	1.410755	0.161826	-5.08393	0.476056	0.461331
Dendritic.cells	ACTB	0.119541	13.9201	1.410386	0.161934	-6.89255	0.407171	0.364708
Dendritic.cells	ACCS	0.524783	2.079594	1.410002	0.162047	-4.69981	0.508329	0.507051
Dendritic.cells	CSTA2	1.249181	1.96893	1.409972	0.162056	-4.5839	0.509421	0.508614
Dendritic.cells	CAB39	-0.13102	6.974308	-1.40924	0.162272	-5.82693	0.463355	0.442703
Dendritic.cells	GM20186	0.336312	3.559404	1.408788	0.162405	-5.19511	0.494728	0.487072
Dendritic.cells	UGP2	0.269627	5.910415	1.408676	0.162438	-5.34119	0.472977	0.456246
Dendritic.cells	KCTD9	0.408323	3.130956	1.408518	0.162485	-4.80933	0.498828	0.493038
Dendritic.cells	MIDN	-0.19291	6.064607	-1.40841	0.162518	-5.50516	0.471594	0.454435
Dendritic.cells	MRPS22	0.43437	3.085855	1.408063	0.162619	-4.76061	0.499315	0.493865
Dendritic.cells	4732440D(	-0.55417	2.03703	-1.40799	0.162639	-4.7517	0.509546	0.508495
Dendritic.cells	5730480H(	0.377117	2.846707	1.40782	0.162691	-4.8626	0.501625	0.497246

Dendritic.cells	TBRG4	0.274869	4.075048	1.407738	0.162715	-5.17415	0.489901	0.480536
Dendritic.cells	LRRC61	-0.57481	2.011331	-1.4074	0.162815	-4.67003	0.509972	0.50902
Dendritic.cells	GM50399	-0.88332	0.166375	-1.40683	0.162982	-4.58531	0.528887	0.535947
Dendritic.cells	ARL5A	0.195099	5.831093	1.406815	0.162988	-5.49487	0.474141	0.45782
Dendritic.cells	TECPR1	-0.27976	4.375591	-1.4064	0.163111	-5.29291	0.487732	0.476788
Dendritic.cells	FUOM	0.770203	2.61981	1.406228	0.163161	-4.64162	0.504514	0.500661
Dendritic.cells	STEAP3	0.702646	1.980258	1.405787	0.163292	-4.56534	0.51106	0.509839
Dendritic.cells	ELP1	-0.20851	4.553493	-1.40555	0.163362	-5.21376	0.486335	0.474671
Dendritic.cells	GGA3	0.285501	3.302395	1.405491	0.16338	-5.01254	0.498164	0.491491
Dendritic.cells	RHOG	0.19055	7.243088	1.405209	0.163463	-5.79765	0.462141	0.440497
Dendritic.cells	ZWINT	-0.22544	5.039001	-1.40496	0.163536	-5.26772	0.481873	0.468483
Dendritic.cells	RAPGEF2	-0.19721	7.429198	-1.40486	0.163566	-5.95926	0.460524	0.438336
Dendritic.cells	AW549877	0.381944	3.352769	1.404847	0.163571	-4.94048	0.497713	0.491009
Dendritic.cells	GPR171	-0.19277	4.027921	-1.40444	0.163693	-5.86169	0.491354	0.482043
Dendritic.cells	GM12703	-0.88334	-0.21463	-1.40433	0.163725	-4.55109	0.533487	0.542417
Dendritic.cells	ENTPD5	0.357554	3.78751	1.404321	0.163726	-4.81517	0.493629	0.485312
Dendritic.cells	CD46	0.556365	2.11028	1.403982	0.163827	-4.7149	0.509975	0.508721
Dendritic.cells	GM29570	0.821401	0.692445	1.403663	0.163922	-4.5478	0.524226	0.529305
Dendritic.cells	SMARCA2	0.149806	6.123463	1.403561	0.163952	-5.73818	0.472184	0.455124
Dendritic.cells	ZFP518A	0.327134	3.684945	1.403327	0.164022	-5.01955	0.494704	0.487167
Dendritic.cells	FAM98C	0.256706	3.737769	1.403018	0.164114	-5.25129	0.494201	0.486487
Dendritic.cells	CAML	-0.23093	4.427078	-1.403	0.16412	-5.19433	0.487706	0.477242
Dendritic.cells	HSP90B1	0.114149	8.976388	1.40299	0.164122	-6.3216	0.447521	0.420478
Dendritic.cells	ZFP729B	-0.2979	3.639068	-1.40297	0.164128	-5.03134	0.49514	0.487825
Dendritic.cells	ATG12	-0.26153	4.873947	-1.40276	0.164189	-5.19565	0.483554	0.471424
Dendritic.cells	NUDT18	0.372263	2.627052	1.402485	0.164272	-4.83502	0.504899	0.501927
Dendritic.cells	CCNL2	0.119979	6.219089	1.402426	0.16429	-5.73632	0.471329	0.454171
Dendritic.cells	TBC1D15	-0.17059	6.062519	-1.40239	0.164301	-5.55288	0.472731	0.456156
Dendritic.cells	GM12905	-0.36692	2.138199	-1.40184	0.164463	-4.82994	0.509882	0.508942
Dendritic.cells	SKIL	-0.15182	7.304614	-1.40176	0.164488	-6.02805	0.461924	0.440784
Dendritic.cells	ACO2	-0.14234	6.116578	-1.40174	0.164495	-5.62627	0.472416	0.455607
Dendritic.cells	ERCC4	0.428584	2.949475	1.401539	0.164554	-4.87641	0.501986	0.497638
Dendritic.cells	CORO1A	0.150667	8.516071	1.401138	0.164673	-6.15778	0.451784	0.426208
Dendritic.cells	B3GALT2	0.714757	0.771125	1.400816	0.164769	-4.66754	0.524047	0.528796
Dendritic.cells	IRF3	0.302397	3.786081	1.400639	0.164822	-5.05323	0.494332	0.486317
Dendritic.cells	SYTL1	-0.48822	1.59739	-1.40042	0.164888	-4.82634	0.51569	0.516953
Dendritic.cells	GM4890	-0.66675	0.373848	-1.40037	0.164903	-4.59332	0.528124	0.534827
Dendritic.cells	GM41077	-0.71114	0.721818	-1.4001	0.164984	-4.61616	0.524579	0.529684
Dendritic.cells	STAT5B	0.181397	6.043742	1.400019	0.165007	-5.60983	0.473489	0.45681
Dendritic.cells	BRCC3	-0.20951	4.874732	-1.39988	0.165047	-5.35774	0.48415	0.472002
Dendritic.cells	ACOT1	-1.01266	2.604682	-1.39955	0.165147	-4.59576	0.505797	0.502856
Dendritic.cells	ZNFX1	0.436335	4.359029	1.399186	0.165256	-5.07421	0.489	0.478901
Dendritic.cells	MSN	0.136752	8.454473	1.39912	0.165276	-6.07322	0.452509	0.427301
Dendritic.cells	MRPL36	0.200325	5.835469	1.399076	0.165289	-5.52938	0.475412	0.459616
Dendritic.cells	USP14	-0.17163	5.793901	-1.39907	0.165289	-5.46026	0.475788	0.460148
Dendritic.cells	SLC25A18	-0.64986	4.491338	-1.39879	0.165373	-4.8646	0.487811	0.477225
Dendritic.cells	ABCC1	0.21677	4.467713	1.398466	0.165471	-5.52685	0.488032	0.477662

Dendritic.cells	RMND5B	-0.18379	4.49308	-1.39838	0.165495	-5.4378	0.487795	0.477333
Dendritic.cells	HGH1	0.431273	1.856316	1.398324	0.165514	-4.83178	0.513234	0.513667
Dendritic.cells	CDCA3	-0.61433	4.895561	-1.39816	0.165562	-5.02444	0.484054	0.472077
Dendritic.cells	E2F7	-0.62296	2.983824	-1.39804	0.165597	-4.77396	0.502157	0.497889
Dendritic.cells	CCDC9	-0.23722	4.238269	-1.39796	0.165622	-5.12111	0.490183	0.48084
Dendritic.cells	HABP4	-0.36106	3.054352	-1.39778	0.165675	-4.91386	0.501499	0.497019
Dendritic.cells	ZFP52	0.338096	3.859767	1.397456	0.165774	-5.03679	0.493842	0.486181
Dendritic.cells	LGALS8	-0.27236	5.110017	-1.39741	0.165786	-5.22773	0.482158	0.469551
Dendritic.cells	KCNMB4	-0.78141	1.763375	-1.39672	0.165995	-4.61428	0.514756	0.515592
Dendritic.cells	FAM162A	0.335304	5.640039	1.396092	0.166182	-5.31322	0.478194	0.463079
Dendritic.cells	LDLRAP1	0.570082	4.099537	1.395575	0.166338	-4.71161	0.492618	0.483525
Dendritic.cells	S100A1	-0.39717	4.086611	-1.39535	0.166405	-4.94353	0.492741	0.483704
Dendritic.cells	DNAAF5	-0.38159	2.988111	-1.39535	0.166405	-4.86897	0.503269	0.498719
Dendritic.cells	RINL	0.461346	3.632551	1.395073	0.166488	-4.82685	0.497058	0.489902
Dendritic.cells	IFI205	0.977422	2.758001	1.394991	0.166513	-4.70547	0.50551	0.501969
Dendritic.cells	SDR42E1	-0.63879	0.45745	-1.3945	0.16666	-4.60746	0.528608	0.535221
Dendritic.cells	ENTR1	-0.2232	4.880508	-1.3941	0.166782	-5.35263	0.485305	0.473413
Dendritic.cells	XPA	0.311031	4.138354	1.393943	0.166828	-5.09775	0.492251	0.483364
Dendritic.cells	HMGCLL1	0.738629	1.17582	1.393798	0.166872	-4.61768	0.52126	0.524931
Dendritic.cells	TNFRSF14	0.729961	0.252225	1.393715	0.166897	-4.63811	0.53073	0.538579
Dendritic.cells	PALLD	-0.28228	3.882236	-1.39359	0.166933	-5.40124	0.494678	0.486938
Dendritic.cells	STK39	-0.63213	3.517793	-1.39351	0.166959	-4.85682	0.498157	0.49194
Dendritic.cells	CALR	-0.14352	7.35156	-1.39342	0.166986	-5.97801	0.463078	0.442146
Dendritic.cells	ZFP36L2	0.153396	8.376464	1.393411	0.166989	-6.21708	0.454257	0.429713
Dendritic.cells	EIF3I	0.149963	6.822191	1.392987	0.167116	-5.7989	0.467725	0.448802
Dendritic.cells	HK1	0.268523	4.85354	1.392919	0.167137	-5.19797	0.485556	0.474129
Dendritic.cells	FUCA1	0.18277	5.963141	1.392726	0.167195	-5.54682	0.475398	0.459801
Dendritic.cells	VIPR1	0.732935	0.964939	1.392712	0.167199	-4.62831	0.523404	0.5284
Dendritic.cells	SCAI	0.284583	4.434969	1.392536	0.167252	-5.25596	0.48946	0.479894
Dendritic.cells	SNX25	0.215215	5.540177	1.392459	0.167276	-5.35237	0.479237	0.465368
Dendritic.cells	PLAG1	0.248917	2.8948	1.392438	0.167282	-5.1824	0.504177	0.500928
Dendritic.cells	MZB1	0.155073	5.956787	1.39232	0.167318	-6.20989	0.475456	0.460066
Dendritic.cells	HDHD2	-0.31353	3.552765	-1.39226	0.167334	-4.94957	0.497822	0.491907
Dendritic.cells	GM10785	0.61637	2.451276	1.392219	0.167348	-4.70983	0.508517	0.507215
Dendritic.cells	METRNL	0.763313	3.205494	1.392178	0.16736	-4.70267	0.501163	0.496684
Dendritic.cells	STX4A	0.175299	4.972734	1.391931	0.167435	-5.42847	0.484536	0.472962
Dendritic.cells	ZFAND1	0.3561	2.727292	1.391556	0.167548	-4.87658	0.506105	0.503593
Dendritic.cells	ATXN7	-0.15661	6.23066	-1.39032	0.167922	-5.78113	0.474086	0.457298
Dendritic.cells	RSF1OS1	0.36285	2.978349	1.390296	0.167929	-4.95804	0.504529	0.500634
Dendritic.cells	SERTAD3	0.290583	4.131362	1.390135	0.167978	-5.27845	0.493467	0.484826
Dendritic.cells	TRADD	0.302207	3.982911	1.389658	0.168122	-5.10953	0.495066	0.486987
Dendritic.cells	KCTD21	-0.81186	0.182578	-1.38945	0.168185	-4.60611	0.532898	0.541336
Dendritic.cells	TIMM22	-0.24565	4.4471	-1.38896	0.168332	-5.18074	0.490678	0.481068
Dendritic.cells	FANCF	-0.63994	1.911025	-1.38892	0.168345	-4.67637	0.515266	0.516241
Dendritic.cells	LDHA	0.177682	8.792149	1.388844	0.168369	-6.09975	0.45197	0.426314
Dendritic.cells	PPARG	0.695874	2.579221	1.388531	0.168464	-4.73965	0.50864	0.5069
Dendritic.cells	CHST2	0.833896	-0.02925	1.388497	0.168474	-4.59865	0.535109	0.545004

Dendritic.cells	MCM6	0.299082	6.155584	1.388478	0.16848	-5.5372	0.474954	0.458872
Dendritic.cells	SLC36A3OS	0.845709	1.010121	1.388362	0.168515	-4.63236	0.524366	0.529523
Dendritic.cells	SBNO1	-0.1351	7.359347	-1.38831	0.16853	-5.85995	0.46427	0.443771
Dendritic.cells	EDIL3	-0.75795	1.492552	-1.38827	0.168542	-4.67541	0.519469	0.522495
Dendritic.cells	LRATD2	0.642239	1.700967	1.387819	0.16868	-4.60417	0.517655	0.519731
Dendritic.cells	SORT1	0.226721	4.067523	1.387294	0.168839	-5.51085	0.494868	0.48674
Dendritic.cells	NXPE4	0.612407	2.181788	1.38709	0.168901	-4.77351	0.51322	0.512959
Dendritic.cells	NAAA	0.572439	3.389339	1.386911	0.168956	-4.76047	0.501387	0.496014
Dendritic.cells	AURKAIP1	0.193349	5.837172	1.38682	0.168983	-5.47032	0.478442	0.463342
Dendritic.cells	6030458C1	-0.3725	2.74674	-1.38657	0.169059	-4.85654	0.507732	0.50497
Dendritic.cells	ACOD1	1.978493	2.318035	1.386149	0.169187	-4.61993	0.512103	0.511152
Dendritic.cells	EARS2	-0.48811	1.644083	-1.38602	0.169228	-4.73511	0.518839	0.520827
Dendritic.cells	RAB19	0.673841	2.561986	1.385967	0.169243	-4.63841	0.509691	0.507702
Dendritic.cells	CYP2AB1	0.883993	-0.30874	1.385718	0.169319	-4.56567	0.539071	0.549903
Dendritic.cells	TXNIP	0.257076	5.791358	1.385019	0.169532	-5.53968	0.479513	0.464335
Dendritic.cells	PKD1L3	0.75421	0.837305	1.385015	0.169533	-4.58768	0.527524	0.53295
Dendritic.cells	ZFP677	-0.60164	0.788621	-1.38481	0.169595	-4.65871	0.528075	0.533727
Dendritic.cells	WDR4	0.385583	2.999114	1.384606	0.169657	-4.84595	0.505964	0.501963
Dendritic.cells	LLGL2	0.485704	2.291993	1.384323	0.169744	-4.78966	0.512985	0.51199
Dendritic.cells	DOP1B	-0.36932	4.289272	-1.38425	0.169767	-5.00758	0.493617	0.484319
Dendritic.cells	PDXDC1	-0.11023	6.616598	-1.38368	0.169939	-5.88797	0.472539	0.454187
Dendritic.cells	TMED7	0.117509	6.308978	1.38345	0.17001	-5.75128	0.4753	0.458143
Dendritic.cells	TRERF1	0.264964	4.396216	1.383415	0.170021	-5.5814	0.492946	0.483201
Dendritic.cells	LOXL3	0.833093	0.451305	1.383061	0.170129	-4.61309	0.53218	0.539408
Dendritic.cells	ATXN1	-0.33565	6.919224	-1.3829	0.170178	-5.39521	0.469967	0.450653
Dendritic.cells	USF2	0.148264	6.021725	1.382822	0.170202	-5.7169	0.478021	0.462055
Dendritic.cells	MAPKAPK3	0.346089	5.306805	1.382044	0.17044	-5.09473	0.485114	0.471668
Dendritic.cells	TCF7L2	-0.17112	7.34951	-1.38165	0.17056	-6.24462	0.466866	0.445714
Dendritic.cells	GM13205	-0.68293	0.234435	-1.3811	0.17073	-4.6215	0.535235	0.543449
Dendritic.cells	ARMCX3	-0.25041	3.573363	-1.38089	0.170793	-5.25594	0.501675	0.495398
Dendritic.cells	FILIP1L	-0.18946	5.411773	-1.38087	0.1708	-5.67127	0.484321	0.470684
Dendritic.cells	DDX54	0.179445	5.820595	1.380701	0.170851	-5.58299	0.480567	0.465426
Dendritic.cells	RPRD1A	0.228598	4.057705	1.380654	0.170866	-5.16662	0.497027	0.488837
Dendritic.cells	ALG5	0.25183	4.2129	1.380632	0.170873	-5.23289	0.495549	0.48673
Dendritic.cells	LZTR1	0.391321	2.612289	1.38047	0.170922	-4.87955	0.511062	0.50893
Dendritic.cells	PPP3CB	-0.14268	5.980209	-1.38042	0.170938	-5.63394	0.479112	0.463391
Dendritic.cells	SETD4	-0.3236	2.964232	-1.38024	0.170992	-4.98042	0.507599	0.503972
Dendritic.cells	AI662270	0.185633	6.090129	1.380197	0.171006	-5.71723	0.478114	0.461975
Dendritic.cells	N4BP2L2	0.096607	6.786437	1.380056	0.171049	-5.93387	0.471851	0.453173
Dendritic.cells	CLEC10A	0.214372	0.930777	1.379823	0.171121	-5.70875	0.528094	0.533472
Dendritic.cells	TBC1D4	0.322425	4.706501	1.379636	0.171178	-5.45607	0.490993	0.480307
Dendritic.cells	CKLF	0.261093	4.804772	1.37948	0.171226	-5.21609	0.490078	0.479035
Dendritic.cells	MST1	-0.86195	0.649079	-1.37917	0.17132	-4.61014	0.531079	0.537768
Dendritic.cells	CNRIP1	-0.62547	1.648634	-1.37915	0.171328	-4.70418	0.520848	0.523039
Dendritic.cells	C330013E1	0.778789	0.677864	1.37816	0.171632	-4.60189	0.531456	0.537834
Dendritic.cells	FOXP4	0.212	4.962815	1.378131	0.171641	-5.56103	0.489252	0.477415
Dendritic.cells	TMEM128	0.200354	5.718829	1.377993	0.171683	-5.51499	0.482253	0.46751

Dendritic.cells	SYNRG	-0.16796	5.053855	-1.37752	0.171829	-5.55511	0.488689	0.476467
Dendritic.cells	ACIN1	0.100948	7.577859	1.377347	0.171882	-5.99476	0.465884	0.444185
Dendritic.cells	QTRT2	0.692234	1.344368	1.377056	0.171972	-4.68391	0.525066	0.528362
Dendritic.cells	AADAT	-0.69865	1.2345	-1.37671	0.17208	-4.62536	0.526358	0.530009
Dendritic.cells	CYSLTR2	0.865874	1.496466	1.37658	0.172118	-4.6125	0.523684	0.526233
Dendritic.cells	ABCC10	-0.59639	0.65899	-1.37639	0.172177	-4.63712	0.532309	0.538728
Dendritic.cells	WDR41	0.315568	4.001344	1.376264	0.172216	-4.9835	0.498961	0.490921
Dendritic.cells	EFCC1	-0.84269	0.001369	-1.37569	0.172393	-4.5764	0.539599	0.548972
Dendritic.cells	2010309G2	0.702985	1.223176	1.375471	0.172461	-4.67602	0.526941	0.530727
Dendritic.cells	EFCAB11	-0.60773	3.410869	-1.37534	0.1725	-4.80825	0.505089	0.499425
Dendritic.cells	CIAO3	0.372813	3.316721	1.375094	0.172577	-4.8908	0.506098	0.500769
Dendritic.cells	ATP8B1	-0.68843	0.813793	-1.37486	0.172649	-4.66391	0.531335	0.536948
Dendritic.cells	VIM	0.255545	8.109275	1.374303	0.172821	-6.01374	0.462368	0.4385
Dendritic.cells	ARL10	0.378243	2.295947	1.374227	0.172845	-4.92467	0.516566	0.515514
Dendritic.cells	TIAL1	-0.14866	5.798885	-1.37361	0.173035	-5.61036	0.483383	0.467805
Dendritic.cells	TTC13	0.243497	3.938924	1.373353	0.173115	-5.2253	0.500972	0.492781
Dendritic.cells	SPTY2D1	-0.14705	5.765398	-1.37293	0.173245	-5.71897	0.48402	0.468492
Dendritic.cells	METRN	-0.52612	2.538002	-1.37269	0.173321	-4.77345	0.515023	0.512628
Dendritic.cells	CYBC1	0.270057	4.562008	1.371719	0.173622	-5.26358	0.496075	0.484905
Dendritic.cells	PACSIN1	0.111978	2.953403	1.371457	0.173703	-6.1538	0.511754	0.507166
Dendritic.cells	ELL	-0.2362	5.024631	-1.37121	0.173781	-5.30338	0.491801	0.478756
Dendritic.cells	ASNS	-0.74652	1.465562	-1.37117	0.173793	-4.59164	0.526717	0.528603
Dendritic.cells	GM20274	0.358843	2.727126	1.371009	0.173842	-4.98492	0.514005	0.510446
Dendritic.cells	PGAP3	0.595655	1.12543	1.370861	0.173888	-4.7282	0.530223	0.533732
Dendritic.cells	6430550D2	0.662412	1.142129	1.370655	0.173952	-4.65338	0.530106	0.533587
Dendritic.cells	ANXA11OS	-0.73963	0.812081	-1.37048	0.174008	-4.62534	0.533553	0.5386
Dendritic.cells	E430024P1	-0.75837	0.53335	-1.37012	0.174119	-4.61941	0.536578	0.542996
Dendritic.cells	PAOX	0.446799	2.987177	1.370057	0.174138	-4.87963	0.511624	0.507204
Dendritic.cells	1700061N1	-0.90942	-0.1438	-1.36977	0.174226	-4.61941	0.543853	0.553459
Dendritic.cells	PDXK	-0.18025	4.441778	-1.36937	0.174351	-5.60605	0.497857	0.487256
Dendritic.cells	GRK6	0.153477	5.764162	1.369124	0.174429	-5.71005	0.485537	0.469743
Dendritic.cells	ITK	-0.71537	4.224474	-1.36857	0.174603	-5.00704	0.500292	0.49056
Dendritic.cells	SMIM10L1	0.178909	4.931338	1.368422	0.174647	-5.54284	0.493569	0.481025
Dendritic.cells	TNIP1	0.308166	4.966974	1.368382	0.17466	-5.32778	0.493233	0.480548
Dendritic.cells	SDHA	-0.15257	5.691569	-1.36818	0.174722	-5.58409	0.486512	0.471038
Dendritic.cells	MAVS	0.417061	2.932268	1.367786	0.174846	-4.85075	0.512978	0.50884
Dendritic.cells	AMACR	-0.66999	2.326832	-1.36769	0.174874	-4.721	0.519017	0.517482
Dendritic.cells	GPR19	-0.52891	2.550401	-1.36764	0.174892	-4.7402	0.516777	0.514274
Dendritic.cells	ANKFY1	-0.17341	5.995175	-1.36754	0.174921	-5.60604	0.483753	0.467257
Dendritic.cells	DCP1A	0.18887	4.850131	1.367267	0.175008	-5.4417	0.494536	0.482463
Dendritic.cells	D6WSU163	0.327427	3.1191	1.366736	0.175173	-4.96964	0.5116	0.506446
Dendritic.cells	DMAC1	0.233518	4.611093	1.366264	0.175321	-5.28763	0.497407	0.486003
Dendritic.cells	ATP6V0A2	-0.2213	4.619482	-1.3659	0.175434	-5.29895	0.497327	0.485949
Dendritic.cells	HERPUD2	-0.16145	5.474289	-1.36579	0.17547	-5.51779	0.489277	0.474524
Dendritic.cells	4931406G0	0.879958	0.347467	1.365779	0.175473	-4.64825	0.540164	0.547252
Dendritic.cells	CBFA2T3	0.187924	5.507245	1.365672	0.175506	-5.76881	0.48897	0.47413
Dendritic.cells	GCAT	0.504037	2.390839	1.365455	0.175574	-4.81777	0.519126	0.517174

Dendritic.cells	RNF6	-0.14553	5.670645	-1.36533	0.175613	-5.66182	0.487452	0.472087
Dendritic.cells	DERL1	0.113984	6.22546	1.365231	0.175644	-5.72369	0.482342	0.464879
Dendritic.cells	REEP6	0.701492	1.791912	1.36511	0.175682	-4.68093	0.525187	0.525967
Dendritic.cells	DUS1L	0.303768	4.064535	1.364862	0.17576	-5.13359	0.502653	0.493812
Dendritic.cells	ZFP236	0.210068	4.729945	1.364806	0.175777	-5.36801	0.496285	0.484749
Dendritic.cells	PHKG2	-0.23011	4.528144	-1.36454	0.175862	-5.2773	0.498268	0.487636
Dendritic.cells	HMGXB3	-0.2468	4.408539	-1.36426	0.175947	-5.23438	0.499411	0.489345
Dendritic.cells	GLDC	-0.73612	1.391092	-1.36408	0.176003	-4.66374	0.529367	0.532209
Dendritic.cells	TSPO2	0.838449	0.283153	1.363991	0.176033	-4.60587	0.540919	0.548873
Dendritic.cells	MTHFSD	0.433644	2.433778	1.363882	0.176067	-4.85346	0.518769	0.517031
Dendritic.cells	IGKV1-110	0.617331	0.050225	1.363687	0.176128	-4.7001	0.543386	0.552472
Dendritic.cells	RBM28	0.189435	5.311789	1.363598	0.176156	-5.4449	0.490864	0.477277
Dendritic.cells	S100A8	1.158707	6.633628	1.363558	0.176169	-5.23065	0.478696	0.460033
Dendritic.cells	RAB11FIP5	-0.61256	0.808074	-1.36337	0.176227	-4.75348	0.535444	0.541
Dendritic.cells	AW112010	-0.83633	7.279978	-1.36292	0.176369	-5.39226	0.473181	0.452017
Dendritic.cells	ADORA3	-0.67489	-0.30137	-1.36262	0.176464	-4.65624	0.547622	0.558252
Dendritic.cells	GLIS1	-0.83559	0.394465	-1.36175	0.176738	-4.60648	0.540921	0.54796
Dendritic.cells	ZFP992	0.339739	3.563813	1.361148	0.176926	-5.08027	0.508944	0.502011
Dendritic.cells	SND1	-0.09713	7.195863	-1.3611	0.176943	-6.17993	0.474915	0.453741
Dendritic.cells	GM12462	0.730336	-0.49881	1.361001	0.176972	-4.60318	0.550731	0.562033
Dendritic.cells	GM46620	0.690973	0.683678	1.360872	0.177013	-4.69537	0.538152	0.543908
Dendritic.cells	CDIPTOS	0.680074	-0.30875	1.360419	0.177156	-4.58939	0.548952	0.559285
Dendritic.cells	ZFP526	-0.71229	0.37218	-1.3602	0.177223	-4.61836	0.541694	0.548828
Dendritic.cells	TRMT112	0.127508	7.277188	1.36016	0.177237	-5.9942	0.474418	0.452908
Dendritic.cells	TCF25	0.109223	6.939299	1.359757	0.177364	-5.89922	0.477668	0.457314
Dendritic.cells	ERCC6	-0.28621	3.56729	-1.35933	0.177499	-5.19514	0.509617	0.502492
Dendritic.cells	GM14698	-0.6303	0.738672	-1.35902	0.177596	-4.67321	0.538323	0.543716
Dendritic.cells	D16ERTD47	-0.25836	4.914897	-1.35886	0.177646	-5.36307	0.496616	0.484178
Dendritic.cells	RTN4RL1	0.364857	2.381813	1.358488	0.177765	-5.25123	0.521411	0.519621
Dendritic.cells	TTBK2	-0.50252	1.314702	-1.35827	0.177835	-4.73158	0.532319	0.535352
Dendritic.cells	ZDHHC21	0.248369	4.271545	1.358176	0.177864	-5.26533	0.502769	0.493092
Dendritic.cells	TMBIM4	0.15739	6.749974	1.358154	0.17787	-5.78668	0.479595	0.460216
Dendritic.cells	IL4I1	0.709377	2.841673	1.357993	0.177921	-4.78399	0.516796	0.513157
Dendritic.cells	PARL	0.253087	4.391466	1.357946	0.177936	-5.17496	0.501615	0.491506
Dendritic.cells	MED27	-0.13213	5.275175	-1.35764	0.178032	-5.66464	0.493213	0.479653
Dendritic.cells	AFG1L	0.367706	3.691074	1.357615	0.178041	-5.09509	0.508405	0.501277
Dendritic.cells	CYB5D2	0.601403	1.870839	1.357514	0.178073	-4.71772	0.5266	0.527312
Dendritic.cells	STK26	0.312981	4.048898	1.357421	0.178102	-5.12478	0.504921	0.496309
Dendritic.cells	PLA2G7	1.186178	4.021195	1.357144	0.17819	-4.78216	0.50519	0.496784
Dendritic.cells	MOSPD3	-0.23687	4.656046	-1.35703	0.178226	-5.2602	0.49908	0.488089
Dendritic.cells	EMC3	0.136573	5.490353	1.356907	0.178265	-5.72092	0.491194	0.476883
Dendritic.cells	PGAP2	0.193282	5.75264	1.356801	0.178299	-5.6756	0.488749	0.47342
Dendritic.cells	NRAS	-0.15256	6.075126	-1.35672	0.178325	-5.63868	0.485764	0.469213
Dendritic.cells	TSC22D1	0.164005	4.872446	1.356672	0.178339	-5.81993	0.497019	0.485209
Dendritic.cells	GM41409	-0.67476	3.031183	-1.35657	0.178371	-4.79841	0.514909	0.510769
Dendritic.cells	CACTIN	-0.26944	3.991663	-1.35634	0.178444	-5.22772	0.505553	0.497392
Dendritic.cells	NAP1L4	0.122462	6.249563	1.355348	0.178759	-5.75237	0.484961	0.467347

Dendritic.cells	CLASP2	-0.13978	6.927855	-1.35518	0.178812	-5.89326	0.478793	0.458712
Dendritic.cells	ABCB1B	-0.22681	3.934965	-1.35503	0.178859	-5.77601	0.506883	0.498634
Dendritic.cells	CCL24	-1.05172	3.596362	-1.35441	0.179056	-4.89828	0.510292	0.503518
Dendritic.cells	SRP9	0.106169	7.516452	1.3543	0.179092	-6.01935	0.473604	0.451463
Dendritic.cells	MRPL51	0.307314	4.486929	1.354276	0.179099	-5.06287	0.501642	0.491191
Dendritic.cells	MRTFB	-0.319	4.475202	-1.35418	0.179129	-5.1218	0.501755	0.491351
Dendritic.cells	NDUFA12	0.220001	5.362792	1.354161	0.179136	-5.3632	0.493318	0.479359
Dendritic.cells	USP48	-0.17736	5.391239	-1.35405	0.179172	-5.49571	0.493051	0.479022
Dendritic.cells	CD3E	-0.81576	3.278256	-1.35327	0.179419	-4.82255	0.514003	0.508422
Dendritic.cells	HIST1H2AP	0.485683	7.597755	1.352234	0.179749	-5.79796	0.473898	0.451116
Dendritic.cells	GM44649	-0.26511	3.949723	-1.35218	0.179765	-5.32933	0.507928	0.499337
Dendritic.cells	YWHAZ	-0.07923	8.482146	-1.35215	0.179775	-6.15315	0.466107	0.440149
Dendritic.cells	TBC1D13	-0.37481	3.495587	-1.35177	0.179898	-4.96925	0.512383	0.505835
Dendritic.cells	RCAN3	0.357861	2.117276	1.351745	0.179905	-4.9887	0.526207	0.525609
Dendritic.cells	PSMD2	0.148463	6.149979	1.351702	0.179919	-5.69213	0.487035	0.469815
Dendritic.cells	GM15445	0.541589	0.994582	1.351669	0.179929	-4.73046	0.53781	0.542291
Dendritic.cells	LMNB2	0.572514	2.474409	1.351182	0.180084	-4.77401	0.522767	0.520557
Dendritic.cells	CD200R2	0.865167	0.789771	1.351001	0.180143	-4.68744	0.540152	0.545557
Dendritic.cells	NMD3	-0.17006	4.999909	-1.35084	0.180194	-5.39775	0.497991	0.485278
Dendritic.cells	ITPRIPL1	0.343473	3.28736	1.350834	0.180196	-4.96071	0.514625	0.50897
Dendritic.cells	CXXC1	0.264858	4.307295	1.350638	0.180258	-5.18608	0.504635	0.494775
Dendritic.cells	EIF4A2	0.161582	5.530319	1.350593	0.180273	-5.56131	0.49298	0.478218
Dendritic.cells	PPP1R14B	0.126064	6.546146	1.350183	0.180403	-6.01331	0.483791	0.465029
Dendritic.cells	HMOX1	-0.68629	4.807778	-1.34967	0.180568	-5.03361	0.500269	0.48825
Dendritic.cells	ACSL1	0.232447	5.758511	1.349531	0.180612	-5.74069	0.491283	0.475496
Dendritic.cells	LYPLA1	0.139412	5.988433	1.34946	0.180635	-5.75498	0.489141	0.472466
Dendritic.cells	CAMSAP1	-0.32961	3.53399	-1.34923	0.180708	-5.07825	0.512644	0.505933
Dendritic.cells	SUZ12	0.13856	6.954117	1.349191	0.180721	-5.84285	0.480279	0.459993
Dendritic.cells	SLK	0.144046	6.477015	1.349068	0.18076	-5.93251	0.484631	0.466179
Dendritic.cells	LYSMD4	0.35554	3.330154	1.348789	0.180849	-5.04821	0.514783	0.508984
Dendritic.cells	BE692007	0.824058	2.816357	1.34836	0.180987	-4.66444	0.520055	0.516557
Dendritic.cells	HELB	0.376577	3.704754	1.348341	0.180993	-4.9783	0.511226	0.503946
Dendritic.cells	FSCN1	-0.60727	3.378992	-1.34814	0.181057	-5.11728	0.514489	0.508587
Dendritic.cells	MAP2K3	-0.22876	6.01457	-1.348	0.181103	-5.61516	0.489195	0.472669
Dendritic.cells	VPS26A	-0.14619	6.273847	-1.34778	0.181172	-5.72684	0.486855	0.469346
Dendritic.cells	RPP21	0.252077	4.873001	1.347444	0.18128	-5.23972	0.500085	0.488112
Dendritic.cells	STK19	-0.25995	4.61763	-1.34728	0.181333	-5.27932	0.502534	0.491627
Dendritic.cells	IRF2	0.183367	6.689517	1.347262	0.181339	-5.66317	0.48311	0.464071
Dendritic.cells	DZIP3	0.378354	3.257554	1.346795	0.181488	-5.03455	0.516023	0.510782
Dendritic.cells	OLFR543	-0.77185	0.201065	-1.34676	0.181498	-4.6175	0.547561	0.556086
Dendritic.cells	TFDP2	0.370269	5.591554	1.345868	0.181786	-5.21844	0.494039	0.478971
Dendritic.cells	ST14	-0.60674	1.167666	-1.34576	0.181819	-4.83628	0.53797	0.541717
Dendritic.cells	KIRREL3	-0.59144	1.272544	-1.34567	0.181851	-4.7514	0.536874	0.540185
Dendritic.cells	RMDN3	0.292675	3.398108	1.345516	0.181899	-5.02997	0.515235	0.509248
Dendritic.cells	EEF1AKMT:	0.384074	3.325831	1.345162	0.182013	-5.02229	0.516144	0.510393
Dendritic.cells	ZBTB33	0.322296	2.982117	1.34491	0.182094	-5.00411	0.519673	0.515297
Dendritic.cells	BAK1	0.262791	4.936771	1.344485	0.182231	-5.34315	0.500776	0.488119

Dendritic.cells	GM15943	-0.82045	0.207344	-1.34432	0.182285	-4.62443	0.548732	0.556731
Dendritic.cells	SLC12A2	-0.58278	3.544469	-1.34407	0.182364	-4.81715	0.514339	0.507524
Dendritic.cells	GM39302	0.369558	0.880898	1.344035	0.182376	-5.10401	0.541569	0.546541
Dendritic.cells	TEDC2	0.659064	0.906107	1.343641	0.182503	-4.65024	0.541427	0.546393
Dendritic.cells	EHD2	-0.70142	1.82262	-1.34357	0.182527	-4.66632	0.531865	0.532661
Dendritic.cells	NAALADL2	-0.54553	1.964286	-1.34331	0.18261	-4.76182	0.530405	0.530653
Dendritic.cells	KAT6A	-0.13484	6.479855	-1.3431	0.182678	-5.88967	0.486416	0.468042
Dendritic.cells	PRCP	0.12439	5.424232	1.34301	0.182706	-5.95488	0.496266	0.482062
Dendritic.cells	EIF5A2	-0.69901	0.577892	-1.34291	0.182739	-4.68285	0.544902	0.551678
Dendritic.cells	DSTYK	0.256759	3.925134	1.342772	0.182783	-5.15225	0.510705	0.502665
Dendritic.cells	SDC1	0.769181	2.216388	1.342746	0.182792	-4.73017	0.52782	0.527139
Dendritic.cells	MAP1S	-0.29946	3.781603	-1.34255	0.182856	-5.16205	0.512164	0.50476
Dendritic.cells	RNF157	0.249726	5.375061	1.341824	0.183089	-5.66299	0.497285	0.483173
Dendritic.cells	RAD54B	0.661019	2.207787	1.341196	0.183292	-4.73651	0.528862	0.52808
Dendritic.cells	HRH2	-0.64284	1.160498	-1.34114	0.183309	-4.70359	0.539725	0.543679
Dendritic.cells	GM14302	-0.57682	0.609708	-1.3406	0.183486	-4.71052	0.545879	0.552333
Dendritic.cells	PDLIM5	-0.20681	6.703681	-1.34041	0.183546	-5.65277	0.48553	0.466243
Dendritic.cells	ZAP70	-0.77845	1.982594	-1.34036	0.183561	-4.67353	0.531498	0.531711
Dendritic.cells	GM16287	0.707082	-0.44109	1.340024	0.183671	-4.61054	0.557255	0.568742
Dendritic.cells	GM4869	-0.69579	1.447403	-1.34002	0.183672	-4.70363	0.537098	0.539688
Dendritic.cells	UHRF1BP1	-0.58446	1.998967	-1.3396	0.183809	-4.80545	0.531613	0.531669
Dendritic.cells	SH3KBP1	0.116478	7.863843	1.339481	0.183847	-6.09976	0.475289	0.451639
Dendritic.cells	RNF168	0.309811	4.161361	1.339149	0.183955	-5.16234	0.509935	0.500761
Dendritic.cells	ZFP715	-0.2625	3.662783	-1.33884	0.184055	-5.16514	0.514843	0.507776
Dendritic.cells	PSMD8	0.149559	6.592353	1.338761	0.184081	-5.82291	0.486853	0.468028
Dendritic.cells	B230369F2	-0.45831	2.457789	-1.33871	0.184097	-4.81851	0.526952	0.52511
Dendritic.cells	PANK2	0.14619	5.327592	1.338452	0.184181	-5.70494	0.498688	0.484889
Dendritic.cells	MRPS12	0.243251	4.774775	1.338424	0.18419	-5.29343	0.503979	0.492416
Dendritic.cells	NDUFA2	0.130684	7.238115	1.338389	0.184201	-5.92714	0.480953	0.459778
Dendritic.cells	DCUN1D2	-0.43269	2.743543	-1.33828	0.184237	-4.93037	0.524048	0.521087
Dendritic.cells	ALDH18A1	-0.51121	3.070395	-1.33786	0.184372	-4.87627	0.520882	0.516507
Dendritic.cells	ERAL1	0.438292	1.918255	1.337767	0.184403	-4.81018	0.532621	0.533335
Dendritic.cells	GM12227	-0.62413	1.113465	-1.3377	0.184424	-4.66155	0.541015	0.545405
Dendritic.cells	PCBD1	-0.70516	3.53173	-1.33743	0.184513	-4.89547	0.516391	0.510135
Dendritic.cells	SSBP1	0.172605	5.592236	1.337104	0.184619	-5.57228	0.496478	0.481795
Dendritic.cells	KAZN	-0.70113	0.348768	-1.33706	0.184633	-4.71143	0.549331	0.557462
Dendritic.cells	DNHD1	-0.56884	1.01891	-1.33683	0.184709	-4.76761	0.542201	0.547279
Dendritic.cells	IMPAD1	0.197222	4.365894	1.336785	0.184723	-5.36533	0.508243	0.498652
Dendritic.cells	SRL	0.327248	0.890591	1.336177	0.18492	-5.0785	0.544001	0.549585
Dendritic.cells	DNAJC10	0.215579	4.533218	1.335683	0.185081	-5.32243	0.507317	0.496815
Dendritic.cells	SPINDOC	-0.2051	4.427162	-1.33556	0.185122	-5.40319	0.508348	0.498344
Dendritic.cells	GM3055	-0.71038	0.319624	-1.33537	0.185182	-4.64705	0.550403	0.558653
Dendritic.cells	MUS81	-0.48175	2.048507	-1.33514	0.185257	-4.82397	0.532197	0.532562
Dendritic.cells	PDE12	-0.2556	3.991564	-1.33513	0.18526	-5.13381	0.512613	0.504538
Dendritic.cells	CWC25	-0.25895	5.255579	-1.33425	0.185549	-5.42481	0.501016	0.487352
Dendritic.cells	SULT2A1	-0.80685	3.901678	-1.33407	0.185608	-4.93523	0.514203	0.506116
Dendritic.cells	PHF11C	-0.55541	1.989779	-1.33373	0.185717	-4.74336	0.533713	0.533935

Dendritic.cells	UBL5	0.108165	8.259007	1.333469	0.185803	-6.13034	0.473603	0.448543
Dendritic.cells	GM15489	-0.8084	0.208522	-1.33339	0.185828	-4.64012	0.55259	0.561183
Dendritic.cells	SPSB2	-0.27501	2.383882	-1.33315	0.185907	-5.14213	0.529791	0.528336
Dendritic.cells	INPP5D	0.12243	8.764735	1.332822	0.186015	-6.40537	0.469363	0.442434
Dendritic.cells	TUBA1A	-0.25929	5.430541	-1.3327	0.186054	-5.58951	0.499821	0.485489
Dendritic.cells	IGIP	0.623632	0.675003	1.332459	0.186133	-4.71362	0.547927	0.554242
Dendritic.cells	ABCD2	-0.68337	2.633823	-1.33162	0.186408	-4.69756	0.528055	0.525192
Dendritic.cells	SMAD4	0.109303	6.23224	1.331455	0.186462	-5.93526	0.492886	0.475209
Dendritic.cells	SCRN2	0.513032	1.43712	1.331169	0.186556	-4.71467	0.540448	0.543129
Dendritic.cells	ZBTB26	0.636603	0.850706	1.331136	0.186567	-4.69699	0.546651	0.552065
Dendritic.cells	CHORDC1	0.184103	5.082056	1.331002	0.186611	-5.45687	0.50379	0.490814
Dendritic.cells	F730311O2	0.826944	-0.31758	1.330973	0.18662	-4.61811	0.559265	0.57032
Dendritic.cells	NFS1	0.262745	4.533914	1.33073	0.1867	-5.1989	0.509123	0.498485
Dendritic.cells	FBXL15	0.421618	2.696607	1.330372	0.186817	-4.88143	0.527441	0.524863
Dendritic.cells	ALYREF2	0.300482	3.243584	1.330293	0.186843	-5.10918	0.521902	0.516968
Dendritic.cells	TTI2	-0.33326	2.749363	-1.33027	0.186851	-4.97374	0.526904	0.524125
Dendritic.cells	ADGRA3	-0.58641	0.459311	-1.33023	0.186865	-4.70328	0.550867	0.558594
Dendritic.cells	HYAL3	-0.74947	0.035974	-1.32979	0.187007	-4.63018	0.55561	0.565348
Dendritic.cells	MYO3B	0.633575	1.289051	1.329763	0.187017	-4.69285	0.5422	0.546012
Dendritic.cells	KRIT1	0.129753	5.937975	1.328248	0.187515	-5.83748	0.496963	0.480184
Dendritic.cells	ZFP282	0.275835	4.010784	1.328144	0.187549	-5.2421	0.515596	0.506666
Dendritic.cells	PCGF2	-0.71008	0.79144	-1.32802	0.187589	-4.66251	0.548736	0.554148
Dendritic.cells	NAGPA	0.283106	3.354165	1.327788	0.187666	-5.12493	0.522232	0.516048
Dendritic.cells	A630001O1	-0.39139	2.282008	-1.32728	0.187835	-4.97642	0.533219	0.531861
Dendritic.cells	CYP2C29	-0.85677	1.011219	-1.32708	0.1879	-4.67197	0.546541	0.551053
Dendritic.cells	DUS3L	-0.31329	3.300011	-1.32694	0.187944	-5.0244	0.522835	0.517076
Dendritic.cells	EEFSEC	0.350246	4.399413	1.326938	0.187946	-5.17038	0.511906	0.501494
Dendritic.cells	PDK4	-0.6127	1.417008	-1.32691	0.187955	-4.78124	0.542243	0.544875
Dendritic.cells	IKBKB	-0.13009	5.778617	-1.32686	0.187971	-5.86041	0.498606	0.482608
Dendritic.cells	FANCG	0.485435	2.222876	1.326416	0.188118	-4.79593	0.534114	0.532893
Dendritic.cells	NGLY1	0.160493	4.849758	1.326081	0.188229	-5.38714	0.507844	0.495485
Dendritic.cells	MDN1	-0.24906	5.788045	-1.32605	0.188237	-5.59968	0.498841	0.482709
Dendritic.cells	GM10101	-0.68512	0.672014	-1.32587	0.188299	-4.65939	0.550523	0.55659
Dendritic.cells	STN1	0.267248	3.886297	1.325774	0.18833	-5.17449	0.517307	0.50906
Dendritic.cells	RXYLT1	-0.24492	3.864731	-1.32551	0.188419	-5.19292	0.517635	0.509391
Dendritic.cells	USP22	0.222611	4.389395	1.325359	0.188467	-5.26668	0.512453	0.502022
Dendritic.cells	ZFP85	0.744724	0.25725	1.325164	0.188531	-4.63976	0.555123	0.56321
Dendritic.cells	CASK	0.22887	5.500078	1.32507	0.188562	-5.556	0.501696	0.486803
Dendritic.cells	PRR33	0.600146	0.255516	1.324789	0.188655	-4.7508	0.555276	0.563299
Dendritic.cells	IRF7	0.331006	6.139097	1.324267	0.188828	-6.04975	0.496088	0.47843
Dendritic.cells	NDRG3	-0.18777	4.407312	-1.32412	0.188877	-5.42679	0.512749	0.502137
Dendritic.cells	GOLGA5	0.187623	4.913418	1.323908	0.188946	-5.44858	0.507865	0.495232
Dendritic.cells	RTN1	0.430977	2.561189	1.323569	0.189058	-5.19866	0.531364	0.528819
Dendritic.cells	ZKSCAN1	0.2124	4.068204	1.323531	0.189071	-5.33902	0.516157	0.507102
Dendritic.cells	PRMT5	0.350161	3.483009	1.323397	0.189116	-5.0074	0.521996	0.515429
Dendritic.cells	5430405H0	0.27788	3.910079	1.323331	0.189137	-5.2555	0.517726	0.509339
Dendritic.cells	ABCE1	-0.21374	5.007871	-1.32314	0.189199	-5.4624	0.506993	0.494062

Dendritic.cells	MPP6	0.160083	6.194697	1.322763	0.189325	-5.76998	0.495876	0.478135
Dendritic.cells	POLR2J	0.199766	4.922519	1.322513	0.189408	-5.38519	0.508126	0.495401
Dendritic.cells	CCNB1IP1	-0.56877	1.863098	-1.32205	0.189563	-4.72476	0.539225	0.539518
Dendritic.cells	IER3	-0.46141	6.13903	-1.32191	0.189609	-5.43128	0.496733	0.479021
Dendritic.cells	GM37233	0.700781	0.010375	1.321786	0.189649	-4.66748	0.559037	0.568076
Dendritic.cells	BICRAL	0.179439	5.023041	1.321665	0.18969	-5.60957	0.507397	0.494164
Dendritic.cells	PGPEP1	0.334408	3.461593	1.321459	0.189758	-5.02225	0.522877	0.516158
Dendritic.cells	TAF1B	0.234789	4.199522	1.321275	0.189819	-5.25524	0.515551	0.505763
Dendritic.cells	ADD1	0.183962	5.795836	1.321055	0.189892	-5.57068	0.500085	0.483854
Dendritic.cells	ALS2	0.161788	4.283789	1.320973	0.189919	-5.60796	0.514732	0.50467
Dendritic.cells	TRIM33	0.144561	6.368235	1.319978	0.19025	-5.78565	0.495135	0.476532
Dendritic.cells	PCOLCE	0.743771	0.911461	1.31994	0.190262	-4.67363	0.549911	0.554661
Dendritic.cells	CSF1R	-0.53018	5.549681	-1.31991	0.190271	-5.30512	0.502892	0.487516
Dendritic.cells	CYB5R4	0.171384	5.869945	1.319879	0.190283	-5.61313	0.499838	0.483192
Dendritic.cells	DHX8	-0.20631	5.129659	-1.31973	0.190334	-5.42286	0.506936	0.493266
Dendritic.cells	TEX261	0.157954	5.229001	1.319591	0.190378	-5.59873	0.505977	0.491947
Dendritic.cells	LSM12	0.129645	6.360706	1.319155	0.190524	-5.83024	0.49543	0.476884
Dendritic.cells	PDCD2L	-0.15167	4.660225	-1.31905	0.190559	-5.55416	0.511734	0.50001
Dendritic.cells	ZFP35	0.392057	2.543067	1.318634	0.190697	-4.89715	0.533218	0.530505
Dendritic.cells	NHLRC2	0.158753	4.994885	1.318223	0.190834	-5.63686	0.508671	0.495618
Dendritic.cells	MTF2	0.183683	5.644163	1.318166	0.190853	-5.52713	0.502414	0.486743
Dendritic.cells	UBA3	0.244781	4.076756	1.318045	0.190893	-5.17324	0.517691	0.508497
Dendritic.cells	UBALD2	0.196498	7.028455	1.317884	0.190947	-5.75281	0.489407	0.468394
Dendritic.cells	RGS1	-0.35976	5.563816	-1.31782	0.190969	-5.60053	0.503183	0.487887
Dendritic.cells	BET1	-0.22946	4.246255	-1.31761	0.191037	-5.34022	0.516011	0.506223
Dendritic.cells	PQBP1	-0.26552	4.6495	-1.31759	0.191045	-5.21618	0.51204	0.500573
Dendritic.cells	PPP1R7	0.213217	4.409711	1.317527	0.191066	-5.25261	0.514397	0.503925
Dendritic.cells	N6AMT1	-0.44	2.597729	-1.31705	0.191224	-4.87753	0.532655	0.530175
Dendritic.cells	HDHD5	0.403168	2.842941	1.317038	0.191229	-4.98361	0.530137	0.526571
Dendritic.cells	CASP8	0.205389	5.513709	1.316988	0.191246	-5.40541	0.503663	0.488847
Dendritic.cells	TLR3	-0.79476	1.534056	-1.31697	0.191252	-4.7092	0.543751	0.546089
Dendritic.cells	GAB1	0.60333	4.473058	1.316424	0.191434	-5.02629	0.514135	0.503422
Dendritic.cells	TMEM79	0.634433	0.778123	1.316136	0.191531	-4.71862	0.552337	0.558012
Dendritic.cells	6430548M	-0.53112	2.407786	-1.31569	0.191679	-4.7956	0.535409	0.533454
Dendritic.cells	LYSMD3	-0.20258	4.927993	-1.31528	0.191817	-5.48383	0.510222	0.49751
Dendritic.cells	NRG4	0.742552	2.017195	1.315007	0.191908	-4.75352	0.539629	0.53967
Dendritic.cells	UTP14B	-0.47446	3.081232	-1.31493	0.191933	-4.84941	0.528637	0.523933
Dendritic.cells	PLPP5	0.285043	3.394162	1.314883	0.191949	-5.1771	0.525458	0.51942
Dendritic.cells	PINK1	0.233525	5.115722	1.314742	0.191997	-5.50598	0.508396	0.495179
Dendritic.cells	POLR3E	-0.21088	3.918387	-1.31468	0.192019	-5.3689	0.520186	0.511965
Dendritic.cells	5430427M	0.663283	1.237849	1.314265	0.192157	-4.7598	0.547868	0.551762
Dendritic.cells	3300005D(	-0.46133	0.756323	-1.31422	0.192171	-4.961	0.553029	0.559218
Dendritic.cells	UPB1	0.178304	3.0781	1.314141	0.192198	-5.85281	0.528679	0.524269
Dendritic.cells	CCDC73	-0.3143	3.100981	-1.31406	0.192224	-5.05172	0.528445	0.523935
Dendritic.cells	CAMK2A	0.451215	0.846737	1.313855	0.192294	-4.7915	0.552055	0.557822
Dendritic.cells	TMX3	-0.15417	5.505888	-1.31381	0.192308	-5.66258	0.504637	0.490011
Dendritic.cells	GDF11	0.718426	1.639394	1.312704	0.19268	-4.67549	0.544343	0.546

Dendritic.cells	CD27	0.447465	3.318035	1.312616	0.19271	-4.96127	0.526949	0.521095
Dendritic.cells	SLC22A1	0.790352	0.740269	1.312612	0.192711	-4.65873	0.553949	0.559812
Dendritic.cells	REC114	-0.42372	3.19631	-1.31237	0.192792	-4.9855	0.52828	0.522948
Dendritic.cells	GM30054	0.591157	1.953461	1.312182	0.192855	-4.81244	0.541173	0.541396
Dendritic.cells	GFRA1	-0.60324	4.918403	-1.31189	0.192955	-5.01388	0.511281	0.49861
Dendritic.cells	ETF1	-0.11195	7.536791	-1.31171	0.193015	-6.11359	0.48655	0.463619
Dendritic.cells	MIR155HG	-0.34816	4.086438	-1.31148	0.19309	-5.52811	0.519537	0.510424
Dendritic.cells	GPC6	0.986191	1.763476	1.311305	0.19315	-4.72168	0.543364	0.544505
Dendritic.cells	HEMGN	0.954669	0.35427	1.311214	0.193181	-4.66338	0.558476	0.566246
Dendritic.cells	CLN5	-0.21818	4.008222	-1.31113	0.193211	-5.3455	0.520317	0.511543
Dendritic.cells	TPCN2	-0.51959	2.597655	-1.31098	0.193258	-4.80039	0.534654	0.53202
Dendritic.cells	CDK5RAP3	0.199773	4.379321	1.31074	0.19334	-5.47008	0.51672	0.506382
Dendritic.cells	SEH1L	0.160929	5.334868	1.31009	0.193559	-5.54829	0.507698	0.493331
Dendritic.cells	ROR1	0.481076	1.374979	1.309765	0.193669	-4.99232	0.547934	0.550791
Dendritic.cells	AMDHD2	0.302412	3.693973	1.309714	0.193686	-5.11256	0.523903	0.516388
Dendritic.cells	FKBP1A	-0.1241	7.336878	-1.30971	0.193687	-6.05631	0.4888	0.466588
Dendritic.cells	RMDN2	0.534009	1.873328	1.309658	0.193705	-4.81385	0.542657	0.543214
Dendritic.cells	CCDC61	0.371689	2.934126	1.309219	0.193853	-4.95509	0.531819	0.527472
Dendritic.cells	XRCC4	0.187873	5.185209	1.30917	0.193869	-5.55794	0.50933	0.495438
Dendritic.cells	KCNN4	0.342364	3.850661	1.308837	0.193982	-5.3319	0.522687	0.514218
Dendritic.cells	SNHG17	0.464736	1.368538	1.308545	0.19408	-4.83	0.548421	0.551053
Dendritic.cells	MRPS17	0.204128	5.224333	1.308512	0.194091	-5.42421	0.509157	0.495002
Dendritic.cells	GRAP2	-0.21802	4.975054	-1.308	0.194263	-5.85329	0.511787	0.498656
Dendritic.cells	TDP2	-0.31751	4.340054	-1.308	0.194263	-5.08639	0.518043	0.50755
Dendritic.cells	PARD3	-0.50676	2.300134	-1.30774	0.19435	-4.98835	0.538918	0.537327
Dendritic.cells	RPA2	0.417427	4.645282	1.307575	0.194407	-5.10201	0.515152	0.503383
Dendritic.cells	BIN3	0.191908	5.271025	1.307346	0.194485	-5.54073	0.509108	0.494764
Dendritic.cells	GM37168	-0.61923	0.65738	-1.307	0.194602	-4.7311	0.55656	0.562672
Dendritic.cells	PUM2	0.095066	7.324014	1.306975	0.19461	-6.05491	0.489725	0.467411
Dendritic.cells	S1PR4	0.309287	4.183137	1.30687	0.194646	-5.25954	0.51986	0.510177
Dendritic.cells	LIMK1	0.384097	2.1198	1.306593	0.19474	-5.02515	0.541088	0.540442
Dendritic.cells	LRIF1	0.233254	4.523869	1.305926	0.194965	-5.20176	0.516872	0.50571
Dendritic.cells	CBX5	0.263558	5.103912	1.305883	0.19498	-5.39323	0.511173	0.497616
Dendritic.cells	NICN1	-0.7088	0.820121	-1.30574	0.195029	-4.67117	0.555223	0.560612
Dendritic.cells	PEX6	0.302359	4.039128	1.305721	0.195035	-5.21398	0.521698	0.512607
Dendritic.cells	TSIX	-2.81043	1.906429	-1.30557	0.195084	-4.85584	0.543624	0.543998
Dendritic.cells	RNF215	0.324418	2.627986	1.305219	0.195205	-4.98056	0.536281	0.533306
Dendritic.cells	HRH1	0.354757	-0.56863	1.304821	0.19534	-5.02224	0.570853	0.582962
Dendritic.cells	MCRIP2	0.510574	2.015335	1.304793	0.195349	-4.77923	0.542815	0.542594
Dendritic.cells	EDC3	-0.27826	3.628178	-1.30386	0.195666	-5.13109	0.526797	0.519185
Dendritic.cells	GM17056	-0.7327	2.866748	-1.3037	0.195719	-4.74948	0.534586	0.530302
Dendritic.cells	RBL1	0.352979	4.466968	1.303587	0.195759	-5.12863	0.518383	0.507241
Dendritic.cells	WDR7	-0.22826	5.188411	-1.30354	0.195775	-5.53977	0.511283	0.497155
Dendritic.cells	9530077CC	-0.7263	1.213471	-1.30308	0.195929	-4.68753	0.552146	0.555458
Dendritic.cells	BMP2K	0.094563	6.936172	1.302829	0.196016	-6.273	0.494737	0.47383
Dendritic.cells	VAMP3	-0.13995	5.320751	-1.3026	0.196093	-5.63975	0.510134	0.495715
Dendritic.cells	PEX26	-0.61106	1.217016	-1.3025	0.196128	-4.77779	0.552108	0.555716

Dendritic.cells	VKORC1	-0.31268	4.701765	-1.30232	0.196189	-5.30641	0.516199	0.504421
Dendritic.cells	LRRC20	0.46507	2.057401	1.302305	0.196194	-4.86856	0.543171	0.542935
Dendritic.cells	GOLGA1	0.239207	3.427325	1.302292	0.196199	-5.2695	0.528982	0.522635
Dendritic.cells	AZIN1	-0.16911	6.940521	-1.30195	0.196315	-5.88446	0.494696	0.474094
Dendritic.cells	CRAMP1L	0.212743	4.984741	1.301771	0.196376	-5.38364	0.513415	0.50069
Dendritic.cells	MAP3K4	0.249437	4.074187	1.301416	0.196497	-5.27073	0.522444	0.513598
Dendritic.cells	ZBTB34	0.298676	3.592868	1.301299	0.196537	-5.17622	0.527299	0.520584
Dendritic.cells	GM34983	0.690012	0.754004	1.300994	0.196641	-4.70899	0.557109	0.563414
Dendritic.cells	GM12992	-0.72516	2.041763	-1.30099	0.196642	-4.71754	0.543336	0.543607
Dendritic.cells	PDS5A	0.105533	7.411745	1.300943	0.196658	-6.08535	0.490321	0.468141
Dendritic.cells	JMY	-0.17063	6.282387	-1.30087	0.196682	-5.89832	0.500893	0.483093
Dendritic.cells	TSR3	0.37689	3.569257	1.300785	0.196712	-5.03921	0.527539	0.520993
Dendritic.cells	TPP2	-0.12224	7.082191	-1.30075	0.196723	-5.92048	0.493375	0.472455
Dendritic.cells	OPHN1	-0.28826	2.997504	-1.30071	0.196736	-5.46763	0.533384	0.529348
Dendritic.cells	TMTC4	-0.63199	1.357794	-1.3007	0.19674	-4.76323	0.550599	0.554042
Dendritic.cells	GM10974	0.490086	0.824708	1.30049	0.196813	-4.83768	0.556411	0.562376
Dendritic.cells	FADS1	-0.37969	3.059286	-1.30026	0.196891	-5.0254	0.532896	0.528609
Dendritic.cells	LARP7	-0.15232	5.919166	-1.29966	0.197097	-5.65939	0.504701	0.488392
Dendritic.cells	CYP27A1	0.27022	2.194427	1.299436	0.197172	-5.36566	0.5421	0.541737
Dendritic.cells	MAP1LC3A	-0.24571	5.476214	-1.29942	0.197177	-5.60682	0.508971	0.494452
Dendritic.cells	GTPBP2	0.221885	4.751688	1.299338	0.197206	-5.43666	0.516058	0.504527
Dendritic.cells	NFATC2IP	-0.43218	3.151572	-1.29933	0.19721	-4.88976	0.532163	0.527504
Dendritic.cells	ZFP395	0.345086	4.289929	1.298997	0.197322	-5.08179	0.520736	0.511136
Dendritic.cells	KANSL2	-0.18929	5.119718	-1.29894	0.197343	-5.49701	0.512536	0.499467
Dendritic.cells	PRSS57	0.478701	-0.03051	1.298743	0.197409	-4.86007	0.566194	0.576418
Dendritic.cells	GM15247	0.908232	0.995367	1.298562	0.197471	-4.65512	0.554974	0.560316
Dendritic.cells	LRRC8B	-0.33532	3.358799	-1.29852	0.197486	-4.964	0.530138	0.524677
Dendritic.cells	WDR81	-0.22789	3.600126	-1.29825	0.197576	-5.35521	0.527779	0.521263
Dendritic.cells	IRAK1BP1	0.836771	0.288762	1.298023	0.197655	-4.65321	0.562778	0.571635
Dendritic.cells	TULP4	-0.15129	5.928036	-1.29767	0.197777	-5.84321	0.504802	0.488816
Dendritic.cells	GNMT	0.600297	4.232803	1.297638	0.197787	-5.15496	0.521403	0.512449
Dendritic.cells	B130055M	0.448552	2.013314	1.297622	0.197793	-4.90302	0.544206	0.545091
Dendritic.cells	AP4S1	0.186333	4.920297	1.297514	0.19783	-5.46057	0.514587	0.502737
Dendritic.cells	ZFP850	-0.5508	1.010206	-1.29736	0.197884	-4.72672	0.554917	0.560509
Dendritic.cells	GM42722	0.452467	2.335621	1.297016	0.198	-5.0133	0.540818	0.540303
Dendritic.cells	ASPA	-0.92568	1.375432	-1.29702	0.198	-4.69351	0.550987	0.55492
Dendritic.cells	RARRES1	0.916564	0.889058	1.297007	0.198003	-4.6705	0.556228	0.56247
Dendritic.cells	ABCG1	-0.20598	6.591271	-1.29632	0.198239	-5.89529	0.49874	0.480136
Dendritic.cells	1500004A1	0.375248	2.078931	1.296305	0.198244	-4.94492	0.543782	0.544384
Dendritic.cells	ZXDB	0.24844	3.1887	1.296302	0.198245	-5.31771	0.53224	0.527836
Dendritic.cells	2310022B0	-0.20319	2.488564	-1.29546	0.198534	-5.34575	0.54007	0.538498
Dendritic.cells	RSPRY1	0.13797	5.833379	1.295396	0.198556	-5.7593	0.506508	0.490651
Dendritic.cells	INO80B	-0.26609	4.146114	-1.2943	0.198934	-5.22784	0.523963	0.5146
Dendritic.cells	TMEM134	0.137956	6.132006	1.293471	0.199217	-5.88484	0.505076	0.487075
Dendritic.cells	LCA5	0.366374	1.313513	1.292905	0.199412	-5.09049	0.554444	0.557208
Dendritic.cells	CHID1	0.30853	2.453126	1.29267	0.199493	-5.03299	0.542322	0.539898
Dendritic.cells	MARK2	0.113028	6.879023	1.292564	0.19953	-5.97967	0.4983	0.477348

Dendritic.cells	GM31728	0.796848	-0.17439	1.292544	0.199537	-4.66923	0.57077	0.580816
Dendritic.cells	CHP2	-1.07309	-0.09865	-1.29214	0.199676	-4.6755	0.570186	0.579749
Dendritic.cells	FAM71E1	0.585467	0.837279	1.291482	0.199903	-4.8356	0.56019	0.564974
Dendritic.cells	FAM199X	0.356544	2.776803	1.291386	0.199936	-5.04741	0.539499	0.535319
Dendritic.cells	ZFP653	-0.33876	3.131364	-1.29138	0.199937	-5.04542	0.535819	0.530065
Dendritic.cells	LSM6	0.154989	6.681324	1.290946	0.200088	-5.9313	0.500941	0.48035
Dendritic.cells	SIAH1A	-0.19391	5.079563	-1.29064	0.200193	-5.52866	0.516567	0.502389
Dendritic.cells	EIF5A	0.123402	9.061608	1.290169	0.200356	-6.35363	0.479305	0.449758
Dendritic.cells	RPGRIP1L	-0.47865	1.613197	-1.28996	0.200427	-4.82901	0.552364	0.553352
Dendritic.cells	DDX3X	-0.14668	7.320412	-1.28988	0.200455	-6.06003	0.495191	0.472087
Dendritic.cells	METTL3	-0.31106	3.319562	-1.28979	0.200488	-5.05831	0.534427	0.527704
Dendritic.cells	PRPF19	0.224466	5.053951	1.289776	0.200492	-5.33832	0.51694	0.502834
Dendritic.cells	CD302	-0.41434	5.847821	-1.28967	0.20053	-5.36326	0.509182	0.491849
Dendritic.cells	AGL	0.346259	3.963587	1.28933	0.200646	-5.20715	0.528026	0.518396
Dendritic.cells	CDV3	0.12371	7.090195	1.288999	0.200761	-6.05617	0.497678	0.475334
Dendritic.cells	CES1G	-0.81624	0.85155	-1.28868	0.200872	-4.70538	0.561056	0.565533
Dendritic.cells	TET3	-0.12868	6.827605	-1.2886	0.200898	-5.97639	0.500215	0.478923
Dendritic.cells	FYN	-0.13785	7.843798	-1.28838	0.200976	-6.60106	0.490733	0.465598
Dendritic.cells	THAP3	0.27465	3.799217	1.288366	0.20098	-5.21128	0.529939	0.521086
Dendritic.cells	DNAJC4	-0.35063	3.061383	-1.28814	0.201057	-5.01369	0.537544	0.531981
Dendritic.cells	TSPAN31	0.18867	4.424978	1.288036	0.201094	-5.63872	0.523634	0.512203
Dendritic.cells	OPA3	-0.20409	5.128547	-1.28789	0.201145	-5.4723	0.516637	0.502293
Dendritic.cells	RRAD	-0.49726	2.552475	-1.28773	0.201199	-4.9105	0.542854	0.539691
Dendritic.cells	RBM4B	-0.14925	5.747385	-1.28749	0.201283	-5.80808	0.510582	0.493878
Dendritic.cells	MVP	0.183961	5.108561	1.287305	0.201348	-5.62722	0.516834	0.502807
Dendritic.cells	GM19585	-0.77394	2.331819	-1.28726	0.201363	-4.81851	0.545177	0.543201
Dendritic.cells	C1GALT1	0.141975	6.353776	1.287226	0.201375	-6.01958	0.504739	0.485675
Dendritic.cells	2310008N1	-0.77981	0.80481	-1.28693	0.201476	-4.66606	0.561731	0.566803
Dendritic.cells	HMG20B	0.127822	5.119833	1.286792	0.201526	-5.77563	0.516853	0.50278
Dendritic.cells	VPS26B	0.244139	3.835261	1.286661	0.201571	-5.32643	0.529726	0.521096
Dendritic.cells	ABLIM2	0.771277	-0.47962	1.286397	0.201663	-4.66352	0.576128	0.587538
Dendritic.cells	RNF139	0.173967	5.218473	1.285992	0.201803	-5.69484	0.516227	0.501598
Dendritic.cells	MSRB1	0.317916	5.958368	1.28577	0.201881	-5.29208	0.509082	0.491448
Dendritic.cells	APRT	0.278784	6.416788	1.285237	0.202066	-5.52421	0.505016	0.485436
Dendritic.cells	H3F3B	0.091218	9.934295	1.284436	0.202345	-6.49133	0.47328	0.440409
Dendritic.cells	CGGBP1	0.12102	6.845343	1.284262	0.202405	-5.9933	0.501424	0.479946
Dendritic.cells	ARL4C	-0.2792	5.243589	-1.28426	0.202407	-5.38441	0.516903	0.501833
Dendritic.cells	PDCD1LG2	0.804517	1.775707	1.283902	0.202531	-4.68102	0.552773	0.552821
Dendritic.cells	3300002A1	-0.61197	0.451891	-1.28371	0.202597	-4.7216	0.5672	0.573598
Dendritic.cells	GNL1	-0.22226	4.299889	-1.28364	0.202623	-5.35679	0.526498	0.515394
Dendritic.cells	MESD	0.226186	4.085411	1.283451	0.202688	-5.26343	0.528711	0.518537
Dendritic.cells	MEX3B	-0.50969	2.233947	-1.28307	0.20282	-4.84321	0.548152	0.546098
Dendritic.cells	EEF1AKNM	0.50452	2.318289	1.282701	0.202949	-4.79013	0.547392	0.544908
Dendritic.cells	SMYD1	0.495828	0.29641	1.282551	0.203002	-4.93795	0.569342	0.57643
Dendritic.cells	2010109A1	-0.43532	2.521995	-1.28251	0.203015	-4.94155	0.545238	0.541876
Dendritic.cells	GRK3	0.364536	2.91909	1.281607	0.203332	-5.15685	0.541457	0.536104
Dendritic.cells	PRELID3B	-0.16872	5.574007	-1.28147	0.20338	-5.62469	0.514584	0.497916

Dendritic.cells	TBL1XR1	0.109109	6.669065	1.281366	0.203415	-6.08252	0.504003	0.482969
Dendritic.cells	NLGN2	-0.73703	0.349554	-1.28136	0.203418	-4.77176	0.569159	0.575796
Dendritic.cells	HECA	0.177205	6.23169	1.280787	0.203618	-5.66033	0.508194	0.489126
Dendritic.cells	G530011O(	0.772612	1.80368	1.280786	0.203618	-4.74818	0.553275	0.553271
Dendritic.cells	ZSWIM3	-0.58936	1.503758	-1.28041	0.203748	-4.79017	0.556506	0.558022
Dendritic.cells	1300017J0.	-0.52018	3.006144	-1.28022	0.203815	-4.91998	0.540548	0.535189
Dendritic.cells	ZFP974	0.702674	0.877863	1.280207	0.203821	-4.74561	0.563325	0.567812
Dendritic.cells	MAT2A	-0.13677	6.522913	-1.28018	0.203831	-5.81991	0.505398	0.485281
Dendritic.cells	DRG2	-0.30856	3.507943	-1.28015	0.203839	-5.0386	0.535345	0.527768
Dendritic.cells	SMG9	-0.23625	4.52517	-1.27995	0.203909	-5.32903	0.524992	0.513048
Dendritic.cells	HRH4	-0.83433	0.27047	-1.27991	0.203925	-4.67068	0.570038	0.577484
Dendritic.cells	CTBP2	-0.42184	3.249836	-1.2799	0.203928	-4.98104	0.538014	0.531586
Dendritic.cells	SACM1L	0.173652	5.504292	1.279824	0.203955	-5.6502	0.515267	0.499252
Dendritic.cells	VTI1B	0.135033	5.690918	1.279794	0.203965	-5.64547	0.51344	0.496665
Dendritic.cells	PPP2R5E	-0.1036	6.820761	-1.27973	0.203987	-6.01106	0.50256	0.481285
Dendritic.cells	RAB11FIP1	0.601382	4.829945	1.279584	0.204039	-4.99547	0.521948	0.50875
Dendritic.cells	NOSIP	-0.1947	5.158105	-1.27911	0.204206	-5.44157	0.518716	0.5043
Dendritic.cells	LRRC75AOS	-0.67196	0.512247	-1.27908	0.204215	-4.72598	0.567395	0.573872
Dendritic.cells	BAD	0.267834	3.912661	1.279015	0.204238	-5.26144	0.531233	0.522103
Dendritic.cells	FUZ	-0.59693	1.08456	-1.279	0.204245	-4.79144	0.561102	0.564826
Dendritic.cells	SMAP2	0.118105	6.821529	1.278842	0.204299	-5.97912	0.502603	0.481522
Dendritic.cells	DDX10	0.143138	5.436521	1.278591	0.204387	-5.726	0.516083	0.5005
Dendritic.cells	ARHGAP5	0.183911	4.809305	1.278183	0.20453	-5.92356	0.522519	0.509494
Dendritic.cells	RAB6B	0.218416	1.697022	1.278067	0.204571	-5.51233	0.554818	0.555621
Dendritic.cells	GM17354	0.485008	1.393239	1.277819	0.204658	-4.83227	0.558118	0.560354
Dendritic.cells	ASTE1	0.364323	2.753642	1.277773	0.204674	-4.99707	0.543595	0.539554
Dendritic.cells	F5	0.714076	4.094976	1.277339	0.204827	-4.98416	0.529952	0.519866
Dendritic.cells	TFCP2	-0.45679	1.971586	-1.27696	0.20496	-4.87632	0.552114	0.551558
Dendritic.cells	RPH3AL	0.688447	1.299189	1.276936	0.204968	-4.68738	0.559371	0.561963
Dendritic.cells	0610010F0	-0.32938	4.458879	-1.27684	0.205001	-5.17787	0.526267	0.514681
Dendritic.cells	EGLN3	0.53666	3.294296	1.276533	0.20511	-5.00711	0.538176	0.53167
Dendritic.cells	AGPAT3	-0.2703	5.164345	-1.27647	0.205132	-5.32051	0.519218	0.504703
Dendritic.cells	AQP9	-0.67186	2.330322	-1.27628	0.205198	-4.7696	0.54829	0.5462
Dendritic.cells	AU020206	0.22171	5.320744	1.276	0.205297	-5.51119	0.517671	0.502695
Dendritic.cells	CDK18	-0.77532	-0.22726	-1.27579	0.20537	-4.68097	0.576278	0.586674
Dendritic.cells	SH3TC1	-0.31276	2.768133	-1.27564	0.205424	-5.14959	0.543667	0.539882
Dendritic.cells	AHCYL1	0.139725	5.179051	1.27535	0.205526	-5.63579	0.519072	0.504844
Dendritic.cells	GM2245	0.344524	1.558462	1.275303	0.205543	-5.21439	0.556559	0.558362
Dendritic.cells	2010320M	-0.38862	2.480423	-1.27522	0.205571	-5.00366	0.5467	0.544231
Dendritic.cells	GM16536	-0.46251	1.530823	-1.27505	0.205632	-4.83687	0.556858	0.558791
Dendritic.cells	SBDS	0.141521	5.561152	1.275028	0.20564	-5.6808	0.515306	0.499512
Dendritic.cells	SEM1	0.101598	9.100384	1.274929	0.205675	-6.35087	0.482085	0.452652
Dendritic.cells	SF3B3	0.128711	6.034328	1.274844	0.205705	-5.8036	0.510692	0.492975
Dendritic.cells	INSR	0.221089	5.770425	1.274734	0.205743	-5.64939	0.513258	0.496611
Dendritic.cells	BABAM2	0.128171	7.216423	1.274719	0.205749	-5.99494	0.4994	0.477016
Dendritic.cells	CTIF	0.20385	2.596638	1.274671	0.205766	-5.37665	0.545472	0.542474
Dendritic.cells	GAK	-0.11767	6.280821	-1.2746	0.205789	-5.87989	0.508309	0.489604

Dendritic.cells	DNAJC5	-0.09576	6.766699	-1.27431	0.205893	-5.97698	0.503656	0.483138
Dendritic.cells	CDKL5	0.592491	2.066071	1.274257	0.205912	-4.85452	0.551104	0.550692
Dendritic.cells	LIG4	-0.72523	1.902715	-1.27421	0.205927	-4.71547	0.552852	0.553204
Dendritic.cells	NECTIN1	-0.74847	1.073651	-1.27404	0.205988	-4.70742	0.561863	0.566189
Dendritic.cells	CWC15	0.126564	6.384372	1.273523	0.20617	-5.83334	0.507672	0.488669
Dendritic.cells	TRIM12A	0.206518	5.048891	1.272462	0.206545	-5.6009	0.521014	0.507587
Dendritic.cells	ABCC5	0.476253	4.301545	1.272405	0.206565	-5.01009	0.528517	0.518257
Dendritic.cells	RNH1	0.18516	5.988026	1.272354	0.206583	-5.78878	0.511781	0.494487
Dendritic.cells	ANP32B	0.146339	8.191649	1.272249	0.206621	-6.18012	0.490947	0.465066
Dendritic.cells	MCAM	-0.53267	1.173297	-1.27221	0.206635	-4.94439	0.561445	0.565372
Dendritic.cells	LRRC41	-0.18578	4.919545	-1.27219	0.206642	-5.58607	0.522303	0.509418
Dendritic.cells	PYGM	-0.46873	4.300481	-1.27215	0.206655	-5.06317	0.528528	0.51828
Dendritic.cells	ALAD	-0.40188	4.057214	-1.2721	0.206672	-5.09751	0.531	0.521815
Dendritic.cells	DNAH7A	0.796421	-0.11769	1.271787	0.206784	-4.68652	0.575939	0.586158
Dendritic.cells	CLIC5	-0.79176	0.878367	-1.27111	0.207024	-4.69983	0.565371	0.570256
Dendritic.cells	MGMT	0.388496	3.273792	1.270962	0.207076	-5.07984	0.539733	0.533586
Dendritic.cells	BLNK	-0.12646	7.200732	-1.27038	0.207283	-6.48502	0.501178	0.47859
Dendritic.cells	MPZL1	-0.58193	2.78686	-1.26982	0.207479	-4.92033	0.545562	0.541417
Dendritic.cells	CNP	0.186592	5.75023	1.26976	0.207502	-5.83621	0.515432	0.498577
Dendritic.cells	CMTM6	-0.18427	4.866252	-1.26876	0.207856	-5.49904	0.524962	0.511456
Dendritic.cells	SYNGR2	0.106014	6.111593	1.268055	0.208107	-6.21727	0.512959	0.494275
Dendritic.cells	4930481A1	0.541222	1.854963	1.268045	0.208111	-4.79888	0.556638	0.556434
Dendritic.cells	HCFC1R1	0.198262	5.33224	1.267863	0.208176	-5.53191	0.520619	0.505117
Dendritic.cells	ARL3	0.214331	4.04145	1.267803	0.208197	-5.45296	0.533634	0.523622
Dendritic.cells	MAN2A2	0.147574	4.595669	1.267731	0.208223	-5.88972	0.527995	0.515612
Dendritic.cells	GM4117	0.558537	0.660809	1.26722	0.208404	-4.79729	0.570082	0.575434
Dendritic.cells	NSF	0.148876	6.770124	1.267009	0.208479	-6.02054	0.506988	0.485585
Dendritic.cells	NIPAL1	0.722432	1.060638	1.266714	0.208584	-4.71363	0.56588	0.569334
Dendritic.cells	FAM221A	0.593714	1.467642	1.266308	0.208729	-4.75487	0.561598	0.563059
Dendritic.cells	FLOT2	0.218112	4.443449	1.266123	0.208795	-5.41016	0.530252	0.518341
Dendritic.cells	PXDC1	-0.74241	2.191629	-1.26594	0.208861	-4.79285	0.553764	0.551883
Dendritic.cells	MYBL1	-0.66442	1.840587	-1.2659	0.208875	-4.71925	0.557545	0.557296
Dendritic.cells	S100PBP	-0.1646	4.405401	-1.26584	0.208896	-5.58591	0.530639	0.518925
Dendritic.cells	SLX4IP	0.21134	4.853222	1.265288	0.209093	-5.50259	0.526482	0.512643
Dendritic.cells	MLEC	-0.16648	5.064037	-1.2648	0.209268	-5.64628	0.524682	0.509915
Dendritic.cells	ICAM2	-0.47978	4.282374	-1.26464	0.209324	-5.27035	0.532605	0.521197
Dendritic.cells	MCTP1	0.369723	5.535347	1.264252	0.209462	-5.682	0.520039	0.503433
Dendritic.cells	STK38	0.17033	6.531625	1.264203	0.20948	-5.74359	0.510296	0.489661
Dendritic.cells	ICK	0.489552	2.147459	1.264081	0.209524	-4.85988	0.555017	0.553207
Dendritic.cells	GPATCH8	-0.09611	7.002079	-1.26405	0.209535	-6.15813	0.50578	0.48329
Dendritic.cells	RNF111	0.128178	6.692893	1.263915	0.209583	-6.00429	0.508742	0.487551
Dendritic.cells	UBXN1	0.119861	6.759331	1.263409	0.209763	-5.90109	0.508423	0.486759
Dendritic.cells	HIST1H2AB	0.996427	1.438759	1.263161	0.209852	-4.70348	0.563163	0.564514
Dendritic.cells	RAB37	0.509891	2.340912	1.262661	0.210031	-4.82613	0.553516	0.550743
Dendritic.cells	TOR1A	0.222412	4.653633	1.262654	0.210034	-5.3607	0.529415	0.516416
Dendritic.cells	ARIH1	-0.11834	8.728541	-1.26262	0.210046	-6.36725	0.490164	0.461048
Dendritic.cells	ATP6V0E	0.104585	7.713213	1.261842	0.210324	-6.17179	0.499951	0.474474

Dendritic.cells	6330409D2	0.718797	0.333222	1.261841	0.210325	-4.69509	0.575989	0.582615
Dendritic.cells	BC035044	0.244005	5.146379	1.26176	0.210354	-5.75315	0.524853	0.509661
Dendritic.cells	POLB	0.162612	5.332268	1.261484	0.210453	-5.68322	0.523047	0.507091
Dendritic.cells	FAM221B	0.696308	0.343919	1.261428	0.210473	-4.71459	0.575925	0.582559
Dendritic.cells	GM5547	-0.16062	1.560402	-1.26061	0.210768	-5.79319	0.562718	0.563663
Dendritic.cells	BC051226	0.410583	2.179737	1.260603	0.210768	-4.89178	0.555997	0.554041
Dendritic.cells	SLIRP	0.197583	5.558884	1.260345	0.210861	-5.6555	0.52105	0.50438
Dendritic.cells	GM19684	0.758121	1.522121	1.260222	0.210905	-4.69131	0.563136	0.564349
Dendritic.cells	DPP10	-0.78746	0.411958	-1.25989	0.211023	-4.73508	0.575443	0.582154
Dendritic.cells	ARHGDIB	0.161212	8.995183	1.259883	0.211027	-6.30112	0.488393	0.458485
Dendritic.cells	PARP3	0.318622	2.362796	1.259839	0.211043	-5.20196	0.554029	0.551434
Dendritic.cells	SLC7A5	-0.18884	6.118367	-1.25968	0.211099	-5.97896	0.515539	0.496752
Dendritic.cells	RORA	-0.26473	5.020685	-1.25948	0.211172	-5.64722	0.526424	0.512256
Dendritic.cells	DPP3	0.220627	4.728026	1.259472	0.211175	-5.43509	0.529376	0.516446
Dendritic.cells	COX7B	0.131791	7.597144	1.259423	0.211192	-6.09982	0.501339	0.476826
Dendritic.cells	AKR1B3	-0.21553	5.402163	-1.25933	0.211225	-5.41452	0.522607	0.506899
Dendritic.cells	KIF9	0.375218	1.680578	1.259324	0.211228	-5.07299	0.561406	0.562205
Dendritic.cells	USB1	0.322891	3.245957	1.259219	0.211266	-5.16965	0.544658	0.538254
Dendritic.cells	MIB2	-0.3645	2.662247	-1.25846	0.211537	-5.04879	0.551364	0.5474
Dendritic.cells	UBE2F	0.148054	6.306137	1.258359	0.211575	-5.86077	0.514205	0.494644
Dendritic.cells	IDH3G	0.300588	5.124044	1.258138	0.211654	-5.26965	0.525896	0.511273
Dendritic.cells	AHR	-0.20186	4.719836	-1.2581	0.211668	-6.00727	0.529973	0.517061
Dendritic.cells	F630028O1	0.621763	1.027185	1.257519	0.211877	-4.79189	0.569528	0.573317
Dendritic.cells	CEP70	0.375266	3.195368	1.257422	0.211912	-5.02121	0.546095	0.539803
Dendritic.cells	STFA1	0.82734	5.975982	1.257089	0.212032	-5.30282	0.517903	0.499683
Dendritic.cells	ZFP719	0.381584	2.119349	1.257023	0.212056	-4.93677	0.557691	0.556299
Dendritic.cells	SNX20	0.21456	5.514815	1.256778	0.212144	-5.54487	0.522548	0.506224
Dendritic.cells	NTM	0.731277	0.913874	1.256655	0.212189	-4.72977	0.570999	0.575396
Dendritic.cells	CDA	-0.63037	0.894078	-1.25633	0.212307	-4.82734	0.571405	0.575883
Dendritic.cells	ACAT3	-0.69807	1.453412	-1.25608	0.212397	-4.76183	0.565309	0.567069
Dendritic.cells	MRPL17	0.173756	5.426857	1.25596	0.212439	-5.6077	0.523677	0.507778
Dendritic.cells	ZDHHC6	0.220854	4.609891	1.255558	0.212584	-5.40078	0.532056	0.519546
Dendritic.cells	FKBP1B	0.560405	0.793011	1.255412	0.212637	-4.80553	0.572778	0.577795
Dendritic.cells	PWWP2A	0.175128	5.141602	1.255339	0.212663	-5.66109	0.526677	0.511968
Dendritic.cells	HSPB6	0.717183	0.40248	1.255234	0.212701	-4.71978	0.577157	0.584122
Dendritic.cells	HTRA2	-0.29455	3.681601	-1.25512	0.212744	-5.03758	0.541617	0.533316
Dendritic.cells	ZFP605	0.534605	1.633403	1.254491	0.21297	-4.87879	0.563807	0.564744
Dendritic.cells	GAS2L3	0.410821	4.330262	1.254445	0.212986	-5.09586	0.535214	0.523925
Dendritic.cells	GNS	0.120717	6.931874	1.254195	0.213077	-6.25155	0.509366	0.487401
Dendritic.cells	CMPK2	0.667105	3.672891	1.254127	0.213102	-4.93726	0.542013	0.533688
Dendritic.cells	TRAPPC6B	-0.11931	6.134079	-1.2541	0.213111	-5.82488	0.517115	0.498347
Dendritic.cells	BIRC3	0.171862	7.111789	1.253837	0.213206	-5.96838	0.507732	0.485055
Dendritic.cells	MED9	-0.35966	3.526994	-1.25357	0.213302	-5.05622	0.543635	0.536069
Dendritic.cells	KAT7	0.137253	5.68662	1.253479	0.213336	-5.76258	0.521624	0.504791
Dendritic.cells	EID2	0.715362	0.075956	1.253449	0.213347	-4.70827	0.581282	0.59004
Dendritic.cells	TLE4	-0.13299	7.814076	-1.25303	0.213497	-6.15072	0.501214	0.475907
Dendritic.cells	ARG1	0.669405	3.498182	1.253002	0.213509	-5.03298	0.544097	0.536661

Dendritic.cells	AARS2	0.61892	0.821033	1.252849	0.213564	-4.75767	0.573077	0.578139
Dendritic.cells	AP3B1	0.100813	7.329007	1.252351	0.213745	-6.14069	0.506132	0.482453
Dendritic.cells	TPP1	-0.18307	5.393356	-1.25207	0.213847	-5.71721	0.525166	0.509212
Dendritic.cells	FBXW9	-0.37934	2.418265	-1.25179	0.213947	-4.94498	0.556184	0.553255
Dendritic.cells	GM15478	0.233159	4.735611	1.251515	0.214048	-5.48847	0.531941	0.518801
Dendritic.cells	DLL4	-0.31557	0.717902	-1.25145	0.21407	-5.34278	0.574874	0.580138
Dendritic.cells	SEC11C	0.121078	7.287381	1.251164	0.214175	-6.14334	0.506786	0.483318
Dendritic.cells	CCL7	-1.33568	1.659903	-1.25107	0.21421	-4.74767	0.564433	0.565262
Dendritic.cells	COPS8	-0.17855	5.091019	-1.25105	0.214218	-5.4665	0.528341	0.513796
Dendritic.cells	PPM1B	0.147616	6.192574	1.250961	0.214249	-5.76228	0.517382	0.498308
Dendritic.cells	BTF3L4	-0.21033	4.269235	-1.25025	0.214506	-5.33646	0.53682	0.525888
Dendritic.cells	MRPL49	0.364379	3.778216	1.250142	0.214546	-5.06756	0.541905	0.533125
Dendritic.cells	SPATS2	-0.46013	2.720159	-1.25009	0.214566	-5.02002	0.55307	0.549053
Dendritic.cells	NCOR1	0.083522	8.098707	1.250064	0.214575	-6.26783	0.49922	0.472711
Dendritic.cells	DAPL1	0.644834	-1.31502	1.25003	0.214587	-4.69463	0.598321	0.614154
Dendritic.cells	ZFP961	0.428543	3.308556	1.250026	0.214589	-4.95495	0.546825	0.540138
Dendritic.cells	TTC28	-0.27076	4.798912	-1.24941	0.214814	-5.52416	0.531838	0.51838
Dendritic.cells	E230016K2	-0.72622	0.143576	-1.24917	0.214898	-4.70605	0.58204	0.590125
Dendritic.cells	SRRD	0.320961	3.465767	1.248863	0.215012	-5.06735	0.545751	0.538133
Dendritic.cells	OTUD5	0.15597	5.568497	1.248855	0.215015	-5.63841	0.524215	0.507539
Dendritic.cells	PAQR7	-0.61033	1.552734	-1.24867	0.215084	-4.8021	0.566375	0.567585
Dendritic.cells	HIST2H2AC	0.878318	2.376405	1.24838	0.215188	-4.79136	0.557541	0.554881
Dendritic.cells	CEP44	0.242634	3.531339	1.248095	0.215291	-5.18256	0.545256	0.537472
Dendritic.cells	SNRPD2	0.127427	6.391824	1.248073	0.215299	-5.93145	0.516266	0.496333
Dendritic.cells	IFNGR1	-0.21168	7.03854	-1.24797	0.215339	-5.70684	0.509993	0.487508
Dendritic.cells	RHOF	0.438376	3.558194	1.247802	0.215398	-5.02922	0.544999	0.537144
Dendritic.cells	TOMM40	0.165922	5.225725	1.247425	0.215536	-5.67775	0.528077	0.512919
Dendritic.cells	BIRC6	0.075187	7.8393	1.246443	0.215894	-6.25938	0.503031	0.477288
Dendritic.cells	PEX11G	-0.47282	2.394748	-1.24638	0.215919	-4.84862	0.558089	0.555418
Dendritic.cells	NUAK2	0.322923	4.289152	1.246292	0.215949	-5.14883	0.538088	0.526929
Dendritic.cells	SIRT2	-0.15445	5.688086	-1.24627	0.215956	-5.69625	0.523903	0.506802
Dendritic.cells	GTF2IRD1	0.41126	3.292146	1.246231	0.215972	-5.08471	0.5485	0.541761
Dendritic.cells	QPRT	-0.62537	2.660001	-1.24605	0.216037	-4.85982	0.555274	0.551451
Dendritic.cells	PLEKHA7	0.396938	2.084938	1.245815	0.216124	-5.06567	0.561585	0.560361
Dendritic.cells	TRP53COR1	-0.78631	1.008827	-1.24544	0.216259	-4.74945	0.573591	0.577509
Dendritic.cells	TMEM163	0.137807	6.026941	1.245335	0.216299	-6.30284	0.520796	0.502297
Dendritic.cells	GM7854	0.620936	0.52802	1.24514	0.21637	-4.83942	0.578991	0.585387
Dendritic.cells	PDK1	0.275448	4.164844	1.245122	0.216377	-5.2233	0.539638	0.529081
Dendritic.cells	TJP1	-0.36841	2.024999	-1.24499	0.216425	-5.11892	0.562377	0.561542
Dendritic.cells	IQCH	-0.90322	0.250926	-1.24483	0.216483	-4.72986	0.582152	0.59001
Dendritic.cells	PLK1	0.769035	3.704872	1.244398	0.216642	-4.94002	0.544721	0.536198
Dendritic.cells	SIDT1	-0.67095	3.376538	-1.24416	0.216729	-4.94382	0.54821	0.541232
Dendritic.cells	IL27RA	-0.66773	1.535873	-1.24409	0.216755	-4.8193	0.568085	0.569711
Dendritic.cells	4921509O0	-0.81575	0.064578	-1.24389	0.216828	-4.70729	0.584624	0.593515
Dendritic.cells	4921516A0	-0.56614	0.76983	-1.2438	0.216861	-4.79917	0.576633	0.582007
Dendritic.cells	DNAJA3	0.300964	3.76841	1.243512	0.216966	-5.18983	0.544247	0.535567
Dendritic.cells	PPP2R3D	0.333638	3.751015	1.243107	0.217115	-5.15074	0.544532	0.535959

Dendritic.cells	SH3BGRL3	0.123413	9.061448	1.243054	0.217134	-6.40471	0.492443	0.462314
Dendritic.cells	RHOT1	-0.13351	5.400436	-1.24299	0.217156	-5.77255	0.527609	0.51193
Dendritic.cells	ENDOG	-0.41178	2.943039	-1.24277	0.217239	-4.90445	0.553159	0.548226
Dendritic.cells	CHEK1	-0.49497	2.722134	-1.2423	0.217413	-4.90157	0.555586	0.55183
Dendritic.cells	GSTT2	-0.43961	3.554147	-1.24218	0.217455	-4.96757	0.546742	0.539248
Dendritic.cells	TECPR2	0.288222	3.905519	1.242104	0.217483	-5.25206	0.543061	0.534024
Dendritic.cells	NES	-0.68613	0.578345	-1.24201	0.217518	-4.82382	0.579201	0.585798
Dendritic.cells	ACBD6	0.139842	5.436733	1.241793	0.217597	-5.74016	0.527383	0.511819
Dendritic.cells	MRPS11	0.314024	3.950885	1.241728	0.217621	-5.21556	0.542588	0.533458
Dendritic.cells	FOXA3	-0.63621	1.425712	-1.24162	0.21766	-4.7782	0.569723	0.572289
Dendritic.cells	FMN1	-0.51968	3.350464	-1.24161	0.217663	-5.03068	0.548891	0.542461
Dendritic.cells	POMT1	-0.56218	2.21731	-1.24148	0.217711	-4.86639	0.561039	0.559874
Dendritic.cells	TMEM205	-0.42858	3.71868	-1.24121	0.217811	-5.09415	0.545141	0.53703
Dendritic.cells	MTCP1	0.611541	0.737147	1.240951	0.217906	-4.79328	0.577573	0.583552
Dendritic.cells	UCK1	-0.42119	3.265642	-1.24091	0.217921	-5.01591	0.549943	0.543938
Dendritic.cells	KIF21A	0.737347	0.515306	1.240749	0.217981	-4.71536	0.580104	0.587158
Dendritic.cells	THEMIS	-0.87227	1.160853	-1.24043	0.218099	-4.75439	0.572904	0.576904
Dendritic.cells	CRYZ	0.620352	1.288432	1.240426	0.2181	-4.74469	0.571484	0.574862
Dendritic.cells	1810062O1	-0.48197	2.0989	-1.23988	0.218301	-4.90018	0.562952	0.562323
Dendritic.cells	ATMIN	0.435111	2.969649	1.239734	0.218354	-4.97761	0.55356	0.54891
Dendritic.cells	NAA38	0.181889	5.467647	1.239445	0.218461	-5.6643	0.527804	0.512105
Dendritic.cells	PPIP5K1	-0.59829	1.776843	-1.23919	0.218553	-4.827	0.566725	0.567582
Dendritic.cells	AMZ2	0.22807	3.901939	1.238889	0.218666	-5.32313	0.543938	0.535048
Dendritic.cells	STXBP2	0.17016	5.31968	1.238675	0.218744	-5.68552	0.529378	0.514413
Dendritic.cells	RBMX	0.205524	4.578527	1.238398	0.218846	-5.43049	0.536926	0.52531
Dendritic.cells	4933406I1	0.561093	4.543564	1.238231	0.218908	-5.03673	0.537285	0.525896
Dendritic.cells	KBTBD4	-0.39833	2.78551	-1.23817	0.21893	-4.96201	0.555764	0.552289
Dendritic.cells	ABCB4	-0.44071	3.391454	-1.23807	0.218966	-5.06887	0.549305	0.543107
Dendritic.cells	CYP2D22	0.636821	0.864397	1.238034	0.218981	-4.77548	0.576871	0.582644
Dendritic.cells	CPSF7	0.138628	5.801172	1.237982	0.219	-5.75413	0.524548	0.507874
Dendritic.cells	HIST1H4M	0.650806	1.423329	1.237562	0.219155	-4.81358	0.57063	0.573783
Dendritic.cells	MBD5	0.11525	6.903175	1.237416	0.219209	-6.13074	0.513709	0.49265
Dendritic.cells	STRADB	0.328243	3.238382	1.237262	0.219266	-5.09292	0.550928	0.545562
Dendritic.cells	USP28	-0.17855	4.553264	-1.23724	0.219274	-5.59427	0.537185	0.525958
Dendritic.cells	RASSF8	-0.47325	2.215433	-1.23724	0.219274	-5.00657	0.561926	0.561309
Dendritic.cells	1700096K1	0.30801	2.613489	1.23721	0.219285	-5.20957	0.557614	0.555132
Dendritic.cells	BCLAF3	0.222549	4.966172	1.237126	0.219316	-5.42195	0.532961	0.519959
Dendritic.cells	GM20337	0.510441	1.078162	1.237057	0.219341	-4.89136	0.574474	0.579401
Dendritic.cells	SRMS	-0.73875	-0.72874	-1.2366	0.21951	-4.71047	0.595434	0.609385
Dendritic.cells	TAF6L	0.31839	2.800016	1.236286	0.219626	-5.11571	0.55599	0.55254
Dendritic.cells	HAUS8	-0.22121	4.722045	-1.23624	0.219642	-5.44244	0.535822	0.523761
Dendritic.cells	MACF1	0.103514	8.000182	1.235916	0.219763	-6.37081	0.503724	0.478226
Dendritic.cells	TOM1	0.138533	5.431971	1.235582	0.219887	-6.0549	0.528828	0.513777
Dendritic.cells	EXOC4	-0.08233	8.03297	-1.23554	0.219902	-6.37922	0.503458	0.477891
Dendritic.cells	FASTK	0.305984	3.15048	1.235461	0.219932	-5.1175	0.55247	0.547486
Dendritic.cells	PDIA4	-0.13332	5.653573	-1.23518	0.220036	-5.85958	0.526731	0.510741
Dendritic.cells	PARP11	0.350943	3.46828	1.234837	0.220163	-5.24645	0.549424	0.542959

Dendritic.cells	SLC25A37	0.442576	5.267981	1.234627	0.220241	-5.18703	0.530817	0.516458
Dendritic.cells	EFEMP2	0.433282	1.944258	1.234185	0.220405	-4.90968	0.565866	0.566736
Dendritic.cells	CIZ1	0.259976	3.940619	1.23411	0.220432	-5.24073	0.544475	0.536142
Dendritic.cells	PRPF6	0.15364	5.300623	1.234109	0.220433	-5.64918	0.530487	0.516224
Dendritic.cells	RABGGTA	-0.45292	2.703471	-1.23406	0.220452	-4.83118	0.557609	0.554923
Dendritic.cells	GATA3	-0.66064	1.116393	-1.23401	0.220469	-4.79334	0.575041	0.579968
Dendritic.cells	ZDHHC24	-0.59176	1.089708	-1.23347	0.220668	-4.82225	0.575563	0.580473
Dendritic.cells	GM26749	0.442811	2.222722	1.233406	0.220693	-4.93878	0.563039	0.562476
Dendritic.cells	5430416N(	-0.29117	3.547711	-1.23317	0.220783	-5.12258	0.548817	0.542245
Dendritic.cells	BTD	0.402455	2.37707	1.233018	0.220837	-5.02017	0.561359	0.560195
Dendritic.cells	GOPC	0.214813	4.545466	1.232961	0.220859	-5.32838	0.538405	0.527393
Dendritic.cells	FAM124A	-0.35776	1.64482	-1.23295	0.220864	-5.11305	0.569385	0.571717
Dendritic.cells	GM50373	0.71099	0.358844	1.232816	0.220912	-4.76072	0.583822	0.592511
Dendritic.cells	NRF1	0.120226	6.474245	1.232704	0.220954	-5.89024	0.518991	0.49982
Dendritic.cells	ZFP821	-0.38313	3.905173	-1.23167	0.221337	-4.99815	0.545879	0.537141
Dendritic.cells	EID2B	0.431921	1.703903	1.231529	0.221391	-4.85936	0.569598	0.571093
Dendritic.cells	TACO1OS	-0.44449	1.705089	-1.23116	0.221528	-4.97378	0.569647	0.57117
Dendritic.cells	DNAH12	-0.80545	1.676699	-1.23108	0.221558	-4.78791	0.569961	0.571652
Dendritic.cells	ZFP770	0.52045	1.760027	1.230941	0.22161	-4.85399	0.56904	0.570403
Dendritic.cells	IFI204	-0.4797	4.390085	-1.23093	0.221614	-5.3107	0.540891	0.530157
Dendritic.cells	DNAJC8	0.098421	6.901934	1.230542	0.221758	-5.95744	0.515797	0.494512
Dendritic.cells	CD200	0.191252	1.910379	1.230491	0.221777	-5.87228	0.567542	0.56823
Dendritic.cells	GNG5	0.119824	8.925654	1.23012	0.221915	-6.34398	0.496725	0.467619
Dendritic.cells	PGRMC1	0.236915	4.647619	1.23001	0.221956	-5.38895	0.538539	0.526755
Dendritic.cells	CPB2	-0.48607	3.209826	-1.22992	0.221991	-4.97157	0.553619	0.548261
Dendritic.cells	PGM2	-0.25149	4.115633	-1.22973	0.22206	-5.32347	0.544103	0.534663
Dendritic.cells	GM37401	0.570051	0.875344	1.229384	0.22219	-4.81523	0.579499	0.585162
Dendritic.cells	RPAIN	-0.27791	3.771433	-1.22916	0.222273	-5.16268	0.547905	0.539945
Dendritic.cells	PNKD	0.227065	4.867546	1.229058	0.222311	-5.49058	0.536514	0.523767
Dendritic.cells	BRAP	0.19001	4.924367	1.228941	0.222355	-5.50839	0.535931	0.522984
Dendritic.cells	ALKBH2	0.308731	2.187068	1.228788	0.222412	-5.06918	0.564918	0.564459
Dendritic.cells	PLAGL2	-0.21406	4.367209	-1.22866	0.222459	-5.43835	0.541675	0.53126
Dendritic.cells	NDUFC2	0.159597	6.741013	1.228582	0.222489	-5.88783	0.517744	0.49731
Dendritic.cells	ZFP422	0.237177	4.395092	1.228434	0.222544	-5.39184	0.541399	0.530905
Dendritic.cells	CSNK1E	0.213824	4.953409	1.228134	0.222656	-5.54276	0.535728	0.522822
Dendritic.cells	MKS1	-0.50514	1.092934	-1.22754	0.222879	-4.89861	0.577151	0.582309
Dendritic.cells	PDE4D	-0.24261	6.428002	-1.22747	0.222904	-5.87172	0.520911	0.501978
Dendritic.cells	HCFC1	-0.21914	4.949877	-1.2274	0.222932	-5.48282	0.535764	0.523113
Dendritic.cells	GM10790	0.239928	-0.77429	1.227334	0.222955	-5.39388	0.598591	0.613339
Dendritic.cells	ABCB10	0.372638	2.977675	1.227096	0.223044	-5.06011	0.556445	0.552675
Dendritic.cells	SOX4	0.39237	5.508952	1.227005	0.223078	-5.54287	0.530082	0.515107
Dendritic.cells	BLMH	0.132411	5.648784	1.226969	0.223092	-5.79974	0.528673	0.513105
Dendritic.cells	CD68	-0.18189	4.872198	-1.22695	0.2231	-5.99365	0.53656	0.524319
Dendritic.cells	SLC25A1	0.277678	3.772843	1.226938	0.223103	-5.30776	0.547987	0.540612
Dendritic.cells	ECI1	0.375168	3.920606	1.226771	0.223166	-4.99121	0.546433	0.538396
Dendritic.cells	CDK9	0.171115	5.578202	1.226737	0.223178	-5.65166	0.529383	0.514116
Dendritic.cells	FAM53A	0.280264	3.797306	1.226499	0.223267	-5.18962	0.547741	0.540317

Dendritic.cells	RNF149	0.24941	5.786169	1.226456	0.223283	-5.78216	0.527305	0.511224
Dendritic.cells	CCDC97	-0.28776	3.33761	-1.22623	0.223369	-5.14866	0.552636	0.547369
Dendritic.cells	ITPK1	0.175654	5.403224	1.226159	0.223394	-5.61708	0.531189	0.516828
Dendritic.cells	ZSCAN12	0.452239	1.093811	1.225852	0.22351	-4.86509	0.577252	0.582736
Dendritic.cells	GINM1	-0.15692	4.990227	-1.22582	0.223521	-5.63135	0.535454	0.522875
Dendritic.cells	STRAP	0.157792	6.174275	1.225624	0.223595	-5.82392	0.523533	0.506015
Dendritic.cells	MVB12A	0.091956	5.948563	1.225388	0.223683	-6.2801	0.525781	0.509322
Dendritic.cells	CCDC181	0.450488	1.514839	1.225358	0.223695	-4.92191	0.572556	0.576213
Dendritic.cells	GM49067	-0.54818	0.406337	-1.22521	0.223752	-4.8012	0.585051	0.594287
Dendritic.cells	BBS7	-0.70239	0.558111	-1.22501	0.223825	-4.7862	0.583321	0.591854
Dendritic.cells	ECSIT	0.220714	4.015765	1.225007	0.223826	-5.41898	0.545551	0.5376
Dendritic.cells	LDB1	-0.25752	4.482765	-1.22451	0.224011	-5.35072	0.540894	0.530891
Dendritic.cells	ZFP618	0.399693	0.531603	1.224265	0.224104	-5.10298	0.583845	0.592624
Dendritic.cells	SERTAD4	-0.76582	-0.0023	-1.22426	0.224108	-4.73199	0.589964	0.601473
Dendritic.cells	KCNK6	-0.38008	2.306704	-1.22425	0.224112	-5.04331	0.564044	0.564099
Dendritic.cells	TAX1BP1	0.112334	8.297296	1.223983	0.22421	-6.27595	0.503304	0.477521
Dendritic.cells	MTSS2	-0.38206	0.842863	-1.22379	0.224284	-5.14492	0.580498	0.58762
Dendritic.cells	ECHDC3	-0.76194	0.833138	-1.22345	0.224412	-4.76082	0.580659	0.587863
Dendritic.cells	2610301B2	-0.41874	1.613471	-1.22342	0.224421	-4.94212	0.571911	0.575254
Dendritic.cells	CLIP1	-0.18927	5.440971	-1.22334	0.224453	-5.64241	0.5313	0.517139
Dendritic.cells	NMRK1	0.285335	3.751312	1.2229	0.224617	-5.25182	0.54904	0.542133
Dendritic.cells	PMPCA	0.277413	4.024403	1.222689	0.224696	-5.24384	0.546238	0.53814
Dendritic.cells	PAIP1	0.129155	5.907952	1.222357	0.224821	-5.97506	0.527103	0.510812
Dendritic.cells	TPMT	-0.4699	2.097645	-1.22223	0.224868	-4.86642	0.567104	0.568003
Dendritic.cells	KMT2C	-0.10674	7.717211	-1.22193	0.224983	-6.26482	0.509413	0.485877
Dendritic.cells	ACTR3B	0.589182	-0.01555	1.221791	0.225034	-4.72506	0.590946	0.602493
Dendritic.cells	SOD3	0.663114	1.286857	1.221779	0.225039	-4.81715	0.576127	0.581091
Dendritic.cells	VAMP8	0.122034	7.209731	1.221673	0.225079	-6.08628	0.514301	0.49281
Dendritic.cells	H2-OA	-0.21585	3.19042	-1.22148	0.22515	-5.79915	0.555328	0.551242
Dendritic.cells	GM15832	-0.55082	1.685015	-1.22128	0.225226	-4.83757	0.571806	0.574846
Dendritic.cells	EXOSC4	0.315421	3.784732	1.220601	0.225483	-5.18059	0.549114	0.542506
Dendritic.cells	HUWE1	-0.116	7.155414	-1.22055	0.225501	-6.20221	0.514974	0.493915
Dendritic.cells	DROSHA	-0.26599	3.121263	-1.22032	0.225587	-5.15492	0.556174	0.55269
Dendritic.cells	ATL1	0.699145	0.498179	1.220284	0.225602	-4.78249	0.585214	0.59452
Dendritic.cells	BRD2	-0.10942	7.085393	-1.2202	0.225632	-6.13205	0.515655	0.495002
Dendritic.cells	UTP20	-0.35695	3.652111	-1.22011	0.225666	-5.2131	0.550516	0.544658
Dendritic.cells	IZUMO4	0.631324	1.453448	1.22006	0.225687	-4.85275	0.574428	0.579041
Dendritic.cells	HMGN1	0.138933	7.26929	1.219889	0.225751	-6.23146	0.51387	0.492579
Dendritic.cells	GM50163	-0.64994	0.458993	-1.21989	0.225752	-4.75477	0.585662	0.595337
Dendritic.cells	D430042O(	-0.21251	3.792114	-1.21968	0.225831	-5.44495	0.549036	0.542743
Dendritic.cells	CCP110	-0.45198	2.37539	-1.21962	0.225852	-4.92398	0.564248	0.564563
Dendritic.cells	SH3BGRL	0.133647	6.824052	1.219613	0.225855	-6.02132	0.518205	0.498812
Dendritic.cells	GM12353	0.563508	1.278675	1.219397	0.225937	-4.85156	0.576383	0.582155
Dendritic.cells	4930579G2	-0.4175	2.431609	-1.21927	0.225985	-4.93367	0.563634	0.563885
Dendritic.cells	GM50209	-0.63908	0.629068	-1.21915	0.226028	-4.75659	0.583722	0.592903
Dendritic.cells	RNF5	0.253314	4.122516	1.219063	0.226063	-5.3353	0.545563	0.538014
Dendritic.cells	NUP88	0.232062	4.421593	1.218869	0.226136	-5.36465	0.542444	0.533554

Dendritic.cells	ELOVL6	0.199993	4.451031	1.218844	0.226146	-5.80964	0.542138	0.533117
Dendritic.cells	MAOA	-0.5536	0.932781	-1.21867	0.226212	-4.92917	0.580319	0.587947
Dendritic.cells	FAM210A	-0.26835	3.636981	-1.21852	0.226268	-5.20015	0.550729	0.545386
Dendritic.cells	GPX7	0.74062	0.610424	1.218286	0.226356	-4.75091	0.58409	0.593401
Dendritic.cells	FAM219B	0.459966	2.367885	1.217957	0.226481	-4.94521	0.56453	0.565194
Dendritic.cells	SCPEP1	0.120032	5.169388	1.217732	0.226566	-6.14381	0.534933	0.522854
Dendritic.cells	METTL7B	-0.7731	2.263028	-1.21766	0.226592	-4.87652	0.565677	0.5669
Dendritic.cells	CARS2	0.207893	3.502896	1.217582	0.226622	-5.26058	0.552295	0.54768
Dendritic.cells	NPM1	-0.13023	8.143673	-1.21757	0.226627	-6.24644	0.505678	0.481307
Dendritic.cells	SULT2B1	-0.43777	2.700547	-1.21741	0.226687	-5.03124	0.560933	0.560043
Dendritic.cells	LPP	-0.1527	7.6885	-1.21662	0.226986	-6.2465	0.510547	0.487627
Dendritic.cells	PFKFB1	0.5911	0.961216	1.216547	0.227014	-4.81227	0.580767	0.588021
Dendritic.cells	FBXL14	0.154432	5.203671	1.216401	0.22707	-5.70088	0.535154	0.522608
Dendritic.cells	PARD6A	-0.36002	2.688858	-1.21627	0.22712	-5.16739	0.561634	0.560544
Dendritic.cells	TMEM198B	0.575897	0.786307	1.215829	0.227286	-4.80968	0.582953	0.591253
Dendritic.cells	NFYC	-0.10087	6.202043	-1.21573	0.227325	-5.92755	0.525259	0.508586
Dendritic.cells	GM19522	0.556896	0.940675	1.215675	0.227345	-4.82866	0.581202	0.58876
Dendritic.cells	SCAMP3	0.170306	4.819715	1.215513	0.227406	-5.57105	0.539295	0.528574
Dendritic.cells	SH3BP2	0.44159	3.288629	1.215186	0.22753	-5.05152	0.555559	0.551669
Dendritic.cells	GM31763	0.464396	3.257819	1.214986	0.227606	-5.03309	0.555952	0.552194
Dendritic.cells	CERS6	0.16161	7.330325	1.214417	0.227822	-6.28728	0.514776	0.493217
Dendritic.cells	ZKSCAN14	0.27564	3.081299	1.214315	0.227861	-5.1771	0.558226	0.555188
Dendritic.cells	GM29394	0.619849	0.417159	1.21396	0.227996	-4.78216	0.58806	0.598093
Dendritic.cells	GM13166	0.686423	-0.19353	1.213758	0.228072	-4.7341	0.595119	0.608416
Dendritic.cells	RBM38	0.175424	6.898184	1.213602	0.228132	-5.97733	0.519175	0.499538
Dendritic.cells	MRNIP	-0.52202	2.538227	-1.21356	0.228148	-4.91885	0.564315	0.564
Dendritic.cells	2900060B1	0.45768	2.614995	1.213437	0.228195	-4.88773	0.563478	0.562809
Dendritic.cells	TMEM30A	-0.10058	6.581779	-1.21303	0.228348	-6.01572	0.522437	0.504096
Dendritic.cells	0610040J0	-0.5073	2.378015	-1.21293	0.228388	-4.95968	0.566227	0.566701
Dendritic.cells	MRPL37	0.198178	4.2912	1.212773	0.228447	-5.46173	0.545737	0.537359
Dendritic.cells	MFGE8	-0.47714	2.292559	-1.21259	0.228518	-4.9995	0.567165	0.568156
Dendritic.cells	TLNRD1	-0.30532	4.581796	-1.21232	0.22862	-5.33946	0.542707	0.533227
Dendritic.cells	EIF4EBP1	0.244753	5.454955	1.212307	0.228625	-5.34676	0.533734	0.520422
Dendritic.cells	NMRAL1	-0.32232	3.904291	-1.21206	0.22872	-5.25703	0.549804	0.54339
Dendritic.cells	ARID5B	0.244753	7.244193	1.212018	0.228735	-5.95999	0.515944	0.495144
Dendritic.cells	MRPL12	0.228444	5.090142	1.212008	0.228738	-5.50725	0.537459	0.525746
Dendritic.cells	AGGF1	0.147762	5.384652	1.211946	0.228762	-5.70659	0.534449	0.521452
Dendritic.cells	CSPRS	0.327127	0.292172	1.21174	0.22884	-5.16363	0.589735	0.600861
Dendritic.cells	ARPC1B	0.104071	8.892807	1.211528	0.228921	-6.4127	0.500374	0.473084
Dendritic.cells	B230219D2	0.100326	6.252623	1.210818	0.229192	-5.9738	0.526255	0.509433
Dendritic.cells	ZBTB16	-0.69719	2.808614	-1.21079	0.229204	-4.89298	0.562121	0.560686
Dendritic.cells	TMEM177	0.695989	0.336385	1.21038	0.229359	-4.74926	0.590009	0.600735
Dendritic.cells	TFB1M	0.49337	1.706255	1.21016	0.229443	-4.86523	0.574481	0.578303
Dendritic.cells	SULT1A1	-0.49027	3.923228	-1.21015	0.229447	-5.10305	0.550402	0.543717
Dendritic.cells	MCEE	-0.2919	4.194477	-1.2095	0.229696	-5.26761	0.547993	0.539865
Dendritic.cells	MANF	0.098048	6.808848	1.209328	0.229761	-6.23827	0.521382	0.502034
Dendritic.cells	ETFB	-0.17848	6.979015	-1.20926	0.229787	-5.96135	0.519709	0.499661

Dendritic.cells	ZFP267	-0.40932	1.251689	-1.20907	0.22986	-4.93019	0.580089	0.586014
Dendritic.cells	GM9949	0.787836	0.483247	1.208961	0.229901	-4.73561	0.588839	0.598684
Dendritic.cells	STX7	0.089392	6.176182	1.208495	0.230079	-6.20685	0.527988	0.511089
Dendritic.cells	ICE2	0.332834	2.160721	1.207868	0.230319	-5.06901	0.570727	0.571715
Dendritic.cells	CNN3	-0.34611	4.531127	-1.20771	0.23038	-5.27991	0.545266	0.535255
Dendritic.cells	COX6B1	0.122248	7.821955	1.207588	0.230426	-6.18224	0.512277	0.488452
Dendritic.cells	PSPH	0.271715	3.906589	1.207082	0.23062	-5.28118	0.55218	0.544892
Dendritic.cells	POLL	-0.60246	1.158885	-1.20691	0.230685	-4.74131	0.582352	0.588223
Dendritic.cells	DAGLA	-0.70824	-0.78197	-1.20676	0.230743	-4.73306	0.604873	0.620864
Dendritic.cells	SOCS5	0.292958	4.029298	1.206245	0.230941	-5.35955	0.551205	0.543188
Dendritic.cells	CFL1	0.118278	10.01399	1.2062	0.230958	-6.59406	0.492369	0.459848
Dendritic.cells	PCMTD2	0.292303	3.680281	1.206062	0.231011	-5.26497	0.55492	0.548529
Dendritic.cells	EAF2	0.271386	3.647314	1.20531	0.2313	-5.26053	0.555789	0.549277
Dendritic.cells	SLC12A9	0.18084	4.242634	1.205118	0.231373	-5.76919	0.54947	0.540251
Dendritic.cells	CDK6	0.210772	6.322112	1.205008	0.231416	-5.98722	0.528112	0.50991
Dendritic.cells	PHF20	-0.11608	6.472356	-1.20494	0.231444	-6.03369	0.526611	0.507816
Dendritic.cells	CASP7	0.342321	4.140272	1.204798	0.231496	-5.17236	0.55055	0.541944
Dendritic.cells	BTBD8	-0.48416	1.572677	-1.2047	0.231535	-4.95783	0.578534	0.582119
Dendritic.cells	ZFP219	0.403739	1.771622	1.204579	0.231581	-4.94426	0.576304	0.578944
Dendritic.cells	ETFBKMT	0.492413	2.261696	1.204424	0.23164	-4.86135	0.570876	0.57121
Dendritic.cells	SAYSD1	0.233001	3.323883	1.20429	0.231692	-5.31192	0.559284	0.554627
Dendritic.cells	GM11579	-0.71883	-0.20636	-1.20397	0.231815	-4.77754	0.599056	0.611846
Dendritic.cells	TM9SF3	-0.08542	7.288714	-1.20395	0.231824	-6.19578	0.518645	0.496712
Dendritic.cells	ANKRD13D	-0.41062	2.738056	-1.20333	0.232061	-4.98852	0.56605	0.564034
Dendritic.cells	FAM8A1	0.294367	3.231881	1.202992	0.232191	-5.18013	0.560681	0.556515
Dendritic.cells	MTPN	0.091826	6.898113	1.20285	0.232246	-6.14535	0.522787	0.50253
Dendritic.cells	RCBTB1	0.222118	3.826201	1.202845	0.232247	-5.42249	0.554303	0.547397
Dendritic.cells	MRPS18C	0.176572	5.696786	1.202811	0.232261	-5.68818	0.534827	0.519622
Dendritic.cells	ZC2HC1A	0.464005	1.741533	1.202745	0.232286	-5.01359	0.577081	0.580078
Dendritic.cells	FBXL3	-0.16447	5.215741	-1.20267	0.232315	-5.63362	0.53975	0.526641
Dendritic.cells	DIDO1	-0.1446	5.734022	-1.2023	0.232457	-5.88519	0.534574	0.519139
Dendritic.cells	SNX14	-0.3355	4.500746	-1.20216	0.23251	-5.14858	0.547305	0.537292
Dendritic.cells	POLR3G	0.481572	2.101279	1.202135	0.232521	-4.94405	0.573203	0.574387
Dendritic.cells	ACAT2	0.3041	2.91084	1.201988	0.232578	-5.15027	0.564311	0.561674
Dendritic.cells	NR4A1	-0.20859	7.413157	-1.20175	0.232668	-6.29426	0.517958	0.495626
Dendritic.cells	GM32296	-0.69688	-0.65404	-1.20161	0.232723	-4.7399	0.604958	0.620386
Dendritic.cells	FAAP24	0.639364	2.002599	1.201393	0.232807	-4.85756	0.574506	0.576364
Dendritic.cells	SERPINB8	0.668349	-0.3114	1.201186	0.232887	-4.74087	0.600998	0.61473
Dendritic.cells	NAV2	0.198964	4.841571	1.20113	0.232909	-5.84219	0.543942	0.532701
Dendritic.cells	NAIP5	-0.3818	2.68586	-1.20083	0.233026	-5.13985	0.567119	0.565775
Dendritic.cells	NELFA	-0.16623	4.630912	-1.20056	0.233128	-5.56208	0.546415	0.535981
Dendritic.cells	SENP5	-0.13668	5.992571	-1.20015	0.233289	-5.94609	0.532435	0.516158
Dendritic.cells	VPS45	0.25793	3.758422	1.200135	0.233293	-5.32938	0.555658	0.54928
Dendritic.cells	HIP1	0.241701	5.505684	1.199873	0.233394	-5.70222	0.537387	0.523252
Dendritic.cells	GADD45B	-0.39938	5.322838	-1.19964	0.233486	-5.32064	0.539262	0.526015
Dendritic.cells	PLSCR2	0.852385	-0.38835	1.199433	0.233564	-4.74058	0.602232	0.616577
Dendritic.cells	HERC1	-0.12902	7.056727	-1.19926	0.23363	-6.12922	0.521818	0.501274

Dendritic.cells	COMTD1	0.329937	2.92368	1.199072	0.233704	-5.09232	0.564666	0.562393
Dendritic.cells	PHF3	0.09853	6.980017	1.19907	0.233705	-6.16772	0.522574	0.502345
Dendritic.cells	CNR2	0.502233	3.101889	1.199024	0.233722	-4.86175	0.562728	0.559611
Dendritic.cells	5330417C2	-0.72294	0.541978	-1.199	0.233732	-4.77493	0.591383	0.600885
Dendritic.cells	TMEM242	-0.1868	4.292008	-1.19876	0.233823	-5.49201	0.549995	0.541501
Dendritic.cells	CDO1	-0.44439	3.756999	-1.19874	0.233832	-5.12743	0.555673	0.549625
Dendritic.cells	MPEG1	0.147323	6.081136	1.198692	0.233851	-6.51419	0.53154	0.51519
Dendritic.cells	TMEM209	0.353361	3.362785	1.198562	0.233901	-5.1727	0.559905	0.555698
Dendritic.cells	ANGPTL6	-0.58866	0.97577	-1.19849	0.233928	-4.82561	0.586404	0.593833
Dendritic.cells	PFKFB2	0.338082	2.465839	1.198318	0.233996	-5.04492	0.569685	0.569739
Dendritic.cells	RABIF	-0.20485	4.708703	-1.19831	0.233999	-5.37768	0.545624	0.535266
Dendritic.cells	HDAC11	-0.71295	0.5008	-1.19797	0.23413	-4.76724	0.592061	0.601889
Dendritic.cells	ABHD17C	-0.25902	4.833665	-1.19782	0.234189	-5.36902	0.544527	0.533598
Dendritic.cells	ITGAE	-0.7229	2.127038	-1.19761	0.234271	-4.8124	0.573727	0.575411
Dendritic.cells	LMNB1	-0.17015	7.679763	-1.19712	0.234458	-6.1499	0.516297	0.493167
Dendritic.cells	TBX2	-0.64089	0.45083	-1.19699	0.234509	-4.81801	0.593084	0.60311
Dendritic.cells	LRRC57	0.454713	2.732576	1.196767	0.234596	-5.03385	0.567463	0.566179
Dendritic.cells	MTCH2	0.147165	5.950226	1.196609	0.234658	-5.80269	0.533554	0.517739
Dendritic.cells	GPX1	0.162788	11.17498	1.196268	0.23479	-6.78688	0.484119	0.44778
Dendritic.cells	PYCR2	0.236168	3.687049	1.195913	0.234928	-5.44985	0.557542	0.551576
Dendritic.cells	GNB4	0.204766	2.895468	1.195688	0.235016	-5.61378	0.566201	0.56395
Dendritic.cells	THAP12	0.213211	4.362104	1.195324	0.235157	-5.35087	0.550545	0.54152
Dendritic.cells	MNAT1	0.182199	4.818214	1.195141	0.235228	-5.65153	0.54576	0.534705
Dendritic.cells	RAB14	0.081184	7.091867	1.195095	0.235246	-6.20463	0.522696	0.501908
Dendritic.cells	RNF8	-0.18209	4.800789	-1.19506	0.23526	-5.55391	0.545941	0.534968
Dendritic.cells	AIF1	-0.43655	4.896333	-1.19488	0.235329	-5.39968	0.544951	0.533634
Dendritic.cells	PHF19	0.446357	1.934693	1.194733	0.235386	-4.89981	0.576933	0.579493
Dendritic.cells	6720427I0	-0.19057	4.894793	-1.19462	0.23543	-5.62904	0.544967	0.533676
Dendritic.cells	SLC25A45	0.3191	3.260909	1.194367	0.235529	-5.11938	0.562327	0.558601
Dendritic.cells	SUV39H1	-0.3328	3.622039	-1.19429	0.23556	-5.07437	0.55843	0.553019
Dendritic.cells	PKP3	0.201214	2.747004	1.194268	0.235567	-5.75564	0.567932	0.566649
Dendritic.cells	KLF9	-0.45734	3.693914	-1.19294	0.236083	-5.07225	0.558758	0.552374
Dendritic.cells	ERO1LB	-0.10276	6.652932	-1.19254	0.236239	-6.28981	0.528294	0.50885
Dendritic.cells	RHBDD3	0.358006	2.194151	1.192376	0.236304	-5.00324	0.575391	0.576111
Dendritic.cells	HSPA8	-0.10848	10.08692	-1.19232	0.236326	-6.60903	0.495431	0.462534
Dendritic.cells	XPO1	0.189838	5.786847	1.192142	0.236396	-5.73192	0.537084	0.521431
Dendritic.cells	PI4K2B	-0.29302	3.903982	-1.19181	0.236526	-5.23856	0.556854	0.549645
Dendritic.cells	NQO1	-0.69402	0.792254	-1.1916	0.236609	-4.77002	0.591423	0.599359
Dendritic.cells	PUM3	0.175514	4.640352	1.191472	0.236657	-5.58871	0.549047	0.538534
Dendritic.cells	NOP14	-0.26715	4.004398	-1.19128	0.236731	-5.31916	0.555781	0.548176
Dendritic.cells	CDK2AP1	-0.14803	5.884299	-1.19128	0.236731	-5.82865	0.536177	0.520226
Dendritic.cells	EMILIN1	-0.25898	2.608435	-1.19125	0.236745	-5.26821	0.570934	0.569888
Dendritic.cells	ACTL6A	0.163233	5.235041	1.191129	0.236791	-5.66375	0.542845	0.529735
Dendritic.cells	SEC61A2	0.246962	3.741527	1.1909	0.23688	-5.30943	0.558599	0.552304
Dendritic.cells	B9D1	-0.67911	0.371188	-1.19086	0.236894	-4.76827	0.596304	0.606562
Dendritic.cells	GSTO2	-0.62808	0.524993	-1.19044	0.237061	-4.8426	0.594808	0.604243
Dendritic.cells	NRDE2	-0.40546	3.371854	-1.19021	0.23715	-5.05872	0.562921	0.558339

Dendritic.cells	TAP1	0.373994	5.446269	1.190107	0.23719	-5.72475	0.540989	0.527046
Dendritic.cells	TGFBRAP1	-0.23652	3.494101	-1.18913	0.237572	-5.27499	0.561598	0.556903
Dendritic.cells	SAG	-0.54484	3.5306	-1.18889	0.237666	-4.97183	0.561203	0.556402
Dendritic.cells	IL23A	0.806168	-0.31658	1.18865	0.23776	-4.76081	0.604733	0.619306
Dendritic.cells	METTL9	-0.20087	5.718204	-1.18863	0.237767	-5.60896	0.538196	0.523629
Dendritic.cells	WDR90	-0.4593	1.959845	-1.18849	0.237821	-4.93711	0.578497	0.581395
Dendritic.cells	ZFP738	0.293257	2.843416	1.188292	0.237901	-5.14809	0.568688	0.567352
Dendritic.cells	RENBP	-0.20739	4.474184	-1.18826	0.237911	-5.60394	0.551127	0.54218
Dendritic.cells	GM10053	0.638281	1.424678	1.188157	0.237954	-4.82031	0.584539	0.590218
Dendritic.cells	WIPF2	0.140043	5.343948	1.188056	0.237993	-5.70651	0.542044	0.529258
Dendritic.cells	WWP1	0.189647	5.227114	1.188043	0.237998	-5.75288	0.543253	0.530982
Dendritic.cells	GM13012	0.387933	2.775114	1.188012	0.23801	-5.01443	0.569439	0.568486
Dendritic.cells	ISCA2	0.205601	4.968036	1.187933	0.238041	-5.57685	0.545946	0.534828
Dendritic.cells	PRKCE	-0.14724	7.233184	-1.18777	0.238105	-6.18898	0.522978	0.502167
Dendritic.cells	ARMCX5	-0.35765	2.433168	-1.18749	0.238216	-5.02976	0.573217	0.574068
Dendritic.cells	DBF4	0.242196	5.205532	1.187358	0.238267	-5.56555	0.543477	0.531442
Dendritic.cells	GM4631	0.46884	0.664941	1.187318	0.238282	-4.90752	0.593249	0.602989
Dendritic.cells	PUS10	0.223344	4.485499	1.187275	0.238299	-5.50709	0.551008	0.542229
Dendritic.cells	CLCC1	0.18455	4.329275	1.187215	0.238323	-5.4337	0.55266	0.544617
Dendritic.cells	TIAM1	0.388413	4.628891	1.187193	0.238332	-5.2072	0.549497	0.5401
Dendritic.cells	UGT3A2	-0.67654	1.174889	-1.18697	0.238421	-4.78705	0.587385	0.594631
Dendritic.cells	GOT1	-0.2133	6.081478	-1.1869	0.238448	-5.79967	0.534494	0.518768
Dendritic.cells	PNN	0.116794	6.310373	1.186566	0.238578	-5.97958	0.532179	0.515598
Dendritic.cells	TRAPPC4	0.152154	5.137813	1.186515	0.238597	-5.722	0.544179	0.532707
Dendritic.cells	PSTK	0.315871	3.061289	1.186475	0.238613	-5.08447	0.566302	0.564403
Dendritic.cells	BAP1	-0.28953	3.565024	-1.18644	0.238629	-5.20266	0.560832	0.556547
Dendritic.cells	NIPA1	0.704192	0.13903	1.186405	0.238641	-4.7552	0.599369	0.612164
Dendritic.cells	SLC25A46	-0.16201	4.587825	-1.18635	0.238664	-5.51656	0.54993	0.540957
Dendritic.cells	GM10135	-0.65436	0.409376	-1.18625	0.2387	-4.77194	0.596214	0.607627
Dendritic.cells	MDFIC	-0.40252	4.328783	-1.18611	0.238756	-5.26392	0.552665	0.544941
Dendritic.cells	D430040D:	-0.66912	-0.34621	-1.1861	0.23876	-4.79388	0.605084	0.620568
Dendritic.cells	ELOA	0.170952	5.789735	1.185963	0.238814	-5.72548	0.537464	0.523265
Dendritic.cells	2810004N:	-0.26479	4.315225	-1.18583	0.238866	-5.31916	0.552809	0.545232
Dendritic.cells	BC065397	0.430069	1.292469	1.185576	0.238966	-4.96239	0.586043	0.593158
Dendritic.cells	KIDINS220	0.150723	5.419693	1.185576	0.238967	-5.77881	0.541262	0.528812
Dendritic.cells	GM29170	-0.36889	1.532706	-1.18556	0.238973	-5.00869	0.583313	0.589211
Dendritic.cells	CELF6	-0.79395	-0.70622	-1.18501	0.239191	-4.76145	0.60979	0.627224
Dendritic.cells	CCDC186	0.190269	4.760025	1.184611	0.239346	-5.56945	0.548683	0.538905
Dendritic.cells	P2RY6	0.361603	2.305897	1.184547	0.239371	-5.20625	0.575219	0.577001
Dendritic.cells	CYP4A31	-0.70551	0.922941	-1.18441	0.239424	-4.81094	0.590883	0.599651
Dendritic.cells	E230013L2	0.639776	-0.72305	1.183768	0.239678	-4.75465	0.610712	0.627961
Dendritic.cells	DGCR6	0.218441	3.650268	1.183599	0.239745	-5.45406	0.56099	0.556191
Dendritic.cells	DNAJA4	-0.64351	0.616749	-1.18335	0.239844	-4.79556	0.594949	0.605263
Dendritic.cells	EXOSC7	0.222528	4.629702	1.183261	0.239878	-5.43141	0.550547	0.541371
Dendritic.cells	YTHDF1	-0.1387	5.78074	-1.18302	0.239971	-5.86486	0.538591	0.524453
Dendritic.cells	HDGF	0.127429	6.998077	1.182847	0.240041	-6.04205	0.526313	0.507032
Dendritic.cells	LRRC29	0.349394	1.690453	1.182832	0.240047	-4.97297	0.582648	0.58774

Dendritic.cells	RFTN1	0.101676	6.737622	1.182733	0.240086	-6.47539	0.528909	0.510727
Dendritic.cells	DNAJC19	0.137274	5.950115	1.182421	0.240209	-5.84549	0.53686	0.522195
Dendritic.cells	ABCB11	-0.62308	1.681529	-1.18231	0.240252	-4.84805	0.582749	0.588061
Dendritic.cells	SLC37A4	0.368689	2.862467	1.182078	0.240344	-5.05334	0.569573	0.569141
Dendritic.cells	GALC	0.33738	3.891293	1.182036	0.240361	-5.209	0.558397	0.553093
Dendritic.cells	ETFA	-0.15934	6.599323	-1.1818	0.240453	-5.94954	0.530294	0.512991
Dendritic.cells	SLC31A1	0.176954	5.429507	1.18165	0.240513	-5.72707	0.542203	0.530035
Dendritic.cells	NFE2L2	-0.1637	7.355477	-1.18155	0.240554	-6.14027	0.522779	0.502368
Dendritic.cells	HVCN1	0.247974	4.117697	1.18152	0.240565	-5.74432	0.555975	0.549754
Dendritic.cells	PIP4K2C	-0.19771	4.238405	-1.18152	0.240565	-5.5569	0.554689	0.54791
Dendritic.cells	ZFP40	0.503667	1.347612	1.181266	0.240665	-4.94508	0.586542	0.593884
Dendritic.cells	DENND1C	0.291225	3.966917	1.181215	0.240685	-5.29095	0.557586	0.552155
Dendritic.cells	SIGMAR1	0.230548	3.549056	1.181202	0.24069	-5.42249	0.562084	0.558614
Dendritic.cells	TMEM170	-0.45135	1.517214	-1.18103	0.240758	-4.91845	0.584612	0.591137
Dendritic.cells	WDR83OS	0.149816	6.074614	1.181022	0.240761	-5.88042	0.535593	0.52073
Dendritic.cells	GPM6B	0.196247	2.439081	1.180584	0.240934	-5.80006	0.574545	0.576234
Dendritic.cells	AP1S2	0.233621	5.081901	1.180223	0.241077	-5.44223	0.546287	0.535493
Dendritic.cells	ELOC	-0.14034	7.093954	-1.17997	0.241178	-5.98398	0.525822	0.506345
Dendritic.cells	ICE1	-0.2432	4.081492	-1.17991	0.241203	-5.4381	0.556849	0.55066
Dendritic.cells	AGT	-0.57664	4.084746	-1.17957	0.241335	-5.25747	0.556814	0.550672
Dendritic.cells	IKBKG	0.312967	3.339723	1.179503	0.241362	-5.12565	0.564849	0.562209
Dendritic.cells	CHTOP	0.174404	5.225925	1.179501	0.241363	-5.69822	0.544788	0.533453
Dendritic.cells	IGFLR1	-0.71207	0.807622	-1.17944	0.241389	-4.80513	0.593259	0.603216
Dendritic.cells	MAGOHB	0.318283	4.560419	1.179325	0.241432	-5.35638	0.55176	0.543429
Dendritic.cells	GREM2	-0.65623	0.454759	-1.17902	0.241552	-4.83064	0.597525	0.609295
Dendritic.cells	FAM168A	0.151461	5.93211	1.178871	0.241612	-5.82043	0.537687	0.523249
Dendritic.cells	GM8113	-0.66317	-0.25687	-1.17831	0.241837	-4.7714	0.606301	0.621719
Dendritic.cells	ATP6V1C1	0.146369	5.189889	1.178154	0.241896	-5.73036	0.545686	0.534343
Dendritic.cells	PAQR3	0.49867	1.469925	1.17795	0.241977	-4.85698	0.586224	0.592636
Dendritic.cells	NCBP3	0.142848	5.506298	1.177852	0.242016	-5.81024	0.542405	0.52971
Dendritic.cells	GM30211	0.454248	4.041214	1.17772	0.242069	-5.32927	0.557814	0.551782
Dendritic.cells	INTS12	0.235145	4.380987	1.177675	0.242087	-5.33907	0.554191	0.546596
Dendritic.cells	C5AR2	0.695743	1.231827	1.177578	0.242125	-4.80066	0.588944	0.596654
Dendritic.cells	SETD7	0.114853	4.9704	1.176678	0.242482	-6.12189	0.548598	0.538019
Dendritic.cells	FLAD1	0.336359	2.957237	1.176486	0.242559	-5.16591	0.570223	0.569074
Dendritic.cells	AI504432	-0.25645	2.840905	-1.17639	0.242597	-5.35033	0.571505	0.570931
Dendritic.cells	SPECC1L	-0.19072	5.413581	-1.17619	0.242677	-5.78829	0.54398	0.531507
Dendritic.cells	SERPING1	-0.4785	3.004813	-1.17617	0.242683	-5.03726	0.569699	0.568363
Dendritic.cells	EYA2	-0.68025	1.389743	-1.17611	0.242707	-4.79752	0.587804	0.594471
Dendritic.cells	FZD7	-0.59107	0.743156	-1.17555	0.24293	-4.80094	0.59567	0.605382
Dendritic.cells	PLEKHB2	-0.18415	4.419141	-1.17521	0.243066	-5.56345	0.555001	0.546672
Dendritic.cells	EPSTI1	0.217532	7.192656	1.174895	0.243191	-6.17134	0.52666	0.506158
Dendritic.cells	A530072M	0.600547	0.764735	1.174651	0.243289	-4.86748	0.595921	0.605424
Dendritic.cells	ZFP580	0.313006	2.166288	1.174311	0.243424	-5.2364	0.580109	0.582392
Dendritic.cells	VIRMA	0.149308	5.71037	1.174072	0.243519	-5.86864	0.542027	0.527735
Dendritic.cells	USP25	0.114345	7.051473	1.173803	0.243626	-6.13003	0.52843	0.508464
Dendritic.cells	BPNT1	-0.24479	3.339718	-1.1738	0.243629	-5.30923	0.567195	0.563811

Dendritic.cells	SLC17A2	-0.63422	1.156237	-1.1736	0.243709	-4.85038	0.591693	0.599162
Dendritic.cells	JAM3	-0.68201	0.244994	-1.17335	0.243806	-4.78802	0.602298	0.614572
Dendritic.cells	FANCL	0.220972	4.144904	1.173332	0.243814	-5.39024	0.558482	0.551418
Dendritic.cells	CSF3R	0.572901	2.933462	1.173312	0.243822	-4.98119	0.571657	0.570324
Dendritic.cells	MBNL1	0.110796	8.978349	1.172847	0.244008	-6.63295	0.50985	0.482069
Dendritic.cells	LDLRAD4	-0.17629	6.109073	-1.17273	0.244056	-6.07024	0.538114	0.522146
Dendritic.cells	GM1673	-0.40994	2.42109	-1.17272	0.244057	-5.04307	0.577537	0.578589
Dendritic.cells	ZFP456	0.630027	0.791994	1.172316	0.244219	-4.81534	0.596235	0.60545
Dendritic.cells	RTEL1	0.459408	2.682882	1.172313	0.24422	-5.00347	0.574749	0.574449
Dendritic.cells	GM36161	0.784409	0.563057	1.171979	0.244354	-4.80609	0.598903	0.609312
Dendritic.cells	SEC31A	0.109129	5.620105	1.171963	0.24436	-5.91012	0.543259	0.529356
Dendritic.cells	ATG101	-0.19227	5.222366	-1.17194	0.244368	-5.57069	0.547392	0.53525
Dendritic.cells	HS3ST1	0.241404	1.500377	1.171241	0.244649	-5.88017	0.588624	0.594004
Dendritic.cells	BOD1	-0.29927	3.243589	-1.17108	0.244714	-5.24042	0.569127	0.56594
Dendritic.cells	YEATS4	0.132996	5.906755	1.170795	0.244827	-5.84146	0.540926	0.525554
Dendritic.cells	BC002059	0.305974	2.758564	1.170698	0.244866	-5.19972	0.574567	0.573719
Dendritic.cells	PHF10	-0.2037	4.930175	-1.1706	0.244906	-5.54712	0.551084	0.540106
Dendritic.cells	UBE2D3	-0.06966	8.966917	-1.17035	0.245006	-6.4559	0.51076	0.482759
Dendritic.cells	MKNK2	0.154538	6.23257	1.17016	0.245081	-5.78424	0.537743	0.520968
Dendritic.cells	ZFP169	0.400773	2.782441	1.17004	0.245129	-5.16516	0.574464	0.573479
Dendritic.cells	MBD6	0.293199	3.632499	1.169667	0.245279	-5.2018	0.565293	0.560088
Dendritic.cells	2700038G2	0.312018	2.577847	1.169602	0.245304	-5.16803	0.57691	0.576777
Dendritic.cells	ISOC2A	-0.40071	2.013655	-1.16866	0.24568	-5.00781	0.583913	0.586164
Dendritic.cells	MROH1	0.20733	4.199162	1.168646	0.245688	-5.58545	0.559812	0.551585
Dendritic.cells	STXBP6	0.263816	2.953526	1.168303	0.245825	-5.78553	0.573536	0.571062
Dendritic.cells	IFT43	-0.45156	1.54627	-1.16783	0.246016	-4.93077	0.589381	0.59396
Dendritic.cells	DMC1	-0.63032	0.152078	-1.16779	0.246032	-4.76856	0.60561	0.617412
Dendritic.cells	GM47200	0.685755	0.11613	1.16754	0.246131	-4.77676	0.606036	0.61804
Dendritic.cells	PRRC2B	-0.11401	6.226887	-1.16751	0.246143	-6.05872	0.538709	0.521433
Dendritic.cells	TPM1	0.201058	5.177563	1.16738	0.246196	-5.77512	0.549569	0.536921
Dendritic.cells	MAP3K5	0.122689	7.255072	1.167342	0.246211	-6.30972	0.528341	0.506748
Dendritic.cells	MYBPC2	-0.24807	2.760882	-1.16734	0.246211	-5.59656	0.575673	0.574276
Dendritic.cells	TAMM41	0.371701	2.732365	1.167323	0.246219	-5.10436	0.575991	0.574735
Dendritic.cells	SCAMP5	-0.48816	1.229049	-1.16721	0.246264	-4.90971	0.593027	0.599314
Dendritic.cells	STAT1	0.337215	6.871703	1.166985	0.246354	-6.19693	0.532258	0.512337
Dendritic.cells	AI847159	-0.67253	-0.25702	-1.16681	0.246423	-4.78164	0.61061	0.624786
Dendritic.cells	CPOX	0.227304	4.570271	1.166417	0.246583	-5.49657	0.556166	0.546454
Dendritic.cells	JAG2	-0.48152	1.093215	-1.16637	0.246602	-4.96795	0.594791	0.601946
Dendritic.cells	OSGIN1	-0.4633	3.869004	-1.16637	0.246603	-5.09005	0.563696	0.557226
Dendritic.cells	GM20559	0.248196	4.344461	1.166041	0.246734	-5.54565	0.558757	0.55007
Dendritic.cells	GDPD3	-0.68592	1.793559	-1.16531	0.247027	-4.80836	0.587449	0.590884
Dendritic.cells	STAM	-0.18311	4.382468	-1.16519	0.247076	-5.51099	0.558835	0.549874
Dendritic.cells	TMEM29	0.198794	4.706351	1.165123	0.247103	-5.54084	0.55538	0.544977
Dendritic.cells	MIEF2	0.525258	1.112018	1.164304	0.247433	-4.86047	0.595869	0.602618
Dendritic.cells	CELF1	-0.07844	7.40812	-1.16425	0.247455	-6.30931	0.528141	0.505773
Dendritic.cells	ZFP982	0.742544	0.056297	1.163746	0.247658	-4.79522	0.608266	0.620778
Dendritic.cells	DNAIC1	-0.67884	0.436784	-1.16373	0.247665	-4.79318	0.603763	0.614256

Dendritic.cells	SP140	0.139441	6.188606	1.163649	0.247697	-6.23211	0.540452	0.523432
Dendritic.cells	PRDX2	0.203325	7.774205	1.163481	0.247765	-6.02201	0.524518	0.500838
Dendritic.cells	NT5E	-0.76299	2.431121	-1.16344	0.24778	-4.79878	0.580808	0.581147
Dendritic.cells	DNAH10	-0.66248	0.024576	-1.16343	0.247787	-4.80253	0.608643	0.621334
Dendritic.cells	TRIM26	0.143957	5.330265	1.163364	0.247812	-5.88478	0.549345	0.536099
Dendritic.cells	ATG16L2	0.160961	5.680082	1.162868	0.248012	-5.80222	0.545911	0.531011
Dendritic.cells	EIF4E	-0.16417	6.950147	-1.16287	0.248012	-6.00973	0.532929	0.512563
Dendritic.cells	NR2C2AP	0.183289	4.44571	1.162439	0.248185	-5.74417	0.559087	0.549637
Dendritic.cells	KCNIP2	-0.59615	1.248736	-1.16236	0.248216	-4.8598	0.594688	0.600755
Dendritic.cells	TMEM62	0.433282	2.375873	1.162202	0.248281	-4.98376	0.581823	0.582224
Dendritic.cells	RAP2C	0.206541	4.944479	1.162164	0.248296	-5.48733	0.553778	0.542059
Dendritic.cells	DEGS2	0.640615	0.674225	1.161816	0.248437	-4.81531	0.601379	0.61055
Dendritic.cells	TRAPPC6A	0.157288	5.057196	1.161693	0.248487	-5.61649	0.552587	0.540525
Dendritic.cells	BEND6	-0.70486	0.170455	-1.16168	0.248493	-4.78319	0.607321	0.619207
Dendritic.cells	UBE2D1	-0.13499	5.377113	-1.16155	0.248543	-5.77014	0.549225	0.535761
Dendritic.cells	TIRAP	0.443159	3.005516	1.161527	0.248554	-4.91956	0.574785	0.57232
Dendritic.cells	SNHG12	-0.4279	3.189103	-1.16134	0.248631	-4.97836	0.572753	0.569486
Dendritic.cells	COG5	0.118867	6.582266	1.161182	0.248693	-6.16441	0.536799	0.518221
Dendritic.cells	DCAKD	0.275779	4.076447	1.160949	0.248788	-5.34341	0.563059	0.555774
Dendritic.cells	ENTPD6	0.319008	2.776877	1.160605	0.248927	-5.11217	0.577328	0.576379
Dendritic.cells	GM29243	-0.65586	-0.26495	-1.16057	0.248943	-4.77563	0.612513	0.62721
Dendritic.cells	PIR	-0.17517	1.198746	-1.16033	0.249037	-5.84757	0.595267	0.602282
Dendritic.cells	SNRNP35	0.308527	3.056704	1.160258	0.249067	-5.18299	0.574218	0.571973
Dendritic.cells	NENF	-0.15652	4.583677	-1.16014	0.249114	-5.7051	0.557612	0.548204
Dendritic.cells	DBN1	0.609596	1.388658	1.160118	0.249124	-4.91977	0.593072	0.59916
Dendritic.cells	DNAJC3	-0.10812	6.725717	-1.16	0.249173	-6.27188	0.535345	0.516508
Dendritic.cells	DEPDC7	-0.69082	1.678465	-1.15993	0.249201	-4.81378	0.589743	0.5944
Dendritic.cells	SAMM50	0.174856	5.177169	1.159777	0.249262	-5.65118	0.551323	0.53933
Dendritic.cells	CRADD	-0.14528	5.224096	-1.15975	0.249273	-5.8019	0.55083	0.538626
Dendritic.cells	UBE2L3	0.092504	7.560477	1.15968	0.249301	-6.2336	0.526985	0.504707
Dendritic.cells	PAPSS2	-0.59822	2.513577	-1.15962	0.249324	-4.92882	0.580275	0.580829
Dendritic.cells	ANKRD39	-0.21516	3.543857	-1.15926	0.249473	-5.35082	0.568904	0.564549
Dendritic.cells	ZFP839	0.40902	1.537463	1.159245	0.249478	-4.9772	0.591413	0.596948
Dendritic.cells	GPX4	0.116457	7.153816	1.15913	0.249524	-6.0572	0.531084	0.510576
Dendritic.cells	GM14410	-0.47219	0.850334	-1.1589	0.249616	-4.92743	0.599373	0.608502
Dendritic.cells	ATE1	-0.21292	3.945361	-1.15856	0.249756	-5.37141	0.56453	0.558476
Dendritic.cells	ZFP938	0.474622	1.450658	1.158371	0.249832	-4.90961	0.592412	0.598756
Dendritic.cells	NHSL1	-0.47752	1.728678	-1.15831	0.249855	-4.9691	0.589221	0.594161
Dendritic.cells	SNHG3	0.250287	5.291632	1.158283	0.249868	-5.58252	0.550171	0.538082
Dendritic.cells	EIF2B2	-0.21933	4.936837	-1.15828	0.24987	-5.51107	0.553909	0.543423
Dendritic.cells	CCT6A	0.155154	5.802009	1.158058	0.249959	-5.80938	0.544851	0.530537
Dendritic.cells	NUP43	-0.38606	2.795528	-1.15803	0.24997	-5.02295	0.577173	0.576843
Dendritic.cells	TEAD2	0.555656	1.154161	1.158028	0.249971	-4.85424	0.595838	0.603777
Dendritic.cells	PPIH	0.19223	5.305306	1.157889	0.250028	-5.62703	0.550028	0.537924
Dendritic.cells	GM9917	-0.62034	0.773036	-1.15778	0.25007	-4.87045	0.600277	0.610204
Dendritic.cells	IPCEF1	-0.59215	4.679593	-1.15724	0.250292	-5.01132	0.557018	0.547583
Dendritic.cells	ANAPC4	0.169628	4.541254	1.156872	0.250441	-5.59712	0.558509	0.549853

Dendritic.cells	NPM3	0.209719	5.097579	1.15676	0.250486	-5.67559	0.5526	0.54146
Dendritic.cells	MAP3K12	0.35475	2.718206	1.156739	0.250495	-5.05986	0.578443	0.578535
Dendritic.cells	IGSF6	0.627457	3.689623	1.156611	0.250547	-5.018	0.567711	0.563162
Dendritic.cells	EME1	-0.4833	2.299791	-1.15651	0.250588	-4.96259	0.583144	0.585391
Dendritic.cells	ARHGEF3	-0.28781	5.916073	-1.15627	0.250686	-5.79143	0.544055	0.529427
Dendritic.cells	PRKCB	0.095822	8.511819	1.156204	0.250712	-6.49071	0.518082	0.492526
Dendritic.cells	GM41555	-0.70807	0.571556	-1.15602	0.250788	-4.79352	0.603064	0.61436
Dendritic.cells	ACADL	0.137753	6.734458	1.155995	0.250797	-6.11446	0.535682	0.517558
Dendritic.cells	CHIC2	0.087461	7.043743	1.15595	0.250816	-6.19983	0.532563	0.513134
Dendritic.cells	BTK	0.159683	6.033608	1.155564	0.250973	-5.88701	0.54307	0.527848
Dendritic.cells	PRKAB1	-0.3933	3.654114	-1.15529	0.251085	-5.05445	0.568474	0.564012
Dendritic.cells	SFT2D2	0.225239	4.677531	1.154707	0.251322	-5.51102	0.557832	0.548377
Dendritic.cells	CERS5	-0.15606	5.514163	-1.15455	0.251384	-5.77937	0.549018	0.535767
Dendritic.cells	LCP1	0.125018	8.858643	1.154106	0.251567	-6.45355	0.515576	0.488295
Dendritic.cells	CLP1	-0.22569	4.608479	-1.15406	0.251586	-5.42063	0.558698	0.54962
Dendritic.cells	POLA2	-0.29508	4.050563	-1.15406	0.251587	-5.31813	0.564704	0.558222
Dendritic.cells	CDC42BPG	-0.55982	2.57838	-1.15369	0.251735	-4.91314	0.581071	0.581816
Dendritic.cells	LDLR	0.268881	4.577033	1.153677	0.251742	-5.66121	0.559148	0.550339
Dendritic.cells	SRSF5	-0.09477	6.955445	-1.15314	0.251961	-6.20809	0.534784	0.515237
Dendritic.cells	SHPK	-0.58142	0.252393	-1.1528	0.252101	-4.84553	0.608559	0.621094
Dendritic.cells	ARHGEF4	-0.83758	0.759185	-1.15258	0.252192	-4.83365	0.602612	0.612428
Dendritic.cells	SMOX	0.36212	5.116761	1.152459	0.252239	-5.47746	0.554007	0.54256
Dendritic.cells	ZFP53	0.193633	4.791451	1.152264	0.252319	-5.64348	0.55746	0.547561
Dendritic.cells	RNF31	0.325331	3.162894	1.152115	0.25238	-5.19167	0.575169	0.572961
Dendritic.cells	TBC1D10B	0.187621	5.147294	1.152047	0.252407	-5.60499	0.553684	0.542177
Dendritic.cells	GMEB1	0.180977	5.025748	1.151437	0.252657	-5.67669	0.55497	0.544176
Dendritic.cells	ESRRA	0.265151	3.981496	1.151273	0.252724	-5.29719	0.566179	0.560267
Dendritic.cells	RUFY2	0.370118	2.954919	1.15118	0.252762	-5.01564	0.577481	0.576516
Dendritic.cells	MKLN1OS	-0.67989	0.018205	-1.15106	0.25281	-4.79209	0.61139	0.62561
Dendritic.cells	GAB2	-0.13333	7.176286	-1.15099	0.252839	-6.48129	0.53278	0.512669
Dendritic.cells	BAZ2A	0.12438	6.97841	1.150847	0.252898	-6.2174	0.534772	0.515529
Dendritic.cells	ARHGEF10	0.336392	2.266878	1.150721	0.25295	-5.42378	0.585215	0.587806
Dendritic.cells	GAPVD1	0.100729	6.832806	1.150608	0.252996	-6.17501	0.536245	0.517702
Dendritic.cells	GXYLT1	0.190934	5.093219	1.15059	0.253003	-5.58933	0.554256	0.543394
Dendritic.cells	HEATR1	-0.25458	4.84445	-1.15032	0.253112	-5.48271	0.556896	0.547284
Dendritic.cells	TRIM30C	0.638427	1.320225	1.150138	0.253189	-4.86571	0.596065	0.603663
Dendritic.cells	GM4924	0.604594	0.278075	1.150134	0.25319	-4.82836	0.608294	0.621392
Dendritic.cells	NCEH1	-0.12188	4.906119	-1.15013	0.253191	-6.29239	0.55624	0.546348
Dendritic.cells	BTNL9	-0.50482	0.689973	-1.15008	0.253211	-5.06272	0.603425	0.614325
Dendritic.cells	GM15956	-0.62828	0.27605	-1.14992	0.253276	-4.79583	0.608318	0.621427
Dendritic.cells	COX6A2	0.148714	2.070766	1.149792	0.25333	-6.23296	0.587443	0.591199
Dendritic.cells	MED29	0.204119	3.806911	1.14977	0.253339	-5.42229	0.568082	0.563323
Dendritic.cells	POLK	-0.29865	3.481233	-1.14971	0.253364	-5.21057	0.571652	0.568452
Dendritic.cells	POLE2	-0.46464	3.014685	-1.14957	0.253421	-5.02097	0.576816	0.575916
Dendritic.cells	LDLRAD3	0.119461	4.591753	1.149434	0.253477	-6.3179	0.559594	0.55123
Dendritic.cells	R3HDM1	0.088415	6.683532	1.149392	0.253494	-6.25822	0.53776	0.520046
Dendritic.cells	GM48236	-0.54288	0.1418	-1.14933	0.253518	-4.96387	0.609915	0.62384

Dendritic.cells	CENPU	0.472096	2.562499	1.148565	0.253833	-4.91833	0.582457	0.583531
Dendritic.cells	FAM129A	0.17983	5.667894	1.148462	0.253875	-5.90192	0.548767	0.535218
Dendritic.cells	TARM1	0.763441	-0.18149	1.148018	0.254058	-4.78972	0.614711	0.630006
Dendritic.cells	CDC42BPA	-0.25365	3.315927	-1.14772	0.254179	-5.45671	0.5745	0.571778
Dendritic.cells	TK2	0.319174	3.825305	1.14718	0.254402	-5.24541	0.569277	0.563885
Dendritic.cells	PIK3CG	0.231241	4.936727	1.146999	0.254477	-5.4765	0.557331	0.546798
Dendritic.cells	GFOD1	-0.14102	6.621215	-1.1467	0.254598	-6.28759	0.539818	0.521812
Dendritic.cells	KLHL12	0.352103	4.259168	1.146601	0.25464	-5.17506	0.564661	0.557318
Dendritic.cells	PI4K2A	0.18452	5.639245	1.146543	0.254664	-5.80845	0.549969	0.536331
Dendritic.cells	CSNK1G1	0.126887	6.535289	1.146139	0.25483	-6.05979	0.540697	0.523182
Dendritic.cells	CCDC85C	-0.55267	0.977037	-1.14608	0.254854	-4.83876	0.601647	0.610697
Dendritic.cells	XPO4	-0.13265	5.623363	-1.14581	0.254965	-5.94076	0.550136	0.536736
Dendritic.cells	GPBP1L1	0.141268	5.913267	1.145752	0.25499	-5.90478	0.547112	0.532421
Dendritic.cells	GM31508	0.667533	0.763062	1.145675	0.255021	-4.80378	0.60416	0.614441
Dendritic.cells	SLC16A7	-0.29598	3.236151	-1.14563	0.255039	-5.3061	0.575877	0.573613
Dendritic.cells	TBC1D1	-0.11013	7.18844	-1.1456	0.255051	-6.4144	0.534067	0.513858
Dendritic.cells	ARIH2	-0.1153	7.527213	-1.14553	0.255082	-6.2361	0.530672	0.509056
Dendritic.cells	LSM8	0.184867	5.264944	1.145214	0.255211	-5.62295	0.553905	0.542266
Dendritic.cells	CTTN	-0.50381	1.717261	-1.14518	0.255224	-4.93009	0.59305	0.598527
Dendritic.cells	RIPOR1	-0.20363	3.960811	-1.14511	0.255256	-5.53807	0.567903	0.562315
Dendritic.cells	ZSWIM8	-0.16661	4.703109	-1.14504	0.255282	-5.62352	0.55988	0.550815
Dendritic.cells	PI16	0.626526	1.135522	1.144592	0.255467	-4.8396	0.600036	0.60837
Dendritic.cells	DOK3	-0.46795	5.36627	-1.1445	0.255507	-5.10635	0.553059	0.54084
Dendritic.cells	APPL1	-0.14762	5.974209	-1.14441	0.255541	-5.98068	0.5467	0.531782
Dendritic.cells	GUCY1A1	-0.70215	-0.00341	-1.14366	0.255852	-4.82231	0.614135	0.628146
Dendritic.cells	GM5086	0.184402	-0.01663	1.143225	0.256031	-5.70042	0.614523	0.62843
Dendritic.cells	CEP104	0.261621	3.105243	1.143176	0.256052	-5.24772	0.578366	0.576205
Dendritic.cells	H6PD	-0.37459	2.776773	-1.14256	0.256307	-5.02868	0.582373	0.581795
Dendritic.cells	A430005L1	0.263284	3.764054	1.142447	0.256353	-5.2826	0.571397	0.566042
Dendritic.cells	PRKG1	-0.34194	3.504872	-1.14231	0.256409	-5.54206	0.574253	0.570188
Dendritic.cells	TMEM126/	0.136144	5.857631	1.14225	0.256434	-5.83895	0.548981	0.534064
Dendritic.cells	CLK2	0.156105	4.63296	1.142194	0.256457	-5.62873	0.561953	0.552587
Dendritic.cells	KLRA5	-0.8175	2.005641	-1.14188	0.256589	-4.91876	0.591213	0.594621
Dendritic.cells	0610009L1	0.387741	2.030404	1.141757	0.256638	-5.09732	0.590929	0.594274
Dendritic.cells	SLCO4A1	0.239306	3.403213	1.14173	0.256649	-5.71376	0.575458	0.572008
Dendritic.cells	HSPA2	-0.46532	3.291574	-1.14143	0.256775	-5.00276	0.576748	0.573856
Dendritic.cells	SEC24C	0.142275	4.859773	1.141391	0.256789	-5.76541	0.559648	0.549347
Dendritic.cells	A730081D(	-0.34958	3.129627	-1.14115	0.256891	-5.22957	0.578552	0.576546
Dendritic.cells	RNF41	-0.19687	4.424549	-1.14087	0.257006	-5.46857	0.564328	0.556193
Dendritic.cells	ATG7	0.222377	5.786606	1.140851	0.257013	-5.62266	0.549847	0.535498
Dendritic.cells	FTX	0.152261	4.311116	1.140747	0.257056	-5.94155	0.565556	0.557989
Dendritic.cells	ZCCHC8	0.173938	5.108019	1.140691	0.257079	-5.59106	0.557001	0.545755
Dendritic.cells	SNRPA1	-0.24944	5.077996	-1.14056	0.257134	-5.51023	0.55732	0.546259
Dendritic.cells	SPATA24	0.479101	2.193104	1.140541	0.257141	-5.00742	0.58912	0.591921
Dendritic.cells	HPS5	0.18204	4.448069	1.140358	0.257217	-5.59861	0.564113	0.55595
Dendritic.cells	ITGA5	-0.41809	3.54178	-1.14012	0.257315	-5.17964	0.574016	0.57023
Dendritic.cells	NCBP2	-0.21274	4.599992	-1.13981	0.257445	-5.46096	0.562475	0.553733

Dendritic.cells	2510017J1(	-0.57377	1.414805	-1.13979	0.257452	-4.91431	0.598127	0.605038
Dendritic.cells	HK1OS	0.657073	0.741866	1.139787	0.257453	-4.80221	0.606014	0.616456
Dendritic.cells	PFKP	0.237156	5.538024	1.139553	0.257551	-5.67336	0.552492	0.539588
Dendritic.cells	GYG	0.244398	6.126163	1.139511	0.257568	-5.6795	0.546351	0.530841
Dendritic.cells	GM44148	0.547989	1.90428	1.139307	0.257653	-4.944	0.592469	0.597091
Dendritic.cells	GM48696	-0.38719	2.476417	-1.13928	0.257662	-5.10787	0.585939	0.587673
Dendritic.cells	NANOS1	0.627961	0.634448	1.139238	0.257681	-4.80907	0.607284	0.618573
Dendritic.cells	GEMIN7	0.151745	5.084337	1.138773	0.257874	-5.66845	0.557596	0.546722
Dendritic.cells	COA5	0.205138	4.468388	1.138284	0.258077	-5.68905	0.564462	0.55638
Dendritic.cells	FXN	0.247461	4.085717	1.138117	0.258146	-5.31709	0.568618	0.562433
Dendritic.cells	D830025C(	0.4503	2.231904	1.137989	0.258199	-5.00177	0.589312	0.592218
Dendritic.cells	SLC4A8	0.748901	0.467759	1.137928	0.258225	-4.79804	0.609876	0.621974
Dendritic.cells	ITGA6	-0.50343	4.818835	-1.13786	0.258254	-5.20038	0.560689	0.551085
Dendritic.cells	NKTR	-0.08845	7.193099	-1.13747	0.258414	-6.29312	0.536142	0.515944
Dendritic.cells	LACC1	0.300882	3.279516	1.137415	0.258438	-5.39063	0.577681	0.575352
Dendritic.cells	MSH6	0.231356	4.38755	1.137139	0.258553	-5.55858	0.565647	0.557992
Dendritic.cells	EDF1	0.103091	7.047067	1.136846	0.258675	-6.1871	0.537896	0.518298
Dendritic.cells	MANSC1	0.530184	0.242039	1.136563	0.258792	-4.97539	0.613226	0.626448
Dendritic.cells	GM28791	-0.26912	3.918458	-1.1364	0.258862	-5.36212	0.571097	0.565616
Dendritic.cells	MMS19	0.150858	4.947005	1.135875	0.259079	-5.73326	0.56031	0.549743
Dendritic.cells	MIS18A	-0.31002	4.400206	-1.13522	0.259352	-5.28944	0.566385	0.558496
Dendritic.cells	LILR4B	0.776628	3.079256	1.135097	0.259402	-4.93671	0.580954	0.579409
Dendritic.cells	CEP85L	0.187639	4.806918	1.135026	0.259432	-5.87055	0.561993	0.552208
Dendritic.cells	ZFP319	0.396437	2.544028	1.134968	0.259456	-5.17376	0.586991	0.588101
Dendritic.cells	JPT2	-0.35135	3.513173	-1.13496	0.259459	-5.19186	0.576116	0.572456
Dendritic.cells	AP1G1	-0.13255	6.456316	-1.13492	0.259476	-6.05092	0.544629	0.527432
Dendritic.cells	EPN1	0.115824	6.109989	1.134456	0.25967	-6.10818	0.548417	0.532723
Dendritic.cells	ST13	-0.10588	6.987135	-1.13443	0.25968	-6.10067	0.539389	0.519872
Dendritic.cells	GM16794	-0.52899	0.41499	-1.13421	0.259774	-4.8681	0.612008	0.62427
Dendritic.cells	TRAPPC9	-0.12713	6.178368	-1.13419	0.25978	-6.044	0.547706	0.531795
Dendritic.cells	PHACTR4	-0.14829	5.677431	-1.13402	0.259852	-5.75896	0.552985	0.539295
Dendritic.cells	ITSN1	-0.16383	5.300914	-1.13371	0.259982	-6.07298	0.55702	0.545121
Dendritic.cells	STK38L	0.150093	4.003603	1.133609	0.260023	-5.77567	0.57102	0.565178
Dendritic.cells	UBXN6	0.178023	4.786672	1.133511	0.260064	-5.73788	0.562516	0.55301
Dendritic.cells	LYAR	0.24178	4.937131	1.133453	0.260088	-5.54636	0.560901	0.550699
Dendritic.cells	ICAM1	0.218616	5.898773	1.133282	0.260159	-5.85862	0.550758	0.5362
Dendritic.cells	GID4	0.179359	4.230623	1.133119	0.260228	-5.56903	0.568614	0.561752
Dendritic.cells	PPP1R18O(	0.428517	0.993134	1.132877	0.260329	-4.9037	0.605364	0.614813
Dendritic.cells	OSBPL2	0.198421	3.979026	1.132841	0.260344	-5.46633	0.571391	0.565825
Dendritic.cells	MAD2L1BP	0.31146	4.194819	1.13214	0.260637	-5.17262	0.5695	0.562657
Dendritic.cells	GM12158	-0.71221	0.549233	-1.13186	0.260754	-4.80072	0.611129	0.622688
Dendritic.cells	HSF2	-0.27324	4.185277	-1.13184	0.260762	-5.33484	0.569604	0.562806
Dendritic.cells	ALPK2	0.587115	1.260876	1.131824	0.260769	-4.8585	0.602716	0.6105
Dendritic.cells	COTL1	0.205992	6.629821	1.131646	0.260843	-5.67629	0.543731	0.525815
Dendritic.cells	DDB2	0.462589	3.43077	1.131427	0.260935	-5.03903	0.578061	0.574859
Dendritic.cells	TIFA	0.151498	6.262161	1.13093	0.261143	-6.30362	0.547751	0.531488
Dendritic.cells	GRPEL2	-0.30067	3.176522	-1.13091	0.26115	-5.157	0.581047	0.579157

Dendritic.cells	GOLGA2	0.18622	3.814242	1.130893	0.261158	-5.54052	0.573957	0.56897
Dendritic.cells	SLC52A2	0.329062	2.391257	1.130255	0.261426	-5.18421	0.590414	0.592158
Dendritic.cells	TIPIN	0.290194	5.238466	1.129544	0.261724	-5.63355	0.559501	0.547324
Dendritic.cells	USP37	-0.13492	6.268931	-1.12933	0.261811	-6.03193	0.548719	0.531917
Dendritic.cells	TMEM60	0.233501	4.112089	1.129164	0.261883	-5.37119	0.571791	0.564883
Dendritic.cells	WDR59	-0.31445	3.072953	-1.12896	0.26197	-5.22694	0.583339	0.581472
Dendritic.cells	LMAN2L	0.15973	4.447735	1.128931	0.261981	-5.6912	0.568124	0.559654
Dendritic.cells	DMAP1	-0.3566	2.496634	-1.12869	0.262084	-5.06227	0.589945	0.591023
Dendritic.cells	GM37240	0.201782	5.162317	1.128601	0.262119	-5.75976	0.560487	0.548764
Dendritic.cells	RABEP1	0.092934	6.897474	1.12824	0.262271	-6.29028	0.542531	0.523009
Dendritic.cells	GSDMD	0.398099	4.268046	1.127784	0.262462	-5.17328	0.570673	0.562951
Dendritic.cells	TTC27	0.279184	3.520631	1.127481	0.26259	-5.29144	0.578993	0.574893
Dendritic.cells	RARG	-0.47531	1.622679	-1.12746	0.262598	-5.02736	0.600637	0.606056
Dendritic.cells	CDK5RAP2	0.221616	4.446752	1.127185	0.262714	-5.42814	0.568879	0.560373
Dendritic.cells	ELOF1	0.215849	5.375272	1.127111	0.262745	-5.51777	0.558876	0.546074
Dendritic.cells	STK35	0.273	3.447827	1.126742	0.2629	-5.21978	0.580125	0.576332
Dendritic.cells	TXLNG	0.158942	5.107247	1.126432	0.263031	-5.7691	0.562126	0.550419
Dendritic.cells	ATP6V0D1	-0.07812	7.228201	-1.12621	0.263123	-6.29074	0.540042	0.518979
Dendritic.cells	JMJD6	-0.1932	4.999369	-1.12569	0.263344	-5.63418	0.56373	0.552517
Dendritic.cells	GM40645	0.677509	0.751306	1.125477	0.263433	-4.83899	0.611993	0.621935
Dendritic.cells	4930430F0	0.425615	1.035161	1.125122	0.263583	-4.94681	0.608648	0.617127
Dendritic.cells	ACRBP	0.502301	1.566441	1.124756	0.263737	-4.91316	0.602392	0.608154
Dendritic.cells	NT5DC2	0.514885	2.432435	1.124353	0.263907	-4.92702	0.592362	0.593842
Dendritic.cells	HERPUD1	-0.13135	7.853564	-1.12434	0.263911	-6.48261	0.53417	0.510669
Dendritic.cells	TK1	0.338074	4.882981	1.124279	0.263938	-5.51172	0.56509	0.554699
Dendritic.cells	SMC5	0.192875	5.613071	1.124077	0.264023	-5.64733	0.557277	0.543591
Dendritic.cells	TRPM4	0.523869	1.109553	1.123864	0.264113	-4.88174	0.607768	0.616174
Dendritic.cells	CALD1	-0.38211	3.777745	-1.12382	0.26413	-5.38004	0.577189	0.572106
Dendritic.cells	SEL1L	-0.13475	5.161227	-1.12381	0.264137	-5.87846	0.562096	0.550493
Dendritic.cells	INHBC	-0.6491	0.808252	-1.1237	0.264184	-4.87192	0.611344	0.621402
Dendritic.cells	SLC2A1	0.19428	4.676925	1.12365	0.264203	-5.6611	0.567321	0.558009
Dendritic.cells	CAP1	0.119364	6.878742	1.123637	0.264209	-6.03356	0.544064	0.524831
Dendritic.cells	ADCY10	-0.6661	0.912909	-1.1236	0.264223	-4.82887	0.610099	0.619598
Dendritic.cells	ARID2	0.133716	6.543762	1.123469	0.26428	-6.04005	0.547521	0.529815
Dendritic.cells	DDX42	0.116578	5.684077	1.123347	0.264331	-5.91859	0.556525	0.542669
Dendritic.cells	MRPL20	0.15047	6.1388	1.123103	0.264434	-5.85509	0.551738	0.535972
Dendritic.cells	TSPAN17	-0.53648	1.115501	-1.12309	0.264441	-4.85741	0.607697	0.616371
Dendritic.cells	SLC18A1	-0.73756	0.497721	-1.12299	0.264484	-4.81186	0.615057	0.627054
Dendritic.cells	MAN2A1	0.152355	7.183028	1.122729	0.264592	-6.13481	0.540949	0.520645
Dendritic.cells	CECR2	-0.25101	6.605628	-1.12259	0.26465	-6.07671	0.54688	0.529144
Dendritic.cells	EFNA1	-0.58277	0.531324	-1.1223	0.264773	-4.91253	0.614654	0.626715
Dendritic.cells	TRIM25	0.114899	6.978283	1.122293	0.264777	-6.53093	0.543043	0.523809
Dendritic.cells	GM44659	-0.41723	0.682551	-1.12209	0.264862	-5.03404	0.612844	0.624115
Dendritic.cells	COPS3	0.11299	5.92883	1.122054	0.264878	-5.99773	0.553942	0.539363
Dendritic.cells	IGKV2-109	-0.55579	-0.87987	-1.12203	0.264888	-4.81473	0.631855	0.651768
Dendritic.cells	1110035H1	0.462436	1.216236	1.121869	0.264956	-5.00887	0.606507	0.614938
Dendritic.cells	EMG1	0.154251	5.937655	1.121736	0.265012	-5.86244	0.553849	0.539236

Dendritic.cells	HMGA2	-0.79628	1.149583	-1.12173	0.265015	-4.96119	0.607294	0.616078
Dendritic.cells	TNKS1BP1	-0.45402	2.080541	-1.12173	0.265016	-4.97938	0.596413	0.600341
Dendritic.cells	LARP4	-0.10351	6.410341	-1.12158	0.265078	-6.20267	0.548905	0.532237
Dendritic.cells	TMEM39A	-0.16843	4.395391	-1.12142	0.265147	-5.67689	0.570387	0.562994
Dendritic.cells	PACRG	-0.54384	-0.43352	-1.12133	0.265184	-4.93479	0.626354	0.643873
Dendritic.cells	CBARP	-0.43042	1.923122	-1.1213	0.265197	-5.10033	0.598236	0.603081
Dendritic.cells	C3	-0.39589	6.327835	-1.1212	0.265238	-5.71827	0.549764	0.533505
Dendritic.cells	FHL1	0.593636	0.58688	1.120954	0.265343	-4.86031	0.614032	0.626017
Dendritic.cells	GALNT12	0.208698	2.991393	1.120833	0.265394	-5.60944	0.58604	0.585574
Dendritic.cells	SRRT	-0.14997	5.528359	-1.12078	0.265415	-5.82113	0.558216	0.54565
Dendritic.cells	PILRB2	-0.62709	2.237136	-1.12064	0.265476	-4.8938	0.594655	0.598035
Dendritic.cells	IL1RAP	-0.26731	4.731572	-1.12053	0.265523	-5.55305	0.566775	0.55798
Dendritic.cells	CHAC2	0.389452	2.893423	1.120363	0.265593	-4.96856	0.587193	0.587336
Dendritic.cells	OARD1	0.246687	4.902059	1.119782	0.265839	-5.41813	0.565377	0.555509
Dendritic.cells	EVI2A	0.12027	5.251447	1.119534	0.265944	-6.21637	0.561619	0.550268
Dendritic.cells	FOSL1	-0.74237	1.662098	-1.11944	0.265984	-4.838	0.601799	0.608078
Dendritic.cells	TMEM38B	0.249714	4.174266	1.119384	0.266008	-5.40403	0.57331	0.567044
Dendritic.cells	CNOT1	-0.09569	7.026328	-1.11927	0.266055	-6.22755	0.543024	0.523825
Dendritic.cells	GM26802	-0.62386	1.069129	-1.11916	0.266102	-4.89011	0.608777	0.618275
Dendritic.cells	TPD52	0.134528	8.000639	1.119007	0.266168	-6.48745	0.533189	0.50983
Dendritic.cells	AI413582	0.179491	4.68032	1.118853	0.266233	-5.76522	0.567834	0.559249
Dendritic.cells	GM11508	0.348298	3.437566	1.1187	0.266298	-5.30001	0.581547	0.578938
Dendritic.cells	MYEF2	0.184033	5.328236	1.118596	0.266343	-5.72931	0.560856	0.549282
Dendritic.cells	TUSC1	0.24109	3.124075	1.118435	0.266411	-5.40715	0.585071	0.584041
Dendritic.cells	4732496C0	-0.34808	0.689806	-1.11813	0.26654	-5.16698	0.613356	0.625072
Dendritic.cells	ARL5B	0.230874	5.460574	1.118078	0.266563	-5.68154	0.559443	0.547419
Dendritic.cells	AHCTF1	-0.15691	5.405997	-1.11773	0.266712	-5.80977	0.560025	0.548345
Dendritic.cells	YWHAG	-0.13543	6.203239	-1.11767	0.266736	-6.02575	0.551602	0.536332
Dendritic.cells	PCSK7	0.14633	5.282369	1.117529	0.266796	-5.90615	0.561346	0.550314
Dendritic.cells	GM11696	0.486421	1.871722	1.117315	0.266887	-4.97679	0.599418	0.605203
Dendritic.cells	GORASP2	-0.10333	5.534187	-1.11704	0.267004	-5.99438	0.55866	0.546642
Dendritic.cells	BCAM	-0.77123	0.298097	-1.11681	0.267102	-4.92372	0.618062	0.632375
Dendritic.cells	PPP3R1	-0.13546	5.5618	-1.1168	0.267107	-5.84766	0.558366	0.546252
Dendritic.cells	DENND2D	0.396031	2.202221	1.116747	0.267128	-5.00977	0.59559	0.599787
Dendritic.cells	TMEM234	0.087703	7.040364	1.116661	0.267165	-6.20087	0.542937	0.524222
Dendritic.cells	USP30	0.327471	2.252852	1.116525	0.267223	-5.13745	0.595006	0.598943
Dendritic.cells	NMT1	-0.0908	6.941125	-1.11631	0.267312	-6.26391	0.543954	0.525673
Dendritic.cells	PCDH9	0.875643	0.472713	1.116284	0.267325	-4.82714	0.615959	0.629317
Dendritic.cells	B4GALT5	-0.16226	5.975954	-1.11611	0.2674	-6.07895	0.553987	0.540013
Dendritic.cells	ID3	0.186602	6.599763	1.116094	0.267406	-6.07834	0.547475	0.530716
Dendritic.cells	YJU2	0.260675	3.454196	1.11603	0.267433	-5.21978	0.58136	0.579295
Dendritic.cells	APIP	0.213935	4.220389	1.116029	0.267434	-5.4073	0.572863	0.567073
Dendritic.cells	TAF11	0.191739	4.933052	1.115604	0.267615	-5.52914	0.565101	0.556065
Dendritic.cells	TRIO	-0.12029	6.069746	-1.11554	0.267643	-6.1969	0.553001	0.538743
Dendritic.cells	CACNA1F	0.693916	-0.45662	1.115525	0.267649	-4.81153	0.62725	0.645953
Dendritic.cells	MRPS24	0.126739	6.031221	1.11535	0.267723	-5.92923	0.553406	0.539329
Dendritic.cells	AP3M1	0.154945	4.881049	1.115231	0.267774	-5.65969	0.565663	0.556889

Dendritic.cells	PPID	0.212487	4.87562	1.115117	0.267822	-5.54118	0.565722	0.557007
Dendritic.cells	UBE2M	0.10716	7.20691	1.114888	0.26792	-6.20404	0.541234	0.522078
Dendritic.cells	PROX2	0.51173	1.188752	1.114847	0.267937	-4.95556	0.607424	0.617259
Dendritic.cells	BEND4	-0.16244	3.557561	-1.11476	0.267976	-5.99859	0.580205	0.577912
Dendritic.cells	TNFRSF11A	-0.451	2.190262	-1.11471	0.267997	-5.12329	0.595727	0.600318
Dendritic.cells	TPD52L2	0.143082	5.433659	1.114667	0.268014	-5.71856	0.55973	0.548518
Dendritic.cells	MIGA2	0.574631	0.892517	1.114633	0.268029	-4.89601	0.610938	0.622384
Dendritic.cells	MCU	-0.18343	6.164566	-1.11463	0.268029	-5.82984	0.552007	0.537473
Dendritic.cells	PER1	0.189343	4.926726	1.114407	0.268125	-5.75977	0.565261	0.556355
Dendritic.cells	PRDX5	0.231898	7.635313	1.113889	0.268346	-6.21783	0.537179	0.516127
Dendritic.cells	LSP1	0.105313	8.230432	1.113836	0.268368	-6.69499	0.531215	0.507655
Dendritic.cells	MCM8	0.50167	1.54016	1.113796	0.268385	-4.92019	0.603615	0.61156
Dendritic.cells	ANKRD40	0.192512	4.238658	1.113397	0.268556	-5.52534	0.573224	0.56751
Dendritic.cells	GOLPH3	0.110476	5.902166	1.113067	0.268697	-5.92748	0.55549	0.541962
Dendritic.cells	AMPD2	-0.45289	2.339378	-1.11251	0.268934	-4.95627	0.59507	0.598608
Dendritic.cells	NACC2	-0.39915	1.77434	-1.1125	0.268937	-5.14265	0.601623	0.608087
Dendritic.cells	HYPK	0.123325	5.8151	1.112317	0.269017	-5.93652	0.556674	0.543458
Dendritic.cells	SESN3	-0.18174	4.83647	-1.11216	0.269086	-5.7469	0.567156	0.558589
Dendritic.cells	CCR5	0.172405	4.693422	1.112149	0.269089	-6.08882	0.568709	0.560817
Dendritic.cells	2310040G2	-0.36354	1.291307	-1.1112	0.269493	-4.95911	0.608088	0.616829
Dendritic.cells	CABIN1	-0.14724	5.4487	-1.11106	0.269557	-5.86165	0.561324	0.549486
Dendritic.cells	GPD2	-0.19999	6.625661	-1.11078	0.269676	-6.11981	0.549055	0.53193
Dendritic.cells	NSFL1C	-0.20269	4.168167	-1.11064	0.269734	-5.43905	0.575387	0.56962
Dendritic.cells	ZFP358	0.297223	3.044691	1.110451	0.269815	-5.2803	0.588021	0.587746
Dendritic.cells	ST3GAL3	0.152984	5.293649	1.110153	0.269943	-5.86554	0.563237	0.552159
Dendritic.cells	SULT2A2	-0.58011	4.037802	-1.11012	0.269958	-5.26116	0.576934	0.571803
Dendritic.cells	QRICH1	-0.08776	6.585338	-1.11002	0.269999	-6.17806	0.549587	0.532655
Dendritic.cells	ERBB3	0.331561	1.113728	1.109838	0.270078	-5.34434	0.610492	0.620269
Dendritic.cells	GTF2I	-0.09429	5.762327	-1.10978	0.270101	-6.27975	0.558233	0.545018
Dendritic.cells	PSMB1	0.10012	7.299863	1.109319	0.270301	-6.2842	0.542484	0.522265
Dendritic.cells	DOCK2	0.096519	9.34967	1.109235	0.270337	-6.65894	0.522073	0.493336
Dendritic.cells	DVL1	-0.40645	2.507773	-1.10887	0.270491	-5.048	0.59468	0.596837
Dendritic.cells	LY6I	1.174052	0.802083	1.10878	0.270532	-4.86381	0.614706	0.625873
Dendritic.cells	TRMT1L	0.153608	4.566129	1.108462	0.270668	-5.61235	0.571754	0.563728
Dendritic.cells	NAPEPLD	0.533556	1.056502	1.108344	0.270719	-4.92821	0.611851	0.621537
Dendritic.cells	AGTPBP1	-0.20115	5.128653	-1.10768	0.271006	-5.69432	0.565647	0.555259
Dendritic.cells	KBTBD8	-0.53958	0.251382	-1.10762	0.271028	-4.88505	0.62154	0.635922
Dendritic.cells	NEDD4	-0.15652	5.933587	-1.10761	0.271032	-6.05798	0.557044	0.542954
Dendritic.cells	ACER2	-0.34509	2.516505	-1.10754	0.271066	-5.2835	0.594766	0.597143
Dendritic.cells	FBXO30	-0.25312	5.032199	-1.10741	0.27112	-5.4904	0.566689	0.556766
Dendritic.cells	TMC7	0.592264	-0.40026	1.107253	0.271188	-4.81632	0.629509	0.647542
Dendritic.cells	SNX11	0.354466	2.539196	1.107194	0.271213	-5.09627	0.594505	0.596766
Dendritic.cells	COG4	0.133246	5.336083	1.107152	0.271231	-5.90264	0.563414	0.552076
Dendritic.cells	HSF5	0.645137	0.277217	1.107141	0.271236	-4.83699	0.621226	0.635483
Dendritic.cells	ELOVL1	0.195901	4.858115	1.1071	0.271254	-5.65661	0.568577	0.559484
Dendritic.cells	GM867	0.680671	-0.30876	1.106849	0.271362	-4.82887	0.628383	0.645968
Dendritic.cells	TRIB1	-0.14799	5.765307	-1.10682	0.271376	-6.18338	0.558829	0.545583

Dendritic.cells	STAU1	0.12565	6.034578	1.106733	0.271412	-6.0337	0.555977	0.541546
Dendritic.cells	SLC22A4	0.664337	0.816422	1.106369	0.271568	-4.83342	0.614961	0.626427
Dendritic.cells	PSEN2	0.207737	5.03423	1.105862	0.271786	-5.46817	0.566976	0.557205
Dendritic.cells	TBL3	0.225433	3.91508	1.105614	0.271893	-5.41743	0.57926	0.574837
Dendritic.cells	H13	0.077679	6.42662	1.10549	0.271946	-6.4204	0.55216	0.536057
Dendritic.cells	ETFDH	0.169596	4.872939	1.1054	0.271985	-5.66386	0.568726	0.559771
Dendritic.cells	CCDC91	0.420767	2.408137	1.105327	0.272017	-5.0294	0.596339	0.599514
Dendritic.cells	SNX17	0.124377	5.853791	1.105319	0.27202	-6.02068	0.558194	0.544692
Dendritic.cells	GM8251	-0.4927	3.392286	-1.1053	0.272029	-5.08607	0.585115	0.583323
Dendritic.cells	SLC22A18	-0.56755	1.143718	-1.10528	0.272037	-4.90035	0.611154	0.620961
Dendritic.cells	SNX1	0.121141	5.483558	1.104883	0.272208	-5.82255	0.562383	0.550452
Dendritic.cells	PDRG1	0.197677	4.496479	1.104407	0.272413	-5.56408	0.573211	0.565868
Dendritic.cells	RAP2B	0.223545	4.614993	1.104404	0.272414	-5.47727	0.571912	0.564004
Dendritic.cells	GM43774	-0.46978	2.238278	-1.10428	0.272469	-4.99341	0.598695	0.602565
Dendritic.cells	SLC4A2	-0.27336	3.559435	-1.10425	0.272481	-5.24701	0.583616	0.58082
Dendritic.cells	CAR12	0.630375	0.013745	1.104129	0.272533	-4.82019	0.625181	0.64101
Dendritic.cells	GM16201	-0.46052	1.818935	-1.10391	0.272627	-5.05602	0.60367	0.609774
Dendritic.cells	MAST2	0.239892	5.283184	1.103755	0.272695	-5.5467	0.564741	0.553815
Dendritic.cells	PLAC8	0.235102	8.2646	1.10356	0.272779	-6.69407	0.533805	0.509803
Dendritic.cells	ARRDC4	0.358607	2.427989	1.103534	0.27279	-5.21179	0.596587	0.59974
Dendritic.cells	RPIA	0.16994	5.458024	1.103422	0.272838	-5.76028	0.562862	0.551297
Dendritic.cells	RTN4	-0.08664	7.592315	-1.10298	0.273027	-6.37124	0.54058	0.519667
Dendritic.cells	TIMM21	-0.36067	2.602996	-1.10289	0.273066	-5.08338	0.594571	0.5971
Dendritic.cells	EYA3	0.126358	5.473081	1.10279	0.273111	-6.06405	0.562701	0.55126
Dendritic.cells	CCDC166	0.554877	0.615517	1.102658	0.273168	-4.87834	0.617969	0.631068
Dendritic.cells	PCSK5	0.611042	0.558557	1.102579	0.273202	-4.91849	0.618656	0.632116
Dendritic.cells	WDPCP	0.284302	3.269533	1.102374	0.273291	-5.3571	0.58697	0.58634
Dendritic.cells	SIRPB1B	0.848099	1.445504	1.102257	0.273341	-4.84066	0.608064	0.616881
Dendritic.cells	NBEAL1	0.189973	4.937374	1.102219	0.273358	-5.68414	0.568481	0.559784
Dendritic.cells	ZFP553	0.316232	2.414705	1.102163	0.273382	-5.14338	0.59674	0.600502
Dendritic.cells	DAZAP1	0.091788	6.776612	1.10207	0.273422	-6.24624	0.548956	0.531856
Dendritic.cells	MAP3K1	-0.08878	7.970553	-1.10182	0.273528	-6.56064	0.536754	0.514476
Dendritic.cells	PTCH1	-0.2249	3.139748	-1.10179	0.273544	-5.6625	0.58844	0.588532
Dendritic.cells	IFNAR1	0.103022	5.246213	1.101783	0.273546	-6.20768	0.565139	0.555018
Dendritic.cells	DDX27	-0.16044	4.974515	-1.10168	0.273592	-5.74471	0.568077	0.559243
Dendritic.cells	LEAP2	0.59972	3.060255	1.101391	0.273716	-5.05567	0.589343	0.589964
Dendritic.cells	TUBGCP6	-0.36434	2.759296	-1.10104	0.273869	-5.13256	0.592777	0.595042
Dendritic.cells	AGTR1A	-0.76727	0.270352	-1.10095	0.273908	-4.84086	0.622146	0.637705
Dendritic.cells	NFATC3	0.143439	7.290076	1.100828	0.273959	-6.07016	0.543663	0.524596
Dendritic.cells	BRI3BP	0.125948	4.756436	1.100815	0.273965	-6.06997	0.57045	0.562935
Dendritic.cells	MGST3	0.377187	3.846236	1.10063	0.274045	-5.26307	0.580491	0.577416
Dendritic.cells	A230072C0	0.44497	1.545767	1.100611	0.274054	-4.98807	0.60688	0.615572
Dendritic.cells	MCFD2	-0.30298	3.702476	-1.10054	0.274083	-5.31849	0.582097	0.579735
Dendritic.cells	ECT2	-0.38301	4.373594	-1.10026	0.274204	-5.48433	0.574646	0.569124
Dendritic.cells	TRAPPC10	-0.14233	5.170629	-1.10026	0.274206	-5.82077	0.565955	0.556635
Dendritic.cells	GPR18	0.195451	4.514903	1.100129	0.274262	-5.82575	0.573093	0.566979
Dendritic.cells	C2CD2	-0.28831	3.079676	-1.10002	0.274311	-5.29661	0.589122	0.590086

Dendritic.cells	PLK2	-0.34551	5.006155	-1.09994	0.274344	-5.5459	0.567734	0.559287
Dendritic.cells	MAT2B	0.164884	5.679223	1.099938	0.274345	-5.7001	0.560498	0.548904
Dendritic.cells	SERP2	-0.4098	0.429349	-1.09935	0.274602	-5.0247	0.620218	0.635528
Dendritic.cells	PDE3B	0.269866	6.955861	1.099252	0.274642	-5.83094	0.5471	0.530032
Dendritic.cells	CD82	-0.13101	5.476033	-1.09921	0.274662	-6.03173	0.56267	0.552334
Dendritic.cells	2810403D2	0.302248	2.774023	1.099031	0.274738	-5.25277	0.592609	0.595579
Dendritic.cells	NLRP1B	-0.5498	1.79452	-1.09892	0.274786	-4.97112	0.603956	0.612051
Dendritic.cells	TSPYL1	0.152821	4.888609	1.098907	0.274792	-5.81854	0.569011	0.561564
Dendritic.cells	RPTOR	-0.10989	6.294365	-1.09864	0.274909	-6.15538	0.553989	0.540081
Dendritic.cells	CLEC1B	-0.4156	3.741016	-1.09858	0.274936	-5.34196	0.581666	0.579868
Dendritic.cells	HAO1	-0.52903	3.070076	-1.09856	0.274942	-5.0634	0.589231	0.590796
Dendritic.cells	RSAD2	0.621361	4.537164	1.0985	0.274969	-5.41675	0.572848	0.567174
Dendritic.cells	RNASE4	-0.34568	5.17058	-1.09848	0.274975	-5.60834	0.565955	0.55726
Dendritic.cells	CCDC88B	0.275127	3.695402	1.098472	0.274981	-5.33831	0.582177	0.58062
Dendritic.cells	COQ3	0.216898	3.500438	1.09831	0.275051	-5.31706	0.584365	0.583781
Dendritic.cells	FBXO22	-0.20136	4.616588	-1.09827	0.275067	-5.48243	0.571978	0.565924
Dendritic.cells	FH1	0.184457	5.484899	1.097763	0.275288	-5.89283	0.562918	0.552558
Dendritic.cells	HYAL1	0.442092	1.311575	1.097513	0.275397	-4.95909	0.610145	0.620682
Dendritic.cells	EFNA5	-0.19299	2.313548	-1.09721	0.275528	-6.12149	0.598566	0.603724
Dendritic.cells	PPP1R15A	-0.24986	7.089434	-1.09701	0.275616	-5.9462	0.54639	0.528581
Dendritic.cells	NUP153	-0.12168	5.848135	-1.09627	0.275937	-5.99815	0.559581	0.547437
Dendritic.cells	TBC1D12	-0.27136	3.768556	-1.09598	0.276066	-5.54655	0.582275	0.580115
Dendritic.cells	LCAT	-0.47155	2.143834	-1.09576	0.276159	-5.05326	0.600824	0.607013
Dendritic.cells	TOMM5	0.168922	5.458336	1.095644	0.27621	-5.8432	0.563746	0.553535
Dendritic.cells	ZFP758	0.309592	2.74078	1.095531	0.276259	-5.18613	0.593924	0.597071
Dendritic.cells	SHLD1	0.330077	3.33904	1.09549	0.276277	-5.17026	0.587108	0.587216
Dendritic.cells	GM38560	0.603394	0.991541	1.095393	0.276319	-4.91015	0.614425	0.626892
Dendritic.cells	IMPA1	-0.16159	5.285622	-1.09538	0.276327	-5.71783	0.565605	0.556305
Dendritic.cells	CD40LG	-0.60664	-0.56972	-1.09533	0.276347	-4.83274	0.633449	0.654663
Dendritic.cells	SCAF11	0.107396	7.16109	1.095278	0.276369	-6.18975	0.545845	0.528009
Dendritic.cells	WDR26	0.08983	7.53429	1.095259	0.276378	-6.35992	0.542023	0.522553
Dendritic.cells	SNAPC1	0.267769	3.850567	1.095222	0.276394	-5.31717	0.581358	0.578984
Dendritic.cells	TNS4	0.668217	-0.27421	1.095197	0.276405	-4.82918	0.629795	0.649319
Dendritic.cells	GM48768	0.607721	0.621002	1.094482	0.276716	-4.84256	0.619368	0.633662
Dendritic.cells	KCNA2	-0.826	0.96787	-1.09434	0.27678	-4.86486	0.615195	0.627645
Dendritic.cells	UQCC3	0.224778	4.016511	1.094274	0.276807	-5.40493	0.579967	0.576638
Dendritic.cells	SFT2D1	0.106564	5.999884	1.094098	0.276884	-6.0032	0.558412	0.545697
Dendritic.cells	PPM1A	-0.09537	6.125745	-1.09385	0.276992	-6.05711	0.55708	0.543805
Dendritic.cells	VPS72	0.189385	4.357985	1.093836	0.276998	-5.53018	0.57618	0.571237
Dendritic.cells	2610027K0	0.512617	0.681173	1.09371	0.277053	-4.917	0.618641	0.632772
Dendritic.cells	KDM1A	0.11481	5.813944	1.09334	0.277214	-5.96767	0.560388	0.548742
Dendritic.cells	ACAD12	0.548711	0.714351	1.093283	0.277239	-4.91884	0.618241	0.632405
Dendritic.cells	PYGL	-0.49116	5.16283	-1.09318	0.277283	-5.16184	0.56738	0.55886
Dendritic.cells	GNB1	-0.06578	8.848488	-1.09316	0.277295	-6.5493	0.529265	0.504369
Dendritic.cells	NR3C2	-0.45608	1.939637	-1.09304	0.277347	-5.14755	0.603685	0.611366
Dendritic.cells	RAD52	-0.24782	3.121528	-1.09292	0.277399	-5.41491	0.590042	0.591638
Dendritic.cells	TSACC	0.458121	1.881856	1.092328	0.277656	-5.14224	0.604362	0.612678

Dendritic.cells	PCF11	-0.13809	6.482932	-1.09199	0.277804	-6.07747	0.553322	0.539102
Dendritic.cells	CDC37L1	0.136799	5.167302	1.091898	0.277845	-5.81848	0.567332	0.559243
Dendritic.cells	CIR1	0.197459	5.515333	1.091888	0.277849	-5.59895	0.563581	0.553851
Dendritic.cells	KLHL22	-0.27834	3.001151	-1.09174	0.277911	-5.31645	0.591414	0.594067
Dendritic.cells	IL13RA1	0.347504	3.558786	1.091734	0.277916	-5.38476	0.585092	0.584921
Dendritic.cells	1110051M	0.247242	3.263469	1.091592	0.277978	-5.3972	0.588429	0.58978
Dendritic.cells	GM16740	0.350779	2.544974	1.091574	0.277986	-5.14775	0.596649	0.601691
Dendritic.cells	RASA2	-0.17204	6.158678	-1.09144	0.278044	-5.99473	0.556732	0.544178
Dendritic.cells	AP3S1	0.121979	7.096627	1.091345	0.278086	-6.10703	0.546942	0.530187
Dendritic.cells	IFIT3B	-0.73744	1.43036	-1.09132	0.278097	-4.95486	0.609684	0.62074
Dendritic.cells	GM7030	-0.68343	2.084686	-1.09112	0.278185	-4.91173	0.60199	0.609618
Dendritic.cells	IGHD	-0.28161	3.245617	-1.09105	0.278217	-5.39264	0.588632	0.590248
Dendritic.cells	SPTA1	0.763553	0.279808	1.091018	0.278229	-4.85247	0.623505	0.64097
Dendritic.cells	BAZ1B	0.132066	6.23949	1.091016	0.27823	-6.03203	0.55588	0.543051
Dendritic.cells	PELP1	0.285187	3.358177	1.091012	0.278232	-5.25573	0.587357	0.588402
Dendritic.cells	TM9SF2	-0.07477	6.683958	-1.09094	0.278266	-6.26922	0.551221	0.536413
Dendritic.cells	ARHGEF37	0.931257	0.616444	1.090703	0.278367	-4.84294	0.619423	0.635129
Dendritic.cells	NCKAP5LO:	-0.65857	0.041134	-1.09051	0.278453	-4.87268	0.626419	0.645415
Dendritic.cells	TMEM126I	0.369809	2.697386	1.09046	0.278473	-5.03594	0.594894	0.599491
Dendritic.cells	EIF6	0.146507	5.835967	1.090281	0.278552	-5.86977	0.560154	0.549348
Dendritic.cells	GLMN	-0.33873	2.819036	-1.09025	0.278567	-5.15697	0.593497	0.597473
Dendritic.cells	TRMU	0.331947	1.548223	1.090131	0.278618	-5.09851	0.608289	0.618996
Dendritic.cells	KCP	-0.66397	0.100139	-1.09009	0.278634	-4.83389	0.625697	0.6444
Dendritic.cells	HMGN3	0.15229	3.255903	1.090068	0.278645	-6.08523	0.588515	0.590285
Dendritic.cells	GM20721	0.202321	3.72758	1.089614	0.278844	-5.6046	0.583465	0.582752
Dendritic.cells	HCK	0.20746	5.693071	1.089529	0.278881	-5.95175	0.561938	0.551731
Dendritic.cells	RPE	0.181058	4.554255	1.089405	0.278936	-5.67554	0.574284	0.569557
Dendritic.cells	IL15RA	0.568869	2.38833	1.089288	0.278987	-4.9285	0.598737	0.604998
Dendritic.cells	GORASP1	-0.59335	0.733344	-1.08878	0.279208	-4.8941	0.618661	0.63375
Dendritic.cells	MYO9A	-0.17197	6.336763	-1.08867	0.279257	-6.11781	0.555438	0.542316
Dendritic.cells	AXIN2	0.596615	-0.02376	1.088364	0.279392	-4.83581	0.627921	0.647285
Dendritic.cells	ARHGAP12	0.151719	5.076663	1.088353	0.279397	-5.89538	0.568954	0.561693
Dendritic.cells	GCHFR	-0.49741	3.389908	-1.08815	0.279488	-5.20543	0.587659	0.588718
Dendritic.cells	ALG14	0.278391	3.562595	1.088126	0.279497	-5.33392	0.585708	0.585896
Dendritic.cells	CACYBP	0.178711	5.736095	1.087845	0.27962	-5.72872	0.561851	0.551566
Dendritic.cells	CCDC117	0.136819	4.699485	1.087765	0.279655	-5.87171	0.57307	0.567719
Dendritic.cells	ARHGEF11	0.178392	4.907611	1.087732	0.27967	-5.78417	0.570794	0.56444
Dendritic.cells	NFKBIL1	0.226876	3.861655	1.087653	0.279705	-5.37148	0.58235	0.581141
Dendritic.cells	EXOSC8	0.225849	5.033192	1.087409	0.279812	-5.59869	0.569432	0.562539
Dendritic.cells	FKBP15	0.125355	5.239594	1.087404	0.279814	-5.98086	0.567194	0.559316
Dendritic.cells	AP5Z1	-0.30681	2.539751	-1.08705	0.279968	-5.16175	0.597589	0.603086
Dendritic.cells	LPXN	-0.23903	5.23201	-1.08695	0.280015	-5.54562	0.567468	0.559605
Dendritic.cells	PPP1R13B	0.161144	5.30235	1.086298	0.2803	-5.85942	0.567176	0.558681
Dendritic.cells	RAB21	-0.0916	6.891793	-1.08535	0.280717	-6.26933	0.55095	0.53486
Dendritic.cells	TM7SF3	0.179195	3.924878	1.085332	0.280725	-5.65294	0.582988	0.580913
Dendritic.cells	MED10	0.139071	4.886924	1.085045	0.280852	-5.87314	0.572413	0.565676
Dendritic.cells	YARS2	0.309769	3.150094	1.085009	0.280867	-5.24456	0.591821	0.5937

Dendritic.cells	TOMT	-0.55595	1.736925	-1.08472	0.280993	-4.98046	0.608384	0.617558
Dendritic.cells	ABCG3	0.280254	4.394356	1.084469	0.281105	-5.59223	0.578104	0.573573
Dendritic.cells	FAM177A	0.458411	1.731422	1.08409	0.281272	-4.98202	0.608822	0.617696
Dendritic.cells	MAP2K5	0.104616	6.143251	1.083675	0.281455	-6.11253	0.559521	0.546494
Dendritic.cells	POLR2L	0.16072	5.414671	1.083461	0.28155	-5.81914	0.567325	0.557733
Dendritic.cells	GPS2	-0.11066	5.859455	-1.08344	0.281561	-5.91019	0.562544	0.550878
Dendritic.cells	DAP	0.107023	6.19848	1.08332	0.281612	-6.40329	0.558935	0.54573
Dendritic.cells	UHMK1	0.145903	4.630018	1.083311	0.281616	-5.75645	0.575889	0.570089
Dendritic.cells	OSBPL1A	-0.30948	3.288159	-1.08301	0.28175	-5.24025	0.590988	0.591835
Dendritic.cells	HAT1	0.113082	6.105162	1.082822	0.281832	-6.24137	0.559988	0.547262
Dendritic.cells	9530082P2	-0.45417	0.699259	-1.08275	0.281863	-4.96003	0.621408	0.636112
Dendritic.cells	NDUFS4	0.110431	6.335519	1.082742	0.281867	-6.09177	0.557547	0.543793
Dendritic.cells	AAR2	-0.18885	4.021304	-1.08262	0.28192	-5.46934	0.582712	0.579991
Dendritic.cells	LZTS2	-0.37405	1.249256	-1.0822	0.282106	-5.23994	0.614788	0.626626
Dendritic.cells	MAD2L1	-0.41503	4.166093	-1.08219	0.282112	-5.16621	0.581096	0.577795
Dendritic.cells	C130026I2	0.170025	2.944263	1.081968	0.282209	-5.95339	0.594921	0.597816
Dendritic.cells	ARHGAP22	0.665831	1.023886	1.081898	0.28224	-4.94755	0.61749	0.630629
Dendritic.cells	GM29707	0.547607	-0.46354	1.08185	0.282261	-4.83908	0.635687	0.657214
Dendritic.cells	AAGAB	0.148764	4.862045	1.081678	0.282337	-5.81629	0.573403	0.56682
Dendritic.cells	10-Sep	0.274096	3.119761	1.081659	0.282345	-5.31045	0.59291	0.594984
Dendritic.cells	PPAN	-0.29242	4.072585	-1.08161	0.282366	-5.34299	0.582139	0.579414
Dendritic.cells	OSBPL3	0.284825	3.353205	1.081546	0.282395	-5.43537	0.590248	0.591137
Dendritic.cells	MATR3	-0.08419	6.50291	-1.08092	0.282674	-6.17565	0.556205	0.541691
Dendritic.cells	SRSF11	0.065432	7.871425	1.080699	0.28277	-6.43595	0.542042	0.521533
Dendritic.cells	UMPS	-0.25737	4.099845	-1.0807	0.282771	-5.31898	0.582278	0.579269
Dendritic.cells	WRAP73	-0.29531	2.923556	-1.08057	0.282827	-5.1529	0.595617	0.598583
Dendritic.cells	ALOX5AP	0.182732	7.325815	1.080374	0.282914	-6.68921	0.5477	0.529631
Dendritic.cells	CALML4	-0.522	1.801954	-1.08021	0.282985	-5.0411	0.6088	0.617746
Dendritic.cells	CHSY1	0.24195	5.079341	1.079961	0.283097	-5.49315	0.57162	0.563983
Dendritic.cells	MBNL2	-0.11624	8.406165	-1.07976	0.283187	-6.43969	0.536786	0.514133
Dendritic.cells	DVL2	0.332847	3.263703	1.079673	0.283224	-5.23448	0.59188	0.593278
Dendritic.cells	IL2RG	0.271876	6.294179	1.079602	0.283256	-6.00222	0.558562	0.545285
Dendritic.cells	RABL2	0.446405	1.106471	1.079516	0.283294	-4.99697	0.617137	0.629959
Dendritic.cells	CTDSP2	-0.19908	4.801724	-1.07943	0.28333	-5.47511	0.574659	0.568432
Dendritic.cells	KLHL24	0.145229	6.228518	1.079299	0.28339	-5.98674	0.55927	0.546367
Dendritic.cells	HAUS4	0.276922	4.204699	1.079131	0.283465	-5.35627	0.581323	0.578078
Dendritic.cells	MAPK11	-0.58576	0.260399	-1.07856	0.283715	-4.87321	0.627772	0.645367
Dendritic.cells	PCK1	-0.6087	4.182878	-1.07851	0.28374	-5.26918	0.581854	0.578694
Dendritic.cells	ASF1A	0.210827	5.210724	1.078451	0.283766	-5.62334	0.570525	0.562384
Dendritic.cells	DAPP1	0.092039	6.482475	1.078221	0.283868	-6.39077	0.556902	0.542854
Dendritic.cells	1810058I24	0.161013	7.005863	1.078123	0.283912	-6.14503	0.55142	0.535002
Dendritic.cells	DCP1B	0.293605	2.830948	1.078014	0.28396	-5.27778	0.597195	0.600993
Dendritic.cells	COPS7B	0.25661	3.34463	1.07797	0.283979	-5.33295	0.591306	0.592488
Dendritic.cells	RNF38	-0.12279	6.163439	-1.07777	0.284067	-6.02696	0.560284	0.547843
Dendritic.cells	MRPL44	0.372299	2.945204	1.077597	0.284145	-5.07844	0.595884	0.599237
Dendritic.cells	SORD	-0.40023	4.176195	-1.07755	0.284164	-5.24792	0.581934	0.57907
Dendritic.cells	ATL2	-0.12438	5.337211	-1.07744	0.284215	-5.93853	0.569156	0.560653

Dendritic.cells	SLFN4	0.900517	0.48225	1.077361	0.28425	-4.84796	0.625065	0.641721
Dendritic.cells	TICAM1	-0.33197	2.819811	-1.0769	0.284455	-5.20458	0.597597	0.601554
Dendritic.cells	SRPR	-0.09534	5.794906	-1.07683	0.284484	-6.09191	0.564472	0.553768
Dendritic.cells	EEF1B2	0.097251	8.556525	1.076325	0.28471	-6.51337	0.536137	0.512981
Dendritic.cells	SAPCD1	0.417893	1.629002	1.076123	0.2848	-5.07929	0.611878	0.622125
Dendritic.cells	IFT122	-0.32013	1.594011	-1.07612	0.284802	-5.15267	0.612294	0.622731
Dendritic.cells	GNG2	-0.09192	6.302982	-1.07581	0.28494	-6.45565	0.559529	0.546315
Dendritic.cells	ZUP1	0.163162	5.168406	1.075416	0.285114	-5.92166	0.571909	0.564007
Dendritic.cells	PPFIA1	-0.14248	5.566923	-1.07537	0.285136	-5.94658	0.567582	0.5578
Dendritic.cells	OTUD6B	0.178166	4.301102	1.07509	0.285259	-5.50874	0.581565	0.577837
Dendritic.cells	TESMIN	-0.65444	0.971475	-1.07503	0.285287	-4.90305	0.620224	0.633904
Dendritic.cells	NINJ2	-0.68716	0.701022	-1.07479	0.285395	-4.88837	0.62362	0.638728
Dendritic.cells	EPB41L5	0.421867	3.379051	1.074106	0.285698	-5.10613	0.592591	0.593053
Dendritic.cells	F2RL2	0.613038	0.129948	1.073967	0.28576	-4.90852	0.631177	0.649163
Dendritic.cells	CNKSR3	-0.23515	4.754523	-1.07376	0.285851	-5.77238	0.577244	0.570891
Dendritic.cells	RHOT2	0.373062	2.679369	1.073432	0.285998	-5.11225	0.600938	0.604947
Dendritic.cells	ZFP36L1	0.11187	7.742233	1.072845	0.28626	-6.47803	0.545987	0.525629
Dendritic.cells	ING3	-0.16507	4.830612	-1.07278	0.286289	-5.64719	0.576963	0.570009
Dendritic.cells	CCL27A	0.363086	1.684593	1.072347	0.286483	-5.12605	0.613189	0.622335
Dendritic.cells	DPY19L1	0.148181	4.584338	1.072293	0.286507	-5.94114	0.579842	0.574101
Dendritic.cells	1300002E1	0.270505	3.19234	1.072233	0.286533	-5.35654	0.595558	0.596806
Dendritic.cells	HIST1H3C	-0.78225	1.047316	-1.07185	0.286704	-4.85814	0.620833	0.633506
Dendritic.cells	GM3435	0.666195	0.205181	1.071792	0.28673	-4.86253	0.631113	0.648505
Dendritic.cells	PANK3	0.158585	4.591077	1.071787	0.286733	-5.69639	0.579767	0.574047
Dendritic.cells	GM45267	0.645186	0.245242	1.071766	0.286742	-4.85163	0.630619	0.647785
Dendritic.cells	NCOA4	0.185765	6.199192	1.071409	0.286902	-6.03049	0.562411	0.549042
Dendritic.cells	ZFP568	-0.25263	4.08691	-1.07132	0.28694	-5.42818	0.585541	0.582321
Dendritic.cells	BC055324	-0.43699	2.245694	-1.07125	0.286974	-5.00881	0.606702	0.612936
Dendritic.cells	ERGIC3	0.123758	5.513051	1.071038	0.287068	-5.98279	0.569803	0.559707
Dendritic.cells	GM14548	0.372484	1.47249	1.070991	0.287088	-5.22499	0.615884	0.626325
Dendritic.cells	PIGM	-0.19872	3.53133	-1.07087	0.287141	-5.48528	0.591839	0.591479
Dendritic.cells	FZD5	-0.36654	2.536377	-1.0707	0.28722	-5.10067	0.603331	0.608083
Dendritic.cells	PSAP	0.095046	9.249596	1.070504	0.287306	-6.95015	0.531122	0.504449
Dendritic.cells	SCARF1	-0.43499	1.587665	-1.07049	0.287312	-5.05973	0.614528	0.624337
Dendritic.cells	TSEN2	0.420654	1.266976	1.070084	0.287494	-5.02278	0.618647	0.630184
Dendritic.cells	RFC5	0.330008	4.185223	1.06979	0.287626	-5.42921	0.584732	0.581082
Dendritic.cells	PHC2	0.142543	5.798035	1.069708	0.287663	-6.07423	0.566997	0.555569
Dendritic.cells	MYO9B	0.123501	5.866728	1.069597	0.287712	-6.07588	0.566257	0.554514
Dendritic.cells	1700109H(	-0.26635	3.650709	-1.06958	0.287721	-5.44992	0.590767	0.589798
Dendritic.cells	GM34084	0.848245	1.964286	1.06948	0.287765	-4.89313	0.610328	0.618127
Dendritic.cells	DNAJC2	0.132263	5.81945	1.069169	0.287904	-5.90705	0.566918	0.55532
Dendritic.cells	PECAM1	0.145243	7.734027	1.069067	0.28795	-6.47258	0.546774	0.526528
Dendritic.cells	TRIM23	0.18416	3.295163	1.068714	0.288108	-5.6044	0.595171	0.595778
Dendritic.cells	PPA1	-0.25293	4.797054	-1.06863	0.288147	-5.53528	0.578257	0.571418
Dendritic.cells	KCNAB1	-0.60487	1.324987	-1.06834	0.288276	-4.9596	0.618473	0.629462
Dendritic.cells	LSM2	0.235539	5.695608	1.068027	0.288416	-5.7357	0.56875	0.557444
Dendritic.cells	C2CD2L	0.32541	2.991873	1.067861	0.28849	-5.19773	0.59903	0.601091

Dendritic.cells	ZFP763	0.606286	0.433803	1.067669	0.288577	-4.85741	0.629527	0.64548
Dendritic.cells	TENM3	-0.45283	1.688761	-1.06763	0.288594	-5.14012	0.614334	0.623364
Dendritic.cells	PLPP2	0.438994	1.875681	1.067477	0.288662	-4.98377	0.612141	0.620207
Dendritic.cells	DYNLT3	-0.16194	4.662522	-1.06727	0.288754	-5.67638	0.580202	0.573988
Dendritic.cells	RABGAP1L	0.0745	7.908574	1.067068	0.288846	-6.91602	0.545584	0.52444
Dendritic.cells	RAD21	0.120822	7.022909	1.067041	0.288858	-6.14052	0.554752	0.53754
Dendritic.cells	SIRT3	0.195556	3.344497	1.066589	0.289061	-5.53553	0.595204	0.59567
Dendritic.cells	INSL6	0.572352	1.68935	1.066558	0.289075	-4.93263	0.614567	0.623733
Dendritic.cells	BCORL1	-0.29695	3.355845	-1.06654	0.289084	-5.37164	0.595074	0.595485
Dendritic.cells	TCAIM	0.362975	1.789758	1.066205	0.289234	-5.1699	0.61353	0.622116
Dendritic.cells	GM13610	0.527851	0.396658	1.066131	0.289267	-4.91157	0.630393	0.646666
Dendritic.cells	CLEC4A1	0.719271	3.158732	1.065893	0.289374	-5.02473	0.597604	0.598913
Dendritic.cells	TMEM144	-0.54798	0.882973	-1.06541	0.289591	-4.97395	0.624909	0.638251
Dendritic.cells	MNT	-0.22383	4.606728	-1.0651	0.289729	-5.55644	0.581642	0.575583
Dendritic.cells	TMEM86A	-0.29168	2.885047	-1.06496	0.289795	-5.37514	0.601217	0.603914
Dendritic.cells	SLC27A4	-0.29708	2.770367	-1.06488	0.289831	-5.14263	0.60255	0.605863
Dendritic.cells	E030030I0(	-0.41536	2.657085	-1.06481	0.28986	-4.955	0.603871	0.607808
Dendritic.cells	DMTF1	0.131734	4.947065	1.064358	0.290065	-5.84761	0.57817	0.570552
Dendritic.cells	COPA	-0.09077	6.661065	-1.064	0.290224	-6.25185	0.559841	0.544101
Dendritic.cells	SPSB4	-0.58641	0.417547	-1.06381	0.29031	-4.92355	0.631307	0.647333
Dendritic.cells	CDC14B	0.157966	3.454363	1.063738	0.290344	-5.97313	0.595203	0.594959
Dendritic.cells	FOXP1	0.076891	9.650428	1.062933	0.290707	-6.74075	0.529866	0.500892
Dendritic.cells	RBPMS2	0.397377	1.246218	1.062786	0.290773	-5.01972	0.621768	0.632881
Dendritic.cells	PLEKHG3	-0.3361	4.378559	-1.06276	0.290787	-5.26546	0.585269	0.580072
Dendritic.cells	HSD11B1	0.170742	4.593839	1.062664	0.290828	-5.98868	0.582861	0.576614
Dendritic.cells	DPYSL3	-0.42876	0.753878	-1.0625	0.290904	-5.126	0.627804	0.641649
Dendritic.cells	XRCC6	0.238155	5.144906	1.061736	0.291247	-5.72787	0.577371	0.56813
Dendritic.cells	1700019L1	0.645087	0.774577	1.061482	0.291362	-4.8877	0.628305	0.641674
Dendritic.cells	CEP83OS	0.370825	2.022979	1.060462	0.291823	-5.15896	0.613967	0.62006
Dendritic.cells	TOMM34	-0.14651	5.601463	-1.06028	0.291903	-5.79544	0.573166	0.561324
Dendritic.cells	MYH9	0.097314	7.80338	1.059986	0.292038	-6.31902	0.549796	0.528005
Dendritic.cells	ZDHHC5	0.122545	5.017726	1.059984	0.292039	-5.83136	0.57958	0.570581
Dendritic.cells	WDR20	0.103864	5.843767	1.059903	0.292076	-6.12835	0.570531	0.55765
Dendritic.cells	CAPZA2	0.071127	7.929486	1.059798	0.292123	-6.40717	0.548496	0.526208
Dendritic.cells	RHOQ	-0.12422	5.771108	-1.05977	0.292137	-6.31183	0.571319	0.558794
Dendritic.cells	ARG2	0.60018	3.174154	1.059665	0.292184	-5.02551	0.600452	0.600686
Dendritic.cells	DUSP8	0.577815	1.274945	1.059641	0.292194	-4.8856	0.62295	0.633263
Dendritic.cells	ITPRIPL2	-0.27588	3.897046	-1.05943	0.292291	-5.40984	0.592162	0.588788
Dendritic.cells	STOML2	0.199479	4.805582	1.059334	0.292333	-5.64168	0.58194	0.57414
Dendritic.cells	GM31645	-0.51326	0.726126	-1.05927	0.29236	-5.00235	0.62965	0.643154
Dendritic.cells	ANPEP	-0.71103	1.184937	-1.05901	0.292478	-4.90547	0.624185	0.635178
Dendritic.cells	ZFP69	-0.39391	2.612118	-1.05867	0.292635	-5.14431	0.607254	0.610629
Dendritic.cells	EIF3C	0.104591	7.172674	1.058657	0.29264	-6.24818	0.556588	0.537833
Dendritic.cells	SHISA8	0.683068	-0.21439	1.058307	0.292798	-4.86748	0.641674	0.660671
Dendritic.cells	AI837181	0.23957	3.127875	1.05813	0.292879	-5.28835	0.601325	0.602072
Dendritic.cells	CSNK1D	0.083612	6.6346	1.058037	0.292921	-6.15437	0.562355	0.546078
Dendritic.cells	SLC37A2	0.150893	3.315046	1.057996	0.292939	-6.02531	0.599159	0.598945

Dendritic.cells	VPS26C	0.278872	4.084075	1.057622	0.293109	-5.32954	0.590363	0.586372
Dendritic.cells	GTF2F1	0.139365	5.030746	1.057498	0.293165	-5.75911	0.579761	0.571152
Dendritic.cells	ATG9A	0.300598	3.85849	1.057464	0.293181	-5.21556	0.592926	0.590102
Dendritic.cells	TYMS	0.412591	4.922548	1.057425	0.293198	-5.41612	0.58096	0.572883
Dendritic.cells	ABHD2	-0.13578	5.471153	-1.05741	0.293205	-5.8983	0.574912	0.5642
Dendritic.cells	PID1	0.322415	5.484883	1.057365	0.293226	-5.58038	0.574762	0.563984
Dendritic.cells	RBPMS	-0.20862	5.842983	-1.05696	0.293408	-5.89739	0.571109	0.558572
Dendritic.cells	ETV1	0.328904	1.377482	1.056784	0.293489	-5.30597	0.622386	0.632527
Dendritic.cells	SLC30A7	0.144521	5.62501	1.056606	0.29357	-5.96403	0.573585	0.562022
Dendritic.cells	FOXO3	0.145991	6.397005	1.056479	0.293627	-6.10443	0.565246	0.550071
Dendritic.cells	MLLT11	0.192876	3.193389	1.056016	0.293838	-5.5252	0.601263	0.601499
Dendritic.cells	GLG1	0.106354	6.951102	1.055207	0.294206	-6.28136	0.560163	0.542025
Dendritic.cells	SMYD4	-0.36897	2.820357	-1.05519	0.294215	-5.23551	0.606161	0.608075
Dendritic.cells	TRIOBP	0.160814	4.877202	1.055018	0.294291	-5.67454	0.582715	0.57426
Dendritic.cells	ATP1B3	-0.15674	7.281647	-1.05435	0.294597	-6.36414	0.556895	0.537224
Dendritic.cells	TM9SF4	-0.17311	4.769644	-1.05433	0.294607	-5.75247	0.584094	0.576139
Dendritic.cells	CRKL	0.140631	5.071994	1.054311	0.294613	-5.81512	0.58073	0.57131
Dendritic.cells	A530013C2	-0.63138	2.431674	-1.05423	0.294651	-4.94894	0.610967	0.614884
Dendritic.cells	FBXO28	0.184229	4.523471	1.054222	0.294654	-5.68919	0.586852	0.580115
Dendritic.cells	GDPGP1	0.470539	2.18615	1.054035	0.294739	-5.12618	0.613943	0.619148
Dendritic.cells	CHN2	-0.53939	5.358074	-1.05389	0.294805	-5.32885	0.577652	0.566874
Dendritic.cells	TUBGCP3	0.187371	3.88521	1.053451	0.295005	-5.46278	0.594316	0.590811
Dendritic.cells	TMEM220	0.578287	0.299081	1.053391	0.295032	-4.89744	0.637083	0.652755
Dendritic.cells	IFITM2	-0.18819	7.227901	-1.0532	0.295118	-6.11736	0.557681	0.538364
Dendritic.cells	CYFIP2	0.138984	6.947339	1.053159	0.295138	-6.17034	0.56064	0.542593
Dendritic.cells	PAIP2	0.09198	7.620191	1.053058	0.295184	-6.26379	0.553579	0.532538
Dendritic.cells	RANBP2	-0.131	6.846596	-1.05301	0.295206	-6.25584	0.561708	0.544155
Dendritic.cells	ISY1	-0.13682	6.241546	-1.05288	0.295263	-6.07577	0.568181	0.55343
Dendritic.cells	HSD17B11	-0.19873	4.796644	-1.05189	0.295717	-5.60729	0.584582	0.576549
Dendritic.cells	CLEC1A	0.499347	0.972503	1.051787	0.295764	-5.08174	0.629372	0.641259
Dendritic.cells	E530011L2	-0.52143	1.095538	-1.05178	0.295768	-5.02058	0.627866	0.639071
Dendritic.cells	TNFAIP2	-0.55196	4.351498	-1.05176	0.295775	-5.21446	0.589585	0.583738
Dendritic.cells	8030462N1	0.090493	6.073398	1.051514	0.295888	-6.09929	0.570536	0.556416
Dendritic.cells	SKA2	0.356554	3.293834	1.051426	0.295928	-5.19502	0.601692	0.601177
Dendritic.cells	PATZ1	0.172145	4.251704	1.051407	0.295937	-5.70799	0.590714	0.585362
Dendritic.cells	ZBTB40	0.280277	3.163496	1.0512	0.296032	-5.29023	0.603206	0.6034
Dendritic.cells	GM49961	-0.56905	0.574285	-1.05108	0.296086	-4.92858	0.634276	0.648433
Dendritic.cells	POLM	-0.35749	3.245007	-1.05108	0.296087	-5.04584	0.602258	0.602033
Dendritic.cells	LMAN1L	-0.62132	1.485183	-1.05048	0.296361	-4.89396	0.623481	0.632537
Dendritic.cells	GSTM5	-0.66331	1.19189	-1.05039	0.2964	-4.89361	0.627047	0.63773
Dendritic.cells	SFMBT2	0.5342	0.60235	1.050237	0.296471	-4.96189	0.63429	0.648272
Dendritic.cells	GM10762	0.311282	2.549426	1.050194	0.296491	-5.21363	0.610749	0.614122
Dendritic.cells	RTF1	0.086325	6.81408	1.050046	0.296559	-6.22791	0.56291	0.545407
Dendritic.cells	SMURF1	0.16428	4.688664	1.049995	0.296582	-5.79633	0.586125	0.578667
Dendritic.cells	ARHGAP29	-0.29475	2.520124	-1.04982	0.296662	-5.43925	0.611148	0.614716
Dendritic.cells	GNAI3	0.103691	6.443605	1.049067	0.297006	-6.06256	0.567381	0.551361
Dendritic.cells	AURKA	-0.42118	3.318106	-1.04905	0.297013	-5.10908	0.602297	0.601481

Dendritic.cells	RBM18	0.177447	4.236008	1.048921	0.297073	-5.54076	0.591773	0.586332
Dendritic.cells	RHBDD1	0.181202	4.175534	1.048721	0.297165	-5.61862	0.592534	0.587386
Dendritic.cells	ZFP287	-0.64314	0.566909	-1.04835	0.297335	-4.87174	0.635641	0.649552
Dendritic.cells	CHM	-0.13502	5.555869	-1.0481	0.297451	-5.97519	0.577451	0.565514
Dendritic.cells	CNOT8	0.093826	5.284943	1.047671	0.297646	-6.11912	0.580713	0.569976
Dendritic.cells	MBTPS2	0.188496	3.917498	1.047081	0.297916	-5.61078	0.596544	0.592373
Dendritic.cells	SLC39A13	-0.32847	2.796524	-1.04615	0.298344	-5.1739	0.61016	0.611484
Dendritic.cells	PRAF2	0.323396	2.133854	1.046105	0.298364	-5.11348	0.618034	0.622858
Dendritic.cells	DALRD3	0.224459	3.729807	1.045986	0.298419	-5.37984	0.599283	0.595809
Dendritic.cells	TMEM159	-0.63986	1.553977	-1.04586	0.298474	-4.90131	0.625026	0.633005
Dendritic.cells	NUFIP2	-0.10314	7.215419	-1.04564	0.298577	-6.32656	0.560797	0.540821
Dendritic.cells	GM38604	-0.3709	1.796583	-1.04556	0.298616	-5.11296	0.622089	0.628857
Dendritic.cells	SLC15A2	-1.13052	4.597147	-1.04533	0.29872	-5.49056	0.589394	0.581828
Dendritic.cells	RMC1	0.160483	4.652621	1.045272	0.298747	-5.8064	0.588769	0.580931
Dendritic.cells	TRAF4	-0.13857	4.196822	-1.04515	0.298803	-6.29372	0.593932	0.588388
Dendritic.cells	ACP6	0.343302	3.140192	1.044951	0.298894	-5.1549	0.606126	0.606005
Dendritic.cells	SLC19A2	0.421261	1.834884	1.044922	0.298908	-5.02667	0.621627	0.62841
Dendritic.cells	ZSWIM9	0.612689	0.544388	1.044901	0.298917	-4.91319	0.637432	0.651351
Dendritic.cells	CDC42SE1	-0.10032	6.18973	-1.04483	0.29895	-6.22238	0.571778	0.556686
Dendritic.cells	FAM110A	-0.23183	3.760358	-1.04445	0.299125	-5.56233	0.598931	0.595693
Dendritic.cells	KLC2	0.357748	2.681752	1.04442	0.299138	-5.20916	0.611515	0.613842
Dendritic.cells	CERCAM	0.473856	0.652443	1.044358	0.299167	-4.97379	0.63609	0.649509
Dendritic.cells	BCKDHA	0.21478	4.820285	1.044258	0.299213	-5.61709	0.586884	0.57843
Dendritic.cells	GM13830	-0.61158	0.287554	-1.04399	0.299336	-4.93005	0.640635	0.656137
Dendritic.cells	YIPF4	0.15018	6.515782	1.043937	0.299361	-6.04816	0.568256	0.55176
Dendritic.cells	WIPF1	-0.11277	7.425545	-1.04392	0.29937	-6.4411	0.558582	0.537962
Dendritic.cells	6030468B1	-0.54793	0.279697	-1.0437	0.299469	-5.03617	0.640734	0.656282
Dendritic.cells	GM26510	0.241072	4.122817	1.043641	0.299497	-5.51525	0.594776	0.589771
Dendritic.cells	PLIN3	-0.25266	3.527331	-1.04339	0.29961	-5.37062	0.601622	0.599627
Dendritic.cells	F13B	-0.55303	1.463355	-1.04339	0.299613	-4.97275	0.626128	0.635055
Dendritic.cells	ZFP667	0.440983	2.726732	1.043265	0.29967	-5.05282	0.610984	0.613134
Dendritic.cells	NDUFAF5	-0.34703	2.563951	-1.04305	0.299769	-5.14086	0.612909	0.615916
Dendritic.cells	MYLPF	0.507954	2.109786	1.042914	0.299831	-5.0757	0.618322	0.623785
Dendritic.cells	RASIP1	-0.37411	1.808725	-1.04282	0.299876	-5.23426	0.621943	0.629068
Dendritic.cells	KDM4B	-0.18996	4.441359	-1.04278	0.299893	-5.74511	0.591155	0.584647
Dendritic.cells	DTWD2	-0.37194	2.777791	-1.04252	0.300011	-5.17675	0.610381	0.612429
Dendritic.cells	LMLN	0.407412	1.750464	1.042505	0.30002	-5.05437	0.622646	0.630178
Dendritic.cells	NR6A1OS	-0.49641	2.067272	-1.04243	0.300056	-5.02812	0.618832	0.624669
Dendritic.cells	SNX29	-0.11943	6.395771	-1.0424	0.30007	-6.41783	0.569549	0.553775
Dendritic.cells	YAF2	0.143179	6.056106	1.042208	0.300157	-5.79186	0.573229	0.559156
Dendritic.cells	ZFP407	-0.09934	7.039039	-1.0422	0.300159	-6.25023	0.562665	0.544062
Dendritic.cells	CSRNP1	-0.1622	6.641086	-1.04217	0.300175	-6.20278	0.566911	0.550123
Dendritic.cells	TMEM161E	0.153862	4.545354	1.041641	0.300418	-5.7389	0.590349	0.583371
Dendritic.cells	JAZF1	-0.43436	3.086872	-1.04066	0.300873	-5.07835	0.607736	0.607817
Dendritic.cells	MFSD14B	0.122137	5.841678	1.040646	0.300877	-6.02608	0.576504	0.562952
Dendritic.cells	MAN2C1OS	0.188178	4.443794	1.040637	0.300881	-5.67884	0.592088	0.585292
Dendritic.cells	PDCD11	-0.18791	4.214015	-1.04018	0.301092	-5.58936	0.594917	0.589221

Dendritic.cells	PHF6	0.170703	4.940138	1.040037	0.301158	-5.75232	0.586705	0.577453
Dendritic.cells	RAVER2	0.506284	1.423822	1.040023	0.301165	-4.95902	0.627855	0.6368
Dendritic.cells	MPV17L2	0.210236	4.459696	1.039927	0.301209	-5.62005	0.592122	0.585288
Dendritic.cells	SDE2	-0.15729	6.013145	-1.03957	0.301372	-5.95099	0.574992	0.560669
Dendritic.cells	MEST	0.267519	3.792937	1.039514	0.3014	-5.76846	0.599908	0.596442
Dendritic.cells	CAPN7	-0.13347	5.340998	-1.03936	0.301472	-5.81584	0.582424	0.571342
Dendritic.cells	TBC1D31	0.251738	4.166685	1.038879	0.301694	-5.53242	0.595981	0.590379
Dendritic.cells	GALNT11	0.160318	4.120023	1.038382	0.301924	-5.73312	0.596606	0.591387
Dendritic.cells	APOLD1	0.431835	1.979983	1.038169	0.302022	-5.24304	0.621753	0.627676
Dendritic.cells	AVPI1	0.281141	2.712104	1.038168	0.302023	-5.3811	0.613004	0.615025
Dendritic.cells	FAH	-0.5454	3.892683	-1.03816	0.302029	-5.2341	0.599216	0.59515
Dendritic.cells	IGF2BP3	0.137929	6.5516	1.038125	0.302043	-6.12872	0.569588	0.552687
Dendritic.cells	GM5544	-0.57721	0.129876	-1.03795	0.302125	-4.88992	0.644554	0.660781
Dendritic.cells	RSL1	0.506331	1.039109	1.037896	0.302148	-5.02237	0.633225	0.644306
Dendritic.cells	CACNA1A	-0.50595	1.424509	-1.03774	0.302223	-4.98902	0.628495	0.637443
Dendritic.cells	COL15A1	-0.49692	0.332157	-1.03763	0.302273	-5.11211	0.642013	0.657081
Dendritic.cells	KCTD18	0.341113	3.044012	1.037565	0.302302	-5.31538	0.609087	0.609372
Dendritic.cells	GM15446	0.353634	2.166881	1.037476	0.302343	-5.21117	0.619505	0.624465
Dendritic.cells	EP300	0.1046	6.696726	1.037298	0.302426	-6.17186	0.568078	0.550533
Dendritic.cells	TARBP1	0.32966	2.687423	1.037005	0.302561	-5.23619	0.613517	0.615674
Dendritic.cells	MIF4GD	0.196633	4.964455	1.036862	0.302628	-5.65231	0.587272	0.577888
Dendritic.cells	SLC30A9	-0.11959	5.265307	-1.03666	0.30272	-5.97011	0.583936	0.573083
Dendritic.cells	PLCXD2	-0.36528	3.46805	-1.0366	0.302751	-5.19881	0.604397	0.602482
Dendritic.cells	PSMB9	0.234757	6.228284	1.036027	0.303015	-6.08124	0.573633	0.558158
Dendritic.cells	ABCA2	0.455422	1.527476	1.035795	0.303123	-5.06208	0.627836	0.63633
Dendritic.cells	PUSL1	0.335239	2.317284	1.035779	0.30313	-5.13351	0.618292	0.622511
Dendritic.cells	NSMCE1	0.186431	4.829662	1.035661	0.303185	-5.69492	0.589113	0.580489
Dendritic.cells	IFI30	0.156463	6.746076	1.035557	0.303233	-6.29422	0.568038	0.55033
Dendritic.cells	COPS5	-0.14343	5.288344	-1.03544	0.303285	-5.81529	0.583977	0.573124
Dendritic.cells	RBM25	0.065847	8.296585	1.035434	0.30329	-6.58639	0.551711	0.527085
Dendritic.cells	GM36199	-0.63907	-0.13106	-1.03516	0.303417	-4.88177	0.648622	0.666342
Dendritic.cells	DYNLRB1	0.115264	6.280147	1.034771	0.303598	-6.04225	0.573444	0.557605
Dendritic.cells	EVI5L	0.300648	2.262161	1.034554	0.303699	-5.25442	0.619408	0.623654
Dendritic.cells	SMIM40	0.496345	0.301755	1.034481	0.303733	-5.00926	0.643479	0.658576
Dendritic.cells	OASL2	0.483361	3.934807	1.034128	0.303897	-5.67101	0.599838	0.595381
Dendritic.cells	PSMB10	-0.18392	5.706129	-1.03406	0.303929	-6.00313	0.579868	0.566735
Dendritic.cells	VPREB1	-0.84181	2.621584	-1.03402	0.303946	-5.00941	0.615213	0.617539
Dendritic.cells	TM4SF5	-0.3619	0.261552	-1.0335	0.304191	-5.3616	0.644418	0.659693
Dendritic.cells	ZFP346	-0.23458	3.658973	-1.03342	0.304225	-5.55486	0.603337	0.60027
Dendritic.cells	PRPF8	0.106769	5.801492	1.033332	0.304267	-6.04901	0.579115	0.565516
Dendritic.cells	KLF3	0.192128	6.281206	1.033211	0.304323	-5.82791	0.573867	0.558028
Dendritic.cells	PCDH7	0.784229	0.520175	1.032936	0.304451	-4.92176	0.641271	0.655069
Dendritic.cells	WDR37	0.113134	5.478401	1.032799	0.304515	-6.09104	0.582775	0.570721
Dendritic.cells	HHEX	0.235023	4.675113	1.032544	0.304634	-5.58049	0.59178	0.583703
Dendritic.cells	IMPG2	-0.65981	0.6613	-1.03254	0.304638	-4.90223	0.639508	0.65264
Dendritic.cells	EPB41	0.187331	8.026595	1.032523	0.304643	-6.2934	0.555375	0.531707
Dendritic.cells	PTPN6	0.110123	6.758702	1.032084	0.304848	-6.40561	0.568997	0.550858

Dendritic.cells	SOCS3	0.239388	4.698376	1.031977	0.304898	-5.89427	0.591733	0.583403
Dendritic.cells	GM11476	-0.37971	2.536389	-1.03193	0.304919	-5.16175	0.616863	0.619586
Dendritic.cells	GM27201	0.260247	2.321008	1.031526	0.305108	-5.29285	0.619644	0.623447
Dendritic.cells	11-Sep	0.127474	6.415184	1.031359	0.305186	-6.41445	0.572896	0.556371
Dendritic.cells	INPP5B	0.158173	4.216748	1.03129	0.305218	-5.809	0.597414	0.591522
Dendritic.cells	LRRC32	-0.44275	1.241998	-1.03124	0.30524	-5.0782	0.632757	0.642557
Dendritic.cells	SERHL	-0.24225	3.492794	-1.03103	0.305341	-5.40381	0.605783	0.60371
Dendritic.cells	CCL12	-0.93437	0.036828	-1.03101	0.30535	-4.91457	0.647803	0.664587
Dendritic.cells	OAZ1	0.096754	9.534615	1.03086	0.305418	-6.71745	0.540364	0.510293
Dendritic.cells	CANX	-0.0737	7.35016	-1.03071	0.305486	-6.46201	0.562888	0.542271
Dendritic.cells	RANGAP1	-0.17173	5.610379	-1.03063	0.305525	-5.84696	0.581736	0.569205
Dendritic.cells	ZFP933	-0.23162	3.490032	-1.0303	0.305681	-5.52207	0.606033	0.603891
Dendritic.cells	GM12367	-0.66877	0.051988	-1.03018	0.305735	-4.89231	0.647844	0.664482
Dendritic.cells	SACS	0.461568	2.596254	1.02986	0.305885	-5.1192	0.616766	0.619228
Dendritic.cells	CTNNA1	-0.11491	6.643473	-1.02968	0.305969	-6.35177	0.570862	0.553285
Dendritic.cells	DDX50	-0.08329	6.369428	-1.02923	0.306177	-6.26046	0.57409	0.557585
Dendritic.cells	MLF1	-0.56744	0.512644	-1.02915	0.306217	-4.91188	0.642592	0.656254
Dendritic.cells	SPOCK2	0.556723	0.349422	1.029012	0.306281	-4.89644	0.64466	0.659276
Dendritic.cells	ZNRF3	-0.18842	5.517512	-1.02871	0.306423	-5.9106	0.583637	0.571085
Dendritic.cells	ZFP386	0.203285	3.889207	1.028068	0.306722	-5.57154	0.602444	0.597841
Dendritic.cells	GM15964	0.730263	0.162356	1.027999	0.306754	-4.88849	0.647572	0.663147
Dendritic.cells	PSMG2	-0.28458	3.919974	-1.02798	0.306763	-5.3428	0.602088	0.59737
Dendritic.cells	TRIM34A	0.370212	3.308515	1.027813	0.306841	-5.31828	0.609259	0.607709
Dendritic.cells	MED21	0.196692	5.21874	1.027504	0.306986	-5.77743	0.587365	0.576429
Dendritic.cells	PAFAH1B2	0.115572	5.489377	1.027498	0.306989	-5.9627	0.584343	0.572102
Dendritic.cells	RAB5B	0.190435	4.382557	1.027308	0.307078	-5.62124	0.59683	0.590102
Dendritic.cells	ELDR	0.166533	3.256101	1.027262	0.307099	-6.14641	0.609892	0.608919
Dendritic.cells	GM10802	0.606086	-0.25172	1.027206	0.307125	-4.88291	0.652903	0.671272
Dendritic.cells	SNX10	0.300947	5.029489	1.027069	0.30719	-5.47201	0.589507	0.579631
Dendritic.cells	ARL8B	-0.0961	5.97123	-1.02657	0.307424	-6.14623	0.5793	0.564864
Dendritic.cells	ARF4	-0.09294	8.479652	-1.02644	0.307485	-6.60609	0.552556	0.526803
Dendritic.cells	AMOT	-0.42695	0.863491	-1.02641	0.307498	-5.10153	0.639158	0.651158
Dendritic.cells	ZCRB1	0.094154	6.149583	1.026251	0.307573	-6.12181	0.577378	0.562122
Dendritic.cells	NCL	-0.13277	7.783001	-1.02604	0.307673	-6.37872	0.559886	0.537174
Dendritic.cells	MASP2	-0.5873	1.894509	-1.02597	0.307703	-5.02181	0.626554	0.632839
Dendritic.cells	D330023K1	0.382889	2.22594	1.025424	0.30796	-5.16986	0.622814	0.627229
Dendritic.cells	1700123M	-0.46476	0.89752	-1.02536	0.30799	-5.00562	0.639095	0.65082
Dendritic.cells	KLRK1	0.500549	3.149025	1.025328	0.308005	-5.26617	0.611801	0.611329
Dendritic.cells	RAC2	0.093117	9.052768	1.025163	0.308083	-6.72174	0.546989	0.518751
Dendritic.cells	KRTCAP3	-0.48503	0.893962	-1.02489	0.308213	-4.95233	0.63914	0.651067
Dendritic.cells	SMG8	-0.30788	2.847118	-1.02488	0.308215	-5.20764	0.615376	0.616657
Dendritic.cells	CHST15	0.192514	3.54275	1.024806	0.308251	-5.64338	0.607178	0.604841
Dendritic.cells	PRRT1	0.32019	0.466797	1.024535	0.308378	-5.31448	0.644485	0.659028
Dendritic.cells	AARSD1	-0.23739	3.977143	-1.02444	0.308424	-5.47921	0.602129	0.597802
Dendritic.cells	ZFP592	0.09943	5.89966	1.024383	0.308449	-6.09926	0.580416	0.566657
Dendritic.cells	DOT1L	0.186753	5.289755	1.024256	0.308509	-5.77664	0.587193	0.576422
Dendritic.cells	GM16066	0.429065	2.012511	1.024117	0.308574	-4.99869	0.625396	0.631497

Dendritic.cells	MED20	0.199377	4.104119	1.023737	0.308752	-5.51936	0.600663	0.595985
Dendritic.cells	SUSD3	0.38535	3.170727	1.023477	0.308875	-5.08384	0.611545	0.611829
Dendritic.cells	TTC25	-0.62382	0.218704	-1.02327	0.30897	-4.88617	0.647614	0.664183
Dendritic.cells	KRAS	-0.0812	7.278938	-1.02316	0.309021	-6.45821	0.565467	0.545761
Dendritic.cells	CZIB	-0.24771	3.658795	-1.02311	0.309045	-5.38011	0.605824	0.603628
Dendritic.cells	WWC1	0.451737	1.216205	1.023084	0.309059	-4.98208	0.635143	0.646058
Dendritic.cells	UBXN8	0.183861	4.404408	1.02291	0.309141	-5.61477	0.597214	0.591257
Dendritic.cells	MSR1	-0.37839	3.816756	-1.02284	0.309176	-5.3161	0.603987	0.601052
Dendritic.cells	TIGD2	0.199643	3.85213	1.022813	0.309187	-5.53024	0.603576	0.600461
Dendritic.cells	IL6RA	-0.15449	5.504339	-1.02274	0.309221	-6.12503	0.584797	0.573487
Dendritic.cells	CERK	0.097791	7.028222	1.022692	0.309244	-6.48491	0.568146	0.549685
Dendritic.cells	1700003F1	-0.46889	2.027827	-1.02267	0.309256	-5.03193	0.62521	0.631764
Dendritic.cells	SLC35A3	0.133731	4.775291	1.022344	0.309407	-5.80413	0.592989	0.585324
Dendritic.cells	ZFP607A	0.364151	1.840055	1.022327	0.309415	-5.07374	0.627491	0.635133
Dendritic.cells	FKBP5	0.192282	5.30259	1.02191	0.309612	-5.96655	0.587049	0.576823
Dendritic.cells	GM26827	0.624338	1.344192	1.021806	0.309661	-4.9166	0.633564	0.644
Dendritic.cells	EZR	-0.09145	7.933064	-1.02178	0.309673	-6.44267	0.558556	0.53611
Dendritic.cells	CHADL	-0.47859	0.647864	-1.02177	0.309678	-5.01665	0.642213	0.656579
Dendritic.cells	2-Sep	-0.36826	2.22603	-1.0217	0.309712	-5.20569	0.622813	0.628437
Dendritic.cells	PSMB5	0.141021	6.258665	1.021214	0.309939	-6.0117	0.576475	0.56189
Dendritic.cells	CENPW	-0.35406	4.349764	-1.02096	0.310061	-5.34718	0.59784	0.592673
Dendritic.cells	NUPR1	0.792359	2.41246	1.0209	0.310087	-5.04614	0.620569	0.625524
Dendritic.cells	IGHJ4	0.412907	0.337645	1.020878	0.310098	-5.16753	0.646111	0.662658
Dendritic.cells	PDZD11	0.219076	4.30203	1.02072	0.310172	-5.56564	0.598387	0.593537
Dendritic.cells	ALDH1B1	-0.26139	1.910302	-1.02071	0.310176	-5.37955	0.626636	0.634376
Dendritic.cells	SAA1	0.979967	-0.05604	1.020607	0.310225	-4.91455	0.6511	0.670031
Dendritic.cells	INTS6	-0.1589	6.588587	-1.02058	0.31024	-6.19736	0.572885	0.556966
Dendritic.cells	ARL5C	0.161096	6.264356	1.020513	0.310269	-6.24623	0.576413	0.562018
Dendritic.cells	GM14325	0.265762	2.324076	1.020498	0.310277	-5.24452	0.621632	0.627159
Dendritic.cells	AKR1B10	0.390726	3.494629	1.020476	0.310287	-5.09943	0.607741	0.607065
Dendritic.cells	CDS1	0.166623	2.788548	1.020453	0.310298	-6.06252	0.616073	0.619109
Dendritic.cells	VCP	-0.08035	7.431716	-1.02041	0.310316	-6.43326	0.563842	0.544061
Dendritic.cells	D430001F1	0.677859	-0.87048	1.020403	0.310321	-4.88772	0.661565	0.685357
Dendritic.cells	NT5C3B	0.239143	3.396774	1.020016	0.310504	-5.38432	0.609136	0.608908
Dendritic.cells	GM20682	0.354756	1.711443	1.019775	0.310617	-5.11034	0.629435	0.63824
Dendritic.cells	CDYL2	0.15548	4.757934	1.019561	0.310719	-6.0184	0.593542	0.586495
Dendritic.cells	CERS2	0.138291	5.262709	1.019413	0.310788	-5.90609	0.587848	0.578326
Dendritic.cells	ZCCHC10	0.244703	4.048466	1.019205	0.310886	-5.41052	0.601665	0.598318
Dendritic.cells	TWNK	0.351856	2.886068	1.01908	0.310946	-5.20492	0.615282	0.618017
Dendritic.cells	STK3	-0.13795	5.985237	-1.01908	0.310947	-6.1174	0.579821	0.566939
Dendritic.cells	PCDH15	-0.41249	1.323083	-1.01907	0.310949	-5.04733	0.634204	0.645468
Dendritic.cells	GM29966	-0.63239	1.411825	-1.01891	0.311026	-5.00514	0.633155	0.643946
Dendritic.cells	PHYH	-0.3091	5.40217	-1.01862	0.311161	-5.66047	0.58639	0.576294
Dendritic.cells	LRRC45	0.321687	2.190744	1.018606	0.311169	-5.2009	0.623722	0.630169
Dendritic.cells	HIST1H2AI	-0.76088	2.588783	-1.01822	0.311353	-5.00668	0.619191	0.623281
Dendritic.cells	MMAB	-0.45968	1.326097	-1.01806	0.311429	-5.04324	0.634557	0.645574
Dendritic.cells	ACLY	0.10728	6.383195	1.017965	0.311472	-6.07838	0.575815	0.560869

Dendritic.cells	PBLD1	-0.56462	1.75083	-1.01738	0.311751	-5.02193	0.629728	0.63833
Dendritic.cells	TLR13	0.592119	1.881588	1.017132	0.311866	-4.95852	0.628132	0.636137
Dendritic.cells	PTPRCAP	0.122644	5.807512	1.016936	0.311959	-6.27834	0.582497	0.570394
Dendritic.cells	MRPL1	0.156485	4.619868	1.016841	0.312004	-5.63235	0.595843	0.589628
Dendritic.cells	TMEM35B	0.419865	2.207613	1.016709	0.312066	-5.14672	0.624175	0.630621
Dendritic.cells	POGLUT3	-0.47699	0.979034	-1.01663	0.312105	-4.98958	0.639249	0.652528
Dendritic.cells	GM13710	-0.73253	1.36556	-1.0165	0.312163	-4.91167	0.634459	0.645558
Dendritic.cells	DENND5B	-0.30562	4.198991	-1.01649	0.312169	-5.33536	0.600667	0.596631
Dendritic.cells	ME1	-0.65341	-0.00982	-1.01646	0.312186	-4.89288	0.651702	0.670693
Dendritic.cells	LYNX1	0.315051	0.898277	1.016328	0.312246	-5.42261	0.640255	0.653994
Dendritic.cells	4932422M	-0.39102	0.972719	-1.01619	0.31231	-5.12374	0.639327	0.652643
Dendritic.cells	CASC3	0.137153	5.292913	1.016006	0.312399	-5.93202	0.588231	0.578736
Dendritic.cells	PKDCC	-0.33518	1.303453	-1.01593	0.312433	-5.27996	0.635225	0.646673
Dendritic.cells	GDPD5	-0.5298	1.750804	-1.01593	0.312437	-4.96911	0.629728	0.638682
Dendritic.cells	TOP1MT	0.398977	1.70028	1.015778	0.312507	-5.14568	0.630375	0.639624
Dendritic.cells	MPC1	0.091703	7.569491	1.01555	0.312615	-6.54099	0.563532	0.543289
Dendritic.cells	MRPS15	0.111461	5.910327	1.015311	0.312728	-6.1174	0.581563	0.569078
Dendritic.cells	RAB31	-0.31145	4.770711	-1.0152	0.312779	-5.32914	0.594333	0.587467
Dendritic.cells	TDP1	-0.31141	3.397629	-1.01482	0.31296	-5.28723	0.610204	0.610452
Dendritic.cells	DBNDD2	-0.40403	2.836435	-1.0148	0.312972	-5.11043	0.616844	0.620061
Dendritic.cells	SAMD8	-0.15456	5.289431	-1.01477	0.312985	-5.87715	0.588476	0.579145
Dendritic.cells	TMEM250-	-0.15884	4.629225	-1.01458	0.313075	-5.87545	0.595944	0.589937
Dendritic.cells	2810001G2	0.316366	2.479465	1.014532	0.313097	-5.11575	0.621115	0.626306
Dendritic.cells	BZW1	-0.06446	7.459579	-1.01453	0.313099	-6.46798	0.564774	0.545237
Dendritic.cells	MFSD2B	0.491968	0.599514	1.014202	0.313254	-4.96372	0.644376	0.660065
Dendritic.cells	NECAB3	-0.51386	1.186088	-1.01413	0.313286	-4.93961	0.637055	0.649408
Dendritic.cells	MVK	0.286312	2.634356	1.01381	0.31344	-5.34226	0.619485	0.623975
Dendritic.cells	MRPL30	0.10475	6.172662	1.013716	0.313485	-6.07511	0.578887	0.565521
Dendritic.cells	SLC25A4	0.115284	7.14787	1.01368	0.313501	-6.42188	0.568309	0.550374
Dendritic.cells	MFAP3	0.123212	5.124697	1.013586	0.313546	-5.97773	0.590545	0.582264
Dendritic.cells	PPP2R5C	-0.06582	7.456887	-1.01334	0.313662	-6.46618	0.565071	0.545731
Dendritic.cells	LY6E	0.114275	9.08245	1.013282	0.313691	-6.83081	0.548138	0.521627
Dendritic.cells	FAM114A2	0.127632	5.166561	1.013124	0.313765	-5.87311	0.590173	0.581731
Dendritic.cells	ZFP207	0.08282	6.934778	1.012647	0.313992	-6.28054	0.571006	0.553901
Dendritic.cells	SYNJ1	-0.1106	6.731012	-1.0125	0.314063	-6.28199	0.573237	0.557065
Dendritic.cells	TLCD2	-0.32094	2.659161	-1.01235	0.314132	-5.32965	0.619689	0.624006
Dendritic.cells	FAM193B	0.198937	4.050857	1.012001	0.314299	-5.55432	0.603511	0.60046
Dendritic.cells	TCEAL9	-0.14645	5.693961	-1.01182	0.314384	-6.03253	0.584901	0.573631
Dendritic.cells	GRM8	-0.13097	1.381099	-1.01172	0.314435	-6.42138	0.635511	0.646768
Dendritic.cells	EIF3J2	-0.40893	1.443832	-1.01148	0.314548	-5.07638	0.634854	0.645697
Dendritic.cells	SET	0.100702	7.946372	1.011311	0.314627	-6.47136	0.56068	0.538869
Dendritic.cells	GBE1	-0.14523	6.483019	-1.01063	0.314949	-6.21128	0.576734	0.561404
Dendritic.cells	ELL2	-0.14047	6.734248	-1.01056	0.314983	-6.49397	0.573999	0.557515
Dendritic.cells	CCDC22	0.258469	3.49155	1.010454	0.315035	-5.34617	0.610639	0.610204
Dendritic.cells	CASP2	0.197242	4.01281	1.010414	0.315054	-5.51192	0.60455	0.601427
Dendritic.cells	MPP7	-0.20869	7.383215	-1.01	0.315252	-6.42078	0.567237	0.547735
Dendritic.cells	NPC1	-0.08494	4.971884	-1.00992	0.315289	-6.53708	0.593781	0.585767

Dendritic.cells	HACD4	-0.47103	3.532004	-1.00977	0.315362	-5.16775	0.610438	0.609738
Dendritic.cells	FAM120C	-0.27786	3.050639	-1.00897	0.315742	-5.40417	0.616239	0.618407
Dendritic.cells	RNF17	0.65491	-0.02892	1.008919	0.315766	-4.95051	0.654232	0.673673
Dendritic.cells	NBDY	0.252439	3.659616	1.008918	0.315767	-5.32031	0.609051	0.608025
Dendritic.cells	PCBD2	-0.21185	5.24057	-1.00886	0.315795	-5.60542	0.590884	0.581867
Dendritic.cells	NOTCH3	0.322049	1.028947	1.008807	0.31582	-5.3969	0.640868	0.654196
Dendritic.cells	BSG	0.128178	7.716678	1.008664	0.315888	-6.39998	0.563826	0.54318
Dendritic.cells	GNAS	0.071517	8.931692	1.008126	0.316145	-6.74614	0.551153	0.525241
Dendritic.cells	PXK	-0.16967	6.270189	-1.00808	0.316169	-5.81972	0.579429	0.565626
Dendritic.cells	CDC40	0.121764	6.047181	1.007966	0.316221	-6.0643	0.581885	0.569149
Dendritic.cells	OSER1	-0.11795	6.59749	-1.00795	0.31623	-6.17939	0.575849	0.560495
Dendritic.cells	ZFAT	-0.24869	3.828322	-1.00786	0.316273	-5.50305	0.607079	0.605413
Dendritic.cells	PEBP1	0.107319	6.440655	1.007846	0.316279	-6.18839	0.57756	0.562948
Dendritic.cells	CDC23	0.278559	2.927739	1.007761	0.31632	-5.24333	0.617702	0.620783
Dendritic.cells	TNFSF14	0.493361	-0.05669	1.007742	0.316328	-4.89956	0.654587	0.674463
Dendritic.cells	MYCT1	-0.31833	1.654516	-1.00759	0.316403	-5.30432	0.63312	0.643168
Dendritic.cells	SPRYD7	0.342898	2.133927	1.007483	0.316452	-5.18121	0.627259	0.634683
Dendritic.cells	PLXNB2	-0.2138	3.959065	-1.00747	0.316459	-5.61382	0.605556	0.603259
Dendritic.cells	CPPED1	0.121854	3.886993	1.007441	0.316472	-5.88303	0.606395	0.604483
Dendritic.cells	AXDND1	-0.65251	1.557778	-1.00741	0.316489	-4.92525	0.63431	0.644945
Dendritic.cells	ZFP120	0.291438	2.536502	1.007322	0.316529	-5.21675	0.62239	0.627645
Dendritic.cells	EGR3	-0.68944	3.533307	-1.00644	0.316949	-5.15052	0.611236	0.610654
Dendritic.cells	F8	-0.25287	3.134673	-1.00633	0.317004	-5.69157	0.615948	0.617465
Dendritic.cells	ROBO3	-0.20673	-0.8339	-1.00611	0.317106	-5.57892	0.665485	0.68944
Dendritic.cells	SOCS2	-0.44358	3.962092	-1.00571	0.317301	-5.20244	0.606351	0.603599
Dendritic.cells	ARPP21	0.479074	1.234602	1.005616	0.317345	-5.28809	0.639183	0.651179
Dendritic.cells	GM3448	0.351725	1.9113	1.005557	0.317373	-5.20691	0.630836	0.639046
Dendritic.cells	GM30948	-0.76125	0.467484	-1.00539	0.317451	-4.91311	0.648808	0.665217
Dendritic.cells	BC024063	0.545515	0.183577	1.005389	0.317453	-4.90115	0.652414	0.67048
Dendritic.cells	HSD3B7	-0.32986	3.537133	-1.00537	0.317463	-5.34846	0.611325	0.610801
Dendritic.cells	MFF	0.123422	5.906654	1.005115	0.317584	-5.9681	0.58436	0.571898
Dendritic.cells	EGFR	0.330853	2.469637	1.004857	0.317708	-5.67917	0.624256	0.62934
Dendritic.cells	ARPP19	0.089797	6.840626	1.004748	0.31776	-6.27793	0.574183	0.557247
Dendritic.cells	RUFY1	-0.08431	5.607628	-1.00469	0.31779	-6.34864	0.587765	0.576708
Dendritic.cells	GM15327	-0.30889	1.294764	-1.0044	0.317928	-5.21361	0.638798	0.650282
Dendritic.cells	ALG8	-0.26459	3.226775	-1.00429	0.31798	-5.41593	0.61534	0.61629
Dendritic.cells	CPNE9	-0.5552	1.867904	-1.00401	0.318116	-5.08162	0.631725	0.640074
Dendritic.cells	CD52	0.139401	9.30175	1.004	0.318118	-6.69907	0.548432	0.520454
Dendritic.cells	DHX9	0.136203	6.492589	1.003948	0.318144	-6.23394	0.578112	0.562776
Dendritic.cells	FGD6	-0.26382	4.002414	-1.00354	0.318338	-5.47799	0.606269	0.60335
Dendritic.cells	ITGB7	0.141654	4.620993	1.00349	0.318363	-6.22282	0.599123	0.593084
Dendritic.cells	INPP5J	0.525179	-1.12022	1.003314	0.318448	-4.90025	0.66971	0.695726
Dendritic.cells	ZFP41	0.54781	0.707145	1.003198	0.318503	-4.92774	0.646196	0.661333
Dendritic.cells	CYP3A16	-0.58401	0.777697	-1.00319	0.318509	-4.93902	0.645307	0.660038
Dendritic.cells	BTBD19	-0.35601	2.725168	-1.00314	0.31853	-5.12995	0.621371	0.625269
Dendritic.cells	PTPRJ	0.087482	8.855581	1.003088	0.318556	-6.77172	0.553046	0.527205
Dendritic.cells	SNX22	0.605808	-0.37781	1.002729	0.318728	-4.90529	0.660179	0.681785

Dendritic.cells	ARMC3	-0.3355	1.900651	-1.00271	0.318735	-5.26479	0.631505	0.639978
Dendritic.cells	IFITM10	-0.70033	3.144158	-1.00244	0.318865	-5.06553	0.616641	0.618274
Dendritic.cells	COX11	0.194432	3.751078	1.002132	0.319015	-5.49063	0.609555	0.608035
Dendritic.cells	KXD1	0.121707	5.765423	1.001927	0.319113	-6.06626	0.586523	0.574954
Dendritic.cells	HIKESHI	-0.15841	5.228907	-1.00188	0.319136	-5.76111	0.592546	0.583623
Dendritic.cells	PUS7	-0.28394	2.95762	-1.00168	0.319233	-5.3119	0.618944	0.621777
Dendritic.cells	NIF3L1	-0.31088	3.000203	-1.00158	0.319282	-5.18901	0.618436	0.621076
Dendritic.cells	FBXO9	0.204898	4.752411	1.001373	0.319379	-5.61511	0.597963	0.591594
Dendritic.cells	SAR1A	-0.13049	5.508767	-1.00136	0.319384	-5.95632	0.589394	0.579268
Dendritic.cells	CCDC162	0.284154	2.876554	1.00122	0.319453	-5.65512	0.619914	0.623353
Dendritic.cells	FBXO7	-0.25669	4.165071	-1.00119	0.319469	-5.3805	0.604728	0.601403
Dendritic.cells	KIF13A	-0.16269	4.605824	-1.00117	0.319476	-5.89184	0.599642	0.59407
Dendritic.cells	ZBTB2	-0.12937	5.709287	-1.00105	0.319537	-5.96746	0.587149	0.576141
Dendritic.cells	TRIB2	-0.48593	2.561151	-1.00097	0.319574	-5.06565	0.623704	0.628908
Dendritic.cells	SPRYD4	0.370643	1.803638	1.000715	0.319696	-5.06638	0.632948	0.642352
Dendritic.cells	ALAS1	-0.3356	3.683631	-1.00071	0.319697	-5.30417	0.610369	0.609624
Dendritic.cells	ABCA6	-0.59904	0.705764	-1.00041	0.31984	-5.00688	0.646786	0.662311
Dendritic.cells	MIEF1	0.172986	4.21125	1.00025	0.319919	-5.80526	0.604425	0.600887
Dendritic.cells	ZFYVE16	-0.21506	3.016629	-1.00004	0.320021	-5.53636	0.618514	0.621213
Dendritic.cells	BTAF1	-0.07813	7.190783	-0.99974	0.320166	-6.52045	0.571161	0.553163
Dendritic.cells	GUF1	0.27599	2.589786	0.999431	0.320314	-5.20698	0.623635	0.628869
Dendritic.cells	5830418P1	0.572813	1.463039	0.999383	0.320337	-5.03178	0.637407	0.648885
Dendritic.cells	BCL7C	0.099025	5.355918	0.999341	0.320357	-6.11742	0.591375	0.582275
Dendritic.cells	FAM126B	0.222039	3.772291	0.999111	0.320467	-5.44187	0.609577	0.608612
Dendritic.cells	MEPCE	0.230992	4.466254	0.999106	0.32047	-5.55721	0.601514	0.596969
Dendritic.cells	RWDD1	0.111498	6.105394	0.999054	0.320495	-6.05628	0.583006	0.57034
Dendritic.cells	CRELD1	-0.32514	1.843254	-0.99904	0.320503	-5.25262	0.632718	0.642163
Dendritic.cells	CREB1	0.108773	6.332794	0.999037	0.320503	-6.09303	0.580498	0.56674
Dendritic.cells	GM10863	0.585221	0.229142	0.998907	0.320566	-4.97028	0.652916	0.671662
Dendritic.cells	MTRR	0.447077	1.505683	0.998803	0.320616	-5.05945	0.636879	0.648263
Dendritic.cells	RNPC3	-0.14447	4.741295	-0.99871	0.320662	-5.71861	0.598356	0.592458
Dendritic.cells	GM11837	-0.65938	0.292319	-0.99842	0.320801	-4.9191	0.652282	0.670612
Dendritic.cells	GM26542	-0.23501	4.802534	-0.99822	0.320895	-5.57187	0.597882	0.591642
Dendritic.cells	PPP5C	0.175452	4.346979	0.997986	0.32101	-5.64239	0.603179	0.599254
Dendritic.cells	CCNT2	0.106583	5.37232	0.997913	0.321045	-6.10573	0.591474	0.582405
Dendritic.cells	LSM11	-0.44799	2.171179	-0.99781	0.321094	-5.08445	0.62901	0.636719
Dendritic.cells	SYVN1	-0.2662	4.228615	-0.99732	0.321333	-5.52459	0.604729	0.601413
Dendritic.cells	EZH2	0.184528	6.663574	0.996939	0.321515	-6.07124	0.577323	0.562084
Dendritic.cells	RNF113A2	0.156929	4.271264	0.996763	0.3216	-5.79892	0.604235	0.600874
Dendritic.cells	GANC	-0.3138	3.377098	-0.99671	0.321625	-5.28751	0.614708	0.616015
Dendritic.cells	SREBF2	0.105539	5.9605	0.996647	0.321656	-6.32531	0.585067	0.57327
Dendritic.cells	ALOX5	0.629526	0.791987	0.996646	0.321656	-4.94355	0.646288	0.661922
Dendritic.cells	CTLA2B	0.580315	3.353706	0.996384	0.321783	-5.20648	0.614985	0.616492
Dendritic.cells	TOB2	-0.12424	6.656821	-0.99632	0.321812	-6.20308	0.577397	0.562342
Dendritic.cells	CCNT1	-0.0837	6.841664	-0.99607	0.321933	-6.31623	0.575383	0.559559
Dendritic.cells	GPS1	0.15542	4.905295	0.9959	0.322017	-5.77083	0.596946	0.590555
Dendritic.cells	RNF25	0.222809	3.103588	0.995751	0.322088	-5.44858	0.617957	0.620925

Dendritic.cells	PLAGL1	-0.58502	1.041672	-0.99549	0.322214	-4.99053	0.643153	0.657586
Dendritic.cells	UROD	-0.2691	4.467162	-0.99537	0.322271	-5.39036	0.601971	0.597863
Dendritic.cells	ECHDC2	-0.56555	1.612612	-0.99521	0.322349	-4.99711	0.636052	0.647299
Dendritic.cells	PLP2	0.132775	5.623922	0.995125	0.322391	-6.2997	0.588822	0.578936
Dendritic.cells	ERLEC1	0.199943	4.341715	0.995119	0.322394	-5.60625	0.603419	0.599975
Dendritic.cells	IMP4	-0.19639	4.46298	-0.99512	0.322396	-5.52388	0.602019	0.597953
Dendritic.cells	NDE1	-0.22867	4.58489	-0.99509	0.322408	-5.56792	0.600615	0.595927
Dendritic.cells	MR1	0.553361	0.68263	0.995048	0.322429	-4.95127	0.647667	0.664245
Dendritic.cells	SAP30	-0.18297	5.143228	-0.99504	0.32243	-5.77166	0.59424	0.586737
Dendritic.cells	FAM57B	-0.55434	0.285081	-0.99497	0.322467	-4.97865	0.652711	0.671673
Dendritic.cells	CCT5	0.112222	6.571584	0.994809	0.322544	-6.28234	0.578328	0.563909
Dendritic.cells	ABHD14B	-0.32751	2.125702	-0.99443	0.322729	-5.24919	0.629752	0.638356
Dendritic.cells	SLC1A2	-0.37959	2.197177	-0.99419	0.322844	-5.16551	0.62888	0.637177
Dendritic.cells	NSUN3	0.285338	2.658689	0.994019	0.322926	-5.22599	0.623289	0.62909
Dendritic.cells	GM37612	-0.42749	0.985122	-0.99397	0.322952	-5.07219	0.643861	0.659057
Dendritic.cells	CDK20	-0.42492	0.593617	-0.99392	0.322977	-5.11889	0.648793	0.666263
Dendritic.cells	SEC61A1	0.092723	5.49084	0.993783	0.323041	-6.17298	0.590316	0.581447
Dendritic.cells	PITRM1	-0.24608	3.560314	-0.99357	0.323143	-5.42601	0.612543	0.613666
Dendritic.cells	DEPTOR	-0.36318	3.469195	-0.99355	0.323151	-5.22466	0.613619	0.615224
Dendritic.cells	PPT2	-0.25031	4.199673	-0.9934	0.323225	-5.455	0.605065	0.602878
Dendritic.cells	TRP53RKA	-0.27819	2.472703	-0.99339	0.323229	-5.27345	0.625535	0.632556
Dendritic.cells	TSHZ1	0.221391	5.060403	0.993332	0.323259	-5.69565	0.595181	0.588604
Dendritic.cells	ZFP827	-0.43737	2.85843	-0.9932	0.323321	-5.1313	0.620888	0.625836
Dendritic.cells	1700037H(	-0.32378	3.714085	-0.9932	0.323323	-5.19914	0.610734	0.611112
Dendritic.cells	SOD1	-0.14934	5.993562	-0.99301	0.323413	-5.99052	0.584699	0.573608
Dendritic.cells	MAP3K8	0.171185	4.64612	0.992877	0.323479	-5.82441	0.599912	0.595567
Dendritic.cells	TCF12	-0.06645	8.463403	-0.99287	0.323484	-6.96579	0.558112	0.535527
Dendritic.cells	MKRN2	-0.14839	4.074932	-0.99271	0.323559	-5.65053	0.606515	0.60515
Dendritic.cells	DHX16	-0.23672	4.087189	-0.9927	0.323565	-5.49271	0.606372	0.604943
Dendritic.cells	IFI35	0.176442	5.333965	0.992669	0.32358	-6.01283	0.592083	0.584307
Dendritic.cells	ESPL1	-0.4211	2.563574	-0.99267	0.323581	-5.07842	0.624436	0.631141
Dendritic.cells	FCGR1	-0.59938	2.432362	-0.99263	0.323598	-5.06312	0.626023	0.633468
Dendritic.cells	GM43623	0.592623	-0.49767	0.992522	0.323652	-4.91244	0.662778	0.687237
Dendritic.cells	OGFRL1	0.172107	5.082967	0.992285	0.323766	-5.92112	0.594924	0.588502
Dendritic.cells	SLC5A11	-0.6007	-0.01556	-0.99223	0.323794	-4.91068	0.656556	0.678172
Dendritic.cells	1110065P2	0.235633	3.514697	0.992201	0.323807	-5.35845	0.613082	0.614798
Dendritic.cells	BANF1	0.131228	6.873409	0.991853	0.323976	-6.2045	0.575239	0.560045
Dendritic.cells	GM39090	0.412899	0.497553	0.991689	0.324056	-5.06918	0.650285	0.668835
Dendritic.cells	EFCAB7	0.432531	0.535523	0.991265	0.324261	-5.02422	0.650104	0.668339
Dendritic.cells	GM11464	0.510274	-0.20123	0.99099	0.324395	-4.9526	0.65962	0.682326
Dendritic.cells	MYOF	0.571756	2.65687	0.990853	0.324461	-5.07752	0.623951	0.630333
Dendritic.cells	ARMH2	0.599671	0.301427	0.990745	0.324514	-4.92852	0.653172	0.673005
Dendritic.cells	NEMP1	-0.40487	3.065406	-0.99054	0.324614	-5.19416	0.619048	0.623304
Dendritic.cells	CD14	0.681754	4.260683	0.990497	0.324634	-5.28719	0.604978	0.602916
Dendritic.cells	FAM20C	-0.67808	1.641237	-0.99048	0.324641	-5.08598	0.636351	0.648491
Dendritic.cells	MEX3A	-0.41936	1.577184	-0.99	0.324874	-5.10041	0.63749	0.649834
Dendritic.cells	AHSA2	-0.22224	3.646243	-0.98986	0.324943	-5.42081	0.612517	0.61358

Dendritic.cells	TLE1	-0.12332	4.060562	-0.98955	0.325095	-5.98972	0.607821	0.606618
Dendritic.cells	MAML3	0.147632	6.974808	0.98941	0.325163	-6.37624	0.575015	0.559346
Dendritic.cells	BUB1B	0.3477	4.016283	0.989252	0.325239	-5.33608	0.608338	0.607396
Dendritic.cells	ANKRD22	0.578598	-0.56253	0.989226	0.325252	-4.91142	0.664866	0.689826
Dendritic.cells	ACADVL	0.199146	4.901912	0.988943	0.32539	-5.68903	0.598257	0.592702
Dendritic.cells	YPEL5	0.140213	6.583092	0.988565	0.325574	-6.12957	0.579603	0.565682
Dendritic.cells	SFMBT1	-0.13126	6.08199	-0.98852	0.325597	-6.07525	0.585133	0.573629
Dendritic.cells	HSH2D	0.301275	3.210107	0.988353	0.325677	-5.34173	0.61823	0.621441
Dendritic.cells	SELENOK	-0.09227	7.584969	-0.98821	0.325747	-6.45441	0.568821	0.55027
Dendritic.cells	SUGP2	-0.19931	4.059588	-0.98797	0.325861	-5.72188	0.608345	0.607053
Dendritic.cells	COX19	-0.22058	4.496433	-0.98759	0.326049	-5.59162	0.603304	0.599716
Dendritic.cells	SDHAF3	0.258143	3.044533	0.987518	0.326083	-5.31815	0.62038	0.624445
Dendritic.cells	HABP2	-0.58823	0.622763	-0.98743	0.326128	-4.95663	0.650225	0.667932
Dendritic.cells	APBB3	0.447333	1.534528	0.98739	0.326146	-5.02941	0.638786	0.65123
Dendritic.cells	VPS28	0.11599	6.686374	0.987161	0.326258	-6.19907	0.578676	0.564326
Dendritic.cells	GM15972	0.6178	-0.2921	0.987119	0.326278	-4.91311	0.66195	0.685209
Dendritic.cells	TMEM67	0.376429	1.688091	0.98702	0.326326	-5.17206	0.636884	0.648562
Dendritic.cells	RALB	-0.21936	4.247517	-0.98687	0.326401	-5.39016	0.606189	0.603989
Dendritic.cells	DEPDC1A	-0.57999	3.001084	-0.98683	0.326418	-5.12684	0.6209	0.625307
Dendritic.cells	ADA	-0.47991	2.074866	-0.98679	0.326438	-5.05563	0.632123	0.641625
Dendritic.cells	MICAL3	-0.32663	3.436889	-0.98654	0.32656	-5.26068	0.615831	0.61778
Dendritic.cells	2810002D1	-0.36133	1.66458	-0.98637	0.326644	-5.04691	0.63736	0.649079
Dendritic.cells	BSN	0.517488	0.764883	0.985997	0.326825	-5.05585	0.648862	0.665711
Dendritic.cells	TAF1A	-0.22252	3.171877	-0.98564	0.327	-5.53373	0.619409	0.622743
Dendritic.cells	CCL21A	-1.46863	0.049265	-0.98562	0.327008	-4.96058	0.65813	0.679204
Dendritic.cells	SDHAF1	0.210593	4.132974	0.985385	0.327124	-5.62269	0.608175	0.606337
Dendritic.cells	GM46224	0.561575	2.389468	0.98479	0.327415	-5.17962	0.629243	0.636737
Dendritic.cells	ZSCAN21	-0.22797	3.394527	-0.98442	0.327593	-5.54146	0.61715	0.619308
Dendritic.cells	AA386476	0.44118	1.390731	0.984347	0.327631	-5.07261	0.641553	0.6548
Dendritic.cells	MS4A1	0.588346	3.936889	0.984337	0.327636	-5.23755	0.610745	0.61003
Dendritic.cells	HMCES	-0.21337	4.456587	-0.9843	0.327652	-5.61233	0.604687	0.60127
Dendritic.cells	GM45669	-0.57255	0.129811	-0.98427	0.327668	-4.97948	0.657514	0.678137
Dendritic.cells	GCLC	-0.19568	6.841762	-0.98405	0.327774	-6.01039	0.577861	0.56271
Dendritic.cells	ERCC8	0.26789	2.45611	0.984012	0.327795	-5.28823	0.628432	0.635785
Dendritic.cells	MSANTD2	-0.11634	4.864755	-0.98401	0.327796	-5.97985	0.599983	0.594566
Dendritic.cells	GOLGA3	-0.18011	4.032459	-0.98388	0.327858	-5.61311	0.609625	0.608548
Dendritic.cells	B930036N1	-0.26027	5.605823	-0.98373	0.327934	-5.72199	0.591563	0.5825
Dendritic.cells	HAUS3	0.194244	4.199727	0.98363	0.327981	-5.50393	0.607672	0.605766
Dendritic.cells	DOCK4	-0.1526	6.427831	-0.98359	0.327999	-6.25419	0.582403	0.569332
Dendritic.cells	LIMD1	0.08543	6.016318	0.98344	0.328075	-6.30773	0.587	0.575959
Dendritic.cells	GM43256	0.526655	0.309245	0.982828	0.328374	-4.9622	0.655697	0.675226
Dendritic.cells	ITGAL	-0.14738	6.272091	-0.98276	0.328409	-6.21413	0.584555	0.572027
Dendritic.cells	IFT22	0.307778	3.654091	0.982538	0.328516	-5.29263	0.614623	0.615348
Dendritic.cells	APOO	0.221781	3.509146	0.982354	0.328606	-5.44625	0.616403	0.617904
Dendritic.cells	BATF2	-0.58681	0.948583	-0.98198	0.328792	-5.05217	0.647898	0.663608
Dendritic.cells	YY1	0.072071	7.144963	0.981963	0.328798	-6.35834	0.575276	0.558512
Dendritic.cells	RBM3	0.090812	9.070642	0.981849	0.328853	-6.68163	0.55489	0.529428

Dendritic.cells	GOT2	0.115153	6.235083	0.981312	0.329116	-6.137	0.585469	0.573195
Dendritic.cells	FCER2A	-0.71208	0.458562	-0.98128	0.329132	-4.95392	0.654352	0.67312
Dendritic.cells	COX7A2L	-0.0959	6.644119	-0.98106	0.329239	-6.21036	0.580952	0.566778
Dendritic.cells	2210408I2:	-0.27713	3.277757	-0.98105	0.329246	-5.41479	0.619529	0.622459
Dendritic.cells	TSPAN7	-0.19589	3.257326	-0.98089	0.329324	-5.77195	0.619773	0.622871
Dendritic.cells	PFKL	0.170467	5.330071	0.980855	0.329341	-5.85237	0.595629	0.587952
Dendritic.cells	HACD1	-0.30168	3.002315	-0.98081	0.329364	-5.35694	0.622829	0.627307
Dendritic.cells	PPWD1	0.145677	4.68019	0.980614	0.329459	-5.68943	0.603138	0.598687
Dendritic.cells	PTOV1	0.177912	4.038474	0.980477	0.329526	-5.649	0.610622	0.609537
Dendritic.cells	GM20513	-0.56665	1.300029	-0.98027	0.329629	-5.14263	0.643903	0.657904
Dendritic.cells	PLAAT3	0.28265	5.19136	0.980075	0.329724	-5.70937	0.597452	0.590454
Dendritic.cells	GIGYF2	0.115907	5.464029	0.979923	0.329798	-6.10305	0.594388	0.586048
Dendritic.cells	GPANK1	-0.241	3.300008	-0.97953	0.329991	-5.38064	0.619809	0.622573
Dendritic.cells	FBL	0.105943	6.091074	0.979408	0.330051	-6.27448	0.587595	0.576051
Dendritic.cells	GRAMD4	0.158111	5.165751	0.979262	0.330122	-6.03873	0.598061	0.591134
Dendritic.cells	INTS9	-0.18339	4.394064	-0.97887	0.330315	-5.62929	0.607049	0.604058
Dendritic.cells	TRMT2A	0.181593	3.456488	0.978798	0.33035	-5.61778	0.618081	0.620042
Dendritic.cells	GEMIN5	0.265752	3.385628	0.978708	0.330394	-5.33575	0.618925	0.621294
Dendritic.cells	CARHSP1	-0.2155	5.430457	-0.97864	0.33043	-5.6744	0.595147	0.586933
Dendritic.cells	UQCRC2	0.099171	6.345631	0.978517	0.330488	-6.14257	0.584888	0.572191
Dendritic.cells	CLEC16A	-0.14644	4.763553	-0.97847	0.330513	-5.91491	0.602771	0.597957
Dendritic.cells	MICALL2	0.383483	1.195719	0.978212	0.330639	-5.19651	0.645868	0.660376
Dendritic.cells	MDP1	0.154589	4.373314	0.977974	0.330756	-5.74385	0.607507	0.604617
Dendritic.cells	A530017D2	-0.29964	2.325165	-0.97781	0.330835	-5.25221	0.631956	0.640074
Dendritic.cells	MRPL42	0.157939	6.521235	0.977767	0.330857	-6.07271	0.583154	0.569532
Dendritic.cells	MEIS1	0.212736	3.866059	0.977658	0.330911	-5.71406	0.613449	0.613226
Dendritic.cells	KPNB1	0.124	5.739818	0.977362	0.331057	-5.97269	0.59196	0.582154
Dendritic.cells	DPF3	-0.52279	1.667384	-0.97713	0.33117	-5.02497	0.640171	0.652086
Dendritic.cells	MAN2B1	-0.13947	6.851978	-0.97711	0.331183	-6.29832	0.579613	0.564473
Dendritic.cells	DGKE	-0.10336	4.880229	-0.97709	0.331189	-6.35837	0.60174	0.596325
Dendritic.cells	MAF1	0.180003	5.342723	0.976929	0.33127	-5.81472	0.596495	0.588719
Dendritic.cells	ZW10	0.257965	4.014837	0.976684	0.331391	-5.38338	0.611842	0.610944
Dendritic.cells	CAMSAP2	0.151894	4.496866	0.976539	0.331462	-5.84982	0.606213	0.602809
Dendritic.cells	CD300A	0.196843	4.623283	0.976458	0.331501	-5.95319	0.604748	0.600718
Dendritic.cells	CLCN3	-0.08373	6.389845	-0.97636	0.331548	-6.50029	0.584744	0.571898
Dendritic.cells	TMEM143	0.352142	1.491704	0.976215	0.331621	-5.13472	0.642406	0.655459
Dendritic.cells	SUPT20	0.135466	5.30473	0.976004	0.331725	-5.88462	0.596927	0.589556
Dendritic.cells	SRSF3	-0.08238	7.532017	-0.97593	0.33176	-6.42738	0.572272	0.554156
Dendritic.cells	PHF5A	0.134336	6.072643	0.975807	0.331823	-6.04429	0.588271	0.577195
Dendritic.cells	DEDD2	0.18783	4.544003	0.975795	0.331828	-5.74251	0.605666	0.602289
Dendritic.cells	PDIK1L	-0.24627	3.065249	-0.97579	0.33183	-5.40458	0.623127	0.627593
Dendritic.cells	PARP9	0.249592	5.150171	0.975436	0.332005	-5.84632	0.598826	0.592271
Dendritic.cells	1810013L2	-0.11985	5.901803	-0.97541	0.332018	-6.08206	0.590317	0.580006
Dendritic.cells	UBTD1	-0.22589	4.402162	-0.97521	0.332119	-5.56702	0.607523	0.604776
Dendritic.cells	SMIM41	0.534338	0.89661	0.975074	0.332184	-4.95857	0.650111	0.666739
Dendritic.cells	GM16867	0.558627	2.522419	0.97499	0.332225	-5.01029	0.629913	0.6373
Dendritic.cells	BICD2	-0.194	4.09822	-0.97461	0.332412	-5.61522	0.611314	0.610081

Dendritic.cells	PRRG1	-0.44819	1.915898	-0.97433	0.33255	-5.13817	0.637685	0.64836
Dendritic.cells	STUB1	-0.13121	5.426852	-0.9743	0.332565	-5.89685	0.596052	0.588015
Dendritic.cells	ADAMTS17	-0.33257	0.355692	-0.9739	0.332764	-5.37906	0.657626	0.677223
Dendritic.cells	KDELR2	-0.11201	5.993878	-0.97352	0.332953	-6.11874	0.590147	0.579125
Dendritic.cells	CHP1	-0.09965	7.01438	-0.97339	0.333016	-6.29779	0.578869	0.562953
Dendritic.cells	GM49692	-0.55469	0.104027	-0.97326	0.333079	-4.92479	0.661157	0.682338
Dendritic.cells	GPR137	0.231383	2.506946	0.973049	0.333184	-5.34776	0.630983	0.638305
Dendritic.cells	KDM8	0.333659	1.814343	0.973023	0.333197	-5.14438	0.639508	0.650718
Dendritic.cells	GUCA1A	-0.52626	1.4794	-0.97289	0.33326	-4.95212	0.643681	0.656826
Dendritic.cells	UST	-0.19087	6.028853	-0.97276	0.333326	-6.4299	0.589788	0.578788
Dendritic.cells	ZFP646	0.246918	3.903418	0.972599	0.333406	-5.49808	0.614223	0.614081
Dendritic.cells	CC2D1A	0.260651	3.044755	0.972568	0.333421	-5.38433	0.624461	0.628933
Dendritic.cells	GM43707	-0.41892	0.407926	-0.97244	0.333482	-5.12719	0.657264	0.676771
Dendritic.cells	CXCR3	-0.25125	1.731497	-0.97208	0.33366	-5.73796	0.64073	0.652419
Dendritic.cells	AIM2	0.173348	5.579386	0.971767	0.333818	-5.95243	0.595028	0.586193
Dendritic.cells	EI24	0.147452	4.630195	0.971709	0.333846	-5.74579	0.605906	0.60189
Dendritic.cells	ZFP141	0.295754	3.367308	0.971674	0.333864	-5.35997	0.620778	0.623422
Dendritic.cells	FANCM	-0.34366	3.67592	-0.97158	0.333912	-5.22515	0.617101	0.61811
Dendritic.cells	GM6710	0.439055	0.761027	0.971508	0.333946	-5.09805	0.652941	0.670295
Dendritic.cells	ENPP2	-0.49569	2.411456	-0.97146	0.333972	-5.07251	0.632341	0.640254
Dendritic.cells	IQCE	0.31877	2.144332	0.971089	0.334153	-5.29289	0.635735	0.645135
Dendritic.cells	PLD1	-0.42784	3.080795	-0.97104	0.334178	-5.20231	0.624328	0.628548
Dendritic.cells	ATXN7L1OS	0.426366	1.337166	0.970818	0.334287	-5.10945	0.645774	0.659858
Dendritic.cells	UNC13A	0.480885	1.183593	0.970755	0.334318	-5.0339	0.647705	0.662703
Dendritic.cells	GM42726	-0.20443	3.69069	-0.97073	0.33433	-5.51395	0.617036	0.618077
Dendritic.cells	BCL9L	0.23993	3.410778	0.970653	0.334369	-5.4418	0.620369	0.622942
Dendritic.cells	RASGRP2	0.107293	6.609517	0.970479	0.334455	-6.28622	0.583666	0.569946
Dendritic.cells	ECM2	0.610892	0.502848	0.970203	0.334592	-4.97426	0.656528	0.675545
Dendritic.cells	PRR14	0.200891	4.524923	0.97005	0.334668	-5.60227	0.607398	0.604056
Dendritic.cells	CARNS1	-0.41025	2.476067	-0.96992	0.33473	-5.21686	0.631832	0.639503
Dendritic.cells	PPME1	-0.1563	4.862972	-0.96985	0.334767	-5.78226	0.603483	0.598405
Dendritic.cells	GM27253	0.187598	1.3478	0.969793	0.334795	-5.74912	0.645811	0.659881
Dendritic.cells	KDM3A	0.142039	5.567098	0.969646	0.334868	-5.91466	0.595461	0.586871
Dendritic.cells	CRY1	0.140173	5.502006	0.969485	0.334948	-6.00825	0.596241	0.588004
Dendritic.cells	HIF1AN	-0.25116	3.373427	-0.96921	0.335082	-5.45395	0.621122	0.624006
Dendritic.cells	SCHIP1	-0.35807	1.591487	-0.96906	0.335159	-5.26588	0.642907	0.655735
Dendritic.cells	SMPD1	0.396977	2.643295	0.96888	0.335248	-5.08796	0.629936	0.63691
Dendritic.cells	SLFN5	0.306876	4.230734	0.968868	0.335254	-5.76582	0.610971	0.609388
Dendritic.cells	LGR5	0.511201	1.073909	0.968814	0.335281	-4.99946	0.64941	0.665345
Dendritic.cells	GPBP1	0.092129	7.723603	0.968609	0.335383	-6.49369	0.571767	0.553007
Dendritic.cells	XXYLT1	0.160467	3.591278	0.96849	0.335441	-5.79351	0.618523	0.620483
Dendritic.cells	LUM	0.582331	0.551186	0.968391	0.335491	-4.98504	0.656059	0.675233
Dendritic.cells	GM16599	0.192894	4.782001	0.968312	0.33553	-5.6441	0.604556	0.600301
Dendritic.cells	SHLD2	0.175097	3.853936	0.968277	0.335547	-5.74876	0.615407	0.616029
Dendritic.cells	C8B	-0.50302	0.571894	-0.96765	0.335857	-4.97631	0.656242	0.675205
Dendritic.cells	XKR6	-0.4548	0.862903	-0.96759	0.335889	-5.04746	0.65253	0.6698
Dendritic.cells	ENPP5	0.544995	0.877362	0.967296	0.336035	-5.02101	0.65252	0.669659

Dendritic.cells	ZFP654	0.136103	6.120389	0.966933	0.336215	-6.13296	0.590063	0.578797
Dendritic.cells	IRAK4	0.176047	4.419459	0.966764	0.336299	-5.66797	0.609514	0.606864
Dendritic.cells	TMPRSS3	-0.55161	-0.48523	-0.96671	0.336326	-4.9244	0.670307	0.695508
Dendritic.cells	2810408I1:	-0.53472	1.398496	-0.96647	0.336444	-4.97055	0.646117	0.660136
Dendritic.cells	LRP6	-0.14889	5.378913	-0.9664	0.33648	-5.99173	0.598442	0.590954
Dendritic.cells	MAG	0.62206	0.11897	0.966278	0.336541	-4.92645	0.662432	0.684055
Dendritic.cells	RAB11B	0.085239	7.147669	0.966133	0.336613	-6.32291	0.578709	0.562585
Dendritic.cells	ACOX1	-0.15236	5.892871	-0.96609	0.336634	-6.05663	0.592618	0.582585
Dendritic.cells	ACAD11	0.288695	2.331168	0.966086	0.336636	-5.27631	0.634531	0.643295
Dendritic.cells	SASH3	0.12853	5.4972	0.96595	0.336704	-5.98381	0.597115	0.589081
Dendritic.cells	GLA	-0.27457	4.568639	-0.96579	0.336781	-5.52271	0.607834	0.604585
Dendritic.cells	GM49085	-0.35707	2.025817	-0.96548	0.336938	-5.10431	0.638547	0.64903
Dendritic.cells	INTS13	0.158505	4.149388	0.965322	0.337017	-5.71651	0.612952	0.611841
Dendritic.cells	FLYWCH1	-0.19381	3.850858	-0.96515	0.3371	-5.58378	0.616475	0.617014
Dendritic.cells	ZDHHC15	0.308079	1.125862	0.965101	0.337127	-5.38689	0.649842	0.665592
Dendritic.cells	FAM133B	-0.13791	4.911343	-0.96494	0.337207	-5.81443	0.604075	0.599095
Dendritic.cells	BRI3	-0.09276	7.328714	-0.96488	0.337237	-6.46873	0.576996	0.560103
Dendritic.cells	SLC26A2	-0.25722	3.742645	-0.96465	0.337349	-5.42065	0.617823	0.619001
Dendritic.cells	S1PR2	-0.53932	1.261742	-0.96433	0.337512	-4.95513	0.648194	0.663249
Dendritic.cells	UMAD1	-0.13651	5.658219	-0.96415	0.3376	-6.01819	0.595595	0.586884
Dendritic.cells	A230083N1	-0.56332	0.546139	-0.96393	0.337708	-4.97354	0.657293	0.676674
Dendritic.cells	CCDC127	-0.1543	4.15296	-0.96382	0.337763	-5.67064	0.612974	0.612159
Dendritic.cells	WDR3	0.188474	4.225869	0.963563	0.337893	-5.56309	0.612117	0.610919
Dendritic.cells	MYG1	-0.20003	3.803891	-0.96343	0.337962	-5.46187	0.617096	0.618196
Dendritic.cells	PKN2	-0.10109	6.774243	-0.9634	0.337976	-6.32321	0.583124	0.569133
Dendritic.cells	TNNT3	0.37812	1.772075	0.96313	0.338109	-5.26245	0.641798	0.654142
Dendritic.cells	SGSM3	0.212312	4.063557	0.963004	0.338172	-5.66401	0.614026	0.61375
Dendritic.cells	FMO1	-0.48261	2.23787	-0.96294	0.338205	-5.21088	0.636028	0.645731
Dendritic.cells	GM26944	-0.4183	0.852005	-0.9629	0.338223	-5.07412	0.653385	0.671088
Dendritic.cells	USP24	0.11031	5.459806	0.962806	0.338271	-6.11894	0.597849	0.590368
Dendritic.cells	ISG20L2	0.139863	5.083016	0.962724	0.338312	-5.91761	0.60216	0.596594
Dendritic.cells	CD300C2	-0.27379	4.335062	-0.96271	0.33832	-5.70668	0.610837	0.609146
Dendritic.cells	MRPS7	0.138064	5.010323	0.962685	0.338331	-5.89886	0.602996	0.597802
Dendritic.cells	SIGLECE	-0.64892	2.293576	-0.96268	0.338332	-5.08591	0.635343	0.64475
Dendritic.cells	BORA	0.29624	3.535011	0.962665	0.338341	-5.29566	0.620296	0.622867
Dendritic.cells	SNN	-0.18242	3.649853	-0.9625	0.338426	-5.84666	0.618927	0.620983
Dendritic.cells	ADAR	0.193241	4.311216	0.962445	0.338451	-5.75011	0.611116	0.609685
Dendritic.cells	SAA3	1.703938	0.738679	0.962417	0.338465	-4.97233	0.654829	0.673375
Dendritic.cells	XIAP	0.083432	6.725324	0.962321	0.338513	-6.30759	0.583663	0.570059
Dendritic.cells	FBXW8	0.217154	3.868261	0.962115	0.338616	-5.48176	0.616418	0.617294
Dendritic.cells	TMTC1	-0.32667	2.714836	-0.96194	0.338701	-5.59188	0.630328	0.637498
Dendritic.cells	GKAP1	-0.26392	3.541031	-0.96162	0.338865	-5.44659	0.6205	0.623131
Dendritic.cells	AC125149.	-0.47792	-0.34803	-0.9615	0.338923	-5.11832	0.669174	0.694317
Dendritic.cells	TMEM51O!	-0.49693	-0.90367	-0.96128	0.339032	-4.9306	0.676498	0.705221
Dendritic.cells	GM42670	0.433535	0.89839	0.961221	0.339063	-5.0834	0.653084	0.670821
Dendritic.cells	BCAS3OS1	-0.20304	3.793281	-0.96122	0.339065	-5.57722	0.617496	0.618934
Dendritic.cells	HMOX2	0.091826	6.471899	0.961076	0.339135	-6.19819	0.586728	0.574483

Dendritic.cells	PIK3C3	0.183399	4.439233	0.960906	0.33922	-5.63167	0.609889	0.607936
Dendritic.cells	ZPBP	0.35444	1.648971	0.960892	0.339227	-5.18136	0.643619	0.657007
Dendritic.cells	SPARC	0.20266	5.322477	0.960572	0.339387	-6.1913	0.599755	0.593225
Dendritic.cells	ARFGAP2	-0.12393	4.901097	-0.96031	0.33952	-5.87297	0.604598	0.600305
Dendritic.cells	GM11998	-0.58168	0.318253	-0.96028	0.339534	-4.94824	0.660596	0.681904
Dendritic.cells	KATNBL1	-0.09533	5.963648	-0.96011	0.339618	-6.25666	0.592483	0.582909
Dendritic.cells	KHDRBS1	0.072932	7.151511	0.96001	0.339668	-6.3629	0.579313	0.563968
Dendritic.cells	FCMR	-0.43886	2.105124	-0.95994	0.339704	-5.10687	0.638027	0.649045
Dendritic.cells	SLC16A13	-0.49496	-0.01818	-0.9598	0.339775	-5.04444	0.664952	0.688513
Dendritic.cells	ADAMTSL1	0.779974	0.587709	0.959684	0.339831	-5.02563	0.657131	0.677077
Dendritic.cells	ATM	0.176262	4.221236	0.95957	0.339888	-5.69175	0.612518	0.612071
Dendritic.cells	NCF2	0.207868	6.576577	0.959504	0.339922	-5.85179	0.585638	0.573228
Dendritic.cells	CREBRF	0.12174	6.98351	0.959388	0.33998	-6.35121	0.581152	0.56681
Dendritic.cells	UBAP2	-0.11229	6.131148	-0.95916	0.340092	-6.10442	0.590602	0.580436
Dendritic.cells	2010016I1	-0.50476	-0.03474	-0.95909	0.340131	-4.97187	0.665167	0.689022
Dendritic.cells	ACOT8	0.193787	4.129949	0.959083	0.340132	-5.58576	0.613591	0.613691
Dendritic.cells	VNN3	0.56356	1.614791	0.958985	0.340182	-5.00268	0.644125	0.658228
Dendritic.cells	PNP	0.192856	5.924728	0.958942	0.340203	-5.87022	0.592921	0.583849
Dendritic.cells	LMO2	0.143056	4.903927	0.958894	0.340227	-6.08464	0.604565	0.60068
Dendritic.cells	ENSA	0.135718	5.388151	0.958692	0.340328	-5.88418	0.599063	0.592733
Dendritic.cells	CNBP	-0.06465	8.132464	-0.95851	0.340421	-6.62151	0.568787	0.549202
Dendritic.cells	HIST1H1C	0.399626	4.106663	0.95849	0.34043	-5.29986	0.613925	0.614332
Dendritic.cells	ID2	-0.20835	6.76154	-0.95821	0.340571	-5.96564	0.583728	0.570692
Dendritic.cells	STPG4	-0.37679	2.37884	-0.95817	0.340588	-5.14028	0.6348	0.644739
Dendritic.cells	EFHD2	0.15426	6.92246	0.957906	0.340722	-6.04025	0.582036	0.568169
Dendritic.cells	ANAPC2	-0.18816	4.497598	-0.95779	0.340783	-5.64374	0.609506	0.607906
Dendritic.cells	KRTCAP2	0.079854	7.062528	0.957591	0.34088	-6.55475	0.580499	0.566054
Dendritic.cells	TMEM186	0.322943	2.111676	0.957392	0.34098	-5.29598	0.63818	0.649753
Dendritic.cells	DIAPH2	0.096604	7.666379	0.957322	0.341015	-6.60208	0.573937	0.55668
Dendritic.cells	DOK1	0.260793	3.614618	0.957304	0.341024	-5.33306	0.619924	0.623147
Dendritic.cells	HIF1A	0.108793	6.608469	0.957281	0.341036	-6.38707	0.5855	0.573317
Dendritic.cells	GM10550	0.480516	0.590308	0.957145	0.341104	-5.01982	0.657339	0.67785
Dendritic.cells	ADO	-0.21322	3.708631	-0.95707	0.341142	-5.47939	0.618804	0.621571
Dendritic.cells	CHD7	-0.12631	6.463817	-0.95656	0.341396	-6.24796	0.587445	0.575845
Dendritic.cells	IFITM3	0.271017	7.709419	0.956307	0.341525	-6.3286	0.573927	0.556275
Dendritic.cells	ST5	-0.35485	2.235439	-0.95616	0.341597	-5.22181	0.637184	0.647863
Dendritic.cells	EIPR1	-0.13367	4.924643	-0.95588	0.341737	-5.87551	0.605202	0.601249
Dendritic.cells	TRAT1	-0.71863	-0.05747	-0.95541	0.341974	-4.9434	0.666779	0.690739
Dendritic.cells	MED18	0.406811	1.412452	0.95498	0.342192	-5.09791	0.648247	0.663311
Dendritic.cells	8-Mar	-0.40621	3.141103	-0.95463	0.34237	-5.12009	0.626963	0.632179
Dendritic.cells	SUN2	-0.12349	6.122993	-0.95462	0.342373	-6.05639	0.592193	0.581825
Dendritic.cells	ZFP971	0.353234	1.911606	0.954583	0.342391	-5.11596	0.642051	0.654171
Dendritic.cells	COX6A1	0.126904	6.768979	0.954086	0.342641	-6.13999	0.585324	0.571596
Dendritic.cells	SH2B3	-0.12615	5.315208	-0.95381	0.342778	-6.02914	0.601841	0.595268
Dendritic.cells	MON1B	0.352431	1.881481	0.953468	0.342953	-5.10319	0.643116	0.655003
Dendritic.cells	CCPG1	0.101174	6.037912	0.953403	0.342985	-6.34368	0.593786	0.583462
Dendritic.cells	CX3CR1	-0.42409	3.268892	-0.95318	0.343099	-5.33026	0.626134	0.630224

Dendritic.cells	PIK3R2	0.38667	2.306399	0.953132	0.343122	-5.13333	0.637885	0.647326
Dendritic.cells	NPHP1	-0.46726	0.652168	-0.95289	0.343242	-5.04855	0.658823	0.677789
Dendritic.cells	ANKRD11	-0.08023	9.391983	-0.95268	0.343347	-6.77581	0.557654	0.531533
Dendritic.cells	GM38394	0.422691	0.848639	0.952668	0.343355	-5.10773	0.656305	0.67417
Dendritic.cells	HNRNPA3	0.083043	8.554364	0.952579	0.343401	-6.64862	0.566424	0.544102
Dendritic.cells	CALCOCO1	0.130273	3.863697	0.952428	0.343477	-6.04336	0.619137	0.620011
Dendritic.cells	TRPC4AP	-0.09875	5.651799	-0.95214	0.343621	-6.10435	0.598385	0.58991
Dendritic.cells	PRKAR1B	-0.61989	-0.37005	-0.95213	0.343625	-4.93881	0.672218	0.697443
Dendritic.cells	FFAR4	0.42814	0.260828	0.951741	0.343823	-5.16175	0.664252	0.685437
Dendritic.cells	SCAF4	0.090812	6.317786	0.951003	0.344195	-6.31979	0.591643	0.579292
Dendritic.cells	GM10353	0.27501	2.745067	0.950901	0.344247	-5.35784	0.633524	0.639905
Dendritic.cells	FLNB	-0.27376	5.398208	-0.95063	0.344384	-6.03559	0.602214	0.594438
Dendritic.cells	ARHGEF10I	0.194381	2.695098	0.950502	0.344448	-5.83682	0.634298	0.640947
Dendritic.cells	ANGPTL2	0.491126	0.84131	0.950343	0.344528	-5.09758	0.657579	0.674895
Dendritic.cells	PPOX	0.287978	3.108757	0.949907	0.344749	-5.271	0.629594	0.633817
Dendritic.cells	RCBTB2	0.179295	5.187796	0.949739	0.344834	-5.70153	0.605026	0.598279
Dendritic.cells	MARF1	0.09746	6.147381	0.949502	0.344954	-6.22343	0.59419	0.582586
Dendritic.cells	5730409E0	0.503805	0.23499	0.949318	0.345047	-5.00755	0.665965	0.686789
Dendritic.cells	E230032D2	0.524373	1.879678	0.948751	0.345333	-4.96526	0.645411	0.656211
Dendritic.cells	ZC3H13	0.120389	5.653384	0.948246	0.345589	-6.05752	0.6006	0.590966
Dendritic.cells	WASHC2	-0.15293	5.655214	-0.94744	0.345996	-5.99702	0.600824	0.591149
Dendritic.cells	MFSD12	-0.12641	2.730413	-0.94741	0.34601	-5.96053	0.63549	0.641285
Dendritic.cells	VPS51	0.209344	3.730024	0.947399	0.346018	-5.5519	0.623362	0.623694
Dendritic.cells	GTPBP10	0.259591	2.397542	0.947349	0.346043	-5.32333	0.639594	0.64725
Dendritic.cells	GM33280	-0.58831	-0.66423	-0.94733	0.346054	-4.95705	0.678901	0.7047
Dendritic.cells	METAP1	0.122284	4.718677	0.947295	0.346071	-5.85888	0.611655	0.606765
Dendritic.cells	KANK2	-0.26886	2.38565	-0.94696	0.346239	-5.45658	0.639873	0.647573
Dendritic.cells	CBX6	0.231072	2.859775	0.946661	0.346392	-5.46807	0.634034	0.639232
Dendritic.cells	IGF2BP1	0.561429	-0.96106	0.946632	0.346407	-4.93829	0.683003	0.710803
Dendritic.cells	D11WSU47	0.369035	1.299867	0.946578	0.346434	-5.19783	0.653496	0.667568
Dendritic.cells	SGTA	-0.15886	4.959007	-0.94655	0.346448	-5.81432	0.608976	0.602966
Dendritic.cells	AHNAK	-0.14386	6.416793	-0.94648	0.346483	-6.64707	0.592322	0.578995
Dendritic.cells	CYCS	0.14042	7.712668	0.945635	0.346912	-6.38005	0.578639	0.558795
Dendritic.cells	NADK2	0.224423	4.994117	0.945462	0.347	-5.54104	0.609276	0.602782
Dendritic.cells	CCT4	-0.09484	6.487167	-0.94499	0.34724	-6.21334	0.592533	0.578414
Dendritic.cells	RIN2	-0.32922	3.687666	-0.94479	0.347341	-5.31739	0.625133	0.625333
Dendritic.cells	AK4	-0.58215	1.048143	-0.94466	0.347406	-4.97992	0.657915	0.673009
Dendritic.cells	DHRS11	0.238542	4.678666	0.943895	0.347795	-5.61268	0.61366	0.608628
Dendritic.cells	CSRP1	-0.17895	5.881401	-0.94388	0.347805	-5.89842	0.599747	0.588586
Dendritic.cells	SLC20A2	0.163519	4.036306	0.943784	0.347852	-5.83251	0.621261	0.619627
Dendritic.cells	YAP1	-0.48123	1.070556	-0.94353	0.347981	-5.19532	0.657931	0.672933
Dendritic.cells	KIF1BP	-0.19128	3.865166	-0.94343	0.348033	-5.59907	0.623306	0.622639
Dendritic.cells	F12	-0.58655	1.691224	-0.94314	0.348177	-5.03364	0.65004	0.661556
Dendritic.cells	SEPHS1	0.179251	4.119783	0.94289	0.348307	-5.48012	0.620266	0.618398
Dendritic.cells	CLEC12A	-0.11825	4.937614	-0.94276	0.348373	-6.49403	0.610629	0.604479
Dendritic.cells	JADE3	0.207023	3.471551	0.942745	0.34838	-5.5108	0.628043	0.629656
Dendritic.cells	FGF1	-0.43284	0.63081	-0.94268	0.348415	-5.0677	0.663592	0.681417

Dendritic.cells	KMT5A	0.132679	5.846575	0.94267	0.348418	-5.90629	0.600144	0.589385
Dendritic.cells	CPSF4	0.201631	4.317677	0.94266	0.348423	-5.63742	0.617917	0.615013
Dendritic.cells	JPX	0.216634	3.810956	0.942487	0.348511	-5.77883	0.623956	0.623818
Dendritic.cells	BRAT1	0.27326	2.183649	0.94236	0.348576	-5.33035	0.643861	0.652804
Dendritic.cells	CRK	-0.10648	6.391053	-0.94232	0.348595	-6.248	0.593976	0.580678
Dendritic.cells	POGZ	0.16169	4.431535	0.942175	0.34867	-5.69678	0.61657	0.613256
Dendritic.cells	PDAP1	0.101004	6.715812	0.942077	0.34872	-6.23792	0.590337	0.575471
Dendritic.cells	ACAA1A	0.135594	5.302507	0.942038	0.34874	-6.00632	0.606392	0.598566
Dendritic.cells	LEMD2	-0.16764	4.232797	-0.94195	0.348784	-5.67071	0.618923	0.616658
Dendritic.cells	AP5B1	-0.40863	1.293299	-0.94191	0.348803	-5.11254	0.655086	0.669197
Dendritic.cells	ATF5	-0.39969	3.267979	-0.94187	0.348823	-5.23287	0.630511	0.633441
Dendritic.cells	ABR	-0.10099	6.594873	-0.94161	0.348961	-6.8298	0.591689	0.577477
Dendritic.cells	USP34	-0.07589	7.703229	-0.94154	0.348992	-6.55018	0.579453	0.559958
Dendritic.cells	BRMS1L	-0.15131	4.269409	-0.9415	0.349016	-5.7602	0.618489	0.616135
Dendritic.cells	PRKAB2	0.208881	3.392055	0.941383	0.349074	-5.60488	0.629005	0.631405
Dendritic.cells	DNAH17	-0.56135	3.275665	-0.94115	0.349191	-5.15834	0.630417	0.633481
Dendritic.cells	POLE	-0.41152	3.301306	-0.94114	0.349196	-5.25017	0.630106	0.633029
Dendritic.cells	PRPF18	-0.17571	4.616004	-0.94111	0.349212	-5.68119	0.614396	0.610301
Dendritic.cells	BCKDK	0.144159	4.559456	0.940986	0.349276	-5.80131	0.615061	0.6113
Dendritic.cells	SOAT1	-0.27031	5.176779	-0.94092	0.349311	-5.55704	0.607848	0.600893
Dendritic.cells	SHPRH	0.152513	4.541567	0.940855	0.349343	-5.83114	0.615272	0.611618
Dendritic.cells	ARF3	0.072968	6.29303	0.94066	0.349442	-6.43726	0.595081	0.58258
Dendritic.cells	ISCU	0.10475	7.465509	0.940457	0.349546	-6.43019	0.582049	0.563879
Dendritic.cells	DDAH2	-0.28552	3.503269	-0.94034	0.349603	-5.39556	0.62766	0.629634
Dendritic.cells	AU022252	0.293109	2.452615	0.94032	0.349615	-5.28317	0.640517	0.64831
Dendritic.cells	CCR7	-0.51976	4.535564	-0.94031	0.34962	-5.29662	0.615343	0.611801
Dendritic.cells	NFIA	-0.17738	5.904	-0.9402	0.349676	-6.07548	0.59949	0.588931
Dendritic.cells	GZMM	-0.47294	2.051803	-0.94	0.349777	-5.04781	0.645591	0.655643
Dendritic.cells	SMPD2	-0.40456	2.04317	-0.9395	0.350035	-5.09629	0.646069	0.655897
Dendritic.cells	DECR2	-0.31635	2.741577	-0.93896	0.350311	-5.29307	0.637794	0.643462
Dendritic.cells	OLFR1033	0.577426	0.583769	0.938781	0.350401	-5.04211	0.665144	0.68334
Dendritic.cells	MFSD4B4	0.498503	-0.0204	0.938391	0.3506	-5.01235	0.673311	0.694969
Dendritic.cells	FAM98A	-0.19439	3.607656	-0.93808	0.350761	-5.55562	0.627719	0.628423
Dendritic.cells	CMYA5	0.379089	0.674059	0.937815	0.350894	-5.36666	0.664429	0.681857
Dendritic.cells	MPV17L	-0.34976	2.006778	-0.93772	0.350941	-5.20667	0.647433	0.657071
Dendritic.cells	ARPC5	0.073412	8.144041	0.937656	0.350976	-6.53186	0.575891	0.553898
Dendritic.cells	NMNAT1	-0.5052	1.326233	-0.93764	0.350983	-5.05463	0.656045	0.669619
Dendritic.cells	R3HCC1L	-0.12661	5.540599	-0.93711	0.351256	-6.0291	0.60529	0.595786
Dendritic.cells	GM50334	0.594548	-0.42372	0.936996	0.351313	-4.95351	0.67925	0.703307
Dendritic.cells	GM6377	-0.30259	3.091942	-0.93658	0.351528	-5.4618	0.634412	0.638074
Dendritic.cells	PDF	0.421696	0.794298	0.93631	0.351664	-5.07357	0.663318	0.680209
Dendritic.cells	GM31323	0.454005	0.948728	0.936268	0.351686	-5.00741	0.661325	0.677308
Dendritic.cells	REXO1	-0.15207	4.912691	-0.93605	0.351797	-5.81341	0.612617	0.60669
Dendritic.cells	GPR174	0.174478	2.267506	0.93602	0.351812	-5.88636	0.644602	0.653043
Dendritic.cells	GM12840	-0.3751	3.766389	-0.936	0.351825	-5.60963	0.626225	0.626371
Dendritic.cells	SLC2A2	-0.52733	1.627926	-0.93577	0.351941	-5.04665	0.652647	0.664798
Dendritic.cells	NUP160	0.188749	5.017011	0.935768	0.351942	-5.77386	0.611397	0.604975

Dendritic.cells	AKAP6	-0.69755	0.299684	-0.93568	0.351985	-5.01394	0.669749	0.689785
Dendritic.cells	OCEL1	0.196897	3.584489	0.935665	0.351994	-5.57402	0.62842	0.629586
Dendritic.cells	GTF3C3	0.288763	3.102011	0.935517	0.35207	-5.33852	0.634288	0.638111
Dendritic.cells	CTSD	-0.15878	7.133555	-0.93546	0.3521	-6.24727	0.587326	0.57039
Dendritic.cells	SNX27	0.119129	5.803763	0.935354	0.352154	-5.97947	0.602301	0.591932
Dendritic.cells	TTC1	0.126556	4.740195	0.935296	0.352184	-5.75403	0.61464	0.609741
Dendritic.cells	GM38190	-0.33632	1.823044	-0.93523	0.352216	-5.19601	0.65018	0.661313
Dendritic.cells	RASSF1	-0.10982	5.940086	-0.93517	0.352248	-6.12184	0.600743	0.589752
Dendritic.cells	TMEM248	0.091161	5.858866	0.934759	0.352459	-6.26746	0.601934	0.59131
Dendritic.cells	ATP5G1	0.107519	7.643771	0.933897	0.352901	-6.47099	0.582601	0.562934
Dendritic.cells	SLC30A1	-0.32488	3.386735	-0.93345	0.353132	-5.32908	0.631982	0.633976
Dendritic.cells	CCDC47	0.123026	5.110399	0.933386	0.353163	-5.94167	0.611436	0.604278
Dendritic.cells	BOP1	-0.18237	4.167475	-0.93322	0.353247	-5.62603	0.622568	0.620389
Dendritic.cells	CCDC90B	0.263959	3.165156	0.933161	0.353278	-5.34445	0.634687	0.637971
Dendritic.cells	CD2BP2	-0.18939	4.011388	-0.93312	0.353299	-5.55596	0.624436	0.623123
Dendritic.cells	B3GNT6	0.475438	-0.72192	0.93285	0.353438	-4.99068	0.684546	0.710789
Dendritic.cells	CFHR1	-0.60311	0.449486	-0.93284	0.353443	-5.00739	0.669043	0.688058
Dendritic.cells	IVD	0.159916	4.435995	0.932769	0.353479	-5.80911	0.619386	0.615856
Dendritic.cells	CASP8AP2	-0.14431	5.182109	-0.93232	0.353709	-5.84454	0.610794	0.603279
Dendritic.cells	CEBPD	0.195752	3.956744	0.932138	0.353804	-5.7379	0.625291	0.624255
Dendritic.cells	TAF13	0.178818	4.566883	0.932078	0.353834	-5.70674	0.618018	0.613768
Dendritic.cells	4930403D(	-0.41998	1.041064	-0.93191	0.35392	-5.10686	0.661568	0.677111
Dendritic.cells	ADIPOR2	0.076569	6.368043	0.931898	0.353927	-6.531	0.597177	0.583835
Dendritic.cells	MICOS10	0.087219	7.131558	0.93182	0.353967	-6.41754	0.588621	0.571557
Dendritic.cells	PRDX1	-0.09858	9.719927	-0.93164	0.354057	-6.8047	0.560814	0.531841
Dendritic.cells	PHF13	-0.22978	3.504231	-0.93164	0.35406	-5.38256	0.630755	0.632286
Dendritic.cells	TMCO3	-0.23068	3.656165	-0.93156	0.354099	-5.40636	0.628914	0.629619
Dendritic.cells	MAMLD1	-0.57576	0.110917	-0.93144	0.354164	-4.95864	0.673693	0.69483
Dendritic.cells	FUNDC2	-0.11706	7.178207	-0.93101	0.354385	-6.35155	0.588346	0.570879
Dendritic.cells	KEL	-0.65581	-0.44363	-0.93094	0.354417	-4.95451	0.681307	0.705705
Dendritic.cells	TOR3A	0.284382	4.075925	0.930361	0.354718	-5.62201	0.624392	0.622562
Dendritic.cells	ANKRD49	0.203604	3.032797	0.930229	0.354785	-5.52342	0.637054	0.640911
Dendritic.cells	SPTSSA	0.094539	6.822761	0.930115	0.354844	-6.42162	0.592565	0.576757
Dendritic.cells	PAX5	-0.33394	5.141192	-0.93004	0.354883	-5.52187	0.611791	0.604407
Dendritic.cells	IQCG	-0.59533	0.050135	-0.93003	0.354887	-4.99417	0.675054	0.696331
Dendritic.cells	TBC1D17	0.211386	3.823308	0.929975	0.354917	-5.56392	0.627429	0.627027
Dendritic.cells	PHLDB3	0.306011	2.97973	0.929812	0.355	-5.25416	0.637754	0.641925
Dendritic.cells	NDUFB8	0.097677	7.487591	0.929586	0.355117	-6.39411	0.585325	0.566224
Dendritic.cells	MCM4	0.238691	5.68459	0.929379	0.355223	-5.80422	0.60572	0.595465
Dendritic.cells	VPS36	0.128344	5.315956	0.92885	0.355496	-5.8869	0.610317	0.601833
Dendritic.cells	HPSE	0.12134	3.426146	0.92875	0.355548	-6.21335	0.632823	0.634359
Dendritic.cells	SLC25A24	0.253731	3.486183	0.92864	0.355604	-5.47939	0.632092	0.633301
Dendritic.cells	CYP2D10	-0.44159	1.738911	-0.92856	0.355645	-5.1315	0.653809	0.664877
Dendritic.cells	RGS2	-0.17613	6.977171	-0.92836	0.355751	-6.01684	0.59141	0.574757
Dendritic.cells	ZFP367	0.21702	5.436634	0.928305	0.355777	-5.81208	0.608942	0.599944
Dendritic.cells	DCUN1D4	-0.31093	2.540378	-0.92801	0.355928	-5.37612	0.643764	0.650427
Dendritic.cells	LSG1	0.114159	4.928986	0.927994	0.355937	-5.99825	0.614868	0.608601

Dendritic.cells	CUL1	0.080555	6.562305	0.927809	0.356033	-6.33018	0.596066	0.581606
Dendritic.cells	MAN2B2	0.220901	3.799809	0.927783	0.356046	-5.60731	0.628318	0.628099
Dendritic.cells	PPP1R2	0.076111	6.908394	0.92774	0.356068	-6.28629	0.592179	0.576053
Dendritic.cells	GATB	-0.28341	3.11047	-0.92764	0.35612	-5.39077	0.636714	0.640294
Dendritic.cells	C8G	-0.45828	3.782028	-0.92727	0.356309	-5.41674	0.628652	0.628614
Dendritic.cells	SDCBP2	0.474913	1.097863	0.926932	0.356485	-5.08421	0.662163	0.677449
Dendritic.cells	CD28	-0.17225	2.935145	-0.92681	0.356548	-6.05075	0.638993	0.643759
Dendritic.cells	DUSP5	0.115387	6.834196	0.926708	0.356601	-6.50008	0.593122	0.577564
Dendritic.cells	VPS8	0.119629	4.586793	0.926637	0.356638	-6.10327	0.619022	0.614867
Dendritic.cells	MMADHC	0.151335	4.299266	0.926622	0.356645	-5.71022	0.622441	0.619808
Dendritic.cells	INPP4B	-0.13898	6.480863	-0.92661	0.356652	-6.59722	0.597098	0.583284
Dendritic.cells	ATP23	0.472432	1.824257	0.92655	0.356683	-5.1493	0.652881	0.663989
Dendritic.cells	GM42937	-0.50193	0.198102	-0.92644	0.356737	-5.05551	0.673882	0.694724
Dendritic.cells	RNGTT	-0.08428	6.852257	-0.9264	0.356762	-6.41923	0.592919	0.577327
Dendritic.cells	MYCBP2	0.098171	7.768752	0.926233	0.356846	-6.58027	0.582817	0.562883
Dendritic.cells	ABHD8	0.351265	2.172201	0.925619	0.357164	-5.16227	0.64886	0.658095
Dendritic.cells	ARHGAP21	-0.12484	5.511128	-0.92554	0.357205	-6.13936	0.608541	0.599722
Dendritic.cells	COX20	0.13509	5.583215	0.925409	0.357272	-6.0331	0.607706	0.598528
Dendritic.cells	CIAO2A	0.12116	6.679979	0.925375	0.35729	-6.1742	0.595192	0.580528
Dendritic.cells	GM35853	0.519791	0.184156	0.925297	0.35733	-4.96263	0.67445	0.695503
Dendritic.cells	ZMIZ1	-0.08786	6.978265	-0.92523	0.357367	-6.41031	0.591847	0.575728
Dendritic.cells	SERINC1	0.092167	6.230989	0.925089	0.357438	-6.23289	0.600296	0.58783
Dendritic.cells	FMC1	0.247851	4.072806	0.924699	0.357639	-5.49066	0.625783	0.624328
Dendritic.cells	IL2RA	-0.65025	2.557963	-0.92457	0.357708	-5.05487	0.644338	0.651229
Dendritic.cells	TTC7B	-0.14084	5.391283	-0.92424	0.357876	-5.96017	0.610321	0.60189
Dendritic.cells	STIM2	-0.11383	6.210089	-0.92422	0.357889	-6.26757	0.600895	0.588324
Dendritic.cells	KRCC1	0.082197	5.9705	0.924119	0.35794	-6.23721	0.603633	0.592334
Dendritic.cells	TOR1AIP1	0.092783	7.42657	0.923504	0.358258	-6.47431	0.587379	0.568843
Dendritic.cells	KIF19A	0.488875	1.071139	0.923305	0.358361	-4.95719	0.663462	0.678946
Dendritic.cells	TPM3	0.057224	8.661561	0.923216	0.358407	-6.66979	0.573937	0.549673
Dendritic.cells	GM26936	0.40107	0.478634	0.923157	0.358438	-5.16691	0.671168	0.690231
Dendritic.cells	LEPROT	0.131761	5.065196	0.923141	0.358446	-5.95771	0.614271	0.607566
Dendritic.cells	PITHD1	0.211376	4.744392	0.923106	0.358464	-5.55328	0.618048	0.613017
Dendritic.cells	RACGAP1	-0.23382	5.205247	-0.92305	0.358492	-5.96732	0.612632	0.605202
Dendritic.cells	PYCARD	0.134847	5.876191	0.9229	0.358571	-6.12609	0.604856	0.594047
Dendritic.cells	9830166KC	-0.44239	-1.08122	-0.92267	0.35869	-4.95517	0.691968	0.720899
Dendritic.cells	RSL1D1	0.114898	6.043414	0.922664	0.358693	-6.17644	0.602938	0.591361
Dendritic.cells	DYNLL2	-0.17837	5.082989	-0.92254	0.358757	-5.8908	0.614062	0.607412
Dendritic.cells	ATP6V1A	-0.11152	6.534998	-0.92253	0.358764	-6.26057	0.597346	0.583347
Dendritic.cells	MRM2	-0.55953	2.071278	-0.92239	0.358834	-5.03113	0.650697	0.660577
Dendritic.cells	SEMA4C	-0.25703	2.202853	-0.92232	0.358871	-5.42623	0.64904	0.658165
Dendritic.cells	NUDCD1	-0.25825	3.226429	-0.92229	0.358885	-5.43671	0.636328	0.639689
Dendritic.cells	B4GALT4	0.300608	1.774117	0.921895	0.359092	-5.29483	0.65473	0.666103
Dendritic.cells	MCCC1	-0.30596	2.50652	-0.92066	0.359736	-5.32804	0.646558	0.653177
Dendritic.cells	VAT1	-0.24453	3.491225	-0.92054	0.359798	-5.49512	0.634394	0.635544
Dendritic.cells	FBXO11	-0.09819	8.797772	-0.91973	0.360217	-6.77349	0.573948	0.548349
Dendritic.cells	NR3C1	0.10152	7.440275	0.919668	0.360249	-6.55969	0.588732	0.569467

Dendritic.cells	TBXAS1	-0.48223	3.468333	-0.91965	0.360258	-5.28946	0.634992	0.636045
Dendritic.cells	ASB5	-0.45505	0.515697	-0.91961	0.36028	-5.04656	0.672401	0.690475
Dendritic.cells	U2AF1L4	-0.3161	3.047054	-0.91951	0.360329	-5.31795	0.640169	0.643627
Dendritic.cells	4933439C1	-0.40439	1.200712	-0.91951	0.360332	-5.12966	0.663487	0.677532
Dendritic.cells	E4F1	0.292914	3.127007	0.919385	0.360396	-5.34834	0.639183	0.64226
Dendritic.cells	LLPH	0.087796	6.447819	0.919302	0.360439	-6.28274	0.599866	0.585563
Dendritic.cells	PIGV	0.20122	2.921169	0.919117	0.360535	-5.44015	0.641795	0.646021
Dendritic.cells	EXOG	0.33578	1.745846	0.918881	0.360658	-5.20228	0.656661	0.667523
Dendritic.cells	IDH2	-0.12673	5.667278	-0.91868	0.36076	-6.10821	0.608973	0.598604
Dendritic.cells	CDH22	-0.16216	-0.85879	-0.91868	0.360761	-6.07286	0.690897	0.717688
Dendritic.cells	2010007H(	-0.47195	0.459338	-0.91849	0.360862	-4.96514	0.673393	0.691954
Dendritic.cells	GTF2H1	-0.11275	5.958143	-0.91831	0.360953	-6.13649	0.605745	0.593828
Dendritic.cells	EHD4	0.116857	6.076676	0.918139	0.361044	-6.22445	0.604414	0.591872
Dendritic.cells	UTP3	-0.1311	5.450096	-0.9179	0.361169	-5.99535	0.611657	0.602366
Dendritic.cells	CHST12	0.135236	5.763881	0.917625	0.361312	-6.23148	0.608016	0.597225
Dendritic.cells	OXSR1	-0.2603	3.496646	-0.91759	0.361332	-5.36611	0.634974	0.636132
Dendritic.cells	AU019990	-0.46523	1.211418	-0.91757	0.361341	-5.16363	0.663691	0.677871
Dendritic.cells	HBP1	-0.14954	5.788072	-0.91747	0.361394	-5.91995	0.607736	0.596881
Dendritic.cells	IRGM1	0.277939	4.93997	0.917414	0.361422	-5.93937	0.617639	0.611153
Dendritic.cells	ADD3	-0.19732	6.047415	-0.91739	0.361432	-5.95883	0.604749	0.592597
Dendritic.cells	NKIRAS1	0.220128	3.432264	0.917187	0.36154	-5.52939	0.635802	0.637408
Dendritic.cells	ATP8A2	0.544996	2.725958	0.917128	0.361571	-5.13187	0.644524	0.650058
Dendritic.cells	HECTD1	-0.07819	7.428886	-0.91681	0.361737	-6.48649	0.58932	0.5703
Dendritic.cells	SARS2	0.251287	2.675724	0.916765	0.361759	-5.28925	0.645281	0.650982
Dendritic.cells	DLG4	-0.35046	3.571009	-0.91663	0.36183	-5.5354	0.634257	0.634984
Dendritic.cells	LMBRD2	-0.15908	4.481892	-0.91631	0.361994	-5.9132	0.623292	0.619179
Dendritic.cells	PPP1CA	0.084472	8.233158	0.916126	0.362093	-6.52396	0.580537	0.55782
Dendritic.cells	HARBI1	-0.43687	1.346342	-0.91586	0.362229	-5.10858	0.662183	0.675745
Dendritic.cells	COX15	0.253898	2.891641	0.915861	0.362231	-5.27166	0.642648	0.647325
Dendritic.cells	5-Mar	0.148384	6.018808	0.915795	0.362266	-6.02282	0.605289	0.593395
Dendritic.cells	IL1RL2	-0.37119	0.726101	-0.91575	0.362287	-5.29347	0.670228	0.687529
Dendritic.cells	IGSF9	-0.19827	0.790152	-0.91565	0.362342	-5.66645	0.669391	0.686307
Dendritic.cells	CD24A	0.274768	7.730753	0.915531	0.362403	-6.05012	0.586033	0.565786
Dendritic.cells	DTD1	-0.33265	3.881415	-0.91552	0.362407	-5.30269	0.630513	0.629783
Dendritic.cells	GM47819	-0.60097	-0.22199	-0.91549	0.362424	-5.01194	0.682752	0.705889
Dendritic.cells	WDR36	-0.16822	4.578146	-0.91528	0.362534	-5.67908	0.622233	0.617771
Dendritic.cells	UBL4A	-0.2419	4.202976	-0.91494	0.362712	-5.5261	0.626867	0.624351
Dendritic.cells	BIN1	-0.16271	5.230633	-0.9149	0.362732	-5.76824	0.614665	0.606747
Dendritic.cells	RBM45	-0.23264	3.10947	-0.91468	0.362847	-5.41906	0.640197	0.643698
Dendritic.cells	TADA3	-0.24503	3.403164	-0.91467	0.362851	-5.38962	0.636584	0.638459
Dendritic.cells	FAM129C	0.276494	3.433215	0.914517	0.362933	-5.37678	0.636257	0.637931
Dendritic.cells	ECD	0.112729	4.899349	0.914382	0.363003	-6.00996	0.618633	0.612422
Dendritic.cells	AK7	0.477469	1.686834	0.913902	0.363254	-5.11271	0.658477	0.669889
Dendritic.cells	NDUFA8	0.148915	5.798653	0.913799	0.363308	-5.97948	0.608436	0.597465
Dendritic.cells	KLHL13	0.567695	0.360663	0.913646	0.363387	-5.0319	0.675742	0.69512
Dendritic.cells	PLS3	-0.18088	2.162516	-0.91332	0.36356	-5.77199	0.652677	0.661211
Dendritic.cells	GBP8	-0.27227	3.65151	-0.91308	0.363685	-5.59532	0.634227	0.634421

Dendritic.cells	CCDC122	-0.3563	1.315452	-0.91301	0.363721	-5.20483	0.663545	0.677041
Dendritic.cells	JUP	-0.3075	3.562597	-0.91287	0.363792	-5.31888	0.635313	0.636039
Dendritic.cells	SH3BP5L	0.350138	2.252113	0.912824	0.363817	-5.16554	0.651592	0.659683
Dendritic.cells	FAM71A	-0.51895	0.237403	-0.9125	0.363987	-5.01471	0.677841	0.697729
Dendritic.cells	ACVR2A	0.127436	4.7385	0.912365	0.364058	-6.28326	0.621353	0.615699
Dendritic.cells	TXN2	0.094879	6.315861	0.912252	0.364117	-6.2753	0.602973	0.589307
Dendritic.cells	LIPO3	0.268317	2.915424	0.91205	0.364223	-5.3801	0.643514	0.64791
Dendritic.cells	ZFP629	0.441106	0.926	0.911808	0.364349	-5.11554	0.668829	0.68479
Dendritic.cells	PNPLA2	0.183732	5.509886	0.911712	0.3644	-5.83955	0.612279	0.602843
Dendritic.cells	PDIA5	0.206202	2.273787	0.911642	0.364436	-5.69804	0.651548	0.659664
Dendritic.cells	TEX264	-0.11762	4.497588	-0.91149	0.364513	-5.95287	0.62423	0.620083
Dendritic.cells	LRRC51	-0.34655	1.76843	-0.91141	0.364556	-5.19213	0.657963	0.669015
Dendritic.cells	FANCC	-0.14654	4.74282	-0.9114	0.36456	-6.02064	0.621308	0.615879
Dendritic.cells	2610008E1	0.266949	2.466966	0.911201	0.364667	-5.43215	0.649116	0.656225
Dendritic.cells	ARMC6	-0.35178	1.405639	-0.91119	0.364673	-5.14049	0.662616	0.675877
Dendritic.cells	DNAL1	-0.40374	1.64713	-0.91114	0.364697	-5.13717	0.659514	0.671355
Dendritic.cells	GGNBP1	0.500152	1.044976	0.910918	0.364816	-5.03465	0.66731	0.682713
Dendritic.cells	HADHB	-0.12016	5.576702	-0.91086	0.364847	-6.05875	0.611528	0.601844
Dendritic.cells	GPHN	0.088978	7.486182	0.910737	0.36491	-6.56085	0.589823	0.570725
Dendritic.cells	ASH2L	-0.17068	4.017187	-0.9105	0.365035	-5.61306	0.630035	0.628597
Dendritic.cells	ST8SIA4	0.075584	7.324778	0.910388	0.365093	-6.96958	0.591618	0.57332
Dendritic.cells	PEX16	0.21251	3.474463	0.91035	0.365113	-5.46964	0.636643	0.638185
Dendritic.cells	PIF1	-0.57659	1.716548	-0.91034	0.365117	-5.04665	0.658654	0.670165
Dendritic.cells	EMC9	0.327866	1.600787	0.909966	0.365314	-5.21602	0.660359	0.672387
Dendritic.cells	ACADSB	0.208308	3.270603	0.909886	0.365356	-5.50231	0.639364	0.641875
Dendritic.cells	TAB3	0.202329	3.680364	0.909765	0.36542	-5.54848	0.63435	0.634598
Dendritic.cells	ZFP652OS	-0.49015	-0.171	-0.90947	0.365574	-4.99839	0.683756	0.706314
Dendritic.cells	SCMH1	0.130827	5.849321	0.909069	0.365785	-6.19487	0.608995	0.597493
Dendritic.cells	BC003965	0.218365	3.701969	0.908959	0.365843	-5.52617	0.634519	0.634347
Dendritic.cells	ARHGEF26	0.538481	0.379311	0.908717	0.36597	-5.01291	0.676803	0.695818
Dendritic.cells	DGAT1	-0.23829	7.095267	-0.90866	0.366	-6.15984	0.594865	0.577246
Dendritic.cells	PLEKHA6	0.263076	1.808761	0.908497	0.366086	-5.40817	0.658257	0.668832
Dendritic.cells	BC017158	-0.4245	1.449949	-0.90841	0.366129	-5.10993	0.66286	0.67555
Dendritic.cells	NOM1	-0.16486	4.141882	-0.90799	0.36635	-5.70234	0.629474	0.627005
Dendritic.cells	BC005624	0.096027	5.761918	0.907972	0.366361	-6.11844	0.610293	0.599362
Dendritic.cells	NBEAL2	-0.27693	2.635242	-0.90767	0.366519	-5.24815	0.648185	0.653979
Dendritic.cells	E2F5	-0.24774	2.69996	-0.90749	0.366613	-5.3546	0.647438	0.652839
Dendritic.cells	GM6712	0.296096	2.305447	0.907292	0.366719	-5.34849	0.652469	0.660127
Dendritic.cells	IST1	-0.1179	5.536178	-0.9072	0.366768	-5.99942	0.613214	0.603396
Dendritic.cells	JUNOS	-0.45884	1.820722	-0.90687	0.366941	-5.09483	0.658762	0.669194
Dendritic.cells	GM43305	0.194545	7.45195	0.906658	0.367052	-6.61273	0.591494	0.572141
Dendritic.cells	CLDN1	-0.61305	1.493829	-0.90662	0.367071	-5.0457	0.662957	0.675335
Dendritic.cells	TPM4	0.088767	7.167543	0.906616	0.367074	-6.45643	0.594671	0.576696
Dendritic.cells	TMEM191(	0.234243	1.703176	0.906372	0.367202	-5.33966	0.6603	0.671483
Dendritic.cells	ADI1	-0.17479	3.749643	-0.90636	0.367209	-5.60356	0.634699	0.634326
Dendritic.cells	GM46560	-0.495	-0.84515	-0.90588	0.367461	-4.96731	0.694081	0.720764
Dendritic.cells	ZBTB22	0.272519	3.076444	0.905877	0.367463	-5.36189	0.64309	0.646369

Dendritic.cells	KAT2B	0.137685	6.221283	0.905773	0.367518	-6.09981	0.605538	0.59221
Dendritic.cells	MRPL46	0.27821	3.264758	0.905726	0.367542	-5.29678	0.640759	0.64301
Dendritic.cells	MED4	-0.1828	4.020834	-0.90569	0.36756	-5.5153	0.631507	0.629639
Dendritic.cells	GM9929	0.269013	1.684976	0.905456	0.367685	-5.35806	0.660661	0.672003
Dendritic.cells	2900076A(	0.244047	2.621325	0.905321	0.367756	-5.36428	0.648783	0.654772
Dendritic.cells	KBTBD3	0.289396	2.727361	0.905225	0.367807	-5.33013	0.647455	0.652883
Dendritic.cells	USP5	0.179194	4.34395	0.905201	0.367819	-5.69513	0.62762	0.624163
Dendritic.cells	PNRC1	-0.0897	8.470962	-0.90511	0.367866	-6.6414	0.580438	0.55643
Dendritic.cells	MTMR1	0.102697	4.622377	0.905015	0.367917	-6.18459	0.624282	0.619361
Dendritic.cells	POC1A	-0.30742	3.229083	-0.9049	0.367977	-5.28642	0.641219	0.643893
Dendritic.cells	TMEM14A	0.527981	0.419553	0.904656	0.368106	-4.99148	0.677273	0.696332
Dendritic.cells	THSD4	-0.74393	0.910314	-0.90415	0.36837	-5.00048	0.670875	0.687162
Dendritic.cells	CIDEB	-0.40259	1.942054	-0.90415	0.368372	-5.13071	0.657555	0.667739
Dendritic.cells	NEDD4L	0.084984	6.886893	0.904113	0.368392	-6.44117	0.59814	0.581905
Dendritic.cells	GM26511	0.408172	2.075738	0.903994	0.368455	-5.13703	0.655853	0.665329
Dendritic.cells	SLC2A12	0.376711	1.619007	0.903909	0.3685	-5.17338	0.661691	0.673831
Dendritic.cells	ARV1	0.254485	2.101251	0.903835	0.368539	-5.4964	0.655529	0.664858
Dendritic.cells	GPLD1	-0.51092	0.934219	-0.90383	0.36854	-5.04097	0.670563	0.686776
Dendritic.cells	GM11110	0.233445	1.970897	0.903574	0.368677	-5.44688	0.657303	0.667379
Dendritic.cells	TRAF2	-0.19475	3.917831	-0.90349	0.368721	-5.59249	0.63306	0.632203
Dendritic.cells	ERG	-0.46879	3.921953	-0.90302	0.368971	-5.44421	0.633306	0.632245
Dendritic.cells	SHOC2	-0.09299	6.344774	-0.90294	0.36901	-6.2814	0.604695	0.591008
Dendritic.cells	ADAM30	-0.52359	0.62147	-0.90281	0.369082	-4.98526	0.675124	0.69304
Dendritic.cells	ARFGEF1	0.101248	6.613928	0.90263	0.369175	-6.32239	0.601662	0.586694
Dendritic.cells	SPCS3	0.104218	4.704887	0.90247	0.369259	-6.08137	0.623914	0.618741
Dendritic.cells	RPP40	0.342205	1.432828	0.902431	0.36928	-5.21855	0.664562	0.677706
Dendritic.cells	EXOC6B	-0.11051	5.502152	-0.90235	0.369325	-6.29378	0.614493	0.605159
Dendritic.cells	EWSR1	-0.07135	7.421625	-0.90205	0.369483	-6.47591	0.592592	0.573747
Dendritic.cells	ACP5	-0.3755	5.117131	-0.90197	0.369524	-5.45571	0.619052	0.611763
Dendritic.cells	RICTOR	0.081679	6.409431	0.90177	0.369629	-6.42153	0.604026	0.59016
Dendritic.cells	MPDU1	0.151798	4.71579	0.901587	0.369726	-5.76003	0.623817	0.618705
Dendritic.cells	ING1	-0.14547	5.135774	-0.90158	0.369728	-5.85004	0.618831	0.611513
Dendritic.cells	KBTBD7	-0.31983	2.137804	-0.90149	0.369777	-5.23474	0.655565	0.664717
Dendritic.cells	PSENEN	0.083482	6.713106	0.901468	0.369789	-6.28684	0.600565	0.58524
Dendritic.cells	LAMTOR3	0.118891	5.240813	0.901432	0.369808	-5.99851	0.617593	0.609727
Dendritic.cells	SVIP	0.490869	1.34626	0.901213	0.369924	-4.98009	0.66582	0.679602
Dendritic.cells	EXOC3L4	0.54331	0.594811	0.900866	0.370107	-5.02047	0.675837	0.69404
Dendritic.cells	XYLT2	-0.31168	1.972195	-0.90068	0.370206	-5.21184	0.657973	0.668059
Dendritic.cells	NCKAP5	-0.52665	1.217214	-0.90067	0.370209	-5.06296	0.667692	0.682226
Dendritic.cells	PHKA1	-0.28334	3.411651	-0.90033	0.370392	-5.38168	0.63992	0.642017
Dendritic.cells	SMO	0.502346	1.198551	0.90028	0.370417	-5.05302	0.667935	0.682772
Dendritic.cells	BLVRA	-0.15279	5.071733	-0.90001	0.37056	-5.8807	0.61987	0.613124
Dendritic.cells	CXADR	0.432022	1.645302	0.899884	0.370626	-5.11046	0.66216	0.674522
Dendritic.cells	CD79A	-0.26642	6.200185	-0.89971	0.370716	-5.77103	0.606703	0.594284
Dendritic.cells	SNX16	-0.26405	2.847666	-0.89961	0.370774	-5.35846	0.646919	0.652448
Dendritic.cells	SWI5	0.097786	7.120072	0.89955	0.370803	-6.47694	0.59624	0.579311
Dendritic.cells	A330023F2	0.34293	3.109027	0.899542	0.370808	-5.24563	0.643664	0.647743

Dendritic.cells	SV2C	0.536413	-0.28271	0.899452	0.370855	-4.98148	0.687522	0.711795
Dendritic.cells	GHDC	-0.3429	1.628326	-0.89938	0.370891	-5.16943	0.662378	0.675007
Dendritic.cells	HIRA	0.118472	5.652742	0.899318	0.370926	-6.12288	0.613044	0.603506
Dendritic.cells	CLK1	0.07928	7.55289	0.899266	0.370954	-6.5433	0.5914	0.572419
Dendritic.cells	GSPT1	-0.07269	6.707096	-0.89915	0.371015	-6.43395	0.600907	0.586075
Dendritic.cells	ICOSL	0.407788	1.327034	0.89914	0.37102	-5.11264	0.666268	0.680725
Dendritic.cells	1600014C1	0.299956	4.275444	0.898707	0.37125	-5.59038	0.629676	0.62719
Dendritic.cells	PADI6	-0.56487	0.058128	-0.8981	0.371574	-4.99955	0.683717	0.705392
Dendritic.cells	ILF2	0.130718	5.763945	0.897941	0.371656	-6.00972	0.612432	0.601927
Dendritic.cells	2300009A0	0.202461	4.080294	0.89792	0.371667	-5.63636	0.632452	0.630812
Dendritic.cells	PSRC1	-0.46069	0.886949	-0.89779	0.371736	-5.05945	0.672768	0.689443
Dendritic.cells	ZFP521	0.191057	2.100385	0.897502	0.371889	-5.99655	0.657259	0.666741
Dendritic.cells	TBCK	0.112104	5.26974	0.897224	0.372036	-6.09628	0.618546	0.610538
Dendritic.cells	CADM4	-0.38912	1.741687	-0.89709	0.372107	-5.27605	0.662029	0.673514
Dendritic.cells	NDUFB5	0.089074	7.037752	0.896817	0.372252	-6.41463	0.598306	0.581247
Dendritic.cells	GM19325	-0.48496	1.67424	-0.89625	0.372552	-5.09475	0.663383	0.675038
Dendritic.cells	F2R	-0.20198	3.599158	-0.89625	0.372555	-5.72186	0.639152	0.639846
Dendritic.cells	NCOA2	0.093912	7.684552	0.895819	0.372782	-6.52027	0.591629	0.571234
Dendritic.cells	TMEM202	0.499945	0.104736	0.895507	0.372948	-4.97688	0.684294	0.705369
Dendritic.cells	FANCB	0.443494	0.677958	0.89535	0.373031	-5.03445	0.67668	0.694265
Dendritic.cells	SLC43A1	0.508988	1.089863	0.895255	0.373082	-5.04692	0.671272	0.686366
Dendritic.cells	WDR44	0.183378	4.70447	0.895134	0.373146	-5.74925	0.626029	0.620711
Dendritic.cells	RCN3	0.340975	1.932131	0.895019	0.373207	-5.20707	0.660376	0.670486
Dendritic.cells	TULP3	0.300695	1.974251	0.894919	0.37326	-5.31082	0.659837	0.669701
Dendritic.cells	CYP2B9	-0.53963	1.9577	-0.89485	0.373297	-5.15527	0.660048	0.670009
Dendritic.cells	PSEN1	0.084848	6.297037	0.894848	0.373298	-6.30745	0.607329	0.593791
Dendritic.cells	LOCKD	-0.37663	4.472202	-0.89483	0.373308	-5.41781	0.628818	0.624737
Dendritic.cells	FAHD1	-0.41353	1.917334	-0.89469	0.373384	-5.13659	0.660597	0.670762
Dendritic.cells	CDKL2	-0.49187	1.163972	-0.89394	0.373781	-5.03162	0.670581	0.685217
Dendritic.cells	KLRA1	-0.74244	0.700337	-0.89392	0.373792	-4.98645	0.676663	0.6941
Dendritic.cells	RNPEPL1	-0.17437	4.75379	-0.89377	0.373873	-5.81069	0.625696	0.620157
Dendritic.cells	9930111J2	0.211397	4.28297	0.893603	0.37396	-5.87467	0.631363	0.628376
Dendritic.cells	SLC4A9	0.519183	0.122757	0.893596	0.373964	-4.97656	0.684334	0.705431
Dendritic.cells	GM16310	-0.42036	1.197906	-0.89349	0.374023	-5.15679	0.670138	0.684705
Dendritic.cells	GM16754	-0.53177	0.04601	-0.89346	0.374035	-4.9817	0.685361	0.706962
Dendritic.cells	HSPH1	0.288826	4.050366	0.893365	0.374087	-5.62715	0.634186	0.632482
Dendritic.cells	NUP93	0.168069	4.586111	0.893349	0.374095	-5.72526	0.627707	0.623122
Dendritic.cells	ERICH1	0.222115	3.61294	0.893211	0.374169	-5.52117	0.63954	0.640295
Dendritic.cells	ITFG2	-0.18698	3.893716	-0.89301	0.374277	-5.67679	0.636097	0.635374
Dendritic.cells	5031425E2	-0.13119	5.390925	-0.89287	0.374352	-6.02146	0.618133	0.609478
Dendritic.cells	PHF2OS1	0.346115	1.058613	0.892822	0.374376	-5.14493	0.671957	0.687546
Dendritic.cells	GSDME	0.240432	4.386903	0.892759	0.37441	-5.60639	0.630106	0.626776
Dendritic.cells	AXIN1	-0.08307	6.046226	-0.89266	0.374461	-6.26174	0.610477	0.5985
Dendritic.cells	MRTFA	0.131195	7.084199	0.892654	0.374466	-6.28965	0.598604	0.581453
Dendritic.cells	APOBEC4	0.520164	0.042895	0.892528	0.374533	-4.97729	0.685403	0.707267
Dendritic.cells	RAB39	0.370548	2.115142	0.892462	0.374568	-5.28597	0.658308	0.667717
Dendritic.cells	ANKRD6	-0.5359	0.95646	-0.89222	0.374697	-5.00643	0.673346	0.689708

Dendritic.cells	E130311K1	0.490483	0.244935	0.89211	0.374756	-5.06096	0.682755	0.703525
Dendritic.cells	CDK2AP2	0.113097	6.467925	0.892081	0.374771	-6.15499	0.605662	0.591709
Dendritic.cells	EEF1E1	-0.16768	4.824221	-0.89168	0.374987	-5.86662	0.625023	0.619576
Dendritic.cells	CAMTA2	-0.20776	3.696678	-0.89165	0.375002	-5.53434	0.638683	0.63933
Dendritic.cells	BNIP3L	0.140241	7.263429	0.891503	0.375079	-6.29305	0.596745	0.578938
Dendritic.cells	DENND4B	-0.17795	3.747946	-0.8914	0.375135	-5.66576	0.638053	0.638461
Dendritic.cells	SUOX	0.361015	1.361958	0.891153	0.375266	-5.10862	0.668183	0.682286
Dendritic.cells	CDKN1B	0.126058	6.470899	0.891111	0.375288	-6.19806	0.605744	0.591881
Dendritic.cells	NCR1	-0.64597	1.170957	-0.89108	0.375306	-5.0381	0.67067	0.685937
Dendritic.cells	STX11	0.341589	4.829435	0.891025	0.375334	-5.34146	0.62496	0.619585
Dendritic.cells	RRP36	-0.17286	3.775241	-0.89086	0.375423	-5.56657	0.637718	0.63809
Dendritic.cells	FRAT1	0.19183	3.463579	0.890854	0.375426	-5.55212	0.641554	0.643648
Dendritic.cells	MYO1B	-0.39802	2.148687	-0.89053	0.375601	-5.26668	0.658191	0.667735
Dendritic.cells	SOD2	0.279911	6.929524	0.890492	0.375619	-6.15757	0.600637	0.584543
Dendritic.cells	FBXW5	-0.19402	3.39907	-0.89007	0.375844	-5.55863	0.642669	0.64498
Dendritic.cells	PRR12	0.209719	3.451612	0.89007	0.375844	-5.48285	0.642019	0.644037
Dendritic.cells	GM45051	0.292828	1.845863	0.889782	0.375997	-5.27879	0.66234	0.67361
Dendritic.cells	RHOV	0.637102	-0.80702	0.889649	0.376069	-4.98072	0.697517	0.725169
Dendritic.cells	PCIF1	0.094057	6.021144	0.889291	0.37626	-6.17494	0.611308	0.599952
Dendritic.cells	EIF2S3Y	2.229371	1.945318	0.88912	0.376351	-5.3035	0.661063	0.672083
Dendritic.cells	H2-DMB1	-0.23302	4.478206	-0.88911	0.376354	-6.13878	0.629562	0.62635
Dendritic.cells	NR1D2	-0.25627	3.478586	-0.88902	0.376404	-5.38359	0.641763	0.644021
Dendritic.cells	FAHD2A	-0.24422	2.845982	-0.88902	0.376405	-5.34811	0.64964	0.655457
Dendritic.cells	RACK1	0.061891	8.637453	0.888941	0.376447	-6.71687	0.58192	0.557873
Dendritic.cells	SIT1	0.30878	2.062249	0.888765	0.376541	-5.38858	0.659566	0.669958
Dendritic.cells	OLFR77	-0.41796	1.376904	-0.88875	0.37655	-5.11068	0.6684	0.682844
Dendritic.cells	RETREG1	-0.17202	6.437217	-0.88869	0.376579	-5.95401	0.606504	0.593157
Dendritic.cells	TPST2	-0.1082	5.593782	-0.88867	0.376592	-6.18138	0.616294	0.60725
Dendritic.cells	BORCS7	0.180089	3.716689	0.888567	0.376647	-5.6205	0.63883	0.639834
Dendritic.cells	1600002D2	-0.66179	0.257645	-0.88827	0.376808	-4.98067	0.683273	0.704491
Dendritic.cells	GM26887	-0.35036	3.10483	-0.88817	0.376857	-5.49878	0.64653	0.650888
Dendritic.cells	YDJC	0.407284	1.21958	0.8881	0.376896	-5.15108	0.670581	0.68596
Dendritic.cells	ZFP566	0.399371	1.254913	0.887874	0.377018	-5.13236	0.670232	0.685372
Dendritic.cells	CAR13	0.481293	1.31675	0.887452	0.377244	-4.99192	0.669679	0.684376
Dendritic.cells	ROPN1L	0.391055	2.420763	0.887258	0.377347	-5.20854	0.655489	0.663738
Dendritic.cells	ZFP7	0.542747	0.286794	0.887229	0.377363	-4.98622	0.683256	0.704288
Dendritic.cells	ABCF2	0.169802	4.411019	0.887082	0.377442	-5.65275	0.630841	0.627995
Dendritic.cells	EME2	-0.32083	0.924802	-0.88695	0.377514	-5.15374	0.674807	0.69197
Dendritic.cells	CD80	0.50816	3.929604	0.886858	0.377561	-5.18012	0.636694	0.636523
Dendritic.cells	TBC1D8B	-0.22093	3.257326	-0.88685	0.377568	-5.46977	0.644983	0.648544
Dendritic.cells	NT5DC1	0.1557	4.5014	0.886667	0.377664	-5.84298	0.629804	0.626521
Dendritic.cells	PRAMEF8	-0.28834	3.188085	-0.88657	0.377718	-5.35431	0.645901	0.649845
Dendritic.cells	PNPLA1	-0.53115	0.231599	-0.88635	0.377831	-5.0137	0.684152	0.705573
Dendritic.cells	IL5RA	-0.50282	0.975245	-0.88624	0.37789	-5.11297	0.674302	0.69116
Dendritic.cells	ILK	0.145039	5.628263	0.88595	0.378048	-5.95829	0.616492	0.607329
Dendritic.cells	GAS2	-0.32362	2.456745	-0.88568	0.378194	-5.20213	0.655186	0.663449
Dendritic.cells	4930404N1	0.487758	0.191025	0.885616	0.378227	-5.02903	0.684695	0.706582

Dendritic.cells	RWDD2B	0.341027	1.849393	0.885602	0.378234	-5.24899	0.662941	0.674768
Dendritic.cells	SEC22B	0.109207	5.449712	0.885501	0.378289	-6.09333	0.618591	0.610477
Dendritic.cells	EHHADH	-0.48242	1.66442	-0.88549	0.378293	-5.05437	0.665326	0.678285
Dendritic.cells	OLFR920	0.509118	0.137933	0.885311	0.378391	-5.01567	0.685406	0.707767
Dendritic.cells	HMCN1	-0.38603	1.920186	-0.88528	0.378405	-5.4644	0.662032	0.673578
Dendritic.cells	LRP8OS2	-0.14869	0.163168	-0.88527	0.378415	-6.1911	0.685068	0.707272
Dendritic.cells	P2RX7	-0.21456	3.223954	-0.88495	0.378582	-5.76581	0.645696	0.649716
Dendritic.cells	NTPCR	-0.19202	3.847172	-0.88484	0.378642	-5.66389	0.637997	0.638545
Dendritic.cells	PJA2	0.143336	4.695864	0.884723	0.378706	-5.98008	0.627699	0.623679
Dendritic.cells	GNA11	0.129416	3.954617	0.884617	0.378763	-6.10735	0.636681	0.636682
Dendritic.cells	SDC4	-0.11751	6.436865	-0.88456	0.378792	-6.60123	0.607238	0.59417
Dendritic.cells	RTF2	0.08935	5.957747	0.883685	0.379263	-6.19742	0.613448	0.602472
Dendritic.cells	TRAF3IP2	0.203551	3.229202	0.883413	0.379409	-5.58726	0.646483	0.650164
Dendritic.cells	ANKRD37	0.311305	3.981977	0.883022	0.379619	-5.46373	0.637442	0.636832
Dendritic.cells	VPS13C	-0.19008	4.373385	-0.88289	0.379688	-5.69714	0.63269	0.629932
Dendritic.cells	ARF4OS	0.382445	0.845404	0.882665	0.379811	-5.10738	0.677367	0.694942
Dendritic.cells	SLC25A47	-0.27173	3.963	-0.88265	0.379817	-5.50446	0.637712	0.63723
Dendritic.cells	PPP3CC	-0.31151	3.993482	-0.88225	0.380035	-5.38264	0.637606	0.63675
Dendritic.cells	SELENOF	0.123815	5.422799	0.881975	0.380182	-6.08785	0.620457	0.611987
Dendritic.cells	NOS2	0.717004	-1.12584	0.881859	0.380244	-4.98348	0.704336	0.734231
Dendritic.cells	HOMEZ	0.345315	1.333922	0.881808	0.380271	-5.1664	0.671288	0.685794
Dendritic.cells	GM28192	0.462258	0.064757	0.88176	0.380297	-4.98784	0.688105	0.710397
Dendritic.cells	API5	0.096436	6.03062	0.881509	0.380433	-6.18257	0.613403	0.60178
Dendritic.cells	LRRC40	0.248234	3.249731	0.881454	0.380462	-5.38917	0.646925	0.650213
Dendritic.cells	BC005537	-0.08744	7.116434	-0.88132	0.380533	-6.42517	0.60095	0.583879
Dendritic.cells	G3BP1	-0.10235	6.728766	-0.88089	0.380764	-6.28675	0.605431	0.590335
Dendritic.cells	ZFP626	0.218774	2.562798	0.880781	0.380824	-5.46119	0.655656	0.662956
Dendritic.cells	SQOR	-0.37132	3.496234	-0.88067	0.380883	-5.24546	0.643952	0.645999
Dendritic.cells	MYADM	-0.15453	5.586342	-0.88064	0.380903	-6.13132	0.618694	0.609506
Dendritic.cells	ZDHHC8	0.158752	3.666743	0.880531	0.380959	-5.79067	0.641843	0.642968
Dendritic.cells	CFLAR	-0.16427	5.971185	-0.88052	0.380962	-6.06415	0.614184	0.603016
Dendritic.cells	TCN2	-0.21487	4.791081	-0.88048	0.380984	-5.7786	0.628152	0.623157
Dendritic.cells	ATPAF2	0.252594	2.92038	0.880318	0.381074	-5.3861	0.651194	0.656489
Dendritic.cells	PTBP1	-0.09728	6.221744	-0.88017	0.381156	-6.27077	0.611359	0.598908
Dendritic.cells	ILVBL	0.187647	3.757211	0.879848	0.381327	-5.62798	0.641009	0.641495
Dendritic.cells	AGFG1	-0.10206	6.147106	-0.87948	0.381527	-6.15175	0.612633	0.600315
Dendritic.cells	A530064D(	0.369278	0.81889	0.879273	0.381637	-5.23435	0.678866	0.69632
Dendritic.cells	UBE4A	-0.1311	5.03345	-0.87905	0.381757	-5.98491	0.625832	0.619347
Dendritic.cells	1110038F1	0.171143	4.590808	0.878991	0.381789	-5.74074	0.63115	0.62705
Dendritic.cells	KLHL14	0.518487	2.717642	0.878761	0.381913	-5.10298	0.654305	0.660743
Dendritic.cells	PSMA6	0.109753	6.941715	0.878709	0.381941	-6.26498	0.603562	0.58748
Dendritic.cells	RIPOR2	0.097243	7.617108	0.878649	0.381973	-6.7339	0.595931	0.576564
Dendritic.cells	ESCO2	0.461561	3.734708	0.878545	0.382029	-5.24081	0.641602	0.642402
Dendritic.cells	TRIM59	0.271493	4.368452	0.878456	0.382077	-5.49435	0.633844	0.63117
Dendritic.cells	SEMA4D	0.109955	6.446214	0.878256	0.382186	-6.61354	0.609243	0.595696
Dendritic.cells	GTF2H5	0.08903	6.547345	0.878224	0.382202	-6.31589	0.608078	0.594021
Dendritic.cells	GM16124	0.32809	2.669782	0.878093	0.382273	-5.35279	0.65491	0.661721

Dendritic.cells	ABI3	0.215185	4.401261	0.877984	0.382332	-5.88251	0.633445	0.630621
Dendritic.cells	CAVIN1	0.415186	1.312054	0.87798	0.382334	-5.24909	0.67238	0.687209
Dendritic.cells	HPS4	-0.19478	4.16681	-0.87768	0.382499	-5.74311	0.636454	0.634859
Dendritic.cells	CHCHD7	0.163353	4.398636	0.877591	0.382544	-5.68917	0.633632	0.630776
Dendritic.cells	APLF	-0.37358	2.05909	-0.87741	0.382639	-5.16134	0.662923	0.673284
Dendritic.cells	PPP1R11	0.162042	4.640461	0.877302	0.3827	-5.82118	0.630768	0.626656
Dendritic.cells	GTF2H2	0.177572	3.759481	0.875786	0.38352	-5.54214	0.642641	0.642723
Dendritic.cells	NFE2L1	0.138497	4.98448	0.875772	0.383527	-6.0156	0.627731	0.621176
Dendritic.cells	MRPS30	0.143353	4.726011	0.875736	0.383547	-5.78075	0.63084	0.625667
Dendritic.cells	EOMES	-0.59086	-0.77158	-0.87528	0.383795	-4.99065	0.702115	0.729094
Dendritic.cells	ACSL3	0.301136	3.828777	0.875061	0.383912	-5.34532	0.642126	0.641654
Dendritic.cells	MPPED2	-0.58986	-0.32287	-0.87485	0.384023	-4.9922	0.695997	0.720209
Dendritic.cells	RAB3IL1	-0.32979	1.73222	-0.87477	0.384068	-5.27907	0.668666	0.680255
Dendritic.cells	ALCAM	0.130135	6.646567	0.874738	0.384087	-6.63609	0.608533	0.593311
Dendritic.cells	NAA10	-0.16979	5.084078	-0.87443	0.384255	-5.84915	0.626871	0.619763
Dendritic.cells	B630019A1	-0.44002	1.596371	-0.87437	0.384287	-5.12034	0.670433	0.682919
Dendritic.cells	PPM1G	-0.08324	6.448565	-0.87419	0.384385	-6.32374	0.610817	0.596692
Dendritic.cells	ZC3H11A	0.333386	2.01149	0.874042	0.384464	-5.24847	0.665053	0.675201
Dendritic.cells	MPLKIP	0.125914	4.793719	0.87401	0.384481	-5.82295	0.630358	0.624907
Dendritic.cells	ABHD17A	0.064317	6.296745	0.87395	0.384513	-6.52266	0.612577	0.599297
Dendritic.cells	GM49602	0.322543	1.89388	0.873581	0.384713	-5.22945	0.666572	0.677417
Dendritic.cells	ATP1B1	0.096818	5.798004	0.873529	0.384741	-6.70817	0.618403	0.607676
Dendritic.cells	PNPLA8	-0.11404	6.018439	-0.87347	0.384776	-6.14757	0.615819	0.603958
Dendritic.cells	LCMT1	0.258468	3.124061	0.87339	0.384816	-5.34528	0.650898	0.654631
Dendritic.cells	SUV39H2	-0.2524	2.381659	-0.87338	0.384821	-5.41404	0.660302	0.668291
Dendritic.cells	GDI1	0.159638	4.991215	0.873358	0.384834	-5.83813	0.627983	0.62148
Dendritic.cells	SMARCAD1	0.110559	4.984115	0.873267	0.384883	-5.99415	0.628069	0.621603
Dendritic.cells	HSPE1	0.112549	7.179751	0.87324	0.384898	-6.46931	0.602437	0.584745
Dendritic.cells	E330020D1	-0.17728	5.11024	-0.87309	0.384978	-6.08646	0.626558	0.619432
Dendritic.cells	D830036C2	0.516175	0.420218	0.873084	0.384982	-5.01642	0.685963	0.705738
Dendritic.cells	SLC35C2	0.079388	4.846147	0.872713	0.385183	-6.29036	0.629888	0.624127
Dendritic.cells	APH1B	-0.39224	2.578014	-0.87268	0.385199	-5.15754	0.657967	0.664795
Dendritic.cells	ERLIN2	0.220155	3.099067	0.872061	0.385537	-5.47948	0.651517	0.655361
Dendritic.cells	GM4107	0.614756	0.916265	0.871947	0.385599	-5.0038	0.679679	0.696365
Dendritic.cells	EPB41L3	-0.54765	2.334313	-0.87193	0.385606	-5.18672	0.661217	0.669451
Dendritic.cells	TAF1C	-0.2971	2.170342	-0.87184	0.385658	-5.25009	0.66332	0.672562
Dendritic.cells	TBCB	0.092297	6.070727	0.871661	0.385754	-6.24472	0.615497	0.60346
Dendritic.cells	PACSIN2	0.12484	5.573303	0.871583	0.385797	-5.88816	0.621344	0.611879
Dendritic.cells	CARF	0.311314	2.216581	0.871505	0.385839	-5.28287	0.662726	0.671794
Dendritic.cells	ZFP995	0.25688	2.383748	0.871373	0.38591	-5.38397	0.660585	0.668681
Dendritic.cells	MTHFR	0.160692	3.640415	0.871366	0.385914	-5.72862	0.644758	0.645707
Dendritic.cells	SH3GL1	-0.11816	4.688824	-0.87115	0.386032	-6.00425	0.63192	0.62718
Dendritic.cells	ASB6	0.252801	2.575022	0.871058	0.386081	-5.39373	0.658145	0.665179
Dendritic.cells	TSC22D2	-0.08144	6.616128	-0.87105	0.386085	-6.43104	0.609169	0.594414
Dendritic.cells	SHMT2	-0.25104	4.430904	-0.87102	0.386101	-5.56981	0.635048	0.631699
Dendritic.cells	ZBTB1	-0.13456	5.505188	-0.87102	0.386102	-6.05812	0.62215	0.613086
Dendritic.cells	ZFP28	0.436998	0.2908	0.870588	0.386337	-5.10423	0.68816	0.708943

Dendritic.cells	MYO7A	-0.24165	3.25231	-0.87047	0.386399	-5.43054	0.649726	0.652973
Dendritic.cells	SMIM15	0.120944	4.858356	0.870392	0.386444	-5.94178	0.630002	0.624448
Dendritic.cells	ITPKC	-0.40025	1.763821	-0.87037	0.386456	-5.15556	0.668705	0.680596
Dendritic.cells	GM11423	0.324514	0.892029	0.870335	0.386474	-5.23165	0.680138	0.697286
Dendritic.cells	PCMT1	-0.08947	6.63523	-0.87003	0.38664	-6.31193	0.609242	0.594423
Dendritic.cells	GM12764	0.370778	1.453299	0.86981	0.38676	-5.2013	0.673036	0.686749
Dendritic.cells	ZFP180	0.238639	3.315586	0.869709	0.386815	-5.36487	0.64921	0.652118
Dendritic.cells	MTA2	0.114441	5.782517	0.869487	0.386935	-6.16316	0.619363	0.608901
Dendritic.cells	GM48089	0.503432	1.135961	0.869374	0.386997	-5.08272	0.677316	0.692951
Dendritic.cells	CCDC180	-0.38463	1.286108	-0.86913	0.387128	-5.20176	0.675466	0.690107
Dendritic.cells	CFH	-0.27968	4.930751	-0.86826	0.387605	-5.7176	0.6303	0.623768
Dendritic.cells	GABPB1	-0.14047	5.380129	-0.86815	0.387663	-5.87489	0.624918	0.616053
Dendritic.cells	IGKC	-0.27392	7.88266	-0.86801	0.38774	-6.60623	0.596058	0.574596
Dendritic.cells	CCNY	-0.08483	6.67252	-0.86773	0.387893	-6.43989	0.60995	0.594342
Dendritic.cells	WIPI1	-0.41028	2.277802	-0.86697	0.388307	-5.17207	0.66391	0.671889
Dendritic.cells	SAMSN1	-0.08846	7.050554	-0.86694	0.388322	-6.69378	0.605988	0.588309
Dendritic.cells	SKINT3	0.503806	0.115469	0.866907	0.38834	-4.99849	0.692435	0.713493
Dendritic.cells	SLC12A8	0.495286	0.657727	0.866798	0.388399	-5.02633	0.685145	0.702892
Dendritic.cells	SPN	0.154443	3.586459	0.866592	0.388512	-5.86572	0.647348	0.647986
Dendritic.cells	PLBD1	0.292267	5.389651	0.866543	0.388538	-5.73564	0.625376	0.616263
Dendritic.cells	C1QTNF6	-0.36065	1.904338	-0.86653	0.388547	-5.34273	0.668733	0.679026
Dendritic.cells	RHOH	0.188305	6.471792	0.866331	0.388653	-6.03294	0.612728	0.598084
Dendritic.cells	SLC17A9	0.403978	2.888147	0.865941	0.388866	-5.19975	0.656375	0.660934
Dendritic.cells	KALRN	-0.32042	2.750236	-0.86593	0.388874	-5.43693	0.658126	0.663474
Dendritic.cells	RAP2A	0.207013	3.974658	0.86567	0.389014	-5.57526	0.642877	0.641334
Dendritic.cells	SETD3	0.08403	5.687067	0.865609	0.389048	-6.31515	0.622175	0.611495
Dendritic.cells	SETDB2	0.157039	4.580351	0.86526	0.389238	-5.81222	0.635639	0.630781
Dendritic.cells	EXD2	0.250559	3.156926	0.865161	0.389292	-5.40337	0.653263	0.656287
Dendritic.cells	RAB2B	-0.20271	3.661593	-0.86508	0.389337	-5.56407	0.646944	0.647155
Dendritic.cells	ARHGAP26	-0.15623	6.685583	-0.86489	0.389438	-6.1606	0.610689	0.59498
Dendritic.cells	RHNO1	0.161314	4.345115	0.864848	0.389463	-5.82063	0.638518	0.635064
Dendritic.cells	AI987944	0.261291	2.764701	0.864743	0.38952	-5.39764	0.658237	0.663629
Dendritic.cells	COPS9	0.102579	6.68605	0.864496	0.389655	-6.26893	0.610803	0.595089
Dendritic.cells	9230114K1	0.217642	2.574674	0.864081	0.389882	-5.43432	0.661073	0.667441
Dendritic.cells	JAM2	-0.38971	1.449339	-0.86386	0.39	-5.34602	0.675725	0.688661
Dendritic.cells	PLCB3	-0.25615	2.612	-0.86376	0.390055	-5.38107	0.660665	0.666761
Dendritic.cells	MARK4	0.158981	4.541737	0.863682	0.390099	-5.83494	0.636583	0.63192
Dendritic.cells	PGS1	-0.12151	4.740858	-0.86332	0.390296	-5.99917	0.634191	0.628645
Dendritic.cells	SRXN1	0.456274	1.094676	0.863207	0.390359	-5.02532	0.680433	0.695758
Dendritic.cells	IL23R	0.531838	-0.9447	0.863204	0.390361	-4.99539	0.708095	0.736273
Dendritic.cells	ULK2	-0.11376	5.457806	-0.86311	0.39041	-6.17617	0.625572	0.616228
Dendritic.cells	OLA1	0.08966	5.835409	0.863092	0.390422	-6.20954	0.621093	0.609784
Dendritic.cells	ARPC1A	-0.08325	6.586291	-0.863	0.390471	-6.40443	0.612312	0.597198
Dendritic.cells	TXNDC12	-0.18452	3.827411	-0.86284	0.390558	-5.54948	0.645441	0.644916
Dendritic.cells	TRP53RKB	0.263561	2.444883	0.862624	0.390678	-5.34714	0.662955	0.670301
Dendritic.cells	MIA2	-0.07206	6.77012	-0.86255	0.390719	-6.46429	0.610299	0.594274
Dendritic.cells	ZFP787	-0.13428	5.128821	-0.86233	0.390838	-5.81553	0.629686	0.622121

Dendritic.cells	GM32916	-0.87031	-0.24915	-0.86226	0.390877	-5.00532	0.698712	0.722491
Dendritic.cells	PTRHD1	0.1951	4.56189	0.861704	0.391181	-5.67271	0.636947	0.632223
Dendritic.cells	HEXDC	-0.42276	1.86348	-0.86147	0.391309	-5.19201	0.671072	0.681649
Dendritic.cells	RBBP4	-0.07435	7.676556	-0.86123	0.39144	-6.52314	0.600561	0.579884
Dendritic.cells	ING2	0.146476	4.920364	0.861049	0.39154	-5.804	0.632764	0.62613
Dendritic.cells	WDR48	-0.15706	4.298768	-0.86098	0.391575	-5.67365	0.640337	0.637062
Dendritic.cells	NAA50	0.085117	6.371464	0.860746	0.391706	-6.28587	0.615533	0.601369
Dendritic.cells	PRRC2A	-0.11468	5.517966	-0.86058	0.391796	-5.99302	0.625593	0.615833
Dendritic.cells	2610507B1	-0.09388	5.431746	-0.86056	0.391808	-6.07942	0.626621	0.617313
Dendritic.cells	PPIL4	0.095114	5.46202	0.860275	0.391964	-6.08784	0.62626	0.616826
Dendritic.cells	TNFRSF18	-0.25855	2.322619	-0.86022	0.391994	-5.39514	0.665189	0.673156
Dendritic.cells	SYS1	0.077676	6.417243	0.860178	0.392018	-6.38484	0.615	0.600652
Dendritic.cells	TCEANC	-0.35711	1.730178	-0.86013	0.392046	-5.23467	0.672872	0.684338
Dendritic.cells	GM10371	0.463082	-0.68835	0.860115	0.392052	-5.00075	0.705378	0.731871
Dendritic.cells	JDP2	-0.29811	5.304027	-0.86011	0.392054	-5.59171	0.628148	0.619559
Dendritic.cells	PIWIL2	-0.53774	0.700548	-0.86	0.392117	-5.03237	0.686494	0.704218
Dendritic.cells	GOLPH3L	0.183315	4.39604	0.859865	0.392189	-5.7214	0.639172	0.635437
Dendritic.cells	RGMB	-0.25458	1.845211	-0.85974	0.392255	-5.48705	0.671413	0.682178
Dendritic.cells	GM10134	-0.56397	0.795093	-0.85929	0.392503	-5.01389	0.685291	0.702538
Dendritic.cells	CCDC92	0.469576	-0.17821	0.859104	0.392606	-5.01006	0.698438	0.721829
Dendritic.cells	GM24362	0.431439	0.694716	0.859067	0.392627	-5.06887	0.686633	0.704548
Dendritic.cells	MTFR2	0.349443	3.615592	0.859058	0.392632	-5.33456	0.648858	0.64959
Dendritic.cells	SMIM12	0.183288	4.0506	0.858638	0.392862	-5.58387	0.643456	0.641792
Dendritic.cells	RFESD	0.319479	2.541257	0.858593	0.392887	-5.28559	0.662446	0.66932
Dendritic.cells	ACOT4	-0.54073	0.046894	-0.85857	0.3929	-5.02562	0.69537	0.717356
Dendritic.cells	PRKCD	0.071138	6.897294	0.858528	0.392923	-6.62347	0.609502	0.592894
Dendritic.cells	PTGER2	-0.50351	1.047913	-0.85847	0.392954	-5.00368	0.681924	0.697696
Dendritic.cells	FNBP1L	-0.34231	3.079155	-0.85841	0.392987	-5.34887	0.655599	0.659384
Dendritic.cells	CCDC125	0.342179	4.497843	0.858363	0.393013	-5.29559	0.637962	0.633857
Dendritic.cells	BCL10	-0.10123	6.452929	-0.85834	0.393027	-6.1729	0.614645	0.600279
Dendritic.cells	CXCL3	0.734116	-0.07498	0.858318	0.393038	-5.00073	0.697029	0.719792
Dendritic.cells	ORMDL1	-0.20969	3.609727	-0.85795	0.393241	-5.47539	0.649086	0.649838
Dendritic.cells	PQLC3	0.12651	3.812077	0.857933	0.393249	-6.08606	0.646565	0.646191
Dendritic.cells	RBMX2	-0.26547	3.368821	-0.8578	0.393321	-5.37683	0.652124	0.654286
Dendritic.cells	DOLPP1	0.306081	2.514642	0.85757	0.393449	-5.27461	0.663081	0.6701
Dendritic.cells	NAXD	-0.1457	4.128354	-0.85728	0.393609	-5.7988	0.642947	0.640808
Dendritic.cells	TJP3	-0.24659	1.707068	-0.85691	0.393813	-5.50022	0.67388	0.685584
Dendritic.cells	GM11973	-0.23909	3.179185	-0.8568	0.393872	-5.58211	0.654956	0.658132
Dendritic.cells	ITGA8	-0.40939	1.726804	-0.85676	0.393896	-5.3359	0.673622	0.68526
Dendritic.cells	KIF5B	0.058337	7.401742	0.856672	0.393942	-6.58682	0.604306	0.585178
Dendritic.cells	ZMPSTE24	0.190241	4.942134	0.856356	0.394116	-5.7726	0.63333	0.626762
Dendritic.cells	MGAT5	0.111469	6.916886	0.856268	0.394164	-6.31593	0.610015	0.59324
Dendritic.cells	ULBP1	0.305501	4.251838	0.855715	0.394469	-5.42115	0.642153	0.639043
Dendritic.cells	PAPSS1	0.145232	4.181725	0.855404	0.39464	-5.76074	0.64313	0.640372
Dendritic.cells	PRDX4	-0.1754	5.118984	-0.85537	0.394657	-5.79722	0.631699	0.62388
Dendritic.cells	CCL2	-0.60011	3.089272	-0.85513	0.394788	-5.31467	0.65689	0.66027
Dendritic.cells	RARS	0.145735	5.121145	0.855047	0.394836	-5.84041	0.631771	0.623995

Dendritic.cells	DCTN2	-0.11642	5.367124	-0.85457	0.395096	-6.04243	0.629059	0.619929
Dendritic.cells	SLFN1	0.783031	2.335521	0.854467	0.395156	-5.10523	0.666789	0.674502
Dendritic.cells	SLC35E1	0.177266	4.084727	0.854343	0.395224	-5.60852	0.644679	0.64246
Dendritic.cells	CXCR2	-0.55014	-0.00657	-0.8542	0.395304	-5.0121	0.697877	0.719852
Dendritic.cells	GSKIP	-0.1161	4.602025	-0.85418	0.395312	-5.87271	0.638319	0.633276
Dendritic.cells	TSPAN13	0.07085	6.684078	0.854034	0.395394	-6.75884	0.613528	0.597661
Dendritic.cells	SH3GLB2	-0.18737	3.721025	-0.85402	0.395404	-5.6178	0.649199	0.64905
Dendritic.cells	GPATCH4	-0.25597	3.338867	-0.85384	0.395499	-5.40607	0.65405	0.656028
Dendritic.cells	RIPK2	0.223164	4.205503	0.853506	0.395685	-5.62968	0.643314	0.640554
Dendritic.cells	4732471J0	-0.32332	1.996323	-0.85345	0.395715	-5.25998	0.671318	0.681165
Dendritic.cells	MSL1	0.135217	5.419802	0.853444	0.395719	-5.96993	0.628552	0.619267
Dendritic.cells	TTI1	-0.33169	2.712153	-0.85332	0.39579	-5.27293	0.662099	0.667819
Dendritic.cells	RIF1	0.133515	5.435862	0.853108	0.395905	-6.02906	0.628437	0.61913
Dendritic.cells	CPT1B	-0.33353	1.536488	-0.85293	0.396004	-5.14917	0.677419	0.690111
Dendritic.cells	CDADC1	0.228359	4.609962	0.852863	0.39604	-5.66405	0.638426	0.63358
Dendritic.cells	TBC1D7	0.248056	2.220043	0.852819	0.396064	-5.28471	0.668495	0.677161
Dendritic.cells	GM8797	0.344017	2.19543	0.852541	0.396217	-5.16376	0.668851	0.677719
Dendritic.cells	SNAI3	0.391014	-0.34486	0.852456	0.396264	-5.16927	0.702774	0.727307
Dendritic.cells	ABRAXAS2	-0.1295	4.960803	-0.85245	0.396265	-5.90158	0.634193	0.627557
Dendritic.cells	ORC1	-0.32845	3.345968	-0.85224	0.396385	-5.31469	0.654148	0.656468
Dendritic.cells	CMSS1	-0.17975	6.448827	-0.85224	0.396386	-6.24234	0.616496	0.602145
Dendritic.cells	TBC1D25	0.259821	2.839206	0.851992	0.39652	-5.44026	0.660574	0.665863
Dendritic.cells	TMEM218	0.214693	1.874833	0.851971	0.396532	-5.46907	0.673021	0.683963
Dendritic.cells	EIF3H	0.075747	7.353901	0.851809	0.396621	-6.51774	0.60605	0.587228
Dendritic.cells	TMEM223	0.160119	4.652686	0.851727	0.396667	-5.72715	0.63794	0.633091
Dendritic.cells	DPP4	0.158696	5.100888	0.851679	0.396693	-5.98908	0.632499	0.625241
Dendritic.cells	KDM5D	1.401835	0.730109	0.851409	0.396842	-5.18525	0.688172	0.706114
Dendritic.cells	PRORP	0.181499	3.853039	0.851183	0.396967	-5.59123	0.647797	0.647467
Dendritic.cells	INIP	0.141492	4.523666	0.851064	0.397033	-5.80472	0.639517	0.635522
Dendritic.cells	FAM118B	-0.20622	3.464677	-0.85082	0.397169	-5.49431	0.652654	0.654548
Dendritic.cells	CABP4	0.547161	-0.22452	0.850809	0.397174	-5.00487	0.701121	0.725222
Dendritic.cells	SVBP	0.090379	5.267752	0.850662	0.397255	-6.35758	0.630489	0.622508
Dendritic.cells	MFSD3	-0.39444	0.642198	-0.85061	0.397283	-5.14339	0.689352	0.707982
Dendritic.cells	UBR5	-0.0827	6.888097	-0.85045	0.397372	-6.49119	0.611396	0.59505
Dendritic.cells	1-Sep	0.081748	6.037433	0.850448	0.397373	-6.54601	0.621323	0.60931
Dendritic.cells	USP32	-0.11695	6.849617	-0.85032	0.397443	-6.31776	0.611841	0.595738
Dendritic.cells	1810059H2	-0.56775	1.799204	-0.85026	0.397475	-5.07798	0.67401	0.68567
Dendritic.cells	HEY1	0.521758	-0.2714	0.850114	0.397558	-5.00931	0.701764	0.72629
Dendritic.cells	SPG11	-0.17748	4.094018	-0.85	0.397623	-5.72272	0.644806	0.643338
Dendritic.cells	SDCCAG8	0.088406	5.914953	0.849866	0.397695	-6.36195	0.62277	0.611561
Dendritic.cells	RGS14	0.242813	3.585563	0.849649	0.397815	-5.51903	0.651138	0.652665
Dendritic.cells	NDST2	0.249981	2.934923	0.849421	0.397941	-5.38479	0.659354	0.664638
Dendritic.cells	CCL6	-0.33248	5.306299	-0.84938	0.397962	-5.67961	0.630026	0.622187
Dendritic.cells	STAMBP	0.205377	3.374422	0.849265	0.398028	-5.60959	0.65379	0.65656
Dendritic.cells	GM42418	-0.20035	11.10924	-0.84925	0.398034	-7.09521	0.565177	0.529424
Dendritic.cells	ERBIN	0.073137	7.975801	0.849231	0.398047	-6.76238	0.599008	0.577626
Dendritic.cells	IDH3B	0.134895	5.850654	0.849176	0.398077	-6.06405	0.623532	0.612828

Dendritic.cells	CELF4	-0.40051	0.6776	-0.84916	0.398088	-5.12724	0.688877	0.707681
Dendritic.cells	ANG	-0.34789	4.211322	-0.84911	0.398111	-5.55818	0.643357	0.641444
Dendritic.cells	TAOK1	0.070602	7.033029	0.84889	0.398235	-6.53365	0.609726	0.592984
Dendritic.cells	TBRG1	-0.14371	5.618457	-0.84885	0.398257	-6.03942	0.626291	0.616803
Dendritic.cells	CACNA1I	0.381959	-0.17458	0.848822	0.398273	-5.00528	0.700437	0.724621
Dendritic.cells	GM5089	0.356624	0.395428	0.848813	0.398278	-5.25095	0.692679	0.713247
Dendritic.cells	FPGS	0.265672	3.013479	0.848638	0.398375	-5.34789	0.658398	0.663187
Dendritic.cells	ZFP39	0.426796	1.023509	0.848343	0.398538	-5.11877	0.684294	0.701025
Dendritic.cells	ALDH7A1	-0.30018	3.227154	-0.84818	0.398629	-5.36043	0.655691	0.659436
Dendritic.cells	IQSEC1	0.126106	6.277452	0.84811	0.398667	-6.09116	0.618541	0.605755
Dendritic.cells	TRIM72	-0.45065	0.692552	-0.84802	0.398717	-5.09357	0.688721	0.707581
Dendritic.cells	PFDN2	0.141177	5.031244	0.847939	0.398762	-5.80332	0.633382	0.627141
Dendritic.cells	MORRBID	-0.34283	5.127394	-0.84789	0.398787	-5.62296	0.632221	0.625465
Dendritic.cells	ETAA1OS	0.439679	0.543342	0.847825	0.398825	-5.00962	0.690728	0.710519
Dendritic.cells	CPNE3	0.125606	5.367342	0.847804	0.398837	-6.12368	0.629334	0.621307
Dendritic.cells	WDR66	-0.35574	1.900032	-0.84767	0.398908	-5.21005	0.672743	0.684256
Dendritic.cells	TCP1	0.106522	6.67677	0.847582	0.398959	-6.3306	0.613889	0.599116
Dendritic.cells	CAAA0111ﾠ	-0.10953	5.624186	-0.84744	0.399036	-6.15216	0.626297	0.616944
Dendritic.cells	ZFP945	-0.21911	3.17689	-0.84713	0.39921	-5.49654	0.656551	0.660521
Dendritic.cells	STAT3	-0.08181	7.950149	-0.84694	0.399314	-6.75773	0.599544	0.578392
Dendritic.cells	GPRIN3	0.427366	0.638034	0.846912	0.399331	-5.14028	0.689693	0.708896
Dendritic.cells	TRIM13	0.475706	0.46615	0.846652	0.399475	-5.05277	0.692031	0.712382
Dendritic.cells	NEPRO	-0.24616	2.709524	-0.8466	0.399504	-5.43664	0.662526	0.66932
Dendritic.cells	TRIM10	0.552226	0.349121	0.846568	0.399521	-5.07385	0.693614	0.714701
Dendritic.cells	MAFG	-0.14233	5.07367	-0.8463	0.39967	-5.88483	0.633116	0.626745
Dendritic.cells	CCL5	-0.54697	7.931718	-0.84609	0.399786	-6.44679	0.599776	0.578834
Dendritic.cells	DHFR	-0.30452	4.331798	-0.84609	0.399787	-5.50314	0.642164	0.639849
Dendritic.cells	NPTN	0.052608	8.043161	0.846082	0.399791	-6.83768	0.598524	0.577042
Dendritic.cells	MIEN1	0.094419	5.845651	0.846027	0.399822	-6.12757	0.623875	0.613456
Dendritic.cells	TNFRSF12A	0.400076	1.479733	0.845723	0.39999	-5.12547	0.678698	0.692797
Dendritic.cells	MBTD1	-0.08665	7.420536	-0.84561	0.400053	-6.59227	0.605736	0.58728
Dendritic.cells	SKP1A	-0.08228	6.639256	-0.84535	0.400199	-6.33991	0.614865	0.600289
Dendritic.cells	4931413K1	0.298105	1.943113	0.844805	0.4005	-5.27654	0.673112	0.684207
Dendritic.cells	TMEM140	-0.32023	3.514222	-0.84477	0.40052	-5.47062	0.652984	0.654958
Dendritic.cells	TRIP6	0.338512	0.834471	0.844614	0.400606	-5.14946	0.687794	0.705652
Dendritic.cells	GM44777	-0.45543	0.565153	-0.84454	0.400649	-5.05166	0.691415	0.710968
Dendritic.cells	SREK1	-0.06973	6.429668	-0.84439	0.400729	-6.38399	0.617668	0.603961
Dendritic.cells	METTL27	0.51338	0.539053	0.844083	0.400901	-5.06864	0.691998	0.711596
Dendritic.cells	ZBED3	0.223886	3.559356	0.843949	0.400976	-5.47068	0.652676	0.654361
Dendritic.cells	PDLIM2	0.198538	3.759145	0.842993	0.401507	-5.6946	0.650938	0.651079
Dendritic.cells	AKTIP	-0.21399	3.102695	-0.84285	0.401586	-5.45763	0.659251	0.663114
Dendritic.cells	TRPS1	0.159785	7.683996	0.842454	0.401807	-6.34101	0.604257	0.583862
Dendritic.cells	NAGA	-0.13951	4.159018	-0.84227	0.40191	-6.04847	0.646097	0.64412
Dendritic.cells	ARFRP1	-0.16268	4.120918	-0.84226	0.401917	-5.61827	0.64657	0.644804
Dendritic.cells	GSTM4	-0.4325	1.121715	-0.8421	0.402006	-5.11822	0.685164	0.700906
Dendritic.cells	P2RX4	0.098474	5.534178	0.84199	0.402065	-6.44489	0.629337	0.620003
Dendritic.cells	GIPC1	-0.15035	4.359749	-0.8419	0.402113	-5.81716	0.643615	0.640626

Dendritic.cells	LAT	0.207696	2.414617	0.841677	0.40224	-5.60383	0.668185	0.676344
Dendritic.cells	TTF2	-0.29969	3.091877	-0.84152	0.40233	-5.31248	0.659499	0.663734
Dendritic.cells	MBOAT1	0.565263	0.682144	0.841464	0.402358	-5.02686	0.691056	0.709721
Dendritic.cells	NEK11	-0.47533	0.082898	-0.84136	0.402418	-5.0331	0.699187	0.721655
Dendritic.cells	GM42997	-0.4534	0.465543	-0.84128	0.402459	-5.11645	0.693982	0.714063
Dendritic.cells	NSUN5	-0.28636	2.124735	-0.84114	0.402541	-5.27886	0.671947	0.681952
Dendritic.cells	VPS25	0.41414	0.881521	0.841018	0.402607	-5.06356	0.688376	0.705929
Dendritic.cells	GAPT	0.168034	1.236779	0.840986	0.402624	-5.92632	0.683632	0.698996
Dendritic.cells	MPND	0.109453	4.813143	0.840942	0.402649	-6.21527	0.638054	0.6328
Dendritic.cells	ACTA2	0.688458	3.320545	0.84068	0.402795	-5.3163	0.656598	0.659752
Dendritic.cells	TBK1	-0.10231	5.964636	-0.84058	0.402852	-6.34135	0.624207	0.61298
Dendritic.cells	TEAD1	0.308445	1.756692	0.840492	0.4029	-5.40481	0.67676	0.68913
Dendritic.cells	TCTN1	0.3796	1.315572	0.840455	0.402921	-5.21831	0.682585	0.697635
Dendritic.cells	COL6A1	0.478756	0.367808	0.840383	0.402961	-5.04774	0.695307	0.716288
Dendritic.cells	COMMD6	-0.13892	4.358964	-0.84036	0.402976	-5.87668	0.643625	0.641045
Dendritic.cells	GRHPR	-0.22479	4.431109	-0.84035	0.40298	-5.73983	0.642736	0.639759
Dendritic.cells	ENHO	-0.33576	2.481211	-0.84021	0.403059	-5.35907	0.667325	0.675435
Dendritic.cells	THUMPD3	0.143716	4.262161	0.84017	0.403079	-5.85849	0.64482	0.6428
Dendritic.cells	GALK2	0.105729	4.897213	0.839979	0.403186	-6.0996	0.637086	0.631584
Dendritic.cells	FBH1	0.19677	3.425128	0.839862	0.403251	-5.49907	0.655334	0.658014
Dendritic.cells	STAG1	-0.05806	8.353909	-0.83976	0.40331	-6.78978	0.596768	0.573664
Dendritic.cells	DHX30	-0.12748	4.509922	-0.83968	0.403352	-5.98345	0.641823	0.638475
Dendritic.cells	FOXD2OS	-0.45155	0.311339	-0.83939	0.403514	-5.06027	0.696175	0.717655
Dendritic.cells	CRACR2A	-0.16337	2.835916	-0.83938	0.403519	-5.96381	0.662861	0.669026
Dendritic.cells	SNX7	0.391652	1.212304	0.83932	0.403554	-5.15021	0.684057	0.699945
Dendritic.cells	RAB24	0.113341	5.118704	0.838159	0.404201	-5.99322	0.635357	0.628195
Dendritic.cells	NEURL1A	0.511821	-0.39095	0.837955	0.404316	-5.04045	0.706915	0.732242
Dendritic.cells	PSMA2	-0.08865	7.21693	-0.83786	0.404371	-6.42758	0.610645	0.592593
Dendritic.cells	A430018G1	-0.31129	1.329295	-0.83767	0.404475	-5.18901	0.683595	0.698117
Dendritic.cells	SSR3	0.083464	6.071068	0.837612	0.404507	-6.36497	0.624035	0.611825
Dendritic.cells	GPATCH2	-0.16607	4.253922	-0.83743	0.404609	-5.76374	0.646049	0.64363
Dendritic.cells	ACBD5	0.073406	6.231959	0.837298	0.404683	-6.49953	0.622133	0.609185
Dendritic.cells	IRAK3	0.539641	4.187779	0.837298	0.404683	-5.19569	0.646869	0.644858
Dendritic.cells	RNF115	0.0731	6.978362	0.837152	0.404764	-6.4553	0.61341	0.596689
Dendritic.cells	NSMCE3	-0.1349	4.271532	-0.83702	0.404836	-5.77004	0.645831	0.643467
Dendritic.cells	PLXNA4	-0.53719	2.166702	-0.83692	0.404893	-5.44335	0.672574	0.682288
Dendritic.cells	GM44987	-0.44463	-0.34886	-0.83688	0.404914	-5.01122	0.706347	0.731693
Dendritic.cells	IFT74	-0.30761	2.322027	-0.83647	0.405148	-5.31105	0.670842	0.679464
Dendritic.cells	CCSER2	-0.14623	4.778998	-0.83618	0.405311	-5.92783	0.639992	0.634642
Dendritic.cells	GM43388	-0.55288	0.386691	-0.83595	0.405438	-5.02491	0.696707	0.717193
Dendritic.cells	VPS35	-0.08771	6.232281	-0.8359	0.405467	-6.27579	0.622525	0.609563
Dendritic.cells	RNPEP	0.19045	5.72187	0.835891	0.405469	-5.86086	0.628588	0.618287
Dendritic.cells	ERI3	0.097672	5.177084	0.835397	0.405746	-6.12342	0.63546	0.627908
Dendritic.cells	GPR89	0.177911	3.742407	0.835318	0.40579	-5.70242	0.653164	0.6535
Dendritic.cells	PARVB	-0.32776	2.578711	-0.83519	0.40586	-5.33116	0.668003	0.675074
Dendritic.cells	ATP5E	0.063942	8.783189	0.835071	0.405929	-6.79195	0.593684	0.56806
Dendritic.cells	DDX58	-0.31536	4.759373	-0.83492	0.406014	-5.42194	0.640622	0.63544

Dendritic.cells	AIMP1	0.087222	6.38044	0.834793	0.406084	-6.29391	0.621173	0.607437
Dendritic.cells	TRNT1	-0.14039	4.772449	-0.83453	0.406234	-5.81986	0.640539	0.635382
Dendritic.cells	CPSF3	-0.14372	4.770083	-0.83451	0.406242	-5.77494	0.640568	0.635424
Dendritic.cells	SRSF6	-0.09264	6.210068	-0.83423	0.406399	-6.32324	0.623253	0.610512
Dendritic.cells	PYROXD1	-0.24993	2.95488	-0.8342	0.406418	-5.37671	0.663317	0.668414
Dendritic.cells	METTL17	-0.25357	2.488504	-0.83418	0.406429	-5.36887	0.669322	0.677142
Dendritic.cells	FAM149B	0.2182	2.992688	0.833858	0.406608	-5.48612	0.663028	0.667769
Dendritic.cells	IL3RA	-0.14715	3.561284	-0.83337	0.406881	-5.88742	0.655893	0.657464
Dendritic.cells	VASP	-0.11769	6.541669	-0.83322	0.406966	-6.27191	0.619619	0.605156
Dendritic.cells	ZFP706	0.063686	7.871583	0.833064	0.407054	-6.63769	0.604282	0.583218
Dendritic.cells	PLEKHB1	-0.41733	0.728002	-0.83306	0.407055	-5.07816	0.692903	0.711466
Dendritic.cells	AKIRIN2	0.10668	5.756269	0.832892	0.40715	-6.1178	0.62892	0.618665
Dendritic.cells	LYRM2	-0.27377	3.377583	-0.83286	0.407165	-5.37247	0.658217	0.661016
Dendritic.cells	QDPR	0.104017	5.245358	0.832672	0.407273	-6.14192	0.635068	0.627586
Dendritic.cells	RCC1	-0.24742	3.989305	-0.83254	0.40735	-5.42459	0.650519	0.649977
Dendritic.cells	GM550	-0.45218	0.859238	-0.83245	0.407396	-5.05635	0.691133	0.709133
Dendritic.cells	TRPM2	-0.48207	2.540831	-0.8324	0.407429	-5.1509	0.668935	0.676749
Dendritic.cells	DEGS1	-0.11471	6.525466	-0.83186	0.407729	-6.31982	0.619809	0.605812
Dendritic.cells	NCK2	-0.33112	4.870391	-0.83175	0.407793	-5.3582	0.639631	0.634457
Dendritic.cells	HIGD1A	0.128411	6.029655	0.831544	0.407907	-6.03861	0.625662	0.614332
Dendritic.cells	KDM6A	-0.18756	6.914035	-0.83149	0.407939	-6.51739	0.615273	0.599417
Dendritic.cells	SYTL3	-0.41284	2.803793	-0.83137	0.408005	-5.35197	0.665543	0.672092
Dendritic.cells	RIC8A	0.15188	3.980741	0.831045	0.408187	-5.76348	0.650626	0.650518
Dendritic.cells	MTERF1A	0.310542	1.405346	0.83095	0.40824	-5.22289	0.683826	0.698855
Dendritic.cells	ZFYVE21	-0.25316	2.542215	-0.83094	0.408243	-5.43476	0.668917	0.677111
Dendritic.cells	BAIAP3	-0.51188	-0.32152	-0.83085	0.408297	-5.01992	0.707253	0.733186
Dendritic.cells	FAR1OS	0.344878	2.788806	0.830798	0.408326	-5.37228	0.665736	0.672486
Dendritic.cells	RYBP	0.110902	5.626574	0.830669	0.408398	-6.17794	0.630473	0.621473
Dendritic.cells	POGLUT1	0.241926	2.752407	0.830618	0.408427	-5.41618	0.666204	0.673231
Dendritic.cells	MCM2	0.251658	4.762793	0.830562	0.408458	-5.79149	0.640948	0.636599
Dendritic.cells	ZCCHC2	-0.16884	5.422407	-0.83054	0.408473	-5.84894	0.632929	0.625015
Dendritic.cells	SERPINB1A	0.59507	3.600711	0.830446	0.408524	-5.11384	0.655396	0.657527
Dendritic.cells	TMA7	0.07361	6.564001	0.830409	0.408544	-6.35011	0.619357	0.605463
Dendritic.cells	GON4L	0.102475	5.15234	0.830367	0.408568	-6.09444	0.636196	0.629732
Dendritic.cells	RAPGEF4	0.417948	2.563789	0.830353	0.408576	-5.17596	0.668638	0.676772
Dendritic.cells	MCTS2	-0.42348	1.612847	-0.83031	0.408599	-5.16833	0.681075	0.694906
Dendritic.cells	EXOC3L2	-0.49519	2.269133	-0.83028	0.408619	-5.38708	0.672461	0.682341
Dendritic.cells	CHMP1A	0.130169	5.305048	0.830201	0.408661	-6.02941	0.634346	0.627061
Dendritic.cells	KCNB1	-0.39054	0.988195	-0.83019	0.408668	-5.21626	0.689399	0.707073
Dendritic.cells	GZMC	-0.96072	1.539474	-0.83014	0.408697	-5.1393	0.682046	0.696362
Dendritic.cells	MS4A8A	0.607748	0.670009	0.830025	0.40876	-5.03242	0.693687	0.713432
Dendritic.cells	GUCD1	-0.24869	3.805941	-0.82991	0.408826	-5.46138	0.652814	0.653857
Dendritic.cells	CARNMT1	0.104613	5.353674	0.829847	0.408861	-6.07862	0.633758	0.626284
Dendritic.cells	CERS4	0.256243	3.651416	0.829794	0.40889	-5.44215	0.654757	0.656675
Dendritic.cells	HNRNPR	0.072793	6.29021	0.829722	0.408931	-6.37047	0.622577	0.610165
Dendritic.cells	HDAC2	-0.13683	5.300553	-0.82942	0.409103	-5.96164	0.63455	0.627303
Dendritic.cells	SLAIN2	-0.10209	5.670672	-0.82915	0.40925	-6.08132	0.630093	0.620946

Dendritic.cells	MCM9	0.136588	4.75152	0.82911	0.409275	-5.93796	0.641237	0.637041
Dendritic.cells	EEF1D	0.088347	7.27018	0.828993	0.409341	-6.43285	0.611298	0.593902
Dendritic.cells	GABPB2	0.078316	6.502447	0.828974	0.409351	-6.39726	0.620225	0.606732
Dendritic.cells	STAT5A	0.173276	3.992851	0.828914	0.409385	-5.72564	0.650628	0.650657
Dendritic.cells	SOGA1	0.130387	3.807296	0.82848	0.40963	-6.14511	0.653151	0.65423
Dendritic.cells	MRPL4	0.120262	5.176328	0.828467	0.409637	-6.00238	0.63625	0.629767
Dendritic.cells	SPTBN5	-0.47683	-0.00378	-0.82837	0.40969	-5.05204	0.703253	0.727345
Dendritic.cells	ZFP984	0.14337	3.878402	0.828032	0.409882	-5.78037	0.652359	0.653004
Dendritic.cells	DTX2	-0.11521	4.396914	-0.82799	0.409903	-6.1098	0.645901	0.643648
Dendritic.cells	POLR2G	0.126912	5.51542	0.827867	0.409975	-5.90119	0.632248	0.623915
Dendritic.cells	GM27188	-0.19513	0.619818	-0.82775	0.410041	-5.89021	0.694849	0.714948
Dendritic.cells	DNAJC9	0.175938	5.617283	0.827647	0.410099	-5.96861	0.631023	0.622158
Dendritic.cells	UBA5	0.096774	4.489487	0.827627	0.41011	-6.0475	0.644757	0.642002
Dendritic.cells	AFF3	0.094081	7.930696	0.827464	0.410202	-6.94854	0.604079	0.583414
Dendritic.cells	MED14	-0.13275	6.321429	-0.8272	0.410352	-6.20822	0.622745	0.610238
Dendritic.cells	CHPF	-0.3717	1.242066	-0.82718	0.410362	-5.13448	0.686592	0.702878
Dendritic.cells	ZHX2	-0.21876	5.614006	-0.8266	0.410691	-5.77371	0.631167	0.622553
Dendritic.cells	ALDH16A1	-0.17622	4.358468	-0.82657	0.410703	-5.67545	0.646485	0.644698
Dendritic.cells	ELAVL1	-0.05555	7.604056	-0.82652	0.410735	-6.60933	0.607849	0.589006
Dendritic.cells	QSOX1	0.140577	4.311358	0.826478	0.410758	-5.93852	0.647069	0.645549
Dendritic.cells	FARS2	0.062373	6.899126	0.826398	0.410803	-6.57181	0.615976	0.600703
Dendritic.cells	RAB40C	0.104623	4.838453	0.826246	0.410889	-6.1486	0.640573	0.636186
Dendritic.cells	PSMC5	0.095642	6.00886	0.826212	0.410908	-6.1981	0.626448	0.61579
Dendritic.cells	NCBP1	-0.09142	5.256901	-0.82612	0.410962	-6.08546	0.635476	0.628863
Dendritic.cells	TSFM	0.162667	4.258618	0.826014	0.41102	-5.699	0.647723	0.646605
Dendritic.cells	DUSP7	0.200856	3.559127	0.825923	0.411071	-5.68851	0.656486	0.659346
Dendritic.cells	BAZ1A	-0.08293	7.694958	-0.8257	0.411195	-6.54548	0.606812	0.587715
Dendritic.cells	BTBD10	-0.09809	5.583956	-0.82561	0.411248	-6.13519	0.631528	0.623289
Dendritic.cells	IL11RA1	-0.25144	2.113905	-0.82559	0.411261	-5.34997	0.675068	0.686481
Dendritic.cells	ARNTL	0.205845	5.38742	0.825408	0.411362	-5.82268	0.633897	0.626751
Dendritic.cells	KLHDC10	0.112526	5.434437	0.825175	0.411493	-6.13157	0.633329	0.625971
Dendritic.cells	CD3D	-0.47392	2.678526	-0.82505	0.411562	-5.18678	0.667731	0.675874
Dendritic.cells	RFC2	0.149535	5.227985	0.82505	0.411564	-5.93063	0.635826	0.629579
Dendritic.cells	4632404H1	0.381963	1.238383	0.825029	0.411576	-5.21967	0.686641	0.703484
Dendritic.cells	GPATCH2L	-0.09966	5.064269	-0.82495	0.41162	-6.07549	0.637816	0.632455
Dendritic.cells	PAK4	0.251583	2.299468	0.824901	0.411648	-5.3658	0.672645	0.683037
Dendritic.cells	GM13684	0.279162	3.516914	0.824869	0.411666	-5.40509	0.657019	0.660291
Dendritic.cells	TNK2	-0.29248	3.209858	-0.82469	0.411767	-5.29147	0.660917	0.666045
Dendritic.cells	TLR1	-0.53236	1.219121	-0.82468	0.411772	-5.02518	0.686899	0.703955
Dendritic.cells	ATG14	-0.20291	3.27883	-0.82462	0.411805	-5.45547	0.660039	0.664792
Dendritic.cells	FAM192A	0.134359	4.607769	0.824487	0.411882	-5.76865	0.643405	0.640682
Dendritic.cells	PDZK1	-0.52718	0.933701	-0.82444	0.411909	-5.05707	0.690726	0.709618
Dendritic.cells	C1S1	-0.37448	1.787052	-0.82413	0.412085	-5.1971	0.6794	0.693126
Dendritic.cells	AP1S1	0.144945	4.706161	0.824115	0.412092	-5.85413	0.642233	0.639057
Dendritic.cells	VPS41	0.110398	5.101022	0.824078	0.412113	-6.05037	0.637406	0.632072
Dendritic.cells	GM48653	-0.49554	-0.16399	-0.82379	0.412276	-5.0298	0.705904	0.731887
Dendritic.cells	RNF167	0.129149	5.334087	0.823487	0.412447	-6.05491	0.634869	0.628159

Dendritic.cells	2700049A(	-0.19785	4.236674	-0.82329	0.412556	-5.64417	0.648331	0.647712
Dendritic.cells	OIT3	-0.21337	2.345496	-0.82323	0.412593	-5.6864	0.672394	0.682708
Dendritic.cells	GPN1	-0.23523	3.042468	-0.82322	0.4126	-5.38313	0.663397	0.6696
Dendritic.cells	MLLT3	0.234422	5.728512	0.822831	0.412818	-5.74713	0.63027	0.621481
Dendritic.cells	IFI47	0.240711	5.277962	0.822695	0.412895	-6.30508	0.635701	0.629398
Dendritic.cells	FAM126A	-0.15072	5.549539	-0.82262	0.41294	-5.98462	0.63242	0.62469
Dendritic.cells	PDHX	0.168583	3.395129	0.822455	0.41303	-5.57607	0.65906	0.663357
Dendritic.cells	ACTR1B	-0.13832	4.215968	-0.8224	0.413061	-5.93121	0.648744	0.648379
Dendritic.cells	FCHSD2	0.133921	6.823892	0.822313	0.413111	-6.4877	0.617319	0.602988
Dendritic.cells	ZFP131	0.096412	5.815875	0.822302	0.413117	-6.2616	0.629224	0.62015
Dendritic.cells	ASXL2	0.07373	6.836454	0.822127	0.413216	-6.6143	0.617182	0.602777
Dendritic.cells	9130019O2	-0.47218	0.355077	-0.82201	0.413284	-5.09166	0.699103	0.721923
Dendritic.cells	RHOC	-0.21124	4.059106	-0.82197	0.413303	-5.69008	0.650709	0.651279
Dendritic.cells	CREB3	0.130021	3.923009	0.821306	0.413681	-5.84678	0.65274	0.653895
Dendritic.cells	NKRF	0.2469	2.983842	0.821253	0.413711	-5.4293	0.664652	0.67121
Dendritic.cells	AGXT	-0.42273	3.279368	-0.82115	0.413769	-5.38461	0.660874	0.665713
Dendritic.cells	PRKRIP1	0.16851	3.964552	0.820882	0.413922	-5.60069	0.65222	0.653252
Dendritic.cells	GM36486	-0.49598	0.805963	-0.82087	0.413928	-5.12469	0.69333	0.713227
Dendritic.cells	FOXN3	-0.07785	8.899803	-0.82081	0.413964	-6.92398	0.59404	0.569411
Dendritic.cells	RREB1	0.140831	6.814971	0.820733	0.414006	-6.32416	0.617743	0.603466
Dendritic.cells	1700086O(	0.326266	1.546442	0.820681	0.414035	-5.17548	0.683412	0.698788
Dendritic.cells	SMTNL2	0.538237	0.4226	0.820608	0.414076	-5.02779	0.698533	0.720963
Dendritic.cells	TMEM154	0.325313	2.583653	0.820552	0.414108	-5.31974	0.66981	0.678953
Dendritic.cells	UGDH	0.200966	4.250685	0.82027	0.414268	-5.59716	0.648805	0.64831
Dendritic.cells	E130307A1	0.151527	4.490339	0.819582	0.414658	-5.72243	0.646347	0.644257
Dendritic.cells	ENO3	-0.39382	2.077848	-0.81929	0.414822	-5.24304	0.677273	0.68907
Dendritic.cells	NSG2	0.390898	0.587521	0.819179	0.414887	-5.12638	0.697196	0.71823
Dendritic.cells	UBE2K	-0.05464	8.214477	-0.81904	0.414964	-6.79834	0.602503	0.580905
Dendritic.cells	9330136K2	-0.27928	1.883233	-0.81869	0.415167	-5.41781	0.680085	0.692929
Dendritic.cells	GM16062	-0.29073	1.874018	-0.81856	0.415241	-5.22526	0.680207	0.693154
Dendritic.cells	SELPLG	0.070711	6.845492	0.818496	0.415274	-6.79298	0.618412	0.603598
Dendritic.cells	SLC9A8	0.129075	4.988676	0.818266	0.415405	-5.98886	0.640722	0.635664
Dendritic.cells	NFIL3	-0.30427	4.515288	-0.8178	0.41567	-5.60928	0.646801	0.644132
Dendritic.cells	BOLL	-0.42695	1.308178	-0.81777	0.415687	-5.16954	0.688109	0.704239
Dendritic.cells	CCDC57	-0.30804	2.546499	-0.81733	0.415936	-5.20761	0.671996	0.680519
Dendritic.cells	NR1D1	-0.52649	1.661874	-0.81725	0.415983	-5.08767	0.683616	0.697465
Dendritic.cells	CCDC25	-0.16214	4.712121	-0.81694	0.416158	-5.70983	0.644577	0.640718
Dendritic.cells	MRPL24	0.085507	5.658173	0.816897	0.416183	-6.20774	0.633048	0.624063
Dendritic.cells	SF3A2	0.129493	5.260106	0.816859	0.416205	-6.03797	0.637866	0.631018
Dendritic.cells	EIF1	-0.08565	9.721973	-0.81685	0.416209	-6.86882	0.58652	0.557329
Dendritic.cells	ZC4H2	0.438786	0.054562	0.816796	0.41624	-5.02546	0.705362	0.729309
Dendritic.cells	CLASRP	0.189372	3.334689	0.81653	0.416391	-5.59936	0.661995	0.665843
Dendritic.cells	GPC5	-0.5377	0.598275	-0.81613	0.416617	-5.07795	0.698333	0.718536
Dendritic.cells	SLC22A14	0.329533	2.744823	0.816041	0.41667	-5.29302	0.669827	0.676945
Dendritic.cells	GTF2H4	0.239279	2.307317	0.815797	0.416809	-5.33492	0.675647	0.685268
Dendritic.cells	NAB1	0.085527	6.555305	0.815388	0.417042	-6.46682	0.622994	0.608932
Dendritic.cells	ABCD4	0.239663	2.782602	0.815333	0.417072	-5.34719	0.669615	0.676368

Dendritic.cells	GM36551	0.350375	-0.80034	0.815229	0.417132	-5.02491	0.717991	0.747187
Dendritic.cells	CTBS	0.211399	2.934998	0.815147	0.417178	-5.47803	0.667647	0.673569
Dendritic.cells	EPHX1	-0.35943	3.053436	-0.81511	0.417202	-5.26294	0.666123	0.671368
Dendritic.cells	SLC35G1	0.486891	1.026313	0.814897	0.417321	-5.0897	0.692855	0.710423
Dendritic.cells	ABHD13	-0.1829	3.557429	-0.81475	0.417405	-5.53454	0.65972	0.662139
Dendritic.cells	SLC22A17	-0.46471	-0.51359	-0.81467	0.417451	-5.03865	0.714006	0.741449
Dendritic.cells	SPHK2	-0.22216	3.388566	-0.81465	0.417464	-5.49114	0.661868	0.665257
Dendritic.cells	SARDH	-0.39663	2.900243	-0.81441	0.4176	-5.37555	0.668252	0.674358
Dendritic.cells	MCTS1	0.08722	6.207891	0.813665	0.418023	-6.27107	0.627458	0.615271
Dendritic.cells	GM3550	-0.29109	1.752212	-0.81355	0.418091	-5.23271	0.683492	0.696493
Dendritic.cells	GNPNAT1	-0.17693	4.157449	-0.81334	0.418207	-5.65367	0.652487	0.651462
Dendritic.cells	IFI206	-0.78016	3.940563	-0.8133	0.41823	-5.13794	0.655209	0.655407
Dendritic.cells	CES2E	0.472487	1.228112	0.812908	0.418455	-5.07387	0.690489	0.706874
Dendritic.cells	FAM135A	0.287449	2.016455	0.812876	0.418473	-5.39388	0.679997	0.691555
Dendritic.cells	GM19951	0.513935	3.23896	0.812778	0.418529	-5.30938	0.664115	0.668477
Dendritic.cells	FBXO46	-0.25764	2.47185	-0.81275	0.418542	-5.41788	0.674026	0.682904
Dendritic.cells	KDM5B	0.226588	5.362074	0.812672	0.418589	-5.7626	0.637629	0.630134
Dendritic.cells	ZFP788	0.257255	2.069958	0.812636	0.418609	-5.30004	0.679293	0.690589
Dendritic.cells	TRIP10	-0.3301	1.355077	-0.81261	0.418624	-5.17465	0.688786	0.70445
Dendritic.cells	FAM92A	0.216518	3.301539	0.812565	0.41865	-5.55585	0.663314	0.667352
Dendritic.cells	B4GALNT1	0.090637	4.892601	0.812541	0.418664	-6.31974	0.643367	0.638453
Dendritic.cells	TFB2M	0.16193	3.953928	0.812453	0.418714	-5.62267	0.655041	0.655399
Dendritic.cells	UBE2J1	-0.07055	6.282597	-0.81229	0.418808	-6.45988	0.62657	0.614303
Dendritic.cells	MRPL38	0.147902	3.863246	0.812274	0.418816	-5.77832	0.656183	0.657103
Dendritic.cells	FBXO31	0.203301	3.549697	0.812238	0.418837	-5.6018	0.660152	0.662877
Dendritic.cells	PPM1L	-0.09965	4.617569	-0.81211	0.418908	-6.44832	0.64676	0.643512
Dendritic.cells	NIPBL	0.061497	8.615732	0.811994	0.418976	-6.80261	0.599657	0.575761
Dendritic.cells	CHRM3	-0.43437	0.831294	-0.81175	0.419115	-5.2521	0.695844	0.715134
Dendritic.cells	TRAPPC3	-0.12466	4.917333	-0.8117	0.419143	-5.98884	0.643063	0.638304
Dendritic.cells	CMAS	0.116184	5.862643	0.811538	0.419236	-6.22893	0.631584	0.621725
Dendritic.cells	FAM20A	-0.38267	1.833508	-0.81153	0.41924	-5.28742	0.682415	0.695492
Dendritic.cells	GM17023	0.577331	-0.64235	0.811445	0.419289	-5.03134	0.716171	0.74499
Dendritic.cells	CCDC88A	0.08515	5.372063	0.811395	0.419318	-6.52268	0.637507	0.630275
Dendritic.cells	4930484I0	-0.30819	1.660784	-0.81135	0.419342	-5.27577	0.684706	0.69884
Dendritic.cells	TRAF3IP1	-0.36837	1.889467	-0.81133	0.419356	-5.23698	0.681674	0.694411
Dendritic.cells	1700030J2	-0.53992	-0.14389	-0.81118	0.419441	-5.03463	0.709258	0.73483
Dendritic.cells	SLC20A1	-0.21553	5.219384	-0.81097	0.419559	-5.66635	0.639402	0.633053
Dendritic.cells	ZFAND6	-0.06674	6.978135	-0.81097	0.41956	-6.51075	0.618416	0.602802
Dendritic.cells	GOLT1B	-0.14725	4.631519	-0.81066	0.419737	-5.90528	0.646668	0.643612
Dendritic.cells	RGP1	-0.1384	2.34849	-0.81058	0.419784	-5.758	0.675722	0.685856
Dendritic.cells	9330175E1	0.552346	1.002962	0.810512	0.419822	-5.05431	0.693608	0.711999
Dendritic.cells	FAM104A	0.094804	6.153765	0.810501	0.419828	-6.24905	0.628181	0.616937
Dendritic.cells	ALPK3	-0.53418	-0.11417	-0.81022	0.419987	-5.02888	0.709014	0.734526
Dendritic.cells	FAM234A	-0.25614	4.099085	-0.81017	0.420019	-5.57703	0.653411	0.653343
Dendritic.cells	CNTRL	0.094383	5.907012	0.809709	0.420282	-6.26367	0.631541	0.62145
Dendritic.cells	RABGEF1	-0.15125	6.01057	-0.80944	0.420433	-6.12811	0.630438	0.619774
Dendritic.cells	VEGFB	-0.15103	3.802219	-0.80906	0.420652	-5.84989	0.657712	0.659226

Dendritic.cells	GGNBP2	-0.06965	7.268609	-0.809	0.420686	-6.53207	0.615713	0.598611
Dendritic.cells	ARHGAP15	0.078186	9.872031	0.80887	0.420761	-6.9609	0.586489	0.556787
Dendritic.cells	LRMDA	-0.20805	7.760594	-0.80886	0.420764	-6.38298	0.610041	0.590472
Dendritic.cells	TRMT10C	0.126593	4.996252	0.808801	0.420801	-5.93738	0.642836	0.637707
Dendritic.cells	PPFIA4	0.089877	2.735956	0.808588	0.420922	-6.46429	0.671454	0.679139
Dendritic.cells	NCLN	0.192891	3.839103	0.808496	0.420975	-5.55648	0.65733	0.658634
Dendritic.cells	APOPT1	0.095075	4.910205	0.808277	0.421101	-6.00774	0.643977	0.639384
Dendritic.cells	ACOT7	-0.19978	4.076826	-0.80814	0.421181	-5.56132	0.654337	0.654422
Dendritic.cells	HDAC7	-0.19592	4.31747	-0.80805	0.42123	-5.65976	0.651323	0.650073
Dendritic.cells	TMCC3	-0.19948	5.475701	-0.808	0.421258	-6.08886	0.637067	0.629447
Dendritic.cells	HIST1H2AE	0.557574	4.491316	0.807971	0.421276	-5.41876	0.649157	0.646947
Dendritic.cells	IPO9	0.094477	4.771136	0.807731	0.421413	-6.1306	0.645795	0.641979
Dendritic.cells	TMEM245	0.141118	4.753996	0.807531	0.421528	-5.92734	0.646006	0.642355
Dendritic.cells	TTC32	0.207189	4.101346	0.807423	0.421589	-5.51131	0.654134	0.654132
Dendritic.cells	MAGED2	0.379692	1.585547	0.807378	0.421616	-5.2166	0.686699	0.70155
Dendritic.cells	LST1	-0.25315	5.23223	-0.80725	0.421691	-5.6986	0.640133	0.633859
Dendritic.cells	HMGB3	0.300498	4.658182	0.807223	0.421705	-5.48765	0.647191	0.644071
Dendritic.cells	TMEM164	0.091262	6.945657	0.807117	0.421765	-6.38642	0.619655	0.604355
Dendritic.cells	U2AF1	0.062177	7.593832	0.806785	0.421955	-6.68085	0.612141	0.593624
Dendritic.cells	CPLX1	-0.5743	-0.57566	-0.80654	0.422095	-5.0397	0.716279	0.745142
Dendritic.cells	LBH	0.085653	6.308404	0.806486	0.422127	-6.32775	0.627176	0.615332
Dendritic.cells	CSTB	-0.13225	6.672627	-0.80619	0.422298	-6.30193	0.622866	0.609171
Dendritic.cells	PCCA	0.153596	4.599387	0.806019	0.422395	-5.91125	0.647928	0.645429
Dendritic.cells	ZFP820	-0.41434	-0.04486	-0.8059	0.422462	-5.10583	0.708877	0.734427
Dendritic.cells	STARD4	0.193388	3.526928	0.805782	0.422531	-5.61725	0.661404	0.665016
Dendritic.cells	2610306M	-0.43279	0.680103	-0.80572	0.422567	-5.11405	0.698914	0.719794
Dendritic.cells	PEAR1	-0.34769	2.352213	-0.80571	0.422574	-5.19075	0.676573	0.687109
Dendritic.cells	AOAH	-0.47915	4.784961	-0.80562	0.422621	-5.45763	0.645632	0.642133
Dendritic.cells	NHLRC3	0.210602	3.16365	0.805539	0.42267	-5.59471	0.666049	0.671773
Dendritic.cells	COMMD1	0.14134	5.141948	0.805472	0.422708	-5.90435	0.641244	0.635818
Dendritic.cells	PSME3	0.171182	5.282258	0.80533	0.42279	-5.81997	0.63953	0.6334
Dendritic.cells	BUD31	-0.12411	5.474604	-0.8052	0.422865	-5.99687	0.63719	0.630078
Dendritic.cells	GM16279	-0.44132	1.054801	-0.805	0.422976	-5.1208	0.693831	0.712554
Dendritic.cells	ETNK2	-0.44452	1.270533	-0.80496	0.423004	-5.08408	0.690925	0.708316
Dendritic.cells	DDHD1	-0.15886	6.276227	-0.80477	0.423111	-6.19211	0.627559	0.6163
Dendritic.cells	PFDN6	0.159069	4.874434	0.804634	0.423189	-5.73503	0.644528	0.640864
Dendritic.cells	RASSF7	-0.40433	0.86906	-0.80448	0.423275	-5.09802	0.696345	0.716416
Dendritic.cells	CSTDC6	0.358171	-1.16323	0.804423	0.42331	-5.0344	0.724577	0.757977
Dendritic.cells	FCRL5	-0.45704	-0.65204	-0.8044	0.423322	-5.03427	0.717351	0.747312
Dendritic.cells	HLTF	0.158504	4.64409	0.804318	0.423371	-5.83796	0.647374	0.64503
Dendritic.cells	TTC39A	0.306699	2.027911	0.804248	0.423411	-5.43771	0.680837	0.693735
Dendritic.cells	SEC16A	-0.12335	4.57627	-0.80406	0.423517	-6.04762	0.648214	0.646249
Dendritic.cells	DMXL2	0.453681	2.146865	0.804026	0.423539	-5.2014	0.679269	0.691444
Dendritic.cells	MTMR3	0.069448	7.886466	0.804	0.423553	-6.66583	0.608786	0.589357
Dendritic.cells	SLC35E3	0.302638	1.909396	0.80399	0.423559	-5.32881	0.682404	0.696024
Dendritic.cells	SPRYD3	-0.1613	3.699854	-0.80399	0.42356	-5.63912	0.659207	0.662207
Dendritic.cells	SCG5	-0.46288	0.801405	-0.80396	0.423575	-5.10742	0.697264	0.717789

Dendritic.cells	GSTM2	-0.35983	1.633616	-0.80395	0.423583	-5.25565	0.686066	0.701381
Dendritic.cells	HSCB	0.211483	4.143832	0.803862	0.423633	-5.61938	0.653609	0.654085
Dendritic.cells	5031425F1	-0.50006	-0.17235	-0.80363	0.423767	-5.03546	0.710772	0.737542
Dendritic.cells	ARHGAP28	-0.40909	0.598833	-0.80333	0.423938	-5.29451	0.700329	0.722118
Dendritic.cells	GHITM	0.117415	7.4577	0.802985	0.424137	-6.49199	0.614129	0.596778
Dendritic.cells	PLK4	0.319698	3.856529	0.802941	0.424162	-5.39639	0.657672	0.659722
Dendritic.cells	TMPRSS6	-0.49145	1.467899	-0.80273	0.424285	-5.09475	0.688849	0.705115
Dendritic.cells	PREPL	-0.30518	1.734988	-0.80245	0.424443	-5.23083	0.685444	0.700064
Dendritic.cells	RAB29	0.187711	3.635461	0.802312	0.424524	-5.70167	0.660756	0.664018
Dendritic.cells	KBTBD11	-0.33644	0.726015	-0.80212	0.424637	-5.41992	0.699123	0.720035
Dendritic.cells	GABARAPL	-0.11278	7.762001	-0.80204	0.42468	-6.59048	0.610938	0.592048
Dendritic.cells	DIRAS2	0.444663	0.718948	0.8019	0.424761	-5.077	0.699248	0.720234
Dendritic.cells	TATDN3	-0.27164	2.490952	-0.8018	0.424818	-5.33707	0.675592	0.685651
Dendritic.cells	H2AFX	-0.20649	6.482628	-0.80147	0.425006	-6.16701	0.626003	0.613634
Dendritic.cells	A630089N(	-0.29042	2.672384	-0.80141	0.42504	-5.33991	0.673357	0.682316
Dendritic.cells	CALHM6	0.50494	3.048969	0.801345	0.42508	-5.37737	0.668478	0.675245
Dendritic.cells	LINS1	0.286939	1.704648	0.801216	0.425154	-5.21546	0.686122	0.701004
Dendritic.cells	APOL9B	-0.48568	0.574633	-0.80101	0.425273	-5.04609	0.701473	0.723428
Dendritic.cells	RBMS2	0.116903	4.872344	0.800442	0.425601	-5.95614	0.645908	0.64207
Dendritic.cells	GM34095	-0.4821	-0.39051	-0.80043	0.425606	-5.04146	0.715185	0.743238
Dendritic.cells	SC5D	0.136036	4.248539	0.8003	0.425682	-5.99352	0.653673	0.653364
Dendritic.cells	USP44	0.411527	-0.6309	0.800217	0.42573	-5.03288	0.718566	0.748294
Dendritic.cells	ORC3	0.115097	4.958044	0.800111	0.425791	-5.99904	0.644857	0.64068
Dendritic.cells	SMARCAL1	-0.23122	2.628016	-0.79988	0.425925	-5.34367	0.674412	0.683635
Dendritic.cells	ARHGAP6	0.152942	3.609197	0.799683	0.426038	-6.1323	0.661769	0.665242
Dendritic.cells	GM43112	0.389876	0.075373	0.799652	0.426056	-5.11415	0.708725	0.733908
Dendritic.cells	E2F4	0.087798	5.334947	0.799576	0.426099	-6.27969	0.640254	0.634057
Dendritic.cells	SENP2	-0.08734	6.656089	-0.79957	0.426104	-6.3886	0.624393	0.611159
Dendritic.cells	CAPN11	-0.69256	1.046105	-0.79945	0.426169	-5.09037	0.69544	0.714469
Dendritic.cells	SLC46A3	0.15521	3.085031	0.799166	0.426336	-5.8629	0.668522	0.675169
Dendritic.cells	ALDOB	-0.3628	4.949636	-0.79904	0.42641	-5.75903	0.645013	0.641024
Dendritic.cells	TGM1	0.519177	0.53017	0.798989	0.426438	-5.04021	0.702497	0.724866
Dendritic.cells	CD200R4	-0.49697	1.662182	-0.79879	0.426554	-5.22023	0.687188	0.702478
Dendritic.cells	VPS39	0.12203	3.331542	0.798605	0.42666	-5.96339	0.665352	0.670608
Dendritic.cells	GM5577	-0.45641	0.20371	-0.7986	0.426661	-5.03619	0.706988	0.731522
Dendritic.cells	AC160336.	-0.34967	1.371003	-0.79852	0.42671	-5.22176	0.691087	0.708186
Dendritic.cells	TM4SF1	-0.29387	2.15291	-0.79835	0.426806	-5.57922	0.680678	0.692959
Dendritic.cells	CDK5R1	-0.34045	2.111423	-0.79831	0.426829	-5.22035	0.681226	0.693763
Dendritic.cells	ZZZ3	-0.09825	5.884403	-0.7982	0.426891	-6.30186	0.633628	0.624644
Dendritic.cells	TNS1	0.124964	2.761945	0.798137	0.426929	-6.21664	0.672704	0.681376
Dendritic.cells	IARS2	-0.15149	4.475268	-0.79808	0.426962	-5.82337	0.650893	0.649656
Dendritic.cells	DDX20	-0.16061	3.596417	-0.79799	0.427016	-5.62796	0.661967	0.665743
Dendritic.cells	CHRNA9	0.449956	2.214161	0.797962	0.427031	-5.18919	0.679871	0.691842
Dendritic.cells	UTY	2.403633	2.644423	0.797656	0.427207	-5.49255	0.67436	0.68369
Dendritic.cells	ARAP2	-0.1305	5.924266	-0.79761	0.427231	-6.30368	0.633267	0.624051
Dendritic.cells	CLINT1	0.06549	8.205941	0.797362	0.427377	-6.73387	0.606644	0.585702
Dendritic.cells	SQSTM1	0.105049	7.466775	0.797341	0.427389	-6.4994	0.61512	0.597876

Dendritic.cells	GM21781	0.332764	1.561921	0.797203	0.427469	-5.21757	0.688749	0.70469
Dendritic.cells	NIT1	0.1863	3.750254	0.797085	0.427537	-5.62516	0.660236	0.663083
Dendritic.cells	HSP90AB1	0.065873	9.925498	0.796801	0.427701	-6.98154	0.587712	0.558431
Dendritic.cells	RNF13	0.078995	6.065838	0.796412	0.427926	-6.47384	0.63203	0.621817
Dendritic.cells	WDFY4	0.13015	7.251372	0.796343	0.427966	-6.45465	0.618017	0.601647
Dendritic.cells	4931428F0	0.384796	0.65018	0.795966	0.428184	-5.13386	0.701758	0.722972
Dendritic.cells	HSD17B4	-0.1356	5.027211	-0.79566	0.428361	-5.86001	0.645065	0.640197
Dendritic.cells	EPS8L1	-0.62945	0.408966	-0.79536	0.428536	-5.07367	0.705449	0.728059
Dendritic.cells	BMF	-0.27652	2.137288	-0.79504	0.428719	-5.42319	0.682242	0.694032
Dendritic.cells	PUS1	-0.19444	3.723827	-0.79477	0.428873	-5.59276	0.661664	0.664188
Dendritic.cells	CBX4	-0.13981	5.468948	-0.79472	0.428905	-6.01176	0.639929	0.632718
Dendritic.cells	IGKV12-46	0.416224	-0.77013	0.794604	0.428972	-5.03757	0.722025	0.75262
Dendritic.cells	MAGED1	-0.21914	2.409871	-0.79459	0.428979	-5.61392	0.67865	0.689
Dendritic.cells	ARID3B	0.144458	3.836648	0.794471	0.429049	-5.94022	0.66023	0.662185
Dendritic.cells	SETBP1	0.096243	4.621756	0.794422	0.429077	-6.82507	0.650364	0.647871
Dendritic.cells	NOTCH4	0.431901	0.210596	0.794402	0.429089	-5.16788	0.708303	0.732419
Dendritic.cells	CXCR4	0.155162	6.146916	0.79402	0.42931	-6.19527	0.63193	0.620963
Dendritic.cells	SPATA2	-0.16914	4.217056	-0.79389	0.429385	-5.81051	0.655628	0.655292
Dendritic.cells	CCAR2	-0.21527	3.432111	-0.79374	0.429473	-5.48625	0.665593	0.669765
Dendritic.cells	KCNRG	-0.36712	2.320944	-0.79363	0.429538	-5.12581	0.680029	0.69082
Dendritic.cells	PRKCQ	0.367401	3.12892	0.793509	0.429606	-5.30903	0.669494	0.675491
Dendritic.cells	GM156	-0.42917	-1.54566	-0.79347	0.429628	-5.03897	0.733323	0.769127
Dendritic.cells	RFXANK	0.209425	2.879474	0.793444	0.429644	-5.5057	0.672724	0.680207
Dendritic.cells	KCTD6	-0.22739	2.981269	-0.79291	0.429952	-5.4734	0.671649	0.678496
Dendritic.cells	4833438C0	-0.24583	2.025244	-0.79284	0.429992	-5.27851	0.684185	0.69682
Dendritic.cells	SMIM24	-0.14533	3.210803	-0.79277	0.430033	-5.79757	0.668681	0.674216
Dendritic.cells	CTTNBP2NI	-0.15842	2.52025	-0.79276	0.430042	-5.84333	0.677658	0.687293
Dendritic.cells	UBR1	-0.11898	5.323458	-0.79257	0.430153	-6.14336	0.642154	0.635728
Dendritic.cells	MAP3K20	-0.32436	3.798494	-0.79251	0.430182	-5.30096	0.661176	0.663301
Dendritic.cells	TMED5	-0.0543	7.227678	-0.79242	0.430234	-6.64919	0.619389	0.602932
Dendritic.cells	ARL1	0.093468	5.538643	0.792182	0.430375	-6.1691	0.639556	0.632045
Dendritic.cells	MGST2	-0.24321	2.953494	-0.79217	0.430383	-5.49661	0.672057	0.6792
Dendritic.cells	4932438A1	-0.10326	7.087671	-0.79208	0.430435	-6.5478	0.621055	0.605404
Dendritic.cells	GM37768	0.291132	2.087377	0.791742	0.43063	-5.30683	0.683581	0.695885
Dendritic.cells	TXK	-0.41169	3.287256	-0.79169	0.430663	-5.28562	0.667911	0.673043
Dendritic.cells	SEC62	0.051277	7.437896	0.791214	0.430937	-6.59386	0.617317	0.599734
Dendritic.cells	FAM50A	-0.10705	5.186332	-0.791	0.431061	-6.06654	0.644226	0.638566
Dendritic.cells	FNDC3A	-0.05915	7.526942	-0.79099	0.431065	-6.92324	0.616284	0.598283
Dendritic.cells	GM26901	-0.39882	0.57091	-0.79095	0.43109	-5.04192	0.704258	0.726037
Dendritic.cells	GMPPB	0.198144	2.629402	0.790577	0.431306	-5.57932	0.676658	0.685728
Dendritic.cells	NEK4	-0.38124	1.202148	-0.79043	0.431393	-5.16961	0.695651	0.71354
Dendritic.cells	2610021A0	0.240316	2.362444	0.790328	0.431451	-5.42503	0.680161	0.690942
Dendritic.cells	NUP210	0.098902	5.041054	0.790053	0.431611	-6.22303	0.646015	0.641404
Dendritic.cells	GM49774	-0.32562	3.174932	-0.79003	0.431623	-5.39265	0.669568	0.6756
Dendritic.cells	TEPSIN	-0.39687	0.762658	-0.79002	0.43163	-5.13075	0.70163	0.722463
Dendritic.cells	URB2	-0.26005	2.571455	-0.78964	0.431848	-5.35973	0.677416	0.687187
Dendritic.cells	BMP2	-0.2633	1.613957	-0.7895	0.431929	-5.52905	0.690104	0.705764

Dendritic.cells	NT5C	0.124293	5.114948	0.789268	0.432067	-6.04092	0.645105	0.640274
Dendritic.cells	GNG4	-0.44942	1.462633	-0.78918	0.432116	-5.12294	0.692136	0.708798
Dendritic.cells	COPS2	0.079645	6.020343	0.789172	0.432123	-6.26415	0.634082	0.624373
Dendritic.cells	IL33	-0.41402	0.25962	-0.78905	0.432191	-5.12222	0.708549	0.732939
Dendritic.cells	ARL2BP	-0.13583	5.086344	-0.78905	0.432194	-6.00258	0.645457	0.640871
Dendritic.cells	EIF2D	0.164437	3.5699	0.788923	0.432267	-5.6755	0.664493	0.66851
Dendritic.cells	CPNE2	-0.26189	3.37335	-0.78889	0.432287	-5.53837	0.667012	0.672177
Dendritic.cells	TSR1	0.180679	3.584651	0.788869	0.432299	-5.71243	0.664304	0.668236
Dendritic.cells	CENPB	0.122067	5.450808	0.788684	0.432407	-6.06316	0.640987	0.634467
Dendritic.cells	CDC20	-0.3967	3.639962	-0.78864	0.432433	-5.35479	0.663598	0.667278
Dendritic.cells	CCDC157	0.331958	1.103128	0.788535	0.432493	-5.24815	0.696992	0.716106
Dendritic.cells	ADAM15	-0.3097	2.475369	-0.78853	0.432497	-5.39153	0.678676	0.689303
Dendritic.cells	RIOX1	-0.17575	3.521185	-0.78843	0.432556	-5.58944	0.665116	0.669565
Dendritic.cells	ASRGL1	-0.2792	3.315534	-0.78823	0.432671	-5.41694	0.667756	0.673453
Dendritic.cells	DNMT1	0.167693	5.753517	0.788129	0.432729	-5.97724	0.637305	0.629306
Dendritic.cells	CPNE5	-0.47121	0.481176	-0.78805	0.432775	-5.05443	0.705492	0.72871
Dendritic.cells	HHAT	0.275742	1.670909	0.787934	0.432843	-5.41524	0.689341	0.705017
Dendritic.cells	EPS8	0.411275	4.963297	0.787916	0.432853	-5.45346	0.646975	0.643304
Dendritic.cells	B3GNT3	-0.34489	0.711737	-0.78779	0.432924	-5.22751	0.702327	0.724059
Dendritic.cells	BATF	-0.14634	4.183315	-0.78778	0.432935	-5.8931	0.656707	0.657425
Dendritic.cells	TCOF1	0.130902	5.717375	0.787771	0.432937	-6.12189	0.637743	0.629939
Dendritic.cells	BAX	0.099358	6.141068	0.787769	0.432939	-6.32976	0.632631	0.622552
Dendritic.cells	ZBTB41	0.193588	2.915286	0.787261	0.433235	-5.55397	0.672931	0.681239
Dendritic.cells	NUP188	-0.13728	4.513741	-0.78696	0.433409	-5.91078	0.652562	0.651714
Dendritic.cells	SNRPG	0.077565	8.018397	0.786799	0.433504	-6.63566	0.610622	0.591158
Dendritic.cells	COL5A3	-0.50234	0.940726	-0.78675	0.433533	-5.06069	0.6992	0.719861
Dendritic.cells	PIAS3	0.216821	2.896492	0.786737	0.43354	-5.37116	0.673175	0.681766
Dendritic.cells	ELMO2	0.201012	3.945024	0.786698	0.433562	-5.59515	0.659718	0.662179
Dendritic.cells	SUPV3L1	-0.20662	3.910174	-0.78668	0.43357	-5.58359	0.66016	0.662822
Dendritic.cells	UBN2	0.071723	7.076175	0.78665	0.43359	-6.6667	0.621538	0.606902
Dendritic.cells	ZKSCAN16	-0.4156	-0.59074	-0.78664	0.433597	-5.04163	0.720432	0.751142
Dendritic.cells	TMEM167	0.069032	6.804318	0.786526	0.433662	-6.47627	0.624736	0.611541
Dendritic.cells	CRYBA4	0.48492	-0.18755	0.786479	0.43369	-5.04424	0.714769	0.742844
Dendritic.cells	GRPEL1	0.112101	5.877727	0.786403	0.433734	-6.22508	0.635802	0.627557
Dendritic.cells	GBP6	0.437216	1.984096	0.78635	0.433765	-5.36938	0.685165	0.699383
Dendritic.cells	KIF24	-0.18813	3.667581	-0.78627	0.433812	-5.58402	0.663245	0.667392
Dendritic.cells	KHDRBS3	-0.34012	1.943216	-0.78613	0.433892	-5.34207	0.685708	0.7002
Dendritic.cells	HIST1H2BG	-0.50713	1.128352	-0.78607	0.433929	-5.04654	0.69665	0.716248
Dendritic.cells	NKG7	-0.37796	4.013022	-0.78607	0.433931	-5.51674	0.658857	0.661028
Dendritic.cells	TSC22D3	0.154627	5.71665	0.78605	0.43394	-6.20589	0.637752	0.63041
Dendritic.cells	B3GALT1	-0.35495	3.129188	-0.78576	0.434109	-5.63128	0.670326	0.677599
Dendritic.cells	MTHFD1	-0.23024	3.79072	-0.78493	0.434591	-5.46546	0.662169	0.665447
Dendritic.cells	ZFP341	-0.25593	1.984672	-0.78488	0.434619	-5.3316	0.685667	0.699735
Dendritic.cells	GM14286	-0.33233	0.211974	-0.78475	0.434698	-5.22489	0.709737	0.735143
Dendritic.cells	DUSP22	0.153643	4.486105	0.784605	0.434782	-5.91234	0.653393	0.6528
Dendritic.cells	KIF7	0.422416	-0.46987	0.78454	0.43482	-5.10889	0.719264	0.749224
Dendritic.cells	GALNT1	0.069993	6.628673	0.784502	0.434842	-6.47732	0.627281	0.614999

Dendritic.cells	ADGRV1	0.514828	0.711845	0.78449	0.434849	-5.05432	0.702848	0.725049
Dendritic.cells	GRHL1	0.466152	1.025537	0.784442	0.434878	-5.07003	0.698565	0.718759
Dendritic.cells	NDUFA9	-0.13497	4.840567	-0.78442	0.434889	-5.85633	0.648977	0.64639
Dendritic.cells	IGF2	-0.48355	4.193244	-0.78436	0.434928	-5.59964	0.657071	0.658158
Dendritic.cells	VCPIP1	-0.07835	6.167501	-0.78415	0.435049	-6.36109	0.632873	0.622983
Dendritic.cells	GM49482	0.469527	-0.71551	0.783976	0.435149	-5.04358	0.722855	0.75446
Dendritic.cells	RNF144B	-0.30576	2.562148	-0.7839	0.435194	-5.2995	0.678157	0.688848
Dendritic.cells	RFXAP	-0.14088	4.179927	-0.78381	0.435244	-5.72657	0.65735	0.658536
Dendritic.cells	HSPA13	-0.15811	3.082179	-0.78354	0.435402	-5.64958	0.67153	0.678996
Dendritic.cells	ASCC3	-0.08237	6.882346	-0.7833	0.435545	-6.62932	0.624645	0.610905
Dendritic.cells	STK40	-0.12779	5.69472	-0.78316	0.435623	-6.02213	0.638891	0.631498
Dendritic.cells	SGPP1	0.115313	5.017924	0.782821	0.435824	-6.16892	0.647216	0.64356
Dendritic.cells	ONECUT2	-0.42976	1.559667	-0.7828	0.435835	-5.17302	0.691811	0.708564
Dendritic.cells	GRINA	0.173236	6.205395	0.782751	0.435865	-6.1704	0.632754	0.622658
Dendritic.cells	HIST1H4N	0.460017	1.07648	0.782715	0.435886	-5.10043	0.698342	0.718189
Dendritic.cells	MYLIP	-0.14606	5.991118	-0.78253	0.435995	-6.04024	0.635404	0.626451
Dendritic.cells	GM15706	0.321455	1.015787	0.781261	0.436736	-5.24921	0.700303	0.719878
Dendritic.cells	TMSB15B1	0.245237	2.422831	0.781177	0.436785	-5.41822	0.681434	0.692314
Dendritic.cells	GALNT10	-0.20391	4.614762	-0.78109	0.436837	-5.64636	0.653283	0.651351
Dendritic.cells	ZFP90	0.288778	2.283533	0.780665	0.437084	-5.37154	0.683567	0.69521
Dendritic.cells	TFE3	0.175864	4.149746	0.780428	0.437223	-5.62858	0.659531	0.660145
Dendritic.cells	FOXJ2	-0.14992	3.829076	-0.78029	0.437304	-5.89602	0.663634	0.666084
Dendritic.cells	GM14305	0.211627	2.260659	0.779979	0.437486	-5.42937	0.684141	0.695835
Dendritic.cells	CSGALNAC	-0.29322	2.818139	-0.77996	0.4375	-5.53795	0.676804	0.685129
Dendritic.cells	CMC1	0.112361	4.601302	0.779258	0.437909	-5.96984	0.654387	0.652109
Dendritic.cells	NCSTN	0.105569	5.576404	0.779166	0.437962	-6.16261	0.64232	0.6347
Dendritic.cells	MYH10	-0.24128	2.573809	-0.77915	0.437971	-5.46443	0.68042	0.69002
Dendritic.cells	GM20069	-0.4795	-0.52038	-0.77888	0.438131	-5.04973	0.722663	0.751984
Dendritic.cells	CCNH	0.118061	5.363256	0.778853	0.438146	-5.96756	0.644933	0.638504
Dendritic.cells	BEND5	0.376621	0.115495	0.778707	0.438232	-5.08742	0.71373	0.738886
Dendritic.cells	WDYHV1	0.150491	4.174037	0.778601	0.438294	-5.73826	0.659767	0.660057
Dendritic.cells	FAM122B	-0.24614	2.015817	-0.77846	0.438378	-5.35894	0.687811	0.70095
Dendritic.cells	BRWD1	-0.0744	6.550877	-0.77845	0.438383	-6.52192	0.63055	0.617835
Dendritic.cells	A530076I1	0.436033	-0.88246	0.778337	0.438448	-5.04812	0.727809	0.759712
Dendritic.cells	5530601H(	-0.20332	3.904215	-0.77829	0.438476	-5.66913	0.663193	0.665106
Dendritic.cells	LY6G2	-0.49397	0.952461	-0.77826	0.438494	-5.13248	0.702172	0.722007
Dendritic.cells	PAK2	0.056854	7.859842	0.777978	0.438659	-6.67867	0.615331	0.595855
Dendritic.cells	C130050O	-0.38812	1.379067	-0.77768	0.438831	-5.16392	0.696709	0.713756
Dendritic.cells	PODXL	-0.37328	0.550401	-0.77749	0.438946	-5.26002	0.708131	0.7305
Dendritic.cells	2310058D1	0.144947	2.785149	0.777263	0.439079	-5.70558	0.678165	0.686576
Dendritic.cells	GM50431	-0.47446	0.122176	-0.77711	0.439167	-5.06427	0.714184	0.739349
Dendritic.cells	GPR107	0.107126	5.057833	0.777065	0.439195	-6.05659	0.649198	0.644543
Dendritic.cells	HADH	-0.18999	5.394105	-0.77672	0.439395	-5.86437	0.645219	0.638645
Dendritic.cells	SUCLG1	-0.12831	5.422184	-0.77666	0.439433	-6.03517	0.644874	0.638166
Dendritic.cells	HSD17B12	-0.09222	6.085483	-0.77641	0.439577	-6.36948	0.636912	0.626545
Dendritic.cells	PLEKHA5	0.157414	4.693302	0.776137	0.43974	-5.88095	0.654185	0.651396
Dendritic.cells	BHLHB9	0.217827	2.203905	0.776035	0.439799	-5.48187	0.686304	0.698144

Dendritic.cells	NDUFA10	0.092877	6.050526	0.775898	0.43988	-6.21894	0.63751	0.627294
Dendritic.cells	PIRB	0.155979	4.941687	0.775675	0.440011	-6.3093	0.651164	0.646999
Dendritic.cells	P3H1	-0.40126	0.585168	-0.77563	0.440035	-5.20124	0.708328	0.730342
Dendritic.cells	MIRT2	-0.40785	-1.00119	-0.77541	0.440168	-5.05065	0.730773	0.763348
Dendritic.cells	PON3	0.224331	3.489649	0.775097	0.440351	-5.61657	0.669759	0.673928
Dendritic.cells	GM2629	0.408495	0.511245	0.775093	0.440353	-5.06039	0.709569	0.732108
Dendritic.cells	FAM13B	-0.07798	6.457749	-0.77483	0.44051	-6.44514	0.632989	0.620532
Dendritic.cells	RASSF4	-0.12048	4.553252	-0.77456	0.440665	-6.22118	0.656432	0.654475
Dendritic.cells	TMEM97	0.216274	3.649444	0.774542	0.440677	-5.52936	0.667917	0.671148
Dendritic.cells	MAPRE1	-0.06109	7.148653	-0.77436	0.440785	-6.53123	0.624878	0.608912
Dendritic.cells	GM10874	-0.44685	-0.06869	-0.77429	0.440823	-5.0571	0.717922	0.744332
Dendritic.cells	FAM107B	0.088787	7.858238	0.774094	0.44094	-6.62669	0.616671	0.597062
Dendritic.cells	GM50071	-0.35753	0.514178	-0.77384	0.44109	-5.14476	0.710029	0.73242
Dendritic.cells	UXT	0.158385	4.452478	0.773634	0.441211	-5.73623	0.658043	0.656496
Dendritic.cells	OSGEPL1	0.237204	2.472736	0.773434	0.441329	-5.34082	0.683609	0.693808
Dendritic.cells	RTN2	0.446375	-0.4318	0.773354	0.441376	-5.07083	0.723377	0.752114
Dendritic.cells	TRAF1	0.355798	3.343827	0.773293	0.441412	-5.47977	0.672209	0.677207
Dendritic.cells	LAMA4	-0.33423	1.088771	-0.773	0.441586	-5.43421	0.702219	0.721147
Dendritic.cells	MOB2	0.097085	5.416589	0.772989	0.441591	-6.1272	0.646034	0.639343
Dendritic.cells	MEGF8	0.343789	0.288303	0.772778	0.441715	-5.18138	0.713264	0.737358
Dendritic.cells	P4HTM	-0.37589	2.773581	-0.77276	0.441726	-5.30474	0.679645	0.688147
Dendritic.cells	ZPR1	-0.13506	4.397865	-0.77266	0.441784	-5.81137	0.658732	0.657753
Dendritic.cells	GM12185	0.240774	3.341306	0.77264	0.441796	-5.55885	0.672241	0.677389
Dendritic.cells	NUS1	-0.10602	4.837828	-0.77252	0.441869	-5.93419	0.653209	0.649805
Dendritic.cells	SLC35A5	0.144158	3.986264	0.772483	0.441889	-5.72184	0.663953	0.66541
Dendritic.cells	1500015A(	0.230588	1.76661	0.772375	0.441952	-5.39021	0.693028	0.707833
Dendritic.cells	NDC80	-0.32902	4.625978	-0.77229	0.442002	-5.53752	0.655861	0.653736
Dendritic.cells	CHCHD10	0.211895	6.141333	0.772153	0.442083	-5.98552	0.637222	0.626802
Dendritic.cells	BOD1L	0.082678	5.740505	0.771716	0.44234	-6.23187	0.64229	0.633988
Dendritic.cells	TMEM158	-0.50334	0.271092	-0.77172	0.442341	-5.10765	0.713755	0.738158
Dendritic.cells	KARS	0.165834	4.273346	0.77142	0.442515	-5.79088	0.660544	0.660509
Dendritic.cells	MTRF1	0.357589	1.208712	0.771343	0.44256	-5.18854	0.700834	0.719326
Dendritic.cells	MAP2K1	0.089125	6.311963	0.771207	0.44264	-6.42505	0.635364	0.624141
Dendritic.cells	ZFP518B	-0.30311	0.418092	-0.77118	0.442655	-5.18613	0.711715	0.735346
Dendritic.cells	MRPL2	0.148822	4.862057	0.771133	0.442684	-5.88922	0.653142	0.64987
Dendritic.cells	TES	-0.16678	5.486918	-0.771	0.44276	-5.88279	0.645402	0.638674
Dendritic.cells	RAP1GAP	-0.44567	0.58551	-0.77096	0.442787	-5.08028	0.709394	0.731965
Dendritic.cells	RCCD1	0.259823	3.170294	0.770748	0.442911	-5.48574	0.674704	0.681267
Dendritic.cells	VEGFC	0.259812	1.200834	0.770506	0.443054	-5.49841	0.700942	0.719752
Dendritic.cells	PDS5B	0.083291	6.377064	0.770339	0.443153	-6.39478	0.634581	0.623206
Dendritic.cells	FAM160A2	-0.37459	2.604929	-0.77033	0.443159	-5.06059	0.682109	0.692197
Dendritic.cells	CLEC4N	-0.44949	4.093598	-0.77031	0.443168	-5.40996	0.662825	0.66411
Dendritic.cells	GPR155	0.191104	3.05644	0.770282	0.443186	-5.73894	0.676187	0.683557
Dendritic.cells	DVL3	-0.1794	3.300094	-0.77026	0.443197	-5.57184	0.673018	0.678939
Dendritic.cells	ZFP36	-0.10478	7.849088	-0.77009	0.443299	-6.74081	0.617208	0.598245
Dendritic.cells	TMF1	0.077369	6.243916	0.770058	0.443318	-6.35869	0.636184	0.625583
Dendritic.cells	INSYN2B	0.148977	4.140533	0.76978	0.443482	-5.87648	0.662382	0.663429

Dendritic.cells	BCAT2	0.131558	4.601375	0.76961	0.443582	-5.89842	0.656617	0.655031
Dendritic.cells	UGGT1	0.070062	5.389379	0.769394	0.44371	-6.44662	0.646847	0.640958
Dendritic.cells	PWWP3A	0.170769	3.715755	0.76936	0.44373	-5.62181	0.667907	0.671529
Dendritic.cells	CHST14	-0.37688	0.872451	-0.76902	0.443933	-5.2054	0.705878	0.726912
Dendritic.cells	ZFP398	0.113778	4.517006	0.768921	0.44399	-6.20878	0.657878	0.656887
Dendritic.cells	PITPNA	0.05361	7.992513	0.7687	0.44412	-6.76402	0.615934	0.596356
Dendritic.cells	AZI2	-0.08283	5.839646	-0.76856	0.444204	-6.23986	0.641485	0.633241
Dendritic.cells	PIGL	-0.21517	2.746364	-0.76851	0.444229	-5.37755	0.680672	0.690177
Dendritic.cells	CFAP20	-0.09785	5.191326	-0.76845	0.444269	-6.11818	0.649453	0.644805
Dendritic.cells	SSBP3	0.117763	5.600863	0.768352	0.444326	-6.07136	0.644405	0.6375
Dendritic.cells	NDUFB2	0.121656	5.815553	0.768344	0.44433	-5.99696	0.641779	0.633701
Dendritic.cells	MTX2	0.137213	4.838218	0.768248	0.444387	-5.82757	0.653847	0.651188
Dendritic.cells	MID1	-0.39758	5.805162	-0.76787	0.444609	-6.11046	0.642111	0.634062
Dendritic.cells	ZSCAN20	-0.3882	1.073033	-0.7678	0.444651	-5.22494	0.703351	0.723288
Dendritic.cells	CNTLN	-0.2233	4.34763	-0.76729	0.444955	-5.64111	0.660517	0.660536
Dendritic.cells	UQCR10	0.093005	6.961957	0.767264	0.444969	-6.44794	0.62847	0.614169
Dendritic.cells	JAKMIP1	0.092295	3.981458	0.76694	0.44516	-6.45975	0.665369	0.667358
Dendritic.cells	9530052E0	0.36952	1.000649	0.766732	0.445284	-5.10592	0.704958	0.725147
Dendritic.cells	PPM1D	-0.11527	4.948971	-0.76638	0.44549	-5.95143	0.653396	0.649725
Dendritic.cells	POR	-0.16349	5.6252	-0.76628	0.445552	-5.88416	0.645026	0.637607
Dendritic.cells	CC2D2B	0.715762	2.70829	0.766133	0.445638	-5.15673	0.682146	0.691531
Dendritic.cells	APOC2	0.356329	4.203003	0.766089	0.445664	-5.47528	0.662793	0.663358
Dendritic.cells	NADK	0.109795	6.266885	0.76604	0.445693	-6.25337	0.637213	0.626318
Dendritic.cells	MAFK	-0.17828	4.524193	-0.76576	0.445859	-5.91039	0.658847	0.657496
Dendritic.cells	GLT1D1	-0.26856	0.611079	-0.7654	0.44607	-5.4667	0.710627	0.733245
Dendritic.cells	BCLAF1	0.058944	7.155376	0.765401	0.446071	-6.58178	0.626714	0.611186
Dendritic.cells	CRLS1	0.179713	3.622878	0.765309	0.446126	-5.54228	0.670344	0.674353
Dendritic.cells	NEMF	0.071631	5.95092	0.76527	0.446149	-6.38187	0.641162	0.632039
Dendritic.cells	B3GALT5	-0.2368	0.779445	-0.76524	0.446164	-5.74959	0.708298	0.72984
Dendritic.cells	SLC25A39	0.138936	6.472882	0.765181	0.446202	-6.24258	0.634847	0.622954
Dendritic.cells	NR2C1	-0.27788	2.276051	-0.76494	0.446341	-5.37376	0.688121	0.700209
Dendritic.cells	GM48027	0.212154	2.815176	0.76472	0.446475	-5.55091	0.681094	0.689787
Dendritic.cells	SNRPC	0.087707	6.245324	0.763887	0.446968	-6.237	0.638419	0.627255
Dendritic.cells	PRDM15	-0.12282	3.462741	-0.76379	0.447026	-5.94424	0.673287	0.677805
Dendritic.cells	EXOC7	0.14228	4.087686	0.763609	0.447133	-5.75771	0.665286	0.666168
Dendritic.cells	EFNB2	-0.337	2.469455	-0.7635	0.447199	-5.37243	0.68636	0.69688
Dendritic.cells	CNOT9	-0.1296	4.541441	-0.76335	0.447285	-5.83997	0.659525	0.6579
Dendritic.cells	SNHG10	0.437879	0.227889	0.763309	0.447311	-5.12454	0.716934	0.74174
Dendritic.cells	NFKBIZ	0.113683	6.516715	0.76323	0.447358	-6.48565	0.635183	0.62273
Dendritic.cells	DIPK2A	-0.15192	4.112992	-0.76249	0.447794	-5.77716	0.665082	0.666136
Dendritic.cells	TAGLN2	0.088186	8.716101	0.762481	0.447802	-7.03263	0.609568	0.586025
Dendritic.cells	TTC39B	-0.2572	4.653747	-0.76225	0.447938	-5.41521	0.658226	0.656257
Dendritic.cells	GRWD1	-0.27175	2.674334	-0.7622	0.447967	-5.33263	0.683769	0.693425
Dendritic.cells	OGFOD3	-0.19973	3.187381	-0.76218	0.447978	-5.54338	0.67703	0.683598
Dendritic.cells	ZFP740	-0.10063	4.76726	-0.76216	0.447991	-6.04974	0.656798	0.65419
Dendritic.cells	YIPF2	0.251924	1.926928	0.76192	0.448136	-5.38111	0.693736	0.708045
Dendritic.cells	PHF8	0.096152	5.563796	0.761913	0.44814	-6.20167	0.646892	0.639894

Dendritic.cells	TMEM238	-0.35108	2.938537	-0.7619	0.44815	-5.24377	0.680288	0.688402
Dendritic.cells	OS9	-0.0811	5.617189	-0.76174	0.448242	-6.3344	0.646235	0.638977
Dendritic.cells	ERCC2	0.250705	1.983619	0.761665	0.448287	-5.36183	0.692973	0.707002
Dendritic.cells	PAF1	-0.11432	4.600235	-0.76156	0.44835	-5.9584	0.6589	0.657402
Dendritic.cells	SCD1	-0.49679	3.037555	-0.76147	0.4484	-5.13913	0.678989	0.686637
Dendritic.cells	RBM15B	0.113582	4.047089	0.761438	0.448422	-5.96205	0.665924	0.667616
Dendritic.cells	ATP5B	0.073751	8.182512	0.761172	0.44858	-6.70893	0.615679	0.595052
Dendritic.cells	PDE7B	-0.33971	5.37371	-0.76114	0.448597	-5.87942	0.649239	0.64346
Dendritic.cells	KCNK5	-0.39333	1.47886	-0.76106	0.448644	-5.1431	0.699797	0.717115
Dendritic.cells	TPT1	-0.05462	10.10831	-0.76103	0.448664	-7.02593	0.594009	0.564032
Dendritic.cells	SIGLECH	0.099326	0.771994	0.761009	0.448676	-6.60184	0.70949	0.731338
Dendritic.cells	NME7	0.139595	3.562527	0.760995	0.448685	-5.73426	0.672155	0.676738
Dendritic.cells	SPTBN1	-0.10865	6.620496	-0.76093	0.448721	-6.36973	0.63405	0.621521
Dendritic.cells	FTL1-PS1	-0.2741	3.902493	-0.76056	0.448944	-5.59093	0.667796	0.670421
Dendritic.cells	ALDH6A1	-0.31546	2.786139	-0.76039	0.449043	-5.42946	0.682313	0.691612
Dendritic.cells	NAA16	0.094566	5.024992	0.76033	0.44908	-6.0842	0.653592	0.649839
Dendritic.cells	SAAL1	-0.21939	3.298168	-0.76027	0.449117	-5.53947	0.675606	0.681856
Dendritic.cells	SLC35D2	-0.23965	4.232	-0.76015	0.449186	-5.53441	0.663585	0.664387
Dendritic.cells	GK	0.228705	4.764608	0.76008	0.449229	-5.66503	0.656852	0.654634
Dendritic.cells	NDUFS7	0.08323	6.320866	0.759894	0.449339	-6.28467	0.637676	0.626932
Dendritic.cells	B230206L0	-0.53144	1.02404	-0.75965	0.449486	-5.13897	0.706037	0.726549
Dendritic.cells	RIT1	0.16849	3.965996	0.758946	0.449903	-5.60834	0.666982	0.669641
Dendritic.cells	GM34471	0.377476	0.232245	0.758896	0.449933	-5.16638	0.717023	0.742901
Dendritic.cells	NKAPD1	0.148128	4.086425	0.758846	0.449963	-5.76439	0.665441	0.667409
Dendritic.cells	PCM1	0.075422	6.494399	0.758687	0.450058	-6.52186	0.635584	0.62418
Dendritic.cells	MITF	0.158752	4.369202	0.75867	0.450068	-6.07849	0.661842	0.662224
Dendritic.cells	GPAT4	0.123475	4.264538	0.758664	0.450071	-5.8009	0.663171	0.664156
Dendritic.cells	SLC7A7	-0.2413	3.785978	-0.75848	0.450179	-5.5401	0.669293	0.673062
Dendritic.cells	ARID3A	0.074668	5.513727	0.758175	0.450362	-6.5615	0.647529	0.641456
Dendritic.cells	LRRC27	-0.42669	0.302993	-0.75813	0.450391	-5.06145	0.716033	0.741497
Dendritic.cells	TMEM41B	-0.13641	4.300772	-0.75812	0.450397	-5.81834	0.662711	0.663487
Dendritic.cells	GLRX3	0.069974	6.625536	0.758027	0.450451	-6.61555	0.634009	0.621905
Dendritic.cells	CTSW	0.361884	2.120663	0.758018	0.450456	-5.38328	0.691156	0.704978
Dendritic.cells	MFSD5	-0.15051	4.282812	-0.75793	0.450507	-5.85111	0.662939	0.663839
Dendritic.cells	DDX17	0.076868	6.357267	0.757916	0.450517	-6.47741	0.637237	0.626589
Dendritic.cells	ZFP87	-0.2188	3.396086	-0.75788	0.450535	-5.49608	0.674333	0.680432
Dendritic.cells	TIMM10	-0.21325	3.050855	-0.75781	0.45058	-5.48573	0.678835	0.687004
Dendritic.cells	PHAX	0.138525	4.599165	0.757768	0.450605	-5.90452	0.658934	0.658038
Dendritic.cells	ZFP983	0.244265	2.38923	0.757675	0.45066	-5.45292	0.68757	0.699813
Dendritic.cells	GTPBP4	-0.07617	5.956432	-0.7576	0.450706	-6.37737	0.6421	0.633676
Dendritic.cells	HAUS1	0.231115	3.65784	0.757587	0.450713	-5.50255	0.670944	0.675549
Dendritic.cells	NOL6	0.221254	2.582305	0.757342	0.450859	-5.46358	0.685007	0.696171
Dendritic.cells	TOR4A	0.315864	1.678835	0.757278	0.450897	-5.28627	0.697105	0.713913
Dendritic.cells	SAA2	0.657653	-1.02347	0.757189	0.45095	-5.07821	0.734865	0.769545
Dendritic.cells	NEIL3	0.285579	4.375558	0.757156	0.45097	-5.60907	0.661762	0.662341
Dendritic.cells	TM2D2	0.091622	5.178235	0.756804	0.45118	-6.20532	0.651683	0.64778
Dendritic.cells	ANK3	-0.47876	1.681131	-0.75672	0.45123	-5.18537	0.697074	0.714

Dendritic.cells	GM33677	-0.51031	-1.06476	-0.75659	0.451307	-5.05967	0.73546	0.770578
Dendritic.cells	SLC6A19	0.444065	-0.35949	0.756453	0.451389	-5.05931	0.725366	0.755678
Dendritic.cells	QK	-0.05199	8.142329	-0.75638	0.45143	-6.82499	0.616161	0.596534
Dendritic.cells	UBE2O	0.218859	4.874388	0.756382	0.451431	-5.79712	0.655475	0.653348
Dendritic.cells	NPFF	-0.33152	0.954229	-0.75633	0.451462	-5.21055	0.706997	0.728627
Dendritic.cells	PRADC1	-0.20269	3.374688	-0.75628	0.451492	-5.52601	0.674611	0.681202
Dendritic.cells	ZFP414	0.224651	3.443642	0.756131	0.451581	-5.48125	0.673715	0.679936
Dendritic.cells	BOLA2	0.12465	5.686348	0.756041	0.451634	-6.06848	0.645405	0.638786
Dendritic.cells	TBC1D2B	-0.18848	4.091379	-0.75602	0.451644	-5.6983	0.665378	0.667789
Dendritic.cells	ASB13	0.21619	3.434394	0.755956	0.451685	-5.60197	0.673835	0.680111
Dendritic.cells	ATP5L	0.062346	8.67785	0.755631	0.451879	-6.78922	0.610022	0.587799
Dendritic.cells	MBD4	0.229057	3.212172	0.755563	0.451919	-5.45687	0.676727	0.684386
Dendritic.cells	VEZT	0.128103	4.178132	0.755483	0.451967	-5.87549	0.664271	0.666234
Dendritic.cells	PIM1	-0.11303	9.488973	-0.75518	0.452149	-6.75046	0.600881	0.574791
Dendritic.cells	OTUD3	-0.26291	2.558441	-0.75509	0.452201	-5.43357	0.685323	0.697127
Dendritic.cells	BLOC1S5	0.277957	1.955013	0.754978	0.452269	-5.34025	0.693379	0.70896
Dendritic.cells	AHDC1	0.120497	4.689967	0.754946	0.452288	-5.87432	0.65779	0.657012
Dendritic.cells	ZFP951	-0.26876	2.641469	-0.75484	0.452351	-5.42938	0.684224	0.695555
Dendritic.cells	JKAMP	0.128176	3.680975	0.75478	0.452387	-5.77823	0.670645	0.675736
Dendritic.cells	CCDC174	-0.10798	5.082834	-0.75472	0.452425	-6.01027	0.65287	0.649895
Dendritic.cells	RPUSD4	0.244035	2.682051	0.754602	0.452493	-5.39176	0.683687	0.694848
Dendritic.cells	NUP107	0.137522	5.353646	0.7546	0.452494	-5.94358	0.649507	0.645057
Dendritic.cells	CDC42EP1	-0.37756	0.752796	-0.75456	0.45252	-5.276	0.709777	0.733129
Dendritic.cells	RECQL	-0.2236	3.363727	-0.75456	0.45252	-5.52726	0.674753	0.681795
Dendritic.cells	CAR5A	-0.40652	0.542195	-0.75434	0.452647	-5.17154	0.712698	0.737565
Dendritic.cells	CYP2J5	-0.42675	1.06057	-0.75422	0.452723	-5.16206	0.705535	0.727035
Dendritic.cells	SLC17A5	-0.19549	3.572742	-0.75412	0.45278	-5.64553	0.672043	0.677983
Dendritic.cells	ATP5K	0.09836	7.486519	0.753917	0.452902	-6.54251	0.623794	0.608016
Dendritic.cells	QARS	0.16042	4.552856	0.753882	0.452923	-5.75726	0.659518	0.659758
Dendritic.cells	NRG1	0.511635	2.541088	0.753881	0.452923	-5.2869	0.685553	0.697746
Dendritic.cells	TEX30	-0.10955	5.223272	-0.75388	0.452926	-6.06057	0.651123	0.647558
Dendritic.cells	RBAK	-0.27853	1.336784	-0.75365	0.453064	-5.35314	0.701753	0.721504
Dendritic.cells	ANKRD46	-0.20057	2.377526	-0.75358	0.453103	-5.36448	0.687726	0.700927
Dendritic.cells	ZFP119B	-0.32186	1.297642	-0.75353	0.453131	-5.21586	0.702288	0.722289
Dendritic.cells	PPP2R5D	0.192722	3.682866	0.753514	0.453143	-5.54171	0.670621	0.675929
Dendritic.cells	SUPT3	0.083867	5.480744	0.753508	0.453147	-6.48549	0.647936	0.642933
Dendritic.cells	ABI1	-0.08391	7.702393	-0.75333	0.453255	-6.65107	0.621268	0.604379
Dendritic.cells	CD48	0.098891	6.795574	0.753187	0.453338	-6.47628	0.631974	0.619845
Dendritic.cells	TVP23B	0.127975	4.042523	0.752871	0.453527	-5.85684	0.666002	0.669263
Dendritic.cells	9030025P2	0.343318	1.737859	0.75286	0.453534	-5.20355	0.696306	0.713576
Dendritic.cells	TUT1	0.1797	2.638669	0.752843	0.453544	-5.48534	0.684261	0.695924
Dendritic.cells	ARMC5	0.159695	3.383082	0.752761	0.453593	-5.62399	0.674501	0.681687
Dendritic.cells	NUP62	-0.22532	4.181003	-0.75268	0.453642	-5.65209	0.664235	0.666733
Dendritic.cells	GM20139	0.391894	-0.63311	0.752669	0.453648	-5.06252	0.729263	0.762187
Dendritic.cells	BC029722	0.215382	3.061908	0.752606	0.453686	-5.49238	0.678691	0.687836
Dendritic.cells	GM10131	-0.44395	1.072727	-0.75257	0.453709	-5.15597	0.705368	0.72695
Dendritic.cells	CFAP410	0.228617	2.119698	0.752454	0.453777	-5.32659	0.691169	0.706141

Dendritic.cells	PURB	-0.06041	6.824936	-0.75229	0.453873	-6.59759	0.631623	0.619503
Dendritic.cells	CHD1L	-0.14215	3.722264	-0.75207	0.454005	-5.75595	0.670113	0.675396
Dendritic.cells	FGD3	0.178499	4.660813	0.752059	0.454013	-5.76235	0.658157	0.65798
Dendritic.cells	PRPF4	0.171597	3.92555	0.752051	0.454018	-5.6865	0.6675	0.671585
Dendritic.cells	TCP11L1	0.290947	2.689841	0.752034	0.454028	-5.30263	0.683584	0.695079
Dendritic.cells	IQCC	-0.33088	1.695654	-0.75187	0.454129	-5.25053	0.696877	0.714563
Dendritic.cells	ANKRD13A	-0.08489	6.215118	-0.75182	0.454154	-6.3169	0.638956	0.630125
Dendritic.cells	KLHL3	-0.24062	2.005874	-0.75179	0.454174	-5.46788	0.692695	0.708437
Dendritic.cells	MFNG	0.18612	3.090104	0.751601	0.454287	-5.55656	0.67833	0.687458
Dendritic.cells	TRIM7	0.422877	0.591087	0.751576	0.454302	-5.09445	0.712027	0.736914
Dendritic.cells	PELO	0.19524	3.125716	0.751244	0.454501	-5.53603	0.678069	0.686915
Dendritic.cells	ST7L	-0.13145	4.451288	-0.75087	0.454725	-5.94476	0.661037	0.662217
Dendritic.cells	TMED9	0.059334	6.609324	0.750854	0.454735	-6.5727	0.634428	0.623605
Dendritic.cells	PPP2R1A	0.101491	6.00443	0.75084	0.454743	-6.20492	0.641743	0.634196
Dendritic.cells	NME1	-0.12372	6.668403	-0.75076	0.45479	-6.369	0.63372	0.622604
Dendritic.cells	AP4B1	0.315147	2.273778	0.750703	0.454825	-5.23447	0.689353	0.70364
Dendritic.cells	KCTD20	-0.11222	4.885027	-0.75023	0.455106	-5.99329	0.655582	0.654453
Dendritic.cells	EID1	-0.08637	5.155191	-0.75009	0.45519	-6.28871	0.652209	0.649622
Dendritic.cells	RAB8B	0.119131	7.724426	0.750065	0.455207	-6.44463	0.621239	0.604781
Dendritic.cells	HDAC9	0.099133	7.563843	0.750009	0.45524	-6.93966	0.623117	0.60749
Dendritic.cells	HIST2H3B	0.417599	1.047683	0.749955	0.455273	-5.1327	0.705971	0.728259
Dendritic.cells	TMCO1	0.070147	6.265444	0.749951	0.455275	-6.40272	0.638581	0.629849
Dendritic.cells	WDR25	-0.24297	1.998808	-0.74984	0.45534	-5.35836	0.693045	0.709296
Dendritic.cells	ZDBF2	0.533401	-0.23932	0.749814	0.455357	-5.07745	0.723929	0.75479
Dendritic.cells	TSR2	-0.24015	2.118125	-0.74976	0.455387	-5.51436	0.691444	0.706946
Dendritic.cells	FCOR	-0.2271	0.865885	-0.74965	0.455455	-5.5456	0.708475	0.732018
Dendritic.cells	GANAB	0.10343	4.730059	0.749571	0.455503	-6.09578	0.657527	0.657457
Dendritic.cells	4930438A(	0.359628	-0.84008	0.749439	0.455582	-5.06599	0.732525	0.767571
Dendritic.cells	TMEM119	0.294594	0.305437	0.74911	0.455779	-5.33395	0.716504	0.743716
Dendritic.cells	ZBTB6	0.245239	1.997204	0.748974	0.455861	-5.40231	0.693307	0.709634
Dendritic.cells	PRKACB	0.093586	5.548308	0.748814	0.455957	-6.16296	0.647565	0.642888
Dendritic.cells	C9ORF72	0.34511	3.893192	0.748698	0.456026	-5.35437	0.668392	0.673206
Dendritic.cells	DNAJB12	-0.12612	4.757611	-0.7487	0.456028	-5.90669	0.657409	0.657201
Dendritic.cells	GM43660	-0.48183	0.127169	-0.74857	0.456101	-5.07592	0.719028	0.747574
Dendritic.cells	ZC3H7B	-0.20007	3.353116	-0.74828	0.456275	-5.56527	0.675471	0.683495
Dendritic.cells	TRIM32	0.397959	1.488698	0.748266	0.456286	-5.19319	0.700285	0.719874
Dendritic.cells	NLN	0.141478	4.446951	0.748178	0.456338	-5.92734	0.661425	0.663025
Dendritic.cells	ABCA8B	-0.42497	1.118002	-0.74779	0.456568	-5.19543	0.705538	0.727484
Dendritic.cells	GM26590	0.22291	1.314817	0.747773	0.456582	-5.36755	0.702842	0.723516
Dendritic.cells	FCRL6	-0.40049	-0.87747	-0.74763	0.456667	-5.06251	0.733626	0.768974
Dendritic.cells	STOM	-0.28624	3.274711	-0.74741	0.456802	-5.36889	0.676791	0.685283
Dendritic.cells	IDE	0.125241	4.161112	0.74727	0.456883	-5.80032	0.665353	0.668647
Dendritic.cells	CXCL1	-0.79438	2.815955	-0.74723	0.456907	-5.26237	0.682808	0.694175
Dendritic.cells	THBS3	-0.42038	1.01596	-0.74708	0.456999	-5.19605	0.707114	0.729846
Dendritic.cells	CHPT1	-0.17051	3.607446	-0.74684	0.457139	-5.64856	0.672624	0.67917
Dendritic.cells	SLC9A3R1	0.102447	6.690174	0.746748	0.457197	-6.27409	0.634205	0.62334
Dendritic.cells	ANO10	0.27677	3.16746	0.746424	0.457392	-5.37875	0.678462	0.687662

Dendritic.cells	EIF4H	0.064651	6.535904	0.746338	0.457443	-6.43325	0.636164	0.626136
Dendritic.cells	PPP6C	-0.06024	6.737344	-0.74632	0.457457	-6.50693	0.633745	0.622635
Dendritic.cells	MAP3K2	0.07879	6.248682	0.746153	0.457554	-6.46567	0.639665	0.631163
Dendritic.cells	COA4	0.275355	2.161432	0.746019	0.457635	-5.30428	0.691816	0.707245
Dendritic.cells	ZFP773	0.473489	0.510873	0.745956	0.457673	-5.08838	0.714379	0.740444
Dendritic.cells	TMEM184I	0.142247	4.707523	0.745873	0.457723	-5.9964	0.658717	0.658899
Dendritic.cells	ARFGEF2	0.088824	6.285468	0.745501	0.457946	-6.41738	0.639303	0.630729
Dendritic.cells	CEP131	0.42124	1.3043	0.745385	0.458016	-5.17977	0.703516	0.724517
Dendritic.cells	EPHA2	-0.11105	2.408212	-0.7453	0.458067	-6.13413	0.688609	0.702649
Dendritic.cells	CWF19L2	0.097906	5.038452	0.745069	0.458206	-6.15751	0.654651	0.653092
Dendritic.cells	EXOSC9	-0.15385	4.169312	-0.74505	0.458218	-5.74547	0.665632	0.669086
Dendritic.cells	ANXA4	0.224235	3.74233	0.745017	0.458237	-5.62073	0.671113	0.677098
Dendritic.cells	ANAPC5	0.065897	6.399962	0.744748	0.458399	-6.48165	0.637918	0.628823
Dendritic.cells	PRKD3	0.129668	5.198034	0.744739	0.458404	-5.97402	0.652661	0.650221
Dendritic.cells	GNAI2	0.047692	9.008515	0.744724	0.458414	-6.90857	0.607412	0.58479
Dendritic.cells	HIBCH	-0.18906	3.478236	-0.74466	0.458452	-5.47732	0.674532	0.682115
Dendritic.cells	RAB20	-0.32395	3.301078	-0.74465	0.458457	-5.39476	0.676837	0.685485
Dendritic.cells	LTO1	-0.11314	3.526077	-0.74434	0.458643	-6.0258	0.674095	0.681363
Dendritic.cells	SCO2	0.215852	2.851352	0.744085	0.458798	-5.48525	0.683061	0.694335
Dendritic.cells	FGF13	-0.36372	3.379693	-0.74387	0.45893	-5.45851	0.676151	0.68427
Dendritic.cells	MTFP1	-0.44403	0.572509	-0.74386	0.458931	-5.071	0.713972	0.739813
Dendritic.cells	4930590J0	-0.35385	1.547542	-0.74366	0.459052	-5.1746	0.700639	0.720109
Dendritic.cells	GPR68	0.428144	1.433054	0.743289	0.459278	-5.20303	0.702372	0.722572
Dendritic.cells	WAPL	-0.0705	7.165135	-0.7432	0.45933	-6.66248	0.629311	0.616034
Dendritic.cells	ZBTB7A	0.06137	7.04476	0.743078	0.459404	-6.59202	0.630742	0.618133
Dendritic.cells	TEDC1	0.326954	1.701406	0.742956	0.459478	-5.20863	0.69872	0.717274
Dendritic.cells	HIST4H4	0.355229	1.887782	0.742781	0.459583	-5.24914	0.696198	0.713622
Dendritic.cells	FADS2	-0.13981	3.530927	-0.74266	0.459655	-6.24393	0.674438	0.681758
Dendritic.cells	PCGF6	0.206887	2.512206	0.742473	0.459769	-5.43203	0.687827	0.701368
Dendritic.cells	SLC15A3	-0.12157	5.005968	-0.74243	0.459797	-6.29811	0.655632	0.654344
Dendritic.cells	EIF2B5	-0.12736	4.703803	-0.74237	0.459829	-5.93944	0.659429	0.659871
Dendritic.cells	NMT2	-0.08585	5.47269	-0.74203	0.460033	-6.24957	0.649822	0.645915
Dendritic.cells	GM44710	-0.31062	2.000891	-0.742	0.460054	-5.34091	0.694672	0.711432
Dendritic.cells	GM14326	-0.21219	2.868013	-0.74199	0.460057	-5.52979	0.683114	0.69448
Dendritic.cells	MAP3K10	-0.22045	2.731125	-0.74199	0.46006	-5.46955	0.684922	0.69713
Dendritic.cells	IGLC1	-0.62778	3.761408	-0.74195	0.460084	-5.48894	0.671455	0.677429
Dendritic.cells	GARS	-0.1088	5.511566	-0.74195	0.460084	-6.08666	0.649341	0.645216
Dendritic.cells	CCL9	0.405663	2.831707	0.74165	0.460265	-5.23746	0.683631	0.695287
Dendritic.cells	PIP5K1A	0.107748	5.595581	0.741594	0.460298	-6.13946	0.648339	0.64381
Dendritic.cells	VANGL2	0.208014	2.225601	0.741557	0.460321	-5.55202	0.691692	0.707135
Dendritic.cells	OSGIN2	-0.24856	3.18431	-0.7415	0.460355	-5.44374	0.678995	0.688529
Dendritic.cells	NUDC	-0.09209	5.698731	-0.74095	0.460688	-6.21126	0.647358	0.642244
Dendritic.cells	SAMD12	-0.52271	0.266619	-0.74095	0.460689	-5.08042	0.718879	0.746995
Dendritic.cells	CLCN4	0.101031	5.289066	0.740662	0.460861	-6.13436	0.652428	0.649708
Dendritic.cells	ACAA2	-0.20208	5.01941	-0.74053	0.460942	-5.82313	0.655794	0.654664
Dendritic.cells	SNAP23	0.082616	6.222753	0.740467	0.460979	-6.40889	0.640948	0.633124
Dendritic.cells	RASL11A	-0.45489	0.28073	-0.74045	0.460988	-5.07431	0.718681	0.746919

Dendritic.cells	RIMS3	-0.44785	1.085548	-0.7404	0.46102	-5.12938	0.707492	0.730435
Dendritic.cells	GRAMD1C	0.288446	2.440736	0.74031	0.461073	-5.34484	0.689126	0.703454
Dendritic.cells	DCLRE1C	0.059117	6.497189	0.740206	0.461136	-6.87865	0.637624	0.628394
Dendritic.cells	MORN2	-0.28975	2.045949	-0.74014	0.461175	-5.29719	0.694415	0.711316
Dendritic.cells	SLC25A5	0.081062	7.608641	0.739904	0.461318	-6.52388	0.624505	0.609374
Dendritic.cells	MFHAS1	0.091868	3.729832	0.739419	0.461611	-6.44964	0.672378	0.678972
Dendritic.cells	PIP5K1C	-0.07031	5.626433	-0.73916	0.461769	-6.60339	0.648419	0.644119
Dendritic.cells	POLR3K	-0.17188	3.885777	-0.73915	0.461774	-5.5701	0.670366	0.676099
Dendritic.cells	PBXIP1	0.138842	4.578633	0.739	0.461864	-6.00388	0.661517	0.663242
Dendritic.cells	NUDT9	-0.20681	4.362001	-0.73885	0.461955	-5.62387	0.664268	0.667317
Dendritic.cells	EML5	-0.17628	3.697759	-0.73883	0.461966	-5.86717	0.672793	0.679773
Dendritic.cells	MFN1	-0.15724	3.297935	-0.73872	0.462031	-5.69975	0.677991	0.68742
Dendritic.cells	DNAJC18	0.154103	3.682567	0.738644	0.462079	-5.77563	0.672989	0.680102
Dendritic.cells	POFUT2	-0.15134	4.257323	-0.73864	0.462083	-5.78238	0.665602	0.669308
Dendritic.cells	RNF11	-0.11357	6.207032	-0.73863	0.462089	-6.18863	0.641307	0.633947
Dendritic.cells	GPC3	-0.36882	1.806193	-0.73863	0.46209	-5.1766	0.697835	0.716542
Dendritic.cells	CRTAM	-0.54218	1.176627	-0.73862	0.462093	-5.11844	0.706425	0.72919
Dendritic.cells	CAPN5	-0.13131	1.546779	-0.73841	0.462219	-6.16639	0.701458	0.721801
Dendritic.cells	REM2	-0.44648	0.836601	-0.73823	0.462327	-5.12977	0.711229	0.736186
Dendritic.cells	TNRC6C	0.067485	7.143837	0.738076	0.462423	-6.60935	0.630145	0.617739
Dendritic.cells	RPP30	0.167351	3.635635	0.737965	0.46249	-5.60215	0.673702	0.681123
Dendritic.cells	C920021L1	-0.26347	2.268205	-0.73787	0.462546	-5.28564	0.691721	0.707544
Dendritic.cells	ADCY4	0.441526	1.413611	0.737678	0.462664	-5.21621	0.703287	0.724555
Dendritic.cells	DCTD	-0.32107	1.018621	-0.73764	0.462685	-5.20252	0.708712	0.732551
Dendritic.cells	DNAJC12	-0.33688	3.069108	-0.73764	0.462687	-5.24534	0.681095	0.691956
Dendritic.cells	SPRED2	-0.09623	6.335393	-0.73759	0.462718	-6.36871	0.639849	0.631822
Dendritic.cells	ATP5F1	0.06112	8.084387	0.737371	0.462849	-6.76034	0.619196	0.601818
Dendritic.cells	VAMP7	0.13629	4.132998	0.737129	0.462996	-5.81868	0.667519	0.671853
Dendritic.cells	ATP5O	0.086847	7.142669	0.736732	0.463236	-6.49301	0.630611	0.617972
Dendritic.cells	MLYCD	0.164582	3.124459	0.736634	0.463295	-5.66717	0.680857	0.691124
Dendritic.cells	GM26789	0.448833	0.271556	0.736508	0.463372	-5.08705	0.719637	0.748222
Dendritic.cells	MYZAP	-0.13317	3.284402	-0.73642	0.463424	-6.03813	0.67877	0.688144
Dendritic.cells	PDCD2	0.162796	3.817161	0.736012	0.463671	-5.69316	0.672061	0.678119
Dendritic.cells	GM47802	-0.44539	0.099495	-0.73569	0.463865	-5.08517	0.722291	0.75198
Dendritic.cells	PTAR1	-0.11007	3.690053	-0.73552	0.463971	-5.9621	0.673705	0.680547
Dendritic.cells	SORBS2	0.45899	1.878636	0.735318	0.464092	-5.21058	0.697697	0.715753
Dendritic.cells	LIG1	0.262121	5.051661	0.735232	0.464144	-5.67339	0.656354	0.655314
Dendritic.cells	TPM2	0.367833	0.689833	0.735225	0.464148	-5.31031	0.714016	0.739843
Dendritic.cells	AEN	-0.19018	3.664015	-0.73495	0.464314	-5.67413	0.674043	0.681131
Dendritic.cells	CDC27	0.084982	6.009717	0.734868	0.464365	-6.34282	0.64449	0.638075
Dendritic.cells	OLR1	0.645994	1.333367	0.734851	0.464375	-5.10666	0.705125	0.726745
Dendritic.cells	SOS2	0.117738	5.600384	0.734795	0.464409	-6.07824	0.649525	0.645388
Dendritic.cells	AIP	0.149571	4.946962	0.734597	0.464529	-5.84475	0.657668	0.657267
Dendritic.cells	PLCL2	0.089557	6.985417	0.734597	0.464529	-6.4613	0.632696	0.621005
Dendritic.cells	ARHGAP45	0.095851	6.710119	0.734576	0.464541	-6.37989	0.635995	0.625782
Dendritic.cells	MTMR6	0.138699	4.718718	0.734549	0.464558	-5.86913	0.660543	0.661456
Dendritic.cells	IFI209	0.182307	5.809713	0.734503	0.464586	-5.99195	0.646944	0.641668

Dendritic.cells	TRAFD1	0.185933	5.468862	0.734348	0.46468	-5.97704	0.651153	0.647799
Dendritic.cells	PLRG1	-0.13945	4.093452	-0.73434	0.464683	-5.7283	0.668504	0.673083
Dendritic.cells	TMEM129	-0.35077	2.084833	-0.73426	0.464733	-5.20176	0.694913	0.711783
Dendritic.cells	RPGR	-0.27052	1.599467	-0.73425	0.464739	-5.31102	0.701488	0.721452
Dendritic.cells	CHMP2B	0.109828	4.813502	0.733974	0.464907	-5.9582	0.659362	0.659801
Dendritic.cells	OSBPL9	0.059631	7.511715	0.733788	0.465019	-6.6404	0.626467	0.612058
Dendritic.cells	PMS2	-0.14854	3.954235	-0.73363	0.465118	-5.74647	0.670309	0.675793
Dendritic.cells	1110020A2	0.412196	0.468534	0.733541	0.465169	-5.08835	0.717121	0.744576
Dendritic.cells	STX5A	0.071126	5.871144	0.733467	0.465214	-6.34497	0.646204	0.640697
Dendritic.cells	ZFP101	0.20526	2.916235	0.73316	0.4654	-5.50302	0.683842	0.695674
Dendritic.cells	CHD1	-0.06779	6.90203	-0.73307	0.465453	-6.47607	0.633707	0.622618
Dendritic.cells	TYW3	0.321823	0.995715	0.733	0.465498	-5.23939	0.70979	0.733829
Dendritic.cells	ENDOU	-0.37765	0.913658	-0.73289	0.465565	-5.23012	0.710925	0.735509
Dendritic.cells	RXRB	0.142824	4.393184	0.732758	0.465644	-5.82655	0.664688	0.667682
Dendritic.cells	DDX56	-0.14688	3.951871	-0.73271	0.465676	-5.75454	0.670339	0.675935
Dendritic.cells	GM9856	-0.27953	1.246414	-0.73263	0.465725	-5.31204	0.706335	0.728767
Dendritic.cells	GADD45A	0.282393	4.876654	0.732594	0.465744	-5.50553	0.658567	0.658767
Dendritic.cells	COMMD4	0.092	5.326168	0.732565	0.465761	-6.24993	0.652941	0.650574
Dendritic.cells	CLCF1	0.220899	2.650892	0.732553	0.465769	-5.58797	0.687356	0.700863
Dendritic.cells	MAP3K11	0.175776	3.876975	0.732478	0.465814	-5.66377	0.671304	0.677356
Dendritic.cells	CENPN	-0.38645	2.920239	-0.73233	0.465903	-5.23399	0.683789	0.695631
Dendritic.cells	U2SURP	-0.06471	6.667671	-0.73227	0.465938	-6.45731	0.63652	0.626747
Dendritic.cells	MFSD11	-0.1638	3.854162	-0.7322	0.465985	-5.77111	0.671599	0.677842
Dendritic.cells	GM17178	-0.27514	1.460682	-0.73209	0.466053	-5.29545	0.703398	0.724517
Dendritic.cells	CLDN11	-0.46769	-0.29485	-0.73197	0.466123	-5.0785	0.727898	0.760713
Dendritic.cells	TAT	-0.41301	1.826535	-0.7319	0.466166	-5.22925	0.698419	0.717209
Dendritic.cells	REST	0.111984	5.400614	0.731835	0.466205	-6.10055	0.652015	0.649332
Dendritic.cells	ERCC3	-0.16799	3.695125	-0.73175	0.466258	-5.59153	0.673655	0.680947
Dendritic.cells	TNR	0.398691	0.036927	0.731575	0.466363	-5.07397	0.723191	0.753855
Dendritic.cells	SCLT1	-0.17729	4.474106	-0.73132	0.466518	-5.78389	0.663658	0.666398
Dendritic.cells	CENPC1	-0.09004	5.265605	-0.73115	0.466618	-6.11127	0.653695	0.651874
Dendritic.cells	DCLRE1A	-0.33611	1.621612	-0.73109	0.46666	-5.18705	0.701203	0.721436
Dendritic.cells	GM15543	-0.38632	0.901403	-0.73103	0.466692	-5.07686	0.711095	0.736019
Dendritic.cells	GIT2	0.058396	7.020155	0.73102	0.4667	-6.65474	0.632296	0.620797
Dendritic.cells	PSMD3	-0.12348	4.642778	-0.73098	0.466726	-5.91954	0.661519	0.663276
Dendritic.cells	MAP4K2	0.134293	5.348887	0.730812	0.466826	-6.05604	0.652658	0.650407
Dendritic.cells	CYC1	0.109041	6.36195	0.730767	0.466854	-6.31654	0.640214	0.632319
Dendritic.cells	9130230N(	-0.45067	0.640779	-0.73071	0.466889	-5.10368	0.714716	0.741415
Dendritic.cells	NLRP1A	-0.48348	0.449959	-0.73069	0.466899	-5.08704	0.717381	0.745354
Dendritic.cells	CCL4	0.134268	7.388292	0.730486	0.467024	-7.16542	0.628009	0.614619
Dendritic.cells	ZFP366	-0.43763	1.840557	-0.73031	0.467134	-5.27341	0.698397	0.717287
Dendritic.cells	1110038B1	0.226191	4.235292	0.729939	0.467357	-5.49572	0.666966	0.671121
Dendritic.cells	LRRC1	0.177963	3.264954	0.729886	0.467389	-5.83813	0.679526	0.689491
Dendritic.cells	NFKBIE	0.184661	4.879022	0.729883	0.467391	-5.82412	0.658797	0.659201
Dendritic.cells	DENND3	0.153616	3.548798	0.729646	0.467535	-5.90114	0.675936	0.684147
Dendritic.cells	1700020L2	0.39804	0.390371	0.729518	0.467613	-5.08309	0.718622	0.746975
Dendritic.cells	DCAF1	0.093332	5.222364	0.729444	0.467658	-6.31049	0.654605	0.65305

Dendritic.cells	MOCS3	0.28762	1.446277	0.729348	0.467717	-5.36819	0.703993	0.725394
Dendritic.cells	GPR55	-0.16537	1.548876	-0.72908	0.467879	-5.78133	0.702673	0.723388
Dendritic.cells	PAFAH1B1	-0.04724	8.064884	-0.72905	0.467897	-6.7926	0.620417	0.603417
Dendritic.cells	MCTP2	0.057868	6.80258	0.728955	0.467956	-7.10955	0.635332	0.62506
Dendritic.cells	TMEM185/	-0.20559	2.540183	-0.72848	0.468243	-5.53135	0.689592	0.703933
Dendritic.cells	NQO2	-0.177	3.942173	-0.72823	0.468399	-5.5741	0.671206	0.67702
Dendritic.cells	MALSU1	0.108689	4.843801	0.728188	0.468423	-6.1248	0.65971	0.660255
Dendritic.cells	PPIL1	-0.25122	4.052063	-0.72815	0.468449	-5.45575	0.669791	0.674968
Dendritic.cells	PPP6R3	0.06006	7.283025	0.728072	0.468493	-6.67416	0.629866	0.616907
Dendritic.cells	HPX	-0.21036	5.670685	-0.72799	0.468545	-6.06208	0.649389	0.645267
Dendritic.cells	ILDR1	-0.50666	1.791756	-0.72792	0.468584	-5.0913	0.699664	0.718838
Dendritic.cells	VOPP1	-0.19278	3.965239	-0.72775	0.468692	-5.71587	0.670967	0.676685
Dendritic.cells	OAS1A	-0.47378	2.355225	-0.72765	0.468748	-5.23635	0.692124	0.707696
Dendritic.cells	GM20275	0.222211	3.071387	0.727356	0.46893	-5.56157	0.682753	0.693824
Dendritic.cells	PIKFYVE	-0.14845	4.426045	-0.72674	0.469304	-5.80045	0.665201	0.668236
Dendritic.cells	POLH	0.199838	4.035992	0.726708	0.469324	-5.55969	0.670195	0.67553
Dendritic.cells	SDR39U1	0.313237	1.131364	0.726666	0.46935	-5.29282	0.70891	0.732368
Dendritic.cells	NOL4L	-0.38859	2.680623	-0.72665	0.469359	-5.25394	0.687924	0.701494
Dendritic.cells	RDH5	-0.29052	2.368316	-0.72665	0.469361	-5.38048	0.692092	0.707614
Dendritic.cells	ADAMTS14	-0.45517	0.543497	-0.72658	0.469403	-5.08099	0.717077	0.744425
Dendritic.cells	ZFP930	-0.24118	2.204594	-0.72641	0.469507	-5.42145	0.69429	0.710902
Dendritic.cells	USP40	-0.22062	3.64171	-0.72639	0.469516	-5.60945	0.675293	0.683041
Dendritic.cells	MRPL3	-0.14079	4.701467	-0.72623	0.469617	-5.90703	0.661703	0.663249
Dendritic.cells	BBS9	-0.20397	4.909527	-0.7261	0.469696	-5.76936	0.659076	0.659432
Dendritic.cells	UCKL1	0.173173	3.705129	0.725896	0.46982	-5.61315	0.674469	0.681987
Dendritic.cells	GM38115	0.180337	3.861728	0.725805	0.469875	-5.63147	0.672442	0.679026
Dendritic.cells	DBI	-0.11164	7.067278	-0.72577	0.469899	-6.39758	0.632619	0.621056
Dendritic.cells	NUFIP1	-0.13012	4.171093	-0.72569	0.469942	-5.80799	0.66846	0.673204
Dendritic.cells	LAPTM5	-0.05279	8.326927	-0.72569	0.469944	-6.93251	0.617824	0.599659
Dendritic.cells	GM47371	0.37346	0.907518	0.725661	0.469963	-5.21802	0.712006	0.737167
Dendritic.cells	PIBF1	0.101766	5.059147	0.725497	0.470063	-6.16003	0.657195	0.656766
Dendritic.cells	COL25A1	0.500334	0.981282	0.725473	0.470078	-5.09516	0.710984	0.735658
Dendritic.cells	CIART	-0.29906	2.328452	-0.72536	0.470149	-5.32888	0.692627	0.708619
Dendritic.cells	9330160F1	0.27771	2.295032	0.725248	0.470215	-5.30604	0.693075	0.709278
Dendritic.cells	STK10	-0.0974	7.315855	-0.72518	0.470259	-6.34752	0.629662	0.616773
Dendritic.cells	SEC23IP	-0.10193	4.499732	-0.72518	0.47026	-5.9065	0.664263	0.667074
Dendritic.cells	DIMT1	0.178833	3.243055	0.725011	0.47036	-5.59671	0.6805	0.690846
Dendritic.cells	MORC2A	-0.13253	4.295558	-0.72497	0.470385	-5.93323	0.666869	0.670906
Dendritic.cells	PTCD3	-0.12148	4.845166	-0.72475	0.470519	-5.98026	0.659907	0.66076
Dendritic.cells	TNPO1	0.066006	7.082293	0.724749	0.47052	-6.6309	0.632459	0.620862
Dendritic.cells	CAMKK2	-0.11445	5.188179	-0.72459	0.470614	-5.95989	0.655645	0.654515
Dendritic.cells	F7	-0.4166	0.719823	-0.72445	0.470702	-5.16226	0.714727	0.741163
Dendritic.cells	TMEM216	0.183284	4.069162	0.724338	0.470771	-5.71601	0.669883	0.675292
Dendritic.cells	HIRIP3	0.248441	3.169999	0.723921	0.471025	-5.40881	0.681607	0.692532
Dendritic.cells	MICALL1	0.160369	2.97359	0.723858	0.471064	-5.65631	0.684195	0.696327
Dendritic.cells	CD72	0.239589	4.334961	0.723788	0.471107	-5.61689	0.666511	0.670443
Dendritic.cells	CLCN7	0.189916	3.608444	0.72373	0.471142	-5.71952	0.675875	0.684141

Dendritic.cells	GM42699	0.434151	0.727533	0.723706	0.471157	-5.13772	0.714667	0.741198
Dendritic.cells	TRUB1	-0.26066	1.825881	-0.72369	0.471164	-5.31246	0.699562	0.718918
Dendritic.cells	GM12979	-0.41588	0.049111	-0.72334	0.471382	-5.08675	0.724435	0.755456
Dendritic.cells	SERPINE2	-0.41903	1.29875	-0.72323	0.471446	-5.11244	0.707001	0.729747
Dendritic.cells	CSF3	0.618297	-0.096	0.723046	0.47156	-5.08017	0.726505	0.758603
Dendritic.cells	SLC1A3	-0.56817	-0.29724	-0.72302	0.471575	-5.07986	0.729371	0.76285
Dendritic.cells	RAD9B	-0.18017	3.275507	-0.72268	0.471785	-5.65722	0.680606	0.690885
Dendritic.cells	CDC42	0.045609	8.943013	0.722551	0.471863	-6.86243	0.611238	0.590118
Dendritic.cells	TRAP1	-0.12331	4.381095	-0.7225	0.471894	-5.79047	0.666297	0.670019
Dendritic.cells	ROCK2	0.070868	7.468662	0.722387	0.471963	-6.61361	0.628347	0.614841
Dendritic.cells	A430073D2	-0.27794	1.815155	-0.72235	0.471983	-5.33261	0.700102	0.719625
Dendritic.cells	TMEM237	0.305086	2.157499	0.722096	0.472141	-5.30783	0.695498	0.712931
Dendritic.cells	JAGN1	0.143379	3.955321	0.722009	0.472194	-5.87376	0.671789	0.67817
Dendritic.cells	EOGT	0.234261	2.532472	0.721981	0.472211	-5.462	0.690468	0.705559
Dendritic.cells	FBXL19	0.246351	2.333257	0.721851	0.472291	-5.43345	0.693135	0.709488
Dendritic.cells	ZFP326	-0.1004	5.21559	-0.72182	0.47231	-6.06387	0.655778	0.6548
Dendritic.cells	YPEL1	-0.25356	2.4444	-0.72163	0.472426	-5.32286	0.691724	0.707358
Dendritic.cells	ESF1	-0.10304	5.001622	-0.72118	0.472699	-5.99236	0.658831	0.65893
Dendritic.cells	2410004B1	0.104646	4.71052	0.721017	0.472801	-5.95114	0.662564	0.664382
Dendritic.cells	MAPK13	0.455246	0.83712	0.720703	0.472994	-5.08635	0.714144	0.740081
Dendritic.cells	TCHP	0.224278	1.792962	0.720699	0.472996	-5.46794	0.700992	0.720682
Dendritic.cells	GM5244	-0.46474	-0.39473	-0.72052	0.473105	-5.08084	0.731606	0.765908
Dendritic.cells	GM16556	0.456304	1.676001	0.720234	0.473281	-5.09377	0.70275	0.723155
Dendritic.cells	IFNGR2	0.105781	5.782195	0.720217	0.473291	-6.41147	0.649411	0.645072
Dendritic.cells	POLRMT	-0.25404	1.91437	-0.71994	0.473459	-5.38469	0.699663	0.718438
Dendritic.cells	VBP1	-0.11389	4.92592	-0.71983	0.473526	-5.90084	0.660252	0.660718
Dendritic.cells	APTX	-0.18362	3.052742	-0.71973	0.473588	-5.44759	0.684432	0.696116
Dendritic.cells	AW554918	0.087383	5.656196	0.719331	0.473834	-6.31893	0.651371	0.647582
Dendritic.cells	SRPK1	-0.06835	6.11152	-0.71908	0.473986	-6.35021	0.64576	0.639474
Dendritic.cells	1110002L0	0.216795	2.8153	0.719075	0.473991	-5.49957	0.687838	0.70092
Dendritic.cells	F2RL1	0.395662	-1.13295	0.718641	0.474257	-5.08154	0.742826	0.782321
Dendritic.cells	KIF2C	0.382325	2.663513	0.718513	0.474335	-5.24973	0.689858	0.704083
Dendritic.cells	TPM3-RS7	-0.35189	0.526188	-0.7185	0.474345	-5.15658	0.719098	0.747176
Dendritic.cells	EIF2S3X	-0.16402	5.323448	-0.71848	0.474353	-6.10634	0.655512	0.653842
Dendritic.cells	HIST1H2AN	-0.54081	3.254826	-0.7184	0.474404	-5.32756	0.682029	0.692595
Dendritic.cells	LEKR1	0.421755	1.047991	0.718367	0.474425	-5.16124	0.711822	0.736425
Dendritic.cells	PPP2R5A	-0.05769	7.861178	-0.71824	0.474503	-6.68743	0.624785	0.609276
Dendritic.cells	FARP1	-0.16866	3.619271	-0.71814	0.474562	-5.82428	0.677259	0.685657
Dendritic.cells	ARMC8	-0.10967	5.019279	-0.71802	0.47464	-5.89964	0.659327	0.659485
Dendritic.cells	SSR2	0.102243	5.189958	0.717815	0.474764	-6.18262	0.657182	0.656422
Dendritic.cells	SRFBP1	-0.1885	3.231687	-0.7178	0.47477	-5.60939	0.682334	0.693194
Dendritic.cells	UBQLN4	0.17021	3.216072	0.717803	0.474771	-5.57033	0.682539	0.693495
Dendritic.cells	TEN1	-0.07797	5.852604	-0.71774	0.474809	-6.31782	0.648943	0.644427
Dendritic.cells	TNRC6B	0.071862	8.234075	0.717729	0.474816	-6.76513	0.620433	0.603092
Dendritic.cells	F930017D2	0.550145	0.898224	0.717529	0.474939	-5.1102	0.713993	0.739703
Dendritic.cells	FAM76A	-0.0914	5.119697	-0.7174	0.475018	-6.18328	0.658172	0.65777
Dendritic.cells	2900093K2	-0.20766	2.884005	-0.71716	0.475167	-5.55745	0.687091	0.699999

Dendritic.cells	PI4KA	-0.07729	6.333121	-0.71714	0.475177	-6.49206	0.643207	0.63592
Dendritic.cells	SNRNP200	0.130071	5.038857	0.716936	0.475304	-5.96804	0.659256	0.659298
Dendritic.cells	CAMTA1	0.085257	5.835211	0.71692	0.475313	-6.25059	0.64933	0.644842
Dendritic.cells	IRF1	0.142647	7.149071	0.716651	0.475478	-6.72953	0.633442	0.621731
Dendritic.cells	MBOAT2	0.454587	0.666194	0.716559	0.475535	-5.21507	0.717399	0.744646
Dendritic.cells	TYW5	0.211117	3.139549	0.716502	0.47557	-5.5406	0.683797	0.695164
Dendritic.cells	CCDC167	0.168614	4.065969	0.716439	0.475608	-5.72497	0.671716	0.677482
Dendritic.cells	GM35867	0.421412	0.456051	0.716318	0.475683	-5.15461	0.720346	0.749036
Dendritic.cells	RHEBL1	-0.35669	1.532009	-0.71624	0.47573	-5.18628	0.705411	0.727003
Dendritic.cells	KCTD14	0.247877	1.055743	0.715964	0.4759	-5.44607	0.712093	0.736855
Dendritic.cells	TRP53	-0.1034	5.681235	-0.71591	0.475933	-6.10016	0.651407	0.647902
Dendritic.cells	CNOT2	0.057902	7.008824	0.71569	0.476069	-6.61177	0.635226	0.624433
Dendritic.cells	SPPL2A	0.106013	6.600738	0.715422	0.476233	-6.44541	0.640143	0.631698
Dendritic.cells	WDR74	0.124264	4.042303	0.715377	0.476261	-5.78685	0.672133	0.678331
Dendritic.cells	WTIP	-0.32146	1.051984	-0.71535	0.47628	-5.2061	0.712145	0.737158
Dendritic.cells	PCYOX1	0.159337	4.579765	0.715331	0.476289	-5.74856	0.665244	0.668259
Dendritic.cells	FCGR4	-0.5546	3.388468	-0.71531	0.476303	-5.31703	0.680637	0.690788
Dendritic.cells	MMP14	-0.28958	3.837765	-0.71451	0.476793	-5.8298	0.675386	0.682395
Dendritic.cells	BMP1	0.417478	0.911089	0.714275	0.476939	-5.16184	0.714796	0.740313
Dendritic.cells	LSAMP	0.416541	0.929492	0.7142	0.476985	-5.1385	0.71454	0.739987
Dendritic.cells	ELF2	0.065786	7.39491	0.714155	0.477013	-6.65888	0.631233	0.618149
Dendritic.cells	IL15	0.397254	3.93993	0.713985	0.477117	-5.37695	0.674165	0.680581
Dendritic.cells	CLK3	-0.1051	4.807812	-0.71385	0.477198	-6.03832	0.663045	0.664346
Dendritic.cells	ATG4B	0.118004	4.800676	0.713792	0.477236	-5.97542	0.663136	0.664499
Dendritic.cells	LIN7C	-0.1015	4.956273	-0.71337	0.477496	-6.07086	0.661318	0.661739
Dendritic.cells	IL20RB	-0.21044	3.81612	-0.71322	0.477586	-5.5528	0.675924	0.683104
Dendritic.cells	HNRNPUL2	0.064187	6.468104	0.71322	0.477588	-6.45681	0.642574	0.634489
Dendritic.cells	GLS2	-0.38911	1.618045	-0.71321	0.477592	-5.21411	0.705252	0.726173
Dendritic.cells	PLEK	0.093356	7.520494	0.713066	0.477683	-6.82151	0.629976	0.616232
Dendritic.cells	PRUNE1	0.166573	3.600837	0.712763	0.47787	-5.66525	0.678874	0.687379
Dendritic.cells	PLCG1	-0.14357	3.409844	-0.71258	0.477982	-5.82006	0.681375	0.691066
Dendritic.cells	EPM2AIP1	0.209208	2.609784	0.712459	0.478057	-5.50992	0.691976	0.70662
Dendritic.cells	SLPI	0.305244	4.206306	0.712458	0.478057	-5.77555	0.671024	0.675916
Dendritic.cells	STYK1	-0.36134	0.538888	-0.71244	0.478065	-5.20264	0.720378	0.748485
Dendritic.cells	PLA2G12A	0.179081	4.277535	0.712096	0.47828	-5.66392	0.670214	0.674577
Dendritic.cells	SPG20	0.174197	2.615271	0.711745	0.478497	-5.6197	0.692013	0.706608
Dendritic.cells	RFFL	0.122863	5.915131	0.711495	0.478651	-6.20607	0.649591	0.644621
Dendritic.cells	MTMR4	-0.22414	2.648419	-0.71147	0.478664	-5.38901	0.69157	0.705979
Dendritic.cells	SF3B4	0.107719	5.494923	0.71147	0.478666	-6.13437	0.654803	0.652207
Dendritic.cells	GM10603	-0.43244	0.220408	-0.71146	0.478673	-5.10214	0.724986	0.755258
Dendritic.cells	SLC35A2	0.200893	3.165869	0.711452	0.478677	-5.5789	0.684695	0.695889
Dendritic.cells	EEF1G	0.071957	7.187877	0.711388	0.478716	-6.61736	0.634132	0.622205
Dendritic.cells	DNAJC21	0.087018	5.890572	0.711367	0.47873	-6.34063	0.649894	0.645091
Dendritic.cells	AKAP8L	0.127255	4.8709	0.711268	0.478791	-6.01137	0.662645	0.663685
Dendritic.cells	HNRNPF	-0.049	9.158537	-0.71122	0.478821	-6.96607	0.611155	0.589022
Dendritic.cells	STRIP2	-0.40741	1.016356	-0.71108	0.478906	-5.16693	0.713853	0.738854
Dendritic.cells	PSMD7	0.102322	5.828246	0.710589	0.479209	-6.21669	0.650763	0.646488

Dendritic.cells	MPHOSPH(	0.150208	3.799303	0.710588	0.47921	-5.66851	0.676499	0.684054
Dendritic.cells	RRAGA	0.11257	5.020687	0.710583	0.479213	-6.04321	0.660853	0.661188
Dendritic.cells	2810013P(	0.191244	4.123121	0.710551	0.479233	-5.748	0.672305	0.677921
Dendritic.cells	CFAP53	-0.44463	0.830789	-0.71044	0.4793	-5.14882	0.716514	0.742988
Dendritic.cells	R3HDM4	0.067996	6.20323	0.710398	0.479327	-6.66914	0.646147	0.63984
Dendritic.cells	OLFM1	0.281235	2.469141	0.710085	0.47952	-5.44623	0.694268	0.709996
Dendritic.cells	WDR53	0.243435	2.715022	0.7095	0.479881	-5.40357	0.691122	0.705339
Dendritic.cells	PDCL	0.171207	3.891934	0.709458	0.479907	-5.6984	0.675625	0.682615
Dendritic.cells	RRM2B	0.14501	4.437435	0.709455	0.479909	-5.79456	0.668591	0.672329
Dendritic.cells	AUP1	0.086381	6.010505	0.70945	0.479912	-6.30318	0.64883	0.64352
Dendritic.cells	PTRH2	-0.15818	4.181735	-0.70936	0.47997	-5.70546	0.671876	0.677131
Dendritic.cells	MRPS36	0.122098	5.569029	0.709238	0.480043	-6.03745	0.654298	0.651522
Dendritic.cells	DUSP28	0.225886	1.99306	0.709121	0.480115	-5.44192	0.700848	0.719745
Dendritic.cells	UBE2E3	-0.06515	7.066891	-0.70912	0.480117	-6.49437	0.635987	0.624957
Dendritic.cells	RERE	0.065276	7.702335	0.708878	0.480265	-6.72312	0.628538	0.614041
Dendritic.cells	DCAF13	0.112512	4.771338	0.70845	0.48053	-5.95428	0.664733	0.666354
Dendritic.cells	CD36	-0.20339	5.712063	-0.70823	0.480667	-6.16869	0.653016	0.64922
Dendritic.cells	RPF2	0.144383	4.402771	0.707928	0.480852	-5.9064	0.669631	0.673385
Dendritic.cells	CENPK	-0.28007	3.283005	-0.70793	0.480854	-5.41407	0.684197	0.694702
Dendritic.cells	ZFP318	-0.25533	3.491008	-0.70734	0.481217	-5.52535	0.681863	0.690934
Dendritic.cells	NVL	-0.09912	4.723925	-0.70721	0.481297	-6.05706	0.665919	0.667679
Dendritic.cells	NAA25	0.15057	3.927641	0.707148	0.481334	-5.88555	0.676161	0.682644
Dendritic.cells	PTBP3	0.05448	8.46802	0.707029	0.481408	-6.76131	0.620389	0.601639
Dendritic.cells	MED16	-0.08886	4.12299	-0.70693	0.48147	-6.16744	0.67363	0.679
Dendritic.cells	BANP	0.11256	4.601582	0.706752	0.481579	-5.93887	0.66748	0.670021
Dendritic.cells	ELK1	0.242884	1.559348	0.706735	0.48159	-5.28171	0.707819	0.729198
Dendritic.cells	RABGGTB	0.174284	3.732854	0.706512	0.481728	-5.60542	0.678697	0.686499
Dendritic.cells	FAM160B1	-0.10563	4.443367	-0.70637	0.481818	-6.07634	0.669505	0.673056
Dendritic.cells	OLFML3	-0.38491	1.284348	-0.7063	0.481857	-5.10488	0.711613	0.734877
Dendritic.cells	ELP6	0.265839	2.398694	0.706251	0.481889	-5.3561	0.696392	0.71246
Dendritic.cells	MIR22HG	-0.15585	4.089128	-0.7061	0.481984	-5.94629	0.674068	0.679725
Dendritic.cells	LRP1	0.219651	4.178873	0.706081	0.481994	-5.64315	0.672908	0.67803
Dendritic.cells	UBE2E1	0.059398	6.229498	0.706065	0.482004	-6.45844	0.647096	0.640406
Dendritic.cells	RSU1	0.075682	5.746299	0.70578	0.48218	-6.36035	0.65312	0.649142
Dendritic.cells	DAPK1	-0.19241	4.932787	-0.70565	0.482264	-5.92045	0.663326	0.664048
Dendritic.cells	RABL3	0.244244	2.218563	0.705623	0.482277	-5.37226	0.698889	0.716146
Dendritic.cells	NOP58	-0.11512	5.838554	-0.70559	0.482295	-6.22315	0.651976	0.647512
Dendritic.cells	REEP3	0.084612	6.727725	0.705173	0.482556	-6.43847	0.641165	0.631803
Dendritic.cells	CEBPB	-0.16857	9.155958	-0.70513	0.482585	-6.81422	0.612618	0.590519
Dendritic.cells	SRP72	-0.05962	6.657086	-0.7051	0.482604	-6.56442	0.642022	0.633047
Dendritic.cells	ASB4	-0.35866	1.68448	-0.70509	0.482607	-5.52214	0.706259	0.727001
Dendritic.cells	DLEU2	0.091681	8.40867	0.704885	0.482735	-6.64612	0.621262	0.602958
Dendritic.cells	WDR5B	0.341353	0.670974	0.704701	0.482848	-5.23854	0.720378	0.747806
Dendritic.cells	GM43936	-0.40602	-1.34803	-0.7047	0.482852	-5.08556	0.749409	0.790883
Dendritic.cells	FUS	-0.0521	7.820328	-0.70463	0.48289	-6.72715	0.628149	0.612911
Dendritic.cells	RTTN	-0.21627	3.609694	-0.70447	0.482991	-5.5328	0.68056	0.689133
Dendritic.cells	AGBL5	0.212847	2.244561	0.704266	0.483118	-5.41882	0.698734	0.715841

Dendritic.cells	SMARCA4	-0.07899	7.5105	-0.70427	0.483118	-6.61512	0.631864	0.618248
Dendritic.cells	STX3	-0.36272	2.314188	-0.70418	0.483174	-5.24434	0.697792	0.71446
Dendritic.cells	MGAT1	0.09317	4.704416	0.703923	0.483331	-6.25568	0.66652	0.668594
Dendritic.cells	ASXL1	-0.0721	7.242343	-0.70385	0.483374	-6.59519	0.635161	0.622996
Dendritic.cells	SLC35B2	-0.13922	4.882082	-0.70361	0.483525	-5.87533	0.664382	0.665402
Dendritic.cells	GM17494	-0.19403	2.389013	-0.70336	0.483679	-5.42518	0.697152	0.713218
Dendritic.cells	RET	0.41071	0.727544	0.702952	0.483932	-5.09583	0.720212	0.74706
Dendritic.cells	IRS1	-0.41936	0.821216	-0.70279	0.484031	-5.09759	0.718898	0.745199
Dendritic.cells	UBR3	-0.07069	6.575859	-0.70277	0.484044	-6.57066	0.643618	0.634953
Dendritic.cells	AA388235	-0.42985	0.991788	-0.70275	0.484056	-5.15174	0.716514	0.741685
Dendritic.cells	TRIM41	0.125963	4.185669	0.702631	0.484132	-5.79595	0.673628	0.678679
Dendritic.cells	PMVK	-0.10457	4.327226	-0.70247	0.484229	-6.13781	0.67183	0.67601
Dendritic.cells	GM17276	0.42633	0.785789	0.702398	0.484276	-5.14816	0.719446	0.746009
Dendritic.cells	TOM1L2	-0.0927	5.471299	-0.702	0.484521	-6.39101	0.657519	0.654969
Dendritic.cells	PACS2	-0.11256	4.036916	-0.70194	0.484559	-5.95727	0.675804	0.681652
Dendritic.cells	TSNAX	-0.11481	4.49606	-0.70145	0.484865	-6.00232	0.670063	0.673085
Dendritic.cells	GPIHBP1	-0.13255	4.095185	-0.70142	0.484884	-6.20314	0.675232	0.680638
Dendritic.cells	RB1CC1	0.083126	6.500368	0.70123	0.485001	-6.4388	0.644971	0.636587
Dendritic.cells	ACSF3	-0.30533	1.59243	-0.70115	0.485052	-5.20985	0.708668	0.729749
Dendritic.cells	SRF	0.165107	2.927184	0.701123	0.485067	-5.49974	0.690585	0.703159
Dendritic.cells	FAM214A	0.071685	5.246692	0.701076	0.485097	-6.76189	0.660519	0.659198
Dendritic.cells	CXCL10	-0.29949	4.788598	-0.70103	0.485125	-6.01317	0.666322	0.667659
Dendritic.cells	AQP11	-0.40576	0.397356	-0.70061	0.485386	-5.11882	0.725649	0.754466
Dendritic.cells	GM42047	0.156566	5.263713	0.700238	0.485617	-6.38608	0.66075	0.659041
Dendritic.cells	UBE2T	0.245232	4.06115	0.70007	0.485722	-5.55799	0.676129	0.681487
Dendritic.cells	TCEA1	0.056171	7.486226	0.700031	0.485746	-6.68538	0.633509	0.619509
Dendritic.cells	GZMA	0.599364	4.671597	0.700004	0.485763	-5.75354	0.668265	0.670005
Dendritic.cells	RSF1	0.063783	7.014648	0.699633	0.485993	-6.64841	0.639373	0.627788
Dendritic.cells	9930104L0	0.253739	1.356879	0.699548	0.486046	-5.35694	0.712634	0.734878
Dendritic.cells	TMEM260	-0.13801	3.273577	-0.69939	0.486146	-5.90633	0.686727	0.696748
Dendritic.cells	PIK3AP1	0.096373	8.295612	0.698863	0.486472	-6.87327	0.624505	0.605986
Dendritic.cells	PPM1H	-0.06793	6.473739	-0.69869	0.486581	-7.03634	0.646295	0.63755
Dendritic.cells	TELO2	-0.31114	2.455163	-0.69853	0.486682	-5.33521	0.697993	0.713023
Dendritic.cells	SZRD1	-0.07322	5.906997	-0.69845	0.486727	-6.26447	0.653279	0.647728
Dendritic.cells	MAB21L3	0.495736	-0.30498	0.698377	0.486775	-5.09549	0.736516	0.769876
Dendritic.cells	STIM1	0.115581	7.712751	0.698029	0.486991	-6.49791	0.631368	0.616017
Dendritic.cells	KLRA7	-0.67582	0.646446	-0.69803	0.486993	-5.11909	0.722954	0.749859
Dendritic.cells	BCL7B	0.085904	5.531179	0.697749	0.487165	-6.23006	0.657965	0.654588
Dendritic.cells	1700048O2	0.512105	-0.24632	0.697484	0.48733	-5.09152	0.735671	0.768734
Dendritic.cells	ZFYVE19	0.160367	3.132938	0.697481	0.487332	-5.65927	0.688914	0.699831
Dendritic.cells	CYP2D9	-0.36596	0.530177	-0.69721	0.487498	-5.15062	0.724595	0.752431
Dendritic.cells	LRP10	0.079943	6.125795	0.697178	0.48752	-6.40943	0.650571	0.643975
Dendritic.cells	EEF2	0.068093	9.105144	0.697159	0.487532	-6.94684	0.615141	0.59273
Dendritic.cells	NME4	-0.33747	2.364464	-0.69713	0.487553	-5.30422	0.699219	0.715047
Dendritic.cells	CD59A	-0.17522	2.984691	-0.69699	0.487639	-5.7479	0.690887	0.702848
Dendritic.cells	CABLES2	0.183381	3.580958	0.696978	0.487645	-5.53766	0.682993	0.691283
Dendritic.cells	SNX12	0.093333	4.718214	0.696914	0.487685	-6.03861	0.668252	0.669746

Dendritic.cells	BRD9	-0.10546	5.185663	-0.69691	0.487686	-6.02845	0.662312	0.661087
Dendritic.cells	HIST1H4H	0.39124	0.513275	0.696786	0.487765	-5.09668	0.724834	0.752887
Dendritic.cells	SS18L1	0.306377	2.325233	0.696698	0.487819	-5.31214	0.69975	0.715954
Dendritic.cells	UBP1	-0.09261	5.448392	-0.69665	0.487848	-6.16169	0.659003	0.656353
Dendritic.cells	R74862	-0.43804	1.054776	-0.69661	0.487877	-5.12086	0.717225	0.74169
Dendritic.cells	CBX7	-0.20357	2.651462	-0.69656	0.487907	-5.41795	0.695348	0.709501
Dendritic.cells	BUB1	-0.34175	3.743439	-0.69646	0.48797	-5.44977	0.680862	0.688305
Dendritic.cells	CHMP5	-0.08998	5.632148	-0.69643	0.487988	-6.15473	0.656702	0.653051
Dendritic.cells	HLCS	0.176088	4.15558	0.696427	0.487988	-5.78025	0.675494	0.680455
Dendritic.cells	POLR2H	0.129068	4.512761	0.696306	0.488064	-5.81524	0.670903	0.673742
Dendritic.cells	BIRC5	-0.30972	5.35755	-0.69594	0.488294	-5.81293	0.66039	0.658151
Dendritic.cells	ENC1	0.186601	3.181392	0.695541	0.488541	-5.61622	0.688659	0.699297
Dendritic.cells	CMC2	-0.24887	4.535117	-0.69549	0.488572	-5.64643	0.670977	0.673441
Dendritic.cells	RASSF3	0.110193	6.834864	0.695341	0.488665	-6.38911	0.642259	0.631675
Dendritic.cells	UHRF2	0.074196	6.43989	0.69534	0.488666	-6.34309	0.647075	0.638659
Dendritic.cells	AGO4	-0.13122	3.167589	-0.69524	0.488731	-5.98205	0.688843	0.699605
Dendritic.cells	ZNRD1AS	0.397612	0.28937	0.695104	0.488813	-5.10807	0.72842	0.757919
Dendritic.cells	MFSD6	0.186928	4.864614	0.694995	0.488881	-5.87871	0.666761	0.667368
Dendritic.cells	WDR38	-0.29293	0.617809	-0.69499	0.488886	-5.21931	0.723767	0.751039
Dendritic.cells	SLC39A9	-0.10489	4.172579	-0.69483	0.488985	-5.88358	0.675706	0.68039
Dendritic.cells	P2RY10	-0.10771	3.930309	-0.69381	0.489617	-6.48551	0.679648	0.685397
Dendritic.cells	TAPBPL	-0.16618	4.125165	-0.69367	0.48971	-5.76903	0.677152	0.681756
Dendritic.cells	REX1BD	0.089085	5.688469	0.693448	0.489846	-6.19091	0.657324	0.65275
Dendritic.cells	ARHGAP27	0.266935	0.465307	0.693347	0.48991	-5.31877	0.726981	0.75483
Dendritic.cells	ANKRD10	-0.07237	5.371393	-0.69318	0.490016	-6.36372	0.661366	0.658624
Dendritic.cells	MBOAT7	-0.1427	4.278521	-0.69304	0.490104	-5.79687	0.675332	0.679026
Dendritic.cells	BBC3	0.203492	2.639565	0.692971	0.490144	-5.49279	0.696983	0.710736
Dendritic.cells	ADCY9	0.244272	3.136828	0.692725	0.490298	-5.49796	0.690418	0.701088
Dendritic.cells	DMXL1	-0.07357	6.604824	-0.69266	0.490341	-6.6809	0.64615	0.636589
Dendritic.cells	PHF20L1	0.069459	7.046788	0.692377	0.490515	-6.58972	0.640778	0.628908
Dendritic.cells	OLFM4	0.516105	-0.01496	0.692261	0.490588	-5.1007	0.734004	0.765444
Dendritic.cells	CDC16	0.12215	3.893321	0.692258	0.490589	-5.89587	0.680437	0.686639
Dendritic.cells	FIGNL1	0.327887	2.757262	0.692148	0.490658	-5.2892	0.695496	0.708732
Dendritic.cells	RNF14	0.081895	5.242687	0.692142	0.490662	-6.20011	0.663087	0.661363
Dendritic.cells	EZH1	0.167781	3.600438	0.692065	0.49071	-5.73523	0.68428	0.692336
Dendritic.cells	HES7	0.380587	-0.49621	0.690872	0.491456	-5.09595	0.74198	0.776072
Dendritic.cells	PPFIBP1	0.134327	3.366596	0.690664	0.491586	-5.87943	0.68842	0.697128
Dendritic.cells	GIMAP5	0.263955	3.319876	0.690141	0.491913	-5.46776	0.689411	0.698221
Dendritic.cells	MEF2D	0.064786	7.288749	0.689945	0.492036	-6.72076	0.63925	0.625246
Dendritic.cells	ULK1	0.208593	3.637915	0.689822	0.492113	-5.4861	0.685275	0.692163
Dendritic.cells	BFAR	0.082535	4.85458	0.689758	0.492153	-6.15357	0.669473	0.669128
Dendritic.cells	TRRAP	-0.11817	4.784536	-0.68935	0.492406	-5.98944	0.67063	0.67058
Dendritic.cells	EIF3G	0.114829	4.982954	0.688827	0.492736	-5.9921	0.668428	0.667122
Dendritic.cells	NAIP2	0.241374	3.006951	0.688634	0.492856	-5.48169	0.694276	0.704926
Dendritic.cells	GIMAP3	-0.50133	2.932139	-0.68849	0.492945	-5.33411	0.695279	0.706442
Dendritic.cells	UNKL	-0.15695	3.970057	-0.68817	0.493149	-5.79689	0.681522	0.686406
Dendritic.cells	LETMD1	0.176328	2.91201	0.688162	0.493153	-5.59059	0.695549	0.706916

Dendritic.cells	TRAC	0.381308	2.266621	0.688059	0.493217	-5.3105	0.704284	0.71972
Dendritic.cells	HIST1H4C	0.415054	0.309171	0.688023	0.493239	-5.09716	0.731611	0.759952
Dendritic.cells	F730043M	0.407125	0.260543	0.687982	0.493265	-5.16583	0.732306	0.760978
Dendritic.cells	TADA2B	-0.17259	3.468615	-0.68797	0.493273	-5.65371	0.688125	0.696052
Dendritic.cells	TACO1	0.253782	3.738306	0.687961	0.493278	-5.59138	0.684563	0.690848
Dendritic.cells	ITGAD	0.163629	0.789813	0.687707	0.493438	-5.95591	0.724845	0.749876
Dendritic.cells	SPEN	-0.0854	5.922479	-0.68769	0.493445	-6.24462	0.656628	0.650094
Dendritic.cells	2310061I0	0.152423	3.521012	0.687407	0.493625	-5.66801	0.687535	0.695133
Dendritic.cells	ABCF3	-0.14245	3.761251	-0.6873	0.493692	-5.71845	0.684365	0.690502
Dendritic.cells	PPP4R1	-0.10987	5.579566	-0.68712	0.493803	-6.0934	0.660968	0.65642
Dendritic.cells	PRPSAP2	-0.14113	4.045546	-0.68708	0.493833	-5.75686	0.680638	0.685059
Dendritic.cells	CNOT6	0.064966	5.947416	0.687041	0.493855	-6.33412	0.656361	0.649732
Dendritic.cells	GABARAPL	-0.1303	3.974288	-0.68703	0.49386	-5.94606	0.681569	0.686419
Dendritic.cells	GCC2	0.107009	5.193926	0.686945	0.493915	-6.10342	0.665843	0.663538
Dendritic.cells	PDCD4	-0.07207	7.282332	-0.68674	0.494043	-6.67166	0.640078	0.626083
Dendritic.cells	JMJD1C	-0.07015	8.048596	-0.68641	0.49425	-6.82957	0.631007	0.612931
Dendritic.cells	ZFP512	0.189136	3.916117	0.686407	0.494252	-5.58256	0.682506	0.687657
Dendritic.cells	PGD	-0.14508	5.716274	-0.68624	0.494358	-5.83632	0.65942	0.654078
Dendritic.cells	AAMDC	0.145418	3.299635	0.686102	0.494444	-5.60906	0.69065	0.699643
Dendritic.cells	VMA21	0.095243	5.176423	0.686098	0.494447	-6.16497	0.666236	0.664033
Dendritic.cells	D5ERTD57	0.094386	5.221315	0.685754	0.494662	-6.21252	0.665665	0.663243
Dendritic.cells	ZFP61	-0.33339	1.174198	-0.68573	0.494676	-5.25307	0.719672	0.742285
Dendritic.cells	GM32569	-0.43266	1.845691	-0.68568	0.49471	-5.23493	0.710343	0.728572
Dendritic.cells	4933433G1	-0.28991	1.434512	-0.68568	0.494712	-5.31934	0.716038	0.736947
Dendritic.cells	FAM217B	0.283066	0.616789	0.685648	0.494729	-5.36368	0.727529	0.75388
Dendritic.cells	SHE	-0.31378	0.946591	-0.68549	0.49483	-5.37219	0.722924	0.747049
Dendritic.cells	NUDT8	0.220466	2.208161	0.68513	0.495054	-5.47351	0.705459	0.721307
Dendritic.cells	GM19705	-0.23934	1.868138	-0.68506	0.4951	-5.58898	0.710125	0.72817
Dendritic.cells	AKT1S1	-0.16044	3.589237	-0.68495	0.495167	-5.63572	0.686897	0.694132
Dendritic.cells	FBXL17	-0.0573	7.560342	-0.68468	0.49534	-6.84476	0.636899	0.621584
Dendritic.cells	KATNB1	-0.28941	2.346793	-0.6846	0.495386	-5.30195	0.703568	0.718659
Dendritic.cells	PDE8A	-0.19328	5.847934	-0.68456	0.495409	-5.87472	0.657855	0.651936
Dendritic.cells	GCC1	-0.23647	2.459454	-0.68456	0.495413	-5.46314	0.702036	0.716411
Dendritic.cells	PDGFB	-0.48331	1.062654	-0.68453	0.495431	-5.12745	0.721328	0.744775
Dendritic.cells	STK32C	0.389251	0.553606	0.684378	0.495526	-5.10159	0.728519	0.75541
Dendritic.cells	GM11944	-0.16773	4.498486	-0.68432	0.495564	-5.8987	0.675011	0.67693
Dendritic.cells	CPED1	-0.37439	2.524781	-0.68429	0.495582	-5.27205	0.701149	0.715163
Dendritic.cells	HNRNPAB	-0.06634	8.05587	-0.68421	0.495634	-6.76579	0.631001	0.613118
Dendritic.cells	IRF5	0.093409	5.351662	0.684154	0.495667	-6.4314	0.664098	0.661069
Dendritic.cells	SGIP1	-0.40223	0.175941	-0.68398	0.495777	-5.10545	0.733978	0.763457
Dendritic.cells	LIMK2	-0.12423	4.779797	-0.68388	0.495838	-5.93866	0.67145	0.671755
Dendritic.cells	RCHY1	0.088275	5.661194	0.683655	0.495981	-6.18981	0.66026	0.655536
Dendritic.cells	IAH1	0.154547	4.521488	0.683511	0.496071	-5.82753	0.674781	0.676682
Dendritic.cells	MRGPRA2E	0.467235	-0.43033	0.683486	0.496087	-5.10322	0.742736	0.776546
Dendritic.cells	ARHGEF39	-0.39172	1.960521	-0.68347	0.4961	-5.2703	0.708923	0.726657
Dendritic.cells	SLC29A3	0.075044	3.68183	0.683137	0.496307	-6.42707	0.685742	0.692686
Dendritic.cells	PBRM1	0.053351	7.416728	0.683126	0.496313	-6.64625	0.638685	0.624283

Dendritic.cells	PROSER1	-0.14774	4.213066	-0.68304	0.496368	-5.79364	0.678781	0.68252
Dendritic.cells	GM16152	0.20573	2.101073	0.682921	0.496443	-5.67408	0.706994	0.723827
Dendritic.cells	RAB12	0.087091	5.073594	0.682899	0.496456	-6.15279	0.667695	0.666364
Dendritic.cells	ATRX	0.058826	7.666179	0.682861	0.49648	-6.73197	0.635696	0.619964
Dendritic.cells	3110009E1	0.241674	2.175408	0.682726	0.496565	-5.33222	0.705977	0.722332
Dendritic.cells	SRCAP	-0.06083	6.652894	-0.68255	0.496679	-6.51766	0.647956	0.637776
Dendritic.cells	ZFP106	0.069283	6.288522	0.682517	0.496696	-6.58729	0.652441	0.644289
Dendritic.cells	B230118H(	-0.20781	2.933488	-0.68241	0.496761	-5.55493	0.695702	0.70739
Dendritic.cells	NR1I3	-0.40425	1.055611	-0.68239	0.496779	-5.177	0.721498	0.745304
Dendritic.cells	CCDC28B	-0.16955	3.504556	-0.68217	0.496915	-5.71534	0.688188	0.696264
Dendritic.cells	ZFP472	-0.12448	3.448072	-0.68089	0.497718	-5.94685	0.689888	0.697655
Dendritic.cells	LATS1	-0.09661	4.725566	-0.68088	0.497727	-6.05348	0.67318	0.673284
Dendritic.cells	GMEB2	0.078717	5.764218	0.680547	0.497937	-6.28619	0.66009	0.654094
Dendritic.cells	6530413G1	0.338575	-0.08741	0.680545	0.497938	-5.09983	0.739026	0.769692
Dendritic.cells	PTK6	-0.48082	-0.3416	-0.67988	0.498359	-5.12564	0.743107	0.775396
Dendritic.cells	LRRC4	0.122733	3.774431	0.679779	0.498421	-6.06887	0.686054	0.691577
Dendritic.cells	NGRN	0.199237	3.173648	0.679756	0.498435	-5.564	0.694036	0.703237
Dendritic.cells	GDE1	0.197069	4.883129	0.679676	0.498485	-5.78197	0.671629	0.670598
Dendritic.cells	CD84	-0.24988	5.366126	-0.67963	0.498517	-5.53229	0.665467	0.66166
Dendritic.cells	SLC35B1	0.077605	5.751128	0.67936	0.498685	-6.46111	0.660704	0.654632
Dendritic.cells	VIL1	-0.45293	0.018277	-0.67931	0.498715	-5.10375	0.738002	0.767799
Dendritic.cells	2310009A(	0.117768	4.692253	0.679157	0.498812	-5.9819	0.674219	0.674272
Dendritic.cells	GM33782	0.381309	0.19699	0.67907	0.498867	-5.10351	0.73547	0.764052
Dendritic.cells	CCNA2	-0.2953	5.520186	-0.67891	0.498966	-5.82121	0.663669	0.658988
Dendritic.cells	GM20492	0.260446	1.798274	0.678848	0.499007	-5.3069	0.712913	0.730929
Dendritic.cells	DRAP1	0.064181	6.508223	0.678621	0.499151	-6.45486	0.651355	0.641273
Dendritic.cells	9830107B1	0.365255	-0.62676	0.6785	0.499227	-5.10518	0.747461	0.782033
Dendritic.cells	NOMO1	-0.13492	3.5373	-0.67847	0.499244	-5.82943	0.689369	0.696584
Dendritic.cells	MAPRE2	-0.07543	6.802434	-0.67822	0.499401	-6.54538	0.64774	0.636116
Dendritic.cells	RNMT	0.104043	4.970618	0.677994	0.499546	-6.04371	0.67068	0.669399
Dendritic.cells	TUFT1	-0.2243	2.2113	-0.67792	0.499595	-5.35747	0.707248	0.722823
Dendritic.cells	IFI207	-0.13004	4.721848	-0.67789	0.499612	-6.52769	0.673877	0.674051
Dendritic.cells	GSTM7	-0.28807	1.02465	-0.67788	0.499618	-5.21108	0.723736	0.747062
Dendritic.cells	MED24	0.182631	3.187102	0.67775	0.4997	-5.52076	0.694035	0.703502
Dendritic.cells	IKZF4	0.351357	-0.0706	0.677629	0.499776	-5.10267	0.739369	0.770167
Dendritic.cells	DOCK5	0.312023	4.189727	0.677557	0.499822	-5.37184	0.680781	0.68416
Dendritic.cells	MRTO4	-0.16155	4.665156	-0.67755	0.499827	-5.848	0.674608	0.675165
Dendritic.cells	SMUG1	-0.21475	1.976529	-0.67753	0.499836	-5.39302	0.710473	0.727611
Dendritic.cells	BICDL1	-0.2911	1.878488	-0.6769	0.500239	-5.30813	0.71231	0.72991
Dendritic.cells	MKNK1	0.08321	4.508378	0.676741	0.500337	-6.22738	0.677114	0.678438
Dendritic.cells	GBF1	-0.04832	6.562441	-0.67667	0.500385	-6.67164	0.651147	0.640749
Dendritic.cells	CTPS2	0.115342	5.066744	0.676577	0.50044	-5.97905	0.669924	0.667994
Dendritic.cells	RCC2	0.069059	6.222377	0.676291	0.500621	-6.46276	0.655509	0.646853
Dendritic.cells	IL18R1	-0.42053	0.978465	-0.67586	0.500893	-5.13159	0.72537	0.748492
Dendritic.cells	IP6K2	-0.14397	3.644965	-0.67556	0.501081	-5.69905	0.688875	0.695036
Dendritic.cells	GALNT4	-0.23843	1.622606	-0.67556	0.501084	-5.30311	0.71634	0.735253
Dendritic.cells	NSMCE4A	0.076129	6.083141	0.675441	0.501159	-6.32194	0.657514	0.649427

Dendritic.cells	RFC1	0.090788	5.999025	0.675391	0.50119	-6.4144	0.658565	0.650949
Dendritic.cells	TMCC2	-0.41181	1.686886	-0.67527	0.501268	-5.26241	0.715446	0.733941
Dendritic.cells	CNOT4	-0.05062	7.523005	-0.67517	0.501332	-6.83469	0.639868	0.62391
Dendritic.cells	COLEC10	0.36199	0.557157	0.67509	0.50138	-5.14449	0.731351	0.757343
Dendritic.cells	IL4	-0.60845	0.330173	-0.67507	0.501392	-5.10998	0.734597	0.762131
Dendritic.cells	ELP5	0.126526	4.633093	0.674969	0.501457	-5.88402	0.67594	0.67618
Dendritic.cells	PNISR	0.068326	5.773836	0.674795	0.501567	-6.30706	0.661409	0.655107
Dendritic.cells	HEATR5B	0.134878	3.730171	0.674706	0.501623	-5.91419	0.687769	0.693476
Dendritic.cells	ARHGAP32	-0.06905	3.193867	-0.67461	0.501684	-6.55856	0.694908	0.703937
Dendritic.cells	GEMIN6	0.261572	2.59595	0.674451	0.501784	-5.34382	0.702977	0.715821
Dendritic.cells	CD8B1	0.13884	0.660348	0.674451	0.501785	-6.18593	0.729903	0.755387
Dendritic.cells	FMNL2	0.089344	7.100621	0.674074	0.502023	-7.01421	0.645224	0.631526
Dendritic.cells	ERP29	0.052576	7.289905	0.673721	0.502246	-6.74306	0.643132	0.628322
Dendritic.cells	TMEM222	-0.13341	4.289191	-0.67328	0.502527	-5.88124	0.681063	0.683196
Dendritic.cells	3110082I1	0.127045	4.199896	0.673225	0.50256	-6.07671	0.68223	0.684896
Dendritic.cells	GM9725	-0.33424	2.030059	-0.67313	0.502621	-5.24601	0.711386	0.727531
Dendritic.cells	CRIP2	0.15482	4.565121	0.673086	0.502648	-5.96947	0.677472	0.677987
Dendritic.cells	NRM	0.171098	5.099836	0.673015	0.502693	-5.88236	0.670583	0.667971
Dendritic.cells	GM16104	0.451773	-0.60575	0.672678	0.502906	-5.10358	0.749113	0.782872
Dendritic.cells	CDKN3	0.300618	4.160534	0.672332	0.503126	-5.58838	0.683162	0.6859
Dendritic.cells	GM11713	-0.38375	2.37115	-0.67214	0.503246	-5.15627	0.707132	0.720971
Dendritic.cells	XRCC3	0.338578	0.159666	0.672129	0.503254	-5.13669	0.73821	0.766664
Dendritic.cells	PRPF3	-0.10172	4.472577	-0.67178	0.503477	-5.93731	0.679087	0.680128
Dendritic.cells	RING1	0.152979	3.585545	0.671712	0.503518	-5.74872	0.690751	0.697137
Dendritic.cells	GPR132	-0.09326	6.00422	-0.67166	0.503548	-6.74752	0.659538	0.651751
Dendritic.cells	TNFRSF23	0.373703	1.669914	0.671634	0.503568	-5.12349	0.716811	0.735302
Dendritic.cells	COX18	0.192792	3.223872	0.67162	0.503576	-5.5255	0.695579	0.7042
Dendritic.cells	BST2	0.067031	6.606914	0.671527	0.503636	-7.13101	0.652048	0.640918
Dendritic.cells	GLT8D1	-0.14157	3.339159	-0.67134	0.503752	-5.7508	0.694036	0.701997
Dendritic.cells	CRMP1	-0.39104	-1.07546	-0.67134	0.503754	-5.10651	0.756278	0.793588
Dendritic.cells	LYRM1	-0.23473	2.700522	-0.67123	0.503821	-5.35794	0.702647	0.714609
Dendritic.cells	MAX	-0.07097	6.499429	-0.67098	0.503984	-6.4674	0.65351	0.64297
Dendritic.cells	SERPINA3G	0.654503	3.597185	0.670621	0.50421	-5.48065	0.690917	0.697191
Dendritic.cells	DGKA	-0.20255	5.023185	-0.67046	0.50431	-5.68991	0.672286	0.670098
Dendritic.cells	UBAP2L	-0.0519	7.113568	-0.67046	0.504315	-6.6452	0.646137	0.632213
Dendritic.cells	CCS	0.135259	4.318203	0.670313	0.504405	-5.91926	0.681415	0.6834
Dendritic.cells	HP	-0.18095	7.365035	-0.67012	0.504526	-6.40601	0.643083	0.627846
Dendritic.cells	ARAP1	-0.16165	5.049234	-0.67007	0.504559	-5.86075	0.671952	0.66966
Dendritic.cells	PRKCSH	-0.09659	4.862373	-0.67001	0.504598	-6.02328	0.674355	0.673152
Dendritic.cells	TJAP1	0.127426	4.452059	0.66998	0.504616	-5.84267	0.67967	0.680886
Dendritic.cells	1810021B2	0.319212	0.427969	0.669762	0.504755	-5.3354	0.734698	0.761549
Dendritic.cells	SEC11A	-0.06177	6.406384	-0.66971	0.504789	-6.4282	0.654836	0.644886
Dendritic.cells	SERPINF1	-0.18097	3.83888	-0.66968	0.504808	-5.99621	0.687717	0.692667
Dendritic.cells	CDNF	-0.34434	0.317972	-0.66948	0.504933	-5.21606	0.736282	0.763989
Dendritic.cells	BLOC1S6	-0.15631	3.751167	-0.66948	0.504937	-5.66317	0.688882	0.694439
Dendritic.cells	HSDL2	-0.10238	4.684002	-0.6692	0.50511	-6.11537	0.676814	0.676735
Dendritic.cells	INO80E	0.106767	4.165534	0.669056	0.505203	-5.80956	0.683611	0.68664

Dendritic.cells	MAP11	0.219198	2.866952	0.668526	0.50554	-5.47475	0.701156	0.712071
Dendritic.cells	RSBN1L	0.063845	7.198782	0.668448	0.505589	-6.6275	0.64551	0.631184
Dendritic.cells	GNB2	0.054901	8.428837	0.668393	0.505624	-6.86211	0.630775	0.609941
Dendritic.cells	PELI1	-0.06558	7.676753	-0.66829	0.505692	-6.67758	0.63973	0.622848
Dendritic.cells	EHD1	-0.13354	6.1755	-0.66829	0.505692	-6.07343	0.658121	0.649429
Dendritic.cells	PFAS	0.21221	4.205445	0.668145	0.505782	-5.70521	0.68336	0.686049
Dendritic.cells	CYP2C69	0.369131	1.432054	0.667895	0.50594	-5.25958	0.721028	0.741146
Dendritic.cells	POLD1	-0.11354	4.276679	-0.66779	0.506005	-6.1093	0.682491	0.684779
Dendritic.cells	GPR84	0.434176	0.260814	0.667774	0.506017	-5.11163	0.737671	0.765675
Dendritic.cells	SLF1	-0.11794	4.44199	-0.6676	0.50613	-6.05958	0.680399	0.681691
Dendritic.cells	ARRDC3	-0.22153	3.403751	-0.66732	0.506306	-5.58244	0.694197	0.701734
Dendritic.cells	IRAK2	0.098091	6.796286	0.667287	0.506327	-6.69597	0.650678	0.638495
Dendritic.cells	MGP	0.452595	0.463003	0.667085	0.506455	-5.16016	0.734936	0.761576
Dendritic.cells	AGBL3	-0.20575	1.72445	-0.66705	0.506477	-5.50155	0.717111	0.735351
Dendritic.cells	RAB11FIP2	0.144479	3.576284	0.666995	0.506513	-5.87665	0.691895	0.698455
Dendritic.cells	ZRANB3	-0.21866	3.148069	-0.66665	0.506733	-5.49961	0.697791	0.706931
Dendritic.cells	AMD1	0.159358	4.581194	0.666423	0.506876	-5.74523	0.678839	0.679309
Dendritic.cells	GM41611	-0.41271	0.284798	-0.66641	0.506882	-5.11151	0.737671	0.765515
Dendritic.cells	C4BP	-0.34099	1.968581	-0.66641	0.506884	-5.32647	0.713891	0.730528
Dendritic.cells	KLHL32	0.405084	1.452777	0.666036	0.507122	-5.20063	0.721236	0.74116
Dendritic.cells	WDHD1	0.207989	4.56722	0.666035	0.507123	-5.67166	0.679172	0.679648
Dendritic.cells	DGKI	-0.38467	0.78	-0.66585	0.507241	-5.50995	0.730822	0.755202
Dendritic.cells	PTTG1IP	-0.09555	4.790183	-0.66548	0.507475	-6.02679	0.676508	0.67562
Dendritic.cells	DCUN1D5	0.052711	6.828224	0.665464	0.507486	-6.55247	0.650802	0.638353
Dendritic.cells	RASSF2	0.174679	4.259468	0.66535	0.507558	-5.69135	0.683417	0.685721
Dendritic.cells	VARS	0.083711	5.713285	0.665205	0.507651	-6.36203	0.664703	0.658552
Dendritic.cells	TUT4	0.102635	7.475638	0.665035	0.507759	-6.41321	0.642907	0.627048
Dendritic.cells	KMT5C	-0.22432	2.877228	-0.66502	0.507768	-5.45541	0.70184	0.712674
Dendritic.cells	NLE1	0.241545	2.257092	0.664955	0.50781	-5.50007	0.710307	0.725073
Dendritic.cells	GM42567	-0.38394	1.12927	-0.66488	0.50786	-5.26492	0.726032	0.748164
Dendritic.cells	SCFD1	-0.09373	4.929173	-0.66438	0.508176	-6.28169	0.67505	0.673194
Dendritic.cells	SRRM2	0.048756	8.62357	0.664129	0.508336	-6.91403	0.629656	0.607486
Dendritic.cells	RAD50	0.110087	4.906644	0.663931	0.508462	-6.0251	0.675505	0.673739
Dendritic.cells	TMEM14C	0.079906	6.950542	0.663889	0.508489	-6.47831	0.649783	0.636479
Dendritic.cells	CBR4	-0.21529	2.335594	-0.6634	0.508802	-5.39776	0.709945	0.723822
Dendritic.cells	OTUD7B	-0.09249	5.17042	-0.66329	0.508871	-6.21685	0.672292	0.668954
Dendritic.cells	XKR8	-0.3768	0.500884	-0.66328	0.508878	-5.14514	0.735719	0.761731
Dendritic.cells	GM13963	-0.42872	-0.29578	-0.66309	0.508996	-5.14204	0.747261	0.778799
Dendritic.cells	ATP5J2	0.074407	7.723844	0.66304	0.50903	-6.65293	0.640561	0.623083
Dendritic.cells	TAF8	0.186558	3.254648	0.663028	0.509037	-5.56376	0.697452	0.705628
Dendritic.cells	ABI2	-0.15233	3.903545	-0.66298	0.509067	-5.84458	0.688797	0.693
Dendritic.cells	LY6G	0.426787	-0.81776	0.662711	0.50924	-5.11267	0.755003	0.790284
Dendritic.cells	GM31718	0.107298	3.725877	0.662596	0.509313	-6.14658	0.691211	0.696609
Dendritic.cells	TBCEL	0.095433	4.594507	0.662562	0.509334	-6.18421	0.679788	0.679986
Dendritic.cells	TNFSF13OS	-0.45096	0.055873	-0.66252	0.509361	-5.13369	0.742203	0.771466
Dendritic.cells	PRMT7	0.217268	3.247895	0.661932	0.509736	-5.61195	0.697867	0.706204
Dendritic.cells	USP38	0.081685	5.889969	0.661839	0.509796	-6.31206	0.663452	0.656134

Dendritic.cells	LTB4R1	0.398938	2.689349	0.661832	0.5098	-5.2672	0.705431	0.717268
Dendritic.cells	SLC39A6	0.114163	4.11285	0.66182	0.509808	-5.95046	0.686353	0.689412
Dendritic.cells	FBXO6	-0.12933	3.653081	-0.66165	0.509916	-5.86222	0.69249	0.69836
Dendritic.cells	CBFA2T2	-0.07855	5.563389	-0.66157	0.509968	-6.36532	0.667631	0.66222
Dendritic.cells	OLFML2B	0.431786	0.874884	0.661363	0.5101	-5.11648	0.730861	0.754522
Dendritic.cells	8030453O2	0.339943	-0.75227	0.661114	0.510259	-5.11043	0.754615	0.789499
Dendritic.cells	KIF23	0.18166	5.173452	0.660943	0.510367	-5.95324	0.672849	0.669592
Dendritic.cells	CCDC85B	-0.12763	3.779227	-0.66089	0.510402	-5.79027	0.691058	0.69609
Dendritic.cells	C730034F0	0.27871	2.636781	0.660701	0.510522	-5.35743	0.706526	0.718629
Dendritic.cells	PTPRS	-0.05875	4.390471	-0.66056	0.51061	-6.74006	0.683108	0.684456
Dendritic.cells	TECR	0.07265	6.763011	0.660203	0.51084	-6.54177	0.653161	0.640807
Dendritic.cells	CFAP126	0.349811	1.069649	0.659932	0.511013	-5.20331	0.728686	0.750963
Dendritic.cells	4-Sep	-0.22602	1.735848	-0.65992	0.51102	-5.57936	0.719311	0.737187
Dendritic.cells	LRFN1	-0.38454	1.097146	-0.65974	0.511136	-5.20444	0.728372	0.750458
Dendritic.cells	SLC24A3	-0.4731	1.280307	-0.65944	0.511325	-5.16275	0.725906	0.746663
Dendritic.cells	GM36371	0.256339	0.884673	0.659404	0.51135	-5.25819	0.731518	0.754915
Dendritic.cells	PMF1	-0.18296	5.697613	-0.65865	0.51183	-5.78347	0.667223	0.660419
Dendritic.cells	ZFAND4	-0.3471	3.4762	-0.65851	0.511924	-5.41979	0.696203	0.702546
Dendritic.cells	MRPL47	0.238074	2.974642	0.658464	0.511951	-5.43	0.702967	0.712419
Dendritic.cells	SMPD5	-0.33676	-0.18019	-0.65841	0.511988	-5.11712	0.747421	0.777726
Dendritic.cells	TPK1	0.096428	4.865838	0.658304	0.512053	-6.23528	0.677889	0.675964
Dendritic.cells	SMYD3	-0.07655	6.67093	-0.65823	0.512098	-6.63699	0.655019	0.642862
Dendritic.cells	DDX24	0.064284	6.717234	0.658038	0.512224	-6.55838	0.654526	0.642034
Dendritic.cells	0610043K1	-0.34886	1.133363	-0.65787	0.512332	-5.2967	0.728661	0.750028
Dendritic.cells	PIGW	0.295977	0.765108	0.657519	0.512555	-5.21158	0.734053	0.75786
Dendritic.cells	PHF1	0.210814	2.695984	0.657339	0.51267	-5.518	0.70705	0.718295
Dendritic.cells	PLOD2	0.142206	1.261605	0.657094	0.512827	-6.0447	0.726991	0.7476
Dendritic.cells	NR1H3	-0.35116	3.325046	-0.65697	0.512906	-5.39855	0.698519	0.705928
Dendritic.cells	TRIM27	-0.07733	5.675636	-0.65693	0.512931	-6.33103	0.667776	0.66122
Dendritic.cells	RPP14	0.196799	2.846249	0.65687	0.512971	-5.49877	0.705001	0.715398
Dendritic.cells	PTGS2OS	0.438219	-1.09526	0.656861	0.512977	-5.11432	0.761252	0.79816
Dendritic.cells	1700094D0	-0.23412	1.476408	-0.65683	0.512994	-5.32669	0.723962	0.743174
Dendritic.cells	MRPL57	0.076105	5.995401	0.656819	0.513003	-6.40617	0.66373	0.65536
Dendritic.cells	DNPH1	0.370587	0.708539	0.656448	0.513241	-5.11457	0.73502	0.75934
Dendritic.cells	PABPN1	0.060032	6.640276	0.65623	0.513381	-6.48288	0.655808	0.643895
Dendritic.cells	ASTN2	0.341291	1.045238	0.656221	0.513386	-5.24236	0.730215	0.75236
Dendritic.cells	ALDH4A1	-0.22467	2.912017	-0.65617	0.513416	-5.51108	0.704256	0.714322
Dendritic.cells	SMIM27	0.144328	3.590898	0.656154	0.513429	-5.70178	0.695101	0.700961
Dendritic.cells	SUCO	-0.09745	6.354348	-0.65599	0.513532	-6.37094	0.659418	0.649125
Dendritic.cells	HIP1R	-0.12735	5.271328	-0.65568	0.513734	-5.96443	0.673217	0.669109
Dendritic.cells	ACSS2	0.24263	3.033515	0.655554	0.513814	-5.42463	0.702748	0.712157
Dendritic.cells	GALT	-0.22062	2.389889	-0.65549	0.513851	-5.46905	0.711544	0.725052
Dendritic.cells	ZFP276	-0.18525	2.479933	-0.65546	0.513872	-5.53145	0.710305	0.723255
Dendritic.cells	TMEM173	0.100227	4.250581	0.6554	0.513912	-6.16876	0.686487	0.688506
Dendritic.cells	PARK7	0.07305	6.806683	0.655089	0.514111	-6.53723	0.654053	0.641226
Dendritic.cells	CBWD1	-0.13896	4.090993	-0.65491	0.514226	-5.8908	0.688842	0.691673
Dendritic.cells	ADGB	-0.41321	2.335955	-0.65429	0.514622	-5.28491	0.712895	0.726376

Dendritic.cells	GLS	-0.06566	7.7257	-0.65423	0.514661	-6.77729	0.643205	0.625205
Dendritic.cells	GM12166	-0.32633	0.781167	-0.65422	0.514667	-5.21929	0.734755	0.758474
Dendritic.cells	GAN	0.103891	4.031697	0.654062	0.51477	-6.19207	0.690017	0.692958
Dendritic.cells	SERINC4	0.351305	0.365548	0.653581	0.515078	-5.18457	0.740936	0.767523
Dendritic.cells	ZFP574	-0.16122	3.401172	-0.65352	0.515118	-5.67508	0.69857	0.705411
Dendritic.cells	4930539J0.	-0.32487	0.706555	-0.65346	0.515155	-5.21869	0.736024	0.760301
Dendritic.cells	ARF6	-0.08141	6.770128	-0.65344	0.51517	-6.48338	0.655068	0.642272
Dendritic.cells	TRP53INP1	0.144937	5.187354	0.653411	0.515187	-5.99251	0.675055	0.671207
Dendritic.cells	MIIP	0.190426	3.057652	0.653172	0.515341	-5.50438	0.703299	0.71226
Dendritic.cells	CCL17	-0.4827	-0.70076	-0.65251	0.515762	-5.11702	0.756645	0.790827
Dendritic.cells	ROCK1	-0.04679	8.134703	-0.65251	0.515763	-6.97137	0.638541	0.618526
Dendritic.cells	2510039O1	-0.09159	5.179646	-0.6525	0.515771	-6.14255	0.675238	0.671559
Dendritic.cells	EIF4G3	-0.05276	7.91677	-0.6525	0.515774	-6.79515	0.641156	0.622289
Dendritic.cells	RGL2	0.187472	3.300422	0.651994	0.516096	-5.57396	0.700015	0.707742
Dendritic.cells	CENPE	0.304979	4.9887	0.651948	0.516126	-5.78856	0.677704	0.675254
Dendritic.cells	METTL8	0.219807	2.52734	0.651927	0.51614	-5.47854	0.710541	0.723129
Dendritic.cells	SLC25A10	0.251562	3.077126	0.651701	0.516285	-5.35611	0.703035	0.712207
Dendritic.cells	BCOR	-0.11106	5.214585	-0.65159	0.516354	-6.10519	0.674788	0.671131
Dendritic.cells	ELP4	-0.09705	4.963596	-0.65151	0.516409	-6.17899	0.678029	0.67586
Dendritic.cells	GM36447	-0.38877	0.209364	-0.6515	0.516416	-5.12229	0.743292	0.771394
Dendritic.cells	IFT88	-0.20758	1.778503	-0.65138	0.516489	-5.44585	0.720925	0.738522
Dendritic.cells	PHIP	0.06085	7.276011	0.651356	0.516506	-6.71225	0.648926	0.633748
Dendritic.cells	ANP32A	0.042268	7.495527	0.651145	0.516642	-6.66208	0.64625	0.629889
Dendritic.cells	KPNA4	0.066107	8.246447	0.651068	0.516691	-6.90316	0.637207	0.616861
Dendritic.cells	PEX19	-0.13013	3.955615	-0.65104	0.516708	-5.82588	0.691247	0.695124
Dendritic.cells	ZFP523	-0.20566	2.19531	-0.65099	0.516741	-5.44879	0.715122	0.730014
Dendritic.cells	4921524J1	0.088491	5.474163	0.650897	0.516801	-6.099	0.671458	0.666351
Dendritic.cells	PSMG4	0.085617	5.042688	0.650846	0.516834	-6.19794	0.677006	0.674417
Dendritic.cells	TWF2	0.098156	5.240324	0.650626	0.516975	-6.15388	0.674457	0.670808
Dendritic.cells	ATOX1	0.070276	8.244709	0.650571	0.51701	-6.75654	0.637227	0.616987
Dendritic.cells	LRRC4C	0.22892	0.648779	0.650548	0.517025	-5.66128	0.736945	0.762192
Dendritic.cells	THRA	0.219229	3.771843	0.650523	0.517041	-5.49045	0.693692	0.698797
Dendritic.cells	RBM34	0.097379	4.465573	0.650386	0.517129	-5.97291	0.684519	0.685485
Dendritic.cells	FURIN	0.129893	5.290193	0.65026	0.517211	-6.14547	0.673816	0.669983
Dendritic.cells	FCRLA	-0.09908	5.107141	-0.65017	0.517271	-6.52257	0.676173	0.673467
Dendritic.cells	HCLS1	0.074711	6.691521	0.649916	0.517432	-6.55881	0.656124	0.644438
Dendritic.cells	CAMK2G	0.113907	4.893224	0.649828	0.517488	-6.04871	0.678941	0.677537
Dendritic.cells	PTP4A1	-0.34055	1.179279	-0.64972	0.517559	-5.2507	0.729369	0.751313
Dendritic.cells	PIM3	-0.16613	4.641176	-0.64958	0.517648	-5.82638	0.682221	0.68236
Dendritic.cells	MAPRE3	-0.30241	1.502586	-0.64957	0.517656	-5.25028	0.724799	0.744619
Dendritic.cells	TFDP1	0.132643	5.887837	0.649556	0.517663	-6.15527	0.666195	0.659083
Dendritic.cells	AMOTL2	-0.34474	0.684568	-0.6495	0.517699	-5.30004	0.736431	0.761736
Dendritic.cells	THYN1	0.158434	3.271604	0.649378	0.517777	-5.64301	0.700404	0.708872
Dendritic.cells	APBB1IP	-0.05463	7.638967	-0.64938	0.517779	-6.8512	0.644509	0.627724
Dendritic.cells	LY75	-0.51651	3.332572	-0.64936	0.517787	-5.15956	0.699581	0.707671
Dendritic.cells	PRDM1	0.286095	2.929068	0.649356	0.517792	-5.44929	0.705047	0.71566
Dendritic.cells	THG1L	0.239162	2.021146	0.649285	0.517838	-5.40608	0.71754	0.733974

Dendritic.cells	SFR1	0.07227	6.44096	0.649277	0.517843	-6.37321	0.659242	0.649022
Dendritic.cells	ETV3	0.194515	4.950898	0.649269	0.517848	-5.83243	0.678193	0.676512
Dendritic.cells	KHNYN	0.122033	4.263958	0.648865	0.518107	-5.87201	0.687296	0.689697
Dendritic.cells	GM48765	0.266355	0.952243	0.648685	0.518223	-5.34664	0.732736	0.756311
Dendritic.cells	CHCHD1	0.091351	5.813268	0.648598	0.518279	-6.24076	0.667263	0.660671
Dendritic.cells	CCL25	0.152178	4.40296	0.648567	0.518299	-5.87745	0.685467	0.687127
Dendritic.cells	BRD1	0.060171	5.991255	0.648558	0.518305	-6.61099	0.665011	0.657405
Dendritic.cells	ANAPC15	0.121667	4.974484	0.648465	0.518365	-5.9635	0.678014	0.676328
Dendritic.cells	TTLL1	-0.29282	1.626512	-0.64843	0.518387	-5.21566	0.72319	0.742364
Dendritic.cells	PHGDH	0.149454	4.379094	0.64798	0.518677	-6.1376	0.685967	0.687678
Dendritic.cells	INTS2	0.173449	4.120259	0.647931	0.518709	-5.73831	0.689381	0.69266
Dendritic.cells	SPRY2	-0.2443	5.158508	-0.64787	0.518748	-5.66125	0.67582	0.672935
Dendritic.cells	SMIM8	0.114424	4.587829	0.647818	0.518782	-5.99181	0.683231	0.683712
Dendritic.cells	ZGRF1	-0.23443	3.956437	-0.64755	0.518952	-5.67548	0.691625	0.695867
Dendritic.cells	LGALS3BP	0.165203	4.826846	0.647536	0.518963	-6.20188	0.680186	0.67921
Dendritic.cells	SCOC	0.142023	3.752146	0.647126	0.519227	-5.82964	0.694614	0.69997
Dendritic.cells	RSAD1	-0.32866	1.298807	-0.64691	0.519364	-5.33169	0.72843	0.749535
Dendritic.cells	FLT3L	0.263628	2.201527	0.646796	0.51944	-5.33232	0.715778	0.73101
Dendritic.cells	SGO1	-0.32389	3.188246	-0.64674	0.519475	-5.41097	0.702257	0.71123
Dendritic.cells	CRELD2	-0.09592	4.759514	-0.64666	0.519526	-6.30446	0.681385	0.680797
Dendritic.cells	ADAMDEC1	-0.40526	0.746267	-0.64637	0.519715	-5.18617	0.736329	0.76127
Dendritic.cells	DHX58OS	0.372514	0.831273	0.646365	0.519717	-5.21178	0.73511	0.759475
Dendritic.cells	ZKSCAN5	0.221043	2.402977	0.646345	0.51973	-5.43733	0.713011	0.727011
Dendritic.cells	DTYMK	0.140918	5.126752	0.646238	0.519799	-6.01048	0.676647	0.673968
Dendritic.cells	ANKRD28	-0.08379	5.835458	-0.64607	0.519906	-6.4386	0.667631	0.660827
Dendritic.cells	ODC1	-0.10816	5.188947	-0.64587	0.520034	-6.13752	0.675985	0.672929
Dendritic.cells	SLC25A26	0.163448	3.795963	0.645195	0.520472	-5.7261	0.69476	0.699742
Dendritic.cells	SFXN2	0.153804	3.18033	0.644988	0.520605	-5.66041	0.703114	0.711918
Dendritic.cells	SRI	0.073437	6.74315	0.644916	0.520651	-6.52302	0.656864	0.644704
Dendritic.cells	GSTP3	0.302599	3.39573	0.644748	0.52076	-5.46814	0.700262	0.707709
Dendritic.cells	GLRX5	0.09291	6.17176	0.644581	0.520867	-6.23845	0.664123	0.655104
Dendritic.cells	GMPS	-0.06569	5.784964	-0.64397	0.52126	-6.40308	0.669163	0.662388
Dendritic.cells	LPCAT1	0.181884	4.107714	0.643967	0.521264	-5.57802	0.690949	0.69405
Dendritic.cells	TMEM132E	-0.34819	0.329919	-0.64394	0.521282	-5.20809	0.743393	0.770922
Dendritic.cells	RAD54L2	0.07854	4.552757	0.64365	0.521468	-6.25021	0.68508	0.685614
Dendritic.cells	6330418K0	0.304015	1.506684	0.643638	0.521476	-5.23753	0.726548	0.746246
Dendritic.cells	ABCA3	0.115515	4.193838	0.643568	0.521522	-5.84171	0.689808	0.692529
Dendritic.cells	LEPR	-0.31637	1.823369	-0.6435	0.521564	-5.42472	0.722094	0.739742
Dendritic.cells	RCC1L	0.18903	3.072184	0.643381	0.521642	-5.57757	0.704855	0.714521
Dendritic.cells	HSPA4	0.04783	7.667014	0.643342	0.521667	-6.75849	0.645775	0.628735
Dendritic.cells	HYLS1	-0.19236	3.183514	-0.64314	0.521797	-5.50963	0.703343	0.712379
Dendritic.cells	HIST2H2BB	0.365133	-0.261	0.64313	0.521804	-5.11822	0.752028	0.783939
Dendritic.cells	SESN2	0.142598	3.371941	0.643108	0.521818	-5.84687	0.700793	0.708654
Dendritic.cells	HIST1H1B	0.473867	5.277543	0.643055	0.521853	-5.63849	0.675659	0.672066
Dendritic.cells	PIGO	-0.27717	1.804475	-0.64303	0.521868	-5.32428	0.722358	0.740257
Dendritic.cells	KCTD4	0.217188	2.200261	0.642688	0.52209	-5.55641	0.717053	0.732223
Dendritic.cells	GM15411	-0.35366	0.212426	-0.64243	0.522255	-5.14174	0.745471	0.773821

Dendritic.cells	MRAS	0.206423	0.476226	0.642323	0.522326	-5.64738	0.741652	0.768185
Dendritic.cells	IKZF2	0.191789	4.781138	0.641916	0.522589	-6.22759	0.682703	0.681689
Dendritic.cells	A430093F1	-0.12892	2.363356	-0.64181	0.522658	-5.97602	0.715221	0.729143
Dendritic.cells	NARF	0.135566	4.893244	0.641661	0.522754	-5.84898	0.68129	0.679612
Dendritic.cells	GM34921	0.366799	0.841113	0.641248	0.52302	-5.14233	0.736883	0.76085
Dendritic.cells	4930435F1	0.369849	0.358699	0.641246	0.523022	-5.21819	0.743849	0.771111
Dendritic.cells	AFF2	-0.45215	0.04795	-0.6412	0.523054	-5.12354	0.748377	0.777791
Dendritic.cells	SLC35E4	-0.23843	0.657751	-0.64071	0.523365	-5.51011	0.739613	0.764834
Dendritic.cells	ARMCX1	0.316794	0.550831	0.640697	0.523376	-5.31701	0.741157	0.767109
Dendritic.cells	1190007I0	-0.18156	3.503332	-0.64061	0.523431	-5.54425	0.699931	0.706669
Dendritic.cells	ARF2	-0.17223	4.470722	-0.64058	0.523453	-5.75335	0.687049	0.687896
Dendritic.cells	MOCS1	-0.17667	3.367675	-0.64054	0.523477	-5.56334	0.701762	0.709342
Dendritic.cells	ZCCHC24	0.10294	3.666755	0.640506	0.5235	-6.18757	0.697733	0.703463
Dendritic.cells	GRIA3	0.089609	3.336555	0.640377	0.523584	-6.79775	0.702204	0.710009
Dendritic.cells	GINS4	-0.17046	3.753755	-0.64029	0.523643	-5.61209	0.696588	0.701857
Dendritic.cells	AFTPH	0.063195	6.658529	0.640061	0.523788	-6.56632	0.659094	0.647394
Dendritic.cells	1600020E0	-0.10449	6.223144	-0.64003	0.523809	-6.34035	0.66455	0.655298
Dendritic.cells	ZFYVE27	0.108209	4.063949	0.639578	0.524101	-5.93898	0.692734	0.695987
Dendritic.cells	ZFP316	-0.3645	0.380007	-0.63945	0.524184	-5.17907	0.74396	0.771057
Dendritic.cells	MYCN	0.378396	-0.18583	0.639197	0.524347	-5.12675	0.752231	0.783376
Dendritic.cells	HSPA14	0.120127	5.034968	0.639195	0.524349	-5.98738	0.679976	0.677525
Dendritic.cells	GM14296	0.241769	0.969349	0.639164	0.524369	-5.28904	0.735461	0.758651
Dendritic.cells	CEP170	-0.06363	5.909007	-0.63904	0.524446	-6.62703	0.66875	0.661237
Dendritic.cells	THAP4	-0.12578	4.091332	-0.63904	0.524449	-5.82223	0.692371	0.695558
Dendritic.cells	SKA3	0.266876	2.738324	0.63897	0.524495	-5.31846	0.710649	0.722244
Dendritic.cells	APOBR	-0.27381	2.373103	-0.63885	0.524574	-5.35719	0.715707	0.729665
Dendritic.cells	ITGB2	0.105858	6.734664	0.638539	0.524773	-6.54213	0.658558	0.646297
Dendritic.cells	4833407H1	0.183602	2.098821	0.637861	0.525213	-5.49786	0.720221	0.735527
Dendritic.cells	KIF5A	0.208872	0.429339	0.637722	0.525303	-5.64657	0.744029	0.770468
Dendritic.cells	AP1AR	0.057111	5.002368	0.637429	0.525493	-6.72971	0.681253	0.678531
Dendritic.cells	GM47754	0.349996	0.667894	0.637257	0.525604	-5.23161	0.740722	0.7655
Dendritic.cells	G730013B(	-0.31986	-0.968	-0.63726	0.525604	-5.12283	0.764802	0.801046
Dendritic.cells	CETN3	0.097988	5.807387	0.637131	0.525686	-6.18824	0.670884	0.663518
Dendritic.cells	SLC19A1	0.350318	1.065999	0.637069	0.525726	-5.1473	0.734999	0.757081
Dendritic.cells	SMAD1	0.133811	3.547772	0.636955	0.5258	-5.82689	0.700533	0.706594
Dendritic.cells	NARS2	-0.09054	4.271035	-0.63668	0.525976	-6.26004	0.691015	0.692619
Dendritic.cells	1700084C0	-0.18136	2.9115	-0.63654	0.52607	-5.65655	0.709371	0.719377
Dendritic.cells	IRGC1	0.22063	0.115465	0.635903	0.526482	-5.49952	0.749193	0.77768
Dendritic.cells	BAIAP2	-0.1511	5.269985	-0.63579	0.526555	-6.00004	0.678182	0.673822
Dendritic.cells	CAST	0.063473	6.266263	0.635754	0.526579	-6.47025	0.665457	0.655389
Dendritic.cells	DSCAM	0.449412	0.029718	0.635692	0.526619	-5.12619	0.750449	0.779565
Dendritic.cells	MOGS	0.122249	4.095452	0.63564	0.526653	-6.00138	0.693593	0.696247
Dendritic.cells	NLRP12	0.393646	-0.12085	0.635627	0.526661	-5.12491	0.752661	0.782829
Dendritic.cells	DNMT3B	-0.20359	2.497904	-0.63561	0.526673	-5.44705	0.715276	0.727894
Dendritic.cells	DMGDH	-0.32962	1.667706	-0.63541	0.526799	-5.2821	0.726958	0.745008
Dendritic.cells	DDX3Y	1.736787	2.707432	0.635325	0.526858	-5.63473	0.712462	0.723786
Dendritic.cells	HS1BP3	0.306519	1.087416	0.635142	0.526976	-5.16532	0.735282	0.757197

Dendritic.cells	FAM189B	-0.24398	2.569109	-0.6349	0.527133	-5.45475	0.714536	0.726699
Dendritic.cells	FKBP8	0.080133	6.067485	0.634842	0.527171	-6.38692	0.668198	0.659277
Dendritic.cells	PHLDB1	-0.27987	1.355652	-0.63473	0.527246	-5.31978	0.731565	0.751654
Dendritic.cells	GAREM1	0.355939	0.757375	0.634495	0.527396	-5.16784	0.740233	0.764293
Dendritic.cells	GM32031	0.180461	2.845461	0.634397	0.52746	-5.63445	0.71084	0.721192
Dendritic.cells	TRMT10A	-0.14798	3.81366	-0.63432	0.527508	-5.73125	0.697702	0.702043
Dendritic.cells	MS4A7	-0.48929	1.921581	-0.63394	0.527759	-5.20261	0.723922	0.740089
Dendritic.cells	FAM49A	0.170385	5.390412	0.633758	0.527875	-6.00736	0.677224	0.67199
Dendritic.cells	CLN3	-0.14553	4.768913	-0.6337	0.527916	-5.97568	0.685306	0.683721
Dendritic.cells	GPSM3	0.093528	6.022347	0.633577	0.527993	-6.21996	0.669133	0.660284
Dendritic.cells	ZBTB43	-0.15177	3.163113	-0.63349	0.528048	-5.67023	0.706769	0.715006
Dendritic.cells	MTHFD2L	-0.16634	3.033816	-0.63334	0.528145	-5.60361	0.708534	0.717619
Dendritic.cells	PAWR	0.367948	0.662659	0.63327	0.528192	-5.12788	0.741888	0.766516
Dendritic.cells	PHF11A	0.364578	1.004104	0.633224	0.528222	-5.22941	0.736969	0.759281
Dendritic.cells	NSA2	0.044228	7.622863	0.633069	0.528323	-6.76856	0.64925	0.631563
Dendritic.cells	LIN52	-0.09381	5.546829	-0.63271	0.528555	-6.29232	0.675425	0.669169
Dendritic.cells	ZFAND3	0.06064	8.233036	0.632664	0.528586	-6.72109	0.64202	0.620966
Dendritic.cells	SYTL2	0.407144	1.257049	0.632573	0.528645	-5.20053	0.733584	0.754024
Dendritic.cells	XLR	0.394978	2.01867	0.632348	0.528791	-5.26432	0.722854	0.738276
Dendritic.cells	CEP85	0.121945	4.470348	0.632312	0.528815	-5.91856	0.689492	0.689595
Dendritic.cells	FHOD1	-0.21423	2.907952	-0.63219	0.528894	-5.40278	0.710523	0.720261
Dendritic.cells	FARP2	0.241373	2.6131	0.632092	0.528958	-5.4308	0.714583	0.726194
Dendritic.cells	IGLC3	-0.1209	4.04289	-0.63204	0.528994	-6.83384	0.695166	0.69787
Dendritic.cells	ADPRH	-0.12322	4.93883	-0.63189	0.529088	-5.934	0.683379	0.680713
Dendritic.cells	TCTEX1D2	0.143007	3.301638	0.631347	0.529443	-5.80597	0.705534	0.712583
Dendritic.cells	DNAAF3	0.365325	-0.6587	0.631298	0.529475	-5.12526	0.762	0.795494
Dendritic.cells	ARPC4	0.054443	7.957802	0.631026	0.529652	-6.78585	0.645857	0.626111
Dendritic.cells	GTF2E1	0.209214	2.399479	0.630854	0.529764	-5.46516	0.718077	0.730926
Dendritic.cells	RNF2	0.077508	5.723956	0.630782	0.529811	-6.26304	0.673693	0.666332
Dendritic.cells	PMP22	-0.3458	1.847479	-0.63074	0.529836	-5.32824	0.725799	0.742232
Dendritic.cells	TNFAIP6	0.471	1.129558	0.630388	0.530066	-5.14609	0.736151	0.757326
Dendritic.cells	ZSWIM4	0.134136	4.579223	0.630351	0.530091	-5.89019	0.688715	0.688034
Dendritic.cells	GM15246	0.3178	1.046088	0.63026	0.53015	-5.31841	0.737348	0.759086
Dendritic.cells	ARVCF	0.371529	1.228411	0.629926	0.530368	-5.15984	0.734736	0.755329
Dendritic.cells	NFATC2	-0.11134	2.489047	-0.62986	0.53041	-6.13359	0.716985	0.729336
Dendritic.cells	SNHG1	-0.11943	5.230493	-0.62971	0.530505	-5.95826	0.680197	0.675791
Dendritic.cells	GLYCTK	-0.32017	0.979545	-0.62964	0.530556	-5.21477	0.738304	0.760643
Dendritic.cells	IFRD2	0.200937	3.30652	0.629609	0.530574	-5.49301	0.705757	0.712985
Dendritic.cells	XPO7	0.073773	6.710919	0.629606	0.530576	-6.53691	0.661341	0.648519
Dendritic.cells	CAPN3	-0.25621	1.422788	-0.62959	0.530586	-5.38743	0.731964	0.751332
Dendritic.cells	MTHFS	0.191969	5.861718	0.629379	0.530724	-6.11114	0.672114	0.664102
Dendritic.cells	B3GNT2	0.075117	6.739389	0.629344	0.530747	-6.51453	0.661026	0.648071
Dendritic.cells	0610009B2	0.186889	3.780562	0.628741	0.531139	-5.65261	0.699824	0.703787
Dendritic.cells	PPARD	-0.06883	5.613407	-0.62853	0.53128	-6.57015	0.675749	0.668814
Dendritic.cells	ARHGAP17	0.05781	7.245574	0.628503	0.531295	-6.8241	0.655182	0.639104
Dendritic.cells	GIMAP1	-0.15744	4.06141	-0.62834	0.531403	-5.73348	0.696108	0.698362
Dendritic.cells	OTOA	-0.36592	0.757069	-0.62825	0.531459	-5.15527	0.742069	0.765584

Dendritic.cells	GM45442	-0.40767	-0.57631	-0.62819	0.531497	-5.1252	0.761657	0.794486
Dendritic.cells	FAM83D	-0.17335	2.774945	-0.62763	0.531864	-5.65686	0.713759	0.724124
Dendritic.cells	DST	0.120261	4.335211	0.627614	0.531875	-6.22473	0.692645	0.693359
Dendritic.cells	1810044D(	0.247161	1.915203	0.627525	0.531932	-5.40248	0.725737	0.741669
Dendritic.cells	D130020L(	-0.34968	0.782996	-0.62752	0.531938	-5.17932	0.741891	0.765376
Dendritic.cells	KLRG1	-0.35158	-1.146	-0.62749	0.531957	-5.1281	0.770415	0.807454
Dendritic.cells	ADAL	-0.18238	2.279066	-0.62697	0.532295	-5.51381	0.72092	0.73443
Dendritic.cells	EIF2S1	0.08106	6.052768	0.626932	0.53232	-6.27156	0.670583	0.661208
Dendritic.cells	ELF4	0.058645	6.785373	0.626873	0.532358	-6.67062	0.661343	0.647858
Dendritic.cells	FTSJ3	-0.18055	4.016619	-0.62649	0.532608	-5.71964	0.697277	0.699814
Dendritic.cells	TMEM107	0.251385	1.616764	0.626463	0.532625	-5.29835	0.730357	0.748126
Dendritic.cells	KNL1	0.246156	5.131736	0.626339	0.532707	-5.89599	0.682551	0.678459
Dendritic.cells	A630023P1	-0.41185	0.306091	-0.62631	0.532727	-5.12839	0.749239	0.775923
Dendritic.cells	RAB43	-0.0895	6.948549	-0.62625	0.532763	-6.52249	0.659414	0.645016
Dendritic.cells	NCAM1	-0.44848	1.275146	-0.62575	0.533093	-5.26701	0.735578	0.755505
Dendritic.cells	ZFP324	-0.31491	0.798242	-0.62557	0.533207	-5.25518	0.74244	0.765586
Dendritic.cells	HEATR9	0.355916	-0.41964	0.625563	0.533213	-5.13027	0.760313	0.79191
Dendritic.cells	FBXL6	0.137282	3.399881	0.625312	0.533377	-5.73916	0.70607	0.712294
Dendritic.cells	EIF3M	0.05063	6.621301	0.625027	0.533563	-6.5049	0.664047	0.6512
Dendritic.cells	MTMR12	-0.08936	5.334196	-0.62466	0.533805	-6.18286	0.680477	0.674974
Dendritic.cells	SH3D21	-0.30425	1.99564	-0.62465	0.533808	-5.2759	0.7256	0.740686
Dendritic.cells	CBX3	0.07057	8.102288	0.624617	0.533831	-6.74311	0.645787	0.624916
Dendritic.cells	PSMD12	0.073951	5.784758	0.624604	0.533839	-6.32241	0.674665	0.66656
Dendritic.cells	SAMD4B	0.093949	5.014196	0.624518	0.533896	-6.08706	0.684644	0.681023
Dendritic.cells	WHAMM	0.133966	4.147659	0.624507	0.533903	-5.81353	0.696094	0.697651
Dendritic.cells	CDK14	0.172279	5.455475	0.623847	0.534334	-6.35174	0.679273	0.672999
Dendritic.cells	TMEM185E	0.140411	4.12483	0.623807	0.534361	-5.75954	0.696776	0.69841
Dendritic.cells	4930404I0!	0.392791	0.086501	0.623771	0.534384	-5.16985	0.753482	0.781411
Dendritic.cells	VARS2	0.289093	1.401859	0.623389	0.534634	-5.32067	0.734465	0.753407
Dendritic.cells	DYNC1H1	-0.04511	6.255557	-0.62335	0.534659	-6.7219	0.669074	0.658224
Dendritic.cells	RRN3	-0.09802	4.400393	-0.62332	0.534676	-6.01425	0.693156	0.693113
Dendritic.cells	FAM193A	-0.06005	6.704133	-0.62332	0.53468	-6.64347	0.663416	0.650055
Dendritic.cells	RALGAPB	-0.08126	4.920242	-0.62278	0.53503	-6.22788	0.686663	0.683339
Dendritic.cells	INPP1	0.230842	4.03687	0.622612	0.535142	-5.43343	0.698421	0.70037
Dendritic.cells	DMD	0.208196	2.419953	0.622514	0.535206	-5.6326	0.720532	0.732621
Dendritic.cells	GM16618	0.281552	0.733543	0.622441	0.535254	-5.24991	0.744525	0.767785
Dendritic.cells	YTHDC2	-0.10177	4.693584	-0.62203	0.535524	-6.08316	0.689893	0.687811
Dendritic.cells	NANS	0.113967	4.873876	0.621988	0.535551	-6.05824	0.687519	0.684371
Dendritic.cells	WBP1	-0.15403	3.813733	-0.6219	0.535607	-5.78404	0.701631	0.704898
Dendritic.cells	DTX3	-0.14807	2.572046	-0.62158	0.535815	-5.76341	0.718764	0.729746
Dendritic.cells	NUP214	0.082761	5.2678	0.621553	0.535835	-6.20851	0.682495	0.677003
Dendritic.cells	GM12216	-0.14613	4.554087	-0.62127	0.536021	-6.01468	0.692023	0.690679
Dendritic.cells	VEGFA	0.218299	4.223513	0.620825	0.536312	-5.61509	0.696559	0.697184
Dendritic.cells	ZMAT2	0.082525	5.466404	0.620755	0.536358	-6.17847	0.680207	0.673474
Dendritic.cells	GM43848	-0.10213	2.767038	-0.62063	0.53644	-6.05876	0.716365	0.726036
Dendritic.cells	7-Mar	0.052819	6.926226	0.620504	0.536522	-6.55127	0.661634	0.646676
Dendritic.cells	GM13481	0.402243	0.231781	0.620368	0.536612	-5.1309	0.752536	0.779051

Dendritic.cells	ZFP955A	0.281091	1.472841	0.620361	0.536616	-5.33315	0.734558	0.752653
Dendritic.cells	PAM	0.12401	4.417759	0.620326	0.536639	-6.00827	0.69397	0.693452
Dendritic.cells	LIG3	0.154248	3.358188	0.620323	0.536641	-5.75584	0.708242	0.714206
Dendritic.cells	FAR1	0.079784	6.64871	0.620219	0.536709	-6.51375	0.665118	0.651707
Dendritic.cells	F830016B0	0.421229	0.444299	0.620106	0.536783	-5.20702	0.749439	0.774513
Dendritic.cells	MCMDC2	0.229094	3.465667	0.619804	0.536981	-5.59257	0.706972	0.712264
Dendritic.cells	C030005K0	0.291651	0.674423	0.619624	0.537099	-5.25646	0.746344	0.769773
Dendritic.cells	DDX55	-0.17263	2.934608	-0.61944	0.537216	-5.60497	0.714333	0.722954
Dendritic.cells	YIPF5	-0.07194	5.24625	-0.61941	0.537238	-6.27285	0.683337	0.677904
Dendritic.cells	STARD10	-0.25042	4.596544	-0.61902	0.537495	-5.77207	0.692121	0.690389
Dendritic.cells	PITPNM2	-0.17779	3.66119	-0.61876	0.537666	-5.63518	0.704658	0.708647
Dendritic.cells	GM28529	0.340663	0.724072	0.618627	0.537753	-5.21233	0.745909	0.769027
Dendritic.cells	H2-T22	0.178969	5.391347	0.61857	0.53779	-6.22142	0.681698	0.675413
Dendritic.cells	INHBA	0.542785	1.050066	0.618519	0.537823	-5.15081	0.741187	0.762116
Dendritic.cells	FTH1	-0.09128	12.5537	-0.61849	0.537845	-7.67786	0.596587	0.55343
Dendritic.cells	SLC44A1	0.070012	4.637679	0.618428	0.537883	-6.54212	0.69158	0.689734
Dendritic.cells	CEP152	0.171645	3.629164	0.618171	0.538052	-5.573	0.705229	0.709471
Dendritic.cells	GM15559	-0.17536	3.148018	-0.61801	0.53816	-5.53619	0.711854	0.719039
Dendritic.cells	SNRPB2	0.064572	6.282619	0.617884	0.53824	-6.40667	0.670433	0.65901
Dendritic.cells	ZFP963	-0.23962	1.15161	-0.61776	0.538325	-5.32997	0.739928	0.760168
Dendritic.cells	USP35	0.411562	0.017078	0.617616	0.538416	-5.1416	0.756483	0.784547
Dendritic.cells	SPICE1	0.286145	1.890422	0.617562	0.538451	-5.23203	0.729382	0.744791
Dendritic.cells	TATDN2	-0.08087	5.004884	-0.61743	0.538538	-6.20308	0.686932	0.682993
Dendritic.cells	IER5	-0.08504	6.692584	-0.61742	0.538546	-6.69073	0.66525	0.651643
Dendritic.cells	MAGT1	0.057925	5.872067	0.617018	0.538809	-6.61907	0.675898	0.666788
Dendritic.cells	JMJD4	-0.24265	1.268434	-0.61696	0.53885	-5.26199	0.738488	0.757909
Dendritic.cells	LMBRD1	-0.08205	6.356577	-0.6165	0.539146	-6.38182	0.670001	0.657971
Dendritic.cells	EIF4B	-0.06437	6.136378	-0.61634	0.539251	-6.44918	0.672813	0.662025
Dendritic.cells	ZFP146	0.127213	3.413704	0.616298	0.539281	-5.85946	0.708764	0.714152
Dendritic.cells	P2RX3	-0.38273	2.219391	-0.61617	0.539369	-5.21763	0.725311	0.738323
Dendritic.cells	MMUT	-0.19361	3.4125	-0.61607	0.53943	-5.61108	0.708787	0.714255
Dendritic.cells	GPRC5C	-0.34435	1.29284	-0.616	0.539479	-5.34052	0.738473	0.75763
Dendritic.cells	APEX1	0.098222	5.465947	0.61532	0.539924	-6.21872	0.68187	0.674771
Dendritic.cells	NAGLU	0.162022	2.6543	0.615293	0.539942	-5.67371	0.719678	0.729695
Dendritic.cells	COMMD10	-0.11275	4.694613	-0.61465	0.540368	-5.97669	0.692342	0.689529
Dendritic.cells	CNOT6L	-0.06195	7.08541	-0.61463	0.540375	-6.69186	0.661599	0.645128
Dendritic.cells	DTX4	0.210473	2.365512	0.614376	0.540545	-5.64183	0.724083	0.735811
Dendritic.cells	SERPINH1	-0.14695	2.557349	-0.61429	0.540599	-6.00518	0.721401	0.731896
Dendritic.cells	N4BP2L1	0.14866	4.595001	0.614023	0.540777	-5.83323	0.693663	0.69155
Dendritic.cells	ANKRD50	-0.13571	2.62264	-0.61391	0.540853	-5.6435	0.720491	0.730568
Dendritic.cells	GM16268	-0.33437	0.706579	-0.6139	0.540859	-5.24217	0.7478	0.770532
Dendritic.cells	AI597479	0.228032	1.688645	0.613578	0.54107	-5.3512	0.733647	0.749841
Dendritic.cells	BAG1	0.060013	6.693589	0.613473	0.541139	-6.50396	0.666513	0.652348
Dendritic.cells	GM42984	0.284378	0.916614	0.613395	0.54119	-5.34965	0.744746	0.766102
Dendritic.cells	TMEM268	0.144505	3.100152	0.613367	0.541209	-5.72915	0.713877	0.720976
Dendritic.cells	RLN3	-0.33033	-0.84713	-0.61336	0.54121	-5.1359	0.770864	0.804534
Dendritic.cells	SLC30A4	-0.17398	1.941057	-0.61322	0.541303	-5.6072	0.730063	0.744648

Dendritic.cells	NBR1	0.079922	5.455137	0.613155	0.541348	-6.20113	0.682364	0.675289
Dendritic.cells	MALT1	0.146693	7.764534	0.613077	0.541399	-6.68424	0.653197	0.63324
Dendritic.cells	HYKK	0.336839	0.618386	0.612969	0.54147	-5.15913	0.749087	0.772559
Dendritic.cells	ATP1A1	-0.05879	6.234757	-0.61289	0.541524	-6.69577	0.672328	0.660822
Dendritic.cells	ST6GAL1	0.128451	5.862113	0.612775	0.541598	-6.43055	0.677101	0.667714
Dendritic.cells	BCAP31	-0.07005	5.901725	-0.61254	0.541756	-6.30896	0.676591	0.667011
Dendritic.cells	CYTIP	0.058852	8.087657	0.612529	0.54176	-7.02754	0.649249	0.627634
Dendritic.cells	EIF3B	-0.07992	5.890853	-0.61251	0.54177	-6.32774	0.676731	0.667213
Dendritic.cells	UBAP1	0.099062	5.688984	0.612448	0.541813	-6.19728	0.679333	0.670974
Dendritic.cells	B430306N(	0.234602	2.692804	0.612447	0.541814	-5.46158	0.719514	0.729327
Dendritic.cells	DNM2	-0.05343	7.193868	-0.61241	0.54184	-6.66749	0.660248	0.643443
Dendritic.cells	SLC28A2	0.311271	2.961181	0.612317	0.5419	-5.29029	0.715794	0.723939
Dendritic.cells	TMX1	0.075516	5.362	0.612235	0.541953	-6.27559	0.683576	0.677158
Dendritic.cells	4931414P1	-0.23325	2.099255	-0.61223	0.541958	-5.45644	0.727827	0.741518
Dendritic.cells	IGSF3	-0.32069	0.826018	-0.61172	0.542291	-5.25287	0.74631	0.768367
Dendritic.cells	PPP1R15B	-0.09861	5.553021	-0.61169	0.542313	-6.17464	0.68132	0.673696
Dendritic.cells	CARD11	0.070461	4.618562	0.611541	0.542411	-6.79901	0.693581	0.691515
Dendritic.cells	SAP130	-0.07542	5.619105	-0.6115	0.542435	-6.38615	0.680464	0.672521
Dendritic.cells	ZMIZ2	0.066583	4.532649	0.611388	0.542511	-6.43906	0.694723	0.693222
Dendritic.cells	HNRNPK	-0.03917	8.860645	-0.61103	0.542749	-6.94307	0.64015	0.614607
Dendritic.cells	PTPRE	-0.07127	5.539856	-0.61098	0.542782	-6.61387	0.681491	0.674125
Dendritic.cells	PPP1R13L	0.408592	-0.0774	0.610969	0.542788	-5.13464	0.759587	0.788104
Dendritic.cells	FBXL12	-0.11289	4.348122	-0.61094	0.542808	-5.96996	0.697183	0.696866
Dendritic.cells	DOK2	-0.27488	2.811456	-0.6109	0.542833	-5.2892	0.718106	0.727312
Dendritic.cells	MEF2B	0.113238	3.605786	0.610879	0.542847	-5.97347	0.707193	0.711413
Dendritic.cells	CRAT	0.218191	3.198294	0.610318	0.543217	-5.46213	0.713038	0.719573
Dendritic.cells	SNAPC5	-0.12264	4.755447	-0.61019	0.543303	-5.86565	0.692033	0.689098
Dendritic.cells	SPIB	-0.08349	4.129765	-0.61017	0.543314	-6.58569	0.700377	0.70121
Dendritic.cells	SPATA21	-0.22085	3.397572	-0.61007	0.54338	-5.51602	0.710305	0.715648
Dendritic.cells	BCL2L14	0.432041	0.459361	0.609894	0.543497	-5.21845	0.751953	0.776624
Dendritic.cells	POGK	-0.19658	2.454489	-0.60985	0.543523	-5.56744	0.723356	0.734735
Dendritic.cells	DIS3	-0.17186	3.258023	-0.60984	0.543535	-5.56506	0.712217	0.718495
Dendritic.cells	SLFN9	0.321391	2.410472	0.609783	0.54357	-5.30277	0.723972	0.735637
Dendritic.cells	PDHB	0.076797	5.703174	0.609537	0.543732	-6.24495	0.679741	0.671228
Dendritic.cells	TNFRSF10E	0.349445	-0.0063	0.60946	0.543783	-5.14445	0.75894	0.786752
Dendritic.cells	UBASH3B	-0.18017	5.794781	-0.60931	0.54388	-6.01947	0.6786	0.669551
Dendritic.cells	NCKAP5L	-0.13866	4.041979	-0.60883	0.544201	-5.91532	0.701851	0.703248
Dendritic.cells	STAG2	0.063219	7.464582	0.608747	0.544253	-6.71478	0.657636	0.639335
Dendritic.cells	PDPK1	-0.08548	6.462559	-0.6087	0.544287	-6.63739	0.670193	0.657419
Dendritic.cells	STEAP4	0.402253	2.205553	0.608579	0.544364	-5.20743	0.727154	0.740113
Dendritic.cells	SERPINA3F	-0.46662	2.19929	-0.60851	0.544407	-5.40971	0.727243	0.740242
Dendritic.cells	MRPL21	0.094435	5.233418	0.608352	0.544514	-6.14835	0.686031	0.680306
Dendritic.cells	DHCR24	0.132394	2.897631	0.60831	0.544541	-5.94116	0.717487	0.726002
Dendritic.cells	NUP37	0.224927	3.351508	0.608186	0.544624	-5.42905	0.711233	0.716892
Dendritic.cells	MPHOSPH1	0.093699	4.827879	0.608119	0.544667	-6.11537	0.691364	0.68803
Dendritic.cells	GM45353	-0.30231	-0.0712	-0.60801	0.544741	-5.14041	0.760104	0.788438
Dendritic.cells	OGFR	0.098809	5.244677	0.607992	0.544751	-6.24966	0.685884	0.680093

Dendritic.cells	MMRN2	-0.18889	1.228883	-0.60794	0.544786	-5.61361	0.741073	0.760482
Dendritic.cells	GM48623	0.357607	-0.24558	0.607876	0.544828	-5.13468	0.762701	0.792262
Dendritic.cells	MXD4	0.107276	5.790486	0.607773	0.544896	-6.32534	0.678797	0.669836
Dendritic.cells	ERMARD	0.160502	3.076068	0.607436	0.545119	-5.62584	0.715122	0.722464
Dendritic.cells	5830432E0	0.213105	1.494203	0.607428	0.545124	-5.53236	0.737365	0.754955
Dendritic.cells	GM50322	0.369999	0.358739	0.607348	0.545176	-5.14977	0.753854	0.779181
Dendritic.cells	CCDC102A	0.151147	2.048525	0.607223	0.545259	-5.88179	0.729474	0.743457
Dendritic.cells	PKNOX1	0.133892	4.569323	0.607173	0.545292	-5.87332	0.694891	0.693122
Dendritic.cells	PRICKLE3	0.194509	2.034653	0.606992	0.545412	-5.51756	0.729745	0.743792
Dendritic.cells	SRGAP2	-0.09118	6.60483	-0.60659	0.545676	-6.41451	0.668798	0.655036
Dendritic.cells	TCEA3	-0.31416	2.275566	-0.60643	0.545784	-5.32855	0.726665	0.738943
Dendritic.cells	PPIE	-0.10764	4.586932	-0.60634	0.545844	-5.92301	0.695032	0.692949
Dendritic.cells	SPRED3	-0.34268	0.719893	-0.60622	0.545919	-5.19409	0.74898	0.77165
Dendritic.cells	9430091E2	-0.19915	2.439837	-0.606	0.546069	-5.47648	0.724485	0.735699
Dendritic.cells	PSMB4	0.076141	6.172049	0.605824	0.546184	-6.33509	0.674533	0.663114
Dendritic.cells	GPD1L	-0.10571	5.296236	-0.60553	0.546378	-6.14734	0.685964	0.679533
Dendritic.cells	RBX1	0.05362	7.700144	0.605417	0.546453	-6.75568	0.65545	0.635559
Dendritic.cells	ATF2	-0.06401	6.46092	-0.60538	0.546475	-6.5653	0.670951	0.657866
Dendritic.cells	IGSF5	0.383405	1.922281	0.60517	0.546617	-5.22491	0.731962	0.746494
Dendritic.cells	ZFP658	0.222176	0.297611	0.605166	0.546619	-5.51713	0.755476	0.780961
Dendritic.cells	PLSCR1	0.260857	4.550033	0.605025	0.546712	-5.48786	0.695813	0.693903
Dendritic.cells	MED6	0.117608	4.540282	0.60493	0.546775	-5.97348	0.695943	0.694108
Dendritic.cells	IIGP1	0.261872	4.709506	0.604921	0.546781	-6.13682	0.693693	0.690846
Dendritic.cells	DHX15	-0.0513	6.794688	-0.60472	0.546916	-6.63756	0.666814	0.651916
Dendritic.cells	SLC24A5	0.130678	4.423305	0.604173	0.547276	-5.85591	0.697768	0.696636
Dendritic.cells	CCNE2	0.309683	3.75287	0.603816	0.547512	-5.44598	0.706804	0.709873
Dendritic.cells	TRAPPC11	-0.13093	3.489165	-0.60366	0.547615	-5.71681	0.710399	0.715129
Dendritic.cells	DNAJC14	0.092914	4.252973	0.603641	0.547628	-5.91494	0.70005	0.700087
Dendritic.cells	CD300E	0.554006	0.628868	0.603631	0.547634	-5.1658	0.750893	0.774322
Dendritic.cells	NREP	0.292997	1.234795	0.603598	0.547657	-5.27599	0.742083	0.761397
Dendritic.cells	PDIA6	0.061194	6.97381	0.603582	0.547667	-6.74533	0.664728	0.649016
Dendritic.cells	TXNL4B	-0.25166	1.740021	-0.60356	0.547679	-5.23828	0.734834	0.750781
Dendritic.cells	RAMAC	0.077625	5.913686	0.603462	0.547747	-6.22734	0.678207	0.668471
Dendritic.cells	PDE1B	0.221008	3.114849	0.603158	0.547948	-5.396	0.715542	0.722778
Dendritic.cells	GM43378	-0.22636	1.243694	-0.60303	0.548035	-5.31942	0.741955	0.761385
Dendritic.cells	HIBADH	0.080914	5.742005	0.60299	0.548059	-6.41643	0.680424	0.671814
Dendritic.cells	EBPL	-0.10922	4.469152	-0.60299	0.54806	-6.04889	0.697156	0.696047
Dendritic.cells	GM37529	0.09833	1.962925	0.602888	0.548127	-6.34442	0.731662	0.746315
Dendritic.cells	ARPC2	0.038307	9.318766	0.602844	0.548156	-6.99888	0.636162	0.608157
Dendritic.cells	ALKBH6	-0.14881	2.755561	-0.60278	0.548197	-5.55689	0.720522	0.730052
Dendritic.cells	LAMP2	-0.05502	7.069003	-0.60276	0.548213	-6.70976	0.663535	0.647455
Dendritic.cells	GM44899	0.271138	0.774696	0.602641	0.54829	-5.27883	0.748761	0.771452
Dendritic.cells	MDH2	0.078312	6.490426	0.602586	0.548326	-6.37369	0.67083	0.658063
Dendritic.cells	CDK7	0.094262	4.595584	0.602534	0.548361	-6.1627	0.69547	0.693716
Dendritic.cells	ODR4	-0.12765	4.042451	-0.60242	0.548438	-5.80477	0.702883	0.704492
Dendritic.cells	F830208F2	0.375113	-0.23725	0.602152	0.548614	-5.1394	0.763704	0.793569
Dendritic.cells	BATF3	0.298762	2.332688	0.602142	0.548621	-5.35041	0.726439	0.738889

Dendritic.cells	AK3	0.133023	4.328071	0.601935	0.548758	-5.76795	0.699043	0.699034
Dendritic.cells	MCL1	-0.07307	8.364811	-0.60176	0.548871	-6.73174	0.647579	0.624744
Dendritic.cells	NECAP1	-0.08538	4.774239	-0.60166	0.54894	-6.11661	0.693097	0.690431
Dendritic.cells	RBSN	0.143076	2.910659	0.601648	0.548948	-5.63796	0.718367	0.727185
Dendritic.cells	FBXO17	-0.23658	0.782245	-0.60151	0.549042	-5.438	0.748651	0.771541
Dendritic.cells	WFDC17	-0.4058	5.906168	-0.60149	0.549054	-5.98589	0.678304	0.669043
Dendritic.cells	9230111E0	0.373414	-0.18455	0.601255	0.549209	-5.13927	0.762917	0.792641
Dendritic.cells	LBHD1	0.303605	0.16876	0.601129	0.549292	-5.14622	0.757667	0.784985
Dendritic.cells	EEPD1	0.049348	4.881821	0.601059	0.549339	-6.99582	0.691673	0.688578
Dendritic.cells	POLR1C	-0.14235	3.945898	-0.60084	0.549484	-5.7931	0.704187	0.706831
Dendritic.cells	INSIG1	-0.06758	5.457849	-0.60063	0.549623	-6.56669	0.684113	0.677748
Dendritic.cells	FOSL2	-0.16051	5.561041	-0.6006	0.549641	-5.9549	0.68277	0.675804
Dendritic.cells	GM36279	-0.18907	2.959709	-0.60059	0.549652	-5.58353	0.717687	0.72655
Dendritic.cells	PEPD	-0.06623	5.308275	-0.60052	0.549699	-6.50393	0.686066	0.680576
Dendritic.cells	DONSON	-0.14391	4.025423	-0.60045	0.54974	-5.70068	0.703113	0.705338
Dendritic.cells	LACTB2	0.125389	3.94655	0.600412	0.549768	-5.83436	0.704179	0.70689
Dendritic.cells	CAGE1	-0.26263	1.94977	-0.60037	0.549797	-5.40951	0.731849	0.747269
Dendritic.cells	GM47507	-0.42162	0.732528	-0.60031	0.549837	-5.14602	0.749377	0.772979
Dendritic.cells	IK	0.060677	6.288849	0.600282	0.549854	-6.42094	0.673396	0.662268
Dendritic.cells	5031434O1	0.245902	0.524887	0.600231	0.549887	-5.34746	0.752418	0.777449
Dendritic.cells	GPR160	0.22941	2.407708	0.600226	0.549891	-5.56662	0.725385	0.737814
Dendritic.cells	ARHGAP4	0.094349	5.057347	0.600084	0.549985	-6.05072	0.689367	0.685368
Dendritic.cells	ADAP2	-0.2305	2.585365	-0.59998	0.550056	-5.59338	0.722906	0.734178
Dendritic.cells	EPHX3	-0.3032	0.657793	-0.59988	0.550122	-5.21546	0.750479	0.7746
Dendritic.cells	BBS2	0.278577	0.404044	0.599827	0.550156	-5.28588	0.754203	0.780077
Dendritic.cells	ODF2	-0.06875	5.483537	-0.59937	0.550462	-6.29455	0.684033	0.677434
Dendritic.cells	DTX1	-0.37983	2.069415	-0.59934	0.550482	-5.17734	0.730424	0.744965
Dendritic.cells	GM15494	-0.40349	0.759331	-0.59896	0.550731	-5.16683	0.749507	0.772682
Dendritic.cells	CSTF3	-0.06268	6.037918	-0.59887	0.55079	-6.4609	0.67708	0.667202
Dendritic.cells	AIDA	0.109914	4.437691	0.598666	0.550926	-5.91229	0.698152	0.697695
Dendritic.cells	MARK3	0.047099	6.304211	0.598246	0.551205	-6.66069	0.673905	0.662458
Dendritic.cells	TMED10	-0.04563	7.769396	-0.5982	0.551235	-6.89588	0.655532	0.635982
Dendritic.cells	FAM89B	0.110948	5.277421	0.598105	0.551299	-6.02357	0.687189	0.681688
Dendritic.cells	TIMM44	0.089927	4.84696	0.598052	0.551334	-6.04278	0.692859	0.689906
Dendritic.cells	MAP3K15	-0.2726	3.326785	-0.598	0.551371	-5.39765	0.713371	0.719725
Dendritic.cells	CRPPA	0.156607	2.552625	0.597725	0.551551	-5.79436	0.724264	0.735472
Dendritic.cells	OSBPL7	-0.12292	3.426262	-0.59751	0.551694	-5.82832	0.712169	0.717864
Dendritic.cells	SYNE3	0.187157	2.767053	0.597485	0.551711	-5.71701	0.721283	0.731152
Dendritic.cells	GM26632	-0.34584	0.366747	-0.59742	0.551756	-5.15153	0.755708	0.781616
Dendritic.cells	FBXO32	0.290911	3.711448	0.597229	0.551881	-5.5469	0.708301	0.712211
Dendritic.cells	TOR1AIP2	0.05989	6.580878	0.597187	0.551909	-6.47746	0.670566	0.657533
Dendritic.cells	TTPA	-0.3494	1.860466	-0.59702	0.552021	-5.27961	0.734104	0.749843
Dendritic.cells	COQ5	0.11545	3.832511	0.596866	0.552122	-6.02898	0.706672	0.709821
Dendritic.cells	SPC24	-0.23293	4.768056	-0.59686	0.552128	-5.842	0.694111	0.691575
Dendritic.cells	UNC45A	-0.10287	4.303611	-0.59678	0.552181	-6.02735	0.700311	0.700575
Dendritic.cells	PDIA3	0.051337	8.546649	0.596284	0.552509	-6.98964	0.646556	0.622659
Dendritic.cells	MTF1	-0.1192	4.234341	-0.59619	0.552572	-5.81588	0.701575	0.702108

Dendritic.cells	ALG3	0.254294	1.77052	0.595886	0.552774	-5.37286	0.735833	0.752039
Dendritic.cells	PLEKHM1	0.130265	4.771883	0.595884	0.552775	-5.77633	0.694483	0.691789
Dendritic.cells	ARID1B	0.046422	8.198201	0.595787	0.55284	-6.88762	0.65087	0.628854
Dendritic.cells	BBX	-0.07376	6.494425	-0.59525	0.553195	-6.58865	0.67243	0.659466
Dendritic.cells	AGAP2	0.131388	2.925575	0.595012	0.553355	-5.82363	0.719927	0.728372
Dendritic.cells	DNAJA2	0.047652	7.208069	0.594928	0.553411	-6.6625	0.663426	0.64652
Dendritic.cells	FAM216A	0.219389	2.582038	0.594882	0.553442	-5.37172	0.724722	0.735366
Dendritic.cells	GM36445	-0.25046	1.003387	-0.59488	0.553443	-5.25612	0.747267	0.768356
Dendritic.cells	GCH1	-0.09514	5.663529	-0.59449	0.553702	-6.39722	0.683291	0.67493
Dendritic.cells	PRPS1	0.166651	3.621673	0.594437	0.553738	-5.60226	0.710514	0.714403
Dendritic.cells	TNFRSF9	0.317609	2.460851	0.594286	0.553838	-5.40226	0.726607	0.737898
Dendritic.cells	ARHGAP27	0.096081	2.032169	0.594223	0.553881	-6.08706	0.732665	0.746748
Dendritic.cells	RNF181	0.130666	4.477136	0.594162	0.553921	-5.88947	0.698942	0.697638
Dendritic.cells	H2-Q7	-0.38114	3.889878	-0.59404	0.554003	-5.58952	0.70686	0.709141
Dendritic.cells	ABCF1	-0.05537	6.430861	-0.59399	0.554036	-6.59083	0.67341	0.660721
Dendritic.cells	RBPJ	0.121896	6.055966	0.593788	0.55417	-6.37597	0.678299	0.667702
Dendritic.cells	PSMD11	-0.03826	7.032517	-0.59363	0.554276	-6.67778	0.665928	0.649794
Dendritic.cells	XBP1	-0.04456	6.135195	-0.59342	0.554413	-6.86091	0.677353	0.666266
Dendritic.cells	PSMC2	0.086162	5.362149	0.593353	0.55446	-6.19022	0.687383	0.680781
Dendritic.cells	TYSND1	-0.16162	2.640407	-0.59329	0.5545	-5.51027	0.724257	0.734362
Dendritic.cells	NAIP1	0.359049	-0.47909	0.593213	0.554553	-5.14392	0.76959	0.800881
Dendritic.cells	ZFP973	-0.258	0.609416	-0.59304	0.554671	-5.31051	0.753465	0.777049
Dendritic.cells	GM49417	-0.39447	1.078235	-0.59277	0.554846	-5.22174	0.746705	0.767145
Dendritic.cells	IFI208	0.391629	2.936618	0.592677	0.55491	-5.28185	0.720281	0.728527
Dendritic.cells	TMEM43	0.169284	3.43413	0.592644	0.554932	-5.57175	0.713404	0.718512
Dendritic.cells	SLC35A1	-0.12318	3.416075	-0.59242	0.55508	-5.72657	0.713675	0.718896
Dendritic.cells	PEX2	0.143782	3.914925	0.59233	0.555142	-5.79479	0.706859	0.709009
Dendritic.cells	B430010I2:	0.326744	-0.41456	0.592327	0.555144	-5.14618	0.768809	0.799672
Dendritic.cells	TCRG-C4	0.367208	0.327974	0.592173	0.555246	-5.15253	0.757763	0.783462
Dendritic.cells	E2F3	0.109247	5.460693	0.59205	0.555328	-6.22088	0.686294	0.679243
Dendritic.cells	NSMF	-0.21611	2.696993	-0.59186	0.555455	-5.39521	0.723676	0.733569
Dendritic.cells	PRPF38B	0.045379	6.611272	0.591756	0.555524	-6.54603	0.671467	0.657845
Dendritic.cells	TLR6	0.379121	1.070509	0.591686	0.555571	-5.14665	0.746873	0.767501
Dendritic.cells	D130040H:	0.234078	2.397794	0.591617	0.555616	-5.3754	0.727876	0.739699
Dendritic.cells	CRISPLD2	0.406328	0.884481	0.591611	0.555621	-5.20527	0.749583	0.771478
Dendritic.cells	FRRS1	0.121049	4.851486	0.591332	0.555807	-5.94759	0.694448	0.690927
Dendritic.cells	RBM43	0.18542	2.961078	0.591261	0.555854	-5.53111	0.720129	0.728258
Dendritic.cells	ARSK	-0.2228	2.705302	-0.59107	0.555985	-5.47693	0.723771	0.733558
Dendritic.cells	JMJD8	0.244963	1.564022	0.59094	0.556068	-5.41181	0.739955	0.757224
Dendritic.cells	CHCHD5	0.187556	3.092559	0.590868	0.556116	-5.54667	0.71838	0.72572
Dendritic.cells	CBX1	0.07411	6.075378	0.590664	0.556253	-6.3482	0.678518	0.667957
Dendritic.cells	RFNG	0.190579	2.228203	0.590627	0.556277	-5.44343	0.730483	0.743445
Dendritic.cells	AP4M1	0.127704	3.581284	0.590497	0.556364	-5.66449	0.711647	0.715988
Dendritic.cells	ZFP296	-0.25517	2.857765	-0.59049	0.556366	-5.42971	0.721642	0.730548
Dendritic.cells	AI506816	0.170827	5.207229	0.590286	0.556505	-5.92846	0.689827	0.684351
Dendritic.cells	COX10	-0.12779	3.775159	-0.59028	0.55651	-5.80819	0.709007	0.712182
Dendritic.cells	TNNI1	-0.39045	-0.907	-0.59012	0.556612	-5.14853	0.776532	0.811134

Dendritic.cells	ZFP950	0.105225	4.197661	0.590097	0.556631	-6.00455	0.703278	0.703859
Dendritic.cells	DNM1L	0.068978	5.639142	0.58992	0.556749	-6.39692	0.684236	0.676246
Dendritic.cells	MPST	0.181649	3.682692	0.589827	0.556811	-5.56369	0.710333	0.714105
Dendritic.cells	MYO1C	-0.0706	5.436164	-0.58948	0.557039	-6.27866	0.687087	0.680197
Dendritic.cells	CD59B	-0.38432	0.413	-0.58916	0.557255	-5.18929	0.757195	0.782312
Dendritic.cells	MET	0.340269	1.991608	0.589106	0.557293	-5.32159	0.734294	0.748757
Dendritic.cells	KIF11	-0.2631	5.441855	-0.58885	0.557467	-5.86522	0.687277	0.680288
Dendritic.cells	ARFIP1	0.141095	3.797972	0.588775	0.557513	-5.7483	0.709242	0.712167
Dendritic.cells	PLXDC2	-0.0903	5.144109	-0.58867	0.557586	-6.9023	0.6912	0.685976
Dendritic.cells	PPP1R12C	0.100136	5.009564	0.588289	0.557838	-5.95454	0.693018	0.688695
Dendritic.cells	ST3GAL6	-0.09441	5.092875	-0.58821	0.557894	-6.27706	0.691916	0.687104
Dendritic.cells	WWP2	0.06537	6.537142	0.588205	0.557894	-6.44767	0.67318	0.660024
Dendritic.cells	NIPA2	0.0736	6.042829	0.588137	0.55794	-6.38454	0.679517	0.669189
Dendritic.cells	POU5F1	-0.22636	1.26222	-0.58813	0.557942	-5.40254	0.744945	0.764394
Dendritic.cells	MAOB	-0.34279	1.627366	-0.5878	0.558164	-5.28067	0.739884	0.756802
Dendritic.cells	ATAD5	0.224266	4.742851	0.587475	0.558382	-5.69872	0.696786	0.694021
Dendritic.cells	RAG2	0.419683	0.168525	0.587463	0.55839	-5.14611	0.761246	0.788162
Dendritic.cells	ACVRL1	-0.26633	2.619629	-0.58745	0.558402	-5.41761	0.725827	0.736264
Dendritic.cells	COX4I2	0.323463	0.956141	0.587376	0.558449	-5.14886	0.749641	0.771111
Dendritic.cells	KPTN	-0.13522	4.098478	-0.58703	0.558681	-5.99885	0.705601	0.706667
Dendritic.cells	SETD1A	-0.10437	4.19796	-0.58695	0.55873	-5.87236	0.704256	0.704734
Dendritic.cells	STK17B	-0.04686	8.129238	-0.58676	0.558862	-7.0375	0.653659	0.631755
Dendritic.cells	MEX3D	0.197339	2.642534	0.586452	0.559066	-5.53105	0.72567	0.736125
Dendritic.cells	PIGF	-0.20438	3.388301	-0.58644	0.559075	-5.47607	0.715302	0.721009
Dendritic.cells	NGP	0.385384	5.022684	0.586312	0.559159	-5.63381	0.693227	0.688978
Dendritic.cells	ERC1	-0.10076	4.872109	-0.58621	0.559229	-6.1917	0.695224	0.691908
Dendritic.cells	PPCDC	-0.11829	3.567947	-0.58603	0.559351	-5.88704	0.712832	0.71751
Dendritic.cells	PHACTR2	0.055637	6.036575	0.585999	0.559369	-6.9182	0.679974	0.669858
Dendritic.cells	SFXN1	-0.10167	5.121606	-0.58593	0.559418	-6.12448	0.69192	0.687141
Dendritic.cells	COLEC12	-0.3158	2.890318	-0.58588	0.55945	-5.46569	0.722205	0.731166
Dendritic.cells	ZFP212	0.124308	3.640876	0.585861	0.559461	-5.71541	0.711833	0.716057
Dendritic.cells	POLE3	0.143319	4.133596	0.585803	0.5595	-5.77511	0.705126	0.706321
Dendritic.cells	ALKBH4	0.181701	2.574916	0.5857	0.559569	-5.50982	0.72662	0.737668
Dendritic.cells	MAPK14	-0.06489	6.656022	-0.58554	0.559678	-6.44537	0.67204	0.658502
Dendritic.cells	MYOM1	-0.28116	1.071853	-0.58547	0.559721	-5.30299	0.748123	0.769207
Dendritic.cells	6720489N1	0.291932	0.964398	0.585314	0.559828	-5.1821	0.74969	0.771557
Dendritic.cells	GM16083	-0.25046	1.977218	-0.5853	0.559836	-5.43022	0.735079	0.750143
Dendritic.cells	STK25	-0.10844	4.440838	-0.58527	0.559856	-5.88159	0.700985	0.700462
Dendritic.cells	LILRA6	0.300446	1.213739	0.585068	0.559992	-5.31713	0.74606	0.766334
Dendritic.cells	SLC12A4	-0.19583	1.745624	-0.58505	0.560004	-5.54418	0.738389	0.755089
Dendritic.cells	PLAUR	0.067262	6.837467	0.585026	0.56002	-7.1549	0.669739	0.655324
Dendritic.cells	ADGRA2	-0.311	0.687625	-0.58471	0.56023	-5.35974	0.753744	0.777748
Dendritic.cells	RUVBL2	-0.17409	3.693884	-0.58468	0.560249	-5.61596	0.711107	0.715379
Dendritic.cells	RNPS1	0.066807	6.167896	0.584492	0.560378	-6.34269	0.678282	0.667833
Dendritic.cells	CCT7	-0.0628	6.427225	-0.58448	0.560386	-6.49073	0.674956	0.663029
Dendritic.cells	AHCY	-0.21945	3.495963	-0.58425	0.560537	-5.49047	0.713821	0.719441
Dendritic.cells	1700012D1	0.208713	1.843803	0.584235	0.56055	-5.47562	0.736983	0.753275

Dendritic.cells	WNT4	0.279544	1.526488	0.584097	0.560642	-5.25545	0.741538	0.75996
Dendritic.cells	REL	-0.07513	8.12408	-0.58406	0.560669	-6.82292	0.653722	0.632485
Dendritic.cells	GM10658	-0.24991	1.776179	-0.58392	0.560764	-5.3473	0.737951	0.754733
Dendritic.cells	TRMT12	-0.23859	1.30746	-0.58384	0.560814	-5.33239	0.744702	0.764665
Dendritic.cells	HGF	-0.30403	2.743326	-0.5837	0.560911	-5.44735	0.724258	0.734748
Dendritic.cells	PTDSS1	-0.07052	5.371518	-0.58368	0.560924	-6.3263	0.68863	0.682921
Dendritic.cells	PHLPP1	-0.08168	7.671226	-0.58365	0.560942	-6.84935	0.6593	0.64057
Dendritic.cells	DOCK3	0.345086	0.039329	0.583527	0.561024	-5.18182	0.763343	0.79213
Dendritic.cells	LARS	0.09962	4.738364	0.583524	0.561026	-6.10108	0.697004	0.695106
Dendritic.cells	BC005561	-0.11144	4.273496	-0.58351	0.561039	-5.90839	0.703237	0.704159
Dendritic.cells	ZBTB7B	-0.24594	2.473173	-0.58328	0.561189	-5.42248	0.728162	0.740411
Dendritic.cells	COQ4	0.188014	2.66674	0.583102	0.561309	-5.47701	0.725446	0.736522
Dendritic.cells	UBTF	-0.05944	5.879519	-0.58291	0.561437	-6.42874	0.682113	0.673583
Dendritic.cells	AKAP8	0.07342	5.39229	0.582827	0.561493	-6.20239	0.688466	0.6828
Dendritic.cells	UQCRC1	0.069669	6.508803	0.582654	0.561609	-6.4483	0.674021	0.66194
Dendritic.cells	RNF135	-0.22178	2.08421	-0.58257	0.561665	-5.39372	0.733672	0.748684
Dendritic.cells	COLGALT1	0.066857	5.889717	0.58257	0.561665	-6.44923	0.681981	0.673448
Dendritic.cells	CAMK2N1	-0.34556	1.714702	-0.5825	0.56171	-5.31431	0.738949	0.756415
Dendritic.cells	PECR	-0.31381	2.35098	-0.5825	0.561711	-5.33982	0.72989	0.74315
Dendritic.cells	GM27241	-0.18079	2.603068	-0.58215	0.561949	-5.68418	0.72644	0.73804
Dendritic.cells	URB1	0.159464	2.176346	0.581982	0.56206	-5.51731	0.732464	0.746865
Dendritic.cells	SEMA4A	0.240794	3.364224	0.581936	0.562091	-5.42205	0.715846	0.722618
Dendritic.cells	ZBTB12	-0.24422	1.665552	-0.58192	0.5621	-5.27319	0.739757	0.757579
Dendritic.cells	SGCE	-0.27947	1.156412	-0.58186	0.562141	-5.39228	0.747115	0.768383
Dendritic.cells	AKR1C12	0.299854	0.756271	0.581806	0.562178	-5.18556	0.75296	0.776972
Dendritic.cells	CFAP45	0.198493	0.728674	0.581676	0.562265	-5.66253	0.753368	0.777605
Dendritic.cells	LRSAM1	0.228366	1.610096	0.581594	0.56232	-5.37412	0.740557	0.758814
Dendritic.cells	GM11655	-0.18113	0.316941	-0.58151	0.562375	-5.62225	0.759444	0.786588
Dendritic.cells	CDON	0.240085	1.952332	0.581256	0.562547	-5.44124	0.735796	0.75178
Dendritic.cells	AURKB	0.284885	4.237652	0.581107	0.562647	-5.55259	0.704111	0.705534
Dendritic.cells	SIRPB1A	0.36091	-0.18195	0.580957	0.562748	-5.1523	0.767127	0.797777
Dendritic.cells	SSBP4	0.096944	4.90464	0.580793	0.562857	-6.01619	0.695235	0.692634
Dendritic.cells	AREL1	0.124814	4.263334	0.580658	0.562948	-5.78155	0.703821	0.705153
Dendritic.cells	CIP2A	-0.24952	3.506565	-0.58061	0.56298	-5.4893	0.714129	0.720171
Dendritic.cells	PDCL3	0.112461	4.536557	0.580555	0.563017	-5.89022	0.700147	0.699825
Dendritic.cells	MOB4	-0.05166	6.832732	-0.58037	0.563141	-6.58364	0.670233	0.656518
Dendritic.cells	RBMS1	-0.05872	7.558573	-0.58035	0.563154	-6.64096	0.661126	0.643386
Dendritic.cells	PEX13	-0.088	5.524698	-0.58	0.56339	-6.18584	0.687111	0.680923
Dendritic.cells	FAF1	0.053439	6.739255	0.579905	0.563453	-6.63459	0.671459	0.658292
Dendritic.cells	DRAM1	0.38458	2.581586	0.579813	0.563516	-5.22445	0.727042	0.739056
Dendritic.cells	CRACR2B	0.279019	1.009115	0.579758	0.563552	-5.3737	0.749569	0.772079
Dendritic.cells	ALPL	0.337061	1.490191	0.579649	0.563625	-5.18485	0.742588	0.761849
Dendritic.cells	PTPN3	0.22897	1.040176	0.579626	0.563641	-5.56225	0.749116	0.771436
Dendritic.cells	COG2	-0.12499	3.567744	-0.57953	0.563706	-5.80399	0.713341	0.719076
Dendritic.cells	VPS53	0.083914	4.462382	0.579527	0.563707	-5.96953	0.701193	0.701391
Dendritic.cells	SETD6	-0.19491	1.423905	-0.57875	0.564229	-5.40308	0.744085	0.763643
Dendritic.cells	SCAF8	-0.04925	6.711338	-0.57873	0.564246	-6.68076	0.672301	0.659159

Dendritic.cells	ENPP4	-0.10226	2.924155	-0.57853	0.56438	-6.12108	0.722858	0.732509
Dendritic.cells	5730522E0	-0.16735	3.610742	-0.57839	0.564473	-5.93521	0.713381	0.71866
Dendritic.cells	MTHFD2	-0.10642	5.486209	-0.57821	0.564595	-6.26675	0.688222	0.682146
Dendritic.cells	GZMB	-0.35289	3.971899	-0.57815	0.564634	-5.69047	0.708446	0.711531
Dendritic.cells	ELMO1	-0.05736	8.719508	-0.57811	0.56466	-6.84751	0.647515	0.623447
Dendritic.cells	PRKCZ	0.285311	0.606888	0.577661	0.564961	-5.19315	0.756167	0.781466
Dendritic.cells	GM525	0.143457	0.742971	0.577565	0.565026	-5.69622	0.754163	0.778518
Dendritic.cells	TMEM69	-0.25481	1.585462	-0.57756	0.565032	-5.32941	0.7419	0.760506
Dendritic.cells	LIN54	-0.08607	6.14118	-0.5775	0.565068	-6.46938	0.679735	0.669955
Dendritic.cells	HINT3	0.089267	4.643869	0.577493	0.565074	-6.18297	0.699407	0.698473
Dendritic.cells	COL1A1	0.330102	2.255466	0.577473	0.565088	-5.3999	0.732322	0.74647
Dendritic.cells	USP47	0.056975	6.472249	0.577384	0.565148	-6.63339	0.675484	0.663828
Dendritic.cells	CXCL9	0.548524	2.080321	0.577302	0.565203	-5.33827	0.734811	0.750187
Dendritic.cells	ACER3	0.055484	6.311004	0.577062	0.565364	-6.76109	0.677606	0.666906
Dendritic.cells	SLC9A6	-0.15334	2.81804	-0.57695	0.565441	-5.59468	0.724456	0.735022
Dendritic.cells	NFXL1	-0.15028	3.364621	-0.57692	0.565458	-5.73953	0.716858	0.723929
Dendritic.cells	BRF1	0.07456	4.828649	0.576821	0.565526	-6.18663	0.696998	0.695023
Dendritic.cells	SLC1A5	-0.08413	6.46392	-0.57675	0.565574	-6.43191	0.675646	0.66409
Dendritic.cells	SLC16A9	-0.41492	0.530927	-0.57659	0.565684	-5.20402	0.757374	0.783307
Dendritic.cells	NOP2	-0.13015	3.635097	-0.57635	0.565845	-5.72491	0.713158	0.718573
Dendritic.cells	GLCE	-0.10377	3.79856	-0.57632	0.565863	-6.0359	0.71092	0.715311
Dendritic.cells	4833439L1	-0.08959	4.862518	-0.57627	0.565899	-6.13004	0.696569	0.694437
Dendritic.cells	POLG	-0.18793	3.808912	-0.57624	0.565917	-5.486	0.710778	0.715107
Dendritic.cells	BAHD1	-0.19281	2.543391	-0.57577	0.566234	-5.4401	0.728643	0.740916
Dendritic.cells	ZEB2OS	-0.09063	4.678362	-0.57569	0.566286	-6.30686	0.699322	0.698178
Dendritic.cells	2810402E2	0.168665	2.412627	0.575422	0.566467	-5.53712	0.730563	0.743719
Dendritic.cells	RHOBTB1	-0.23559	3.180961	-0.57541	0.566477	-5.55874	0.7198	0.727994
Dendritic.cells	TBC1D22A	0.058389	5.871527	0.575317	0.566538	-6.55231	0.683661	0.675465
Dendritic.cells	BEND3	-0.1892	2.456588	-0.57506	0.566711	-5.57614	0.730081	0.742876
Dendritic.cells	ATP2A1	-0.08592	1.448126	-0.57442	0.567145	-6.35761	0.744907	0.764175
Dendritic.cells	BCAP29	0.081807	4.765797	0.57432	0.567209	-6.24004	0.698739	0.696858
Dendritic.cells	AGAP3	-0.1169	4.127556	-0.57431	0.567216	-5.97622	0.707334	0.70935
Dendritic.cells	CD163L1	0.393129	0.60908	0.574175	0.567307	-5.15656	0.75721	0.782284
Dendritic.cells	FGFRL1	0.352057	0.299565	0.573628	0.567676	-5.19289	0.762205	0.789185
Dendritic.cells	TRIB3	0.339873	1.057747	0.573522	0.567748	-5.26606	0.75103	0.772749
Dendritic.cells	ZFP516	0.106073	4.66128	0.573307	0.567892	-6.08711	0.70065	0.699159
Dendritic.cells	INPPL1	0.152833	2.471527	0.573049	0.568066	-5.63085	0.730945	0.743252
Dendritic.cells	EIF5B	0.052307	7.158051	0.572874	0.568184	-6.62383	0.668326	0.652412
Dendritic.cells	4933432I0	0.348954	0.408312	0.572824	0.568218	-5.17603	0.760853	0.787182
Dendritic.cells	RNASEH1	0.150027	2.84733	0.57269	0.568308	-5.57406	0.725657	0.735629
Dendritic.cells	NIP7	-0.09338	4.635807	-0.57261	0.568364	-5.93711	0.701129	0.699911
Dendritic.cells	TGFBR3	0.136733	3.337964	0.57239	0.568511	-6.00001	0.718821	0.725667
Dendritic.cells	NR1H2	0.123907	4.589379	0.57235	0.568538	-5.92191	0.701752	0.700827
Dendritic.cells	GM29488	0.349028	0.172443	0.57234	0.568544	-5.15529	0.764365	0.792411
Dendritic.cells	UBXN4	0.033304	6.814787	0.572274	0.568589	-6.82984	0.67267	0.658724
Dendritic.cells	NOLC1	-0.10215	5.264292	-0.57215	0.56867	-6.17947	0.692782	0.687841
Dendritic.cells	SIPA1L2	-0.15845	5.103799	-0.5718	0.56891	-5.79216	0.69512	0.691054

Dendritic.cells	CD207	0.380883	-0.64958	0.5716	0.569043	-5.15277	0.777032	0.810941
Dendritic.cells	TDRD3	-0.08929	4.059195	-0.57151	0.569104	-6.13064	0.709172	0.711466
Dendritic.cells	GAB3	0.138342	4.746655	0.571492	0.569116	-5.94135	0.699892	0.697981
Dendritic.cells	ATG4D	0.115407	3.954415	0.570951	0.569481	-5.88449	0.710889	0.713778
Dendritic.cells	CNPY4	-0.19569	2.433697	-0.57078	0.569598	-5.42587	0.73204	0.744657
Dendritic.cells	RPN1	-0.06692	5.957169	-0.57077	0.569607	-6.47274	0.684208	0.675098
Dendritic.cells	SGPL1	-0.07383	5.922758	-0.57072	0.569638	-6.4497	0.684656	0.675745
Dendritic.cells	TMCC1	-0.09143	7.946033	-0.57067	0.569673	-6.68268	0.658998	0.638758
Dendritic.cells	MRI1	0.09366	3.963231	0.570231	0.569967	-5.99584	0.710969	0.713735
Dendritic.cells	PIGC	0.162513	2.730331	0.570119	0.570043	-5.55417	0.728058	0.738666
Dendritic.cells	PAXX	0.119768	3.270058	0.569996	0.570126	-5.78889	0.720514	0.727687
Dendritic.cells	SMIM19	0.080542	4.75168	0.569859	0.570218	-6.17304	0.700307	0.698318
Dendritic.cells	RERG	0.2883	0.522461	0.569841	0.570231	-5.35537	0.759953	0.78548
Dendritic.cells	PDSS2	-0.08951	5.377267	-0.56984	0.570234	-6.24903	0.691993	0.686261
Dendritic.cells	CCDC93	0.15475	3.72019	0.56972	0.570312	-5.69298	0.714297	0.718692
Dendritic.cells	NIPSNAP1	-0.20249	3.134643	-0.56966	0.570355	-5.49365	0.722398	0.730501
Dendritic.cells	PCBP4	0.25352	0.472145	0.568562	0.571095	-5.35423	0.761412	0.786969
Dendritic.cells	ADRM1	-0.09919	5.477684	-0.56843	0.571183	-6.14407	0.691318	0.684685
Dendritic.cells	KRT8	0.317525	1.975849	0.568411	0.571197	-5.31128	0.739462	0.754765
Dendritic.cells	FASN	0.173441	3.14635	0.568227	0.571321	-5.64492	0.722912	0.730612
Dendritic.cells	COX16	0.053615	6.153691	0.567992	0.57148	-6.42133	0.682492	0.671942
Dendritic.cells	ARSA	0.193338	1.69399	0.567981	0.571487	-5.46304	0.743517	0.760728
Dendritic.cells	GM38832	-0.31229	0.763984	-0.56796	0.5715	-5.1842	0.757091	0.780643
Dendritic.cells	ADSL	-0.12231	4.033205	-0.56792	0.57153	-5.80344	0.71068	0.712806
Dendritic.cells	DUT	-0.17894	5.882801	-0.56774	0.57165	-6.02401	0.686011	0.677032
Dendritic.cells	ZFP704	-0.23823	3.792086	-0.56769	0.571685	-5.57932	0.713979	0.717627
Dendritic.cells	GM19265	0.315139	0.22098	0.567591	0.571752	-5.15978	0.765155	0.792566
Dendritic.cells	UPF3B	0.099339	4.979585	0.567574	0.571763	-6.05127	0.697917	0.694325
Dendritic.cells	CRYL1	0.135253	3.73595	0.567567	0.571767	-5.77223	0.71475	0.718782
Dendritic.cells	ARMC10	0.147693	3.275459	0.567515	0.571803	-5.63488	0.721114	0.72807
Dendritic.cells	VPS29	0.055822	6.521761	0.567321	0.571934	-6.524	0.677753	0.665219
Dendritic.cells	SLC16A1	-0.22037	3.905544	-0.56729	0.571957	-5.41976	0.712429	0.715491
Dendritic.cells	VHL	-0.11681	3.486608	-0.56722	0.572002	-5.73973	0.718192	0.723892
Dendritic.cells	PSMB8	0.085988	7.077604	0.567109	0.572077	-6.76172	0.670684	0.655027
Dendritic.cells	SARNP	-0.04312	7.797226	-0.56692	0.572205	-6.80588	0.661674	0.642044
Dendritic.cells	FAM171B	0.34547	0.217464	0.566902	0.572217	-5.25576	0.765238	0.792768
Dendritic.cells	LAT2	-0.06885	4.773693	-0.56654	0.572461	-6.52888	0.700814	0.698472
Dendritic.cells	MNS1	0.192159	2.821224	0.566387	0.572566	-5.49001	0.727613	0.737475
Dendritic.cells	CACUL1	0.073963	6.117834	0.566264	0.572649	-6.46363	0.683098	0.672817
Dendritic.cells	PSMC3	0.067124	6.11628	0.566162	0.572718	-6.34807	0.683119	0.672848
Dendritic.cells	BST1	0.294509	3.315218	0.566073	0.572778	-5.39259	0.720712	0.727411
Dendritic.cells	DHCR7	0.126582	2.184949	0.566018	0.572815	-5.76078	0.736624	0.750671
Dendritic.cells	2010310CC	-0.31379	1.298313	-0.56587	0.572917	-5.38598	0.749412	0.76943
Dendritic.cells	ADAM3	-0.31333	-0.17326	-0.56554	0.573138	-5.1578	0.771233	0.801603
Dendritic.cells	ZFP991	0.183787	2.80432	0.565414	0.573225	-5.59924	0.727851	0.737995
Dendritic.cells	DDX59	-0.18796	1.892836	-0.56539	0.573243	-5.49826	0.740807	0.756942
Dendritic.cells	ALKBH8	0.123567	3.835022	0.565348	0.573269	-5.74652	0.713539	0.71713

Dendritic.cells	EPHA1	0.306381	0.461905	0.5653	0.573302	-5.25801	0.761723	0.787642
Dendritic.cells	NUCKS1	0.079926	6.576295	0.565294	0.573306	-6.47915	0.67719	0.664439
Dendritic.cells	GM50340	-0.25522	1.533078	-0.56524	0.573339	-5.36169	0.745999	0.764549
Dendritic.cells	USPL1	0.121906	3.533846	0.565203	0.573367	-5.7899	0.717684	0.723179
Dendritic.cells	RCN1	0.160123	3.060814	0.565139	0.573411	-5.63176	0.724256	0.732779
Dendritic.cells	SYF2	-0.05598	5.903221	-0.56497	0.573526	-6.35997	0.685887	0.67705
Dendritic.cells	TMEM214	0.080893	4.338367	0.56493	0.573552	-6.09719	0.706679	0.707198
Dendritic.cells	CUL4A	-0.07569	5.002429	-0.56492	0.573561	-6.18138	0.697758	0.694244
Dendritic.cells	TAF9B	-0.26302	0.956005	-0.56471	0.5737	-5.30885	0.754421	0.777002
Dendritic.cells	ERP27	0.257201	1.896721	0.564688	0.573716	-5.31274	0.740752	0.756958
Dendritic.cells	PRDM16	-0.36113	0.306557	-0.56464	0.573748	-5.18513	0.764036	0.791149
Dendritic.cells	BRIP1OS	-0.08801	5.408086	-0.56443	0.573889	-6.15029	0.692405	0.686539
Dendritic.cells	ETV5	-0.21778	3.199847	-0.56419	0.574053	-5.52634	0.722343	0.730086
Dendritic.cells	SDCBP	-0.05801	7.674968	-0.56395	0.574215	-6.68745	0.66333	0.644573
Dendritic.cells	ATP10A	0.163337	3.430551	0.563932	0.574228	-5.76302	0.719139	0.725414
Dendritic.cells	SHROOM4	-0.2922	0.86592	-0.56393	0.574233	-5.40189	0.755773	0.779032
Dendritic.cells	CDH23	0.191264	2.820164	0.563852	0.574282	-5.55812	0.727655	0.737839
Dendritic.cells	1700102H2	0.29505	0.529845	0.563579	0.574467	-5.20837	0.760741	0.786463
Dendritic.cells	CASTOR2	0.140246	3.643855	0.563394	0.574593	-6.09386	0.716193	0.721252
Dendritic.cells	FAN1	0.24751	1.159783	0.563379	0.574603	-5.36408	0.751461	0.772839
Dendritic.cells	C330018D2	0.206222	2.325358	0.563287	0.574665	-5.37656	0.734651	0.748201
Dendritic.cells	MYCBP	0.145973	4.22905	0.56314	0.574765	-5.6794	0.708188	0.709683
Dendritic.cells	AMFR	0.046565	6.011988	0.563126	0.574774	-6.60063	0.684497	0.675317
Dendritic.cells	PPIL6	0.304234	0.219778	0.563113	0.574783	-5.21361	0.76536	0.793375
Dendritic.cells	GM49041	0.275079	1.334735	0.563013	0.574851	-5.32515	0.748908	0.76922
Dendritic.cells	APCS	0.192292	4.833138	0.562858	0.574956	-5.85129	0.700044	0.69792
Dendritic.cells	CUL3	-0.04275	7.198802	-0.56275	0.575028	-6.69912	0.669301	0.653491
Dendritic.cells	ZNRD1	0.096713	5.076686	0.562538	0.575173	-6.01728	0.696794	0.693326
Dendritic.cells	H2-EB2	0.349447	0.547733	0.562517	0.575187	-5.16931	0.760476	0.786401
Dendritic.cells	VTA1	0.061183	5.707573	0.5625	0.575199	-6.39825	0.688468	0.681255
Dendritic.cells	LRRC8A	0.084245	5.427019	0.562469	0.57522	-6.18194	0.692155	0.686597
Dendritic.cells	ALYREF	-0.07096	8.216984	-0.56245	0.575236	-6.82056	0.65662	0.63529
Dendritic.cells	MORF4L1	0.029986	8.201561	0.562318	0.575322	-6.90593	0.65681	0.635563
Dendritic.cells	NT5C2	0.081783	5.867578	0.562228	0.575383	-6.46586	0.686377	0.678226
Dendritic.cells	GORAB	-0.22253	1.673364	-0.56222	0.575388	-5.36031	0.743997	0.762188
Dendritic.cells	PARVG	-0.08394	5.071901	-0.56215	0.575439	-6.21253	0.696858	0.693446
Dendritic.cells	XRCC5	-0.26849	2.083305	-0.56201	0.575534	-5.22417	0.738104	0.753581
Dendritic.cells	XIST	-1.81406	4.173151	-0.56185	0.575643	-5.98176	0.708947	0.711014
Dendritic.cells	NEDD1	-0.16314	3.041246	-0.56166	0.57577	-5.63234	0.724556	0.733768
Dendritic.cells	GM32743	-0.31876	-0.48509	-0.56145	0.575909	-5.19207	0.775983	0.809335
Dendritic.cells	GM26771	0.357046	-0.2163	0.561424	0.575929	-5.15892	0.771911	0.803323
Dendritic.cells	GM49662	-0.36623	2.537113	-0.56142	0.57593	-5.34092	0.731647	0.744162
Dendritic.cells	DCAF7	-0.05989	5.327488	-0.56137	0.575962	-6.35949	0.693469	0.688581
Dendritic.cells	PDHA1	0.093671	5.115043	0.561275	0.57603	-6.07253	0.696285	0.692666
Dendritic.cells	ATP6V0A1	0.129012	4.202735	0.561162	0.576107	-5.81592	0.708545	0.710483
Dendritic.cells	NEK10	-0.35614	1.324395	-0.56112	0.576135	-5.1986	0.749059	0.769705
Dendritic.cells	PRDM9	0.253477	1.146615	0.561075	0.576166	-5.31712	0.751654	0.773517

Dendritic.cells	STARD9	-0.1313	3.529605	-0.56105	0.576182	-5.8972	0.717769	0.723919
Dendritic.cells	H2-Q6	-0.4879	3.115257	-0.56059	0.576496	-5.29817	0.723834	0.732402
Dendritic.cells	ARMC1	-0.09182	4.827556	-0.56021	0.576753	-5.98162	0.700515	0.698368
Dendritic.cells	LNPK	0.124955	4.386002	0.560187	0.576769	-5.87943	0.70646	0.707011
Dendritic.cells	NR4A2	-0.13902	6.380914	-0.56014	0.576799	-6.51781	0.680109	0.668823
Dendritic.cells	WASL	-0.05706	5.483088	-0.55999	0.576903	-6.36698	0.691807	0.685796
Dendritic.cells	GM12689	0.316999	-0.48996	0.559876	0.57698	-5.15747	0.776496	0.809704
Dendritic.cells	DCTN5	0.10657	4.598222	0.559815	0.577021	-5.92947	0.703594	0.702925
Dendritic.cells	GALNT16	0.267591	0.933714	0.559802	0.57703	-5.24639	0.755203	0.778352
Dendritic.cells	RRAS2	-0.09533	5.198697	-0.5596	0.577168	-6.22907	0.695612	0.691275
Dendritic.cells	TUBG1	0.210094	3.393021	0.55953	0.577215	-5.59379	0.720112	0.726907
Dendritic.cells	DAG1	-0.07355	4.867236	-0.55946	0.577263	-6.42177	0.700028	0.697683
Dendritic.cells	RTL6	0.269952	0.292529	0.559205	0.577435	-5.34133	0.764839	0.792371
Dendritic.cells	GM13402	-0.29301	0.307958	-0.55897	0.577592	-5.26596	0.764609	0.792032
Dendritic.cells	1700008J0	-0.23995	1.842472	-0.55896	0.5776	-5.40053	0.742108	0.758975
Dendritic.cells	PSTPIP2	0.18677	4.482064	0.558923	0.577627	-5.87977	0.705284	0.705235
Dendritic.cells	CD86	0.158162	6.31281	0.558885	0.577653	-6.27343	0.681107	0.670191
Dendritic.cells	FLVCR1	-0.11447	4.670527	-0.55873	0.57776	-5.91289	0.702795	0.701634
Dendritic.cells	ATG13	0.09535	4.765899	0.558464	0.577939	-6.11805	0.701652	0.699831
Dendritic.cells	CES2A	-0.34127	0.898465	-0.55825	0.578087	-5.21263	0.756083	0.779327
Dendritic.cells	TRIM5	0.173959	3.589282	0.558136	0.578162	-5.62339	0.717694	0.723155
Dendritic.cells	MYO1D	-0.19311	3.025547	-0.55807	0.578208	-5.52065	0.725532	0.734605
Dendritic.cells	CYREN	-0.13663	3.374354	-0.55805	0.57822	-5.80127	0.72067	0.727515
Dendritic.cells	CASC1	-0.3142	1.810453	-0.55772	0.578447	-5.26906	0.743002	0.760018
Dendritic.cells	GM43061	0.284013	0.306464	0.557614	0.578517	-5.27397	0.765083	0.792487
Dendritic.cells	ZFP619	-0.1716	2.21582	-0.55712	0.578851	-5.49883	0.737382	0.751665
Dendritic.cells	B3GALT6	0.256935	1.806345	0.557096	0.578869	-5.37141	0.743259	0.760272
Dendritic.cells	EIF4A3	0.068527	5.877476	0.55702	0.578921	-6.35073	0.68734	0.678868
Dendritic.cells	TDRP	0.357129	-0.36439	0.556982	0.578947	-5.21355	0.775383	0.807549
Dendritic.cells	PLA1A	-0.33084	1.841769	-0.55691	0.578993	-5.22691	0.742748	0.759558
Dendritic.cells	FAM49B	-0.03884	9.086243	-0.55662	0.579192	-7.05262	0.647135	0.620903
Dendritic.cells	TMC4	-0.22011	1.556285	-0.55661	0.579198	-5.29273	0.746943	0.76565
Dendritic.cells	R3HCC1	-0.22868	2.353768	-0.55655	0.579241	-5.42641	0.73548	0.748886
Dendritic.cells	CDS2	0.101594	4.708784	0.556291	0.579417	-5.96079	0.702888	0.701463
Dendritic.cells	NDUFS8	0.058539	6.232258	0.556276	0.579427	-6.4959	0.682787	0.672333
Dendritic.cells	DNAH8	0.210283	3.758702	0.556149	0.579514	-5.53174	0.715812	0.720306
Dendritic.cells	1110046J0	0.304185	0.031359	0.556137	0.579522	-5.20778	0.769472	0.798943
Dendritic.cells	NPEPPS	0.06291	7.184287	0.555909	0.579677	-6.66245	0.670608	0.654844
Dendritic.cells	SELENBP1	0.248052	3.795416	0.555831	0.57973	-5.4845	0.715307	0.719632
Dendritic.cells	D830050J1	0.257951	1.312085	0.555811	0.579744	-5.3138	0.750496	0.771092
Dendritic.cells	NR1H4	-0.28655	1.386536	-0.55578	0.579763	-5.31483	0.749411	0.769513
Dendritic.cells	IL27	0.398181	0.053926	0.555195	0.580163	-5.16317	0.769577	0.79862
Dendritic.cells	A330032B1	0.377349	-0.15579	0.555036	0.580271	-5.15861	0.772738	0.803381
Dendritic.cells	YIF1B	-0.09112	4.886916	-0.55493	0.580343	-6.10175	0.700903	0.698315
Dendritic.cells	GM26801	-0.34592	0.557742	-0.55476	0.580457	-5.19474	0.762044	0.787695
Dendritic.cells	PAH	-0.29446	3.508909	-0.55475	0.58047	-5.61658	0.719676	0.725645
Dendritic.cells	P4HB	0.049907	7.267668	0.554451	0.58067	-6.76758	0.669941	0.653638

Dendritic.cells	A530041M	0.182468	2.069945	0.554436	0.58068	-5.46265	0.739961	0.755378
Dendritic.cells	MED12L	0.079309	3.141983	0.554401	0.580704	-6.29662	0.724782	0.733192
Dendritic.cells	CTDP1	-0.08702	4.660739	-0.55431	0.580765	-6.05172	0.703941	0.7029
Dendritic.cells	USP7	0.049399	6.215443	0.554181	0.580854	-6.54327	0.683399	0.673141
Dendritic.cells	UGT2B5	-0.31877	1.786068	-0.55404	0.580951	-5.3582	0.744047	0.761451
Dendritic.cells	FNTA	0.061035	5.505108	0.554012	0.580969	-6.3118	0.692686	0.68659
Dendritic.cells	TMEM9B	0.061762	5.558526	0.553923	0.58103	-6.35954	0.691982	0.68557
Dendritic.cells	ARFGAP3	-0.11276	3.55174	-0.55388	0.581059	-5.91127	0.719083	0.724957
Dendritic.cells	PREP	-0.10902	5.298922	-0.55385	0.581078	-5.99617	0.695413	0.690543
Dendritic.cells	ANXA3	0.26047	3.542549	0.553483	0.58133	-5.47647	0.719275	0.725205
Dendritic.cells	CCDC69	0.1523	3.297575	0.553482	0.581331	-5.6834	0.722677	0.730166
Dendritic.cells	NUDT16L1	0.12069	4.234234	0.553248	0.58149	-5.90019	0.70978	0.711402
Dendritic.cells	UIMC1	0.060402	5.853468	0.553219	0.58151	-6.43181	0.688174	0.680045
Dendritic.cells	TMEM251	-0.08137	4.619848	-0.55321	0.581516	-6.19203	0.704556	0.703806
Dendritic.cells	VPS9D1	0.157973	3.05216	0.553126	0.581573	-5.62682	0.726105	0.735193
Dendritic.cells	PGP	-0.11437	5.374059	-0.5531	0.581589	-6.01347	0.694481	0.689183
Dendritic.cells	CLPB	-0.09179	4.010407	-0.55266	0.581892	-5.96	0.713127	0.715899
Dendritic.cells	GM15728	0.319754	0.500791	0.552401	0.582068	-5.182	0.763303	0.789403
Dendritic.cells	FRA10AC1	0.136821	3.408508	0.552331	0.582115	-5.70495	0.721457	0.728101
Dendritic.cells	GM17268	-0.39672	0.383225	-0.55214	0.582245	-5.19809	0.765056	0.791998
Dendritic.cells	LRRC59	-0.06288	5.556125	-0.55212	0.582257	-6.36052	0.692387	0.685862
Dendritic.cells	FBXO47	-0.31633	1.069812	-0.55211	0.582269	-5.2501	0.754885	0.777039
Dendritic.cells	TSEN34	0.1239	4.195681	0.552049	0.582307	-5.85471	0.710624	0.712336
Dendritic.cells	RASL11B	0.252781	0.665224	0.551767	0.5825	-5.3279	0.76089	0.785821
Dendritic.cells	PLA2G4A	-0.23442	3.294743	-0.55163	0.582592	-5.51635	0.72307	0.730423
Dendritic.cells	WDR82	0.068807	4.82833	0.551626	0.582596	-6.14215	0.702096	0.699901
Dendritic.cells	ACBD4	-0.17137	1.764413	-0.55162	0.582599	-5.58788	0.744792	0.762183
Dendritic.cells	GRAMD1A	-0.15588	4.325204	-0.55153	0.582658	-5.72144	0.70889	0.709788
Dendritic.cells	GM6225	0.181192	2.958455	0.551434	0.582727	-5.63018	0.727779	0.737339
Dendritic.cells	PPIB	0.051049	7.605608	0.551325	0.582801	-6.80855	0.666095	0.647872
Dendritic.cells	FBXO44	0.330968	0.220079	0.55094	0.583064	-5.20166	0.767805	0.795699
Dendritic.cells	ZFP385A	-0.09017	3.938147	-0.55074	0.583203	-6.1825	0.71444	0.717585
Dendritic.cells	GM16230	0.365751	0.238127	0.550524	0.583348	-5.17554	0.767543	0.795395
Dendritic.cells	PRDX3	-0.10375	5.400912	-0.55051	0.58336	-6.0492	0.694723	0.689006
Dendritic.cells	GM37494	-0.10868	3.825168	-0.5505	0.583368	-5.93504	0.715992	0.719902
Dendritic.cells	KCTD10	-0.11285	4.043279	-0.5504	0.583433	-5.87433	0.712999	0.715545
Dendritic.cells	EBNA1BP2	-0.13371	4.017915	-0.55036	0.583463	-5.78452	0.713346	0.71605
Dendritic.cells	MAPKAPK5	0.170735	2.128701	0.550126	0.58362	-5.46736	0.739895	0.754777
Dendritic.cells	SPATA48	-0.23021	2.190427	-0.54998	0.583719	-5.40613	0.739011	0.753507
Dendritic.cells	UBE2CBP	0.33122	1.761179	0.549975	0.583723	-5.32468	0.745187	0.762554
Dendritic.cells	TBC1D5	0.053834	7.293343	0.549678	0.583926	-6.83295	0.67032	0.653781
Dendritic.cells	POLR2B	0.082349	4.911643	0.549662	0.583937	-6.09635	0.701307	0.698601
Dendritic.cells	CNNM2	-0.13714	5.427728	-0.54961	0.583976	-6.262	0.694436	0.688635
Dendritic.cells	TPPP	0.348689	0.182012	0.549602	0.583978	-5.27418	0.768459	0.796801
Dendritic.cells	CEP135	-0.10269	3.853066	-0.5495	0.58405	-5.82202	0.715685	0.719492
Dendritic.cells	BCCIP	0.06487	5.436859	0.549357	0.584145	-6.23008	0.694358	0.68846
Dendritic.cells	UBE2Q2	-0.0636	5.51991	-0.54912	0.584306	-6.39282	0.693374	0.686903

Dendritic.cells	DDX49	0.08127	4.208396	0.548996	0.584392	-6.05152	0.710998	0.712474
Dendritic.cells	EXTL3	0.158141	3.56619	0.548478	0.584746	-5.65622	0.72018	0.72559
Dendritic.cells	PTPN4	-0.06958	5.463527	-0.54819	0.584946	-6.51444	0.694645	0.68835
Dendritic.cells	IKBIP	0.155343	1.938543	0.548089	0.585012	-5.6478	0.743359	0.759314
Dendritic.cells	SNX15	0.076571	4.880351	0.547981	0.585086	-6.25212	0.702428	0.699636
Dendritic.cells	PPM1J	-0.30926	-0.35731	-0.54764	0.585319	-5.16448	0.777527	0.809385
Dendritic.cells	PIN1	0.084132	5.245371	0.547629	0.585326	-6.11507	0.697681	0.692628
Dendritic.cells	HIST1H4D	-0.34233	1.04631	-0.54736	0.585514	-5.2232	0.756669	0.778598
Dendritic.cells	CCDC141	0.293284	1.120838	0.546801	0.585893	-5.22567	0.755817	0.777119
Dendritic.cells	A930024E(	-0.21803	1.108621	-0.5468	0.585893	-5.50572	0.755997	0.777383
Dendritic.cells	NEMP2	0.130227	2.854914	0.54679	0.5859	-5.67859	0.730828	0.740541
Dendritic.cells	GBP5	0.490066	2.014589	0.546501	0.586098	-5.18958	0.742937	0.758052
Dendritic.cells	TBC1D10C	-0.12289	4.736281	-0.54645	0.586133	-6.05646	0.704994	0.702773
Dendritic.cells	CAR1	0.447125	0.491473	0.546285	0.586246	-5.18132	0.765278	0.790875
Dendritic.cells	AC154200.	-0.24754	0.660722	-0.54626	0.586265	-5.25831	0.762756	0.787168
Dendritic.cells	DCAF10	-0.06932	5.193751	-0.54613	0.586354	-6.28034	0.69889	0.693957
Dendritic.cells	1700025G(	0.135167	4.957756	0.545908	0.586504	-6.09675	0.702147	0.698592
Dendritic.cells	NDUFS6	0.08213	5.716521	0.545806	0.586574	-6.25277	0.69207	0.684016
Dendritic.cells	MFSD7A	0.317236	-0.44923	0.545636	0.58669	-5.19893	0.779695	0.812012
Dendritic.cells	PARP1	0.082993	5.30881	0.545342	0.586891	-6.29675	0.69766	0.691896
Dendritic.cells	PIGN	0.104734	4.40092	0.545278	0.586935	-6.07624	0.709871	0.709607
Dendritic.cells	AK5	0.30381	0.348906	0.545089	0.587065	-5.23417	0.767815	0.794289
Dendritic.cells	PRKCA	-0.04908	7.401898	-0.54504	0.587099	-7.22273	0.670573	0.652771
Dendritic.cells	NRBF2	0.108577	4.314912	0.544882	0.587207	-5.88865	0.711132	0.711449
Dendritic.cells	4930595D1	-0.28407	1.248821	-0.54426	0.587636	-5.23905	0.754924	0.774908
Dendritic.cells	SRPRB	0.083547	4.104103	0.544174	0.587692	-6.09698	0.714394	0.715764
Dendritic.cells	SLC25A22	0.207497	2.495595	0.544029	0.587791	-5.56972	0.736884	0.748534
Dendritic.cells	P2RY12	0.088196	2.616799	0.543964	0.587836	-6.38865	0.735158	0.746034
Dendritic.cells	TRAF5	0.124181	5.308971	0.543806	0.587943	-5.91218	0.698118	0.692225
Dendritic.cells	IGFBP1	0.343018	4.147259	0.543779	0.587962	-5.73165	0.713803	0.714978
Dendritic.cells	HMGCR	0.066233	4.712579	0.543754	0.587979	-6.42959	0.706114	0.703814
Dendritic.cells	GM27010	0.254571	2.340877	0.543496	0.588156	-5.25056	0.739189	0.751934
Dendritic.cells	ADAM17	-0.07695	6.061586	-0.54344	0.588196	-6.56337	0.688284	0.677981
Dendritic.cells	NEIL1	0.151928	3.000738	0.543356	0.588252	-5.72949	0.729821	0.738317
Dendritic.cells	GM15472	-0.31558	1.795978	-0.54323	0.588337	-5.26453	0.747062	0.763515
Dendritic.cells	GIT1	-0.10375	3.282244	-0.54282	0.588617	-5.78072	0.726076	0.732701
Dendritic.cells	TMEM208	-0.07629	4.89435	-0.54282	0.588623	-6.06733	0.703956	0.700551
Dendritic.cells	WDR76	0.17106	4.504143	0.542729	0.588682	-5.74816	0.70923	0.708246
Dendritic.cells	PHF2	-0.05632	5.076145	-0.54238	0.588919	-6.40025	0.70172	0.697171
Dendritic.cells	IRS2	-0.11719	5.572327	-0.54229	0.588986	-6.21432	0.695116	0.687627
Dendritic.cells	GM12655	0.291274	0.353725	0.54209	0.58912	-5.27733	0.768754	0.795094
Dendritic.cells	4833408A1	-0.30659	0.127774	-0.54209	0.589121	-5.28155	0.772154	0.800096
Dendritic.cells	GEN1	-0.24888	2.372171	-0.54183	0.589296	-5.45099	0.739201	0.751822
Dendritic.cells	SELENOS	-0.04832	6.113957	-0.5417	0.589388	-6.69002	0.688028	0.677531
Dendritic.cells	HPF1	0.085054	5.467524	0.541669	0.589409	-6.38029	0.696536	0.689836
Dendritic.cells	TRIR	-0.06274	6.300182	-0.54151	0.589521	-6.41876	0.685603	0.674041
Dendritic.cells	RBM12B1	-0.27563	0.935508	-0.54123	0.589711	-5.28792	0.760113	0.782578

Dendritic.cells	PRMT6	0.134584	2.45108	0.541164	0.589755	-5.64864	0.738073	0.750312
Dendritic.cells	SLC23A1	0.206296	1.528872	0.541125	0.589782	-5.43637	0.751389	0.769799
Dendritic.cells	DYNLT1B	0.240117	1.188553	0.54103	0.589847	-5.40284	0.756378	0.77712
Dendritic.cells	BET1L	-0.10238	3.860623	-0.54101	0.58986	-5.92303	0.718282	0.721462
Dendritic.cells	CEP128	-0.07768	6.336302	-0.541	0.589869	-6.58456	0.685134	0.673413
Dendritic.cells	SUMF1	-0.08921	4.240634	-0.54085	0.589973	-6.17443	0.713062	0.713871
Dendritic.cells	FN3KRP	0.237738	1.481688	0.540833	0.589983	-5.37509	0.752079	0.770815
Dendritic.cells	TRMT2B	0.117868	3.750232	0.540792	0.590011	-5.86911	0.719808	0.723684
Dendritic.cells	TSN	0.051654	6.360058	0.540648	0.59011	-6.45364	0.684825	0.673008
Dendritic.cells	DUSP16	0.086673	6.833204	0.540619	0.590129	-6.6149	0.678723	0.664205
Dendritic.cells	CYBA	0.058678	8.55691	0.540386	0.59029	-6.90762	0.657158	0.633158
Dendritic.cells	IL2RB	-0.27607	3.130268	-0.54035	0.590317	-5.54817	0.728522	0.736418
Dendritic.cells	MAP1LC3B	0.072282	7.434486	0.540049	0.590521	-6.61932	0.671154	0.65331
Dendritic.cells	SP3OS	0.117006	4.205373	0.540008	0.590549	-5.8421	0.713632	0.714761
Dendritic.cells	SERPINB2	0.914089	0.991964	0.539993	0.590559	-5.18881	0.759371	0.781583
Dendritic.cells	H1FX	-0.28478	2.127581	-0.53994	0.590596	-5.34524	0.742802	0.757299
Dendritic.cells	AGPAT4	0.182716	5.023605	0.539506	0.590894	-5.81791	0.70282	0.698739
Dendritic.cells	FBXO21	-0.13731	3.292952	-0.53919	0.591112	-5.67449	0.726711	0.733362
Dendritic.cells	SLMAP	-0.0578	6.393027	-0.53912	0.591159	-6.56804	0.684906	0.672729
Dendritic.cells	WDR78	-0.28613	0.561969	-0.53889	0.59132	-5.24402	0.766324	0.791261
Dendritic.cells	CDC123	0.079071	5.404958	0.538829	0.591359	-6.24399	0.697965	0.691537
Dendritic.cells	TRABD	0.055456	5.363153	0.53861	0.591509	-6.56653	0.698579	0.692343
Dendritic.cells	FEN1	0.137597	4.525584	0.538566	0.59154	-5.88403	0.709847	0.708676
Dendritic.cells	RBM8A	0.071032	5.709751	0.538279	0.591737	-6.25965	0.693988	0.685772
Dendritic.cells	PRR7	0.148558	3.334702	0.538252	0.591755	-5.71987	0.726275	0.732642
Dendritic.cells	SGTB	0.294878	0.3418	0.538222	0.591776	-5.18813	0.769692	0.796204
Dendritic.cells	CDCA7	0.242889	3.246784	0.538137	0.591835	-5.43259	0.727506	0.734458
Dendritic.cells	MYL4	-0.26365	4.638008	-0.53807	0.59188	-5.64819	0.708324	0.706573
Dendritic.cells	ABCA9	0.389844	0.787541	0.537941	0.591969	-5.17775	0.763029	0.786506
Dendritic.cells	RECQL5	0.066807	3.722047	0.537892	0.592003	-6.37115	0.72088	0.724897
Dendritic.cells	TESPA1	0.124744	2.504605	0.537729	0.592115	-5.97172	0.738065	0.749935
Dendritic.cells	FGL2	0.382611	4.054695	0.53754	0.592245	-5.45588	0.716421	0.718343
Dendritic.cells	CASR	0.326543	-0.61039	0.537343	0.59238	-5.16841	0.784316	0.817836
Dendritic.cells	EIF3J1	-0.04882	7.268033	-0.53734	0.592385	-6.67968	0.67395	0.656912
Dendritic.cells	PJA1	-0.12082	3.699202	-0.53696	0.592643	-5.81718	0.721536	0.725667
Dendritic.cells	MON1A	-0.11069	3.876268	-0.53691	0.592676	-5.86426	0.719084	0.722099
Dendritic.cells	NUF2	-0.24626	3.365247	-0.53668	0.592836	-5.45738	0.726271	0.732564
Dendritic.cells	CASP3	0.107384	4.814976	0.536623	0.592876	-5.89207	0.706343	0.703609
Dendritic.cells	SH3GLB1	0.050095	8.546317	0.536481	0.592974	-6.86652	0.658232	0.634228
Dendritic.cells	ITGA1	-0.17573	3.828562	-0.53643	0.593007	-5.85087	0.719825	0.723235
Dendritic.cells	WDR91	-0.09178	4.750458	-0.53622	0.593154	-6.16774	0.707227	0.704935
Dendritic.cells	SNRPA	-0.08194	5.131803	-0.53621	0.593158	-6.06039	0.702093	0.697491
Dendritic.cells	PSMB7	0.068624	5.624583	0.535979	0.593319	-6.25573	0.695531	0.688079
Dendritic.cells	COP1	-0.05141	7.053619	-0.5359	0.593372	-6.82279	0.676951	0.661274
Dendritic.cells	ZCCHC17	-0.0735	5.162664	-0.53559	0.593585	-6.17995	0.70168	0.697155
Dendritic.cells	UBE4BOS1	-0.32345	0.735049	-0.53558	0.593595	-5.22457	0.764269	0.788527
Dendritic.cells	GBP9	0.243074	3.036901	0.535548	0.593616	-5.43104	0.730895	0.739652

Dendritic.cells	RAB5A	-0.04651	6.913368	-0.53546	0.593674	-6.6616	0.678745	0.664004
Dendritic.cells	NBAS	-0.07555	4.535139	-0.5353	0.593786	-6.24867	0.710147	0.709456
Dendritic.cells	GSG1L	-0.35066	-0.16326	-0.5352	0.593854	-5.17715	0.777793	0.808455
Dendritic.cells	4921511C1	-0.15804	2.59393	-0.53519	0.593864	-5.65025	0.737176	0.748834
Dendritic.cells	GM11457	0.35597	0.10252	0.535092	0.593929	-5.1881	0.773762	0.802532
Dendritic.cells	TSPAN4	0.167641	3.356981	0.535061	0.593951	-5.5359	0.726399	0.733122
Dendritic.cells	LMBR1	0.194909	1.883516	0.534938	0.594036	-5.45491	0.747389	0.763827
Dendritic.cells	TIMM13	0.056827	6.732513	0.534919	0.594049	-6.59788	0.681068	0.667401
Dendritic.cells	HOTAIRM1	0.249994	0.698917	0.534889	0.594069	-5.43308	0.764807	0.789389
Dendritic.cells	RASGRP1	-0.25767	4.119019	-0.5346	0.594267	-5.42024	0.715944	0.717812
Dendritic.cells	CAPNS1	0.04521	7.25812	0.534519	0.594324	-6.76034	0.67445	0.657769
Dendritic.cells	GNGT2	0.11857	5.675602	0.534353	0.594439	-6.27655	0.694964	0.687399
Dendritic.cells	POU2F2	0.149722	5.772063	0.534326	0.594457	-6.12972	0.69369	0.685556
Dendritic.cells	SEC23B	-0.0597	5.223375	-0.53428	0.59449	-6.37231	0.700976	0.696109
Dendritic.cells	GM48678	-0.15143	3.349644	-0.534	0.594678	-5.78197	0.726656	0.733463
Dendritic.cells	ANO6	0.090302	6.75402	0.533843	0.59479	-6.38332	0.680937	0.667146
Dendritic.cells	YLPM1	0.062251	5.660547	0.533748	0.594856	-6.44569	0.695204	0.687768
Dendritic.cells	MIRT1	0.10831	4.950639	0.533737	0.594863	-6.16139	0.704676	0.701496
Dendritic.cells	TMEM183A	-0.0599	5.16956	-0.5336	0.59496	-6.27591	0.701737	0.697234
Dendritic.cells	MAP3K13	0.297759	0.779749	0.533579	0.594972	-5.2404	0.763766	0.787789
Dendritic.cells	OAT	-0.11771	5.245936	-0.53353	0.595005	-5.96304	0.700716	0.695753
Dendritic.cells	LTC4S	-0.51172	1.066227	-0.53344	0.595069	-5.22078	0.759516	0.781586
Dendritic.cells	ASNSD1	0.076961	5.360445	0.533368	0.595117	-6.14842	0.699188	0.693593
Dendritic.cells	5033406O(	-0.30008	0.605012	-0.53309	0.59531	-5.24484	0.766536	0.791798
Dendritic.cells	GM13212	0.143181	3.205264	0.532695	0.595581	-5.73041	0.728906	0.736692
Dendritic.cells	YRDC	-0.07989	5.035051	-0.53266	0.595606	-6.22665	0.703758	0.700112
Dendritic.cells	ALG9	0.119943	3.375018	0.532639	0.59562	-5.79912	0.726526	0.733226
Dendritic.cells	RNF144A	0.155576	3.619246	0.532131	0.59597	-5.62802	0.723118	0.728436
Dendritic.cells	MSI2	0.04355	7.509903	0.532077	0.596007	-6.94863	0.671508	0.653663
Dendritic.cells	G6PC3	0.146353	3.106653	0.531948	0.596096	-5.72098	0.730294	0.738901
Dendritic.cells	SAP30L	-0.10263	4.309459	-0.53191	0.596121	-5.80776	0.713596	0.714576
Dendritic.cells	CPN1	-0.25918	1.600653	-0.53187	0.596149	-5.32873	0.751896	0.770505
Dendritic.cells	BSDC1	-0.11981	5.056629	-0.53183	0.596176	-5.99556	0.703469	0.699867
Dendritic.cells	TAB2	0.051709	6.780443	0.531794	0.596203	-6.5751	0.680807	0.667072
Dendritic.cells	SLC10A3	-0.17784	2.160549	-0.53173	0.596249	-5.60284	0.743774	0.758604
Dendritic.cells	ZFP202	0.245825	0.604939	0.531598	0.596338	-5.4348	0.76661	0.792125
Dendritic.cells	PHF14	0.052587	6.331947	0.531595	0.59634	-6.57919	0.686609	0.67546
Dendritic.cells	PROCR	0.424335	0.279761	0.531545	0.596374	-5.23367	0.77149	0.799309
Dendritic.cells	MTERF2	-0.23347	2.099595	-0.53151	0.596395	-5.34309	0.744653	0.759898
Dendritic.cells	GM43331	-0.09677	2.057137	-0.53148	0.59642	-5.97344	0.745266	0.760798
Dendritic.cells	DGCR2	-0.07822	4.849233	-0.5312	0.596612	-6.24816	0.706372	0.703961
Dendritic.cells	ITSN2	0.049252	6.989544	0.531134	0.596657	-6.75555	0.67823	0.663239
Dendritic.cells	TNFRSF13C	0.141328	3.92598	0.531057	0.596711	-5.81951	0.718979	0.722287
Dendritic.cells	ZFP709	0.187519	1.431477	0.530878	0.596834	-5.33972	0.754505	0.774182
Dendritic.cells	RSPO3	-0.35156	0.723734	-0.53075	0.596924	-5.41409	0.764971	0.789555
Dendritic.cells	ACOT11	0.162101	1.943725	0.530535	0.597071	-5.54597	0.747038	0.763236
Dendritic.cells	TRIM21	0.233886	2.57157	0.530457	0.597125	-5.42962	0.73801	0.750029

Dendritic.cells	MAP4K3	0.10315	4.283134	0.530401	0.597163	-6.09038	0.714082	0.715166
Dendritic.cells	NDUFV2	0.070415	6.136177	0.530187	0.597311	-6.42382	0.689284	0.679255
Dendritic.cells	WSB2	0.1034	4.493162	0.530185	0.597313	-5.97548	0.711214	0.711046
Dendritic.cells	SIRPA	0.0635	5.832373	0.530175	0.59732	-6.8256	0.693272	0.685024
Dendritic.cells	SLC10A7	0.070098	5.908741	0.529934	0.597486	-6.32699	0.692266	0.683642
Dendritic.cells	TMEM120E	0.209071	3.044848	0.529923	0.597493	-5.58945	0.731293	0.74037
Dendritic.cells	IFIT1BL2	-0.32659	-0.46837	-0.5299	0.597507	-5.16785	0.782996	0.816286
Dendritic.cells	FRMD8OS	-0.29769	0.736632	-0.52961	0.597708	-5.22153	0.764779	0.789486
Dendritic.cells	TEP1	0.084023	3.914158	0.529533	0.597763	-6.1888	0.719156	0.722737
Dendritic.cells	COX6C	-0.04492	8.594079	-0.52953	0.597768	-6.91448	0.658115	0.634459
Dendritic.cells	RP9	0.047078	6.834563	0.529391	0.597861	-6.5737	0.68023	0.666303
Dendritic.cells	GM50232	-0.2356	1.372215	-0.52921	0.597987	-5.41459	0.755374	0.775686
Dendritic.cells	BMS1	0.096053	4.737717	0.529002	0.598129	-6.02312	0.707893	0.706357
Dendritic.cells	CIB2	0.130543	2.152566	0.528993	0.598136	-5.79495	0.74402	0.75904
Dendritic.cells	ATP8A1	0.051014	7.601268	0.528942	0.598171	-6.82788	0.670473	0.652233
Dendritic.cells	MFAP4	-0.28605	1.50654	-0.5289	0.598203	-5.35732	0.753405	0.772795
Dendritic.cells	NPHS1	-0.29933	0.439647	-0.52884	0.598242	-5.22555	0.769221	0.796042
Dendritic.cells	EIF2B3	0.150175	2.996786	0.528741	0.59831	-5.69364	0.731972	0.741424
Dendritic.cells	TMEM161A	-0.1257	2.853132	-0.52868	0.598349	-5.82527	0.734005	0.744393
Dendritic.cells	MARS	-0.17288	3.10364	-0.52866	0.598369	-5.5654	0.730464	0.739232
Dendritic.cells	CLU	-0.2695	5.440247	-0.52864	0.598382	-6.18118	0.698464	0.692684
Dendritic.cells	POT1A	-0.14285	3.012767	-0.52833	0.598596	-5.6511	0.731746	0.741187
Dendritic.cells	PRCC	0.071099	5.107381	0.528324	0.598598	-6.21341	0.702911	0.699212
Dendritic.cells	LAMP1	0.053132	7.757865	0.528307	0.59861	-6.78368	0.668503	0.649477
Dendritic.cells	MPO	0.83675	1.827356	0.528198	0.598685	-5.21416	0.748726	0.76603
Dendritic.cells	TMLHE	0.230878	2.31306	0.527443	0.599207	-5.51418	0.74171	0.755943
Dendritic.cells	BROX	-0.07014	4.85097	-0.52735	0.599274	-6.13819	0.706362	0.704398
Dendritic.cells	XAB2	-0.12872	3.922858	-0.52733	0.599286	-5.79097	0.719036	0.722833
Dendritic.cells	ZDHHC9	0.086801	4.094804	0.527314	0.599296	-6.17273	0.716666	0.719382
Dendritic.cells	GM4221	-0.18833	1.846721	-0.52728	0.599322	-5.50679	0.748445	0.765809
Dendritic.cells	POSTN	0.317238	1.260435	0.527143	0.599414	-5.25663	0.757018	0.778391
Dendritic.cells	LRRC58	-0.05345	5.945766	-0.52714	0.599416	-6.51205	0.69178	0.683252
Dendritic.cells	ZC3H14	-0.05182	5.656337	-0.52708	0.599458	-6.47823	0.695596	0.68878
Dendritic.cells	GLIPR2	-0.17946	4.6029	-0.52706	0.599475	-5.83372	0.709721	0.709279
Dendritic.cells	MS4A4C	0.265732	3.224383	0.527023	0.599497	-5.80819	0.728766	0.73702
Dendritic.cells	DSCAML1	0.318155	-0.596	0.526966	0.599537	-5.17123	0.784956	0.819558
Dendritic.cells	NRXN1	-0.37331	2.142242	-0.52688	0.599599	-5.2863	0.744168	0.759543
Dendritic.cells	ENDOD1	0.151929	2.901638	0.526853	0.599615	-5.60706	0.733318	0.743668
Dendritic.cells	ITPR2	-0.05392	6.912603	-0.52685	0.59962	-6.71192	0.679229	0.665106
Dendritic.cells	PLEKHM2	0.11762	5.202185	0.526819	0.599639	-6.1319	0.70164	0.697544
Dendritic.cells	PTPN9	0.073562	5.706865	0.526738	0.599695	-6.35786	0.694928	0.687835
Dendritic.cells	LYPLA2	0.081627	4.945046	0.526657	0.599751	-6.10866	0.705093	0.70258
Dendritic.cells	GIMAP6	-0.11188	5.672059	-0.52632	0.599981	-6.36552	0.695473	0.688613
Dendritic.cells	NABP1	-0.13304	4.743721	-0.52627	0.600016	-5.87473	0.707898	0.706644
Dendritic.cells	VPS11	-0.08736	4.072729	-0.52627	0.600021	-6.01237	0.717057	0.719967
Dendritic.cells	NECTIN4	0.208502	-0.02307	0.52579	0.60035	-5.46928	0.77664	0.807016
Dendritic.cells	ZFP81	0.187265	1.873403	0.525519	0.600538	-5.46188	0.748619	0.765635

Dendritic.cells	MAP2K2	-0.04171	7.299123	-0.52537	0.600641	-6.75301	0.674802	0.658363
Dendritic.cells	OSTC	0.049614	6.387429	0.52534	0.600662	-6.59154	0.686523	0.675301
Dendritic.cells	MYNN	0.080982	4.303762	0.525167	0.600782	-6.04911	0.714385	0.715668
Dendritic.cells	PTMS	-0.05269	6.015415	-0.52509	0.600835	-6.82065	0.691432	0.682392
Dendritic.cells	FCSK	0.276431	0.928399	0.52477	0.601056	-5.23336	0.762677	0.786197
Dendritic.cells	DELE1	0.12502	3.426108	0.524755	0.601067	-5.66183	0.726654	0.73345
Dendritic.cells	CTSL	0.044797	6.758061	0.524625	0.601157	-7.06007	0.681913	0.668533
Dendritic.cells	IL12RB1	-0.33773	0.500116	-0.52433	0.601359	-5.21251	0.769278	0.795743
Dendritic.cells	FASTKD1	0.167153	2.189988	0.524105	0.601517	-5.54496	0.744523	0.759315
Dendritic.cells	LCMT2	0.155001	2.687418	0.523876	0.601675	-5.54599	0.737391	0.748897
Dendritic.cells	RBM14	-0.10944	3.712533	-0.52387	0.601681	-5.77586	0.722961	0.727838
Dendritic.cells	CENPJ	-0.16637	3.331867	-0.5238	0.601729	-5.50207	0.728277	0.735589
Dendritic.cells	PIK3R4	0.155012	3.189781	0.523677	0.601813	-5.62492	0.730275	0.738508
Dendritic.cells	CAMK2D	-0.07984	7.214604	-0.52363	0.601847	-6.68648	0.676317	0.660251
Dendritic.cells	AAK1	0.067519	6.392459	0.523504	0.601933	-6.51113	0.686905	0.67557
Dendritic.cells	NCAPD2	0.182515	4.81058	0.523442	0.601976	-5.81859	0.707898	0.705998
Dendritic.cells	TM2D1	-0.04919	5.944576	-0.52325	0.60211	-6.53417	0.692824	0.684092
Dendritic.cells	RALY	0.065715	6.422986	0.523178	0.602159	-6.40246	0.686567	0.675041
Dendritic.cells	1700034P1	-0.28366	1.464772	-0.52306	0.602242	-5.37226	0.75516	0.774909
Dendritic.cells	AZGP1	-0.23505	3.926277	-0.52296	0.602308	-5.68839	0.720079	0.723643
Dendritic.cells	EDNRB	-0.37055	2.175551	-0.52274	0.602465	-5.61262	0.744869	0.759805
Dendritic.cells	ENTPD7	-0.07669	4.693758	-0.52271	0.602483	-6.24842	0.709613	0.708387
Dendritic.cells	GM17106	0.110039	3.395192	0.522448	0.602665	-6.05579	0.727665	0.734531
Dendritic.cells	RBM6	0.042007	7.184868	0.522122	0.602891	-6.82025	0.677132	0.661094
Dendritic.cells	ATP5G3	0.061039	8.015505	0.521771	0.603135	-6.81	0.666737	0.646022
Dendritic.cells	CXCL13	0.405171	-1.21101	0.521663	0.603209	-5.16937	0.796221	0.834974
Dendritic.cells	SWT1	0.06895	5.244266	0.521622	0.603238	-6.28673	0.702615	0.697833
Dendritic.cells	TMOD3	0.049772	7.218311	0.521615	0.603243	-6.7661	0.676804	0.660517
Dendritic.cells	ITGA3	0.269517	-0.28405	0.52151	0.603315	-5.1838	0.781896	0.81383
Dendritic.cells	BCL2A1B	0.274804	5.877283	0.52139	0.603399	-5.68465	0.694227	0.685726
Dendritic.cells	SPATA6	-0.14265	4.52297	-0.52115	0.603568	-5.7559	0.712479	0.712148
Dendritic.cells	UBE2G2	-0.09165	4.661055	-0.521	0.603671	-5.95407	0.710598	0.709424
Dendritic.cells	NSD2	-0.06824	6.699324	-0.52093	0.603716	-6.52844	0.683575	0.670288
Dendritic.cells	CHD4	0.04889	7.633169	0.520828	0.603789	-6.7412	0.671646	0.6531
Dendritic.cells	ZFYVE9	-0.24137	2.603777	-0.52081	0.6038	-5.4773	0.739284	0.751238
Dendritic.cells	TMEM127	-0.08654	4.290896	-0.52069	0.603881	-5.97464	0.715672	0.716785
Dendritic.cells	MLXIPL	0.389227	0.355448	0.520453	0.604049	-5.17869	0.772447	0.799708
Dendritic.cells	NAIP6	-0.14423	2.310523	-0.52035	0.604123	-5.7199	0.743645	0.75742
Dendritic.cells	1700001K1	-0.26744	1.052633	-0.51998	0.604374	-5.32753	0.762144	0.78442
Dendritic.cells	PKP4	0.107164	5.958306	0.519983	0.604375	-6.28643	0.693488	0.684341
Dendritic.cells	CENPX	0.066382	6.347296	0.519924	0.604416	-6.4588	0.68839	0.676976
Dendritic.cells	THAP7	0.122623	3.607354	0.519651	0.604605	-5.85633	0.72537	0.730705
Dendritic.cells	NUDCD3	0.043736	5.998557	0.51965	0.604606	-6.58529	0.692958	0.683649
Dendritic.cells	KCNJ2	0.247405	-0.01521	0.519566	0.604664	-5.17941	0.778188	0.80816
Dendritic.cells	8-Sep	0.188529	1.954462	0.519466	0.604734	-5.44849	0.748905	0.765154
Dendritic.cells	SURF4	0.058983	5.782118	0.519366	0.604803	-6.54669	0.695814	0.687872
Dendritic.cells	PROK2	0.421918	-0.61756	0.519356	0.60481	-5.176	0.787414	0.821841

Dendritic.cells	RNASEH2C	-0.12137	4.998911	-0.51921	0.604913	-5.79304	0.706276	0.703042
Dendritic.cells	HIPK1	-0.06716	6.269668	-0.51917	0.60494	-6.48864	0.689403	0.678604
Dendritic.cells	PLEC	0.067997	4.805597	0.518857	0.605157	-6.41195	0.708896	0.706946
Dendritic.cells	MFSD14A	-0.03719	6.156841	-0.51885	0.605158	-6.79142	0.690886	0.680845
Dendritic.cells	LSM14A	-0.04223	6.620649	-0.51881	0.605188	-6.63355	0.684842	0.67211
Dendritic.cells	ZFP871	-0.06998	5.249328	-0.51878	0.605209	-6.36301	0.702915	0.698267
Dendritic.cells	ANXA7	0.059374	5.409471	0.518392	0.605479	-6.4191	0.701009	0.69522
Dendritic.cells	RRP1	0.044948	6.518197	0.518118	0.60567	-6.59392	0.686545	0.674161
Dendritic.cells	WARS	0.115255	3.723103	0.517805	0.605888	-5.93527	0.724231	0.728773
Dendritic.cells	PDPR	-0.10861	3.937711	-0.51773	0.605939	-6.00523	0.72125	0.724433
Dendritic.cells	FAS	-0.21812	3.936884	-0.51768	0.605976	-5.55408	0.721262	0.724467
Dendritic.cells	THOP1	-0.24207	2.236401	-0.51756	0.606058	-5.32629	0.745313	0.759601
Dendritic.cells	C130036L2	-0.1767	1.354595	-0.51755	0.606063	-5.47851	0.758177	0.778452
Dendritic.cells	ANAPC10	-0.08078	4.581596	-0.51727	0.60626	-6.07699	0.712554	0.711681
Dendritic.cells	DLGAP1	-0.26344	1.398192	-0.51665	0.606689	-5.29913	0.757947	0.777735
Dendritic.cells	ZSWIM7	-0.13462	3.432711	-0.51632	0.606922	-5.74389	0.728686	0.734977
Dendritic.cells	RGS9	-0.33163	0.03543	-0.51627	0.606957	-5.17619	0.778351	0.807799
Dendritic.cells	MED13	0.050738	7.722854	0.516238	0.606977	-6.83488	0.671559	0.652276
Dendritic.cells	GCA	-0.36572	1.099128	-0.51623	0.60698	-5.18038	0.762369	0.784295
Dendritic.cells	GM5617	0.11572	3.238502	0.516124	0.607056	-5.87455	0.731418	0.739022
Dendritic.cells	TOPBP1	0.102385	5.680028	0.516008	0.607137	-6.19256	0.698003	0.690517
Dendritic.cells	CENPI	0.186831	2.997583	0.515913	0.607203	-5.60309	0.734824	0.744061
Dendritic.cells	PBX4	-0.29851	0.976518	-0.51589	0.607219	-5.19562	0.764191	0.787072
Dendritic.cells	SLC23A2	-0.07464	5.113974	-0.51583	0.607263	-6.34936	0.705572	0.701519
Dendritic.cells	GRIK5	0.324296	0.167717	0.515805	0.607278	-5.19421	0.776342	0.804978
Dendritic.cells	SMARCB1	-0.05987	5.410318	-0.51572	0.60734	-6.26708	0.701596	0.695796
Dendritic.cells	TTC19	-0.06291	4.958514	-0.51568	0.607366	-6.47315	0.707669	0.704615
Dendritic.cells	GM15726	0.366228	2.074088	0.515622	0.607405	-5.32506	0.748067	0.76351
Dendritic.cells	FAM20B	0.097995	3.958304	0.515557	0.60745	-5.98966	0.721358	0.72453
Dendritic.cells	NEURL1B	-0.23095	0.43588	-0.51537	0.607582	-5.29949	0.77237	0.799155
Dendritic.cells	PSMB6	0.063891	6.304312	0.514817	0.607966	-6.4762	0.689973	0.678905
Dendritic.cells	CXCR5	0.309742	2.183855	0.514724	0.608031	-5.26542	0.746689	0.761401
Dendritic.cells	LARGE1	-0.06443	5.482959	-0.51472	0.608036	-6.79141	0.700824	0.694609
Dendritic.cells	FAM131A	-0.30375	0.401139	-0.51446	0.608217	-5.26882	0.77303	0.800085
Dendritic.cells	WDR77	0.126904	3.895337	0.514393	0.608261	-5.75982	0.722435	0.726011
Dendritic.cells	POU2AF1	-0.24342	4.320007	-0.5143	0.608322	-5.52717	0.716572	0.71748
Dendritic.cells	PROSER3	-0.23734	1.105594	-0.5142	0.608393	-5.3055	0.762489	0.784583
Dendritic.cells	PIGA	0.215276	1.918295	0.514176	0.608412	-5.34797	0.750543	0.767069
Dendritic.cells	TMEM71	0.23564	3.858441	0.514162	0.608422	-5.38571	0.722948	0.726768
Dendritic.cells	SPSB3	-0.10457	3.587341	-0.51402	0.608523	-5.77813	0.726726	0.732345
Dendritic.cells	ETOHD2	0.154493	2.615429	0.514003	0.608532	-5.52622	0.740478	0.752419
Dendritic.cells	MELK	-0.26006	3.016502	-0.51396	0.608562	-5.39998	0.734764	0.744089
Dendritic.cells	EAPP	-0.05982	5.602282	-0.5139	0.608603	-6.27499	0.699234	0.692439
Dendritic.cells	CROCC	-0.2563	1.025643	-0.51384	0.608649	-5.25925	0.763676	0.786483
Dendritic.cells	PIGH	0.187832	1.827535	0.513736	0.608718	-5.49421	0.751866	0.769195
Dendritic.cells	GM42659	0.105972	4.567867	0.513682	0.608756	-5.92147	0.713178	0.712739
Dendritic.cells	BAG6	0.082435	5.438014	0.513524	0.608865	-6.24865	0.701425	0.695692

Dendritic.cells	MEFV	0.341592	-0.03339	0.513506	0.608879	-5.18078	0.77962	0.810025
Dendritic.cells	RNASEH2B	0.12205	4.594626	0.51319	0.609098	-5.85737	0.712891	0.71229
Dendritic.cells	SLC18A2	-0.15477	2.251616	-0.51319	0.6091	-5.56671	0.745792	0.760275
Dendritic.cells	RAC3	0.331481	-0.22085	0.513125	0.609144	-5.17584	0.782569	0.814341
Dendritic.cells	ITCH	0.052322	7.100446	0.512755	0.609401	-6.7609	0.679917	0.664384
Dendritic.cells	RAP1GDS1	-0.06923	6.711436	-0.51271	0.609434	-6.4351	0.684929	0.671621
Dendritic.cells	ZFP655	-0.08675	4.453763	-0.51214	0.609833	-6.06044	0.71535	0.715313
Dendritic.cells	BMI1	-0.11512	3.545427	-0.51208	0.609874	-5.8347	0.727936	0.733628
Dendritic.cells	CCNE1	-0.21958	3.530373	-0.51188	0.610013	-5.44408	0.728146	0.733998
Dendritic.cells	SPC25	-0.21952	4.372308	-0.51183	0.610047	-5.62189	0.716467	0.717001
Dendritic.cells	ASPM	0.270213	3.870548	0.511807	0.610062	-5.61783	0.723399	0.727085
Dendritic.cells	METTL18	0.214225	1.271701	0.511557	0.610237	-5.39537	0.760773	0.781638
Dendritic.cells	PRMT9	0.133271	4.074852	0.511501	0.610275	-5.76951	0.720654	0.723015
Dendritic.cells	UNC119B	0.132514	3.551645	0.511393	0.610351	-5.72652	0.727937	0.733659
Dendritic.cells	9530068E0	0.096232	4.851397	0.511321	0.610401	-6.04772	0.710015	0.707621
Dendritic.cells	ZFP994	0.151926	2.481603	0.511174	0.610503	-5.5342	0.743153	0.755901
Dendritic.cells	DET1	0.216768	2.186379	0.511097	0.610557	-5.39237	0.747412	0.762159
Dendritic.cells	UFD1	0.068937	5.129038	0.510436	0.611018	-6.17999	0.706724	0.702383
Dendritic.cells	1600012H(	-0.12347	2.771016	-0.51037	0.611067	-5.6511	0.739464	0.750065
Dendritic.cells	UPF3A	0.07918	4.634592	0.510044	0.611292	-5.99818	0.713618	0.712205
Dendritic.cells	NAA80	0.160707	2.402845	0.509919	0.611379	-5.50581	0.744965	0.757899
Dendritic.cells	CAMP	-0.38144	4.463959	-0.50965	0.611565	-5.565	0.716118	0.715702
Dendritic.cells	SMYD5	-0.23553	2.056268	-0.50934	0.61178	-5.38942	0.75029	0.765381
Dendritic.cells	RSL24D1	0.065562	5.674542	0.509267	0.611834	-6.3169	0.699908	0.692081
Dendritic.cells	DESI1	0.078183	5.729904	0.509181	0.611894	-6.36983	0.699171	0.691028
Dendritic.cells	POLD4	0.078025	5.76477	0.508907	0.612085	-6.219	0.698839	0.690398
Dendritic.cells	GM9993	-0.22464	1.16551	-0.50883	0.612142	-5.37576	0.763526	0.784645
Dendritic.cells	GM37305	-0.21924	1.078554	-0.50856	0.612325	-5.33924	0.764966	0.786664
Dendritic.cells	MAEA	0.074479	5.40999	0.508218	0.612566	-6.13295	0.70391	0.697418
Dendritic.cells	CHMP7	0.174062	2.616172	0.508082	0.612661	-5.49767	0.74272	0.753872
Dendritic.cells	LMAN1	-0.09334	4.627609	-0.508	0.61272	-6.09354	0.714529	0.712848
Dendritic.cells	DNAJC24	0.139275	3.89302	0.507665	0.612953	-5.73931	0.724727	0.727653
Dendritic.cells	SLC25A51	-0.08173	5.697758	-0.50764	0.61297	-6.25078	0.700148	0.692008
Dendritic.cells	SLC25A42	-0.21488	1.940911	-0.50759	0.613002	-5.44136	0.75256	0.76828
Dendritic.cells	HIST1H3G	-0.30204	1.46486	-0.50753	0.613049	-5.24457	0.759548	0.778516
Dendritic.cells	TBL1X	-0.04859	7.155308	-0.50736	0.613166	-6.78816	0.681086	0.664498
Dendritic.cells	SKIV2L	-0.11823	3.938774	-0.50735	0.61317	-5.78978	0.72409	0.726746
Dendritic.cells	RAP1B	-0.03807	8.543309	-0.50695	0.613454	-6.87682	0.663696	0.639376
Dendritic.cells	TSPAN12	-0.27145	1.194761	-0.50685	0.61352	-5.33151	0.763689	0.784466
Dendritic.cells	CCDC43	0.130447	3.103767	0.506833	0.613534	-5.65504	0.735959	0.743911
Dendritic.cells	RIIAD1	-0.24779	0.993222	-0.50681	0.61355	-5.25088	0.766691	0.788871
Dendritic.cells	MBIP	0.120583	3.658726	0.506434	0.613813	-5.81045	0.728195	0.732663
Dendritic.cells	NOB1	-0.07873	4.287446	-0.50643	0.613818	-6.09041	0.719455	0.719954
Dendritic.cells	UBL3	0.035719	7.408809	0.506405	0.613833	-6.89083	0.678024	0.660034
Dendritic.cells	CSTF1	-0.11898	3.243385	-0.50636	0.613867	-5.72138	0.734042	0.741206
Dendritic.cells	TMUB1	0.105931	3.138278	0.506263	0.613932	-5.74213	0.735531	0.74339
Dendritic.cells	GM31462	0.274273	0.152102	0.50567	0.614347	-5.22043	0.77988	0.807863

Dendritic.cells	CCDC106	-0.25421	0.51131	-0.50554	0.614438	-5.29319	0.774461	0.799901
Dendritic.cells	HELZ	-0.05501	6.25998	-0.50539	0.614542	-6.572	0.693356	0.681743
Dendritic.cells	MBD1	-0.08875	4.580853	-0.5053	0.614603	-6.06163	0.715894	0.714383
Dendritic.cells	B4GALT6	0.190268	2.991751	0.504929	0.614865	-5.57687	0.738258	0.746803
Dendritic.cells	ADAM10	-0.05057	7.403441	-0.50487	0.614903	-6.64639	0.678686	0.660514
Dendritic.cells	C4B	0.393119	2.480976	0.50483	0.614934	-5.34898	0.74558	0.757523
Dendritic.cells	9930021J0	-0.05108	6.407459	-0.50473	0.615004	-6.64973	0.691574	0.679107
Dendritic.cells	PSMD14	0.043888	6.618882	0.504231	0.615353	-6.65185	0.689129	0.675237
Dendritic.cells	SRP54A	-0.10391	3.599318	-0.50396	0.615546	-5.80787	0.730155	0.734527
Dendritic.cells	DIP2C	-0.08135	6.172429	-0.50377	0.615678	-6.72914	0.695197	0.683811
Dendritic.cells	ZFP354C	-0.21232	1.777029	-0.5036	0.615792	-5.45638	0.756466	0.77283
Dendritic.cells	GM43466	-0.1665	1.497294	-0.50337	0.615956	-5.60895	0.760707	0.778947
Dendritic.cells	TRIM8	-0.06365	5.616506	-0.5027	0.616427	-6.38149	0.703145	0.694798
Dendritic.cells	NKAIN2	0.305257	1.350359	0.502614	0.616486	-5.31272	0.763317	0.782372
Dendritic.cells	WRNIP1	-0.10374	3.743434	-0.50214	0.616819	-5.80188	0.728797	0.732131
Dendritic.cells	TAF5	0.102318	4.146651	0.502115	0.616835	-5.89039	0.723175	0.723961
Dendritic.cells	NDUFAF6	-0.14374	2.973661	-0.50195	0.61695	-5.59761	0.739685	0.748045
Dendritic.cells	SHFL	0.256269	1.832835	0.501824	0.617039	-5.28303	0.756197	0.772169
Dendritic.cells	TNFRSF19	-0.31026	0.886333	-0.50175	0.617089	-5.18371	0.770242	0.792753
Dendritic.cells	MAP4K1	0.053466	4.801156	0.501668	0.617148	-6.46462	0.714166	0.710962
Dendritic.cells	DOLK	-0.16546	1.544978	-0.50165	0.617161	-5.40762	0.760436	0.778376
Dendritic.cells	GM15738	0.258653	0.402397	0.501541	0.617237	-5.22993	0.777545	0.803493
Dendritic.cells	CKS2	-0.11864	5.959802	-0.50152	0.617254	-6.15484	0.698571	0.688407
Dendritic.cells	BTG1	0.050679	9.314237	0.501516	0.617255	-7.11495	0.655893	0.627047
Dendritic.cells	FAM102B	0.104582	4.23575	0.501465	0.61729	-6.01855	0.72194	0.722256
Dendritic.cells	CMIP	-0.05002	9.635584	-0.50138	0.617347	-7.17339	0.651992	0.62151
Dendritic.cells	8430429K0	-0.18249	1.679457	-0.50133	0.617388	-5.38325	0.758452	0.77557
Dendritic.cells	CDK8	-0.09533	6.559883	-0.50123	0.617455	-6.57777	0.690669	0.677106
Dendritic.cells	ATP6V1G1	0.04454	7.177612	0.501225	0.617459	-6.75526	0.682658	0.665562
Dendritic.cells	ZFP335	0.13593	2.842169	0.501092	0.617552	-5.6265	0.741565	0.750962
Dendritic.cells	NCOA5	0.097387	4.146435	0.501066	0.61757	-5.99674	0.723178	0.724197
Dendritic.cells	RMDN1	-0.09796	4.528231	-0.50084	0.617726	-6.09129	0.717971	0.716606
Dendritic.cells	UNC45B	0.237958	0.423376	0.500634	0.617873	-5.45466	0.777298	0.80332
Dendritic.cells	SORBS3	-0.20745	0.937867	-0.50061	0.617887	-5.44192	0.76954	0.791924
Dendritic.cells	4933408B1	-0.23461	1.941966	-0.50049	0.617974	-5.34089	0.754668	0.770143
Dendritic.cells	ZKSCAN17	-0.13114	3.498798	-0.5004	0.618037	-5.65758	0.732303	0.737508
Dendritic.cells	CUX2	-0.21095	1.039079	-0.50038	0.618054	-5.40463	0.768025	0.789718
Dendritic.cells	HOPX	0.225114	4.034194	0.500321	0.618093	-5.49209	0.724804	0.7266
Dendritic.cells	DARS	-0.08136	5.511648	-0.50011	0.618243	-6.21214	0.70471	0.697379
Dendritic.cells	DENND1B	0.053354	7.299486	0.499904	0.618385	-6.76244	0.681305	0.663559
Dendritic.cells	ATG16L1	0.072596	5.446329	0.499757	0.618488	-6.29733	0.705647	0.698725
Dendritic.cells	MEI4	-0.22721	1.201602	-0.49968	0.618541	-5.52552	0.765768	0.786314
Dendritic.cells	CDR2	0.266181	2.488604	0.499637	0.618572	-5.27465	0.746884	0.758678
Dendritic.cells	LGALS1	0.066871	6.036335	0.499384	0.618749	-6.88645	0.697774	0.687365
Dendritic.cells	POPDC3	-0.36159	0.894439	-0.49934	0.618777	-5.19614	0.770361	0.793078
Dendritic.cells	TXNL1	-0.05917	6.955558	-0.49927	0.618827	-6.59514	0.685738	0.670017
Dendritic.cells	HAUS6	0.112106	4.556972	0.499214	0.618869	-5.93083	0.717734	0.716313

Dendritic.cells	RBMXL1	-0.07764	5.042632	-0.49911	0.618939	-6.07307	0.711101	0.706718
Dendritic.cells	KLHDC4	-0.12631	3.945597	-0.49891	0.619079	-5.72759	0.726198	0.728701
Dendritic.cells	GM8066	0.221058	0.498967	0.498794	0.619163	-5.34647	0.776324	0.801996
Dendritic.cells	PRODH	0.16925	2.780405	0.498647	0.619266	-5.75119	0.742682	0.752795
Dendritic.cells	FRS2	-0.04932	5.84525	-0.4986	0.619299	-6.51558	0.700312	0.691249
Dendritic.cells	GDF15	-0.37899	1.508239	-0.49857	0.619318	-5.26745	0.761216	0.779918
Dendritic.cells	ZFP707	-0.16905	1.958642	-0.49852	0.619354	-5.50635	0.75459	0.770219
Dendritic.cells	H2-T23	0.082949	6.358381	0.498437	0.619414	-6.73979	0.693526	0.681485
Dendritic.cells	BRPF1	-0.09418	4.929241	-0.49842	0.619426	-6.04614	0.712642	0.70915
Dendritic.cells	MAP2K4	0.066554	6.321239	0.498275	0.619528	-6.55297	0.694034	0.682268
Dendritic.cells	4930503L1	-0.1468	2.576564	-0.4982	0.619577	-5.59771	0.745636	0.757227
Dendritic.cells	H2-M3	-0.13599	4.138363	-0.49804	0.619692	-5.96641	0.723593	0.7251
Dendritic.cells	GM10634	0.300254	0.651485	0.497791	0.619867	-5.18534	0.774236	0.79905
Dendritic.cells	CDKL1	-0.30317	1.100722	-0.49758	0.620012	-5.30103	0.767587	0.789184
Dendritic.cells	ITM2B	0.048218	10.00633	0.497369	0.620164	-7.23297	0.648026	0.615972
Dendritic.cells	GM28417	-0.20023	1.320479	-0.49736	0.620172	-5.46805	0.764345	0.784379
Dendritic.cells	FRMD6	0.147036	3.47737	0.497245	0.620251	-5.79065	0.733112	0.738736
Dendritic.cells	CHD6	0.059224	5.997264	0.49704	0.620394	-6.54838	0.698709	0.688756
Dendritic.cells	CITED2	-0.12199	6.149916	-0.49673	0.620614	-5.98089	0.696836	0.685913
Dendritic.cells	GM29994	0.200146	1.150146	0.496662	0.62066	-5.35325	0.767156	0.788289
Dendritic.cells	STRN4	0.076629	4.492619	0.496472	0.620794	-6.10888	0.719204	0.718334
Dendritic.cells	CENPQ	-0.10724	5.314475	-0.49646	0.620801	-6.06663	0.707998	0.702086
Dendritic.cells	TAGAP1	-0.18748	2.214287	-0.49637	0.620862	-5.54906	0.751472	0.765371
Dendritic.cells	HNRNPA1	0.051074	7.807246	0.496209	0.620978	-6.84412	0.675388	0.655047
Dendritic.cells	YIPF6	0.108061	3.457527	0.496155	0.621016	-5.91541	0.733652	0.739365
Dendritic.cells	POLR2M	0.056928	5.403452	0.495968	0.621148	-6.31904	0.706807	0.700391
Dendritic.cells	TLR7	0.074366	2.963787	0.49595	0.62116	-6.52452	0.740668	0.749602
Dendritic.cells	ITGAV	0.101069	6.550452	0.4957	0.621336	-6.29403	0.691579	0.678456
Dendritic.cells	MED23	-0.10127	3.692708	-0.49565	0.621374	-5.82583	0.730339	0.734653
Dendritic.cells	KLRB1F	0.343735	0.424065	0.495508	0.621471	-5.20393	0.778101	0.804581
Dendritic.cells	KIFC5B	-0.25136	1.787655	-0.49547	0.621495	-5.25101	0.757722	0.77468
Dendritic.cells	OAS1C	-0.28005	1.903745	-0.49533	0.621593	-5.25477	0.756018	0.772235
Dendritic.cells	SMARCC1	-0.05559	6.606315	-0.49529	0.621621	-6.63601	0.690848	0.67751
Dendritic.cells	MANEA	0.109645	3.728612	0.495276	0.621634	-5.89111	0.729835	0.73402
Dendritic.cells	MMP11	-0.26884	0.128652	-0.49523	0.621668	-5.23761	0.782603	0.811313
Dendritic.cells	GM4788	-0.27785	1.237901	-0.49497	0.621851	-5.29394	0.765926	0.786799
Dendritic.cells	IFT20	-0.06764	5.442542	-0.49496	0.621855	-6.28655	0.706342	0.699938
Dendritic.cells	CEP68	0.104146	4.299523	0.494682	0.622052	-5.97318	0.722059	0.722663
Dendritic.cells	CEP72	0.180504	2.208429	0.494502	0.622178	-5.45255	0.751754	0.765998
Dendritic.cells	CRYBB3	0.287455	0.578324	0.494481	0.622193	-5.21903	0.775957	0.801496
Dendritic.cells	ZBED4	-0.05177	4.650997	-0.49444	0.622223	-6.52776	0.717215	0.715666
Dendritic.cells	SPCS2	0.035946	7.595587	0.494181	0.622404	-6.93902	0.678334	0.659412
Dendritic.cells	GM16150	0.233789	0.174831	0.494137	0.622435	-5.32651	0.782191	0.810615
Dendritic.cells	ITIH5	-0.28508	1.943297	-0.49395	0.622565	-5.20863	0.755776	0.771783
Dendritic.cells	FEM1B	-0.09815	4.728435	-0.49388	0.622613	-6.01288	0.716294	0.714233
Dendritic.cells	ZFP317	-0.15223	2.898111	-0.49377	0.62269	-5.55774	0.741948	0.751609
Dendritic.cells	CMTM3	0.101736	3.593966	0.493661	0.62277	-5.99939	0.732063	0.737252

Dendritic.cells	MADD	0.077872	5.091113	0.493527	0.622864	-6.20417	0.711356	0.707161
Dendritic.cells	LRP5	0.133884	4.078112	0.493448	0.62292	-5.81879	0.725284	0.727405
Dendritic.cells	E2F1	0.127664	4.409532	0.493381	0.622967	-5.89999	0.720689	0.720726
Dendritic.cells	MRPL15	0.072297	5.337746	0.49316	0.623123	-6.20372	0.708018	0.702375
Dendritic.cells	CD34	0.333928	0.953495	0.493141	0.623136	-5.37626	0.770465	0.793484
Dendritic.cells	UNC5CL	-0.28555	0.465408	-0.49303	0.623213	-5.32193	0.777831	0.804313
Dendritic.cells	6-Sep	0.079015	5.624977	0.493022	0.623219	-6.39431	0.704155	0.696781
Dendritic.cells	DMTN	-0.26899	0.637339	-0.49283	0.623356	-5.25767	0.775314	0.800562
Dendritic.cells	MRM3	-0.19528	2.005465	-0.49209	0.623873	-5.48214	0.755505	0.770935
Dendritic.cells	CD151	0.152118	2.758187	0.491956	0.62397	-5.59852	0.744614	0.75502
Dendritic.cells	LIAS	0.094107	4.310022	0.491674	0.624168	-5.94161	0.722855	0.723146
Dendritic.cells	TTLL5	-0.08411	4.471737	-0.49099	0.624651	-6.0669	0.721073	0.720048
Dendritic.cells	MEGF9	-0.15797	4.103779	-0.49067	0.624872	-5.75518	0.726178	0.72746
Dendritic.cells	SSB	0.043448	6.803522	0.490627	0.624906	-6.60751	0.689782	0.674789
Dendritic.cells	PRKACA	-0.08646	4.156404	-0.49049	0.625005	-5.98483	0.725445	0.726396
Dendritic.cells	GM20457	-0.27866	-0.16272	-0.49046	0.625022	-5.19381	0.788793	0.819065
Dendritic.cells	GTDC1	-0.09109	6.409058	-0.4904	0.625065	-6.17645	0.694948	0.68224
Dendritic.cells	2610307P1	0.227897	4.550996	0.490345	0.625104	-5.76765	0.71998	0.718461
Dendritic.cells	CTDSP1	0.082698	5.3236	0.490341	0.625107	-6.16199	0.709431	0.703172
Dendritic.cells	PHF11D	0.331353	1.34229	0.490195	0.62521	-5.28632	0.765977	0.785561
Dendritic.cells	MPV17	-0.09161	4.075124	-0.49012	0.625261	-5.90055	0.726577	0.728064
Dendritic.cells	PANX1	0.071133	4.208683	0.490073	0.625296	-6.19401	0.724718	0.725367
Dendritic.cells	FDX1	0.101806	4.692703	0.489906	0.625414	-6.08226	0.718088	0.715686
Dendritic.cells	YEATS2	0.085311	4.376137	0.489662	0.625586	-5.9988	0.722576	0.722065
Dendritic.cells	SETD1B	-0.06356	5.026478	-0.48949	0.625707	-6.382	0.713676	0.70911
Dendritic.cells	GNG7	-0.2888	-0.40318	-0.48943	0.625748	-5.18277	0.792748	0.824666
Dendritic.cells	SEMA4G	-0.26099	0.909795	-0.48926	0.625872	-5.31555	0.772682	0.79518
Dendritic.cells	RAD18	-0.14861	4.103468	-0.48918	0.625925	-5.7163	0.726398	0.727598
Dendritic.cells	1810055G(	-0.1731	2.268587	-0.48912	0.625969	-5.4133	0.752556	0.765708
Dendritic.cells	HASPIN	-0.22115	2.299155	-0.48905	0.626016	-5.34401	0.75211	0.765058
Dendritic.cells	LFNG	-0.14775	4.413378	-0.4888	0.626191	-5.61024	0.722191	0.721409
Dendritic.cells	GALNS	0.107692	3.757313	0.488745	0.626233	-5.86932	0.731341	0.734723
Dendritic.cells	TATDN1	0.11104	3.630234	0.488624	0.626318	-5.77752	0.733134	0.737326
Dendritic.cells	KIF2A	0.058131	6.338703	0.488552	0.626368	-6.41647	0.696177	0.683777
Dendritic.cells	BFSP2	0.231861	2.440103	0.488221	0.626602	-5.43306	0.750292	0.762341
Dendritic.cells	KPNA2	0.15254	4.937072	0.488211	0.626609	-5.80583	0.715116	0.711167
Dendritic.cells	SIRT7	-0.06927	4.918849	-0.48801	0.62675	-6.06576	0.71545	0.711532
Dendritic.cells	TBC1D20	-0.06148	5.789234	-0.48778	0.62691	-6.47297	0.703784	0.694497
Dendritic.cells	SOX6	0.330273	1.905309	0.487553	0.627073	-5.33864	0.758425	0.773802
Dendritic.cells	TMEM165	0.061244	5.311537	0.487096	0.627396	-6.27684	0.710486	0.703928
Dendritic.cells	GM46440	0.26854	0.637929	0.486919	0.62752	-5.27421	0.777532	0.801695
Dendritic.cells	PPP1R21	0.09979	4.557871	0.486916	0.627523	-6.00169	0.72079	0.718891
Dendritic.cells	BMP6	0.11019	0.450706	0.486902	0.627533	-6.06281	0.780377	0.805876
Dendritic.cells	CHCHD3	-0.03436	6.768694	-0.48687	0.627552	-6.73691	0.691104	0.675984
Dendritic.cells	INTS10	-0.13311	3.410048	-0.48668	0.627689	-5.64912	0.736866	0.74226
Dendritic.cells	GNL3L	-0.06741	4.467102	-0.48663	0.627725	-6.08367	0.722056	0.72074
Dendritic.cells	5033421B(	-0.33384	-0.4422	-0.48658	0.627763	-5.18792	0.79413	0.826128

Dendritic.cells	DNMT3A	-0.05497	6.044028	-0.48638	0.627898	-6.64724	0.700743	0.689796
Dendritic.cells	DPM1	0.051337	5.979025	0.486028	0.62815	-6.49868	0.701815	0.691124
Dendritic.cells	ARHGAP30	0.038366	7.139253	0.485873	0.628259	-6.80447	0.686622	0.669157
Dendritic.cells	COQ6	-0.1456	2.285167	-0.48548	0.628536	-5.49525	0.753541	0.76609
Dendritic.cells	KLRB1B	-0.28491	1.981821	-0.48535	0.628626	-5.44008	0.757983	0.772615
Dendritic.cells	TXNDC11	0.05608	5.714713	0.48534	0.628636	-6.50505	0.705525	0.696383
Dendritic.cells	STRN3	-0.04086	7.654054	-0.48525	0.628696	-6.96387	0.680124	0.659783
Dendritic.cells	FGL1	0.205565	3.523532	0.485238	0.628708	-5.5906	0.735738	0.740182
Dendritic.cells	PLEKHA1	0.086698	4.707996	0.485042	0.628846	-6.33994	0.719282	0.716226
Dendritic.cells	NLRC5	0.158637	5.064856	0.484952	0.62891	-6.21615	0.714394	0.709165
Dendritic.cells	LRMP	0.086101	6.432878	0.484782	0.62903	-6.25136	0.696109	0.682738
Dendritic.cells	CAR9	-0.2295	0.285801	-0.484	0.629583	-5.32556	0.784064	0.810325
Dendritic.cells	TRA2B	-0.03881	7.832072	-0.484	0.629585	-6.85425	0.678413	0.656873
Dendritic.cells	GLIPR1	0.07618	5.059395	0.483839	0.629697	-6.31245	0.71498	0.709583
Dendritic.cells	FAM43A	-0.07465	4.098571	-0.48383	0.6297	-6.25424	0.728251	0.728818
Dendritic.cells	E130317F2	0.183736	0.77534	0.483747	0.629762	-5.38772	0.776612	0.799385
Dendritic.cells	PIDD1	0.255391	0.824038	0.483503	0.629934	-5.22555	0.776004	0.798368
Dendritic.cells	ACTN1	-0.08021	5.757216	-0.48341	0.63	-6.35707	0.705654	0.696
Dendritic.cells	GM16552	-0.15998	1.517	-0.48327	0.630096	-5.52342	0.765616	0.783192
Dendritic.cells	1810024B0	-0.15078	2.692562	-0.48321	0.630138	-5.60031	0.748371	0.758006
Dendritic.cells	ZDHHC16	0.127257	2.888345	0.483067	0.630242	-5.54992	0.74559	0.753889
Dendritic.cells	WDR18	0.068866	4.82244	0.482582	0.630585	-6.24657	0.718455	0.714431
Dendritic.cells	CYP4B1	-0.24189	0.266849	-0.48258	0.630586	-5.45469	0.784607	0.810924
Dendritic.cells	DUSP2	0.18939	5.991936	0.482569	0.630595	-5.76855	0.702624	0.691545
Dendritic.cells	CACNB1	0.253243	0.846674	0.482558	0.630602	-5.31112	0.775783	0.797972
Dendritic.cells	ILF3	-0.05144	6.541502	-0.48251	0.630634	-6.63188	0.695342	0.681044
Dendritic.cells	TCRG-C2	-0.4234	0.685448	-0.48238	0.630731	-5.22373	0.778261	0.801553
Dendritic.cells	PNRC2	-0.06504	5.35165	-0.4821	0.630924	-6.29755	0.711411	0.704076
Dendritic.cells	RAE1	0.0776	4.525674	0.481958	0.631027	-6.08441	0.722766	0.72049
Dendritic.cells	DRC7	0.293469	-0.15036	0.48165	0.631244	-5.19096	0.79141	0.820556
Dendritic.cells	GM42701	-0.22935	0.844253	-0.48161	0.631272	-5.36858	0.776192	0.798209
Dendritic.cells	B9D2	0.109262	4.930502	0.481337	0.631466	-5.98282	0.717416	0.71253
Dendritic.cells	MRGBP	0.108348	3.37422	0.481165	0.631588	-5.82268	0.739159	0.744152
Dendritic.cells	FBF1	-0.2914	1.539498	-0.48111	0.631626	-5.32201	0.765869	0.783109
Dendritic.cells	COA6	0.101969	4.110702	0.481111	0.631626	-5.87412	0.728766	0.729055
Dendritic.cells	SPTLC2	0.04761	6.245992	0.480923	0.631759	-6.50481	0.69975	0.687042
Dendritic.cells	SMG1	0.036476	7.499087	0.480356	0.632161	-6.85047	0.683746	0.663629
Dendritic.cells	MN1	-0.07913	0.308399	-0.48012	0.632325	-6.34404	0.785073	0.810649
Dendritic.cells	UBALD1	0.07774	4.983613	0.479921	0.632469	-6.21048	0.717284	0.711893
Dendritic.cells	IKZF3	0.148056	5.866917	0.479861	0.632511	-5.95154	0.705315	0.694599
Dendritic.cells	MRPL34	0.07094	5.428125	0.479802	0.632553	-6.12539	0.711228	0.703137
Dendritic.cells	5830448L0	0.222375	0.904406	0.479613	0.632687	-5.30765	0.776116	0.79749
Dendritic.cells	ACOX2	-0.28533	1.030725	-0.47926	0.63294	-5.27781	0.774437	0.794844
Dendritic.cells	QSER1	0.102896	4.323503	0.479094	0.633055	-5.89641	0.726745	0.725303
Dendritic.cells	KLHDC3	-0.08111	4.338451	-0.479	0.633121	-5.91579	0.726537	0.725029
Dendritic.cells	TMEM132A	0.226781	0.400445	0.478615	0.633394	-5.31796	0.784324	0.809005
Dendritic.cells	CCDC115	-0.098	4.152653	-0.47837	0.633568	-5.84533	0.72946	0.728973

Dendritic.cells	0610039K1	0.300235	0.022539	0.478278	0.633633	-5.1895	0.790234	0.817715
Dendritic.cells	LRRC18	-0.22728	1.588071	-0.47822	0.633675	-5.41978	0.766491	0.782942
Dendritic.cells	COG3	-0.08228	4.292574	-0.47778	0.633989	-6.09325	0.727602	0.726279
Dendritic.cells	CDC34	0.063664	6.050691	0.477687	0.634052	-6.3834	0.703602	0.691587
Dendritic.cells	GPKOW	-0.0737	4.488998	-0.47768	0.634055	-6.01398	0.724869	0.722319
Dendritic.cells	TKFC	0.132324	3.130684	0.477638	0.634087	-5.83941	0.744041	0.750147
Dendritic.cells	MRPS6	-0.05573	6.008603	-0.47759	0.63412	-6.55147	0.704164	0.6924
Dendritic.cells	USP10	-0.09837	4.567936	-0.47753	0.634162	-5.99604	0.723774	0.72074
Dendritic.cells	ZFP687	0.144639	2.919042	0.47724	0.634369	-5.53001	0.74725	0.754686
Dendritic.cells	CKB	-0.19135	4.578345	-0.47703	0.634515	-5.69856	0.723879	0.720647
Dendritic.cells	CAPZA1	0.032906	7.715586	0.476686	0.634762	-6.82069	0.682252	0.660431
Dendritic.cells	FBXO33	-0.06283	5.984659	-0.47649	0.634901	-6.37948	0.704935	0.693058
Dendritic.cells	OGA	0.052687	6.76343	0.476126	0.635159	-6.57843	0.694611	0.678327
Dendritic.cells	MRPL55	0.094458	4.367452	0.47594	0.635291	-5.9392	0.727023	0.725135
Dendritic.cells	GM34225	0.217687	-0.06364	0.475904	0.635317	-5.43586	0.792178	0.820203
Dendritic.cells	1810006J0	-0.27329	-0.01116	-0.4759	0.635321	-5.19284	0.791366	0.81901
Dendritic.cells	NUDT21	0.058169	6.148811	0.475732	0.635439	-6.44124	0.702742	0.690069
Dendritic.cells	RAB4B	-0.05215	5.9254	-0.47561	0.635525	-6.43632	0.705729	0.694419
Dendritic.cells	PIGS	-0.08386	4.078714	-0.47548	0.635615	-5.98811	0.73106	0.731097
Dendritic.cells	EGFEM1	-0.09189	1.229611	-0.47547	0.635625	-6.51614	0.772447	0.79139
Dendritic.cells	HIST1H2AF	0.324599	0.617707	0.475445	0.635642	-5.19408	0.781708	0.804957
Dendritic.cells	SAT1	-0.0658	8.474503	-0.47543	0.635651	-6.84217	0.672627	0.646893
Dendritic.cells	A430072P0	0.320113	0.354986	0.475226	0.635798	-5.19295	0.785726	0.810925
Dendritic.cells	GYPC	-0.18698	3.080733	-0.47511	0.635879	-5.58149	0.745235	0.751811
Dendritic.cells	SH3PXD2B	-0.30733	1.543779	-0.47497	0.635982	-5.34109	0.767744	0.784695
Dendritic.cells	GM41790	-0.30978	-0.09939	-0.47489	0.636034	-5.21573	0.792732	0.821351
Dendritic.cells	TMEM98	-0.23647	0.620631	-0.47488	0.636042	-5.31661	0.781664	0.805097
Dendritic.cells	AI467606	0.08207	4.111903	0.474871	0.63605	-6.27347	0.730594	0.730609
Dendritic.cells	CPSF2	-0.0822	5.649423	-0.4747	0.636171	-6.14968	0.709441	0.700034
Dendritic.cells	ACTN4	-0.04724	6.545001	-0.47467	0.63619	-6.50229	0.697487	0.682796
Dendritic.cells	ARHGEF18	0.121514	5.591269	0.474664	0.636197	-5.9189	0.710227	0.701169
Dendritic.cells	GNPDA2	-0.12256	3.100129	-0.47453	0.636292	-5.62479	0.744956	0.751517
Dendritic.cells	ACOT2	-0.09787	4.416307	-0.47451	0.636309	-5.82396	0.726343	0.724492
Dendritic.cells	ZFR	0.038348	6.532381	0.4745	0.636313	-6.71643	0.697653	0.683044
Dendritic.cells	HOOK1	-0.13526	2.667358	-0.4744	0.636385	-5.67109	0.751212	0.760629
Dendritic.cells	CISH	0.248184	2.887974	0.474201	0.636526	-5.38146	0.748104	0.755985
Dendritic.cells	APOOL	0.10313	3.780654	0.473818	0.636797	-5.83377	0.735352	0.737518
Dendritic.cells	GM13431	-0.27136	0.492651	-0.47379	0.63682	-5.21456	0.783718	0.80812
Dendritic.cells	TSTD3	-0.21456	1.784277	-0.47372	0.63687	-5.28543	0.764265	0.779634
Dendritic.cells	TBC1D10A	-0.13461	3.56507	-0.47371	0.636872	-5.6008	0.738407	0.741957
Dendritic.cells	DPP6	-0.3117	-0.15893	-0.47369	0.636889	-5.19171	0.793757	0.822869
Dendritic.cells	DDX1	0.094771	4.964571	0.473629	0.636932	-6.05129	0.718857	0.71362
Dendritic.cells	A130010J1	-0.20109	1.72998	-0.47347	0.637044	-5.40085	0.765125	0.780879
Dendritic.cells	C230037L1	0.21052	0.378737	0.473142	0.637278	-5.33933	0.785723	0.810908
Dendritic.cells	TNFSF8	0.330839	-0.51498	0.473036	0.637353	-5.19249	0.799583	0.831314
Dendritic.cells	NUDT4	0.102919	5.949353	0.472856	0.637481	-5.92857	0.705809	0.694563
Dendritic.cells	OTUB2	-0.23754	1.885457	-0.47271	0.637586	-5.29052	0.763147	0.77778

Dendritic.cells	GM15492	-0.23834	0.742028	-0.47249	0.637742	-5.27286	0.780414	0.802973
Dendritic.cells	DDX43	0.281733	0.153087	0.472123	0.638002	-5.20081	0.789443	0.816372
Dendritic.cells	DBT	0.111243	3.826101	0.472062	0.638045	-5.90236	0.735187	0.737157
Dendritic.cells	P4HA2	-0.26368	0.108055	-0.47197	0.63811	-5.23093	0.790138	0.817485
Dendritic.cells	RNASET2B	-0.0717	5.139167	-0.47188	0.638172	-6.48113	0.71693	0.710752
Dendritic.cells	MBNL3	0.127849	4.597519	0.471866	0.638185	-5.8671	0.724389	0.72155
Dendritic.cells	HPCAL1	0.061635	6.117607	0.471773	0.638251	-6.47909	0.703705	0.691652
Dendritic.cells	SCP2	-0.08747	7.526193	-0.47166	0.638334	-6.6714	0.685226	0.665055
Dendritic.cells	BHLHE41	-0.31569	1.739361	-0.47162	0.638357	-5.19859	0.765428	0.78127
Dendritic.cells	TLR8	-0.26717	0.054908	-0.47162	0.63836	-5.20358	0.790959	0.818694
Dendritic.cells	BMPR2	0.068625	5.519027	0.471473	0.638464	-6.50931	0.711757	0.703305
Dendritic.cells	LTBR	-0.12533	2.974023	-0.47144	0.638488	-5.73476	0.747351	0.754936
Dendritic.cells	TANGO2	0.134023	3.93274	0.471342	0.638557	-5.9141	0.733684	0.735061
Dendritic.cells	MKLN1	-0.04232	7.159858	-0.47095	0.63884	-6.79785	0.689998	0.67194
Dendritic.cells	ENY2	-0.06351	5.972518	-0.47094	0.63884	-6.34713	0.705675	0.694522
Dendritic.cells	GM43111	-0.24525	-0.1905	-0.47083	0.63892	-5.26916	0.794796	0.824372
Dendritic.cells	4930522L1	-0.12598	3.684756	-0.47068	0.639028	-5.72683	0.737218	0.740192
Dendritic.cells	9530034E1	-0.20492	0.330435	-0.47065	0.639051	-5.36703	0.786746	0.812539
Dendritic.cells	VPS4A	-0.08621	4.241466	-0.47065	0.639053	-5.94238	0.729378	0.728811
Dendritic.cells	NCAPG	0.239198	3.22832	0.470563	0.639112	-5.44785	0.743725	0.749653
Dendritic.cells	TLN2	-0.2886	1.298032	-0.47046	0.639185	-5.22666	0.772051	0.791009
Dendritic.cells	SLC46A1	-0.32487	0.761766	-0.47046	0.639186	-5.23311	0.780154	0.802882
Dendritic.cells	STRADA	0.086467	4.58715	0.470101	0.63944	-6.08164	0.724703	0.721992
Dendritic.cells	ZFP931	-0.16648	1.514653	-0.4701	0.639444	-5.45245	0.768956	0.786419
Dendritic.cells	CYP2R1	0.20448	1.009263	0.469541	0.639839	-5.42264	0.776906	0.797766
Dendritic.cells	STARD3NL	0.070057	5.861943	0.469363	0.639966	-6.27703	0.707618	0.697019
Dendritic.cells	PAPLN	-0.24471	0.894565	-0.46914	0.640126	-5.27052	0.778643	0.800391
Dendritic.cells	MCOLN1	0.111587	2.728161	0.468983	0.640236	-5.72943	0.751427	0.760668
Dendritic.cells	SECISBP2L	0.069735	4.964176	0.468893	0.6403	-6.29855	0.719825	0.714776
Dendritic.cells	ADGRL1	0.161669	2.476047	0.468854	0.640328	-5.51028	0.755099	0.766023
Dendritic.cells	ECHDC1	-0.11386	3.820316	-0.46883	0.640342	-5.86084	0.735776	0.737901
Dendritic.cells	KLHL8	-0.22274	2.402928	-0.46874	0.640407	-5.39881	0.756168	0.767587
Dendritic.cells	HEMK1	0.242021	1.147753	0.4687	0.640438	-5.25718	0.774814	0.794843
Dendritic.cells	CASP12	0.270699	-0.47839	0.468603	0.640506	-5.19428	0.799805	0.831559
Dendritic.cells	MOCOS	-0.28528	1.324581	-0.46859	0.640517	-5.28576	0.772154	0.790958
Dendritic.cells	ZFP84	0.122547	3.7569	0.468506	0.640576	-5.7771	0.736673	0.73922
Dendritic.cells	PLA2G4C	0.297679	0.890136	0.468386	0.640661	-5.19361	0.77871	0.80059
Dendritic.cells	ZFP157	0.153663	2.446699	0.468364	0.640677	-5.60609	0.755528	0.76669
Dendritic.cells	4930445E1	0.239317	-0.41363	0.468256	0.640754	-5.24161	0.798804	0.830104
Dendritic.cells	GBP3	-0.2454	3.228626	-0.46742	0.641348	-5.51646	0.744826	0.750422
Dendritic.cells	FTCD	-0.28438	1.403237	-0.46725	0.641471	-5.31613	0.771619	0.78956
Dendritic.cells	GM29114	0.232111	-1.28439	0.467234	0.641482	-5.19333	0.813224	0.850702
Dendritic.cells	ZFP703	-0.17909	4.364442	-0.46704	0.641621	-5.57429	0.728782	0.727132
Dendritic.cells	TXNRD3	0.193423	1.950944	0.466995	0.641652	-5.36539	0.763499	0.777635
Dendritic.cells	UBA1	-0.05766	6.00691	-0.46674	0.641831	-6.41004	0.706386	0.694632
Dendritic.cells	ATP6AP1	0.058319	5.990589	0.466635	0.641909	-6.41138	0.706605	0.694948
Dendritic.cells	CHKB	0.090349	4.306938	0.466605	0.64193	-5.88289	0.729673	0.728297

Dendritic.cells	GM39121	0.261143	-0.94202	0.466366	0.642101	-5.19342	0.80805	0.842674
Dendritic.cells	POLR3A	0.114666	3.30507	0.466098	0.642291	-5.86018	0.744122	0.748892
Dendritic.cells	CFB	-0.41486	4.663824	-0.46575	0.64254	-5.76692	0.725163	0.721244
Dendritic.cells	ZER1	0.107157	3.262662	0.465569	0.642669	-5.7501	0.745012	0.749952
Dendritic.cells	TRPV2	0.06608	4.873594	0.465343	0.64283	-6.32394	0.722434	0.717138
Dendritic.cells	TMEM11	-0.07752	5.070903	-0.46494	0.643121	-6.11529	0.719819	0.713236
Dendritic.cells	NNT	0.112988	2.482711	0.464905	0.643142	-5.75359	0.756535	0.766515
Dendritic.cells	VSIG10L	-0.22558	0.298869	-0.46489	0.643154	-5.19936	0.789342	0.814477
Dendritic.cells	HDLBP	-0.03697	6.655734	-0.46484	0.643191	-6.72655	0.698465	0.682463
Dendritic.cells	HAUS7	0.126038	2.992661	0.464711	0.643281	-5.63796	0.749144	0.755736
Dendritic.cells	TMEM39B	0.107795	4.225197	0.464305	0.64357	-5.87461	0.73184	0.730267
Dendritic.cells	TCERG1	0.044401	6.296507	0.464211	0.643638	-6.55721	0.703499	0.689347
Dendritic.cells	KLC1	-0.0597	4.702224	-0.46398	0.643802	-6.24736	0.725245	0.720634
Dendritic.cells	NOCT	-0.14864	5.04776	-0.46394	0.643831	-5.8073	0.720472	0.713735
Dendritic.cells	CD63	-0.17883	4.105165	-0.46385	0.643894	-5.7084	0.733589	0.732737
Dendritic.cells	RBM24	0.314962	1.008251	0.463595	0.644077	-5.24069	0.778887	0.798713
Dendritic.cells	MRRF	0.116361	3.454685	0.463448	0.644182	-5.73185	0.742845	0.746258
Dendritic.cells	INAFM2	0.078974	3.225336	0.463406	0.644212	-6.13248	0.746134	0.751039
Dendritic.cells	DPYD	-0.24032	3.567148	-0.46337	0.644234	-5.65669	0.741238	0.743929
Dendritic.cells	TRAF3	-0.0689	6.856964	-0.46335	0.644253	-6.59916	0.69616	0.678825
Dendritic.cells	GM5431	0.295033	0.742737	0.463035	0.644477	-5.29168	0.783049	0.80479
Dendritic.cells	4933407K1	-0.14999	1.39879	-0.46302	0.644488	-5.50168	0.773113	0.790254
Dendritic.cells	CTBP1	-0.04696	6.500976	-0.46242	0.644914	-6.6612	0.701053	0.685798
Dendritic.cells	RINT1	0.081853	3.771156	0.462392	0.644936	-6.00067	0.738537	0.739946
Dendritic.cells	RAB27B	-0.25968	0.615378	-0.46235	0.644968	-5.33075	0.785087	0.807808
Dendritic.cells	PRELID2	0.226373	2.621255	0.462299	0.645003	-5.48321	0.755092	0.764009
Dendritic.cells	INTS8	-0.06967	4.693172	-0.46221	0.645063	-6.13983	0.725593	0.721215
Dendritic.cells	GM11613	-0.23961	2.020755	-0.46218	0.645089	-5.44224	0.763922	0.776888
Dendritic.cells	THAP1	0.138699	2.879636	0.462171	0.645094	-5.58703	0.751332	0.75855
Dendritic.cells	ARMC9	0.130363	2.484131	0.462088	0.645154	-5.59539	0.757098	0.766967
Dendritic.cells	2900089D1	0.16286	2.58395	0.461977	0.645232	-5.59227	0.755637	0.764841
Dendritic.cells	TACC1	-0.02767	7.703473	-0.46197	0.645238	-7.09966	0.685334	0.66326
Dendritic.cells	MIS12	-0.15608	3.322859	-0.46163	0.645481	-5.58181	0.74514	0.749363
Dendritic.cells	GM44284	-0.2639	-0.05378	-0.46124	0.645757	-5.23752	0.795762	0.82318
Dendritic.cells	TOR1B	-0.08508	3.704769	-0.46113	0.645836	-5.99156	0.739805	0.741517
Dendritic.cells	LENG1	0.122145	2.925078	0.461015	0.64592	-5.62651	0.751003	0.757793
Dendritic.cells	PEG3	-0.26225	1.53048	-0.46094	0.645974	-5.28729	0.771565	0.787768
Dendritic.cells	ADIPOR1	0.07895	7.346972	0.46089	0.64601	-6.42947	0.690248	0.670039
Dendritic.cells	PCNX	-0.05364	5.518161	-0.46052	0.64627	-6.59551	0.714571	0.705119
Dendritic.cells	UTRN	0.050382	7.31422	0.460498	0.646289	-6.89703	0.690674	0.670729
Dendritic.cells	5430414B1	-0.26843	0.449396	-0.46046	0.646315	-5.25777	0.78798	0.811884
Dendritic.cells	TIMM9	-0.08269	3.882337	-0.46043	0.646339	-5.99426	0.737284	0.737976
Dendritic.cells	YKT6	0.086555	4.483546	0.460394	0.646364	-5.92345	0.72883	0.725729
Dendritic.cells	MAP2K7	-0.07828	4.294187	-0.46038	0.646374	-6.00118	0.731479	0.729564
Dendritic.cells	AK2	0.063856	5.995761	0.460168	0.646525	-6.39165	0.708112	0.69581
Dendritic.cells	EMC4	-0.07455	4.771124	-0.46015	0.646536	-6.20407	0.72483	0.719942
Dendritic.cells	TMEM181/	-0.04637	4.58903	-0.46007	0.646593	-6.52694	0.72736	0.723617

Dendritic.cells	PGM2L1	0.114358	5.192591	0.46002	0.646631	-5.95784	0.719019	0.711566
Dendritic.cells	IFT52	-0.06978	4.249963	-0.45991	0.646709	-6.08073	0.7321	0.730507
Dendritic.cells	GGCT	-0.20225	3.249782	-0.45987	0.646735	-5.46446	0.746314	0.751128
Dendritic.cells	NT5M	-0.08162	3.572881	-0.45965	0.646893	-6.00696	0.741744	0.744504
Dendritic.cells	LCORL	-0.04452	6.985276	-0.45961	0.646922	-6.84677	0.695027	0.677036
Dendritic.cells	SNUPN	0.119049	2.593487	0.459338	0.647119	-5.61276	0.756007	0.765153
Dendritic.cells	TSC2	0.119154	3.109391	0.459262	0.647173	-5.66347	0.748512	0.754288
Dendritic.cells	MCPT8	-0.56556	-0.5048	-0.45919	0.647224	-5.19864	0.803003	0.833995
Dendritic.cells	TOE1	-0.15077	2.490022	-0.45905	0.647322	-5.53712	0.757557	0.767445
Dendritic.cells	ZXDC	-0.08675	4.004625	-0.45873	0.647554	-5.98454	0.735806	0.735802
Dendritic.cells	MAU2	0.055365	6.192162	0.458723	0.647559	-6.65771	0.705719	0.69233
Dendritic.cells	B3GALT4	0.209332	1.404829	0.458656	0.647607	-5.43745	0.773716	0.790982
Dendritic.cells	4632428C0	0.263364	0.110114	0.458538	0.647691	-5.19512	0.793489	0.819961
Dendritic.cells	KCTD7	0.240049	0.387211	0.458362	0.647817	-5.19606	0.789206	0.813722
Dendritic.cells	RTCA	0.090707	4.282973	0.458296	0.647864	-5.95754	0.731887	0.73018
Dendritic.cells	KIF18A	-0.14898	3.977118	-0.45817	0.647954	-5.75297	0.736195	0.736484
Dendritic.cells	DHX29	-0.10635	3.415036	-0.45815	0.647968	-5.79298	0.744196	0.748095
Dendritic.cells	ZFP260	0.060514	4.245881	0.45815	0.647969	-6.21304	0.732408	0.730995
Dendritic.cells	UBA6	0.06157	5.172539	0.458049	0.648041	-6.3266	0.719546	0.712412
Dendritic.cells	BTBD7	0.035397	7.004982	0.457826	0.648201	-6.76589	0.694974	0.677058
Dendritic.cells	HMBS	-0.14765	4.451319	-0.45775	0.648257	-5.72149	0.729555	0.726954
Dendritic.cells	RTCB	0.067829	5.290693	0.457601	0.648362	-6.2378	0.717946	0.710177
Dendritic.cells	A430090L1	-0.2343	0.628221	-0.45759	0.648369	-5.20477	0.785532	0.808519
Dendritic.cells	A930001A2	0.280083	-0.71301	0.457553	0.648396	-5.19626	0.806401	0.839187
Dendritic.cells	ACO1	-0.09904	3.712371	-0.45737	0.648529	-5.82152	0.74005	0.742151
Dendritic.cells	ZFP507	0.224072	2.176566	0.457209	0.648642	-5.33343	0.762372	0.774558
Dendritic.cells	ARHGAP19	-0.20363	4.256309	-0.45707	0.648741	-5.55851	0.732448	0.731033
Dendritic.cells	IWS1	0.051286	5.729671	0.456819	0.648922	-6.41661	0.71225	0.701718
Dendritic.cells	NOC3L	0.139167	3.216026	0.456397	0.649223	-5.76008	0.747606	0.752696
Dendritic.cells	CAPN15	0.069654	4.710463	0.456287	0.649302	-6.20825	0.726455	0.722031
Dendritic.cells	ATF7	-0.05051	6.314357	-0.45617	0.649388	-6.63105	0.704605	0.690519
Dendritic.cells	H1F0	0.074584	6.001224	0.456162	0.649392	-6.56166	0.708802	0.696565
Dendritic.cells	PLXDC1	0.070541	3.94302	0.455948	0.649545	-6.62014	0.737318	0.737717
Dendritic.cells	DHX36	0.052773	5.392248	0.455655	0.649755	-6.28494	0.717205	0.708569
Dendritic.cells	GM20300	0.161131	1.343674	0.455584	0.649806	-5.44549	0.775363	0.793061
Dendritic.cells	GM26839	-0.23044	0.656622	-0.45548	0.649878	-5.32147	0.785806	0.808344
Dendritic.cells	ZFP975	0.272663	0.810489	0.455457	0.649897	-5.3094	0.783453	0.804897
Dendritic.cells	VAMP5	-0.12776	4.717071	-0.45541	0.649927	-5.86301	0.726509	0.722037
Dendritic.cells	LYZ2	0.293834	7.170673	0.455083	0.650165	-6.20648	0.69356	0.674443
Dendritic.cells	UFSP2	0.077775	4.93604	0.45494	0.650267	-6.13975	0.723609	0.717747
Dendritic.cells	PADI4	0.285977	1.890862	0.454926	0.650277	-5.38542	0.76731	0.781213
Dendritic.cells	FAM151B	0.213554	1.753415	0.454869	0.650318	-5.39471	0.769359	0.784204
Dendritic.cells	IFIH1	0.156504	3.912874	0.454709	0.650433	-5.8447	0.737986	0.738511
Dendritic.cells	HERC6	0.135119	4.719873	0.454483	0.650595	-6.05614	0.726763	0.722107
Dendritic.cells	SLC25A20	-0.09199	5.597573	-0.45437	0.650677	-6.1645	0.714709	0.704694
Dendritic.cells	SUGT1	-0.05294	5.949959	-0.45423	0.650777	-6.36318	0.709973	0.697863
Dendritic.cells	ADCY3	-0.21151	3.001893	-0.45406	0.650902	-5.64545	0.751274	0.757545

Dendritic.cells	CYB5B	0.046574	5.866247	0.453814	0.651075	-6.54067	0.711255	0.699593
Dendritic.cells	ZFP874A	-0.16842	2.146516	-0.45375	0.651119	-5.45753	0.7639	0.775929
Dendritic.cells	MYSM1	0.053294	5.388465	0.453345	0.651411	-6.43495	0.717947	0.709053
Dendritic.cells	LATS2	-0.04132	5.889771	-0.45331	0.651433	-6.71707	0.711131	0.699226
Dendritic.cells	CCDC114	-0.18869	1.271818	-0.45322	0.6515	-5.36676	0.777195	0.795106
Dendritic.cells	AKR1A1	0.043996	7.188669	0.452821	0.651787	-6.74943	0.693959	0.674431
Dendritic.cells	PCDHGC4	0.272432	0.256002	0.452799	0.651803	-5.20536	0.792845	0.817894
Dendritic.cells	FAM3A	-0.12203	2.785704	-0.45276	0.65183	-5.68035	0.754822	0.762381
Dendritic.cells	GPR141B	-0.22217	-0.65516	-0.45264	0.651913	-5.20227	0.807102	0.838868
Dendritic.cells	GM13708	0.113832	3.088737	0.452482	0.65203	-5.84831	0.750419	0.75605
Dendritic.cells	PITPNB	0.047454	5.507244	0.45244	0.65206	-6.42906	0.716423	0.70683
Dendritic.cells	SH2B1	0.085446	3.699274	0.452066	0.652329	-6.0948	0.741646	0.743465
Dendritic.cells	CAMKK1	0.224991	0.590902	0.451988	0.652385	-5.30584	0.78768	0.810579
Dendritic.cells	HEBP1	-0.18416	4.67467	-0.45191	0.652439	-5.82458	0.727899	0.723552
Dendritic.cells	GM4876	0.184197	1.628342	0.451855	0.65248	-5.35504	0.771935	0.787549
Dendritic.cells	GMIP	-0.05434	5.964525	-0.45185	0.652482	-6.40452	0.71022	0.69803
Dendritic.cells	FAM83F	-0.10898	1.041009	-0.45183	0.652501	-6.01032	0.780801	0.800508
Dendritic.cells	COASY	-0.1449	3.053875	-0.4517	0.652593	-5.60358	0.750924	0.756938
Dendritic.cells	LRAT	-0.27538	-0.03713	-0.45154	0.652706	-5.19982	0.797399	0.824865
Dendritic.cells	PRKCI	-0.13125	3.113961	-0.4515	0.652738	-5.70316	0.750054	0.755729
Dendritic.cells	BUD23	0.101274	4.09892	0.451433	0.652783	-5.95292	0.735974	0.735317
Dendritic.cells	DYNC1I2	0.040083	6.441227	0.451245	0.652918	-6.57775	0.703828	0.688978
Dendritic.cells	SPRED1	0.108821	4.710293	0.450988	0.653103	-6.03372	0.727403	0.723076
Dendritic.cells	3300002I0	0.182778	1.970632	0.450974	0.653113	-5.43739	0.766824	0.780353
Dendritic.cells	UBAC1	-0.08768	4.029628	-0.45095	0.653131	-5.8639	0.736953	0.736918
Dendritic.cells	E230016M	0.190875	2.465953	0.45088	0.65318	-5.45514	0.759502	0.769715
Dendritic.cells	1110019D1	-0.21519	1.751896	-0.45074	0.653283	-5.28754	0.770085	0.7852
Dendritic.cells	CACFD1	0.100025	3.174111	0.45065	0.653345	-5.79148	0.749184	0.754748
Dendritic.cells	RASD1	0.193795	3.88428	0.450644	0.65335	-5.59873	0.739013	0.739979
Dendritic.cells	GM43149	0.201301	0.774872	0.450597	0.653383	-5.3734	0.78486	0.806815
Dendritic.cells	SPAST	-0.05722	5.207046	-0.45023	0.653643	-6.34004	0.720534	0.713312
Dendritic.cells	GM47167	0.139056	2.927116	0.450195	0.653672	-5.59205	0.752763	0.760043
Dendritic.cells	FAM98B	0.092959	4.474383	0.450165	0.653693	-5.89609	0.730695	0.728011
Dendritic.cells	ARHGEF1	0.043474	7.10928	0.450077	0.653757	-6.73081	0.694999	0.676519
Dendritic.cells	TMBIM6	0.04026	9.142023	0.450068	0.653763	-7.12554	0.66903	0.639312
Dendritic.cells	GM15886	-0.15032	1.881502	-0.45006	0.653772	-5.42837	0.768151	0.78247
Dendritic.cells	RAB3GAP1	0.030657	6.044858	0.449949	0.653849	-6.83878	0.709138	0.69688
Dendritic.cells	2310009B1	-0.07491	4.473004	-0.44993	0.653861	-6.03876	0.730714	0.728053
Dendritic.cells	MAD1L1	-0.06614	5.227133	-0.44989	0.653888	-6.13986	0.720258	0.712927
Dendritic.cells	MRPL58	0.072901	5.199269	0.44948	0.654185	-6.23261	0.72083	0.713588
Dendritic.cells	SHROOM2	-0.16773	1.506317	-0.44947	0.654196	-5.70025	0.773971	0.790808
Dendritic.cells	CEP350	-0.04986	6.505585	-0.44921	0.654381	-6.66525	0.703194	0.688106
Dendritic.cells	PIMREG	0.251353	2.104196	0.449193	0.654392	-5.40918	0.765083	0.777778
Dendritic.cells	SLC37A3	0.124169	3.658041	0.449135	0.654433	-5.61917	0.74247	0.744883
Dendritic.cells	GFER	-0.07485	4.406699	-0.44894	0.65457	-5.97925	0.731892	0.729566
Dendritic.cells	CDKN1A	-0.14153	5.267608	-0.44864	0.65479	-5.88658	0.719947	0.712367
Dendritic.cells	TOP3A	0.111166	3.860539	0.448546	0.654857	-5.84352	0.739603	0.740858

Dendritic.cells	CHSY3	-0.26255	1.978293	-0.44854	0.654864	-5.43762	0.766972	0.780685
Dendritic.cells	TMEM80	0.099734	3.276451	0.448467	0.654914	-5.73563	0.747963	0.753006
Dendritic.cells	ZFP952	0.140157	2.251647	0.4481	0.655177	-5.58163	0.76292	0.774835
Dendritic.cells	GARNL3	-0.10044	2.487215	-0.44805	0.655214	-5.98026	0.759449	0.769782
Dendritic.cells	INAFM1	0.172634	1.866251	0.448032	0.655226	-5.45596	0.76864	0.783189
Dendritic.cells	VDAC1	0.050669	6.677223	0.447992	0.655255	-6.64564	0.700931	0.685044
Dendritic.cells	VPS50	-0.0667	4.131766	-0.44796	0.655275	-6.14836	0.735761	0.735351
Dendritic.cells	ERAP1	-0.07031	5.228177	-0.44794	0.65529	-6.24117	0.720489	0.713245
Dendritic.cells	ZFP335OS	-0.08685	4.456083	-0.44768	0.655481	-6.07602	0.7312	0.728821
Dendritic.cells	IDI1	0.055055	3.90584	0.447598	0.655538	-6.44248	0.738959	0.740069
Dendritic.cells	2310022A1	0.124113	2.998253	0.447581	0.65555	-5.65667	0.751986	0.758998
Dendritic.cells	B230307C2	-0.08956	3.741811	-0.4475	0.65561	-5.9449	0.741292	0.743455
Dendritic.cells	ZYG11B	-0.06237	5.883084	-0.44747	0.655632	-6.37839	0.711562	0.700434
Dendritic.cells	IP6K1	0.046885	6.598351	0.447449	0.655645	-6.72263	0.701978	0.686624
Dendritic.cells	ZFP382	0.194467	2.008207	0.447374	0.655699	-5.42904	0.766527	0.780188
Dendritic.cells	CHKA	-0.06437	6.940391	-0.44732	0.655734	-6.53083	0.697455	0.680117
Dendritic.cells	SNAPC2	-0.14012	3.015281	-0.44685	0.656075	-5.63155	0.751937	0.75876
Dendritic.cells	BVHT	0.248965	-0.0458	0.446824	0.656095	-5.307	0.798015	0.826134
Dendritic.cells	GM42658	0.185295	1.544714	0.44675	0.656148	-5.418	0.773657	0.790441
Dendritic.cells	GM1043	-0.19909	1.915295	-0.44667	0.656203	-5.6078	0.768111	0.782369
Dendritic.cells	SPDEF	-0.28051	-0.4296	-0.44662	0.656245	-5.20348	0.80403	0.835032
Dendritic.cells	NDUFA6	0.053527	6.56293	0.446416	0.656388	-6.55428	0.702714	0.6875
Dendritic.cells	GM14321	-0.2446	-0.49943	-0.44621	0.656533	-5.19998	0.805275	0.836728
Dendritic.cells	SLC35B3	-0.05898	4.482615	-0.44617	0.656566	-6.15609	0.731152	0.728531
Dendritic.cells	CD274	0.335243	5.805413	0.445943	0.656729	-5.85944	0.712938	0.70219
Dendritic.cells	UXS1	0.086484	4.429155	0.445878	0.656775	-6.02262	0.731911	0.729616
Dendritic.cells	HOMER2	0.271973	0.275337	0.445695	0.656907	-5.20813	0.793177	0.818949
Dendritic.cells	POLR3H	0.102422	3.509866	0.445661	0.656931	-5.8151	0.744947	0.748525
Dendritic.cells	CCDC146	0.214901	2.867299	0.445601	0.656974	-5.43881	0.754233	0.762028
Dendritic.cells	ZFP780B	-0.1059	3.329247	-0.44559	0.65698	-5.86881	0.747542	0.752297
Dendritic.cells	KCTD11	-0.22126	0.546292	-0.44532	0.657176	-5.31254	0.789147	0.81289
Dendritic.cells	TRGV2	-0.30252	-0.51519	-0.44502	0.657395	-5.20373	0.805876	0.837232
Dendritic.cells	MCM3	0.123356	5.484871	0.44488	0.657494	-6.11202	0.717638	0.708603
Dendritic.cells	KIF16B	-0.07531	4.843803	-0.44468	0.657634	-6.19264	0.72655	0.721404
Dendritic.cells	TCF3	0.065608	6.601305	0.444569	0.657717	-6.42077	0.702684	0.686973
Dendritic.cells	PUM1	0.032297	7.56821	0.444376	0.657857	-6.87073	0.69	0.668752
Dendritic.cells	TPRA1	-0.14375	2.802051	-0.44436	0.657867	-5.55787	0.755658	0.763629
Dendritic.cells	GM49797	0.079352	4.882769	0.443833	0.658247	-6.05528	0.726387	0.720857
Dendritic.cells	4931423N1	0.256267	0.653443	0.443347	0.658597	-5.25741	0.788374	0.81093
Dendritic.cells	COQ7	0.124064	4.79596	0.443346	0.658598	-5.7947	0.727739	0.722679
Dendritic.cells	H19	-0.20322	6.408472	-0.44326	0.658664	-6.39177	0.705746	0.690983
Dendritic.cells	KCNIP3	0.298575	0.193763	0.443242	0.658674	-5.27347	0.795478	0.821381
Dendritic.cells	URGCP	-0.08306	4.434418	-0.44318	0.658719	-6.11857	0.732792	0.730023
Dendritic.cells	UTP18	-0.06332	5.338906	-0.44305	0.658813	-6.2644	0.720265	0.711886
Dendritic.cells	WBP11	0.043976	6.346343	0.442618	0.659123	-6.56409	0.706825	0.692303
Dendritic.cells	SURF2	0.111036	3.331878	0.442577	0.659153	-5.73013	0.748742	0.75291
Dendritic.cells	CREB3L1	-0.17884	2.035248	-0.4422	0.659424	-5.45699	0.767949	0.780672

Dendritic.cells	PLEKHO1	0.05204	5.499121	0.442143	0.659465	-6.52905	0.718485	0.70895
Dendritic.cells	MAPKBP1	-0.14686	3.047991	-0.44203	0.659544	-5.68728	0.753066	0.75903
Dendritic.cells	LGALS9	0.079885	7.157478	0.441892	0.659646	-6.44211	0.696306	0.677045
Dendritic.cells	MINPP1	0.062478	4.734583	0.441656	0.659816	-6.20331	0.729118	0.724304
Dendritic.cells	ELMOD2	0.115563	2.807903	0.441641	0.659827	-5.73297	0.756628	0.764201
Dendritic.cells	RAB13	-0.19891	1.635625	-0.44158	0.65987	-5.42333	0.774004	0.789524
Dendritic.cells	TLN1	0.046098	7.109295	0.441484	0.65994	-6.78017	0.696963	0.677968
Dendritic.cells	TLE5	0.039331	7.209326	0.44113	0.660196	-6.84724	0.695848	0.676144
Dendritic.cells	ZFP266	0.07098	4.029092	0.440829	0.660413	-6.01472	0.739328	0.738821
Dendritic.cells	LETM2	-0.12566	3.131534	-0.44076	0.660465	-5.67003	0.752208	0.757514
Dendritic.cells	FBXW4	0.084912	4.272711	0.440756	0.660465	-6.13866	0.735881	0.733834
Dendritic.cells	ORC4	0.052688	5.046376	0.440562	0.660605	-6.31227	0.725147	0.718245
Dendritic.cells	MIER3	0.07045	4.078409	0.440356	0.660754	-6.17646	0.738732	0.737885
Dendritic.cells	WDR27	0.22442	0.499373	0.440286	0.660804	-5.22169	0.791733	0.815123
Dendritic.cells	CCSER1	-0.31071	1.697174	-0.44013	0.660915	-5.32366	0.773488	0.788459
Dendritic.cells	PWWP2B	0.184774	2.082717	0.440105	0.660935	-5.40961	0.767725	0.780053
Dendritic.cells	SMAD5	0.088667	3.702902	0.440064	0.660965	-5.82868	0.74408	0.745675
Dendritic.cells	STAC2	0.105356	1.824947	0.439824	0.661138	-6.0872	0.77158	0.785663
Dendritic.cells	RPN2	-0.0457	6.097946	-0.43979	0.661163	-6.61459	0.710806	0.697579
Dendritic.cells	DTL	0.134203	4.961318	0.439681	0.661241	-6.01272	0.726355	0.720007
Dendritic.cells	DSTN	-0.07521	6.886463	-0.43968	0.661243	-6.37488	0.700274	0.682433
Dendritic.cells	FOXK2	0.060097	4.920069	0.439555	0.661332	-6.24033	0.726951	0.720875
Dendritic.cells	AKIP1	0.137817	3.24519	0.439456	0.661403	-5.63919	0.750702	0.755313
Dendritic.cells	FXR2	-0.06573	5.267562	-0.439	0.661729	-6.28496	0.722411	0.714089
Dendritic.cells	ZFP280B	-0.17449	1.826966	-0.43894	0.661778	-5.46233	0.771858	0.785857
Dendritic.cells	MAFF	-0.17169	3.876198	-0.4385	0.662091	-5.63642	0.742184	0.742435
Dendritic.cells	ARMCX4	-0.18697	1.19399	-0.43793	0.662503	-5.47664	0.78202	0.800098
Dendritic.cells	BC024978	-0.17787	1.865186	-0.43787	0.662547	-5.48215	0.771888	0.785311
Dendritic.cells	OAZ2	0.069946	4.485342	0.437844	0.662567	-6.068	0.733863	0.7301
Dendritic.cells	HEXIM2	-0.2406	0.998422	-0.43755	0.662778	-5.26257	0.785172	0.804539
Dendritic.cells	HKDC1	0.31136	0.37121	0.437441	0.662858	-5.22779	0.794847	0.818715
Dendritic.cells	NTMT1	-0.06321	3.588232	-0.43728	0.662977	-6.01139	0.74682	0.748688
Dendritic.cells	VPS4B	-0.04476	6.231035	-0.43716	0.66306	-6.5167	0.710049	0.695619
Dendritic.cells	RBFA	-0.08694	4.826424	-0.43712	0.663091	-6.07194	0.729293	0.723366
Dendritic.cells	MGL2	0.251084	-0.18973	0.436925	0.663231	-5.63591	0.80369	0.831718
Dendritic.cells	AKT1	-0.05822	6.230392	-0.43688	0.663266	-6.47257	0.710099	0.695741
Dendritic.cells	TGDS	0.129818	3.1411	0.436578	0.663482	-5.57698	0.753492	0.758327
Dendritic.cells	MEAK7	0.227546	0.666682	0.436009	0.663893	-5.28511	0.790865	0.812526
Dendritic.cells	ACOT12	-0.24076	0.798615	-0.43601	0.663894	-5.26263	0.788833	0.809554
Dendritic.cells	LYVE1	-0.16871	2.42534	-0.43565	0.664153	-5.88475	0.764352	0.773837
Dendritic.cells	WAC	-0.0328	7.414892	-0.43553	0.664236	-6.86312	0.694875	0.673496
Dendritic.cells	PAQR4	-0.25975	1.484926	-0.43551	0.664254	-5.32977	0.77842	0.794337
Dendritic.cells	SCCPDH	0.139053	2.465776	0.435202	0.664476	-5.53899	0.763754	0.773088
Dendritic.cells	HADHA	-0.05527	5.770414	-0.43518	0.664491	-6.34491	0.716833	0.705162
Dendritic.cells	PDZD2	0.206282	3.46937	0.435146	0.664517	-5.52662	0.749102	0.751816
Dendritic.cells	A930014D(	0.206725	-1.05861	0.435138	0.664523	-5.20175	0.818063	0.852594
Dendritic.cells	THOC1	-0.05117	5.704802	-0.43503	0.664602	-6.4727	0.717728	0.70647

Dendritic.cells	IFT46	-0.05752	4.502973	-0.43501	0.664614	-6.22238	0.734381	0.730507
Dendritic.cells	FOCAD	-0.11508	2.908895	-0.43501	0.664618	-5.81334	0.757241	0.763644
Dendritic.cells	GM15345	-0.26204	2.98417	-0.43463	0.664891	-5.41182	0.756333	0.762107
Dendritic.cells	GM16090	-0.29555	-0.96132	-0.43449	0.664993	-5.20324	0.81671	0.850378
Dendritic.cells	GM43768	-0.2254	0.70894	-0.43443	0.665033	-5.31333	0.790466	0.811885
Dendritic.cells	GNPDA1	0.099268	4.395098	0.434306	0.665125	-5.87643	0.736087	0.732824
Dendritic.cells	BPHL	-0.11259	3.663858	-0.43423	0.665181	-5.77254	0.746493	0.747915
Dendritic.cells	CCL8	-0.21922	-1.46778	-0.43419	0.665208	-5.20046	0.824868	0.862495
Dendritic.cells	HIST1H2BN	-0.25554	2.139918	-0.43405	0.665311	-5.31335	0.768783	0.780304
Dendritic.cells	SNHG20	0.13664	2.355617	0.434013	0.665336	-5.47658	0.765578	0.775638
Dendritic.cells	PLSCR4	-0.2227	0.494112	-0.43393	0.665398	-5.34034	0.793785	0.816849
Dendritic.cells	HDAC10	0.148494	1.608346	0.433796	0.665493	-5.51983	0.776783	0.791995
Dendritic.cells	MEN1	0.141896	2.866329	0.433264	0.665878	-5.5819	0.758333	0.764871
Dendritic.cells	TMEM104	-0.14657	3.273099	-0.43324	0.665892	-5.60088	0.752406	0.756264
Dendritic.cells	SMAD2	0.039728	6.426685	0.433127	0.665977	-6.62163	0.7084	0.692716
Dendritic.cells	NOC4L	-0.10154	3.728892	-0.43298	0.666084	-5.7615	0.745833	0.746788
Dendritic.cells	SPIDR	-0.06606	5.228567	-0.43278	0.666226	-6.23941	0.724717	0.71629
Dendritic.cells	GMNN	0.151333	5.558334	0.432649	0.666323	-5.84468	0.720177	0.709752
Dendritic.cells	SUMO2	0.03177	8.656817	0.432631	0.666336	-7.01754	0.679305	0.651103
Dendritic.cells	CTSZ	0.04739	6.598627	0.432544	0.6664	-6.92441	0.706098	0.689521
Dendritic.cells	GM43728	-0.22749	-0.48628	-0.43237	0.666522	-5.21893	0.809439	0.83973
Dendritic.cells	ANP32E	0.051643	7.305672	0.432052	0.666755	-6.7738	0.696737	0.676133
Dendritic.cells	SMARCD1	0.080339	4.031896	0.432003	0.666791	-5.88337	0.741504	0.740662
Dendritic.cells	THOC3	-0.10054	4.102819	-0.43194	0.666834	-5.82346	0.740495	0.739212
Dendritic.cells	GM36198	-0.13945	2.976425	-0.43188	0.666878	-5.64687	0.756723	0.762758
Dendritic.cells	PMEL	0.262211	0.339511	0.43182	0.666923	-5.21515	0.796475	0.820762
Dendritic.cells	RANBP17	0.120982	1.134101	0.431639	0.667054	-5.81589	0.784234	0.802912
Dendritic.cells	TUBB3	0.187686	0.736309	0.431156	0.667404	-5.39148	0.790334	0.812009
Dendritic.cells	MARVELD2	-0.24441	1.369704	-0.4311	0.667441	-5.25934	0.780648	0.797862
Dendritic.cells	GM16153	-0.18212	2.536009	-0.43094	0.66756	-5.42957	0.76319	0.772412
Dendritic.cells	DPF1	0.27555	0.292792	0.430791	0.667668	-5.24136	0.797202	0.822095
Dendritic.cells	SELENOM	0.204195	2.384919	0.430705	0.667731	-5.45327	0.765424	0.775664
Dendritic.cells	RNASEL	-0.14943	3.944163	-0.43062	0.66779	-5.76777	0.742754	0.742733
Dendritic.cells	ALG6	-0.17583	2.044219	-0.4306	0.667809	-5.46894	0.770492	0.783048
Dendritic.cells	KPNA3	0.04734	6.307968	0.430567	0.667831	-6.58897	0.709995	0.69543
Dendritic.cells	IFT140	0.113518	3.507073	0.430523	0.667863	-5.76696	0.749023	0.751823
Dendritic.cells	ZFP408	-0.08864	3.716601	-0.4305	0.667883	-5.93621	0.746009	0.747452
Dendritic.cells	MPPE1	0.104602	4.363196	0.43048	0.667894	-5.83364	0.736806	0.734121
Dendritic.cells	NAPG	-0.08533	4.542195	-0.43045	0.667918	-5.99044	0.734285	0.730472
Dendritic.cells	MOAP1	-0.18632	0.547771	-0.43044	0.667926	-5.26461	0.793245	0.816296
Dendritic.cells	CCL22	-0.39377	-0.37257	-0.4304	0.667954	-5.20984	0.807639	0.837415
Dendritic.cells	SHLD3	-0.1413	2.343634	-0.43038	0.667964	-5.57473	0.766036	0.776555
Dendritic.cells	TMTC3	0.10172	2.514058	0.430356	0.667984	-5.71363	0.763514	0.772883
Dendritic.cells	SLC3A1	-0.23784	0.899213	-0.43031	0.668018	-5.22604	0.787829	0.808367
Dendritic.cells	BNIP1	-0.11315	3.526643	-0.43022	0.66808	-5.80457	0.748741	0.751445
Dendritic.cells	CREB5	-0.31865	2.17534	-0.43021	0.668088	-5.29109	0.768537	0.78023
Dendritic.cells	HERC3	0.07655	4.345366	0.430126	0.668151	-6.16345	0.737058	0.734538

Dendritic.cells	GM42917	0.189966	1.691044	0.430114	0.668159	-5.37723	0.77579	0.790832
Dendritic.cells	ANKRD61	0.180735	0.799732	0.429799	0.668387	-5.4176	0.789446	0.810754
Dendritic.cells	P2RY1	-0.20228	0.13392	-0.42955	0.668568	-5.33709	0.799769	0.825934
Dendritic.cells	ZFP3	0.203205	0.562138	0.42951	0.668597	-5.33503	0.793111	0.816173
Dendritic.cells	MINK1	0.055825	4.171023	0.429504	0.668601	-6.38602	0.739609	0.738242
Dendritic.cells	MACROD2	-0.16778	2.251559	-0.42946	0.668634	-5.59695	0.767489	0.778741
Dendritic.cells	GM10382	0.197852	0.439288	0.429418	0.668664	-5.41104	0.795014	0.818969
Dendritic.cells	LENG8	-0.07538	4.564084	-0.42935	0.668711	-6.09885	0.734059	0.730215
Dendritic.cells	KNTC1	0.206361	2.818917	0.42926	0.668778	-5.42947	0.759113	0.766589
Dendritic.cells	AAAS	-0.12902	3.794127	-0.42905	0.668931	-5.68482	0.745023	0.746128
Dendritic.cells	IL10RB	-0.0513	5.555085	-0.42902	0.668952	-6.49891	0.720342	0.710435
Dendritic.cells	F9	0.233958	0.896192	0.428922	0.669024	-5.29381	0.788007	0.808746
Dendritic.cells	LSM3	0.085248	5.624923	0.428833	0.669088	-6.14701	0.719385	0.709055
Dendritic.cells	WDR70	0.042874	6.1341	0.428643	0.669225	-6.58179	0.712503	0.699074
Dendritic.cells	IBA57	0.153311	1.934328	0.428588	0.669265	-5.42621	0.772313	0.785774
Dendritic.cells	CHTF8	0.178321	1.341638	0.428363	0.669429	-5.4156	0.781329	0.798964
Dendritic.cells	EDC4	-0.14283	2.984777	-0.42802	0.669677	-5.61301	0.756848	0.763319
Dendritic.cells	KRR1	0.095244	3.94276	0.428003	0.66969	-5.86379	0.743016	0.743239
Dendritic.cells	IL1RN	0.379372	2.526967	0.427969	0.669715	-5.37028	0.763573	0.773102
Dendritic.cells	GM17745	-0.24225	0.962497	-0.42788	0.66978	-5.21546	0.787115	0.807507
Dendritic.cells	DGUOK	-0.08019	4.460054	-0.42787	0.66979	-6.11215	0.73568	0.732653
Dendritic.cells	PDCD6	-0.05918	6.13963	-0.42762	0.669968	-6.43258	0.712497	0.699183
Dendritic.cells	OSBPL8	0.044249	7.62098	0.427524	0.670037	-6.90271	0.692841	0.670937
Dendritic.cells	SERPINA10	0.228033	0.966902	0.427508	0.670049	-5.27337	0.787048	0.80741
Dendritic.cells	TENT4A	-0.1175	3.460072	-0.42728	0.670211	-5.73234	0.749945	0.753362
Dendritic.cells	VPS52	-0.07867	3.923327	-0.42724	0.670242	-5.86059	0.743294	0.743712
Dendritic.cells	BCO2	0.219113	0.643324	0.427144	0.670313	-5.2228	0.792026	0.814799
Dendritic.cells	2510009E0	-0.13304	2.702942	-0.42707	0.670366	-5.63566	0.760979	0.769469
Dendritic.cells	PPP1R10	-0.05699	6.277719	-0.42707	0.670369	-6.53642	0.710634	0.696592
Dendritic.cells	1700027J0	-0.24399	2.481407	-0.42701	0.67041	-5.42112	0.764246	0.774231
Dendritic.cells	KPNA6	-0.07503	3.896998	-0.4269	0.670486	-5.99132	0.74367	0.744364
Dendritic.cells	SLC25A15	0.141996	2.488304	0.426899	0.67049	-5.53866	0.764144	0.774118
Dendritic.cells	HMG20A	0.070791	4.897858	0.42675	0.670599	-6.18975	0.729544	0.723929
Dendritic.cells	KMT5B	0.047792	5.91383	0.426716	0.670623	-6.50184	0.715558	0.703739
Dendritic.cells	TMSB15B2	0.136613	2.47439	0.426493	0.670786	-5.63241	0.764457	0.774464
Dendritic.cells	TIMMDC1	0.075977	4.271519	0.426217	0.670985	-6.00077	0.738592	0.736695
Dendritic.cells	ERCC6L2	-0.09174	3.372895	-0.42604	0.671114	-5.94452	0.751526	0.755392
Dendritic.cells	ZFP292	0.043183	7.046023	0.425902	0.671215	-6.77311	0.700717	0.682036
Dendritic.cells	GNA14	-0.29274	-0.0617	-0.42571	0.671354	-5.26307	0.803472	0.831178
Dendritic.cells	PRF1	-0.27864	0.377462	-0.4254	0.671579	-5.22085	0.796664	0.821166
Dendritic.cells	GM49169	0.25931	-0.42801	0.425354	0.671612	-5.20979	0.809307	0.839727
Dendritic.cells	FCGR3	0.230195	4.189291	0.42535	0.671615	-5.47423	0.739991	0.738583
Dendritic.cells	GFOD2	-0.1333	2.309358	-0.42515	0.671764	-5.662	0.767295	0.778279
Dendritic.cells	EHBP1L1	0.048243	6.149657	0.42514	0.671767	-6.55562	0.712827	0.699386
Dendritic.cells	ANGEL2	0.055821	5.052327	0.424938	0.671914	-6.22254	0.72787	0.721158
Dendritic.cells	ABITRAM	-0.11675	2.584915	-0.4248	0.672014	-5.62798	0.763215	0.77242
Dendritic.cells	KLF8	0.125441	0.979838	0.424782	0.672027	-5.91569	0.787363	0.807665

Dendritic.cells	PLPBP	-0.08398	4.145683	-0.42475	0.672053	-5.92941	0.74061	0.739592
Dendritic.cells	GM44174	0.253903	-0.56348	0.424707	0.672082	-5.21976	0.811457	0.843016
Dendritic.cells	NDRG1	-0.16927	2.72107	-0.42433	0.672355	-5.61226	0.761406	0.769604
Dendritic.cells	IL6ST	-0.07771	4.326252	-0.42428	0.672392	-6.32904	0.738241	0.736001
Dendritic.cells	NAA60	-0.07666	4.697726	-0.42399	0.672601	-6.03441	0.733105	0.728554
Dendritic.cells	ROMO1	0.044224	6.595619	0.423971	0.672617	-6.57706	0.707106	0.691062
Dendritic.cells	PHF7	0.102016	3.28527	0.423685	0.672824	-5.83399	0.753297	0.757908
Dendritic.cells	CBR1	-0.11684	4.350162	-0.42363	0.672864	-5.84179	0.738037	0.735784
Dendritic.cells	RAB6A	0.041789	6.637549	0.423592	0.672892	-6.63327	0.70658	0.690394
Dendritic.cells	MAK16	-0.05652	5.072635	-0.42355	0.672923	-6.27892	0.727908	0.721139
Dendritic.cells	ERLIN1	-0.04546	4.765624	-0.42313	0.673227	-6.61737	0.732376	0.727346
Dendritic.cells	GMDS	-0.03896	6.245209	-0.42298	0.673334	-6.77459	0.71203	0.698043
Dendritic.cells	MOCS2	0.068886	5.094585	0.422924	0.673377	-6.19529	0.727788	0.720767
Dendritic.cells	SNX2	0.037634	7.314048	0.422792	0.673474	-6.64744	0.697793	0.677583
Dendritic.cells	SLC25A40	-0.12874	3.227708	-0.42276	0.673495	-5.64322	0.754325	0.759215
Dendritic.cells	NAXE	-0.06561	5.283838	-0.42263	0.673589	-6.25935	0.725165	0.717015
Dendritic.cells	PRPSAP1	0.079287	4.34081	0.422606	0.673608	-5.92959	0.738357	0.736097
Dendritic.cells	AFG3L2	-0.06574	4.593409	-0.42242	0.673744	-6.0059	0.734793	0.73097
Dendritic.cells	LTBP1	-0.41862	0.730726	-0.42231	0.673821	-5.27063	0.791743	0.813933
Dendritic.cells	CDC42BPB	-0.07	3.262023	-0.4223	0.673829	-6.34799	0.753826	0.758621
Dendritic.cells	MRPL11	-0.08821	4.26095	-0.42228	0.673844	-5.91386	0.739488	0.737824
Dendritic.cells	RSRP1	-0.05675	6.653226	-0.42214	0.673948	-6.50102	0.706588	0.690343
Dendritic.cells	CTSG	-0.45864	-0.2691	-0.42173	0.674247	-5.20994	0.807628	0.836947
Dendritic.cells	6530402F1	0.241989	-0.17553	0.421688	0.674276	-5.21041	0.806151	0.834777
Dendritic.cells	4933423P2	0.147724	1.804113	0.421592	0.674346	-5.52442	0.775649	0.790124
Dendritic.cells	2810454H(	0.155458	2.514759	0.421395	0.67449	-5.48816	0.765105	0.774747
Dendritic.cells	SLC25A19	0.084054	3.390435	0.421329	0.674538	-6.00151	0.752281	0.756101
Dendritic.cells	MTREX	-0.04951	5.477446	-0.4207	0.674998	-6.36479	0.723009	0.713454
Dendritic.cells	A430033K(	-0.23563	0.6406	-0.42066	0.675026	-5.28439	0.7937	0.816204
Dendritic.cells	PCP4L1	-0.28686	1.36121	-0.42059	0.675077	-5.2696	0.782641	0.800026
Dendritic.cells	ZFP428	0.178703	2.058083	0.420587	0.675077	-5.38281	0.772124	0.784678
Dendritic.cells	TUG1	-0.04447	6.098897	-0.42035	0.675249	-6.59093	0.714517	0.701223
Dendritic.cells	HIST1H1A	-0.29127	3.773403	-0.42017	0.675384	-5.51693	0.746974	0.748153
Dendritic.cells	WDR60	0.246797	0.883885	0.420097	0.675434	-5.22228	0.789945	0.810751
Dendritic.cells	RRS1	-0.08401	4.476059	-0.42007	0.675455	-6.0098	0.73697	0.733664
Dendritic.cells	KBTBD2	-0.05393	5.176548	-0.42007	0.675456	-6.20079	0.727168	0.719496
Dendritic.cells	TRIM65	0.096468	3.22435	0.419779	0.675665	-5.88688	0.754911	0.759734
Dendritic.cells	PLD2	-0.15808	1.042444	-0.41972	0.675712	-5.53486	0.78751	0.807253
Dendritic.cells	TOP2A	-0.13291	7.615909	-0.41971	0.675715	-6.57131	0.694335	0.672311
Dendritic.cells	CHIL5	0.211127	-0.02332	0.419653	0.675757	-5.24879	0.804055	0.831499
Dendritic.cells	GM28809	0.244821	-0.12461	0.419652	0.675758	-5.21107	0.805649	0.833839
Dendritic.cells	TPX2	-0.17855	5.047457	-0.41949	0.675878	-5.86141	0.728961	0.722164
Dendritic.cells	ADCK1	-0.09993	3.101425	-0.41944	0.675915	-5.86599	0.756702	0.762358
Dendritic.cells	TMEM131	0.053035	6.606387	0.419411	0.675933	-6.5076	0.70768	0.691493
Dendritic.cells	KLF13	0.031704	8.183848	0.419374	0.67596	-7.13892	0.686975	0.661788
Dendritic.cells	PLGRKT	-0.06593	5.796951	-0.41911	0.676151	-6.32756	0.718699	0.70731
Dendritic.cells	CYP7B1	0.226697	0.01895	0.418962	0.67626	-5.4598	0.803473	0.830664

Dendritic.cells	GNG11	-0.09078	3.831566	-0.41881	0.676372	-6.22693	0.746215	0.747151
Dendritic.cells	TGFBI	0.188937	4.412001	0.418736	0.676425	-5.6876	0.73795	0.735177
Dendritic.cells	EFR3A	-0.04446	5.780301	-0.41872	0.676438	-6.49619	0.718927	0.707698
Dendritic.cells	UBB	0.049755	10.85501	0.418487	0.676606	-7.22678	0.653819	0.614546
Dendritic.cells	AI839979	0.252167	0.274557	0.418479	0.676612	-5.26967	0.79947	0.824861
Dendritic.cells	UAP1	0.071495	5.112787	0.418311	0.676734	-6.18953	0.728127	0.721014
Dendritic.cells	GM20528	-0.24722	-0.01428	-0.4183	0.676744	-5.21267	0.803995	0.8315
Dendritic.cells	SLC26A11	-0.19694	3.200265	-0.41829	0.676751	-5.42012	0.755338	0.760435
Dendritic.cells	SALL2	-0.23022	0.090711	-0.41818	0.676826	-5.26162	0.802347	0.829083
Dendritic.cells	ARMH3	0.055398	5.427651	0.418076	0.676905	-6.37586	0.723768	0.714736
Dendritic.cells	RGS10	-0.03867	5.509055	-0.41806	0.676915	-6.80874	0.722647	0.713118
Dendritic.cells	TM6SF1	0.083477	6.252773	0.417921	0.677018	-6.21767	0.712511	0.698516
Dendritic.cells	EIF2AK3	0.054266	6.399228	0.417754	0.67714	-6.59372	0.710537	0.695674
Dendritic.cells	COMMD8	0.067938	5.188265	0.417652	0.677214	-6.11497	0.727083	0.719574
Dendritic.cells	BCL2L2	0.11809	1.276497	0.417641	0.677222	-5.6559	0.784015	0.802305
Dendritic.cells	SLC25A34	0.241393	-0.47643	0.417372	0.677418	-5.20933	0.811302	0.84244
Dendritic.cells	TLR12	-0.12353	2.045397	-0.41737	0.677419	-5.95358	0.772397	0.785454
Dendritic.cells	SUCLA2	-0.05542	5.593683	-0.41734	0.677441	-6.31054	0.721488	0.711608
Dendritic.cells	ZNHIT2	-0.07739	3.310699	-0.41728	0.677484	-5.93115	0.753736	0.758308
Dendritic.cells	YBX3	0.047061	6.314911	0.417214	0.677533	-6.87447	0.711673	0.697471
Dendritic.cells	ATXN1L	0.117796	2.959513	0.417081	0.67763	-5.66271	0.758891	0.765776
Dendritic.cells	RPRD1B	0.062203	5.480381	0.416428	0.678106	-6.28159	0.723493	0.714103
Dendritic.cells	TBL2	-0.10555	2.392625	-0.41636	0.678157	-5.61098	0.767694	0.778183
Dendritic.cells	TSPAN6	0.222496	1.103606	0.416187	0.678282	-5.38278	0.787189	0.806595
Dendritic.cells	CHRNB1	-0.15978	0.775917	-0.41612	0.678332	-5.527	0.792229	0.81399
Dendritic.cells	COL3A1	0.221033	3.678331	0.415708	0.678631	-5.69245	0.74902	0.750989
Dendritic.cells	DCAF15	-0.11524	3.456621	-0.41571	0.678631	-5.70832	0.752223	0.755637
Dendritic.cells	FAM76B	-0.04628	5.401156	-0.41569	0.678643	-6.42289	0.724718	0.715824
Dendritic.cells	METTL6	-0.08859	4.505421	-0.41563	0.67869	-5.95099	0.737225	0.733927
Dendritic.cells	PDPN	0.324628	-0.21403	0.415446	0.678822	-5.21305	0.807868	0.836935
Dendritic.cells	GNE	0.037769	4.467546	0.415261	0.678957	-6.69517	0.737832	0.734815
Dendritic.cells	MXD1	-0.07463	6.036212	-0.415	0.679145	-6.28053	0.716088	0.703428
Dendritic.cells	DHX32	-0.10003	3.380004	-0.41494	0.67919	-5.73438	0.753408	0.75741
Dendritic.cells	LSR	0.226162	1.400914	0.414913	0.679211	-5.3679	0.782825	0.800266
Dendritic.cells	FNDC5	-0.24908	0.474524	-0.41491	0.679215	-5.24557	0.797077	0.821128
Dendritic.cells	D130062J1	-0.18652	1.642184	-0.41482	0.679278	-5.37181	0.779164	0.794947
Dendritic.cells	ADAT1	-0.15126	2.003267	-0.41479	0.679303	-5.5508	0.773723	0.787007
Dendritic.cells	PRMT1	0.050803	5.803475	0.414602	0.679437	-6.4397	0.719331	0.708058
Dendritic.cells	FFAR1	0.20971	-0.22509	0.414343	0.679626	-5.20883	0.808146	0.837326
Dendritic.cells	ZFP959	-0.11596	3.241052	-0.41431	0.679651	-5.64511	0.75552	0.760433
Dendritic.cells	CDCA8	-0.20284	5.109425	-0.41414	0.679778	-5.80625	0.728922	0.721899
Dendritic.cells	ABCB1A	0.220478	2.010725	0.414119	0.67979	-5.51474	0.773706	0.786908
Dendritic.cells	PIAS1	0.034471	7.42613	0.414043	0.679845	-6.80586	0.697605	0.676839
Dendritic.cells	COG1	-0.08003	3.677624	-0.41402	0.679864	-5.88005	0.749196	0.751286
Dendritic.cells	ATAD3A	-0.1002	4.10261	-0.41391	0.679943	-5.91521	0.743115	0.742474
Dendritic.cells	DNAL4	-0.14731	1.951429	-0.41367	0.680121	-5.52971	0.774612	0.788332
Dendritic.cells	ZBTB38	0.04744	5.068147	0.413558	0.680199	-6.58152	0.729511	0.722846

Dendritic.cells	PYGO2	-0.14003	3.374512	-0.41348	0.680255	-5.71349	0.753595	0.757743
Dendritic.cells	4930549G2	0.125526	2.714781	0.413256	0.68042	-5.5908	0.763249	0.771832
Dendritic.cells	PMM1	0.140082	3.07058	0.413208	0.680455	-5.58029	0.758023	0.76423
Dendritic.cells	AB124611	0.107384	5.452214	0.41316	0.68049	-5.87926	0.724188	0.715211
Dendritic.cells	NPHP3	-0.15534	1.097931	-0.41315	0.680497	-5.53777	0.787564	0.807318
Dendritic.cells	6030443J0	-0.20017	0.848488	-0.41305	0.680572	-5.31927	0.791399	0.812952
Dendritic.cells	WBP4	-0.05797	5.270226	-0.41302	0.68059	-6.24612	0.726704	0.718865
Dendritic.cells	GAS7	0.065344	6.417925	0.41297	0.680629	-6.81811	0.711025	0.696258
Dendritic.cells	REV3L	-0.04303	6.713202	-0.41284	0.680721	-6.66381	0.707088	0.690563
Dendritic.cells	A930029G2	0.187798	1.230907	0.412622	0.680882	-5.38665	0.785643	0.804394
Dendritic.cells	DPH5	0.106842	3.732552	0.412518	0.680959	-5.77224	0.748528	0.75036
Dendritic.cells	UBA2	0.040771	6.457603	0.41246	0.681001	-6.60973	0.710594	0.695543
Dendritic.cells	SNTB2	-0.10172	5.607555	-0.41236	0.681074	-6.06209	0.72216	0.7122
Dendritic.cells	UNC13D	-0.0988	3.31115	-0.41215	0.681228	-5.83352	0.754725	0.759212
Dendritic.cells	EMSY	-0.0511	5.828738	-0.41182	0.681471	-6.44686	0.71927	0.707828
Dendritic.cells	MAPKAP1	0.047082	5.764174	0.41163	0.681607	-6.49745	0.720153	0.709101
Dendritic.cells	TBC1D19	-0.13641	1.749793	-0.41151	0.681691	-5.47828	0.77792	0.792908
Dendritic.cells	ABHD16A	0.06138	4.808556	0.411386	0.681785	-6.23292	0.733395	0.72822
Dendritic.cells	GBP7	0.255736	4.443583	0.411377	0.681791	-5.61034	0.738535	0.735656
Dendritic.cells	NAB2	0.184732	3.217989	0.411294	0.681852	-5.40545	0.756136	0.761182
Dendritic.cells	BECN1	-0.05317	5.896307	-0.41113	0.681972	-6.36342	0.718347	0.706509
Dendritic.cells	RTP4	-0.26428	4.174749	-0.41094	0.682108	-5.65654	0.742351	0.741254
Dendritic.cells	GM26737	0.241968	0.478605	0.410852	0.682175	-5.21268	0.797406	0.821492
Dendritic.cells	BCL7A	0.112706	4.80999	0.41084	0.682184	-5.95773	0.733374	0.728262
Dendritic.cells	MED25	-0.06362	4.496149	-0.41081	0.682205	-6.11532	0.737792	0.734654
Dendritic.cells	TTLL4	-0.09169	3.000367	-0.41078	0.682227	-5.8514	0.759317	0.765882
Dendritic.cells	COBLL1	0.052016	5.069977	0.410691	0.682293	-6.61566	0.729741	0.723044
Dendritic.cells	GM15788	0.229214	0.240256	0.41056	0.682388	-5.30312	0.801125	0.827044
Dendritic.cells	TFAP4	0.142028	2.748617	0.410392	0.682511	-5.62342	0.763017	0.77139
Dendritic.cells	MAZ	0.074089	6.241133	0.410385	0.682516	-6.44753	0.713661	0.699932
Dendritic.cells	MTFR1L	0.067087	4.250609	0.410239	0.682623	-6.03513	0.741271	0.739837
Dendritic.cells	ATP2A3	0.048256	5.210591	0.410235	0.682626	-6.55125	0.727786	0.720328
Dendritic.cells	TAF12	0.066636	5.021001	0.410195	0.682655	-6.12191	0.730424	0.724149
Dendritic.cells	4930453N2	0.077944	4.481233	0.410145	0.682691	-5.982	0.738002	0.735118
Dendritic.cells	CNIH1	0.062908	5.17624	0.410099	0.682725	-6.24413	0.728263	0.721042
Dendritic.cells	SNW1	-0.03517	6.240739	-0.41002	0.682779	-6.60888	0.713666	0.700006
Dendritic.cells	4732465J0	0.259188	1.227323	0.409767	0.682968	-5.21958	0.785997	0.804886
Dendritic.cells	SLC43A2	0.097375	6.179865	0.409524	0.683145	-6.25444	0.714654	0.701321
Dendritic.cells	MMP19	0.279192	1.213067	0.409404	0.683233	-5.24221	0.786256	0.805269
Dendritic.cells	TRIP12	-0.03311	7.435654	-0.40906	0.683486	-6.85185	0.697897	0.677272
Dendritic.cells	CDIPT	0.069687	5.093616	0.409036	0.683502	-6.16195	0.729578	0.722903
Dendritic.cells	RHBDF2	-0.04153	4.650027	-0.40903	0.683508	-6.61004	0.735789	0.731885
Dendritic.cells	SCLY	0.103402	3.466279	0.408913	0.683592	-5.73103	0.752699	0.756401
Dendritic.cells	GPN2	0.130783	2.713988	0.408906	0.683597	-5.53908	0.763702	0.772399
Dendritic.cells	OSBPL10	-0.16403	0.973543	-0.40885	0.683639	-5.51052	0.78993	0.810705
Dendritic.cells	CKS1B	0.125583	5.377173	0.408738	0.68372	-6.10374	0.725643	0.717231
Dendritic.cells	BAZ2B	-0.03456	8.430417	-0.40874	0.683721	-7.00831	0.684995	0.658791

Dendritic.cells	AGFG2	0.090921	4.605897	0.408605	0.683818	-5.90677	0.736411	0.732817
Dendritic.cells	ZFP579	-0.14496	1.862923	-0.4086	0.683823	-5.53925	0.776391	0.790933
Dendritic.cells	PDE1A	-0.266	0.441701	-0.4084	0.683965	-5.21421	0.798248	0.822821
Dendritic.cells	RASA1	0.049934	6.030636	0.40822	0.684099	-6.47502	0.716826	0.704358
Dendritic.cells	ENKD1	-0.19105	1.3576	-0.40789	0.684342	-5.31281	0.784323	0.802249
Dendritic.cells	SNHG4.1	0.258003	0.471422	0.407853	0.684368	-5.2749	0.797979	0.822243
Dendritic.cells	FCF1	0.044484	6.136138	0.407804	0.684404	-6.49338	0.715497	0.702385
Dendritic.cells	HCAR2	0.341635	2.377294	0.407216	0.684834	-5.34253	0.76917	0.780047
Dendritic.cells	SART1	0.067547	4.872457	0.407203	0.684843	-6.13513	0.733123	0.727722
Dendritic.cells	LIX1L	0.184958	0.803407	0.407179	0.68486	-5.36397	0.793046	0.814928
Dendritic.cells	EDEM1	0.046374	6.015715	0.40715	0.684882	-6.61888	0.71733	0.704929
Dendritic.cells	EDARADD	0.170298	3.159788	0.407081	0.684933	-5.41382	0.757629	0.763284
Dendritic.cells	GSK3A	-0.05637	5.557032	-0.40679	0.685148	-6.27369	0.723768	0.71402
Dendritic.cells	RNF20	0.059871	5.755436	0.406565	0.68531	-6.33944	0.721139	0.710121
Dendritic.cells	CXCL2	0.248694	7.158496	0.406354	0.685465	-6.44465	0.702267	0.682926
Dendritic.cells	RAB5C	-0.04666	6.358629	-0.40609	0.685661	-6.49606	0.712964	0.698355
Dendritic.cells	HMGXB4	0.079978	4.148587	0.406071	0.685672	-5.94747	0.743652	0.742674
Dendritic.cells	TNIP3	0.407146	1.037658	0.405966	0.685748	-5.22566	0.789751	0.809824
Dendritic.cells	ZDHHC2	-0.16232	2.276539	-0.40581	0.685862	-5.54638	0.770979	0.782464
Dendritic.cells	CARM1	-0.06932	4.727042	-0.40578	0.685884	-6.05355	0.735457	0.730892
Dendritic.cells	POGLUT2	-0.18421	1.00166	-0.40562	0.686	-5.40078	0.790305	0.810721
Dendritic.cells	CAPG	-0.07935	5.264343	-0.40554	0.68606	-6.45541	0.727949	0.720064
Dendritic.cells	GSS	-0.12435	3.399727	-0.40551	0.686084	-5.67931	0.754435	0.758432
Dendritic.cells	SLC5A6	-0.20555	0.868812	-0.40537	0.686188	-5.32288	0.792352	0.813738
Dendritic.cells	GM4013	-0.12209	1.984122	-0.40534	0.686209	-5.54339	0.77536	0.788908
Dendritic.cells	SDHB	0.044964	7.274793	0.405251	0.686272	-6.81704	0.70073	0.680898
Dendritic.cells	CKAP2	0.167056	3.667324	0.405232	0.686286	-5.63114	0.750559	0.75283
Dendritic.cells	GSAP	0.13232	5.642393	0.405185	0.68632	-5.95131	0.722726	0.712557
Dendritic.cells	GATAD2B	-0.03933	7.433227	-0.40512	0.686369	-6.83353	0.698642	0.677914
Dendritic.cells	YOD1	-0.10198	4.084266	-0.40492	0.686518	-5.94553	0.744658	0.744158
Dendritic.cells	RAP1A	0.019373	8.772339	0.40477	0.686624	-7.27433	0.681444	0.653126
Dendritic.cells	GNL2	-0.06763	4.675227	-0.40459	0.686754	-6.11345	0.736309	0.732032
Dendritic.cells	EIF3F	0.035921	7.589953	0.404445	0.686862	-6.88856	0.696702	0.674979
Dendritic.cells	CSF1	-0.26199	2.480595	-0.40423	0.687021	-5.45667	0.768068	0.778188
Dendritic.cells	METTL26	-0.09561	4.639923	-0.40412	0.6871	-5.95883	0.736807	0.732827
Dendritic.cells	COPRS	-0.21866	0.717795	-0.4041	0.687117	-5.24472	0.79482	0.817295
Dendritic.cells	TADA1	0.06173	4.679062	0.403974	0.687207	-6.16552	0.736255	0.732045
Dendritic.cells	CINP	-0.1626	3.163035	-0.40388	0.687274	-5.53031	0.75801	0.763637
Dendritic.cells	ATF6	-0.03481	6.979377	-0.40382	0.687321	-6.92639	0.704761	0.686683
Dendritic.cells	EGR2	-0.2977	2.237621	-0.4038	0.687335	-5.24973	0.771689	0.783561
Dendritic.cells	CCNF	0.214776	3.438612	0.40375	0.687372	-5.42716	0.753996	0.757827
Dendritic.cells	KCNK13	-0.25158	2.912398	-0.40375	0.687372	-5.31803	0.761685	0.769004
Dendritic.cells	ARID5A	-0.06297	4.681741	-0.40355	0.687516	-6.30958	0.736252	0.732126
Dendritic.cells	ZFP142	-0.09853	3.207748	-0.40346	0.687587	-5.74581	0.757393	0.76279
Dendritic.cells	CENPT	0.125501	2.566821	0.403425	0.68761	-5.51666	0.766825	0.776512
Dendritic.cells	NATD1	-0.15958	3.25628	-0.40315	0.68781	-5.52946	0.756743	0.761845
Dendritic.cells	SSBP2	0.049421	6.284595	0.403119	0.687834	-6.81596	0.714173	0.700263

Dendritic.cells	GM15448	0.194792	-0.00747	0.402957	0.687953	-5.24043	0.806253	0.834215
Dendritic.cells	PPM1N	-0.27014	0.159456	-0.40265	0.688174	-5.22004	0.803627	0.830409
Dendritic.cells	AP2A2	0.041663	5.931782	0.402459	0.688318	-6.58155	0.71897	0.707237
Dendritic.cells	MTOR	0.052248	4.766611	0.402455	0.688321	-6.31619	0.735115	0.730555
Dendritic.cells	CTSA	-0.05055	6.259221	-0.40241	0.688351	-6.75354	0.714516	0.700819
Dendritic.cells	LLGL1	0.112331	2.998446	0.402397	0.688363	-5.66803	0.760515	0.767405
Dendritic.cells	NDST1	-0.06946	4.570663	-0.4021	0.688582	-6.29841	0.737876	0.734551
Dendritic.cells	ATG4A	-0.10016	4.314693	-0.40209	0.688587	-5.91303	0.741503	0.739804
Dendritic.cells	CCDC71L	0.098592	4.455272	0.402012	0.688645	-5.92129	0.739508	0.736914
Dendritic.cells	ABL2	-0.05426	6.21523	-0.40196	0.688681	-6.58667	0.715113	0.701678
Dendritic.cells	GM26724	-0.16493	1.517501	-0.40191	0.688719	-5.47854	0.782642	0.799669
Dendritic.cells	PTGES2	0.140254	2.422157	0.401907	0.688722	-5.49534	0.769033	0.779806
Dendritic.cells	USP42	-0.07863	3.500066	-0.40182	0.688784	-5.89344	0.753198	0.75677
Dendritic.cells	SMAD6	0.173926	2.733914	0.401805	0.688797	-5.65117	0.76441	0.773075
Dendritic.cells	ZFP874B	0.171527	2.178101	0.401766	0.688826	-5.47012	0.772675	0.785118
Dendritic.cells	ATRNL1	-0.04481	6.820202	-0.40163	0.688927	-6.91938	0.707005	0.690002
Dendritic.cells	AP4E1	-0.10492	3.305374	-0.40145	0.68906	-5.83386	0.756135	0.760927
Dendritic.cells	PHEX	0.212462	1.372943	0.40128	0.689182	-5.42894	0.785017	0.802949
Dendritic.cells	ZDHHC4	0.066698	4.474275	0.400841	0.689505	-6.13739	0.739562	0.736672
Dendritic.cells	PLIN2	0.110642	5.831377	0.400811	0.689527	-6.0988	0.720658	0.709358
Dendritic.cells	MUTYH	0.202102	0.370874	0.400568	0.689704	-5.24162	0.800667	0.825715
Dendritic.cells	SRP54C	0.157614	1.755516	0.40056	0.68971	-5.47346	0.779374	0.794555
Dendritic.cells	TAF5L	0.065061	4.562119	0.400491	0.689761	-6.11915	0.738319	0.734887
Dendritic.cells	ZFP869	0.096003	4.026546	0.400364	0.689854	-5.87239	0.745939	0.745931
Dendritic.cells	AGTRAP	0.097855	4.534904	0.400176	0.689992	-6.10185	0.738704	0.735517
Dendritic.cells	GLRX2	0.055261	5.111058	0.400095	0.690052	-6.24074	0.730613	0.723837
Dendritic.cells	PSMG1	-0.10217	3.325411	-0.40005	0.690087	-5.67667	0.756066	0.760723
Dendritic.cells	NUSAP1	-0.17939	5.649629	-0.40003	0.690099	-5.90658	0.723153	0.713065
Dendritic.cells	ADRB2	0.168581	4.714467	0.400015	0.69011	-5.78795	0.73617	0.731873
Dendritic.cells	HINT1	0.039646	7.995864	0.399921	0.690179	-6.86893	0.691801	0.66798
Dendritic.cells	SUPT7L	0.133451	2.767637	0.399792	0.690274	-5.55316	0.764247	0.772648
Dendritic.cells	ORMDL3	0.114861	3.803807	0.399769	0.690291	-5.73342	0.749138	0.750689
Dendritic.cells	RAPGEF3	-0.16894	1.358645	-0.39966	0.690369	-5.49433	0.785418	0.803542
Dendritic.cells	PSMF1	0.084576	4.460205	0.399372	0.690583	-5.9155	0.739793	0.737233
Dendritic.cells	RELA	-0.06519	5.114793	-0.39934	0.690606	-6.18997	0.730591	0.723926
Dendritic.cells	LRRFIP2	0.060092	5.900501	0.399285	0.690646	-6.2032	0.719742	0.708268
Dendritic.cells	COA7	-0.10287	3.404389	-0.39922	0.69069	-5.62857	0.754949	0.759252
Dendritic.cells	PLCB2	0.091148	3.320212	0.399171	0.69073	-5.89314	0.756174	0.761047
Dendritic.cells	B4GALT1	0.042102	7.137524	0.398947	0.690894	-6.66431	0.703184	0.684362
Dendritic.cells	BTBD1	0.041198	6.497876	0.398398	0.691297	-6.62814	0.711839	0.696779
Dendritic.cells	PPP2R3C	0.085803	4.333149	0.398343	0.691337	-5.87541	0.741809	0.740062
Dendritic.cells	CEP290	-0.10259	2.468766	-0.39833	0.691347	-5.74492	0.768929	0.779468
Dendritic.cells	CCT6B	-0.24562	0.315333	-0.39831	0.69136	-5.22959	0.8018	0.827537
Dendritic.cells	MRPL27	-0.09581	4.210901	-0.39822	0.691431	-5.8622	0.74355	0.7426
Dendritic.cells	SNRPE	0.047633	7.064225	0.398183	0.691455	-6.59523	0.704261	0.685893
Dendritic.cells	A330040F1	-0.32582	2.508264	-0.39794	0.691636	-5.30849	0.768342	0.77868
Dendritic.cells	GRIPAP1	0.052809	5.594976	0.397825	0.691718	-6.30777	0.724144	0.714594

Dendritic.cells	DUSP12	0.088517	3.283921	0.397747	0.691776	-5.86258	0.75692	0.762087
Dendritic.cells	GM43445	-0.16061	1.896237	-0.39773	0.691787	-5.41187	0.777505	0.792077
Dendritic.cells	FLOT1	-0.08085	4.575358	-0.39757	0.691906	-6.03774	0.738375	0.735239
Dendritic.cells	GM9887	0.161062	1.686181	0.397513	0.691948	-5.47139	0.780681	0.796778
Dendritic.cells	ARHGEF2	0.059696	5.586467	0.397508	0.691951	-6.21604	0.724261	0.714832
Dendritic.cells	CHD2	0.037533	7.754934	0.397454	0.69199	-6.98137	0.695165	0.672956
Dendritic.cells	LRCH3	0.034636	6.497112	0.397386	0.69204	-6.84156	0.711849	0.696936
Dendritic.cells	PSD4	-0.08444	4.541706	-0.39725	0.692139	-5.96509	0.738851	0.73597
Dendritic.cells	ZFP51	0.09863	2.86166	0.397202	0.692176	-5.70898	0.763111	0.771197
Dendritic.cells	RDH16F2	-0.25912	1.257357	-0.39713	0.692229	-5.30354	0.787214	0.806394
Dendritic.cells	JHY	-0.27109	-0.37714	-0.39691	0.692391	-5.21885	0.812837	0.843838
Dendritic.cells	GM14023	0.237527	1.123999	0.396737	0.692517	-5.23698	0.789431	0.809475
Dendritic.cells	CDT1	-0.10513	4.370678	-0.39659	0.692623	-5.70736	0.741447	0.739628
Dendritic.cells	KCNJ10	-0.20282	-0.1202	-0.39645	0.69273	-5.21684	0.808839	0.838036
Dendritic.cells	PEX14	0.052821	5.405291	0.396446	0.692731	-6.39586	0.726932	0.718672
Dendritic.cells	SMURF2	0.041864	6.82379	0.396052	0.693021	-6.70747	0.707819	0.690857
Dendritic.cells	ESYT2	-0.04604	6.802347	-0.39601	0.693055	-6.58364	0.708106	0.69127
Dendritic.cells	CEP57L1	0.106013	3.287731	0.395841	0.693176	-5.78738	0.757301	0.762321
Dendritic.cells	RALGAPA1	0.049035	6.84207	0.395494	0.693431	-6.78408	0.707686	0.690573
Dendritic.cells	XPOT	-0.07099	4.512589	-0.39534	0.693546	-6.09413	0.73975	0.736911
Dendritic.cells	LMO1	0.153988	1.523387	0.39527	0.693595	-5.6372	0.783669	0.800814
Dendritic.cells	CPSF1	-0.10654	3.349532	-0.39525	0.693609	-5.69688	0.756461	0.761159
Dendritic.cells	ZKSCAN6	0.12441	2.942031	0.395181	0.693661	-5.67456	0.762429	0.76986
Dendritic.cells	MAPK6	0.072524	5.88266	0.395143	0.693689	-6.36041	0.720667	0.709356
Dendritic.cells	BCL2	0.119846	5.016128	0.395096	0.693724	-6.13609	0.732661	0.726686
Dendritic.cells	SEC14L2	-0.19832	1.70477	-0.39502	0.693781	-5.3915	0.780913	0.796834
Dendritic.cells	DLGAP5	0.18763	3.248568	0.394925	0.693849	-5.55082	0.757934	0.763364
Dendritic.cells	CEBPG	-0.04635	5.586056	-0.39478	0.693957	-6.41591	0.724785	0.715294
Dendritic.cells	HCCS	0.053953	4.627385	0.394451	0.694198	-6.22671	0.738351	0.734703
Dendritic.cells	SH2D3C	0.082408	4.693449	0.394177	0.694399	-6.13909	0.737525	0.733442
Dendritic.cells	CABLES1	-0.07893	4.513404	-0.39405	0.69449	-6.62949	0.74007	0.737145
Dendritic.cells	HARS	0.054561	5.114979	0.393945	0.69457	-6.22199	0.731608	0.724903
Dendritic.cells	EVA1B	-0.11649	3.639669	-0.39394	0.694572	-5.72465	0.752586	0.755292
Dendritic.cells	TTC5	-0.06049	4.64029	-0.3938	0.694675	-6.11035	0.738311	0.734571
Dendritic.cells	RBM22	-0.04173	5.603703	-0.39344	0.694942	-6.37003	0.725025	0.715212
Dendritic.cells	AC149090.	0.084316	5.49949	0.393404	0.694968	-6.61338	0.726464	0.71729
Dendritic.cells	SLC35F2	-0.21212	0.357488	-0.39313	0.695166	-5.28633	0.802334	0.827655
Dendritic.cells	TMEM50B	-0.06771	4.107732	-0.39298	0.695282	-6.35071	0.746133	0.745717
Dendritic.cells	CEP19	-0.19062	2.707827	-0.39294	0.695311	-5.37192	0.766524	0.775339
Dendritic.cells	GNG12	-0.03367	6.49518	-0.39285	0.695377	-6.94496	0.712936	0.697786
Dendritic.cells	GPNMB	0.166391	0.898381	0.392752	0.695448	-5.6834	0.793916	0.815383
Dendritic.cells	RBCK1	0.047003	5.550838	0.392743	0.695455	-6.44795	0.725833	0.716393
Dendritic.cells	ODF2L	0.108989	2.682465	0.392363	0.695734	-5.69585	0.767066	0.775949
Dendritic.cells	MFN2	0.082171	3.671067	0.392235	0.695829	-5.84349	0.752582	0.754897
Dendritic.cells	PLEKHA2	-0.03627	7.455833	-0.39215	0.695888	-6.98506	0.700278	0.679407
Dendritic.cells	SMG7	-0.04167	6.385921	-0.39215	0.695894	-6.59388	0.714568	0.699947
Dendritic.cells	RAB7	0.03182	7.931545	0.391992	0.696007	-6.89146	0.694047	0.670497

Dendritic.cells	PSMC3IP	-0.15803	2.412129	-0.39196	0.696029	-5.49213	0.771087	0.781837
Dendritic.cells	GM29417	-0.11878	1.098886	-0.39182	0.696132	-5.65045	0.790994	0.810941
Dendritic.cells	STX12	-0.0499	5.552332	-0.39179	0.696154	-6.40009	0.72597	0.71644
Dendritic.cells	D730003I1	-0.11009	2.591958	-0.3916	0.696299	-5.70673	0.768492	0.778027
Dendritic.cells	CAP2	-0.25111	0.071585	-0.3915	0.696367	-5.22014	0.807089	0.834471
Dendritic.cells	SF3A1	-0.05708	4.851981	-0.39084	0.696858	-6.15721	0.736156	0.730748
Dendritic.cells	PRNP	0.172519	2.012971	0.390778	0.696901	-5.39161	0.777518	0.790825
Dendritic.cells	SOCS1	-0.18179	5.152296	-0.39075	0.696921	-5.74988	0.731946	0.724673
Dendritic.cells	COL20A1	-0.1133	1.340253	-0.39063	0.697009	-5.58076	0.78774	0.805752
Dendritic.cells	LRRC43	0.242164	-0.12202	0.390531	0.697083	-5.21682	0.810527	0.839145
Dendritic.cells	CETN4	-0.20996	-0.08195	-0.39045	0.697141	-5.21969	0.809892	0.838212
Dendritic.cells	ANKRD52	-0.07996	3.881446	-0.39041	0.697172	-5.923	0.749975	0.750768
Dendritic.cells	ABL1	-0.06046	5.653787	-0.39032	0.69724	-6.35496	0.724985	0.714656
Dendritic.cells	ATP9B	-0.03775	5.858499	-0.38989	0.697552	-6.60823	0.722376	0.710709
Dendritic.cells	METTL14	-0.09985	3.09472	-0.38983	0.697596	-5.78725	0.761639	0.767543
Dendritic.cells	UBQLN1	-0.04832	5.517917	-0.38977	0.697646	-6.3031	0.727071	0.717482
Dendritic.cells	CCR3	0.377396	-0.02075	0.389516	0.697831	-5.31213	0.809261	0.837088
Dendritic.cells	PDSS1	0.09575	4.315269	0.389457	0.697874	-5.93574	0.744068	0.742021
Dendritic.cells	CTR9	0.062035	4.518969	0.389161	0.698093	-6.0817	0.741291	0.737907
Dendritic.cells	FBXO38	-0.04436	5.443447	-0.38911	0.698131	-6.40444	0.728317	0.71918
Dendritic.cells	GNASAS1	-0.19885	0.682179	-0.38892	0.69827	-5.31764	0.798362	0.82111
Dendritic.cells	TMEM175	0.099597	3.375009	0.388918	0.698272	-5.83544	0.757758	0.76188
Dendritic.cells	ARC	0.2441	0.905214	0.38867	0.698455	-5.22827	0.7949	0.816141
Dendritic.cells	TERF2	-0.05043	4.800606	-0.38861	0.698498	-6.2902	0.737307	0.732333
Dendritic.cells	TNNI2	0.060226	2.310806	0.38856	0.698536	-6.30014	0.773493	0.784871
Dendritic.cells	SBNO2	0.045859	5.566821	0.388557	0.698538	-6.48674	0.726608	0.716876
Dendritic.cells	FEZ2	-0.08842	3.693286	-0.38845	0.698619	-5.7679	0.753144	0.755256
Dendritic.cells	ZFP263	-0.07591	5.167548	-0.38814	0.698843	-6.20441	0.732319	0.724993
Dendritic.cells	SLC10A1	-0.15424	3.248903	-0.38789	0.699026	-5.61051	0.759768	0.76488
Dendritic.cells	L1CAM	0.112788	3.644556	0.387663	0.699197	-5.90566	0.754002	0.756544
Dendritic.cells	HPRT	0.057345	6.310323	0.387537	0.69929	-6.34834	0.716576	0.702434
Dendritic.cells	TRP53I11	-0.05499	4.565294	-0.38748	0.699332	-6.43993	0.740796	0.737402
Dendritic.cells	SYNCRIP	-0.03415	7.375906	-0.38738	0.699406	-6.84229	0.702297	0.681903
Dendritic.cells	EPS8L2	0.225869	0.893203	0.387336	0.699438	-5.29039	0.79526	0.816697
Dendritic.cells	CFAP43	-0.07587	3.09234	-0.38722	0.699525	-5.99266	0.762065	0.768267
Dendritic.cells	BC049715	-0.22472	0.604594	-0.38715	0.699579	-5.35701	0.799746	0.82328
Dendritic.cells	PCNX3	-0.09761	3.30596	-0.38703	0.699665	-5.74448	0.758933	0.763744
Dendritic.cells	FUT10	-0.21788	-0.01434	-0.38693	0.699736	-5.22709	0.80947	0.837612
Dendritic.cells	ARHGAP9	-0.06923	5.14536	-0.38691	0.699755	-6.18522	0.732629	0.725654
Dendritic.cells	MYO18A	0.052065	4.439945	0.386779	0.699849	-6.32701	0.742577	0.74006
Dendritic.cells	SEC22A	-0.09484	3.176208	-0.38673	0.699888	-5.7618	0.760834	0.766555
Dendritic.cells	RAD51D	-0.08924	2.984907	-0.38658	0.699999	-5.83216	0.763647	0.77069
Dendritic.cells	AUNIP	-0.18932	1.893508	-0.38652	0.70004	-5.30632	0.779945	0.794442
Dendritic.cells	GPR65	0.13579	4.250918	0.386441	0.700098	-5.85984	0.745272	0.74401
Dendritic.cells	COL11A2	0.222567	0.72136	0.386393	0.700134	-5.30109	0.797928	0.820744
Dendritic.cells	COPG1	0.053346	5.141092	0.386329	0.700181	-6.36531	0.732688	0.725799
Dendritic.cells	PTPRO	-0.10211	2.363394	-0.38619	0.700281	-6.03965	0.772876	0.784129

Dendritic.cells	CCDC66	-0.12255	2.265203	-0.38618	0.70029	-5.55064	0.774346	0.786273
Dendritic.cells	MLLT6	0.160199	3.270712	0.386175	0.700295	-5.63119	0.759449	0.764585
Dendritic.cells	SMOC1	-0.18427	2.28252	-0.38612	0.700339	-5.58764	0.774087	0.785895
Dendritic.cells	ORC5	0.113692	3.474755	0.385858	0.700528	-5.68007	0.756527	0.760262
Dendritic.cells	STYX	0.05723	4.526153	0.385858	0.700529	-6.15415	0.741407	0.738336
Dendritic.cells	FKBP4	-0.04967	5.691682	-0.3856	0.700718	-6.45657	0.725222	0.714828
Dendritic.cells	SEC61G	0.04013	10.02398	0.385271	0.700962	-7.30942	0.668767	0.633679
Dendritic.cells	SESTD1	0.201676	2.300083	0.385011	0.701154	-5.48908	0.774344	0.785717
Dendritic.cells	TCEANC2	-0.06984	4.247598	-0.3849	0.701237	-5.97339	0.745836	0.744312
Dendritic.cells	ARMCX2	-0.11459	1.41393	-0.38467	0.701404	-5.76018	0.787898	0.8054
Dendritic.cells	ECE1	-0.06412	5.613294	-0.38432	0.701661	-6.53907	0.726925	0.716589
Dendritic.cells	GM10130	-0.14767	1.783227	-0.38413	0.701802	-5.4737	0.782489	0.797181
Dendritic.cells	HSP90AA1	0.040531	7.657631	0.384124	0.701809	-6.83788	0.699366	0.676946
Dendritic.cells	MARCO	-0.25095	5.795483	-0.38382	0.702034	-6.09795	0.724528	0.712998
Dendritic.cells	NDUFA5	-0.0728	5.432033	-0.38373	0.702102	-6.2495	0.729556	0.720279
Dendritic.cells	ASL	0.087313	4.484774	0.383659	0.702152	-5.99574	0.742877	0.739526
Dendritic.cells	BTBD6	0.202142	1.542046	0.383612	0.702187	-5.29521	0.786277	0.802615
Dendritic.cells	GTSF2	-0.24891	0.091247	-0.38336	0.70237	-5.22325	0.808845	0.83568
Dendritic.cells	BAMBI	-0.12004	3.967331	-0.38332	0.702404	-5.84992	0.750307	0.750313
Dendritic.cells	ARL2	0.102126	3.356926	0.383306	0.702413	-5.78134	0.759168	0.763167
Dendritic.cells	LYZL4	-0.20477	-1.24765	-0.38307	0.702589	-5.21706	0.830402	0.86739
Dendritic.cells	FOXRED2	-0.12024	1.168266	-0.38285	0.702752	-5.6322	0.792098	0.811217
Dendritic.cells	RUSC2	0.220943	0.909936	0.382795	0.70279	-5.33561	0.796092	0.817072
Dendritic.cells	PHC3	0.053448	4.499127	0.382766	0.702812	-6.3224	0.742753	0.739444
Dendritic.cells	RAG1	0.259844	1.672599	0.382708	0.702854	-5.27274	0.784371	0.799975
Dendritic.cells	RFX7	-0.03808	7.368214	-0.38267	0.702882	-6.95552	0.703363	0.682711
Dendritic.cells	VPS35L	0.046735	5.162292	0.382331	0.703133	-6.29727	0.733588	0.726123
Dendritic.cells	PDE11A	-0.2617	0.072949	-0.3817	0.703602	-5.24508	0.809782	0.83682
Dendritic.cells	FAM210B	0.111951	3.914295	0.381573	0.703694	-5.72591	0.751673	0.752073
Dendritic.cells	SLC36A3	0.212722	0.101985	0.381544	0.703715	-5.26381	0.809322	0.836158
Dendritic.cells	CDKN2C	-0.14602	3.918847	-0.38152	0.703732	-5.7086	0.751607	0.751977
Dendritic.cells	SEC63	-0.03071	7.09953	-0.38108	0.704058	-6.8272	0.707668	0.688375
Dendritic.cells	GM17231	0.122628	2.734245	0.380923	0.704174	-5.77142	0.769204	0.777334
Dendritic.cells	RNF114	-0.0654	5.645299	-0.38083	0.704239	-6.29147	0.727434	0.716836
Dendritic.cells	GM34466	-0.19702	-1.10689	-0.38081	0.704257	-5.21812	0.828973	0.864842
Dendritic.cells	FBXO36	-0.21052	1.228268	-0.38075	0.704301	-5.32515	0.791996	0.810573
Dendritic.cells	ACER1	-0.2804	-0.7216	-0.3806	0.704414	-5.23059	0.822768	0.855654
Dendritic.cells	TDO2	-0.23444	3.218699	-0.38047	0.704511	-5.60009	0.762077	0.766928
Dendritic.cells	PCGF5	0.059421	6.06196	0.380384	0.704572	-6.52998	0.721727	0.708574
Dendritic.cells	KLRI1	0.234018	-0.19768	0.380135	0.704757	-5.22424	0.814372	0.843413
Dendritic.cells	HIPK2	0.049571	6.550097	0.380059	0.704813	-6.64031	0.715081	0.69909
Dendritic.cells	USP19	0.080751	4.704682	0.37982	0.70499	-6.02473	0.740637	0.735966
Dendritic.cells	ZFP944	0.081068	4.397621	0.379774	0.705024	-5.96926	0.745003	0.742283
Dendritic.cells	CHST8	0.265295	-0.80735	0.379762	0.705032	-5.22401	0.824152	0.857819
Dendritic.cells	HSPD1	0.057549	7.46114	0.379678	0.705095	-6.68726	0.702893	0.681601
Dendritic.cells	DDOST	0.036533	5.788445	0.379486	0.705237	-6.65234	0.725487	0.714092
Dendritic.cells	FARSA	-0.05153	4.90554	-0.37945	0.70526	-6.23667	0.737798	0.731862

Dendritic.cells	MAPK4	-0.10915	0.274036	-0.37941	0.705294	-5.82065	0.806898	0.832467
Dendritic.cells	HRAS	-0.05699	4.842094	-0.3794	0.705298	-6.19318	0.738693	0.733156
Dendritic.cells	TAF4	0.068092	4.075399	0.379345	0.705341	-5.97789	0.749621	0.748971
Dendritic.cells	FAM129B	0.139819	3.14002	0.379316	0.705363	-5.75838	0.763234	0.768723
Dendritic.cells	ARHGAP27	0.074623	3.664658	0.379135	0.705497	-6.02634	0.75563	0.757582
Dendritic.cells	ZBTB10	-0.0609	4.077959	-0.37904	0.705565	-6.37557	0.749653	0.748937
Dendritic.cells	FOXJ3	-0.03552	5.947043	-0.37853	0.705942	-6.60378	0.723545	0.711115
Dendritic.cells	ATXN7L3	-0.07074	4.200833	-0.37853	0.705944	-5.96899	0.748069	0.746539
Dendritic.cells	RAD54L	-0.15465	2.724058	-0.37836	0.70607	-5.46744	0.769644	0.777885
Dendritic.cells	NAT9	0.104148	3.244632	0.378317	0.706102	-5.74585	0.76195	0.7667
Dendritic.cells	SHQ1	-0.11921	2.60612	-0.37811	0.706255	-5.67352	0.771401	0.780487
Dendritic.cells	SRSF7	-0.05477	6.098296	-0.37798	0.706349	-6.49042	0.72147	0.708199
Dendritic.cells	CCRL2	0.303151	4.741765	0.377938	0.706383	-5.59924	0.740359	0.735457
Dendritic.cells	HELLS	-0.14313	4.919254	-0.37788	0.706427	-5.85658	0.737851	0.731832
Dendritic.cells	FASTKD5	0.169696	0.844458	0.377745	0.706526	-5.35441	0.798236	0.819686
Dendritic.cells	TOX	-0.18916	4.351569	-0.3777	0.706559	-5.74407	0.74591	0.743521
Dendritic.cells	ATP2C1	0.042588	5.85373	0.377624	0.706615	-6.5086	0.724829	0.713083
Dendritic.cells	2310016D2	0.191494	-0.3578	0.377557	0.706665	-5.37477	0.8172	0.847562
Dendritic.cells	MTURN	-0.19174	2.270409	-0.37755	0.706671	-5.31618	0.776428	0.787889
Dendritic.cells	PUS3	0.105852	2.37825	0.377509	0.7067	-5.62292	0.774809	0.785535
Dendritic.cells	GM45435	-0.19542	0.920103	-0.37742	0.706769	-5.45706	0.797061	0.818044
Dendritic.cells	PAN3	0.025975	8.767803	0.377145	0.70697	-7.14626	0.68617	0.657621
Dendritic.cells	CAPSL	-0.24892	0.928873	-0.37708	0.70702	-5.29255	0.796979	0.817946
Dendritic.cells	LIX1	-0.1962	0.723268	-0.37688	0.707162	-5.33691	0.800179	0.822666
Dendritic.cells	NOC2L	-0.06283	5.059461	-0.37683	0.7072	-6.26712	0.735928	0.729192
Dendritic.cells	PIGP	0.060602	4.420664	0.376806	0.707221	-6.22453	0.744974	0.742275
Dendritic.cells	CAND1	0.047098	5.132169	0.376793	0.70723	-6.30745	0.734908	0.727718
Dendritic.cells	PSMG3	0.11634	3.202758	0.37664	0.707343	-5.65664	0.762628	0.767925
Dendritic.cells	PGAP1	-0.18853	3.785443	-0.37659	0.707377	-5.4446	0.754121	0.755583
Dendritic.cells	DAP3	0.066673	5.039126	0.376468	0.707471	-6.05924	0.736229	0.72967
Dendritic.cells	GM42595	0.163371	0.917394	0.376402	0.70752	-5.41016	0.797174	0.818314
Dendritic.cells	INTS7	0.034164	5.677826	0.376279	0.707611	-6.58652	0.727345	0.716827
Dendritic.cells	NEGR1	-0.18205	-0.13284	-0.37608	0.70776	-5.52797	0.81371	0.842628
Dendritic.cells	PRPF38A	-0.06632	5.176932	-0.37607	0.707768	-6.1172	0.73432	0.726964
Dendritic.cells	PRKAR2B	-0.09734	3.305356	-0.37597	0.70784	-5.8795	0.761152	0.765853
Dendritic.cells	MRPS2	-0.15694	2.843371	-0.37582	0.707955	-5.5488	0.767966	0.77578
Dendritic.cells	ATP6V0C	-0.03253	8.851286	-0.37578	0.707978	-7.12218	0.685139	0.656318
Dendritic.cells	E130309D0	0.084481	3.983364	0.375695	0.708043	-5.84227	0.751289	0.751581
Dendritic.cells	BRD8	0.042227	6.236165	0.375613	0.708105	-6.42554	0.719674	0.70592
Dendritic.cells	LHPP	0.116309	3.112672	0.375525	0.70817	-5.60801	0.763985	0.770052
Dendritic.cells	ANKRD24	0.160328	1.054272	0.37544	0.708232	-5.39354	0.795078	0.81541
Dendritic.cells	MIER2	0.140652	2.002333	0.375336	0.708309	-5.48737	0.780576	0.794204
Dendritic.cells	CLIP2	0.106887	2.886513	0.375142	0.708453	-5.60241	0.767417	0.774912
Dendritic.cells	TCIRG1	0.043944	5.455039	0.375048	0.708523	-6.64759	0.730524	0.721448
Dendritic.cells	LMTK2	-0.04499	5.616268	-0.37488	0.708645	-6.50737	0.728296	0.718216
Dendritic.cells	ELOVL5	-0.05224	6.195778	-0.37483	0.708688	-6.53843	0.720322	0.706722
Dendritic.cells	PSMA5	-0.05542	5.884469	-0.37472	0.708766	-6.43126	0.724591	0.712882

Dendritic.cells	A230056P1	-0.19447	0.407448	-0.37466	0.708809	-5.29343	0.805274	0.8302
Dendritic.cells	FAM117B	-0.04202	7.11596	-0.37418	0.709167	-6.88017	0.707964	0.688903
Dendritic.cells	CDKN2AIP	-0.07377	4.567595	-0.37405	0.709261	-5.94473	0.743093	0.739582
Dendritic.cells	PER3	-0.16027	1.517982	-0.37405	0.709265	-5.42647	0.788122	0.805066
Dendritic.cells	CCDC126	0.146319	1.899321	0.373981	0.709314	-5.54344	0.782309	0.796577
Dendritic.cells	CSRP2	-0.07928	5.20682	-0.37397	0.709322	-6.0942	0.734071	0.726537
Dendritic.cells	ZBTB18	-0.06861	3.894799	-0.37397	0.709323	-6.04146	0.752743	0.753563
Dendritic.cells	SLC17A3	-0.21665	0.779648	-0.37392	0.70936	-5.31015	0.799528	0.821766
Dendritic.cells	HBQ1A	-0.19106	-1.39639	-0.37334	0.709792	-5.22533	0.834731	0.873144
Dendritic.cells	ZFP330	-0.06457	4.432352	-0.37324	0.709862	-6.03378	0.745402	0.742631
Dendritic.cells	NFE2L3	0.155161	2.246069	0.373059	0.709997	-5.48275	0.777474	0.789256
Dendritic.cells	STX17	-0.08971	4.034327	-0.37302	0.710028	-5.91982	0.75112	0.750968
Dendritic.cells	GM43329	-0.06724	3.105098	-0.37285	0.710151	-6.23053	0.764672	0.770641
Dendritic.cells	PYGB	0.061002	4.748164	0.372777	0.710207	-6.11935	0.740916	0.736193
Dendritic.cells	ARFGAP1	-0.05389	4.620741	-0.37277	0.710213	-6.23226	0.742724	0.738809
Dendritic.cells	KCNIP4	0.287019	0.608323	0.372476	0.71043	-5.26089	0.802774	0.82614
Dendritic.cells	UQCRB	-0.04297	8.047525	-0.3724	0.710486	-6.81687	0.696168	0.671693
Dendritic.cells	MINDY1	-0.09592	3.987424	-0.37223	0.710609	-5.72774	0.751997	0.75208
Dendritic.cells	ZFAND2A	0.098182	3.630572	0.372054	0.710743	-5.67804	0.757241	0.759604
Dendritic.cells	TRMT11	-0.07321	3.646744	-0.37192	0.710844	-5.95302	0.75704	0.759292
Dendritic.cells	CYP7A1	-0.23742	0.739983	-0.37158	0.711096	-5.24451	0.80095	0.82321
Dendritic.cells	CHTF18	-0.18543	1.800347	-0.37151	0.711144	-5.36806	0.7846	0.799332
Dendritic.cells	AKNA	0.077707	4.948701	0.371395	0.711232	-5.90956	0.738438	0.732318
Dendritic.cells	PLEKHG6	0.165326	-0.11425	0.371323	0.711285	-5.23562	0.81442	0.843066
Dendritic.cells	HSPBP1	-0.07817	3.934559	-0.37122	0.711361	-5.88052	0.752923	0.753339
Dendritic.cells	GM47828	0.204705	0.334612	0.370874	0.711619	-5.23175	0.807308	0.832684
Dendritic.cells	FOXM1	-0.13987	3.10354	-0.37086	0.711625	-5.62041	0.765064	0.771
Dendritic.cells	TMEM229/	-0.21307	-1.03017	-0.37085	0.711635	-5.22151	0.829162	0.864813
Dendritic.cells	HAVCR2	0.191742	2.301711	0.3707	0.711747	-5.56847	0.777012	0.78844
Dendritic.cells	CNDP2	-0.06866	5.051865	-0.37068	0.711762	-6.16876	0.736984	0.730351
Dendritic.cells	VPS16	0.060656	4.436967	0.370591	0.711828	-6.01963	0.745702	0.742985
Dendritic.cells	CPS1	0.184366	4.481272	0.370451	0.711933	-5.89927	0.74507	0.74207
Dendritic.cells	ARHGAP25	-0.06161	6.214201	-0.37043	0.711951	-6.34586	0.720864	0.707118
Dendritic.cells	CD300LB	0.25064	1.973752	0.370362	0.711999	-5.29708	0.781965	0.79569
Dendritic.cells	KLC4	0.122585	2.884186	0.370361	0.712	-5.55441	0.76831	0.775795
Dendritic.cells	GRSF1	0.048797	5.185543	0.370171	0.71214	-6.21359	0.735107	0.727662
Dendritic.cells	CENPF	0.172341	5.050794	0.370141	0.712163	-5.95767	0.736999	0.730397
Dendritic.cells	ATIC	0.06932	4.493549	0.370103	0.712191	-6.18498	0.744894	0.741816
Dendritic.cells	GM36723	-0.2566	3.324777	-0.36998	0.712285	-5.44816	0.761808	0.766373
Dendritic.cells	COG8	-0.05903	4.299473	-0.36992	0.712324	-6.06514	0.74767	0.745863
Dendritic.cells	SEC24A	-0.05575	6.616568	-0.36989	0.712348	-6.64875	0.715391	0.699268
Dendritic.cells	CEP76	-0.12376	2.990307	-0.36967	0.712514	-5.57843	0.766789	0.77355
Dendritic.cells	OAS2	-0.27842	1.571395	-0.36943	0.712687	-5.24057	0.788149	0.804725
Dendritic.cells	LAPTM4B	-0.0908	3.501356	-0.36943	0.712694	-6.14193	0.759273	0.762668
Dendritic.cells	RBBP9	0.16866	1.214794	0.36925	0.712824	-5.438	0.793632	0.812767
Dendritic.cells	UBE2Q1	-0.03547	6.334643	-0.36893	0.713059	-6.61538	0.719268	0.70489
Dendritic.cells	TUBB6	-0.14578	3.749371	-0.36881	0.713154	-5.67302	0.755659	0.757491

Dendritic.cells	SPATA13	-0.08409	5.147731	-0.36878	0.713171	-6.0857	0.735687	0.728572
Dendritic.cells	1700007L1	0.143932	1.663099	0.368703	0.713231	-5.49084	0.786746	0.80275
Dendritic.cells	SPP1	0.306356	2.353951	0.368678	0.713249	-5.43043	0.776279	0.787477
Dendritic.cells	BBOF1	-0.17736	1.302277	-0.36858	0.713322	-5.32328	0.792282	0.810868
Dendritic.cells	DSEL	0.165659	0.375304	0.368541	0.713351	-5.30728	0.806722	0.832021
Dendritic.cells	GM43647	0.230933	-0.94495	0.368541	0.713351	-5.22532	0.827833	0.863059
Dendritic.cells	GM30198	-0.18603	1.865066	-0.36846	0.713415	-5.49692	0.783668	0.798328
Dendritic.cells	S100A13	0.047524	6.040644	0.368323	0.713513	-6.47743	0.72329	0.710804
Dendritic.cells	GIGYF1	0.063692	4.441868	0.368284	0.713541	-6.08501	0.745682	0.743177
Dendritic.cells	DR1	-0.04785	4.953577	-0.36821	0.7136	-6.26929	0.738419	0.732677
Dendritic.cells	E2F8	0.147439	3.686572	0.368085	0.71369	-5.59249	0.756572	0.758978
Dendritic.cells	RIPK3	0.137323	3.285039	0.368084	0.71369	-5.5205	0.762443	0.767506
Dendritic.cells	CACNA1E	-0.0577	4.907691	-0.36806	0.71371	-7.03302	0.739066	0.733625
Dendritic.cells	DNAJB1	-0.05462	6.228259	-0.3679	0.713825	-6.59629	0.72072	0.707195
Dendritic.cells	SNHG8	-0.08638	4.013183	-0.3678	0.713901	-5.81638	0.751838	0.752205
Dendritic.cells	ALPK1	-0.05637	4.802266	-0.36772	0.713961	-6.59337	0.740557	0.7359
Dendritic.cells	FKBP9	0.183276	1.265181	0.3676	0.71405	-5.44519	0.792854	0.812003
Dendritic.cells	TAPBP	-0.04928	6.597076	-0.36745	0.714161	-6.87005	0.715703	0.700051
Dendritic.cells	PRRC2C	0.029332	7.744202	0.367414	0.714188	-6.89407	0.700393	0.67805
Dendritic.cells	RBM5	-0.03074	6.623423	-0.36728	0.714288	-6.74094	0.715347	0.699575
Dendritic.cells	TRIM3	0.092848	2.274801	0.367152	0.714383	-5.78526	0.777469	0.789614
Dendritic.cells	CDK1	-0.1697	5.506863	-0.36714	0.71439	-5.88691	0.730668	0.72169
Dendritic.cells	TMEM168	-0.0575	4.583494	-0.36701	0.714487	-6.21277	0.743663	0.740519
Dendritic.cells	MCPH1	-0.06345	4.913551	-0.36695	0.714531	-6.13872	0.738984	0.733746
Dendritic.cells	CATSPERE2	-0.12281	1.174644	-0.36695	0.714534	-5.53172	0.794252	0.814175
Dendritic.cells	B3GLCT	0.115035	3.311117	0.366711	0.714711	-5.67341	0.762174	0.767305
Dendritic.cells	MBLAC1	-0.19177	-0.19049	-0.36635	0.71498	-5.22822	0.816037	0.845773
Dendritic.cells	RUVBL1	0.06446	4.77265	0.366222	0.715074	-6.0704	0.741292	0.736768
Dendritic.cells	GM48302	0.129858	1.63727	0.366168	0.715114	-5.70056	0.787476	0.803929
Dendritic.cells	FKBPL	0.160046	1.019839	0.366071	0.715187	-5.4004	0.796992	0.817863
Dendritic.cells	BLVRB	0.086664	7.092023	0.36579	0.715395	-6.55302	0.709442	0.690738
Dendritic.cells	MYO1G	-0.06255	5.563064	-0.36565	0.715501	-6.3678	0.730297	0.720847
Dendritic.cells	SLC25A27	0.201796	0.260835	0.365448	0.71565	-5.23114	0.808982	0.835414
Dendritic.cells	HECTD4	0.04708	5.548933	0.365403	0.715683	-6.47122	0.730494	0.721158
Dendritic.cells	GM43065	0.224013	0.153169	0.365314	0.715749	-5.23585	0.810685	0.837926
Dendritic.cells	POLA1	-0.0895	5.968201	-0.36517	0.715854	-6.2634	0.724694	0.712831
Dendritic.cells	ZFP955B	-0.12462	2.201747	-0.365	0.715986	-5.5825	0.779009	0.791624
Dendritic.cells	JOSD2	0.068144	4.09679	0.364996	0.715986	-5.98222	0.751055	0.750967
Dendritic.cells	LGALS4	-0.06842	2.563012	-0.36495	0.716017	-6.22776	0.77358	0.78371
Dendritic.cells	ZC3H12A	-0.07271	5.128189	-0.36494	0.716028	-6.14461	0.736376	0.729712
Dendritic.cells	CDC37	0.031115	6.605127	0.364833	0.716107	-6.69634	0.715997	0.700316
Dendritic.cells	METAP1D	-0.09455	3.768583	-0.36481	0.716127	-5.82696	0.755805	0.757858
Dendritic.cells	MMP25	-0.22906	0.499533	-0.36471	0.716197	-5.23358	0.805222	0.82997
Dendritic.cells	NPAT	0.074678	4.485178	0.364645	0.716247	-6.04622	0.745484	0.742892
Dendritic.cells	BRK1	0.034368	6.271039	0.364489	0.716363	-6.63348	0.720588	0.706948
Dendritic.cells	KIFC1	0.200648	3.200672	0.364345	0.71647	-5.53064	0.764204	0.770077
Dendritic.cells	SCFD2	-0.03851	5.78946	-0.36407	0.716677	-6.67536	0.727346	0.716611

Dendritic.cells	WHRN	-0.08748	3.066048	-0.364	0.716731	-6.01068	0.766298	0.773054
Dendritic.cells	ADH5	0.052583	6.03865	0.363887	0.716811	-6.48658	0.723911	0.711671
Dendritic.cells	GM43260	-0.12884	2.252383	-0.36375	0.716911	-5.53418	0.778446	0.790794
Dendritic.cells	CENPP	0.09864	5.39603	0.363657	0.716982	-6.15134	0.732813	0.724552
Dendritic.cells	ADSSL1	0.05175	4.426295	0.363539	0.71707	-6.32537	0.746517	0.744393
Dendritic.cells	SLC22A15	0.114708	3.077716	0.363454	0.717134	-5.63033	0.766125	0.772874
Dendritic.cells	TIGAR	-0.12889	1.851972	-0.3634	0.717173	-5.54995	0.784511	0.799676
Dendritic.cells	GTF3A	-0.05401	4.569389	-0.36338	0.717191	-6.14078	0.744474	0.741445
Dendritic.cells	IKBKE	-0.26554	3.116112	-0.36309	0.717408	-5.3559	0.765656	0.772093
Dendritic.cells	TBCC	-0.08657	3.579816	-0.36307	0.717421	-5.83876	0.758846	0.762194
Dendritic.cells	FABP7	0.299447	1.989157	0.36282	0.717605	-5.28887	0.782613	0.796724
Dendritic.cells	USP39	-0.08283	4.614508	-0.36278	0.717638	-5.98011	0.744009	0.740617
Dendritic.cells	KIF20A	-0.20117	3.768581	-0.36261	0.717761	-5.56456	0.756221	0.758331
Dendritic.cells	RAB9	-0.06253	4.887523	-0.36254	0.717815	-6.02166	0.740175	0.735076
Dendritic.cells	DPF2	0.048788	5.127706	0.362403	0.717916	-6.2977	0.73682	0.730177
Dendritic.cells	KLHDC2	0.07665	4.860142	0.361633	0.718489	-5.87913	0.741115	0.735762
Dendritic.cells	PCNP	-0.03483	6.359657	-0.36135	0.718698	-6.54159	0.720378	0.705741
Dendritic.cells	CNOT11	-0.0723	4.112527	-0.3613	0.718739	-5.95833	0.75192	0.751316
Dendritic.cells	MMP9	0.2702	1.381978	0.361	0.718961	-5.27475	0.792734	0.810728
Dendritic.cells	MID1IP1	-0.11931	3.917342	-0.36092	0.71902	-5.65939	0.754821	0.755549
Dendritic.cells	SIAH1B	0.14927	1.91846	0.360918	0.719022	-5.40703	0.784518	0.798753
Dendritic.cells	USP6NL	0.046676	5.563085	0.360691	0.719191	-6.4656	0.731495	0.721792
Dendritic.cells	ACAP1	-0.07138	3.923819	-0.3606	0.71926	-6.15044	0.75479	0.755513
Dendritic.cells	MB21D2	0.149374	0.883088	0.360438	0.71938	-5.57759	0.800534	0.822235
Dendritic.cells	CTSO	-0.05047	4.26707	-0.36039	0.719416	-6.47364	0.749834	0.74839
Dendritic.cells	ALDH1A1	-0.24313	-5.25E-05	-0.36037	0.719426	-5.23084	0.81445	0.842657
Dendritic.cells	TFEB	-0.05906	5.158966	-0.36004	0.719678	-6.21506	0.737267	0.730103
Dendritic.cells	5730455P1	0.101481	2.914853	0.360014	0.719695	-5.61913	0.769721	0.777159
Dendritic.cells	LEO1	-0.06412	4.138442	-0.35987	0.719804	-6.14802	0.751805	0.75116
Dendritic.cells	TBPL1	-0.05494	4.928003	-0.35985	0.719817	-6.17031	0.740525	0.73483
Dendritic.cells	9130230L2	0.122754	3.687563	0.359589	0.720012	-6.00485	0.758478	0.760702
Dendritic.cells	DSN1	-0.13283	2.622227	-0.35941	0.720145	-5.4961	0.774289	0.783615
Dendritic.cells	CPT2	0.150302	2.829472	0.359211	0.720294	-5.51121	0.77128	0.779165
Dendritic.cells	ZFP866	-0.10222	2.993086	-0.35894	0.720493	-5.70147	0.768917	0.775715
Dendritic.cells	FAM122A	-0.0615	3.563965	-0.35887	0.720546	-6.10173	0.760504	0.763506
Dendritic.cells	NANP	-0.12528	2.821135	-0.35878	0.720616	-5.61139	0.771474	0.779456
Dendritic.cells	RDH11	0.091441	3.215453	0.358552	0.720785	-5.76564	0.765626	0.771017
Dendritic.cells	PEX10	-0.16964	0.82136	-0.35854	0.720797	-5.34709	0.801997	0.824084
Dendritic.cells	RPF1	0.050382	5.149471	0.358453	0.720859	-6.20157	0.737744	0.730609
Dendritic.cells	IPO11	0.058183	4.478076	0.358444	0.720866	-6.21208	0.747274	0.744393
Dendritic.cells	SLAIN1	0.075862	3.6132	0.358368	0.720922	-5.91703	0.759784	0.762549
Dendritic.cells	TRAIP	0.147789	2.119414	0.358192	0.721053	-5.48564	0.782019	0.794905
Dendritic.cells	SLCO5A1	-0.16658	-0.20706	-0.35814	0.72109	-5.22791	0.818263	0.847972
Dendritic.cells	CDH17	0.20951	0.13748	0.358053	0.721157	-5.25745	0.81277	0.839903
Dendritic.cells	ENGASE	0.227922	1.496503	0.35801	0.721189	-5.25093	0.79153	0.80879
Dendritic.cells	CEP120	-0.04935	5.494784	-0.3578	0.721348	-6.32484	0.732961	0.723637
Dendritic.cells	WDR33	-0.02513	7.186641	-0.35768	0.721435	-6.88836	0.709838	0.690359

Dendritic.cells	SCIMP	0.050267	3.702449	0.357628	0.721474	-6.70157	0.75854	0.760675
Dendritic.cells	ITM2A	-0.1462	2.544803	-0.35756	0.721522	-5.574	0.775666	0.785574
Dendritic.cells	GRK5	0.152064	5.747033	0.357361	0.721673	-5.84356	0.729534	0.718626
Dendritic.cells	TXNL4A	0.058409	4.968748	0.357132	0.721844	-6.16743	0.740537	0.734417
Dendritic.cells	1700047M	-0.22057	-0.15234	-0.35698	0.72196	-5.23677	0.817657	0.846806
Dendritic.cells	TCF19	0.146535	3.199871	0.356861	0.722046	-5.59923	0.766108	0.77148
Dendritic.cells	SLC39A7	0.052318	4.500813	0.356615	0.72223	-6.20022	0.747194	0.744047
Dendritic.cells	ARFIP2	-0.12048	2.138474	-0.35655	0.722277	-5.50942	0.781988	0.794596
Dendritic.cells	TSG101	0.044465	5.622255	0.356546	0.722281	-6.4265	0.731368	0.721176
Dendritic.cells	IPMK	0.064914	5.867681	0.356511	0.722307	-6.16707	0.727963	0.716265
Dendritic.cells	TNFRSF1A	0.060808	5.171275	0.356487	0.722326	-6.34056	0.73768	0.730288
Dendritic.cells	FBXL2	-0.09118	3.231347	-0.35622	0.722527	-5.82604	0.765733	0.770889
Dendritic.cells	EMID1	0.073845	3.280299	0.356188	0.722548	-6.02848	0.765011	0.769839
Dendritic.cells	TTC7	0.040641	5.873227	0.355995	0.722692	-6.70275	0.728046	0.716279
Dendritic.cells	1810030O(	-0.05957	4.824335	-0.35565	0.722953	-6.14242	0.742876	0.737563
Dendritic.cells	MAPK3	-0.07413	4.874121	-0.35564	0.722956	-5.99574	0.74217	0.736542
Dendritic.cells	PPP4R3B	-0.02768	6.842276	-0.3554	0.723135	-6.78522	0.715022	0.697349
Dendritic.cells	GM38843	-0.2177	0.985485	-0.35526	0.723241	-5.24643	0.800123	0.82085
Dendritic.cells	RGS5	0.26989	1.107497	0.355182	0.723299	-5.30213	0.798225	0.818072
Dendritic.cells	NUDT22	0.100182	2.557891	0.35514	0.723331	-5.66621	0.776075	0.785743
Dendritic.cells	SDHC	-0.05464	5.09505	-0.35462	0.723721	-6.21935	0.739412	0.732283
Dendritic.cells	SLC25A43	0.202869	-0.21871	0.354539	0.723779	-5.2284	0.819449	0.848891
Dendritic.cells	PVRIG	-0.18009	-0.53207	-0.35451	0.7238	-5.22799	0.82449	0.856302
Dendritic.cells	2610206C1	-0.17667	0.40853	-0.35429	0.723968	-5.33468	0.809478	0.834241
Dendritic.cells	CUL7	0.122831	1.968845	0.354037	0.724154	-5.60754	0.785273	0.798846
Dendritic.cells	WDR35	-0.20219	0.509893	-0.35403	0.724161	-5.28171	0.807878	0.831896
Dendritic.cells	PPP1R42	0.219647	0.580161	0.353995	0.724186	-5.27188	0.806772	0.830275
Dendritic.cells	KLHL5	0.065393	4.645979	0.353984	0.724194	-5.97063	0.745795	0.741504
Dendritic.cells	WTAP	0.032845	6.931297	0.353941	0.724225	-6.65351	0.714089	0.695796
Dendritic.cells	GPX3	0.23121	1.463214	0.352941	0.724973	-5.33951	0.793762	0.810453
Dendritic.cells	SASS6	-0.09244	4.42508	-0.35235	0.725416	-5.8533	0.750044	0.746533
Dendritic.cells	ZFP846	0.115351	2.431015	0.352162	0.725554	-5.59081	0.779479	0.789215
Dendritic.cells	PMPCB	-0.06385	4.974651	-0.35174	0.725873	-6.11967	0.74246	0.735397
Dendritic.cells	CFAP298	-0.09672	3.642504	-0.35173	0.725878	-5.7051	0.761664	0.763178
Dendritic.cells	ZBTB8OS	0.059112	5.027468	0.351593	0.72598	-6.16679	0.741744	0.734375
Dendritic.cells	CASP9	-0.1072	2.447605	-0.35138	0.726142	-5.53052	0.779561	0.78913
Dendritic.cells	FDXR	0.094086	2.625449	0.351136	0.726322	-5.74093	0.777003	0.785307
Dendritic.cells	LRRCC1	-0.07115	3.748019	-0.35081	0.726569	-5.82272	0.760553	0.76126
Dendritic.cells	ADAM8	-0.20744	2.602795	-0.35054	0.726765	-5.33061	0.777669	0.786021
Dendritic.cells	HGSNAT	0.114945	4.642285	0.350296	0.72695	-5.8397	0.747811	0.742742
Dendritic.cells	CD69	-0.05605	5.620451	-0.35028	0.726965	-6.85127	0.733981	0.722788
Dendritic.cells	NUPL2	0.108282	2.367649	0.350017	0.727158	-5.68694	0.781403	0.791371
Dendritic.cells	TWISTNB	-0.04911	5.520145	-0.34969	0.727403	-6.27821	0.7355	0.724984
Dendritic.cells	SDHD	0.060775	5.858408	0.349674	0.727415	-6.27112	0.730783	0.718188
Dendritic.cells	H2AFV	-0.04862	8.095101	-0.34961	0.727461	-6.84707	0.700576	0.674835
Dendritic.cells	ZNHIT6	-0.10212	3.231636	-0.34952	0.727532	-5.73726	0.76847	0.772669
Dendritic.cells	CACNA1D	0.065455	3.396539	0.3495	0.727545	-6.29387	0.766032	0.769131

Dendritic.cells	CDKAL1	0.035422	6.364518	0.349448	0.727584	-6.68437	0.723798	0.708139
Dendritic.cells	TMEM18	-0.13657	1.593876	-0.34936	0.727652	-5.46681	0.793215	0.808676
Dendritic.cells	HAX1	-0.07989	4.020737	-0.34929	0.727704	-5.88984	0.756892	0.755887
Dendritic.cells	DHX58	0.158672	2.999964	0.349089	0.727853	-5.58219	0.771958	0.777666
Dendritic.cells	ACE	0.288751	0.341503	0.349034	0.727893	-5.24593	0.812848	0.837306
Dendritic.cells	FAM173A	0.061862	4.68182	0.348951	0.727956	-6.09048	0.747408	0.742104
Dendritic.cells	ERCC5	-0.06975	2.909402	-0.34871	0.728134	-5.95156	0.773424	0.779655
Dendritic.cells	HIVEP3	0.041305	5.144836	0.34852	0.728278	-6.75557	0.740966	0.732683
Dendritic.cells	BTF3	0.026723	8.999494	0.348435	0.728342	-7.08096	0.689011	0.658164
Dendritic.cells	RSRC2	-0.02851	6.479986	-0.34839	0.728372	-6.63636	0.722398	0.70598
Dendritic.cells	SELP	0.241726	1.452771	0.348064	0.728619	-5.38191	0.795606	0.812073
Dendritic.cells	CYB5RL	0.150513	0.380261	0.348033	0.728643	-5.3855	0.812402	0.836631
Dendritic.cells	WIZ	-0.06948	3.836067	-0.34777	0.728839	-6.08563	0.759785	0.760052
Dendritic.cells	KLHL28	0.083737	3.44241	0.34776	0.728847	-5.78072	0.765561	0.768425
Dendritic.cells	STXBP5	-0.06354	5.383452	-0.34775	0.728856	-6.30896	0.737615	0.728008
Dendritic.cells	AMY1	-0.13801	2.627056	-0.34771	0.728887	-5.56416	0.7777	0.786055
Dendritic.cells	SATB1	-0.04457	7.143818	-0.34761	0.728956	-7.00145	0.713407	0.693213
Dendritic.cells	PRR5L	-0.08869	2.756439	-0.34752	0.729028	-6.15544	0.775758	0.783273
Dendritic.cells	ATRN	0.05433	5.965865	0.347301	0.729191	-6.43627	0.729488	0.716426
Dendritic.cells	NCAPG2	0.095877	5.068495	0.347241	0.729235	-6.00826	0.74206	0.734581
Dendritic.cells	COMMD9	0.111371	2.646144	0.347106	0.729337	-5.61147	0.777413	0.785842
Dendritic.cells	CYP3A25	0.182377	1.898523	0.347008	0.72941	-5.45566	0.78875	0.802354
Dendritic.cells	INO80DOS	-0.10609	4.442013	-0.34699	0.729423	-5.94821	0.751003	0.747538
Dendritic.cells	KIT	-0.12457	3.453389	-0.3468	0.729569	-5.8833	0.765399	0.768393
Dendritic.cells	RAB11FIP3	0.09801	2.230172	0.346741	0.72961	-5.71296	0.783696	0.794996
Dendritic.cells	AIFM1	0.063489	4.370662	0.346727	0.72962	-6.02012	0.75203	0.74903
Dendritic.cells	UNC119	0.05624	5.47027	0.346446	0.729831	-6.23676	0.736396	0.726487
Dendritic.cells	HPS1	-0.07678	3.445228	-0.34638	0.729884	-5.96605	0.765519	0.768629
Dendritic.cells	LCOR	-0.03236	7.538405	-0.3463	0.729939	-6.891	0.708126	0.685873
Dendritic.cells	CSNK2A2	-0.03438	5.948451	-0.34628	0.729954	-6.51231	0.729729	0.716919
Dendritic.cells	B230208H1	-0.24403	0.861264	-0.34622	0.729997	-5.23573	0.804817	0.82592
Dendritic.cells	FAM83E	0.167796	1.10583	0.345934	0.730214	-5.33775	0.800993	0.820432
Dendritic.cells	ALMS1	-0.11373	3.824261	-0.3458	0.730317	-5.64853	0.759958	0.760708
Dendritic.cells	G6PC	-0.20101	1.911843	-0.34568	0.730404	-5.47469	0.788546	0.802294
Dendritic.cells	GM27216	0.218596	0.912751	0.34562	0.730449	-5.27527	0.80401	0.824897
Dendritic.cells	LTN1	0.038609	5.03701	0.345587	0.730474	-6.46426	0.742506	0.735488
Dendritic.cells	ELP2	-0.06254	4.144774	-0.34556	0.730493	-5.99393	0.755295	0.753985
Dendritic.cells	GM49864	-0.18433	0.452692	-0.34555	0.730505	-5.30417	0.811254	0.835505
Dendritic.cells	SGO2A	0.197692	3.063462	0.345485	0.730551	-5.46219	0.771173	0.777018
Dendritic.cells	9-Sep	-0.04354	5.640387	-0.34548	0.730555	-6.59908	0.734015	0.723236
Dendritic.cells	IGBP1	-0.06262	4.749526	-0.34548	0.730557	-6.08404	0.746596	0.741398
Dendritic.cells	LSM10	0.107008	3.07773	0.345409	0.730608	-5.63836	0.770961	0.77671
Dendritic.cells	POLR2D	-0.0549	5.184758	-0.34531	0.730679	-6.26562	0.740417	0.732492
Dendritic.cells	SCIN	0.252962	0.090871	0.345162	0.730792	-5.24204	0.817057	0.843984
Dendritic.cells	KLRB1A	-0.22939	-0.08222	-0.34493	0.73097	-5.23865	0.819845	0.848111
Dendritic.cells	MYBBP1A	-0.0749	4.85707	-0.34483	0.731042	-6.12879	0.745126	0.739283
Dendritic.cells	ALOX15	0.391502	-0.82153	0.344801	0.731063	-5.23004	0.831798	0.86569

Dendritic.cells	USP21	0.103007	3.140041	0.344769	0.731087	-5.67473	0.770101	0.775484
Dendritic.cells	MSS51	-0.11219	2.436698	-0.3441	0.73159	-5.6416	0.78102	0.790942
Dendritic.cells	NFATC1	-0.0352	5.77824	-0.34396	0.731696	-6.72728	0.732516	0.720713
Dendritic.cells	TMA16	-0.10266	3.964843	-0.34394	0.731705	-5.85101	0.758346	0.75803
Dendritic.cells	RNF10	0.041588	6.51711	0.343918	0.731724	-6.57868	0.722321	0.706039
Dendritic.cells	ISYNA1	0.071009	4.852057	0.343601	0.731962	-5.99056	0.745636	0.739616
Dendritic.cells	MRPL19	-0.08002	3.923908	-0.34356	0.731995	-5.83329	0.759015	0.758986
Dendritic.cells	CCDC62	0.132552	2.858429	0.343487	0.732047	-5.62958	0.774753	0.781839
Dendritic.cells	STARD7	-0.05387	5.358518	-0.34345	0.732073	-6.22881	0.738463	0.729285
Dendritic.cells	MOB1A	0.037418	5.970142	0.343181	0.732277	-6.5409	0.730054	0.717002
Dendritic.cells	CYB561D1	0.157827	1.785161	0.34293	0.732465	-5.40086	0.791294	0.805621
Dendritic.cells	GM26674	-0.17846	-1.15959	-0.34268	0.732656	-5.22796	0.838151	0.874305
Dendritic.cells	PRG4	0.514455	1.084056	0.342628	0.732691	-5.2387	0.802186	0.821531
Dendritic.cells	TRIM68	0.140833	0.946883	0.342608	0.732706	-5.42173	0.804331	0.824671
Dendritic.cells	MS4A6D	0.23788	2.866447	0.34221	0.733005	-5.40801	0.775089	0.781851
Dendritic.cells	CCDC163	-0.15314	2.446867	-0.34217	0.733032	-5.49809	0.781401	0.79104
Dendritic.cells	TUBB5	-0.05698	9.849372	-0.3421	0.733084	-7.12625	0.679091	0.643774
Dendritic.cells	CHFR	-0.03761	5.641975	-0.34204	0.73313	-6.49314	0.734921	0.723764
Dendritic.cells	GYS1	0.140405	3.38284	0.341702	0.733386	-5.58921	0.767603	0.77088
Dendritic.cells	COQ8A	-0.12953	2.303584	-0.34143	0.733587	-5.48668	0.783911	0.794469
Dendritic.cells	ITGA2	-0.17426	0.926385	-0.34118	0.733777	-5.35765	0.805293	0.82555
Dendritic.cells	CAPN10	-0.11987	2.598593	-0.34106	0.733867	-5.52718	0.779599	0.788053
Dendritic.cells	SLC25A14	0.108434	2.078186	0.340954	0.733947	-5.53186	0.787504	0.799541
Dendritic.cells	SESN1	0.048007	5.939971	0.340848	0.734027	-6.44885	0.731246	0.718068
Dendritic.cells	NECAB2	-0.21527	0.442881	-0.3407	0.734135	-5.24968	0.812974	0.836747
Dendritic.cells	CNEP1R1	0.05558	4.587392	0.340488	0.734297	-6.11954	0.750359	0.745656
Dendritic.cells	ASPDH	0.20382	1.294554	0.340424	0.734345	-5.31478	0.799596	0.817184
Dendritic.cells	RUNDC3B	-0.06768	3.848947	-0.34039	0.734374	-6.37612	0.761062	0.761142
Dendritic.cells	GM41496	-0.16442	0.985043	-0.34037	0.734382	-5.45715	0.804427	0.824242
Dendritic.cells	AKR1C14	-0.17793	0.911524	-0.34011	0.734578	-5.29829	0.805626	0.825984
Dendritic.cells	F11	0.185181	0.954836	0.33996	0.734693	-5.33207	0.804947	0.825011
Dendritic.cells	MOSPD2	-0.07835	4.445481	-0.33995	0.734704	-5.95291	0.752444	0.748678
Dendritic.cells	NCAPH2	0.049847	5.344606	0.339663	0.734916	-6.25704	0.739627	0.730178
Dendritic.cells	9030404E1	-0.21439	-0.16276	-0.33964	0.734935	-5.23223	0.822698	0.851021
Dendritic.cells	TGFBR2	-0.02729	7.292661	-0.33959	0.734969	-6.98886	0.712821	0.691643
Dendritic.cells	PROS1	-0.09227	2.741429	-0.33955	0.735001	-5.89167	0.777524	0.78506
Dendritic.cells	SMARCA5	0.02941	7.448521	0.339451	0.735075	-6.80117	0.710733	0.688678
Dendritic.cells	RAB3D	-0.08417	3.11921	-0.33933	0.735169	-5.93228	0.771874	0.776901
Dendritic.cells	RAB11FIP4	-0.18016	-0.78929	-0.33931	0.735179	-5.23189	0.832852	0.866009
Dendritic.cells	IFFO1	0.109346	2.880878	0.33912	0.735324	-5.79686	0.775432	0.78211
Dendritic.cells	HIST1H1E	-0.225	4.203103	-0.33906	0.73537	-5.70011	0.755948	0.753855
Dendritic.cells	MMP13	-0.27165	-1.01302	-0.33904	0.73538	-5.23085	0.836513	0.871441
Dendritic.cells	FAIM	0.075782	3.865848	0.338885	0.7355	-5.89209	0.760858	0.760965
Dendritic.cells	ZC3H3	-0.07425	3.468484	-0.33885	0.735526	-5.82259	0.766696	0.769429
Dendritic.cells	TIMM23	0.028566	6.942518	0.338784	0.735576	-6.77688	0.717544	0.698538
Dendritic.cells	9530062KC	-0.18548	0.793704	-0.33873	0.735618	-5.29296	0.807477	0.828911
Dendritic.cells	GM15860	0.178639	0.373729	0.338649	0.735677	-5.26175	0.814119	0.838635

Dendritic.cells	GM16272	0.186924	0.363126	0.338447	0.735829	-5.25793	0.814378	0.838937
Dendritic.cells	GM43727	0.185167	-0.59426	0.338097	0.736091	-5.23521	0.82994	0.861581
Dendritic.cells	MCOLN2	0.085564	3.161941	0.338013	0.736155	-5.80889	0.771484	0.77624
Dendritic.cells	GLIS3	0.210333	3.068689	0.337966	0.73619	-5.52498	0.772873	0.778284
Dendritic.cells	TAOK2	0.064582	4.127104	0.337843	0.736282	-6.02031	0.757314	0.755721
Dendritic.cells	EXO1	0.179439	2.019131	0.337754	0.73635	-5.37163	0.788743	0.801384
Dendritic.cells	CD53	0.028441	7.920856	0.337503	0.736538	-6.94716	0.704745	0.680022
Dendritic.cells	GPATCH1	0.085168	2.73622	0.337503	0.736538	-5.74422	0.777924	0.785594
Dendritic.cells	H2-Q4	-0.10556	4.933329	-0.33725	0.736731	-6.03708	0.745888	0.73904
Dendritic.cells	SLC13A3	-0.22798	1.11728	-0.33698	0.736928	-5.28458	0.80301	0.821816
Dendritic.cells	CC2D2A	-0.20912	0.81427	-0.3367	0.737141	-5.25705	0.807839	0.828804
Dendritic.cells	CLEC9A	0.082571	2.519945	0.336612	0.737207	-6.21096	0.781524	0.79043
Dendritic.cells	NLRC4	-0.13812	3.885651	-0.33661	0.737207	-5.49561	0.761215	0.760958
Dendritic.cells	BIRC2	-0.05222	5.673482	-0.33639	0.737375	-6.30869	0.735733	0.72408
Dendritic.cells	BHMT	-0.13578	5.13251	-0.33617	0.737542	-6.04642	0.743387	0.735092
Dendritic.cells	MYH13	-0.05504	-1.61994	-0.33616	0.737546	-6.11029	0.847286	0.886811
Dendritic.cells	ATP5C1	0.024229	8.358211	0.335925	0.737724	-7.05056	0.699509	0.671996
Dendritic.cells	KRT81	-0.1765	-1.53173	-0.33585	0.737777	-5.23204	0.846046	0.884715
Dendritic.cells	HERC2	-0.04136	5.98752	-0.33559	0.737972	-6.53373	0.7316	0.717876
Dendritic.cells	GM49173	-0.24222	-0.87955	-0.33513	0.738319	-5.23264	0.835444	0.869065
Dendritic.cells	RNFT1	0.053308	4.495375	0.335113	0.738334	-6.13489	0.752732	0.748471
Dendritic.cells	KIF13B	0.042709	6.510852	0.334993	0.738424	-6.56289	0.724391	0.70763
Dendritic.cells	NEO1	-0.21035	0.660859	-0.33499	0.738424	-5.28674	0.810655	0.832701
Dendritic.cells	ASH1L	-0.02958	7.367632	-0.33493	0.73847	-6.89073	0.712769	0.690959
Dendritic.cells	GM47469	-0.16429	0.520329	-0.33493	0.73847	-5.3829	0.81288	0.835966
Dendritic.cells	ARHGAP35	-0.05002	5.237019	-0.33485	0.738534	-6.34881	0.742138	0.733215
Dendritic.cells	ITGB2L	-0.17011	-0.61932	-0.33469	0.73865	-5.2355	0.831194	0.862903
Dendritic.cells	ANXA11	0.049922	5.969579	0.334684	0.738656	-6.34283	0.731863	0.718446
Dendritic.cells	TOLLIP	-0.07715	3.832789	-0.33465	0.738682	-5.91639	0.762361	0.762479
Dendritic.cells	IL10	-0.20044	2.999738	-0.33453	0.738775	-5.50523	0.774715	0.780361
Dendritic.cells	LCT	-0.20033	0.170332	-0.33441	0.738863	-5.23122	0.818499	0.844251
Dendritic.cells	VDR	0.103522	0.938817	0.334158	0.739052	-5.84822	0.806346	0.82647
Dendritic.cells	XYLT1	0.058598	7.355246	0.334108	0.739089	-6.63963	0.712997	0.691345
Dendritic.cells	PARP2	0.053706	4.841287	0.333985	0.739182	-6.08921	0.747832	0.741489
Dendritic.cells	DGKZ	-0.05688	6.2774	-0.33392	0.739232	-6.39209	0.727665	0.712428
Dendritic.cells	ABHD11	-0.09345	3.541149	-0.33391	0.739234	-5.74675	0.766716	0.768814
Dendritic.cells	ACTN2	-0.20414	-0.1007	-0.33378	0.739336	-5.23576	0.822906	0.850749
Dendritic.cells	GM14085	-0.18013	-1.35884	-0.33347	0.739566	-5.23305	0.843547	0.881111
Dendritic.cells	GM36756	0.172113	0.926199	0.33345	0.739584	-5.28947	0.806674	0.826987
Dendritic.cells	GABBR1	0.20112	2.766527	0.333385	0.739633	-5.50228	0.778381	0.785775
Dendritic.cells	CSNK1G3	0.033311	6.624241	0.333207	0.739767	-6.67241	0.723081	0.705852
Dendritic.cells	RBBP5	-0.08927	3.457865	-0.33272	0.740133	-5.73197	0.768234	0.770963
Dendritic.cells	FMNL1	0.033752	6.443857	0.332677	0.740166	-6.75331	0.725645	0.70947
Dendritic.cells	GPR27	0.120985	-1.60473	0.332644	0.74019	-5.23147	0.847717	0.887352
Dendritic.cells	CCDC152	0.130529	3.284464	0.332512	0.74029	-5.68209	0.770804	0.774729
Dendritic.cells	9130401M	-0.06213	4.02313	-0.3325	0.740302	-5.98439	0.75993	0.758966
Dendritic.cells	AKAP1	-0.12998	1.799595	-0.33246	0.740326	-5.46396	0.793261	0.807395

Dendritic.cells	VPREB2	-0.20913	-0.41404	-0.33243	0.740348	-5.23222	0.828242	0.85859
Dendritic.cells	DYNC1LI2	-0.05142	4.672528	-0.33234	0.740423	-6.2087	0.750535	0.745399
Dendritic.cells	NADSYN1	-0.14528	1.092531	-0.33211	0.74059	-5.39898	0.804282	0.823505
Dendritic.cells	1110032A0	0.059795	3.592833	0.332061	0.740629	-6.04271	0.766283	0.768198
Dendritic.cells	PPT1	-0.05686	6.003515	-0.33197	0.740701	-6.33133	0.73177	0.718338
Dendritic.cells	CDCA2	0.149732	3.79635	0.331718	0.740887	-5.59455	0.76329	0.763945
Dendritic.cells	XPNPEP1	0.059272	4.365592	0.331709	0.740894	-6.04193	0.754996	0.751936
Dendritic.cells	CHAF1B	-0.13112	3.382455	-0.33159	0.740983	-5.53415	0.769393	0.772815
Dendritic.cells	MMP12	0.283079	-0.05819	0.331586	0.740986	-5.23722	0.822541	0.850367
Dendritic.cells	ARMC2	-0.18399	-0.1641	-0.33155	0.741017	-5.23562	0.824247	0.85287
Dendritic.cells	FOXN2	0.035519	6.146142	0.331423	0.741109	-6.54116	0.729798	0.71563
Dendritic.cells	TTC21B	-0.1245	1.614885	-0.33124	0.741248	-5.43841	0.796161	0.811822
Dendritic.cells	PPP1R16B	0.034108	6.504104	0.331139	0.741323	-7.03582	0.724864	0.708551
Dendritic.cells	POMT2	-0.1035	1.813643	-0.33111	0.741348	-5.61944	0.793095	0.807349
Dendritic.cells	DDX23	0.043536	4.71777	0.331075	0.741371	-6.34224	0.749929	0.74468
Dendritic.cells	GM4356	-0.18238	0.868166	-0.33064	0.741701	-5.34344	0.80803	0.828957
Dendritic.cells	4930444A1	0.103453	3.751804	0.330622	0.741712	-5.78771	0.764157	0.76508
Dendritic.cells	ERP44	0.025336	6.074446	0.330494	0.741808	-6.65543	0.731016	0.717202
Dendritic.cells	HAGHL	0.105917	2.911144	0.3302	0.74203	-5.7137	0.776822	0.783216
Dendritic.cells	AIRN	0.12213	5.663175	0.329986	0.742191	-5.9601	0.736993	0.725473
Dendritic.cells	CLMN	0.185372	0.334512	0.329557	0.742514	-5.31869	0.816799	0.841499
Dendritic.cells	GM9750	0.210633	0.476616	0.329487	0.742567	-5.24174	0.814537	0.83821
Dendritic.cells	PARP16	-0.18279	1.117003	-0.32948	0.742571	-5.28836	0.804435	0.823433
Dendritic.cells	MS4A6B	0.196385	4.588359	0.329385	0.742644	-5.61792	0.752283	0.747645
Dendritic.cells	POLR2E	-0.07185	4.876402	-0.3292	0.742784	-6.10614	0.74815	0.741698
Dendritic.cells	VCPKMT	-0.11716	2.877826	-0.32918	0.742798	-5.592	0.777436	0.784122
Dendritic.cells	HYOU1	-0.04398	4.380576	-0.32916	0.742811	-6.3659	0.755283	0.752009
Dendritic.cells	HDGFL2	-0.04548	4.979057	-0.32904	0.742901	-6.29635	0.746684	0.739625
Dendritic.cells	PSMD6	0.055547	5.199485	0.329039	0.742904	-6.21119	0.743549	0.7351
Dendritic.cells	RELT	0.090428	3.728351	0.328759	0.743115	-5.78844	0.764798	0.765863
Dendritic.cells	CTU1	-0.16454	0.850388	-0.32867	0.743186	-5.36894	0.808622	0.829688
Dendritic.cells	BTBD11	-0.18399	4.309098	-0.3286	0.743235	-5.58006	0.756318	0.753614
Dendritic.cells	PBK	0.147823	3.69023	0.328596	0.743238	-5.63079	0.765359	0.766707
Dendritic.cells	FANCE	-0.08751	2.916052	-0.32858	0.743252	-5.76508	0.776862	0.783403
Dendritic.cells	PREX1	0.052957	6.43388	0.328574	0.743255	-6.44752	0.726307	0.710328
Dendritic.cells	FBXO45	0.08568	3.654199	0.328299	0.743461	-5.7285	0.765953	0.767502
Dendritic.cells	OSM	0.232057	2.305551	0.328106	0.743607	-5.35797	0.786152	0.796899
Dendritic.cells	ASB8	-0.07016	3.607063	-0.32806	0.743645	-5.91717	0.766648	0.768561
Dendritic.cells	WDSUB1	0.097393	2.44185	0.328024	0.743669	-5.61687	0.784081	0.793884
Dendritic.cells	CHMP1B	0.054795	4.698125	0.327943	0.74373	-6.13233	0.750767	0.745571
Dendritic.cells	LMO4	0.053819	7.245458	0.327624	0.743971	-6.7373	0.715317	0.694585
Dendritic.cells	TLR2	-0.13988	3.875493	-0.3276	0.743991	-5.62823	0.762702	0.762903
Dendritic.cells	GM16091	-0.13379	2.306149	-0.32757	0.744015	-5.47644	0.786143	0.796958
Dendritic.cells	B230217O1	0.129728	1.757632	0.327312	0.744206	-5.59088	0.794547	0.809279
Dendritic.cells	PLA2G12B	0.167576	1.0508	0.327162	0.744319	-5.39384	0.80554	0.825374
Dendritic.cells	TRDV2-2	0.128799	-1.43025	0.326994	0.744445	-5.23457	0.845592	0.884213
Dendritic.cells	HNRNPH1	-0.03832	6.424984	-0.32688	0.74453	-6.58423	0.72649	0.710758

Dendritic.cells	KLRI2	0.18612	0.447224	0.326569	0.744766	-5.43934	0.815072	0.83935
Dendritic.cells	TBCA	0.030291	7.625569	0.326562	0.744771	-6.90307	0.710218	0.687396
Dendritic.cells	GFI1	0.160408	1.55053	0.326512	0.744809	-5.35127	0.797749	0.814014
Dendritic.cells	GCFC2	0.102599	2.248325	0.326445	0.74486	-5.55535	0.787024	0.798374
Dendritic.cells	CDK4	-0.04662	5.844705	-0.3264	0.744893	-6.42366	0.734532	0.722334
Dendritic.cells	CCDC15	0.120108	2.810819	0.326383	0.744906	-5.58314	0.778508	0.785981
Dendritic.cells	RSPH3A	0.107169	2.558451	0.32634	0.744939	-5.64886	0.782314	0.791518
Dendritic.cells	FZR1	-0.07016	4.900739	-0.32634	0.744942	-6.06364	0.747865	0.741569
Dendritic.cells	RRP15	0.072836	3.823389	0.326316	0.744956	-5.88178	0.763466	0.764145
Dendritic.cells	DECR1	-0.116	3.696206	-0.3263	0.744968	-5.73719	0.765335	0.766854
Dendritic.cells	CAD	0.158563	1.795668	0.326287	0.744978	-5.38981	0.793961	0.808486
Dendritic.cells	PRPF40A	-0.02388	7.709067	-0.32618	0.745056	-6.90766	0.709105	0.685828
Dendritic.cells	DCPS	-0.05656	5.004121	-0.32613	0.745099	-6.15527	0.746389	0.739474
Dendritic.cells	PRXL2A	0.155884	4.184431	0.325924	0.745252	-5.69151	0.758192	0.756571
Dendritic.cells	TRIM62	0.104705	1.410517	0.325911	0.745262	-5.56434	0.799922	0.817259
Dendritic.cells	MPHOSPH8	0.048414	4.79616	0.3259	0.74527	-6.16457	0.749361	0.743796
Dendritic.cells	GOLGA7	0.035213	5.94	0.325757	0.745378	-6.49121	0.733238	0.720518
Dendritic.cells	TMEM176B	-0.07655	5.475623	-0.32567	0.745445	-6.23243	0.739742	0.729902
Dendritic.cells	MFAP2	-0.18801	0.586159	-0.32541	0.745638	-5.34702	0.813038	0.836316
Dendritic.cells	ADAM9	0.050196	5.178783	0.325192	0.745804	-6.34014	0.744159	0.736094
Dendritic.cells	CD160	0.206072	1.916487	0.32492	0.746009	-5.43277	0.792431	0.806074
Dendritic.cells	SUCLG2	0.064364	4.835404	0.324908	0.746019	-6.12272	0.74911	0.743194
Dendritic.cells	HSPA4L	0.090151	4.348791	0.324842	0.746068	-5.81035	0.75612	0.75333
Dendritic.cells	PHB2	-0.03305	6.570723	-0.3247	0.746178	-6.66871	0.724828	0.708168
Dendritic.cells	ULK3	0.115235	2.219052	0.32459	0.746258	-5.55494	0.78785	0.79935
Dendritic.cells	POU6F1	-0.12202	2.610644	-0.32446	0.746359	-5.54033	0.781934	0.79073
Dendritic.cells	SLC4A7	-0.04418	5.767425	-0.32419	0.746562	-6.55794	0.736128	0.724291
Dendritic.cells	DEPDC1B	-0.17444	2.954227	-0.32383	0.746834	-5.48303	0.777111	0.783352
Dendritic.cells	CYP4F18	-0.11724	3.551567	-0.32333	0.74721	-5.62421	0.76832	0.770489
Dendritic.cells	4632427E1	-0.05948	4.404143	-0.32316	0.747333	-6.10117	0.755844	0.752428
Dendritic.cells	PRKAG1	-0.03821	5.515312	-0.32314	0.747353	-6.33268	0.73997	0.729517
Dendritic.cells	ZSCAN26	-0.04588	4.652438	-0.32305	0.747424	-6.37707	0.752259	0.74727
Dendritic.cells	SWAP70	-0.08354	5.61218	-0.32296	0.747491	-6.09313	0.738607	0.727568
Dendritic.cells	COL23A1	0.158832	1.376373	0.322907	0.747528	-5.4521	0.801343	0.818574
Dendritic.cells	SDK1	0.102426	3.195716	0.322831	0.747586	-6.0213	0.773605	0.77819
Dendritic.cells	GM29093	0.091189	1.269702	0.322749	0.747647	-5.62276	0.803007	0.821004
Dendritic.cells	B230354K1	0.145623	1.756956	0.322731	0.747661	-5.46478	0.79544	0.809961
Dendritic.cells	ZFP729A	-0.09568	3.051831	-0.32273	0.747663	-5.71412	0.775755	0.781312
Dendritic.cells	PLEKHA4	0.128421	2.406918	0.322673	0.747705	-5.59932	0.785483	0.79548
Dendritic.cells	FIP1L1	-0.02492	6.639254	-0.32244	0.747883	-6.68233	0.724451	0.707095
Dendritic.cells	ARID4B	-0.02594	8.259309	-0.32235	0.747947	-7.04949	0.7027	0.675933
Dendritic.cells	CCND1	-0.03696	4.140959	-0.32217	0.748085	-6.90937	0.75977	0.758109
Dendritic.cells	IFI213	0.225023	3.327221	0.322115	0.748126	-5.59917	0.771751	0.775474
Dendritic.cells	HAVCR1	0.054584	-0.3843	0.322077	0.748155	-6.35327	0.829459	0.859731
Dendritic.cells	MPG	-0.06098	3.63806	-0.32188	0.748305	-5.97969	0.767229	0.768831
Dendritic.cells	LANCL2	0.101112	2.807486	0.321525	0.748571	-5.64974	0.779704	0.78685
Dendritic.cells	ZADH2	0.084619	3.381757	0.321519	0.748576	-5.69672	0.771115	0.774378

Dendritic.cells	NFRKB	0.045166	4.478568	0.321261	0.748771	-6.18593	0.755039	0.751147
Dendritic.cells	TEF	0.082183	3.230001	0.321235	0.748791	-5.77918	0.773373	0.777708
Dendritic.cells	GNB1L	0.095945	3.308245	0.321194	0.748821	-5.68231	0.772207	0.77602
Dendritic.cells	5430401H(	-0.21617	-0.47304	-0.32112	0.748874	-5.25955	0.831089	0.861997
Dendritic.cells	FXYD2	0.206998	-0.41192	0.321112	0.748883	-5.23876	0.830095	0.860537
Dendritic.cells	TMEM101	0.114348	2.322989	0.320893	0.749049	-5.53427	0.787145	0.797607
Dendritic.cells	LRP1B	-0.16545	1.219155	-0.32024	0.749545	-5.27539	0.804608	0.822637
Dendritic.cells	BID	0.078715	3.803317	0.320091	0.749655	-5.8569	0.765381	0.765646
Dendritic.cells	RCOR2	0.190837	0.194015	0.32	0.749723	-5.27541	0.820849	0.846459
Dendritic.cells	PTBP2	0.033161	6.156832	0.319828	0.749853	-6.72103	0.731741	0.717122
Dendritic.cells	NUMA1	0.043917	5.705606	0.319826	0.749855	-6.33173	0.738039	0.726188
Dendritic.cells	GRB14	-0.1674	1.939569	-0.31973	0.749927	-5.46996	0.793427	0.80646
Dendritic.cells	DENND1A	0.033281	7.497877	0.319543	0.750069	-6.76294	0.713436	0.690891
Dendritic.cells	ASB2	0.205234	2.906427	0.319508	0.750095	-5.35126	0.77872	0.785093
Dendritic.cells	STARD3	-0.05305	4.116736	-0.31935	0.750211	-6.24451	0.760788	0.759122
Dendritic.cells	FPR1	0.25113	2.992339	0.319334	0.750226	-5.49751	0.77743	0.78325
Dendritic.cells	PDE6H	0.124709	2.093346	0.319256	0.750286	-5.44737	0.791065	0.803083
Dendritic.cells	FMN2	0.207819	2.359589	0.319238	0.750299	-5.39684	0.786996	0.797159
Dendritic.cells	ZFP831	0.154648	3.063289	0.319174	0.750347	-5.47121	0.776366	0.781707
Dendritic.cells	ABCB6	-0.14714	1.101522	-0.31892	0.750537	-5.27589	0.806488	0.825599
Dendritic.cells	PVT1	0.152099	5.282726	0.318779	0.750646	-5.75526	0.744041	0.734985
Dendritic.cells	IGHM	0.066044	8.158942	0.318745	0.750672	-7.09851	0.704669	0.678439
Dendritic.cells	CABP1	0.198682	-0.13612	0.318734	0.75068	-5.23461	0.8262	0.854526
Dendritic.cells	BYSL	0.069269	3.67185	0.318554	0.750816	-5.7793	0.767353	0.768735
Dendritic.cells	AVEN	0.055789	4.926701	0.318535	0.75083	-6.2785	0.749114	0.742347
Dendritic.cells	DSG2	-0.18574	0.740688	-0.31848	0.750871	-5.28462	0.812177	0.834024
Dendritic.cells	BRD7	0.029754	6.307707	0.318174	0.751103	-6.58772	0.729812	0.714412
Dendritic.cells	ITM2C	-0.0372	5.637577	-0.31806	0.751192	-6.72599	0.739158	0.727895
Dendritic.cells	ZCCHC9	0.037992	5.404138	0.31803	0.751212	-6.39292	0.742451	0.732642
Dendritic.cells	RAD51C	0.134192	2.245463	0.317883	0.751323	-5.39685	0.788954	0.799984
Dendritic.cells	EEF2KMT	0.067113	3.145957	0.317774	0.751406	-5.93278	0.775355	0.780208
Dendritic.cells	PLOD3	0.112563	3.277379	0.317204	0.751837	-5.58823	0.77353	0.777409
Dendritic.cells	GM34455	-0.13521	2.623429	-0.31714	0.751888	-5.58666	0.783356	0.791682
Dendritic.cells	LCN4	-0.16709	-1.05402	-0.31689	0.752077	-5.23683	0.841545	0.876808
Dendritic.cells	SRPK3	0.110991	1.292575	0.316702	0.752216	-5.60557	0.803836	0.821548
Dendritic.cells	GM16158	0.155948	0.586451	0.316656	0.752251	-5.45076	0.814968	0.83783
Dendritic.cells	ZFP628	-0.10693	2.635685	-0.31665	0.752258	-5.65844	0.783171	0.791443
Dendritic.cells	KRT83	-0.19825	-0.26141	-0.31646	0.752402	-5.23905	0.828578	0.857861
Dendritic.cells	ANKLE1	-0.16272	0.64315	-0.31643	0.752425	-5.25102	0.814067	0.836592
Dendritic.cells	ABCA13	0.259278	0.73813	0.316317	0.752507	-5.24941	0.812561	0.83442
Dendritic.cells	ATP5MD	-0.0306	8.035566	-0.31627	0.752544	-6.9126	0.706599	0.681103
Dendritic.cells	OLFR1259	0.208813	-1.34501	0.316256	0.752554	-5.23796	0.846365	0.884083
Dendritic.cells	GM27017	0.115082	3.311249	0.316213	0.752586	-5.60758	0.773025	0.776838
Dendritic.cells	TMEM167E	0.081397	3.755594	0.316081	0.752686	-5.83963	0.766442	0.767322
Dendritic.cells	FGFR1OP	-0.03734	4.601111	-0.31592	0.752807	-6.58246	0.754111	0.749543
Dendritic.cells	IGSF8	0.03839	4.266842	0.315862	0.752852	-6.52193	0.758956	0.756579
Dendritic.cells	UCHL1	-0.18456	0.862756	-0.31569	0.752981	-5.29028	0.81059	0.831759

Dendritic.cells	SPIN1	-0.03118	5.805516	-0.31563	0.753027	-6.62224	0.736984	0.7249
Dendritic.cells	2010009K1	0.237639	0.112521	0.315628	0.753029	-5.25893	0.822542	0.849267
Dendritic.cells	ISOC2B	0.101867	2.519105	0.315498	0.753127	-5.6906	0.784938	0.794335
Dendritic.cells	MITD1	0.084378	4.433197	0.315414	0.753191	-5.85771	0.75654	0.753133
Dendritic.cells	MFAP1B	0.046575	4.945536	0.315231	0.753329	-6.1877	0.749161	0.74251
Dendritic.cells	TXNDC15	0.042134	4.911319	0.315168	0.753377	-6.44479	0.749651	0.743231
Dendritic.cells	DNASE1L1	-0.08464	3.048154	-0.31513	0.753404	-5.80312	0.776957	0.782789
Dendritic.cells	GM6787	0.171189	0.188055	0.314973	0.753524	-5.26359	0.82133	0.847616
Dendritic.cells	CDH5	-0.06952	4.480665	-0.31497	0.753528	-6.43937	0.755852	0.752249
Dendritic.cells	DYRK1B	0.104352	1.826196	0.314885	0.753591	-5.53333	0.795546	0.809951
Dendritic.cells	PPM1F	-0.10766	2.206568	-0.31486	0.753607	-5.57898	0.789701	0.80143
Dendritic.cells	TGTP1	0.250916	-0.20272	0.314846	0.75362	-5.237	0.827627	0.856904
Dendritic.cells	PLD4	-0.03159	6.186695	-0.31484	0.753625	-7.17966	0.731669	0.717394
Dendritic.cells	COIL	-0.05091	3.741294	-0.31482	0.753637	-6.19221	0.766653	0.767932
Dendritic.cells	ACBD3	0.034615	5.707973	0.314621	0.75379	-6.46276	0.738366	0.72705
Dendritic.cells	KCNQ5	0.074764	7.024096	0.314467	0.753907	-6.31399	0.720184	0.700923
Dendritic.cells	INO80C	0.090402	3.401246	0.314316	0.754022	-5.64677	0.771701	0.7753
Dendritic.cells	P2RY10B	-0.08878	3.709802	-0.31431	0.754025	-5.78139	0.767132	0.768671
Dendritic.cells	CAVIN2	0.079251	2.454343	0.31425	0.754071	-5.97121	0.785938	0.796009
Dendritic.cells	TMEM259	0.055679	4.879001	0.314195	0.754113	-6.26796	0.750129	0.744071
Dendritic.cells	AEBP2	0.046946	5.787827	0.314084	0.754197	-6.37175	0.737246	0.72548
Dendritic.cells	SNX33	0.138746	0.759185	0.313968	0.754285	-5.38475	0.812243	0.834472
Dendritic.cells	LARP1	-0.02542	6.798018	-0.31386	0.754364	-6.92918	0.723265	0.705427
Dendritic.cells	P4HA3	-0.18287	0.308298	-0.31364	0.754535	-5.23641	0.819419	0.845046
Dendritic.cells	GM49625	-0.06201	2.147374	-0.31356	0.754593	-6.10196	0.790623	0.802948
Dendritic.cells	YAE1D1	0.092404	3.240113	0.313534	0.754614	-5.8148	0.774101	0.778909
Dendritic.cells	NDUFA7	0.024469	7.909207	0.313507	0.754634	-6.93408	0.708289	0.683976
Dendritic.cells	DDX47	-0.0347	5.550552	-0.31339	0.754722	-6.41362	0.740581	0.730393
Dendritic.cells	ASAP2	-0.11888	3.068411	-0.31334	0.754758	-5.62351	0.776668	0.782639
Dendritic.cells	RAPGEF6	0.029364	8.049527	0.313326	0.754771	-6.92354	0.706428	0.68131
Dendritic.cells	RLF	-0.03175	6.699162	-0.31316	0.754895	-6.75319	0.72464	0.707457
Dendritic.cells	ZFP354B	-0.13999	-0.81695	-0.31311	0.754934	-5.23524	0.837685	0.871904
Dendritic.cells	ZFP143	0.070687	3.815014	0.312867	0.755118	-5.81754	0.765722	0.766657
Dendritic.cells	GM50386	-0.16838	0.179027	-0.31253	0.755373	-5.23944	0.821666	0.848308
Dendritic.cells	B3GALNT1	0.116255	0.351359	0.312513	0.755387	-5.72612	0.818906	0.844259
Dendritic.cells	PMM2	0.041358	4.561806	0.312504	0.755394	-6.23279	0.754855	0.750976
Dendritic.cells	DDHD2	0.044149	4.784447	0.312213	0.755614	-6.39048	0.751647	0.746365
Dendritic.cells	ADCY6	0.179325	0.433361	0.312133	0.755674	-5.25439	0.817596	0.842372
Dendritic.cells	AP5S1	0.119798	2.440536	0.312098	0.755701	-5.49148	0.786316	0.796671
Dendritic.cells	PLXNB3	0.176986	0.164762	0.312037	0.755747	-5.23867	0.821895	0.848678
Dendritic.cells	PKN1	0.02365	6.766934	0.312	0.755775	-6.88429	0.723845	0.70629
Dendritic.cells	SNU13	-0.03016	6.96176	-0.31187	0.755874	-6.73756	0.721186	0.702513
Dendritic.cells	YBX1	0.031269	8.398771	0.311644	0.756044	-6.95989	0.701974	0.67499
Dendritic.cells	CHAF1A	0.114714	4.186029	0.311577	0.756096	-5.74929	0.76031	0.758975
Dendritic.cells	GTF2IRD2	0.049942	3.944401	0.311513	0.756144	-6.24223	0.763845	0.764097
Dendritic.cells	MAP3K7	-0.03776	5.219048	-0.31135	0.756269	-6.36526	0.745434	0.73746
Dendritic.cells	APON	0.187255	1.166034	0.311323	0.756288	-5.35884	0.806006	0.825482

Dendritic.cells	S1PR5	-0.18132	-0.65106	-0.3112	0.756382	-5.24148	0.835118	0.868242
Dendritic.cells	BICD1	-0.13014	0.681795	-0.31104	0.756499	-5.52088	0.813644	0.836738
Dendritic.cells	BC049352	0.171707	1.083027	0.311034	0.756507	-5.33124	0.807309	0.827464
Dendritic.cells	PHF23	0.05707	5.240241	0.310993	0.756538	-6.23281	0.745133	0.737097
Dendritic.cells	THTPA	0.118725	1.360701	0.310993	0.756538	-5.46125	0.80296	0.82111
Dendritic.cells	GM17491	0.095736	1.415352	0.310965	0.756559	-5.51731	0.802107	0.819877
Dendritic.cells	TRAK1	-0.02709	7.291138	-0.31086	0.756641	-6.88069	0.716721	0.696206
Dendritic.cells	CLEC14A	0.090985	2.196348	0.310716	0.756748	-5.94155	0.790042	0.80229
Dendritic.cells	ZMYM1	0.084655	3.329996	0.310699	0.756761	-5.7267	0.772927	0.777384
Dendritic.cells	PNP2	-0.19948	0.616927	-0.31056	0.75687	-5.2453	0.814674	0.83831
Dendritic.cells	PADI2	-0.05326	2.631853	-0.31051	0.756901	-6.41975	0.783412	0.792656
Dendritic.cells	CGNL1	0.119226	1.622928	0.310313	0.757053	-5.72461	0.798878	0.815218
Dendritic.cells	GLRA1	-0.22849	1.25067	-0.31025	0.7571	-5.26216	0.80468	0.823696
Dendritic.cells	NSRP1	0.043916	5.229533	0.31021	0.757132	-6.30122	0.745285	0.737381
Dendritic.cells	ACOT9	-0.05325	4.620859	-0.31009	0.757224	-6.13473	0.754002	0.75
Dendritic.cells	CTSS	0.048306	7.501211	0.309858	0.757398	-7.11221	0.713892	0.692239
Dendritic.cells	IGLC2	-0.20734	3.740399	-0.30982	0.757423	-5.99178	0.766845	0.768658
Dendritic.cells	GM15879	-0.17318	0.515986	-0.30973	0.757495	-5.284	0.816279	0.840766
Dendritic.cells	GM45716	-0.10695	3.20355	-0.30962	0.757576	-5.57221	0.774813	0.780255
Dendritic.cells	SHROOM3	0.201641	0.981918	0.309584	0.757606	-5.3135	0.8089	0.829972
Dendritic.cells	DNAJC13	0.024109	6.16837	0.309357	0.757778	-7.02372	0.732094	0.718489
Dendritic.cells	NEU1	-0.08644	4.426111	-0.30934	0.75779	-5.71867	0.756819	0.754198
Dendritic.cells	OPTN	-0.09662	4.651784	-0.30924	0.757866	-5.79046	0.753556	0.749479
Dendritic.cells	TBCE	0.044165	5.119789	0.309172	0.757918	-6.23476	0.746847	0.739777
Dendritic.cells	GM46652	0.184654	-0.73309	0.309017	0.758035	-5.23839	0.836462	0.870515
Dendritic.cells	SMIM7	-0.04747	4.932589	-0.30897	0.758073	-6.18166	0.749521	0.743664
Dendritic.cells	MRAP	0.199209	1.214744	0.308874	0.758144	-5.31647	0.805242	0.824697
Dendritic.cells	URM1	-0.06098	4.343407	-0.30881	0.758189	-5.93327	0.75802	0.755979
Dendritic.cells	SCAMP1	0.108562	2.970331	0.308492	0.758433	-5.67346	0.778306	0.785522
Dendritic.cells	GM29340	0.168334	-0.6731	0.308438	0.758474	-5.23948	0.835479	0.869198
Dendritic.cells	DDX21	-0.03872	6.399043	-0.30843	0.758484	-6.63896	0.728901	0.71402
Dendritic.cells	RAB1B	0.041257	5.58788	0.308408	0.758498	-6.41868	0.740214	0.730329
Dendritic.cells	UCHL5	-0.03622	5.728606	-0.30828	0.758591	-6.5377	0.738235	0.727513
Dendritic.cells	0610040B1	-0.10962	2.051019	-0.30808	0.758749	-5.56584	0.79227	0.80594
Dendritic.cells	CWC22	-0.0605	3.900011	-0.30802	0.758789	-6.04245	0.764496	0.765531
Dendritic.cells	PTCD1	-0.09347	2.585716	-0.30802	0.758794	-5.61791	0.784111	0.794045
Dendritic.cells	USP9X	-0.02939	7.387216	-0.30788	0.758895	-6.94749	0.715425	0.694724
Dendritic.cells	TPGS2	0.068916	3.805381	0.307732	0.75901	-5.88264	0.765888	0.767613
Dendritic.cells	ANXA5	-0.03924	6.137389	-0.30759	0.759117	-6.671	0.732525	0.7194
Dendritic.cells	BSCL2	0.039487	4.542466	0.3075	0.759186	-6.32563	0.755135	0.75207
Dendritic.cells	ADAM33	0.174827	-0.9471	0.307457	0.759218	-5.23794	0.839978	0.876028
Dendritic.cells	SAMD1	0.069448	4.976188	0.307176	0.759431	-5.93725	0.748897	0.743121
Dendritic.cells	GM29264	0.146973	0.505466	0.306964	0.759593	-5.33328	0.816447	0.841563
Dendritic.cells	F8A	0.08871	2.551871	0.306963	0.759594	-5.73043	0.784624	0.795035
Dendritic.cells	SERF1	-0.08563	3.137334	-0.30688	0.759656	-5.62526	0.775802	0.782191
Dendritic.cells	ATXN3	0.044258	5.054807	0.306861	0.759671	-6.23086	0.747774	0.74154
Dendritic.cells	DLAT	0.059051	4.282042	0.306855	0.759675	-5.92978	0.758912	0.757665

Dendritic.cells	ZBTB46	0.095576	3.021217	0.306678	0.759809	-5.77618	0.777542	0.784722
Dendritic.cells	SBF1	-0.04756	4.474225	-0.30656	0.759898	-6.15173	0.756122	0.753627
Dendritic.cells	PKD1	0.063276	3.003757	0.306366	0.760046	-6.02794	0.777804	0.785114
Dendritic.cells	PPP1R9B	-0.05696	4.375184	-0.30632	0.760084	-6.177	0.757558	0.755729
Dendritic.cells	RFLNB	-0.1719	3.463749	-0.30628	0.760111	-5.38	0.770938	0.775145
Dendritic.cells	CELSR2	-0.19634	-0.09555	-0.30625	0.760135	-5.23882	0.826088	0.855753
Dendritic.cells	MYC	-0.20154	3.533741	-0.30602	0.76031	-5.40695	0.7699	0.77364
Dendritic.cells	DLG3	0.156822	0.571754	0.305984	0.760336	-5.3242	0.815392	0.840052
Dendritic.cells	LRWD1	-0.06873	3.781422	-0.30594	0.760373	-5.78884	0.766241	0.768346
Dendritic.cells	TRAF7	0.048616	4.585124	0.305893	0.760405	-6.11076	0.754518	0.751355
Dendritic.cells	GM9530	0.164256	-0.31965	0.305581	0.760642	-5.34473	0.829717	0.861215
Dendritic.cells	BC028528	-0.07265	4.105365	-0.30558	0.760646	-5.89361	0.761488	0.76154
Dendritic.cells	PTPN1	0.032682	7.078745	0.305541	0.760673	-6.8898	0.719596	0.701041
Dendritic.cells	TNRC6A	0.031387	6.648204	0.30545	0.760741	-6.68979	0.725471	0.709494
Dendritic.cells	BRIP1	0.091086	4.898207	0.305298	0.760856	-5.94401	0.750014	0.744917
Dendritic.cells	TLL2	-0.19138	0.725481	-0.30487	0.761185	-5.37561	0.812951	0.836682
Dendritic.cells	RABAC1	0.03528	5.988734	0.304802	0.761234	-6.5376	0.734595	0.722721
Dendritic.cells	PDE4DIP	-0.04853	3.94998	-0.30478	0.76125	-6.22915	0.763763	0.764927
Dendritic.cells	GM17021	-0.08191	0.125797	-0.30474	0.761279	-5.96522	0.822521	0.850732
Dendritic.cells	HIGD2A	-0.03069	6.065754	-0.3047	0.76131	-6.49254	0.733521	0.721191
Dendritic.cells	KTN1	0.03343	5.826158	0.304547	0.761427	-6.49608	0.736867	0.726072
Dendritic.cells	EAR2	0.195747	3.55304	0.304493	0.761468	-5.41106	0.769614	0.773506
Dendritic.cells	GSTCD	0.080889	3.564196	0.304344	0.761581	-5.83823	0.769449	0.77332
Dendritic.cells	ATG2A	0.060925	5.710857	0.304334	0.761589	-6.18438	0.738484	0.728463
Dendritic.cells	GM17484	-0.17607	1.264186	-0.30417	0.761714	-5.32687	0.804468	0.824435
Dendritic.cells	POLR1E	0.12232	2.067812	0.304159	0.761721	-5.48496	0.792012	0.806223
Dendritic.cells	CST7	-0.17368	2.120473	-0.30414	0.761735	-5.40771	0.791205	0.805043
Dendritic.cells	XRN2	-0.02213	7.516217	-0.30408	0.761779	-6.89477	0.713691	0.692787
Dendritic.cells	BORCS6	0.07946	4.007961	0.303903	0.761916	-5.81136	0.762913	0.763871
Dendritic.cells	CBLL1	0.0448	4.917555	0.303896	0.761921	-6.23225	0.749737	0.744773
Dendritic.cells	TMPO	0.052939	7.067829	0.303849	0.761957	-6.53619	0.719744	0.701509
Dendritic.cells	STAR	-0.14221	1.465058	-0.30383	0.761968	-5.48776	0.801332	0.819874
Dendritic.cells	SNAP29	0.035039	5.5062	0.303671	0.762092	-6.47772	0.741366	0.732734
Dendritic.cells	GM31522	0.13459	-1.00368	0.303669	0.762094	-5.23999	0.840911	0.87809
Dendritic.cells	XNDC1	0.073923	3.594505	0.303526	0.762203	-5.85499	0.769	0.772805
Dendritic.cells	MBP	0.04659	5.172136	0.303519	0.762207	-6.45225	0.746101	0.739602
Dendritic.cells	DDX11	-0.10885	2.934033	-0.30349	0.762232	-5.60593	0.778852	0.787139
Dendritic.cells	BCL6	-0.07547	5.992679	-0.30348	0.762238	-6.30664	0.73454	0.722898
Dendritic.cells	8030456M	-0.13478	1.090368	-0.30345	0.762258	-5.42607	0.807193	0.82854
Dendritic.cells	JRKL	-0.12766	1.537523	-0.30344	0.762267	-5.50189	0.800205	0.818308
Dendritic.cells	KRT10	-0.10312	2.100333	-0.30343	0.762279	-5.5856	0.791513	0.805605
Dendritic.cells	IGKV9-124	0.149404	-1.01492	0.303132	0.762501	-5.23664	0.841265	0.878426
Dendritic.cells	IL12B	-0.31627	-0.29714	-0.30294	0.762647	-5.24523	0.8296	0.861132
Dendritic.cells	IQCK	-0.15875	0.158994	-0.3026	0.762907	-5.32408	0.822313	0.8504
Dendritic.cells	FDXACB1	0.101043	2.215747	0.302536	0.762954	-5.49622	0.790058	0.803172
Dendritic.cells	2410006H1	0.043128	7.535004	0.302504	0.762978	-6.96292	0.713721	0.692645
Dendritic.cells	STX6	-0.03824	5.2207	-0.30246	0.76301	-6.49399	0.745706	0.738745

Dendritic.cells	TESC	0.171855	1.561476	0.30226	0.763164	-5.39226	0.800149	0.817977
Dendritic.cells	EDRF1	0.051352	4.217889	0.302235	0.763183	-6.22264	0.760147	0.759711
Dendritic.cells	LRIG1	0.183988	0.178544	0.302199	0.76321	-5.31071	0.821999	0.85001
Dendritic.cells	NEB	0.130023	1.258562	0.301897	0.763439	-5.46669	0.805042	0.824903
Dendritic.cells	OSBPL5	0.170533	0.3884	0.301637	0.763637	-5.30359	0.818941	0.845172
Dendritic.cells	SART3	-0.04017	5.374984	-0.30155	0.763703	-6.35697	0.743791	0.735716
Dendritic.cells	HILPDA	-0.14761	4.798635	-0.30142	0.763804	-5.78265	0.752049	0.747655
Dendritic.cells	WDR61	-0.04566	4.80713	-0.30112	0.764029	-6.12203	0.752079	0.747587
Dendritic.cells	E230014E1	-0.14993	-1.38289	-0.30092	0.764181	-5.23947	0.848139	0.887989
Dendritic.cells	LYPLAL1	-0.16258	1.634017	-0.30065	0.764385	-5.4091	0.799666	0.816753
Dendritic.cells	POC1B	0.060561	4.642223	0.300578	0.764442	-5.9266	0.7546	0.751194
Dendritic.cells	NOX1	0.136723	1.548335	0.30056	0.764456	-5.52319	0.800998	0.818716
Dendritic.cells	CHAMP1	0.087778	2.843874	0.299897	0.76496	-5.68212	0.781356	0.789875
Dendritic.cells	PPP1R18	0.032591	6.994866	0.299868	0.764982	-6.72161	0.721796	0.703684
Dendritic.cells	SERPINA7	0.180799	0.531403	0.299749	0.765072	-5.29509	0.817234	0.842341
Dendritic.cells	STK11	-0.03475	5.696969	-0.29963	0.765166	-6.49206	0.739766	0.729623
Dendritic.cells	ZFP691	-0.0454	3.075803	-0.29961	0.765177	-6.38478	0.777866	0.784846
Dendritic.cells	STRBP	-0.02932	7.195368	-0.29945	0.765297	-6.93862	0.719071	0.699813
Dendritic.cells	FAM220A	-0.13695	2.03012	-0.29941	0.765329	-5.51727	0.793757	0.808008
Dendritic.cells	ANKS3	0.050002	4.082767	0.299303	0.765412	-6.1836	0.762939	0.763158
Dendritic.cells	CDC6	0.135634	3.269665	0.299287	0.765423	-5.51866	0.774964	0.780624
Dendritic.cells	NR2F6	0.091842	3.501892	0.299238	0.765461	-5.71326	0.771505	0.775616
Dendritic.cells	MRS2	0.051556	3.939507	0.299184	0.765502	-6.07273	0.76504	0.766242
Dendritic.cells	JUN	0.08619	7.097589	0.299132	0.765541	-6.73313	0.720398	0.701772
Dendritic.cells	UBE2C	-0.12728	6.746366	-0.29902	0.765627	-6.31739	0.725191	0.708682
Dendritic.cells	GM4316	0.148414	0.707324	0.298878	0.765735	-5.40826	0.814435	0.838374
Dendritic.cells	MSANTD4	-0.07333	2.89703	-0.29882	0.765776	-5.73221	0.780555	0.788867
Dendritic.cells	GOLIM4	-0.04966	5.140893	-0.29882	0.765782	-6.50165	0.747645	0.741107
Dendritic.cells	UQCRQ	0.032043	7.858453	0.298531	0.765998	-6.93945	0.710277	0.687148
Dendritic.cells	RNF145	0.074916	5.159727	0.298383	0.766111	-5.89019	0.7475	0.740761
Dendritic.cells	HMGB2	0.063272	9.132267	0.298349	0.766137	-6.90452	0.693568	0.663219
Dendritic.cells	NUP133	-0.07506	3.691763	-0.29814	0.766295	-5.73285	0.768819	0.77171
Dendritic.cells	TRAPPC13	-0.05465	3.808587	-0.29813	0.766307	-6.01623	0.767094	0.769205
Dendritic.cells	SMIM11	-0.04516	4.805042	-0.2981	0.76633	-6.16515	0.752581	0.748163
Dendritic.cells	SPAG5	-0.14125	3.137614	-0.29783	0.766534	-5.51375	0.777205	0.783714
Dendritic.cells	PDXP	-0.16253	1.557173	-0.29767	0.766651	-5.35587	0.801398	0.818969
Dendritic.cells	RAF1	0.037575	5.986456	0.29749	0.76679	-6.52384	0.736013	0.724021
Dendritic.cells	DPY30	0.055005	5.414831	0.297194	0.767016	-6.23692	0.744059	0.735677
Dendritic.cells	RNF126	0.046118	4.767243	0.297163	0.767039	-6.25396	0.753313	0.749069
Dendritic.cells	NXPH4	0.185171	-0.60095	0.297145	0.767053	-5.24182	0.835873	0.869628
Dendritic.cells	ALDH3B1	0.057656	3.605843	0.297068	0.767111	-6.13163	0.770283	0.773707
Dendritic.cells	NLK	0.038997	5.966033	0.297055	0.767121	-6.58559	0.736298	0.724507
Dendritic.cells	STOML1	0.081949	2.251865	0.297015	0.767152	-5.8041	0.790682	0.803413
Dendritic.cells	MTRF1L	0.079108	3.1332	0.296752	0.767352	-5.80307	0.77746	0.783991
Dendritic.cells	PARP10	-0.12643	3.565506	-0.29627	0.767721	-5.54436	0.771313	0.774772
Dendritic.cells	RYR1	-0.11137	1.831874	-0.29586	0.768028	-5.68818	0.797767	0.813147
Dendritic.cells	GM20712	0.204821	0.063822	0.295724	0.768134	-5.24535	0.825714	0.854111

Dendritic.cells	IQSEC2	0.08824	2.267987	0.295714	0.768142	-5.94862	0.791051	0.803375
Dendritic.cells	0610010K1	0.041205	5.617735	0.295513	0.768295	-6.37435	0.741766	0.7319
Dendritic.cells	F630040K0	0.177304	0.611295	0.295464	0.768332	-5.32402	0.816936	0.841266
Dendritic.cells	TRPM7	0.028797	7.284405	0.295396	0.768384	-6.84244	0.718723	0.698716
Dendritic.cells	SRD5A3	0.050503	4.511422	0.295376	0.768399	-6.27905	0.757599	0.754793
Dendritic.cells	9130019P1	-0.19648	0.265967	-0.29531	0.768451	-5.24983	0.82246	0.849383
Dendritic.cells	FIG4	0.041587	4.603641	0.29504	0.768655	-6.18021	0.756381	0.752947
Dendritic.cells	WFDC18	0.144677	-0.88323	0.294856	0.768795	-5.24288	0.841297	0.876992
Dendritic.cells	PCID2	-0.04867	4.736836	-0.29482	0.768822	-6.07817	0.754456	0.750163
Dendritic.cells	AMBRA1	0.024743	7.654516	0.294781	0.768852	-6.99289	0.713848	0.691654
Dendritic.cells	SAT2	0.199897	-0.24549	0.294674	0.768934	-5.24719	0.830866	0.861683
Dendritic.cells	IPPK	-0.07698	3.400734	-0.29444	0.769115	-5.82291	0.774178	0.778675
Dendritic.cells	AKR1B8	0.118657	1.592252	0.294239	0.769265	-5.61625	0.801752	0.818861
Dendritic.cells	SOX5OS4	-0.1821	-0.23518	-0.29423	0.769271	-5.24153	0.830831	0.861491
Dendritic.cells	CD3EAP	0.077175	3.231663	0.294068	0.769395	-5.82402	0.776715	0.782398
Dendritic.cells	TTC41	0.123076	0.966475	0.293989	0.769456	-5.50438	0.811569	0.833263
Dendritic.cells	CNST	0.066462	3.943862	0.293872	0.769544	-5.77825	0.766144	0.767068
Dendritic.cells	PQLC2	0.06088	3.590318	0.293868	0.769547	-6.07485	0.771368	0.774653
Dendritic.cells	WRB	-0.08684	1.437969	-0.29359	0.769762	-5.7354	0.804257	0.822499
Dendritic.cells	PRPS2	0.040496	4.382206	0.293561	0.769781	-6.35151	0.75982	0.757824
Dendritic.cells	PRLR	-0.1192	3.152323	-0.29333	0.769958	-5.85185	0.778107	0.784235
Dendritic.cells	GM26810	0.164039	0.423092	0.293122	0.770116	-5.36079	0.820485	0.846086
Dendritic.cells	GFM1	0.070376	3.89287	0.29306	0.770163	-5.81669	0.767149	0.768275
Dendritic.cells	NEBL	0.18769	1.182115	0.292979	0.770224	-5.39986	0.808439	0.828436
Dendritic.cells	POC5	-0.08289	2.510705	-0.29279	0.770368	-5.68829	0.787942	0.798435
Dendritic.cells	TAP2	-0.06793	5.110651	-0.29254	0.770556	-6.20141	0.749607	0.74276
Dendritic.cells	KIF4	0.127734	4.394266	0.292419	0.770651	-5.76038	0.759947	0.757727
Dendritic.cells	NDUFAF1	-0.09839	2.81597	-0.29234	0.770708	-5.54561	0.783378	0.791772
Dendritic.cells	E130308A1	-0.05478	4.522919	-0.29225	0.770781	-6.09395	0.758077	0.75504
Dendritic.cells	PRR16	0.148749	1.720127	0.292237	0.77079	-5.5017	0.800179	0.816272
Dendritic.cells	APEH	-0.07717	3.695339	-0.29195	0.771005	-5.87101	0.770279	0.772672
Dendritic.cells	GINS2	0.117957	3.493231	0.291925	0.771028	-5.62765	0.77328	0.77703
Dendritic.cells	HEG1	-0.03872	5.971346	-0.29183	0.771101	-6.68733	0.737488	0.725239
Dendritic.cells	G2E3	-0.06087	3.938116	-0.29178	0.771138	-5.93969	0.766693	0.767481
Dendritic.cells	RTN3	-0.03358	8.060675	-0.29132	0.771487	-6.85269	0.7091	0.684324
Dendritic.cells	UAP1L1	-0.05885	3.734573	-0.29131	0.771494	-6.00658	0.769828	0.771903
Dendritic.cells	B2M	0.049109	9.94338	0.291184	0.771593	-7.2776	0.684667	0.649415
Dendritic.cells	DYNLL1	-0.0269	7.867224	-0.29104	0.771706	-6.86966	0.711678	0.688034
Dendritic.cells	PLATR25	0.081409	2.729731	0.291012	0.771724	-5.74623	0.784885	0.793808
Dendritic.cells	SPATC1	0.137624	-1.06407	0.290982	0.771747	-5.24236	0.845088	0.882018
Dendritic.cells	OASL1	-0.20968	2.957835	-0.29096	0.77176	-5.55246	0.781435	0.788797
Dendritic.cells	EGF	-0.10517	0.634522	-0.2907	0.771963	-5.5378	0.817611	0.841474
Dendritic.cells	AA465934	-0.11004	1.66043	-0.29018	0.772359	-5.50769	0.801652	0.817933
Dendritic.cells	DAZAP2	0.028274	7.70346	0.290173	0.772364	-6.80427	0.714172	0.691319
Dendritic.cells	CD5	0.051322	2.693985	0.290072	0.772441	-6.21446	0.78576	0.794785
Dendritic.cells	IL6	-0.29992	1.106723	-0.29007	0.772441	-5.27687	0.810328	0.830643
Dendritic.cells	LETM1	-0.04579	4.54276	-0.28998	0.772508	-6.13755	0.758304	0.754948

Dendritic.cells	TXNDC17	-0.04119	6.116505	-0.28987	0.772595	-6.47082	0.73591	0.722585
Dendritic.cells	DDB1	-0.0399	5.611857	-0.28968	0.772738	-6.44005	0.743006	0.73282
Dendritic.cells	TLK2	0.024482	6.825344	0.289679	0.77274	-6.78387	0.726105	0.708476
Dendritic.cells	CDC45	-0.12474	3.268129	-0.28956	0.772829	-5.55288	0.777141	0.782222
Dendritic.cells	UBE3B	-0.04434	4.424846	-0.28944	0.772921	-6.1504	0.760064	0.757452
Dendritic.cells	ARHGEF17	0.179586	-0.37609	0.289369	0.772976	-5.24104	0.834175	0.865598
Dendritic.cells	NDUFAF2	-0.05516	4.440034	-0.28922	0.773093	-6.05089	0.759888	0.757137
Dendritic.cells	UBE2J2	0.027346	6.638057	0.289088	0.773191	-6.64078	0.728766	0.712232
Dendritic.cells	TACC2	-0.0681	3.088667	-0.28899	0.773268	-5.99454	0.779908	0.786208
Dendritic.cells	GM17018	0.085198	3.620053	0.288797	0.773413	-5.67513	0.771965	0.774728
Dendritic.cells	KCTD3	-0.0776	3.747291	-0.28857	0.773585	-5.70931	0.770078	0.771997
Dendritic.cells	GPATCH3	0.081683	3.369567	0.288529	0.773618	-5.78074	0.775697	0.780154
Dendritic.cells	HBQ1B	-0.19469	-0.16343	-0.28845	0.773677	-5.25573	0.830782	0.860645
Dendritic.cells	TLK1	0.027749	6.854126	0.288306	0.773787	-6.76877	0.725796	0.708033
Dendritic.cells	APOL7C	-0.21496	-0.99321	-0.2883	0.773788	-5.24331	0.844395	0.880679
Dendritic.cells	FOXR1	0.051083	-0.87975	0.288303	0.77379	-6.15407	0.842518	0.877914
Dendritic.cells	EEF1AKMT	0.084451	2.582886	0.288219	0.773853	-5.6109	0.787564	0.797417
Dendritic.cells	C6	-0.1813	2.802964	-0.28816	0.773899	-5.54622	0.784222	0.79255
Dendritic.cells	SSR1	-0.02219	6.554701	-0.28801	0.774012	-6.87399	0.729916	0.713988
Dendritic.cells	VWA8	0.063361	4.975158	0.287992	0.774027	-5.98085	0.752169	0.746086
Dendritic.cells	CCDC34	0.101379	4.840796	0.287872	0.774118	-5.87843	0.754124	0.748897
Dendritic.cells	HDAC6	0.101654	2.206503	0.287609	0.774319	-5.57386	0.793478	0.805892
Dendritic.cells	MOB3C	0.055128	3.356469	0.287015	0.774773	-6.06008	0.77629	0.780728
Dendritic.cells	PPP6R2	-0.04005	4.368985	-0.28689	0.77487	-6.2883	0.761333	0.759056
Dendritic.cells	PTGER4	-0.05109	4.681638	-0.28686	0.774893	-6.40709	0.756789	0.752478
Dendritic.cells	AGMAT	0.178976	1.438737	0.286831	0.774913	-5.32796	0.805642	0.82352
Dendritic.cells	MDM1	0.112109	3.41542	0.286745	0.774979	-5.56884	0.775409	0.77947
Dendritic.cells	PDE4A	0.077023	3.81993	0.286736	0.774986	-6.06279	0.769398	0.770745
Dendritic.cells	1200007C1	0.186157	-0.38465	0.286606	0.775084	-5.24623	0.834844	0.866326
Dendritic.cells	RTL8B	0.095214	3.297356	0.286449	0.775204	-5.7177	0.77721	0.782122
Dendritic.cells	HNRNPL	0.019577	8.426349	0.286359	0.775273	-7.07641	0.705057	0.678063
Dendritic.cells	FAM160B2	0.067255	3.264944	0.286274	0.775338	-5.79648	0.777696	0.782837
Dendritic.cells	LUC7L	-0.03063	5.622585	-0.28613	0.775446	-6.48491	0.743357	0.733112
Dendritic.cells	GM30541	-0.15538	0.952964	-0.2861	0.775469	-5.24772	0.81333	0.834825
Dendritic.cells	PGM3	-0.06085	2.89728	-0.28604	0.775513	-5.82439	0.783231	0.790933
Dendritic.cells	DDX39B	0.02923	7.251694	0.285907	0.775618	-6.81773	0.720782	0.700682
Dendritic.cells	TGTP2	0.227515	0.773598	0.285851	0.775661	-5.29344	0.816185	0.839098
Dendritic.cells	PRKAR2A	0.104102	5.222556	0.285707	0.77577	-5.97398	0.749082	0.741442
Dendritic.cells	HEPACAM2	0.208973	0.779743	0.285625	0.775833	-5.24928	0.81612	0.838975
Dendritic.cells	GM26520	-0.09549	2.362216	-0.28544	0.775975	-5.91576	0.791446	0.802907
Dendritic.cells	CD300LF	0.062219	3.385271	0.285401	0.776004	-6.3148	0.775964	0.78039
Dendritic.cells	ZFC3H1	-0.02629	6.914629	-0.2849	0.776389	-6.79446	0.725731	0.707409
Dendritic.cells	RAB3A	-0.11625	2.619056	-0.28479	0.776471	-5.50334	0.787843	0.797255
Dendritic.cells	PRC1	-0.11143	5.285692	-0.28471	0.776531	-6.0828	0.748513	0.74024
Dendritic.cells	SAV1	-0.04134	4.735258	-0.28451	0.776685	-6.26968	0.756456	0.751717
Dendritic.cells	THSD1	-0.10356	1.60027	-0.28427	0.776871	-5.57607	0.803588	0.820237
Dendritic.cells	REV1	0.042805	5.326262	0.284242	0.77689	-6.31222	0.747968	0.73946

Dendritic.cells	EMP1	0.121673	1.978152	0.284202	0.77692	-5.74585	0.797716	0.811666
Dendritic.cells	RUSC1	-0.06397	3.288288	-0.28417	0.776948	-6.00225	0.777766	0.78262
Dendritic.cells	UBE2V1	0.024868	6.983076	0.283935	0.777124	-6.79644	0.724826	0.706126
Dendritic.cells	DKC1	-0.05936	4.56874	-0.28382	0.777214	-6.06937	0.75887	0.755239
Dendritic.cells	1700028E1	0.107167	1.36693	0.283747	0.777268	-5.45668	0.80724	0.825595
Dendritic.cells	CRIPT	0.031503	6.218829	0.283597	0.777383	-6.50395	0.735382	0.721329
Dendritic.cells	USP33	-0.05949	4.710834	-0.28356	0.777409	-5.98524	0.75681	0.752258
Dendritic.cells	METTL15	0.069797	3.231154	0.28352	0.777441	-5.83567	0.778623	0.783885
Dendritic.cells	DICER1	0.035621	4.846948	0.28343	0.777509	-6.31278	0.754843	0.749432
Dendritic.cells	OIP5OS1	-0.03684	6.02273	-0.28327	0.777636	-6.44873	0.738123	0.725319
Dendritic.cells	KNOP1	-0.04571	4.90183	-0.28323	0.777661	-6.17048	0.754051	0.74831
Dendritic.cells	ATG10	0.031412	5.64086	0.28316	0.777716	-6.6996	0.7435	0.733077
Dendritic.cells	FICD	-0.12778	0.917558	-0.28287	0.777939	-5.43888	0.81433	0.836085
Dendritic.cells	F3	-0.23138	0.113979	-0.28283	0.777971	-5.24613	0.827197	0.854961
Dendritic.cells	ZFP91	0.024306	6.894237	0.282789	0.778	-6.7832	0.726043	0.708008
Dendritic.cells	ZBTB24	-0.05126	3.32852	-0.28269	0.778077	-5.97248	0.777163	0.781895
Dendritic.cells	LIMS2	0.154792	0.424817	0.282637	0.778115	-5.33058	0.822191	0.847627
Dendritic.cells	PTGES3	0.023823	7.460041	0.282581	0.778159	-6.90524	0.718341	0.696955
Dendritic.cells	TNF	0.137532	4.405834	0.282574	0.778164	-5.9236	0.761241	0.758807
Dendritic.cells	RNF169	0.042456	6.380124	0.282355	0.778331	-6.60457	0.733138	0.718304
Dendritic.cells	LITAF	-0.02424	8.523937	-0.28234	0.778343	-7.05544	0.704152	0.676696
Dendritic.cells	GM47428	-0.16582	0.207827	-0.28229	0.778382	-5.30055	0.825682	0.852885
Dendritic.cells	KCND1	-0.17409	-0.19065	-0.28224	0.778422	-5.24425	0.832138	0.862388
Dendritic.cells	GCNT1	0.102844	2.451925	0.282181	0.778464	-5.52182	0.790429	0.801363
Dendritic.cells	TASP1	-0.05855	3.918389	-0.28205	0.778563	-6.16957	0.768422	0.769336
Dendritic.cells	PRIM2	0.066583	5.536205	0.281801	0.778754	-6.21301	0.745016	0.735513
Dendritic.cells	MPHOSPH9	0.05962	4.258614	0.281763	0.778783	-6.00939	0.763427	0.762146
Dendritic.cells	RAB11FIP4	0.126592	1.161785	0.28168	0.778847	-5.37931	0.810504	0.830727
Dendritic.cells	GSDMC4	-0.14646	0.743558	-0.28144	0.779029	-5.28161	0.817133	0.840529
Dendritic.cells	MINDY2	0.031401	6.610817	0.281405	0.779057	-6.59463	0.729976	0.71395
Dendritic.cells	RBBP6	0.020963	7.269431	0.281265	0.779164	-6.97888	0.720956	0.700989
Dendritic.cells	PAIP2B	-0.0692	3.682629	-0.28126	0.77917	-5.78564	0.771919	0.774596
Dendritic.cells	FADD	-0.12193	2.421277	-0.28123	0.77919	-5.5044	0.790934	0.802263
Dendritic.cells	MGAT2	-0.02792	5.873385	-0.28121	0.779204	-6.64976	0.740254	0.728771
Dendritic.cells	GM6034	-0.19222	0.50272	-0.28092	0.779429	-5.2528	0.821036	0.846294
Dendritic.cells	2210016L2	-0.06038	3.97848	-0.2809	0.779447	-6.01227	0.767595	0.768331
Dendritic.cells	CTPS	0.070197	3.704287	0.280827	0.779499	-5.8784	0.77165	0.774236
Dendritic.cells	CCNB1	0.160055	4.032241	0.280776	0.779538	-5.64531	0.766803	0.767215
Dendritic.cells	ANXA1	0.132731	6.030034	0.280592	0.779679	-5.91338	0.738172	0.725791
Dendritic.cells	NFYB	-0.05794	5.103926	-0.28043	0.779804	-6.0208	0.751352	0.744756
Dendritic.cells	MAP3K3	0.035537	6.795781	0.28019	0.779986	-6.65453	0.727606	0.71052
Dendritic.cells	CCDC6	-0.05687	4.540905	-0.28018	0.779993	-6.06933	0.759495	0.756553
Dendritic.cells	HIST1H2AG	-0.18366	2.011327	-0.28008	0.780073	-5.35039	0.797435	0.811758
Dendritic.cells	GM41556	-0.14358	1.259442	-0.28004	0.780098	-5.30957	0.809164	0.828909
Dendritic.cells	TTC38	0.084836	2.505389	0.279848	0.780248	-5.75589	0.789879	0.800749
Dendritic.cells	FASL	-0.16707	1.037842	-0.27981	0.780276	-5.32509	0.812699	0.834096
Dendritic.cells	MED26	-0.05273	4.429093	-0.27955	0.780478	-6.16506	0.761255	0.75908

Dendritic.cells	YTHDF2	-0.02819	6.035232	-0.27933	0.780645	-6.57423	0.73829	0.725957
Dendritic.cells	FAM111A	-0.06883	5.742953	-0.2793	0.78067	-5.94956	0.742402	0.731889
Dendritic.cells	CENPM	0.101445	3.916181	0.27925	0.780705	-5.74788	0.768782	0.770071
Dendritic.cells	BCL9	0.079371	3.399574	0.279223	0.780726	-5.75623	0.77646	0.781222
Dendritic.cells	RRM1	0.075179	5.449593	0.278879	0.780989	-6.10969	0.746598	0.73798
Dendritic.cells	GPR108	-0.05056	4.177602	-0.27886	0.781004	-6.1018	0.764975	0.76458
Dendritic.cells	CASS4	-0.10979	3.04899	-0.27869	0.78113	-5.77136	0.781767	0.789053
Dendritic.cells	KLK8	-0.11838	1.605933	-0.27853	0.781254	-5.41632	0.803915	0.821362
Dendritic.cells	GM16337	0.124218	1.967439	0.278407	0.78135	-5.4617	0.798295	0.813169
Dendritic.cells	AGMO	-0.08444	3.650343	-0.27838	0.78137	-5.96947	0.772763	0.775978
Dendritic.cells	RABEP2	0.048412	4.414258	0.278344	0.781398	-6.12882	0.761512	0.759653
Dendritic.cells	NMNAT3	0.089559	3.105259	0.278344	0.781398	-5.72707	0.780919	0.787838
Dendritic.cells	ARMC7	-0.07705	3.924467	-0.27834	0.781401	-5.77737	0.768701	0.77008
Dendritic.cells	PIP5K1B	-0.06186	5.568352	-0.27819	0.781516	-6.21186	0.744955	0.735673
Dendritic.cells	TTYH3	-0.05792	5.141527	-0.27804	0.781627	-6.09369	0.751042	0.744485
Dendritic.cells	MOV10	-0.08926	3.628623	-0.27783	0.781789	-5.67067	0.773133	0.77653
Dendritic.cells	FAAP20	0.069037	3.120662	0.27771	0.781883	-5.78231	0.780735	0.787636
Dendritic.cells	ERCC1	-0.0714	3.315868	-0.27768	0.781906	-5.75626	0.777802	0.783374
Dendritic.cells	TCTN2	-0.15269	-0.37598	-0.27759	0.781977	-5.24464	0.835644	0.868044
Dendritic.cells	PRM1	-0.15427	-0.44781	-0.27749	0.782054	-5.24501	0.83682	0.869788
Dendritic.cells	ARL14EP	0.06718	3.524764	0.277422	0.782103	-5.85222	0.774679	0.778885
Dendritic.cells	LRP4	0.114683	2.477171	0.27737	0.782143	-5.69596	0.7905	0.801928
Dendritic.cells	ZFP235	0.108298	1.919758	0.277358	0.782152	-5.52349	0.799082	0.814455
Dendritic.cells	GDPD1	-0.11985	1.600801	-0.27693	0.78248	-5.56257	0.804309	0.821779
Dendritic.cells	MECOM	-0.15473	0.770813	-0.27672	0.782642	-5.67538	0.817498	0.840979
Dendritic.cells	FBXO4	-0.07841	3.431156	-0.27647	0.782832	-5.66176	0.776497	0.781023
Dendritic.cells	CEP89	-0.12166	2.805458	-0.27633	0.782941	-5.52757	0.785924	0.794758
Dendritic.cells	PPP2R3A	-0.07531	5.003266	-0.27628	0.782977	-6.06283	0.753434	0.747602
Dendritic.cells	MAP4	-0.02841	6.676158	-0.27625	0.782999	-6.72309	0.729858	0.713581
Dendritic.cells	FBXL22	0.142596	2.248825	0.275988	0.783202	-5.36485	0.794555	0.807198
Dendritic.cells	MRPS18B	-0.07103	3.873974	-0.27576	0.783376	-5.77822	0.770031	0.771543
Dendritic.cells	TSC1	-0.04551	4.710552	-0.27574	0.783388	-6.2307	0.757779	0.753786
Dendritic.cells	PPP1R37	0.042319	4.592802	0.275655	0.783457	-6.23208	0.759488	0.756261
Dendritic.cells	LRBA	-0.02993	6.139874	-0.27551	0.783564	-6.93178	0.737424	0.72438
Dendritic.cells	EPS15L1	-0.02624	6.525246	-0.27543	0.783631	-6.74098	0.732058	0.71665
Dendritic.cells	ADK	0.034517	6.968234	0.275313	0.783719	-6.97419	0.725955	0.707866
Dendritic.cells	SLBP	-0.03849	7.147492	-0.27528	0.783742	-6.58668	0.723504	0.704342
Dendritic.cells	SLC25A36	0.029206	6.285868	0.274801	0.78411	-6.68432	0.735385	0.721556
Dendritic.cells	IDH3A	0.045803	4.865883	0.274795	0.784115	-6.24226	0.755532	0.750653
Dendritic.cells	CETN2	-0.02926	5.798667	-0.27452	0.784328	-6.52737	0.742218	0.731442
Dendritic.cells	MYO1F	0.06265	5.259228	0.274295	0.784498	-6.23488	0.74988	0.742511
Dendritic.cells	UNC50	0.052298	4.105649	0.274261	0.784524	-5.97998	0.766613	0.766743
Dendritic.cells	SAMD3	0.186773	0.184815	0.274257	0.784527	-5.25562	0.827107	0.855052
Dendritic.cells	SLC46A2	0.128358	-0.09426	0.274163	0.784599	-5.36713	0.83163	0.861701
Dendritic.cells	SQLE	0.054927	1.97366	0.274085	0.784658	-6.17649	0.798804	0.813597
Dendritic.cells	NDUFS2	0.034225	6.235293	0.274038	0.784695	-6.58244	0.73609	0.722606
Dendritic.cells	TNFSF10	-0.10955	2.307037	-0.27398	0.784736	-5.53737	0.79366	0.806108

Dendritic.cells	KDM4A	0.04622	4.161515	0.273721	0.784937	-6.15033	0.765791	0.765636
Dendritic.cells	NDUFV1	-0.04163	5.20278	-0.2736	0.785027	-6.29875	0.750688	0.743789
Dendritic.cells	ZFP777	0.066848	2.809648	0.273556	0.785064	-5.7514	0.785983	0.795017
Dendritic.cells	SAC3D1	0.07652	3.030557	0.27355	0.785068	-5.64149	0.782638	0.790146
Dendritic.cells	GM16341	-0.1505	0.863635	-0.27344	0.78515	-5.28093	0.816226	0.839247
Dendritic.cells	NXF7	-0.13935	-1.34285	-0.27343	0.785161	-5.24383	0.852228	0.892225
Dendritic.cells	TMEM63A	-0.05558	3.49863	-0.27342	0.785166	-6.18267	0.775609	0.779955
Dendritic.cells	ITGB1BP1	-0.04825	4.649919	-0.27325	0.7853	-6.05853	0.758659	0.755436
Dendritic.cells	KCMF1	-0.01955	6.987433	-0.27317	0.785358	-6.82913	0.725692	0.707869
Dendritic.cells	1700120C1	-0.11197	1.482076	-0.27309	0.785421	-5.39214	0.806463	0.825093
Dendritic.cells	METTL16	-0.03829	4.533537	-0.27302	0.785476	-6.27633	0.760351	0.757949
Dendritic.cells	CDC42EP4	-0.11021	2.155265	-0.27284	0.785612	-5.43375	0.795997	0.809832
Dendritic.cells	PDE4B	0.036181	8.527906	0.272716	0.785707	-7.01061	0.704998	0.678237
Dendritic.cells	RIOK3	0.028174	7.578172	0.27262	0.785781	-6.84987	0.717661	0.696402
Dendritic.cells	THAP8	0.187334	-0.29985	0.272604	0.785793	-5.25287	0.834981	0.867018
Dendritic.cells	GM17173	-0.15597	0.150476	-0.2726	0.785798	-5.24825	0.827662	0.856251
Dendritic.cells	CHMP6	-0.07537	3.712475	-0.27256	0.785823	-5.8014	0.772425	0.775533
Dendritic.cells	TGM2	0.113174	4.674438	0.272474	0.785893	-5.90924	0.758303	0.755077
Dendritic.cells	ZFP775	-0.12313	0.81684	-0.27247	0.785894	-5.41715	0.816971	0.840599
Dendritic.cells	RAD23A	0.04382	5.807004	0.272363	0.785978	-6.3836	0.7421	0.731665
Dendritic.cells	H2-D1	0.051041	8.452787	0.272247	0.786067	-6.9744	0.705989	0.679726
Dendritic.cells	TXNRD2	0.0916	3.532111	0.272063	0.786208	-5.67875	0.77511	0.779529
Dendritic.cells	SH3BP4	0.040394	1.369949	0.271777	0.786427	-6.51009	0.808222	0.827846
Dendritic.cells	RAB22A	0.032308	5.60028	0.271505	0.786636	-6.45862	0.745024	0.735948
Dendritic.cells	GPRASP1	-0.04807	3.93667	-0.27136	0.786744	-6.06584	0.769104	0.770832
Dendritic.cells	BOK	-0.12947	1.039523	-0.27135	0.786752	-5.47123	0.813435	0.835511
Dendritic.cells	MRPL32	-0.03949	5.68103	-0.27131	0.786783	-6.36993	0.74388	0.734295
Dendritic.cells	POLR2A	0.026434	6.200996	0.271157	0.786902	-6.56685	0.736569	0.723742
Dendritic.cells	KCNG3	-0.16971	0.517714	-0.27107	0.786972	-5.25228	0.821749	0.847709
Dendritic.cells	MRPL9	-0.04267	4.787977	-0.27098	0.787039	-6.0821	0.756658	0.752781
Dendritic.cells	UGT1A7C	0.159214	-0.45047	0.270915	0.787088	-5.25005	0.837447	0.870795
Dendritic.cells	GM47917	-0.12304	0.467794	-0.27079	0.787185	-5.37182	0.82255	0.848884
Dendritic.cells	DUSP6	0.091798	4.428378	0.270684	0.787265	-5.72608	0.761883	0.760354
Dendritic.cells	SIRT6	-0.09349	2.652878	-0.27067	0.787278	-5.5931	0.788368	0.798865
Dendritic.cells	USP53	0.047405	4.118641	0.270661	0.787282	-6.11009	0.766421	0.766938
Dendritic.cells	EFL1	0.04219	4.5955	0.270621	0.787313	-6.1873	0.759449	0.756825
Dendritic.cells	GNAT3	-0.1597	-0.04367	-0.27057	0.787349	-5.24697	0.830808	0.861052
Dendritic.cells	FPGT	0.128719	1.345954	0.270551	0.787367	-5.4846	0.8086	0.828466
Dendritic.cells	BAG5	0.064564	3.637068	0.270501	0.787405	-5.99463	0.773546	0.777336
Dendritic.cells	ZSCAN2	-0.14696	1.067334	-0.27039	0.787488	-5.37227	0.812995	0.834935
Dendritic.cells	OVGP1	0.141656	0.492644	0.270349	0.787522	-5.26042	0.822151	0.84837
Dendritic.cells	HNRNPDL	0.01972	7.857159	0.270186	0.787647	-6.96766	0.713909	0.691202
Dendritic.cells	TMEM184(	-0.06514	3.390595	-0.27016	0.787663	-5.78128	0.777225	0.782722
Dendritic.cells	CEBPE	0.166077	0.2028	0.270119	0.787698	-5.25085	0.826816	0.855274
Dendritic.cells	QPCT	-0.14042	2.264701	-0.26996	0.787824	-5.35056	0.794311	0.807708
Dendritic.cells	PRXL2B	-0.09242	2.256934	-0.26982	0.787929	-5.54046	0.794431	0.807899
Dendritic.cells	GM10521	-0.14397	0.308847	-0.2697	0.788017	-5.2468	0.825106	0.852837

Dendritic.cells	ZBTB42	-0.09064	1.544195	-0.26962	0.788084	-5.50952	0.80549	0.824067
Dendritic.cells	LYPD6B	0.184479	0.004013	0.269508	0.788166	-5.25027	0.830034	0.860083
Dendritic.cells	PDCD7	0.045378	4.505243	0.26931	0.788318	-6.1156	0.760763	0.758905
Dendritic.cells	GM47448	-0.18087	-0.89373	-0.26924	0.788371	-5.24467	0.844751	0.881769
Dendritic.cells	MRPL33	0.041096	6.142277	0.2692	0.788403	-6.39131	0.73739	0.725094
Dendritic.cells	UCP1	-0.13651	-1.00774	-0.26917	0.788425	-5.24748	0.846642	0.88456
Dendritic.cells	MUL1	-0.07396	2.950066	-0.26916	0.788435	-5.77022	0.783855	0.792472
Dendritic.cells	ARMCX6	0.076088	0.977785	0.26902	0.788541	-5.68983	0.814413	0.83714
Dendritic.cells	JPT1	-0.02712	7.832843	-0.26892	0.788615	-6.91063	0.714235	0.691761
Dendritic.cells	ELF1	0.018931	8.010369	0.268759	0.788742	-7.03329	0.711861	0.688352
Dendritic.cells	FNDC7	0.163944	-0.36573	0.268721	0.78877	-5.25102	0.836059	0.868952
Dendritic.cells	SF3A3	-0.04802	4.984502	-0.26868	0.788798	-6.14611	0.753822	0.748847
Dendritic.cells	PLEKHA3	-0.05118	3.98944	-0.26858	0.788877	-5.99351	0.768325	0.76988
Dendritic.cells	GM11131	0.145118	0.815809	0.268578	0.78888	-5.34766	0.816987	0.840915
Dendritic.cells	GLYR1	0.026799	6.736527	0.268529	0.788918	-6.67053	0.729139	0.713197
Dendritic.cells	FAM45A	-0.04832	3.979967	-0.26848	0.788955	-5.99438	0.768464	0.770083
Dendritic.cells	TDG	-0.0404	5.074746	-0.26848	0.788955	-6.2385	0.752524	0.746968
Dendritic.cells	CYTH4	0.022499	5.757599	0.268463	0.788969	-6.91768	0.742797	0.732902
Dendritic.cells	PDGFA	-0.05877	2.114128	-0.26834	0.789066	-6.24606	0.796631	0.811114
Dendritic.cells	EPG5	-0.03955	4.420072	-0.26828	0.789107	-6.29879	0.762005	0.760707
Dendritic.cells	FIRRE	-0.07726	2.608551	-0.26808	0.789266	-5.88108	0.789067	0.800064
Dendritic.cells	EIF2AK1	0.038459	5.326731	0.268036	0.789296	-6.30636	0.748938	0.741783
Dendritic.cells	PSIP1	0.039077	5.994205	0.267827	0.789457	-6.31309	0.739487	0.728136
Dendritic.cells	PHTF1	0.051687	4.68503	0.267795	0.789481	-6.18186	0.758172	0.755165
Dendritic.cells	MYPOPOS	0.111332	1.654931	0.267792	0.789483	-5.60389	0.803783	0.821587
Dendritic.cells	CS	0.027398	6.103857	0.267598	0.789632	-6.55104	0.738015	0.725957
Dendritic.cells	TMEM201	0.094591	2.102835	0.267424	0.789766	-5.55115	0.7969	0.811557
Dendritic.cells	ZMYND15	-0.20349	0.109664	-0.2673	0.789862	-5.28287	0.82842	0.857801
Dendritic.cells	SLC39A1	-0.03083	6.161792	-0.26727	0.789881	-6.57198	0.737204	0.7249
Dendritic.cells	GGT5	-0.17039	0.649699	-0.26724	0.789905	-5.25499	0.819733	0.845036
Dendritic.cells	EML2	-0.07523	2.655573	-0.26704	0.790057	-5.8346	0.788442	0.799278
Dendritic.cells	MRFAP1	-0.02349	6.452488	-0.26703	0.790068	-6.7029	0.733175	0.719117
Dendritic.cells	GM15441	-0.11011	1.344167	-0.26684	0.790216	-5.43174	0.80875	0.828982
Dendritic.cells	SKP2	-0.08886	3.310393	-0.26675	0.790281	-5.62906	0.778544	0.784881
Dendritic.cells	CUL9	0.077449	2.579779	0.266727	0.7903	-5.768	0.789602	0.800997
Dendritic.cells	1700006J1	0.18079	-0.71534	0.26658	0.790413	-5.25475	0.84193	0.877787
Dendritic.cells	RRAS	0.070387	4.338868	0.266565	0.790425	-6.01792	0.763307	0.762748
Dendritic.cells	RGL1	-0.04642	5.711711	-0.26623	0.79068	-6.56536	0.74364	0.734266
Dendritic.cells	KCNH7	0.15963	-0.13979	0.266208	0.790699	-5.25011	0.832589	0.864025
Dendritic.cells	ZNRF1	0.03229	6.496008	0.266176	0.790723	-6.65893	0.732655	0.718409
Dendritic.cells	SNRNP70	-0.01546	7.511442	-0.26583	0.790989	-6.93024	0.718874	0.698408
Dendritic.cells	PYROXD2	0.149933	0.182818	0.26571	0.791081	-5.24917	0.827499	0.856359
Dendritic.cells	CHCHD6	-0.05458	2.968098	-0.2657	0.791087	-5.91397	0.783923	0.792568
Dendritic.cells	CRYZL1	-0.06126	4.570122	-0.26563	0.791142	-5.86836	0.760149	0.758034
Dendritic.cells	EEA1	0.037315	6.055281	0.265534	0.791216	-6.40391	0.738933	0.727359
Dendritic.cells	SLAMF1	0.173678	0.884468	0.264869	0.791727	-5.25593	0.816652	0.840035
Dendritic.cells	CCND2	0.052948	5.400515	0.264848	0.791743	-6.34389	0.748557	0.740861

Dendritic.cells	SLC35F6	0.06967	2.998461	0.264557	0.791967	-5.76753	0.783886	0.792123
Dendritic.cells	NDC1	-0.07654	3.748037	-0.26452	0.791995	-5.73183	0.772648	0.775778
Dendritic.cells	DMAC2	0.069509	3.093682	0.264462	0.792039	-5.81934	0.782447	0.790068
Dendritic.cells	CFDP1	0.0319	5.986865	0.264432	0.792062	-6.483	0.740289	0.728941
Dendritic.cells	IKZF5	-0.0531	3.804869	-0.26434	0.792132	-5.97537	0.771805	0.774601
Dendritic.cells	EIF4EBP3	-0.09254	2.80817	-0.26422	0.792226	-5.6289	0.786794	0.796423
Dendritic.cells	BCAT1	0.187556	0.852062	0.264023	0.792377	-5.24952	0.817265	0.840967
Dendritic.cells	CHUK	0.032982	5.037427	0.263952	0.792431	-6.43722	0.753849	0.748554
Dendritic.cells	VDAC3	-0.02705	7.191815	-0.26364	0.792669	-6.72485	0.723657	0.705076
Dendritic.cells	SELENOH	0.076338	5.20549	0.263476	0.792797	-6.07037	0.751435	0.745238
Dendritic.cells	ECM1	-0.15045	3.680244	-0.26337	0.792876	-5.66131	0.773713	0.777588
Dendritic.cells	GM47230	-0.13635	0.668652	-0.26336	0.792884	-5.36615	0.820192	0.845505
Dendritic.cells	ERF	-0.0926	3.477368	-0.2633	0.792929	-5.69002	0.77674	0.78199
Dendritic.cells	A230059L0	0.170433	0.47539	0.263289	0.79294	-5.29087	0.82329	0.850058
Dendritic.cells	DENND6B	0.124767	0.94417	0.263239	0.792979	-5.37018	0.8158	0.839075
Dendritic.cells	SNX18	-0.02389	6.77742	-0.26296	0.793193	-7.11895	0.729339	0.71338
Dendritic.cells	IPO8	-0.04419	4.420007	-0.26296	0.793194	-6.08696	0.762804	0.76177
Dendritic.cells	NT5C3	0.05788	4.85257	0.262778	0.793333	-6.00875	0.756516	0.752682
Dendritic.cells	ART2B	0.20578	0.097769	0.262739	0.793363	-5.25009	0.829383	0.85908
Dendritic.cells	DDX6	0.021681	8.314732	0.262697	0.793396	-7.04307	0.708556	0.683526
Dendritic.cells	A930005H1	-0.07942	3.220496	-0.26261	0.793464	-5.57155	0.780593	0.787666
Dendritic.cells	SNX13	0.038078	5.413359	0.262589	0.793478	-6.48976	0.748463	0.741026
Dendritic.cells	MTMR10	-0.08882	2.897646	-0.26257	0.793497	-5.73239	0.785471	0.794773
Dendritic.cells	ZFP597	-0.08599	2.862663	-0.26252	0.79353	-5.62962	0.786001	0.795546
Dendritic.cells	LAPTM4A	0.024263	7.210016	0.262264	0.793728	-6.79722	0.723477	0.704889
Dendritic.cells	ECHS1	-0.0576	5.549982	-0.26224	0.793746	-6.20597	0.746589	0.738239
Dendritic.cells	GM19557	0.119787	-0.95554	0.261973	0.793952	-5.25008	0.846782	0.884736
Dendritic.cells	HIST2H4	0.145659	0.951972	0.261792	0.794091	-5.3743	0.815792	0.839165
Dendritic.cells	GNG3	-0.12385	1.449153	-0.26155	0.794275	-5.41758	0.807939	0.827701
Dendritic.cells	GVIN1	-0.14894	3.037272	-0.26148	0.79433	-5.63933	0.783468	0.791943
Dendritic.cells	ZMAT3	-0.09663	1.902429	-0.26144	0.794363	-5.70478	0.80086	0.817339
Dendritic.cells	MTR	0.04664	4.54186	0.261293	0.794474	-6.08675	0.761134	0.759467
Dendritic.cells	DDX39	0.038274	6.372696	0.261289	0.794478	-6.60688	0.73505	0.721727
Dendritic.cells	SRM	-0.06596	4.842685	-0.26127	0.794494	-6.23649	0.756767	0.753134
Dendritic.cells	ANKMY2	-0.0391	3.602393	-0.26127	0.794496	-6.1945	0.774983	0.779588
Dendritic.cells	CD2	0.104768	3.660392	0.261233	0.794521	-5.59308	0.774119	0.778333
Dendritic.cells	NCOR2	-0.04453	4.956649	-0.26116	0.794577	-6.13209	0.75512	0.75077
Dendritic.cells	ITGB1	0.024561	6.871704	0.26107	0.794645	-6.82203	0.728144	0.711836
Dendritic.cells	PTDSS2	0.067526	3.06586	0.260913	0.794766	-5.7307	0.783036	0.79141
Dendritic.cells	BC050972	-0.16519	-0.27973	-0.26091	0.794771	-5.24986	0.835646	0.868514
Dendritic.cells	DDX19A	0.055689	4.239703	0.260767	0.794879	-5.94239	0.765554	0.76599
Dendritic.cells	NUTF2	-0.10419	1.951713	-0.26052	0.795067	-5.55815	0.800095	0.816394
Dendritic.cells	IGF2R	0.094004	4.985937	0.260458	0.795116	-5.64281	0.754698	0.750314
Dendritic.cells	LRG1	0.174137	2.460703	0.260398	0.795162	-5.44139	0.792245	0.804942
Dendritic.cells	ZBTB49	-0.08956	1.051258	-0.26037	0.795188	-5.45937	0.814217	0.837106
Dendritic.cells	BRCA2	0.089131	3.555566	0.260356	0.795194	-5.71228	0.775682	0.780793
Dendritic.cells	EMC2	-0.03229	5.762132	-0.26019	0.795325	-6.3836	0.743673	0.734324

Dendritic.cells	YIPF1	0.031402	5.234712	0.259846	0.795587	-6.37521	0.751233	0.745212
Dendritic.cells	AKIRIN1	0.024978	6.287656	0.259724	0.795681	-6.56674	0.736343	0.723725
Dendritic.cells	RTN4IP1	-0.11453	1.927586	-0.25964	0.795747	-5.47394	0.800586	0.817121
Dendritic.cells	ANAPC1	0.039613	4.721072	0.259532	0.795828	-6.25112	0.758639	0.756016
Dendritic.cells	NUP85	0.071217	4.345133	0.259439	0.7959	-5.87968	0.764119	0.763969
Dendritic.cells	GM49521	-0.13065	0.473447	-0.25944	0.795902	-5.32558	0.823558	0.850812
Dendritic.cells	NUP50	0.037523	5.304963	0.259243	0.79605	-6.25743	0.750227	0.743829
Dendritic.cells	CCDC112	-0.13989	1.151644	-0.25923	0.796062	-5.35137	0.812746	0.834938
Dendritic.cells	TREM2	0.224343	0.639882	0.259162	0.796112	-5.25488	0.820889	0.8469
Dendritic.cells	SHC4	-0.14284	0.470109	-0.25909	0.796171	-5.27499	0.823612	0.850905
Dendritic.cells	AOX3	0.161603	0.998188	0.259041	0.796206	-5.3399	0.815177	0.838533
Dendritic.cells	MLF2	-0.02895	5.85253	-0.25885	0.796351	-6.5785	0.742449	0.732607
Dendritic.cells	CAMK2B	-0.06199	3.706454	-0.25877	0.796412	-6.24755	0.773546	0.77771
Dendritic.cells	TIGIT	0.15829	-0.09862	0.25873	0.796445	-5.25554	0.832812	0.864474
Dendritic.cells	N4BP1	0.05879	5.4056	0.25873	0.796445	-6.1485	0.74879	0.741782
Dendritic.cells	CCNDBP1	0.054719	5.692836	0.258646	0.796509	-6.26721	0.744706	0.735899
Dendritic.cells	SLCO4C1	0.13523	-1.1708	0.258302	0.796774	-5.25166	0.850666	0.89068
Dendritic.cells	ZMYND19	0.079269	3.409984	0.258248	0.796816	-5.82454	0.778134	0.784258
Dendritic.cells	ELOVL7	-0.15673	-0.19718	-0.25789	0.797091	-5.24992	0.834807	0.867062
Dendritic.cells	OLFR1369-	-0.1827	-0.76388	-0.25778	0.797179	-5.24969	0.844139	0.880817
Dendritic.cells	BRD4	0.015566	8.256086	0.257594	0.797319	-7.02756	0.709889	0.685423
Dendritic.cells	CHEK2	-0.0816	3.145946	-0.25743	0.797447	-5.5736	0.782329	0.790197
Dendritic.cells	RSPH3B	0.051752	3.01938	0.257387	0.797478	-5.98274	0.784241	0.792984
Dendritic.cells	ASF1B	0.103126	4.954931	0.257325	0.797525	-5.8694	0.755629	0.751396
Dendritic.cells	GBP2	-0.26699	4.147241	-0.2573	0.797546	-5.53333	0.767404	0.768482
Dendritic.cells	ISCA1	-0.05497	6.018689	-0.25705	0.797738	-6.34979	0.740476	0.729501
Dendritic.cells	PAN2	0.065529	3.190864	0.25694	0.797822	-5.71845	0.781651	0.789261
Dendritic.cells	RAB11A	0.021708	7.145444	0.256927	0.797832	-6.78326	0.724857	0.706987
Dendritic.cells	GPSM1	0.077461	2.672071	0.256896	0.797856	-5.68676	0.789519	0.800735
Dendritic.cells	GIMAP1OS	-0.1487	0.862202	-0.25678	0.797947	-5.25698	0.817744	0.842101
Dendritic.cells	PNPLA6	-0.07012	2.760588	-0.25675	0.79797	-5.68468	0.78817	0.798819
Dendritic.cells	UTP4	0.037409	5.102474	0.25664	0.798053	-6.30224	0.753504	0.74844
Dendritic.cells	GM30025	0.09821	1.597885	0.256454	0.798196	-5.57855	0.806124	0.825155
Dendritic.cells	GM1604B	0.135746	1.489369	0.256418	0.798224	-5.43083	0.807826	0.827648
Dendritic.cells	TRIP11	0.026534	6.069875	0.256376	0.798256	-6.6728	0.739757	0.728607
Dendritic.cells	FAM89A	-0.10296	0.879902	-0.25629	0.798324	-5.43381	0.817462	0.841817
Dendritic.cells	SMG6	0.018645	8.045885	0.256207	0.798386	-7.036	0.71269	0.689641
Dendritic.cells	MAT1A	-0.10734	4.65605	-0.25605	0.798509	-5.98909	0.759971	0.757946
Dendritic.cells	DHRS7B	0.070134	3.137991	0.256008	0.798539	-5.74197	0.782461	0.790668
Dendritic.cells	SNHG9	-0.09281	4.309776	-0.25562	0.798839	-6.01301	0.765123	0.765453
Dendritic.cells	RFK	-0.0466	5.042963	-0.25556	0.798884	-6.16613	0.754466	0.74999
Dendritic.cells	RNF125	-0.11358	3.852901	-0.2555	0.798929	-5.55269	0.771862	0.775253
Dendritic.cells	SNRNP40	0.027294	5.982356	0.255479	0.798946	-6.55879	0.741091	0.730632
Dendritic.cells	PPIL3	-0.04856	4.605985	-0.25528	0.7991	-6.00367	0.760794	0.759228
Dendritic.cells	C130013H(	-0.08895	0.498295	-0.25522	0.799148	-5.5038	0.823682	0.851116
Dendritic.cells	LPAR2	-0.09394	2.187833	-0.25513	0.799212	-5.40334	0.797064	0.81208
Dendritic.cells	NELFCD	0.054173	4.117826	0.254845	0.799435	-5.91901	0.767945	0.769685

Dendritic.cells	KLRA2	0.186966	2.524963	0.254775	0.799488	-5.39862	0.791879	0.804566
Dendritic.cells	TTLL12	0.118046	2.217401	0.254767	0.799494	-5.57319	0.796607	0.811477
Dendritic.cells	JAK1	0.021472	8.273816	0.254724	0.799528	-6.973	0.709753	0.685596
Dendritic.cells	SINHCAF	-0.05215	4.557551	-0.25469	0.799555	-5.9881	0.7615	0.760321
Dendritic.cells	EIF4G1	0.02308	6.604378	0.254566	0.799649	-6.73942	0.732405	0.718206
Dendritic.cells	DIS3L2	0.02846	5.906117	0.254563	0.799652	-6.58555	0.742165	0.732305
Dendritic.cells	GTPBP8	0.07745	2.317318	0.254503	0.799698	-5.62824	0.795067	0.809225
Dendritic.cells	CPA6	-0.15446	-0.07229	-0.25402	0.800068	-5.25352	0.833177	0.864899
Dendritic.cells	CD320	0.0931	1.856288	0.253954	0.800121	-5.44301	0.802458	0.819809
Dendritic.cells	LSM5	0.045066	5.652829	0.25389	0.80017	-6.24621	0.745984	0.73764
Dendritic.cells	GTPBP3	0.086591	2.225197	0.253743	0.800283	-5.57267	0.79674	0.81147
Dendritic.cells	LEF1OS1	-0.1316	-0.45613	-0.25372	0.800305	-5.24842	0.839458	0.874212
Dendritic.cells	AW011738	-0.12368	2.812185	-0.2532	0.800698	-5.52112	0.787994	0.798422
Dendritic.cells	SNRPB	0.02476	7.300883	0.253203	0.800699	-6.81152	0.723297	0.704643
Dendritic.cells	IRGQ	-0.09492	2.194038	-0.25295	0.800892	-5.59526	0.797595	0.812337
Dendritic.cells	CSAD	-0.05338	3.965874	-0.25278	0.801029	-6.10083	0.770858	0.773302
Dendritic.cells	NUDT7	-0.11945	1.74453	-0.25204	0.801593	-5.42634	0.804775	0.822715
Dendritic.cells	THAP2	0.076724	3.329281	0.251966	0.801652	-5.70904	0.780482	0.787267
Dendritic.cells	CMKLR1	0.070705	2.151939	0.251846	0.801744	-6.16155	0.798441	0.81349
Dendritic.cells	TRDJ1	-0.12236	-1.29553	-0.25181	0.801773	-5.25258	0.853996	0.895198
Dendritic.cells	TTK	0.116372	2.313922	0.251458	0.802043	-5.48865	0.79594	0.809893
Dendritic.cells	FUT11	0.05551	3.493603	0.251343	0.802132	-5.97208	0.778016	0.783745
Dendritic.cells	AGA	0.06326	3.27288	0.251251	0.802203	-5.88922	0.781331	0.788574
Dendritic.cells	PARPBP	-0.17029	2.056928	-0.25123	0.802218	-5.32589	0.799913	0.815702
Dendritic.cells	NRBP1	-0.0301	5.827937	-0.2512	0.802244	-6.4683	0.744035	0.734436
Dendritic.cells	9930022D1	0.160347	0.319301	0.25119	0.80225	-5.2567	0.827418	0.856049
Dendritic.cells	CCNL1	0.023228	7.09056	0.251152	0.802279	-6.78143	0.726458	0.709069
Dendritic.cells	MOB3B	0.023796	4.961752	0.251077	0.802337	-6.8949	0.756417	0.752369
Dendritic.cells	BCS1L	-0.11752	1.699946	-0.25099	0.802405	-5.45773	0.805471	0.823844
Dendritic.cells	KLHL9	0.039777	4.65586	0.250876	0.802492	-6.24157	0.760853	0.758822
Dendritic.cells	BCAS3	-0.02573	7.334935	-0.25085	0.802516	-6.92076	0.72312	0.704297
Dendritic.cells	APPL2	0.088116	3.358029	0.250803	0.802549	-5.7146	0.78005	0.786748
Dendritic.cells	RRP9	-0.07699	2.59462	-0.25068	0.80264	-5.6074	0.791629	0.803636
Dendritic.cells	NOL12	0.040922	4.012955	0.250597	0.802707	-6.01485	0.770287	0.772538
Dendritic.cells	ZFP830	-0.06253	3.541014	-0.25058	0.802717	-5.78568	0.777307	0.782752
Dendritic.cells	METTL2	-0.06008	3.27705	-0.25048	0.802796	-5.80251	0.781268	0.788523
Dendritic.cells	MRPL50	0.054407	4.009842	0.250481	0.802796	-5.85592	0.770333	0.772605
Dendritic.cells	SYK	0.02636	8.336181	0.250471	0.802804	-7.10513	0.709657	0.684943
Dendritic.cells	GM4070	0.151036	2.887167	0.250297	0.802939	-5.53419	0.787167	0.797214
Dendritic.cells	DNTTIP1	-0.0336	4.812219	-0.25016	0.803041	-6.22535	0.758581	0.755638
Dendritic.cells	TRIM56	-0.05772	3.810038	-0.25008	0.803104	-5.85799	0.773295	0.777014
Dendritic.cells	ATPIF1	0.032905	7.745006	0.250061	0.80312	-6.7763	0.717565	0.696395
Dendritic.cells	IFIT2	0.168912	2.721315	0.250058	0.803123	-5.5824	0.789692	0.800913
Dendritic.cells	SYPL	-0.03542	5.860238	-0.25002	0.803149	-6.39207	0.743578	0.733909
Dendritic.cells	ZRANB1	0.024234	6.142303	0.249912	0.803235	-6.64391	0.739607	0.728197
Dendritic.cells	A130014A(	0.106345	1.887317	0.249856	0.803278	-5.42393	0.802548	0.819756
Dendritic.cells	PAGR1A	-0.13318	0.395669	-0.24946	0.803581	-5.35686	0.826281	0.854569

Dendritic.cells	SYAP1	0.031523	5.053275	0.249393	0.803635	-6.33049	0.755183	0.750741
Dendritic.cells	ACYP1	0.066828	3.847714	0.249284	0.803719	-5.7871	0.772825	0.776394
Dendritic.cells	CTNND2	-0.02961	1.726999	-0.24888	0.804033	-6.88229	0.805142	0.823691
Dendritic.cells	NTN4	-0.12012	0.706687	-0.24887	0.804041	-5.50888	0.821283	0.847378
Dendritic.cells	CACNB4	-0.16326	1.615307	-0.24887	0.804042	-5.34179	0.80689	0.826253
Dendritic.cells	TCP11	0.096968	0.648485	0.248694	0.804174	-5.48547	0.822216	0.848759
Dendritic.cells	PRKAG2	0.02735	5.050385	0.248591	0.804253	-6.68333	0.755225	0.750981
Dendritic.cells	IMMP2L	0.038907	6.534254	0.248558	0.804279	-6.82218	0.734219	0.720588
Dendritic.cells	RUNDC1	-0.06037	2.994317	-0.2484	0.8044	-5.84661	0.785631	0.795213
Dendritic.cells	FUBP1	-0.01957	6.678668	-0.24836	0.804428	-6.76403	0.732216	0.717701
Dendritic.cells	1700113A1	-0.08615	2.10081	-0.2483	0.804482	-5.59877	0.799325	0.815223
Dendritic.cells	SIK1	-0.01951	6.868808	-0.24828	0.804495	-7.08876	0.72959	0.713912
Dendritic.cells	MXD3	0.142762	1.735683	0.248254	0.804514	-5.31509	0.805006	0.823541
Dendritic.cells	GPR34	0.176464	0.588061	0.248173	0.804576	-5.28423	0.823185	0.850271
Dendritic.cells	RCAN1	-0.06199	3.708543	-0.24805	0.804673	-5.84267	0.774895	0.779651
Dendritic.cells	TRAPPC2	0.057727	4.171105	0.247946	0.804751	-5.91288	0.768041	0.769696
Dendritic.cells	ILRUN	-0.03393	5.988088	-0.24789	0.804795	-6.51161	0.741861	0.731744
Dendritic.cells	FIGNL2	-0.15713	-0.67701	-0.24789	0.804796	-5.25053	0.843797	0.880736
Dendritic.cells	CLN6	-0.0579	3.482895	-0.24759	0.805025	-5.96315	0.778267	0.784649
Dendritic.cells	FAM78B	0.116204	2.115372	0.247572	0.80504	-5.45059	0.7991	0.815073
Dendritic.cells	PDZD8	0.028413	5.970732	0.247501	0.805094	-6.7043	0.742105	0.732148
Dendritic.cells	HTRA3	0.137236	0.502839	0.24743	0.805149	-5.27526	0.824555	0.852426
Dendritic.cells	PPCS	-0.06765	2.732539	-0.24741	0.805166	-5.68317	0.789613	0.801202
Dendritic.cells	SERPINI1	0.066361	3.178888	0.247366	0.805199	-5.89113	0.782839	0.791315
Dendritic.cells	NXT2	0.073303	2.785909	0.247353	0.805208	-5.58872	0.788799	0.800014
Dendritic.cells	RIC8B	-0.03584	4.502862	-0.24707	0.805426	-6.29715	0.763304	0.76278
Dendritic.cells	TMED1	0.078932	2.452472	0.246991	0.805488	-5.61596	0.794037	0.807593
Dendritic.cells	ABCC4	-0.04574	4.55376	-0.24688	0.805571	-6.08878	0.762573	0.761736
Dendritic.cells	SDC2	0.109229	2.267759	0.246759	0.805667	-5.55349	0.796918	0.811764
Dendritic.cells	ANLN	0.104056	3.747041	0.246454	0.805902	-5.69414	0.774529	0.779034
Dendritic.cells	GM614	0.137571	-0.15476	0.246388	0.805953	-5.25156	0.835438	0.868275
Dendritic.cells	MRPL16	0.054649	3.919331	0.246378	0.80596	-5.89387	0.771968	0.775307
Dendritic.cells	DNPEP	0.051291	4.077287	0.246256	0.806055	-5.99844	0.769631	0.771906
Dendritic.cells	AGPAT1	-0.06447	3.479015	-0.24618	0.806115	-5.8414	0.778533	0.784887
Dendritic.cells	TMEM116	-0.12522	2.038927	-0.24588	0.806347	-5.3858	0.800498	0.817042
Dendritic.cells	PTER	-0.10738	2.433342	-0.24571	0.806472	-5.51513	0.794407	0.808147
Dendritic.cells	BBIP1	0.030253	5.888435	0.245672	0.806506	-6.53193	0.743465	0.734054
Dendritic.cells	AOPEP	0.029826	6.329542	0.245404	0.806712	-6.586	0.737268	0.725123
Dendritic.cells	MRM1	-0.08053	2.067052	-0.24538	0.806734	-5.57595	0.800062	0.816454
Dendritic.cells	GH	0.122298	-0.76502	0.245372	0.806737	-5.25023	0.845479	0.883256
Dendritic.cells	TNFRSF26	0.140055	2.617995	0.24537	0.806739	-5.34891	0.791574	0.804039
Dendritic.cells	FHL2	0.179841	0.017257	0.245314	0.806782	-5.2547	0.832633	0.864311
Dendritic.cells	GM13091	0.136394	0.904415	0.245263	0.806821	-5.30252	0.818343	0.843285
Dendritic.cells	CCAR1	0.019402	6.820278	0.245181	0.806885	-6.79356	0.730454	0.715322
Dendritic.cells	HIST1H2AK	-0.14962	0.954719	-0.24515	0.806907	-5.31063	0.817542	0.842134
Dendritic.cells	TSEN54	-0.06412	2.874509	-0.24509	0.806955	-5.72308	0.787661	0.798371
Dendritic.cells	TPRKB	0.070973	3.157978	0.245059	0.806978	-5.73624	0.783364	0.792099

Dendritic.cells	SHTN1	-0.09249	3.187667	-0.24482	0.807161	-5.66646	0.783024	0.791514
Dendritic.cells	GM20404	-0.11789	1.661785	-0.24459	0.807344	-5.35123	0.806517	0.825836
Dendritic.cells	MTPAP	0.048406	4.284578	0.244582	0.807347	-6.07222	0.766709	0.767725
Dendritic.cells	MECR	0.049148	3.581314	0.244405	0.807483	-6.04009	0.777136	0.782941
Dendritic.cells	ARHGAP11	-0.05919	4.784218	-0.24434	0.807534	-6.01738	0.75941	0.757165
Dendritic.cells	DPH3	0.03321	5.480129	0.244185	0.807654	-6.28782	0.749392	0.742646
Dendritic.cells	DFFA	0.072055	2.939835	0.244175	0.807661	-5.70266	0.786804	0.797056
Dendritic.cells	CDK13	-0.02057	7.414024	-0.24407	0.807745	-6.96202	0.722446	0.703748
Dendritic.cells	ZFP59	-0.13985	0.230401	-0.24398	0.807809	-5.28045	0.829317	0.859486
Dendritic.cells	FXYD4	-0.0812	3.001862	-0.2438	0.80795	-5.70936	0.785863	0.795786
Dendritic.cells	CPLANE1	-0.05178	4.78519	-0.2437	0.808028	-6.14233	0.759396	0.757253
Dendritic.cells	HEXA	-0.0335	5.752202	-0.24367	0.808048	-6.553	0.745522	0.737138
Dendritic.cells	PDK2	-0.08886	2.10287	-0.24359	0.808116	-5.58537	0.799646	0.815935
Dendritic.cells	KLHL2	0.032334	5.570335	0.243502	0.808181	-6.52335	0.748106	0.74088
Dendritic.cells	GM20342	0.069758	3.499425	0.24342	0.808244	-5.77279	0.778362	0.784844
Dendritic.cells	DUBR	0.089282	2.378269	0.243413	0.808249	-5.56398	0.795392	0.80971
Dendritic.cells	1110012L1	0.096133	1.829365	0.243031	0.808544	-5.47557	0.804121	0.822278
Dendritic.cells	CCDC82	-0.04414	4.323551	-0.24289	0.808651	-6.1197	0.766384	0.767172
Dendritic.cells	CCDC173	-0.12083	0.767334	-0.2428	0.808724	-5.34705	0.82094	0.846952
Dendritic.cells	GM12743	0.078468	2.382516	0.242623	0.808859	-5.70065	0.795622	0.80983
Dendritic.cells	SURF1	0.044827	4.36834	0.242566	0.808903	-6.13918	0.765764	0.766309
Dendritic.cells	ZDHHC23	-0.0737	2.221064	-0.24236	0.80906	-5.69823	0.798164	0.813507
Dendritic.cells	EMB	-0.09208	5.68619	-0.24232	0.809096	-6.01437	0.746783	0.738721
Dendritic.cells	NMI	-0.06309	5.064624	-0.24196	0.809374	-6.01095	0.755875	0.751705
Dendritic.cells	ITGAM	-0.13108	4.619395	-0.24172	0.809553	-5.66544	0.762437	0.761137
Dendritic.cells	SHCBP1L	-0.11891	0.854389	-0.2416	0.809648	-5.34573	0.819984	0.845198
Dendritic.cells	STXBP4	0.100937	2.23619	0.241454	0.809762	-5.57116	0.798308	0.813354
Dendritic.cells	ACTG1	-0.02649	11.44474	-0.24106	0.81007	-7.52292	0.670979	0.629609
Dendritic.cells	GM4285	0.084368	1.640385	0.241032	0.810088	-5.56112	0.807607	0.827061
Dendritic.cells	DMWD	0.078898	2.228423	0.24097	0.810137	-5.74835	0.798449	0.813666
Dendritic.cells	NID1	-0.06897	2.394724	-0.24095	0.810149	-5.8648	0.795882	0.809915
Dendritic.cells	PTGS2OS2	-0.14073	-0.48663	-0.24086	0.810219	-5.25868	0.841813	0.877413
Dendritic.cells	MEG3	-0.19464	1.040515	-0.24055	0.810465	-5.3363	0.817082	0.841027
Dendritic.cells	DPM2	0.052646	3.936241	0.240454	0.810535	-5.92582	0.772573	0.775952
Dendritic.cells	ZFP932	-0.05844	2.788655	-0.24035	0.810617	-5.68795	0.789842	0.80113
Dendritic.cells	METTL25	0.03767	4.28478	0.240198	0.810733	-6.12725	0.767423	0.768461
Dendritic.cells	NUAK1	0.129449	3.219187	0.240166	0.810758	-5.43007	0.783307	0.791592
Dendritic.cells	CARD9	0.129388	0.997223	0.239981	0.810901	-5.35676	0.817771	0.842039
Dendritic.cells	TMED8	0.049564	2.954093	0.239953	0.810922	-5.96309	0.787323	0.797452
Dendritic.cells	PBLD2	-0.11852	0.872415	-0.23977	0.811061	-5.31349	0.819761	0.845014
Dendritic.cells	LRRFIP1	0.022787	7.249941	0.239728	0.811096	-6.87438	0.72536	0.707625
Dendritic.cells	E430018J2	-0.11654	0.653485	-0.23938	0.811367	-5.36029	0.823267	0.85032
Dendritic.cells	ARMT1	-0.06126	3.297769	-0.23924	0.811473	-5.67829	0.782122	0.790075
Dendritic.cells	TMEM87A	0.030658	5.236916	0.23923	0.811481	-6.47007	0.753577	0.748561
Dendritic.cells	PKNOX2	0.149712	-0.82371	0.239089	0.81159	-5.25243	0.847391	0.885926
Dendritic.cells	GM21887	-0.11461	2.133944	-0.23901	0.811652	-5.49953	0.799911	0.816072
Dendritic.cells	L3MBTL2	-0.04013	3.445434	-0.23898	0.811671	-6.07129	0.7799	0.786836

Dendritic.cells	USP16	0.031868	5.208005	0.238931	0.811712	-6.42195	0.753993	0.749164
Dendritic.cells	HNRNPC	-0.01516	7.730209	-0.23893	0.811715	-6.97235	0.718835	0.698365
Dendritic.cells	DNAJC11	-0.03829	4.486406	-0.23876	0.811843	-6.19838	0.764464	0.76439
Dendritic.cells	GTF3C1	0.038881	4.779184	0.238585	0.81198	-6.2032	0.760193	0.758196
Dendritic.cells	CMC4	0.061666	3.064546	0.238477	0.812063	-5.74071	0.785647	0.795256
Dendritic.cells	BRCA1	0.09479	4.143927	0.238427	0.812101	-5.76649	0.769499	0.771723
Dendritic.cells	THAP11	0.053592	4.457867	0.238301	0.812199	-5.93882	0.764882	0.765009
Dendritic.cells	RPA1	0.047977	4.922792	0.238277	0.812218	-6.12924	0.758109	0.755171
Dendritic.cells	GPAA1	0.049052	3.764843	0.238233	0.812252	-5.86647	0.775122	0.779909
Dendritic.cells	SAP25	0.107225	2.354647	0.238057	0.812388	-5.49923	0.796499	0.811175
Dendritic.cells	FOXO4	0.089963	2.699869	0.238049	0.812394	-5.52711	0.791199	0.803424
Dendritic.cells	INTS4	-0.0412	3.920928	-0.23795	0.812467	-6.14719	0.772801	0.776617
Dendritic.cells	A930007I1	-0.11658	4.385631	-0.23787	0.812536	-5.64404	0.765941	0.766653
Dendritic.cells	GM14471	-0.11301	0.24931	-0.23785	0.812551	-5.38522	0.829785	0.8601
Dendritic.cells	ATL3	0.030768	5.439187	0.23782	0.812571	-6.35926	0.750677	0.744494
Dendritic.cells	POLDIP2	0.048533	4.278307	0.237744	0.81263	-6.05162	0.767518	0.768963
Dendritic.cells	ABCC2	-0.10395	2.212583	-0.23768	0.812679	-5.51386	0.798694	0.814457
Dendritic.cells	1700110K1	-0.1418	-0.57203	-0.23765	0.812701	-5.30799	0.843222	0.879947
Dendritic.cells	LYN	0.021601	10.03311	0.237554	0.812777	-7.3901	0.688633	0.655163
Dendritic.cells	FEM1C	0.034266	7.117684	0.23754	0.812788	-6.6463	0.72717	0.710521
Dendritic.cells	GM47662	0.130446	-0.79475	0.237505	0.812814	-5.25402	0.84691	0.885398
Dendritic.cells	SOX5	0.096885	4.684841	0.237434	0.81287	-5.93989	0.761566	0.760312
Dendritic.cells	2010315B0	-0.07524	2.024895	-0.23741	0.812889	-5.5833	0.801604	0.818718
Dendritic.cells	GIMAP7	-0.14299	1.458503	-0.23737	0.812917	-5.34711	0.810465	0.831714
Dendritic.cells	RAPGEFL1	0.062821	2.784487	0.237308	0.812967	-5.9547	0.789906	0.801618
Dendritic.cells	TRBC2	0.163799	3.943953	0.23704	0.813174	-5.71061	0.772544	0.77622
Dendritic.cells	GM49463	-0.14834	-0.05016	-0.23695	0.813247	-5.2576	0.834748	0.867372
Dendritic.cells	TTPAL	0.052473	4.234118	0.236917	0.81327	-5.85702	0.768254	0.769976
Dendritic.cells	PTGES	0.181736	0.477516	0.236818	0.813345	-5.25764	0.826194	0.85476
Dendritic.cells	GM40787	0.146709	0.205726	0.236553	0.81355	-5.25409	0.830711	0.861345
Dendritic.cells	GSN	0.025464	5.844895	0.236352	0.813706	-6.97437	0.745101	0.736359
Dendritic.cells	BEX4	0.133486	0.397887	0.236326	0.813727	-5.25652	0.8276	0.856836
Dendritic.cells	EMC10	0.024149	5.502896	0.236061	0.813931	-6.60986	0.749965	0.743483
Dendritic.cells	LRIG3	-0.13201	0.14201	-0.23596	0.814013	-5.33968	0.831745	0.863079
Dendritic.cells	ALKBH7	0.061104	3.373231	0.235914	0.814045	-5.89016	0.781191	0.788949
Dendritic.cells	ZFP503	-0.09048	0.884395	-0.23583	0.814111	-5.55811	0.819786	0.845488
Dendritic.cells	HDDC3	0.098493	0.928077	0.235805	0.814129	-5.49062	0.819089	0.844472
Dendritic.cells	RBM44	-0.15537	0.135835	-0.23579	0.814141	-5.25421	0.831846	0.863258
Dendritic.cells	ZFP608	0.035623	6.820434	0.235624	0.814269	-6.89482	0.731454	0.716795
Dendritic.cells	TSPYL3	-0.11208	0.960124	-0.23551	0.814361	-5.49652	0.818577	0.843744
Dendritic.cells	PTS	0.029825	5.573329	0.235479	0.814381	-6.42264	0.74896	0.742119
Dendritic.cells	TMEM156	0.050175	3.855526	0.235403	0.81444	-6.02284	0.773976	0.778472
Dendritic.cells	PIGG	0.099496	0.963956	0.235384	0.814455	-5.4815	0.818516	0.843658
Dendritic.cells	DPP9	-0.0452	4.420233	-0.23518	0.814616	-6.07753	0.765658	0.766401
Dendritic.cells	NOP53	-0.03317	5.740416	-0.23505	0.814716	-6.46236	0.746605	0.738744
Dendritic.cells	1700017B0	-0.02466	5.285252	-0.235	0.814751	-6.82277	0.753104	0.748173
Dendritic.cells	ZFP598	0.065169	3.44196	0.234919	0.814815	-5.75353	0.780181	0.787568

Dendritic.cells	CD180	0.029101	5.032672	0.234836	0.814879	-6.75981	0.756742	0.753467
Dendritic.cells	SEMA4F	0.126065	-0.41218	0.234742	0.814952	-5.25774	0.840834	0.876639
Dendritic.cells	UVRAG	-0.02119	8.569242	-0.23465	0.815019	-7.26279	0.707834	0.682869
Dendritic.cells	ZFP511	-0.04798	3.816412	-0.23462	0.815046	-5.86928	0.774581	0.779468
Dendritic.cells	NCOA3	-0.02082	6.960263	-0.23428	0.815306	-6.75169	0.729716	0.714199
Dendritic.cells	DNMBP	0.052116	3.488772	0.233987	0.815536	-5.97221	0.779812	0.786713
Dendritic.cells	SH3RF1	-0.0349	4.357213	-0.23374	0.815723	-6.46063	0.767021	0.767978
Dendritic.cells	MIGA1	-0.05541	2.677211	-0.2334	0.815993	-5.7676	0.792423	0.804814
Dendritic.cells	INTS5	0.062	2.704825	0.23316	0.816176	-5.6875	0.792039	0.804269
Dendritic.cells	VAV2	-0.03974	4.977829	-0.23305	0.81626	-6.4149	0.75819	0.75499
Dendritic.cells	UBE2B	0.026798	8.25823	0.232979	0.816316	-6.92372	0.712578	0.689099
Dendritic.cells	TTC4	-0.0536	3.541793	-0.23293	0.816351	-5.80101	0.779357	0.785786
Dendritic.cells	FBXO5	0.112033	4.811103	0.232782	0.816469	-5.80617	0.760609	0.758509
Dendritic.cells	POU5F2	0.089755	1.687999	0.23275	0.816493	-5.50919	0.807795	0.827366
Dendritic.cells	ZFP362	0.039306	3.9339	0.232609	0.816603	-6.12328	0.773503	0.777316
Dendritic.cells	EHMT2	0.035846	4.951712	0.232515	0.816676	-6.27537	0.758568	0.755621
Dendritic.cells	CTSB	0.027866	8.190873	0.232423	0.816747	-7.23781	0.713477	0.690467
Dendritic.cells	FEM1A	0.052622	2.924816	0.232315	0.81683	-5.82839	0.788681	0.799472
Dendritic.cells	ENO1B	0.110306	1.327345	0.23225	0.816881	-5.365	0.813476	0.835784
Dendritic.cells	TEX10	-0.03816	5.266363	-0.23225	0.816884	-6.35824	0.754027	0.749031
Dendritic.cells	MYO1E	-0.09594	6.198716	-0.23212	0.816982	-6.00426	0.7408	0.729858
Dendritic.cells	LZIC	0.05153	3.63991	0.231669	0.817331	-5.91039	0.777948	0.783838
Dendritic.cells	KIF20B	-0.11274	4.170808	-0.23165	0.817349	-5.72293	0.770054	0.772341
Dendritic.cells	SHARPIN	0.041776	4.530571	0.231495	0.817466	-6.06001	0.764763	0.764645
Dendritic.cells	TRAJ18	0.102986	-1.3552	0.231486	0.817472	-5.25451	0.857333	0.900507
Dendritic.cells	ADNP2	0.071502	2.990665	0.231479	0.817478	-5.75306	0.787741	0.798127
Dendritic.cells	TMCO4	0.062693	4.054604	0.231471	0.817484	-5.86593	0.771773	0.774843
Dendritic.cells	GM31814	0.120324	-0.06084	0.231406	0.817534	-5.25809	0.835863	0.868762
Dendritic.cells	TCTN3	0.117667	1.366808	0.231339	0.817586	-5.37925	0.812916	0.835003
Dendritic.cells	TPST1	-0.10175	3.472726	-0.23108	0.81779	-5.49663	0.780564	0.787622
Dendritic.cells	HSD17B10	0.041831	5.540491	0.231009	0.817842	-6.26206	0.750262	0.743568
Dendritic.cells	TCTA	-0.07574	2.098287	-0.2308	0.818005	-5.55398	0.801657	0.818324
Dendritic.cells	TARDBP	0.027885	5.771119	0.230706	0.818076	-6.47736	0.747062	0.738831
Dendritic.cells	PILRB1	-0.13531	2.049283	-0.23035	0.818354	-5.35664	0.802419	0.819543
Dendritic.cells	TTC17	0.025772	5.324345	0.230337	0.818362	-6.5426	0.753443	0.748154
Dendritic.cells	RECK	-0.14976	1.480341	-0.23025	0.818432	-5.32517	0.811328	0.832608
Dendritic.cells	SLC5A10	-0.14118	-0.1778	-0.23006	0.818574	-5.25596	0.837988	0.871909
Dendritic.cells	EID3	0.151462	0.925233	0.230038	0.818594	-5.2717	0.820138	0.845612
Dendritic.cells	SDF2	0.028746	5.410417	0.229984	0.818635	-6.4288	0.752208	0.746419
Dendritic.cells	GDAP2	-0.03658	4.778409	-0.22998	0.818641	-6.22547	0.761337	0.759669
Dendritic.cells	DENR	0.025463	6.279481	0.229975	0.818643	-6.55831	0.739887	0.728576
Dendritic.cells	MRPL41	0.048973	4.003451	0.229851	0.818739	-5.94855	0.77275	0.776234
Dendritic.cells	ZFP213	-0.07065	2.162406	-0.22973	0.818832	-5.67096	0.800698	0.817055
Dendritic.cells	ZFP808	-0.07764	1.973033	-0.22965	0.818893	-5.58029	0.803642	0.821388
Dendritic.cells	CCDC18	-0.08365	2.722921	-0.22952	0.818993	-5.55045	0.792061	0.80447
Dendritic.cells	AFG3L1	0.025612	4.862083	0.229305	0.819162	-6.3317	0.760155	0.757976
Dendritic.cells	ASCC2	0.034633	4.605255	0.229076	0.819339	-6.1515	0.763898	0.763433

Dendritic.cells	TRIM28	0.036963	5.460856	0.229044	0.819364	-6.32818	0.75152	0.745463
Dendritic.cells	PXN	-0.0338	6.266447	-0.22893	0.81945	-6.49052	0.740103	0.728948
Dendritic.cells	USP50	0.063988	3.371827	0.228796	0.819556	-5.74471	0.782206	0.790142
Dendritic.cells	2610001J0	0.052824	3.690163	0.228738	0.819601	-5.89682	0.777428	0.783175
Dendritic.cells	CENPO	-0.08248	2.773597	-0.22845	0.819828	-5.48956	0.791286	0.803461
Dendritic.cells	GM43661	0.113985	2.981343	0.228259	0.819972	-5.51393	0.788118	0.798879
Dendritic.cells	NEK9	-0.03606	5.27603	-0.22825	0.819978	-6.3567	0.754172	0.749451
Dendritic.cells	1700029H1	-0.08763	1.58753	-0.22806	0.820128	-5.51991	0.809678	0.830456
Dendritic.cells	TYW1	0.057495	3.875123	0.228043	0.820139	-5.83346	0.774669	0.779259
Dendritic.cells	SOX12	0.107324	0.585357	0.227984	0.820185	-5.37708	0.825627	0.853903
Dendritic.cells	TFRC	-0.0288	6.38061	-0.22794	0.820222	-6.6449	0.738504	0.726758
Dendritic.cells	2900097C1	0.029931	4.909122	0.227821	0.820312	-6.35777	0.759472	0.757153
Dendritic.cells	IGHMBP2	-0.06158	2.850148	-0.22767	0.820432	-5.71523	0.790116	0.801838
Dendritic.cells	GM20470	0.106578	1.403571	0.22752	0.820545	-5.41157	0.812577	0.834769
Dendritic.cells	TFPT	-0.06087	2.965912	-0.22739	0.820645	-5.66961	0.788352	0.799283
Dendritic.cells	SIAE	-0.06339	2.612072	-0.22733	0.82069	-5.77389	0.79376	0.80719
Dendritic.cells	NRAP	0.122125	-1.09404	0.227279	0.820732	-5.25235	0.853204	0.894693
Dendritic.cells	AV099323	0.127639	1.209882	0.227171	0.820815	-5.33624	0.815644	0.839278
Dendritic.cells	THNSL1	0.10853	1.077135	0.227089	0.820878	-5.4433	0.817753	0.842385
Dendritic.cells	2410002F2	0.036247	3.766412	0.226968	0.820973	-6.20175	0.776289	0.781696
Dendritic.cells	BARD1	0.088434	3.42386	0.22677	0.821126	-5.6434	0.781422	0.78924
Dendritic.cells	GM16541	-0.06651	2.915994	-0.22674	0.82115	-5.72262	0.789112	0.800471
Dendritic.cells	MAP7	-0.06222	3.639692	-0.22669	0.821184	-5.79195	0.778183	0.784513
Dendritic.cells	ZFYVE1	0.040903	4.797159	0.226682	0.821194	-6.22704	0.761099	0.759641
Dendritic.cells	LY9	-0.03965	4.262049	-0.22649	0.821341	-6.18798	0.768937	0.771139
Dendritic.cells	STX18	0.035002	4.637177	0.225969	0.821747	-6.28494	0.763431	0.76325
Dendritic.cells	GM17036	-0.08912	2.038724	-0.22591	0.821791	-5.58117	0.80262	0.820477
Dendritic.cells	PPP1R1C	-0.15751	0.781145	-0.2257	0.821954	-5.29752	0.822481	0.849728
Dendritic.cells	FASTKD2	-0.06076	2.952537	-0.22569	0.821965	-5.64536	0.788556	0.799952
Dendritic.cells	RSPH10B	-0.12678	0.413611	-0.22564	0.822001	-5.38382	0.828398	0.858445
Dendritic.cells	GM45370	-0.13897	-1.29914	-0.22521	0.822333	-5.2525	0.856645	0.900275
Dendritic.cells	MOB1B	-0.02235	6.203813	-0.22503	0.822474	-6.58197	0.740983	0.7308
Dendritic.cells	NFKBIB	-0.03518	5.622292	-0.22499	0.822504	-6.41359	0.749214	0.742731
Dendritic.cells	VPS37C	-0.03977	3.950923	-0.2247	0.822734	-6.03081	0.773541	0.778177
Dendritic.cells	HTR1F	0.084257	1.429334	0.224644	0.822774	-5.47793	0.81217	0.834717
Dendritic.cells	A730036I1	-0.15457	-0.20546	-0.22462	0.822797	-5.25606	0.838479	0.873485
Dendritic.cells	MYB	0.054994	5.137938	0.224528	0.822864	-6.14873	0.756161	0.752916
Dendritic.cells	GADD45G	-0.09744	4.465474	-0.22452	0.822872	-5.70914	0.765945	0.767144
Dendritic.cells	GM47283	-0.05725	7.990664	-0.22421	0.823111	-6.94417	0.716429	0.695465
Dendritic.cells	BASP1	-0.03838	5.646818	-0.22418	0.823136	-6.89288	0.748864	0.742355
Dendritic.cells	SLIT2	-0.08751	0.983319	-0.22417	0.823138	-5.75797	0.819248	0.845197
Dendritic.cells	PROSCOS	-0.13976	0.287878	-0.22404	0.82324	-5.31875	0.830434	0.86171
Dendritic.cells	SDF4	-0.02135	6.506152	-0.22398	0.823291	-6.6351	0.73675	0.724836
Dendritic.cells	NRP1	-0.03324	4.442319	-0.22394	0.823318	-6.78129	0.766285	0.767703
Dendritic.cells	KREMEN1	-0.04002	3.039966	-0.22392	0.823334	-6.095	0.787227	0.798257
Dendritic.cells	TNPO2	-0.04749	4.122122	-0.22392	0.823336	-6.0158	0.771003	0.774576
Dendritic.cells	GIMAP9	0.074519	3.560572	0.223891	0.823359	-5.7138	0.779369	0.786776

Dendritic.cells	PANK4	-0.06171	3.423163	-0.22383	0.823407	-5.75098	0.781433	0.789801
Dendritic.cells	QRSL1	0.05956	3.18623	0.223828	0.823408	-5.84884	0.785009	0.795025
Dendritic.cells	GM48742	0.109863	0.618325	0.223796	0.823433	-5.34752	0.825096	0.853865
Dendritic.cells	HPS6	0.098717	1.22115	0.223698	0.823509	-5.41561	0.815465	0.839721
Dendritic.cells	4930430E1	-0.15325	0.409202	-0.22362	0.823569	-5.29115	0.828469	0.858874
Dendritic.cells	CREG1	0.033551	7.514034	0.2236	0.823584	-6.75659	0.722871	0.704828
Dendritic.cells	1600002KC	0.053972	3.214423	0.223599	0.823585	-5.79836	0.784582	0.794444
Dendritic.cells	ST3GAL1	0.044016	5.796993	0.223536	0.823634	-6.53109	0.746728	0.739348
Dendritic.cells	RNF219	0.061959	3.193241	0.223531	0.823638	-5.81529	0.784903	0.794924
Dendritic.cells	GM26549	-0.06433	3.018851	-0.22352	0.823647	-5.70482	0.787547	0.79879
Dendritic.cells	GM26916	0.108323	0.934105	0.223499	0.823663	-5.2929	0.820034	0.846462
Dendritic.cells	AHRR	-0.1214	-0.36283	-0.22347	0.823689	-5.25998	0.841065	0.877493
Dendritic.cells	ARRB2	0.04358	5.195443	0.223454	0.823698	-6.15877	0.755332	0.751841
Dendritic.cells	CLEC4A4	0.103247	-1.00355	0.223438	0.82371	-5.25765	0.85169	0.893223
Dendritic.cells	D2HGDH	0.075167	2.08867	0.22343	0.823716	-5.58757	0.801843	0.819732
Dendritic.cells	RSBN1	-0.02831	5.326631	-0.22325	0.823854	-6.49891	0.753444	0.749137
Dendritic.cells	PDLIM7	-0.07774	3.638312	-0.22314	0.823939	-5.59915	0.778204	0.785188
Dendritic.cells	KIF15	0.085657	4.90741	0.223124	0.823954	-6.00873	0.759497	0.757935
Dendritic.cells	PPP6R1	-0.01915	5.572544	-0.22308	0.823985	-6.6479	0.749923	0.744029
Dendritic.cells	GM44699	-0.11677	0.559613	-0.22292	0.824114	-5.28064	0.826042	0.855372
Dendritic.cells	UBE3A	0.01775	6.387077	0.222877	0.824145	-6.80991	0.738413	0.727357
Dendritic.cells	SPIRE1	0.118594	2.681156	0.222855	0.824162	-5.51846	0.7927	0.806391
Dendritic.cells	GM20324	-0.08353	2.098382	-0.22279	0.824211	-5.44947	0.801692	0.819567
Dendritic.cells	LGALS2	0.120177	-0.89826	0.222754	0.824241	-5.2548	0.849934	0.890681
Dendritic.cells	RANBP3	0.032299	4.303701	0.222731	0.824259	-6.20169	0.768323	0.770792
Dendritic.cells	SLC38A10	0.030323	5.326136	0.222612	0.824351	-6.38058	0.753452	0.749171
Dendritic.cells	MINDY3	-0.02642	5.598106	-0.22252	0.824422	-6.52935	0.749559	0.74352
Dendritic.cells	NUDCD2	0.051334	4.534449	0.22248	0.824454	-6.01269	0.764934	0.765878
Dendritic.cells	TMEM106C	0.044468	3.205819	0.2223	0.824593	-5.9937	0.784712	0.79478
Dendritic.cells	H2-DMB2	0.079319	3.016521	0.222285	0.824605	-5.71413	0.787583	0.798977
Dendritic.cells	GM15675	-0.12084	2.317552	-0.22223	0.824645	-5.37058	0.798296	0.814668
Dendritic.cells	C230066G2	-0.12073	0.025289	-0.22206	0.824777	-5.35113	0.834705	0.868267
Dendritic.cells	ZFP560	0.045766	4.00302	0.221944	0.824869	-5.95192	0.772768	0.777438
Dendritic.cells	FBXO48	0.100935	0.24175	0.221742	0.825026	-5.38858	0.831182	0.863189
Dendritic.cells	FAM25C	-0.13304	1.148741	-0.22167	0.825085	-5.34337	0.816615	0.841717
Dendritic.cells	ZFP189	-0.09489	1.120206	-0.22166	0.825088	-5.43654	0.817068	0.842385
Dendritic.cells	MAP3K14	0.047182	5.189544	0.221548	0.825176	-6.18249	0.755417	0.75222
Dendritic.cells	SLC45A4	0.060341	3.337998	0.221537	0.825186	-5.69783	0.782716	0.792004
Dendritic.cells	FLII	0.026652	5.969326	0.221467	0.82524	-6.54033	0.744287	0.736068
Dendritic.cells	TRDV4	-0.12334	-1.36214	-0.22127	0.825389	-5.25676	0.857705	0.902484
Dendritic.cells	TTC37	-0.05164	3.798842	-0.22119	0.825454	-6.06943	0.775805	0.781971
Dendritic.cells	GM26930	0.113233	0.305585	0.22099	0.82561	-5.45357	0.830147	0.8618
Dendritic.cells	MMACHC	0.13176	0.470101	0.220979	0.825618	-5.2838	0.827485	0.857873
Dendritic.cells	PXMP4	0.05355	3.831766	0.220964	0.82563	-5.90411	0.775314	0.781322
Dendritic.cells	ENTHD1	0.146457	0.023444	0.220896	0.825683	-5.27761	0.834735	0.868576
Dendritic.cells	SLC25A3	0.020604	7.921066	0.22088	0.825695	-6.87961	0.717365	0.697254
Dendritic.cells	PCMTD1	-0.03148	5.924056	-0.22078	0.825772	-6.49376	0.744932	0.73715

Dendritic.cells	SLC5A3	0.061698	3.816287	0.220291	0.826152	-6.02786	0.775753	0.781802
Dendritic.cells	KATNA1	0.045425	4.864725	0.220168	0.826248	-6.01355	0.76032	0.759321
Dendritic.cells	TMED3	-0.02038	5.491352	-0.22012	0.826286	-6.9023	0.751285	0.746209
Dendritic.cells	GM11290	-0.12077	3.432889	-0.21986	0.826486	-5.53756	0.781496	0.790215
Dendritic.cells	MALAT1	0.017046	14.72453	0.219746	0.826575	-8.07985	0.633498	0.577342
Dendritic.cells	NAGK	0.049501	3.956557	0.219733	0.826586	-5.94721	0.773665	0.778787
Dendritic.cells	UQCRFS1	0.022665	7.164384	0.219716	0.826599	-6.82134	0.727841	0.712254
Dendritic.cells	PLSCR3	-0.05911	4.301027	-0.21967	0.826637	-5.85368	0.768568	0.771356
Dendritic.cells	ZFP275	0.084921	1.581356	0.219622	0.826672	-5.45803	0.809992	0.831979
Dendritic.cells	TRAPPC1	0.041578	5.432947	0.21951	0.826759	-6.31088	0.752121	0.747441
Dendritic.cells	SYNC	0.077807	1.973724	0.219365	0.826871	-5.46556	0.803847	0.823017
Dendritic.cells	BTBD2	0.052944	3.477347	0.219172	0.827021	-5.8696	0.780827	0.789364
Dendritic.cells	OXLD1	-0.08168	1.813163	-0.21906	0.827108	-5.54784	0.806355	0.826763
Dendritic.cells	PPP2R2D	-0.02726	5.459388	-0.21897	0.827174	-6.38291	0.751742	0.746993
Dendritic.cells	TOMM20	-0.0201	6.725117	-0.2189	0.827231	-6.82345	0.733902	0.721128
Dendritic.cells	KIF14	-0.1304	2.55406	-0.21881	0.827301	-5.43835	0.794864	0.809904
Dendritic.cells	GM14295	0.086079	1.289141	0.218786	0.827321	-5.4298	0.814606	0.838892
Dendritic.cells	POFUT1	0.047358	3.458205	0.21876	0.827341	-5.90501	0.781115	0.789785
Dendritic.cells	SPDYA	0.127836	-0.02911	0.21875	0.827349	-5.25985	0.835817	0.870171
Dendritic.cells	INVS	0.069153	2.809082	0.218651	0.827426	-5.71498	0.790955	0.804185
Dendritic.cells	DNAJC25	0.042393	3.856746	0.218318	0.827685	-5.97544	0.77515	0.781238
Dendritic.cells	CCDC71	-0.06981	2.73695	-0.2182	0.82778	-5.62995	0.792058	0.806026
Dendritic.cells	GM16014	-0.13607	-0.09363	-0.21814	0.827819	-5.28015	0.836872	0.871982
Dendritic.cells	GAS5	0.027667	6.391612	0.21812	0.827838	-6.63974	0.738548	0.728065
Dendritic.cells	ZFP683	-0.12953	-1.15907	-0.21808	0.827868	-5.25836	0.854522	0.898136
Dendritic.cells	LIPH	-0.1332	-0.05189	-0.21807	0.827881	-5.26234	0.836189	0.87098
Dendritic.cells	TDRD7	-0.07236	3.385152	-0.21796	0.827962	-5.6792	0.782215	0.79164
Dendritic.cells	COPE	0.021841	6.316581	0.217947	0.827973	-6.70557	0.739599	0.729606
Dendritic.cells	JCAD	-0.07795	1.404554	-0.21776	0.82812	-5.56274	0.812854	0.836512
Dendritic.cells	KAT2A	0.067594	3.199202	0.217422	0.82838	-5.6636	0.785151	0.795896
Dendritic.cells	NLRC3	0.0998	1.662225	0.217206	0.828548	-5.37575	0.808852	0.830685
Dendritic.cells	SCRG1	0.12092	-0.98095	0.217177	0.828571	-5.25926	0.85168	0.893929
Dendritic.cells	INO80D	-0.0234	5.995336	-0.21709	0.828638	-6.53685	0.74424	0.736325
Dendritic.cells	2610507I0	-0.13334	0.583661	-0.21691	0.828779	-5.29042	0.82601	0.855954
Dendritic.cells	MCRIP1	0.022809	5.919349	0.216835	0.828837	-6.50149	0.745315	0.737884
Dendritic.cells	MOK	0.090622	0.377087	0.216754	0.828899	-5.61776	0.829346	0.860878
Dendritic.cells	FSTL1	0.10253	2.107543	0.216745	0.828906	-5.53828	0.801895	0.820464
Dendritic.cells	FAM172A	-0.01747	7.557353	-0.21669	0.828946	-7.02183	0.722593	0.704998
Dendritic.cells	ZFP54	-0.10723	0.794625	-0.21651	0.829091	-5.39344	0.82262	0.851018
Dendritic.cells	CTH	0.125073	3.512521	0.216493	0.829102	-5.63497	0.780426	0.789075
Dendritic.cells	CBX2	0.122693	-0.18673	0.216439	0.829144	-5.25594	0.838533	0.874521
Dendritic.cells	PLPP6	0.058247	2.754142	0.216433	0.829148	-5.81206	0.791924	0.805902
Dendritic.cells	GM48086	-0.09353	1.511775	-0.21622	0.829318	-5.43519	0.811219	0.834233
Dendritic.cells	TNFRSF25	0.102681	-1.26707	0.216086	0.829419	-5.25692	0.856474	0.901117
Dendritic.cells	CLEC4A2	-0.11656	3.416946	-0.21598	0.829501	-5.46432	0.781863	0.79118
Dendritic.cells	2310010J1	-0.05194	3.692212	-0.21595	0.829525	-6.01478	0.777732	0.785143
Dendritic.cells	CXCR6	-0.13071	1.255073	-0.21592	0.829544	-5.34527	0.815278	0.840205

Dendritic.cells	TPGS1	0.046491	4.393149	0.215844	0.829607	-6.02917	0.767337	0.769974
Dendritic.cells	PIGB	0.06475	2.703863	0.215758	0.829673	-5.69367	0.792694	0.807064
Dendritic.cells	RRM2	0.08274	6.880861	0.215662	0.829748	-6.37664	0.731864	0.718521
Dendritic.cells	NUDT15	-0.09165	1.647281	-0.21559	0.829801	-5.40382	0.809087	0.831183
Dendritic.cells	GM11520	-0.10226	1.043872	-0.21558	0.829809	-5.32992	0.818636	0.845238
Dendritic.cells	NEK6	0.019455	3.873937	0.215363	0.82998	-6.86172	0.775094	0.781294
Dendritic.cells	SEPHS2	0.048104	6.39868	0.215135	0.830157	-6.36069	0.738639	0.728265
Dendritic.cells	ZFP12	0.081455	1.346551	0.215109	0.830178	-5.46427	0.813906	0.838192
Dendritic.cells	ST8SIA1	0.047852	0.877835	0.215028	0.830241	-6.29791	0.821365	0.849179
Dendritic.cells	THOC6	-0.05126	4.164901	-0.21503	0.830242	-5.88947	0.770776	0.774992
Dendritic.cells	CPQ	-0.03504	5.056734	-0.21468	0.830514	-6.45594	0.757829	0.756051
Dendritic.cells	FADS3	-0.03525	0.659123	-0.21448	0.830666	-6.29348	0.82498	0.854438
Dendritic.cells	ZFP281	-0.04362	4.496464	-0.21442	0.830715	-6.05152	0.765991	0.767949
Dendritic.cells	CHURC1	0.025595	5.66012	0.214388	0.830738	-6.44954	0.749165	0.743469
Dendritic.cells	CCNK	-0.03386	4.857081	-0.21439	0.83074	-6.17926	0.760725	0.760278
Dendritic.cells	PHOSPHO2	-0.04239	3.724911	-0.21434	0.830774	-5.90731	0.777417	0.784619
Dendritic.cells	TSSC4	0.061001	3.902903	0.213892	0.831124	-5.7286	0.774914	0.780772
Dendritic.cells	HNRNPH2	0.019392	6.435152	0.213842	0.831162	-6.7002	0.738369	0.727631
Dendritic.cells	WASHC1	0.036	4.182087	0.213721	0.831256	-6.05685	0.770772	0.774747
Dendritic.cells	ZFP946	0.074416	1.39583	0.21353	0.831405	-5.55294	0.813391	0.837183
Dendritic.cells	GM42836	-0.13049	-0.40469	-0.21346	0.831459	-5.26516	0.84247	0.8801
Dendritic.cells	GM31812	0.12804	0.191942	0.21346	0.83146	-5.26391	0.832699	0.86565
Dendritic.cells	DLG1	-0.01931	7.219142	-0.21343	0.831486	-6.87558	0.727513	0.711937
Dendritic.cells	SERPINA1E	-0.12184	5.030433	-0.21336	0.83154	-6.09174	0.758358	0.756678
Dendritic.cells	FYTTD1	-0.02578	5.267108	-0.21322	0.831645	-6.33474	0.754941	0.751724
Dendritic.cells	SLC39A14	0.033204	3.169335	0.213181	0.831676	-6.44038	0.785932	0.796936
Dendritic.cells	ATXN2	0.020723	6.28335	0.213129	0.831717	-6.77591	0.740495	0.730766
Dendritic.cells	EBP	0.041124	4.791273	0.213003	0.831815	-6.17641	0.761857	0.761761
Dendritic.cells	FAAP100	0.034005	3.097229	0.212772	0.831994	-6.23012	0.787131	0.79854
Dendritic.cells	SVIL	-0.02222	6.860609	-0.21271	0.832041	-6.8041	0.732549	0.719118
Dendritic.cells	ZDHHC7	0.051055	3.834677	0.212464	0.832234	-5.84581	0.776053	0.78232
Dendritic.cells	DNLZ	0.04629	3.991855	0.21246	0.832237	-6.00982	0.773713	0.778905
Dendritic.cells	PIFO	0.097126	1.246923	0.212273	0.832383	-5.56939	0.815878	0.840753
Dendritic.cells	SPG7	0.032404	4.467655	0.212112	0.832508	-6.09711	0.766686	0.768764
Dendritic.cells	HEATR6	0.035395	4.812778	0.212055	0.832552	-6.16389	0.761639	0.761437
Dendritic.cells	TAF1D	0.026024	6.027224	0.212041	0.832563	-6.45469	0.74422	0.736122
Dendritic.cells	PMS1	0.06212	2.650773	0.211911	0.832664	-5.72405	0.793966	0.808659
Dendritic.cells	SHCBP1	0.082581	4.69138	0.211832	0.832726	-5.89786	0.763409	0.764035
Dendritic.cells	MRPS28	-0.05204	6.885185	-0.21168	0.832841	-6.55589	0.732227	0.718775
Dendritic.cells	EIF5	0.01992	8.418516	0.211627	0.832885	-7.02199	0.711429	0.688745
Dendritic.cells	TENM2	-0.15028	-0.35252	-0.21163	0.832885	-5.27251	0.841744	0.879058
Dendritic.cells	GM41335	-0.15555	0.493808	-0.21159	0.832912	-5.2573	0.827937	0.858651
Dendritic.cells	A2ML1	-0.1152	2.471948	-0.21142	0.833043	-5.47997	0.796746	0.81273
Dendritic.cells	GM26787	0.115493	1.300668	0.211302	0.833138	-5.40697	0.815056	0.839694
Dendritic.cells	LRCH4	0.046269	4.260162	0.211167	0.833243	-5.98233	0.769769	0.77338
Dendritic.cells	NISCH	-0.02174	6.194239	-0.21116	0.833245	-6.66921	0.741893	0.732839
Dendritic.cells	GZMK	0.114243	-1.47202	0.210903	0.833448	-5.2564	0.860457	0.906957

Dendritic.cells	GJB1	-0.10786	1.59761	-0.21086	0.833482	-5.4619	0.810366	0.832905
Dendritic.cells	IL1R2	-0.06611	3.320344	-0.21074	0.833572	-6.25328	0.783801	0.793947
Dendritic.cells	DANCR	0.085137	1.545559	0.210564	0.833712	-5.50083	0.811186	0.834158
Dendritic.cells	IRAK1	-0.02401	5.224747	-0.21055	0.833725	-6.49106	0.755699	0.752992
Dendritic.cells	MDM2	-0.03676	6.41707	-0.21054	0.833729	-6.5406	0.738766	0.728407
Dendritic.cells	GM42962	0.104741	-0.60259	0.210309	0.833911	-5.26105	0.845907	0.885451
Dendritic.cells	EDEM3	0.034468	5.974164	0.210157	0.834029	-6.5754	0.744997	0.737536
Dendritic.cells	6230400D1	0.069742	2.163361	0.210146	0.834037	-5.62374	0.801521	0.820055
Dendritic.cells	MTX1	0.042811	4.556924	0.210123	0.834055	-6.07199	0.765405	0.767222
Dendritic.cells	TGS1	0.024256	5.214453	0.210002	0.83415	-6.46206	0.755848	0.753316
Dendritic.cells	SMS	0.017212	6.477089	0.209962	0.83418	-6.91056	0.737927	0.727295
Dendritic.cells	ZFP112	-0.11334	0.391108	-0.20991	0.834218	-5.3357	0.829629	0.861467
Dendritic.cells	USP15	-0.0187	7.650516	-0.20991	0.83422	-6.99794	0.721772	0.703922
Dendritic.cells	MICU2	0.028878	4.939969	0.209285	0.834707	-6.4226	0.760167	0.759249
Dendritic.cells	ANO8	-0.10429	0.904446	-0.20916	0.834804	-5.40346	0.821743	0.849464
Dendritic.cells	SLC2A8	0.079574	2.226604	0.209136	0.834823	-5.46189	0.800907	0.818814
Dendritic.cells	RGS13	-0.13485	-1.05629	-0.20895	0.834965	-5.257	0.853855	0.896961
Dendritic.cells	SMCHD1	0.02716	7.994517	0.208912	0.834997	-6.86601	0.717456	0.697371
Dendritic.cells	CHIL3	-0.30034	2.447415	-0.20887	0.835029	-5.29381	0.79749	0.813801
Dendritic.cells	SPTLC1	-0.03608	4.232218	-0.20866	0.835191	-6.12223	0.770619	0.774373
Dendritic.cells	LENG9	0.070576	2.352054	0.208415	0.835384	-5.61996	0.799168	0.816008
Dendritic.cells	POLR3C	0.027973	5.018329	0.207843	0.83583	-6.27698	0.759547	0.757843
Dendritic.cells	SUPT5	0.023334	6.05341	0.207713	0.835931	-6.59832	0.744726	0.736328
Dendritic.cells	RIMS4	-0.1306	-0.99611	-0.20768	0.835953	-5.25761	0.853429	0.895755
Dendritic.cells	RGS7BP	-0.08304	1.883105	-0.20735	0.836217	-5.83344	0.806992	0.826972
Dendritic.cells	CLCN5	0.026379	5.160349	0.207255	0.836287	-6.67493	0.757665	0.754912
Dendritic.cells	TBC1D16	-0.0517	2.452428	-0.20695	0.836523	-6.09258	0.798283	0.814008
Dendritic.cells	IER2	-0.03488	8.170209	-0.20688	0.836579	-6.85574	0.715877	0.694249
Dendritic.cells	PIP4P2	0.056327	3.706325	0.206603	0.836795	-5.86136	0.779281	0.786122
Dendritic.cells	NPL	-0.08079	2.535175	-0.20655	0.836836	-5.74562	0.797083	0.812159
Dendritic.cells	MDH1	0.020785	8.120076	0.206502	0.836873	-6.96783	0.716619	0.695231
Dendritic.cells	TMEM147	0.028092	4.948794	0.206167	0.837134	-6.41351	0.761029	0.759459
Dendritic.cells	SSX2IP	0.073568	2.481205	0.206042	0.837232	-5.72416	0.798008	0.813459
Dendritic.cells	KLHL26	-0.04171	2.727028	-0.206	0.837266	-6.08312	0.794224	0.807915
Dendritic.cells	ACSBG1	-0.11877	-0.5064	-0.20597	0.837291	-5.2609	0.845803	0.883848
Dendritic.cells	SLC7A2	-0.1495	2.094729	-0.20592	0.837325	-5.41446	0.804002	0.822249
Dendritic.cells	PER2	0.091691	2.222784	0.205643	0.837542	-5.51853	0.80214	0.819364
Dendritic.cells	LYZ1	0.134336	-0.68152	0.205454	0.83769	-5.26235	0.848847	0.888308
Dendritic.cells	1810020O(	-0.14436	-0.23349	-0.20533	0.837789	-5.26043	0.841434	0.877385
Dendritic.cells	GT(ROSA)2	0.044722	4.594685	0.205296	0.837813	-6.06931	0.766324	0.767176
Dendritic.cells	KLHL15	-0.03681	4.413728	-0.20507	0.837989	-6.08909	0.768984	0.771118
Dendritic.cells	SAE1	-0.02638	6.342657	-0.20497	0.838065	-6.56207	0.741232	0.730834
Dendritic.cells	PPP3CA	-0.01763	8.812084	-0.20495	0.838083	-7.09267	0.707605	0.68229
Dendritic.cells	TMEM94	-0.06028	2.610495	-0.20492	0.838103	-5.58461	0.796144	0.810837
Dendritic.cells	FN1	-0.13617	5.580946	-0.20458	0.838372	-6.04457	0.752033	0.746605
Dendritic.cells	MLST8	0.09126	2.044303	0.20434	0.838558	-5.45291	0.804919	0.823828
Dendritic.cells	CUEDC2	0.027886	5.245055	0.204326	0.838569	-6.40138	0.756861	0.753624

Dendritic.cells	CIT	0.091198	4.461996	0.204205	0.838663	-5.83335	0.768273	0.770236
Dendritic.cells	TUBD1	0.075395	2.095647	0.204172	0.838689	-5.53427	0.804118	0.822656
Dendritic.cells	GM28050	-0.10577	0.410517	-0.20405	0.838782	-5.32966	0.830911	0.862106
Dendritic.cells	CLDN15	0.106714	0.423974	0.20392	0.838885	-5.26152	0.830693	0.861784
Dendritic.cells	AIFM2	0.120427	1.347203	0.203841	0.838946	-5.2687	0.815887	0.839965
Dendritic.cells	SYCE2	-0.06421	4.258668	-0.20382	0.838964	-5.94993	0.771273	0.774621
Dendritic.cells	GM22146	-0.07835	2.073446	-0.2038	0.838975	-5.59364	0.804464	0.823178
Dendritic.cells	ANKRD33B	-0.1158	4.882196	-0.20357	0.83916	-5.72157	0.762122	0.761291
Dendritic.cells	GM4707	-0.09089	2.866085	-0.20324	0.839414	-5.56152	0.792222	0.805229
Dendritic.cells	UBE2V2	0.027365	5.614121	0.20308	0.839539	-6.37933	0.751558	0.745935
Dendritic.cells	ZBTB37	-0.05464	2.901016	-0.20307	0.839544	-5.78486	0.791687	0.804447
Dendritic.cells	POLR3D	0.049348	3.27535	0.202911	0.839671	-5.91143	0.785991	0.796112
Dendritic.cells	SLC38A7	-0.06338	2.59539	-0.2028	0.839758	-5.74683	0.796376	0.811315
Dendritic.cells	RAD17	0.027449	5.023722	0.202794	0.839762	-6.30702	0.760064	0.758298
Dendritic.cells	CASC4	-0.06153	2.64714	-0.20279	0.839764	-5.72801	0.79558	0.810148
Dendritic.cells	FAM53C	-0.0409	3.981286	-0.20277	0.839777	-5.98165	0.775389	0.780624
Dendritic.cells	SF3B2	-0.01747	7.285583	-0.20274	0.839804	-6.80425	0.728144	0.71202
Dendritic.cells	NME6	0.063675	2.576439	0.202624	0.839894	-5.70986	0.796668	0.811751
Dendritic.cells	SAFB2	0.018727	5.993263	0.202574	0.839933	-6.63123	0.746161	0.738111
Dendritic.cells	CCNJL	-0.08637	1.734552	-0.2024	0.840072	-5.49674	0.80977	0.830983
Dendritic.cells	4931406P1	-0.02277	5.594349	-0.20231	0.840139	-6.55196	0.751841	0.746366
Dendritic.cells	GM17227	0.053287	2.746669	0.20219	0.840233	-5.77513	0.794051	0.80793
Dendritic.cells	VAMP1	0.044081	3.997211	0.202035	0.840354	-5.95575	0.775152	0.7803
Dendritic.cells	COL5A2	0.097665	1.225753	0.202003	0.840379	-5.54778	0.817816	0.842827
Dendritic.cells	EXO5	0.079966	2.006793	0.201972	0.840403	-5.60663	0.805504	0.824727
Dendritic.cells	TRA2A	0.01807	7.075692	0.201944	0.840425	-6.82936	0.731031	0.716212
Dendritic.cells	SLC31A2	-0.07347	3.169586	-0.20193	0.840436	-5.6985	0.787595	0.79848
Dendritic.cells	GM15787	0.071196	2.641968	0.201886	0.840469	-5.68764	0.79566	0.810287
Dendritic.cells	MYLK	0.111212	1.606214	0.201841	0.840505	-5.33172	0.81179	0.833962
Dendritic.cells	ABCB7	0.023026	5.334608	0.201821	0.84052	-6.47213	0.75557	0.751783
Dendritic.cells	KLRA6	0.128996	-0.71608	0.2018	0.840537	-5.26189	0.849422	0.889504
Dendritic.cells	GGCX	-0.0751	1.807957	-0.20178	0.840554	-5.55701	0.808617	0.829299
Dendritic.cells	SPINT1	-0.09708	-1.0651	-0.20164	0.840663	-5.26298	0.855254	0.898152
Dendritic.cells	TMEM68	0.036204	3.807022	0.201511	0.840762	-6.06055	0.777989	0.784441
Dendritic.cells	MED1	0.018671	5.883036	0.201466	0.840797	-6.56576	0.747725	0.740392
Dendritic.cells	ZFP644	0.018369	6.331418	0.20146	0.840801	-6.77919	0.74139	0.731206
Dendritic.cells	SAA4	0.12571	0.640047	0.20136	0.84088	-5.3142	0.827206	0.856652
Dendritic.cells	ADHFE1	-0.08429	2.690792	-0.20088	0.84125	-5.51118	0.795131	0.809298
Dendritic.cells	RDH10	-0.06759	3.207433	-0.20088	0.841254	-5.6749	0.78724	0.797749
Dendritic.cells	CAPN2	-0.03089	4.14309	-0.20079	0.841322	-6.2642	0.7732	0.777251
Dendritic.cells	CLASP1	0.026895	6.373608	0.20068	0.84141	-6.45133	0.741004	0.730455
Dendritic.cells	IGTP	-0.11624	3.443365	-0.20061	0.841462	-5.62408	0.78367	0.792536
Dendritic.cells	1700097N(	0.083342	2.346622	0.20037	0.841652	-5.58692	0.800554	0.817148
Dendritic.cells	5031439G(	0.024598	5.696635	0.2001	0.841862	-6.63187	0.750696	0.744523
Dendritic.cells	AI480526	-0.06484	2.064128	-0.20009	0.841873	-5.67613	0.80495	0.823722
Dendritic.cells	PRKN	-0.05617	3.428362	-0.20002	0.841923	-6.0819	0.784009	0.793055
Dendritic.cells	H2-OB	-0.11195	4.007971	-0.1999	0.842018	-5.5431	0.775319	0.780389

Dendritic.cells	UTP23	0.045445	3.577367	0.199794	0.842101	-5.82934	0.781763	0.7898
Dendritic.cells	NFYA	0.045542	4.152707	0.199622	0.842234	-5.93923	0.773169	0.777254
Dendritic.cells	INMT	0.131384	0.639157	0.199537	0.842301	-5.28247	0.827563	0.857031
Dendritic.cells	IMPDH2	-0.03517	5.196107	-0.19947	0.842354	-6.18927	0.757888	0.755002
Dendritic.cells	UVSSA	-0.03549	3.709393	-0.19947	0.842355	-6.15474	0.77978	0.786903
Dendritic.cells	PLEKHN1	-0.07172	1.876111	-0.19941	0.842403	-5.56459	0.80789	0.828074
Dendritic.cells	D330050I1	-0.08032	1.118758	-0.19934	0.842451	-5.50606	0.819867	0.845689
Dendritic.cells	PPP2CB	0.025365	5.60171	0.199213	0.842554	-6.33938	0.752053	0.746525
Dendritic.cells	APAF1	0.029342	5.726771	0.19918	0.842579	-6.434	0.750266	0.74393
Dendritic.cells	IL1R1	0.090864	2.051839	0.198961	0.84275	-5.65245	0.805223	0.824084
Dendritic.cells	2010013B2	-0.04353	3.545259	-0.19884	0.842845	-6.12184	0.782325	0.790566
Dendritic.cells	SLC7A6OS	-0.02391	4.789888	-0.19876	0.842907	-6.45927	0.763867	0.763664
Dendritic.cells	SLC8A1	-0.03245	6.330185	-0.19871	0.842949	-7.07883	0.741796	0.731608
Dendritic.cells	COG6	0.055246	3.137898	0.198616	0.843019	-5.75464	0.788489	0.799606
Dendritic.cells	OSBP	-0.02725	5.016429	-0.19829	0.843276	-6.3762	0.760688	0.75891
Dendritic.cells	GALNT3	0.086807	1.767982	0.198263	0.843294	-5.56829	0.809795	0.830709
Dendritic.cells	LPAR1	0.165481	0.763125	0.19803	0.843476	-5.26928	0.825887	0.854299
Dendritic.cells	SPACA9	0.062164	1.758151	0.197858	0.84361	-5.64964	0.810117	0.831008
Dendritic.cells	CRLF2	0.035093	5.010644	0.197708	0.843728	-6.24338	0.76097	0.759142
Dendritic.cells	GM26759	-0.0328	3.123372	-0.19754	0.843858	-6.41383	0.789096	0.800122
Dendritic.cells	TBKBP1	-0.10274	1.449005	-0.19736	0.844	-5.39509	0.815166	0.838321
Dendritic.cells	UBE3C	0.021692	5.784905	0.19716	0.844155	-6.61585	0.750016	0.743132
Dendritic.cells	1600022D1	-0.09488	-0.42787	-0.19697	0.844305	-5.26011	0.845703	0.883331
Dendritic.cells	GM28403	-0.10048	1.458517	-0.19691	0.84435	-5.45873	0.815147	0.838254
Dendritic.cells	RBM19	-0.0528	2.925866	-0.19675	0.844471	-5.7842	0.792315	0.804742
Dendritic.cells	FZD6	-0.11734	0.693274	-0.19671	0.844505	-5.3302	0.827393	0.856269
Dendritic.cells	FAM91A1	0.021687	5.007003	0.196417	0.844735	-6.43289	0.761331	0.759543
Dendritic.cells	GM20732	0.030182	4.58993	0.196386	0.844758	-6.23972	0.767426	0.768413
Dendritic.cells	PRSS30	-0.04951	-0.33737	-0.19635	0.844785	-5.8192	0.844282	0.881222
Dendritic.cells	TNFSF11	0.114484	0.706959	0.196266	0.844852	-5.56186	0.827234	0.856044
Dendritic.cells	FFAR2	0.1294	-0.53401	0.19579	0.845224	-5.26172	0.84777	0.886041
Dendritic.cells	RIDA	-0.08902	4.316478	-0.19579	0.845226	-5.85539	0.771668	0.774285
Dendritic.cells	PLAA	0.018063	6.069326	0.195584	0.845384	-6.70982	0.746369	0.73742
Dendritic.cells	GAR1	0.048222	4.430651	0.195361	0.845559	-5.96663	0.770155	0.771852
Dendritic.cells	RND1	-0.10768	1.145338	-0.19505	0.845804	-5.31675	0.820632	0.845748
Dendritic.cells	ACSM1	0.13655	1.026687	0.19499	0.845848	-5.32568	0.822529	0.848541
Dendritic.cells	2410131K1	0.07186	2.169111	0.194975	0.84586	-5.60941	0.804479	0.82202
Dendritic.cells	2500004C0	-0.08027	1.043374	-0.19497	0.845867	-5.43446	0.822262	0.848147
Dendritic.cells	SMCO4	-0.05321	4.10439	-0.19485	0.845958	-5.86458	0.775023	0.778967
Dendritic.cells	POLD3	0.037144	4.695421	0.194482	0.846245	-6.11291	0.766296	0.766309
Dendritic.cells	SLC30A5	0.02245	5.799283	0.194444	0.846274	-6.65222	0.750334	0.743117
Dendritic.cells	DCSTAMP	-0.0875	-1.33737	-0.19441	0.846298	-5.26405	0.861464	0.906169
Dendritic.cells	PAXBP1	-0.02695	5.312121	-0.1944	0.846308	-6.52296	0.757324	0.753265
Dendritic.cells	SRBD1	-0.0428	4.393343	-0.1944	0.84631	-6.00012	0.770741	0.77278
Dendritic.cells	CD19	0.08842	3.122136	0.193684	0.846868	-5.4924	0.790198	0.80071
Dendritic.cells	NACA	-0.01578	9.080227	-0.19368	0.846869	-7.15477	0.70576	0.678348
Dendritic.cells	BPTF	-0.01812	7.356344	-0.19353	0.846984	-6.88183	0.728948	0.711715

Dendritic.cells	XDH	-0.05057	4.807401	-0.19343	0.847068	-6.4207	0.765085	0.764055
Dendritic.cells	THOC2	-0.0151	7.106814	-0.19333	0.847145	-6.83232	0.732399	0.716676
Dendritic.cells	NUDT2	-0.04333	3.238769	-0.19315	0.847283	-5.87288	0.788484	0.798152
Dendritic.cells	GM28960	0.105276	-0.82667	0.193038	0.847372	-5.26277	0.853363	0.893588
Dendritic.cells	S1PR1	-0.07454	4.061514	-0.19294	0.847451	-5.71277	0.776104	0.780115
Dendritic.cells	RRP12	-0.05792	2.60063	-0.19275	0.847595	-5.68971	0.798258	0.812493
Dendritic.cells	ATRIP	-0.06327	2.825397	-0.19273	0.847611	-5.71505	0.794799	0.807429
Dendritic.cells	KIF1A	0.096941	-0.59877	0.19273	0.847613	-5.318	0.849561	0.888008
Dendritic.cells	MMS22L	-0.05303	4.820836	-0.19269	0.847648	-6.10403	0.764897	0.76382
Dendritic.cells	CR1L	0.020906	5.693128	0.192533	0.847766	-6.45828	0.752321	0.74551
Dendritic.cells	4921531C2	0.088543	1.642005	0.19223	0.848003	-5.43752	0.81343	0.834579
Dendritic.cells	LONP1	-0.04733	3.653778	-0.19213	0.848084	-5.82896	0.782414	0.789161
Dendritic.cells	GM32089	0.102293	-1.09921	0.191867	0.848286	-5.26299	0.858294	0.900611
Dendritic.cells	KDM5A	-0.01796	7.014524	-0.19169	0.848423	-6.83398	0.734042	0.718765
Dendritic.cells	1700094J0	0.112653	0.092196	0.191521	0.848556	-5.36017	0.838629	0.871463
Dendritic.cells	MGARP	0.125577	-0.49619	0.191216	0.848795	-5.25976	0.848413	0.885859
Dendritic.cells	MDM4	0.019635	5.749077	0.19117	0.84883	-6.56209	0.751973	0.744639
Dendritic.cells	GM17435	-0.09443	1.133905	-0.19113	0.848859	-5.3613	0.821838	0.846672
Dendritic.cells	SYT14	-0.13879	0.008242	-0.19106	0.848918	-5.26528	0.840082	0.87358
Dendritic.cells	FAM185A	0.05349	2.579686	0.19091	0.849034	-5.80207	0.799149	0.813344
Dendritic.cells	GM10143	-0.0884	1.245831	-0.19082	0.849105	-5.38649	0.820093	0.844119
Dendritic.cells	GM40841	0.109753	0.40148	0.190373	0.849453	-5.36729	0.833938	0.864291
Dendritic.cells	RMND1	0.041123	3.79439	0.190293	0.849515	-5.84887	0.780875	0.786487
Dendritic.cells	NOP56	0.037786	4.591451	0.19001	0.849737	-6.03618	0.769028	0.76926
Dendritic.cells	TMEM135	0.028369	5.7512	0.189938	0.849793	-6.4473	0.752199	0.744826
Dendritic.cells	EPO	0.102065	0.415776	0.189922	0.849805	-5.31208	0.833706	0.863989
Dendritic.cells	5830411N(	0.102801	-1.25694	0.18984	0.849869	-5.26239	0.861455	0.90503
Dendritic.cells	INTS14	-0.05047	4.691853	-0.18979	0.849907	-6.07064	0.767551	0.767129
Dendritic.cells	WASHC5	-0.03506	4.472587	-0.18972	0.849966	-6.17834	0.77078	0.771835
Dendritic.cells	USP49	0.043131	4.534917	0.189602	0.850055	-6.13467	0.76986	0.770501
Dendritic.cells	SCYL3	-0.03503	3.642548	-0.18959	0.850063	-6.00565	0.783159	0.789878
Dendritic.cells	E430024I0ɛ	-0.07013	1.898133	-0.18932	0.850274	-5.50655	0.810055	0.829213
Dendritic.cells	PVR	0.035429	3.063552	0.189311	0.850283	-6.24286	0.791995	0.802766
Dendritic.cells	CD96	-0.12341	0.686804	-0.18925	0.850333	-5.38033	0.829364	0.857604
Dendritic.cells	RIN3	0.042525	5.640444	0.189083	0.850461	-6.00113	0.753884	0.747233
Dendritic.cells	MGME1	0.08123	2.312135	0.188991	0.850533	-5.49386	0.803634	0.819807
Dendritic.cells	RALYL	-0.14679	0.59788	-0.18848	0.850931	-5.26293	0.830858	0.859857
Dendritic.cells	IZUMO1R	-0.1304	0.001693	-0.18846	0.850949	-5.26535	0.840586	0.874207
Dendritic.cells	GM43113	0.112269	-0.69248	0.188457	0.850951	-5.26347	0.852084	0.891204
Dendritic.cells	TMEM63B	0.058525	3.925117	0.188419	0.85098	-5.74371	0.779018	0.783874
Dendritic.cells	4931403E2	0.064653	1.501737	0.188387	0.851005	-5.54305	0.816366	0.838534
Dendritic.cells	GM16316	0.086624	1.803894	0.188379	0.851011	-5.41467	0.81159	0.831521
Dendritic.cells	COX7A2	0.018665	8.039242	0.188354	0.851031	-6.91667	0.720487	0.699048
Dendritic.cells	CITED4	-0.13513	-0.2568	-0.1881	0.851227	-5.26558	0.84497	0.880613
Dendritic.cells	FKTN	0.063338	1.595478	0.187742	0.851509	-5.68703	0.815082	0.836517
Dendritic.cells	CDC26	0.021803	5.383294	0.187687	0.851552	-6.29842	0.757772	0.752847
Dendritic.cells	ADAM23	-0.04919	3.184267	-0.18728	0.851872	-6.2082	0.7904	0.800514

Dendritic.cells	CLMP	-0.09969	1.140521	-0.18721	0.851924	-5.39977	0.822324	0.847324
Dendritic.cells	RRP1B	0.051064	4.132089	0.18719	0.85194	-5.92335	0.776121	0.779675
Dendritic.cells	EPC1	-0.02035	6.811099	-0.18714	0.85198	-6.75795	0.737517	0.723656
Dendritic.cells	TESK2	0.069821	4.072329	0.187119	0.851996	-5.70436	0.777012	0.78099
Dendritic.cells	MAGOH	-0.02685	6.296682	-0.18707	0.852032	-6.41696	0.744731	0.734118
Dendritic.cells	DNASE2A	0.041563	4.427932	0.187016	0.852076	-5.98408	0.771731	0.77334
Dendritic.cells	ACAD8	0.053672	2.476536	0.186893	0.852173	-5.68589	0.801279	0.816509
Dendritic.cells	OSCP1	-0.07976	3.25254	-0.18678	0.85226	-5.67262	0.789361	0.799098
Dendritic.cells	PCGF1	0.089496	1.17851	0.186467	0.852506	-5.3379	0.821716	0.846601
Dendritic.cells	NUDT6	-0.05939	2.183509	-0.18639	0.852563	-5.67502	0.805837	0.823278
Dendritic.cells	GM50019	-0.11377	-0.06748	-0.18638	0.852575	-5.26347	0.841931	0.876405
Dendritic.cells	ADM	0.118834	1.330377	0.186302	0.852635	-5.34982	0.819292	0.843036
Dendritic.cells	EPHA4	-0.0867	0.083978	-0.1861	0.852795	-5.57444	0.839443	0.872759
Dendritic.cells	SCAP	0.030553	4.277309	0.186031	0.852847	-6.36006	0.773962	0.776718
Dendritic.cells	EPB41L1	0.111193	1.011227	0.185959	0.852903	-5.34547	0.824396	0.8506
Dendritic.cells	AP2A1	0.032083	4.542647	0.185815	0.853015	-6.25767	0.770038	0.771046
Dendritic.cells	GM28112	-0.10979	0.043725	-0.18566	0.853135	-5.34244	0.840103	0.873809
Dendritic.cells	NEDD9	0.026732	8.003683	0.185647	0.853147	-6.89695	0.721146	0.700238
Dendritic.cells	CD83	0.090841	5.198788	0.185512	0.853253	-5.94146	0.760442	0.757142
Dendritic.cells	PFN2	0.095341	1.339141	0.185352	0.853377	-5.40888	0.819153	0.843002
Dendritic.cells	PI4KB	0.024108	5.414205	0.185341	0.853386	-6.3802	0.757326	0.752642
Dendritic.cells	RABGAP1	0.022692	5.920481	0.18531	0.85341	-6.55051	0.750066	0.742108
Dendritic.cells	CLK4	-0.02105	6.012894	-0.18529	0.853424	-6.54386	0.748751	0.740201
Dendritic.cells	METTL22	0.070363	1.382795	0.185154	0.853532	-5.519	0.818458	0.841986
Dendritic.cells	ANAPC11	0.024807	5.923842	0.184987	0.853663	-6.54523	0.750019	0.742044
Dendritic.cells	MTHFSL	-0.03589	5.211119	-0.18475	0.853845	-6.09477	0.760263	0.75694
Dendritic.cells	GM29019	0.056085	0.361473	0.184661	0.853918	-5.71227	0.834905	0.866242
Dendritic.cells	PCLAF	0.060151	7.982096	0.184655	0.853923	-6.76689	0.721438	0.700745
Dendritic.cells	UQCRH	0.013142	8.592145	0.184593	0.853971	-7.07829	0.713248	0.68895
Dendritic.cells	1700056E2	-0.08219	1.466557	-0.18451	0.854039	-5.46562	0.817126	0.840055
Dendritic.cells	MAML2	0.019168	7.972039	0.184455	0.854079	-7.20522	0.721574	0.700941
Dendritic.cells	GM43914	-0.06336	0.384486	-0.18442	0.854104	-5.68487	0.834531	0.865689
Dendritic.cells	GM28694	-0.10857	-0.23311	-0.18438	0.854137	-5.26016	0.844663	0.880655
Dendritic.cells	CCDC59	0.019315	5.676842	0.18438	0.854137	-6.47988	0.753548	0.747188
Dendritic.cells	LRRC10B	0.116159	0.595128	0.184258	0.854233	-5.29932	0.831108	0.860641
Dendritic.cells	EIF3L	-0.02369	5.672102	-0.18414	0.854329	-6.43126	0.753616	0.747314
Dendritic.cells	1190005I0(	-0.08049	1.185776	-0.18408	0.854373	-5.38627	0.8216	0.846682
Dendritic.cells	NCK1	0.027327	6.174664	0.18405	0.854396	-6.44922	0.746456	0.736945
Dendritic.cells	CEP41	0.06931	1.886422	0.183915	0.854501	-5.52543	0.810492	0.830361
Dendritic.cells	TRIM39	0.045309	2.873416	0.183865	0.85454	-5.75312	0.795156	0.807878
Dendritic.cells	MTG2	0.050185	3.031494	0.183816	0.854579	-5.71428	0.792733	0.804336
Dendritic.cells	KLF2	0.033994	8.937753	0.183757	0.854625	-7.20936	0.708664	0.682403
Dendritic.cells	SIGLECG	-0.03037	4.076853	-0.18366	0.8547	-6.28293	0.776944	0.781274
Dendritic.cells	ZC3H15	0.015589	7.266033	0.183582	0.854762	-6.83098	0.731214	0.7149
Dendritic.cells	ZFP664	0.030352	4.050718	0.18354	0.854795	-6.17104	0.777334	0.781843
Dendritic.cells	CSRNP2	-0.07475	2.107884	-0.18352	0.854808	-5.49947	0.807019	0.825266
Dendritic.cells	ARHGAP1	-0.02695	4.473602	-0.18324	0.85503	-6.13335	0.771192	0.772744

Dendritic.cells	CDKL3	0.054358	3.044103	0.182996	0.85522	-5.88211	0.792789	0.804166
Dendritic.cells	FAM149A	0.106028	-0.42534	0.182589	0.855539	-5.26576	0.848359	0.885657
Dendritic.cells	HELQ	-0.04508	2.411841	-0.1824	0.855687	-5.80133	0.802839	0.818567
Dendritic.cells	IMP3	0.023177	5.264975	0.182254	0.855801	-6.36331	0.760034	0.756099
Dendritic.cells	NFAM1	-0.02731	4.570884	-0.18215	0.855881	-6.41291	0.77018	0.770872
Dendritic.cells	KDM4C	0.018564	6.252217	0.182052	0.855958	-6.76443	0.7459	0.735637
Dendritic.cells	GCN1	0.028026	4.575371	0.181805	0.856152	-6.15689	0.770114	0.770816
Dendritic.cells	GM44702	-0.11742	0.088947	-0.1818	0.856156	-5.30587	0.839971	0.873228
Dendritic.cells	GM14798	0.038905	3.718232	0.181653	0.856271	-5.93711	0.782883	0.789427
Dendritic.cells	ABCA7	-0.03239	3.89815	-0.18164	0.856284	-6.04723	0.780181	0.785484
Dendritic.cells	MAN1C1	-0.01858	4.292733	-0.18113	0.856679	-6.83971	0.774295	0.77701
Dendritic.cells	EIF4E3	0.040752	4.420461	0.180931	0.856836	-6.05304	0.772402	0.774279
Dendritic.cells	SSRP1	0.027625	6.322976	0.180865	0.856887	-6.48259	0.744901	0.734351
Dendritic.cells	D3ERTD751	-0.07769	1.61523	-0.18073	0.856996	-5.46342	0.815361	0.837205
Dendritic.cells	SDHAF4	0.03198	4.365947	0.180725	0.856997	-6.12633	0.773209	0.775529
Dendritic.cells	APOBEC1	0.053434	6.087599	0.180356	0.857286	-6.17248	0.748233	0.739345
Dendritic.cells	SECISBP2	0.025106	4.786541	0.180255	0.857365	-6.32726	0.767009	0.766618
Dendritic.cells	FPR2	-0.17105	1.833327	-0.18015	0.85745	-5.31995	0.811916	0.83227
Dendritic.cells	QRFP	0.108514	-0.61178	0.179976	0.857583	-5.26278	0.851565	0.890742
Dendritic.cells	GM44751	-0.078	1.310612	-0.17987	0.85767	-5.48045	0.820202	0.844451
Dendritic.cells	THOC7	0.019599	6.572603	0.179841	0.857689	-6.58049	0.741388	0.72943
Dendritic.cells	FAM118A	0.075136	2.069936	0.179811	0.857712	-5.41343	0.808199	0.826813
Dendritic.cells	RPA3	-0.03811	4.97161	-0.17976	0.857749	-6.13384	0.764301	0.762678
Dendritic.cells	CGRRF1	-0.04679	3.907331	-0.17955	0.857919	-5.72887	0.780043	0.785611
Dendritic.cells	EXOC1	-0.03565	4.095264	-0.17953	0.857931	-6.04803	0.777233	0.781513
Dendritic.cells	GM16973	0.064468	1.894858	0.1795	0.857956	-5.59152	0.810947	0.830848
Dendritic.cells	4930526L0	0.098311	-0.8055	0.179292	0.858118	-5.26206	0.854804	0.895539
Dendritic.cells	HIST1H3D	-0.10474	1.963471	-0.17928	0.858125	-5.34292	0.809869	0.829265
Dendritic.cells	ZFP593	0.054556	3.359126	0.179265	0.85814	-5.77044	0.788313	0.797689
Dendritic.cells	CREB3L2	0.034657	3.55229	0.179255	0.858148	-6.24363	0.785387	0.793412
Dendritic.cells	FUT8	0.022644	5.850748	0.179151	0.858229	-6.56398	0.751606	0.744238
Dendritic.cells	PDP1	0.057384	2.180234	0.179132	0.858244	-5.56717	0.806473	0.824282
Dendritic.cells	RHOB	-0.05794	5.891039	-0.17911	0.858258	-6.27439	0.751031	0.743403
Dendritic.cells	ZFP110	-0.03683	4.039873	-0.17911	0.85826	-6.01917	0.77806	0.782719
Dendritic.cells	SSPN	0.095972	-0.41075	0.179049	0.858309	-5.26233	0.84822	0.885789
Dendritic.cells	TBC1D23	-0.01943	5.852371	-0.17888	0.858443	-6.62007	0.751583	0.744273
Dendritic.cells	CCDC86	-0.02966	5.146452	-0.17867	0.858602	-6.24527	0.761754	0.759082
Dendritic.cells	4833419F2	-0.08541	1.323387	-0.17867	0.858606	-5.47685	0.819998	0.84427
Dendritic.cells	GM11099	-0.1148	-0.70994	-0.17863	0.858641	-5.26175	0.853205	0.893296
Dendritic.cells	KHSRP	0.02069	5.998752	0.178563	0.858689	-6.51708	0.749496	0.741284
Dendritic.cells	KCTD12	-0.01721	6.383969	-0.17843	0.85879	-6.95722	0.74404	0.733376
Dendritic.cells	BUD13	0.035315	3.714639	0.178427	0.858796	-5.97241	0.782937	0.789949
Dendritic.cells	FNIP2	-0.04502	5.343736	-0.17841	0.858807	-6.6191	0.758894	0.754926
Dendritic.cells	ZFP239	0.101305	-0.06752	0.1783	0.858896	-5.26138	0.842543	0.877543
Dendritic.cells	E2F2	0.071519	4.99405	0.178123	0.859034	-5.69287	0.763974	0.762344
Dendritic.cells	GM49101	0.109426	-0.65342	0.178076	0.859071	-5.26395	0.85226	0.891947
Dendritic.cells	HIST1H3E	0.066968	3.610481	0.178009	0.859123	-5.6658	0.784508	0.792295

Dendritic.cells	TMEM70	-0.04052	3.930301	-0.17787	0.85923	-5.96376	0.779699	0.785292
Dendritic.cells	UNG	0.046883	3.369876	0.177856	0.859243	-6.09682	0.78815	0.797635
Dendritic.cells	IFI27	-0.07763	4.998783	-0.1777	0.859361	-5.80683	0.763905	0.762304
Dendritic.cells	BTRC	0.026744	5.211768	0.177594	0.859448	-6.38217	0.760806	0.757828
Dendritic.cells	HJURP	-0.0392	4.895427	-0.17756	0.859474	-6.02256	0.765414	0.764531
Dendritic.cells	GM826	0.13469	-0.25558	0.177262	0.859708	-5.26433	0.845648	0.882299
Dendritic.cells	MMGT1	-0.05346	2.857236	-0.17716	0.859786	-5.71546	0.795981	0.809202
Dendritic.cells	AP1M1	0.030587	4.979621	0.177083	0.859848	-6.1117	0.764184	0.762788
Dendritic.cells	GSTK1	-0.05936	2.449061	-0.17691	0.859984	-5.64346	0.802287	0.818484
Dendritic.cells	PTGR2	0.037889	3.734192	0.17679	0.860077	-5.95218	0.782643	0.789771
Dendritic.cells	6530409C1	0.055442	1.661527	0.176652	0.860186	-5.8075	0.814628	0.836688
Dendritic.cells	SSH2	-0.02102	8.396317	-0.17661	0.860215	-7.08676	0.716383	0.693686
Dendritic.cells	PIN4	0.028377	4.949389	0.176563	0.860255	-6.10762	0.764626	0.763569
Dendritic.cells	EXOC3	0.025811	5.06994	0.176439	0.860353	-6.30219	0.762868	0.761018
Dendritic.cells	NOL9	-0.03983	3.728213	-0.17643	0.860362	-5.92831	0.782733	0.789978
Dendritic.cells	FBXW7	-0.0199	6.448567	-0.17628	0.860478	-6.76555	0.743131	0.732362
Dendritic.cells	RAD9A	0.075606	2.338849	0.176263	0.86049	-5.54288	0.804	0.821112
Dendritic.cells	ZBP1	0.121036	3.640152	0.176165	0.860567	-5.65285	0.78406	0.791916
Dendritic.cells	DBP	-0.07294	2.187718	-0.17615	0.860582	-5.61706	0.806357	0.82457
Dendritic.cells	PTPN21	-0.09389	0.813665	-0.17601	0.860689	-5.3375	0.828175	0.856698
Dendritic.cells	GM28875	-0.03806	4.612323	-0.17601	0.86069	-6.15894	0.76957	0.770802
Dendritic.cells	VMN2R19	0.111013	-0.79625	0.175998	0.860698	-5.26403	0.854649	0.895841
Dendritic.cells	SMARCE1	-0.01627	6.302182	-0.17592	0.860763	-6.64161	0.745194	0.735379
Dendritic.cells	HSPE1-RS1	0.106491	0.18545	0.175823	0.860835	-5.34169	0.838388	0.871799
Dendritic.cells	CACNA2D1	-0.12814	0.864423	-0.17573	0.860911	-5.27046	0.827357	0.855566
Dendritic.cells	RAD23B	0.015309	6.360558	0.175684	0.860944	-6.6423	0.74437	0.734253
Dendritic.cells	ART3	0.07901	0.944132	0.175631	0.860985	-5.47266	0.826073	0.853683
Dendritic.cells	RFT1	-0.05959	2.53189	-0.1756	0.861012	-5.67503	0.801002	0.816824
Dendritic.cells	PPP1R12B	-0.02249	5.292478	-0.17558	0.861025	-6.45986	0.759636	0.756419
Dendritic.cells	GCSAM	0.103948	-0.31734	0.175549	0.86105	-5.26767	0.846671	0.884109
Dendritic.cells	C1RL	-0.10412	1.632594	-0.17552	0.861069	-5.31735	0.815086	0.837507
Dendritic.cells	CDCA4	0.038354	4.453152	0.175203	0.861321	-5.97932	0.772045	0.774371
Dendritic.cells	GRIK4	-0.10502	0.238216	-0.17516	0.861353	-5.41248	0.837662	0.870675
Dendritic.cells	GM13889	0.076816	1.14195	0.174629	0.86177	-5.57781	0.823274	0.849224
Dendritic.cells	NRD1	0.017509	6.185357	0.174589	0.861802	-6.73285	0.747189	0.738038
Dendritic.cells	EXT2	-0.03103	3.849599	-0.17456	0.861821	-6.12245	0.781266	0.787623
Dendritic.cells	CCPG1OS	0.056531	1.576044	0.17442	0.861934	-5.58339	0.816367	0.839068
Dendritic.cells	NAA40	-0.0222	4.909944	-0.17433	0.862001	-6.35759	0.765563	0.764768
Dendritic.cells	CACNB3	0.120989	-0.407	0.174229	0.862084	-5.32801	0.848558	0.88662
Dendritic.cells	CALCRL	0.036967	5.617508	0.174184	0.862119	-6.45965	0.755304	0.749872
Dendritic.cells	VAC14	0.021105	4.624685	0.173837	0.862391	-6.35728	0.769846	0.770958
Dendritic.cells	CD22	0.062971	2.780412	0.173783	0.862433	-5.62317	0.797639	0.811561
Dendritic.cells	APH1A	0.022693	5.433388	0.173775	0.86244	-6.55652	0.75805	0.753795
Dendritic.cells	MED13L	0.015561	7.196724	0.17351	0.862647	-6.92344	0.733253	0.717763
Dendritic.cells	FBN1	-0.07373	0.898383	-0.17335	0.862773	-5.52598	0.827441	0.855293
Dendritic.cells	MRPS23	-0.03885	4.411962	-0.17322	0.862871	-6.00007	0.773117	0.775672
Dendritic.cells	AP5M1	-0.03957	3.584616	-0.17321	0.862884	-5.90629	0.785497	0.793741

Dendritic.cells	MEX3C	-0.01879	5.133199	-0.17308	0.862983	-6.57026	0.762529	0.760262
Dendritic.cells	FAM169B	0.036944	3.62896	0.173044	0.863013	-6.23349	0.784827	0.792772
Dendritic.cells	RHBDL3	-0.10269	0.747589	-0.17294	0.863093	-5.31567	0.829884	0.858925
Dendritic.cells	NAMPT	0.03252	6.301782	0.172803	0.863202	-6.65737	0.745808	0.735961
Dendritic.cells	RAD51	-0.07584	3.891445	-0.17239	0.863524	-5.69084	0.781087	0.787214
Dendritic.cells	ATP13A2	0.020394	5.610722	0.172382	0.863531	-6.79851	0.755824	0.750424
Dendritic.cells	GBA2	0.051245	2.547922	0.17213	0.863728	-5.61459	0.801698	0.817238
Dendritic.cells	SCPEP1OS	0.077699	0.502575	0.171839	0.863957	-5.5193	0.834321	0.86505
Dendritic.cells	EPM2A	0.073437	1.526051	0.171783	0.864001	-5.54842	0.81786	0.8408
Dendritic.cells	GGA2	-0.05407	4.120751	-0.17153	0.864198	-5.74241	0.77795	0.782252
Dendritic.cells	SLAMF9	0.024932	2.637226	0.171426	0.86428	-6.70628	0.800501	0.81522
Dendritic.cells	ARL11	-0.07075	1.439587	-0.17142	0.864287	-5.4467	0.819303	0.842823
Dendritic.cells	CLEC4B1	0.09509	-0.61033	0.170526	0.864986	-5.26793	0.853137	0.892366
Dendritic.cells	FCHO2	0.018245	6.902577	0.170393	0.86509	-6.76502	0.738159	0.724107
Dendritic.cells	TEC	-0.03095	5.751113	-0.17036	0.865118	-6.31066	0.754442	0.747696
Dendritic.cells	EXOSC3	-0.02729	5.047755	-0.17031	0.865156	-6.21357	0.764621	0.762482
Dendritic.cells	PRR11	0.080522	2.847187	0.170251	0.865201	-5.52952	0.797628	0.81064
Dendritic.cells	WDR1	-0.01868	7.335798	-0.17007	0.865345	-6.85114	0.732153	0.715445
Dendritic.cells	C130046K2	-0.08512	0.716514	-0.17001	0.865391	-5.38872	0.8313	0.860136
Dendritic.cells	CEP55	0.076924	3.678611	0.169715	0.865622	-5.77787	0.784949	0.792244
Dendritic.cells	C1QBP	0.025208	6.055545	0.169609	0.865705	-6.59407	0.750092	0.741535
Dendritic.cells	GM47863	0.097246	0.552776	0.169587	0.865723	-5.3348	0.833959	0.864198
Dendritic.cells	CBR2	0.124521	-1.3198	0.169489	0.865799	-5.26176	0.86509	0.910292
Dendritic.cells	GM17160	0.08068	0.089084	0.169426	0.865849	-5.37015	0.841543	0.875417
Dendritic.cells	RBFOX3	-0.10681	-0.24977	-0.16931	0.865941	-5.27966	0.847137	0.883696
Dendritic.cells	EGLN2	0.032644	4.887876	0.169296	0.865951	-6.18929	0.76696	0.766065
Dendritic.cells	GALNT7	-0.02354	6.060844	-0.16912	0.866088	-6.58924	0.750016	0.741455
Dendritic.cells	EXOSC1	0.031144	4.175365	0.16907	0.866128	-6.04241	0.777494	0.781414
Dendritic.cells	ABTB2	-0.04348	7.412469	-0.16903	0.866156	-6.55665	0.731097	0.714074
Dendritic.cells	MPP1	0.033221	6.203718	0.168987	0.866193	-6.27032	0.747986	0.738511
Dendritic.cells	RGS3	0.074219	2.862627	0.168946	0.866225	-5.6036	0.79739	0.810487
Dendritic.cells	TAF1	0.022168	5.699019	0.168939	0.86623	-6.48418	0.75519	0.748962
Dendritic.cells	PIK3C2A	-0.02449	6.854141	-0.16893	0.866235	-6.55831	0.738835	0.725259
Dendritic.cells	GM43713	-0.0672	2.135076	-0.1689	0.86626	-5.61301	0.808692	0.827055
Dendritic.cells	PYM1	-0.03163	4.692796	-0.16861	0.866491	-5.98245	0.769959	0.770299
Dendritic.cells	STOML3	-0.09057	0.359863	-0.16853	0.866552	-5.3107	0.837248	0.868943
Dendritic.cells	SH3D19	-0.06375	2.244921	-0.16821	0.8668	-5.62815	0.807229	0.824664
Dendritic.cells	GM29282	-0.05958	1.712401	-0.16812	0.866869	-5.71979	0.815607	0.836965
Dendritic.cells	ZFX	0.014376	6.339182	0.16805	0.866927	-6.69444	0.746304	0.735849
Dendritic.cells	GM50218	0.107909	0.559001	0.168004	0.866963	-5.26534	0.834121	0.864221
Dendritic.cells	GM2396	0.086473	-0.85737	0.16786	0.867077	-5.26547	0.85759	0.898902
Dendritic.cells	BCL2L1	-0.06292	6.15449	-0.1673	0.867519	-5.91238	0.749213	0.739777
Dendritic.cells	SPEF2	0.067162	1.371663	0.167208	0.867588	-5.57387	0.821344	0.84509
Dendritic.cells	TNPO3	-0.01622	6.365771	-0.16714	0.867645	-6.70609	0.746218	0.735448
Dendritic.cells	C2CD3	-0.02132	4.657214	-0.16697	0.867778	-6.33246	0.770894	0.771289
Dendritic.cells	PBDC1	0.035612	5.348225	0.16689	0.867838	-6.3066	0.760788	0.756594
Dendritic.cells	MPC2	0.023235	6.800876	0.166812	0.867899	-6.60538	0.740101	0.726613

Dendritic.cells	FCHO1	0.041154	3.772111	0.16673	0.867963	-5.9771	0.784092	0.79054
Dendritic.cells	5830408C2	0.03306	2.989299	0.166667	0.868012	-6.02559	0.796004	0.807971
Dendritic.cells	TMC6	-0.02798	4.632332	-0.16661	0.868057	-6.13802	0.771261	0.771857
Dendritic.cells	FPR3	0.106681	-0.4789	0.166571	0.868088	-5.26563	0.851545	0.889723
Dendritic.cells	FHIT	0.017243	6.167904	0.166387	0.868232	-7.03073	0.749085	0.73959
Dendritic.cells	CRNKL1	0.024523	5.293781	0.166278	0.868318	-6.26234	0.761653	0.75781
Dendritic.cells	NEURL4	-0.06712	2.445552	-0.16609	0.868464	-5.62669	0.804562	0.820323
Dendritic.cells	ZFAND5	0.01462	7.203792	0.165889	0.868623	-6.81741	0.734705	0.718566
Dendritic.cells	FAM72A	0.081926	1.069392	0.165625	0.86883	-5.38172	0.826515	0.852313
Dendritic.cells	RNF26	-0.04813	3.515898	-0.16559	0.868862	-5.79444	0.788278	0.796256
Dendritic.cells	ZFP617	0.064413	2.165547	0.165293	0.86909	-5.55479	0.809252	0.826765
Dendritic.cells	RFX3	-0.01763	6.233922	-0.16493	0.869373	-6.7323	0.748698	0.738356
Dendritic.cells	TBC1D9	0.061828	3.953614	0.16474	0.869525	-5.86521	0.78203	0.786778
Dendritic.cells	FCRLS	0.152836	-1.03209	0.164712	0.869547	-5.26394	0.861563	0.903684
Dendritic.cells	ZBTB9	-0.0446	2.511721	-0.16442	0.869772	-5.76101	0.804095	0.819009
Dendritic.cells	EHMT1	-0.01789	6.063885	-0.16442	0.869779	-6.65727	0.751167	0.74191
Dendritic.cells	KANSL1	0.014465	8.964116	0.164325	0.86985	-7.16839	0.71129	0.684377
Dendritic.cells	BCL3	0.023369	5.236561	0.164313	0.86986	-6.63283	0.763084	0.759199
Dendritic.cells	PSMA1	-0.01784	6.9166	-0.16422	0.869935	-6.70299	0.739141	0.72451
Dendritic.cells	NDUFA3	0.015022	8.000282	0.164054	0.870063	-6.99442	0.724269	0.702982
Dendritic.cells	NINJ1	0.042999	5.750418	0.163807	0.870257	-6.16477	0.755737	0.748472
Dendritic.cells	ZFP800	0.02207	5.365723	0.163792	0.870268	-6.39321	0.761292	0.756531
Dendritic.cells	UTP6	-0.02855	4.42275	-0.16373	0.870315	-6.17119	0.775134	0.776673
Dendritic.cells	NIM1K	0.04615	2.88905	0.163512	0.870489	-5.8592	0.798419	0.810595
Dendritic.cells	CSF2RB	-0.02274	4.838003	-0.16344	0.870544	-6.80154	0.769074	0.767829
Dendritic.cells	VAPB	-0.02832	5.031604	-0.16301	0.870886	-6.21268	0.766473	0.763829
Dendritic.cells	ZFP747	-0.06667	1.605909	-0.16281	0.871042	-5.44827	0.818834	0.840275
Dendritic.cells	PROM1	-0.11431	0.403745	-0.16264	0.871169	-5.27502	0.838227	0.868842
Dendritic.cells	SDAD1	-0.03361	4.473042	-0.16254	0.871249	-6.0672	0.774771	0.775899
Dendritic.cells	BCL2L13	0.028946	5.133362	0.162518	0.871269	-6.23371	0.765052	0.761767
Dendritic.cells	ARHGAP33	0.090691	0.549492	0.162454	0.871319	-5.33461	0.835846	0.865375
Dendritic.cells	GM16286	0.019249	5.859685	0.162221	0.871502	-6.48469	0.754627	0.746549
Dendritic.cells	OLFR56	-0.06427	2.067818	-0.16215	0.87156	-5.77872	0.811619	0.82964
Dendritic.cells	FRAT2	-0.03869	4.805593	-0.16198	0.87169	-6.03383	0.769963	0.768818
Dendritic.cells	CFL2	0.026946	4.631141	0.161852	0.871792	-6.25274	0.772536	0.772563
Dendritic.cells	NONO	-0.01538	6.614635	-0.16184	0.871798	-6.6854	0.743922	0.731058
Dendritic.cells	HK3	-0.11032	2.677613	-0.16162	0.871974	-5.42102	0.802151	0.81575
Dendritic.cells	GM19696	-0.10251	0.016291	-0.1615	0.872065	-5.26402	0.844747	0.878371
Dendritic.cells	SLC35B4	0.053798	2.825471	0.161482	0.872082	-5.62086	0.799862	0.812415
Dendritic.cells	PPAT	-0.04017	4.107428	-0.1614	0.872146	-5.90454	0.780362	0.783945
Dendritic.cells	DNAJC27	0.050899	2.442699	0.16134	0.872194	-5.64156	0.805803	0.821111
Dendritic.cells	OGDH	0.012614	6.625732	0.160815	0.872606	-6.72982	0.744023	0.730948
Dendritic.cells	MAN1B1	0.026152	4.927787	0.160751	0.872656	-6.47211	0.768432	0.766331
Dendritic.cells	DGAT2	-0.05978	3.862389	-0.16073	0.872674	-5.63653	0.78428	0.789398
Dendritic.cells	CLOCK	-0.0215	5.071637	-0.16042	0.872914	-6.45605	0.766345	0.763298
Dendritic.cells	TGIF2	0.043573	3.122183	0.160382	0.872946	-5.76446	0.795558	0.805858
Dendritic.cells	ZFP790	-0.05304	2.226902	-0.16037	0.872957	-5.62654	0.809444	0.826179

Dendritic.cells	WDTC1	0.049702	3.851337	0.160323	0.872993	-5.69247	0.784468	0.789672
Dendritic.cells	1700052K1	-0.06322	1.140287	-0.16024	0.873056	-5.46647	0.826703	0.851514
Dendritic.cells	3-Sep	-0.07813	-1.00287	-0.16001	0.873241	-5.29373	0.862171	0.903753
Dendritic.cells	SLIT1	-0.09949	1.464859	-0.15986	0.87336	-5.27509	0.821652	0.843957
Dendritic.cells	UBQLN2	-0.02841	4.031371	-0.15965	0.873524	-6.05242	0.781918	0.785831
Dendritic.cells	AY036118	0.084395	4.212426	0.159604	0.873557	-5.8377	0.779207	0.781881
Dendritic.cells	E130215H2	-0.09255	-0.13171	-0.15956	0.873592	-5.27911	0.847646	0.882245
Dendritic.cells	HEYL	-0.09614	-0.20551	-0.15929	0.873801	-5.26271	0.848907	0.884059
Dendritic.cells	GM42941	-0.06431	1.267028	-0.15927	0.87382	-5.48054	0.824869	0.848647
Dendritic.cells	FSHR	0.065657	0.419984	0.159182	0.873888	-5.35713	0.838595	0.868889
Dendritic.cells	RILPL2	0.016307	7.158884	0.159135	0.873926	-6.98294	0.736771	0.720347
Dendritic.cells	HIPK3	0.021925	5.445686	0.159044	0.873997	-6.39443	0.761085	0.755578
Dendritic.cells	ANKRD54	0.037631	3.089479	0.158942	0.874077	-5.78664	0.796255	0.806833
Dendritic.cells	SERPINA3N	0.064134	2.557516	0.158853	0.874147	-5.52876	0.804478	0.818866
Dendritic.cells	LARS2	0.05433	5.571824	0.158712	0.874258	-6.21871	0.759259	0.752974
Dendritic.cells	AGPAT2	0.042053	4.355109	0.1585	0.874424	-6.05473	0.777111	0.778928
Dendritic.cells	XRCC1	0.036414	4.144273	0.158431	0.874478	-5.94581	0.780259	0.783513
Dendritic.cells	AFF4	-0.01587	7.842009	-0.15826	0.87461	-7.00455	0.727363	0.706871
Dendritic.cells	ANXA10	-0.08043	0.643116	-0.15805	0.874774	-5.3845	0.834953	0.863678
Dendritic.cells	B4GALT3	0.026549	3.874826	0.158005	0.874813	-6.06378	0.784306	0.789463
Dendritic.cells	ZCWPW2	-0.08569	1.016841	-0.15795	0.874858	-5.39098	0.828895	0.854773
Dendritic.cells	A530032D1	0.049169	0.594014	0.157901	0.874895	-5.84442	0.835753	0.86488
Dendritic.cells	FAM204A	0.023879	4.894011	0.157832	0.874949	-6.20474	0.769138	0.767408
Dendritic.cells	MAMDC2	0.097198	0.366618	0.157713	0.875043	-5.28665	0.839469	0.870399
Dendritic.cells	4930578M	0.072393	0.63262	0.157698	0.875054	-5.44671	0.835124	0.863994
Dendritic.cells	NDUFAF4	0.034581	3.867148	0.157628	0.875109	-5.99676	0.784422	0.789679
Dendritic.cells	NIPSNAP3E	-0.02523	5.573981	-0.15754	0.87518	-6.30234	0.759228	0.753035
Dendritic.cells	APMAP	-0.03226	4.080552	-0.15741	0.875279	-6.02853	0.781214	0.785002
Dendritic.cells	WDR46	-0.04719	3.429733	-0.15721	0.875439	-5.7604	0.79105	0.79935
Dendritic.cells	C530005A1	0.069897	0.742343	0.157156	0.87548	-5.35577	0.833339	0.861366
Dendritic.cells	RAB34	0.100277	0.235405	0.157151	0.875484	-5.29785	0.841622	0.873576
Dendritic.cells	DIS3L	-0.0433	2.828005	-0.15713	0.875504	-5.7213	0.800284	0.812845
Dendritic.cells	VSIR	0.036689	4.468275	0.157096	0.875527	-6.06458	0.775428	0.776576
Dendritic.cells	EIF1AX	0.02082	6.060349	0.156901	0.875681	-6.4942	0.752242	0.742915
Dendritic.cells	PTEN	-0.01239	8.288084	-0.15675	0.875798	-7.06189	0.721308	0.698251
Dendritic.cells	STMN1	0.042913	7.897729	0.156542	0.875963	-6.78872	0.726603	0.705933
Dendritic.cells	PDCD1	0.111039	0.743001	0.156318	0.876139	-5.40479	0.833328	0.861464
Dendritic.cells	GM17382	0.090162	0.002886	0.156185	0.876243	-5.30028	0.845455	0.879348
Dendritic.cells	D230025D1	-0.0309	4.57771	-0.15612	0.876292	-6.08357	0.773805	0.774316
Dendritic.cells	UPRT	0.077346	0.606501	0.155982	0.876403	-5.33617	0.835549	0.864781
Dendritic.cells	PEX1	-0.05131	3.071405	-0.15592	0.87645	-5.72561	0.796532	0.807527
Dendritic.cells	MOSPD1	-0.02933	4.817896	-0.15586	0.876497	-6.28906	0.770258	0.76922
Dendritic.cells	CALM1	-0.01204	9.782329	-0.15584	0.876516	-7.24082	0.701515	0.669954
Dendritic.cells	IRF2BP2	0.01097	7.59206	0.155747	0.876587	-7.21213	0.730787	0.712049
Dendritic.cells	UBFD1	-0.02497	4.520398	-0.15567	0.876651	-6.23935	0.774654	0.775617
Dendritic.cells	ATP13A1	-0.02645	3.809105	-0.15563	0.87668	-6.08572	0.785297	0.791126
Dendritic.cells	EPOR	0.102622	-0.66556	0.155609	0.876696	-5.26928	0.856587	0.895881

Dendritic.cells	ALDH9A1	0.022973	5.005152	0.155598	0.876705	-6.26529	0.767507	0.765221
Dendritic.cells	TMUB2	0.040199	3.181282	0.15549	0.876789	-5.77822	0.794846	0.805085
Dendritic.cells	GM15859	-0.05702	1.208039	-0.15543	0.876839	-5.4564	0.825816	0.850511
Dendritic.cells	GCSH	-0.04266	4.428205	-0.15519	0.877028	-5.84638	0.776023	0.777673
Dendritic.cells	GM14858	0.080287	2.296719	0.155169	0.877042	-5.50165	0.808549	0.825191
Dendritic.cells	DSE	-0.03911	3.834005	-0.15511	0.877085	-6.14434	0.784922	0.790673
Dendritic.cells	ZFP26	0.026466	3.995742	0.155059	0.877129	-6.10316	0.782487	0.787128
Dendritic.cells	ASB7	0.040352	3.936004	0.155047	0.877138	-5.96111	0.783385	0.788438
Dendritic.cells	PIGK	-0.02297	4.298401	-0.15498	0.877192	-6.232	0.777956	0.780537
Dendritic.cells	CD1D1	-0.06789	3.932282	-0.15495	0.877216	-5.61671	0.783441	0.788534
Dendritic.cells	ZRANB2	-0.02124	5.45391	-0.15477	0.877352	-6.30846	0.760977	0.755835
Dendritic.cells	HIST1H3A	0.081363	0.953909	0.154694	0.877415	-5.31861	0.829923	0.856665
Dendritic.cells	SLC39A10	0.027497	3.903206	0.154378	0.877664	-6.15199	0.78389	0.789322
Dendritic.cells	ESPN	0.096605	-0.17599	0.154362	0.877676	-5.26567	0.848429	0.884095
Dendritic.cells	RILPL1	-0.02887	2.31814	-0.15429	0.877734	-6.22561	0.808225	0.824915
Dendritic.cells	IQGAP2	-0.01334	7.075634	-0.15425	0.877767	-7.10621	0.737939	0.72261
Dendritic.cells	TMEM265	0.039296	3.958769	0.154204	0.8778	-5.86753	0.783054	0.788113
Dendritic.cells	PA2G4	0.02223	6.688076	0.154194	0.877808	-6.68032	0.743359	0.730449
Dendritic.cells	CEP250	-0.03351	4.458141	-0.15397	0.877988	-6.04053	0.775589	0.777266
Dendritic.cells	HMGCS1	0.019963	4.453186	0.153904	0.878036	-6.57124	0.775663	0.777382
Dendritic.cells	CLDND1	-0.01525	4.825073	-0.15387	0.878062	-6.60941	0.770163	0.769376
Dendritic.cells	DPP8	-0.01726	5.667042	-0.15384	0.878089	-6.54151	0.757895	0.751566
Dendritic.cells	TRDMT1	-0.05411	2.342131	-0.15356	0.878306	-5.54056	0.807863	0.824458
Dendritic.cells	MBTPS1	0.022574	4.639311	0.153379	0.878449	-6.23607	0.772917	0.773458
Dendritic.cells	MTA1	0.024281	4.55479	0.153312	0.878501	-6.24487	0.774168	0.775284
Dendritic.cells	ZBTB45	-0.03842	2.916254	-0.15327	0.878538	-5.80529	0.798946	0.811455
Dendritic.cells	NAPRT	-0.10044	0.897988	-0.15324	0.878558	-5.28166	0.830841	0.858288
Dendritic.cells	GM3604	-0.07415	0.595301	-0.15318	0.878606	-5.32882	0.835757	0.865534
Dendritic.cells	IPO5	-0.02993	5.437174	-0.15316	0.878618	-6.28582	0.761232	0.756471
Dendritic.cells	XYLB	0.084351	0.922746	0.153088	0.878678	-5.2921	0.830441	0.857708
Dendritic.cells	CYTH1	-0.01647	8.072227	-0.15281	0.878893	-6.99721	0.724267	0.703016
Dendritic.cells	GM43251	-0.10665	-0.97845	-0.15281	0.878895	-5.26486	0.861901	0.904226
Dendritic.cells	MIB1	0.015733	6.189415	0.152791	0.878911	-6.73248	0.75044	0.740847
Dendritic.cells	C79798	0.075937	1.77973	0.152322	0.87928	-5.31421	0.816928	0.837718
Dendritic.cells	GTSE1	-0.11385	1.404909	-0.15232	0.879283	-5.30345	0.822897	0.846491
Dendritic.cells	DDX31	0.039252	3.220713	0.152222	0.879359	-5.80143	0.794471	0.804835
Dendritic.cells	GART	0.033595	4.367941	0.152161	0.879406	-6.08544	0.777144	0.779544
Dendritic.cells	MCUR1	0.029257	4.462065	0.151991	0.879541	-6.09817	0.775765	0.777524
Dendritic.cells	ZMYM2	0.016372	5.970893	0.151758	0.879723	-6.71874	0.753758	0.745575
Dendritic.cells	ZFP638	0.015032	6.22747	0.151674	0.87979	-6.6943	0.750097	0.740286
Dendritic.cells	XCR1	0.108307	0.412393	0.151657	0.879803	-5.27527	0.838984	0.870271
Dendritic.cells	EFHC1	-0.08331	-0.05796	-0.15154	0.879894	-5.27621	0.846728	0.881727
Dendritic.cells	AP2M1	0.017261	6.941455	0.151482	0.879941	-6.73731	0.740033	0.725736
Dendritic.cells	GOSR1	-0.02831	4.182554	-0.15146	0.879957	-6.02595	0.779933	0.783673
Dendritic.cells	VRK2	0.02105	5.691956	0.151378	0.880023	-6.527	0.757765	0.751434
Dendritic.cells	TNFRSF4	-0.10082	0.30724	-0.15119	0.880171	-5.27995	0.840708	0.872895
Dendritic.cells	SMG5	-0.01846	5.55464	-0.15114	0.880207	-6.45511	0.759747	0.754364

Dendritic.cells	PATL1	0.023179	5.222719	0.150945	0.880363	-6.2856	0.764568	0.761409
Dendritic.cells	NUMBL	0.0745	1.04252	0.150859	0.880431	-5.40815	0.828742	0.855315
Dendritic.cells	PIANP	0.081294	-0.06426	0.150857	0.880432	-5.27104	0.846832	0.882008
Dendritic.cells	HELZ2	-0.04693	3.832468	-0.15085	0.880435	-5.84977	0.785193	0.791461
Dendritic.cells	RRP8	-0.03547	3.730434	-0.15067	0.880582	-5.91492	0.786745	0.793795
Dendritic.cells	GTF2F2	0.015482	5.881638	0.150655	0.880591	-6.63307	0.755047	0.747654
Dendritic.cells	PDGFC	0.060914	1.879122	0.150435	0.880764	-5.9447	0.815453	0.835746
Dendritic.cells	ZFP429	-0.04827	2.448075	-0.15038	0.880805	-5.59316	0.806513	0.822639
Dendritic.cells	EXOSC2	-0.04288	2.945711	-0.1501	0.881028	-5.68119	0.798931	0.811393
Dendritic.cells	SLC25A32	-0.04065	3.120627	-0.14933	0.881632	-5.72944	0.79672	0.807664
Dendritic.cells	DCAF11	-0.02672	4.559305	-0.14899	0.881902	-6.14118	0.775113	0.776029
Dendritic.cells	GM35154	-0.08341	1.526848	-0.14893	0.881948	-5.42508	0.821811	0.844337
Dendritic.cells	SLC22A23	-0.04202	3.017213	-0.14889	0.881981	-6.18793	0.798432	0.810064
Dendritic.cells	CLDN10	-0.10302	0.159414	-0.14869	0.882135	-5.2662	0.844078	0.877081
Dendritic.cells	LPIN2	0.020087	6.528551	0.148577	0.882226	-6.67817	0.746679	0.73471
Dendritic.cells	EFCAB5	-0.08459	0.504613	-0.14836	0.882399	-5.32227	0.838523	0.86882
Dendritic.cells	WNT5B	0.05318	1.562043	0.147958	0.882713	-5.598	0.821662	0.843762
Dendritic.cells	SMC1B	-0.10043	-0.03473	-0.14774	0.882885	-5.26725	0.847653	0.882051
Dendritic.cells	FNDC9	-0.04754	2.564671	-0.14772	0.8829	-5.50961	0.805866	0.82059
Dendritic.cells	SIPA1	-0.02207	5.681522	-0.14766	0.882947	-6.28184	0.759087	0.752405
Dendritic.cells	WDR75	0.036605	3.575364	0.147322	0.883213	-5.83371	0.790434	0.797865
Dendritic.cells	INTU	0.068486	0.880465	0.146917	0.883532	-5.62058	0.832783	0.859983
Dendritic.cells	GABARAP	0.011737	8.497974	0.146836	0.883596	-7.06583	0.719937	0.695722
Dendritic.cells	SNRNP48	-0.02221	4.916287	-0.14683	0.883603	-6.26035	0.770368	0.768663
Dendritic.cells	NELFB	0.02502	4.551192	0.146659	0.883735	-6.09511	0.775766	0.776515
Dendritic.cells	GM26917	-0.0221	5.74366	-0.14655	0.883825	-6.6738	0.758314	0.75116
Dendritic.cells	GUSB	-0.01701	5.468903	-0.14649	0.883866	-6.56242	0.76229	0.756928
Dendritic.cells	G430095P1	-0.07946	0.465518	-0.14643	0.883917	-5.28749	0.839548	0.869951
Dendritic.cells	TWSG1	0.043119	3.230079	0.146395	0.883943	-5.79407	0.795708	0.805598
Dendritic.cells	RPUSD1	-0.05841	1.47218	-0.14635	0.883977	-5.48538	0.82325	0.84596
Dendritic.cells	ZZEF1	0.015284	5.867775	0.14635	0.883978	-6.62946	0.756527	0.748568
Dendritic.cells	P2RY13	0.055857	1.100765	0.146262	0.884047	-5.79317	0.829218	0.854753
Dendritic.cells	GM42702	-0.05195	2.365724	-0.14603	0.884232	-5.64234	0.809105	0.8253
Dendritic.cells	POP4	0.034974	4.085123	0.146005	0.88425	-5.96535	0.782728	0.786748
Dendritic.cells	PIP4P1	0.012582	5.856707	0.145745	0.884455	-6.65775	0.756686	0.748911
Dendritic.cells	CD37	0.014643	7.409059	0.145728	0.884468	-6.99836	0.734792	0.717236
Dendritic.cells	SYBU	-0.09998	0.330557	-0.14569	0.884496	-5.30711	0.841762	0.873348
Dendritic.cells	SECTM1A	0.086962	-1.13086	0.145655	0.884526	-5.26388	0.866189	0.909478
Dendritic.cells	MTSS1	0.019318	6.472133	0.145631	0.884544	-6.86427	0.747905	0.736189
Dendritic.cells	ADAM22	-0.09184	0.796933	-0.14557	0.884593	-5.29722	0.83414	0.862111
Dendritic.cells	MATK	0.043871	2.36611	0.145506	0.884643	-5.83488	0.809099	0.825319
Dendritic.cells	UBXN2A	0.020233	5.233503	0.145468	0.884672	-6.33148	0.765717	0.762031
Dendritic.cells	UBAP1L	0.068746	1.134289	0.145175	0.884903	-5.30006	0.828739	0.85423
Dendritic.cells	PSMC6	0.016961	6.141841	0.145097	0.884964	-6.50041	0.752657	0.74314
Dendritic.cells	FMNL3	-0.02605	4.258409	-0.14507	0.884983	-6.23785	0.780189	0.783145
Dendritic.cells	NDUFB11	-0.0138	7.55899	-0.14502	0.885026	-6.88347	0.732778	0.714401
Dendritic.cells	LSM1	-0.01902	5.251939	-0.14469	0.885286	-6.33566	0.765598	0.76182

Dendritic.cells	HMGN5	-0.03014	4.876393	-0.14463	0.885335	-6.07171	0.771106	0.769837
Dendritic.cells	A930001M	-0.02751	3.967402	-0.14461	0.885348	-6.15638	0.784652	0.789565
Dendritic.cells	GPR137C	0.055553	2.390141	0.144358	0.885547	-5.5451	0.808911	0.825019
Dendritic.cells	CLSTN1	-0.05209	1.774497	-0.14433	0.88557	-5.57289	0.818621	0.839278
Dendritic.cells	GM27008	-0.06081	1.012976	-0.14421	0.885663	-5.4539	0.83083	0.857285
Dendritic.cells	SMARCC2	0.014033	6.020787	0.144199	0.885671	-6.61107	0.754507	0.745788
Dendritic.cells	DCXR	0.033924	4.317514	0.14408	0.885765	-6.00367	0.779428	0.782013
Dendritic.cells	PPFIBP2	-0.03546	4.285147	-0.14402	0.885813	-6.33672	0.779912	0.78272
Dendritic.cells	HNRNPM	-0.01067	7.703885	-0.1438	0.885983	-6.93787	0.730959	0.711696
Dendritic.cells	ADCK2	-0.04149	2.282482	-0.1437	0.886064	-5.66467	0.810664	0.827633
Dendritic.cells	CCNB2	-0.05537	5.882225	-0.14358	0.886156	-6.22841	0.756556	0.748757
Dendritic.cells	SFXN3	-0.02958	3.370072	-0.14349	0.886229	-6.08396	0.793812	0.803017
Dendritic.cells	RHEB	0.011115	7.039284	0.143405	0.886297	-6.93605	0.740162	0.725053
Dendritic.cells	IGKV1-35	0.08123	-0.53717	0.143381	0.886316	-5.26529	0.856434	0.895121
Dendritic.cells	2310057M	0.031407	2.710086	0.143083	0.88655	-5.81791	0.804108	0.817964
Dendritic.cells	ASPH	0.02465	4.664011	0.143026	0.886595	-6.58233	0.774454	0.774674
Dendritic.cells	KDM2A	-0.00989	7.465818	-0.1429	0.886698	-6.92862	0.734375	0.716545
Dendritic.cells	EPRS	-0.02307	6.581496	-0.14243	0.887061	-6.66628	0.746879	0.734541
Dendritic.cells	TSKU	-0.08806	-0.3765	-0.14243	0.887062	-5.26757	0.854073	0.891369
Dendritic.cells	NKIRAS2	0.032706	4.042562	0.142399	0.887088	-5.85811	0.783914	0.788339
Dendritic.cells	4930417O1	-0.08824	0.963159	-0.1419	0.887482	-5.30664	0.832309	0.858941
Dendritic.cells	DPY19L4	0.017996	4.417697	0.141722	0.887622	-6.44832	0.778563	0.780269
Dendritic.cells	CWF19L1	-0.05186	2.200341	-0.14153	0.88777	-5.57975	0.812546	0.829925
Dendritic.cells	GM17259	-0.06136	1.365713	-0.14145	0.887838	-5.51072	0.825815	0.849406
Dendritic.cells	MAP2	-0.07633	0.187932	-0.14142	0.88786	-5.35229	0.844989	0.877651
Dendritic.cells	AP1G2	-0.05747	2.809774	-0.14133	0.887928	-5.61048	0.803023	0.815976
Dendritic.cells	ABAT	0.07304	1.196429	0.141272	0.887976	-5.47979	0.828538	0.853431
Dendritic.cells	C030006K1	0.052195	1.498775	0.141025	0.888171	-5.6254	0.823682	0.846328
Dendritic.cells	ATN1	0.040827	3.221914	0.141014	0.88818	-5.7855	0.796662	0.806727
Dendritic.cells	GTF3C5	-0.05496	2.258469	-0.14101	0.88818	-5.55163	0.811632	0.82864
Dendritic.cells	CARD10	0.061766	1.098314	0.141007	0.888185	-5.43064	0.830122	0.855798
Dendritic.cells	DDX46	0.017115	6.040748	0.140799	0.888349	-6.53914	0.75491	0.745877
Dendritic.cells	RDH12	-0.07227	2.275253	-0.14062	0.888493	-5.50803	0.811452	0.828329
Dendritic.cells	RC3H1	-0.01191	6.887392	-0.14053	0.888562	-6.88768	0.742905	0.72856
Dendritic.cells	KAT5	0.047655	2.921057	0.140525	0.888565	-5.67442	0.801382	0.813606
Dendritic.cells	ARHGEF40	-0.07842	0.550928	-0.14032	0.888729	-5.35316	0.839193	0.869057
Dendritic.cells	SYDE1	0.066565	0.308678	0.140225	0.888802	-5.34894	0.843169	0.874922
Dendritic.cells	IER5L	-0.04268	2.89009	-0.14014	0.888868	-5.75344	0.801941	0.814348
Dendritic.cells	ZC3H12C	-0.01614	4.431325	-0.13995	0.889019	-7.14873	0.778585	0.780166
Dendritic.cells	ENG	-0.03374	4.005662	-0.13976	0.889171	-6.25568	0.784997	0.789494
Dendritic.cells	MZT1	0.017845	5.278837	0.139682	0.889229	-6.32322	0.766104	0.762003
Dendritic.cells	HEXB	0.020603	5.741415	0.139633	0.889268	-6.43797	0.759385	0.752252
Dendritic.cells	GM27003	-0.0306	4.204875	-0.13936	0.889483	-5.94911	0.782127	0.78519
Dendritic.cells	IFIT3	0.097703	3.112471	0.139253	0.889567	-5.63055	0.798746	0.809454
Dendritic.cells	SH2D2A	-0.07064	2.764079	-0.13907	0.889709	-5.51267	0.804169	0.817356
Dendritic.cells	GM1123	0.073761	0.265276	0.139024	0.889747	-5.30262	0.844172	0.876144
Dendritic.cells	HMMR	0.077947	4.333361	0.138811	0.889915	-5.70643	0.780329	0.7825

Dendritic.cells	TOX4	-0.01394	5.903687	-0.13843	0.890218	-6.55949	0.757464	0.749097
Dendritic.cells	MYO15	-0.06797	1.234713	-0.13804	0.890521	-5.45867	0.828649	0.853066
Dendritic.cells	XK	-0.07602	1.158214	-0.13786	0.890662	-5.3085	0.829883	0.854881
Dendritic.cells	P2RY14	0.013873	3.467146	0.137836	0.890684	-7.02614	0.793604	0.801716
Dendritic.cells	WDR45	-0.04141	2.960786	-0.13783	0.890685	-5.64216	0.801389	0.813091
Dendritic.cells	ACOXL	-0.08827	1.285897	-0.13775	0.890754	-5.31045	0.827824	0.851854
Dendritic.cells	HIST1H3I	0.088254	1.296054	0.137664	0.890819	-5.27804	0.827661	0.851613
Dendritic.cells	B930095G1	-0.07391	0.087994	-0.13766	0.890822	-5.4179	0.847385	0.88067
Dendritic.cells	WDR62	0.050008	3.526858	0.137658	0.890824	-5.63831	0.792692	0.800385
Dendritic.cells	CAPZB	0.009196	8.722615	0.137591	0.890876	-7.14282	0.718301	0.692611
Dendritic.cells	FAP	0.095432	0.744801	0.137426	0.891006	-5.38303	0.836592	0.864777
Dendritic.cells	IL7R	0.020504	4.966863	0.137271	0.891129	-6.86994	0.771103	0.769011
Dendritic.cells	FXR1	-0.0136	6.109299	-0.13715	0.891221	-6.61088	0.754513	0.744985
Dendritic.cells	KTI12	-0.03373	3.941057	-0.13715	0.891223	-5.992	0.786404	0.791338
Dendritic.cells	ITPA	-0.02767	4.105721	-0.13696	0.891376	-5.90358	0.783922	0.787775
Dendritic.cells	1700037C1	-0.0291	3.065274	-0.13679	0.891511	-6.02107	0.799774	0.810949
Dendritic.cells	DCK	0.034005	5.832657	0.136677	0.891597	-6.04486	0.758487	0.750827
Dendritic.cells	BOLA1	-0.03117	3.767483	-0.13661	0.891647	-6.00459	0.789031	0.795258
Dendritic.cells	PRDM11	-0.03682	3.444862	-0.13653	0.891714	-5.88394	0.793944	0.802431
Dendritic.cells	CENPH	0.062406	3.059161	0.136526	0.891716	-5.55051	0.799869	0.811089
Dendritic.cells	DOP1A	-0.02324	4.09244	-0.13647	0.891763	-6.26915	0.784122	0.788098
Dendritic.cells	FBXW11	0.014693	7.259166	0.136391	0.891823	-6.9481	0.738285	0.721595
Dendritic.cells	PLCD3	0.028206	1.122441	0.136316	0.891882	-6.10871	0.830461	0.855963
Dendritic.cells	ZBTB39	-0.05459	1.975209	-0.13623	0.891946	-5.55159	0.816816	0.835927
Dendritic.cells	EXOSC10	-0.01964	4.93082	-0.13617	0.891994	-6.37309	0.771634	0.769957
Dendritic.cells	GLRX	-0.0347	6.214402	-0.13613	0.892028	-6.10224	0.75301	0.742928
Dendritic.cells	MFSD1	-0.02305	5.149534	-0.13609	0.892063	-6.20076	0.768419	0.765291
Dendritic.cells	THEM4	-0.06094	2.270582	-0.13567	0.89239	-5.50497	0.812381	0.829252
Dendritic.cells	BTG3	0.014583	5.347109	0.135587	0.892456	-6.60184	0.765743	0.761208
Dendritic.cells	UPP1	0.096429	0.646262	0.135413	0.892593	-5.51389	0.838496	0.867555
Dendritic.cells	SAP30BP	0.018118	4.838744	0.135194	0.892765	-6.36213	0.773352	0.772068
Dendritic.cells	PIGZ	0.085072	0.017052	0.135088	0.892849	-5.2697	0.848966	0.882843
Dendritic.cells	MRE11A	0.022036	4.349103	0.13482	0.893061	-6.05215	0.780766	0.782704
Dendritic.cells	MRPS25	0.028206	4.238863	0.134564	0.893263	-6.00614	0.782532	0.785219
Dendritic.cells	RHAG	0.096933	-0.3642	0.134133	0.893602	-5.27503	0.855834	0.892458
Dendritic.cells	GM48099	0.089653	2.773722	0.133832	0.893839	-5.57495	0.80521	0.81807
Dendritic.cells	ITPRID2	0.02162	5.08881	0.133803	0.893862	-6.33744	0.770191	0.767023
Dendritic.cells	PIAS2	-0.01704	6.02482	-0.13378	0.893878	-6.58662	0.756589	0.747293
Dendritic.cells	ASMT	-0.08144	1.052723	-0.13366	0.893977	-5.27685	0.832567	0.858189
Dendritic.cells	4930477G0	0.088904	-0.38448	0.1332	0.894338	-5.26684	0.856498	0.893277
Dendritic.cells	FANCD2	-0.04366	2.80256	-0.13316	0.894373	-5.6814	0.805013	0.81758
Dendritic.cells	HEATR3	-0.02403	4.273336	-0.13255	0.894848	-6.19068	0.782896	0.784883
Dendritic.cells	BRDT	-0.06129	1.567286	-0.13233	0.895024	-5.43557	0.82488	0.846316
Dendritic.cells	PSMC4	-0.01995	5.46131	-0.13228	0.895062	-6.35448	0.765322	0.759413
Dendritic.cells	UBA7	0.035746	3.784743	0.13228	0.895064	-5.96733	0.790275	0.795696
Dendritic.cells	LRP12	-0.05373	2.191669	-0.13187	0.895389	-5.68867	0.815179	0.8318
Dendritic.cells	TLCD1	0.04815	1.624021	0.131628	0.895578	-5.52627	0.824286	0.845031

Dendritic.cells	GM49189	0.058715	0.779988	0.131525	0.895659	-5.43533	0.837933	0.865083
Dendritic.cells	ATP11B	0.015391	7.035822	0.131481	0.895694	-6.79102	0.743099	0.726893
Dendritic.cells	POU2F1	-0.02171	5.39401	-0.13128	0.895853	-6.49106	0.766616	0.760943
Dendritic.cells	FLNA	-0.0311	6.097597	-0.13127	0.895861	-6.22681	0.756428	0.746182
Dendritic.cells	POLQ	0.049286	3.166551	0.130964	0.896102	-5.58693	0.800068	0.80967
Dendritic.cells	STK24	0.012556	7.291795	0.130885	0.896164	-6.81267	0.739546	0.721856
Dendritic.cells	TIAM2	0.081459	2.628932	0.13063	0.896365	-5.39433	0.808416	0.82196
Dendritic.cells	NPHP4	0.075753	0.297045	0.130577	0.896407	-5.29014	0.845892	0.876991
Dendritic.cells	RYR2	-0.10386	0.059974	-0.13055	0.896427	-5.26673	0.849818	0.882785
Dendritic.cells	HEATR5A	-0.02126	5.656048	-0.13049	0.896474	-6.45712	0.762806	0.75555
Dendritic.cells	COL4A3BP	-0.01581	6.352484	-0.13043	0.89652	-6.60663	0.752786	0.741042
Dendritic.cells	CISD1	-0.02886	5.119405	-0.13042	0.896528	-6.21673	0.770647	0.766923
Dendritic.cells	CYP20A1	0.021333	4.104318	0.130403	0.896544	-6.14441	0.785764	0.78891
Dendritic.cells	MYO16	0.083448	-0.30441	0.130345	0.896589	-5.27098	0.855895	0.891774
Dendritic.cells	LARP4B	-0.01105	7.283403	-0.13016	0.896738	-6.89067	0.739725	0.722158
Dendritic.cells	2810405F1	-0.06794	0.687122	-0.12993	0.896914	-5.35313	0.839647	0.867692
Dendritic.cells	MTG1	0.047897	2.186733	0.129681	0.897114	-5.62727	0.81563	0.832292
Dendritic.cells	CDC7	-0.04407	2.873973	-0.12956	0.897208	-5.66872	0.80486	0.816534
Dendritic.cells	SCAPER	0.01607	6.110801	0.129506	0.897251	-6.63158	0.75649	0.746184
Dendritic.cells	UBR7	0.024481	4.241439	0.129265	0.897442	-6.13394	0.783955	0.786045
Dendritic.cells	PSME4	0.013869	7.130098	0.129234	0.897466	-6.83742	0.742044	0.725307
Dendritic.cells	DNAJB14	-0.01681	4.692261	-0.12917	0.89752	-6.60303	0.777215	0.776242
Dendritic.cells	GM15337	-0.06693	2.140023	-0.12915	0.897532	-5.45018	0.816368	0.833373
Dendritic.cells	MEF2A	0.01111	7.727346	0.128855	0.897765	-7.06771	0.733882	0.713374
Dendritic.cells	TLE3	-0.01523	5.003886	-0.12872	0.897869	-6.55083	0.772766	0.769572
Dendritic.cells	PATL2	-0.05566	2.023827	-0.12822	0.898267	-5.54788	0.818409	0.83631
Dendritic.cells	GM12971	0.049704	0.967071	0.128187	0.898292	-5.49557	0.835388	0.861237
Dendritic.cells	WDR13	0.026046	2.570105	0.128086	0.898372	-5.91432	0.809798	0.823701
Dendritic.cells	ZSCAN29	0.024243	3.485733	0.128052	0.898398	-6.04537	0.795616	0.802983
Dendritic.cells	ZBTB48	-0.06626	1.123594	-0.12799	0.898446	-5.39797	0.832846	0.8575
Dendritic.cells	CCT8	-0.01391	6.762623	-0.12797	0.898463	-6.66806	0.747392	0.732975
Dendritic.cells	PPP1CB	-0.0154	7.803763	-0.12797	0.898465	-6.81863	0.732877	0.712038
Dendritic.cells	M6PR	0.011936	6.293265	0.127915	0.898507	-6.85186	0.754061	0.742636
Dendritic.cells	GM43462	-0.02532	3.23183	-0.12784	0.898568	-6.14479	0.799517	0.808722
Dendritic.cells	TONSL	0.067344	1.456718	0.127775	0.898618	-5.31159	0.827468	0.849653
Dendritic.cells	PREB	-0.02255	4.736405	-0.12751	0.898825	-6.09186	0.77678	0.775605
Dendritic.cells	PTP4A3	-0.01452	5.77385	-0.12726	0.89902	-6.63128	0.761563	0.753572
Dendritic.cells	PIGU	0.027713	4.215968	0.127191	0.899078	-6.0165	0.784561	0.786981
Dendritic.cells	ANKS1	0.019267	5.759857	0.127143	0.899116	-6.49061	0.761766	0.753884
Dendritic.cells	CLUH	-0.03187	3.417508	-0.12678	0.899399	-5.8796	0.796693	0.804694
Dendritic.cells	MED12	-0.03144	3.781462	-0.12675	0.899424	-5.84703	0.791134	0.796585
Dendritic.cells	IL18BP	0.078364	3.740937	0.126751	0.899426	-5.65525	0.79175	0.797484
Dendritic.cells	SLC45A1	-0.05572	0.608482	-0.1267	0.899468	-5.36082	0.841279	0.870058
Dendritic.cells	ASTL	0.081627	1.424213	0.126671	0.899488	-5.27386	0.828023	0.850562
Dendritic.cells	TLR11	-0.06533	-0.67266	-0.12657	0.89957	-5.27341	0.862615	0.901546
Dendritic.cells	OCIAD2	-0.06456	0.485681	-0.12642	0.899684	-5.41372	0.843297	0.873037
Dendritic.cells	E030042O2	0.076928	0.180529	0.126423	0.899684	-5.3353	0.848336	0.880465

Dendritic.cells	GM10184	0.073295	1.11236	0.126216	0.899848	-5.32382	0.833061	0.858015
Dendritic.cells	LRRC25	-0.04396	3.856093	-0.12621	0.89985	-5.91525	0.79	0.794978
Dendritic.cells	AFMID	-0.01944	4.371552	-0.12617	0.899887	-6.4141	0.782224	0.783654
Dendritic.cells	H2-K1	-0.02769	8.786019	-0.12617	0.899888	-7.1185	0.719557	0.693039
Dendritic.cells	SRSF1	0.015901	6.067856	0.126126	0.899919	-6.5294	0.757322	0.747506
Dendritic.cells	2310001H1	0.038734	4.065364	0.126082	0.899953	-5.85023	0.786831	0.790375
Dendritic.cells	GM28981	0.072099	0.488246	0.12584	0.900144	-5.2769	0.843281	0.873045
Dendritic.cells	E130102H2	-0.05773	1.232168	-0.12579	0.900182	-5.50801	0.831147	0.855189
Dendritic.cells	KHDC4	-0.01373	6.448578	-0.12577	0.900198	-6.64647	0.751899	0.739654
Dendritic.cells	FES	-0.01896	4.284767	-0.1256	0.900333	-6.46188	0.783561	0.785603
Dendritic.cells	VPS33A	0.019292	4.291318	0.125575	0.900354	-6.22905	0.783462	0.785465
Dendritic.cells	TUBE1	-0.06663	1.254723	-0.12543	0.900468	-5.32454	0.830832	0.854694
Dendritic.cells	ANKRD16	0.034339	3.026656	0.125257	0.900605	-5.76703	0.802838	0.813604
Dendritic.cells	MILR1	-0.0225	4.330073	-0.12488	0.900898	-6.14069	0.782962	0.784643
Dendritic.cells	GM527	0.072921	0.481195	0.12488	0.900902	-5.3619	0.843496	0.873282
Dendritic.cells	LAD1	-0.09257	-0.99444	-0.12473	0.901021	-5.27086	0.868201	0.909778
Dendritic.cells	PPP2R2A	-0.00962	7.180276	-0.12467	0.901067	-6.84247	0.741663	0.724792
Dendritic.cells	NEURL2	0.052827	0.801683	0.124554	0.901159	-5.58966	0.838241	0.865577
Dendritic.cells	TBC1D9B	0.019654	4.555284	0.124535	0.901174	-6.18718	0.779591	0.779769
Dendritic.cells	PSD	-0.05687	1.633137	-0.12452	0.90119	-5.42359	0.824791	0.845809
Dendritic.cells	TNKS2	-0.00999	7.072389	-0.12425	0.9014	-6.8481	0.743173	0.726996
Dendritic.cells	2610318N(	-0.0742	1.172871	-0.12424	0.901405	-5.33736	0.832204	0.856697
Dendritic.cells	TMEM241	0.028307	4.310016	0.124209	0.901432	-6.09336	0.783263	0.785111
Dendritic.cells	USP4	-0.01115	5.660629	-0.12411	0.901513	-6.54652	0.763318	0.756139
Dendritic.cells	GALM	0.06333	2.214948	0.124103	0.901515	-5.40807	0.815536	0.832237
Dendritic.cells	LRRK2	0.053804	4.404591	0.124041	0.901564	-5.68376	0.781845	0.783046
Dendritic.cells	PLA2G6	-0.05818	1.28393	-0.12375	0.901793	-5.48003	0.830408	0.854093
Dendritic.cells	HDAC8	0.01913	6.530622	0.123696	0.901837	-6.59786	0.75082	0.738076
Dendritic.cells	LRRN3	0.074786	-0.11923	0.123651	0.901873	-5.27423	0.853447	0.888048
Dendritic.cells	EIF1AD	0.021646	4.923218	0.123592	0.901919	-6.24461	0.774125	0.771892
Dendritic.cells	STK11IP	-0.03332	2.312647	-0.12339	0.902075	-5.70425	0.813994	0.83007
Dendritic.cells	SOCS4	0.017243	4.684801	0.123322	0.902132	-6.30344	0.777662	0.77707
Dendritic.cells	KLHL11	0.038883	2.776613	0.123267	0.902175	-5.69777	0.806723	0.819451
Dendritic.cells	CDK17	0.019184	6.780519	0.123264	0.902178	-6.44346	0.74728	0.733028
Dendritic.cells	NUDT5	-0.02511	5.148841	-0.12326	0.902182	-6.09704	0.770797	0.767096
Dendritic.cells	RNF44	0.020296	4.833037	0.123093	0.902313	-6.20559	0.775483	0.773891
Dendritic.cells	MIPOL1	0.022783	4.257052	0.122742	0.90259	-6.06157	0.784082	0.786493
Dendritic.cells	SEMA7A	-0.03164	2.017527	-0.12274	0.902591	-5.99255	0.818686	0.837057
Dendritic.cells	ZMYM4	0.014258	5.733892	0.122703	0.902621	-6.59526	0.762277	0.754813
Dendritic.cells	ZFP322A	-0.03659	3.041434	-0.12268	0.902638	-5.75011	0.802632	0.813555
Dendritic.cells	CWC27	0.014471	5.662216	0.122677	0.902642	-6.56754	0.763317	0.756321
Dendritic.cells	DHX38	0.025873	4.560036	0.122282	0.902953	-6.02126	0.779702	0.779986
Dendritic.cells	PLCL1	-0.03662	5.328139	-0.12226	0.90297	-6.18577	0.768345	0.763485
Dendritic.cells	SLC16A12	-0.0745	0.427781	-0.12216	0.903051	-5.29275	0.84458	0.874999
Dendritic.cells	SEPSECS	0.030717	3.143326	0.121838	0.903304	-5.91439	0.801241	0.811429
Dendritic.cells	PRDM4	-0.03444	3.039382	-0.12183	0.903309	-5.68239	0.802849	0.813779
Dendritic.cells	NHEJ1	-0.05456	3.867688	-0.12171	0.903408	-5.78623	0.790146	0.795252

Dendritic.cells	RNF19A	0.019425	5.375931	0.121635	0.903464	-6.31112	0.767666	0.762566
Dendritic.cells	MPZL3	0.044746	1.727945	0.121596	0.903495	-5.68808	0.823488	0.844036
Dendritic.cells	ST6GALNA(	-0.02939	3.867281	-0.12141	0.90364	-5.96226	0.790152	0.795349
Dendritic.cells	IDS	0.037459	2.698638	0.121293	0.903734	-5.70195	0.808149	0.821646
Dendritic.cells	KIF22	-0.05843	4.043139	-0.12128	0.903747	-5.71306	0.787488	0.791474
Dendritic.cells	TMEM19	0.024688	3.958743	0.121247	0.90377	-5.95145	0.788765	0.793336
Dendritic.cells	RNF113A1	0.067401	0.42846	0.121072	0.903908	-5.34398	0.844583	0.875172
Dendritic.cells	EML6	0.035413	2.764743	0.12106	0.903918	-6.12583	0.807117	0.820149
Dendritic.cells	VGLL4	-0.01666	6.477378	-0.12104	0.903933	-6.5156	0.751772	0.739627
Dendritic.cells	ASAH2	-0.04048	2.945427	-0.1206	0.904278	-5.76919	0.804548	0.816127
Dendritic.cells	EPB41L4AC	0.036137	3.25392	0.120263	0.904548	-5.88609	0.799949	0.809231
Dendritic.cells	UBXN2B	-0.03763	2.842475	-0.11989	0.904845	-5.64967	0.806519	0.818694
Dendritic.cells	RTL8A	-0.03373	3.632481	-0.11976	0.904948	-5.87524	0.794331	0.800922
Dendritic.cells	HAUS2	-0.03033	3.755191	-0.11971	0.904983	-5.86554	0.792458	0.798191
Dendritic.cells	GM13822	0.087627	1.17949	0.119594	0.905076	-5.44064	0.832965	0.857474
Dendritic.cells	GPAM	0.047922	3.696477	0.119404	0.905226	-5.68107	0.793382	0.799504
Dendritic.cells	H2-KE6	0.017884	5.147514	0.119363	0.905259	-6.45635	0.771632	0.767854
Dendritic.cells	FBXW2	-0.01135	6.193608	-0.1193	0.905305	-6.63835	0.756428	0.745814
Dendritic.cells	RAB44	-0.08029	1.487929	-0.11909	0.905475	-5.33686	0.828051	0.850155
Dendritic.cells	SWSAP1	0.056176	0.278314	0.119044	0.90551	-5.42808	0.847794	0.879206
Dendritic.cells	SELENON	-0.03224	3.694822	-0.11893	0.905602	-5.93423	0.793478	0.799541
Dendritic.cells	APH1C	-0.03953	4.351444	-0.11859	0.905866	-5.86614	0.783603	0.785096
Dendritic.cells	RRAGD	-0.0436	2.299081	-0.11857	0.905883	-5.61789	0.815209	0.831235
Dendritic.cells	ENTPD1	-0.02254	6.9129	-0.11856	0.905895	-6.59399	0.74633	0.73106
Dendritic.cells	UPP2	0.063879	1.652112	0.118276	0.906117	-5.45758	0.825516	0.846392
Dendritic.cells	SIPA1L1	0.023098	7.327522	0.118256	0.906133	-6.6328	0.740531	0.72273
Dendritic.cells	VRK3	-0.01854	4.98755	-0.11825	0.906137	-6.21323	0.774139	0.771379
Dendritic.cells	AVL9	-0.01508	6.010486	-0.11813	0.906232	-6.59092	0.759205	0.74972
Dendritic.cells	RHBDD2	0.036706	2.284757	0.118078	0.906273	-5.65144	0.81545	0.831655
Dendritic.cells	DGCR8	0.027888	3.779114	0.117847	0.906457	-5.8838	0.792335	0.797866
Dendritic.cells	TIPARP	0.018659	7.437182	0.117638	0.906621	-6.7537	0.739061	0.720618
Dendritic.cells	FKRP	-0.03454	2.332289	-0.11759	0.906657	-5.70505	0.814763	0.83064
Dendritic.cells	4930513N1	-0.05403	0.475363	-0.11757	0.906672	-5.4324	0.844709	0.874629
Dendritic.cells	DYRK2	0.019008	4.884934	0.11751	0.906722	-6.22585	0.775718	0.773695
Dendritic.cells	KLF12	-0.04584	3.585919	-0.11737	0.906837	-5.82435	0.795285	0.802235
Dendritic.cells	KNSTRN	0.052778	3.271497	0.117349	0.90685	-5.70626	0.800115	0.809288
Dendritic.cells	COL1A2	0.056378	2.442649	0.117061	0.907077	-5.58478	0.813108	0.828231
Dendritic.cells	GM15564	-0.05316	2.474453	-0.11705	0.907087	-5.52659	0.812607	0.827499
Dendritic.cells	MS4A3	0.090494	-0.35863	0.116703	0.90736	-5.27157	0.858838	0.895306
Dendritic.cells	GM26756	-0.06296	0.354794	-0.11653	0.907495	-5.47407	0.846945	0.877754
Dendritic.cells	CDC25B	-0.05388	4.400381	-0.11645	0.907561	-5.62049	0.783171	0.784383
Dendritic.cells	TRMT61B	-0.03705	4.178074	-0.11639	0.907611	-6.02552	0.786516	0.789252
Dendritic.cells	FAM136A	-0.02656	4.090791	-0.11626	0.907709	-5.99513	0.787834	0.791189
Dendritic.cells	A930037H(	-0.0505	3.242306	-0.11624	0.907728	-5.69727	0.800797	0.810093
Dendritic.cells	DPAGT1	-0.03067	3.285854	-0.11619	0.907764	-5.86719	0.800125	0.809112
Dendritic.cells	ALG10B	0.027967	2.952601	0.115901	0.907994	-5.84162	0.805425	0.816703
Dendritic.cells	FCNB	0.062633	-0.23854	0.115597	0.908234	-5.27389	0.85713	0.892482

Dendritic.cells	OSGEP	-0.01566	4.907558	-0.11527	0.908493	-6.36781	0.775996	0.773499
Dendritic.cells	TMEM150E	0.074462	1.12138	0.115058	0.90866	-5.30571	0.834802	0.859412
Dendritic.cells	IFIT1	0.09904	2.761439	0.115054	0.908663	-5.48662	0.808668	0.821106
Dendritic.cells	KCNJ16	-0.09864	-0.02269	-0.11503	0.908681	-5.27027	0.853641	0.887153
Dendritic.cells	5830487J0	0.05963	-0.01733	0.114975	0.908725	-5.27962	0.853551	0.887021
Dendritic.cells	ABCA5	0.031666	0.444818	0.114721	0.908927	-5.96422	0.845913	0.875737
Dendritic.cells	MYBL2	0.048768	3.080165	0.114393	0.909185	-5.57192	0.803737	0.813894
Dendritic.cells	GM4951	0.048599	3.770894	0.114387	0.90919	-6.04901	0.793117	0.7984
Dendritic.cells	HACL1	0.035832	3.523051	0.11432	0.909244	-5.84634	0.796907	0.803926
Dendritic.cells	RBBP8	-0.01409	6.609426	-0.11429	0.90927	-6.60434	0.751316	0.737749
Dendritic.cells	CYTH2	-0.02561	3.933028	-0.11418	0.909352	-5.98139	0.79065	0.794818
Dendritic.cells	PSMC1	-0.01407	5.889053	-0.11394	0.909546	-6.40389	0.761647	0.752745
Dendritic.cells	BACH2	-0.01737	8.964274	-0.11391	0.909571	-7.03795	0.718821	0.690996
Dendritic.cells	2700081O1	-0.02972	3.137169	-0.11386	0.90961	-5.85472	0.802854	0.812672
Dendritic.cells	PPP2R5B	-0.05962	1.645269	-0.11385	0.909613	-5.42273	0.826373	0.8471
Dendritic.cells	SIMC1	-0.02401	5.702765	-0.11385	0.909613	-6.17585	0.764349	0.75666
Dendritic.cells	AP2S1	0.011668	6.932961	0.113833	0.909628	-6.72813	0.746736	0.731184
Dendritic.cells	GM15417	-0.0449	2.477041	-0.11366	0.909767	-5.51565	0.813216	0.827758
Dendritic.cells	LMTK3	0.062817	-0.70138	0.113451	0.90993	-5.27018	0.865237	0.904194
Dendritic.cells	ATAD2	-0.02405	6.309192	-0.11314	0.910176	-6.43476	0.75582	0.744131
Dendritic.cells	RNF141	-0.02215	4.511433	-0.11292	0.910348	-6.01474	0.782155	0.782362
Dendritic.cells	RBM48	0.029741	3.037834	0.112879	0.910382	-5.72629	0.804628	0.815115
Dendritic.cells	SNHG4	0.030502	3.683196	0.112872	0.910388	-5.80769	0.794687	0.800608
Dendritic.cells	NPAS2	0.059671	0.089778	0.112856	0.9104	-5.41321	0.852046	0.884712
Dendritic.cells	PCBP2	0.007991	8.511828	0.1127	0.910524	-7.06036	0.725167	0.699918
Dendritic.cells	MKI67	-0.04636	6.498445	-0.11261	0.910598	-6.30299	0.753158	0.740264
Dendritic.cells	SLC15A4	-0.01268	4.804844	-0.1123	0.910841	-6.70553	0.777938	0.776046
Dendritic.cells	HBS1L	-0.01229	5.694465	-0.11225	0.910879	-6.5722	0.764852	0.757062
Dendritic.cells	TMEM176A	-0.03099	4.388779	-0.11179	0.91124	-5.97264	0.784297	0.785142
Dendritic.cells	NINL	-0.05747	0.999292	-0.11178	0.91125	-5.36536	0.837385	0.862762
Dendritic.cells	HS3ST3B1	0.018765	3.380647	0.111772	0.911258	-6.4271	0.799635	0.807483
Dendritic.cells	YWHAB	-0.00912	7.88619	-0.11134	0.911596	-6.92165	0.734082	0.712307
Dendritic.cells	SLC25A25	-0.02029	4.951297	-0.11128	0.911644	-6.16665	0.776036	0.772949
Dendritic.cells	SEC22C	0.040956	2.04121	0.111177	0.911728	-5.55648	0.820747	0.838162
Dendritic.cells	LMO7	-0.09947	0.761873	-0.11098	0.911887	-5.33798	0.841414	0.868536
Dendritic.cells	RASGRP4	0.044065	1.734055	0.110853	0.911985	-5.6053	0.825652	0.845387
Dendritic.cells	KCNAB2	0.027836	4.287693	0.110781	0.912041	-5.93797	0.785955	0.787413
Dendritic.cells	UBE2H	0.01203	8.574917	0.110778	0.912043	-7.01797	0.724677	0.698832
Dendritic.cells	GSTP2	0.060967	0.861498	0.110778	0.912044	-5.27368	0.839782	0.866136
Dendritic.cells	MIS18BP1	-0.04585	3.964435	-0.11075	0.912069	-5.71962	0.790845	0.79453
Dendritic.cells	RETNLA	0.229663	-0.77811	0.110409	0.912336	-5.27262	0.867243	0.90652
Dendritic.cells	MRPS33	-0.01037	6.714474	-0.11029	0.912433	-6.73788	0.750563	0.736022
Dendritic.cells	GPN3	-0.02468	3.982026	-0.11023	0.912474	-5.85096	0.790684	0.794195
Dendritic.cells	CDK16	0.022799	3.39772	0.110225	0.912481	-5.91085	0.799619	0.807216
Dendritic.cells	NR5A2	-0.04329	1.145394	-0.11002	0.912643	-5.53823	0.835303	0.85939
Dendritic.cells	DPH7	-0.04971	1.586984	-0.10999	0.912667	-5.48255	0.828162	0.848909
Dendritic.cells	AMIGO2	-0.06853	0.934211	-0.10968	0.912916	-5.37061	0.838774	0.864497

Dendritic.cells	IMPDH1	-0.0385	4.326873	-0.10963	0.912953	-5.73509	0.785534	0.786654
Dendritic.cells	EGFL6	-0.06679	0.548891	-0.1095	0.913053	-5.28088	0.845097	0.873798
Dendritic.cells	CIAO1	-0.02579	3.481337	-0.10948	0.913072	-5.77334	0.798397	0.805386
Dendritic.cells	EXOC5	0.010489	6.376842	0.109351	0.913172	-6.67271	0.755436	0.743032
Dendritic.cells	TIMP2	-0.02161	4.873204	-0.10926	0.913241	-6.59438	0.777363	0.774804
Dendritic.cells	BSPRY	0.064059	0.341542	0.109091	0.913377	-5.28023	0.848524	0.878912
Dendritic.cells	RAN	0.016482	8.400943	0.109041	0.913417	-6.96102	0.727194	0.702367
Dendritic.cells	GAS2L1	-0.04086	1.765951	-0.10896	0.91348	-5.59008	0.825318	0.844822
Dendritic.cells	BRIX1	-0.00991	6.144863	-0.10883	0.913585	-6.63683	0.758765	0.747941
Dendritic.cells	CSNK1G2	-0.01389	5.468875	-0.10872	0.913674	-6.40169	0.768578	0.762169
Dendritic.cells	RPRD2	-0.01323	5.916624	-0.1087	0.913688	-6.56495	0.76206	0.752727
Dendritic.cells	NOA1	-0.03317	3.137205	-0.1087	0.913689	-5.70102	0.803709	0.813275
Dendritic.cells	NUTF2-PS1	0.053762	2.848853	0.108665	0.913715	-5.60879	0.808194	0.819826
Dendritic.cells	BZW2	0.011767	5.869307	0.10831	0.913995	-6.74359	0.762792	0.753794
Dendritic.cells	PRKCH	-0.02283	6.999653	-0.10808	0.91418	-6.74789	0.746638	0.730468
Dendritic.cells	CDK2	-0.03124	4.350301	-0.108	0.914244	-5.83809	0.785229	0.786382
Dendritic.cells	PCNA	-0.01905	6.710032	-0.10797	0.914264	-6.55732	0.750733	0.736381
Dendritic.cells	PARG	0.011909	5.560263	0.107869	0.914344	-6.5202	0.767289	0.760335
Dendritic.cells	ATXN7L2	-0.04096	2.480154	-0.10757	0.914582	-5.58763	0.814024	0.828414
Dendritic.cells	AP2B1	-0.00939	6.179239	-0.10749	0.914646	-6.7246	0.758317	0.747375
Dendritic.cells	RASGEF1B	-0.02862	5.547786	-0.10747	0.914663	-6.36995	0.767471	0.76063
Dendritic.cells	6-Mar	-0.00997	6.3538	-0.10745	0.914671	-6.68285	0.755812	0.743752
Dendritic.cells	CCDC136	-0.07344	0.119291	-0.10745	0.914674	-5.30822	0.852267	0.88459
Dendritic.cells	TOP2B	-0.01329	6.593853	-0.10741	0.914709	-6.71463	0.752385	0.738798
Dendritic.cells	PARP14	-0.0346	5.480462	-0.10736	0.914749	-6.26763	0.768455	0.762067
Dendritic.cells	AUTS2	0.02775	4.397144	0.107342	0.914761	-6.50926	0.784524	0.7854
Dendritic.cells	RRP7A	-0.02554	3.942345	-0.1072	0.914873	-5.93861	0.791399	0.795444
Dendritic.cells	PDCD6IP	-0.00814	6.988379	-0.10716	0.914903	-6.74994	0.746797	0.730781
Dendritic.cells	COMT	0.017153	5.36738	0.107022	0.915014	-6.30794	0.770113	0.764563
Dendritic.cells	AFDN	-0.01875	3.267689	-0.10699	0.915041	-6.24454	0.801739	0.810595
Dendritic.cells	PPIA	0.010438	10.92524	0.106698	0.915271	-7.49272	0.69399	0.655064
Dendritic.cells	RSPH1	0.075936	1.855808	0.106694	0.915273	-5.33741	0.823931	0.843101
Dendritic.cells	ZRSR1	-0.04069	2.211301	-0.10668	0.915288	-5.53287	0.818272	0.834808
Dendritic.cells	PAK1IP1	0.01011	5.901653	0.106598	0.915349	-6.56516	0.762323	0.753325
Dendritic.cells	ZFP623	0.060384	0.124749	0.106578	0.915365	-5.31671	0.852176	0.884635
Dendritic.cells	IFT27	-0.02853	3.758551	-0.10627	0.915606	-5.89149	0.794265	0.799675
Dendritic.cells	ATPAF1	-0.02216	3.553171	-0.10624	0.915629	-5.9808	0.797409	0.804259
Dendritic.cells	SENP7	-0.01399	5.356774	-0.10617	0.915686	-6.47112	0.770333	0.764878
Dendritic.cells	GM16845	-0.03207	2.527304	-0.10601	0.915817	-5.75978	0.81335	0.827578
Dendritic.cells	TRAPPC12	-0.01873	4.201517	-0.106	0.915821	-6.06468	0.787538	0.789909
Dendritic.cells	ACP2	-0.04109	3.54465	-0.10593	0.915877	-5.61053	0.79754	0.8045
Dendritic.cells	ST6GALNA(	0.05738	0.651842	0.10579	0.915988	-5.31838	0.84356	0.871911
Dendritic.cells	ERO1L	-0.02632	5.171152	-0.10554	0.916183	-6.12339	0.773195	0.76895
Dendritic.cells	3830403N1	0.079427	0.704685	0.105461	0.916249	-5.32454	0.842798	0.87067
Dendritic.cells	TRIM24	-0.01195	5.087563	-0.10533	0.916354	-6.63401	0.774436	0.770761
Dendritic.cells	UPF1	-0.01237	5.35091	-0.10523	0.916431	-6.37732	0.770556	0.765159
Dendritic.cells	MRPS5	0.018746	4.612666	0.105076	0.916553	-6.12753	0.781498	0.78111

Dendritic.cells	TFR2	-0.04786	1.213769	-0.10491	0.916683	-5.41967	0.834491	0.858616
Dendritic.cells	1700126G(	-0.04542	1.104608	-0.10481	0.916762	-5.53381	0.836266	0.861235
Dendritic.cells	GNAL	-0.04519	1.371741	-0.1047	0.916851	-5.41707	0.831931	0.854889
Dendritic.cells	GRK2	0.010497	7.206149	0.104551	0.916969	-6.77659	0.74393	0.726771
Dendritic.cells	IFI214	-0.06584	1.667368	-0.10452	0.916996	-5.33166	0.827166	0.847894
Dendritic.cells	NPRL3	-0.02195	2.892647	-0.10445	0.91705	-6.01406	0.807772	0.81949
Dendritic.cells	XPO5	0.023042	3.603677	0.104314	0.917156	-5.91828	0.796777	0.80344
Dendritic.cells	ZFP68	-0.01725	3.920899	-0.1043	0.917166	-6.1212	0.791933	0.796378
Dendritic.cells	SAMD10	0.043444	1.732501	0.104264	0.917196	-5.49437	0.826121	0.846365
Dendritic.cells	SPPL3	0.008099	7.282604	0.103996	0.917408	-6.9307	0.742859	0.725315
Dendritic.cells	BMX	0.070781	0.02253	0.103986	0.917416	-5.28231	0.854103	0.88764
Dendritic.cells	KAT6B	-0.01098	5.844837	-0.10397	0.917428	-6.72175	0.763347	0.754936
Dendritic.cells	PPIG	0.007921	6.974522	0.10378	0.917579	-6.81661	0.747187	0.731564
Dendritic.cells	SH3BP1	-0.02137	5.21446	-0.10352	0.917782	-6.13185	0.772564	0.768304
Dendritic.cells	GM46367	-0.04857	2.175914	-0.10345	0.917837	-5.47753	0.819048	0.836095
Dendritic.cells	ABCD1	-0.01473	4.771292	-0.10343	0.917853	-6.32021	0.77913	0.777842
Dendritic.cells	TRUB2	-0.02253	4.526877	-0.1034	0.917879	-5.96929	0.782782	0.783152
Dendritic.cells	SOCS6	-0.01885	3.829946	-0.10327	0.917979	-6.03173	0.793318	0.798493
Dendritic.cells	ZMAT1	0.040658	2.090961	0.103253	0.917996	-5.54726	0.820397	0.838073
Dendritic.cells	KANTR	0.029363	2.529607	0.103253	0.917996	-5.81901	0.813459	0.827911
Dendritic.cells	RMI1	-0.02239	3.629414	-0.10325	0.917998	-5.85399	0.796382	0.802962
Dendritic.cells	SGSH	-0.04505	1.453355	-0.10316	0.91807	-5.46385	0.830613	0.853085
Dendritic.cells	LRRC75A	-0.04337	2.6203	-0.10285	0.918312	-5.60251	0.812184	0.825937
Dendritic.cells	SMN1	-0.01811	4.851728	-0.10266	0.918462	-6.22147	0.7781	0.776219
Dendritic.cells	KLHL23	-0.05909	0.171341	-0.10264	0.918485	-5.29636	0.851806	0.884116
Dendritic.cells	LTV1	-0.02296	4.232009	-0.10233	0.918724	-6.01381	0.787513	0.789726
Dendritic.cells	MLX	0.021221	3.767938	0.102266	0.918778	-5.98974	0.794562	0.799993
Dendritic.cells	GM43258	0.046144	0.724157	0.101909	0.91906	-5.43475	0.842994	0.870661
Dendritic.cells	SREBF1	-0.02159	3.760907	-0.10182	0.919134	-5.98989	0.794852	0.80016
Dendritic.cells	SF3B5	0.012217	6.277364	0.101476	0.919402	-6.57027	0.757725	0.746065
Dendritic.cells	ZNRD2	0.024179	4.037669	0.100576	0.920115	-5.92877	0.791173	0.794191
Dendritic.cells	FAM13A	-0.06262	1.15315	-0.10057	0.920123	-5.32492	0.836548	0.860532
Dendritic.cells	GM26782	-0.02421	2.991571	-0.10046	0.92021	-5.74451	0.807266	0.81765
Dendritic.cells	SPAG7	0.018285	4.55728	0.100439	0.920223	-6.18327	0.783331	0.782786
Dendritic.cells	ADPGK	0.016009	4.903066	0.100422	0.920237	-6.59992	0.778168	0.775287
Dendritic.cells	IFIT1BL1	0.079448	0.159661	0.100386	0.920265	-5.27264	0.852911	0.884605
Dendritic.cells	PPP1R3B	-0.0425	2.491892	-0.10013	0.920471	-5.43816	0.81511	0.829128
Dendritic.cells	LYSMD2	0.042572	1.265146	0.099971	0.920594	-5.57323	0.83474	0.857932
Dendritic.cells	GM1604A	-0.03812	2.804564	-0.09993	0.920627	-5.67593	0.810198	0.821986
Dendritic.cells	VPS13B	-0.00797	8.050399	-0.09992	0.920631	-7.05999	0.73317	0.710312
Dendritic.cells	GM16174	-0.03717	0.995634	-0.09967	0.920828	-5.55431	0.83913	0.864412
Dendritic.cells	DARS2	0.047294	2.849125	0.099622	0.92087	-5.53869	0.809501	0.821001
Dendritic.cells	PCCB	0.027156	3.081613	0.099524	0.920947	-5.79685	0.805877	0.815708
Dendritic.cells	ZFP160	0.026326	2.950859	0.099442	0.921013	-5.79972	0.807913	0.818683
Dendritic.cells	PEX5	0.01799	4.071805	0.099246	0.921168	-6.05409	0.790667	0.793606
Dendritic.cells	PTH1R	-0.0705	0.264703	-0.09922	0.921184	-5.27475	0.851176	0.882218
Dendritic.cells	ABHD12	-0.01595	5.584506	-0.09898	0.921379	-6.31179	0.768132	0.760946

Dendritic.cells	POP1	0.028591	2.708556	0.098941	0.921409	-5.72044	0.811703	0.824373
Dendritic.cells	GOSR2	0.013523	5.28486	0.098934	0.921415	-6.43829	0.772529	0.767332
Dendritic.cells	NT5DC3	-0.01502	5.099601	-0.09887	0.921465	-6.34934	0.775264	0.771319
Dendritic.cells	GFPT1	0.017565	5.095805	0.098753	0.921558	-6.41192	0.77532	0.771411
Dendritic.cells	UTP25	-0.03112	2.617836	-0.09874	0.921569	-5.64013	0.813127	0.826487
Dendritic.cells	ERMP1	0.021093	3.633634	0.098702	0.921598	-5.89192	0.797352	0.803456
Dendritic.cells	CNBD2	-0.03337	3.889219	-0.09868	0.921618	-5.72383	0.793444	0.797761
Dendritic.cells	ELANE	0.164275	2.340078	0.098626	0.921658	-5.31177	0.817508	0.832897
Dendritic.cells	TEFM	-0.05761	1.160891	-0.09844	0.921808	-5.29846	0.836472	0.860672
Dendritic.cells	GM26535	0.056186	-0.60061	0.098392	0.921844	-5.27251	0.865742	0.903825
Dendritic.cells	HLX	-0.03697	3.117664	-0.09824	0.921961	-5.53251	0.805391	0.815114
Dendritic.cells	NEFH	-0.06195	-0.09246	-0.09761	0.922465	-5.27163	0.857482	0.891338
Dendritic.cells	PTPN14	0.047018	0.524362	0.097564	0.9225	-5.47796	0.847214	0.876206
Dendritic.cells	WDR43	-0.01122	6.033494	-0.09756	0.922503	-6.67833	0.76191	0.751708
Dendritic.cells	CUL2	-0.0147	4.968394	-0.09752	0.922537	-6.34996	0.777521	0.774332
Dendritic.cells	SNF8	0.010739	5.731869	0.097368	0.922655	-6.55348	0.766326	0.758047
Dendritic.cells	RLIM	0.00804	6.654275	0.097175	0.922808	-6.74555	0.753045	0.738828
Dendritic.cells	MS4A4A	0.058725	0.617812	0.097052	0.922905	-5.27549	0.84572	0.873936
Dendritic.cells	VILL	0.056103	1.228935	0.097027	0.922925	-5.27513	0.835713	0.859225
Dendritic.cells	TGFB3	0.054901	0.567314	0.096878	0.923042	-5.37699	0.846554	0.875199
Dendritic.cells	RRBP1	0.006932	7.951353	0.096757	0.923138	-7.1687	0.734871	0.712692
Dendritic.cells	TAB1	-0.02988	3.004453	-0.09668	0.923202	-5.72432	0.807449	0.817917
Dendritic.cells	LZTS1	0.021402	1.417164	0.096627	0.923242	-6.13104	0.83266	0.854827
Dendritic.cells	TMX4	0.01994	4.420261	0.096604	0.923259	-6.164	0.785763	0.786316
Dendritic.cells	PIK3R1	-0.01344	7.694648	-0.0964	0.923418	-6.87228	0.73842	0.717814
Dendritic.cells	BACH2IT1	0.048193	-0.26225	0.096348	0.923462	-5.26914	0.860385	0.895666
Dendritic.cells	2200002D(	-0.0547	1.337585	-0.09635	0.923463	-5.42222	0.833949	0.856749
Dendritic.cells	MMD	0.028398	4.477186	0.096206	0.923575	-5.8834	0.78494	0.7851
Dendritic.cells	2210408F2	-0.02105	2.856305	-0.09602	0.923721	-6.25938	0.809831	0.821359
Dendritic.cells	IL18RAP	0.061112	1.983582	0.095913	0.923807	-5.41235	0.823624	0.841551
Dendritic.cells	JCHAIN	-0.12989	-0.4625	-0.09581	0.923889	-5.27432	0.863837	0.900739
Dendritic.cells	RNF214	-0.01412	4.961908	-0.09579	0.923905	-6.32748	0.777729	0.774647
Dendritic.cells	LNX2	0.027812	3.592505	0.095643	0.924021	-5.75576	0.798456	0.804744
Dendritic.cells	EPC2	0.011219	6.14343	0.095053	0.924488	-6.60083	0.760792	0.749636
Dendritic.cells	NOL7	0.008319	6.906907	0.09459	0.924854	-6.74298	0.750116	0.733972
Dendritic.cells	1110002J0	0.063059	-0.51304	0.094491	0.924933	-5.27406	0.865383	0.902197
Dendritic.cells	ZFP119A	-0.03072	2.125414	-0.09427	0.925109	-5.54919	0.8221	0.838495
Dendritic.cells	GEM	-0.01992	5.207345	-0.09416	0.925193	-6.38768	0.774789	0.769619
Dendritic.cells	2610002M	0.029646	4.208089	0.0941	0.925243	-5.68646	0.78974	0.791328
Dendritic.cells	SMIM26	0.018891	3.847983	0.0936	0.925639	-6.01487	0.795411	0.799335
Dendritic.cells	CAAP1	0.014503	4.575271	0.093597	0.925641	-6.23889	0.784393	0.783316
Dendritic.cells	ZMIZ1OS1	0.055704	0.352543	0.093539	0.925687	-5.37362	0.85115	0.880917
Dendritic.cells	BCL2A1D	0.065848	4.122114	0.093451	0.925757	-5.50047	0.791235	0.793273
Dendritic.cells	FAM214B	0.035669	3.11159	0.093285	0.925888	-5.66319	0.80677	0.815899
Dendritic.cells	CTSC	0.019898	7.322442	0.093265	0.925904	-6.78972	0.744521	0.725655
Dendritic.cells	CD38	0.026525	5.882237	0.093083	0.926048	-6.38563	0.765143	0.75537
Dendritic.cells	PNPO	0.028533	3.744874	0.092709	0.926345	-5.78086	0.797226	0.801744

Dendritic.cells	NMNAT2	0.060303	2.111738	0.09251	0.926502	-5.50653	0.822761	0.839098
Dendritic.cells	COPZ1	0.00927	6.366584	0.092475	0.92653	-6.6515	0.75831	0.745392
Dendritic.cells	NDUFAB1	0.0145	6.268706	0.092369	0.926614	-6.51099	0.759717	0.747436
Dendritic.cells	TBC1D24	0.038015	1.799284	0.092368	0.926614	-5.50774	0.827761	0.846431
Dendritic.cells	TOPORS	-0.01572	5.749708	-0.09208	0.926842	-6.4076	0.767321	0.758344
Dendritic.cells	DPH6	-0.01722	4.47347	-0.09197	0.926932	-6.20864	0.78624	0.785775
Dendritic.cells	ZFP24	-0.01517	4.243197	-0.09196	0.926936	-6.13063	0.789717	0.790828
Dendritic.cells	H3F3A	-0.00792	10.71708	-0.09186	0.927018	-7.32054	0.699196	0.660461
Dendritic.cells	MRPS31	-0.02096	3.653241	-0.09164	0.927188	-5.83526	0.798716	0.803926
Dendritic.cells	GM47096	0.042453	1.734536	0.091551	0.927262	-5.37921	0.828889	0.847997
Dendritic.cells	HMBOX1	-0.01284	5.578159	-0.09153	0.927281	-6.49424	0.769829	0.761982
Dendritic.cells	GM47689	-0.01901	3.369026	-0.09136	0.927413	-5.91976	0.803098	0.81031
Dendritic.cells	GUK1	0.016539	4.908743	0.091329	0.927438	-6.12613	0.77972	0.776317
Dendritic.cells	SEC24B	0.010807	6.179882	0.091273	0.927483	-6.67057	0.761078	0.749337
Dendritic.cells	JOSD1	0.018951	3.99961	0.091154	0.927576	-5.94769	0.793417	0.796246
Dendritic.cells	TMEM38A	-0.03601	1.868955	-0.09114	0.927585	-5.53268	0.82673	0.844869
Dendritic.cells	MYDGF	-0.01262	4.485984	-0.09096	0.927728	-6.26564	0.786062	0.785567
Dendritic.cells	STAT4	0.028447	5.177658	0.090944	0.927743	-6.13216	0.775738	0.770585
Dendritic.cells	GM5165	0.024394	2.638407	0.090555	0.928051	-5.78798	0.814683	0.827047
Dendritic.cells	CLNS1A	0.011378	5.340846	0.090516	0.928082	-6.36492	0.773489	0.767125
Dendritic.cells	XPR1	0.010759	6.866995	0.090116	0.928399	-6.78289	0.751609	0.735229
Dendritic.cells	FGFR1	0.030248	1.974297	0.089919	0.928555	-6.02281	0.825516	0.842537
Dendritic.cells	LYL1	0.030438	3.172684	0.089743	0.928694	-5.74684	0.806664	0.81495
Dendritic.cells	CLEC4A3	0.058914	3.297848	0.089651	0.928768	-5.52276	0.804721	0.812123
Dendritic.cells	IGLV1	-0.05415	-0.83837	-0.08946	0.928919	-5.27068	0.872193	0.911038
Dendritic.cells	RDH14	-0.0188	3.731822	-0.08929	0.929052	-5.91319	0.798086	0.80239
Dendritic.cells	GM35769	-0.04682	1.096204	-0.08919	0.929134	-5.34214	0.839844	0.863425
Dendritic.cells	NPEPL1	-0.01239	5.049543	-0.08916	0.929159	-6.40082	0.778187	0.773504
Dendritic.cells	ATP6V1G2	0.034982	1.237127	0.089097	0.929206	-5.54243	0.837544	0.860051
Dendritic.cells	A530088E(	-0.03243	1.760356	-0.08901	0.929273	-5.65111	0.829072	0.847645
Dendritic.cells	ZC3H18	-0.01272	5.270525	-0.08888	0.929378	-6.36801	0.77494	0.768804
Dendritic.cells	OTUD4	-0.0128	5.091742	-0.08853	0.929657	-6.32782	0.777654	0.772726
Dendritic.cells	UTP15	0.018717	3.757308	0.088499	0.92968	-5.90155	0.797791	0.801971
Dendritic.cells	F10	-0.05412	4.54109	-0.08826	0.929873	-5.81233	0.785884	0.78471
Dendritic.cells	TACC3	-0.03327	5.072041	-0.08817	0.929944	-5.96604	0.777947	0.773196
Dendritic.cells	TTLL11	-0.02919	1.608719	-0.08802	0.930057	-5.6846	0.831616	0.851453
Dendritic.cells	NAIF1	-0.037	1.251393	-0.08789	0.930166	-5.45437	0.837412	0.859962
Dendritic.cells	1700021F0	-0.02503	3.209032	-0.08775	0.930273	-5.71295	0.806256	0.814419
Dendritic.cells	USO1	0.007975	5.739311	0.087672	0.930336	-6.78011	0.768119	0.759054
Dendritic.cells	INTS11	-0.02027	3.989645	-0.08761	0.930389	-5.97396	0.794237	0.796942
Dendritic.cells	ZMYM3	-0.03115	2.31007	-0.08758	0.930407	-5.65071	0.820382	0.835067
Dendritic.cells	MACO1	-0.00779	6.27208	-0.08742	0.930534	-6.83189	0.760388	0.747929
Dendritic.cells	RDX	0.006999	6.872179	0.087259	0.930663	-6.79285	0.751803	0.735537
Dendritic.cells	ANKRD13C	0.0119	5.868682	0.087257	0.930664	-6.5008	0.766232	0.756376
Dendritic.cells	SLX4	0.030337	1.820472	0.087222	0.930692	-5.48777	0.828205	0.846581
Dendritic.cells	ACADM	-0.02133	4.767805	-0.08712	0.930777	-6.06142	0.782482	0.779929
Dendritic.cells	SLC25A23	-0.02393	2.25448	-0.08711	0.930784	-5.83788	0.821266	0.836428

Dendritic.cells	ATP6V1B2	0.011131	5.98524	0.087104	0.930786	-6.40922	0.764538	0.753937
Dendritic.cells	DDX28	0.034043	1.558733	0.087057	0.930823	-5.43991	0.832424	0.852764
Dendritic.cells	GCNT7	-0.02276	2.712006	-0.087	0.930865	-5.81124	0.814028	0.825852
Dendritic.cells	SIRT1	0.01306	5.043074	0.086942	0.930914	-6.19265	0.778377	0.773975
Dendritic.cells	GGA1	0.01334	4.730426	0.086691	0.931113	-6.18521	0.783047	0.780741
Dendritic.cells	IGFBP3	-0.05594	0.690335	-0.08664	0.931152	-5.30483	0.846618	0.87359
Dendritic.cells	SLA	-0.01435	5.280678	-0.08663	0.931165	-6.47122	0.774861	0.768872
Dendritic.cells	PRDX6	0.009727	7.110346	0.086538	0.931234	-6.70029	0.748437	0.730688
Dendritic.cells	CSNK2B	0.008682	7.247784	0.086475	0.931284	-6.84875	0.7465	0.727898
Dendritic.cells	TMEM117	0.062196	-0.04691	0.08635	0.931384	-5.2758	0.858916	0.891655
Dendritic.cells	PCTP	0.037218	2.252585	0.086055	0.931618	-5.41863	0.821467	0.836525
Dendritic.cells	UFM1	-0.01103	5.448609	-0.08576	0.931854	-6.43799	0.772677	0.765355
Dendritic.cells	CCDC192	0.054256	-0.69514	0.085521	0.93204	-5.27546	0.870292	0.907974
Dendritic.cells	TMEM56	-0.03954	2.147941	-0.08483	0.932586	-5.55372	0.823746	0.839229
Dendritic.cells	TXLNA	-0.01301	4.941372	-0.08481	0.932602	-6.25977	0.780633	0.776465
Dendritic.cells	9030622O2	0.053173	1.05914	0.084438	0.932899	-5.36296	0.841422	0.865024
Dendritic.cells	TMC3	0.053554	-1.08655	0.084434	0.932902	-5.26869	0.877461	0.918094
Dendritic.cells	ESS2	0.022135	2.988114	0.084409	0.932922	-5.69939	0.810538	0.819853
Dendritic.cells	TMOD1	-0.04459	1.823905	-0.08421	0.933079	-5.40448	0.829007	0.846832
Dendritic.cells	NLRX1	-0.04057	1.80919	-0.08419	0.933098	-5.44492	0.829244	0.847179
Dendritic.cells	POLE4	-0.00844	6.652817	-0.08416	0.933118	-6.78444	0.755708	0.740343
Dendritic.cells	IDUA	-0.025	2.137056	-0.08371	0.933473	-5.65538	0.824188	0.839608
Dendritic.cells	RBMS3	0.03245	3.072419	0.083585	0.933575	-5.86746	0.809417	0.818067
Dendritic.cells	5330439KC	0.047508	0.538036	0.08353	0.933619	-5.33297	0.850216	0.87778
Dendritic.cells	SON	0.006083	7.67203	0.083457	0.933677	-6.95494	0.741507	0.719756
Dendritic.cells	2010300CC	0.044575	-0.0882	0.083396	0.933725	-5.27797	0.860678	0.893196
Dendritic.cells	CCDC80	-0.03437	2.683481	-0.08333	0.933781	-5.69333	0.815519	0.827005
Dendritic.cells	CYP4A10	-0.0598	1.117903	-0.08315	0.933922	-5.40785	0.840725	0.863811
Dendritic.cells	LYRM9	-0.03296	2.696887	-0.08299	0.934051	-5.51131	0.815415	0.826734
Dendritic.cells	1600010M	0.021494	4.96425	0.082823	0.93418	-5.98843	0.780657	0.776181
Dendritic.cells	MIA3	0.008296	5.849323	0.08279	0.934206	-6.64343	0.767603	0.757289
Dendritic.cells	CHAC1	-0.04962	0.269965	-0.08267	0.934298	-5.29862	0.854816	0.884422
Dendritic.cells	OMA1	-0.02283	3.410326	-0.08252	0.934419	-5.8015	0.804297	0.81052
Dendritic.cells	TPRN	-0.02677	2.21301	-0.08248	0.934449	-5.72643	0.823115	0.837973
Dendritic.cells	DLD	0.013563	4.599821	0.082353	0.934552	-6.08662	0.786149	0.784158
Dendritic.cells	UBE2G1	-0.00667	7.307993	-0.08219	0.934683	-6.91354	0.74674	0.727238
Dendritic.cells	CRYBG1	-0.03302	4.169377	-0.08211	0.934748	-5.57075	0.79266	0.793656
Dendritic.cells	PUF60	0.01069	5.949591	0.082095	0.934756	-6.52941	0.766174	0.755281
Dendritic.cells	CRP	0.043352	2.076546	0.082005	0.934828	-5.48775	0.825307	0.841259
Dendritic.cells	TGFB1	0.006567	8.800362	0.081773	0.935012	-7.20598	0.726189	0.697735
Dendritic.cells	EIF4E2	-0.01082	5.989442	-0.08174	0.935035	-6.43086	0.765645	0.754528
Dendritic.cells	GPR180	0.020196	2.964205	0.081517	0.935215	-5.83936	0.811404	0.82083
Dendritic.cells	SNRPF	0.010178	6.76677	0.081392	0.935315	-6.65487	0.754561	0.738438
Dendritic.cells	E230029CC	-0.03348	3.508508	-0.08105	0.935588	-5.65897	0.80313	0.808548
Dendritic.cells	PRG3	-0.0635	1.171663	-0.08082	0.935771	-5.28891	0.840364	0.862837
Dendritic.cells	PXYLP1	-0.02314	2.352535	-0.08076	0.935812	-5.79972	0.821323	0.834973
Dendritic.cells	NOL8	0.01818	4.078757	0.080628	0.93592	-6.00607	0.794471	0.795818

Dendritic.cells	TRMT5	-0.03935	1.213824	-0.08025	0.936217	-5.39539	0.839908	0.861827
Dendritic.cells	PPP1CC	0.00717	7.584523	0.080006	0.936414	-6.8681	0.743544	0.721818
Dendritic.cells	MMP27	0.046326	-0.20195	0.079767	0.936603	-5.2746	0.863651	0.896426
Dendritic.cells	SLC22A5	0.026234	3.042195	0.079626	0.936714	-5.83334	0.810907	0.819174
Dendritic.cells	SLC25A11	0.012725	5.162778	0.079362	0.936924	-6.32827	0.778571	0.772276
Dendritic.cells	NAA35	0.012221	4.851078	0.079271	0.936997	-6.222	0.783219	0.779022
Dendritic.cells	CKAP4	0.023128	4.030233	0.079222	0.937035	-5.93045	0.795632	0.79704
Dendritic.cells	N4BP2	0.013298	5.310122	0.079163	0.937082	-6.33357	0.776386	0.769144
Dendritic.cells	THRAP3	0.006521	8.006361	0.079066	0.937159	-7.01385	0.737794	0.71354
Dendritic.cells	AFP	-0.06673	4.371399	-0.079	0.937208	-5.85519	0.790442	0.789531
Dendritic.cells	GTF2H3	0.026703	2.299191	0.078992	0.937218	-5.63895	0.822637	0.836393
Dendritic.cells	OST4	0.008316	8.049265	0.078872	0.937313	-7.0204	0.737209	0.712696
Dendritic.cells	CISD2	0.008741	6.732173	0.078795	0.937374	-6.67102	0.755715	0.739334
Dendritic.cells	GM13986	0.072197	1.376917	0.078476	0.937626	-5.28395	0.837582	0.858222
Dendritic.cells	DYNC2H1	-0.01837	3.842041	-0.07843	0.937664	-5.97764	0.798601	0.801351
Dendritic.cells	APOL7E	-0.04369	-0.10248	-0.0784	0.937686	-5.29054	0.862095	0.894215
Dendritic.cells	GLB1	0.010985	5.221188	0.078103	0.937923	-6.41471	0.777925	0.771175
Dendritic.cells	CD79B	-0.01632	6.976941	-0.07787	0.93811	-6.56775	0.752462	0.7344
Dendritic.cells	AASDH	-0.0305	2.290623	-0.07781	0.938153	-5.61771	0.823037	0.83672
Dendritic.cells	CCDC191	-0.02988	1.993228	-0.07774	0.938211	-5.53397	0.827793	0.843668
Dendritic.cells	TENT4B	-0.00809	6.368937	-0.07769	0.938248	-6.71702	0.761163	0.746937
Dendritic.cells	PRKRA	0.015113	3.39958	0.077137	0.938689	-5.99199	0.805787	0.811358
Dendritic.cells	INCENP	-0.02388	5.454754	-0.07708	0.938731	-6.04119	0.77468	0.766255
Dendritic.cells	ZBTB17	-0.01575	3.917458	-0.07702	0.938784	-6.07985	0.7978	0.799773
Dendritic.cells	KMT2E	-0.00521	8.273581	-0.07681	0.938948	-7.161	0.734518	0.708487
Dendritic.cells	SPATA2L	0.036651	0.521707	0.076635	0.939087	-5.46506	0.852029	0.879027
Dendritic.cells	EIF3K	0.00588	7.871661	0.07663	0.939091	-7.01185	0.740072	0.716456
Dendritic.cells	TMEM87B	0.010562	5.127611	0.076589	0.939123	-6.34982	0.779527	0.773333
Dendritic.cells	RALGPS2	-0.01196	5.773254	-0.0765	0.939198	-6.50381	0.769998	0.759574
Dendritic.cells	PAFAH2	-0.04292	0.619176	-0.07635	0.939316	-5.36567	0.850411	0.876722
Dendritic.cells	B230217C1	-0.03714	1.478169	-0.07625	0.939396	-5.33448	0.836308	0.85607
Dendritic.cells	VAPA	0.004878	8.028548	0.076198	0.939433	-7.01838	0.737897	0.713416
Dendritic.cells	GM32036	-0.01888	3.651119	-0.07619	0.939439	-5.75345	0.801895	0.805869
Dendritic.cells	HIST1H2BJ	-0.041	3.30978	-0.07618	0.939449	-5.54787	0.807182	0.81356
Dendritic.cells	INTS6L	0.012131	5.229737	0.075972	0.939613	-6.37808	0.778009	0.771248
Dendritic.cells	GM14636	0.040824	2.274283	0.075823	0.939731	-5.57212	0.823491	0.837413
Dendritic.cells	GM38134	-0.0343	1.108316	-0.07579	0.939754	-5.48089	0.842345	0.864999
Dendritic.cells	GYS2	-0.03745	0.768164	-0.07575	0.939789	-5.35394	0.847944	0.873211
Dendritic.cells	SOWAHC	-0.02369	2.917305	-0.0757	0.939825	-5.91627	0.813316	0.822569
Dendritic.cells	MCM3AP	-0.01471	3.885875	-0.07536	0.940098	-5.91522	0.798284	0.800765
Dendritic.cells	ACADS	0.015111	4.490963	0.075351	0.940106	-6.03756	0.789072	0.787391
Dendritic.cells	CCDC189	-0.04714	0.183787	-0.07534	0.940117	-5.28259	0.857668	0.887572
Dendritic.cells	GM30239	-0.03791	-0.09316	-0.07525	0.940185	-5.27195	0.862323	0.894445
Dendritic.cells	PRRG2	0.02412	2.322172	0.075137	0.940275	-5.6198	0.822728	0.836414
Dendritic.cells	PRMT3	-0.01613	4.041123	-0.07506	0.940334	-6.04009	0.795908	0.797366
Dendritic.cells	CST3	0.013658	9.157123	0.075037	0.940355	-7.25923	0.722507	0.691533
Dendritic.cells	HIST1H3F	0.051055	0.34671	0.07502	0.940368	-5.27485	0.854944	0.883631

Dendritic.cells	UBXN11	-0.03055	1.770334	-0.0749	0.940467	-5.41934	0.831584	0.849388
Dendritic.cells	GM37982	-0.01692	2.996043	-0.07469	0.940633	-6.25092	0.812089	0.820982
Dendritic.cells	ZBTB11	-0.00769	7.088839	-0.07456	0.940736	-6.81417	0.751059	0.732613
Dendritic.cells	NIT2	-0.02224	3.588667	-0.07435	0.9409	-5.82444	0.802867	0.807651
Dendritic.cells	AHCYL2	0.01188	6.28947	0.074204	0.941016	-6.48037	0.762497	0.749166
Dendritic.cells	TMEM221	-0.02102	-0.26558	-0.07415	0.941058	-5.87023	0.865244	0.898988
Dendritic.cells	TRAPPC8	0.007311	6.225642	0.074095	0.941102	-6.73354	0.763421	0.750511
Dendritic.cells	NTNG2	-0.03102	3.05713	-0.07407	0.941118	-5.71665	0.811132	0.819711
Dendritic.cells	MRPL39	0.015031	3.661118	0.074047	0.94114	-5.91269	0.801749	0.80605
Dendritic.cells	THADA	0.009005	5.881845	0.073923	0.941238	-6.64126	0.768419	0.757729
Dendritic.cells	SMPD3	-0.04718	-0.20687	-0.07385	0.941299	-5.27288	0.864251	0.897537
Dendritic.cells	PODNL1	-0.03724	-1.02387	-0.07367	0.941437	-5.27972	0.878194	0.918141
Dendritic.cells	REPS1	-0.00871	5.964436	-0.07356	0.941526	-6.52528	0.767214	0.756039
Dendritic.cells	G3BP2	-0.00613	6.759047	-0.07352	0.94156	-6.71264	0.755751	0.7395
Dendritic.cells	MORF4L2	0.008936	5.404884	0.073434	0.941627	-6.47597	0.775424	0.767939
Dendritic.cells	RAP1GAP2	0.012589	4.692548	0.073424	0.941635	-6.66258	0.786041	0.78332
Dendritic.cells	CEACAM10	-0.03865	-1.04799	-0.07335	0.941696	-5.27487	0.878609	0.918826
Dendritic.cells	EEF1A1	-0.00551	10.55641	-0.07328	0.94175	-7.38649	0.704036	0.665395
Dendritic.cells	SPATA32	0.045768	0.244996	0.073259	0.941765	-5.30963	0.856652	0.88646
Dendritic.cells	STIL	0.032272	4.156047	0.073106	0.941887	-5.72165	0.794203	0.79518
Dendritic.cells	YIPF3	0.010893	4.904587	0.07287	0.942074	-6.26344	0.782995	0.778802
Dendritic.cells	PLXNA1	-0.04321	0.899006	-0.07267	0.942234	-5.33901	0.845938	0.870615
Dendritic.cells	GM21859	-0.052	1.780794	-0.0726	0.942286	-5.38017	0.831557	0.84954
Dendritic.cells	OAS3	0.060744	2.402295	0.072596	0.942291	-5.41791	0.821601	0.834986
Dendritic.cells	ACSL5	-0.01046	6.00653	-0.07236	0.942477	-6.54351	0.76678	0.755328
Dendritic.cells	CCT2	-0.00783	6.286032	-0.07234	0.942496	-6.60377	0.762725	0.749473
Dendritic.cells	GM7072	-0.0154	3.842908	-0.0721	0.942683	-6.06041	0.799234	0.802274
Dendritic.cells	EXT1	0.012469	7.749502	0.071619	0.943067	-6.83543	0.742246	0.71967
Dendritic.cells	WIPI2	0.011052	5.276718	0.07159	0.94309	-6.25608	0.77781	0.770956
Dendritic.cells	PIPOX	-0.03579	2.011866	-0.07145	0.943199	-5.47224	0.828251	0.844279
Dendritic.cells	TXNRD1	0.014894	5.871273	0.071273	0.943341	-6.19682	0.769143	0.758312
Dendritic.cells	MOSMO	0.012025	4.576833	0.071119	0.943463	-6.21099	0.78841	0.786174
Dendritic.cells	ADAMTS10	0.020273	3.015513	0.070951	0.943597	-5.73521	0.812442	0.821141
Dendritic.cells	KMT2B	0.01559	3.888591	0.07081	0.943709	-5.9391	0.798898	0.801436
Dendritic.cells	PDE6D	-0.01519	3.812865	-0.07066	0.94383	-5.83415	0.800062	0.803151
Dendritic.cells	ENOPH1	-0.02135	2.8863	-0.07062	0.943861	-5.70306	0.814471	0.824128
Dendritic.cells	PIH1D1	-0.01198	5.06576	-0.07055	0.943911	-6.23101	0.781089	0.775623
Dendritic.cells	ILKAP	0.008131	5.651644	0.070538	0.943925	-6.5575	0.772416	0.763074
Dendritic.cells	MRPL14	-0.01293	5.601025	-0.07042	0.944021	-6.32533	0.77316	0.764159
Dendritic.cells	BACE1	-0.0206	2.654059	-0.07036	0.944065	-5.68851	0.818134	0.829478
Dendritic.cells	NRIP1	0.010173	6.353871	0.07023	0.944169	-6.67654	0.762211	0.748309
Dendritic.cells	GLP2R	0.030541	0.940346	0.070072	0.944294	-5.49412	0.845874	0.86995
Dendritic.cells	DDC	-0.03647	1.455602	-0.06995	0.944394	-5.39059	0.837451	0.857584
Dendritic.cells	FRMD5	-0.04218	2.390592	-0.06987	0.944457	-5.68387	0.822401	0.835591
Dendritic.cells	9330111N(	-0.04229	0.392898	-0.0696	0.944666	-5.31433	0.855079	0.883302
Dendritic.cells	DERL2	-0.01109	4.925658	-0.06952	0.944729	-6.22678	0.783369	0.778684
Dendritic.cells	MANBAL	-0.01044	4.531107	-0.06902	0.945131	-6.15253	0.789577	0.787379

Dendritic.cells	FMR1	-0.00841	5.971489	-0.06887	0.945245	-6.5347	0.768213	0.756468
Dendritic.cells	5930430L0	-0.04094	0.229599	-0.06877	0.945328	-5.27482	0.85814	0.887483
Dendritic.cells	GM28198	-0.02064	3.85447	-0.06869	0.945394	-5.86131	0.799921	0.802425
Dendritic.cells	RAI1	-0.00687	6.004853	-0.06849	0.945551	-6.83694	0.767726	0.755803
Dendritic.cells	CAT	-0.01205	6.72379	-0.06845	0.945578	-6.68637	0.75734	0.740837
Dendritic.cells	GTF2A1	0.008697	5.65525	0.06828	0.945717	-6.58629	0.772845	0.763241
Dendritic.cells	SKAP2	-0.00502	7.235487	-0.06827	0.945721	-7.09255	0.750062	0.730392
Dendritic.cells	MTIF3	-0.01974	3.10156	-0.06824	0.945751	-5.78481	0.811601	0.81947
Dendritic.cells	GM48293	0.040262	-0.15995	0.067704	0.946174	-5.27529	0.864995	0.897373
Dendritic.cells	ZSWIM1	-0.03299	1.4089	-0.06764	0.946221	-5.48516	0.838933	0.859124
Dendritic.cells	AIMP2	0.023567	3.206699	0.067424	0.946396	-5.63364	0.810285	0.817296
Dendritic.cells	DENND6A	0.009035	5.102952	0.067298	0.946496	-6.39102	0.781338	0.775296
Dendritic.cells	RUFY3	0.010188	5.26954	0.067207	0.946569	-6.36814	0.778858	0.771709
Dendritic.cells	DNAJC16	0.021169	2.30093	0.067163	0.946603	-5.75336	0.82459	0.83817
Dendritic.cells	TGFB1I1	-0.03022	0.90381	-0.06702	0.946714	-5.52944	0.847272	0.871395
Dendritic.cells	THUMPD1	-0.01171	4.425949	-0.06699	0.946738	-6.13755	0.791518	0.790079
Dendritic.cells	PDP2	0.012361	3.724878	0.066884	0.946825	-6.08449	0.802241	0.805648
Dendritic.cells	MCM7	0.021515	5.384269	0.066833	0.946865	-6.07992	0.777157	0.76929
Dendritic.cells	PPP4C	0.006703	6.80668	0.0665	0.94713	-6.70468	0.756614	0.739446
Dendritic.cells	GRAMD3	-0.01634	6.080829	-0.06594	0.947577	-6.21204	0.767274	0.754569
Dendritic.cells	OXNAD1	-0.02534	1.886539	-0.06593	0.947578	-5.46307	0.831612	0.847991
Dendritic.cells	OFCC1	-0.03915	-0.63548	-0.0659	0.947608	-5.27283	0.873529	0.909508
Dendritic.cells	SS18L2	-0.0159	3.817204	-0.06583	0.947664	-5.90363	0.801177	0.803649
Dendritic.cells	RPAP1	0.020886	2.431442	0.065745	0.947729	-5.63087	0.822878	0.835253
Dendritic.cells	RABEPK	0.017249	3.296184	0.06537	0.948027	-5.7854	0.809293	0.815399
Dendritic.cells	NTAN1	0.005571	6.261702	0.065185	0.948174	-6.71055	0.764686	0.750791
Dendritic.cells	CYP3A13	0.038257	0.779768	0.065166	0.948189	-5.3161	0.849747	0.874491
Dendritic.cells	UBOX5	0.027466	1.390449	0.065052	0.94828	-5.45859	0.839706	0.859779
Dendritic.cells	ZBTB20	0.005726	8.246328	0.065024	0.948301	-7.22624	0.736622	0.71044
Dendritic.cells	TAF2	-0.01101	4.171185	-0.06491	0.948394	-6.06217	0.795792	0.795783
Dendritic.cells	NCF4	0.016995	5.316123	0.064832	0.948454	-6.02102	0.778556	0.770819
Dendritic.cells	CHPF2	-0.02129	2.64648	-0.0648	0.948478	-5.69459	0.819506	0.830272
Dendritic.cells	SH3YL1	-0.03194	1.030881	-0.06473	0.948538	-5.3061	0.845601	0.868412
Dendritic.cells	AKAP17B	0.028978	1.696468	0.064616	0.948626	-5.45836	0.834729	0.852497
Dendritic.cells	FBP1	-0.02633	5.143064	-0.06461	0.948628	-6.1147	0.78113	0.774542
Dendritic.cells	COPB2	-0.0082	5.438302	-0.06458	0.948657	-6.33226	0.776746	0.768201
Dendritic.cells	MYO10	0.013875	5.227281	0.064128	0.949013	-6.33181	0.780106	0.772812
Dendritic.cells	RNF128	-0.03036	2.499728	-0.06397	0.949136	-5.45574	0.822077	0.833758
Dendritic.cells	STAM2	-0.0106	5.475543	-0.06395	0.949156	-6.44578	0.776424	0.767489
Dendritic.cells	ICMT	-0.02196	2.429223	-0.06364	0.949402	-5.63195	0.823222	0.835445
Dendritic.cells	POLR2I	-0.00946	5.116859	-0.06364	0.949403	-6.23649	0.781773	0.775237
Dendritic.cells	DYNLT1A	0.016735	4.14434	0.063105	0.949826	-5.85327	0.796458	0.796606
Dendritic.cells	CRY2	-0.01901	2.553643	-0.06306	0.949858	-5.67191	0.821243	0.832657
Dendritic.cells	MICAL1	-0.01654	3.487265	-0.06304	0.949876	-5.79469	0.80658	0.811308
Dendritic.cells	TSHZ3	-0.02869	2.187789	-0.06289	0.949999	-5.68957	0.827079	0.841173
Dendritic.cells	NR6A1	-0.01183	5.241175	-0.06288	0.950005	-6.34483	0.779921	0.77265
Dendritic.cells	TESK1	-0.01211	3.781202	-0.06274	0.950113	-6.07596	0.802032	0.804731

Dendritic.cells	FAM53B	-0.01733	4.652028	-0.06261	0.950216	-5.95801	0.788748	0.78548
Dendritic.cells	ZC3H4	-0.00965	4.818849	-0.06247	0.950327	-6.14486	0.786236	0.781859
Dendritic.cells	CYP4V3	-0.03334	2.78654	-0.06228	0.950481	-5.49723	0.817554	0.827355
Dendritic.cells	TIMM29	0.015834	3.296538	0.062264	0.950494	-5.84556	0.809548	0.815699
Dendritic.cells	USP46	-0.0205	3.281753	-0.06212	0.95061	-5.71783	0.809779	0.816035
Dendritic.cells	INKA1	0.017879	2.703982	0.062029	0.950679	-5.77628	0.818859	0.829258
Dendritic.cells	HES6	-0.01066	4.105884	-0.06194	0.95075	-6.18721	0.797046	0.797531
Dendritic.cells	1700102PC	-0.03132	0.812589	-0.0619	0.950781	-5.42125	0.849477	0.874024
Dendritic.cells	SLC35D1	0.014764	4.114625	0.061896	0.950785	-5.87618	0.796912	0.797337
Dendritic.cells	MZT2	0.018928	2.206691	0.061848	0.950824	-5.57018	0.826776	0.840807
Dendritic.cells	IL21R	0.010107	5.150614	0.061673	0.950962	-6.63658	0.781269	0.774671
Dendritic.cells	GM21860	-0.04766	-0.14186	-0.06152	0.951088	-5.28924	0.865454	0.897493
Dendritic.cells	CSE1L	0.008751	5.5229	0.0615	0.9511	-6.40196	0.775745	0.766683
Dendritic.cells	ZBTB4	0.015303	3.655496	0.061455	0.951136	-5.87453	0.803973	0.807591
Dendritic.cells	TET1	-0.02385	1.135429	-0.06143	0.951155	-5.66953	0.844153	0.86622
Dendritic.cells	HOOK2	0.019067	3.558904	0.061366	0.951206	-5.81012	0.805469	0.809765
Dendritic.cells	CXXC5	0.006313	5.11182	0.061296	0.951262	-6.73593	0.781848	0.775508
Dendritic.cells	PDE3A	-0.04554	0.411945	-0.06122	0.951318	-5.29173	0.85614	0.883803
Dendritic.cells	ANGPT1	0.032684	0.973826	0.06122	0.951322	-5.42857	0.846813	0.870118
Dendritic.cells	ELN	0.034892	2.40021	0.061155	0.951374	-5.54436	0.823684	0.836294
Dendritic.cells	RNF121	-0.01284	4.41156	-0.06112	0.951404	-6.10143	0.792388	0.790774
Dendritic.cells	EFCAB14	0.010002	4.713606	0.061024	0.951478	-6.31417	0.787819	0.784153
Dendritic.cells	FLRT3	0.037545	-0.84839	0.060925	0.951557	-5.28926	0.87751	0.915304
Dendritic.cells	FHOD3	-0.04101	-0.11029	-0.0609	0.951577	-5.27113	0.86492	0.896757
Dendritic.cells	FRG2F1	-0.02323	0.702787	-0.06058	0.951832	-5.48623	0.851297	0.876776
Dendritic.cells	ZFP958	0.022973	2.267977	0.060545	0.951858	-5.56886	0.825795	0.839454
Dendritic.cells	NCAPH	-0.02716	4.175802	-0.0605	0.951897	-5.69077	0.795977	0.796056
Dendritic.cells	TRP53I13	-0.0115	3.021819	-0.06047	0.951921	-6.02965	0.813848	0.822035
Dendritic.cells	SUMO3	-0.00792	5.724941	-0.06021	0.952125	-6.4815	0.772768	0.762492
Dendritic.cells	NDNF	-0.03445	-0.47189	-0.06003	0.952271	-5.27746	0.871061	0.905927
Dendritic.cells	FAM167A	0.040966	1.837572	0.059908	0.952364	-5.29365	0.832714	0.849655
Dendritic.cells	SYNJ2	0.019233	2.324633	0.059801	0.952449	-5.63572	0.82489	0.838226
Dendritic.cells	ING5	-0.01428	3.456639	-0.0598	0.952452	-5.83862	0.807056	0.81224
Dendritic.cells	KAT8	0.013572	3.624929	0.059732	0.952504	-5.92106	0.804446	0.808445
Dendritic.cells	MFSD8	0.01307	2.979793	0.05973	0.952506	-5.83073	0.814509	0.823088
Dendritic.cells	NPRL2	0.01513	2.790262	0.059728	0.952507	-5.78438	0.817495	0.82744
Dendritic.cells	KIF1C	0.017322	3.254466	0.059517	0.952675	-5.69623	0.810205	0.816881
Dendritic.cells	STAP1	-0.01393	4.891894	-0.05951	0.95268	-6.04317	0.785139	0.780487
Dendritic.cells	UBXN7	0.007739	5.704222	0.059305	0.952843	-6.47577	0.773073	0.763084
Dendritic.cells	PLA2G15	0.023388	3.738633	0.059267	0.952873	-5.64401	0.802689	0.806015
Dendritic.cells	C530008M	0.037157	0.74516	0.059255	0.952883	-5.30199	0.850594	0.875978
Dendritic.cells	CTCF	-0.00451	7.101448	-0.05917	0.952951	-6.84946	0.752883	0.733998
Dendritic.cells	NAP1L1	0.007708	7.439515	0.059123	0.952988	-6.81064	0.748103	0.727123
Dendritic.cells	BEX3	-0.00967	4.990762	-0.05912	0.952991	-6.35877	0.783657	0.778422
Dendritic.cells	ARL16	0.018273	2.250651	0.058966	0.953112	-5.56561	0.826093	0.840109
Dendritic.cells	OGFOD1	0.013269	3.611469	0.058769	0.953269	-5.94388	0.804675	0.808899
Dendritic.cells	RB1	0.006622	7.240705	0.058763	0.953273	-6.83557	0.750928	0.731167

Dendritic.cells	PDE1C	0.017434	3.989093	0.05872	0.953308	-6.25189	0.798855	0.800458
Dendritic.cells	BLM	0.021039	3.897628	0.058514	0.953471	-5.83964	0.800328	0.802578
Dendritic.cells	SLC25A28	-0.00844	4.806244	-0.05836	0.953596	-6.38176	0.786512	0.782603
Dendritic.cells	LTF	0.043197	2.942911	0.058178	0.953738	-5.43107	0.815179	0.824258
Dendritic.cells	CNTD1	-0.02939	0.620711	-0.05818	0.953739	-5.38858	0.852755	0.879221
Dendritic.cells	ZFP868	-0.01588	3.676008	-0.05813	0.953779	-5.80665	0.803745	0.807622
Dendritic.cells	NXN	0.009933	5.325793	0.057842	0.954005	-6.46477	0.77875	0.771405
Dendritic.cells	ATP6V1E1	0.0064	7.59223	0.057763	0.954068	-6.77847	0.74604	0.724236
Dendritic.cells	CNOT3	0.00751	5.46868	0.057697	0.95412	-6.45538	0.776633	0.768343
Dendritic.cells	ZDHHC18	0.011433	5.57552	0.057657	0.954152	-6.177	0.775055	0.766061
Dendritic.cells	AREG	-0.03362	1.634087	-0.05748	0.954291	-5.9994	0.836102	0.854869
Dendritic.cells	GABPA	0.00939	4.662072	0.057467	0.954303	-6.17357	0.788684	0.785804
Dendritic.cells	NDEL1	0.006174	6.927832	0.05739	0.954364	-6.78065	0.755437	0.737786
Dendritic.cells	LSMEM1	0.030478	4.06136	0.057263	0.954465	-5.69198	0.797816	0.799108
Dendritic.cells	GM45902	-0.02156	1.585805	-0.05716	0.954546	-5.53075	0.836887	0.856111
Dendritic.cells	CD40	-0.03894	2.17854	-0.05714	0.95456	-5.35711	0.827319	0.842119
Dendritic.cells	RECQL4	0.030848	-0.06717	0.057142	0.954561	-5.2709	0.864287	0.89633
Dendritic.cells	AMMECR1	-0.01229	4.787165	-0.05698	0.954687	-6.07463	0.786813	0.783192
Dendritic.cells	MRVI1	-0.03155	0.497468	-0.05694	0.954725	-5.31464	0.854823	0.882454
Dendritic.cells	GM47350	-0.02147	1.355941	-0.05684	0.954798	-5.49797	0.840648	0.861661
Dendritic.cells	GM15892	-0.02671	3.443855	-0.0567	0.954909	-5.56601	0.807391	0.813071
Dendritic.cells	TUSC2	-0.01488	3.017242	-0.05616	0.955338	-5.82218	0.814269	0.822798
Dendritic.cells	ZDHHC17	-0.00947	4.015064	-0.05609	0.955394	-6.17599	0.798779	0.800265
Dendritic.cells	ZFP809	0.013429	3.64912	0.056086	0.9554	-5.98276	0.804416	0.808457
Dendritic.cells	GAL3ST1	-0.03332	-0.29084	-0.05602	0.955451	-5.27254	0.868352	0.902016
Dendritic.cells	AP1B1	-0.00736	5.190975	-0.05588	0.955562	-6.45394	0.781034	0.774539
Dendritic.cells	CTNNA3	-0.04133	1.300067	-0.05564	0.955751	-5.39317	0.841896	0.863135
Dendritic.cells	CTNNBIP1	0.009215	4.359655	0.055517	0.955852	-6.31	0.793593	0.79273
Dendritic.cells	PKMYT1	-0.0214	3.261877	-0.0554	0.955942	-5.61663	0.810515	0.817363
Dendritic.cells	DDIAS	0.022357	2.134705	0.055012	0.956253	-5.52474	0.828365	0.843404
Dendritic.cells	FOXRED1	0.018745	2.831724	0.054969	0.956287	-5.69667	0.81727	0.827211
Dendritic.cells	CCDC14	-0.03157	0.809905	-0.0549	0.956343	-5.32448	0.849968	0.875036
Dendritic.cells	TXNDC9	0.007655	5.512196	0.054871	0.956365	-6.31295	0.776311	0.767742
Dendritic.cells	ACPP	0.036179	2.739914	0.054745	0.956466	-5.33411	0.818721	0.829346
Dendritic.cells	TXN1	0.009033	7.304482	0.054728	0.956479	-6.71037	0.750401	0.730391
Dendritic.cells	LTB	0.024447	3.446775	0.054562	0.95661	-5.58886	0.807633	0.813221
Dendritic.cells	MAP2K3OS	0.038458	-0.12812	0.054227	0.956877	-5.27594	0.865676	0.89822
Dendritic.cells	SPNS1	-0.01149	3.498213	-0.05419	0.956906	-5.98124	0.806834	0.812101
Dendritic.cells	GSTO1	0.01119	4.594828	0.054079	0.956994	-5.90803	0.790027	0.787718
Dendritic.cells	VPS13A	0.004511	6.447058	0.054019	0.957042	-6.87063	0.762651	0.748128
Dendritic.cells	SF3B6	-0.00476	7.294759	-0.05401	0.957047	-6.81734	0.750539	0.73068
Dendritic.cells	INPP4A	-0.0043	6.032154	-0.05394	0.957104	-7.10763	0.768674	0.756837
Dendritic.cells	PAPOLG	-0.01582	3.005898	-0.0538	0.957218	-5.74256	0.814526	0.823354
Dendritic.cells	ACAP3	0.016356	2.238471	0.053764	0.957245	-5.58211	0.826701	0.841118
Dendritic.cells	ASAH1	0.004958	6.301867	0.053602	0.957373	-6.94124	0.764752	0.751186
Dendritic.cells	GM16364.1	0.020744	1.279847	0.053417	0.95752	-5.74277	0.842227	0.863845
Dendritic.cells	NOS1AP	-0.01726	3.163666	-0.0532	0.957696	-5.93252	0.812051	0.819775

Dendritic.cells	GM50013	-0.01941	1.85467	-0.05309	0.957777	-5.57464	0.832875	0.850177
Dendritic.cells	PPNR	-0.01956	1.051959	-0.05298	0.957868	-5.69217	0.84597	0.869397
Dendritic.cells	LASP1	0.009186	5.455474	0.052899	0.957932	-6.1567	0.77715	0.76917
Dendritic.cells	AW209491	0.027066	1.448036	0.052868	0.957957	-5.4404	0.839478	0.859898
Dendritic.cells	CRYBG2	0.030613	0.051796	0.052746	0.958053	-5.27305	0.862637	0.893926
Dendritic.cells	HAP1	-0.02963	-0.45019	-0.05254	0.958219	-5.27585	0.871149	0.906524
Dendritic.cells	ZFP280C	0.008519	4.164903	0.05253	0.958225	-6.22902	0.796562	0.797384
Dendritic.cells	TMEM243	-0.00653	6.427837	-0.05248	0.958267	-6.42552	0.762929	0.7487
Dendritic.cells	TIPRL	0.007461	5.525539	0.052458	0.958282	-6.3264	0.776114	0.767746
Dendritic.cells	EVI2	0.01722	3.35768	0.052313	0.958398	-5.81549	0.80902	0.815547
Dendritic.cells	SSH3	0.025883	1.522336	0.052156	0.958522	-5.4444	0.838266	0.858321
Dendritic.cells	CSF2RB2	-0.00768	2.359571	-0.05208	0.958579	-6.60979	0.824765	0.838578
Dendritic.cells	CD177	-0.03385	0.17299	-0.05182	0.958786	-5.28513	0.860596	0.891148
Dendritic.cells	KCNC3	-0.02803	0.561324	-0.05172	0.958867	-5.30064	0.854097	0.881601
Dendritic.cells	GPR183	0.007214	3.488292	0.051692	0.958891	-6.7791	0.806988	0.812701
Dendritic.cells	AP3D1	-0.00604	4.873786	-0.05162	0.958946	-6.31753	0.785823	0.781969
Dendritic.cells	NSUN2	0.007491	5.0541	0.051484	0.959056	-6.3495	0.783121	0.778078
Dendritic.cells	NFKBIA	-0.01048	8.130992	-0.05136	0.959157	-6.67027	0.73884	0.714214
Dendritic.cells	CLUAP1	-0.00839	3.885137	-0.05117	0.959303	-6.14559	0.800852	0.803822
Dendritic.cells	SPTBN4	0.028141	-0.18469	0.051074	0.959382	-5.27424	0.866634	0.9001
Dendritic.cells	RIC1	-0.00865	6.710291	-0.05101	0.959429	-6.58137	0.758862	0.743024
Dendritic.cells	MRPL10	0.010275	4.602348	0.050996	0.959444	-6.09461	0.789913	0.787934
Dendritic.cells	SNHG6	0.015055	3.414627	0.050957	0.959475	-5.77249	0.808133	0.814416
Dendritic.cells	SLC36A1	0.013623	2.851189	0.050913	0.95951	-5.69286	0.816963	0.827284
Dendritic.cells	CNPPD1	0.008744	5.686802	0.050903	0.959518	-6.33925	0.773736	0.7645
Dendritic.cells	TNFRSF1B	-0.01188	4.903674	-0.05079	0.959605	-6.16581	0.785374	0.781352
Dendritic.cells	PAXIP1	-0.01107	4.269449	-0.05078	0.959615	-6.023	0.794967	0.795269
Dendritic.cells	EIF3E	0.004468	7.095265	0.050667	0.959705	-6.81462	0.753366	0.735103
Dendritic.cells	IPO7	-0.00825	5.656347	-0.05061	0.959753	-6.43972	0.774184	0.765149
Dendritic.cells	IGF1OS	-0.03295	-1.03209	-0.0505	0.959839	-5.27313	0.88114	0.921502
Dendritic.cells	DHX35	0.016459	2.187953	0.050444	0.959883	-5.65615	0.827511	0.842687
Dendritic.cells	ZFP943	0.008443	4.280266	0.050422	0.9599	-6.06696	0.794802	0.79503
Dendritic.cells	MEIG1	0.0269	-0.47443	0.050287	0.960008	-5.34663	0.871562	0.907386
Dendritic.cells	CCDC28A	0.019255	2.307492	0.050106	0.960151	-5.48603	0.825597	0.839923
Dendritic.cells	SZT2	0.01403	2.318426	0.05009	0.960164	-5.68806	0.825423	0.839668
Dendritic.cells	FAM114A1	-0.02237	2.398626	-0.04996	0.960265	-5.5321	0.824142	0.837797
Dendritic.cells	RALA	0.005862	5.871704	0.04992	0.960299	-6.57477	0.77102	0.760605
Dendritic.cells	MCM5	0.021603	4.910563	0.049875	0.960334	-5.91185	0.785271	0.781233
Dendritic.cells	UTP14A	-0.00698	5.238791	-0.04986	0.96035	-6.38908	0.780366	0.774127
Dendritic.cells	ZMAT4	0.041807	0.381713	0.049464	0.960661	-5.27741	0.857096	0.886203
Dendritic.cells	PTGIS	-0.04092	-0.12832	-0.0494	0.960708	-5.27524	0.865679	0.898838
Dendritic.cells	INPP5A	0.005929	6.256163	0.049381	0.960727	-6.81339	0.765415	0.752599
Dendritic.cells	PCBP1	-0.00439	8.199098	-0.04936	0.960747	-7.00584	0.737899	0.712977
Dendritic.cells	ZFP60	0.020206	1.431044	0.049266	0.960819	-5.49374	0.839755	0.860745
Dendritic.cells	KLRA4	0.035314	-1.21319	0.049214	0.96086	-5.27415	0.884276	0.926287
Dendritic.cells	FBXL12OS	0.02501	0.679534	0.049054	0.960987	-5.28313	0.852131	0.878905
Dendritic.cells	GM10563	0.013686	3.607105	0.049004	0.961027	-5.84495	0.805145	0.810196

Dendritic.cells	GBA	-0.00793	4.090254	-0.04886	0.96114	-6.24766	0.797704	0.799374
Dendritic.cells	MAD2L2	0.012321	3.472942	0.048861	0.961141	-5.87127	0.807226	0.813227
Dendritic.cells	CDAN1	-0.01559	2.943919	-0.04886	0.961145	-5.70182	0.815501	0.825286
Dendritic.cells	WBP2	-0.007	5.599979	-0.04881	0.961182	-6.42372	0.775015	0.766474
Dendritic.cells	SNX21	-0.01771	2.718125	-0.04839	0.961513	-5.63079	0.819065	0.830502
Dendritic.cells	SUB1	-0.00399	8.911934	-0.04838	0.961523	-7.23688	0.728137	0.698989
Dendritic.cells	EIF2B1	0.011265	3.62049	0.048232	0.96164	-5.84177	0.804937	0.809935
Dendritic.cells	COL4A4	-0.02129	1.009618	-0.04812	0.961732	-5.4746	0.846668	0.870961
Dendritic.cells	ZSCAN18	-0.02689	0.363679	-0.04803	0.961801	-5.41903	0.857398	0.886745
Dendritic.cells	ZHX3	0.007484	4.441922	0.047927	0.961882	-6.40771	0.792343	0.791688
Dendritic.cells	CD226	-0.03313	1.697205	-0.04789	0.961909	-5.37163	0.835424	0.854509
Dendritic.cells	PLTP	0.007115	5.244881	0.047746	0.962026	-7.1047	0.780276	0.774202
Dendritic.cells	CDH24	0.016171	2.179776	0.047723	0.962044	-5.61615	0.827642	0.843136
Dendritic.cells	ABRAXAS1	-0.01627	2.604808	-0.04772	0.962046	-5.66971	0.820862	0.833229
Dendritic.cells	NCAPD3	0.009996	5.127768	0.047685	0.962075	-6.23007	0.782021	0.77673
Dendritic.cells	HMGB1	0.004845	9.807497	0.047574	0.962163	-7.28291	0.716122	0.681889
Dendritic.cells	POLD2	-0.01457	3.401936	-0.04749	0.962229	-5.83146	0.808331	0.814968
Dendritic.cells	PSMD13	-0.00511	6.203145	-0.04746	0.962252	-6.61103	0.766185	0.753834
Dendritic.cells	TEX2	0.003266	6.723879	0.047457	0.962256	-7.38024	0.758668	0.742984
Dendritic.cells	HMGN2	0.00878	7.53605	0.047426	0.96228	-6.72728	0.747138	0.726379
Dendritic.cells	ADRB1	0.036893	0.735849	0.047316	0.962368	-5.28003	0.851196	0.87771
Dendritic.cells	SAFB	-0.00534	6.192591	-0.04724	0.962425	-6.61322	0.766338	0.754117
Dendritic.cells	ZDHHC14	-0.00646	7.022709	-0.04707	0.962567	-7.13969	0.754398	0.736901
Dendritic.cells	DDA1	-0.00714	5.082621	-0.04701	0.962612	-6.34295	0.782695	0.777801
Dendritic.cells	SPRTN	0.010701	3.419835	0.04695	0.962659	-5.97175	0.808052	0.814646
Dendritic.cells	FZD1	-0.02613	-0.15494	-0.04693	0.962676	-5.27902	0.86613	0.899741
Dendritic.cells	COL5A1	-0.02694	0.493409	-0.04693	0.962677	-5.35445	0.85523	0.883695
Dendritic.cells	ANXA2	-0.00823	5.910919	-0.04689	0.962709	-6.60242	0.770446	0.76007
Dendritic.cells	HNRNPA2B	-0.00413	9.287708	-0.04685	0.962741	-7.18246	0.723062	0.691891
Dendritic.cells	ETFRF1	0.015068	3.163487	0.046708	0.962851	-5.74089	0.812054	0.820478
Dendritic.cells	EAF1	-0.01111	4.101569	-0.04668	0.96287	-5.94089	0.797531	0.799336
Dendritic.cells	AGK	-0.0119	3.121713	-0.04636	0.963129	-5.81778	0.812739	0.821506
Dendritic.cells	GM47071	0.025339	1.555335	0.046311	0.963167	-5.45005	0.837761	0.858083
Dendritic.cells	GM20033	0.027221	0.595708	0.046232	0.963229	-5.3302	0.853557	0.881286
Dendritic.cells	UBE4B	-0.00662	5.48847	-0.04594	0.963459	-6.36075	0.776691	0.769187
Dendritic.cells	KYAT1	0.021001	1.503706	0.045913	0.963483	-5.41836	0.838601	0.859376
Dendritic.cells	MED22	-0.01442	2.384243	-0.04584	0.963541	-5.70953	0.824402	0.838601
Dendritic.cells	RANBP10	-0.00766	5.698692	-0.04576	0.963605	-6.37931	0.77359	0.764705
Dendritic.cells	PIAS4	-0.00845	4.224377	-0.04575	0.963614	-6.05105	0.795684	0.796744
Dendritic.cells	SNRNP27	0.005263	5.842837	0.045735	0.963625	-6.49891	0.771472	0.761643
Dendritic.cells	FNTB	-0.01394	2.971515	-0.04572	0.963639	-5.66998	0.815098	0.825013
Dendritic.cells	TACSTD2	0.028276	-0.35025	0.045434	0.963864	-5.27528	0.869615	0.904833
Dendritic.cells	METTL5	0.010883	3.739598	0.045256	0.964005	-5.83443	0.803295	0.807622
Dendritic.cells	RCN2	0.008036	5.075901	0.045052	0.964168	-6.29035	0.78299	0.778157
Dendritic.cells	ADGRG5	0.02317	0.105614	0.045034	0.964182	-5.48318	0.861944	0.8935
Dendritic.cells	4933421O1	0.01304	2.357702	0.045	0.964209	-5.8238	0.825	0.839303
Dendritic.cells	IL12RB2	0.010454	3.672057	0.044871	0.964312	-6.24413	0.804364	0.809167

Dendritic.cells	NCDN	-0.01177	2.826122	-0.04473	0.964424	-5.86232	0.81762	0.828439
Dendritic.cells	CASTOR1	-0.02578	0.626653	-0.04445	0.96465	-5.30023	0.853391	0.880734
Dendritic.cells	GM17387	0.023101	0.498836	0.044259	0.964798	-5.356	0.855521	0.883898
Dendritic.cells	PRTN3	-0.05106	3.6691	-0.0442	0.964847	-5.64957	0.804544	0.809323
Dendritic.cells	MAPK8	-0.00574	5.232129	-0.04405	0.964961	-6.42365	0.780814	0.774896
Dendritic.cells	GM49980	0.009707	6.157222	0.044014	0.964993	-6.76374	0.767195	0.755195
Dendritic.cells	BACE2	-0.01694	1.120623	-0.04393	0.965055	-5.66612	0.845218	0.868801
Dendritic.cells	CMTM4	0.015573	3.141567	0.043797	0.965165	-5.61271	0.81276	0.821364
Dendritic.cells	IL1B	0.025581	5.12575	0.043706	0.965237	-5.94712	0.7824	0.777262
Dendritic.cells	CYP4A14	-0.02681	1.833194	-0.04367	0.965268	-5.4615	0.833594	0.851846
Dendritic.cells	LMBR1L	0.009049	4.517308	0.043619	0.965307	-6.11601	0.791554	0.790562
Dendritic.cells	PTMA	0.004509	11.35991	0.043559	0.965354	-7.45679	0.696247	0.653515
Dendritic.cells	STRN	0.006523	5.530644	0.043468	0.965426	-6.41606	0.776385	0.768571
Dendritic.cells	GZF1	-0.01588	2.752325	-0.04327	0.96558	-5.60145	0.818893	0.830416
Dendritic.cells	HIC2	-0.01488	2.712677	-0.0432	0.965642	-5.66923	0.819521	0.831333
Dendritic.cells	GM45820	0.013098	1.425778	0.043119	0.965704	-5.68289	0.840219	0.861618
Dendritic.cells	ENPP1	0.022872	2.31349	0.043047	0.965761	-5.45742	0.825873	0.840623
Dendritic.cells	SCNN1A	0.028845	0.542515	0.043003	0.965796	-5.27969	0.854796	0.883046
Dendritic.cells	ZFP811	0.027518	-1.02506	0.042614	0.966105	-5.27047	0.88163	0.922342
Dendritic.cells	RWDD2A	-0.02104	0.48641	-0.04219	0.96644	-5.38985	0.856172	0.884577
Dendritic.cells	SNHG14	-0.01408	1.410261	-0.04178	0.96677	-5.56403	0.84105	0.862265
Dendritic.cells	FAAH	-0.02193	1.451163	-0.04173	0.966806	-5.33282	0.840382	0.861286
Dendritic.cells	CAPN1	0.009952	4.246316	0.041674	0.966853	-6.0341	0.796225	0.796855
Dendritic.cells	BCL2L12	0.008771	4.178233	0.041614	0.9669	-6.01482	0.797265	0.798367
Dendritic.cells	BLOC1S3	-0.01545	2.381624	-0.04115	0.967267	-5.61095	0.825441	0.839376
Dendritic.cells	RANBP1	0.006067	7.239938	0.04096	0.96742	-6.78321	0.75225	0.733256
Dendritic.cells	ZFP410	-0.00679	4.138179	-0.0409	0.96747	-6.16546	0.797964	0.799387
Dendritic.cells	ANK	0.011768	3.446389	0.040875	0.967488	-5.96688	0.808646	0.814927
Dendritic.cells	CYLD	0.004348	6.329731	0.040799	0.967548	-6.68325	0.765301	0.752073
Dendritic.cells	NDUFS1	-0.00441	5.752161	-0.04065	0.967669	-6.47481	0.773737	0.764288
Dendritic.cells	MKKS	-0.01513	1.995441	-0.04055	0.967743	-5.51465	0.831639	0.848528
Dendritic.cells	PIGQ	0.01021	3.519745	0.040212	0.968015	-5.78804	0.807504	0.813302
Dendritic.cells	RDM1	0.009497	5.028258	0.040188	0.968034	-6.18327	0.784484	0.779859
Dendritic.cells	OGG1	0.010361	3.189389	0.040175	0.968044	-5.72232	0.81266	0.820813
Dendritic.cells	GM42031	-0.01724	4.172175	-0.04012	0.968092	-6.09129	0.797444	0.798667
Dendritic.cells	AS3MT	-0.00971	2.69274	-0.03983	0.968318	-5.97775	0.820489	0.832274
Dendritic.cells	ICA1L	0.019723	1.590667	0.03963	0.968477	-5.45246	0.838198	0.858232
Dendritic.cells	TMEM106I	0.006937	4.565283	0.039579	0.968518	-6.18623	0.791459	0.790068
Dendritic.cells	CEBPZ	-0.00425	6.141756	-0.03957	0.968525	-6.57492	0.768033	0.756143
Dendritic.cells	VIPAS39	0.007373	4.131233	0.039528	0.968559	-5.95622	0.79807	0.799672
Dendritic.cells	HTR2B	-0.01676	0.855091	-0.03952	0.968568	-5.49944	0.850278	0.875954
Dendritic.cells	CDC25A	0.010553	3.793744	0.039476	0.9686	-5.90494	0.80326	0.807219
Dendritic.cells	RAB23	0.019535	1.279801	0.039424	0.968641	-5.37287	0.843278	0.865679
Dendritic.cells	CUX1	0.003277	7.953864	0.039322	0.968722	-7.06324	0.742221	0.718946
Dendritic.cells	IVNS1ABP	-0.00384	6.027762	-0.03932	0.968724	-6.80408	0.769697	0.758546
Dendritic.cells	NFKB1	-0.00545	8.743793	-0.0392	0.968816	-7.12409	0.731334	0.703326
Dendritic.cells	ECI2	0.007141	4.557161	0.039164	0.968848	-6.24954	0.791582	0.790259

Dendritic.cells	PRDM2	-0.00486	5.970241	-0.03916	0.968848	-6.60158	0.770538	0.759774
Dendritic.cells	LPIN1	-0.01221	2.690777	-0.03915	0.968862	-5.90796	0.82052	0.832389
Dendritic.cells	SLC52A3	-0.01241	0.451484	-0.03902	0.968961	-5.75845	0.856996	0.885893
Dendritic.cells	FHAD1	-0.02141	0.624492	-0.03886	0.969089	-5.43497	0.854109	0.881664
Dendritic.cells	4930532G1	0.023336	1.429337	0.038837	0.969108	-5.42337	0.840829	0.862169
Dendritic.cells	TBP	-0.01122	3.21522	-0.03881	0.969132	-5.77412	0.812255	0.820396
Dendritic.cells	CCNG2	0.009355	5.189545	0.038655	0.969253	-6.06012	0.782111	0.776556
Dendritic.cells	ISOC1	0.006425	4.993923	0.038236	0.969586	-6.24766	0.785116	0.780872
Dendritic.cells	NANOS3	-0.01494	0.427569	-0.03821	0.969609	-5.52104	0.857524	0.886618
Dendritic.cells	DCLRE1B	0.010636	2.770832	0.038174	0.969635	-5.67161	0.819374	0.830714
Dendritic.cells	NDUFAF7	-0.00796	4.007139	-0.03816	0.969644	-5.95486	0.800093	0.802622
Dendritic.cells	COQ10B	-0.00487	5.908947	-0.03746	0.970207	-6.46936	0.771809	0.76135
Dendritic.cells	PABPC1	-0.00426	10.47512	-0.03721	0.9704	-7.32185	0.708567	0.670563
Dendritic.cells	PSAT1	0.01113	4.218142	0.03719	0.970417	-5.96751	0.797127	0.798105
Dendritic.cells	STRIP1	0.007384	4.156745	0.037078	0.970507	-6.04026	0.798066	0.79947
Dendritic.cells	TMEFF1	-0.02386	-0.59624	-0.03705	0.970527	-5.3147	0.875156	0.912377
Dendritic.cells	POP5	0.00635	4.525116	0.036811	0.970718	-6.01138	0.792451	0.791314
Dendritic.cells	CAMKMT	-0.00679	4.501622	-0.03681	0.97072	-6.26487	0.792808	0.791832
Dendritic.cells	TMBIM1	0.010869	2.851295	0.036767	0.970753	-5.80443	0.818375	0.829047
Dendritic.cells	SUPT6	-0.00474	5.808893	-0.03666	0.970841	-6.5391	0.773278	0.763544
Dendritic.cells	EPHB2	0.00585	1.372664	0.036548	0.970928	-6.5157	0.842164	0.863856
Dendritic.cells	SLC43A3	-0.00958	3.813488	-0.03655	0.970928	-5.93142	0.803344	0.807157
Dendritic.cells	SLC2A9	0.019268	2.879571	0.036404	0.971042	-5.37587	0.817929	0.828456
Dendritic.cells	CDC73	0.003659	6.174072	0.036373	0.971067	-6.69253	0.767935	0.755864
Dendritic.cells	GM32401	0.017871	2.445198	0.036267	0.971152	-5.42448	0.824825	0.838558
Dendritic.cells	NEURL3	-0.01333	4.301516	-0.03626	0.97116	-5.67046	0.795854	0.796344
Dendritic.cells	CBY1	0.011534	2.846037	0.036185	0.971216	-5.59343	0.818459	0.829264
Dendritic.cells	FAM57A	0.017769	1.118395	0.036129	0.971261	-5.43587	0.84634	0.87008
Dendritic.cells	TRIM30B	-0.01671	1.730639	-0.03593	0.97142	-5.52451	0.836328	0.855401
Dendritic.cells	SLC11A2	0.016057	3.918763	0.035791	0.97153	-5.51113	0.801721	0.804887
Dendritic.cells	ZFP563	0.014001	0.800774	0.035667	0.971628	-5.41915	0.851591	0.877791
Dendritic.cells	DXO	-0.01139	3.302702	-0.03565	0.971638	-5.74649	0.81128	0.818805
Dendritic.cells	NF2	-0.00532	4.781261	-0.03556	0.971717	-6.35446	0.788577	0.785793
Dendritic.cells	RPP38	0.01479	1.226405	0.035435	0.971813	-5.58095	0.844563	0.867472
Dendritic.cells	NDUFA4	-0.00421	8.596899	-0.0354	0.971842	-7.03689	0.733698	0.70661
Dendritic.cells	WDR5	-0.00714	4.681603	-0.0353	0.971924	-6.05651	0.790082	0.787976
Dendritic.cells	LUC7L2	-0.00204	8.527172	-0.03528	0.971936	-7.19864	0.734654	0.707982
Dendritic.cells	AMMECR1I	0.006121	4.488762	0.035262	0.971951	-6.21696	0.793003	0.792217
Dendritic.cells	4932438H2	0.023365	-0.27879	0.035169	0.972024	-5.28313	0.869735	0.904495
Dendritic.cells	HIST2H2BE	0.014058	0.814865	0.034825	0.972298	-5.54879	0.851503	0.877524
Dendritic.cells	GNPTG	-0.00841	3.368723	-0.03466	0.972428	-5.8015	0.810387	0.817406
Dendritic.cells	DIAPH3	-0.01088	6.026174	-0.03451	0.972549	-6.38417	0.770224	0.759148
Dendritic.cells	RCL1	-0.00737	4.24025	-0.0344	0.972633	-5.97727	0.796925	0.797846
Dendritic.cells	PIK3CA	0.004234	6.128996	0.034348	0.972677	-6.60338	0.768723	0.756978
Dendritic.cells	SCRN3	0.017492	1.928035	0.034314	0.972704	-5.36834	0.833273	0.850858
Dendritic.cells	NCKAP1L	-0.00372	6.137906	-0.03421	0.972786	-6.52189	0.768593	0.756794
Dendritic.cells	XAF1	0.018979	4.033526	0.034072	0.972897	-5.76063	0.800093	0.802457

Dendritic.cells	TRAF6	-0.00451	5.683882	-0.03403	0.972932	-6.46622	0.775251	0.766424
Dendritic.cells	SLC35C1	0.011345	2.749333	0.033966	0.972981	-5.63495	0.820129	0.831655
Dendritic.cells	MYBPC3	-0.01954	1.123244	-0.03375	0.97315	-5.28217	0.846405	0.870162
Dendritic.cells	4930589L2	-0.01766	-0.68311	-0.03362	0.973253	-5.27536	0.876797	0.914929
Dendritic.cells	CSPP1	0.004002	6.098729	0.033436	0.973402	-6.67552	0.769164	0.757739
Dendritic.cells	CAMK1D	-0.00366	8.628354	-0.03337	0.973457	-7.1827	0.733392	0.706226
Dendritic.cells	C1GALT1C1	0.006838	4.044817	0.033289	0.973519	-6.06299	0.799919	0.802334
Dendritic.cells	ACVR2B	0.008528	1.53111	0.033179	0.973607	-5.99192	0.839718	0.860502
Dendritic.cells	AHI1	-0.00734	3.003332	-0.03315	0.973633	-5.98118	0.816117	0.825986
Dendritic.cells	PLEKHF2	0.004362	5.470642	0.033006	0.973744	-6.37801	0.778404	0.771217
Dendritic.cells	NDUFA1	0.003636	6.729501	0.032915	0.973817	-6.71185	0.76003	0.744664
Dendritic.cells	ERCC6L	0.01424	3.034059	0.032901	0.973828	-5.57456	0.815633	0.825344
Dendritic.cells	NLRP3	0.016969	4.527197	0.032884	0.973841	-5.86014	0.792555	0.79175
Dendritic.cells	1700030K0	-0.01561	1.730931	-0.03285	0.973867	-5.45662	0.836466	0.855804
Dendritic.cells	FAM222A	-0.01046	2.331884	-0.03283	0.973885	-5.64972	0.826778	0.841623
Dendritic.cells	HAUS5	-0.01162	2.831524	-0.03274	0.973952	-5.58155	0.818828	0.830008
Dendritic.cells	GM31243	-0.01831	2.779029	-0.03203	0.974518	-5.53413	0.819772	0.831316
Dendritic.cells	CCHCR1	0.014404	1.536464	0.031844	0.974668	-5.47754	0.839747	0.860602
Dendritic.cells	IFI211	-0.0173	2.960201	-0.03171	0.974773	-5.6631	0.816909	0.827195
Dendritic.cells	AMIGO1	0.015508	0.613629	0.03171	0.974775	-5.33153	0.854968	0.882952
Dendritic.cells	ACVR1B	0.010902	2.543644	0.031679	0.974799	-5.56663	0.823511	0.836836
Dendritic.cells	RITA1	0.013307	1.367418	0.031582	0.974877	-5.39053	0.842511	0.864654
Dendritic.cells	MCRS1	0.006532	4.318538	0.031532	0.974916	-5.97297	0.79584	0.796509
Dendritic.cells	CEBPZOS	-0.0068	4.521965	-0.03144	0.974988	-5.98902	0.792744	0.792012
Dendritic.cells	KIF3A	0.010705	2.472633	0.031421	0.975004	-5.56471	0.824643	0.83849
Dendritic.cells	TRDC	0.02501	1.265066	0.031282	0.975115	-5.34931	0.844189	0.867117
Dendritic.cells	2510002D2	-0.00912	3.199654	-0.0312	0.975184	-5.73985	0.813144	0.821702
Dendritic.cells	IL31RA	0.011677	3.109325	0.031152	0.975219	-5.60626	0.814562	0.82377
Dendritic.cells	CEP295	-0.00692	3.949897	-0.03113	0.975238	-5.9237	0.801489	0.804724
Dendritic.cells	CAMK4	0.017941	3.194525	0.031079	0.975277	-5.65372	0.813225	0.821819
Dendritic.cells	THA1	-0.01679	0.09883	-0.03088	0.975432	-5.36823	0.863603	0.895662
Dendritic.cells	BICC1	-0.02061	0.332453	-0.03071	0.975569	-5.28551	0.859672	0.889872
Dendritic.cells	MCAT	-0.01028	2.553855	-0.03066	0.97561	-5.6233	0.823348	0.836598
Dendritic.cells	NUBP2	-0.00623	4.451117	-0.03064	0.975628	-6.14906	0.793821	0.793575
Dendritic.cells	UBE2W	0.002113	6.081677	0.030577	0.975676	-6.74791	0.76952	0.758357
Dendritic.cells	ALKBH5	-0.00313	7.148219	-0.03044	0.975783	-6.79664	0.75415	0.73617
Dendritic.cells	SLC22A27	-0.01832	1.124082	-0.03044	0.975788	-5.36358	0.846508	0.870521
Dendritic.cells	SP4	-0.00507	5.254382	-0.0304	0.975813	-6.33704	0.781727	0.776026
Dendritic.cells	GM36660	-0.0153	-0.14918	-0.03039	0.975827	-5.33764	0.8678	0.901847
Dendritic.cells	PDE5A	-0.00779	2.569114	-0.03018	0.975994	-6.08069	0.823105	0.836243
Dendritic.cells	GM10851	0.009162	4.366594	0.029993	0.97614	-5.79424	0.795107	0.795445
Dendritic.cells	SPATA7	-0.01173	1.309288	-0.02992	0.9762	-5.51023	0.843463	0.866052
Dendritic.cells	DCAF6	-0.00463	5.908083	-0.02987	0.976241	-6.57955	0.77206	0.762031
Dendritic.cells	POLR1A	0.007282	4.926327	0.029863	0.976244	-5.9904	0.786637	0.783146
Dendritic.cells	GBP2B	0.045805	0.300434	0.029578	0.97647	-5.28025	0.860209	0.890663
Dendritic.cells	EMD	-0.00315	6.102062	-0.02936	0.976644	-6.59331	0.769222	0.757926
Dendritic.cells	APEX2	0.005742	4.215093	0.02935	0.976652	-6.05366	0.79742	0.798806

Dendritic.cells	SP2	0.005363	4.742762	0.029312	0.976681	-6.13092	0.789402	0.787159
Dendritic.cells	DHX33	0.00745	3.32737	0.029309	0.976684	-5.80669	0.811145	0.818787
Dendritic.cells	ALDH3A2	-0.00677	3.596521	-0.0292	0.976769	-5.84347	0.806952	0.812678
Dendritic.cells	LONP2	0.002888	5.87764	0.029162	0.976801	-6.56757	0.772507	0.762677
Dendritic.cells	CYB5R1	0.009311	3.659051	0.028952	0.976968	-5.62805	0.805982	0.811313
Dendritic.cells	TMC8	0.006597	3.820781	0.028901	0.977009	-5.97797	0.80348	0.807685
Dendritic.cells	TAX1BP3	-0.00735	4.090035	-0.02885	0.977052	-5.88568	0.799336	0.801656
Dendritic.cells	STX8	0.002354	6.745581	0.028747	0.977131	-6.78587	0.759904	0.744528
Dendritic.cells	NEK8	0.009387	2.113602	0.028673	0.97719	-5.65766	0.830395	0.846978
Dendritic.cells	SERAC1	-0.00644	2.177244	-0.02862	0.977233	-6.09286	0.829372	0.845496
Dendritic.cells	PRPF40B	0.015354	0.534618	0.028616	0.977236	-5.45131	0.856287	0.884985
Dendritic.cells	SPEF1	0.014977	0.343281	0.028543	0.977294	-5.35206	0.85949	0.889704
Dendritic.cells	ZFP689	0.011414	1.407041	0.028513	0.977317	-5.51021	0.841862	0.8638
Dendritic.cells	TM9SF1	0.004342	5.042685	0.028299	0.977487	-6.3419	0.784968	0.780703
Dendritic.cells	TPR	-0.0026	7.524789	-0.02807	0.977668	-6.84733	0.748973	0.728578
Dendritic.cells	DEF8	0.008778	2.04717	0.027929	0.977781	-5.64338	0.831634	0.848567
Dendritic.cells	DUSP11	0.002726	6.33182	0.027894	0.977809	-6.6105	0.766034	0.75318
Dendritic.cells	NASP	-0.00412	6.308139	-0.02778	0.977903	-6.46544	0.766393	0.753684
Dendritic.cells	CYB5R3	-0.0044	4.85016	-0.02768	0.977978	-6.27646	0.78796	0.784926
Dendritic.cells	KCNK10	0.014511	0.557275	0.02725	0.978322	-5.41849	0.856338	0.884619
Dendritic.cells	SLC14A1	-0.0076	3.335927	-0.02696	0.978554	-5.86729	0.81155	0.818887
Dendritic.cells	OXA1L	-0.00419	4.658758	-0.02679	0.978691	-6.22409	0.791248	0.789285
Dendritic.cells	4930509H(	-0.00872	2.090644	-0.02651	0.97891	-5.57009	0.831494	0.847759
Dendritic.cells	LSM14B	-0.00581	3.604671	-0.02634	0.979047	-5.84853	0.807586	0.812795
Dendritic.cells	4930562C1	-0.01507	0.146658	-0.02617	0.979184	-5.27747	0.863666	0.894806
Dendritic.cells	SNX9	-0.00217	6.331978	-0.02588	0.97941	-7.07286	0.766741	0.753385
Dendritic.cells	SAMD9L	-0.00584	5.184732	-0.02583	0.97945	-6.16925	0.78365	0.777843
Dendritic.cells	MTO1	0.004789	3.553315	0.025668	0.97958	-6.06374	0.808582	0.813989
Dendritic.cells	3110056K(	-0.00391	4.723448	-0.02501	0.980102	-6.18532	0.790941	0.788029
Dendritic.cells	HIST1H1D	0.017175	1.531522	0.025	0.980111	-5.28569	0.841154	0.861209
Dendritic.cells	GM26631	-0.01119	1.219353	-0.02483	0.980243	-5.43818	0.846325	0.868751
Dendritic.cells	MON2	-0.00291	5.7408	-0.02465	0.98039	-6.55918	0.775801	0.766068
Dendritic.cells	GM12089	-0.00958	0.306607	-0.02462	0.980413	-5.48722	0.861529	0.89107
Dendritic.cells	A1CF	0.014428	0.569704	0.024479	0.980526	-5.36411	0.857117	0.884584
Dendritic.cells	CRTC1	0.00503	3.126748	0.024399	0.980589	-6.14322	0.815636	0.823897
Dendritic.cells	MFSD4A	-0.01089	2.041521	-0.02432	0.98065	-5.46567	0.832933	0.849146
Dendritic.cells	SIGLEC1	0.017878	0.449661	0.02426	0.9807	-5.29886	0.859127	0.887549
Dendritic.cells	ATP5A1	0.002098	7.857813	0.023915	0.980974	-6.98159	0.745535	0.722312
Dendritic.cells	URAH	-0.01175	3.327083	-0.02312	0.981604	-5.6665	0.812981	0.819517
Dendritic.cells	EIF4G2	-0.00185	7.71796	-0.02312	0.981609	-6.89383	0.747792	0.725228
Dendritic.cells	GM12596	0.010189	2.627238	0.023086	0.981634	-5.46402	0.824036	0.83563
Dendritic.cells	ATRAID	0.003531	4.57765	0.023031	0.981677	-6.25019	0.793687	0.791484
Dendritic.cells	GM29666	0.014554	0.244861	0.022839	0.98183	-5.29821	0.863087	0.89284
Dendritic.cells	RFC4	0.007335	4.603054	0.022637	0.981991	-5.92344	0.793302	0.790953
Dendritic.cells	SCNM1	0.00535	3.621272	0.022593	0.982026	-5.86169	0.808389	0.812866
Dendritic.cells	PURG	0.003909	3.260975	0.022369	0.982204	-6.23775	0.814017	0.821065
Dendritic.cells	ELK4	0.003023	5.882489	0.022342	0.982225	-6.45031	0.77418	0.763284

Dendritic.cells	COPS7A	0.00436	4.237724	0.022206	0.982334	-6.09352	0.798873	0.799049
Dendritic.cells	SH2B2	0.002641	4.550186	0.022171	0.982362	-6.72694	0.794105	0.792129
Dendritic.cells	AR	0.010341	0.218656	0.022073	0.982439	-5.63226	0.863529	0.893555
Dendritic.cells	CLSPN	-0.00864	4.411842	-0.02188	0.98259	-5.80215	0.796212	0.795233
Dendritic.cells	FIZ1	0.003299	4.553694	0.021876	0.982596	-6.16178	0.794051	0.792099
Dendritic.cells	AKR7A5	-0.00524	4.13514	-0.02185	0.982615	-5.9095	0.800447	0.801381
Dendritic.cells	MAIP1	-0.00497	3.588251	-0.0218	0.982658	-5.88983	0.808903	0.813684
Dendritic.cells	PEG13	-0.00645	2.114243	-0.0215	0.982895	-5.74434	0.83226	0.847743
Dendritic.cells	DCTN1	0.0029	4.987161	0.021466	0.982922	-6.33928	0.787497	0.782609
Dendritic.cells	ERMAP	-0.00782	0.529243	-0.02146	0.982925	-5.76516	0.85831	0.885915
Dendritic.cells	SMC2	0.006635	5.816825	0.02141	0.982967	-6.21134	0.775146	0.764739
Dendritic.cells	CDH2	-0.0105	1.947183	-0.0213	0.983056	-5.42711	0.834959	0.851703
Dendritic.cells	ZNRF2	0.002063	5.763077	0.021234	0.983107	-6.70998	0.775939	0.765903
Dendritic.cells	PPP1R8	-0.00418	4.114233	-0.02122	0.98312	-6.04486	0.800768	0.801879
Dendritic.cells	DSCC1	0.009101	2.435007	0.021206	0.983129	-5.48972	0.827106	0.840234
Dendritic.cells	MMRN1	0.020351	-0.22843	0.021027	0.983272	-5.28961	0.87117	0.904794
Dendritic.cells	BACH1	-0.00308	5.9952	-0.0206	0.983608	-6.68585	0.772736	0.761106
Dendritic.cells	4930557K0	-0.0085	1.483636	-0.02051	0.983684	-5.53062	0.842738	0.862911
Dendritic.cells	DNAH1	0.012235	-0.10849	0.020497	0.983693	-5.27255	0.869306	0.901922
Dendritic.cells	MICOS13	0.002253	5.948201	0.020398	0.983772	-6.60731	0.77343	0.762117
Dendritic.cells	CSPG5	0.013639	0.28413	0.019847	0.98421	-5.29988	0.862676	0.89227
Dendritic.cells	GM15265	0.008581	1.797023	0.019691	0.984334	-5.49493	0.837639	0.855575
Dendritic.cells	ADAMTS7	-0.01176	0.676356	-0.01968	0.984344	-5.30361	0.8561	0.882636
Dendritic.cells	CORO1C	-0.00187	6.202322	-0.01965	0.984369	-6.78439	0.769718	0.756861
Dendritic.cells	IL1RL1	0.012638	-0.28284	0.019624	0.984388	-5.27567	0.87229	0.906452
Dendritic.cells	ESYT1	-0.00303	5.654914	-0.01961	0.984399	-6.40208	0.777763	0.768486
Dendritic.cells	CGAS	-0.00843	2.747045	-0.01958	0.984422	-5.61988	0.82237	0.833279
Dendritic.cells	FBXO25	-0.00644	2.127104	-0.01946	0.984515	-5.48167	0.832294	0.847802
Dendritic.cells	5930403N2	-0.01189	-0.48768	-0.01946	0.98452	-5.27693	0.875795	0.911663
Dendritic.cells	PNO1	0.003956	4.446222	0.019346	0.984609	-6.09604	0.795919	0.794841
Dendritic.cells	DYRK3	0.004031	2.520243	0.019337	0.984616	-6.28924	0.825983	0.838602
Dendritic.cells	TSPAN2	-0.00206	3.159011	-0.01927	0.984667	-6.67603	0.815856	0.823858
Dendritic.cells	USP12	-0.00257	6.069178	-0.01905	0.984841	-6.59421	0.771699	0.759804
Dendritic.cells	ZBTB8A	0.006421	1.978565	0.01894	0.984931	-5.57633	0.834732	0.851416
Dendritic.cells	SARDHOS	0.011812	0.147254	0.018939	0.984932	-5.39924	0.865025	0.895854
Dendritic.cells	GMPPA	-0.0043	3.453598	-0.01832	0.985423	-5.95977	0.81161	0.817264
Dendritic.cells	HIST1H2AC	-0.01197	1.907677	-0.01821	0.985513	-5.32868	0.836226	0.853205
Dendritic.cells	NACC1	0.002862	4.380772	0.018081	0.985615	-6.21303	0.797283	0.796481
Dendritic.cells	KIN	-0.00323	4.642417	-0.01806	0.985634	-6.06288	0.793299	0.790701
Dendritic.cells	GM50012	0.004746	1.643382	0.017835	0.985811	-5.78881	0.840536	0.859561
Dendritic.cells	FBXO42	-0.00223	5.871653	-0.01781	0.985827	-6.61944	0.774928	0.764148
Dendritic.cells	ZFPM1	0.005776	4.248218	0.017603	0.985995	-5.7393	0.79932	0.799553
Dendritic.cells	1810046K0	-0.01046	-0.14929	-0.01754	0.986047	-5.27526	0.870423	0.90354
Dendritic.cells	PLEKHG5	0.006342	1.864551	0.017502	0.986076	-5.726	0.836935	0.854396
Dendritic.cells	DCN	0.008318	3.138043	0.017488	0.986087	-5.68214	0.816569	0.824665
Dendritic.cells	TAGLN	0.01123	2.304796	0.017276	0.986255	-5.51361	0.829904	0.844017
Dendritic.cells	GM4673	-0.00452	2.578427	-0.01693	0.986531	-5.76262	0.825691	0.837631

Dendritic.cells	DNAJB6	-0.00129	8.310874	-0.01652	0.986858	-7.09148	0.740452	0.714203
Dendritic.cells	SAP18B	0.005223	2.771863	0.016508	0.986866	-5.68955	0.822766	0.833259
Dendritic.cells	GOLGA4	0.001778	5.30004	0.016365	0.98698	-6.52654	0.783822	0.776658
Dendritic.cells	H2-DMA	-0.00284	5.184297	-0.01623	0.987087	-6.73436	0.785563	0.779195
Dendritic.cells	VPS33B	0.004324	2.932531	0.016112	0.987181	-5.74828	0.82026	0.829612
Dendritic.cells	ATPSCKMT	0.003272	3.937455	0.01603	0.987246	-6.16914	0.804539	0.806758
Dendritic.cells	SLC4A4	0.007262	2.065431	0.015892	0.987357	-5.5682	0.834134	0.849918
Dendritic.cells	DENND5A	-0.00162	6.395889	-0.01587	0.987376	-6.76414	0.767676	0.753417
Dendritic.cells	TERF2IP	-0.00345	3.58313	-0.0158	0.987433	-5.87771	0.810038	0.814782
Dendritic.cells	PPP4R2	-0.00162	6.318519	-0.01556	0.987624	-6.71296	0.76886	0.755045
Dendritic.cells	AKT2	-0.0022	5.234185	-0.01552	0.987655	-6.32453	0.784875	0.778187
Dendritic.cells	FNBP1	0.001565	8.248905	0.015353	0.987785	-7.18585	0.741447	0.715574
Dendritic.cells	CENPV	-0.00846	3.40694	-0.01526	0.98786	-5.43556	0.812899	0.818824
Dendritic.cells	ABHD5	0.004604	3.453436	0.015154	0.987943	-5.72229	0.812173	0.817765
Dendritic.cells	ARL8A	0.001704	6.227073	0.015071	0.98801	-6.66024	0.770241	0.757001
Dendritic.cells	KMT2A	0.001953	6.28827	0.01488	0.988162	-6.60173	0.769409	0.755712
Dendritic.cells	UNK	0.002428	4.287183	0.014715	0.988293	-6.33733	0.79933	0.799008
Dendritic.cells	ECPAS	0.00146	6.743157	0.014675	0.988324	-6.76462	0.762814	0.746202
Dendritic.cells	NETO2	0.006917	3.22493	0.0146	0.988384	-5.53548	0.815822	0.822981
Dendritic.cells	WDR89	-0.00482	2.643388	-0.01434	0.988588	-5.59044	0.825085	0.836404
Dendritic.cells	SELENOI	0.003814	3.422495	0.014288	0.988633	-5.84213	0.812774	0.81847
Dendritic.cells	CYP39A1	0.008199	1.483874	0.014081	0.988797	-5.28991	0.843838	0.863823
Dendritic.cells	4930505N2	0.007212	1.214516	0.014032	0.988836	-5.32018	0.848269	0.870313
Dendritic.cells	WDR55	-0.00408	2.979495	-0.01401	0.98885	-5.66335	0.819745	0.828633
Dendritic.cells	IFT80	-0.00432	3.626112	-0.01391	0.988936	-5.86976	0.809595	0.813897
Dendritic.cells	IREB2	-0.00176	5.493255	-0.01387	0.988963	-6.49538	0.781169	0.772675
Dendritic.cells	SNAI1	0.00657	0.389835	0.013753	0.989058	-5.35111	0.862012	0.890554
Dendritic.cells	PCGF3	0.002366	3.998741	0.013634	0.989153	-6.09878	0.803817	0.805529
Dendritic.cells	NUP205	0.00222	4.408914	0.013482	0.989274	-6.25453	0.797518	0.796424
Dendritic.cells	ACYP2	-0.00432	3.398712	-0.01345	0.989297	-5.7181	0.813146	0.819134
Dendritic.cells	HIST3H2A	0.004946	2.104979	0.01335	0.989378	-5.67671	0.833728	0.849154
Dendritic.cells	IL16	0.004596	4.60831	0.013138	0.989548	-5.84917	0.794479	0.79202
Dendritic.cells	XPNPEP3	0.002947	3.250052	0.01306	0.989609	-5.90775	0.815479	0.822534
Dendritic.cells	QSOX2	0.005446	0.948725	0.012968	0.989683	-5.48938	0.852669	0.876886
Dendritic.cells	IGKV1-135	0.007957	-0.02384	0.012949	0.989698	-5.27617	0.869005	0.90089
Dendritic.cells	DDRGK1	-0.00164	5.272107	-0.01278	0.989833	-6.4073	0.784468	0.77759
Dendritic.cells	YTHDF3	-0.00111	7.017555	-0.01251	0.990049	-6.86768	0.758919	0.740752
Dendritic.cells	TSPYL2	-0.00334	2.8065	-0.01249	0.990063	-5.88655	0.822488	0.832878
Dendritic.cells	USP3	0.001389	6.873279	0.012434	0.990107	-6.73359	0.760988	0.743752
Dendritic.cells	VPS18	-0.00197	4.834066	-0.01212	0.990354	-6.2596	0.791056	0.787216
Dendritic.cells	PBX1	-0.00259	5.465012	-0.01207	0.9904	-6.37722	0.781589	0.773509
Dendritic.cells	PPHLN1	-0.002	4.932629	-0.01193	0.990508	-6.30084	0.789567	0.785059
Dendritic.cells	RC3H2	0.001198	5.728871	0.011824	0.990593	-6.60355	0.777674	0.767848
Dendritic.cells	GM39556	-0.00368	3.384757	-0.01172	0.990674	-5.88382	0.813365	0.819622
Dendritic.cells	REEP1	-0.00416	0.98372	-0.01163	0.990748	-5.72185	0.852089	0.87621
Dendritic.cells	VWA5A	-0.0034	3.406572	-0.01158	0.990788	-5.83717	0.813023	0.819124
Dendritic.cells	TUBA4A	0.002744	4.513714	0.011576	0.99079	-6.02809	0.795919	0.794268

Dendritic.cells	SENP3	-0.00228	4.324023	-0.0115	0.990852	-6.03635	0.798817	0.798473
Dendritic.cells	KLRC2	-0.00716	1.332802	-0.01148	0.990865	-5.42527	0.84632	0.867753
Dendritic.cells	ZCWPW1	-0.0035	2.765843	-0.01147	0.990873	-5.72382	0.823134	0.833857
Dendritic.cells	MTUS1	0.005203	3.721745	0.011412	0.990921	-5.67355	0.808107	0.811971
Dendritic.cells	PNPLA7	0.00171	6.039516	0.011404	0.990927	-6.56496	0.773098	0.761235
Dendritic.cells	CIAPIN1	-0.00209	4.643928	-0.01136	0.990964	-6.19151	0.793937	0.791401
Dendritic.cells	SYT11	-0.00631	1.412759	-0.01124	0.991056	-5.35946	0.845005	0.865853
Dendritic.cells	NAA15	0.000839	6.743192	0.011169	0.991114	-6.87457	0.762861	0.74649
Dendritic.cells	SF3B1	0.000783	8.256825	0.011094	0.991173	-6.99495	0.741447	0.715698
Dendritic.cells	TIMM8A1	-0.00287	4.226992	-0.01056	0.991597	-5.9189	0.800528	0.800795
Dendritic.cells	1700016PC	0.005071	3.898827	0.010487	0.991656	-5.76808	0.805586	0.808143
Dendritic.cells	ADGRG3	0.004354	2.366781	0.010468	0.991671	-5.55774	0.829743	0.843335
Dendritic.cells	CSDE1	0.000632	7.576149	0.009964	0.992073	-6.99254	0.751433	0.729574
Dendritic.cells	RFX5	-0.0036	1.943489	-0.00976	0.992237	-5.55568	0.836873	0.853439
Dendritic.cells	EIF4A1	0.000887	7.526831	0.009748	0.992244	-6.85723	0.752149	0.73061
Dendritic.cells	DHODH	-0.00308	2.109646	-0.0094	0.992518	-5.62722	0.834231	0.849598
Dendritic.cells	IL4RA	0.001684	4.883706	0.009193	0.992686	-6.36346	0.790854	0.786488
Dendritic.cells	PARD6G	0.005153	1.981117	0.009119	0.992745	-5.42792	0.836312	0.852645
Dendritic.cells	HIST1H2BB	0.004972	1.116611	0.009118	0.992746	-5.29524	0.850476	0.87338
Dendritic.cells	ARF1	-0.00066	8.02586	-0.00912	0.992747	-7.02501	0.745178	0.720615
Dendritic.cells	NGDN	0.001008	5.424919	0.008546	0.993201	-6.48305	0.782729	0.77486
Dendritic.cells	DAXX	0.001946	4.352332	0.008522	0.993219	-6.08406	0.798937	0.798354
Dendritic.cells	EPN2	-0.00448	1.664749	-0.00842	0.9933	-5.48139	0.841462	0.860355
Dendritic.cells	COQ10A	0.001849	3.31768	0.008276	0.993415	-5.8604	0.814981	0.821708
Dendritic.cells	CHMP2A	-0.00083	6.544809	-0.00819	0.993486	-6.67403	0.76626	0.751109
Dendritic.cells	ALG1	-0.00199	3.312271	-0.00804	0.9936	-5.93184	0.815066	0.821857
Dendritic.cells	AGXT2	-0.00444	1.612637	-0.00804	0.993601	-5.43497	0.842314	0.861639
Dendritic.cells	HOMER3	0.001939	3.103095	0.008004	0.993632	-5.80044	0.81836	0.826654
Dendritic.cells	MAST3	-0.00203	4.169613	-0.00799	0.993644	-5.9507	0.801741	0.802479
Dendritic.cells	RASA3	0.000658	6.702935	0.007944	0.99368	-7.01672	0.763971	0.747833
Dendritic.cells	RBM39	-0.00044	9.586582	-0.00794	0.993684	-7.28598	0.723801	0.690168
Dendritic.cells	SUSD1	-0.00207	5.183728	-0.00785	0.993752	-5.99188	0.786336	0.780176
Dendritic.cells	GTF3C4	-0.00248	2.57436	-0.00785	0.993755	-5.66601	0.82676	0.83894
Dendritic.cells	ZFP639	0.001657	4.128657	0.007754	0.993831	-5.97798	0.802371	0.803449
Dendritic.cells	I830077J02	0.001285	2.758256	0.00772	0.993858	-6.49809	0.823826	0.83468
Dendritic.cells	CPSF6	0.000569	6.68211	0.007501	0.994032	-6.76685	0.764272	0.748332
Dendritic.cells	EVL	0.001276	6.485099	0.007472	0.994055	-6.49804	0.767126	0.752448
Dendritic.cells	ZFP513	-0.00227	2.534011	-0.00746	0.994066	-5.69821	0.827405	0.839919
Dendritic.cells	0610009E0	0.002941	0.843747	0.007399	0.994113	-5.45949	0.855007	0.88034
Dendritic.cells	ZFP942	0.001277	4.309464	0.007356	0.994147	-6.24394	0.799594	0.799459
Dendritic.cells	MASTL	0.001714	3.782	0.007183	0.994285	-5.9463	0.807783	0.811311
Dendritic.cells	FBRS	0.000998	4.965395	0.006995	0.994434	-6.38446	0.789682	0.785044
Dendritic.cells	ZC3HAV1	-0.00075	8.43854	-0.0068	0.99459	-6.95067	0.7395	0.712721
Dendritic.cells	ZMYM5	-0.00089	5.521459	-0.00679	0.994594	-6.41243	0.781352	0.77301
Dendritic.cells	RNF4	-0.00066	5.964365	-0.00676	0.994621	-6.59554	0.774799	0.763536
Dendritic.cells	TMX2	0.001784	3.46498	0.006703	0.994667	-5.819	0.812736	0.818551
Dendritic.cells	EHBP1	-0.00221	3.52414	-0.00627	0.995015	-5.7795	0.811857	0.817318

Dendritic.cells	TMEM170B	0.000935	4.646846	0.006262	0.995017	-6.36118	0.794551	0.792173
Dendritic.cells	HECTD2	0.003078	0.291184	0.00622	0.995051	-5.38463	0.864389	0.894193
Dendritic.cells	WRAP53	0.001731	2.788872	0.006168	0.995092	-5.69925	0.82345	0.834226
Dendritic.cells	P2RX1	-0.00362	0.81074	-0.00615	0.995103	-5.35781	0.855673	0.881385
Dendritic.cells	ZC3H7A	0.000495	6.883008	0.006075	0.995166	-6.93607	0.761479	0.744375
Dendritic.cells	ZC3H6	-0.00141	3.379852	-0.0059	0.995303	-5.95176	0.81415	0.820652
Dendritic.cells	LCP2	-0.00213	5.109024	-0.00581	0.995374	-5.9103	0.787597	0.782108
Dendritic.cells	MSH5	0.001563	2.9838	0.005711	0.995456	-6.02838	0.820391	0.829791
Dendritic.cells	CUL4B	-0.00138	4.524951	-0.00561	0.995534	-5.95236	0.79644	0.794955
Dendritic.cells	MTERF3	-0.00121	4.014483	-0.00552	0.995607	-5.96879	0.804274	0.806332
Dendritic.cells	MED30	0.00082	5.369422	0.005466	0.995651	-6.34188	0.783696	0.776484
Dendritic.cells	SPOPL	0.000804	4.42179	0.005337	0.995754	-6.28383	0.798018	0.797241
Dendritic.cells	RARA	0.000777	5.561492	0.005277	0.995802	-6.49313	0.780836	0.772343
Dendritic.cells	SLC7A6	-0.00079	4.274758	-0.00499	0.996031	-6.24932	0.800395	0.800578
Dendritic.cells	ARHGAP18	0.000879	6.528672	0.004226	0.996637	-6.53432	0.767052	0.7519
Dendritic.cells	GSK3B	-0.0003	8.105386	-0.00422	0.996646	-7.0252	0.74461	0.719614
Dendritic.cells	METTL21A	-0.00113	1.888583	-0.0041	0.996737	-5.69233	0.838424	0.855545
Dendritic.cells	GM12359	0.001528	1.671224	0.004035	0.99679	-5.53468	0.841969	0.860736
Dendritic.cells	YIF1A	-0.00071	4.016242	-0.00402	0.9968	-6.0347	0.80469	0.80638
Dendritic.cells	NCS1	-0.00238	0.210797	-0.00394	0.996864	-5.29994	0.866261	0.896374
Dendritic.cells	4833417C1	-0.00191	0.707506	-0.00376	0.997007	-5.35298	0.857965	0.884163
Dendritic.cells	CLPTM1L	0.000529	4.883326	0.003521	0.997198	-6.32779	0.791584	0.787243
Dendritic.cells	ZFP799	0.001769	0.939029	0.00332	0.997359	-5.29382	0.854251	0.878532
Dendritic.cells	ZMYND11	0.000308	6.466516	0.003265	0.997402	-6.7575	0.768142	0.753315
Dendritic.cells	ABCA8A	0.00224	1.028779	0.003121	0.997517	-5.33998	0.85278	0.876394
Dendritic.cells	WASHC3	-0.00051	4.517912	-0.00296	0.997647	-6.08547	0.7972	0.795367
Dendritic.cells	GM15563	0.001478	1.923249	0.002905	0.997689	-5.43066	0.838085	0.854928
Dendritic.cells	BRPF3	-0.00044	4.451036	-0.00283	0.997751	-6.15286	0.798221	0.796872
Dendritic.cells	LAMTOR5	-0.00042	5.527925	-0.00273	0.997824	-6.30351	0.781972	0.773366
Dendritic.cells	PIEZO1	0.000595	5.046187	0.002677	0.99787	-6.03911	0.789188	0.783822
Dendritic.cells	CHRAC1	0.000321	5.264928	0.002483	0.998024	-6.36995	0.785901	0.779138
Dendritic.cells	LIMCH1	-0.00113	0.57747	-0.00244	0.998061	-5.55595	0.860313	0.887644
Dendritic.cells	A730063M	-0.00101	1.218514	-0.00232	0.998152	-5.57005	0.849637	0.87201
Dendritic.cells	UBE2D2A	-0.00012	8.209532	-0.00231	0.998164	-7.07922	0.743357	0.717849
Dendritic.cells	CENPA	-0.00043	6.681539	-0.0021	0.998325	-6.64902	0.765045	0.749085
Dendritic.cells	DNAJB11	0.000203	6.073023	0.001999	0.998409	-6.65912	0.773915	0.761889
Dendritic.cells	LBR	-0.00015	7.219631	-0.0018	0.99857	-6.85546	0.757313	0.737994
Dendritic.cells	DISC1	0.000275	3.43956	0.001774	0.998588	-6.33045	0.813884	0.819885
Dendritic.cells	NAE1	0.000433	3.958187	0.001756	0.998603	-5.8033	0.805807	0.808137
Dendritic.cells	ZFP445	-0.00023	4.93802	-0.00175	0.998612	-6.34915	0.790824	0.786393
Dendritic.cells	CDK12	-0.00014	6.896517	-0.0014	0.998883	-6.82396	0.762043	0.744713
Dendritic.cells	KLK1	0.000228	-0.20745	0.001396	0.998889	-6.63717	0.873723	0.907408
Dendritic.cells	PLCB4	0.000186	3.876337	0.00105	0.999165	-6.47146	0.807297	0.8101
Dendritic.cells	SLAMF7	-0.00038	5.151332	-0.00103	0.999183	-5.87177	0.787827	0.781853
Dendritic.cells	ABCG2	0.000279	3.149803	0.00085	0.999324	-5.65331	0.818722	0.826652
Dendritic.cells	UBN1	4.70E-05	6.944041	0.000601	0.999522	-6.83995	0.761613	0.743852
Dendritic.cells	H2AFZ	7.45E-05	10.35837	0.000514	0.999591	-7.24758	0.714585	0.676446

Dendritic.cells	SP1	4.35E-05	5.924217	0.000394	0.999686	-6.53478	0.776481	0.765274
