# Supplementary material for: Immune landscape in liver of neonatal mice with phlebotomy-induced anemia
Source: Pediatr Res. 2025 Sep 17;99(4):1602–12. doi: 10.1038/s41390-025-04361-x (PMC12659965; doi:10.1038/s41390-025-04361-x)
Supplement: Supplementary file 6 — Table S6 [file 41390_2025_4361_MOESM6_ESM.pdf]

immunecell	gene	logFC	AveExpr	t	P.Value	B	adj.P.Val. Within	adj.P.Val. Between
Erythroid.cells	PRR13	1.955721	6.510778	15.38789	9.77E-27	49.63369	8.93E-23	3.98E-22
Erythroid.cells	TCEA1	1.61837	7.486226	15.16738	2.47E-26	49.24521	1.09E-22	6.54E-22
Erythroid.cells	UBE2B	1.657523	8.25823	13.54462	2.78E-23	42.51146	7.99E-20	5.40E-19
Erythroid.cells	SERINC1	1.425522	6.230989	12.92407	4.44E-22	39.62306	1.02E-18	7.30E-18
Erythroid.cells	REXO2	2.171194	6.270041	11.94444	3.84E-20	34.98693	7.07E-17	4.50E-16
Erythroid.cells	GLRX5	1.445705	6.17176	11.68041	1.30E-19	34.12157	2.00E-16	1.19E-15
Erythroid.cells	GM32051	4.226578	-0.14913	11.00041	3.07E-18	28.91087	5.04E-15	2.32E-14
Erythroid.cells	CPOX	1.842923	4.570271	10.88497	5.28E-18	30.24555	6.44E-15	3.24E-14
Erythroid.cells	YIPF4	1.380014	6.515782	10.82295	7.06E-18	30.2374	7.17E-15	3.60E-14
Erythroid.cells	PRDX3	1.68311	5.400912	10.43151	4.45E-17	28.27577	4.23E-14	2.08E-13
Erythroid.cells	TPT1	0.968373	10.10831	10.24701	1.06E-16	27.46335	7.82E-14	4.13E-13
Erythroid.cells	GNG5	1.179036	8.925654	10.1577	1.62E-16	27.10051	1.14E-13	6.19E-13
Erythroid.cells	MT1	3.524079	6.314344	10.01916	3.13E-16	26.4656	2.21E-13	1.17E-12
Erythroid.cells	GCLM	1.339749	6.090034	9.940514	4.54E-16	26.14596	3.01E-13	1.63E-12
Erythroid.cells	UBE2F	1.388728	6.306137	9.86104	6.62E-16	25.74709	4.06E-13	2.17E-12
Erythroid.cells	BMP2K	1.295956	6.936172	9.760772	1.07E-15	25.30203	6.00E-13	3.23E-12
Erythroid.cells	MLLT3	1.868764	5.728512	9.680524	1.56E-15	24.90737	8.61E-13	4.64E-12
Erythroid.cells	UROD	1.631041	4.467162	9.554385	2.84E-15	24.22417	1.55E-12	8.16E-12
Erythroid.cells	EEF1D	1.177533	7.27018	9.346136	7.63E-15	23.38509	3.58E-12	1.97E-11
Erythroid.cells	D5ERTD57!	1.719403	5.221315	9.285395	1.02E-14	22.86656	4.87E-12	2.69E-11
Erythroid.cells	UBA52	1.34624	10.38927	9.240587	1.26E-14	22.70097	4.81E-12	2.80E-11
Erythroid.cells	RAD23A	1.283062	5.807004	9.042333	3.24E-14	21.97486	1.38E-11	7.68E-11
Erythroid.cells	CMAS	1.357148	5.862643	8.981307	4.32E-14	21.69006	1.76E-11	9.70E-11
Erythroid.cells	AZIN1	1.191155	6.940521	8.915079	5.92E-14	21.3618	2.16E-11	1.22E-10
Erythroid.cells	YPEL3	1.457956	7.069109	8.912105	6.01E-14	21.36255	2.15E-11	1.21E-10
Erythroid.cells	ELOC	1.126253	7.093954	8.806461	9.92E-14	20.87564	3.42E-11	1.89E-10
Erythroid.cells	PTBP3	0.87116	8.46802	8.711698	1.56E-13	20.3925	4.93E-11	2.73E-10
Erythroid.cells	MGST3	2.10885	3.846236	8.695986	1.68E-13	20.19288	6.00E-11	3.27E-10
Erythroid.cells	PPOX	2.353153	3.108757	8.680416	1.81E-13	19.33757	6.39E-11	3.52E-10
Erythroid.cells	CALCRL	2.007826	5.617508	8.65096	2.08E-13	20.14692	6.52E-11	3.69E-10
Erythroid.cells	GPX4	1.061982	7.153816	8.494373	4.36E-13	19.43845	1.26E-10	6.59E-10
Erythroid.cells	OAZ1	1.008955	9.534615	8.482944	4.60E-13	19.18328	1.19E-10	6.39E-10
Erythroid.cells	CHP1	0.986616	7.01438	8.430004	5.92E-13	19.11967	1.61E-10	8.65E-10
Erythroid.cells	TAX1BP1	0.923228	8.297296	8.386108	7.28E-13	18.83238	1.84E-10	1.01E-09
Erythroid.cells	CENPQ	1.557344	5.314475	8.323745	9.79E-13	18.63046	2.66E-10	1.42E-09
Erythroid.cells	SSBP2	1.888398	6.284595	8.306239	1.06E-12	18.53852	2.72E-10	1.48E-09
Erythroid.cells	UBE2H	1.055184	8.574917	8.226795	1.55E-12	18.07975	3.56E-10	1.89E-09
Erythroid.cells	HTATIP2	1.532178	4.480259	8.036438	3.80E-12	17.19595	9.80E-10	4.89E-09
Erythroid.cells	GHITM	1.058566	7.4577	8.026336	3.99E-12	17.11391	9.04E-10	4.65E-09
Erythroid.cells	PABPC1	1.053547	10.47512	8.02058	4.09E-12	16.89546	8.17E-10	4.33E-09
Erythroid.cells	MAT2B	1.171439	5.679223	8.006038	4.39E-12	17.18116	1.01E-09	5.23E-09
Erythroid.cells	MT2	4.533572	2.001416	7.970002	5.20E-12	15.52585	1.32E-09	6.67E-09
Erythroid.cells	PSMD4	0.894454	6.691408	7.836806	9.72E-12	16.41593	2.05E-09	1.08E-08
Erythroid.cells	RALY	1.189193	6.422986	7.784636	1.24E-11	16.18461	2.59E-09	1.34E-08

Erythroid.cells	TXNRD2	1.66984	3.532111	7.746376	1.49E-11	15.92456	3.34E-09	1.71E-08
Erythroid.cells	MPP1	1.052762	6.203718	7.72096	1.67E-11	15.84525	3.36E-09	1.70E-08
Erythroid.cells	FTL1	1.089729	11.26205	7.697559	1.87E-11	15.33288	3.09E-09	1.63E-08
Erythroid.cells	YPEL5	1.085213	6.583092	7.686477	1.97E-11	15.69663	3.70E-09	1.93E-08
Erythroid.cells	GLO1	1.446621	5.029475	7.683977	1.99E-11	15.71263	3.91E-09	2.01E-08
Erythroid.cells	ARF5	0.942669	8.486216	7.660429	2.22E-11	15.37973	3.80E-09	2.02E-08
Erythroid.cells	YOD1	1.528471	4.084266	7.6314	2.55E-11	15.47168	4.96E-09	2.56E-08
Erythroid.cells	CYB5R1	2.018745	3.659051	7.62384	2.64E-11	14.93559	5.11E-09	2.64E-08
Erythroid.cells	SMAP1	0.880383	6.875842	7.616073	2.73E-11	15.35728	4.66E-09	2.48E-08
Erythroid.cells	COPS2	1.015745	6.020343	7.589763	3.09E-11	15.29837	5.32E-09	2.81E-08
Erythroid.cells	DSTN	1.213885	6.886463	7.575594	3.30E-11	15.2174	5.42E-09	2.90E-08
Erythroid.cells	RFK	1.247135	5.042963	7.560449	3.55E-11	15.16625	6.09E-09	3.20E-08
Erythroid.cells	ATPIF1	1.051607	7.745006	7.403233	7.37E-11	14.34969	1.13E-08	5.81E-08
Erythroid.cells	EIF5	0.788825	8.418516	7.389114	7.88E-11	14.16982	1.16E-08	5.97E-08
Erythroid.cells	SDCBP	1.004045	7.674968	7.33535	1.01E-10	14.11742	1.50E-08	7.67E-08
Erythroid.cells	TALDO1	0.833534	8.310001	7.308306	1.15E-10	13.8901	1.64E-08	8.46E-08
Erythroid.cells	FOXO3	1.346666	6.397005	7.262867	1.41E-10	13.83575	2.13E-08	1.08E-07
Erythroid.cells	IFT20	1.201334	5.442542	7.249596	1.50E-10	13.76478	2.30E-08	1.17E-07
Erythroid.cells	SELENOW	1.205979	7.046449	7.239297	1.58E-10	13.70759	2.25E-08	1.16E-07
Erythroid.cells	DERL1	0.835867	6.22546	7.219587	1.73E-10	13.6441	2.49E-08	1.28E-07
Erythroid.cells	GALNT1	0.885807	6.628673	7.165614	2.22E-10	13.38463	3.11E-08	1.59E-07
Erythroid.cells	PAIP2	0.748163	7.620191	7.103609	2.95E-10	13.04415	3.94E-08	2.03E-07
Erythroid.cells	SNHG1	1.283535	5.230493	7.048369	3.81E-10	12.86993	5.43E-08	2.70E-07
Erythroid.cells	SELENOT	0.980785	6.182995	6.983984	5.12E-10	12.59963	6.95E-08	3.45E-07
Erythroid.cells	MAP1LC3B	0.951482	7.434486	6.960975	5.69E-10	12.34133	7.30E-08	3.63E-07
Erythroid.cells	UBE2R2	0.837058	7.144409	6.940139	6.25E-10	12.29607	7.99E-08	3.94E-07
Erythroid.cells	WDR26	0.807858	7.53429	6.921109	6.82E-10	12.23633	8.48E-08	4.22E-07
Erythroid.cells	RNF115	0.846315	6.978362	6.917763	6.93E-10	12.27639	8.66E-08	4.32E-07
Erythroid.cells	CAT	1.165394	6.72379	6.913286	7.07E-10	12.13482	8.79E-08	4.41E-07
Erythroid.cells	SLC23A1	2.855592	1.528872	6.89983	7.52E-10	11.03934	1.10E-07	5.38E-07
Erythroid.cells	EIF2AK1	1.078669	5.326731	6.863054	8.90E-10	12.06778	1.13E-07	5.64E-07
Erythroid.cells	COX6B2	2.602029	1.691068	6.777383	1.32E-09	10.94922	1.87E-07	8.82E-07
Erythroid.cells	CREG1	0.966266	7.514034	6.758933	1.43E-09	11.3822	1.64E-07	7.93E-07
Erythroid.cells	EIF3J1	0.800383	7.268033	6.698028	1.89E-09	11.27182	2.15E-07	1.01E-06
Erythroid.cells	ELOB	0.774764	8.913307	6.691467	1.94E-09	11.06609	2.07E-07	9.88E-07
Erythroid.cells	1810058I2	0.950694	7.005863	6.669289	2.15E-09	11.02544	2.41E-07	1.13E-06
Erythroid.cells	MKRN1	0.889083	7.149614	6.64579	2.39E-09	10.79664	2.64E-07	1.22E-06
Erythroid.cells	ATG4A	1.387039	4.314693	6.621766	2.66E-09	11.00945	3.20E-07	1.45E-06
Erythroid.cells	CLCN3	0.989349	6.389845	6.618448	2.70E-09	10.97127	2.99E-07	1.38E-06
Erythroid.cells	UBA1	1.14616	6.00691	6.596778	2.98E-09	10.90696	3.30E-07	1.51E-06
Erythroid.cells	SMIM4	1.392758	5.255277	6.57823	3.24E-09	10.73194	3.64E-07	1.66E-06
Erythroid.cells	SEC62	0.585914	7.437896	6.541491	3.83E-09	10.56723	3.93E-07	1.79E-06
Erythroid.cells	RAMAC	0.867167	5.913686	6.539195	3.87E-09	10.64095	4.15E-07	1.87E-06
Erythroid.cells	SELENOK	0.850485	7.584969	6.536756	3.91E-09	10.53248	3.91E-07	1.80E-06
Erythroid.cells	ISCU	0.82408	7.465509	6.528757	4.05E-09	10.41737	4.03E-07	1.85E-06
Erythroid.cells	GABARAPL	0.926437	7.762001	6.524897	4.12E-09	10.29183	4.01E-07	1.86E-06
Erythroid.cells	FRG1	0.851061	6.660706	6.512043	4.37E-09	10.51165	4.35E-07	2.02E-06

Erythroid.cells	GM10076	1.388356	6.671496	6.510434	4.40E-09	10.50577	4.35E-07	2.02E-06
Erythroid.cells	DYNLL1	0.817762	7.867224	6.479552	5.06E-09	10.28681	4.75E-07	2.16E-06
Erythroid.cells	RFESD	2.23894	2.541257	6.450141	5.77E-09	9.814293	6.43E-07	2.82E-06
Erythroid.cells	FAM117A	1.228818	5.965212	6.443829	5.94E-09	10.19801	5.77E-07	2.59E-06
Erythroid.cells	RNF114	1.238508	5.645299	6.443395	5.95E-09	10.24853	5.83E-07	2.61E-06
Erythroid.cells	FAM126A	1.082826	5.549539	6.416679	6.70E-09	10.10509	6.53E-07	2.92E-06
Erythroid.cells	CD24A	1.204948	7.730753	6.392907	7.46E-09	9.697651	6.67E-07	3.00E-06
Erythroid.cells	TOP1	0.952358	8.354587	6.376565	8.02E-09	9.799291	6.95E-07	3.12E-06
Erythroid.cells	CHMP4B	0.726852	7.889271	6.35295	8.91E-09	9.740396	7.77E-07	3.46E-06
Erythroid.cells	GPX1	0.892895	11.17498	6.323306	1.02E-08	8.923741	7.84E-07	3.58E-06
Erythroid.cells	SMDT1	0.709707	7.773748	6.321666	1.02E-08	9.562741	8.80E-07	3.94E-06
Erythroid.cells	PNPO	1.141466	3.744874	6.317224	1.05E-08	9.704347	1.02E-06	4.44E-06
Erythroid.cells	SLC25A51	0.941536	5.697758	6.289938	1.18E-08	9.539347	1.07E-06	4.71E-06
Erythroid.cells	TMEM86B	1.59922	3.467162	6.270585	1.29E-08	9.361457	1.24E-06	5.38E-06
Erythroid.cells	ACOT2	1.425767	4.416307	6.240651	1.47E-08	9.229034	1.36E-06	5.93E-06
Erythroid.cells	SNAP23	1.141519	6.222753	6.231679	1.53E-08	9.35021	1.32E-06	5.79E-06
Erythroid.cells	CAR2	1.294883	5.171918	6.218782	1.62E-08	9.125598	1.43E-06	6.23E-06
Erythroid.cells	UBB	0.783137	10.85501	6.201126	1.75E-08	8.580639	1.27E-06	5.67E-06
Erythroid.cells	CIR1	0.867543	5.515333	6.188505	1.85E-08	9.116796	1.59E-06	6.90E-06
Erythroid.cells	GPCPD1	0.889223	7.329375	6.180044	1.92E-08	9.021872	1.54E-06	6.78E-06
Erythroid.cells	COPZ1	0.852575	6.366584	6.157098	2.13E-08	9.010051	1.74E-06	7.67E-06
Erythroid.cells	RAP1A	0.537843	8.772339	6.129349	2.40E-08	8.673363	1.80E-06	8.04E-06
Erythroid.cells	UBE2O	1.069638	4.874388	6.089028	2.87E-08	8.628348	2.43E-06	1.05E-05
Erythroid.cells	CARHSP1	1.051038	5.430457	6.055876	3.32E-08	8.574263	2.73E-06	1.18E-05
Erythroid.cells	MRPL32	0.983406	5.68103	6.054849	3.34E-08	8.589257	2.70E-06	1.17E-05
Erythroid.cells	FABP4	1.815886	6.966529	6.046072	3.47E-08	8.457545	2.67E-06	1.16E-05
Erythroid.cells	UROS	1.556202	2.669254	6.032037	3.69E-08	8.231369	3.25E-06	1.37E-05
Erythroid.cells	BCL2L1	1.193989	6.15449	6.031045	3.70E-08	8.387824	2.88E-06	1.24E-05
Erythroid.cells	BSG	0.905567	7.716678	6.025262	3.80E-08	8.237655	2.78E-06	1.21E-05
Erythroid.cells	CALCOCO1	1.642612	3.863697	6.012735	4.01E-08	8.305386	3.32E-06	1.42E-05
Erythroid.cells	MFF	0.799349	5.906654	6.004905	4.15E-08	8.351175	3.18E-06	1.38E-05
Erythroid.cells	YME1L1	0.723166	6.181942	6.002866	4.19E-08	8.385857	3.15E-06	1.37E-05
Erythroid.cells	UOX	2.020453	5.010934	5.983483	4.56E-08	8.275193	3.54E-06	1.51E-05
Erythroid.cells	MAP4K5	1.441705	4.52083	5.976257	4.71E-08	8.270816	3.68E-06	1.58E-05
Erythroid.cells	COX7B	0.79096	7.597144	5.961303	5.02E-08	8.02513	3.51E-06	1.53E-05
Erythroid.cells	TRAK2	1.078599	5.153104	5.86834	7.53E-08	7.835033	5.68E-06	2.35E-05
Erythroid.cells	PSMC6	0.850314	6.141841	5.839283	8.54E-08	7.690114	6.17E-06	2.58E-05
Erythroid.cells	ATP6V0D1	0.650813	7.228201	5.793128	1.04E-07	7.437277	7.21E-06	2.97E-05
Erythroid.cells	ATF5	1.456911	3.267979	5.774218	1.13E-07	7.454189	8.89E-06	3.58E-05
Erythroid.cells	TCEAL8	1.46236	3.331023	5.770278	1.15E-07	7.327265	8.95E-06	3.61E-05
Erythroid.cells	GDA	1.403626	3.902395	5.722597	1.41E-07	7.027935	1.07E-05	4.25E-05
Erythroid.cells	H3F3A	0.591575	10.71708	5.705152	1.52E-07	6.559495	9.06E-06	3.74E-05
Erythroid.cells	ATP5L	0.615098	8.67785	5.698409	1.57E-07	6.807429	9.92E-06	4.06E-05
Erythroid.cells	EIF1	0.713047	9.721973	5.692891	1.60E-07	6.607764	9.73E-06	4.03E-05
Erythroid.cells	MTFR1	1.304644	4.079368	5.666411	1.80E-07	6.998826	1.31E-05	5.24E-05
Erythroid.cells	UBE2D3	0.462512	8.966917	5.665011	1.81E-07	6.655238	1.11E-05	4.59E-05
Erythroid.cells	DHRS11	1.022712	4.678666	5.622355	2.17E-07	6.777688	1.53E-05	6.06E-05

Erythroid.cells	UCP2	0.729425	9.571709	5.618353	2.21E-07	6.270207	1.31E-05	5.34E-05
Erythroid.cells	PRXL2A	1.153262	4.184431	5.60563	2.33E-07	6.6097	1.65E-05	6.52E-05
Erythroid.cells	VBP1	0.988758	4.92592	5.599892	2.39E-07	6.749926	1.63E-05	6.47E-05
Erythroid.cells	ADIPOR1	0.715476	7.346972	5.597105	2.41E-07	6.375639	1.51E-05	6.11E-05
Erythroid.cells	ZFAND6	0.634335	6.978135	5.569371	2.72E-07	6.47408	1.71E-05	6.85E-05
Erythroid.cells	MPC2	0.805107	6.800876	5.563274	2.79E-07	6.409636	1.74E-05	7.01E-05
Erythroid.cells	SNX15	0.946198	4.880351	5.5629	2.79E-07	6.584676	1.86E-05	7.40E-05
Erythroid.cells	OIP5OS1	0.813701	6.02273	5.561531	2.81E-07	6.519592	1.79E-05	7.19E-05
Erythroid.cells	VCP	0.660631	7.431716	5.557296	2.86E-07	6.407885	1.72E-05	7.01E-05
Erythroid.cells	BSDC1	1.098855	5.056629	5.540975	3.06E-07	6.483487	1.99E-05	7.88E-05
Erythroid.cells	NECAP2	1.05316	5.345804	5.530343	3.21E-07	6.47274	2.03E-05	8.11E-05
Erythroid.cells	DNAJB2	1.461129	2.766414	5.530225	3.21E-07	6.414634	2.22E-05	8.71E-05
Erythroid.cells	AHSA1	1.196253	5.071608	5.518151	3.38E-07	6.421581	2.14E-05	8.46E-05
Erythroid.cells	BC005537	0.807377	7.116434	5.517282	3.39E-07	6.311576	1.99E-05	7.99E-05
Erythroid.cells	NDFIP1	0.748621	7.521506	5.516025	3.41E-07	6.25173	1.97E-05	7.90E-05
Erythroid.cells	SIAH1A	1.008284	5.079563	5.506343	3.55E-07	6.370749	2.21E-05	8.74E-05
Erythroid.cells	PITHD1	1.003561	4.744392	5.493359	3.75E-07	6.315092	2.35E-05	9.14E-05
Erythroid.cells	RAB6A	0.757297	6.637549	5.486458	3.86E-07	6.205274	2.25E-05	8.91E-05
Erythroid.cells	NT5C3	0.881406	4.85257	5.465337	4.22E-07	6.145109	2.60E-05	0.000101
Erythroid.cells	PABPC4	0.815822	5.897724	5.419001	5.12E-07	5.938605	3.03E-05	0.000115
Erythroid.cells	SLC48A1	0.761543	5.074284	5.416632	5.18E-07	5.940234	3.13E-05	0.000119
Erythroid.cells	GABARAP	0.576099	8.497974	5.376805	6.12E-07	5.500996	3.26E-05	0.000126
Erythroid.cells	OST4	0.770456	8.049265	5.37531	6.15E-07	5.546162	3.31E-05	0.000128
Erythroid.cells	FZR1	1.084889	4.900739	5.366031	6.40E-07	5.80757	3.80E-05	0.000144
Erythroid.cells	ADAM10	0.691829	7.403441	5.365504	6.41E-07	5.642051	3.49E-05	0.000134
Erythroid.cells	STOM	1.266084	3.274711	5.364004	6.45E-07	5.81747	4.02E-05	0.000151
Erythroid.cells	NBR1	0.903284	5.455137	5.306674	8.19E-07	5.584052	4.71E-05	0.000176
Erythroid.cells	CCNDBP1	0.830398	5.692836	5.305182	8.24E-07	5.354419	4.67E-05	0.000176
Erythroid.cells	CNPPD1	0.999356	5.686802	5.296834	8.53E-07	5.510223	4.80E-05	0.000181
Erythroid.cells	PPP1R15A	1.13181	7.089434	5.275822	9.31E-07	5.234663	4.97E-05	0.000188
Erythroid.cells	SLC4A1	1.248187	2.002694	5.263177	9.81E-07	5.33553	6.20E-05	0.000226
Erythroid.cells	SPECC1	1.235444	4.276803	5.258528	1.00E-06	5.289174	5.81E-05	0.000215
Erythroid.cells	RNF11	0.715097	6.207032	5.246755	1.05E-06	5.133664	5.67E-05	0.00021
Erythroid.cells	EIF3C	0.680116	7.172674	5.237476	1.09E-06	5.123226	5.67E-05	0.000211
Erythroid.cells	PLAAT3	1.125865	5.19136	5.235922	1.10E-06	5.167273	6.07E-05	0.000223
Erythroid.cells	ANK1	2.070699	1.273318	5.226886	1.14E-06	5.260504	7.15E-05	0.000257
Erythroid.cells	RRAGA	1.00608	5.020687	5.225313	1.15E-06	5.271733	6.28E-05	0.000231
Erythroid.cells	UBL7	1.059336	5.130384	5.224903	1.15E-06	5.269419	6.26E-05	0.000231
Erythroid.cells	NCOA7	0.972999	5.299685	5.200019	1.27E-06	5.121919	6.86E-05	0.00025
Erythroid.cells	STK40	0.985689	5.69472	5.141668	1.62E-06	4.932345	8.55E-05	0.000297
Erythroid.cells	BHMT	1.618572	5.13251	5.122165	1.75E-06	4.7863	9.38E-05	0.000322
Erythroid.cells	SWT1	0.942972	5.244266	5.114091	1.81E-06	4.843126	9.61E-05	0.000329
Erythroid.cells	RNF10	0.639081	6.51711	5.101421	1.91E-06	4.539071	9.63E-05	0.000332
Erythroid.cells	NDUFB11	0.649559	7.55899	5.099126	1.93E-06	4.527593	9.33E-05	0.000324
Erythroid.cells	UXT	1.107008	4.452478	5.073183	2.14E-06	4.700068	0.000115	0.000387
Erythroid.cells	TUBA4A	1.146898	4.513714	5.068148	2.18E-06	4.672864	0.000116	0.000394
Erythroid.cells	NDUFA7	0.558096	7.909207	5.048682	2.36E-06	4.305439	0.000111	0.000383

Erythroid.cells	GNAS	0.548299	8.931692	5.043413	2.42E-06	4.13404	0.000109	0.000378
Erythroid.cells	TSPAN33	1.078271	2.55857	5.030739	2.54E-06	4.453338	0.000142	0.00047
Erythroid.cells	PCMTD2	1.461047	3.680281	5.020247	2.65E-06	4.456695	0.000142	0.000471
Erythroid.cells	MYL6	0.621462	9.689609	5.013903	2.72E-06	3.926343	0.000118	0.000407
Erythroid.cells	EPB42	1.454397	-0.1647	5.00867	2.78E-06	4.454128	0.000168	0.000544
Erythroid.cells	RMC1	1.032408	4.652621	5.008599	2.78E-06	4.449702	0.000142	0.000476
Erythroid.cells	SUPT4A	0.742731	6.836136	4.999451	2.89E-06	4.22317	0.000136	0.000463
Erythroid.cells	AHI1	1.861166	3.003332	4.983198	3.08E-06	4.063216	0.000165	0.000541
Erythroid.cells	CEP76	1.523062	2.990307	4.980115	3.12E-06	4.27751	0.000165	0.000544
Erythroid.cells	HIPK1	0.843034	6.269668	4.979542	3.13E-06	4.222009	0.000147	0.000496
Erythroid.cells	HAGH	0.813615	5.358255	4.979271	3.13E-06	4.144623	0.000152	0.000509
Erythroid.cells	PNPLA7	1.088481	6.039516	4.973315	3.21E-06	4.275645	0.000151	0.000508
Erythroid.cells	GTPBP2	1.130329	4.751688	4.960882	3.37E-06	4.278924	0.000165	0.000547
Erythroid.cells	RNF123	1.285819	3.804311	4.954718	3.46E-06	4.255224	0.000174	0.000573
Erythroid.cells	HMBS	1.079184	4.451319	4.953932	3.47E-06	4.206206	0.00017	0.000564
Erythroid.cells	TRAF3	-1.09929	6.856964	-4.94908	3.54E-06	4.130007	0.000159	0.000535
Erythroid.cells	PITRM1	1.401996	3.560314	4.942348	3.63E-06	4.16535	0.000182	0.000598
Erythroid.cells	WBP2	0.818562	5.599979	4.940159	3.67E-06	4.110446	0.00017	0.000568
Erythroid.cells	ISCA1	0.778265	6.018689	4.923528	3.92E-06	3.789689	0.000179	0.000595
Erythroid.cells	D1ERTD62:	0.953327	5.295192	4.882772	4.61E-06	3.966813	0.000214	0.000691
Erythroid.cells	NFE2	1.57091	1.59738	4.881256	4.64E-06	3.942923	0.000244	0.000769
Erythroid.cells	S100A1	1.300089	4.086611	4.872349	4.81E-06	3.949402	0.000231	0.000738
Erythroid.cells	MPST	1.345612	3.682692	4.866187	4.93E-06	3.912376	0.000239	0.000756
Erythroid.cells	PRKAR2B	1.510453	3.305356	4.849274	5.27E-06	3.837006	0.000257	0.000809
Erythroid.cells	KLRA9	-3.08124	-0.30711	-4.84649	5.33E-06	3.200616	0.000292	0.000902
Erythroid.cells	KIF2A	0.659938	6.338703	4.845815	5.34E-06	3.685938	0.000232	0.000751
Erythroid.cells	SH3GLB1	0.510334	8.546317	4.845102	5.36E-06	3.312941	0.000216	0.000707
Erythroid.cells	TPRGL	0.64723	6.927644	4.841239	5.44E-06	3.586849	0.00023	0.000747
Erythroid.cells	HADHB	0.855987	5.576702	4.833432	5.61E-06	3.754167	0.000248	0.000796
Erythroid.cells	ATF4	0.916159	6.443614	4.8174	5.98E-06	3.623945	0.000255	0.000817
Erythroid.cells	ATG12	0.882873	4.873947	4.816209	6.01E-06	3.701891	0.000269	0.000855
Erythroid.cells	DCUN1D1	0.774691	5.787878	4.793632	6.57E-06	3.54541	0.000284	0.000888
Erythroid.cells	TRGV2	-3.16927	-0.51519	-4.77794	6.99E-06	2.911883	0.000373	0.001106
Erythroid.cells	PSME3	0.771814	5.282258	4.775457	7.06E-06	3.42465	0.000307	0.000946
Erythroid.cells	ASL	1.178266	4.484774	4.739982	8.12E-06	3.464223	0.00036	0.001073
Erythroid.cells	TPM1	0.987525	5.177563	4.739964	8.12E-06	3.370783	0.000352	0.001052
Erythroid.cells	ZNHIT2	-1.53407	3.310699	-4.73325	8.34E-06	3.214675	0.000383	0.001131
Erythroid.cells	AXIN1	-0.7456	6.046226	-4.71908	8.82E-06	3.345245	0.000367	0.001088
Erythroid.cells	SERPINA3K	3.043456	1.878609	4.705029	9.32E-06	3.26723	0.000446	0.001284
Erythroid.cells	PRDX6	0.609346	7.110346	4.697891	9.58E-06	3.018489	0.000381	0.001135
Erythroid.cells	KLRA6	-3.17581	-0.71608	-4.69477	9.70E-06	2.469462	0.000503	0.001419
Erythroid.cells	XBP1	0.825773	6.135195	4.674078	1.05E-05	3.155763	0.000429	0.001242
Erythroid.cells	EVI5	0.976164	5.166502	4.657008	1.12E-05	3.081508	0.000472	0.001353
Erythroid.cells	ABTB1	1.256841	4.303875	4.65474	1.13E-05	3.159466	0.000488	0.001394
Erythroid.cells	UQCRH	0.452881	8.592145	4.653195	1.14E-05	2.674542	0.000422	0.00124
Erythroid.cells	RANBP10	0.796191	5.698692	4.647809	1.16E-05	2.972276	0.000474	0.001367
Erythroid.cells	RAB18	0.690531	5.748093	4.629343	1.25E-05	2.99121	0.000506	0.001452

Erythroid.cells	FGFR1	3.073019	1.974297	4.626009	1.27E-05	2.724855	0.00058	0.001617
Erythroid.cells	COX6A1	0.714885	6.768979	4.625665	1.27E-05	2.835619	0.000492	0.001415
Erythroid.cells	PRNP	1.46356	2.012971	4.616421	1.32E-05	3.001612	0.000597	0.001672
Erythroid.cells	SKP1A	0.578885	6.639256	4.603883	1.38E-05	2.754701	0.000533	0.001531
Erythroid.cells	FAM220A	1.100983	2.03012	4.602778	1.39E-05	2.917855	0.000624	0.001743
Erythroid.cells	DCAF6	1.074333	5.908083	4.598381	1.41E-05	2.886778	0.000553	0.001583
Erythroid.cells	ACTR1A	-0.74484	6.140647	-4.5868	1.48E-05	2.86213	0.000572	0.001638
Erythroid.cells	TSPYL1	0.8671	4.888609	4.584699	1.49E-05	2.866703	0.000599	0.001703
Erythroid.cells	UBE2E3	0.506936	7.066891	4.567012	1.59E-05	2.535904	0.000593	0.001694
Erythroid.cells	SNCA	0.976754	4.248926	4.554671	1.67E-05	2.254782	0.000682	0.001906
Erythroid.cells	SEC23A	0.910936	4.425257	4.552095	1.69E-05	2.776342	0.000682	0.001899
Erythroid.cells	MSH3	-1.08157	5.159864	-4.53392	1.81E-05	2.731808	0.00071	0.001942
Erythroid.cells	ST3GAL5	0.865188	6.23691	4.52446	1.88E-05	2.423169	0.000705	0.001936
Erythroid.cells	GNMT	1.698781	4.232803	4.523639	1.88E-05	2.636786	0.000755	0.002051
Erythroid.cells	FECH	0.789752	5.217188	4.523016	1.89E-05	2.27057	0.00073	0.001996
Erythroid.cells	TTC33	1.275867	3.849347	4.519925	1.91E-05	2.677014	0.000771	0.00209
Erythroid.cells	ZFYVE28	2.492826	0.624372	4.51669	1.93E-05	1.733471	0.000869	0.002309
Erythroid.cells	AP2M1	0.680511	6.941455	4.512278	1.97E-05	2.397896	0.000708	0.001965
Erythroid.cells	IFRD2	1.151074	3.30652	4.485303	2.18E-05	2.566775	0.000883	0.002375
Erythroid.cells	PRDX2	0.610856	7.774205	4.484996	2.18E-05	1.919669	0.000758	0.002097
Erythroid.cells	CRLF3	0.846192	6.899396	4.475298	2.26E-05	2.271285	0.000807	0.002213
Erythroid.cells	TMEM256	0.664201	6.982152	4.465759	2.35E-05	2.15048	0.000831	0.002282
Erythroid.cells	LMO2	0.755305	4.903927	4.463288	2.37E-05	2.261413	0.000898	0.002432
Erythroid.cells	USP45	0.96419	4.074097	4.439506	2.59E-05	2.379501	0.001007	0.002698
Erythroid.cells	PAM16	0.84896	4.987535	4.432061	2.67E-05	2.344924	0.001	0.002671
Erythroid.cells	DNAJB6	0.491868	8.310874	4.429209	2.70E-05	1.887016	0.000899	0.002452
Erythroid.cells	FHL1	2.601286	0.58688	4.421177	2.78E-05	1.773226	0.001203	0.003099
Erythroid.cells	FIS1	0.560281	7.530663	4.416697	2.83E-05	1.923767	0.00096	0.002592
Erythroid.cells	HOMER2	2.628224	0.275337	4.403597	2.97E-05	1.791132	0.00129	0.003307
Erythroid.cells	ATP2B4	1.295022	4.169834	4.394235	3.08E-05	2.216822	0.001164	0.003059
Erythroid.cells	ADK	0.916505	6.968234	4.366484	3.42E-05	1.849315	0.00117	0.003065
Erythroid.cells	SLC25A39	0.659865	6.472882	4.356191	3.55E-05	1.648955	0.001232	0.003201
Erythroid.cells	C1GALT1	-0.83253	6.353776	-4.34265	3.74E-05	1.906446	0.001297	0.003336
Erythroid.cells	PRXL2C	0.933963	4.932963	4.339529	3.78E-05	2.046451	0.001371	0.003488
Erythroid.cells	SNRNP25	1.136017	3.939991	4.33878	3.79E-05	2.063233	0.001419	0.003592
Erythroid.cells	ARL5C	-1.04667	6.264356	-4.33515	3.85E-05	1.849158	0.001323	0.003404
Erythroid.cells	FGFR1OP2	0.636501	6.180589	4.333162	3.87E-05	1.844801	0.001331	0.003421
Erythroid.cells	TFRC	0.786419	6.38061	4.323965	4.01E-05	1.830403	0.001359	0.003485
Erythroid.cells	ESCO2	1.452135	3.734708	4.323859	4.01E-05	1.99123	0.001488	0.003751
Erythroid.cells	PMP22	2.771752	1.847479	4.320015	4.07E-05	1.679062	0.001605	0.003998
Erythroid.cells	KDM7A	0.59722	7.129544	4.307546	4.26E-05	1.551457	0.001398	0.003544
Erythroid.cells	GYPA	0.865634	3.611578	4.303942	4.32E-05	1.402932	0.001592	0.003946
Erythroid.cells	EPB41	0.727106	8.026595	4.301136	4.37E-05	1.293438	0.001378	0.003517
Erythroid.cells	ATP6V1G1	0.596634	7.177612	4.298536	4.41E-05	1.653494	0.001427	0.00363
Erythroid.cells	PINK1	0.863787	5.115722	4.297355	4.43E-05	1.790957	0.001533	0.003855
Erythroid.cells	DACH1	2.860999	1.877933	4.288545	4.58E-05	1.605901	0.001765	0.004323
Erythroid.cells	DDB1	0.853853	5.611857	4.279178	4.74E-05	1.781634	0.001599	0.004014

Erythroid.cells	CTH	1.72835	3.512521	4.278525	4.75E-05	1.834676	0.001719	0.004261
Erythroid.cells	FGF23	3.694461	0.594781	4.265344	4.99E-05	1.320111	0.001989	0.0048
Erythroid.cells	VOPP1	0.896377	3.965239	4.257283	5.14E-05	1.675278	0.001818	0.004481
Erythroid.cells	COX7A1	2.417723	0.986976	4.252607	5.23E-05	1.160326	0.002043	0.004943
Erythroid.cells	FBP1	1.36862	5.143064	4.251164	5.26E-05	1.556277	0.001774	0.004415
Erythroid.cells	SNRPA	-0.84496	5.131803	-4.24963	5.29E-05	1.745144	0.001778	0.004427
Erythroid.cells	SLC30A9	0.659068	5.265307	4.233053	5.62E-05	1.578034	0.001875	0.004599
Erythroid.cells	SLC39A4	-2.56363	1.149584	-4.2291	5.71E-05	1.148102	0.002185	0.005217
Erythroid.cells	HBQ1B	1.141481	-0.16343	4.218706	5.93E-05	1.592075	0.002363	0.005579
Erythroid.cells	AMFR	0.631718	6.011988	4.218298	5.94E-05	1.542701	0.00191	0.004701
Erythroid.cells	RBM33	0.659302	5.898903	4.209255	6.14E-05	1.497886	0.001976	0.004839
Erythroid.cells	CAMP	2.442483	4.463959	4.206747	6.20E-05	1.609509	0.002088	0.005059
Erythroid.cells	CRIPT	0.550476	6.218829	4.202964	6.28E-05	1.361281	0.001986	0.004853
Erythroid.cells	GPBP1	0.52055	7.723603	4.194031	6.49E-05	1.083645	0.001943	0.004756
Erythroid.cells	AUH	0.745052	5.641378	4.180777	6.82E-05	1.387978	0.002183	0.005223
Erythroid.cells	NCOA4	0.740832	6.199192	4.167631	7.15E-05	1.095582	0.00224	0.005309
Erythroid.cells	PEF1	1.391812	3.347699	4.157926	7.41E-05	1.41904	0.002551	0.005891
Erythroid.cells	CEBPD	1.470135	3.956744	4.153595	7.53E-05	1.432651	0.00253	0.005864
Erythroid.cells	SSBP3	0.734553	5.600863	4.152218	7.57E-05	1.292053	0.002395	0.00561
Erythroid.cells	CMIP	-0.65278	9.635584	-4.14167	7.87E-05	0.77313	0.002161	0.005161
Erythroid.cells	PPP1CB	0.508875	7.803763	4.139353	7.93E-05	0.800155	0.002309	0.005457
Erythroid.cells	VKORC1L1	0.862092	5.560377	4.138831	7.95E-05	1.336274	0.002493	0.005809
Erythroid.cells	2610002M	1.07545	4.208089	4.1297	8.22E-05	1.362419	0.002689	0.006194
Erythroid.cells	KCMF1	0.490266	6.987433	4.128989	8.24E-05	1.115603	0.002445	0.005737
Erythroid.cells	UBE2M	0.510536	7.20691	4.11307	8.73E-05	0.909435	0.002562	0.005905
Erythroid.cells	TIAM1	1.315261	4.628891	4.112188	8.76E-05	1.30199	0.002799	0.006359
Erythroid.cells	H19	1.745685	6.408472	4.111087	8.79E-05	0.906749	0.002635	0.006066
Erythroid.cells	SQSTM1	0.665594	7.466775	4.110172	8.82E-05	0.863932	0.002542	0.005899
Erythroid.cells	ACADSB	1.228693	3.270603	4.102812	9.06E-05	1.26245	0.003005	0.00677
Erythroid.cells	H2AFJ	0.564999	7.521162	4.089008	9.52E-05	0.895109	0.002722	0.006238
Erythroid.cells	XPO7	0.631729	6.710919	4.086877	9.60E-05	0.903395	0.002811	0.006415
Erythroid.cells	DCAF12	0.523864	6.638146	4.073959	0.000101	0.716505	0.002943	0.006653
Erythroid.cells	CTSF	1.595579	1.860603	4.068191	0.000103	1.168868	0.00353	0.007709
Erythroid.cells	ANXA7	0.738232	5.409471	4.065535	0.000104	1.063385	0.003144	0.007029
Erythroid.cells	LY6I	-3.92735	0.802083	-4.0605	0.000106	0.969842	0.00374	0.008097
Erythroid.cells	CD36	1.152616	5.712063	4.058706	0.000106	0.834887	0.003169	0.007098
Erythroid.cells	2-Mar	0.522107	6.581903	4.050263	0.00011	0.583353	0.003161	0.007085
Erythroid.cells	PQLC1	0.8703	4.395337	4.047127	0.000111	1.089399	0.003435	0.007589
Erythroid.cells	SNX22	2.069245	-0.37781	4.04575	0.000111	0.924617	0.004057	0.008698
Erythroid.cells	GM867	2.03327	-0.30876	4.040397	0.000114	0.946974	0.004113	0.008794
Erythroid.cells	CHIC2	0.481041	7.043743	4.035895	0.000115	0.764468	0.003236	0.007251
Erythroid.cells	HSPA4L	1.179669	4.348791	4.033329	0.000116	1.055208	0.003571	0.007857
Erythroid.cells	NOS2	-4.33399	-1.12584	-4.02762	0.000119	0.131398	0.004388	0.009273
Erythroid.cells	RMND5A	0.580917	6.766957	4.025391	0.00012	0.701978	0.00336	0.007482
Erythroid.cells	MARVELD1	2.131016	1.253094	4.011528	0.000126	0.648288	0.004256	0.009069
Erythroid.cells	SRP14	0.443239	7.234027	4.010527	0.000126	0.619787	0.003467	0.007683
Erythroid.cells	CD209A	2.736465	0.102824	4.009024	0.000127	0.737131	0.00444	0.009393

Erythroid.cells	C330018D2	1.455044	2.325358	4.004934	0.000129	0.897608	0.004161	0.008938
Erythroid.cells	KLF3	0.736442	6.281206	3.9993	0.000132	0.684341	0.003694	0.008108
Erythroid.cells	LSM12	0.565231	6.360706	3.992075	0.000135	0.67902	0.003769	0.008221
Erythroid.cells	GM2682	1.486637	2.755703	3.972085	0.000145	0.801134	0.004567	0.009596
Erythroid.cells	MOB3B	0.95468	4.961752	3.951617	0.000156	0.730844	0.004539	0.009552
Erythroid.cells	GSTO1	0.840428	4.594828	3.948838	0.000157	0.76973	0.004628	0.009725
Erythroid.cells	ABCG2	1.182738	3.149803	3.937307	0.000164	0.754262	0.005052	0.010501
Erythroid.cells	SLC22A15	1.351248	3.077716	3.933741	0.000166	0.726695	0.005113	0.010587
Erythroid.cells	HNRNPH2	0.59506	6.435152	3.931654	0.000167	0.551336	0.004577	0.0097
Erythroid.cells	TWF2	-0.96229	5.240324	-3.92963	0.000169	0.703396	0.004788	0.010091
Erythroid.cells	FBXO9	0.67606	4.752411	3.927779	0.00017	0.514751	0.004886	0.010277
Erythroid.cells	P3H2	2.040405	1.190669	3.925394	0.000171	0.716732	0.005554	0.011419
Erythroid.cells	RGL2	1.574503	3.300422	3.923194	0.000172	0.625716	0.005189	0.010806
Erythroid.cells	DAPK1	1.101853	4.932787	3.918948	0.000175	0.565549	0.004966	0.010446
Erythroid.cells	1700037H(	1.119562	3.714085	3.915337	0.000177	0.68513	0.005228	0.010914
Erythroid.cells	HIST1H4N	2.98254	1.07648	3.913829	0.000178	0.075258	0.005726	0.011779
Erythroid.cells	FMO5	1.326816	3.247756	3.913723	0.000178	0.680208	0.005313	0.011089
Erythroid.cells	GLUL	0.769618	6.384395	3.908237	0.000182	0.352296	0.00485	0.010289
Erythroid.cells	ZFP60	2.343253	1.431044	3.898858	0.000188	0.159884	0.005926	0.01213
Erythroid.cells	FAM210B	0.652684	3.914295	3.88534	0.000197	0.334301	0.005688	0.011735
Erythroid.cells	B630019A1	1.874038	1.596371	3.883886	0.000198	0.502635	0.006175	0.012563
Erythroid.cells	METAP2	0.470317	7.3726	3.875291	0.000204	0.083974	0.005204	0.010909
Erythroid.cells	CLEC4A2	1.734938	3.416946	3.865108	0.000211	0.52336	0.006159	0.012485
Erythroid.cells	HAAO	1.138426	3.64437	3.856483	0.000218	0.482766	0.006278	0.01266
Erythroid.cells	CAST	0.611492	6.266263	3.855737	0.000219	0.305302	0.005738	0.011792
Erythroid.cells	SULT1A1	1.235933	3.923228	3.853818	0.00022	0.430918	0.006242	0.012646
Erythroid.cells	NADK2	0.62571	4.994117	3.844943	0.000227	0.200457	0.006189	0.012554
Erythroid.cells	YTHDF1	-0.76123	5.78074	-3.84077	0.00023	0.358942	0.006095	0.012438
Erythroid.cells	TRIM23	1.223771	3.295163	3.838228	0.000232	0.42964	0.006679	0.013422
Erythroid.cells	FOXP4	-0.96955	4.962815	-3.83613	0.000234	0.360927	0.006335	0.01288
Erythroid.cells	OTUD5	0.589769	5.568497	3.819929	0.000248	0.206508	0.006544	0.013188
Erythroid.cells	CACNB2	2.386092	4.420818	3.819225	0.000248	0.384301	0.006807	0.013632
Erythroid.cells	TRDV2-2	-1.98276	-1.43025	-3.81418	0.000253	-0.36495	0.008452	0.01625
Erythroid.cells	LZIC	-1.27396	3.63991	-3.81168	0.000255	0.344614	0.007138	0.014203
Erythroid.cells	RNF141	1.017138	4.511433	3.805617	0.00026	0.34619	0.007055	0.01411
Erythroid.cells	CYSLTR1	1.794274	2.70023	3.804568	0.000261	0.304155	0.007515	0.014868
Erythroid.cells	PKIG	-0.60516	7.198634	-3.80317	0.000262	-0.06498	0.006453	0.013154
Erythroid.cells	FCNB	2.136948	-0.23854	3.800748	0.000265	0.009781	0.008382	0.016276
Erythroid.cells	PBRM1	0.4178	7.416728	3.793527	0.000271	-0.13735	0.006587	0.013371
Erythroid.cells	PIP4K2A	0.66592	7.239298	3.786581	0.000278	-0.0953	0.00677	0.01367
Erythroid.cells	ASB1	1.540679	1.712368	3.781576	0.000283	0.201937	0.008305	0.016103
Erythroid.cells	E2F5	1.466383	2.69996	3.778154	0.000286	0.16529	0.0081	0.015814
Erythroid.cells	FIP1L1	0.426246	6.639254	3.775547	0.000289	-0.05488	0.00712	0.014265
Erythroid.cells	OTUB1	0.582011	5.728978	3.768074	0.000296	0.112706	0.007517	0.014914
Erythroid.cells	M1AP	1.875087	1.320039	3.761394	0.000303	0.0735	0.008905	0.017151
Erythroid.cells	NDUFA11	0.596219	6.910675	3.761358	0.000303	-0.16795	0.007348	0.01468
Erythroid.cells	TNNI2	1.468509	2.310806	3.758369	0.000306	0.169011	0.008671	0.016827

Erythroid.cells	CRYZ	2.034934	1.288432	3.757593	0.000307	-0.05588	0.008982	0.017328
Erythroid.cells	NME7	-1.13461	3.562527	-3.75263	0.000312	0.166623	0.008426	0.016457
Erythroid.cells	ZFP52	-1.22898	3.859767	-3.74742	0.000318	0.171165	0.008468	0.016538
Erythroid.cells	BAG1	0.509848	6.693589	3.730434	0.000337	-0.2002	0.008123	0.015834
Erythroid.cells	PLCE1	2.091436	1.279254	3.716732	0.000353	0.057859	0.010228	0.01905
Erythroid.cells	APCS	0.923127	4.833138	3.715502	0.000355	-0.20451	0.009064	0.017282
Erythroid.cells	CMTM7	-0.52932	7.897065	-3.71173	0.000359	-0.40872	0.008222	0.016013
Erythroid.cells	HEPACAM2	1.812785	0.779743	3.711058	0.00036	0.057216	0.010499	0.019519
Erythroid.cells	SOD1	0.685339	5.993562	3.711043	0.00036	-0.19663	0.008774	0.016884
Erythroid.cells	F930017D2	1.298101	0.898224	3.708475	0.000363	-0.02791	0.01052	0.019524
Erythroid.cells	CHCHD4	1.240985	3.820424	3.702015	0.000372	0.032812	0.009691	0.018319
Erythroid.cells	MXI1	0.634132	7.063981	3.701482	0.000372	-0.52184	0.008671	0.016749
Erythroid.cells	RGS18	1.77185	3.108266	3.698697	0.000376	-0.02972	0.009986	0.018795
Erythroid.cells	CLTA	-0.42838	8.442682	-3.69806	0.000377	-0.53132	0.008317	0.016208
Erythroid.cells	SH3BGRL	0.595817	6.824052	3.697624	0.000377	-0.31659	0.00879	0.01697
Erythroid.cells	PHACTR4	-0.63103	5.677431	-3.69219	0.000384	-0.15782	0.009289	0.017671
Erythroid.cells	RHOA	0.300637	9.258828	3.691406	0.000385	-0.72026	0.00822	0.016028
Erythroid.cells	KLRA7	-2.70138	0.646446	-3.6906	0.000386	-0.02252	0.011048	0.020413
Erythroid.cells	FAAH	1.651037	1.451163	3.689729	0.000387	-0.05767	0.01075	0.019997
Erythroid.cells	EIF2S2	0.457624	7.925804	3.683581	0.000396	-0.56478	0.008767	0.016952
Erythroid.cells	IFI27	1.094429	4.998783	3.677643	0.000404	-0.23734	0.009863	0.018623
Erythroid.cells	TRMT5	-2.49077	1.213824	-3.67132	0.000412	-0.50494	0.01145	0.021026
Erythroid.cells	SPINT1	-1.89588	-1.0651	-3.66949	0.000415	-0.37208	0.012433	0.022463
Erythroid.cells	STRADB	0.861767	3.238382	3.667702	0.000418	-0.11616	0.010756	0.020002
Erythroid.cells	S100A13	0.649331	6.040644	3.658934	0.00043	-0.33206	0.010038	0.018913
Erythroid.cells	TENT5C	0.768032	5.86321	3.657804	0.000432	-0.79731	0.010112	0.019032
Erythroid.cells	RHBDD2	1.655332	2.284757	3.654813	0.000436	-0.24882	0.011524	0.021149
Erythroid.cells	TPCN1	1.143157	4.187411	3.653364	0.000438	-0.15204	0.010819	0.020136
Erythroid.cells	NUDT16	1.57986	2.966991	3.651339	0.000441	-0.1899	0.011332	0.020908
Erythroid.cells	RNF150	1.852493	2.909798	3.647444	0.000447	-0.1269	0.011454	0.021151
Erythroid.cells	MRPS9	-0.75711	4.889322	-3.64716	0.000448	-0.17758	0.0107	0.020017
Erythroid.cells	CRIP2	1.137872	4.565121	3.646554	0.000448	-0.18046	0.01082	0.020208
Erythroid.cells	YAF2	-0.59622	6.056106	-3.64493	0.000451	-0.32022	0.010312	0.019472
Erythroid.cells	TBCEL	0.781208	4.594507	3.642578	0.000455	-0.2477	0.010901	0.0204
Erythroid.cells	IDH3G	0.869882	5.124044	3.641656	0.000456	-0.24988	0.010712	0.020131
Erythroid.cells	VGLL4	-0.60853	6.477378	-3.63492	0.000466	-0.47819	0.010436	0.01965
Erythroid.cells	HSCB	0.73467	4.143832	3.627155	0.000479	-0.38612	0.011577	0.021251
Erythroid.cells	UQCRQ	0.532181	7.858453	3.625246	0.000482	-0.79133	0.010217	0.019245
Erythroid.cells	GSS	1.111697	3.399727	3.625042	0.000482	-0.20072	0.011904	0.021789
Erythroid.cells	ELOA	0.694444	5.789735	3.618424	0.000493	-0.3918	0.011164	0.020702
Erythroid.cells	ZFHX3	1.013417	5.163754	3.618283	0.000493	-0.26534	0.011406	0.021066
Erythroid.cells	AC154200.	2.012941	0.660722	3.617374	0.000495	-0.51017	0.013327	0.023901
Erythroid.cells	ABCC9	2.364819	1.277502	3.615857	0.000497	-0.42466	0.013082	0.023542
Erythroid.cells	PCBP2	0.366551	8.511828	3.608458	0.00051	-0.87642	0.010438	0.019659
Erythroid.cells	DCTN2	0.683841	5.367124	3.601881	0.000521	-0.40832	0.011853	0.021827
Erythroid.cells	NAGA	1.099716	4.159018	3.600581	0.000524	-0.28882	0.01238	0.022624
Erythroid.cells	UPP1	-3.45472	0.646262	-3.59921	0.000526	-0.46586	0.014003	0.024998

Erythroid.cells	TRIM59	0.877101	4.368452	3.592903	0.000537	-0.43026	0.01254	0.022874
Erythroid.cells	ISG15	1.10534	6.408101	3.592462	0.000538	-1.0213	0.011692	0.021633
Erythroid.cells	UBR4	0.516714	6.056739	3.588664	0.000545	-0.53761	0.011957	0.022038
Erythroid.cells	RIC1	-0.79139	6.710291	-3.58564	0.00055	-0.62632	0.011783	0.02176
Erythroid.cells	RAMP1	0.786776	4.507186	3.578176	0.000564	-0.37402	0.012998	0.023526
Erythroid.cells	SMOX	0.674574	5.116761	3.574735	0.000571	-0.88991	0.012845	0.023322
Erythroid.cells	ADGRG3	1.876811	2.366781	3.573665	0.000573	-0.44436	0.014137	0.025231
Erythroid.cells	GYPC	1.297683	3.080733	3.570614	0.000579	-0.36123	0.013902	0.024879
Erythroid.cells	AIMP1	-0.54564	6.38044	-3.56963	0.000581	-0.63297	0.012424	0.022738
Erythroid.cells	ZFP407	-0.52689	7.039039	-3.56488	0.00059	-0.7167	0.012313	0.022573
Erythroid.cells	CFB	-2.16592	4.663824	-3.5588	0.000602	-0.6369	0.013599	0.024458
Erythroid.cells	RET	-2.27809	0.727544	-3.55595	0.000608	-0.6754	0.015685	0.027378
Erythroid.cells	TMEM243	-0.51758	6.427837	-3.55463	0.00061	-0.68339	0.012897	0.023441
Erythroid.cells	SMTN	1.85363	2.149503	3.554454	0.000611	-0.54752	0.014939	0.026405
Erythroid.cells	GSTP3	1.168811	3.39573	3.552818	0.000614	-0.49856	0.014356	0.025603
Erythroid.cells	PI4KA	-0.64975	6.333121	-3.5471	0.000626	-0.67461	0.013198	0.023899
Erythroid.cells	TSPO2	0.890744	0.283153	3.546056	0.000628	-0.58941	0.016271	0.02834
Erythroid.cells	ATP6V0A1	0.92744	4.202735	3.543229	0.000634	-0.46855	0.014316	0.025556
Erythroid.cells	HSPA8	-0.47235	10.08692	-3.53755	0.000646	-1.30562	0.011904	0.022036
Erythroid.cells	PPARG	1.547295	2.579221	3.53467	0.000652	-0.45401	0.015504	0.027266
Erythroid.cells	RNF122	1.926733	2.64878	3.533082	0.000655	-0.56088	0.015513	0.027332
Erythroid.cells	SLC27A4	-1.33921	2.770367	-3.53189	0.000658	-0.54066	0.015448	0.02731
Erythroid.cells	CDH1	2.411485	1.38751	3.53172	0.000658	-0.69446	0.016202	0.028383
Erythroid.cells	TRIM10	1.022224	0.349121	3.531031	0.00066	-0.61803	0.016794	0.029226
Erythroid.cells	CYP2E1	-1.29317	4.291137	-3.52828	0.000666	-0.61147	0.014761	0.026329
Erythroid.cells	TMEM50A	0.471351	7.711684	3.523089	0.000678	-0.9985	0.013327	0.024203
Erythroid.cells	STARD10	1.117031	4.596544	3.519602	0.000685	-0.72477	0.014965	0.02657
Erythroid.cells	DNAJB4	0.928834	3.700143	3.518087	0.000689	-0.51745	0.015476	0.027316
Erythroid.cells	CCT7	0.515399	6.427225	3.514841	0.000696	-0.86804	0.014214	0.025503
Erythroid.cells	PFKFB4	1.537992	3.15878	3.513276	0.0007	-0.57177	0.015949	0.027986
Erythroid.cells	CRPPA	1.76106	2.552625	3.507746	0.000713	-0.59907	0.016548	0.028866
Erythroid.cells	AP3M1	0.688102	4.881049	3.504427	0.000721	-0.62146	0.015408	0.02727
Erythroid.cells	SHMT2	0.951608	4.430904	3.498867	0.000734	-0.66133	0.015902	0.027926
Erythroid.cells	ZFP971	1.446784	1.911606	3.496877	0.000739	-0.6183	0.017418	0.030097
Erythroid.cells	S100G	2.352763	0.605317	3.492057	0.00075	-0.66528	0.018472	0.031556
Erythroid.cells	IKZF5	1.004008	3.804869	3.49029	0.000755	-0.58086	0.016589	0.029
Erythroid.cells	PTPA	0.548113	5.833043	3.48941	0.000757	-0.85388	0.015473	0.027451
Erythroid.cells	ENTPD7	-1.046	4.693758	-3.48921	0.000758	-0.58868	0.01609	0.028342
Erythroid.cells	UQCRB	0.486822	8.047525	3.487463	0.000762	-1.24115	0.014396	0.025896
Erythroid.cells	TRIB3	2.095112	1.057747	3.486711	0.000764	-0.65792	0.018306	0.031507
Erythroid.cells	FBXL5	-0.98375	6.173273	-3.48565	0.000766	-0.80432	0.015362	0.027362
Erythroid.cells	GOT1	0.766379	6.081478	3.485208	0.000768	-0.91016	0.01541	0.027443
Erythroid.cells	MEMO1	0.468081	6.363616	3.480753	0.000779	-1.07156	0.015453	0.02754
Erythroid.cells	HAVCR2	1.29645	2.301711	3.477588	0.000787	-0.69674	0.017915	0.031104
Erythroid.cells	GADD45A	0.688898	4.876654	3.475264	0.000793	-1.00423	0.016476	0.029108
Erythroid.cells	AW112010	-1.50364	7.279978	-3.47478	0.000794	-1.22027	0.015174	0.027224
Erythroid.cells	BNIP3L	0.474144	7.263429	3.470339	0.000806	-1.40966	0.015372	0.027541

Erythroid.cells	ODR4	-0.99197	4.042451	-3.4673	0.000814	-0.6496	0.017301	0.030281
Erythroid.cells	4931406G(	1.812361	0.347467	3.464602	0.000821	-0.65195	0.01976	0.033685
Erythroid.cells	CYSTM1	1.965658	3.36709	3.46431	0.000822	-0.6499	0.017806	0.030989
Erythroid.cells	SIN3B	0.514692	6.294475	3.460523	0.000832	-0.9242	0.016269	0.028804
Erythroid.cells	GSTP2	2.044772	0.861498	3.458664	0.000837	-0.8954	0.019687	0.03362
Erythroid.cells	ZWINT	0.66814	5.039001	3.458072	0.000839	-0.81624	0.017049	0.029967
Erythroid.cells	USP14	0.498742	5.793901	3.456212	0.000844	-0.96878	0.016675	0.029491
Erythroid.cells	BCL2L11	-0.91981	7.053365	-3.45564	0.000846	-1.05164	0.015972	0.028505
Erythroid.cells	CDO1	1.091988	3.756999	3.451513	0.000857	-0.77361	0.018088	0.031562
Erythroid.cells	UBXN6	0.93205	4.786672	3.446984	0.00087	-0.75179	0.017681	0.030945
Erythroid.cells	ERP27	-2.10225	1.896721	-3.44319	0.000881	-0.94244	0.019732	0.033798
Erythroid.cells	CYP39A1	1.693019	1.483874	3.438632	0.000894	-0.76493	0.020272	0.034573
Erythroid.cells	TM4SF5	-2.65772	0.261552	-3.43589	0.000902	-0.9914	0.021264	0.035967
Erythroid.cells	RALGDS	-1.69989	3.822267	-3.43563	0.000903	-0.73128	0.018808	0.032578
Erythroid.cells	CD81	0.674724	7.604661	3.433617	0.000908	-1.29423	0.016595	0.029478
Erythroid.cells	HTR1F	-1.95959	1.429334	-3.43064	0.000917	-1.03942	0.020672	0.035215
Erythroid.cells	CNBP	0.352646	8.132464	3.42901	0.000922	-1.38813	0.016476	0.029294
Erythroid.cells	BCL2A1B	-1.07081	5.877283	-3.4254	0.000933	-1.09669	0.017969	0.031457
Erythroid.cells	TSPAN15	1.605723	2.388192	3.423014	0.00094	-0.83018	0.020366	0.034773
Erythroid.cells	RHD	1.305202	1.667344	3.42251	0.000942	-0.78666	0.020878	0.035499
Erythroid.cells	TRMT1	0.860642	4.050324	3.413405	0.00097	-0.80419	0.019769	0.033906
Erythroid.cells	2310010J1	1.156547	3.692212	3.412126	0.000974	-0.81003	0.020026	0.03433
Erythroid.cells	LPIN1	1.594388	2.690777	3.411952	0.000975	-0.79807	0.020729	0.0353
Erythroid.cells	RNPS1	-0.53873	6.167896	-3.41011	0.00098	-1.06357	0.018467	0.032149
Erythroid.cells	GSTK1	1.370943	2.449061	3.404679	0.000998	-0.82596	0.021313	0.036125
Erythroid.cells	SERPINA1E	1.561236	5.030433	3.401655	0.001008	-1.18374	0.019653	0.033847
Erythroid.cells	XCR1	1.518707	0.412393	3.40078	0.00101	-0.83196	0.02306	0.038559
Erythroid.cells	SLC25A47	0.97005	3.963	3.391004	0.001043	-0.95343	0.021016	0.035576
Erythroid.cells	LCN2	2.497282	3.398879	3.389785	0.001047	-0.87257	0.021468	0.036244
Erythroid.cells	PSENEN	0.418611	6.713106	3.388443	0.001051	-1.29029	0.019205	0.033115
Erythroid.cells	WDR25	-1.74868	1.998808	-3.38643	0.001058	-1.06247	0.022683	0.037919
Erythroid.cells	CYFIP2	-0.59221	6.947339	-3.3856	0.001061	-1.32851	0.01915	0.0331
Erythroid.cells	PDHB	0.580489	5.703174	3.38156	0.001075	-1.09973	0.020204	0.034595
Erythroid.cells	PCNP	0.437724	6.359657	3.377999	0.001087	-1.24935	0.019943	0.034241
Erythroid.cells	SASH3	-0.68418	5.4972	-3.37412	0.001101	-1.06097	0.020757	0.035282
Erythroid.cells	PARP10	-1.43343	3.565506	-3.37024	0.001115	-0.90953	0.022374	0.03757
Erythroid.cells	SPIRE1	1.226272	2.681156	3.369598	0.001117	-0.98731	0.023065	0.038558
Erythroid.cells	ZFP385A	0.990408	3.938147	3.36958	0.001117	-0.94706	0.022089	0.037233
Erythroid.cells	MAN2A2	1.097276	4.595669	3.363576	0.001139	-0.94178	0.021972	0.037029
Erythroid.cells	NEPRO	-1.51109	2.709524	-3.36202	0.001145	-0.98071	0.023515	0.039087
Erythroid.cells	CHCHD7	0.706002	4.398636	3.359936	0.001152	-1.04737	0.022293	0.037514
Erythroid.cells	RAB5A	0.444222	6.913368	3.354894	0.001171	-1.37629	0.020744	0.035374
Erythroid.cells	NRN1	1.382296	3.109984	3.348248	0.001196	-1.04	0.024097	0.039856
Erythroid.cells	RAB21	0.460002	6.891793	3.346655	0.001202	-1.38826	0.02123	0.03603
Erythroid.cells	RNF20	0.5424	5.755436	3.343557	0.001214	-1.35802	0.022238	0.037447
Erythroid.cells	CDV3	-0.53118	7.090195	-3.3431	0.001216	-1.40597	0.021244	0.036104
Erythroid.cells	HIST2H2AA	1.622659	2.225914	3.341223	0.001224	-0.99582	0.025209	0.041409

Erythroid.cells	FBXO30	0.736448	5.032199	3.338954	0.001232	-1.20946	0.02301	0.038504
Erythroid.cells	BCL2	1.079553	5.016128	3.335648	0.001245	-1.24797	0.023222	0.038825
Erythroid.cells	NDUFA2	0.44666	7.238115	3.33337	0.001255	-1.53283	0.021635	0.0366
Erythroid.cells	TPP2	-0.44936	7.082191	-3.32758	0.001278	-1.46513	0.022104	0.037108
Erythroid.cells	DCAF17	0.90977	4.054876	3.327103	0.00128	-1.0472	0.024522	0.040401
Erythroid.cells	NGP	1.689983	5.022684	3.326221	0.001283	-1.19555	0.023741	0.039358
Erythroid.cells	SMG6	-0.38955	8.045885	-3.32281	0.001297	-1.64743	0.0216	0.036418
Erythroid.cells	KLHL20	1.395064	3.27084	3.314964	0.00133	-1.06126	0.026034	0.042387
Erythroid.cells	EZH1	1.245509	3.600438	3.313015	0.001339	-1.06411	0.025814	0.042206
Erythroid.cells	PLPPR1	1.884711	1.149205	3.312355	0.001341	-1.15627	0.028089	0.045205
Erythroid.cells	RHOH	-0.77044	6.471792	-3.31227	0.001342	-1.45598	0.023391	0.038982
Erythroid.cells	UBE2I	-0.34149	7.624242	-3.30898	0.001356	-1.61851	0.022681	0.03804
Erythroid.cells	BTG3	0.540324	5.347109	3.305304	0.001372	-1.37503	0.024759	0.040845
Erythroid.cells	GM43774	-1.90236	2.238278	-3.30408	0.001377	-1.21911	0.027608	0.044614
Erythroid.cells	FN1	1.380909	5.580946	3.303002	0.001382	-1.63202	0.024648	0.040719
Erythroid.cells	BECN1	0.461895	5.896307	3.301914	0.001387	-1.51494	0.024421	0.040449
Erythroid.cells	GNG4	-2.13695	1.462633	-3.29959	0.001397	-1.22512	0.028601	0.045857
Erythroid.cells	TCP11L2	0.71189	6.196085	3.296169	0.001412	-1.52435	0.024523	0.0405
Erythroid.cells	TGIF1	-0.78817	6.736671	-3.29449	0.00142	-1.50535	0.024156	0.040055
Erythroid.cells	CHMP3	0.476599	5.884611	3.291178	0.001434	-1.35486	0.025086	0.04132
Erythroid.cells	LEAP2	1.316834	3.060255	3.283892	0.001468	-1.21749	0.028233	0.045441
Erythroid.cells	RHAG	1.678131	-0.3642	3.273106	0.001519	-1.17037	0.032812	0.051161
Erythroid.cells	GBP6	-2.49438	1.984096	-3.27128	0.001527	-1.2171	0.030376	0.048118
Erythroid.cells	MMP14	-1.88462	3.837765	-3.26911	0.001538	-1.21578	0.028634	0.045879
Erythroid.cells	GLT1D1	2.143951	0.611079	3.268577	0.001541	-1.3539	0.032002	0.050252
Erythroid.cells	PIWIL2	-2.42495	0.700548	-3.26641	0.001551	-1.39603	0.032063	0.050334
Erythroid.cells	HSDL2	0.749725	4.684002	3.263896	0.001563	-1.32282	0.028122	0.04529
Erythroid.cells	CALM2	-0.37737	8.565355	-3.26179	0.001574	-1.90178	0.02471	0.04086
Erythroid.cells	SLC19A1	-2.25615	1.065999	-3.26163	0.001575	-1.48566	0.031963	0.050342
Erythroid.cells	BRAT1	-1.67687	2.183649	-3.2592	0.001587	-1.33514	0.030933	0.04906
Erythroid.cells	EIF3K	0.373677	7.871661	3.256645	0.001599	-1.85851	0.02558	0.042143
Erythroid.cells	UBALD2	0.545191	7.028455	3.256167	0.001602	-1.75103	0.026328	0.043144
Erythroid.cells	CD9	0.940018	6.033746	3.255797	0.001604	-1.55458	0.02724	0.044355
Erythroid.cells	FAM214B	0.911833	3.11159	3.249454	0.001636	-1.36439	0.030659	0.048829
Erythroid.cells	FGL1	1.023178	3.523532	3.24894	0.001639	-1.37683	0.030227	0.048293
Erythroid.cells	ABHD4	0.912	2.787772	3.245054	0.001659	-1.26712	0.031305	0.049632
Erythroid.cells	ALAS2	0.776034	4.928547	3.244689	0.001661	-2.15057	0.029082	0.046782
Erythroid.cells	4930503L1	1.33886	2.576564	3.241055	0.00168	-1.27512	0.031836	0.050354
Erythroid.cells	TNFRSF13E	0.900454	4.518479	3.238414	0.001694	-1.31179	0.029972	0.048004
Erythroid.cells	RAB11A	0.39159	7.145444	3.237527	0.001698	-1.77949	0.027418	0.044682
Erythroid.cells	PIP5K1C	-0.7643	5.626433	-3.23665	0.001703	-1.42664	0.028908	0.046651
Erythroid.cells	ZFP691	1.145053	3.075803	3.234393	0.001715	-1.28017	0.031723	0.050285
Erythroid.cells	PCK2	0.990554	3.244845	3.228033	0.001749	-1.34692	0.032114	0.050877
Erythroid.cells	TBXAS1	1.481533	3.468333	3.227327	0.001753	-1.34383	0.031881	0.050607
Erythroid.cells	PANX1	-0.86893	4.208683	-3.21158	0.001842	-1.36545	0.03253	0.051256
Erythroid.cells	UBTD1	-1.08099	4.402162	-3.21155	0.001842	-1.35002	0.032315	0.050981
Erythroid.cells	WAS	-0.6646	5.349391	-3.20911	0.001856	-1.45801	0.031463	0.049873

Erythroid.cells	MTA3	0.624854	6.109959	3.206386	0.001872	-1.73046	0.030833	0.048995
Erythroid.cells	RWDD2A	-2.15202	0.48641	-3.20608	0.001873	-1.56061	0.037416	0.057327
Erythroid.cells	SERPINE1	1.872477	0.805177	3.204718	0.001881	-1.40765	0.037098	0.056973
Erythroid.cells	CCS	0.767358	4.318203	3.203719	0.001887	-1.5065	0.032912	0.051781
Erythroid.cells	C920021L1	1.339404	2.268205	3.203144	0.00189	-1.3825	0.035318	0.054863
Erythroid.cells	XPA	0.827229	4.138354	3.202594	0.001894	-1.45421	0.033116	0.052124
Erythroid.cells	DYNLT3	0.673961	4.662522	3.196846	0.001928	-1.49154	0.033052	0.052015
Erythroid.cells	CCDC93	1.200201	3.72019	3.195945	0.001933	-1.37264	0.034176	0.053496
Erythroid.cells	PI4K2A	0.740559	5.639245	3.193456	0.001948	-1.61821	0.032187	0.050947
Erythroid.cells	SELENOP	0.708502	8.699951	3.191804	0.001958	-2.24747	0.029089	0.046942
Erythroid.cells	TNPO1	-0.48588	7.082293	-3.18967	0.001971	-1.85213	0.03089	0.049303
Erythroid.cells	AGBL1	-1.30276	4.663698	-3.18848	0.001978	-1.59227	0.033592	0.052865
Erythroid.cells	SPTB	1.37702	1.120897	3.188223	0.00198	-1.40401	0.037951	0.058365
Erythroid.cells	VWF	2.314487	1.565048	3.186843	0.001989	-1.41025	0.037468	0.057778
Erythroid.cells	GLS2	1.612503	1.618045	3.185025	0.002	-1.40329	0.037482	0.057839
Erythroid.cells	BTBD7	-0.41051	7.004982	-3.18502	0.002	-1.8497	0.031148	0.049787
Erythroid.cells	ACTA2	2.302766	3.320545	3.180861	0.002026	-1.45122	0.035741	0.055463
Erythroid.cells	CDK2AP1	-0.60445	5.884299	-3.18004	0.002031	-1.66604	0.032755	0.051727
Erythroid.cells	GMIP	-0.61745	5.964525	-3.17925	0.002036	-1.61925	0.032689	0.05169
Erythroid.cells	SARNP	0.350586	7.797226	3.178274	0.002042	-2.06842	0.030743	0.049187
Erythroid.cells	PSEN2	-0.63804	5.03423	-3.17464	0.002065	-1.59415	0.034115	0.053443
Erythroid.cells	BTLA	1.170903	3.948652	3.173732	0.002071	-1.55693	0.03545	0.05514
Erythroid.cells	TRIM36	-1.36728	3.146179	-3.17127	0.002087	-1.43882	0.036601	0.05671
Erythroid.cells	GBP2	-2.62401	4.147241	-3.17096	0.002089	-1.48737	0.035362	0.055158
Erythroid.cells	FBXW4	1.046361	4.272711	3.170668	0.002091	-1.47634	0.035209	0.054981
Erythroid.cells	SRCAP	-0.45295	6.652894	-3.17005	0.002095	-1.82585	0.032456	0.051538
Erythroid.cells	ERMP1	-0.99081	3.633634	-3.16228	0.002145	-1.45966	0.036744	0.057142
Erythroid.cells	TSC22D1	0.79729	4.872446	3.162134	0.002146	-1.64843	0.035212	0.055211
Erythroid.cells	NSMCE2	-0.41663	7.428191	-3.16175	0.002149	-1.95974	0.032261	0.051433
Erythroid.cells	RGS5	2.545387	1.107497	3.158914	0.002168	-1.53218	0.04037	0.061656
Erythroid.cells	APEX2	0.740564	4.215093	3.158081	0.002173	-1.58072	0.036303	0.056631
Erythroid.cells	MFSD1	-0.72925	5.149534	-3.15723	0.002179	-1.60073	0.03519	0.055255
Erythroid.cells	COL5A2	2.232573	1.225753	3.155696	0.002189	-1.57423	0.040401	0.061696
Erythroid.cells	NARF	0.693785	4.893244	3.15093	0.002222	-1.67305	0.035943	0.056184
Erythroid.cells	WIPI1	1.136358	2.277802	3.150744	0.002223	-1.52645	0.039328	0.06043
Erythroid.cells	CSGALNAC	0.851827	4.10884	3.150698	0.002223	-1.51759	0.036925	0.057429
Erythroid.cells	TAF15	0.40035	6.905531	3.150384	0.002225	-1.97016	0.033548	0.053128
Erythroid.cells	PLXNB3	-2.26765	0.164762	-3.14942	0.002232	-1.77514	0.042302	0.064194
Erythroid.cells	CHAC2	0.66183	2.893423	3.149384	0.002232	-1.63689	0.038502	0.059506
Erythroid.cells	TGFB1	-0.41803	8.800362	-3.14036	0.002295	-2.27475	0.032228	0.051302
Erythroid.cells	DGLUCY	1.218495	2.944912	3.140318	0.002295	-1.51575	0.039387	0.060383
Erythroid.cells	FAF2	-0.6681	5.155771	-3.13911	0.002304	-1.67194	0.036547	0.05689
Erythroid.cells	MTHFD2	0.683955	5.486209	3.138853	0.002305	-1.89693	0.036135	0.056398
Erythroid.cells	ALAD	0.885868	4.057214	3.137128	0.002318	-1.67214	0.038011	0.058912
Erythroid.cells	DAPL1	1.966501	-1.31502	3.136785	0.00232	-1.756	0.045748	0.068447
Erythroid.cells	TXLNA	-0.71181	4.941372	-3.13666	0.002321	-1.64331	0.036874	0.057529
Erythroid.cells	PIGQ	0.767972	3.519745	3.136202	0.002324	-1.60757	0.03872	0.059917

Erythroid.cells	UBXN2A	0.602543	5.233503	3.133893	0.002341	-1.73658	0.036704	0.057365
Erythroid.cells	OSER1	-0.54357	6.59749	-3.13327	0.002345	-1.93674	0.035038	0.055301
Erythroid.cells	CPSF2	-0.64023	5.649423	-3.13211	0.002354	-1.7891	0.036264	0.056932
Erythroid.cells	CD5	1.119345	2.693985	3.126417	0.002395	-1.55812	0.04078	0.062538
Erythroid.cells	GCNT1	1.291086	2.451925	3.125552	0.002401	-1.55688	0.041163	0.063072
Erythroid.cells	MGL2	3.097453	-0.18973	3.123693	0.002415	-1.6398	0.045275	0.068223
Erythroid.cells	INPP5J	-1.99672	-1.12022	-3.12042	0.002439	-1.79588	0.047062	0.070366
Erythroid.cells	STX8	-0.40974	6.745581	-3.11928	0.002448	-1.99667	0.035899	0.056664
Erythroid.cells	NUDT4	0.516833	5.949353	3.11914	0.002449	-1.97467	0.036891	0.057934
Erythroid.cells	RABEP2	-0.78343	4.414258	-3.11906	0.002449	-1.65346	0.038887	0.060463
Erythroid.cells	IGFBP1	1.478998	4.147259	3.11888	0.002451	-1.85224	0.039245	0.060914
Erythroid.cells	RNMT	0.651317	4.970618	3.117459	0.002461	-1.74363	0.03817	0.059712
Erythroid.cells	RBM34	-0.70225	4.465573	-3.11744	0.002462	-1.64497	0.038838	0.060557
Erythroid.cells	CDKN2C	1.121291	3.918847	3.117131	0.002464	-1.66357	0.039575	0.061498
Erythroid.cells	TMEM40	2.029834	0.497552	3.115622	0.002475	-1.6769	0.044612	0.067766
Erythroid.cells	DAZAP2	0.366942	7.70346	3.115084	0.002479	-2.30072	0.034826	0.055513
Erythroid.cells	ESRRA	-0.95718	3.981496	-3.11494	0.00248	-1.59908	0.039564	0.061573
Erythroid.cells	2810002D1	-1.54453	1.66458	-3.11283	0.002496	-1.71485	0.043058	0.065959
Erythroid.cells	HINT3	0.797985	4.643869	3.111513	0.002506	-1.69632	0.038953	0.060922
Erythroid.cells	TATDN1	0.831716	3.630234	3.111006	0.00251	-1.63876	0.040334	0.062727
Erythroid.cells	TNFRSF21	0.988726	4.10783	3.110253	0.002516	-1.68484	0.039693	0.062006
Erythroid.cells	CNOT1	-0.44037	7.026328	-3.10966	0.002521	-2.04913	0.035913	0.057188
Erythroid.cells	ARL4A	0.698657	4.453783	3.109315	0.002523	-1.76031	0.039223	0.061434
Erythroid.cells	HACE1	0.801375	4.490352	3.107801	0.002535	-1.71655	0.039211	0.061541
Erythroid.cells	KIZ	1.034199	3.359508	3.107461	0.002538	-1.59786	0.040766	0.063512
Erythroid.cells	GM4013	1.154926	1.984122	3.107452	0.002538	-1.60375	0.042744	0.065989
Erythroid.cells	GABPB2	-0.46519	6.502447	-3.10128	0.002586	-2.01663	0.037232	0.058898
Erythroid.cells	NSMAF	0.861201	4.903953	3.097612	0.002615	-1.73755	0.039707	0.061991
Erythroid.cells	SPARC	1.071436	5.322477	3.096259	0.002626	-1.93638	0.039241	0.061452
Erythroid.cells	SOCS1	-1.18683	5.152296	-3.09417	0.002642	-1.82334	0.039606	0.06195
Erythroid.cells	HS6ST1	0.923729	4.156867	3.094095	0.002643	-1.68169	0.040984	0.063691
Erythroid.cells	TRAFD1	0.887054	5.468862	3.092237	0.002658	-1.91196	0.039293	0.061557
Erythroid.cells	SDE2	-0.69918	6.013145	-3.0921	0.002659	-1.89393	0.038567	0.060632
Erythroid.cells	CETN4	-1.85317	-0.08195	-3.09047	0.002672	-1.78064	0.047733	0.072026
Erythroid.cells	TCF7L1	1.520838	3.097343	3.08951	0.00268	-1.64374	0.042833	0.066036
Erythroid.cells	UBE2D2A	-0.25505	8.209532	-3.08568	0.002711	-2.33864	0.036312	0.057649
Erythroid.cells	EZR	-0.41601	7.933064	-3.08126	0.002748	-2.30341	0.037092	0.058701
Erythroid.cells	COL1A2	1.524938	2.442649	3.079577	0.002762	-1.67202	0.044821	0.068531
Erythroid.cells	COL3A1	1.511813	3.678331	3.079469	0.002763	-1.77035	0.042954	0.066215
Erythroid.cells	ZBTB44	0.515034	5.853417	3.079429	0.002763	-2.09616	0.039862	0.062325
Erythroid.cells	UIMC1	-0.55811	5.853468	-3.07861	0.00277	-1.92977	0.039899	0.062433
Erythroid.cells	ABHD5	0.98503	3.453436	3.077493	0.002779	-1.7029	0.043408	0.066865
Erythroid.cells	TJAP1	-0.77871	4.452059	-3.07356	0.002813	-1.7591	0.042356	0.065574
Erythroid.cells	IL12RB2	-0.9592	3.672057	-3.07323	0.002815	-1.85894	0.043508	0.067061
Erythroid.cells	ATP5H	0.345563	7.946365	3.071158	0.002833	-2.40432	0.037758	0.059832
Erythroid.cells	APOC2	0.869276	4.203003	3.068717	0.002854	-2.04049	0.043175	0.06663
Erythroid.cells	ARHGAP24	-0.68371	6.825889	-3.06689	0.00287	-2.2194	0.039604	0.062043

Erythroid.cells	HPS6	-1.74102	1.22115	-3.06651	0.002873	-1.79289	0.048018	0.072578
Erythroid.cells	EID2B	1.358663	1.703903	3.065058	0.002886	-1.75231	0.0473	0.071789
Erythroid.cells	PNPLA8	0.517651	6.018439	3.064984	0.002886	-2.10557	0.040778	0.063667
Erythroid.cells	ZFAND1	1.068478	2.727292	3.062427	0.002909	-1.71024	0.045944	0.070098
Erythroid.cells	PIK3C2A	-0.55688	6.854141	-3.06075	0.002924	-2.18114	0.040016	0.062626
Erythroid.cells	PRKAR1A	-0.32884	7.625008	-3.05984	0.002932	-2.32696	0.039023	0.061367
Erythroid.cells	DMTN	1.370721	0.637339	3.054625	0.002978	-1.72959	0.050324	0.075369
Erythroid.cells	NQO1	1.525404	0.792254	3.052975	0.002993	-1.73769	0.050229	0.07528
Erythroid.cells	E2F2	0.615954	4.99405	3.051662	0.003005	-2.20762	0.043569	0.067136
Erythroid.cells	EPS15	0.487962	6.183956	3.051016	0.003011	-2.09555	0.041845	0.06504
Erythroid.cells	GPIHBP1	1.091851	4.095185	3.048625	0.003032	-1.84793	0.045211	0.069241
Erythroid.cells	TRAF6	-0.58863	5.683882	-3.04082	0.003104	-2.03801	0.043762	0.067072
Erythroid.cells	LONP1	-0.94199	3.653778	-3.03931	0.003118	-1.78984	0.047065	0.071091
Erythroid.cells	NCAM1	2.302422	1.275146	3.038661	0.003124	-1.78243	0.051108	0.076062
Erythroid.cells	NDUFA3	0.399829	8.000282	3.028507	0.003221	-2.52935	0.041762	0.064288
Erythroid.cells	RALB	0.718348	4.247517	3.026541	0.00324	-1.87661	0.047692	0.071612
Erythroid.cells	HAL	1.570063	2.215151	3.026088	0.003244	-1.81404	0.051148	0.07581
Erythroid.cells	MEF2D	-0.49679	7.288749	-3.02546	0.003251	-2.32675	0.042988	0.065933
Erythroid.cells	MANBAL	-0.69698	4.531107	-3.02459	0.003259	-1.87368	0.047274	0.071288
Erythroid.cells	H1F0	0.776128	6.001224	3.024286	0.003262	-2.17814	0.044949	0.068443
Erythroid.cells	PAQR9	0.843558	4.029832	3.021859	0.003286	-2.08201	0.048374	0.072662
Erythroid.cells	B4GAT1	-1.8436	1.65015	-3.01856	0.003318	-1.93542	0.052949	0.078011
Erythroid.cells	USP4	0.443162	5.660629	3.016607	0.003338	-2.08662	0.046326	0.070004
Erythroid.cells	RSPH3A	-1.47769	2.558451	-3.01505	0.003353	-1.84474	0.051703	0.076518
Erythroid.cells	IDH1	0.62531	4.88393	3.013108	0.003373	-2.08733	0.047936	0.071996
Erythroid.cells	ST18	-2.34741	0.283819	-3.01179	0.003386	-1.904	0.05628	0.08196
Erythroid.cells	ISOC2B	1.430914	2.519105	3.011447	0.003389	-1.84162	0.052103	0.077083
Erythroid.cells	PKIB	1.015393	5.453591	3.009398	0.00341	-2.25044	0.047322	0.07127
Erythroid.cells	PROK2	-2.92482	-0.61756	-3.00882	0.003416	-2.00957	0.058345	0.084466
Erythroid.cells	E2F1	-1.03723	4.409532	-3.00681	0.003437	-1.91416	0.049174	0.073728
Erythroid.cells	KHK	0.812636	4.3658	3.006034	0.003445	-1.98174	0.049248	0.073832
Erythroid.cells	THBS1	1.406159	4.967156	3.005917	0.003446	-2.2841	0.048241	0.072607
Erythroid.cells	GOLGA2	0.969103	3.814242	3.005843	0.003447	-1.85278	0.05019	0.074974
Erythroid.cells	GZMK	-2.3303	-1.47202	-3.00515	0.003454	-2.19956	0.06023	0.08688
Erythroid.cells	ATP5D	0.366149	7.898894	3.004996	0.003455	-2.57406	0.043635	0.066966
Erythroid.cells	TMBIM6	0.396924	9.142023	3.004639	0.003459	-2.77507	0.041825	0.064726
Erythroid.cells	IFI209	-0.80937	5.809713	-3.00092	0.003498	-2.20042	0.047261	0.071539
Erythroid.cells	DUSP19	1.497816	2.048701	3.000854	0.003498	-1.91115	0.053786	0.079432
Erythroid.cells	IL2RG	-0.98444	6.294179	-2.99967	0.003511	-2.31996	0.04658	0.070743
Erythroid.cells	IL6ST	1.061247	4.326252	2.997331	0.003535	-1.95892	0.050065	0.075082
Erythroid.cells	NSMCE1	0.739322	4.829662	2.997141	0.003537	-2.02778	0.049207	0.074061
Erythroid.cells	CPS1	1.169748	4.481272	2.993054	0.00358	-2.23633	0.050336	0.075371
Erythroid.cells	ITSN1	0.836242	5.300914	2.988897	0.003625	-2.15249	0.049476	0.074351
Erythroid.cells	ATP5B	0.391148	8.182512	2.987293	0.003642	-2.69194	0.044981	0.068792
Erythroid.cells	GM37982	1.503097	2.996043	2.986609	0.003649	-1.8977	0.053766	0.079581
Erythroid.cells	PARG	-0.56879	5.560263	-2.98487	0.003668	-2.11588	0.049414	0.074306
Erythroid.cells	KRT10	1.523012	2.100333	2.98287	0.00369	-1.94631	0.05591	0.082115

Erythroid.cells	SMPDL3A	0.879793	5.614644	2.981789	0.003702	-2.2421	0.049633	0.074631
Erythroid.cells	NCK2	0.826222	4.870391	2.981242	0.003708	-2.12265	0.050927	0.076299
Erythroid.cells	PIN4	0.546069	4.949389	2.980128	0.00372	-2.12697	0.050885	0.076282
Erythroid.cells	PCK1	1.197945	4.182878	2.979386	0.003728	-2.21426	0.052284	0.077982
Erythroid.cells	PRPF38B	-0.35714	6.611272	-2.97347	0.003794	-2.35998	0.048838	0.073635
Erythroid.cells	GALNT10	0.791364	4.614762	2.972992	0.0038	-2.0907	0.052298	0.077899
Erythroid.cells	NAAA	0.843521	3.389339	2.972197	0.003809	-2.10061	0.054549	0.080711
Erythroid.cells	CCDC86	-0.71703	5.146452	-2.97213	0.003809	-2.08795	0.051352	0.076858
Erythroid.cells	TNKS2	-0.38558	7.072389	-2.97164	0.003815	-2.45218	0.048074	0.072916
Erythroid.cells	PTPN2	-0.42847	6.833794	-2.97103	0.003822	-2.39874	0.048468	0.073484
Erythroid.cells	GM13708	1.316331	3.088737	2.970464	0.003828	-1.93437	0.055117	0.081654
Erythroid.cells	RBBP8	-0.51289	6.609426	-2.97001	0.003833	-2.3699	0.048841	0.074096
Erythroid.cells	PREX1	-0.61695	6.43388	-2.9697	0.003837	-2.34748	0.049136	0.074509
Erythroid.cells	KCTD12	0.73838	6.383969	2.969521	0.003839	-2.19071	0.04922	0.074635
Erythroid.cells	SPINT2	0.728401	4.563442	2.968261	0.003853	-2.09093	0.052513	0.078668
Erythroid.cells	NEK11	-2.25275	0.082898	-2.96317	0.003912	-2.17436	0.062122	0.089784
Erythroid.cells	TBK1	-0.68311	5.964636	-2.96252	0.003919	-2.22527	0.050759	0.076274
Erythroid.cells	ARID5B	-0.75278	7.244193	-2.96207	0.003924	-2.5424	0.048584	0.073637
Erythroid.cells	LYRM4	0.671982	4.142278	2.95998	0.003949	-2.07935	0.054156	0.080519
Erythroid.cells	EEF1AKMT4	1.130523	2.582886	2.959873	0.00395	-1.96103	0.057143	0.084089
Erythroid.cells	8030462N1	0.409434	6.073398	2.959692	0.003952	-2.31774	0.050684	0.07632
Erythroid.cells	DNAJA1	-0.35542	7.461326	-2.95943	0.003955	-2.56382	0.048334	0.073467
Erythroid.cells	CLDN1	1.74629	1.493829	2.956866	0.003985	-1.96663	0.059697	0.087194
Erythroid.cells	PSEN1	-0.48725	6.297037	-2.95512	0.004006	-2.31575	0.0508	0.076525
Erythroid.cells	ETFBKMT	1.232367	2.261696	2.954137	0.004017	-1.97323	0.058445	0.085836
Erythroid.cells	SERPINB8	1.496662	-0.3114	2.95337	0.004026	-1.99025	0.063881	0.092313
Erythroid.cells	TRIM56	0.865165	3.810038	2.953154	0.004029	-2.01752	0.055419	0.082326
Erythroid.cells	DNAAF2	-1.12898	2.845924	-2.95254	0.004036	-1.97665	0.057313	0.084638
Erythroid.cells	MAT1A	0.99557	4.65605	2.950135	0.004065	-2.37729	0.054162	0.080827
Erythroid.cells	IL6	2.761767	1.106723	2.948605	0.004083	-1.99267	0.061396	0.089451
Erythroid.cells	ELP5	0.692814	4.633093	2.947174	0.0041	-2.14241	0.054523	0.081227
Erythroid.cells	CDKN1B	0.532236	6.470899	2.946755	0.004105	-2.48983	0.051193	0.077175
Erythroid.cells	FCF1	-0.47468	6.136138	-2.94458	0.004132	-2.36749	0.052044	0.07815
Erythroid.cells	HBB-BT	0.881924	8.105747	2.943944	0.004139	-3.43725	0.048681	0.07402
Erythroid.cells	MIR155HG	-1.37775	4.086438	-2.94163	0.004168	-2.11948	0.056127	0.083043
Erythroid.cells	ENTPD6	-1.12319	2.776877	-2.94143	0.00417	-2.00362	0.058714	0.086156
Erythroid.cells	FFAR4	1.50452	0.260828	2.94049	0.004182	-2.00546	0.064128	0.092516
Erythroid.cells	BCLAF3	-0.67233	4.966172	-2.93774	0.004216	-2.19042	0.054901	0.081658
Erythroid.cells	COX4I1	0.323099	9.086822	2.936669	0.004229	-2.94021	0.04775	0.072968
Erythroid.cells	TTC39A	1.364178	2.027911	2.936369	0.004232	-2.02552	0.060822	0.08884
Erythroid.cells	REST	-0.6495	5.400614	-2.93425	0.004259	-2.25409	0.054424	0.081127
Erythroid.cells	GM36445	-1.76244	1.003387	-2.93376	0.004265	-2.17827	0.063323	0.091753
Erythroid.cells	SEPHS2	0.598949	6.39868	2.928358	0.004333	-2.64156	0.053322	0.0797
Erythroid.cells	GNAL	-1.76296	1.371741	-2.92819	0.004335	-2.15327	0.063383	0.091671
Erythroid.cells	CEP162	-1.04698	3.080056	-2.92742	0.004345	-2.0374	0.059812	0.08753
Erythroid.cells	SLC9A3R2	1.351667	2.701144	2.925165	0.004374	-2.04971	0.060917	0.08879
Erythroid.cells	PPP2R5B	1.381198	1.645269	2.924349	0.004384	-2.04729	0.063242	0.091505

Erythroid.cells	APON	1.832786	1.166034	2.922868	0.004403	-2.05621	0.064489	0.092994
Erythroid.cells	ACBD4	1.504807	1.764413	2.922184	0.004412	-2.0772	0.063214	0.091603
Erythroid.cells	AMN1	1.023708	3.42505	2.919653	0.004445	-2.06216	0.060062	0.087961
Erythroid.cells	FOCAD	1.330696	2.908895	2.918951	0.004454	-2.05678	0.061183	0.089376
Erythroid.cells	TRAM2	-0.73981	4.061754	-2.91724	0.004476	-2.15699	0.058892	0.086796
Erythroid.cells	PANK1	1.090659	3.430383	2.916947	0.00448	-2.11789	0.060185	0.088389
Erythroid.cells	TIFA	-0.75079	6.262161	-2.9168	0.004482	-2.52441	0.054609	0.081705
Erythroid.cells	IFITM6	1.642373	3.518134	2.916622	0.004484	-2.12457	0.060003	0.088199
Erythroid.cells	CARS2	-0.79723	3.502896	-2.91565	0.004497	-2.0711	0.060035	0.088355
Erythroid.cells	EIF4EBP1	0.541589	5.454955	2.915631	0.004497	-2.38517	0.056141	0.083682
Erythroid.cells	ARG1	1.277508	3.498182	2.915263	0.004502	-2.24366	0.060045	0.088442
Erythroid.cells	MICOS13	0.470954	5.948201	2.913771	0.004522	-2.48123	0.055368	0.082788
Erythroid.cells	LMNA	0.901401	4.617164	2.910655	0.004563	-2.32669	0.058333	0.086437
Erythroid.cells	PAN3	-0.34022	8.767803	-2.91064	0.004563	-2.89145	0.050612	0.077006
Erythroid.cells	RIOX1	-0.93997	3.521185	-2.90858	0.004591	-2.09088	0.060858	0.08945
Erythroid.cells	GABPB1	-0.59909	5.380129	-2.90682	0.004614	-2.3254	0.057311	0.085151
Erythroid.cells	UBE2Q1	-0.47048	6.334643	-2.90521	0.004636	-2.45425	0.055631	0.083177
Erythroid.cells	SLC7A5	0.565792	6.118367	2.904911	0.00464	-2.73587	0.056044	0.08372
Erythroid.cells	UGP2	0.575952	5.910415	2.904407	0.004647	-2.52972	0.056454	0.084297
Erythroid.cells	EEF1A1	0.313122	10.55641	2.900221	0.004704	-3.23758	0.048712	0.074603
Erythroid.cells	CXCL9	-2.17094	2.080321	-2.89956	0.004713	-2.15504	0.065146	0.09454
Erythroid.cells	TBC1D9B	-0.72272	4.555284	-2.89877	0.004724	-2.19619	0.059885	0.088361
Erythroid.cells	A130014A(	1.536816	1.887317	2.897794	0.004737	-2.14274	0.065747	0.095316
Erythroid.cells	PCOLCE	1.919304	0.911461	2.89622	0.004759	-2.17246	0.068143	0.098195
Erythroid.cells	SUCLG2	0.688192	4.835404	2.896088	0.004761	-2.34697	0.05953	0.088056
Erythroid.cells	SIK3	-0.49663	10.09847	-2.89573	0.004766	-3.13998	0.049737	0.076092
Erythroid.cells	LARGE1	1.016034	5.482959	2.889932	0.004847	-2.30964	0.059128	0.087487
Erythroid.cells	CLP1	-0.73056	4.608479	-2.88832	0.004869	-2.24714	0.06093	0.089845
Erythroid.cells	AP3S1	0.415623	7.096627	2.888311	0.004869	-2.7281	0.05595	0.083832
Erythroid.cells	PRUNE2	1.79737	0.638929	2.888266	0.00487	-2.19284	0.069859	0.100332
Erythroid.cells	GTF2B	-0.51525	6.738906	-2.88819	0.004871	-2.61261	0.056639	0.084671
Erythroid.cells	HSPBP1	0.803416	3.934559	2.88731	0.004884	-2.18894	0.062372	0.091598
Erythroid.cells	P2RY14	1.136162	3.467146	2.886593	0.004894	-2.2052	0.063383	0.092805
Erythroid.cells	RAPSN	-1.59282	0.715904	-2.8864	0.004897	-2.21513	0.069689	0.100211
Erythroid.cells	RAC1	0.309659	7.82414	2.886348	0.004897	-2.85571	0.05459	0.082228
Erythroid.cells	ATP6V1D	0.379118	6.650485	2.883823	0.004933	-2.62892	0.057168	0.085382
Erythroid.cells	GPR183	1.197196	3.488292	2.882789	0.004948	-2.22118	0.063787	0.093315
Erythroid.cells	HABP4	0.956226	3.054352	2.88259	0.004951	-2.16137	0.064747	0.094448
Erythroid.cells	FPR3	-2.26169	-0.4789	-2.88011	0.004987	-2.29119	0.073574	0.104596
Erythroid.cells	ITGA8	1.692204	1.726804	2.878154	0.005015	-2.15345	0.068425	0.098722
Erythroid.cells	KBTBD4	-1.21658	2.78551	-2.878	0.005017	-2.15285	0.065973	0.095868
Erythroid.cells	KAT7	-0.5201	5.68662	-2.87754	0.005024	-2.42973	0.059716	0.088467
Erythroid.cells	PLCB1	0.919395	4.622522	2.876789	0.005035	-2.55237	0.061993	0.091209
Erythroid.cells	PSMF1	0.563984	4.460205	2.875311	0.005056	-2.44535	0.062514	0.09186
Erythroid.cells	ZBTB5	-1.35222	2.463409	-2.87495	0.005062	-2.1671	0.066961	0.09717
Erythroid.cells	AI662270	0.453976	6.090129	2.87437	0.00507	-2.80777	0.059125	0.08793
Erythroid.cells	GM525	1.700452	0.742971	2.874013	0.005075	-2.29172	0.071068	0.102034

Erythroid.cells	DDX39	0.576443	6.372696	2.871645	0.00511	-2.67139	0.058884	0.087546
Erythroid.cells	NGDN	-0.56486	5.424919	-2.87036	0.005129	-2.40598	0.060979	0.089988
Erythroid.cells	MYO7A	-1.14618	3.25231	-2.86756	0.005171	-2.17631	0.066107	0.095922
Erythroid.cells	ATP5MD	0.378248	8.035566	2.86699	0.005179	-2.97221	0.05611	0.084041
Erythroid.cells	HIGD2A	0.388499	6.065754	2.866949	0.00518	-2.61376	0.060018	0.088778
Erythroid.cells	TMCC2	1.007425	1.686886	2.863661	0.005229	-2.38542	0.070208	0.100998
Erythroid.cells	TNS1	1.482466	2.761945	2.863543	0.005231	-2.18486	0.067654	0.098026
Erythroid.cells	ARHGAP42	1.407022	1.752056	2.862545	0.005246	-2.18909	0.070051	0.100942
Erythroid.cells	IL21R	-0.79465	5.150614	-2.8624	0.005249	-2.52667	0.062319	0.091854
Erythroid.cells	SERPINI1	-1.28592	3.178888	-2.86209	0.005253	-2.18929	0.06669	0.097102
Erythroid.cells	RELB	-0.63251	6.088989	-2.86196	0.005255	-2.56576	0.060346	0.089561
Erythroid.cells	ZDHHC5	-0.56325	5.017726	-2.86162	0.00526	-2.35002	0.062604	0.092324
Erythroid.cells	DUS4L	1.456719	1.929292	2.860855	0.005272	-2.21744	0.069624	0.100769
Erythroid.cells	PLA2G12A	0.700717	4.277535	2.860677	0.005275	-2.38533	0.064216	0.094425
Erythroid.cells	PTER	1.224096	2.433342	2.860432	0.005278	-2.20866	0.068425	0.099399
Erythroid.cells	SAC3D1	-1.0358	3.030557	-2.86005	0.005284	-2.19341	0.067031	0.097784
Erythroid.cells	GM31597	-1.27992	2.208627	-2.85827	0.005311	-2.20538	0.069228	0.100267
Erythroid.cells	ADORA2A	-1.18762	3.148747	-2.85732	0.005326	-2.24253	0.067121	0.097841
Erythroid.cells	MAP11	1.327876	2.866952	2.855114	0.00536	-2.20731	0.068124	0.098945
Erythroid.cells	GM26511	-1.5393	2.075738	-2.85334	0.005387	-2.2196	0.070279	0.101425
Erythroid.cells	HERPUD2	0.531906	5.474289	2.852685	0.005397	-2.44982	0.062567	0.09239
Erythroid.cells	NEK7	0.476307	6.476805	2.849103	0.005453	-2.69241	0.061006	0.090331
Erythroid.cells	NPHP1	2.092168	0.652168	2.846487	0.005494	-2.37167	0.07501	0.106811
Erythroid.cells	CLN6	1.0803	3.482895	2.845982	0.005502	-2.24212	0.068044	0.098787
Erythroid.cells	SRRM1	-0.32368	7.865839	-2.8456	0.005509	-2.93201	0.058552	0.087469
Erythroid.cells	BIRC2	-0.5959	5.673482	-2.84369	0.005539	-2.53552	0.063312	0.093335
Erythroid.cells	TAGLN	1.885448	2.304796	2.843375	0.005544	-2.23645	0.071087	0.102561
Erythroid.cells	EEF2	0.404094	9.105144	2.843223	0.005546	-3.18626	0.05631	0.084877
Erythroid.cells	GBA	-0.90744	4.090254	-2.84205	0.005565	-2.27205	0.066973	0.09782
Erythroid.cells	PLOD1	1.236122	3.410314	2.841546	0.005573	-2.25457	0.068558	0.099743
Erythroid.cells	SMPDL3B	-1.83776	1.9506	-2.84112	0.00558	-2.24007	0.072094	0.103909
Erythroid.cells	ZFP704	1.312807	3.792086	2.840334	0.005592	-2.2824	0.067664	0.098884
Erythroid.cells	MARCKSL1	-0.73231	7.792982	-2.84014	0.005596	-2.91613	0.058994	0.088495
Erythroid.cells	PRKCB	-0.40612	8.511819	-2.8397	0.005603	-3.00798	0.057566	0.086815
Erythroid.cells	EPG5	0.818407	4.420072	2.839627	0.005604	-2.31211	0.066218	0.09729
Erythroid.cells	RCAN1	0.993303	3.708543	2.837301	0.005641	-2.26669	0.068147	0.09968
Erythroid.cells	BLOC1S1	0.552062	6.24963	2.837229	0.005642	-2.69889	0.062455	0.092873
Erythroid.cells	OSBPL2	-0.81538	3.979026	-2.83679	0.00565	-2.28508	0.067516	0.098996
Erythroid.cells	KHDRBS1	-0.33557	7.151511	-2.83646	0.005655	-2.80862	0.060557	0.090661
Erythroid.cells	CCL25	-0.97199	4.40296	-2.83605	0.005661	-2.33175	0.066539	0.097942
Erythroid.cells	P2RY1	-1.82535	0.13392	-2.83487	0.005681	-2.33919	0.077255	0.110487
Erythroid.cells	COL4A1	1.306511	3.330895	2.834159	0.005692	-2.29957	0.069249	0.101198
Erythroid.cells	TAT	1.483265	1.826535	2.830513	0.005752	-2.26891	0.07361	0.106205
Erythroid.cells	GM45509	-1.83034	1.640462	-2.82892	0.005778	-2.31746	0.074335	0.107029
Erythroid.cells	GLB1L	1.249873	2.192734	2.827763	0.005797	-2.27652	0.073087	0.10554
Erythroid.cells	PI4K2B	0.701125	3.903982	2.827192	0.005807	-2.44634	0.068935	0.100718
Erythroid.cells	GM10143	1.735402	1.245831	2.824287	0.005855	-2.35153	0.076086	0.108919

Erythroid.cells	STXBP6	1.310662	2.953526	2.822754	0.005881	-2.38622	0.071899	0.10414
Erythroid.cells	IBTK	0.667362	4.642613	2.822662	0.005883	-2.53315	0.067841	0.099364
Erythroid.cells	RFTN2	-1.66051	2.237638	-2.82074	0.005915	-2.30351	0.074013	0.106637
Erythroid.cells	FLII	-0.53458	5.969326	-2.81934	0.005939	-2.63447	0.065286	0.096361
Erythroid.cells	GM16552	-1.5811	1.517	-2.8167	0.005984	-2.35582	0.076496	0.109477
Erythroid.cells	KATNB1	-1.39161	2.346793	-2.81646	0.005988	-2.29465	0.074339	0.107012
Erythroid.cells	IWS1	-0.49859	5.729671	-2.81623	0.005992	-2.60803	0.066176	0.097422
Erythroid.cells	AKT1S1	0.861536	3.589237	2.81573	0.006	-2.33179	0.071243	0.10345
Erythroid.cells	PCNX3	1.002528	3.30596	2.813647	0.006036	-2.30774	0.072284	0.104696
Erythroid.cells	COLQ	2.008847	0.244853	2.811227	0.006078	-2.46979	0.080793	0.114549
Erythroid.cells	COX7C	0.32185	8.585633	2.809688	0.006104	-3.20392	0.060866	0.091084
Erythroid.cells	HECTD4	0.492461	5.548933	2.808079	0.006132	-2.75464	0.067754	0.099284
Erythroid.cells	AAMP	0.392679	5.908148	2.805681	0.006174	-2.68732	0.067305	0.098506
Erythroid.cells	TFAM	-0.71703	4.293459	-2.80451	0.006195	-2.45244	0.071286	0.103197
Erythroid.cells	HSPA12B	2.004843	0.480164	2.804139	0.006202	-2.47159	0.081296	0.114741
Erythroid.cells	LHFP	1.867616	1.237889	2.803115	0.00622	-2.35774	0.079336	0.112636
Erythroid.cells	ALDH3A2	0.831116	3.596521	2.801375	0.00625	-2.36901	0.07342	0.105881
Erythroid.cells	RTL8A	0.870577	3.632481	2.800576	0.006265	-2.45206	0.073363	0.105939
Erythroid.cells	REEP3	0.509225	6.727725	2.800395	0.006268	-2.80916	0.065971	0.097224
Erythroid.cells	DNAJC16	-1.48364	2.30093	-2.79928	0.006288	-2.34204	0.07696	0.110131
Erythroid.cells	GGNBP2	-0.3735	7.268609	-2.7982	0.006307	-2.94273	0.065016	0.095986
Erythroid.cells	CD83	0.976712	5.198788	2.797735	0.006315	-2.81614	0.069803	0.101746
Erythroid.cells	ATF6B	-0.80254	4.456866	-2.79661	0.006336	-2.44098	0.071751	0.104035
Erythroid.cells	GEMIN5	-0.99189	3.385628	-2.79565	0.006353	-2.35545	0.074559	0.107361
Erythroid.cells	CLTB	0.716046	4.396264	2.794093	0.006381	-2.51083	0.07219	0.104715
Erythroid.cells	ACOT1	1.796896	2.604682	2.793973	0.006383	-2.34517	0.076781	0.110084
Erythroid.cells	ARPC1A	0.421346	6.586291	2.792754	0.006405	-2.87737	0.067122	0.098653
Erythroid.cells	CIAO3	-0.99106	3.316721	-2.79206	0.006418	-2.35684	0.075156	0.108123
Erythroid.cells	TMEM170I	0.825523	4.646846	2.790062	0.006454	-2.43825	0.071868	0.104654
Erythroid.cells	SMIM7	0.571653	4.932589	2.789997	0.006456	-2.5822	0.071167	0.103825
Erythroid.cells	HNRNPF	-0.30053	9.158537	-2.7899	0.006457	-3.28481	0.061595	0.092303
Erythroid.cells	TNFSF11	2.535002	0.706959	2.789273	0.006469	-2.39936	0.082315	0.116822
Erythroid.cells	PELI2	1.402622	2.831902	2.789127	0.006472	-2.35746	0.076498	0.110121
Erythroid.cells	ELP1	-0.66542	4.553493	-2.78901	0.006474	-2.43993	0.072099	0.104973
Erythroid.cells	CALHM6	-2.09978	3.048969	-2.78881	0.006477	-2.36504	0.075929	0.109505
Erythroid.cells	ELK1	-1.39538	1.559348	-2.78848	0.006483	-2.41591	0.079929	0.114194
Erythroid.cells	SMARCA4	-0.43356	7.5105	-2.78737	0.006504	-3.07216	0.065283	0.096964
Erythroid.cells	DHX15	-0.39833	6.794688	-2.78487	0.00655	-2.87293	0.067299	0.099338
Erythroid.cells	MED29	0.715466	3.806911	2.782583	0.006593	-2.45125	0.074962	0.108473
Erythroid.cells	CEP41	-1.57009	1.886422	-2.78215	0.006601	-2.41621	0.080096	0.114485
Erythroid.cells	BLVRB	0.582406	7.092023	2.780707	0.006628	-3.27471	0.067179	0.099314
Erythroid.cells	ARHGEF12	0.862597	3.963016	2.778992	0.00666	-2.54381	0.074999	0.10862
Erythroid.cells	GM42962	1.441702	-0.60259	2.778667	0.006666	-2.41053	0.087786	0.123315
Erythroid.cells	SCAMP2	-0.48205	6.357233	-2.77847	0.00667	-2.7408	0.069083	0.101648
Erythroid.cells	HIST1H1C	0.958252	4.106663	2.778115	0.006676	-2.5641	0.074629	0.10827
Erythroid.cells	SMIM5	2.147396	0.166279	2.777424	0.006689	-2.49711	0.085557	0.120952
Erythroid.cells	RAB10	0.412953	7.670572	2.774544	0.006744	-3.12113	0.06657	0.098452

Erythroid.cells	CROT	-0.5991	5.259869	-2.77395	0.006755	-2.82916	0.072335	0.105377
Erythroid.cells	PHF14	-0.45172	6.331947	-2.77355	0.006763	-2.83834	0.069728	0.102346
Erythroid.cells	YWHAE	-0.26932	8.88218	-2.77113	0.006809	-3.29298	0.064278	0.095738
Erythroid.cells	PPNR	-1.91461	1.051959	-2.77017	0.006828	-2.46088	0.084132	0.119253
Erythroid.cells	NCOR1	0.288682	8.098707	2.770159	0.006828	-3.17609	0.066051	0.098019
Erythroid.cells	NDUFA12	0.534603	5.362792	2.769174	0.006847	-2.72754	0.072651	0.106
Erythroid.cells	ESAM	1.481511	2.065322	2.768249	0.006865	-2.4024	0.081499	0.116345
Erythroid.cells	OLA1	-0.46482	5.835409	-2.7675	0.006879	-2.75527	0.071662	0.104891
Erythroid.cells	SLC35B3	-0.61639	4.482615	-2.76672	0.006895	-2.51833	0.075149	0.109004
Erythroid.cells	STK35	-0.89616	3.447827	-2.76508	0.006927	-2.4279	0.078145	0.112544
Erythroid.cells	SYNRG	0.661174	5.053855	2.764194	0.006944	-2.56973	0.073991	0.107832
Erythroid.cells	1110032A(	0.736083	3.592833	2.763853	0.006951	-2.5088	0.077801	0.11238
Erythroid.cells	ZBTB7A	-0.38805	7.04476	-2.76355	0.006956	-2.95999	0.069113	0.102141
Erythroid.cells	RCN3	1.357273	1.932131	2.763299	0.006961	-2.41501	0.082381	0.117783
Erythroid.cells	BIRC6	-0.27356	7.8393	-2.76231	0.006981	-3.13809	0.067375	0.100096
Erythroid.cells	NDUFB2	0.468819	5.815553	2.759965	0.007027	-2.85854	0.072464	0.106358
Erythroid.cells	GM6377	0.999194	3.091942	2.759684	0.007033	-2.53581	0.079573	0.114793
Erythroid.cells	CCDC146	-1.50036	2.867299	-2.75906	0.007045	-2.42106	0.080191	0.11561
Erythroid.cells	TPM4	-0.41632	7.167543	-2.75882	0.00705	-3.05451	0.069188	0.102586
Erythroid.cells	GT(ROSA)2	0.787765	4.594685	2.758352	0.007059	-2.58158	0.075564	0.110283
Erythroid.cells	ABHD15	1.3791	2.977881	2.758229	0.007061	-2.42423	0.079886	0.115358
Erythroid.cells	TCF3	-0.47103	6.601305	-2.75793	0.007067	-2.99977	0.07054	0.104291
Erythroid.cells	ZBTB2	-0.53907	5.709287	-2.7579	0.007068	-2.73918	0.072729	0.106913
Erythroid.cells	LPCAT1	0.774959	4.107714	2.754932	0.007127	-2.55079	0.077399	0.112542
Erythroid.cells	PRM1	-1.99677	-0.44781	-2.75371	0.007152	-2.58021	0.090774	0.127917
Erythroid.cells	1700094D(	1.452179	1.476408	2.753015	0.007166	-2.48536	0.084993	0.121478
Erythroid.cells	PAPLN	-1.93746	0.894565	-2.75271	0.007172	-2.53316	0.086717	0.123518
Erythroid.cells	GM17227	-1.11174	2.746669	-2.75166	0.007193	-2.43755	0.081503	0.117445
Erythroid.cells	OSGIN1	-0.89782	3.869004	-2.75053	0.007216	-2.62499	0.078577	0.113916
Erythroid.cells	ATP1B1	-0.78347	5.798004	-2.74959	0.007235	-2.84152	0.073657	0.108072
Erythroid.cells	ATP5J2	0.409629	7.723844	2.748259	0.007262	-3.21927	0.069114	0.102635
Erythroid.cells	PPP3CB	0.404364	5.980209	2.747861	0.00727	-2.89594	0.073361	0.107835
Erythroid.cells	RAB4A	1.18932	1.578469	2.747519	0.007277	-2.46087	0.085351	0.121959
Erythroid.cells	MLYCD	0.883494	3.124459	2.747252	0.007283	-2.48544	0.080923	0.116872
Erythroid.cells	PLCB4	1.069458	3.876337	2.746824	0.007291	-2.5458	0.078865	0.114528
Erythroid.cells	MTX2	0.554706	4.838218	2.746206	0.007304	-2.7184	0.07635	0.111596
Erythroid.cells	SDHA	0.467035	5.691569	2.74517	0.007325	-2.79485	0.074222	0.109195
Erythroid.cells	NLRP1B	1.306793	1.79452	2.745077	0.007327	-2.46612	0.084869	0.121713
Erythroid.cells	BOLA3	0.463894	5.736487	2.742072	0.007389	-3.05963	0.074654	0.109599
Erythroid.cells	EIF4H	-0.3788	6.535904	-2.74169	0.007397	-2.93567	0.072638	0.107249
Erythroid.cells	GM44751	1.494812	1.310612	2.740074	0.007431	-2.47054	0.087233	0.124369
Erythroid.cells	COX20	0.546608	5.583215	2.739602	0.00744	-2.856	0.075325	0.110516
Erythroid.cells	SAG	-1.38002	3.5306	-2.73644	0.007506	-2.48268	0.08137	0.117636
Erythroid.cells	LRRC1	1.163037	3.264954	2.736127	0.007513	-2.50844	0.082118	0.118571
Erythroid.cells	ITPRIPL1	0.919067	3.28736	2.736067	0.007514	-2.49486	0.082054	0.118497
Erythroid.cells	CLPX	0.493055	5.328169	2.734825	0.00754	-2.83266	0.076681	0.112182
Erythroid.cells	ANK	0.894301	3.446389	2.733548	0.007567	-2.64943	0.082008	0.118358

Erythroid.cells	IQGAP2	0.502213	7.075634	2.732739	0.007585	-3.06237	0.072494	0.107085
Erythroid.cells	DHRS4	0.65608	4.374568	2.732025	0.0076	-2.76915	0.079568	0.115584
Erythroid.cells	GM19684	-1.68342	1.522121	-2.73179	0.007605	-2.49957	0.08778	0.12516
Erythroid.cells	9-Sep	0.689501	5.640387	2.731198	0.007617	-2.75022	0.07623	0.111715
Erythroid.cells	MED11	-0.93677	3.312748	-2.73057	0.007631	-2.50095	0.082633	0.11936
Erythroid.cells	DNAH17	-1.81005	3.275665	-2.73015	0.00764	-2.48755	0.082748	0.119541
Erythroid.cells	RHOD	1.485271	1.328394	2.729222	0.007659	-2.49357	0.088629	0.126342
Erythroid.cells	DNAL1	1.500224	1.64713	2.728553	0.007674	-2.49463	0.08773	0.125396
Erythroid.cells	BTBD3	1.858198	1.337187	2.727643	0.007693	-2.52612	0.088805	0.126625
Erythroid.cells	9230116N1	-1.81129	0.920334	-2.72538	0.007742	-2.56735	0.090566	0.128597
Erythroid.cells	GBP8	-1.29759	3.65151	-2.72338	0.007785	-2.53082	0.082739	0.119651
Erythroid.cells	STK17B	0.418428	8.129238	2.723284	0.007787	-3.2552	0.070971	0.105638
Erythroid.cells	SPICE1	1.301648	1.890422	2.722679	0.007801	-2.50087	0.087969	0.125816
Erythroid.cells	THAP1	-1.10465	2.879636	-2.72216	0.007812	-2.50289	0.085054	0.122473
Erythroid.cells	ELOF1	-0.56119	5.375272	-2.71738	0.007917	-2.8337	0.078982	0.115426
Erythroid.cells	STRN4	0.721692	4.492619	2.717198	0.007921	-2.61369	0.081412	0.118334
Erythroid.cells	OLFML3	1.577753	1.284348	2.716625	0.007933	-2.53844	0.090968	0.129432
Erythroid.cells	IFITM2	0.539438	7.227901	2.716161	0.007944	-3.27198	0.074165	0.109805
Erythroid.cells	KANSL1	-0.37912	8.964116	-2.71589	0.00795	-3.42443	0.069901	0.104649
Erythroid.cells	ALDH1B1	1.216532	1.910302	2.714176	0.007988	-2.51851	0.089358	0.12776
Erythroid.cells	GM32089	-1.74702	-1.09921	-2.71351	0.008002	-2.60528	0.099221	0.139008
Erythroid.cells	TMEM181A	-0.71848	4.58903	-2.71287	0.008017	-2.61962	0.081613	0.118811
Erythroid.cells	GM19951	1.356094	3.23896	2.712155	0.008033	-2.67378	0.085572	0.123498
Erythroid.cells	URAH	1.055763	3.327083	2.711626	0.008044	-2.74128	0.085349	0.123199
Erythroid.cells	BLNK	-0.60635	7.200732	-2.70915	0.0081	-3.22278	0.075166	0.111109
Erythroid.cells	ANAPC2	-0.75584	4.497598	-2.70691	0.008151	-2.65635	0.082876	0.120278
Erythroid.cells	GFOD2	1.302906	2.309358	2.7066	0.008158	-2.53484	0.089359	0.127829
Erythroid.cells	GM20721	0.851148	3.72758	2.70603	0.00817	-2.62172	0.085146	0.122991
Erythroid.cells	PPIG	-0.33015	6.974522	-2.70449	0.008205	-3.12044	0.076418	0.112592
Erythroid.cells	RNF14	0.446359	5.242687	2.703672	0.008224	-2.91458	0.081189	0.118342
Erythroid.cells	IFFO2	-1.12291	3.364648	-2.70241	0.008253	-2.5596	0.086816	0.12479
Erythroid.cells	NAIF1	-1.60267	1.251393	-2.70168	0.00827	-2.59292	0.093468	0.132475
Erythroid.cells	KCTD20	0.598608	4.885027	2.700633	0.008294	-2.75841	0.082632	0.119925
Erythroid.cells	GNPNAT1	0.613144	4.157449	2.698459	0.008344	-2.78459	0.085136	0.122853
Erythroid.cells	SARS	0.417764	5.792679	2.698139	0.008351	-2.97058	0.080488	0.117386
Erythroid.cells	LGMN	0.630686	7.188159	2.697317	0.00837	-3.20317	0.07683	0.113075
Erythroid.cells	APOC1	0.924578	7.949726	2.695999	0.008401	-3.51245	0.075055	0.111014
Erythroid.cells	SEMA4B	-0.73855	5.763505	-2.69357	0.008458	-2.89243	0.081343	0.118417
Erythroid.cells	RAB11B	0.334003	7.147669	2.692729	0.008477	-3.23016	0.077642	0.114129
Erythroid.cells	CDR2	0.728316	2.488604	2.692449	0.008484	-2.8292	0.091109	0.129989
Erythroid.cells	CTNND2	1.440368	1.726999	2.692172	0.00849	-2.58091	0.093533	0.132851
Erythroid.cells	MTFR1L	0.648739	4.250609	2.691717	0.008501	-2.69218	0.085768	0.123896
Erythroid.cells	SERF2	0.263804	9.682679	2.691102	0.008516	-3.64796	0.071281	0.10658
Erythroid.cells	NUAK1	-1.23363	3.219187	-2.69063	0.008527	-2.61447	0.088953	0.127667
Erythroid.cells	CD79B	-0.59629	6.976941	-2.68972	0.008548	-3.29555	0.078308	0.115168
Erythroid.cells	FOXJ2	1.011211	3.829076	2.688827	0.008569	-2.6123	0.087362	0.12588
Erythroid.cells	CFL1	-0.40153	10.01399	-2.68694	0.008614	-3.66527	0.070947	0.106309

Erythroid.cells	PHACTR2	0.73341	6.036575	2.686893	0.008615	-2.91144	0.081255	0.118752
Erythroid.cells	PSMC1	-0.4451	5.889053	-2.68488	0.008663	-2.96992	0.082038	0.119526
Erythroid.cells	D430020J0	-2.22664	0.440007	-2.6836	0.008694	-2.72543	0.099212	0.139395
Erythroid.cells	IQSEC2	-1.62206	2.267987	-2.68302	0.008708	-2.58548	0.093203	0.13266
Erythroid.cells	EAF1	0.821896	4.101569	2.681915	0.008735	-2.69039	0.087649	0.12635
Erythroid.cells	FKBP9	1.914471	1.265181	2.681678	0.00874	-2.61909	0.096646	0.13678
Erythroid.cells	NDUFB4	0.40397	6.598671	2.6806	0.008766	-3.18889	0.080559	0.118129
Erythroid.cells	ANXA3	1.143139	3.542549	2.680471	0.008769	-2.68669	0.089468	0.128622
Erythroid.cells	1810037I1	0.506452	6.482621	2.679778	0.008786	-3.10134	0.080953	0.118662
Erythroid.cells	1700030J2	-2.08169	-0.14389	-2.67941	0.008795	-2.65086	0.101689	0.142775
Erythroid.cells	SRP72	0.35755	6.657086	2.679025	0.008804	-3.1861	0.080476	0.118255
Erythroid.cells	WDR27	-1.76327	0.499373	-2.67595	0.00888	-2.69974	0.100144	0.141268
Erythroid.cells	SCP2	0.564465	7.526193	2.675716	0.008885	-3.45915	0.078657	0.116236
Erythroid.cells	COMMD8	0.571418	5.188265	2.675156	0.008899	-2.85234	0.085211	0.124112
Erythroid.cells	CCL6	0.974646	5.306299	2.674862	0.008906	-2.96511	0.084867	0.123733
Erythroid.cells	QDPR	0.536636	5.245358	2.674505	0.008915	-2.9172	0.085045	0.124044
Erythroid.cells	HDAC1	-0.44662	6.039987	-2.67432	0.008919	-2.98379	0.082759	0.121353
Erythroid.cells	FOLR2	1.555737	3.655517	2.673999	0.008927	-2.67086	0.08982	0.129774
Erythroid.cells	WDR92	1.145178	3.083733	2.673569	0.008938	-2.62004	0.091622	0.13199
Erythroid.cells	VSIG4	1.564977	4.823941	2.672024	0.008976	-2.80619	0.086582	0.126099
Erythroid.cells	IL4RA	-0.81496	4.883706	-2.67147	0.00899	-2.81736	0.08645	0.125936
Erythroid.cells	SPATA13	-0.72315	5.147731	-2.67	0.009026	-2.89883	0.085933	0.125275
Erythroid.cells	GTF2A2	0.453401	5.923594	2.667713	0.009083	-3.0423	0.08412	0.123088
Erythroid.cells	CEP120	-0.55415	5.494784	-2.66586	0.009129	-2.91361	0.085713	0.12492
Erythroid.cells	EEF1B2	0.349948	8.556525	2.665515	0.009138	-3.54651	0.077195	0.114727
Erythroid.cells	KHDC4	-0.45544	6.448578	-2.66344	0.00919	-3.09717	0.083348	0.122135
Erythroid.cells	CBS	1.316011	1.874066	2.662523	0.009214	-2.65074	0.097689	0.13891
Erythroid.cells	ACER2	1.326646	2.516505	2.660529	0.009264	-2.6392	0.095979	0.136972
Erythroid.cells	DDIT3	0.828538	4.008692	2.65913	0.0093	-2.74981	0.091268	0.131698
Erythroid.cells	ID1	1.085618	4.07039	2.659062	0.009302	-2.77682	0.091075	0.131475
Erythroid.cells	SRSF10	-0.35363	6.4448	-2.65878	0.009309	-3.15818	0.08395	0.123123
Erythroid.cells	USP19	0.680501	4.704682	2.658714	0.00931	-2.87581	0.089111	0.129235
Erythroid.cells	GCKR	1.536126	1.581851	2.657695	0.009336	-2.6393	0.099405	0.141085
Erythroid.cells	COX14	0.478901	5.656597	2.657103	0.009352	-3.03077	0.086408	0.126085
Erythroid.cells	A630023P1	2.031189	0.306091	2.656712	0.009362	-2.69642	0.103885	0.146404
Erythroid.cells	TRAK1	-0.37882	7.291138	-2.6566	0.009364	-3.31221	0.081706	0.120562
Erythroid.cells	OAZ2	0.570996	4.485342	2.655672	0.009388	-2.8562	0.090094	0.130568
Erythroid.cells	PALLD	1.015836	3.882236	2.651352	0.0095	-2.77924	0.09292	0.133629
Erythroid.cells	MBD6	0.87957	3.632499	2.651251	0.009503	-2.69969	0.093722	0.134572
Erythroid.cells	POLR3A	1.045394	3.30507	2.650261	0.009529	-2.68689	0.094949	0.136035
Erythroid.cells	GM29170	-1.36311	1.532706	-2.64611	0.009638	-2.69067	0.101983	0.14379
Erythroid.cells	DUSP3	0.508155	5.553941	2.645656	0.009649	-3.07399	0.088829	0.128666
Erythroid.cells	HKDC1	-2.10904	0.37121	-2.64329	0.009712	-2.69718	0.106726	0.1492
Erythroid.cells	NARS	0.43286	6.298026	2.643072	0.009718	-3.20872	0.087037	0.126538
Erythroid.cells	ZBTB18	0.771551	3.894799	2.641303	0.009765	-2.76754	0.094886	0.135669
Erythroid.cells	RTF1	-0.3386	6.81408	-2.63973	0.009807	-3.25966	0.085895	0.125308
Erythroid.cells	LMO4	-0.63892	7.245458	-2.63966	0.009809	-3.32316	0.084638	0.123812

Erythroid.cells	KLHL36	-1.48702	2.076404	-2.63914	0.009823	-2.68291	0.101077	0.143107
Erythroid.cells	GM26549	-1.05742	3.018851	-2.63912	0.009823	-2.68628	0.097848	0.139405
Erythroid.cells	MGARP	-2.3353	-0.49619	-2.63854	0.009839	-2.81052	0.110462	0.153806
Erythroid.cells	KATNAL1	-1.96239	0.973387	-2.63844	0.009842	-2.77536	0.104997	0.147668
Erythroid.cells	RAB2A	-0.24773	8.094947	-2.6384	0.009843	-3.47853	0.082219	0.121122
Erythroid.cells	BPGM	0.486833	4.812429	2.638064	0.009852	-3.57809	0.091995	0.132749
Erythroid.cells	PIAS1	-0.34482	7.42613	-2.63764	0.009863	-3.3616	0.084117	0.123536
Erythroid.cells	PRPF40A	-0.30405	7.709067	-2.63754	0.009866	-3.42164	0.083308	0.122567
Erythroid.cells	SKAP2	-0.41229	7.235487	-2.63545	0.009922	-3.28115	0.084992	0.124585
Erythroid.cells	ZRSR1	1.256174	2.211301	2.635415	0.009923	-2.68611	0.100995	0.143284
Erythroid.cells	SUDS3	0.501572	5.515338	2.634944	0.009936	-3.1253	0.090147	0.130696
Erythroid.cells	GM19325	-1.79359	1.67424	-2.63473	0.009942	-2.70406	0.102883	0.145451
Erythroid.cells	UFC1	0.385336	5.792898	2.633623	0.009972	-3.09608	0.089477	0.12993
Erythroid.cells	SLC38A2	-0.32741	8.351985	-2.63298	0.009989	-3.55952	0.082048	0.121099
Erythroid.cells	AK2	-0.49153	5.995761	-2.63254	0.010001	-3.16437	0.08894	0.129419
Erythroid.cells	FAM118A	1.254465	2.069936	2.632117	0.010013	-2.69433	0.101792	0.144412
Erythroid.cells	ISG20	0.539531	4.233394	2.631544	0.010028	-3.52586	0.094485	0.136045
Erythroid.cells	ABCB1A	-1.11006	2.010725	-2.63153	0.010029	-2.85708	0.102	0.144723
Erythroid.cells	PTGS2OS	-2.2169	-1.09526	-2.62912	0.010095	-2.78727	0.114182	0.158424
Erythroid.cells	TARM1	-1.8417	-0.18149	-2.62812	0.010122	-2.71717	0.11083	0.154602
Erythroid.cells	MRC1	1.424892	5.240936	2.626964	0.010154	-3.01807	0.09215	0.133105
Erythroid.cells	CASTOR1	1.619753	0.626653	2.624482	0.010223	-2.72147	0.108643	0.151821
Erythroid.cells	NTMT1	-0.66517	3.588232	-2.62415	0.010232	-2.76432	0.098099	0.139859
Erythroid.cells	CALM1	-0.29421	9.782329	-2.62295	0.010266	-3.8046	0.079521	0.117912
Erythroid.cells	ATAD1	-0.40528	5.731262	-2.62275	0.010271	-3.0626	0.091311	0.132036
Erythroid.cells	ARIH2	0.389136	7.527213	2.621638	0.010302	-3.45304	0.086047	0.125899
Erythroid.cells	FBXO25	-0.99702	2.127104	-2.62119	0.010315	-2.71614	0.103602	0.146384
Erythroid.cells	FBH1	0.877188	3.425128	2.619614	0.010359	-2.74493	0.099402	0.141565
Erythroid.cells	GBP3	-1.49412	3.228626	-2.61878	0.010383	-2.75717	0.100209	0.142525
Erythroid.cells	ABCB10	0.846082	2.977675	2.618083	0.010402	-2.82181	0.101173	0.143708
Erythroid.cells	GPR171	1.082105	4.027921	2.617408	0.010422	-2.8783	0.097582	0.139696
Erythroid.cells	AKAP12	-0.7307	6.169014	-2.61716	0.010428	-3.36765	0.090669	0.131639
Erythroid.cells	TG	1.132785	1.91376	2.616703	0.010441	-2.73788	0.104952	0.148278
Erythroid.cells	PRKG1	1.295857	3.504872	2.616513	0.010447	-2.83563	0.099354	0.141859
Erythroid.cells	GPC3	1.147368	1.806193	2.616358	0.010451	-2.74989	0.105342	0.148752
Erythroid.cells	CPPED1	0.702444	3.886993	2.613389	0.010536	-2.80768	0.098756	0.141176
Erythroid.cells	COL1A1	1.600347	2.255466	2.612407	0.010564	-2.75347	0.104644	0.147922
Erythroid.cells	RANBP3	-0.65052	4.303701	-2.61171	0.010584	-2.85758	0.0976	0.139866
Erythroid.cells	BRD4	0.228608	8.256086	2.611414	0.010592	-3.65329	0.085247	0.125354
Erythroid.cells	GTF2H1	-0.51671	5.958143	-2.60976	0.01064	-3.12138	0.09254	0.134012
Erythroid.cells	FAM129A	0.669759	5.667894	2.607429	0.010707	-3.11253	0.09397	0.135582
Erythroid.cells	GM11714	-1.50093	1.498822	-2.60676	0.010727	-2.75248	0.108558	0.152397
Erythroid.cells	RTL8B	0.841562	3.297356	2.605927	0.010751	-2.90674	0.102108	0.14509
Erythroid.cells	TBCE	-0.5207	5.119789	-2.6058	0.010755	-3.03129	0.095908	0.137919
Erythroid.cells	CCM2	-0.36246	7.391127	-2.60304	0.010835	-3.51448	0.089313	0.130044
Erythroid.cells	ADPRHL2	-0.81353	3.241714	-2.60234	0.010856	-2.78239	0.103049	0.146085
Erythroid.cells	EXOC8	-1.38281	1.951234	-2.60209	0.010863	-2.76592	0.107734	0.151487

Erythroid.cells	VCAN	1.67756	0.76602	2.600776	0.010902	-2.77342	0.112401	0.156861
Erythroid.cells	H2-OB	0.851615	4.007971	2.600667	0.010905	-3.10839	0.10052	0.143338
Erythroid.cells	NDUFA6	0.398456	6.56293	2.600342	0.010915	-3.4206	0.092084	0.133516
Erythroid.cells	TIMM17B	0.433072	5.161183	2.600158	0.01092	-3.07512	0.096616	0.138829
Erythroid.cells	TNFRSF12A	1.453776	1.479733	2.598228	0.010977	-2.7676	0.110139	0.154195
Erythroid.cells	MRAS	1.804072	0.476226	2.597768	0.010991	-2.79208	0.114056	0.158603
Erythroid.cells	NAA38	0.535689	5.467647	2.595944	0.011045	-3.15049	0.096377	0.138249
Erythroid.cells	IFI47	-1.54886	5.277962	-2.59552	0.011058	-3.04894	0.097006	0.13899
Erythroid.cells	TBX2	2.02273	0.45083	2.595474	0.011059	-2.84551	0.114549	0.158962
Erythroid.cells	NFATC3	-0.38819	7.290076	-2.59258	0.011146	-3.5481	0.091174	0.132115
Erythroid.cells	SNX24	0.969921	4.299252	2.591914	0.011166	-2.98825	0.101104	0.143755
Erythroid.cells	GM42726	0.829466	3.69069	2.590424	0.011211	-2.83443	0.103563	0.146521
Erythroid.cells	CHKA	-0.48317	6.940391	-2.58886	0.011258	-3.41471	0.092867	0.134186
Erythroid.cells	INSIG2	0.590083	4.405764	2.58883	0.011259	-3.07441	0.101297	0.143996
Erythroid.cells	PRDX1	0.35274	9.719927	2.587897	0.011287	-3.99129	0.084588	0.124438
Erythroid.cells	POLR2D	-0.59793	5.184758	-2.58764	0.011295	-3.07769	0.098758	0.141192
Erythroid.cells	GAS5	0.466125	6.391612	2.58713	0.011311	-3.36973	0.094801	0.136653
Erythroid.cells	RAB30	1.195757	2.141089	2.586201	0.011339	-2.83856	0.109816	0.15403
Erythroid.cells	HAO1	0.957877	3.070076	2.585801	0.011351	-2.97696	0.106357	0.150203
Erythroid.cells	VWA5A	0.834424	3.406572	2.585758	0.011353	-2.93447	0.105132	0.148803
Erythroid.cells	HSPA1B	2.467361	4.531616	2.584176	0.011401	-2.95773	0.10139	0.144555
Erythroid.cells	CCDC17	-1.93854	0.893924	-2.58417	0.011401	-2.85116	0.114924	0.159946
Erythroid.cells	GSE1	-0.5148	5.664856	-2.58251	0.011452	-3.18256	0.09787	0.140471
Erythroid.cells	HPCAL1	0.548577	6.117607	2.581668	0.011478	-3.21023	0.096446	0.138818
Erythroid.cells	IQSEC1	-0.48246	6.277452	-2.58152	0.011483	-3.29835	0.095919	0.138216
Erythroid.cells	SLC25A20	0.568855	5.597573	2.580898	0.011502	-3.26633	0.098214	0.141002
Erythroid.cells	RBM25	-0.26843	8.296585	-2.58072	0.011508	-3.67188	0.089556	0.130796
Erythroid.cells	GLDC	1.43468	1.391092	2.579507	0.011545	-2.80441	0.113776	0.158725
Erythroid.cells	TRF	0.808663	9.658999	2.578839	0.011566	-4.07086	0.085776	0.126176
Erythroid.cells	STARD3	0.875403	4.116736	2.577541	0.011606	-2.88604	0.103853	0.147475
Erythroid.cells	ASB7	-1.07311	3.936004	-2.57752	0.011607	-2.85095	0.1045	0.148219
Erythroid.cells	ATP13A3	0.338815	7.315105	2.576817	0.011629	-3.45826	0.093161	0.135018
Erythroid.cells	NMI	-0.73939	5.064624	-2.57536	0.011675	-3.09225	0.100929	0.144144
Erythroid.cells	H2-KE6	0.620282	5.147514	2.574903	0.011689	-3.13766	0.100675	0.143921
Erythroid.cells	PIGYL	0.757348	4.270837	2.574301	0.011708	-2.93591	0.103762	0.147612
Erythroid.cells	ASS1	0.714094	6.535258	2.573834	0.011722	-3.6492	0.096008	0.138628
Erythroid.cells	NGLY1	0.454551	4.849758	2.573673	0.011727	-3.04442	0.101718	0.145307
Erythroid.cells	TMED8	-0.98792	2.954093	-2.57353	0.011732	-2.82587	0.108571	0.153215
Erythroid.cells	MAP4K1	-0.52394	4.801156	-2.57231	0.011771	-3.11208	0.102133	0.145827
Erythroid.cells	NIPAL3	0.961402	3.198166	2.570203	0.011837	-2.88007	0.108433	0.153033
Erythroid.cells	HPD	0.943317	4.805638	2.567808	0.011913	-3.37963	0.103119	0.146927
Erythroid.cells	CCL9	0.895969	2.831707	2.567367	0.011928	-3.04742	0.110363	0.155259
Erythroid.cells	AUP1	0.430789	6.010505	2.567331	0.011929	-3.34659	0.098944	0.142112
Erythroid.cells	LTF	1.493278	2.942911	2.565878	0.011975	-2.91624	0.110272	0.155216
Erythroid.cells	LBHD1	-1.75912	0.16876	-2.56526	0.011995	-2.93504	0.121436	0.167798
Erythroid.cells	ALG12	-1.62303	1.395336	-2.56215	0.012095	-2.85525	0.117272	0.163065
Erythroid.cells	ZFP874B	1.302953	2.178101	2.560636	0.012144	-2.84449	0.114318	0.159951

Erythroid.cells	FCNA	1.937971	4.555419	2.560622	0.012145	-3.01784	0.105337	0.149713
Erythroid.cells	CYB5A	0.614554	7.336341	2.560599	0.012146	-3.76907	0.095764	0.13856
Erythroid.cells	OGFRL1	0.697937	5.082967	2.559016	0.012197	-3.14584	0.103793	0.147853
Erythroid.cells	SMG1	-0.32318	7.499087	-2.55853	0.012213	-3.56711	0.095591	0.138335
Erythroid.cells	KATNBL1	-0.45223	5.963648	-2.55714	0.012258	-3.29568	0.101031	0.144769
Erythroid.cells	TRADD	-0.83281	3.982911	-2.5562	0.012289	-2.94326	0.108269	0.15323
Erythroid.cells	FBXO36	1.79306	1.228268	2.555778	0.012303	-2.87058	0.119047	0.165524
Erythroid.cells	IRGM2	-1.44636	2.731293	-2.5557	0.012306	-2.87226	0.113035	0.158758
Erythroid.cells	RHOQ	-0.67281	5.771108	-2.55534	0.012318	-3.24233	0.101831	0.145899
Erythroid.cells	FBXO42	-0.53111	5.871653	-2.55471	0.012338	-3.28064	0.101562	0.145649
Erythroid.cells	GYG	-0.60241	6.126163	-2.55401	0.012362	-3.35928	0.100714	0.144712
Erythroid.cells	EXOSC3	-0.54863	5.047755	-2.55392	0.012364	-3.14233	0.104508	0.149131
Erythroid.cells	ATP2C1	0.506757	5.85373	2.553495	0.012378	-3.20807	0.101673	0.145926
Erythroid.cells	ICOS	1.094051	2.283057	2.553202	0.012388	-2.97246	0.114953	0.16123
Erythroid.cells	BCL10	-0.41721	6.452929	-2.55288	0.012399	-3.36276	0.099608	0.143587
Erythroid.cells	CHD1	-0.36396	6.90203	-2.55243	0.012414	-3.45104	0.098123	0.141933
Erythroid.cells	ATP5E	0.282587	8.783189	2.551602	0.012441	-3.87415	0.092128	0.134725
Erythroid.cells	GM2A	0.429486	7.22888	2.551338	0.01245	-3.64254	0.097144	0.140681
Erythroid.cells	ABCB4	0.873677	3.391454	2.549701	0.012504	-3.04466	0.1112	0.156867
Erythroid.cells	METTL7A1	0.852973	2.456994	2.54628	0.012619	-2.93139	0.115788	0.161717
Erythroid.cells	TMEM29	0.549586	4.706351	2.544684	0.012673	-3.17945	0.107527	0.152179
Erythroid.cells	A930001A2	-1.89704	-0.71301	-2.54397	0.012697	-2.92493	0.1297	0.177014
Erythroid.cells	HELQ	-1.0786	2.411841	-2.54316	0.012724	-2.87609	0.116441	0.162453
Erythroid.cells	CCL24	2.125242	3.596362	2.54302	0.012729	-2.97424	0.111787	0.157224
Erythroid.cells	H3F3B	-0.27749	9.934295	-2.54292	0.012732	-4.00254	0.090003	0.131806
Erythroid.cells	CD1D1	-0.93842	3.932282	-2.54279	0.012737	-3.04941	0.110502	0.155769
Erythroid.cells	DDRGK1	-0.48625	5.272107	-2.54156	0.012779	-3.2283	0.105786	0.150367
Erythroid.cells	PRPF18	0.578238	4.616004	2.539894	0.012835	-3.15147	0.108582	0.153438
Erythroid.cells	MYLPF	1.508272	2.109786	2.539485	0.012849	-2.88562	0.11839	0.164592
Erythroid.cells	LZTS1	-1.6988	1.417164	-2.53862	0.012879	-2.9088	0.121425	0.167934
Erythroid.cells	DIPK1A	0.742297	5.446127	2.538219	0.012893	-3.20559	0.105728	0.150221
Erythroid.cells	BC147527	-1.65537	1.109158	-2.53752	0.012917	-2.90819	0.12287	0.169626
Erythroid.cells	VILL	-1.40157	1.228935	-2.53709	0.012931	-2.88727	0.122398	0.169113
Erythroid.cells	PTMS	0.477644	6.015415	2.535815	0.012975	-3.50811	0.104002	0.148314
Erythroid.cells	SEC63	-0.3411	7.09953	-2.53577	0.012977	-3.55871	0.100214	0.143904
Erythroid.cells	NOL7	-0.31897	6.906907	-2.53464	0.013016	-3.56125	0.101093	0.144919
Erythroid.cells	MED13	-0.39133	7.722854	-2.53381	0.013045	-3.67876	0.09838	0.14184
Erythroid.cells	FAH	0.951292	3.892683	2.533739	0.013047	-3.24887	0.112179	0.157801
Erythroid.cells	OPHN1	1.277878	2.997504	2.530816	0.013149	-2.94359	0.11649	0.162459
Erythroid.cells	ALKBH3	0.892114	3.490416	2.529431	0.013197	-2.96342	0.114779	0.160581
Erythroid.cells	SRRT	-0.49255	5.528359	-2.52916	0.013207	-3.29699	0.107017	0.151725
Erythroid.cells	LYZ2	1.080395	7.170673	2.529027	0.013211	-3.867	0.101166	0.144944
Erythroid.cells	SLC22A23	1.190667	3.017213	2.527023	0.013282	-3.04796	0.11699	0.163011
Erythroid.cells	DNAJC7	-0.40702	7.510277	-2.52702	0.013282	-3.67074	0.100279	0.143847
Erythroid.cells	DAB2	1.539844	4.009728	2.527006	0.013282	-3.07372	0.113062	0.15857
Erythroid.cells	TPR	-0.34031	7.524789	-2.52403	0.013387	-3.66042	0.100838	0.144379
Erythroid.cells	RIOK3	0.300624	7.578172	2.523909	0.013392	-3.91261	0.100654	0.144187

Erythroid.cells	ELK4	-0.48232	5.882489	-2.52375	0.013397	-3.34442	0.106666	0.151198
Erythroid.cells	ATF1	-0.32678	6.564036	-2.52336	0.013411	-3.49098	0.104225	0.14839
Erythroid.cells	MRPL23	0.368332	6.069484	2.521418	0.01348	-3.41788	0.10632	0.150956
Erythroid.cells	PPP1CC	-0.32524	7.584523	-2.52123	0.013487	-3.69913	0.100951	0.144743
Erythroid.cells	ARHGAP32	1.056474	3.193867	2.520934	0.013498	-2.94284	0.117358	0.163588
Erythroid.cells	ITPA	-0.63761	4.105721	-2.52087	0.0135	-3.05347	0.113733	0.159489
Erythroid.cells	CCL7	2.529179	1.659903	2.520633	0.013508	-2.92981	0.123728	0.170718
Erythroid.cells	GM29264	-1.865	0.505466	-2.51925	0.013558	-3.02146	0.129034	0.176547
Erythroid.cells	TBC1D15	0.436356	6.062519	2.51918	0.01356	-3.40133	0.106574	0.15126
Erythroid.cells	DANCR	1.457644	1.545559	2.518341	0.013591	-2.93238	0.124656	0.171629
Erythroid.cells	HERPUD1	-0.50369	7.853564	-2.51534	0.013699	-3.77595	0.101042	0.144685
Erythroid.cells	KDM4C	-0.48076	6.252217	-2.51522	0.013703	-3.40684	0.106727	0.151281
Erythroid.cells	CEBPA	1.03047	2.906324	2.513727	0.013757	-3.05158	0.120098	0.166452
Erythroid.cells	GID8	0.478191	5.195402	2.513091	0.01378	-3.29846	0.111102	0.156378
Erythroid.cells	DBR1	-0.93974	3.262038	-2.51195	0.013822	-2.97323	0.118983	0.1653
Erythroid.cells	GM50218	1.864454	0.559001	2.511663	0.013833	-2.96502	0.130606	0.178264
Erythroid.cells	UBQLN4	-0.8344	3.216072	-2.5087	0.013941	-2.98321	0.119938	0.166446
Erythroid.cells	IRF3	-0.81904	3.786081	-2.5086	0.013945	-3.02074	0.117608	0.163827
Erythroid.cells	OXA1L	-0.61292	4.658758	-2.50791	0.01397	-3.14407	0.114243	0.160077
Erythroid.cells	TMEM115	-0.75189	3.447731	-2.50725	0.013995	-2.99183	0.119209	0.165768
Erythroid.cells	FCRL5	1.866606	-0.65204	2.506897	0.014008	-3.02077	0.137329	0.185818
Erythroid.cells	MED10	-0.60356	4.886924	-2.50635	0.014028	-3.17771	0.113533	0.159449
Erythroid.cells	OLFM4	1.934758	-0.01496	2.505908	0.014044	-2.95297	0.134466	0.18282
Erythroid.cells	CABP4	-2.16614	-0.22452	-2.50528	0.014067	-3.03669	0.135552	0.183997
Erythroid.cells	HEATR5A	0.652538	5.656048	2.503592	0.01413	-3.30714	0.111104	0.156674
Erythroid.cells	STAT5B	0.483543	6.043742	2.502539	0.014169	-3.44861	0.109775	0.155309
Erythroid.cells	TGFB2	2.64034	0.46572	2.502374	0.014176	-2.99819	0.133007	0.181408
Erythroid.cells	PTTG1	0.374752	5.78	2.502166	0.014183	-3.67114	0.110772	0.156524
Erythroid.cells	CBR1	0.638744	4.350162	2.501216	0.014219	-3.29998	0.116538	0.163072
Erythroid.cells	CUL4A	0.436757	5.002429	2.500616	0.014241	-3.36941	0.114041	0.160305
Erythroid.cells	QSOX1	0.671244	4.311358	2.498705	0.014313	-3.18754	0.117272	0.163639
Erythroid.cells	R3HDM4	0.572116	6.20323	2.498069	0.014337	-3.36521	0.109994	0.155324
Erythroid.cells	KPNA6	-0.71502	3.896998	-2.49572	0.014426	-3.06558	0.119598	0.166248
Erythroid.cells	CTDSPL2	-0.47176	5.892856	-2.49539	0.014438	-3.43713	0.111677	0.157316
Erythroid.cells	ITGAE	1.155544	2.127038	2.495392	0.014438	-3.05161	0.127114	0.174698
Erythroid.cells	ARID3B	0.947202	3.836648	2.492367	0.014554	-3.04174	0.120703	0.167246
Erythroid.cells	ARID3A	-0.65969	5.513727	-2.49141	0.01459	-3.24187	0.114045	0.15971
Erythroid.cells	SYNCRIP	-0.35093	7.375906	-2.49141	0.01459	-3.71661	0.107003	0.151641
Erythroid.cells	PTPN23	-0.98169	3.647447	-2.49025	0.014635	-3.01418	0.121845	0.168499
Erythroid.cells	SLCO4A1	-1.20888	3.403213	-2.49	0.014644	-3.04777	0.122873	0.1697
Erythroid.cells	4933412E1	-1.49458	1.908564	-2.48933	0.01467	-2.98191	0.129489	0.176905
Erythroid.cells	JDP2	0.713027	5.304027	2.488048	0.01472	-3.42696	0.115474	0.16124
Erythroid.cells	IGF2	1.219057	4.193244	2.487833	0.014728	-3.41384	0.119964	0.166333
Erythroid.cells	PXDC1	1.583444	2.191629	2.486909	0.014764	-2.9932	0.128728	0.176087
Erythroid.cells	SLC7A6OS	-0.59939	4.789888	-2.48649	0.01478	-3.19978	0.11775	0.163903
Erythroid.cells	ORMDL3	0.769014	3.803807	2.485701	0.01481	-3.15058	0.121962	0.168639
Erythroid.cells	GSDMC4	-1.49849	0.743558	-2.48501	0.014837	-3.00307	0.135622	0.183784

Erythroid.cells	DOCK5	0.738655	4.189727	2.484812	0.014845	-3.31117	0.120437	0.167032
Erythroid.cells	NIPA1	1.491409	0.13903	2.484239	0.014867	-2.99253	0.138514	0.187081
Erythroid.cells	ENDOG	0.794598	2.943039	2.484101	0.014873	-3.07341	0.125745	0.173072
Erythroid.cells	CENPO	0.989552	2.773597	2.483502	0.014896	-3.01033	0.126576	0.174117
Erythroid.cells	PBLD1	1.294027	1.75083	2.482778	0.014924	-3.03358	0.131167	0.179263
Erythroid.cells	PIR	1.417709	1.198746	2.482482	0.014936	-2.9948	0.13369	0.182114
Erythroid.cells	LECT2	-1.03497	3.632928	-2.48235	0.014941	-3.29737	0.122932	0.170194
Erythroid.cells	MIRT2	-1.66901	-1.00119	-2.48204	0.014953	-3.05298	0.14424	0.19363
Erythroid.cells	EFHC1	-1.86664	-0.05796	-2.4818	0.014963	-3.07862	0.139617	0.188677
Erythroid.cells	TANK	0.572056	6.451404	2.480251	0.015023	-3.5493	0.11196	0.157693
Erythroid.cells	GM31323	-1.62679	0.948728	-2.47646	0.015173	-3.04727	0.136523	0.185009
Erythroid.cells	FTL1-PS1	0.930664	3.902493	2.476097	0.015188	-3.22493	0.123328	0.170492
Erythroid.cells	PNPLA2	0.55933	5.509886	2.474587	0.015248	-3.46952	0.116944	0.163385
Erythroid.cells	PIGN	0.799715	4.40092	2.474426	0.015254	-3.17168	0.121482	0.168539
Erythroid.cells	EMC8	-0.60056	4.721373	-2.47433	0.015258	-3.2396	0.120152	0.167043
Erythroid.cells	TMEM248	0.445783	5.858866	2.474018	0.01527	-3.44738	0.115554	0.161922
Erythroid.cells	SLC39A9	-0.62737	4.172579	-2.47376	0.015281	-3.11678	0.122439	0.169797
Erythroid.cells	ADAMTS1	1.475406	2.937185	2.472476	0.015332	-3.06937	0.128082	0.176102
Erythroid.cells	DDX54	-0.47373	5.820595	-2.47137	0.015376	-3.48318	0.11624	0.162774
Erythroid.cells	NFKBIA	-0.53979	8.130992	-2.47037	0.015416	-3.90306	0.107554	0.152925
Erythroid.cells	PPP1CA	-0.31281	8.233158	-2.47025	0.015421	-3.92659	0.10718	0.152498
Erythroid.cells	MSN	-0.35869	8.454473	-2.46909	0.015468	-3.9518	0.106609	0.151716
Erythroid.cells	TSC2	-0.90118	3.109391	-2.46852	0.015491	-3.05077	0.128039	0.17614
Erythroid.cells	ZBTB16	0.878212	2.808614	2.46848	0.015493	-3.32755	0.129372	0.17762
Erythroid.cells	RASGRP4	1.1601	1.734055	2.46683	0.01556	-3.05572	0.134724	0.183433
Erythroid.cells	GM43696	-1.10069	2.459243	-2.46575	0.015603	-3.03396	0.131659	0.180055
Erythroid.cells	PARP4	-0.55976	5.335625	-2.46464	0.015649	-3.34296	0.119259	0.16652
Erythroid.cells	SLF2	-0.47637	6.314028	-2.4646	0.01565	-3.48868	0.115326	0.162046
Erythroid.cells	SENP7	0.538092	5.356774	2.464405	0.015658	-3.37794	0.119172	0.166459
Erythroid.cells	NDUFC1	0.39146	7.367214	2.464026	0.015674	-3.85863	0.111246	0.15743
Erythroid.cells	FAM104A	0.363267	6.153765	2.463843	0.015681	-3.68168	0.115961	0.162839
Erythroid.cells	GPAT3	-0.55185	5.178486	-2.4636	0.015691	-3.47266	0.119904	0.167408
Erythroid.cells	LRP10	-0.47394	6.125795	-2.4635	0.015695	-3.50761	0.116072	0.163071
Erythroid.cells	RAB28	0.694604	4.828065	2.46313	0.01571	-3.21703	0.121355	0.169084
Erythroid.cells	GM30025	1.459341	1.597885	2.462998	0.015716	-3.03451	0.135628	0.185
Erythroid.cells	EHBP1L1	-0.46117	6.149657	-2.46094	0.0158	-3.52202	0.11646	0.163436
Erythroid.cells	RESF1	-0.49856	6.500817	-2.46079	0.015806	-3.59525	0.115068	0.161847
Erythroid.cells	MFN1	0.831272	3.297935	2.45933	0.015867	-3.09982	0.128829	0.177302
Erythroid.cells	RCC2	-0.40606	6.222377	-2.45873	0.015891	-3.58151	0.116561	0.163536
Erythroid.cells	PADI6	-2.29359	0.058128	-2.45844	0.015903	-3.14329	0.144108	0.19416
Erythroid.cells	CAPN10	-1.10018	2.598593	-2.45807	0.015919	-3.04992	0.132016	0.181002
Erythroid.cells	SNAP47	0.868782	2.473639	2.457976	0.015923	-3.09851	0.132585	0.181633
Erythroid.cells	MAP2K2	0.286855	7.299123	2.457383	0.015947	-3.84601	0.11243	0.158922
Erythroid.cells	BCL3	-0.72491	5.236561	-2.45635	0.01599	-3.35795	0.120886	0.168523
Erythroid.cells	ARHGAP26	0.469947	6.685583	2.455091	0.016043	-3.81543	0.115255	0.16216
Erythroid.cells	ZFP444	0.920656	3.63547	2.454707	0.016059	-3.11778	0.127972	0.176618
Erythroid.cells	EMILIN2	1.189141	4.009199	2.454629	0.016062	-3.32152	0.126338	0.174804

Erythroid.cells	BCKDHB	0.706316	3.929893	2.454384	0.016072	-3.27988	0.126683	0.175258
Erythroid.cells	ROBO1	1.373884	2.137687	2.45394	0.016091	-3.07848	0.134796	0.184362
Erythroid.cells	CCL21A	4.596158	0.049265	2.449059	0.016296	-3.07918	0.146562	0.196548
Erythroid.cells	GNS	-0.48861	6.931874	-2.44886	0.016304	-3.68858	0.115666	0.162318
Erythroid.cells	SLC36A1	-0.87126	2.851189	-2.44845	0.016322	-3.08735	0.133096	0.181931
Erythroid.cells	EFEMP2	1.193738	1.944258	2.447871	0.016346	-3.06154	0.137355	0.186697
Erythroid.cells	CCL17	2.164529	-0.70076	2.447753	0.016351	-3.08895	0.150485	0.200939
Erythroid.cells	PBX1	0.64893	5.465012	2.447154	0.016377	-3.56577	0.121781	0.169339
Erythroid.cells	CAMK2B	-1.07889	3.706454	-2.44626	0.016415	-3.21701	0.129564	0.17805
Erythroid.cells	GCAT	1.042957	2.390839	2.445011	0.016468	-3.1155	0.135902	0.185059
Erythroid.cells	A2ML1	1.056281	2.471948	2.444636	0.016484	-3.21443	0.135548	0.184767
Erythroid.cells	GM29394	-1.79281	0.417159	-2.44429	0.016499	-3.11989	0.145519	0.195707
Erythroid.cells	EIF2AK3	-0.45401	6.399228	-2.44399	0.016511	-3.71169	0.118451	0.165736
Erythroid.cells	TMEM131L	-0.38799	7.26323	-2.44365	0.016526	-3.88936	0.115015	0.161878
Erythroid.cells	ZDHHC16	-0.83887	2.888345	-2.44327	0.016543	-3.09157	0.133673	0.182931
Erythroid.cells	ASB13	0.921672	3.434394	2.442425	0.016579	-3.17267	0.131278	0.180208
Erythroid.cells	UROC1	1.253222	1.760508	2.442387	0.01658	-3.12579	0.139072	0.188797
Erythroid.cells	KMT2E	-0.28974	8.273581	-2.44208	0.016593	-3.97411	0.11122	0.157534
Erythroid.cells	CHSY3	1.278252	1.978293	2.440552	0.016659	-3.17682	0.138473	0.188144
Erythroid.cells	ALKBH8	-0.6694	3.835022	-2.43967	0.016698	-3.21897	0.130075	0.178946
Erythroid.cells	PDE6H	-1.24184	2.093346	-2.43942	0.016708	-3.07765	0.138115	0.187888
Erythroid.cells	ACOT8	0.721955	4.129949	2.438723	0.016738	-3.2105	0.128815	0.177609
Erythroid.cells	SMARCD2	-0.40789	5.611886	-2.43837	0.016754	-3.49681	0.122425	0.170518
Erythroid.cells	HNRNPM	-0.28174	7.703885	-2.43836	0.016754	-3.88962	0.113969	0.160871
Erythroid.cells	TMEM175	0.969411	3.375009	2.43788	0.016775	-3.13286	0.132266	0.181654
Erythroid.cells	SORBS3	1.629448	0.937867	2.437519	0.016791	-3.09705	0.143881	0.194488
Erythroid.cells	USP31	-0.90166	3.715776	-2.43671	0.016826	-3.17434	0.130918	0.180287
Erythroid.cells	PIGF	-0.88584	3.388301	-2.43566	0.016871	-3.17242	0.132657	0.182143
Erythroid.cells	FGFRL1	1.836973	0.299565	2.434583	0.016919	-3.10484	0.147771	0.198719
Erythroid.cells	MRPL30	0.352986	6.172662	2.434528	0.016921	-3.66334	0.120729	0.168768
Erythroid.cells	HIST3H2BA	1.090707	0.997637	2.433703	0.016957	-3.09614	0.144303	0.19517
Erythroid.cells	SLC25A13	0.544818	4.679744	2.433671	0.016959	-3.5551	0.127116	0.17617
Erythroid.cells	TBL1X	-0.38486	7.155308	-2.4335	0.016966	-3.8196	0.116779	0.164451
Erythroid.cells	CCSER1	1.108566	1.697174	2.432948	0.01699	-3.29019	0.140951	0.191608
Erythroid.cells	ERP44	-0.32623	6.074446	-2.43266	0.017003	-3.59211	0.121254	0.169699
Erythroid.cells	FNDC5	1.372455	0.474524	2.427797	0.017218	-3.10616	0.148721	0.199795
Erythroid.cells	ZMYM5	0.439212	5.521459	2.427605	0.017226	-3.54553	0.125001	0.173671
Erythroid.cells	AFP	1.449259	4.371399	2.427324	0.017239	-3.62055	0.130035	0.179354
Erythroid.cells	SCAF11	-0.34232	7.16109	-2.42528	0.01733	-3.84136	0.118633	0.166341
Erythroid.cells	ZFP945	0.880527	3.17689	2.425238	0.017332	-3.16426	0.136011	0.185851
Erythroid.cells	GOLIM4	0.809705	5.140893	2.424846	0.017349	-3.37839	0.127163	0.176063
Erythroid.cells	CHD4	-0.33642	7.633169	-2.42399	0.017388	-3.90399	0.116908	0.164438
Erythroid.cells	SHARPIN	-0.5986	4.530571	-2.42377	0.017397	-3.3206	0.130016	0.179288
Erythroid.cells	SYNE1	0.919369	4.964609	2.423074	0.017429	-3.4113	0.128226	0.177285
Erythroid.cells	1110017D1	-2.10921	0.201816	-2.41999	0.017568	-3.15472	0.152142	0.203203
Erythroid.cells	HDAC9	0.695162	7.563843	2.419409	0.017594	-3.8802	0.118126	0.165619
Erythroid.cells	ISOC1	-0.53021	4.993923	-2.4193	0.017599	-3.48098	0.128991	0.177903

Erythroid.cells	SGSM2	-0.78777	3.786605	-2.41919	0.017604	-3.2441	0.134455	0.183999
Erythroid.cells	UBE2C	0.790115	6.746366	2.418368	0.017641	-4.03646	0.121637	0.169585
Erythroid.cells	VDAC1	-0.41925	6.677223	-2.41793	0.017661	-3.7675	0.12197	0.170004
Erythroid.cells	LOCKD	0.809845	4.472202	2.416226	0.017739	-3.48645	0.132027	0.181196
Erythroid.cells	NIPSNAP1	0.775046	3.134643	2.41583	0.017757	-3.29386	0.138279	0.188069
Erythroid.cells	ERRFI1	0.547469	5.004787	2.413504	0.017863	-3.6739	0.130346	0.179083
Erythroid.cells	G2E3	0.748789	3.938116	2.412578	0.017906	-3.23596	0.135432	0.184831
Erythroid.cells	SETD7	0.664956	4.9704	2.411749	0.017944	-3.35782	0.130832	0.179688
Erythroid.cells	PRF1	-1.40658	0.377462	-2.41163	0.017949	-3.14176	0.153257	0.204171
Erythroid.cells	BC050972	-1.95156	-0.27973	-2.4104	0.018006	-3.17502	0.157151	0.208154
Erythroid.cells	1700109H(	-0.96516	3.650709	-2.4091	0.018066	-3.21256	0.137587	0.186952
Erythroid.cells	FAM76B	-0.46093	5.401156	-2.40875	0.018083	-3.49784	0.129576	0.178181
Erythroid.cells	RAB9	-0.48497	4.887523	-2.40797	0.018119	-3.46139	0.132045	0.180939
Erythroid.cells	5-Mar	0.373902	6.018808	2.407358	0.018147	-3.92637	0.127123	0.175494
Erythroid.cells	4932438H2	1.408198	-0.27879	2.40618	0.018202	-3.15295	0.15826	0.209443
Erythroid.cells	LARP1B	0.490171	5.599705	2.404465	0.018283	-3.64729	0.129727	0.178362
Erythroid.cells	SH3BP4	1.359627	1.369949	2.404123	0.018299	-3.14786	0.150069	0.20072
Erythroid.cells	EMP1	1.5561	1.978152	2.403624	0.018322	-3.16983	0.147031	0.197507
Erythroid.cells	YPEL1	-1.07692	2.4444	-2.40149	0.018422	-3.15533	0.145369	0.195507
Erythroid.cells	NXT1	-0.71152	4.399059	-2.39989	0.018498	-3.34839	0.136351	0.185558
Erythroid.cells	ITM2B	0.345823	10.00633	2.399648	0.01851	-4.40869	0.112578	0.158777
Erythroid.cells	LTV1	-0.76541	4.232009	-2.39821	0.018578	-3.31069	0.137498	0.186861
Erythroid.cells	PTGS1	0.974942	3.986721	2.398032	0.018586	-3.29171	0.138663	0.18816
Erythroid.cells	CLIC5	-1.61875	0.878367	-2.39772	0.018601	-3.15628	0.154346	0.205168
Erythroid.cells	TAPBPL	-0.79594	4.125165	-2.3969	0.01864	-3.31481	0.138057	0.187546
Erythroid.cells	PRKCE	-0.43379	7.233184	-2.39688	0.018641	-3.98834	0.124104	0.172001
Erythroid.cells	MTMR3	0.300952	7.886466	2.396714	0.018649	-4.0849	0.121367	0.168909
Erythroid.cells	PTK2	0.858164	4.164046	2.394072	0.018775	-3.32618	0.138704	0.187964
Erythroid.cells	MFSD7A	-1.50503	-0.44923	-2.39368	0.018794	-3.16379	0.162621	0.213747
Erythroid.cells	1700012D1	1.334038	1.843803	2.393299	0.018813	-3.16426	0.150246	0.200704
Erythroid.cells	NFAM1	0.707781	4.570884	2.393183	0.018818	-3.3806	0.136783	0.186056
Erythroid.cells	CYP8B1	1.075473	1.074088	2.391579	0.018895	-3.22142	0.154725	0.205527
Erythroid.cells	GM20324	1.006447	2.098382	2.391381	0.018905	-3.18073	0.149354	0.199783
Erythroid.cells	BEND4	-1.06791	3.557561	-2.39092	0.018927	-3.25015	0.142032	0.191836
Erythroid.cells	CAMK1D	0.392498	8.628354	2.390678	0.018939	-4.25435	0.119389	0.166582
Erythroid.cells	TRP53I11	-0.66669	4.565294	-2.39036	0.018954	-3.44617	0.137195	0.186573
Erythroid.cells	CD209D	2.233779	-0.32218	2.38999	0.018972	-3.2073	0.162366	0.213857
Erythroid.cells	TMC4	1.16903	1.556285	2.389811	0.018981	-3.17092	0.152172	0.203016
Erythroid.cells	ARMC6	-1.36707	1.405639	-2.38976	0.018984	-3.1798	0.152965	0.203868
Erythroid.cells	TRAP1	0.508487	4.381095	2.388598	0.01904	-3.42419	0.138373	0.187906
Erythroid.cells	INHBA	-1.7591	1.050066	-2.38456	0.019237	-3.20964	0.156689	0.207409
Erythroid.cells	9130230N(	1.999973	0.640779	2.383553	0.019286	-3.22616	0.159208	0.210161
Erythroid.cells	SFSWAP	-0.36971	5.786795	-2.38139	0.019393	-3.63876	0.134002	0.182449
Erythroid.cells	SDC4	-0.61576	6.436865	-2.37757	0.019582	-3.865	0.132234	0.180243
Erythroid.cells	OAS1A	-1.7018	2.355225	-2.37599	0.019661	-3.21095	0.152497	0.202464
Erythroid.cells	SLC25A28	-0.64882	4.806244	-2.37596	0.019663	-3.41552	0.140164	0.189115
Erythroid.cells	COMMD2	-0.52023	4.927522	-2.37579	0.019671	-3.48889	0.139582	0.188516

Erythroid.cells	RBM39	-0.20536	9.586582	-2.37545	0.019688	-4.34795	0.119056	0.165605
Erythroid.cells	TUSC3	0.562094	4.978446	2.374741	0.019724	-3.53659	0.139504	0.188394
Erythroid.cells	GM50012	-1.12728	1.643382	-2.37345	0.019789	-3.20265	0.156872	0.207112
Erythroid.cells	FRMD8OS	1.557637	0.736632	2.372847	0.019819	-3.20474	0.161988	0.212511
Erythroid.cells	UCK2	0.634976	5.87449	2.37253	0.019835	-3.70913	0.135746	0.184312
Erythroid.cells	FGD4	0.915958	4.134435	2.371967	0.019863	-3.45002	0.144201	0.193607
Erythroid.cells	MBNL1	0.3967	8.978349	2.371549	0.019884	-4.26904	0.122217	0.169252
Erythroid.cells	ELAVL1	0.245248	7.604056	2.370433	0.019941	-4.05521	0.128349	0.176093
Erythroid.cells	ANG	0.775628	4.211322	2.369874	0.019969	-3.67966	0.14427	0.193638
Erythroid.cells	BC004004	0.499115	5.147988	2.368826	0.020022	-3.52586	0.139941	0.188903
Erythroid.cells	RDX	0.29177	6.872179	2.368627	0.020033	-3.92129	0.131914	0.180101
Erythroid.cells	SLC9A3R1	-0.41206	6.690174	-2.36772	0.020078	-3.87053	0.132946	0.18131
Erythroid.cells	CHIL3	2.446262	2.447415	2.365618	0.020186	-3.31114	0.15443	0.204552
Erythroid.cells	ALDOB	0.849101	4.949636	2.365546	0.02019	-3.84916	0.141692	0.190792
Erythroid.cells	SVBP	0.507306	5.267752	2.364977	0.020219	-3.6324	0.140252	0.18912
Erythroid.cells	KDM3A	-0.49798	5.567098	-2.3647	0.020233	-3.62501	0.138819	0.187591
Erythroid.cells	E130317F2	-1.50654	0.77534	-2.36411	0.020263	-3.25365	0.163838	0.214518
Erythroid.cells	PYROXD2	1.666632	0.182818	2.363732	0.020283	-3.24587	0.167261	0.218163
Erythroid.cells	LUM	1.798997	0.551186	2.362944	0.020323	-3.22848	0.165358	0.216231
Erythroid.cells	RPE	0.671027	4.554255	2.361918	0.020376	-3.42087	0.144326	0.193668
Erythroid.cells	PIGC	0.863443	2.730331	2.361104	0.020418	-3.27584	0.153878	0.204021
Erythroid.cells	PSMG1	-0.76844	3.325411	-2.36061	0.020444	-3.30011	0.150779	0.200703
Erythroid.cells	FLOT2	0.716323	4.443449	2.360474	0.020451	-3.36505	0.145092	0.194558
Erythroid.cells	CST7	-1.10003	2.120473	-2.36005	0.020473	-3.34369	0.157187	0.207676
Erythroid.cells	E230032D2	-1.55782	1.879678	-2.3597	0.020491	-3.23387	0.158497	0.209189
Erythroid.cells	GZMC	1.970175	1.539474	2.35955	0.020499	-3.39299	0.160368	0.211233
Erythroid.cells	ZFAND3	-0.30791	8.233036	-2.3592	0.020517	-4.16605	0.127452	0.175397
Erythroid.cells	DMAC2L	1.010024	2.257897	2.358983	0.020528	-3.24872	0.156444	0.20717
Erythroid.cells	TNFRSF9	-0.92547	2.460851	-2.35853	0.020552	-3.48459	0.155353	0.206054
Erythroid.cells	PPIL2	-0.47718	5.124237	-2.35834	0.020562	-3.5534	0.141754	0.191334
Erythroid.cells	PDIA5	0.914914	2.273787	2.358153	0.020571	-3.3388	0.156358	0.207129
Erythroid.cells	FOS	0.557191	8.489188	2.357013	0.020631	-4.28653	0.126548	0.174555
Erythroid.cells	SMTNL2	1.913585	0.4226	2.356669	0.020649	-3.25354	0.166939	0.218591
Erythroid.cells	CLEC9A	0.906975	2.519945	2.356664	0.020649	-3.49792	0.155287	0.206214
Erythroid.cells	ARMC3	-1.38026	1.900651	-2.35618	0.020674	-3.2324	0.158665	0.209921
Erythroid.cells	UBAP2L	-0.3043	7.113568	-2.35603	0.020682	-3.94092	0.132653	0.181598
Erythroid.cells	MFSD14B	0.5012	5.841678	2.354843	0.020744	-3.61405	0.138791	0.188434
Erythroid.cells	COQ7	-0.62596	4.79596	-2.35461	0.020756	-3.57299	0.14386	0.194065
Erythroid.cells	BCKDK	-0.6185	4.559456	-2.35442	0.020766	-3.42225	0.145034	0.195412
Erythroid.cells	WEE1	-0.85544	3.947727	-2.35423	0.020777	-3.37773	0.148116	0.198768
Erythroid.cells	CCDC189	1.638093	0.183787	2.35376	0.020801	-3.23971	0.168714	0.220727
Erythroid.cells	ABCC3	1.278456	2.689429	2.352902	0.020846	-3.33481	0.154969	0.206103
Erythroid.cells	NCOA5	-0.75599	4.146435	-2.3526	0.020862	-3.38053	0.147395	0.197955
Erythroid.cells	CECR2	-0.66763	6.605628	-2.35208	0.02089	-4.04067	0.135556	0.185001
Erythroid.cells	SMC3	-0.35604	6.498954	-2.35118	0.020937	-3.89216	0.136138	0.185777
Erythroid.cells	RYK	1.1754	2.042505	2.351166	0.020938	-3.25486	0.158668	0.210308
Erythroid.cells	HOOK2	-0.94709	3.558904	-2.35092	0.020951	-3.34219	0.150593	0.201636

Erythroid.cells	ACSL5	-0.48656	6.00653	-2.3507	0.020962	-3.80106	0.138453	0.1884
Erythroid.cells	GM46224	1.269835	2.389468	2.350035	0.020998	-3.38255	0.156933	0.208561
Erythroid.cells	KLRA4	-2.23184	-1.21319	-2.34974	0.021013	-3.28694	0.177717	0.230579
Erythroid.cells	PTGIS	2.204312	-0.12832	2.349163	0.021044	-3.251	0.171311	0.223904
Erythroid.cells	MAT2A	-0.38077	6.522913	-2.34864	0.021071	-3.82599	0.136346	0.18615
Erythroid.cells	EXOC6	0.46552	6.194009	2.348238	0.021093	-3.74866	0.137891	0.187912
Erythroid.cells	CD44	0.446206	8.587659	2.348012	0.021105	-4.22775	0.127066	0.175862
Erythroid.cells	ITGAM	1.110193	4.619395	2.3478	0.021116	-3.49776	0.145543	0.196465
Erythroid.cells	ACTL6A	-0.47536	5.235041	-2.34741	0.021137	-3.62567	0.142541	0.193203
Erythroid.cells	ATP13A2	-0.6817	5.610722	-2.34684	0.021167	-3.59741	0.140815	0.191192
Erythroid.cells	GGACT	0.678136	3.170013	2.345041	0.021264	-3.37099	0.153573	0.205195
Erythroid.cells	COX7A2	0.303566	8.039242	2.345008	0.021266	-4.2488	0.129963	0.179185
Erythroid.cells	SOD3	1.41688	1.286857	2.344619	0.021286	-3.26162	0.163872	0.216227
Erythroid.cells	RP9	-0.30658	6.834563	-2.34453	0.021291	-3.93793	0.135422	0.185295
Erythroid.cells	RFX2	0.813783	3.630115	2.34421	0.021308	-3.41751	0.15116	0.202645
Erythroid.cells	ASPDH	1.315036	1.294554	2.344035	0.021318	-3.28226	0.163829	0.216305
Erythroid.cells	FAS	-0.83292	3.936884	-2.34348	0.021348	-3.51545	0.149678	0.201098
Erythroid.cells	HSD17B11	-0.59006	4.796644	-2.34295	0.021376	-3.54684	0.145413	0.196458
Erythroid.cells	MACO1	-0.39787	6.27208	-2.34224	0.021414	-3.82647	0.138388	0.18866
Erythroid.cells	SLBP	-0.38865	7.147492	-2.34131	0.021464	-4.05528	0.13452	0.184355
Erythroid.cells	ARHGAP5	0.742311	4.809305	2.341048	0.021479	-3.6278	0.14574	0.19683
Erythroid.cells	SLFN4	-1.84626	0.48225	-2.33892	0.021594	-3.27051	0.169952	0.222642
Erythroid.cells	SLC1A2	-1.18227	2.197177	-2.3386	0.021611	-3.27866	0.160207	0.21237
Erythroid.cells	CDK5RAP1	-1.67378	2.44604	-2.33761	0.021666	-3.28018	0.158999	0.211266
Erythroid.cells	EIF4EBP3	0.998008	2.80817	2.337594	0.021666	-3.32442	0.157027	0.209149
Erythroid.cells	GM38843	1.394683	0.985485	2.337367	0.021679	-3.27152	0.167213	0.220122
Erythroid.cells	POLR2C	0.459421	5.080792	2.336754	0.021712	-3.70139	0.145278	0.196476
Erythroid.cells	IFIT2	-2.13568	2.721315	-2.33666	0.021717	-3.28149	0.157558	0.209811
Erythroid.cells	RC3H1	-0.34938	6.887392	-2.33549	0.021781	-3.96139	0.136866	0.187141
Erythroid.cells	SELP	1.893632	1.452771	2.334765	0.021821	-3.27559	0.165158	0.217894
Erythroid.cells	SPTSSA	0.382708	6.822761	2.333949	0.021866	-4.0036	0.137509	0.187894
Erythroid.cells	GM30541	1.200243	0.952964	2.332827	0.021927	-3.28069	0.168617	0.221619
Erythroid.cells	FAM173A	-0.57778	4.68182	-2.33197	0.021974	-3.54585	0.148441	0.200075
Erythroid.cells	TMEM202	-1.62509	0.104736	-2.33188	0.02198	-3.29812	0.173798	0.22723
Erythroid.cells	GMPPB	1.004922	2.629402	2.331535	0.021999	-3.30196	0.15933	0.211835
Erythroid.cells	COPA	-0.34466	6.661065	-2.3309	0.022034	-3.91321	0.138851	0.18939
Erythroid.cells	GM36198	1.055579	2.976425	2.330201	0.022072	-3.31877	0.157745	0.210021
Erythroid.cells	RIDA	0.822119	4.316478	2.329061	0.022136	-3.77288	0.150965	0.202668
Erythroid.cells	NDUFA1	0.349169	6.729501	2.328595	0.022162	-4.04321	0.139042	0.189642
Erythroid.cells	CGRRF1	0.56826	3.907331	2.328307	0.022178	-3.52976	0.153181	0.20516
Erythroid.cells	2810402E2	-0.99916	2.412627	-2.32734	0.022231	-3.30039	0.161551	0.214143
Erythroid.cells	ERN1	-0.47811	7.053542	-2.32672	0.022266	-4.02445	0.137873	0.188348
Erythroid.cells	MORC3	-0.47567	5.857789	-2.32614	0.022299	-3.7234	0.143727	0.194821
Erythroid.cells	ABHD18	0.913906	3.182254	2.325905	0.022312	-3.38731	0.157565	0.209934
Erythroid.cells	UBE2J2	-0.31887	6.638057	-2.32495	0.022365	-3.96552	0.140175	0.190875
Erythroid.cells	HBB-BS	0.976652	11.91128	2.324212	0.022406	-5.15857	0.117299	0.165012
Erythroid.cells	DDX6	-0.30827	8.314732	-2.32377	0.022432	-4.23674	0.132586	0.182416

Erythroid.cells	HOMER1	-0.61087	6.109837	-2.32322	0.022462	-3.77217	0.14306	0.193974
Erythroid.cells	WDR62	-0.89564	3.526858	-2.3226	0.022497	-3.40683	0.156461	0.208679
Erythroid.cells	IDUA	-1.09824	2.137056	-2.32164	0.022552	-3.29638	0.164418	0.217157
Erythroid.cells	CD8A	-1.41283	1.360615	-2.32097	0.022589	-3.3604	0.169046	0.222017
Erythroid.cells	ACP2	-0.87181	3.54465	-2.3205	0.022615	-3.43953	0.156866	0.209017
Erythroid.cells	CD59A	0.644185	2.984691	2.319372	0.02268	-3.60939	0.160207	0.21268
Erythroid.cells	DHX58OS	-1.72309	0.831273	-2.31924	0.022687	-3.30244	0.172556	0.225801
Erythroid.cells	GALM	1.062844	2.214948	2.318865	0.022708	-3.36695	0.164554	0.217399
Erythroid.cells	USP10	0.5428	4.567936	2.317498	0.022786	-3.69209	0.152168	0.204009
Erythroid.cells	GM10134	-1.56467	0.795093	-2.31623	0.022858	-3.30539	0.173717	0.226877
Erythroid.cells	WRB	-1.4266	1.437969	-2.31583	0.022882	-3.31001	0.169936	0.222994
Erythroid.cells	TRAPPC12	0.626834	4.201517	2.315617	0.022894	-3.47067	0.154506	0.206523
Erythroid.cells	HADHA	-0.3763	5.770414	-2.31497	0.02293	-3.87997	0.146478	0.197874
Erythroid.cells	PRRG2	0.978215	2.322172	2.314872	0.022936	-3.33178	0.164915	0.217837
Erythroid.cells	IL12A	-1.21292	2.215059	-2.31438	0.022964	-3.35469	0.165614	0.21864
Erythroid.cells	SHROOM3	1.606989	0.981918	2.313227	0.023031	-3.31412	0.173126	0.22666
Erythroid.cells	FAM98B	-0.59595	4.474383	-2.31311	0.023038	-3.5505	0.153503	0.205696
Erythroid.cells	SGMS1	0.49037	7.102996	2.31199	0.023102	-4.09817	0.140575	0.191356
Erythroid.cells	HSPA1A	2.10749	3.985226	2.310957	0.023162	-3.47565	0.156733	0.209033
Erythroid.cells	MACROD1	1.065938	2.861084	2.310507	0.023188	-3.37365	0.162988	0.215784
Erythroid.cells	EAR2	1.067301	3.55304	2.309462	0.023248	-3.52034	0.159459	0.211938
Erythroid.cells	ALDOC	-1.58007	0.884667	-2.30879	0.023287	-3.31878	0.174873	0.228378
Erythroid.cells	PLD3	0.680672	4.413779	2.30865	0.023295	-3.63564	0.154854	0.207027
Erythroid.cells	PCSK5	1.516231	0.558557	2.308528	0.023302	-3.31971	0.176852	0.230472
Erythroid.cells	MYL4	-0.91155	4.638008	-2.30675	0.023406	-3.71066	0.154139	0.206307
Erythroid.cells	ARL6IP1	0.331922	7.879749	2.306708	0.023408	-4.24662	0.137945	0.188504
Erythroid.cells	SHKBP1	0.734388	4.068341	2.306435	0.023424	-3.44535	0.157186	0.209683
Erythroid.cells	ZDHHC12	-1.4793	1.360226	-2.30604	0.023447	-3.32224	0.172556	0.226229
Erythroid.cells	CSMD3	1.315917	1.302147	2.305802	0.023461	-3.32267	0.172902	0.22662
Erythroid.cells	TTI2	-0.86223	2.749363	-2.30567	0.023469	-3.36202	0.164486	0.217683
Erythroid.cells	GSTM7	1.12144	1.02465	2.30528	0.023492	-3.32718	0.174618	0.228374
Erythroid.cells	CHURC1	0.394005	5.66012	2.304641	0.023529	-3.83757	0.149007	0.200882
Erythroid.cells	PAH	1.010708	3.508909	2.303819	0.023577	-3.67858	0.160656	0.213576
Erythroid.cells	TPGS2	-0.70547	3.805381	-2.30318	0.023615	-3.47462	0.159174	0.212031
Erythroid.cells	BHLHB9	-1.02076	2.203905	-2.30248	0.023656	-3.34773	0.168378	0.221945
Erythroid.cells	EID2	-1.83413	0.075956	-2.30172	0.023701	-3.36844	0.18141	0.235683
Erythroid.cells	PHTF2	-0.48619	5.998759	-2.30107	0.023739	-3.87132	0.147951	0.199981
Erythroid.cells	MED12L	0.946995	3.141983	2.301039	0.023741	-3.38869	0.163206	0.216534
Erythroid.cells	CDT1	-0.64579	4.370678	-2.30087	0.023751	-3.534	0.156452	0.20926
Erythroid.cells	DGKG	0.902694	2.506906	2.300653	0.023764	-3.50119	0.166816	0.220473
Erythroid.cells	GM14286	-1.72082	0.211974	-2.30041	0.023778	-3.37386	0.18056	0.235072
Erythroid.cells	EED	-0.32472	6.114274	-2.29919	0.02385	-3.89495	0.147715	0.199751
Erythroid.cells	RALA	0.437834	5.871704	2.298772	0.023875	-3.83489	0.149004	0.201254
Erythroid.cells	DCLRE1B	0.74986	2.770832	2.297749	0.023936	-3.42438	0.166071	0.219684
Erythroid.cells	SORBS1	-0.61151	4.747678	-2.29662	0.024004	-3.73221	0.155486	0.20819
Erythroid.cells	PPDPF	0.415307	5.630234	2.295765	0.024055	-3.82758	0.151067	0.203408
Erythroid.cells	ATP5MPL	0.276371	8.484004	2.295399	0.024077	-4.4229	0.13706	0.187955

Erythroid.cells	GM43328	-1.25727	2.238918	-2.29389	0.024167	-3.34698	0.170147	0.224072
Erythroid.cells	SYNPO	1.773847	0.219102	2.293752	0.024175	-3.36081	0.182428	0.237007
Erythroid.cells	ANXA5	0.454482	6.137389	2.293707	0.024178	-4.00441	0.14881	0.201035
Erythroid.cells	LMO1	1.15461	1.523387	2.29349	0.024191	-3.40895	0.174398	0.228654
Erythroid.cells	DUSP12	0.774631	3.283921	2.292916	0.024225	-3.44812	0.164229	0.217851
Erythroid.cells	CENPV	0.9247	3.40694	2.292714	0.024238	-3.53695	0.163535	0.217139
Erythroid.cells	ZFP212	-0.68197	3.640876	-2.29224	0.024266	-3.45543	0.162307	0.215897
Erythroid.cells	ST6GAL1	0.758881	5.862113	2.29114	0.024332	-3.90703	0.150698	0.203288
Erythroid.cells	MRPS33	-0.32892	6.714474	-2.28966	0.024422	-4.05086	0.146803	0.198922
Erythroid.cells	POLR3G	-1.24147	2.101279	-2.28802	0.024522	-3.36276	0.172588	0.226674
Erythroid.cells	TMEM134	0.412909	6.132006	2.287661	0.024543	-3.93708	0.150258	0.202691
Erythroid.cells	GPR89	-0.72728	3.742407	-2.28752	0.024552	-3.52078	0.163104	0.21664
Erythroid.cells	SLC25A43	-1.67264	-0.21871	-2.28609	0.024639	-3.379	0.187255	0.242136
Erythroid.cells	SPRTN	-0.85044	3.419835	-2.28597	0.024647	-3.44641	0.165172	0.218852
Erythroid.cells	ABHD10	-0.65883	3.649409	-2.28596	0.024647	-3.47729	0.163872	0.217458
Erythroid.cells	GNE	-0.58162	4.467546	-2.28561	0.024668	-3.61154	0.159326	0.212664
Erythroid.cells	AA467197	-2.00827	0.801131	-2.28533	0.024685	-3.36169	0.180776	0.235547
Erythroid.cells	NONO	-0.33589	6.614635	-2.28519	0.024694	-4.0088	0.148017	0.200392
Erythroid.cells	COMT	0.454797	5.36738	2.284975	0.024707	-3.86238	0.15448	0.207541
Erythroid.cells	SLMAP	-0.41277	6.393027	-2.28479	0.024718	-3.94848	0.149144	0.201751
Erythroid.cells	CEP19	1.149128	2.707827	2.283853	0.024776	-3.39491	0.169521	0.223996
Erythroid.cells	CCAR2	0.854009	3.432111	2.283664	0.024788	-3.43124	0.165345	0.219587
Erythroid.cells	CCAR1	-0.29234	6.820278	-2.2829	0.024834	-4.08544	0.147377	0.200139
Erythroid.cells	PPP2R1A	-0.4515	6.00443	-2.28264	0.02485	-3.87518	0.15155	0.204821
Erythroid.cells	LRCH4	-0.72156	4.260162	-2.28209	0.024884	-3.53256	0.160997	0.215118
Erythroid.cells	ZFP35	-0.96324	2.543067	-2.28186	0.024898	-3.38718	0.170799	0.225679
Erythroid.cells	NFIA	0.567317	5.904	2.281524	0.024919	-3.98963	0.152175	0.205628
Erythroid.cells	RACK1	0.239116	8.637453	2.281311	0.024932	-4.42292	0.138606	0.19064
Erythroid.cells	ZFP445	-0.51878	4.93802	-2.28103	0.02495	-3.66112	0.15731	0.211362
Erythroid.cells	ALG1	-0.89819	3.312271	-2.28052	0.024981	-3.44795	0.166452	0.22133
Erythroid.cells	1700061G1	-1.56354	1.070717	-2.27987	0.025022	-3.37571	0.179996	0.235759
Erythroid.cells	5430401H0	1.213533	-0.47304	2.279239	0.02506	-3.43614	0.19002	0.246346
Erythroid.cells	VIPR1	1.484173	0.964939	2.278475	0.025108	-3.37084	0.180878	0.237037
Erythroid.cells	SERPINA1D	0.872565	5.558333	2.27839	0.025113	-4.15885	0.154428	0.208603
Erythroid.cells	DPH6	0.679466	4.47347	2.278312	0.025118	-3.61421	0.160287	0.215
Erythroid.cells	GM17354	-1.26656	1.393239	-2.27754	0.025166	-3.37218	0.178447	0.234416
Erythroid.cells	POP1	-0.9381	2.708556	-2.27716	0.02519	-3.41321	0.170586	0.226129
Erythroid.cells	FNDC9	-0.72683	2.564671	-2.27626	0.025246	-3.46143	0.171705	0.227192
Erythroid.cells	TOM1L1	1.314462	0.714609	2.275612	0.025286	-3.38263	0.183192	0.239386
Erythroid.cells	ERCC4	-0.97545	2.949475	-2.27501	0.025323	-3.43705	0.169744	0.225025
Erythroid.cells	5430427O1	0.981942	3.846602	2.274614	0.025348	-3.45481	0.164639	0.219563
Erythroid.cells	SLC38A9	-0.58203	5.16902	-2.2741	0.025381	-3.74145	0.157351	0.211694
Erythroid.cells	FPR1	-1.9699	2.992339	-2.27403	0.025385	-3.43496	0.169578	0.224916
Erythroid.cells	GM33104	1.354876	0.440079	2.272332	0.025492	-3.40156	0.185836	0.241928
Erythroid.cells	MAN1C1	0.849794	4.292733	2.27115	0.025566	-3.5274	0.163064	0.217606
Erythroid.cells	TBC1D13	0.892209	3.495587	2.270494	0.025607	-3.45781	0.167596	0.222595
Erythroid.cells	HSD17B2	-0.96535	2.446494	-2.27032	0.025618	-3.49938	0.173762	0.229188

Erythroid.cells	PACS2	0.61728	4.036916	2.270254	0.025623	-3.58015	0.164504	0.219281
Erythroid.cells	CAPZB	-0.2458	8.722615	-2.26991	0.025644	-4.41645	0.140127	0.192562
Erythroid.cells	METTL14	-0.91519	3.09472	-2.26989	0.025645	-3.44272	0.169925	0.225219
Erythroid.cells	GTF2A1	0.528285	5.65525	2.269445	0.025674	-3.81165	0.155612	0.209894
Erythroid.cells	TMEM68	-0.70455	3.807022	-2.26941	0.025676	-3.50519	0.16581	0.220976
Erythroid.cells	FBN1	1.535999	0.898383	2.269095	0.025696	-3.38928	0.183292	0.239731
Erythroid.cells	RBM41	-0.71092	3.907296	-2.26848	0.025735	-3.54435	0.165239	0.220561
Erythroid.cells	CCND1	0.879972	4.140959	2.268126	0.025757	-3.64234	0.163916	0.219259
Erythroid.cells	3830406C1	-0.55274	4.453206	-2.26781	0.025778	-3.66944	0.162167	0.217448
Erythroid.cells	MYZAP	-0.87242	3.284402	-2.26772	0.025783	-3.52064	0.168819	0.224642
Erythroid.cells	VWCE	1.72263	0.865529	2.26765	0.025788	-3.41081	0.1835	0.240289
Erythroid.cells	IL1RL1	1.864753	-0.28284	2.267493	0.025798	-3.40838	0.190921	0.248088
Erythroid.cells	IL33	1.730354	0.25962	2.267328	0.025808	-3.41057	0.187378	0.244382
Erythroid.cells	GPD1L	-0.52866	5.296236	-2.26636	0.02587	-3.8532	0.15779	0.212709
Erythroid.cells	EDA	1.688871	1.445596	2.265972	0.025894	-3.40243	0.180149	0.236894
Erythroid.cells	FAM241A	0.569826	5.59878	2.265905	0.025899	-3.83741	0.156161	0.211056
Erythroid.cells	RPH3AL	1.128103	1.299189	2.265431	0.025929	-3.41667	0.181157	0.238031
Erythroid.cells	POLE3	-0.69744	4.133596	-2.26447	0.02599	-3.60645	0.164589	0.220255
Erythroid.cells	YIPF2	1.117769	1.926928	2.262827	0.026096	-3.40645	0.178189	0.234598
Erythroid.cells	LRRC40	0.799341	3.249731	2.261763	0.026164	-3.49811	0.170332	0.226346
Erythroid.cells	OAS1G	-1.78392	-0.24881	-2.26158	0.026176	-3.40837	0.192177	0.249503
Erythroid.cells	GM26916	-1.40591	0.934105	-2.26149	0.026181	-3.40968	0.184487	0.241442
Erythroid.cells	FEZ2	0.649127	3.693286	2.261493	0.026181	-3.54957	0.167751	0.22361
Erythroid.cells	PPFIA4	0.947381	2.735956	2.261336	0.026192	-3.49727	0.173372	0.229713
Erythroid.cells	GM36199	1.443486	-0.13106	2.260343	0.026256	-3.40383	0.191743	0.248935
Erythroid.cells	SUGCT	0.97931	2.835527	2.259511	0.026309	-3.48528	0.17326	0.229641
Erythroid.cells	BVHT	-1.82779	-0.0458	-2.25915	0.026333	-3.41837	0.191366	0.248877
Erythroid.cells	UAP1L1	-0.80874	3.734573	-2.25897	0.026345	-3.49747	0.16798	0.224072
Erythroid.cells	COX8A	0.254746	9.008145	2.258912	0.026348	-4.56107	0.140238	0.193488
Erythroid.cells	CLEC10A	1.56677	0.930777	2.258014	0.026406	-3.41125	0.185313	0.242505
Erythroid.cells	GATAD1	0.402023	5.768843	2.257615	0.026432	-3.88492	0.156947	0.212112
Erythroid.cells	ZBTB4	0.800395	3.655496	2.257096	0.026466	-3.51762	0.168785	0.2251
Erythroid.cells	SPSB2	-0.98995	2.383882	-2.25698	0.026473	-3.41998	0.176341	0.23322
Erythroid.cells	WDR18	0.533187	4.82244	2.256651	0.026495	-3.73702	0.162149	0.217919
Erythroid.cells	BCL2A1D	-0.87438	4.122114	-2.25644	0.026509	-3.86811	0.166098	0.222209
Erythroid.cells	NMB	-1.15937	1.642171	-2.25622	0.026523	-3.41778	0.180909	0.238117
Erythroid.cells	EMC3	-0.42299	5.490353	-2.25601	0.026536	-3.85474	0.158475	0.213949
Erythroid.cells	MRPL13	0.494538	4.834872	2.254218	0.026654	-3.8007	0.16259	0.218386
Erythroid.cells	PLGRKT	-0.51248	5.796951	-2.25374	0.026685	-3.88857	0.157312	0.212747
Erythroid.cells	PREX2	1.273725	2.374714	2.25365	0.026691	-3.46378	0.176952	0.234006
Erythroid.cells	LY6C1	1.966773	-0.32417	2.253564	0.026697	-3.4198	0.194223	0.252225
Erythroid.cells	TAB1	0.906818	3.004453	2.253449	0.026704	-3.49235	0.173154	0.229943
Erythroid.cells	CDK14	0.808848	5.455475	2.251948	0.026803	-4.0935	0.159235	0.215153
Erythroid.cells	NAPA	0.339921	5.92916	2.251943	0.026803	-4.01027	0.15667	0.212334
Erythroid.cells	HMG20B	0.461221	5.119833	2.251832	0.02681	-3.75778	0.161079	0.217173
Erythroid.cells	PKN2	0.315131	6.774243	2.251791	0.026813	-4.29051	0.152202	0.207397
Erythroid.cells	GSR	0.539616	6.441975	2.251748	0.026816	-4.07938	0.153942	0.209324

Erythroid.cells	CD34	1.351492	0.953495	2.251733	0.026817	-3.53889	0.185925	0.24386
Erythroid.cells	PCGF3	-0.60653	3.998741	-2.24993	0.026935	-3.6126	0.16804	0.224561
Erythroid.cells	USP7	-0.34042	6.215443	-2.24961	0.026957	-4.00211	0.155756	0.211149
Erythroid.cells	ITPR2	-0.38016	6.912603	-2.24856	0.027026	-4.16426	0.152284	0.207371
Erythroid.cells	HMGXB4	-0.64072	4.148587	-2.24842	0.027035	-3.61219	0.167424	0.223958
Erythroid.cells	PURA	-0.39399	5.343404	-2.24831	0.027043	-3.84758	0.160693	0.216673
Erythroid.cells	IL6RA	0.657842	5.504339	2.247341	0.027107	-3.8542	0.159972	0.215908
Erythroid.cells	MAP2K3	0.51936	6.01457	2.247086	0.027124	-4.02891	0.157198	0.212902
Erythroid.cells	FCHSD2	0.588888	6.823892	2.246911	0.027135	-4.16752	0.152903	0.208204
Erythroid.cells	EXOC1	-0.7041	4.095264	-2.24687	0.027138	-3.58806	0.167902	0.224637
Erythroid.cells	L3MBTL1	-1.73982	0.787342	-2.24597	0.027198	-3.44185	0.188292	0.246447
Erythroid.cells	KANSL3	-0.49413	4.995066	-2.24592	0.027201	-3.78381	0.162893	0.219232
Erythroid.cells	CHFR	-0.40572	5.641975	-2.24585	0.027206	-3.91488	0.159318	0.215334
Erythroid.cells	DBI	0.37385	7.067278	2.245424	0.027234	-4.37951	0.151796	0.207116
Erythroid.cells	NSUN4	0.845265	3.189091	2.24511	0.027255	-3.51239	0.173428	0.2308
Erythroid.cells	D030028A(	0.916842	2.795422	2.24439	0.027303	-3.54078	0.175914	0.233505
Erythroid.cells	ZRANB2	0.385559	5.45391	2.244287	0.02731	-3.94683	0.16055	0.216885
Erythroid.cells	ZFP672	0.580748	4.004158	2.244073	0.027325	-3.65507	0.168746	0.225818
Erythroid.cells	ZFP790	1.065886	2.226902	2.243423	0.027368	-3.44853	0.179463	0.237494
Erythroid.cells	GM48512	-1.42885	1.068935	-2.24341	0.027369	-3.43348	0.186776	0.245263
Erythroid.cells	TBC1D8	0.637961	5.179428	2.242988	0.027397	-3.87481	0.162198	0.218921
Erythroid.cells	KDM2A	-0.25583	7.465818	-2.24161	0.02749	-4.26059	0.150326	0.205787
Erythroid.cells	TMEM234	0.255595	7.040364	2.241555	0.027493	-4.26736	0.152527	0.208239
Erythroid.cells	THUMPD3	-0.6513	4.262161	-2.24018	0.027586	-3.64143	0.168217	0.225417
Erythroid.cells	BLOC1S5	1.201397	1.955013	2.239958	0.027601	-3.44293	0.182125	0.24039
Erythroid.cells	8-Mar	0.61837	3.141103	2.238443	0.027703	-3.77815	0.175372	0.23296
Erythroid.cells	CTSB	0.461112	8.190873	2.236876	0.02781	-4.53783	0.147929	0.202901
Erythroid.cells	SPINDOC	-0.58722	4.427162	-2.23655	0.027832	-3.68727	0.168273	0.225419
Erythroid.cells	PXMP4	0.668158	3.831766	2.236354	0.027845	-3.66962	0.171752	0.229222
Erythroid.cells	GM16023	1.101069	1.834877	2.23624	0.027853	-3.452	0.183982	0.242336
Erythroid.cells	HBS1L	-0.39615	5.694465	-2.23552	0.027902	-3.97168	0.161294	0.217796
Erythroid.cells	PMM2	-0.4752	4.561806	-2.23471	0.027957	-3.74421	0.167917	0.224973
Erythroid.cells	RMDN3	-0.67938	3.398108	-2.23376	0.028022	-3.56521	0.175068	0.232821
Erythroid.cells	AK5	-1.68861	0.348906	-2.23254	0.028105	-3.45735	0.194896	0.253912
Erythroid.cells	ABRACL	-0.39751	7.550325	-2.23216	0.028131	-4.29991	0.152162	0.207913
Erythroid.cells	UFD1	0.41576	5.129038	2.232124	0.028134	-3.88335	0.165312	0.222411
Erythroid.cells	GARS	-0.45828	5.511566	-2.23046	0.028248	-3.91394	0.16372	0.22052
Erythroid.cells	ZFP296	-0.98124	2.857765	-2.2289	0.028356	-3.57468	0.179622	0.237874
Erythroid.cells	NIPBL	-0.26703	8.615732	-2.22867	0.028372	-4.50809	0.147456	0.202716
Erythroid.cells	GM15336	1.082111	1.682133	2.228651	0.028373	-3.47566	0.18705	0.245862
Erythroid.cells	SLC36A4	1.013273	3.21113	2.228266	0.0284	-3.53937	0.17745	0.23572
Erythroid.cells	DGAT1	-0.58312	7.095267	-2.22825	0.028401	-4.40477	0.155308	0.211563
Erythroid.cells	NUCKS1	0.450197	6.576295	2.228103	0.028411	-4.14456	0.158089	0.214641
Erythroid.cells	NMRK1	0.745439	3.751312	2.228097	0.028411	-3.61217	0.174181	0.232203
Erythroid.cells	VCAM1	1.377842	4.334733	2.227701	0.028439	-3.71606	0.170782	0.228583
Erythroid.cells	D330050I1	-1.43548	1.118758	-2.22439	0.028669	-3.46545	0.192133	0.250902
Erythroid.cells	EIF4G2	0.252522	7.71796	2.223989	0.028697	-4.39685	0.153165	0.208894

Erythroid.cells	SLC24A3	1.666885	1.280307	2.223919	0.028702	-3.47402	0.191065	0.249878
Erythroid.cells	ZFAND5	0.291177	7.203792	2.223816	0.028709	-4.29429	0.155878	0.211932
Erythroid.cells	CCDC88B	-0.73411	3.695402	-2.22314	0.028756	-3.63744	0.17599	0.233849
Erythroid.cells	GUSB	0.47277	5.468903	2.222831	0.028778	-3.89377	0.165612	0.22263
Erythroid.cells	SLC2A6	-1.2832	1.781297	-2.2221	0.028829	-3.48614	0.18818	0.247012
Erythroid.cells	VPS51	0.76669	3.730024	2.221965	0.028838	-3.59236	0.175962	0.234013
Erythroid.cells	UBE2J1	-0.34859	6.282597	-2.22132	0.028884	-4.0875	0.161357	0.218163
Erythroid.cells	SMARCA5	-0.28026	7.448521	-2.22087	0.028915	-4.34413	0.155124	0.211328
Erythroid.cells	XRCC2	-1.43336	0.802718	-2.21991	0.028983	-3.47474	0.195261	0.254546
Erythroid.cells	FRS2	-0.38013	5.84525	-2.21945	0.029015	-3.99985	0.164238	0.22131
Erythroid.cells	EBNA1BP2	-0.71566	4.017915	-2.21896	0.02905	-3.65867	0.174918	0.232994
Erythroid.cells	ZFP952	1.025619	2.251647	2.218845	0.029058	-3.49493	0.185885	0.244747
Erythroid.cells	FOSB	0.638668	6.636378	2.21795	0.029121	-4.16523	0.160142	0.216898
Erythroid.cells	KCTD14	1.073944	1.055743	2.215645	0.029284	-3.51596	0.194988	0.253717
Erythroid.cells	ZC3H12A	-0.63607	5.128189	-2.21469	0.029352	-3.80902	0.169683	0.226834
Erythroid.cells	PPM1A	0.301366	6.125745	2.214652	0.029355	-4.10511	0.163978	0.22062
Erythroid.cells	PARVB	1.029301	2.578711	2.213409	0.029444	-3.56547	0.185612	0.244005
Erythroid.cells	CBR2	2.414204	-1.3198	2.213086	0.029467	-3.53682	0.212343	0.271937
Erythroid.cells	HAT1	-0.46803	6.105162	-2.2128	0.029487	-4.03874	0.164429	0.221223
Erythroid.cells	MYO1C	-0.41597	5.436164	-2.21269	0.029495	-3.93673	0.168243	0.225391
Erythroid.cells	TPCN2	-1.10277	2.597655	-2.21219	0.02953	-3.50542	0.185491	0.244018
Erythroid.cells	NOL11	-0.64892	4.89374	-2.21197	0.029547	-3.79799	0.171404	0.228976
Erythroid.cells	ERP29	-0.29241	7.289905	-2.21187	0.029553	-4.32748	0.157898	0.21421
Erythroid.cells	CCDC28B	-0.8525	3.504556	-2.21183	0.029557	-3.59387	0.179787	0.238014
Erythroid.cells	FBXL12	-0.64926	4.348122	-2.21003	0.029685	-3.7103	0.175301	0.233255
Erythroid.cells	GM43560	-1.35558	0.578606	-2.20966	0.029712	-3.50825	0.199635	0.259111
Erythroid.cells	GGA3	-0.72533	3.302395	-2.20949	0.029724	-3.57836	0.18174	0.240238
Erythroid.cells	SHC4	-1.63624	0.470109	-2.20841	0.029802	-3.50486	0.200778	0.260105
Erythroid.cells	PRKAR2A	0.721416	5.222556	2.208189	0.029818	-4.03135	0.170468	0.227978
Erythroid.cells	GSDMD	-0.80469	4.268046	-2.2078	0.029846	-3.71457	0.176207	0.234176
Erythroid.cells	GTF2E1	1.046437	2.399479	2.206447	0.029944	-3.51682	0.188418	0.247142
Erythroid.cells	FOXO4	0.830367	2.699869	2.20538	0.030022	-3.61375	0.186848	0.245475
Erythroid.cells	IL1F9	-1.57782	-0.11555	-2.20356	0.030154	-3.49797	0.206623	0.266234
Erythroid.cells	SOX12	1.549255	0.585357	2.203379	0.030167	-3.50911	0.201683	0.261173
Erythroid.cells	ZFP764	1.371391	1.074799	2.203152	0.030184	-3.49922	0.198305	0.257661
Erythroid.cells	CCDC12	0.306178	6.939585	2.202956	0.030198	-4.23704	0.162099	0.218885
Erythroid.cells	CCDC166	1.588501	0.615517	2.202254	0.030249	-3.5133	0.201695	0.261336
Erythroid.cells	CASP3	-0.58272	4.814976	-2.20104	0.030338	-3.7647	0.17494	0.232818
Erythroid.cells	RBBP6	-0.30061	7.269431	-2.19991	0.030421	-4.32494	0.161176	0.217657
Erythroid.cells	PPP4R3A	-0.26121	6.59174	-2.19853	0.030523	-4.21624	0.165409	0.222226
Erythroid.cells	VTI1A	-0.34516	7.233705	-2.19802	0.03056	-4.30571	0.161921	0.21839
Erythroid.cells	RNF168	-0.73542	4.161361	-2.19763	0.030589	-3.69976	0.179913	0.238021
Erythroid.cells	PLEKHM1	-0.54489	4.771883	-2.19737	0.030608	-3.81468	0.176179	0.234122
Erythroid.cells	HSPH1	0.946578	4.050366	2.197256	0.030617	-3.75052	0.180601	0.238902
Erythroid.cells	IVNS1ABP	0.43369	6.027762	2.196226	0.030693	-4.05927	0.16907	0.226412
Erythroid.cells	FAU	0.182231	11.4506	2.195721	0.03073	-5.04087	0.140652	0.194732
Erythroid.cells	4921511C1	1.016304	2.59393	2.195329	0.030759	-3.55474	0.190396	0.249171

Erythroid.cells	CCDC130	-1.23202	2.056293	-2.19516	0.030772	-3.52488	0.193957	0.252957
Erythroid.cells	HDHD2	-0.67141	3.552765	-2.19468	0.030807	-3.64021	0.184297	0.242719
Erythroid.cells	SIPA1L2	-0.65058	5.103799	-2.19448	0.030822	-3.86454	0.174731	0.232482
Erythroid.cells	TMEM176A	0.581062	4.388779	2.192976	0.030934	-3.98056	0.179618	0.237615
Erythroid.cells	SGTA	-0.53362	4.959007	-2.19218	0.030994	-3.87954	0.176369	0.234115
Erythroid.cells	MMADHC	0.511094	4.299266	2.190869	0.031091	-3.79896	0.18086	0.238941
Erythroid.cells	TSPAN14	-0.38692	6.835826	-2.19027	0.031137	-4.24273	0.165795	0.222846
Erythroid.cells	GM12353	1.408744	1.278675	2.189765	0.031174	-3.52105	0.200691	0.260265
Erythroid.cells	SBNO1	-0.32076	7.359347	-2.18966	0.031182	-4.33633	0.162854	0.21975
Erythroid.cells	ZBTB37	-0.89119	2.901016	-2.18952	0.031193	-3.58613	0.189774	0.248787
Erythroid.cells	PLEKHA2	-0.37023	7.455833	-2.1895	0.031194	-4.38924	0.162318	0.219161
Erythroid.cells	KPNA1	0.335874	6.778024	2.189434	0.031199	-4.33618	0.166123	0.223336
Erythroid.cells	C1QC	1.02747	6.384084	2.188515	0.031268	-4.18939	0.168584	0.226101
Erythroid.cells	MYBPC2	-1.06944	2.760882	-2.18801	0.031306	-3.61362	0.190927	0.250144
Erythroid.cells	PRKAB1	0.764812	3.654114	2.18796	0.03131	-3.68536	0.185144	0.244004
Erythroid.cells	EPB41L3	1.597815	2.334313	2.187949	0.031311	-3.56383	0.193754	0.25313
Erythroid.cells	ALG14	0.777386	3.562595	2.187284	0.031361	-3.66833	0.185917	0.244827
Erythroid.cells	DDX24	-0.34431	6.717234	-2.1864	0.031427	-4.24664	0.167097	0.224279
Erythroid.cells	NOL12	-0.5388	4.012955	-2.18573	0.031478	-3.72184	0.183528	0.242016
Erythroid.cells	BLMH	-0.36752	5.648784	-2.18485	0.031545	-4.07321	0.173768	0.231417
Erythroid.cells	EID3	-1.66259	0.925233	-2.18374	0.031629	-3.53042	0.204642	0.264212
Erythroid.cells	1300017J0	0.750384	3.006144	2.183564	0.031643	-3.81241	0.190473	0.249439
Erythroid.cells	GM45902	1.173372	1.585805	2.183539	0.031644	-3.53558	0.20003	0.259489
Erythroid.cells	KRI1	-0.58125	4.367396	-2.18348	0.031649	-3.73609	0.181759	0.240182
Erythroid.cells	PTTG1IP	0.446477	4.790183	2.182869	0.031695	-3.90637	0.179267	0.237486
Erythroid.cells	NPAT	-0.63458	4.485178	-2.18269	0.031709	-3.81095	0.181155	0.239511
Erythroid.cells	CCDC102A	1.095226	2.048525	2.182034	0.031759	-3.59457	0.197025	0.256428
Erythroid.cells	STFA2	1.315518	2.469209	2.181937	0.031766	-3.61741	0.194189	0.253445
Erythroid.cells	FRAT1	0.705493	3.463579	2.181921	0.031768	-3.66085	0.187651	0.24653
Erythroid.cells	ZFP566	-1.49227	1.254913	-2.18124	0.03182	-3.53703	0.202708	0.26237
Erythroid.cells	WIPF1	0.412608	7.425545	2.180739	0.031858	-4.37461	0.164018	0.221015
Erythroid.cells	GM16272	-1.60819	0.363126	-2.18067	0.031863	-3.54614	0.209086	0.269001
Erythroid.cells	RYBP	-0.50869	5.626574	-2.1803	0.031892	-3.97278	0.174466	0.23241
Erythroid.cells	EML2	-1.09081	2.655573	-2.1801	0.031906	-3.57307	0.193226	0.252445
Erythroid.cells	COL4A4	1.417525	1.009618	2.179614	0.031944	-3.53721	0.204633	0.264342
Erythroid.cells	HSPA2	-0.89813	3.291574	-2.17925	0.031972	-3.66182	0.18921	0.248157
Erythroid.cells	MSH2	-0.59328	4.128132	-2.17796	0.032071	-3.79872	0.184304	0.24288
Erythroid.cells	LYN	-0.33552	10.03311	-2.17763	0.032097	-4.82381	0.150655	0.206146
Erythroid.cells	GTF3C2	-0.38242	5.474786	-2.17642	0.03219	-3.99936	0.176321	0.23428
Erythroid.cells	NCS1	-1.82011	0.210797	-2.17633	0.032197	-3.56017	0.211361	0.271218
Erythroid.cells	SUPT6	-0.44762	5.808893	-2.17617	0.032209	-4.07524	0.174313	0.232111
Erythroid.cells	LGR4	1.111118	2.632615	2.17501	0.032299	-3.62961	0.194851	0.25391
Erythroid.cells	MALSU1	0.595102	4.843801	2.174099	0.032369	-3.84783	0.18087	0.239075
Erythroid.cells	VEGFC	1.311453	1.200834	2.173409	0.032423	-3.55858	0.205163	0.264829
Erythroid.cells	SERPINH1	1.119422	2.557349	2.173332	0.032429	-3.63892	0.195787	0.255052
Erythroid.cells	PARP11	-0.97044	3.46828	-2.17313	0.032444	-3.67469	0.18974	0.248719
Erythroid.cells	AKT1	-0.42993	6.230392	-2.17238	0.032502	-4.16364	0.172782	0.23045

Erythroid.cells	TRIM39	-0.78898	2.873416	-2.17174	0.032552	-3.61378	0.194087	0.253214
Erythroid.cells	LRP5	0.710139	4.078112	2.171114	0.032601	-3.81727	0.186278	0.245032
Erythroid.cells	ACTR2	-0.27375	8.066808	-2.1711	0.032603	-4.53136	0.162491	0.21928
Erythroid.cells	COL25A1	-1.50793	0.981282	-2.17058	0.032643	-3.56501	0.207305	0.267223
Erythroid.cells	LTBP1	2.60391	0.730726	2.170441	0.032654	-3.55245	0.209106	0.269153
Erythroid.cells	GM45353	-1.55908	-0.0712	-2.17027	0.032667	-3.58605	0.214976	0.275234
Erythroid.cells	STAG1	-0.25197	8.353909	-2.16995	0.032692	-4.58388	0.160981	0.217836
Erythroid.cells	MBD2	-0.30557	7.044678	-2.16859	0.032799	-4.35761	0.168762	0.226266
Erythroid.cells	DOK2	-0.95466	2.811456	-2.16842	0.032812	-3.63272	0.195156	0.254598
Erythroid.cells	PKD2	1.316522	0.719693	2.16744	0.032889	-3.55787	0.210125	0.270244
Erythroid.cells	HTRA2	-0.57602	3.681601	-2.16671	0.032946	-3.73834	0.189953	0.249078
Erythroid.cells	CYP4B1	1.452191	0.266849	2.16631	0.032978	-3.58018	0.213768	0.274067
Erythroid.cells	CCL5	-1.18797	7.931718	-2.16571	0.033025	-4.91784	0.164411	0.221582
Erythroid.cells	SIRT2	0.392514	5.688086	2.165316	0.033056	-4.09488	0.177591	0.235916
Erythroid.cells	PDXDC1	-0.31006	6.616598	-2.16496	0.033085	-4.24855	0.172067	0.229968
Erythroid.cells	OSBPL8	-0.36961	7.62098	-2.16467	0.033107	-4.47191	0.166261	0.223704
Erythroid.cells	LRCH3	-0.37697	6.497112	-2.16451	0.03312	-4.23285	0.172772	0.23086
Erythroid.cells	SMCHD1	-0.36915	7.994517	-2.16406	0.033155	-4.58645	0.164159	0.221507
Erythroid.cells	PAXX	0.754518	3.270058	2.164026	0.033159	-3.6564	0.193019	0.25264
Erythroid.cells	DCN	0.988212	3.138043	2.1636	0.033192	-3.80031	0.193985	0.253738
Erythroid.cells	FHOD1	-0.86717	2.907952	-2.163	0.03324	-3.6304	0.195697	0.255535
Erythroid.cells	PLCG1	0.780149	3.409844	2.162229	0.033301	-3.68391	0.192502	0.252178
Erythroid.cells	SCAMP5	1.230756	1.229049	2.162022	0.033317	-3.56951	0.207528	0.268031
Erythroid.cells	BCL6	0.657684	5.992679	2.161817	0.033334	-4.16215	0.176161	0.234759
Erythroid.cells	RAD51B	0.700467	6.152694	2.161664	0.033346	-4.21192	0.175198	0.233756
Erythroid.cells	ST5	1.047108	2.235439	2.161308	0.033374	-3.61757	0.200449	0.260815
Erythroid.cells	SLC10A1	0.72296	3.248903	2.161228	0.033381	-3.89121	0.193571	0.253578
Erythroid.cells	D130062J1	-1.26795	1.642184	-2.16083	0.033413	-3.57179	0.204671	0.265298
Erythroid.cells	ADGRL4	1.076101	3.201942	2.159876	0.033489	-3.69002	0.194291	0.254358
Erythroid.cells	NELFE	0.525048	4.393275	2.159518	0.033517	-3.85297	0.186543	0.246228
Erythroid.cells	FAM13A	1.535718	1.15315	2.158221	0.033621	-3.57512	0.208999	0.269849
Erythroid.cells	ZFP335	-0.85255	2.842169	-2.15796	0.033641	-3.63177	0.197176	0.257508
Erythroid.cells	SNIP1	-0.61749	3.633076	-2.15794	0.033643	-3.72511	0.19188	0.251903
Erythroid.cells	EMC2	-0.37351	5.762132	-2.15726	0.033698	-4.11715	0.178438	0.237633
Erythroid.cells	DUSP6	0.749649	4.428378	2.157149	0.033707	-3.82663	0.186796	0.246667
Erythroid.cells	TAF1	0.448509	5.699019	2.156828	0.033733	-4.08184	0.178824	0.238171
Erythroid.cells	LAPTM4A	0.285095	7.210016	2.156785	0.033736	-4.46911	0.169811	0.22836
Erythroid.cells	ADAT1	1.202287	2.003267	2.156125	0.033789	-3.58769	0.203265	0.264165
Erythroid.cells	AIP	0.504595	4.946962	2.155432	0.033845	-4.01437	0.183845	0.24349
Erythroid.cells	IFT46	-0.50011	4.502973	-2.15514	0.033869	-3.8656	0.18667	0.246587
Erythroid.cells	IGIP	1.494842	0.675003	2.155045	0.033876	-3.5785	0.212984	0.2743
Erythroid.cells	MEAF6	-0.43288	4.941914	-2.15442	0.033927	-3.9595	0.184047	0.243801
Erythroid.cells	CPNE2	-0.99279	3.37335	-2.15389	0.03397	-3.6993	0.19438	0.254784
Erythroid.cells	CD69	-0.79397	5.620451	-2.15344	0.034006	-4.12685	0.180009	0.239377
Erythroid.cells	ANKRD46	-0.80962	2.377526	-2.15325	0.034021	-3.60587	0.201242	0.26199
Erythroid.cells	HS3ST1	2.018314	1.500377	2.152899	0.03405	-3.58149	0.207423	0.268502
Erythroid.cells	GM38948	-1.31413	0.463761	-2.15278	0.03406	-3.58125	0.214978	0.276346

Erythroid.cells	KLHL3	0.997265	2.005874	2.152313	0.034097	-3.63709	0.203851	0.2649
Erythroid.cells	SLC35G1	-1.60255	1.026313	-2.15228	0.0341	-3.5816	0.210857	0.272211
Erythroid.cells	CGGBP1	-0.33618	6.845343	-2.15198	0.034124	-4.32404	0.172654	0.231589
Erythroid.cells	ZFP119A	-0.94353	2.125414	-2.15105	0.0342	-3.60469	0.203378	0.264375
Erythroid.cells	RABAC1	0.409444	5.988734	2.150161	0.034272	-4.13616	0.178192	0.237706
Erythroid.cells	1-Mar	1.187163	1.457886	2.150059	0.034281	-3.62977	0.208241	0.269662
Erythroid.cells	ACP1	0.327449	5.923129	2.150025	0.034283	-4.37322	0.178593	0.238154
Erythroid.cells	CORO1C	-0.39451	6.202322	-2.14986	0.034297	-4.17585	0.176893	0.236311
Erythroid.cells	EIPR1	0.430597	4.924643	2.148835	0.03438	-4.03209	0.185161	0.2452
Erythroid.cells	GNA11	0.780397	3.954617	2.147817	0.034464	-3.77472	0.191794	0.252179
Erythroid.cells	ITGB5	0.957421	3.058168	2.147415	0.034497	-3.76607	0.197882	0.258645
Erythroid.cells	FUNDC2	0.317638	7.178207	2.146693	0.034556	-4.62387	0.171886	0.230783
Erythroid.cells	SLAIN1	-0.59798	3.6132	-2.14648	0.034573	-3.83862	0.194242	0.254947
Erythroid.cells	CD72	-0.75213	4.334961	-2.14648	0.034573	-3.90001	0.189481	0.249876
Erythroid.cells	LPAR6	0.672506	4.523596	2.145692	0.034638	-3.95563	0.188505	0.248785
Erythroid.cells	TTC21B	1.087905	1.614885	2.1447	0.03472	-3.60067	0.208737	0.270098
Erythroid.cells	CASK	0.551801	5.500078	2.144411	0.034744	-4.08469	0.182644	0.242533
Erythroid.cells	REC114	-0.49701	3.19631	-2.14405	0.034773	-4.12206	0.19775	0.25868
Erythroid.cells	HNRNPAB	-0.3123	8.05587	-2.14377	0.034796	-4.60697	0.167409	0.226045
Erythroid.cells	HIF1A	-0.44564	6.608469	-2.14357	0.034813	-4.28373	0.175895	0.235343
Erythroid.cells	NDUFA5	0.488806	5.432033	2.142873	0.034871	-4.18844	0.183332	0.24339
Erythroid.cells	FTCD	1.277394	1.403237	2.141491	0.034985	-3.66247	0.211169	0.272677
Erythroid.cells	STIMATE	0.645509	4.159047	2.140102	0.0351	-3.8279	0.192399	0.252865
Erythroid.cells	CLEC4A4	-1.21268	-1.00355	-2.13986	0.035121	-3.60314	0.229887	0.291947
Erythroid.cells	FBXO31	-0.81364	3.549697	-2.13979	0.035127	-3.72657	0.196474	0.257257
Erythroid.cells	CCNYL1	0.58681	4.635175	2.139783	0.035127	-3.89425	0.189277	0.249601
Erythroid.cells	BPTF	-0.31738	7.356344	-2.1394	0.035159	-4.47343	0.172492	0.23145
Erythroid.cells	B4GALT6	1.005411	2.991751	2.139141	0.035181	-3.70694	0.200368	0.261437
Erythroid.cells	GRB10	1.100094	3.090696	2.138366	0.035245	-3.81025	0.199877	0.260961
Erythroid.cells	INTS6L	0.522032	5.229737	2.138273	0.035253	-4.05091	0.185706	0.245895
Erythroid.cells	CLEC4F	1.729071	5.458864	2.13641	0.035409	-4.07534	0.184961	0.245008
Erythroid.cells	BGN	0.971683	2.420901	2.136187	0.035428	-3.70014	0.205329	0.26672
Erythroid.cells	PNRC1	-0.31377	8.470962	-2.13576	0.035463	-4.68415	0.166937	0.225446
Erythroid.cells	ODC1	0.474801	5.188947	2.135233	0.035508	-4.13791	0.1868	0.247143
Erythroid.cells	SSR4	0.334237	7.573816	2.135188	0.035511	-4.54342	0.172156	0.231267
Erythroid.cells	ANGPT2	-1.70078	0.571482	-2.13499	0.035528	-3.62493	0.218993	0.281068
Erythroid.cells	RBM38	0.371882	6.898184	2.134481	0.035571	-4.55317	0.17621	0.235878
Erythroid.cells	FABP1	0.766259	7.428716	2.134444	0.035574	-4.76001	0.173044	0.232421
Erythroid.cells	TEAD2	1.303805	1.154161	2.133858	0.035623	-3.61266	0.2148	0.276851
Erythroid.cells	ZC3H15	0.255113	7.266033	2.133598	0.035645	-4.5449	0.17411	0.233655
Erythroid.cells	PKDCC	1.099708	1.303453	2.133051	0.035691	-3.65485	0.213696	0.275959
Erythroid.cells	NIP7	-0.46265	4.635807	-2.13305	0.035692	-3.94172	0.190529	0.251527
Erythroid.cells	GM14798	-0.64851	3.718232	-2.13284	0.035709	-3.80103	0.196635	0.258074
Erythroid.cells	SLC30A4	1.002479	1.941057	2.132553	0.035733	-3.64903	0.209048	0.271229
Erythroid.cells	FANCL	0.555163	4.144904	2.132245	0.03576	-3.87874	0.193771	0.255159
Erythroid.cells	PLEKHG5	1.276822	1.864551	2.132169	0.035766	-3.6354	0.2096	0.271871
Erythroid.cells	HIST1H2BC	0.594104	5.260139	2.132112	0.035771	-4.21599	0.186489	0.247359

Erythroid.cells	CST3	0.478721	9.157123	2.130698	0.03589	-5.07533	0.163696	0.222255
Erythroid.cells	CYP3A25	1.115553	1.898523	2.130092	0.035942	-3.72867	0.210069	0.272085
Erythroid.cells	TXNRD1	-0.48757	5.871273	-2.12998	0.035952	-4.18571	0.183245	0.243648
Erythroid.cells	INPP5F	0.65509	4.366787	2.129561	0.035987	-3.92944	0.193039	0.254136
Erythroid.cells	CYP4A14	1.19073	1.833194	2.128827	0.03605	-3.74864	0.210656	0.272883
Erythroid.cells	UST	0.865014	6.028853	2.128633	0.036066	-4.25688	0.182356	0.242903
Erythroid.cells	ETV3	0.590056	4.950898	2.128313	0.036093	-4.18406	0.189223	0.25034
Erythroid.cells	PTPRCAP	-0.47246	5.807512	-2.12829	0.036095	-4.26394	0.183744	0.24444
Erythroid.cells	MAPRE1	-0.26652	7.148653	-2.12827	0.036097	-4.42928	0.175501	0.235482
Erythroid.cells	INO80	-0.3894	6.666636	-2.12814	0.036108	-4.347	0.178417	0.238663
Erythroid.cells	RAB29	0.82757	3.635461	2.12787	0.036131	-3.75524	0.197989	0.259788
Erythroid.cells	NRIP1	0.461018	6.353871	2.127647	0.03615	-4.34664	0.18035	0.240942
Erythroid.cells	TNFAIP8L2	-0.74036	3.852483	-2.12716	0.036192	-3.78482	0.196638	0.258471
Erythroid.cells	IFT22	-0.74157	3.654091	-2.1267	0.036231	-3.81107	0.19805	0.260013
Erythroid.cells	GM15987	0.901829	2.537923	2.126554	0.036244	-3.83322	0.20581	0.268218
Erythroid.cells	LDLRAP1	-0.70795	4.099537	-2.12617	0.036276	-3.86905	0.195048	0.256981
Erythroid.cells	CCT8	0.304221	6.762623	2.125893	0.0363	-4.46231	0.178022	0.238697
Erythroid.cells	ZFP652	0.537544	5.654413	2.125794	0.036309	-4.11062	0.184908	0.246196
Erythroid.cells	CWF19L1	1.0549	2.200341	2.125618	0.036324	-3.65723	0.208228	0.271105
Erythroid.cells	FN3KRP	0.826197	1.481688	2.12537	0.036345	-3.79421	0.213462	0.27667
Erythroid.cells	DDT	0.425526	5.541533	2.124931	0.036383	-4.34537	0.185725	0.247311
Erythroid.cells	MLLT1	0.831403	2.735176	2.123697	0.036489	-3.67848	0.205021	0.267799
Erythroid.cells	CD160	-1.03512	1.916487	-2.1231	0.03654	-3.86729	0.211074	0.274086
Erythroid.cells	EFTUD2	-0.45596	5.105706	-2.12261	0.036582	-4.0626	0.18925	0.25089
Erythroid.cells	TFPT	-0.73451	2.965912	-2.12213	0.036624	-3.72256	0.203821	0.266464
Erythroid.cells	NPHP3	-1.40669	1.097931	-2.12173	0.036658	-3.62992	0.217467	0.280774
Erythroid.cells	ACOXL	1.107896	1.285897	2.121307	0.036695	-3.72889	0.21616	0.279498
Erythroid.cells	ARHGDIB	0.386968	8.995183	2.120485	0.036766	-4.79059	0.166134	0.225767
Erythroid.cells	KCTD3	-0.60505	3.747291	-2.12033	0.036779	-3.8657	0.198825	0.261307
Erythroid.cells	WRAP73	-0.76977	2.923556	-2.12	0.036808	-3.70485	0.204596	0.26747
Erythroid.cells	ANAPC10	-0.51725	4.581596	-2.11922	0.036876	-3.93749	0.193504	0.255672
Erythroid.cells	CABIN1	-0.43751	5.4487	-2.11885	0.036908	-4.14465	0.187832	0.249627
Erythroid.cells	GM15559	-0.78062	3.148018	-2.11876	0.036916	-3.73434	0.203286	0.26615
Erythroid.cells	A930007I1	-0.91817	4.385631	-2.11839	0.036948	-3.93239	0.19483	0.257256
Erythroid.cells	PRPS2	-0.58269	4.382206	-2.11826	0.036959	-3.90518	0.194853	0.257289
Erythroid.cells	KLHL24	-0.4102	6.228518	-2.11638	0.037123	-4.29636	0.183527	0.244925
Erythroid.cells	HSPB11	0.829475	2.925359	2.116339	0.037126	-3.76495	0.205578	0.268512
Erythroid.cells	NTAN1	-0.30806	6.261702	-2.11588	0.037167	-4.28627	0.18342	0.244848
Erythroid.cells	PCGF6	0.791296	2.512206	2.114873	0.037255	-3.71295	0.208968	0.272237
Erythroid.cells	MAGED2	1.327251	1.585547	2.114755	0.037265	-3.65318	0.215752	0.279342
Erythroid.cells	CNR2	0.669126	3.101889	2.113889	0.037341	-3.87636	0.205073	0.26812
Erythroid.cells	DAPK2	0.645651	2.818088	2.113085	0.037412	-4.01257	0.207368	0.270517
Erythroid.cells	EBAG9	0.601781	4.117531	2.111981	0.037509	-3.87071	0.198708	0.261272
Erythroid.cells	CDH5	1.008539	4.480665	2.111447	0.037556	-3.92834	0.19638	0.258721
Erythroid.cells	LRRC14	-1.07497	2.339055	-2.11123	0.037575	-3.67114	0.211401	0.27464
Erythroid.cells	KLRI1	-1.23079	-0.19768	-2.11038	0.03765	-3.67655	0.231013	0.295161
Erythroid.cells	HSF2	0.582296	4.185277	2.110273	0.03766	-3.97175	0.198619	0.261313

Erythroid.cells	RIC8B	-0.56222	4.502862	-2.10915	0.037759	-3.93112	0.196878	0.259423
Erythroid.cells	TPK1	0.546964	4.865838	2.10823	0.037841	-4.04951	0.194754	0.257078
Erythroid.cells	ZFP954	-1.17811	2.050779	-2.10742	0.037913	-3.67477	0.214768	0.278343
Erythroid.cells	SERPINA1B	0.750346	7.994808	2.107191	0.037933	-4.89461	0.17514	0.23591
Erythroid.cells	CCDC25	-0.47063	4.712121	-2.10715	0.037937	-4.06296	0.19597	0.258491
Erythroid.cells	GM49521	-1.44197	0.473447	-2.10619	0.038022	-3.65508	0.227137	0.290998
Erythroid.cells	DMAC1	0.532343	4.611093	2.106022	0.038037	-3.98376	0.196961	0.259406
Erythroid.cells	STK11IP	-0.90446	2.312647	-2.10569	0.038067	-3.68574	0.213228	0.276649
Erythroid.cells	TM4SF4	0.991209	2.168937	2.103969	0.038221	-3.81862	0.215037	0.27832
Erythroid.cells	GMEB1	-0.527	5.025748	-2.10344	0.038268	-4.03918	0.195043	0.257199
Erythroid.cells	CNTRL	-0.40031	5.907012	-2.10314	0.038295	-4.23915	0.189269	0.251014
Erythroid.cells	4933432I0:	-1.4808	0.408312	-2.10151	0.038442	-3.66349	0.22936	0.293136
Erythroid.cells	TRAF7	0.512682	4.585124	2.101468	0.038445	-3.94723	0.198619	0.260993
Erythroid.cells	DHDDS	-0.57383	4.630539	-2.10111	0.038477	-3.98231	0.198371	0.260789
Erythroid.cells	SMIM14	-0.27893	7.74729	-2.10064	0.03852	-4.58924	0.178391	0.239219
Erythroid.cells	NFKB1	-0.45172	8.743793	-2.09942	0.03863	-4.78774	0.172766	0.233068
Erythroid.cells	TKFC	0.812523	3.130684	2.099362	0.038635	-3.86949	0.209396	0.272484
Erythroid.cells	ARPC3	0.242098	8.451234	2.097511	0.038803	-4.75248	0.175161	0.235461
Erythroid.cells	BPNT1	0.654119	3.339718	2.097016	0.038848	-3.8389	0.208818	0.271579
Erythroid.cells	HAX1	0.530038	4.020737	2.096447	0.0389	-4.0333	0.204142	0.266688
Erythroid.cells	PRPSAP1	0.496563	4.34081	2.095676	0.03897	-3.99022	0.201908	0.264431
Erythroid.cells	GGTA1	0.602999	5.365487	2.095598	0.038977	-4.1055	0.194928	0.256995
Erythroid.cells	SPARCL1	2.130627	0.3267	2.09556	0.03898	-3.6724	0.231862	0.295657
Erythroid.cells	SPON1	-1.42776	1.404705	-2.09555	0.038981	-3.71082	0.223394	0.286935
Erythroid.cells	PSMA6	-0.3456	6.941715	-2.09455	0.039073	-4.49517	0.184999	0.246228
Erythroid.cells	4933434E2	0.476966	5.269667	2.094332	0.039092	-4.10692	0.195904	0.257992
Erythroid.cells	IL1R1	1.242328	2.051839	2.094054	0.039118	-3.75966	0.218836	0.282212
Erythroid.cells	DAXX	-0.7101	4.352332	-2.09393	0.039129	-3.93306	0.202173	0.264718
Erythroid.cells	IRF1	-0.79585	7.149071	-2.09299	0.039215	-4.47496	0.183998	0.245004
Erythroid.cells	RABGEF1	-0.56118	6.01057	-2.09263	0.039248	-4.24995	0.191367	0.253
Erythroid.cells	SPTA1	0.724176	0.279808	2.091734	0.03933	-4.08598	0.233206	0.297137
Erythroid.cells	ZFP683	-1.53714	-1.15907	-2.0917	0.039333	-3.67731	0.245087	0.309235
Erythroid.cells	PI16	1.044343	1.135522	2.091303	0.03937	-3.75315	0.226419	0.290222
Erythroid.cells	CD99L2	0.838065	2.142314	2.091171	0.039382	-3.7515	0.218691	0.282236
Erythroid.cells	UBE2G1	-0.27778	7.307993	-2.09053	0.039441	-4.55639	0.183147	0.244574
Erythroid.cells	CFAP77	-1.56093	0.931954	-2.09039	0.039454	-3.68026	0.228015	0.292052
Erythroid.cells	TDO2	0.999151	3.218699	2.090357	0.039457	-4.07747	0.21073	0.274061
Erythroid.cells	RRP12	0.874996	2.60063	2.090293	0.039463	-3.75441	0.215264	0.278814
Erythroid.cells	GLRX2	0.401565	5.111058	2.09026	0.039466	-4.1693	0.197457	0.259999
Erythroid.cells	GM14548	1.109701	1.47249	2.090183	0.039473	-3.73571	0.223802	0.287698
Erythroid.cells	PAPSS1	-0.52237	4.181725	-2.08978	0.03951	-3.96592	0.203947	0.266919
Erythroid.cells	GM20508	1.430671	0.064606	2.089558	0.03953	-3.68249	0.235043	0.299389
Erythroid.cells	TTPA	1.057508	1.860466	2.088631	0.039616	-3.82532	0.221281	0.285258
Erythroid.cells	ETHE1	0.461163	4.997365	2.087774	0.039695	-4.1677	0.198918	0.261776
Erythroid.cells	FNBP1	0.36004	8.248905	2.08758	0.039713	-4.78476	0.177975	0.239156
Erythroid.cells	GBP5	-1.67028	2.014589	-2.08722	0.039746	-3.74508	0.220487	0.284614
Erythroid.cells	DNAJA2	-0.2509	7.208069	-2.08688	0.039778	-4.5713	0.184522	0.246412

Erythroid.cells	RAPGEF4	0.84375	2.563789	2.086001	0.03986	-3.89741	0.216744	0.280704
Erythroid.cells	ZFYVE19	-0.75492	3.132938	-2.08425	0.040023	-3.79731	0.213296	0.276771
Erythroid.cells	ANK2	1.524698	2.998552	2.083571	0.040086	-3.82871	0.214512	0.278031
Erythroid.cells	GM15446	-1.1514	2.166881	-2.08185	0.040247	-3.71543	0.221453	0.284908
Erythroid.cells	PDZD8	-0.48024	5.970732	-2.08175	0.040256	-4.23239	0.194308	0.256322
Erythroid.cells	MRPS5	0.497999	4.612666	2.081253	0.040303	-4.06457	0.203707	0.266328
Erythroid.cells	ADGRA3	1.446388	0.459311	2.080353	0.040387	-3.69874	0.235415	0.299208
Erythroid.cells	PRPF4	-0.66596	3.92555	-2.07897	0.040517	-3.90797	0.209285	0.272143
Erythroid.cells	USB1	-0.84073	3.245957	-2.07877	0.040536	-3.81684	0.214237	0.277399
Erythroid.cells	IFI208	-1.26455	2.936618	-2.07875	0.040538	-3.81205	0.216531	0.279796
Erythroid.cells	ANKRD17	-0.28565	7.985576	-2.07868	0.040545	-4.68507	0.182109	0.243131
Erythroid.cells	MFGE8	1.060192	2.292559	2.078376	0.040573	-3.77678	0.221432	0.284969
Erythroid.cells	M6PR	-0.39189	6.293265	-2.07815	0.040594	-4.34178	0.19299	0.255035
Erythroid.cells	TLL2	-1.81608	0.725481	-2.07757	0.040649	-3.69878	0.233926	0.297993
Erythroid.cells	RIPK1	-0.43542	5.973193	-2.07719	0.040685	-4.28009	0.195319	0.257657
Erythroid.cells	PDPN	1.848093	-0.21403	2.07704	0.040699	-3.70101	0.241689	0.306071
Erythroid.cells	MLF1	1.509599	0.512644	2.076673	0.040734	-3.70021	0.235712	0.300054
Erythroid.cells	GM35188	1.331315	1.73277	2.076574	0.040743	-3.72303	0.225994	0.290075
Erythroid.cells	FBXO7	0.443045	4.165071	2.076242	0.040775	-4.20474	0.207888	0.271203
Erythroid.cells	MYO16	-1.46798	-0.30441	-2.07579	0.040818	-3.70552	0.24265	0.307309
Erythroid.cells	DUSP1	0.497839	7.412842	2.075208	0.040873	-4.66256	0.186188	0.248201
Erythroid.cells	1810006J0	1.121084	-0.01116	2.075118	0.040881	-3.73778	0.240331	0.305141
Erythroid.cells	CHMP5	0.357409	5.632148	2.074581	0.040932	-4.29732	0.198033	0.260957
Erythroid.cells	SF3B2	-0.26841	7.285583	-2.07431	0.040958	-4.58248	0.187156	0.249295
Erythroid.cells	LMBR1L	-0.60386	4.517308	-2.07224	0.041155	-4.03582	0.206665	0.269824
Erythroid.cells	GRK5	0.614472	5.747033	2.07193	0.041185	-4.41967	0.198166	0.260813
Erythroid.cells	BCL2L12	-0.62708	4.178233	-2.07089	0.041285	-3.95048	0.209497	0.272695
Erythroid.cells	SLC9A9	0.601032	7.120003	2.070734	0.041299	-4.5999	0.189399	0.25127
Erythroid.cells	FCGRT	0.703128	4.823452	2.069811	0.041388	-4.18792	0.205238	0.268088
Erythroid.cells	ATP6V0C	0.278074	8.851286	2.069058	0.04146	-4.91046	0.179011	0.239826
Erythroid.cells	NFIB	0.939352	3.880029	2.068783	0.041487	-4.07596	0.212217	0.275523
Erythroid.cells	CCR1	1.134297	2.32125	2.068696	0.041495	-3.85675	0.223919	0.287772
Erythroid.cells	BNIP3	0.662971	5.086642	2.068488	0.041515	-4.28945	0.203599	0.266504
Erythroid.cells	HBA-A2	0.755321	9.98555	2.066954	0.041663	-5.61026	0.172764	0.23292
Erythroid.cells	RAB1A	-0.27497	7.08553	-2.06609	0.041746	-4.54111	0.190989	0.252717
Erythroid.cells	GPR84	-1.47069	0.260814	-2.06573	0.041782	-3.72271	0.241589	0.305704
Erythroid.cells	HCLS1	-0.41247	6.691521	-2.06508	0.041844	-4.41866	0.193834	0.25582
Erythroid.cells	VCPIP1	0.329413	6.167501	2.064284	0.041921	-4.40231	0.197606	0.259751
Erythroid.cells	PPP4C	-0.31978	6.80668	-2.06332	0.042015	-4.47449	0.193509	0.255555
Erythroid.cells	CEBPG	0.377054	5.586056	2.063223	0.042024	-4.27571	0.201769	0.264417
Erythroid.cells	TEX9	1.108679	1.820323	2.063196	0.042027	-3.75808	0.229674	0.293629
Erythroid.cells	OLFR1259	1.697083	-1.34501	2.063005	0.042045	-3.7206	0.256193	0.320646
Erythroid.cells	UBXN11	-0.97282	1.770334	-2.06274	0.042071	-3.75254	0.230094	0.294189
Erythroid.cells	SPOP	0.241322	7.366582	2.062255	0.042118	-4.68529	0.189977	0.251865
Erythroid.cells	NEMF	-0.33902	5.95092	-2.06154	0.042188	-4.331	0.199486	0.262194
Erythroid.cells	NAMPT	-0.64316	6.301782	-2.06139	0.042203	-4.38098	0.197103	0.259645
Erythroid.cells	FAM122B	0.89131	2.015817	2.06136	0.042206	-3.77727	0.228387	0.292539

Erythroid.cells	WDR36	-0.48297	4.578146	-2.06119	0.042223	-4.09811	0.209106	0.272416
Erythroid.cells	URB2	-0.9326	2.571455	-2.06098	0.042242	-3.77989	0.224055	0.288093
Erythroid.cells	ANGPT1	1.374823	0.973826	2.060239	0.042315	-3.74119	0.236871	0.301327
Erythroid.cells	ZFP207	-0.26177	6.934778	-2.0599	0.042348	-4.5471	0.192982	0.255373
Erythroid.cells	TACO1OS	0.940725	1.705089	2.059897	0.042349	-3.81573	0.23097	0.295381
Erythroid.cells	LRRC4C	1.777169	0.648779	2.059876	0.042351	-3.72502	0.239544	0.304178
Erythroid.cells	MRPS26	-0.37933	5.167613	-2.05829	0.042506	-4.18155	0.205672	0.268733
Erythroid.cells	GM15728	-1.45713	0.500791	-2.05801	0.042533	-3.72814	0.241565	0.30613
Erythroid.cells	GM28960	-1.20772	-0.82667	-2.05591	0.042741	-3.74002	0.254	0.318349
Erythroid.cells	SH3GLB2	-0.65779	3.721025	-2.05543	0.042788	-3.93336	0.217258	0.280685
Erythroid.cells	SLA	0.623507	5.280678	2.054838	0.042846	-4.14642	0.206099	0.268794
Erythroid.cells	HBA-A1	0.784756	10.46682	2.054011	0.042928	-5.65853	0.172685	0.232815
Erythroid.cells	E330020D1	0.696888	5.11024	2.053948	0.042934	-4.20895	0.207319	0.270238
Erythroid.cells	SCRN2	0.999577	1.43712	2.053638	0.042965	-3.75104	0.235259	0.299404
Erythroid.cells	CXCL16	-0.79745	3.468405	-2.05354	0.042975	-4.09833	0.219354	0.282974
Erythroid.cells	DHX36	-0.35546	5.392248	-2.05344	0.042984	-4.23842	0.205322	0.268252
Erythroid.cells	TMEM176I	0.497512	5.475623	2.053419	0.042986	-4.46456	0.204736	0.26763
Erythroid.cells	AASDHPPT	0.544212	3.841771	2.053288	0.043	-4.01752	0.216554	0.280148
Erythroid.cells	ZFHX2	1.003478	2.765643	2.052296	0.043098	-3.82851	0.224877	0.289002
Erythroid.cells	ZFP788	0.895839	2.069958	2.052227	0.043105	-3.77594	0.230333	0.294674
Erythroid.cells	VPS4B	-0.33344	6.231035	-2.05212	0.043116	-4.38329	0.199637	0.26249
Erythroid.cells	ABTB2	-0.59455	7.412469	-2.05193	0.043135	-4.65354	0.191731	0.254032
Erythroid.cells	TCP11	-1.41888	0.648485	-2.05191	0.043136	-3.74003	0.241911	0.306635
Erythroid.cells	RTN1	1.025044	2.561189	2.050877	0.043239	-3.95202	0.226892	0.291204
Erythroid.cells	SOD2	-0.67799	6.929524	-2.05046	0.04328	-4.69892	0.195289	0.25801
Erythroid.cells	CD33	1.221515	2.518088	2.050296	0.043297	-3.8319	0.22723	0.291787
Erythroid.cells	ERO1LB	-0.33142	6.652932	-2.05006	0.043321	-4.58565	0.197145	0.260112
Erythroid.cells	BRD1	-0.40444	5.991255	-2.05001	0.043326	-4.31885	0.201662	0.264948
Erythroid.cells	LTBP4	1.289244	2.297056	2.049189	0.043407	-3.80376	0.229284	0.294022
Erythroid.cells	CSPG5	-2.01236	0.28413	-2.04893	0.043434	-3.74971	0.245798	0.311036
Erythroid.cells	LAT	0.750856	2.414617	2.048196	0.043507	-3.92015	0.228511	0.293389
Erythroid.cells	RALGPS1	-0.57178	4.92479	-2.04803	0.043523	-4.14064	0.209605	0.273605
Erythroid.cells	ARHGEF26	1.595463	0.379311	2.048028	0.043524	-3.74268	0.245132	0.310474
Erythroid.cells	STAM2	-0.55517	5.475543	-2.04771	0.043555	-4.22724	0.205727	0.269558
Erythroid.cells	TRUB2	-0.59079	4.526877	-2.04632	0.043695	-4.01982	0.213115	0.277285
Erythroid.cells	EFCAB11	0.943104	3.410869	2.045721	0.043755	-3.93693	0.221522	0.286281
Erythroid.cells	POMT2	1.006927	1.813643	2.045204	0.043807	-3.77671	0.234054	0.299405
Erythroid.cells	9330111N(	-1.57638	0.392898	-2.04516	0.043811	-3.74719	0.245815	0.31146
Erythroid.cells	BTK	-0.40252	6.033608	-2.04504	0.043823	-4.43053	0.202441	0.26623
Erythroid.cells	SAA3	-3.55524	0.738679	-2.04487	0.043841	-3.79655	0.242898	0.308483
Erythroid.cells	ZMAT2	0.357412	5.466404	2.04469	0.043859	-4.34466	0.206416	0.270468
Erythroid.cells	NUP37	-0.75745	3.351508	-2.04446	0.043882	-3.93259	0.221975	0.286868
Erythroid.cells	IL13RA1	0.801152	3.558786	2.044451	0.043883	-4.05042	0.220397	0.285218
Erythroid.cells	SATB1	0.449966	7.143818	2.044201	0.043909	-4.73926	0.19491	0.258191
Erythroid.cells	SERP1	0.272896	7.981742	2.043903	0.043939	-4.80975	0.189449	0.252334
Erythroid.cells	CYTH4	0.465849	5.757599	2.043271	0.044003	-4.23962	0.204435	0.268639
Erythroid.cells	2310009B1	-0.47546	4.473004	-2.04324	0.044005	-4.10842	0.21365	0.27842

Erythroid.cells	BMT2	-0.43096	6.027835	-2.04322	0.044008	-4.32715	0.202551	0.266626
Erythroid.cells	KLHL9	0.531418	4.65586	2.043007	0.044029	-4.09528	0.212312	0.277058
Erythroid.cells	ZEB2OS	-0.59659	4.678362	-2.04215	0.044116	-4.15673	0.212439	0.277068
Erythroid.cells	SPN	0.67566	3.586459	2.041705	0.044161	-3.98352	0.220565	0.285863
Erythroid.cells	ATP5F1	0.253385	8.084387	2.041644	0.044167	-4.84464	0.18906	0.25222
Erythroid.cells	XRN2	-0.23432	7.516217	-2.04136	0.044196	-4.71006	0.192761	0.256291
Erythroid.cells	PBDC1	-0.63876	5.348225	-2.04123	0.044209	-4.22169	0.20761	0.272243
Erythroid.cells	PHF10	0.474689	4.930175	2.040646	0.044269	-4.20331	0.21061	0.275613
Erythroid.cells	ERO1L	-0.64452	5.171152	-2.04062	0.044271	-4.18156	0.208875	0.27377
Erythroid.cells	RAB7	-0.25767	7.931545	-2.04056	0.044277	-4.75018	0.190048	0.253536
Erythroid.cells	ZFP874A	0.833433	2.146516	2.040475	0.044286	-3.8637	0.23178	0.297856
Erythroid.cells	PLD1	0.968457	3.080795	2.040038	0.044331	-3.91543	0.224553	0.29039
Erythroid.cells	P4HTM	1.030309	2.773581	2.039428	0.044393	-3.90358	0.227058	0.292993
Erythroid.cells	SLC25A18	-0.76245	4.491338	-2.03939	0.044397	-4.21479	0.214027	0.279324
Erythroid.cells	SP100	0.434211	7.43248	2.038557	0.044481	-4.71027	0.193785	0.257524
Erythroid.cells	ARHGAP6	0.852846	3.609197	2.037695	0.044569	-4.02955	0.221258	0.286627
Erythroid.cells	PCMTD1	0.41817	5.924056	2.037059	0.044635	-4.41383	0.204549	0.268919
Erythroid.cells	SMC5	-0.41558	5.613071	-2.03602	0.044741	-4.35111	0.207053	0.271706
Erythroid.cells	ARHGEF2	-0.42625	5.586467	-2.03581	0.044762	-4.24996	0.207242	0.271975
Erythroid.cells	XPNPEP3	-0.7567	3.250052	-2.03568	0.044776	-3.87284	0.224566	0.290263
Erythroid.cells	FAM219B	0.992193	2.367885	2.035357	0.044809	-3.82549	0.231495	0.297563
Erythroid.cells	ORAI2	-0.48202	5.87495	-2.03504	0.044841	-4.28722	0.205203	0.270014
Erythroid.cells	THRAP3	-0.25845	8.006361	-2.03496	0.04485	-4.81652	0.19078	0.254459
Erythroid.cells	KMT2B	-0.64321	3.888591	-2.0349	0.044856	-3.96731	0.219686	0.285365
Erythroid.cells	TNFAIP3	-0.46026	6.763033	-2.03443	0.044905	-4.59915	0.199111	0.263545
Erythroid.cells	ACYP1	0.657568	3.847714	2.034357	0.044912	-3.99185	0.220054	0.285826
Erythroid.cells	CIZ1	0.603719	3.940619	2.033298	0.045021	-3.98931	0.219778	0.285418
Erythroid.cells	ATR	0.764144	3.862209	2.032569	0.045096	-4.0103	0.220529	0.286303
Erythroid.cells	DYM	0.395116	5.781233	2.03231	0.045123	-4.32317	0.206466	0.271442
Erythroid.cells	NUP85	-0.63901	4.345133	-2.03218	0.045137	-4.09997	0.216898	0.28256
Erythroid.cells	RGS10	0.460735	5.509055	2.031623	0.045194	-4.29889	0.208402	0.273779
Erythroid.cells	GM47371	1.467751	0.907518	2.031104	0.045248	-3.77118	0.244167	0.311358
Erythroid.cells	TFR2	1.045366	1.213769	2.030925	0.045266	-3.81315	0.2416	0.308824
Erythroid.cells	TOP3B	-0.62739	3.549805	-2.03076	0.045283	-3.92025	0.222912	0.289406
Erythroid.cells	UTP4	-0.47926	5.102474	-2.03062	0.045298	-4.17593	0.211329	0.277182
Erythroid.cells	SEMA4F	1.059696	-0.41218	2.030614	0.045299	-3.80265	0.25555	0.323112
Erythroid.cells	GM48099	1.232278	2.773722	2.030604	0.0453	-4.01369	0.228949	0.295742
Erythroid.cells	TMC6	-0.51511	4.632332	-2.03043	0.045317	-4.08772	0.214768	0.280885
Erythroid.cells	ALDH3B1	0.816493	3.605843	2.030429	0.045318	-3.90799	0.222483	0.289026
Erythroid.cells	ERCC6	-0.7236	3.56729	-2.02968	0.045395	-3.9606	0.22305	0.289596
Erythroid.cells	COPS8	-0.39463	5.091019	-2.02928	0.045437	-4.22042	0.211677	0.277615
Erythroid.cells	LRCH1	-0.3353	7.432191	-2.02924	0.045441	-4.69374	0.195371	0.260097
Erythroid.cells	GM48960	1.139009	1.466163	2.028402	0.045528	-3.79427	0.240075	0.30752
Erythroid.cells	RPP14	0.849725	2.846249	2.028272	0.045542	-3.85343	0.228921	0.295956
Erythroid.cells	APC	-0.36616	6.723147	-2.02812	0.045558	-4.52498	0.200388	0.265755
Erythroid.cells	SEMA6D	1.081894	3.713919	2.026961	0.045679	-4.01846	0.222665	0.289347
Erythroid.cells	IFITM3	0.590795	7.709419	2.026122	0.045766	-4.99223	0.194445	0.259019

Erythroid.cells	TSTD3	0.86593	1.784277	2.025861	0.045794	-3.86462	0.238337	0.305456
Erythroid.cells	WDR90	1.008784	1.959845	2.025528	0.045829	-3.82312	0.236964	0.304097
Erythroid.cells	SCYL2	0.504245	4.409687	2.024946	0.045889	-4.10157	0.217982	0.284249
Erythroid.cells	SRBD1	0.571421	4.393343	2.024262	0.045961	-4.11573	0.21834	0.284537
Erythroid.cells	CAB39L	0.542457	4.624571	2.023983	0.045991	-4.12416	0.216646	0.282736
Erythroid.cells	GM29666	-1.765	0.244861	-2.02314	0.046079	-3.78164	0.252213	0.319756
Erythroid.cells	EGLN1	-0.4294	5.631732	-2.02274	0.046122	-4.33428	0.209517	0.275364
Erythroid.cells	ATG14	0.638928	3.27883	2.022576	0.046139	-3.9431	0.227159	0.294002
Erythroid.cells	MAP3K15	-0.997	3.326785	-2.02251	0.046145	-3.90627	0.226784	0.29361
Erythroid.cells	ERI2	1.291376	1.310882	2.022441	0.046153	-3.78754	0.243102	0.310569
Erythroid.cells	UTP15	0.613839	3.757308	2.021568	0.046245	-3.96815	0.223652	0.290346
Erythroid.cells	YWHAZ	-0.17924	8.482146	-2.02112	0.046292	-4.90226	0.190238	0.254735
Erythroid.cells	GON4L	-0.43127	5.15234	-2.02083	0.046323	-4.22148	0.213184	0.279522
Erythroid.cells	TTPAL	-0.50254	4.234118	-2.02066	0.046341	-4.11379	0.220014	0.286759
Erythroid.cells	GM49085	-0.92009	2.025817	-2.02064	0.046343	-3.82984	0.237395	0.30493
Erythroid.cells	2410006H1	0.465926	7.535004	2.020475	0.04636	-4.79066	0.196485	0.261583
Erythroid.cells	SNX13	0.537496	5.413359	2.020409	0.046367	-4.24898	0.211283	0.277498
Erythroid.cells	CCDC71L	0.474534	4.455272	2.020358	0.046373	-4.33314	0.218349	0.284999
Erythroid.cells	ZSWIM8	-0.48489	4.703109	-2.01999	0.046412	-4.12294	0.216575	0.28304
Erythroid.cells	ATP5G1	0.314934	7.643771	2.019732	0.046439	-4.86104	0.195848	0.260792
Erythroid.cells	TMEM159	-1.34227	1.553977	-2.01897	0.04652	-3.808	0.241469	0.309188
Erythroid.cells	MRGPRA2A	-1.37301	-1.27662	-2.0185	0.046569	-3.7893	0.266258	0.334596
Erythroid.cells	SPTBN4	1.497512	-0.18469	2.018385	0.046582	-3.78945	0.256404	0.324618
Erythroid.cells	MINPP1	-0.43721	4.734583	-2.01828	0.046592	-4.2132	0.216424	0.283121
Erythroid.cells	CNOT2	-0.26739	7.008824	-2.01818	0.046603	-4.65139	0.200198	0.265751
Erythroid.cells	ATXN7L1	0.375601	6.345264	2.018169	0.046605	-4.50997	0.204795	0.270706
Erythroid.cells	SIVA1	-0.52154	5.470666	-2.01814	0.046608	-4.34155	0.211025	0.277378
Erythroid.cells	2610008E1	0.951164	2.466966	2.017914	0.046632	-3.86862	0.233996	0.301588
Erythroid.cells	ZFP131	-0.42568	5.815875	-2.01614	0.046821	-4.33154	0.209297	0.2751
Erythroid.cells	ARAP3	-0.76687	3.537048	-2.0151	0.046931	-4.04075	0.226762	0.293448
Erythroid.cells	PON2	-0.38837	6.020273	-2.01473	0.046971	-4.47129	0.208244	0.273951
Erythroid.cells	AXDND1	-1.35402	1.557778	-2.01465	0.04698	-3.82347	0.242786	0.310208
Erythroid.cells	MEFV	-1.41158	-0.03339	-2.01313	0.047142	-3.81198	0.257257	0.3248
Erythroid.cells	KRAS	-0.2912	7.278938	-2.01182	0.047283	-4.6691	0.200562	0.26537
Erythroid.cells	EIF4A2	0.375517	5.530319	2.010905	0.047382	-4.3378	0.213045	0.278815
Erythroid.cells	ZFP758	0.767376	2.74078	2.010732	0.047401	-3.91543	0.234487	0.301324
Erythroid.cells	LDHB	0.921513	3.00278	2.010662	0.047408	-3.98976	0.23238	0.299142
Erythroid.cells	NUSAP1	0.601831	5.649629	2.010571	0.047418	-4.65291	0.212175	0.277904
Erythroid.cells	RETREG1	0.403827	6.437217	2.010546	0.04742	-4.59184	0.206527	0.271876
Erythroid.cells	ZBP1	-1.32057	3.640152	-2.00998	0.047482	-4.07868	0.227511	0.294135
Erythroid.cells	PSMA4	-0.30523	6.877207	-2.00947	0.047536	-4.66781	0.203597	0.268939
Erythroid.cells	COL6A1	1.429961	0.367808	2.009432	0.047541	-3.80167	0.254682	0.322329
Erythroid.cells	UPB1	0.800882	3.0781	2.009098	0.047577	-4.06076	0.231956	0.299011
Erythroid.cells	LRP6	0.551469	5.378913	2.009061	0.047581	-4.23374	0.214318	0.280463
Erythroid.cells	RFLNB	0.873901	3.463749	2.008755	0.047614	-4.027	0.228897	0.295891
Erythroid.cells	DRG2	0.595319	3.507943	2.008748	0.047615	-4.00346	0.228549	0.295527
Erythroid.cells	IRS1	-1.29486	0.821216	-2.00833	0.047659	-3.81571	0.250843	0.318593

Erythroid.cells	SERPING1	0.714303	3.004813	2.007726	0.047725	-4.12819	0.232862	0.300075
Erythroid.cells	CASC3	-0.45936	5.292913	-2.0073	0.047771	-4.26623	0.215302	0.281733
Erythroid.cells	SLC45A3	1.387974	1.111286	2.007198	0.047783	-3.82182	0.248634	0.316518
Erythroid.cells	GATD3A	0.611506	3.544084	2.006925	0.047812	-4.06288	0.22867	0.295875
Erythroid.cells	ATG4C	0.646774	3.11799	2.006341	0.047876	-4.04458	0.232247	0.299573
Erythroid.cells	PIK3AP1	-0.46791	8.295612	-2.00594	0.047919	-4.87991	0.194568	0.259417
Erythroid.cells	SH3D19	0.936373	2.244921	2.005478	0.04797	-3.92243	0.23948	0.307165
Erythroid.cells	NXT2	-0.71879	2.785909	-2.00524	0.047996	-3.918	0.235057	0.302687
Erythroid.cells	2410004B1	-0.43966	4.71052	-2.00493	0.048029	-4.17283	0.219999	0.287021
Erythroid.cells	C1QTNF12	-1.04635	2.21932	-2.00484	0.048039	-3.85769	0.239692	0.307641
Erythroid.cells	POLL	1.149259	1.158885	2.004813	0.048042	-3.81276	0.248621	0.316844
Erythroid.cells	RTN4	0.257726	7.592315	2.004627	0.048062	-4.78007	0.199323	0.26491
Erythroid.cells	NOSIP	-0.40374	5.158105	-2.00357	0.048178	-4.29916	0.217065	0.283928
Erythroid.cells	STXBP3	-0.45632	5.278015	-2.00335	0.048202	-4.32077	0.216176	0.282983
Erythroid.cells	HPS3	0.616256	4.626547	2.002536	0.048291	-4.11308	0.221292	0.288516
Erythroid.cells	POLD2	-0.83192	3.401936	-2.0023	0.048317	-3.99037	0.230809	0.298575
Erythroid.cells	SFT2D1	-0.31108	5.999884	-2.00203	0.048347	-4.4581	0.211107	0.277788
Erythroid.cells	CYP4A10	1.519583	1.117903	2.001987	0.048351	-3.87802	0.249714	0.318204
Erythroid.cells	DNAJC5	-0.25232	6.766699	-2.00187	0.048364	-4.58127	0.205638	0.271921
Erythroid.cells	GREM2	1.359849	0.454759	2.001489	0.048406	-3.8276	0.255547	0.324259
Erythroid.cells	LAPTM5	-0.26599	8.326927	-2.00108	0.04845	-4.89091	0.195008	0.260574
Erythroid.cells	HLCS	0.712065	4.15558	2.001029	0.048456	-4.08754	0.224947	0.292671
Erythroid.cells	APOBR	0.910242	2.373103	2.00095	0.048465	-3.94101	0.239182	0.307566
Erythroid.cells	SELENOS	0.361	6.113957	2.000713	0.048491	-4.46869	0.210341	0.277237
Erythroid.cells	GM35769	1.165656	1.096204	2.000437	0.048521	-3.83946	0.250008	0.318851
Erythroid.cells	GRAP2	0.486478	4.975054	2.000097	0.048559	-4.63413	0.218818	0.286318
Erythroid.cells	TMEM132E	-1.61374	0.329919	-1.99849	0.048736	-3.81583	0.257603	0.32647
Erythroid.cells	ZFP54	-1.47888	0.794625	-1.99797	0.048793	-3.81587	0.253682	0.322495
Erythroid.cells	RASGEF1B	0.721307	5.547786	1.997444	0.048851	-4.41509	0.215405	0.282702
Erythroid.cells	SS18L2	0.587837	3.817204	1.997339	0.048863	-4.11579	0.228597	0.296654
Erythroid.cells	INPP4B	0.590567	6.480863	1.997331	0.048864	-4.66804	0.208628	0.275452
Erythroid.cells	EXOG	-1.07736	1.745846	-1.9967	0.048934	-3.84165	0.245681	0.314462
Erythroid.cells	MAP2K6	0.806669	3.533157	1.996598	0.048945	-4.03025	0.231008	0.299211
Erythroid.cells	DR1	0.395285	4.953577	1.996394	0.048968	-4.28775	0.220001	0.287646
Erythroid.cells	ANKRA2	-0.59202	3.792457	-1.9958	0.049033	-4.02542	0.229111	0.297182
Erythroid.cells	RAC2	-0.32025	9.052768	-1.99569	0.049046	-4.98181	0.191363	0.256704
Erythroid.cells	AP3B1	-0.26161	7.329007	-1.9939	0.049245	-4.75029	0.203674	0.269924
Erythroid.cells	TTC36	0.687654	5.028124	1.993545	0.049284	-4.61516	0.220446	0.287873
Erythroid.cells	CCDC9	-0.4706	4.238269	-1.99316	0.049327	-4.13391	0.226602	0.294429
Erythroid.cells	MFSD12	-0.78206	2.730413	-1.99207	0.049448	-3.91829	0.239136	0.307482
Erythroid.cells	TSTA3	-0.60611	4.100613	-1.99189	0.049469	-4.08729	0.228121	0.296024
Erythroid.cells	UBXN1	0.257297	6.759331	1.991416	0.049522	-4.70564	0.208363	0.275064
Erythroid.cells	SUPT16	-0.26518	7.068147	-1.99099	0.049569	-4.72919	0.206252	0.27282
Erythroid.cells	COX17	0.301995	6.919214	1.990657	0.049607	-4.73459	0.207305	0.274026
Erythroid.cells	PLATR25	0.907397	2.729731	1.990633	0.04961	-3.89692	0.239375	0.307919
Erythroid.cells	GM42702	-1.035	2.365724	-1.98935	0.049754	-3.88431	0.242989	0.31154
Erythroid.cells	AU041133	-1.18296	1.264215	-1.98899	0.049794	-3.8318	0.252482	0.321388

Erythroid.cells	GM47601	-1.33133	0.02249	-1.98876	0.04982	-3.83256	0.263556	0.332757
Erythroid.cells	GM36551	-1.22624	-0.80034	-1.98823	0.049879	-3.83772	0.271351	0.340591
Erythroid.cells	HLTF	0.566222	4.64409	1.987304	0.049984	-4.19629	0.225091	0.293082
Erythroid.cells	USP25	0.265336	7.051473	1.987252	0.04999	-4.82423	0.207266	0.274083
Erythroid.cells	GMPS	-0.33123	5.784964	-1.98724	0.049991	-4.47858	0.21645	0.28392
Erythroid.cells	MAPK7	-0.85544	3.195275	-1.98691	0.050028	-3.94301	0.236591	0.305245
Erythroid.cells	TMEM216	-0.70627	4.069162	-1.98681	0.050039	-4.08118	0.229583	0.297911
Erythroid.cells	RPRD1B	0.4179	5.480381	1.986664	0.050056	-4.39578	0.218722	0.286458
Erythroid.cells	AVPI1	0.631242	2.712104	1.986439	0.050081	-4.14328	0.240572	0.309463
Erythroid.cells	BAHD1	-0.82276	2.543391	-1.98587	0.050146	-3.92439	0.242174	0.311118
Erythroid.cells	ARL11	0.95003	1.439587	1.984725	0.050275	-3.89373	0.252102	0.321358
Erythroid.cells	NOP10	0.299922	7.15867	1.984403	0.050312	-4.78774	0.207181	0.274116
Erythroid.cells	LRIF1	0.45179	4.523869	1.98287	0.050486	-4.18426	0.227348	0.295248
Erythroid.cells	SAMSN1	0.42879	7.050554	1.982844	0.050489	-4.66947	0.208488	0.275194
Erythroid.cells	FBXO46	0.971053	2.47185	1.982417	0.050537	-3.90129	0.244061	0.312766
Erythroid.cells	ITFG2	-0.62882	3.893716	-1.98229	0.050552	-4.10456	0.232401	0.300698
Erythroid.cells	CES1G	-1.43609	0.85155	-1.98179	0.050609	-3.86408	0.258263	0.32746
Erythroid.cells	5830448L0	0.804763	0.904406	1.981451	0.050648	-3.99149	0.25787	0.327065
Erythroid.cells	D10WSU1(	-0.7337	3.652731	-1.98023	0.050787	-4.03697	0.235101	0.303347
Erythroid.cells	VASP	-0.44522	6.541669	-1.97904	0.050924	-4.52204	0.213386	0.280244
Erythroid.cells	CYYR1	1.187039	2.282713	1.978282	0.05101	-3.93422	0.247317	0.315728
Erythroid.cells	VPS13B	-0.25777	8.050399	-1.978	0.051043	-4.91554	0.202929	0.268944
Erythroid.cells	SULT2A2	0.784569	4.037802	1.977849	0.05106	-4.45291	0.232828	0.300751
Erythroid.cells	LCP1	-0.32021	8.858643	-1.97726	0.051127	-5.05185	0.197586	0.263096
Erythroid.cells	F13A1	1.350608	2.512117	1.977018	0.051155	-4.20553	0.245617	0.314041
Erythroid.cells	CRYL1	0.687863	3.73595	1.974618	0.051432	-4.05145	0.236633	0.304471
Erythroid.cells	PPP1R18OS	-1.13742	0.993134	-1.97444	0.051453	-3.8495	0.260092	0.328691
Erythroid.cells	MAGT1	0.393623	5.872067	1.973251	0.05159	-4.4538	0.220385	0.28741
Erythroid.cells	FRMD4B	0.751216	5.267906	1.972104	0.051723	-4.39684	0.225475	0.292638
Erythroid.cells	CYTH1	-0.32392	8.072227	-1.97185	0.051753	-4.93956	0.204872	0.270681
Erythroid.cells	MYOF	-1.01477	2.65687	-1.97092	0.051861	-4.05964	0.247087	0.315027
Erythroid.cells	SLC24A1	0.96314	1.860788	1.970102	0.051956	-3.9318	0.254311	0.322311
Erythroid.cells	APOL11B	1.073349	-0.04187	1.969351	0.052044	-4.40555	0.271905	0.340295
Erythroid.cells	DLEU2	-0.32262	8.40867	-1.96904	0.05208	-5.01568	0.203446	0.269114
Erythroid.cells	STXBP5	0.603436	5.383452	1.968186	0.05218	-4.28998	0.225827	0.29305
Erythroid.cells	NMT1	-0.27338	6.941125	-1.96816	0.052182	-4.72255	0.214095	0.280614
Erythroid.cells	CLASP1	-0.35547	6.373608	-1.96802	0.0522	-4.55983	0.218293	0.285093
Erythroid.cells	NEDD4	0.455563	5.933587	1.967615	0.052247	-4.56189	0.221706	0.288717
Erythroid.cells	FSCN1	1.060871	3.378992	1.967384	0.052274	-4.23015	0.242053	0.309996
Erythroid.cells	IMMP1L	0.401247	4.995599	1.967089	0.052308	-4.3716	0.229016	0.29644
Erythroid.cells	HSPA12A	-1.55744	0.150275	-1.96639	0.05239	-3.86095	0.270914	0.339452
Erythroid.cells	KTN1	-0.32549	5.826158	-1.966	0.052436	-4.52857	0.222922	0.290001
Erythroid.cells	USP37	-0.37808	6.268931	-1.96551	0.052494	-4.57911	0.219709	0.286595
Erythroid.cells	HIVEP1	-0.41718	6.40877	-1.96505	0.052547	-4.57772	0.218785	0.285551
Erythroid.cells	GM527	-1.57839	0.481195	-1.96464	0.052596	-3.86495	0.268403	0.336869
Erythroid.cells	GCNT7	0.813811	2.712006	1.964425	0.052622	-3.96536	0.248532	0.316671
Erythroid.cells	XK	1.230876	1.158214	1.963929	0.05268	-3.88246	0.262349	0.330724

Erythroid.cells	NPRL3	0.694174	2.892647	1.963741	0.052702	-4.04806	0.247121	0.315245
Erythroid.cells	PRKCSH	-0.40731	4.862373	-1.96358	0.052721	-4.31575	0.230934	0.298497
Erythroid.cells	GM42477	-1.15124	1.273145	-1.96324	0.052762	-3.87711	0.261311	0.329903
Erythroid.cells	SNRNP40	-0.33193	5.982356	-1.96318	0.052769	-4.53554	0.222231	0.289388
Erythroid.cells	EPHX3	-1.31145	0.657793	-1.96262	0.052834	-3.87001	0.267131	0.335692
Erythroid.cells	ARHGAP18	0.53415	6.528672	1.962126	0.052893	-4.65399	0.21829	0.285212
Erythroid.cells	LARS	0.535451	4.738364	1.962049	0.052902	-4.25819	0.232109	0.299788
Erythroid.cells	PXMP2	0.720369	4.067436	1.962006	0.052907	-4.45885	0.237522	0.30544
Erythroid.cells	KIF5B	-0.22478	7.401742	-1.96165	0.052949	-4.8132	0.211944	0.278412
Erythroid.cells	BORCS8	0.448626	4.590375	1.961173	0.053006	-4.33788	0.233516	0.301197
Erythroid.cells	AFAP1	0.762673	1.955044	1.960328	0.053106	-4.00923	0.255829	0.324504
Erythroid.cells	4930595D1	-1.2449	1.248821	-1.96013	0.05313	-3.88509	0.262138	0.330997
Erythroid.cells	2810408I1	1.190648	1.398496	1.960099	0.053133	-3.90125	0.260787	0.329623
Erythroid.cells	LRRC45	0.886787	2.190744	1.959628	0.053189	-3.94331	0.253758	0.322661
Erythroid.cells	EPB41L4B	1.060573	1.976143	1.959475	0.053207	-3.95134	0.255643	0.324648
Erythroid.cells	ARID4B	-0.25683	8.259309	-1.95943	0.053212	-4.98832	0.20607	0.272563
Erythroid.cells	GSTM5	0.900593	1.19189	1.959343	0.053223	-4.07305	0.262653	0.331802
Erythroid.cells	TMEM123	-0.40048	6.484305	-1.95932	0.053226	-4.61236	0.218951	0.286367
Erythroid.cells	ZSCAN21	-0.77375	3.394527	-1.95851	0.053323	-4.03729	0.243783	0.312349
Erythroid.cells	UQCR11	0.303918	7.224799	1.958136	0.053367	-4.90505	0.213814	0.28087
Erythroid.cells	ADAM15	0.887923	2.475369	1.957861	0.0534	-4.054	0.251678	0.320654
Erythroid.cells	KCTD2	0.699749	3.355478	1.957807	0.053406	-4.04156	0.244164	0.312898
Erythroid.cells	CARM1	0.43276	4.727042	1.956809	0.053525	-4.32229	0.233312	0.301632
Erythroid.cells	NFIL3	0.712428	4.515288	1.956645	0.053545	-4.3673	0.235016	0.303497
Erythroid.cells	SETD3	0.339409	5.687067	1.95569	0.053659	-4.52791	0.226132	0.294154
Erythroid.cells	GSN	0.576235	5.844895	1.954939	0.053749	-4.43934	0.225045	0.293153
Erythroid.cells	ADRB2	0.824479	4.714467	1.954803	0.053766	-4.30197	0.233944	0.302526
Erythroid.cells	2300009A0	0.564217	4.080294	1.95473	0.053774	-4.24303	0.239099	0.307913
Erythroid.cells	HPGD	0.80233	5.673887	1.954314	0.053824	-4.6835	0.226368	0.294686
Erythroid.cells	HPX	0.509569	5.670685	1.954236	0.053834	-4.80781	0.226393	0.294712
Erythroid.cells	TIMM8B	0.456022	5.242586	1.954222	0.053835	-4.47406	0.229741	0.298246
Erythroid.cells	SNRNP35	0.785444	3.056704	1.953331	0.053943	-3.99605	0.248052	0.317259
Erythroid.cells	DEPTOR	0.651327	3.469195	1.95295	0.053988	-4.20938	0.244644	0.31374
Erythroid.cells	GTF3C3	-0.82006	3.102011	-1.95277	0.05401	-4.01417	0.247756	0.31703
Erythroid.cells	NOTCH4	1.437098	0.210596	1.951988	0.054104	-3.89378	0.27409	0.343797
Erythroid.cells	SPTY2D1	-0.38497	5.765398	-1.95163	0.054148	-4.47507	0.226457	0.29465
Erythroid.cells	DYRK3	0.868676	2.520243	1.951438	0.054171	-4.0952	0.253182	0.322506
Erythroid.cells	TSLP	1.628292	-0.44209	1.950923	0.054233	-3.88233	0.280581	0.350168
Erythroid.cells	ARID1B	-0.23699	8.198201	-1.9508	0.054248	-5.01152	0.208496	0.275398
Erythroid.cells	CARMIL1	-0.69017	4.075468	-1.94964	0.054388	-4.18168	0.240626	0.309337
Erythroid.cells	GM32031	0.860806	2.845461	1.949107	0.054453	-4.00241	0.251152	0.320322
Erythroid.cells	D430042O0	-0.63458	3.792114	-1.94901	0.054465	-4.09737	0.243099	0.311999
Erythroid.cells	CDC42BPA	0.767572	3.315927	1.948825	0.054487	-4.07979	0.247116	0.316193
Erythroid.cells	TTLL5	-0.51386	4.471737	-1.9485	0.054527	-4.24613	0.237552	0.306256
Erythroid.cells	ALCAM	0.498043	6.646567	1.948197	0.054563	-4.87998	0.220532	0.28837
Erythroid.cells	KIFAP3	0.664778	2.849059	1.946985	0.054711	-4.03202	0.251815	0.321033
Erythroid.cells	WDR44	0.522893	4.70447	1.946218	0.054804	-4.33203	0.236405	0.30512

Erythroid.cells	SUMO1	-0.16171	7.992553	-1.9461	0.054819	-4.98772	0.211238	0.278441
Erythroid.cells	CSNK1G1	-0.35229	6.535289	-1.94608	0.054821	-4.64408	0.222022	0.289968
Erythroid.cells	9230114K1	-0.78746	2.574674	-1.94585	0.054849	-3.97088	0.254397	0.323846
Erythroid.cells	ZC3H10	0.674364	2.921486	1.945201	0.054928	-4.04317	0.251595	0.320979
Erythroid.cells	PRXL2B	-0.78023	2.256934	-1.94506	0.054945	-4.0104	0.257422	0.326998
Erythroid.cells	NEU1	-0.56298	4.426111	-1.94463	0.054998	-4.21363	0.239025	0.308059
Erythroid.cells	TOMT	1.342417	1.736925	1.944209	0.05505	-3.91695	0.262345	0.33211
Erythroid.cells	E230013L2	-1.45085	-0.72305	-1.94389	0.055089	-3.89281	0.285674	0.355586
Erythroid.cells	PTCD1	-0.81657	2.585716	-1.94274	0.05523	-3.98431	0.255386	0.324687
Erythroid.cells	CPNE1	-0.33559	6.038278	-1.94113	0.055428	-4.55839	0.226905	0.295471
Erythroid.cells	ADAM30	-1.22953	0.62147	-1.94112	0.055429	-3.91929	0.273398	0.343526
Erythroid.cells	PRKAR1B	-1.77494	-0.37005	-1.94102	0.055441	-3.89748	0.28292	0.353111
Erythroid.cells	GM38832	1.025953	0.763984	1.940961	0.055449	-3.96294	0.272057	0.342169
Erythroid.cells	GM15492	-1.36609	0.742028	-1.9409	0.055456	-3.8973	0.272263	0.342378
Erythroid.cells	ROCK1	0.251003	8.134703	1.940851	0.055462	-5.00608	0.211215	0.278722
Erythroid.cells	CX3CR1	0.983067	3.268892	1.940795	0.055469	-4.14112	0.249548	0.31915
Erythroid.cells	ADSSL1	-0.58282	4.426295	-1.94077	0.055472	-4.1885	0.239809	0.309033
Erythroid.cells	MTF1	0.5015	4.234341	1.940661	0.055486	-4.2344	0.241396	0.310689
Erythroid.cells	FAM199X	0.84751	2.776803	1.940575	0.055496	-4.00196	0.253813	0.32355
Erythroid.cells	CRELD2	-0.61262	4.759514	-1.94034	0.055525	-4.27061	0.237097	0.30618
Erythroid.cells	SP3OS	0.557731	4.205373	1.939569	0.05562	-4.21464	0.241964	0.311166
Erythroid.cells	TRERF1	0.646362	4.396216	1.938815	0.055713	-4.43657	0.240525	0.309717
Erythroid.cells	GM36723	0.721516	3.324777	1.938696	0.055728	-4.50974	0.249554	0.319136
Erythroid.cells	CLEC2G	1.343393	0.015976	1.93857	0.055744	-3.91333	0.279718	0.349907
Erythroid.cells	SYK	-0.33701	8.336181	-1.93836	0.05577	-5.0478	0.210178	0.277641
Erythroid.cells	2010013B2	0.841148	3.545259	1.938313	0.055776	-4.07104	0.247667	0.317251
Erythroid.cells	GM43707	-1.59435	0.407926	-1.93678	0.055966	-3.90271	0.276779	0.346832
Erythroid.cells	SAMD1	-0.4594	4.976188	-1.93615	0.056044	-4.39937	0.236707	0.305685
Erythroid.cells	GM26756	-1.51252	0.354794	-1.93584	0.056082	-3.91786	0.277608	0.347753
Erythroid.cells	PROSCOS	-1.6465	0.287878	-1.93566	0.056105	-3.9043	0.27825	0.348416
Erythroid.cells	GM11998	-1.59308	0.318253	-1.93534	0.056144	-3.90329	0.278034	0.348148
Erythroid.cells	DNAIC1	1.406705	0.436784	1.934195	0.056287	-3.91062	0.277482	0.34743
Erythroid.cells	DDX58	-0.61067	4.759373	-1.93364	0.056356	-4.37277	0.23928	0.308278
Erythroid.cells	CIAO1	0.561603	3.481337	1.932694	0.056475	-4.13124	0.250445	0.319749
Erythroid.cells	NDRG2	0.770781	2.964877	1.931375	0.05664	-4.26056	0.255572	0.324675
Erythroid.cells	GM20406	-1.31652	-0.53168	-1.93074	0.05672	-3.91052	0.288459	0.357966
Erythroid.cells	RPUSD4	0.814059	2.682051	1.930589	0.056739	-4.02234	0.258183	0.327438
Erythroid.cells	TNFRSF26	1.085977	2.617995	1.930429	0.056759	-4.00325	0.258753	0.328083
Erythroid.cells	ASNS	0.824137	1.465562	1.930396	0.056763	-4.06892	0.269241	0.338766
Erythroid.cells	TAF7	-0.39912	5.1473	-1.93014	0.056795	-4.39993	0.237225	0.305853
Erythroid.cells	TENM4	1.510339	1.760128	1.929709	0.056849	-3.92965	0.266692	0.336221
Erythroid.cells	ADH5	0.359312	6.03865	1.927767	0.057094	-4.74702	0.230998	0.299021
Erythroid.cells	RIPK2	0.620167	4.205503	1.92775	0.057096	-4.28436	0.245997	0.314677
Erythroid.cells	OXCT1	0.361403	5.719852	1.927565	0.05712	-4.54923	0.233536	0.301687
Erythroid.cells	GM15518	1.20572	0.80065	1.927189	0.057167	-3.96176	0.276727	0.346038
Erythroid.cells	RP2	-0.47162	5.226456	-1.92676	0.057221	-4.3706	0.237688	0.306109
Erythroid.cells	ANKRD27	0.736285	3.098779	1.926385	0.057269	-4.05333	0.255724	0.324854

Erythroid.cells	GM43914	0.951525	0.384486	1.926355	0.057273	-4.01417	0.280817	0.350312
Erythroid.cells	NKAPD1	0.554894	4.086425	1.926264	0.057284	-4.20741	0.24718	0.316066
Erythroid.cells	ZFP36	0.438987	7.849088	1.926045	0.057312	-4.96295	0.217296	0.284699
Erythroid.cells	NR1D1	1.369584	1.661874	1.925583	0.05737	-3.95905	0.268799	0.338344
Erythroid.cells	RINL	0.693391	3.632551	1.92552	0.057378	-4.15966	0.251155	0.320297
Erythroid.cells	METTL9	-0.42035	5.718204	-1.92487	0.057461	-4.51781	0.234027	0.302437
Erythroid.cells	GM10802	-1.4387	-0.25172	-1.92346	0.057641	-3.92086	0.288215	0.357458
Erythroid.cells	CYP4V3	-0.87265	2.78654	-1.92319	0.057675	-4.18216	0.25954	0.328556
Erythroid.cells	CDKN1A	0.55604	5.267608	1.923053	0.057692	-4.53493	0.238318	0.306642
Erythroid.cells	MYO10	-0.64181	5.227281	-1.92271	0.057736	-4.4099	0.238727	0.30711
Erythroid.cells	REPS2	1.30245	0.905504	1.922445	0.057769	-3.94417	0.277066	0.34645
Erythroid.cells	BCL11B	0.928754	1.080616	1.922183	0.057803	-4.23175	0.275436	0.344826
Erythroid.cells	TLE5	0.273652	7.209326	1.921225	0.057925	-4.9286	0.223502	0.291149
Erythroid.cells	OTULINL	0.506674	5.347398	1.920691	0.057994	-4.56103	0.238392	0.30676
Erythroid.cells	PSMA3	-0.2212	7.755055	-1.91951	0.058145	-5.00249	0.219906	0.28707
Erythroid.cells	RFWD3	-0.36124	5.6362	-1.91942	0.058156	-4.58021	0.236438	0.304522
Erythroid.cells	NLRC3	-0.90215	1.662225	-1.91935	0.058166	-4.01691	0.271074	0.340129
Erythroid.cells	SFXN3	0.634528	3.370072	1.919013	0.058209	-4.20318	0.255659	0.324452
Erythroid.cells	RAD18	0.598569	4.103468	1.918773	0.058239	-4.31706	0.249313	0.317952
Erythroid.cells	BTBD1	0.312425	6.497876	1.918344	0.058294	-4.72633	0.229771	0.297517
Erythroid.cells	YRDC	-0.4813	5.035051	-1.91794	0.058346	-4.38019	0.241694	0.310005
Erythroid.cells	VRK2	0.460345	5.691956	1.91764	0.058385	-4.49856	0.236351	0.304505
Erythroid.cells	ARCN1	0.372412	5.873425	1.917472	0.058407	-4.54026	0.234885	0.303015
Erythroid.cells	ZFP251	-0.90645	2.407099	-1.91674	0.058501	-4.0144	0.264848	0.333888
Erythroid.cells	BATF2	-1.66851	0.948583	-1.91667	0.05851	-3.93852	0.278512	0.347713
Erythroid.cells	4632404H1	1.36552	1.238383	1.915692	0.058636	-3.94222	0.276212	0.345272
Erythroid.cells	ARHGAP17	-0.33306	7.245574	-1.91551	0.05866	-4.85507	0.224697	0.292121
Erythroid.cells	0610012G(	0.385611	5.039442	1.914623	0.058774	-4.44559	0.2427	0.310881
Erythroid.cells	UTP23	-0.59511	3.577367	-1.91401	0.058854	-4.14631	0.255269	0.32405
Erythroid.cells	WNK1	0.284475	9.07644	1.913823	0.058878	-5.21703	0.211469	0.278099
Erythroid.cells	U2SURP	-0.26348	6.667671	-1.91381	0.058879	-4.7596	0.229584	0.297381
Erythroid.cells	IGLV3	1.567626	-0.70552	1.913734	0.058889	-3.93287	0.295904	0.365088
Erythroid.cells	DOK3	-0.49373	5.36627	-1.91341	0.058932	-4.59845	0.24012	0.308467
Erythroid.cells	BRMS1L	-0.51248	4.269409	-1.91308	0.058974	-4.25437	0.249408	0.31808
Erythroid.cells	EPHA4	1.81865	0.083978	1.912227	0.059085	-3.93487	0.288427	0.3577
Erythroid.cells	1700017B(	-0.51836	5.285252	-1.9122	0.059089	-4.43769	0.241122	0.309528
Erythroid.cells	PEX10	-1.32242	0.82136	-1.91186	0.059133	-3.93799	0.281264	0.350536
Erythroid.cells	GM16310	-1.37817	1.197906	-1.91153	0.059175	-3.94605	0.277714	0.347047
Erythroid.cells	E130215H2	1.528141	-0.13171	1.911012	0.059243	-3.93607	0.290925	0.360293
Erythroid.cells	IL1R2	1.21116	3.320344	1.910883	0.05926	-4.18977	0.258271	0.327355
Erythroid.cells	GM28529	-1.54122	0.724072	-1.91004	0.05937	-3.93786	0.282858	0.352238
Erythroid.cells	CYCS	0.351588	7.712668	1.909485	0.059442	-5.07479	0.222643	0.290145
Erythroid.cells	SYT14	0.729342	0.008242	1.909166	0.059484	-4.34814	0.29015	0.359567
Erythroid.cells	MPLKIP	-0.4042	4.793719	-1.90912	0.05949	-4.35662	0.246045	0.314767
Erythroid.cells	MRE11A	-0.44078	4.349103	-1.90869	0.059546	-4.28479	0.24986	0.318817
Erythroid.cells	TTBK2	1.001286	1.314702	1.908683	0.059547	-3.96841	0.277386	0.346896
Erythroid.cells	SLC40A1	0.722242	5.835325	1.908427	0.059581	-4.66997	0.237467	0.30601

Erythroid.cells	GMFG	-0.37353	7.424355	-1.90801	0.059636	-4.87352	0.224954	0.292911
Erythroid.cells	CNOT4	-0.25586	7.523005	-1.90793	0.059646	-4.95884	0.224197	0.292121
Erythroid.cells	LIMS2	1.430155	0.424817	1.907481	0.059705	-3.94587	0.286196	0.356125
Erythroid.cells	RTN3	-0.30568	8.060675	-1.90732	0.059726	-5.03781	0.220161	0.287976
Erythroid.cells	RNF113A2	-0.54057	4.271264	-1.90727	0.059733	-4.27873	0.250668	0.320028
Erythroid.cells	PAFAH1B3	-0.43566	5.814072	-1.90532	0.059989	-4.69124	0.238657	0.307385
Erythroid.cells	ARF4OS	-1.24079	0.845404	-1.90451	0.060095	-3.94851	0.283542	0.353262
Erythroid.cells	ITGB2L	1.122803	-0.61932	1.90404	0.060157	-3.95805	0.298375	0.368004
Erythroid.cells	LIFR	0.738913	3.842745	1.903935	0.060171	-4.34639	0.255821	0.325086
Erythroid.cells	CD200	1.132773	1.910379	1.903723	0.060199	-4.04504	0.273438	0.343047
Erythroid.cells	PLEKHA5	0.430329	4.693302	1.902561	0.060353	-4.575	0.248989	0.317945
Erythroid.cells	BATF3	0.768073	2.332688	1.901282	0.060522	-4.2503	0.270702	0.339984
Erythroid.cells	NDUFB9	0.272944	7.069533	1.900725	0.060596	-4.96708	0.230203	0.298149
Erythroid.cells	PITPNC1	-0.3371	8.799744	-1.90062	0.06061	-5.21735	0.217008	0.284132
Erythroid.cells	ABCC5	-0.73557	4.301545	-1.90042	0.060637	-4.273	0.253122	0.322094
Erythroid.cells	SNAPC3	-0.50668	4.825524	-1.89979	0.06072	-4.38638	0.248843	0.317731
Erythroid.cells	EI24	0.395373	4.630195	1.89956	0.060751	-4.43519	0.250538	0.319535
Erythroid.cells	MECR	0.619921	3.581314	1.898707	0.060865	-4.21243	0.260113	0.329384
Erythroid.cells	RC3H2	-0.33443	5.728871	-1.8983	0.060919	-4.58298	0.241733	0.310418
Erythroid.cells	ARMC9	0.786423	2.484131	1.897937	0.060967	-4.04596	0.270357	0.339832
Erythroid.cells	TRIR	0.298248	6.300182	1.897192	0.061067	-4.76769	0.23742	0.305819
Erythroid.cells	PDK3	-0.61398	4.945028	-1.89612	0.061211	-4.39168	0.249115	0.317941
Erythroid.cells	CEP97	0.680171	2.922474	1.896003	0.061226	-4.17864	0.267059	0.336352
Erythroid.cells	GRIPAP1	-0.37356	5.594976	-1.89583	0.061249	-4.53274	0.243621	0.31224
Erythroid.cells	UNC119	0.385385	5.47027	1.895679	0.061269	-4.5982	0.244666	0.313326
Erythroid.cells	PARD3	0.962053	2.300134	1.895111	0.061345	-4.14667	0.273014	0.342398
Erythroid.cells	GRB7	1.549978	0.183341	1.895016	0.061358	-3.9584	0.293699	0.363174
Erythroid.cells	PRKCD	-0.32458	6.897294	-1.89461	0.061413	-4.78325	0.233249	0.301462
Erythroid.cells	NUDT14	-0.46096	4.342372	-1.89387	0.061512	-4.34582	0.254905	0.32399
Erythroid.cells	TGFBR3	0.835365	3.337964	1.893256	0.061595	-4.19923	0.26404	0.33327
Erythroid.cells	CDIPTOS	-1.25776	-0.30875	-1.89304	0.061624	-3.9602	0.299429	0.368909
Erythroid.cells	2310040G2	0.721366	1.291307	1.892988	0.061631	-4.04367	0.283338	0.352894
Erythroid.cells	2410022M	1.004295	1.591485	1.892748	0.061663	-3.99682	0.280448	0.35002
Erythroid.cells	RTCB	-0.39364	5.290693	-1.89232	0.061721	-4.54697	0.247055	0.315888
Erythroid.cells	CTU1	-1.39585	0.850388	-1.89158	0.061821	-3.96693	0.288059	0.357491
Erythroid.cells	LANCL1	0.516717	3.448045	1.891553	0.061824	-4.21641	0.263386	0.33258
Erythroid.cells	HDDC2	0.572548	3.817561	1.891283	0.061861	-4.2399	0.260059	0.329258
Erythroid.cells	CEP170B	-1.33392	0.457754	-1.89124	0.061867	-3.96272	0.29199	0.361514
Erythroid.cells	AREL1	-0.50271	4.263334	-1.89051	0.061965	-4.31934	0.256313	0.325626
Erythroid.cells	GINS4	-0.6211	3.753755	-1.89043	0.061977	-4.21723	0.260843	0.330305
Erythroid.cells	BPHL	0.516692	3.663858	1.889932	0.062043	-4.37482	0.261651	0.331207
Erythroid.cells	TMF1	0.297838	6.243916	1.889928	0.062044	-4.73173	0.239474	0.308253
Erythroid.cells	FMC1	0.547698	4.072806	1.889902	0.062048	-4.37903	0.257997	0.327459
Erythroid.cells	C1QA	0.988468	5.480946	1.889156	0.062149	-4.57935	0.246115	0.315111
Erythroid.cells	2010001A1	1.088439	1.389339	1.888877	0.062187	-3.99053	0.28338	0.353146
Erythroid.cells	SLC25A46	0.373149	4.587825	1.887656	0.062352	-4.40719	0.254368	0.323464
Erythroid.cells	RBM27	-0.28721	6.399999	-1.88751	0.062372	-4.75705	0.239041	0.307551

Erythroid.cells	NLRX1	-1.08974	1.80919	-1.88683	0.062465	-4.03171	0.280204	0.34977
Erythroid.cells	SERPINE2	1.107325	1.29875	1.886517	0.062507	-4.03152	0.285256	0.354935
Erythroid.cells	NR1H4	1.098647	1.386536	1.886002	0.062578	-4.05264	0.284595	0.354251
Erythroid.cells	CPB2	0.585455	3.209826	1.885435	0.062655	-4.41566	0.267479	0.336933
Erythroid.cells	SPTAN1	-0.35047	6.559592	-1.88479	0.062743	-4.78225	0.238554	0.307166
Erythroid.cells	PPFIBP2	0.656404	4.285147	1.884739	0.06275	-4.55953	0.257908	0.327245
Erythroid.cells	SUSD6	-0.33105	7.690279	-1.88429	0.062811	-4.9723	0.229511	0.297716
Erythroid.cells	PUS7L	-1.02328	1.839535	-1.88405	0.062844	-4.02857	0.280567	0.350414
Erythroid.cells	TIMP3	1.376347	2.197691	1.884043	0.062845	-4.0827	0.277124	0.346942
Erythroid.cells	ATP11A	-0.75579	2.676915	-1.88403	0.062847	-4.15607	0.272585	0.342348
Erythroid.cells	HSPA4	-0.22214	7.667014	-1.88283	0.063012	-5.02447	0.23014	0.298431
Erythroid.cells	COG7	-0.78224	2.604364	-1.88257	0.063047	-4.08229	0.273799	0.343703
Erythroid.cells	NCF2	0.442132	6.576577	1.882569	0.063048	-4.75175	0.238879	0.307727
Erythroid.cells	GM48742	-1.33542	0.618325	-1.88225	0.063091	-3.97639	0.293292	0.363329
Erythroid.cells	PLCB2	0.738342	3.320212	1.881599	0.063181	-4.14311	0.267473	0.337339
Erythroid.cells	GM4890	1.334337	0.373848	1.880726	0.063301	-3.97635	0.296516	0.366537
Erythroid.cells	MFSD2B	1.107846	0.599514	1.880432	0.063341	-4.00804	0.294249	0.364387
Erythroid.cells	FAM171A1	1.207584	1.565475	1.880148	0.063381	-4.00997	0.284602	0.354861
Erythroid.cells	ETFDH	0.374636	4.872939	1.880101	0.063387	-4.55964	0.253975	0.323675
Erythroid.cells	SRPK3	-0.99804	1.292575	-1.87966	0.063448	-4.04394	0.28745	0.357638
Erythroid.cells	ITM2A	0.825915	2.544803	1.8793	0.063498	-4.20313	0.275405	0.345549
Erythroid.cells	GM13610	-1.2906	0.396658	-1.87885	0.06356	-3.98352	0.296654	0.36699
Erythroid.cells	EXTL3	0.649822	3.56619	1.878816	0.063565	-4.25086	0.265946	0.336036
Erythroid.cells	TRAF3IP3	-0.48105	5.030914	-1.87833	0.063632	-4.41097	0.252998	0.32273
Erythroid.cells	DNMBP	0.693887	3.488772	1.878238	0.063644	-4.18687	0.26677	0.336887
Erythroid.cells	TMEM33	0.348126	5.133185	1.877006	0.063815	-4.56529	0.252431	0.322254
Erythroid.cells	CARD10	0.943021	1.098314	1.87674	0.063852	-4.05939	0.290047	0.360657
Erythroid.cells	ATRX	-0.25837	7.666179	-1.87654	0.06388	-5.02878	0.231466	0.300468
Erythroid.cells	NID1	0.90732	2.394724	1.876521	0.063882	-4.11737	0.277365	0.347953
Erythroid.cells	CSK	-0.3144	6.929767	-1.87644	0.063893	-4.86855	0.237363	0.306722
Erythroid.cells	FLT3	0.708545	2.584668	1.876257	0.063919	-4.31767	0.275555	0.346194
Erythroid.cells	SPA17	-1.13916	0.979282	-1.87615	0.063934	-3.99693	0.291241	0.362003
Erythroid.cells	UQCC1	0.550315	3.934429	1.876136	0.063936	-4.29557	0.263044	0.333442
Erythroid.cells	EXT1	0.398871	7.749502	1.874845	0.064115	-5.16451	0.231361	0.300418
Erythroid.cells	NOP2	0.608919	3.635097	1.87451	0.064162	-4.2021	0.266487	0.336999
Erythroid.cells	CLCN5	0.598501	5.160349	1.874282	0.064194	-4.40428	0.252901	0.323055
Erythroid.cells	RAB3IL1	0.850658	1.73222	1.872605	0.064428	-4.1383	0.285487	0.356175
Erythroid.cells	ITGA1	0.617406	3.828562	1.871982	0.064515	-4.5515	0.2658	0.336179
Erythroid.cells	RBM18	0.438973	4.236008	1.871875	0.06453	-4.36451	0.262102	0.332388
Erythroid.cells	EME1	0.966077	2.299791	1.871377	0.0646	-4.08197	0.280228	0.350901
Erythroid.cells	GM20732	-0.4897	4.58993	-1.87138	0.0646	-4.36362	0.25899	0.329238
Erythroid.cells	GABBR1	1.305722	2.766527	1.871207	0.064624	-4.12359	0.275757	0.346376
Erythroid.cells	PIP5K1A	-0.40955	5.595581	-1.87073	0.06469	-4.58491	0.250358	0.32037
Erythroid.cells	EPS15L1	-0.29977	6.525246	-1.87048	0.064725	-4.81867	0.242518	0.312266
Erythroid.cells	CD300C	1.251879	0.72621	1.870342	0.064745	-4.00228	0.296051	0.366963
Erythroid.cells	GM43329	0.865028	3.105098	1.869862	0.064812	-4.13332	0.272821	0.343698
Erythroid.cells	ADNP	-0.31047	6.50802	-1.86974	0.064829	-4.78459	0.242736	0.312636

Erythroid.cells	NOMO1	-0.67198	3.5373	-1.86963	0.064845	-4.18217	0.268792	0.33959
Erythroid.cells	PSD4	-0.55065	4.541706	-1.86882	0.064959	-4.35129	0.259847	0.330443
Erythroid.cells	GM15564	0.648971	2.474453	1.868682	0.064978	-4.37519	0.279005	0.350041
Erythroid.cells	CDC23	-0.69636	2.927739	-1.86825	0.065039	-4.1361	0.274682	0.345684
Erythroid.cells	MRPL57	0.341956	5.995401	1.868166	0.065051	-4.73464	0.247205	0.317421
Erythroid.cells	NCR1	-0.94167	1.170957	-1.86812	0.065058	-4.28381	0.291833	0.363022
Erythroid.cells	H2-K1	-0.61446	8.786019	-1.86794	0.065083	-5.25994	0.224729	0.293699
Erythroid.cells	ZRANB1	0.2964	6.142303	1.867879	0.065091	-4.75347	0.245964	0.316126
Erythroid.cells	LITAF	-0.25623	8.523937	-1.86745	0.065152	-5.16976	0.226744	0.295999
Erythroid.cells	KDELR1	0.29259	6.091298	1.867376	0.065163	-4.76601	0.246394	0.316765
Erythroid.cells	CSDE1	0.191998	7.576149	1.86721	0.065186	-5.058	0.234194	0.303996
Erythroid.cells	SIRT6	0.847305	2.652878	1.866671	0.065262	-4.11136	0.277295	0.34883
Erythroid.cells	NKG7	-0.55661	4.013022	-1.86649	0.065287	-4.80612	0.264612	0.335963
Erythroid.cells	IGF1	0.646684	5.292109	1.866338	0.065309	-4.80512	0.253238	0.324245
Erythroid.cells	4930481A1	0.983564	1.854963	1.866307	0.065313	-4.058	0.285028	0.356791
Erythroid.cells	PFDN5	0.213785	7.413663	1.866191	0.06533	-5.0338	0.235497	0.30571
Erythroid.cells	TTC5	0.422813	4.64029	1.866097	0.065343	-4.42339	0.258968	0.330292
Erythroid.cells	F11R	-0.64437	3.432174	-1.86593	0.065367	-4.3996	0.269952	0.341587
Erythroid.cells	HAUS8	-0.50585	4.722045	-1.86586	0.065377	-4.39589	0.258242	0.32956
Erythroid.cells	HOOK1	0.719999	2.667358	1.865735	0.065394	-4.18429	0.277157	0.349026
Erythroid.cells	TELO2	-0.98982	2.455163	-1.86474	0.065535	-4.1161	0.279679	0.351351
Erythroid.cells	UHRF2	-0.28362	6.43989	-1.8639	0.065654	-4.79924	0.244238	0.314665
Erythroid.cells	NAA20	-0.36288	5.164245	-1.86344	0.06572	-4.51893	0.255308	0.326221
Erythroid.cells	POLR3H	-0.60557	3.509866	-1.8626	0.06584	-4.25127	0.270626	0.341855
Erythroid.cells	IFI214	-1.23429	1.667368	-1.86215	0.065903	-4.06545	0.288433	0.360073
Erythroid.cells	MAN2B1	0.422957	6.851978	1.862028	0.065921	-4.87802	0.241363	0.311728
Erythroid.cells	DNAJB11	-0.31856	6.073023	-1.86192	0.065936	-4.72453	0.247885	0.318572
Erythroid.cells	SLC50A1	-0.45478	5.247798	-1.86152	0.065993	-4.52435	0.255116	0.326068
Erythroid.cells	JAKMIP1	-0.64094	3.981458	-1.86132	0.066022	-4.37051	0.266467	0.337786
Erythroid.cells	PRPF38A	-0.41602	5.176932	-1.86086	0.066088	-4.55332	0.255896	0.326915
Erythroid.cells	SLC35E4	1.118711	0.657751	1.860066	0.066201	-4.06829	0.299259	0.370953
Erythroid.cells	UQCC3	0.511013	4.016511	1.860057	0.066202	-4.33619	0.266549	0.337875
Erythroid.cells	GM10053	1.119077	1.424678	1.859739	0.066248	-4.06702	0.291498	0.363213
Erythroid.cells	COL13A1	1.442843	0.62777	1.859595	0.066269	-4.00943	0.299626	0.371416
Erythroid.cells	IDI1	-0.62156	3.90584	-1.85803	0.066493	-4.26335	0.2684	0.339773
Erythroid.cells	GM15445	-1.17272	0.994582	-1.85787	0.066516	-4.01727	0.296722	0.368433
Erythroid.cells	SLC7A2	-1.10741	2.094729	-1.85703	0.066637	-4.26114	0.286076	0.357852
Erythroid.cells	AASS	1.180884	1.208483	1.856132	0.066766	-4.07941	0.295341	0.367107
Erythroid.cells	MCM4	-0.53148	5.68459	-1.85596	0.06679	-4.70298	0.253183	0.324178
Erythroid.cells	DEGS2	-1.29785	0.674225	-1.85586	0.066805	-4.0247	0.300837	0.372699
Erythroid.cells	NAALADL2	0.811925	1.964286	1.855373	0.066875	-4.16848	0.287872	0.35975
Erythroid.cells	NARS2	-0.55841	4.271035	-1.85528	0.066889	-4.38334	0.265893	0.337413
Erythroid.cells	GM13986	1.427595	1.376917	1.85505	0.066922	-4.14709	0.293789	0.365751
Erythroid.cells	IGKV2-109	1.299633	-0.87987	1.854799	0.066958	-4.00914	0.317638	0.38944
Erythroid.cells	ADAM9	-0.45756	5.178783	-1.85436	0.067021	-4.54963	0.257929	0.329261
Erythroid.cells	SUZ12	-0.26936	6.954117	-1.85359	0.067133	-4.97233	0.242914	0.313701
Erythroid.cells	DPP9	-0.53022	4.420233	-1.85357	0.067136	-4.37437	0.264958	0.33663

Erythroid.cells	RSRC1	0.314712	6.605429	1.853413	0.067158	-4.84202	0.245829	0.316781
Erythroid.cells	TRPM2	0.926684	2.540831	1.852965	0.067223	-4.23847	0.28282	0.354968
Erythroid.cells	CGAS	-0.93141	2.747045	-1.85239	0.067307	-4.1914	0.280858	0.353035
Erythroid.cells	GM16853	-0.92728	2.351019	-1.85237	0.06731	-4.10739	0.284717	0.356945
Erythroid.cells	CD47	0.2203	8.628324	1.852323	0.067316	-5.24929	0.229586	0.299736
Erythroid.cells	GM20186	0.823725	3.559404	1.852154	0.067341	-4.25139	0.273111	0.345183
Erythroid.cells	TRIM35	0.430214	5.607952	1.851709	0.067405	-4.71686	0.254639	0.326211
Erythroid.cells	SMC6	-0.28135	7.579287	-1.85146	0.067441	-5.05132	0.238029	0.30888
Erythroid.cells	MCTP1	0.665302	5.535347	1.851438	0.067445	-4.84627	0.255274	0.326965
Erythroid.cells	FOXN2	-0.31405	6.146142	-1.8499	0.067669	-4.7425	0.250717	0.321928
Erythroid.cells	IRGM1	-0.8777	4.93997	-1.84938	0.067745	-4.53954	0.261493	0.332995
Erythroid.cells	ADAM23	0.834739	3.184267	1.848897	0.067815	-4.36952	0.277939	0.349859
Erythroid.cells	GM1043	-1.12191	1.915295	-1.84856	0.067864	-4.16237	0.290394	0.362511
Erythroid.cells	PLEK	0.447294	7.520494	1.848352	0.067894	-5.04768	0.239555	0.310149
Erythroid.cells	NUDCD3	-0.28735	5.998557	-1.84829	0.067904	-4.7211	0.252356	0.323572
Erythroid.cells	TBL2	-0.72905	2.392625	-1.84734	0.068042	-4.09922	0.286121	0.35813
Erythroid.cells	FAM53A	-0.5337	3.797306	-1.84608	0.068227	-4.33747	0.273184	0.344863
Erythroid.cells	MDH2	0.311052	6.490426	1.845957	0.068245	-4.89024	0.249069	0.320017
Erythroid.cells	BC052040	0.685135	4.165796	1.845751	0.068275	-4.32002	0.269745	0.341484
Erythroid.cells	PRR33	-1.42138	0.255516	-1.84554	0.068306	-4.02335	0.308661	0.380775
Erythroid.cells	MORC2A	0.582242	4.295558	1.845439	0.068321	-4.34435	0.268544	0.34033
Erythroid.cells	ZC3H12B	-1.52476	1.084598	-1.84456	0.06845	-4.02267	0.300401	0.37248
Erythroid.cells	PSMB10	-0.53217	5.706129	-1.84435	0.068481	-4.67259	0.256248	0.327577
Erythroid.cells	SPPL2A	-0.42491	6.600738	-1.84265	0.068731	-4.86556	0.249323	0.320132
Erythroid.cells	1500011B(	0.557656	4.65787	1.841742	0.068866	-4.47165	0.26688	0.338333
Erythroid.cells	SHROOM2	1.319935	1.506317	1.84161	0.068885	-4.07469	0.297468	0.369398
Erythroid.cells	DNAJB13	0.960719	2.103334	1.841234	0.068941	-4.10238	0.291526	0.363428
Erythroid.cells	KLHDC4	-0.60319	3.945597	-1.84087	0.068995	-4.2988	0.273711	0.345378
Erythroid.cells	ZFYVE26	-0.50897	5.03643	-1.84055	0.069042	-4.45833	0.263644	0.335156
Erythroid.cells	UBE2Z	-0.34699	5.437928	-1.84044	0.069059	-4.62026	0.260037	0.331431
Erythroid.cells	BRCC3	0.413057	4.874732	1.840311	0.069077	-4.55297	0.265112	0.336674
Erythroid.cells	GM49961	-1.33131	0.574285	-1.83981	0.069152	-4.04328	0.307634	0.379546
Erythroid.cells	2210016L2	-0.60605	3.97848	-1.83964	0.069176	-4.33645	0.273578	0.345267
Erythroid.cells	NDUFS4	-0.28369	6.335519	-1.83939	0.069213	-4.87123	0.252327	0.323414
Erythroid.cells	ONECUT2	0.987151	1.559667	1.839264	0.069233	-4.18158	0.297356	0.369411
Erythroid.cells	SPAG1	-1.23165	1.4147	-1.83863	0.069327	-4.05841	0.298893	0.37117
Erythroid.cells	A530017D2	0.853179	2.325165	1.838628	0.069327	-4.11845	0.289654	0.361893
Erythroid.cells	UQCR10	0.292158	6.961957	1.838526	0.069342	-5.06942	0.247015	0.318077
Erythroid.cells	YARS2	0.727518	3.150094	1.838366	0.069366	-4.21868	0.281539	0.353717
Erythroid.cells	CLEC1A	1.169315	0.972503	1.838268	0.069381	-4.1119	0.303488	0.375833
Erythroid.cells	DMD	0.912655	2.419953	1.836956	0.069576	-4.20111	0.289295	0.361444
Erythroid.cells	PID1	0.387317	5.484883	1.836902	0.069584	-5.06911	0.260357	0.331898
Erythroid.cells	PTGR1	-0.75162	3.377249	-1.83661	0.069628	-4.29541	0.279912	0.352067
Erythroid.cells	HDAC3	-0.45377	4.707537	-1.83659	0.069631	-4.42326	0.267397	0.339269
Erythroid.cells	G3BP1	-0.31118	6.728766	-1.83618	0.069692	-4.90959	0.249616	0.320823
Erythroid.cells	KCND1	1.366931	-0.19065	1.835942	0.069728	-4.04596	0.316718	0.38902
Erythroid.cells	C130013H(	-1.16198	0.498295	-1.83561	0.069778	-4.03615	0.309273	0.381702

Erythroid.cells	CAPRIN1	-0.21885	7.460311	-1.83556	0.069784	-5.0633	0.243455	0.314487
Erythroid.cells	SLC41A3	0.819122	1.73389	1.835437	0.069803	-4.15489	0.296364	0.368857
Erythroid.cells	SLC25A19	0.713792	3.390435	1.83522	0.069835	-4.21707	0.279941	0.352344
Erythroid.cells	AMT	0.980876	1.565291	1.83496	0.069874	-4.17912	0.298162	0.370812
Erythroid.cells	PSMD5	0.520608	3.835237	1.834117	0.070001	-4.33997	0.27611	0.348337
Erythroid.cells	IFIT1BL1	-1.59591	0.159661	-1.83396	0.070025	-4.06898	0.313409	0.385813
Erythroid.cells	GALNT2	-0.42682	5.565067	-1.83358	0.070081	-4.68532	0.260185	0.331997
Erythroid.cells	6720489N1	-1.11431	0.964398	-1.83348	0.070096	-4.05732	0.304825	0.37734
Erythroid.cells	ELP6	-0.86587	2.398694	-1.8334	0.070108	-4.15571	0.290113	0.362633
Erythroid.cells	NXF1	-0.4029	5.180351	-1.83253	0.070239	-4.55248	0.263928	0.335902
Erythroid.cells	PCBD1	0.720932	3.53173	1.832375	0.070262	-4.59893	0.279312	0.351716
Erythroid.cells	STYK1	0.979078	0.538888	1.832251	0.070281	-4.14731	0.30967	0.382259
Erythroid.cells	CD40	-1.07671	2.17854	-1.83216	0.070294	-4.21691	0.292639	0.365232
Erythroid.cells	MYOM1	1.138879	1.071853	1.83193	0.070329	-4.0864	0.304057	0.376735
Erythroid.cells	NEURL1B	1.058603	0.43588	1.831175	0.070443	-4.07529	0.311186	0.383793
Erythroid.cells	TMEM221	1.476528	-0.26558	1.830694	0.070515	-4.03942	0.319018	0.39152
Erythroid.cells	TLR11	0.820221	-0.67266	1.83028	0.070578	-4.16302	0.323694	0.396084
Erythroid.cells	NET1	0.622759	4.035875	1.82996	0.070626	-4.41991	0.275205	0.347585
Erythroid.cells	RSL24D1	-0.34804	5.674542	-1.82975	0.070658	-4.70369	0.260149	0.332128
Erythroid.cells	ELK3	-0.43594	5.288608	-1.8297	0.070666	-4.6616	0.263615	0.33572
Erythroid.cells	GM20707	-0.8785	1.898927	-1.82949	0.070697	-4.0846	0.296239	0.368991
Erythroid.cells	GTF2H5	-0.27564	6.547345	-1.82892	0.070784	-4.93004	0.25271	0.32438
Erythroid.cells	IRF2BP2	-0.29292	7.59206	-1.82714	0.071053	-5.02999	0.244677	0.315679
Erythroid.cells	CPT1A	-0.33509	5.446894	-1.82674	0.071113	-4.70722	0.263369	0.335259
Erythroid.cells	BLCAP	0.748212	3.009547	1.82646	0.071156	-4.18309	0.286383	0.358885
Erythroid.cells	CLNK	0.860192	1.404175	1.826364	0.071171	-4.34553	0.302678	0.375269
Erythroid.cells	PLTP	1.007674	5.244881	1.826311	0.071179	-4.50488	0.2652	0.337253
Erythroid.cells	GM1604A	-1.01956	2.804564	-1.82611	0.071209	-4.15334	0.288423	0.361062
Erythroid.cells	ATP1A3	0.979675	1.709416	1.825267	0.071337	-4.19356	0.299942	0.372546
Erythroid.cells	PLP2	0.531311	5.623922	1.824333	0.07148	-4.68122	0.262574	0.334394
Erythroid.cells	PROCR	-1.7141	0.279761	-1.82323	0.071648	-4.08982	0.316238	0.388458
Erythroid.cells	YWHAQ	-0.21859	7.746706	-1.82263	0.07174	-5.14965	0.244879	0.315751
Erythroid.cells	IL2RA	-1.07918	2.557963	-1.82162	0.071895	-4.27685	0.293114	0.36515
Erythroid.cells	CFAP53	-1.46423	0.830789	-1.82044	0.072075	-4.07306	0.311738	0.383617
Erythroid.cells	6530402F1	-1.24162	-0.17553	-1.8203	0.072097	-4.0707	0.322759	0.394542
Erythroid.cells	RNF144B	0.706033	2.562148	1.820032	0.072138	-4.30061	0.29372	0.365695
Erythroid.cells	MRPS12	0.445829	4.774775	1.819754	0.072181	-4.54341	0.272209	0.343888
Erythroid.cells	CDPF1	-0.99631	2.220471	-1.81964	0.072199	-4.15104	0.297218	0.369266
Erythroid.cells	COPE	0.289923	6.316581	1.819164	0.072271	-4.9141	0.25835	0.329627
Erythroid.cells	LAT2	-0.49047	4.773693	-1.8186	0.072358	-4.58988	0.272519	0.344201
Erythroid.cells	NRBP1	-0.32353	5.827937	-1.81857	0.072363	-4.76763	0.262839	0.334248
Erythroid.cells	VPS4A	-0.47844	4.241466	-1.81789	0.072467	-4.41643	0.277757	0.349534
Erythroid.cells	FKBP8	0.339693	6.067485	1.817853	0.072473	-4.85	0.260886	0.332221
Erythroid.cells	KCTD12B	1.21743	0.963206	1.817436	0.072538	-4.09757	0.311123	0.383082
Erythroid.cells	TCN2	0.610315	4.791081	1.816967	0.07261	-4.53294	0.272801	0.344519
Erythroid.cells	AP1B1	0.427135	5.190975	1.816895	0.072621	-4.57678	0.269081	0.340705
Erythroid.cells	XPO5	0.597403	3.603677	1.81613	0.07274	-4.30468	0.284478	0.356338

Erythroid.cells	LY6G5B	-1.16088	1.345565	-1.81601	0.072758	-4.07848	0.307498	0.379435
Erythroid.cells	CMTR2	-1.1296	1.0685	-1.81539	0.072854	-4.08464	0.310719	0.382548
Erythroid.cells	TLE1	0.466116	4.060562	1.815042	0.072908	-4.47907	0.280283	0.352147
Erythroid.cells	MAPK8IP3	-0.46484	4.521476	-1.81475	0.072954	-4.44033	0.275878	0.347777
Erythroid.cells	HNRNPDL	-0.20952	7.857159	-1.81464	0.07297	-5.18549	0.246098	0.316936
Erythroid.cells	ICA1	1.11705	2.463147	1.814581	0.07298	-4.1666	0.296128	0.368278
Erythroid.cells	NCAPH2	0.379517	5.344606	1.81452	0.072989	-4.64574	0.268191	0.339898
Erythroid.cells	ELL2	0.498783	6.734248	1.813899	0.073086	-4.97688	0.255866	0.327265
Erythroid.cells	TEX45	-1.57788	0.222756	-1.81376	0.073108	-4.06151	0.320102	0.392284
Erythroid.cells	NOXRED1	-1.2575	0.546984	-1.81371	0.073115	-4.07762	0.316539	0.388769
Erythroid.cells	RAB7B	0.712455	2.804284	1.813517	0.073145	-4.53214	0.29284	0.365161
Erythroid.cells	ADCY9	0.828225	3.136828	1.813324	0.073175	-4.25499	0.289512	0.361849
Erythroid.cells	FNIP1	-0.32445	7.760209	-1.81169	0.073429	-5.11792	0.247782	0.318859
Erythroid.cells	CRYBG1	0.549949	4.169377	1.811596	0.073444	-4.51804	0.280219	0.352414
Erythroid.cells	AK7	-1.23318	1.686834	-1.8113	0.07349	-4.11932	0.305306	0.377746
Erythroid.cells	PHLDB3	-0.69674	2.97973	-1.81108	0.073524	-4.26586	0.29202	0.364511
Erythroid.cells	GM28707	1.343331	1.154193	1.810888	0.073555	-4.07516	0.311	0.383587
Erythroid.cells	SGK3	0.433123	5.592861	1.810378	0.073634	-4.72763	0.266962	0.339063
Erythroid.cells	STX16	0.415954	5.756821	1.810368	0.073636	-4.68818	0.265466	0.337519
Erythroid.cells	TRMT13	0.587802	3.89491	1.810316	0.073644	-4.36171	0.282992	0.355475
Erythroid.cells	GEM	-0.51915	5.207345	-1.80989	0.07371	-4.76536	0.270551	0.342835
Erythroid.cells	ZFP646	-0.63918	3.903418	-1.80973	0.073736	-4.37258	0.282946	0.355508
Erythroid.cells	AKAP13	0.243126	8.500476	1.809712	0.073738	-5.30777	0.241736	0.312805
Erythroid.cells	SERINC5	-0.46691	5.287482	-1.80939	0.073789	-4.70961	0.269889	0.342186
Erythroid.cells	GNB1	-0.16795	8.848488	-1.80896	0.073856	-5.35556	0.239084	0.310043
Erythroid.cells	EXOSC8	-0.48284	5.033192	-1.80867	0.073902	-4.61504	0.272462	0.344943
Erythroid.cells	NDUFAF8	0.356608	5.052226	1.808208	0.073974	-4.65409	0.272446	0.344951
Erythroid.cells	SERPINB6A	0.574004	5.570318	1.807742	0.074047	-4.61403	0.26775	0.340093
Erythroid.cells	FBXO21	-0.58575	3.292952	-1.80766	0.07406	-4.339	0.289544	0.36235
Erythroid.cells	AMOT	1.139405	0.863491	1.807342	0.07411	-4.11313	0.314933	0.387737
Erythroid.cells	2610206C1	-1.34781	0.40853	-1.80692	0.074177	-4.07482	0.320007	0.392926
Erythroid.cells	BCR	-0.38205	5.853797	-1.80677	0.0742	-4.82617	0.265314	0.337699
Erythroid.cells	SMIM1	1.01975	1.759237	1.806669	0.074216	-4.20873	0.305434	0.378455
Erythroid.cells	SUOX	0.821504	1.361958	1.80643	0.074253	-4.17201	0.309687	0.382767
Erythroid.cells	OLFR56	-1.2922	2.067818	-1.80614	0.074299	-4.14711	0.302308	0.375428
Erythroid.cells	A930006K0	0.688536	2.074746	1.805386	0.074418	-4.25954	0.302604	0.375553
Erythroid.cells	TPM3	-0.17124	8.661561	-1.80447	0.074562	-5.32248	0.241648	0.312987
Erythroid.cells	TNS4	-1.37112	-0.27421	-1.80412	0.074617	-4.08413	0.328461	0.401375
Erythroid.cells	HIST1H2BE	0.97485	2.079102	1.804083	0.074624	-4.17059	0.30284	0.375977
Erythroid.cells	SKAP1	0.478773	4.523681	1.803978	0.07464	-5.02679	0.2784	0.351254
Erythroid.cells	APBA3	-0.8628	2.107298	-1.80396	0.074643	-4.17717	0.302546	0.375683
Erythroid.cells	GMEB2	-0.33355	5.764218	-1.80373	0.07468	-4.74211	0.266796	0.33933
Erythroid.cells	CASZ1	0.666532	3.474262	1.803687	0.074686	-4.32336	0.28863	0.361665
Erythroid.cells	ALDH1L1	0.661754	3.098676	1.803036	0.074789	-4.48445	0.292677	0.365709
Erythroid.cells	SCRN3	0.879729	1.928035	1.802832	0.074822	-4.21424	0.304741	0.377871
Erythroid.cells	CPEB4	0.339454	6.51399	1.802052	0.074945	-4.99031	0.260572	0.332752
Erythroid.cells	TRIM21	-0.97446	2.57157	-1.80199	0.074955	-4.20258	0.298361	0.371364

Erythroid.cells	MRPL47	-0.75049	2.974642	-1.80134	0.075058	-4.28012	0.294536	0.367388
Erythroid.cells	HMGXB3	-0.47547	4.408539	-1.80097	0.075117	-4.48988	0.280377	0.353122
Erythroid.cells	PELI1	-0.26458	7.676753	-1.80096	0.075119	-5.16254	0.250681	0.322412
Erythroid.cells	TMEM60	0.474342	4.112089	1.800118	0.075253	-4.47299	0.283643	0.356471
Erythroid.cells	COX6C	0.205954	8.594079	1.799875	0.075291	-5.41279	0.243332	0.314738
Erythroid.cells	FCRLS	2.299759	-1.03209	1.798837	0.075457	-4.07982	0.339116	0.411947
Erythroid.cells	2310039H(	0.445871	4.246315	1.798727	0.075474	-4.57895	0.282687	0.35577
Erythroid.cells	ERI1	-0.41836	5.459943	-1.79868	0.075482	-4.71634	0.271149	0.343962
Erythroid.cells	MAPT	-1.04415	1.597756	-1.79854	0.075503	-4.12808	0.309684	0.383016
Erythroid.cells	CLIC1	-0.27463	8.942892	-1.79839	0.075528	-5.39879	0.240724	0.312241
Erythroid.cells	GM43647	1.30012	-0.94495	1.798277	0.075546	-4.10676	0.338097	0.411083
Erythroid.cells	CABYR	-1.2673	1.135391	-1.79798	0.075594	-4.12316	0.314743	0.388136
Erythroid.cells	KLHL12	-0.50837	4.259168	-1.7973	0.075701	-4.56171	0.282846	0.356039
Erythroid.cells	CLEC14A	1.010541	2.196348	1.797263	0.075708	-4.22786	0.303662	0.377071
Erythroid.cells	MSH6	-0.55906	4.38755	-1.79594	0.07592	-4.50912	0.282281	0.355288
Erythroid.cells	TMEM101	-0.82945	2.322989	-1.79568	0.075962	-4.2016	0.303124	0.376317
Erythroid.cells	MAPK3	0.452024	4.874121	1.795075	0.076059	-4.59368	0.277844	0.350792
Erythroid.cells	H1FX	1.048466	2.127581	1.794992	0.076072	-4.21407	0.305385	0.378676
Erythroid.cells	SALL2	-1.45573	0.090711	-1.794	0.076231	-4.0862	0.328182	0.401149
Erythroid.cells	CCDC152	0.719637	3.284464	1.79364	0.07629	-4.45515	0.294069	0.367178
Erythroid.cells	COL9A3	-1.51074	0.618437	-1.79345	0.07632	-4.09438	0.32239	0.395478
Erythroid.cells	RASD1	-0.63366	3.88428	-1.79308	0.076379	-4.58675	0.288141	0.361347
Erythroid.cells	GM15417	0.718227	2.477041	1.792974	0.076397	-4.30025	0.302445	0.375802
Erythroid.cells	BARD1	0.693482	3.42386	1.792641	0.07645	-4.42508	0.292837	0.366151
Erythroid.cells	INTS9	-0.47676	4.394064	-1.79229	0.076508	-4.49741	0.283332	0.356501
Erythroid.cells	GPATCH8	-0.23904	7.002079	-1.79191	0.076569	-5.07771	0.259211	0.331629
Erythroid.cells	4930403D(	-1.16311	1.041064	-1.79156	0.076625	-4.12098	0.318268	0.391472
Erythroid.cells	VPS26C	-0.57795	4.084075	-1.791	0.076715	-4.41959	0.286748	0.359835
Erythroid.cells	SIGMAR1	0.526261	3.549056	1.790941	0.076725	-4.45632	0.292074	0.365243
Erythroid.cells	TAP2	-0.60361	5.110651	-1.79065	0.076772	-4.61066	0.276876	0.349862
Erythroid.cells	VPS72	-0.45378	4.357985	-1.79026	0.076835	-4.48938	0.284256	0.357452
Erythroid.cells	ZFP983	-0.89647	2.38923	-1.79	0.076877	-4.19625	0.304235	0.377637
Erythroid.cells	TRNT1	-0.4196	4.772449	-1.78949	0.076961	-4.57182	0.280483	0.353566
Erythroid.cells	GK	-0.48518	4.764608	-1.78931	0.07699	-4.74092	0.280559	0.353704
Erythroid.cells	KIF18A	0.671382	3.977118	1.788962	0.077046	-4.46898	0.288266	0.361686
Erythroid.cells	2310022B(	0.570894	2.488564	1.788427	0.077133	-4.30326	0.303424	0.377038
Erythroid.cells	LIMD2	-0.32612	7.333227	-1.78841	0.077136	-5.11584	0.256937	0.329474
Erythroid.cells	NUBPL	0.685982	2.913085	1.788346	0.077146	-4.27729	0.299018	0.372618
Erythroid.cells	GM44699	-1.2107	0.559613	-1.78815	0.077178	-4.10407	0.3243	0.397873
Erythroid.cells	ZFP623	1.424113	0.124749	1.78806	0.077192	-4.09679	0.329205	0.402749
Erythroid.cells	TUBB6	0.654879	3.749371	1.788043	0.077195	-4.54016	0.290533	0.364144
Erythroid.cells	COMTD1	-0.77444	2.92368	-1.78751	0.077281	-4.20835	0.299131	0.372764
Erythroid.cells	CDK11B	-0.31571	6.82853	-1.78637	0.077468	-5.00802	0.262136	0.334699
Erythroid.cells	SMARCC1	-0.317	6.606315	-1.78617	0.0775	-5.00301	0.264147	0.336828
Erythroid.cells	GM49417	1.428375	1.078235	1.785867	0.077549	-4.14257	0.31953	0.392935
Erythroid.cells	XRCC3	1.258752	0.159666	1.785514	0.077607	-4.09896	0.329945	0.403192
Erythroid.cells	2500002B1	0.599036	1.915535	1.784996	0.077692	-4.39752	0.31077	0.384147

Erythroid.cells	SNX2	-0.21114	7.314048	-1.78465	0.077747	-5.14639	0.258203	0.330651
Erythroid.cells	PARP9	-0.67327	5.150171	-1.7846	0.077757	-4.66423	0.278062	0.351205
Erythroid.cells	SNF8	-0.31968	5.731869	-1.78432	0.077802	-4.79966	0.272627	0.345674
Erythroid.cells	ATM	-0.48865	4.221236	-1.78404	0.077848	-4.51106	0.2872	0.360621
Erythroid.cells	CCDC117	-0.42304	4.699485	-1.7835	0.077937	-4.5851	0.282716	0.355928
Erythroid.cells	GSTP1	0.330782	6.928594	1.783352	0.077961	-5.18578	0.261922	0.334511
Erythroid.cells	SGMS2	-0.72216	3.643674	-1.78312	0.077999	-4.64478	0.293201	0.366665
Erythroid.cells	ZFP653	0.678992	3.131364	1.782806	0.07805	-4.29548	0.298502	0.371948
Erythroid.cells	UBL4A	0.54977	4.202976	1.782486	0.078103	-4.52579	0.287735	0.361076
Erythroid.cells	NCBP1	-0.32401	5.256901	-1.78239	0.078119	-4.67312	0.277506	0.350671
Erythroid.cells	ETL4	0.93134	2.396305	1.782137	0.07816	-4.24982	0.306245	0.37981
Erythroid.cells	ZNRF1	-0.34412	6.496008	-1.78186	0.078205	-4.9449	0.266014	0.338897
Erythroid.cells	SAP30BP	-0.40562	4.838744	-1.78178	0.078219	-4.60549	0.281565	0.354907
Erythroid.cells	AKIP1	0.70462	3.24519	1.781467	0.07827	-4.32892	0.297507	0.371068
Erythroid.cells	EPAS1	0.720285	3.448567	1.781254	0.078305	-4.42973	0.295454	0.369046
Erythroid.cells	CD38	-0.71596	5.882237	-1.7798	0.078544	-4.77948	0.272491	0.345321
Erythroid.cells	CFAP126	-1.15584	1.069649	-1.77956	0.078585	-4.15178	0.321607	0.394877
Erythroid.cells	TIAL1	0.286228	5.798885	1.779227	0.078639	-4.86523	0.273397	0.346358
Erythroid.cells	TSEN2	1.045654	1.266976	1.778882	0.078696	-4.14025	0.31964	0.392955
Erythroid.cells	RHOJ	1.020245	1.992484	1.77845	0.078767	-4.22892	0.311907	0.385307
Erythroid.cells	CDC5L	-0.26836	5.768771	-1.77792	0.078855	-4.8098	0.274125	0.347095
Erythroid.cells	TMEM141	0.888379	2.239657	1.776888	0.079026	-4.32701	0.309909	0.383309
Erythroid.cells	FRRS1	0.460696	4.851486	1.776753	0.079048	-4.6364	0.283272	0.356506
Erythroid.cells	MMRN2	1.184313	1.228883	1.776712	0.079055	-4.13246	0.320903	0.394306
Erythroid.cells	ALOX15	2.748188	-0.82153	1.776569	0.079079	-4.1083	0.344443	0.417445
Erythroid.cells	RFXANK	0.712759	2.879474	1.775649	0.079232	-4.27325	0.303624	0.376971
Erythroid.cells	GTF2H2	-0.45814	3.759481	-1.77529	0.079291	-4.45901	0.294677	0.368024
Erythroid.cells	TMEM218	0.85343	1.874833	1.775044	0.079332	-4.17575	0.314487	0.387918
Erythroid.cells	COG3	0.519147	4.292574	1.774408	0.079438	-4.4844	0.289646	0.362785
Erythroid.cells	DLG1	-0.26145	7.219142	-1.77392	0.07952	-5.12776	0.262174	0.33462
Erythroid.cells	UQCRC2	0.241387	6.345631	1.773331	0.079617	-5.04174	0.270308	0.343058
Erythroid.cells	SMARCC2	-0.2819	6.020787	-1.77322	0.079636	-4.87005	0.273333	0.346185
Erythroid.cells	GM43149	1.213927	0.774872	1.77281	0.079704	-4.12944	0.327403	0.400673
Erythroid.cells	TBC1D2	-0.96597	1.45751	-1.77267	0.079728	-4.23134	0.319781	0.393201
Erythroid.cells	FAM91A1	0.358795	5.007003	1.772606	0.079738	-4.62604	0.283002	0.356254
Erythroid.cells	FOXP1	-0.19368	9.650428	-1.77237	0.079778	-5.59545	0.241492	0.313135
Erythroid.cells	LIMK2	-0.48314	4.779797	-1.77236	0.079779	-4.54982	0.285219	0.358595
Erythroid.cells	IL4	1.650525	0.330173	1.771766	0.079879	-4.17805	0.3327	0.406091
Erythroid.cells	POLR1D	-0.23765	6.995878	-1.77162	0.079904	-5.10129	0.264546	0.337374
Erythroid.cells	MOSPD1	0.472758	4.817896	1.771499	0.079923	-4.71126	0.285044	0.358486
Erythroid.cells	NDUFS6	0.342122	5.716521	1.771199	0.079974	-4.88567	0.27639	0.34965
Erythroid.cells	ULK2	0.436902	5.457806	1.77114	0.079983	-4.68832	0.278853	0.352179
Erythroid.cells	ABCF3	-0.52966	3.761251	-1.77078	0.080044	-4.40932	0.295587	0.369346
Erythroid.cells	DTNBP1	-0.24389	6.683213	-1.77068	0.08006	-4.96607	0.26739	0.340505
Erythroid.cells	ALYREF	-0.3142	8.216984	-1.77062	0.08007	-5.34927	0.253743	0.326299
Erythroid.cells	HGSNAT	-0.70239	4.642285	-1.77019	0.080143	-4.5024	0.286923	0.360598
Erythroid.cells	WDR41	0.52418	4.001344	1.76956	0.080248	-4.423	0.293394	0.367244

Erythroid.cells	FABP5	0.453463	7.278075	1.769469	0.080264	-5.3614	0.262219	0.335228
Erythroid.cells	RRAD	-0.78012	2.552475	-1.76945	0.080267	-4.34663	0.308398	0.382353
Erythroid.cells	TENT2	-0.26622	6.624422	-1.76935	0.080283	-4.98914	0.268146	0.341386
Erythroid.cells	TNFRSF11A	0.90003	2.190262	1.768488	0.080428	-4.31591	0.312588	0.38646
Erythroid.cells	MMP12	1.573955	-0.05819	1.768479	0.08043	-4.17831	0.337806	0.41137
Erythroid.cells	4930403P2	-1.18009	0.612375	-1.76822	0.080474	-4.12279	0.330075	0.403899
Erythroid.cells	IPO9	-0.41055	4.771136	-1.76817	0.080482	-4.56974	0.286023	0.359789
Erythroid.cells	USF3	0.468842	4.977969	1.767499	0.080595	-4.6191	0.284232	0.357927
Erythroid.cells	DICER1	-0.37554	4.846948	-1.7671	0.080662	-4.61761	0.285514	0.359393
Erythroid.cells	MRPL44	-0.65737	2.945204	-1.76709	0.080664	-4.34592	0.304812	0.378927
Erythroid.cells	CPEB2	0.431527	5.412476	1.766856	0.080703	-4.69529	0.280026	0.353819
Erythroid.cells	FAM83F	-1.11296	1.041009	-1.76681	0.080712	-4.19287	0.325495	0.399575
Erythroid.cells	USP32	0.331274	6.849617	1.766728	0.080725	-5.09605	0.266576	0.339942
Erythroid.cells	IFT88	0.868162	1.778503	1.765309	0.080965	-4.19821	0.318144	0.392116
Erythroid.cells	ITPR3	-0.55884	4.288295	-1.76466	0.081075	-4.56602	0.292095	0.36595
Erythroid.cells	SCCPDH	0.761787	2.465776	1.764416	0.081116	-4.2521	0.311047	0.385013
Erythroid.cells	PCED1A	-0.75753	2.508638	-1.7635	0.081271	-4.27664	0.310971	0.385018
Erythroid.cells	RECQL	-0.74962	3.363727	-1.76332	0.081302	-4.32065	0.301947	0.375967
Erythroid.cells	SCRIB	-0.55222	3.392621	-1.7633	0.081305	-4.39345	0.301646	0.375664
Erythroid.cells	EME2	-0.98969	0.924802	-1.7629	0.081373	-4.14612	0.328585	0.402452
Erythroid.cells	CCNL2	-0.24904	6.219089	-1.76262	0.08142	-4.95441	0.273935	0.34736
Erythroid.cells	SLC35B2	-0.45548	4.882082	-1.76231	0.081473	-4.63304	0.28687	0.36057
Erythroid.cells	MAPK1IP1I	-0.26707	6.060276	-1.7617	0.081576	-4.89675	0.275757	0.34905
Erythroid.cells	ERLIN2	-0.6557	3.099067	-1.76141	0.081627	-4.33449	0.305361	0.379083
Erythroid.cells	MED6	-0.5018	4.540282	-1.76104	0.081689	-4.57476	0.290706	0.364253
Erythroid.cells	ASNA1	0.418427	4.877847	1.760681	0.08175	-4.65044	0.287468	0.360981
Erythroid.cells	GNPTG	-0.61536	3.368723	-1.76017	0.081837	-4.33182	0.302924	0.376569
Erythroid.cells	CD247	0.574849	3.01096	1.760098	0.08185	-4.65341	0.306678	0.380336
Erythroid.cells	TIMM9	-0.48364	3.882337	-1.75989	0.081885	-4.50155	0.297638	0.37124
Erythroid.cells	FAM49B	-0.19218	9.086243	-1.75966	0.081924	-5.48743	0.249114	0.321264
Erythroid.cells	RNASET2B	-0.58113	5.139167	-1.75943	0.081963	-4.62537	0.285118	0.358647
Erythroid.cells	TTLL3	-0.6175	4.027206	-1.75927	0.081991	-4.44112	0.29622	0.369935
Erythroid.cells	SFT2D3	-0.64663	2.864525	-1.75904	0.082031	-4.29463	0.30835	0.382235
Erythroid.cells	HDGFL2	-0.3957	4.979057	-1.75819	0.082175	-4.6603	0.287024	0.360771
Erythroid.cells	PIF1	1.280771	1.716548	1.757954	0.082216	-4.20333	0.321132	0.395087
Erythroid.cells	TIMM23	-0.23433	6.942518	-1.75761	0.082274	-5.12257	0.26835	0.341739
Erythroid.cells	THSD4	1.779821	0.910314	1.757513	0.082292	-4.15805	0.33019	0.404243
Erythroid.cells	MEF2C	0.353384	7.538963	1.757363	0.082317	-5.25498	0.262936	0.336171
Erythroid.cells	HILPDA	-0.8304	4.798635	-1.75736	0.082318	-4.63668	0.288807	0.362821
Erythroid.cells	NOP14	0.544976	4.004398	1.757262	0.082335	-4.49518	0.296798	0.370935
Erythroid.cells	PTEN	-0.21672	8.288084	-1.75706	0.08237	-5.36518	0.256301	0.329302
Erythroid.cells	ARSG	0.71644	1.867658	1.75697	0.082385	-4.26536	0.319463	0.39373
Erythroid.cells	FLNA	0.480544	6.097597	1.756428	0.082478	-4.85422	0.276435	0.350216
Erythroid.cells	GFRA1	-0.54952	4.918403	-1.75571	0.0826	-4.91065	0.288113	0.36205
Erythroid.cells	ID3	0.440426	6.599763	1.75563	0.082615	-5.04335	0.271978	0.3455
Erythroid.cells	SLC16A3	0.683917	3.935418	1.7553	0.082672	-4.55216	0.29809	0.372153
Erythroid.cells	ADD3	0.436472	6.047415	1.755139	0.082699	-4.97982	0.277244	0.350948

Erythroid.cells	CCDC85C	0.962207	0.977037	1.754981	0.082727	-4.20063	0.330081	0.404195
Erythroid.cells	RHOBTB2	0.770364	2.943311	1.754456	0.082817	-4.29	0.30867	0.382888
Erythroid.cells	HMCN1	1.37394	1.920186	1.753822	0.082926	-4.24056	0.320054	0.394152
Erythroid.cells	GM12971	-1.11301	0.967071	-1.75253	0.08315	-4.15343	0.331444	0.405371
Erythroid.cells	SLC16A13	-1.47768	-0.01818	-1.75247	0.08316	-4.13819	0.342911	0.416616
Erythroid.cells	ZFP275	0.893694	1.581356	1.752251	0.083198	-4.20738	0.324525	0.398586
Erythroid.cells	CSTB	-0.39589	6.672627	-1.75175	0.083284	-5.06229	0.272441	0.346084
Erythroid.cells	ZFP668	-0.49963	3.829078	-1.75172	0.08329	-4.48492	0.30036	0.374594
Erythroid.cells	GM13710	-1.45903	1.36556	-1.75171	0.083292	-4.17959	0.326967	0.401161
Erythroid.cells	OAS1C	1.062071	1.903745	1.751399	0.083345	-4.22786	0.321043	0.395327
Erythroid.cells	FAM78A	-0.8373	2.648934	-1.75103	0.083409	-4.2727	0.31299	0.387326
Erythroid.cells	TRPM4	0.947214	1.109553	1.750917	0.083429	-4.21388	0.330053	0.404293
Erythroid.cells	TMEM56	0.82046	2.147941	1.750553	0.083492	-4.39742	0.318526	0.392927
Erythroid.cells	FERMT3	-0.30578	7.235899	-1.75044	0.083511	-5.10241	0.267466	0.341061
Erythroid.cells	B4GALT1	-0.2618	7.137524	-1.74985	0.083614	-5.15187	0.268602	0.342284
Erythroid.cells	TSACC	1.133811	1.881856	1.749295	0.08371	-4.2383	0.321997	0.396455
Erythroid.cells	PPP2R2D	-0.32764	5.459388	-1.74886	0.083785	-4.79068	0.284864	0.35908
Erythroid.cells	AUTS2	0.86626	4.397144	1.748535	0.083842	-4.68178	0.295507	0.370016
Erythroid.cells	AP1G1	-0.3392	6.456316	-1.74842	0.083863	-4.95675	0.275357	0.34947
Erythroid.cells	GALNS	-0.60504	3.757313	-1.74801	0.083934	-4.41838	0.302229	0.376886
Erythroid.cells	CCDC59	-0.28004	5.676842	-1.74776	0.083977	-4.85839	0.282988	0.357364
Erythroid.cells	SLC38A10	-0.38517	5.326136	-1.74702	0.084106	-4.73066	0.286751	0.361022
Erythroid.cells	TMEM64	0.454334	5.051385	1.746505	0.084196	-4.80872	0.289674	0.36398
Erythroid.cells	LY9	-0.51949	4.262049	-1.74619	0.084251	-4.53626	0.297723	0.372159
Erythroid.cells	RAB12	-0.374	5.073594	-1.74569	0.084338	-4.68208	0.289732	0.364089
Erythroid.cells	ANKRD11	-0.22526	9.391983	-1.74546	0.08438	-5.55327	0.250021	0.322966
Erythroid.cells	EGFR	1.148557	2.469637	1.744765	0.084501	-4.392	0.317265	0.391849
Erythroid.cells	SYN1	-1.38853	0.783746	-1.74452	0.084544	-4.16965	0.336313	0.410796
Erythroid.cells	GM47730	-1.39634	-0.65913	-1.74406	0.084624	-4.14255	0.353595	0.427713
Erythroid.cells	GAS6	1.085181	1.545619	1.744033	0.084629	-4.22331	0.327682	0.402317
Erythroid.cells	AS3MT	0.750094	2.69274	1.743252	0.084766	-4.35049	0.315307	0.389924
Erythroid.cells	TUBA1C	0.354325	7.576145	1.743146	0.084785	-5.3107	0.266664	0.340366
Erythroid.cells	MAP2K3OS	-1.53215	-0.12812	-1.74301	0.084809	-4.16458	0.347535	0.421716
Erythroid.cells	ALDH8A1	0.866782	2.197617	1.74224	0.084944	-4.43793	0.321025	0.395669
Erythroid.cells	ANGPTL4	1.082508	1.23193	1.742205	0.08495	-4.20989	0.331897	0.40644
Erythroid.cells	MAD2L1BP	0.450121	4.194819	1.742054	0.084977	-4.61937	0.299694	0.374312
Erythroid.cells	IFI206	-1.16031	3.940563	-1.74155	0.085065	-4.54111	0.302457	0.377135
Erythroid.cells	IGTP	-0.96197	3.443365	-1.7415	0.085074	-4.51532	0.307676	0.382411
Erythroid.cells	ATP6V1B2	0.315585	5.98524	1.740824	0.085193	-4.86059	0.282249	0.35646
Erythroid.cells	PIGU	0.557194	4.215968	1.740465	0.085257	-4.50681	0.300035	0.374622
Erythroid.cells	GM42982	-0.87405	1.514483	-1.73974	0.085385	-4.23177	0.329572	0.404183
Erythroid.cells	ZSCAN18	1.564993	0.363679	1.739709	0.08539	-4.15618	0.342924	0.417315
Erythroid.cells	ADPRH	-0.46774	4.93883	-1.73941	0.085443	-4.63776	0.293003	0.367573
Erythroid.cells	GM37612	-1.21978	0.985122	-1.73908	0.0855	-4.16734	0.335767	0.410449
Erythroid.cells	NKAIN2	1.181937	1.350359	1.739021	0.085512	-4.26215	0.331562	0.406324
Erythroid.cells	PRAMEF8	0.781013	3.188085	1.738728	0.085564	-4.32853	0.311289	0.386203
Erythroid.cells	GM4876	0.794499	1.628342	1.738325	0.085635	-4.27568	0.328637	0.403533

Erythroid.cells	KRT81	-1.27085	-1.53173	-1.73802	0.08569	-4.15337	0.366624	0.440533
Erythroid.cells	OCIAD1	-0.28128	5.885315	-1.73736	0.085805	-4.91614	0.284238	0.358622
Erythroid.cells	HEXA	0.431029	5.752202	1.737127	0.085847	-4.80835	0.285538	0.360024
Erythroid.cells	CLASP2	-0.2769	6.927855	-1.73702	0.085866	-5.14716	0.274273	0.348472
Erythroid.cells	UBR1	-0.41742	5.323458	-1.73654	0.085951	-4.79772	0.289951	0.364461
Erythroid.cells	UGCG	-0.34354	6.436724	-1.73637	0.085981	-5.12875	0.279097	0.353405
Erythroid.cells	GM15478	-0.50815	4.735611	-1.7362	0.086011	-4.63747	0.295861	0.370594
Erythroid.cells	FAM234A	0.658793	4.099085	1.734914	0.086241	-4.55191	0.303085	0.377471
Erythroid.cells	UBE2G2	-0.39818	4.661055	-1.73452	0.086311	-4.65451	0.297287	0.371879
Erythroid.cells	DBN1	1.023617	1.388658	1.734376	0.086337	-4.30451	0.332745	0.40736
Erythroid.cells	GM36486	1.196225	0.805963	1.734302	0.08635	-4.24938	0.339504	0.41403
Erythroid.cells	MTMR10	0.798803	2.897646	1.734062	0.086393	-4.38022	0.315878	0.390704
Erythroid.cells	2610021A0	-0.79407	2.362444	-1.73404	0.086396	-4.28961	0.321757	0.396564
Erythroid.cells	POSTN	1.123039	1.260435	1.733915	0.086419	-4.25969	0.334221	0.408978
Erythroid.cells	NTM	1.153145	0.913874	1.73371	0.086455	-4.2237	0.338265	0.412948
Erythroid.cells	TRAPPC2	0.505272	4.171105	1.733444	0.086503	-4.57108	0.302415	0.377311
Erythroid.cells	ANGPTL1	1.242564	0.376343	1.732869	0.086606	-4.18307	0.344943	0.419482
Erythroid.cells	ATP11C	-0.24923	6.869185	-1.73272	0.086633	-5.13659	0.275911	0.35018
Erythroid.cells	ZFP706	-0.2002	7.871583	-1.73239	0.086691	-5.35032	0.266707	0.340673
Erythroid.cells	VSIG10	1.162506	0.47062	1.731554	0.086841	-4.19792	0.344404	0.418953
Erythroid.cells	TUBB2A	0.442206	5.517234	1.730906	0.086957	-4.97238	0.289666	0.364282
Erythroid.cells	TNK2	0.666239	3.209858	1.730841	0.086969	-4.40135	0.313569	0.388448
Erythroid.cells	LMTK3	-1.23333	-0.70138	-1.73072	0.08699	-4.17264	0.358865	0.433094
Erythroid.cells	NDUFA13	0.228227	7.128419	1.730376	0.087052	-5.24539	0.274219	0.348406
Erythroid.cells	ADAM19	0.538743	5.568234	1.730137	0.087095	-4.95931	0.289305	0.363997
Erythroid.cells	RSBN1	-0.40376	5.326631	-1.7293	0.087245	-4.75337	0.292111	0.366926
Erythroid.cells	COQ9	0.550372	3.205352	1.729	0.087299	-4.50173	0.31429	0.389305
Erythroid.cells	RBM5	-0.24682	6.623423	-1.72863	0.087365	-5.07653	0.279606	0.354149
Erythroid.cells	GM35853	0.909343	0.184156	1.727856	0.087506	-4.28242	0.349219	0.424022
Erythroid.cells	DDX55	-0.71189	2.934608	-1.72778	0.087519	-4.3523	0.317612	0.392817
Erythroid.cells	AP3S2	-0.5933	3.43138	-1.72774	0.087526	-4.42443	0.312226	0.387425
Erythroid.cells	LRMDA	-0.51569	7.760594	-1.72713	0.087636	-5.34423	0.269264	0.343664
Erythroid.cells	LIPH	-1.48229	-0.05189	-1.72705	0.08765	-4.16629	0.352211	0.427116
Erythroid.cells	CLINT1	-0.21977	8.205941	-1.72689	0.08768	-5.40094	0.265204	0.339483
Erythroid.cells	NXPE3	0.60895	3.424156	1.726789	0.087698	-4.45221	0.312423	0.387832
Erythroid.cells	SUSD3	0.610704	3.170727	1.726702	0.087714	-4.45693	0.315161	0.390587
Erythroid.cells	SLC25A30	-0.53774	3.550303	-1.72633	0.087782	-4.49424	0.311181	0.386539
Erythroid.cells	ARL8A	-0.3366	6.227073	-1.72595	0.087849	-4.93528	0.283859	0.358855
Erythroid.cells	TSC22D3	0.545653	5.71665	1.725891	0.08786	-4.86337	0.288866	0.364009
Erythroid.cells	TTC23	1.050572	1.101964	1.725848	0.087868	-4.22811	0.338579	0.413892
Erythroid.cells	CD96	-1.01226	0.686804	-1.72469	0.088078	-4.38411	0.344165	0.419026
Erythroid.cells	EGR3	1.101293	3.533307	1.724222	0.088163	-4.51532	0.312188	0.387264
Erythroid.cells	GORASP2	0.303683	5.534187	1.723849	0.08823	-4.80773	0.291476	0.366392
Erythroid.cells	WDR45	0.587676	2.960786	1.723838	0.088232	-4.43019	0.318432	0.393598
Erythroid.cells	USE1	0.393467	5.084893	1.723394	0.088313	-4.74174	0.296081	0.371085
Erythroid.cells	MGME1	-0.90933	2.312135	-1.72337	0.088317	-4.29424	0.325713	0.400852
Erythroid.cells	LIN54	-0.39664	6.14118	-1.72292	0.088399	-5.01607	0.285715	0.36045

Erythroid.cells	PNPLA6	-0.66573	2.760588	-1.72248	0.088479	-4.34372	0.321083	0.396265
Erythroid.cells	CHRAC1	-0.33608	5.264928	-1.72182	0.088599	-4.79936	0.294856	0.369776
Erythroid.cells	ZBTB14	-0.56649	3.004063	-1.7217	0.08862	-4.38387	0.318687	0.393809
Erythroid.cells	ACAT2	0.628313	2.91084	1.721201	0.088712	-4.3958	0.319906	0.395026
Erythroid.cells	GRHPR	0.47495	4.431109	1.721069	0.088736	-4.86635	0.303604	0.378736
Erythroid.cells	LETM2	0.66395	3.131534	1.720609	0.08882	-4.4006	0.317551	0.392711
Erythroid.cells	ZFP982	-1.60322	0.056297	-1.72051	0.088838	-4.1744	0.353082	0.427752
Erythroid.cells	DYNC2H1	-0.58107	3.842041	-1.72046	0.088847	-4.51833	0.30988	0.385042
Erythroid.cells	IRAK3	-0.8233	4.187779	-1.72021	0.088893	-4.6023	0.30627	0.381402
Erythroid.cells	1810024B0	0.746062	2.692562	1.719683	0.088989	-4.35199	0.32262	0.397734
Erythroid.cells	KDM4D	1.11364	0.328103	1.7196	0.089004	-4.23302	0.350036	0.424748
Erythroid.cells	GAPVD1	0.247011	6.832806	1.719394	0.089042	-5.14961	0.279887	0.354463
Erythroid.cells	NBEAL1	0.462306	4.937374	1.719189	0.089079	-4.69383	0.298692	0.373717
Erythroid.cells	CLEC12A	-0.62719	4.937614	-1.71899	0.089116	-4.73853	0.29871	0.37378
Erythroid.cells	VAMP4	-0.31905	6.154005	-1.71801	0.089295	-4.94406	0.286881	0.361782
Erythroid.cells	MED17	-0.33483	4.867425	-1.71791	0.089313	-4.68769	0.299822	0.374984
Erythroid.cells	SLC25A22	0.866037	2.495595	1.717844	0.089326	-4.38176	0.325312	0.400607
Erythroid.cells	GM16794	-1.19044	0.41499	-1.71723	0.089439	-4.18775	0.34979	0.424761
Erythroid.cells	SMCR8	0.688549	3.28105	1.717098	0.089462	-4.40061	0.316874	0.392317
Erythroid.cells	MEX3D	-0.79017	2.642534	-1.71696	0.089488	-4.35238	0.32392	0.399377
Erythroid.cells	AIG1	-0.46801	4.903663	-1.7167	0.089536	-4.80118	0.299736	0.375092
Erythroid.cells	PON3	-0.70378	3.489649	-1.71468	0.089908	-4.4766	0.315764	0.390873
Erythroid.cells	WDR55	-0.62387	2.979495	-1.71466	0.08991	-4.39305	0.321358	0.396461
Erythroid.cells	GM30239	-1.09551	-0.09316	-1.7144	0.089959	-4.19539	0.357359	0.431841
Erythroid.cells	DMGDH	0.862061	1.667706	1.714019	0.090029	-4.39547	0.336328	0.411301
Erythroid.cells	DHX35	0.817014	2.187953	1.714002	0.090032	-4.30677	0.330349	0.405395
Erythroid.cells	BCL6B	-1.32223	0.649778	-1.71379	0.090071	-4.18568	0.348382	0.4231
Erythroid.cells	TRIM8	0.377035	5.616506	1.712861	0.090243	-4.81339	0.294092	0.368873
Erythroid.cells	MATN2	1.208231	0.980441	1.712668	0.090279	-4.21305	0.344983	0.419758
Erythroid.cells	JUP	0.655478	3.562597	1.712494	0.090311	-4.47672	0.31561	0.390724
Erythroid.cells	IRGC1	-1.2516	0.115465	-1.71227	0.090353	-4.19556	0.355485	0.430063
Erythroid.cells	G6PDX	-0.53027	4.072997	-1.71205	0.090393	-4.51836	0.310185	0.385311
Erythroid.cells	9430015G1	-0.89795	1.911053	-1.7117	0.090459	-4.25969	0.334186	0.409437
Erythroid.cells	RECQL4	-1.34593	-0.06717	-1.7115	0.090494	-4.18061	0.357798	0.432651
Erythroid.cells	PHF11A	-0.97778	1.004104	-1.71138	0.090516	-4.34555	0.344808	0.419975
Erythroid.cells	B230354K1	1.032956	1.756956	1.711337	0.090525	-4.27313	0.335967	0.41129
Erythroid.cells	ELMSAN1	0.395277	6.924696	1.711133	0.090563	-5.15278	0.281331	0.356292
Erythroid.cells	UMAD1	0.385501	5.658219	1.710718	0.09064	-4.86486	0.293938	0.369307
Erythroid.cells	DDX21	-0.33456	6.399043	-1.7105	0.090681	-5.07623	0.286572	0.36188
Erythroid.cells	CRTAP	0.711787	3.004923	1.710207	0.090735	-4.38349	0.322	0.397855
Erythroid.cells	JTB	0.271966	5.842584	1.710068	0.090761	-4.92204	0.292087	0.367661
Erythroid.cells	TSPAN4	0.593578	3.356981	1.710059	0.090762	-4.47627	0.318121	0.394001
Erythroid.cells	NCK1	-0.36019	6.174664	-1.70929	0.090905	-4.95968	0.289136	0.364565
Erythroid.cells	ETFB	0.300717	6.979015	1.708931	0.090972	-5.3255	0.281398	0.35666
Erythroid.cells	NT5C3B	-0.53928	3.396774	-1.70856	0.091041	-4.49851	0.318233	0.394164
Erythroid.cells	ARL6IP6	-0.35744	5.278997	-1.70855	0.091044	-4.82757	0.298299	0.374044
Erythroid.cells	OTULIN	-0.23947	6.57022	-1.7082	0.091108	-5.10482	0.285494	0.360837

Erythroid.cells	TIPRL	-0.31555	5.525539	-1.70745	0.091248	-4.9152	0.296248	0.371711
Erythroid.cells	FBXO3	0.304722	5.012921	1.707082	0.091317	-4.87706	0.301627	0.377179
Erythroid.cells	4932422M	1.069217	0.972719	1.706357	0.091452	-4.23456	0.34704	0.422352
Erythroid.cells	GM42997	-1.42551	0.465543	-1.70599	0.091521	-4.19935	0.353313	0.428415
Erythroid.cells	ZDHHC13	0.50758	3.693777	1.705759	0.091564	-4.51923	0.316145	0.391709
Erythroid.cells	ITPR1	0.325344	6.992018	1.705213	0.091666	-5.22242	0.282544	0.357568
Erythroid.cells	TSHZ1	-0.45222	5.060403	-1.70458	0.091785	-4.85407	0.3021	0.377601
Erythroid.cells	PSAP	0.289673	9.249596	1.70451	0.091798	-5.69293	0.261805	0.336068
Erythroid.cells	TMEM108	-0.66961	5.137851	-1.70414	0.091868	-4.90911	0.301425	0.376958
Erythroid.cells	FRYL	-0.24134	7.564295	-1.70317	0.09205	-5.33999	0.277737	0.352776
Erythroid.cells	REXO5	-0.81706	1.950184	-1.7031	0.092063	-4.31947	0.336777	0.412475
Erythroid.cells	STX4A	-0.35237	4.972734	-1.7026	0.092157	-4.75926	0.303508	0.379395
Erythroid.cells	RIN2	0.610439	3.687666	1.702567	0.092163	-4.58383	0.317213	0.393212
Erythroid.cells	C1QB	0.713578	6.51237	1.702327	0.092208	-5.15916	0.287906	0.363594
Erythroid.cells	CC2D2A	-1.13433	0.81427	-1.70229	0.092214	-4.26957	0.350237	0.426056
Erythroid.cells	HACD4	0.751215	3.532004	1.702191	0.092233	-4.55822	0.318917	0.395076
Erythroid.cells	AKR7A5	0.405441	4.13514	1.70213	0.092245	-4.7767	0.312369	0.388503
Erythroid.cells	E330009J0	-0.84581	2.552592	-1.70185	0.092298	-4.40247	0.329856	0.406131
Erythroid.cells	IFIH1	-0.67115	3.912874	-1.70174	0.092317	-4.68992	0.314765	0.39109
Erythroid.cells	PAFAH1B1	0.175785	8.064884	1.701696	0.092327	-5.42205	0.273033	0.348402
Erythroid.cells	PTDSS2	0.473791	3.06586	1.701399	0.092382	-4.58792	0.324161	0.400505
Erythroid.cells	SGIP1	-1.19708	0.175941	-1.70093	0.092471	-4.22797	0.358294	0.434186
Erythroid.cells	CTNNA3	1.510099	1.300067	1.700848	0.092486	-4.27649	0.344658	0.42087
Erythroid.cells	PCGF5	-0.38827	6.06196	-1.70037	0.092576	-5.07248	0.292755	0.368892
Erythroid.cells	TMEM18	-0.97735	1.593876	-1.70023	0.092603	-4.25682	0.341378	0.417768
Erythroid.cells	GGPS1	0.255399	5.393996	1.700066	0.092634	-4.98822	0.299535	0.375872
Erythroid.cells	PSMC4	-0.36499	5.46131	-1.69989	0.092668	-4.87933	0.298853	0.375222
Erythroid.cells	PSMB5	0.303433	6.258665	1.699543	0.092733	-5.11433	0.290901	0.36705
Erythroid.cells	RBBP7	0.299854	6.2357	1.698909	0.092853	-5.09075	0.291201	0.367562
Erythroid.cells	PCX	0.690142	2.70013	1.6988	0.092873	-4.55703	0.32881	0.40562
Erythroid.cells	TEX2	-0.34274	6.723879	-1.69865	0.092901	-5.15864	0.286375	0.362684
Erythroid.cells	RAB3IP	-0.47624	4.587906	-1.69855	0.092921	-4.7343	0.308133	0.384906
Erythroid.cells	DLGAP1	0.984338	1.398192	1.698483	0.092933	-4.30776	0.343906	0.420621
Erythroid.cells	EXT2	0.515627	3.849599	1.698424	0.092945	-4.54954	0.316053	0.392902
Erythroid.cells	ASNSD1	0.346501	5.360445	1.698251	0.092977	-4.82083	0.300072	0.376744
Erythroid.cells	OVCA2	-1.52351	0.174482	-1.69776	0.093071	-4.19881	0.358784	0.435446
Erythroid.cells	PDCD2	-0.49245	3.817161	-1.69769	0.093084	-4.59715	0.316442	0.393543
Erythroid.cells	RAB34	1.383998	0.235405	1.69757	0.093106	-4.22444	0.35803	0.434809
Erythroid.cells	1700102P0	1.216474	0.812589	1.697388	0.093141	-4.23598	0.350966	0.427951
Erythroid.cells	HIRIP3	0.62234	3.169999	1.697368	0.093145	-4.50226	0.323569	0.400802
Erythroid.cells	ACADVL	0.359146	4.901912	1.696701	0.093271	-4.96506	0.305003	0.382294
Erythroid.cells	KIT	0.760997	3.453389	1.696629	0.093285	-4.62921	0.320575	0.398032
Erythroid.cells	GLP2R	-1.14761	0.940346	-1.69663	0.093285	-4.23641	0.349583	0.42684
Erythroid.cells	PARP8	0.34504	6.68237	1.696477	0.093314	-5.14063	0.286947	0.363867
Erythroid.cells	PPP2R5E	-0.22287	6.820761	-1.6958	0.093442	-5.18906	0.28562	0.362567
Erythroid.cells	FPR2	-1.43711	1.833327	-1.69554	0.093492	-4.38597	0.339011	0.416552
Erythroid.cells	PMM1	-0.72367	3.07058	-1.69552	0.093496	-4.41234	0.32486	0.402462

Erythroid.cells	4732496C0	-1.15446	0.689806	-1.69546	0.093508	-4.21979	0.352654	0.430001
Erythroid.cells	AKR1D1	0.795583	2.587501	1.695354	0.093527	-4.57875	0.330311	0.407907
Erythroid.cells	PPM1H	0.42815	6.473739	1.695318	0.093534	-5.18316	0.289032	0.366094
Erythroid.cells	DACH2	-1.41987	0.599965	-1.69518	0.09356	-4.22005	0.353749	0.43114
Erythroid.cells	SENP2	0.298605	6.656089	1.695101	0.093576	-5.08803	0.287234	0.364322
Erythroid.cells	KDM8	-0.78798	1.814343	-1.69446	0.093698	-4.31122	0.339424	0.417037
Erythroid.cells	HYAL3	1.316119	0.035974	1.694454	0.093699	-4.21034	0.360908	0.438156
Erythroid.cells	A630001O1	-0.79594	2.282008	-1.69398	0.09379	-4.35724	0.333995	0.411729
Erythroid.cells	CYP2C69	0.99717	1.432054	1.693876	0.093809	-4.36794	0.34393	0.42161
Erythroid.cells	GM15411	1.184665	0.212426	1.693866	0.093811	-4.22779	0.358716	0.436137
Erythroid.cells	OLFR1369-	1.621263	-0.76388	1.693829	0.093818	-4.20984	0.371016	0.448111
Erythroid.cells	APOL7C	1.652661	-0.99321	1.693509	0.093879	-4.21534	0.373972	0.451162
Erythroid.cells	SLC35E2	0.471886	3.898481	1.693491	0.093883	-4.56385	0.315917	0.393825
Erythroid.cells	INMT	1.214899	0.639157	1.692622	0.094049	-4.27527	0.353941	0.431741
Erythroid.cells	IQCH	1.34326	0.250926	1.692418	0.094088	-4.28798	0.358717	0.436538
Erythroid.cells	PGAM5	-0.48489	3.934831	-1.69235	0.094101	-4.57729	0.315939	0.394079
Erythroid.cells	EVI2	0.79408	3.35768	1.692099	0.094149	-4.45056	0.322329	0.400585
Erythroid.cells	NKRF	-0.68462	2.983842	-1.69077	0.094403	-4.43134	0.327246	0.405319
Erythroid.cells	PMVK	-0.51729	4.327226	-1.69065	0.094426	-4.60993	0.312465	0.390489
Erythroid.cells	ANK3	1.137831	1.681131	1.689115	0.094721	-4.35882	0.34323	0.420843
Erythroid.cells	TMED7	0.235105	6.308978	1.688905	0.094762	-5.07251	0.292769	0.370066
Erythroid.cells	MIR142HG	-0.25836	8.019491	-1.68861	0.094818	-5.38125	0.276185	0.352949
Erythroid.cells	PSMD2	0.260825	6.149979	1.688494	0.094841	-5.11556	0.29441	0.37184
Erythroid.cells	SLC43A1	0.87915	1.089863	1.688339	0.094871	-4.40414	0.350384	0.42815
Erythroid.cells	FAAP20	0.592304	3.120662	1.687931	0.094949	-4.46376	0.326818	0.404762
Erythroid.cells	TOR1AIP1	-0.26795	7.42657	-1.68782	0.09497	-5.30042	0.281939	0.359041
Erythroid.cells	1810021B2	1.484862	0.427969	1.687337	0.095064	-4.21805	0.358788	0.436421
Erythroid.cells	MFAP4	1.028269	1.50654	1.687258	0.095079	-4.36755	0.345678	0.423567
Erythroid.cells	NR2C2	-0.28044	6.925422	-1.68694	0.09514	-5.20612	0.286976	0.364423
Erythroid.cells	H2-DMA	0.602762	5.184297	1.686873	0.095153	-4.91499	0.304614	0.38254
Erythroid.cells	ZFP112	-1.3147	0.391108	-1.68612	0.095299	-4.22517	0.359444	0.437396
Erythroid.cells	AGFG1	0.26763	6.147106	1.68584	0.095353	-5.09254	0.294854	0.372732
Erythroid.cells	WTAP	-0.22581	6.931297	-1.68581	0.095359	-5.21464	0.287046	0.364683
Erythroid.cells	GIT2	-0.23912	7.020155	-1.68581	0.095359	-5.21555	0.286176	0.363782
Erythroid.cells	YJU2	-0.51918	3.454196	-1.68558	0.095403	-4.48935	0.323419	0.401846
Erythroid.cells	FAP	-1.4528	0.744801	-1.68557	0.095405	-4.281	0.355082	0.433287
Erythroid.cells	SRGAP2	0.371764	6.60483	1.6855	0.095419	-5.08845	0.29027	0.36812
Erythroid.cells	L3MBTL3	-0.47981	5.067059	-1.68461	0.095591	-4.76613	0.306429	0.384534
Erythroid.cells	ST6GALNA(	0.497185	5.127632	1.684433	0.095625	-5.0469	0.305799	0.383949
Erythroid.cells	NRF1	-0.23729	6.474245	-1.684	0.095709	-5.1446	0.292113	0.369935
Erythroid.cells	RNF185	-0.40088	5.117108	-1.68392	0.095724	-4.75779	0.306022	0.384222
Erythroid.cells	GM26802	1.063998	1.069129	1.683564	0.095794	-4.31984	0.351842	0.430143
Erythroid.cells	PYCARD	-0.41067	5.876191	-1.68351	0.095804	-4.93016	0.298207	0.376295
Erythroid.cells	SMARCB1	-0.28375	5.410318	-1.68205	0.096088	-4.92109	0.30351	0.381715
Erythroid.cells	PCOLCE2	1.191234	1.678726	1.681923	0.096113	-4.33978	0.34509	0.423552
Erythroid.cells	FOXK2	0.380525	4.920069	1.681899	0.096117	-4.75703	0.308659	0.387026
Erythroid.cells	HTRA3	1.000839	0.502839	1.681898	0.096118	-4.29614	0.359381	0.437617

Erythroid.cells	CYP4F13	0.712409	2.829707	1.68171	0.096154	-4.41852	0.331667	0.410288
Erythroid.cells	GM11131	-1.20105	0.815809	-1.6814	0.096215	-4.25966	0.355519	0.434018
Erythroid.cells	PPM1J	1.042072	-0.35731	1.681385	0.096218	-4.27793	0.370214	0.448384
Erythroid.cells	ZC3HAV1	0.220751	8.43854	1.681304	0.096233	-5.62613	0.273657	0.35109
Erythroid.cells	RPP40	-1.08667	1.432828	-1.68114	0.096265	-4.25425	0.348029	0.426729
Erythroid.cells	SPATC1	-1.09931	-1.06407	-1.68071	0.096349	-4.2218	0.379362	0.457598
Erythroid.cells	SAMD4	1.131514	3.187151	1.680622	0.096366	-4.50008	0.327609	0.406663
Erythroid.cells	SDF4	-0.23899	6.506152	-1.68039	0.096412	-5.14033	0.292326	0.370858
Erythroid.cells	SGPP1	0.462136	5.017924	1.680293	0.09643	-4.77473	0.307624	0.386553
Erythroid.cells	PPIC	0.752253	2.745716	1.680263	0.096436	-4.42713	0.332628	0.411788
Erythroid.cells	YIPF6	0.628847	3.457527	1.680234	0.096442	-4.49734	0.324574	0.403713
Erythroid.cells	1700007L1	0.99568	1.663099	1.679893	0.096509	-4.28518	0.3454	0.424373
Erythroid.cells	HNRNPLL	0.416153	4.231786	1.679635	0.096559	-4.81903	0.316196	0.39516
Erythroid.cells	LLPH	-0.24794	6.447819	-1.67908	0.096668	-5.14854	0.29305	0.37175
Erythroid.cells	LRRC10B	-1.19846	0.595128	-1.67902	0.09668	-4.28657	0.358409	0.437497
Erythroid.cells	FOXRED2	1.032382	1.168266	1.678947	0.096693	-4.25934	0.351389	0.430587
Erythroid.cells	PEG3	0.91102	1.53048	1.678903	0.096702	-4.4011	0.347025	0.426275
Erythroid.cells	EXOC5	-0.26555	6.376842	-1.6788	0.096722	-5.06889	0.293763	0.372514
Erythroid.cells	TSPAN2	0.670718	3.159011	1.678689	0.096744	-4.43032	0.328083	0.407442
Erythroid.cells	REX1BD	0.28796	5.688469	1.678493	0.096782	-5.02817	0.300792	0.379784
Erythroid.cells	ARHGAP27	-0.84486	2.032169	-1.67814	0.096852	-4.30791	0.341228	0.420523
Erythroid.cells	SELENOF	0.376819	5.422799	1.677633	0.096951	-4.90909	0.303852	0.382804
Erythroid.cells	GNPDA2	-0.59874	3.100129	-1.6775	0.096976	-4.4304	0.329103	0.408398
Erythroid.cells	NFYC	-0.21754	6.202043	-1.67654	0.097165	-5.10026	0.296322	0.374955
Erythroid.cells	PEX14	-0.34349	5.405291	-1.67611	0.097249	-4.94871	0.30469	0.383466
Erythroid.cells	CABP1	-1.50769	-0.13612	-1.67574	0.097322	-4.2222	0.368769	0.447457
Erythroid.cells	NAGPA	-0.56785	3.354165	-1.67552	0.097365	-4.49638	0.326947	0.406104
Erythroid.cells	GNPTAB	-0.49431	4.589659	-1.67536	0.097397	-4.71056	0.313348	0.392378
Erythroid.cells	USP46	0.673967	3.281753	1.675351	0.097399	-4.50701	0.327763	0.406925
Erythroid.cells	BC017158	-1.00858	1.449949	-1.67528	0.097412	-4.30846	0.349123	0.428211
Erythroid.cells	MPZL1	0.960369	2.78686	1.675039	0.09746	-4.42454	0.333453	0.412633
Erythroid.cells	D830025C(	0.878043	2.231904	1.674733	0.09752	-4.35278	0.339954	0.419201
Erythroid.cells	SPIN1	-0.30714	5.805516	-1.67462	0.097542	-4.96359	0.300649	0.379532
Erythroid.cells	PPHLN1	-0.4655	4.932629	-1.67415	0.097636	-4.73972	0.309983	0.389061
Erythroid.cells	CCNE1	-0.64271	3.530373	-1.6734	0.097784	-4.59493	0.325598	0.404695
Erythroid.cells	NHLRC2	0.371123	4.994885	1.67322	0.097819	-4.81114	0.309613	0.388595
Erythroid.cells	CCDC171	0.576214	3.57202	1.672935	0.097875	-4.52388	0.325131	0.404428
Erythroid.cells	VCL	0.357564	6.049702	1.672903	0.097882	-5.08519	0.298614	0.377473
Erythroid.cells	GCFC2	0.693514	2.248325	1.672863	0.097889	-4.38436	0.340297	0.419604
Erythroid.cells	POR	-0.38143	5.6252	-1.67165	0.098129	-4.99732	0.303632	0.382437
Erythroid.cells	LETM1	-0.39326	4.54276	-1.67142	0.098175	-4.72685	0.31517	0.39425
Erythroid.cells	KLHDC2	-0.33388	4.860142	-1.67084	0.09829	-4.8993	0.312014	0.391017
Erythroid.cells	ATP5J	0.236846	7.873083	1.670676	0.098322	-5.49706	0.281437	0.359611
Erythroid.cells	CGNL1	1.073211	1.622928	1.670117	0.098433	-4.3632	0.348901	0.428116
Erythroid.cells	WDR20	-0.30695	5.843767	-1.66993	0.09847	-4.97287	0.301754	0.380729
Erythroid.cells	4930455G(	0.751494	1.816178	1.66993	0.09847	-4.5321	0.346583	0.425884
Erythroid.cells	GM614	-1.26483	-0.15476	-1.66972	0.098513	-4.23755	0.370979	0.4499

Erythroid.cells	DECR1	0.542134	3.696206	1.669712	0.098513	-4.78636	0.324849	0.404231
Erythroid.cells	SMARCD1	-0.42741	4.031896	-1.66906	0.098643	-4.62355	0.321435	0.400745
Erythroid.cells	GM35154	0.972305	1.526848	1.668852	0.098684	-4.41042	0.350437	0.429697
Erythroid.cells	CCDC32	0.621048	3.31271	1.667897	0.098874	-4.48355	0.330025	0.409051
Erythroid.cells	MRS2	-0.48287	3.939507	-1.66777	0.0989	-4.59173	0.322984	0.401977
Erythroid.cells	PHGDH	-0.63436	4.379094	-1.66722	0.099009	-4.78922	0.318384	0.397246
Erythroid.cells	CHD9	0.383887	6.065045	1.666876	0.099078	-5.02724	0.3006	0.379155
Erythroid.cells	MSL2	-0.31189	6.010868	-1.66665	0.099122	-4.99679	0.301195	0.379809
Erythroid.cells	JAGN1	-0.54947	3.955321	-1.66611	0.099231	-4.60899	0.323463	0.402338
Erythroid.cells	ACAA1A	-0.36703	5.302507	-1.66569	0.099314	-4.94088	0.308947	0.387708
Erythroid.cells	ANXA1	0.58359	6.030034	1.665592	0.099334	-5.13147	0.301337	0.379934
Erythroid.cells	SPOPL	-0.43471	4.42179	-1.66544	0.099364	-4.704	0.318432	0.397336
Erythroid.cells	VDAC3	0.222477	7.191815	1.6647	0.099513	-5.38706	0.289691	0.368082
Erythroid.cells	MORF4L2	0.332011	5.404884	1.664645	0.099524	-4.91614	0.307968	0.386856
Erythroid.cells	GM34095	-1.40544	-0.39051	-1.66463	0.099526	-4.2401	0.376013	0.4545
Erythroid.cells	EMC1	0.496473	3.622778	1.664614	0.09953	-4.56013	0.327413	0.406531
Erythroid.cells	GSTZ1	0.42665	4.205829	1.664466	0.09956	-4.87693	0.320913	0.400032
Erythroid.cells	IQGAP3	1.00138	2.400987	1.663784	0.099696	-4.40405	0.341677	0.420746
Erythroid.cells	FRG2F1	1.030969	0.702787	1.663707	0.099712	-4.26442	0.36229	0.441081
Erythroid.cells	GEMIN7	0.32225	5.084337	1.663698	0.099714	-4.86117	0.311551	0.390472
Erythroid.cells	OTUD4	-0.36439	5.091742	-1.66337	0.099778	-4.85604	0.311508	0.390423
Erythroid.cells	CCNI	0.219538	6.783841	1.663217	0.09981	-5.32521	0.293961	0.372507
Erythroid.cells	SLC12A6	-0.29886	8.133974	-1.66315	0.099823	-5.51858	0.280714	0.358796
Erythroid.cells	CAPZA1	-0.18083	7.715586	-1.6624	0.099974	-5.37773	0.285089	0.363196
Erythroid.cells	1700030K0	1.010347	1.730931	1.661836	0.100088	-4.3328	0.350403	0.4292
Erythroid.cells	MAFB	0.63002	4.60884	1.661356	0.100184	-4.90368	0.317406	0.396271
Erythroid.cells	NRG4	0.933626	2.017195	1.661337	0.100188	-4.46998	0.347022	0.425902
Erythroid.cells	CTBP2	0.677289	3.249836	1.66123	0.100209	-4.51354	0.332593	0.411578
Erythroid.cells	GM9856	-1.0666	1.246414	-1.66102	0.100251	-4.27392	0.356372	0.435198
Erythroid.cells	ZFPL1	0.49085	3.466605	1.66082	0.100292	-4.56734	0.33012	0.409196
Erythroid.cells	KRTCAP2	0.256865	7.062528	1.660545	0.100347	-5.30101	0.291807	0.37032
Erythroid.cells	CNN3	-0.49719	4.531127	-1.66023	0.10041	-4.84929	0.318255	0.397453
Erythroid.cells	GM10874	1.197676	-0.06869	1.659923	0.100473	-4.26845	0.372922	0.451677
Erythroid.cells	CKB	0.531334	4.578345	1.659888	0.10048	-4.93165	0.317739	0.396931
Erythroid.cells	TIMM44	-0.34545	4.84696	-1.65987	0.100484	-4.83729	0.314821	0.393974
Erythroid.cells	4930523C0	-0.26157	6.126408	-1.65973	0.100513	-5.22213	0.301306	0.380187
Erythroid.cells	MATR3	-0.21463	6.50291	-1.65972	0.100514	-5.16344	0.297446	0.376223
Erythroid.cells	GM1604B	-1.29776	1.489369	-1.65965	0.100528	-4.28437	0.353397	0.432549
Erythroid.cells	GPR160	0.900112	2.407708	1.659008	0.100658	-4.44316	0.342713	0.421936
Erythroid.cells	CDH23	0.709282	2.820164	1.658427	0.100775	-4.49644	0.338161	0.417439
Erythroid.cells	GM43062	-0.8327	1.638658	-1.65763	0.100936	-4.3124	0.352482	0.431635
Erythroid.cells	PIANP	0.698991	-0.06426	1.657518	0.100959	-4.47709	0.37382	0.452542
Erythroid.cells	FAM149A	1.003593	-0.42534	1.657447	0.100973	-4.32919	0.37851	0.45712
Erythroid.cells	AI987944	-0.71574	2.764701	-1.65741	0.100981	-4.44536	0.339062	0.418362
Erythroid.cells	CRYBA4	-1.31644	-0.18755	-1.65716	0.101031	-4.26262	0.375479	0.454222
Erythroid.cells	ADIPOR2	0.285646	6.368043	1.65655	0.101155	-5.14783	0.299912	0.378692
Erythroid.cells	STX5A	-0.2691	5.871144	-1.65607	0.101254	-5.02913	0.305257	0.384199

Erythroid.cells	HHEX	0.540422	4.675113	1.655519	0.101365	-4.70786	0.31829	0.397436
Erythroid.cells	ZFP456	-1.17601	0.791994	-1.65499	0.101472	-4.28399	0.36408	0.442824
Erythroid.cells	FOSL2	0.432201	5.561041	1.654868	0.101497	-5.06805	0.308962	0.387859
Erythroid.cells	CD274	-1.00741	5.805413	-1.65442	0.101589	-5.07073	0.306522	0.385421
Erythroid.cells	MAPK1	-0.17839	7.693924	-1.65419	0.101636	-5.44892	0.287347	0.365799
Erythroid.cells	CKM	1.513272	0.1057	1.653912	0.101692	-4.29998	0.372976	0.451917
Erythroid.cells	MBNL3	-0.47856	4.597519	-1.65388	0.101699	-4.84867	0.319493	0.398855
Erythroid.cells	PIM3	-0.52262	4.641176	-1.65384	0.101706	-4.7766	0.319015	0.398371
Erythroid.cells	PRKAG2	0.398395	5.050385	1.653581	0.101759	-4.89074	0.314626	0.393925
Erythroid.cells	USP15	0.217608	7.650516	1.653341	0.101808	-5.5363	0.287876	0.366549
Erythroid.cells	MAVS	0.599921	2.932268	1.651892	0.102104	-4.52639	0.339293	0.418787
Erythroid.cells	1500015A(	-0.81394	1.76661	-1.65178	0.102128	-4.33676	0.353202	0.432586
Erythroid.cells	CEP128	0.39315	6.336302	1.651265	0.102232	-5.15534	0.301855	0.381109
Erythroid.cells	NIN	0.381663	5.920519	1.650966	0.102293	-4.99761	0.306185	0.385665
Erythroid.cells	TLR6	-1.12669	1.070509	-1.65094	0.102298	-4.32926	0.361786	0.441381
Erythroid.cells	OARD1	0.357423	4.902059	1.650811	0.102325	-4.95903	0.31707	0.396781
Erythroid.cells	ATP6V1H	-0.34577	6.803114	-1.65079	0.10233	-5.19931	0.297072	0.376327
Erythroid.cells	B3GALT6	-1.11503	1.806345	-1.65061	0.102367	-4.31628	0.352718	0.43251
Erythroid.cells	PRR14L	-0.2844	6.50182	-1.65052	0.102384	-5.17488	0.30015	0.379559
Erythroid.cells	PXK	-0.31348	6.270189	-1.65016	0.102459	-5.11991	0.302539	0.382134
Erythroid.cells	FAM3A	0.655657	2.785704	1.650109	0.102469	-4.46254	0.34101	0.421054
Erythroid.cells	ICE2	0.72138	2.160721	1.650078	0.102475	-4.39209	0.348434	0.428437
Erythroid.cells	1700016P(	0.721688	3.898827	1.650015	0.102488	-4.8086	0.328191	0.408212
Erythroid.cells	IKBKG	0.498615	3.339723	1.649202	0.102655	-4.61927	0.334875	0.414919
Erythroid.cells	GAREM1	1.065499	0.757375	1.649124	0.102671	-4.32468	0.366055	0.445811
Erythroid.cells	AGRP	1.097972	1.935634	1.649073	0.102682	-4.36041	0.351473	0.43145
Erythroid.cells	CENPC1	-0.30468	5.265605	-1.64877	0.102743	-4.92364	0.313514	0.393346
Erythroid.cells	SCAND1	0.232934	7.018464	1.647946	0.102913	-5.27711	0.295463	0.374762
Erythroid.cells	SIMC1	-0.36449	5.702765	-1.64793	0.102916	-5.06594	0.309076	0.388744
Erythroid.cells	POLA2	-0.53911	4.050563	-1.64793	0.102917	-4.68596	0.327116	0.407041
Erythroid.cells	MLXIP	-0.28241	6.94185	-1.64766	0.102973	-5.30334	0.296249	0.37566
Erythroid.cells	HIF1AN	0.652622	3.373427	1.647546	0.102996	-4.52266	0.334838	0.414907
Erythroid.cells	IKZF2	-0.53616	4.781138	-1.64739	0.103027	-5.26699	0.319017	0.399052
Erythroid.cells	TMEM265	0.555942	3.958769	1.64727	0.103052	-4.61178	0.328162	0.40831
Erythroid.cells	APOLD1	1.189574	1.979983	1.646759	0.103158	-4.35685	0.351551	0.431456
Erythroid.cells	FCOR	0.862364	0.865885	1.64636	0.10324	-4.39844	0.365369	0.445237
Erythroid.cells	NECAB2	-1.35092	0.442881	-1.64602	0.10331	-4.27961	0.370743	0.450599
Erythroid.cells	CBX4	-0.41775	5.468948	-1.64597	0.103321	-4.91466	0.311831	0.39182
Erythroid.cells	GM50013	-0.87045	1.85467	-1.64582	0.103351	-4.36219	0.353115	0.433304
Erythroid.cells	POLR2F	0.312938	5.381223	1.64568	0.10338	-4.94909	0.312771	0.392807
Erythroid.cells	YWHAB	-0.19553	7.88619	-1.6454	0.103438	-5.4772	0.287082	0.366452
Erythroid.cells	GM12064	0.93111	0.96957	1.64538	0.103442	-4.3526	0.364064	0.444225
Erythroid.cells	PCID2	-0.36663	4.736836	-1.64526	0.103466	-4.79748	0.319766	0.400048
Erythroid.cells	NCOR2	-0.36134	4.956649	-1.64516	0.103488	-4.87557	0.317361	0.397607
Erythroid.cells	ANGPTL8	-0.88862	1.825356	-1.6451	0.103499	-4.50855	0.353473	0.433803
Erythroid.cells	PPAT	0.47969	4.107428	1.6443	0.103665	-4.73922	0.327178	0.407577
Erythroid.cells	SLC25A15	0.603239	2.488304	1.643446	0.103842	-4.54403	0.346411	0.42668

Erythroid.cells	ST6GALNA(	-0.65612	3.214358	-1.64293	0.103949	-4.55027	0.337924	0.418356
Erythroid.cells	CHMP1B	0.364392	4.698125	1.642904	0.103955	-4.87076	0.321114	0.401431
Erythroid.cells	PSMA1	-0.25212	6.9166	-1.64278	0.103981	-5.30397	0.297604	0.377388
Erythroid.cells	POLR2J	-0.34908	4.922519	-1.64271	0.103996	-4.85968	0.318649	0.398931
Erythroid.cells	HIPK3	0.359785	5.445686	1.642273	0.104086	-4.91432	0.313053	0.393279
Erythroid.cells	GM20513	1.072786	1.300029	1.642272	0.104086	-4.48367	0.361058	0.441361
Erythroid.cells	SUPT3	0.418391	5.480744	1.641771	0.10419	-4.9176	0.312769	0.393137
Erythroid.cells	CXXC1	-0.45681	4.307295	-1.64158	0.10423	-4.70411	0.325628	0.406197
Erythroid.cells	ADCY6	1.031635	0.433361	1.641512	0.104244	-4.338	0.372132	0.452404
Erythroid.cells	MECOM	1.646429	0.770813	1.641487	0.104249	-4.34188	0.367822	0.448184
Erythroid.cells	LENG9	-0.75703	2.352054	-1.64103	0.104345	-4.43433	0.348473	0.429077
Erythroid.cells	IQCC	-0.981	1.695654	-1.64088	0.104375	-4.35095	0.356449	0.437013
Erythroid.cells	TMEM189	-0.41727	6.387922	-1.64076	0.104401	-5.09128	0.30335	0.383555
Erythroid.cells	PRSS30	-0.99953	-0.33737	-1.64049	0.104457	-4.29154	0.382446	0.46257
Erythroid.cells	SNRPC	-0.25858	6.245324	-1.6399	0.10458	-5.1545	0.305165	0.385413
Erythroid.cells	IGHMBP2	-0.62864	2.850148	-1.63935	0.104694	-4.4777	0.343181	0.4238
Erythroid.cells	HSP90B1	-0.23852	8.976388	-1.63896	0.104776	-5.64817	0.278341	0.357502
Erythroid.cells	OTC	0.670973	3.438444	1.638309	0.104911	-4.87449	0.336583	0.417361
Erythroid.cells	LYRM1	-0.61132	2.700522	-1.63821	0.104932	-4.52271	0.345245	0.426018
Erythroid.cells	CCDC51	0.763674	1.810129	1.638184	0.104937	-4.39011	0.356006	0.436698
Erythroid.cells	COL4A2	0.818494	2.620499	1.638026	0.10497	-4.50674	0.346199	0.427019
Erythroid.cells	ERI3	0.346485	5.177084	1.637973	0.104982	-4.86305	0.31706	0.397698
Erythroid.cells	ACPP	0.845067	2.739914	1.637156	0.105153	-4.5545	0.34523	0.425882
Erythroid.cells	MCPT8	2.628975	-0.5048	1.636567	0.105276	-4.28503	0.386456	0.46627
Erythroid.cells	HP	-0.38478	7.365035	-1.6364	0.10531	-5.69151	0.294817	0.374697
Erythroid.cells	2010016I1{	1.050516	-0.03474	1.635923	0.105411	-4.32266	0.380481	0.46054
Erythroid.cells	CCR2	0.586051	3.092746	1.635622	0.105474	-4.99604	0.341651	0.422295
Erythroid.cells	BTBD2	0.557481	3.477347	1.635493	0.105501	-4.59466	0.337158	0.417861
Erythroid.cells	CELF1	-0.18763	7.40812	-1.63463	0.105682	-5.39522	0.294927	0.374715
Erythroid.cells	NAP1L4	-0.23039	6.249563	-1.63438	0.105736	-5.16932	0.306848	0.387046
Erythroid.cells	NINJ2	1.296398	0.701022	1.634337	0.105744	-4.32301	0.371386	0.45165
Erythroid.cells	CTNNA1	0.313563	6.643473	1.63412	0.10579	-5.28815	0.302738	0.382826
Erythroid.cells	SF3B6	-0.21007	7.294759	-1.63408	0.105798	-5.38467	0.296072	0.375948
Erythroid.cells	ZFP709	-0.76475	1.431477	-1.63405	0.105804	-4.35478	0.362141	0.442579
Erythroid.cells	ALOX5	1.105486	0.791987	1.633666	0.105885	-4.37634	0.370287	0.450663
Erythroid.cells	IL23A	-1.51789	-0.31658	-1.63361	0.105898	-4.28239	0.384734	0.464747
Erythroid.cells	LMNB1	-0.32141	7.679763	-1.63349	0.105923	-5.47646	0.292256	0.372076
Erythroid.cells	PMS2	-0.4664	3.954235	-1.63289	0.106048	-4.68347	0.332344	0.412925
Erythroid.cells	ZFP319	-0.95014	2.544028	-1.63168	0.106305	-4.41077	0.349598	0.430037
Erythroid.cells	VPS54	0.323045	6.506686	1.631419	0.106359	-5.16686	0.305106	0.3852
Erythroid.cells	PDE1B	-0.64382	3.114849	-1.63138	0.106368	-4.52727	0.342791	0.423342
Erythroid.cells	TNFRSF13C	-0.46901	3.92598	-1.63107	0.106432	-4.85618	0.333452	0.414015
Erythroid.cells	UBE4A	-0.39644	5.03345	-1.63005	0.106649	-4.87271	0.321328	0.401659
Erythroid.cells	REEP6	0.799909	1.791912	1.630017	0.106656	-4.51992	0.35925	0.43957
Erythroid.cells	PBX2	-0.491	4.930622	-1.63001	0.106657	-4.7728	0.322464	0.40281
Erythroid.cells	GM13684	-0.5974	3.516914	-1.62966	0.10673	-4.64967	0.338521	0.419052
Erythroid.cells	HINT1	0.22106	7.995864	1.629635	0.106737	-5.579	0.290349	0.36993

Erythroid.cells	SLPI	0.962114	4.206306	1.629506	0.106764	-4.71844	0.330589	0.411098
Erythroid.cells	GBP4	-1.10316	3.363964	-1.62927	0.106814	-4.61189	0.340308	0.420947
Erythroid.cells	JOSD2	0.422817	4.09679	1.629217	0.106825	-4.7362	0.331836	0.412475
Erythroid.cells	RASSF3	0.348189	6.834864	1.62896	0.10688	-5.28597	0.302094	0.382306
Erythroid.cells	LMAN2L	-0.44911	4.447735	-1.62882	0.10691	-4.68962	0.327857	0.408594
Erythroid.cells	ERG	1.005287	3.921953	1.628465	0.106985	-4.67076	0.333837	0.414691
Erythroid.cells	FKBP1B	0.798598	0.793011	1.628325	0.107014	-4.44901	0.371847	0.45244
Erythroid.cells	DNAJB14	0.458346	4.692261	1.627828	0.10712	-4.85262	0.325115	0.406112
Erythroid.cells	CLK3	-0.37973	4.807812	-1.62778	0.10713	-4.83494	0.323827	0.404828
Erythroid.cells	COQ3	-0.45454	3.500438	-1.62752	0.107186	-4.59261	0.338713	0.419939
Erythroid.cells	EMC6	0.282871	5.61708	1.627431	0.107204	-5.09944	0.314959	0.395943
Erythroid.cells	WDR81	0.53141	3.600126	1.627397	0.107212	-4.61382	0.337553	0.418809
Erythroid.cells	MYCL	0.718645	1.305739	1.627234	0.107246	-4.59441	0.365325	0.446404
Erythroid.cells	AP3M2	0.712855	2.489505	1.627221	0.107249	-4.43158	0.350713	0.431952
Erythroid.cells	TBC1D5	0.292981	7.293343	1.627188	0.107256	-5.33564	0.297397	0.37789
Erythroid.cells	RAB3GAP2	0.385644	5.259956	1.627077	0.10728	-4.88801	0.31884	0.399899
Erythroid.cells	MBL1	0.820402	1.455107	1.626987	0.107299	-4.47505	0.363448	0.444555
Erythroid.cells	3-Mar	0.406737	6.392222	1.626941	0.107309	-5.4219	0.306704	0.38749
Erythroid.cells	PRMT6	-0.66202	2.45108	-1.62605	0.107499	-4.44258	0.351341	0.432677
Erythroid.cells	NRD1	0.289771	6.185357	1.626035	0.107502	-5.14828	0.309028	0.389965
Erythroid.cells	SMURF1	0.455459	4.688664	1.625922	0.107526	-4.77789	0.325306	0.40663
Erythroid.cells	NLRP12	1.174943	-0.12085	1.625728	0.107567	-4.32797	0.383944	0.464914
Erythroid.cells	INTS11	-0.54936	3.989645	-1.62569	0.107574	-4.64971	0.333215	0.414713
Erythroid.cells	CEACAM16	-1.16123	1.137316	-1.62563	0.107587	-4.31673	0.367625	0.448953
Erythroid.cells	DVL2	-0.72275	3.263703	-1.62561	0.107592	-4.50297	0.341643	0.423177
Erythroid.cells	ADPRM	-0.53945	3.321315	-1.62534	0.10765	-4.55296	0.341041	0.422593
Erythroid.cells	DEK	-0.2527	7.842	-1.62515	0.107691	-5.54367	0.292098	0.372667
Erythroid.cells	PABPC1L	-0.84199	2.906471	-1.62465	0.107796	-4.46881	0.346204	0.427672
Erythroid.cells	FANCA	0.639149	3.05493	1.623942	0.107948	-4.5793	0.344817	0.426167
Erythroid.cells	AKAP8L	0.414579	4.8709	1.623487	0.108046	-4.90321	0.324135	0.405336
Erythroid.cells	EIF4EBP2	-0.20685	6.850744	-1.62309	0.108131	-5.28986	0.303017	0.383813
Erythroid.cells	RTKN2	1.080647	1.228878	1.622906	0.10817	-4.34386	0.367633	0.448821
Erythroid.cells	ABHD17B	-0.26133	6.542955	-1.6225	0.108256	-5.16668	0.30639	0.387219
Erythroid.cells	KAT2A	0.631752	3.199202	1.622209	0.108319	-4.5409	0.343755	0.42503
Erythroid.cells	ZFP428	-0.76727	2.058083	-1.62165	0.108439	-4.45354	0.357822	0.438903
Erythroid.cells	SULT2A1	0.695479	3.901678	1.621364	0.108501	-5.0277	0.335898	0.416999
Erythroid.cells	GM20300	0.795282	1.343674	1.621073	0.108563	-4.39185	0.366942	0.447921
Erythroid.cells	GM34471	-1.23575	0.232245	-1.62085	0.108611	-4.30216	0.381341	0.462022
Erythroid.cells	GM10101	-1.17052	0.672014	-1.62046	0.108695	-4.30669	0.375725	0.456602
Erythroid.cells	MPND	0.457132	4.813143	1.620328	0.108723	-4.82335	0.325781	0.40696
Erythroid.cells	TTC9C	0.332964	4.558959	1.620199	0.108751	-4.8514	0.328638	0.409879
Erythroid.cells	CWC25	-0.46216	5.255579	-1.62005	0.108783	-4.90829	0.320871	0.402036
Erythroid.cells	HRH2	-1.02806	1.160498	-1.61985	0.108826	-4.36261	0.369477	0.450631
Erythroid.cells	QTRT2	1.134409	1.344368	1.619527	0.108896	-4.35741	0.367192	0.448441
Erythroid.cells	ANKRD12	-0.2873	7.518738	-1.61947	0.108909	-5.39814	0.297019	0.377645
Erythroid.cells	DUSP8	0.704932	1.274945	1.618576	0.1091	-4.54977	0.368526	0.449466
Erythroid.cells	ST3GAL4	0.316851	6.423085	1.618427	0.109133	-5.18111	0.308736	0.389502

Erythroid.cells	ROGDI	0.47236	4.014028	1.618375	0.109144	-4.78186	0.33534	0.41652
Erythroid.cells	PDE4D	0.432957	6.428002	1.617644	0.109302	-5.28573	0.308887	0.38949
Erythroid.cells	CIAO2B	-0.41459	4.566233	-1.61748	0.109337	-4.85064	0.329252	0.410211
Erythroid.cells	SECTM1A	-1.46915	-1.13086	-1.61732	0.109371	-4.2809	0.400707	0.480616
Erythroid.cells	NEIL3	0.59918	4.375558	1.617188	0.1094	-4.88769	0.331416	0.412468
Erythroid.cells	NSG2	0.90087	0.587521	1.617039	0.109432	-4.3992	0.377622	0.458313
Erythroid.cells	SLC35A3	-0.35642	4.775291	-1.61701	0.109439	-4.82289	0.326896	0.407924
Erythroid.cells	UBE4BOS1	-1.21971	0.735049	-1.6169	0.109463	-4.34254	0.375703	0.456496
Erythroid.cells	PPM1N	-1.29608	0.159456	-1.61679	0.109485	-4.33342	0.383244	0.463927
Erythroid.cells	XXYLT1	-0.57668	3.591278	-1.61606	0.109645	-4.59069	0.340703	0.421917
Erythroid.cells	CHD8	-0.28639	5.733086	-1.61602	0.109652	-5.03439	0.316539	0.397497
Erythroid.cells	PPM1M	0.450008	4.583696	1.61599	0.109659	-4.78093	0.329274	0.410422
Erythroid.cells	BHMT2	0.765334	2.060069	1.615645	0.109734	-4.63038	0.35919	0.440292
Erythroid.cells	NUP155	-0.33813	5.174039	-1.61563	0.109738	-4.9552	0.322698	0.403746
Erythroid.cells	MFAP1B	0.338824	4.945536	1.615255	0.109819	-4.90162	0.32538	0.406451
Erythroid.cells	TTC1	-0.30861	4.740195	-1.61484	0.109908	-4.85407	0.327848	0.408867
Erythroid.cells	ALOX5AP	0.52534	7.325815	1.614432	0.109997	-5.58871	0.30019	0.380505
Erythroid.cells	RBM26	-0.23087	6.821937	-1.61417	0.110055	-5.28678	0.305405	0.385979
Erythroid.cells	RARB	1.17475	1.586463	1.614126	0.110064	-4.44261	0.365608	0.446518
Erythroid.cells	HNRNPL	0.169034	8.426349	1.613998	0.110091	-5.65865	0.289127	0.369122
Erythroid.cells	PLS3	0.824275	2.162516	1.613475	0.110205	-4.44055	0.358599	0.439705
Erythroid.cells	COX6B1	0.219542	7.821955	1.61343	0.110215	-5.5803	0.295297	0.375623
Erythroid.cells	PLEKHG3	0.521853	4.378559	1.613254	0.110253	-4.77532	0.332274	0.413446
Erythroid.cells	VPS36	0.313689	5.315956	1.612544	0.110408	-4.97543	0.321772	0.402963
Erythroid.cells	LAMB3	0.5549	4.067884	1.612454	0.110427	-4.8121	0.335865	0.417229
Erythroid.cells	NAT8L	-1.05798	0.332665	-1.61242	0.110434	-4.35454	0.38201	0.462901
Erythroid.cells	RHOBTB1	0.689765	3.180961	1.612336	0.110453	-4.69903	0.346272	0.427681
Erythroid.cells	GM19585	-0.64951	2.331819	-1.61229	0.110462	-4.87577	0.356551	0.437907
Erythroid.cells	ATP2B1	0.257767	8.327101	1.612288	0.110463	-5.61595	0.290285	0.370606
Erythroid.cells	PRR5	0.678761	3.479528	1.611913	0.110545	-4.62148	0.342881	0.424269
Erythroid.cells	NR1I3	1.032336	1.055611	1.611553	0.110624	-4.42149	0.372872	0.454025
Erythroid.cells	ATXN2	-0.29538	6.28335	-1.61129	0.11068	-5.16381	0.311509	0.392657
Erythroid.cells	CDYL	-0.29855	5.659184	-1.61129	0.110681	-5.02997	0.318242	0.39954
Erythroid.cells	BC024063	-1.17276	0.183577	-1.61106	0.110731	-4.30897	0.384325	0.465297
Erythroid.cells	HSD17B10	0.316405	5.540491	1.610789	0.11079	-5.19301	0.319606	0.400939
Erythroid.cells	ZFP729B	0.555259	3.639068	1.610675	0.110815	-4.57724	0.341179	0.422769
Erythroid.cells	TAB3	0.437806	3.680364	1.610557	0.110841	-4.77339	0.340694	0.422319
Erythroid.cells	BMP1	1.172776	0.911089	1.610412	0.110873	-4.36387	0.374814	0.456128
Erythroid.cells	IFNG	-0.96455	1.241502	-1.60987	0.110991	-4.58554	0.370821	0.452082
Erythroid.cells	USP3	-0.26122	6.873279	-1.60918	0.111142	-5.34696	0.305558	0.38679
Erythroid.cells	ZEB1	0.37084	6.848689	1.609118	0.111156	-5.41594	0.305815	0.387059
Erythroid.cells	TIMM13	0.247442	6.732513	1.609056	0.111169	-5.35695	0.307033	0.388313
Erythroid.cells	PTBP2	-0.31668	6.156832	-1.60895	0.111192	-5.13689	0.313143	0.394587
Erythroid.cells	APBA1	0.568236	4.384633	1.608868	0.11121	-4.86544	0.332774	0.414543
Erythroid.cells	SPEF2	-1.05578	1.371663	-1.60884	0.111217	-4.36522	0.36916	0.450785
Erythroid.cells	9930022D1	-1.20632	0.319301	-1.60882	0.11122	-4.34839	0.382815	0.464156
Erythroid.cells	NUFIP1	-0.42695	4.171093	-1.60857	0.111276	-4.72932	0.335291	0.417099

Erythroid.cells	PAM	0.446471	4.417759	1.607876	0.111428	-4.89486	0.332813	0.414335
Erythroid.cells	PCCA	0.448235	4.599387	1.607662	0.111475	-4.8792	0.330781	0.412323
Erythroid.cells	KLHL7	0.39515	4.739495	1.60662	0.111704	-4.89249	0.329731	0.41104
Erythroid.cells	DCTN6	-0.3598	5.142412	-1.60646	0.111739	-4.9105	0.325202	0.406498
Erythroid.cells	B3GALT4	-1.10929	1.404829	-1.60637	0.111759	-4.37125	0.369848	0.451074
Erythroid.cells	IGKC	-0.67509	7.88266	-1.60564	0.111918	-5.67498	0.296361	0.376847
Erythroid.cells	SENP8	-1.2303	0.568146	-1.6056	0.111929	-4.34055	0.381029	0.461907
Erythroid.cells	SLC22A14	0.813194	2.744823	1.60539	0.111974	-4.49235	0.353508	0.434929
Erythroid.cells	TSEN15	0.631928	2.776415	1.60512	0.112034	-4.53221	0.353169	0.43463
Erythroid.cells	SCO1	0.847606	1.930574	1.605017	0.112056	-4.43895	0.363616	0.444994
Erythroid.cells	PWP2	-0.93025	1.891751	-1.60403	0.112275	-4.41096	0.364702	0.445781
Erythroid.cells	0610005C1	0.705426	1.911141	1.603837	0.112316	-4.5657	0.364483	0.445543
Erythroid.cells	STAMBPL1	-0.34172	6.55028	-1.60305	0.112491	-5.25182	0.311159	0.391879
Erythroid.cells	CEP295	-0.47525	3.949897	-1.6026	0.112589	-4.70829	0.340372	0.421529
Erythroid.cells	AGO1	-0.52949	3.874896	-1.60247	0.112618	-4.69845	0.341251	0.422454
Erythroid.cells	CD79A	-0.36205	6.200185	-1.60229	0.112659	-5.40992	0.315094	0.396087
Erythroid.cells	AKR1B8	1.007428	1.592252	1.601341	0.112869	-4.42775	0.369236	0.450708
Erythroid.cells	CMKLR1	-1.03633	2.151939	-1.6013	0.112878	-4.5308	0.362178	0.443749
Erythroid.cells	FNBP4	-0.26799	6.074991	-1.6012	0.112901	-5.1646	0.316486	0.397851
Erythroid.cells	CSTA3	-1.46325	0.791872	-1.60119	0.112901	-4.41533	0.379576	0.460843
Erythroid.cells	CD177	1.100374	0.17299	1.601193	0.112901	-4.40291	0.387773	0.468831
Erythroid.cells	FUBP1	-0.20883	6.678668	-1.60115	0.112911	-5.29552	0.310013	0.39122
Erythroid.cells	COL27A1	1.012637	2.314283	1.601146	0.112912	-4.51416	0.360157	0.44175
Erythroid.cells	RPF2	-0.50703	4.402771	-1.60077	0.112994	-4.77556	0.335226	0.416973
Erythroid.cells	CKAP2	0.685596	3.667324	1.600695	0.113012	-4.71801	0.343811	0.425619
Erythroid.cells	SLC35E3	-0.91627	1.909396	-1.60061	0.113031	-4.41767	0.365274	0.446949
Erythroid.cells	PTPN4	0.375798	5.463527	1.600303	0.113099	-5.07473	0.323334	0.404927
Erythroid.cells	MTMR12	-0.32941	5.334196	-1.59956	0.113264	-5.03616	0.325148	0.406697
Erythroid.cells	ELF1	-0.18784	8.010369	-1.59893	0.113404	-5.55377	0.296977	0.377723
Erythroid.cells	COLEC11	0.812566	1.589372	1.598424	0.113517	-4.48153	0.370363	0.451868
Erythroid.cells	PTGES	-1.21681	0.477516	-1.59825	0.113556	-4.42876	0.38485	0.466114
Erythroid.cells	TMEM185E	-0.47758	4.12483	-1.59812	0.113584	-4.73295	0.339392	0.421176
Erythroid.cells	REXO4	-0.42507	4.291897	-1.59811	0.113587	-4.76335	0.337449	0.419222
Erythroid.cells	GM32569	-0.98352	1.845691	-1.59791	0.113632	-4.542	0.367108	0.448745
Erythroid.cells	1700019L1	-1.15019	0.774577	-1.59779	0.113657	-4.37771	0.380928	0.462327
Erythroid.cells	A930029G2	1.004536	1.230907	1.597481	0.113727	-4.38506	0.37507	0.456515
Erythroid.cells	PEAK1	-0.34885	6.584571	-1.59736	0.113754	-5.36308	0.312012	0.393351
Erythroid.cells	FGG	0.412764	6.166651	1.596932	0.113849	-5.51366	0.316592	0.398024
Erythroid.cells	GINM1	-0.32109	4.990227	-1.59692	0.113852	-4.95458	0.329624	0.411276
Erythroid.cells	4933439C1	-1.06914	1.200712	-1.59647	0.113953	-4.36222	0.375642	0.457075
Erythroid.cells	0610040B1	-0.80006	2.051019	-1.59637	0.113975	-4.45797	0.364785	0.446399
Erythroid.cells	BCL7A	-0.4322	4.80999	-1.59627	0.113996	-5.06517	0.331743	0.413412
Erythroid.cells	CIB1	-0.33416	5.732242	-1.59619	0.114014	-5.08457	0.32141	0.402932
Erythroid.cells	PRKRIP1	-0.43463	3.964552	-1.5958	0.114103	-4.71523	0.341586	0.423377
Erythroid.cells	CCNK	-0.36207	4.857081	-1.59579	0.114104	-4.89105	0.331269	0.412988
Erythroid.cells	UQCRFS1	0.235218	7.164384	1.595231	0.114229	-5.49094	0.306335	0.38745
Erythroid.cells	BRIX1	-0.22431	6.144863	-1.59445	0.114405	-5.22713	0.317601	0.398888

Erythroid.cells	HOPX	-0.50728	4.034194	-1.59393	0.114521	-4.94189	0.341635	0.423243
Erythroid.cells	H2-T22	-0.65589	5.391347	-1.59388	0.114531	-5.08735	0.326077	0.407552
Erythroid.cells	GM15708	-0.69186	3.198583	-1.5937	0.114572	-4.5705	0.351618	0.433264
Erythroid.cells	USP8	0.308676	5.372996	1.593326	0.114656	-5.02322	0.326397	0.407988
Erythroid.cells	GM15848	-1.26298	-0.86297	-1.59311	0.114705	-4.31638	0.404627	0.485216
Erythroid.cells	PAG1	-0.37518	7.038518	-1.59297	0.114736	-5.39291	0.308302	0.389502
Erythroid.cells	MOB1B	0.226967	6.203813	1.592866	0.114759	-5.26417	0.317234	0.398659
Erythroid.cells	RASGRP3	0.705187	3.586569	1.592783	0.114778	-4.7425	0.347059	0.428805
Erythroid.cells	NFKBIE	-0.50074	4.879022	-1.59202	0.114949	-4.90994	0.332286	0.413828
Erythroid.cells	CSRNP2	-0.85362	2.107884	-1.59185	0.114988	-4.44295	0.365532	0.447001
Erythroid.cells	CIRBP	0.277292	7.218243	1.59158	0.115048	-5.39395	0.306698	0.38777
Erythroid.cells	STK32C	-0.97623	0.553606	-1.59155	0.115055	-4.39981	0.385668	0.46677
Erythroid.cells	TNFSF13	0.855304	1.863945	1.591545	0.115056	-4.56229	0.36862	0.450081
Erythroid.cells	GM26740	0.340943	7.068845	1.591141	0.115147	-5.37872	0.308412	0.389484
Erythroid.cells	D430001F1	-1.57804	-0.87048	-1.59094	0.115193	-4.31175	0.405297	0.48579
Erythroid.cells	SPPL3	-0.2094	7.282604	-1.59085	0.115213	-5.42558	0.306167	0.387243
Erythroid.cells	MICALL1	-0.57539	2.97359	-1.58986	0.115436	-4.56883	0.355535	0.436927
Erythroid.cells	PDXP	-1.11367	1.557173	-1.58897	0.115637	-4.39265	0.373843	0.454887
Erythroid.cells	GATA3	0.714721	1.116393	1.588647	0.11571	-4.63364	0.379571	0.460662
Erythroid.cells	LEMD2	0.426882	4.232797	1.588545	0.115733	-4.77208	0.340933	0.422443
Erythroid.cells	TDRD7	0.568589	3.385152	1.588467	0.115751	-4.70443	0.35102	0.432547
Erythroid.cells	ZFAND2B	0.406136	4.313639	1.588272	0.115795	-4.85999	0.339987	0.421557
Erythroid.cells	AGT	0.649088	4.084746	1.588181	0.115815	-5.09766	0.342673	0.424301
Erythroid.cells	PEA15A	-0.48969	3.90143	-1.58783	0.115895	-4.78074	0.344839	0.426622
Erythroid.cells	ZFP944	-0.45779	4.397621	-1.58779	0.115904	-4.81359	0.339007	0.420778
Erythroid.cells	UBAP1L	-0.93906	1.134289	-1.58778	0.115907	-4.36579	0.379337	0.460759
Erythroid.cells	CBFB	-0.19625	6.809866	-1.5876	0.115947	-5.34729	0.312112	0.393483
Erythroid.cells	BMYC	0.74723	3.380337	1.58728	0.116019	-4.66433	0.351209	0.432946
Erythroid.cells	LMBR1	0.81743	1.883516	1.586923	0.1161	-4.45212	0.369881	0.45152
Erythroid.cells	RNF34	-0.42428	4.455799	-1.58684	0.116119	-4.79677	0.338534	0.420353
Erythroid.cells	FUOM	0.695668	2.61981	1.586705	0.11615	-4.71098	0.360612	0.442394
Erythroid.cells	PDE4DIP	-0.46791	3.94998	-1.58655	0.116184	-4.76214	0.344472	0.426345
Erythroid.cells	POLD3	-0.41361	4.695421	-1.58578	0.11636	-4.88693	0.336169	0.417842
Erythroid.cells	APLF	0.771709	2.05909	1.585393	0.116447	-4.50594	0.368233	0.449706
Erythroid.cells	PCM1	-0.2747	6.494399	-1.58527	0.116475	-5.28478	0.316175	0.397512
Erythroid.cells	LRRC8C	-0.36735	6.412612	-1.58513	0.116507	-5.25712	0.317062	0.398465
Erythroid.cells	CRY2	-0.64161	2.553643	-1.58482	0.116578	-4.54187	0.362021	0.443728
Erythroid.cells	PF4	1.312594	4.278945	1.584361	0.116682	-4.92957	0.34115	0.423075
Erythroid.cells	GAL3ST1	1.299673	-0.29084	1.583895	0.116788	-4.33204	0.399354	0.480473
Erythroid.cells	CXCL10	-1.01092	4.788598	-1.58389	0.116789	-4.9197	0.335229	0.417194
Erythroid.cells	DOLK	-0.76269	1.544978	-1.58368	0.116838	-4.40766	0.374832	0.45662
Erythroid.cells	BEX4	1.036844	0.397887	1.583393	0.116903	-4.38248	0.389968	0.471548
Erythroid.cells	BRI3BP	0.402393	4.756436	1.583225	0.116941	-4.94655	0.335599	0.417781
Erythroid.cells	FAM131A	-1.24248	0.401139	-1.58321	0.116944	-4.37897	0.389925	0.471575
Erythroid.cells	NFS1	0.374574	4.533914	1.583085	0.116973	-4.91544	0.338174	0.420405
Erythroid.cells	CSPRS	1.031156	0.292172	1.58262	0.117079	-4.36114	0.391394	0.473088
Erythroid.cells	MFSD14A	0.25769	6.156841	1.58256	0.117093	-5.15552	0.319861	0.40188

Erythroid.cells	2510017J1	-1.07458	1.414805	-1.58254	0.117096	-4.40825	0.376519	0.458566
Erythroid.cells	ZFP993	-0.91476	1.824761	-1.58254	0.117098	-4.43919	0.371233	0.453371
Erythroid.cells	TOR1AIP2	-0.23086	6.580878	-1.58239	0.117131	-5.31201	0.315252	0.397167
Erythroid.cells	SLC6A6	-0.27628	7.287991	-1.58235	0.117142	-5.39874	0.307721	0.389425
Erythroid.cells	CYREN	0.629457	3.374354	1.582337	0.117143	-4.6155	0.351932	0.434249
Erythroid.cells	TLCD1	0.745038	1.624021	1.582083	0.117202	-4.47881	0.373812	0.455912
Erythroid.cells	PRRG4	1.303699	0.01648	1.582028	0.117214	-4.33024	0.395138	0.476729
Erythroid.cells	AGTRAP	-0.68765	4.534904	-1.58203	0.117214	-4.725	0.338163	0.420446
Erythroid.cells	PROX2	-1.14091	1.188752	-1.58193	0.117235	-4.36489	0.379467	0.46146
Erythroid.cells	ARFGEF2	-0.34341	6.285468	-1.58156	0.11732	-5.1434	0.318456	0.400562
Erythroid.cells	BZW1	-0.16813	7.459579	-1.58153	0.117329	-5.49252	0.305922	0.387684
Erythroid.cells	HIC2	0.755452	2.712677	1.581381	0.117362	-4.52542	0.360043	0.442468
Erythroid.cells	CD63	0.543308	4.105165	1.581375	0.117363	-4.96076	0.343194	0.425651
Erythroid.cells	TTC27	-0.56176	3.520631	-1.58117	0.117411	-4.65228	0.350164	0.432655
Erythroid.cells	ITGAX	0.551999	3.098724	1.58116	0.117412	-4.86868	0.355287	0.437765
Erythroid.cells	HNF4A	1.055181	0.691681	1.580745	0.117507	-4.42368	0.386033	0.468278
Erythroid.cells	PTMA	-0.22377	11.35991	-1.58072	0.117513	-6.15599	0.26793	0.348029
Erythroid.cells	ATG4D	0.450149	3.954415	1.580719	0.117513	-4.79222	0.344978	0.427662
Erythroid.cells	IARS2	-0.43058	4.475268	-1.58032	0.117605	-4.85276	0.33902	0.421646
Erythroid.cells	TXNDC15	0.390094	4.911319	1.579839	0.117715	-4.95681	0.334195	0.416745
Erythroid.cells	PPP1R8	0.448574	4.114233	1.579651	0.117758	-4.78079	0.343499	0.426107
Erythroid.cells	F730311O2	-1.3409	-0.31758	-1.57906	0.117893	-4.34008	0.400542	0.482258
Erythroid.cells	TBC1D14	0.339294	5.680933	1.578766	0.117961	-5.04428	0.32588	0.408216
Erythroid.cells	GFM2	-0.54006	3.590998	-1.57792	0.118154	-4.66342	0.350428	0.432976
Erythroid.cells	GNPDA1	0.466494	4.395098	1.577807	0.118181	-4.80141	0.340871	0.423409
Erythroid.cells	BICC1	1.235954	0.332453	1.577806	0.118182	-4.3908	0.392093	0.474047
Erythroid.cells	ATAT1	-0.6413	2.722421	-1.57773	0.118199	-4.56224	0.361066	0.443583
Erythroid.cells	RNF4	-0.24833	5.964365	-1.57682	0.118409	-5.16907	0.323481	0.405606
Erythroid.cells	ALAS1	-0.55891	3.683631	-1.57598	0.118603	-4.78552	0.35024	0.432558
Erythroid.cells	L3MBTL2	-0.48474	3.445434	-1.57591	0.118617	-4.65162	0.353123	0.435435
Erythroid.cells	PEMT	0.543431	3.483706	1.574889	0.118854	-4.94544	0.35319	0.43541
Erythroid.cells	VPREB3	-0.49753	6.013944	-1.57483	0.118868	-5.41338	0.323797	0.405797
Erythroid.cells	MBLAC2	0.694568	2.64789	1.574654	0.118908	-4.53975	0.363503	0.445654
Erythroid.cells	USP11	-0.73897	2.611991	-1.57454	0.118935	-4.5111	0.363953	0.446144
Erythroid.cells	IL10RB	0.350753	5.555085	1.573491	0.119177	-5.04273	0.32946	0.411424
Erythroid.cells	NOL4L	-0.8186	2.680623	-1.57338	0.119204	-4.61402	0.363679	0.445714
Erythroid.cells	SUGP1	-0.37677	4.615277	-1.57321	0.119242	-4.89115	0.34026	0.422395
Erythroid.cells	PRG4	2.093883	1.084056	1.573103	0.119267	-4.52317	0.384263	0.466047
Erythroid.cells	MYO1E	-0.43553	6.198716	-1.57267	0.119367	-5.44826	0.322405	0.404271
Erythroid.cells	HSPE1	0.281458	7.179751	1.57259	0.119386	-5.5567	0.311764	0.393407
Erythroid.cells	STXBP1	-0.56478	3.786972	-1.57221	0.119473	-4.77224	0.350386	0.432532
Erythroid.cells	GM20342	0.617241	3.499425	1.571366	0.11967	-4.63721	0.354312	0.436278
Erythroid.cells	RAI14	0.740182	2.228896	1.571268	0.119692	-4.6363	0.370162	0.451984
Erythroid.cells	AFMID	0.417615	4.371552	1.570277	0.119923	-4.96597	0.344198	0.426051
Erythroid.cells	PFAS	0.527981	4.205445	1.570102	0.119963	-4.93009	0.346168	0.428026
Erythroid.cells	MMD	0.444822	4.477186	1.57004	0.119978	-4.98878	0.342951	0.424801
Erythroid.cells	GM11084	-0.58641	3.368087	-1.56992	0.120006	-4.60197	0.356284	0.438118

Erythroid.cells	PLEKHJ1	-0.25533	5.969804	-1.56991	0.120007	-5.16262	0.325828	0.40751
Erythroid.cells	TMEM179E	-0.27245	5.864754	-1.56944	0.120117	-5.11415	0.327003	0.408906
Erythroid.cells	FMNL3	0.539985	4.258409	1.569401	0.120127	-4.74033	0.345539	0.427607
Erythroid.cells	ANKRD23	-1.12655	0.33175	-1.56937	0.120134	-4.35782	0.395609	0.476939
Erythroid.cells	MOB2	0.279645	5.416589	1.569194	0.120175	-5.1368	0.332067	0.414066
Erythroid.cells	LAMTOR5	0.320915	5.527925	1.569147	0.120186	-5.07685	0.330801	0.412786
Erythroid.cells	GRAMD4	-0.38391	5.165751	-1.56908	0.120201	-5.16065	0.334937	0.416975
Erythroid.cells	PLXNC1	0.450911	5.427157	1.568594	0.120315	-5.13831	0.332165	0.414127
Erythroid.cells	ZFP524	0.587015	3.672126	1.568243	0.120397	-4.69842	0.352945	0.434962
Erythroid.cells	BORCS5	-0.45314	4.024358	-1.56765	0.120535	-4.76308	0.348996	0.430892
Erythroid.cells	SFMBT2	1.106759	0.60235	1.567145	0.120653	-4.39698	0.392947	0.474086
Erythroid.cells	NPR1	1.110492	1.263007	1.566613	0.120777	-4.40925	0.384374	0.465754
Erythroid.cells	5830487J0	1.05462	-0.01733	1.566036	0.120913	-4.37016	0.401969	0.482799
Erythroid.cells	KIRREL3	0.961032	1.272544	1.565914	0.120941	-4.44748	0.384464	0.465811
Erythroid.cells	UBAC1	0.311497	4.029628	1.565872	0.120951	-5.00669	0.349627	0.431425
Erythroid.cells	PIK3R4	-0.65345	3.189781	-1.56449	0.121274	-4.59146	0.360734	0.442241
Erythroid.cells	PIMREG	1.058417	2.104196	1.563226	0.121572	-4.51142	0.375292	0.456214
Erythroid.cells	GCLC	-0.33039	6.841762	-1.5627	0.121697	-5.47497	0.31916	0.399967
Erythroid.cells	HIKESHI	-0.32746	5.228907	-1.56232	0.121786	-5.04929	0.337441	0.418393
Erythroid.cells	FRAT2	-0.42847	4.805593	-1.56214	0.121828	-4.99224	0.342399	0.423429
Erythroid.cells	CRIP1	0.325484	8.825299	1.561008	0.122094	-5.85053	0.298868	0.379002
Erythroid.cells	ISG20L2	-0.34696	5.083016	-1.56094	0.12211	-5.03745	0.33966	0.420631
Erythroid.cells	PNN	-0.24134	6.310373	-1.56091	0.122116	-5.29254	0.325665	0.406505
Erythroid.cells	GM36447	-1.34814	0.209364	-1.56018	0.122289	-4.35057	0.402195	0.482097
Erythroid.cells	MTG2	0.53441	3.031494	1.559745	0.122393	-4.66023	0.364914	0.445839
Erythroid.cells	AP5M1	0.526523	3.584616	1.559741	0.122394	-4.69171	0.358031	0.43903
Erythroid.cells	ACVR1B	-0.68596	2.543644	-1.55947	0.122458	-4.53354	0.371098	0.451942
Erythroid.cells	GM30211	-0.63362	4.041214	-1.55944	0.122466	-5.01079	0.352452	0.433497
Erythroid.cells	EFHD2	-0.26753	6.92246	-1.55941	0.122473	-5.4831	0.319292	0.400084
Erythroid.cells	ACACA	0.405391	5.153719	1.559039	0.12256	-5.01077	0.339379	0.42046
Erythroid.cells	UBE2E1	-0.22176	6.229498	-1.5587	0.12264	-5.25726	0.327212	0.408102
Erythroid.cells	GNB5	-0.81472	0.819995	-1.55833	0.122728	-4.47012	0.394224	0.474533
Erythroid.cells	PHC1	-0.48141	3.5385	-1.55829	0.122738	-4.66033	0.35896	0.439985
Erythroid.cells	TNIP1	-0.5116	4.966974	-1.55812	0.122776	-4.9577	0.34177	0.422858
Erythroid.cells	PPP1R7	-0.35608	4.409711	-1.55777	0.122859	-4.85386	0.348424	0.429512
Erythroid.cells	F3	-1.6204	0.113979	-1.55775	0.122865	-4.38235	0.404018	0.484003
Erythroid.cells	NPM1	-0.24169	8.143673	-1.55733	0.122964	-5.6681	0.30677	0.387172
Erythroid.cells	GM44752	0.625147	2.484975	1.557128	0.123013	-4.59673	0.372518	0.453245
Erythroid.cells	SPCS1	0.196433	7.852067	1.556612	0.123135	-5.64483	0.31008	0.390552
Erythroid.cells	CUL7	-0.89355	1.968845	-1.55648	0.123167	-4.46217	0.379463	0.460105
Erythroid.cells	ANKDD1A	1.132438	2.346094	1.55624	0.123223	-4.63765	0.374622	0.455378
Erythroid.cells	4921516A0	-0.94981	0.76983	-1.55582	0.123322	-4.43323	0.395679	0.475999
Erythroid.cells	DNASE1L1	0.608835	3.048154	1.555721	0.123347	-4.65382	0.365784	0.446796
Erythroid.cells	WARS	0.599767	3.723103	1.55555	0.123388	-4.69299	0.357385	0.438509
Erythroid.cells	GM28875	-0.44713	4.612323	-1.55546	0.123408	-4.99138	0.346625	0.427809
Erythroid.cells	GM11520	0.933155	1.043872	1.555331	0.12344	-4.41827	0.391954	0.472479
Erythroid.cells	INIP	-0.38977	4.523666	-1.55511	0.123493	-4.87001	0.347733	0.428954

Erythroid.cells	NSD2	-0.27419	6.699324	-1.55474	0.123581	-5.43458	0.322874	0.403866
Erythroid.cells	ZFP354B	1.035693	-0.81695	1.554246	0.123698	-4.34953	0.418363	0.497891
Erythroid.cells	ATP9A	1.0945	0.837325	1.554238	0.1237	-4.42834	0.395136	0.475551
Erythroid.cells	SMYD5	0.922662	2.056268	1.554017	0.123753	-4.49527	0.378917	0.45981
Erythroid.cells	FXYD1	0.639451	2.943369	1.553593	0.123854	-4.86066	0.367647	0.448778
Erythroid.cells	ITGB6	-1.45982	-0.27325	-1.55352	0.123871	-4.37402	0.410792	0.490737
Erythroid.cells	EPM2A	0.949417	1.526051	1.553011	0.123993	-4.47976	0.386172	0.466947
Erythroid.cells	IFI204	-0.7081	4.390085	-1.55292	0.124015	-4.97204	0.349902	0.431187
Erythroid.cells	ROPN1L	0.819294	2.420763	1.552858	0.12403	-4.56536	0.37444	0.455475
Erythroid.cells	GPD1	0.776395	1.749644	1.552751	0.124055	-4.60355	0.383205	0.464054
Erythroid.cells	CCDC122	-0.80081	1.315452	-1.55262	0.124086	-4.52188	0.388988	0.469719
Erythroid.cells	SERPINA6	-1.1564	1.127419	-1.55208	0.124215	-4.52473	0.391815	0.472286
Erythroid.cells	KIF19A	-0.93135	1.071139	-1.55138	0.124383	-4.42843	0.392933	0.473304
Erythroid.cells	DNAJC17	-0.43134	3.568748	-1.55131	0.124399	-4.71451	0.360524	0.441546
Erythroid.cells	HIPK2	0.312463	6.550097	1.550858	0.124508	-5.3583	0.32565	0.40644
Erythroid.cells	PAFAH1B2	-0.29623	5.489377	-1.54976	0.12477	-5.06136	0.338271	0.418989
Erythroid.cells	IL1RAP	0.510454	4.731572	1.549685	0.124789	-4.95165	0.347184	0.427935
Erythroid.cells	TEDC1	0.861132	1.701406	1.549282	0.124886	-4.47168	0.385537	0.465797
Erythroid.cells	CCNA2	0.572532	5.520186	1.549021	0.124949	-5.27324	0.338072	0.418947
Erythroid.cells	MTERF3	0.465546	4.014483	1.548518	0.12507	-4.80116	0.356013	0.437055
Erythroid.cells	CEP57L1	-0.61534	3.287731	-1.54845	0.125086	-4.65959	0.365027	0.445987
Erythroid.cells	BMF	-0.79802	2.137288	-1.54827	0.125129	-4.55512	0.379785	0.460514
Erythroid.cells	RBAK	-0.91052	1.336784	-1.54824	0.125136	-4.45794	0.390416	0.470876
Erythroid.cells	AI837181	-0.50954	3.127875	-1.54821	0.125145	-4.63011	0.367042	0.447998
Erythroid.cells	GM47662	-1.08751	-0.79475	-1.54817	0.125154	-4.39115	0.42023	0.499587
Erythroid.cells	SLC37A3	-0.42546	3.658041	-1.5481	0.12517	-4.83174	0.360405	0.441466
Erythroid.cells	MPP6	0.294037	6.194697	1.547937	0.12521	-5.27174	0.330358	0.411405
Erythroid.cells	STAT5A	0.441308	3.992851	1.547297	0.125364	-4.87663	0.356449	0.437669
Erythroid.cells	FAM45A	0.421592	3.979967	1.547261	0.125373	-4.77585	0.356607	0.437826
Erythroid.cells	TMEM242	-0.36865	4.292008	-1.54726	0.125373	-4.8945	0.352802	0.43404
Erythroid.cells	SLC2A8	0.68261	2.226604	1.546882	0.125464	-4.54679	0.378965	0.45984
Erythroid.cells	RFX1	-0.4887	3.263376	-1.54659	0.125535	-4.63544	0.365771	0.446845
Erythroid.cells	ANAPC7	-0.37909	4.359074	-1.54553	0.125791	-4.87621	0.352861	0.433727
Erythroid.cells	ARSK	0.754572	2.705302	1.545354	0.125833	-4.58745	0.373541	0.45418
Erythroid.cells	PSMB8	-0.40587	7.077604	-1.54468	0.125995	-5.45913	0.321803	0.402492
Erythroid.cells	DUS2	-0.57766	3.353616	-1.54417	0.126118	-4.73929	0.365914	0.44663
Erythroid.cells	PCBP3	0.629621	2.616922	1.543913	0.126181	-4.61262	0.375349	0.455906
Erythroid.cells	NR2F2	0.739728	3.13223	1.543833	0.126201	-4.75911	0.368745	0.449441
Erythroid.cells	B3GNT6	-1.12672	-0.72192	-1.54292	0.126422	-4.38465	0.421702	0.500703
Erythroid.cells	MRPS22	0.508704	3.085855	1.542895	0.126428	-4.71234	0.369789	0.450441
Erythroid.cells	ZFP68	0.436031	3.920899	1.542471	0.126531	-4.7689	0.359504	0.440215
Erythroid.cells	KPNA2	0.462593	4.937072	1.541531	0.126759	-5.05774	0.347638	0.428268
Erythroid.cells	MIR22HG	0.56257	4.089128	1.541469	0.126774	-4.84431	0.357915	0.438515
Erythroid.cells	GM4952	0.686122	2.667628	1.541114	0.126861	-4.83289	0.375944	0.456281
Erythroid.cells	CAND1	-0.30683	5.132169	-1.54083	0.12693	-5.038	0.345394	0.42607
Erythroid.cells	UTRN	0.301589	7.31422	1.540796	0.126938	-5.5118	0.320526	0.400957
Erythroid.cells	SDHC	0.323619	5.09505	1.540762	0.126946	-5.08688	0.345834	0.42651

Erythroid.cells	FLT1	0.767248	4.243341	1.540505	0.127009	-5.07518	0.356175	0.436861
Erythroid.cells	PROX1OS	1.00646	0.612207	1.540221	0.127078	-4.49168	0.403752	0.483438
Erythroid.cells	ENPP1	0.873735	2.31349	1.539914	0.127153	-4.58839	0.380755	0.461248
Erythroid.cells	ZFP292	-0.25541	7.046023	-1.53977	0.127187	-5.46476	0.323642	0.404333
Erythroid.cells	FLYWCH1	-0.45792	3.850858	-1.53968	0.127209	-4.80041	0.361123	0.441981
Erythroid.cells	EPRS	0.336153	6.581496	1.539607	0.127228	-5.46837	0.328825	0.409638
Erythroid.cells	CCDC80	0.768468	2.683481	1.538922	0.127395	-4.68808	0.376318	0.45677
Erythroid.cells	GM11772	0.926939	0.302935	1.538638	0.127464	-4.4506	0.408596	0.488056
Erythroid.cells	DHRS13	-1.09075	0.959658	-1.53853	0.127489	-4.46562	0.399438	0.479251
Erythroid.cells	ESD	-0.26297	7.081837	-1.53794	0.127635	-5.59726	0.32389	0.404389
Erythroid.cells	FOXRED1	-0.76842	2.831724	-1.53781	0.127667	-4.5694	0.374761	0.455196
Erythroid.cells	MYLK	0.832078	1.606214	1.537682	0.127698	-4.55966	0.390931	0.471053
Erythroid.cells	PLEKHF2	0.264443	5.470642	1.537494	0.127744	-5.22279	0.342287	0.423115
Erythroid.cells	RAP1GAP	-1.22219	0.58551	-1.53683	0.127905	-4.39543	0.405223	0.484877
Erythroid.cells	DCLRE1A	-0.798	1.621612	-1.53668	0.127943	-4.52281	0.390991	0.471118
Erythroid.cells	SNX27	-0.28269	5.803763	-1.53665	0.127949	-5.1575	0.338605	0.419362
Erythroid.cells	CDC45	0.6641	3.268129	1.536466	0.127995	-4.73767	0.369423	0.450113
Erythroid.cells	NOC4L	-0.44085	3.728892	-1.53644	0.128002	-4.80581	0.363611	0.444381
Erythroid.cells	SNRPD1	-0.25811	7.17842	-1.53629	0.128039	-5.5222	0.323045	0.403748
Erythroid.cells	TRIM72	-1.18915	0.692552	-1.5361	0.128084	-4.40665	0.403733	0.483671
Erythroid.cells	PWP1	0.48505	3.903059	1.535935	0.128125	-4.75081	0.361443	0.442448
Erythroid.cells	8430429KC	-0.76472	1.679457	-1.53562	0.128204	-4.48201	0.390216	0.470808
Erythroid.cells	MCMDC2	-0.68828	3.465667	-1.53556	0.128216	-4.74771	0.366924	0.447991
Erythroid.cells	AY036118	0.638106	4.212426	1.535548	0.12822	-5.17816	0.357619	0.438777
Erythroid.cells	SCO2	0.587553	2.851352	1.535248	0.128294	-4.66454	0.374807	0.455853
Erythroid.cells	SNHG10	1.308297	0.227889	1.535056	0.128341	-4.39283	0.410305	0.490379
Erythroid.cells	HEY1	1.327351	-0.2714	1.535051	0.128342	-4.38257	0.41744	0.497221
Erythroid.cells	DTX1	-0.75349	2.069415	-1.53477	0.12841	-4.68619	0.38514	0.466015
Erythroid.cells	ZCCHC17	-0.29792	5.162664	-1.53363	0.128691	-5.09999	0.346919	0.42809
Erythroid.cells	TMED1	-0.67699	2.452472	-1.53325	0.128786	-4.57329	0.380985	0.461772
Erythroid.cells	DNAJC18	-0.48631	3.682567	-1.5329	0.128871	-4.79778	0.365321	0.446288
Erythroid.cells	TMEM156	0.547393	3.855526	1.531925	0.129111	-4.73824	0.363731	0.444382
Erythroid.cells	SLC25A10	0.548988	3.077126	1.531503	0.129216	-4.75685	0.373667	0.454319
Erythroid.cells	METTL3	-0.51892	3.319562	-1.53146	0.129226	-4.68918	0.370561	0.451285
Erythroid.cells	MCCC2	0.820789	2.436244	1.531398	0.129241	-4.64688	0.382009	0.462547
Erythroid.cells	RRBP1	-0.20573	7.951353	-1.53069	0.129417	-5.64504	0.316368	0.396936
Erythroid.cells	RTL6	-1.36052	0.292529	-1.53055	0.12945	-4.38191	0.411606	0.491236
Erythroid.cells	SNAP29	-0.27839	5.5062	-1.5304	0.129489	-5.19408	0.343968	0.424953
Erythroid.cells	ZFP771	0.391584	4.065583	1.530242	0.129527	-4.87166	0.361412	0.442351
Erythroid.cells	CYP3A13	0.92275	0.779768	1.53024	0.129528	-4.52392	0.40474	0.484676
Erythroid.cells	SRRM2	-0.17896	8.62357	-1.53017	0.129546	-5.76621	0.309201	0.389641
Erythroid.cells	JCHAIN	-2.77497	-0.4625	-1.52968	0.129667	-4.40009	0.422755	0.501824
Erythroid.cells	APOPT1	0.281744	4.910205	1.529268	0.129768	-5.02459	0.35148	0.432347
Erythroid.cells	CHMP7	0.695633	2.616172	1.528631	0.129926	-4.58075	0.380695	0.461031
Erythroid.cells	GM9733	0.921212	0.76406	1.528463	0.129968	-4.68481	0.405808	0.48541
Erythroid.cells	AR	1.078859	0.218656	1.528335	0.129999	-4.52572	0.413521	0.492842
Erythroid.cells	APOL8	0.632987	2.828251	1.527924	0.130101	-4.65947	0.377941	0.458555

Erythroid.cells	HCFC1R1	0.317892	5.33224	1.527829	0.130125	-5.15476	0.346766	0.427719
Erythroid.cells	ZFP810	0.713065	2.914739	1.527744	0.130146	-4.59476	0.376817	0.457509
Erythroid.cells	COA6	0.423928	4.110702	1.527724	0.130151	-4.87867	0.361624	0.442533
Erythroid.cells	MTHFD1L	-0.32191	5.661426	-1.52717	0.130288	-5.25198	0.343139	0.424097
Erythroid.cells	HARS2	0.507334	2.848743	1.526883	0.13036	-4.6427	0.37807	0.458675
Erythroid.cells	FBXO28	-0.44397	4.523471	-1.52642	0.130474	-4.89245	0.357115	0.437795
Erythroid.cells	TMEM30A	0.197002	6.581779	1.526131	0.130547	-5.41725	0.332872	0.41358
Erythroid.cells	ZFP395	0.444854	4.289929	1.525774	0.130636	-4.91517	0.360132	0.440861
Erythroid.cells	NFX1	-0.33488	4.900546	-1.52568	0.13066	-5.00927	0.352657	0.43343
Erythroid.cells	FAM71F2	-0.98929	2.260769	-1.52563	0.130671	-4.55976	0.386187	0.466468
Erythroid.cells	LRRC49	0.935722	1.020554	1.524941	0.130843	-4.47575	0.403145	0.483031
Erythroid.cells	ZBED3	0.500722	3.559356	1.524934	0.130845	-4.77077	0.369368	0.450105
Erythroid.cells	GDPD5	0.780479	1.750804	1.5249	0.130853	-4.60006	0.393115	0.473327
Erythroid.cells	AFF2	1.179849	0.04795	1.524893	0.130855	-4.47462	0.41691	0.496253
Erythroid.cells	GM13012	-0.63087	2.775114	-1.5248	0.130879	-4.62961	0.379477	0.460064
Erythroid.cells	4930557J0	-0.97903	1.834427	-1.52438	0.130982	-4.57863	0.392032	0.472393
Erythroid.cells	AA465934	0.833727	1.66043	1.524382	0.130982	-4.50158	0.394392	0.474684
Erythroid.cells	1600022D1	-0.97836	-0.42787	-1.52429	0.131005	-4.40596	0.42387	0.503033
Erythroid.cells	STON2	0.662412	3.518101	1.523892	0.131105	-4.73897	0.370118	0.450859
Erythroid.cells	TRIP6	0.824432	0.834471	1.523534	0.131194	-4.47757	0.406153	0.485829
Erythroid.cells	GM13402	1.157429	0.307958	1.522956	0.131339	-4.42743	0.413941	0.493224
Erythroid.cells	DNAJC25	0.392872	3.856746	1.522724	0.131397	-4.88613	0.366278	0.446993
Erythroid.cells	SLFN9	-0.85003	2.410472	-1.52266	0.131411	-4.61494	0.384979	0.465361
Erythroid.cells	PSTK	0.517861	3.061289	1.521947	0.131591	-4.68809	0.376813	0.457342
Erythroid.cells	GAB1	0.580294	4.473058	1.521857	0.131614	-5.11734	0.358951	0.439722
Erythroid.cells	UBE2N	-0.18491	7.48412	-1.5217	0.131653	-5.57997	0.323776	0.404387
Erythroid.cells	NHSL2	0.795216	2.487556	1.521283	0.131757	-4.71347	0.384541	0.464921
Erythroid.cells	RDH12	-0.94254	2.275253	-1.52095	0.13184	-4.58281	0.387411	0.467826
Erythroid.cells	CC2D2B	-1.40177	2.70829	-1.52093	0.131846	-4.61194	0.381672	0.462225
Erythroid.cells	ZFP773	0.803849	0.510873	1.519475	0.132211	-4.64495	0.412623	0.49208
Erythroid.cells	E130311K1	-1.21309	0.244935	-1.51922	0.132275	-4.42544	0.416429	0.495727
Erythroid.cells	LACTB2	0.415167	3.94655	1.519137	0.132296	-4.90957	0.366544	0.447247
Erythroid.cells	PLBD1	0.391299	5.389651	1.519115	0.132302	-5.46963	0.348819	0.429637
Erythroid.cells	PNO1	0.438108	4.446222	1.518767	0.132389	-4.92698	0.360302	0.441211
Erythroid.cells	QRICH1	-0.20169	6.585338	-1.51874	0.132395	-5.40027	0.334818	0.415702
Erythroid.cells	PREP	-0.32902	5.298922	-1.51866	0.132416	-5.18214	0.349906	0.430895
Erythroid.cells	F5	0.610713	4.094976	1.518613	0.132428	-5.14813	0.364678	0.445582
Erythroid.cells	AOX3	1.016608	0.998188	1.518382	0.132486	-4.54875	0.405806	0.485707
Erythroid.cells	STK25	0.366347	4.440838	1.517898	0.132608	-4.94912	0.360537	0.441521
Erythroid.cells	FGB	0.391026	5.797972	1.517848	0.132621	-5.56114	0.34413	0.425151
Erythroid.cells	CFAP97	-0.48583	3.29046	-1.51778	0.132637	-4.68263	0.375088	0.455886
Erythroid.cells	TRIM41	-0.41034	4.185669	-1.51701	0.13283	-4.82213	0.364143	0.444992
Erythroid.cells	BUB3	-0.30467	5.956108	-1.51669	0.132913	-5.29158	0.342768	0.423732
Erythroid.cells	MAPRE3	0.826534	1.502586	1.516581	0.13294	-4.5421	0.399506	0.479658
Erythroid.cells	PRCC	-0.30773	5.107381	-1.51599	0.133089	-5.09697	0.353189	0.434174
Erythroid.cells	G6PC	0.877071	1.911843	1.515262	0.133273	-4.7193	0.394473	0.474697
Erythroid.cells	CD28	0.507709	2.935145	1.515122	0.133308	-5.05307	0.380804	0.461393

Erythroid.cells	SPATA5	0.353055	5.905372	1.515111	0.133311	-5.22692	0.343858	0.424782
Erythroid.cells	YKT6	-0.39535	4.483546	-1.5151	0.133315	-4.8843	0.36105	0.441933
Erythroid.cells	MSL3	-0.3546	4.998465	-1.51494	0.133354	-5.01962	0.354726	0.435672
Erythroid.cells	ING2	0.327181	4.920364	1.514603	0.13344	-5.07805	0.35581	0.436744
Erythroid.cells	HEYL	1.285251	-0.20551	1.514288	0.13352	-4.40237	0.424663	0.503726
Erythroid.cells	ST7L	-0.46072	4.451288	-1.51416	0.133552	-4.86849	0.361693	0.44258
Erythroid.cells	DCP1B	0.614844	2.830948	1.51376	0.133653	-4.66636	0.38259	0.463117
Erythroid.cells	SDC2	0.715596	2.267759	1.51354	0.133709	-4.7169	0.390088	0.470499
Erythroid.cells	ATG13	-0.39844	4.765899	-1.51341	0.133743	-5.01091	0.357956	0.438941
Erythroid.cells	SPR	0.431262	3.527522	1.513356	0.133756	-4.93081	0.373524	0.454335
Erythroid.cells	ZFP747	0.797404	1.605909	1.513159	0.133806	-4.5215	0.399131	0.47934
Erythroid.cells	ZFP410	-0.39918	4.138179	-1.51275	0.133909	-4.89899	0.365925	0.446893
Erythroid.cells	PLK3	-0.5206	4.483011	-1.51263	0.13394	-4.97953	0.361614	0.44267
Erythroid.cells	BCL9L	0.571671	3.410778	1.512412	0.133995	-4.70169	0.375197	0.456168
Erythroid.cells	UBE2D1	-0.27732	5.377113	-1.51239	0.134001	-5.13444	0.350682	0.431871
Erythroid.cells	GM16973	0.823545	1.894858	1.512005	0.134099	-4.52722	0.395408	0.475964
Erythroid.cells	IKZF3	-0.34351	5.866917	-1.51197	0.134107	-5.48149	0.344925	0.426156
Erythroid.cells	CLDN5	-1.20646	1.006603	-1.51166	0.134186	-4.44405	0.407841	0.487951
Erythroid.cells	STRIP1	-0.45078	4.156745	-1.51144	0.134243	-4.84373	0.36595	0.447057
Erythroid.cells	GNG10	0.270083	6.940589	1.511273	0.134285	-5.50342	0.33264	0.413799
Erythroid.cells	ZADH2	0.449301	3.381757	1.511137	0.13432	-4.81392	0.375844	0.456941
Erythroid.cells	CRLS1	0.41855	3.622878	1.510768	0.134414	-4.8383	0.372897	0.454116
Erythroid.cells	ATAD2B	-0.24565	6.740991	-1.50996	0.134619	-5.44573	0.335481	0.416653
Erythroid.cells	TEP1	0.504272	3.914158	1.509164	0.134822	-4.814	0.369877	0.450913
Erythroid.cells	MED1	0.230802	5.883036	1.50913	0.134831	-5.24123	0.345701	0.426864
Erythroid.cells	ZFP692	-0.68127	2.030033	-1.50911	0.134835	-4.5494	0.394672	0.475179
Erythroid.cells	FZD5	0.643405	2.536377	1.509045	0.134853	-4.63639	0.387844	0.46855
Erythroid.cells	ATN1	0.577272	3.221914	1.508263	0.135052	-4.74421	0.37925	0.460109
Erythroid.cells	ACTG1	-0.2518	11.44474	-1.50781	0.135167	-6.25615	0.286549	0.366111
Erythroid.cells	PTPRB	0.809503	3.708559	1.507685	0.1352	-4.89667	0.373155	0.454065
Erythroid.cells	CCDC106	-1.14089	0.51131	-1.50732	0.135295	-4.43481	0.416786	0.496277
Erythroid.cells	CHID1	-0.5674	2.453126	-1.5072	0.135324	-4.62805	0.389783	0.470205
Erythroid.cells	OASL1	-1.17507	2.957835	-1.50691	0.135398	-4.7243	0.383141	0.463685
Erythroid.cells	GRIA3	0.947989	3.336555	1.506805	0.135425	-4.76597	0.378177	0.458861
Erythroid.cells	ZSWIM3	-1.04562	1.503758	-1.50626	0.135564	-4.46508	0.403137	0.483104
Erythroid.cells	TMEM67	-0.8329	1.688091	-1.50456	0.136002	-4.55253	0.401671	0.481443
Erythroid.cells	VPS45	0.499932	3.758422	1.504536	0.136007	-4.80331	0.374021	0.454503
Erythroid.cells	ARNT	-0.36518	5.869604	-1.50413	0.136111	-5.22893	0.348037	0.428743
Erythroid.cells	AP1S1	-0.38531	4.706161	-1.50387	0.136179	-4.9953	0.36229	0.442947
Erythroid.cells	CD207	-1.32715	-0.64958	-1.50363	0.136241	-4.43024	0.435736	0.514162
Erythroid.cells	TAF12	-0.33625	5.021001	-1.50358	0.136253	-5.06248	0.358394	0.439174
Erythroid.cells	RAF1	-0.30281	5.986456	-1.50301	0.136399	-5.26399	0.347	0.427689
Erythroid.cells	DYNLL2	0.408573	5.082989	1.50203	0.136653	-5.13704	0.358486	0.438769
Erythroid.cells	EIF2B1	-0.45581	3.62049	-1.50136	0.136825	-4.80395	0.377335	0.457301
Erythroid.cells	GM15489	-1.36706	0.208522	-1.50016	0.137137	-4.41624	0.425291	0.503131
Erythroid.cells	MAF1	0.378256	5.342723	1.499805	0.137228	-5.13983	0.356507	0.436323
Erythroid.cells	NR3C2	0.892119	1.939637	1.499411	0.13733	-4.62183	0.400907	0.479823

Erythroid.cells	FBL	0.297535	6.091074	1.499356	0.137344	-5.31097	0.347582	0.427482
Erythroid.cells	AEN	0.527368	3.664015	1.499031	0.137428	-4.84214	0.377915	0.457554
Erythroid.cells	NELFB	-0.36698	4.551192	-1.49878	0.137494	-4.9488	0.366607	0.446494
Erythroid.cells	MPP5	-0.34967	5.379551	-1.49868	0.13752	-5.13037	0.356329	0.436377
Erythroid.cells	ZBTB33	-0.58389	2.982117	-1.49837	0.1376	-4.64722	0.387059	0.466568
Erythroid.cells	CCDC134	0.595141	3.407824	1.49821	0.137641	-4.72615	0.381436	0.461162
Erythroid.cells	UBQLN1	0.271265	5.517917	1.497905	0.13772	-5.17583	0.354868	0.434941
Erythroid.cells	CHCHD10	0.362472	6.141333	1.497573	0.137806	-5.54316	0.347489	0.427563
Erythroid.cells	TERF2	0.348055	4.800606	1.497077	0.137935	-4.99004	0.364081	0.443938
Erythroid.cells	RNF170	-0.49542	3.319753	-1.49675	0.13802	-4.73753	0.383167	0.46269
Erythroid.cells	SEPHS1	0.333618	4.119783	1.496692	0.138035	-4.9331	0.372765	0.452504
Erythroid.cells	MUTYH	-1.10376	0.370874	-1.4965	0.138085	-4.42145	0.424217	0.502289
Erythroid.cells	SRM	0.588684	4.842685	1.496067	0.138197	-5.02613	0.363698	0.443657
Erythroid.cells	FAM210A	-0.44628	3.636981	-1.49603	0.138207	-4.81711	0.379088	0.458802
Erythroid.cells	RBFOX3	1.297022	-0.24977	1.4957	0.138293	-4.43402	0.433459	0.51119
Erythroid.cells	ECHDC2	0.816337	1.612612	1.49562	0.138314	-4.66247	0.406471	0.485423
Erythroid.cells	SLC43A2	-0.42223	6.179865	-1.49559	0.138323	-5.32772	0.347397	0.427487
Erythroid.cells	GM47230	-1.17248	0.668652	-1.49557	0.138327	-4.44497	0.419929	0.498322
Erythroid.cells	RNF25	-0.56446	3.103588	-1.49529	0.1384	-4.68188	0.38621	0.465756
Erythroid.cells	EDARADD	-0.49933	3.159788	-1.49467	0.138562	-4.87222	0.385809	0.465303
Erythroid.cells	EOGT	0.707923	2.532472	1.494329	0.13865	-4.62571	0.39431	0.473621
Erythroid.cells	CYP2C29	0.981609	1.011219	1.494156	0.138695	-4.60532	0.415549	0.494085
Erythroid.cells	COQ6	-0.60098	2.285167	-1.49403	0.138728	-4.61079	0.397685	0.476942
Erythroid.cells	CYP2D9	0.932639	0.530177	1.493992	0.138738	-4.51199	0.422506	0.50078
Erythroid.cells	DCAF8	0.221958	5.699638	1.493666	0.138823	-5.27399	0.353754	0.433826
Erythroid.cells	GM12802	0.89087	0.658153	1.493438	0.138883	-4.46391	0.420791	0.499153
Erythroid.cells	PSMD9	0.275445	5.225742	1.493374	0.1389	-5.20504	0.359553	0.439599
Erythroid.cells	COL14A1	0.832562	1.863173	1.493122	0.138965	-4.65119	0.403736	0.482764
Erythroid.cells	WDR45B	-0.22252	6.330115	-1.49226	0.139191	-5.3475	0.346736	0.426675
Erythroid.cells	FRA10AC1	0.506715	3.408508	1.491692	0.139339	-4.78058	0.383625	0.463074
Erythroid.cells	FNTB	0.589738	2.971515	1.490718	0.139595	-4.68637	0.389811	0.469033
Erythroid.cells	GM48226	0.666545	2.396833	1.490601	0.139626	-4.62953	0.397606	0.47659
Erythroid.cells	FBXO17	1.121397	0.782245	1.490563	0.139635	-4.45307	0.420378	0.498465
Erythroid.cells	NEFH	-1.25269	-0.09246	-1.4905	0.139653	-4.44441	0.433266	0.510718
Erythroid.cells	ST6GALNA(	1.065909	0.651842	1.490447	0.139666	-4.46606	0.422274	0.500274
Erythroid.cells	MBD3	-0.28817	5.696238	-1.49028	0.139709	-5.23984	0.354968	0.434776
Erythroid.cells	RBM45	-0.56081	3.10947	-1.49018	0.139736	-4.69689	0.387964	0.467256
Erythroid.cells	NOCT	0.445958	5.04776	1.489606	0.139886	-5.14713	0.363244	0.442952
Erythroid.cells	GLT8D1	-0.52345	3.339159	-1.48901	0.140043	-4.73552	0.385373	0.464798
Erythroid.cells	OLFM1	0.504239	2.469141	1.488833	0.14009	-5.00065	0.397095	0.476215
Erythroid.cells	HSD17B13	0.668135	2.671988	1.488812	0.140095	-4.87176	0.39433	0.473536
Erythroid.cells	UBA3	0.376752	4.076756	1.488611	0.140148	-4.90573	0.375717	0.455443
Erythroid.cells	GM6787	-1.14276	0.188055	-1.48861	0.140148	-4.43804	0.429608	0.507445
Erythroid.cells	MAN2C1	-0.49386	3.509599	-1.48853	0.14017	-4.75089	0.383119	0.462708
Erythroid.cells	CEP83	-0.29814	5.662564	-1.48833	0.140222	-5.20893	0.355813	0.435923
Erythroid.cells	KLRA1	-1.22254	0.700337	-1.48823	0.140248	-4.57493	0.422084	0.500503
Erythroid.cells	FMNL2	0.46396	7.100621	1.487754	0.140374	-5.558	0.338812	0.419002

Erythroid.cells	CHIL1	-0.96745	-0.07877	-1.4877	0.140388	-4.51949	0.433709	0.511624
Erythroid.cells	LRP1	0.435023	4.178873	1.487657	0.140399	-5.11354	0.374508	0.454519
Erythroid.cells	PRKCQ	0.566853	3.12892	1.486746	0.140639	-4.95116	0.388843	0.468156
Erythroid.cells	APOC4	0.507931	5.632488	1.48647	0.140712	-5.5884	0.356811	0.436706
Erythroid.cells	GM17231	0.852837	2.734245	1.486427	0.140724	-4.61164	0.394188	0.473393
Erythroid.cells	NLRC5	-0.71866	5.064856	-1.4859	0.140863	-5.14009	0.363865	0.443748
Erythroid.cells	2700062C0	-0.58524	2.646093	-1.48588	0.140867	-4.65551	0.395429	0.474648
Erythroid.cells	PIP5K1B	0.386631	5.568352	1.485839	0.140879	-5.27301	0.357634	0.437572
Erythroid.cells	GM20069	-0.98294	-0.52038	-1.48578	0.140895	-4.5298	0.441081	0.518291
Erythroid.cells	HPS4	-0.51638	4.16681	-1.48565	0.140928	-4.9027	0.375265	0.454982
Erythroid.cells	DPYD	0.607949	3.567148	1.485353	0.141007	-5.15398	0.38317	0.46274
Erythroid.cells	UBA6	0.332462	5.172539	1.485249	0.141035	-5.12583	0.362603	0.44252
Erythroid.cells	SYTL3	-0.59395	2.803793	-1.48494	0.141116	-5.04435	0.393467	0.472816
Erythroid.cells	POLD1	-0.4701	4.276679	-1.48474	0.14117	-4.9442	0.374021	0.4539
Erythroid.cells	UHRF1BP1l	0.297196	5.734451	1.48462	0.141201	-5.27	0.355766	0.435848
Erythroid.cells	ATG16L2	-0.3147	5.680082	-1.4845	0.141232	-5.23211	0.35643	0.436571
Erythroid.cells	APAF1	-0.33083	5.726771	-1.48425	0.141299	-5.23326	0.35586	0.436039
Erythroid.cells	6030443J0	-1.02343	0.848488	-1.48414	0.141328	-4.47556	0.420912	0.499478
Erythroid.cells	ZFP511	-0.39517	3.816412	-1.48412	0.141333	-4.8688	0.379988	0.45991
Erythroid.cells	GM11523	1.460946	-0.95948	1.483699	0.141445	-4.43447	0.448259	0.525299
Erythroid.cells	CUEDC1	0.753874	2.113054	1.48327	0.141559	-4.61165	0.403371	0.482394
Erythroid.cells	STOX2	0.896899	3.196694	1.482956	0.141642	-4.8068	0.388713	0.46819
Erythroid.cells	GLYAT	0.692694	2.069154	1.4824	0.14179	-4.78386	0.40435	0.483203
Erythroid.cells	MTFMT	-0.52238	2.527907	-1.48235	0.141802	-4.65723	0.398006	0.477078
Erythroid.cells	AEBP2	-0.29957	5.787827	-1.48203	0.141887	-5.30695	0.355934	0.43573
Erythroid.cells	ST3GAL3	0.32265	5.293649	1.481612	0.141999	-5.21299	0.362034	0.441766
Erythroid.cells	GM45871	-0.96904	0.985003	-1.48161	0.142	-4.50338	0.419918	0.498021
Erythroid.cells	USP35	-1.29566	0.017078	-1.48153	0.14202	-4.46104	0.434186	0.511618
Erythroid.cells	KANSL1L	-0.432	7.202302	-1.48144	0.142046	-5.53093	0.339129	0.418958
Erythroid.cells	5730522E0	0.597951	3.610742	1.481249	0.142095	-4.98771	0.383617	0.463104
Erythroid.cells	GIPC1	-0.42555	4.359749	-1.47988	0.14246	-4.92695	0.374325	0.454071
Erythroid.cells	TSR1	-0.55064	3.584651	-1.47985	0.142468	-4.79895	0.384436	0.463973
Erythroid.cells	CUX1	-0.20057	7.953864	-1.4793	0.142615	-5.75136	0.330973	0.410899
Erythroid.cells	CADM1	0.520774	5.440386	1.479204	0.14264	-5.56154	0.360691	0.440707
Erythroid.cells	FNDC7	-0.94286	-0.36573	-1.47919	0.142645	-4.55161	0.440544	0.517923
Erythroid.cells	IPPK	-0.55986	3.400734	-1.47891	0.142719	-4.77186	0.386877	0.466555
Erythroid.cells	CDC25A	0.548599	3.793744	1.478905	0.14272	-4.83616	0.381681	0.46148
Erythroid.cells	ALDH9A1	0.307054	5.005152	1.478808	0.142746	-5.14491	0.366118	0.446215
Erythroid.cells	BRI3	-0.23638	7.328714	-1.47872	0.142769	-5.60451	0.338113	0.418285
Erythroid.cells	NECTIN1	0.709043	1.073651	1.47869	0.142777	-4.74115	0.419188	0.49782
Erythroid.cells	RNGTT	-0.21626	6.852257	-1.47839	0.142857	-5.55412	0.343664	0.423926
Erythroid.cells	SPATA2	-0.52252	4.217056	-1.47834	0.14287	-4.86842	0.376165	0.456196
Erythroid.cells	TYW3	-1.02016	0.995715	-1.47825	0.142894	-4.4794	0.420316	0.498977
Erythroid.cells	BST1	-0.6949	3.315218	-1.478	0.14296	-4.8422	0.388018	0.467888
Erythroid.cells	UNKL	-0.48104	3.970057	-1.47799	0.142964	-4.91131	0.379373	0.459438
Erythroid.cells	GM26901	0.929854	0.57091	1.477895	0.142989	-4.47765	0.426525	0.505015
Erythroid.cells	MFHAS1	0.599372	3.729832	1.477637	0.143058	-4.83088	0.382521	0.462595

Erythroid.cells	PHF19	-0.72739	1.934693	-1.4775	0.143094	-4.57574	0.40692	0.486298
Erythroid.cells	ATP11B	-0.26639	7.035822	-1.47737	0.14313	-5.55155	0.341514	0.421942
Erythroid.cells	TRMT44	-0.79399	1.793941	-1.47736	0.143133	-4.58568	0.408899	0.488213
Erythroid.cells	MAP2	1.249846	0.187932	1.477313	0.143145	-4.44237	0.432202	0.51049
Erythroid.cells	VPS28	-0.25031	6.686374	-1.47728	0.143155	-5.50101	0.34562	0.426069
Erythroid.cells	TRIM33	-0.2493	6.368235	-1.4771	0.143203	-5.40323	0.349403	0.42993
Erythroid.cells	SECISBP2L	0.392055	4.964176	1.477017	0.143224	-5.05745	0.366633	0.447104
Erythroid.cells	ASXL2	-0.25593	6.836454	-1.4769	0.143254	-5.48354	0.34385	0.424423
Erythroid.cells	WDR24	-0.79271	1.796393	-1.47688	0.14326	-4.56282	0.408865	0.488332
Erythroid.cells	UTP14B	-0.60452	3.081232	-1.47663	0.143327	-4.73823	0.391236	0.471269
Erythroid.cells	GPR137C	0.763196	2.390141	1.4759	0.143523	-4.60324	0.400964	0.480621
Erythroid.cells	LRRC8D	-0.27959	7.366735	-1.47576	0.14356	-5.579	0.337997	0.418481
Erythroid.cells	NDUFS3	0.247552	6.050235	1.475665	0.143586	-5.44868	0.353567	0.434123
Erythroid.cells	BFAR	-0.31449	4.85458	-1.47566	0.143587	-5.04392	0.368368	0.448817
Erythroid.cells	CENPE	0.683569	4.9887	1.474936	0.143782	-5.1932	0.367075	0.447471
Erythroid.cells	SAP18	0.200222	7.043708	1.474756	0.14383	-5.58796	0.342145	0.422631
Erythroid.cells	DERL2	-0.32383	4.925658	-1.47453	0.14389	-5.12614	0.367952	0.448323
Erythroid.cells	RBMXL1	-0.30502	5.042632	-1.47404	0.144022	-5.12457	0.366604	0.447069
Erythroid.cells	SNX6	-0.22192	6.568189	-1.47401	0.144029	-5.38386	0.347929	0.428478
Erythroid.cells	EXOSC10	-0.3807	4.93082	-1.47391	0.144058	-5.03768	0.368014	0.448483
Erythroid.cells	GAN	-0.49856	4.031697	-1.47378	0.144093	-4.91367	0.379561	0.459889
Erythroid.cells	SERPINB2	2.204316	0.991964	1.473302	0.144221	-4.66738	0.421735	0.500629
Erythroid.cells	KLRA3	-1.22914	-0.66191	-1.47299	0.144305	-4.52774	0.446626	0.524281
Erythroid.cells	DEGS1	0.297026	6.525466	1.472772	0.144364	-5.47634	0.34874	0.429349
Erythroid.cells	F11	0.864414	0.954836	1.472688	0.144386	-4.61119	0.422378	0.501361
Erythroid.cells	VPS13D	-0.3003	6.149191	-1.4726	0.144409	-5.33885	0.353262	0.433897
Erythroid.cells	GABPA	-0.35967	4.662072	-1.47244	0.144454	-5.01157	0.371768	0.452312
Erythroid.cells	APBB3	-0.85832	1.534528	-1.47144	0.144721	-4.55171	0.414691	0.493713
Erythroid.cells	ANKRD55	1.03981	0.009583	1.471036	0.144832	-4.4871	0.43732	0.515336
Erythroid.cells	DDX10	-0.27792	5.436521	-1.47067	0.144931	-5.22245	0.362922	0.443317
Erythroid.cells	LYSMD2	-0.93902	1.265146	-1.47036	0.145015	-4.5668	0.41908	0.497979
Erythroid.cells	FAM13B	-0.26026	6.457749	-1.46925	0.145313	-5.3855	0.351118	0.431492
Erythroid.cells	PNP	0.322903	5.924728	1.46916	0.145339	-5.42415	0.357586	0.437957
Erythroid.cells	CASP8AP2	-0.31923	5.182109	-1.46907	0.145363	-5.1503	0.366809	0.447133
Erythroid.cells	ZYG11B	-0.2861	5.883084	-1.46856	0.145502	-5.34913	0.358258	0.438562
Erythroid.cells	UBE2L6	0.310207	4.64931	1.468543	0.145506	-5.81441	0.373749	0.453887
Erythroid.cells	GM47428	-0.96606	0.207827	-1.46823	0.145591	-4.56495	0.43563	0.513641
Erythroid.cells	PIGX	-0.32098	5.308726	-1.46816	0.145608	-5.17221	0.365449	0.445727
Erythroid.cells	ZBTB17	-0.50594	3.917458	-1.46684	0.145968	-4.84746	0.383787	0.46406
Erythroid.cells	CCDC71	0.627164	2.73695	1.466758	0.14599	-4.68375	0.399704	0.479589
Erythroid.cells	PCBP1	-0.18881	8.199098	-1.46666	0.146018	-5.82628	0.331436	0.411945
Erythroid.cells	ZFP277	-0.29225	5.309649	-1.46643	0.146079	-5.17318	0.365866	0.446483
Erythroid.cells	RIOK1	-0.25388	6.007255	-1.46641	0.146084	-5.34234	0.357219	0.437895
Erythroid.cells	OSGEP	-0.34267	4.907558	-1.4664	0.146087	-5.07545	0.37095	0.451509
Erythroid.cells	FABP7	1.135072	1.989157	1.466284	0.146119	-4.67209	0.41014	0.489716
Erythroid.cells	CRKL	0.315994	5.071994	1.466259	0.146125	-5.11374	0.368862	0.449458
Erythroid.cells	RFX7	0.260996	7.368214	1.466227	0.146134	-5.64525	0.340969	0.421625

Erythroid.cells	RGMB	0.748791	1.845211	1.466067	0.146178	-4.64886	0.41218	0.491684
Erythroid.cells	MIB2	-0.64722	2.662247	-1.46597	0.146203	-4.68271	0.400734	0.480639
Erythroid.cells	2310022A1	-0.54682	2.998253	-1.46574	0.146266	-4.75188	0.396123	0.476247
Erythroid.cells	SP2	0.321619	4.742762	1.465654	0.14629	-5.16959	0.373055	0.453706
Erythroid.cells	6530409C1	-0.85153	1.661527	-1.46548	0.146336	-4.60597	0.4148	0.494383
Erythroid.cells	MAGOHB	-0.48458	4.560419	-1.46535	0.146372	-5.04465	0.375399	0.456139
Erythroid.cells	BRD2	-0.21764	7.085393	-1.46514	0.14643	-5.56822	0.34428	0.425248
Erythroid.cells	TNFSF12	-0.57233	2.295218	-1.46513	0.146432	-4.78955	0.405835	0.485898
Erythroid.cells	RPAIN	0.425691	3.771433	1.464864	0.146505	-4.87478	0.385811	0.466392
Erythroid.cells	ASGR1	0.526746	3.372794	1.464601	0.146577	-5.10019	0.391229	0.471615
Erythroid.cells	PPP2R5D	-0.46406	3.682866	-1.46438	0.146637	-4.85603	0.387133	0.467624
Erythroid.cells	MRPL10	-0.39612	4.602348	-1.46398	0.146747	-5.04321	0.375259	0.456026
Erythroid.cells	KLF5	1.123648	0.36413	1.463806	0.146793	-4.53668	0.434257	0.513122
Erythroid.cells	RAB11FIP5	-0.99067	0.808074	-1.46371	0.146819	-4.55273	0.427653	0.506836
Erythroid.cells	GOLPH3L	-0.44297	4.39604	-1.46346	0.146888	-4.97935	0.378007	0.458767
Erythroid.cells	PLXNB2	0.457697	3.959065	1.462615	0.147119	-4.97071	0.384229	0.464499
Erythroid.cells	HC	0.689337	2.358321	1.462051	0.147273	-4.86469	0.406155	0.485774
Erythroid.cells	TARBP2	0.507316	3.242608	1.461993	0.147288	-4.80669	0.393969	0.473979
Erythroid.cells	WBP4	0.293756	5.270226	1.461819	0.147336	-5.20042	0.367448	0.448082
Erythroid.cells	UTP6	-0.4083	4.42275	-1.46165	0.147381	-4.9774	0.378298	0.458799
Erythroid.cells	CLEC2I	0.728838	3.177972	1.461591	0.147399	-4.79004	0.394847	0.474972
Erythroid.cells	VDR	-0.98578	0.938817	-1.46156	0.147406	-4.57101	0.426535	0.505482
Erythroid.cells	RBPMS	0.368272	5.842983	1.461403	0.14745	-5.41193	0.360315	0.441013
Erythroid.cells	TADA3	0.51652	3.403164	1.461126	0.147526	-4.78871	0.391829	0.472042
Erythroid.cells	GBA2	-0.5924	2.547922	-1.4611	0.147533	-4.6628	0.403542	0.483409
Erythroid.cells	FAM98A	-0.44741	3.607656	-1.46056	0.147681	-4.8648	0.389345	0.469649
Erythroid.cells	ZC3H3	0.460757	3.468484	1.460452	0.147711	-4.80999	0.391215	0.471492
Erythroid.cells	GANC	-0.62248	3.377098	-1.4601	0.147806	-4.74566	0.392597	0.472861
Erythroid.cells	PKNOX1	-0.4371	4.569323	-1.4598	0.14789	-4.99902	0.376942	0.457605
Erythroid.cells	SMIM19	0.354257	4.75168	1.459643	0.147932	-5.04902	0.3746	0.455355
Erythroid.cells	SNRPD2	0.236184	6.391824	1.45935	0.148013	-5.45606	0.354214	0.435131
Erythroid.cells	OS9	-0.28472	5.617189	-1.4586	0.14822	-5.25098	0.364154	0.444881
Erythroid.cells	ARMCX3	0.537506	3.573363	1.457932	0.148402	-4.78862	0.390702	0.471115
Erythroid.cells	KYNU	0.427732	3.853994	1.457921	0.148405	-5.13121	0.386949	0.46745
Erythroid.cells	GGCT	-0.58968	3.249782	-1.45789	0.148414	-4.87904	0.395078	0.475377
Erythroid.cells	DUS3L	-0.46319	3.300011	-1.45772	0.148461	-4.82675	0.394395	0.474761
Erythroid.cells	NCOA3	-0.20495	6.960263	-1.45769	0.148469	-5.53456	0.347843	0.428769
Erythroid.cells	KIF3C	-0.67702	2.145051	-1.45767	0.148476	-4.65721	0.410404	0.490261
Erythroid.cells	THUMPD2	-0.64723	1.928752	-1.45729	0.14858	-4.62835	0.413559	0.493352
Erythroid.cells	PLAC8	-0.59914	8.2646	-1.45728	0.148582	-5.85024	0.332759	0.413594
Erythroid.cells	RAB27A	0.456661	4.50985	1.456914	0.148682	-4.96608	0.378553	0.45925
Erythroid.cells	DUSP2	-0.41328	5.991936	-1.45676	0.148725	-5.39407	0.359794	0.440686
Erythroid.cells	PRPSAP2	0.419633	4.045546	1.456437	0.148814	-4.91877	0.384783	0.465371
Erythroid.cells	TOE1	-0.63209	2.490022	-1.45615	0.148893	-4.68899	0.406059	0.485991
Erythroid.cells	SEMA4C	-0.66761	2.202853	-1.45549	0.149077	-4.68331	0.410497	0.490225
Erythroid.cells	CYB5R3	0.299199	4.85016	1.455029	0.149203	-5.25186	0.374816	0.455624
Erythroid.cells	MRVI1	0.976933	0.497468	1.455006	0.149209	-4.53484	0.435443	0.514257

Erythroid.cells	IQCB1	-0.321	4.824078	-1.45498	0.149217	-5.17036	0.375152	0.455955
Erythroid.cells	IGKV1-135	-1.01301	-0.02384	-1.45483	0.149257	-4.54124	0.443352	0.521762
Erythroid.cells	SEMA4A	-0.44924	3.364224	-1.4547	0.149294	-5.06303	0.394462	0.474871
Erythroid.cells	PRUNE1	-0.50447	3.600837	-1.45405	0.149473	-4.81686	0.391631	0.472101
Erythroid.cells	SPC24	0.585641	4.768056	1.453844	0.14953	-5.20642	0.376247	0.457096
Erythroid.cells	HMOX2	-0.21637	6.471899	-1.45374	0.149558	-5.43735	0.354897	0.435963
Erythroid.cells	HDAC10	0.789102	1.608346	1.45346	0.149637	-4.57314	0.419592	0.499225
Erythroid.cells	KLF9	0.537752	3.693914	1.452563	0.149885	-4.98536	0.391048	0.47134
Erythroid.cells	HBEGF	0.895683	2.789221	1.452307	0.149956	-4.76675	0.403506	0.483479
Erythroid.cells	ZFP472	-0.522	3.448072	-1.45184	0.150086	-4.82181	0.394497	0.474859
Erythroid.cells	STARD8	0.58669	3.359704	1.451756	0.150109	-4.80245	0.395699	0.476028
Erythroid.cells	4930438A(	0.876312	-0.84008	1.451694	0.150126	-4.5033	0.457386	0.534958
Erythroid.cells	ZFYVE27	-0.42843	4.063949	-1.45154	0.150168	-4.88416	0.386227	0.466829
Erythroid.cells	ZMYM2	0.295476	5.970893	1.451461	0.150191	-5.33434	0.361756	0.442693
Erythroid.cells	FAM57B	-1.23306	0.285081	-1.45141	0.150205	-4.48045	0.439955	0.518547
Erythroid.cells	FBXL22	-0.78794	2.248825	-1.4511	0.150292	-4.6645	0.411215	0.491146
Erythroid.cells	GPRIN3	0.958266	0.638034	1.451022	0.150313	-4.57207	0.434712	0.513685
Erythroid.cells	AFDN	0.4842	3.267689	1.450506	0.150456	-4.90955	0.397309	0.477574
Erythroid.cells	ZFP219	0.695011	1.771622	1.449938	0.150614	-4.60906	0.418558	0.49815
Erythroid.cells	AGFG2	-0.35862	4.605897	-1.44982	0.150646	-5.12398	0.379647	0.460395
Erythroid.cells	IL15RA	-0.69423	2.38833	-1.44973	0.150671	-4.76166	0.409752	0.489729
Erythroid.cells	ARHGAP11	0.417088	4.784218	1.449649	0.150694	-5.12205	0.377329	0.458188
Erythroid.cells	GM50386	0.838765	0.179027	1.449099	0.150847	-4.572	0.442257	0.521098
Erythroid.cells	EDIL3	0.828899	1.492552	1.449042	0.150863	-4.72647	0.422655	0.502428
Erythroid.cells	KCTD1	-0.68927	1.719442	-1.44903	0.150866	-4.66329	0.41936	0.49927
Erythroid.cells	ACSM1	1.083285	1.026687	1.449028	0.150867	-4.61844	0.429504	0.508975
Erythroid.cells	LLGL1	-0.55153	2.998446	-1.44814	0.151114	-4.76018	0.401744	0.482047
Erythroid.cells	OLFR1033	1.156943	0.583769	1.448016	0.15115	-4.55703	0.43663	0.515544
Erythroid.cells	TNFSF9	0.652295	3.435421	1.447977	0.15116	-4.98848	0.395746	0.47625
Erythroid.cells	CCDC28A	0.629382	2.307492	1.447759	0.151221	-4.69196	0.41144	0.491506
Erythroid.cells	EXO1	0.862104	2.019131	1.447653	0.151251	-4.64748	0.415551	0.495503
Erythroid.cells	GHDC	-0.81599	1.628326	-1.44754	0.151284	-4.56272	0.421189	0.500916
Erythroid.cells	USP24	0.30526	5.459806	1.447218	0.151372	-5.22196	0.369259	0.450321
Erythroid.cells	PODXL	1.108083	0.550401	1.447125	0.151398	-4.53286	0.437258	0.516267
Erythroid.cells	NUDT15	-0.72994	1.647281	-1.44698	0.151439	-4.61979	0.42102	0.500834
Erythroid.cells	EMC10	0.276665	5.502896	1.446566	0.151554	-5.26462	0.368735	0.449982
Erythroid.cells	TMTC1	1.09164	2.714836	1.446538	0.151562	-4.65245	0.405827	0.48629
Erythroid.cells	ZFP763	1.034368	0.433803	1.446537	0.151563	-4.51285	0.439046	0.518118
Erythroid.cells	SENP6	-0.20564	6.681311	-1.4458	0.151768	-5.47753	0.354535	0.435677
Erythroid.cells	DLAT	-0.34554	4.282042	-1.44558	0.151829	-5.02601	0.384997	0.465829
Erythroid.cells	IAH1	0.347344	4.521488	1.445207	0.151935	-5.22899	0.38201	0.462828
Erythroid.cells	UBE3A	0.220016	6.387077	1.445013	0.151989	-5.42842	0.358369	0.439528
Erythroid.cells	FH1	0.335066	5.484899	1.444814	0.152045	-5.40046	0.369661	0.450794
Erythroid.cells	CCDC88A	0.352208	5.372063	1.444473	0.152141	-5.26637	0.371164	0.452305
Erythroid.cells	FABP2	-0.54533	3.257265	-1.44443	0.152151	-5.10159	0.399145	0.479729
Erythroid.cells	CSE1L	-0.29473	5.5229	-1.44406	0.152256	-5.29153	0.369338	0.450576
Erythroid.cells	PPP1R1C	-1.0652	0.781145	-1.44381	0.152325	-4.61359	0.434821	0.51419

Erythroid.cells	RAB35	-0.34641	4.720448	-1.44375	0.152344	-5.04545	0.379651	0.460828
Erythroid.cells	TIMD4	0.786634	3.658253	1.443746	0.152345	-4.94452	0.39377	0.474656
Erythroid.cells	A630052C1	1.139459	0.189023	1.443548	0.1524	-4.4966	0.443849	0.52283
Erythroid.cells	ABCB1B	-0.60792	3.934965	-1.44318	0.152504	-5.05006	0.390248	0.471193
Erythroid.cells	TEX30	-0.30445	5.223272	-1.44271	0.152637	-5.22213	0.373581	0.454763
Erythroid.cells	TTC28	0.517772	4.798912	1.442424	0.152716	-5.05715	0.379163	0.460261
Erythroid.cells	MRPS7	0.306779	5.010323	1.442124	0.1528	-5.21592	0.37644	0.457668
Erythroid.cells	2810403D2	0.612632	2.774023	1.441875	0.152871	-4.7321	0.406534	0.487144
Erythroid.cells	GM12743	-0.69033	2.382516	-1.44175	0.152904	-4.70452	0.412056	0.492495
Erythroid.cells	MVB12B	0.476383	4.074675	1.441481	0.152982	-4.96624	0.388737	0.469871
Erythroid.cells	TUT7	0.1904	7.206797	1.441461	0.152987	-5.65178	0.349158	0.430636
Erythroid.cells	KLC1	0.332253	4.702224	1.441414	0.153	-5.05634	0.380444	0.461758
Erythroid.cells	FBXO10	-0.80388	1.355085	-1.44123	0.153053	-4.56686	0.426916	0.506835
Erythroid.cells	USP38	-0.27422	5.889969	-1.4412	0.153061	-5.34562	0.365253	0.446759
Erythroid.cells	TBC1D8B	-0.50253	3.257326	-1.44108	0.153095	-4.82091	0.399824	0.480783
Erythroid.cells	JUN	0.61921	7.097589	1.440994	0.153119	-5.61271	0.350464	0.432076
Erythroid.cells	GM42556	1.000988	0.793712	1.440676	0.153208	-4.56177	0.435267	0.514972
Erythroid.cells	AGTPBP1	-0.39878	5.128653	-1.44059	0.153234	-5.13889	0.374914	0.456473
Erythroid.cells	WASF2	0.154098	8.064634	1.440393	0.153288	-5.84806	0.339081	0.420658
Erythroid.cells	ACTN4	-0.19695	6.545001	-1.44035	0.153299	-5.53419	0.357151	0.438833
Erythroid.cells	NUP98	-0.24306	7.461994	-1.44031	0.15331	-5.68098	0.346128	0.427773
Erythroid.cells	NPEPL1	0.325709	5.049543	1.439985	0.153403	-5.18582	0.37604	0.45755
Erythroid.cells	CREBL2	0.56909	3.233581	1.439633	0.153502	-4.79989	0.400264	0.481272
Erythroid.cells	OIP5	0.640953	2.207869	1.439631	0.153503	-4.7461	0.414661	0.495201
Erythroid.cells	TMEM260	0.620909	3.273577	1.43956	0.153523	-4.74352	0.399713	0.480736
Erythroid.cells	HIST1H4H	1.00963	0.513275	1.439143	0.153641	-4.5205	0.439626	0.519303
Erythroid.cells	SLC12A9	0.502045	4.242634	1.439006	0.15368	-4.95452	0.386608	0.468201
Erythroid.cells	MINDY2	-0.22835	6.610817	-1.43898	0.153686	-5.52275	0.356448	0.438328
Erythroid.cells	MFAP1A	-0.30152	4.832089	-1.4389	0.15371	-5.12239	0.378858	0.460604
Erythroid.cells	HIST2H2BB	-1.10358	-0.261	-1.43881	0.153736	-4.49153	0.451537	0.530701
Erythroid.cells	NR2C1	0.699558	2.276051	1.438775	0.153745	-4.70357	0.413687	0.494586
Erythroid.cells	MGAM	-1.17898	0.078138	-1.43824	0.153896	-4.52357	0.446511	0.525969
Erythroid.cells	DNAJB5	0.926722	1.175316	1.43812	0.15393	-4.54007	0.429917	0.510187
Erythroid.cells	KIF1B	0.331027	5.385583	1.437996	0.153966	-5.27529	0.371921	0.453822
Erythroid.cells	ZFP87	0.489933	3.396086	1.437937	0.153982	-4.8899	0.398237	0.479698
Erythroid.cells	TCAIM	0.763043	1.789758	1.437608	0.154075	-4.64393	0.4209	0.501779
Erythroid.cells	TBC1D23	0.302822	5.852371	1.437584	0.154082	-5.24278	0.366016	0.448144
Erythroid.cells	AHDC1	-0.3151	4.689967	-1.43752	0.154101	-5.10696	0.380909	0.462921
Erythroid.cells	PDRG1	-0.38049	4.496479	-1.43721	0.154189	-5.04546	0.383449	0.465511
Erythroid.cells	TCIM	1.028266	1.634355	1.437137	0.154209	-4.62928	0.423162	0.504112
Erythroid.cells	POLR3C	-0.30027	5.018329	-1.43711	0.154216	-5.13552	0.376638	0.458815
Erythroid.cells	MMP25	-0.97105	0.499533	-1.43683	0.154295	-4.59025	0.440176	0.520435
Erythroid.cells	SNHG4	-0.50653	3.683196	-1.43645	0.154402	-4.86317	0.394499	0.476427
Erythroid.cells	SHROOM4	1.103286	0.86592	1.436452	0.154403	-4.5892	0.434726	0.515252
Erythroid.cells	KIDINS220	0.324242	5.419693	1.43606	0.154514	-5.17094	0.371826	0.454131
Erythroid.cells	GM4117	0.808062	0.660809	1.43574	0.154605	-4.61669	0.438143	0.518545
Erythroid.cells	DUSP7	-0.61755	3.559127	-1.43563	0.154636	-4.79909	0.396483	0.478398

Erythroid.cells	PEX12	0.750958	1.86574	1.435437	0.154691	-4.64776	0.420344	0.50158
Erythroid.cells	THOC7	0.204299	6.572603	1.434982	0.15482	-5.57228	0.357771	0.440227
Erythroid.cells	DSCAM	1.133403	0.029718	1.434816	0.154868	-4.57102	0.448123	0.528181
Erythroid.cells	NOM1	-0.42556	4.141882	-1.43458	0.154935	-4.92506	0.38894	0.471149
Erythroid.cells	ALG3	-0.83556	1.77052	-1.43449	0.154962	-4.62512	0.422036	0.503278
Erythroid.cells	CHPF2	-0.65892	2.64648	-1.4342	0.155043	-4.72805	0.409502	0.49127
Erythroid.cells	PDCD1	-1.01005	0.743001	-1.43398	0.155105	-4.73653	0.437288	0.518016
Erythroid.cells	SMAD2	-0.22547	6.426685	-1.43392	0.155123	-5.4325	0.359634	0.442251
Erythroid.cells	CD86	0.416448	6.31281	1.433908	0.155126	-5.63821	0.361039	0.443655
Erythroid.cells	LPXN	-0.34565	5.23201	-1.43373	0.155177	-5.27345	0.374693	0.457231
Erythroid.cells	HS1BP3	-0.80783	1.087416	-1.43344	0.155258	-4.57858	0.432271	0.513207
Erythroid.cells	DNAJC14	0.346437	4.252973	1.433036	0.155374	-4.95206	0.387711	0.469956
Erythroid.cells	NDUFB7	0.241105	6.480862	1.433026	0.155377	-5.53993	0.359186	0.441693
Erythroid.cells	TTLL4	-0.5583	3.000367	-1.43286	0.155424	-4.75265	0.404808	0.486687
Erythroid.cells	TMEM201	-0.75927	2.102835	-1.43234	0.155572	-4.61441	0.417724	0.499202
Erythroid.cells	HRH4	-1.05004	0.27047	-1.43232	0.155578	-4.57359	0.444987	0.525275
Erythroid.cells	4833408A1	-1.16204	0.127774	-1.43209	0.155644	-4.53508	0.447262	0.527448
Erythroid.cells	CREBRF	-0.28158	6.98351	-1.4319	0.155699	-5.61866	0.353347	0.435932
Erythroid.cells	2010007H(	-0.80089	0.459338	-1.43158	0.15579	-4.58923	0.442287	0.522764
Erythroid.cells	SLC39A13	0.584125	2.796524	1.431525	0.155805	-4.75068	0.408033	0.4899
Erythroid.cells	GM17056	0.71177	2.866748	1.431397	0.155842	-4.84254	0.407047	0.488978
Erythroid.cells	PTCH1	0.667351	3.139748	1.430861	0.155995	-4.80063	0.403491	0.485461
Erythroid.cells	JAM2	0.999822	1.449339	1.430533	0.156089	-4.65831	0.427699	0.508962
Erythroid.cells	CLIC4	-0.35054	7.449524	-1.43033	0.156147	-5.75658	0.34804	0.430779
Erythroid.cells	AP2A2	0.271577	5.931782	1.430261	0.156167	-5.32657	0.366588	0.449378
Erythroid.cells	FZD7	0.822935	0.743156	1.430243	0.156172	-4.60109	0.438249	0.51916
Erythroid.cells	TOMM7	0.180888	7.561253	1.43021	0.156181	-5.74505	0.346714	0.429449
Erythroid.cells	PHF11B	-0.60138	4.313119	-1.42999	0.156245	-5.09029	0.387529	0.470139
Erythroid.cells	AW011738	0.535573	2.812185	1.429955	0.156254	-5.05443	0.408069	0.490197
Erythroid.cells	GAPDH	0.26665	10.59184	1.429441	0.156402	-6.28545	0.312817	0.395013
Erythroid.cells	LBP	0.483857	3.292366	1.429365	0.156423	-5.02134	0.401471	0.484013
Erythroid.cells	DMTF1	-0.30666	4.947065	-1.42932	0.156436	-5.14268	0.37927	0.46225
Erythroid.cells	ST3GAL2	0.500369	3.085043	1.429209	0.156468	-4.9133	0.404347	0.486891
Erythroid.cells	SRGAP3	0.601246	4.261174	1.428985	0.156532	-5.03878	0.388312	0.471294
Erythroid.cells	CTIF	-0.47562	2.596638	-1.42879	0.156588	-4.79903	0.411207	0.493736
Erythroid.cells	4931428F0	-1.06521	0.65018	-1.42857	0.156652	-4.50714	0.439761	0.521273
Erythroid.cells	FAM20C	0.966214	1.641237	1.428355	0.156713	-4.81444	0.424977	0.507135
Erythroid.cells	MTMR14	-0.33642	5.427711	-1.42832	0.156724	-5.23867	0.373066	0.456433
Erythroid.cells	MRPL37	-0.37871	4.2912	-1.4283	0.156728	-4.99726	0.387911	0.471105
Erythroid.cells	PPP1R10	0.318566	6.277719	1.428273	0.156737	-5.38133	0.362355	0.445767
Erythroid.cells	PITPNB	-0.2435	5.507244	-1.42818	0.156763	-5.31724	0.372049	0.455469
Erythroid.cells	R74862	-1.11186	1.054776	-1.42798	0.156821	-4.55038	0.433715	0.515624
Erythroid.cells	DCP1A	-0.3352	4.850131	-1.42768	0.156908	-5.11554	0.380586	0.464091
Erythroid.cells	VMAC	0.838905	1.5263	1.427569	0.156939	-4.58138	0.426723	0.509091
Erythroid.cells	SMIM20	-0.31926	4.914811	-1.42754	0.156946	-5.18098	0.379742	0.46329
Erythroid.cells	UBE4B	-0.28941	5.48847	-1.42708	0.157079	-5.29356	0.37234	0.456132
Erythroid.cells	HECW2	0.743432	3.162677	1.427016	0.157097	-4.83181	0.403323	0.486638

Erythroid.cells	TES	-0.32615	5.486918	-1.42702	0.157098	-5.37202	0.37236	0.456152
Erythroid.cells	SPDEF	-1.27181	-0.4296	-1.42701	0.157099	-4.49111	0.456529	0.53772
Erythroid.cells	SLC35D1	0.385631	4.114625	1.426858	0.157143	-5.04132	0.390338	0.473982
Erythroid.cells	MAGOH	-0.25522	6.296682	-1.42621	0.157328	-5.47284	0.362516	0.44627
Erythroid.cells	IER5L	-0.58823	2.89009	-1.42518	0.157625	-4.8096	0.408128	0.491074
Erythroid.cells	GM10552	0.85095	1.54086	1.425121	0.157643	-4.63198	0.427558	0.509871
Erythroid.cells	ASPSCR1	-0.32975	4.685187	-1.42496	0.157688	-5.11435	0.383704	0.467233
Erythroid.cells	BCAT1	-1.06787	0.852062	-1.42444	0.157838	-4.60864	0.438012	0.520017
Erythroid.cells	CMSS1	0.368183	6.448827	1.424405	0.157849	-5.65218	0.361314	0.445031
Erythroid.cells	GM33782	-0.98122	0.19699	-1.42436	0.157862	-4.54193	0.44803	0.529562
Erythroid.cells	HTATSF1	-0.32454	4.697996	-1.42409	0.157941	-5.11292	0.383765	0.467303
Erythroid.cells	TMEM220	-0.97564	0.299081	-1.42371	0.158051	-4.56176	0.446712	0.528208
Erythroid.cells	CHST11	-0.43303	6.242747	-1.42363	0.158074	-5.54741	0.364084	0.447732
Erythroid.cells	HIP1R	-0.30396	5.271328	-1.4235	0.158112	-5.34311	0.376411	0.460032
Erythroid.cells	SKI	0.339969	5.372605	1.423275	0.158176	-5.22887	0.375163	0.458796
Erythroid.cells	WWP1	0.3811	5.227114	1.423011	0.158252	-5.21689	0.377129	0.460711
Erythroid.cells	RARA	0.351706	5.561492	1.421973	0.158552	-5.26184	0.373309	0.456719
Erythroid.cells	CD19	-0.49851	3.122136	-1.42185	0.158587	-5.02456	0.405953	0.488881
Erythroid.cells	MTG1	0.681036	2.186733	1.42178	0.158608	-4.7468	0.419249	0.501804
Erythroid.cells	UBR3	0.243885	6.575859	1.421758	0.158614	-5.55009	0.36056	0.4441
Erythroid.cells	TRAPPC11	0.433989	3.489165	1.421242	0.158764	-4.88543	0.401096	0.484113
Erythroid.cells	LNPK	0.432503	4.386002	1.421016	0.15883	-5.02912	0.388914	0.472181
Erythroid.cells	LENG1	0.531976	2.925078	1.420986	0.158838	-4.75657	0.408962	0.491773
Erythroid.cells	TMEM273	0.742397	1.19098	1.420885	0.158868	-4.68523	0.434157	0.516125
Erythroid.cells	LAGE3	-0.32883	4.771468	-1.42072	0.158915	-5.17623	0.383817	0.467286
Erythroid.cells	SLC25A12	0.331208	5.092687	1.419962	0.159135	-5.12016	0.379999	0.463421
Erythroid.cells	TADA2B	-0.50766	3.468615	-1.41978	0.159187	-4.86785	0.401814	0.484948
Erythroid.cells	NAPRT	0.910957	0.897988	1.419718	0.159206	-4.66473	0.439044	0.52088
Erythroid.cells	PPP1R14B	-0.25968	6.546146	-1.41961	0.159237	-5.51793	0.361534	0.445152
Erythroid.cells	IFNAR2	0.233663	7.128736	1.419448	0.159285	-5.67106	0.354418	0.43807
Erythroid.cells	RSBN1L	-0.20541	7.198782	-1.41931	0.159325	-5.69762	0.353572	0.437254
Erythroid.cells	NREP	-0.86657	1.234795	-1.41878	0.159479	-4.62308	0.434278	0.516441
Erythroid.cells	RHPN2	0.853893	0.915486	1.418517	0.159555	-4.59544	0.43909	0.521087
Erythroid.cells	TMA16	-0.5416	3.964843	-1.41841	0.159587	-4.97917	0.395293	0.478753
Erythroid.cells	ARL16	0.558295	2.250651	1.418404	0.159588	-4.71472	0.419326	0.502139
Erythroid.cells	GJA1	1.087824	1.208053	1.418037	0.159695	-4.6013	0.434862	0.516991
Erythroid.cells	PTPRE	0.393633	5.539856	1.417853	0.159749	-5.23561	0.37467	0.458292
Erythroid.cells	FAM107B	0.231502	7.858238	1.417007	0.159995	-5.83335	0.346425	0.42986
Erythroid.cells	GM11837	0.877116	0.292319	1.416971	0.160006	-4.69791	0.449284	0.53057
Erythroid.cells	SSR3	0.243873	6.071068	1.416934	0.160016	-5.44715	0.368253	0.451786
Erythroid.cells	HHAT	0.871688	1.670909	1.416779	0.160062	-4.61543	0.428424	0.510709
Erythroid.cells	SPRYD3	-0.40506	3.699854	-1.41659	0.160117	-4.92768	0.39954	0.482763
Erythroid.cells	MFSD8	-0.49508	2.979793	-1.4162	0.160231	-4.7614	0.409637	0.492602
Erythroid.cells	PSMB4	0.22986	6.172049	1.416089	0.160263	-5.53223	0.367094	0.450736
Erythroid.cells	SEC61A2	-0.43621	3.741527	-1.41591	0.160316	-4.92372	0.399036	0.482323
Erythroid.cells	HGD	0.663106	2.465599	1.415686	0.160381	-4.95307	0.416958	0.499778
Erythroid.cells	S1PR5	0.879797	-0.65106	1.415671	0.160385	-4.61473	0.464303	0.544971

Erythroid.cells	TRP53BP1	-0.47043	3.888271	-1.4156	0.160407	-4.92092	0.397027	0.480392
Erythroid.cells	MYCBP	0.377855	4.22905	1.415542	0.160423	-5.08844	0.392402	0.475857
Erythroid.cells	SH3TC1	0.547028	2.768133	1.415351	0.160478	-4.82524	0.412643	0.4956
Erythroid.cells	METTL27	0.968674	0.539053	1.415259	0.160505	-4.63455	0.445618	0.527332
Erythroid.cells	NDRG3	0.326324	4.407312	1.414693	0.160671	-5.10441	0.390063	0.473859
Erythroid.cells	SPI1	-0.27401	6.411888	-1.41451	0.160725	-5.53062	0.364145	0.448234
Erythroid.cells	PPP1R12B	-0.31225	5.292478	-1.41441	0.160754	-5.23349	0.378386	0.462423
Erythroid.cells	CDK18	1.221414	-0.22726	1.414389	0.16076	-4.50897	0.457625	0.539135
Erythroid.cells	CTTNBP2NI	0.562956	2.52025	1.414346	0.160772	-4.84175	0.416235	0.499518
Erythroid.cells	TMEM238	0.536792	2.938537	1.414238	0.160804	-4.9583	0.41028	0.493756
Erythroid.cells	SH2B1	0.567619	3.699274	1.414216	0.16081	-4.8321	0.399676	0.483414
Erythroid.cells	LZTS2	1.035968	1.249256	1.414103	0.160843	-4.57221	0.434884	0.517502
Erythroid.cells	AAR2	0.365369	4.021304	1.413206	0.161106	-4.96907	0.395638	0.479403
Erythroid.cells	SFI1	0.346154	5.320089	1.413182	0.161113	-5.26116	0.378376	0.46238
Erythroid.cells	NRG2	0.782847	1.685011	1.413122	0.161131	-4.75384	0.42879	0.511602
Erythroid.cells	GPHN	-0.22378	7.486182	-1.41305	0.161151	-5.76187	0.351336	0.435328
Erythroid.cells	6720427I0	0.36968	4.894793	1.412655	0.161268	-5.14226	0.384124	0.468032
Erythroid.cells	IRF5	0.355721	5.351662	1.412419	0.161337	-5.34717	0.378217	0.462165
Erythroid.cells	SIAH1B	0.667186	1.91846	1.411776	0.161526	-4.71028	0.426027	0.508749
Erythroid.cells	MYH10	0.604541	2.573809	1.41135	0.161651	-4.79502	0.416591	0.499761
Erythroid.cells	WAPL	0.218674	7.165135	1.411116	0.16172	-5.72192	0.355843	0.439853
Erythroid.cells	VIPAS39	0.36153	4.131233	1.410999	0.161754	-4.9754	0.394847	0.478617
Erythroid.cells	CDKN2AIPI	0.382427	4.272414	1.410994	0.161755	-5.02121	0.392935	0.47674
Erythroid.cells	GM12227	-0.8609	1.113465	-1.41099	0.161756	-4.57667	0.438108	0.520524
Erythroid.cells	LAMC1	-0.39138	4.523339	-1.41073	0.161833	-5.23003	0.389617	0.473539
Erythroid.cells	CALCA	-1.141	-0.96013	-1.41064	0.161859	-4.58973	0.470691	0.551522
Erythroid.cells	POLR1C	-0.45396	3.945898	-1.4103	0.161959	-4.99052	0.397513	0.481286
Erythroid.cells	BC051537	-1.24297	0.044835	-1.41025	0.161973	-4.51419	0.454733	0.536443
Erythroid.cells	PSD	-0.86495	1.633137	-1.409	0.162342	-4.60458	0.43133	0.513768
Erythroid.cells	1700126G(	0.913312	1.104608	1.408714	0.162427	-4.61627	0.439267	0.521517
Erythroid.cells	TRMT12	0.820317	1.30746	1.408453	0.162504	-4.6033	0.436203	0.518727
Erythroid.cells	GM49336	0.323084	4.881295	1.408358	0.162532	-5.14852	0.385702	0.469655
Erythroid.cells	HOOK3	-0.25205	6.076111	-1.40821	0.162576	-5.40449	0.370216	0.454364
Erythroid.cells	WDR38	-0.90127	0.617809	-1.40803	0.162629	-4.54605	0.44671	0.528949
Erythroid.cells	SCN4A	-1.12379	0.410123	-1.40794	0.162656	-4.52345	0.449924	0.532038
Erythroid.cells	ZKSCAN8	0.925633	0.632724	1.407927	0.162659	-4.56172	0.44648	0.52876
Erythroid.cells	ZFP341	0.671845	1.984672	1.407815	0.162692	-4.66671	0.426132	0.509311
Erythroid.cells	DCAF13	-0.33979	4.771338	-1.40774	0.162716	-5.10362	0.387161	0.471312
Erythroid.cells	BCL2L14	0.919203	0.459361	1.407677	0.162733	-4.77643	0.44916	0.53138
Erythroid.cells	NUCB2	0.424419	4.304904	1.407392	0.162817	-4.90132	0.393522	0.477552
Erythroid.cells	CYB5R4	0.281092	5.869945	1.406779	0.162998	-5.33501	0.373192	0.457325
Erythroid.cells	GAB2	0.36037	7.176286	1.406752	0.163006	-5.63509	0.356875	0.440992
Erythroid.cells	RLIM	-0.19269	6.654275	-1.40628	0.163145	-5.53561	0.363523	0.44748
Erythroid.cells	PICALM	0.195753	8.404518	1.406066	0.163209	-5.94476	0.342484	0.42628
Erythroid.cells	VMN2R19	-1.13104	-0.79625	-1.40554	0.163364	-4.54638	0.47019	0.550769
Erythroid.cells	GM42567	1.076064	1.12927	1.4052	0.163466	-4.62577	0.440017	0.522189
Erythroid.cells	XRCC1	-0.41547	4.144273	-1.40519	0.16347	-5.03608	0.396607	0.480185

Erythroid.cells	THBD	0.682071	3.145231	1.40489	0.163558	-4.96844	0.410596	0.493853
Erythroid.cells	TBC1D10C	0.490574	4.736281	1.404386	0.163707	-5.09335	0.388994	0.472639
Erythroid.cells	RABIF	-0.30098	4.708703	-1.40412	0.163787	-5.14869	0.389456	0.473137
Erythroid.cells	MOSPD2	0.446871	4.445481	1.403796	0.163882	-5.02369	0.393127	0.476814
Erythroid.cells	DHRS7B	0.522878	3.137991	1.403374	0.164008	-4.84181	0.411338	0.494628
Erythroid.cells	CTDP1	0.330257	4.660739	1.403357	0.164013	-5.12403	0.390351	0.474104
Erythroid.cells	OCIAD2	-1.05562	0.485681	-1.40273	0.164201	-4.56559	0.450875	0.532626
Erythroid.cells	ZFP335OS	-0.41625	4.456083	-1.40268	0.164214	-5.08387	0.393229	0.476948
Erythroid.cells	USP22	0.356374	4.389395	1.402459	0.16428	-5.03279	0.394131	0.477906
Erythroid.cells	ACE	0.975736	0.341503	1.402426	0.16429	-4.78012	0.453125	0.534843
Erythroid.cells	PLK2	-0.5223	5.006155	-1.4024	0.164298	-5.2597	0.38587	0.46977
Erythroid.cells	NUDT18	-0.5149	2.627052	-1.40237	0.164305	-4.78582	0.418773	0.501927
Erythroid.cells	FUT8	-0.29096	5.850748	-1.40208	0.164393	-5.38946	0.374904	0.458944
Erythroid.cells	SULT2A5	-0.76163	2.02078	-1.40203	0.164408	-4.85906	0.427675	0.510575
Erythroid.cells	FBXW17	0.793365	1.479346	1.401424	0.164588	-4.66436	0.435891	0.518404
Erythroid.cells	MBNL2	0.217249	8.406165	1.40138	0.164601	-5.93701	0.343672	0.427513
Erythroid.cells	ETFA	0.228656	6.599323	1.401362	0.164606	-5.73171	0.365545	0.449564
Erythroid.cells	MRPS14	0.201522	6.892734	1.401213	0.164651	-5.66838	0.361895	0.445906
Erythroid.cells	RBM8A	-0.2561	5.709751	-1.40116	0.164667	-5.37975	0.376854	0.460837
Erythroid.cells	1810044D(	0.722993	1.915203	1.400698	0.164804	-4.72087	0.429641	0.512277
Erythroid.cells	U2AF2	-0.24124	6.245648	-1.40047	0.164873	-5.47688	0.370279	0.454227
Erythroid.cells	TINAGL1	0.777302	2.066409	1.400227	0.164945	-4.79896	0.42752	0.510257
Erythroid.cells	FGFR2	-0.29244	5.987704	-1.40015	0.164969	-5.42941	0.373601	0.457504
Erythroid.cells	GM20743	-1.17449	0.031876	-1.39957	0.16514	-4.53642	0.458725	0.540122
Erythroid.cells	GM26520	-1.0124	2.362216	-1.39954	0.165149	-4.67701	0.423286	0.506258
Erythroid.cells	CD48	-0.27641	6.795574	-1.39936	0.165204	-5.64328	0.363498	0.447493
Erythroid.cells	GM9725	-0.7605	2.030059	-1.39933	0.165211	-4.72918	0.428161	0.51096
Erythroid.cells	CHIL5	1.000732	-0.02332	1.399179	0.165258	-4.53384	0.459601	0.540954
Erythroid.cells	PSMG2	-0.46222	3.919974	-1.39915	0.165267	-4.99227	0.401181	0.484789
Erythroid.cells	VPS26B	-0.43622	3.835261	-1.39907	0.16529	-4.98222	0.402352	0.485934
Erythroid.cells	IL2RB	-0.45717	3.130268	-1.39892	0.165336	-5.38471	0.412249	0.495606
Erythroid.cells	GLCE	-0.4818	3.79856	-1.39868	0.165407	-4.93053	0.402949	0.486543
Erythroid.cells	SPRY1	1.061875	1.345776	1.397779	0.165677	-4.58679	0.439092	0.521203
Erythroid.cells	TMEM37	0.499526	3.751452	1.397198	0.165851	-5.13709	0.404483	0.487649
Erythroid.cells	ZFP408	-0.48057	3.716601	-1.3968	0.16597	-4.89858	0.404968	0.488283
Erythroid.cells	SERPINA7	0.947609	0.531403	1.396603	0.166029	-4.64411	0.451965	0.533593
Erythroid.cells	FRMD6	0.607759	3.47737	1.396484	0.166065	-4.89952	0.408315	0.491672
Erythroid.cells	ANKHD1	-0.20751	7.647791	-1.39638	0.166097	-5.76986	0.353914	0.437822
Erythroid.cells	CMC4	0.45654	3.064546	1.396077	0.166187	-4.89942	0.41416	0.497495
Erythroid.cells	NSMF	0.573873	2.696993	1.396009	0.166207	-4.80345	0.419436	0.502611
Erythroid.cells	TCEAL9	0.32103	5.693961	1.395436	0.16638	-5.38763	0.378379	0.462537
Erythroid.cells	PFKFB3	-0.33198	6.273061	-1.39538	0.166396	-5.51652	0.370946	0.45514
Erythroid.cells	MRPL11	-0.36768	4.26095	-1.39537	0.166401	-5.10084	0.397458	0.481363
Erythroid.cells	AZI2	-0.23569	5.839646	-1.39535	0.166404	-5.39568	0.376494	0.460665
Erythroid.cells	USP18	0.706619	2.737474	1.395347	0.166406	-4.91283	0.418852	0.502209
Erythroid.cells	TMEM69	0.838423	1.585462	1.395206	0.166448	-4.63638	0.43582	0.518605
Erythroid.cells	ENY2	0.235002	5.972518	1.395066	0.166491	-5.53473	0.374784	0.459007

Erythroid.cells	GM42701	-1.01848	0.844253	-1.3948	0.16657	-4.5802	0.44711	0.529453
Erythroid.cells	PIM1	-0.24686	9.488973	-1.39479	0.166572	-6.15318	0.332397	0.416259
Erythroid.cells	KCTD9	0.485928	3.130956	1.394716	0.166596	-4.87492	0.413214	0.496875
Erythroid.cells	DEXI	0.51767	2.91114	1.394653	0.166615	-4.87209	0.416353	0.499932
Erythroid.cells	SURF4	-0.28954	5.782118	-1.39462	0.166625	-5.39504	0.377237	0.461539
Erythroid.cells	TAP1	-0.66534	5.446269	-1.39457	0.16664	-5.31923	0.381607	0.465875
Erythroid.cells	NENF	0.321849	4.583677	1.394435	0.16668	-5.16435	0.393075	0.477198
Erythroid.cells	ITIH5	0.673182	1.943297	1.394392	0.166693	-4.80196	0.430475	0.513591
Erythroid.cells	RBCK1	-0.27571	5.550838	-1.39411	0.166778	-5.34277	0.380252	0.46466
Erythroid.cells	E2F4	-0.28512	5.334947	-1.39411	0.166779	-5.30107	0.383079	0.467461
Erythroid.cells	CBX7	-0.53243	2.651462	-1.39375	0.166885	-4.77344	0.420274	0.50384
Erythroid.cells	ZFP236	0.358202	4.729945	1.393366	0.167002	-5.10415	0.391288	0.475623
Erythroid.cells	3300002I0	-0.69455	1.970632	-1.39323	0.167043	-4.73329	0.430271	0.513603
Erythroid.cells	ZFP644	-0.22641	6.331418	-1.39315	0.167066	-5.53136	0.370378	0.454905
Erythroid.cells	FBXO6	-0.41557	3.653081	-1.3931	0.167083	-5.01142	0.406044	0.490144
Erythroid.cells	AW549877	0.546568	3.352769	1.393063	0.167093	-4.87005	0.410262	0.494258
Erythroid.cells	PNISR	-0.22641	5.773836	-1.39193	0.167434	-5.37836	0.3782	0.46253
Erythroid.cells	SH2D4B	-0.60878	4.439997	-1.3916	0.167536	-5.06103	0.396057	0.480154
Erythroid.cells	OCRL	0.669943	2.602911	1.391351	0.16761	-4.80662	0.421903	0.505431
Erythroid.cells	SELENOI	0.491588	3.422495	1.391243	0.167643	-4.93339	0.410162	0.494084
Erythroid.cells	SEC61B	0.19098	8.272683	1.3912	0.167656	-5.92266	0.347359	0.431687
Erythroid.cells	GM6034	-1.22973	0.50272	-1.39099	0.16772	-4.56006	0.453665	0.535967
Erythroid.cells	SNRNP27	0.221329	5.842837	1.390544	0.167854	-5.48742	0.377679	0.462153
Erythroid.cells	SLX4	-0.64356	1.820472	-1.39045	0.167882	-4.66818	0.433709	0.516933
Erythroid.cells	GM553	-1.07338	0.241543	-1.38994	0.168037	-4.57107	0.458162	0.540282
Erythroid.cells	NLE1	-0.79316	2.257092	-1.38994	0.168038	-4.71084	0.427383	0.510855
Erythroid.cells	EDNRB	1.443757	2.175551	1.389851	0.168064	-4.7806	0.428586	0.512044
Erythroid.cells	ATL2	0.259994	5.337211	1.389043	0.168308	-5.35256	0.384889	0.469242
Erythroid.cells	TLR4	0.67566	3.122783	1.388752	0.168397	-4.85293	0.415447	0.499199
Erythroid.cells	CDK5R1	-0.73671	2.111423	-1.38833	0.168524	-4.7059	0.43037	0.513563
Erythroid.cells	KMO	0.484336	3.038738	1.388195	0.168566	-5.02621	0.416836	0.50056
Erythroid.cells	DAZAP1	-0.19585	6.776612	-1.38811	0.168593	-5.63336	0.366638	0.451109
Erythroid.cells	PJA1	-0.4466	3.699202	-1.38712	0.168893	-4.97072	0.407979	0.491755
Erythroid.cells	SCOC	0.355138	3.752146	1.387027	0.16892	-5.20556	0.407237	0.491031
Erythroid.cells	GPATCH11	0.43308	3.800176	1.386979	0.168935	-4.97379	0.406565	0.490375
Erythroid.cells	ZCCHC2	-0.36891	5.422407	-1.38678	0.168996	-5.24595	0.384529	0.468731
Erythroid.cells	DUT	-0.41047	5.882801	-1.38655	0.169066	-5.54551	0.378507	0.462762
Erythroid.cells	TCEA2	0.972882	0.335442	1.386375	0.169119	-4.56473	0.458142	0.540018
Erythroid.cells	ALPL	-0.76872	1.490191	-1.38626	0.169153	-4.73767	0.440243	0.523041
Erythroid.cells	TOLLIP	0.472172	3.832789	1.386217	0.169167	-4.96051	0.406109	0.490054
Erythroid.cells	HYOU1	0.412037	4.380576	1.386129	0.169194	-5.0079	0.398532	0.482642
Erythroid.cells	TATDN2	-0.3101	5.004884	-1.38607	0.16921	-5.20272	0.390077	0.474333
Erythroid.cells	PARP1	-0.33098	5.30881	-1.38579	0.169295	-5.32563	0.386132	0.470374
Erythroid.cells	GM41335	-1.31858	0.493808	-1.38554	0.169372	-4.5606	0.455862	0.537793
Erythroid.cells	ACTN1	0.366855	5.757216	1.38529	0.169449	-5.33796	0.380402	0.464684
Erythroid.cells	ANKLE2	-0.3245	5.177792	-1.38449	0.169691	-5.24941	0.388417	0.472441
Erythroid.cells	WDR47	-0.55481	3.140773	-1.38448	0.169696	-4.83139	0.416591	0.500002

Erythroid.cells	ABCA9	0.939743	0.787541	1.384302	0.16975	-4.72917	0.451837	0.533852
Erythroid.cells	TOX4	-0.22884	5.903687	-1.38415	0.169798	-5.40362	0.378914	0.463059
Erythroid.cells	1810026B0	0.20542	6.570259	1.383685	0.169938	-5.586	0.370559	0.454773
Erythroid.cells	PRDM4	-0.50661	3.039382	-1.38356	0.169977	-4.83555	0.418321	0.501782
Erythroid.cells	LILRA6	0.780752	1.213739	1.383452	0.17001	-4.76654	0.445495	0.527926
Erythroid.cells	GAPT	0.834016	1.236779	1.383278	0.170063	-4.69489	0.445174	0.527656
Erythroid.cells	LYSMD3	0.326234	4.927993	1.38303	0.170139	-5.24104	0.392127	0.476262
Erythroid.cells	VEZF1	-0.22096	6.00178	-1.3828	0.170209	-5.49178	0.378015	0.462311
Erythroid.cells	NDUFAB1	0.269218	6.268706	1.382282	0.170367	-5.61469	0.374833	0.459118
Erythroid.cells	CAMTA1	-0.2455	5.835211	-1.38134	0.170656	-5.43227	0.380996	0.464971
Erythroid.cells	ARMH2	-1.17844	0.301427	-1.38088	0.170796	-4.54797	0.461213	0.542572
Erythroid.cells	CAAA0114	0.610729	2.131904	1.380497	0.170914	-4.76555	0.433037	0.515775
Erythroid.cells	DTX2	-0.39799	4.396914	-1.38038	0.170951	-5.09561	0.400556	0.484274
Erythroid.cells	ERCC1	0.479966	3.315868	1.380279	0.170981	-4.89001	0.41573	0.499065
Erythroid.cells	2210016F1	-0.29988	4.954824	-1.38027	0.170983	-5.21924	0.392953	0.47681
Erythroid.cells	FAM217B	-0.887	0.616789	-1.38005	0.171052	-4.64466	0.456274	0.538035
Erythroid.cells	MPI	-0.65068	2.225319	-1.38	0.171066	-4.71495	0.431644	0.514537
Erythroid.cells	IL12B	-1.13101	-0.29714	-1.37986	0.171111	-4.84292	0.470902	0.551971
Erythroid.cells	ARL1	-0.24806	5.538643	-1.37975	0.171143	-5.3924	0.38516	0.469292
Erythroid.cells	CUL2	-0.32035	4.968394	-1.37888	0.171409	-5.26975	0.393273	0.47703
Erythroid.cells	NUP88	-0.37532	4.421593	-1.37877	0.171444	-5.08154	0.400728	0.484392
Erythroid.cells	MTX3	-1.02433	0.591443	-1.37821	0.171616	-4.5939	0.457441	0.539126
Erythroid.cells	LRIG2	-0.40288	4.256808	-1.37819	0.171623	-5.06985	0.403166	0.486881
Erythroid.cells	SERTAD4	-1.09032	-0.0023	-1.37801	0.171678	-4.58997	0.466915	0.548142
Erythroid.cells	CBWD1	-0.438	4.090993	-1.378	0.17168	-5.06681	0.405471	0.489184
Erythroid.cells	DLL4	-1.18842	0.717902	-1.37788	0.171717	-4.55562	0.455449	0.537379
Erythroid.cells	TOMM6	0.186296	7.550466	1.377585	0.171809	-5.83218	0.360141	0.44446
Erythroid.cells	PARP14	-0.59955	5.480462	-1.37749	0.171837	-5.31423	0.386579	0.470845
Erythroid.cells	SCAMP1	0.545786	2.970331	1.377113	0.171954	-4.96849	0.421414	0.505006
Erythroid.cells	HECA	0.237174	6.23169	1.376912	0.172016	-5.56788	0.376754	0.46118
Erythroid.cells	OSTM1	-0.35085	4.762239	-1.3768	0.17205	-5.09756	0.396226	0.480467
Erythroid.cells	SIRT7	-0.27626	4.918849	-1.37679	0.172053	-5.19117	0.394101	0.478377
Erythroid.cells	MARF1	-0.22033	6.147381	-1.37678	0.172058	-5.55547	0.377843	0.462291
Erythroid.cells	IKBKE	-0.86758	3.116112	-1.37674	0.172069	-4.89957	0.419304	0.502991
Erythroid.cells	ARSB	0.479659	4.938464	1.376689	0.172085	-5.36034	0.393836	0.478115
Erythroid.cells	LRBA	0.306176	6.139874	1.376353	0.172188	-5.58503	0.378009	0.46254
Erythroid.cells	GM12367	-1.21171	0.051988	-1.37632	0.172198	-4.56213	0.466127	0.547883
Erythroid.cells	9830166K0	0.965876	-1.08122	1.375943	0.172315	-4.52918	0.484945	0.565572
Erythroid.cells	ST6GALNA(	-0.50385	3.867281	-1.37522	0.172537	-4.95575	0.409199	0.49314
Erythroid.cells	LARP7	-0.23889	5.919166	-1.37521	0.17254	-5.4311	0.381364	0.46576
Erythroid.cells	TFEB	0.331423	5.158966	1.375078	0.172582	-5.24771	0.391439	0.475737
Erythroid.cells	TNKS	0.29826	6.066905	1.374839	0.172656	-5.46198	0.379513	0.463942
Erythroid.cells	PRELID3B	-0.26128	5.574007	-1.37457	0.172738	-5.39996	0.386015	0.470514
Erythroid.cells	TBC1D22A	-0.28001	5.871527	-1.37452	0.172754	-5.37021	0.382098	0.466647
Erythroid.cells	SLC35F6	0.494662	2.998461	1.374233	0.172843	-4.89607	0.42186	0.505538
Erythroid.cells	GM37065	-0.68174	2.706239	-1.37398	0.17292	-4.91206	0.426217	0.509737
Erythroid.cells	MR1	-1.07197	0.68263	-1.37369	0.17301	-4.57262	0.457116	0.539353

Erythroid.cells	C3AR1	0.887529	2.290307	1.373609	0.173036	-4.91933	0.432455	0.515789
Erythroid.cells	GXYLT1	0.330041	5.093219	1.373249	0.173147	-5.21738	0.392765	0.477256
Erythroid.cells	GNL1	-0.3706	4.299889	-1.37321	0.173159	-5.08198	0.403614	0.48792
Erythroid.cells	RTEL1	0.690353	2.682882	1.373126	0.173186	-4.78636	0.42671	0.510416
Erythroid.cells	SLC35E1	0.414098	4.084727	1.372733	0.173308	-4.97518	0.4068	0.491027
Erythroid.cells	GM14295	-0.72761	1.289141	-1.37226	0.173455	-4.65548	0.448201	0.530922
Erythroid.cells	FXYD5	0.327025	6.974009	1.371807	0.173595	-5.75717	0.368865	0.453371
Erythroid.cells	MGAT2	-0.25609	5.873385	-1.3713	0.173752	-5.40787	0.383225	0.467576
Erythroid.cells	IL1RN	-1.10724	2.526967	-1.37125	0.173767	-4.89856	0.42994	0.513218
Erythroid.cells	FBXO45	0.436737	3.654199	1.370425	0.174024	-4.96705	0.413941	0.497674
Erythroid.cells	TMEM167E	0.423205	3.755594	1.370162	0.174105	-5.07545	0.412499	0.49644
Erythroid.cells	PWWP2A	-0.32153	5.141602	-1.36984	0.174205	-5.27334	0.393315	0.4778
Erythroid.cells	TMEM109	0.477483	3.841384	1.369832	0.174208	-5.03057	0.411284	0.49541
Erythroid.cells	RBM4	0.299982	4.655948	1.369671	0.174258	-5.18493	0.399928	0.48436
Erythroid.cells	TRIM32	0.931887	1.488698	1.369507	0.174309	-4.66723	0.446008	0.528975
Erythroid.cells	SDCCAG8	-0.27381	5.914953	-1.36945	0.174326	-5.41945	0.383021	0.467676
Erythroid.cells	RMND1	-0.37663	3.79439	-1.36945	0.174327	-5.01088	0.411949	0.496116
Erythroid.cells	GM41790	-1.20825	-0.09939	-1.36942	0.174335	-4.57424	0.471138	0.552827
Erythroid.cells	CD37	-0.24349	7.409059	-1.36914	0.174422	-5.77036	0.363934	0.448718
Erythroid.cells	LARP4	-0.23352	6.410341	-1.36912	0.174429	-5.55556	0.376577	0.461368
Erythroid.cells	GM17382	-1.14802	0.002886	-1.36908	0.174442	-4.55314	0.469477	0.551377
Erythroid.cells	CSF2RB	0.493121	4.838003	1.368963	0.174479	-5.23961	0.397435	0.482052
Erythroid.cells	AGMAT	0.794741	1.438737	1.368887	0.174502	-4.81224	0.446777	0.529893
Erythroid.cells	SLC31A1	-0.30344	5.429507	-1.36877	0.174538	-5.36879	0.389449	0.474277
Erythroid.cells	SAP18B	-0.58543	2.771863	-1.36771	0.17487	-4.82522	0.427421	0.511116
Erythroid.cells	VTN	0.483932	4.267725	1.367457	0.174948	-5.42752	0.405995	0.49029
Erythroid.cells	PVRIG	-0.89906	-0.53207	-1.36742	0.174961	-4.57942	0.479053	0.56027
Erythroid.cells	TMEM251	-0.39272	4.619848	-1.36713	0.175051	-5.07264	0.401172	0.485587
Erythroid.cells	PECR	0.681114	2.35098	1.367041	0.175078	-4.92469	0.43375	0.517233
Erythroid.cells	ABT1	0.450565	3.534654	1.366939	0.17511	-4.90919	0.416423	0.50052
Erythroid.cells	RRP15	0.426485	3.823389	1.366407	0.175276	-4.99684	0.412601	0.496754
Erythroid.cells	GPSM2	0.610131	2.440784	1.366271	0.175319	-4.86874	0.432721	0.516258
Erythroid.cells	CNPY4	0.59221	2.433697	1.365735	0.175486	-4.77935	0.433108	0.516447
Erythroid.cells	C8G	0.560829	3.782028	1.365644	0.175515	-5.32574	0.41346	0.497465
Erythroid.cells	GM15738	0.99518	0.402397	1.365508	0.175558	-4.57435	0.46455	0.546556
Erythroid.cells	AZGP1	0.474448	3.926277	1.364789	0.175783	-5.3944	0.411847	0.495716
Erythroid.cells	GM47071	0.939797	1.555335	1.364436	0.175893	-4.7012	0.447073	0.529701
Erythroid.cells	OPRM1	0.582015	2.803916	1.363902	0.176061	-5.00328	0.4285	0.511736
Erythroid.cells	PDE3B	0.294787	6.955861	1.36383	0.176083	-5.87562	0.371577	0.455887
Erythroid.cells	GM32296	-1.01308	-0.65404	-1.36311	0.176308	-4.58918	0.483083	0.563536
Erythroid.cells	MCM3AP	-0.37921	3.885875	-1.36311	0.176309	-4.97461	0.413078	0.496739
Erythroid.cells	CRMP1	0.976826	-1.07546	1.363105	0.176311	-4.60419	0.490166	0.570158
Erythroid.cells	DYNLT1A	0.419401	4.14434	1.362785	0.176411	-5.08411	0.40956	0.493303
Erythroid.cells	1700052K1	-0.82695	1.140287	-1.36256	0.176481	-4.61884	0.454291	0.536384
Erythroid.cells	FKBPL	-0.84765	1.019839	-1.36232	0.176557	-4.64311	0.456272	0.538291
Erythroid.cells	FAM78B	0.659334	2.115372	1.361663	0.176764	-4.84804	0.439684	0.522374
Erythroid.cells	ZER1	0.444226	3.262662	1.361554	0.176798	-4.92569	0.422644	0.506

Erythroid.cells	CLDN34C1	-1.09457	0.809812	-1.3615	0.176815	-4.5851	0.45994	0.541745
Erythroid.cells	GM1123	-0.93585	0.265276	-1.36125	0.176894	-4.60366	0.468767	0.550157
Erythroid.cells	GRIP1	-0.74997	2.360437	-1.36102	0.176965	-4.9158	0.436113	0.519123
Erythroid.cells	1600020E0	0.248026	6.223144	1.360944	0.17699	-5.77023	0.381899	0.466215
Erythroid.cells	IGHM	-0.44794	8.158942	-1.36075	0.177052	-6.0226	0.357494	0.441815
Erythroid.cells	GM27010	-0.59505	2.340877	-1.36054	0.177118	-4.82593	0.436472	0.519566
Erythroid.cells	RALGAPA2	-0.31624	6.409122	-1.36041	0.177157	-5.63575	0.379532	0.463972
Erythroid.cells	TMED4	0.441646	3.553347	1.360251	0.177209	-4.9766	0.418621	0.502374
Erythroid.cells	IPCEF1	0.452168	4.679593	1.360203	0.177224	-5.33157	0.402722	0.48687
Erythroid.cells	CERS4	-0.45169	3.651416	-1.3595	0.177446	-5.06018	0.417638	0.501265
Erythroid.cells	THADA	-0.27893	5.881845	-1.35907	0.17758	-5.48256	0.387045	0.471203
Erythroid.cells	CHMP2A	0.191942	6.544809	1.358872	0.177644	-5.69226	0.378404	0.462698
Erythroid.cells	MTOR	-0.31563	4.766611	-1.35865	0.177715	-5.20308	0.402256	0.486243
Erythroid.cells	ASAP2	0.494309	3.068411	1.357945	0.177937	-5.07557	0.426882	0.509941
Erythroid.cells	GM43258	-0.96648	0.724157	-1.35773	0.178005	-4.59511	0.462859	0.544385
Erythroid.cells	ATP5K	0.232988	7.486519	1.357636	0.178034	-5.89305	0.366879	0.451015
Erythroid.cells	NUMB	-0.23881	6.956562	-1.35739	0.178113	-5.69179	0.37366	0.457718
Erythroid.cells	MED20	-0.39306	4.104119	-1.35706	0.178215	-5.02999	0.412102	0.495645
Erythroid.cells	PARP16	0.93569	1.117003	1.357063	0.178216	-4.63829	0.456772	0.538583
Erythroid.cells	MTLN	0.448496	3.060484	1.35666	0.178343	-4.94718	0.427376	0.510306
Erythroid.cells	APOA2	0.44826	8.419445	1.356081	0.178527	-6.22353	0.355898	0.439766
Erythroid.cells	1810062O1	-0.70011	2.0989	-1.35604	0.17854	-4.7535	0.442055	0.524349
Erythroid.cells	GM4070	0.791296	2.887167	1.3558	0.178616	-4.97558	0.430273	0.513013
Erythroid.cells	FAM20B	-0.43777	3.958304	-1.35569	0.17865	-5.01188	0.4147	0.498014
Erythroid.cells	SAE1	-0.23851	6.342657	-1.35538	0.178748	-5.62379	0.38224	0.466133
Erythroid.cells	ADGRE5	0.252188	7.153842	1.355121	0.178831	-5.66506	0.371866	0.455854
Erythroid.cells	STIM1	-0.28088	7.712751	-1.35487	0.178912	-5.86087	0.364914	0.448864
Erythroid.cells	BRMS1	-0.39251	4.236998	-1.35458	0.179002	-5.08806	0.411171	0.494588
Erythroid.cells	ZFP658	-1.16566	0.297611	-1.35415	0.17914	-4.55724	0.471116	0.551877
Erythroid.cells	PGM2L1	0.344558	5.192591	1.354135	0.179144	-5.39249	0.398028	0.481636
Erythroid.cells	SPATA6	0.392729	4.52297	1.353593	0.179316	-5.19641	0.407553	0.490955
Erythroid.cells	PHIP	-0.208	7.276011	-1.3535	0.179345	-5.77929	0.370864	0.454773
Erythroid.cells	HSPA9	0.206772	6.483768	1.353145	0.179459	-5.68832	0.381166	0.465095
Erythroid.cells	TBC1D22B	0.457188	3.778303	1.353069	0.179483	-4.97149	0.418249	0.501477
Erythroid.cells	FAM214A	0.43068	5.246692	1.352784	0.179574	-5.26939	0.397781	0.481476
Erythroid.cells	1190005I0	0.763397	1.185776	1.352154	0.179775	-4.66783	0.457777	0.539236
Erythroid.cells	FAM129C	-0.40834	3.433215	-1.35213	0.179783	-5.13166	0.423656	0.506566
Erythroid.cells	FAM110B	1.019642	0.653098	1.351446	0.18	-4.64281	0.466531	0.547488
Erythroid.cells	FGFBP3	-1.2212	0.028033	-1.35134	0.180034	-4.59103	0.476709	0.557138
Erythroid.cells	KRIT1	-0.24038	5.937975	-1.35097	0.180153	-5.48098	0.388952	0.472758
Erythroid.cells	SLK	0.250542	6.477015	1.35096	0.180155	-5.64345	0.381838	0.465716
Erythroid.cells	RHBDD3	0.559211	2.194151	1.350936	0.180163	-4.78436	0.442377	0.524674
Erythroid.cells	PACRG	-1.17815	-0.43352	-1.35079	0.180209	-4.55948	0.484368	0.564417
Erythroid.cells	WDHD1	-0.4111	4.56722	-1.35042	0.180327	-5.26868	0.407682	0.491292
Erythroid.cells	ZC3H18	-0.30972	5.270525	-1.35035	0.180352	-5.28794	0.397956	0.481766
Erythroid.cells	HIST1H1D	0.935499	1.531522	1.350293	0.180368	-4.72349	0.452602	0.534615
Erythroid.cells	COLEC12	0.693342	2.890318	1.350244	0.180384	-5.04416	0.431891	0.514819

Erythroid.cells	A530064D(	-0.83112	0.81889	-1.34991	0.180492	-4.70999	0.463869	0.545512
Erythroid.cells	C6	0.994376	2.802964	1.349901	0.180494	-4.86122	0.433193	0.516239
Erythroid.cells	NEO1	1.047998	0.660859	1.349617	0.180584	-4.64829	0.466406	0.547941
Erythroid.cells	HERC3	0.421343	4.345366	1.349576	0.180597	-5.10774	0.410801	0.494576
Erythroid.cells	SLC6A13	0.510906	2.762131	1.349559	0.180603	-5.03825	0.433802	0.516853
Erythroid.cells	GM13431	0.925179	0.492651	1.349549	0.180606	-4.6451	0.469123	0.550506
Erythroid.cells	MOB4	-0.17925	6.832732	-1.34945	0.180637	-5.71082	0.377219	0.46149
Erythroid.cells	B9D1	1.035632	0.371188	1.349437	0.180642	-4.59883	0.471094	0.55237
Erythroid.cells	AXL	0.713409	3.708037	1.34933	0.180676	-5.0703	0.419903	0.503482
Erythroid.cells	2310001H1	0.524039	4.065364	1.348608	0.180907	-5.03465	0.415174	0.498756
Erythroid.cells	GLMP	-0.33541	5.839868	-1.34853	0.180934	-5.42337	0.390637	0.474741
Erythroid.cells	PPP2R3D	-0.49508	3.751015	-1.34813	0.181059	-4.97918	0.419881	0.503349
Erythroid.cells	COQ4	0.610682	2.66674	1.347624	0.181222	-4.7775	0.436143	0.51896
Erythroid.cells	KCTD7	-0.93642	0.387211	-1.34723	0.181348	-4.5897	0.472039	0.553006
Erythroid.cells	PPFIBP1	0.449146	3.366596	1.346984	0.181428	-5.00792	0.425999	0.509145
Erythroid.cells	ZFP992	-0.53571	3.563813	-1.34691	0.181451	-4.93174	0.423117	0.506386
Erythroid.cells	GM5547	-0.75554	1.560402	-1.34651	0.181579	-4.69954	0.453573	0.535619
Erythroid.cells	GAK	-0.21197	6.280821	-1.34632	0.181642	-5.52292	0.385664	0.469775
Erythroid.cells	GM43305	-0.44607	7.45195	-1.34606	0.181726	-5.94606	0.370598	0.454787
Erythroid.cells	HYPK	0.218679	5.8151	1.345941	0.181763	-5.53556	0.391946	0.475999
Erythroid.cells	H2-Q4	-0.49978	4.933329	-1.34575	0.181826	-5.30213	0.404029	0.487841
Erythroid.cells	NDUFV3	0.223542	7.098589	1.34536	0.181949	-5.79626	0.375313	0.459456
Erythroid.cells	ACOT11	-0.5795	1.943725	-1.34477	0.182138	-4.81089	0.448374	0.53043
Erythroid.cells	LRRCC1	0.353765	3.748019	1.344547	0.182211	-5.03895	0.421428	0.504527
Erythroid.cells	TMEM8	0.63923	2.000346	1.344358	0.182272	-4.78131	0.447622	0.529677
Erythroid.cells	LNCPPARA	0.9957	0.60974	1.344096	0.182356	-4.62971	0.469691	0.550672
Erythroid.cells	BAIAP2	-0.39703	5.269985	-1.34398	0.182393	-5.3598	0.400059	0.483749
Erythroid.cells	KLHDC1	0.619153	2.152219	1.3437	0.182484	-4.79949	0.44535	0.527756
Erythroid.cells	SMU1	-0.23729	5.608875	-1.34358	0.182521	-5.42468	0.395436	0.479395
Erythroid.cells	MCM9	-0.35126	4.75152	-1.34358	0.182521	-5.21568	0.407242	0.490975
Erythroid.cells	NFKBID	-0.32453	6.350031	-1.34349	0.182551	-5.55433	0.385521	0.469617
Erythroid.cells	ELANE	1.678672	2.340078	1.343105	0.182676	-4.9541	0.442677	0.5252
Erythroid.cells	CPSF1	-0.47241	3.349532	-1.34287	0.182752	-4.94248	0.427558	0.510769
Erythroid.cells	RFTN1	-0.27681	6.737622	-1.34283	0.182764	-5.67339	0.380622	0.464798
Erythroid.cells	SEM1	0.155235	9.100384	1.342022	0.183025	-6.14637	0.351566	0.435481
Erythroid.cells	GM26787	-1.04442	1.300668	-1.34174	0.183116	-4.64653	0.459449	0.541233
Erythroid.cells	GTF3C4	-0.60819	2.57436	-1.34169	0.183131	-4.80026	0.439707	0.522425
Erythroid.cells	ABCB7	0.234818	5.334608	1.341231	0.183281	-5.44951	0.399938	0.484071
Erythroid.cells	F9	0.777164	0.896192	1.341153	0.183306	-4.76497	0.465962	0.547686
Erythroid.cells	MAML2	-0.27327	7.972039	-1.34115	0.183308	-5.91545	0.365431	0.449803
Erythroid.cells	GC	0.489815	5.287073	1.341106	0.183321	-5.71277	0.400591	0.484728
Erythroid.cells	RFX5	-0.70824	1.943489	-1.3407	0.183454	-4.72582	0.44954	0.532197
Erythroid.cells	EPHB2	1.048806	1.372664	1.340676	0.18346	-4.62642	0.458479	0.540708
Erythroid.cells	UBALD1	0.366472	4.983613	1.34042	0.183543	-5.21211	0.404887	0.489113
Erythroid.cells	GNAT3	-1.08917	-0.04367	-1.34037	0.183561	-4.58283	0.48145	0.562498
Erythroid.cells	GSTM2	-0.68961	1.633616	-1.34014	0.183633	-4.84403	0.45437	0.536902
Erythroid.cells	LEPROTL1	0.347376	5.362087	1.340138	0.183634	-5.39274	0.399662	0.483994

Erythroid.cells	PDXK	0.40048	4.441778	1.340054	0.183662	-5.13669	0.412492	0.496554
Erythroid.cells	PHF2	-0.26842	5.076145	-1.33907	0.18398	-5.29547	0.404211	0.488169
Erythroid.cells	CYP2D22	0.853014	0.864397	1.338853	0.184051	-4.69318	0.467366	0.548867
Erythroid.cells	TUBE1	0.805244	1.254723	1.338566	0.184144	-4.70467	0.461214	0.543067
Erythroid.cells	ATF7IP	-0.25734	6.540023	-1.33821	0.184258	-5.62553	0.384575	0.468831
Erythroid.cells	EIF3J2	0.726649	1.443832	1.338132	0.184285	-4.73202	0.458215	0.540305
Erythroid.cells	KPNA4	0.221988	8.246447	1.337986	0.184332	-5.99219	0.3628	0.447162
Erythroid.cells	VAPB	0.342408	5.031604	1.3378	0.184393	-5.24556	0.404981	0.489077
Erythroid.cells	ITGB3BP	0.416446	3.52337	1.337763	0.184404	-4.97181	0.426529	0.510046
Erythroid.cells	PIK3CD	-0.29747	6.624599	-1.33775	0.184409	-5.61267	0.383464	0.46786
Erythroid.cells	FMN2	-0.73471	2.359589	-1.33769	0.184428	-4.99	0.443971	0.526836
Erythroid.cells	GVIN1	0.843267	3.037272	1.337412	0.184519	-4.99017	0.43382	0.517202
Erythroid.cells	CANX	0.170372	7.35016	1.337308	0.184552	-5.78916	0.37415	0.458725
Erythroid.cells	ADAMTS7	-1.01398	0.676356	-1.33689	0.184688	-4.63361	0.470854	0.552484
Erythroid.cells	ERBB3	-0.9742	1.113728	-1.33624	0.184901	-4.66336	0.464137	0.546167
Erythroid.cells	FOPNL	0.335265	4.468642	1.336105	0.184944	-5.20847	0.413487	0.497525
Erythroid.cells	4930473A(	-0.90128	0.38363	-1.33606	0.18496	-4.6079	0.475982	0.557377
Erythroid.cells	TDG	-0.29101	5.074746	-1.33596	0.184992	-5.30659	0.40497	0.489239
Erythroid.cells	GM49602	0.654092	1.89388	1.335465	0.185152	-4.77725	0.452102	0.534746
Erythroid.cells	SMAD6	-0.69427	2.733914	-1.33505	0.185288	-4.97722	0.439404	0.522536
Erythroid.cells	GM13091	-0.99438	0.904415	-1.33494	0.185323	-4.62488	0.468019	0.549835
Erythroid.cells	YAE1D1	0.556579	3.240113	1.334747	0.185386	-4.96739	0.431859	0.515314
Erythroid.cells	MAST4	-0.3948	6.39259	-1.33441	0.185495	-5.69195	0.387695	0.472118
Erythroid.cells	GPI1	0.207626	7.844302	1.334141	0.185584	-5.92564	0.368996	0.453495
Erythroid.cells	FUT10	-1.01997	-0.01434	-1.33405	0.185614	-4.59828	0.48342	0.564324
Erythroid.cells	UBP1	0.268334	5.448392	1.333766	0.185706	-5.38572	0.400625	0.484928
Erythroid.cells	ZFP429	-0.57698	2.448075	-1.33362	0.185753	-4.80244	0.444175	0.527213
Erythroid.cells	BBS4	0.692155	2.313596	1.3326	0.186087	-4.73379	0.446941	0.529527
Erythroid.cells	GALNT6	-0.55942	3.115592	-1.33233	0.186175	-5.02804	0.434815	0.517944
Erythroid.cells	SLC36A3	0.910497	0.101985	1.332271	0.186195	-4.66407	0.48244	0.563238
Erythroid.cells	ASGR2	0.751005	1.698173	1.331984	0.186289	-4.88655	0.456709	0.538904
Erythroid.cells	ADSS	-0.21288	6.271707	-1.3313	0.186512	-5.61165	0.390635	0.474699
Erythroid.cells	RSL1	-0.99322	1.039109	-1.33043	0.186798	-4.64964	0.468275	0.549608
Erythroid.cells	KIF1BP	0.424966	3.865166	1.32979	0.187008	-5.02242	0.425151	0.508259
Erythroid.cells	ENOPH1	-0.54712	2.8863	-1.32963	0.18706	-4.87778	0.43972	0.522314
Erythroid.cells	HNRNPA0	-0.17136	7.214278	-1.3296	0.187069	-5.79033	0.379027	0.463037
Erythroid.cells	PHF5A	-0.24839	6.072643	-1.3287	0.187365	-5.598	0.394265	0.478345
Erythroid.cells	APOH	0.45664	4.983199	1.328615	0.187394	-5.65546	0.409273	0.493111
Erythroid.cells	LRRK1	0.384088	5.612519	1.328567	0.18741	-5.36451	0.400531	0.484546
Erythroid.cells	CDC16	-0.39782	3.893321	-1.32855	0.187415	-5.05315	0.424891	0.5083
Erythroid.cells	MORN3	-0.82244	1.323497	-1.32851	0.187429	-4.69383	0.464225	0.545962
Erythroid.cells	SLC9A5	-1.0207	0.362304	-1.32842	0.187457	-4.60694	0.479884	0.560734
Erythroid.cells	TTL	0.623946	1.740811	1.328381	0.187471	-4.79096	0.457591	0.539666
Erythroid.cells	SPINK10	0.9301	0.791956	1.328272	0.187507	-4.65123	0.472819	0.554093
Erythroid.cells	GM15859	0.720731	1.208039	1.328125	0.187555	-4.66633	0.466078	0.547742
Erythroid.cells	KLRG1	-0.92487	-1.146	-1.32787	0.187639	-4.63545	0.505542	0.584763
Erythroid.cells	ATP6V1G2	-0.83912	1.237127	-1.32783	0.187652	-4.67161	0.465611	0.547341

Erythroid.cells	SF3A3	-0.3186	4.984502	-1.32782	0.187655	-5.29858	0.409255	0.493154
Erythroid.cells	APBB2	0.669668	4.02877	1.327446	0.187779	-5.10396	0.422916	0.506559
Erythroid.cells	NECTIN3	0.788866	1.220532	1.32744	0.187781	-4.70756	0.465878	0.547711
Erythroid.cells	CRYBG2	-1.04925	0.051796	-1.32744	0.187781	-4.59874	0.485057	0.565779
Erythroid.cells	SEC61A1	-0.24052	5.49084	-1.32733	0.187816	-5.41014	0.402206	0.486393
Erythroid.cells	RNF215	0.498411	2.627986	1.327069	0.187903	-4.84213	0.443914	0.526834
Erythroid.cells	PDCD2L	-0.28549	4.660225	-1.32655	0.188072	-5.21025	0.414168	0.497938
Erythroid.cells	PPP1R12A	-0.17151	8.052992	-1.32649	0.188093	-5.95504	0.368753	0.453061
Erythroid.cells	CFP	0.452241	5.518079	1.325997	0.188256	-5.40743	0.402409	0.486384
Erythroid.cells	EFCAB14	0.39353	4.713606	1.325859	0.188302	-5.16442	0.413681	0.497413
Erythroid.cells	ACSL4	-0.27917	6.189637	-1.32543	0.188444	-5.51506	0.393385	0.477467
Erythroid.cells	HIST1H2BJ	-0.641	3.30978	-1.32542	0.188447	-5.06004	0.434278	0.517319
Erythroid.cells	SLC23A2	0.370918	5.113974	1.325112	0.188548	-5.25324	0.408291	0.492058
Erythroid.cells	IMMP2L	0.343878	6.534254	1.324884	0.188624	-5.7405	0.38896	0.473026
Erythroid.cells	GOLGA7	0.193239	5.94	1.324603	0.188717	-5.63864	0.397062	0.480941
Erythroid.cells	TMEM9	0.640474	2.576914	1.324251	0.188833	-4.87906	0.445888	0.528296
Erythroid.cells	GM12248	-0.63645	2.619411	-1.32371	0.189012	-4.86343	0.445488	0.527963
Erythroid.cells	GM39469	0.503341	2.328802	1.323633	0.189037	-4.85388	0.449972	0.532248
Erythroid.cells	TRIM11	-0.29797	5.06784	-1.32353	0.18907	-5.34792	0.40951	0.493186
Erythroid.cells	TANC2	0.466553	5.521032	1.323412	0.18911	-5.30581	0.403192	0.487002
Erythroid.cells	SLC15A3	0.463073	5.005968	1.322806	0.189311	-5.24908	0.410608	0.494223
Erythroid.cells	IGFBP2	0.629251	4.701389	1.322728	0.189337	-5.62327	0.414924	0.498432
Erythroid.cells	WDR12	0.407143	4.283459	1.322709	0.189343	-5.14284	0.420926	0.504265
Erythroid.cells	COL5A3	-0.90731	0.940726	-1.3224	0.189445	-4.72543	0.472448	0.553467
Erythroid.cells	AGGF1	-0.26233	5.384652	-1.32206	0.189558	-5.3578	0.40546	0.489157
Erythroid.cells	CORO7	-0.24093	6.215775	-1.32188	0.189617	-5.57202	0.394073	0.477999
Erythroid.cells	ARHGAP12	0.360395	5.076663	1.321866	0.189623	-5.24409	0.409768	0.493401
Erythroid.cells	HMGA1	-0.39226	4.670399	-1.32182	0.18964	-5.27467	0.415524	0.499027
Erythroid.cells	SLC8B1	0.341108	4.721857	1.32172	0.189671	-5.36754	0.41479	0.498325
Erythroid.cells	LONP2	-0.20037	5.87764	-1.32144	0.189764	-5.54801	0.398712	0.482585
Erythroid.cells	SMARCA2	0.242182	6.123463	1.321376	0.189785	-5.57464	0.395368	0.479317
Erythroid.cells	FCER2A	0.968326	0.458562	1.321245	0.189829	-4.7853	0.480467	0.561221
Erythroid.cells	OSBPL1A	0.422869	3.288159	1.321137	0.189865	-5.09148	0.435803	0.518801
Erythroid.cells	SRP9	0.161678	7.516452	1.320629	0.190033	-5.87633	0.377224	0.461359
Erythroid.cells	AHR	0.429364	4.719836	1.319988	0.190247	-5.49955	0.415501	0.49891
Erythroid.cells	GM31508	0.92123	0.763062	1.319765	0.190321	-4.67597	0.476168	0.556969
Erythroid.cells	RASSF7	0.790328	0.86906	1.319751	0.190325	-4.71233	0.474429	0.555332
Erythroid.cells	RFC2	-0.32183	5.227985	-1.31883	0.190633	-5.3812	0.408741	0.492264
Erythroid.cells	2010110K1	-1.23044	-0.03523	-1.31873	0.190666	-4.60139	0.489984	0.569871
Erythroid.cells	ABCA8B	0.8399	1.118002	1.318651	0.190691	-4.80061	0.470861	0.551944
Erythroid.cells	FTSJ1	0.410654	3.078297	1.318461	0.190755	-4.95244	0.440091	0.522693
Erythroid.cells	VPS9D1	0.515075	3.05216	1.318409	0.190772	-4.93543	0.440488	0.523073
Erythroid.cells	SNRPG	-0.18335	8.018397	-1.31831	0.190807	-5.99329	0.371523	0.455573
Erythroid.cells	MDP1	-0.32826	4.373314	-1.31826	0.190823	-5.1947	0.420915	0.504255
Erythroid.cells	FER	-0.5347	3.489418	-1.31746	0.191088	-5.01489	0.434412	0.517042
Erythroid.cells	RNF41	-0.34128	4.424549	-1.31724	0.191161	-5.13402	0.42073	0.50385
Erythroid.cells	NR1H3	0.65112	3.325046	1.31691	0.191272	-5.08048	0.437009	0.519555

Erythroid.cells	BTBD11	0.544984	4.309098	1.316902	0.191275	-5.30966	0.422465	0.505519
Erythroid.cells	MRPL39	0.355521	3.661118	1.316224	0.191501	-5.08336	0.432335	0.514998
Erythroid.cells	ADAM12	0.951677	0.815122	1.316124	0.191534	-4.70712	0.476894	0.557439
Erythroid.cells	LRRC59	-0.24348	5.556125	-1.31605	0.191559	-5.44716	0.405089	0.488564
Erythroid.cells	FCGR3	0.576836	4.189291	1.315729	0.191666	-5.15774	0.424696	0.507655
Erythroid.cells	SLAMF8	-0.91131	0.718707	-1.31499	0.191912	-4.88442	0.479153	0.5595
Erythroid.cells	FGD3	0.399124	4.660813	1.314757	0.191992	-5.22045	0.418364	0.50149
Erythroid.cells	MPC1	0.179165	7.569491	1.314667	0.192022	-6.0411	0.378694	0.462485
Erythroid.cells	DDR1	0.790394	0.137297	1.314509	0.192075	-4.68059	0.488953	0.568753
Erythroid.cells	1700056N1	-0.63626	2.033678	-1.31434	0.19213	-4.76878	0.45801	0.539648
Erythroid.cells	ZFC3H1	-0.20536	6.914629	-1.31404	0.192232	-5.71691	0.387341	0.471165
Erythroid.cells	GARNL3	-0.70124	2.487215	-1.31404	0.192232	-4.79274	0.450944	0.532957
Erythroid.cells	ARL5A	0.255961	5.831093	1.31369	0.192349	-5.5359	0.402051	0.485595
Erythroid.cells	TXN1	-0.29363	7.304482	-1.31368	0.192354	-5.81163	0.382283	0.466079
Erythroid.cells	4833439L1	0.288932	4.862518	1.313551	0.192396	-5.37196	0.415634	0.498867
Erythroid.cells	CASP8	-0.2566	5.513709	-1.31303	0.192569	-5.43457	0.406725	0.490049
Erythroid.cells	SRI	-0.23603	6.74315	-1.31255	0.192731	-5.68441	0.390194	0.473726
Erythroid.cells	CEP70	-0.52579	3.195368	-1.31237	0.192791	-4.9279	0.440766	0.522962
Erythroid.cells	SLC30A6	-0.45503	3.417011	-1.31213	0.192873	-4.97319	0.437504	0.519816
Erythroid.cells	BACH2	-0.24571	8.964274	-1.31197	0.192926	-6.2349	0.361838	0.445504
Erythroid.cells	LSM11	-0.78113	2.171179	-1.31137	0.19313	-4.76532	0.457092	0.538368
Erythroid.cells	SMG5	-0.25319	5.55464	-1.31108	0.193227	-5.4366	0.406915	0.489989
Erythroid.cells	CLPTM1	-0.23899	5.552635	-1.31106	0.193231	-5.43746	0.406943	0.490016
Erythroid.cells	GM16014	-1.09468	-0.09363	-1.31059	0.193392	-4.63377	0.494554	0.573485
Erythroid.cells	ARHGEF40	0.82926	0.550928	1.310403	0.193454	-4.75661	0.48367	0.563411
Erythroid.cells	TREM1	0.718739	1.639916	1.310287	0.193493	-4.90911	0.465831	0.546697
Erythroid.cells	HIST3H2A	0.738471	2.104979	1.310211	0.193518	-4.80534	0.45842	0.539697
Erythroid.cells	FCMR	-0.54678	2.105124	-1.31007	0.193564	-5.01301	0.458418	0.539724
Erythroid.cells	MAGED1	0.656668	2.409871	1.309896	0.193624	-4.8557	0.453628	0.53517
Erythroid.cells	F2	0.492471	4.237274	1.309857	0.193637	-5.51108	0.425972	0.508638
Erythroid.cells	NLN	0.409507	4.446951	1.309194	0.193861	-5.15831	0.423308	0.505725
Erythroid.cells	AKIRIN1	-0.18646	6.287656	-1.30856	0.194075	-5.62556	0.397687	0.480559
Erythroid.cells	MXD3	-0.7551	1.735683	-1.30853	0.194084	-4.81445	0.465056	0.545458
Erythroid.cells	PTGS2OS2	-0.88418	-0.48663	-1.30836	0.194142	-4.6913	0.502171	0.580236
Erythroid.cells	SH3PXD2B	0.647027	1.543779	1.308209	0.194193	-5.09809	0.468199	0.548519
Erythroid.cells	FASTK	0.453499	3.15048	1.307799	0.194332	-4.97041	0.443158	0.524749
Erythroid.cells	SLC2A9	0.585766	2.879571	1.307605	0.194397	-4.96483	0.447312	0.528737
Erythroid.cells	SELPLG	0.266411	6.845492	1.30759	0.194402	-5.6919	0.390372	0.473474
Erythroid.cells	BABAM1	0.260938	5.569892	1.307188	0.194538	-5.44874	0.407998	0.490735
Erythroid.cells	AVIL	-0.6907	2.092681	-1.30698	0.19461	-4.83005	0.459827	0.540671
Erythroid.cells	MBD4	0.512694	3.212172	1.30686	0.194649	-4.94664	0.442429	0.524151
Erythroid.cells	MBD5	-0.22298	6.903175	-1.3068	0.194668	-5.70499	0.389789	0.472988
Erythroid.cells	DDX20	-0.38878	3.596417	-1.30627	0.194849	-5.02168	0.436876	0.518868
Erythroid.cells	POLR2I	-0.25026	5.116859	-1.30621	0.19487	-5.41474	0.414635	0.497367
Erythroid.cells	MYH9	-0.1726	7.80338	-1.30581	0.195004	-5.92413	0.378385	0.461606
Erythroid.cells	PIEZO1	-0.3313	5.046187	-1.30538	0.19515	-5.33746	0.415963	0.498537
Erythroid.cells	STXBP4	0.714963	2.23619	1.305377	0.195151	-4.84763	0.458184	0.539086

Erythroid.cells	ARMCX5	0.509803	2.433168	1.305217	0.195206	-4.89667	0.455084	0.536203
Erythroid.cells	ACY1	0.77896	1.799123	1.304859	0.195327	-4.76081	0.465141	0.545865
Erythroid.cells	KCNIP4	1.115125	0.608323	1.304838	0.195334	-4.74025	0.484651	0.564228
Erythroid.cells	HEMK1	-0.81087	1.147753	-1.30477	0.195358	-4.71661	0.475711	0.555837
Erythroid.cells	RELCH	-0.20819	6.238997	-1.30474	0.195367	-5.60754	0.399296	0.48242
Erythroid.cells	ESR1	-0.50149	3.611322	-1.30432	0.195509	-5.2118	0.437211	0.519244
Erythroid.cells	SETDB2	-0.33268	4.580351	-1.30389	0.195657	-5.28079	0.422953	0.505606
Erythroid.cells	AGXT	0.545729	3.279368	1.303795	0.195688	-5.30017	0.442306	0.524249
Erythroid.cells	RHOV	-1.18557	-0.80702	-1.3037	0.19572	-4.65063	0.509265	0.587277
Erythroid.cells	MRPS18B	0.369912	3.873974	1.303562	0.195767	-5.16229	0.433348	0.515683
Erythroid.cells	TTC37	0.492445	3.798842	1.303548	0.195772	-5.09333	0.43447	0.516762
Erythroid.cells	SNRK	0.341155	4.865356	1.303438	0.195809	-5.29449	0.418833	0.501664
Erythroid.cells	USP44	0.97731	-0.6309	1.303335	0.195844	-4.59627	0.506177	0.584489
Erythroid.cells	TRIM12A	-0.37367	5.048891	-1.30317	0.195902	-5.278	0.416233	0.499201
Erythroid.cells	RASSF2	0.447267	4.259468	1.302826	0.196017	-5.09857	0.427746	0.510489
Erythroid.cells	POMGNT1	-0.56418	2.312495	-1.30269	0.196064	-4.82111	0.457396	0.538956
Erythroid.cells	DPH5	0.419765	3.732552	1.302686	0.196065	-5.09791	0.435567	0.518078
Erythroid.cells	CTNS	0.499307	3.606042	1.302388	0.196166	-5.00006	0.437596	0.520042
Erythroid.cells	ATAD5	-0.44418	4.742851	-1.30215	0.196247	-5.38797	0.420837	0.503909
Erythroid.cells	CHAF1A	-0.4901	4.186029	-1.30168	0.196407	-5.20909	0.428966	0.511947
Erythroid.cells	DNAJC30	-0.36173	3.959336	-1.30167	0.196411	-5.17075	0.432322	0.515187
Erythroid.cells	WDR70	-0.21059	6.1341	-1.3016	0.196433	-5.62162	0.401227	0.484933
Erythroid.cells	ARL8B	-0.21778	5.97123	-1.30143	0.196492	-5.52388	0.403472	0.487178
Erythroid.cells	SMOC1	-0.72245	2.28252	-1.30132	0.19653	-4.98173	0.458017	0.539911
Erythroid.cells	ZFPM2	0.852933	2.777013	1.301182	0.196577	-4.95097	0.450279	0.5326
Erythroid.cells	MIEF2	-0.81007	1.112018	-1.30115	0.196586	-4.69094	0.476886	0.557834
Erythroid.cells	HSPA13	-0.44607	3.082179	-1.30098	0.196645	-4.93376	0.445571	0.528101
Erythroid.cells	IDH2	0.251778	5.667278	1.300892	0.196676	-5.61349	0.407697	0.491437
Erythroid.cells	DDOST	-0.24955	5.788445	-1.3007	0.196741	-5.47774	0.406007	0.489782
Erythroid.cells	APH1B	0.610068	2.578014	1.300666	0.196753	-4.90953	0.453377	0.535561
Erythroid.cells	CMBL	0.649719	2.192141	1.300612	0.196771	-5.01683	0.459446	0.541346
Erythroid.cells	TCRG-C2	-0.8353	0.685448	-1.30047	0.196818	-5.02141	0.483959	0.564555
Erythroid.cells	AKR1B10	-0.47718	3.494629	-1.30046	0.196822	-5.03349	0.439289	0.522138
Erythroid.cells	P4HB	-0.18671	7.267668	-1.30026	0.196891	-5.87487	0.385998	0.470174
Erythroid.cells	CNOT11	-0.3962	4.112527	-1.30016	0.196925	-5.08464	0.430087	0.513344
Erythroid.cells	GM17491	0.626528	1.415352	1.299962	0.196993	-4.72212	0.471991	0.553419
Erythroid.cells	PPP1R15B	-0.30459	5.553021	-1.29987	0.197024	-5.42578	0.409358	0.493274
Erythroid.cells	SEC14L1	0.356456	4.618739	1.299712	0.197078	-5.24043	0.42272	0.506397
Erythroid.cells	MCUR1	-0.36227	4.462065	-1.2994	0.197185	-5.2036	0.425121	0.508611
Erythroid.cells	RTL4	1.058344	0.402884	1.299277	0.197227	-4.70705	0.488939	0.569407
Erythroid.cells	ALPK1	-0.46965	4.802266	-1.29917	0.197264	-5.27268	0.420182	0.503868
Erythroid.cells	SLC39A3	0.681983	1.91724	1.298988	0.197326	-4.79518	0.46409	0.546015
Erythroid.cells	DNAJA3	0.413766	3.76841	1.29878	0.197396	-5.10707	0.43543	0.518634
Erythroid.cells	PAXBP1	0.315137	5.312121	1.298572	0.197468	-5.42959	0.412939	0.496912
Erythroid.cells	SLC38A4	0.515353	3.153421	1.298456	0.197507	-5.3065	0.444744	0.527692
Erythroid.cells	KCNIP3	-1.10335	0.193763	-1.29835	0.197545	-4.70401	0.492537	0.572964
Erythroid.cells	TMEM42	0.475704	2.617603	1.298344	0.197546	-4.90264	0.453028	0.535642

Erythroid.cells	GM16158	0.924368	0.586451	1.298212	0.197591	-4.7246	0.485908	0.566846
Erythroid.cells	PJA2	0.380137	4.695864	1.298048	0.197647	-5.24066	0.421798	0.505701
Erythroid.cells	GM5431	-1.04807	0.742737	-1.29777	0.197742	-4.71945	0.483423	0.564622
Erythroid.cells	PBXIP1	-0.40698	4.578633	-1.29759	0.197803	-5.22703	0.423585	0.507541
Erythroid.cells	RGS16	-0.84837	1.351563	-1.29755	0.197817	-4.79619	0.473372	0.555212
Erythroid.cells	WWOX	0.239223	6.961551	1.297043	0.197991	-5.7467	0.390605	0.475029
Erythroid.cells	TMEM106E	0.33653	4.565283	1.296832	0.198063	-5.25723	0.424041	0.507863
Erythroid.cells	POLR2H	-0.3094	4.512761	-1.29681	0.198069	-5.25057	0.424807	0.508606
Erythroid.cells	NATD1	-0.53657	3.25628	-1.29661	0.198138	-5.02171	0.443577	0.526779
Erythroid.cells	PPIE	-0.29901	4.586932	-1.29643	0.1982	-5.29128	0.423735	0.50769
Erythroid.cells	FICD	-0.88824	0.917558	-1.29642	0.198204	-4.68976	0.480822	0.562242
Erythroid.cells	SSNA1	-0.23384	5.922585	-1.29611	0.19831	-5.57952	0.404842	0.489174
Erythroid.cells	UGDH	0.389171	4.250685	1.296044	0.198333	-5.17157	0.428752	0.51249
Erythroid.cells	GPAT4	0.300565	4.264538	1.295805	0.198415	-5.192	0.428632	0.512372
Erythroid.cells	STAT4	0.386323	5.177658	1.295391	0.198557	-5.60708	0.415605	0.499691
Erythroid.cells	ZBTB7B	0.663339	2.473173	1.295216	0.198617	-4.88863	0.456128	0.538785
Erythroid.cells	GPATCH3	-0.51166	3.369567	-1.29513	0.198648	-4.9905	0.442261	0.525515
Erythroid.cells	GINS2	-0.57833	3.493231	-1.29475	0.198777	-5.03821	0.440525	0.523834
Erythroid.cells	CD7	-0.48456	4.199176	-1.29455	0.198845	-5.63473	0.429957	0.513729
Erythroid.cells	GM48293	-0.99412	-0.15995	-1.29448	0.198871	-4.65237	0.49967	0.579971
Erythroid.cells	NBEA	0.660586	2.602645	1.294433	0.198887	-4.89016	0.454244	0.53713
Erythroid.cells	SOCS4	-0.31414	4.684801	-1.29425	0.19895	-5.23428	0.422884	0.506942
Erythroid.cells	MGP	0.992211	0.463003	1.294103	0.199	-4.75471	0.4891	0.570162
Erythroid.cells	GRSF1	0.239075	5.185543	1.29382	0.199097	-5.39409	0.415688	0.500013
Erythroid.cells	GM10382	0.989508	0.439288	1.293687	0.199143	-4.66014	0.4895	0.570574
Erythroid.cells	SDAD1	-0.35017	4.473042	-1.29367	0.199149	-5.27047	0.425984	0.51003
Erythroid.cells	LCLAT1	-0.45858	3.99918	-1.29355	0.199192	-5.08704	0.432979	0.516851
Erythroid.cells	LST1	0.411726	5.23223	1.293426	0.199233	-5.42737	0.415023	0.499433
Erythroid.cells	GPR155	-0.56357	3.05644	-1.29335	0.19926	-4.9828	0.447256	0.530613
Erythroid.cells	FAM160B2	-0.41325	3.264944	-1.2927	0.199484	-5.02501	0.444373	0.527841
Erythroid.cells	MGLL	0.554609	3.495334	1.29262	0.19951	-5.09906	0.440863	0.524475
Erythroid.cells	NOP9	-0.32423	4.555827	-1.29256	0.199532	-5.23399	0.425077	0.509242
Erythroid.cells	A930037H(	-0.69441	3.242306	-1.29224	0.199641	-4.98808	0.444721	0.528306
Erythroid.cells	DHX34	-0.60656	2.203437	-1.2921	0.19969	-4.83484	0.460926	0.543832
Erythroid.cells	TMUB1	0.418025	3.138278	1.291912	0.199754	-4.95241	0.446317	0.529921
Erythroid.cells	APOBEC1	-0.36974	6.087599	-1.29181	0.199789	-5.65014	0.403318	0.488154
Erythroid.cells	LANCL2	0.519589	2.807486	1.291744	0.199812	-4.9581	0.451431	0.53482
Erythroid.cells	FAM110A	0.48015	3.760358	1.291712	0.199824	-5.07118	0.436863	0.520826
Erythroid.cells	TSPO	0.28079	7.399271	1.291586	0.199867	-5.98606	0.385622	0.470663
Erythroid.cells	AI480526	0.648553	2.064128	1.291337	0.199953	-4.82656	0.463145	0.546036
Erythroid.cells	MAP3K14	0.330293	5.189544	1.291238	0.199987	-5.46711	0.415929	0.500553
Erythroid.cells	SLC25A3	-0.16697	7.921066	-1.29082	0.200133	-6.00535	0.378813	0.464109
Erythroid.cells	TBC1D20	-0.27976	5.789234	-1.29074	0.200157	-5.50119	0.407462	0.492532
Erythroid.cells	SGO2A	0.688069	3.063462	1.290495	0.200244	-5.03834	0.447468	0.531426
Erythroid.cells	PRDM10	-0.31329	5.213796	-1.29041	0.200273	-5.40434	0.415583	0.500553
Erythroid.cells	NF2	-0.34193	4.781261	-1.29035	0.200293	-5.2615	0.4218	0.506641
Erythroid.cells	LSM14B	-0.37489	3.604671	-1.29019	0.200348	-5.06507	0.439209	0.523534

Erythroid.cells	GOT2	0.196394	6.235083	1.290186	0.20035	-5.78629	0.401286	0.486568
Erythroid.cells	PSMG3	-0.47179	3.202758	-1.29009	0.200383	-5.03855	0.445327	0.529422
Erythroid.cells	LRRC63	-0.93276	0.544146	-1.28998	0.200421	-4.74465	0.488081	0.570033
Erythroid.cells	SUMO2	-0.14217	8.656817	-1.28992	0.200444	-6.13292	0.369431	0.454851
Erythroid.cells	TM9SF2	-0.16155	6.683958	-1.28991	0.200444	-5.70009	0.395167	0.480526
Erythroid.cells	RIF1	0.28429	5.435862	1.289908	0.200446	-5.49253	0.412429	0.497516
Erythroid.cells	QRFP	0.968292	-0.61178	1.28958	0.20056	-4.67161	0.508081	0.588622
Erythroid.cells	GAS7	-0.39715	6.417925	-1.28951	0.200582	-5.68191	0.398879	0.484104
Erythroid.cells	POLG2	0.315558	5.080463	1.289332	0.200646	-5.3539	0.417633	0.502526
Erythroid.cells	CCZ1	-0.21658	6.158644	-1.28876	0.200843	-5.59187	0.402754	0.487899
Erythroid.cells	SEMA6B	0.801515	1.154578	1.28868	0.200871	-4.75013	0.4784	0.560792
Erythroid.cells	9530052E0	-0.79197	1.000649	-1.28849	0.200935	-4.69815	0.480997	0.563259
Erythroid.cells	TOR1A	-0.31162	4.653633	-1.28808	0.201077	-5.27362	0.424342	0.508942
Erythroid.cells	PEX7	0.30632	4.45855	1.287462	0.201293	-5.23096	0.427505	0.511921
Erythroid.cells	BTBD9	-0.19435	8.508871	-1.28741	0.20131	-6.04907	0.372171	0.457353
Erythroid.cells	FANCE	0.524488	2.916052	1.286639	0.201579	-4.92961	0.45115	0.534597
Erythroid.cells	EPN2	0.919764	1.664749	1.286584	0.201598	-4.76859	0.471033	0.553527
Erythroid.cells	9330151L1	-0.77422	0.96571	-1.28658	0.201598	-4.71632	0.482532	0.564384
Erythroid.cells	ACTR10	0.180006	6.186686	1.286466	0.201639	-5.6518	0.403206	0.488089
Erythroid.cells	PDIA4	-0.24518	5.653573	-1.28614	0.201754	-5.50096	0.410644	0.495559
Erythroid.cells	HMGCS2	0.496357	4.456909	1.286104	0.201765	-5.59375	0.427862	0.512346
Erythroid.cells	1110008P1	0.302587	5.226277	1.285959	0.201815	-5.37306	0.416707	0.501503
Erythroid.cells	PRRC2A	-0.25297	5.517966	-1.2859	0.201834	-5.42631	0.412558	0.497479
Erythroid.cells	FADD	-0.66787	2.421277	-1.28583	0.201861	-4.88347	0.458912	0.542286
Erythroid.cells	MEAK7	-0.95583	0.666682	-1.28561	0.201935	-4.66542	0.487544	0.569421
Erythroid.cells	A930005H1	-0.39818	3.220496	-1.28558	0.201947	-5.05144	0.44645	0.530411
Erythroid.cells	SLAMF7	-0.33274	5.151332	-1.28519	0.202082	-5.63828	0.417969	0.502846
Erythroid.cells	HNRNPK	0.118656	8.860645	1.28489	0.202187	-6.19803	0.368303	0.453659
Erythroid.cells	ZFAT	-0.50702	3.828322	-1.28475	0.202234	-5.03207	0.437547	0.521939
Erythroid.cells	SHLD2	0.40107	3.853936	1.284328	0.202382	-5.15537	0.437257	0.521626
Erythroid.cells	ZFP142	-0.44283	3.207748	-1.28429	0.202394	-4.99567	0.447089	0.531091
Erythroid.cells	ARPC5	0.146359	8.144041	1.284183	0.202433	-6.03156	0.377495	0.462937
Erythroid.cells	CHMP1A	0.309461	5.305048	1.284126	0.202453	-5.40306	0.415994	0.501043
Erythroid.cells	COL23A1	-0.89384	1.376373	-1.28389	0.202534	-4.75271	0.476228	0.558992
Erythroid.cells	XKR5	-0.8704	0.190356	-1.28387	0.202544	-4.65961	0.496128	0.577722
Erythroid.cells	ANKRD13C	0.25015	5.868682	1.283531	0.20266	-5.59958	0.408046	0.493384
Erythroid.cells	CENPJ	-0.43729	3.331867	-1.2835	0.202669	-5.03685	0.445199	0.529494
Erythroid.cells	SLC31A2	-0.5742	3.169586	-1.28316	0.20279	-5.01422	0.447693	0.532049
Erythroid.cells	PGLYRP1	0.449841	4.959034	1.282858	0.202895	-5.49154	0.420978	0.506218
Erythroid.cells	PUM3	0.321239	4.640352	1.282848	0.202898	-5.2351	0.425611	0.510729
Erythroid.cells	SF3B1	-0.1312	8.256825	-1.28279	0.202917	-6.06157	0.37606	0.461845
Erythroid.cells	TXNDC11	-0.2543	5.714713	-1.28271	0.202946	-5.54219	0.410205	0.49576
Erythroid.cells	CD3E	-0.43298	3.278256	-1.28259	0.202989	-5.59561	0.446021	0.530578
Erythroid.cells	PVT1	0.499232	5.282726	1.282087	0.203164	-5.43821	0.416328	0.501921
Erythroid.cells	GM20712	1.184325	0.063822	1.281815	0.203259	-4.65152	0.498309	0.580377
Erythroid.cells	ACOT12	0.767075	0.798615	1.281647	0.203317	-4.79445	0.485827	0.568656
Erythroid.cells	RNF213	-0.49871	6.151249	-1.28164	0.203318	-5.62602	0.404115	0.489978

Erythroid.cells	GM17092	-0.65211	2.1249	-1.28163	0.203324	-4.82817	0.4641	0.548077
Erythroid.cells	GPBP1L1	0.231456	5.913267	1.281565	0.203346	-5.62262	0.407423	0.493235
Erythroid.cells	CDC26	0.204114	5.383294	1.281565	0.203346	-5.46187	0.414894	0.500567
Erythroid.cells	GM15787	0.643344	2.641968	1.281543	0.203354	-4.90182	0.455903	0.540252
Erythroid.cells	CLIP2	-0.45261	2.886513	-1.28153	0.20336	-4.96701	0.452078	0.53659
Erythroid.cells	GOLGA4	0.251605	5.30004	1.281515	0.203364	-5.40843	0.41608	0.501728
Erythroid.cells	LYST	0.248966	6.729745	1.281476	0.203377	-5.74885	0.396192	0.48215
Erythroid.cells	CDC14B	0.518332	3.454363	1.280605	0.203682	-5.06522	0.44379	0.528499
Erythroid.cells	TEX264	-0.30975	4.497588	-1.28052	0.203711	-5.22744	0.428151	0.5134
Erythroid.cells	SBDS	0.216115	5.561152	1.280361	0.203767	-5.5194	0.412803	0.49845
Erythroid.cells	SSPN	-0.88934	-0.41075	-1.28026	0.203801	-4.66279	0.507073	0.58849
Erythroid.cells	LIMD1	-0.20122	6.016318	-1.28014	0.203844	-5.65257	0.406413	0.492173
Erythroid.cells	ADGRL3	0.860558	3.47827	1.280117	0.203853	-5.19491	0.443425	0.528204
Erythroid.cells	PWWP3A	-0.36824	3.715755	-1.27984	0.203948	-5.13273	0.439872	0.524738
Erythroid.cells	RSRP1	-0.24247	6.653226	-1.27978	0.203971	-5.69826	0.397697	0.483535
Erythroid.cells	CTDSPL	0.584533	3.831098	1.279669	0.204009	-5.13397	0.43813	0.523076
Erythroid.cells	B3GAT3	-0.37131	4.307364	-1.27946	0.204081	-5.22216	0.431073	0.516267
Erythroid.cells	SOGA1	-0.54706	3.807296	-1.2793	0.20414	-5.03214	0.438581	0.523597
Erythroid.cells	MKLN1OS	0.951539	0.018205	1.27887	0.204289	-4.68627	0.500035	0.58184
Erythroid.cells	MTPAP	0.390308	4.284578	1.278675	0.204358	-5.16868	0.431658	0.516784
Erythroid.cells	PPP1R3E	0.687256	1.031561	1.278511	0.204415	-4.71811	0.482845	0.565783
Erythroid.cells	UNC13B	0.953958	0.758993	1.278507	0.204417	-4.69298	0.487409	0.570083
Erythroid.cells	TBC1D10B	-0.29974	5.147294	-1.27803	0.204583	-5.35805	0.419198	0.504761
Erythroid.cells	TMEM198[illegible]	-0.86039	0.786307	-1.27803	0.204585	-4.69791	0.487115	0.569861
Erythroid.cells	CSNK1A1	0.12966	7.684452	1.277864	0.204642	-5.99096	0.384328	0.470357
Erythroid.cells	MOCS3	-0.83514	1.446277	-1.2778	0.204665	-4.73988	0.476147	0.559528
Erythroid.cells	TUSC2	0.53796	3.017242	1.277344	0.204825	-4.92012	0.451049	0.535694
Erythroid.cells	NTN4	1.025475	0.706687	1.277299	0.204841	-4.70042	0.488456	0.571232
Erythroid.cells	SLCO5A1	0.730706	-0.20706	1.27728	0.204847	-4.6898	0.504111	0.585907
Erythroid.cells	SPATA21	-0.52268	3.397572	-1.27727	0.204851	-5.05589	0.445181	0.530055
Erythroid.cells	SCARB2	0.260476	6.538743	1.27712	0.204904	-5.65575	0.399677	0.485709
Erythroid.cells	AXIN2	-0.85983	-0.02376	-1.27691	0.204976	-4.69211	0.50093	0.582993
Erythroid.cells	GAS2L1	0.578487	1.765951	1.276829	0.205006	-4.93344	0.470926	0.554722
Erythroid.cells	TUT1	0.458237	2.638669	1.276725	0.205042	-4.89729	0.456969	0.541452
Erythroid.cells	B230118H[illegible]	-0.55703	2.933488	-1.27664	0.205073	-4.95039	0.452352	0.537034
Erythroid.cells	SNTB1	0.496007	4.264888	1.276565	0.205099	-5.44445	0.432097	0.5175
Erythroid.cells	KALRN	0.695498	2.750236	1.276365	0.205169	-4.94047	0.455221	0.539813
Erythroid.cells	ATF3	0.464781	6.1843	1.276285	0.205197	-5.73148	0.404561	0.490645
Erythroid.cells	RHOG	-0.26852	7.243088	-1.27618	0.205232	-5.78756	0.390169	0.476433
Erythroid.cells	EIF1AX	-0.23322	6.060349	-1.27572	0.205394	-5.64506	0.406516	0.492582
Erythroid.cells	ARHGAP15	0.18062	9.872031	1.2756	0.205438	-6.34993	0.356951	0.443032
Erythroid.cells	GM36862	-1.06707	0.340355	-1.27509	0.205618	-4.6415	0.495248	0.577579
Erythroid.cells	ZC3H7A	-0.19539	6.883008	-1.27501	0.205644	-5.76368	0.395457	0.481464
Erythroid.cells	IER3	0.434272	6.13903	1.274773	0.205729	-5.74731	0.405737	0.49154
Erythroid.cells	BMI1	-0.45026	3.545427	-1.27461	0.205788	-5.03823	0.443558	0.528342
Erythroid.cells	SUV39H1	-0.4081	3.622039	-1.27445	0.205845	-5.07291	0.442418	0.527278
Erythroid.cells	KLHL13	1.116938	0.360663	1.274033	0.205991	-4.68091	0.495312	0.577512

Erythroid.cells	TBC1D2B	0.382482	4.091379	1.273899	0.206038	-5.26369	0.435556	0.520676
Erythroid.cells	UTP18	0.263259	5.338906	1.273414	0.206209	-5.46536	0.417536	0.503152
Erythroid.cells	ACRBP	-0.7733	1.566441	-1.27317	0.206293	-4.74015	0.475413	0.558952
Erythroid.cells	MED21	0.355283	5.21874	1.27303	0.206344	-5.39336	0.419261	0.504994
Erythroid.cells	PADI4	1.011062	1.890862	1.273014	0.20635	-4.81262	0.470123	0.553991
Erythroid.cells	JHY	-1.14834	-0.37714	-1.27293	0.206379	-4.66375	0.508402	0.59004
Erythroid.cells	PCCB	0.466174	3.081613	1.27258	0.206504	-5.03086	0.451398	0.536155
Erythroid.cells	RFC1	-0.29234	5.999025	-1.27231	0.206598	-5.57598	0.408437	0.494336
Erythroid.cells	4930505N2	0.701086	1.214516	1.272209	0.206635	-4.79799	0.481509	0.564725
Erythroid.cells	PHLDB1	0.660998	1.355652	1.271948	0.206727	-4.8705	0.479282	0.562592
Erythroid.cells	RUSC1	0.48121	3.288288	1.27177	0.20679	-5.05708	0.448414	0.533218
Erythroid.cells	IFIT3B	-1.02955	1.43036	-1.2716	0.206852	-4.82478	0.478066	0.561504
Erythroid.cells	DNMT1	-0.31166	5.753517	-1.27155	0.206867	-5.59951	0.412	0.497882
Erythroid.cells	G530011O(	-0.888	1.80368	-1.27141	0.206918	-4.81882	0.471966	0.55573
Erythroid.cells	SRP54A	0.376941	3.599318	1.270849	0.207116	-5.08509	0.443986	0.528861
Erythroid.cells	CLMN	-0.84554	0.334512	-1.27066	0.207181	-4.77375	0.496933	0.579176
Erythroid.cells	CPSF7	-0.22236	5.801172	-1.27007	0.207391	-5.53398	0.411927	0.497774
Erythroid.cells	SRPK2	-0.23605	7.096447	-1.26996	0.20743	-5.79855	0.394064	0.480144
Erythroid.cells	GM26930	-1.18833	0.305585	-1.26996	0.207432	-4.63685	0.497714	0.579988
Erythroid.cells	CUTC	0.371457	3.519969	1.268613	0.207909	-5.12505	0.446317	0.531063
Erythroid.cells	TENT4B	-0.21451	6.368937	-1.26857	0.207926	-5.70325	0.404734	0.490583
Erythroid.cells	METTL18	0.790855	1.271701	1.268313	0.208016	-4.74756	0.482273	0.565471
Erythroid.cells	ERCC6L2	0.480697	3.372895	1.267998	0.208128	-5.01818	0.448582	0.53343
Erythroid.cells	NPRL2	-0.48439	2.790262	-1.26795	0.208144	-4.93847	0.457673	0.542148
Erythroid.cells	BAZ1A	-0.18619	7.694958	-1.26794	0.208147	-5.96459	0.386797	0.472962
Erythroid.cells	FIZ1	-0.2801	4.553694	-1.26791	0.208159	-5.27875	0.430732	0.516184
Erythroid.cells	RAD9A	-0.7842	2.338849	-1.26789	0.208165	-4.79909	0.464847	0.548998
Erythroid.cells	CEP63	-0.35943	4.291786	-1.26768	0.208241	-5.23053	0.434692	0.520012
Erythroid.cells	DIP2C	0.445884	6.172429	1.267459	0.208319	-5.55954	0.407596	0.493601
Erythroid.cells	CREBZF	0.296905	4.715073	1.267257	0.208391	-5.30725	0.428545	0.514073
Erythroid.cells	COL5A1	0.92227	0.493409	1.266778	0.208562	-4.73579	0.495928	0.578345
Erythroid.cells	CDC25B	0.343168	4.400381	1.266432	0.208685	-5.5809	0.433622	0.518947
Erythroid.cells	HEATR3	-0.37822	4.273336	-1.26632	0.208725	-5.2138	0.435519	0.520785
Erythroid.cells	LOXL3	-1.1436	0.451305	-1.2659	0.208874	-4.66288	0.497078	0.579228
Erythroid.cells	ZFP760	0.78525	1.032725	1.265521	0.20901	-4.70789	0.487415	0.570101
Erythroid.cells	GDI2	0.138157	8.891579	1.26535	0.209071	-6.20108	0.372243	0.458138
Erythroid.cells	CAMK2N1	0.762683	1.714702	1.265041	0.209181	-4.95102	0.476266	0.559566
Erythroid.cells	MAGI3	-0.31222	6.598022	-1.26492	0.209224	-5.75158	0.402678	0.488535
Erythroid.cells	OGT	0.201053	6.649131	1.264216	0.209475	-5.74025	0.402373	0.488062
Erythroid.cells	NXN	-0.40657	5.325793	-1.26378	0.209632	-5.34327	0.421212	0.506498
Erythroid.cells	DEPP1	0.845717	1.043364	1.263722	0.209652	-4.81168	0.488113	0.570601
Erythroid.cells	DDI2	0.2479	5.943537	1.26352	0.209724	-5.59229	0.412425	0.497923
Erythroid.cells	ASB2	0.711988	2.906427	1.263418	0.20976	-5.00355	0.457789	0.541865
Erythroid.cells	CALU	0.297074	4.640586	1.262978	0.209918	-5.29014	0.431389	0.516491
Erythroid.cells	ECI1	0.297412	3.920606	1.262933	0.209934	-5.36626	0.442198	0.526946
Erythroid.cells	ZBTB20	0.21296	8.246328	1.262808	0.209979	-6.04411	0.381305	0.467202
Erythroid.cells	DHRS7	-0.54889	4.016547	-1.26267	0.210028	-5.13128	0.440741	0.525656

Erythroid.cells	NECAP1	-0.28732	4.774239	-1.26253	0.210077	-5.32474	0.429414	0.514695
Erythroid.cells	FAIM	-0.38195	3.865848	-1.26253	0.21008	-5.16038	0.443031	0.527875
Erythroid.cells	CYB561D1	-0.72812	1.785161	-1.26224	0.210182	-4.81236	0.47608	0.559506
Erythroid.cells	PRMT5	-0.43809	3.483009	-1.26148	0.210453	-5.06449	0.449373	0.533877
Erythroid.cells	CCDC84	0.558336	2.649641	1.26145	0.210465	-4.90334	0.462456	0.5464
Erythroid.cells	5830411N(	0.84175	-1.25694	1.261419	0.210476	-4.70395	0.529201	0.609008
Erythroid.cells	NUP210	0.266348	5.041054	1.261092	0.210593	-5.49911	0.42609	0.511432
Erythroid.cells	FBXO5	-0.4747	4.811103	-1.26092	0.210655	-5.53161	0.429501	0.514749
Erythroid.cells	1700001K1	0.887737	1.052633	1.260525	0.210797	-4.75656	0.489074	0.571717
Erythroid.cells	SIGLEC1	1.078777	0.449661	1.260057	0.210965	-4.74995	0.499652	0.581492
Erythroid.cells	CPM	-0.44407	4.160192	-1.25984	0.211041	-5.40663	0.439744	0.524535
Erythroid.cells	EOMES	-0.94916	-0.77158	-1.25956	0.211143	-4.75638	0.52132	0.601735
Erythroid.cells	ZFP949	0.527416	2.594224	1.259521	0.211157	-4.96493	0.464155	0.548042
Erythroid.cells	CAPN1	0.440448	4.246316	1.259089	0.211312	-5.17661	0.43873	0.523535
Erythroid.cells	PEPD	0.277888	5.308275	1.258305	0.211594	-5.48761	0.423346	0.508579
Erythroid.cells	MSRB3	0.634866	2.604277	1.258284	0.211602	-4.96432	0.464597	0.548329
Erythroid.cells	SAP130	-0.27131	5.619105	-1.25826	0.211609	-5.50749	0.418857	0.504196
Erythroid.cells	TWNK	0.53515	2.886068	1.257735	0.211799	-4.99806	0.460426	0.544365
Erythroid.cells	CCDC191	0.600826	1.993228	1.257535	0.211871	-4.88818	0.474875	0.558129
Erythroid.cells	SERPINA1A	0.448575	8.012506	1.257276	0.211965	-6.27816	0.386342	0.472126
Erythroid.cells	NUDT19	-0.34118	4.544356	-1.25696	0.21208	-5.32706	0.435196	0.520027
Erythroid.cells	ALDH2	0.225693	6.861181	1.256704	0.212171	-5.87772	0.402058	0.487624
Erythroid.cells	ZFP574	-0.44786	3.401172	-1.25652	0.212238	-5.05326	0.452755	0.537024
Erythroid.cells	PSMA2	0.180713	7.21693	1.256443	0.212265	-5.94292	0.397208	0.482939
Erythroid.cells	WDR33	-0.14988	7.186641	-1.25631	0.212312	-5.90001	0.397624	0.483386
Erythroid.cells	EXOSC7	0.298272	4.629702	1.255864	0.212474	-5.36448	0.434167	0.519184
Erythroid.cells	SNRPB	-0.18303	7.300883	-1.25575	0.212514	-5.92034	0.396197	0.482023
Erythroid.cells	ZZZ3	-0.26134	5.884403	-1.2557	0.212531	-5.58425	0.415873	0.501435
Erythroid.cells	CNNM3	0.484551	3.157652	1.255637	0.212556	-4.98574	0.456714	0.540975
Erythroid.cells	ANKRD10	-0.24066	5.371393	-1.25532	0.21267	-5.49454	0.423391	0.508703
Erythroid.cells	BC024386	0.694451	1.614053	1.254804	0.212857	-4.93861	0.482148	0.564993
Erythroid.cells	CLEC4A3	0.633171	3.297848	1.254486	0.212972	-5.24996	0.455089	0.539197
Erythroid.cells	RPTOR	-0.21589	6.294365	-1.2544	0.213003	-5.69853	0.410592	0.496055
Erythroid.cells	GM11099	-1.14195	-0.70994	-1.25427	0.21305	-4.65045	0.522569	0.60281
Erythroid.cells	CYP1A2	-0.70551	1.633956	-1.25405	0.21313	-4.92416	0.481992	0.564844
Erythroid.cells	SEC61G	0.158824	10.02398	1.253974	0.213157	-6.65537	0.361595	0.447276
Erythroid.cells	PLXNA4	1.186774	2.166702	1.253519	0.213321	-4.95106	0.473441	0.556743
Erythroid.cells	GPT2	0.695079	1.738624	1.25332	0.213394	-4.87533	0.480481	0.563451
Erythroid.cells	ROMO1	0.182131	6.595619	1.253296	0.213402	-5.81609	0.406611	0.49222
Erythroid.cells	LZTR1	0.510673	2.612289	1.253057	0.213489	-4.94812	0.466226	0.550052
Erythroid.cells	ENSA	-0.25256	5.388151	-1.25297	0.213519	-5.47767	0.423784	0.509158
Erythroid.cells	SLC20A2	0.406658	4.036306	1.252902	0.213545	-5.17938	0.443924	0.528684
Erythroid.cells	PFKL	0.305902	5.330071	1.252818	0.213575	-5.47937	0.42463	0.509986
Erythroid.cells	SEPSECS	0.441404	3.143326	1.252461	0.213705	-5.12395	0.457776	0.542054
Erythroid.cells	MAPK4	0.995185	0.274036	1.252369	0.213738	-4.67423	0.50539	0.587076
Erythroid.cells	INSIG1	-0.3011	5.457849	-1.25213	0.213826	-5.49398	0.422772	0.508292
Erythroid.cells	KRT18	0.522938	3.557982	1.252056	0.213851	-5.42497	0.451289	0.535904

Erythroid.cells	NAA25	-0.46153	3.927641	-1.25195	0.21389	-5.12702	0.445586	0.530463
Erythroid.cells	KCNC3	-0.83365	0.561324	-1.25189	0.213911	-4.73093	0.500403	0.582533
Erythroid.cells	RNF166	0.246937	5.468282	1.251874	0.213918	-5.46249	0.422621	0.508209
Erythroid.cells	RAD54L2	-0.31668	4.552757	-1.25176	0.213959	-5.25909	0.436114	0.52135
Erythroid.cells	ZSCAN22	0.847626	1.316109	1.251726	0.213971	-4.7476	0.487536	0.570485
Erythroid.cells	KRTCAP3	-0.79674	0.893962	-1.25145	0.214072	-4.72846	0.494758	0.577308
Erythroid.cells	GM43112	0.723325	0.075373	1.251399	0.21409	-4.79616	0.508939	0.590596
Erythroid.cells	MITD1	-0.39147	4.433197	-1.25126	0.214139	-5.25727	0.437979	0.52329
Erythroid.cells	PHF7	0.470407	3.28527	1.251106	0.214196	-5.03676	0.455643	0.540278
Erythroid.cells	2610307P1	0.546938	4.550996	1.250734	0.214331	-5.42444	0.43629	0.521676
Erythroid.cells	PTPRM	0.569052	5.186994	1.250487	0.214421	-5.47023	0.426866	0.512596
Erythroid.cells	ENDOU	-0.94257	0.913658	-1.25043	0.214443	-4.74042	0.494524	0.577323
Erythroid.cells	ACAD12	-0.9175	0.714351	-1.25035	0.21447	-4.70836	0.497938	0.580535
Erythroid.cells	PDE3A	1.08559	0.411945	1.25022	0.214518	-4.75289	0.503163	0.585431
Erythroid.cells	PICK1	0.664832	1.538716	1.250151	0.214543	-4.85427	0.483973	0.56739
Erythroid.cells	FKRP	-0.56017	2.332289	-1.2501	0.214561	-4.8933	0.470909	0.555008
Erythroid.cells	SIGIRR	0.468203	2.175284	1.25004	0.214583	-4.96806	0.473464	0.557437
Erythroid.cells	SMIM27	0.371484	3.590898	1.249926	0.214625	-5.13826	0.450933	0.535911
Erythroid.cells	GM15892	-0.53779	3.443855	-1.24946	0.214795	-5.15201	0.453485	0.538305
Erythroid.cells	SLAIN2	-0.23549	5.670672	-1.24925	0.214871	-5.49698	0.420149	0.505998
Erythroid.cells	RNF24	-0.38669	4.233052	-1.24912	0.214917	-5.22178	0.441408	0.526716
Erythroid.cells	ZMYM3	-0.65862	2.31007	-1.24874	0.215058	-4.87119	0.471827	0.555717
Erythroid.cells	D330023K1	0.703777	2.22594	1.247724	0.215427	-4.84438	0.47391	0.557425
Erythroid.cells	PLCL2	-0.2232	6.985417	-1.24753	0.215496	-5.83462	0.402479	0.48836
Erythroid.cells	FBXO34	-0.25455	5.78838	-1.24743	0.215535	-5.54623	0.419314	0.504946
Erythroid.cells	TMEM87A	-0.27894	5.236916	-1.24694	0.215711	-5.45512	0.427498	0.512973
Erythroid.cells	TGTP1	-1.31767	-0.20272	-1.24666	0.215815	-4.6838	0.515599	0.596877
Erythroid.cells	TFPI	0.464589	3.468648	1.246635	0.215824	-5.07899	0.454282	0.538981
Erythroid.cells	BNIP1	-0.45642	3.526643	-1.24659	0.215842	-5.10794	0.453376	0.538111
Erythroid.cells	CYP2C37	-0.58935	1.772648	-1.24657	0.215848	-4.97949	0.481618	0.565015
Erythroid.cells	HOXB4	0.660276	1.361865	1.246174	0.215992	-4.84613	0.488494	0.571664
Erythroid.cells	QARS	-0.33906	4.552856	-1.24606	0.216033	-5.29943	0.43766	0.523155
Erythroid.cells	ACAD10	0.722728	1.501708	1.246025	0.216047	-4.80761	0.486143	0.569501
Erythroid.cells	D430040D1	1.094906	-0.34621	1.245935	0.21608	-4.65454	0.518163	0.599486
Erythroid.cells	CPT2	0.512102	2.829472	1.245896	0.216094	-5.09797	0.464393	0.548863
Erythroid.cells	LMO7	-1.15933	0.761873	-1.24583	0.216118	-4.86298	0.498714	0.581348
Erythroid.cells	CACNB4	-0.9123	1.615307	-1.24551	0.216234	-4.83687	0.484328	0.567847
Erythroid.cells	MTREX	-0.23353	5.477446	-1.24548	0.216246	-5.49647	0.424063	0.509988
Erythroid.cells	CDK9	-0.25192	5.578202	-1.24532	0.216305	-5.5196	0.422627	0.508611
Erythroid.cells	GM550	0.870408	0.859238	1.245046	0.216405	-4.72902	0.497173	0.580027
Erythroid.cells	HEATR6	-0.32043	4.812778	-1.24499	0.216425	-5.28292	0.433886	0.51963
Erythroid.cells	ARNTL	-0.32352	5.38742	-1.24485	0.216477	-5.6004	0.425412	0.511466
Erythroid.cells	ADD1	0.231254	5.795836	1.244814	0.21649	-5.63186	0.419496	0.505683
Erythroid.cells	ACLY	0.199223	6.383195	1.244668	0.216543	-5.67123	0.411157	0.497517
Erythroid.cells	ALG2	0.45881	3.062397	1.244483	0.216611	-4.94097	0.460872	0.545838
Erythroid.cells	GDF11	-0.64306	1.639394	-1.24422	0.216707	-4.92814	0.484153	0.567996
Erythroid.cells	MAPK13	0.698746	0.83712	1.242961	0.217168	-4.91428	0.4987	0.581222

Erythroid.cells	ZSCAN20	-1.00548	1.073033	-1.24232	0.217402	-4.715	0.495087	0.577708
Erythroid.cells	PAFAH2	0.98353	0.619176	1.241779	0.217602	-4.71597	0.503179	0.585214
Erythroid.cells	ZFP90	-0.68896	2.283533	-1.24162	0.217659	-4.86148	0.475103	0.558802
Erythroid.cells	VEGFB	0.412698	3.802219	1.241476	0.217713	-5.14953	0.450895	0.535723
Erythroid.cells	GM26771	-0.90185	-0.2163	-1.24144	0.217728	-4.76176	0.517906	0.599034
Erythroid.cells	CTDSP2	0.261202	4.801724	1.241226	0.217805	-5.40422	0.435669	0.521082
Erythroid.cells	OGFOD1	-0.40472	3.611469	-1.24108	0.217858	-5.1315	0.453864	0.538707
Erythroid.cells	SH2B2	-0.41587	4.550186	-1.241	0.217887	-5.24887	0.439449	0.524835
Erythroid.cells	POU3F1	-1.1244	0.061626	-1.24092	0.217919	-4.6948	0.512959	0.5946
Erythroid.cells	GM26982	-0.55937	1.652146	-1.2409	0.217926	-4.84556	0.48556	0.568903
Erythroid.cells	D230025D1	-0.32968	4.57771	-1.24074	0.217983	-5.32571	0.439058	0.524433
Erythroid.cells	NUDT3	0.24591	5.213023	1.240514	0.218067	-5.43182	0.429662	0.515389
Erythroid.cells	IGFBP4	0.538443	5.679023	1.240174	0.218192	-5.75551	0.422964	0.508859
Erythroid.cells	NFKBIB	-0.28665	5.622292	-1.24001	0.218251	-5.55852	0.423787	0.509757
Erythroid.cells	ASAP1	0.206839	7.200045	1.239651	0.218385	-5.93372	0.401507	0.487932
Erythroid.cells	FAF1	-0.18454	6.739255	-1.23962	0.218398	-5.82292	0.407882	0.494247
Erythroid.cells	AKAP1	-0.64019	1.799595	-1.23958	0.21841	-4.84864	0.483341	0.567085
Erythroid.cells	MCF2L	1.005193	0.46165	1.239364	0.21849	-4.74255	0.506179	0.588633
Erythroid.cells	STX7	-0.20666	6.176182	-1.23936	0.218492	-5.63336	0.41582	0.502118
Erythroid.cells	RUBCNL	-0.51112	3.191015	-1.23921	0.218546	-5.06183	0.460713	0.545691
Erythroid.cells	WIZ	0.428882	3.836067	1.239147	0.218571	-5.15635	0.450598	0.535983
Erythroid.cells	DNAJA4	0.467337	0.616749	1.239132	0.218576	-5.16003	0.503476	0.586175
Erythroid.cells	ZC3H8	0.552176	2.13952	1.238756	0.218715	-4.90534	0.477913	0.561984
Erythroid.cells	ELF2	-0.18598	7.39491	-1.23847	0.218819	-5.92327	0.399122	0.485661
Erythroid.cells	GALNT4	0.583413	1.622606	1.238296	0.218884	-4.83216	0.486686	0.570385
Erythroid.cells	RAD52	0.482359	3.121528	1.238116	0.218951	-5.0265	0.462227	0.547186
Erythroid.cells	EPB41L5	-0.43024	3.379051	-1.23785	0.21905	-5.23133	0.458211	0.543345
Erythroid.cells	SPACA6	-0.7833	1.094819	-1.23766	0.219119	-4.77918	0.495747	0.578994
Erythroid.cells	SVIP	0.525847	1.34626	1.237335	0.219239	-5.01726	0.491464	0.575037
Erythroid.cells	GM43063	-0.80651	0.779437	-1.23726	0.219268	-4.74581	0.501172	0.584164
Erythroid.cells	POLR2A	-0.19175	6.200996	-1.23714	0.219312	-5.65057	0.415894	0.502388
Erythroid.cells	ZFP229	-0.98639	-0.01049	-1.23701	0.21936	-4.67989	0.515028	0.597211
Erythroid.cells	SLC39A11	0.434349	4.419343	1.236903	0.219398	-5.18721	0.442107	0.528059
Erythroid.cells	A230056P1	-0.94227	0.407448	-1.23682	0.219431	-4.69112	0.507649	0.590404
Erythroid.cells	YIPF5	-0.24516	5.24625	-1.2366	0.21951	-5.45383	0.429731	0.516122
Erythroid.cells	RNPEP	0.348813	5.72187	1.23658	0.219518	-5.4983	0.422778	0.509333
Erythroid.cells	NCL	-0.23277	7.783001	-1.23654	0.219534	-6.02487	0.393998	0.480952
Erythroid.cells	SLC46A2	-0.92722	-0.09426	-1.23645	0.219565	-4.68081	0.51652	0.5988
Erythroid.cells	ERCC3	-0.36598	3.695125	-1.23645	0.219567	-5.13541	0.453255	0.538936
Erythroid.cells	SLC38A3	0.646234	1.479322	1.23624	0.219643	-4.97292	0.489284	0.573208
Erythroid.cells	SLC25A38	0.391537	3.517886	1.235676	0.219852	-5.1612	0.456434	0.541857
Erythroid.cells	ACADM	0.296593	4.767805	1.23553	0.219906	-5.58486	0.437254	0.523365
Erythroid.cells	LMNB2	0.661902	2.474409	1.235209	0.220025	-4.91223	0.473157	0.557991
Erythroid.cells	GM19265	-0.95313	0.22098	-1.23518	0.220037	-4.67845	0.511408	0.594049
Erythroid.cells	ZFP263	-0.35389	5.167548	-1.23516	0.220043	-5.4228	0.431297	0.517699
Erythroid.cells	PELO	0.418596	3.125716	1.234832	0.220165	-5.08975	0.462722	0.548159
Erythroid.cells	IL23R	-1.06108	-0.9447	-1.23472	0.220208	-4.67346	0.532486	0.613796

Erythroid.cells	MIR99AHG	0.660093	3.430837	1.234715	0.220208	-5.18302	0.457887	0.543538
Erythroid.cells	P4HA1	0.426541	5.786072	1.23423	0.220388	-5.49843	0.422478	0.509267
Erythroid.cells	1810030O(	-0.31029	4.824335	-1.23422	0.22039	-5.33495	0.43665	0.523087
Erythroid.cells	DNTT	1.158006	1.449877	1.233951	0.220491	-4.8272	0.490487	0.574625
Erythroid.cells	FAM83D	-0.53361	2.774945	-1.23392	0.220502	-4.9903	0.468585	0.553838
Erythroid.cells	CAMTA2	-0.40364	3.696678	-1.2338	0.220546	-5.12778	0.453949	0.539834
Erythroid.cells	SPRY2	-0.3288	5.158508	-1.23325	0.220751	-5.68341	0.432024	0.518444
Erythroid.cells	SUV39H2	0.568008	2.381659	1.232953	0.220862	-4.91871	0.475378	0.560124
Erythroid.cells	CARS	-0.45083	4.118134	-1.23294	0.220867	-5.19866	0.447793	0.533704
Erythroid.cells	WWP2	0.201613	6.537142	1.232639	0.220978	-5.74357	0.412178	0.498946
Erythroid.cells	ST14	-0.99987	1.167666	-1.23258	0.221001	-4.75698	0.495756	0.579349
Erythroid.cells	SHQ1	0.583755	2.60612	1.232499	0.22103	-4.94278	0.471766	0.556638
Erythroid.cells	THEMIS	0.572818	1.160853	1.232231	0.22113	-5.22641	0.495873	0.579573
Erythroid.cells	MSTO1	-0.51789	2.681663	-1.2321	0.221178	-4.98006	0.470539	0.555674
Erythroid.cells	KCNJ10	-0.81244	-0.1202	-1.23207	0.221188	-4.71047	0.518294	0.600663
Erythroid.cells	SURF6	0.386305	3.185009	1.23188	0.22126	-5.03966	0.462452	0.547998
Erythroid.cells	FUZ	0.830039	1.08456	1.231848	0.221272	-4.77438	0.49718	0.580958
Erythroid.cells	USP40	-0.46877	3.64171	-1.23178	0.221297	-5.17688	0.45524	0.54108
Erythroid.cells	RASSF5	-0.29967	5.485464	-1.2313	0.221477	-5.43476	0.42756	0.514093
Erythroid.cells	TBC1D17	0.391956	3.823308	1.231129	0.221539	-5.18984	0.452716	0.538488
Erythroid.cells	GM16675	-0.88476	1.196561	-1.2308	0.221662	-4.79282	0.495775	0.579456
Erythroid.cells	RHOB	0.518715	5.891039	1.230637	0.221723	-5.54529	0.421867	0.5086
Erythroid.cells	FLRT3	0.886371	-0.84839	1.230434	0.221798	-4.7696	0.532085	0.613508
Erythroid.cells	TMEM263	-0.3494	3.98713	-1.23032	0.221842	-5.2848	0.450364	0.536421
Erythroid.cells	ZMAT4	0.936106	0.381713	1.230269	0.22186	-4.91684	0.509957	0.593
Erythroid.cells	FOXR1	0.973552	-0.87975	1.22997	0.221971	-4.63614	0.532755	0.614227
Erythroid.cells	GM37401	0.794516	0.875344	1.229923	0.221989	-4.77086	0.501428	0.585028
Erythroid.cells	ENTHD1	-0.93751	0.023444	-1.22955	0.222126	-4.78314	0.51645	0.599156
Erythroid.cells	RNF180	0.674286	2.247716	1.229529	0.222136	-5.00016	0.478296	0.563251
Erythroid.cells	AA388235	0.847219	0.991788	1.229494	0.222148	-4.83127	0.499471	0.583261
Erythroid.cells	SDHAF2	-0.31862	4.655098	-1.22901	0.222329	-5.31881	0.440471	0.526891
Erythroid.cells	POP5	0.246469	4.525116	1.228977	0.222342	-5.38815	0.442442	0.528812
Erythroid.cells	CRP	0.581404	2.076546	1.228677	0.222453	-5.11922	0.481477	0.566221
Erythroid.cells	ADCK5	0.660671	1.510315	1.228491	0.222523	-4.85418	0.490972	0.575232
Erythroid.cells	NRAS	-0.21123	6.075126	-1.2283	0.222594	-5.63799	0.419641	0.506661
Erythroid.cells	CPNE5	0.97243	0.481176	1.228087	0.222673	-4.70984	0.508723	0.592099
Erythroid.cells	PLA1A	0.582389	1.841769	1.22802	0.222699	-5.07222	0.485391	0.57016
Erythroid.cells	CDK6	0.357069	6.322112	1.227831	0.222769	-5.71628	0.416106	0.503426
Erythroid.cells	TEK	0.704862	2.175307	1.227823	0.222772	-4.93512	0.479841	0.565013
Erythroid.cells	PTP4A3	0.202182	5.77385	1.227764	0.222794	-5.78705	0.423995	0.511181
Erythroid.cells	NSUN2	0.279246	5.0541	1.227728	0.222808	-5.44092	0.434593	0.521528
Erythroid.cells	SOAT1	0.373375	5.176779	1.227529	0.222882	-5.43693	0.432772	0.519772
Erythroid.cells	PANK2	-0.26265	5.327592	-1.22748	0.222901	-5.4537	0.430538	0.517594
Erythroid.cells	IK	-0.18831	6.288849	-1.22725	0.222987	-5.70197	0.416663	0.504013
Erythroid.cells	GALNT7	0.255012	6.060844	1.226985	0.223085	-5.72155	0.420028	0.507252
Erythroid.cells	1700047M	0.874749	-0.15234	1.226784	0.223161	-4.77587	0.520268	0.602936
Erythroid.cells	POM121	-0.28138	4.883475	-1.22642	0.223297	-5.35372	0.437572	0.524234

Erythroid.cells	2310015A1	0.643465	2.091938	1.225975	0.223463	-4.86356	0.481832	0.566776
Erythroid.cells	INF2	0.543682	3.252642	1.225897	0.223493	-5.04372	0.462944	0.548784
Erythroid.cells	GTF2F2	-0.2201	5.881638	-1.22588	0.223498	-5.61515	0.422967	0.510058
Erythroid.cells	GM17484	-0.89255	1.264186	-1.22555	0.223622	-4.78572	0.495962	0.580133
Erythroid.cells	URI1	0.243892	5.89939	1.225239	0.223739	-5.63043	0.422995	0.510079
Erythroid.cells	RASGRP1	-0.45166	4.119019	-1.22488	0.223873	-5.30428	0.449831	0.536096
Erythroid.cells	ARPP21	1.09919	1.234602	1.224227	0.224119	-4.81262	0.497175	0.581164
Erythroid.cells	URB1	-0.51396	2.176346	-1.22408	0.224173	-4.88592	0.481286	0.566213
Erythroid.cells	PRKCI	0.539764	3.113961	1.224006	0.224202	-4.99038	0.465987	0.551637
Erythroid.cells	4931413K1	-0.62228	1.943113	-1.22396	0.224219	-4.86566	0.485172	0.569898
Erythroid.cells	MYPOPOS	0.826533	1.654931	1.223739	0.224302	-4.82756	0.490018	0.574509
Erythroid.cells	TAPBP	-0.33292	6.597076	-1.22373	0.224304	-5.75587	0.413465	0.500687
Erythroid.cells	EIF2S3Y	3.366288	1.945318	1.223578	0.224362	-5.11402	0.485164	0.569972
Erythroid.cells	BBS5	-0.8337	0.624034	-1.22301	0.224576	-4.80728	0.50786	0.591384
Erythroid.cells	ATE1	0.339892	3.945361	1.222921	0.224609	-5.16554	0.452931	0.539213
Erythroid.cells	B2M	-0.31102	9.94338	-1.22291	0.224614	-6.4173	0.368962	0.456312
Erythroid.cells	HARS	-0.24037	5.114979	-1.2229	0.224618	-5.4841	0.435091	0.52194
Erythroid.cells	SGK1	-0.33309	5.355259	-1.2229	0.224619	-5.5449	0.431519	0.51846
Erythroid.cells	1810009A1	-0.5207	2.243882	-1.22277	0.224667	-4.90097	0.480264	0.565426
Erythroid.cells	RRP8	0.404849	3.730434	1.222499	0.224768	-5.15627	0.456402	0.542704
Erythroid.cells	NCKAP5LO:	-1.11073	0.041134	-1.22239	0.224809	-4.68494	0.51831	0.601349
Erythroid.cells	CANT1	-0.3903	3.891787	-1.22214	0.224902	-5.14928	0.453971	0.540387
Erythroid.cells	METTL23	-0.21231	5.675126	-1.22182	0.225023	-5.57874	0.427102	0.514302
Erythroid.cells	GTF3C5	-0.61477	2.258469	-1.22163	0.225094	-4.91913	0.480344	0.565636
Erythroid.cells	MGST1	-0.27208	7.132587	-1.22149	0.225147	-6.1851	0.406316	0.493951
Erythroid.cells	EFR3A	0.207976	5.780301	1.221467	0.225156	-5.64213	0.425564	0.512901
Erythroid.cells	MAPKBP1	-0.59128	3.047991	-1.2214	0.22518	-5.00625	0.467454	0.553437
Erythroid.cells	NKIRAS2	-0.35072	4.042562	-1.22107	0.225305	-5.20263	0.451774	0.538455
Erythroid.cells	LYRM7	-0.79199	1.247217	-1.22107	0.225305	-4.77201	0.497444	0.58199
Erythroid.cells	DESI1	-0.27591	5.729904	-1.22098	0.22534	-5.6415	0.426345	0.513793
Erythroid.cells	USP36	-0.31327	4.970109	-1.22073	0.225433	-5.36906	0.437665	0.524856
Erythroid.cells	MCRIP2	0.525802	2.015335	1.220509	0.225517	-4.9707	0.484506	0.569888
Erythroid.cells	ZFP362	0.364242	3.9339	1.220484	0.225527	-5.16634	0.453529	0.540276
Erythroid.cells	PANK3	-0.29511	4.591077	-1.22041	0.225553	-5.32095	0.443399	0.530495
Erythroid.cells	NSL1	0.598197	2.25699	1.219984	0.225715	-4.92407	0.480665	0.56629
Erythroid.cells	ZKSCAN5	0.571182	2.402977	1.21986	0.225762	-4.92885	0.478253	0.564035
Erythroid.cells	ZFP652OS	-0.87138	-0.171	-1.21983	0.225774	-4.73319	0.522672	0.605862
Erythroid.cells	CDKL3	-0.62473	3.044103	-1.21961	0.225854	-4.98209	0.467878	0.554158
Erythroid.cells	ZMYND11	-0.20034	6.466516	-1.21914	0.226035	-5.73418	0.41625	0.504026
Erythroid.cells	IARS	-0.3049	5.056202	-1.21893	0.226114	-5.4465	0.43691	0.52421
Erythroid.cells	MFSD13A	-0.63083	2.008416	-1.21883	0.226149	-4.90743	0.485218	0.570607
Erythroid.cells	SLC25A32	0.41196	3.120627	1.218373	0.226324	-5.09861	0.46724	0.553355
Erythroid.cells	TICRR	0.529504	3.009192	1.218071	0.226438	-5.12098	0.469179	0.555198
Erythroid.cells	APBB1IP	-0.19805	7.638967	-1.21791	0.226497	-5.92846	0.400312	0.488073
Erythroid.cells	COPS4	-0.22798	5.581159	-1.2176	0.226615	-5.54367	0.429651	0.516895
Erythroid.cells	GM15247	0.943909	0.995367	1.217443	0.226675	-4.80783	0.50313	0.587231
Erythroid.cells	METTL17	-0.5101	2.488504	-1.21726	0.226746	-4.97242	0.477877	0.563438

Erythroid.cells	NME6	0.571119	2.576439	1.217118	0.226798	-4.95551	0.476432	0.562125
Erythroid.cells	TSPAN13	0.243962	6.684078	1.216989	0.226847	-5.77574	0.413744	0.501417
Erythroid.cells	ZSCAN25	-0.63438	1.792602	-1.21697	0.226854	-4.89294	0.489481	0.574539
Erythroid.cells	AGMO	0.442747	3.650343	1.216807	0.226916	-5.36931	0.459169	0.545603
Erythroid.cells	FAM167B	0.65548	2.127548	1.216592	0.226997	-4.90966	0.48397	0.569257
Erythroid.cells	IGFBP7	0.368142	5.173401	1.21627	0.227119	-5.64466	0.43591	0.523093
Erythroid.cells	SEC24D	0.348421	4.470665	1.216234	0.227133	-5.31144	0.446556	0.533443
Erythroid.cells	DMAC2	0.47168	3.093682	1.215867	0.227272	-5.06375	0.468409	0.554501
Erythroid.cells	C230066G2	-0.99382	0.025289	-1.21563	0.227362	-4.71167	0.520744	0.60392
Erythroid.cells	MAEA	-0.24038	5.40999	-1.21558	0.227382	-5.50164	0.4326	0.519955
Erythroid.cells	MFN2	0.359397	3.671067	1.21522	0.227517	-5.15299	0.459377	0.545822
Erythroid.cells	BSPRY	-0.70177	0.341542	-1.21515	0.227546	-4.88042	0.515252	0.598768
Erythroid.cells	RPAP3	0.364045	3.841822	1.214831	0.227665	-5.23313	0.456834	0.543358
Erythroid.cells	DHPS	-0.29544	4.486343	-1.21469	0.22772	-5.32241	0.446842	0.533789
Erythroid.cells	PDE6D	0.319274	3.812865	1.214212	0.2279	-5.20069	0.457578	0.544054
Erythroid.cells	IL31RA	0.407588	3.109325	1.213427	0.228199	-5.24633	0.468982	0.555122
Erythroid.cells	SH2D1A	-0.60706	0.051779	-1.21339	0.228214	-4.98218	0.52114	0.604373
Erythroid.cells	5330439K0	-0.92745	0.538036	-1.21331	0.228243	-4.74485	0.512464	0.596297
Erythroid.cells	HSPA5	-0.17297	8.355671	-1.21326	0.228263	-6.15169	0.391804	0.47976
Erythroid.cells	GM14325	0.46701	2.324076	1.213142	0.228307	-4.95362	0.481842	0.56749
Erythroid.cells	CSNK2A1	0.151183	6.59773	1.213124	0.228313	-5.81678	0.416047	0.503848
Erythroid.cells	FHL3	-0.77652	2.081558	-1.21288	0.228406	-4.84243	0.485887	0.571406
Erythroid.cells	ZFP503	-0.70432	0.884395	-1.21284	0.228421	-4.84388	0.506374	0.590751
Erythroid.cells	ATG5	-0.20628	5.691584	-1.21278	0.228443	-5.55859	0.429161	0.516813
Erythroid.cells	PIGP	0.324257	4.420664	1.212704	0.228474	-5.35209	0.448297	0.535445
Erythroid.cells	RNF43	0.612314	1.563577	1.21265	0.228494	-5.06423	0.494645	0.579761
Erythroid.cells	4930589L2	-0.81628	-0.68311	-1.21208	0.22871	-4.73072	0.534764	0.617274
Erythroid.cells	BC028528	-0.31291	4.105365	-1.21205	0.228722	-5.39775	0.453375	0.540358
Erythroid.cells	RBMS3	0.606604	3.072419	1.211988	0.228746	-5.19066	0.469779	0.556116
Erythroid.cells	ECM1	0.579317	3.680244	1.211913	0.228775	-5.39063	0.460053	0.546789
Erythroid.cells	EIF3I	0.189462	6.822191	1.211666	0.228869	-5.91368	0.413128	0.501084
Erythroid.cells	GM34983	-0.88816	0.754004	-1.21139	0.228973	-4.7597	0.509113	0.593371
Erythroid.cells	UBIAD1	0.495337	1.941616	1.210819	0.229191	-4.9088	0.489044	0.57437
Erythroid.cells	WDR59	-0.493	3.072953	-1.21032	0.229384	-5.04245	0.470547	0.556735
Erythroid.cells	FYTTD1	-0.21934	5.267108	-1.21021	0.229424	-5.50464	0.436361	0.523752
Erythroid.cells	ADGRA2	-0.82605	0.687625	-1.21009	0.229471	-4.89173	0.510885	0.594905
Erythroid.cells	EXOC3L4	-0.82469	0.594811	-1.21002	0.229497	-4.84154	0.512525	0.596459
Erythroid.cells	P2RY10	0.446586	3.930309	1.209782	0.229587	-5.42834	0.456866	0.543679
Erythroid.cells	DCAF4	-0.55693	2.008291	-1.20977	0.229593	-4.89301	0.48813	0.573541
Erythroid.cells	REEP1	0.907404	0.98372	1.209723	0.22961	-4.7558	0.505691	0.590134
Erythroid.cells	REM2	-0.8767	0.836601	-1.20911	0.229845	-4.81348	0.508473	0.59279
Erythroid.cells	SNRNP70	-0.11426	7.511442	-1.20902	0.229878	-6.01226	0.404257	0.492352
Erythroid.cells	GLCCI1	-0.23644	7.348661	-1.20896	0.229901	-5.9525	0.406511	0.494588
Erythroid.cells	MCOLN1	0.452065	2.728161	1.208806	0.22996	-5.00806	0.476363	0.562524
Erythroid.cells	KLHL14	-0.508	2.717642	-1.20865	0.230019	-5.25369	0.476536	0.562735
Erythroid.cells	SSH3	-0.77727	1.522336	-1.20857	0.230052	-4.84354	0.496583	0.581753
Erythroid.cells	THRB	0.540165	3.815098	1.208505	0.230076	-5.32974	0.458868	0.545811

Erythroid.cells	GPC6	0.900597	1.763476	1.208415	0.23011	-4.99435	0.49247	0.577867
Erythroid.cells	CD2AP	0.26978	6.24029	1.20834	0.230138	-5.7353	0.422218	0.510189
Erythroid.cells	DCTN1	-0.2751	4.987161	-1.20828	0.230162	-5.38641	0.440754	0.528325
Erythroid.cells	BACH1	0.324641	5.9952	1.208054	0.230248	-5.64788	0.425827	0.513764
Erythroid.cells	BBIP1	0.236465	5.888435	1.207869	0.230319	-5.64593	0.427388	0.515377
Erythroid.cells	GM31763	0.563085	3.257819	1.20785	0.230326	-5.08126	0.467808	0.554533
Erythroid.cells	BTF3L4	-0.28701	4.269235	-1.20732	0.23053	-5.30536	0.452122	0.539339
Erythroid.cells	SORBS2	0.791206	1.878636	1.207154	0.230592	-4.97031	0.490917	0.576497
Erythroid.cells	GM47664	-0.40256	3.774581	-1.20707	0.230624	-5.15937	0.459882	0.546898
Erythroid.cells	GALNT16	-0.75307	0.933714	-1.20657	0.230815	-4.78886	0.507267	0.591994
Erythroid.cells	KYAT3	0.419717	3.603362	1.206543	0.230827	-5.44787	0.462674	0.549638
Erythroid.cells	CACNA1E	0.574479	4.907691	1.206491	0.230847	-5.55385	0.442389	0.530067
Erythroid.cells	WDR78	0.941133	0.561969	1.206446	0.230864	-4.72327	0.513817	0.598167
Erythroid.cells	AKTIP	0.436304	3.102695	1.206377	0.23089	-5.04319	0.470717	0.557377
Erythroid.cells	GBP7	-0.64237	4.443583	-1.20622	0.230951	-5.37038	0.449528	0.53696
Erythroid.cells	GM44702	1.084166	0.088947	1.205737	0.231136	-4.72478	0.522415	0.606294
Erythroid.cells	3110001I2	-0.4678	2.677347	-1.20563	0.231179	-4.99287	0.477789	0.564216
Erythroid.cells	GM43330	-0.71503	1.526899	-1.20562	0.23118	-4.81386	0.49712	0.582557
Erythroid.cells	RBM17	0.245053	5.789508	1.205622	0.23118	-5.58338	0.42932	0.5174
Erythroid.cells	MESD	0.315575	4.085411	1.205242	0.231326	-5.23287	0.455274	0.54262
Erythroid.cells	ZFP516	0.34578	4.66128	1.205202	0.231341	-5.37889	0.446353	0.533991
Erythroid.cells	RNASEH2A	-0.34627	4.54522	-1.20515	0.231362	-5.35026	0.448136	0.535719
Erythroid.cells	2810004N2	0.340658	4.315225	1.204855	0.231475	-5.31198	0.451821	0.539275
Erythroid.cells	TMX1	0.235807	5.362	1.204612	0.231568	-5.55557	0.435959	0.523885
Erythroid.cells	SPAG7	0.339225	4.55728	1.204109	0.231761	-5.32889	0.448456	0.535942
Erythroid.cells	RAB23	0.765821	1.279801	1.203772	0.231891	-4.8023	0.502139	0.587209
Erythroid.cells	TCF7L2	-0.33893	7.34951	-1.20375	0.231899	-5.917	0.407623	0.495954
Erythroid.cells	ABI1	-0.215	7.702393	-1.20353	0.231983	-6.00108	0.402806	0.491191
Erythroid.cells	ABCA13	-1.01056	0.73813	-1.20333	0.23206	-4.86886	0.511768	0.596278
Erythroid.cells	GATD1	0.329968	4.394103	1.202973	0.232198	-5.29497	0.45138	0.538804
Erythroid.cells	PLEKHA6	-0.53704	1.808761	-1.20275	0.232282	-4.99185	0.493415	0.578995
Erythroid.cells	4732440D0	-0.73652	2.03703	-1.20272	0.232296	-4.85794	0.489547	0.575332
Erythroid.cells	ZFP800	-0.25351	5.365723	-1.20242	0.232412	-5.52085	0.436664	0.524463
Erythroid.cells	LAPTM4B	0.49615	3.501356	1.202343	0.232441	-5.27915	0.465553	0.552403
Erythroid.cells	CMYA5	-1.1201	0.674059	-1.20222	0.23249	-4.74046	0.513214	0.597627
Erythroid.cells	BC005561	-0.3118	4.273496	-1.20213	0.232524	-5.33792	0.453353	0.540701
Erythroid.cells	CEP68	0.382163	4.299523	1.20169	0.232693	-5.26515	0.453186	0.540543
Erythroid.cells	DIDO1	-0.25612	5.734022	-1.20146	0.23278	-5.58589	0.431411	0.519484
Erythroid.cells	RIOK2	-0.30666	4.466641	-1.20125	0.232863	-5.2941	0.45059	0.538261
Erythroid.cells	DNAJC27	0.513157	2.442699	1.201231	0.23287	-4.98105	0.483094	0.569445
Erythroid.cells	F830016B0	1.059338	0.444299	1.201215	0.232876	-4.78865	0.517573	0.601965
Erythroid.cells	SERTAD1	-0.30329	5.61417	-1.20082	0.233026	-5.60157	0.433262	0.521492
Erythroid.cells	GM49189	-0.79835	0.779988	-1.20079	0.233041	-4.772	0.511698	0.596562
Erythroid.cells	CLEC4D	0.787161	2.949351	1.200633	0.2331	-5.25201	0.474817	0.56166
Erythroid.cells	PARP2	-0.25251	4.841287	-1.20048	0.23316	-5.4388	0.444906	0.53287
Erythroid.cells	MDFIC	-0.42632	4.328783	-1.20039	0.233194	-5.42639	0.452808	0.540564
Erythroid.cells	PCP4L1	0.87993	1.36121	1.200367	0.233203	-4.88398	0.501537	0.587085

Erythroid.cells	GM13205	0.88497	0.234435	1.200278	0.233238	-4.72986	0.521426	0.605762
Erythroid.cells	CD59B	1.064035	0.413	1.200057	0.233323	-4.74384	0.518303	0.602824
Erythroid.cells	MCFD2	0.405856	3.702476	1.199903	0.233382	-5.25199	0.462738	0.550187
Erythroid.cells	PNLDC1	-0.89445	0.924032	-1.19974	0.233444	-4.76208	0.50924	0.594461
Erythroid.cells	PRLR	0.576096	3.152323	1.199702	0.23346	-5.29613	0.471584	0.558758
Erythroid.cells	GM15972	-1.02579	-0.2921	-1.19956	0.233517	-4.71436	0.531076	0.614924
Erythroid.cells	TRAPPC2L	0.232422	5.811456	1.19937	0.233588	-5.65172	0.430409	0.518936
Erythroid.cells	NPTN	-0.14421	8.043161	-1.19935	0.233596	-6.08012	0.398788	0.487678
Erythroid.cells	RCOR2	0.990834	0.194015	1.19918	0.233662	-4.73049	0.522281	0.606718
Erythroid.cells	RSAD2	0.349491	4.537164	1.198736	0.233834	-6.16025	0.449715	0.537818
Erythroid.cells	PRRC2C	-0.14945	7.744202	-1.19868	0.233854	-6.05905	0.402936	0.491883
Erythroid.cells	ANAPC13	0.194641	5.65737	1.198625	0.233877	-5.69109	0.432753	0.521314
Erythroid.cells	TMEM147	0.281836	4.948794	1.198419	0.233956	-5.46304	0.443402	0.5317
Erythroid.cells	SUMO3	-0.24239	5.724941	-1.19836	0.233978	-5.61398	0.431752	0.520334
Erythroid.cells	EFNA1	0.902109	0.531324	1.198331	0.23399	-4.77618	0.516267	0.601209
Erythroid.cells	CDIP1	-0.22671	5.935336	-1.19831	0.233999	-5.6509	0.42865	0.517295
Erythroid.cells	JADE2	-0.54234	3.270443	-1.19815	0.234059	-5.08732	0.469768	0.557175
Erythroid.cells	HNRNPA1	-0.19067	7.807246	-1.19762	0.234265	-6.07522	0.40237	0.491267
Erythroid.cells	1700112J1	-0.93	0.638697	-1.19742	0.234345	-4.77219	0.514797	0.599801
Erythroid.cells	NFKBIZ	0.338571	6.516715	1.197317	0.234383	-5.69509	0.42056	0.509343
Erythroid.cells	PSME2	-0.3098	7.278608	-1.19693	0.234533	-6.0048	0.409879	0.498788
Erythroid.cells	OXNAD1	-0.56967	1.886539	-1.19655	0.234679	-4.89756	0.493265	0.579727
Erythroid.cells	TXN2	0.183319	6.315861	1.196517	0.234693	-5.8514	0.4236	0.512495
Erythroid.cells	GLB1	-0.28898	5.221188	-1.19651	0.234698	-5.4544	0.439796	0.528355
Erythroid.cells	CRIM1	0.386606	5.191827	1.196468	0.234713	-5.65419	0.44024	0.528793
Erythroid.cells	SLC39A10	0.381517	3.903206	1.19632	0.23477	-5.19389	0.460167	0.548177
Erythroid.cells	HPSE	-0.43728	3.426146	-1.19626	0.234795	-5.22996	0.467782	0.555525
Erythroid.cells	DNTTIP2	-0.23859	5.107858	-1.19617	0.234828	-5.46892	0.44151	0.53016
Erythroid.cells	DPH2	0.878102	0.879573	1.195974	0.234904	-4.75885	0.510768	0.596363
Erythroid.cells	EML5	-0.48691	3.697759	-1.19564	0.235034	-5.2046	0.463654	0.551513
Erythroid.cells	STARD9	0.463305	3.529605	1.195451	0.235107	-5.15935	0.466399	0.554178
Erythroid.cells	SLC35C2	0.27601	4.846147	1.19521	0.235201	-5.33691	0.445852	0.534272
Erythroid.cells	UBTD2	0.454718	3.37685	1.195015	0.235277	-5.15232	0.469002	0.556611
Erythroid.cells	DHFR	-0.40439	4.331798	-1.19491	0.235318	-5.44475	0.453847	0.542074
Erythroid.cells	ECPAS	-0.1867	6.743157	-1.19464	0.235421	-5.86687	0.417885	0.506908
Erythroid.cells	ZMAT1	-0.68969	2.090961	-1.19459	0.235441	-4.85944	0.490309	0.57698
Erythroid.cells	SIGLECH	1.17254	0.771994	1.194441	0.2355	-4.74123	0.513163	0.598595
Erythroid.cells	EPB41L4AC	0.535301	3.25392	1.194099	0.235633	-5.11307	0.471252	0.55879
Erythroid.cells	GM16618	0.75399	0.733543	1.193754	0.235767	-4.789	0.514152	0.599473
Erythroid.cells	CUL1	-0.16929	6.562305	-1.19372	0.235779	-5.82384	0.420753	0.509731
Erythroid.cells	GM5086	0.745554	-0.01663	1.192508	0.236253	-4.79155	0.528598	0.612574
Erythroid.cells	GM15545	-0.71845	1.102359	-1.19178	0.236536	-4.76426	0.509079	0.594194
Erythroid.cells	EHD1	-0.27521	6.1755	-1.19143	0.236674	-5.70287	0.427728	0.516025
Erythroid.cells	PMPCA	0.347229	4.024403	1.191178	0.236772	-5.28065	0.460548	0.547877
Erythroid.cells	FXYD4	-0.57636	3.001862	-1.1911	0.236802	-5.00796	0.477043	0.563707
Erythroid.cells	CYBA	-0.1991	8.55691	-1.19101	0.236837	-6.1724	0.394352	0.482994
Erythroid.cells	TCERG1	-0.17465	6.296507	-1.19021	0.23715	-5.79762	0.426483	0.514684

Erythroid.cells	ZFP715	-0.37736	3.662783	-1.19004	0.237218	-5.15159	0.466879	0.553908
Erythroid.cells	GM19466	-0.92992	0.661272	-1.18985	0.237289	-4.74703	0.517824	0.602151
Erythroid.cells	4732465J0	0.701277	1.227323	1.189294	0.237508	-4.93964	0.50816	0.593074
Erythroid.cells	CRYBB3	0.768405	0.578324	1.189177	0.237554	-4.85401	0.51967	0.603928
Erythroid.cells	GM9949	0.810673	0.483247	1.189078	0.237593	-4.8656	0.521379	0.605531
Erythroid.cells	ADPGK	0.520367	4.903066	1.188858	0.237679	-5.22411	0.447827	0.535547
Erythroid.cells	MAST3	0.420685	4.169613	1.188291	0.237901	-5.22229	0.459441	0.546796
Erythroid.cells	PCYT2	0.294103	4.635801	1.18821	0.237933	-5.51742	0.45214	0.539747
Erythroid.cells	FCGR2B	-0.37848	4.881357	-1.18799	0.238018	-5.56102	0.448343	0.53612
Erythroid.cells	NRXN1	0.766628	2.142242	1.18796	0.238031	-5.06303	0.492656	0.578581
Erythroid.cells	CHST12	-0.3301	5.763881	-1.18793	0.238043	-5.59266	0.434972	0.523108
Erythroid.cells	FAM89A	-0.56365	0.879902	-1.18789	0.238057	-4.93428	0.514583	0.599258
Erythroid.cells	TRIM5	-0.48182	3.589282	-1.18778	0.2381	-5.11773	0.468704	0.55578
Erythroid.cells	GM42031	-0.64748	4.172175	-1.18747	0.238223	-5.43299	0.459546	0.546952
Erythroid.cells	ASH1L	-0.17317	7.367632	-1.18733	0.238277	-6.00393	0.411874	0.5004
Erythroid.cells	GM17251	0.643493	2.365949	1.187095	0.23837	-4.94009	0.489026	0.575265
Erythroid.cells	PKMYT1	0.48424	3.261877	1.187073	0.238379	-5.15211	0.474164	0.561105
Erythroid.cells	TBCK	-0.26389	5.26974	-1.187	0.238407	-5.50188	0.442545	0.530606
Erythroid.cells	SDR39U1	0.710499	1.131364	1.186526	0.238593	-4.87487	0.510597	0.595517
Erythroid.cells	GM47469	-0.9556	0.520329	-1.18636	0.238658	-4.73024	0.521521	0.605745
Erythroid.cells	N4BP3	0.463797	2.955418	1.185965	0.238813	-5.0822	0.479732	0.566218
Erythroid.cells	DUSP10	0.373054	4.392133	1.185409	0.239032	-5.42864	0.456925	0.544178
Erythroid.cells	GMFB	-0.25715	5.451593	-1.18516	0.239128	-5.49329	0.440695	0.528491
Erythroid.cells	CREB3L3	-0.63403	1.62088	-1.1849	0.239234	-4.98259	0.50282	0.588039
Erythroid.cells	DYRK1A	-0.19855	7.419436	-1.18488	0.23924	-6.00008	0.412021	0.500406
Erythroid.cells	PLAGL2	-0.34169	4.367209	-1.18471	0.239306	-5.26999	0.457484	0.544831
Erythroid.cells	D730003I1	0.514277	2.591958	1.184367	0.239442	-5.00337	0.486418	0.572466
Erythroid.cells	RMDN1	0.35945	4.528231	1.184334	0.239455	-5.37098	0.455066	0.542436
Erythroid.cells	RAD9B	0.437145	3.275507	1.184034	0.239573	-5.13655	0.475231	0.561827
Erythroid.cells	EMC4	-0.30783	4.771124	-1.18392	0.239618	-5.42784	0.451409	0.538993
Erythroid.cells	DPF1	-1.07797	0.292792	-1.18381	0.23966	-4.73033	0.526713	0.610506
Erythroid.cells	RNF121	-0.35736	4.41156	-1.18295	0.240002	-5.33945	0.457582	0.544856
Erythroid.cells	GM47096	0.682941	1.734536	1.182733	0.240086	-4.84588	0.501784	0.587026
Erythroid.cells	TTC3	0.266859	5.355192	1.182663	0.240113	-5.55961	0.443012	0.530805
Erythroid.cells	CHEK2	0.412975	3.145946	1.182375	0.240227	-5.11014	0.477961	0.564588
Erythroid.cells	EDEM1	-0.24884	6.015715	-1.18235	0.240239	-5.66751	0.433093	0.521242
Erythroid.cells	TOP2B	-0.22239	6.593853	-1.18233	0.240244	-5.86936	0.424604	0.51292
Erythroid.cells	GM27003	-0.33464	4.204875	-1.18172	0.240486	-5.3022	0.461082	0.5484
Erythroid.cells	RPAP1	0.517385	2.431442	1.181696	0.240495	-4.97264	0.490105	0.576146
Erythroid.cells	JMJD8	-0.72575	1.564022	-1.18156	0.240548	-4.87318	0.504985	0.590227
Erythroid.cells	CHRNA9	-0.78827	2.214161	-1.18128	0.24066	-4.93227	0.49379	0.579748
Erythroid.cells	DPY19L3	-0.59299	2.757574	-1.181	0.240771	-4.98154	0.484629	0.571105
Erythroid.cells	NCKAP1	0.468597	2.425386	1.180873	0.24082	-5.07042	0.490208	0.576438
Erythroid.cells	ABHD13	0.362489	3.557429	1.18086	0.240825	-5.1515	0.471465	0.558566
Erythroid.cells	CCDC58	-0.30561	4.247902	-1.18073	0.240876	-5.38013	0.460401	0.547943
Erythroid.cells	MLKL	0.629803	2.335281	1.180644	0.240911	-5.00875	0.491732	0.5779
Erythroid.cells	DNAJC11	-0.29547	4.486406	-1.18063	0.240914	-5.38206	0.456642	0.544324

Erythroid.cells	GM20470	0.710168	1.403571	1.180503	0.240967	-4.86676	0.507788	0.593121
Erythroid.cells	TDRKH	-0.74152	1.697145	-1.18043	0.240997	-4.83945	0.502672	0.588317
Erythroid.cells	SIDT1	-0.45633	3.376538	-1.18021	0.241082	-5.48952	0.474409	0.561524
Erythroid.cells	ARHGAP29	0.588871	2.520124	1.180134	0.241112	-5.04722	0.48861	0.575064
Erythroid.cells	OXR1	-0.20944	6.336267	-1.18006	0.241143	-5.83384	0.428569	0.517115
Erythroid.cells	GDF15	0.803983	1.508239	1.180043	0.241148	-5.05302	0.505958	0.591472
Erythroid.cells	GFM1	-0.32335	3.89287	-1.18001	0.24116	-5.31494	0.466055	0.553513
Erythroid.cells	CMTM8	0.391002	4.192364	1.179867	0.241218	-5.44379	0.46128	0.548972
Erythroid.cells	MCTS2	0.760802	1.612847	1.179721	0.241276	-4.89207	0.504135	0.589865
Erythroid.cells	PTPN13	0.739428	0.415506	1.179661	0.2413	-4.91791	0.525403	0.60982
Erythroid.cells	OAF	0.402293	3.151249	1.179451	0.241383	-5.29322	0.478102	0.565165
Erythroid.cells	TMCC3	-0.44658	5.475701	-1.17945	0.241384	-5.56761	0.441395	0.529756
Erythroid.cells	CWF19L2	-0.27498	5.038452	-1.17932	0.241434	-5.45596	0.448072	0.536245
Erythroid.cells	FOXJ3	-0.19737	5.947043	-1.17874	0.241666	-5.67923	0.434481	0.523082
Erythroid.cells	DNAH7A	-0.99335	-0.11769	-1.17871	0.241677	-4.75029	0.535363	0.619187
Erythroid.cells	DOCK8	0.21874	7.656272	1.178663	0.241695	-6.01794	0.40981	0.498775
Erythroid.cells	GM43581	0.598185	2.093907	1.178633	0.241707	-4.97876	0.496025	0.582291
Erythroid.cells	RNF181	0.342152	4.477136	1.178282	0.241846	-5.37687	0.456989	0.545051
Erythroid.cells	RGL1	0.400733	5.711711	1.17815	0.241898	-5.52843	0.43803	0.526636
Erythroid.cells	ENTR1	-0.30137	4.880508	-1.17814	0.241903	-5.42386	0.450701	0.538965
Erythroid.cells	GSTT2	0.421516	3.554147	1.178121	0.24191	-5.22242	0.471725	0.559235
Erythroid.cells	MICU3	0.426338	3.81398	1.177888	0.242002	-5.19613	0.467615	0.555258
Erythroid.cells	UNC13A	0.785537	1.183593	1.177359	0.242212	-4.82972	0.512163	0.597544
Erythroid.cells	SPATA48	-0.6395	2.190427	-1.17717	0.242286	-4.94145	0.494682	0.58117
Erythroid.cells	GM13889	0.804674	1.14195	1.177069	0.242327	-4.86874	0.512899	0.598405
Erythroid.cells	ACBD5	0.190715	6.231959	1.177018	0.242347	-5.81342	0.430527	0.519384
Erythroid.cells	ZFP777	0.422012	2.809648	1.176933	0.242381	-5.05518	0.484239	0.571293
Erythroid.cells	RARRES1	0.905402	0.889058	1.176917	0.242388	-4.87576	0.517395	0.602624
Erythroid.cells	TCEANC2	0.296505	4.247598	1.176846	0.242416	-5.32245	0.460861	0.548894
Erythroid.cells	ABAT	0.783422	1.196429	1.176146	0.242694	-4.91063	0.512167	0.597673
Erythroid.cells	FARS2	0.14869	6.899126	1.176082	0.242719	-5.96982	0.421	0.509977
Erythroid.cells	2810013P0	0.438355	4.123121	1.176013	0.242747	-5.27408	0.463046	0.55098
Erythroid.cells	GM29488	-0.95392	0.172443	-1.17596	0.242766	-4.74705	0.530594	0.614949
Erythroid.cells	GM17023	-0.87302	-0.64235	-1.17592	0.242784	-4.8768	0.545735	0.629009
Erythroid.cells	GLUD1	0.142864	7.653063	1.175779	0.24284	-6.2364	0.410293	0.499412
Erythroid.cells	ANKRD52	-0.35164	3.881446	-1.17575	0.242852	-5.23796	0.46691	0.5547
Erythroid.cells	CWC27	-0.24181	5.662216	-1.17562	0.242901	-5.60997	0.439218	0.527925
Erythroid.cells	DNAJC13	-0.26203	6.16837	-1.17549	0.242954	-5.61737	0.431676	0.520609
Erythroid.cells	GM4258	0.424826	4.929368	1.175005	0.243148	-5.49967	0.450684	0.539037
Erythroid.cells	NAV1	0.434506	3.595293	1.174855	0.243207	-5.37523	0.471833	0.559472
Erythroid.cells	9930111J2	-0.82531	1.279459	-1.17477	0.243242	-4.84975	0.511031	0.596745
Erythroid.cells	GABARAPL	0.344362	3.974288	1.174349	0.243409	-5.34998	0.465951	0.553822
Erythroid.cells	RAB31	0.358363	4.770711	1.173926	0.243577	-5.38684	0.453598	0.541753
Erythroid.cells	AMZ1	0.585137	3.157688	1.17372	0.243659	-5.15211	0.479539	0.566715
Erythroid.cells	LRRC8B	0.322911	3.358799	1.173197	0.243868	-5.25183	0.476365	0.563614
Erythroid.cells	ADGRV1	-0.88596	0.711845	-1.17318	0.243876	-4.82876	0.52188	0.606657
Erythroid.cells	ASB6	0.516166	2.575022	1.173144	0.243889	-4.99641	0.489398	0.576039

Erythroid.cells	3110040N1	0.431469	3.553549	1.17304	0.243931	-5.2086	0.473182	0.560607
Erythroid.cells	PP2D1	-0.84079	1.690903	-1.17296	0.243962	-4.8613	0.504545	0.590456
Erythroid.cells	ERICH1	-0.42917	3.61294	-1.17265	0.244087	-5.14631	0.472295	0.559719
Erythroid.cells	LYZ1	-0.82231	-0.68152	-1.1724	0.244184	-4.86359	0.547694	0.630723
Erythroid.cells	TTC25	0.991185	0.218704	1.172148	0.244286	-4.73102	0.53093	0.615165
Erythroid.cells	UPF1	0.194845	5.35091	1.172014	0.24434	-5.62567	0.444918	0.533338
Erythroid.cells	AMDHD1	0.542985	2.26974	1.171997	0.244347	-5.15851	0.494657	0.581088
Erythroid.cells	ANKRD6	0.793903	0.95646	1.171877	0.244395	-4.86023	0.517579	0.602691
Erythroid.cells	AGO3	-0.24449	5.940327	-1.17165	0.244484	-5.65354	0.436017	0.524766
Erythroid.cells	PPP6R2	0.285775	4.368985	1.171571	0.244517	-5.38613	0.460174	0.548262
Erythroid.cells	RNASET2A	-0.3617	5.439463	-1.17127	0.244635	-5.52842	0.443569	0.532339
Erythroid.cells	GHR	0.500053	4.215001	1.17119	0.244669	-5.67187	0.462616	0.550817
Erythroid.cells	MACROD2	0.59432	2.251559	1.171041	0.244729	-5.0637	0.494967	0.581775
Erythroid.cells	CD8B1	0.745116	0.660348	1.171017	0.244738	-4.99891	0.522896	0.608079
Erythroid.cells	CETN2	0.195454	5.798667	1.170972	0.244756	-5.70493	0.438139	0.527094
Erythroid.cells	SIT1	-0.6487	2.062249	-1.17096	0.244761	-4.97109	0.498208	0.584853
Erythroid.cells	USP54	-0.4591	2.915478	-1.17061	0.244903	-5.06231	0.483772	0.571257
Erythroid.cells	GM15832	-0.6199	1.685015	-1.17043	0.244971	-4.94557	0.504731	0.59121
Erythroid.cells	SMURF2	-0.19794	6.82379	-1.17036	0.245002	-5.89756	0.423028	0.512429
Erythroid.cells	GM10642	0.926982	0.270003	1.170288	0.24503	-4.76383	0.52999	0.614913
Erythroid.cells	TMEM161/	0.508734	2.853132	1.170288	0.24503	-5.03708	0.484812	0.572314
Erythroid.cells	CHD7	-0.22656	6.463817	-1.17013	0.245092	-5.89948	0.42827	0.517625
Erythroid.cells	SSR2	-0.26576	5.189958	-1.17011	0.245101	-5.56778	0.447382	0.536312
Erythroid.cells	C130036L2	-0.74608	1.354595	-1.16992	0.245176	-4.77712	0.510517	0.596713
Erythroid.cells	AGO4	0.517178	3.167589	1.169747	0.245247	-5.05616	0.47959	0.567381
Erythroid.cells	ADAMTS17	1.00314	0.355692	1.169713	0.24526	-4.75321	0.528425	0.613508
Erythroid.cells	RFC4	-0.39468	4.603054	-1.16965	0.245287	-5.46934	0.456488	0.54516
Erythroid.cells	ZDHHC3	-0.27609	5.048938	-1.16953	0.245332	-5.45413	0.449552	0.538491
Erythroid.cells	RDH10	0.472183	3.207433	1.169425	0.245375	-5.13734	0.478933	0.566824
Erythroid.cells	EIF3D	-0.23212	5.480464	-1.16937	0.245398	-5.6279	0.442945	0.532092
Erythroid.cells	ZWILCH	0.61202	3.028209	1.16934	0.24541	-5.08405	0.481898	0.569678
Erythroid.cells	6330418K0	-0.69642	1.506684	-1.16904	0.24553	-4.86787	0.507845	0.594359
Erythroid.cells	CLEC4E	0.864388	2.622943	1.168943	0.245569	-5.20767	0.488672	0.576228
Erythroid.cells	CXCR2	-0.84688	-0.00657	-1.16894	0.245569	-4.8461	0.535076	0.619907
Erythroid.cells	ZFP619	0.531214	2.21582	1.168713	0.245661	-4.94192	0.495577	0.582878
Erythroid.cells	JMJD1C	0.20195	8.048596	1.168555	0.245724	-6.11846	0.405697	0.495533
Erythroid.cells	GM49774	-0.6104	3.174932	-1.16855	0.245726	-5.07709	0.479469	0.567533
Erythroid.cells	ARSA	0.608344	1.69399	1.168434	0.245772	-4.89592	0.504575	0.591397
Erythroid.cells	ZFP639	-0.32737	4.128657	-1.16839	0.245792	-5.32064	0.463992	0.552668
Erythroid.cells	GMPR2	0.382035	3.866899	1.168368	0.245799	-5.22296	0.468187	0.556709
Erythroid.cells	GPR137B	-0.36454	5.316315	-1.16817	0.245878	-5.46994	0.445447	0.534747
Erythroid.cells	RAB24	0.242955	5.118704	1.168107	0.245904	-5.50977	0.448477	0.53774
Erythroid.cells	PROCA1	-0.52067	2.988273	-1.16803	0.245934	-5.05487	0.482561	0.570596
Erythroid.cells	NFATC2	0.48851	2.489047	1.167914	0.245981	-5.13789	0.490932	0.578592
Erythroid.cells	COPZ2	0.47795	2.183281	1.16761	0.246103	-5.09817	0.496133	0.583533
Erythroid.cells	TIE1	-0.69616	1.482911	-1.16761	0.246105	-4.88552	0.508262	0.595006
Erythroid.cells	ZFP580	0.596007	2.166288	1.1676	0.246107	-4.94573	0.496424	0.583809

Erythroid.cells	8030456M	-0.71022	1.090368	-1.16725	0.246248	-4.86381	0.51539	0.601616
Erythroid.cells	GM44649	-0.41884	3.949723	-1.16713	0.246296	-5.24517	0.467035	0.555681
Erythroid.cells	PLXNA4OS:	0.76133	0.51532	1.166815	0.246423	-4.86775	0.525869	0.611536
Erythroid.cells	SH3RF1	0.356663	4.357213	1.166726	0.246458	-5.39572	0.460666	0.549591
Erythroid.cells	RBM14	0.318106	3.712533	1.16654	0.246533	-5.25992	0.471046	0.559612
Erythroid.cells	GSK3B	-0.13165	8.105386	-1.16638	0.246597	-6.13143	0.405255	0.495238
Erythroid.cells	DMXL1	-0.24041	6.604824	-1.16595	0.246769	-5.84831	0.426645	0.516574
Erythroid.cells	CCDC18	-0.50018	2.722921	-1.16587	0.246803	-5.05194	0.487491	0.575539
Erythroid.cells	ZFP113	-0.80559	1.151185	-1.16584	0.246815	-4.81693	0.514639	0.60126
Erythroid.cells	COIL	0.358527	3.741294	1.165807	0.246828	-5.24317	0.470696	0.559472
Erythroid.cells	RUNDC3B	-0.38315	3.848947	-1.1651	0.247113	-5.53774	0.469332	0.55814
Erythroid.cells	ERCC2	-0.55292	1.983619	-1.16508	0.24712	-4.94585	0.500473	0.587869
Erythroid.cells	IL27	-0.97217	0.053926	-1.16494	0.247179	-4.8247	0.534961	0.620284
Erythroid.cells	SLC10A3	-0.65818	2.160549	-1.16453	0.24734	-4.92743	0.497603	0.585213
Erythroid.cells	CFDP1	-0.20403	5.986865	-1.16452	0.247347	-5.73782	0.436271	0.526098
Erythroid.cells	EGLN2	-0.31256	4.887876	-1.16412	0.247505	-5.47985	0.453234	0.542588
Erythroid.cells	RBP4	0.386621	8.031076	1.163661	0.247692	-6.39273	0.407237	0.497263
Erythroid.cells	FAM89B	-0.28098	5.277421	-1.16349	0.247763	-5.53494	0.44751	0.536889
Erythroid.cells	MCM2	0.44871	4.762793	1.163098	0.247919	-5.48559	0.455687	0.544704
Erythroid.cells	RBM6	-0.1629	7.184868	-1.16264	0.248105	-5.99071	0.419423	0.509281
Erythroid.cells	GALK2	-0.27209	4.897213	-1.16263	0.248108	-5.44755	0.453615	0.542772
Erythroid.cells	ITGA2B	0.650394	0.938407	1.162557	0.248138	-4.90208	0.519867	0.605957
Erythroid.cells	MASP1	0.701717	1.673868	1.162496	0.248163	-4.94565	0.506841	0.593698
Erythroid.cells	CPLANE2	-0.88421	0.331465	-1.16237	0.248211	-4.77611	0.530873	0.616257
Erythroid.cells	GM40787	0.840683	0.205726	1.162192	0.248285	-4.82297	0.533183	0.618413
Erythroid.cells	PPP2CA	-0.11155	7.762516	-1.16217	0.248295	-6.11188	0.41123	0.50116
Erythroid.cells	GM12158	0.723542	0.549233	1.162106	0.24832	-4.92412	0.526897	0.612585
Erythroid.cells	MICAL2	0.612259	1.966497	1.162016	0.248356	-5.06925	0.501752	0.58894
Erythroid.cells	PHYKPL	0.337415	3.470266	1.161079	0.248735	-5.19186	0.477057	0.565154
Erythroid.cells	MCEE	0.316665	4.194477	1.160851	0.248827	-5.43369	0.465342	0.553997
Erythroid.cells	H2AFX	0.332583	6.482628	1.160818	0.24884	-5.96203	0.430212	0.519814
Erythroid.cells	SON	-0.13647	7.67203	-1.16058	0.248937	-6.06867	0.413102	0.502958
Erythroid.cells	PPIA	-0.17746	10.92524	-1.16053	0.248956	-6.63205	0.369836	0.459448
Erythroid.cells	PDS5A	-0.15252	7.411745	-1.16033	0.249039	-6.03201	0.41679	0.506657
Erythroid.cells	TMEM132E	-0.88355	0.984501	-1.16031	0.249046	-4.86743	0.519795	0.605877
Erythroid.cells	FEM1A	-0.43337	2.924816	-1.16	0.249173	-5.04118	0.486242	0.57415
Erythroid.cells	PPCS	0.412876	2.732539	1.159971	0.249183	-5.09996	0.489473	0.57723
Erythroid.cells	TRIM13	-0.85656	0.46615	-1.15964	0.249317	-4.80059	0.529272	0.614838
Erythroid.cells	4930402H2	-0.33512	4.453202	-1.15958	0.249343	-5.37165	0.461337	0.550311
Erythroid.cells	RNF103	-0.33747	4.305165	-1.15957	0.249347	-5.29802	0.46369	0.552583
Erythroid.cells	SULT2B1	-0.38077	2.700547	-1.15933	0.249444	-5.37126	0.490014	0.57794
Erythroid.cells	CATSPERE2	0.681495	1.174644	1.159194	0.249498	-4.81523	0.516487	0.602998
Erythroid.cells	ANKRD40	0.293971	4.238658	1.159167	0.249509	-5.36151	0.464751	0.553737
Erythroid.cells	GM14698	-0.84047	0.738672	-1.15916	0.249514	-4.76963	0.524317	0.610345
Erythroid.cells	CLEC4G	-0.49601	3.468481	-1.15881	0.249654	-5.24091	0.477405	0.565808
Erythroid.cells	OLFR920	-0.79961	0.137933	-1.15856	0.249756	-4.84101	0.535582	0.620834
Erythroid.cells	SLC1A5	0.206949	6.46392	1.158237	0.249886	-5.98343	0.430818	0.520825

Erythroid.cells	2-Sep	-0.61803	2.22603	-1.1582	0.2499	-4.97512	0.498352	0.586043
Erythroid.cells	CHN2	0.365281	5.358074	1.158146	0.249923	-5.88463	0.447457	0.537128
Erythroid.cells	MAP3K7	-0.22597	5.219048	-1.15814	0.249927	-5.55161	0.449596	0.539211
Erythroid.cells	LDHA	0.197641	8.792149	1.15802	0.249975	-6.35432	0.397901	0.488168
Erythroid.cells	SUGP2	0.422265	4.059588	1.157816	0.250057	-5.25228	0.467863	0.556906
Erythroid.cells	DNAH2	-0.70839	0.783062	-1.15779	0.250067	-4.88185	0.523785	0.610034
Erythroid.cells	GCDH	0.479323	3.116618	1.157283	0.250274	-5.29575	0.483467	0.571929
Erythroid.cells	GM10851	-0.34349	4.366594	-1.1572	0.250306	-5.42024	0.463119	0.552392
Erythroid.cells	GM19557	-0.64629	-0.95554	-1.15714	0.250333	-4.80342	0.556394	0.640466
Erythroid.cells	ANP32A	-0.11424	7.495527	-1.15711	0.250345	-6.04686	0.416038	0.506327
Erythroid.cells	GM49164	-0.78831	0.920748	-1.15688	0.250439	-4.83604	0.521588	0.608108
Erythroid.cells	TXNL4B	-0.5757	1.740021	-1.15643	0.250622	-4.89698	0.507326	0.594593
Erythroid.cells	AOPEP	-0.20593	6.329542	-1.15621	0.250711	-5.83321	0.433333	0.52337
Erythroid.cells	MAP3K9	0.848687	0.308951	1.155736	0.250903	-4.83166	0.533078	0.618948
Erythroid.cells	RTL5	-0.68205	0.491888	-1.15569	0.25092	-4.84971	0.529722	0.615813
Erythroid.cells	KAT14	-0.3231	3.838268	-1.15569	0.250922	-5.21577	0.472013	0.5611
Erythroid.cells	RALGAPB	0.270795	4.920242	1.155687	0.250923	-5.46073	0.454785	0.544453
Erythroid.cells	CAVIN1	0.831747	1.312054	1.155621	0.25095	-4.89608	0.514938	0.601942
Erythroid.cells	PIGW	-0.7343	0.765108	-1.15548	0.251005	-4.80308	0.524749	0.611173
Erythroid.cells	NDUFB1-PS	0.127277	8.902498	1.155421	0.251031	-6.35657	0.396892	0.487338
Erythroid.cells	C030005K0	-0.79569	0.674423	-1.1552	0.251123	-4.80269	0.526488	0.612744
Erythroid.cells	ILK	-0.25597	5.628263	-1.15484	0.251266	-5.64571	0.443984	0.534022
Erythroid.cells	LCORL	-0.21174	6.985276	-1.15477	0.251295	-5.91573	0.423827	0.514244
Erythroid.cells	TRIM30C	-0.74478	1.320225	-1.15475	0.251303	-4.92395	0.514924	0.602056
Erythroid.cells	PDCD10	-0.16024	6.713222	-1.15467	0.251338	-5.88144	0.427789	0.518177
Erythroid.cells	CDKN2AIP	0.269351	4.567595	1.15451	0.251402	-5.45596	0.460444	0.550073
Erythroid.cells	EIF3A	-0.13965	7.379667	-1.15445	0.251425	-6.04692	0.418153	0.508686
Erythroid.cells	TFB2M	0.302447	3.953928	1.154219	0.251521	-5.29218	0.470349	0.559703
Erythroid.cells	GM8113	0.973888	-0.25687	1.15382	0.251684	-4.72307	0.544089	0.629414
Erythroid.cells	NUDT13	-0.54785	2.358115	-1.15364	0.251756	-4.97818	0.497194	0.585391
Erythroid.cells	FIRRE	0.641365	2.608551	1.153306	0.251893	-4.984	0.493097	0.58146
Erythroid.cells	PIAS2	-0.23063	6.02482	-1.15259	0.252185	-5.75228	0.438867	0.528974
Erythroid.cells	1810055G0	0.472814	2.268587	1.152315	0.252298	-5.02693	0.499353	0.587366
Erythroid.cells	TEN1	-0.20657	5.852604	-1.15219	0.252347	-5.68477	0.441465	0.531621
Erythroid.cells	QPCTL	-0.43718	3.010203	-1.15218	0.252352	-5.02366	0.486755	0.575391
Erythroid.cells	AC160336.	-0.7629	1.371003	-1.15218	0.252353	-4.83809	0.515052	0.602225
Erythroid.cells	CRAT	0.39325	3.198294	1.152059	0.252403	-5.27903	0.483616	0.572402
Erythroid.cells	EIF2B2	-0.28507	4.936837	-1.15178	0.252518	-5.50445	0.455679	0.545447
Erythroid.cells	GFRA2	0.613593	2.545769	1.150985	0.252842	-5.03644	0.495274	0.583193
Erythroid.cells	PCIF1	0.177245	6.021144	1.150882	0.252884	-5.79645	0.439516	0.529452
Erythroid.cells	STYX	-0.27011	4.526153	-1.15051	0.253035	-5.40302	0.462836	0.552081
Erythroid.cells	BIN1	-0.25592	5.230633	-1.1504	0.253082	-5.56168	0.451776	0.541404
Erythroid.cells	GRK4	-0.48102	3.108893	-1.15006	0.253222	-5.08023	0.486028	0.574372
Erythroid.cells	PAK1IP1	0.161405	5.901653	1.149989	0.253249	-5.78755	0.441563	0.531413
Erythroid.cells	SLC16A6	0.388881	4.554893	1.14982	0.253319	-5.43711	0.462446	0.551716
Erythroid.cells	LRRC58	0.203133	5.945766	1.149713	0.253363	-5.71027	0.440896	0.530761
Erythroid.cells	ARL3	0.345276	4.04145	1.149678	0.253377	-5.30578	0.470679	0.559658

Erythroid.cells	2610037D(	0.261472	5.44268	1.149573	0.25342	-5.60801	0.448566	0.538281
Erythroid.cells	NDUFB10	0.171951	6.731387	1.149462	0.253465	-5.9937	0.429195	0.519353
Erythroid.cells	TCF7	0.460199	2.524319	1.149386	0.253497	-5.41543	0.495914	0.583873
Erythroid.cells	DEAF1	-0.379	3.281829	-1.14867	0.25379	-5.16634	0.483611	0.571925
Erythroid.cells	PADI2	0.474795	2.631853	1.14851	0.253856	-5.25895	0.494595	0.58241
Erythroid.cells	HIST1H2BN	-0.64501	2.139918	-1.14836	0.253916	-5.06072	0.503078	0.590465
Erythroid.cells	KMT5C	0.491407	2.877228	1.14816	0.254	-5.08094	0.490527	0.578597
Erythroid.cells	RAP2C	-0.28647	4.944479	-1.14804	0.25405	-5.42577	0.456869	0.546314
Erythroid.cells	UBFD1	-0.30576	4.520398	-1.14741	0.254307	-5.38663	0.463946	0.553013
Erythroid.cells	SLC39A12	1.142116	0.492802	1.147095	0.254437	-4.84085	0.533006	0.618669
Erythroid.cells	CENPK	0.489948	3.283005	1.146959	0.254493	-5.19237	0.484116	0.572559
Erythroid.cells	RBM3	-0.15533	9.070642	-1.14695	0.254498	-6.3803	0.397086	0.487368
Erythroid.cells	PTCD3	-0.26451	4.845166	-1.14688	0.254524	-5.53929	0.4588	0.548204
Erythroid.cells	CCR3	1.650648	-0.02075	1.146795	0.25456	-4.77749	0.542541	0.62766
Erythroid.cells	ZFP821	-0.33328	3.905173	-1.14673	0.254587	-5.34452	0.473862	0.562785
Erythroid.cells	GP9	-0.87774	0.659096	-1.14652	0.254672	-4.96781	0.529969	0.616029
Erythroid.cells	IGFLR1	0.861452	0.807622	1.146442	0.254706	-4.85353	0.527259	0.613524
Erythroid.cells	CAPN3	-0.69708	1.422788	-1.14637	0.254736	-4.89814	0.516185	0.603127
Erythroid.cells	MASTL	-0.38573	3.782	-1.14619	0.254811	-5.25489	0.475911	0.56486
Erythroid.cells	CCDC14	0.861977	0.809905	1.146107	0.254843	-4.81167	0.527244	0.613527
Erythroid.cells	LYVE1	0.837165	2.42534	1.145773	0.254981	-5.0525	0.498783	0.586698
Erythroid.cells	HIST1H3D	0.683154	1.963471	1.145737	0.254996	-4.9856	0.506788	0.594282
Erythroid.cells	MLLT6	-0.57008	3.270712	-1.14554	0.255077	-5.16705	0.484533	0.573092
Erythroid.cells	IGLL1	-0.68568	3.916663	-1.14488	0.255348	-5.54882	0.474268	0.563073
Erythroid.cells	TGM2	0.509756	4.674438	1.144763	0.255397	-5.49047	0.462075	0.551321
Erythroid.cells	CDC25C	0.620684	2.030722	1.144629	0.255452	-5.02286	0.506093	0.593416
Erythroid.cells	TEC	0.252171	5.751113	1.144563	0.255479	-5.74382	0.445316	0.535053
Erythroid.cells	GAS8	-0.54803	1.529564	-1.14446	0.255522	-4.92551	0.514916	0.60177
Erythroid.cells	SAP25	0.65162	2.354647	1.144323	0.255578	-4.98154	0.50049	0.588156
Erythroid.cells	MVK	-0.51063	2.634356	-1.14392	0.255745	-5.05654	0.495799	0.583737
Erythroid.cells	LRIG1	0.871253	0.178544	1.143881	0.255761	-4.85265	0.539632	0.624966
Erythroid.cells	BLOC1S3	0.582374	2.381624	1.143832	0.255781	-5.00457	0.500135	0.587855
Erythroid.cells	ORAI1	-0.21407	6.491763	-1.14364	0.255861	-5.8482	0.434271	0.524324
Erythroid.cells	ZBTB46	0.406468	3.021217	1.143515	0.255912	-5.32046	0.489235	0.577504
Erythroid.cells	B4GALT7	-0.45932	2.869796	-1.14339	0.255962	-5.05665	0.491794	0.579945
Erythroid.cells	ARV1	-0.62395	2.101251	-1.1433	0.256001	-4.9622	0.504993	0.592498
Erythroid.cells	RAB44	0.678685	1.487929	1.143113	0.256077	-5.08129	0.515788	0.602715
Erythroid.cells	TNNT3	-0.67517	1.772075	-1.14303	0.25611	-4.98359	0.510757	0.59802
Erythroid.cells	ADAP1	-0.3589	4.152492	-1.14284	0.256192	-5.35185	0.470557	0.5598
Erythroid.cells	CNIH4	0.231732	5.763142	1.142812	0.256202	-5.65573	0.445245	0.535256
Erythroid.cells	P2RY6	0.508414	2.305897	1.142714	0.256242	-5.15129	0.501443	0.589365
Erythroid.cells	STARD7	0.243652	5.358518	1.14269	0.256252	-5.60316	0.451466	0.541372
Erythroid.cells	TCTN3	-0.73595	1.366808	-1.14242	0.256363	-4.88691	0.518076	0.605073
Erythroid.cells	ITGAD	-0.94141	0.789813	-1.1421	0.256495	-4.81281	0.528669	0.614926
Erythroid.cells	6530413G1	0.827506	-0.08741	1.14149	0.256748	-4.73245	0.545336	0.630338
Erythroid.cells	VAV3	0.232678	7.363151	1.141405	0.256783	-6.17583	0.422084	0.512365
Erythroid.cells	FHIT	-0.2647	6.167904	-1.14105	0.256931	-5.85978	0.439869	0.529833

Erythroid.cells	EARS2	0.544203	1.644083	1.140375	0.25721	-4.96253	0.514302	0.601055
Erythroid.cells	TLCD2	-0.49857	2.659161	-1.14001	0.257361	-5.1503	0.496616	0.584406
Erythroid.cells	SRPRB	-0.31401	4.104103	-1.13999	0.257371	-5.31956	0.472522	0.561375
Erythroid.cells	IER2	0.249667	8.170209	1.139837	0.257433	-6.22271	0.411098	0.501345
Erythroid.cells	GM43623	0.826297	-0.49767	1.139779	0.257457	-4.82597	0.553765	0.638034
Erythroid.cells	HNRNPUL1	-0.1386	7.28107	-1.13978	0.257457	-6.04021	0.423766	0.513907
Erythroid.cells	KLC2	-0.5467	2.681752	-1.13976	0.257465	-5.05425	0.49623	0.584099
Erythroid.cells	GM15503	-0.64555	0.81285	-1.1395	0.257571	-4.8408	0.529277	0.615407
Erythroid.cells	NCBP3	-0.22874	5.506298	-1.13942	0.257608	-5.61871	0.450318	0.540088
Erythroid.cells	PYGL	0.302451	5.16283	1.139402	0.257613	-5.79323	0.455655	0.545277
Erythroid.cells	GM16201	-0.58997	1.818935	-1.1391	0.25774	-5.04793	0.511374	0.598619
Erythroid.cells	CCT3	0.19323	5.801241	1.138985	0.257786	-5.7374	0.445922	0.53583
Erythroid.cells	GPAM	-0.42519	3.696477	-1.13852	0.257979	-5.37433	0.479618	0.568404
Erythroid.cells	WDR73	-0.42902	2.976485	-1.13785	0.258259	-5.09611	0.49206	0.580079
Erythroid.cells	GH	-0.81045	-0.76502	-1.13777	0.258288	-4.74334	0.559861	0.643617
Erythroid.cells	GM15327	0.608441	1.294764	1.137077	0.258579	-4.88573	0.521789	0.608159
Erythroid.cells	GPX7	-0.91749	0.610424	-1.13707	0.258581	-4.80748	0.534259	0.619831
Erythroid.cells	ZFP266	-0.28475	4.029092	-1.1369	0.258654	-5.32622	0.474881	0.563726
Erythroid.cells	ZHX2	-0.30889	5.614006	-1.13687	0.258665	-5.69003	0.449728	0.539395
Erythroid.cells	UBXN2B	0.457156	2.842475	1.136774	0.258705	-5.08893	0.494676	0.582672
Erythroid.cells	CYP2A12	0.50241	2.85261	1.136486	0.258824	-5.40843	0.494641	0.58262
Erythroid.cells	CRADD	0.24658	5.224096	1.136245	0.258925	-5.55289	0.455942	0.545428
Erythroid.cells	TMEM38B	0.31492	4.174266	1.136141	0.258968	-5.41702	0.472682	0.561641
Erythroid.cells	MCL1	-0.18558	8.364811	-1.13606	0.259	-6.22805	0.409505	0.499801
Erythroid.cells	1700120C1	0.589759	1.482076	1.135983	0.259034	-4.90936	0.518609	0.605334
Erythroid.cells	ZFP664	0.331071	4.050718	1.135082	0.259409	-5.29226	0.475008	0.563955
Erythroid.cells	FARSA	0.239398	4.90554	1.135073	0.259412	-5.51022	0.46126	0.550694
Erythroid.cells	GADD45GII	0.236401	5.128075	1.135026	0.259432	-5.6586	0.45775	0.547293
Erythroid.cells	9130230L2	-0.5858	3.687563	-1.13496	0.25946	-5.31544	0.480978	0.569684
Erythroid.cells	ARHGDIA	-0.19227	7.188393	-1.13495	0.259465	-6.00801	0.426563	0.516784
Erythroid.cells	TRAF1	-0.45832	3.343827	-1.13488	0.259492	-5.49193	0.486702	0.575176
Erythroid.cells	CCL2	0.75479	3.089272	1.134437	0.259678	-5.31386	0.491247	0.579454
Erythroid.cells	RNF6	-0.20945	5.670645	-1.13408	0.259827	-5.66355	0.449724	0.539383
Erythroid.cells	AI504432	0.404814	2.840905	1.133634	0.260013	-5.1955	0.495868	0.583762
Erythroid.cells	DIO1	0.609942	0.977554	1.13359	0.260031	-4.9689	0.528774	0.614804
Erythroid.cells	MCMBP	-0.16257	7.089729	-1.13249	0.260489	-5.99251	0.429192	0.519015
Erythroid.cells	RPA2	-0.35637	4.645282	-1.13247	0.2605	-5.53054	0.466693	0.555566
Erythroid.cells	ZPBP	0.628414	1.648971	1.131982	0.260703	-4.92334	0.517551	0.60395
Erythroid.cells	IGFALS	-0.56241	1.779759	-1.13188	0.260746	-5.04765	0.515221	0.601758
Erythroid.cells	DNAJC19	0.174696	5.950115	1.131853	0.260757	-5.87179	0.446386	0.535823
Erythroid.cells	IRF2BPL	0.399406	4.091669	1.131721	0.260812	-5.40715	0.47579	0.564305
Erythroid.cells	GM36279	0.446881	2.959709	1.131638	0.260847	-5.18939	0.494689	0.582372
Erythroid.cells	GM10125	-0.56331	2.003063	-1.13161	0.26086	-4.99504	0.511269	0.598075
Erythroid.cells	TM9SF1	-0.27366	5.042685	-1.13091	0.261153	-5.53353	0.460929	0.549842
Erythroid.cells	SPSB1	-0.77509	1.685361	-1.13073	0.261228	-4.90548	0.517441	0.603773
Erythroid.cells	ALDH7A1	0.41475	3.227154	1.130516	0.261317	-5.28545	0.490742	0.578526
Erythroid.cells	INO80B	0.32494	4.146114	1.13023	0.261436	-5.34261	0.475589	0.563939

Erythroid.cells	VMP1	0.23365	7.164309	1.13013	0.261478	-6.04775	0.42884	0.518534
Erythroid.cells	ZNHIT6	0.419221	3.231636	1.129755	0.261635	-5.22207	0.490878	0.578686
Erythroid.cells	FLT4	0.594276	2.321562	1.129754	0.261636	-5.04056	0.506511	0.593524
Erythroid.cells	ART2B	1.096936	0.097769	1.129674	0.261669	-4.8195	0.546904	0.631387
Erythroid.cells	FUT4	-0.76116	-0.16835	-1.12953	0.261731	-4.85317	0.551983	0.636124
Erythroid.cells	CALM3	-0.17468	7.10732	-1.12872	0.26207	-6.02329	0.430255	0.519826
Erythroid.cells	GPC4	0.665391	1.1587	1.128567	0.262133	-4.97677	0.527882	0.613419
Erythroid.cells	HIBADH	0.228116	5.742005	1.128227	0.262276	-5.81934	0.45102	0.540107
Erythroid.cells	TMEM229/	-0.94642	-1.03017	-1.12799	0.262377	-4.74204	0.569517	0.652133
Erythroid.cells	MAP4	-0.18338	6.676158	-1.12796	0.262387	-5.9328	0.43682	0.526362
Erythroid.cells	PRDM9	-0.72628	1.146615	-1.1278	0.262455	-4.86427	0.528283	0.614011
Erythroid.cells	ZFP217	0.306003	4.011596	1.127654	0.262517	-5.28157	0.478628	0.567055
Erythroid.cells	GM42917	0.601333	1.691044	1.127628	0.262528	-4.97225	0.518453	0.604867
Erythroid.cells	SCAF1	0.276026	4.690272	1.127553	0.262559	-5.43712	0.467593	0.556468
Erythroid.cells	STX1A	0.752138	1.792697	1.127414	0.262618	-4.90737	0.516659	0.603199
Erythroid.cells	WFDC21	1.058683	2.947804	1.126805	0.262874	-5.30104	0.496888	0.5844
Erythroid.cells	CCDC90B	0.39857	3.165156	1.126476	0.263012	-5.18849	0.493272	0.580991
Erythroid.cells	CPEB1	0.775491	0.696287	1.126274	0.263097	-4.84019	0.537101	0.622383
Erythroid.cells	DFFA	-0.4612	2.939835	-1.12621	0.263122	-5.10174	0.497114	0.58478
Erythroid.cells	VCPKMT	-0.46958	2.877826	-1.12612	0.263162	-5.15373	0.498177	0.585839
Erythroid.cells	ESCO1	-0.21924	5.77786	-1.12611	0.263165	-5.71257	0.450922	0.540408
Erythroid.cells	SPIB	-0.41241	4.129765	-1.12601	0.26321	-5.45763	0.47717	0.565843
Erythroid.cells	GM50232	-0.74666	1.372215	-1.12581	0.263291	-4.9017	0.524777	0.611103
Erythroid.cells	NUTF2	-0.64753	1.951713	-1.12571	0.263336	-4.96341	0.514392	0.601412
Erythroid.cells	NAB2	-0.42727	3.217989	-1.12535	0.263487	-5.26202	0.49257	0.580759
Erythroid.cells	GM12166	0.784458	0.781167	1.12517	0.263562	-4.83901	0.535742	0.621496
Erythroid.cells	ARRDC3	0.477713	3.403751	1.124988	0.263639	-5.21492	0.489431	0.577791
Erythroid.cells	SMC2	0.349702	5.816825	1.124954	0.263653	-5.83337	0.450498	0.540254
Erythroid.cells	CCDC85B	0.303881	3.779227	1.124941	0.263659	-5.32938	0.483148	0.571785
Erythroid.cells	PCBP4	0.80771	0.472145	1.124844	0.263699	-4.82844	0.541487	0.626882
Erythroid.cells	C5AR1	0.620429	4.179898	1.124426	0.263876	-5.44122	0.476732	0.565639
Erythroid.cells	UTP14A	-0.26602	5.238791	-1.12428	0.263936	-5.52294	0.459706	0.549231
Erythroid.cells	COX10	0.344965	3.775159	1.124246	0.263952	-5.29718	0.483414	0.572071
Erythroid.cells	TOMM70A	-0.18812	5.752051	-1.12381	0.264135	-5.75503	0.451916	0.541512
Erythroid.cells	CIDEB	0.488438	1.942054	1.123587	0.26423	-5.14353	0.515274	0.602129
Erythroid.cells	PAX5	-0.30075	5.141192	-1.12342	0.264301	-5.83094	0.461606	0.550913
Erythroid.cells	TRDMT1	-0.51356	2.342131	-1.1233	0.264351	-5.00593	0.508264	0.595574
Erythroid.cells	LAMTOR2	0.152845	6.577213	1.122769	0.264575	-5.94312	0.439658	0.529495
Erythroid.cells	THUMPD1	-0.27965	4.425949	-1.12276	0.264578	-5.44564	0.47332	0.562176
Erythroid.cells	BCAR1	0.826255	0.776645	1.122161	0.264832	-4.84156	0.537068	0.622484
Erythroid.cells	PSMD1	-0.18069	6.546401	-1.12214	0.264841	-5.90073	0.4404	0.530166
Erythroid.cells	A530013C2	0.568059	2.431674	1.121988	0.264905	-5.18378	0.507298	0.594507
Erythroid.cells	FAM126B	-0.34779	3.772291	-1.12149	0.265115	-5.25474	0.484708	0.572795
Erythroid.cells	ZFP687	-0.42865	2.919042	-1.12134	0.265178	-5.09426	0.499182	0.586606
Erythroid.cells	COA5	0.38183	4.468388	1.121141	0.265264	-5.36361	0.473338	0.561851
Erythroid.cells	MYO19	-0.71012	1.357151	-1.12098	0.26533	-4.90165	0.526907	0.612752
Erythroid.cells	RETREG3	-0.21525	5.527566	-1.12079	0.265413	-5.72302	0.456485	0.545662

Erythroid.cells	CCDC162	-0.6596	2.876554	-1.12073	0.265436	-5.1341	0.500036	0.587457
Erythroid.cells	GM33280	-1.08228	-0.66423	-1.12042	0.26557	-4.73521	0.565193	0.648326
Erythroid.cells	IGF1R	0.315565	6.357541	1.12027	0.265633	-5.71148	0.443852	0.533361
Erythroid.cells	USP34	-0.14552	7.703229	-1.12016	0.26568	-6.14817	0.423896	0.513805
Erythroid.cells	KLHL21	0.509363	2.689731	1.119927	0.265778	-5.13378	0.503546	0.590786
Erythroid.cells	2610027K0	-0.70576	0.681173	-1.1197	0.265874	-4.86862	0.539762	0.624713
Erythroid.cells	ABHD12	0.256038	5.584506	1.119208	0.266083	-5.61784	0.456132	0.545281
Erythroid.cells	NAP1L1	-0.20282	7.439515	-1.11896	0.266186	-6.11632	0.428075	0.517877
Erythroid.cells	MRPS21	0.142661	7.034242	1.118893	0.266216	-6.04025	0.434044	0.523769
Erythroid.cells	CNPY2	0.289912	4.719728	1.118797	0.266257	-5.53454	0.469871	0.558636
Erythroid.cells	FUS	-0.132	7.820328	-1.11876	0.266273	-6.1426	0.422545	0.512439
Erythroid.cells	COBLL1	0.28007	5.069977	1.11873	0.266286	-5.67396	0.464255	0.553221
Erythroid.cells	DTWD1	0.534073	1.928598	1.118627	0.266329	-4.99158	0.517251	0.603782
Erythroid.cells	GM17435	0.694013	1.133905	1.118223	0.266501	-4.89478	0.531873	0.617361
Erythroid.cells	FLNB	0.500076	5.398208	1.117817	0.266673	-5.6905	0.459442	0.548463
Erythroid.cells	TMEM41B	0.307897	4.300772	1.117758	0.266699	-5.34673	0.477085	0.565478
Erythroid.cells	ASPA	0.867283	1.375432	1.117472	0.26682	-4.96321	0.527803	0.61353
Erythroid.cells	GM16541	-0.44655	2.915994	-1.1169	0.267064	-5.13835	0.500759	0.587997
Erythroid.cells	ADAT2	-0.73352	1.285306	-1.11679	0.267111	-4.87505	0.529717	0.615271
Erythroid.cells	CD3D	-0.39305	2.678526	-1.11671	0.267144	-5.54244	0.504872	0.591894
Erythroid.cells	VKORC1	0.278944	4.701765	1.116588	0.267196	-5.71328	0.470926	0.559488
Erythroid.cells	AP1AR	-0.29667	5.002368	-1.11658	0.267198	-5.52184	0.46609	0.554826
Erythroid.cells	YWHAG	-0.2189	6.203239	-1.11634	0.2673	-5.80904	0.447377	0.536583
Erythroid.cells	PRKAG1	-0.19589	5.515312	-1.11601	0.267443	-5.64389	0.458139	0.546999
Erythroid.cells	SGF29	-0.24954	4.671195	-1.1159	0.267489	-5.4906	0.471605	0.560064
Erythroid.cells	GM45267	0.657205	0.245242	1.115881	0.267497	-4.97746	0.549294	0.633416
Erythroid.cells	EPHX2	0.525349	2.477792	1.115562	0.267633	-5.26506	0.508691	0.595387
Erythroid.cells	IKBKB	0.215589	5.778617	1.115396	0.267704	-5.70806	0.454124	0.543135
Erythroid.cells	CUL5	-0.18586	5.877947	-1.11539	0.267708	-5.74339	0.452581	0.541635
Erythroid.cells	SNW1	-0.15513	6.240739	-1.11528	0.267751	-5.85641	0.446991	0.536202
Erythroid.cells	PRR11	0.542839	2.847187	1.115084	0.267836	-5.17723	0.502302	0.589357
Erythroid.cells	PCNX	0.273145	5.518161	1.114851	0.267935	-5.62227	0.458238	0.547212
Erythroid.cells	MAST2	-0.25329	5.283184	-1.11476	0.267972	-5.72555	0.461946	0.550809
Erythroid.cells	LGALS3BP	-0.48356	4.826846	-1.11456	0.268061	-5.54393	0.469239	0.557898
Erythroid.cells	TRAIP	0.539731	2.119414	1.114455	0.268104	-5.07715	0.515059	0.601559
Erythroid.cells	GM9929	0.614101	1.684976	1.114441	0.26811	-4.91332	0.522833	0.608868
Erythroid.cells	HIRA	-0.22379	5.652742	-1.11443	0.268114	-5.75136	0.456128	0.545223
Erythroid.cells	ATP2A1	0.822923	1.448126	1.114293	0.268174	-4.83248	0.527144	0.612941
Erythroid.cells	PRRG1	0.70086	1.915898	1.11407	0.268269	-4.97356	0.518738	0.605169
Erythroid.cells	STARD3NL	-0.21065	5.861943	-1.11403	0.268287	-5.79442	0.452915	0.542233
Erythroid.cells	TBC1D9	-0.36964	3.953614	-1.11362	0.268462	-5.63343	0.483811	0.571974
Erythroid.cells	PHYH	0.286717	5.40217	1.112964	0.268741	-5.953	0.460602	0.549578
Erythroid.cells	GM19967	-0.66269	1.377066	-1.11277	0.268824	-4.8848	0.529033	0.614817
Erythroid.cells	SHB	0.392962	4.498582	1.112691	0.268857	-5.39407	0.475114	0.563682
Erythroid.cells	PATL2	-0.68563	2.023827	-1.11265	0.268876	-4.9591	0.517362	0.603863
Erythroid.cells	ZFYVE9	0.454441	2.603777	1.112624	0.268886	-5.35834	0.507123	0.59421
Erythroid.cells	6330562C2	-0.67522	1.275201	-1.11256	0.268912	-4.91591	0.530895	0.616577

Erythroid.cells	SLTM	-0.15644	6.63584	-1.11211	0.269104	-5.95487	0.441764	0.531295
Erythroid.cells	MTCH1	-0.17276	6.297903	-1.11171	0.269278	-5.8437	0.446903	0.536463
Erythroid.cells	SETD4	-0.37787	2.964232	-1.11169	0.269284	-5.1896	0.501118	0.588627
Erythroid.cells	PGAP1	-0.42159	3.785443	-1.11169	0.269286	-5.39739	0.487152	0.575328
Erythroid.cells	LXN	-0.35024	3.855864	-1.11154	0.26935	-5.28412	0.485973	0.574294
Erythroid.cells	MTIF3	0.443	3.10156	1.111472	0.269379	-5.17017	0.498754	0.586476
Erythroid.cells	D130020L(	0.740798	0.782996	1.111405	0.269407	-4.89689	0.540263	0.62554
Erythroid.cells	GM26590	0.562945	1.314817	1.111337	0.269436	-4.89035	0.530438	0.616372
Erythroid.cells	GM36738	0.443992	3.095803	1.110408	0.269834	-5.17529	0.499499	0.586911
Erythroid.cells	PROM1	0.927182	0.403745	1.110198	0.269924	-4.86567	0.548176	0.632576
Erythroid.cells	CAMK1	0.431598	2.966119	1.110004	0.270007	-5.24722	0.501831	0.589113
Erythroid.cells	NPC2	0.190558	7.639426	1.109929	0.270039	-6.14857	0.427503	0.51726
Erythroid.cells	VAMP2	0.259791	4.421644	1.109726	0.270126	-5.40413	0.477321	0.565745
Erythroid.cells	NDUFV2	0.198444	6.136177	1.10972	0.270129	-5.91373	0.450054	0.539375
Erythroid.cells	DPM3	0.181277	6.309203	1.109557	0.270199	-5.8918	0.44743	0.536783
Erythroid.cells	GPKOW	-0.25769	4.488998	-1.109	0.270437	-5.39709	0.476576	0.564806
Erythroid.cells	ELN	0.629906	2.40021	1.108867	0.270495	-5.21344	0.512096	0.598626
Erythroid.cells	RBIS	-0.23552	5.209656	-1.10872	0.270556	-5.64213	0.464928	0.553668
Erythroid.cells	PDCD4	0.173311	7.282332	1.108679	0.270575	-6.15371	0.433074	0.522632
Erythroid.cells	NDUFS1	-0.16533	5.752161	-1.10847	0.270664	-5.81011	0.456411	0.545418
Erythroid.cells	THTPA	-0.59455	1.360701	-1.10834	0.27072	-4.92981	0.530848	0.616305
Erythroid.cells	B230369F2	-0.52874	2.457789	-1.10816	0.270797	-5.00462	0.511141	0.597791
Erythroid.cells	GM47644	-0.7838	1.060844	-1.10815	0.270801	-4.93663	0.536369	0.621462
Erythroid.cells	ANKRD33B	-0.51479	4.882196	-1.10799	0.270869	-5.57453	0.470271	0.558908
Erythroid.cells	DIAPH1	-0.16154	7.355352	-1.10733	0.271154	-6.06392	0.432453	0.521897
Erythroid.cells	MRM2	0.717051	2.071278	1.107067	0.271268	-5.00196	0.518571	0.604596
Erythroid.cells	FANCM	-0.3874	3.67592	-1.10696	0.271316	-5.31045	0.490688	0.578205
Erythroid.cells	PDGFB	0.766048	1.062654	1.106887	0.271345	-5.00673	0.53693	0.621836
Erythroid.cells	KDM1B	-0.4003	3.6758	-1.1066	0.27147	-5.26728	0.490788	0.578387
Erythroid.cells	GM3235	-0.87879	0.645113	-1.10653	0.271497	-4.84643	0.544833	0.629281
Erythroid.cells	WLS	0.388666	4.263574	1.106305	0.271596	-5.40699	0.480969	0.569177
Erythroid.cells	CYFIP1	-0.28448	5.64309	-1.1063	0.271596	-5.63231	0.458722	0.547733
Erythroid.cells	GM13391	-0.70283	0.765079	-1.10606	0.271703	-4.90807	0.542701	0.627461
Erythroid.cells	SMAGP	0.388337	3.61538	1.105894	0.271772	-5.33301	0.491955	0.579651
Erythroid.cells	TMEM209	-0.47298	3.362785	-1.10519	0.272076	-5.17445	0.496715	0.583802
Erythroid.cells	MIS18A	0.332089	4.400206	1.105033	0.272143	-5.45525	0.479338	0.567215
Erythroid.cells	TBC1D31	0.377685	4.166685	1.104431	0.272403	-5.3861	0.483574	0.571086
Erythroid.cells	SLC35B1	0.214182	5.751128	1.104262	0.272476	-5.82263	0.45799	0.546444
Erythroid.cells	TYMS	0.410555	4.922548	1.104182	0.27251	-5.63602	0.471196	0.559214
Erythroid.cells	GM2245	0.575437	1.558462	1.103655	0.272738	-5.07939	0.529391	0.614362
Erythroid.cells	NIPSNAP2	0.241212	4.810103	1.10319	0.272938	-5.45973	0.473591	0.561429
Erythroid.cells	RDH13	-0.65692	1.398416	-1.1027	0.27315	-4.90173	0.532913	0.61748
Erythroid.cells	MCM6	-0.30394	6.155584	-1.10226	0.273339	-5.88827	0.452485	0.541105
Erythroid.cells	IFFO1	0.582882	2.880878	1.102197	0.273367	-5.0996	0.506362	0.592758
Erythroid.cells	COG2	-0.38823	3.567744	-1.10216	0.273384	-5.19541	0.494529	0.581545
Erythroid.cells	CLCN7	-0.4428	3.608444	-1.10204	0.273435	-5.25068	0.493837	0.580894
Erythroid.cells	GUK1	0.257432	4.908743	1.101972	0.273465	-5.5468	0.472251	0.560245

Erythroid.cells	CHPT1	-0.31416	3.607446	-1.10194	0.27348	-5.36958	0.493854	0.58091
Erythroid.cells	ZFP869	0.37972	4.026546	1.101935	0.273481	-5.28643	0.486786	0.574174
Erythroid.cells	POLR2L	0.239676	5.414671	1.101521	0.27366	-5.67829	0.464306	0.552494
Erythroid.cells	GM42670	-0.78511	0.89839	-1.10146	0.273686	-4.84294	0.542402	0.626461
Erythroid.cells	5830428M	0.489707	2.548294	1.101307	0.273752	-5.12211	0.512431	0.598463
Erythroid.cells	IGKV1-110	0.779643	0.050225	1.101114	0.273836	-4.84869	0.55859	0.641551
Erythroid.cells	SORCS2	-0.43357	3.3545	-1.10104	0.273868	-5.4221	0.498432	0.585269
Erythroid.cells	NAIP6	0.505846	2.310523	1.1009	0.273928	-5.08909	0.516689	0.602561
Erythroid.cells	THOC1	-0.20255	5.704802	-1.10072	0.274005	-5.78486	0.459775	0.548294
Erythroid.cells	NSF	-0.20506	6.770124	-1.10069	0.274019	-6.01246	0.443304	0.532278
Erythroid.cells	5031439G(	-0.25489	5.696635	-1.10039	0.274149	-5.69288	0.46004	0.548496
Erythroid.cells	MAP4K4	-0.20992	7.459224	-1.09991	0.274357	-6.16706	0.433262	0.522377
Erythroid.cells	DNMT3A	0.264832	6.044028	1.099817	0.274398	-5.68621	0.454752	0.543383
Erythroid.cells	TNFAIP8	-0.18482	7.424897	-1.0998	0.274407	-6.07983	0.433771	0.522896
Erythroid.cells	STRN	-0.26376	5.530644	-1.09971	0.274445	-5.62443	0.462824	0.551234
Erythroid.cells	SERINC4	-0.86633	0.365548	-1.0995	0.274534	-4.81035	0.552903	0.636529
Erythroid.cells	SLC7A1	0.344494	5.141375	1.099482	0.274543	-5.59289	0.469046	0.557359
Erythroid.cells	SNX11	0.494448	2.539196	1.099302	0.274621	-5.04062	0.512961	0.59935
Erythroid.cells	KHNYN	0.30678	4.263958	1.09927	0.274635	-5.37421	0.483397	0.571277
Erythroid.cells	PHACTR1	0.69139	1.519212	1.098603	0.274924	-4.93511	0.531708	0.616833
Erythroid.cells	MMP11	-0.92125	0.128652	-1.09858	0.274932	-4.77465	0.557849	0.641124
Erythroid.cells	FAM222B	0.298733	5.370089	1.098447	0.274992	-5.61891	0.465736	0.554176
Erythroid.cells	RRAS	-0.36206	4.338868	-1.09808	0.27515	-5.50627	0.482583	0.570336
Erythroid.cells	CC2D1A	-0.43045	3.044755	-1.09796	0.275202	-5.16418	0.504554	0.591322
Erythroid.cells	PTPRS	-0.34998	4.390471	-1.09793	0.275218	-5.39724	0.481728	0.569586
Erythroid.cells	POLR1A	0.303695	4.926327	1.097916	0.275222	-5.53437	0.472943	0.561153
Erythroid.cells	NPFF	-0.73742	0.954229	-1.09679	0.27571	-4.84956	0.542884	0.6271
Erythroid.cells	ZFP1	0.363673	3.874226	1.096727	0.275739	-5.27917	0.490915	0.578209
Erythroid.cells	KBTBD7	0.516509	2.137804	1.096681	0.275759	-5.04484	0.521166	0.606841
Erythroid.cells	NEAT1	0.241067	8.674976	1.096662	0.275767	-6.34554	0.416511	0.505896
Erythroid.cells	NID2	0.63253	2.19946	1.096627	0.275783	-5.06725	0.520059	0.605825
Erythroid.cells	CERCAM	0.692966	0.652443	1.096317	0.275917	-4.88214	0.548572	0.632566
Erythroid.cells	HIST1H4C	0.863557	0.309171	1.096296	0.275926	-4.81943	0.555112	0.638624
Erythroid.cells	DNAJC4	0.406865	3.061383	1.096101	0.276011	-5.17375	0.504846	0.591644
Erythroid.cells	ENGASE	0.697214	1.496503	1.096061	0.276029	-4.99762	0.532824	0.617959
Erythroid.cells	4632428C(	0.825875	0.110114	1.096039	0.276038	-4.81698	0.55894	0.642219
Erythroid.cells	IER3IP1	0.154798	6.363638	1.095908	0.276095	-5.88208	0.450721	0.539683
Erythroid.cells	TNFAIP2	0.452707	4.351498	1.095711	0.276181	-5.59022	0.482929	0.570849
Erythroid.cells	RFC5	-0.42449	4.185223	-1.09569	0.276191	-5.42433	0.485697	0.573497
Erythroid.cells	AIDA	-0.28378	4.437691	-1.09561	0.276226	-5.43117	0.481501	0.569498
Erythroid.cells	DHRS3	0.369956	4.170119	1.095154	0.276424	-5.50162	0.486144	0.573857
Erythroid.cells	MELK	0.522677	3.016502	1.095128	0.276435	-5.23825	0.505829	0.592579
Erythroid.cells	CTLA2A	0.375873	4.28991	1.095013	0.276485	-5.68563	0.484148	0.571958
Erythroid.cells	NUDT16L1	0.321871	4.234234	1.094721	0.276612	-5.43716	0.485186	0.572983
Erythroid.cells	APOB	0.388496	4.836413	1.094275	0.276807	-5.92721	0.475251	0.563627
Erythroid.cells	NOA1	-0.41824	3.137205	-1.09415	0.276862	-5.19172	0.503847	0.59096
Erythroid.cells	NFATC1	-0.22843	5.77824	-1.09407	0.276895	-5.75265	0.46014	0.549084

Erythroid.cells	RBMS1	-0.15306	7.558573	-1.09399	0.276931	-6.14534	0.43295	0.52254
Erythroid.cells	ZFP963	-0.652	1.15161	-1.09391	0.276965	-4.88063	0.539543	0.624524
Erythroid.cells	DPP4	0.261482	5.100888	1.093887	0.276976	-5.76372	0.470956	0.559544
Erythroid.cells	PDS5B	-0.17982	6.377064	-1.09388	0.276979	-5.9375	0.450798	0.540013
Erythroid.cells	DCAF10	0.213007	5.193751	1.093855	0.27699	-5.67763	0.469457	0.5581
Erythroid.cells	ETS2	-0.31731	5.865691	-1.09369	0.27706	-5.67737	0.458799	0.547785
Erythroid.cells	MARS	-0.45603	3.10364	-1.09338	0.277198	-5.16483	0.504631	0.591732
Erythroid.cells	GRK6	0.236886	5.764162	1.092691	0.277498	-5.65835	0.460888	0.549783
Erythroid.cells	SUGT1	-0.18484	5.949959	-1.09268	0.277502	-5.76641	0.457963	0.546946
Erythroid.cells	RAPGEFL1	-0.50728	2.784487	-1.09244	0.277605	-5.16497	0.510625	0.597441
Erythroid.cells	UTP3	-0.23968	5.450096	-1.09241	0.277621	-5.66004	0.465912	0.55472
Erythroid.cells	CES1D	-0.60939	1.90006	-1.09221	0.277709	-5.15538	0.526503	0.612368
Erythroid.cells	SMN1	-0.29264	4.851728	-1.09201	0.277794	-5.50116	0.475706	0.564149
Erythroid.cells	GM42869	-0.63054	1.342309	-1.09154	0.278001	-4.90019	0.537105	0.622169
Erythroid.cells	C8B	0.645489	0.571894	1.091362	0.278079	-4.95201	0.551591	0.635722
Erythroid.cells	EYA1	-0.65163	2.426875	-1.0913	0.278106	-5.06645	0.517401	0.603778
Erythroid.cells	ATG10	0.247401	5.64086	1.091088	0.278199	-5.70989	0.463306	0.552177
Erythroid.cells	1700123O2	0.252591	4.989927	1.091029	0.278224	-5.59625	0.473768	0.562275
Erythroid.cells	IGF2BP2	-0.64662	1.457318	-1.09079	0.278331	-4.96636	0.535078	0.620447
Erythroid.cells	NOX1	-0.78467	1.548335	-1.09066	0.278387	-4.91641	0.533401	0.618914
Erythroid.cells	ADAM3	-0.71835	-0.17326	-1.09053	0.278441	-4.87675	0.566058	0.649266
Erythroid.cells	5930403N2	-0.94856	-0.48768	-1.09035	0.278522	-4.78442	0.572238	0.654954
Erythroid.cells	DOHH	0.273771	4.347216	1.090331	0.27853	-5.51641	0.484388	0.57259
Erythroid.cells	PRELID2	0.636636	2.621255	1.090286	0.27855	-5.11918	0.51403	0.600767
Erythroid.cells	1110002L0	-0.48736	2.8153	-1.0901	0.278633	-5.09065	0.510605	0.597566
Erythroid.cells	SEC24A	-0.25606	6.616568	-1.08998	0.278684	-5.94468	0.448119	0.537588
Erythroid.cells	MED13L	0.168738	7.196724	1.08996	0.278693	-6.06187	0.439317	0.52898
Erythroid.cells	ZBTB41	-0.40706	2.915286	-1.08978	0.278772	-5.16126	0.508905	0.596047
Erythroid.cells	VPS37B	-0.2258	7.797142	-1.08966	0.278826	-6.30908	0.430454	0.520335
Erythroid.cells	RBM43	0.43466	2.961078	1.088857	0.279176	-5.16808	0.508169	0.595674
Erythroid.cells	ZFP946	0.555207	1.39583	1.088813	0.279195	-4.9745	0.536341	0.622193
Erythroid.cells	NR2C2AP	0.339665	4.44571	1.088784	0.279208	-5.43322	0.482866	0.571582
Erythroid.cells	GDI1	0.26596	4.991215	1.088772	0.279213	-5.60104	0.473903	0.562968
Erythroid.cells	PGK1	0.221761	7.765914	1.08852	0.279324	-6.23762	0.430962	0.521164
Erythroid.cells	CD300A	0.373506	4.623283	1.088457	0.279351	-5.58195	0.479929	0.568813
Erythroid.cells	TM6SF1	0.228815	6.252773	1.088432	0.279363	-5.91706	0.453842	0.543585
Erythroid.cells	NCAPG2	-0.32902	5.068495	-1.08827	0.279432	-5.64866	0.472648	0.561841
Erythroid.cells	CBARP	0.571453	1.923122	1.088254	0.279441	-5.08719	0.526675	0.613231
Erythroid.cells	PTPN14	-0.88121	0.524362	-1.08824	0.279447	-4.83146	0.552717	0.637534
Erythroid.cells	CLYBL	0.306964	4.489599	1.088152	0.279486	-5.50623	0.482138	0.570972
Erythroid.cells	SELENOO	0.416674	3.136064	1.088142	0.27949	-5.17263	0.505116	0.592891
Erythroid.cells	ZFP710	-0.24668	6.297621	-1.08813	0.279497	-5.9101	0.453146	0.542943
Erythroid.cells	DDX42	-0.18512	5.684077	-1.08738	0.279826	-5.72865	0.463135	0.552437
Erythroid.cells	GM15494	-0.89362	0.759331	-1.08733	0.279846	-4.88441	0.548683	0.633551
Erythroid.cells	SLC44A2	0.261263	6.242042	1.087143	0.279929	-5.78343	0.454366	0.543997
Erythroid.cells	EIF2B4	-0.25983	4.102435	-1.0871	0.279948	-5.40101	0.48898	0.57741
Erythroid.cells	MAGI1	0.532165	4.373842	1.087065	0.279963	-5.56616	0.48444	0.573079

Erythroid.cells	IFRD1	-0.21419	8.532379	-1.08663	0.280153	-6.36358	0.420382	0.510573
Erythroid.cells	GSTA3	-0.47925	4.0016	-1.08649	0.280216	-5.70091	0.490921	0.579253
Erythroid.cells	PPP6C	0.139727	6.737344	1.086341	0.280281	-5.99259	0.446949	0.536801
Erythroid.cells	SLC28A2	-0.49454	2.961181	-1.0863	0.280298	-5.22358	0.508817	0.596299
Erythroid.cells	BMP8A	-0.66897	2.321943	-1.08604	0.280415	-5.03712	0.520273	0.607134
Erythroid.cells	HSD17B7	-0.57868	1.697013	-1.08577	0.280532	-4.95321	0.531733	0.61788
Erythroid.cells	RAG1	-0.66387	1.672599	-1.08542	0.280686	-5.10836	0.53238	0.618467
Erythroid.cells	NUPL2	0.521274	2.367649	1.085177	0.280793	-5.06933	0.519853	0.606659
Erythroid.cells	SMLR1	0.510133	1.822706	1.084714	0.280997	-5.19446	0.529712	0.616087
Erythroid.cells	MSMO1	-0.38809	3.610529	-1.08469	0.281008	-5.24092	0.498072	0.586198
Erythroid.cells	ZBTB9	-0.48173	2.511721	-1.08467	0.281018	-5.05627	0.517279	0.604395
Erythroid.cells	CAPN11	-1.13021	1.046105	-1.0846	0.281048	-4.92234	0.544096	0.629528
Erythroid.cells	GSAP	-0.3272	5.642393	-1.08457	0.28106	-5.77429	0.464495	0.553962
Erythroid.cells	POLB	-0.23059	5.332268	-1.08448	0.281099	-5.65332	0.469462	0.55879
Erythroid.cells	EXTL2	0.604564	2.165568	1.084303	0.281178	-5.00987	0.523487	0.610285
Erythroid.cells	LYL1	0.45482	3.172684	1.084259	0.281198	-5.25091	0.505635	0.593426
Erythroid.cells	FEM1C	0.209257	7.117684	1.084203	0.281222	-6.05584	0.441619	0.531708
Erythroid.cells	CDH22	-1.18671	-0.85879	-1.08402	0.281302	-4.73698	0.581138	0.663776
Erythroid.cells	FDX2	0.278465	4.192147	1.083828	0.281388	-5.42838	0.488253	0.576942
Erythroid.cells	CCNH	-0.2148	5.363256	-1.08381	0.281396	-5.67898	0.469006	0.558438
Erythroid.cells	B3GLCT	-0.44173	3.311117	-1.08366	0.281463	-5.19782	0.503308	0.591322
Erythroid.cells	NCOA2	-0.17121	7.684552	-1.08347	0.281548	-6.18228	0.433271	0.523579
Erythroid.cells	HFE	0.319027	3.452689	1.082957	0.281772	-5.51579	0.501181	0.589143
Erythroid.cells	ZFP74	-0.53864	2.132709	-1.08292	0.28179	-4.99011	0.524495	0.611192
Erythroid.cells	DESI2	-0.14895	6.03278	-1.08259	0.281933	-5.82918	0.458802	0.548405
Erythroid.cells	ADAMTS14	0.742923	0.543497	1.082466	0.281989	-4.9328	0.554198	0.63891
Erythroid.cells	SLC35C1	0.453929	2.749333	1.082312	0.282057	-5.13956	0.513599	0.600958
Erythroid.cells	GM16104	1.075025	-0.60575	1.082153	0.282127	-4.7711	0.576632	0.65974
Erythroid.cells	1-Mar	0.568633	4.477654	1.08215	0.282128	-5.5155	0.483948	0.572805
Erythroid.cells	SART3	-0.21742	5.374984	-1.08198	0.282204	-5.68161	0.469266	0.558709
Erythroid.cells	TMED5	-0.13304	7.227678	-1.08197	0.282209	-6.05664	0.440423	0.530622
Erythroid.cells	MRPL43	-0.23112	5.608686	-1.08164	0.282353	-5.72486	0.46566	0.555159
Erythroid.cells	RAB3GAP1	-0.16536	6.044858	-1.08155	0.282392	-5.82217	0.458751	0.548464
Erythroid.cells	RAP2A	0.307527	3.974658	1.08118	0.282557	-5.44836	0.492707	0.581145
Erythroid.cells	KDM1A	-0.18139	5.813944	-1.08111	0.282589	-5.77596	0.462555	0.552172
Erythroid.cells	ATPAF2	0.407931	2.92038	1.080501	0.282858	-5.19274	0.511309	0.598687
Erythroid.cells	TMC3	-1.06719	-1.08655	-1.08005	0.283057	-4.75028	0.587433	0.669372
Erythroid.cells	CCPG1OS	0.573901	1.576044	1.079891	0.283128	-4.92806	0.535884	0.621733
Erythroid.cells	GMCL1	-0.22935	4.636774	-1.07959	0.28326	-5.55998	0.482426	0.571036
Erythroid.cells	GM17103	0.771289	1.028835	1.079133	0.283464	-4.92078	0.546554	0.631463
Erythroid.cells	SHFL	0.612565	1.832835	1.078967	0.283537	-5.01425	0.531648	0.617554
Erythroid.cells	DPH7	-0.6646	1.586984	-1.07878	0.28362	-4.96418	0.536238	0.62189
Erythroid.cells	ASCC2	0.223352	4.605255	1.078388	0.283794	-5.56117	0.483375	0.571944
Erythroid.cells	MIA2	0.149341	6.77012	1.078185	0.283884	-6.01218	0.448801	0.538525
Erythroid.cells	B230208H1	0.819394	0.861264	1.078139	0.283905	-4.94781	0.549909	0.634755
Erythroid.cells	CLDN11	0.8298	-0.29485	1.078017	0.283959	-4.8811	0.572303	0.655523
Erythroid.cells	E2F7	0.540886	2.983824	1.077958	0.283985	-5.2197	0.511101	0.598466

Erythroid.cells	SYTL2	0.579943	1.257049	1.077822	0.284045	-5.20088	0.54245	0.62797
Erythroid.cells	XRCC4	0.270111	5.185209	1.07775	0.284077	-5.54428	0.473846	0.562969
Erythroid.cells	ALDH18A1	-0.53624	3.070395	-1.07773	0.284087	-5.14279	0.509579	0.597101
Erythroid.cells	GCH1	0.253328	5.663529	1.077634	0.284128	-5.87419	0.466135	0.555564
Erythroid.cells	A430072P0	-0.87846	0.354986	-1.07747	0.284203	-4.8775	0.559604	0.643984
Erythroid.cells	MAPKAPK3	-0.26572	5.306805	-1.07743	0.284219	-5.68437	0.471873	0.561128
Erythroid.cells	ERF	0.444816	3.477368	1.077251	0.284299	-5.2736	0.50249	0.590472
Erythroid.cells	LGALS4	0.609069	2.563012	1.077243	0.284302	-5.02228	0.518564	0.605685
Erythroid.cells	METTL15	0.38177	3.231154	1.077104	0.284364	-5.24379	0.506788	0.594599
Erythroid.cells	INTS3	0.312573	3.898156	1.07641	0.284672	-5.32978	0.495598	0.584006
Erythroid.cells	CCDC167	0.337543	4.065969	1.076397	0.284678	-5.39293	0.492747	0.581285
Erythroid.cells	MED4	-0.28526	4.020834	-1.07633	0.284706	-5.34199	0.493512	0.582015
Erythroid.cells	GEN1	-0.67445	2.372171	-1.07626	0.28474	-5.0373	0.522334	0.60934
Erythroid.cells	TIMELESS	0.414781	3.491901	1.075967	0.284869	-5.33299	0.502676	0.590743
Erythroid.cells	ANKRD13D	-0.49082	2.738056	-1.07568	0.284998	-5.0867	0.515894	0.603393
Erythroid.cells	ARFIP2	-0.49589	2.138474	-1.0756	0.285034	-5.02657	0.526665	0.613571
Erythroid.cells	RRM2B	-0.2933	4.437435	-1.07541	0.285117	-5.45433	0.486594	0.575601
Erythroid.cells	TMSB15B1	0.472616	2.422831	1.075215	0.285204	-5.11211	0.521528	0.608825
Erythroid.cells	GM12655	-0.90258	0.353725	-1.07521	0.285207	-4.82505	0.560116	0.644858
Erythroid.cells	GM20492	0.525222	1.798274	1.075194	0.285213	-5.02326	0.53288	0.61949
Erythroid.cells	LASP1	0.220837	5.455474	1.075159	0.285229	-5.68024	0.469881	0.559562
Erythroid.cells	RHBDF2	0.326693	4.650027	1.075079	0.285264	-5.34544	0.483053	0.572251
Erythroid.cells	EML1	0.701083	1.303195	1.074737	0.285417	-4.96768	0.542058	0.628132
Erythroid.cells	NAA40	-0.24003	4.909944	-1.07471	0.28543	-5.52508	0.47876	0.568165
Erythroid.cells	HMOX1	-0.47734	4.807778	-1.07466	0.285449	-5.68192	0.480443	0.569783
Erythroid.cells	ERLEC1	0.266443	4.341715	1.074564	0.285494	-5.56611	0.488197	0.577279
Erythroid.cells	ZBTB8A	0.51653	1.978565	1.07451	0.285518	-5.01096	0.529577	0.616532
Erythroid.cells	PAN2	0.360849	3.190864	1.074373	0.285579	-5.20858	0.507912	0.596101
Erythroid.cells	ZFP101	0.421926	2.916235	1.074331	0.285598	-5.15822	0.512738	0.600673
Erythroid.cells	ERGIC1	0.30951	5.084699	1.074179	0.285665	-5.58012	0.475897	0.565536
Erythroid.cells	1700123M	-0.71759	0.89752	-1.07412	0.28569	-4.88278	0.5497	0.635418
Erythroid.cells	QK	-0.12707	8.142329	-1.07383	0.285821	-6.25079	0.428636	0.519511
Erythroid.cells	CNOT9	-0.26446	4.541441	-1.07369	0.285885	-5.4619	0.484869	0.574317
Erythroid.cells	DDHD2	-0.28998	4.784447	-1.07368	0.285886	-5.51077	0.480839	0.570444
Erythroid.cells	SMIM8	0.259866	4.587829	1.073657	0.285898	-5.58265	0.484097	0.573586
Erythroid.cells	RTF2	0.168999	5.957747	1.073497	0.285969	-5.83406	0.461871	0.55219
Erythroid.cells	MTO1	0.355524	3.553315	1.073441	0.285994	-5.24308	0.501626	0.590415
Erythroid.cells	ZFP617	0.545257	2.165547	1.073271	0.28607	-5.06222	0.526211	0.613692
Erythroid.cells	NDUFS7	0.15628	6.320866	1.073188	0.286107	-5.97167	0.456183	0.546675
Erythroid.cells	GM11655	-0.8735	0.316941	-1.073	0.28619	-4.79317	0.56092	0.646141
Erythroid.cells	SRSF1	-0.19173	6.067856	-1.07278	0.286291	-5.87918	0.460197	0.550709
Erythroid.cells	PYROXD1	0.40643	2.95488	1.072734	0.28631	-5.18864	0.51214	0.600556
Erythroid.cells	NUP43	0.436769	2.795528	1.072683	0.286332	-5.14427	0.514959	0.603234
Erythroid.cells	HSD3B3	0.566641	2.014179	1.072518	0.286406	-5.22777	0.52902	0.6166
Erythroid.cells	MS4A6C	0.459182	5.165825	1.072454	0.286435	-5.78211	0.474657	0.564845
Erythroid.cells	XAB2	0.356075	3.922858	1.072136	0.286577	-5.33798	0.49552	0.584812
Erythroid.cells	ZFP820	0.858514	-0.04486	1.071875	0.286693	-4.80841	0.568156	0.653007

Erythroid.cells	PPT2	0.312538	4.199673	1.071872	0.286695	-5.47196	0.490826	0.580323
Erythroid.cells	CAR9	-0.75155	0.285801	-1.07175	0.286748	-4.87324	0.561706	0.647056
Erythroid.cells	H2-AA	0.431503	8.331767	1.071576	0.286827	-6.60983	0.426075	0.517306
Erythroid.cells	SNHG4.1	-0.88558	0.471422	-1.07157	0.286828	-4.88535	0.558118	0.643765
Erythroid.cells	TTC39C	-0.45326	2.230936	-1.07148	0.286872	-5.31229	0.525246	0.61312
Erythroid.cells	GM15956	-0.82376	0.27605	-1.07116	0.287011	-4.81558	0.562027	0.647417
Erythroid.cells	DLD	0.222297	4.599821	1.070945	0.287109	-5.5617	0.484237	0.574138
Erythroid.cells	ACTR3	-0.11822	8.876057	-1.07074	0.287199	-6.4024	0.418347	0.509718
Erythroid.cells	7-Sep	0.107581	8.277792	1.070685	0.287225	-6.33805	0.42696	0.518274
Erythroid.cells	GM14963	-0.88162	0.898614	-1.07068	0.287229	-4.88808	0.550078	0.636406
Erythroid.cells	XRCC6	-0.29348	5.144906	-1.07066	0.287237	-5.767	0.475259	0.565503
Erythroid.cells	PLSCR2	0.980065	-0.38835	1.070536	0.287292	-4.84823	0.575082	0.659552
Erythroid.cells	PLEKHA4	0.613769	2.406918	1.07002	0.287523	-5.05854	0.522488	0.610558
Erythroid.cells	STAT1	-0.50742	6.871703	-1.06995	0.287556	-6.06803	0.448212	0.539208
Erythroid.cells	GM16287	-0.73495	-0.44109	-1.06985	0.287599	-4.84488	0.576445	0.660823
Erythroid.cells	LRRC41	-0.27297	4.919545	-1.06974	0.287648	-5.53467	0.47922	0.569325
Erythroid.cells	MST1	0.751021	0.649079	1.06892	0.288015	-4.98667	0.555276	0.64126
Erythroid.cells	GET4	-0.23773	5.110437	-1.06891	0.28802	-5.63703	0.476199	0.566424
Erythroid.cells	LYPLA2	0.253925	4.945046	1.068871	0.288037	-5.53239	0.47891	0.569038
Erythroid.cells	BIN3	-0.26038	5.271025	-1.06883	0.288055	-5.58028	0.473582	0.563897
Erythroid.cells	AIFM1	0.247344	4.370662	1.068755	0.28809	-5.54993	0.488452	0.578209
Erythroid.cells	PLPP5	-0.36053	3.394162	-1.06875	0.28809	-5.29	0.505135	0.594139
Erythroid.cells	TRIM47	0.501147	2.41312	1.068736	0.288098	-5.07513	0.522495	0.610578
Erythroid.cells	TXNL1	0.170211	6.955558	1.068678	0.288124	-6.11187	0.44703	0.538064
Erythroid.cells	DCAF7	0.195189	5.327488	1.068598	0.28816	-5.68587	0.472665	0.563045
Erythroid.cells	MIRT1	0.363407	4.950639	1.068042	0.288409	-5.50589	0.479027	0.569127
Erythroid.cells	EXOSC2	-0.40065	2.945711	-1.06801	0.288424	-5.16717	0.51322	0.601787
Erythroid.cells	G0S2	0.402211	3.133285	1.067984	0.288435	-5.41375	0.509915	0.598655
Erythroid.cells	AMIGO2	0.918908	0.934211	1.06748	0.288661	-4.8843	0.550335	0.636649
Erythroid.cells	ZBTB40	0.386517	3.163496	1.067463	0.288669	-5.23143	0.509623	0.598386
Erythroid.cells	ZFP398	0.340622	4.517006	1.067282	0.28875	-5.46568	0.486495	0.576318
Erythroid.cells	MAMLD1	0.743536	0.110917	1.066968	0.288891	-4.93322	0.566441	0.651546
Erythroid.cells	DCPS	0.258941	5.004121	1.066644	0.289037	-5.5819	0.478638	0.56877
Erythroid.cells	CAAP1	-0.26525	4.575271	-1.06646	0.289121	-5.49358	0.485739	0.575637
Erythroid.cells	EFNA5	0.846506	2.313548	1.066427	0.289134	-5.05372	0.525058	0.613029
Erythroid.cells	FAM207A	-0.2757	4.223482	-1.06641	0.289142	-5.4365	0.491647	0.581302
Erythroid.cells	GM49864	-0.84884	0.452692	-1.06632	0.289181	-4.82667	0.55987	0.645608
Erythroid.cells	CDC34	0.190293	6.050691	1.066217	0.289229	-5.90008	0.461765	0.552554
Erythroid.cells	RIN3	0.224051	5.640444	1.066046	0.289305	-5.77151	0.468303	0.558901
Erythroid.cells	ARID5A	-0.29116	4.681741	-1.066	0.289327	-5.55157	0.483966	0.574023
Erythroid.cells	RETNLG	0.695354	3.313557	1.065871	0.289384	-5.39225	0.507282	0.596338
Erythroid.cells	TFG	-0.18548	5.864765	-1.06577	0.289428	-5.8082	0.464718	0.555477
Erythroid.cells	MPV17	0.275725	4.075124	1.065437	0.289579	-5.41449	0.494336	0.584023
Erythroid.cells	HEATR9	-0.66611	-0.41964	-1.06516	0.289702	-4.94389	0.577343	0.661885
Erythroid.cells	SLC7A7	-0.42403	3.785978	-1.0645	0.290001	-5.30855	0.499792	0.589205
Erythroid.cells	4930590J0	-0.62215	1.547542	-1.06438	0.290053	-4.9723	0.53986	0.62714
Erythroid.cells	GANAB	0.252706	4.730059	1.064138	0.290164	-5.55128	0.483834	0.574125

Erythroid.cells	SLC10A7	0.201011	5.908741	1.063988	0.290231	-5.81198	0.46466	0.555618
Erythroid.cells	4930581F2	0.376827	3.634004	1.06394	0.290253	-5.24317	0.502412	0.591939
Erythroid.cells	3110056K0	0.239545	4.723448	1.063803	0.290315	-5.53985	0.483944	0.574277
Erythroid.cells	DOP1A	0.340035	4.09244	1.063716	0.290354	-5.39177	0.494552	0.584493
Erythroid.cells	NRGN	-0.4693	3.663682	-1.06353	0.290437	-5.52788	0.501899	0.591555
Erythroid.cells	RASGRF2	-0.68639	0.116081	-1.06347	0.290463	-4.89577	0.567201	0.65288
Erythroid.cells	TGFBR2	-0.16538	7.292661	-1.06343	0.290484	-6.08474	0.44317	0.534726
Erythroid.cells	FLVCR1	0.319259	4.670527	1.063375	0.290508	-5.41084	0.484825	0.575241
Erythroid.cells	BIRC3	-0.21209	7.111789	-1.06319	0.290593	-6.06657	0.445917	0.537502
Erythroid.cells	KDM4A	0.284365	4.161515	1.063132	0.290617	-5.45688	0.493379	0.583513
Erythroid.cells	NDUFA9	0.227951	4.840567	1.06301	0.290672	-5.63952	0.482002	0.572624
Erythroid.cells	DDX3Y	3.56406	2.707432	1.062932	0.290708	-5.3424	0.518698	0.607665
Erythroid.cells	OPA1	0.23834	4.835691	1.062894	0.290725	-5.56191	0.482082	0.572723
Erythroid.cells	SH3KBP1	-0.14804	7.863843	-1.06258	0.290868	-6.21375	0.434751	0.526467
Erythroid.cells	CYC1	0.202999	6.36195	1.062289	0.290997	-6.02928	0.457722	0.548955
Erythroid.cells	SMIM10L1	0.21713	4.931338	1.062253	0.291014	-5.72137	0.480732	0.571261
Erythroid.cells	NRARP	-0.59778	1.780032	-1.06198	0.291139	-5.08124	0.535823	0.623799
Erythroid.cells	3110082I1	-0.41326	4.199896	-1.06197	0.291144	-5.33128	0.492982	0.583187
Erythroid.cells	GBP9	-0.5248	3.036901	-1.0619	0.291173	-5.19643	0.513109	0.602394
Erythroid.cells	POU2AF1	-0.32304	4.320007	-1.06169	0.291267	-5.74314	0.491026	0.58133
Erythroid.cells	GRB14	0.523829	1.939569	1.061398	0.2914	-5.273	0.533115	0.621243
Erythroid.cells	RAB5IF	-0.15834	7.307335	-1.06114	0.291516	-6.11371	0.443432	0.535061
Erythroid.cells	NUBP2	-0.32338	4.451117	-1.06105	0.291558	-5.47385	0.489027	0.579348
Erythroid.cells	BIVM	-0.63085	0.882383	-1.06098	0.291591	-4.90772	0.552997	0.63983
Erythroid.cells	AMOTL2	-0.83335	0.684568	-1.06058	0.291772	-4.92037	0.557035	0.643517
Erythroid.cells	LY6G	0.786073	-0.81776	1.060239	0.291923	-4.92028	0.586792	0.670997
Erythroid.cells	GM26839	0.802685	0.656622	1.06015	0.291964	-4.86551	0.557666	0.644099
Erythroid.cells	NBEAL2	0.406469	2.635242	1.060137	0.29197	-5.13589	0.520879	0.609632
Erythroid.cells	SAMD3	0.618144	0.184815	1.059949	0.292055	-5.12504	0.566892	0.652677
Erythroid.cells	REV1	-0.22443	5.326262	-1.0596	0.292212	-5.72197	0.474976	0.56576
Erythroid.cells	AGBL5	-0.45336	2.244561	-1.05956	0.292229	-5.0744	0.528087	0.616442
Erythroid.cells	SMIM12	-0.2857	4.0506	-1.05951	0.292255	-5.4207	0.49625	0.586232
Erythroid.cells	C2CD2L	0.400521	2.991873	1.059307	0.292346	-5.23482	0.514663	0.603877
Erythroid.cells	PSMD14	-0.15213	6.618882	-1.05923	0.29238	-5.98225	0.4544	0.545913
Erythroid.cells	6-Sep	0.236132	5.624977	1.059098	0.29244	-5.8891	0.470135	0.561331
Erythroid.cells	ZNHIT1	0.209398	5.422819	1.059074	0.292451	-5.7157	0.473406	0.564508
Erythroid.cells	ZFP628	0.518796	2.635685	1.05892	0.292521	-5.11119	0.521052	0.610139
Erythroid.cells	EPC2	-0.19477	6.14343	-1.05816	0.292866	-5.86971	0.462227	0.553597
Erythroid.cells	H2-EB1	0.444958	6.754688	1.058117	0.292884	-6.39159	0.452656	0.544255
Erythroid.cells	CACNA1D	-0.48734	3.396539	-1.05812	0.292885	-5.23847	0.507949	0.597588
Erythroid.cells	MAB21L3	-0.75337	-0.30498	-1.05779	0.293035	-5.01006	0.577312	0.662414
Erythroid.cells	COL4A3BP	-0.19008	6.352484	-1.05742	0.2932	-5.92775	0.459266	0.550524
Erythroid.cells	TGFA	0.67631	0.796415	1.057287	0.293261	-5.02024	0.55601	0.642593
Erythroid.cells	PXN	-0.2334	6.266447	-1.05715	0.293324	-5.82493	0.460659	0.551908
Erythroid.cells	TRDC	-0.53633	1.265066	-1.05697	0.293403	-5.4403	0.547148	0.63445
Erythroid.cells	TPRN	0.574226	2.21301	1.056889	0.293442	-5.03199	0.529549	0.617968
Erythroid.cells	1700021F0	-0.34676	3.209032	-1.05671	0.293522	-5.30586	0.511737	0.601117

Erythroid.cells	1110012L1	0.528348	1.829365	1.056544	0.293598	-5.04418	0.536653	0.624632
Erythroid.cells	CLN8	0.354387	3.202024	1.056495	0.29362	-5.4826	0.51186	0.601234
Erythroid.cells	OSBP	-0.26417	5.016429	-1.05621	0.29375	-5.53202	0.48096	0.57172
Erythroid.cells	CD52	-0.21483	9.30175	-1.05618	0.293765	-6.51796	0.415442	0.507437
Erythroid.cells	ESPN	-0.86822	-0.17599	-1.056	0.293845	-4.84824	0.575175	0.660543
Erythroid.cells	4930453N2	0.245693	4.481233	1.055833	0.293921	-5.57692	0.489882	0.580333
Erythroid.cells	GM32036	-0.31947	3.651119	-1.05583	0.293922	-5.29397	0.504066	0.593898
Erythroid.cells	GM50019	-0.8815	-0.06748	-1.05575	0.293957	-4.82936	0.573024	0.6586
Erythroid.cells	ZFP146	-0.3648	3.413704	-1.05563	0.294015	-5.2874	0.508201	0.597931
Erythroid.cells	SP140	-0.29084	6.188606	-1.05554	0.294053	-5.79283	0.462013	0.553516
Erythroid.cells	PDE7A	0.196539	6.703412	1.055368	0.294132	-6.06259	0.453955	0.545712
Erythroid.cells	PTPMT1	0.251049	4.267152	1.055222	0.294199	-5.52129	0.493512	0.584076
Erythroid.cells	MANEA	-0.38032	3.728612	-1.05505	0.294279	-5.28223	0.502736	0.592917
Erythroid.cells	GM41409	-0.65587	3.031183	-1.05502	0.294293	-5.13198	0.514951	0.604536
Erythroid.cells	SNHG16	0.566035	2.227913	1.054976	0.29431	-5.03273	0.529402	0.618195
Erythroid.cells	AGXT2	0.572776	1.612637	1.054823	0.29438	-5.17004	0.540756	0.62891
Erythroid.cells	PPM1G	-0.15866	6.448565	-1.05475	0.294412	-5.95513	0.457933	0.54974
Erythroid.cells	INSR	-0.2729	5.770425	-1.05456	0.294502	-5.7595	0.46871	0.560238
Erythroid.cells	EEPD1	-0.37954	4.881821	-1.05451	0.294522	-5.4574	0.483219	0.574296
Erythroid.cells	WFS1	0.557256	1.279726	1.054396	0.294575	-4.93493	0.547027	0.634876
Erythroid.cells	FCGR1	0.620079	2.432362	1.054245	0.294643	-5.2144	0.525743	0.614872
Erythroid.cells	SLC12A7	-0.40356	3.37429	-1.05361	0.294931	-5.31245	0.509364	0.599232
Erythroid.cells	TMEM39B	-0.32259	4.225197	-1.0533	0.295072	-5.43319	0.494671	0.585276
Erythroid.cells	NDUFAF5	-0.44798	2.563951	-1.05327	0.295089	-5.13669	0.523779	0.612966
Erythroid.cells	KLRA5	-0.49497	2.005641	-1.05319	0.295122	-5.53045	0.533956	0.622585
Erythroid.cells	PHKB	0.266136	5.588989	1.053183	0.295127	-5.64063	0.472043	0.563497
Erythroid.cells	HAUS6	-0.30242	4.556972	-1.05291	0.295251	-5.52889	0.489186	0.580003
Erythroid.cells	H2-DMB2	0.374069	3.016521	1.052745	0.295327	-5.45106	0.51585	0.605444
Erythroid.cells	TM2D2	0.236957	5.178235	1.052594	0.295396	-5.60093	0.478933	0.570107
Erythroid.cells	RAB11FIP4	0.534565	1.161785	1.052137	0.295604	-5.05044	0.550143	0.637757
Erythroid.cells	AP2A1	-0.30918	4.542647	-1.05178	0.295766	-5.48497	0.489674	0.580641
Erythroid.cells	TMEM53	0.654334	0.637469	1.051654	0.295824	-4.99164	0.560189	0.647255
Erythroid.cells	COMMD6	0.282069	4.358964	1.051634	0.295833	-5.4763	0.492775	0.583617
Erythroid.cells	4930522L1	-0.33167	3.684756	-1.05158	0.295856	-5.39515	0.504332	0.594672
Erythroid.cells	DOCK9	-0.37702	4.197961	-1.05151	0.295891	-5.505	0.495509	0.586238
Erythroid.cells	GEMIN8	-0.4977	2.284507	-1.05145	0.295919	-5.05676	0.529248	0.618295
Erythroid.cells	SELL	0.372434	5.472373	1.051435	0.295924	-5.60661	0.474294	0.565805
Erythroid.cells	DPH3	0.186442	5.480129	1.051375	0.295951	-5.76736	0.474167	0.565682
Erythroid.cells	FBXL2	-0.40665	3.231347	-1.05124	0.296011	-5.2403	0.512272	0.602262
Erythroid.cells	ALB	0.422867	9.441682	1.051145	0.296056	-6.72376	0.414174	0.50666
Erythroid.cells	FRMD8	0.319046	4.222806	1.050968	0.296137	-5.42131	0.495103	0.585925
Erythroid.cells	RCAN3	0.501741	2.117276	1.050553	0.296327	-5.05827	0.532326	0.621506
Erythroid.cells	MRTFB	0.296927	4.475202	1.050418	0.296388	-5.52381	0.490827	0.582067
Erythroid.cells	LAMTOR1	-0.16309	6.503458	-1.05036	0.296415	-5.93914	0.457846	0.55011
Erythroid.cells	HUS1	0.586583	1.653586	1.05028	0.296452	-4.97402	0.540906	0.629611
Erythroid.cells	COX15	-0.36135	2.891641	-1.05022	0.29648	-5.1838	0.518309	0.608304
Erythroid.cells	MEGF11	0.762972	0.651843	1.050127	0.296522	-4.92356	0.55993	0.647409

Erythroid.cells	TPRA1	0.413739	2.802051	1.050101	0.296534	-5.20194	0.519911	0.609848
Erythroid.cells	MGST2	-0.48694	2.953494	-1.05004	0.296559	-5.15535	0.517206	0.607284
Erythroid.cells	FYN	0.263886	7.843798	1.050038	0.296562	-6.28454	0.437347	0.529996
Erythroid.cells	GRCC10	0.159229	6.427807	1.049889	0.296631	-5.96299	0.459062	0.551359
Erythroid.cells	CCHCR1	0.634685	1.536464	1.049572	0.296776	-4.9945	0.543219	0.631888
Erythroid.cells	GM13547	-0.99285	0.514665	-1.04951	0.296802	-4.85564	0.562715	0.650095
Erythroid.cells	KIF21B	0.285885	4.865707	1.049453	0.29683	-5.54158	0.484397	0.576014
Erythroid.cells	PDE2A	-0.29949	6.050057	-1.04932	0.296889	-5.86433	0.465121	0.557381
Erythroid.cells	XPOT	-0.28273	4.512589	-1.04913	0.296977	-5.50268	0.490314	0.581813
Erythroid.cells	GM4673	-0.43306	2.578427	-1.04904	0.297021	-5.12207	0.524057	0.613988
Erythroid.cells	RRP36	-0.29242	3.775241	-1.04883	0.297114	-5.33066	0.502902	0.593939
Erythroid.cells	IDNK	-0.21717	5.52475	-1.04873	0.297161	-5.74825	0.473571	0.565733
Erythroid.cells	PIRA2	0.517611	3.589246	1.048695	0.297177	-5.3267	0.50613	0.59705
Erythroid.cells	LUZP1	-0.23215	5.787075	-1.04868	0.297184	-5.8352	0.469331	0.561617
Erythroid.cells	PLPP3	0.543305	4.693864	1.048245	0.297383	-5.57866	0.487514	0.57917
Erythroid.cells	CDKL4	-0.666	1.561956	-1.048	0.297495	-5.02967	0.543132	0.632007
Erythroid.cells	RANBP2	0.212311	6.846596	1.047879	0.297551	-6.03343	0.45294	0.545663
Erythroid.cells	MYBPC3	0.606432	1.123244	1.047712	0.297627	-5.02467	0.551475	0.639928
Erythroid.cells	RPGRIP1	-0.18211	6.55945	-1.0474	0.29777	-5.97563	0.457452	0.550203
Erythroid.cells	SMAD3	-0.24865	6.315623	-1.04739	0.297774	-5.95687	0.461286	0.553951
Erythroid.cells	QRSL1	-0.41997	3.18623	-1.04738	0.297777	-5.23493	0.513618	0.60437
Erythroid.cells	VPS25	-0.65554	0.881521	-1.04719	0.297867	-4.92032	0.556169	0.644467
Erythroid.cells	EFNB2	0.609494	2.469455	1.047015	0.297947	-5.12995	0.526546	0.616753
Erythroid.cells	PURG	0.400218	3.260975	1.046947	0.297978	-5.24685	0.512384	0.603324
Erythroid.cells	MIEN1	-0.17271	5.845651	-1.04668	0.2981	-5.81091	0.468964	0.561531
Erythroid.cells	GM14634	-0.61377	1.834434	-1.04652	0.298174	-5.02231	0.538373	0.627928
Erythroid.cells	COX11	-0.27857	3.751078	-1.04621	0.298316	-5.39333	0.50399	0.595456
Erythroid.cells	4931406P1	-0.20257	5.594349	-1.04612	0.298357	-5.75815	0.473072	0.565673
Erythroid.cells	CNOT3	-0.2293	5.46868	-1.046	0.298412	-5.66937	0.475115	0.567656
Erythroid.cells	TMEM14C	-0.17089	6.950542	-1.046	0.298414	-6.10435	0.451606	0.544713
Erythroid.cells	SMAD1	0.357192	3.547772	1.045902	0.298457	-5.30277	0.507528	0.598858
Erythroid.cells	MTMR6	-0.29309	4.718718	-1.04583	0.298489	-5.47766	0.487503	0.579655
Erythroid.cells	TUBGCP6	-0.47352	2.759296	-1.04574	0.298533	-5.13846	0.521495	0.612194
Erythroid.cells	SLC39A8	0.575953	2.726982	1.045557	0.298616	-5.13038	0.522133	0.612795
Erythroid.cells	TTC7	-0.24414	5.873227	-1.04507	0.298841	-5.78113	0.468803	0.561578
Erythroid.cells	AFF3	0.228235	7.930696	1.045012	0.298866	-6.35102	0.436954	0.53035
Erythroid.cells	QPRT	0.444102	2.660001	1.04486	0.298936	-5.37755	0.523541	0.614155
Erythroid.cells	ARHGEF10I	0.38899	2.695098	1.044786	0.29897	-5.43429	0.522908	0.613555
Erythroid.cells	GM20033	0.807851	0.595708	1.044543	0.299082	-4.89757	0.562176	0.650486
Erythroid.cells	TRIM62	-0.53911	1.410517	-1.04429	0.299197	-4.98125	0.546588	0.635987
Erythroid.cells	GM3336	0.67731	1.732452	1.044269	0.299208	-5.08539	0.540551	0.630322
Erythroid.cells	GPR34	0.695616	0.588061	1.044195	0.299242	-5.09667	0.562324	0.650686
Erythroid.cells	TRRAP	0.30023	4.784536	1.044066	0.299301	-5.45582	0.486643	0.579006
Erythroid.cells	NKTR	-0.14934	7.193099	-1.04402	0.299322	-6.0468	0.448098	0.541454
Erythroid.cells	EML6	0.573635	2.764743	1.043995	0.299334	-5.28408	0.521655	0.612485
Erythroid.cells	GIMAP1	-0.28474	4.06141	-1.04395	0.299355	-5.55674	0.498886	0.590779
Erythroid.cells	LOXL2	0.653064	1.406795	1.043741	0.29945	-4.99094	0.546658	0.636054

Erythroid.cells	2700081O1	-0.39807	3.137169	-1.04364	0.299496	-5.25312	0.515007	0.606173
Erythroid.cells	NEDD9	0.221898	8.003683	1.043633	0.2995	-6.23804	0.435867	0.529376
Erythroid.cells	REPIN1	0.667429	1.082403	1.0435	0.299562	-4.94306	0.552811	0.641813
Erythroid.cells	1110059G1	-0.29268	3.928256	-1.04332	0.299646	-5.37795	0.501176	0.592974
Erythroid.cells	SMPD1	0.460008	2.643295	1.04325	0.299677	-5.15404	0.523842	0.614559
Erythroid.cells	SUCLA2	0.174852	5.593683	1.043234	0.299684	-5.88266	0.473316	0.56611
Erythroid.cells	BDP1	-0.21731	5.529011	-1.043	0.29979	-5.72691	0.474426	0.56713
Erythroid.cells	MCRS1	-0.27286	4.318538	-1.04294	0.299822	-5.47835	0.494557	0.586605
Erythroid.cells	ZFP597	-0.45804	2.862663	-1.04247	0.300036	-5.14812	0.520247	0.610989
Erythroid.cells	EBF1	-0.28268	8.683852	-1.04211	0.300201	-6.56792	0.426327	0.519719
Erythroid.cells	TICAM2	0.527595	1.274799	1.041136	0.300651	-5.07245	0.550464	0.639114
Erythroid.cells	SOS1	-0.18415	5.916592	-1.04101	0.300711	-5.82409	0.469226	0.561692
Erythroid.cells	BASP1	-0.40504	5.646818	-1.04091	0.300753	-5.83112	0.473585	0.565956
Erythroid.cells	RALBP1	0.159092	6.268527	1.040741	0.300834	-5.9844	0.46365	0.556301
Erythroid.cells	IL12RB1	-0.8021	0.500116	-1.04016	0.301101	-4.94604	0.565847	0.65333
Erythroid.cells	TMEM229I	-0.37046	4.03146	-1.03983	0.301256	-5.30181	0.501109	0.592345
Erythroid.cells	1810034E1	0.546511	1.895787	1.03981	0.301264	-5.05106	0.539354	0.628607
Erythroid.cells	MRPL4	-0.21904	5.176328	-1.03931	0.301493	-5.69946	0.481839	0.573967
Erythroid.cells	MFSD11	-0.36867	3.854162	-1.03928	0.301507	-5.33507	0.504231	0.595486
Erythroid.cells	ZMYND8	-0.19764	6.108671	-1.03915	0.301571	-5.80944	0.46668	0.55926
Erythroid.cells	F10	-0.39584	4.54109	-1.03913	0.301578	-5.97904	0.492464	0.584208
Erythroid.cells	GALT	-0.46108	2.389889	-1.03909	0.301597	-5.16391	0.530305	0.620247
Erythroid.cells	TMCO6	-0.42137	2.683081	-1.03908	0.3016	-5.12973	0.524974	0.615209
Erythroid.cells	AKIRIN2	-0.19525	5.756269	-1.03873	0.301761	-5.80586	0.472419	0.564873
Erythroid.cells	4833438C0	-0.42978	2.025244	-1.03867	0.30179	-5.06135	0.537092	0.626708
Erythroid.cells	TBCB	-0.18088	6.070727	-1.03854	0.301852	-5.85409	0.467356	0.560055
Erythroid.cells	NCKAP5	0.757408	1.217214	1.038379	0.301925	-4.97662	0.552272	0.641115
Erythroid.cells	FBXL14	-0.23202	5.203671	-1.038	0.302099	-5.60566	0.481458	0.573822
Erythroid.cells	GM12596	-0.43591	2.627238	-1.03792	0.302135	-5.24019	0.526062	0.616479
Erythroid.cells	CALD1	0.386023	3.777745	1.037818	0.302185	-5.61147	0.505632	0.597057
Erythroid.cells	C1GALT1C1	0.319768	4.044817	1.037795	0.302196	-5.4171	0.501009	0.592635
Erythroid.cells	GM16230	-0.96929	0.238127	-1.03775	0.302216	-4.83841	0.571255	0.658798
Erythroid.cells	PHTF1	-0.31378	4.68503	-1.03772	0.302232	-5.51917	0.490107	0.582167
Erythroid.cells	BC030867	0.598051	2.099983	1.03769	0.302244	-5.14917	0.53571	0.625585
Erythroid.cells	STEAP4	-0.61469	2.205553	-1.03767	0.302254	-5.17933	0.533763	0.623751
Erythroid.cells	TACSTD2	-0.81401	-0.35025	-1.03757	0.302299	-4.88554	0.58298	0.669643
Erythroid.cells	ZDHHC18	-0.2291	5.57552	-1.03707	0.30253	-5.79456	0.475639	0.568121
Erythroid.cells	TGFBRAP1	-0.31666	3.494101	-1.0369	0.302612	-5.28443	0.51095	0.60203
Erythroid.cells	FOSL1	-0.63448	1.662098	-1.03667	0.302719	-5.12427	0.544295	0.633518
Erythroid.cells	N4BP2L2	-0.12755	6.786437	-1.03661	0.302742	-6.03998	0.45641	0.54932
Erythroid.cells	UBA2	-0.1608	6.457603	-1.03643	0.302829	-5.9718	0.46163	0.55449
Erythroid.cells	RSPO3	1.047559	0.723734	1.036287	0.302894	-4.96426	0.562287	0.650443
Erythroid.cells	ING5	-0.36975	3.456639	-1.03614	0.302962	-5.22441	0.511732	0.602877
Erythroid.cells	GM49439	0.768149	-0.46286	1.035864	0.303091	-4.89173	0.585803	0.672431
Erythroid.cells	TTC13	-0.32761	3.938924	-1.03571	0.30316	-5.32668	0.503311	0.595054
Erythroid.cells	NDUFA4	0.16239	8.596899	1.035604	0.303211	-6.47829	0.429119	0.522698
Erythroid.cells	TCTN1	-0.74595	1.315572	-1.03559	0.303218	-4.92604	0.550919	0.640105

Erythroid.cells	GM21188	-0.6387	1.578983	-1.03559	0.30322	-5.23997	0.545936	0.635436
Erythroid.cells	MANF	-0.16729	6.808848	-1.03554	0.303239	-6.00659	0.456125	0.549369
Erythroid.cells	LRRC43	-0.91114	-0.12202	-1.03533	0.303339	-4.82491	0.57902	0.666175
Erythroid.cells	TIAM2	0.568727	2.628932	1.035166	0.303414	-5.24231	0.526593	0.617153
Erythroid.cells	CATSPER2	0.597654	1.800753	1.035063	0.303462	-5.01665	0.541843	0.631531
Erythroid.cells	HDAC5	-0.31391	4.378269	-1.03503	0.303479	-5.49982	0.495828	0.587843
Erythroid.cells	1110038F1	-0.27817	4.590808	-1.03475	0.303607	-5.53603	0.492348	0.584457
Erythroid.cells	CORO1B	0.336939	4.252422	1.034542	0.303704	-5.44062	0.498155	0.590057
Erythroid.cells	F630028O1	-0.65226	1.027185	-1.03445	0.303748	-5.00469	0.556697	0.645441
Erythroid.cells	TST	0.415357	3.470042	1.03436	0.303789	-5.60646	0.511743	0.60308
Erythroid.cells	NDFIP2	-0.17654	5.951714	-1.03392	0.303992	-5.87888	0.470152	0.562912
Erythroid.cells	NAA35	-0.23211	4.851078	-1.03373	0.304082	-5.59605	0.48824	0.58053
Erythroid.cells	MAFF	0.402453	3.876198	1.033719	0.304087	-5.45806	0.504875	0.5965
Erythroid.cells	CREB3L2	-0.44267	3.55229	-1.03328	0.30429	-5.26704	0.510532	0.602055
Erythroid.cells	SOX5	0.362736	4.684841	1.033219	0.30432	-5.83079	0.491036	0.583388
Erythroid.cells	FASL	-0.46908	1.037842	-1.03317	0.304344	-5.31713	0.55675	0.645707
Erythroid.cells	MSANTD2	-0.2399	4.864755	-1.03311	0.304371	-5.54128	0.488011	0.580502
Erythroid.cells	UBE2V1	0.138444	6.983076	1.033107	0.304372	-6.13833	0.453845	0.547257
Erythroid.cells	JAK1	0.12416	8.273816	1.033081	0.304384	-6.37307	0.43428	0.527952
Erythroid.cells	GM4566	-0.6101	1.718905	-1.03292	0.304459	-5.03712	0.543831	0.633613
Erythroid.cells	CSNK1G3	-0.1527	6.624241	-1.03285	0.30449	-6.06489	0.459457	0.552781
Erythroid.cells	LAMA4	0.865725	1.088771	1.032572	0.304621	-4.9388	0.555878	0.644936
Erythroid.cells	C1RL	-0.51329	1.632594	-1.03252	0.304644	-5.21206	0.545546	0.635263
Erythroid.cells	CNOT6	-0.14958	5.947416	-1.03236	0.304718	-5.87622	0.470348	0.563419
Erythroid.cells	CAPN2	-0.25617	4.14309	-1.03215	0.304819	-5.56051	0.500413	0.592467
Erythroid.cells	2700049A0	-0.32485	4.236674	-1.03212	0.304829	-5.44778	0.498806	0.590925
Erythroid.cells	PLK4	-0.40341	3.856529	-1.03201	0.304881	-5.43281	0.505372	0.597235
Erythroid.cells	API5	-0.18009	6.03062	-1.03154	0.305101	-5.83745	0.469107	0.562291
Erythroid.cells	PGM3	-0.31547	2.89728	-1.03144	0.305147	-5.27897	0.522426	0.613594
Erythroid.cells	2310058D1	0.341961	2.785149	1.031375	0.305178	-5.22023	0.524447	0.615524
Erythroid.cells	TERF1	-0.20107	5.254483	-1.03132	0.305202	-5.75076	0.481757	0.574661
Erythroid.cells	UBXN8	-0.27434	4.404408	-1.03093	0.305385	-5.48245	0.496027	0.588616
Erythroid.cells	TDP1	-0.39949	3.397629	-1.03087	0.305414	-5.28788	0.513503	0.605343
Erythroid.cells	GM44686	-0.37063	3.136727	-1.03068	0.305503	-5.24294	0.518135	0.609753
Erythroid.cells	TIMMDC1	0.263398	4.271519	1.030671	0.305506	-5.48546	0.498297	0.590797
Erythroid.cells	A530088E0	-0.56522	1.760356	-1.03054	0.305569	-5.0703	0.543304	0.633577
Erythroid.cells	ZC3H13	-0.20635	5.653384	-1.03049	0.305589	-5.78601	0.475211	0.568545
Erythroid.cells	ZFP740	-0.23284	4.76726	-1.03048	0.305594	-5.55518	0.489882	0.582745
Erythroid.cells	SPNS1	0.361014	3.498213	1.030305	0.305677	-5.27201	0.511728	0.603753
Erythroid.cells	KSR2	-0.70225	2.695294	-1.03023	0.305712	-5.12984	0.526073	0.617423
Erythroid.cells	SLIRP	-0.2281	5.558884	-1.03017	0.305738	-5.82381	0.476753	0.570124
Erythroid.cells	YEATS4	-0.1802	5.906755	-1.03017	0.305739	-5.82264	0.471102	0.564629
Erythroid.cells	ZFP322A	-0.41808	3.041434	-1.02993	0.30585	-5.23182	0.519838	0.611583
Erythroid.cells	GM16093	-0.52481	2.640752	-1.02991	0.305861	-5.12154	0.527063	0.618447
Erythroid.cells	HMGN1	0.196098	7.26929	1.029836	0.305896	-6.18728	0.449642	0.543702
Erythroid.cells	IGSF5	0.514484	1.922281	1.029779	0.305923	-5.3055	0.540278	0.630972
Erythroid.cells	GK5	0.348871	4.404631	1.029736	0.305943	-5.51072	0.496023	0.588865

Erythroid.cells	CNP	-0.24142	5.75023	-1.02943	0.306088	-5.90104	0.473782	0.567227
Erythroid.cells	STT3B	-0.14581	6.666062	-1.02924	0.306174	-5.98709	0.459207	0.553
Erythroid.cells	CCDC83	-0.8873	0.420205	-1.02893	0.30632	-4.98332	0.569439	0.657982
Erythroid.cells	ZFP959	-0.36466	3.241052	-1.02849	0.306526	-5.25984	0.516903	0.608537
Erythroid.cells	DPY30	0.244995	5.414831	1.028402	0.306566	-5.76682	0.479694	0.572844
Erythroid.cells	NUCB1	0.22098	5.383551	1.02817	0.306674	-5.78475	0.48026	0.573444
Erythroid.cells	SGCE	0.804735	1.156412	1.028116	0.3067	-4.93285	0.555473	0.645018
Erythroid.cells	KIF16B	-0.29178	4.843803	-1.02792	0.306792	-5.53545	0.489306	0.582245
Erythroid.cells	ALG5	0.294305	4.2129	1.027244	0.307108	-5.45951	0.500426	0.592921
Erythroid.cells	5330417C2	-0.80362	0.541978	-1.02718	0.307137	-4.91656	0.567907	0.656649
Erythroid.cells	LDB2	0.538351	3.160304	1.026833	0.3073	-5.29407	0.51907	0.610737
Erythroid.cells	AVL9	-0.20987	6.010486	-1.02672	0.307353	-5.8694	0.470663	0.564186
Erythroid.cells	RNASEH2C	0.236483	4.998911	1.026525	0.307444	-5.66475	0.487341	0.58037
Erythroid.cells	RCBTB2	0.242389	5.187796	1.026313	0.307544	-5.65527	0.484269	0.577377
Erythroid.cells	CIT	0.441318	4.461996	1.025965	0.307707	-5.62045	0.496671	0.58921
Erythroid.cells	4930430F0	-0.55333	1.035161	-1.0256	0.307877	-4.96835	0.559113	0.648284
Erythroid.cells	FBXW8	0.316	3.868261	1.02545	0.307948	-5.37325	0.507144	0.599215
Erythroid.cells	COG1	-0.2973	3.677624	-1.02519	0.308068	-5.33695	0.510519	0.602468
Erythroid.cells	DTX4	0.569198	2.365512	1.025184	0.308073	-5.15155	0.534116	0.624867
Erythroid.cells	PARN	0.31721	3.875182	1.024958	0.308179	-5.37393	0.507153	0.599227
Erythroid.cells	PSMD8	0.163654	6.592353	1.024587	0.308353	-6.08535	0.462194	0.55577
Erythroid.cells	CTNND1	0.327601	4.294756	1.02423	0.308521	-5.54355	0.500173	0.592611
Erythroid.cells	ALKBH6	0.343772	2.755561	1.024212	0.30853	-5.20637	0.527379	0.618547
Erythroid.cells	BBS9	0.341768	4.909527	1.024153	0.308557	-5.59477	0.48972	0.58257
Erythroid.cells	COX16	0.142237	6.153691	1.023517	0.308856	-5.93603	0.469643	0.563069
Erythroid.cells	NEK6	0.319874	3.873937	1.023233	0.308989	-5.43751	0.508006	0.600009
Erythroid.cells	2500004C0	0.594647	1.043374	1.023031	0.309084	-4.96058	0.560117	0.649168
Erythroid.cells	SLC25A17	-0.19549	5.308731	-1.02249	0.30934	-5.7877	0.48363	0.576695
Erythroid.cells	VPS50	0.270807	4.131766	1.022449	0.309358	-5.44943	0.50358	0.595904
Erythroid.cells	MOCOS	-0.64917	1.324581	-1.02234	0.309409	-5.09942	0.554709	0.644298
Erythroid.cells	ZFP598	0.35598	3.44196	1.022285	0.309435	-5.32357	0.515672	0.607454
Erythroid.cells	DYNLRB1	0.154961	6.280147	1.022251	0.309451	-6.0015	0.467793	0.561308
Erythroid.cells	SLC37A4	0.341792	2.862467	1.022229	0.309461	-5.35611	0.526064	0.617328
Erythroid.cells	GSPT1	-0.14188	6.707096	-1.02219	0.309478	-6.03792	0.461007	0.554675
Erythroid.cells	DUBR	0.478755	2.378269	1.021983	0.309577	-5.14275	0.534914	0.62573
Erythroid.cells	RGN	0.37872	4.650255	1.021979	0.309579	-5.93972	0.494687	0.587395
Erythroid.cells	GIMAP5	-0.37701	3.319876	-1.02197	0.309583	-5.45395	0.517844	0.609552
Erythroid.cells	ARMC5	-0.33104	3.383082	-1.02153	0.30979	-5.26167	0.516944	0.608606
Erythroid.cells	EEF1AKNM	0.450025	2.318289	1.02144	0.309833	-5.1355	0.536256	0.626928
Erythroid.cells	MRPL45	-0.30253	4.340705	-1.02136	0.309871	-5.50111	0.500195	0.592647
Erythroid.cells	JMJD6	-0.25336	4.999369	-1.02123	0.309933	-5.63552	0.489023	0.581939
Erythroid.cells	PIGL	-0.36523	2.746364	-1.02059	0.310234	-5.18524	0.528641	0.619835
Erythroid.cells	IFT57	0.4026	2.891644	1.020566	0.310244	-5.23323	0.526002	0.617335
Erythroid.cells	SMC4	0.180134	7.287715	1.020561	0.310247	-6.22573	0.452347	0.546231
Erythroid.cells	KCNB1	-0.7958	0.988195	-1.02039	0.310327	-4.90844	0.561682	0.650901
Erythroid.cells	CLCC1	0.236405	4.329275	1.020279	0.31038	-5.47812	0.500616	0.593179
Erythroid.cells	ARF2	0.320343	4.470722	1.020244	0.310396	-5.54393	0.498189	0.590864

Erythroid.cells	H2-T23	0.307818	6.358381	1.020201	0.310417	-6.01441	0.466955	0.560638
Erythroid.cells	SOX18	0.82406	0.720608	1.019776	0.310617	-4.90724	0.567167	0.656056
Erythroid.cells	ZFP273	-0.73229	0.440289	-1.01923	0.310875	-4.8648	0.573064	0.66146
Erythroid.cells	E230029CC	0.421223	3.508508	1.018636	0.311155	-5.40706	0.51592	0.607486
Erythroid.cells	NT5E	0.480036	2.431121	1.01851	0.311214	-5.33536	0.535437	0.625986
Erythroid.cells	PLG	0.390332	4.004656	1.018305	0.311312	-5.76806	0.507223	0.599204
Erythroid.cells	PHB	0.273562	4.493205	1.018235	0.311344	-5.61893	0.498777	0.59111
Erythroid.cells	ANKRD16	0.394829	3.026656	1.018067	0.311424	-5.22716	0.524585	0.615757
Erythroid.cells	TLK2	-0.14354	6.825344	-1.01805	0.311431	-6.05938	0.460451	0.553971
Erythroid.cells	CAR3	-0.61692	3.476221	-1.01791	0.311497	-5.64559	0.516553	0.608152
Erythroid.cells	GUF1	0.37111	2.589786	1.017803	0.311549	-5.17134	0.532566	0.62341
Erythroid.cells	RNASEH2B	-0.2855	4.594626	-1.0173	0.311786	-5.5714	0.497239	0.589826
Erythroid.cells	THAP2	0.368897	3.329281	1.017232	0.311818	-5.33421	0.519353	0.611011
Erythroid.cells	ZFP148	-0.1441	6.830997	-1.0172	0.311836	-6.04991	0.460545	0.554262
Erythroid.cells	NUP107	-0.25477	5.353646	-1.01714	0.31186	-5.65948	0.48445	0.577552
Erythroid.cells	ZFP553	-0.41724	2.414705	-1.01662	0.312108	-5.14313	0.536079	0.627
Erythroid.cells	SGO1	0.53372	3.188246	1.016468	0.31218	-5.30488	0.521983	0.613652
Erythroid.cells	NCKAP1L	0.171918	6.137906	1.016455	0.312186	-5.83584	0.471692	0.56528
Erythroid.cells	IFT27	-0.37128	3.758551	-1.01644	0.312193	-5.36891	0.511838	0.60399
Erythroid.cells	DGAT2	0.290141	3.862389	1.016397	0.312213	-5.62945	0.510013	0.602247
Erythroid.cells	GM12905	-0.44809	2.138199	-1.01637	0.312226	-5.04402	0.541212	0.63184
Erythroid.cells	TSG101	0.194268	5.622255	1.016158	0.312327	-5.81672	0.48011	0.573454
Erythroid.cells	ABI2	-0.35399	3.903545	-1.01614	0.312334	-5.4201	0.509301	0.601558
Erythroid.cells	CBX8	-0.56439	1.617418	-1.01568	0.312555	-5.07333	0.551294	0.641133
Erythroid.cells	TEF	-0.37175	3.230001	-1.01561	0.312586	-5.26627	0.521493	0.613015
Erythroid.cells	GM43768	-0.77734	0.70894	-1.01504	0.312855	-4.8968	0.568904	0.657795
Erythroid.cells	POMT1	-0.57586	2.21731	-1.01502	0.312867	-5.09931	0.540058	0.63079
Erythroid.cells	ZFP507	-0.54276	2.176566	-1.01496	0.312895	-5.11428	0.540817	0.631506
Erythroid.cells	CDCA7L	-0.45547	3.766654	-1.01495	0.312901	-5.32203	0.511998	0.604179
Erythroid.cells	GM38134	-0.63459	1.108316	-1.01491	0.31292	-5.00619	0.561116	0.650539
Erythroid.cells	DVL3	-0.34043	3.300094	-1.0149	0.312921	-5.27861	0.520285	0.612075
Erythroid.cells	NEB	0.645711	1.258562	1.014751	0.312994	-4.98036	0.558252	0.647878
Erythroid.cells	PQBP1	-0.26312	4.6495	-1.01415	0.313278	-5.54822	0.496922	0.589762
Erythroid.cells	KTI12	0.347678	3.941057	1.014139	0.313284	-5.44553	0.50917	0.601507
Erythroid.cells	CSRNP1	-0.25727	6.641086	-1.01413	0.313287	-6.00339	0.464125	0.557952
Erythroid.cells	GRIK5	0.849915	0.167717	1.013979	0.31336	-4.8871	0.579899	0.668149
Erythroid.cells	OLFR164	0.782124	-0.16043	1.013955	0.313371	-4.88031	0.586507	0.67427
Erythroid.cells	CCDC82	-0.30319	4.323551	-1.01382	0.313435	-5.43672	0.502531	0.595324
Erythroid.cells	GNG2	0.223948	6.302982	1.013535	0.31357	-5.8456	0.469539	0.563477
Erythroid.cells	MYB	-0.27576	5.137938	-1.01352	0.313579	-5.81523	0.48867	0.582053
Erythroid.cells	LCMT1	-0.37611	3.124061	-1.01351	0.313583	-5.24859	0.523701	0.615611
Erythroid.cells	MMAB	-0.67213	1.326097	-1.01323	0.313716	-4.96176	0.557303	0.647291
Erythroid.cells	TAF8	-0.38021	3.254648	-1.01311	0.313771	-5.2694	0.521462	0.613527
Erythroid.cells	A630001G2	-0.23167	5.126842	-1.01299	0.313828	-5.72018	0.48896	0.582396
Erythroid.cells	PPID	-0.24541	4.87562	-1.01294	0.313853	-5.65588	0.493194	0.586504
Erythroid.cells	CXCR4	0.267907	6.146916	1.012754	0.313941	-5.95359	0.472188	0.566163
Erythroid.cells	CFLAR	-0.27864	5.971185	-1.01268	0.313977	-5.81366	0.475039	0.56895

Erythroid.cells	NFKB2	-0.3232	5.096214	-1.01232	0.314148	-5.62596	0.489645	0.583103
Erythroid.cells	SPATA32	0.782721	0.244996	1.012053	0.314274	-4.94293	0.578693	0.667463
Erythroid.cells	GATAD2A	-0.14365	7.859185	-1.01194	0.314326	-6.28017	0.445465	0.540059
Erythroid.cells	CCP110	0.475302	2.37539	1.011822	0.314384	-5.13248	0.537685	0.629135
Erythroid.cells	POLDIP3	-0.17048	6.078814	-1.01181	0.31439	-5.89037	0.473422	0.567517
Erythroid.cells	PMEPA1	0.38658	4.361383	1.01175	0.314418	-5.6177	0.502159	0.595311
Erythroid.cells	STAB2	0.525562	5.148934	1.011681	0.314451	-5.7237	0.48876	0.582401
Erythroid.cells	AGPAT5	-0.20499	5.644905	-1.01153	0.314523	-5.71828	0.480515	0.574446
Erythroid.cells	ITPRID2	0.25259	5.08881	1.011224	0.314669	-5.67775	0.489769	0.58351
Erythroid.cells	CDK17	-0.17597	6.780519	-1.01115	0.314702	-6.08423	0.462187	0.556665
Erythroid.cells	IQGAP1	-0.13577	9.247795	-1.01113	0.314714	-6.48549	0.424882	0.519734
Erythroid.cells	CERKL	-0.51271	1.852174	-1.01106	0.314745	-5.18304	0.547472	0.638519
Erythroid.cells	CYP51	-0.35371	3.916341	-1.01102	0.314767	-5.39616	0.509901	0.602886
Erythroid.cells	ABCA6	0.709047	0.705764	1.010968	0.31479	-5.06117	0.569561	0.659187
Erythroid.cells	SCFD2	0.215507	5.78946	1.010884	0.31483	-5.806	0.47814	0.572297
Erythroid.cells	NPC1	-0.31759	4.971884	-1.01066	0.314938	-5.52435	0.491813	0.585527
Erythroid.cells	ZFP81	0.535929	1.873403	1.010539	0.314995	-5.03774	0.547153	0.638285
Erythroid.cells	KBTBD2	-0.1845	5.176548	-1.01046	0.315031	-5.69876	0.48837	0.582245
Erythroid.cells	PA2G4	-0.21117	6.688076	-1.01013	0.315189	-6.0719	0.463803	0.558416
Erythroid.cells	IGSF8	0.329263	4.266842	1.010099	0.315204	-5.39582	0.503959	0.597355
Erythroid.cells	CHEK1	0.505756	2.722134	1.010023	0.315241	-5.14173	0.531474	0.623615
Erythroid.cells	DTD2	0.379875	3.491192	1.009721	0.315384	-5.3556	0.517737	0.610448
Erythroid.cells	EXOSC9	0.270574	4.169312	1.009117	0.315672	-5.51945	0.506181	0.599364
Erythroid.cells	PRPF31	-0.30033	3.79385	-1.00774	0.316327	-5.39234	0.513721	0.606001
Erythroid.cells	PET100	0.185681	5.786929	1.007661	0.316367	-5.86442	0.479737	0.573304
Erythroid.cells	ATIC	0.313944	4.493549	1.006618	0.316865	-5.52731	0.50221	0.59455
Erythroid.cells	C530005A1	-0.62868	0.742343	-1.00651	0.316917	-4.91439	0.57149	0.659929
Erythroid.cells	METTL22	-0.55494	1.382795	-1.00631	0.317014	-5.02049	0.558998	0.648291
Erythroid.cells	FGFR1OP	0.277209	4.601111	1.006243	0.317045	-5.5758	0.500358	0.592772
Erythroid.cells	GPSM1	-0.42494	2.672071	-1.0062	0.317065	-5.19582	0.534693	0.625467
Erythroid.cells	TMTC2	0.409517	4.574445	1.005909	0.317204	-5.84518	0.500956	0.593312
Erythroid.cells	CD300E	-1.01304	0.628868	-1.00567	0.317319	-5.018	0.573917	0.662229
Erythroid.cells	SH2B3	-0.23993	5.315208	-1.00563	0.317338	-5.66408	0.488398	0.581294
Erythroid.cells	DXO	-0.40279	3.302702	-1.00543	0.317433	-5.31074	0.523374	0.614799
Erythroid.cells	GM43773	-0.69265	1.074002	-1.00536	0.317469	-4.94401	0.565167	0.654104
Erythroid.cells	CYTIP	0.170433	8.087657	1.005344	0.317475	-6.39968	0.444206	0.538129
Erythroid.cells	AK6	0.248947	4.761906	1.004813	0.317729	-5.61172	0.49782	0.590448
Erythroid.cells	CDK2AP2	0.177656	6.467925	1.00474	0.317764	-5.97347	0.469536	0.563059
Erythroid.cells	CABLES1	-0.42636	4.513404	-1.00472	0.317776	-5.73302	0.502087	0.594547
Erythroid.cells	GM43259	-0.76707	1.452797	-1.00468	0.317791	-4.96544	0.557893	0.647386
Erythroid.cells	ACER1	1.11367	-0.7216	1.004629	0.317817	-4.82614	0.601383	0.687669
Erythroid.cells	AU022252	0.404952	2.452615	1.004625	0.317819	-5.22424	0.538987	0.629638
Erythroid.cells	PDAP1	-0.14753	6.715812	-1.00438	0.317936	-6.11787	0.465667	0.559228
Erythroid.cells	GNG7	0.883197	-0.40318	1.004051	0.318094	-4.82284	0.595129	0.681746
Erythroid.cells	SZRD1	-0.15591	5.906997	-1.00394	0.318149	-5.87599	0.478908	0.572027
Erythroid.cells	CASC4	0.385769	2.64714	1.003802	0.318213	-5.30407	0.535705	0.626428
Erythroid.cells	ANKMY2	0.311723	3.602393	1.00358	0.31832	-5.32085	0.518462	0.610087

Erythroid.cells	RAPGEF2	-0.23813	7.429198	-1.00335	0.318428	-6.16957	0.454784	0.548514
Erythroid.cells	MCM3	-0.32278	5.484871	-1.00316	0.31852	-5.8185	0.486142	0.579037
Erythroid.cells	GTF2H3	-0.48802	2.299191	-1.00292	0.318636	-5.1154	0.542537	0.632824
Erythroid.cells	TFDP2	0.167934	5.591554	1.00273	0.318728	-6.30687	0.484526	0.57751
Erythroid.cells	ITGB7	0.279354	4.620993	1.002557	0.318811	-5.81513	0.500988	0.593342
Erythroid.cells	VNN3	-0.51752	1.614791	-1.00235	0.318908	-5.23906	0.555698	0.645175
Erythroid.cells	DHDH	0.370191	2.654165	1.001975	0.31909	-5.33243	0.536359	0.626961
Erythroid.cells	OGG1	0.357959	3.189389	1.001564	0.319288	-5.21367	0.526803	0.617815
Erythroid.cells	1700102H2	0.690978	0.529845	1.00128	0.319424	-4.92987	0.577552	0.665353
Erythroid.cells	ALMS1	0.341073	3.824261	1.001127	0.319498	-5.43328	0.515577	0.607136
Erythroid.cells	CYP2AB1	-0.71761	-0.30874	-1.00097	0.319572	-4.98533	0.594544	0.681104
Erythroid.cells	GM42067	0.663531	0.558955	1.000932	0.319591	-4.90291	0.576996	0.664908
Erythroid.cells	1500004A1	0.429055	2.078931	1.000697	0.319704	-5.13797	0.547622	0.637454
Erythroid.cells	HSF1	-0.23507	4.997353	-1.00056	0.319769	-5.64309	0.495323	0.58776
Erythroid.cells	HMMR	0.496458	4.333361	1.000147	0.319969	-5.5859	0.506874	0.598848
Erythroid.cells	ATG4B	-0.24501	4.800676	-1.00014	0.319972	-5.61037	0.498801	0.591108
Erythroid.cells	UBASH3B	0.25873	5.794781	1.000044	0.320018	-6.08105	0.482076	0.574986
Erythroid.cells	UXS1	0.294204	4.429155	0.999971	0.320054	-5.49319	0.505208	0.597302
Erythroid.cells	STRBP	-0.15844	7.195368	-0.9995	0.320281	-6.26092	0.459635	0.553205
Erythroid.cells	ZFP622	-0.22214	5.377893	-0.9994	0.320327	-5.71697	0.489147	0.581919
Erythroid.cells	ENO1	0.187765	8.066335	0.999348	0.320353	-6.36745	0.446169	0.539958
Erythroid.cells	NBAS	-0.27492	4.535139	-0.9993	0.320378	-5.52061	0.503505	0.59576
Erythroid.cells	WASHC4	0.182601	5.792598	0.999208	0.320421	-5.79036	0.48224	0.575328
Erythroid.cells	LRATD2	-0.47214	1.700967	-0.99915	0.320448	-5.11799	0.555114	0.644708
Erythroid.cells	KANTR	0.485167	2.529607	0.998733	0.32065	-5.14232	0.539653	0.630068
Erythroid.cells	GM7030	-0.62727	2.084686	-0.99873	0.320653	-5.16059	0.547993	0.637912
Erythroid.cells	TMSB4X	0.150975	12.24836	0.998507	0.320759	-6.99309	0.387296	0.480867
Erythroid.cells	ARMC1	0.209635	4.827556	0.998442	0.32079	-5.65908	0.498686	0.591141
Erythroid.cells	SLFN3	0.623711	1.4477	0.998212	0.320901	-5.00127	0.560243	0.64952
Erythroid.cells	ECSIT	-0.28269	4.015765	-0.99812	0.320945	-5.48064	0.51281	0.604742
Erythroid.cells	IFIT1BL2	-0.88772	-0.46837	-0.9981	0.320956	-4.84263	0.598552	0.685011
Erythroid.cells	AK3	0.242924	4.328071	0.997923	0.32104	-5.60969	0.507386	0.599584
Erythroid.cells	SLC22A5	-0.49829	3.042195	-0.99748	0.321252	-5.24457	0.530602	0.621575
Erythroid.cells	D030056L2	-0.3385	3.888031	-0.99721	0.321383	-5.45307	0.515506	0.607157
Erythroid.cells	KLC4	0.392642	2.884186	0.997096	0.321439	-5.24086	0.533636	0.624383
Erythroid.cells	HERC2	0.199466	5.98752	0.996678	0.321641	-5.87779	0.479889	0.572839
Erythroid.cells	TBRG4	0.317573	4.075048	0.996183	0.32188	-5.43231	0.512709	0.604319
Erythroid.cells	ZFP287	-0.65845	0.566909	-0.99608	0.32193	-5.01961	0.578598	0.666288
Erythroid.cells	ITGB1BP1	-0.23633	4.649919	-0.99598	0.32198	-5.5819	0.50268	0.594769
Erythroid.cells	CHPF	0.52288	1.242066	0.99591	0.322012	-5.07571	0.565272	0.653911
Erythroid.cells	5033421B0	-0.8814	-0.4422	-0.99559	0.322167	-4.90511	0.599266	0.685239
Erythroid.cells	CARD6	-0.46734	3.06005	-0.9955	0.322211	-5.24598	0.531068	0.621699
Erythroid.cells	IFI207	0.390984	4.721848	0.995401	0.322258	-5.82046	0.501567	0.593639
Erythroid.cells	GM8251	-0.45601	3.392286	-0.9953	0.322306	-5.36625	0.525028	0.61604
Erythroid.cells	GM47350	-0.5267	1.355941	-0.99521	0.322352	-5.02578	0.563199	0.651933
Erythroid.cells	ADAP2OS	-0.66991	0.724917	-0.99494	0.32248	-5.03711	0.57568	0.66352
Erythroid.cells	TTYH3	-0.26187	5.141527	-0.9949	0.322501	-5.67327	0.494463	0.586814

Erythroid.cells	ZFP606	-0.40696	2.487995	-0.99427	0.322807	-5.15712	0.542085	0.632026
Erythroid.cells	FAM72A	0.680833	1.069392	0.994046	0.322913	-4.95211	0.569265	0.657496
Erythroid.cells	ERBIN	-0.15341	7.975801	-0.9939	0.322986	-6.30459	0.449066	0.542553
Erythroid.cells	GPR18	-0.29274	4.514903	-0.99379	0.323035	-5.69761	0.505563	0.597461
Erythroid.cells	PLAA	-0.16406	6.069326	-0.99377	0.323046	-5.92365	0.479308	0.572157
Erythroid.cells	WHRN	-0.46467	3.066048	-0.99363	0.323116	-5.25223	0.531398	0.622152
Erythroid.cells	ANKLE1	0.639501	0.64315	0.993529	0.323164	-4.93302	0.577702	0.665514
Erythroid.cells	DYNC1LI1	0.153802	6.412559	0.993511	0.323172	-5.99907	0.473708	0.566828
Erythroid.cells	SLC4A8	0.748805	0.467759	0.993449	0.323202	-4.98163	0.58121	0.668802
Erythroid.cells	ZBTB11OS1	-0.44458	2.065369	-0.99326	0.323294	-5.107	0.55004	0.639789
Erythroid.cells	IDE	0.237051	4.161112	0.993168	0.323338	-5.50744	0.511748	0.603563
Erythroid.cells	KPNA3	-0.17051	6.307968	-0.99316	0.323341	-6.00681	0.475407	0.568547
Erythroid.cells	MRPS18A	-0.22003	5.09387	-0.99288	0.323476	-5.7125	0.495703	0.588189
Erythroid.cells	PALM	-0.35854	4.231998	-0.99253	0.323649	-5.44769	0.510596	0.602577
Erythroid.cells	DCTN3	-0.17557	5.963361	-0.99245	0.323685	-5.91535	0.48114	0.574229
Erythroid.cells	ZXDB	-0.39677	3.1887	-0.99243	0.323696	-5.2358	0.529256	0.620337
Erythroid.cells	PROS1	0.394584	2.741429	0.992411	0.323706	-5.37466	0.537471	0.628104
Erythroid.cells	WASHC5	-0.29917	4.472587	-0.9924	0.323711	-5.50764	0.506392	0.598562
Erythroid.cells	ZFP827	-0.50399	2.85843	-0.99129	0.324251	-5.21458	0.536117	0.62655
Erythroid.cells	CHST2	-0.80194	-0.02925	-0.9902	0.324779	-4.93617	0.593028	0.679407
Erythroid.cells	TCIRG1	-0.27273	5.455039	-0.99019	0.324786	-5.66169	0.490963	0.583301
Erythroid.cells	GM49169	-0.82383	-0.42801	-0.99009	0.32483	-4.87957	0.60125	0.687009
Erythroid.cells	BLOC1S2	-0.24882	4.833886	-0.99001	0.324872	-5.62789	0.501541	0.593543
Erythroid.cells	GM17655	-0.59646	1.183959	-0.98972	0.325013	-5.0102	0.568797	0.657095
Erythroid.cells	AKAP8	-0.17971	5.39229	-0.98962	0.32506	-5.78451	0.4921	0.58454
Erythroid.cells	SLC44A1	0.29603	4.637679	0.989493	0.325122	-5.57038	0.505013	0.596951
Erythroid.cells	MYO9B	-0.21631	5.866728	-0.98947	0.325132	-5.78996	0.48416	0.576871
Erythroid.cells	AFF1	-0.14009	8.730515	-0.98936	0.325185	-6.49732	0.439036	0.532672
Erythroid.cells	SLC25A37	0.165884	5.267981	0.989183	0.325273	-6.38574	0.494259	0.586606
Erythroid.cells	IL18	0.33737	3.422293	0.98886	0.32543	-5.44912	0.526796	0.617632
Erythroid.cells	ZFP330	0.237385	4.432352	0.988718	0.325499	-5.56586	0.508838	0.60055
Erythroid.cells	ACSF3	0.500489	1.59243	0.988505	0.325603	-5.08295	0.561181	0.649963
Erythroid.cells	DOCK2	-0.14039	9.34967	-0.98842	0.325643	-6.55019	0.430144	0.523783
Erythroid.cells	CDC37L1	0.20595	5.167302	0.988067	0.325816	-5.68433	0.496403	0.58862
Erythroid.cells	TTC38	0.441144	2.505389	0.987932	0.325882	-5.23749	0.544018	0.633881
Erythroid.cells	LPIN2	-0.20102	6.528551	-0.98775	0.325971	-6.0549	0.473821	0.566729
Erythroid.cells	9530082P2	-0.63066	0.699259	-0.9875	0.32609	-4.93534	0.579016	0.666515
Erythroid.cells	RBBP5	0.331376	3.457865	0.987479	0.326103	-5.3619	0.526489	0.617305
Erythroid.cells	SPSB4	0.716429	0.417547	0.987473	0.326106	-4.9713	0.584674	0.671749
Erythroid.cells	CASP1	-0.40147	3.51676	-0.98722	0.326229	-5.3454	0.525459	0.616383
Erythroid.cells	GM47200	-0.83257	0.11613	-0.98721	0.326236	-4.86327	0.590831	0.677491
Erythroid.cells	RXRA	0.342805	3.1994	0.987107	0.326284	-5.39831	0.531231	0.62185
Erythroid.cells	GZMB	-0.38058	3.971899	-0.98669	0.326485	-6.04537	0.517486	0.60864
Erythroid.cells	MTA2	-0.2117	5.782517	-0.98665	0.326507	-5.83238	0.486295	0.578722
Erythroid.cells	CLK1	-0.13977	7.55289	-0.98653	0.326567	-6.24472	0.457727	0.5509
Erythroid.cells	ZFP276	0.407915	2.479933	0.986363	0.326647	-5.1873	0.54482	0.63453
Erythroid.cells	IPO7	-0.23815	5.656347	-0.98612	0.326767	-5.83161	0.48847	0.580964

Erythroid.cells	NAB1	-0.17812	6.555305	-0.98609	0.326782	-6.03298	0.473659	0.566619
Erythroid.cells	DENND4B	0.323813	3.747946	0.986025	0.326812	-5.36933	0.521559	0.612707
Erythroid.cells	MBOAT1	0.604095	0.682144	0.985902	0.326871	-5.15474	0.579704	0.667274
Erythroid.cells	CRAMP1L	-0.22792	4.984741	-0.98579	0.326927	-5.64256	0.499874	0.592044
Erythroid.cells	COPG1	-0.23772	5.141092	-0.98547	0.327082	-5.6776	0.497287	0.589558
Erythroid.cells	S1PR4	-0.33201	4.183137	-0.98536	0.327136	-5.51748	0.513922	0.605491
Erythroid.cells	FURIN	0.276477	5.290193	0.98535	0.327141	-5.79692	0.494749	0.587116
Erythroid.cells	OGA	-0.15682	6.76343	-0.98498	0.327323	-6.10302	0.470537	0.563723
Erythroid.cells	DHX33	-0.3376	3.32737	-0.98494	0.327341	-5.32421	0.529431	0.620307
Erythroid.cells	GM48768	-0.71211	0.621002	-0.98467	0.327474	-4.92152	0.581354	0.668878
Erythroid.cells	NT5DC1	0.278673	4.5014	0.98432	0.327644	-5.55081	0.508781	0.60052
Erythroid.cells	BCL2L13	-0.23426	5.133362	-0.98423	0.32769	-5.74271	0.497859	0.590053
Erythroid.cells	FERMT2	0.348627	3.451103	0.983955	0.327823	-5.50499	0.527622	0.61844
Erythroid.cells	MLH1	0.331341	2.750298	0.98372	0.327938	-5.24393	0.540531	0.630685
Erythroid.cells	GM43660	-0.75531	0.127169	-0.98371	0.327944	-4.96786	0.591723	0.678377
Erythroid.cells	PGPEP1L	-0.75142	-0.29843	-0.98353	0.328033	-4.87989	0.600483	0.686506
Erythroid.cells	IRS2	-0.29011	5.572327	-0.9835	0.328044	-5.83117	0.490558	0.58311
Erythroid.cells	COQ8A	-0.40183	2.303584	-0.98302	0.328282	-5.25972	0.549205	0.638774
Erythroid.cells	ANO10	0.433444	3.16746	0.982924	0.328327	-5.26167	0.533101	0.623646
Erythroid.cells	ATXN7L2	-0.46504	2.480154	-0.98283	0.328372	-5.1999	0.545873	0.635695
Erythroid.cells	SNX20	-0.23947	5.514815	-0.98237	0.328599	-5.74459	0.491906	0.584351
Erythroid.cells	ZFP974	0.718888	0.877863	0.982209	0.328677	-4.99139	0.57703	0.664865
Erythroid.cells	DECR2	0.349009	2.741577	0.982173	0.328695	-5.41995	0.541111	0.631296
Erythroid.cells	BCO2	0.546845	0.643324	0.982117	0.328722	-5.03286	0.581721	0.669218
Erythroid.cells	LFNG	-0.28226	4.413378	-0.98207	0.328744	-5.54085	0.510862	0.602615
Erythroid.cells	PSRC1	-0.641	0.886949	-0.98203	0.328765	-5.00428	0.576849	0.664715
Erythroid.cells	DUSP5	-0.22442	6.834196	-0.98182	0.328868	-6.10242	0.470246	0.563438
Erythroid.cells	MED16	0.278028	4.12299	0.981626	0.328963	-5.43207	0.516133	0.607735
Erythroid.cells	TMEM51OS	-0.72434	-0.90367	-0.98147	0.329039	-4.86125	0.61386	0.698933
Erythroid.cells	RASAL2	0.323108	5.468995	0.981004	0.329267	-5.77356	0.49312	0.585653
Erythroid.cells	ARL4C	0.265584	5.243589	0.980793	0.329371	-5.74245	0.496965	0.58937
Erythroid.cells	IMPACT	-0.26529	5.540939	-0.98077	0.329383	-5.70613	0.491922	0.584511
Erythroid.cells	ST8SIA6	0.733718	1.211567	0.980452	0.329539	-5.15033	0.571132	0.659408
Erythroid.cells	RNF38	-0.17483	6.163439	-0.98025	0.329639	-5.93814	0.481761	0.574636
Erythroid.cells	SNAPC2	-0.42459	3.015281	-0.98008	0.32972	-5.23366	0.53681	0.627321
Erythroid.cells	MIF	0.227257	7.650894	0.979995	0.329763	-6.34153	0.457897	0.551401
Erythroid.cells	LRMP	-0.19008	6.432878	-0.9796	0.329957	-6.05407	0.477534	0.570579
Erythroid.cells	MYBL1	0.56081	1.840587	0.979544	0.329984	-5.07395	0.559186	0.648365
Erythroid.cells	DDX46	-0.17481	6.040748	-0.97936	0.330072	-5.95045	0.484043	0.576859
Erythroid.cells	B930036N1	-0.31626	5.605823	-0.97915	0.330179	-5.77523	0.491393	0.583976
Erythroid.cells	MRPS35	-0.23611	4.266456	-0.97887	0.330316	-5.55572	0.514584	0.606207
Erythroid.cells	PLEKHN1	0.478939	1.876111	0.978846	0.330327	-5.12668	0.558729	0.647892
Erythroid.cells	ITIH3	0.364441	3.636116	0.978287	0.330602	-5.71741	0.526186	0.617028
Erythroid.cells	FAM49A	0.340641	5.390412	0.978218	0.330635	-5.73425	0.495409	0.587636
Erythroid.cells	TOM1	-0.28813	5.431971	-0.97765	0.330916	-5.65796	0.494988	0.587069
Erythroid.cells	FKBP11	0.399471	1.995525	0.977619	0.33093	-5.201	0.557099	0.645982
Erythroid.cells	SURF1	0.274075	4.36834	0.977462	0.331007	-5.5594	0.513437	0.604786

Erythroid.cells	GM27216	0.636906	0.912751	0.976998	0.331236	-5.09876	0.578575	0.665905
Erythroid.cells	MRNIP	0.495614	2.538227	0.976982	0.331244	-5.20366	0.547032	0.636487
Erythroid.cells	MED15	-0.19714	5.758733	-0.97664	0.331413	-5.85621	0.489878	0.581993
Erythroid.cells	MEIS2	0.523456	2.894046	0.976533	0.331465	-5.3303	0.540559	0.630307
Erythroid.cells	MYO18A	0.247199	4.439945	0.976189	0.331634	-5.58392	0.512741	0.603857
Erythroid.cells	ADORA2B	-0.56846	0.37813	-0.97562	0.331915	-5.17098	0.590013	0.676329
Erythroid.cells	ATL3	0.19244	5.439187	0.975613	0.331918	-5.76314	0.495655	0.587521
Erythroid.cells	POLRMT	-0.52388	1.91437	-0.97552	0.331964	-5.07458	0.559551	0.648073
Erythroid.cells	4930426D0	-0.70459	-0.21569	-0.97541	0.332018	-4.94075	0.602235	0.687572
Erythroid.cells	SCML4	0.286675	4.659948	0.975379	0.332033	-5.68876	0.509091	0.600411
Erythroid.cells	AOAH	-0.49788	4.784961	-0.9749	0.332267	-5.67313	0.507187	0.598457
Erythroid.cells	ZFP182	-0.33453	4.211679	-0.97457	0.332431	-5.46137	0.517449	0.608178
Erythroid.cells	BRWD3	0.266558	5.109603	0.974006	0.332711	-5.64332	0.502078	0.593315
Erythroid.cells	JADE1	0.355814	4.165478	0.973458	0.332981	-5.43126	0.51889	0.60938
Erythroid.cells	SMIM24	-0.3695	3.210803	-0.97315	0.333135	-5.24116	0.536216	0.625928
Erythroid.cells	DDX43	0.621013	0.153087	0.973067	0.333175	-5.0436	0.595849	0.681451
Erythroid.cells	SERPINA3N	0.327608	2.557516	0.973026	0.333195	-5.4848	0.548418	0.637408
Erythroid.cells	IRAK4	-0.26173	4.419459	-0.97297	0.333225	-5.53886	0.514381	0.605244
Erythroid.cells	CYP27A1	0.419381	2.194427	0.972934	0.33324	-5.30464	0.555324	0.643899
Erythroid.cells	CAR8	0.480076	2.375063	0.972922	0.333246	-5.31226	0.551878	0.640672
Erythroid.cells	EIF4G1	-0.14186	6.604378	-0.97274	0.333336	-6.08609	0.477301	0.569588
Erythroid.cells	0610040J0	0.407712	2.378015	0.97245	0.33348	-5.38574	0.552034	0.640716
Erythroid.cells	CD164L2	-0.64756	1.352514	-0.97228	0.333565	-5.03423	0.571962	0.659276
Erythroid.cells	PCDH15	0.51598	1.323083	0.972034	0.333685	-5.07321	0.572659	0.659872
Erythroid.cells	GM33370	0.558441	0.508404	0.97174	0.333831	-5.08556	0.589153	0.674994
Erythroid.cells	GM28501	0.641158	0.476037	0.971593	0.333904	-4.95897	0.589848	0.675621
Erythroid.cells	ME2	-0.23973	5.963937	-0.97125	0.334073	-5.82511	0.488501	0.58007
Erythroid.cells	DHX29	-0.343	3.415036	-0.97113	0.334131	-5.31247	0.533202	0.622729
Erythroid.cells	TENT4A	-0.35346	3.460072	-0.97061	0.334391	-5.36042	0.532707	0.622091
Erythroid.cells	SCPEP1OS	-0.68101	0.502575	-0.97026	0.334563	-5.00414	0.589999	0.67555
Erythroid.cells	MTHFD2L	0.361483	3.033816	0.970235	0.334576	-5.27861	0.540682	0.62965
Erythroid.cells	PRG3	-0.77381	1.171663	-0.97003	0.334676	-5.08717	0.57653	0.663111
Erythroid.cells	ZFP560	0.282372	4.00302	0.970027	0.334679	-5.45173	0.522946	0.612895
Erythroid.cells	4930579G2	-0.40714	2.431609	-0.96987	0.334759	-5.2037	0.552016	0.640294
Erythroid.cells	SRPR	-0.15892	5.794906	-0.96981	0.334786	-5.85267	0.491744	0.583078
Erythroid.cells	SENP5	-0.19665	5.992571	-0.96974	0.33482	-5.88113	0.488424	0.579906
Erythroid.cells	CYBC1	0.324157	4.562008	0.969494	0.334944	-5.46441	0.513058	0.603594
Erythroid.cells	P2RX1	0.783248	0.81074	0.969447	0.334967	-4.94864	0.583828	0.669978
Erythroid.cells	MXRA7	0.578776	1.654737	0.969316	0.335032	-5.09803	0.567093	0.654527
Erythroid.cells	PPP1R11	0.266991	4.640461	0.968985	0.335196	-5.60568	0.511867	0.602427
Erythroid.cells	GM17494	-0.36355	2.389013	-0.96836	0.335505	-5.18784	0.553523	0.641615
Erythroid.cells	SBNO2	0.209961	5.566821	0.967735	0.335817	-5.76269	0.49659	0.587594
Erythroid.cells	CELA1	0.495376	2.24977	0.967659	0.335854	-5.24763	0.556591	0.644444
Erythroid.cells	HMBOX1	0.225049	5.578159	0.966999	0.336182	-5.79899	0.496803	0.587601
Erythroid.cells	GM13212	-0.42479	3.205264	-0.96675	0.336308	-5.23448	0.539126	0.627722
Erythroid.cells	KRT222	-0.70823	-0.05515	-0.96649	0.336435	-4.94103	0.603425	0.687331
Erythroid.cells	SLCO2A1	0.724657	2.568296	0.96575	0.336803	-5.22456	0.551638	0.639194

Erythroid.cells	B130055M	0.499426	2.013314	0.96575	0.336803	-5.11848	0.562293	0.649135
Erythroid.cells	9030622O2	0.658914	1.05914	0.965619	0.336869	-5.11296	0.58113	0.666657
Erythroid.cells	TGOLN1	-0.20762	5.839155	-0.96548	0.336937	-5.85921	0.493021	0.583682
Erythroid.cells	ATP2A3	0.220433	5.210591	0.965373	0.336991	-5.78362	0.503765	0.594012
Erythroid.cells	GM45669	0.840456	0.129811	0.965216	0.337069	-4.90586	0.60015	0.684236
Erythroid.cells	CCND2	-0.2535	5.400515	-0.96459	0.337382	-5.90373	0.500857	0.591083
Erythroid.cells	FAR1	0.193828	6.64871	0.964564	0.337394	-6.05696	0.479891	0.570895
Erythroid.cells	MCCC1	0.456493	2.50652	0.964372	0.33749	-5.21188	0.553264	0.640689
Erythroid.cells	5430405H(	-0.36392	3.910079	-0.96435	0.3375	-5.35371	0.527165	0.616151
Erythroid.cells	PPP5C	0.256875	4.346979	0.963811	0.337769	-5.52786	0.51964	0.608862
Erythroid.cells	CRTC1	-0.43081	3.126748	-0.96366	0.337845	-5.25028	0.541953	0.629893
Erythroid.cells	GM15448	-0.55924	-0.00747	-0.96351	0.337921	-4.99143	0.603847	0.687235
Erythroid.cells	GM10563	-0.35279	3.607105	-0.96333	0.33801	-5.402	0.533088	0.621591
Erythroid.cells	PIP4K2C	-0.28528	4.238405	-0.96327	0.338039	-5.4945	0.521639	0.610764
Erythroid.cells	IMPDH2	-0.21962	5.196107	-0.9632	0.338072	-5.75632	0.50476	0.594696
Erythroid.cells	HNRNPC	-0.10102	7.730209	-0.9631	0.338122	-6.29287	0.462826	0.554188
Erythroid.cells	CD302	-0.2455	5.847821	-0.96297	0.338188	-6.1712	0.493606	0.584008
Erythroid.cells	EPB41L2	-0.17595	6.975502	-0.96289	0.338229	-6.13504	0.474915	0.565958
Erythroid.cells	SLC25A16	0.284184	3.724214	0.962382	0.338482	-5.47403	0.531261	0.619731
Erythroid.cells	WDR75	-0.31696	3.575364	-0.96208	0.338635	-5.39167	0.534146	0.622346
Erythroid.cells	HEXB	0.200519	5.741415	0.961768	0.338789	-5.90534	0.495907	0.586038
Erythroid.cells	CYB5RL	0.681926	0.380261	0.961736	0.338805	-4.90942	0.596418	0.6803
Erythroid.cells	TRMT11	0.284337	3.646744	0.96168	0.338833	-5.44583	0.532898	0.621273
Erythroid.cells	EGR2	0.609116	2.237621	0.961175	0.339086	-5.23787	0.559643	0.64631
Erythroid.cells	MOSPD3	0.241193	4.656046	0.96112	0.339113	-5.59599	0.514951	0.604267
Erythroid.cells	PPP1R2	0.112568	6.908394	0.961015	0.339166	-6.1548	0.476693	0.567544
Erythroid.cells	DUSP18	0.735916	0.621447	0.960959	0.339193	-4.99388	0.591732	0.676028
Erythroid.cells	PLA2G7	0.6135	4.021195	0.960828	0.339259	-5.64724	0.52633	0.615076
Erythroid.cells	GM50373	-0.85531	0.358844	-0.96053	0.339407	-4.88847	0.597151	0.681048
Erythroid.cells	RNASEL	0.397343	3.944163	0.960521	0.339412	-5.49337	0.527732	0.616431
Erythroid.cells	DUSP11	-0.13662	6.33182	-0.9605	0.339422	-6.03677	0.486216	0.576794
Erythroid.cells	LSM14A	0.128243	6.620649	0.960281	0.339533	-6.07486	0.481515	0.572244
Erythroid.cells	SAMD12	0.728669	0.266619	0.960034	0.339656	-5.05225	0.599282	0.683026
Erythroid.cells	FAR1OS	0.487248	2.788806	0.959777	0.339785	-5.31084	0.549472	0.636849
Erythroid.cells	RABEP1	-0.13958	6.897474	-0.95962	0.339866	-6.15628	0.4772	0.568099
Erythroid.cells	ECD	0.218173	4.899349	0.959495	0.339926	-5.63464	0.511018	0.60063
Erythroid.cells	TMEM163	-0.23248	6.026941	-0.95943	0.339956	-6.09629	0.491633	0.582095
Erythroid.cells	CAPZA2	-0.09774	7.929486	-0.95927	0.340037	-6.34598	0.460707	0.552095
Erythroid.cells	SF3A2	-0.23315	5.260106	-0.95917	0.340088	-5.7515	0.504769	0.594684
Erythroid.cells	SMO	0.673208	1.198551	0.958867	0.340241	-5.02461	0.580677	0.665908
Erythroid.cells	GAA	0.366104	3.191942	0.958516	0.340416	-5.32399	0.542312	0.630171
Erythroid.cells	TBX21	-0.36865	2.437202	-0.95837	0.340489	-5.62829	0.556628	0.643598
Erythroid.cells	AGA	-0.40334	3.27288	-0.95807	0.340642	-5.27215	0.540914	0.628902
Erythroid.cells	ENAH	0.603429	0.744942	0.95806	0.340645	-5.11402	0.590173	0.674693
Erythroid.cells	PIK3R3	-0.49951	3.43068	-0.9577	0.340824	-5.36688	0.538167	0.626229
Erythroid.cells	RNF111	-0.16716	6.692893	-0.95762	0.340867	-6.05793	0.481165	0.571908
Erythroid.cells	MALT1	-0.29382	7.764534	-0.95716	0.341095	-6.41914	0.464102	0.555189

Erythroid.cells	HERC1	0.162875	7.056727	0.957037	0.341158	-6.18618	0.475473	0.56627
Erythroid.cells	PLCL1	0.340892	5.328139	0.956765	0.341295	-5.82478	0.504581	0.594305
Erythroid.cells	STUB1	0.182863	5.426852	0.956603	0.341376	-5.82159	0.502875	0.592712
Erythroid.cells	NUP35	-0.35769	3.291207	-0.95657	0.341392	-5.34643	0.54117	0.629015
Erythroid.cells	GM14636	0.662366	2.274283	0.956354	0.341501	-5.18014	0.560555	0.647127
Erythroid.cells	GM19696	-0.84245	0.016291	-0.95605	0.341654	-4.89152	0.606116	0.689121
Erythroid.cells	XPR1	-0.1855	6.866995	-0.9559	0.34173	-6.10677	0.478863	0.5696
Erythroid.cells	TRIM16	-0.47792	2.341778	-0.95586	0.341749	-5.24502	0.559383	0.646065
Erythroid.cells	CINP	0.419439	3.163035	0.955751	0.341804	-5.33506	0.543779	0.631497
Erythroid.cells	PLEKHG6	0.511293	-0.11425	0.955583	0.341889	-4.98476	0.608855	0.691755
Erythroid.cells	GPR141	0.527445	2.440611	0.955518	0.341921	-5.58208	0.557481	0.644402
Erythroid.cells	BORCS7	-0.28715	3.716689	-0.95546	0.341952	-5.43936	0.533515	0.621945
Erythroid.cells	NR4A1	0.281979	7.413157	0.955053	0.342155	-6.28449	0.470172	0.561298
Erythroid.cells	EXOC6B	-0.23606	5.502152	-0.95501	0.342178	-5.81613	0.501953	0.591969
Erythroid.cells	TMEM117	-0.88912	-0.04691	-0.95481	0.342276	-4.93483	0.607682	0.690746
Erythroid.cells	4833445I0	0.618372	0.48418	0.954529	0.342418	-4.97284	0.59664	0.680638
Erythroid.cells	CD53	-0.13425	7.920856	-0.9545	0.342435	-6.2951	0.462115	0.553422
Erythroid.cells	ABCE1	-0.22722	5.007871	-0.95441	0.342476	-5.74106	0.510562	0.600171
Erythroid.cells	GCSAM	0.530615	-0.31734	0.954055	0.342657	-5.14388	0.613383	0.696121
Erythroid.cells	SNX8	-0.30514	5.468943	-0.95405	0.342657	-5.86239	0.502549	0.592663
Erythroid.cells	TMEFF1	0.754914	-0.59624	0.953935	0.342717	-4.99452	0.61932	0.701562
Erythroid.cells	MUL1	0.371935	2.950066	0.95366	0.342856	-5.3067	0.547999	0.635773
Erythroid.cells	AKAP7	0.27439	3.374989	0.953586	0.342893	-5.49795	0.54004	0.628307
Erythroid.cells	ING3	0.220514	4.830612	0.95318	0.343098	-5.62168	0.513679	0.603416
Erythroid.cells	DNAL4	-0.51485	1.951429	-0.95315	0.343114	-5.08908	0.567187	0.653747
Erythroid.cells	ASB5	-0.65129	0.515697	-0.95307	0.343153	-4.97773	0.595992	0.680354
Erythroid.cells	ZEB2	-0.22215	9.36649	-0.95306	0.343159	-6.63354	0.439912	0.531848
Erythroid.cells	KLRD1	-0.30641	3.767425	-0.95304	0.34317	-5.95002	0.532796	0.621541
Erythroid.cells	MIER1	-0.14002	6.946828	-0.95299	0.343191	-6.16902	0.477747	0.568892
Erythroid.cells	EBI3	-0.33054	4.387447	-0.95293	0.343222	-5.51287	0.521559	0.610912
Erythroid.cells	MANBA	-0.27488	4.766674	-0.95292	0.343228	-5.5513	0.514808	0.604497
Erythroid.cells	GM15965	-0.51098	2.027609	-0.95286	0.343261	-5.17589	0.565699	0.652381
Erythroid.cells	PISD	-0.26234	4.8584	-0.9528	0.343287	-5.67002	0.513189	0.602974
Erythroid.cells	LCK	-0.26194	3.728178	-0.95275	0.343317	-5.72413	0.533516	0.622286
Erythroid.cells	MN1	0.859106	0.308399	0.952596	0.343392	-4.86771	0.600272	0.684405
Erythroid.cells	D16ERTD4	-0.24766	4.914897	-0.95254	0.343421	-5.75731	0.512194	0.602122
Erythroid.cells	KDM6B	-0.17377	8.152763	-0.95241	0.343485	-6.3925	0.458474	0.550233
Erythroid.cells	COPS9	0.156498	6.68605	0.952334	0.343524	-6.11964	0.482027	0.573161
Erythroid.cells	MID2	0.79363	0.27499	0.952293	0.343545	-4.90662	0.600965	0.685064
Erythroid.cells	NSDHL	-0.45161	2.406095	-0.95215	0.343618	-5.18548	0.558366	0.645678
Erythroid.cells	NR5A2	0.534571	1.145394	0.952062	0.343661	-5.08285	0.583178	0.668708
Erythroid.cells	LIPT2	0.557625	1.430834	0.952025	0.343679	-5.07296	0.577463	0.663447
Erythroid.cells	PPP3CC	0.292405	3.993482	0.951812	0.343787	-5.66413	0.528738	0.617855
Erythroid.cells	FCRLA	-0.28166	5.107141	-0.95153	0.343931	-5.88893	0.508891	0.599078
Erythroid.cells	1810046KC	0.652318	-0.14929	0.951413	0.343989	-5.00486	0.609913	0.693364
Erythroid.cells	LIMK1	-0.47586	2.1198	-0.9514	0.343996	-5.16793	0.563976	0.651008
Erythroid.cells	SF1	-0.12013	7.215593	-0.95135	0.344021	-6.18625	0.473439	0.564952

Erythroid.cells	MLLT10	0.116112	7.464033	0.951146	0.344123	-6.25116	0.469438	0.561076
Erythroid.cells	TMEM39A	-0.24943	4.395391	-0.95112	0.344136	-5.56852	0.521483	0.611121
Erythroid.cells	IZUMO1R	0.652462	0.001693	0.951096	0.344148	-5.10707	0.606741	0.690527
Erythroid.cells	TK1	0.365003	4.882981	0.95093	0.344232	-5.69034	0.512868	0.602931
Erythroid.cells	WDR34	-0.76981	1.224098	-0.95063	0.344386	-4.97686	0.581837	0.667638
Erythroid.cells	ILVBL	-0.29068	3.757211	-0.95041	0.344495	-5.47271	0.533204	0.622318
Erythroid.cells	N4BP2L1	0.286963	4.595001	0.950338	0.344531	-5.61292	0.518066	0.607987
Erythroid.cells	ZDBF2	-0.80554	-0.23932	-0.95028	0.344563	-4.99575	0.611986	0.695458
Erythroid.cells	MOK	-0.85959	0.377087	-0.95027	0.344564	-4.89733	0.599098	0.683662
Erythroid.cells	RNPEPL1	-0.27263	4.75379	-0.94999	0.344708	-5.63897	0.515331	0.60543
Erythroid.cells	SAMHD1	-0.27797	7.45997	-0.94995	0.344726	-6.26031	0.469712	0.5615
Erythroid.cells	BCL11A	0.295075	5.196907	0.94971	0.344849	-5.81451	0.507634	0.598136
Erythroid.cells	SUMF1	0.289585	4.240634	0.949631	0.344888	-5.54738	0.524583	0.614272
Erythroid.cells	CARNMT1	0.183396	5.353674	0.949359	0.345026	-5.81071	0.505004	0.595636
Erythroid.cells	EFL1	-0.22559	4.5955	-0.94908	0.345166	-5.63235	0.51832	0.608453
Erythroid.cells	OFD1	-0.30204	3.31561	-0.94902	0.345198	-5.32786	0.541643	0.630519
Erythroid.cells	DHODH	0.469598	2.109646	0.948978	0.345218	-5.11313	0.564621	0.652029
Erythroid.cells	ZC3H12C	0.342052	4.431325	0.948916	0.34525	-5.84827	0.521252	0.611241
Erythroid.cells	B430306N(	0.501332	2.692804	0.948875	0.34527	-5.17914	0.553386	0.641539
Erythroid.cells	EMG1	-0.17845	5.937655	-0.94833	0.345549	-5.97917	0.495247	0.586307
Erythroid.cells	PPARD	0.247943	5.613407	0.948299	0.345562	-5.73192	0.500782	0.591624
Erythroid.cells	G6PC3	-0.40615	3.106653	-0.94821	0.345607	-5.27993	0.545834	0.63439
Erythroid.cells	BACH2OS	-0.55897	1.526179	-0.94795	0.345741	-5.06784	0.576527	0.662992
Erythroid.cells	MPV17L2	-0.28778	4.459696	-0.94783	0.345797	-5.56833	0.521138	0.611047
Erythroid.cells	HES1	0.314934	5.161335	0.947472	0.345981	-5.77067	0.508924	0.599333
Erythroid.cells	NECAB3	-0.60876	1.186088	-0.947	0.346218	-4.99947	0.583822	0.669522
Erythroid.cells	SLC43A3	0.321179	3.813488	0.94684	0.346301	-5.50777	0.533288	0.622416
Erythroid.cells	ZSWIM6	-0.16376	8.155524	-0.94648	0.346481	-6.39637	0.459582	0.55164
Erythroid.cells	GM36975	0.400891	3.276788	0.946442	0.346503	-5.40172	0.543229	0.631904
Erythroid.cells	DDAH1	0.369832	2.481818	0.946385	0.346531	-5.37567	0.558309	0.646075
Erythroid.cells	DAG1	0.271127	4.867236	0.946334	0.346557	-5.64083	0.514321	0.6046
Erythroid.cells	TRIM25	0.229079	6.978283	0.946233	0.346609	-6.11679	0.478431	0.570113
Erythroid.cells	CWC15	-0.13577	6.384372	-0.94622	0.346615	-6.07863	0.488252	0.579618
Erythroid.cells	COA3	0.181341	5.812938	0.946218	0.346616	-5.95819	0.497903	0.588913
Erythroid.cells	ZFP384	-0.22554	4.90083	-0.94586	0.346797	-5.64631	0.513854	0.604179
Erythroid.cells	DENND1A	-0.13869	7.497877	-0.94576	0.346847	-6.28175	0.470127	0.562076
Erythroid.cells	EXO5	0.59022	2.006793	0.945735	0.346861	-5.08419	0.567665	0.654901
Erythroid.cells	GM20682	-0.4661	1.711443	-0.94533	0.347068	-5.1033	0.573731	0.660465
Erythroid.cells	SLC22A17	0.766197	-0.51359	0.945072	0.347198	-4.89755	0.619668	0.702708
Erythroid.cells	MZT1	-0.15886	5.278837	-0.94481	0.347331	-5.87669	0.507684	0.59824
Erythroid.cells	GM48086	-0.52651	1.511775	-0.9447	0.347386	-5.08576	0.577959	0.664389
Erythroid.cells	PLEKHO1	0.194566	5.499121	0.944331	0.347574	-5.89136	0.50406	0.594742
Erythroid.cells	ASCC3	-0.18302	6.882346	-0.94386	0.347813	-6.14129	0.480938	0.572411
Erythroid.cells	PON1	-0.33655	4.304512	-0.94384	0.347824	-5.92295	0.525381	0.615
Erythroid.cells	MRPL1	-0.19121	4.619868	-0.94338	0.348057	-5.68008	0.519918	0.609704
Erythroid.cells	APEH	0.323766	3.695339	0.943205	0.348147	-5.4881	0.536708	0.625657
Erythroid.cells	CMTM3	-0.32746	3.593966	-0.943	0.348251	-5.45213	0.538583	0.627515

Erythroid.cells	NUP205	-0.2584	4.408914	-0.94285	0.348325	-5.58907	0.523699	0.613441
Erythroid.cells	BEND5	0.594459	0.115495	0.94285	0.348327	-4.97147	0.607212	0.691223
Erythroid.cells	ADGRE4	0.487391	3.108019	0.942754	0.348376	-5.44734	0.547667	0.636058
Erythroid.cells	FAM25C	0.568165	1.148741	0.94264	0.348434	-5.18422	0.585936	0.671687
Erythroid.cells	CDC37	-0.13044	6.605127	-0.9426	0.348455	-6.09724	0.485706	0.577086
Erythroid.cells	UPF3B	0.215059	4.979585	0.942285	0.348614	-5.76649	0.513535	0.603968
Erythroid.cells	TET1	-0.67913	1.135429	-0.94216	0.348678	-5.01364	0.586206	0.672139
Erythroid.cells	AC166172.	-0.64394	0.939575	-0.94211	0.348704	-4.98982	0.590181	0.675806
Erythroid.cells	HAP1	-0.72603	-0.45019	-0.94209	0.348712	-4.91157	0.61919	0.702387
Erythroid.cells	CLN5	-0.26416	4.008222	-0.94203	0.348744	-5.50035	0.530963	0.620525
Erythroid.cells	TEAD1	0.581751	1.756692	0.942002	0.348759	-5.11925	0.573776	0.660634
Erythroid.cells	ATXN7L3B	0.149291	5.941589	0.941949	0.348786	-5.98915	0.496868	0.58801
Erythroid.cells	GM16286	-0.16451	5.859685	-0.94187	0.348824	-5.9336	0.498264	0.589352
Erythroid.cells	COPS6	0.183443	5.552293	0.941764	0.348879	-5.92484	0.503542	0.594419
Erythroid.cells	CUEDC2	-0.21563	5.245055	-0.94169	0.348917	-5.73909	0.508877	0.599567
Erythroid.cells	GM5544	0.632567	0.129876	0.941324	0.349104	-5.00019	0.60711	0.691351
Erythroid.cells	USF2	0.175859	6.021725	0.941256	0.349138	-5.92899	0.495669	0.586862
Erythroid.cells	COMMD9	0.388692	2.646144	0.941077	0.34923	-5.27219	0.556694	0.644697
Erythroid.cells	ZFP942	-0.2793	4.309464	-0.94073	0.349408	-5.55692	0.525809	0.615638
Erythroid.cells	DONSON	0.273958	4.025423	0.940591	0.349477	-5.50791	0.530968	0.62055
Erythroid.cells	GM37494	0.300498	3.825168	0.94059	0.349478	-5.456	0.534638	0.624019
Erythroid.cells	PAQR3	-0.41427	1.469925	-0.94055	0.3495	-5.21155	0.579828	0.666267
Erythroid.cells	KSR1	0.28112	4.69672	0.940263	0.349645	-5.68754	0.518994	0.609062
Erythroid.cells	AADAT	0.478994	1.2345	0.940099	0.349728	-5.19426	0.584741	0.670675
Erythroid.cells	LUC7L2	0.091669	8.527172	0.939926	0.349816	-6.49099	0.455271	0.547522
Erythroid.cells	MRTO4	0.276404	4.665156	0.939886	0.349837	-5.68501	0.519586	0.609664
Erythroid.cells	MED27	0.174963	5.275175	0.939807	0.349877	-5.75865	0.508818	0.599434
Erythroid.cells	MED18	0.566355	1.412452	0.939636	0.349964	-5.04556	0.581218	0.667498
Erythroid.cells	E430024I0[illegible]	-0.46265	1.898133	-0.93947	0.35005	-5.12439	0.571615	0.658553
Erythroid.cells	RRNAD1	0.288235	3.384404	0.939284	0.350144	-5.37868	0.543144	0.631973
Erythroid.cells	MRGBP	0.335706	3.37422	0.939115	0.35023	-5.34092	0.543352	0.632152
Erythroid.cells	TVP23B	-0.25663	4.042523	-0.93905	0.350262	-5.50753	0.531002	0.620535
Erythroid.cells	ZFP994	0.38408	2.481603	0.93819	0.350703	-5.20888	0.560889	0.648392
Erythroid.cells	CBX2	0.760472	-0.18673	0.938142	0.350727	-4.88793	0.614981	0.698293
Erythroid.cells	0610009E0	0.588802	0.843747	0.937942	0.350829	-4.96411	0.593562	0.678639
Erythroid.cells	ZGPAT	-0.25477	3.668613	-0.93785	0.350879	-5.44118	0.538494	0.627382
Erythroid.cells	NUB1	0.174388	5.534309	0.937628	0.35099	-5.84294	0.505149	0.595635
Erythroid.cells	GM13008	-0.61951	1.087607	-0.93744	0.351086	-4.9995	0.588746	0.674218
Erythroid.cells	MRPS24	0.150211	6.031221	0.937347	0.351134	-6.0205	0.49667	0.587609
Erythroid.cells	TFIP11	-0.32765	3.322157	-0.93669	0.351468	-5.36884	0.545478	0.633935
Erythroid.cells	GPRASP1	0.26757	3.93667	0.936385	0.351626	-5.48153	0.534065	0.623292
Erythroid.cells	PLAGL1	0.580784	1.041672	0.936335	0.351651	-5.17404	0.590088	0.675551
Erythroid.cells	GGA1	-0.22283	4.730426	-0.93631	0.351666	-5.59913	0.519693	0.609671
Erythroid.cells	2900093K2	-0.43284	2.884005	-0.93622	0.35171	-5.2197	0.55377	0.641849
Erythroid.cells	MXD1	0.184679	6.036212	0.93617	0.351736	-6.08939	0.496929	0.587963
Erythroid.cells	CSTF2T	-0.34827	3.017029	-0.93614	0.351751	-5.27423	0.551238	0.639526
Erythroid.cells	EXOSC1	-0.24351	4.175365	-0.93584	0.351904	-5.55628	0.529844	0.619317

Erythroid.cells	SPEF1	-0.72867	0.343281	-0.93574	0.351954	-4.92498	0.60465	0.688951
Erythroid.cells	AKR1C13	-0.44579	1.84784	-0.9352	0.352231	-5.26641	0.574424	0.660869
Erythroid.cells	RRN3	-0.2303	4.400393	-0.93463	0.352525	-5.6016	0.526244	0.615887
Erythroid.cells	DDIAS	-0.47292	2.134705	-0.93458	0.352553	-5.17954	0.56893	0.655967
Erythroid.cells	FBXO33	-0.186	5.984659	-0.93457	0.352558	-5.91054	0.498399	0.589317
Erythroid.cells	ABL1	0.215613	5.653787	0.934541	0.35257	-5.86373	0.504083	0.594776
Erythroid.cells	EIF5A	0.132004	9.061608	0.934397	0.352644	-6.61002	0.448691	0.54102
Erythroid.cells	CETN3	-0.19222	5.807387	-0.93438	0.352651	-5.92388	0.501436	0.59231
Erythroid.cells	DENND2A	-0.57582	1.374468	-0.93396	0.352869	-5.1088	0.584315	0.670207
Erythroid.cells	GM16341	-0.57502	0.863635	-0.93361	0.353049	-5.06558	0.59489	0.679974
Erythroid.cells	CSNK1D	-0.11359	6.6346	-0.93354	0.353084	-6.10322	0.487807	0.579124
Erythroid.cells	TSR3	-0.34002	3.569257	-0.9334	0.353156	-5.43182	0.541948	0.630764
Erythroid.cells	RNF40	-0.27048	4.021973	-0.93297	0.353374	-5.44444	0.533758	0.622995
Erythroid.cells	BLZF1	0.325715	3.200504	0.932933	0.353395	-5.33431	0.54906	0.637401
Erythroid.cells	TMSB10	-0.14597	10.73818	-0.93285	0.35344	-6.96447	0.424354	0.516794
Erythroid.cells	GM20559	-0.34693	4.344461	-0.93234	0.353698	-5.50506	0.52818	0.617512
Erythroid.cells	OSBPL9	-0.11798	7.511715	-0.93221	0.353767	-6.28987	0.473883	0.565409
Erythroid.cells	JMY	-0.21362	6.282387	-0.93169	0.354035	-6.00814	0.494358	0.58523
Erythroid.cells	EDEM3	-0.24103	5.974164	-0.93165	0.354052	-5.9737	0.499605	0.590274
Erythroid.cells	GLG1	-0.15547	6.951102	-0.93144	0.354165	-6.15784	0.483175	0.574436
Erythroid.cells	RPN1	-0.19479	5.957169	-0.93139	0.354186	-5.89341	0.499896	0.590553
Erythroid.cells	ARMCX4	0.63493	1.19399	0.931383	0.354192	-5.03598	0.588902	0.674242
Erythroid.cells	TAOK1	-0.13367	7.033029	-0.93136	0.354202	-6.17518	0.481824	0.573132
Erythroid.cells	INTS8	-0.21815	4.693172	-0.93126	0.354254	-5.65055	0.522049	0.611726
Erythroid.cells	PIGK	0.253558	4.298401	0.931193	0.354289	-5.50204	0.529179	0.618509
Erythroid.cells	ARHGEF17	0.82841	-0.37609	0.930681	0.354553	-4.89052	0.622071	0.704418
Erythroid.cells	ZFP512B	0.500225	2.377698	0.930173	0.354814	-5.18098	0.565909	0.652716
Erythroid.cells	AUNIP	-0.49355	1.893508	-0.93009	0.354858	-5.14117	0.575434	0.661564
Erythroid.cells	PDCD5	-0.14047	6.418317	-0.93009	0.354858	-6.11436	0.492553	0.583288
Erythroid.cells	PLEKHA3	-0.25068	3.98944	-0.92988	0.354963	-5.53383	0.535362	0.624139
Erythroid.cells	CR1L	-0.15653	5.693128	-0.92976	0.355029	-5.87524	0.504942	0.595229
Erythroid.cells	WFDC17	0.633559	5.906168	0.929716	0.35505	-5.97143	0.501269	0.591709
Erythroid.cells	BC031181	0.152211	5.703047	0.929672	0.355072	-5.92754	0.504771	0.595064
Erythroid.cells	NSMCE3	-0.22289	4.271532	-0.92932	0.355255	-5.56208	0.530292	0.619361
Erythroid.cells	ARMCX1	0.766074	0.550831	0.929289	0.35527	-4.9597	0.602831	0.686877
Erythroid.cells	ENO1B	-0.54362	1.327345	-0.92923	0.355298	-5.0709	0.58689	0.672235
Erythroid.cells	TIMM29	0.336226	3.296538	0.928828	0.355507	-5.36444	0.548618	0.636631
Erythroid.cells	CEP250	-0.26989	4.458141	-0.92871	0.355569	-5.60856	0.527145	0.616398
Erythroid.cells	CIC	0.242208	4.821881	0.928541	0.355655	-5.64483	0.520647	0.610229
Erythroid.cells	TMEM144	-0.69123	0.882973	-0.92842	0.355718	-5.00807	0.596299	0.680952
Erythroid.cells	ZFP326	0.190018	5.21559	0.928319	0.35577	-5.77341	0.513668	0.603647
Erythroid.cells	VSIG10L	-0.61026	0.298869	-0.92803	0.355918	-4.92854	0.608605	0.692225
Erythroid.cells	EIF3M	-0.11205	6.621301	-0.92786	0.356008	-6.13018	0.489691	0.580642
Erythroid.cells	GM17745	0.567855	0.962497	0.927627	0.356126	-5.07089	0.594963	0.679698
Erythroid.cells	SQOR	-0.28865	3.496234	-0.92741	0.35624	-5.67756	0.545207	0.633621
Erythroid.cells	FUT11	-0.3399	3.493603	-0.92732	0.356285	-5.37637	0.545256	0.633671
Erythroid.cells	CDC42SE2	-0.13939	7.179375	-0.9272	0.356346	-6.19262	0.4805	0.571893

Erythroid.cells	APH1A	-0.22675	5.433388	-0.92719	0.356353	-5.79188	0.5101	0.600379
Erythroid.cells	KIFC5B	-0.50271	1.787655	-0.92711	0.356396	-5.13648	0.578265	0.664508
Erythroid.cells	GM43111	0.589106	-0.1905	0.927034	0.356433	-5.05359	0.619124	0.702073
Erythroid.cells	RBM28	-0.19015	5.311789	-0.92649	0.356712	-5.7991	0.512557	0.602613
Erythroid.cells	FLCN	-0.29513	4.338996	-0.92618	0.356876	-5.53488	0.53013	0.619298
Erythroid.cells	GM1673	0.3901	2.42109	0.925862	0.357038	-5.32233	0.566478	0.653391
Erythroid.cells	DHX8	-0.20452	5.129659	-0.92573	0.357107	-5.76109	0.516107	0.606038
Erythroid.cells	SLAMF6	-0.28197	4.602908	-0.92529	0.357332	-5.72269	0.525779	0.615088
Erythroid.cells	CACNB3	-0.7835	-0.407	-0.92509	0.357439	-5.04106	0.624931	0.707006
Erythroid.cells	RSF1OS1	-0.35266	2.978349	-0.9249	0.357535	-5.34396	0.556048	0.643579
Erythroid.cells	FHAD1	-0.58641	0.624492	-0.92482	0.357579	-5.1649	0.603066	0.687154
Erythroid.cells	USP47	-0.16268	6.472249	-0.9248	0.357585	-6.05908	0.493179	0.583981
Erythroid.cells	MCM5	-0.39231	4.910563	-0.92471	0.357635	-5.75908	0.520301	0.609937
Erythroid.cells	NIPAL1	0.536008	1.060638	0.924339	0.357826	-5.16071	0.594262	0.67891
Erythroid.cells	RABL2	0.572768	1.106471	0.924229	0.357883	-5.02342	0.593323	0.67809
Erythroid.cells	RBL1	-0.26734	4.466968	-0.92386	0.358073	-5.68588	0.528473	0.617658
Erythroid.cells	ARRB2	-0.25923	5.195443	-0.92385	0.358077	-5.66958	0.515417	0.605259
Erythroid.cells	NOD1	-0.43858	3.387607	-0.92364	0.358187	-5.40062	0.548458	0.636507
Erythroid.cells	PDSS2	-0.22941	5.377267	-0.92363	0.358192	-5.79728	0.512212	0.602214
Erythroid.cells	AAK1	-0.17387	6.392459	-0.92341	0.358308	-6.10366	0.494699	0.585508
Erythroid.cells	RCL1	0.215833	4.24025	0.923362	0.358332	-5.7243	0.532607	0.621662
Erythroid.cells	ANLN	0.406513	3.747041	0.923341	0.358342	-5.51561	0.541717	0.630255
Erythroid.cells	KLHL23	0.767454	0.171341	0.923312	0.358357	-4.91143	0.612787	0.696125
Erythroid.cells	B4GALT5	-0.26073	5.975954	-0.92323	0.358398	-5.87841	0.501806	0.592337
Erythroid.cells	GM36371	0.53927	0.884673	0.923194	0.358419	-4.98774	0.597882	0.682472
Erythroid.cells	SPAST	-0.20462	5.207046	-0.92311	0.35846	-5.73687	0.515212	0.605172
Erythroid.cells	FBF1	-0.68369	1.539498	-0.92204	0.359019	-5.10583	0.584953	0.670454
Erythroid.cells	NRROS	0.161595	7.067702	0.92202	0.359027	-6.17303	0.483758	0.574851
Erythroid.cells	2900089D1	-0.43405	2.58395	-0.92197	0.359054	-5.27915	0.564264	0.651263
Erythroid.cells	TUBA1B	-0.2325	7.795232	-0.92196	0.359056	-6.38916	0.471886	0.563327
Erythroid.cells	GM42659	-0.25774	4.567867	-0.92193	0.359074	-5.59709	0.527031	0.616283
Erythroid.cells	REEP5	-0.12632	7.566136	-0.92187	0.359107	-6.29997	0.47559	0.566939
Erythroid.cells	EVA1A	0.497415	1.060527	0.92184	0.359121	-5.1612	0.5947	0.679459
Erythroid.cells	ZFP12	-0.48585	1.346551	-0.92177	0.359155	-5.07452	0.58886	0.674117
Erythroid.cells	KCNH7	0.749751	-0.13979	0.920658	0.359734	-4.92932	0.620715	0.70301
Erythroid.cells	SCN1B	-0.55531	1.834445	-0.92053	0.3598	-5.15377	0.579831	0.665518
Erythroid.cells	GM44899	0.608524	0.774696	0.920386	0.359876	-4.9963	0.601424	0.685408
Erythroid.cells	GM14085	0.639671	-1.35884	0.920294	0.359923	-4.95861	0.647406	0.727199
Erythroid.cells	AFG3L1	-0.17979	4.862083	-0.92029	0.359923	-5.65967	0.522451	0.611751
Erythroid.cells	CCDC107	0.223703	4.620705	0.920197	0.359974	-5.68905	0.5268	0.6159
Erythroid.cells	RSPH3B	-0.34346	3.01938	-0.91981	0.360175	-5.3016	0.556792	0.644033
Erythroid.cells	CEP89	0.446157	2.805458	0.919787	0.360187	-5.31787	0.560909	0.647877
Erythroid.cells	LILRA5	-0.59991	1.497471	-0.9194	0.360387	-5.20065	0.586881	0.672031
Erythroid.cells	BORA	0.321028	3.535011	0.919176	0.360504	-5.43667	0.547089	0.635081
Erythroid.cells	KDM5A	-0.13895	7.014524	-0.91907	0.36056	-6.21163	0.485531	0.576451
Erythroid.cells	TGFBI	0.31086	4.412001	0.918528	0.360842	-5.86526	0.530836	0.619927
Erythroid.cells	PSMB9	-0.32258	6.228284	-0.91834	0.360937	-6.01561	0.49877	0.589369

Erythroid.cells	RXRB	-0.25576	4.393184	-0.91823	0.360995	-5.58689	0.53118	0.620272
Erythroid.cells	INCENP	0.265995	5.454754	0.918223	0.361	-5.93891	0.512169	0.602209
Erythroid.cells	GM2396	-0.60355	-0.85737	-0.91803	0.361101	-4.96596	0.636584	0.717769
Erythroid.cells	MUC13	-0.77052	1.034673	-0.91798	0.361126	-4.97848	0.596327	0.681081
Erythroid.cells	GLOD4	-0.19987	5.050654	-0.91777	0.361235	-5.72688	0.51932	0.609152
Erythroid.cells	FOXO1	-0.14581	7.035015	-0.9177	0.361274	-6.29379	0.485191	0.576452
Erythroid.cells	FAM151B	-0.56736	1.753415	-0.91764	0.361305	-5.11381	0.581722	0.667737
Erythroid.cells	ENOX2	0.243398	5.852601	0.917574	0.361338	-5.88642	0.505231	0.595752
Erythroid.cells	FAM8A1	0.320385	3.231881	0.917387	0.361436	-5.37061	0.552827	0.64091
Erythroid.cells	FPGS	0.326002	3.013479	0.917321	0.36147	-5.39671	0.557	0.644846
Erythroid.cells	MTX1	0.248589	4.556924	0.917198	0.361534	-5.66121	0.5282	0.617765
Erythroid.cells	APOBEC3	-0.18051	6.862598	-0.91713	0.361569	-6.20339	0.48806	0.579356
Erythroid.cells	ZDHHC2	0.468823	2.276539	0.917094	0.361588	-5.25594	0.571324	0.658234
Erythroid.cells	PMF1	-0.23683	5.697613	-0.91708	0.361596	-5.89004	0.507922	0.598455
Erythroid.cells	SERPINA1C	0.341925	7.348457	0.917065	0.361604	-6.54413	0.480022	0.571573
Erythroid.cells	AMPD3	0.485177	2.578161	0.916987	0.361644	-5.23601	0.565416	0.652735
Erythroid.cells	SREBF1	-0.29239	3.760907	-0.91697	0.361653	-5.46165	0.542854	0.631605
Erythroid.cells	PDP2	-0.26442	3.724878	-0.91696	0.36166	-5.51373	0.543527	0.632238
Erythroid.cells	DCBLD2	-0.53684	1.32551	-0.91694	0.361668	-5.02975	0.590373	0.675869
Erythroid.cells	ECH1	0.180423	5.784916	0.916941	0.361668	-6.05319	0.506404	0.597002
Erythroid.cells	RGCC	0.289555	5.613883	0.916918	0.36168	-5.8553	0.509382	0.599851
Erythroid.cells	ERAL1	0.426866	1.918255	0.916865	0.361708	-5.14313	0.578425	0.664824
Erythroid.cells	NEK8	0.425005	2.113602	0.916448	0.361925	-5.21181	0.574801	0.661319
Erythroid.cells	CDNF	-0.77358	0.317972	-0.91587	0.362226	-4.91351	0.611928	0.695325
Erythroid.cells	STK26	-0.27608	4.048898	-0.91565	0.362341	-5.57974	0.538088	0.626846
Erythroid.cells	KRT83	-0.69769	-0.26141	-0.91564	0.362345	-4.99698	0.624294	0.706636
Erythroid.cells	GM17259	0.5685	1.365713	0.915592	0.362371	-5.08906	0.590195	0.67542
Erythroid.cells	BBS7	0.752731	0.558111	0.915105	0.362626	-4.9799	0.606993	0.690882
Erythroid.cells	BMP2	0.603712	1.613957	0.915058	0.36265	-5.09831	0.585275	0.670917
Erythroid.cells	1700008J0	-0.56204	1.842472	-0.91486	0.362753	-5.10174	0.580681	0.666743
Erythroid.cells	NLRP3	0.446386	4.527197	0.914836	0.362766	-5.74805	0.529416	0.618755
Erythroid.cells	RAPGEF6	-0.12892	8.049527	-0.91475	0.362811	-6.40253	0.469271	0.560998
Erythroid.cells	HACD3	0.172099	4.907611	0.914699	0.362838	-5.85701	0.522544	0.612276
Erythroid.cells	LY6D	-0.26024	5.594266	-0.91448	0.362954	-6.03498	0.510377	0.600687
Erythroid.cells	MAP3K12	0.351956	2.718206	0.914469	0.362957	-5.29983	0.563414	0.650748
Erythroid.cells	TECR	-0.15685	6.763011	-0.91443	0.362977	-6.15768	0.490352	0.581456
Erythroid.cells	IL4I1	-0.49773	2.841673	-0.91442	0.362982	-5.38252	0.561023	0.648517
Erythroid.cells	SLAMF1	0.575276	0.884468	0.914308	0.363042	-5.16271	0.600203	0.684812
Erythroid.cells	ZFP707	0.443018	1.958642	0.913981	0.363212	-5.17084	0.578553	0.664797
Erythroid.cells	PKD1L2	-0.54612	1.173969	-0.91386	0.363274	-5.22041	0.594439	0.679507
Erythroid.cells	SUCO	0.204331	6.354348	0.913412	0.36351	-6.02131	0.497562	0.5884
Erythroid.cells	AP3D1	-0.17839	4.873786	-0.91326	0.36359	-5.67197	0.523471	0.613161
Erythroid.cells	KDM5D	1.982136	0.730109	0.913248	0.363596	-5.12285	0.603769	0.688063
Erythroid.cells	COPG2	0.22956	5.004811	0.913233	0.363604	-5.69576	0.521122	0.610928
Erythroid.cells	PLEKHA8	0.521707	0.886546	0.913168	0.363637	-5.05005	0.600518	0.68508
Erythroid.cells	TMED3	0.246928	5.491352	0.912742	0.36386	-5.7264	0.512733	0.602838
Erythroid.cells	LRFN1	-0.69786	1.097146	-0.91261	0.363931	-5.03442	0.596473	0.681243

Erythroid.cells	FLOT1	-0.26553	4.575358	-0.91192	0.364292	-5.61905	0.52958	0.61869
Erythroid.cells	FEM1B	0.26103	4.728435	0.911767	0.364371	-5.62927	0.526838	0.616073
Erythroid.cells	MRFAP1	0.12609	6.452488	0.911661	0.364426	-6.1319	0.496599	0.587233
Erythroid.cells	RO60	-0.39644	2.493379	-0.91132	0.364606	-5.19647	0.569021	0.655694
Erythroid.cells	FAM189A1	-0.4745	2.327494	-0.91116	0.364688	-5.44202	0.572283	0.658775
Erythroid.cells	PPP4R3B	-0.11955	6.842276	-0.91106	0.364738	-6.17301	0.490079	0.580991
Erythroid.cells	FNTA	-0.15782	5.505108	-0.91099	0.364779	-5.86571	0.513044	0.603045
Erythroid.cells	GM43445	-0.46693	1.896237	-0.91088	0.364834	-5.14214	0.580856	0.666751
Erythroid.cells	SP110	-0.24976	5.924125	-0.91088	0.364838	-5.86351	0.505728	0.596051
Erythroid.cells	SCD1	0.426332	3.037555	0.910852	0.36485	-5.4892	0.558453	0.645919
Erythroid.cells	GM35867	-0.76046	0.456051	-0.9106	0.364981	-4.97064	0.610498	0.694026
Erythroid.cells	MEX3A	-0.55046	1.577184	-0.91053	0.365017	-5.09093	0.587329	0.672743
Erythroid.cells	ACD	-0.2133	4.940696	-0.91049	0.365041	-5.73402	0.523118	0.612643
Erythroid.cells	BTG1	0.140877	9.314237	0.910224	0.365179	-6.65447	0.450595	0.542445
Erythroid.cells	SCAP	0.295209	4.277309	0.910117	0.365235	-5.57777	0.535302	0.62412
Erythroid.cells	SEC22A	0.312382	3.176208	0.909673	0.365468	-5.37915	0.556229	0.643563
Erythroid.cells	SLC41A1	0.382371	2.387756	0.909457	0.365582	-5.28465	0.571544	0.657847
Erythroid.cells	TBL1XR1	0.15112	6.669065	0.909451	0.365584	-6.08039	0.493378	0.583947
Erythroid.cells	AGRN	-0.61454	1.254784	-0.90938	0.365625	-5.09194	0.594317	0.678897
Erythroid.cells	3300002A1	0.621338	0.451891	0.909175	0.365729	-4.97649	0.611101	0.694254
Erythroid.cells	OSTF1	0.11283	7.480967	0.908895	0.365877	-6.25881	0.48006	0.571004
Erythroid.cells	MSL1	-0.2181	5.419802	-0.90879	0.365929	-5.77066	0.515151	0.604765
Erythroid.cells	H2-D1	-0.24522	8.452787	-0.90851	0.366079	-6.48932	0.464518	0.555919
Erythroid.cells	TFEC	0.482078	2.368464	0.90842	0.366126	-5.32766	0.572284	0.658518
Erythroid.cells	HAUS5	0.364577	2.831524	0.907997	0.366348	-5.32629	0.563462	0.65029
Erythroid.cells	A430035B1	0.497016	3.071834	0.907834	0.366434	-5.2835	0.558818	0.646028
Erythroid.cells	PFKFB2	0.346709	2.465839	0.907816	0.366443	-5.2753	0.570606	0.657015
Erythroid.cells	BTBD10	-0.17114	5.583956	-0.90766	0.366527	-5.86713	0.51264	0.602451
Erythroid.cells	SHTN1	0.281434	3.187667	0.907475	0.366623	-5.71475	0.556701	0.644041
Erythroid.cells	KNTC1	0.460277	2.818917	0.907235	0.366749	-5.31229	0.563898	0.650791
Erythroid.cells	TMEM184E	0.287194	4.707523	0.907159	0.366789	-5.63376	0.528429	0.617488
Erythroid.cells	PPP6R3	-0.12616	7.283025	-0.90699	0.366875	-6.26464	0.483801	0.574792
Erythroid.cells	NCLN	0.288404	3.839103	0.906959	0.366894	-5.44878	0.54444	0.632628
Erythroid.cells	0610030E2	0.236096	4.591291	0.906819	0.366967	-5.62638	0.530571	0.619554
Erythroid.cells	EBP	-0.23309	4.791273	-0.90671	0.367027	-5.77016	0.526947	0.616121
Erythroid.cells	SART1	-0.21765	4.872457	-0.90657	0.367096	-5.71018	0.525499	0.614747
Erythroid.cells	RAB19	-0.35961	2.561986	-0.90643	0.367173	-5.4259	0.568971	0.655613
Erythroid.cells	GZMA	-0.4994	4.671597	-0.90637	0.367203	-6.24799	0.529136	0.618255
Erythroid.cells	VANGL2	-0.41555	2.225601	-0.90617	0.367309	-5.23518	0.57561	0.661849
Erythroid.cells	PAIP1	0.175314	5.907952	0.906124	0.367333	-5.96693	0.507165	0.597381
Erythroid.cells	ORC2	-0.23594	4.279461	-0.90598	0.367409	-5.61293	0.536318	0.625087
Erythroid.cells	GM47802	-0.78487	0.099495	-0.90595	0.367423	-4.91996	0.619424	0.702123
Erythroid.cells	ERAP1	-0.2098	5.228177	-0.90576	0.367525	-5.81108	0.519192	0.608812
Erythroid.cells	VAMP3	0.165759	5.320751	0.905574	0.367623	-5.80185	0.517607	0.607283
Erythroid.cells	ACTR5	-0.3122	3.477256	-0.90545	0.367689	-5.41374	0.551475	0.639324
Erythroid.cells	TRIM26	0.217749	5.330265	0.905097	0.367874	-5.75664	0.517638	0.607267
Erythroid.cells	KLF10	-0.22768	5.248269	-0.90485	0.368006	-5.73597	0.519207	0.608735

Erythroid.cells	CLEC5A	-0.56421	0.512385	-0.90469	0.368089	-5.16306	0.611221	0.694535
Erythroid.cells	NSUN5	0.421847	2.124735	0.904444	0.368218	-5.18427	0.578187	0.664198
Erythroid.cells	ZCCHC14	0.565445	1.412865	0.904397	0.368243	-5.09548	0.592558	0.677473
Erythroid.cells	SNRPN	-0.89891	-0.6475	-0.90416	0.36837	-4.89641	0.636241	0.717525
Erythroid.cells	MBP	0.218912	5.172136	0.904079	0.36841	-5.90222	0.52064	0.610365
Erythroid.cells	PLXDC1	0.452312	3.94302	0.90392	0.368494	-5.46165	0.543095	0.631649
Erythroid.cells	OTUB2	0.464322	1.885457	0.903877	0.368517	-5.21778	0.582977	0.668859
Erythroid.cells	SET	-0.13431	7.946372	-0.90387	0.368519	-6.42824	0.473489	0.565035
Erythroid.cells	GLIPR2	-0.34506	4.6029	-0.90382	0.368549	-5.70444	0.530916	0.620152
Erythroid.cells	PER2	0.522841	2.222784	0.903529	0.3687	-5.1824	0.576388	0.662721
Erythroid.cells	GM42699	-0.70196	0.727533	-0.90319	0.368878	-5.02015	0.6071	0.690942
Erythroid.cells	TAF13	0.257663	4.566883	0.902939	0.369012	-5.60158	0.531949	0.620997
Erythroid.cells	CFL2	0.214847	4.631141	0.902909	0.369027	-5.7724	0.530776	0.619894
Erythroid.cells	ACOX1	0.183637	5.892871	0.902783	0.369094	-6.11261	0.508306	0.598514
Erythroid.cells	TRIM2	-0.4575	2.325336	-0.90257	0.369204	-5.26974	0.574664	0.661098
Erythroid.cells	PLXNA1	-0.66248	0.899006	-0.90253	0.369226	-5.05054	0.603643	0.687833
Erythroid.cells	CD163	0.878154	1.13156	0.90209	0.36946	-5.10515	0.59897	0.683496
Erythroid.cells	TNFRSF14	0.66966	0.252225	0.901974	0.369522	-5.0278	0.617429	0.700394
Erythroid.cells	DIRAS2	-0.57171	0.718948	-0.90197	0.369525	-5.08877	0.607561	0.691375
Erythroid.cells	ARRDC1	0.282704	4.471227	0.901942	0.369538	-5.54908	0.533887	0.622863
Erythroid.cells	PHF1	-0.40935	2.695984	-0.90175	0.369638	-5.27136	0.567584	0.654423
Erythroid.cells	CHP2	0.96993	-0.09865	0.90139	0.36983	-4.99558	0.625204	0.707345
Erythroid.cells	GM39556	0.466009	3.384757	0.901354	0.369849	-5.29985	0.554434	0.642091
Erythroid.cells	F830208F2	-0.71714	-0.23725	-0.90116	0.369952	-4.97628	0.628235	0.710151
Erythroid.cells	STRADA	0.252713	4.58715	0.901089	0.369989	-5.6422	0.532004	0.621039
Erythroid.cells	SLC9A8	-0.2249	4.988676	-0.90101	0.370031	-5.72786	0.524718	0.614154
Erythroid.cells	GM44127	0.825995	-0.52381	0.900676	0.370208	-4.92428	0.634693	0.71601
Erythroid.cells	CMPK1	0.113806	6.881961	0.900111	0.370506	-6.22924	0.49219	0.582974
Erythroid.cells	SNX10	0.2777	5.029489	0.900026	0.370551	-5.76833	0.524443	0.613846
Erythroid.cells	TACC2	-0.34732	3.088667	-0.89997	0.370581	-5.43893	0.560633	0.647929
Erythroid.cells	GM16573	0.445611	1.655105	0.899799	0.370672	-5.18806	0.589028	0.674341
Erythroid.cells	RPA3	0.242899	4.97161	0.899676	0.370736	-5.77002	0.525486	0.614975
Erythroid.cells	MBTPS2	-0.2792	3.917498	-0.89967	0.370738	-5.48713	0.544868	0.633287
Erythroid.cells	GM5089	-0.69214	0.395428	-0.8994	0.370883	-4.97468	0.615198	0.698524
Erythroid.cells	GCA	0.619427	1.099128	0.899236	0.37097	-5.16295	0.600435	0.685027
Erythroid.cells	TEX14	-0.28135	5.64791	-0.89903	0.371079	-5.87667	0.513434	0.603637
Erythroid.cells	PECAM1	-0.21313	7.734027	-0.899	0.371096	-6.34003	0.478069	0.56958
Erythroid.cells	1110004F1	0.116812	6.204	0.898963	0.371114	-6.03283	0.503743	0.594363
Erythroid.cells	GM15265	-0.52564	1.797023	-0.89874	0.371231	-5.12618	0.586152	0.671959
Erythroid.cells	RDH5	-0.42069	2.368316	-0.89874	0.371231	-5.33375	0.57472	0.661369
Erythroid.cells	INTS7	-0.15098	5.677826	-0.89872	0.371241	-5.90944	0.512907	0.603174
Erythroid.cells	SPATA1	0.25802	3.467697	0.898574	0.37132	-5.4317	0.553365	0.641485
Erythroid.cells	AMBP	0.283828	4.730423	0.898478	0.371371	-6.07689	0.529856	0.619382
Erythroid.cells	4921531C2	-0.56398	1.642005	-0.89831	0.371458	-5.08066	0.589294	0.674989
Erythroid.cells	SSH2	0.176926	8.396317	0.898273	0.37148	-6.45138	0.467391	0.559321
Erythroid.cells	TXNDC5	0.225996	5.179694	0.898266	0.371484	-5.81742	0.521746	0.611713
Erythroid.cells	ZC3H4	-0.19652	4.818849	-0.89823	0.3715	-5.67186	0.528249	0.61789

Erythroid.cells	AP1M1	-0.20113	4.979621	-0.89803	0.371611	-5.7332	0.525421	0.615177
Erythroid.cells	FRMPD4	0.700043	0.351798	0.897408	0.371938	-5.03426	0.616557	0.699943
Erythroid.cells	GNB4	-0.36881	2.895468	-0.89737	0.371958	-5.36046	0.564771	0.652175
Erythroid.cells	SGCZ	-0.48132	1.627249	-0.89712	0.372092	-5.27927	0.590007	0.67566
Erythroid.cells	CCDC73	0.284226	3.100981	0.89703	0.372139	-5.44861	0.560788	0.648528
Erythroid.cells	STX3	0.418598	2.314188	0.896733	0.372296	-5.38733	0.576197	0.662957
Erythroid.cells	CARMIL2	-0.37758	2.704609	-0.89667	0.37233	-5.31763	0.568496	0.655844
Erythroid.cells	TMEM204	0.535179	1.038857	0.896626	0.372353	-5.02231	0.602107	0.686948
Erythroid.cells	PRSS57	0.61568	-0.03051	0.89652	0.37241	-4.98513	0.62475	0.707676
Erythroid.cells	TNFSF13OS	-0.77953	0.055873	-0.89652	0.372411	-5.00002	0.622889	0.705982
Erythroid.cells	ATP6V0A2	-0.21627	4.619482	-0.89646	0.372439	-5.67256	0.532251	0.621866
Erythroid.cells	SERPINB1A	0.480389	3.600711	0.896456	0.372444	-5.48841	0.551224	0.639752
Erythroid.cells	TGFBR1	-0.23075	6.24383	-0.89641	0.37247	-5.90922	0.503409	0.594389
Erythroid.cells	2700038G2	0.414797	2.577847	0.896003	0.372684	-5.18715	0.571064	0.658365
Erythroid.cells	FAM177A	0.488309	1.731422	0.895945	0.372715	-5.13461	0.587973	0.674073
Erythroid.cells	ARAP1	0.27939	5.049234	0.895736	0.372826	-5.68853	0.524528	0.614721
Erythroid.cells	BAD	0.284796	3.912661	0.895532	0.372934	-5.50745	0.545416	0.634508
Erythroid.cells	USP50	0.301501	3.371827	0.895302	0.373057	-5.46017	0.55566	0.644166
Erythroid.cells	ALKBH5	0.13025	7.148219	0.895277	0.37307	-6.30474	0.48814	0.579886
Erythroid.cells	TSC22D4	-0.15717	6.684783	-0.89507	0.373182	-6.11744	0.495937	0.587415
Erythroid.cells	GM44710	-0.52605	2.000891	-0.89494	0.373247	-5.16805	0.582535	0.669205
Erythroid.cells	4930578M	-0.65311	0.63262	-0.89487	0.373287	-4.98819	0.610694	0.695139
Erythroid.cells	CCDC163	0.48239	2.446867	0.894737	0.373357	-5.25607	0.573647	0.660957
Erythroid.cells	D930030I0	-0.64741	0.408475	-0.89473	0.373363	-5.04561	0.615438	0.699479
Erythroid.cells	PTGES2	0.374098	2.422157	0.894718	0.373367	-5.27558	0.574136	0.661411
Erythroid.cells	MICU1	-0.16019	5.891562	-0.89466	0.3734	-5.9509	0.509591	0.600532
Erythroid.cells	DALRD3	-0.25664	3.729807	-0.89464	0.37341	-5.48292	0.548858	0.637781
Erythroid.cells	ZFP143	-0.2707	3.815014	-0.89462	0.37342	-5.45824	0.547251	0.63627
Erythroid.cells	ORMDL2	0.194025	5.109617	0.894571	0.373445	-5.81922	0.523442	0.613771
Erythroid.cells	RUVBL2	-0.31867	3.693884	-0.89456	0.37345	-5.46949	0.549536	0.638442
Erythroid.cells	ENDOD1	0.279985	2.901638	0.894316	0.373581	-5.51584	0.564846	0.652788
Erythroid.cells	ALS2	0.231837	4.283789	0.894013	0.373742	-5.60575	0.53867	0.628257
Erythroid.cells	WDFY4	0.183349	7.251372	0.893961	0.37377	-6.39777	0.486575	0.578445
Erythroid.cells	SLC30A1	-0.36039	3.386735	-0.89387	0.37382	-5.44939	0.555549	0.644162
Erythroid.cells	PTPRD	0.446233	2.563535	0.893824	0.373843	-5.47839	0.571525	0.659112
Erythroid.cells	GM43466	0.565453	1.497294	0.893646	0.373938	-5.06766	0.592926	0.679006
Erythroid.cells	IL1A	-0.54962	2.787687	-0.89362	0.373949	-5.37518	0.567128	0.655075
Erythroid.cells	GM9917	-0.64097	0.773036	-0.89347	0.374029	-5.06147	0.607934	0.692835
Erythroid.cells	RAI1	-0.18327	6.004853	-0.89342	0.37406	-5.99371	0.507778	0.598988
Erythroid.cells	MED25	-0.2053	4.496149	-0.89302	0.374272	-5.68111	0.534981	0.624809
Erythroid.cells	RUNX1	0.153068	9.201324	0.892746	0.374417	-6.64438	0.455572	0.548168
Erythroid.cells	TTLL11	-0.55808	1.608719	-0.89257	0.374512	-5.0518	0.591032	0.677167
Erythroid.cells	UFSP2	-0.22118	4.93604	-0.89256	0.374517	-5.74438	0.527074	0.617292
Erythroid.cells	CITED4	0.713679	-0.2568	0.892215	0.374699	-5.07195	0.630556	0.713335
Erythroid.cells	5530601H0	-0.30645	3.904215	-0.89199	0.374822	-5.56125	0.546382	0.635533
Erythroid.cells	NEMP1	0.429858	3.065406	0.89123	0.375225	-5.34501	0.562907	0.650721
Erythroid.cells	GM29570	-0.64652	0.692445	-0.89061	0.375554	-5.00586	0.611349	0.695279

Erythroid.cells	FKBP7	0.528412	1.867273	0.890257	0.375744	-5.13981	0.58723	0.673054
Erythroid.cells	PLEC	0.2354	4.805597	0.890067	0.375845	-5.73051	0.530746	0.620217
Erythroid.cells	HIST1H3G	-0.58957	1.46486	-0.89004	0.375859	-5.13392	0.595437	0.680634
Erythroid.cells	NMNAT2	-0.39963	2.111738	-0.89	0.375879	-5.6966	0.582301	0.668504
Erythroid.cells	UAP1	-0.21414	5.112787	-0.88986	0.375958	-5.80644	0.525212	0.615038
Erythroid.cells	ACYP2	-0.33486	3.398712	-0.88973	0.376026	-5.4392	0.557103	0.645171
Erythroid.cells	ROBO3	-0.75142	-0.8339	-0.88943	0.376184	-4.84649	0.644857	0.725868
Erythroid.cells	SSRP1	-0.17285	6.322976	-0.88924	0.376285	-6.12045	0.504087	0.594959
Erythroid.cells	UNC45A	-0.26238	4.303611	-0.889	0.376418	-5.55959	0.540324	0.629402
Erythroid.cells	DSEL	-0.58632	0.375304	-0.88894	0.376449	-4.96889	0.61865	0.702049
Erythroid.cells	P2RY13	-0.70528	1.100765	-0.88856	0.376651	-5.07467	0.603444	0.688117
Erythroid.cells	KREMEN1	-0.32923	3.039966	-0.88842	0.376727	-5.32709	0.56442	0.652021
Erythroid.cells	POLR2K	-0.13177	6.77475	-0.88841	0.376734	-6.17205	0.496502	0.58764
Erythroid.cells	HADH	0.191665	5.394105	0.888343	0.376767	-6.1322	0.520547	0.610691
Erythroid.cells	TMEM106/	-0.35025	3.645492	-0.88832	0.376781	-5.47395	0.552777	0.64116
Erythroid.cells	LYSMD1	0.451886	1.591133	0.888168	0.37686	-5.10435	0.593359	0.678873
Erythroid.cells	GM5608	-0.58992	0.923456	-0.88786	0.377026	-5.06965	0.60728	0.691713
Erythroid.cells	RPP38	-0.62539	1.226405	-0.88786	0.377028	-5.04799	0.600965	0.685915
Erythroid.cells	PKD1	-0.35674	3.003757	-0.88764	0.377144	-5.33976	0.565337	0.652939
Erythroid.cells	OSGIN2	-0.3552	3.18431	-0.88754	0.377197	-5.39953	0.561833	0.649675
Erythroid.cells	SNX7	0.496761	1.212304	0.887244	0.377355	-5.16638	0.601392	0.686415
Erythroid.cells	GM4788	0.506924	1.237901	0.887211	0.377373	-5.24611	0.600861	0.685927
Erythroid.cells	RNF31	-0.36566	3.162894	-0.88718	0.377386	-5.33311	0.562284	0.650187
Erythroid.cells	PLCXD2	-0.27409	3.46805	-0.88697	0.3775	-5.65496	0.556496	0.644723
Erythroid.cells	SERPINA3M	0.315759	3.688152	0.886799	0.377593	-5.82877	0.552344	0.640828
Erythroid.cells	BAG2	-0.54148	1.435408	-0.88668	0.377658	-5.07276	0.596924	0.682303
Erythroid.cells	CLCN6	-0.34755	3.649755	-0.88661	0.377697	-5.40743	0.553074	0.64155
Erythroid.cells	GTF2I	0.175537	5.762327	0.886271	0.377876	-5.92688	0.514405	0.604977
Erythroid.cells	SOX5OS4	0.681205	-0.23518	0.88621	0.377909	-5.00216	0.632406	0.714781
Erythroid.cells	1600002D2	0.864242	0.257645	0.886171	0.377929	-4.97217	0.621735	0.705097
Erythroid.cells	CEP85L	0.290479	4.806918	0.886143	0.377944	-5.71056	0.531546	0.62132
Erythroid.cells	HJURP	-0.2341	4.895427	-0.88593	0.378059	-5.73063	0.530017	0.619848
Erythroid.cells	SLA2	-0.35848	2.746588	-0.88567	0.378197	-5.42657	0.570799	0.658122
Erythroid.cells	GM26885	0.477761	2.317843	0.885423	0.37833	-5.28958	0.579415	0.666098
Erythroid.cells	CASP12	-0.65458	-0.47839	-0.885	0.378555	-4.98683	0.638184	0.720104
Erythroid.cells	PRR5L	0.486899	2.756439	0.885003	0.378556	-5.37901	0.570788	0.658212
Erythroid.cells	5031425F1	-0.7361	-0.17235	-0.88491	0.378607	-4.96219	0.631475	0.714027
Erythroid.cells	IFI203	-0.26667	6.302394	-0.88482	0.378656	-6.05395	0.505327	0.596358
Erythroid.cells	GM16364.1	0.604926	1.279847	0.884636	0.378753	-5.14706	0.6006	0.685854
Erythroid.cells	GIGYF2	0.188036	5.464029	0.88438	0.37889	-5.87237	0.520056	0.610565
Erythroid.cells	EXOC3L2	0.744018	2.269133	0.884372	0.378894	-5.27284	0.580454	0.667338
Erythroid.cells	KHSRP	-0.15081	5.998752	-0.8841	0.379039	-6.0189	0.51061	0.601624
Erythroid.cells	METTL16	-0.21506	4.533537	-0.8841	0.379039	-5.65651	0.536935	0.626672
Erythroid.cells	MADD	0.216377	5.091113	0.884091	0.379045	-5.76819	0.526752	0.61702
Erythroid.cells	D11WSU47	0.575901	1.299867	0.883982	0.379104	-5.06071	0.600185	0.68571
Erythroid.cells	IMPAD1	0.213406	4.365894	0.883849	0.379175	-5.62745	0.540037	0.629722
Erythroid.cells	GM8369	0.356453	3.324129	0.883686	0.379262	-5.63305	0.559738	0.648299

Erythroid.cells	TIFAB	0.435798	2.73524	0.883595	0.379311	-5.40898	0.571205	0.659068
Erythroid.cells	CCNT2	-0.17955	5.37232	-0.88341	0.379409	-5.83951	0.521694	0.612514
Erythroid.cells	IGHJ4	-0.68385	0.337645	-0.88335	0.379442	-5.00288	0.620452	0.704615
Erythroid.cells	MAN1A2	-0.13257	6.392544	-0.88319	0.379527	-6.10396	0.50377	0.595454
Erythroid.cells	LTN1	-0.19263	5.03701	-0.88307	0.379592	-5.74633	0.527731	0.6184
Erythroid.cells	RASAL3	0.2277	4.10056	0.883064	0.379596	-5.6373	0.544985	0.634733
Erythroid.cells	MAOB	0.499146	1.627366	0.883018	0.379621	-5.31606	0.593442	0.679939
Erythroid.cells	ARF6	-0.156	6.770128	-0.88301	0.379627	-6.21489	0.497303	0.58927
Erythroid.cells	CASD1	-0.24949	4.363906	-0.88289	0.379689	-5.55993	0.540074	0.630098
Erythroid.cells	CIITA	0.462493	2.478401	0.882874	0.379699	-5.4336	0.576282	0.66404
Erythroid.cells	MRPL35	-0.18621	5.21625	-0.88281	0.379734	-5.90067	0.524495	0.615331
Erythroid.cells	MYG1	-0.24434	3.803891	-0.88269	0.379797	-5.50044	0.550573	0.640058
Erythroid.cells	APOO	0.266077	3.509146	0.882436	0.379934	-5.50054	0.556185	0.645344
Erythroid.cells	PDK4	-0.53411	1.417008	-0.88233	0.379989	-5.18008	0.597764	0.68404
Erythroid.cells	C2CD2	-0.3237	3.079676	-0.88218	0.38007	-5.42351	0.564469	0.653165
Erythroid.cells	WASHC2	-0.24157	5.655214	-0.88215	0.380087	-5.7954	0.516657	0.607979
Erythroid.cells	KLRB1C	-0.40157	1.954564	-0.8821	0.380116	-5.65612	0.586783	0.673943
Erythroid.cells	TCOF1	0.207418	5.717375	0.881958	0.380191	-5.95484	0.515558	0.606988
Erythroid.cells	LSM6	-0.16516	6.681324	-0.88186	0.380244	-6.18199	0.498816	0.590964
Erythroid.cells	SLC35F5	0.386574	3.11698	0.881666	0.380348	-5.37115	0.563744	0.65262
Erythroid.cells	CCDC136	0.576189	0.119291	0.881566	0.380402	-5.21996	0.625147	0.709323
Erythroid.cells	SREBF2	0.195612	5.9605	0.881557	0.380407	-5.96186	0.51128	0.602956
Erythroid.cells	MCUB	0.437199	1.557619	0.881492	0.380442	-5.42674	0.594872	0.681543
Erythroid.cells	SNAPIN	0.25251	4.083623	0.881452	0.380463	-5.5073	0.545302	0.635298
Erythroid.cells	BC003965	-0.30214	3.701969	-0.88139	0.380496	-5.4601	0.552507	0.642093
Erythroid.cells	TSPAN12	0.563728	1.194761	0.881269	0.380562	-5.17223	0.602365	0.688507
Erythroid.cells	DPM2	0.270543	3.936241	0.88123	0.380583	-5.51312	0.548073	0.637963
Erythroid.cells	FAM71D	-0.7585	0.095273	-0.88109	0.38066	-4.94409	0.625666	0.70989
Erythroid.cells	GM44659	-0.62724	0.682551	-0.88105	0.380678	-4.97168	0.613108	0.69843
Erythroid.cells	METTL21A	0.372105	1.888583	0.880613	0.380915	-5.20974	0.5884	0.675565
Erythroid.cells	SNX25	0.178923	5.540177	0.880513	0.380969	-5.89211	0.518946	0.610296
Erythroid.cells	PCNX4	0.437516	2.141019	0.880285	0.381091	-5.17906	0.583378	0.670876
Erythroid.cells	RILPL1	0.506495	2.31814	0.880214	0.38113	-5.12856	0.579827	0.667609
Erythroid.cells	MAMDC2	-0.60246	0.366618	-0.87995	0.381273	-5.09766	0.620326	0.70489
Erythroid.cells	MOB3C	0.340574	3.356469	0.879775	0.381366	-5.34034	0.559561	0.648694
Erythroid.cells	SNX4	-0.13909	6.329814	-0.87978	0.381366	-6.04947	0.505256	0.597164
Erythroid.cells	PAK2	-0.10208	7.859842	-0.87956	0.38148	-6.38874	0.479585	0.572364
Erythroid.cells	FCHSD1	-0.51942	1.15994	-0.8794	0.381569	-5.07717	0.603669	0.689681
Erythroid.cells	SIRT4	0.738584	0.56797	0.879354	0.381593	-4.98367	0.61613	0.701146
Erythroid.cells	ADAM11	-0.65537	-1.14352	-0.87907	0.381748	-4.88004	0.653645	0.73535
Erythroid.cells	MAP3K10	-0.35051	2.731125	-0.87904	0.38176	-5.33595	0.571839	0.660331
Erythroid.cells	SSBP4	-0.20755	4.90464	-0.87902	0.381774	-5.71988	0.530648	0.621584
Erythroid.cells	COA7	-0.25561	3.404389	-0.87894	0.381815	-5.4637	0.558734	0.648134
Erythroid.cells	PIGA	-0.42148	1.918295	-0.8785	0.382053	-5.22369	0.588354	0.675696
Erythroid.cells	PHRF1	-0.161	5.804424	-0.87836	0.382127	-5.95169	0.514753	0.606435
Erythroid.cells	RITA1	-0.44982	1.367418	-0.87831	0.382154	-5.12106	0.59964	0.686122
Erythroid.cells	TIMM10	0.299169	3.050855	0.878147	0.382244	-5.44827	0.565871	0.654743

Erythroid.cells	GM43388	-0.56394	0.386691	-0.87798	0.382337	-5.18744	0.620325	0.705101
Erythroid.cells	ACSS2	0.32902	3.033515	0.877859	0.3824	-5.47254	0.566209	0.655111
Erythroid.cells	BUB1	0.403903	3.743439	0.877798	0.382432	-5.59171	0.552541	0.642313
Erythroid.cells	VPREB1	-0.60931	2.621584	-0.87776	0.382455	-5.45368	0.574301	0.662703
Erythroid.cells	UCHL1	-0.54708	0.862756	-0.87764	0.382518	-5.15833	0.610219	0.69596
Erythroid.cells	DMPK	0.447991	2.786398	0.877548	0.382568	-5.38955	0.571053	0.659729
Erythroid.cells	DCUN1D2	0.415087	2.743543	0.877423	0.382635	-5.28324	0.571912	0.660589
Erythroid.cells	TRMT112	-0.13392	7.277188	-0.87733	0.382688	-6.29725	0.489502	0.582303
Erythroid.cells	FAM120B	-0.27127	4.124917	-0.87719	0.382763	-5.51508	0.545388	0.635721
Erythroid.cells	ESS2	0.299286	2.988114	0.876754	0.382996	-5.34885	0.567353	0.656303
Erythroid.cells	CAR5A	0.599139	0.542195	0.876723	0.383013	-5.10163	0.617284	0.702478
Erythroid.cells	NISCH	0.159446	6.194239	0.876587	0.383086	-6.00522	0.508213	0.600324
Erythroid.cells	GM49067	-0.63777	0.406337	-0.87607	0.383368	-4.94868	0.620341	0.705426
Erythroid.cells	H2-Q7	-0.52659	3.889878	-0.87604	0.383383	-5.67865	0.550153	0.640313
Erythroid.cells	PIGO	0.498072	1.804475	0.875893	0.383462	-5.15813	0.591123	0.678638
Erythroid.cells	RAMP3	0.511027	0.947281	0.875887	0.383465	-5.27318	0.608866	0.694998
Erythroid.cells	PEX11G	0.347853	2.394748	0.875835	0.383493	-5.33488	0.579215	0.66759
Erythroid.cells	YDJC	-0.59956	1.21958	-0.87583	0.383494	-5.05828	0.603171	0.68976
Erythroid.cells	RIIAD1	-0.54652	0.993222	-0.87568	0.383579	-5.05659	0.607948	0.694123
Erythroid.cells	AP4S1	0.191259	4.920297	0.875506	0.383671	-5.74671	0.531103	0.622357
Erythroid.cells	GOSR2	0.192709	5.28486	0.875408	0.383724	-5.85499	0.524499	0.616097
Erythroid.cells	SBF1	-0.21183	4.474225	-0.87528	0.383794	-5.65138	0.539324	0.630177
Erythroid.cells	VTI1B	0.144055	5.690918	0.875081	0.383901	-5.88737	0.517336	0.609239
Erythroid.cells	PTRH2	-0.25041	4.181735	-0.87481	0.384049	-5.58525	0.544978	0.635444
Erythroid.cells	TRP53INP2	0.250058	4.134264	0.874699	0.384108	-5.62158	0.545874	0.636294
Erythroid.cells	GPR68	0.501034	1.433054	0.87448	0.384226	-5.30919	0.5992	0.686022
Erythroid.cells	ZFP839	0.420357	1.537463	0.874355	0.384294	-5.18116	0.597054	0.684038
Erythroid.cells	FRMD4A	0.344375	3.969412	0.874266	0.384342	-5.61596	0.549075	0.639319
Erythroid.cells	DET1	0.457546	2.186379	0.873839	0.384573	-5.21203	0.583888	0.67193
Erythroid.cells	CLK4	-0.15739	6.012894	-0.87378	0.384603	-5.98447	0.51192	0.604052
Erythroid.cells	LGALS9	0.171967	7.157478	0.873698	0.38465	-6.32456	0.492258	0.585107
Erythroid.cells	GM10521	-0.64381	0.308847	-0.87358	0.384712	-4.94855	0.622964	0.707915
Erythroid.cells	STAG2	0.142034	7.464582	0.873557	0.384726	-6.32789	0.48712	0.580127
Erythroid.cells	CAMKK2	-0.17423	5.188179	-0.8735	0.384756	-5.86301	0.526602	0.618082
Erythroid.cells	UBE2L3	0.10458	7.560477	0.873478	0.384769	-6.37092	0.485527	0.57858
Erythroid.cells	SLC20A1	-0.2383	5.219384	-0.8732	0.38492	-5.82297	0.526039	0.617545
Erythroid.cells	TSEN34	-0.26805	4.195681	-0.87315	0.384948	-5.58455	0.544863	0.635397
Erythroid.cells	APOM	0.308512	4.854579	0.873141	0.384952	-6.13607	0.532667	0.623849
Erythroid.cells	GRINA	0.177276	6.205395	0.873004	0.385025	-6.42552	0.508555	0.600847
Erythroid.cells	NLRC4	-0.29401	3.885651	-0.87295	0.385054	-5.58974	0.550702	0.640958
Erythroid.cells	GM5577	0.663776	0.20371	0.872671	0.385206	-4.97049	0.62523	0.710138
Erythroid.cells	IGKV1-35	-0.6895	-0.53717	-0.87265	0.385219	-4.9427	0.641432	0.724887
Erythroid.cells	FIG4	0.181612	4.603641	0.872636	0.385225	-5.72725	0.537278	0.628358
Erythroid.cells	CDC42EP4	-0.36945	2.155265	-0.87258	0.385253	-5.30393	0.584515	0.672686
Erythroid.cells	KIF5A	-0.66595	0.429339	-0.8723	0.385405	-5.03376	0.62038	0.705788
Erythroid.cells	TAF10	0.133797	6.856819	0.872295	0.38541	-6.22378	0.497345	0.590223
Erythroid.cells	WDR13	0.310596	2.570105	0.872278	0.38542	-5.3075	0.576219	0.665021

Erythroid.cells	ZFP84	0.310683	3.7569	0.871784	0.385687	-5.47722	0.553427	0.643547
Erythroid.cells	MAP7D3	-0.66045	0.126284	-0.87172	0.385724	-5.0134	0.627221	0.711931
Erythroid.cells	GEMIN2	0.293743	3.260952	0.871565	0.385806	-5.44615	0.562978	0.652558
Erythroid.cells	PDCL	0.289851	3.891934	0.871483	0.385851	-5.51888	0.550886	0.641199
Erythroid.cells	HMGN3	0.384497	3.255903	0.871263	0.38597	-5.46421	0.563109	0.652662
Erythroid.cells	CD164	0.173721	6.379209	0.871161	0.386026	-6.01903	0.505846	0.598348
Erythroid.cells	SETD2	-0.14351	6.922415	-0.8711	0.386058	-6.19193	0.496531	0.589367
Erythroid.cells	DDX1	-0.21544	4.964571	-0.87099	0.386121	-5.83193	0.530983	0.622394
Erythroid.cells	BAG3	0.300874	2.967773	0.870934	0.386149	-5.52997	0.568723	0.657973
Erythroid.cells	FAM169B	0.33928	3.62896	0.870284	0.386502	-5.5625	0.556343	0.646187
Erythroid.cells	BOLA1	0.282842	3.767483	0.870197	0.386549	-5.56448	0.553698	0.643721
Erythroid.cells	ACIN1	0.095895	7.577859	0.869871	0.386726	-6.40475	0.486053	0.579091
Erythroid.cells	TOR4A	-0.51997	1.678835	-0.86969	0.386822	-5.13076	0.595256	0.68249
Erythroid.cells	DCAKD	0.272925	4.076447	0.869462	0.386949	-5.59694	0.548125	0.638518
Erythroid.cells	CD320	0.387871	1.856288	0.869429	0.386967	-5.19662	0.591677	0.679199
Erythroid.cells	3110009E1	0.375366	2.175408	0.869229	0.387075	-5.24999	0.585253	0.673255
Erythroid.cells	HCFC1	-0.22407	4.949877	-0.86916	0.387111	-5.73663	0.531967	0.623233
Erythroid.cells	ILF3	-0.15262	6.541502	-0.86876	0.387329	-6.13344	0.503804	0.596317
Erythroid.cells	TUFT1	-0.34331	2.2113	-0.86873	0.387349	-5.28886	0.584624	0.672697
Erythroid.cells	SLC16A9	0.761751	0.530927	0.868632	0.3874	-5.07619	0.619518	0.7049
Erythroid.cells	IFIT3	-0.64288	3.112471	-0.8685	0.387471	-5.47056	0.566752	0.656145
Erythroid.cells	RGS3	-0.38019	2.862627	-0.86849	0.387475	-5.49692	0.571649	0.660722
Erythroid.cells	ANKRD39	0.248392	3.543857	0.86844	0.387505	-5.46965	0.558398	0.648316
Erythroid.cells	RAB3D	0.385569	3.11921	0.868185	0.387643	-5.3485	0.566706	0.656065
Erythroid.cells	MAP1LC3A	0.243936	5.476214	0.868102	0.387689	-5.92477	0.52261	0.614407
Erythroid.cells	CALHM2	0.322354	3.088827	0.868027	0.387729	-5.34109	0.5673	0.656622
Erythroid.cells	BHLHE41	0.475859	1.739361	0.867802	0.387852	-5.31953	0.594393	0.68178
Erythroid.cells	DCUN1D3	-0.25593	4.424811	-0.86768	0.387918	-5.6318	0.541898	0.632729
Erythroid.cells	CDON	0.434125	1.952332	0.867618	0.387952	-5.30903	0.590044	0.677794
Erythroid.cells	GIMAP3	0.441853	2.932139	0.867359	0.388093	-5.73005	0.570579	0.659619
Erythroid.cells	ARMH3	0.187787	5.427651	0.867156	0.388204	-5.86634	0.523749	0.615452
Erythroid.cells	RPGR	0.481644	1.599467	0.866893	0.388347	-5.10662	0.597622	0.684681
Erythroid.cells	ID2	0.17968	6.76154	0.866732	0.388435	-6.3368	0.500478	0.593156
Erythroid.cells	MAFK	0.308229	4.524193	0.866627	0.388492	-5.6741	0.540377	0.631292
Erythroid.cells	TAF4	0.237635	4.075399	0.866579	0.388518	-5.53693	0.548777	0.639228
Erythroid.cells	ASTE1	0.359091	2.753642	0.866263	0.38869	-5.29293	0.574382	0.663298
Erythroid.cells	VAC14	-0.19705	4.624685	-0.8662	0.388726	-5.65686	0.538574	0.629644
Erythroid.cells	MRPL12	0.19552	5.090142	0.866194	0.388728	-5.9195	0.530035	0.621538
Erythroid.cells	AC142100.	-0.67377	0.335219	-0.86592	0.388879	-5.04087	0.624351	0.709535
Erythroid.cells	MNDAL	-0.25762	6.786829	-0.8659	0.388886	-6.17097	0.5001	0.592962
Erythroid.cells	TMEM250-	0.236874	4.629225	0.865854	0.388914	-5.72612	0.53849	0.629681
Erythroid.cells	NUP214	0.186165	5.2678	0.865792	0.388948	-5.81758	0.526814	0.61861
Erythroid.cells	PRKD3	0.19517	5.198034	0.865554	0.389077	-5.87428	0.528131	0.619879
Erythroid.cells	PPARA	0.391034	2.784821	0.865515	0.389099	-5.55922	0.573825	0.662955
Erythroid.cells	A430093F1	-0.43739	2.363356	-0.86527	0.389231	-5.23891	0.582307	0.67092
Erythroid.cells	MREG	-0.42631	3.199109	-0.86516	0.389294	-5.59047	0.565782	0.655517
Erythroid.cells	KCNIP2	-0.59673	1.248736	-0.8651	0.389323	-5.07103	0.605126	0.692061

Erythroid.cells	ZMYM4	0.170627	5.733892	0.864928	0.389419	-5.96757	0.518646	0.610944
Erythroid.cells	LMF2	0.32405	3.103696	0.864563	0.389619	-5.40799	0.567825	0.657469
Erythroid.cells	4930445E1	-0.62038	-0.41363	-0.86446	0.389674	-4.98454	0.641075	0.724995
Erythroid.cells	NUDT5	0.20611	5.148841	0.864439	0.389686	-5.83448	0.529273	0.62111
Erythroid.cells	LGALS8	-0.21528	5.110017	-0.86437	0.389724	-5.80834	0.529979	0.621813
Erythroid.cells	NEIL1	-0.36449	3.000738	-0.86413	0.389854	-5.38616	0.569933	0.659544
Erythroid.cells	MRPL53	0.21732	4.291701	0.864053	0.389897	-5.76698	0.545172	0.636305
Erythroid.cells	SLFN8	0.512518	3.326481	0.86377	0.390052	-5.37235	0.56372	0.653638
Erythroid.cells	ZMAT5	-0.2121	4.547775	-0.86366	0.390111	-5.62464	0.54054	0.631821
Erythroid.cells	TSPAN18	0.567855	1.764014	0.863101	0.390417	-5.15241	0.595291	0.682936
Erythroid.cells	MIB1	0.172283	6.189415	0.862928	0.390512	-5.97944	0.511322	0.603874
Erythroid.cells	CDK2	-0.27689	4.350301	-0.86255	0.390719	-5.6301	0.544764	0.635688
Erythroid.cells	MAJIN	-0.70663	0.644865	-0.86254	0.390722	-5.0791	0.618948	0.704688
Erythroid.cells	ZFP958	-0.40954	2.267977	-0.86237	0.390817	-5.27105	0.585304	0.673671
Erythroid.cells	NLK	0.191657	5.966033	0.862244	0.390886	-5.9981	0.51545	0.607811
Erythroid.cells	NELFA	-0.19196	4.630912	-0.86186	0.391097	-5.69252	0.539821	0.63099
Erythroid.cells	UGT1A7C	-0.59724	-0.45047	-0.86176	0.391153	-5.05418	0.643144	0.726736
Erythroid.cells	TDRP	0.70335	-0.36439	0.861576	0.391251	-5.05312	0.641235	0.725105
Erythroid.cells	ACOD1	-1.16466	2.318035	-0.86143	0.391334	-5.30853	0.584543	0.673094
Erythroid.cells	FAM102B	-0.29215	4.23575	-0.86114	0.391492	-5.57215	0.547201	0.638177
Erythroid.cells	GM33524	0.655313	0.491882	0.861033	0.391549	-5.01905	0.622554	0.708198
Erythroid.cells	BORCS6	-0.28675	4.007961	-0.86102	0.391558	-5.52025	0.551503	0.642236
Erythroid.cells	RDH7	0.376797	2.864625	0.860979	0.391579	-5.64712	0.573637	0.663001
Erythroid.cells	SH2D2A	-0.29404	2.764079	-0.86087	0.391636	-5.75453	0.575628	0.664863
Erythroid.cells	ARHGAP10	-0.34125	5.105594	-0.86078	0.391687	-5.80105	0.531094	0.622924
Erythroid.cells	ASPRV1	-0.62903	1.33931	-0.86035	0.391926	-5.22063	0.60461	0.691794
Erythroid.cells	SERPINF1	0.321784	3.83888	0.860222	0.391993	-5.78812	0.55472	0.645349
Erythroid.cells	SREK1	-0.11719	6.429668	-0.86016	0.392027	-6.13014	0.50753	0.600444
Erythroid.cells	ZFP948	-0.41526	2.965976	-0.86015	0.392032	-5.39083	0.571638	0.661218
Erythroid.cells	TMED9	-0.12202	6.609324	-0.86008	0.392071	-6.16152	0.504419	0.597453
Erythroid.cells	TNNI1	-0.76701	-0.907	-0.85989	0.392177	-4.97479	0.653365	0.736341
Erythroid.cells	ALG6	-0.49023	2.044219	-0.85989	0.392178	-5.17159	0.590087	0.678407
Erythroid.cells	CYP2C70	0.328512	4.144913	0.859825	0.392211	-5.99635	0.548913	0.6399
Erythroid.cells	CRYZL2	0.326675	2.609637	0.859807	0.392221	-5.32953	0.578699	0.66783
Erythroid.cells	RNF19A	-0.19608	5.375931	-0.85974	0.392257	-5.86047	0.526191	0.618343
Erythroid.cells	PRTN3	0.931306	3.6691	0.859724	0.392266	-5.59133	0.557969	0.648431
Erythroid.cells	RNPC3	0.152774	4.741295	0.859653	0.392305	-5.88625	0.537778	0.629394
Erythroid.cells	EMCN	0.483654	1.650943	0.859485	0.392397	-5.20929	0.598145	0.685985
Erythroid.cells	S1PR2	-0.49044	1.261742	-0.85944	0.392424	-5.18599	0.60623	0.69346
Erythroid.cells	LY86	0.21737	6.704851	0.859253	0.392525	-6.20852	0.502773	0.596041
Erythroid.cells	SNX3	0.096226	7.730038	0.859234	0.392535	-6.47472	0.485465	0.579276
Erythroid.cells	ASF1B	-0.31416	4.954931	-0.85913	0.392593	-5.86849	0.533848	0.625819
Erythroid.cells	SRA1	0.167347	5.239409	0.859	0.392664	-5.91194	0.528661	0.620882
Erythroid.cells	ZFP119B	-0.48591	1.297642	-0.85891	0.39271	-5.12602	0.605479	0.692845
Erythroid.cells	NDUFC2	0.143084	6.741013	0.858837	0.392753	-6.27009	0.502151	0.595466
Erythroid.cells	HIST1H3C	0.674749	1.047316	0.858821	0.392762	-5.12966	0.610732	0.697688
Erythroid.cells	4933433G1	-0.49928	1.434512	-0.85866	0.392851	-5.15812	0.602627	0.690236

Erythroid.cells	PLK1	0.487571	3.704872	0.858603	0.392881	-5.57367	0.557283	0.647998
Erythroid.cells	D130040H:	-0.39876	2.397794	-0.85853	0.392922	-5.28171	0.582939	0.671996
Erythroid.cells	SYDE2	0.667606	-0.10373	0.858271	0.393064	-5.00962	0.635629	0.720401
Erythroid.cells	TRIM7	0.480092	0.591087	0.857861	0.393289	-5.20632	0.620721	0.706779
Erythroid.cells	AMOTL1	0.554945	1.696223	0.857797	0.393324	-5.12015	0.597496	0.685443
Erythroid.cells	COQ5	0.313051	3.832511	0.857692	0.393382	-5.50189	0.555106	0.645928
Erythroid.cells	GM16576	-0.58441	1.513376	-0.85759	0.393437	-5.11026	0.601277	0.689017
Erythroid.cells	PRKCG	0.379375	4.448629	0.857271	0.393613	-5.66667	0.543472	0.63512
Erythroid.cells	TMEM267	0.473331	1.734822	0.857167	0.39367	-5.17632	0.596701	0.684962
Erythroid.cells	HSPBAP1	0.326166	3.764205	0.857006	0.393759	-5.53112	0.556412	0.647415
Erythroid.cells	ENO3	0.447412	2.077848	0.856899	0.393818	-5.34874	0.589685	0.678553
Erythroid.cells	ATRNL1	-0.19295	6.820202	-0.85681	0.393869	-6.19394	0.501032	0.594696
Erythroid.cells	PILRA	-0.41228	3.3927	-0.85676	0.393894	-5.4244	0.56357	0.654233
Erythroid.cells	MPPE1	0.26212	4.363196	0.856756	0.393896	-5.59666	0.545069	0.636799
Erythroid.cells	TNFSF8	0.679772	-0.51498	0.85675	0.393899	-5.08199	0.644886	0.729274
Erythroid.cells	RFX3	-0.15881	6.233922	-0.85655	0.394007	-6.09249	0.511186	0.604543
Erythroid.cells	MTSS2	-0.66431	0.842863	-0.85648	0.394047	-5.00305	0.61535	0.702393
Erythroid.cells	1500009L1	-0.54336	-0.02505	-0.85639	0.394099	-5.03176	0.634067	0.719579
Erythroid.cells	TXNL4A	0.199826	4.968748	0.856257	0.39417	-5.77867	0.533849	0.626304
Erythroid.cells	CCNE2	-0.38625	3.75287	-0.85618	0.394215	-5.60516	0.556629	0.647884
Erythroid.cells	FSHR	-0.54496	0.419984	-0.85617	0.394217	-4.96521	0.624399	0.710789
Erythroid.cells	R3HDM1	0.118904	6.683532	0.856154	0.394227	-6.219	0.50338	0.597125
Erythroid.cells	PTBP1	-0.15484	6.221744	-0.8559	0.394367	-6.05989	0.5114	0.604919
Erythroid.cells	PRR16	0.459348	1.720127	0.855875	0.39438	-5.38296	0.597004	0.68565
Erythroid.cells	TONSL	-0.4989	1.456718	-0.85587	0.394383	-5.13752	0.602453	0.690689
Erythroid.cells	MET	-0.38976	1.991608	-0.85558	0.394544	-5.55658	0.591599	0.680572
Erythroid.cells	KBTBD11	0.610031	0.726015	0.855372	0.394657	-5.16519	0.618093	0.704967
Erythroid.cells	AMPD2	-0.41029	2.339378	-0.85511	0.394803	-5.23001	0.58475	0.674188
Erythroid.cells	CHDH	0.606861	1.157054	0.855029	0.394846	-5.10214	0.609086	0.696752
Erythroid.cells	PTGES3	0.114505	7.460041	0.854339	0.395226	-6.374	0.49073	0.584841
Erythroid.cells	CENPS	-0.37854	3.529733	-0.85422	0.395293	-5.51132	0.561531	0.652461
Erythroid.cells	PARVA	0.480736	1.329681	0.8542	0.395303	-5.21084	0.60576	0.693629
Erythroid.cells	COL18A1	0.385656	2.743841	0.854191	0.395308	-5.5588	0.576934	0.666885
Erythroid.cells	POGLUT3	0.540607	0.979034	0.854173	0.395317	-5.07982	0.613134	0.700419
Erythroid.cells	IBA57	0.356046	1.934328	0.854002	0.395412	-5.27165	0.593257	0.682125
Erythroid.cells	ZFP592	0.139411	5.89966	0.853905	0.395465	-5.99977	0.517638	0.610892
Erythroid.cells	GM9844	-0.32856	2.309102	-0.85388	0.39548	-5.38285	0.585642	0.675093
Erythroid.cells	CHD3	-0.25167	4.533669	-0.85301	0.395959	-5.73785	0.542993	0.634903
Erythroid.cells	AV099323	0.611921	1.209882	0.852997	0.395966	-5.07571	0.608846	0.696403
Erythroid.cells	SLC15A4	-0.24004	4.804844	-0.85282	0.396061	-5.72319	0.538012	0.630166
Erythroid.cells	CLK2	-0.1881	4.63296	-0.85266	0.396151	-5.69792	0.541199	0.633259
Erythroid.cells	EMB	0.226191	5.68619	0.852595	0.396187	-6.27441	0.521988	0.614978
Erythroid.cells	SIGLECG	0.219976	4.076853	0.852521	0.396229	-5.76608	0.551642	0.643156
Erythroid.cells	SLC4A9	0.667823	0.122757	0.852231	0.396389	-4.97579	0.63233	0.717997
Erythroid.cells	LRRN3	-0.75038	-0.11923	-0.85216	0.396426	-4.93676	0.637636	0.72286
Erythroid.cells	GM17749	-0.37449	4.634129	-0.85166	0.396701	-5.82239	0.541476	0.633419
Erythroid.cells	ANXA6	0.173946	6.174347	0.851515	0.396784	-6.1182	0.513612	0.606831

Erythroid.cells	EAPP	-0.14147	5.602282	-0.8515	0.39679	-5.95134	0.52378	0.616574
Erythroid.cells	BACE1	-0.35108	2.654059	-0.85149	0.396795	-5.29464	0.579649	0.669301
Erythroid.cells	PYGM	-0.34826	4.300481	-0.85118	0.396968	-5.597	0.547719	0.639453
Erythroid.cells	ERCC8	-0.34511	2.45611	-0.85113	0.396997	-5.28021	0.583616	0.673138
Erythroid.cells	GM28694	-0.6932	-0.23311	-0.85106	0.397038	-4.96044	0.640362	0.725377
Erythroid.cells	CSF3	-0.9086	-0.096	-0.85094	0.397101	-5.02373	0.637337	0.72264
Erythroid.cells	SLC16A7	0.285086	3.236151	0.850686	0.397242	-5.57254	0.568144	0.658724
Erythroid.cells	CAPN5	0.582134	1.546779	0.85054	0.397322	-5.13485	0.602204	0.690411
Erythroid.cells	TOMM20	0.137099	6.725117	0.850522	0.397332	-6.1851	0.50402	0.597756
Erythroid.cells	LIAS	0.227175	4.310022	0.8505	0.397345	-5.63957	0.54754	0.639328
Erythroid.cells	9930104L0	0.516172	1.356879	0.850423	0.397387	-5.06903	0.606162	0.694065
Erythroid.cells	LACTB	-0.18353	5.139278	-0.8504	0.397399	-5.89258	0.532166	0.624738
Erythroid.cells	SAP30L	-0.19851	4.309459	-0.85022	0.397501	-5.65674	0.54755	0.639419
Erythroid.cells	SRXN1	0.404191	1.094676	0.850126	0.397551	-5.28828	0.611671	0.699259
Erythroid.cells	POLR2B	-0.18701	4.911643	-0.8501	0.397568	-5.77054	0.53634	0.628818
Erythroid.cells	0610043K1	-0.55812	1.133363	-0.85009	0.397573	-5.20465	0.610855	0.698508
Erythroid.cells	LMF1	-0.31553	3.239428	-0.85001	0.397614	-5.41805	0.56808	0.658819
Erythroid.cells	CCDC157	-0.53143	1.103128	-0.84962	0.397833	-5.10391	0.611549	0.69933
Erythroid.cells	LARP4B	0.12416	7.283403	0.849605	0.397839	-6.25529	0.494537	0.588836
Erythroid.cells	TCF12	-0.13075	8.463403	-0.84933	0.397991	-6.52337	0.475021	0.569841
Erythroid.cells	RGS1	0.275556	5.563816	0.849187	0.398071	-6.17093	0.524519	0.617742
Erythroid.cells	MCPH1	0.201294	4.913551	0.84884	0.398263	-5.79686	0.536354	0.629027
Erythroid.cells	SENP3	0.244356	4.324023	0.848827	0.39827	-5.58704	0.547326	0.639435
Erythroid.cells	DNAJC12	-0.32706	3.069108	-0.84866	0.398364	-5.50965	0.571473	0.662161
Erythroid.cells	CFHR2	0.363074	2.56401	0.848653	0.398366	-5.58175	0.581503	0.671531
Erythroid.cells	TMEM65	-0.24799	4.9205	-0.84865	0.39837	-5.76959	0.536226	0.628905
Erythroid.cells	DENND5A	0.158319	6.395889	0.848614	0.398388	-6.06614	0.509777	0.603596
Erythroid.cells	WDCP	-0.33765	3.079235	-0.8486	0.398398	-5.375	0.571274	0.661975
Erythroid.cells	YIF1B	0.198617	4.886916	0.848578	0.398408	-5.80705	0.536845	0.629494
Erythroid.cells	GTF2E2	0.159337	5.440806	0.848552	0.398422	-5.93978	0.526737	0.619861
Erythroid.cells	TSTD1	-0.39946	2.589608	-0.84854	0.398428	-5.46522	0.580991	0.671053
Erythroid.cells	IQCK	0.700715	0.158994	0.848165	0.398637	-4.95373	0.631971	0.718141
Erythroid.cells	TCP11L1	0.329061	2.689841	0.848156	0.398642	-5.43706	0.579137	0.669368
Erythroid.cells	9330159M	-0.53441	1.076034	-0.84798	0.39874	-5.10194	0.612298	0.700083
Erythroid.cells	ERG28	-0.15678	5.118614	-0.84793	0.398765	-5.87681	0.532746	0.625618
Erythroid.cells	SERF1	0.295314	3.137334	0.847442	0.399037	-5.37263	0.570606	0.661162
Erythroid.cells	TECPR2	-0.28436	3.905519	-0.84716	0.399191	-5.53477	0.555857	0.647263
Erythroid.cells	ASMT	-0.55118	1.052723	-0.84702	0.399273	-5.13641	0.613315	0.700738
Erythroid.cells	TMEM94	0.310902	2.610495	0.846475	0.399573	-5.31443	0.581593	0.671188
Erythroid.cells	NDUFS5	0.138108	6.478554	0.8463	0.39967	-6.21839	0.509284	0.602704
Erythroid.cells	LY6C2	-0.42714	5.53324	-0.84558	0.400068	-6.06386	0.526438	0.618961
Erythroid.cells	NRXN3	0.573307	1.145579	0.845564	0.400078	-5.17258	0.612245	0.699324
Erythroid.cells	HOGA1	0.349728	2.370342	0.84523	0.400264	-5.47352	0.587032	0.676024
Erythroid.cells	TMEM259	0.236004	4.879001	0.845157	0.400304	-5.77919	0.53849	0.63044
Erythroid.cells	LPCAT3	-0.20624	5.162712	-0.84498	0.400404	-5.82953	0.533271	0.625515
Erythroid.cells	CRY1	-0.2047	5.502006	-0.84495	0.400417	-5.86884	0.5271	0.619634
Erythroid.cells	CD300LG	0.460987	1.259921	0.844928	0.400432	-5.24507	0.609948	0.697257

Erythroid.cells	KLRK1	-0.34267	3.149025	-0.84481	0.400499	-5.80497	0.571511	0.661639
Erythroid.cells	CDCA2	0.343659	3.79635	0.844457	0.400694	-5.6311	0.558995	0.649899
Erythroid.cells	GM43462	-0.36082	3.23183	-0.84444	0.400701	-5.35735	0.569961	0.660189
Erythroid.cells	NAA80	0.337559	2.402845	0.844409	0.40072	-5.31909	0.586471	0.675591
Erythroid.cells	PIK3IP1	0.399672	2.920799	0.844135	0.400872	-5.35895	0.576232	0.665999
Erythroid.cells	1700010I1	-0.51194	1.215796	-0.84404	0.400925	-5.06997	0.611119	0.698359
Erythroid.cells	SLCO1B2	0.303005	4.25024	0.843921	0.400991	-6.0276	0.550481	0.641901
Erythroid.cells	PPP6R1	0.146134	5.572544	0.843737	0.401094	-5.88935	0.526053	0.618767
Erythroid.cells	AURKAIP1	0.153749	5.837172	0.843715	0.401106	-6.04292	0.521302	0.614227
Erythroid.cells	SLC37A2	-0.41584	3.315046	-0.84342	0.401268	-5.33061	0.568595	0.659072
Erythroid.cells	TBXA2R	0.585728	2.36173	0.843325	0.401322	-5.17817	0.587576	0.676813
Erythroid.cells	ANKS1B	0.502399	1.030517	0.843271	0.401353	-5.18966	0.615183	0.702327
Erythroid.cells	MAPKAPK5	0.366861	2.128701	0.842837	0.401594	-5.22421	0.592546	0.681325
Erythroid.cells	RABGAP1	-0.16528	5.920481	-0.84269	0.401677	-6.00718	0.520123	0.613162
Erythroid.cells	1700037C1	0.350094	3.065274	0.842688	0.401677	-5.33465	0.57373	0.663882
Erythroid.cells	FXYD2	-0.6946	-0.41192	-0.84259	0.401734	-5.01556	0.646849	0.731246
Erythroid.cells	PRPS1L3	0.389004	2.045409	0.842357	0.401861	-5.26136	0.59433	0.683115
Erythroid.cells	GLIS1	-0.73693	0.394465	-0.84229	0.401898	-4.97744	0.629168	0.7152
Erythroid.cells	TIGD2	0.26794	3.85213	0.841654	0.402252	-5.48118	0.558872	0.649836
Erythroid.cells	DCTN5	0.207251	4.598222	0.841562	0.402304	-5.73662	0.544722	0.636483
Erythroid.cells	ATP6AP2	-0.1141	6.938045	-0.84147	0.402354	-6.21594	0.502742	0.596343
Erythroid.cells	ZFP865	-0.33276	3.265059	-0.84138	0.402406	-5.39929	0.570277	0.660569
Erythroid.cells	ARHGEF7	0.248552	4.675352	0.841079	0.402573	-5.6846	0.543356	0.635232
Erythroid.cells	ZFP65	-0.48152	1.662117	-0.84108	0.402574	-5.207	0.602749	0.690782
Erythroid.cells	NDUFV1	0.169211	5.20278	0.840822	0.402716	-5.96463	0.533721	0.626011
Erythroid.cells	NUF2	-0.38009	3.365247	-0.84064	0.402819	-5.49509	0.568582	0.658938
Erythroid.cells	BET1L	-0.24841	3.860623	-0.84026	0.403031	-5.55332	0.559163	0.650003
Erythroid.cells	GM49463	-0.7335	-0.05016	-0.84008	0.403128	-4.96689	0.639874	0.724709
Erythroid.cells	ENKD1	-0.4899	1.3576	-0.84003	0.403156	-5.12088	0.609524	0.696944
Erythroid.cells	PDLIM5	0.159097	6.703681	0.839959	0.403197	-6.23116	0.5072	0.600606
Erythroid.cells	TENM2	0.8463	-0.35252	0.839842	0.403262	-4.96536	0.64659	0.730856
Erythroid.cells	A1CF	0.56322	0.569704	0.839727	0.403326	-5.17111	0.626326	0.712408
Erythroid.cells	RPP25L	-0.25574	3.672149	-0.83969	0.403348	-5.55892	0.562801	0.653538
Erythroid.cells	BATF	0.274733	4.183315	0.839424	0.403495	-5.56808	0.553052	0.644406
Erythroid.cells	ADARB1	0.426221	3.009129	0.839412	0.403502	-5.35339	0.575853	0.66581
Erythroid.cells	GLS	-0.14034	7.7257	-0.83921	0.403613	-6.38632	0.489911	0.583934
Erythroid.cells	TIMP2	0.371131	4.873204	0.838825	0.40383	-5.73015	0.540386	0.632356
Erythroid.cells	NME3	0.737617	0.405363	0.838439	0.404046	-4.9792	0.630468	0.716152
Erythroid.cells	TYK2	0.250007	3.992109	0.838409	0.404062	-5.52486	0.557152	0.648177
Erythroid.cells	EML3	-0.24326	4.139794	-0.83832	0.40411	-5.62376	0.55433	0.645547
Erythroid.cells	AI847159	0.559642	-0.25702	0.838222	0.404166	-5.09742	0.645055	0.729474
Erythroid.cells	SMNDC1	-0.12571	6.059801	-0.83794	0.404325	-6.0596	0.519018	0.611993
Erythroid.cells	8-Sep	0.378145	1.954462	0.837881	0.404357	-5.28927	0.597697	0.686064
Erythroid.cells	S100A10	0.183625	6.601647	0.83787	0.404363	-6.34144	0.509479	0.60283
Erythroid.cells	GNG11	0.306501	3.831566	0.837679	0.40447	-5.71879	0.560311	0.651182
Erythroid.cells	SGSM3	0.284626	4.063557	0.837614	0.404506	-5.6088	0.555859	0.646991
Erythroid.cells	CCT2	-0.13696	6.286032	-0.83753	0.404555	-6.10537	0.515043	0.608176

Erythroid.cells	PPT1	0.172725	6.003515	0.837308	0.404677	-6.10574	0.520137	0.613071
Erythroid.cells	5033406O(	0.539473	0.605012	0.837127	0.404778	-5.16188	0.626393	0.712466
Erythroid.cells	GM20457	0.671768	-0.16272	0.836924	0.404892	-4.96596	0.643311	0.727899
Erythroid.cells	GM47448	0.86668	-0.89373	0.836618	0.405063	-4.90974	0.659946	0.742921
Erythroid.cells	TOMM22	0.125236	6.905252	0.836493	0.405133	-6.26271	0.504615	0.598088
Erythroid.cells	NTNG2	-0.41041	3.05713	-0.8364	0.405187	-5.48319	0.575868	0.665723
Erythroid.cells	P4HA2	-0.61737	0.108055	-0.83625	0.405268	-5.03148	0.637564	0.722623
Erythroid.cells	PCSK7	-0.19986	5.282369	-0.83561	0.405627	-5.84356	0.533892	0.626024
Erythroid.cells	DNAJC15	-0.16247	5.723768	-0.83544	0.405724	-6.06063	0.525924	0.618423
Erythroid.cells	SEMA4G	0.546536	0.909795	0.834945	0.405999	-5.16408	0.621022	0.707245
Erythroid.cells	PKN1	0.128836	6.766934	0.834826	0.406066	-6.13856	0.507756	0.600931
Erythroid.cells	CAR13	-0.46613	1.31675	-0.8344	0.406303	-5.17603	0.612642	0.699534
Erythroid.cells	LRAT	0.627711	-0.03713	0.834278	0.406373	-5.0422	0.641952	0.726367
Erythroid.cells	TRAPPC5	0.237373	4.379372	0.834212	0.40641	-5.64828	0.551311	0.642435
Erythroid.cells	RABEPK	-0.29583	3.296184	-0.83404	0.406508	-5.43283	0.572265	0.662134
Erythroid.cells	DPY19L1	0.230566	4.584338	0.833978	0.406541	-5.74804	0.547468	0.638816
Erythroid.cells	TPGS1	-0.24055	4.393149	-0.83383	0.406622	-5.68433	0.5511	0.642247
Erythroid.cells	PDE4B	-0.16276	8.527906	-0.83367	0.406714	-6.56847	0.478382	0.572491
Erythroid.cells	ITGB2	-0.20829	6.734664	-0.83365	0.406727	-6.24627	0.508583	0.60179
Erythroid.cells	ANXA10	-0.60137	0.643116	-0.83352	0.406797	-5.0516	0.62713	0.712988
Erythroid.cells	CD5L	0.461913	6.068559	0.833291	0.406926	-6.04109	0.520424	0.61314
Erythroid.cells	INPP5B	-0.2485	4.216748	-0.83277	0.407221	-5.60819	0.554895	0.645782
Erythroid.cells	KRCC1	0.126996	5.9705	0.832411	0.40742	-6.04	0.522663	0.615136
Erythroid.cells	LRP8	-0.53649	2.371033	-0.83231	0.407474	-5.15036	0.5915	0.67997
Erythroid.cells	COA4	-0.38353	2.161432	-0.83187	0.407723	-5.26954	0.595915	0.684049
Erythroid.cells	PTCD2	0.211405	4.58679	0.831863	0.407727	-5.71625	0.548191	0.639408
Erythroid.cells	COMMD1	0.192049	5.141948	0.831807	0.407759	-5.86306	0.53784	0.629637
Erythroid.cells	BCL2A1A	-0.40194	2.399522	-0.83167	0.407837	-5.71042	0.591045	0.67962
Erythroid.cells	FBXO8	0.182935	4.606605	0.831647	0.407849	-5.7764	0.547818	0.639137
Erythroid.cells	P2RY10B	-0.29376	3.709802	-0.83133	0.408027	-5.53326	0.56497	0.655365
Erythroid.cells	CACFD1	0.273407	3.174111	0.831284	0.408053	-5.44371	0.575483	0.665208
Erythroid.cells	LGALSL	0.484074	1.281509	0.831223	0.408087	-5.19262	0.614277	0.70118
Erythroid.cells	TRPS1	0.170413	7.683996	0.831051	0.408184	-6.52111	0.493019	0.586795
Erythroid.cells	CACNA1B	0.680952	0.056619	0.830991	0.408217	-5.00168	0.640806	0.725527
Erythroid.cells	IFITM1	-0.7387	3.489377	-0.83098	0.408224	-5.61444	0.569271	0.659499
Erythroid.cells	SASS6	0.254296	4.42508	0.830807	0.408321	-5.67655	0.551245	0.642551
Erythroid.cells	PNKD	0.197902	4.867546	0.830791	0.40833	-5.88256	0.54293	0.634685
Erythroid.cells	GALE	0.336674	2.330073	0.83076	0.408347	-5.32232	0.592461	0.681134
Erythroid.cells	MOGS	-0.27704	4.095452	-0.83038	0.408559	-5.5924	0.557682	0.648535
Erythroid.cells	RARS2	-0.27433	3.441715	-0.83025	0.408636	-5.48585	0.570366	0.660442
Erythroid.cells	BMPR2	0.235406	5.519027	0.830195	0.408664	-5.88279	0.531076	0.623336
Erythroid.cells	STK3	-0.1746	5.985237	-0.83011	0.408714	-6.10761	0.522657	0.615331
Erythroid.cells	RAC3	-0.71761	-0.22085	-0.82994	0.408807	-4.99358	0.647157	0.731294
Erythroid.cells	ISOC2A	0.366699	2.013655	0.829912	0.408824	-5.29958	0.599127	0.687284
Erythroid.cells	GM16153	-0.39661	2.536009	-0.82975	0.408917	-5.31701	0.588436	0.677373
Erythroid.cells	BLOC1S4	-0.25076	3.695064	-0.82973	0.408924	-5.50911	0.565415	0.655878
Erythroid.cells	GM50322	-0.66831	0.358739	-0.82966	0.408967	-5.01292	0.634335	0.719625

Erythroid.cells	NQO2	0.212652	3.942173	0.829338	0.409147	-5.67715	0.560791	0.651502
Erythroid.cells	TSHZ3	-0.51876	2.187789	-0.82903	0.409319	-5.33261	0.595713	0.684146
Erythroid.cells	AMBRA1	-0.1182	7.654516	-0.829	0.409337	-6.42025	0.493796	0.587582
Erythroid.cells	TRAF5	-0.17981	5.308971	-0.82891	0.409386	-6.00911	0.53507	0.627249
Erythroid.cells	GM20404	-0.48184	1.661785	-0.82884	0.409426	-5.14684	0.606616	0.694265
Erythroid.cells	ZFP62	-0.22274	4.580193	-0.82879	0.409455	-5.66759	0.548626	0.640132
Erythroid.cells	ALYREF2	0.291414	3.243584	0.828524	0.409605	-5.44705	0.574434	0.664564
Erythroid.cells	GM32250	-0.60769	-0.2746	-0.82839	0.409681	-5.00469	0.648546	0.732831
Erythroid.cells	BRD8	-0.12122	6.236165	-0.82834	0.40971	-6.14656	0.518333	0.611456
Erythroid.cells	1700086O(	0.437883	1.546442	0.828281	0.409742	-5.14716	0.609034	0.696713
Erythroid.cells	DFFB	-0.34269	2.981393	-0.82811	0.409836	-5.3741	0.579644	0.669499
Erythroid.cells	GM46620	0.664277	0.683678	0.827936	0.409936	-5.03349	0.627439	0.713669
Erythroid.cells	LATS1	-0.19665	4.725566	-0.82793	0.409942	-5.70265	0.545894	0.637786
Erythroid.cells	POGK	-0.40907	2.454489	-0.82789	0.409963	-5.28498	0.590262	0.679395
Erythroid.cells	CTSC	0.262527	7.322442	0.827785	0.410021	-6.31264	0.499428	0.593307
Erythroid.cells	SIRPB1C	0.501929	1.778958	0.827734	0.41005	-5.4598	0.60417	0.692288
Erythroid.cells	LDLRAD4	0.215927	6.109073	0.827606	0.410122	-6.12088	0.520594	0.613697
Erythroid.cells	MFSD5	-0.25398	4.282812	-0.82739	0.410242	-5.63219	0.554261	0.645775
Erythroid.cells	2410002F2	-0.27276	3.766412	-0.82721	0.410343	-5.47091	0.564191	0.655224
Erythroid.cells	RCCD1	-0.39606	3.170294	-0.82711	0.410402	-5.36355	0.575885	0.666189
Erythroid.cells	LRRC32	0.4527	1.241998	0.827107	0.410403	-5.24593	0.615465	0.702886
Erythroid.cells	DROSHA	0.267748	3.121263	0.827025	0.410449	-5.40224	0.576858	0.667116
Erythroid.cells	1700094J0	0.739408	0.092196	0.827001	0.410463	-4.99741	0.640383	0.725711
Erythroid.cells	DNPH1	0.524897	0.708539	0.82695	0.410492	-5.10752	0.626901	0.713403
Erythroid.cells	AFAP1L1	0.600357	1.508026	0.826863	0.410541	-5.1261	0.609842	0.697748
Erythroid.cells	SMPD2	0.392402	2.04317	0.826855	0.410545	-5.29526	0.59869	0.687444
Erythroid.cells	ZCCHC24	0.30203	3.666755	0.826322	0.410846	-5.53234	0.56643	0.657276
Erythroid.cells	MTUS2	0.707076	0.654472	0.826268	0.410876	-5.0462	0.628406	0.714699
Erythroid.cells	CPQ	0.208514	5.056734	0.826051	0.410999	-5.96792	0.540097	0.632383
Erythroid.cells	LACC1	-0.349	3.279516	-0.82595	0.411057	-5.48814	0.574125	0.664496
Erythroid.cells	VPS52	0.217459	3.923327	0.825586	0.411261	-5.53356	0.561547	0.652779
Erythroid.cells	GM43331	-0.38898	2.057137	-0.82554	0.411285	-5.19492	0.598821	0.687565
Erythroid.cells	SMIM40	0.592893	0.301755	0.825509	0.411305	-5.06524	0.636212	0.721931
Erythroid.cells	USP33	0.224263	4.710834	0.825464	0.41133	-5.69471	0.546553	0.638651
Erythroid.cells	ELP2	0.2178	4.144774	0.825374	0.411381	-5.61442	0.557288	0.648818
Erythroid.cells	ZFP746	0.253399	3.717554	0.825329	0.411406	-5.51372	0.565536	0.65658
Erythroid.cells	TOP1MT	0.504876	1.70028	0.825182	0.411489	-5.15478	0.606236	0.694511
Erythroid.cells	PLIN3	-0.29195	3.527331	-0.82506	0.411559	-5.45005	0.56925	0.660099
Erythroid.cells	MEGF9	0.301513	4.103779	0.825041	0.411569	-5.66262	0.558074	0.649594
Erythroid.cells	PRKD2	0.237347	4.348606	0.824951	0.41162	-5.6519	0.553397	0.645182
Erythroid.cells	CCDC97	-0.272	3.33761	-0.82466	0.411784	-5.45038	0.573129	0.663688
Erythroid.cells	PDE10A	0.497968	2.555984	0.824341	0.411964	-5.32518	0.588871	0.678372
Erythroid.cells	PRIM1	-0.29691	4.178781	-0.82422	0.412035	-5.74673	0.556881	0.648481
Erythroid.cells	FAM102A	0.254765	4.182226	0.824078	0.412113	-5.69416	0.556815	0.648447
Erythroid.cells	HMGCS1	-0.23254	4.453186	-0.82395	0.412187	-5.75098	0.551653	0.643667
Erythroid.cells	EDC4	0.364535	2.984777	0.823758	0.412294	-5.36711	0.580236	0.670581
Erythroid.cells	INSL6	-0.51078	1.68935	-0.82365	0.412353	-5.19099	0.60673	0.695192

Erythroid.cells	PLIN2	-0.22222	5.831377	-0.82365	0.412353	-6.10961	0.52617	0.61951
Erythroid.cells	TARSL2	0.429443	1.415252	0.82364	0.41236	-5.15851	0.612494	0.700509
Erythroid.cells	RASIP1	-0.46356	1.808725	-0.82358	0.412392	-5.25918	0.604237	0.69289
Erythroid.cells	OLFR543	0.640975	0.201065	0.82352	0.412428	-5.01183	0.638708	0.724545
Erythroid.cells	NRG1	0.413833	2.541088	0.823437	0.412475	-5.70529	0.589173	0.678937
Erythroid.cells	ANKRD50	-0.28871	2.62264	-0.8232	0.412608	-5.33196	0.587605	0.677466
Erythroid.cells	SLC1A3	-0.86472	-0.29724	-0.82313	0.412648	-4.9993	0.649887	0.734736
Erythroid.cells	CRYBG3	-0.26001	4.474287	-0.8229	0.412777	-5.65532	0.551412	0.643565
Erythroid.cells	FARP2	0.378782	2.6131	0.82283	0.412819	-5.36233	0.587882	0.677775
Erythroid.cells	SLC15A2	-1.18516	4.597147	-0.82269	0.412896	-5.65549	0.549117	0.641441
Erythroid.cells	SKA3	-0.34364	2.738324	-0.82255	0.412977	-5.40405	0.585415	0.67555
Erythroid.cells	A330040F1	-0.62016	2.508264	-0.82218	0.413185	-5.35234	0.590145	0.67995
Erythroid.cells	CCL22	0.680875	-0.37257	0.822093	0.413235	-5.27164	0.651822	0.736604
Erythroid.cells	UPRT	0.536872	0.606501	0.822069	0.413249	-5.06641	0.630155	0.716857
Erythroid.cells	RBM4B	-0.15729	5.747385	-0.82207	0.413251	-6.05006	0.527959	0.621334
Erythroid.cells	JKAMP	0.227691	3.680975	0.821905	0.413342	-5.51766	0.566837	0.658188
Erythroid.cells	MATK	0.401472	2.36611	0.821556	0.41354	-5.34013	0.593271	0.6828
Erythroid.cells	THOC5	0.261152	3.564433	0.821433	0.413609	-5.48188	0.569286	0.660441
Erythroid.cells	AHSG	0.289984	9.130873	0.821363	0.413649	-6.88882	0.470524	0.565657
Erythroid.cells	XPO1	-0.18016	5.786847	-0.82119	0.413749	-6.0306	0.527449	0.620832
Erythroid.cells	IL27RA	-0.46691	1.535873	-0.82111	0.41379	-5.32447	0.610501	0.698788
Erythroid.cells	POLN	-0.39806	3.191009	-0.82109	0.413804	-5.46511	0.57665	0.667352
Erythroid.cells	TUBGCP3	0.20928	3.88521	0.820843	0.413943	-5.57182	0.563096	0.654697
Erythroid.cells	BTD	-0.39074	2.37707	-0.82068	0.414038	-5.29552	0.593109	0.682836
Erythroid.cells	GLE1	-0.20558	4.4454	-0.82061	0.414078	-5.67057	0.552356	0.644658
Erythroid.cells	RIMS4	0.733513	-0.99611	0.820583	0.414091	-4.96385	0.666336	0.749904
Erythroid.cells	RBM42	-0.12505	6.063078	-0.82051	0.414133	-6.08214	0.522534	0.616299
Erythroid.cells	TLR1	-0.54528	1.219121	-0.82038	0.414208	-5.17761	0.617273	0.705293
Erythroid.cells	PLRG1	0.22523	4.093452	0.820256	0.414276	-5.56229	0.559078	0.651154
Erythroid.cells	USP5	-0.24681	4.34395	-0.82022	0.414297	-5.61484	0.554285	0.64663
Erythroid.cells	FAM160A2	0.339017	2.604929	0.819665	0.414611	-5.32691	0.588623	0.678942
Erythroid.cells	NUP50	0.164837	5.304963	0.819657	0.414616	-5.89142	0.536432	0.629793
Erythroid.cells	ZFP850	-0.48058	1.010206	-0.81961	0.41464	-5.08184	0.621901	0.709737
Erythroid.cells	NOD2	-0.43339	1.576786	-0.81957	0.414663	-5.35567	0.609862	0.698651
Erythroid.cells	UBE2K	-0.09347	8.214477	-0.81953	0.414687	-6.51798	0.485618	0.580811
Erythroid.cells	CYP7B1	0.798957	0.01895	0.819407	0.414757	-5.01712	0.643572	0.729602
Erythroid.cells	GSTM1	0.256846	4.059268	0.819157	0.4149	-5.90236	0.559964	0.652134
Erythroid.cells	MID1	-0.5739	5.805162	-0.81913	0.414917	-6.02309	0.527387	0.621196
Erythroid.cells	RANBP6	0.423895	1.620455	0.818638	0.415194	-5.168	0.609302	0.698009
Erythroid.cells	ORC6	0.207428	5.216442	0.818525	0.415258	-5.93279	0.538381	0.631597
Erythroid.cells	SLC4A2	0.279877	3.559435	0.818364	0.41535	-5.4374	0.569926	0.661462
Erythroid.cells	CTBP1	-0.14066	6.500976	-0.81834	0.415363	-6.16897	0.515195	0.609456
Erythroid.cells	ELOVL7	0.641798	-0.19718	0.818307	0.415382	-5.02285	0.648753	0.73426
Erythroid.cells	ZFP825	-0.36559	2.050646	-0.81813	0.41548	-5.297	0.60039	0.689838
Erythroid.cells	DAGLA	-0.70668	-0.78197	-0.81802	0.415542	-4.94669	0.662064	0.746302
Erythroid.cells	AHCTF1	-0.17721	5.405997	-0.81783	0.415654	-5.92028	0.534978	0.628356
Erythroid.cells	COX5B	0.118561	8.103976	0.817791	0.415675	-6.57842	0.487818	0.582892

Erythroid.cells	RASSF1	-0.14895	5.940086	-0.8172	0.416012	-6.04361	0.525437	0.619184
Erythroid.cells	MRPL18	-0.15577	6.633228	-0.81715	0.416041	-6.24778	0.513113	0.60735
Erythroid.cells	SNHG15	-0.30287	3.481407	-0.81711	0.416063	-5.49527	0.571731	0.663046
Erythroid.cells	TMEM245	0.212422	4.753996	0.81704	0.416101	-5.78522	0.547256	0.639972
Erythroid.cells	SS18L1	-0.43084	2.325233	-0.81701	0.41612	-5.28942	0.594958	0.684719
Erythroid.cells	TARS2	-0.24326	3.819829	-0.81697	0.416144	-5.57741	0.565113	0.656831
Erythroid.cells	MARVELD2	0.502853	1.369704	0.816827	0.416223	-5.19035	0.614889	0.703152
Erythroid.cells	DAD1	0.11998	6.948328	0.816768	0.416256	-6.32666	0.507613	0.602046
Erythroid.cells	JMJD7	-0.40271	1.757778	-0.81661	0.416349	-5.21775	0.606765	0.695662
Erythroid.cells	TMEM106(	0.316766	3.205819	0.816182	0.416589	-5.34845	0.577461	0.668293
Erythroid.cells	SIAH2	-0.18973	4.793136	-0.81596	0.416714	-5.78085	0.546787	0.639464
Erythroid.cells	FAM3C	0.175562	5.36557	0.81582	0.416796	-5.85922	0.536149	0.629358
Erythroid.cells	TM2D1	0.133652	5.944576	0.815778	0.416819	-6.04815	0.525611	0.619294
Erythroid.cells	DHX32	0.265069	3.380004	0.815758	0.416831	-5.46863	0.574009	0.665121
Erythroid.cells	PITPNM1	-0.26966	3.730007	-0.81572	0.416854	-5.5	0.567137	0.658674
Erythroid.cells	HIST1H2BH	0.669117	0.17295	0.815561	0.416943	-4.99028	0.641134	0.727188
Erythroid.cells	CCL27A	0.390035	1.684593	0.815491	0.416983	-5.26501	0.608544	0.697289
Erythroid.cells	LSR	0.412281	1.400914	0.815418	0.417024	-5.4203	0.614528	0.702847
Erythroid.cells	F630040K0	-0.56921	0.611295	-0.81519	0.417152	-5.16653	0.631613	0.718494
Erythroid.cells	ZFP799	-0.52491	0.939029	-0.81466	0.417454	-5.08269	0.624677	0.712147
Erythroid.cells	ZFP420	-0.61742	0.275776	-0.81466	0.417456	-5.01472	0.639144	0.725386
Erythroid.cells	FYB	0.227039	6.869674	0.814553	0.417517	-6.33456	0.509454	0.603815
Erythroid.cells	THAP8	0.773638	-0.29985	0.814485	0.417556	-4.9889	0.651975	0.737066
Erythroid.cells	ACER3	0.160898	6.311004	0.814484	0.417556	-6.14093	0.519287	0.61328
Erythroid.cells	NOB1	0.210922	4.287446	0.814375	0.417618	-5.65723	0.556618	0.648821
Erythroid.cells	GM43065	-0.50191	0.153169	-0.81428	0.417674	-5.20492	0.641855	0.727904
Erythroid.cells	PEX5	0.220569	4.071805	0.814202	0.417717	-5.60849	0.560759	0.65277
Erythroid.cells	MON1A	-0.24779	3.876268	-0.81382	0.417936	-5.56528	0.564668	0.656524
Erythroid.cells	GM28198	-0.30207	3.85447	-0.81377	0.417962	-5.59239	0.565091	0.656931
Erythroid.cells	GM41496	-0.63247	0.985043	-0.81366	0.418026	-5.07044	0.623824	0.711501
Erythroid.cells	SERPINA3G	-0.78435	3.597185	-0.81364	0.418035	-5.61245	0.570115	0.661651
Erythroid.cells	ALKBH1	0.191661	4.99535	0.813445	0.418148	-5.78514	0.543377	0.636403
Erythroid.cells	E030030I0(	-0.34144	2.657085	-0.81335	0.418203	-5.27708	0.588882	0.679236
Erythroid.cells	PDPR	-0.28235	3.937711	-0.81326	0.418251	-5.58935	0.563486	0.655474
Erythroid.cells	SEC16A	0.241281	4.57627	0.813234	0.418268	-5.66269	0.551253	0.643941
Erythroid.cells	SYMPK	-0.22963	4.293778	-0.81284	0.418494	-5.62391	0.556855	0.649107
Erythroid.cells	CEACAM10	-0.52374	-1.04799	-0.81232	0.41879	-5.06169	0.669631	0.753176
Erythroid.cells	E130102H2	0.575165	1.232168	0.812293	0.418805	-5.10586	0.618935	0.706991
Erythroid.cells	GM16152	0.409864	2.101073	0.812284	0.41881	-5.36134	0.600661	0.690119
Erythroid.cells	MYADM	0.234723	5.586342	0.812268	0.418819	-5.95297	0.532812	0.626327
Erythroid.cells	TTC19	0.211626	4.958514	0.812018	0.418962	-5.79764	0.544526	0.637463
Erythroid.cells	DDX5	-0.07557	9.318404	-0.81185	0.419057	-6.70315	0.469191	0.564701
Erythroid.cells	EYA2	0.382262	1.389743	0.811698	0.419145	-5.41893	0.615765	0.704124
Erythroid.cells	GM29019	-0.60084	0.361473	-0.8116	0.419198	-4.97389	0.63801	0.724526
Erythroid.cells	ATF2	-0.14247	6.46092	-0.81155	0.419228	-6.17322	0.51724	0.611455
Erythroid.cells	NSA2	-0.08839	7.622863	-0.81134	0.419347	-6.45175	0.497172	0.591993
Erythroid.cells	DLC1	0.352527	4.570858	0.81074	0.419692	-5.83748	0.552275	0.644661

Erythroid.cells	KNG2	0.361286	2.382731	0.810661	0.419737	-5.54838	0.595467	0.685174
Erythroid.cells	HSPA14	-0.20542	5.034968	-0.81063	0.419757	-5.82453	0.543542	0.636439
Erythroid.cells	BOP1	-0.23198	4.167475	-0.81051	0.419823	-5.61135	0.559997	0.652013
Erythroid.cells	A330032B1	0.691213	-0.15579	0.810405	0.419884	-5.04452	0.649998	0.735315
Erythroid.cells	RIPK3	-0.32472	3.285039	-0.8102	0.42	-5.44153	0.577305	0.668311
Erythroid.cells	LGALS3	0.212388	6.142988	0.810153	0.420027	-6.31615	0.523335	0.617205
Erythroid.cells	TPST2	-0.17337	5.593782	-0.80994	0.420152	-5.94894	0.533343	0.626789
Erythroid.cells	ZKSCAN3	0.169792	4.82207	0.809865	0.420192	-5.76485	0.547655	0.640428
Erythroid.cells	EID1	0.171885	5.155191	0.809403	0.420457	-5.90016	0.54167	0.634735
Erythroid.cells	ERMARD	-0.31736	3.076068	-0.80934	0.420493	-5.37629	0.581805	0.672572
Erythroid.cells	S100A16	0.341029	2.552707	0.808952	0.420715	-5.41428	0.592575	0.682582
Erythroid.cells	HGH1	0.429014	1.856316	0.808693	0.420863	-5.18298	0.606972	0.695929
Erythroid.cells	GM12253	-0.71834	-0.86999	-0.80866	0.420879	-4.91497	0.666867	0.750652
Erythroid.cells	KAT6A	-0.14009	6.479855	-0.80864	0.420891	-6.17096	0.517791	0.611903
Erythroid.cells	HPS1	-0.30571	3.445228	-0.80861	0.420908	-5.46554	0.574638	0.665857
Erythroid.cells	NAT10	0.317331	3.270157	0.808483	0.420983	-5.41449	0.578136	0.669145
Erythroid.cells	SETD6	0.369609	1.423905	0.808367	0.421049	-5.22654	0.616134	0.704458
Erythroid.cells	GM45715	-0.50286	1.039938	-0.80815	0.421172	-5.07596	0.624352	0.712097
Erythroid.cells	ANKS1	-0.21248	5.759857	-0.808	0.421261	-5.89304	0.530755	0.624487
Erythroid.cells	SBF2	0.219392	5.368073	0.807931	0.421299	-5.92901	0.537933	0.631347
Erythroid.cells	SAA2	-0.93389	-1.02347	-0.80784	0.421349	-5.02739	0.670457	0.75415
Erythroid.cells	TRPC1	0.660733	-0.17084	0.807812	0.421367	-4.95994	0.651003	0.736531
Erythroid.cells	ORMDL1	0.255612	3.609727	0.807773	0.421389	-5.50861	0.571433	0.663071
Erythroid.cells	GM16754	0.607431	0.04601	0.807648	0.421461	-5.0478	0.646168	0.732185
Erythroid.cells	4930430E1	-0.62722	0.409202	-0.80739	0.421608	-5.14602	0.638138	0.724854
Erythroid.cells	MYO9A	0.218386	6.336763	0.807314	0.421652	-6.10459	0.520404	0.614664
Erythroid.cells	DOCK7	0.250996	3.830588	0.807218	0.421707	-5.69145	0.567146	0.65908
Erythroid.cells	SCAI	0.245708	4.434969	0.807148	0.421747	-5.73597	0.555484	0.64811
Erythroid.cells	PRORSD1	-0.19079	4.586531	-0.80713	0.421758	-5.77742	0.552599	0.645381
Erythroid.cells	E230016M	0.381745	2.465953	0.806953	0.421859	-5.37554	0.594493	0.684634
Erythroid.cells	ZW10	0.242478	4.014837	0.806728	0.421988	-5.63004	0.563625	0.655816
Erythroid.cells	ST8SIA1	0.521253	0.877835	0.806605	0.422059	-5.28712	0.627965	0.715641
Erythroid.cells	TCRG-C4	-0.38699	0.327974	-0.80638	0.422189	-5.41407	0.639998	0.72676
Erythroid.cells	POLA1	-0.22862	5.968201	-0.80634	0.422211	-6.1132	0.527071	0.621234
Erythroid.cells	MIER2	0.400436	2.002333	0.806228	0.422275	-5.24701	0.604071	0.693727
Erythroid.cells	LATS2	-0.15545	5.889771	-0.80619	0.422298	-6.01532	0.52849	0.622593
Erythroid.cells	LIG4	-0.47647	1.902715	-0.80618	0.422302	-5.24526	0.606149	0.695651
Erythroid.cells	INTS12	0.232493	4.380987	0.806162	0.422312	-5.58218	0.556575	0.649307
Erythroid.cells	DYSF	0.624539	2.592245	0.80562	0.422623	-5.31767	0.592163	0.682537
Erythroid.cells	DMXL2	-0.44705	2.146865	-0.80556	0.422655	-5.41084	0.601323	0.691043
Erythroid.cells	EVA1B	-0.28246	3.639669	-0.80555	0.422662	-5.56972	0.571187	0.662933
Erythroid.cells	GM27253	-0.55151	1.3478	-0.80538	0.42276	-5.06021	0.618153	0.706653
Erythroid.cells	MID1IP1	0.271483	3.917342	0.805323	0.422794	-5.62697	0.565783	0.657929
Erythroid.cells	SNAI3	0.612849	-0.34486	0.805038	0.422957	-5.02985	0.655446	0.740787
Erythroid.cells	CCDC43	-0.28376	3.103767	-0.80502	0.42297	-5.42489	0.581933	0.673094
Erythroid.cells	PRDX5	-0.24032	7.635313	-0.80462	0.423196	-6.40506	0.498277	0.593452
Erythroid.cells	HMGCL	0.168559	5.187114	0.804598	0.42321	-6.02301	0.541833	0.63531

Erythroid.cells	SLC22A4	0.361679	0.816422	0.804465	0.423287	-5.45545	0.629825	0.717467
Erythroid.cells	ZC3H6	0.302176	3.379852	0.804439	0.423301	-5.47304	0.576556	0.66811
Erythroid.cells	TMEM104	-0.33563	3.273099	-0.80423	0.423422	-5.43127	0.578679	0.670118
Erythroid.cells	SEC24C	-0.18432	4.859773	-0.80418	0.423452	-5.75048	0.547955	0.641168
Erythroid.cells	TSPYL3	-0.60624	0.960124	-0.80409	0.423504	-5.09768	0.626709	0.714631
Erythroid.cells	IKZF4	0.551613	-0.0706	0.804064	0.423516	-5.016	0.649411	0.735378
Erythroid.cells	HSPE1-RS1	0.686784	0.18545	0.803822	0.423656	-5.04065	0.643818	0.730192
Erythroid.cells	SEC23B	-0.16179	5.223375	-0.80373	0.423711	-5.87537	0.541262	0.634765
Erythroid.cells	NEK10	-0.55863	1.324395	-0.80352	0.423831	-5.18207	0.61907	0.707585
Erythroid.cells	UCK1	0.309682	3.265642	0.803375	0.423913	-5.5192	0.579004	0.670505
Erythroid.cells	APMAP	-0.25675	4.080552	-0.80335	0.423929	-5.56211	0.562996	0.655482
Erythroid.cells	KLHDC3	-0.18365	4.338451	-0.80304	0.424103	-5.67539	0.55813	0.650902
Erythroid.cells	CENPB	-0.17932	5.450808	-0.80301	0.42412	-5.9439	0.537216	0.631057
Erythroid.cells	RUBCN	-0.24208	3.888841	-0.80289	0.424191	-5.63123	0.566843	0.659129
Erythroid.cells	ARPC4	-0.10753	7.957802	-0.8027	0.424299	-6.47876	0.49314	0.588602
Erythroid.cells	KMT5A	-0.14047	5.846575	-0.80234	0.424509	-6.05785	0.530249	0.624379
Erythroid.cells	MMUT	0.27954	3.4125	0.801918	0.424751	-5.62045	0.576497	0.668266
Erythroid.cells	TOMM34	-0.1536	5.601463	-0.8019	0.424758	-5.95877	0.534732	0.628763
Erythroid.cells	MRPL22	0.239092	3.858759	0.801878	0.424773	-5.66549	0.567713	0.660018
Erythroid.cells	CHD6	-0.16108	5.997264	-0.80187	0.424776	-6.09164	0.527527	0.621872
Erythroid.cells	CCSER2	-0.20528	4.778998	-0.80164	0.424909	-5.83465	0.550039	0.643384
Erythroid.cells	MIF4GD	0.194934	4.964455	0.801641	0.42491	-5.86306	0.546547	0.640071
Erythroid.cells	SNTB2	-0.21544	5.607555	-0.80155	0.424963	-5.99126	0.53462	0.628743
Erythroid.cells	TRA2A	-0.11917	7.075692	-0.80154	0.42497	-6.28029	0.508413	0.603562
Erythroid.cells	SMG7	-0.12942	6.385921	-0.80127	0.425125	-6.19091	0.52059	0.61538
Erythroid.cells	C4B	0.504295	2.480976	0.801202	0.425162	-5.5647	0.595336	0.686034
Erythroid.cells	ACP5	-0.29599	5.117131	-0.80119	0.425171	-5.86988	0.54373	0.637508
Erythroid.cells	RPP21	0.191568	4.873001	0.80067	0.425469	-5.80236	0.548575	0.641968
Erythroid.cells	DGKH	0.241334	4.909539	0.800629	0.425493	-5.8186	0.547887	0.641315
Erythroid.cells	CPNE9	-0.59569	1.867904	-0.80034	0.425659	-5.20259	0.608506	0.698076
Erythroid.cells	PTPRC	-0.13128	8.915439	-0.7998	0.425969	-6.65339	0.478163	0.573897
Erythroid.cells	TRAF3IP2	0.278226	3.229202	0.799583	0.426096	-5.51315	0.581063	0.672331
Erythroid.cells	TCF4	0.177103	7.694523	0.799187	0.426324	-6.32285	0.498603	0.593864
Erythroid.cells	GM7072	0.278688	3.842908	0.799131	0.426356	-5.54575	0.568963	0.66108
Erythroid.cells	C5AR2	0.511967	1.231827	0.799105	0.426371	-5.20919	0.622524	0.710874
Erythroid.cells	BCL7B	-0.15514	5.531179	-0.7991	0.426377	-5.96214	0.53691	0.630729
Erythroid.cells	APPBP2OS	0.430282	0.985935	0.799008	0.426427	-5.14287	0.627829	0.715748
Erythroid.cells	FKTN	0.482697	1.595478	0.798936	0.426469	-5.16146	0.614763	0.70375
Erythroid.cells	FAM83E	-0.50049	1.10583	-0.79882	0.426536	-5.12818	0.625251	0.713423
Erythroid.cells	TYW5	-0.31505	3.139549	-0.79848	0.426729	-5.4507	0.582977	0.6742
Erythroid.cells	MLLT11	-0.28307	3.193389	-0.79841	0.42677	-5.39337	0.581898	0.673191
Erythroid.cells	CBX5	0.207989	5.103912	0.798407	0.426774	-5.90234	0.544907	0.638328
Erythroid.cells	C1D	-0.12675	5.843961	-0.79822	0.426884	-6.06768	0.531251	0.625347
Erythroid.cells	PATJ	0.365736	3.348038	0.798147	0.426924	-5.63499	0.578808	0.670355
Erythroid.cells	BLM	-0.33262	3.897628	-0.79804	0.426988	-5.63911	0.567966	0.660186
Erythroid.cells	EIF1A	0.176437	5.711772	0.797849	0.427096	-6.01196	0.533664	0.627717
Erythroid.cells	CBX1	0.139114	6.075378	0.797752	0.427152	-6.13283	0.527055	0.621421

Erythroid.cells	MYSM1	-0.16464	5.388465	-0.79753	0.427282	-5.92641	0.539613	0.63343
Erythroid.cells	SPATA24	-0.43177	2.193104	-0.79737	0.427374	-5.29076	0.6023	0.692402
Erythroid.cells	LAMTOR3	-0.17289	5.240813	-0.79711	0.427521	-5.85379	0.542353	0.636133
Erythroid.cells	TMEM241	-0.27337	4.310016	-0.79695	0.427612	-5.65452	0.55997	0.652896
Erythroid.cells	PER1	0.231203	4.926726	0.796954	0.427613	-5.78952	0.548232	0.641783
Erythroid.cells	USP30	0.345941	2.252852	0.796889	0.42765	-5.29689	0.601061	0.691357
Erythroid.cells	CCR5	-0.27448	4.693422	-0.79685	0.427671	-5.863	0.552641	0.645965
Erythroid.cells	DNLZ	-0.2467	3.991855	-0.79681	0.427696	-5.64052	0.566128	0.658704
Erythroid.cells	SLC8A1	0.253385	6.330185	0.796804	0.4277	-6.37408	0.522476	0.61719
Erythroid.cells	GM38115	0.232949	3.861728	0.796744	0.427734	-5.66096	0.568667	0.661094
Erythroid.cells	MARS2	-0.28685	2.690445	-0.79673	0.427741	-5.38662	0.592065	0.682994
Erythroid.cells	NOP53	-0.16601	5.740416	-0.79668	0.427773	-6.04565	0.53314	0.627421
Erythroid.cells	GM3604	-0.51898	0.595301	-0.79665	0.427788	-5.06179	0.636433	0.723965
Erythroid.cells	PDGFA	0.482236	2.114128	0.796431	0.427915	-5.30075	0.603942	0.694105
Erythroid.cells	PIGZ	-0.6	0.017052	-0.7964	0.427932	-5.08585	0.649266	0.735744
Erythroid.cells	MMS19	-0.18113	4.947005	-0.79638	0.427945	-5.78416	0.54785	0.64149
Erythroid.cells	GM37240	0.226954	5.162317	0.796284	0.428	-5.86884	0.543816	0.63769
Erythroid.cells	LRIG3	-0.63592	0.14201	-0.79614	0.428084	-5.04401	0.646503	0.733238
Erythroid.cells	TMEM79	0.562375	0.778123	0.796003	0.428162	-5.07176	0.632461	0.720407
Erythroid.cells	DPY19L4	0.213091	4.417697	0.795749	0.428309	-5.68525	0.557929	0.651131
Erythroid.cells	CCDC57	0.339796	2.546499	0.795717	0.428328	-5.31998	0.595039	0.685938
Erythroid.cells	CCDC141	0.449478	1.120838	0.795615	0.428387	-5.23769	0.625024	0.7137
Erythroid.cells	GTPBP8	0.348581	2.317318	0.795608	0.428391	-5.28739	0.599757	0.690353
Erythroid.cells	PKP4	0.225034	5.958306	0.795558	0.428419	-6.04751	0.5292	0.623834
Erythroid.cells	LRRC8A	-0.16847	5.427019	-0.7954	0.42851	-5.93883	0.538969	0.633214
Erythroid.cells	TENT5A	0.228244	5.169086	0.795144	0.428659	-5.8801	0.543877	0.637825
Erythroid.cells	GM48653	-0.67415	-0.16399	-0.79496	0.428767	-4.99221	0.6536	0.739866
Erythroid.cells	PSKH1	0.251426	3.273493	0.794573	0.428989	-5.43561	0.580528	0.672644
Erythroid.cells	GPR141B	0.329451	-0.65516	0.794536	0.429011	-5.3451	0.664781	0.750225
Erythroid.cells	PCLAF	-0.29242	7.982096	-0.79413	0.429246	-6.59768	0.493995	0.590038
Erythroid.cells	OGFOD3	-0.2898	3.187381	-0.79403	0.429303	-5.45362	0.582252	0.674286
Erythroid.cells	0610010K1	-0.15874	5.617735	-0.79402	0.429312	-6.00522	0.535602	0.63018
Erythroid.cells	CSRP2	0.176956	5.20682	0.793996	0.429324	-6.04863	0.543205	0.637437
Erythroid.cells	ZC4H2	-0.59373	0.054562	-0.79396	0.429345	-4.992	0.648686	0.73566
Erythroid.cells	GM30054	-0.44996	1.953461	-0.79379	0.429445	-5.29232	0.607541	0.697887
Erythroid.cells	PAXIP1	-0.24088	4.269449	-0.79368	0.429506	-5.65474	0.560976	0.654337
Erythroid.cells	KCTD10	0.236095	4.043279	0.793543	0.429586	-5.61217	0.565355	0.658502
Erythroid.cells	PAQR7	-0.44652	1.552734	-0.79354	0.429587	-5.26215	0.615996	0.70574
Erythroid.cells	AKR1C6	-0.32726	4.841164	-0.79326	0.42975	-6.17525	0.550066	0.64418
Erythroid.cells	LDLR	-0.29932	4.577033	-0.79323	0.429765	-5.78635	0.555078	0.648945
Erythroid.cells	FBXO38	0.148376	5.443447	0.793159	0.429809	-5.93832	0.538813	0.633511
Erythroid.cells	GM12089	-0.48363	0.306607	-0.79313	0.429823	-5.08555	0.643066	0.730808
Erythroid.cells	ULK4	0.31728	2.667963	0.793074	0.429858	-5.40823	0.592762	0.684388
Erythroid.cells	CELF4	0.544476	0.6776	0.793067	0.429862	-5.08067	0.634882	0.723318
Erythroid.cells	VPS13A	-0.12458	6.447058	-0.793	0.4299	-6.18873	0.520599	0.616067
Erythroid.cells	TRIM37	0.206368	4.93302	0.792734	0.430055	-5.82955	0.548334	0.64266
Erythroid.cells	GM15472	-0.36077	1.795978	-0.79272	0.430063	-5.52076	0.61085	0.701276

Erythroid.cells	USP12	0.178701	6.069178	0.792713	0.430067	-6.03353	0.527379	0.62265
Erythroid.cells	MYO5A	0.250194	5.016572	0.79265	0.430104	-5.81206	0.546763	0.641183
Erythroid.cells	PSMD11	-0.0805	7.032517	-0.79258	0.430146	-6.34268	0.510277	0.606188
Erythroid.cells	DQX1	0.952286	1.215444	0.792553	0.43016	-5.09286	0.623206	0.7127
Erythroid.cells	CXADR	0.422232	1.645302	0.79255	0.430161	-5.29617	0.614032	0.704237
Erythroid.cells	ABHD6	-0.34784	2.548845	-0.79238	0.430262	-5.31358	0.595261	0.686805
Erythroid.cells	PPTC7	0.191203	5.148669	0.791965	0.430501	-5.88448	0.544415	0.638996
Erythroid.cells	CD6	0.397858	0.929158	0.791711	0.430648	-5.37964	0.629538	0.71865
Erythroid.cells	FCER1G	-0.22218	8.628675	-0.79167	0.430673	-6.67048	0.483338	0.580058
Erythroid.cells	G730013B(	-0.55735	-0.968	-0.79166	0.430679	-4.9889	0.672157	0.757498
Erythroid.cells	PLPP2	0.328805	1.875681	0.791605	0.43071	-5.38134	0.609313	0.700019
Erythroid.cells	GM31814	-0.43212	-0.06084	-0.79154	0.430746	-5.18043	0.651426	0.738715
Erythroid.cells	CCDC62	0.426166	2.858429	0.791518	0.43076	-5.33744	0.589021	0.681155
Erythroid.cells	PHC2	0.193082	5.798035	0.791498	0.430772	-5.99058	0.532424	0.627642
Erythroid.cells	BNIP2	0.123192	6.377564	0.791189	0.430951	-6.1678	0.522107	0.617701
Erythroid.cells	CS	-0.12502	6.103857	-0.79094	0.431097	-6.10198	0.527128	0.622461
Erythroid.cells	CNTROB	0.411818	2.044211	0.790638	0.431271	-5.26231	0.606074	0.696964
Erythroid.cells	SLC1A4	0.484585	0.947937	0.790603	0.431291	-5.17266	0.629433	0.718535
Erythroid.cells	AHNAK	0.240587	6.416793	0.790552	0.431321	-6.34783	0.52151	0.617127
Erythroid.cells	DARS	0.182001	5.511648	0.790497	0.431353	-5.93972	0.537936	0.632881
Erythroid.cells	RNF167	0.18751	5.334087	0.790455	0.431377	-5.92804	0.541223	0.636043
Erythroid.cells	STEAP3	-0.3591	1.980258	-0.7903	0.431469	-5.33194	0.607461	0.698324
Erythroid.cells	CISD2	0.128716	6.732173	0.790133	0.431564	-6.23652	0.515999	0.611876
Erythroid.cells	IGF1OS	-0.71021	-1.03209	-0.78996	0.431667	-4.99945	0.674158	0.759363
Erythroid.cells	RYR1	0.443902	1.831874	0.789442	0.431965	-5.32513	0.610977	0.701502
Erythroid.cells	HIGD1A	-0.13733	6.029655	-0.78942	0.431979	-6.19986	0.528858	0.624159
Erythroid.cells	MED8	-0.18121	5.008062	-0.78906	0.432187	-5.86488	0.547906	0.642269
Erythroid.cells	ADAMTSL1	0.855856	0.587709	0.788864	0.432302	-5.11984	0.638034	0.726303
Erythroid.cells	TMPRSS6	-0.42022	1.467899	-0.78883	0.432324	-5.39706	0.618945	0.708777
Erythroid.cells	PMPCB	-0.16761	4.974651	-0.78834	0.432609	-5.96821	0.548853	0.643017
Erythroid.cells	KCTD13	0.338039	2.619439	0.78801	0.432798	-5.30891	0.595349	0.686665
Erythroid.cells	TMEM120/	0.248356	3.799392	0.787826	0.432905	-5.58087	0.571714	0.664487
Erythroid.cells	ADAR	-0.28153	4.311216	-0.78769	0.432986	-5.60185	0.561771	0.655089
Erythroid.cells	MFAP2	0.657276	0.586159	0.787205	0.433267	-5.064	0.639039	0.72682
Erythroid.cells	TBC1D10A	0.256174	3.56507	0.786865	0.433465	-5.52242	0.576859	0.669179
Erythroid.cells	GM20528	-0.50281	-0.01428	-0.78666	0.433585	-5.16269	0.652646	0.739225
Erythroid.cells	TCF21	-0.59106	0.477537	-0.78659	0.433626	-5.09392	0.641657	0.729214
Erythroid.cells	ALDH6A1	0.348973	2.786139	0.786561	0.433642	-5.56902	0.592547	0.683892
Erythroid.cells	DCLRE1C	0.180893	6.497189	0.786301	0.433793	-6.05716	0.521663	0.616832
Erythroid.cells	MIS12	0.319812	3.322859	0.786202	0.433851	-5.45765	0.581724	0.673825
Erythroid.cells	ABCD3	0.196636	4.193442	0.786022	0.433956	-5.80328	0.56456	0.657717
Erythroid.cells	6430550D2	-0.49035	1.142129	-0.786	0.433967	-5.15452	0.627136	0.715974
Erythroid.cells	RAD54B	0.44544	2.207787	0.785996	0.433972	-5.31104	0.604504	0.695076
Erythroid.cells	MRPS36	-0.16317	5.569029	-0.78594	0.434005	-6.04363	0.538516	0.633019
Erythroid.cells	CLCF1	-0.37895	2.650892	-0.78567	0.434159	-5.36831	0.595366	0.686675
Erythroid.cells	THEM6	-0.32236	3.448768	-0.78567	0.434164	-5.46761	0.579231	0.6716
Erythroid.cells	GLIS3	0.509024	3.068689	0.785583	0.434212	-5.48097	0.58686	0.67874

Erythroid.cells	LHPP	-0.28024	3.112672	-0.78547	0.43428	-5.48913	0.585972	0.677921
Erythroid.cells	BOD1	-0.28198	3.243589	-0.78542	0.434308	-5.51225	0.583337	0.675457
Erythroid.cells	ANGPTL2	0.653953	0.84131	0.785285	0.434386	-5.06128	0.633732	0.722177
Erythroid.cells	GM20139	-0.55376	-0.63311	-0.78519	0.43444	-5.0127	0.666829	0.752353
Erythroid.cells	HIST1H3I	-0.53567	1.296054	-0.78509	0.434502	-5.18148	0.623872	0.713172
Erythroid.cells	SERINC3	0.081866	9.707046	0.784626	0.43477	-6.85583	0.467845	0.564511
Erythroid.cells	RAB3A	-0.36043	2.619056	-0.78444	0.434878	-5.38139	0.596357	0.687613
Erythroid.cells	FRY	0.184539	6.378637	0.784411	0.434896	-6.16251	0.524101	0.619295
Erythroid.cells	RAB5B	0.234757	4.382557	0.784034	0.435115	-5.62552	0.561452	0.654784
Erythroid.cells	CRTC3	0.176999	5.658838	0.783849	0.435224	-5.97636	0.537387	0.631964
Erythroid.cells	TAOK3	0.101956	7.012234	0.783839	0.43523	-6.29121	0.513055	0.608596
Erythroid.cells	SPPL2B	0.383437	2.17345	0.783494	0.435431	-5.3233	0.605873	0.696378
Erythroid.cells	PAGR1A	-0.66125	0.395669	-0.78336	0.435512	-5.00476	0.644198	0.73169
Erythroid.cells	SAFB	-0.13216	6.192591	-0.78305	0.43569	-6.16656	0.5277	0.622835
Erythroid.cells	GM10847	0.60227	0.165978	0.78304	0.435696	-5.02219	0.649327	0.736505
Erythroid.cells	ATXN1	-0.17537	6.919224	-0.78293	0.435758	-6.34254	0.51474	0.610373
Erythroid.cells	TUBD1	0.387258	2.095647	0.782933	0.435759	-5.25847	0.6075	0.69807
Erythroid.cells	PRPF8	0.133607	5.801492	0.782918	0.435767	-6.03295	0.534818	0.629665
Erythroid.cells	CSTA2	-0.6143	1.96893	-0.78291	0.43577	-5.40186	0.61016	0.700534
Erythroid.cells	ZFP367	-0.21568	5.436634	-0.78286	0.435804	-6.01541	0.54155	0.636094
Erythroid.cells	RAB39	0.437784	2.115142	0.782787	0.435844	-5.33023	0.607092	0.697708
Erythroid.cells	XRCC5	0.333169	2.083305	0.782621	0.435941	-5.38315	0.607814	0.698397
Erythroid.cells	CD84	0.251139	5.366126	0.782317	0.436118	-5.80859	0.543028	0.637528
Erythroid.cells	TNFSF13B	0.494576	0.855901	0.782191	0.436192	-5.21667	0.634239	0.722764
Erythroid.cells	1700006J1	-0.76765	-0.71534	-0.78217	0.436206	-4.98127	0.669598	0.754979
Erythroid.cells	9130401M	-0.20334	4.02313	-0.78197	0.436322	-5.67574	0.56874	0.661862
Erythroid.cells	HIST1H2AP	0.303366	7.597755	0.781824	0.436406	-6.56761	0.503197	0.59919
Erythroid.cells	ELF4	-0.13403	6.785373	-0.7811	0.436833	-6.21723	0.517788	0.613155
Erythroid.cells	KIF20B	0.352433	4.170808	0.781015	0.43688	-5.76022	0.56636	0.659451
Erythroid.cells	PRKDC	-0.19977	4.69925	-0.78086	0.436968	-5.75885	0.556208	0.649904
Erythroid.cells	ZFP518A	-0.27855	3.684945	-0.7804	0.437237	-5.49886	0.576223	0.66874
Erythroid.cells	CAMK2A	-0.45584	0.846737	-0.78019	0.437363	-5.06431	0.63548	0.72371
Erythroid.cells	5830418P1	-0.48564	1.463039	-0.78016	0.437379	-5.31958	0.622108	0.711423
Erythroid.cells	GPSM3	-0.17496	6.022347	-0.7799	0.437532	-5.9608	0.531916	0.62667
Erythroid.cells	ESRRG	0.459711	1.226258	0.779815	0.437583	-5.25703	0.627325	0.716149
Erythroid.cells	ACTR1B	-0.23452	4.215968	-0.77973	0.43763	-5.66143	0.565935	0.659041
Erythroid.cells	CREB5	-0.49556	2.17534	-0.77946	0.43779	-5.43333	0.607266	0.697611
Erythroid.cells	SUFU	0.170026	5.025085	0.778956	0.438086	-5.88898	0.55085	0.644544
Erythroid.cells	GOLM1	-0.29178	3.2957	-0.77882	0.438163	-5.47216	0.584618	0.676328
Erythroid.cells	4930477G0	-0.74161	-0.38448	-0.77867	0.438251	-4.98504	0.663791	0.749215
Erythroid.cells	LRRC28	0.343128	3.874144	0.778449	0.438382	-5.58164	0.573203	0.665633
Erythroid.cells	GDE1	-0.26641	4.883129	-0.77839	0.438416	-5.793	0.553669	0.647212
Erythroid.cells	CTCF	-0.09136	7.101448	-0.77823	0.438512	-6.37303	0.513196	0.60848
Erythroid.cells	FETUB	0.318275	3.175824	0.77803	0.438628	-5.7662	0.587276	0.678833
Erythroid.cells	UBN2	-0.12902	7.076175	-0.77793	0.438689	-6.33446	0.513713	0.609018
Erythroid.cells	ABCA7	-0.21331	3.89815	-0.77771	0.438816	-5.61395	0.572954	0.665458
Erythroid.cells	ZBTB26	0.505599	0.850706	0.777376	0.439012	-5.12555	0.6366	0.724508

Erythroid.cells	SCG5	0.60012	0.801405	0.777017	0.439223	-5.09642	0.637844	0.725569
Erythroid.cells	SPATS2L	0.592932	0.9742	0.776993	0.439237	-5.13997	0.634051	0.722105
Erythroid.cells	IMPA1	0.158505	5.285622	0.776751	0.439379	-5.93848	0.546701	0.640564
Erythroid.cells	INSYN2B	-0.24446	4.140533	-0.77652	0.439513	-5.65761	0.568642	0.661403
Erythroid.cells	FBXO11	-0.13445	8.797772	-0.77649	0.439533	-6.66613	0.484864	0.580985
Erythroid.cells	TK2	-0.3094	3.825305	-0.77632	0.439633	-5.481	0.57484	0.667277
Erythroid.cells	TBCA	0.10775	7.625569	0.776305	0.439641	-6.50741	0.504635	0.60029
Erythroid.cells	PHF8	0.143354	5.563796	0.776121	0.439749	-6.06776	0.541521	0.6358
Erythroid.cells	SYNGR2	0.165166	6.111593	0.776115	0.439752	-6.05173	0.53145	0.626176
Erythroid.cells	GM47863	-0.52152	0.552776	-0.77605	0.43979	-5.17539	0.643479	0.730924
Erythroid.cells	RBP1	0.286634	3.578118	0.775476	0.440128	-5.7789	0.58012	0.672124
Erythroid.cells	PPA1	0.185599	4.797054	0.775367	0.440192	-6.01747	0.556321	0.649743
Erythroid.cells	PLXND1	0.245571	3.5086	0.77493	0.440449	-5.5759	0.581781	0.673601
Erythroid.cells	MED23	0.232796	3.692708	0.774661	0.440607	-5.54218	0.578178	0.670296
Erythroid.cells	CES2A	0.503128	0.898465	0.7745	0.440701	-5.23651	0.636628	0.724511
Erythroid.cells	CCNG2	-0.20695	5.189545	-0.77418	0.440889	-5.91677	0.549192	0.643108
Erythroid.cells	TADA2A	0.31237	2.820969	0.774093	0.440941	-5.3891	0.595792	0.686932
Erythroid.cells	EBPL	-0.18002	4.469152	-0.77398	0.441007	-5.93732	0.562947	0.656136
Erythroid.cells	LIPO3	0.314424	2.915424	0.773966	0.441016	-5.42638	0.593857	0.685129
Erythroid.cells	SYBU	-0.62359	0.330557	-0.7739	0.441052	-5.14718	0.649232	0.736163
Erythroid.cells	CTSG	0.939179	-0.2691	0.773883	0.441064	-5.14705	0.662815	0.748517
Erythroid.cells	ZNHIT3	0.250863	3.532147	0.77386	0.441078	-5.54135	0.581381	0.673473
Erythroid.cells	GM34455	0.392765	2.623429	0.77368	0.441184	-5.43138	0.59986	0.690763
Erythroid.cells	GM19710	-0.4023	2.595654	-0.77368	0.441184	-5.41282	0.600435	0.691297
Erythroid.cells	5730480H(	-0.3009	2.846707	-0.77367	0.441192	-5.38333	0.595264	0.686486
Erythroid.cells	CTPS2	-0.16806	5.066744	-0.77331	0.441403	-5.92046	0.551704	0.645495
Erythroid.cells	RNF146	0.173468	5.30614	0.773045	0.441557	-5.91423	0.547311	0.641312
Erythroid.cells	GM44067	0.415891	1.717425	0.772789	0.441708	-5.26568	0.619297	0.708657
Erythroid.cells	VAT1	-0.31265	3.491225	-0.77278	0.441717	-5.46582	0.582581	0.674543
Erythroid.cells	SPOCK2	-0.55174	0.349422	-0.77267	0.441779	-5.05466	0.649234	0.736129
Erythroid.cells	IER5	0.20416	6.692584	0.772568	0.441838	-6.19429	0.521966	0.617081
Erythroid.cells	ARHGAP21	0.186294	5.511128	0.772447	0.44191	-5.95279	0.543523	0.637774
Erythroid.cells	CCNB2	0.300749	5.882225	0.772395	0.44194	-6.18037	0.536652	0.63123
Erythroid.cells	HYAL2	0.433321	1.859326	0.772286	0.442004	-5.22933	0.616283	0.706046
Erythroid.cells	GM13184	-0.39929	2.666577	-0.77215	0.442086	-5.32488	0.599403	0.690452
Erythroid.cells	YBX3	0.155035	6.314911	0.771683	0.44236	-6.34577	0.528892	0.623941
Erythroid.cells	2610044O:	-0.29607	2.24732	-0.77166	0.442371	-5.30475	0.60824	0.698734
Erythroid.cells	KIFC3	-0.39737	1.625707	-0.77158	0.442418	-5.31091	0.621418	0.710968
Erythroid.cells	TEX10	-0.19921	5.266363	-0.77145	0.442496	-5.90425	0.548246	0.6425
Erythroid.cells	TEFM	-0.51094	1.160891	-0.77142	0.442512	-5.16238	0.631463	0.720238
Erythroid.cells	SOS2	-0.1682	5.600384	-0.77142	0.442513	-5.99858	0.542	0.636553
Erythroid.cells	LYRM9	0.318285	2.696887	0.771298	0.442587	-5.43303	0.598911	0.690171
Erythroid.cells	STAT2	-0.32495	5.210285	-0.77099	0.442768	-5.84597	0.549428	0.643611
Erythroid.cells	MFNG	-0.2775	3.090104	-0.77095	0.44279	-5.43972	0.590968	0.682735
Erythroid.cells	ETF1	-0.11278	7.536791	-0.77086	0.442845	-6.38967	0.507367	0.603259
Erythroid.cells	CLNS1A	-0.14324	5.340846	-0.77028	0.443188	-5.94667	0.547325	0.641447
Erythroid.cells	LTA4H	-0.22305	5.724021	-0.77015	0.443265	-5.95009	0.5402	0.634682

Erythroid.cells	AGPAT2	0.21597	4.355109	0.770055	0.44332	-5.96704	0.566189	0.659359
Erythroid.cells	TNIK	0.236282	4.035038	0.769184	0.443834	-5.96042	0.573042	0.665645
Erythroid.cells	SOCS3	0.300804	4.698376	0.768838	0.444039	-5.86978	0.560251	0.653579
Erythroid.cells	ABHD17C	0.188538	4.833665	0.768823	0.444047	-5.88456	0.557654	0.651122
Erythroid.cells	KCNG2	0.590051	-0.23458	0.768429	0.44428	-5.0655	0.664325	0.749816
Erythroid.cells	GM41555	-0.65833	0.571556	-0.76821	0.444411	-5.02264	0.646185	0.733235
Erythroid.cells	DSE	0.335023	3.834005	0.768122	0.444461	-5.6034	0.577459	0.669682
Erythroid.cells	TTI1	-0.36336	2.712153	-0.76796	0.444556	-5.3776	0.600215	0.691016
Erythroid.cells	U2AF1L4	0.34428	3.047054	0.767869	0.444611	-5.42108	0.593331	0.684657
Erythroid.cells	SNRPA1	-0.19668	5.077996	-0.76781	0.444648	-5.93764	0.553313	0.647031
Erythroid.cells	MVD	-0.37102	2.348846	-0.76751	0.444824	-5.32057	0.607938	0.698251
Erythroid.cells	ADAMTS9	0.537461	2.970839	0.767405	0.444885	-5.51985	0.595053	0.686311
Erythroid.cells	GM9828	0.493347	1.076181	0.766987	0.445133	-5.18452	0.635493	0.723519
Erythroid.cells	B3GALNT1	0.633272	0.351359	0.766557	0.445387	-5.08631	0.651815	0.738346
Erythroid.cells	TCP1	-0.13664	6.67677	-0.76643	0.445461	-6.30071	0.52436	0.619211
Erythroid.cells	ZNFX1	-0.31813	4.359029	-0.76633	0.445521	-5.68474	0.567737	0.660484
Erythroid.cells	PTPRG	0.42949	2.191113	0.766298	0.44554	-5.47073	0.611727	0.70155
Erythroid.cells	GPR19	0.356822	2.550401	0.766172	0.445615	-5.3326	0.604198	0.694575
Erythroid.cells	CAND2	0.517735	0.775357	0.76605	0.445687	-5.07559	0.642344	0.729704
Erythroid.cells	GM29243	-0.47666	-0.26495	-0.76598	0.445726	-5.16252	0.665835	0.751084
Erythroid.cells	CTLA2B	0.450821	3.353706	0.765902	0.445774	-5.5898	0.587712	0.679253
Erythroid.cells	CENPA	0.214074	6.681539	0.765815	0.445826	-6.30237	0.524275	0.619168
Erythroid.cells	PTPN12	0.177751	6.049828	0.765748	0.445865	-6.07018	0.535736	0.630154
Erythroid.cells	CCNB1IP1	0.417746	1.863098	0.765613	0.445946	-5.20618	0.618684	0.708096
Erythroid.cells	SLC19A2	-0.34373	1.834884	-0.76556	0.445977	-5.34866	0.619287	0.708679
Erythroid.cells	GPN3	-0.20931	3.982026	-0.76526	0.446153	-5.63134	0.575296	0.667649
Erythroid.cells	HK1OS	0.44089	0.741866	0.765058	0.446275	-5.24653	0.643268	0.730708
Erythroid.cells	RLF	-0.13999	6.699162	-0.76493	0.446352	-6.18176	0.524106	0.619103
Erythroid.cells	TCEANC	0.510983	1.730178	0.764903	0.446366	-5.1368	0.621702	0.710917
Erythroid.cells	INTS1	0.31363	3.347025	0.764861	0.446391	-5.45073	0.588013	0.679655
Erythroid.cells	PTH1R	0.685089	0.264703	0.764577	0.446559	-5.07221	0.654052	0.740536
Erythroid.cells	ANKRD54	0.246929	3.089479	0.764551	0.446575	-5.48069	0.59334	0.684617
Erythroid.cells	PSMD10	-0.25246	3.584601	-0.76437	0.446679	-5.59104	0.583317	0.675302
Erythroid.cells	HIST1H4M	0.529041	1.423329	0.764351	0.446693	-5.18233	0.628418	0.717149
Erythroid.cells	VTA1	-0.14397	5.707573	-0.7641	0.446845	-5.98198	0.54241	0.636645
Erythroid.cells	4930562C1	-0.63254	0.146658	-0.76393	0.446945	-4.99218	0.656929	0.743218
Erythroid.cells	TUBA1A	-0.21447	5.430541	-0.76365	0.447112	-6.06047	0.547695	0.641722
Erythroid.cells	TRAPPC1	-0.19493	5.432947	-0.76362	0.447124	-6.00606	0.54765	0.641679
Erythroid.cells	KCNQ1	-0.5608	1.396097	-0.76356	0.447164	-5.18066	0.629265	0.717969
Erythroid.cells	3300005D(	0.609888	0.756323	0.763339	0.447293	-5.04852	0.643396	0.730936
Erythroid.cells	ADAM32	0.628622	0.784382	0.763264	0.447338	-5.13206	0.642773	0.730385
Erythroid.cells	CDC42EP3	-0.18656	5.142958	-0.76309	0.44744	-6.01811	0.553252	0.647092
Erythroid.cells	ADGRG5	0.488834	0.105614	0.762918	0.447543	-5.26531	0.658145	0.744482
Erythroid.cells	CAAA0111ε	0.151722	5.624186	0.762594	0.447736	-6.04766	0.544353	0.638687
Erythroid.cells	DAP3	-0.15844	5.039126	-0.76258	0.447743	-5.93468	0.555389	0.649175
Erythroid.cells	CLUAP1	-0.22267	3.885137	-0.76224	0.447944	-5.57289	0.577945	0.670449
Erythroid.cells	NUPR1	0.407541	2.41246	0.762173	0.447985	-5.76428	0.608005	0.698494

Erythroid.cells	NAGLU	0.32252	2.6543	0.762169	0.447988	-5.39235	0.602959	0.693811
Erythroid.cells	ZPR1	-0.19201	4.397865	-0.76203	0.448073	-5.71567	0.567881	0.660981
Erythroid.cells	GM9967	-0.74197	0.569425	-0.76177	0.448226	-5.02376	0.648093	0.735302
Erythroid.cells	STN1	-0.21445	3.886297	-0.76164	0.448301	-5.65291	0.578102	0.670562
Erythroid.cells	DNAJC24	0.262427	3.89302	0.761301	0.448503	-5.61155	0.578013	0.670492
Erythroid.cells	ACSL3	-0.25797	3.828777	-0.76121	0.448554	-5.65939	0.579291	0.671722
Erythroid.cells	PARD6G	0.460293	1.981117	0.7612	0.448563	-5.33114	0.617353	0.707152
Erythroid.cells	CRISPLD2	0.614679	0.884481	0.761195	0.448566	-5.17872	0.641157	0.729035
Erythroid.cells	GSG1L	-0.72678	-0.16326	-0.76097	0.448701	-4.99148	0.664887	0.750576
Erythroid.cells	WBP1L	-0.18467	5.329247	-0.76062	0.448907	-5.89961	0.550464	0.644316
Erythroid.cells	FASTKD3	-0.42089	1.993657	-0.75982	0.449386	-5.24184	0.61792	0.70719
Erythroid.cells	CYP2J5	0.487519	1.06057	0.759786	0.449404	-5.25091	0.638133	0.725771
Erythroid.cells	LGR5	-0.49412	1.073909	-0.75968	0.449465	-5.17234	0.637844	0.725539
Erythroid.cells	METTL4	-0.2804	2.874732	-0.75943	0.449616	-5.424	0.599567	0.690186
Erythroid.cells	GRASP	0.268505	3.781204	0.759006	0.449868	-5.66742	0.581399	0.673094
Erythroid.cells	FADS3	-0.50387	0.659123	-0.75838	0.45024	-5.07805	0.647784	0.734201
Erythroid.cells	UPP2	0.423882	1.652112	0.758194	0.450351	-5.39475	0.625964	0.714221
Erythroid.cells	SDHB	-0.12742	7.274793	-0.75819	0.450352	-6.45259	0.516025	0.610766
Erythroid.cells	TSPAN31	0.191797	4.424978	0.75818	0.45036	-5.88791	0.568962	0.661197
Erythroid.cells	MAN1A	0.150237	8.000333	0.758051	0.450436	-6.58342	0.5034	0.598556
Erythroid.cells	ADO	0.226095	3.708631	0.75786	0.45055	-5.60316	0.58315	0.674539
Erythroid.cells	HNRNPA2E	-0.09731	9.287708	-0.75784	0.450562	-6.77378	0.481804	0.577519
Erythroid.cells	SIRT1	0.152733	5.043074	0.757836	0.450565	-5.8799	0.557012	0.649943
Erythroid.cells	FRMD5	0.480086	2.390592	0.75774	0.450622	-5.60416	0.610227	0.699751
Erythroid.cells	BLK	-0.22983	4.074052	-0.75763	0.450687	-5.82343	0.575867	0.667768
Erythroid.cells	COLEC10	0.482628	0.557157	0.757389	0.450831	-5.15347	0.650069	0.736459
Erythroid.cells	MYBL2	-0.33383	3.080165	-0.75735	0.450852	-5.50968	0.595902	0.686596
Erythroid.cells	COL11A2	-0.62828	0.72136	-0.75727	0.450904	-5.05976	0.646394	0.733161
Erythroid.cells	WDR66	-0.42485	1.900032	-0.75726	0.450909	-5.24258	0.620635	0.709536
Erythroid.cells	OSM	0.425372	2.305551	0.75694	0.451098	-5.5544	0.612157	0.701635
Erythroid.cells	AIM2	0.211426	5.579386	0.756892	0.451127	-5.94748	0.546981	0.640544
Erythroid.cells	ZFP830	-0.24643	3.541014	-0.75669	0.451247	-5.53932	0.586737	0.678017
Erythroid.cells	A430090L1	-0.45395	0.628221	-0.75656	0.451327	-5.13035	0.648727	0.735228
Erythroid.cells	CCDC127	0.191184	4.15296	0.756478	0.451373	-5.65511	0.574529	0.666584
Erythroid.cells	FSTL1	0.439684	2.107543	0.756302	0.451479	-5.34893	0.616472	0.705641
Erythroid.cells	HIST1H3F	-0.67015	0.34671	-0.75626	0.451506	-5.0553	0.655087	0.74103
Erythroid.cells	UTY	2.776723	2.644423	0.755969	0.451677	-5.56916	0.605322	0.695224
Erythroid.cells	PALB2	0.356578	2.125531	0.755652	0.451866	-5.31697	0.616418	0.705388
Erythroid.cells	ALKBH4	-0.32863	2.574916	-0.75556	0.451921	-5.33974	0.606944	0.696628
Erythroid.cells	VMA21	-0.16376	5.176423	-0.75451	0.452549	-5.93232	0.555623	0.648273
Erythroid.cells	CD200R1	0.343969	2.827999	0.754334	0.452653	-5.51913	0.602351	0.69216
Erythroid.cells	NEDD1	-0.32189	3.041246	-0.75429	0.45268	-5.40587	0.597943	0.688073
Erythroid.cells	MRPL34	0.135227	5.428125	0.754135	0.452772	-6.01765	0.550846	0.643856
Erythroid.cells	EPSTI1	-0.20828	7.192656	-0.7541	0.452791	-6.42829	0.518552	0.612995
Erythroid.cells	AP2B1	-0.1216	6.179239	-0.75391	0.452904	-6.09888	0.536846	0.630551
Erythroid.cells	TINF2	-0.24386	4.054948	-0.75391	0.452908	-5.64965	0.577445	0.668965
Erythroid.cells	C430049B0	0.469545	1.844436	0.753866	0.452933	-5.19395	0.623121	0.711395

Erythroid.cells	REPS1	-0.13329	5.964436	-0.75384	0.452951	-6.10466	0.54081	0.634333
Erythroid.cells	N4BP2	-0.17718	5.310122	-0.75344	0.453188	-5.97972	0.553156	0.646
Erythroid.cells	GM44174	0.484773	-0.56348	0.753313	0.453263	-5.19599	0.677216	0.760596
Erythroid.cells	FAM136A	0.221835	4.090791	0.753297	0.453273	-5.73129	0.576814	0.668304
Erythroid.cells	DDA1	-0.1817	5.082621	-0.75323	0.453313	-5.86862	0.557491	0.650111
Erythroid.cells	POU6F1	0.336115	2.610644	0.753225	0.453316	-5.40544	0.606962	0.696411
Erythroid.cells	HEATR1	-0.22599	4.84445	-0.75311	0.453385	-5.7947	0.562068	0.654489
Erythroid.cells	PYM1	-0.16592	4.692796	-0.7529	0.453513	-5.81128	0.565003	0.657258
Erythroid.cells	TMSB15B2	-0.35153	2.47439	-0.75289	0.453516	-5.35726	0.609819	0.699111
Erythroid.cells	ACACB	-0.68608	0.257652	-0.7527	0.45363	-4.9875	0.658284	0.743581
Erythroid.cells	GM42941	0.398844	1.267028	0.752638	0.453667	-5.23882	0.635744	0.723024
Erythroid.cells	GLIPR1	0.203836	5.059395	0.752461	0.453773	-5.87488	0.557936	0.650676
Erythroid.cells	HMGB1	-0.11302	9.807497	-0.75231	0.453861	-6.86488	0.474418	0.570165
Erythroid.cells	RNF217	-0.35702	2.071962	-0.75224	0.453908	-5.59857	0.618337	0.707126
Erythroid.cells	ALG13	-0.1572	4.514642	-0.75212	0.453975	-5.77106	0.568472	0.660663
Erythroid.cells	PCYT1A	0.214755	5.255992	0.752057	0.454014	-5.85347	0.554184	0.647168
Erythroid.cells	MYCN	-0.63136	-0.18583	-0.75204	0.454023	-5.00843	0.668442	0.752895
Erythroid.cells	ATXN3	0.160851	5.054807	0.752012	0.454041	-5.86874	0.558024	0.650802
Erythroid.cells	STRN3	-0.11538	7.654054	-0.75198	0.454059	-6.45663	0.510515	0.605373
Erythroid.cells	GM26810	-0.66551	0.423092	-0.75173	0.45421	-5.0323	0.65465	0.740334
Erythroid.cells	PIP4K2B	-0.22712	4.178712	-0.75165	0.454255	-5.60487	0.575174	0.666931
Erythroid.cells	A630072M	0.303744	3.151561	0.751334	0.454447	-5.49554	0.596028	0.686402
Erythroid.cells	ACCS	0.365244	2.079594	0.75125	0.454497	-5.29025	0.618454	0.707197
Erythroid.cells	NANOS3	-0.55453	0.427569	-0.75006	0.455208	-5.01837	0.655578	0.740911
Erythroid.cells	HLF	0.49817	1.023669	0.750054	0.455214	-5.20777	0.642227	0.728745
Erythroid.cells	LRG1	0.414613	2.460703	0.749854	0.455333	-5.59772	0.611174	0.700243
Erythroid.cells	RANBP9	0.129986	6.962828	0.749727	0.455409	-6.27731	0.523629	0.617789
Erythroid.cells	TMEM43	-0.27157	3.43413	-0.74969	0.455433	-5.48927	0.591024	0.681563
Erythroid.cells	NACA	0.089863	9.080227	0.749685	0.455435	-6.75978	0.487144	0.582443
Erythroid.cells	CWC22	-0.23675	3.900011	-0.74919	0.455734	-5.61769	0.581818	0.672958
Erythroid.cells	DDX19B	-0.24269	3.529612	-0.74915	0.455755	-5.53477	0.58928	0.679939
Erythroid.cells	KRT80	-0.33394	0.161146	-0.74914	0.45576	-5.31853	0.661855	0.746682
Erythroid.cells	VIRMA	-0.15225	5.71037	-0.74884	0.455942	-6.06098	0.546899	0.639994
Erythroid.cells	PLXDC2	0.414899	5.144109	0.748344	0.456239	-5.79964	0.557863	0.650243
Erythroid.cells	GOLGA3	0.217561	4.032459	0.748108	0.45638	-5.61719	0.579578	0.67074
Erythroid.cells	CASP4	-0.35378	4.029936	-0.74787	0.456524	-5.71007	0.579629	0.670889
Erythroid.cells	MED7	-0.21675	3.583194	-0.74785	0.456536	-5.60613	0.588605	0.679282
Erythroid.cells	PBX3	-0.18018	5.496226	-0.74784	0.456543	-6.04261	0.551164	0.644055
Erythroid.cells	HMCES	-0.22074	4.456587	-0.7478	0.456568	-5.75538	0.57119	0.662968
Erythroid.cells	ARHGAP19	0.309179	4.256309	0.747782	0.456576	-5.68544	0.575135	0.666675
Erythroid.cells	MZB1	-0.21955	5.956787	-0.74769	0.456631	-6.19093	0.542531	0.635895
Erythroid.cells	COPS3	-0.1231	5.92883	-0.74764	0.456661	-6.11093	0.543051	0.636397
Erythroid.cells	RASL11A	0.570747	0.28073	0.747117	0.456975	-5.09296	0.659958	0.744899
Erythroid.cells	MAP3K1	-0.10703	7.970553	-0.74693	0.457088	-6.56806	0.506779	0.601481
Erythroid.cells	FEN1	0.23702	4.525584	0.746475	0.457361	-5.78945	0.570424	0.662135
Erythroid.cells	TMOD1	0.31427	1.823905	0.74647	0.457364	-5.56739	0.626026	0.713823
Erythroid.cells	TOM1L2	0.195206	5.471299	0.746186	0.457535	-5.9459	0.552292	0.644973

Erythroid.cells	CYBB	0.274675	7.155496	0.746154	0.457554	-6.31123	0.521346	0.615429
Erythroid.cells	CARF	0.372089	2.216581	0.74595	0.457677	-5.30688	0.617793	0.706166
Erythroid.cells	DPYSL2	-0.14181	6.279623	-0.74585	0.457736	-6.24119	0.537281	0.630691
Erythroid.cells	NAT2	0.310087	2.532818	0.745596	0.457889	-5.43499	0.611224	0.700154
Erythroid.cells	TRIM27	-0.1524	5.675636	-0.74539	0.458015	-5.98308	0.548641	0.641545
Erythroid.cells	YTHDC1	-0.09	7.312924	-0.74538	0.458019	-6.39576	0.518743	0.612959
Erythroid.cells	MCM10	-0.3548	3.112366	-0.74529	0.458075	-5.41398	0.599158	0.68902
Erythroid.cells	PLPP6	-0.3227	2.754142	-0.74497	0.458267	-5.38879	0.606751	0.695986
Erythroid.cells	NOTCH2	0.128697	7.16468	0.744898	0.458309	-6.36579	0.521511	0.615577
Erythroid.cells	SKIL	-0.14466	7.304614	-0.74456	0.458512	-6.39948	0.519041	0.613235
Erythroid.cells	PSPH	0.22346	3.906589	0.744507	0.458544	-5.67135	0.583172	0.674099
Erythroid.cells	EVI2A	-0.20721	5.251447	-0.7445	0.458549	-5.88889	0.556843	0.64933
Erythroid.cells	CAPN15	-0.18778	4.710463	-0.74432	0.458654	-5.80584	0.567281	0.659267
Erythroid.cells	PSIP1	-0.12459	5.994205	-0.74425	0.458702	-6.18769	0.542841	0.636155
Erythroid.cells	EFCAB2	-0.38994	2.668273	-0.74414	0.458762	-5.33575	0.608569	0.697869
Erythroid.cells	NDC1	0.2607	3.748037	0.744117	0.458779	-5.56439	0.586361	0.677214
Erythroid.cells	RAD1	-0.33546	2.477895	-0.74404	0.458825	-5.35316	0.612574	0.701598
Erythroid.cells	DDX23	-0.18666	4.71777	-0.744	0.45885	-5.75581	0.567138	0.659221
Erythroid.cells	SLC25A53	0.259391	3.336328	0.74384	0.458945	-5.48547	0.594776	0.685106
Erythroid.cells	H2AFY	0.095157	7.697828	0.743684	0.459039	-6.51225	0.512199	0.606826
Erythroid.cells	SNHG14	-0.38598	1.410261	-0.74341	0.459205	-5.19095	0.635724	0.722994
Erythroid.cells	RALYL	0.644985	0.59788	0.743398	0.459212	-5.16621	0.653799	0.739496
Erythroid.cells	PLA2G4C	-0.54046	0.890136	-0.74244	0.459787	-5.14712	0.647795	0.733716
Erythroid.cells	DOCK4	0.175897	6.427831	0.74242	0.459801	-6.29341	0.535451	0.628947
Erythroid.cells	TMEM11	-0.16799	5.070903	-0.74227	0.459888	-5.90354	0.560944	0.65318
Erythroid.cells	GM21781	0.42069	1.561921	0.742084	0.460004	-5.21311	0.632952	0.720167
Erythroid.cells	NAIP1	0.580447	-0.47909	0.742067	0.460014	-5.06709	0.679154	0.76216
Erythroid.cells	PHLPP2	0.243948	4.283952	0.742017	0.460044	-5.63894	0.576313	0.667648
Erythroid.cells	NXPE4	0.349152	2.181788	0.741979	0.460067	-5.52404	0.619566	0.707863
Erythroid.cells	PACSIN1	0.34797	2.953403	0.741965	0.460075	-5.40947	0.60331	0.692832
Erythroid.cells	BLVRA	-0.19094	5.071733	-0.74176	0.460196	-5.85294	0.561004	0.653221
Erythroid.cells	SLC4A1AP	0.190454	4.278134	0.741475	0.46037	-5.71197	0.576651	0.667885
Erythroid.cells	ZFP41	-0.51703	0.707145	-0.74137	0.460436	-5.10702	0.652162	0.737671
Erythroid.cells	RNF145	0.17761	5.159727	0.740634	0.460878	-5.94904	0.559867	0.652054
Erythroid.cells	NOTCH1	-0.17264	5.292946	-0.74062	0.460887	-5.85095	0.557314	0.649641
Erythroid.cells	HTR2B	-0.5047	0.855091	-0.74009	0.461208	-5.11005	0.649627	0.735274
Erythroid.cells	LIPC	0.280785	2.510562	0.740053	0.461228	-5.641	0.613575	0.702219
Erythroid.cells	GM12703	0.641131	-0.21463	0.739882	0.461332	-5.03946	0.674133	0.757518
Erythroid.cells	GM10184	-0.515	1.11236	-0.73938	0.461632	-5.16655	0.644285	0.730239
Erythroid.cells	R3HDM2	0.11103	6.193799	0.739033	0.461844	-6.12697	0.541147	0.634072
Erythroid.cells	PKM	-0.15351	8.60779	-0.73877	0.462004	-6.64138	0.498396	0.592964
Erythroid.cells	MTHFD1	0.21241	3.79072	0.738525	0.462151	-5.73707	0.587771	0.678098
Erythroid.cells	C9	0.658878	0.60037	0.738404	0.462225	-5.19708	0.656094	0.741032
Erythroid.cells	IST1	-0.14566	5.536178	-0.73837	0.462245	-5.97813	0.553569	0.645965
Erythroid.cells	VAMP5	-0.23704	4.717071	-0.73831	0.462284	-5.81117	0.569351	0.660865
Erythroid.cells	RAP1GDS1	-0.13426	6.711436	-0.73827	0.462304	-6.25201	0.531728	0.625171
Erythroid.cells	GM8066	-0.53477	0.498967	-0.73823	0.462328	-5.0607	0.658394	0.743121

Erythroid.cells	A430018G1	0.378768	1.329295	0.737926	0.462513	-5.20234	0.639838	0.726244
Erythroid.cells	ANXA11	0.163078	5.969579	0.737822	0.462577	-5.99689	0.545443	0.638286
Erythroid.cells	MRPL21	0.163819	5.233418	0.73781	0.462584	-5.96355	0.559386	0.651503
Erythroid.cells	GM26812	-0.70224	0.020142	-0.7377	0.462651	-4.97361	0.669415	0.753146
Erythroid.cells	TJP3	0.403204	1.707068	0.737697	0.462652	-5.26773	0.631554	0.71867
Erythroid.cells	SMUG1	0.3251	1.976529	0.737398	0.462833	-5.29525	0.62572	0.713333
Erythroid.cells	PRMT3	0.230565	4.041123	0.737334	0.462872	-5.67275	0.582778	0.673515
Erythroid.cells	RBM12B1	0.532778	0.935508	0.737293	0.462896	-5.11906	0.648599	0.734273
Erythroid.cells	NEDD8	0.082661	7.48397	0.73725	0.462922	-6.48649	0.517912	0.611966
Erythroid.cells	CNOT6L	0.121394	7.08541	0.737168	0.462972	-6.37585	0.525013	0.618829
Erythroid.cells	4931406CC	0.240978	3.540099	0.737107	0.463009	-5.66709	0.592908	0.683039
Erythroid.cells	RBM24	-0.52671	1.008251	-0.73694	0.463111	-5.25794	0.647013	0.73295
Erythroid.cells	BRCA1	-0.29497	4.143927	-0.73687	0.463154	-5.76235	0.580757	0.671767
Erythroid.cells	ATP1B3	-0.18621	7.281647	-0.73655	0.463344	-6.37349	0.521628	0.615644
Erythroid.cells	UEVLD	0.332695	2.944551	0.736332	0.463478	-5.41627	0.605335	0.6947
Erythroid.cells	D930016D(	-0.28656	2.876865	-0.73631	0.46349	-5.42228	0.606747	0.69601
Erythroid.cells	TTC32	0.209218	4.101346	0.73627	0.463516	-5.69465	0.581711	0.672693
Erythroid.cells	RAD51C	-0.32713	2.245463	-0.73625	0.463529	-5.37091	0.620093	0.708341
Erythroid.cells	STX17	0.23386	4.034327	0.735916	0.46373	-5.70604	0.583225	0.674036
Erythroid.cells	NRIP3	0.688471	0.094317	0.735741	0.463836	-5.06718	0.668067	0.752088
Erythroid.cells	TRPM7	-0.11552	7.284405	-0.73568	0.463872	-6.39627	0.521733	0.615747
Erythroid.cells	CEP350	-0.13829	6.505585	-0.73554	0.463956	-6.22932	0.535815	0.629243
Erythroid.cells	GLMN	0.300255	2.819036	0.735475	0.463997	-5.4273	0.608137	0.697243
Erythroid.cells	GM17276	-0.49821	0.785789	-0.73544	0.464018	-5.23103	0.652308	0.73779
Erythroid.cells	IPO5	-0.18807	5.437174	-0.73517	0.464182	-6.01797	0.555919	0.648318
Erythroid.cells	CTSZ	-0.17369	6.598627	-0.735	0.464284	-6.24262	0.534283	0.627692
Erythroid.cells	ERC1	-0.21678	4.872109	-0.7349	0.464346	-5.82529	0.566862	0.658608
Erythroid.cells	CCDC91	0.321017	2.408137	0.734795	0.464409	-5.41208	0.617015	0.705351
Erythroid.cells	GM12236	-0.52818	1.087964	-0.73459	0.464533	-5.23903	0.645789	0.731751
Erythroid.cells	CENPF	0.348494	5.050794	0.734565	0.464548	-5.98378	0.563427	0.65537
Erythroid.cells	ZC3H7B	0.260206	3.353116	0.733887	0.464959	-5.54923	0.597688	0.687176
Erythroid.cells	ASAH1	0.152357	6.301867	0.733788	0.46502	-6.13173	0.540137	0.633023
Erythroid.cells	OAT	0.172643	5.245936	0.733719	0.465061	-6.01786	0.560044	0.651916
Erythroid.cells	NRP1	0.303134	4.442319	0.733661	0.465097	-5.80001	0.575713	0.666665
Erythroid.cells	RRP7A	0.255735	3.942345	0.733387	0.465262	-5.57739	0.58582	0.676076
Erythroid.cells	PIGM	0.225956	3.53133	0.733221	0.465364	-5.54598	0.594162	0.683866
Erythroid.cells	MRPL52	-0.11561	7.102833	-0.7332	0.465374	-6.44411	0.525651	0.619144
Erythroid.cells	BCORL1	0.309842	3.355845	0.733039	0.465474	-5.50615	0.597815	0.687217
Erythroid.cells	CD151	-0.29868	2.758187	-0.73273	0.465663	-5.44621	0.610417	0.698763
Erythroid.cells	IFNGR1	0.119953	7.03854	0.732486	0.46581	-6.48452	0.527022	0.620287
Erythroid.cells	DBNL	-0.13177	6.127196	-0.73242	0.465851	-6.11871	0.543716	0.636226
Erythroid.cells	NDUFS2	0.131058	6.235293	0.732314	0.465914	-6.25001	0.541707	0.634314
Erythroid.cells	ARID2	0.131116	6.543762	0.732155	0.46601	-6.24604	0.536016	0.628942
Erythroid.cells	DNMT3B	-0.29871	2.497904	-0.73214	0.466022	-5.39767	0.615938	0.70391
Erythroid.cells	GM50020	0.5232	-0.11132	0.732119	0.466032	-5.05481	0.67396	0.756839
Erythroid.cells	TMPO	-0.14161	7.067829	-0.73186	0.466192	-6.46092	0.526608	0.619875
Erythroid.cells	ITGA3	0.415857	-0.28405	0.731652	0.466316	-5.19216	0.678151	0.760599

Erythroid.cells	DIS3L2	-0.13196	5.906117	-0.73165	0.466318	-6.13439	0.547978	0.640315
Erythroid.cells	MICAL3	-0.2534	3.436889	-0.73147	0.466428	-5.63454	0.596479	0.685904
Erythroid.cells	HAGHL	-0.318	2.911144	-0.73146	0.466435	-5.47604	0.607374	0.696012
Erythroid.cells	CCR9	0.44758	2.410766	0.731116	0.466642	-5.28208	0.617948	0.705779
Erythroid.cells	HSP90AA1	-0.11618	7.657631	-0.73111	0.466643	-6.49056	0.516124	0.609879
Erythroid.cells	FMO1	0.388908	2.23787	0.731026	0.466696	-5.54359	0.621641	0.70918
Erythroid.cells	H2-Q10	-0.23275	4.038149	-0.73096	0.466734	-5.96439	0.584279	0.674534
Erythroid.cells	CAPG	0.273352	5.264343	0.730957	0.466738	-5.89632	0.560181	0.651897
Erythroid.cells	DYNLT1C	-0.46651	1.100031	-0.73081	0.466829	-5.13039	0.646567	0.732017
Erythroid.cells	FZD1	0.40379	-0.15494	0.730233	0.467178	-5.26147	0.675615	0.758225
Erythroid.cells	GFPT1	0.21744	5.095805	0.73009	0.467265	-5.9057	0.56384	0.655235
Erythroid.cells	GM4924	0.570448	0.278075	0.729965	0.467342	-5.04087	0.665611	0.749181
Erythroid.cells	LSM10	-0.2712	3.07773	-0.72992	0.467371	-5.51308	0.60435	0.693092
Erythroid.cells	TRIB2	0.335651	2.561151	0.729614	0.467555	-5.50816	0.615307	0.703212
Erythroid.cells	THAP7	0.266704	3.607354	0.72959	0.467569	-5.56789	0.593535	0.683039
Erythroid.cells	PSMB2	0.098424	7.058131	0.729382	0.467696	-6.40427	0.527303	0.620512
Erythroid.cells	SESN2	0.289293	3.371941	0.729211	0.4678	-5.4989	0.598393	0.68762
Erythroid.cells	BUD23	0.234461	4.09892	0.729187	0.467814	-5.69907	0.583613	0.673848
Erythroid.cells	C2CD5	0.179513	4.918041	0.729166	0.467828	-5.87692	0.567417	0.658656
Erythroid.cells	ACAP2	-0.12879	7.320314	-0.72905	0.467899	-6.37889	0.522613	0.616052
Erythroid.cells	PTPRK	0.366394	3.178658	0.728853	0.468018	-5.64672	0.602481	0.691459
Erythroid.cells	VPS35	0.118218	6.232281	0.728401	0.468293	-6.18088	0.542621	0.635195
Erythroid.cells	IRF8	0.204509	6.49623	0.728376	0.468308	-6.22801	0.53774	0.630545
Erythroid.cells	YIPF3	0.152467	4.904587	0.728364	0.468315	-5.92866	0.567895	0.659114
Erythroid.cells	PRAM1	-0.26987	3.279629	-0.72829	0.468358	-5.49094	0.600525	0.689609
Erythroid.cells	KIN	0.171148	4.642417	0.728127	0.46846	-5.78965	0.573054	0.663967
Erythroid.cells	MTIF2	-0.18881	4.285566	-0.72807	0.468495	-5.71952	0.580124	0.670595
Erythroid.cells	TMPRSS3	0.60351	-0.48523	0.727973	0.468554	-5.0008	0.683828	0.765734
Erythroid.cells	CP	-0.22451	4.588111	-0.7277	0.468722	-6.12053	0.574259	0.665054
Erythroid.cells	GSTO2	0.556468	0.524993	0.727555	0.468808	-5.11684	0.660579	0.744626
Erythroid.cells	SPRED1	0.246715	4.710293	0.727153	0.469053	-5.79582	0.572113	0.662895
Erythroid.cells	1600002K0	0.254198	3.214423	0.727058	0.469111	-5.47671	0.602313	0.691076
Erythroid.cells	OSGEPL1	-0.28556	2.472736	-0.72684	0.469246	-5.37507	0.617994	0.705544
Erythroid.cells	NDUFA8	0.143489	5.798653	0.726747	0.469301	-6.18729	0.551228	0.643182
Erythroid.cells	CDC40	-0.13682	6.047181	-0.72613	0.469675	-6.11932	0.546778	0.638871
Erythroid.cells	DNAAF3	-0.55938	-0.6587	-0.72612	0.469681	-5.0559	0.688799	0.769828
Erythroid.cells	DGCR6	0.23608	3.650268	0.726079	0.469708	-5.58661	0.59368	0.682959
Erythroid.cells	POLR2M	0.130036	5.403452	0.725853	0.469846	-5.99729	0.558978	0.650496
Erythroid.cells	RARRES2	-0.28099	3.7462	-0.72581	0.469874	-5.9557	0.591724	0.681213
Erythroid.cells	RARG	0.352731	1.622679	0.725787	0.469886	-5.4366	0.636636	0.722662
Erythroid.cells	TNFAIP8L1	0.285659	2.396525	0.725755	0.469906	-5.4623	0.619874	0.707279
Erythroid.cells	ARL6IP4	-0.14201	5.733017	-0.72545	0.470094	-6.06999	0.552848	0.644559
Erythroid.cells	RDM1	0.236329	5.028258	0.725226	0.470228	-5.86567	0.56644	0.657331
Erythroid.cells	ESYT2	0.112873	6.802347	0.725166	0.470265	-6.36236	0.533032	0.625653
Erythroid.cells	FKBP4	0.14178	5.691682	0.72496	0.470391	-6.1118	0.553767	0.645331
Erythroid.cells	LBR	0.094498	7.219631	0.724639	0.470587	-6.4204	0.525551	0.618525
Erythroid.cells	GM28375	-0.22046	3.206287	-0.72462	0.470598	-5.54868	0.603135	0.691635

Erythroid.cells	CYB561D2	-0.24736	3.329402	-0.72459	0.470614	-5.52079	0.600584	0.68927
Erythroid.cells	SNAI1	-0.50763	0.389835	-0.72452	0.470659	-5.05904	0.664651	0.74796
Erythroid.cells	RAD17	-0.15611	5.023722	-0.72449	0.47068	-5.87075	0.566603	0.657535
Erythroid.cells	ABCB8	0.265706	2.240197	0.724015	0.470968	-5.32929	0.623746	0.710677
Erythroid.cells	TPM2	0.595937	0.689833	0.723963	0.470999	-5.13701	0.658014	0.741968
Erythroid.cells	HPRT	0.128255	6.310323	0.723889	0.471045	-6.23462	0.542327	0.634565
Erythroid.cells	CNOT7	0.12934	5.288607	0.723854	0.471066	-5.92857	0.561655	0.652875
Erythroid.cells	GPR108	-0.20793	4.177602	-0.7236	0.471221	-5.69668	0.583586	0.673459
Erythroid.cells	THAP11	-0.1932	4.457867	-0.72354	0.471259	-5.79232	0.577992	0.668236
Erythroid.cells	TRIM17	-0.36976	2.197819	-0.72345	0.471313	-5.28579	0.62475	0.711651
Erythroid.cells	NMNAT1	0.491451	1.326233	0.72314	0.471503	-5.2128	0.643933	0.729199
Erythroid.cells	SELENOM	0.359005	2.384919	0.723116	0.471517	-5.47697	0.620848	0.70805
Erythroid.cells	CD68	-0.26309	4.872198	-0.72293	0.471631	-5.85946	0.569996	0.660754
Erythroid.cells	TERF2IP	-0.22595	3.58313	-0.72282	0.471698	-5.54268	0.59583	0.684927
Erythroid.cells	TRMT10B	-0.41712	1.681609	-0.72252	0.47188	-5.20147	0.636277	0.722275
Erythroid.cells	NBDY	-0.2118	3.659616	-0.7225	0.471894	-5.62785	0.594362	0.68361
Erythroid.cells	EXOC2	0.143352	5.578749	0.722157	0.472103	-6.00545	0.556573	0.648173
Erythroid.cells	PIK3C2B	0.332569	2.667746	0.721916	0.472251	-5.46647	0.615153	0.702918
Erythroid.cells	PFN1	-0.08996	10.31807	-0.72191	0.472258	-6.93695	0.473488	0.568124
Erythroid.cells	GM10785	0.372973	2.451276	0.721856	0.472288	-5.38361	0.619758	0.707163
Erythroid.cells	ST13	0.095381	6.987135	0.721839	0.472298	-6.38565	0.53037	0.623289
Erythroid.cells	TMA7	0.098104	6.564001	0.721677	0.472397	-6.26322	0.538148	0.630728
Erythroid.cells	RELT	-0.25865	3.728351	-0.72147	0.472523	-5.58702	0.593167	0.682574
Erythroid.cells	ADAL	0.321639	2.279066	0.721457	0.472532	-5.31571	0.623522	0.710658
Erythroid.cells	RNASEH1	0.261221	2.84733	0.721239	0.472665	-5.42779	0.611528	0.699611
Erythroid.cells	ZFP809	-0.26631	3.64912	-0.72105	0.472781	-5.57734	0.594933	0.684284
Erythroid.cells	XLR	0.463492	2.01867	0.720978	0.472825	-5.38819	0.6293	0.716074
Erythroid.cells	BOLL	0.467418	1.308178	0.720614	0.473048	-5.25721	0.645038	0.730514
Erythroid.cells	GM29282	0.413659	1.712401	0.720591	0.473062	-5.31036	0.636106	0.722353
Erythroid.cells	MPDU1	-0.17544	4.71579	-0.72054	0.473094	-5.79305	0.573624	0.664467
Erythroid.cells	KIF13A	0.229953	4.605824	0.720396	0.473182	-5.7321	0.575829	0.666536
Erythroid.cells	SLC16A10	0.159891	7.024829	0.720277	0.473255	-6.57906	0.530032	0.623136
Erythroid.cells	SYNJ2BP	-0.12941	5.520643	-0.72002	0.473413	-6.05735	0.558103	0.64984
Erythroid.cells	GM43113	0.518753	-0.69248	0.720004	0.473421	-5.12353	0.691298	0.772373
Erythroid.cells	FAM135A	0.321851	2.016455	0.719838	0.473524	-5.4724	0.629603	0.716433
Erythroid.cells	LYZL4	0.588629	-1.24765	0.719713	0.4736	-4.975	0.704694	0.784427
Erythroid.cells	ST8SIA4	0.153959	7.324778	0.719606	0.473665	-6.43946	0.52469	0.618068
Erythroid.cells	ACTR3B	0.446987	-0.01555	0.719605	0.473666	-5.08796	0.675337	0.758079
Erythroid.cells	TTLL12	0.441418	2.217401	0.71935	0.473822	-5.35284	0.625373	0.712618
Erythroid.cells	9330175E1	-0.53762	1.002962	-0.71924	0.473891	-5.2191	0.652126	0.737094
Erythroid.cells	MAP3K3	-0.14062	6.795781	-0.71917	0.473932	-6.26129	0.534364	0.627379
Erythroid.cells	PIK3CG	-0.1901	4.936727	-0.719	0.474038	-5.83915	0.569572	0.66074
Erythroid.cells	BAZ2B	-0.0952	8.430417	-0.71867	0.474239	-6.63949	0.505561	0.599619
Erythroid.cells	CMC1	-0.15404	4.601302	-0.71835	0.474437	-5.87843	0.576425	0.66712
Erythroid.cells	MRC2	-0.4894	1.416971	-0.71834	0.47444	-5.20237	0.643225	0.728914
Erythroid.cells	FBXL6	-0.24696	3.399881	-0.71824	0.474505	-5.50913	0.600738	0.68984
Erythroid.cells	DUSP16	-0.17541	6.833204	-0.71817	0.474543	-6.33933	0.53397	0.626965

Erythroid.cells	GM12940	-0.20553	4.51886	-0.71788	0.474721	-5.80556	0.578073	0.668801
Erythroid.cells	ZFP871	0.175891	5.249328	0.71786	0.474736	-5.91394	0.563755	0.655337
Erythroid.cells	RPRD2	-0.13704	5.916624	-0.71769	0.47484	-6.12528	0.551003	0.643279
Erythroid.cells	SHISA5	-0.17272	6.572889	-0.71768	0.474846	-6.33457	0.538758	0.631633
Erythroid.cells	DNAJC9	-0.18503	5.617283	-0.71765	0.474865	-6.10781	0.556685	0.648663
Erythroid.cells	KCNG3	-0.46765	0.517714	-0.71754	0.474933	-5.23406	0.663515	0.747561
Erythroid.cells	SLC25A34	-0.57128	-0.47643	-0.71737	0.475037	-5.03974	0.686687	0.768502
Erythroid.cells	CCDC92	0.49651	-0.17821	0.717297	0.475082	-5.10479	0.679651	0.76217
Erythroid.cells	NUP93	0.17913	4.586111	0.717291	0.475085	-5.83214	0.576739	0.667614
Erythroid.cells	TMEM88	-0.37809	2.768173	-0.71706	0.475228	-5.45497	0.614071	0.702259
Erythroid.cells	MRPS11	0.233793	3.950885	0.716784	0.475396	-5.69204	0.589692	0.679586
Erythroid.cells	5430416N(	-0.21424	3.547711	-0.71671	0.475441	-5.59646	0.597927	0.687277
Erythroid.cells	EIF6	-0.13168	5.835967	-0.7165	0.475571	-6.14139	0.552818	0.644898
Erythroid.cells	GPR137	0.27612	2.506946	0.716327	0.475677	-5.35308	0.619899	0.707537
Erythroid.cells	UNC50	-0.19962	4.105649	-0.71605	0.475845	-5.6575	0.586841	0.676914
Erythroid.cells	GSTT1	0.338027	2.240293	0.715739	0.476039	-5.59174	0.625892	0.713061
Erythroid.cells	TMEM119	-0.48531	0.305437	-0.71571	0.476059	-5.13788	0.6691	0.752447
Erythroid.cells	DCUN1D5	-0.08805	6.828224	-0.71532	0.476297	-6.33436	0.534836	0.62771
Erythroid.cells	SRPK1	-0.10428	6.11152	-0.7152	0.476368	-6.18322	0.548121	0.640417
Erythroid.cells	KLRI2	-0.43694	0.447224	-0.71512	0.476417	-5.41451	0.666086	0.749711
Erythroid.cells	TNFSF14	-0.40577	-0.05669	-0.71496	0.476518	-5.15804	0.677834	0.76032
Erythroid.cells	TOP3A	-0.26306	3.860539	-0.71455	0.47677	-5.57158	0.592444	0.681941
Erythroid.cells	POLR3F	-0.20785	3.882542	-0.71439	0.47687	-5.65807	0.592047	0.681588
Erythroid.cells	KCNQ1OT1	0.16627	6.399833	0.713946	0.477141	-6.23545	0.543158	0.635534
Erythroid.cells	CYB5D2	0.393042	1.870839	0.713916	0.47716	-5.29578	0.634649	0.720905
Erythroid.cells	GM49980	-0.25096	6.157222	-0.71388	0.477184	-6.30256	0.547689	0.639851
Erythroid.cells	BRIP1OS	0.151848	5.408086	0.713847	0.477202	-6.00223	0.561934	0.653336
Erythroid.cells	CCDC61	0.279411	2.934126	0.713516	0.477405	-5.43511	0.611998	0.700031
Erythroid.cells	ZSWIM9	-0.63494	0.544388	-0.71287	0.477806	-5.03214	0.66466	0.748329
Erythroid.cells	EPN1	0.131756	6.109989	0.712813	0.477838	-6.16152	0.548814	0.640999
Erythroid.cells	FBXO48	-0.52237	0.24175	-0.71276	0.477874	-5.05224	0.671641	0.75466
Erythroid.cells	SMIM15	0.160657	4.858356	0.712686	0.477917	-5.8457	0.572885	0.663758
Erythroid.cells	SLC35B4	-0.28752	2.825471	-0.71266	0.477932	-5.459	0.614374	0.702394
Erythroid.cells	FAM117B	-0.13942	7.11596	-0.71264	0.477943	-6.39945	0.530245	0.623323
Erythroid.cells	PKD1L3	-0.45678	0.837305	-0.71238	0.478103	-5.1865	0.657973	0.742308
Erythroid.cells	TSGA10	-0.25442	2.968133	-0.71222	0.478202	-5.51702	0.611363	0.699611
Erythroid.cells	SLC23A3	-0.58555	1.461516	-0.7122	0.478216	-5.20765	0.643952	0.729545
Erythroid.cells	PTPN1	-0.14797	7.078745	-0.71218	0.478231	-6.28052	0.53092	0.623969
Erythroid.cells	PPIP5K1	-0.43465	1.776843	-0.71215	0.478248	-5.26247	0.636986	0.723178
Erythroid.cells	2610203C2	-0.5522	0.354453	-0.71213	0.478258	-5.09247	0.669032	0.752328
Erythroid.cells	CERK	-0.1289	7.028222	-0.71213	0.478261	-6.37406	0.531837	0.624848
Erythroid.cells	MON1B	0.351624	1.881481	0.712045	0.478312	-5.24252	0.634692	0.721081
Erythroid.cells	ADA	-0.4375	2.074866	-0.71196	0.478363	-5.26166	0.630474	0.717233
Erythroid.cells	GM16556	-0.43358	1.676001	-0.71172	0.478511	-5.3079	0.639206	0.725307
Erythroid.cells	ACAD8	-0.29872	2.476536	-0.7117	0.478522	-5.36976	0.621805	0.70934
Erythroid.cells	PIGH	-0.39824	1.827535	-0.71166	0.478547	-5.25297	0.635874	0.722261
Erythroid.cells	SCFD1	-0.20139	4.929173	-0.71157	0.478606	-5.82926	0.571494	0.662548

Erythroid.cells	5730455P1	-0.25756	2.914853	-0.71148	0.478661	-5.45315	0.612486	0.700766
Erythroid.cells	PGS1	0.183487	4.740858	0.711317	0.478761	-5.78442	0.575201	0.666082
Erythroid.cells	PDF	0.479331	0.794298	0.711225	0.478817	-5.11866	0.658951	0.743373
Erythroid.cells	ZFP951	0.346594	2.641469	0.711144	0.478867	-5.40707	0.618281	0.706217
Erythroid.cells	MAOA	0.540498	0.932781	0.711037	0.478933	-5.15256	0.655809	0.740572
Erythroid.cells	KYAT1	0.385853	1.503706	0.711011	0.478949	-5.27415	0.643016	0.728917
Erythroid.cells	PRKAB2	0.265744	3.392055	0.710628	0.479185	-5.54508	0.602727	0.691851
Erythroid.cells	ZFP386	0.22041	3.889207	0.710517	0.479254	-5.65429	0.592516	0.682355
Erythroid.cells	XPC	-0.2517	3.385386	-0.71023	0.47943	-5.49512	0.602945	0.692081
Erythroid.cells	SEC14L2	0.35486	1.70477	0.710226	0.479433	-5.45119	0.638892	0.725199
Erythroid.cells	LILR4B	0.392715	3.079256	0.710141	0.479486	-5.64552	0.609333	0.698026
Erythroid.cells	ZFP775	0.460781	0.81684	0.710014	0.479564	-5.16111	0.658794	0.743376
Erythroid.cells	TRP53RKB	-0.33296	2.444883	-0.70961	0.479813	-5.31156	0.622989	0.710659
Erythroid.cells	SIL1	-0.19137	5.149956	-0.70949	0.479887	-5.88592	0.567639	0.659124
Erythroid.cells	ARRDC2	0.392194	1.79539	0.709489	0.479888	-5.25769	0.637096	0.72361
Erythroid.cells	CCT6B	0.575628	0.315333	0.709355	0.479971	-5.08504	0.67048	0.753984
Erythroid.cells	PRICKLE1	0.394381	3.386121	0.709323	0.47999	-5.52553	0.603117	0.692299
Erythroid.cells	D8ERTD738	0.088664	7.358725	0.709228	0.480049	-6.46803	0.526291	0.619855
Erythroid.cells	PIK3CB	0.251537	4.688415	0.709082	0.480139	-5.82187	0.576742	0.667741
Erythroid.cells	CKAP4	0.269098	4.030233	0.708855	0.480279	-5.68838	0.590013	0.680132
Erythroid.cells	B930095G1	0.545581	0.087994	0.70879	0.48032	-5.14406	0.675894	0.758928
Erythroid.cells	LAG3	-0.40878	1.429969	-0.70866	0.480397	-5.3407	0.645326	0.731201
Erythroid.cells	GM21887	0.434849	2.133944	0.708553	0.480466	-5.31476	0.62986	0.71707
Erythroid.cells	TAGAP1	0.405053	2.214287	0.708377	0.480574	-5.3057	0.628133	0.71552
Erythroid.cells	PLOD3	0.277903	3.277379	0.708312	0.480615	-5.5393	0.605546	0.694679
Erythroid.cells	2810006K2	0.355078	2.570884	0.708183	0.480694	-5.42125	0.620461	0.708541
Erythroid.cells	GM17018	-0.2431	3.620053	-0.70815	0.480715	-5.57354	0.598446	0.688164
Erythroid.cells	GM15133	0.398368	1.788244	0.707997	0.480809	-5.27874	0.637475	0.724166
Erythroid.cells	2010315B0	-0.31429	2.024895	-0.70786	0.480896	-5.32131	0.63233	0.719479
Erythroid.cells	VPS33B	0.281945	2.932531	0.707386	0.481187	-5.40178	0.613153	0.701734
Erythroid.cells	SYPL	-0.13552	5.860238	-0.70713	0.481348	-6.12941	0.554521	0.646887
Erythroid.cells	ZDHHC24	-0.4963	1.089708	-0.70712	0.481349	-5.14707	0.653434	0.738706
Erythroid.cells	CEACAM1	0.215858	3.675486	0.706945	0.48146	-5.71573	0.597743	0.68751
Erythroid.cells	TMEM41A	0.404752	1.51992	0.706899	0.481488	-5.25734	0.643827	0.72999
Erythroid.cells	MSI2	0.103194	7.509903	0.706565	0.481695	-6.51862	0.524109	0.617981
Erythroid.cells	PPP1R14A	0.441688	0.601813	0.706457	0.481761	-5.24638	0.664552	0.748943
Erythroid.cells	GPC1	-0.50929	0.351919	-0.70626	0.481884	-5.19301	0.670309	0.754158
Erythroid.cells	SRGAP1	-0.39428	2.559229	-0.70613	0.481963	-5.39605	0.621165	0.709286
Erythroid.cells	DELE1	-0.21863	3.426108	-0.70612	0.481968	-5.55151	0.602895	0.692384
Erythroid.cells	SLC9A6	-0.26679	2.81804	-0.70611	0.481974	-5.43609	0.615651	0.704198
Erythroid.cells	GNAI2	-0.0722	9.008515	-0.70607	0.482002	-6.72338	0.498005	0.59274
Erythroid.cells	GM2788	0.487603	0.569462	0.706059	0.482008	-5.15478	0.665294	0.749616
Erythroid.cells	FBXW5	-0.23632	3.39907	-0.70604	0.482017	-5.5405	0.603456	0.692906
Erythroid.cells	WDR49	-0.54543	0.34569	-0.70594	0.482083	-5.09118	0.670462	0.754343
Erythroid.cells	SNX5	-0.10399	7.890887	-0.70583	0.482148	-6.50423	0.51735	0.611507
Erythroid.cells	AMMECR1L	0.199946	4.488762	0.705737	0.482207	-5.75618	0.581275	0.672283
Erythroid.cells	6030458C1	0.265047	2.74674	0.705212	0.482532	-5.42137	0.617432	0.70584

Erythroid.cells	CKS1B	0.241409	5.377173	0.705148	0.482571	-6.08084	0.564044	0.656011
Erythroid.cells	SWAP70	-0.18713	5.61218	-0.70513	0.482582	-6.11855	0.559516	0.651733
Erythroid.cells	GM12979	-0.54313	0.049111	-0.7049	0.482725	-5.07069	0.677766	0.760829
Erythroid.cells	WSB1	-0.12015	6.461454	-0.70469	0.482853	-6.24064	0.543596	0.63654
Erythroid.cells	MAZ	-0.17352	6.241133	-0.70467	0.48287	-6.22964	0.547712	0.640456
Erythroid.cells	GM11464	-0.5543	-0.20123	-0.70431	0.483093	-5.0298	0.683691	0.766168
Erythroid.cells	FXR1	-0.10896	6.109299	-0.70427	0.483117	-6.18805	0.550194	0.642841
Erythroid.cells	DST	-0.28665	4.335211	-0.70426	0.483121	-5.7063	0.584726	0.675388
Erythroid.cells	PPRC1	-0.2298	3.956149	-0.70424	0.483132	-5.5878	0.592396	0.682552
Erythroid.cells	ERBB4	0.506972	0.909465	0.704182	0.48317	-5.26699	0.657971	0.742907
Erythroid.cells	C130046K2	-0.51345	0.716514	-0.70358	0.483542	-5.11456	0.662568	0.74714
Erythroid.cells	CPSF4	-0.20721	4.317677	-0.70356	0.483554	-5.76252	0.585255	0.675939
Erythroid.cells	CHST3	0.21576	4.312271	0.70352	0.48358	-5.98313	0.585364	0.676053
Erythroid.cells	SDK1	0.39578	3.195716	0.70344	0.48363	-5.54788	0.608285	0.697391
Erythroid.cells	FANCF	0.400716	1.911025	0.703379	0.483668	-5.29757	0.635817	0.722761
Erythroid.cells	AMZ2	-0.19752	3.901939	-0.70337	0.483673	-5.66063	0.593681	0.683819
Erythroid.cells	DBP	-0.47123	2.187718	-0.70302	0.483889	-5.23408	0.629886	0.717347
Erythroid.cells	SRGN	0.103874	9.652655	0.702978	0.483916	-6.86249	0.487771	0.582783
Erythroid.cells	JAZF1	-0.26483	3.086872	-0.70295	0.483932	-5.62583	0.610672	0.699632
Erythroid.cells	RAB4B	0.126655	5.9254	0.702566	0.484172	-6.06905	0.554094	0.646592
Erythroid.cells	HRH1	0.511438	-0.56863	0.702533	0.484193	-5.02226	0.69295	0.774557
Erythroid.cells	GM2000	-0.23414	4.799183	-0.70228	0.484349	-5.87223	0.575987	0.667261
Erythroid.cells	RAD51	0.303278	3.891445	0.702198	0.4844	-5.71965	0.594239	0.68434
Erythroid.cells	WDFY3	0.211982	5.666809	0.702012	0.484516	-6.05113	0.559093	0.651414
Erythroid.cells	SAA1	0.772095	-0.05604	0.701992	0.484528	-5.20516	0.680872	0.763797
Erythroid.cells	DNAAF5	0.26599	2.988111	0.701915	0.484576	-5.45826	0.613004	0.70184
Erythroid.cells	SERPINB6B	-0.3076	2.936635	-0.70179	0.484651	-5.79849	0.614092	0.70287
Erythroid.cells	PSMC3IP	0.346292	2.412129	0.70174	0.484685	-5.38671	0.62529	0.713203
Erythroid.cells	MFSD3	-0.50763	0.642198	-0.70161	0.484762	-5.10325	0.664655	0.749234
Erythroid.cells	CRK	-0.12819	6.391053	-0.70149	0.484841	-6.22182	0.545392	0.638496
Erythroid.cells	TRBC2	-0.26775	3.943953	-0.70148	0.484844	-6.2956	0.593167	0.683509
Erythroid.cells	PDCL3	0.191419	4.536557	0.70117	0.485038	-5.7339	0.581286	0.67236
Erythroid.cells	PCNT	-0.16205	5.233516	-0.70094	0.485178	-5.97176	0.567541	0.65944
Erythroid.cells	EHD4	-0.15778	6.076676	-0.70091	0.4852	-6.09116	0.55137	0.64414
Erythroid.cells	SERPINB9	-0.26226	3.483496	-0.7009	0.485203	-5.97784	0.60272	0.692358
Erythroid.cells	IFT43	-0.37034	1.54627	-0.7009	0.485207	-5.25038	0.644327	0.730686
Erythroid.cells	MOV10	-0.259	3.628623	-0.70075	0.485301	-5.58116	0.59976	0.68964
Erythroid.cells	ROBO2	0.590779	0.744725	0.700483	0.485465	-5.17878	0.662576	0.747241
Erythroid.cells	DDAH2	-0.2639	3.503269	-0.70031	0.485574	-5.60717	0.602473	0.692131
Erythroid.cells	TMEM62	-0.32028	2.375873	-0.70025	0.485611	-5.39017	0.626326	0.714193
Erythroid.cells	TMOD3	-0.11187	7.218311	-0.70021	0.485636	-6.39943	0.530392	0.624146
Erythroid.cells	PRDM16	-0.60663	0.306557	-0.69999	0.485772	-5.08602	0.672684	0.756476
Erythroid.cells	HECTD3	0.271383	2.789878	0.699901	0.485827	-5.41376	0.617463	0.706028
Erythroid.cells	WRNIP1	-0.19456	3.743434	-0.69985	0.48586	-5.65106	0.597523	0.687558
Erythroid.cells	MAP3K11	-0.22858	3.876975	-0.69981	0.48588	-5.63588	0.594785	0.685007
Erythroid.cells	TLR7	0.373561	2.963787	0.699532	0.486056	-5.46255	0.613922	0.702767
Erythroid.cells	HIST2H2AC	0.461048	2.376405	0.699373	0.486155	-5.47333	0.626524	0.714356

Erythroid.cells	GM38190	0.33357	1.823044	0.69909	0.486331	-5.33803	0.638744	0.725549
Erythroid.cells	CEP85	0.177509	4.470348	0.698933	0.486428	-5.85493	0.583122	0.674059
Erythroid.cells	GM15232	-0.40258	1.187425	-0.69885	0.486482	-5.27721	0.652928	0.738534
Erythroid.cells	5031425E2	0.159698	5.390925	0.698642	0.486609	-5.99197	0.564981	0.657069
Erythroid.cells	DCAF1	-0.17213	5.222364	-0.69855	0.486666	-5.94666	0.568258	0.660173
Erythroid.cells	MYEF2	0.147697	5.328236	0.698499	0.486698	-6.10117	0.566197	0.65823
Erythroid.cells	TREML2	-0.25565	3.901235	-0.69849	0.486705	-5.61486	0.594643	0.684904
Erythroid.cells	DYRK2	-0.17428	4.884934	-0.69827	0.486839	-5.82685	0.574965	0.666458
Erythroid.cells	SH3BGR	0.66708	-0.59926	0.697893	0.487076	-4.95248	0.69481	0.776236
Erythroid.cells	FASTKD1	-0.31549	2.189988	-0.69782	0.487122	-5.37176	0.631045	0.718373
Erythroid.cells	CHML	-0.36905	1.690018	-0.69761	0.487253	-5.29305	0.642109	0.728486
Erythroid.cells	PER3	-0.42364	1.517982	-0.69739	0.48739	-5.20953	0.645931	0.732021
Erythroid.cells	ZDHHC20	-0.09716	6.740107	-0.69735	0.487411	-6.28712	0.539804	0.633015
Erythroid.cells	5830432E0	-0.41441	1.494203	-0.69733	0.487429	-5.26035	0.646461	0.732513
Erythroid.cells	OLFML2B	0.531077	0.874884	0.696964	0.487654	-5.16452	0.660505	0.745309
Erythroid.cells	PPIP5K2	0.172749	4.496011	0.696846	0.487727	-5.79922	0.583047	0.673926
Erythroid.cells	UBL3	0.090966	7.408809	0.696745	0.48779	-6.41933	0.527669	0.621475
Erythroid.cells	PFDN6	0.160389	4.874434	0.696674	0.487834	-5.90467	0.575517	0.666926
Erythroid.cells	2810405F1	0.488796	0.687122	0.696609	0.487875	-5.13945	0.664799	0.749303
Erythroid.cells	ZBTB49	-0.36912	1.051258	-0.69634	0.488042	-5.1533	0.656496	0.741828
Erythroid.cells	CENPW	0.242059	4.349764	0.696192	0.488135	-5.82779	0.585984	0.676877
Erythroid.cells	ZFP738	0.260867	2.843416	0.696149	0.488161	-5.42929	0.617157	0.705855
Erythroid.cells	MPG	0.192987	3.63806	0.69611	0.488186	-5.68241	0.600502	0.690419
Erythroid.cells	CACYBP	-0.13713	5.736095	-0.69605	0.488225	-6.13481	0.558753	0.651251
Erythroid.cells	EPS8L1	-0.76036	0.408966	-0.69603	0.488233	-5.08434	0.671213	0.755269
Erythroid.cells	ERMAP	0.457636	0.529243	0.695871	0.488335	-5.2698	0.668432	0.752751
Erythroid.cells	RREB1	-0.17556	6.814971	-0.69569	0.488445	-6.28823	0.538489	0.632025
Erythroid.cells	EXOC7	-0.19197	4.087686	-0.69565	0.488471	-5.67759	0.591287	0.681881
Erythroid.cells	KAT2B	0.139403	6.221283	0.695615	0.488494	-6.24185	0.549542	0.642558
Erythroid.cells	ZFP64	-0.19874	5.317866	-0.69555	0.488534	-5.9723	0.566825	0.658924
Erythroid.cells	ROR1	-0.44457	1.374979	-0.69545	0.488597	-5.30071	0.649204	0.735323
Erythroid.cells	XYLT1	-0.13788	7.355246	-0.69525	0.488723	-6.58216	0.528635	0.622625
Erythroid.cells	SCNN1A	-0.48708	0.542515	-0.69522	0.488741	-5.24492	0.668126	0.75257
Erythroid.cells	DDX49	-0.17316	4.208396	-0.6952	0.488751	-5.71651	0.588838	0.679637
Erythroid.cells	GM26801	-0.49958	0.557742	-0.69507	0.488836	-5.20458	0.667775	0.752293
Erythroid.cells	NDUFAF4	0.207161	3.867148	0.695057	0.488842	-5.73032	0.595788	0.686157
Erythroid.cells	HARBI1	-0.44465	1.346342	-0.69503	0.488857	-5.22447	0.649846	0.735992
Erythroid.cells	SCAF4	0.124448	6.317786	0.694781	0.489014	-6.16269	0.547811	0.640974
Erythroid.cells	RNF26	0.270914	3.515898	0.694724	0.48905	-5.54933	0.603122	0.692998
Erythroid.cells	ITPRIP	0.275461	3.468077	0.694576	0.489142	-5.50919	0.604122	0.693997
Erythroid.cells	SNHG8	0.196065	4.013183	0.694526	0.489174	-5.71619	0.592899	0.68355
Erythroid.cells	VPREB2	0.571431	-0.41404	0.694357	0.489279	-5.08279	0.690737	0.773135
Erythroid.cells	PLCB3	-0.30201	2.612	-0.69402	0.489492	-5.391	0.622351	0.71096
Erythroid.cells	TTC17	-0.15104	5.324345	-0.6939	0.489565	-5.93575	0.566931	0.659308
Erythroid.cells	CCDC88C	0.154467	5.294187	0.693863	0.489587	-6.07523	0.567517	0.659861
Erythroid.cells	ACO2	-0.10644	6.116578	-0.69385	0.489593	-6.19735	0.551741	0.644925
Erythroid.cells	RBMX2	-0.2625	3.368821	-0.6937	0.489691	-5.53451	0.606358	0.696258

Erythroid.cells	MAP2K5	-0.11385	6.143251	-0.69364	0.489727	-6.18444	0.551254	0.644512
Erythroid.cells	PDHA1	0.154891	5.115043	0.693481	0.489826	-5.95555	0.57108	0.663254
Erythroid.cells	CNPY3	-0.13198	5.300686	-0.69335	0.489905	-5.95031	0.567476	0.659842
Erythroid.cells	RAD51D	0.270516	2.984907	0.692826	0.490235	-5.44203	0.614838	0.704037
Erythroid.cells	SPOUT1	0.313407	2.468742	0.692717	0.490303	-5.35127	0.625869	0.714239
Erythroid.cells	METTL1	0.277486	3.431367	0.692636	0.490353	-5.58028	0.60546	0.695392
Erythroid.cells	TDRD3	-0.20314	4.059195	-0.69234	0.49054	-5.70587	0.592677	0.683465
Erythroid.cells	RENBP	-0.20741	4.474184	-0.69212	0.490675	-5.78563	0.584373	0.675673
Erythroid.cells	GZMM	0.405821	2.051803	0.69174	0.490913	-5.28408	0.635305	0.722926
Erythroid.cells	BOK	0.538602	1.039523	0.691685	0.490947	-5.12855	0.657881	0.743561
Erythroid.cells	CCR4	0.522643	-1.08113	0.691642	0.490974	-5.05728	0.70786	0.788631
Erythroid.cells	RANBP1	-0.14327	7.239938	-0.69157	0.491018	-6.46446	0.531626	0.625729
Erythroid.cells	SLC17A5	0.267682	3.572742	0.691408	0.491121	-5.55687	0.602879	0.692993
Erythroid.cells	GM12216	-0.2524	4.554087	-0.69115	0.491285	-5.79617	0.582876	0.67442
Erythroid.cells	SERBP1	0.083887	8.529561	0.691107	0.491309	-6.70814	0.508741	0.603768
Erythroid.cells	CCNG1	0.15376	4.873028	0.690991	0.491382	-5.9397	0.576526	0.66846
Erythroid.cells	HEBP1	0.217997	4.67467	0.690826	0.491485	-6.17134	0.580466	0.672161
Erythroid.cells	SSB	-0.08949	6.803522	-0.69082	0.491485	-6.36513	0.539617	0.633481
Erythroid.cells	UBTF	-0.11784	5.879519	-0.69073	0.491542	-6.10795	0.556961	0.650013
Erythroid.cells	PLEKHM3	0.190624	6.068485	0.690593	0.491631	-6.06257	0.553366	0.646668
Erythroid.cells	CHST10	0.422817	0.375693	0.690516	0.491679	-5.23944	0.67313	0.757676
Erythroid.cells	PEX26	-0.44461	1.217016	-0.69039	0.491759	-5.23447	0.653864	0.740215
Erythroid.cells	NR2F6	-0.22275	3.501892	-0.69034	0.491787	-5.71582	0.60435	0.694651
Erythroid.cells	GM13963	-0.62924	-0.29578	-0.69026	0.491837	-5.06855	0.688919	0.772004
Erythroid.cells	SIPA1	-0.13917	5.681522	-0.69016	0.491899	-6.01557	0.560753	0.653798
Erythroid.cells	ZFP950	-0.2022	4.197661	-0.69009	0.491947	-5.71795	0.590059	0.681371
Erythroid.cells	TPI1	0.144781	6.690385	0.690029	0.491983	-6.40215	0.54171	0.63571
Erythroid.cells	CCT4	0.102184	6.487167	0.689992	0.492006	-6.28618	0.54549	0.639326
Erythroid.cells	DTX3	0.292928	2.572046	0.68993	0.492045	-5.43881	0.624015	0.712967
Erythroid.cells	GM48236	0.531731	0.1418	0.689882	0.492075	-5.14586	0.678588	0.7628
Erythroid.cells	9430038I0	0.163814	4.248537	0.689587	0.49226	-5.74796	0.589178	0.680561
Erythroid.cells	ARFRP1	0.177812	4.120918	0.689408	0.492372	-5.69493	0.591809	0.68306
Erythroid.cells	GM16062	-0.308	1.874018	-0.68931	0.492433	-5.32515	0.639418	0.727144
Erythroid.cells	PSME2B	-0.34198	2.815452	-0.68925	0.492473	-5.41099	0.619004	0.708354
Erythroid.cells	EPM2AIP1	0.300503	2.609784	0.689079	0.492578	-5.40518	0.623463	0.712531
Erythroid.cells	9130019P1	0.484751	0.265967	0.688916	0.49268	-5.24163	0.676024	0.760576
Erythroid.cells	DNAJC10	-0.16635	4.533218	-0.68874	0.49279	-5.80849	0.583646	0.675573
Erythroid.cells	GSKIP	0.150298	4.602025	0.688536	0.492918	-5.79021	0.582286	0.674353
Erythroid.cells	SIRPB1A	-0.39545	-0.18195	-0.68844	0.49298	-5.34115	0.686652	0.770312
Erythroid.cells	FAM57A	-0.46878	1.118395	-0.68808	0.493205	-5.18877	0.65651	0.743063
Erythroid.cells	GPATCH4	-0.25451	3.338867	-0.68807	0.49321	-5.55759	0.608137	0.69857
Erythroid.cells	MAPKAPK2	-0.13283	7.207807	-0.688	0.493256	-6.35018	0.532549	0.627243
Erythroid.cells	STOML1	0.36562	2.251865	0.687983	0.493265	-5.29163	0.63134	0.720016
Erythroid.cells	ORC3	-0.14983	4.958044	-0.68798	0.493269	-5.92686	0.575211	0.667786
Erythroid.cells	BMS1	-0.16969	4.737717	-0.68795	0.493283	-5.87974	0.579579	0.671894
Erythroid.cells	PRPF3	-0.15973	4.472577	-0.68776	0.493406	-5.76496	0.584957	0.676872
Erythroid.cells	RAB11FIP4	0.492477	-0.78929	0.68759	0.493511	-5.06797	0.70136	0.783498

Erythroid.cells	KLHL26	0.298417	2.727028	0.687389	0.493637	-5.42648	0.621285	0.710661
Erythroid.cells	E530011L2	-0.55595	1.095538	-0.68732	0.49368	-5.11434	0.657238	0.743637
Erythroid.cells	4933411E0	-0.50274	0.555657	-0.68719	0.493762	-5.12631	0.669631	0.754872
Erythroid.cells	MIS18BP1	0.267832	3.964435	0.68706	0.493843	-5.78883	0.595432	0.686608
Erythroid.cells	NLRP1A	-0.5121	0.449959	-0.68678	0.494018	-5.20235	0.672265	0.757154
Erythroid.cells	SVIL	-0.12295	6.860609	-0.6864	0.494255	-6.31173	0.539308	0.633537
Erythroid.cells	RNF169	0.155904	6.380124	0.686356	0.494285	-6.25506	0.548248	0.642087
Erythroid.cells	I730030J21	-0.46908	0.707146	-0.68626	0.494344	-5.1755	0.66639	0.751873
Erythroid.cells	MRPS18C	0.139321	5.696786	0.686091	0.494451	-6.09399	0.561233	0.654482
Erythroid.cells	USP2	0.342653	2.711696	0.686063	0.494469	-5.38271	0.621876	0.711181
Erythroid.cells	ATP6V1F	-0.09179	6.902317	-0.68604	0.494481	-6.3865	0.538539	0.63288
Erythroid.cells	ANXA4	-0.21572	3.74233	-0.68602	0.494493	-5.84941	0.600198	0.691082
Erythroid.cells	CSAD	0.21123	3.965874	0.685827	0.494617	-5.78993	0.595601	0.686826
Erythroid.cells	GM48027	0.298716	2.815176	0.685566	0.49478	-5.43405	0.619663	0.709229
Erythroid.cells	LAX1	0.302772	2.319766	0.685393	0.494889	-5.57461	0.630332	0.719072
Erythroid.cells	TET2	0.136653	6.024646	0.685383	0.494895	-6.12411	0.554963	0.648621
Erythroid.cells	ZBED4	0.176147	4.650997	0.685364	0.494907	-5.80149	0.58174	0.673907
Erythroid.cells	ZYX	0.176227	6.244314	0.685325	0.494932	-6.18503	0.550803	0.644667
Erythroid.cells	BCL7C	-0.12127	5.355918	-0.68526	0.494974	-6.06239	0.567832	0.660811
Erythroid.cells	SPNS2	-0.39345	1.813184	-0.68513	0.495056	-5.26741	0.641438	0.729273
Erythroid.cells	ITM2C	0.157693	5.637577	0.685032	0.495116	-6.13063	0.562374	0.655659
Erythroid.cells	LZTFL1	-0.1919	5.075487	-0.68495	0.495168	-5.95115	0.573323	0.666001
Erythroid.cells	ZDHHC8	-0.22361	3.666743	-0.68487	0.495219	-5.62639	0.601761	0.692664
Erythroid.cells	MMGT2	-0.23952	3.166256	-0.68483	0.495244	-5.48292	0.612216	0.702381
Erythroid.cells	CDH24	0.329054	2.179776	0.684799	0.495262	-5.33368	0.633381	0.721922
Erythroid.cells	CCDC77	0.218481	3.291762	0.68465	0.495355	-5.58442	0.609618	0.700014
Erythroid.cells	GM11290	0.359053	3.432889	0.684525	0.495434	-5.60719	0.606688	0.69727
Erythroid.cells	TSKU	-0.55022	-0.3765	-0.68424	0.495615	-5.093	0.692033	0.775192
Erythroid.cells	2010309G2	-0.37509	1.223176	-0.68415	0.495671	-5.399	0.654852	0.741532
Erythroid.cells	GM11755	-0.64977	-0.115	-0.68404	0.495739	-5.0216	0.685823	0.769625
Erythroid.cells	ADAM22	0.400724	0.796933	0.683684	0.495963	-5.36779	0.664787	0.750525
Erythroid.cells	KLRC3	0.366709	-1.20661	0.68349	0.496084	-5.17139	0.712484	0.793439
Erythroid.cells	BAP1	-0.22921	3.565024	-0.68317	0.496285	-5.57713	0.604354	0.694942
Erythroid.cells	FAHD2A	0.232333	2.845982	0.683068	0.49635	-5.5258	0.619501	0.708985
Erythroid.cells	CDCA7	0.306371	3.246784	0.683002	0.496391	-5.57223	0.61101	0.701123
Erythroid.cells	AGPS	0.111215	6.813953	0.68299	0.496399	-6.28553	0.540601	0.634847
Erythroid.cells	SRD5A3	-0.21866	4.511422	-0.68297	0.496413	-5.74428	0.585004	0.676875
Erythroid.cells	FUCA2	-0.17681	4.624115	-0.68279	0.496524	-5.77813	0.582743	0.674755
Erythroid.cells	CDCA3	0.284867	4.895561	0.682777	0.496533	-5.99661	0.577336	0.669675
Erythroid.cells	LEFTY1	0.520359	0.408138	0.682743	0.496555	-5.07531	0.673842	0.75869
Erythroid.cells	HK1	-0.17395	4.85354	-0.68254	0.49668	-5.87354	0.578216	0.670539
Erythroid.cells	FBXW11	0.130135	7.259166	0.68242	0.496758	-6.41178	0.532479	0.627141
Erythroid.cells	IKZF1	0.102173	8.378161	0.682383	0.496781	-6.64045	0.512529	0.607911
Erythroid.cells	ABCA1	0.226629	5.836161	0.682299	0.496834	-6.16699	0.55905	0.652505
Erythroid.cells	INHBC	0.459525	0.808252	0.682142	0.496932	-5.25795	0.664705	0.75047
Erythroid.cells	MAPK1IP1	-0.3047	2.304416	-0.68187	0.497104	-5.32595	0.631413	0.719955
Erythroid.cells	ETS1	0.116788	7.418769	0.681671	0.497229	-6.53256	0.529754	0.624489

Erythroid.cells	CTSH	0.184519	5.779307	0.681617	0.497263	-6.12956	0.560322	0.653666
Erythroid.cells	CIPC	0.266843	2.655218	0.681487	0.497345	-5.50327	0.623838	0.71308
Erythroid.cells	ECHDC1	0.226011	3.820316	0.681337	0.497439	-5.6922	0.599311	0.690361
Erythroid.cells	SLCO4C1	0.441693	-1.1708	0.681182	0.497537	-5.12829	0.711891	0.793152
Erythroid.cells	DNAJC2	-0.12065	5.81945	-0.68115	0.497556	-6.13684	0.559551	0.653083
Erythroid.cells	TWSG1	0.269143	3.230079	0.681082	0.4976	-5.55492	0.611608	0.701897
Erythroid.cells	KCNA2	0.587572	0.96787	0.680911	0.497707	-5.23096	0.661215	0.747467
Erythroid.cells	TRMU	0.335941	1.548223	0.68091	0.497708	-5.23489	0.648106	0.73551
Erythroid.cells	NAXD	0.175905	4.128354	0.680748	0.49781	-5.77513	0.592996	0.684571
Erythroid.cells	WTIP	-0.37008	1.051984	-0.68074	0.497817	-5.27181	0.659298	0.745723
Erythroid.cells	MTBP	-0.25804	3.256156	-0.68071	0.497831	-5.56856	0.611059	0.701391
Erythroid.cells	KEL	0.389923	-0.44363	0.680483	0.497977	-5.44973	0.694355	0.777336
Erythroid.cells	SMIM3	-0.17975	4.840554	-0.68034	0.498064	-5.9212	0.578793	0.671196
Erythroid.cells	STAP1	0.188318	4.891894	0.680234	0.498134	-5.90308	0.577785	0.670251
Erythroid.cells	D6WSU163	-0.24274	3.1191	-0.67986	0.498371	-5.48425	0.61432	0.704304
Erythroid.cells	HIST1H2BN	0.410719	1.240034	0.679466	0.498617	-5.24518	0.655675	0.742158
Erythroid.cells	FGF1	0.418876	0.63081	0.679082	0.49886	-5.21032	0.66982	0.754905
Erythroid.cells	MIER3	-0.20489	4.078409	-0.67902	0.498896	-5.69291	0.594785	0.685911
Erythroid.cells	TMEM35B	-0.39679	2.207613	-0.67893	0.498957	-5.31942	0.634359	0.722626
Erythroid.cells	WDR3	0.180309	4.225869	0.678532	0.499207	-5.73465	0.591824	0.68336
Erythroid.cells	TAF5	-0.18989	4.146651	-0.67852	0.499211	-5.73271	0.593438	0.684869
Erythroid.cells	WWC2	-0.18095	5.107898	-0.6785	0.499224	-5.91699	0.574163	0.666786
Erythroid.cells	A930015D0	0.190662	4.760503	0.678479	0.49924	-5.83179	0.581052	0.673267
Erythroid.cells	ZFP369	0.25825	2.919936	0.678245	0.499388	-5.52638	0.619025	0.708715
Erythroid.cells	TMCO4	0.219851	4.054604	0.678047	0.499513	-5.74175	0.595319	0.686687
Erythroid.cells	CENPU	0.28847	2.562499	0.677996	0.499545	-5.44523	0.626694	0.715797
Erythroid.cells	APPL1	0.143321	5.974209	0.6779	0.499605	-6.16497	0.557357	0.650954
Erythroid.cells	DHX9	-0.14586	6.492589	-0.67779	0.499673	-6.30149	0.54755	0.641659
Erythroid.cells	CNTLN	-0.23302	4.34763	-0.67776	0.499697	-5.77916	0.589352	0.681143
Erythroid.cells	POGLUT1	-0.26548	2.752407	-0.67766	0.499758	-5.4827	0.622607	0.712061
Erythroid.cells	UBC	-0.12051	8.132317	-0.67764	0.49977	-6.59108	0.51772	0.61303
Erythroid.cells	LDB1	0.185725	4.482765	0.677635	0.499773	-5.83157	0.586622	0.678586
Erythroid.cells	RAP1B	-0.07187	8.543309	-0.67742	0.499908	-6.68413	0.510595	0.606133
Erythroid.cells	NDE1	-0.18563	4.58489	-0.67727	0.500003	-5.89003	0.584697	0.676807
Erythroid.cells	GM156	-0.50453	-1.54566	-0.67706	0.500136	-5.05939	0.722424	0.802591
Erythroid.cells	GM20274	0.301485	2.727126	0.676797	0.500302	-5.39835	0.62351	0.712907
Erythroid.cells	MS4A4A	-0.36946	0.617812	-0.67632	0.500602	-5.36702	0.670655	0.755997
Erythroid.cells	NUS1	-0.13609	4.837828	-0.67631	0.500609	-5.87952	0.579928	0.672274
Erythroid.cells	TRIP13	-0.34649	2.772205	-0.67625	0.500647	-5.50062	0.62263	0.712054
Erythroid.cells	PPP1R37	-0.15625	4.592802	-0.67622	0.500667	-5.8995	0.584829	0.676876
Erythroid.cells	CELSR2	0.586883	-0.09555	0.676211	0.500672	-5.07044	0.687378	0.771117
Erythroid.cells	DCXR	0.166934	4.317514	0.675875	0.500884	-5.98698	0.590552	0.68211
Erythroid.cells	SCLY	-0.21248	3.466279	-0.6758	0.500931	-5.62851	0.608096	0.698473
Erythroid.cells	SNTA1	0.338863	1.865421	0.675621	0.501045	-5.35999	0.642646	0.730307
Erythroid.cells	FIGNL1	0.334983	2.757262	0.675338	0.501223	-5.46473	0.623339	0.712417
Erythroid.cells	NDUFB6	0.13544	6.317819	0.675112	0.501366	-6.33478	0.551582	0.645198
Erythroid.cells	PIGB	0.310176	2.703863	0.675068	0.501394	-5.36622	0.624494	0.713476

Erythroid.cells	ARHGEF1	-0.10191	7.10928	-0.675	0.501435	-6.41455	0.536848	0.631137
Erythroid.cells	CCDC180	-0.42734	1.286108	-0.67495	0.501469	-5.24793	0.655779	0.742161
Erythroid.cells	TRMT10A	0.214983	3.81366	0.674395	0.50182	-5.6533	0.601316	0.692001
Erythroid.cells	CAMK4	0.221832	3.194525	0.674386	0.501826	-6.19482	0.614266	0.704027
Erythroid.cells	GM14302	0.463703	0.609708	0.674361	0.501842	-5.10465	0.671526	0.756479
Erythroid.cells	CDCA4	-0.18037	4.453152	-0.67414	0.501982	-5.82619	0.588258	0.679831
Erythroid.cells	IFT74	-0.33019	2.322027	-0.67411	0.501998	-5.39043	0.633027	0.721364
Erythroid.cells	GNA13	-0.12071	7.201206	-0.67405	0.502036	-6.43313	0.535386	0.62976
Erythroid.cells	STRAP	0.121956	6.174275	0.673819	0.502184	-6.2523	0.554617	0.648082
Erythroid.cells	SIK2	-0.12005	7.126745	-0.67347	0.502405	-6.36021	0.536835	0.631278
Erythroid.cells	SDC1	-0.35475	2.216388	-0.67339	0.502453	-5.56211	0.635437	0.723756
Erythroid.cells	UNC45B	0.561984	0.423376	0.673232	0.502556	-5.13695	0.675987	0.76073
Erythroid.cells	TRAPPC4	-0.14641	5.137813	-0.6731	0.50264	-5.92494	0.574679	0.667263
Erythroid.cells	4930509H(	-0.28666	2.090644	-0.67304	0.502677	-5.36964	0.638198	0.726313
Erythroid.cells	MED30	-0.14576	5.369422	-0.67298	0.502712	-6.01151	0.570131	0.662976
Erythroid.cells	ARL6	-0.29838	1.698649	-0.67295	0.502735	-5.35094	0.646883	0.734275
Erythroid.cells	THEMIS2	0.269493	4.692319	0.672901	0.502766	-5.73931	0.583536	0.675608
Erythroid.cells	RRAS2	0.171715	5.198697	0.672871	0.502784	-6.0162	0.57348	0.66615
Erythroid.cells	TMEM140	-0.3281	3.514222	-0.67275	0.502863	-5.59024	0.607657	0.698165
Erythroid.cells	EEA1	-0.13254	6.055281	-0.67273	0.502874	-6.13402	0.556882	0.650486
Erythroid.cells	ACSBG1	-0.4123	-0.5064	-0.67268	0.502906	-5.24106	0.698042	0.780718
Erythroid.cells	PPP1R16A	-0.26842	2.885752	-0.6726	0.502955	-5.46802	0.620948	0.710538
Erythroid.cells	SNN	-0.26683	3.649853	-0.67178	0.503474	-5.62781	0.605334	0.695882
Erythroid.cells	EIF2AK2	-0.24118	4.927436	-0.67175	0.503496	-5.80519	0.579329	0.67157
Erythroid.cells	PITPNA	-0.07935	7.992513	-0.67156	0.503617	-6.56289	0.521666	0.616661
Erythroid.cells	DDX47	0.112594	5.550552	0.671508	0.503648	-6.08416	0.567109	0.660052
Erythroid.cells	BC065397	-0.40552	1.292469	-0.67114	0.503881	-5.17678	0.656826	0.743146
Erythroid.cells	MAGI2	0.436307	0.831391	0.670893	0.504037	-5.20041	0.667489	0.752798
Erythroid.cells	MNAT1	0.175138	4.818214	0.670465	0.504309	-5.85682	0.582097	0.673932
Erythroid.cells	JAG2	-0.35839	1.093215	-0.67026	0.504441	-5.35075	0.661816	0.747535
Erythroid.cells	PHLDA1	-0.27959	4.035457	-0.67019	0.504485	-5.79719	0.598015	0.688812
Erythroid.cells	POLH	-0.20234	4.035992	-0.67005	0.504572	-5.73726	0.598004	0.688817
Erythroid.cells	ABCC4	-0.17636	4.55376	-0.67002	0.504591	-5.8112	0.587456	0.67896
Erythroid.cells	RNF5	0.193726	4.122516	0.669686	0.504803	-5.7534	0.596407	0.68722
Erythroid.cells	GM3550	0.308588	1.752212	0.669399	0.504986	-5.32216	0.647222	0.734157
Erythroid.cells	PTPRJ	0.105243	8.855581	0.669389	0.504992	-6.7167	0.507293	0.602468
Erythroid.cells	CRYZL1	-0.17623	4.570122	-0.66867	0.505449	-5.80039	0.587662	0.679107
Erythroid.cells	6230400D1	-0.32133	2.163361	-0.66859	0.505497	-5.35012	0.638415	0.726143
Erythroid.cells	CTLA4	-0.37457	2.060232	-0.66854	0.50553	-5.51513	0.640689	0.728228
Erythroid.cells	EGLN3	0.335978	3.294296	0.668443	0.505592	-5.60171	0.61402	0.703654
Erythroid.cells	ACAA1B	0.360385	3.318751	0.668402	0.505619	-5.89	0.613503	0.703176
Erythroid.cells	KDM5B	0.191372	5.362074	0.668396	0.505622	-6.07679	0.5719	0.664298
Erythroid.cells	LTO1	0.239388	3.526077	0.668348	0.505653	-5.52684	0.609141	0.699137
Erythroid.cells	IPO4	-0.27436	2.384797	-0.66817	0.505763	-5.35098	0.633573	0.721699
Erythroid.cells	INAFM1	-0.34211	1.866251	-0.66814	0.505783	-5.32579	0.645002	0.73218
Erythroid.cells	C530008M	0.485588	0.74516	0.667887	0.505946	-5.20594	0.670511	0.755426
Erythroid.cells	4833418N(	0.420883	1.533706	0.667867	0.505958	-5.24886	0.652512	0.739062

Erythroid.cells	DSCC1	-0.31588	2.435007	-0.66775	0.50603	-5.42627	0.632561	0.720785
Erythroid.cells	PATL1	-0.15426	5.222719	-0.66754	0.506168	-5.93189	0.574816	0.667043
Erythroid.cells	POU5F2	-0.35298	1.687999	-0.66739	0.506258	-5.29294	0.649165	0.736014
Erythroid.cells	HACD1	0.278547	3.002315	0.667157	0.50641	-5.54661	0.620414	0.709657
Erythroid.cells	SAAL1	-0.24996	3.298168	-0.66707	0.506466	-5.59268	0.614127	0.703859
Erythroid.cells	AIF1	0.242107	4.896333	0.667042	0.506483	-6.10987	0.581293	0.673236
Erythroid.cells	HEXIM2	0.480164	0.998422	0.666933	0.506552	-5.15216	0.664795	0.750385
Erythroid.cells	CYP2R1	-0.46218	1.009263	-0.66689	0.50658	-5.17026	0.664546	0.750165
Erythroid.cells	CENPM	0.248417	3.916181	0.666727	0.506683	-5.78302	0.601205	0.691981
Erythroid.cells	QSOX2	0.442895	0.948725	0.66666	0.506725	-5.15224	0.665936	0.751509
Erythroid.cells	ADCY7	-0.15731	5.583087	-0.66659	0.506768	-5.95075	0.567756	0.660604
Erythroid.cells	BRWD1	0.114618	6.550877	0.666573	0.506781	-6.27529	0.549242	0.643043
Erythroid.cells	NOL10	0.166127	4.91748	0.666494	0.506831	-5.87479	0.580871	0.672979
Erythroid.cells	D2HGDH	-0.32858	2.08867	-0.66628	0.506965	-5.31154	0.640351	0.728114
Erythroid.cells	PACC1	-0.18962	5.421952	-0.66618	0.507032	-5.99501	0.570993	0.66364
Erythroid.cells	TTK	0.377264	2.313922	0.66604	0.50712	-5.36716	0.635441	0.723626
Erythroid.cells	TRAT1	0.493762	-0.05747	0.665864	0.507231	-5.31221	0.689628	0.772964
Erythroid.cells	FBXL18	0.401988	2.985602	0.665564	0.507422	-5.43683	0.62091	0.710419
Erythroid.cells	TUSC1	0.223006	3.124075	0.665479	0.507477	-5.61775	0.617956	0.707687
Erythroid.cells	SIRPA	0.198208	5.832373	0.665449	0.507496	-6.10322	0.56305	0.656299
Erythroid.cells	IFI44	-0.65949	0.579228	-0.66543	0.50751	-5.15118	0.674633	0.759567
Erythroid.cells	B020010K1	-0.48151	1.134374	-0.66526	0.507615	-5.19681	0.66183	0.748017
Erythroid.cells	RAPH1	0.151589	5.055385	0.66525	0.507623	-6.12007	0.578256	0.670717
Erythroid.cells	GNA12	0.190425	4.652134	0.66511	0.507712	-5.79578	0.586319	0.678326
Erythroid.cells	ATG3	-0.10231	6.261249	-0.66506	0.507744	-6.21131	0.554838	0.648609
Erythroid.cells	TMEM143	0.35434	1.491704	0.665024	0.507766	-5.24643	0.653721	0.740667
Erythroid.cells	ATL1	0.509626	0.498179	0.664949	0.507814	-5.16678	0.676523	0.761395
Erythroid.cells	AKNA	-0.15295	4.948701	-0.6649	0.507846	-5.91136	0.580378	0.672749
Erythroid.cells	CFAP20	-0.13701	5.191326	-0.66476	0.507936	-5.97275	0.575598	0.66822
Erythroid.cells	NAF1	-0.19806	3.658508	-0.66465	0.508001	-5.65252	0.606734	0.697363
Erythroid.cells	APTX	0.199379	3.052742	0.664437	0.50814	-5.55848	0.619614	0.709273
Erythroid.cells	SPG11	-0.21382	4.094018	-0.66416	0.508316	-5.69212	0.597943	0.68912
Erythroid.cells	DTL	-0.22614	4.961318	-0.66396	0.508441	-6.02822	0.580462	0.672714
Erythroid.cells	FNDC3B	0.136566	6.549271	0.663633	0.508652	-6.25909	0.549828	0.643696
Erythroid.cells	RIC8A	0.195366	3.980741	0.663528	0.508719	-5.67827	0.600479	0.691427
Erythroid.cells	KAT6B	0.141116	5.844837	0.663512	0.508729	-6.07518	0.563254	0.656462
Erythroid.cells	JAML	0.305495	2.748338	0.663401	0.5088	-5.61161	0.626512	0.71556
Erythroid.cells	CNTD1	-0.457	0.620711	-0.66315	0.508957	-5.17203	0.674343	0.759202
Erythroid.cells	MRPL17	0.124695	5.426857	0.66298	0.509068	-6.10792	0.571561	0.664228
Erythroid.cells	GM4129	0.32436	1.28611	0.662471	0.509393	-5.39652	0.659417	0.745572
Erythroid.cells	DDX3X	0.119979	7.320412	0.662405	0.509435	-6.4567	0.535945	0.630348
Erythroid.cells	ZFP975	0.551786	0.810489	0.662286	0.50951	-5.16124	0.67035	0.755574
Erythroid.cells	MRPS6	-0.13268	6.008603	-0.66211	0.509621	-6.1678	0.560619	0.65389
Erythroid.cells	LIX1L	-0.40527	0.803407	-0.66198	0.509708	-5.21865	0.670616	0.755843
Erythroid.cells	RBM44	0.55714	0.135835	0.661826	0.509804	-5.11593	0.686298	0.770015
Erythroid.cells	POLR2G	-0.12593	5.51542	-0.66146	0.510038	-6.0549	0.570414	0.663142
Erythroid.cells	CDC42BPB	0.24153	3.262023	0.661402	0.510074	-5.71703	0.616338	0.706069

Erythroid.cells	RHOT1	-0.12599	5.400436	-0.66101	0.510326	-6.00722	0.572849	0.665374
Erythroid.cells	PRPF4B	-0.11122	6.312389	-0.66096	0.510356	-6.25971	0.555223	0.648693
Erythroid.cells	GTF2F1	0.133552	5.030746	0.660715	0.510513	-5.92398	0.580272	0.67231
Erythroid.cells	ITPRIPL2	0.230207	3.897046	0.660608	0.510581	-5.69493	0.603331	0.693893
Erythroid.cells	BBC3	-0.27599	2.639565	-0.66043	0.510696	-5.4286	0.630089	0.718651
Erythroid.cells	EIF4E3	-0.22858	4.420461	-0.66027	0.510795	-5.69862	0.59268	0.683956
Erythroid.cells	WSB2	0.193623	4.493162	0.660056	0.510934	-5.75802	0.591283	0.682702
Erythroid.cells	USP21	-0.28806	3.140041	-0.65997	0.510987	-5.42551	0.619453	0.708942
Erythroid.cells	ZFP317	0.253	2.898111	0.659791	0.511103	-5.5015	0.624705	0.713783
Erythroid.cells	NR4A3	0.183319	6.012984	0.65968	0.511174	-6.40874	0.561314	0.654526
Erythroid.cells	GM4707	0.317277	2.866085	0.659565	0.511247	-5.54047	0.625422	0.714443
Erythroid.cells	IMPA2	-0.1815	4.492173	-0.65933	0.511395	-5.8423	0.591468	0.68286
Erythroid.cells	ARGLU1	-0.06702	7.597485	-0.65928	0.511431	-6.54866	0.531783	0.626307
Erythroid.cells	IL10	-0.44524	2.999738	-0.65904	0.511585	-5.47504	0.622741	0.711857
Erythroid.cells	2510039O1	-0.14371	5.179646	-0.65871	0.511791	-5.95965	0.577836	0.67001
Erythroid.cells	PRIMPOL	0.2368	3.486578	0.658645	0.511835	-5.59539	0.612455	0.702343
Erythroid.cells	CAMK2D	-0.1491	7.214604	-0.65864	0.511839	-6.467	0.538938	0.633109
Erythroid.cells	WDR1	-0.11739	7.335798	-0.65851	0.51192	-6.42179	0.536711	0.630976
Erythroid.cells	GM21859	0.4732	1.780794	0.658477	0.511942	-5.40036	0.649528	0.73647
Erythroid.cells	SRC	-0.4359	0.980292	-0.65833	0.512039	-5.20425	0.667716	0.753108
Erythroid.cells	KIF17	-0.47046	1.692096	-0.65813	0.512163	-5.24138	0.651518	0.738401
Erythroid.cells	NAA50	0.100024	6.371464	0.657909	0.512306	-6.26534	0.554707	0.648293
Erythroid.cells	FGGY	0.263733	3.459854	0.657881	0.512324	-5.69555	0.613019	0.70304
Erythroid.cells	GM41442	-0.51002	-0.19949	-0.65774	0.512414	-5.13784	0.695474	0.778358
Erythroid.cells	PPM1E	0.215488	4.964118	0.657708	0.512434	-6.08919	0.582126	0.674258
Erythroid.cells	C2CD3	0.148545	4.657214	0.657527	0.512551	-5.83539	0.588294	0.680104
Erythroid.cells	TFCP2	0.340515	1.971586	0.657496	0.51257	-5.29792	0.645268	0.732882
Erythroid.cells	GM41077	-0.44694	0.721818	-0.65746	0.512594	-5.1957	0.6737	0.758782
Erythroid.cells	TNPO2	-0.21217	4.122122	-0.65742	0.512622	-5.68184	0.599211	0.690325
Erythroid.cells	DEDD2	0.196605	4.544003	0.657287	0.512704	-5.81746	0.590586	0.682311
Erythroid.cells	GALNT3	0.434101	1.767982	0.657265	0.512718	-5.27334	0.649815	0.737106
Erythroid.cells	CCL12	0.767091	0.036828	0.657216	0.512749	-5.15646	0.689821	0.773441
Erythroid.cells	IL15	0.330122	3.93993	0.657158	0.512786	-5.71636	0.602977	0.693933
Erythroid.cells	RBMS2	-0.14251	4.872344	-0.657	0.512884	-5.92264	0.58398	0.676153
Erythroid.cells	CLEC4B1	0.372837	-0.61033	0.656946	0.512922	-5.28427	0.705432	0.787502
Erythroid.cells	RNASEK	0.100711	6.735066	0.656592	0.513149	-6.31021	0.548041	0.642057
Erythroid.cells	GAS2	0.249225	2.456745	0.656204	0.513397	-5.52892	0.634949	0.723412
Erythroid.cells	3010003L2	0.398557	1.349271	0.656203	0.513398	-5.2677	0.659666	0.746031
Erythroid.cells	TMEM150A	0.292148	2.327871	0.656005	0.513524	-5.56468	0.637858	0.726104
Erythroid.cells	CTC1	0.221213	3.25694	0.655854	0.513621	-5.53347	0.617802	0.707652
Erythroid.cells	SCPEP1	-0.17053	5.169388	-0.65576	0.513683	-5.97057	0.578495	0.670997
Erythroid.cells	PZP	0.23561	4.39586	0.655676	0.513735	-6.17276	0.594068	0.685617
Erythroid.cells	KLHL5	0.147586	4.645979	0.655574	0.513801	-5.87643	0.588991	0.680904
Erythroid.cells	CLEC1B	0.315607	3.741016	0.655355	0.513941	-5.71271	0.607696	0.698349
Erythroid.cells	AREG	-0.70151	1.634087	-0.65511	0.514099	-5.48692	0.65357	0.740504
Erythroid.cells	MNS1	0.235711	2.821224	0.654924	0.514217	-5.60727	0.627374	0.716549
Erythroid.cells	CCNF	0.286232	3.438612	0.654866	0.514254	-5.69316	0.614179	0.704354

Erythroid.cells	EIF2B5	0.167519	4.703803	0.654813	0.514288	-5.86095	0.588034	0.68001
Erythroid.cells	GM26881	-0.50397	0.245715	-0.6545	0.51449	-5.14858	0.685747	0.769786
Erythroid.cells	MYCBP2	-0.1141	7.768752	-0.65444	0.514526	-6.55254	0.529517	0.624416
Erythroid.cells	GM14471	-0.51914	0.24931	-0.65443	0.514537	-5.05814	0.685662	0.769709
Erythroid.cells	FZD4	0.388002	1.267832	0.654209	0.514675	-5.26612	0.662077	0.748289
Erythroid.cells	PTPN18	-0.0988	7.11924	-0.65411	0.514739	-6.37443	0.541481	0.635868
Erythroid.cells	FBXL3	0.127615	5.215741	0.65396	0.514835	-6.02943	0.577977	0.670501
Erythroid.cells	GM15964	0.445486	0.162356	0.65388	0.514886	-5.33881	0.687857	0.771733
Erythroid.cells	GM37168	0.402063	0.65738	0.653635	0.515043	-5.24098	0.676201	0.761329
Erythroid.cells	EIF3B	-0.1251	5.890853	-0.65357	0.515083	-6.1759	0.56475	0.658179
Erythroid.cells	UBA7	0.261917	3.784743	0.653556	0.515094	-5.64652	0.607103	0.697921
Erythroid.cells	NNT	0.275708	2.482711	0.653516	0.51512	-5.40109	0.634939	0.723668
Erythroid.cells	UVSSA	0.218811	3.709393	0.653267	0.515279	-5.62092	0.608741	0.699445
Erythroid.cells	TLNRD1	0.206966	4.581796	0.653246	0.515293	-5.8528	0.590755	0.68267
Erythroid.cells	ANAPC1	0.162966	4.721072	0.652937	0.515491	-5.85161	0.588092	0.680104
Erythroid.cells	CEP152	-0.21678	3.629164	-0.65276	0.515608	-5.61697	0.610617	0.701132
Erythroid.cells	LRP4	0.355765	2.477171	0.652708	0.515638	-5.51775	0.635327	0.72398
Erythroid.cells	ARHGAP4	-0.15285	5.057347	-0.65253	0.515752	-5.88182	0.581382	0.67385
Erythroid.cells	STK19	-0.18078	4.61763	-0.65248	0.515784	-5.83985	0.590227	0.682149
Erythroid.cells	C730034F0	-0.33102	2.636781	-0.65241	0.51583	-5.41676	0.631855	0.720816
Erythroid.cells	SLC35A4	0.234366	3.561213	0.65199	0.516099	-5.5686	0.612306	0.702625
Erythroid.cells	ATP8A1	-0.10733	7.601268	-0.65183	0.516205	-6.47769	0.533144	0.627892
Erythroid.cells	GGCX	-0.32871	1.807957	-0.65174	0.516258	-5.34965	0.650477	0.737776
Erythroid.cells	TRAF2	-0.19593	3.917831	-0.65145	0.516448	-5.70381	0.605037	0.695826
Erythroid.cells	TFB1M	-0.32526	1.706255	-0.65133	0.516521	-5.33311	0.652934	0.740006
Erythroid.cells	GM28379	-0.51244	0.264238	-0.65116	0.516635	-5.12679	0.686249	0.770254
Erythroid.cells	ANGPTL6	0.428054	0.97577	0.651155	0.516635	-5.20211	0.669599	0.755184
Erythroid.cells	ECHS1	0.138142	5.549982	0.650893	0.516804	-6.3089	0.572181	0.664946
Erythroid.cells	GM9993	0.420785	1.16551	0.650563	0.517015	-5.21037	0.665549	0.75133
Erythroid.cells	MAPRE2	-0.11767	6.802434	-0.65049	0.517065	-6.33514	0.548306	0.642262
Erythroid.cells	METRNL	-0.30627	3.205494	-0.65002	0.517366	-5.67901	0.620625	0.710106
Erythroid.cells	THOC6	0.194729	4.164901	0.649934	0.51742	-5.735	0.600474	0.6914
Erythroid.cells	ANKS3	-0.20525	4.082767	-0.64985	0.517473	-5.6682	0.602172	0.692997
Erythroid.cells	JUNOS	-0.41167	1.820722	-0.64954	0.517671	-5.27836	0.651084	0.738063
Erythroid.cells	HEMGN	0.43058	0.35427	0.649418	0.517752	-5.42093	0.68488	0.768754
Erythroid.cells	ZFP324	0.488979	0.798242	0.649364	0.517786	-5.13652	0.674463	0.759334
Erythroid.cells	2610301B2	0.329328	1.613471	0.649319	0.517816	-5.29439	0.655755	0.742339
Erythroid.cells	SLC35D2	0.207553	4.232	0.64913	0.517937	-5.82015	0.599241	0.690239
Erythroid.cells	GM50240	0.388033	1.572659	0.649084	0.517966	-5.23489	0.656716	0.743238
Erythroid.cells	MAP2K1	-0.12783	6.311963	-0.64893	0.518067	-6.23784	0.55801	0.651446
Erythroid.cells	ENDOV	0.338363	2.177707	0.648796	0.518152	-5.35782	0.643203	0.730952
Erythroid.cells	TUBB2B	0.28386	2.9596	0.648628	0.51826	-5.5489	0.626105	0.715282
Erythroid.cells	PRKN	0.304795	3.428362	0.648357	0.518434	-5.66019	0.616081	0.706077
Erythroid.cells	B230219D2	0.092397	6.252623	0.648299	0.518472	-6.20747	0.559145	0.652694
Erythroid.cells	SEC22C	-0.35007	2.04121	-0.64826	0.5185	-5.27904	0.646237	0.733857
Erythroid.cells	PTGER2	0.39825	1.047913	0.64823	0.518516	-5.26854	0.668763	0.7544
Erythroid.cells	F7	-0.36371	0.719823	-0.64821	0.518527	-5.41686	0.676379	0.761309

Erythroid.cells	MPV17L	0.335454	2.006778	0.648153	0.518566	-5.34523	0.647004	0.734563
Erythroid.cells	BAIAP2L1	-0.22248	3.098445	-0.64799	0.51867	-5.6495	0.623119	0.712629
Erythroid.cells	GM26632	0.476479	0.366747	0.64799	0.518671	-5.13673	0.684673	0.768847
Erythroid.cells	AP5S1	0.294914	2.440536	0.647749	0.518826	-5.41982	0.63744	0.725811
Erythroid.cells	GSTA4	0.341072	1.583658	0.647642	0.518895	-5.39408	0.656553	0.743317
Erythroid.cells	GM11808	0.112186	7.262783	0.647553	0.518952	-6.58426	0.540186	0.634675
Erythroid.cells	PPP4R2	-0.11174	6.318519	-0.64755	0.518955	-6.25616	0.557917	0.65158
Erythroid.cells	AFG1L	0.256973	3.691074	0.647441	0.519024	-5.64247	0.610573	0.701076
Erythroid.cells	DEPDC1B	0.328101	2.954227	0.647377	0.519065	-5.61128	0.626258	0.715634
Erythroid.cells	KLRC1	-0.26267	1.444197	-0.64658	0.519579	-5.72527	0.660295	0.746604
Erythroid.cells	GM16151	0.484891	-0.28838	0.646472	0.519648	-5.07757	0.700998	0.783446
Erythroid.cells	METTL6	-0.18422	4.505421	-0.64638	0.519706	-5.79949	0.59424	0.685699
Erythroid.cells	TGS1	-0.13665	5.214453	-0.6458	0.520083	-5.94166	0.5803	0.672479
Erythroid.cells	FANCC	-0.18725	4.74282	-0.64554	0.520249	-5.90267	0.589892	0.681413
Erythroid.cells	DAB2IP	-0.36402	2.225574	-0.64524	0.520441	-5.30367	0.643325	0.730991
Erythroid.cells	CCDC138	0.244641	4.628632	0.645174	0.520485	-5.80519	0.592261	0.683714
Erythroid.cells	LIN37	-0.21673	3.646925	-0.64499	0.520605	-5.63197	0.612591	0.702734
Erythroid.cells	B4GALT4	0.318	1.774117	0.644814	0.520717	-5.35553	0.653418	0.74036
Erythroid.cells	CHSY1	-0.15014	5.079341	-0.64479	0.52073	-6.04884	0.583166	0.675309
Erythroid.cells	SECISBP2	-0.1521	4.786541	-0.64473	0.520774	-5.88074	0.589058	0.680836
Erythroid.cells	BRIP1	-0.20187	4.898207	-0.64468	0.520804	-5.98101	0.586804	0.678723
Erythroid.cells	BRD3	0.132591	5.712362	0.644591	0.520861	-6.11631	0.57064	0.663537
Erythroid.cells	SDF2L1	0.201147	4.888426	0.644588	0.520863	-5.86063	0.587001	0.678933
Erythroid.cells	ZCRB1	-0.09612	6.149583	-0.64437	0.521006	-6.20976	0.562155	0.655583
Erythroid.cells	ECE1	0.175571	5.613294	0.644347	0.521019	-6.1761	0.572582	0.665445
Erythroid.cells	MYL9	0.394151	1.95475	0.644336	0.521026	-5.56591	0.64936	0.736763
Erythroid.cells	PFKP	-0.16179	5.538024	-0.64425	0.521084	-6.16295	0.574061	0.666859
Erythroid.cells	TXNIP	0.187825	5.791358	0.644063	0.521202	-6.08099	0.569159	0.662241
Erythroid.cells	HAUS2	-0.22337	3.755191	-0.64397	0.521264	-5.62456	0.610383	0.700893
Erythroid.cells	GM11508	-0.26949	3.437566	-0.64361	0.521495	-5.64804	0.617291	0.707234
Erythroid.cells	MBTPS1	-0.14792	4.639311	-0.64336	0.521654	-5.86481	0.592416	0.684028
Erythroid.cells	ZFAS1	0.179391	5.058651	0.642829	0.521999	-6.02422	0.584264	0.676232
Erythroid.cells	MALAT1	-0.08932	14.72453	-0.6426	0.522147	-7.56593	0.420843	0.516936
Erythroid.cells	PDIK1L	0.256509	3.065249	0.641909	0.522593	-5.47449	0.626151	0.715034
Erythroid.cells	GM31462	0.456231	0.152102	0.641908	0.522594	-5.14797	0.692331	0.775336
Erythroid.cells	DZIP3	0.283073	3.257554	0.641886	0.522608	-5.50658	0.622019	0.71122
Erythroid.cells	BTBD8	-0.43291	1.572677	-0.64157	0.522814	-5.20188	0.659376	0.745413
Erythroid.cells	4930594M	-0.5598	-0.02762	-0.64143	0.522902	-5.05852	0.696821	0.779376
Erythroid.cells	ARAP2	0.170429	5.924266	0.641327	0.522969	-6.1894	0.567719	0.660515
Erythroid.cells	DENND1C	-0.22695	3.966917	-0.6413	0.522987	-5.65224	0.607178	0.697505
Erythroid.cells	MAIP1	-0.20514	3.588251	-0.64115	0.523084	-5.62137	0.615157	0.704894
Erythroid.cells	AI839979	0.347578	0.274557	0.640991	0.523186	-5.45327	0.689609	0.773001
Erythroid.cells	SLC3A2	0.125949	6.772545	0.640899	0.523246	-6.41678	0.551485	0.645171
Erythroid.cells	PARP12	-0.25212	3.018486	-0.64086	0.52327	-5.68347	0.627341	0.716248
Erythroid.cells	GPR107	-0.1375	5.057833	-0.6408	0.523307	-5.97044	0.584859	0.67673
Erythroid.cells	MIR17HG	-0.34094	2.192534	-0.64058	0.523454	-5.331	0.645557	0.732913
Erythroid.cells	IRF7	-0.2576	6.139097	-0.64048	0.523516	-6.49496	0.563667	0.656676

Erythroid.cells	PRRC2B	-0.10481	6.226887	-0.64007	0.52378	-6.27161	0.562107	0.655221
Erythroid.cells	SLC46A3	0.260338	3.085031	0.64	0.523828	-5.53335	0.626157	0.715126
Erythroid.cells	HPS5	0.171535	4.448069	0.639929	0.523873	-5.78016	0.597475	0.688538
Erythroid.cells	RTP4	-0.40632	4.174749	-0.63985	0.523927	-5.78309	0.603114	0.693804
Erythroid.cells	VWA8	0.145449	4.975158	0.639821	0.523943	-6.03103	0.586757	0.678516
Erythroid.cells	ELMOD2	-0.28673	2.807903	-0.6397	0.524024	-5.39231	0.632185	0.720743
Erythroid.cells	EGR1	0.242103	6.545199	0.639496	0.524154	-6.35048	0.556109	0.649534
Erythroid.cells	LMBRD1	-0.11816	6.356577	-0.63911	0.524405	-6.29376	0.559716	0.653048
Erythroid.cells	NMRAL1	-0.22025	3.904291	-0.63909	0.524417	-5.74545	0.608858	0.699188
Erythroid.cells	ARHGAP28	0.459585	0.598833	0.639017	0.524464	-5.32311	0.682328	0.766498
Erythroid.cells	BACH2IT1	0.447151	-0.26225	0.638898	0.524541	-5.06619	0.702918	0.78507
Erythroid.cells	MB21D2	0.460483	0.883088	0.638809	0.524599	-5.21985	0.675666	0.760527
Erythroid.cells	SUPT5	-0.11775	6.05341	-0.63874	0.524641	-6.18228	0.565557	0.658634
Erythroid.cells	KEAP1	0.131746	5.115335	0.638722	0.524655	-5.98332	0.584045	0.676062
Erythroid.cells	IL18RAP	-0.35541	1.983582	-0.63871	0.524665	-5.56353	0.650495	0.737621
Erythroid.cells	EPHB4	0.538654	0.794939	0.638642	0.524707	-5.20619	0.677724	0.76241
Erythroid.cells	IFITM10	0.386697	3.144158	0.638295	0.524931	-5.64368	0.62519	0.714331
Erythroid.cells	MRPS23	-0.18396	4.411962	-0.63812	0.525046	-5.82232	0.598557	0.689627
Erythroid.cells	NR3C1	-0.11546	7.440275	-0.63804	0.525094	-6.53993	0.539564	0.633882
Erythroid.cells	SUN2	-0.1223	6.122993	-0.63706	0.525731	-6.16718	0.56505	0.657713
Erythroid.cells	PXDN	0.406875	0.993778	0.636955	0.5258	-5.20736	0.674101	0.758602
Erythroid.cells	TRAC	-0.25283	2.266621	-0.63681	0.525893	-5.84512	0.645185	0.732312
Erythroid.cells	VAV2	-0.18921	4.977829	-0.63651	0.526091	-5.9533	0.587878	0.679182
Erythroid.cells	CELF2	-0.08243	8.597509	-0.63631	0.526217	-6.70082	0.519489	0.614144
Erythroid.cells	AAAS	0.222855	3.794127	0.636174	0.526306	-5.67209	0.612389	0.702037
Erythroid.cells	RAE1	-0.16594	4.525674	-0.63609	0.526364	-5.7873	0.59718	0.68791
Erythroid.cells	FGL2	-0.29105	4.054695	-0.6358	0.52655	-6.02415	0.607063	0.697008
Erythroid.cells	LRSAM1	-0.37259	1.610096	-0.63568	0.52663	-5.23348	0.660391	0.746086
Erythroid.cells	DDX41	0.162861	4.356626	0.63562	0.526666	-5.80339	0.600795	0.691203
Erythroid.cells	TMEM183/	0.103335	5.16956	0.635355	0.526838	-6.10418	0.584366	0.675842
Erythroid.cells	DCAF11	0.146476	4.559305	0.635047	0.527038	-5.95833	0.59674	0.68751
Erythroid.cells	TNFSF10	0.294366	2.307037	0.635047	0.527038	-5.48165	0.644834	0.731976
Erythroid.cells	TNRC6C	-0.09365	7.143837	-0.63503	0.527052	-6.45829	0.546159	0.639787
Erythroid.cells	PILRB2	0.346622	2.237136	0.634931	0.527113	-5.45152	0.64639	0.733439
Erythroid.cells	CTNNB1	0.091109	6.277504	0.634889	0.527141	-6.23922	0.562588	0.655432
Erythroid.cells	PUS7	-0.25104	2.95762	-0.63459	0.527333	-5.51469	0.630541	0.71893
Erythroid.cells	ABCB11	0.319732	1.681529	0.634586	0.527337	-5.48389	0.658893	0.744901
Erythroid.cells	CDK13	0.092556	7.414024	0.634527	0.527375	-6.49925	0.541141	0.635068
Erythroid.cells	TEPSIN	0.41792	0.762658	0.634523	0.527378	-5.20552	0.680118	0.764178
Erythroid.cells	WDR46	0.260373	3.429733	0.634326	0.527506	-5.53577	0.620454	0.709584
Erythroid.cells	YAP1	0.528791	1.070556	0.634125	0.527636	-5.22159	0.673103	0.757761
Erythroid.cells	ANP32B	-0.10244	8.191649	-0.63402	0.527708	-6.69695	0.527116	0.62158
Erythroid.cells	NUDT1	0.273257	3.00659	0.633647	0.527947	-5.56755	0.629866	0.718104
Erythroid.cells	KMT5B	-0.11319	5.91383	-0.63351	0.528035	-6.18564	0.57002	0.662303
Erythroid.cells	LNCPINT	-0.15119	8.328661	-0.63311	0.528293	-6.73705	0.524942	0.619278
Erythroid.cells	DNPEP	-0.19306	4.077287	-0.63304	0.528339	-5.71932	0.607203	0.697081
Erythroid.cells	OAS3	0.371544	2.402295	0.633043	0.52834	-5.76578	0.643245	0.730337

Erythroid.cells	FAM168B	-0.09407	6.280422	-0.63291	0.528428	-6.19684	0.562991	0.655635
Erythroid.cells	TICAM1	0.265526	2.819811	0.632844	0.528469	-5.49767	0.634058	0.721937
Erythroid.cells	HACL1	0.209542	3.523051	0.6328	0.528497	-5.84586	0.61889	0.707948
Erythroid.cells	WDR53	-0.27127	2.715022	-0.63273	0.528542	-5.47345	0.636351	0.724044
Erythroid.cells	TRMO	-0.22214	3.013486	-0.63251	0.528688	-5.59492	0.629931	0.718085
Erythroid.cells	GM46652	0.519845	-0.73309	0.632345	0.528793	-5.08118	0.716852	0.796902
Erythroid.cells	ZFP36L2	-0.11419	8.376464	-0.63217	0.528906	-6.74189	0.524161	0.618562
Erythroid.cells	RPN2	-0.11792	6.097946	-0.63208	0.528965	-6.18894	0.5666	0.659065
Erythroid.cells	BC051226	-0.25603	2.179737	-0.63207	0.528971	-5.43008	0.64829	0.734999
Erythroid.cells	COX18	0.238366	3.223872	0.632054	0.528983	-5.53123	0.625384	0.713959
Erythroid.cells	CRACR2B	0.44339	1.009115	0.631863	0.529107	-5.25153	0.675083	0.759345
Erythroid.cells	FAAP100	0.251378	3.097229	0.631583	0.529289	-5.46343	0.628335	0.716622
Erythroid.cells	RAPGEF3	-0.44667	1.358645	-0.63144	0.52938	-5.19191	0.66718	0.752145
Erythroid.cells	ACAD11	0.264842	2.331168	0.631347	0.529442	-5.44203	0.645177	0.732082
Erythroid.cells	TUBB4B	-0.16997	6.422022	-0.63117	0.529558	-6.35961	0.560583	0.653401
Erythroid.cells	BABAM2	0.094941	7.216423	0.631149	0.529572	-6.45319	0.545557	0.639116
Erythroid.cells	LPL	0.194614	5.132487	0.630923	0.529719	-6.22366	0.585998	0.677378
Erythroid.cells	WNT5B	0.347874	1.562043	0.630785	0.529809	-5.29396	0.662618	0.748144
Erythroid.cells	GATAD2B	-0.09816	7.433227	-0.63064	0.5299	-6.50115	0.541607	0.635379
Erythroid.cells	PUS10	-0.18353	4.485499	-0.6306	0.529926	-5.82105	0.599163	0.689719
Erythroid.cells	ANAPC16	0.103319	5.656735	0.630324	0.530109	-6.17624	0.575554	0.667701
Erythroid.cells	TIGIT	0.348227	-0.09862	0.630182	0.530201	-5.39451	0.701708	0.783613
Erythroid.cells	TNFAIP6	0.387042	1.129558	0.63017	0.530209	-5.49031	0.672579	0.75734
Erythroid.cells	PDSS1	0.218096	4.315269	0.630167	0.530211	-5.7378	0.602678	0.69312
Erythroid.cells	NT5C2	0.171589	5.867578	0.6301	0.530254	-6.06451	0.57141	0.663804
Erythroid.cells	HGF	0.306136	2.743326	0.630095	0.530258	-5.69983	0.636176	0.724112
Erythroid.cells	6030468B1	-0.51718	0.279697	-0.62986	0.530408	-5.18087	0.692701	0.775518
Erythroid.cells	GLRX3	-0.10615	6.625536	-0.62979	0.530456	-6.36148	0.55685	0.650047
Erythroid.cells	CDC42EP2	-0.30136	3.08028	-0.62945	0.530679	-5.60456	0.629118	0.717554
Erythroid.cells	GTF2IRD2	-0.20937	3.944401	-0.62931	0.530771	-5.65513	0.61072	0.700511
Erythroid.cells	PDCD7	0.166981	4.505243	0.628914	0.531027	-5.77285	0.59928	0.689733
Erythroid.cells	FIBP	0.157608	4.462532	0.62863	0.531212	-5.79638	0.600237	0.690577
Erythroid.cells	POGLUT2	0.401927	1.00166	0.628622	0.531218	-5.23742	0.676232	0.760359
Erythroid.cells	PCMT1	0.094077	6.63523	0.628346	0.531397	-6.37082	0.557179	0.650073
Erythroid.cells	MYD88	-0.19346	4.609439	-0.62829	0.531434	-5.79732	0.597254	0.687789
Erythroid.cells	EIF4E	-0.12	6.950147	-0.62828	0.531437	-6.43298	0.55121	0.644398
Erythroid.cells	VAPA	0.063521	8.028548	0.628117	0.531546	-6.62175	0.531279	0.625396
Erythroid.cells	TMUB2	-0.21868	3.181282	-0.6281	0.531558	-5.56927	0.627321	0.715744
Erythroid.cells	MAP2K4	-0.14616	6.321239	-0.62798	0.531639	-6.18658	0.56322	0.655892
Erythroid.cells	HEXIM1	-0.16796	5.48831	-0.62763	0.531861	-6.04064	0.579673	0.671452
Erythroid.cells	WDR83OS	0.114816	6.074614	0.627582	0.531895	-6.26137	0.56814	0.660599
Erythroid.cells	SPACA9	0.309163	1.758151	0.627365	0.532037	-5.33125	0.659117	0.744966
Erythroid.cells	2700097O(	-0.20513	3.090234	-0.6273	0.532076	-5.71911	0.629542	0.717873
Erythroid.cells	SNRPE	-0.09585	7.064225	-0.6266	0.532537	-6.48552	0.549428	0.642889
Erythroid.cells	ANGEL2	-0.12932	5.052327	-0.62657	0.532556	-5.9306	0.588629	0.679926
Erythroid.cells	CCL3	0.338135	5.069599	0.62657	0.532556	-6.08388	0.58828	0.679599
Erythroid.cells	SCD2	-0.23041	4.726396	-0.62646	0.53263	-5.81604	0.595253	0.686123

Erythroid.cells	GM10658	0.348293	1.776179	0.626416	0.532656	-5.31856	0.658881	0.744824
Erythroid.cells	9530034E1	0.430139	0.330435	0.626222	0.532783	-5.14901	0.692586	0.775423
Erythroid.cells	2210408I2	-0.26949	3.277757	-0.62613	0.532844	-5.53079	0.625656	0.714475
Erythroid.cells	WDR7	-0.18275	5.188411	-0.62611	0.532858	-5.92881	0.585887	0.677471
Erythroid.cells	HK3	-0.42516	2.677613	-0.62611	0.532859	-5.47635	0.638722	0.726504
Erythroid.cells	CDKN2D	0.118758	6.09877	0.626069	0.532882	-6.27066	0.567883	0.660525
Erythroid.cells	SKA2	0.236633	3.293834	0.626057	0.53289	-5.63687	0.62531	0.714166
Erythroid.cells	RNF220	0.149986	5.723291	0.625842	0.533031	-6.0422	0.575291	0.667552
Erythroid.cells	GM10353	0.255938	2.745067	0.625792	0.533063	-5.5096	0.637299	0.725251
Erythroid.cells	TRIM34A	-0.31185	3.308515	-0.62564	0.533166	-5.55869	0.625097	0.713975
Erythroid.cells	GM37768	-0.31281	2.087377	-0.62555	0.533223	-5.36441	0.651955	0.738645
Erythroid.cells	FAM32A	0.10897	5.312501	0.625347	0.533354	-6.09033	0.583566	0.675355
Erythroid.cells	MRPL46	0.207471	3.264758	0.625258	0.533412	-5.63771	0.626117	0.714979
Erythroid.cells	FMN1	0.295555	3.350464	0.625085	0.533525	-5.6592	0.624333	0.713276
Erythroid.cells	CBX6	-0.25816	2.859775	-0.62486	0.53367	-5.46521	0.635024	0.723081
Erythroid.cells	KMT2D	0.107965	5.945956	0.624708	0.533771	-6.16446	0.571132	0.663575
Erythroid.cells	NUDT21	-0.10723	6.148811	-0.62464	0.533819	-6.27607	0.567176	0.659838
Erythroid.cells	HIST1H2AB	0.476561	1.438759	0.62447	0.533927	-5.37423	0.666907	0.752267
Erythroid.cells	XIAP	0.088485	6.725324	0.62444	0.533947	-6.33981	0.556094	0.649354
Erythroid.cells	COASY	0.229597	3.053875	0.62441	0.533966	-5.62059	0.630793	0.719228
Erythroid.cells	ZBTB1	-0.14615	5.505188	-0.62434	0.534011	-6.08232	0.579827	0.671833
Erythroid.cells	GM15726	-0.50383	2.074088	-0.62427	0.53406	-5.3711	0.652453	0.7392
Erythroid.cells	GM16279	-0.45396	1.054801	-0.62402	0.534221	-5.20164	0.675812	0.760568
Erythroid.cells	GM26759	-0.32156	3.123372	-0.62399	0.534244	-5.43975	0.629296	0.718066
Erythroid.cells	SPTBN1	-0.12228	6.620496	-0.62392	0.534287	-6.40082	0.558101	0.651484
Erythroid.cells	TOB2	0.116467	6.656821	0.62381	0.534359	-6.35383	0.557408	0.650875
Erythroid.cells	PUS3	-0.25269	2.37825	-0.62357	0.534515	-5.41641	0.645659	0.733231
Erythroid.cells	CRCP	0.129791	4.449755	0.623487	0.53457	-5.91588	0.60123	0.692162
Erythroid.cells	SLC25A27	0.442271	0.260835	0.623447	0.534596	-5.13647	0.69459	0.777667
Erythroid.cells	4931414P1	0.336513	2.099255	0.623416	0.534616	-5.37235	0.651898	0.738942
Erythroid.cells	POLG	-0.19382	3.808912	-0.62341	0.534621	-5.72184	0.614623	0.704619
Erythroid.cells	SFMBT1	-0.12874	6.08199	-0.62327	0.534714	-6.18168	0.56852	0.661445
Erythroid.cells	NAPEPLD	0.418335	1.056502	0.622914	0.534944	-5.20993	0.676026	0.760876
Erythroid.cells	TMEM214	-0.15749	4.338367	-0.62276	0.535048	-5.76917	0.603762	0.694498
Erythroid.cells	NPAS2	0.489906	0.089778	0.62256	0.535176	-5.1565	0.698967	0.781614
Erythroid.cells	AVEN	0.169375	4.926701	0.622559	0.535177	-5.96049	0.591683	0.683247
Erythroid.cells	H2-EB2	0.377582	0.547733	0.622426	0.535264	-5.34533	0.688002	0.771757
Erythroid.cells	LTC4S	0.619463	1.066227	0.622372	0.535299	-5.34145	0.675799	0.760747
Erythroid.cells	TUBG1	-0.27874	3.393021	-0.62228	0.535357	-5.62646	0.623715	0.713094
Erythroid.cells	EIF2A	0.105958	5.395584	0.622252	0.535378	-6.07621	0.582238	0.674439
Erythroid.cells	ORC1	-0.25791	3.345968	-0.62217	0.535434	-5.61732	0.624726	0.714066
Erythroid.cells	CORO1A	-0.10427	8.516071	-0.62197	0.535559	-6.74991	0.523382	0.618387
Erythroid.cells	FAM120C	0.262515	3.050639	0.621713	0.53573	-5.54295	0.631315	0.720075
Erythroid.cells	PROSER1	-0.18692	4.213066	-0.62158	0.535817	-5.7612	0.606593	0.697173
Erythroid.cells	DUSP22	0.172842	4.486105	0.621368	0.535956	-5.93048	0.600966	0.691986
Erythroid.cells	ACAT3	0.362889	1.453412	0.621325	0.535984	-5.39146	0.667122	0.752899
Erythroid.cells	PMAIP1	0.208957	4.983149	0.621139	0.536106	-6.06958	0.590794	0.68253

Erythroid.cells	MLX	-0.19263	3.767938	-0.62099	0.536202	-5.67672	0.615989	0.706065
Erythroid.cells	GM6225	0.271753	2.958455	0.620887	0.536272	-5.56341	0.633393	0.722166
Erythroid.cells	WASL	-0.10598	5.483088	-0.62085	0.536294	-6.07809	0.580746	0.673161
Erythroid.cells	COX4I2	0.412993	0.956141	0.620833	0.536307	-5.17722	0.678667	0.763508
Erythroid.cells	KNL1	0.249166	5.131736	0.620616	0.536449	-6.11745	0.587789	0.679792
Erythroid.cells	TMEM132A	0.396662	0.400445	0.62056	0.536485	-5.22031	0.691811	0.775412
Erythroid.cells	MVP	-0.15795	5.108561	-0.6205	0.536524	-5.93774	0.588257	0.680243
Erythroid.cells	1700010K2	0.371382	0.709295	0.62041	0.536584	-5.17804	0.684474	0.76879
Erythroid.cells	GRAMD2	-0.38557	0.533275	-0.62027	0.536675	-5.2306	0.688646	0.772562
Erythroid.cells	1-Sep	-0.10977	6.037433	-0.62026	0.536679	-6.36359	0.569816	0.662881
Erythroid.cells	MINDY1	0.178316	3.987424	0.620147	0.536756	-5.74232	0.611356	0.701848
Erythroid.cells	AKAP10	0.154119	5.860095	0.620123	0.536772	-6.08368	0.573289	0.666196
Erythroid.cells	RND3	0.261757	2.943599	0.619974	0.53687	-5.6673	0.633761	0.722577
Erythroid.cells	MED19	0.160608	3.778351	0.619717	0.537038	-5.72274	0.615933	0.706104
Erythroid.cells	GM16638	-0.35082	2.040296	-0.61951	0.537176	-5.2509	0.654022	0.741131
Erythroid.cells	UBE2T	-0.23754	4.06115	-0.6194	0.537247	-5.75075	0.610069	0.700642
Erythroid.cells	ZFP239	-0.50251	-0.06752	-0.61924	0.537349	-5.06276	0.703386	0.785912
Erythroid.cells	CHCHD3	-0.07283	6.768694	-0.61921	0.537373	-6.41057	0.555971	0.649818
Erythroid.cells	FANCB	-0.42798	0.677958	-0.61895	0.537538	-5.1415	0.685627	0.769883
Erythroid.cells	MAG	-0.51642	0.11897	-0.61888	0.537586	-5.12686	0.698987	0.781947
Erythroid.cells	MLH3	-0.31578	2.01272	-0.61877	0.537656	-5.34368	0.654781	0.741901
Erythroid.cells	ARHGEF5	-0.52668	0.327235	-0.61848	0.537847	-5.10283	0.694089	0.77757
Erythroid.cells	GPN1	0.217612	3.042468	0.618471	0.537855	-5.55254	0.632044	0.721046
Erythroid.cells	SNAPC4	-0.35671	1.856185	-0.6181	0.538095	-5.3239	0.658637	0.74534
Erythroid.cells	RACGAP1	0.227296	5.205247	0.617866	0.538252	-6.08137	0.586993	0.679076
Erythroid.cells	MS4A8A	0.436453	0.670009	0.617843	0.538267	-5.36169	0.686203	0.77039
Erythroid.cells	GPR35	0.312156	2.516821	0.617687	0.538369	-5.56345	0.643855	0.731873
Erythroid.cells	EYA3	-0.14834	5.473081	-0.61762	0.538414	-6.00578	0.581624	0.674096
Erythroid.cells	RND1	0.369774	1.145338	0.617576	0.538442	-5.3096	0.675038	0.760374
Erythroid.cells	CSNK2B	-0.09325	7.247784	-0.61744	0.53853	-6.50913	0.547364	0.641649
Erythroid.cells	MEGF8	-0.40864	0.288303	-0.61719	0.538698	-5.16461	0.695337	0.778777
Erythroid.cells	BDH1	0.217239	3.282333	0.617185	0.538699	-5.81416	0.627129	0.716587
Erythroid.cells	CNDP2	-0.16881	5.051865	-0.61716	0.538716	-5.92132	0.590118	0.682153
Erythroid.cells	TNRC18	-0.10839	5.931838	-0.61707	0.538774	-6.12674	0.572575	0.665649
Erythroid.cells	SASH1	0.225399	5.303699	0.616965	0.538843	-6.02333	0.585048	0.677423
Erythroid.cells	SPIDR	-0.14829	5.228567	-0.61677	0.538971	-5.95371	0.586631	0.678912
Erythroid.cells	TRAPPC10	-0.13445	5.170629	-0.61633	0.539263	-5.96869	0.58805	0.680067
Erythroid.cells	HIST1H2AI	-0.40606	2.588783	-0.61576	0.539632	-5.58147	0.642983	0.730884
Erythroid.cells	USP42	-0.18295	3.500066	-0.61572	0.539663	-5.64221	0.623119	0.712587
Erythroid.cells	LCA5	-0.39718	1.313513	-0.61543	0.53985	-5.218	0.672043	0.757394
Erythroid.cells	PRKACA	-0.16815	4.156404	-0.61534	0.539912	-5.74767	0.609351	0.699824
Erythroid.cells	ETOHD2	0.23929	2.615429	0.615158	0.540031	-5.47124	0.642553	0.730496
Erythroid.cells	GM26532	0.209562	5.294849	0.615138	0.540044	-5.89683	0.585996	0.678008
Erythroid.cells	SNX12	0.139228	4.718214	0.614931	0.54018	-5.83517	0.597732	0.689019
Erythroid.cells	CIART	0.318986	2.328452	0.614921	0.540187	-5.4108	0.648963	0.736396
Erythroid.cells	MMP27	0.462755	-0.20195	0.614707	0.540327	-5.13047	0.708278	0.790091
Erythroid.cells	ASPM	0.320521	3.870548	0.614286	0.540604	-5.801	0.615619	0.705636

Erythroid.cells	GGNBP1	0.414614	1.044976	0.6142	0.540661	-5.24606	0.678574	0.763323
Erythroid.cells	SLC39A1	-0.11247	6.161792	-0.61414	0.540697	-6.21709	0.569057	0.662033
Erythroid.cells	FBXL4	0.271201	2.64655	0.614052	0.540758	-5.44437	0.642113	0.730082
Erythroid.cells	COX7A2L	0.088589	6.644119	0.613983	0.540803	-6.36608	0.559739	0.653202
Erythroid.cells	MMGT1	0.258629	2.857236	0.613926	0.540841	-5.49014	0.637469	0.725816
Erythroid.cells	1700020L2	-0.41749	0.390371	-0.61388	0.540868	-5.16341	0.694081	0.777327
Erythroid.cells	OAS2	-0.43741	1.571395	-0.61371	0.540985	-5.37884	0.666428	0.752279
Erythroid.cells	PTPN9	0.137799	5.706865	0.613568	0.541076	-6.09501	0.578089	0.670517
Erythroid.cells	CDK19	0.119284	6.390727	0.613409	0.541181	-6.31483	0.564742	0.657871
Erythroid.cells	SNX1	-0.10194	5.483558	-0.61332	0.541237	-6.07795	0.582572	0.674715
Erythroid.cells	KCNQ5	0.118903	7.024096	0.613164	0.541342	-6.62249	0.552662	0.646427
Erythroid.cells	RWDD2B	0.351141	1.849393	0.6131	0.541385	-5.31101	0.660181	0.74656
Erythroid.cells	UBLCP1	-0.13634	4.773952	-0.61299	0.541457	-5.9525	0.596973	0.688228
Erythroid.cells	SINHCAF	-0.13682	4.557551	-0.61287	0.541535	-5.99521	0.601444	0.69241
Erythroid.cells	EPS8	0.184524	4.963297	0.612595	0.541716	-6.30854	0.593254	0.684667
Erythroid.cells	GM28050	-0.46434	0.410517	-0.61233	0.541889	-5.1115	0.694075	0.777124
Erythroid.cells	SLC16A12	0.430327	0.427781	0.612234	0.541954	-5.23402	0.693661	0.77677
Erythroid.cells	RDH9	-0.41334	0.805989	-0.61213	0.542025	-5.29479	0.684664	0.768696
Erythroid.cells	NUDT7	0.305288	1.74453	0.612045	0.542079	-5.4584	0.662848	0.748921
Erythroid.cells	AHSA2	0.176603	3.646243	0.61201	0.542102	-5.70223	0.620814	0.710334
Erythroid.cells	NME2	0.105355	8.557653	0.611857	0.542203	-6.80156	0.524738	0.61961
Erythroid.cells	RSL1D1	-0.12015	6.043414	-0.61174	0.542279	-6.21464	0.571819	0.664548
Erythroid.cells	CZIB	0.190449	3.658795	0.611608	0.542367	-5.69143	0.620638	0.710202
Erythroid.cells	UNC5CL	0.436071	0.465408	0.611478	0.542452	-5.30232	0.692894	0.776182
Erythroid.cells	DHRS1	0.186489	4.762169	0.611085	0.542711	-5.97348	0.597714	0.68888
Erythroid.cells	ACBD3	-0.11138	5.707973	-0.61107	0.542718	-6.08465	0.578629	0.670977
Erythroid.cells	PODNL1	0.392768	-1.02387	0.610813	0.54289	-5.14917	0.729643	0.809065
Erythroid.cells	PPA2	0.154656	4.580694	0.610761	0.542925	-5.93897	0.601452	0.692369
Erythroid.cells	PDE1C	-0.38072	3.989093	-0.61074	0.542937	-5.62247	0.613807	0.703865
Erythroid.cells	CCL8	-0.48869	-1.46778	-0.61071	0.542957	-5.00266	0.740915	0.819087
Erythroid.cells	CSF2RA	0.143836	5.196056	0.610471	0.543116	-6.09479	0.588941	0.680634
Erythroid.cells	AP4B1	0.290936	2.273778	0.6104	0.543162	-5.41324	0.65122	0.738318
Erythroid.cells	TRIB1	0.173133	5.765307	0.610333	0.543207	-6.11998	0.577554	0.669951
Erythroid.cells	RCBTB1	-0.17788	3.826201	-0.61009	0.543367	-5.74617	0.617321	0.707162
Erythroid.cells	DHX58	0.332582	2.999964	0.610069	0.543381	-5.53136	0.635128	0.723609
Erythroid.cells	RUNX2	0.317993	4.240789	0.60994	0.543466	-5.77925	0.608583	0.699104
Erythroid.cells	E430024P1	-0.48013	0.53335	-0.60978	0.543574	-5.1338	0.691518	0.775032
Erythroid.cells	TM7SF2	0.319159	1.754706	0.609753	0.543589	-5.33302	0.66298	0.749173
Erythroid.cells	AGPAT3	0.137893	5.164345	0.609653	0.543655	-6.09371	0.589582	0.681373
Erythroid.cells	TBPL1	0.121889	4.928003	0.60964	0.543664	-6.03641	0.594384	0.685871
Erythroid.cells	GM16283	0.421396	0.252287	0.609549	0.543724	-5.12181	0.69826	0.781113
Erythroid.cells	COMMD10	-0.15512	4.694613	-0.6095	0.543756	-5.90957	0.599167	0.690345
Erythroid.cells	CATSPERG1	-0.47969	0.160917	-0.60941	0.543816	-5.1777	0.700467	0.783109
Erythroid.cells	6430590AC	0.29234	1.647577	0.609176	0.54397	-5.2898	0.665514	0.751522
Erythroid.cells	ABCD1	-0.15791	4.771292	-0.60901	0.544083	-5.81847	0.597664	0.689048
Erythroid.cells	CHKB	0.156323	4.306938	0.608983	0.544097	-5.78355	0.607275	0.698021
Erythroid.cells	GM42595	-0.40866	0.917394	-0.60894	0.544128	-5.17966	0.682494	0.767047

Erythroid.cells	ARL10	0.263463	2.295947	0.608757	0.544247	-5.44072	0.650869	0.738255
Erythroid.cells	MKNK2	-0.1005	6.23257	-0.60842	0.544471	-6.30002	0.568606	0.661642
Erythroid.cells	GM44777	-0.4415	0.565153	-0.60841	0.544474	-5.15326	0.691032	0.774633
Erythroid.cells	TRAM1	0.082444	7.121393	0.608337	0.544524	-6.45756	0.551576	0.645471
Erythroid.cells	SARAF	-0.12552	5.365255	-0.60822	0.544599	-6.11307	0.585778	0.677813
Erythroid.cells	1700029H1	0.336759	1.58753	0.608118	0.544668	-5.29532	0.6671	0.75294
Erythroid.cells	4-Sep	0.362147	1.735848	0.607976	0.544762	-5.35788	0.663696	0.749842
Erythroid.cells	MAP3K20	0.197875	3.798494	0.607935	0.544789	-5.83277	0.618175	0.70803
Erythroid.cells	XKR6	0.37273	0.862903	0.60782	0.544865	-5.26784	0.684007	0.768253
Erythroid.cells	WASHC1	0.165319	4.182087	0.607694	0.544949	-5.69472	0.610115	0.700511
Erythroid.cells	TMPRSS5	-0.42586	0.722493	-0.60755	0.545045	-5.16808	0.687392	0.771316
Erythroid.cells	DAPK3	0.148838	4.42231	0.607403	0.54514	-5.83925	0.605128	0.695901
Erythroid.cells	PTPRO	0.28645	2.363394	0.607324	0.545193	-5.6371	0.649565	0.736962
Erythroid.cells	KANSL2	-0.13077	5.119718	-0.60728	0.545222	-6.00352	0.590808	0.682551
Erythroid.cells	HNRNPU	-0.08038	8.26781	-0.60715	0.545306	-6.68074	0.53051	0.625315
Erythroid.cells	ANO6	-0.13706	6.75402	-0.60706	0.54537	-6.30693	0.558658	0.652234
Erythroid.cells	RNF13	0.121436	6.065838	0.606793	0.545543	-6.1687	0.572025	0.664911
Erythroid.cells	CCRL2	-0.34776	4.741765	-0.60671	0.545596	-6.03037	0.598604	0.689888
Erythroid.cells	SCUBE2	-0.52878	-0.26058	-0.60668	0.54562	-5.14787	0.711219	0.79283
Erythroid.cells	IGHD	-0.20521	3.245617	-0.60652	0.545721	-5.76077	0.630206	0.719244
Erythroid.cells	MTMR9	0.165319	3.972449	0.606516	0.545726	-5.69868	0.614641	0.704842
Erythroid.cells	SNRNP200	-0.15719	5.038857	-0.6063	0.545872	-5.94038	0.59262	0.6843
Erythroid.cells	C130026I2	-0.30709	2.944263	-0.60599	0.546077	-5.49959	0.636962	0.725448
Erythroid.cells	SYNJ1	-0.10411	6.731012	-0.60583	0.546177	-6.4115	0.559307	0.652875
Erythroid.cells	FAM192A	0.134579	4.607769	0.605776	0.546215	-5.86465	0.601537	0.692642
Erythroid.cells	CCDC6	0.172853	4.540905	0.605714	0.546257	-5.84909	0.602921	0.693946
Erythroid.cells	GM13822	-0.58592	1.17949	-0.60564	0.546306	-5.27595	0.67692	0.761992
Erythroid.cells	PEBP1	0.099439	6.440655	0.605529	0.546379	-6.33282	0.564892	0.658223
Erythroid.cells	PPP1R9B	0.194533	4.375184	0.605411	0.546457	-5.77093	0.606364	0.697197
Erythroid.cells	1600014C1	-0.23032	4.275444	-0.60536	0.546492	-5.90989	0.608446	0.699135
Erythroid.cells	SLC16A1	-0.16727	3.905544	-0.60535	0.546499	-5.92252	0.616233	0.70637
Erythroid.cells	METAP1D	0.185287	3.768583	0.605114	0.546654	-5.79604	0.619226	0.709128
Erythroid.cells	CAV2	0.251128	2.771749	0.605051	0.546695	-5.53951	0.640844	0.729095
Erythroid.cells	N6AMT1	0.262578	2.597729	0.60476	0.546888	-5.46757	0.644852	0.7327
Erythroid.cells	NRM	-0.17007	5.099836	-0.60451	0.547052	-6.06658	0.59179	0.683512
Erythroid.cells	ZFP593	0.241179	3.359126	0.604246	0.547227	-5.60163	0.628411	0.717531
Erythroid.cells	ABCC1	0.215523	4.467713	0.603462	0.547746	-5.75386	0.605408	0.695929
Erythroid.cells	PRKCA	-0.14368	7.401898	-0.60323	0.547903	-6.47747	0.547553	0.641458
Erythroid.cells	CLEC2D	0.237914	4.7558	0.603197	0.547922	-5.98369	0.599501	0.690497
Erythroid.cells	NEK4	0.369482	1.202148	0.603039	0.548027	-5.28575	0.677537	0.762265
Erythroid.cells	SPRYD7	-0.27856	2.133927	-0.60301	0.548043	-5.4225	0.656109	0.742775
Erythroid.cells	SLC25A40	-0.22347	3.227708	-0.60283	0.548166	-5.60833	0.631916	0.720517
Erythroid.cells	PHKA1	0.223786	3.411651	0.60257	0.548337	-5.66015	0.627958	0.71695
Erythroid.cells	GADD45G	0.240836	4.465474	0.602503	0.548381	-5.86792	0.605608	0.696236
Erythroid.cells	KCNRG	-0.25863	2.320944	-0.6024	0.548451	-5.48962	0.651998	0.739057
Erythroid.cells	RCC1L	-0.24434	3.072184	-0.60233	0.548497	-5.54025	0.63534	0.723776
Erythroid.cells	BAMBI	0.247518	3.967331	0.602321	0.548502	-5.73139	0.616067	0.705969

Erythroid.cells	MDGA1	-0.46182	0.010829	-0.6019	0.548779	-5.16938	0.706372	0.788183
Erythroid.cells	OASL2	-0.34753	3.934807	-0.60171	0.54891	-6.04959	0.61703	0.706754
Erythroid.cells	PCYOX1	-0.16827	4.579765	-0.60166	0.548941	-5.84208	0.603502	0.694179
Erythroid.cells	SOX6	0.319942	1.905309	0.601581	0.548993	-5.70035	0.661701	0.747822
Erythroid.cells	GPD2	0.148876	6.625661	0.601238	0.54922	-6.48593	0.562789	0.655866
Erythroid.cells	KCNMB4	-0.38767	1.763375	-0.60092	0.54943	-5.3271	0.66529	0.751013
Erythroid.cells	RHOF	-0.23843	3.558194	-0.60077	0.549529	-5.67935	0.625397	0.714514
Erythroid.cells	NCAPG	0.289072	3.22832	0.600761	0.549536	-5.64824	0.632539	0.721103
Erythroid.cells	NASP	-0.11513	6.308139	-0.60064	0.549617	-6.28435	0.569059	0.661892
Erythroid.cells	PPP2R5A	-0.07078	7.861178	-0.6006	0.549646	-6.63559	0.539638	0.633887
Erythroid.cells	GTF3A	0.139749	4.569389	0.600379	0.54979	-5.84683	0.604118	0.694741
Erythroid.cells	BHLHE40	-0.15725	6.323106	-0.6002	0.549908	-6.40081	0.568877	0.661636
Erythroid.cells	SLC6A19	-0.49706	-0.35949	-0.60014	0.54995	-5.07857	0.716006	0.796768
Erythroid.cells	GOLGB1	-0.1297	5.4427	-0.60006	0.549999	-6.00836	0.586302	0.678054
Erythroid.cells	ZFP414	0.217949	3.443642	0.599859	0.550135	-5.62162	0.628072	0.716868
Erythroid.cells	GAB3	0.209916	4.746655	0.599767	0.550196	-5.8179	0.600556	0.691346
Erythroid.cells	SIK1	-0.10956	6.868808	-0.59953	0.55035	-6.35798	0.558467	0.65175
Erythroid.cells	GPT	0.344673	1.203309	0.599522	0.550358	-5.35753	0.678538	0.76304
Erythroid.cells	IRF9	-0.24286	4.515749	-0.59928	0.550517	-5.85677	0.605488	0.695963
Erythroid.cells	IL10RA	-0.18472	4.971561	-0.59905	0.55067	-5.90649	0.596181	0.687221
Erythroid.cells	TEDC2	0.391917	0.906107	0.598675	0.550921	-5.18751	0.685849	0.769705
Erythroid.cells	SLIT2	-0.51988	0.983319	-0.59865	0.550939	-5.18063	0.684023	0.768056
Erythroid.cells	DDX52	0.135397	4.930462	0.59852	0.551024	-5.92853	0.597095	0.688216
Erythroid.cells	BRAF	-0.12049	7.588384	-0.59845	0.551072	-6.52577	0.545154	0.639161
Erythroid.cells	GTSE1	-0.4846	1.404909	-0.59842	0.551092	-5.30084	0.674145	0.759188
Erythroid.cells	SEC22B	-0.122	5.449712	-0.59839	0.551107	-6.06223	0.58655	0.678382
Erythroid.cells	ANAPC15	-0.15618	4.974484	-0.59812	0.551287	-5.92053	0.596321	0.687461
Erythroid.cells	TMED2	0.074786	8.136615	0.597725	0.551552	-6.67211	0.535227	0.629601
Erythroid.cells	GM45606	-0.49439	0.477564	-0.59769	0.551576	-5.12672	0.696299	0.779136
Erythroid.cells	STIP1	0.145491	5.419076	0.597653	0.551599	-6.04077	0.587361	0.679097
Erythroid.cells	PHF6	-0.14703	4.940138	-0.59751	0.551697	-5.95887	0.597094	0.688244
Erythroid.cells	NINL	0.410305	0.999292	0.59721	0.551894	-5.20165	0.683872	0.768006
Erythroid.cells	TMEM173	0.182791	4.250581	0.597148	0.551935	-5.78925	0.611406	0.701604
Erythroid.cells	ABCG3	-0.2639	4.394356	-0.59707	0.551984	-5.72282	0.608393	0.698802
Erythroid.cells	CD200R4	0.37642	1.662182	0.597065	0.55199	-5.53396	0.668409	0.753988
Erythroid.cells	GM26670	-0.39413	0.402458	-0.59705	0.552001	-5.10183	0.698107	0.780842
Erythroid.cells	DHX30	0.167936	4.509922	0.596939	0.552074	-5.81102	0.605982	0.696557
Erythroid.cells	PRX	0.417211	1.104861	0.596918	0.552087	-5.19678	0.681385	0.765756
Erythroid.cells	GM46430	0.275063	1.969969	0.596909	0.552094	-5.32776	0.661352	0.747564
Erythroid.cells	MVB12A	0.114372	5.948563	0.59664	0.552273	-6.17867	0.576874	0.669265
Erythroid.cells	DTNB	0.152288	5.293687	0.596609	0.552293	-6.05684	0.589973	0.68159
Erythroid.cells	NCKAP5L	-0.21751	4.041979	-0.5963	0.552501	-5.72205	0.616054	0.705844
Erythroid.cells	TRAPPC6A	0.11886	5.057196	0.595952	0.55273	-5.96924	0.595061	0.686303
Erythroid.cells	KAZN	-0.50895	0.348768	-0.59587	0.552787	-5.12559	0.699828	0.782338
Erythroid.cells	SLC2A3	0.23828	4.519005	0.595724	0.552881	-5.72994	0.606161	0.696676
Erythroid.cells	MKLN1	-0.0899	7.159858	-0.5957	0.552899	-6.47022	0.553712	0.647293
Erythroid.cells	TOPORS	-0.15141	5.749708	-0.59545	0.553065	-6.09789	0.581093	0.673261

Erythroid.cells	RNF8	-0.13119	4.800789	-0.59538	0.553112	-5.96136	0.600323	0.691302
Erythroid.cells	USP16	-0.1373	5.208005	-0.59526	0.553189	-5.99352	0.59199	0.683513
Erythroid.cells	IFT80	-0.2437	3.626112	-0.59526	0.553192	-5.68319	0.625059	0.714283
Erythroid.cells	1700028E1	0.326992	1.36693	0.595205	0.553227	-5.24857	0.675663	0.760614
Erythroid.cells	INPP1	0.173729	4.03687	0.595192	0.553235	-5.88198	0.61629	0.706174
Erythroid.cells	COG6	-0.23056	3.137898	-0.59489	0.553438	-5.54248	0.635774	0.724082
Erythroid.cells	DBT	-0.18269	3.826101	-0.5947	0.553565	-5.82865	0.620893	0.71035
Erythroid.cells	FAM221B	0.433296	0.343919	0.594612	0.553622	-5.17119	0.70008	0.782561
Erythroid.cells	CCN1	0.485413	1.098666	0.594598	0.553631	-5.27783	0.682077	0.766328
Erythroid.cells	XYLB	0.363552	0.922746	0.594567	0.553652	-5.27322	0.686231	0.770083
Erythroid.cells	ASAH2	0.269087	2.945427	0.594404	0.55376	-5.57376	0.640016	0.727973
Erythroid.cells	AGK	0.227023	3.121713	0.594265	0.553852	-5.53004	0.636143	0.724457
Erythroid.cells	ILRUN	0.116271	5.988088	0.59421	0.553889	-6.26567	0.576489	0.6689
Erythroid.cells	MMP9	0.455589	1.381978	0.594176	0.553912	-5.35966	0.675457	0.760367
Erythroid.cells	CEBPZOS	0.148693	4.521965	0.594077	0.553978	-5.92028	0.606235	0.696774
Erythroid.cells	INO80C	-0.19659	3.401246	-0.59369	0.554233	-5.62979	0.630227	0.718913
Erythroid.cells	PIM2	0.224938	3.150665	0.593662	0.554254	-5.59645	0.635687	0.723943
Erythroid.cells	IL1B	-0.27836	5.12575	-0.59357	0.554315	-6.2282	0.593957	0.685225
Erythroid.cells	ADTRP	-0.30072	1.835711	-0.59315	0.554593	-5.49413	0.665365	0.751018
Erythroid.cells	MOSMO	-0.15123	4.576833	-0.59312	0.554614	-5.89433	0.60545	0.695871
Erythroid.cells	SPSB3	0.150187	3.587341	0.592666	0.554918	-5.76721	0.626622	0.715455
Erythroid.cells	C030006K1	-0.37661	1.498775	-0.59249	0.555035	-5.25126	0.673379	0.75827
Erythroid.cells	GM38604	-0.29893	1.796583	-0.59244	0.555071	-5.3532	0.666497	0.752019
Erythroid.cells	GM24362	-0.4319	0.694716	-0.59237	0.555118	-5.13709	0.692324	0.775413
Erythroid.cells	CDKL5	0.369069	2.066071	0.592246	0.555198	-5.36281	0.660332	0.746493
Erythroid.cells	SKIV2L	0.17898	3.938774	0.592117	0.555283	-5.73925	0.619092	0.708631
Erythroid.cells	H2AFV	-0.10629	8.095101	-0.592	0.555362	-6.73012	0.536935	0.631213
Erythroid.cells	EEF1AKMT:	-0.25115	3.325831	-0.59193	0.555411	-5.56564	0.632287	0.720834
Erythroid.cells	BOLA2	-0.13077	5.686348	-0.59185	0.555461	-6.13785	0.583034	0.675005
Erythroid.cells	MTERF4	0.298355	1.913823	0.591744	0.555532	-5.36617	0.663808	0.749721
Erythroid.cells	DCTD	-0.39596	1.018621	-0.59162	0.555614	-5.15355	0.684628	0.768608
Erythroid.cells	GM21860	0.621106	-0.14186	0.591615	0.555618	-5.12346	0.712613	0.793776
Erythroid.cells	RNF187	-0.11054	6.12757	-0.5916	0.555625	-6.22873	0.574285	0.666764
Erythroid.cells	CDC42	-0.05467	8.943013	-0.59153	0.555677	-6.82091	0.521646	0.616524
Erythroid.cells	YES1	0.231644	4.740029	0.591336	0.555804	-5.96363	0.602347	0.693074
Erythroid.cells	NUMA1	0.112649	5.705606	0.591208	0.555889	-6.13844	0.582742	0.67472
Erythroid.cells	GM20219	0.432151	0.646352	0.590895	0.556098	-5.14452	0.693775	0.776788
Erythroid.cells	SPTLC2	0.094966	6.245992	0.590572	0.556314	-6.22089	0.572298	0.664791
Erythroid.cells	MPHOSPH9	-0.17105	4.258614	-0.59057	0.556315	-5.82424	0.612683	0.702609
Erythroid.cells	TMEM230	-0.13299	4.742854	-0.59029	0.556503	-5.86399	0.602615	0.693255
Erythroid.cells	GM15879	0.468931	0.515986	0.590268	0.556517	-5.11922	0.697068	0.779734
Erythroid.cells	MYC	0.315614	3.533741	0.590233	0.55654	-5.6868	0.628192	0.716983
Erythroid.cells	PDIA3	-0.08307	8.546649	-0.58989	0.556768	-6.75626	0.52914	0.623672
Erythroid.cells	SNHG6	0.217654	3.414627	0.589889	0.55677	-5.64125	0.630841	0.719444
Erythroid.cells	NR1D2	0.211681	3.478586	0.589864	0.556786	-5.61233	0.629454	0.718165
Erythroid.cells	PES1	0.130659	4.691666	0.589621	0.556949	-5.93567	0.60384	0.69439
Erythroid.cells	ITPK1	0.120232	5.403224	0.589541	0.557002	-6.0817	0.589271	0.680805

Erythroid.cells	SLC25A5	-0.08471	7.608641	-0.58932	0.557152	-6.60458	0.546455	0.640342
Erythroid.cells	DDX18	0.137948	4.987355	0.58924	0.557203	-5.9441	0.597766	0.688838
Erythroid.cells	ZFP608	0.165839	6.820434	0.589227	0.557212	-6.38703	0.561373	0.654566
Erythroid.cells	RNF128	-0.23933	2.499728	-0.58891	0.557425	-5.6531	0.651277	0.738336
Erythroid.cells	LRRFIP2	0.106109	5.900501	0.588716	0.557553	-6.18111	0.57943	0.671682
Erythroid.cells	LIPE	0.171648	3.783042	0.588652	0.557596	-5.74242	0.623127	0.712465
Erythroid.cells	ACVRL1	-0.25962	2.619629	-0.58858	0.557647	-5.65542	0.648592	0.735938
Erythroid.cells	IGBP1	-0.14375	4.749526	-0.58856	0.557655	-5.93192	0.602767	0.693585
Erythroid.cells	TMEM240	0.511853	0.240106	0.588531	0.557677	-5.1183	0.704078	0.786259
Erythroid.cells	GPANK1	-0.20293	3.300008	-0.58821	0.557892	-5.58681	0.633676	0.722262
Erythroid.cells	TAGLN2	-0.1292	8.716101	-0.5881	0.557965	-6.76485	0.526378	0.621211
Erythroid.cells	GM26510	-0.20953	4.122817	-0.588	0.558032	-5.70199	0.61599	0.705962
Erythroid.cells	POLK	-0.20872	3.481233	-0.58797	0.558054	-5.60403	0.629735	0.718699
Erythroid.cells	TMTC4	0.355213	1.357794	0.587862	0.558123	-5.34224	0.677546	0.76248
Erythroid.cells	FDXR	0.240404	2.625449	0.587796	0.558167	-5.49217	0.648569	0.736087
Erythroid.cells	METRN	-0.2954	2.538002	-0.58762	0.558283	-5.41928	0.650526	0.737933
Erythroid.cells	NES	-0.496	0.578345	-0.58741	0.558426	-5.18862	0.696019	0.779245
Erythroid.cells	TREM3	0.376154	2.200561	0.587353	0.558464	-5.56956	0.658136	0.744896
Erythroid.cells	ACADL	0.098356	6.734458	0.58733	0.558479	-6.51847	0.563213	0.656587
Erythroid.cells	LONRF1	0.243679	3.091293	0.587284	0.55851	-5.57832	0.638245	0.726679
Erythroid.cells	PCBD2	0.133914	5.24057	0.587107	0.558628	-6.08616	0.592789	0.684517
Erythroid.cells	ZFP991	-0.28058	2.80432	-0.58702	0.558689	-5.47455	0.644585	0.732579
Erythroid.cells	SMS	0.10446	6.477089	0.587	0.5587	-6.24914	0.568195	0.661391
Erythroid.cells	RRAGD	-0.30068	2.299081	-0.58684	0.558807	-5.40736	0.655905	0.743039
Erythroid.cells	ESF1	-0.11504	5.001622	-0.58683	0.558813	-6.01695	0.597671	0.689207
Erythroid.cells	STK38	-0.10532	6.531625	-0.58681	0.558824	-6.32412	0.567135	0.660468
Erythroid.cells	B130034C1	0.327558	1.038829	0.586583	0.558979	-5.18912	0.685127	0.769621
Erythroid.cells	GPRC5C	0.40282	1.29284	0.586316	0.559157	-5.39238	0.679148	0.764311
Erythroid.cells	TAF4B	-0.19433	5.130162	-0.58628	0.559183	-6.03988	0.595112	0.686939
Erythroid.cells	TRPV4	-0.46175	-0.51202	-0.58611	0.559295	-5.10793	0.722811	0.803653
Erythroid.cells	MAP3K8	0.178862	4.64612	0.586065	0.559325	-5.83671	0.605085	0.696297
Erythroid.cells	ZBTB45	0.215179	2.916254	0.585977	0.559384	-5.54517	0.642182	0.730639
Erythroid.cells	GPR157	-0.29413	2.137362	-0.58596	0.559397	-5.34931	0.659652	0.74664
Erythroid.cells	GM4107	0.41341	0.916265	0.585886	0.559444	-5.34672	0.688031	0.772415
Erythroid.cells	PRODH	0.347984	2.780405	0.585853	0.559467	-5.44239	0.645194	0.733407
Erythroid.cells	AMDHD2	-0.18965	3.693973	-0.58578	0.559513	-5.67165	0.625218	0.715017
Erythroid.cells	CD22	0.252754	2.780412	0.585679	0.559583	-5.55088	0.645204	0.733464
Erythroid.cells	CRTC2	-0.18985	4.134514	-0.58546	0.559727	-5.78294	0.615915	0.706398
Erythroid.cells	MRPL24	-0.09738	5.658173	-0.58535	0.559801	-6.16394	0.584536	0.677092
Erythroid.cells	LMLN	-0.31878	1.750464	-0.58512	0.559958	-5.30555	0.668746	0.754987
Erythroid.cells	CKAP5	0.140737	6.118962	0.584772	0.56019	-6.25387	0.575651	0.668678
Erythroid.cells	2610507B1	-0.10497	5.431746	-0.58451	0.560369	-6.03929	0.589374	0.681662
Erythroid.cells	RERG	0.483762	0.522461	0.584495	0.560376	-5.1706	0.697907	0.7814
Erythroid.cells	ERCC5	0.215408	2.909402	0.584418	0.560428	-5.52793	0.642757	0.731267
Erythroid.cells	IRAK2	-0.14254	6.796286	-0.5844	0.560441	-6.498	0.562461	0.656278
Erythroid.cells	SLC9A1	-0.12503	5.416377	-0.58407	0.560659	-6.00564	0.589784	0.682002
Erythroid.cells	GM16268	-0.47493	0.706579	-0.58392	0.56076	-5.15087	0.693602	0.7775

Erythroid.cells	OPA3	-0.14	5.128547	-0.58383	0.560822	-5.955	0.595637	0.687548
Erythroid.cells	CMPK2	0.268943	3.672891	0.583672	0.560927	-5.82273	0.626189	0.715988
Erythroid.cells	POLR3GL	0.178625	3.878267	0.583636	0.560951	-5.66503	0.621781	0.711907
Erythroid.cells	OFCC1	-0.4667	-0.63548	-0.5835	0.56104	-5.11685	0.726499	0.807099
Erythroid.cells	DLG4	-0.34829	3.571009	-0.58333	0.561155	-5.59689	0.628388	0.718127
Erythroid.cells	ATXN1L	-0.23164	2.959513	-0.5832	0.561243	-5.51152	0.641756	0.730512
Erythroid.cells	SERTAD3	-0.21525	4.131362	-0.58312	0.561295	-5.74809	0.616393	0.707108
Erythroid.cells	POLE	0.275666	3.301306	0.583009	0.561371	-5.64021	0.634248	0.723647
Erythroid.cells	CHST8	0.593215	-0.80735	0.582844	0.561482	-5.06992	0.730825	0.811232
Erythroid.cells	GM8797	0.212412	2.19543	0.582804	0.561509	-5.61782	0.658877	0.746329
Erythroid.cells	CDKL1	-0.35521	1.100722	-0.58278	0.561528	-5.46306	0.684231	0.769388
Erythroid.cells	GM11457	0.529973	0.10252	0.582774	0.561529	-5.12709	0.708218	0.791013
Erythroid.cells	ZFP703	-0.2153	4.364442	-0.58252	0.561699	-5.7752	0.611474	0.70266
Erythroid.cells	ACAA2	0.140478	5.01941	0.582465	0.561736	-6.28085	0.597872	0.689965
Erythroid.cells	SARDHOS	-0.61526	0.147254	-0.58239	0.56179	-5.07382	0.707125	0.790105
Erythroid.cells	TM4SF1	0.364225	2.15291	0.582228	0.561895	-5.47626	0.659843	0.747326
Erythroid.cells	SRSF4	-0.08917	6.225555	-0.58215	0.561948	-6.28825	0.573652	0.667254
Erythroid.cells	SLC25A44	0.22692	3.239164	0.582115	0.56197	-5.53592	0.635606	0.725094
Erythroid.cells	F730043M	0.466217	0.260543	0.582103	0.561978	-5.18289	0.704365	0.787684
Erythroid.cells	GZF1	0.261397	2.752325	0.58207	0.562001	-5.49634	0.646353	0.734982
Erythroid.cells	HINT2	0.14152	4.356845	0.582032	0.562026	-5.95274	0.611634	0.702895
Erythroid.cells	CD93	0.195791	3.89899	0.582032	0.562027	-5.82311	0.621338	0.71191
Erythroid.cells	IL17RA	0.150166	5.907729	0.581804	0.562179	-6.13861	0.580025	0.673233
Erythroid.cells	TRDV4	-0.41504	-1.36214	-0.58167	0.562272	-5.15205	0.745122	0.824039
Erythroid.cells	GM31522	-0.35131	-1.00368	-0.58116	0.56261	-5.10951	0.736317	0.816129
Erythroid.cells	KLHL18	-0.17801	4.310869	-0.58042	0.563105	-5.75636	0.613505	0.704264
Erythroid.cells	MNT	-0.17604	4.606728	-0.58031	0.563181	-5.83028	0.607304	0.698491
Erythroid.cells	CLN3	-0.21888	4.768913	-0.58023	0.563233	-5.78066	0.603931	0.695362
Erythroid.cells	SMPD5	-0.42444	-0.18019	-0.58005	0.563357	-5.08403	0.716304	0.797964
Erythroid.cells	SF3B3	-0.0921	6.034328	-0.57984	0.563496	-6.24168	0.578344	0.671339
Erythroid.cells	BST2	0.183439	6.606914	0.579777	0.56354	-6.30073	0.567116	0.660722
Erythroid.cells	TIPARP	0.124377	7.437182	0.579766	0.563547	-6.54457	0.551246	0.645626
Erythroid.cells	A230083N1	-0.49104	0.546139	-0.5785	0.564395	-5.12064	0.699343	0.782566
Erythroid.cells	MAP2K7	-0.14794	4.294187	-0.5785	0.564399	-5.79842	0.614613	0.705107
Erythroid.cells	SSX2IP	0.292795	2.481205	0.578233	0.564577	-5.50865	0.654187	0.741644
Erythroid.cells	CLDND1	0.124745	4.825073	0.577985	0.564744	-5.95103	0.603505	0.694869
Erythroid.cells	SDF2	-0.11696	5.410417	-0.57797	0.564751	-6.07035	0.591502	0.683637
Erythroid.cells	RPF1	-0.11112	5.149471	-0.57796	0.564763	-6.04379	0.596822	0.688622
Erythroid.cells	GM12689	-0.43446	-0.48996	-0.57796	0.564763	-5.12896	0.724816	0.805516
Erythroid.cells	4930404I05	0.481133	0.086501	0.577951	0.564766	-5.17238	0.71053	0.792736
Erythroid.cells	RMI1	-0.18115	3.629414	-0.57792	0.564788	-5.60136	0.628826	0.718402
Erythroid.cells	PARD6A	-0.2766	2.688858	-0.57781	0.564859	-5.4953	0.649523	0.737511
Erythroid.cells	ATMIN	0.23698	2.969649	0.577676	0.564951	-5.58929	0.643271	0.731788
Erythroid.cells	LIG1	-0.19279	5.051661	-0.57754	0.565041	-6.06651	0.598829	0.690588
Erythroid.cells	CTSL	0.11305	6.758061	0.577511	0.565062	-6.46155	0.564818	0.658547
Erythroid.cells	ISY1	-0.11405	6.241546	-0.57746	0.565097	-6.29612	0.574891	0.668086
Erythroid.cells	GNGT2	-0.19671	5.675602	-0.57744	0.565107	-6.0841	0.586148	0.678696

Erythroid.cells	ADAMTS6	0.154964	5.940983	0.577212	0.565263	-6.23787	0.580892	0.673782
Erythroid.cells	ITPKA	-0.4246	0.529225	-0.57718	0.565282	-5.17123	0.699814	0.783249
Erythroid.cells	RSPRY1	-0.10832	5.833379	-0.57702	0.565394	-6.11559	0.583089	0.675839
Erythroid.cells	ADAP2	-0.26308	2.585365	-0.57679	0.56555	-5.67699	0.651991	0.739795
Erythroid.cells	ZDHHC4	0.157319	4.474275	0.576739	0.565581	-5.82812	0.61096	0.701913
Erythroid.cells	GUCA1A	-0.28499	1.4794	-0.5767	0.56561	-5.45005	0.677333	0.762897
Erythroid.cells	FAM193A	-0.09735	6.704133	-0.57635	0.565845	-6.36882	0.566135	0.65973
Erythroid.cells	KCNK5	0.32691	1.47886	0.576295	0.56588	-5.36268	0.677519	0.762985
Erythroid.cells	RFC3	-0.15476	4.31335	-0.57584	0.566188	-5.85424	0.614714	0.705302
Erythroid.cells	ARL15	0.096086	7.591924	0.575825	0.566196	-6.60476	0.549401	0.643778
Erythroid.cells	CABCOCO1	-0.41846	0.315412	-0.57552	0.566403	-5.12422	0.705696	0.788328
Erythroid.cells	2310057M	0.207723	2.710086	0.57539	0.566489	-5.46344	0.649775	0.737595
Erythroid.cells	HOMER3	0.202524	3.103095	0.574704	0.566951	-5.54241	0.64149	0.729756
Erythroid.cells	TXLNG	-0.12623	5.107247	-0.57458	0.567035	-6.04448	0.598801	0.690164
Erythroid.cells	NEK1	-0.23203	3.697931	-0.57422	0.567276	-5.62462	0.628712	0.717902
Erythroid.cells	CYP3A44	0.29695	2.308322	0.57379	0.567567	-5.73737	0.659648	0.74633
Erythroid.cells	NEBL	-0.39207	1.182115	-0.57362	0.567681	-5.44933	0.685773	0.770076
Erythroid.cells	TSTD2	0.179176	3.84075	0.573563	0.56772	-5.7295	0.625743	0.715197
Erythroid.cells	POU2F2	-0.18614	5.772063	-0.57353	0.567739	-6.20969	0.585591	0.67776
Erythroid.cells	MEPCE	-0.18192	4.466254	-0.57352	0.56775	-5.81521	0.61243	0.702852
Erythroid.cells	SNAPC5	0.142438	4.755447	0.573515	0.567752	-5.92424	0.606377	0.697217
Erythroid.cells	ST7	-0.1334	5.144851	-0.57271	0.568297	-6.16354	0.598833	0.690062
Erythroid.cells	WDR91	-0.15206	4.750458	-0.57259	0.568373	-5.97096	0.60701	0.697681
Erythroid.cells	2010320M	0.244454	2.480423	0.572377	0.56852	-5.51306	0.656361	0.743187
Erythroid.cells	RRAGB	0.433505	0.125251	0.572356	0.568533	-5.16036	0.711919	0.79345
Erythroid.cells	TMEM150I	0.342115	1.12138	0.572205	0.568635	-5.43867	0.687903	0.771854
Erythroid.cells	UBQLN2	-0.15539	4.031371	-0.57206	0.568731	-5.75158	0.622316	0.711894
Erythroid.cells	EGF	-0.40372	0.634522	-0.57197	0.568797	-5.12275	0.699605	0.782451
Erythroid.cells	MTHFS	0.207894	5.861718	0.571816	0.568898	-6.21856	0.584434	0.676625
Erythroid.cells	TARDBP	0.106963	5.771119	0.571698	0.568977	-6.16431	0.586251	0.678354
Erythroid.cells	ANKRD22	0.400218	-0.56253	0.571665	0.568999	-5.20518	0.729147	0.808986
Erythroid.cells	DGKI	0.618289	0.78	0.570988	0.569457	-5.2596	0.696532	0.779651
Erythroid.cells	GM16091	0.298573	2.306149	0.570929	0.569496	-5.38767	0.660812	0.747282
Erythroid.cells	COG8	-0.13482	4.299473	-0.57089	0.569525	-5.84432	0.616992	0.706979
Erythroid.cells	ARPC5L	-0.089	6.300784	-0.57073	0.56963	-6.33271	0.576095	0.66867
Erythroid.cells	FDXACB1	-0.22915	2.215747	-0.57059	0.569727	-5.45173	0.662964	0.749208
Erythroid.cells	MYO1D	0.214569	3.025547	0.570273	0.569939	-5.66078	0.644876	0.732604
Erythroid.cells	NANOS1	0.400026	0.634448	0.5702	0.569988	-5.1847	0.700302	0.782958
Erythroid.cells	STK24	-0.08052	7.291795	-0.56982	0.570247	-6.5268	0.55721	0.650641
Erythroid.cells	ULBP1	-0.20095	4.251838	-0.56979	0.570265	-5.84345	0.618398	0.708102
Erythroid.cells	TXNDC12	-0.15904	3.827411	-0.56967	0.570346	-5.73208	0.62751	0.716553
Erythroid.cells	PCTP	0.236584	2.252585	0.569498	0.570462	-5.52543	0.662529	0.748713
Erythroid.cells	SELENON	-0.22975	3.694822	-0.56942	0.570514	-5.65773	0.630426	0.71929
Erythroid.cells	NAXE	0.124521	5.283838	0.569265	0.57062	-6.10163	0.596973	0.688201
Erythroid.cells	DEF6	0.120786	5.457612	0.569174	0.570681	-6.07393	0.593425	0.684899
Erythroid.cells	ZCCHC18	-0.34469	1.04036	-0.56878	0.570948	-5.29688	0.691057	0.774631
Erythroid.cells	SRP54C	0.30153	1.755516	0.568584	0.57108	-5.36692	0.674215	0.759403

Erythroid.cells	TRAJ18	-0.32694	-1.3552	-0.5685	0.571134	-5.15059	0.750649	0.827883
Erythroid.cells	TRAPPC3	0.139919	4.917333	0.568445	0.571174	-5.9445	0.604696	0.69545
Erythroid.cells	PLA2G12B	0.365129	1.0508	0.568376	0.571221	-5.3251	0.690808	0.774422
Erythroid.cells	CLPP	-0.12908	4.725598	-0.56818	0.571355	-6.00508	0.60869	0.699195
Erythroid.cells	BCAS2	0.07423	6.380106	0.568155	0.57137	-6.38452	0.575118	0.667719
Erythroid.cells	MED26	-0.17506	4.429093	-0.56811	0.571399	-5.82404	0.614922	0.70499
Erythroid.cells	MBOAT7	0.175043	4.278521	0.568094	0.571411	-5.73397	0.618112	0.707951
Erythroid.cells	UGT2B5	0.328029	1.786068	0.567948	0.57151	-5.55668	0.673505	0.758783
Erythroid.cells	MCM7	-0.18472	5.384269	-0.56784	0.571584	-6.15666	0.595083	0.686495
Erythroid.cells	NCAPD2	0.194833	4.81058	0.567759	0.571638	-6.04583	0.606916	0.697547
Erythroid.cells	PPP1R3B	0.216162	2.491892	0.567738	0.571652	-5.60719	0.657315	0.744041
Erythroid.cells	TWISTNB	0.110574	5.520145	0.567566	0.571768	-6.10892	0.592372	0.683954
Erythroid.cells	PMEL	-0.47346	0.339511	-0.56737	0.571901	-5.12	0.708132	0.790045
Erythroid.cells	SEMA7A	0.297841	2.017527	0.56714	0.572056	-5.44418	0.668296	0.754087
Erythroid.cells	ISYNA1	-0.14199	4.852057	-0.56709	0.572089	-5.94996	0.606184	0.696896
Erythroid.cells	SHOC2	-0.09635	6.344774	-0.56708	0.572099	-6.28384	0.575939	0.668527
Erythroid.cells	B230217O1	0.358828	1.757632	0.566999	0.572152	-5.32199	0.674313	0.759564
Erythroid.cells	COX6A2	0.520323	2.070766	0.566905	0.572215	-5.18967	0.667071	0.75298
Erythroid.cells	TPX2	0.217535	5.047457	0.566626	0.572404	-6.12603	0.602265	0.693142
Erythroid.cells	HDAC8	-0.11434	6.530622	-0.56623	0.57267	-6.43262	0.572488	0.665129
Erythroid.cells	EEF2K	0.156629	4.644155	0.56623	0.572672	-5.96258	0.610742	0.700995
Erythroid.cells	CLPTM1L	-0.14851	4.883326	-0.56621	0.572687	-5.8827	0.605746	0.696343
Erythroid.cells	NBN	-0.19839	3.4476	-0.56607	0.572779	-5.64748	0.636393	0.724737
Erythroid.cells	TXNDC16	0.157421	5.44957	0.565969	0.572849	-6.16798	0.594089	0.685495
Erythroid.cells	4930404N1	-0.44029	0.191025	-0.56596	0.572854	-5.14412	0.712021	0.793451
Erythroid.cells	CHST15	0.203216	3.54275	0.56578	0.572977	-5.63175	0.634338	0.722915
Erythroid.cells	1110059E2	0.16119	4.393524	0.565692	0.573036	-5.79607	0.616049	0.706011
Erythroid.cells	PLSCR1	0.191755	4.550033	0.565649	0.573065	-5.94995	0.612745	0.702947
Erythroid.cells	DIP2A	-0.25534	2.079809	-0.5653	0.573301	-5.43573	0.667224	0.753017
Erythroid.cells	BC002059	0.239744	2.758564	0.565016	0.573494	-5.4806	0.651797	0.738981
Erythroid.cells	PASK	0.450462	1.295986	0.5648	0.57364	-5.24473	0.685508	0.769679
Erythroid.cells	CD82	0.127302	5.476033	0.564779	0.573654	-6.09805	0.593664	0.685176
Erythroid.cells	ELAVL3	-0.51521	1.03829	-0.56457	0.573796	-5.1727	0.691631	0.775234
Erythroid.cells	PTP4A1	-0.3705	1.179279	-0.56455	0.573806	-5.31393	0.688274	0.772204
Erythroid.cells	STAP2	0.425647	0.504981	0.564535	0.57382	-5.1947	0.70448	0.786804
Erythroid.cells	MRPL41	0.161797	4.003451	0.564476	0.57386	-5.7826	0.624459	0.71389
Erythroid.cells	ZFP579	-0.30587	1.862923	-0.56442	0.573899	-5.3347	0.672232	0.757721
Erythroid.cells	NOL9	-0.1743	3.728213	-0.56437	0.573934	-5.73777	0.630398	0.719424
Erythroid.cells	GRN	0.135629	7.188808	0.564275	0.573996	-6.41401	0.559853	0.65335
Erythroid.cells	INTS4	-0.17511	3.920928	-0.56422	0.574034	-5.71512	0.626234	0.715581
Erythroid.cells	INO80E	-0.13138	4.165534	-0.56405	0.574149	-5.75589	0.620989	0.710726
Erythroid.cells	MAP4K3	-0.18181	4.283134	-0.56403	0.574158	-5.83591	0.618483	0.708403
Erythroid.cells	MEF2B	-0.19052	3.605786	-0.56394	0.574222	-5.68056	0.633059	0.721885
Erythroid.cells	TMX4	0.175807	4.420261	0.563922	0.574235	-5.8655	0.615576	0.705704
Erythroid.cells	TUG1	0.104062	6.098897	0.563887	0.574258	-6.22526	0.581124	0.673483
Erythroid.cells	ZFP354C	0.312634	1.777029	0.563856	0.57428	-5.41115	0.674227	0.759556
Erythroid.cells	FBXO32	0.300837	3.711448	0.563698	0.574386	-5.70598	0.630805	0.719843

Erythroid.cells	VPS35L	0.108565	5.162292	0.563613	0.574444	-6.02029	0.600128	0.691387
Erythroid.cells	EIF4B	-0.0943	6.136378	-0.56339	0.574597	-6.2669	0.580445	0.672903
Erythroid.cells	RCOR1	-0.10233	6.85813	-0.56333	0.574635	-6.38756	0.566283	0.659518
Erythroid.cells	RBM48	-0.20084	3.037834	-0.5633	0.57466	-5.54531	0.645632	0.733519
Erythroid.cells	MZT2	0.219052	2.206691	0.562915	0.574918	-5.44465	0.664614	0.750831
Erythroid.cells	ATP23	-0.36309	1.824257	-0.56227	0.575355	-5.39091	0.673737	0.758998
Erythroid.cells	GYS2	0.316901	0.768164	0.562265	0.575358	-5.32267	0.698739	0.781602
Erythroid.cells	SLX1B	-0.19259	3.074893	-0.56226	0.575362	-5.56372	0.645316	0.733052
Erythroid.cells	GM17529	0.438055	0.365187	0.562053	0.575502	-5.1514	0.708621	0.790429
Erythroid.cells	DOCK1	0.165662	4.079995	0.56186	0.575633	-5.94326	0.623464	0.71286
Erythroid.cells	UBE2E2	0.219644	3.465951	0.561759	0.575702	-5.89725	0.636773	0.725147
Erythroid.cells	SNHG17	0.286293	1.368538	0.561669	0.575763	-5.33952	0.684504	0.768704
Erythroid.cells	CHAC1	0.429195	0.269965	0.561631	0.575789	-5.18844	0.710956	0.792521
Erythroid.cells	F2R	0.17959	3.599158	0.561532	0.575855	-5.86423	0.633861	0.722488
Erythroid.cells	POLD4	-0.10773	5.76477	-0.5615	0.57588	-6.21862	0.588425	0.68022
Erythroid.cells	PUS1	0.184274	3.723827	0.561262	0.576039	-5.70246	0.631254	0.720016
Erythroid.cells	TMEM63A	-0.26062	3.49863	-0.56116	0.57611	-5.54434	0.636173	0.724543
Erythroid.cells	SNX14	0.163188	4.500746	0.560782	0.576365	-5.85498	0.614701	0.704682
Erythroid.cells	NOC2L	-0.14725	5.059461	-0.5607	0.57642	-5.99092	0.603019	0.693822
Erythroid.cells	ZFP512	0.181845	3.916117	0.560669	0.576441	-5.73418	0.627179	0.716259
Erythroid.cells	AK8	0.289816	2.179586	0.560521	0.576542	-5.38078	0.665826	0.751714
Erythroid.cells	GM49101	0.445901	-0.65342	0.560494	0.57656	-5.15405	0.734216	0.813247
Erythroid.cells	RB1	0.089625	7.240705	0.560482	0.576568	-6.58462	0.559612	0.652944
Erythroid.cells	PSME1	-0.11906	8.384368	-0.5604	0.576621	-6.76854	0.538192	0.632494
Erythroid.cells	ABHD14A	-0.3357	1.44344	-0.55982	0.577016	-5.27774	0.68308	0.767405
Erythroid.cells	GIGYF1	-0.14966	4.441868	-0.55976	0.577057	-5.82886	0.616066	0.705994
Erythroid.cells	DDIT4	0.313175	3.744278	0.559732	0.577077	-5.69059	0.63102	0.719836
Erythroid.cells	ZNRD1	0.124533	5.076686	0.559663	0.577124	-6.01365	0.60278	0.693627
Erythroid.cells	KXD1	-0.10954	5.765423	-0.55963	0.577144	-6.18871	0.588707	0.680453
Erythroid.cells	TBC1D12	0.235632	3.768556	0.559632	0.577145	-5.71449	0.630493	0.719349
Erythroid.cells	ANAPC11	0.116836	5.923842	0.559584	0.577178	-6.22284	0.58552	0.677459
Erythroid.cells	ATG101	0.121944	5.222366	0.559443	0.577274	-6.05154	0.599774	0.690819
Erythroid.cells	SMC1A	-0.09881	6.748393	-0.55941	0.577299	-6.44371	0.569222	0.662084
Erythroid.cells	ARHGAP27	-0.20698	3.664658	-0.55906	0.577532	-5.60159	0.632827	0.721464
Erythroid.cells	FILIP1	0.405607	0.904589	0.558966	0.577598	-5.22177	0.695983	0.779017
Erythroid.cells	FFAR2	-0.45796	-0.53401	-0.55896	0.577604	-5.18489	0.731425	0.810754
Erythroid.cells	XRN1	-0.1051	5.924264	-0.55891	0.577634	-6.23062	0.585581	0.67748
Erythroid.cells	BAG4	-0.14667	4.226706	-0.55884	0.577682	-5.82968	0.620713	0.710291
Erythroid.cells	1700084C0	-0.24277	2.9115	-0.55864	0.577823	-5.55083	0.649536	0.736853
Erythroid.cells	ZCCHC10	-0.16926	4.048466	-0.55844	0.577954	-5.76552	0.624672	0.713947
Erythroid.cells	NELFCD	-0.14947	4.117826	-0.55827	0.578072	-5.82271	0.623184	0.712575
Erythroid.cells	4930486L2	-0.35585	0.484692	-0.55817	0.578136	-5.30462	0.706307	0.788347
Erythroid.cells	ZFAND4	-0.32529	3.4762	-0.55766	0.578483	-5.60224	0.637091	0.725632
Erythroid.cells	CDA	0.396814	0.894078	0.55763	0.578506	-5.27861	0.696396	0.77966
Erythroid.cells	AGBL3	0.301596	1.72445	0.557619	0.578514	-5.30919	0.676728	0.761876
Erythroid.cells	NOP16	-0.16026	4.283714	-0.55758	0.578544	-5.87173	0.619641	0.70954
Erythroid.cells	LCN4	-0.37333	-1.05402	-0.55753	0.578572	-5.16726	0.744852	0.822997

Erythroid.cells	TECPR1	0.16481	4.375591	0.557523	0.578579	-5.95833	0.617687	0.707732
Erythroid.cells	TNFRSF1A	-0.13818	5.171275	-0.55745	0.578629	-6.1339	0.601038	0.692228
Erythroid.cells	MRPS16	0.109306	5.607422	0.557448	0.57863	-6.19956	0.592113	0.683874
Erythroid.cells	ZFP866	0.229621	2.993086	0.557171	0.578818	-5.51786	0.647776	0.735587
Erythroid.cells	CCPG1	-0.11998	6.037912	-0.55716	0.578828	-6.19718	0.583441	0.675814
Erythroid.cells	CASP2	-0.15084	4.01281	-0.55711	0.578863	-5.77828	0.625439	0.715009
Erythroid.cells	ITSN2	0.092094	6.989544	0.557062	0.578892	-6.42172	0.564745	0.658153
Erythroid.cells	SHCBP1	0.209198	4.69138	0.556888	0.579011	-6.04252	0.611022	0.701662
Erythroid.cells	DPAGT1	0.208509	3.285854	0.556887	0.579012	-5.64248	0.641279	0.729655
Erythroid.cells	RAB11FIP1	-0.1643	4.829945	-0.55678	0.579084	-6.25503	0.608121	0.698971
Erythroid.cells	NFE2L2	-0.1128	7.355477	-0.55672	0.579126	-6.55251	0.557726	0.651534
Erythroid.cells	DIAPH2	0.101339	7.666379	0.556707	0.579134	-6.5715	0.551836	0.64592
Erythroid.cells	ACADS	0.123669	4.490963	0.556182	0.579491	-6.03267	0.615504	0.705773
Erythroid.cells	TUT4	-0.09524	7.475638	-0.55591	0.579677	-6.62426	0.555678	0.649554
Erythroid.cells	SRMS	-0.51924	-0.72874	-0.55591	0.579679	-5.04013	0.736843	0.816036
Erythroid.cells	CAPRIN2	-0.29056	3.181523	-0.55575	0.579786	-5.54157	0.64386	0.732054
Erythroid.cells	ZFP280C	0.159281	4.164903	0.555656	0.57985	-5.78472	0.622441	0.712298
Erythroid.cells	LEF1	0.178258	5.638758	0.555644	0.579858	-6.4152	0.591728	0.683663
Erythroid.cells	DLG2	-0.40937	2.402232	-0.5556	0.579888	-5.32577	0.661378	0.748104
Erythroid.cells	AP1G2	-0.27116	2.809774	-0.55552	0.579942	-5.54269	0.652156	0.739671
Erythroid.cells	ALS2CL	0.410224	0.148166	0.555515	0.579946	-5.20868	0.714865	0.796458
Erythroid.cells	SPRED2	-0.11388	6.335393	-0.55531	0.580088	-6.29079	0.577851	0.670595
Erythroid.cells	LRRC51	-0.2936	1.76843	-0.55499	0.5803	-5.35325	0.676256	0.761578
Erythroid.cells	PPM1B	0.082655	6.192574	0.55482	0.580419	-6.39686	0.580885	0.67341
Erythroid.cells	SYS1	-0.08482	6.417243	-0.55473	0.580477	-6.33134	0.576433	0.669211
Erythroid.cells	MAPKAP1	-0.10832	5.764174	-0.5544	0.580704	-6.15551	0.58964	0.681583
Erythroid.cells	LPCAT4	-0.23694	3.121289	-0.55426	0.580798	-5.72674	0.645725	0.733681
Erythroid.cells	EP300	-0.08785	6.696726	-0.554	0.580979	-6.35646	0.571254	0.664186
Erythroid.cells	TTR	0.210134	9.202897	0.553876	0.581062	-7.08759	0.52445	0.619441
Erythroid.cells	TAF1C	-0.25729	2.170342	-0.5538	0.581112	-5.39916	0.667375	0.753371
Erythroid.cells	ENHO	0.243315	2.481211	0.553621	0.581236	-5.67357	0.660309	0.746938
Erythroid.cells	SCYL3	0.158873	3.642548	0.553483	0.58133	-5.70578	0.634424	0.72319
Erythroid.cells	A730036I1	-0.50319	-0.20546	-0.55342	0.581375	-5.14239	0.724447	0.804834
Erythroid.cells	SCARF1	0.332297	1.587665	0.553374	0.581404	-5.3089	0.680968	0.765737
Erythroid.cells	C87436	0.166115	3.905756	0.553213	0.581514	-5.76354	0.628708	0.717936
Erythroid.cells	HVCN1	-0.24797	4.117697	-0.55319	0.581532	-5.7982	0.624142	0.713713
Erythroid.cells	MMP19	0.308464	1.213067	0.553006	0.581655	-5.54548	0.68987	0.773811
Erythroid.cells	SEL1L	-0.12361	5.161227	-0.55285	0.58176	-5.99255	0.602203	0.693319
Erythroid.cells	RAD21	-0.08686	7.022909	-0.5528	0.581794	-6.47853	0.565	0.6583
Erythroid.cells	CDC27	-0.10713	6.009717	-0.55277	0.581818	-6.20283	0.584935	0.677133
Erythroid.cells	JPT2	-0.21528	3.513173	-0.55264	0.581902	-5.65764	0.637317	0.725875
Erythroid.cells	COPS7A	0.154912	4.237724	0.55237	0.582088	-5.8507	0.621667	0.71149
Erythroid.cells	ZFR	0.079906	6.532381	0.552348	0.582104	-6.35375	0.574613	0.667459
Erythroid.cells	GPATCH1	0.217591	2.73622	0.552319	0.582124	-5.49013	0.654634	0.741848
Erythroid.cells	DMWD	0.284147	2.228423	0.552061	0.582299	-5.46462	0.66627	0.752413
Erythroid.cells	H6PD	0.210047	2.776773	0.551991	0.582347	-5.54885	0.653798	0.741025
Erythroid.cells	GM27241	0.262365	2.603068	0.551761	0.582504	-5.54811	0.657723	0.744614

Erythroid.cells	ZDHHC21	0.160358	4.271545	0.551752	0.58251	-5.80404	0.621019	0.710834
Erythroid.cells	MRPS27	-0.20748	3.208477	-0.55167	0.58257	-5.61022	0.64415	0.732178
Erythroid.cells	LPP	-0.11341	7.6885	-0.55166	0.582571	-6.59256	0.552415	0.646333
Erythroid.cells	TESPA1	0.254707	2.504605	0.551482	0.582694	-5.57012	0.66002	0.746675
Erythroid.cells	MRPL51	0.132596	4.486929	0.551399	0.582751	-5.95153	0.616497	0.706632
Erythroid.cells	GM39326	0.346616	1.289251	0.551046	0.582992	-5.28022	0.688385	0.772418
Erythroid.cells	SDHAF4	-0.13524	4.365947	-0.55098	0.583036	-5.92574	0.61917	0.709062
Erythroid.cells	GM12840	0.338615	3.766389	0.550935	0.583068	-5.72072	0.632067	0.720994
Erythroid.cells	ANKRD26	-0.27372	2.576265	-0.55089	0.583101	-5.45977	0.658504	0.74528
Erythroid.cells	RSF1OS2	0.307916	1.663513	0.550751	0.583193	-5.38387	0.679588	0.764457
Erythroid.cells	EEF1E1	0.157806	4.824221	0.550461	0.583391	-5.94507	0.609607	0.700157
Erythroid.cells	IL11RA1	0.224278	2.113905	0.550412	0.583425	-5.48471	0.6692	0.755003
Erythroid.cells	PPP2R3A	-0.17263	5.003266	-0.55037	0.583456	-6.0598	0.605871	0.696676
Erythroid.cells	4930417O1	0.285289	0.963159	0.550196	0.583572	-5.53931	0.696361	0.779596
Erythroid.cells	ZFP811	-0.5576	-1.02506	-0.54983	0.583822	-5.03137	0.745895	0.823905
Erythroid.cells	PLEKHO2	0.118033	6.069061	0.549801	0.583842	-6.19901	0.584219	0.676428
Erythroid.cells	PDLIM2	-0.21408	3.759145	-0.5498	0.583843	-5.66106	0.632435	0.721354
Erythroid.cells	GM44987	0.426404	-0.34886	0.549757	0.583872	-5.07708	0.728678	0.808601
Erythroid.cells	GM39090	-0.38068	0.497553	-0.54968	0.583925	-5.16321	0.70769	0.789851
Erythroid.cells	SC5D	-0.14777	4.248539	-0.54931	0.584179	-5.96242	0.62195	0.711616
Erythroid.cells	HS2ST1	0.116196	5.689893	0.549275	0.584202	-6.08036	0.591925	0.683627
Erythroid.cells	SUPV3L1	0.188557	3.910174	0.549219	0.58424	-5.71684	0.629228	0.71835
Erythroid.cells	ITPKC	-0.32087	1.763821	-0.54922	0.584242	-5.355	0.677509	0.762546
Erythroid.cells	LSM4	0.086379	6.704001	0.549045	0.584359	-6.43599	0.571773	0.664628
Erythroid.cells	SLC33A1	0.175259	3.794664	0.54887	0.584478	-5.7329	0.631852	0.720775
Erythroid.cells	UMPS	-0.1541	4.099845	-0.54873	0.584571	-5.79797	0.625287	0.714739
Erythroid.cells	SETD5	-0.0798	6.628233	-0.54856	0.584688	-6.37796	0.573395	0.666264
Erythroid.cells	4833403J1	-0.43463	0.509888	-0.54841	0.584795	-5.24662	0.707755	0.789974
Erythroid.cells	CIP2A	0.239841	3.506565	0.547922	0.585126	-5.70942	0.638578	0.72691
Erythroid.cells	MYO15	-0.40507	1.234713	-0.54718	0.585631	-5.22502	0.6911	0.774605
Erythroid.cells	MED22	0.261574	2.384243	0.547101	0.585688	-5.43544	0.664239	0.750254
Erythroid.cells	LTBR	-0.18063	2.974023	-0.54679	0.585903	-5.73087	0.650989	0.738091
Erythroid.cells	HMGA2	0.60861	1.149583	0.546552	0.586063	-5.21157	0.693251	0.776498
Erythroid.cells	ACP6	-0.19778	3.140192	-0.54652	0.586087	-5.63692	0.647274	0.734687
Erythroid.cells	KCNK6	-0.2462	2.306704	-0.54648	0.586116	-5.47755	0.666129	0.751919
Erythroid.cells	KLRA17	-0.36292	0.224958	-0.54647	0.586117	-5.21613	0.715733	0.796696
Erythroid.cells	2010109A1	-0.26573	2.521995	-0.54636	0.586194	-5.44564	0.661205	0.747456
Erythroid.cells	ZSWIM1	0.386588	1.4089	0.54631	0.586229	-5.26977	0.687076	0.770973
Erythroid.cells	OSBPL11	-0.14711	5.368512	-0.5461	0.58637	-5.99205	0.59958	0.690598
Erythroid.cells	KIF20A	0.278855	3.768581	0.546044	0.586411	-5.78547	0.633457	0.722067
Erythroid.cells	FDFT1	-0.16769	3.796269	-0.54599	0.586447	-5.7797	0.632854	0.721538
Erythroid.cells	IQCN	-0.38792	1.791223	-0.54556	0.586743	-5.30559	0.678377	0.763029
Erythroid.cells	TLK1	-0.08333	6.854126	-0.54527	0.58694	-6.45604	0.570163	0.662761
Erythroid.cells	GM45716	-0.18973	3.20355	-0.54506	0.587085	-5.69592	0.646269	0.733697
Erythroid.cells	KCTD17	-0.34098	0.958849	-0.54506	0.587086	-5.3874	0.698267	0.780948
Erythroid.cells	CHCHD5	0.204115	3.092559	0.544861	0.587221	-5.64579	0.648744	0.736067
Erythroid.cells	CCDC137	-0.21269	2.673176	-0.54459	0.587404	-5.48754	0.658183	0.744773

Erythroid.cells	ADAM8	-0.2427	2.602795	-0.54456	0.587429	-5.70476	0.659781	0.746251
Erythroid.cells	PFKFB1	0.327812	0.961216	0.544504	0.587465	-5.31612	0.69821	0.781111
Erythroid.cells	GM2629	0.324793	0.511245	0.544491	0.587474	-5.26756	0.709139	0.790934
Erythroid.cells	NECTIN4	-0.43872	-0.02307	-0.54441	0.587531	-5.11877	0.722342	0.802765
Erythroid.cells	DNAJC8	0.060512	6.901934	0.544382	0.587549	-6.48287	0.569232	0.662112
Erythroid.cells	GM50399	0.530616	0.166375	0.544284	0.587616	-5.11115	0.717633	0.798557
Erythroid.cells	4833417C1	-0.37402	0.707506	-0.54412	0.587725	-5.20023	0.704351	0.786667
Erythroid.cells	KDM5C	0.123902	6.827805	0.544085	0.587752	-6.45382	0.570677	0.663496
Erythroid.cells	ZFP91	-0.07283	6.894237	-0.54403	0.587792	-6.48823	0.569382	0.662277
Erythroid.cells	MIOS	-0.16328	3.665658	-0.54397	0.587833	-5.69317	0.636072	0.724577
Erythroid.cells	2210408F2	-0.2418	2.856305	-0.54386	0.587909	-5.69809	0.654044	0.741126
Erythroid.cells	RNASE4	0.148754	5.17058	0.543822	0.587933	-6.42404	0.604015	0.694897
Erythroid.cells	GALC	0.194341	3.891293	0.543745	0.587985	-5.74051	0.631154	0.720089
Erythroid.cells	BMPR1A	-0.32114	2.310682	-0.54353	0.588131	-5.47876	0.666489	0.752563
Erythroid.cells	6430548M	0.233278	2.407786	0.543519	0.588141	-5.56025	0.664262	0.750533
Erythroid.cells	SLC37A1	0.232793	3.202303	0.543415	0.588212	-5.57738	0.646337	0.734158
Erythroid.cells	KLF16	-0.20191	3.164065	-0.54312	0.588414	-5.56457	0.647314	0.735009
Erythroid.cells	IMPG2	0.450309	0.6613	0.543065	0.588451	-5.19017	0.705655	0.787984
Erythroid.cells	DGCR8	0.165512	3.779114	0.542683	0.588714	-5.75164	0.633903	0.722691
Erythroid.cells	CASTOR2	-0.28581	3.643855	-0.54268	0.588714	-5.64671	0.636859	0.725417
Erythroid.cells	SERP2	0.307316	0.429349	0.541911	0.589243	-5.28267	0.712056	0.793493
Erythroid.cells	DAAM1	0.129862	4.764899	0.54162	0.589443	-6.03054	0.613412	0.703462
Erythroid.cells	TRMT6	-0.15305	4.373913	-0.54136	0.58962	-5.78175	0.621732	0.711205
Erythroid.cells	MCTP2	-0.1298	6.80258	-0.54133	0.589639	-6.38719	0.572056	0.664707
Erythroid.cells	JADE3	0.18567	3.471551	0.541307	0.589657	-5.61615	0.641329	0.729293
Erythroid.cells	FAN1	-0.36054	1.159783	-0.54108	0.589812	-5.24872	0.694627	0.777742
Erythroid.cells	MEN1	0.241631	2.866329	0.540896	0.589939	-5.4863	0.655006	0.741701
Erythroid.cells	PTPRF	0.299267	1.276101	0.540554	0.590174	-5.29181	0.69206	0.775304
Erythroid.cells	MINK1	0.155196	4.171023	0.540535	0.590187	-5.82117	0.626376	0.715322
Erythroid.cells	NECTIN2	-0.23735	2.109704	-0.54045	0.590249	-5.48936	0.672446	0.757572
Erythroid.cells	ARHGAP39	0.214534	4.38051	0.54034	0.590321	-5.77849	0.621891	0.711235
Erythroid.cells	CHADL	-0.39316	0.647864	-0.54004	0.590529	-5.18575	0.707291	0.789089
Erythroid.cells	CRLF2	0.153778	5.010644	0.539988	0.590563	-5.9127	0.608621	0.698917
Erythroid.cells	F13B	0.299613	1.463355	0.539859	0.590651	-5.45826	0.687664	0.771421
Erythroid.cells	FASN	0.224916	3.14635	0.539759	0.59072	-5.60768	0.648909	0.736176
Erythroid.cells	GM50209	0.392857	0.629068	0.539723	0.590745	-5.16277	0.70775	0.789551
Erythroid.cells	RELN	-0.21707	3.647841	-0.53966	0.59079	-5.99138	0.637804	0.725986
Erythroid.cells	JMJD4	-0.28166	1.268434	-0.53963	0.590807	-5.27525	0.692304	0.775653
Erythroid.cells	MPHOSPH:	0.138902	4.827879	0.539538	0.590872	-5.90727	0.612454	0.70254
Erythroid.cells	TRIM65	-0.208	3.22435	-0.53925	0.591072	-5.59515	0.64732	0.734761
Erythroid.cells	CPT1B	-0.30223	1.536488	-0.53887	0.591328	-5.24717	0.686207	0.770126
Erythroid.cells	FAM20A	0.321276	1.833508	0.53887	0.591331	-5.46099	0.679213	0.763794
Erythroid.cells	RMND5B	-0.14021	4.49308	-0.53879	0.591389	-5.83909	0.619785	0.709318
Erythroid.cells	ETNK2	0.307063	1.270533	0.538678	0.591463	-5.39143	0.692532	0.775839
Erythroid.cells	WFDC18	-0.34432	-0.88323	-0.53856	0.591547	-5.15835	0.745991	0.823634
Erythroid.cells	FAM216A	-0.2234	2.582038	-0.53855	0.591551	-5.51008	0.661912	0.74806
Erythroid.cells	TMEM107	-0.28636	1.616764	-0.53824	0.591765	-5.31798	0.684485	0.768497

Erythroid.cells	HNRNPUL2	0.076243	6.468104	0.538016	0.591918	-6.36861	0.57943	0.671499
Erythroid.cells	VAMP8	0.082735	7.209731	0.537719	0.592122	-6.50534	0.565053	0.65788
Erythroid.cells	ITGB1	0.084869	6.871704	0.537564	0.592229	-6.45144	0.571662	0.664151
Erythroid.cells	MPEG1	0.167626	6.081136	0.537181	0.592492	-6.41642	0.58754	0.679035
Erythroid.cells	TRIM24	-0.1252	5.087563	-0.53698	0.59263	-6.04462	0.607917	0.698157
Erythroid.cells	HIBCH	-0.14112	3.478236	-0.53697	0.592634	-5.79159	0.64249	0.730143
Erythroid.cells	FDPS	0.189413	4.623809	0.536465	0.592984	-5.90111	0.617712	0.707402
Erythroid.cells	TSPAN9	0.216651	3.290989	0.536401	0.593028	-5.77081	0.646683	0.734149
Erythroid.cells	SMARCE1	-0.07297	6.302182	-0.53639	0.593039	-6.38375	0.583159	0.67512
Erythroid.cells	RFNG	0.224073	2.228203	0.536355	0.59306	-5.46521	0.670798	0.756177
Erythroid.cells	MSS51	0.262924	2.436698	0.536343	0.593068	-5.43514	0.665995	0.751805
Erythroid.cells	FANCG	-0.25922	2.222876	-0.53616	0.593194	-5.39922	0.670921	0.756317
Erythroid.cells	ATG2A	0.133669	5.710857	0.536046	0.593273	-6.12114	0.595094	0.686412
Erythroid.cells	DYNC1I2	0.077652	6.441227	0.536017	0.593293	-6.30552	0.58039	0.672602
Erythroid.cells	GM16066	0.263948	2.012511	0.536015	0.593294	-5.37876	0.675804	0.760828
Erythroid.cells	4931403E2	-0.27541	1.501737	-0.536	0.593303	-5.3145	0.687815	0.771709
Erythroid.cells	WDR61	0.107117	4.80713	0.5359	0.593373	-6.00477	0.613842	0.703953
Erythroid.cells	IGFBP3	0.391546	0.690335	0.535601	0.593579	-5.29164	0.707524	0.789507
Erythroid.cells	GM45442	0.532172	-0.57631	0.535305	0.593783	-5.0529	0.739202	0.817771
Erythroid.cells	SYCE2	-0.2224	4.258668	-0.53524	0.59383	-5.81186	0.625709	0.714937
Erythroid.cells	DDX60	-0.36397	2.866859	-0.53506	0.593952	-5.50809	0.656405	0.743188
Erythroid.cells	LENG8	-0.1475	4.564084	-0.53501	0.593989	-5.86351	0.619177	0.708904
Erythroid.cells	FCGR4	-0.37614	3.388468	-0.535	0.593993	-5.71328	0.644721	0.732483
Erythroid.cells	10-Sep	-0.18231	3.119761	-0.53482	0.594114	-5.67808	0.650713	0.738029
Erythroid.cells	SARDH	0.274062	2.900243	0.534819	0.594117	-5.71985	0.65565	0.74255
Erythroid.cells	SPG21	-0.0911	6.057126	-0.53466	0.594229	-6.27793	0.58826	0.680108
Erythroid.cells	GIT1	-0.15753	3.282244	-0.53463	0.59425	-5.62358	0.647083	0.734724
Erythroid.cells	RRM2	0.182696	6.880861	0.534601	0.594268	-6.61817	0.571907	0.664689
Erythroid.cells	GM15246	0.427276	1.046088	0.534533	0.594315	-5.20819	0.698935	0.781887
Erythroid.cells	ATPSCKMT	-0.18716	3.937455	-0.53433	0.594454	-5.76448	0.632667	0.721478
Erythroid.cells	2610318N(	-0.38014	1.172871	-0.53396	0.594706	-5.2886	0.695895	0.779232
Erythroid.cells	MRPL55	0.136825	4.367452	0.533956	0.594712	-5.90841	0.623384	0.712978
Erythroid.cells	ILKAP	-0.10404	5.651644	-0.53395	0.594715	-6.15548	0.596501	0.687938
Erythroid.cells	AKT2	-0.10794	5.234185	-0.53378	0.594831	-6.08903	0.605103	0.69598
Erythroid.cells	WDR82	0.106627	4.82833	0.533726	0.59487	-5.9447	0.613592	0.703889
Erythroid.cells	AARSD1	-0.17209	3.977143	-0.53372	0.594877	-5.76726	0.631805	0.720766
Erythroid.cells	LAP3	-0.15097	4.901491	-0.53367	0.594912	-6.13382	0.612053	0.702457
Erythroid.cells	CACTIN	-0.16871	3.991663	-0.53355	0.594995	-5.77004	0.631489	0.720475
Erythroid.cells	RBM15B	-0.15518	4.047089	-0.53352	0.59501	-5.71945	0.630287	0.719364
Erythroid.cells	IZUMO4	-0.35433	1.453448	-0.53351	0.595021	-5.35168	0.68919	0.77318
Erythroid.cells	F12	0.316163	1.691224	0.533405	0.595092	-5.51564	0.68357	0.768136
Erythroid.cells	AIRN	-0.16674	5.663175	-0.53322	0.59522	-6.27608	0.596338	0.687818
Erythroid.cells	FAM167A	0.380134	1.837572	0.532859	0.595468	-5.34297	0.680413	0.765244
Erythroid.cells	ZFP458	-0.43749	0.876726	-0.53266	0.595607	-5.19049	0.703361	0.785964
Erythroid.cells	MTMR2	0.105793	5.396075	0.532658	0.595607	-6.09815	0.602024	0.69311
Erythroid.cells	GM13561	0.26206	1.307424	0.532564	0.595671	-5.40179	0.692985	0.776678
Erythroid.cells	FBLIM1	-0.39055	0.928973	-0.53215	0.595956	-5.32069	0.702313	0.785021

Erythroid.cells	CTDNEP1	-0.12324	5.516749	-0.53196	0.59609	-6.09289	0.599725	0.690959
Erythroid.cells	DIAPH3	0.161181	6.026174	0.531836	0.596173	-6.52993	0.589344	0.681228
Erythroid.cells	CD4	0.393696	1.246276	0.531829	0.596178	-5.29494	0.694665	0.778131
Erythroid.cells	HK2	0.187736	5.335441	0.531721	0.596253	-6.0255	0.603466	0.694455
Erythroid.cells	ZFP867	0.349747	0.61031	0.531451	0.596439	-5.17696	0.710081	0.79201
Erythroid.cells	CKS2	0.138511	5.959802	0.531376	0.596491	-6.28937	0.590685	0.682527
Erythroid.cells	ATF6	0.088301	6.979377	0.531079	0.596696	-6.46864	0.570428	0.663427
Erythroid.cells	TMEM38A	0.313769	1.868955	0.531046	0.596718	-5.29771	0.679903	0.764824
Erythroid.cells	E130309D(	-0.15026	3.983364	-0.53105	0.596719	-5.79699	0.632152	0.721134
Erythroid.cells	CD180	0.150186	5.032672	0.530877	0.596835	-6.09069	0.609768	0.700375
Erythroid.cells	LRRC27	-0.39842	0.302993	-0.53085	0.596852	-5.1469	0.717654	0.798833
Erythroid.cells	KLF4	0.127885	6.447469	0.530847	0.596856	-6.39858	0.580902	0.673324
Erythroid.cells	B3GNT8	0.288626	2.202747	0.530793	0.596893	-5.48179	0.672123	0.757759
Erythroid.cells	PEX11A	0.353355	0.072757	0.530725	0.59694	-5.28177	0.723382	0.803955
Erythroid.cells	PPP1R21	0.158085	4.557871	0.530634	0.597003	-5.86699	0.619792	0.709694
Erythroid.cells	STRIP2	0.278891	1.016356	0.530574	0.597044	-5.52561	0.700198	0.783163
Erythroid.cells	SRFBP1	-0.21774	3.231687	-0.53056	0.597055	-5.54724	0.648716	0.736378
Erythroid.cells	MSRA	0.088997	6.629496	0.530549	0.597061	-6.41471	0.577295	0.66992
Erythroid.cells	MRPL36	-0.10874	5.835469	-0.53027	0.597253	-6.24383	0.593302	0.684955
Erythroid.cells	TAGAP	0.240431	4.269177	0.530151	0.597336	-5.79286	0.626071	0.715494
Erythroid.cells	CDK10	-0.23406	2.234353	-0.52977	0.597597	-5.45057	0.671498	0.757292
Erythroid.cells	TMEM128	-0.11208	5.718829	-0.52973	0.597627	-6.18469	0.595679	0.687309
Erythroid.cells	STAMBP	0.202318	3.374422	0.529717	0.597636	-5.63263	0.645639	0.733658
Erythroid.cells	TSFM	-0.135	4.258618	-0.5297	0.59765	-5.88432	0.626298	0.715824
Erythroid.cells	CCDC112	-0.36768	1.151644	-0.52969	0.597658	-5.26669	0.697048	0.780436
Erythroid.cells	SPRYD4	-0.24184	1.803638	-0.52968	0.597664	-5.40129	0.681545	0.766422
Erythroid.cells	FAM124A	-0.29459	1.64482	-0.52949	0.597791	-5.3435	0.685289	0.769831
Erythroid.cells	ZFP955B	-0.25841	2.201747	-0.52948	0.597798	-5.43029	0.672254	0.758003
Erythroid.cells	ARHGEF4	-0.5121	0.759185	-0.52931	0.597914	-5.21831	0.706554	0.78901
Erythroid.cells	ITGAL	-0.13523	6.272091	-0.5293	0.597924	-6.28005	0.584494	0.676818
Erythroid.cells	IGLC1	0.314234	3.761408	0.529216	0.597982	-6.1709	0.637099	0.725816
Erythroid.cells	HDAC7	-0.14324	4.31747	-0.52907	0.598084	-5.96725	0.625074	0.714669
Erythroid.cells	GLRA1	0.390674	1.25067	0.528915	0.598189	-5.37988	0.694765	0.77831
Erythroid.cells	PCF11	-0.10783	6.482932	-0.52856	0.598438	-6.30798	0.580446	0.672882
Erythroid.cells	KIF18B	-0.2565	2.84215	-0.52852	0.598462	-5.6155	0.657757	0.744661
Erythroid.cells	ZGRF1	-0.23054	3.956437	-0.52845	0.598514	-5.8003	0.633009	0.721926
Erythroid.cells	ZFP605	-0.31812	1.633403	-0.52843	0.598522	-5.35518	0.685742	0.770117
Erythroid.cells	GNG3	-0.32241	1.449153	-0.52825	0.598652	-5.32966	0.690137	0.774125
Erythroid.cells	THY1	0.23859	1.573983	0.528222	0.598669	-5.68177	0.687172	0.771444
Erythroid.cells	5830408C2	0.204166	2.989299	0.527886	0.598901	-5.53452	0.654618	0.74172
Erythroid.cells	FAM219A	-0.23451	4.171512	-0.52782	0.598945	-5.69474	0.628523	0.717755
Erythroid.cells	SIPA1L1	-0.11921	7.327522	-0.52756	0.599125	-6.60624	0.564191	0.657457
Erythroid.cells	CENPX	-0.09626	6.347296	-0.52717	0.599395	-6.35245	0.583468	0.675656
Erythroid.cells	GNPAT	0.157541	4.15461	0.526973	0.599532	-5.75164	0.629056	0.718185
Erythroid.cells	SHANK3	0.389579	1.430051	0.526972	0.599533	-5.31561	0.690949	0.77473
Erythroid.cells	RAB10OS	0.139353	4.429445	0.5269	0.599583	-5.9031	0.623142	0.712712
Erythroid.cells	APP	0.14392	5.938302	0.526876	0.599599	-6.38459	0.591699	0.683394

Erythroid.cells	SLC5A3	-0.25331	3.816287	-0.52683	0.599634	-5.6567	0.636417	0.72498
Erythroid.cells	ELP4	0.129264	4.963596	0.526815	0.599641	-6.01548	0.611814	0.702192
Erythroid.cells	SULT1D1	0.243876	2.290136	0.526656	0.599751	-5.7108	0.670757	0.756452
Erythroid.cells	ADAMDEC1	0.395399	0.746267	0.526581	0.599803	-5.295	0.707447	0.789654
Erythroid.cells	TRIO	0.113798	6.069746	0.526434	0.599905	-6.24366	0.58904	0.680994
Erythroid.cells	CATSPERD	-0.40583	1.329786	-0.52637	0.599949	-5.22873	0.693344	0.777027
Erythroid.cells	TRIP4	-0.16113	4.852518	-0.52633	0.599979	-5.97633	0.614151	0.704505
Erythroid.cells	ALG9	-0.17964	3.375018	-0.52621	0.600061	-5.65522	0.646154	0.734111
Erythroid.cells	SSR1	0.082245	6.554701	0.526138	0.60011	-6.33104	0.57934	0.671924
Erythroid.cells	KIF7	0.436405	-0.46987	0.526102	0.600134	-5.1169	0.737785	0.816889
Erythroid.cells	CEP192	0.121099	5.27108	0.52594	0.600247	-6.03849	0.605391	0.696464
Erythroid.cells	JAG1	0.330197	1.3378	0.525938	0.600248	-5.48811	0.693152	0.777006
Erythroid.cells	GM4356	0.359338	0.868166	0.525585	0.600492	-5.3051	0.704617	0.787319
Erythroid.cells	BACE2	0.371815	1.120623	0.525501	0.60055	-5.2599	0.698504	0.781822
Erythroid.cells	ZFP973	0.367395	0.609416	0.525491	0.600557	-5.17779	0.710938	0.793003
Erythroid.cells	CDK5RAP3	-0.13684	4.379321	-0.52527	0.600707	-5.8993	0.624429	0.714151
Erythroid.cells	1200007C1	-0.39525	-0.38465	-0.52501	0.600888	-5.24836	0.735932	0.815322
Erythroid.cells	GM11613	0.348231	2.020755	0.524938	0.60094	-5.42059	0.677305	0.762664
Erythroid.cells	A3GALT2	0.244188	2.675415	0.524838	0.601009	-5.58453	0.662193	0.748904
Erythroid.cells	HMGB2	0.126667	9.132267	0.524834	0.601012	-6.93318	0.530789	0.625717
Erythroid.cells	YY1	0.059365	7.144963	0.524502	0.601242	-6.52286	0.568162	0.661393
Erythroid.cells	GM49482	0.450934	-0.71551	0.524306	0.601378	-5.09754	0.744625	0.823045
Erythroid.cells	SMYD2	-0.21934	3.028651	-0.52428	0.601394	-5.63427	0.654392	0.741736
Erythroid.cells	TNPO3	-0.08381	6.365771	-0.52386	0.601686	-6.34198	0.5836	0.676016
Erythroid.cells	VDAC2	-0.07799	7.488132	-0.52376	0.601758	-6.56845	0.561628	0.655221
Erythroid.cells	CEBPB	0.1502	9.155958	0.523732	0.601775	-6.99545	0.53059	0.625507
Erythroid.cells	KPTN	0.226251	4.098478	0.523607	0.601862	-5.68608	0.630813	0.720093
Erythroid.cells	GM43936	0.477058	-1.34803	0.523533	0.601913	-5.03646	0.761158	0.837759
Erythroid.cells	SLC27A1	-0.28153	2.972091	-0.52344	0.601979	-5.48876	0.655743	0.743063
Erythroid.cells	GM4869	-0.38429	1.447403	-0.52334	0.602048	-5.24959	0.69113	0.77526
Erythroid.cells	TARS	-0.1385	4.374393	-0.52328	0.602088	-5.89773	0.624858	0.714659
Erythroid.cells	PRADC1	-0.1813	3.374688	-0.52322	0.602133	-5.65824	0.646717	0.734823
Erythroid.cells	COQ10A	0.158893	3.31768	0.523181	0.602157	-5.6843	0.647987	0.735989
Erythroid.cells	SLC22A1	0.351251	0.740269	0.523081	0.602227	-5.31694	0.708202	0.790666
Erythroid.cells	STOML3	-0.39784	0.359863	-0.52304	0.602256	-5.14979	0.717563	0.799062
Erythroid.cells	IGSF6	0.262552	3.689623	0.522991	0.602289	-5.76714	0.639746	0.728411
Erythroid.cells	ORC5	0.183657	3.474755	0.522767	0.602444	-5.69121	0.644564	0.732835
Erythroid.cells	IGKV12-46	0.368436	-0.77013	0.522712	0.602482	-5.14099	0.746199	0.824572
Erythroid.cells	EVL	-0.11028	6.485099	-0.52245	0.602663	-6.40272	0.581399	0.673991
Erythroid.cells	TBC1D16	0.278772	2.452428	0.522259	0.602796	-5.52602	0.667837	0.754121
Erythroid.cells	SRL	-0.36844	0.890591	-0.52216	0.602868	-5.12996	0.704799	0.78763
Erythroid.cells	SNRPB2	0.082312	6.282619	0.522118	0.602894	-6.30629	0.585482	0.67791
Erythroid.cells	TJP1	0.254359	2.024999	0.521683	0.603196	-5.48968	0.677992	0.763233
Erythroid.cells	PRCP	0.11648	5.424232	0.521624	0.603237	-6.10158	0.603176	0.694346
Erythroid.cells	YBEY	-0.2618	1.366271	-0.52144	0.603362	-5.32407	0.693579	0.777415
Erythroid.cells	LRRC61	-0.27456	2.011331	-0.52143	0.60337	-5.37954	0.678319	0.7636
Erythroid.cells	SUB1	0.067289	8.911934	0.521289	0.603469	-6.90652	0.535442	0.630224

Erythroid.cells	E2F3	-0.14255	5.460693	-0.52095	0.603705	-6.12376	0.602633	0.693806
Erythroid.cells	ASCC1	-0.14997	3.774861	-0.52081	0.603798	-5.74221	0.638561	0.727154
Erythroid.cells	LSP1	-0.09248	8.230432	-0.52074	0.60385	-6.80412	0.548172	0.642334
Erythroid.cells	HES7	0.391498	-0.49621	0.520678	0.603893	-5.13327	0.739889	0.8188
Erythroid.cells	CTNNBL1	-0.11131	5.139211	-0.52039	0.604091	-6.01634	0.609426	0.700084
Erythroid.cells	AW209491	-0.35087	1.448036	-0.52034	0.604128	-5.31229	0.691984	0.775801
Erythroid.cells	GM15886	-0.2384	1.881502	-0.51998	0.604375	-5.34858	0.681825	0.766557
Erythroid.cells	CBLB	-0.08735	7.336459	-0.51987	0.604452	-6.69583	0.565346	0.658589
Erythroid.cells	HIP1	-0.15513	5.505684	-0.51984	0.604476	-6.13699	0.601908	0.693026
Erythroid.cells	FITM2	-0.28419	0.918829	-0.51982	0.604486	-5.26376	0.704849	0.787349
Erythroid.cells	CFH	0.137922	4.930751	0.519741	0.604543	-6.3749	0.6139	0.704243
Erythroid.cells	ICE1	-0.17306	4.081492	-0.51945	0.604747	-5.76232	0.632224	0.721195
Erythroid.cells	AMPD1	-0.254	2.911186	-0.51911	0.60498	-5.56466	0.658391	0.745122
Erythroid.cells	GEMIN6	0.233867	2.59595	0.518983	0.605069	-5.50785	0.665607	0.751764
Erythroid.cells	SLC39A7	-0.1324	4.500813	-0.51885	0.605159	-5.84829	0.623405	0.71297
Erythroid.cells	MEX3C	-0.11694	5.133199	-0.5179	0.605819	-5.99121	0.61055	0.700722
Erythroid.cells	HDC	0.400331	3.642871	0.517899	0.605822	-5.76866	0.642633	0.730402
Erythroid.cells	SHLD1	0.163269	3.33904	0.517791	0.605897	-5.77048	0.649388	0.736604
Erythroid.cells	ASPH	0.188426	4.664011	0.517721	0.605946	-5.98395	0.620465	0.709935
Erythroid.cells	FYCO1	0.158655	4.137271	0.517476	0.606116	-5.77931	0.631801	0.720505
Erythroid.cells	GRIK4	-0.50169	0.238216	-0.51741	0.606165	-5.15038	0.722675	0.803026
Erythroid.cells	A530076I1	-0.39273	-0.88246	-0.51738	0.606185	-5.13946	0.751191	0.828387
Erythroid.cells	MRPS10	-0.13038	4.162492	-0.51733	0.606221	-5.89894	0.631253	0.72003
Erythroid.cells	ARMC2	-0.39205	-0.1641	-0.51726	0.606269	-5.13614	0.732784	0.812061
Erythroid.cells	HMGCLL1	-0.29528	1.17582	-0.51717	0.606331	-5.42098	0.699661	0.782421
Erythroid.cells	ISCA2	0.127129	4.968036	0.517045	0.606416	-6.03533	0.614045	0.704084
Erythroid.cells	TMCC1	-0.11801	7.946033	-0.51679	0.606591	-6.61149	0.554613	0.648175
Erythroid.cells	TBC1D19	-0.25424	1.749793	-0.51675	0.60662	-5.32297	0.686055	0.770172
Erythroid.cells	CLEC16A	-0.13737	4.763553	-0.51653	0.606771	-5.94523	0.618521	0.708228
Erythroid.cells	RBM15	-0.0943	5.696792	-0.51639	0.606871	-6.2125	0.599029	0.690083
Erythroid.cells	PEX1	0.216493	3.071405	0.516375	0.606881	-5.62471	0.655583	0.7424
Erythroid.cells	HABP2	-0.36331	0.622763	-0.51619	0.607013	-5.29449	0.713422	0.794763
Erythroid.cells	MLEC	-0.12246	5.064037	-0.51599	0.607151	-5.97453	0.612316	0.702461
Erythroid.cells	AW554918	0.106314	5.656196	0.515884	0.607223	-6.17574	0.600012	0.690992
Erythroid.cells	ZFP787	-0.09969	5.128821	-0.51561	0.607416	-6.07962	0.611012	0.701291
Erythroid.cells	OPTN	-0.16033	4.651784	-0.5156	0.607423	-5.94749	0.621102	0.710666
Erythroid.cells	MRPL16	-0.14218	3.919331	-0.51543	0.60754	-5.82916	0.636937	0.725367
Erythroid.cells	LSM3	-0.12311	5.624923	-0.51542	0.607546	-6.17311	0.600702	0.691732
Erythroid.cells	ATP6V1C1	-0.11418	5.189889	-0.51536	0.607589	-5.96675	0.609733	0.700176
Erythroid.cells	HMGN2	-0.12118	7.53605	-0.51514	0.607743	-6.60895	0.56271	0.655955
Erythroid.cells	KNOP1	-0.11249	4.90183	-0.51506	0.607793	-6.0333	0.615829	0.705906
Erythroid.cells	PARP3	0.246214	2.362796	0.514905	0.607904	-5.48063	0.672044	0.757621
Erythroid.cells	MS4A4C	-0.35983	3.224383	-0.5149	0.607908	-5.7469	0.652391	0.739679
Erythroid.cells	AFTPH	-0.08308	6.658529	-0.51485	0.607945	-6.40441	0.579844	0.672243
Erythroid.cells	LRPPRC	-0.11261	5.200579	-0.51466	0.608077	-6.14383	0.609572	0.700091
Erythroid.cells	GM38560	0.348138	0.991541	0.514545	0.608155	-5.31826	0.704621	0.787073
Erythroid.cells	CHCHD6	0.175415	2.968098	0.514531	0.608165	-5.59546	0.658201	0.744988

Erythroid.cells	A930014D(	-0.36211	-1.05861	-0.51444	0.608227	-5.0808	0.756308	0.833148
Erythroid.cells	SAV1	-0.12955	4.735258	-0.51432	0.608313	-5.89877	0.619388	0.709234
Erythroid.cells	UTP25	0.213253	2.617836	0.514261	0.608352	-5.50874	0.666191	0.752282
Erythroid.cells	PILRB1	0.281305	2.049283	0.514003	0.608532	-5.53149	0.679473	0.764359
Erythroid.cells	GDAP2	0.126875	4.778409	0.513893	0.608609	-5.9458	0.61856	0.708505
Erythroid.cells	MRRF	-0.16246	3.454685	-0.51386	0.608632	-5.72883	0.64736	0.735103
Erythroid.cells	TPST1	-0.19474	3.472726	-0.51368	0.608756	-5.75303	0.646995	0.734799
Erythroid.cells	ART3	-0.33857	0.944132	-0.51361	0.608802	-5.27488	0.705917	0.788333
Erythroid.cells	RGS13	-0.45362	-1.05629	-0.51349	0.608892	-5.13287	0.756399	0.833333
Erythroid.cells	EIF2B3	0.218932	2.996786	0.513445	0.608921	-5.56601	0.657683	0.744606
Erythroid.cells	VAMP7	0.140793	4.132998	0.513316	0.60901	-5.82082	0.632493	0.721481
Erythroid.cells	PAK1	-0.19202	5.334473	-0.51282	0.609356	-6.21329	0.607174	0.697847
Erythroid.cells	IL7R	0.186156	4.966863	0.512571	0.60953	-6.1286	0.614882	0.705063
Erythroid.cells	2410131K1	-0.28493	2.169111	-0.51251	0.60957	-5.38028	0.677015	0.762172
Erythroid.cells	STK4	-0.07175	7.52595	-0.51246	0.609608	-6.60615	0.563295	0.656624
Erythroid.cells	ZBTB11	-0.0845	7.088839	-0.51234	0.609688	-6.47313	0.57177	0.664712
Erythroid.cells	GM14296	-0.28055	0.969349	-0.51229	0.609728	-5.25984	0.705621	0.788108
Erythroid.cells	AK4	0.307482	1.048143	0.512284	0.60973	-5.43752	0.703705	0.786385
Erythroid.cells	AHCYL1	0.093417	5.179051	0.512072	0.609877	-6.05402	0.61042	0.701015
Erythroid.cells	METTL5	0.143937	3.739598	0.512059	0.609887	-5.80127	0.64137	0.729674
Erythroid.cells	ALPK3	-0.47537	-0.11417	-0.51203	0.609905	-5.11562	0.732516	0.812178
Erythroid.cells	SDHAF1	0.15736	4.132974	0.511876	0.610014	-5.85725	0.632752	0.721791
Erythroid.cells	TMEM203	0.204648	3.168142	0.51186	0.610025	-5.6057	0.654109	0.741434
Erythroid.cells	CYP2D26	0.231913	3.247258	0.511601	0.610205	-5.96495	0.652426	0.739856
Erythroid.cells	ADORA3	0.299214	-0.30137	0.511547	0.610243	-5.35786	0.737375	0.816535
Erythroid.cells	ICAM1	-0.14675	5.898773	-0.51122	0.610469	-6.21942	0.595785	0.687302
Erythroid.cells	SCRG1	-0.34576	-0.98095	-0.51098	0.610637	-5.21949	0.755148	0.832254
Erythroid.cells	CCDC126	0.311137	1.899321	0.510827	0.610745	-5.33675	0.683681	0.768305
Erythroid.cells	PRDM2	-0.1018	5.970241	-0.51081	0.610761	-6.25758	0.594377	0.686022
Erythroid.cells	UGT2B34	-0.26109	1.748323	-0.51061	0.610897	-5.57098	0.68725	0.771601
Erythroid.cells	SUPT20	0.113101	5.30473	0.510575	0.610921	-6.01658	0.608095	0.698911
Erythroid.cells	TRIM30A	-0.19722	6.169574	-0.51052	0.61096	-6.18699	0.590332	0.682296
Erythroid.cells	VPS26A	0.089507	6.273847	0.510478	0.610989	-6.26922	0.588228	0.680326
Erythroid.cells	CDK20	0.366568	0.593617	0.51042	0.61103	-5.22357	0.715186	0.796824
Erythroid.cells	SLC26A10	0.370977	0.914242	0.510209	0.611177	-5.28052	0.707384	0.789825
Erythroid.cells	TCTA	0.226372	2.098287	0.51015	0.611218	-5.42377	0.679074	0.764257
Erythroid.cells	BRAP	0.111298	4.924367	0.509879	0.611407	-6.023	0.616204	0.706557
Erythroid.cells	RTRAF	0.077057	7.336334	0.509777	0.611478	-6.61302	0.567346	0.66068
Erythroid.cells	MGAT1	-0.13759	4.704416	-0.50969	0.61154	-5.9321	0.620876	0.710928
Erythroid.cells	3-Sep	0.237698	-1.00287	0.509657	0.611561	-5.43272	0.755867	0.833133
Erythroid.cells	4732471J0	0.271369	1.996323	0.509609	0.611595	-5.39062	0.68153	0.766567
Erythroid.cells	HSH2D	0.213342	3.210107	0.509457	0.611701	-5.61129	0.653641	0.741163
Erythroid.cells	BIRC5	0.213181	5.35755	0.509392	0.611747	-6.21752	0.607135	0.698197
Erythroid.cells	RAB13	-0.30572	1.635625	-0.50913	0.611929	-5.36827	0.690147	0.774448
Erythroid.cells	GM26542	-0.14302	4.802534	-0.50902	0.612005	-5.99232	0.618865	0.709137
Erythroid.cells	FAM189B	-0.2323	2.569109	-0.50888	0.612105	-5.58689	0.66829	0.754617
Erythroid.cells	SNU13	0.074195	6.96176	0.508856	0.612121	-6.50167	0.574728	0.66777

Erythroid.cells	KLHDC10	0.117125	5.434437	0.508835	0.612135	-6.0783	0.605588	0.696777
Erythroid.cells	CDYL2	0.16582	4.757934	0.508691	0.612236	-5.91738	0.619814	0.710109
Erythroid.cells	ZFP467	-0.2299	2.303773	-0.50866	0.612255	-5.55059	0.674429	0.760308
Erythroid.cells	RSPH1	-0.3475	1.855808	-0.5085	0.612367	-5.47008	0.684926	0.769855
Erythroid.cells	TFE3	0.146862	4.149746	0.508493	0.612374	-5.79615	0.632903	0.72226
Erythroid.cells	TAF11	0.114	4.933052	0.508354	0.612471	-5.98741	0.616131	0.706712
Erythroid.cells	COL20A1	-0.27361	1.340253	-0.50785	0.612825	-5.24247	0.697584	0.781161
Erythroid.cells	CEP112	0.352638	1.570821	0.507594	0.613002	-5.30969	0.692184	0.776225
Erythroid.cells	SDC3	0.217786	4.594842	0.507482	0.613081	-6.00778	0.623754	0.713595
Erythroid.cells	B9D2	-0.13887	4.930502	-0.50732	0.613193	-6.03195	0.616654	0.706958
Erythroid.cells	CYB561A3	-0.12258	5.573777	-0.50693	0.613466	-6.11527	0.603322	0.694472
Erythroid.cells	STAR	0.360312	1.465058	0.506921	0.613472	-5.28802	0.694935	0.778582
Erythroid.cells	TMEM87B	-0.11367	5.127611	-0.50686	0.613516	-6.00407	0.612628	0.703165
Erythroid.cells	RCC1	-0.16029	3.989305	-0.50658	0.613709	-5.79128	0.637165	0.725894
Erythroid.cells	LUC7L3	-0.06893	6.706417	-0.50649	0.613771	-6.43448	0.580462	0.673051
Erythroid.cells	ELOVL2	0.240449	2.450205	0.506429	0.613816	-5.774	0.671837	0.757717
Erythroid.cells	INO80D	-0.08432	5.995336	-0.5063	0.613907	-6.256	0.594767	0.686549
Erythroid.cells	HSD11B1	0.141953	4.593839	0.506202	0.613975	-6.12381	0.624063	0.713893
Erythroid.cells	PFN2	0.340774	1.339141	0.506147	0.614013	-5.30549	0.698076	0.781545
Erythroid.cells	IFI211	-0.2488	2.960201	-0.50607	0.614066	-5.86753	0.660136	0.747127
Erythroid.cells	RHOU	0.304997	1.27725	0.505901	0.614185	-5.52998	0.699632	0.782934
Erythroid.cells	LRRK2	0.160591	4.404591	0.505694	0.61433	-6.09154	0.628273	0.717762
Erythroid.cells	PUM1	-0.05927	7.56821	-0.50546	0.614495	-6.61888	0.563793	0.657349
Erythroid.cells	PQLC2	-0.18652	3.590318	-0.50535	0.614573	-5.69509	0.646174	0.734345
Erythroid.cells	GBP2B	-0.84308	0.300434	-0.50534	0.61458	-5.31492	0.723785	0.804675
Erythroid.cells	SPG7	-0.11112	4.467655	-0.50482	0.614944	-5.89083	0.627059	0.716782
Erythroid.cells	SMYD1	0.417637	0.29641	0.504743	0.614995	-5.15625	0.723986	0.804944
Erythroid.cells	PPP2R5C	-0.05575	7.456887	-0.50473	0.615005	-6.57601	0.566018	0.659555
Erythroid.cells	ZFP433	0.384248	0.355661	0.504639	0.615068	-5.18467	0.722506	0.803657
Erythroid.cells	GM5914	-0.17848	3.331997	-0.50457	0.615117	-5.67888	0.652035	0.739854
Erythroid.cells	ZCWPW2	0.376456	1.016841	0.504499	0.615166	-5.24871	0.706204	0.789066
Erythroid.cells	DCTPP1	-0.14483	5.089369	-0.50442	0.615221	-6.09435	0.613813	0.70455
Erythroid.cells	FGD5	0.3609	1.663977	0.504405	0.615231	-5.35119	0.690611	0.775003
Erythroid.cells	NFKBIL1	-0.14697	3.861655	-0.50427	0.615326	-5.73003	0.640259	0.729108
Erythroid.cells	KLRC2	0.239928	1.332802	0.504234	0.615351	-5.76334	0.698546	0.782237
Erythroid.cells	PGAP3	-0.35203	1.12543	-0.50421	0.615368	-5.24	0.703562	0.786765
Erythroid.cells	UNC13D	-0.20331	3.31115	-0.50407	0.615466	-5.55676	0.65253	0.740408
Erythroid.cells	GCC2	-0.12533	5.193926	-0.50391	0.615578	-6.00461	0.611639	0.702636
Erythroid.cells	NAIP5	0.228392	2.68586	0.5039	0.615585	-5.59104	0.666733	0.753434
Erythroid.cells	GM26749	-0.2246	2.222722	-0.50333	0.615983	-5.51684	0.677828	0.763416
Erythroid.cells	NICN1	0.344504	0.820121	0.503162	0.616102	-5.24206	0.711487	0.793807
Erythroid.cells	MAPK11	0.398148	0.260399	0.503008	0.61621	-5.13589	0.725368	0.806324
Erythroid.cells	ATP6V1A	0.102356	6.534998	0.502831	0.616333	-6.29469	0.58453	0.677251
Erythroid.cells	UBE2Q2	-0.10196	5.51991	-0.50281	0.616351	-6.11996	0.605215	0.696664
Erythroid.cells	ETV1	-0.32425	1.377482	-0.50273	0.616407	-5.30419	0.697934	0.781775
Erythroid.cells	TGM1	-0.37577	0.53017	-0.50264	0.616467	-5.26562	0.718644	0.800402
Erythroid.cells	SLC5A11	-0.41898	-0.01556	-0.50261	0.616488	-5.13814	0.732313	0.81262

Erythroid.cells	KLHL32	-0.38815	1.452777	-0.5024	0.616634	-5.28665	0.696123	0.780227
Erythroid.cells	NDUFB5	-0.07361	7.037752	-0.50234	0.616675	-6.55464	0.574564	0.667931
Erythroid.cells	GM15345	0.262444	2.98417	0.502289	0.616713	-5.72238	0.660329	0.747725
Erythroid.cells	ARPC2	-0.04687	9.318766	-0.50218	0.616792	-6.92379	0.531571	0.626893
Erythroid.cells	VPS13C	0.151433	4.373385	0.502109	0.616839	-5.86961	0.629511	0.719406
Erythroid.cells	XNDC1	-0.171	3.594505	-0.50207	0.616865	-5.70551	0.6466	0.735172
Erythroid.cells	GPM6B	-0.31812	2.439081	-0.50194	0.616959	-5.37378	0.672879	0.759253
Erythroid.cells	C230037L1	-0.36025	0.378737	-0.50166	0.617157	-5.16279	0.722543	0.804007
Erythroid.cells	PML	-0.19622	5.273859	-0.50164	0.61717	-6.02724	0.610456	0.701667
Erythroid.cells	WASHC3	0.119767	4.517912	0.50127	0.617427	-5.89406	0.626703	0.716697
Erythroid.cells	STAM	-0.11246	4.382468	-0.50112	0.617529	-5.94352	0.629665	0.719452
Erythroid.cells	NFU1	0.142086	4.549473	0.500875	0.617704	-5.91521	0.626129	0.716177
Erythroid.cells	KDM4B	-0.15411	4.441359	-0.5008	0.617754	-5.89378	0.628459	0.718351
Erythroid.cells	PCDHGC4	0.402581	0.256002	0.500757	0.617786	-5.16229	0.725959	0.807026
Erythroid.cells	CAPNS1	0.069692	7.25812	0.500638	0.61787	-6.53597	0.570649	0.664213
Erythroid.cells	B3GNT2	0.089358	6.739389	0.500466	0.617991	-6.43969	0.580913	0.673886
Erythroid.cells	AMER1	-0.20481	2.080672	-0.50022	0.618165	-5.43648	0.681807	0.767198
Erythroid.cells	PBK	0.211014	3.69023	0.499864	0.618412	-5.84956	0.645038	0.733703
Erythroid.cells	NDUFS8	-0.08126	6.232258	-0.49984	0.618431	-6.3474	0.591137	0.683577
Erythroid.cells	CIAPIN1	-0.13081	4.643928	-0.49982	0.618441	-5.97534	0.624233	0.714482
Erythroid.cells	TAZ	0.138659	4.057371	0.499728	0.618508	-5.74917	0.636944	0.726271
Erythroid.cells	A530041M	0.23322	2.069945	0.49961	0.618591	-5.40807	0.682059	0.767574
Erythroid.cells	ITK	0.155275	4.224474	0.499566	0.618621	-6.48718	0.633296	0.722921
Erythroid.cells	PSTPIP2	-0.20013	4.482064	-0.49942	0.618723	-5.97581	0.627714	0.717761
Erythroid.cells	GYS1	0.242551	3.38284	0.499414	0.618728	-5.60385	0.651898	0.74006
Erythroid.cells	MBLAC1	-0.37939	-0.19049	-0.49932	0.618792	-5.10436	0.737395	0.817329
Erythroid.cells	PHF23	-0.1329	5.240241	-0.49926	0.618838	-6.04467	0.611584	0.702794
Erythroid.cells	DHX37	-0.21709	2.67509	-0.49921	0.618874	-5.485	0.667981	0.754826
Erythroid.cells	ZFP97	-0.30873	1.263623	-0.49908	0.618964	-5.27519	0.701294	0.785063
Erythroid.cells	SETX	-0.10596	5.905752	-0.49906	0.618973	-6.21762	0.597786	0.689938
Erythroid.cells	PACSIN2	0.085739	5.573303	0.499017	0.619007	-6.20315	0.604637	0.696353
Erythroid.cells	CTTN	-0.28548	1.717261	-0.49885	0.619123	-5.3619	0.690463	0.775304
Erythroid.cells	FOXA3	0.261722	1.425712	0.498702	0.619228	-5.4751	0.697488	0.78166
Erythroid.cells	KHDRBS3	0.299601	1.943216	0.498451	0.619404	-5.44026	0.685273	0.770584
Erythroid.cells	ZNRF2	0.094115	5.763077	0.498004	0.619718	-6.18521	0.601125	0.693054
Erythroid.cells	DERA	-0.10462	4.534652	-0.49796	0.619749	-5.97938	0.627008	0.717193
Erythroid.cells	INAFM2	-0.19578	3.225336	-0.49779	0.619869	-5.58173	0.655947	0.743871
Erythroid.cells	UBASH3A	-0.26721	2.009253	-0.49764	0.619975	-5.54244	0.684054	0.769505
Erythroid.cells	AP5B1	-0.31111	1.293299	-0.49756	0.620032	-5.29144	0.701157	0.784972
Erythroid.cells	PMS1	-0.21803	2.650773	-0.49736	0.620172	-5.50324	0.669176	0.755898
Erythroid.cells	DEPDC1A	0.287467	3.001084	0.497226	0.620264	-5.6661	0.661149	0.748594
Erythroid.cells	AARS	0.110865	5.020707	0.497161	0.62031	-6.12921	0.616794	0.707709
Erythroid.cells	DENND6A	-0.11356	5.102952	-0.49708	0.620364	-6.00533	0.615055	0.7061
Erythroid.cells	MRPL14	0.10529	5.601025	0.496837	0.620537	-6.31195	0.604651	0.696425
Erythroid.cells	GLIS2	-0.28652	1.3513	-0.49675	0.620596	-5.36091	0.699857	0.783894
Erythroid.cells	ATP1A1	-0.09734	6.234757	-0.49662	0.620693	-6.3077	0.591662	0.684295
Erythroid.cells	MPRIP	0.10756	5.481388	0.496607	0.620699	-6.1931	0.607137	0.6988

Erythroid.cells	SLAMF9	-0.29703	2.637226	-0.49659	0.620708	-5.51244	0.669505	0.756364
Erythroid.cells	EFNB1	0.276583	1.912975	0.496514	0.620764	-5.37883	0.686429	0.771772
Erythroid.cells	TNKS1BP1	0.25202	2.080541	0.496203	0.620982	-5.42525	0.682491	0.768235
Erythroid.cells	PDCD1LG2	-0.24828	1.775707	-0.49619	0.620994	-5.59274	0.689703	0.774773
Erythroid.cells	H2-Q6	-0.32197	3.115257	-0.49612	0.621039	-5.7901	0.658588	0.74644
Erythroid.cells	METTL8	-0.23582	2.52734	-0.49611	0.621047	-5.48112	0.672061	0.758748
Erythroid.cells	DISC1	-0.1591	3.43956	-0.49601	0.621116	-5.7661	0.651276	0.739744
Erythroid.cells	ATCAYOS	0.444911	1.220314	0.495914	0.621186	-5.26233	0.703044	0.786843
Erythroid.cells	VRK3	0.103374	4.98755	0.495763	0.621292	-6.06983	0.617527	0.708628
Erythroid.cells	OXLD1	0.234747	1.813163	0.495734	0.621312	-5.47341	0.688812	0.774066
Erythroid.cells	DDX59	0.254246	1.892836	0.495671	0.621356	-5.3816	0.686923	0.77236
Erythroid.cells	RNF144A	0.178606	3.619246	0.495497	0.621478	-5.68324	0.647322	0.736191
Erythroid.cells	2200002D(	0.35724	1.337585	0.495005	0.621824	-5.33549	0.700588	0.784622
Erythroid.cells	MIIP	-0.18979	3.057652	-0.49467	0.622061	-5.56675	0.660439	0.748075
Erythroid.cells	AQR	-0.09864	4.812338	-0.49442	0.622235	-5.96887	0.621869	0.712459
Erythroid.cells	LSAMP	0.332495	0.929492	0.494244	0.622359	-5.32428	0.71084	0.793805
Erythroid.cells	4932438A1	-0.10696	7.087671	-0.49416	0.622415	-6.48843	0.575231	0.668801
Erythroid.cells	GM10135	-0.36311	0.409376	-0.49411	0.622454	-5.18893	0.723718	0.805354
Erythroid.cells	ZSWIM7	0.177547	3.432711	0.494064	0.622486	-5.69297	0.652076	0.740449
Erythroid.cells	LMBRD2	-0.16358	4.481892	-0.49355	0.622849	-5.83197	0.62927	0.719257
Erythroid.cells	DCAF15	0.186402	3.456621	0.493329	0.623003	-5.62556	0.651946	0.740104
Erythroid.cells	GGT5	-0.37357	0.649699	-0.49308	0.623178	-5.24763	0.718314	0.800208
Erythroid.cells	BTRC	0.111276	5.211768	0.492949	0.623271	-6.13193	0.613921	0.704864
Erythroid.cells	DNAJC21	-0.09525	5.890572	-0.49273	0.623424	-6.27133	0.599881	0.691754
Erythroid.cells	AASDH	-0.2766	2.290623	-0.49215	0.623835	-5.42442	0.679288	0.764846
Erythroid.cells	4833407H1	-0.19953	2.098821	-0.49205	0.623901	-5.49463	0.683796	0.76895
Erythroid.cells	SAMD4B	0.112037	5.014196	0.491757	0.62411	-6.00467	0.618574	0.709108
Erythroid.cells	HNRNPA3	-0.06519	8.554364	-0.49173	0.62413	-6.81561	0.548034	0.642561
Erythroid.cells	RNF227	-0.26918	1.852006	-0.49172	0.624137	-5.40852	0.689688	0.774328
Erythroid.cells	GM27017	-0.19335	3.311249	-0.49153	0.624269	-5.69736	0.655895	0.743546
Erythroid.cells	GSK3A	0.095213	5.557032	0.491499	0.624291	-6.15724	0.607186	0.698488
Erythroid.cells	DCLK2	0.21496	2.72479	0.491303	0.62443	-5.59495	0.669323	0.755811
Erythroid.cells	DMC1	-0.36446	0.152078	-0.49125	0.624464	-5.1413	0.731439	0.811826
Erythroid.cells	USP20	-0.18863	2.877475	-0.49088	0.624727	-5.51021	0.666024	0.752713
Erythroid.cells	MRPL20	0.083616	6.1388	0.490606	0.62492	-6.36623	0.595439	0.687393
Erythroid.cells	TACC3	0.184622	5.072041	0.490524	0.624978	-6.09513	0.617621	0.708114
Erythroid.cells	LDHC	0.478554	0.610466	0.490489	0.625003	-5.16392	0.720194	0.801673
Erythroid.cells	E130308A1	0.126821	4.522919	0.490327	0.625117	-5.95405	0.629378	0.719022
Erythroid.cells	GM14326	-0.19765	2.868013	-0.49031	0.625131	-5.59165	0.66625	0.752911
Erythroid.cells	HIST2H2BE	0.335722	0.814865	0.490183	0.625219	-5.23191	0.71513	0.797157
Erythroid.cells	DHRS9	0.292817	0.196917	0.490143	0.625247	-5.37398	0.73055	0.810953
Erythroid.cells	MAP7D1	-0.10775	5.36793	-0.49014	0.62525	-6.01732	0.611382	0.702325
Erythroid.cells	SLCO3A1	0.174037	4.38978	0.489488	0.625709	-6.07248	0.632623	0.721791
Erythroid.cells	CSNK1E	-0.13377	4.953409	-0.48945	0.625734	-5.96753	0.620494	0.71056
Erythroid.cells	GM49692	-0.37044	0.104027	-0.48926	0.625872	-5.16755	0.733335	0.813172
Erythroid.cells	ANXA2	0.12572	5.910919	0.489039	0.626025	-6.35883	0.600465	0.691893
Erythroid.cells	PUM2	0.057858	7.324014	0.488993	0.626058	-6.55186	0.572123	0.665203

Erythroid.cells	ITGA4	0.094218	7.657286	0.488909	0.626117	-6.68698	0.565648	0.65909
Erythroid.cells	NDUFA10	0.077228	6.050526	0.488834	0.626169	-6.37336	0.597599	0.689261
Erythroid.cells	RBMX	-0.12482	4.578527	-0.48872	0.626253	-5.88309	0.628552	0.718119
Erythroid.cells	OSBPL3	-0.13078	3.353205	-0.4887	0.626265	-6.09516	0.655603	0.743032
Erythroid.cells	PTPN22	0.107263	5.324165	0.488646	0.626302	-6.51772	0.612667	0.703366
Erythroid.cells	PREB	0.114386	4.736405	0.488604	0.626332	-5.95878	0.625153	0.71497
Erythroid.cells	PCGF1	-0.27952	1.17851	-0.48811	0.626681	-5.31492	0.706777	0.789557
Erythroid.cells	FUCA1	0.090557	5.963141	0.488087	0.626696	-6.2865	0.599512	0.691143
Erythroid.cells	TESC	0.249803	1.561476	0.488054	0.62672	-5.6287	0.6975	0.781199
Erythroid.cells	EFCAB8	-0.27142	1.374064	-0.48805	0.626721	-5.35546	0.702024	0.785278
Erythroid.cells	GM49173	0.494191	-0.87955	0.487876	0.626845	-5.12775	0.758825	0.835971
Erythroid.cells	DHX57	-0.1459	3.871504	-0.4878	0.626898	-5.76442	0.644145	0.732581
Erythroid.cells	UTP20	0.201412	3.652111	0.487786	0.626909	-5.71021	0.649025	0.737068
Erythroid.cells	PSMB1	-0.06755	7.299863	-0.48756	0.627066	-6.60353	0.572783	0.665926
Erythroid.cells	TMEM258	0.08087	6.825258	0.48749	0.627117	-6.493	0.582152	0.674785
Erythroid.cells	STAG3	-0.47626	0.607181	-0.48732	0.627241	-5.12734	0.720946	0.802267
Erythroid.cells	SEC23IP	-0.1061	4.499732	-0.48723	0.627303	-5.87196	0.630466	0.719959
Erythroid.cells	TMEM86A	0.220294	2.885047	0.487214	0.627313	-5.57118	0.666479	0.753049
Erythroid.cells	DUSP4	0.270426	0.909125	0.486823	0.627589	-5.3155	0.713573	0.79569
Erythroid.cells	DCAF5	0.109423	5.52135	0.486737	0.627649	-6.13163	0.608827	0.699894
Erythroid.cells	ZFP638	-0.08272	6.22747	-0.48668	0.62769	-6.30795	0.594271	0.686269
Erythroid.cells	REEP4	0.164071	4.356559	0.48661	0.627739	-5.82812	0.633666	0.722962
Erythroid.cells	RBX1	0.065109	7.700144	0.486535	0.627792	-6.66233	0.56509	0.658704
Erythroid.cells	TYROBP	0.100174	9.095091	0.486458	0.627846	-6.891	0.538854	0.633638
Erythroid.cells	C030034I2:	0.197257	2.630863	0.486448	0.627853	-5.53242	0.672437	0.75854
Erythroid.cells	PLA2G6	-0.34282	1.28393	-0.48635	0.627923	-5.2694	0.704409	0.787485
Erythroid.cells	ZFP637	0.227749	2.315939	0.485918	0.628227	-5.48814	0.68004	0.7653
Erythroid.cells	SLC26A11	0.22323	3.200265	0.485581	0.628465	-5.62686	0.659791	0.746815
Erythroid.cells	RPAP2	-0.17809	3.02458	-0.48525	0.628699	-5.58878	0.663795	0.750529
Erythroid.cells	MDM1	-0.18758	3.41542	-0.48525	0.628701	-5.73165	0.654923	0.742412
Erythroid.cells	HIST2H4	-0.40121	0.951972	-0.48522	0.628723	-5.20116	0.712966	0.795057
Erythroid.cells	HDHD5	0.197205	2.842941	0.485125	0.628788	-5.63345	0.667961	0.754377
Erythroid.cells	PHPT1	-0.13665	4.330971	-0.48509	0.628815	-5.88586	0.634621	0.723783
Erythroid.cells	AGAP2	0.195205	2.925575	0.485069	0.628827	-5.5684	0.666062	0.752651
Erythroid.cells	FAM92A	0.17832	3.301539	0.484748	0.629054	-5.68995	0.65763	0.744921
Erythroid.cells	DENND4A	-0.08532	10.03042	-0.4847	0.629085	-7.07418	0.522429	0.617707
Erythroid.cells	BAHCC1	0.301045	1.235974	0.484402	0.629299	-5.33597	0.70625	0.789059
Erythroid.cells	POLDIP2	0.134608	4.278307	0.484253	0.629404	-5.91388	0.635985	0.725106
Erythroid.cells	CEPT1	-0.13366	5.374134	-0.48418	0.629453	-6.11695	0.612498	0.703347
Erythroid.cells	STARD5	0.146472	4.526318	0.483946	0.629621	-5.85236	0.630587	0.720192
Erythroid.cells	DPF3	0.280426	1.667384	0.483904	0.62965	-5.45313	0.695817	0.779814
Erythroid.cells	KCTD4	-0.24923	2.200261	-0.48386	0.629682	-5.49239	0.683148	0.768346
Erythroid.cells	TYSND1	-0.17196	2.640407	-0.48375	0.629761	-5.55103	0.672864	0.758998
Erythroid.cells	IDH3A	-0.11926	4.865883	-0.48367	0.629819	-6.03266	0.623276	0.713462
Erythroid.cells	ATP13A1	0.148658	3.809105	0.483644	0.629834	-5.67377	0.646328	0.734764
Erythroid.cells	SEMA5A	0.352749	1.106724	0.483638	0.629839	-5.32816	0.709407	0.792114
Erythroid.cells	GPS2	0.068042	5.859455	0.483443	0.629977	-6.30482	0.602391	0.694022

Erythroid.cells	XPO4	0.095968	5.623363	0.483421	0.629992	-6.19354	0.607285	0.698599
Erythroid.cells	MRAP	-0.35539	1.214744	-0.4834	0.630005	-5.35471	0.706768	0.789765
Erythroid.cells	NSMCE4A	0.076303	6.083141	0.483244	0.630117	-6.34944	0.597805	0.689743
Erythroid.cells	SRR	0.185397	2.599008	0.483201	0.630148	-5.55845	0.673839	0.759981
Erythroid.cells	ZC3HC1	0.124808	4.34425	0.483068	0.630242	-5.89017	0.63458	0.723955
Erythroid.cells	RRAGC	0.104065	5.819399	0.482989	0.630298	-6.11199	0.603251	0.694826
Erythroid.cells	TMEM50B	-0.20256	4.107732	-0.48278	0.630444	-5.71369	0.639844	0.728787
Erythroid.cells	4933423P2	-0.26175	1.804113	-0.48267	0.630521	-5.3506	0.692686	0.777021
Erythroid.cells	SNHG20	-0.18729	2.355617	-0.48202	0.630985	-5.49258	0.679946	0.765321
Erythroid.cells	SLC25A26	-0.17038	3.795963	-0.48195	0.631035	-5.73892	0.647046	0.735273
Erythroid.cells	CHCHD2	0.053013	8.94643	0.481876	0.631085	-6.91926	0.542465	0.63707
Erythroid.cells	MAD2L2	-0.16097	3.472942	-0.48184	0.631107	-5.73824	0.654277	0.741914
Erythroid.cells	GPATCH2	0.144512	4.253922	0.481758	0.631169	-5.83802	0.636936	0.725994
Erythroid.cells	KITL	0.409509	1.956944	0.481664	0.631235	-5.45795	0.689356	0.773904
Erythroid.cells	ZFP58	0.30559	1.194809	0.481575	0.631298	-5.28272	0.707719	0.790476
Erythroid.cells	FCRL1	0.20746	2.73778	0.481501	0.63135	-5.68571	0.671051	0.757306
Erythroid.cells	GM16740	0.221744	2.544974	0.481449	0.631387	-5.48236	0.675524	0.761397
Erythroid.cells	PIP4P1	-0.07482	5.856707	-0.48043	0.632111	-6.22534	0.603412	0.694671
Erythroid.cells	PRRC1	0.128056	4.219989	0.480251	0.632235	-5.83881	0.638281	0.727079
Erythroid.cells	HSD17B6	-0.327	0.767255	-0.48024	0.632243	-5.37331	0.718916	0.800357
Erythroid.cells	ELMOD3	-0.26097	3.437093	-0.48022	0.632254	-5.605	0.655703	0.743098
Erythroid.cells	STOML2	-0.117	4.805582	-0.4801	0.632339	-6.06512	0.625568	0.715356
Erythroid.cells	GRAP	0.143396	4.536304	0.480061	0.632369	-5.95327	0.63138	0.720759
Erythroid.cells	RNF126	-0.12301	4.767243	-0.47987	0.632505	-5.94826	0.626462	0.716194
Erythroid.cells	POFUT2	-0.13868	4.257323	-0.47928	0.632922	-5.84666	0.63761	0.726619
Erythroid.cells	HGS	0.136075	4.094551	0.479229	0.632959	-5.80976	0.641188	0.729926
Erythroid.cells	E030042O2	-0.46107	0.180529	-0.47921	0.632973	-5.10933	0.733796	0.813848
Erythroid.cells	CXCL2	0.317502	7.158496	0.479142	0.63302	-6.58199	0.577252	0.670254
Erythroid.cells	CCDC115	-0.13598	4.152653	-0.47913	0.633032	-5.77782	0.639908	0.728755
Erythroid.cells	DNAH12	0.325675	1.676699	0.47907	0.633072	-5.47059	0.696868	0.780735
Erythroid.cells	NUFIP2	0.077108	7.215419	0.479001	0.633121	-6.52213	0.57613	0.669223
Erythroid.cells	GM11342	-0.28472	2.120734	-0.47888	0.633203	-5.47616	0.686279	0.771181
Erythroid.cells	CELF6	-0.46026	-0.70622	-0.4788	0.633264	-5.12798	0.756614	0.834166
Erythroid.cells	RPIA	-0.10593	5.458024	-0.47879	0.633269	-6.13581	0.611858	0.702787
Erythroid.cells	RAB8B	-0.08651	7.724426	-0.47876	0.63329	-6.73163	0.566201	0.659825
Erythroid.cells	GPR27	0.25673	-1.60473	0.478262	0.633644	-5.09587	0.780817	0.855454
Erythroid.cells	LRPAP1	0.126107	4.499631	0.477922	0.633885	-5.96609	0.63265	0.722058
Erythroid.cells	ARID1A	-0.07141	6.658177	-0.47792	0.633889	-6.40014	0.587516	0.679945
Erythroid.cells	YARS	-0.1346	5.391202	-0.4777	0.63404	-6.1455	0.613579	0.704402
Erythroid.cells	PWWP2B	0.228845	2.082717	0.477643	0.634083	-5.49382	0.687534	0.772335
Erythroid.cells	GM16196	-0.27673	1.751417	-0.47763	0.63409	-5.33444	0.695434	0.779482
Erythroid.cells	TRABD	0.111048	5.363153	0.47762	0.6341	-6.05343	0.614169	0.704955
Erythroid.cells	C2	0.286709	2.413631	0.47757	0.634135	-5.54499	0.679736	0.765263
Erythroid.cells	5730409E0	-0.35439	0.23499	-0.47724	0.634366	-5.21182	0.732894	0.813137
Erythroid.cells	JPX	-0.21384	3.810956	-0.47721	0.634388	-5.7128	0.647893	0.736188
Erythroid.cells	GRPEL1	0.093976	5.877727	0.477102	0.634467	-6.34864	0.603509	0.695027
Erythroid.cells	NT5M	0.15208	3.572881	0.477087	0.634477	-5.69363	0.653221	0.741082

Erythroid.cells	NOP56	-0.12295	4.591451	-0.47699	0.634544	-5.93825	0.630743	0.720397
Erythroid.cells	RNF130	-0.07747	7.282781	-0.47686	0.634642	-6.51307	0.575211	0.668435
Erythroid.cells	HIST1H2AF	0.364902	0.617707	0.47666	0.63478	-5.29433	0.723374	0.804629
Erythroid.cells	KLHL28	0.164439	3.44241	0.476587	0.634832	-5.63628	0.656251	0.743873
Erythroid.cells	ADI1	0.123004	3.749643	0.476494	0.634898	-5.84784	0.64935	0.737548
Erythroid.cells	2310008N1	-0.31813	0.80481	-0.47639	0.634972	-5.31452	0.718717	0.800513
Erythroid.cells	ARRB1	0.120921	3.996665	0.476246	0.635074	-5.9143	0.643857	0.73258
Erythroid.cells	GM12992	0.27909	2.041763	0.476221	0.635092	-5.46109	0.688692	0.773536
Erythroid.cells	MBOAT2	0.391076	0.666194	0.476069	0.6352	-5.3273	0.722164	0.803746
Erythroid.cells	GGH	-0.15845	4.555539	-0.47605	0.635211	-5.9746	0.631607	0.721343
Erythroid.cells	ANKRD9	-0.23845	2.821177	-0.47588	0.635334	-5.51719	0.670458	0.757019
Erythroid.cells	GM22146	-0.24987	2.073446	-0.47583	0.635367	-5.46298	0.687959	0.772927
Erythroid.cells	4930532G1	-0.37065	1.429337	-0.4757	0.63546	-5.32581	0.703413	0.786913
Erythroid.cells	IFI35	0.14466	5.333965	0.475586	0.635542	-6.11113	0.614968	0.705958
Erythroid.cells	CISH	0.203305	2.887974	0.475573	0.635552	-5.7707	0.668917	0.755694
Erythroid.cells	NF1	0.084309	6.550388	0.475244	0.635785	-6.36418	0.589993	0.682545
Erythroid.cells	LRRC47	-0.11856	4.227098	-0.47519	0.635822	-5.84152	0.638934	0.728168
Erythroid.cells	SLC46A1	0.3955	0.761766	0.475046	0.635926	-5.27963	0.719964	0.801893
Erythroid.cells	STIL	-0.19134	4.156047	-0.47501	0.635952	-5.93375	0.640497	0.729682
Erythroid.cells	TNFRSF25	-0.30101	-1.26707	-0.47485	0.636062	-5.16515	0.772212	0.84838
Erythroid.cells	9430060I0	0.337031	1.308677	0.474829	0.63608	-5.27351	0.706503	0.789857
Erythroid.cells	NDNF	-0.28869	-0.47189	-0.47461	0.636238	-5.33792	0.751404	0.82993
Erythroid.cells	ZFPM1	0.125324	4.248218	0.47446	0.636341	-6.06803	0.638603	0.727894
Erythroid.cells	PLEKHB2	0.135166	4.419141	0.474261	0.636483	-5.79748	0.63494	0.724448
Erythroid.cells	GM47819	0.442982	-0.22199	0.473995	0.636672	-5.17824	0.745192	0.824276
Erythroid.cells	FOXN3	0.07675	8.899803	0.473947	0.636706	-6.86854	0.544788	0.639547
Erythroid.cells	AI182371	0.211001	2.439191	0.473856	0.63677	-5.77852	0.679814	0.765565
Erythroid.cells	AHCYL2	0.09513	6.28947	0.473344	0.637135	-6.39692	0.595754	0.687887
Erythroid.cells	PIBF1	-0.1193	5.059147	-0.47332	0.637154	-5.98956	0.621419	0.71187
Erythroid.cells	NRBF2	-0.12747	4.314912	-0.47326	0.637194	-5.87795	0.637509	0.726777
Erythroid.cells	GM31728	-0.37542	-0.17439	-0.47322	0.637226	-5.21522	0.744198	0.823408
Erythroid.cells	PPM1K	0.163144	3.579795	0.473071	0.637328	-5.82093	0.653863	0.74189
Erythroid.cells	UQCC2	0.095038	6.141014	0.472847	0.637488	-6.36515	0.59882	0.690867
Erythroid.cells	SYF2	0.066826	5.903221	0.472832	0.637498	-6.30749	0.603718	0.695456
Erythroid.cells	NAPG	-0.12895	4.542195	-0.47281	0.637516	-5.90997	0.632581	0.722323
Erythroid.cells	LAD1	0.445583	-0.99444	0.472528	0.637714	-5.20196	0.765773	0.842605
Erythroid.cells	BID	-0.16332	3.803317	-0.47163	0.638354	-5.71951	0.649537	0.737679
Erythroid.cells	CYP7A1	-0.3102	0.739983	-0.47148	0.638462	-5.38112	0.721866	0.803305
Erythroid.cells	IMPDH1	-0.16546	4.326873	-0.47137	0.638536	-5.86785	0.637949	0.727042
Erythroid.cells	ENTPD5	0.119709	3.78751	0.471207	0.638654	-5.81749	0.64989	0.738078
Erythroid.cells	GPR55	-0.2383	1.548876	-0.47107	0.638749	-5.44285	0.701996	0.785498
Erythroid.cells	ARL4D	-0.28521	1.705958	-0.47105	0.638767	-5.41426	0.698202	0.782075
Erythroid.cells	GALNT12	-0.18975	2.991393	-0.47097	0.638819	-5.63005	0.667945	0.754612
Erythroid.cells	ZFP280D	0.101107	5.625842	0.470957	0.638831	-6.1813	0.610127	0.701258
Erythroid.cells	MDC1	-0.18015	3.159139	-0.4709	0.638871	-5.59513	0.664098	0.751098
Erythroid.cells	TRBC1	0.14099	3.183542	0.470706	0.63901	-6.30883	0.66354	0.750588
Erythroid.cells	DRAM2	-0.11405	4.847911	-0.47068	0.639027	-6.05404	0.626632	0.716611

Erythroid.cells	APOBEC4	-0.36334	0.042895	-0.47068	0.639029	-5.17199	0.739449	0.81905
Erythroid.cells	CYP2D10	-0.23286	1.738911	-0.47017	0.639391	-5.57665	0.697733	0.781532
Erythroid.cells	RUNDC1	-0.17663	2.994317	-0.47008	0.639456	-5.60937	0.668188	0.754717
Erythroid.cells	ACOT7	-0.11707	4.076826	-0.46969	0.639731	-5.98713	0.64391	0.732477
Erythroid.cells	LIMS1	0.079177	7.288787	0.469681	0.639739	-6.5162	0.576776	0.669824
Erythroid.cells	CHMP6	0.162912	3.712475	0.469567	0.639821	-5.75678	0.652047	0.739992
Erythroid.cells	B3GNTL1	0.210663	2.673331	0.469365	0.639964	-5.54087	0.675816	0.761706
Erythroid.cells	DLG3	-0.32494	0.571754	-0.46936	0.639968	-5.23856	0.726621	0.807525
Erythroid.cells	AHRR	-0.31897	-0.36283	-0.46913	0.640128	-5.21592	0.750488	0.828822
Erythroid.cells	LSMEM1	0.226396	4.06136	0.469134	0.640129	-5.91038	0.644319	0.732916
Erythroid.cells	CPA6	0.327642	-0.07229	0.469021	0.640209	-5.27758	0.743014	0.822207
Erythroid.cells	RIOX2	-0.12644	3.769866	-0.46892	0.640284	-5.73211	0.650837	0.738938
Erythroid.cells	SLC16A4	0.250444	1.428027	0.468492	0.640586	-5.36199	0.705787	0.788687
Erythroid.cells	1700096K1	-0.22326	2.613489	-0.46815	0.640827	-5.47115	0.677686	0.763207
Erythroid.cells	ANKZF1	-0.15112	2.742515	-0.46798	0.640951	-5.55788	0.67468	0.760504
Erythroid.cells	CSTDC6	-0.25789	-1.16323	-0.46791	0.641003	-5.22229	0.772027	0.847634
Erythroid.cells	EPCAM	0.256137	2.160039	0.467897	0.64101	-5.55027	0.68836	0.772925
Erythroid.cells	SHPK	-0.32033	0.252393	-0.46782	0.641066	-5.24361	0.735193	0.815022
Erythroid.cells	ELOVL1	-0.1256	4.858115	-0.46771	0.641146	-5.98692	0.627333	0.717066
Erythroid.cells	HIST1H1E	0.270136	4.203103	0.467572	0.641241	-6.01079	0.64161	0.73033
Erythroid.cells	HDGF	-0.07039	6.998077	-0.46754	0.641265	-6.54459	0.58298	0.675671
Erythroid.cells	ITIH4	0.160633	3.988196	0.46743	0.641342	-6.16237	0.646381	0.734726
Erythroid.cells	B3GALT5	-0.28506	0.779445	-0.4672	0.641503	-5.54179	0.722045	0.803363
Erythroid.cells	MEI4	-0.346	1.201602	-0.46683	0.641771	-5.34647	0.711602	0.794001
Erythroid.cells	2010009K1	-0.51188	0.112521	-0.46677	0.641813	-5.12898	0.738863	0.818382
Erythroid.cells	4930444A1	0.167573	3.751804	0.46676	0.64182	-5.84209	0.651738	0.739637
Erythroid.cells	WARS2	-0.17267	3.512477	-0.46674	0.641837	-5.69585	0.657127	0.74458
Erythroid.cells	ZFP651	0.255297	1.578642	0.466709	0.641856	-5.34447	0.702406	0.785725
Erythroid.cells	AURKA	0.186464	3.318106	0.466666	0.641887	-5.71372	0.661538	0.748618
Erythroid.cells	LILRB4A	-0.16196	4.163584	-0.46643	0.642054	-6.09074	0.642574	0.731209
Erythroid.cells	AGAP1	0.163089	4.225875	0.466418	0.642063	-5.92539	0.641199	0.729942
Erythroid.cells	TASP1	-0.16561	3.918389	-0.46637	0.642097	-5.83636	0.648014	0.736216
Erythroid.cells	ZMYM1	0.164703	3.329996	0.466318	0.642135	-5.66523	0.661267	0.748371
Erythroid.cells	ZFP618	-0.36198	0.531603	-0.46582	0.642492	-5.18956	0.728581	0.809054
Erythroid.cells	ZFP46	0.194392	1.77659	0.465677	0.642592	-5.36587	0.697949	0.781575
Erythroid.cells	NIT2	0.149932	3.588667	0.465435	0.642764	-5.88963	0.655709	0.743222
Erythroid.cells	TRIP11	-0.08687	6.069875	-0.46534	0.642834	-6.26886	0.60216	0.693647
Erythroid.cells	AMIGO1	0.310348	0.613629	0.465323	0.642845	-5.20598	0.726526	0.807345
Erythroid.cells	PSMD6	0.10718	5.199485	0.465304	0.642858	-6.10284	0.6204	0.710661
Erythroid.cells	ARMC8	0.097465	5.019279	0.465265	0.642886	-5.98365	0.624249	0.714235
Erythroid.cells	CHORDC1	0.096501	5.082056	0.465117	0.642991	-6.06325	0.622944	0.712991
Erythroid.cells	1700110K1	-0.45047	-0.57203	-0.46484	0.64319	-5.08732	0.757019	0.834399
Erythroid.cells	MFAP3	-0.10479	5.124697	-0.46483	0.643193	-6.02525	0.622102	0.712182
Erythroid.cells	NCEH1	0.171408	4.906119	0.464617	0.643348	-5.88075	0.626876	0.716555
Erythroid.cells	PUSL1	-0.19799	2.317284	-0.46451	0.643426	-5.4866	0.685278	0.770081
Erythroid.cells	IPP	-0.19588	2.465742	-0.46436	0.643528	-5.52129	0.68178	0.766908
Erythroid.cells	RETREG2	0.111174	4.884018	0.464329	0.643554	-6.07354	0.627355	0.716996

Erythroid.cells	MLST8	0.270079	2.044303	0.464142	0.643687	-5.37084	0.691757	0.775949
Erythroid.cells	SLC4A4	0.218777	2.065431	0.464132	0.643694	-5.6653	0.691253	0.775493
Erythroid.cells	NFAT5	-0.09067	7.501438	-0.46407	0.643741	-6.58695	0.573569	0.666608
Erythroid.cells	NDUFB3	0.084021	5.72376	0.46395	0.643824	-6.2822	0.609556	0.700428
Erythroid.cells	CFI	-0.1809	3.743943	-0.4633	0.644286	-6.12046	0.652809	0.740296
Erythroid.cells	FBXW9	-0.18653	2.418265	-0.46325	0.644322	-5.50905	0.683299	0.768124
Erythroid.cells	ANTXR2	0.162007	7.654982	0.463166	0.644383	-6.71612	0.570906	0.663977
Erythroid.cells	SLC7A11	-0.24362	4.278698	-0.46305	0.644469	-6.14145	0.640915	0.729416
Erythroid.cells	AHCY	0.151411	3.495963	0.462989	0.64451	-5.89604	0.658403	0.745481
Erythroid.cells	CTSK	0.345673	0.234472	0.46285	0.644609	-5.2281	0.736807	0.816277
Erythroid.cells	DRC7	0.39839	-0.15036	0.462709	0.64471	-5.13386	0.746705	0.825072
Erythroid.cells	BAIAP3	-0.32677	-0.32152	-0.46254	0.644834	-5.28236	0.751135	0.829077
Erythroid.cells	HDLBP	0.063028	6.655734	0.462413	0.644921	-6.44674	0.590806	0.68285
Erythroid.cells	0610039K1	0.421367	0.022539	0.462378	0.644946	-5.11377	0.742263	0.821213
Erythroid.cells	ZFP873	-0.27071	0.995152	-0.46234	0.644973	-5.26166	0.717755	0.799332
Erythroid.cells	PSME4	-0.07573	7.130098	-0.46212	0.64513	-6.57096	0.581355	0.673919
Erythroid.cells	VPS11	-0.13678	4.072729	-0.46204	0.645188	-5.74119	0.645603	0.733804
Erythroid.cells	R3HCC1	0.218446	2.353768	0.461983	0.645228	-5.56235	0.684962	0.769753
Erythroid.cells	GM34086	0.183321	2.658244	0.461776	0.645376	-5.70338	0.677899	0.763348
Erythroid.cells	GM40645	0.266125	0.751306	0.461173	0.645807	-5.47532	0.72432	0.804985
Erythroid.cells	APOA1	0.1702	8.277086	0.461173	0.645807	-6.98136	0.559366	0.652897
Erythroid.cells	SESTD1	-0.24174	2.300083	-0.46095	0.645964	-5.67708	0.686736	0.771092
Erythroid.cells	SDR42E1	0.274759	0.45745	0.46057	0.646238	-5.27986	0.732046	0.811701
Erythroid.cells	MYNN	-0.10654	4.303762	-0.46033	0.64641	-5.93538	0.641267	0.72937
Erythroid.cells	CEP170	0.096691	5.909007	0.460204	0.6465	-6.24221	0.606894	0.697493
Erythroid.cells	RUSC2	0.311423	0.909936	0.460167	0.646526	-5.38211	0.720804	0.801569
Erythroid.cells	TTLL1	-0.24584	1.626512	-0.46006	0.646604	-5.36415	0.703216	0.785784
Erythroid.cells	PYGO2	0.215226	3.374512	0.45959	0.646939	-5.63169	0.662193	0.748625
Erythroid.cells	RABL6	0.097154	5.136693	0.459402	0.647073	-5.98401	0.623275	0.712865
Erythroid.cells	TMEM51	-0.16083	3.130512	-0.45933	0.647128	-5.85225	0.667779	0.753807
Erythroid.cells	MEA1	0.10069	5.276389	0.45904	0.647332	-6.09012	0.620295	0.710182
Erythroid.cells	CTR9	0.109317	4.518969	0.459025	0.647342	-5.91328	0.636637	0.725316
Erythroid.cells	NFIC	-0.12736	4.464453	-0.45891	0.647427	-5.88995	0.637831	0.726444
Erythroid.cells	TRIAP1	-0.12991	4.111675	-0.45888	0.647444	-5.84694	0.645612	0.733613
Erythroid.cells	FBXL7	0.265156	3.621991	0.458773	0.647523	-5.83089	0.656577	0.74368
Erythroid.cells	PVR	-0.19654	3.063552	-0.45869	0.647583	-5.62664	0.66932	0.755326
Erythroid.cells	HSD17B4	0.091597	5.027211	0.458577	0.647663	-6.1528	0.625622	0.715168
Erythroid.cells	BEX3	0.108614	4.990762	0.458568	0.64767	-6.14409	0.626405	0.715894
Erythroid.cells	RAG2	0.385065	0.168525	0.458411	0.647782	-5.26195	0.739601	0.818677
Erythroid.cells	GADD45B	-0.15728	5.322838	-0.45839	0.647798	-6.10796	0.619307	0.709345
Erythroid.cells	PRPF40B	0.405221	0.534618	0.458373	0.647809	-5.18129	0.730311	0.810399
Erythroid.cells	F8A	0.197499	2.551871	0.458358	0.64782	-5.54944	0.681222	0.766213
Erythroid.cells	RNF139	-0.1106	5.218473	-0.45836	0.647821	-6.08704	0.621529	0.711409
Erythroid.cells	RHEBL1	-0.28101	1.532009	-0.45834	0.647835	-5.34205	0.705602	0.788252
Erythroid.cells	CHD1L	0.141376	3.722264	0.458304	0.647858	-5.70776	0.654316	0.741664
Erythroid.cells	GBF1	0.065226	6.562441	0.457698	0.648293	-6.36531	0.593885	0.685467
Erythroid.cells	CEBPZ	0.077402	6.141756	0.457617	0.64835	-6.27553	0.6025	0.693547

Erythroid.cells	ARL13B	0.142909	3.775076	0.457344	0.648546	-5.72129	0.65358	0.740857
Erythroid.cells	JAK2	0.091421	6.674709	0.457191	0.648655	-6.52433	0.591686	0.683416
Erythroid.cells	TESK1	0.146263	3.781202	0.457003	0.64879	-5.74418	0.653442	0.740764
Erythroid.cells	KCNAB1	0.313791	1.324987	0.4569	0.648864	-5.38436	0.711151	0.793167
Erythroid.cells	RAB11FIP3	-0.20498	2.230172	-0.45669	0.649016	-5.4916	0.689293	0.773522
Erythroid.cells	BMX	0.31597	0.02253	0.456595	0.649082	-5.38569	0.743852	0.822499
Erythroid.cells	1700066M	0.228604	1.288003	0.456561	0.649106	-5.37381	0.712059	0.794105
Erythroid.cells	LUC7L	0.075114	5.622585	0.456496	0.649153	-6.25038	0.613397	0.703917
Erythroid.cells	ZFP629	-0.34926	0.926	-0.45636	0.649248	-5.21726	0.721009	0.802181
Erythroid.cells	SLC2A12	-0.25948	1.619007	-0.45636	0.64925	-5.38414	0.703974	0.786881
Erythroid.cells	ZFX	-0.06551	6.339182	-0.45632	0.649281	-6.34574	0.59852	0.690022
Erythroid.cells	CD55B	0.42187	0.178504	0.456269	0.649315	-5.15102	0.739857	0.819005
Erythroid.cells	EIF3L	-0.08375	5.672102	-0.45625	0.649329	-6.24565	0.612356	0.702957
Erythroid.cells	ZFP568	-0.13881	4.08691	-0.45616	0.64939	-5.86685	0.646608	0.734694
Erythroid.cells	BC029722	0.17688	3.061908	0.456078	0.649452	-5.60969	0.669821	0.755988
Erythroid.cells	TMEM167	-0.06202	6.804318	-0.45604	0.649478	-6.49821	0.589069	0.68121
Erythroid.cells	SRSF7	0.101638	6.098296	0.455867	0.649603	-6.28479	0.603514	0.694758
Erythroid.cells	RASA3	0.08187	6.702935	0.455679	0.649738	-6.39959	0.591151	0.683179
Erythroid.cells	CD200R2	0.26023	0.789771	0.455658	0.649753	-5.64696	0.724451	0.805351
Erythroid.cells	ABR	-0.15237	6.594873	-0.45562	0.649777	-6.29839	0.593341	0.685245
Erythroid.cells	RAB33B	-0.12606	4.405753	-0.45548	0.649879	-5.7784	0.639634	0.728306
Erythroid.cells	BICD1	0.340316	0.681795	0.455111	0.650145	-5.21847	0.727373	0.807892
Erythroid.cells	SPDYA	-0.35623	-0.02911	-0.45501	0.650215	-5.17217	0.745446	0.824009
Erythroid.cells	DNAJC1	0.071978	6.915183	0.454991	0.650231	-6.49078	0.58705	0.679269
Erythroid.cells	FGD2	0.16964	3.69567	0.454692	0.650445	-5.83587	0.655752	0.742989
Erythroid.cells	COL15A1	0.40797	0.332157	0.454488	0.650591	-5.19661	0.736394	0.815818
Erythroid.cells	MMRN1	0.608835	-0.22843	0.454485	0.650593	-5.16092	0.750787	0.82861
Erythroid.cells	RIPOR2	-0.09419	7.617108	-0.45435	0.65069	-6.64563	0.573313	0.666168
Erythroid.cells	AGO2	0.06923	7.576219	0.454149	0.650834	-6.61719	0.574184	0.666973
Erythroid.cells	BTAF1	-0.06721	7.190783	-0.45362	0.651215	-6.53424	0.581943	0.674332
Erythroid.cells	IFT122	0.235371	1.594011	0.453599	0.651229	-5.32517	0.705316	0.787991
Erythroid.cells	BBS2	0.326223	0.404044	0.453531	0.651277	-5.21071	0.734883	0.814494
Erythroid.cells	GM16150	-0.34774	0.174831	-0.45348	0.651312	-5.17286	0.740722	0.819693
Erythroid.cells	DENND2D	0.196083	2.202221	0.453461	0.651328	-5.49775	0.690677	0.774796
Erythroid.cells	ZFP36L1	0.080389	7.742233	0.453242	0.651485	-6.67357	0.571166	0.664088
Erythroid.cells	SIKE1	-0.11228	4.570739	-0.45287	0.651755	-5.92406	0.636846	0.725421
Erythroid.cells	MPO	0.645979	1.827356	0.45285	0.651766	-5.52571	0.699923	0.782958
Erythroid.cells	ITGB1BP2	0.332971	0.43994	0.452708	0.651868	-5.17024	0.734288	0.813758
Erythroid.cells	LRP1B	0.224394	1.219155	0.452473	0.652037	-5.46458	0.714917	0.796394
Erythroid.cells	TPMT	0.173738	2.097645	0.451628	0.652643	-5.61512	0.694075	0.77734
Erythroid.cells	ZFP358	0.18569	3.044691	0.451583	0.652675	-5.5974	0.671792	0.757143
Erythroid.cells	ACAP3	0.18531	2.238471	0.45155	0.652699	-5.45571	0.690714	0.774331
Erythroid.cells	SGSH	0.252726	1.453355	0.451169	0.652972	-5.38616	0.709896	0.791608
Erythroid.cells	CNOT10	0.09847	5.238486	0.451079	0.653037	-6.05378	0.623189	0.712515
Erythroid.cells	ROCK2	-0.07077	7.468662	-0.45088	0.653182	-6.55821	0.57745	0.669662
Erythroid.cells	GNL3	-0.11301	5.420028	-0.45068	0.653323	-6.10627	0.619433	0.70901
Erythroid.cells	CRELD1	-0.24543	1.843254	-0.45064	0.653352	-5.38832	0.700541	0.783209

Erythroid.cells	STK16	0.121815	4.37442	0.450467	0.653476	-5.97621	0.642093	0.729985
Erythroid.cells	PPP1R18	-0.07817	6.994866	-0.45043	0.653503	-6.45402	0.586929	0.678645
Erythroid.cells	EGFEM1	0.437661	1.229611	0.450283	0.653609	-5.29403	0.715575	0.796777
Erythroid.cells	NIM1K	-0.2105	2.88905	-0.45022	0.653656	-5.55446	0.675785	0.760849
Erythroid.cells	GM42658	-0.25511	1.544714	-0.45012	0.653723	-5.36891	0.707839	0.78983
Erythroid.cells	PPIL4	0.076591	5.46202	0.449888	0.653892	-6.1787	0.618681	0.70828
Erythroid.cells	POMP	-0.06747	7.268227	-0.44979	0.653965	-6.61466	0.581592	0.673577
Erythroid.cells	MDH1	0.062799	8.120076	0.44955	0.654135	-6.81349	0.564968	0.657863
Erythroid.cells	EHD2	0.260718	1.82262	0.449533	0.654147	-5.41525	0.701262	0.783886
Erythroid.cells	CACNA1A	0.265148	1.424509	0.449307	0.65431	-5.39324	0.711029	0.792613
Erythroid.cells	UBE2S	0.087928	7.624597	0.44904	0.654501	-6.71146	0.574658	0.66704
Erythroid.cells	LRP2BP	-0.29753	1.991491	-0.44902	0.654513	-5.42495	0.69726	0.780263
Erythroid.cells	USP9X	-0.07413	7.387216	-0.44899	0.654537	-6.60269	0.579336	0.671473
Erythroid.cells	FBXW7	-0.0913	6.448567	-0.44886	0.654634	-6.35161	0.598232	0.689266
Erythroid.cells	4833419F2	-0.29886	1.323387	-0.44884	0.654643	-5.34303	0.713514	0.79492
Erythroid.cells	ALDOA	0.111851	8.900702	0.448806	0.65467	-6.88485	0.550195	0.643794
Erythroid.cells	GFI1	-0.25171	1.55053	-0.44821	0.655098	-5.40656	0.708337	0.790073
Erythroid.cells	ABCD4	0.179726	2.782602	0.447964	0.655275	-5.50207	0.678998	0.763483
Erythroid.cells	FCRL6	0.374925	-0.87747	0.447872	0.655341	-5.06816	0.770401	0.845192
Erythroid.cells	USP49	-0.15183	4.534917	-0.44777	0.655412	-5.91754	0.639278	0.727203
Erythroid.cells	TOMM40	-0.09596	5.225725	-0.44753	0.655588	-6.12277	0.624399	0.713371
Erythroid.cells	ARHGEF37	-0.28004	0.616444	-0.44736	0.655712	-5.67087	0.731804	0.810953
Erythroid.cells	ACVR1	0.166705	3.707524	0.446913	0.656031	-5.83704	0.657842	0.744252
Erythroid.cells	SWSAP1	-0.34926	0.278314	-0.44678	0.656125	-5.16643	0.740397	0.818742
Erythroid.cells	ARL2BP	0.110752	5.086344	0.446748	0.656149	-6.10114	0.627399	0.716267
Erythroid.cells	WIPI2	0.089721	5.276718	0.446746	0.656151	-6.15119	0.623314	0.712481
Erythroid.cells	CSTDC4	-0.34015	3.661016	-0.44667	0.656208	-5.84261	0.658895	0.745284
Erythroid.cells	GM10790	0.376274	-0.77429	0.446595	0.65626	-5.0395	0.767805	0.843053
Erythroid.cells	NPLOC4	0.093674	5.537568	0.446588	0.656265	-6.17584	0.617762	0.707366
Erythroid.cells	FCHO2	0.075754	6.902577	0.446577	0.656273	-6.47631	0.58955	0.680989
Erythroid.cells	RPP30	0.130508	3.635635	0.446498	0.656329	-5.7607	0.659471	0.745842
Erythroid.cells	ABLIM1	0.087426	6.121137	0.446442	0.65637	-6.44897	0.605529	0.696006
Erythroid.cells	MAX	-0.07338	6.499429	-0.44643	0.656379	-6.40759	0.597737	0.688716
Erythroid.cells	H2-AB1	0.192556	6.764416	0.446308	0.656466	-6.75707	0.592361	0.683673
Erythroid.cells	ZCWPW1	0.206074	2.765843	0.446061	0.656643	-5.49349	0.679551	0.764182
Erythroid.cells	GFER	0.091724	4.406699	0.445997	0.65669	-6.02056	0.642247	0.730072
Erythroid.cells	2310011J0	-0.12119	4.446593	-0.44585	0.656797	-5.90377	0.641367	0.729262
Erythroid.cells	GM34466	0.347498	-1.10689	0.445773	0.656851	-5.09263	0.776713	0.850957
Erythroid.cells	TRIM14	0.160944	4.686216	0.445762	0.656859	-5.90767	0.636109	0.724414
Erythroid.cells	SELENBP1	0.127311	3.795416	0.445703	0.656902	-6.17126	0.655888	0.742601
Erythroid.cells	TSHZ2	0.225879	3.393266	0.445614	0.656966	-5.80161	0.665026	0.750958
Erythroid.cells	PPP1R3F	-0.30665	0.867721	-0.44557	0.656999	-5.23775	0.72552	0.805601
Erythroid.cells	ZFP526	0.319001	0.37218	0.445226	0.657244	-5.20817	0.738239	0.81684
Erythroid.cells	MORN2	-0.21396	2.045949	-0.44494	0.657449	-5.4848	0.696959	0.779761
Erythroid.cells	NINJ1	-0.12008	5.750418	-0.44476	0.657582	-6.29721	0.613661	0.703395
Erythroid.cells	GM16867	0.125866	2.522419	0.444528	0.657747	-6.15628	0.685741	0.76957
Erythroid.cells	MKS1	-0.29961	1.092934	-0.44447	0.657792	-5.26526	0.720393	0.80079

Erythroid.cells	CLMP	-0.29045	1.140521	-0.44439	0.657844	-5.37089	0.719211	0.799732
Erythroid.cells	ZFP157	-0.21219	2.446699	-0.44424	0.657956	-5.51041	0.687533	0.771256
Erythroid.cells	CCDC34	0.156695	4.840796	0.444218	0.65797	-6.05869	0.633172	0.721546
Erythroid.cells	FTSJ3	0.156146	4.016619	0.444077	0.658072	-5.85996	0.651396	0.738357
Erythroid.cells	MMP8	0.321238	1.517071	0.443892	0.658205	-5.56263	0.709989	0.79161
Erythroid.cells	THOC3	-0.12432	4.102819	-0.44387	0.658223	-5.88049	0.649488	0.736678
Erythroid.cells	MRPS30	-0.09871	4.726011	-0.44365	0.658377	-6.00316	0.635733	0.724015
Erythroid.cells	NFXL1	0.179134	3.364621	0.443604	0.658412	-5.6365	0.666199	0.751975
Erythroid.cells	BRCA2	-0.17774	3.555566	-0.44359	0.658425	-5.73841	0.661835	0.74799
Erythroid.cells	CD2	-0.13645	3.660392	-0.4434	0.658559	-6.00301	0.659521	0.745879
Erythroid.cells	SGTB	0.31553	0.3418	0.443209	0.658697	-5.21682	0.73948	0.818015
Erythroid.cells	PHF3	-0.06209	6.980017	-0.44309	0.658779	-6.50666	0.588551	0.680091
Erythroid.cells	ABCA8A	0.353104	1.028779	0.443082	0.658789	-5.37667	0.722151	0.802572
Erythroid.cells	SLC14A1	0.175788	3.335927	0.443015	0.658837	-5.69552	0.666943	0.752703
Erythroid.cells	HPGDS	0.20566	3.647133	0.442855	0.658952	-5.69602	0.659839	0.746231
Erythroid.cells	PDE8B	0.331991	-0.24267	0.442832	0.658969	-5.1738	0.754556	0.831441
Erythroid.cells	HOTAIRM1	0.326875	0.698917	0.442623	0.65912	-5.30845	0.730509	0.810086
Erythroid.cells	CES1C	-0.18341	4.023969	-0.44254	0.65918	-6.18549	0.651421	0.738558
Erythroid.cells	PHOSPHO2	0.116121	3.724911	0.442422	0.659264	-5.80898	0.658169	0.74476
Erythroid.cells	LY6A	-0.39368	4.339309	-0.44231	0.659343	-5.89563	0.64441	0.732167
Erythroid.cells	ATG2B	-0.10426	4.861013	-0.44225	0.659389	-6.01836	0.632964	0.72162
Erythroid.cells	WDPCP	-0.18566	3.269533	-0.4421	0.659495	-5.62178	0.668606	0.754344
Erythroid.cells	PPM1L	0.137683	4.617569	0.441526	0.65991	-6.02047	0.638346	0.726668
Erythroid.cells	LETMD1	0.168148	2.91201	0.441513	0.65992	-5.59173	0.676918	0.761991
Erythroid.cells	GM11423	0.287572	0.892029	0.441509	0.659923	-5.23488	0.725748	0.806007
Erythroid.cells	GM11944	-0.15033	4.498486	-0.4415	0.659929	-5.95823	0.640963	0.72908
Erythroid.cells	STFA3	-0.3299	2.751543	-0.4415	0.659931	-5.68753	0.68067	0.7654
Erythroid.cells	ARF3	0.072695	6.29303	0.441457	0.65996	-6.29067	0.602699	0.69355
Erythroid.cells	ZBTB43	-0.15555	3.163113	-0.44141	0.659994	-5.64676	0.67109	0.756693
Erythroid.cells	HELZ	-0.08231	6.25998	-0.44128	0.660085	-6.32284	0.603382	0.69422
Erythroid.cells	PRDX4	-0.09845	5.118984	-0.44114	0.660187	-6.21543	0.627451	0.71666
Erythroid.cells	BCAP31	-0.07476	5.901725	-0.44114	0.660192	-6.26135	0.610832	0.701211
Erythroid.cells	SLC39A14	0.163865	3.169335	0.441062	0.660245	-5.7835	0.670946	0.756634
Erythroid.cells	CCDC15	0.209842	2.810819	0.440519	0.660636	-5.53974	0.679549	0.764356
Erythroid.cells	GM4951	-0.25611	3.770894	-0.44045	0.660683	-5.90538	0.657458	0.744207
Erythroid.cells	ARFIP1	-0.14399	3.797972	-0.44043	0.660701	-5.76677	0.656845	0.743646
Erythroid.cells	LRRC75AOS	-0.33719	0.512247	-0.44002	0.661	-5.20215	0.735874	0.814848
Erythroid.cells	AMACR	-0.21965	2.326832	-0.4397	0.66123	-5.61813	0.691394	0.774775
Erythroid.cells	CCDC192	-0.33504	-0.69514	-0.43958	0.661316	-5.2553	0.767418	0.842652
Erythroid.cells	PSMG4	-0.09893	5.042688	-0.4393	0.661519	-6.03798	0.629823	0.718475
Erythroid.cells	TULP4	-0.08748	5.928036	-0.43909	0.661667	-6.3012	0.610987	0.701058
Erythroid.cells	SORT1	-0.16205	4.067523	-0.43902	0.661716	-5.82195	0.65128	0.738348
Erythroid.cells	EIF4ENIF1	-0.08584	5.556503	-0.43901	0.661725	-6.16264	0.618818	0.708368
Erythroid.cells	GUCD1	0.142899	3.805941	0.439005	0.661729	-5.84078	0.657166	0.743743
Erythroid.cells	MAN2A1	-0.08306	7.183028	-0.43883	0.661853	-6.58433	0.585355	0.677064
Erythroid.cells	BC048403	-0.20918	1.255612	-0.43845	0.662132	-5.34715	0.717721	0.798582
Erythroid.cells	GM32916	-0.65255	-0.24915	-0.4384	0.662163	-5.11974	0.755989	0.832672

Erythroid.cells	NAPSA	-0.09062	6.459379	-0.43815	0.662344	-6.55446	0.600131	0.691034
Erythroid.cells	MCAT	0.179552	2.553855	0.438145	0.662349	-5.58393	0.6863	0.770388
Erythroid.cells	FADS1	-0.15774	3.059286	-0.43812	0.662365	-5.74513	0.674454	0.759632
Erythroid.cells	CDK8	-0.11627	6.559883	-0.43812	0.662366	-6.52805	0.598071	0.689103
Erythroid.cells	DSTYK	-0.12218	3.925134	-0.438	0.662457	-5.79367	0.654679	0.741573
Erythroid.cells	UBAC2	-0.06766	6.679645	-0.43777	0.662622	-6.50818	0.595737	0.686853
Erythroid.cells	IL3RA	-0.14776	3.561284	-0.43739	0.662893	-5.76137	0.66323	0.749233
Erythroid.cells	CCDC50	0.079945	5.994315	0.437238	0.663005	-6.26767	0.610112	0.700196
Erythroid.cells	FAM50A	0.094446	5.186332	0.437016	0.663165	-6.07248	0.627309	0.716207
Erythroid.cells	SEC31A	-0.06949	5.620105	-0.43697	0.663197	-6.21723	0.618044	0.707634
Erythroid.cells	RAB11FIP2	0.16731	3.576284	0.43655	0.663502	-5.70598	0.663234	0.7492
Erythroid.cells	PDE7B	-0.24095	5.37371	-0.43643	0.663591	-6.10434	0.623536	0.712674
Erythroid.cells	DEDD	0.116138	4.437275	0.436061	0.663856	-5.85524	0.644095	0.731637
Erythroid.cells	MTAP	-0.13771	3.671485	-0.43598	0.663914	-5.79887	0.66128	0.747399
Erythroid.cells	CBL	0.072617	7.22396	0.435656	0.664148	-6.48538	0.585578	0.677085
Erythroid.cells	ZFP940	-0.27017	0.734633	-0.43549	0.664271	-5.24767	0.731975	0.811069
Erythroid.cells	GM46367	-0.23937	2.175914	-0.43498	0.664639	-5.47917	0.696777	0.779372
Erythroid.cells	ANKFY1	-0.08258	5.995175	-0.4349	0.664694	-6.28143	0.611044	0.700782
Erythroid.cells	TRAPPC6B	0.063795	6.134079	0.434657	0.664871	-6.32861	0.608147	0.698132
Erythroid.cells	WDFY2	0.128815	5.19857	0.434606	0.664907	-6.00143	0.627968	0.716554
Erythroid.cells	NAT9	-0.16364	3.244632	-0.43456	0.664941	-5.67061	0.671593	0.756604
Erythroid.cells	SLC2A1	-0.13007	4.676925	-0.43453	0.664961	-5.90073	0.639315	0.727036
Erythroid.cells	OSCP1	0.254153	3.25254	0.434227	0.665182	-5.54957	0.671449	0.756525
Erythroid.cells	CFAP43	0.160516	3.09234	0.434178	0.665217	-5.6265	0.675163	0.759904
Erythroid.cells	PARPBP	0.285603	2.056928	0.434168	0.665224	-5.50115	0.699686	0.782107
Erythroid.cells	SLC26A2	-0.1578	3.742645	-0.43406	0.665302	-5.71136	0.660219	0.746277
Erythroid.cells	ADCK2	-0.18227	2.282482	-0.43402	0.665329	-5.48591	0.694266	0.777217
Erythroid.cells	NAIP2	0.175026	3.006951	0.433827	0.665471	-5.70836	0.677151	0.761783
Erythroid.cells	TDP2	0.115442	4.340054	0.433664	0.665589	-5.93658	0.646796	0.734039
Erythroid.cells	TTC7B	0.098908	5.391283	0.433563	0.665662	-6.14039	0.623866	0.712901
Erythroid.cells	C79798	-0.2111	1.77973	-0.43345	0.665744	-5.46623	0.706406	0.788315
Erythroid.cells	TMLHE	-0.27022	2.31306	-0.43341	0.66577	-5.46885	0.693535	0.776724
Erythroid.cells	OMA1	-0.15879	3.410326	-0.43341	0.665775	-5.69295	0.667812	0.753373
Erythroid.cells	CNNM4	-0.12739	4.077084	-0.43331	0.665848	-5.8157	0.652669	0.73953
Erythroid.cells	TTYH2	0.201132	3.075271	0.433249	0.665889	-5.65146	0.67556	0.760438
Erythroid.cells	QTRT1	-0.15477	4.980734	-0.43319	0.665929	-5.99355	0.632718	0.72116
Erythroid.cells	TUFM	0.10647	4.731912	0.433116	0.665985	-6.09448	0.638147	0.726184
Erythroid.cells	PIK3R5	0.122215	4.45137	0.433006	0.666065	-5.99715	0.64434	0.731909
Erythroid.cells	ARHGAP35	0.100709	5.237019	0.432885	0.666152	-6.12863	0.627191	0.716117
Erythroid.cells	IPO13	-0.21485	2.264795	-0.43282	0.666197	-5.45904	0.694705	0.777873
Erythroid.cells	BCAS3	0.073598	7.334935	0.432684	0.666298	-6.60867	0.58375	0.675501
Erythroid.cells	KIF14	0.262722	2.55406	0.432471	0.666453	-5.57402	0.68792	0.77172
Erythroid.cells	TRPV2	-0.11306	4.873594	-0.43241	0.666497	-5.99597	0.635161	0.723479
Erythroid.cells	RER1	0.070883	6.413933	0.432263	0.666603	-6.42204	0.602514	0.69311
Erythroid.cells	EFCC1	-0.29981	0.001369	-0.43218	0.666664	-5.30521	0.751283	0.828447
Erythroid.cells	TRIM12C	0.14169	4.726648	0.432099	0.666721	-5.92871	0.638401	0.726446
Erythroid.cells	GM15543	-0.28775	0.901403	-0.43163	0.667062	-5.22159	0.72848	0.808119

Erythroid.cells	THOP1	-0.23267	2.236401	-0.43161	0.667075	-5.44853	0.695694	0.778683
Erythroid.cells	PFDN2	0.09062	5.031244	0.431598	0.667084	-6.07871	0.631916	0.720417
Erythroid.cells	TNR	0.311384	0.036927	0.431416	0.667216	-5.18444	0.750622	0.827872
Erythroid.cells	PGAP2	-0.10741	5.75264	-0.43132	0.667282	-6.1904	0.616533	0.706206
Erythroid.cells	4930539J0	0.321294	0.706555	0.431226	0.667354	-5.20326	0.733475	0.812671
Erythroid.cells	GM20275	-0.18582	3.071387	-0.43107	0.667468	-5.63755	0.676062	0.760986
Erythroid.cells	CXCL13	-0.52056	-1.21101	-0.43101	0.667508	-5.07614	0.783713	0.857109
Erythroid.cells	TAPT1	0.097881	5.802801	0.430758	0.667692	-6.26511	0.615548	0.70528
Erythroid.cells	DIS3L	0.16611	2.828005	0.43072	0.66772	-5.57571	0.681806	0.766163
Erythroid.cells	ACY3	-0.22615	2.04257	-0.43063	0.667784	-5.60495	0.700513	0.783084
Erythroid.cells	SNX33	-0.27266	0.759185	-0.43054	0.667854	-5.26745	0.732226	0.811517
Erythroid.cells	MGA	-0.09354	5.917554	-0.43049	0.667889	-6.24247	0.613132	0.703031
Erythroid.cells	GM29707	-0.28684	-0.46354	-0.43005	0.668207	-5.18955	0.763928	0.839596
Erythroid.cells	MYL12A	-0.06369	7.929477	-0.43002	0.66823	-6.71753	0.572466	0.664789
Erythroid.cells	ABCG1	-0.11565	6.591271	-0.42987	0.668339	-6.36499	0.599249	0.690085
Erythroid.cells	CC2D1B	-0.14126	3.659138	-0.42982	0.668371	-5.66324	0.662685	0.748787
Erythroid.cells	GM26789	-0.3416	0.271556	-0.42977	0.66841	-5.21296	0.744781	0.822735
Erythroid.cells	PDCD6	-0.08671	6.13963	-0.42976	0.668415	-6.31069	0.608587	0.698829
Erythroid.cells	C9ORF72	-0.16829	3.893192	-0.42965	0.668498	-5.8874	0.657371	0.743973
Erythroid.cells	EZH2	0.092622	6.663574	0.429607	0.668527	-6.56755	0.597769	0.688751
Erythroid.cells	RRP1	-0.05941	6.518197	-0.42948	0.668616	-6.42591	0.600758	0.691544
Erythroid.cells	BUB1B	0.157103	4.016283	0.429413	0.668667	-5.89086	0.654603	0.741435
Erythroid.cells	DLST	-0.0702	5.852364	-0.42893	0.66902	-6.24335	0.61475	0.704579
Erythroid.cells	CDK1	0.166182	5.506863	0.428907	0.669034	-6.26962	0.622074	0.711389
Erythroid.cells	BBOX1	0.263239	0.92351	0.428776	0.669129	-5.42473	0.728376	0.808133
Erythroid.cells	MRPL33	-0.08455	6.142277	-0.42876	0.669143	-6.33303	0.608674	0.698915
Erythroid.cells	LEO1	0.135875	4.138442	0.42866	0.669213	-5.81894	0.652003	0.739012
Erythroid.cells	FOXK1	-0.10423	4.627409	-0.4286	0.669255	-5.95948	0.641138	0.729045
Erythroid.cells	9530077C0	-0.29415	1.213471	-0.42852	0.669313	-5.30904	0.721124	0.801707
Erythroid.cells	GM31645	-0.31603	0.726126	-0.42826	0.669504	-5.26121	0.733356	0.812778
Erythroid.cells	CCT5	-0.08025	6.571584	-0.42823	0.669527	-6.43204	0.599793	0.690783
Erythroid.cells	LGALS2	0.325733	-0.89826	0.428167	0.669571	-5.14197	0.775665	0.850251
Erythroid.cells	GM9530	0.291026	-0.31965	0.428154	0.66958	-5.32232	0.760319	0.836717
Erythroid.cells	IGLV1	-0.35548	-0.83837	-0.42812	0.669603	-5.16085	0.774062	0.848841
Erythroid.cells	CD226	0.171142	1.697205	0.427975	0.66971	-5.99295	0.709233	0.791147
Erythroid.cells	SLC25A14	-0.18817	2.078186	-0.42776	0.669867	-5.44321	0.699981	0.782853
Erythroid.cells	BTG2	0.091613	7.421641	0.427633	0.669958	-6.59846	0.582652	0.67467
Erythroid.cells	9530062K0	-0.32718	0.793704	-0.42753	0.670035	-5.20672	0.731696	0.811303
Erythroid.cells	FUNDC1	0.10932	4.65588	0.427458	0.670085	-5.93076	0.640555	0.72867
Erythroid.cells	EGFL6	0.2861	0.548891	0.427432	0.670104	-5.33524	0.737906	0.816839
Erythroid.cells	CPEB3	-0.14342	4.234811	-0.42727	0.670221	-6.04974	0.64989	0.737292
Erythroid.cells	SNAPC1	0.133743	3.850567	0.426945	0.670457	-5.79179	0.658533	0.745317
Erythroid.cells	DENND6B	0.276893	0.94417	0.426921	0.670475	-5.27817	0.727906	0.808069
Erythroid.cells	TOX	-0.13633	4.351569	-0.42689	0.6705	-6.38784	0.647287	0.735006
Erythroid.cells	COLGALT2	-0.42998	-0.12576	-0.42687	0.67051	-5.11596	0.755296	0.832436
Erythroid.cells	RAVER2	-0.26119	1.423822	-0.42674	0.670603	-5.33639	0.715958	0.79739
Erythroid.cells	SRSF5	-0.06023	6.955445	-0.42668	0.670652	-6.49382	0.592009	0.683625

Erythroid.cells	PLPP1	-0.15398	4.327383	-0.42664	0.670682	-6.03124	0.647825	0.735524
Erythroid.cells	CLASRP	0.160194	3.334689	0.426402	0.670851	-5.61985	0.670327	0.756149
Erythroid.cells	NUDT8	-0.19856	2.208161	-0.42635	0.670889	-5.50182	0.696852	0.780227
Erythroid.cells	TCRG-C1	-0.2326	0.880703	-0.42632	0.67091	-5.73555	0.729502	0.809551
Erythroid.cells	ZFP956	-0.25893	1.264702	-0.42625	0.670964	-5.28582	0.719899	0.80096
Erythroid.cells	POLE4	0.068812	6.652817	0.426177	0.671015	-6.47777	0.598169	0.68944
Erythroid.cells	EMP3	0.083678	7.421524	0.426157	0.671029	-6.55582	0.582655	0.674843
Erythroid.cells	2010310CC	0.342378	1.298313	0.426157	0.671029	-5.35019	0.719065	0.800212
Erythroid.cells	SPAG5	-0.20555	3.137614	-0.42595	0.671182	-5.67594	0.674978	0.76041
Erythroid.cells	FILIP1L	0.115816	5.411773	0.425676	0.671378	-6.10018	0.624321	0.71382
Erythroid.cells	TMEM237	0.219874	2.157499	0.425578	0.671449	-5.49525	0.698263	0.781482
Erythroid.cells	C4BP	0.219486	1.968581	0.425539	0.671478	-5.64559	0.702826	0.785603
Erythroid.cells	HDAC11	0.33767	0.5008	0.425218	0.671711	-5.21995	0.739511	0.818394
Erythroid.cells	ANKRD42	0.384641	0.346625	0.425075	0.671815	-5.17907	0.743457	0.821933
Erythroid.cells	SHF	-0.21542	1.832889	-0.42504	0.671839	-5.39985	0.706289	0.788704
Erythroid.cells	2310061I0	0.129771	3.521012	0.424923	0.671926	-5.74265	0.666384	0.752563
Erythroid.cells	RNF125	-0.12765	3.852901	-0.42487	0.671966	-6.08489	0.658818	0.745644
Erythroid.cells	PIPOX	0.206864	2.011866	0.424745	0.672055	-5.63503	0.701968	0.784828
Erythroid.cells	BCKDHA	-0.10686	4.820285	-0.42452	0.672218	-6.10104	0.637389	0.725875
Erythroid.cells	ARHGEF39	0.286642	1.960521	0.42416	0.67248	-5.46067	0.703518	0.786132
Erythroid.cells	GM11713	-0.21445	2.37115	-0.42335	0.673065	-5.56049	0.694119	0.77746
Erythroid.cells	FAM220A.1	0.208685	2.200036	0.4233	0.673104	-5.52982	0.698225	0.781168
Erythroid.cells	PIGV	-0.14851	2.921169	-0.42323	0.673152	-5.55348	0.681089	0.765656
Erythroid.cells	LYPLAL1	0.27242	1.634017	0.422802	0.673466	-5.43467	0.71225	0.793622
Erythroid.cells	SACS	-0.21468	2.596254	-0.42268	0.673553	-5.6084	0.689031	0.77268
Erythroid.cells	ZBTB48	-0.31111	1.123594	-0.42251	0.673678	-5.25687	0.724932	0.805028
Erythroid.cells	RIPOR1	-0.13814	3.960811	-0.42228	0.673849	-5.75009	0.657419	0.744014
Erythroid.cells	CYTH3	-0.11196	4.88921	-0.42218	0.673915	-6.0908	0.636777	0.725067
Erythroid.cells	KLC3	-0.36925	0.19256	-0.42187	0.674143	-5.14426	0.748608	0.826293
Erythroid.cells	FPGT	0.281781	1.345954	0.421808	0.674189	-5.37021	0.719392	0.800243
Erythroid.cells	WRN	-0.08772	5.831312	-0.42171	0.67426	-6.21921	0.616527	0.706351
Erythroid.cells	CISD1	0.102314	5.119405	0.42165	0.674304	-6.29282	0.631765	0.720491
Erythroid.cells	SRP19	-0.07653	6.048783	-0.42165	0.674306	-6.3133	0.61195	0.702088
Erythroid.cells	LIMA1	0.173649	4.507655	0.421644	0.674308	-6.00852	0.645177	0.732866
Erythroid.cells	CD74	0.158806	11.27972	0.421633	0.674316	-7.43302	0.51209	0.607009
Erythroid.cells	GM15860	-0.29339	0.373729	-0.4216	0.674337	-5.21988	0.74394	0.822155
Erythroid.cells	RCHY1	0.081696	5.661194	0.421473	0.674432	-6.18365	0.620133	0.709783
Erythroid.cells	FOXM1	-0.18743	3.10354	-0.42141	0.674479	-5.67935	0.677102	0.762161
Erythroid.cells	SEMA4D	-0.11528	6.446214	-0.42141	0.674481	-6.38954	0.603678	0.694443
Erythroid.cells	PREPL	-0.21203	1.734988	-0.42117	0.674655	-5.40456	0.709915	0.791729
Erythroid.cells	EEF1G	-0.06743	7.187877	-0.42106	0.674733	-6.57735	0.588663	0.680252
Erythroid.cells	SMYD4	0.210108	2.820357	0.420604	0.675065	-5.56677	0.684114	0.768217
Erythroid.cells	RIPPLY3	0.38263	0.048429	0.420443	0.675182	-5.17019	0.752757	0.829741
Erythroid.cells	LTB	0.164686	3.446775	0.420442	0.675182	-5.8556	0.669517	0.754952
Erythroid.cells	RAMP2	0.199405	3.013058	0.419969	0.675527	-5.68586	0.679869	0.76417
Erythroid.cells	JOSD1	-0.11739	3.99961	-0.41967	0.675745	-5.82779	0.657322	0.743518
Erythroid.cells	TRA2B	-0.05233	7.832072	-0.41954	0.675838	-6.69629	0.57644	0.668296

Erythroid.cells	EMSY	-0.08143	5.828738	-0.41948	0.675881	-6.25444	0.617318	0.706625
Erythroid.cells	1600010M	-0.12958	4.96425	-0.41939	0.675949	-5.98111	0.635899	0.723841
Erythroid.cells	CLCN4	0.093987	5.289066	0.419116	0.676148	-6.08227	0.628916	0.717383
Erythroid.cells	CLDN15	0.248624	0.423974	0.41911	0.676152	-5.30464	0.743619	0.82133
Erythroid.cells	UGT2B36	-0.19404	2.198922	-0.41879	0.676388	-5.71537	0.699623	0.78187
Erythroid.cells	PLEKHM2	0.137771	5.202185	0.418573	0.676543	-6.0524	0.631025	0.719222
Erythroid.cells	LRRC75A	-0.21735	2.6203	-0.41845	0.676632	-5.56781	0.689616	0.772804
Erythroid.cells	ZFP260	0.099473	4.245881	0.418305	0.676739	-5.91217	0.652094	0.738626
Erythroid.cells	NPM3	0.111588	5.097579	0.418238	0.676787	-6.11201	0.633294	0.721319
Erythroid.cells	FAM122A	0.142057	3.563965	0.418226	0.676796	-5.68143	0.667568	0.752778
Erythroid.cells	EIF2S1	-0.06923	6.052768	-0.4181	0.676891	-6.38383	0.612899	0.702404
Erythroid.cells	IMP4	0.100264	4.46298	0.418028	0.67694	-5.96182	0.647258	0.734199
Erythroid.cells	SLC41A2	-0.19637	2.900939	-0.41781	0.677096	-5.69908	0.683042	0.766824
Erythroid.cells	IL18BP	0.266292	3.740937	0.417781	0.67712	-5.78153	0.663575	0.749109
Erythroid.cells	PDE4C	0.328546	3.131277	0.417672	0.6772	-5.58354	0.677659	0.761973
Erythroid.cells	KCTD11	-0.30488	0.546292	-0.41724	0.677516	-5.22317	0.741008	0.818883
Erythroid.cells	A430005L1	0.123219	3.764054	0.417201	0.677543	-5.81074	0.663213	0.748801
Erythroid.cells	GJB1	0.233616	1.59761	0.417138	0.677588	-5.54174	0.714606	0.795327
Erythroid.cells	PEX6	-0.13689	4.039128	-0.4171	0.67762	-5.84584	0.656968	0.743112
Erythroid.cells	KIF23	0.146942	5.173452	0.416816	0.677823	-6.0834	0.63199	0.720126
Erythroid.cells	DUSP23	0.181372	2.373334	0.416538	0.678026	-5.51664	0.695953	0.778598
Erythroid.cells	INTU	0.373635	0.880465	0.416406	0.678122	-5.234	0.732728	0.811588
Erythroid.cells	LAMP1	-0.05999	7.757865	-0.41639	0.678135	-6.7308	0.578544	0.670233
Erythroid.cells	PINX1	0.158202	3.022074	0.416345	0.678167	-5.66284	0.680571	0.764676
Erythroid.cells	PLSCR4	0.298883	0.494112	0.416192	0.678278	-5.28263	0.742565	0.820366
Erythroid.cells	POC1B	-0.10313	4.642223	-0.41613	0.678321	-5.92959	0.643683	0.731
Erythroid.cells	DHX38	0.120168	4.560036	0.416093	0.67835	-5.89137	0.645503	0.732675
Erythroid.cells	TAF1B	0.110345	4.199522	0.41544	0.678826	-5.86251	0.653945	0.740299
Erythroid.cells	1700113A1	0.210273	2.10081	0.415215	0.67899	-5.44477	0.702992	0.784847
Erythroid.cells	1700027J0	0.214679	2.481407	0.415199	0.679002	-5.72772	0.69383	0.776583
Erythroid.cells	PEX3	-0.11102	3.770542	-0.41482	0.679276	-5.83452	0.663839	0.749251
Erythroid.cells	MPHOSPH8	-0.08893	4.79616	-0.41465	0.679402	-6.02378	0.640846	0.728196
Erythroid.cells	TBCC	-0.13772	3.579816	-0.4146	0.679442	-5.74822	0.66821	0.753272
Erythroid.cells	CSTF1	0.147163	3.243385	0.414538	0.679484	-5.61808	0.675992	0.760357
Erythroid.cells	RALGPS2	0.088636	5.773254	0.414465	0.679538	-6.36263	0.619719	0.708679
Erythroid.cells	SLC12A8	-0.30955	0.657727	-0.41441	0.679578	-5.23718	0.739025	0.817056
Erythroid.cells	LEKR1	0.298516	1.047991	0.414328	0.679638	-5.33396	0.729137	0.808247
Erythroid.cells	NCOA1	0.080984	7.07231	0.41422	0.679716	-6.50624	0.592766	0.683508
Erythroid.cells	CCNL1	0.06141	7.09056	0.414197	0.679733	-6.51163	0.592396	0.683161
Erythroid.cells	GM26936	-0.3238	0.478634	-0.41398	0.67989	-5.19884	0.743616	0.821168
Erythroid.cells	SETDB1	0.107235	4.832864	0.413972	0.679897	-5.98192	0.640046	0.727512
Erythroid.cells	ESPL1	0.174689	2.563574	0.413914	0.679939	-5.63651	0.692015	0.774961
Erythroid.cells	COQ10B	0.076629	5.908947	0.413718	0.680082	-6.28778	0.616921	0.706117
Erythroid.cells	FAM53C	-0.12676	3.981286	-0.41353	0.680219	-5.76749	0.659132	0.74505
Erythroid.cells	HDAC4	-0.11946	5.402881	-0.4135	0.680242	-6.09307	0.627723	0.716143
Erythroid.cells	GM50431	-0.32287	0.122176	-0.41344	0.680286	-5.23264	0.752916	0.829437
Erythroid.cells	ZSCAN12	0.236309	1.093811	0.413156	0.680493	-5.27566	0.728143	0.80741

Erythroid.cells	HSDL1	-0.10793	4.167902	-0.41311	0.680527	-5.79226	0.654972	0.741261
Erythroid.cells	VPS8	-0.12947	4.586793	-0.41309	0.680542	-5.83784	0.645611	0.732669
Erythroid.cells	MTMR1	0.111473	4.622377	0.412973	0.680626	-5.91601	0.64484	0.731947
Erythroid.cells	B430010I2	-0.3048	-0.41456	-0.41287	0.680704	-5.18645	0.767099	0.841969
Erythroid.cells	MTF2	0.077675	5.644163	0.412589	0.680906	-6.24183	0.622755	0.711446
Erythroid.cells	GM16599	-0.09562	4.782001	-0.41245	0.681012	-6.10573	0.64149	0.728758
Erythroid.cells	CAMK2G	0.11313	4.893224	0.412118	0.68125	-5.98523	0.639113	0.726505
Erythroid.cells	TFDP1	-0.1084	5.887837	-0.41187	0.681435	-6.27746	0.617676	0.706664
Erythroid.cells	GCN1	0.09734	4.575371	0.411773	0.681503	-5.92608	0.646128	0.732963
Erythroid.cells	SLC29A3	0.143304	3.68183	0.411607	0.681624	-5.71896	0.666284	0.751421
Erythroid.cells	2810001G2	0.152623	2.479465	0.411451	0.681737	-5.55079	0.694449	0.776982
Erythroid.cells	EIF5A2	0.288502	0.577892	0.411429	0.681753	-5.30214	0.741527	0.819151
Erythroid.cells	FKBP3	0.08614	6.130579	0.411374	0.681794	-6.39288	0.61256	0.701904
Erythroid.cells	KLF13	0.059924	8.183848	0.411367	0.681799	-6.74253	0.57105	0.662912
Erythroid.cells	PSMD12	-0.07372	5.784758	-0.41132	0.681831	-6.27401	0.619862	0.708695
Erythroid.cells	MICOS10	0.057813	7.131558	0.411313	0.681838	-6.61328	0.591936	0.682615
Erythroid.cells	NAA30	0.117086	3.918894	0.411296	0.681851	-5.81161	0.660873	0.746479
Erythroid.cells	NTHL1	0.242194	0.544589	0.411207	0.681916	-5.27034	0.74238	0.819922
Erythroid.cells	IFIT1	-0.23195	2.761439	-0.41117	0.681944	-6.02032	0.687735	0.770925
Erythroid.cells	TMX3	-0.08558	5.505888	-0.41076	0.68224	-6.11427	0.62601	0.714293
Erythroid.cells	MYO3B	-0.22564	1.289051	-0.41054	0.682405	-5.41825	0.723772	0.803301
Erythroid.cells	SORD	0.127799	4.176195	0.410526	0.682414	-6.09548	0.655254	0.741313
Erythroid.cells	CSF1	-0.32592	2.480595	-0.41044	0.682476	-5.49546	0.694636	0.777162
Erythroid.cells	ANAPC5	-0.05987	6.399962	-0.41041	0.682494	-6.43105	0.607123	0.696843
Erythroid.cells	KLHL6	-0.09283	6.109159	-0.41034	0.682551	-6.40539	0.613199	0.702524
Erythroid.cells	ZFP846	-0.19127	2.431015	-0.41019	0.68266	-5.48594	0.695868	0.77827
Erythroid.cells	ADRM1	0.091449	5.477684	0.409884	0.682882	-6.22611	0.626769	0.715072
Erythroid.cells	PITPNM2	-0.12937	3.66119	-0.40984	0.682917	-5.87842	0.667127	0.752183
Erythroid.cells	SPATA7	0.234215	1.309288	0.409382	0.68325	-5.33969	0.723726	0.803119
Erythroid.cells	4930484I0	0.215067	1.660784	0.409112	0.683447	-5.4271	0.715141	0.795355
Erythroid.cells	TMEM164	-0.06972	6.945657	-0.40891	0.683597	-6.47148	0.59646	0.686573
Erythroid.cells	GORASP1	0.302624	0.733344	0.40871	0.683741	-5.25353	0.738571	0.816136
Erythroid.cells	CCDC148	0.390563	1.762244	0.408345	0.684007	-5.43747	0.712958	0.793189
Erythroid.cells	CHST14	-0.30254	0.872451	-0.40826	0.684068	-5.25704	0.735185	0.813051
Erythroid.cells	BANF1	-0.07417	6.873409	-0.40825	0.68408	-6.54531	0.598126	0.688024
Erythroid.cells	PTPN3	0.265261	1.040176	0.407873	0.684353	-5.43013	0.731162	0.809356
Erythroid.cells	DDX28	0.184051	1.558733	0.407375	0.684717	-5.46195	0.718473	0.797936
Erythroid.cells	MCAM	0.31811	1.173297	0.407201	0.684845	-5.28312	0.728091	0.806597
Erythroid.cells	GM34680	-0.35516	0.042973	-0.40718	0.684858	-5.18704	0.757061	0.832316
Erythroid.cells	SNX30	0.092834	5.755716	0.406985	0.685003	-6.28058	0.621885	0.710076
Erythroid.cells	FBXO44	0.354492	0.220079	0.406964	0.685018	-5.17019	0.752445	0.828271
Erythroid.cells	TARBP1	0.176589	2.687423	0.406774	0.685157	-5.59296	0.691053	0.773386
Erythroid.cells	PRR12	0.135455	3.451612	0.406761	0.685167	-5.68835	0.673105	0.757117
Erythroid.cells	BCAS3OS1	0.132672	3.793281	0.406733	0.685187	-5.76858	0.665239	0.749952
Erythroid.cells	CEP95	-0.10348	4.108354	-0.4067	0.685213	-5.87178	0.65807	0.743407
Erythroid.cells	SHISA8	0.316836	-0.21439	0.406385	0.685442	-5.27305	0.763861	0.838375
Erythroid.cells	SPG20	0.17014	2.615271	0.406364	0.685457	-5.52775	0.692812	0.774988

Erythroid.cells	FAM129B	-0.16841	3.14002	-0.40611	0.68564	-5.87154	0.680402	0.763812
Erythroid.cells	CSGALNAC	-0.23532	2.818139	-0.4061	0.685649	-5.58278	0.687986	0.770682
Erythroid.cells	UBE2A	-0.0529	6.786705	-0.40604	0.685693	-6.49392	0.600348	0.690096
Erythroid.cells	MRPS28	0.126206	6.885185	0.405993	0.685729	-6.51384	0.598329	0.688209
Erythroid.cells	DAPP1	-0.07297	6.482475	-0.40575	0.685904	-6.40291	0.606629	0.696035
Erythroid.cells	AFG3L2	-0.08198	4.593409	-0.4057	0.685947	-6.0043	0.647227	0.733621
Erythroid.cells	ATP10A	0.151433	3.430551	0.40567	0.685965	-5.83367	0.673631	0.757763
Erythroid.cells	IPO8	-0.09941	4.420007	-0.40551	0.686084	-5.92382	0.651094	0.737198
Erythroid.cells	GM28192	0.223663	0.064757	0.405424	0.686146	-5.36908	0.756534	0.832101
Erythroid.cells	PTPN6	-0.093	6.758702	-0.4054	0.686163	-6.35029	0.600923	0.690731
Erythroid.cells	ITFG1	-0.07745	5.454025	-0.40529	0.686242	-6.11989	0.628386	0.716303
Erythroid.cells	AU040320	0.120684	4.589216	0.405017	0.686443	-5.87637	0.64732	0.733771
Erythroid.cells	NUMBL	-0.26474	1.04252	-0.40491	0.686522	-5.32875	0.731425	0.809851
Erythroid.cells	FAM71A	0.283461	0.237403	0.404904	0.686526	-5.30826	0.752037	0.828162
Erythroid.cells	KARS	0.130272	4.273346	0.404897	0.686531	-5.85632	0.654384	0.740251
Erythroid.cells	OSBPL7	-0.14048	3.426262	-0.40488	0.686543	-5.67397	0.67373	0.757911
Erythroid.cells	TGFB3	-0.32469	0.567314	-0.4048	0.686605	-5.24422	0.74352	0.820624
Erythroid.cells	RDH11	-0.15757	3.215453	-0.40458	0.686765	-5.59815	0.678637	0.762386
Erythroid.cells	MAP4K2	0.115472	5.348887	0.404515	0.686811	-6.05701	0.630657	0.718432
Erythroid.cells	TRMT2A	0.141458	3.456488	0.404447	0.686861	-5.64781	0.67303	0.75729
Erythroid.cells	CMAH	0.135671	5.561409	0.404439	0.686866	-6.1947	0.626077	0.714193
Erythroid.cells	VSIR	0.131732	4.468275	0.404303	0.686966	-5.92277	0.650015	0.736285
Erythroid.cells	CCDC186	-0.1041	4.760025	-0.40427	0.686987	-5.95995	0.643533	0.730337
Erythroid.cells	P2RX7	-0.18463	3.223954	-0.40427	0.686991	-5.71164	0.678439	0.762238
Erythroid.cells	GAMT	0.141854	4.228598	0.403993	0.687193	-6.26595	0.655391	0.741282
Erythroid.cells	AKR1C14	0.233681	0.911524	0.403973	0.687208	-5.39743	0.734739	0.812922
Erythroid.cells	URM1	-0.1121	4.343407	-0.40392	0.687247	-5.82023	0.65281	0.738946
Erythroid.cells	RARS	-0.09257	5.121145	-0.40384	0.687303	-6.07271	0.635604	0.723139
Erythroid.cells	1810059H2	-0.25296	1.799204	-0.40384	0.687307	-5.55479	0.712578	0.793167
Erythroid.cells	MTHFSL	0.083283	5.211119	0.403585	0.687493	-6.21594	0.633755	0.721409
Erythroid.cells	RIT1	0.108462	3.965996	0.403466	0.68758	-5.85625	0.661471	0.746863
Erythroid.cells	ZFP213	-0.2027	2.162406	-0.40299	0.687932	-5.41985	0.704096	0.785412
Erythroid.cells	HOMEZ	-0.23997	1.333922	-0.40296	0.68795	-5.29351	0.724505	0.80371
Erythroid.cells	MICALL2	0.232861	1.195719	0.402445	0.688328	-5.42971	0.728065	0.806852
Erythroid.cells	TOP2A	0.135493	7.615909	0.402369	0.688383	-6.76638	0.583973	0.674845
Erythroid.cells	KCNAB2	0.130747	4.287693	0.402354	0.688395	-5.86315	0.654508	0.74036
Erythroid.cells	INTS13	0.107889	4.149388	0.402273	0.688454	-5.86349	0.657627	0.743216
Erythroid.cells	PHLPP1	0.088461	7.671226	0.40223	0.688486	-6.74153	0.582871	0.673806
Erythroid.cells	GM15441	-0.22919	1.344167	-0.4022	0.688505	-5.3387	0.724345	0.80353
Erythroid.cells	HELZ2	0.176366	3.832468	0.402182	0.688521	-5.7033	0.664833	0.7498
Erythroid.cells	PTP4A2	-0.03807	8.699413	-0.40211	0.688572	-6.86482	0.562791	0.6548
Erythroid.cells	CDC42EP1	0.319935	0.752796	0.401782	0.688814	-5.28208	0.739398	0.816842
Erythroid.cells	S100A8	-0.22566	6.633628	-0.40178	0.688815	-6.68435	0.604009	0.693553
Erythroid.cells	TTC4	-0.11593	3.541793	-0.40142	0.689077	-5.77447	0.671813	0.755938
Erythroid.cells	CIAO2A	-0.06778	6.679979	-0.40126	0.689196	-6.51513	0.603269	0.69271
Erythroid.cells	LPAR2	0.177241	2.187833	0.400949	0.689425	-5.41969	0.704046	0.785061
Erythroid.cells	P2RY12	-0.22726	2.616799	-0.40088	0.689473	-5.52727	0.693714	0.775751

Erythroid.cells	TSPAN6	0.320877	1.103606	0.400848	0.689499	-5.27138	0.730873	0.809081
Erythroid.cells	VEZT	-0.10939	4.178132	-0.40055	0.689719	-5.86304	0.657536	0.74277
Erythroid.cells	GM5535	-0.40987	0.151841	-0.4005	0.689755	-5.12212	0.755419	0.830767
Erythroid.cells	CYP3A11	-0.17992	3.352158	-0.40029	0.689909	-6.00531	0.676573	0.76008
Erythroid.cells	TRIT1	-0.12052	4.076961	-0.40009	0.690053	-5.80359	0.65994	0.744977
Erythroid.cells	ZDHHC23	-0.17147	2.221064	-0.40007	0.690073	-5.5734	0.703486	0.784455
Erythroid.cells	GRB2	-0.06049	7.898248	-0.39992	0.690177	-6.66516	0.578996	0.669797
Erythroid.cells	PLXNA2	0.229924	2.31233	0.3996	0.690415	-5.54635	0.701489	0.782533
Erythroid.cells	TMEM219	0.100207	4.624458	0.399504	0.690485	-6.00316	0.647839	0.73382
Erythroid.cells	TNS3	-0.10874	5.235143	-0.39913	0.690762	-6.27067	0.634592	0.721552
Erythroid.cells	TSIX	1.419265	1.906429	0.398843	0.690971	-5.32078	0.711636	0.791615
Erythroid.cells	CAR1	0.33961	0.491473	0.398829	0.690981	-5.39671	0.747242	0.823358
Erythroid.cells	RHNO1	-0.11926	4.345115	-0.3988	0.691001	-5.89796	0.654324	0.739689
Erythroid.cells	PLEKHA7	0.212289	2.084938	0.39819	0.69145	-5.47033	0.707642	0.787849
Erythroid.cells	GM50334	0.376888	-0.42372	0.398036	0.691563	-5.12209	0.771638	0.844724
Erythroid.cells	PPIL1	-0.13184	4.052063	-0.39796	0.691621	-5.88616	0.661297	0.74594
Erythroid.cells	TMEM116	0.236295	2.038927	0.397944	0.691631	-5.42471	0.708765	0.78891
Erythroid.cells	ZMPSTE24	-0.12393	4.942134	-0.39775	0.691774	-5.96749	0.641451	0.727713
Erythroid.cells	RAPGEF5	0.189135	5.011787	0.397593	0.691889	-6.01825	0.639963	0.72636
Erythroid.cells	PRPF6	-0.07779	5.300623	-0.3972	0.692174	-6.12117	0.6337	0.720584
Erythroid.cells	CHTF8	-0.23544	1.341638	-0.39719	0.692187	-5.34607	0.726204	0.804491
Erythroid.cells	GTF3C1	0.094855	4.779184	0.397088	0.692259	-6.03074	0.645144	0.731137
Erythroid.cells	2610001J0	0.136858	3.690163	0.397037	0.692297	-5.71305	0.669751	0.753639
Erythroid.cells	SAYSD1	0.124579	3.323883	0.396979	0.69234	-5.7089	0.678247	0.76136
Erythroid.cells	SCAF8	-0.05827	6.711338	-0.39686	0.692426	-6.48142	0.603801	0.692864
Erythroid.cells	TTF1	-0.14037	3.466533	-0.39678	0.692483	-5.69917	0.674925	0.758406
Erythroid.cells	SLC16A2	0.157612	2.626868	0.396718	0.692531	-5.75156	0.694725	0.776357
Erythroid.cells	TNIP2	0.116156	3.816648	0.396707	0.69254	-5.79559	0.666843	0.751077
Erythroid.cells	TMEM71	0.141482	3.858441	0.396273	0.692859	-5.87026	0.666112	0.750295
Erythroid.cells	HIST1H3B	-0.29225	1.995292	-0.39603	0.69304	-5.55735	0.710256	0.790225
Erythroid.cells	ZFP438	0.142228	3.204144	0.395987	0.693068	-5.69452	0.68128	0.764096
Erythroid.cells	CEP164	0.126493	4.052145	0.395979	0.693075	-5.76151	0.66169	0.746275
Erythroid.cells	PARVG	-0.10483	5.071901	-0.39586	0.693164	-5.98478	0.63891	0.725389
Erythroid.cells	MAPK14	-0.062	6.656022	-0.39584	0.693175	-6.44615	0.605151	0.694092
Erythroid.cells	ZBTB42	-0.17974	1.544195	-0.395	0.693796	-5.43251	0.721969	0.800399
Erythroid.cells	ZFP523	-0.18522	2.19531	-0.39477	0.693967	-5.44683	0.706046	0.786079
Erythroid.cells	TXNDC17	0.077483	6.116505	0.394483	0.694174	-6.36619	0.617095	0.704867
Erythroid.cells	GM26944	0.257548	0.852005	0.394358	0.694266	-5.27256	0.739625	0.81605
Erythroid.cells	1810041H1	-0.18549	1.76748	-0.39433	0.694289	-5.63591	0.716629	0.795552
Erythroid.cells	ACAP1	0.149131	3.923819	0.394284	0.694321	-5.76961	0.665332	0.749258
Erythroid.cells	LY6G2	-0.27756	0.952461	-0.39405	0.694492	-5.42818	0.737117	0.813894
Erythroid.cells	CAMKMT	-0.12064	4.501622	-0.39398	0.694541	-5.94832	0.652293	0.737387
Erythroid.cells	PLAUR	0.124791	6.837467	0.393947	0.694569	-6.49647	0.602096	0.690961
Erythroid.cells	2310016D2	0.231531	-0.3578	0.393674	0.69477	-5.45379	0.771339	0.844118
Erythroid.cells	FGA	0.105198	5.727993	0.39356	0.694854	-6.59442	0.6255	0.712725
Erythroid.cells	MTTP	0.169656	2.566189	0.393538	0.69487	-5.67401	0.697316	0.778287
Erythroid.cells	PSTPIP1	0.126125	4.472927	0.39333	0.695023	-5.93387	0.653112	0.738085

Erythroid.cells	DDX27	0.08893	4.974515	0.393124	0.695174	-6.07978	0.642036	0.727921
Erythroid.cells	TBP	0.16019	3.21522	0.392915	0.695328	-5.62338	0.682154	0.764471
Erythroid.cells	MS4A3	0.377409	-0.35863	0.392508	0.695628	-5.24573	0.771901	0.844358
Erythroid.cells	CDIPT	0.094228	5.093616	0.392393	0.695712	-6.08186	0.639707	0.725588
Erythroid.cells	ZRANB3	0.152109	3.148069	0.392333	0.695756	-5.70519	0.683957	0.765963
Erythroid.cells	RASAL1	0.304764	0.712454	0.392015	0.69599	-5.30244	0.744032	0.819657
Erythroid.cells	CNST	0.095009	3.943862	0.391951	0.696037	-5.91492	0.665621	0.749217
Erythroid.cells	TGIF2	-0.14447	3.122183	-0.39185	0.696114	-5.65907	0.684711	0.766579
Erythroid.cells	PTK2B	-0.07367	7.134222	-0.39177	0.696169	-6.56393	0.596647	0.685579
Erythroid.cells	WBP11	-0.06168	6.346343	-0.39163	0.696272	-6.38165	0.612975	0.700828
Erythroid.cells	GM14023	0.260304	1.123999	0.391547	0.696335	-5.34988	0.733574	0.810437
Erythroid.cells	ENPEP	-0.29578	0.401804	-0.39107	0.696684	-5.25994	0.752316	0.826976
Erythroid.cells	PROZ	0.17733	1.915226	0.391039	0.696709	-5.73243	0.714037	0.792934
Erythroid.cells	IFT140	-0.12433	3.507073	-0.39091	0.696808	-5.84132	0.675933	0.758619
Erythroid.cells	RANBP17	0.275442	1.134101	0.390846	0.696851	-5.26781	0.733538	0.810372
Erythroid.cells	PCGF2	0.253358	0.79144	0.390631	0.69701	-5.33346	0.742264	0.818132
Erythroid.cells	MAN2C1OS	-0.11147	4.443794	-0.39056	0.697063	-5.93463	0.654507	0.739108
Erythroid.cells	TSPAN17	-0.21246	1.115501	-0.39051	0.697095	-5.38473	0.734009	0.8108
Erythroid.cells	MKNK1	-0.10595	4.508378	-0.39046	0.697138	-5.86489	0.653056	0.73778
Erythroid.cells	ZSCAN26	0.105047	4.652438	0.390414	0.697169	-5.98385	0.649833	0.734828
Erythroid.cells	2010300C0	-0.27029	-0.0882	-0.39036	0.697211	-5.21857	0.765152	0.838389
Erythroid.cells	NR4A2	-0.11145	6.380914	-0.39012	0.697383	-6.70799	0.612466	0.700383
Erythroid.cells	PURB	0.056806	6.824936	0.390123	0.697384	-6.46688	0.603231	0.691779
Erythroid.cells	PCYOX1L	0.207436	1.613818	0.390036	0.697448	-5.41658	0.721536	0.79979
Erythroid.cells	GM42047	0.180123	5.263713	0.389779	0.697638	-6.0954	0.636483	0.722578
Erythroid.cells	SLC17A2	-0.23637	1.156237	-0.38942	0.6979	-5.45092	0.733343	0.810176
Erythroid.cells	FAM204A	0.079592	4.894011	0.389343	0.697958	-6.09698	0.644784	0.730183
Erythroid.cells	TENM3	-0.21895	1.688761	-0.38925	0.698026	-5.54915	0.719994	0.79832
Erythroid.cells	SERPINF2	0.146469	3.929119	0.389001	0.698211	-6.11479	0.666635	0.750212
Erythroid.cells	HCK	-0.12201	5.693071	-0.38862	0.698494	-6.22082	0.627578	0.714361
Erythroid.cells	QPCT	-0.13517	2.264701	-0.38849	0.698586	-5.88067	0.706094	0.785878
Erythroid.cells	RHOT2	0.171009	2.679369	0.388464	0.698607	-5.56289	0.696076	0.776866
Erythroid.cells	5430427M	0.275474	1.237849	0.388433	0.698629	-5.34206	0.731548	0.808634
Erythroid.cells	CXCR3	0.211416	1.731497	0.388213	0.698791	-5.66578	0.719196	0.797702
Erythroid.cells	HIST1H4I	0.135744	4.409355	0.388167	0.698826	-6.06484	0.655848	0.740449
Erythroid.cells	BAZ2A	-0.0725	6.97841	-0.38811	0.698867	-6.50984	0.60056	0.689361
Erythroid.cells	TMEM80	-0.12726	3.276451	-0.38809	0.698881	-5.63793	0.681908	0.764191
Erythroid.cells	XDH	-0.19518	4.807401	-0.38789	0.699033	-5.98022	0.64702	0.732411
Erythroid.cells	MTM1	-0.14371	4.433358	-0.38764	0.699212	-5.87038	0.655428	0.740115
Erythroid.cells	UBE3B	0.090002	4.424846	0.387509	0.699311	-5.96359	0.65562	0.740291
Erythroid.cells	KCTD21	-0.29789	0.182578	-0.38749	0.699321	-5.28552	0.758826	0.832973
Erythroid.cells	PDCD11	0.111597	4.214015	0.387463	0.699344	-5.83502	0.660388	0.744648
Erythroid.cells	CCNO	0.309871	0.174468	0.387298	0.699466	-5.23333	0.759098	0.83316
Erythroid.cells	NFE2L3	0.20425	2.246069	0.387157	0.69957	-5.49036	0.706757	0.786568
Erythroid.cells	CD300LF	-0.20228	3.385271	-0.38709	0.699622	-5.6998	0.67956	0.762044
Erythroid.cells	SRP68	0.092485	3.983827	0.386899	0.699761	-5.78211	0.665724	0.749501
Erythroid.cells	DNASE1L3	0.118582	6.30437	0.386883	0.699772	-6.54996	0.614767	0.702613

Erythroid.cells	LCAT	0.176539	2.143834	0.386774	0.699853	-5.67225	0.709283	0.788884
Erythroid.cells	CCNC	0.104378	4.398502	0.386588	0.69999	-5.97694	0.656364	0.740959
Erythroid.cells	EVI5L	-0.18168	2.262161	-0.38647	0.70008	-5.4734	0.706448	0.78634
Erythroid.cells	SEC11A	-0.05292	6.406384	-0.38643	0.700106	-6.43669	0.612682	0.700677
Erythroid.cells	GM20536	0.178466	2.340392	0.386329	0.700181	-5.51012	0.704555	0.784631
Erythroid.cells	TLN1	-0.06947	7.109295	-0.38616	0.700303	-6.51623	0.59818	0.687099
Erythroid.cells	CAVIN2	0.17701	2.454343	0.385863	0.700525	-5.63355	0.702004	0.782154
Erythroid.cells	ZBTB25	0.140982	3.709262	0.385652	0.700681	-5.7582	0.672395	0.755365
Erythroid.cells	1700048O2	0.395583	-0.24632	0.38557	0.700742	-5.16057	0.770656	0.843172
Erythroid.cells	EIF3E	-0.05051	7.095265	-0.3854	0.700866	-6.60007	0.59872	0.687475
Erythroid.cells	SCYL1	-0.12547	4.072604	-0.38523	0.700993	-5.82205	0.664156	0.747852
Erythroid.cells	BTBD19	-0.17603	2.725168	-0.38494	0.701203	-5.49754	0.695765	0.776503
Erythroid.cells	CUL3	-0.04579	7.198802	-0.38494	0.70121	-6.59012	0.596727	0.685587
Erythroid.cells	B3GALNT2	0.132452	3.643954	0.384697	0.701386	-5.77569	0.674122	0.756964
Erythroid.cells	CYP2A22	0.209679	1.632221	0.384692	0.701389	-5.5918	0.722502	0.800528
Erythroid.cells	GM13919	0.151622	3.111975	0.384557	0.701489	-5.73476	0.686579	0.768259
Erythroid.cells	4930526L0	0.276017	-0.8055	0.384508	0.701525	-5.20662	0.785933	0.856588
Erythroid.cells	MLXIPL	0.334839	0.355448	0.384453	0.701566	-5.29881	0.755049	0.829438
Erythroid.cells	KLHL8	0.199249	2.402928	0.384143	0.701795	-5.56701	0.703623	0.783562
Erythroid.cells	CHERP	-0.08244	5.016827	-0.38405	0.701865	-6.04915	0.643115	0.728588
Erythroid.cells	DEPDC7	0.179249	1.678465	0.384017	0.701888	-5.68555	0.72142	0.799506
Erythroid.cells	CYB5D1	0.307331	0.775244	0.383943	0.701943	-5.21978	0.744257	0.819841
Erythroid.cells	1810020O(	0.340667	-0.23349	0.383909	0.701968	-5.23503	0.770634	0.843123
Erythroid.cells	SOCS2	0.139331	3.962092	0.383725	0.702104	-5.98814	0.666915	0.750364
Erythroid.cells	POLR3E	-0.11568	3.918387	-0.38361	0.702191	-5.77443	0.667937	0.751277
Erythroid.cells	CENPH	-0.19035	3.059161	-0.38333	0.702397	-5.64058	0.688101	0.769516
Erythroid.cells	UFL1	-0.10923	3.965196	-0.38326	0.702446	-5.7951	0.666978	0.750357
Erythroid.cells	CRNKL1	0.072161	5.293781	0.38265	0.702897	-6.21079	0.637572	0.723307
Erythroid.cells	NDUFB8	-0.05447	7.487591	-0.38205	0.703342	-6.69904	0.591666	0.680742
Erythroid.cells	NEURL3	0.122929	4.301516	0.382038	0.70335	-5.95529	0.659917	0.743881
Erythroid.cells	TIMM10B	0.070112	6.181583	0.382024	0.70336	-6.34452	0.618692	0.705949
Erythroid.cells	RABGAP1L	0.066896	7.908574	0.381923	0.703435	-6.75043	0.583231	0.672842
Erythroid.cells	MORF4L1	0.03248	8.201561	0.381573	0.703693	-6.77835	0.57753	0.667465
Erythroid.cells	RSU1	0.074651	5.746299	0.381561	0.703702	-6.19614	0.628103	0.714679
Erythroid.cells	CASR	-0.27725	-0.61039	-0.38149	0.703756	-5.27588	0.781826	0.852858
Erythroid.cells	SAA4	-0.25112	0.640047	-0.38125	0.703936	-5.42845	0.748908	0.82384
Erythroid.cells	APOE	0.1232	9.241961	0.381098	0.704045	-7.13724	0.55755	0.648534
Erythroid.cells	COG5	0.070061	6.582266	0.380937	0.704164	-6.43349	0.610541	0.698379
Erythroid.cells	PHKA2	-0.14246	3.438409	-0.38067	0.704363	-5.71966	0.68011	0.762253
Erythroid.cells	CSF3R	0.184448	2.933462	0.38057	0.704435	-5.73747	0.692037	0.773038
Erythroid.cells	INO80DOS	0.14894	4.442013	0.380564	0.70444	-5.94179	0.657037	0.741256
Erythroid.cells	DCSTAMP	0.192454	-1.33737	0.380461	0.704516	-5.32469	0.801923	0.870383
Erythroid.cells	BUD31	0.071348	5.474604	0.380258	0.704666	-6.29615	0.634154	0.720291
Erythroid.cells	ADGRL2	0.18179	4.208198	0.380131	0.70476	-5.84751	0.662339	0.746187
Erythroid.cells	KCTD6	0.149119	2.981269	0.380099	0.704784	-5.63953	0.690899	0.772105
Erythroid.cells	HELLS	-0.13677	4.919254	-0.38006	0.704815	-6.14097	0.646355	0.731563
Erythroid.cells	LONRF3	-0.19685	1.525709	-0.37996	0.704887	-5.49943	0.726447	0.80402

Erythroid.cells	AQP1	0.182446	2.964821	0.379824	0.704987	-5.70432	0.69129	0.772479
Erythroid.cells	CAPSL	-0.29581	0.928873	-0.37978	0.70502	-5.35866	0.741562	0.817465
Erythroid.cells	PBX4	0.254273	0.976518	0.379739	0.70505	-5.32157	0.740344	0.816384
Erythroid.cells	ZFP768	0.272301	0.794694	0.379725	0.70506	-5.31896	0.745005	0.820518
Erythroid.cells	YIF1A	-0.09059	4.016242	-0.37962	0.70514	-5.9242	0.666724	0.750205
Erythroid.cells	PSMA5	0.079659	5.884469	0.379599	0.705153	-6.33473	0.625306	0.712185
Erythroid.cells	TMEM192	-0.09622	4.694631	-0.37927	0.705398	-5.96981	0.651408	0.736172
Erythroid.cells	NMT2	-0.07398	5.47269	-0.37922	0.705435	-6.18867	0.634243	0.720397
Erythroid.cells	IREB2	-0.07936	5.493255	-0.37915	0.705488	-6.18544	0.633796	0.719984
Erythroid.cells	ALG8	-0.16141	3.226775	-0.37889	0.705677	-5.61353	0.685133	0.766934
Erythroid.cells	SFXN2	-0.11318	3.18033	-0.37887	0.705693	-5.81134	0.68623	0.767926
Erythroid.cells	ADCY3	0.24665	3.001893	0.378814	0.705734	-5.60161	0.690459	0.771774
Erythroid.cells	NABP2	0.08654	4.980112	0.37877	0.705767	-6.04702	0.645054	0.730437
Erythroid.cells	HSF5	-0.30459	0.277217	-0.37848	0.705982	-5.19687	0.758489	0.832592
Erythroid.cells	CNNM2	-0.14185	5.427728	-0.37835	0.706074	-6.19076	0.635222	0.721498
Erythroid.cells	LEF1OS1	-0.28546	-0.45613	-0.37831	0.706104	-5.13466	0.777941	0.849739
Erythroid.cells	DPP8	-0.07223	5.667042	-0.37828	0.706131	-6.21151	0.63003	0.716707
Erythroid.cells	NFATC2IP	0.141685	3.151572	0.378248	0.706153	-5.68063	0.68691	0.768695
Erythroid.cells	WDR83	0.146282	2.732947	0.378148	0.706227	-5.62498	0.696885	0.777738
Erythroid.cells	UNC93B1	-0.05988	7.659349	-0.37814	0.706234	-6.65582	0.588517	0.678078
Erythroid.cells	RAP1GAP2	-0.17091	4.692548	-0.37811	0.706258	-5.90551	0.651455	0.736461
Erythroid.cells	CTSS	0.114255	7.501211	0.377943	0.706379	-6.68317	0.591704	0.681073
Erythroid.cells	CCT6A	-0.0719	5.802009	-0.37793	0.70639	-6.30062	0.627122	0.714065
Erythroid.cells	OPLAH	-0.2264	1.500611	-0.37789	0.706417	-5.35409	0.72713	0.804855
Erythroid.cells	AMY1	-0.15503	2.627056	-0.37781	0.706478	-5.74717	0.699432	0.780059
Erythroid.cells	PPAN	0.125896	4.072585	0.377515	0.706696	-5.85845	0.665624	0.749362
Erythroid.cells	SAPCD2	0.198367	1.790194	0.377357	0.706813	-5.50993	0.720056	0.798514
Erythroid.cells	NXPH4	-0.31454	-0.60095	-0.37734	0.706828	-5.19428	0.782007	0.853333
Erythroid.cells	FXN	0.096235	4.085717	0.377031	0.707054	-5.93975	0.665434	0.749206
Erythroid.cells	SAPCD1	0.215077	1.629002	0.376996	0.70708	-5.40858	0.72419	0.8022
Erythroid.cells	SNX17	-0.07484	5.853791	-0.37655	0.707408	-6.2454	0.626437	0.713306
Erythroid.cells	TOB1	-0.09235	5.050855	-0.37637	0.707543	-6.12425	0.643929	0.729432
Erythroid.cells	GGA2	-0.11096	4.120751	-0.37636	0.707553	-5.98379	0.664835	0.748564
Erythroid.cells	CAD	0.2168	1.795668	0.376343	0.707563	-5.44163	0.720259	0.798584
Erythroid.cells	RHEB	-0.05186	7.039284	-0.37625	0.707632	-6.5569	0.601528	0.690177
Erythroid.cells	AP4E1	-0.15372	3.305374	-0.37603	0.707798	-5.66732	0.683846	0.765838
Erythroid.cells	FANCD2	0.157516	2.80256	0.375902	0.70789	-5.63216	0.695815	0.776642
Erythroid.cells	MYCT1	-0.20501	1.654516	-0.37568	0.708055	-5.44857	0.724003	0.801886
Erythroid.cells	NEK2	0.176542	3.092142	0.375573	0.708134	-5.74622	0.689016	0.770492
Erythroid.cells	RAB27B	0.206066	0.615378	0.375383	0.708275	-5.58803	0.750482	0.825411
Erythroid.cells	ZFP28	0.302729	0.2908	0.375347	0.708301	-5.18937	0.758939	0.832883
Erythroid.cells	4921524J1	-0.0637	5.474163	-0.37522	0.708395	-6.26615	0.634909	0.721064
Erythroid.cells	YBX1	-0.05411	8.398771	-0.3749	0.70863	-6.8281	0.574557	0.664687
Erythroid.cells	MARK3	-0.0537	6.304211	-0.37487	0.708658	-6.4292	0.617175	0.704591
Erythroid.cells	COPS5	0.070556	5.288344	0.37484	0.708677	-6.21653	0.639042	0.724804
Erythroid.cells	LYNX1	-0.27123	0.898277	-0.37472	0.708766	-5.3292	0.743325	0.818961
Erythroid.cells	CCDC114	-0.23127	1.271818	-0.37438	0.709014	-5.28365	0.733921	0.810538

Erythroid.cells	DGKA	0.094027	5.023185	0.374374	0.709022	-6.24659	0.645004	0.730212
Erythroid.cells	AI413582	0.117602	4.68032	0.374276	0.709095	-5.90806	0.652643	0.73722
Erythroid.cells	PLD4	0.101141	6.186695	0.374208	0.709146	-6.43036	0.619779	0.706954
Erythroid.cells	ETNK1	0.073436	5.999727	0.373987	0.709309	-6.29185	0.623846	0.710674
Erythroid.cells	DCUN1D4	-0.20328	2.540378	-0.37371	0.709516	-5.5028	0.702742	0.782601
Erythroid.cells	SLC25A42	-0.20019	1.940911	-0.37361	0.709588	-5.50617	0.71742	0.7958
Erythroid.cells	KCP	-0.28755	0.100139	-0.37335	0.709781	-5.23797	0.764569	0.837649
Erythroid.cells	WAC	0.04693	7.414892	0.37328	0.709834	-6.63161	0.594561	0.68344
Erythroid.cells	AP1S2	0.092094	5.081901	0.373204	0.70989	-6.07018	0.644007	0.729334
Erythroid.cells	PRKCZ	-0.20712	0.606888	-0.37288	0.710132	-5.37173	0.751309	0.826063
Erythroid.cells	ELOVL5	-0.07254	6.195778	-0.3728	0.710192	-6.45975	0.619877	0.707113
Erythroid.cells	SPHK2	0.133505	3.388566	0.372792	0.710195	-5.71324	0.682603	0.764586
Erythroid.cells	MAP3K2	0.073207	6.248682	0.37276	0.710219	-6.31355	0.618755	0.706072
Erythroid.cells	H2-DMB1	0.143042	4.478206	0.372739	0.710235	-6.33421	0.657498	0.741747
Erythroid.cells	MRPS17	0.073096	5.224333	0.372675	0.710282	-6.20503	0.640867	0.726499
Erythroid.cells	DEPDC5	-0.08309	4.978079	-0.37255	0.710376	-6.10615	0.646331	0.731547
Erythroid.cells	GM31243	0.185085	2.779029	0.372204	0.710632	-5.81835	0.697276	0.777799
Erythroid.cells	GM14029	0.223294	0.297438	0.371919	0.710843	-5.42425	0.75959	0.833388
Erythroid.cells	ELP3	0.151408	2.716115	0.371885	0.710868	-5.56237	0.698789	0.779209
Erythroid.cells	SDCBP2	0.233279	1.097863	0.371814	0.710921	-5.41887	0.738891	0.815066
Erythroid.cells	BICRA	-0.07345	5.82128	-0.37178	0.710948	-6.32589	0.628056	0.714693
Erythroid.cells	VIL1	-0.32804	0.018277	-0.37174	0.710975	-5.20508	0.766947	0.839871
Erythroid.cells	MYL6B	0.242785	0.862198	0.371684	0.711017	-5.2694	0.744925	0.82042
Erythroid.cells	1110019D1	0.190733	1.751896	0.37144	0.711199	-5.45603	0.722521	0.800433
Erythroid.cells	TPRKB	-0.13168	3.157978	-0.37129	0.711309	-5.74541	0.688388	0.769789
Erythroid.cells	PRDM15	-0.12918	3.462741	-0.37111	0.711447	-5.67835	0.681271	0.763307
Erythroid.cells	GNB2	0.047343	8.428837	0.371002	0.711524	-6.8305	0.574698	0.66478
Erythroid.cells	DBNDD2	-0.16361	2.836435	-0.37057	0.711843	-5.61949	0.696378	0.776827
Erythroid.cells	ZFP667	-0.16616	2.726732	-0.37012	0.712178	-5.61668	0.699255	0.779201
Erythroid.cells	ZNRF3	-0.08983	5.517512	-0.3699	0.712345	-6.33275	0.635286	0.720995
Erythroid.cells	GM29994	-0.21283	1.150146	-0.36989	0.71235	-5.31751	0.73832	0.814146
Erythroid.cells	4930556J2	-0.15447	2.185085	-0.36968	0.712505	-5.56123	0.71243	0.79107
Erythroid.cells	PDE12	-0.09438	3.991564	-0.36962	0.712553	-5.87095	0.669465	0.752294
Erythroid.cells	ERGIC3	0.077045	5.513051	0.369611	0.712556	-6.15316	0.635383	0.721096
Erythroid.cells	ASF1A	-0.07871	5.210724	-0.36961	0.712558	-6.27701	0.642007	0.727191
Erythroid.cells	TPPP3	-0.21525	1.071021	-0.36906	0.712964	-5.60626	0.740569	0.816043
Erythroid.cells	NOTCH3	0.268727	1.028947	0.369046	0.712976	-5.31933	0.741645	0.816998
Erythroid.cells	TRIP12	-0.04713	7.435654	-0.36895	0.713043	-6.66634	0.595104	0.683613
Erythroid.cells	GM7854	-0.29137	0.52802	-0.36882	0.713145	-5.26589	0.75458	0.828469
Erythroid.cells	GM16536	-0.20123	1.530823	-0.36877	0.713179	-5.34025	0.728913	0.805707
Erythroid.cells	SAT2	-0.33703	-0.24549	-0.36877	0.713182	-5.19628	0.775004	0.846448
Erythroid.cells	GNASAS1	0.261476	0.682179	0.368647	0.713272	-5.26725	0.750599	0.82493
Erythroid.cells	SLFN5	0.187637	4.230734	0.368125	0.71366	-5.94264	0.664509	0.747569
Erythroid.cells	ZFAND2A	0.111941	3.630572	0.368034	0.713728	-5.77845	0.678367	0.760183
Erythroid.cells	FTO	0.05894	6.592827	0.367944	0.713794	-6.43943	0.612795	0.700047
Erythroid.cells	TBC1D1	0.0747	7.18844	0.367649	0.714014	-6.51145	0.600568	0.68861
Erythroid.cells	CCDC47	0.075896	5.110399	0.367171	0.714369	-6.11153	0.645129	0.7297

Erythroid.cells	KLK8	-0.15972	1.605933	-0.36705	0.71446	-5.5831	0.727837	0.804472
Erythroid.cells	AKR1B3	0.075902	5.402163	0.366974	0.714515	-6.17038	0.638714	0.723833
Erythroid.cells	B230217C1	-0.18382	1.478169	-0.36646	0.714899	-5.46022	0.731375	0.807468
Erythroid.cells	GM10138	0.2014	2.471458	0.366349	0.71498	-5.45453	0.706764	0.78548
Erythroid.cells	EWSR1	0.043287	7.421625	0.366218	0.715077	-6.66693	0.596353	0.684389
Erythroid.cells	CLEC4A1	-0.1934	3.158732	-0.36599	0.715248	-5.88163	0.690358	0.770642
Erythroid.cells	9530068E0	0.100906	4.851397	0.365851	0.71535	-5.98667	0.651328	0.735198
Erythroid.cells	CSNK1G2	0.070862	5.468875	0.365801	0.715388	-6.18352	0.637672	0.722668
Erythroid.cells	PSMD7	-0.07369	5.828246	-0.3656	0.715536	-6.3179	0.62993	0.715553
Erythroid.cells	CD209F	0.488281	2.767776	0.365479	0.715627	-5.56522	0.699795	0.77919
Erythroid.cells	KDELR2	0.063133	5.993878	0.365356	0.715718	-6.36987	0.626365	0.712282
Erythroid.cells	CFHR1	0.275649	0.449486	0.365313	0.71575	-5.37086	0.758048	0.831062
Erythroid.cells	YWHAH	-0.05887	7.687722	-0.36509	0.715915	-6.69	0.591111	0.679515
Erythroid.cells	SH2D3C	0.120289	4.693449	0.365027	0.715963	-5.95818	0.65494	0.738597
Erythroid.cells	AKAP6	0.358213	0.299684	0.364978	0.715999	-5.28866	0.761979	0.834588
Erythroid.cells	HRG	-0.14772	3.61876	-0.36497	0.716002	-6.11023	0.679589	0.761025
Erythroid.cells	PLSCR3	0.111346	4.301027	0.364191	0.716585	-5.92939	0.664308	0.746894
Erythroid.cells	GM47689	-0.10928	3.369026	-0.36406	0.716679	-5.75732	0.685974	0.766557
Erythroid.cells	1190007I0	-0.11735	3.503332	-0.3639	0.7168	-5.78038	0.682862	0.763713
Erythroid.cells	GM29966	-0.24401	1.411825	-0.36346	0.717131	-5.51593	0.734	0.80952
Erythroid.cells	MEX3B	-0.18389	2.233947	-0.36344	0.71714	-5.49528	0.713482	0.791228
Erythroid.cells	ZFP780B	0.140979	3.329247	0.363434	0.717148	-5.68444	0.687064	0.767484
Erythroid.cells	TXNDC9	-0.06461	5.512196	-0.3634	0.717172	-6.25943	0.637413	0.722238
Erythroid.cells	CAGE1	-0.20803	1.94977	-0.36324	0.717295	-5.49389	0.72052	0.79755
Erythroid.cells	CEP44	-0.10025	3.531339	-0.3632	0.71732	-5.75504	0.682314	0.763223
Erythroid.cells	PTK6	-0.3773	-0.3416	-0.36287	0.717565	-5.15787	0.780007	0.850006
Erythroid.cells	ATRN	0.08465	5.965865	0.362445	0.717884	-6.28699	0.627933	0.713336
Erythroid.cells	IL18R1	-0.19813	0.978465	-0.36228	0.718007	-5.56117	0.74548	0.819514
Erythroid.cells	ADH1	0.155872	3.503888	0.362226	0.718048	-6.07445	0.683333	0.763938
Erythroid.cells	GNA14	-0.39398	-0.0617	-0.36222	0.71805	-5.1369	0.772736	0.843527
Erythroid.cells	ATP7A	-0.07614	5.8288	-0.36202	0.718203	-6.21908	0.630945	0.716127
Erythroid.cells	SOCS6	0.103354	3.829946	0.361956	0.718248	-5.79424	0.67577	0.757111
Erythroid.cells	ULK1	-0.11334	3.637915	-0.3618	0.718366	-5.83576	0.680297	0.761185
Erythroid.cells	E2F8	-0.13261	3.686572	-0.36127	0.718758	-5.90016	0.679421	0.760287
Erythroid.cells	MCEMP1	-0.22997	1.650063	-0.36124	0.718779	-5.6553	0.728802	0.804611
Erythroid.cells	SLC29A1	-0.09906	5.215066	-0.36115	0.718847	-6.12646	0.64466	0.728678
Erythroid.cells	ELAC2	-0.13919	2.817476	-0.36087	0.719054	-5.60607	0.700127	0.779049
Erythroid.cells	SLC45A1	0.235113	0.608482	0.360852	0.719071	-5.21174	0.755549	0.828384
Erythroid.cells	PSMB6	0.062595	6.304312	0.360785	0.719121	-6.46224	0.621096	0.707006
Erythroid.cells	FAAP24	0.225208	2.002599	0.360467	0.719358	-5.47069	0.720182	0.797044
Erythroid.cells	AIFM2	-0.18308	1.347203	-0.36027	0.719507	-5.55539	0.736647	0.811747
Erythroid.cells	TAF1D	-0.06226	6.027224	-0.36023	0.719533	-6.32551	0.627118	0.712647
Erythroid.cells	MASP2	-0.20879	1.894509	-0.36016	0.71959	-5.60262	0.722871	0.799515
Erythroid.cells	TPP1	-0.09103	5.393356	-0.36011	0.719624	-6.1361	0.640894	0.725342
Erythroid.cells	COQ2	0.100256	4.467496	0.360089	0.71964	-5.91985	0.661593	0.744281
Erythroid.cells	FASTKD5	-0.22449	0.844458	-0.35992	0.719763	-5.30136	0.749597	0.823203
Erythroid.cells	RAB1B	-0.07511	5.58788	-0.35978	0.719868	-6.21542	0.636715	0.721454

Erythroid.cells	NSD3	-0.04662	8.849458	-0.35956	0.720035	-6.93269	0.569594	0.658891
Erythroid.cells	PARK7	0.058457	6.806683	0.359555	0.720038	-6.56387	0.610715	0.697436
Erythroid.cells	LZTS3	0.253711	0.323606	0.359111	0.720368	-5.23572	0.763414	0.835392
Erythroid.cells	XAF1	0.209272	4.033526	0.359109	0.720369	-5.89084	0.671779	0.753516
Erythroid.cells	SLC17A3	0.241069	0.779648	0.359104	0.720373	-5.39675	0.75149	0.82488
Erythroid.cells	TMEM19	-0.10116	3.958743	-0.35807	0.721148	-5.82868	0.673842	0.755251
Erythroid.cells	TASOR	0.076728	5.742445	0.358015	0.721186	-6.2638	0.633811	0.718665
Erythroid.cells	EIF4A1	-0.0477	7.526831	-0.35795	0.721231	-6.66642	0.596273	0.683837
Erythroid.cells	SNHG9	0.182752	4.309776	0.357896	0.721274	-5.90045	0.665759	0.747908
Erythroid.cells	SP1	-0.06055	5.924217	-0.35785	0.721312	-6.30046	0.629875	0.715037
Erythroid.cells	SUPT7L	-0.14133	2.767637	-0.35778	0.721357	-5.64011	0.702042	0.780702
Erythroid.cells	ATP6V0E	0.046788	7.713213	0.357767	0.72137	-6.69745	0.592491	0.680299
Erythroid.cells	E4F1	0.152726	3.127007	0.357764	0.721373	-5.63621	0.693407	0.772936
Erythroid.cells	ZFP932	-0.11998	2.788655	-0.35762	0.72148	-5.59556	0.701534	0.780264
Erythroid.cells	SMIM26	-0.09928	3.847983	-0.35761	0.721489	-5.89638	0.676414	0.7576
Erythroid.cells	SLC36A3OS	0.236351	1.010121	0.357576	0.721513	-5.48971	0.745904	0.819814
Erythroid.cells	SNX19	-0.12668	2.862011	-0.35754	0.72154	-5.59147	0.699764	0.778683
Erythroid.cells	A230059L0	0.209617	0.47539	0.357376	0.721662	-5.54941	0.759847	0.832122
Erythroid.cells	MDN1	-0.0974	5.788045	-0.3573	0.72172	-6.2793	0.632861	0.717812
Erythroid.cells	TIMM50	0.088358	4.827954	0.357094	0.721872	-6.08214	0.65413	0.737301
Erythroid.cells	AGAP3	0.123715	4.127556	0.356721	0.72215	-5.81229	0.670258	0.751817
Erythroid.cells	A330023F2	0.143536	3.109027	0.35662	0.722226	-5.71538	0.694171	0.773432
Erythroid.cells	CDK7	-0.10408	4.595584	-0.35648	0.722327	-5.92511	0.659602	0.742086
Erythroid.cells	GM43728	-0.22942	-0.48628	-0.35633	0.722439	-5.27891	0.785895	0.854751
Erythroid.cells	TSSC4	0.112232	3.902903	0.356045	0.722655	-5.82933	0.675517	0.756644
Erythroid.cells	5430431A1	0.184297	1.423829	0.35582	0.722823	-5.3962	0.735746	0.810746
Erythroid.cells	SH3BGRL2	-0.1983	2.700678	-0.35571	0.722908	-5.63386	0.70406	0.782468
Erythroid.cells	SNRPF	-0.06032	6.76677	-0.35562	0.722976	-6.54391	0.61231	0.698746
Erythroid.cells	MANSC1	-0.2917	0.242039	-0.35559	0.722997	-5.22579	0.766377	0.837817
Erythroid.cells	VAV1	-0.06981	6.364742	-0.35528	0.723228	-6.30588	0.620792	0.706746
Erythroid.cells	HIST1H3E	0.136563	3.610481	0.355187	0.723295	-5.83017	0.682347	0.763066
Erythroid.cells	MARCKS	0.072779	7.242888	0.355176	0.723304	-6.64045	0.602424	0.689678
Erythroid.cells	ADHFE1	-0.14712	2.690792	-0.35513	0.72334	-5.70315	0.7043	0.782843
Erythroid.cells	TLR8	-0.21297	0.054908	-0.35512	0.723347	-5.389	0.771344	0.842337
Erythroid.cells	APEX1	-0.08607	5.465947	-0.35506	0.72339	-6.18317	0.640209	0.724654
Erythroid.cells	RSRC2	-0.04708	6.479986	-0.35504	0.723403	-6.40866	0.618348	0.704486
Erythroid.cells	GCNT2	0.102864	4.580489	0.35493	0.723487	-6.24066	0.659966	0.742803
Erythroid.cells	9130019O2	-0.31976	0.355077	-0.3548	0.723587	-5.16503	0.763391	0.835463
Erythroid.cells	CFAP410	0.137643	2.119698	0.354781	0.723598	-5.52353	0.718299	0.795487
Erythroid.cells	CPLX2	-0.14105	3.378009	-0.35476	0.723618	-5.93551	0.687828	0.768125
Erythroid.cells	1110065P2	-0.10197	3.514697	-0.35472	0.723646	-5.80736	0.6846	0.765214
Erythroid.cells	2410018L1	-0.21029	0.727626	-0.35469	0.723667	-5.36987	0.753636	0.826871
Erythroid.cells	NR6A1OS	-0.18734	2.067272	-0.35456	0.723765	-5.55191	0.719599	0.796663
Erythroid.cells	UBXN7	0.06763	5.704222	0.35455	0.723771	-6.29203	0.634999	0.719977
Erythroid.cells	DGCR2	-0.09008	4.849233	-0.35444	0.723855	-6.04383	0.653911	0.73732
Erythroid.cells	LRWD1	-0.10034	3.781422	-0.35436	0.723916	-5.79313	0.678355	0.759565
Erythroid.cells	PLEKHB1	-0.22547	0.728002	-0.35424	0.724003	-5.31075	0.753653	0.826873

Erythroid.cells	TGDS	0.126019	3.1411	0.354155	0.724066	-5.66987	0.693484	0.77322
Erythroid.cells	CREBBP	0.044925	7.888027	0.35387	0.724279	-6.75669	0.589437	0.677511
Erythroid.cells	HNMT	0.235793	-0.38409	0.35355	0.724518	-5.22939	0.783496	0.852922
Erythroid.cells	PDZD2	-0.14966	3.46937	-0.35341	0.72462	-5.88083	0.686025	0.766304
Erythroid.cells	RNF2	0.064445	5.723956	0.35326	0.724735	-6.25911	0.634901	0.71973
Erythroid.cells	A730081D(	0.16414	3.129627	0.353231	0.724756	-5.62349	0.694096	0.773638
Erythroid.cells	MAPK8	-0.07568	5.232129	-0.35281	0.725074	-6.12597	0.645879	0.729807
Erythroid.cells	PPP2R1B	-0.10207	4.339805	-0.35278	0.725092	-5.91249	0.665977	0.748163
Erythroid.cells	4930414N(	-0.15665	3.331114	-0.35234	0.725424	-5.65649	0.689739	0.769612
Erythroid.cells	MICU2	-0.08679	4.939969	-0.35219	0.725535	-6.06824	0.652652	0.735991
Erythroid.cells	GM47917	0.216482	0.467794	0.352117	0.725588	-5.3128	0.761341	0.833477
Erythroid.cells	MTRF1L	-0.14228	3.1332	-0.35201	0.725672	-5.64017	0.694498	0.773977
Erythroid.cells	ENTPD1	-0.09701	6.9129	-0.35175	0.72586	-6.4227	0.610104	0.696765
Erythroid.cells	TRP53COR:	0.255545	1.008827	0.351557	0.726007	-5.39428	0.747477	0.821256
Erythroid.cells	RBM12B2	-0.16319	2.316248	-0.35119	0.726283	-5.4679	0.714723	0.79209
Erythroid.cells	SRF	-0.11026	2.927184	-0.35109	0.726355	-5.6346	0.699835	0.778743
Erythroid.cells	UBE3C	-0.06775	5.784905	-0.35101	0.726417	-6.27243	0.634376	0.719236
Erythroid.cells	ELAC1	0.154979	1.710286	0.3509	0.726498	-5.45394	0.729826	0.805649
Erythroid.cells	LCOR	0.053095	7.538405	0.350803	0.72657	-6.66245	0.597453	0.685027
Erythroid.cells	ALG11	-0.13926	2.610001	-0.35063	0.726698	-5.52648	0.707599	0.785852
Erythroid.cells	GMNN	-0.10068	5.558334	-0.35053	0.726773	-6.30111	0.639398	0.723952
Erythroid.cells	NAE1	-0.09864	3.958187	-0.3503	0.726949	-5.85626	0.675618	0.757021
Erythroid.cells	PPBP	-0.29256	0.842206	-0.35012	0.727084	-5.54633	0.752212	0.825602
Erythroid.cells	PHF20L1	-0.05841	7.046788	-0.3501	0.727094	-6.51559	0.607728	0.694698
Erythroid.cells	GLA	-0.12352	4.568639	-0.34971	0.727388	-5.9364	0.661622	0.744445
Erythroid.cells	CXCL12	0.13747	2.838235	0.349655	0.727429	-5.83451	0.702204	0.781172
Erythroid.cells	GM49662	0.182131	2.537113	0.349586	0.727481	-5.83632	0.709527	0.787743
Erythroid.cells	TAF3	-0.06749	5.653075	-0.34951	0.727539	-6.24474	0.637451	0.722306
Erythroid.cells	IGSF9	-0.23226	0.790152	-0.34937	0.727644	-5.26046	0.753592	0.826938
Erythroid.cells	GTF2H4	-0.14013	2.307317	-0.34928	0.727707	-5.52193	0.715169	0.792794
Erythroid.cells	ZFP26	0.095914	3.995742	0.349261	0.727724	-5.84776	0.674779	0.756413
Erythroid.cells	ABRAXAS1	-0.1665	2.604808	-0.3492	0.72777	-5.5449	0.707874	0.786262
Erythroid.cells	ARMCX6	-0.193	0.977785	-0.34905	0.727883	-5.3204	0.748728	0.82264
Erythroid.cells	TMED10	0.04832	7.769396	0.34902	0.727904	-6.67424	0.592933	0.680981
Erythroid.cells	ZFP626	0.142838	2.562798	0.348916	0.727982	-5.5443	0.708899	0.787181
Erythroid.cells	TCF19	-0.15085	3.199871	-0.34878	0.728087	-5.77986	0.693513	0.773377
Erythroid.cells	RAB6B	0.210249	1.697022	0.348756	0.728102	-5.34332	0.730377	0.806387
Erythroid.cells	NCF1	-0.12005	5.301832	-0.34861	0.728213	-5.98799	0.645176	0.729456
Erythroid.cells	ARHGAP30	-0.05008	7.139253	-0.34841	0.72836	-6.52057	0.605832	0.693138
Erythroid.cells	HDAC6	0.172371	2.206503	0.348208	0.728512	-5.48572	0.717658	0.79522
Erythroid.cells	EP400	0.049269	6.870294	0.348188	0.728526	-6.52552	0.611428	0.698415
Erythroid.cells	RASA4	0.155717	4.228858	0.348165	0.728544	-5.86878	0.669393	0.75171
Erythroid.cells	ZAP70	0.138845	1.982594	0.348158	0.728549	-5.87862	0.72322	0.800187
Erythroid.cells	GM14858	-0.20794	2.296719	-0.34814	0.72856	-5.55178	0.71543	0.793228
Erythroid.cells	DDX31	0.128352	3.220713	0.348142	0.728561	-5.66246	0.693016	0.773099
Erythroid.cells	GRK3	0.128938	2.91909	0.347991	0.728674	-5.85339	0.700251	0.779642
Erythroid.cells	GM21762	0.303995	-1.20491	0.347909	0.728735	-5.03955	0.807338	0.874275

Erythroid.cells	QSER1	0.09278	4.323503	0.347818	0.728804	-5.98134	0.667219	0.74981
Erythroid.cells	LAYN	0.222719	0.629576	0.347804	0.728814	-5.40444	0.757781	0.830931
Erythroid.cells	ABHD14B	0.156039	2.125702	0.347676	0.72891	-5.58672	0.719688	0.7971
Erythroid.cells	ITGAV	-0.0824	6.550452	-0.34751	0.729031	-6.45423	0.618224	0.704773
Erythroid.cells	SYNC	-0.1562	1.973724	-0.34732	0.729179	-5.44741	0.723602	0.800606
Erythroid.cells	MGAT4A	0.090756	5.666978	0.346828	0.729544	-6.12617	0.637542	0.722475
Erythroid.cells	RHBDD1	0.098426	4.175534	0.346689	0.729648	-5.85973	0.671036	0.753109
Erythroid.cells	1110002J0	-0.26601	-0.51304	-0.34666	0.729672	-5.31609	0.788764	0.857953
Erythroid.cells	TLE4	0.056444	7.814076	0.346271	0.729962	-6.69582	0.592554	0.680636
Erythroid.cells	CEP55	-0.17732	3.678611	-0.34622	0.730002	-5.85453	0.682785	0.763685
Erythroid.cells	POLR3D	-0.13411	3.27535	-0.34612	0.730072	-5.68122	0.692329	0.772331
Erythroid.cells	PTS	-0.06687	5.573329	-0.3458	0.730315	-6.22592	0.639899	0.724556
Erythroid.cells	GM11973	-0.14996	3.179185	-0.34559	0.730474	-5.69822	0.69477	0.77452
Erythroid.cells	I830077J02	0.170466	2.758256	0.345434	0.730589	-5.65571	0.704916	0.783644
Erythroid.cells	MLF2	-0.0609	5.85253	-0.34541	0.730605	-6.35468	0.633804	0.718977
Erythroid.cells	TMEM131	0.056526	6.606387	0.345274	0.730709	-6.52999	0.617651	0.704067
Erythroid.cells	ATXN10	0.059869	6.66392	0.345205	0.73076	-6.45116	0.616436	0.702955
Erythroid.cells	VEGFA	0.108199	4.223513	0.34516	0.730794	-6.07729	0.670252	0.752374
Erythroid.cells	B3GALT1	0.193596	3.129188	0.345114	0.730828	-5.89757	0.695967	0.77564
Erythroid.cells	ZFP451	-0.079	5.019065	-0.34501	0.730904	-6.1085	0.652183	0.73591
Erythroid.cells	ARMC10	0.128061	3.275459	0.344736	0.731111	-5.63613	0.692471	0.772569
Erythroid.cells	CTSA	-0.08955	6.259221	-0.3447	0.731137	-6.31071	0.625035	0.711002
Erythroid.cells	F2RL2	-0.23744	0.129948	-0.34465	0.731179	-5.36529	0.771813	0.843184
Erythroid.cells	POU5F1	-0.20955	1.26222	-0.3446	0.731214	-5.33484	0.74223	0.817103
Erythroid.cells	EIF1B	-0.05945	5.828759	-0.34457	0.731233	-6.29821	0.63432	0.719608
Erythroid.cells	CEP72	-0.1671	2.208429	-0.34453	0.731265	-5.48026	0.718399	0.795903
Erythroid.cells	CNRIP1	0.229577	1.648634	0.344472	0.73131	-5.34207	0.732401	0.808393
Erythroid.cells	GM43661	0.162403	2.981343	0.343698	0.73189	-5.75329	0.70001	0.779128
Erythroid.cells	PDK1	0.093983	4.164844	0.343471	0.732059	-5.92946	0.672163	0.753926
Erythroid.cells	METTL2	0.114528	3.27705	0.343279	0.732203	-5.70728	0.693008	0.772801
Erythroid.cells	4930599N2	-0.17721	1.733523	-0.34321	0.732254	-5.52057	0.730867	0.806711
Erythroid.cells	DCBLD1	0.160656	3.33755	0.343209	0.732256	-5.69047	0.691567	0.771512
Erythroid.cells	ZBTB34	0.129248	3.592868	0.342849	0.732526	-5.69214	0.685564	0.766132
Erythroid.cells	TXNRD3	0.182761	1.950944	0.342706	0.732633	-5.40684	0.725457	0.801967
Erythroid.cells	GINS1	-0.15089	3.197409	-0.34265	0.732673	-5.76168	0.694959	0.774646
Erythroid.cells	INPP5A	-0.07802	6.256163	-0.34265	0.732677	-6.37613	0.625662	0.711419
Erythroid.cells	CD55	0.145567	4.863884	0.3426	0.732713	-6.05518	0.656257	0.73954
Erythroid.cells	ABHD16A	-0.08355	4.808556	-0.34243	0.732842	-6.02981	0.657505	0.740699
Erythroid.cells	IFI205	-0.14151	2.758001	-0.34231	0.732929	-6.18381	0.705556	0.784198
Erythroid.cells	TET3	-0.05559	6.827605	-0.34226	0.732969	-6.50956	0.613546	0.700218
Erythroid.cells	UACA	0.202257	2.51718	0.342255	0.732971	-5.47898	0.711434	0.789475
Erythroid.cells	DCP2	-0.09237	5.103401	-0.34224	0.732985	-6.01346	0.650883	0.734656
Erythroid.cells	IL16	-0.12844	4.60831	-0.34183	0.733287	-5.96214	0.662254	0.745015
Erythroid.cells	H2AFZ	-0.06267	10.35837	-0.34126	0.733717	-7.20429	0.544437	0.63499
Erythroid.cells	FAM168A	-0.06633	5.93211	-0.34115	0.733799	-6.28745	0.633131	0.718225
Erythroid.cells	PPIF	-0.11621	3.577224	-0.34112	0.733819	-5.78358	0.686459	0.766885
Erythroid.cells	PQLC3	0.120393	3.812077	0.34086	0.734018	-5.80193	0.680991	0.761889

Erythroid.cells	BCL9	0.130356	3.399574	0.340645	0.734179	-5.69861	0.690726	0.77072
Erythroid.cells	PAKAP.1	-0.12327	4.53559	-0.34059	0.734221	-5.95899	0.664263	0.746733
Erythroid.cells	GPATCH2L	-0.07457	5.064269	-0.34058	0.73423	-6.02424	0.652312	0.735823
Erythroid.cells	SCIN	0.278178	0.090871	0.340508	0.734282	-5.35182	0.774208	0.844964
Erythroid.cells	CYP4F18	0.124615	3.551567	0.340375	0.734381	-5.83665	0.687122	0.767467
Erythroid.cells	4930469K1	0.226575	1.311112	0.340335	0.734412	-5.44872	0.742276	0.816813
Erythroid.cells	TIMD2	0.200878	0.986598	0.34032	0.734423	-5.42454	0.750634	0.824214
Erythroid.cells	4921509O(	0.270405	0.064578	0.340119	0.734573	-5.28789	0.774991	0.845638
Erythroid.cells	TSPAN5	-0.08334	5.373256	-0.34004	0.734635	-6.21879	0.645499	0.729588
Erythroid.cells	STAB1	-0.16993	3.176342	-0.33945	0.735074	-5.78692	0.696478	0.775648
Erythroid.cells	HSBP1	-0.06497	6.003464	-0.33902	0.735397	-6.38787	0.632046	0.717002
Erythroid.cells	NUDT9	0.099586	4.362001	0.338984	0.735426	-6.01387	0.668669	0.750528
Erythroid.cells	CEP135	0.089459	3.853066	0.338963	0.735441	-5.82476	0.680472	0.761235
Erythroid.cells	TRAF3IP1	-0.1963	1.889467	-0.33892	0.735471	-5.47445	0.728086	0.803974
Erythroid.cells	SH3BP2	0.157384	3.288629	0.33892	0.735474	-5.66672	0.693818	0.773287
Erythroid.cells	CDH17	0.224076	0.13748	0.338871	0.73551	-5.36175	0.773463	0.844072
Erythroid.cells	STON1	-0.25933	1.087662	-0.33812	0.736074	-5.28885	0.748764	0.82228
Erythroid.cells	PHAX	-0.09228	4.599165	-0.33785	0.736278	-5.98544	0.663472	0.745855
Erythroid.cells	TMEM120E	-0.17162	3.044848	-0.33783	0.736295	-5.66428	0.699908	0.778827
Erythroid.cells	GM17036	0.190015	2.038724	0.337718	0.736376	-5.46741	0.724599	0.800948
Erythroid.cells	PDLIM7	0.114601	3.638312	0.337582	0.736478	-5.81811	0.685755	0.766121
Erythroid.cells	EHD3	0.148426	3.517645	0.337529	0.736518	-5.76493	0.688608	0.768698
Erythroid.cells	DIPK2A	-0.09675	4.112992	-0.33749	0.736549	-5.89583	0.67465	0.756066
Erythroid.cells	NUDT22	-0.13347	2.557891	-0.33748	0.736555	-5.59696	0.711747	0.7895
Erythroid.cells	ACAD9	0.142072	2.542008	0.337408	0.736609	-5.55838	0.712137	0.789867
Erythroid.cells	CLPB	0.088637	4.010407	0.337333	0.736666	-5.8593	0.677034	0.758273
Erythroid.cells	SCARB1	0.078518	4.995715	0.337285	0.736702	-6.1221	0.654498	0.737757
Erythroid.cells	HAVCR1	-0.27755	-0.3843	-0.33719	0.736773	-5.18702	0.787798	0.856792
Erythroid.cells	TMEM97	-0.11289	3.649444	-0.33705	0.736877	-5.81643	0.685493	0.765929
Erythroid.cells	S100A4	0.138475	2.87429	0.336902	0.73699	-6.05309	0.704031	0.782639
Erythroid.cells	LYSMD4	0.129238	3.330154	0.336768	0.73709	-5.72508	0.693066	0.772809
Erythroid.cells	PLLP	0.294254	-0.20457	0.336749	0.737104	-5.17257	0.782923	0.85258
Erythroid.cells	RRP1B	-0.11563	4.132089	-0.33671	0.737136	-5.90707	0.674208	0.755752
Erythroid.cells	BFSP2	-0.16212	2.440103	-0.33669	0.737145	-5.69232	0.714642	0.792183
Erythroid.cells	OCEL1	-0.11874	3.584489	-0.33659	0.737226	-5.69256	0.687026	0.767362
Erythroid.cells	PIGT	-0.07701	5.640537	-0.33653	0.737268	-6.1933	0.640179	0.724696
Erythroid.cells	MRI1	-0.09104	3.963231	-0.33641	0.737362	-5.8666	0.678134	0.759388
Erythroid.cells	APRT	-0.07497	6.416788	-0.33629	0.73745	-6.48886	0.623379	0.709273
Erythroid.cells	AQP9	0.153473	2.330322	0.336034	0.737641	-5.72015	0.717351	0.79475
Erythroid.cells	NADK	0.07026	6.266885	0.335927	0.737721	-6.36495	0.626586	0.712275
Erythroid.cells	ARL6IP5	0.051161	7.048458	0.335771	0.737839	-6.61342	0.610053	0.696982
Erythroid.cells	TSN	-0.04427	6.360058	-0.33577	0.737842	-6.47455	0.624591	0.710468
Erythroid.cells	SFT2D2	0.094323	4.677531	0.335725	0.737873	-5.9895	0.661688	0.744541
Erythroid.cells	CUL4B	-0.09707	4.524951	-0.33563	0.737946	-5.98651	0.665166	0.74771
Erythroid.cells	BCLAF1	-0.04061	7.155376	-0.33561	0.737957	-6.62102	0.607828	0.694912
Erythroid.cells	LCT	-0.27789	0.170332	-0.3356	0.737964	-5.18066	0.772854	0.843956
Erythroid.cells	METTL25	-0.0848	4.28478	-0.33557	0.737986	-5.88984	0.670678	0.752726

Erythroid.cells	GM15563	0.188486	1.923249	0.335548	0.738007	-5.5102	0.72749	0.803842
Erythroid.cells	ZCCHC7	0.084402	6.744533	0.335546	0.738008	-6.45719	0.616427	0.702905
Erythroid.cells	LYPLA1	0.05709	5.988433	0.33502	0.738403	-6.35338	0.632821	0.717997
Erythroid.cells	CACNB1	-0.26167	0.846674	-0.33501	0.738412	-5.24982	0.755292	0.828415
Erythroid.cells	GPNMB	-0.22583	0.898381	-0.33453	0.738768	-5.51734	0.754216	0.827309
Erythroid.cells	SLC4A7	-0.07718	5.767425	-0.3344	0.738872	-6.28742	0.63786	0.722542
Erythroid.cells	OVGP1	-0.23758	0.492644	-0.33438	0.738881	-5.20835	0.764853	0.836731
Erythroid.cells	GMPPA	-0.1232	3.453598	-0.33426	0.738978	-5.72625	0.690637	0.770646
Erythroid.cells	SMPD4	0.133511	3.209019	0.334144	0.739062	-5.68216	0.696476	0.775914
Erythroid.cells	LY6E	-0.06776	9.08245	-0.3341	0.739097	-6.95642	0.569588	0.658903
Erythroid.cells	CALR	-0.05575	7.35156	-0.33389	0.739256	-6.65669	0.604282	0.691444
Erythroid.cells	CD244A	-0.12596	3.429777	-0.3338	0.739318	-5.89802	0.691283	0.771234
Erythroid.cells	SKA1	0.176364	2.918501	0.333494	0.739551	-5.69593	0.703716	0.782427
Erythroid.cells	SLC12A2	0.138903	3.544469	0.333039	0.739893	-5.80803	0.688969	0.769098
Erythroid.cells	BANK1	0.107238	6.09331	0.33266	0.740178	-6.49681	0.631308	0.716428
Erythroid.cells	ZCCHC9	-0.06375	5.404138	-0.33266	0.740181	-6.17557	0.646399	0.730307
Erythroid.cells	VPS18	-0.08797	4.834066	-0.33265	0.740184	-5.98422	0.65917	0.74199
Erythroid.cells	ZDHHC6	-0.08603	4.609891	-0.33251	0.740288	-5.97632	0.664291	0.74667
Erythroid.cells	ACTR6	0.108035	3.522286	0.332407	0.740369	-5.78029	0.689603	0.769622
Erythroid.cells	TRMT61A	-0.17933	1.593744	-0.33234	0.740417	-5.39535	0.736984	0.812061
Erythroid.cells	NCOA6	0.062597	5.600455	0.33214	0.74057	-6.18975	0.64216	0.726438
Erythroid.cells	SP4	0.080985	5.254382	0.33198	0.74069	-6.16031	0.649836	0.733477
Erythroid.cells	FAM149B	0.127738	2.992688	0.331909	0.740744	-5.63579	0.702379	0.781148
Erythroid.cells	FBRS	0.089454	4.965395	0.331857	0.740783	-5.96425	0.656313	0.739446
Erythroid.cells	NXPE2	0.117854	1.56591	0.331377	0.741144	-5.93577	0.738076	0.812916
Erythroid.cells	ZRSR2	0.062761	5.063714	0.331132	0.741328	-6.07642	0.654393	0.737542
Erythroid.cells	GRWD1	-0.15037	2.674334	-0.3311	0.741355	-5.54599	0.710439	0.788255
Erythroid.cells	MCTS1	0.052186	6.207891	0.331055	0.741387	-6.41398	0.629219	0.714418
Erythroid.cells	INTS2	0.113853	4.120259	0.330927	0.741483	-5.88543	0.675949	0.757185
Erythroid.cells	YTHDC2	0.089785	4.693584	0.330825	0.74156	-5.97716	0.662762	0.745246
Erythroid.cells	ELOVL6	0.108693	4.451031	0.330688	0.741662	-6.05868	0.668308	0.750334
Erythroid.cells	FZD6	0.252783	0.693274	0.330593	0.741734	-5.30564	0.760677	0.832995
Erythroid.cells	VHL	0.098865	3.486608	0.330592	0.741735	-5.72934	0.690844	0.770749
Erythroid.cells	EPS8L2	0.239042	0.893203	0.330511	0.741796	-5.33487	0.755446	0.82841
Erythroid.cells	MTUS1	-0.1193	3.721745	-0.33042	0.741862	-6.06941	0.685277	0.765748
Erythroid.cells	HIST1H2BB	-0.20389	1.116611	-0.33024	0.742003	-5.35079	0.749699	0.823285
Erythroid.cells	HCCS	0.075147	4.627385	0.330161	0.742059	-6.00472	0.66432	0.746686
Erythroid.cells	CASS4	-0.18225	3.04899	-0.33004	0.742148	-5.6807	0.701382	0.780229
Erythroid.cells	GM47283	-0.12069	7.990664	-0.32998	0.742199	-6.80904	0.592066	0.679958
Erythroid.cells	FAM185A	-0.1416	2.579686	-0.32987	0.742278	-5.61393	0.712813	0.790513
Erythroid.cells	MED31	0.116549	3.491551	0.329774	0.742351	-5.68048	0.690777	0.770732
Erythroid.cells	KLF11	-0.09654	3.555665	-0.32972	0.742393	-5.73656	0.689255	0.769377
Erythroid.cells	GM6710	-0.23143	0.761027	-0.32944	0.742601	-5.29919	0.759098	0.831621
Erythroid.cells	MRPS15	0.060909	5.910327	0.329185	0.742794	-6.31003	0.635939	0.720653
Erythroid.cells	SMG9	-0.08925	4.52517	-0.32878	0.7431	-5.93265	0.667047	0.749064
Erythroid.cells	DHX16	0.106652	4.087189	0.328765	0.743111	-5.85334	0.677164	0.758252
Erythroid.cells	GCHFR	-0.128	3.389908	-0.32869	0.743169	-6.07088	0.693604	0.773124

Erythroid.cells	SULF2	-0.12755	2.585463	-0.32832	0.743448	-5.83115	0.713271	0.790666
Erythroid.cells	BC049715	0.276001	0.604594	0.327863	0.74379	-5.28246	0.763708	0.835494
Erythroid.cells	SLF1	0.099556	4.44199	0.32786	0.743792	-5.94966	0.669128	0.750924
Erythroid.cells	BLOC1S6	-0.10543	3.751167	-0.32785	0.743799	-5.75992	0.685211	0.765502
Erythroid.cells	ECM2	-0.30703	0.502848	-0.32772	0.743897	-5.19316	0.766395	0.837888
Erythroid.cells	NCF4	0.079784	5.316123	0.327714	0.743903	-6.28282	0.649344	0.732897
Erythroid.cells	DDX17	-0.06039	6.357267	-0.32767	0.743938	-6.37179	0.626583	0.711961
Erythroid.cells	PRC1	0.135433	5.285692	0.327609	0.743982	-6.25927	0.650022	0.733534
Erythroid.cells	GART	-0.11701	4.367941	-0.32758	0.744	-5.83206	0.670833	0.752518
Erythroid.cells	UBE2W	0.040692	6.081677	0.327487	0.744074	-6.36783	0.632524	0.717491
Erythroid.cells	DDHD1	0.092915	6.276227	0.327288	0.744224	-6.35036	0.628324	0.713653
Erythroid.cells	GM34225	-0.29337	-0.06364	-0.32723	0.744264	-5.16618	0.781533	0.851289
Erythroid.cells	MSH5	0.186458	2.9838	0.327003	0.744438	-5.54422	0.703551	0.782208
Erythroid.cells	EHHADH	0.18191	1.66442	0.326976	0.744459	-5.50792	0.736283	0.811441
Erythroid.cells	TBKBP1	-0.18938	1.449005	-0.32682	0.744573	-5.48529	0.741775	0.816314
Erythroid.cells	PHYHD1	-0.13875	2.730001	-0.32679	0.744599	-5.65759	0.709728	0.78775
Erythroid.cells	BAZ1B	-0.06233	6.23949	-0.32677	0.74461	-6.36887	0.629114	0.714438
Erythroid.cells	TCF25	0.040677	6.939299	0.326743	0.744635	-6.57736	0.614229	0.700654
Erythroid.cells	ADM	0.257178	1.330377	0.326715	0.744655	-5.35387	0.744817	0.81901
Erythroid.cells	GPR132	-0.08814	6.00422	-0.32612	0.745107	-6.5123	0.634531	0.719169
Erythroid.cells	PDLIM4	-0.20821	2.040162	-0.32595	0.745231	-5.56485	0.727233	0.803079
Erythroid.cells	MTR	0.08776	4.54186	0.325743	0.745389	-5.91901	0.667309	0.749117
Erythroid.cells	NOL6	0.142848	2.582305	0.325533	0.745547	-5.54033	0.713861	0.791166
Erythroid.cells	ZFP689	0.183671	1.407041	0.325494	0.745576	-5.39187	0.743382	0.817449
Erythroid.cells	POP7	-0.07767	4.908142	-0.32546	0.745599	-6.08006	0.658972	0.741582
Erythroid.cells	PPIH	-0.07015	5.305306	-0.32525	0.745758	-6.21592	0.65013	0.733505
Erythroid.cells	DGUOK	-0.09989	4.460054	-0.32503	0.745926	-5.92124	0.669286	0.750993
Erythroid.cells	PRAF2	0.123544	2.133854	0.324967	0.745973	-5.58047	0.725082	0.801208
Erythroid.cells	NACC2	0.176488	1.77434	0.324879	0.74604	-5.50777	0.734126	0.809265
Erythroid.cells	DOK1	0.091001	3.614618	0.324873	0.746045	-5.90432	0.689028	0.768882
Erythroid.cells	POLE2	-0.14503	3.014685	-0.32461	0.746242	-5.68007	0.703527	0.781854
Erythroid.cells	EIF4A3	-0.05908	5.877476	-0.32436	0.746428	-6.31579	0.637654	0.722034
Erythroid.cells	DYNLT1F	0.106221	5.068561	0.324304	0.746474	-5.99761	0.655592	0.738499
Erythroid.cells	RAB20	-0.1439	3.301078	-0.32429	0.746485	-5.79983	0.696659	0.775738
Erythroid.cells	TIMM17A	-0.06426	5.135955	-0.32405	0.746669	-6.1431	0.654133	0.737182
Erythroid.cells	MTPN	-0.0424	6.898113	-0.3239	0.746778	-6.52854	0.615812	0.701906
Erythroid.cells	ZSWIM4	-0.10132	4.579223	-0.32383	0.746833	-5.90557	0.666758	0.748715
Erythroid.cells	SEMA6A	-0.24608	2.821738	-0.32363	0.74698	-5.61508	0.708314	0.786263
Erythroid.cells	MIGA2	-0.24813	0.892517	-0.3236	0.747004	-5.24144	0.757038	0.829583
Erythroid.cells	GM34084	0.190866	1.964286	0.323563	0.747033	-5.75514	0.729558	0.805238
Erythroid.cells	KLRB1F	-0.20775	0.424065	-0.32354	0.747049	-5.51881	0.769379	0.840453
Erythroid.cells	NMNAT3	0.133075	3.105259	0.323481	0.747095	-5.72068	0.701432	0.780094
Erythroid.cells	INPP5K	-0.07157	5.371477	-0.32327	0.747253	-6.12893	0.648928	0.732429
Erythroid.cells	HNRNPD	-0.03731	7.908504	-0.32321	0.7473	-6.7808	0.594976	0.682484
Erythroid.cells	KLK1B27	0.215454	-1.42677	0.322881	0.747548	-5.05825	0.820393	0.884788
Erythroid.cells	SFR1	0.04906	6.44096	0.322822	0.747593	-6.47253	0.625696	0.710973
Erythroid.cells	RECQL5	-0.1221	3.722047	-0.32272	0.747673	-5.65302	0.686893	0.766876

Erythroid.cells	SWI5	0.054093	7.120072	0.322658	0.747716	-6.64964	0.61133	0.697682
Erythroid.cells	ACTR8	0.110027	3.537225	0.322498	0.747837	-5.72677	0.691287	0.770866
Erythroid.cells	SLC30A7	0.076796	5.62501	0.322451	0.747873	-6.16906	0.643444	0.727346
Erythroid.cells	LEPROT	-0.07512	5.065196	-0.32216	0.748089	-6.09451	0.65592	0.738853
Erythroid.cells	WDTC1	0.105186	3.851337	0.322109	0.74813	-5.83773	0.683856	0.764245
Erythroid.cells	EXOC3	-0.07508	5.06994	-0.32191	0.748284	-6.06196	0.655814	0.73879
Erythroid.cells	MRPS34	0.066771	4.826949	0.321887	0.748298	-6.14898	0.661307	0.743805
Erythroid.cells	RSAD1	0.234353	1.298807	0.321874	0.748308	-5.37798	0.746723	0.820513
Erythroid.cells	PGLYRP2	0.131341	2.088786	0.321857	0.748321	-5.80763	0.726652	0.802693
Erythroid.cells	ZFP53	0.088622	4.791451	0.321801	0.748363	-6.00647	0.662114	0.744552
Erythroid.cells	GM26887	0.194321	3.10483	0.321612	0.748506	-5.66002	0.701653	0.780361
Erythroid.cells	MRM3	0.174077	2.005465	0.321569	0.748538	-5.49362	0.728742	0.804591
Erythroid.cells	TADA1	-0.07603	4.679062	-0.32149	0.748598	-6.01923	0.664675	0.746929
Erythroid.cells	CERS5	0.066612	5.514163	0.321465	0.748617	-6.2114	0.645894	0.729765
Erythroid.cells	A530040E1	0.238347	0.013043	0.32116	0.748847	-5.26932	0.780779	0.85045
Erythroid.cells	ANP32E	-0.05525	7.305672	-0.32077	0.749143	-6.70417	0.607744	0.694318
Erythroid.cells	4933421O1	-0.15777	2.357702	-0.32068	0.74921	-5.53386	0.720265	0.796837
Erythroid.cells	NADSYN1	0.208025	1.092531	0.320665	0.749221	-5.29191	0.752389	0.82536
Erythroid.cells	PRODH2	-0.17	2.255274	-0.32034	0.749469	-5.73215	0.722986	0.79922
Erythroid.cells	A130010J1	0.193764	1.72998	0.320051	0.749685	-5.37002	0.736345	0.811102
Erythroid.cells	LRRC4	0.113562	3.774431	0.319878	0.749815	-5.84436	0.686311	0.766292
Erythroid.cells	MCRIP1	0.05135	5.919349	0.319812	0.749866	-6.3084	0.637583	0.721879
Erythroid.cells	CXCR5	0.18045	2.183855	0.319658	0.749982	-5.58964	0.725	0.801
Erythroid.cells	SAMD10	0.180213	1.732501	0.319231	0.750305	-5.41421	0.736494	0.811181
Erythroid.cells	ETFRF1	0.120225	3.163487	0.319191	0.750335	-5.78327	0.701055	0.779531
Erythroid.cells	GM30198	0.210893	1.865066	0.319134	0.750378	-5.49751	0.733134	0.808197
Erythroid.cells	GCC1	0.151622	2.459454	0.319048	0.750443	-5.5689	0.718265	0.794975
Erythroid.cells	TAF9B	-0.21965	0.956005	-0.31904	0.750448	-5.29068	0.756491	0.828903
Erythroid.cells	TANGO2	0.128792	3.93274	0.318558	0.750813	-5.8789	0.682775	0.763174
Erythroid.cells	ECT2	0.141593	4.373594	0.318449	0.750895	-6.01837	0.672505	0.753883
Erythroid.cells	MAP3K5	0.063037	7.255072	0.318356	0.750966	-6.56125	0.609268	0.695802
Erythroid.cells	GM15337	0.191584	2.140023	0.318256	0.751041	-5.49942	0.726253	0.80226
Erythroid.cells	CBLL1	-0.07278	4.917555	-0.31773	0.751436	-6.04845	0.660056	0.742683
Erythroid.cells	TM2D3	-0.08207	4.227452	-0.31771	0.751456	-5.96361	0.675892	0.757091
Erythroid.cells	NUP160	-0.07992	5.017011	-0.31761	0.751532	-6.1775	0.657807	0.740658
Erythroid.cells	SZT2	0.141928	2.318426	0.317584	0.75155	-5.46096	0.7218	0.798425
Erythroid.cells	ANO8	0.225884	0.904446	0.317527	0.751592	-5.30038	0.757876	0.830431
Erythroid.cells	OTUD3	0.151132	2.558441	0.317448	0.751652	-5.59078	0.715855	0.793114
Erythroid.cells	SUN1	-0.09363	3.918531	-0.31739	0.751694	-5.7949	0.683109	0.763661
Erythroid.cells	PPP2R2A	-0.03887	7.180276	-0.31736	0.751717	-6.59383	0.610827	0.697373
Erythroid.cells	MKRN2	0.075399	4.074932	0.317269	0.751787	-5.87637	0.679445	0.760344
Erythroid.cells	DISP1	0.109545	3.443949	0.317246	0.751805	-5.73796	0.694354	0.773814
Erythroid.cells	CTSW	0.12532	2.120663	0.317229	0.751818	-5.99257	0.726738	0.802829
Erythroid.cells	DGKZ	-0.07257	6.2774	-0.3172	0.751839	-6.35606	0.629987	0.715124
Erythroid.cells	PTAR1	0.0933	3.690053	0.317176	0.751858	-5.77942	0.688499	0.768533
Erythroid.cells	SERGEF	0.097483	4.018452	0.317033	0.751966	-5.88195	0.680766	0.76154
Erythroid.cells	DDX56	0.090731	3.951871	0.31701	0.751984	-5.86513	0.682326	0.762953

Erythroid.cells	P3H1	0.225694	0.585168	0.31697	0.752013	-5.35465	0.766275	0.837827
Erythroid.cells	C77080	0.448952	0.694235	0.316884	0.752079	-5.2424	0.763395	0.835294
Erythroid.cells	ACAT1	-0.071	6.480379	-0.31683	0.752117	-6.67781	0.625625	0.711109
Erythroid.cells	KMT2A	-0.06158	6.28827	-0.31681	0.752131	-6.41486	0.629753	0.714923
Erythroid.cells	RASL11B	-0.24402	0.665224	-0.31675	0.752178	-5.20946	0.76416	0.835986
Erythroid.cells	CPLX1	-0.34589	-0.57566	-0.31673	0.752195	-5.12538	0.797613	0.865278
Erythroid.cells	SLFN1	-0.25632	2.335521	-0.31652	0.752355	-5.67384	0.721463	0.798136
Erythroid.cells	PPP1R35	0.110828	3.395179	0.315646	0.753015	-5.72226	0.695996	0.775206
Erythroid.cells	ATP8B4	0.098123	4.781456	0.315602	0.753048	-6.35835	0.663601	0.745865
Erythroid.cells	MRPL3	-0.09003	4.701467	-0.31556	0.753079	-5.98646	0.665427	0.74753
Erythroid.cells	EML4	0.051014	7.138312	0.315545	0.753091	-6.58912	0.612122	0.698498
Erythroid.cells	MPP7	0.106618	7.383215	0.315466	0.753151	-6.64255	0.607021	0.693752
Erythroid.cells	UBR5	0.05218	6.888097	0.315417	0.753189	-6.52729	0.61738	0.70338
Erythroid.cells	CDKN1C	-0.15198	3.302414	-0.31461	0.753797	-5.86051	0.698653	0.777401
Erythroid.cells	PHF13	0.10093	3.504231	0.314557	0.753839	-5.7233	0.693816	0.773069
Erythroid.cells	MTERF1A	-0.1815	1.405346	-0.31452	0.75387	-5.32764	0.745858	0.819541
Erythroid.cells	SLC38A6	0.109573	4.233853	0.314253	0.754069	-5.85903	0.676622	0.757554
Erythroid.cells	DNA2	0.088351	3.583362	0.314168	0.754134	-5.93501	0.69193	0.771394
Erythroid.cells	GPR65	-0.12535	4.250918	-0.31414	0.754152	-6.00048	0.676225	0.757194
Erythroid.cells	CPNE3	0.081084	5.367342	0.314094	0.75419	-6.13617	0.650792	0.734023
Erythroid.cells	CACNA2D1	0.263048	0.864423	0.314074	0.754204	-5.33033	0.759911	0.831976
Erythroid.cells	SOX7	-0.23117	0.442832	-0.31399	0.754269	-5.26277	0.771051	0.841827
Erythroid.cells	HBQ1A	0.12193	-1.39639	0.313546	0.754604	-5.66949	0.821901	0.885955
Erythroid.cells	TMEM205	0.098542	3.71868	0.313395	0.754719	-6.07158	0.688958	0.768605
Erythroid.cells	ZFP59	0.252998	0.230401	0.313361	0.754744	-5.20065	0.776999	0.846875
Erythroid.cells	TMEM268	0.121884	3.100152	0.313228	0.754846	-5.63489	0.703782	0.781981
Erythroid.cells	IMP3	-0.06032	5.264975	-0.31299	0.755028	-6.17412	0.65331	0.736292
Erythroid.cells	MACF1	0.047118	8.000182	0.3129	0.755093	-6.75166	0.594946	0.682337
Erythroid.cells	DPP6	-0.25263	-0.15893	-0.31271	0.755234	-5.27393	0.787515	0.85625
Erythroid.cells	ITGA5	0.135499	3.54178	0.312554	0.755355	-5.80163	0.693164	0.772603
Erythroid.cells	RAB14	0.037581	7.091867	0.31242	0.755457	-6.55098	0.613688	0.699927
Erythroid.cells	ITGB3	0.128183	2.991713	0.312374	0.755492	-5.70209	0.706415	0.784549
Erythroid.cells	DAND5	0.164166	3.96611	0.312232	0.755599	-5.84854	0.683119	0.763562
Erythroid.cells	ZFP85	-0.25339	0.25725	-0.31215	0.755661	-5.18476	0.776279	0.846501
Erythroid.cells	LMAN2	-0.04919	6.112382	-0.31213	0.755674	-6.36186	0.634604	0.719276
Erythroid.cells	1700061N1	0.318503	-0.1438	0.31212	0.755684	-5.18114	0.787104	0.855975
Erythroid.cells	APPL2	0.132749	3.358029	0.312112	0.75569	-5.75389	0.697561	0.776594
Erythroid.cells	ATXN7	-0.06139	6.23066	-0.31201	0.755769	-6.38697	0.632039	0.716914
Erythroid.cells	XIST	1.925522	4.173151	0.311889	0.755859	-5.72061	0.678273	0.759224
Erythroid.cells	KAT5	-0.14004	2.921057	-0.31188	0.755865	-5.61499	0.708136	0.786144
Erythroid.cells	GM27188	-0.30606	0.619818	-0.31172	0.75599	-5.16902	0.766623	0.838102
Erythroid.cells	PLAG1	-0.11464	2.8948	-0.31157	0.756102	-5.62818	0.708777	0.786743
Erythroid.cells	SHLD3	-0.13987	2.343634	-0.31149	0.756165	-5.56152	0.722365	0.798897
Erythroid.cells	ZDHHC7	-0.09632	3.834677	-0.31141	0.756221	-5.82171	0.686214	0.766434
Erythroid.cells	LIN7C	0.06946	4.956273	0.311344	0.756272	-6.08285	0.660269	0.742875
Erythroid.cells	MSRB1	0.065326	5.958368	0.31131	0.756298	-6.43106	0.637962	0.722436
Erythroid.cells	VAMP1	0.090894	3.997211	0.311169	0.756404	-5.89274	0.682389	0.763022

Erythroid.cells	PHLDB2	0.159402	2.509693	0.311159	0.756412	-5.66985	0.718243	0.795266
Erythroid.cells	NETO2	0.140192	3.22493	0.311157	0.756414	-5.75775	0.700765	0.779598
Erythroid.cells	ANKRD37	0.123694	3.981977	0.311107	0.756452	-5.89891	0.682747	0.763366
Erythroid.cells	SYT11	0.193478	1.412759	0.310851	0.756646	-5.43964	0.746004	0.819941
Erythroid.cells	HIST1H4D	0.234764	1.04631	0.310413	0.756977	-5.35062	0.755496	0.828376
Erythroid.cells	NUBP1	0.064824	5.473714	0.310306	0.757059	-6.24471	0.648714	0.732386
Erythroid.cells	NOLC1	-0.08382	5.264292	-0.31031	0.757059	-6.13713	0.653391	0.736668
Erythroid.cells	RNF44	0.07556	4.833037	0.310298	0.757064	-6.03551	0.663134	0.745566
Erythroid.cells	TSEN54	-0.10912	2.874509	-0.31025	0.757103	-5.66802	0.709343	0.787333
Erythroid.cells	FLI1	-0.04959	8.191063	-0.31022	0.757123	-6.77057	0.591144	0.679029
Erythroid.cells	RHOC	0.097207	4.059106	0.309967	0.757315	-6.01466	0.681006	0.761845
Erythroid.cells	MTSS1	0.071372	6.472133	0.30994	0.757336	-6.53239	0.626898	0.712347
Erythroid.cells	EEF1AKMT	-0.09361	4.588498	-0.30991	0.757355	-6.00038	0.668727	0.750702
Erythroid.cells	LAIR1	-0.17885	4.246709	-0.30987	0.757392	-5.8358	0.676628	0.757887
Erythroid.cells	ANXA11OS	-0.20392	0.812081	-0.3098	0.75744	-5.36367	0.761628	0.833869
Erythroid.cells	GIMAP7	-0.15383	1.458503	-0.30977	0.757467	-5.70452	0.744827	0.819027
Erythroid.cells	NRP2	-0.16083	3.300945	-0.30966	0.757552	-5.83038	0.699003	0.778143
Erythroid.cells	SERPINA3F	0.330039	2.19929	0.30959	0.757601	-5.46402	0.72604	0.802359
Erythroid.cells	GPR180	-0.11522	2.964205	-0.30954	0.75764	-5.65941	0.707155	0.785475
Erythroid.cells	RB1CC1	-0.05397	6.500368	-0.30947	0.757691	-6.50429	0.626292	0.711852
Erythroid.cells	ATOX1	0.04283	8.244709	0.309329	0.757799	-6.92439	0.590095	0.67814
Erythroid.cells	EIF2D	-0.10094	3.5699	-0.30917	0.757917	-5.74399	0.692623	0.772391
Erythroid.cells	1700097N(	-0.15797	2.346622	-0.30911	0.757964	-5.60276	0.722426	0.799129
Erythroid.cells	H2-OA	-0.14238	3.19042	-0.30894	0.758092	-5.81243	0.701785	0.780612
Erythroid.cells	ZC3H14	-0.05467	5.656337	-0.30853	0.758405	-6.24121	0.644975	0.728935
Erythroid.cells	GDPGP1	-0.1866	2.18615	-0.30845	0.758468	-5.52524	0.72672	0.802846
Erythroid.cells	DNASE2A	-0.0864	4.427932	-0.30834	0.75855	-5.97491	0.672763	0.754316
Erythroid.cells	PYGB	-0.0728	4.748164	-0.30815	0.758693	-6.03165	0.665468	0.74767
Erythroid.cells	GTPBP4	0.052126	5.956432	0.307968	0.758831	-6.35258	0.638507	0.722954
Erythroid.cells	AFM	0.137672	2.294567	0.307858	0.758915	-5.74753	0.724173	0.800519
Erythroid.cells	RAP2B	-0.08442	4.614993	-0.30732	0.759322	-5.96188	0.668859	0.750592
Erythroid.cells	MKI67	0.113851	6.498445	0.307194	0.759418	-6.61159	0.627027	0.712281
Erythroid.cells	RCOR3	-0.11951	2.937283	-0.30711	0.759484	-5.67065	0.708594	0.786519
Erythroid.cells	YPEL2	-0.09956	4.572346	-0.30705	0.759525	-6.04607	0.669839	0.751554
Erythroid.cells	TSPYL2	0.146652	2.8065	0.306918	0.759628	-5.55206	0.711794	0.789423
Erythroid.cells	GM6712	0.154685	2.305447	0.306641	0.759838	-5.51803	0.72419	0.800507
Erythroid.cells	FTX	-0.1026	4.311116	-0.30664	0.75984	-5.8971	0.675879	0.757057
Erythroid.cells	FKBP15	0.074611	5.239594	0.306569	0.759892	-6.0395	0.654669	0.73774
Erythroid.cells	KIF21A	0.200627	0.515306	0.306564	0.759896	-5.37935	0.770323	0.841338
Erythroid.cells	LRR1	-0.17306	1.435876	-0.30652	0.759929	-5.42645	0.746234	0.820095
Erythroid.cells	LHFPL2	-0.14292	2.227897	-0.30645	0.759984	-5.6375	0.726128	0.802235
Erythroid.cells	CXCL1	0.294736	2.815955	0.306311	0.760088	-5.7629	0.711562	0.789243
Erythroid.cells	PAPSS2	0.115917	2.513577	0.306197	0.760175	-5.948	0.719014	0.795912
Erythroid.cells	2510002D2	0.11002	3.199654	0.306187	0.760182	-5.73922	0.702221	0.780865
Erythroid.cells	UBL5	0.035894	8.259007	0.30609	0.760256	-6.85143	0.59043	0.678287
Erythroid.cells	IGF2R	-0.0821	4.985937	-0.30601	0.760313	-6.1367	0.660393	0.742992
Erythroid.cells	GM46560	-0.25233	-0.84515	-0.30553	0.760683	-5.12766	0.807694	0.873858

Erythroid.cells	SDHAF3	-0.10604	3.044533	-0.30528	0.760871	-5.68975	0.706335	0.784357
Erythroid.cells	AMD2	-0.22498	0.044662	-0.30522	0.760914	-5.17905	0.783334	0.852571
Erythroid.cells	ITIH2	-0.13262	3.79432	-0.30515	0.760968	-6.17111	0.688341	0.768203
Erythroid.cells	GM26674	-0.24281	-1.15959	-0.30496	0.761117	-5.11878	0.816685	0.881574
Erythroid.cells	HSPD1	0.056542	7.46114	0.304807	0.76123	-6.76256	0.607128	0.693696
Erythroid.cells	9430091E2	0.141349	2.439837	0.304424	0.76152	-5.5181	0.721532	0.797855
Erythroid.cells	ARVCF	-0.21067	1.228411	-0.30405	0.761802	-5.34625	0.752481	0.825259
Erythroid.cells	4933408B1	0.181176	1.941966	0.304019	0.761828	-5.41601	0.734186	0.809053
Erythroid.cells	POC5	0.128083	2.510705	0.303541	0.762191	-5.55759	0.72004	0.796463
Erythroid.cells	RNF216	-0.04439	6.677461	-0.3034	0.762295	-6.50586	0.624026	0.709247
Erythroid.cells	ZMYM6	0.16129	2.526746	0.303383	0.762311	-5.52646	0.719643	0.796111
Erythroid.cells	SURF2	0.101962	3.331878	0.303249	0.762413	-5.74162	0.699961	0.778497
Erythroid.cells	BRD7	-0.04363	6.307707	-0.30322	0.762432	-6.41782	0.631973	0.716607
Erythroid.cells	PGM2	0.087689	4.115633	0.303016	0.76259	-5.90686	0.681341	0.761721
Erythroid.cells	TOMM5	-0.06871	5.458336	-0.30299	0.762608	-6.25622	0.650638	0.733775
Erythroid.cells	SNED1	0.185754	1.751149	0.302985	0.762613	-5.50689	0.739144	0.81351
Erythroid.cells	FAM114A2	0.059003	5.166561	0.302972	0.762623	-6.15297	0.657184	0.73976
Erythroid.cells	GID4	0.079572	4.230623	0.302965	0.762628	-5.90301	0.678653	0.759287
Erythroid.cells	CASP6	0.096468	3.628567	0.302808	0.762747	-5.82758	0.692896	0.772168
Erythroid.cells	E130307A1	0.075164	4.490339	0.302583	0.762919	-5.95808	0.672757	0.753908
Erythroid.cells	CHAF1B	-0.12612	3.382455	-0.30247	0.763002	-5.71633	0.698897	0.777529
Erythroid.cells	KIF2C	-0.1589	2.663513	-0.3023	0.763133	-5.65027	0.716475	0.793292
Erythroid.cells	GM45820	0.15894	1.425778	0.302185	0.763221	-5.39358	0.747728	0.821068
Erythroid.cells	AP5Z1	-0.12533	2.539751	-0.30206	0.763316	-5.53264	0.719553	0.796032
Erythroid.cells	GM12592	0.111675	3.124779	0.301969	0.763385	-5.71093	0.705198	0.783187
Erythroid.cells	MRPL54	-0.05826	5.906363	-0.30191	0.763429	-6.35759	0.640928	0.724836
Erythroid.cells	HS3ST3B1	0.117808	3.380647	0.301827	0.763493	-5.82742	0.699013	0.777646
Erythroid.cells	NUDCD1	-0.11568	3.226429	-0.30167	0.763613	-5.73023	0.702781	0.781057
Erythroid.cells	DOCK3	0.208323	0.039329	0.301577	0.763683	-5.35176	0.784434	0.853401
Erythroid.cells	ZFP382	-0.16614	2.008207	-0.30142	0.763803	-5.48788	0.732958	0.808019
Erythroid.cells	GM49797	-0.07263	4.882769	-0.30123	0.763949	-6.06221	0.663959	0.745987
Erythroid.cells	ZFP282	0.101174	4.010784	0.301181	0.763984	-5.82349	0.684154	0.764327
Erythroid.cells	FBXL19	0.143657	2.333257	0.300796	0.764276	-5.55265	0.725044	0.800977
Erythroid.cells	PRIM2	0.084355	5.536205	0.300059	0.764837	-6.28359	0.649696	0.732824
Erythroid.cells	PKD2L2	-0.14286	1.170955	-0.30005	0.764845	-5.3786	0.75501	0.827455
Erythroid.cells	PCNA	-0.06731	6.710032	-0.30003	0.764862	-6.53049	0.624093	0.709262
Erythroid.cells	GM17173	-0.20617	0.150476	-0.30002	0.764867	-5.29361	0.782079	0.85124
Erythroid.cells	AGBL2	-0.2226	0.26749	-0.29968	0.765122	-5.19388	0.779115	0.848548
Erythroid.cells	DBF4	0.078561	5.205532	0.299548	0.765225	-6.20399	0.657297	0.739674
Erythroid.cells	UBA5	0.070101	4.489487	0.299052	0.765603	-5.91709	0.673839	0.754639
Erythroid.cells	CSRP1	-0.0715	5.881401	-0.29902	0.765629	-6.30862	0.642411	0.725955
Erythroid.cells	SLC18A1	-0.24815	0.497721	-0.29891	0.765711	-5.26435	0.773189	0.843269
Erythroid.cells	ATRAID	-0.06914	4.57765	-0.29873	0.76585	-6.05505	0.671801	0.752845
Erythroid.cells	CALML4	-0.17616	1.801954	-0.29868	0.765888	-5.54441	0.739163	0.813307
Erythroid.cells	S100A9	0.173266	6.814997	0.298548	0.765985	-6.62513	0.622203	0.707398
Erythroid.cells	TRIM30D	-0.14508	4.544173	-0.29853	0.765998	-5.88214	0.672574	0.753564
Erythroid.cells	ZCCHC4	-0.10554	3.455439	-0.29852	0.766004	-5.81694	0.698231	0.776749

Erythroid.cells	TRIM69	-0.18276	2.05026	-0.2984	0.766101	-5.47518	0.732862	0.807711
Erythroid.cells	KCNK13	0.159267	2.912398	0.298374	0.766118	-5.68903	0.711408	0.788574
Erythroid.cells	ANKRD61	0.191378	0.799732	0.298097	0.766329	-5.3222	0.765197	0.836333
Erythroid.cells	TRAF4	0.122614	4.196822	0.297912	0.766469	-5.94057	0.680675	0.760949
Erythroid.cells	TIRAP	-0.11421	3.005516	-0.29786	0.76651	-5.65393	0.709156	0.7866
Erythroid.cells	2900026A0	0.125632	3.595436	0.297842	0.766523	-5.94402	0.6949	0.773793
Erythroid.cells	MIGA1	0.113669	2.677211	0.297701	0.76663	-5.55605	0.717221	0.793856
Erythroid.cells	CREB3L1	-0.1481	2.035248	-0.29769	0.766642	-5.54107	0.733267	0.808157
Erythroid.cells	AU019990	0.209459	1.211418	0.297623	0.766689	-5.42089	0.754403	0.826876
Erythroid.cells	HDGFL3	-0.16803	1.511028	-0.29761	0.766696	-5.52866	0.746645	0.82002
Erythroid.cells	MTURN	-0.13378	2.270409	-0.29744	0.76683	-5.6483	0.727409	0.802918
Erythroid.cells	CDC20	0.1442	3.639962	0.297224	0.766993	-5.84714	0.693932	0.772937
Erythroid.cells	2610507I0	-0.22059	0.583661	-0.2972	0.767008	-5.32292	0.771032	0.84149
Erythroid.cells	ERGIC2	0.0417	6.017059	0.296587	0.767478	-6.36457	0.639821	0.723563
Erythroid.cells	FXR2	-0.06925	5.267562	-0.29659	0.767479	-6.15156	0.656475	0.73882
Erythroid.cells	CREB3	-0.0852	3.923009	-0.29626	0.767727	-5.80751	0.687668	0.767102
Erythroid.cells	CYP4A31	0.191075	0.922941	0.296094	0.767853	-5.46476	0.762576	0.83392
Erythroid.cells	AKR1E1	0.109377	2.622552	0.296037	0.767896	-5.68864	0.719163	0.795451
Erythroid.cells	CENPL	0.099765	3.393274	0.295972	0.767945	-5.81421	0.700327	0.778576
Erythroid.cells	UBOX5	-0.16801	1.390449	-0.29578	0.768091	-5.38848	0.75042	0.823207
Erythroid.cells	REL	-0.05469	8.12408	-0.29573	0.768131	-6.84036	0.595546	0.682544
Erythroid.cells	PRKRA	-0.08972	3.39958	-0.29547	0.768328	-5.7823	0.700233	0.778515
Erythroid.cells	AI467606	0.105588	4.111903	0.29545	0.768343	-5.89063	0.683283	0.763233
Erythroid.cells	NSUN6	0.100203	3.821373	0.295414	0.76837	-5.8315	0.690144	0.76943
Erythroid.cells	PRMT7	-0.14046	3.247895	-0.29534	0.768423	-5.66747	0.703899	0.781808
Erythroid.cells	PTPRA	-0.04104	6.521638	-0.295	0.768685	-6.4693	0.629156	0.713797
Erythroid.cells	CD27	-0.10517	3.318035	-0.29497	0.768708	-5.88408	0.702298	0.780378
Erythroid.cells	KANK2	-0.15592	2.38565	-0.29489	0.768767	-5.50658	0.725219	0.800884
Erythroid.cells	ADAM33	-0.24355	-0.9471	-0.29464	0.768956	-5.15561	0.81362	0.878619
Erythroid.cells	CHRNE	0.209908	-0.75209	0.294524	0.769048	-5.21213	0.808158	0.873911
Erythroid.cells	FHOD3	-0.28092	-0.11029	-0.29447	0.769093	-5.17239	0.790443	0.858492
Erythroid.cells	RGS12	-0.13511	2.240588	-0.29443	0.769119	-5.5275	0.728854	0.804215
Erythroid.cells	CAMKK1	-0.2245	0.590902	-0.2944	0.76914	-5.22263	0.771537	0.841943
Erythroid.cells	EDF1	0.040227	7.047067	0.294244	0.769261	-6.62935	0.61795	0.70353
Erythroid.cells	MYBBP1A	-0.08695	4.85707	-0.29418	0.769308	-6.09151	0.666102	0.74777
Erythroid.cells	SPTBN5	-0.23809	-0.00378	-0.29406	0.769401	-5.21748	0.787541	0.856013
Erythroid.cells	EXD2	0.111715	3.156926	0.294051	0.769408	-5.68594	0.706204	0.784026
Erythroid.cells	C1QTNF6	0.163541	1.904338	0.294031	0.769423	-5.60585	0.737352	0.811822
Erythroid.cells	EMC9	-0.13112	1.600787	-0.29375	0.769635	-5.56888	0.745164	0.818752
Erythroid.cells	POT1A	-0.11236	3.012767	-0.29374	0.769649	-5.65517	0.709768	0.787225
Erythroid.cells	IQCG	-0.27565	0.050135	-0.29365	0.769711	-5.20015	0.786131	0.854812
Erythroid.cells	CDKAL1	-0.04926	6.364518	-0.2936	0.769752	-6.45562	0.632593	0.71713
Erythroid.cells	MSR1	0.112919	3.816756	0.293399	0.769905	-5.94997	0.690448	0.769868
Erythroid.cells	NUDC	-0.05468	5.698731	-0.2933	0.769979	-6.29054	0.647236	0.730573
Erythroid.cells	DDC	0.179025	1.455602	0.293242	0.770025	-5.43353	0.748961	0.822146
Erythroid.cells	YTHDF2	-0.04755	6.035232	-0.29302	0.770193	-6.35293	0.639815	0.723794
Erythroid.cells	PDE4A	0.12452	3.81993	0.292996	0.770212	-5.86812	0.690372	0.769836

Erythroid.cells	TCTEX1D2	-0.10443	3.301638	-0.29298	0.770223	-5.76917	0.702795	0.78102
Erythroid.cells	CNOT8	0.054776	5.284943	0.292637	0.770486	-6.14851	0.65662	0.739139
Erythroid.cells	ACTB	0.039069	13.9201	0.292594	0.770518	-7.64096	0.489635	0.581419
Erythroid.cells	HLX	-0.11075	3.117664	-0.29212	0.770878	-5.71244	0.707596	0.785166
Erythroid.cells	C1RA	0.149905	1.359965	0.292013	0.770961	-5.49341	0.751792	0.824524
Erythroid.cells	PSMA7	-0.04375	7.683799	-0.29198	0.770988	-6.75121	0.605028	0.691473
Erythroid.cells	ZDHHC17	-0.08603	4.015064	-0.29196	0.771001	-5.85051	0.68608	0.765827
Erythroid.cells	SIPA1L3	0.073965	5.201146	0.291704	0.771196	-6.15172	0.658729	0.740982
Erythroid.cells	RASA2	0.075992	6.158678	0.291546	0.771316	-6.29568	0.637456	0.72153
Erythroid.cells	PIK3R1	0.056587	7.694648	0.291479	0.771367	-6.75651	0.604841	0.691329
Erythroid.cells	SMCO4	0.086896	4.10439	0.291464	0.77138	-5.99569	0.684018	0.763997
Erythroid.cells	KLRE1	-0.12818	1.626605	-0.29132	0.771491	-5.92272	0.744955	0.818516
Erythroid.cells	CAB39	-0.04392	6.974308	-0.2912	0.771581	-6.53809	0.61991	0.705339
Erythroid.cells	GM12359	-0.15411	1.671224	-0.29114	0.771623	-5.43019	0.743809	0.81752
Erythroid.cells	5031434O1	-0.21338	0.524887	-0.29109	0.771668	-5.21005	0.773822	0.843976
Erythroid.cells	GM11696	-0.1677	1.871722	-0.29108	0.771669	-5.44197	0.738684	0.812976
Erythroid.cells	S1PR1	0.092982	4.061514	0.290869	0.771833	-6.09936	0.685057	0.764957
Erythroid.cells	DNAJB9	-0.07378	5.168187	-0.29086	0.77184	-6.05067	0.659503	0.741751
Erythroid.cells	EHBP1	-0.11313	3.52414	-0.29047	0.77214	-5.88252	0.697909	0.776565
Erythroid.cells	MAPK6	0.08113	5.88266	0.290388	0.7722	-6.23343	0.643607	0.727227
Erythroid.cells	THRA	-0.10277	3.771843	-0.29039	0.772201	-5.82423	0.691986	0.77124
Erythroid.cells	PAQR5	-0.26091	0.502008	-0.29033	0.772247	-5.16663	0.774547	0.844662
Erythroid.cells	YTHDF3	-0.0451	7.017555	-0.2903	0.772263	-6.52338	0.619085	0.704625
Erythroid.cells	CLDN3	0.167304	1.461002	0.290138	0.77239	-5.51849	0.749335	0.822479
Erythroid.cells	SPECC1L	0.078552	5.413581	0.290088	0.772428	-6.17266	0.654043	0.736847
Erythroid.cells	PRELID1	-0.04303	7.529242	-0.28998	0.772511	-6.71329	0.608358	0.694719
Erythroid.cells	MITF	0.116725	4.369202	0.289766	0.772674	-5.93559	0.677921	0.758614
Erythroid.cells	SPC25	0.105639	4.372308	0.289756	0.772681	-6.08588	0.677849	0.758549
Erythroid.cells	MRPS25	-0.0808	4.238863	-0.28955	0.772836	-5.94341	0.680965	0.761372
Erythroid.cells	SNX32	0.112697	2.812618	0.289362	0.772981	-5.61592	0.71522	0.79221
Erythroid.cells	TRP53INP1	-0.0761	5.187354	-0.28935	0.772994	-6.23728	0.659139	0.741543
Erythroid.cells	WRAP53	-0.11388	2.788872	-0.28925	0.773066	-5.58645	0.715805	0.792733
Erythroid.cells	NEGR1	0.262875	-0.13284	0.289198	0.773107	-5.25217	0.791715	0.859788
Erythroid.cells	CAMSAP1	0.10416	3.53399	0.288866	0.77336	-5.76067	0.697675	0.776571
Erythroid.cells	HSD3B7	-0.08836	3.537133	-0.28878	0.773429	-6.07269	0.6976	0.776518
Erythroid.cells	GM17160	0.206667	0.089084	0.288758	0.773442	-5.23193	0.785671	0.854628
Erythroid.cells	GM47167	-0.11274	2.927116	-0.28859	0.77357	-5.66091	0.712405	0.789818
Erythroid.cells	ICMT	0.139543	2.429223	0.288591	0.77357	-5.52426	0.724731	0.800832
Erythroid.cells	FARP1	-0.09979	3.619271	-0.28859	0.773572	-5.90503	0.695631	0.774752
Erythroid.cells	USP48	0.058427	5.391239	0.288583	0.773576	-6.17027	0.654545	0.737464
Erythroid.cells	CCDC173	-0.2117	0.767334	-0.28854	0.773612	-5.23569	0.767488	0.838692
Erythroid.cells	EIF2S3X	-0.09704	5.323448	-0.2885	0.77364	-6.18793	0.656069	0.738858
Erythroid.cells	KLF8	0.223973	0.979838	0.288268	0.773817	-5.34063	0.761879	0.833756
Erythroid.cells	PIP4P2	0.103554	3.706325	0.288211	0.77386	-5.82091	0.69355	0.772877
Erythroid.cells	8030453O2	0.208874	-0.75227	0.288174	0.773888	-5.17856	0.808833	0.874817
Erythroid.cells	PIGG	0.188816	0.963956	0.288166	0.773894	-5.32152	0.762297	0.834124
Erythroid.cells	MYO1B	0.149048	2.148687	0.288132	0.77392	-5.66984	0.731773	0.807105

Erythroid.cells	SYAP1	0.056399	5.053275	0.287963	0.774049	-6.14189	0.662179	0.744466
Erythroid.cells	GM43061	0.22549	0.306464	0.287951	0.774058	-5.23188	0.779796	0.849528
Erythroid.cells	GDPD1	-0.19089	1.600801	-0.28777	0.774199	-5.41466	0.745775	0.819541
Erythroid.cells	MS4A1	-0.11182	3.936889	-0.28756	0.774356	-6.26382	0.688113	0.768032
Erythroid.cells	2900076A0	-0.11641	2.621325	-0.28748	0.774415	-5.562	0.719992	0.796667
Erythroid.cells	1700034P1	0.220742	1.464772	0.287461	0.774432	-5.37556	0.749282	0.822702
Erythroid.cells	TNFRSF19	0.181665	0.886333	0.287399	0.774479	-5.42505	0.764388	0.836052
Erythroid.cells	ZFP189	-0.17855	1.120206	-0.28735	0.774518	-5.3278	0.758243	0.83066
Erythroid.cells	ZFP39	0.196365	1.023509	0.287144	0.774674	-5.33382	0.760839	0.833015
Erythroid.cells	GTF2IRD1	0.137881	3.292146	0.287086	0.774719	-5.64435	0.703606	0.782103
Erythroid.cells	YEATS2	-0.07532	4.376137	-0.28699	0.774788	-5.92666	0.677856	0.758883
Erythroid.cells	0610009B2	-0.09475	3.780562	-0.28672	0.775001	-5.93214	0.692008	0.771596
Erythroid.cells	ZFP655	0.076148	4.453763	0.286385	0.775253	-5.95995	0.676236	0.75735
Erythroid.cells	RNF113A1	0.241061	0.42846	0.286356	0.775276	-5.19922	0.776843	0.847053
Erythroid.cells	CDK5	-0.09788	3.72259	-0.28635	0.775277	-5.77458	0.693452	0.772921
Erythroid.cells	1700025G0	0.083575	4.957756	0.28615	0.775433	-6.2858	0.664705	0.746808
Erythroid.cells	POPDC3	0.257909	0.894439	0.285935	0.775597	-5.28401	0.764583	0.836256
Erythroid.cells	TMEM126B	-0.10408	2.697386	-0.2859	0.775622	-5.66432	0.718492	0.795384
Erythroid.cells	MFSD4A	0.148735	2.041521	0.285504	0.775926	-5.5155	0.735121	0.810068
Erythroid.cells	SNX18	-0.06861	6.77742	-0.2853	0.776083	-6.40212	0.624735	0.710071
Erythroid.cells	ZFP27	0.180361	1.016419	0.28529	0.776089	-5.31328	0.761581	0.833525
Erythroid.cells	2900060B1	-0.13432	2.614995	-0.28517	0.776178	-5.49657	0.720733	0.797295
Erythroid.cells	TLR12	0.197737	2.045397	0.285171	0.77618	-5.46427	0.735022	0.810025
Erythroid.cells	EXOC4	-0.03399	8.03297	-0.28489	0.776394	-6.77198	0.598585	0.685728
Erythroid.cells	NPEPPS	-0.05285	7.184287	-0.28484	0.776435	-6.53796	0.616183	0.702119
Erythroid.cells	TREML4	0.152505	2.758058	0.284771	0.776486	-5.75027	0.71728	0.794179
Erythroid.cells	PIN1	-0.05975	5.245371	-0.28447	0.776714	-6.16384	0.65862	0.741121
Erythroid.cells	GM29585	-0.21195	1.247477	-0.28437	0.776792	-5.27661	0.755792	0.828319
Erythroid.cells	GALNT18	0.204353	2.025829	0.284151	0.776959	-5.56177	0.735799	0.81059
Erythroid.cells	NSFL1C	-0.07208	4.168167	-0.28405	0.777037	-5.91206	0.683477	0.763688
Erythroid.cells	GM26827	-0.16867	1.344192	-0.28395	0.777113	-5.50961	0.753303	0.826095
Erythroid.cells	ARID4A	0.048492	7.062741	0.283715	0.777292	-6.58448	0.618906	0.70458
Erythroid.cells	GM37233	0.219158	0.010375	0.283639	0.77735	-5.22248	0.788796	0.857291
Erythroid.cells	TACC1	-0.03395	7.703473	-0.28363	0.777356	-6.73045	0.605507	0.692125
Erythroid.cells	PRR3	0.082527	3.987386	0.283551	0.777417	-5.90879	0.687738	0.76756
Erythroid.cells	TRAPPC9	0.054201	6.178368	0.283538	0.777427	-6.36461	0.63792	0.722142
Erythroid.cells	ZFP770	-0.15653	1.760027	-0.28352	0.777443	-5.43901	0.742574	0.816623
Erythroid.cells	DOT1L	0.073336	5.289755	0.283154	0.77772	-6.15226	0.657827	0.740278
Erythroid.cells	GM16316	-0.12883	1.803894	-0.28252	0.778204	-5.61471	0.742046	0.815801
Erythroid.cells	HAUS3	-0.06997	4.199727	-0.28207	0.778549	-5.94361	0.683436	0.763336
Erythroid.cells	PTOV1	-0.07703	4.038474	-0.28206	0.778559	-5.92376	0.687236	0.766769
Erythroid.cells	ACSF2	0.08523	4.286847	0.281974	0.778622	-5.98212	0.681393	0.761501
Erythroid.cells	TRNAU1AP	-0.0847	4.126118	-0.28182	0.778741	-5.93855	0.685168	0.76496
Erythroid.cells	CDC42SE1	-0.05225	6.18973	-0.28172	0.778819	-6.32695	0.638326	0.722268
Erythroid.cells	PPIL3	0.067344	4.605985	0.281708	0.778825	-6.01939	0.673961	0.754827
Erythroid.cells	MIEF1	0.0934	4.21125	0.28166	0.778862	-5.87564	0.683166	0.763173
Erythroid.cells	PALD1	-0.17526	1.292537	-0.28138	0.779078	-5.44843	0.755564	0.827826

Erythroid.cells	ADGRG1	-0.19048	1.720983	-0.28114	0.779259	-5.49378	0.744585	0.81807
Erythroid.cells	CHTOP	-0.06425	5.225925	-0.28076	0.779548	-6.15999	0.660174	0.74214
Erythroid.cells	MEIG1	0.241712	-0.47443	0.28038	0.779841	-5.15475	0.80366	0.869701
Erythroid.cells	KRT8	-0.14317	1.975849	-0.28026	0.779934	-5.70319	0.738512	0.812514
Erythroid.cells	TMEM26	-0.16988	2.190341	-0.2799	0.780211	-5.53998	0.733246	0.807796
Erythroid.cells	CCNJ	-0.17873	2.015982	-0.27984	0.780258	-5.40045	0.737667	0.811722
Erythroid.cells	WDR19	0.211125	0.508741	0.279576	0.780456	-5.25063	0.777172	0.846516
Erythroid.cells	MAU2	-0.06011	6.192162	-0.27929	0.780672	-6.40418	0.639235	0.722793
Erythroid.cells	CARD19	-0.07053	5.613475	-0.27922	0.780728	-6.24298	0.652038	0.734532
Erythroid.cells	ATPAF1	-0.09377	3.553171	-0.27887	0.780992	-5.74269	0.700022	0.777984
Erythroid.cells	AATF	-0.06544	4.995367	-0.27865	0.781164	-6.09867	0.666169	0.747445
Erythroid.cells	MDM2	-0.06745	6.41707	-0.27859	0.781209	-6.45126	0.634481	0.718438
Erythroid.cells	SLC5A6	-0.20247	0.868812	-0.27857	0.781224	-5.27556	0.767884	0.838359
Erythroid.cells	BCCIP	0.044883	5.436859	0.278528	0.781258	-6.28343	0.656153	0.738314
Erythroid.cells	MRPL38	-0.07734	3.863246	-0.27818	0.781523	-5.89689	0.692769	0.771435
Erythroid.cells	GM43727	0.200966	-0.59426	0.277938	0.781709	-5.23076	0.807895	0.873218
Erythroid.cells	EEFSEC	0.08769	4.399413	0.277828	0.781793	-6.03073	0.680134	0.760048
Erythroid.cells	RMDN2	0.142248	1.873328	0.277821	0.781798	-5.53039	0.741934	0.815386
Erythroid.cells	BSCL2	0.067995	4.542466	0.277677	0.781909	-5.95922	0.676799	0.757082
Erythroid.cells	IRAK1BP1	0.250593	0.288762	0.277599	0.781968	-5.19266	0.783632	0.852178
Erythroid.cells	C1S1	0.121942	1.787052	0.277462	0.782073	-5.69331	0.744145	0.817447
Erythroid.cells	HAO2	0.19235	1.393032	0.277237	0.782245	-5.56927	0.754328	0.826506
Erythroid.cells	TMX2	-0.09996	3.46498	-0.27717	0.782299	-5.73714	0.702345	0.780226
Erythroid.cells	VPS53	-0.0588	4.462382	-0.27686	0.782534	-5.97104	0.678664	0.758895
Erythroid.cells	KANK1	-0.16442	1.172928	-0.27682	0.782567	-5.42762	0.760078	0.831619
Erythroid.cells	TLN2	-0.16806	1.298032	-0.27679	0.782591	-5.4905	0.756804	0.828735
Erythroid.cells	PRPS1	-0.08841	3.621673	-0.27665	0.782697	-5.8386	0.698568	0.776893
Erythroid.cells	TMEM161I	0.069005	4.545354	0.27662	0.782718	-5.96943	0.676732	0.757174
Erythroid.cells	LNPEP	-0.04417	7.335044	-0.27656	0.782761	-6.59707	0.615043	0.700595
Erythroid.cells	FAM83G	0.200305	0.263823	0.276459	0.782841	-5.18407	0.784307	0.852908
Erythroid.cells	SFXN1	-0.06703	5.121606	-0.27633	0.782942	-6.17533	0.663473	0.745143
Erythroid.cells	RETSAT	-0.13074	1.405172	-0.2763	0.782961	-5.40437	0.754012	0.826313
Erythroid.cells	OGFOD2	-0.08296	3.850672	-0.27625	0.783001	-5.83422	0.693087	0.771977
Erythroid.cells	THAP12	0.065442	4.362104	0.276229	0.783017	-5.96055	0.681006	0.761065
Erythroid.cells	PPP2R3C	-0.0721	4.333149	-0.27609	0.783121	-5.97009	0.681684	0.761679
Erythroid.cells	SLC12A4	-0.15443	1.745624	-0.27598	0.783205	-5.4411	0.745209	0.81857
Erythroid.cells	ESYT1	0.059822	5.654914	0.275957	0.783225	-6.28219	0.651446	0.734198
Erythroid.cells	FAM98C	-0.09977	3.737769	-0.27593	0.783247	-5.75838	0.695783	0.774451
Erythroid.cells	GM11476	0.12704	2.536389	0.275777	0.783363	-5.61946	0.725168	0.800798
Erythroid.cells	HCFC2	-0.0856	3.758586	-0.27577	0.783369	-5.82497	0.695285	0.774025
Erythroid.cells	GM5150	-0.16232	2.574091	-0.27577	0.783372	-5.70636	0.724227	0.799959
Erythroid.cells	RBSN	-0.0981	2.910659	-0.27559	0.783503	-5.63494	0.715878	0.792507
Erythroid.cells	GM43813	-0.08711	4.535806	-0.27557	0.78352	-6.01838	0.676954	0.757462
Erythroid.cells	TIA1	-0.04679	5.466514	-0.27537	0.783671	-6.23285	0.655668	0.738088
Erythroid.cells	MDM4	-0.04791	5.749077	-0.27537	0.783671	-6.27533	0.649346	0.732304
Erythroid.cells	GM10634	0.169471	0.651485	0.275351	0.783689	-5.4295	0.773881	0.843873
Erythroid.cells	SHCBP1L	-0.17922	0.854389	-0.27489	0.784045	-5.31676	0.768761	0.839242

Erythroid.cells	GM49625	0.146718	2.147374	0.274586	0.784275	-5.44717	0.735376	0.809656
Erythroid.cells	SUCLG1	0.053829	5.422184	0.274463	0.784369	-6.37117	0.657047	0.73915
Erythroid.cells	CDK5RAP2	0.071911	4.446752	0.274335	0.784467	-5.96075	0.679423	0.759497
Erythroid.cells	PYCRL	0.083731	3.541294	0.274229	0.784549	-5.8043	0.70091	0.778881
Erythroid.cells	CASC1	-0.17314	1.810453	-0.27412	0.784629	-5.47548	0.743977	0.817297
Erythroid.cells	ZBTB12	-0.13705	1.665552	-0.27412	0.78463	-5.4423	0.747704	0.820596
Erythroid.cells	GM5244	-0.23207	-0.39473	-0.27356	0.785059	-5.23154	0.803182	0.869043
Erythroid.cells	SIN3A	-0.05301	5.871572	-0.27308	0.785427	-6.29171	0.647522	0.730228
Erythroid.cells	PLA2G4A	-0.12584	3.294743	-0.27299	0.785498	-5.77026	0.707452	0.784538
Erythroid.cells	GM42984	-0.21696	0.916614	-0.27294	0.785538	-5.25278	0.767894	0.838167
Erythroid.cells	ZC3H12D	-0.11677	3.479104	-0.27268	0.785731	-5.76265	0.703076	0.780579
Erythroid.cells	MAFG	-0.059	5.07367	-0.27261	0.785787	-6.19582	0.66558	0.746691
Erythroid.cells	MIPEP	-0.09311	2.843457	-0.27232	0.786014	-5.64091	0.718772	0.794585
Erythroid.cells	EIF4E2	-0.04724	5.989442	-0.27223	0.786078	-6.37958	0.645128	0.727969
Erythroid.cells	PDE1A	0.213117	0.441701	0.272045	0.786222	-5.29947	0.780921	0.849466
Erythroid.cells	PHF21A	0.052048	7.069098	0.271786	0.786421	-6.56329	0.621791	0.706436
Erythroid.cells	HPN	0.116815	2.642028	0.271745	0.786452	-5.76177	0.723853	0.799078
Erythroid.cells	ZCCHC3	-0.16984	0.70709	-0.27166	0.786516	-5.35541	0.773807	0.843233
Erythroid.cells	DENND3	0.109695	3.548798	0.271623	0.786545	-5.75106	0.701601	0.779171
Erythroid.cells	XYLT2	-0.12986	1.972195	-0.27154	0.786612	-5.46943	0.740759	0.814132
Erythroid.cells	OSBPL10	-0.19354	0.973543	-0.27131	0.786789	-5.29099	0.766723	0.837044
Erythroid.cells	MRPL9	-0.05359	4.787977	-0.2713	0.786792	-6.10634	0.672341	0.752781
Erythroid.cells	IGSF3	-0.20849	0.826018	-0.27117	0.786895	-5.27424	0.770637	0.840481
Erythroid.cells	ARRDC4	0.121999	2.427989	0.27108	0.786962	-5.66526	0.729211	0.803878
Erythroid.cells	SLC25A33	0.09648	3.983563	0.271009	0.787016	-5.82381	0.691186	0.769827
Erythroid.cells	ACO1	0.078339	3.712371	0.271006	0.787018	-5.85166	0.697663	0.775659
Erythroid.cells	SLC25A36	-0.04951	6.285868	-0.27091	0.78709	-6.4018	0.638678	0.722034
Erythroid.cells	SLC39A6	0.078148	4.11285	0.270859	0.787131	-5.88017	0.68812	0.767062
Erythroid.cells	BRDT	0.161571	1.567286	0.270451	0.787444	-5.41088	0.751285	0.823415
Erythroid.cells	CFAP36	-0.07261	4.102137	-0.2704	0.787486	-5.90084	0.688473	0.767354
Erythroid.cells	LRRC25	-0.1145	3.856093	-0.27032	0.787546	-5.94114	0.694323	0.772628
Erythroid.cells	GM39302	-0.21102	0.880898	-0.27019	0.787641	-5.24347	0.76929	0.839285
Erythroid.cells	UPF2	0.044858	6.075856	0.270036	0.787762	-6.40078	0.643381	0.726401
Erythroid.cells	DDB2	-0.10179	3.43077	-0.27	0.787786	-5.8593	0.704558	0.781908
Erythroid.cells	LYRM2	-0.10439	3.377583	-0.26968	0.788033	-5.72843	0.705849	0.783102
Erythroid.cells	LIMCH1	0.214702	0.57747	0.269473	0.788194	-5.29489	0.77739	0.8465
Erythroid.cells	OXSR1	-0.09453	3.496646	-0.26944	0.788219	-5.7502	0.702963	0.780511
Erythroid.cells	LRRC3	-0.15329	0.375073	-0.26935	0.788284	-5.42756	0.782841	0.85127
Erythroid.cells	MYLIP	0.066745	5.991118	0.269259	0.788358	-6.2736	0.645252	0.728152
Erythroid.cells	ZFP961	0.10411	3.308556	0.269141	0.788448	-5.72481	0.707529	0.784607
Erythroid.cells	AFF4	-0.04351	7.842009	-0.2691	0.788482	-6.75778	0.60568	0.691585
Erythroid.cells	DCK	0.058004	5.832657	0.269083	0.788493	-6.39895	0.648765	0.731372
Erythroid.cells	DOLPP1	0.121119	2.514642	0.269062	0.788509	-5.58044	0.727142	0.802129
Erythroid.cells	GM16083	0.178255	1.977218	0.269048	0.78852	-5.39487	0.740738	0.814207
Erythroid.cells	BRK1	0.041513	6.271039	0.268925	0.788614	-6.42802	0.639095	0.7225
Erythroid.cells	FBXW2	0.037776	6.193608	0.268905	0.78863	-6.47465	0.640792	0.724059
Erythroid.cells	GMDS	0.052089	6.245209	0.268796	0.788713	-6.34602	0.63966	0.723019

Erythroid.cells	ZFP120	-0.10509	2.536502	-0.26875	0.788749	-5.59214	0.726595	0.801642
Erythroid.cells	CES2E	-0.15211	1.228112	-0.26862	0.788849	-5.5257	0.760128	0.831338
Erythroid.cells	IGF2BP3	-0.05446	6.5516	-0.26859	0.788874	-6.45899	0.632986	0.716878
Erythroid.cells	MAPK9	-0.06257	4.751185	-0.2683	0.789094	-6.07076	0.673288	0.753727
Erythroid.cells	ARHGEF15	-0.22504	0.580885	-0.26824	0.789141	-5.25858	0.777298	0.846419
Erythroid.cells	GM42937	-0.20137	0.198102	-0.26823	0.789148	-5.31452	0.787639	0.855463
Erythroid.cells	CDC42BPG	0.138132	2.57838	0.268173	0.78919	-5.59456	0.725547	0.800716
Erythroid.cells	MBL2	0.102184	4.246085	0.268095	0.789251	-6.32001	0.685075	0.764426
Erythroid.cells	SHPRH	0.070427	4.541567	0.267942	0.789368	-6.00574	0.678154	0.758185
Erythroid.cells	AGPAT4	0.076994	5.023605	0.267938	0.789371	-6.31772	0.66702	0.748079
Erythroid.cells	DGKE	-0.07955	4.880229	-0.26791	0.789394	-6.03995	0.670311	0.751071
Erythroid.cells	PIK3R6	-0.16522	2.596377	-0.26786	0.789429	-5.61151	0.725097	0.800359
Erythroid.cells	LARP1	-0.04323	6.798018	-0.26782	0.789464	-6.51177	0.627672	0.712023
Erythroid.cells	ZFP346	0.098687	3.658973	0.267455	0.789741	-5.81195	0.699231	0.777159
Erythroid.cells	LMAN1	0.066898	4.627609	0.267346	0.789825	-6.13016	0.676342	0.756515
Erythroid.cells	WDYHV1	0.073642	4.174037	0.267207	0.789932	-5.89676	0.68698	0.766154
Erythroid.cells	B4GALNT1	-0.0559	4.892601	-0.26715	0.789979	-6.24454	0.670228	0.750993
Erythroid.cells	UPF3A	0.054694	4.634592	0.266979	0.790107	-6.06539	0.676198	0.756459
Erythroid.cells	9030404E1	0.234406	-0.16276	0.266957	0.790124	-5.19335	0.79776	0.864395
Erythroid.cells	DKC1	0.072967	4.56874	0.266623	0.790381	-6.0583	0.677891	0.757883
Erythroid.cells	RELL1	-0.05026	6.866963	-0.26649	0.790484	-6.47849	0.626563	0.710948
Erythroid.cells	VARS2	0.179139	1.401859	0.266296	0.790631	-5.35784	0.756104	0.827799
Erythroid.cells	1810013L2	0.05151	5.901803	0.266192	0.790711	-6.31946	0.647683	0.73039
Erythroid.cells	ASTN2	0.188917	1.045238	0.265984	0.790871	-5.34219	0.765563	0.836083
Erythroid.cells	TRMT10C	0.057729	4.996252	0.265567	0.791191	-6.14776	0.668402	0.74912
Erythroid.cells	DENND2C	-0.10874	2.008648	-0.26538	0.791336	-5.63901	0.740844	0.814097
Erythroid.cells	CABLES2	0.08477	3.580958	0.265206	0.791468	-5.7787	0.701814	0.779294
Erythroid.cells	FMNL1	-0.04688	6.443857	-0.26504	0.791599	-6.47536	0.63613	0.71965
Erythroid.cells	AKAP17B	-0.14161	1.696468	-0.26498	0.791644	-5.49438	0.748892	0.821292
Erythroid.cells	ARF4	-0.03816	8.479652	-0.26498	0.791645	-6.88736	0.593404	0.680005
Erythroid.cells	GM42836	-0.20126	-0.40469	-0.26473	0.791835	-5.28139	0.805318	0.87064
Erythroid.cells	GM50333	-0.15748	1.191453	-0.26468	0.791876	-5.34191	0.762143	0.832903
Erythroid.cells	CYP2B9	0.158263	1.9577	0.264563	0.791962	-5.68474	0.742278	0.815387
Erythroid.cells	CYP3A16	-0.15912	0.777697	-0.26409	0.792325	-5.47353	0.773344	0.842706
Erythroid.cells	NDUFAF7	-0.07132	4.007139	-0.26408	0.792331	-5.91751	0.691897	0.770324
Erythroid.cells	MRPL40	0.070695	4.651456	0.263982	0.792408	-6.04495	0.676751	0.756641
Erythroid.cells	RTN2	0.211322	-0.4318	0.263711	0.792617	-5.21663	0.806473	0.871579
Erythroid.cells	TTC14	0.042274	5.803768	0.263321	0.792916	-6.33129	0.650817	0.732954
Erythroid.cells	NCAPH	-0.10762	4.175802	-0.26304	0.793135	-5.97618	0.688278	0.766965
Erythroid.cells	TCHP	-0.13676	1.792962	-0.2629	0.793242	-5.447	0.747138	0.81954
Erythroid.cells	LSM2	0.068734	5.695608	0.262829	0.793294	-6.33488	0.653287	0.735222
Erythroid.cells	LGALS1	-0.08079	6.036335	-0.26282	0.793299	-6.40743	0.645703	0.72828
Erythroid.cells	TMEM208	0.053284	4.89435	0.262735	0.793366	-6.06774	0.671495	0.751807
Erythroid.cells	AC149090.	-0.1218	5.49949	-0.26267	0.79342	-6.15132	0.657695	0.739251
Erythroid.cells	NT5DC3	-0.06712	5.099601	-0.2625	0.793545	-6.06307	0.66678	0.747527
Erythroid.cells	TPBGL	-0.2222	0.559425	-0.2624	0.793621	-5.24196	0.779626	0.848155
Erythroid.cells	KANK3	0.146495	2.000717	0.262382	0.793638	-5.58589	0.741805	0.814858

Erythroid.cells	STX12	-0.05434	5.552332	-0.26233	0.793674	-6.21533	0.656504	0.738174
Erythroid.cells	PRR14	-0.07495	4.524923	-0.26188	0.794024	-5.95015	0.68021	0.759761
Erythroid.cells	SPP1	0.200393	2.353951	0.261875	0.794027	-5.74828	0.732978	0.807072
Erythroid.cells	CERS2	-0.05593	5.262709	-0.26183	0.794059	-6.1863	0.663196	0.744322
Erythroid.cells	FLT3L	0.117028	2.201527	0.261493	0.794321	-5.61563	0.736839	0.810569
Erythroid.cells	ACOT13	-0.05372	4.43558	-0.26147	0.794335	-6.09939	0.682301	0.761717
Erythroid.cells	GM16124	-0.1354	2.669782	-0.26136	0.794419	-5.6023	0.725044	0.800082
Erythroid.cells	GM27008	-0.15671	1.012976	-0.26132	0.794451	-5.35459	0.767674	0.837795
Erythroid.cells	NFRKB	0.0603	4.478568	0.261303	0.794467	-5.97219	0.681294	0.760807
Erythroid.cells	PDIA6	-0.04656	6.97381	-0.26126	0.794501	-6.58589	0.625444	0.709749
Erythroid.cells	SLC22A18	0.138107	1.143718	0.261216	0.794533	-5.54265	0.764219	0.834764
Erythroid.cells	STX11	-0.0912	4.829435	-0.26088	0.794792	-5.98079	0.673249	0.753503
Erythroid.cells	NUTF2-PS1	-0.15057	2.848853	-0.26086	0.79481	-5.6699	0.720709	0.796203
Erythroid.cells	GM10131	-0.20269	1.072727	-0.26074	0.794898	-5.33664	0.766244	0.836523
Erythroid.cells	NUP54	-0.06214	4.859149	-0.26057	0.795027	-6.05228	0.672585	0.752941
Erythroid.cells	GM49359	0.121348	2.260358	0.260507	0.795078	-5.53425	0.735499	0.80942
Erythroid.cells	AU020206	-0.06024	5.320744	-0.26046	0.795115	-6.23432	0.662014	0.743353
Erythroid.cells	MAD2L1	0.091453	4.166093	0.260035	0.795442	-5.96647	0.688912	0.767709
Erythroid.cells	RIMS3	0.191117	1.085548	0.259954	0.795504	-5.36164	0.766046	0.836392
Erythroid.cells	PUF60	-0.04892	5.949591	-0.25986	0.795578	-6.38596	0.648006	0.730527
Erythroid.cells	GM10603	0.15351	0.220408	0.259828	0.7956	-5.49012	0.789268	0.85673
Erythroid.cells	IL5RA	-0.18591	0.975245	-0.25982	0.795609	-5.42299	0.768968	0.838959
Erythroid.cells	SOCS7	-0.07243	4.207792	-0.25967	0.795725	-5.91513	0.687926	0.766894
Erythroid.cells	LEMD3	-0.0518	5.687224	-0.25962	0.795762	-6.27278	0.653859	0.735951
Erythroid.cells	PFDN1	-0.04932	5.109296	-0.25952	0.795837	-6.20294	0.66695	0.747889
Erythroid.cells	PAICS	0.045848	6.310596	0.259299	0.796007	-6.50997	0.640043	0.723292
Erythroid.cells	CORO2B	0.176576	1.197161	0.259288	0.796015	-5.28342	0.763101	0.833883
Erythroid.cells	ARHGAP23	0.090644	3.694446	0.259189	0.796092	-5.88378	0.700178	0.777934
Erythroid.cells	POLI	-0.15794	1.527716	-0.25895	0.796272	-5.32484	0.754448	0.826293
Erythroid.cells	NUDT2	0.082967	3.238769	0.258866	0.79634	-5.76381	0.711245	0.787896
Erythroid.cells	PDZD11	0.080052	4.30203	0.258609	0.796538	-5.92276	0.685701	0.764953
Erythroid.cells	CHST7	-0.20907	0.725254	-0.25853	0.796602	-5.2886	0.775631	0.844994
Erythroid.cells	TRPC4AP	0.046369	5.651799	0.258519	0.796607	-6.23135	0.654654	0.736774
Erythroid.cells	ZFP110	0.082561	4.039873	0.258397	0.796701	-5.82777	0.691909	0.770613
Erythroid.cells	CPED1	-0.11813	2.524781	-0.25838	0.796713	-5.81452	0.728953	0.803795
Erythroid.cells	PGD	-0.0565	5.716274	-0.25834	0.796744	-6.23767	0.653208	0.735492
Erythroid.cells	PRKACB	0.05031	5.548308	0.258298	0.796777	-6.22055	0.656981	0.738939
Erythroid.cells	ALDH1A1	0.134921	-5.25E-05	0.258271	0.796798	-5.66944	0.795299	0.862227
Erythroid.cells	RNH1	-0.0552	5.988026	-0.25821	0.796848	-6.42252	0.647153	0.729948
Erythroid.cells	ADGRG6	-0.2122	0.969217	-0.2582	0.796853	-5.43078	0.769128	0.839332
Erythroid.cells	SAMD9L	-0.07423	5.184732	-0.25782	0.797142	-6.14711	0.66541	0.746558
Erythroid.cells	GM38973	-0.15392	1.613034	-0.25738	0.797486	-5.46447	0.752697	0.824612
Erythroid.cells	ZFYVE1	0.074564	4.797159	0.257284	0.797558	-6.00914	0.674555	0.754703
Erythroid.cells	ANKIB1	-0.04896	5.807453	-0.25711	0.79769	-6.30462	0.651627	0.733804
Erythroid.cells	SELENOH	0.082785	5.20549	0.256896	0.797856	-6.21145	0.665298	0.746221
Erythroid.cells	RANGAP1	0.057113	5.610379	0.256706	0.798002	-6.29746	0.656186	0.737916
Erythroid.cells	PRKAA1	-0.08109	4.45764	-0.25642	0.798226	-5.94275	0.682802	0.762051

Erythroid.cells	TM9SF3	0.030538	7.288714	0.256049	0.798508	-6.61952	0.619837	0.704381
Erythroid.cells	PIDD1	0.163638	0.824038	0.255841	0.798668	-5.33696	0.774143	0.843251
Erythroid.cells	GM10371	-0.11294	-0.68835	-0.25557	0.798878	-5.61674	0.815787	0.879531
Erythroid.cells	PHF12	0.046885	6.186244	0.255214	0.799151	-6.36128	0.643999	0.726558
Erythroid.cells	ACVR2B	-0.16085	1.53111	-0.25514	0.79921	-5.39567	0.755797	0.827043
Erythroid.cells	CDC7	-0.11028	2.873973	-0.25505	0.79928	-5.65817	0.721608	0.796754
Erythroid.cells	GM31718	-0.09585	3.725877	-0.25496	0.799347	-5.77374	0.700754	0.778112
Erythroid.cells	FAM120A	0.037879	6.352078	0.254759	0.799501	-6.42148	0.64042	0.723269
Erythroid.cells	ATP6AP1	0.047857	5.990589	0.254572	0.799645	-6.33529	0.648424	0.730584
Erythroid.cells	LRP12	0.157696	2.191669	0.25447	0.799723	-5.5232	0.738884	0.812058
Erythroid.cells	TKT	0.050908	6.932084	0.254445	0.799743	-6.49609	0.627862	0.711684
Erythroid.cells	STK11	-0.04574	5.696969	-0.25415	0.799971	-6.3225	0.655112	0.736658
Erythroid.cells	CSPP1	0.052597	6.098729	0.254015	0.800074	-6.35485	0.646181	0.728494
Erythroid.cells	LYPD6B	0.178406	0.004013	0.253681	0.800331	-5.42768	0.797111	0.863126
Erythroid.cells	EXOSC4	0.081985	3.784732	0.253652	0.800353	-5.86511	0.699695	0.777018
Erythroid.cells	GM32401	-0.12965	2.445198	-0.25356	0.800424	-5.5779	0.732723	0.806523
Erythroid.cells	P2RX3	-0.12599	2.219391	-0.25348	0.800486	-5.74423	0.738448	0.811621
Erythroid.cells	KAT8	-0.08516	3.624929	-0.25331	0.800613	-5.76507	0.703552	0.780561
Erythroid.cells	CFAP298	0.079226	3.642504	0.253304	0.800621	-5.8446	0.703127	0.78018
Erythroid.cells	GM36161	0.155088	0.563057	0.253128	0.800757	-5.5919	0.781873	0.849954
Erythroid.cells	ZFP316	-0.19919	0.380007	-0.25308	0.800795	-5.26096	0.786829	0.854286
Erythroid.cells	NPHP4	-0.19836	0.297045	-0.25307	0.800802	-5.23671	0.789086	0.856256
Erythroid.cells	RETNLA	0.635466	-0.77811	0.25281	0.801002	-5.30016	0.819019	0.88227
Erythroid.cells	GM10863	0.220391	0.229142	0.252701	0.801086	-5.22964	0.791021	0.857995
Erythroid.cells	POGZ	-0.06412	4.431535	-0.25268	0.801103	-5.96069	0.684378	0.763376
Erythroid.cells	MEST	0.113048	3.792937	0.252478	0.801258	-5.97526	0.69957	0.777135
Erythroid.cells	ARPP19	-0.03469	6.840626	-0.2524	0.801318	-6.57228	0.630125	0.713926
Erythroid.cells	THSD1	0.147776	1.60027	0.252324	0.801376	-5.39855	0.754461	0.826016
Erythroid.cells	TKTL1	-0.15574	1.290296	-0.2522	0.80147	-5.34693	0.762572	0.833167
Erythroid.cells	MARCO	-0.17584	5.795483	-0.25219	0.801481	-6.31934	0.653081	0.735009
Erythroid.cells	GPLD1	0.15379	0.934219	0.252128	0.801528	-5.47975	0.772	0.841451
Erythroid.cells	SPCS3	0.05865	4.704887	0.252037	0.801597	-6.01968	0.677982	0.757668
Erythroid.cells	ING1	0.056359	5.135774	0.251895	0.801707	-6.14179	0.668026	0.748672
Erythroid.cells	DENND1B	-0.0446	7.299486	-0.25185	0.801743	-6.60535	0.620318	0.704905
Erythroid.cells	LPGAT1	0.059587	6.245156	0.251784	0.801792	-6.35723	0.643097	0.725905
Erythroid.cells	ELMO2	0.080657	3.945024	0.251632	0.80191	-5.87734	0.695934	0.773951
Erythroid.cells	SLC52A2	0.12159	2.391257	0.251495	0.802015	-5.51239	0.734177	0.808153
Erythroid.cells	PPARGC1B	-0.11442	2.799015	-0.2514	0.802092	-5.63616	0.723934	0.799039
Erythroid.cells	SLC25A1	0.079676	3.772843	0.251351	0.802126	-5.90947	0.700068	0.777678
Erythroid.cells	CBFA2T3	0.056408	5.507245	0.251273	0.802186	-6.4242	0.65958	0.741023
Erythroid.cells	ZSCAN29	-0.08813	3.485733	-0.25122	0.802226	-5.68858	0.707018	0.783917
Erythroid.cells	EFCAB5	0.201636	0.504613	0.25105	0.802358	-5.23942	0.783602	0.851663
Erythroid.cells	DYNLT1B	0.169779	1.188553	0.25097	0.80242	-5.36352	0.765321	0.835637
Erythroid.cells	MGAT4B	0.075889	3.847759	0.250676	0.802646	-5.92719	0.69845	0.776099
Erythroid.cells	FAM222A	0.109548	2.331884	0.249822	0.803304	-5.56477	0.736365	0.80969
Erythroid.cells	ZFP995	-0.10868	2.383748	-0.2498	0.80332	-5.5783	0.735049	0.808524
Erythroid.cells	ZDHHC1	-0.13011	1.415954	-0.24953	0.803532	-5.405	0.760103	0.830689

Erythroid.cells	RTN4IP1	-0.14118	1.927586	-0.24939	0.803635	-5.47863	0.746807	0.818959
Erythroid.cells	TMCO1	0.036542	6.265444	0.24938	0.803645	-6.45837	0.643353	0.725827
Erythroid.cells	WDR5	0.065283	4.681603	0.249077	0.803879	-6.02171	0.679273	0.758661
Erythroid.cells	USP28	-0.06755	4.553264	-0.24906	0.803895	-5.94718	0.682274	0.761381
Erythroid.cells	KIF3A	-0.10179	2.472633	-0.24905	0.803901	-5.59868	0.732915	0.806764
Erythroid.cells	SPDL1	-0.11306	2.833783	-0.24901	0.803933	-5.65968	0.723852	0.798706
Erythroid.cells	ZC2HC1A	-0.15092	1.741533	-0.2488	0.804092	-5.4535	0.751643	0.823364
Erythroid.cells	PSD3	-0.07449	5.794857	-0.2488	0.804095	-6.29393	0.653833	0.73554
Erythroid.cells	CDC14A	0.067569	5.803629	0.248597	0.804249	-6.29261	0.653706	0.735396
Erythroid.cells	HEATR5B	-0.09564	3.730171	-0.24845	0.804363	-5.73001	0.701987	0.779092
Erythroid.cells	GM34921	-0.18614	0.841113	-0.24827	0.8045	-5.26198	0.775549	0.844269
Erythroid.cells	PTPN7	0.079906	4.151708	0.247911	0.804778	-5.98063	0.692121	0.770126
Erythroid.cells	DPP7	0.141054	1.957266	0.247726	0.804921	-5.51676	0.746457	0.818664
Erythroid.cells	SLC22A21	0.129468	1.348839	0.247665	0.804968	-5.58205	0.762287	0.832627
Erythroid.cells	ATG9A	0.072543	3.85849	0.247504	0.805092	-5.89131	0.699145	0.77649
Erythroid.cells	ATP8B2	-0.08591	2.774077	-0.24744	0.805143	-5.67282	0.725736	0.800277
Erythroid.cells	TUBGCP5	-0.08501	3.976604	-0.24735	0.805213	-5.7345	0.69631	0.773941
Erythroid.cells	MARK4	-0.07342	4.541737	-0.24711	0.805394	-5.95143	0.682915	0.761955
Erythroid.cells	MMACHC	-0.19882	0.470101	-0.24708	0.805418	-5.24429	0.78576	0.853305
Erythroid.cells	ZFP51	0.087643	2.86166	0.246993	0.805486	-5.66007	0.72355	0.798453
Erythroid.cells	RBFOX2	0.125902	2.758111	0.246846	0.805599	-5.73358	0.726135	0.800768
Erythroid.cells	PPP1R9A	0.108881	3.383193	0.246844	0.805601	-5.83244	0.710672	0.786962
Erythroid.cells	PDHX	-0.07541	3.395129	-0.24683	0.805613	-5.72937	0.71038	0.786701
Erythroid.cells	ANKRD13A	0.044348	6.215118	0.246673	0.805733	-6.35881	0.644819	0.727306
Erythroid.cells	XPNPEP1	-0.06018	4.365592	-0.24659	0.805797	-6.00959	0.687061	0.765737
Erythroid.cells	PDE11A	0.198292	0.072949	0.246539	0.805837	-5.34821	0.796608	0.862806
Erythroid.cells	SNRPD3	-0.03996	6.518599	-0.24632	0.806008	-6.51573	0.638155	0.721187
Erythroid.cells	BBX	-0.05211	6.494425	-0.2463	0.806022	-6.45754	0.638683	0.721673
Erythroid.cells	MED28	0.041265	6.023126	0.246258	0.806053	-6.37176	0.649074	0.731204
Erythroid.cells	BEND6	0.197475	0.170455	0.246178	0.806115	-5.24846	0.793931	0.860497
Erythroid.cells	SH3BP5	0.057426	6.380773	0.246131	0.806152	-6.40272	0.641172	0.723981
Erythroid.cells	RUVBL1	0.056279	4.77265	0.24604	0.806222	-6.09674	0.67752	0.757131
Erythroid.cells	SLC35A2	-0.10226	3.165869	-0.246	0.806255	-5.6635	0.716009	0.791768
Erythroid.cells	H2-M3	-0.11009	4.138363	-0.24556	0.806595	-5.82952	0.692677	0.770734
Erythroid.cells	CROCC	-0.17027	1.025643	-0.24547	0.806661	-5.28115	0.77109	0.840431
Erythroid.cells	SLC25A4	-0.04557	7.14787	-0.24538	0.806734	-6.66843	0.624776	0.70879
Erythroid.cells	UGGT1	0.049555	5.389379	0.245215	0.806858	-6.18026	0.663582	0.744394
Erythroid.cells	SETD1B	0.064658	5.026478	0.245068	0.806972	-6.03137	0.671897	0.751958
Erythroid.cells	GM41556	-0.14519	1.259442	-0.24505	0.806982	-5.40117	0.764934	0.83504
Erythroid.cells	BET1	-0.06348	4.246255	-0.24483	0.807157	-6.00511	0.690237	0.768536
Erythroid.cells	MRPL49	0.079384	3.778216	0.244696	0.807259	-5.88184	0.701464	0.778622
Erythroid.cells	KRR1	-0.07841	3.94276	-0.24452	0.807393	-5.81789	0.697561	0.775065
Erythroid.cells	ASRGL1	0.096096	3.315534	0.244208	0.807636	-5.80057	0.712905	0.788767
Erythroid.cells	APH1C	-0.08154	4.351444	-0.24412	0.807703	-6.07898	0.687951	0.766376
Erythroid.cells	AP1S3	0.059216	5.57812	0.244065	0.807746	-6.40126	0.659583	0.740652
Erythroid.cells	ZNRD2	0.071249	4.037669	0.243831	0.807927	-5.95891	0.695432	0.773175
Erythroid.cells	ZFP280B	-0.13524	1.826966	-0.24379	0.80796	-5.45238	0.750446	0.822218

Erythroid.cells	VPS16	-0.05648	4.436967	-0.24363	0.808078	-5.96706	0.685952	0.764629
Erythroid.cells	IPMK	-0.05152	5.867681	-0.24358	0.808117	-6.29885	0.653086	0.734772
Erythroid.cells	WDR74	0.059258	4.042303	0.243277	0.808354	-5.92189	0.695321	0.773111
Erythroid.cells	PHF11D	0.177125	1.34229	0.243197	0.808416	-5.50309	0.763098	0.833408
Erythroid.cells	CUL9	-0.11885	2.579779	-0.2432	0.808417	-5.53784	0.731222	0.805221
Erythroid.cells	AKR1C12	-0.14786	0.756271	-0.24319	0.808424	-5.36009	0.778687	0.847087
Erythroid.cells	ZUP1	0.067089	5.168406	0.243187	0.808424	-6.12654	0.668937	0.749251
Erythroid.cells	GM29340	-0.18101	-0.6731	-0.2431	0.80849	-5.2111	0.818083	0.881417
Erythroid.cells	PFDN4	0.057283	5.046958	0.243035	0.808541	-6.17822	0.671731	0.75186
Erythroid.cells	EMD	-0.04233	6.102062	-0.24295	0.808608	-6.33701	0.647863	0.730105
Erythroid.cells	ERH	-0.04293	7.372533	-0.24261	0.808873	-6.70125	0.620387	0.704818
Erythroid.cells	1110035H1	-0.16118	1.216236	-0.24237	0.809054	-5.33762	0.766518	0.83655
Erythroid.cells	STARD13	0.179767	1.070842	0.242325	0.80909	-5.42461	0.770374	0.839936
Erythroid.cells	B3GALT2	-0.18401	0.771125	-0.24232	0.809096	-5.33973	0.778384	0.846959
Erythroid.cells	STAU2	0.108787	2.900653	0.242272	0.809131	-5.64748	0.723272	0.798274
Erythroid.cells	TBC1D32	-0.12949	2.141265	-0.24224	0.809154	-5.5451	0.742449	0.815317
Erythroid.cells	NHLRC3	-0.10136	3.16365	-0.24219	0.809197	-5.67272	0.716751	0.792478
Erythroid.cells	RAB5C	0.039699	6.358629	0.241952	0.809378	-6.44717	0.642363	0.725106
Erythroid.cells	MRPL28	0.044687	6.016074	0.241858	0.80945	-6.41284	0.649945	0.732088
Erythroid.cells	NUP188	-0.06929	4.513741	-0.24173	0.809548	-5.95096	0.684348	0.763379
Erythroid.cells	ILDR1	-0.12677	1.791756	-0.24155	0.809689	-5.71135	0.751648	0.823467
Erythroid.cells	APOL7E	0.127125	-0.10248	0.241458	0.809759	-5.51648	0.802429	0.867952
Erythroid.cells	RWDD4A	-0.06899	3.983066	-0.24135	0.809843	-5.92217	0.697019	0.774787
Erythroid.cells	ENPP2	0.110502	2.411456	0.241156	0.809993	-5.74917	0.735798	0.809506
Erythroid.cells	SMAP2	0.035802	6.821529	0.241132	0.810011	-6.54054	0.632381	0.716
Erythroid.cells	DNM2	0.033222	7.193868	0.240891	0.810197	-6.62315	0.624472	0.708651
Erythroid.cells	ZC3H11A	0.132655	2.01149	0.24071	0.810337	-5.45715	0.746139	0.818623
Erythroid.cells	CCSAP	0.109055	2.176115	0.240683	0.810358	-5.54841	0.741916	0.814889
Erythroid.cells	USPL1	-0.07847	3.533846	-0.24053	0.810474	-5.76507	0.708045	0.784691
Erythroid.cells	DGKD	-0.03539	7.687633	-0.24046	0.810528	-6.79044	0.614067	0.699005
Erythroid.cells	DIP2B	0.037068	7.880088	0.240074	0.810829	-6.75151	0.610208	0.695271
Erythroid.cells	CDKN3	0.106502	4.160534	0.23998	0.810901	-6.00939	0.693129	0.771123
Erythroid.cells	CCND3	-0.04972	7.994145	-0.23991	0.810952	-6.83376	0.607838	0.693067
Erythroid.cells	CCNQ	0.08533	3.060749	0.239578	0.811212	-5.71125	0.719975	0.795197
Erythroid.cells	TRP53BP2	-0.07237	3.957204	-0.2395	0.811271	-5.90301	0.698103	0.775643
Erythroid.cells	CEP290	-0.09888	2.468766	-0.23948	0.811291	-5.59667	0.734808	0.808451
Erythroid.cells	DSN1	-0.10433	2.622227	-0.23937	0.811376	-5.60188	0.730947	0.805039
Erythroid.cells	SMAD7	0.068634	5.164939	0.239219	0.81149	-6.09186	0.66978	0.750063
Erythroid.cells	RBM7	-0.04907	5.855126	-0.23889	0.811741	-6.31668	0.654224	0.735788
Erythroid.cells	GM26782	0.079374	2.991571	0.238787	0.811823	-5.66175	0.721868	0.796856
Erythroid.cells	GM13830	-0.22088	0.287554	-0.23872	0.811874	-5.18537	0.792427	0.859064
Erythroid.cells	PPP2CB	-0.04665	5.60171	-0.23868	0.811908	-6.16134	0.659932	0.741031
Erythroid.cells	EIF2AK4	0.069479	4.653928	0.23826	0.812231	-6.02743	0.68191	0.760845
Erythroid.cells	MTRF1	-0.14395	1.208712	-0.23821	0.812267	-5.3986	0.767807	0.83737
Erythroid.cells	RNF219	-0.1028	3.193241	-0.23816	0.812307	-5.64156	0.71704	0.792413
Erythroid.cells	GM43251	-0.22994	-0.97845	-0.23781	0.812578	-5.19965	0.828106	0.889897
Erythroid.cells	GM16845	0.11311	2.527304	0.237811	0.812578	-5.56348	0.733741	0.80734

Erythroid.cells	PXYLP1	0.115439	2.352535	0.237578	0.812758	-5.53432	0.738174	0.811293
Erythroid.cells	PLBD2	-0.06733	4.310294	-0.23756	0.812775	-5.91512	0.690069	0.76828
Erythroid.cells	NGRN	0.095961	3.173648	0.237516	0.812806	-5.68789	0.717586	0.792972
Erythroid.cells	STAT6	-0.05901	5.031546	-0.23749	0.812825	-6.03988	0.673184	0.753009
Erythroid.cells	TAB2	0.034711	6.780443	0.237246	0.813015	-6.53441	0.634026	0.717258
Erythroid.cells	ECSCR	0.170428	1.207762	0.237159	0.813082	-5.35671	0.7679	0.837597
Erythroid.cells	GIMAP6	0.065691	5.672059	0.237097	0.81313	-6.46226	0.658554	0.739762
Erythroid.cells	IGFBP6	-0.17983	-0.02144	-0.23699	0.813215	-5.36992	0.801184	0.866681
Erythroid.cells	TTC39B	-0.0651	4.653747	-0.23689	0.81329	-6.06891	0.681974	0.761034
Erythroid.cells	SERPINA11	0.113977	1.813558	0.23687	0.813305	-5.6487	0.752019	0.823612
Erythroid.cells	CXXC5	0.051133	5.11182	0.236782	0.813374	-6.23374	0.671332	0.751391
Erythroid.cells	CLTC	0.028339	7.90636	0.236709	0.81343	-6.75395	0.610102	0.695184
Erythroid.cells	TBC1D4	0.088223	4.706501	0.236686	0.813448	-6.14725	0.680739	0.759961
Erythroid.cells	RAB43	0.047345	6.948549	0.236599	0.813515	-6.60688	0.630392	0.714011
Erythroid.cells	PPME1	-0.05441	4.862972	-0.23653	0.813572	-6.11142	0.677091	0.756716
Erythroid.cells	SYNE3	0.114831	2.767053	0.23644	0.813639	-5.68894	0.727705	0.802172
Erythroid.cells	NRAP	-0.19494	-1.09404	-0.23615	0.813864	-5.128	0.831577	0.893104
Erythroid.cells	RNFT1	-0.05684	4.495375	-0.23606	0.813935	-6.00848	0.685826	0.764632
Erythroid.cells	STT3A	0.038478	6.169392	0.235838	0.814103	-6.36115	0.647629	0.729877
Erythroid.cells	SLC25A24	-0.09034	3.486183	-0.23538	0.814456	-5.7744	0.710376	0.786515
Erythroid.cells	HPF1	-0.06254	5.467524	-0.23526	0.814548	-6.23186	0.663644	0.744333
Erythroid.cells	CHTF18	0.140856	1.800347	0.234913	0.81482	-5.45795	0.752878	0.824363
Erythroid.cells	TOR3A	-0.11116	4.075925	-0.23491	0.814825	-5.83507	0.696129	0.773794
Erythroid.cells	SLC38A7	0.115937	2.59539	0.234802	0.814906	-5.56048	0.732524	0.806355
Erythroid.cells	ZFP61	0.156438	1.174198	0.234646	0.815026	-5.37356	0.769317	0.838898
Erythroid.cells	MCM8	0.140446	1.54016	0.234577	0.81508	-5.37554	0.759665	0.830416
Erythroid.cells	ENKUR	0.18089	0.339833	0.234573	0.815082	-5.24658	0.791795	0.858572
Erythroid.cells	FAM172A	-0.0345	7.557353	-0.2345	0.815135	-6.67267	0.617837	0.702391
Erythroid.cells	NCAPD3	0.063803	5.127768	0.234475	0.815159	-6.19635	0.671425	0.751543
Erythroid.cells	GM17268	0.179538	0.383225	0.23445	0.815178	-5.44264	0.79061	0.857555
Erythroid.cells	NPHS1	-0.19347	0.439647	-0.23427	0.815317	-5.22052	0.789138	0.856256
Erythroid.cells	GM17021	-0.21232	0.125797	-0.23404	0.815493	-5.17506	0.79784	0.8638
Erythroid.cells	SDHD	0.046096	5.858408	0.23369	0.815766	-6.42647	0.655113	0.736555
Erythroid.cells	SYDE1	-0.16831	0.308678	-0.23344	0.815956	-5.2124	0.793131	0.859559
Erythroid.cells	THYN1	-0.08278	3.271604	-0.23327	0.816093	-5.70252	0.716157	0.791662
Erythroid.cells	TMEM223	-0.05437	4.652686	-0.23274	0.816498	-6.0808	0.683202	0.761861
Erythroid.cells	ZFP831	-0.10069	3.063289	-0.23267	0.816556	-5.8118	0.721589	0.79635
Erythroid.cells	PIK3R2	0.108553	2.306399	0.23259	0.816617	-5.59023	0.740653	0.813328
Erythroid.cells	HDDC3	-0.15954	0.928077	-0.23234	0.816812	-5.29658	0.776728	0.845107
Erythroid.cells	MSRB2	0.171393	1.023579	0.232306	0.816837	-5.29973	0.774173	0.842868
Erythroid.cells	RXYLT1	0.061316	3.864731	0.232301	0.816841	-5.89849	0.70197	0.778833
Erythroid.cells	7-Mar	-0.03015	6.926226	-0.23214	0.816965	-6.54887	0.632029	0.715224
Erythroid.cells	EPB41L1	-0.17011	1.011227	-0.23207	0.817023	-5.37596	0.774542	0.843195
Erythroid.cells	ZFP938	0.123401	1.450658	0.231867	0.817177	-5.43397	0.762886	0.833004
Erythroid.cells	PRPF19	0.048876	5.053951	0.231852	0.817189	-6.1649	0.673899	0.753545
Erythroid.cells	ZFP82	-0.19023	0.121707	-0.23179	0.817233	-5.18931	0.798694	0.864326
Erythroid.cells	MTCP1	0.163059	0.737147	0.230958	0.817881	-5.30903	0.782426	0.849866

Erythroid.cells	COQ8B	-0.09412	3.190292	-0.23091	0.817919	-5.71266	0.718969	0.793837
Erythroid.cells	CREM	0.065401	6.0952	0.230663	0.81811	-6.42248	0.650792	0.732194
Erythroid.cells	APPBP2	-0.03989	6.248663	-0.23053	0.818215	-6.36104	0.647408	0.729103
Erythroid.cells	MYO1G	0.058938	5.563064	0.230269	0.818415	-6.2617	0.662839	0.743199
Erythroid.cells	NCKIPSD	-0.08631	2.952621	-0.23026	0.818424	-5.63952	0.72506	0.799209
Erythroid.cells	ITGA9	0.093703	5.58915	0.230089	0.818554	-6.18508	0.662247	0.742714
Erythroid.cells	ABI3	-0.10426	4.401261	-0.22999	0.81863	-5.88803	0.689809	0.767687
Erythroid.cells	PRAG1	-0.12282	1.923732	-0.22993	0.818678	-5.53222	0.75123	0.82247
Erythroid.cells	COX19	0.065361	4.496433	0.229809	0.818771	-6.04043	0.687557	0.765655
Erythroid.cells	KBTBD8	-0.17257	0.251382	-0.22967	0.818881	-5.21369	0.795857	0.861628
Erythroid.cells	YLPM1	0.044899	5.660547	0.22961	0.818925	-6.30312	0.660628	0.741294
Erythroid.cells	PTGER4	0.075259	4.681638	0.2295	0.81901	-6.077	0.683197	0.761792
Erythroid.cells	DNAH1	0.191016	-0.10849	0.229433	0.819063	-5.19549	0.805809	0.870319
Erythroid.cells	EIF3H	-0.03088	7.353901	-0.2294	0.819089	-6.69287	0.623431	0.707176
Erythroid.cells	PGPEP1	0.07387	3.461593	0.229341	0.819134	-5.80651	0.712465	0.788106
Erythroid.cells	KIF22	0.100718	4.043139	0.229283	0.819178	-6.01249	0.698353	0.775454
Erythroid.cells	COPS7B	0.081047	3.34463	0.229109	0.819314	-5.71621	0.71539	0.790695
Erythroid.cells	WDR60	0.162937	0.883885	0.229027	0.819377	-5.31514	0.778724	0.846688
Erythroid.cells	GNA15	0.108449	3.584316	0.228936	0.819447	-5.64529	0.709513	0.785459
Erythroid.cells	CTSD	-0.05225	7.133555	-0.22872	0.819618	-6.61087	0.628248	0.711587
Erythroid.cells	MED24	-0.07994	3.187102	-0.22865	0.819668	-5.69755	0.71935	0.794236
Erythroid.cells	ACSS1	0.074163	3.946222	0.228421	0.819846	-5.97815	0.700896	0.777673
Erythroid.cells	CYB5B	-0.04078	5.866247	-0.22826	0.819972	-6.32239	0.65621	0.737237
Erythroid.cells	TPD52L2	0.037594	5.433659	0.228152	0.820055	-6.29977	0.666015	0.746173
Erythroid.cells	GM14321	0.168223	-0.49943	0.228106	0.82009	-5.22311	0.817041	0.880002
Erythroid.cells	ZCCHC8	0.048629	5.108019	0.227929	0.820228	-6.11365	0.673553	0.752969
Erythroid.cells	PNRC2	0.048597	5.35165	0.227268	0.82074	-6.18539	0.668202	0.747933
Erythroid.cells	AAMDC	0.065382	3.299635	0.22725	0.820754	-5.7541	0.717027	0.791896
Erythroid.cells	PGRMC1	-0.04846	4.647619	-0.22719	0.820804	-6.22962	0.684548	0.762737
Erythroid.cells	ZBTB8OS	-0.05234	5.027468	-0.22716	0.820824	-6.14167	0.675677	0.754713
Erythroid.cells	CNBD2	0.087202	3.889219	0.226948	0.820988	-5.81758	0.702707	0.779029
Erythroid.cells	HRAS	0.046547	4.842094	0.226833	0.821078	-6.18106	0.680085	0.758684
Erythroid.cells	CEP104	-0.08141	3.105243	-0.22672	0.821165	-5.64843	0.721956	0.796268
Erythroid.cells	CHCHD1	0.043928	5.813268	0.226391	0.82142	-6.35641	0.657824	0.738556
Erythroid.cells	ZZEF1	-0.04307	5.867775	-0.22634	0.821457	-6.27593	0.656597	0.737438
Erythroid.cells	OTUD6B	-0.05187	4.301102	-0.22634	0.82146	-5.96616	0.692868	0.770313
Erythroid.cells	SUMF2	0.097721	2.684259	0.226298	0.821492	-5.59821	0.732513	0.805777
Erythroid.cells	MED14	-0.04807	6.321429	-0.2261	0.821648	-6.49243	0.646473	0.728258
Erythroid.cells	GATA4	-0.14365	1.621315	-0.22609	0.821652	-5.39033	0.759853	0.830021
Erythroid.cells	PIKFYVE	0.065359	4.426045	0.225943	0.821767	-5.93826	0.6899	0.767749
Erythroid.cells	HMGCR	-0.06193	4.712579	-0.2259	0.821802	-6.01204	0.683142	0.761651
Erythroid.cells	PLD2	-0.13469	1.042444	-0.22582	0.821859	-5.41643	0.775181	0.843543
Erythroid.cells	SLC18A2	0.101782	2.251616	0.225686	0.821967	-5.53213	0.743516	0.815701
Erythroid.cells	BAG5	-0.09046	3.637068	-0.22561	0.822023	-5.75949	0.708872	0.784859
Erythroid.cells	ENG	-0.09648	4.005662	-0.22552	0.822097	-5.93717	0.699942	0.776864
Erythroid.cells	DDX51	0.141491	1.37149	0.225322	0.822249	-5.35081	0.766429	0.835955
Erythroid.cells	SPIC	-0.17831	1.863637	-0.22529	0.822275	-5.56868	0.753529	0.824598

Erythroid.cells	KDM6A	-0.08924	6.914035	-0.22526	0.822294	-6.53062	0.633496	0.716472
Erythroid.cells	KLRB1A	-0.134	-0.08222	-0.22525	0.822306	-5.55551	0.80587	0.870393
Erythroid.cells	2510046G1	0.09158	2.620896	0.225162	0.822373	-5.62134	0.734114	0.807435
Erythroid.cells	GREB1L	0.125444	1.947775	0.225021	0.822482	-5.59147	0.751381	0.822694
Erythroid.cells	GM3448	-0.12652	1.9113	-0.22481	0.822644	-5.44238	0.752344	0.823596
Erythroid.cells	DMAP1	-0.08984	2.496634	-0.2248	0.822651	-5.61164	0.737315	0.810299
Erythroid.cells	ANKRD24	-0.13721	1.054272	-0.22473	0.822712	-5.34742	0.774919	0.843461
Erythroid.cells	NRDE2	0.091552	3.371854	0.224173	0.82314	-5.72981	0.715714	0.790886
Erythroid.cells	RTN4RL1	-0.11781	2.381813	-0.22411	0.823188	-5.7192	0.740541	0.812977
Erythroid.cells	ZFP688	-0.1179	1.695397	-0.22402	0.823259	-5.4794	0.758277	0.82867
Erythroid.cells	SLC11A2	-0.08075	3.918763	-0.22383	0.823404	-5.90719	0.702437	0.778982
Erythroid.cells	SCHIP1	0.134791	1.591487	0.223613	0.823574	-5.45975	0.76116	0.831123
Erythroid.cells	AKR1A1	-0.03268	7.188669	-0.22323	0.823872	-6.67158	0.62811	0.711291
Erythroid.cells	ATF7	-0.04198	6.314357	-0.22318	0.823911	-6.43776	0.647179	0.728816
Erythroid.cells	RLN3	0.123637	-0.84713	0.223173	0.823916	-5.43221	0.828144	0.889402
Erythroid.cells	GM49041	0.131988	1.334735	0.222893	0.824133	-5.48544	0.768191	0.837151
Erythroid.cells	KDM3B	0.036848	6.131762	0.222761	0.824235	-6.37049	0.65138	0.732545
Erythroid.cells	INTS5	-0.08479	2.704825	-0.22253	0.824416	-5.59559	0.732824	0.805871
Erythroid.cells	STXBP2	0.055876	5.31968	0.222509	0.824431	-6.07322	0.669812	0.749306
Erythroid.cells	GM48855	-0.10822	1.463573	-0.22224	0.82464	-5.4514	0.764986	0.834241
Erythroid.cells	SLC25A23	0.102578	2.25448	0.222089	0.824757	-5.58101	0.744404	0.816123
Erythroid.cells	ECHDC3	0.15389	0.833138	0.222068	0.824773	-5.43753	0.78181	0.849008
Erythroid.cells	GOSR1	0.06166	4.182554	0.221668	0.825083	-5.87817	0.696676	0.773605
Erythroid.cells	XCL1	0.098848	2.335401	0.221606	0.825131	-6.09803	0.742415	0.814404
Erythroid.cells	AKR1C20	-0.10456	2.839499	-0.22157	0.825162	-5.94149	0.729631	0.803065
Erythroid.cells	SLFN2	-0.06863	6.335056	-0.22153	0.825188	-6.39735	0.64708	0.728609
Erythroid.cells	GRM8	0.218093	1.381099	0.221406	0.825287	-5.27344	0.767253	0.836305
Erythroid.cells	GJB2	-0.10862	1.638244	-0.2214	0.825289	-5.68426	0.760477	0.83035
Erythroid.cells	PRORP	0.062131	3.853039	0.221173	0.825468	-5.88623	0.704708	0.780793
Erythroid.cells	TRAPPC8	0.038769	6.225642	0.221015	0.82559	-6.39553	0.649635	0.73095
Erythroid.cells	ZFP984	-0.06769	3.878402	-0.22073	0.825808	-5.78844	0.704263	0.780365
Erythroid.cells	UNK	-0.0709	4.287183	-0.2203	0.826147	-5.91833	0.694503	0.771622
Erythroid.cells	TRIM30B	0.125987	1.730639	0.220254	0.826181	-5.54256	0.758416	0.8285
Erythroid.cells	EGFL7	0.090568	3.872106	0.220242	0.82619	-5.91819	0.704486	0.780588
Erythroid.cells	PGM1	-0.05338	4.592397	-0.22023	0.826202	-6.18088	0.687258	0.765094
Erythroid.cells	LARS2	-0.06686	5.571824	-0.22017	0.826249	-6.56902	0.664539	0.744519
Erythroid.cells	GM30948	-0.20232	0.467484	-0.22	0.82638	-5.32098	0.792205	0.858121
Erythroid.cells	TTC8	0.139496	0.78875	0.219943	0.826422	-5.38008	0.783468	0.8505
Erythroid.cells	METAP1	0.045092	4.718677	0.219876	0.826474	-6.0702	0.684283	0.762435
Erythroid.cells	ZMYND15	0.177918	0.109664	0.219637	0.82666	-5.44125	0.802053	0.866693
Erythroid.cells	H2-T24	-0.14325	1.828462	-0.21963	0.826662	-5.44569	0.755862	0.826284
Erythroid.cells	N4BP1	-0.06629	5.4056	-0.21963	0.826665	-6.12746	0.668338	0.747998
Erythroid.cells	MGRN1	0.052274	5.682428	0.219264	0.82695	-6.26328	0.662196	0.742356
Erythroid.cells	NHEJ1	0.125262	3.867688	0.218909	0.827226	-5.77855	0.704846	0.780836
Erythroid.cells	SAMM50	0.046534	5.177169	0.218898	0.827234	-6.19274	0.673838	0.752886
Erythroid.cells	ETV5	0.110643	3.199847	0.218847	0.827274	-5.63158	0.721232	0.795483
Erythroid.cells	HSD17B12	0.044831	6.085483	0.218796	0.827313	-6.35835	0.653177	0.734085

Erythroid.cells	GM11579	-0.13743	-0.20636	-0.21842	0.827605	-5.4806	0.811361	0.874599
Erythroid.cells	ZFP281	-0.06301	4.496464	-0.21821	0.827771	-5.95881	0.689975	0.76751
Erythroid.cells	SLIT1	-0.15611	1.464859	-0.21818	0.827789	-5.35266	0.765899	0.835046
Erythroid.cells	SMG8	-0.08754	2.847118	-0.21812	0.827836	-5.62046	0.730259	0.80356
Erythroid.cells	TSC22D2	0.034901	6.616128	0.218001	0.827931	-6.50418	0.641617	0.723546
Erythroid.cells	ITCH	0.037751	7.100446	0.217916	0.827997	-6.59562	0.631078	0.713857
Erythroid.cells	MOB1A	-0.04085	5.970142	-0.21783	0.828064	-6.29332	0.655967	0.736701
Erythroid.cells	NTPCR	0.074568	3.847172	0.217721	0.828148	-5.82394	0.705574	0.781581
Erythroid.cells	BC035044	0.072922	5.146379	0.217491	0.828327	-6.22181	0.674812	0.753891
Erythroid.cells	CTNNAL1	-0.1293	1.924908	-0.21734	0.828445	-5.47907	0.753914	0.824624
Erythroid.cells	INTS10	0.077817	3.410048	0.217289	0.828484	-5.7201	0.716309	0.791255
Erythroid.cells	TMTC3	0.078583	2.514058	0.217102	0.828629	-5.62248	0.738757	0.811241
Erythroid.cells	KLHL2	-0.05116	5.570335	-0.2169	0.828784	-6.21661	0.665069	0.745089
Erythroid.cells	STAU1	0.040006	6.034578	0.21679	0.828872	-6.38692	0.65457	0.735521
Erythroid.cells	NCDN	0.092844	2.826122	0.216743	0.828908	-5.63076	0.730857	0.804229
Erythroid.cells	G430095P1	-0.10854	0.465518	-0.21666	0.82897	-5.54403	0.79285	0.858776
Erythroid.cells	SV2C	-0.16296	-0.28271	-0.21662	0.829003	-5.28338	0.8136	0.876821
Erythroid.cells	AMMECR1	-0.056	4.787165	-0.21641	0.829166	-6.1202	0.683185	0.761574
Erythroid.cells	GM5617	-0.07566	3.238502	-0.21637	0.829195	-5.80117	0.720552	0.795119
Erythroid.cells	PLPBP	-0.06304	4.145683	-0.21618	0.829347	-5.86627	0.69841	0.775296
Erythroid.cells	NHSL1	0.121687	1.728678	0.216056	0.829442	-5.47028	0.759033	0.829218
Erythroid.cells	PHKG2	-0.05746	4.528144	-0.21598	0.8295	-5.95379	0.68929	0.767086
Erythroid.cells	UBR2	-0.03521	6.362518	-0.21585	0.829604	-6.42743	0.64726	0.728895
Erythroid.cells	ANKRD28	-0.04715	5.835458	-0.21572	0.829702	-6.35913	0.659052	0.739714
Erythroid.cells	BBOF1	-0.13267	1.302277	-0.21568	0.829738	-5.36433	0.770281	0.839183
Erythroid.cells	THEM4	0.102624	2.270582	0.215406	0.829947	-5.65924	0.744983	0.816935
Erythroid.cells	EPHA2	-0.10013	2.408212	-0.21529	0.830036	-5.56183	0.741457	0.813814
Erythroid.cells	GM42418	0.064703	11.10924	0.215273	0.83005	-7.44413	0.550658	0.638823
Erythroid.cells	RBM19	-0.08243	2.925866	-0.2152	0.830105	-5.68788	0.72835	0.80218
Erythroid.cells	MTFR2	0.092032	3.615592	0.215167	0.830133	-5.82267	0.71126	0.786931
Erythroid.cells	TACO1	-0.09375	3.738306	-0.21514	0.830151	-5.86618	0.708264	0.784248
Erythroid.cells	RNF157	0.071458	5.375061	0.215102	0.830183	-6.20564	0.669538	0.749318
Erythroid.cells	VPS39	0.075678	3.331542	0.215003	0.830261	-5.70753	0.718247	0.793176
Erythroid.cells	TNFAIP1	-0.07752	3.775763	-0.21493	0.830314	-5.83774	0.707352	0.783431
Erythroid.cells	MBIP	0.074093	3.658726	0.214931	0.830316	-5.80111	0.710206	0.785987
Erythroid.cells	DNHD1	0.149671	1.01891	0.214912	0.830331	-5.30859	0.777849	0.845857
Erythroid.cells	LY96	0.065254	4.287348	0.214755	0.830453	-5.95844	0.695018	0.772394
Erythroid.cells	TAF6	-0.07283	3.851886	-0.21467	0.830515	-5.85411	0.705502	0.781838
Erythroid.cells	CTSE	0.045647	5.492809	0.21434	0.830775	-6.44566	0.666839	0.74694
Erythroid.cells	TATDN3	0.089543	2.490952	0.214319	0.830792	-5.63611	0.739346	0.812024
Erythroid.cells	TREM2	0.175965	0.639882	0.214144	0.830928	-5.50358	0.788092	0.854927
Erythroid.cells	MRPS31	-0.06079	3.653241	-0.21414	0.83093	-5.851	0.71034	0.786215
Erythroid.cells	AURKB	0.090931	4.237652	0.214019	0.831025	-6.04742	0.696206	0.773531
Erythroid.cells	MCOLN2	-0.08158	3.161941	-0.21396	0.831067	-5.69868	0.722453	0.797038
Erythroid.cells	GM14221	-0.1381	1.910062	-0.21375	0.831237	-5.58076	0.7543	0.825299
Erythroid.cells	HMGN5	-0.0505	4.876393	-0.21367	0.831293	-6.1804	0.681095	0.759917
Erythroid.cells	CYP2C23	-0.14193	0.860959	-0.2136	0.83135	-5.4035	0.782101	0.849717

Erythroid.cells	BAG6	-0.04633	5.438014	-0.21359	0.831357	-6.30861	0.668094	0.748129
Erythroid.cells	FAM111A	-0.04931	5.742953	-0.21352	0.831412	-6.26687	0.661145	0.741805
Erythroid.cells	POLR1E	-0.10881	2.067812	-0.21349	0.831433	-5.51807	0.750208	0.821689
Erythroid.cells	CLUH	0.073669	3.417508	0.213301	0.831583	-5.80599	0.716125	0.791439
Erythroid.cells	SH3D21	-0.12119	1.99564	-0.21312	0.831721	-5.49758	0.752078	0.823383
Erythroid.cells	NCALD	0.109012	2.377149	0.212929	0.831872	-5.59598	0.742251	0.814694
Erythroid.cells	CD14	0.121745	4.260683	0.212901	0.831894	-6.11404	0.695655	0.773096
Erythroid.cells	PARP6	0.090133	2.991541	0.212893	0.831901	-5.65125	0.726705	0.800889
Erythroid.cells	KDSR	-0.06435	3.963038	-0.21289	0.831903	-5.85113	0.70281	0.779527
Erythroid.cells	GM13427	-0.1045	2.062778	-0.21286	0.831929	-5.51037	0.750339	0.82185
Erythroid.cells	NAA16	-0.04169	5.024992	-0.21279	0.831977	-6.15043	0.677629	0.756823
Erythroid.cells	ADAMTS1C	-0.07849	3.015513	-0.21261	0.832118	-5.71364	0.726105	0.800357
Erythroid.cells	NOP58	0.048319	5.838554	0.212566	0.832155	-6.36883	0.658982	0.739874
Erythroid.cells	WDR6	0.077686	3.277321	0.212535	0.832179	-5.77329	0.719589	0.794549
Erythroid.cells	DSCAML1	0.164281	-0.596	0.212527	0.832185	-5.26238	0.822451	0.884831
Erythroid.cells	TMEM70	0.06414	3.930301	0.212443	0.83225	-5.93005	0.703602	0.780241
Erythroid.cells	ANAPC4	-0.05024	4.541254	-0.21244	0.83225	-6.00715	0.68898	0.767084
Erythroid.cells	CDKL2	0.137459	1.163972	0.212408	0.832278	-5.39654	0.773965	0.84265
Erythroid.cells	TNRC6B	-0.03143	8.234075	-0.21233	0.832338	-6.86305	0.607169	0.692167
Erythroid.cells	NT5C	0.050416	5.114948	0.212309	0.832354	-6.17076	0.67554	0.75496
Erythroid.cells	ARL2	-0.07903	3.356926	-0.21203	0.832568	-5.75305	0.71771	0.792925
Erythroid.cells	L1CAM	0.104087	3.644556	0.211954	0.83263	-5.7076	0.710641	0.786606
Erythroid.cells	CTSO	-0.07151	4.26707	-0.2119	0.83267	-5.91491	0.69559	0.773094
Erythroid.cells	GM3435	-0.1795	0.205181	-0.2117	0.832827	-5.23748	0.80019	0.865559
Erythroid.cells	KNG1	-0.05948	6.039457	-0.21154	0.832951	-6.70946	0.654609	0.735909
Erythroid.cells	KLRA2	-0.12735	2.524963	-0.21149	0.832992	-5.79489	0.738647	0.811533
Erythroid.cells	CTBS	0.078355	2.934998	0.211433	0.833036	-5.66759	0.728286	0.802329
Erythroid.cells	UBE2V2	0.039543	5.614121	0.21114	0.833264	-6.30327	0.664281	0.744772
Erythroid.cells	KLHL42	-0.10037	2.445253	-0.21111	0.833289	-5.47956	0.740745	0.813439
Erythroid.cells	HIST1H2AE	0.106715	4.491316	0.211066	0.833322	-6.2166	0.690381	0.768425
Erythroid.cells	ARFGAP3	-0.07308	3.55174	-0.2104	0.83384	-5.77561	0.713433	0.788888
Erythroid.cells	HAUS7	0.075773	2.992661	0.209924	0.83421	-5.68615	0.727471	0.801371
Erythroid.cells	SCNM1	-0.06419	3.621272	-0.20987	0.834253	-5.83817	0.711899	0.787476
Erythroid.cells	TIGAR	-0.11113	1.851972	-0.20976	0.834336	-5.46292	0.756639	0.82723
Erythroid.cells	ABCB6	0.115319	1.101522	0.209719	0.83437	-5.35728	0.776483	0.844671
Erythroid.cells	SCAPER	-0.04398	6.110801	-0.20969	0.834393	-6.35193	0.653577	0.734786
Erythroid.cells	LIX1	-0.15787	0.723268	-0.20955	0.834501	-5.28616	0.786721	0.85365
Erythroid.cells	CSF1R	0.08134	5.549681	0.20937	0.834641	-6.39136	0.666321	0.746449
Erythroid.cells	TASOR2	0.047089	5.373883	0.209263	0.834724	-6.20853	0.67035	0.750132
Erythroid.cells	RPGRIP1L	0.117858	1.613197	0.20919	0.834781	-5.38033	0.762956	0.832898
Erythroid.cells	DNM1L	-0.04127	5.639142	-0.20905	0.834892	-6.28337	0.66428	0.744629
Erythroid.cells	ARL14EP	-0.07199	3.524764	-0.209	0.834933	-5.77642	0.714324	0.789771
Erythroid.cells	ERC2	0.188958	0.862583	0.208969	0.834953	-5.44239	0.782975	0.850452
Erythroid.cells	ZMIZ1OS1	-0.17348	0.352543	-0.20877	0.83511	-5.29759	0.796919	0.862584
Erythroid.cells	GM26631	0.126937	1.219353	0.208686	0.835173	-5.38266	0.77343	0.842064
Erythroid.cells	WDR5B	0.157931	0.670974	0.208419	0.835381	-5.27335	0.788207	0.855038
Erythroid.cells	ADGRF5	0.127941	2.598167	0.208356	0.83543	-5.62761	0.737518	0.810451

Erythroid.cells	EMID1	0.071861	3.280299	0.20835	0.835435	-5.82925	0.720393	0.795216
Erythroid.cells	CEBPE	0.143644	0.2028	0.208317	0.835461	-5.36697	0.80105	0.866248
Erythroid.cells	ARL5B	-0.05952	5.460574	-0.20828	0.835486	-6.19693	0.668391	0.748413
Erythroid.cells	CITED2	0.046818	6.149916	0.208111	0.835621	-6.40578	0.652835	0.734229
Erythroid.cells	UVRAG	0.03683	8.569242	0.2077	0.835941	-6.84938	0.601231	0.686528
Erythroid.cells	NUDT6	-0.09973	2.183509	-0.20729	0.83626	-5.5331	0.748637	0.820107
Erythroid.cells	TLR2	0.081769	3.875493	0.207044	0.836451	-5.95773	0.706344	0.782407
Erythroid.cells	PIFO	-0.13442	1.246923	-0.20699	0.836495	-5.48773	0.773301	0.84175
Erythroid.cells	RECK	0.153258	1.480341	0.206633	0.836772	-5.42497	0.767251	0.836331
Erythroid.cells	CUX2	-0.12626	1.039079	-0.20644	0.836922	-5.39503	0.779022	0.846649
Erythroid.cells	ACOX3	-0.05883	4.372027	-0.20644	0.836925	-5.94335	0.694526	0.77169
Erythroid.cells	GM44284	0.167967	-0.05378	0.206376	0.836972	-5.22941	0.808974	0.872754
Erythroid.cells	5430414B1	-0.17495	0.449396	-0.20632	0.837014	-5.233	0.795041	0.860675
Erythroid.cells	A430073D2	0.110251	1.815155	0.206185	0.83712	-5.47456	0.758474	0.82865
Erythroid.cells	RFFL	0.05141	5.915131	0.206091	0.837194	-6.32206	0.658734	0.739313
Erythroid.cells	TFAP4	0.100659	2.748617	0.205883	0.837356	-5.59854	0.734486	0.80743
Erythroid.cells	PLA2G15	0.086697	3.738633	0.205823	0.837403	-5.79375	0.709875	0.785492
Erythroid.cells	HUWE1	0.033476	7.155414	0.205781	0.837435	-6.63974	0.631381	0.714221
Erythroid.cells	GOPC	-0.04599	4.545466	-0.20562	0.837557	-6.03096	0.690497	0.768087
Erythroid.cells	SLU7	0.046079	4.955244	0.205388	0.837741	-6.05124	0.680854	0.759501
Erythroid.cells	EPOP	-0.12327	1.472765	-0.20534	0.837779	-5.40665	0.767568	0.836782
Erythroid.cells	KLHL25	0.072471	2.493825	0.205268	0.837835	-5.72254	0.741015	0.813366
Erythroid.cells	ZFP366	0.108365	1.840557	0.205252	0.837847	-5.81187	0.757892	0.828276
Erythroid.cells	UCP1	0.140935	-1.00774	0.204853	0.838158	-5.22085	0.836435	0.896554
Erythroid.cells	IFI213	0.160561	3.327221	0.204534	0.838406	-5.7471	0.720406	0.794946
Erythroid.cells	COLGALT1	-0.04244	5.889717	-0.20439	0.838517	-6.26514	0.659738	0.740219
Erythroid.cells	LAMTOR4	0.043142	6.205335	0.204263	0.838618	-6.39146	0.652666	0.733743
Erythroid.cells	ADGRE1	0.104665	4.711233	0.203896	0.838903	-6.08036	0.687148	0.764923
Erythroid.cells	SH3PXD2A	0.055821	5.753145	0.203781	0.838993	-6.24128	0.663016	0.743054
Erythroid.cells	TMEM126A	-0.03355	5.857631	-0.20373	0.839031	-6.38911	0.660646	0.740895
Erythroid.cells	BOD1L	0.035956	5.740505	0.203482	0.839226	-6.26423	0.663363	0.743315
Erythroid.cells	TRP53RKA	0.087157	2.472703	0.203374	0.83931	-5.54728	0.742231	0.814088
Erythroid.cells	FAM171B	-0.18415	0.217464	-0.20327	0.839391	-5.25819	0.802286	0.866739
Erythroid.cells	CNN2	-0.0457	6.538205	-0.20323	0.839421	-6.54883	0.645484	0.726989
Erythroid.cells	9330136K2	-0.11105	1.883233	-0.20301	0.839595	-5.5106	0.757468	0.827542
Erythroid.cells	EFNA2	-0.17308	1.10216	-0.203	0.839598	-5.36509	0.778156	0.845703
Erythroid.cells	STK10	-0.03231	7.315855	-0.2029	0.83968	-6.66833	0.628547	0.71142
Erythroid.cells	ILF2	-0.03919	5.763945	-0.20284	0.839728	-6.3546	0.662831	0.74283
Erythroid.cells	CCR7	-0.07191	4.535564	-0.20279	0.839764	-6.3241	0.691369	0.768673
Erythroid.cells	CXCL3	-0.21809	-0.07498	-0.20263	0.839891	-5.27777	0.810428	0.873812
Erythroid.cells	HTT	-0.04446	5.362069	-0.20253	0.839966	-6.17395	0.672028	0.751196
Erythroid.cells	RABGGTA	0.073538	2.703471	0.202464	0.840019	-5.6644	0.736353	0.808892
Erythroid.cells	GM43256	-0.16762	0.309245	-0.20242	0.84005	-5.18776	0.799747	0.864547
Erythroid.cells	TBRG1	0.044852	5.618457	0.202365	0.840096	-6.35572	0.666145	0.745866
Erythroid.cells	GM48302	0.123922	1.63727	0.202271	0.840169	-5.48757	0.763923	0.833243
Erythroid.cells	2310009A0	-0.05406	4.692253	-0.20218	0.840243	-6.01371	0.687663	0.765348
Erythroid.cells	PBLD2	0.117184	0.872415	0.202037	0.840352	-5.39232	0.78439	0.851141

Erythroid.cells	ECI2	-0.05219	4.557161	-0.20191	0.840448	-6.13801	0.690913	0.768232
Erythroid.cells	BTBD6	0.118225	1.542046	0.201631	0.840668	-5.45335	0.766614	0.835451
Erythroid.cells	TRMT61B	0.089406	4.178074	0.201569	0.840717	-5.9383	0.700079	0.776381
Erythroid.cells	ARF1	-0.02308	8.02586	-0.2012	0.841002	-6.8029	0.613711	0.697541
Erythroid.cells	GIMAP8	-0.08561	2.465426	-0.20118	0.841019	-5.82991	0.742678	0.814301
Erythroid.cells	ZFP532	0.142378	1.223667	0.201171	0.841027	-5.40791	0.775171	0.842903
Erythroid.cells	TRMT1L	0.04278	4.566129	0.201019	0.841146	-6.01708	0.690925	0.768128
Erythroid.cells	SARS2	-0.07055	2.675724	-0.2007	0.841392	-5.6472	0.737511	0.809644
Erythroid.cells	ZFP719	0.091545	2.119349	0.200279	0.841722	-5.47655	0.752021	0.822371
Erythroid.cells	SPP2	0.085353	3.372982	0.200018	0.841926	-6.08962	0.720259	0.794278
Erythroid.cells	KCNN4	-0.07198	3.850661	-0.2	0.841938	-6.06864	0.708517	0.783789
Erythroid.cells	GM16090	0.19802	-0.96132	0.199973	0.841961	-5.17162	0.836418	0.895926
Erythroid.cells	PAOX	-0.08667	2.987177	-0.19968	0.842189	-5.67089	0.729965	0.802877
Erythroid.cells	MERTK	-0.10091	3.774359	-0.19962	0.84224	-5.93461	0.710453	0.785482
Erythroid.cells	HNRNPR	-0.02803	6.29021	-0.19959	0.842261	-6.47851	0.651678	0.732351
Erythroid.cells	REV3L	0.031258	6.713202	0.199436	0.842379	-6.57598	0.642321	0.723777
Erythroid.cells	CD163L1	0.152619	0.60908	0.199385	0.842419	-5.37342	0.792363	0.857747
Erythroid.cells	WHAMM	-0.05401	4.147659	-0.19924	0.84253	-5.99689	0.701401	0.777363
Erythroid.cells	ATG7	-0.04923	5.786606	-0.19907	0.842668	-6.24672	0.663028	0.742692
Erythroid.cells	SAP30	-0.05085	5.143228	-0.19894	0.84277	-6.15819	0.677821	0.756162
Erythroid.cells	PTK7	-0.18674	1.073787	-0.1989	0.842795	-5.28214	0.779756	0.846803
Erythroid.cells	HIST2H3B	0.143983	1.047683	0.19871	0.842946	-5.3299	0.780458	0.847443
Erythroid.cells	SIRT5	-0.10743	1.958147	-0.19863	0.843012	-5.45174	0.756327	0.826279
Erythroid.cells	C130050O1	-0.10572	1.379067	-0.19857	0.843058	-5.54764	0.771585	0.839705
Erythroid.cells	STK38L	0.056972	4.003603	0.198467	0.843135	-5.88757	0.704884	0.780599
Erythroid.cells	LYAR	-0.05446	4.937131	-0.19845	0.843151	-6.14455	0.682633	0.760578
Erythroid.cells	RAB8A	0.040601	5.454242	0.198403	0.843185	-6.19983	0.670627	0.749706
Erythroid.cells	TWF1	-0.03817	5.464598	-0.19839	0.843194	-6.23398	0.670389	0.74949
Erythroid.cells	MCC	0.103532	2.092188	0.198301	0.843265	-5.60841	0.75284	0.823246
Erythroid.cells	KLRB1B	0.081841	1.981821	0.198006	0.843495	-6.06967	0.755839	0.825851
Erythroid.cells	THNSL1	0.138305	1.077135	0.197816	0.843643	-5.34626	0.779799	0.846885
Erythroid.cells	VPS37A	-0.05078	5.2037	-0.19774	0.843701	-6.11806	0.676532	0.755064
Erythroid.cells	SLC25A21	0.106562	1.867089	0.197707	0.843728	-5.90051	0.758836	0.828534
Erythroid.cells	TMBIM1	0.080418	2.851295	0.197563	0.843841	-5.72628	0.733527	0.806202
Erythroid.cells	GM29114	0.125626	-1.28439	0.197551	0.84385	-5.26467	0.846048	0.904322
Erythroid.cells	PLCD3	-0.13634	1.122441	-0.19736	0.843996	-5.27037	0.778581	0.845963
Erythroid.cells	NOL8	-0.06483	4.078757	-0.19712	0.844187	-5.87424	0.703185	0.779302
Erythroid.cells	SSU72	0.02774	6.693565	0.197059	0.844234	-6.49812	0.642863	0.724599
Erythroid.cells	EPHX1	0.094249	3.053436	0.196956	0.844314	-5.67894	0.728438	0.801888
Erythroid.cells	TOMM40L	-0.08553	1.733558	-0.19689	0.844367	-5.4791	0.762338	0.83187
Erythroid.cells	CYP2C68	0.083031	2.791797	0.196517	0.844657	-5.95449	0.735031	0.807815
Erythroid.cells	POLM	0.060348	3.245007	0.196374	0.844768	-5.80554	0.723649	0.797721
Erythroid.cells	CASP7	-0.06458	4.140272	-0.19625	0.844864	-5.91897	0.7017	0.778092
Erythroid.cells	MTERF2	0.104947	2.099595	0.196186	0.844915	-5.49011	0.752776	0.823546
Erythroid.cells	PSMD13	-0.03254	6.203145	-0.19616	0.844937	-6.41734	0.653745	0.734665
Erythroid.cells	KDM2B	-0.04929	7.133876	-0.19613	0.844958	-6.76913	0.633257	0.715874
Erythroid.cells	CDS2	-0.05109	4.708784	-0.19606	0.845017	-5.99351	0.688125	0.765875

Erythroid.cells	GRAMD1B	0.062842	5.536445	0.196039	0.845029	-6.18595	0.668854	0.748429
Erythroid.cells	PPP1R13B	-0.04635	5.30235	-0.19604	0.845029	-6.2114	0.674246	0.753323
Erythroid.cells	ZFP677	-0.13045	0.788621	-0.19589	0.845144	-5.28703	0.787603	0.854105
Erythroid.cells	GM50071	-0.13623	0.514178	-0.19587	0.845161	-5.24981	0.795099	0.860639
Erythroid.cells	SYTL1	0.122213	1.59739	0.195861	0.845169	-5.4017	0.765927	0.835125
Erythroid.cells	NRXN2	-0.16518	2.095803	-0.19583	0.845191	-5.40843	0.752875	0.823633
Erythroid.cells	CISD3	-0.0733	3.000972	-0.19569	0.845302	-5.80975	0.729755	0.803157
Erythroid.cells	CDCA8	0.080222	5.109425	0.195671	0.845317	-6.27562	0.678724	0.757381
Erythroid.cells	CCDC124	-0.03533	5.595782	-0.19567	0.84532	-6.27306	0.667494	0.747195
Erythroid.cells	TGTP2	-0.18729	0.773598	-0.19553	0.845427	-5.36228	0.78804	0.854475
Erythroid.cells	CEP57	-0.04389	5.139583	-0.19545	0.845492	-6.16193	0.678047	0.756757
Erythroid.cells	FKBP2	-0.04467	5.611404	-0.19521	0.845675	-6.29797	0.667203	0.746893
Erythroid.cells	TRAPPC13	0.057265	3.808587	0.195197	0.845687	-5.83767	0.70982	0.785333
Erythroid.cells	RCN2	-0.05266	5.075901	-0.19511	0.845758	-6.11756	0.679574	0.758144
Erythroid.cells	GM26724	-0.11302	1.517501	-0.19496	0.845869	-5.45409	0.768154	0.837046
Erythroid.cells	FBXL8	-0.10561	1.99347	-0.19458	0.84617	-5.47638	0.755777	0.826141
Erythroid.cells	0610040F0	0.124783	0.269406	0.194526	0.846211	-5.29116	0.802103	0.866676
Erythroid.cells	ZFP563	-0.10998	0.800774	-0.1945	0.846227	-5.31613	0.787524	0.853982
Erythroid.cells	CHRNB1	-0.14687	0.775917	-0.19426	0.846422	-5.24582	0.788314	0.854609
Erythroid.cells	GALNT11	0.055924	4.120023	0.19405	0.846582	-5.88555	0.702515	0.778776
Erythroid.cells	GM14966	-0.07496	2.847703	-0.19398	0.846638	-5.61801	0.733958	0.806842
Erythroid.cells	ARIH1	0.029084	8.728541	0.193944	0.846665	-6.92662	0.600004	0.685014
Erythroid.cells	2-Mar	0.032763	5.868752	0.193894	0.846704	-6.46153	0.661585	0.741771
Erythroid.cells	WNT4	0.102776	1.526488	0.193691	0.846862	-5.49906	0.768239	0.837041
Erythroid.cells	9830107B1	-0.12029	-0.62676	-0.19359	0.846939	-5.34651	0.827525	0.888632
Erythroid.cells	TSPYL4	-0.11577	0.723815	-0.19324	0.847214	-5.31725	0.789949	0.855957
Erythroid.cells	GSDME	-0.06824	4.386903	-0.19323	0.847218	-5.94737	0.69629	0.773052
Erythroid.cells	TM7SF3	0.056482	3.924878	0.193046	0.847366	-5.90141	0.707502	0.783057
Erythroid.cells	RASA1	0.037205	6.030636	0.19287	0.847503	-6.34112	0.658216	0.738511
Erythroid.cells	ZFYVE21	0.078044	2.542215	0.192456	0.847826	-5.70095	0.742134	0.813852
Erythroid.cells	PHB2	-0.02941	6.570723	-0.19239	0.847876	-6.55132	0.646228	0.727548
Erythroid.cells	TBCCD1	-0.08607	2.589728	-0.19229	0.847955	-5.58971	0.74092	0.812854
Erythroid.cells	CPLANE1	0.056174	4.78519	0.192178	0.848043	-6.08976	0.68702	0.764695
Erythroid.cells	GM14455	0.110429	1.315653	0.1921	0.848105	-5.37973	0.774195	0.842183
Erythroid.cells	SPEN	0.036408	5.922479	0.192068	0.848129	-6.29374	0.660733	0.740867
Erythroid.cells	GDPD3	0.10168	1.793559	0.192038	0.848153	-5.61302	0.761537	0.831076
Erythroid.cells	GOLGA1	0.06337	3.427325	0.192024	0.848164	-5.74817	0.719853	0.794155
Erythroid.cells	POMK	0.130397	1.068205	0.191833	0.848313	-5.34696	0.780862	0.84804
Erythroid.cells	FHL2	-0.14233	0.017257	-0.1918	0.848337	-5.45099	0.809712	0.873159
Erythroid.cells	HNRNPH1	0.034685	6.424984	0.191446	0.848615	-6.43992	0.6496	0.730732
Erythroid.cells	POLQ	-0.07896	3.166551	-0.19134	0.848696	-5.71915	0.726502	0.800133
Erythroid.cells	MPPED2	0.168257	-0.32287	0.191335	0.848702	-5.2556	0.819425	0.881604
Erythroid.cells	PDK2	0.087687	2.10287	0.191232	0.848782	-5.61594	0.753629	0.824166
Erythroid.cells	GPS1	-0.04222	4.905295	-0.19112	0.848872	-6.14234	0.684363	0.76235
Erythroid.cells	INVS	0.085335	2.809082	0.190466	0.849381	-5.63539	0.735788	0.808348
Erythroid.cells	CIB2	0.089964	2.152566	0.190201	0.849588	-5.55328	0.752624	0.823244
Erythroid.cells	RPUSD1	-0.11119	1.47218	-0.19019	0.849593	-5.3744	0.770492	0.838964

Erythroid.cells	INKA1	0.089325	2.703982	0.190168	0.849614	-5.55397	0.738456	0.810715
Erythroid.cells	STIM2	-0.04147	6.210089	-0.19012	0.849652	-6.36075	0.654652	0.735336
Erythroid.cells	6330409D2	0.141828	0.333222	0.190117	0.849653	-5.26824	0.801382	0.865931
Erythroid.cells	SETBP1	0.072017	4.621756	0.190062	0.849696	-6.25048	0.691306	0.7686
Erythroid.cells	TJP2	-0.05648	3.845594	-0.19	0.849742	-6.04282	0.709998	0.785398
Erythroid.cells	EXOSC5	-0.04048	5.376885	-0.18975	0.849938	-6.21423	0.673666	0.752638
Erythroid.cells	GPR182	0.094902	2.557152	0.189743	0.849946	-5.64377	0.742256	0.814081
Erythroid.cells	A930001M	-0.06561	3.967402	-0.18939	0.850221	-5.82427	0.707252	0.782802
Erythroid.cells	TMBIM4	0.031184	6.749974	0.189253	0.850328	-6.58019	0.642896	0.724479
Erythroid.cells	GM42722	0.096148	2.335621	0.188885	0.850616	-5.64775	0.748347	0.819309
Erythroid.cells	KLHL11	0.085221	2.776613	0.188649	0.8508	-5.59505	0.73716	0.80933
Erythroid.cells	GPC5	-0.15999	0.598275	-0.18854	0.850888	-5.32281	0.79469	0.859848
Erythroid.cells	HERC6	0.067069	4.719873	0.187851	0.851424	-6.20521	0.689852	0.766869
Erythroid.cells	TULP3	-0.09944	1.974251	-0.18781	0.851455	-5.47776	0.758223	0.82775
Erythroid.cells	RANGRF	-0.12879	1.262196	-0.18762	0.851603	-5.37434	0.777105	0.844341
Erythroid.cells	TAF6L	0.078309	2.800016	0.18759	0.851628	-5.59349	0.736975	0.809015
Erythroid.cells	GM4285	0.093582	1.640385	0.187409	0.851769	-5.48424	0.767085	0.835601
Erythroid.cells	ABCC10	-0.12481	0.65899	-0.18733	0.85183	-5.24093	0.793507	0.858729
Erythroid.cells	IDH3B	-0.03733	5.850654	-0.18723	0.851907	-6.42508	0.663677	0.743251
Erythroid.cells	S100A6	-0.0632	7.580063	-0.18701	0.852078	-6.94445	0.625578	0.70837
Erythroid.cells	SEH1L	-0.03419	5.334868	-0.18692	0.852153	-6.22571	0.675547	0.754073
Erythroid.cells	HES6	0.054108	4.105884	0.186915	0.852155	-5.91907	0.704671	0.780318
Erythroid.cells	MRM1	-0.08651	2.067052	-0.18645	0.852518	-5.5072	0.756009	0.825948
Erythroid.cells	KIF15	-0.07716	4.90741	-0.18643	0.852537	-6.18433	0.685613	0.763182
Erythroid.cells	1110046J0	-0.15938	0.031359	-0.18618	0.852733	-5.17256	0.811026	0.874035
Erythroid.cells	STAT3	-0.03111	7.950149	-0.18615	0.852756	-6.806	0.617801	0.701223
Erythroid.cells	CKLF	0.052143	4.804772	0.186121	0.852776	-5.9742	0.688033	0.765392
Erythroid.cells	FAM193B	0.051219	4.050857	0.186058	0.852826	-5.94487	0.70609	0.781625
Erythroid.cells	HCST	0.046276	5.375869	0.186052	0.85283	-6.39147	0.674678	0.75332
Erythroid.cells	PSMC5	-0.03028	6.00886	-0.18605	0.852833	-6.43146	0.660196	0.74016
Erythroid.cells	RELA	-0.04353	5.114793	-0.18583	0.853003	-6.14662	0.680749	0.758862
Erythroid.cells	HIST1H1A	0.094688	3.773403	0.185806	0.853023	-6.08964	0.71286	0.787733
Erythroid.cells	NT5DC2	-0.08222	2.432435	-0.18579	0.853039	-5.67467	0.746547	0.817678
Erythroid.cells	WDFY1	0.058766	4.332443	0.185273	0.85344	-5.90407	0.699559	0.775657
Erythroid.cells	ARHGAP45	0.033102	6.710119	0.185072	0.853596	-6.56864	0.64484	0.725977
Erythroid.cells	RNF138	0.031147	5.857254	0.184997	0.853655	-6.38265	0.663948	0.743421
Erythroid.cells	HSPB6	0.135878	0.40248	0.184872	0.853753	-5.31187	0.801103	0.865245
Erythroid.cells	ZFP609	0.034367	6.067152	0.184676	0.853906	-6.37847	0.659274	0.739113
Erythroid.cells	DENND4C	0.042104	5.068456	0.18445	0.854083	-6.20176	0.682265	0.759952
Erythroid.cells	CPN1	0.090938	1.600653	0.184376	0.854141	-5.63457	0.768754	0.83693
Erythroid.cells	GPR146	0.0652	3.278936	0.184367	0.854148	-5.75715	0.725553	0.798763
Erythroid.cells	WDR48	-0.0444	4.298768	-0.18419	0.854284	-5.98457	0.700596	0.77643
Erythroid.cells	PSMC2	-0.03785	5.362149	-0.18394	0.854479	-6.25326	0.675573	0.753812
Erythroid.cells	ENC1	-0.07215	3.181392	-0.18357	0.854772	-5.70735	0.728247	0.800988
Erythroid.cells	EIF4G3	-0.02392	7.91677	-0.18355	0.854786	-6.76871	0.619112	0.702068
Erythroid.cells	CLSPN	0.063805	4.411842	0.183464	0.854854	-6.14131	0.698067	0.774023
Erythroid.cells	NCBP2	-0.04619	4.599992	-0.18344	0.854871	-6.01888	0.693569	0.769983

Erythroid.cells	PABPN1	-0.02451	6.640276	-0.18322	0.855048	-6.55477	0.646776	0.727515
Erythroid.cells	EPC1	-0.03103	6.811099	-0.18312	0.855125	-6.57048	0.643007	0.724093
Erythroid.cells	PDZK1	0.12192	0.933701	0.182924	0.855277	-5.47335	0.787002	0.852765
Erythroid.cells	BRF1	0.038988	4.828649	0.182899	0.855296	-6.09513	0.688213	0.765213
Erythroid.cells	GM48383	-0.10701	1.758143	-0.18288	0.855312	-5.47049	0.764932	0.833466
Erythroid.cells	ARHGEF3	0.065658	5.916073	0.182567	0.855555	-6.31703	0.663019	0.742474
Erythroid.cells	EAF2	-0.05679	3.647314	-0.18249	0.855613	-5.85766	0.716738	0.790904
Erythroid.cells	GBE1	-0.0446	6.483019	-0.18242	0.855669	-6.4274	0.65027	0.730874
Erythroid.cells	PTPN11	0.03739	5.063935	0.182345	0.85573	-6.09133	0.682681	0.760328
Erythroid.cells	SLC35A5	-0.05524	3.986264	-0.18232	0.855747	-5.77445	0.708431	0.783483
Erythroid.cells	GM17066	0.102996	1.747467	0.182319	0.85575	-5.41328	0.765219	0.833829
Erythroid.cells	ARMCX2	0.115032	1.41393	0.182034	0.855973	-5.38357	0.774073	0.841654
Erythroid.cells	FBXL20	0.05462	5.495646	0.181852	0.856115	-6.24577	0.672644	0.751318
Erythroid.cells	DYNC1H1	0.030614	6.255557	0.181752	0.856194	-6.31827	0.655353	0.73559
Erythroid.cells	HIST1H2AK	0.136629	0.954719	0.18175	0.856195	-5.35525	0.786437	0.852486
Erythroid.cells	GTF3C6	-0.03481	5.226124	-0.18158	0.856327	-6.20672	0.678892	0.756992
Erythroid.cells	PAWR	0.119612	0.662659	0.181546	0.856355	-5.36274	0.794404	0.859448
Erythroid.cells	ADRB1	0.15317	0.735849	0.181528	0.856369	-5.40228	0.7924	0.857703
Erythroid.cells	GM36756	0.112138	0.926199	0.181436	0.856441	-5.3762	0.787211	0.853228
Erythroid.cells	GM48678	-0.07706	3.349644	-0.1814	0.856468	-5.76132	0.724118	0.797628
Erythroid.cells	ABCA5	0.148587	0.444818	0.181214	0.856614	-5.24399	0.800401	0.864737
Erythroid.cells	ZFR2	0.115251	0.817601	0.181186	0.856636	-5.31731	0.790167	0.855831
Erythroid.cells	RNF7	-0.03229	6.742111	-0.1811	0.856701	-6.53132	0.644531	0.725795
Erythroid.cells	SMC1B	0.163855	-0.03473	0.181063	0.856733	-5.23852	0.813764	0.876369
Erythroid.cells	SPCS2	-0.02365	7.595587	-0.181	0.85678	-6.71523	0.626003	0.708776
Erythroid.cells	PROX1	0.108766	2.15604	0.180959	0.856814	-5.65131	0.754514	0.824632
Erythroid.cells	HDAC2	0.040091	5.300553	0.180718	0.857003	-6.23844	0.67716	0.755612
Erythroid.cells	CYLD	-0.03141	6.329731	-0.18072	0.857004	-6.43584	0.653691	0.734268
Erythroid.cells	MBD1	0.048938	4.580853	0.180691	0.857024	-5.99792	0.6941	0.770904
Erythroid.cells	NKAP	0.037261	4.989943	0.180454	0.857209	-6.09491	0.684509	0.762252
Erythroid.cells	MTHFR	0.053007	3.640415	0.180358	0.857284	-5.87603	0.717008	0.791468
Erythroid.cells	DDX39B	-0.02863	7.251694	-0.18015	0.857449	-6.65621	0.633524	0.71577
Erythroid.cells	TCTN2	-0.14152	-0.37598	-0.18008	0.857498	-5.18677	0.823573	0.884936
Erythroid.cells	TMEM165	-0.03772	5.311537	-0.17991	0.857638	-6.1281	0.677038	0.755495
Erythroid.cells	ITGA2	0.091758	0.926385	0.179724	0.85778	-5.60253	0.787361	0.853514
Erythroid.cells	MRPL58	0.041064	5.199269	0.179715	0.857787	-6.22853	0.679651	0.75786
Erythroid.cells	A730063M	-0.13577	1.218514	-0.17945	0.857995	-5.30901	0.779464	0.846614
Erythroid.cells	SLC7A8	0.109614	2.86439	0.179425	0.858015	-5.75321	0.736464	0.808745
Erythroid.cells	SF3B5	0.031953	6.277364	0.1794	0.858034	-6.42335	0.654993	0.73545
Erythroid.cells	SRSF6	-0.03352	6.210068	-0.17938	0.85805	-6.39006	0.656504	0.73683
Erythroid.cells	GM36839	0.08537	2.346299	0.179368	0.85806	-5.53702	0.749729	0.820484
Erythroid.cells	COG4	-0.03718	5.336083	-0.17929	0.858117	-6.20606	0.676469	0.754979
Erythroid.cells	ZHX1	0.057403	4.078556	0.178837	0.858475	-5.86256	0.706562	0.781962
Erythroid.cells	HIST1H2AN	0.112229	3.254826	0.178676	0.858601	-5.92255	0.726883	0.80012
Erythroid.cells	GM15675	-0.09965	2.317552	-0.17864	0.85863	-5.56486	0.750735	0.821256
Erythroid.cells	RPUSD3	0.132847	0.640562	0.178267	0.858921	-5.32516	0.795649	0.860463
Erythroid.cells	SLC25A45	-0.06767	3.260909	-0.17811	0.859047	-5.66215	0.726963	0.800025

Erythroid.cells	GM47754	0.129847	0.667894	0.177809	0.85928	-5.35673	0.795099	0.859863
Erythroid.cells	GAR1	0.055005	4.430651	0.177616	0.859431	-5.99377	0.698429	0.774486
Erythroid.cells	EPO	-0.14023	0.415776	-0.17751	0.859516	-5.21589	0.802051	0.865968
Erythroid.cells	CBR3	-0.11457	0.624626	-0.17745	0.85956	-5.47875	0.796289	0.860982
Erythroid.cells	NEK3	0.11449	1.761696	0.177443	0.859566	-5.47272	0.765652	0.834206
Erythroid.cells	GM11707	-0.10975	0.863582	-0.17709	0.859845	-5.40932	0.789883	0.855317
Erythroid.cells	BTNL9	0.143524	0.689973	0.177074	0.859855	-5.33248	0.79463	0.859452
Erythroid.cells	ARHGAP22	-0.10214	1.023886	-0.17665	0.860184	-5.67663	0.785639	0.851645
Erythroid.cells	CAPN7	-0.03366	5.340998	-0.17649	0.860315	-6.18907	0.67715	0.755297
Erythroid.cells	RING1	-0.06594	3.585545	-0.17643	0.860361	-5.72428	0.71925	0.793121
Erythroid.cells	BEND3	-0.09288	2.456588	-0.17642	0.860369	-5.52227	0.747763	0.818427
Erythroid.cells	CCNY	0.029492	6.67252	0.176227	0.860518	-6.51851	0.646954	0.72781
Erythroid.cells	NDC80	-0.06638	4.625978	-0.17622	0.860526	-6.14837	0.693976	0.770482
Erythroid.cells	JCAD	0.100871	1.404554	0.176197	0.860542	-5.38999	0.775387	0.842719
Erythroid.cells	MOCS1	0.054486	3.367675	0.176123	0.8606	-5.86081	0.724663	0.797944
Erythroid.cells	CD3EAP	0.072399	3.231663	0.175951	0.860735	-5.67186	0.728064	0.800998
Erythroid.cells	RRP9	0.07583	2.59462	0.175932	0.860749	-5.54706	0.744215	0.81532
Erythroid.cells	RASGRP2	-0.03463	6.609517	-0.17589	0.860782	-6.41977	0.64835	0.729113
Erythroid.cells	KIF13B	-0.03423	6.510852	-0.17561	0.861003	-6.43485	0.650543	0.731191
Erythroid.cells	ICAM2	-0.08777	4.282374	-0.17556	0.861041	-5.98875	0.702218	0.777991
Erythroid.cells	KLF2	0.055436	8.937753	0.175555	0.861045	-6.93058	0.598827	0.683434
Erythroid.cells	PTDSS1	-0.03618	5.371518	-0.17551	0.861081	-6.20594	0.676441	0.754756
Erythroid.cells	IFNAR1	0.040561	5.246213	0.175394	0.861171	-6.15784	0.679356	0.757401
Erythroid.cells	VRK1	0.040723	5.309256	0.175304	0.861242	-6.14399	0.677888	0.756095
Erythroid.cells	MKKS	-0.07842	1.995441	-0.17528	0.861261	-5.56234	0.759745	0.829143
Erythroid.cells	TAF5L	0.042731	4.562119	0.174855	0.861593	-6.03375	0.695711	0.772125
Erythroid.cells	ZFP595	-0.0732	1.84184	-0.17461	0.861783	-5.46077	0.764116	0.832865
Erythroid.cells	TESK2	0.070742	4.072329	0.174493	0.861877	-5.86011	0.707638	0.782755
Erythroid.cells	GM28112	0.097577	0.043725	0.174218	0.862093	-5.59671	0.813163	0.875602
Erythroid.cells	ARFGAP1	-0.04259	4.620741	-0.17408	0.862201	-6.02387	0.694517	0.771009
Erythroid.cells	CPSF4L	-0.11267	0.499907	-0.17395	0.862299	-5.27524	0.800456	0.864637
Erythroid.cells	CTDSP1	0.039094	5.3236	0.173917	0.862328	-6.23087	0.67796	0.756085
Erythroid.cells	SH3BP5L	0.079435	2.252113	0.173894	0.862346	-5.57156	0.753502	0.823562
Erythroid.cells	CAML	0.045801	4.427078	0.173473	0.862676	-5.91117	0.699222	0.775312
Erythroid.cells	ZNRD1AS	0.121854	0.28937	0.173242	0.862857	-5.31411	0.806375	0.869889
Erythroid.cells	TSC1	-0.04577	4.710552	-0.17321	0.862879	-6.04485	0.692446	0.769252
Erythroid.cells	GIMAP9	-0.05705	3.560572	-0.17316	0.862922	-5.96371	0.72037	0.794281
Erythroid.cells	CYP4F16	0.09488	1.441368	0.173079	0.862985	-5.56737	0.774943	0.842503
Erythroid.cells	D3ERTD75:	-0.10092	1.61523	-0.17301	0.863037	-5.42147	0.770309	0.838449
Erythroid.cells	S100A11	-0.03164	7.270251	-0.17292	0.863109	-6.74915	0.634309	0.71637
Erythroid.cells	KIF9	0.096994	1.680578	0.172915	0.863113	-5.45161	0.768575	0.836944
Erythroid.cells	SLC7A6	-0.04812	4.274758	-0.1729	0.863128	-5.94053	0.702892	0.778671
Erythroid.cells	MYO6	0.060086	3.1857	0.172877	0.863143	-5.89105	0.729725	0.802634
Erythroid.cells	CEP83OS	-0.09598	2.022979	-0.17261	0.863351	-5.46267	0.759673	0.829128
Erythroid.cells	SHANK2	0.099767	1.410698	0.172254	0.863632	-5.48481	0.776045	0.843466
Erythroid.cells	MICAL1	-0.06124	3.487265	-0.17219	0.863682	-5.73615	0.722452	0.796142
Erythroid.cells	ZFP169	-0.08341	2.782441	-0.17197	0.863855	-5.6893	0.740199	0.811934

Erythroid.cells	USF1	-0.04692	4.934416	-0.17196	0.86386	-6.08032	0.687393	0.764734
Erythroid.cells	SEC11C	0.027248	7.287381	0.171862	0.863938	-6.66742	0.634168	0.716244
Erythroid.cells	GM15952	-0.10768	1.028007	-0.17184	0.863955	-5.40999	0.78636	0.852518
Erythroid.cells	ZFP329	0.064034	2.511051	0.171652	0.864103	-5.61267	0.747219	0.818086
Erythroid.cells	SHC1	0.041813	4.384809	0.171472	0.864244	-6.00527	0.700611	0.776524
Erythroid.cells	USP53	0.049547	4.118641	0.171315	0.864367	-5.8969	0.707059	0.782317
Erythroid.cells	SND1	0.024505	7.195863	0.171277	0.864397	-6.64778	0.636269	0.718091
Erythroid.cells	SERTAD2	-0.03843	6.12155	-0.17103	0.864589	-6.46428	0.660183	0.739935
Erythroid.cells	POLR3B	0.045843	5.512738	0.17049	0.865014	-6.23816	0.67431	0.752675
Erythroid.cells	COX5A	0.025717	8.546847	0.170338	0.865134	-6.92873	0.607876	0.691716
Erythroid.cells	TMEM184(	-0.05358	3.390595	-0.17028	0.865176	-5.78921	0.725315	0.798476
Erythroid.cells	ACOX2	0.113311	1.030725	0.170272	0.865185	-5.47119	0.78678	0.85263
Erythroid.cells	BSN	0.138181	0.764883	0.170239	0.865211	-5.30052	0.794032	0.858949
Erythroid.cells	PPP1R16B	-0.03681	6.504104	-0.16982	0.865536	-6.59065	0.651983	0.732288
Erythroid.cells	PHKG1	-0.11794	1.107052	-0.16965	0.865672	-5.40094	0.784999	0.850995
Erythroid.cells	GOLT1B	-0.05104	4.631519	-0.16912	0.866086	-5.96745	0.695547	0.771554
Erythroid.cells	RABL3	0.074918	2.218563	0.169002	0.866181	-5.58072	0.755794	0.825137
Erythroid.cells	KLHL22	-0.06662	3.001151	-0.16866	0.866448	-5.687	0.735693	0.807432
Erythroid.cells	MYO1F	-0.04601	5.259228	-0.16859	0.866504	-6.32971	0.680743	0.758247
Erythroid.cells	MEIS1	0.063325	3.866059	0.168576	0.866515	-5.88407	0.714116	0.788231
Erythroid.cells	MTCH2	-0.0278	5.950226	-0.1685	0.866577	-6.45059	0.664804	0.743814
Erythroid.cells	BTF3	0.018186	8.999494	0.168359	0.866685	-7.03829	0.599058	0.683308
Erythroid.cells	ABITRAM	-0.06777	2.584915	-0.16816	0.866845	-5.58234	0.746318	0.816903
Erythroid.cells	CD3G	-0.05448	3.343766	-0.16812	0.86687	-6.39511	0.727066	0.799838
Erythroid.cells	DIABLO	-0.04611	4.259159	-0.16807	0.866914	-5.9497	0.704529	0.779718
Erythroid.cells	2310033P(	-0.04774	4.144026	-0.16806	0.866923	-5.9186	0.707323	0.782221
Erythroid.cells	HECTD2	0.103581	0.291184	0.168036	0.866939	-5.39934	0.807769	0.870671
Erythroid.cells	FAM118B	-0.0554	3.464677	-0.16799	0.866973	-5.76433	0.724046	0.797151
Erythroid.cells	SH3BGRL3	-0.02514	9.061448	-0.16771	0.867196	-7.0022	0.597881	0.682219
Erythroid.cells	GLTP	-0.02614	6.759436	-0.16765	0.86724	-6.51873	0.646732	0.727363
Erythroid.cells	CD101	0.10106	0.741284	0.167447	0.867401	-5.4567	0.795511	0.85997
Erythroid.cells	SNHG5	-0.08519	2.416156	-0.16698	0.867769	-5.53013	0.751065	0.82092
Erythroid.cells	KLK1	0.196264	-0.20745	0.166957	0.867785	-5.11373	0.822229	0.88299
Erythroid.cells	RASSF8	0.08612	2.215433	0.166829	0.867885	-5.63275	0.756304	0.825524
Erythroid.cells	TTC41	-0.12171	0.966475	-0.16661	0.868055	-5.28608	0.789648	0.854713
Erythroid.cells	PHF2OS1	-0.09958	1.058613	-0.16651	0.868137	-5.32133	0.787141	0.852547
Erythroid.cells	ASB3	-0.03797	4.711015	-0.16649	0.868155	-6.08963	0.694107	0.770188
Erythroid.cells	C330013E1	-0.12098	0.677864	-0.16638	0.868238	-5.32265	0.797565	0.861633
Erythroid.cells	DIMT1	0.057878	3.243055	0.166156	0.868414	-5.74275	0.730058	0.802325
Erythroid.cells	MAST1	0.144557	1.020113	0.166121	0.868441	-5.36903	0.788202	0.853481
Erythroid.cells	GM32743	-0.13611	-0.48509	-0.16605	0.868498	-5.21826	0.830246	0.889897
Erythroid.cells	PLEKHA1	-0.0581	4.707996	-0.16584	0.868664	-6.03784	0.694192	0.770269
Erythroid.cells	GRAMD1C	-0.07561	2.440736	-0.16575	0.868734	-5.65302	0.750515	0.820433
Erythroid.cells	PDGFC	-0.1395	1.879122	-0.16573	0.868745	-5.5147	0.765187	0.833346
Erythroid.cells	IGLC2	-0.11829	3.740399	-0.16559	0.868854	-6.20261	0.717669	0.791297
Erythroid.cells	NR6A1	0.044308	5.241175	0.165494	0.868933	-6.2368	0.681601	0.758923
Erythroid.cells	GLRX	-0.0359	6.214402	-0.16549	0.868938	-6.49753	0.659237	0.738636

Erythroid.cells	GM28809	0.121315	-0.12461	0.165465	0.868956	-5.28476	0.819975	0.881046
Erythroid.cells	11-Sep	-0.04164	6.415184	-0.16532	0.869067	-6.47817	0.654735	0.734541
Erythroid.cells	TMEM98	-0.1236	0.620631	-0.16526	0.869117	-5.27771	0.799162	0.863034
Erythroid.cells	AADAC	-0.07874	2.701819	-0.16502	0.869302	-5.93333	0.743906	0.814623
Erythroid.cells	TM9SF4	0.046431	4.769644	0.164378	0.869809	-6.00226	0.693153	0.769131
Erythroid.cells	GNAI3	-0.02424	6.443605	-0.16427	0.869891	-6.44717	0.65449	0.734113
Erythroid.cells	DPP10	0.125532	0.411958	0.164201	0.869947	-5.38497	0.805422	0.86823
Erythroid.cells	GM42829	0.080653	2.205641	0.164096	0.87003	-5.51297	0.757091	0.826016
Erythroid.cells	ARHGEF10	-0.09936	2.266878	-0.16405	0.870066	-5.60563	0.755494	0.82461
Erythroid.cells	MAK16	-0.03386	5.072635	-0.16381	0.870254	-6.17533	0.686071	0.76273
Erythroid.cells	RCE1	0.053686	3.757774	0.163543	0.870464	-5.79515	0.717895	0.791236
Erythroid.cells	TAOK2	-0.04781	4.127104	-0.16322	0.870716	-5.89892	0.708911	0.783222
Erythroid.cells	GM46218	-0.12728	0.58606	-0.16321	0.870728	-5.22592	0.800917	0.864269
Erythroid.cells	ASXL1	-0.0266	7.242343	-0.16315	0.870773	-6.61955	0.637109	0.718179
Erythroid.cells	CLEC4N	-0.08533	4.093598	-0.1629	0.870966	-5.99153	0.709828	0.784068
Erythroid.cells	GM14410	0.1068	0.850334	0.16253	0.871259	-5.29765	0.793837	0.858152
Erythroid.cells	ELMO1	-0.02267	8.719508	-0.16253	0.871261	-6.91406	0.605948	0.689397
Erythroid.cells	EFCAB7	-0.11272	0.535523	-0.16249	0.871288	-5.25048	0.802511	0.865695
Erythroid.cells	ALDH4A1	0.062428	2.912017	0.162305	0.871436	-5.81539	0.739362	0.810383
Erythroid.cells	MRPL19	0.049275	3.923908	0.162186	0.87153	-5.86382	0.714055	0.787887
Erythroid.cells	TNFRSF4	0.116357	0.30724	0.16217	0.871542	-5.41794	0.80886	0.871232
Erythroid.cells	PNKP	-0.04888	4.959191	-0.16209	0.871608	-6.0288	0.6891	0.765515
Erythroid.cells	SRSF9	0.024552	6.428408	0.162007	0.87167	-6.44863	0.655252	0.734848
Erythroid.cells	IDS	-0.06586	2.698638	-0.16196	0.871703	-5.67665	0.744816	0.815246
Erythroid.cells	JUNB	0.030032	9.343714	0.161878	0.871771	-7.08419	0.593212	0.677591
Erythroid.cells	ARHGAP31	-0.04047	6.152696	-0.16177	0.871854	-6.47098	0.661476	0.740543
Erythroid.cells	GPX3	-0.11296	1.463214	-0.16158	0.872007	-5.53279	0.777308	0.843765
Erythroid.cells	NEK9	-0.04224	5.27603	-0.16123	0.872279	-6.12566	0.681723	0.758852
Erythroid.cells	FBXO22	0.03632	4.616588	0.161178	0.872321	-6.08667	0.697333	0.772932
Erythroid.cells	SOAT2	-0.08949	0.810649	-0.16113	0.872355	-5.51793	0.795014	0.859265
Erythroid.cells	GSTM4	0.099856	1.121715	0.160973	0.872482	-5.44525	0.786525	0.85192
Erythroid.cells	UQCRC1	0.025297	6.508803	0.160859	0.872571	-6.5826	0.653524	0.733332
Erythroid.cells	CSNK2A2	-0.02501	5.948451	-0.16079	0.872624	-6.36728	0.666186	0.744862
Erythroid.cells	THAP3	0.048682	3.799217	0.160709	0.872689	-5.88712	0.717204	0.790788
Erythroid.cells	FFAR1	0.114491	-0.22509	0.160651	0.872735	-5.19891	0.823957	0.884404
Erythroid.cells	SMAD4	0.025852	6.23224	0.16061	0.872767	-6.38639	0.659741	0.739023
Erythroid.cells	FAM160B1	-0.04633	4.443367	-0.16058	0.872791	-5.92238	0.701495	0.77676
Erythroid.cells	ZSCAN2	0.12352	1.067334	0.160572	0.872797	-5.3173	0.788002	0.853235
Erythroid.cells	PDCD6IP	-0.01759	6.988379	-0.16044	0.872897	-6.62941	0.642912	0.723634
Erythroid.cells	GM37305	-0.09379	1.078554	-0.15963	0.873534	-5.38942	0.788199	0.853091
Erythroid.cells	LPAR1	0.133254	0.763125	0.159589	0.873569	-5.47386	0.796827	0.860599
Erythroid.cells	ATP6V0B	0.020093	8.046898	0.159488	0.873648	-6.78883	0.620469	0.702724
Erythroid.cells	COMMD7	-0.02764	5.29863	-0.1593	0.873796	-6.17529	0.681695	0.758597
Erythroid.cells	HASPIN	-0.07652	2.299155	-0.15913	0.87393	-5.59242	0.755829	0.82468
Erythroid.cells	GM17106	0.068235	3.395192	0.15904	0.874	-5.74749	0.727826	0.799959
Erythroid.cells	GLYCTK	-0.09864	0.979545	-0.15884	0.874155	-5.40414	0.791026	0.855586
Erythroid.cells	ZKSCAN16	-0.11541	-0.59074	-0.15863	0.874321	-5.21452	0.835095	0.893708

Erythroid.cells	MRGPRA2E	-0.12	-0.43033	-0.15857	0.874367	-5.385	0.830482	0.889752
Erythroid.cells	GM48796	-0.10879	0.350478	-0.15853	0.874398	-5.27846	0.80839	0.870681
Erythroid.cells	MS4A6B	-0.06116	4.588359	-0.15853	0.874399	-6.29593	0.698568	0.773887
Erythroid.cells	PLS1	0.106051	0.910051	0.158498	0.874426	-5.37098	0.792925	0.857255
Erythroid.cells	MRPL50	0.036446	4.009842	0.158226	0.87464	-6.05466	0.712713	0.786454
Erythroid.cells	C330007PC	-0.03561	5.50907	-0.15803	0.87479	-6.23052	0.67699	0.754341
Erythroid.cells	ZFP180	-0.05173	3.315586	-0.15797	0.874839	-5.76056	0.729989	0.801846
Erythroid.cells	KCNJ2	0.091756	-0.01521	0.157669	0.875077	-5.31067	0.818926	0.879673
Erythroid.cells	DEFB1	0.109208	0.86149	0.157405	0.875285	-5.48149	0.794512	0.85852
Erythroid.cells	MRPL2	0.039003	4.862057	0.157359	0.875321	-6.16631	0.692256	0.768109
Erythroid.cells	KCNJ16	0.180014	-0.02269	0.157328	0.875345	-5.23959	0.819138	0.879856
Erythroid.cells	CMTM6	-0.03712	4.866252	-0.15731	0.875361	-6.04293	0.692156	0.768019
Erythroid.cells	ZFP808	0.079783	1.973033	0.157153	0.875483	-5.46242	0.764622	0.832405
Erythroid.cells	TAMM41	0.071935	2.732365	0.15715	0.875485	-5.60643	0.74487	0.815011
Erythroid.cells	NFIX	-0.0513	3.676603	-0.15705	0.875563	-5.99505	0.721045	0.793877
Erythroid.cells	ETV6	0.029024	8.156803	0.156963	0.875632	-6.83944	0.618442	0.700801
Erythroid.cells	KLHL4	-0.12884	0.018315	-0.15689	0.875693	-5.27768	0.817979	0.878865
Erythroid.cells	ADCY10	0.118398	0.912909	0.156863	0.875711	-5.30908	0.793103	0.85731
Erythroid.cells	NOSTRIN	-0.05525	3.426931	-0.15656	0.875946	-6.00353	0.727332	0.799491
Erythroid.cells	TMEM185A	0.068506	2.540183	0.156502	0.875994	-5.59721	0.749886	0.819459
Erythroid.cells	IFNGR2	-0.04342	5.782195	-0.15645	0.876033	-6.28198	0.670806	0.748766
Erythroid.cells	CXCR6	-0.07003	1.255073	-0.15639	0.876086	-5.82858	0.783864	0.849289
Erythroid.cells	MRPL48	-0.02704	5.282039	-0.15631	0.876148	-6.23743	0.68241	0.759281
Erythroid.cells	GM9887	-0.0946	1.686181	-0.15614	0.876282	-5.40508	0.772348	0.83918
Erythroid.cells	POLR3K	0.042757	3.885777	0.155841	0.876514	-5.89052	0.716122	0.78944
Erythroid.cells	PPP1R13L	-0.14058	-0.0774	-0.15564	0.876675	-5.23031	0.820996	0.881383
Erythroid.cells	TRP53I13	0.05687	3.021819	0.155623	0.876685	-5.65377	0.737759	0.808649
Erythroid.cells	BUD13	0.047482	3.714639	0.15517	0.877041	-5.80663	0.720506	0.793273
Erythroid.cells	INTS6	-0.03799	6.588587	-0.15504	0.877144	-6.50399	0.652841	0.732313
Erythroid.cells	RTTN	0.057124	3.609694	0.155039	0.877144	-5.811	0.723112	0.795631
Erythroid.cells	ABCF2	-0.0391	4.411019	-0.15504	0.877144	-5.99278	0.703459	0.778082
Erythroid.cells	ANKRD49	0.0582	3.032797	0.154962	0.877205	-5.65652	0.737615	0.808518
Erythroid.cells	URGCP	0.050542	4.434418	0.154815	0.87732	-5.91612	0.702901	0.777595
Erythroid.cells	RRM1	0.046268	5.449593	0.154768	0.877357	-6.27874	0.678826	0.755926
Erythroid.cells	PGLS	-0.02267	7.788311	-0.15449	0.877572	-6.74891	0.626725	0.708383
Erythroid.cells	KIF4	-0.05908	4.394266	-0.1543	0.877726	-6.14772	0.704053	0.778612
Erythroid.cells	DOCK10	-0.02397	8.80921	-0.15413	0.87786	-7.01417	0.605349	0.688596
Erythroid.cells	CHUK	-0.03383	5.037427	-0.15411	0.877872	-6.15904	0.688674	0.764825
Erythroid.cells	MINDY3	0.0323	5.598106	0.153968	0.877986	-6.23423	0.675586	0.752986
Erythroid.cells	RINT1	0.044792	3.771156	0.153872	0.878061	-5.86262	0.719339	0.792281
Erythroid.cells	MMAA	0.083797	1.748976	0.153623	0.878257	-5.49758	0.771238	0.838159
Erythroid.cells	SIRT3	0.044177	3.344497	0.153618	0.878261	-5.88109	0.729982	0.801778
Erythroid.cells	MAN2B2	0.058578	3.799809	0.153593	0.878281	-5.80124	0.718634	0.791684
Erythroid.cells	COP1	-0.02776	7.053619	-0.15342	0.878414	-6.57766	0.642774	0.723174
Erythroid.cells	LLGL2	0.067688	2.291993	0.15337	0.878456	-5.63465	0.75696	0.825665
Erythroid.cells	DNTTIP1	-0.0292	4.812219	-0.15318	0.878608	-6.15066	0.694093	0.769748
Erythroid.cells	WDR35	0.126881	0.509893	0.153174	0.87861	-5.23253	0.804968	0.867589

Erythroid.cells	LIN52	0.034901	5.546829	0.152849	0.878866	-6.3038	0.676927	0.754212
Erythroid.cells	AC125149.	-0.12926	-0.34803	-0.15281	0.8789	-5.27692	0.829308	0.888557
Erythroid.cells	ZFP235	-0.07985	1.919758	-0.15267	0.879005	-5.51292	0.766903	0.83434
Erythroid.cells	CDK4	0.03098	5.844705	0.152459	0.879172	-6.36727	0.670148	0.74809
Erythroid.cells	WWTR1	0.092753	2.126919	0.152258	0.879331	-5.54566	0.761603	0.829685
Erythroid.cells	USO1	0.029016	5.739311	0.152106	0.87945	-6.29477	0.672676	0.750343
Erythroid.cells	ENPP4	-0.06363	2.924155	-0.152	0.879529	-5.70318	0.741012	0.811524
Erythroid.cells	TMEM14A	-0.10753	0.419553	-0.1518	0.87969	-5.33915	0.807873	0.870073
Erythroid.cells	BAX	0.030061	6.141068	0.151784	0.879703	-6.43111	0.663491	0.742051
Erythroid.cells	4833420G1	-0.03529	5.494494	-0.15172	0.879752	-6.33273	0.678358	0.755543
Erythroid.cells	PPP1R12C	-0.03373	5.009564	-0.15163	0.879826	-6.09532	0.689741	0.765826
Erythroid.cells	ZBTB6	0.076914	1.997204	0.151454	0.879963	-5.48127	0.76513	0.832799
Erythroid.cells	UHRF1	-0.05475	4.598357	-0.15127	0.880104	-6.15694	0.699614	0.774709
Erythroid.cells	SOWAHC	0.067571	2.917305	0.1512	0.880162	-5.81273	0.741263	0.811814
Erythroid.cells	DAP	0.031299	6.19848	0.151161	0.880193	-6.49588	0.66225	0.740963
Erythroid.cells	STFA2L1	-0.10225	2.448793	-0.15105	0.88028	-5.77038	0.753338	0.8225
Erythroid.cells	OGDH	-0.01854	6.625732	-0.1506	0.880635	-6.53633	0.652812	0.732325
Erythroid.cells	SAR1A	0.029375	5.508767	0.150347	0.880833	-6.28878	0.678274	0.755467
Erythroid.cells	SMIM11	0.03324	4.805042	0.150322	0.880853	-6.07726	0.694853	0.770419
Erythroid.cells	DOCK6	-0.08023	1.914885	-0.15024	0.880919	-5.51096	0.767527	0.834944
Erythroid.cells	SEC24B	0.02996	6.179882	0.150143	0.880994	-6.39969	0.662852	0.741539
Erythroid.cells	HCAR2	-0.11492	2.377294	-0.15006	0.881058	-5.6921	0.755388	0.824324
Erythroid.cells	UFM1	-0.0291	5.448609	-0.14996	0.881135	-6.27657	0.679675	0.756826
Erythroid.cells	SLC25A25	-0.03373	4.951297	-0.14979	0.881269	-6.20154	0.691373	0.767428
Erythroid.cells	PRKCH	-0.03634	6.999653	-0.14971	0.881336	-6.85437	0.644516	0.724924
Erythroid.cells	MYH13	-0.11696	-1.61994	-0.14955	0.881457	-5.06675	0.867141	0.921236
Erythroid.cells	PSMC3	-0.02337	6.11628	-0.14955	0.881462	-6.47224	0.664297	0.742996
Erythroid.cells	ACSL1	0.034541	5.758511	0.149508	0.881494	-6.58516	0.672491	0.750434
Erythroid.cells	FBRSL1	-0.02786	5.766788	-0.14949	0.881507	-6.25577	0.6723	0.750261
Erythroid.cells	RAB22A	-0.02952	5.60028	-0.14949	0.881508	-6.23751	0.676149	0.753747
Erythroid.cells	MGMT	0.063691	3.273792	0.149106	0.881809	-5.73402	0.732549	0.804297
Erythroid.cells	PTAFR	0.071002	4.645313	0.149087	0.881824	-6.15476	0.698801	0.774173
Erythroid.cells	CMTR1	0.043651	5.166737	0.149015	0.881881	-6.18949	0.686402	0.763016
Erythroid.cells	CSTDC5	0.084606	5.46156	0.148586	0.882218	-6.3305	0.679699	0.756863
Erythroid.cells	ABCA3	-0.03854	4.193838	-0.14835	0.882408	-5.9186	0.710035	0.784109
Erythroid.cells	TMC8	0.053031	3.820781	0.147959	0.882712	-5.78467	0.719337	0.792382
Erythroid.cells	GLYR1	-0.0229	6.736527	-0.14767	0.882938	-6.51054	0.650863	0.730627
Erythroid.cells	ADAM17	-0.04025	6.061586	-0.14761	0.882985	-6.30567	0.666076	0.744486
Erythroid.cells	IGKV1-117	0.091781	-0.39916	0.1475	0.883073	-5.28887	0.832008	0.891015
Erythroid.cells	KIF1A	0.091932	-0.59877	0.147403	0.883149	-5.35294	0.837763	0.895962
Erythroid.cells	ARHGAP27	-0.08863	0.465307	-0.14729	0.883238	-5.35626	0.80754	0.869932
Erythroid.cells	AA386476	0.099805	1.390731	0.147215	0.883297	-5.33757	0.782156	0.847854
Erythroid.cells	RNASE6	0.048984	4.491549	0.147191	0.883317	-6.12309	0.702939	0.777784
Erythroid.cells	SPRED3	-0.10687	0.719893	-0.14718	0.883322	-5.3306	0.800474	0.863803
Erythroid.cells	GM17477	0.097311	0.61424	0.147073	0.883409	-5.31466	0.803398	0.866342
Erythroid.cells	GFOD1	-0.03292	6.621215	-0.1469	0.883548	-6.58763	0.653436	0.73303
Erythroid.cells	TNFRSF18	0.048349	2.322619	0.146843	0.88359	-5.86576	0.75742	0.826193

Erythroid.cells	TRIOBP	0.03495	4.877202	0.14677	0.883647	-6.01698	0.69369	0.7695
Erythroid.cells	TMEM91	-0.07517	2.459733	-0.14676	0.883659	-5.56423	0.753849	0.823049
Erythroid.cells	JRKL	-0.08778	1.537523	-0.14671	0.883692	-5.44907	0.778205	0.844425
Erythroid.cells	9030025P2	0.079104	1.737859	0.146677	0.883721	-5.4968	0.772846	0.839736
Erythroid.cells	XLR4A	-0.09061	0.802973	-0.14658	0.883799	-5.37678	0.798187	0.861837
Erythroid.cells	UBAP1	-0.03276	5.688984	-0.14625	0.884059	-6.29983	0.674683	0.752318
Erythroid.cells	PDP1	-0.05786	2.180234	-0.14613	0.884148	-5.60687	0.7612	0.829531
Erythroid.cells	NEURL1A	-0.13716	-0.39095	-0.14613	0.884151	-5.17298	0.83183	0.890941
Erythroid.cells	CDH2	0.063797	1.947183	0.146029	0.884231	-5.73812	0.767341	0.834962
Erythroid.cells	NANP	0.061384	2.821135	0.145925	0.884312	-5.7181	0.744573	0.814913
Erythroid.cells	NUAK2	0.040808	4.289152	0.145922	0.884315	-6.0197	0.707895	0.782293
Erythroid.cells	STMN1	0.047243	7.897729	0.145838	0.884382	-6.87484	0.625592	0.707546
Erythroid.cells	SLC25A11	-0.03483	5.162778	-0.14574	0.884462	-6.17959	0.686971	0.763521
Erythroid.cells	CARD11	0.051655	4.618562	0.145579	0.884585	-6.12297	0.699928	0.775177
Erythroid.cells	CCDC69	0.05385	3.297575	0.145508	0.884641	-5.77509	0.732457	0.804208
Erythroid.cells	B3GNT3	-0.10759	0.711737	-0.1455	0.884645	-5.26516	0.800756	0.864153
Erythroid.cells	GM43713	0.084413	2.135076	0.145115	0.88495	-5.50737	0.762445	0.830805
Erythroid.cells	JAM3	-0.1188	0.244994	-0.14509	0.88497	-5.23871	0.813826	0.875601
Erythroid.cells	SNX9	-0.03108	6.331978	-0.14507	0.884985	-6.38643	0.660035	0.739216
Erythroid.cells	OXSM	-0.06904	1.788374	-0.14502	0.885021	-5.48985	0.771615	0.838863
Erythroid.cells	BRPF1	0.03907	4.929241	0.14496	0.885072	-6.05826	0.692554	0.768665
Erythroid.cells	WNT2	-0.12248	1.077377	-0.14484	0.88517	-5.34431	0.790798	0.855611
Erythroid.cells	GM43848	0.059779	2.767038	0.144473	0.885456	-5.61644	0.746185	0.816398
Erythroid.cells	1300002E1	0.055815	3.19234	0.144437	0.885484	-5.72251	0.735336	0.806797
Erythroid.cells	FAM221A	-0.08502	1.467642	-0.14406	0.885783	-5.4044	0.780511	0.846535
Erythroid.cells	HSP90AB1	0.0174	9.925498	0.144039	0.885797	-7.16341	0.584148	0.669008
Erythroid.cells	2900097C1	-0.03289	4.909122	-0.14396	0.885857	-6.08385	0.69331	0.769263
Erythroid.cells	CPD	0.045556	4.333844	0.143854	0.885943	-5.98519	0.707158	0.781691
Erythroid.cells	DTD1	-0.05447	3.881415	-0.14374	0.886036	-5.85631	0.718261	0.79162
Erythroid.cells	POC1A	0.055691	3.229083	0.143494	0.886227	-5.74504	0.7346	0.806174
Erythroid.cells	ARHGAP33	-0.11916	0.549492	-0.14338	0.88632	-5.22831	0.805707	0.86853
Erythroid.cells	VPS41	-0.03234	5.101022	-0.14322	0.886439	-6.11331	0.688817	0.765286
Erythroid.cells	GM38394	0.098456	0.848639	0.143109	0.88653	-5.32965	0.79743	0.861404
Erythroid.cells	SLC45A4	0.0521	3.337998	0.143096	0.88654	-5.67718	0.731851	0.803799
Erythroid.cells	HNRNPH3	-0.02182	5.447268	-0.14303	0.886591	-6.29801	0.680682	0.757965
Erythroid.cells	GATC	-0.0691	2.698485	-0.14296	0.886645	-5.65052	0.748147	0.818228
Erythroid.cells	PEG13	0.076804	2.114243	0.142954	0.886651	-5.45089	0.763364	0.831632
Erythroid.cells	CCNB1	0.074728	4.032241	0.142643	0.886897	-5.97908	0.714718	0.788475
Erythroid.cells	GRHL1	-0.10706	1.025537	-0.14228	0.887179	-5.30963	0.79292	0.857375
Erythroid.cells	LSG1	0.031037	4.928986	0.142008	0.887397	-6.0999	0.693282	0.769209
Erythroid.cells	DENND5B	-0.03744	4.198991	-0.14196	0.887432	-6.15824	0.710888	0.785
Erythroid.cells	HECTD1	-0.01915	7.428886	-0.14153	0.887774	-6.68865	0.636569	0.717526
Erythroid.cells	PARL	-0.03463	4.391466	-0.14148	0.887814	-6.01803	0.706391	0.780839
Erythroid.cells	THG1L	-0.07669	2.021146	-0.14137	0.8879	-5.46728	0.766462	0.83407
Erythroid.cells	A930024E0	-0.08485	1.108621	-0.14113	0.88809	-5.49382	0.791044	0.855544
Erythroid.cells	AGPAT1	-0.05341	3.479015	-0.14103	0.88817	-5.75417	0.728987	0.800977
Erythroid.cells	MRTFA	-0.0278	7.084199	-0.14099	0.888197	-6.63406	0.64418	0.724492

Erythroid.cells	SPATA2L	0.099214	0.521707	0.1408	0.888348	-5.35093	0.807254	0.869649
Erythroid.cells	WDR4	-0.05237	2.999114	-0.14078	0.888365	-5.65664	0.741151	0.811777
Erythroid.cells	CSTF3	0.024767	6.037918	0.140601	0.888505	-6.36688	0.667684	0.745976
Erythroid.cells	BYSL	-0.03917	3.67185	-0.14059	0.888514	-5.82123	0.72419	0.796765
Erythroid.cells	BCDIN3D	0.063123	2.223563	0.140206	0.888816	-5.48775	0.76137	0.829586
Erythroid.cells	HERC4	0.024309	7.008496	0.140195	0.888825	-6.5836	0.645992	0.726144
Erythroid.cells	MTRR	-0.08979	1.505683	-0.13999	0.888989	-5.37926	0.780478	0.846317
Erythroid.cells	SERHL	-0.04803	3.492794	-0.1399	0.88906	-5.77324	0.728825	0.800829
Erythroid.cells	MSANTD4	-0.05039	2.89703	-0.13989	0.889068	-5.64699	0.743928	0.814214
Erythroid.cells	CKAP2L	-0.04958	4.357364	-0.13962	0.889282	-5.99934	0.70753	0.781803
Erythroid.cells	LRP8OS2	0.131727	0.163168	0.139613	0.889283	-5.24456	0.817559	0.878505
Erythroid.cells	INPP5D	0.023767	8.764735	0.139347	0.889493	-6.91694	0.608577	0.691592
Erythroid.cells	TYW1	-0.04636	3.875123	-0.13926	0.889559	-5.8253	0.719465	0.792457
Erythroid.cells	ZKSCAN14	0.048871	3.081299	0.139117	0.889674	-5.68143	0.739427	0.810163
Erythroid.cells	ALDH16A1	-0.03791	4.358468	-0.13893	0.889821	-5.99492	0.70766	0.781908
Erythroid.cells	FDX1	0.03427	4.692703	0.138817	0.889911	-6.26631	0.69958	0.774712
Erythroid.cells	SRSF11	0.012989	7.871425	0.138816	0.889912	-6.82721	0.627439	0.709102
Erythroid.cells	RAB37	-0.06177	2.340912	-0.13842	0.890223	-5.6126	0.758577	0.827183
Erythroid.cells	TNFRSF23	-0.07968	1.669914	-0.13835	0.890278	-5.45917	0.776331	0.842763
Erythroid.cells	TRMT2B	-0.0466	3.750232	-0.13814	0.890444	-5.82777	0.722642	0.795425
Erythroid.cells	CBFA2T2	0.029523	5.563389	0.138067	0.890501	-6.24575	0.67901	0.756273
Erythroid.cells	DRG1	0.019355	6.40915	0.137921	0.890617	-6.49676	0.659617	0.738674
Erythroid.cells	AMD1	0.037875	4.581194	0.137844	0.890677	-6.05209	0.70229	0.777238
Erythroid.cells	MROH1	-0.04755	4.199162	-0.13783	0.890689	-5.84703	0.711571	0.785548
Erythroid.cells	PDZD4	0.106596	1.698737	0.137786	0.890723	-5.27086	0.77556	0.842135
Erythroid.cells	DTX3L	0.051575	4.876216	0.137777	0.89073	-6.17978	0.695211	0.770881
Erythroid.cells	ALKBH7	0.051889	3.373231	0.137726	0.89077	-5.79503	0.732077	0.803814
Erythroid.cells	ACKR3	0.132292	0.835269	0.137688	0.8908	-5.37703	0.799014	0.862586
Erythroid.cells	CTNNBIP1	0.041537	4.359655	0.137653	0.890827	-5.97304	0.707657	0.782046
Erythroid.cells	NAT8F1	-0.08069	1.619362	-0.13753	0.890921	-5.57763	0.777686	0.843998
Erythroid.cells	CSF2RB2	0.069505	2.359571	0.137497	0.890951	-5.67383	0.758089	0.826809
Erythroid.cells	AARS2	0.098948	0.821033	0.137323	0.891088	-5.27593	0.799465	0.862984
Erythroid.cells	WBP1	0.048844	3.813733	0.136753	0.891537	-5.85237	0.721359	0.794234
Erythroid.cells	SLC35F2	-0.10314	0.357488	-0.1367	0.891581	-5.27677	0.812632	0.874341
Erythroid.cells	MFSD4B4	-0.11243	-0.0204	-0.13667	0.891605	-5.21357	0.823305	0.883561
Erythroid.cells	MEF2A	-0.0214	7.727346	-0.13658	0.891674	-6.72515	0.630812	0.712252
Erythroid.cells	FAM53B	-0.04029	4.652028	-0.13627	0.891918	-6.12704	0.701005	0.775917
Erythroid.cells	SCMH1	0.031477	5.849321	0.135862	0.892239	-6.37554	0.672962	0.750605
Erythroid.cells	VARS	0.027033	5.713285	0.1358	0.892288	-6.37059	0.676107	0.753475
Erythroid.cells	LAMP2	-0.02027	7.069003	-0.13562	0.892432	-6.64466	0.645467	0.725578
Erythroid.cells	PPFIA1	0.03002	5.566923	0.135466	0.892551	-6.25145	0.67953	0.756574
Erythroid.cells	DDX19A	-0.03613	4.239703	-0.13534	0.892654	-5.99345	0.711211	0.785051
Erythroid.cells	GIMAP1OS	0.068862	0.862202	0.135336	0.892654	-5.58307	0.79898	0.862368
Erythroid.cells	HIST4H4	-0.08035	1.887782	-0.13527	0.892707	-5.50175	0.771204	0.838136
Erythroid.cells	NDUFAF3	-0.03663	4.160988	-0.13521	0.892749	-5.95589	0.713138	0.786773
Erythroid.cells	SRRD	0.043318	3.465767	0.134939	0.892967	-5.79354	0.730516	0.802166
Erythroid.cells	FKBP5	0.042532	5.30259	0.134592	0.89324	-6.21254	0.68592	0.762307

Erythroid.cells	PRDM11	-0.04968	3.444862	-0.13459	0.893243	-5.82782	0.731137	0.802764
Erythroid.cells	COMMD4	-0.02862	5.326168	-0.13452	0.893299	-6.23881	0.685366	0.761813
Erythroid.cells	KCTD5	-0.04702	3.640452	-0.13427	0.893492	-5.76307	0.72631	0.798439
Erythroid.cells	SLC2A2	0.071472	1.627926	0.134214	0.893538	-5.65107	0.778458	0.844409
Erythroid.cells	SH2D1B1	-0.07138	1.695197	-0.13388	0.893802	-5.67488	0.776757	0.842953
Erythroid.cells	PHF11C	0.059945	1.989779	0.133741	0.893911	-5.62608	0.768906	0.836119
Erythroid.cells	SNX21	-0.06161	2.718125	-0.13365	0.893985	-5.64296	0.749844	0.819361
Erythroid.cells	ODF2	-0.0245	5.483537	-0.13356	0.894051	-6.25208	0.681839	0.758698
Erythroid.cells	EPOR	0.127151	-0.66556	0.13345	0.894141	-5.18869	0.842706	0.900209
Erythroid.cells	IL1RL2	-0.11121	0.726101	-0.13338	0.894196	-5.27406	0.803169	0.866127
Erythroid.cells	PERP	-0.07603	1.228461	-0.13323	0.894316	-5.5633	0.789365	0.854157
Erythroid.cells	RAD51AP1	-0.06957	3.61991	-0.13313	0.894393	-5.85834	0.72692	0.799197
Erythroid.cells	SGCB	-0.0825	2.212559	-0.13311	0.894408	-5.44369	0.763023	0.831108
Erythroid.cells	B230206L0	0.105023	1.02404	0.1331	0.894417	-5.46691	0.794953	0.859022
Erythroid.cells	MTMR4	0.050297	2.648419	0.133059	0.894449	-5.66432	0.751647	0.821094
Erythroid.cells	HIST1H2AC	-0.08773	1.907677	-0.13295	0.894534	-5.5226	0.771097	0.838183
Erythroid.cells	MYDGF	-0.03065	4.485984	-0.13265	0.89477	-6.01528	0.70563	0.780233
Erythroid.cells	GMPR	0.075167	1.923101	0.132605	0.894807	-5.52613	0.770716	0.83792
Erythroid.cells	CCDC181	-0.07539	1.514839	-0.13252	0.894872	-5.4057	0.781646	0.847483
Erythroid.cells	FTH1	-0.03328	12.5537	-0.13241	0.894957	-7.58232	0.536069	0.623456
Erythroid.cells	SIRPB1B	0.083276	1.445504	0.13235	0.895008	-5.66885	0.783517	0.849178
Erythroid.cells	ZFP868	-0.04475	3.676008	-0.13228	0.895065	-5.83558	0.725557	0.798107
Erythroid.cells	TSPAN7	0.0533	3.257326	0.132171	0.895149	-5.80587	0.736087	0.807478
Erythroid.cells	PTGR2	0.040284	3.734192	0.132149	0.895167	-5.86599	0.724106	0.796835
Erythroid.cells	GATB	-0.05421	3.11047	-0.13184	0.895414	-5.73524	0.739819	0.810811
Erythroid.cells	TRIM68	-0.08323	0.946883	-0.13167	0.895545	-5.32189	0.797115	0.861082
Erythroid.cells	HYLS1	0.047258	3.183514	0.131666	0.895548	-5.75001	0.73796	0.809165
Erythroid.cells	PCDH7	0.112406	0.520175	0.131637	0.89557	-5.4356	0.808942	0.871348
Erythroid.cells	PPWD1	0.027807	4.68019	0.131604	0.895597	-6.0482	0.700938	0.776151
Erythroid.cells	MRPS2	-0.05866	2.843371	-0.13158	0.895616	-5.76897	0.746655	0.816858
Erythroid.cells	RBL2	-0.03431	4.765126	-0.13154	0.895649	-6.07825	0.698896	0.774318
Erythroid.cells	2610020C0	-0.02803	4.983149	-0.13127	0.895861	-6.14107	0.693694	0.769697
Erythroid.cells	ACTN2	-0.09742	-0.1007	-0.1311	0.895991	-5.31462	0.826482	0.886609
Erythroid.cells	ABCA2	-0.07592	1.527476	-0.13104	0.896039	-5.48845	0.781316	0.847411
Erythroid.cells	TRUB1	0.063857	1.825881	0.13104	0.896042	-5.48701	0.773316	0.840411
Erythroid.cells	4930549G2	-0.05312	2.714781	-0.13096	0.896105	-5.62709	0.749981	0.819911
Erythroid.cells	COPB1	0.019094	6.220287	0.130872	0.896174	-6.43633	0.664884	0.743713
Erythroid.cells	1110020A2	-0.09413	0.468534	-0.13085	0.896192	-5.28969	0.810397	0.872759
Erythroid.cells	TMEM127	0.034906	4.290896	0.130675	0.896329	-5.88823	0.710387	0.784812
Erythroid.cells	NKIRAS1	0.04534	3.432264	0.130664	0.896338	-5.80788	0.731679	0.803793
Erythroid.cells	BC024978	-0.07124	1.865186	-0.13057	0.896413	-5.52154	0.772268	0.839604
Erythroid.cells	CBY1	0.048958	2.846037	0.130517	0.896454	-5.66478	0.746597	0.817015
Erythroid.cells	CHMP2B	-0.02869	4.813502	-0.13033	0.896603	-6.08345	0.697806	0.773501
Erythroid.cells	BAK1	0.037848	4.936771	0.130019	0.896847	-6.06464	0.69496	0.770951
Erythroid.cells	ATP5O	0.020249	7.142669	0.12998	0.896877	-6.72863	0.644379	0.725033
Erythroid.cells	ABHD8	0.05972	2.172201	0.12948	0.897272	-5.56777	0.764369	0.832628
Erythroid.cells	9330020H0	0.060583	2.471892	0.129455	0.897292	-5.55059	0.756514	0.825717

Erythroid.cells	ZDHHC15	0.078295	1.125862	0.129439	0.897304	-5.4691	0.792459	0.8572
Erythroid.cells	PLEKHG2	0.045854	4.157105	0.129389	0.897343	-5.94622	0.713879	0.787892
Erythroid.cells	XPO6	0.025886	5.572883	0.129276	0.897433	-6.20484	0.680006	0.757443
Erythroid.cells	INPP4A	0.032232	6.032154	0.129242	0.89746	-6.22923	0.669386	0.747819
Erythroid.cells	GM19522	-0.09104	0.940675	-0.12911	0.897564	-5.28283	0.79754	0.861621
Erythroid.cells	PYCR2	0.048192	3.687049	0.129012	0.897641	-5.76739	0.725513	0.798289
Erythroid.cells	GM46440	0.093028	0.637929	0.128984	0.897663	-5.34939	0.805917	0.86892
Erythroid.cells	ULK3	0.066636	2.219052	0.128965	0.897678	-5.48948	0.763136	0.831566
Erythroid.cells	SYVN1	0.047928	4.228615	0.128892	0.897736	-5.91237	0.712126	0.786353
Erythroid.cells	ATP6V1E1	0.017968	7.59223	0.128525	0.898025	-6.79166	0.634728	0.716138
Erythroid.cells	E230016K2	0.089098	0.143576	0.12849	0.898053	-5.34525	0.819948	0.881038
Erythroid.cells	MECP2	0.023008	5.847525	0.128349	0.898165	-6.33208	0.673774	0.751858
Erythroid.cells	ERCC6L	0.056552	3.034059	0.128202	0.89828	-5.77398	0.742158	0.813139
Erythroid.cells	BANP	-0.03136	4.601582	-0.12814	0.898328	-6.00406	0.703203	0.778428
Erythroid.cells	CBR4	0.050398	2.335594	0.128108	0.898354	-5.63419	0.760234	0.829091
Erythroid.cells	ADSL	-0.03668	4.033205	-0.12783	0.898572	-5.91552	0.717136	0.790886
Erythroid.cells	TPM3-RS7	-0.08948	0.526188	-0.12779	0.898609	-5.27386	0.809269	0.871904
Erythroid.cells	TAF2	0.032306	4.171185	0.127737	0.898647	-5.91852	0.713742	0.78786
Erythroid.cells	A530072M	-0.09522	0.764735	-0.12732	0.89898	-5.32072	0.802806	0.86625
Erythroid.cells	ADNP2	0.058382	2.990665	0.127272	0.899014	-5.64271	0.743492	0.814271
Erythroid.cells	NABP1	0.035139	4.743721	0.127217	0.899057	-6.15389	0.699989	0.775504
Erythroid.cells	GM15614	-0.05811	2.981808	-0.12692	0.899289	-5.73736	0.743763	0.814509
Erythroid.cells	PNP2	0.092655	0.616927	0.126868	0.899333	-5.37068	0.80696	0.869854
Erythroid.cells	MORN1	0.078117	1.227517	0.126838	0.899357	-5.35718	0.790132	0.855224
Erythroid.cells	GIN1	0.030457	3.954097	0.126761	0.899417	-5.88824	0.719287	0.792767
Erythroid.cells	DDX50	0.018288	6.369428	0.126681	0.89948	-6.45768	0.662074	0.741213
Erythroid.cells	ZBTB24	-0.04113	3.32852	-0.1266	0.899543	-5.69943	0.734937	0.806689
Erythroid.cells	ABHD17A	0.019175	6.296745	0.126277	0.899799	-6.43133	0.66386	0.742758
Erythroid.cells	FAM76A	0.027101	5.119697	0.126162	0.899891	-6.16751	0.691209	0.767501
Erythroid.cells	GM40841	-0.11159	0.40148	-0.12606	0.899973	-5.24158	0.813177	0.875139
Erythroid.cells	BIK	-0.08058	1.76969	-0.12596	0.900053	-5.5422	0.775683	0.842498
Erythroid.cells	SCLT1	0.041703	4.474106	0.125802	0.900174	-5.97404	0.706719	0.781444
Erythroid.cells	ZFP518B	0.075439	0.418092	0.125609	0.900326	-5.28881	0.812719	0.87478
Erythroid.cells	ZFP141	0.054189	3.367308	0.125569	0.900358	-5.6971	0.734138	0.805926
Erythroid.cells	ATRIP	-0.05866	2.825397	-0.12548	0.90043	-5.63521	0.747966	0.818172
Erythroid.cells	TRP53	-0.02387	5.681235	-0.12546	0.900445	-6.32061	0.678037	0.755655
Erythroid.cells	FAM162A	0.03366	5.640039	0.125381	0.900506	-6.38114	0.678996	0.756522
Erythroid.cells	GTDC1	0.024946	6.409058	0.125168	0.900674	-6.46302	0.661341	0.740497
Erythroid.cells	CTPS	-0.04204	3.704287	-0.12511	0.90072	-5.86782	0.725674	0.798407
Erythroid.cells	MARK2	0.018129	6.879023	0.124889	0.900895	-6.54829	0.650792	0.730892
Erythroid.cells	C330011M	-0.08433	0.592367	-0.12482	0.900951	-5.25767	0.807844	0.870594
Erythroid.cells	RSPH10B	0.108432	0.413611	0.124793	0.900971	-5.25725	0.812845	0.874926
Erythroid.cells	PAK4	0.053337	2.299468	0.12473	0.90102	-5.58683	0.761644	0.830256
Erythroid.cells	ZFP934	0.055196	2.990341	0.12455	0.901163	-5.64269	0.743729	0.81448
Erythroid.cells	CHM	0.028902	5.555869	0.124477	0.90122	-6.18135	0.680958	0.758347
Erythroid.cells	TRIM44	0.022229	6.053928	0.124473	0.901223	-6.41865	0.669433	0.747904
Erythroid.cells	TNRC6A	-0.02026	6.648204	-0.12429	0.901371	-6.52318	0.65595	0.735632

Erythroid.cells	GM47507	0.098378	0.732528	0.124203	0.901436	-5.37744	0.803945	0.867241
Erythroid.cells	MAN1B1	-0.04251	4.927787	-0.12416	0.901471	-6.00469	0.695792	0.771724
Erythroid.cells	FIGNL2	-0.10568	-0.67701	-0.12415	0.901477	-5.23683	0.844041	0.901838
Erythroid.cells	NVL	-0.02941	4.723925	-0.12404	0.901568	-6.0321	0.70068	0.776116
Erythroid.cells	KIF1C	0.041585	3.254466	0.123997	0.901599	-5.79039	0.736995	0.808518
Erythroid.cells	TIMM21	0.050212	2.602996	0.123898	0.901678	-5.64052	0.753719	0.823309
Erythroid.cells	MMS22L	0.040776	4.820836	0.123771	0.901777	-6.18393	0.698352	0.774058
Erythroid.cells	SLC9A7	0.026475	5.115655	0.123762	0.901785	-6.39185	0.69132	0.767731
Erythroid.cells	D830050J1	-0.07342	1.312085	-0.12353	0.901966	-5.43886	0.788079	0.85348
Erythroid.cells	SIGLECE	0.085481	2.293576	0.123431	0.902046	-5.65695	0.761851	0.83051
Erythroid.cells	ATP8B1	0.07543	0.813793	0.123357	0.902104	-5.46683	0.801748	0.865398
Erythroid.cells	TRIM3	-0.05476	2.274801	-0.12321	0.902224	-5.56379	0.762344	0.830981
Erythroid.cells	PROC	-0.04832	2.923674	-0.12313	0.902286	-5.97605	0.745489	0.816127
Erythroid.cells	TRIM28	0.025813	5.460856	0.123055	0.902343	-6.33698	0.683227	0.760482
Erythroid.cells	BCOR	-0.03101	5.214585	-0.12305	0.902351	-6.17157	0.689024	0.765714
Erythroid.cells	DPF2	0.026384	5.127706	0.122789	0.902553	-6.14752	0.691133	0.76766
Erythroid.cells	NIPA2	-0.02359	6.042829	-0.1227	0.902626	-6.37566	0.669783	0.748349
Erythroid.cells	TMEM191(	0.055285	1.703176	0.122685	0.902635	-5.44173	0.777577	0.844404
Erythroid.cells	SETD1A	-0.03017	4.19796	-0.12214	0.903064	-5.95788	0.713738	0.787912
Erythroid.cells	RGS19	-0.03408	5.393744	-0.12192	0.903241	-6.10006	0.685023	0.762148
Erythroid.cells	LCMT2	0.047036	2.687418	0.121878	0.903272	-5.65002	0.751824	0.821786
Erythroid.cells	SREK1IP1	0.035779	4.172778	0.121832	0.903309	-5.96405	0.714356	0.788512
Erythroid.cells	KIFC1	0.064009	3.200672	0.121821	0.903317	-5.84914	0.738651	0.810135
Erythroid.cells	AGTR1A	0.098807	0.270352	0.121773	0.903355	-5.37197	0.817195	0.878897
Erythroid.cells	GPR174	0.054611	2.267506	0.121762	0.903364	-5.7193	0.762782	0.831452
Erythroid.cells	PPP3R1	0.023729	5.5618	0.121305	0.903724	-6.23293	0.681148	0.758711
Erythroid.cells	RNF19B	0.031572	5.758355	0.121215	0.903796	-6.26797	0.676573	0.754581
Erythroid.cells	GM20045	-0.07149	1.764585	-0.1211	0.903885	-5.442	0.776195	0.84328
Erythroid.cells	AI597479	0.063582	1.688645	0.121012	0.903956	-5.4271	0.77823	0.845067
Erythroid.cells	TGFB1I1	0.087101	0.90381	0.120885	0.904056	-5.34929	0.799592	0.863713
Erythroid.cells	RAPGEF1	0.021601	6.775482	0.120864	0.904073	-6.49704	0.653415	0.733532
Erythroid.cells	2610035D1	-0.04804	4.872984	-0.12084	0.904089	-6.0701	0.697439	0.773432
Erythroid.cells	PSPC1	-0.02717	5.702286	-0.12079	0.90413	-6.28978	0.677875	0.755789
Erythroid.cells	INPPL1	-0.0507	2.471527	-0.12064	0.904253	-5.59519	0.757506	0.826942
Erythroid.cells	DPP3	-0.03192	4.728026	-0.12053	0.904336	-6.03747	0.700919	0.776645
Erythroid.cells	RFXAP	-0.02977	4.179927	-0.12044	0.904404	-5.94931	0.714245	0.788603
Erythroid.cells	RBKS	0.045059	3.630745	0.120444	0.904405	-5.7971	0.727863	0.80075
Erythroid.cells	CDCA5	-0.05543	3.028781	-0.12041	0.904429	-5.77332	0.743103	0.814275
Erythroid.cells	CCDC125	0.040305	4.497843	0.120379	0.904456	-6.00001	0.706483	0.78166
Erythroid.cells	BCAP29	-0.03214	4.765797	-0.11996	0.904789	-6.03078	0.700112	0.776024
Erythroid.cells	MAF	0.044272	5.654779	0.11974	0.904961	-6.27481	0.679078	0.75708
Erythroid.cells	CDC73	0.021185	6.174072	0.11962	0.905056	-6.37936	0.667101	0.746219
Erythroid.cells	BE692007	-0.05548	2.816357	-0.11913	0.905444	-5.83166	0.748667	0.819325
Erythroid.cells	SNX29	0.028622	6.395771	0.119057	0.9055	-6.47066	0.662056	0.741628
Erythroid.cells	HYKK	0.075779	0.618386	0.11905	0.905506	-5.3787	0.807625	0.870943
Erythroid.cells	5330438D1	-0.02825	4.842586	-0.11896	0.905574	-6.18427	0.698268	0.774401
Erythroid.cells	ICOSL	-0.0608	1.327034	-0.11894	0.905591	-5.54981	0.788113	0.853965

Erythroid.cells	DNAJC3	-0.0209	6.725717	-0.11889	0.905629	-6.55722	0.654623	0.734847
Erythroid.cells	GAS2L3	0.03999	4.330262	0.118839	0.905672	-6.01781	0.710666	0.785524
Erythroid.cells	PAIP2B	0.037183	3.682629	0.118834	0.905676	-5.82673	0.72667	0.799812
Erythroid.cells	0610010F0	-0.03678	4.458879	-0.11879	0.90571	-5.99739	0.707532	0.782717
Erythroid.cells	D17H6S53E	0.036354	4.009289	0.118781	0.905718	-5.80175	0.718551	0.792573
Erythroid.cells	WIPF2	0.02214	5.343948	0.118768	0.905729	-6.18927	0.686355	0.763667
Erythroid.cells	FBXL12OS	-0.07438	0.679534	-0.11873	0.905758	-5.31378	0.805922	0.869466
Erythroid.cells	WDR11	-0.04211	3.124121	-0.11871	0.905776	-5.72517	0.740774	0.812342
Erythroid.cells	1700056E2	-0.0728	1.466557	-0.11866	0.905813	-5.4186	0.784328	0.850681
Erythroid.cells	NUP210L	-0.04561	6.407383	-0.11848	0.90596	-6.43767	0.661831	0.741459
Erythroid.cells	USHBP1	0.082928	1.085061	0.118413	0.906009	-5.32203	0.794766	0.859827
Erythroid.cells	KCNK10	0.087477	0.557275	0.118164	0.906206	-5.35207	0.809488	0.872533
Erythroid.cells	PIAS4	-0.03118	4.224377	-0.11799	0.906344	-5.94553	0.713397	0.788008
Erythroid.cells	LINS1	-0.06164	1.704648	-0.11792	0.906396	-5.37633	0.778065	0.84525
Erythroid.cells	ABCC2	-0.04991	2.212583	-0.11763	0.906626	-5.78071	0.764557	0.83341
Erythroid.cells	UBR7	-0.03535	4.241439	-0.11759	0.906659	-5.93751	0.712979	0.787667
Erythroid.cells	RMI2	0.043907	3.3602	0.117515	0.906719	-5.83207	0.734922	0.807236
Erythroid.cells	PCDH9	-0.11654	0.472713	-0.11747	0.906757	-5.30692	0.811855	0.874723
Erythroid.cells	HSPG2	0.052888	2.741833	0.117447	0.906773	-5.68926	0.750739	0.821265
Erythroid.cells	TNFRSF10E	0.086053	-0.0063	0.117256	0.906923	-5.2836	0.825394	0.886496
Erythroid.cells	CMTM4	-0.05005	3.141567	-0.11701	0.907119	-5.6668	0.740474	0.812284
Erythroid.cells	MON2	0.023289	5.7408	0.116968	0.907151	-6.2883	0.677211	0.755591
Erythroid.cells	MOCS2	-0.02322	5.094585	-0.11693	0.907178	-6.32837	0.692389	0.769308
Erythroid.cells	NME1	0.025953	6.668403	0.116763	0.907313	-6.59566	0.656036	0.736367
Erythroid.cells	GM27201	-0.04939	2.321008	-0.11669	0.907371	-5.53807	0.761705	0.831082
Erythroid.cells	GUCY1A1	0.093656	-0.00341	0.116546	0.907484	-5.30763	0.825311	0.886525
Erythroid.cells	GM48765	0.075754	0.952243	0.116114	0.907826	-5.34907	0.798528	0.863358
Erythroid.cells	KNSTRN	0.064095	3.271497	0.115888	0.908004	-5.74731	0.737169	0.809489
Erythroid.cells	WWC1	0.055524	1.216205	0.115856	0.90803	-5.5384	0.791287	0.857101
Erythroid.cells	TANGO6	0.034273	4.382985	0.115827	0.908052	-5.95989	0.709518	0.784837
Erythroid.cells	WDR86	-0.06751	0.234245	-0.11577	0.908099	-5.3246	0.818567	0.880805
Erythroid.cells	ZBTB39	-0.06458	1.975209	-0.11553	0.908291	-5.486	0.77084	0.839258
Erythroid.cells	UHMK1	0.025614	4.630018	0.115508	0.908304	-6.08468	0.703522	0.779498
Erythroid.cells	SELENBP2	-0.07178	1.116284	-0.11548	0.90833	-5.52393	0.79402	0.859534
Erythroid.cells	PFKM	0.067741	1.17862	0.115391	0.908397	-5.41239	0.792314	0.858047
Erythroid.cells	SRSF3	0.014676	7.532017	0.115307	0.908463	-6.73864	0.636954	0.719
Erythroid.cells	RORA	0.030974	5.020685	0.115276	0.908488	-6.42159	0.694147	0.771067
Erythroid.cells	POP4	0.037416	4.085123	0.115219	0.908533	-5.92079	0.71682	0.791412
Erythroid.cells	ACBD6	-0.02166	5.436733	-0.11516	0.908577	-6.23657	0.684308	0.762187
Erythroid.cells	PDE8A	-0.03245	5.847934	-0.11505	0.908668	-6.33628	0.674729	0.753511
Erythroid.cells	ARAF	0.030599	4.478585	0.115003	0.908704	-5.91783	0.707191	0.782791
Erythroid.cells	NDUFAF1	0.041963	2.81597	0.11499	0.908714	-5.7278	0.748824	0.819861
Erythroid.cells	UTP11	0.026798	4.845019	0.114958	0.908739	-6.08104	0.698346	0.774847
Erythroid.cells	APOL9B	0.076687	0.574633	0.114913	0.908775	-5.40473	0.809004	0.872574
Erythroid.cells	ABCB9	-0.03525	3.155352	-0.11488	0.908799	-6.04111	0.740123	0.812166
Erythroid.cells	SMIM41	-0.07769	0.89661	-0.11473	0.908916	-5.35173	0.800063	0.864818
Erythroid.cells	NDOR1	-0.04547	3.086341	-0.11473	0.90892	-5.70631	0.741883	0.813738

Erythroid.cells	MCU	-0.02575	6.164566	-0.11472	0.90893	-6.37525	0.667449	0.746917
Erythroid.cells	NAV2	-0.0362	4.841571	-0.11443	0.90916	-6.16211	0.698429	0.774956
Erythroid.cells	GM4316	-0.07873	0.707324	-0.11441	0.909171	-5.38046	0.805307	0.869394
Erythroid.cells	TBC1D30	0.08759	1.06093	0.114316	0.909247	-5.40407	0.795538	0.860896
Erythroid.cells	ADGRL1	0.044392	2.476047	0.114258	0.909292	-5.71809	0.757646	0.8277
Erythroid.cells	PRR7	0.048756	3.334702	0.114211	0.90933	-5.68237	0.735567	0.808163
Erythroid.cells	IMMT	-0.0175	6.049157	-0.11419	0.909345	-6.43991	0.670093	0.749347
Erythroid.cells	A	0.038095	4.78007	0.114075	0.909437	-6.03693	0.699906	0.77633
Erythroid.cells	ARHGAP1	0.02608	4.473602	0.11401	0.909489	-5.96451	0.707313	0.783
Erythroid.cells	SSBP1	-0.02153	5.592236	-0.11387	0.9096	-6.31317	0.680669	0.758992
Erythroid.cells	G3BP2	-0.01441	6.759047	-0.1138	0.909655	-6.54598	0.654005	0.734732
Erythroid.cells	SERPIND1	-0.04705	2.832849	-0.11363	0.909785	-5.97756	0.748389	0.819614
Erythroid.cells	TMEM177	-0.09325	0.336385	-0.11353	0.909868	-5.22378	0.815685	0.87853
Erythroid.cells	PLOD2	0.080638	1.261605	0.113508	0.909886	-5.40398	0.790049	0.856241
Erythroid.cells	SEC13	0.022161	5.361757	0.113427	0.909949	-6.26138	0.68607	0.763932
Erythroid.cells	FAM114A1	0.051556	2.398626	0.113424	0.909952	-5.73464	0.75967	0.8296
Erythroid.cells	ERLIN1	0.028443	4.765624	0.113415	0.909959	-6.12148	0.700253	0.776716
Erythroid.cells	TMC7	-0.08859	-0.40026	-0.11314	0.910174	-5.17565	0.836831	0.896778
Erythroid.cells	ZFP943	-0.02683	4.280266	-0.11293	0.910339	-5.97035	0.712145	0.787413
Erythroid.cells	PSMD3	-0.02823	4.642778	-0.1128	0.910442	-6.03754	0.703329	0.779507
Erythroid.cells	NFE2L1	-0.03058	4.98448	-0.11275	0.910483	-6.10868	0.695125	0.772127
Erythroid.cells	ZHX3	0.033169	4.441922	0.112747	0.910486	-6.03683	0.708199	0.783878
Erythroid.cells	LIPA	0.026534	5.64326	0.112678	0.910541	-6.36902	0.679591	0.758098
Erythroid.cells	NIPSNAP3E	-0.02104	5.573981	-0.11243	0.910735	-6.40234	0.681298	0.759591
Erythroid.cells	THBS3	0.081879	1.01596	0.11214	0.910966	-5.41194	0.79709	0.862302
Erythroid.cells	EIF1AD	-0.02865	4.923218	-0.11214	0.91097	-6.12605	0.69675	0.773493
Erythroid.cells	ZFP426	-0.04621	2.769149	-0.11202	0.91106	-5.61206	0.750345	0.821292
Erythroid.cells	SLC30A5	-0.02373	5.799283	-0.11189	0.911162	-6.31743	0.676153	0.754908
Erythroid.cells	GM12462	-0.08774	-0.49881	-0.11176	0.911266	-5.20536	0.839923	0.899397
Erythroid.cells	H13	-0.01811	6.42662	-0.11155	0.911432	-6.44692	0.661779	0.741841
Erythroid.cells	GM47828	0.066789	0.334612	0.111549	0.911434	-5.41176	0.816096	0.878839
Erythroid.cells	CDK12	-0.01785	6.896517	-0.11154	0.911444	-6.58729	0.651225	0.732199
Erythroid.cells	OSBPL5	-0.08393	0.3884	-0.11135	0.911592	-5.30743	0.814649	0.877532
Erythroid.cells	4931423N1	0.08503	0.653443	0.110749	0.912067	-5.30522	0.807464	0.871155
Erythroid.cells	RBM47	-0.02533	6.404832	-0.11074	0.912075	-6.60252	0.66252	0.742343
Erythroid.cells	WDR77	-0.03749	3.895337	-0.11074	0.912075	-5.81883	0.722102	0.796059
Erythroid.cells	GM1976	-0.04812	2.701657	-0.11065	0.912145	-5.60858	0.752391	0.822944
Erythroid.cells	DNAH10	-0.09398	0.024576	-0.11036	0.912371	-5.20709	0.825326	0.886559
Erythroid.cells	PIAS3	-0.04018	2.896492	-0.11024	0.912467	-5.61563	0.747488	0.818552
Erythroid.cells	GOLPH3	0.016197	5.902166	0.10987	0.912761	-6.391	0.674141	0.752891
Erythroid.cells	PLCG2	0.016767	7.02313	0.109562	0.913005	-6.55964	0.648766	0.729767
Erythroid.cells	ZFP729A	0.044889	3.051831	0.109538	0.913024	-5.69236	0.7435	0.815069
Erythroid.cells	DEF8	0.049709	2.04717	0.109521	0.913037	-5.53918	0.769689	0.838166
Erythroid.cells	PSAT1	0.038794	4.218142	0.109505	0.91305	-6.04125	0.714255	0.789038
Erythroid.cells	SLC3A1	0.066396	0.899213	0.109471	0.913077	-5.41644	0.800781	0.865338
Erythroid.cells	PRKX	-0.02252	5.379346	-0.10946	0.913087	-6.23972	0.686334	0.76394
Erythroid.cells	CNEP1R1	-0.02686	4.587392	-0.10937	0.91316	-6.02157	0.705249	0.780987

Erythroid.cells	GORAB	0.054805	1.673364	0.109361	0.913164	-5.50209	0.779675	0.84694
Erythroid.cells	SAR1B	-0.01756	5.553451	-0.10935	0.91317	-6.35283	0.682248	0.760262
Erythroid.cells	LMTK2	-0.02379	5.616268	-0.10908	0.913389	-6.2356	0.680846	0.758976
Erythroid.cells	GM19605	0.074649	1.260305	0.10884	0.913576	-5.36357	0.790941	0.856827
Erythroid.cells	ZKSCAN1	-0.02895	4.068204	-0.10877	0.913633	-5.93348	0.718015	0.792469
Erythroid.cells	MAP3K13	-0.07375	0.779749	-0.10865	0.913727	-5.39237	0.804167	0.868364
Erythroid.cells	ARG2	-0.05991	3.174154	-0.10853	0.913824	-5.73241	0.740446	0.812469
Erythroid.cells	TXK	0.03321	3.287256	0.10848	0.913861	-6.26988	0.737568	0.809921
Erythroid.cells	IIGP1	-0.06754	4.709506	-0.10817	0.914108	-6.20012	0.702365	0.778551
Erythroid.cells	4933433G1	-0.08852	0.742117	-0.10801	0.914236	-5.31052	0.805213	0.869389
Erythroid.cells	FNIP2	-0.0398	5.343736	-0.10798	0.914257	-6.50507	0.687239	0.764932
Erythroid.cells	GM31812	0.084953	0.191942	0.107865	0.914347	-5.262	0.820653	0.882772
Erythroid.cells	LRRFIP1	-0.01681	7.249941	-0.10784	0.914368	-6.65825	0.643818	0.725396
Erythroid.cells	NEURL4	0.05925	2.445552	0.107838	0.914369	-5.58621	0.759265	0.829186
Erythroid.cells	CREB1	0.017264	6.332794	0.107789	0.914408	-6.44905	0.664335	0.744163
Erythroid.cells	PRRT1	-0.06432	0.466797	-0.10776	0.914429	-5.44632	0.812902	0.876079
Erythroid.cells	SNRNP48	-0.02441	4.916287	-0.10774	0.914445	-6.11988	0.697395	0.774105
Erythroid.cells	ATP2A2	0.01583	6.456942	0.107719	0.914462	-6.49096	0.661517	0.741603
Erythroid.cells	TOPBP1	-0.02766	5.680028	-0.1076	0.914557	-6.31072	0.679359	0.757827
Erythroid.cells	TMCO3	-0.03594	3.656165	-0.10759	0.914567	-5.75607	0.728263	0.801748
Erythroid.cells	SERAC1	-0.06121	2.177244	-0.10744	0.914682	-5.47864	0.766319	0.83543
Erythroid.cells	NSRP1	0.02343	5.229533	0.107333	0.914768	-6.16871	0.689937	0.76741
Erythroid.cells	NR1H2	-0.03059	4.589379	-0.10729	0.914799	-6.0515	0.705269	0.781213
Erythroid.cells	NAGK	-0.03286	3.956557	-0.10723	0.914847	-5.91305	0.720777	0.795129
Erythroid.cells	GUCY2C	-0.06152	-0.14698	-0.10685	0.915148	-5.36968	0.830485	0.891304
Erythroid.cells	MOB3A	0.03032	4.775701	0.10681	0.915181	-6.00221	0.700915	0.777304
Erythroid.cells	MS4A6D	0.061014	2.866447	0.106451	0.915466	-5.84111	0.748635	0.819734
Erythroid.cells	OTUD1	-0.04077	3.007254	-0.10629	0.915594	-5.7124	0.745013	0.816569
Erythroid.cells	BICDL1	0.048625	1.878488	0.106137	0.915714	-5.65919	0.774565	0.842614
Erythroid.cells	EPHA1	0.080896	0.461905	0.106104	0.915741	-5.31252	0.813366	0.876452
Erythroid.cells	PGGT1B	0.024821	4.818529	0.106053	0.915781	-6.07601	0.700021	0.776454
Erythroid.cells	TOGARAM1	0.022917	5.297825	0.106037	0.915793	-6.16463	0.688599	0.766165
Erythroid.cells	GNG12	0.020013	6.49518	0.105538	0.916188	-6.4828	0.661097	0.741121
Erythroid.cells	E2F6	0.048095	2.266229	0.105476	0.916237	-5.5414	0.764487	0.833693
Erythroid.cells	CLU	-0.05029	5.440247	-0.10537	0.916323	-6.6231	0.68543	0.76323
Erythroid.cells	ALG10B	0.040856	2.952601	0.105357	0.916331	-5.64327	0.746619	0.817948
Erythroid.cells	RBBP4	0.012744	7.676556	0.105	0.916614	-6.80003	0.635079	0.717273
Erythroid.cells	PLVAP	0.06649	1.394862	0.104533	0.916984	-5.42957	0.787997	0.85434
Erythroid.cells	SLC8A2	0.090556	1.394752	0.104518	0.916995	-5.42261	0.788	0.854343
Erythroid.cells	SOX4	-0.03907	5.508952	-0.10442	0.917072	-6.35813	0.68398	0.761937
Erythroid.cells	CD300LD	0.057502	3.173108	0.104391	0.917096	-5.75859	0.741149	0.813122
Erythroid.cells	SLC35A1	0.031302	3.416075	0.104333	0.917141	-5.78069	0.734976	0.807646
Erythroid.cells	ABHD11	-0.03569	3.541149	-0.10428	0.917186	-5.83983	0.731819	0.80484
Erythroid.cells	5930430L0	-0.08325	0.229599	-0.10423	0.917223	-5.24218	0.820335	0.882449
Erythroid.cells	DARS2	-0.05497	2.849125	-0.10422	0.917235	-5.66568	0.749465	0.820487
Erythroid.cells	SFPQ	-0.01249	8.189863	-0.10394	0.917449	-6.85797	0.624061	0.70723
Erythroid.cells	ZFP654	0.022598	6.120389	0.103862	0.917514	-6.41023	0.669796	0.749168

Erythroid.cells	HAUS4	-0.03072	4.204699	-0.10381	0.917557	-5.9952	0.715307	0.790207
Erythroid.cells	L2HGDH	0.052761	1.767651	0.103769	0.917587	-5.5316	0.777929	0.84564
Erythroid.cells	UHRF1BP1	0.060553	1.998967	0.103762	0.917593	-5.53904	0.771748	0.840219
Erythroid.cells	STX6	0.024968	5.2207	0.103709	0.917635	-6.15446	0.690777	0.768171
Erythroid.cells	ZFP607A	0.0466	1.840055	0.103563	0.917751	-5.52655	0.776013	0.843939
Erythroid.cells	PPM1D	0.020217	4.948971	0.10349	0.917809	-6.22244	0.697272	0.774004
Erythroid.cells	TBC1D25	0.050132	2.839206	0.103152	0.918076	-5.57739	0.749758	0.820837
Erythroid.cells	KPNB1	-0.01945	5.739818	-0.10311	0.918111	-6.29565	0.678621	0.757193
Erythroid.cells	THOC2	0.012636	7.106814	0.103081	0.918132	-6.65003	0.647599	0.728927
Erythroid.cells	ANPEP	-0.05232	1.184937	-0.10302	0.918179	-5.78898	0.793765	0.859535
Erythroid.cells	COPB2	0.017296	5.438302	0.102823	0.918336	-6.30126	0.685674	0.763634
Erythroid.cells	FAM174A	-0.02703	6.050716	-0.10275	0.91839	-6.32374	0.67143	0.750732
Erythroid.cells	NUP62	-0.03948	4.181003	-0.10267	0.918458	-5.91431	0.715925	0.790848
Erythroid.cells	ZFP513	-0.04743	2.534011	-0.10265	0.91847	-5.5506	0.757683	0.827935
Erythroid.cells	MUS81	-0.05167	2.048507	-0.10254	0.918558	-5.52025	0.770469	0.839189
Erythroid.cells	TNNT1	-0.06203	1.520318	-0.10253	0.918571	-5.38594	0.784633	0.851602
Erythroid.cells	SP3	0.01496	6.891028	0.102388	0.91868	-6.57565	0.652394	0.733377
Erythroid.cells	ITGA6	0.03953	4.818835	0.102206	0.918825	-6.14698	0.700407	0.776941
Erythroid.cells	SSH1	0.031256	3.775324	0.102025	0.918968	-5.86448	0.725983	0.799839
Erythroid.cells	MED12	-0.02802	3.781462	-0.10199	0.918999	-5.98925	0.72583	0.799702
Erythroid.cells	GPAA1	-0.02905	3.764843	-0.10185	0.919108	-5.82057	0.726245	0.800072
Erythroid.cells	ORAI3	0.035186	3.868765	0.10183	0.919123	-5.84233	0.723653	0.797762
Erythroid.cells	VSTM4	0.089888	0.147061	0.101793	0.919152	-5.27708	0.822717	0.884724
Erythroid.cells	RSPH9	0.062933	1.514419	0.101787	0.919156	-5.48761	0.784793	0.851753
Erythroid.cells	FCSK	0.070604	0.928399	0.101733	0.919199	-5.31911	0.800823	0.865736
Erythroid.cells	POT1B	0.034575	4.421725	0.101619	0.919289	-5.97329	0.71004	0.785591
Erythroid.cells	IGKV9-124	0.077474	-1.01492	0.101366	0.91949	-5.13657	0.856484	0.913772
Erythroid.cells	NAA60	-0.02543	4.697726	-0.10135	0.919503	-6.04463	0.703393	0.779623
Erythroid.cells	ABCD2	0.036796	2.633823	0.101183	0.919635	-5.92852	0.755191	0.82573
Erythroid.cells	UBN1	-0.01309	6.944041	-0.10105	0.919736	-6.58703	0.651306	0.732406
Erythroid.cells	RBFA	-0.03034	4.826424	-0.10094	0.919824	-6.0072	0.700325	0.776894
Erythroid.cells	FBXO4	-0.03067	3.431156	-0.10093	0.919838	-5.85745	0.734737	0.807659
Erythroid.cells	SH3YL1	-0.04618	1.030881	-0.10064	0.920066	-5.56719	0.798135	0.86346
Erythroid.cells	TANC1	0.043688	3.838336	0.100531	0.92015	-5.85352	0.724538	0.798609
Erythroid.cells	ZFP422	0.026169	4.395092	0.100267	0.920359	-6.06038	0.710801	0.786361
Erythroid.cells	AKAP11	0.028326	4.648059	0.100246	0.920376	-6.02585	0.70465	0.780843
Erythroid.cells	TBC1D24	-0.05246	1.799284	-0.10024	0.920377	-5.51737	0.777255	0.845251
Erythroid.cells	EIF5B	-0.01332	7.158051	-0.10022	0.9204	-6.66096	0.646579	0.728142
Erythroid.cells	CERS6	-0.02469	7.330325	-0.10016	0.920442	-6.65609	0.642784	0.724658
Erythroid.cells	GM34961	-0.073	0.869528	-0.10016	0.920445	-5.34035	0.802592	0.867374
Erythroid.cells	GNL2	-0.02438	4.675227	-0.10005	0.920527	-6.06941	0.704001	0.780285
Erythroid.cells	TNIP3	-0.07375	1.037658	-0.09989	0.920654	-5.75014	0.798005	0.86338
Erythroid.cells	LRRC18	0.057527	1.588071	0.099676	0.920827	-5.56214	0.783079	0.850298
Erythroid.cells	FMR1	-0.01848	5.971489	-0.09957	0.920912	-6.36974	0.673503	0.752661
Erythroid.cells	CDC123	-0.02143	5.404958	-0.09945	0.921004	-6.25393	0.686726	0.764629
Erythroid.cells	HYAL1	-0.0554	1.311575	-0.0993	0.921121	-5.39186	0.79065	0.856927
Erythroid.cells	VPS29	-0.0148	6.521761	-0.09918	0.921221	-6.51795	0.660996	0.741287

Erythroid.cells	CDAN1	0.041408	2.943919	0.099075	0.921303	-5.68324	0.747416	0.818945
Erythroid.cells	ATP9B	0.017172	5.858499	0.09875	0.92156	-6.35317	0.676301	0.755204
Erythroid.cells	ACOT9	-0.02772	4.620859	-0.09857	0.921705	-5.9487	0.705639	0.781679
Erythroid.cells	GPALPP1	-0.02965	3.737889	-0.09856	0.921712	-5.80501	0.727386	0.801128
Erythroid.cells	AACS	-0.03299	3.861959	-0.09852	0.921744	-5.77943	0.724288	0.798366
Erythroid.cells	BC049352	0.065199	1.083027	0.098243	0.921962	-5.42038	0.797084	0.862557
Erythroid.cells	FGR	0.049641	4.732993	0.098236	0.921968	-6.01407	0.702936	0.77928
Erythroid.cells	1110006O2	0.071733	0.319918	0.098136	0.922047	-5.22318	0.818358	0.881047
Erythroid.cells	PPIL6	-0.08215	0.219778	-0.09807	0.922101	-5.19648	0.821193	0.88353
Erythroid.cells	CD300LB	-0.06077	1.973752	-0.09805	0.922118	-5.62949	0.772965	0.841508
Erythroid.cells	NDST1	-0.03437	4.570663	-0.09778	0.92233	-5.93611	0.706913	0.782946
Erythroid.cells	STX18	-0.02734	4.637177	-0.09758	0.922486	-5.98065	0.7053	0.781505
Erythroid.cells	F2RL1	0.06962	-1.13295	0.097574	0.922491	-5.26631	0.860522	0.917431
Erythroid.cells	TRDJ1	-0.05576	-1.29553	-0.09754	0.922517	-5.34113	0.865368	0.921573
Erythroid.cells	SERPINC1	-0.02993	5.285455	-0.09736	0.922661	-6.55164	0.689777	0.767527
Erythroid.cells	KIF3B	-0.03333	3.495441	-0.09735	0.92267	-5.72632	0.733538	0.806739
Erythroid.cells	PIK3CA	0.018528	6.128996	0.097329	0.922686	-6.42101	0.670116	0.749712
Erythroid.cells	PLAU	-0.08045	0.814983	-0.09718	0.922806	-5.3884	0.804587	0.869163
Erythroid.cells	GM48623	-0.08001	-0.24558	-0.09708	0.922882	-5.20814	0.834597	0.895133
Erythroid.cells	TSR2	-0.05694	2.118125	-0.09695	0.922987	-5.45958	0.769246	0.838248
Erythroid.cells	RAD54L	-0.04348	2.724058	-0.0968	0.923101	-5.66115	0.753349	0.824307
Erythroid.cells	TIMM8A1	0.029654	4.226992	0.096675	0.923203	-6.03866	0.715374	0.790558
Erythroid.cells	ZKSCAN6	0.045706	2.942031	0.096634	0.923235	-5.60466	0.747713	0.819338
Erythroid.cells	TROAP	0.058612	1.774645	0.096426	0.923401	-5.45731	0.778411	0.846382
Erythroid.cells	TMEM160	-0.01878	5.666014	-0.09642	0.923408	-6.28301	0.680892	0.759519
Erythroid.cells	MRPL42	-0.01918	6.521235	-0.0964	0.923418	-6.58208	0.661235	0.741648
Erythroid.cells	RUFY3	0.023101	5.26954	0.096211	0.92357	-6.16575	0.690274	0.767972
Erythroid.cells	SIAE	0.042688	2.612072	0.095827	0.923875	-5.60144	0.756516	0.826998
Erythroid.cells	RWDD3	0.06416	0.507573	0.095693	0.923981	-5.32285	0.813479	0.876813
Erythroid.cells	RGP1	-0.03936	2.34849	-0.09564	0.924025	-5.50182	0.763423	0.833083
Erythroid.cells	ZBTB10	0.036022	4.077959	0.095433	0.924187	-5.86842	0.719312	0.793975
Erythroid.cells	AP4M1	-0.02719	3.581284	-0.09543	0.924191	-5.79995	0.731706	0.805026
Erythroid.cells	PIRB	-0.04354	4.941687	-0.09534	0.924263	-6.05339	0.69828	0.775118
Erythroid.cells	TRIP10	0.047723	1.355077	0.095141	0.924418	-5.47696	0.790059	0.856451
Erythroid.cells	JUND	0.016275	10.62781	0.095123	0.924432	-7.28463	0.575132	0.661686
Erythroid.cells	GM826	0.088975	-0.25558	0.09497	0.924553	-5.30502	0.83523	0.895668
Erythroid.cells	VPS33A	0.026868	4.291318	0.094938	0.924579	-5.89334	0.714066	0.789324
Erythroid.cells	GM10130	-0.04988	1.783227	-0.09468	0.924782	-5.48044	0.778476	0.846454
Erythroid.cells	ZFP106	0.018825	6.288522	0.094498	0.924927	-6.40101	0.666777	0.746719
Erythroid.cells	NFYA	-0.02919	4.152707	-0.09443	0.924981	-5.90608	0.717476	0.792466
Erythroid.cells	PTPN21	0.066856	0.813665	0.094362	0.925035	-5.32199	0.804958	0.86961
Erythroid.cells	LRRC20	-0.04704	2.057401	-0.09407	0.925268	-5.5282	0.771151	0.840124
Erythroid.cells	RICTOR	-0.01597	6.409431	-0.09405	0.925281	-6.45206	0.664023	0.744288
Erythroid.cells	RUNX3	0.023344	5.712717	0.094043	0.925288	-6.50125	0.680061	0.758866
Erythroid.cells	ETAA1	0.03278	3.334503	0.094025	0.925302	-5.78568	0.737958	0.810803
Erythroid.cells	SLC27A2	0.035331	3.949769	0.094004	0.925319	-6.25942	0.7225	0.797039
Erythroid.cells	PSMB7	0.015914	5.624583	0.093865	0.925429	-6.36494	0.682119	0.760731

Erythroid.cells	FBXL15	-0.03886	2.696607	-0.09367	0.925581	-5.64282	0.754348	0.825357
Erythroid.cells	ETAA1OS	-0.06078	0.543342	-0.09359	0.925646	-5.29646	0.812505	0.876262
Erythroid.cells	FNBP1L	-0.04151	3.079155	-0.09356	0.925668	-5.80415	0.744474	0.81662
Erythroid.cells	TSPOAP1	-0.04821	2.650845	-0.09351	0.925708	-5.60891	0.755538	0.826409
Erythroid.cells	ELL	0.024167	5.024631	0.093489	0.925726	-6.05571	0.696304	0.773578
Erythroid.cells	CDADC1	-0.03215	4.609962	-0.09335	0.925836	-6.0051	0.70629	0.782574
Erythroid.cells	FAHD1	0.041272	1.917334	0.093253	0.925913	-5.72052	0.774884	0.843455
Erythroid.cells	AW146154	-0.04874	2.334177	-0.09311	0.926029	-5.55588	0.763828	0.833767
Erythroid.cells	GM28403	0.061787	1.458517	0.093058	0.926068	-5.46243	0.787244	0.854314
Erythroid.cells	DNAJB1	-0.02327	6.228259	-0.09305	0.926071	-6.39609	0.668154	0.748128
Erythroid.cells	MIDN	-0.01872	6.064607	-0.09297	0.926138	-6.33999	0.67191	0.751544
Erythroid.cells	P4HA3	0.073744	0.308298	0.092912	0.926184	-5.22925	0.819125	0.882083
Erythroid.cells	PPIB	-0.01279	7.605608	-0.09285	0.926231	-6.81324	0.637416	0.720032
Erythroid.cells	CHD2	-0.01554	7.754934	-0.09271	0.926343	-6.73852	0.634203	0.717059
Erythroid.cells	CACUL1	0.019554	6.117834	0.092178	0.926765	-6.3911	0.670775	0.750612
Erythroid.cells	IYD	0.060623	0.499692	0.092148	0.926789	-5.37991	0.813837	0.877587
Erythroid.cells	PAQR4	-0.06521	1.484926	-0.09189	0.92699	-5.46721	0.786631	0.8539
Erythroid.cells	GM47889	-0.0602	1.560566	-0.09179	0.927071	-5.41379	0.784581	0.852108
Erythroid.cells	RBM22	-0.01441	5.603703	-0.09176	0.927093	-6.30622	0.682698	0.761441
Erythroid.cells	STFA1	0.049696	5.975982	0.091709	0.927137	-6.47779	0.674042	0.753589
Erythroid.cells	GM14305	0.036924	2.260659	0.091654	0.92718	-5.57359	0.765867	0.835683
Erythroid.cells	BC005624	0.015511	5.761918	0.09157	0.927247	-6.30765	0.679005	0.758094
Erythroid.cells	GM45894	0.035705	2.290972	0.091541	0.92727	-5.57922	0.765068	0.834979
Erythroid.cells	RRS1	-0.02611	4.476059	-0.09148	0.927319	-5.99305	0.70964	0.785724
Erythroid.cells	PRICKLE3	0.047264	2.034653	0.09125	0.927501	-5.48282	0.771858	0.840971
Erythroid.cells	EDC3	-0.02803	3.628178	-0.09121	0.927528	-5.81495	0.730633	0.804508
Erythroid.cells	GKAP1	-0.03249	3.541031	-0.09117	0.92756	-5.86389	0.732828	0.806468
Erythroid.cells	LSM8	0.019875	5.264944	0.0911	0.927619	-6.20646	0.690677	0.7687
Erythroid.cells	OSTC	0.013371	6.387429	0.091024	0.927679	-6.53876	0.664611	0.745063
Erythroid.cells	LPCAT2	-0.03266	4.726381	-0.09102	0.927681	-6.03025	0.703564	0.780327
Erythroid.cells	PCDH17	0.050376	2.459624	0.090981	0.927714	-5.69231	0.760634	0.831135
Erythroid.cells	ZFP521	0.068235	2.100385	0.090962	0.927729	-5.46255	0.770111	0.83948
Erythroid.cells	NME4	0.051535	2.364464	0.090484	0.928108	-5.58278	0.763305	0.833373
Erythroid.cells	DNAH8	-0.03121	3.758702	-0.09038	0.928191	-5.97901	0.727524	0.801665
Erythroid.cells	ABHD2	0.015249	5.471153	0.090287	0.928263	-6.42107	0.685963	0.764347
Erythroid.cells	KLKB1	0.042225	1.848942	0.090284	0.928266	-5.70176	0.776992	0.845405
Erythroid.cells	PKP3	0.039779	2.747004	0.09024	0.928301	-5.77718	0.753309	0.824553
Erythroid.cells	ICA1L	-0.05135	1.590667	-0.08989	0.928581	-5.54541	0.784085	0.851575
Erythroid.cells	NOC3L	-0.04082	3.216026	-0.08985	0.928612	-5.72031	0.741371	0.813949
Erythroid.cells	RUFY1	-0.01791	5.607628	-0.08959	0.928818	-6.24424	0.682893	0.761556
Erythroid.cells	LCP2	-0.02992	5.109024	-0.08953	0.928863	-6.23181	0.694674	0.772212
Erythroid.cells	ACVR2A	0.033093	4.7385	0.089522	0.928869	-5.99489	0.703567	0.780218
Erythroid.cells	ATP5G3	0.014381	8.015505	0.089437	0.928937	-6.87718	0.628909	0.712192
Erythroid.cells	TMEM129	0.046179	2.084833	0.089372	0.928988	-5.56292	0.770848	0.840009
Erythroid.cells	OTUD7B	-0.02122	5.17042	-0.0891	0.929207	-6.15604	0.693291	0.770905
Erythroid.cells	BROX	-0.01923	4.85097	-0.08879	0.929449	-6.04511	0.700935	0.777866
Erythroid.cells	ABLIM2	-0.06654	-0.47962	-0.08867	0.929549	-5.29867	0.842279	0.90214

Erythroid.cells	C1QBP	0.020077	6.055545	0.088472	0.929702	-6.43735	0.672567	0.752269
Erythroid.cells	PRMT9	-0.02926	4.074852	-0.08847	0.929707	-5.90093	0.719879	0.794914
Erythroid.cells	CACNA1I	-0.05078	-0.17458	-0.08833	0.929812	-5.27731	0.833453	0.894636
Erythroid.cells	MEIS3	-0.06246	0.488054	-0.0883	0.929836	-5.29989	0.8146	0.878335
Erythroid.cells	RUFY2	-0.03082	2.954919	-0.08824	0.929882	-5.73505	0.748165	0.820125
Erythroid.cells	SESN1	0.018169	5.939971	0.08822	0.929902	-6.37808	0.675236	0.754737
Erythroid.cells	IRF2	-0.01594	6.689517	-0.08819	0.929927	-6.51876	0.658127	0.73915
Erythroid.cells	HIVEP3	0.028423	5.144836	0.088168	0.929942	-6.08426	0.6939	0.771631
Erythroid.cells	UCHL3	0.017274	6.235223	0.088141	0.929964	-6.43256	0.668441	0.748558
Erythroid.cells	SHE	-0.06598	0.946591	-0.08798	0.930093	-5.37326	0.801832	0.867268
Erythroid.cells	HEXDC	0.057438	1.86348	0.087924	0.930136	-5.47366	0.776865	0.845448
Erythroid.cells	IL20RB	0.033216	3.81612	0.087736	0.930285	-5.78482	0.726393	0.800776
Erythroid.cells	STAC2	-0.05563	1.824947	-0.08765	0.930356	-5.51925	0.777961	0.846407
Erythroid.cells	NDEL1	0.015664	6.927832	0.087284	0.930643	-6.52355	0.653007	0.734399
Erythroid.cells	PTRHD1	0.024971	4.56189	0.087052	0.930827	-6.03646	0.708185	0.784413
Erythroid.cells	ZFP446	0.05424	1.164128	0.087034	0.930841	-5.36106	0.796099	0.862167
Erythroid.cells	LSS	0.064516	0.604735	0.086939	0.930916	-5.25054	0.811619	0.875669
Erythroid.cells	GM26535	0.062915	-0.60061	0.086753	0.931064	-5.28333	0.846111	0.905455
Erythroid.cells	JAK3	0.040435	2.778845	0.086748	0.931068	-5.66137	0.752988	0.824317
Erythroid.cells	GATM	0.028059	4.530012	0.086671	0.931129	-6.15131	0.708961	0.785109
Erythroid.cells	CYSLTR2	0.042622	1.496466	0.086477	0.931283	-5.76673	0.787023	0.854241
Erythroid.cells	AP2S1	-0.01298	6.932961	-0.08647	0.93129	-6.60401	0.652906	0.734305
Erythroid.cells	R3HCC1L	0.01856	5.540599	0.086319	0.931408	-6.2508	0.68479	0.763329
Erythroid.cells	MTFP1	0.06225	0.572509	0.086248	0.931464	-5.32313	0.812522	0.876483
Erythroid.cells	DDX11	-0.04142	2.934033	-0.08606	0.931615	-5.60724	0.748973	0.820818
Erythroid.cells	NCSTN	0.018657	5.576404	0.086019	0.931646	-6.24601	0.68395	0.762616
Erythroid.cells	LSM1	-0.01718	5.251939	-0.08597	0.931683	-6.18227	0.691604	0.769536
Erythroid.cells	DHCR24	0.0316	2.897631	0.085904	0.931737	-5.86008	0.749913	0.82165
Erythroid.cells	PIK3C3	0.022606	4.439233	0.085888	0.931749	-6.0032	0.711176	0.787145
Erythroid.cells	ASH2L	-0.02427	4.017187	-0.08571	0.931889	-5.85317	0.721621	0.796476
Erythroid.cells	POLR2E	0.025086	4.876402	0.085302	0.932214	-6.11132	0.700788	0.777799
Erythroid.cells	PRMT1	0.016029	5.803475	0.085181	0.93231	-6.34903	0.678851	0.757981
Erythroid.cells	SPNS3	-0.03502	3.650358	-0.08517	0.93232	-5.902	0.730951	0.804802
Erythroid.cells	ALPK2	-0.04421	1.260876	-0.08507	0.932396	-5.56515	0.793689	0.860119
Erythroid.cells	IFI30	0.020835	6.746076	0.084903	0.93253	-6.59422	0.657304	0.738404
Erythroid.cells	GM5165	-0.0343	2.638407	-0.0848	0.932616	-5.65059	0.756883	0.827859
Erythroid.cells	JARID2	-0.01272	8.144583	-0.08466	0.932726	-6.90748	0.626646	0.710231
Erythroid.cells	EIF3G	0.018824	4.982954	0.084473	0.932871	-6.18237	0.698243	0.775574
Erythroid.cells	9230111E0	0.068438	-0.18455	0.084456	0.932885	-5.26205	0.834309	0.895415
Erythroid.cells	GM43260	-0.04153	2.252383	-0.08444	0.932897	-5.52403	0.76702	0.836803
Erythroid.cells	E430018J2	-0.05688	0.653485	-0.08429	0.933015	-5.32271	0.810515	0.874826
Erythroid.cells	CRACR2A	0.04097	2.835916	0.084266	0.933035	-5.68394	0.751751	0.823331
Erythroid.cells	AI506816	-0.02571	5.207229	-0.08423	0.933064	-6.28293	0.692889	0.770747
Erythroid.cells	TSNAX	-0.02213	4.49606	-0.08394	0.933293	-6.02411	0.710053	0.786245
Erythroid.cells	A430033K0	0.061619	0.6406	0.083796	0.933408	-5.33611	0.810916	0.87524
Erythroid.cells	GSTCD	-0.03226	3.564196	-0.0835	0.933646	-5.76715	0.733173	0.806952
Erythroid.cells	BCAM	-0.08984	0.298097	-0.08348	0.93366	-5.27982	0.820561	0.883651

Erythroid.cells	PATZ1	0.025156	4.251704	0.08337	0.933746	-5.89971	0.71604	0.791685
Erythroid.cells	HINFP	0.028083	3.494398	0.083333	0.933776	-5.78073	0.734936	0.808555
Erythroid.cells	PHF20	-0.01425	6.472356	-0.08331	0.933796	-6.43299	0.663522	0.744229
Erythroid.cells	WDR43	0.016178	6.033494	0.083286	0.933812	-6.41303	0.673569	0.753378
Erythroid.cells	ELDR	0.047738	3.256101	0.08327	0.933825	-5.70392	0.740989	0.813934
Erythroid.cells	LDAH	0.021702	4.225642	0.083256	0.933836	-5.96706	0.716682	0.792267
Erythroid.cells	FOXD2OS	-0.06195	0.311339	-0.08282	0.934181	-5.27289	0.820425	0.883407
Erythroid.cells	VPS37C	0.023325	3.950923	0.082676	0.934296	-5.85211	0.723728	0.798387
Erythroid.cells	ZFP748	0.045476	1.938501	0.08257	0.93438	-5.51097	0.775675	0.844374
Erythroid.cells	CCDC66	0.036097	2.265203	0.082341	0.934562	-5.55234	0.767077	0.83679
Erythroid.cells	CD46	0.041105	2.11028	0.082152	0.934712	-5.5682	0.771249	0.840435
Erythroid.cells	A630089N(	0.039233	2.672384	0.081787	0.935001	-5.59461	0.756627	0.827495
Erythroid.cells	SH3GL1	-0.02081	4.688824	-0.08154	0.935197	-6.00861	0.706011	0.782356
Erythroid.cells	RASSF4	-0.02517	4.553252	-0.08135	0.935346	-6.09332	0.70935	0.785376
Erythroid.cells	SMIM13	-0.02667	3.919727	-0.08128	0.9354	-5.79753	0.724967	0.799348
Erythroid.cells	EMILIN1	-0.02811	2.608435	-0.08076	0.935815	-5.72796	0.758594	0.829052
Erythroid.cells	USP6NL	-0.01669	5.563085	-0.0806	0.935939	-6.31507	0.685322	0.763603
Erythroid.cells	MAP7	0.02685	3.639692	0.080522	0.936004	-5.8656	0.732131	0.805602
Erythroid.cells	DVL1	0.037254	2.507773	0.080187	0.93627	-5.58905	0.76123	0.831376
Erythroid.cells	XLR4B	-0.04123	1.285179	-0.08019	0.93627	-5.46487	0.794009	0.86012
Erythroid.cells	GM10501	0.041714	2.138944	0.080143	0.936304	-5.46463	0.770969	0.839947
Erythroid.cells	MEG3	-0.07518	1.040515	-0.08004	0.936385	-5.43918	0.800741	0.865987
Erythroid.cells	RGS9	0.063975	0.03543	0.080037	0.936389	-5.29921	0.829011	0.890496
Erythroid.cells	GM28417	0.055546	1.320479	0.079933	0.936471	-5.33271	0.793042	0.859277
Erythroid.cells	GM16174	-0.04875	0.995634	-0.07989	0.936508	-5.35892	0.801982	0.867067
Erythroid.cells	IP6K1	-0.01401	6.598351	-0.07989	0.936508	-6.56055	0.661445	0.741909
Erythroid.cells	NDUFAF2	-0.0191	4.440034	-0.0798	0.936578	-6.11203	0.712258	0.787881
Erythroid.cells	MTA1	0.020069	4.55479	0.079668	0.936682	-6.05105	0.709455	0.785398
Erythroid.cells	STX2	0.021238	3.698442	0.07965	0.936696	-5.91159	0.730652	0.804344
Erythroid.cells	SESN3	0.019336	4.83647	0.0796	0.936735	-6.20721	0.702624	0.779273
Erythroid.cells	TUBGCP2	0.023696	3.994036	0.079529	0.936791	-5.89044	0.723261	0.797789
Erythroid.cells	PAPOLA	0.008945	7.055885	0.079492	0.936821	-6.61107	0.651175	0.732604
Erythroid.cells	PKNOX2	0.069719	-0.82371	0.079318	0.936959	-5.20873	0.85403	0.912126
Erythroid.cells	NUP133	-0.02243	3.691763	-0.07912	0.937114	-5.89325	0.730866	0.804598
Erythroid.cells	ARFGEF1	-0.0146	6.613928	-0.0791	0.937133	-6.48274	0.661134	0.741737
Erythroid.cells	SLC24A5	0.019577	4.423305	0.07897	0.937235	-6.13674	0.712712	0.788391
Erythroid.cells	RALGAPA1	0.018012	6.84207	0.078959	0.937243	-6.50746	0.655994	0.73705
Erythroid.cells	ZMIZ1	0.012115	6.978265	0.078622	0.937511	-6.6048	0.653068	0.734342
Erythroid.cells	METTL26	0.018162	4.639923	0.078555	0.937564	-6.29716	0.70756	0.783745
Erythroid.cells	RERE	-0.01129	7.702335	-0.07834	0.937732	-6.77804	0.63714	0.7197
Erythroid.cells	PEX2	-0.02595	3.914925	-0.07832	0.93775	-5.90295	0.725445	0.799727
Erythroid.cells	RAET1E	-0.02284	4.113795	-0.07819	0.937853	-6.05064	0.720501	0.795312
Erythroid.cells	TMEM135	0.016455	5.7512	0.078137	0.937896	-6.37335	0.681117	0.759873
Erythroid.cells	HIST1H3A	0.049941	0.953909	0.077882	0.938098	-5.36774	0.803386	0.868392
Erythroid.cells	CYTH2	0.025225	3.933028	0.077848	0.938125	-5.87724	0.725005	0.799346
Erythroid.cells	GM15943	-0.06498	0.207344	-0.0778	0.938161	-5.2633	0.82436	0.886583
Erythroid.cells	FANCI	-0.03268	2.266344	-0.07772	0.93823	-5.59267	0.767828	0.837285

Erythroid.cells	OLFR77	-0.04528	1.376904	-0.07766	0.938273	-5.46193	0.791745	0.858246
Erythroid.cells	RPA1	0.019838	4.922792	0.077511	0.938392	-6.1658	0.700792	0.777657
Erythroid.cells	TMEM59	-0.01139	6.649746	-0.07732	0.938542	-6.55907	0.660516	0.741172
Erythroid.cells	CCNJL	0.045127	1.734552	0.077192	0.938645	-5.45978	0.782071	0.849817
Erythroid.cells	FARSB	0.016918	5.008108	0.077179	0.938655	-6.16164	0.698743	0.775833
Erythroid.cells	DNAJB12	0.018206	4.757611	0.077152	0.938677	-6.09368	0.704778	0.781263
Erythroid.cells	KLHL15	-0.02013	4.413728	-0.07691	0.938865	-5.98853	0.713205	0.788842
Erythroid.cells	PLEKHG1	0.023684	4.398533	0.076884	0.93889	-6.18414	0.713577	0.789176
Erythroid.cells	APOF	0.025449	3.847555	0.07662	0.939099	-6.25239	0.727324	0.801384
Erythroid.cells	TPD52	0.016529	8.000639	0.076537	0.939165	-6.77886	0.630858	0.713895
Erythroid.cells	SLC13A3	-0.05159	1.11728	-0.07621	0.939427	-5.53015	0.79922	0.864703
Erythroid.cells	TESMIN	-0.05529	0.971475	-0.07614	0.939484	-5.41432	0.803251	0.868213
Erythroid.cells	GIMAP4	0.027375	4.091849	0.075982	0.939605	-6.13968	0.72141	0.796069
Erythroid.cells	SIDT2	-0.01861	5.063847	-0.07566	0.939863	-6.1292	0.697806	0.774865
Erythroid.cells	GM16337	-0.0405	1.967439	-0.07565	0.939867	-5.55124	0.776259	0.844589
Erythroid.cells	EHMT1	0.013902	6.063885	0.075493	0.939993	-6.40013	0.674321	0.753628
Erythroid.cells	GALK1	0.018242	5.140655	0.075226	0.940205	-6.26508	0.696033	0.773299
Erythroid.cells	SACM1L	0.01633	5.504292	0.075201	0.940225	-6.23666	0.687403	0.76551
Erythroid.cells	DOP1B	0.022778	4.289272	0.075176	0.940244	-6.00834	0.716685	0.791846
Erythroid.cells	PRMT2	0.038178	1.665423	0.074931	0.940439	-5.49216	0.78456	0.851868
Erythroid.cells	PEX13	-0.01572	5.524698	-0.07456	0.940734	-6.27392	0.687174	0.76522
Erythroid.cells	APLP2	-0.01602	5.805079	-0.07439	0.940868	-6.44654	0.680645	0.759312
Erythroid.cells	NLGN2	-0.0645	0.349554	-0.07425	0.940982	-5.31346	0.821266	0.883768
Erythroid.cells	ZKSCAN17	0.023434	3.498798	0.074193	0.941025	-5.82933	0.736763	0.809686
Erythroid.cells	KLF6	-0.01752	7.581942	-0.07402	0.941165	-6.72196	0.640547	0.722702
Erythroid.cells	HTR7	0.034402	0.754108	0.073803	0.941334	-6.09409	0.809922	0.87392
Erythroid.cells	CPSF6	-0.00889	6.68211	-0.07368	0.941431	-6.55621	0.660552	0.741064
Erythroid.cells	RAB40C	-0.0185	4.838453	-0.07368	0.941433	-6.06526	0.703642	0.780095
Erythroid.cells	KLF7	0.02162	6.159891	0.073621	0.941478	-6.27611	0.672466	0.75192
Erythroid.cells	USP39	-0.02191	4.614508	-0.07345	0.941615	-6.02632	0.709075	0.785022
Erythroid.cells	GM20234	-0.03578	2.059362	-0.07335	0.941692	-5.51291	0.77426	0.842878
Erythroid.cells	CD80	0.027292	3.929604	0.072951	0.94201	-6.12575	0.725965	0.800211
Erythroid.cells	SH3BP1	0.017465	5.21446	0.072874	0.942071	-6.23315	0.694619	0.772112
Erythroid.cells	SQLE	-0.0397	1.97366	-0.07283	0.942104	-5.52638	0.776551	0.844984
Erythroid.cells	CMC2	0.026472	4.535117	0.072745	0.942173	-6.08953	0.711011	0.786859
Erythroid.cells	CPNE8	0.03708	2.649239	0.072715	0.942197	-5.72195	0.758678	0.82927
Erythroid.cells	SBK1	0.022328	3.374185	0.072439	0.942416	-5.93684	0.739971	0.812754
Erythroid.cells	FBXL17	0.011206	7.560342	0.072361	0.942478	-6.71755	0.641019	0.723352
Erythroid.cells	GM3055	0.054464	0.319624	0.072344	0.942492	-5.26135	0.822161	0.884781
Erythroid.cells	GTSF2	0.06437	0.091247	0.072277	0.942545	-5.23006	0.82867	0.89044
Erythroid.cells	RGS7BP	0.064332	1.883105	0.072213	0.942596	-5.37867	0.77898	0.847206
Erythroid.cells	KIF24	0.022405	3.667581	0.072202	0.942604	-5.86184	0.732538	0.806178
Erythroid.cells	RFT1	-0.0348	2.53189	-0.07201	0.942753	-5.598	0.761752	0.832104
Erythroid.cells	DYNC1LI2	0.017867	4.672528	0.071894	0.942849	-6.04855	0.707663	0.783984
Erythroid.cells	FGF13	0.035226	3.379693	0.071798	0.942925	-5.93211	0.739831	0.812732
Erythroid.cells	OGFR	0.018748	5.244677	0.071779	0.94294	-6.21397	0.693899	0.771618
Erythroid.cells	GM43672	0.042611	1.700182	0.071636	0.943054	-5.48216	0.78391	0.851627

Erythroid.cells	ITPKB	0.012693	7.462345	0.071556	0.943117	-6.75747	0.643168	0.725445
Erythroid.cells	CD209G	-0.11874	1.089148	-0.07126	0.943349	-5.38889	0.80061	0.866225
Erythroid.cells	PPM1F	0.036146	2.206568	0.071201	0.943399	-5.4985	0.770341	0.839734
Erythroid.cells	SMAD5	0.020364	3.702902	0.071152	0.943437	-5.82635	0.731648	0.80552
Erythroid.cells	SAT1	-0.01308	8.474503	-0.07111	0.94347	-6.92098	0.621339	0.705339
Erythroid.cells	MGAT5	0.013345	6.916886	0.070909	0.94363	-6.56277	0.655269	0.736638
Erythroid.cells	PHETA1	-0.0533	0.450779	-0.07088	0.943651	-5.29156	0.818447	0.881823
Erythroid.cells	IRF2BP1	0.019668	4.152538	0.070776	0.943736	-5.93782	0.720422	0.795577
Erythroid.cells	CSTF2	0.020683	4.139659	0.07052	0.943939	-5.95605	0.720741	0.795879
Erythroid.cells	ZFP69	-0.0377	2.612118	-0.07041	0.944027	-5.53861	0.759649	0.830446
Erythroid.cells	GM43126	-0.0509	0.276018	-0.07033	0.944086	-5.32999	0.8234	0.886148
Erythroid.cells	GM4221	0.041148	1.846721	0.070216	0.944181	-5.42268	0.779958	0.848311
Erythroid.cells	SGPL1	-0.01627	5.922758	-0.0702	0.944191	-6.2862	0.677951	0.757345
Erythroid.cells	DPYS	0.02911	2.829004	0.070159	0.944225	-5.98894	0.753994	0.825451
Erythroid.cells	IGLC3	-0.04255	4.04289	-0.07013	0.944251	-6.14858	0.723143	0.798034
Erythroid.cells	TMEM138	0.027757	2.910493	0.07011	0.944264	-5.61527	0.75188	0.823582
Erythroid.cells	PGAM1	0.0143	6.892134	0.070082	0.944287	-6.6042	0.655824	0.737179
Erythroid.cells	BCS1L	-0.04339	1.699946	-0.07005	0.944308	-5.45957	0.783916	0.85178
Erythroid.cells	GM13166	0.0547	-0.19353	0.070048	0.944313	-5.2329	0.836859	0.897772
Erythroid.cells	ATP5A1	-0.0091	7.857813	-0.06997	0.944374	-6.83962	0.634543	0.717628
Erythroid.cells	STARD4	0.023539	3.526928	0.069938	0.944401	-5.83979	0.736092	0.809577
Erythroid.cells	TCF20	-0.01186	7.561094	-0.06991	0.944424	-6.71588	0.641003	0.723579
Erythroid.cells	TTF2	0.026831	3.091877	0.069723	0.944571	-5.77683	0.747198	0.819459
Erythroid.cells	CDS1	-0.03841	2.788548	-0.06967	0.944613	-5.66253	0.755045	0.826404
Erythroid.cells	DPYSL3	-0.05511	0.753878	-0.06962	0.944657	-5.27521	0.809928	0.874492
Erythroid.cells	C8A	0.044459	0.525618	0.06956	0.944701	-5.40686	0.816335	0.880053
Erythroid.cells	FAM133B	-0.01481	4.911343	-0.06947	0.944769	-6.12251	0.701883	0.778995
Erythroid.cells	FASTKD2	-0.0227	2.952537	-0.06934	0.944878	-5.71651	0.750792	0.822672
Erythroid.cells	DTYMK	0.020022	5.126752	0.069302	0.944905	-6.18279	0.696712	0.774365
Erythroid.cells	E230014E1	-0.04959	-1.38289	-0.06923	0.944959	-5.19206	0.871949	0.927943
Erythroid.cells	CEP131	0.049577	1.3043	0.069165	0.945014	-5.41404	0.794689	0.861282
Erythroid.cells	FUBP3	0.020324	4.08675	0.069088	0.945075	-5.9453	0.722053	0.797153
Erythroid.cells	DENR	0.011618	6.279481	0.068806	0.945299	-6.41643	0.669725	0.750023
Erythroid.cells	DYRK1B	0.031379	1.826196	0.068764	0.945333	-5.51697	0.780518	0.848979
Erythroid.cells	CENPP	0.020927	5.39603	0.06868	0.9454	-6.34465	0.690312	0.768719
Erythroid.cells	NEDD4L	-0.0106	6.886893	-0.06862	0.945444	-6.60477	0.655948	0.737465
Erythroid.cells	TOR1B	-0.02201	3.704769	-0.06862	0.945449	-5.81113	0.731609	0.805775
Erythroid.cells	AKAP9	0.012935	6.105265	0.068504	0.945539	-6.40634	0.673745	0.753693
Erythroid.cells	POU2F1	0.018986	5.39401	0.068389	0.94563	-6.23949	0.690382	0.768791
Erythroid.cells	CPTP	-0.0291	2.248177	-0.06831	0.945696	-5.54349	0.769268	0.839153
Erythroid.cells	IFI27L2A	0.034762	7.373467	0.068181	0.945796	-6.62336	0.645169	0.727603
Erythroid.cells	4930557KC	0.043145	1.483636	0.0676	0.946257	-5.38197	0.790167	0.857358
Erythroid.cells	KBTBD3	-0.02949	2.727361	-0.06739	0.946421	-5.6407	0.757015	0.828272
Erythroid.cells	IFT172	-0.02741	2.410455	-0.06732	0.946479	-5.65463	0.765327	0.835621
Erythroid.cells	AGL	-0.02688	3.963587	-0.06725	0.946532	-5.85114	0.725478	0.80028
Erythroid.cells	IVD	-0.01632	4.435995	-0.06715	0.946617	-6.13519	0.713792	0.789826
Erythroid.cells	B4GALT3	0.018495	3.874826	0.067045	0.946697	-5.84628	0.727696	0.802292

Erythroid.cells	DIS3	-0.02309	3.258023	-0.06698	0.946748	-5.77719	0.743306	0.816182
Erythroid.cells	CDK16	-0.02086	3.39772	-0.06694	0.946782	-5.77016	0.73974	0.813016
Erythroid.cells	HMG20A	-0.01719	4.897858	-0.06653	0.947102	-6.09371	0.702623	0.779805
Erythroid.cells	S100PBP	-0.01644	4.405401	-0.06641	0.947199	-5.98884	0.714609	0.790581
Erythroid.cells	GM45370	-0.06361	-1.29914	-0.06637	0.947233	-5.13791	0.869944	0.926373
Erythroid.cells	LNX2	0.021296	3.592505	0.066291	0.947295	-5.89774	0.734866	0.808687
Erythroid.cells	SULT2A8	0.050011	0.571296	0.066223	0.94735	-5.42183	0.81553	0.879554
Erythroid.cells	DGKQ	-0.03742	1.470676	-0.06614	0.947419	-5.41951	0.790607	0.85785
Erythroid.cells	CORO2A	-0.01887	5.527432	-0.06612	0.947431	-6.1833	0.687607	0.766285
Erythroid.cells	GM12764	0.040472	1.453299	0.066094	0.947452	-5.42971	0.791081	0.858265
Erythroid.cells	XKR8	-0.0482	0.500884	-0.06579	0.947692	-5.31111	0.81766	0.881276
Erythroid.cells	GRK2	0.009847	7.206149	0.065426	0.947983	-6.62978	0.649469	0.731308
Erythroid.cells	CD300C2	0.025539	4.335062	0.065127	0.94822	-6.08452	0.716706	0.792161
Erythroid.cells	LRRC42	0.020577	3.272197	0.065116	0.948229	-5.81217	0.743393	0.815944
Erythroid.cells	GM10550	0.047518	0.590308	0.064965	0.948349	-5.28805	0.815453	0.879105
Erythroid.cells	UGT3A2	-0.03693	1.174889	-0.0647	0.94856	-5.53211	0.799283	0.864945
Erythroid.cells	NIF3L1	0.023638	3.000203	0.064108	0.949029	-5.69829	0.750717	0.822231
Erythroid.cells	F8	0.032209	3.134673	0.063958	0.949148	-5.76367	0.747249	0.819161
Erythroid.cells	REXO1	-0.01516	4.912691	-0.06369	0.949362	-6.11191	0.702934	0.779654
Erythroid.cells	ADCY4	0.042152	1.413611	0.063612	0.949423	-5.56406	0.792919	0.859371
Erythroid.cells	ARFGAP2	0.012455	4.901097	0.063578	0.94945	-6.16139	0.703213	0.779914
Erythroid.cells	HMGB3	-0.0191	4.658182	-0.06354	0.949477	-6.22078	0.709104	0.78521
Erythroid.cells	NUDCD2	-0.01783	4.534449	-0.06343	0.949564	-6.08659	0.712125	0.787935
Erythroid.cells	DAGLB	0.019539	4.366228	0.06323	0.949726	-5.97915	0.716253	0.791677
Erythroid.cells	HIST1H2BG	-0.04528	1.128352	-0.06315	0.949786	-5.39004	0.800761	0.866312
Erythroid.cells	ARMT1	-0.01931	3.297769	-0.06311	0.949818	-5.76895	0.743065	0.815597
Erythroid.cells	GRAMD1A	-0.02046	4.325204	-0.06307	0.94985	-5.96724	0.717263	0.792606
Erythroid.cells	GM48696	0.028875	2.476417	0.063033	0.949882	-5.64289	0.764387	0.834448
Erythroid.cells	TIMM22	0.015848	4.4471	0.062857	0.950022	-5.99899	0.714265	0.789921
Erythroid.cells	ZFP202	-0.05248	0.604939	-0.06269	0.950156	-5.28087	0.815359	0.879038
Erythroid.cells	ZFP267	-0.03403	1.251689	-0.06245	0.950346	-5.36973	0.797361	0.863427
Erythroid.cells	HOXA7	-0.0554	-0.09629	-0.06239	0.950395	-5.18717	0.83534	0.896359
Erythroid.cells	CDH13	0.050502	2.030566	0.062382	0.9504	-5.56579	0.776225	0.844935
Erythroid.cells	HBP1	-0.01272	5.788072	-0.06235	0.950421	-6.3554	0.682139	0.761044
Erythroid.cells	ZFP24	-0.01575	4.243197	-0.06228	0.950481	-5.96764	0.719288	0.794489
Erythroid.cells	FKBP1A	-0.00879	7.336878	-0.06228	0.950482	-6.76063	0.646927	0.728927
Erythroid.cells	NEMP2	0.021739	2.854914	0.062166	0.950571	-5.69591	0.754483	0.825783
Erythroid.cells	RHBDL3	-0.04868	0.747589	-0.06206	0.950657	-5.30591	0.811354	0.875603
Erythroid.cells	STPG4	0.030397	2.37884	0.062023	0.950684	-5.55648	0.766961	0.836791
Erythroid.cells	ACOT4	-0.05193	0.046894	-0.06194	0.950748	-5.2805	0.83122	0.892804
Erythroid.cells	ATXN2L	0.012184	5.47603	0.061931	0.950758	-6.23937	0.689476	0.767684
Erythroid.cells	SLC17A9	0.033188	2.888147	0.061927	0.950761	-5.61315	0.75362	0.82502
Erythroid.cells	NFYB	0.012984	5.103926	0.061922	0.950765	-6.28403	0.698334	0.775679
Erythroid.cells	NPL	0.034951	2.535175	0.061731	0.950916	-5.65857	0.76284	0.833161
Erythroid.cells	GM15706	0.041798	1.015787	0.061437	0.95115	-5.305	0.803878	0.869104
Erythroid.cells	VIM	-0.0158	8.109275	-0.06125	0.951297	-6.87432	0.630094	0.713424
Erythroid.cells	NHP2	-0.01432	5.748278	-0.06124	0.951304	-6.33912	0.68307	0.761887

Erythroid.cells	PGP	0.014567	5.374059	0.061235	0.95131	-6.2672	0.691891	0.769867
Erythroid.cells	ZFP397	0.017581	3.944609	0.061212	0.951329	-5.82894	0.72671	0.801119
Erythroid.cells	MDK	-0.03915	1.436872	-0.06107	0.951445	-5.43708	0.792283	0.858995
Erythroid.cells	SPAG9	0.011611	9.266573	0.061063	0.951447	-7.06853	0.605743	0.690818
Erythroid.cells	IKBIP	-0.0314	1.938543	-0.06087	0.951598	-5.48177	0.778692	0.847153
Erythroid.cells	UNC119B	0.019264	3.551645	0.060854	0.951613	-5.89418	0.736601	0.809981
Erythroid.cells	EMC7	0.008982	6.059195	0.060841	0.951623	-6.40789	0.675831	0.755369
Erythroid.cells	SUSD1	0.01592	5.183728	0.060833	0.95163	-6.23625	0.696424	0.774007
Erythroid.cells	RAN	0.012156	8.400943	0.060446	0.951937	-6.93238	0.623925	0.707724
Erythroid.cells	OIT3	-0.031	2.345496	-0.06023	0.952109	-5.61168	0.767924	0.837687
Erythroid.cells	ZFYVE16	0.023839	3.016629	0.060102	0.95221	-5.67823	0.750371	0.822218
Erythroid.cells	NIT1	0.017947	3.750254	0.060091	0.952219	-5.92777	0.731661	0.805603
Erythroid.cells	RNF214	0.014894	4.961908	0.060003	0.952289	-6.07226	0.70182	0.778907
Erythroid.cells	LEPR	0.039455	1.823369	0.059955	0.952327	-5.57056	0.781872	0.84999
Erythroid.cells	METTL7B	-0.03416	2.263028	-0.0599	0.952373	-5.81057	0.77011	0.839663
Erythroid.cells	PRPF39	-0.01319	5.43876	-0.05988	0.952388	-6.20967	0.69043	0.768639
Erythroid.cells	ZBTB21	-0.01923	3.713914	-0.05979	0.952454	-5.83465	0.732576	0.806445
Erythroid.cells	LRRC57	-0.03022	2.732576	-0.05972	0.952516	-5.63598	0.757749	0.828769
Erythroid.cells	EEF2KMT	-0.02058	3.145957	-0.0595	0.952685	-5.75736	0.747037	0.819351
Erythroid.cells	GM41611	0.044163	0.284798	0.059387	0.952778	-5.32954	0.824507	0.887193
Erythroid.cells	HIST1H1B	-0.029	5.277543	-0.05939	0.952779	-6.45941	0.694259	0.772169
Erythroid.cells	ZC3HAV1L	0.024171	3.053639	0.059198	0.952928	-5.77931	0.749416	0.821541
Erythroid.cells	OLR1	0.042183	1.333367	0.059132	0.95298	-5.69668	0.7952	0.861796
Erythroid.cells	CAR12	-0.04435	0.013745	-0.0591	0.953006	-5.2574	0.832259	0.893967
Erythroid.cells	GM37529	0.036933	1.962925	0.058933	0.953139	-5.50607	0.778119	0.846857
Erythroid.cells	DOCK11	0.010383	7.111908	0.058902	0.953163	-6.60178	0.651987	0.733793
Erythroid.cells	FAM43A	-0.01991	4.098571	-0.05881	0.953235	-5.86091	0.722949	0.798007
Erythroid.cells	JPT1	0.008395	7.832843	0.058552	0.953441	-6.84676	0.636131	0.719291
Erythroid.cells	CHRM3	0.046124	0.831294	0.058508	0.953476	-5.41982	0.809098	0.874017
Erythroid.cells	MMP13	-0.06768	-1.01302	-0.05834	0.953606	-5.18813	0.8623	0.919991
Erythroid.cells	TIPIN	-0.01909	5.238466	-0.05832	0.953625	-6.25871	0.69519	0.773242
Erythroid.cells	PDLIM1	0.015284	4.944927	0.058227	0.953699	-6.22286	0.70223	0.779587
Erythroid.cells	GULO	-0.02799	1.982596	-0.05809	0.953806	-5.71688	0.777591	0.846593
Erythroid.cells	NDST2	0.021672	2.934923	0.057999	0.953881	-5.71954	0.752486	0.824485
Erythroid.cells	BICD2	-0.01797	4.09822	-0.05796	0.953913	-5.88761	0.722957	0.798222
Erythroid.cells	A530032D1	0.040894	0.594014	0.05792	0.953943	-5.36653	0.815752	0.87992
Erythroid.cells	SF3A1	0.012145	4.851981	0.057907	0.953954	-6.12499	0.704474	0.781648
Erythroid.cells	GM10762	0.02444	2.549426	0.057673	0.954139	-5.61365	0.762546	0.833374
Erythroid.cells	GTPBP6	0.024343	2.84005	0.057661	0.954149	-5.66114	0.754949	0.826664
Erythroid.cells	LIG3	0.022946	3.358188	0.057564	0.954226	-5.73419	0.741599	0.814832
Erythroid.cells	ZFP930	-0.02761	2.204594	-0.05756	0.954233	-5.55381	0.771662	0.841405
Erythroid.cells	PEAR1	0.024242	2.352213	0.057527	0.954255	-5.69518	0.767746	0.83796
Erythroid.cells	CNIH1	-0.01239	5.17624	-0.05748	0.954292	-6.24001	0.696676	0.774635
Erythroid.cells	6-Mar	0.008867	6.3538	0.057407	0.954351	-6.43507	0.669117	0.749668
Erythroid.cells	LSM5	-0.01253	5.652829	-0.05719	0.954521	-6.31655	0.685451	0.764506
Erythroid.cells	CBX3	0.008738	8.102288	0.056862	0.954784	-6.88189	0.6305	0.714169
Erythroid.cells	SMYD3	-0.01149	6.67093	-0.05648	0.955087	-6.54913	0.662219	0.74323

Erythroid.cells	IP6K2	0.016135	3.644965	0.056449	0.955112	-5.87315	0.734677	0.808551
Erythroid.cells	TCEA3	-0.02352	2.275566	-0.05612	0.955373	-5.89148	0.770307	0.839948
Erythroid.cells	PIGS	0.015872	4.078714	0.055845	0.955591	-5.88431	0.724047	0.798896
Erythroid.cells	NMD3	-0.01053	4.999909	-0.05565	0.955745	-6.12901	0.701491	0.778712
Erythroid.cells	BMP6	0.052574	0.450706	0.055594	0.955791	-5.16808	0.820482	0.883743
Erythroid.cells	SHMT1	-0.01856	4.012421	-0.05558	0.955803	-5.99905	0.725699	0.800419
Erythroid.cells	SKP2	-0.02133	3.310393	-0.05534	0.95599	-5.75458	0.74349	0.816268
Erythroid.cells	ATP5C1	0.006112	8.358211	0.055315	0.956012	-6.93286	0.625398	0.709266
Erythroid.cells	CAP1	-0.00873	6.878742	-0.05515	0.95614	-6.51198	0.657836	0.739082
Erythroid.cells	FCHO1	0.017204	3.772111	0.054849	0.956382	-6.0009	0.731941	0.805847
Erythroid.cells	KCTD18	0.026012	3.044012	0.054647	0.956543	-5.70098	0.750572	0.822375
Erythroid.cells	RAD23B	-0.00723	6.360558	-0.05443	0.956718	-6.48977	0.669772	0.749867
Erythroid.cells	A230072C0	0.028706	1.545767	0.054421	0.956722	-5.48285	0.79035	0.857379
Erythroid.cells	GM48089	-0.04437	1.135961	-0.05439	0.956745	-5.31314	0.801605	0.867199
Erythroid.cells	BZW2	-0.01102	5.869307	-0.0543	0.956819	-6.38269	0.681137	0.760224
Erythroid.cells	4933406I1	-0.01812	4.543564	-0.05379	0.957223	-6.23579	0.713085	0.788862
Erythroid.cells	D130043K2	0.033776	1.054045	0.053563	0.957404	-5.42545	0.804243	0.869281
Erythroid.cells	TEX261	0.010775	5.229001	0.053338	0.957583	-6.18555	0.696621	0.773962
Erythroid.cells	PGRMC2	-0.0145	3.628957	-0.0533	0.957609	-5.82825	0.735994	0.809212
Erythroid.cells	UBXN4	-0.00622	6.814787	-0.05305	0.957813	-6.55008	0.659876	0.740576
Erythroid.cells	9930021J0	0.009543	6.407459	0.052953	0.957889	-6.45657	0.669139	0.74904
Erythroid.cells	GM19705	-0.03392	1.868138	-0.0528	0.958009	-5.49095	0.782137	0.849926
Erythroid.cells	SLC12A3	0.03015	1.874484	0.052757	0.958045	-5.59234	0.781965	0.849791
Erythroid.cells	APIP	0.013294	4.220389	0.052591	0.958177	-5.97037	0.721328	0.796135
Erythroid.cells	MAML1	0.011821	5.435368	0.052347	0.95837	-6.21716	0.69194	0.769719
Erythroid.cells	SLC52A3	0.031515	0.451484	0.051868	0.958751	-5.41833	0.821737	0.884285
Erythroid.cells	RNF149	-0.01741	5.786169	-0.05175	0.958844	-6.27493	0.683902	0.762336
Erythroid.cells	RABGGTB	0.015817	3.732854	0.051446	0.959086	-5.86444	0.733923	0.807236
Erythroid.cells	MBTD1	0.00878	7.420536	0.051352	0.959161	-6.6914	0.646756	0.728477
Erythroid.cells	ALKBH2	0.020769	2.187068	0.05129	0.95921	-5.55201	0.774053	0.842707
Erythroid.cells	PAPOLG	0.020395	3.005898	0.051238	0.959251	-5.69772	0.752519	0.823731
Erythroid.cells	ANKRD44	-0.00855	8.18547	-0.05105	0.9594	-6.86769	0.630091	0.713132
Erythroid.cells	PPCDC	-0.01913	3.567947	-0.051	0.959442	-5.7112	0.738099	0.810949
Erythroid.cells	ARMC7	0.016814	3.924467	0.050914	0.959509	-5.88163	0.729102	0.802942
Erythroid.cells	FHDC1	0.033205	0.748905	0.050695	0.959683	-5.42229	0.813423	0.877072
Erythroid.cells	CNKSR3	0.018733	4.754523	0.050666	0.959706	-6.07209	0.708597	0.784603
Erythroid.cells	GM50163	0.035084	0.458993	0.050561	0.959789	-5.31735	0.821605	0.884161
Erythroid.cells	LIN9	0.014607	4.133885	0.050375	0.959937	-5.99323	0.72387	0.798276
Erythroid.cells	ZDHHC9	-0.01667	4.094804	-0.05014	0.960127	-5.87997	0.724844	0.799176
Erythroid.cells	CHAMP1	0.020617	2.843874	0.050113	0.960145	-5.64366	0.75673	0.827485
Erythroid.cells	PROSER3	0.032089	1.105594	0.050025	0.960215	-5.35122	0.803471	0.868459
Erythroid.cells	CD40LG	-0.02825	-0.56972	-0.04992	0.960295	-5.46294	0.851314	0.909801
Erythroid.cells	GM4631	0.033775	0.664941	0.049841	0.960361	-5.26926	0.815784	0.879154
Erythroid.cells	ATG16L1	0.01143	5.446329	0.049828	0.960372	-6.22107	0.69197	0.769667
Erythroid.cells	MRPL15	-0.00951	5.337746	-0.04979	0.960405	-6.30955	0.694553	0.771997
Erythroid.cells	GTPBP1	-0.01408	4.641527	-0.04937	0.960734	-5.98957	0.711352	0.787201
Erythroid.cells	RDH14	-0.01382	3.731822	-0.04931	0.960787	-5.885	0.733949	0.807389

Erythroid.cells	MYL12B	-0.0064	8.183707	-0.04928	0.960806	-6.8483	0.630129	0.713282
Erythroid.cells	TMEM186	-0.02666	2.111676	-0.04919	0.960878	-5.51346	0.776067	0.844612
Erythroid.cells	HAUS1	0.017483	3.65784	0.049176	0.96089	-5.85328	0.73582	0.809053
Erythroid.cells	GM13481	-0.041	0.231781	-0.04912	0.960935	-5.2742	0.828075	0.8899
Erythroid.cells	RCSD1	-0.00992	7.091061	-0.04912	0.960937	-6.618	0.654078	0.735308
Erythroid.cells	CDC6	0.020917	3.269665	0.04908	0.960966	-5.83485	0.745716	0.81784
Erythroid.cells	4632427E1	-0.01394	4.404143	-0.04907	0.960971	-5.97851	0.717177	0.792421
Erythroid.cells	IQCE	0.025534	2.144332	0.04902	0.961014	-5.53314	0.775194	0.843845
Erythroid.cells	AIMP2	-0.01849	3.206699	-0.04899	0.961035	-5.79137	0.747334	0.819274
Erythroid.cells	GM28791	0.015539	3.918458	0.048833	0.961162	-5.92732	0.729294	0.803206
Erythroid.cells	STK39	-0.01999	3.517793	-0.04871	0.961259	-6.05021	0.739434	0.812227
Erythroid.cells	GRTP1	-0.02936	0.35364	-0.04853	0.961402	-5.38479	0.824704	0.886912
Erythroid.cells	2610306M	0.033435	0.680103	0.048344	0.961551	-5.37056	0.815462	0.878921
Erythroid.cells	SFN	-0.02777	2.056108	-0.04832	0.961569	-5.54573	0.777655	0.845947
Erythroid.cells	ZMIZ2	0.013021	4.532649	0.048112	0.961736	-5.98847	0.714109	0.789662
Erythroid.cells	MTDH	-0.00517	7.470279	-0.04795	0.961867	-6.6777	0.64574	0.727679
Erythroid.cells	NSD1	-0.00637	7.273059	-0.04794	0.961871	-6.66828	0.650105	0.731684
Erythroid.cells	DUS1L	0.014604	4.064535	0.047941	0.961871	-5.95158	0.725691	0.800048
Erythroid.cells	CEP78	-0.01834	3.001386	-0.0479	0.961903	-5.63683	0.752732	0.824071
Erythroid.cells	BRPF3	0.009906	4.451036	0.047739	0.962032	-6.12392	0.716114	0.791502
Erythroid.cells	GM26737	-0.0379	0.478605	-0.04774	0.962034	-5.24131	0.821154	0.883951
Erythroid.cells	TMEM9B	0.009078	5.558526	0.047179	0.962477	-6.22537	0.689625	0.767548
Erythroid.cells	ATAD2	0.012236	6.309192	0.047159	0.962492	-6.49203	0.672113	0.751674
Erythroid.cells	9930111J2	0.021213	4.28297	0.046981	0.962634	-5.88817	0.720548	0.79531
Erythroid.cells	TBC1D7	0.017967	2.220043	0.046748	0.96282	-5.57832	0.773613	0.842295
Erythroid.cells	SYNE2	-0.0149	5.373755	-0.04672	0.962844	-6.12448	0.69409	0.771522
Erythroid.cells	D830036C2	0.034794	0.420218	0.046652	0.962896	-5.32211	0.823173	0.885492
Erythroid.cells	GM43378	0.024768	1.243694	0.046324	0.963156	-5.38803	0.800262	0.865546
Erythroid.cells	ICK	-0.02305	2.147459	-0.04588	0.963509	-5.53081	0.775744	0.84414
Erythroid.cells	IRGQ	-0.02455	2.194038	-0.04576	0.963603	-5.53221	0.774499	0.843049
Erythroid.cells	ABRAXAS2	-0.01031	4.960803	-0.04572	0.963635	-6.11618	0.704171	0.780577
Erythroid.cells	PAQR8	0.037799	0.223677	0.045607	0.963726	-5.26618	0.828984	0.890511
Erythroid.cells	1110038B1	0.013749	4.235292	0.045569	0.963757	-5.99274	0.721941	0.796525
Erythroid.cells	MFSD10	0.01274	4.464143	0.045535	0.963784	-6.04829	0.716286	0.791467
Erythroid.cells	UCHL5	0.009425	5.728606	0.045404	0.963888	-6.27517	0.685866	0.764109
Erythroid.cells	LDLRAD3	0.013897	4.591753	0.045382	0.963905	-6.16398	0.713152	0.78868
Erythroid.cells	RWDD1	-0.00746	6.105394	-0.04534	0.963938	-6.41432	0.677067	0.756135
Erythroid.cells	ST3GAL1	0.013292	5.796993	0.045339	0.96394	-6.41262	0.68426	0.762656
Erythroid.cells	ASB4	-0.04041	1.68448	-0.0448	0.964365	-5.46946	0.788461	0.855161
Erythroid.cells	SERPINA10	-0.02587	0.966902	-0.04472	0.964428	-5.48207	0.808225	0.872385
Erythroid.cells	CELSR1	0.032937	1.626552	0.044705	0.964443	-5.49343	0.790038	0.856542
Erythroid.cells	ARHGEF18	-0.01015	5.591269	-0.04448	0.964618	-6.37777	0.689343	0.767126
Erythroid.cells	ZDHHC14	0.014059	7.022709	0.044456	0.964641	-6.56442	0.656373	0.737158
Erythroid.cells	TMEM154	-0.02137	2.583653	-0.04419	0.964851	-5.66086	0.76454	0.834138
Erythroid.cells	SKINT3	-0.02507	0.115469	-0.044	0.965005	-5.49562	0.832492	0.893393
Erythroid.cells	GM29093	-0.02294	1.269702	-0.04399	0.965012	-5.36605	0.799976	0.865209
Erythroid.cells	SNHG12	-0.01683	3.189103	-0.04392	0.965066	-5.74176	0.748762	0.820241

Erythroid.cells	4933407K1	0.024529	1.39879	0.043625	0.965301	-5.34807	0.796555	0.862208
Erythroid.cells	ABL2	0.010223	6.21523	0.043517	0.965387	-6.37101	0.674975	0.754061
Erythroid.cells	KATNA1	0.010037	4.864725	0.043268	0.965585	-6.16438	0.707061	0.783004
Erythroid.cells	NOS1AP	0.019614	3.163666	0.042895	0.965882	-5.84935	0.749824	0.820992
Erythroid.cells	SORL1	0.013216	5.66068	0.042792	0.965964	-6.35368	0.688178	0.765867
Erythroid.cells	CAMSAP2	-0.01147	4.496866	-0.04253	0.966172	-6.02812	0.716281	0.791126
Erythroid.cells	EHMT2	0.010409	4.951712	0.042511	0.966187	-6.09931	0.705178	0.781172
Erythroid.cells	SF3B4	0.009389	5.494923	0.042383	0.966289	-6.25744	0.692176	0.769501
Erythroid.cells	D330041H(	0.019361	2.247493	0.042102	0.966513	-5.55358	0.773988	0.842329
Erythroid.cells	GM15283	-0.01474	4.261918	-0.04204	0.966565	-5.96445	0.722134	0.796427
Erythroid.cells	NEURL2	-0.02807	0.801683	-0.04197	0.966615	-5.4255	0.813568	0.876907
Erythroid.cells	TAF1A	-0.01644	3.171877	-0.04197	0.966615	-5.71756	0.749728	0.820984
Erythroid.cells	SMARCAD1	0.008838	4.984115	0.041674	0.966853	-6.11263	0.704516	0.780656
Erythroid.cells	RTCA	-0.01175	4.282973	-0.04166	0.966866	-5.96117	0.721689	0.796043
Erythroid.cells	GM44148	0.025309	1.90428	0.041559	0.966944	-5.49803	0.783289	0.850517
Erythroid.cells	DTWD2	0.018101	2.777791	0.041302	0.967149	-5.69791	0.760099	0.830158
Erythroid.cells	TUBB3	0.026197	0.736309	0.041148	0.967271	-5.36533	0.815542	0.878651
Erythroid.cells	PCED1B	0.01374	4.966857	0.041121	0.967292	-6.14473	0.704975	0.781115
Erythroid.cells	SENP1	0.008538	5.013417	0.041038	0.967358	-6.12576	0.703849	0.780122
Erythroid.cells	SLC49A4	0.013784	6.30055	0.041033	0.967362	-6.30544	0.673465	0.752662
Erythroid.cells	MILR1	-0.01228	4.330073	-0.04087	0.967495	-5.90358	0.720609	0.795128
Erythroid.cells	GM15788	-0.03623	0.240256	-0.04077	0.967568	-5.21046	0.829679	0.890892
Erythroid.cells	ZBTB22	0.017081	3.076444	0.040507	0.967781	-5.65575	0.752476	0.823396
Erythroid.cells	MIPOL1	0.010353	4.257052	0.040286	0.967956	-5.99919	0.722548	0.796783
Erythroid.cells	MFSD6	-0.01192	4.864614	-0.04022	0.968008	-6.25831	0.707623	0.78342
Erythroid.cells	ARC	-0.03169	0.905214	-0.04017	0.96805	-5.30321	0.810995	0.874632
Erythroid.cells	RYR2	-0.04521	0.059974	-0.04005	0.968142	-5.20209	0.835008	0.895412
Erythroid.cells	MXD4	-0.01049	5.790486	-0.04001	0.968178	-6.36746	0.6855	0.763493
Erythroid.cells	MPHOSPH(	-0.011	3.799303	-0.03945	0.968622	-5.88148	0.734291	0.807094
Erythroid.cells	A230072E1	-0.03338	0.278064	-0.0393	0.968743	-5.23167	0.829102	0.890103
Erythroid.cells	AAGAB	-0.0097	4.862045	-0.03918	0.968835	-6.06484	0.708005	0.78358
Erythroid.cells	PANK4	0.013326	3.423163	0.038807	0.969132	-5.80317	0.74401	0.815663
Erythroid.cells	GM28981	-0.02574	0.488246	-0.03868	0.969234	-5.36092	0.823237	0.885037
Erythroid.cells	ZMYND19	0.016733	3.409984	0.038445	0.96942	-5.7666	0.744348	0.816031
Erythroid.cells	3830403N1	0.036571	0.704685	0.038358	0.969489	-5.34116	0.817109	0.87981
Erythroid.cells	CRBN	0.01027	4.060609	0.038277	0.969553	-5.94499	0.727873	0.801422
Erythroid.cells	PPP3CA	0.005043	8.812084	0.038267	0.969561	-6.93991	0.618631	0.702135
Erythroid.cells	SPATS2	-0.01731	2.720159	-0.03823	0.969594	-5.75983	0.762241	0.831883
Erythroid.cells	TMEM158	0.03279	0.271092	0.03814	0.969662	-5.32106	0.829432	0.890479
Erythroid.cells	ARHGEF6	-0.00893	5.643537	-0.03802	0.96976	-6.21645	0.689368	0.766913
Erythroid.cells	TOR2A	0.01166	3.670287	0.037984	0.969786	-5.82996	0.73771	0.810225
Erythroid.cells	ZMAT3	-0.02189	1.902429	-0.03787	0.969877	-5.56416	0.784031	0.851079
Erythroid.cells	CTU2	-0.01309	3.206943	-0.03784	0.969902	-5.76767	0.749569	0.820755
Erythroid.cells	FAM234B	0.020205	2.096184	0.037388	0.97026	-5.5282	0.77881	0.846578
Erythroid.cells	9330160F1	0.017921	2.295032	0.037314	0.970319	-5.55166	0.77349	0.841953
Erythroid.cells	MAP1S	0.013106	3.781603	0.037165	0.970437	-5.81917	0.734891	0.807862
Erythroid.cells	TMEM222	-0.01105	4.289191	-0.03713	0.970468	-5.95195	0.722176	0.796528

Erythroid.cells	ZFP933	0.014065	3.490032	0.037105	0.970485	-5.77433	0.7423	0.814443
Erythroid.cells	PHC3	0.009559	4.499127	0.03662	0.97087	-6.03553	0.716984	0.791899
Erythroid.cells	HELB	-0.0125	3.704754	-0.03658	0.9709	-5.85302	0.736836	0.809604
Erythroid.cells	GOLGA5	-0.00847	4.913418	-0.03657	0.970909	-6.067	0.706855	0.782819
Erythroid.cells	NAA10	-0.00915	5.084078	-0.03646	0.971	-6.21486	0.702726	0.779129
Erythroid.cells	PNPT1	0.010555	4.198204	0.036256	0.97116	-5.96601	0.724438	0.798637
Erythroid.cells	SNUPN	0.014034	2.593487	0.036189	0.971213	-5.57531	0.765575	0.83511
Erythroid.cells	ORC4	0.006986	5.046376	0.036152	0.971243	-6.18724	0.703636	0.78001
Erythroid.cells	ST3GAL6	0.010428	5.092875	0.036112	0.971275	-6.12355	0.702514	0.779005
Erythroid.cells	SMARCAL1	-0.01376	2.628016	-0.03592	0.971427	-5.59879	0.764665	0.834319
Erythroid.cells	ADGB	0.022971	2.335955	0.035899	0.971444	-5.66669	0.7724	0.841124
Erythroid.cells	IRF4	0.010977	4.497863	0.03577	0.971547	-6.12846	0.717016	0.792017
Erythroid.cells	PTGS2	0.026088	2.215449	0.035669	0.971627	-5.81603	0.775615	0.843948
Erythroid.cells	COMMD3	0.006706	5.85015	0.035549	0.971722	-6.36786	0.684503	0.762753
Erythroid.cells	LBH	0.005961	6.308404	0.035508	0.971754	-6.4481	0.673842	0.753084
Erythroid.cells	SCIMP	-0.01865	3.702449	-0.03551	0.971757	-5.74536	0.736895	0.809747
Erythroid.cells	GINS3	0.016915	2.330816	0.035389	0.971849	-5.59102	0.772537	0.841244
Erythroid.cells	TMEM168	0.010002	4.583494	0.035359	0.971873	-5.95598	0.714909	0.790131
Erythroid.cells	USP1	0.007679	5.737466	0.035344	0.971886	-6.35455	0.687152	0.76515
Erythroid.cells	CAP2	-0.02631	0.071585	-0.03529	0.971925	-5.35524	0.835165	0.895743
Erythroid.cells	ATXN7L1OS	-0.02252	1.337166	-0.03527	0.971942	-5.40602	0.799467	0.864807
Erythroid.cells	RAB2B	0.01178	3.661593	0.035221	0.971983	-5.81802	0.737931	0.810668
Erythroid.cells	CCDC22	0.01272	3.49155	0.035193	0.972005	-5.72951	0.742261	0.814513
Erythroid.cells	EDRF1	-0.01069	4.217889	-0.03518	0.972017	-5.95371	0.723948	0.798213
Erythroid.cells	LSM7	0.005867	6.346432	0.035163	0.972029	-6.52005	0.672965	0.752287
Erythroid.cells	PELP1	0.012053	3.358177	0.035053	0.972117	-5.7792	0.745677	0.817578
Erythroid.cells	ADCK1	-0.0142	3.101425	-0.03495	0.9722	-5.69185	0.752297	0.823462
Erythroid.cells	PAF1	-0.00844	4.600235	-0.0349	0.972236	-6.02495	0.714498	0.789819
Erythroid.cells	PDPK1	-0.00947	6.462559	-0.03484	0.972286	-6.42126	0.670295	0.749923
Erythroid.cells	GNAQ	-0.00616	7.882192	-0.03468	0.97241	-6.73568	0.638552	0.720889
Erythroid.cells	PRDM1	0.01708	2.929068	0.034684	0.97241	-5.76038	0.756776	0.827458
Erythroid.cells	4930435F1	-0.0268	0.358699	-0.03457	0.972498	-5.34811	0.82694	0.888768
Erythroid.cells	RAD50	0.00865	4.906644	0.034324	0.972696	-6.07966	0.707081	0.783182
Erythroid.cells	SLC5A10	-0.02986	-0.1778	-0.03428	0.97273	-5.20465	0.842462	0.902111
Erythroid.cells	ATXN7L3	-0.00944	4.200833	-0.03412	0.972862	-5.90579	0.724436	0.798726
Erythroid.cells	DRAP1	-0.00491	6.508223	-0.03407	0.972901	-6.51671	0.669307	0.749034
Erythroid.cells	PPP1R42	0.024718	0.580161	0.034025	0.972934	-5.39398	0.820701	0.883337
Erythroid.cells	CCNT1	0.004668	6.841664	0.033714	0.973181	-6.56538	0.661833	0.742157
Erythroid.cells	GM17387	0.024417	0.498836	0.033351	0.97347	-5.29044	0.823292	0.885476
Erythroid.cells	SNHG3	-0.00944	5.291632	-0.03333	0.973489	-6.21231	0.69804	0.774964
Erythroid.cells	ARPC1B	-0.0044	8.892807	-0.03315	0.973628	-6.96358	0.617241	0.701098
Erythroid.cells	NUP153	0.005954	5.848135	0.033054	0.973706	-6.36365	0.684895	0.763138
Erythroid.cells	TPPP	-0.03427	0.182012	-0.03274	0.973957	-5.20817	0.832504	0.893431
Erythroid.cells	TLR13	0.014943	1.881588	0.032727	0.973966	-5.78983	0.785082	0.852227
Erythroid.cells	1600012H(	-0.01167	2.771016	-0.03261	0.974062	-5.64524	0.761397	0.831462
Erythroid.cells	ARHGAP25	-0.00683	6.214201	-0.03216	0.974416	-6.42123	0.676623	0.755511
Erythroid.cells	SNX16	0.012928	2.847666	0.032118	0.974451	-5.66617	0.759578	0.82973

Erythroid.cells	PEX11B	0.011323	3.446594	0.031715	0.974771	-5.74004	0.744207	0.816075
Erythroid.cells	PNPLA1	-0.02832	0.231599	-0.03165	0.974822	-5.20563	0.831454	0.892367
Erythroid.cells	HACD2	-0.00551	5.732831	-0.03148	0.974959	-6.37806	0.687997	0.765758
Erythroid.cells	ENPP5	-0.02309	0.877362	-0.03123	0.975153	-5.38087	0.813124	0.876508
Erythroid.cells	HIC1	0.02111	2.158311	0.031223	0.975162	-5.61947	0.777977	0.845852
Erythroid.cells	DLGAP4	-0.00848	5.015503	-0.03121	0.975171	-6.13742	0.705137	0.781204
Erythroid.cells	GM26917	0.007951	5.74366	0.031177	0.975199	-6.41659	0.687742	0.765527
Erythroid.cells	RNF135	-0.01611	2.08421	-0.03115	0.975217	-5.49345	0.779967	0.847596
Erythroid.cells	CARNS1	0.01977	2.476067	0.030984	0.975352	-5.49398	0.769503	0.83841
Erythroid.cells	MAD1L1	-0.00624	5.227133	-0.03098	0.975358	-6.17747	0.700034	0.776615
Erythroid.cells	BRD9	0.006803	5.185663	0.030835	0.975471	-6.14454	0.701055	0.777512
Erythroid.cells	ME1	-0.02438	-0.00982	-0.0307	0.975579	-5.3101	0.838443	0.898366
Erythroid.cells	MRPL27	0.008383	4.210901	0.030658	0.975612	-6.05326	0.724923	0.798899
Erythroid.cells	ASTL	-0.02077	1.424213	-0.03027	0.975916	-5.4273	0.798142	0.863291
Erythroid.cells	WDR76	0.009666	4.504143	0.030082	0.976069	-6.10403	0.717883	0.792405
Erythroid.cells	APOA5	-0.01735	1.793969	-0.0298	0.976293	-5.66608	0.788212	0.854464
Erythroid.cells	ZXDC	0.009267	4.004625	0.029406	0.976607	-5.84365	0.730583	0.803499
Erythroid.cells	SPTLC1	0.008071	4.232218	0.029219	0.976756	-5.93897	0.724888	0.798425
Erythroid.cells	LAS1L	0.008183	4.172574	0.029213	0.976761	-5.9194	0.726376	0.799752
Erythroid.cells	MND1	0.01372	2.222735	0.029125	0.97683	-5.61135	0.77681	0.844338
Erythroid.cells	TAX1BP3	0.009432	4.090035	0.029079	0.976867	-5.89589	0.72844	0.801599
Erythroid.cells	IRAK1	0.005906	5.224747	0.028843	0.977055	-6.18989	0.700642	0.776729
Erythroid.cells	KIF11	0.011035	5.441855	0.028813	0.977079	-6.42042	0.695443	0.772048
Erythroid.cells	P2RX4	-0.00802	5.534178	-0.02867	0.977192	-6.19278	0.693255	0.770072
Erythroid.cells	PGGHG	0.012405	2.820106	0.028578	0.977265	-5.66704	0.761046	0.830513
Erythroid.cells	FADS2	-0.01214	3.530927	-0.02852	0.977314	-5.97421	0.742646	0.814254
Erythroid.cells	ABCF1	0.004453	6.430861	0.028227	0.977545	-6.49725	0.672389	0.751106
Erythroid.cells	TLR3	-0.01357	1.534056	-0.02809	0.977657	-5.81372	0.795704	0.860758
Erythroid.cells	FES	0.010114	4.284767	0.027833	0.977858	-5.87189	0.72388	0.797358
Erythroid.cells	NAA15	-0.00374	6.743192	-0.02772	0.977949	-6.54596	0.665367	0.744626
Erythroid.cells	GM12185	0.012172	3.341306	0.027604	0.97804	-5.74805	0.747784	0.818611
Erythroid.cells	HIST1H2AG	-0.01836	2.011327	-0.0272	0.978361	-5.56637	0.782929	0.849589
Erythroid.cells	1110051M	-0.01056	3.263469	-0.02719	0.978371	-5.69346	0.749871	0.820508
Erythroid.cells	ZFP318	-0.01172	3.491008	-0.02713	0.978418	-5.81844	0.744021	0.815336
Erythroid.cells	MAML3	0.006607	6.974808	0.027103	0.978439	-6.62767	0.6602	0.739983
Erythroid.cells	CASP9	0.009986	2.447605	0.02679	0.978688	-5.62922	0.771348	0.839376
Erythroid.cells	CENPI	-0.01253	2.997583	-0.02672	0.978746	-5.71729	0.75687	0.826632
Erythroid.cells	RBM10	0.006076	4.779494	0.026363	0.979027	-6.08751	0.711879	0.786646
Erythroid.cells	ITIH1	0.012494	2.749811	0.026328	0.979055	-5.92317	0.763357	0.832359
Erythroid.cells	TUBB5	0.005694	9.849372	0.026302	0.979076	-7.17489	0.598676	0.683125
Erythroid.cells	SMPD3	-0.0231	-0.20687	-0.0263	0.979078	-5.22637	0.845332	0.903627
Erythroid.cells	DLGAP5	0.013324	3.248568	0.026261	0.979108	-5.78539	0.750358	0.820889
Erythroid.cells	UNG	-0.013	3.369876	-0.02612	0.979218	-5.75543	0.747231	0.818123
Erythroid.cells	2510009E0	-0.01063	2.702942	-0.0261	0.979233	-5.70388	0.764591	0.833445
Erythroid.cells	GM39121	-0.01919	-0.94202	-0.02581	0.979468	-5.25169	0.867212	0.922268
Erythroid.cells	COTL1	0.004852	6.629821	0.025707	0.979549	-6.50777	0.668243	0.747178
Erythroid.cells	B3GNT5	0.010852	2.546338	0.025549	0.979675	-5.79759	0.768902	0.837134

Erythroid.cells	PACS1	-0.00586	5.972963	-0.02529	0.979881	-6.38265	0.683555	0.761069
Erythroid.cells	GM29417	-0.01438	1.098886	-0.02512	0.980014	-5.39384	0.808345	0.871623
Erythroid.cells	TMEM199	-0.00829	3.379806	-0.02506	0.980065	-5.74954	0.747227	0.818058
Erythroid.cells	ZFP40	-0.017	1.347612	-0.02504	0.980077	-5.36396	0.801438	0.865622
Erythroid.cells	HGFAC	0.013507	1.432763	0.024722	0.980332	-5.59128	0.799096	0.863627
Erythroid.cells	THAP4	-0.00745	4.091332	-0.0247	0.980352	-5.85356	0.729168	0.802066
Erythroid.cells	SAFB2	0.004023	5.993263	0.024673	0.980371	-6.34333	0.683088	0.760703
Erythroid.cells	SRSF2	-0.00364	7.941182	-0.02466	0.980382	-6.81358	0.639076	0.720552
Erythroid.cells	EIF3F	-0.00347	7.589953	-0.0245	0.980511	-6.75803	0.64682	0.72763
Erythroid.cells	IFT52	-0.00634	4.249963	-0.0244	0.980591	-5.91345	0.725248	0.798541
Erythroid.cells	POLR1B	0.011113	2.442843	0.024234	0.98072	-5.60891	0.771816	0.839722
Erythroid.cells	MAP3K4	-0.00715	4.074187	-0.02409	0.980835	-5.90043	0.729667	0.802505
Erythroid.cells	PHEX	-0.01901	1.372943	-0.02408	0.980842	-5.37689	0.800823	0.865125
Erythroid.cells	SLC27A5	0.013974	1.457834	0.023907	0.98098	-5.59652	0.798534	0.863129
Erythroid.cells	FLAD1	0.010032	2.957237	0.023782	0.98108	-5.67438	0.758329	0.827917
Erythroid.cells	GM33677	-0.0232	-1.06476	-0.02356	0.981259	-5.18311	0.871313	0.925788
Erythroid.cells	SS18	-0.00423	5.946104	-0.02345	0.981348	-6.4088	0.684405	0.761842
Erythroid.cells	NDUFAF6	-0.00765	2.973661	-0.02291	0.981773	-5.80037	0.758265	0.827627
Erythroid.cells	UBE2CBP	-0.01597	1.761179	-0.0228	0.981864	-5.53401	0.790638	0.856017
Erythroid.cells	GPN2	-0.00913	2.713988	-0.02268	0.981959	-5.63148	0.765108	0.833647
Erythroid.cells	CCL4	0.010009	7.388292	0.022506	0.982095	-6.80639	0.651714	0.731948
Erythroid.cells	YIPF1	0.004383	5.234712	0.02233	0.982235	-6.18347	0.701583	0.777175
Erythroid.cells	CFAP45	0.016949	0.728674	0.022275	0.982278	-5.34872	0.81933	0.880957
Erythroid.cells	SATB2	0.013266	1.643339	0.022259	0.982292	-5.52851	0.793875	0.85885
Erythroid.cells	GM13483	0.012632	1.85821	0.022216	0.982326	-5.5082	0.788013	0.853736
Erythroid.cells	WDR89	0.00871	2.643388	0.022116	0.982405	-5.67252	0.766976	0.835326
Erythroid.cells	NSUN3	0.008161	2.658689	0.021621	0.982799	-5.64494	0.766767	0.835003
Erythroid.cells	ZFP955A	0.013373	1.472841	0.021553	0.982853	-5.44383	0.798766	0.862994
Erythroid.cells	MS4A7	-0.01595	1.921581	-0.02152	0.982876	-5.60415	0.786498	0.852296
Erythroid.cells	MS4A4B	0.006452	3.207847	0.021147	0.983176	-6.24376	0.752575	0.822389
Erythroid.cells	PPARGC1A	-0.01482	1.419055	-0.02096	0.983328	-5.47333	0.800485	0.864351
Erythroid.cells	FNDC3A	-0.00354	7.526942	-0.02088	0.983386	-6.67648	0.648996	0.729268
Erythroid.cells	PPP4R1	-0.00452	5.579566	-0.02077	0.983477	-6.27044	0.693731	0.769916
Erythroid.cells	GM45051	0.010075	1.845863	0.020553	0.983648	-5.50144	0.788879	0.854266
Erythroid.cells	UBAP2	-0.00348	6.131148	-0.02014	0.983977	-6.42405	0.680985	0.758287
Erythroid.cells	TSPAN3	-0.00617	4.739337	-0.02	0.984089	-6.05161	0.714289	0.788284
Erythroid.cells	EFCAB9	0.012132	0.443216	0.019942	0.984135	-5.30457	0.828224	0.888338
Erythroid.cells	GCSH	0.004679	4.428205	0.019207	0.984719	-6.20925	0.722339	0.795286
Erythroid.cells	DHCR7	-0.00886	2.184949	-0.01903	0.984863	-5.48505	0.780381	0.846544
Erythroid.cells	TSPAN32	-0.00772	2.972272	-0.01883	0.985017	-5.79841	0.759557	0.828207
Erythroid.cells	ZFP7	0.014773	0.286794	0.01869	0.985131	-5.27881	0.833268	0.89239
Erythroid.cells	MOAP1	0.010727	0.547771	0.018648	0.985164	-5.3123	0.825794	0.885947
Erythroid.cells	SLC38A1	0.003415	7.937408	0.018317	0.985428	-6.77373	0.64072	0.721365
Erythroid.cells	GM30881	-0.0092	1.986079	-0.01821	0.985515	-5.5071	0.785906	0.851347
Erythroid.cells	LY75	0.007902	3.332572	0.018142	0.985567	-6.07094	0.750281	0.820059
Erythroid.cells	NXF7	-0.01423	-1.34285	-0.01805	0.985642	-5.14217	0.881611	0.933776
Erythroid.cells	HORMAD2	0.014864	1.357831	0.01804	0.985648	-5.4225	0.803122	0.866353

Erythroid.cells	SLX4IP	-0.00464	4.853222	-0.01778	0.985855	-6.09076	0.712134	0.786133
Erythroid.cells	GNL3L	0.003614	4.467102	0.017731	0.985894	-6.07082	0.721641	0.794652
Erythroid.cells	PDZRN3	0.011449	0.932125	0.017296	0.98624	-5.43715	0.815257	0.876841
Erythroid.cells	TNFRSF1B	0.005436	4.903674	0.017289	0.986245	-6.12541	0.711048	0.785132
Erythroid.cells	BRF2	-0.00701	2.326101	-0.01716	0.986347	-5.5988	0.777009	0.843519
Erythroid.cells	HIVEP2	0.003803	8.386816	0.016838	0.986604	-6.9589	0.631306	0.712614
Erythroid.cells	PI4KB	-0.00329	5.414205	-0.01675	0.986678	-6.24219	0.698849	0.774119
Erythroid.cells	CLDN10	0.014139	0.159414	0.016375	0.986972	-5.29867	0.837498	0.896023
Erythroid.cells	MPZL3	-0.00947	1.727945	-0.01632	0.987018	-5.51822	0.793368	0.857822
Erythroid.cells	CRTAM	0.011104	1.176627	0.016311	0.987023	-5.59918	0.808602	0.871067
Erythroid.cells	RPRD1A	-0.00416	4.057705	-0.01627	0.987053	-5.89844	0.732196	0.804006
Erythroid.cells	BCAR3	0.005772	4.496691	0.01625	0.987072	-6.23109	0.721231	0.794249
Erythroid.cells	PSMB3	-0.00227	7.724991	-0.01555	0.98763	-6.80651	0.64601	0.726055
Erythroid.cells	APOOL	0.004477	3.780654	0.015537	0.987639	-5.9132	0.73952	0.810319
Erythroid.cells	PDE5A	-0.00834	2.569114	-0.0154	0.987745	-5.63331	0.771045	0.83813
Erythroid.cells	GM11110	-0.00703	1.970897	-0.01526	0.987858	-5.53395	0.787141	0.852194
Erythroid.cells	ATP8A2	0.007516	2.725958	0.015155	0.987943	-5.86479	0.766929	0.834486
Erythroid.cells	RILPL2	-0.00302	7.158884	-0.0148	0.988222	-6.56548	0.658722	0.737616
Erythroid.cells	GM20337	-0.01016	1.078162	-0.01478	0.988239	-5.33241	0.811823	0.873603
Erythroid.cells	EDEM2	0.004604	4.515035	0.014727	0.988283	-5.94102	0.721193	0.793956
Erythroid.cells	CPSF3	0.003328	4.770083	0.0147	0.988305	-6.0886	0.714902	0.788339
Erythroid.cells	MED9	0.00494	3.526994	0.014371	0.988566	-5.83297	0.746226	0.816093
Erythroid.cells	HP1BP3	0.002522	6.529858	0.014331	0.988598	-6.51039	0.673146	0.750647
Erythroid.cells	U2AF1	0.001772	7.593832	0.014013	0.988851	-6.76587	0.649221	0.728751
Erythroid.cells	TBCD	-0.00331	4.783335	-0.01388	0.988959	-6.12706	0.714837	0.788098
Erythroid.cells	PARD3B	-0.00612	4.55014	-0.01363	0.989152	-6.02925	0.720665	0.79326
Erythroid.cells	SLC22A27	0.00841	1.124082	0.013533	0.989233	-5.54438	0.810921	0.872607
Erythroid.cells	AATK	0.010686	0.48294	0.013462	0.989289	-5.30857	0.829066	0.888299
Erythroid.cells	CCDC174	0.002892	5.082834	0.013112	0.989568	-6.11449	0.70766	0.781626
Erythroid.cells	GM17178	0.00734	1.460682	0.013086	0.989589	-5.40146	0.801623	0.864501
Erythroid.cells	TMEM63B	0.004085	3.925117	0.013084	0.989591	-5.98783	0.736375	0.807226
Erythroid.cells	CADM4	-0.00908	1.741687	-0.01296	0.989686	-5.43812	0.793908	0.857775
Erythroid.cells	IGF2BP1	0.011485	-0.96106	0.01284	0.989784	-5.15979	0.871555	0.924661
Erythroid.cells	RBBP9	0.008436	1.214794	0.01267	0.98992	-5.39387	0.80848	0.870549
Erythroid.cells	COPRS	-0.00746	0.717795	-0.01264	0.989947	-5.43774	0.822466	0.882652
Erythroid.cells	ODF2L	-0.00555	2.682465	-0.01252	0.990038	-5.57571	0.768583	0.835751
Erythroid.cells	ARHGAP9	-0.0034	5.14536	-0.01248	0.990075	-6.09554	0.706166	0.780391
Erythroid.cells	CYP20A1	-0.00329	4.104318	-0.01223	0.990271	-5.92265	0.731876	0.803371
Erythroid.cells	BIN2	0.003035	5.165118	0.012182	0.990308	-6.17135	0.705688	0.779994
Erythroid.cells	GM7160	-0.00678	2.542262	-0.01188	0.990546	-5.55806	0.772305	0.839059
Erythroid.cells	TBL3	-0.00349	3.91508	-0.01187	0.990558	-5.88626	0.736654	0.807614
Erythroid.cells	UCKL1	-0.00383	3.705129	-0.01186	0.990566	-5.81156	0.741993	0.812347
Erythroid.cells	RDH16F2	0.007438	1.257357	0.011714	0.99068	-5.59768	0.807293	0.869574
Erythroid.cells	MTHFSD	0.005349	2.433778	0.011702	0.99069	-5.57957	0.775198	0.841595
Erythroid.cells	AKT3	0.002373	6.643383	0.011657	0.990725	-6.58717	0.670831	0.748545
Erythroid.cells	TMEM170	-0.00677	1.517214	-0.01161	0.990763	-5.36509	0.800088	0.863317
Erythroid.cells	GTPBP10	0.004813	2.397542	0.011559	0.990803	-5.58175	0.776166	0.842443

Erythroid.cells	RCN1	0.00439	3.060814	0.011416	0.990917	-5.73008	0.758663	0.827043
Erythroid.cells	FBXO47	0.008589	1.069812	0.011195	0.991093	-5.34037	0.812651	0.874144
Erythroid.cells	CD2BP2	-0.00314	4.011388	-0.01095	0.991285	-5.87868	0.734331	0.805488
Erythroid.cells	PEX19	-0.00292	3.955615	-0.01095	0.991285	-5.9939	0.735741	0.80674
Erythroid.cells	RGS2	-0.0023	6.977171	-0.01088	0.991346	-6.57875	0.663319	0.741686
Erythroid.cells	SYNJ2	-0.00485	2.324633	-0.01072	0.991474	-5.56016	0.778239	0.844313
Erythroid.cells	ATP5G2	0.001347	8.844228	0.010566	0.991593	-6.99806	0.622383	0.704212
Erythroid.cells	SCAMP3	0.002317	4.819715	0.010509	0.991639	-6.09393	0.714215	0.78769
Erythroid.cells	GM45435	0.008896	0.920103	0.0104	0.991726	-5.31672	0.816869	0.877959
Erythroid.cells	SLC11A1	-0.0048	3.505984	-0.01039	0.991733	-5.9327	0.74721	0.817046
Erythroid.cells	POFUT1	-0.00341	3.458205	-0.01035	0.991767	-5.77332	0.74844	0.81814
Erythroid.cells	GNB1L	-0.0036	3.308245	-0.01012	0.991946	-5.79899	0.752343	0.821607
Erythroid.cells	KMT2C	-0.00146	7.717211	-0.01009	0.991972	-6.75584	0.646786	0.726712
Erythroid.cells	NIFK	-0.00263	4.620947	-0.01	0.992041	-6.04783	0.719136	0.792134
Erythroid.cells	RBPMS2	-0.0045	1.246218	-0.00993	0.992102	-5.54264	0.80776	0.870109
Erythroid.cells	SAMD8	-0.00249	5.289431	-0.00979	0.992215	-6.12732	0.702828	0.777565
Erythroid.cells	ATAD3A	-0.00314	4.10261	-0.00973	0.99226	-5.96447	0.732069	0.803684
Erythroid.cells	GM9750	0.007455	0.476616	0.009591	0.99237	-5.32587	0.829531	0.888988
Erythroid.cells	THA1	0.007727	0.09883	0.009515	0.992429	-5.2553	0.840421	0.898366
Erythroid.cells	INPP5E	-0.00399	2.260693	-0.00918	0.992698	-5.5311	0.780166	0.846042
Erythroid.cells	PDE6C	-0.00612	0.436862	-0.00896	0.992871	-5.40553	0.83089	0.890073
Erythroid.cells	LMAN1L	0.005723	1.485183	0.008855	0.992955	-5.44663	0.801367	0.864526
Erythroid.cells	BC055324	0.004226	2.245694	0.008737	0.993048	-5.55753	0.780624	0.846461
Erythroid.cells	AQP11	0.006107	0.397356	0.008721	0.993061	-5.3547	0.832024	0.891072
Erythroid.cells	ING4	-0.00281	4.08954	-0.00861	0.993146	-5.86601	0.732617	0.804182
Erythroid.cells	RSF1	0.001323	7.014648	0.008432	0.993291	-6.60619	0.662748	0.74134
Erythroid.cells	BICRAL	0.002086	5.023041	0.008207	0.99347	-6.10159	0.709615	0.783616
Erythroid.cells	DRAM1	-0.00489	2.581586	-0.00812	0.993536	-5.64795	0.771779	0.838725
Erythroid.cells	B230307C2	0.002591	3.741811	0.007855	0.993751	-5.81045	0.741661	0.812127
Erythroid.cells	GTPBP3	-0.00378	2.225197	-0.00776	0.993828	-5.51152	0.78143	0.847146
Erythroid.cells	GRAMD3	0.001528	6.080829	0.007492	0.99404	-6.64943	0.684531	0.760965
Erythroid.cells	ANGPTL7	0.006962	-0.05517	0.007239	0.994241	-5.17304	0.845628	0.902617
Erythroid.cells	CENPT	-0.0029	2.566821	-0.00711	0.994343	-5.57102	0.772512	0.839215
Erythroid.cells	LTB4R1	-0.00327	2.689349	-0.00691	0.994506	-5.88016	0.769323	0.836388
Erythroid.cells	OTOA	0.005098	0.757069	0.006705	0.994665	-5.29568	0.822338	0.882561
Erythroid.cells	DHX40	0.001604	7.808142	0.006643	0.994715	-6.73201	0.64543	0.725347
Erythroid.cells	WDSUB1	0.002644	2.44185	0.006632	0.994724	-5.62753	0.775912	0.842198
Erythroid.cells	HEG1	-0.00157	5.971346	-0.00649	0.994838	-6.3894	0.687298	0.763448
Erythroid.cells	WDR37	-0.00131	5.478401	-0.00611	0.99514	-6.21314	0.699162	0.77406
Erythroid.cells	1700003F1	-0.00336	2.027827	-0.00597	0.995251	-5.50378	0.787269	0.852104
Erythroid.cells	IPO11	-0.00153	4.478076	-0.00569	0.995469	-6.03334	0.723588	0.795992
Erythroid.cells	SFXN5	-0.00241	3.363367	-0.00567	0.995488	-5.74054	0.751866	0.821074
Erythroid.cells	GM2449	-0.00395	1.279792	-0.00559	0.995551	-5.39506	0.807845	0.870056
Erythroid.cells	ZFP160	0.002173	2.950859	0.00547	0.995648	-5.66662	0.762621	0.830554
Erythroid.cells	CLOCK	-0.00131	5.071637	-0.00546	0.995653	-6.15139	0.708988	0.782948
Erythroid.cells	ZFP3	0.003778	0.562138	0.005404	0.9957	-5.30211	0.828104	0.887575
Erythroid.cells	BCAT2	0.001417	4.601375	0.005344	0.995748	-6.00736	0.72053	0.793266

Erythroid.cells	CACNA1F	0.004468	-0.45662	0.005288	0.995793	-5.23573	0.857752	0.913028
Erythroid.cells	CARD9	0.002914	0.997223	0.004825	0.996161	-5.49296	0.815911	0.877003
Erythroid.cells	GRPEL2	-0.00156	3.176522	-0.00462	0.996322	-5.74783	0.756858	0.82544
Erythroid.cells	CYHR1	0.001168	4.48568	0.004515	0.996407	-6.02126	0.723533	0.795906
Erythroid.cells	NACC1	-0.00122	4.380772	-0.00445	0.99646	-5.92974	0.726146	0.798234
Erythroid.cells	RNF17	-0.00402	-0.02892	-0.00436	0.996533	-5.27299	0.845332	0.90235
Erythroid.cells	PIH1D1	-0.00106	5.06576	-0.00435	0.996537	-6.13116	0.709262	0.783156
Erythroid.cells	GM10974	-0.00268	0.824708	-0.0043	0.99658	-5.35481	0.820784	0.881216
Erythroid.cells	TLE3	0.001036	5.003886	0.004242	0.996625	-6.10186	0.71077	0.784507
Erythroid.cells	AB124611	-0.00114	5.452214	-0.0041	0.996737	-6.13391	0.69992	0.774776
Erythroid.cells	NANS	0.001153	4.873876	0.004081	0.996753	-6.07384	0.71395	0.787351
Erythroid.cells	2810454H(	-0.00183	2.514759	-0.004	0.99682	-5.59594	0.774308	0.840782
Erythroid.cells	FSD2	0.003014	-0.73763	0.003515	0.997204	-5.15421	0.866277	0.92038
Erythroid.cells	KLF12	-0.00127	3.585919	-0.00351	0.997204	-6.13933	0.746267	0.816192
Erythroid.cells	ZFP931	-0.00168	1.514653	-0.00345	0.997257	-5.49355	0.801474	0.864592
Erythroid.cells	RAB32	-0.0009	4.344985	-0.00334	0.997339	-6.12759	0.727039	0.799129
Erythroid.cells	ASB8	-0.00104	3.607063	-0.00325	0.997411	-5.83342	0.745725	0.815732
Erythroid.cells	RGS14	0.001252	3.585563	0.00319	0.997462	-5.79406	0.746276	0.816247
Erythroid.cells	CLIP1	-0.00073	5.440971	-0.00315	0.997492	-6.23018	0.70019	0.775159
Erythroid.cells	4930513N1	0.002348	0.475363	0.003042	0.99758	-5.23322	0.830742	0.889984
Erythroid.cells	DCTN4	0.000448	6.423452	0.002984	0.997626	-6.50949	0.67701	0.754269
Erythroid.cells	0610009L1	-0.00153	2.030404	-0.00291	0.997687	-5.5128	0.787344	0.852385
Erythroid.cells	C3	0.000793	6.327835	0.002877	0.997711	-6.82055	0.67923	0.756305
Erythroid.cells	FAM71E1	-0.00222	0.837279	-0.00285	0.997731	-5.31193	0.820428	0.881128
Erythroid.cells	RBPJ	-0.00081	6.055966	-0.00283	0.997749	-6.47712	0.685584	0.762051
Erythroid.cells	CEMIP2	0.000708	5.494454	0.002556	0.997967	-6.23732	0.698982	0.774147
Erythroid.cells	PEX16	-0.00071	3.474463	-0.00243	0.998068	-5.88176	0.749216	0.818954
Erythroid.cells	CENPN	0.001086	2.920239	0.002356	0.998125	-5.79258	0.763649	0.83171
Erythroid.cells	BCL2L2	0.001175	1.276497	0.002266	0.998197	-5.43087	0.808173	0.870624
Erythroid.cells	DSG2	-0.00146	0.740688	-0.00223	0.998226	-5.43395	0.823256	0.883681
Erythroid.cells	MIA3	0.000324	5.849323	0.001744	0.998612	-6.29154	0.690743	0.766692
Erythroid.cells	GM50340	0.000942	1.533078	0.001485	0.998819	-5.42452	0.801298	0.864591
Erythroid.cells	DPM1	-0.00024	5.979025	-0.00146	0.99884	-6.44058	0.68768	0.76398
Erythroid.cells	INTS14	-0.0005	4.691853	-0.00135	0.998924	-6.02002	0.718726	0.791854
Erythroid.cells	KDR	0.000683	3.318647	0.001345	0.99893	-5.82723	0.753477	0.822705
Erythroid.cells	ZBTB38	0.000318	5.068147	0.001285	0.998977	-6.16367	0.7095	0.783614
Erythroid.cells	CLSTN1	-0.00059	1.774497	-0.00109	0.999131	-5.45672	0.794685	0.858877
Erythroid.cells	ARHGEF11	0.0003	4.907611	0.001051	0.999164	-6.06962	0.713448	0.787176
Erythroid.cells	MORRBID	0.000374	5.127394	0.00093	0.99926	-6.20654	0.708087	0.782374
Erythroid.cells	TUBGCP4	0.000203	4.42498	0.000744	0.999408	-6.00446	0.725375	0.79785
Erythroid.cells	CUTA	-0.00011	6.189562	-0.00068	0.99946	-6.44498	0.682765	0.759603
Erythroid.cells	TNF	0.0004	4.405834	0.000678	0.99946	-6.01894	0.725852	0.798276
Erythroid.cells	NDRG1	-0.00025	2.72107	-0.00049	0.99961	-5.71569	0.769175	0.836636
Erythroid.cells	GM36660	-0.0003	-0.14918	-0.0004	0.999682	-5.22343	0.849239	0.906092
Erythroid.cells	LRRC29	0.000167	1.690453	0.00038	0.999698	-5.45409	0.796993	0.86096
Erythroid.cells	FGD6	-8.32E-05	4.002414	-0.00025	0.999802	-5.96025	0.735989	0.807352
Erythroid.cells	DUSP28	9.36E-05	1.99306	0.000192	0.999848	-5.51478	0.788719	0.853774

Erythroid.cells	SOCS5	-4.34E-05	4.029298	-0.00012	0.999905	-5.90781	0.735309	0.806777
