# Supplementary material for: Immune landscape in liver of neonatal mice with phlebotomy-induced anemia
Source: Pediatr Res. 2025 Sep 17;99(4):1602–12. doi: 10.1038/s41390-025-04361-x (PMC12659965; doi:10.1038/s41390-025-04361-x)
Supplement: Supplementary file 7 — Table S7 [file 41390_2025_4361_MOESM7_ESM.pdf]

immunecell	gene	logFC	AveExpr	t	P.Value	B	adj.P.Val.Within	adj.P.Val.Between
B.cells	CYP2E1	-2.44339	4.291137	-8.60749	2.55E-13	19.64278	2.95E-09	4.52E-10
B.cells	MT1	1.711751	6.314344	6.804963	1.16E-09	11.76597	6.48E-06	6.89E-07
B.cells	GM10076	0.897891	6.671496	6.562097	3.49E-09	10.66358	1.29E-05	1.70E-06
B.cells	MT2	2.73263	2.001416	6.418601	6.65E-09	9.451084	1.99E-05	3.22E-06
B.cells	SORL1	0.938018	5.66068	6.130362	2.39E-08	8.820276	5.41E-05	8.76E-06
B.cells	CROT	-0.94521	5.259869	-6.05799	3.29E-08	8.609442	6.23E-05	1.18E-05
B.cells	FABP2	-1.79591	3.257265	-5.92875	5.79E-08	7.945699	9.14E-05	1.99E-05
B.cells	FABP4	1.266686	6.966529	5.911805	6.23E-08	7.854642	8.62E-05	1.91E-05
B.cells	SERPINA6	-3.53801	1.127419	-5.88111	7.12E-08	6.449294	9.61E-05	2.52E-05
B.cells	LECT2	-1.75052	3.632928	-5.47712	4.01E-07	6.244954	0.00045	0.0001
B.cells	HBB-BT	1.887461	8.105747	5.464087	4.24E-07	5.733097	0.000419	9.19E-05
B.cells	HSD17B2	-1.72854	2.446494	-5.29367	8.65E-07	5.233697	0.000857	0.0002
B.cells	FGF23	3.682291	0.594781	5.253984	1.02E-06	4.10356	0.000916	0.000241
B.cells	SNCA	1.92506	4.248926	5.247612	1.05E-06	5.364216	0.000864	0.000222
B.cells	MBL2	-1.55951	4.246085	-5.18225	1.37E-06	5.109672	0.000938	0.000273
B.cells	FADS1	-1.44568	3.059286	-5.17414	1.42E-06	4.843095	0.000956	0.000287
B.cells	SERPINA3K	2.586409	1.878609	5.164136	1.48E-06	4.874943	0.000975	0.000307
B.cells	JCHAIN	-5.84851	-0.4625	-5.15199	1.55E-06	3.71585	0.001012	0.000342
B.cells	SLC27A2	-1.49697	3.949769	-5.14046	1.63E-06	4.955448	0.000943	0.000312
B.cells	GYPA	1.863196	3.611578	5.137888	1.64E-06	4.943126	0.000948	0.000318
B.cells	GSTA3	-1.78277	4.0016	-5.12885	1.71E-06	4.91135	0.000942	0.000326
B.cells	CYP3A11	-1.80958	3.352158	-5.11494	1.81E-06	4.822273	0.000962	0.000347
B.cells	APOA1	-1.4371	8.277086	-5.07677	2.11E-06	4.268766	0.000994	0.000344
B.cells	CES1G	-2.78813	0.85155	-5.02722	2.58E-06	3.502547	0.001311	0.000499
B.cells	CHAC1	-2.66636	0.269965	-4.94154	3.64E-06	2.964672	0.001795	0.000656
B.cells	OSGIN1	-1.24909	3.869004	-4.89748	4.35E-06	4.033591	0.001944	0.000685
B.cells	SLC16A3	1.525014	3.935418	4.885761	4.56E-06	3.930886	0.00196	0.000712
B.cells	KNG1	-1.04138	6.039457	-4.86882	4.88E-06	3.722954	0.001956	0.000704
B.cells	H2-Q10	-1.17257	4.038149	-4.85082	5.24E-06	3.87032	0.002094	0.000789
B.cells	HBA-A1	1.853615	10.46682	4.841978	5.43E-06	3.016211	0.001895	0.000676
B.cells	PON1	-1.30855	4.304512	-4.80225	6.35E-06	3.664447	0.002296	0.000904
B.cells	SERPINF2	-1.36976	3.929119	-4.79907	6.43E-06	3.682774	0.00231	0.000922
B.cells	HBA-A2	1.809057	9.98555	4.793945	6.56E-06	2.880184	0.002099	0.00079
B.cells	CES1C	-1.53993	4.023969	-4.76451	7.37E-06	3.548935	0.002482	0.001013
B.cells	UBA52	0.488749	10.38927	4.760523	7.49E-06	2.750377	0.002244	0.000858
B.cells	SPTA1	2.360648	0.279808	4.727498	8.53E-06	2.901592	0.002917	0.001251
B.cells	SERPINC1	-1.08938	5.285455	-4.72053	8.77E-06	3.264301	0.002693	0.00111
B.cells	BNIP3	0.926955	5.086642	4.675458	1.05E-05	3.115037	0.003138	0.001278
B.cells	CYP2C37	-1.68179	1.772648	-4.64774	1.17E-05	2.829974	0.003591	0.001524
B.cells	CFH	-0.9579	4.930751	-4.62857	1.26E-05	2.970605	0.003536	0.001485
B.cells	ALAS2	1.524904	4.928547	4.625847	1.27E-05	2.919263	0.003536	0.00149
B.cells	EPB42	2.013189	-0.1647	4.602149	1.39E-05	2.43311	0.004105	0.001854
B.cells	AHSG	-1.23628	9.130873	-4.57379	1.55E-05	2.221272	0.00386	0.001575
B.cells	VKORC1	-0.85224	4.701765	-4.55198	1.69E-05	2.757887	0.004344	0.001884

B.cells	CYP1A2	-1.95816	1.633956	-4.54797	1.71E-05	2.417386	0.004562	0.002067
B.cells	KLF4	-0.61037	6.447469	-4.5416	1.76E-05	2.417285	0.004225	0.001849
B.cells	TTR	-1.32511	9.202897	-4.53818	1.78E-05	2.080762	0.004046	0.001724
B.cells	CAR3	-2.22376	3.476221	-4.51848	1.92E-05	2.681676	0.004678	0.002121
B.cells	GUCY2C	1.905122	-0.14698	4.507671	2.00E-05	1.840368	0.005061	0.002426
B.cells	HBB-BS	1.731823	11.91128	4.473333	2.28E-05	1.485816	0.004673	0.001935
B.cells	RGS10	-0.6079	5.509055	-4.43299	2.66E-05	2.261814	0.005852	0.002629
B.cells	VTN	-1.20181	4.267725	-4.43011	2.69E-05	2.32702	0.005969	0.002742
B.cells	EEA1	0.472017	6.055281	4.425218	2.74E-05	2.107999	0.005802	0.002639
B.cells	OSER1	-0.4624	6.59749	-4.396	3.06E-05	1.946458	0.006306	0.002843
B.cells	SDHAF1	0.674328	4.132974	4.37452	3.32E-05	2.109045	0.006924	0.003257
B.cells	CD40LG	-2.07041	-0.56972	-4.37191	3.35E-05	1.414392	0.007464	0.00374
B.cells	HOGA1	-1.41753	2.370342	-4.36537	3.43E-05	2.046204	0.007171	0.003494
B.cells	TMEM192	0.541935	4.694631	4.350136	3.64E-05	1.983638	0.007191	0.003425
B.cells	AKR1C6	-1.37062	4.841164	-4.33817	3.80E-05	1.931199	0.007376	0.003507
B.cells	FGFR2	-0.50897	5.987704	-4.32632	3.98E-05	1.763476	0.007446	0.003502
B.cells	ZDHHC18	0.526015	5.57552	4.310079	4.22E-05	1.737983	0.007802	0.003689
B.cells	APOF	-1.11001	3.847555	-4.30678	4.28E-05	1.928206	0.008019	0.003891
B.cells	ITIH2	-1.44054	3.79432	-4.28871	4.57E-05	1.875917	0.008449	0.004098
B.cells	HRG	-1.32567	3.61876	-4.24417	5.40E-05	1.733835	0.009692	0.004681
B.cells	CCND3	0.545577	7.994145	4.244059	5.40E-05	1.162171	0.009043	0.004144
B.cells	INSIG1	-0.57275	5.457849	-4.23373	5.61E-05	1.526927	0.009631	0.004567
B.cells	TPI1	0.562154	6.690385	4.216582	5.98E-05	1.272676	0.009912	0.004638
B.cells	TMEM205	-1.0064	3.71868	-4.21102	6.10E-05	1.629814	0.01045	0.005113
B.cells	PXK	0.424985	6.270189	4.20352	6.27E-05	1.245071	0.010167	0.004845
B.cells	SGCZ	-1.70623	1.627249	-4.19288	6.52E-05	1.373603	0.011151	0.005644
B.cells	5730522E0	-1.137	3.610742	-4.19073	6.57E-05	1.559031	0.010803	0.005363
B.cells	CD300LG	-1.69956	1.259921	-4.17592	6.94E-05	1.233784	0.011678	0.005984
B.cells	1-Mar	0.908069	4.477654	4.155295	7.48E-05	1.178461	0.011785	0.005749
B.cells	LRRK2	0.592451	4.404591	4.151916	7.58E-05	1.114527	0.011799	0.0058
B.cells	CRELD2	-0.69041	4.759514	-4.13573	8.04E-05	1.287696	0.012281	0.005993
B.cells	GIGYF2	0.461418	5.464029	4.128502	8.25E-05	1.119168	0.012208	0.005991
B.cells	XBP1	-0.44536	6.135195	-4.12707	8.30E-05	1.081516	0.012079	0.005886
B.cells	SERPIND1	-1.31134	2.832849	-4.12311	8.42E-05	1.346254	0.012749	0.006515
B.cells	TGM2	1.285881	4.674438	4.098694	9.20E-05	1.239625	0.013358	0.006586
B.cells	MAP2K1	0.434957	6.311963	4.091634	9.43E-05	0.866291	0.013186	0.006408
B.cells	C1RA	-1.60845	1.359965	-4.07862	9.89E-05	0.882137	0.014771	0.00762
B.cells	HSD17B6	-2.16418	0.767255	-4.06617	0.000103	0.716486	0.015408	0.007991
B.cells	CCNB1IP1	-1.43668	1.863098	-4.047	0.000111	0.872968	0.015942	0.008143
B.cells	SDF2L1	-0.67565	4.888426	-4.04516	0.000112	0.971595	0.015192	0.007523
B.cells	AVIL	-1.2513	2.092681	-4.007	0.000128	0.927836	0.017913	0.008937
B.cells	CORO1B	0.624788	4.252422	4.002742	0.00013	0.837957	0.017306	0.008514
B.cells	RGN	-1.148	4.650255	-4.00187	0.00013	0.805654	0.017197	0.008426
B.cells	ITGA4	0.455118	7.657286	3.971416	0.000145	0.232344	0.018072	0.00838
B.cells	RARRES2	-1.17097	3.7462	-3.96083	0.000151	0.78194	0.01974	0.009621
B.cells	SERPINA11	-1.47018	1.813558	-3.94916	0.000157	0.671984	0.020985	0.010503
B.cells	GM20406	1.938451	-0.53168	3.935183	0.000165	0.099337	0.022643	0.011664

B.cells	FADS2	-1.28078	3.530927	-3.92261	0.000173	0.708283	0.021941	0.010737
B.cells	HSD3B7	-0.9273	3.537133	-3.915	0.000177	0.668364	0.022294	0.010974
B.cells	ENO1	0.466999	8.066335	3.910068	0.000181	0.033245	0.02089	0.009768
B.cells	LCN2	1.916128	3.398879	3.904447	0.000184	0.639443	0.022702	0.011292
B.cells	NRROS	0.361686	7.067702	3.894777	0.000191	0.062856	0.021929	0.01049
B.cells	IFNGR2	0.53604	5.782195	3.875681	0.000204	0.199077	0.023686	0.0114
B.cells	CXCL12	-1.13537	2.838235	-3.8716	0.000207	0.548033	0.024922	0.012466
B.cells	CDH2	-1.31189	1.947183	-3.8604	0.000215	0.45931	0.025712	0.013195
B.cells	APOE	-0.94432	9.241961	-3.85743	0.000217	-0.29784	0.022905	0.010833
B.cells	SLC4A1	1.483019	2.002694	3.856866	0.000218	0.497524	0.025689	0.013251
B.cells	APOA2	-0.98149	8.419445	-3.85532	0.000219	-0.22374	0.023203	0.011114
B.cells	CALM2	-0.28248	8.565355	-3.82667	0.000242	-0.30041	0.025135	0.011935
B.cells	SNX25	0.421673	5.540177	3.826168	0.000242	0.119317	0.026364	0.012998
B.cells	TMEM176I	-0.71239	5.475623	-3.81912	0.000248	0.173166	0.026788	0.013237
B.cells	UGT2B34	-1.51623	1.748323	-3.81185	0.000255	0.281307	0.02888	0.01497
B.cells	IDI1	-0.72711	3.90584	-3.80388	0.000262	0.319018	0.028416	0.014395
B.cells	JAK2	0.441453	6.674709	3.799361	0.000266	-0.20366	0.027368	0.01345
B.cells	TANK	0.476519	6.451404	3.790061	0.000275	-0.16623	0.028047	0.013849
B.cells	KIT	-1.00851	3.453389	-3.78615	0.000278	0.225834	0.029415	0.015194
B.cells	HBQ1B	1.823602	-0.16343	3.785384	0.000279	0.091584	0.031169	0.016824
B.cells	RBP4	-0.96337	8.031076	-3.78229	0.000282	-0.40444	0.027405	0.013481
B.cells	ADTRP	-1.50306	1.835711	-3.77833	0.000286	0.152146	0.030383	0.016199
B.cells	GPI1	0.370294	7.844302	3.769389	0.000295	-0.38514	0.028232	0.014039
B.cells	SERPINA1A	-1.02704	8.012506	-3.76166	0.000303	-0.47081	0.028666	0.014237
B.cells	RNASE4	-0.77571	5.17058	-3.74952	0.000316	-0.11169	0.030559	0.015872
B.cells	ITIH1	-1.36631	2.749811	-3.74607	0.00032	0.167366	0.03176	0.017115
B.cells	NOTCH1	-0.45254	5.292946	-3.74423	0.000322	-0.06417	0.0305	0.015995
B.cells	PRKRA	-0.7661	3.39958	-3.74382	0.000322	0.160379	0.031432	0.016861
B.cells	DBI	-0.45183	7.067278	-3.74375	0.000322	-0.37019	0.029655	0.015224
B.cells	F2	-1.08042	4.237274	-3.73052	0.000337	-0.01266	0.031901	0.016955
B.cells	SERPINA1C	-1.06191	7.348457	-3.72662	0.000342	-0.49593	0.030367	0.01568
B.cells	LYST	0.407584	6.729745	3.725728	0.000343	-0.40619	0.030665	0.015987
B.cells	GULO	-1.38097	1.982596	-3.72549	0.000343	0.058192	0.03307	0.018245
B.cells	APOC4	-0.97875	5.632488	-3.72185	0.000347	-0.26558	0.031202	0.016651
B.cells	FFAR4	1.612913	0.260828	3.721299	0.000348	-0.0592	0.033996	0.019359
B.cells	GC	-1.03371	5.287073	-3.71372	0.000357	-0.23952	0.031943	0.017148
B.cells	SULT2A5	-1.57715	2.02078	-3.7043	0.000369	0.001099	0.033961	0.019142
B.cells	PSEN2	0.383511	5.03423	3.703955	0.000369	-0.27761	0.032368	0.017614
B.cells	CLDN3	-1.64052	1.461002	-3.70335	0.00037	-0.12486	0.034266	0.019483
B.cells	TMEM181/	-0.49958	4.58903	-3.70186	0.000372	-0.09936	0.032598	0.017931
B.cells	IRAK2	0.542247	6.796286	3.699711	0.000374	-0.53153	0.031477	0.016929
B.cells	PGK1	0.484364	7.765914	3.696553	0.000379	-0.62165	0.03105	0.016551
B.cells	ACAA1B	-1.63876	3.318751	-3.69407	0.000382	-0.0021	0.033318	0.018833
B.cells	FOLR2	-1.42699	3.655517	-3.69264	0.000384	0.00152	0.033139	0.018683
B.cells	HSPA5	-0.33741	8.355671	-3.68463	0.000394	-0.71146	0.03134	0.016709
B.cells	HPGD	-1.08518	5.673887	-3.68285	0.000397	-0.33487	0.032695	0.018064
B.cells	HSD3B3	-1.56875	2.014179	-3.67916	0.000402	-0.07995	0.03484	0.02015

B.cells	TCF21	-2.16582	0.477537	-3.67466	0.000408	-0.5639	0.035998	0.021264
B.cells	LMNA	-0.66448	4.617164	-3.67007	0.000414	-0.32493	0.033826	0.019177
B.cells	HSP90B1	-0.34019	8.976388	-3.66929	0.000415	-0.85208	0.031576	0.01699
B.cells	MACF1	0.331964	8.000182	3.656344	0.000434	-0.78458	0.033266	0.017974
B.cells	MAPK1	-0.2458	7.693924	-3.65216	0.00044	-0.76204	0.033588	0.018299
B.cells	ASGR1	-1.00313	3.372794	-3.64677	0.000448	-0.17022	0.035969	0.02089
B.cells	2010300CC	-1.82443	-0.0882	-3.64517	0.000451	-0.62643	0.03802	0.023095
B.cells	UTP14B	-0.74889	3.081232	-3.63636	0.000464	-0.19323	0.036137	0.021571
B.cells	RTL8B	-0.91751	3.297356	-3.63617	0.000464	-0.16768	0.036013	0.021444
B.cells	PROC	-1.09701	2.923674	-3.63546	0.000466	-0.16704	0.036228	0.021693
B.cells	CFI	-1.085	3.743943	-3.6352	0.000466	-0.24953	0.035757	0.021203
B.cells	APOBEC3	0.430977	6.862598	3.634992	0.000466	-0.69354	0.034031	0.01944
B.cells	SERPINF1	-1.02441	3.83888	-3.63128	0.000472	-0.23121	0.035703	0.021287
B.cells	TMEM176A	-0.75019	4.388779	-3.62961	0.000475	-0.27528	0.035392	0.02102
B.cells	CBFA2T3	-0.47054	5.507245	-3.62918	0.000476	-0.60883	0.034769	0.020376
B.cells	ALB	-1.11848	9.441682	-3.62713	0.000479	-1.09595	0.032678	0.018338
B.cells	FOSB	-0.61623	6.636378	-3.62191	0.000487	-0.67896	0.034153	0.020066
B.cells	CIDEB	-1.23319	1.942054	-3.62048	0.00049	-0.25035	0.036801	0.022953
B.cells	SPP2	-1.18498	3.372982	-3.62029	0.00049	-0.24502	0.035969	0.02206
B.cells	AI413582	0.521355	4.68032	3.619484	0.000491	-0.43953	0.035228	0.021308
B.cells	APOL8	-0.87275	2.828251	-3.6184	0.000493	-0.21286	0.036283	0.022481
B.cells	CYP2D26	-1.27078	3.247258	-3.61733	0.000495	-0.2446	0.036041	0.022242
B.cells	PCMTD1	0.439943	5.924056	3.617086	0.000495	-0.58898	0.03454	0.020645
B.cells	PYGM	-0.73412	4.300481	-3.61583	0.000497	-0.37625	0.035442	0.021644
B.cells	FAM162A	0.604391	5.640039	3.600709	0.000523	-0.58416	0.036278	0.02171
B.cells	ASPH	0.632766	4.664011	3.595029	0.000533	-0.57999	0.037279	0.022592
B.cells	ASPRV1	1.868711	1.33931	3.593561	0.000536	-0.3781	0.039311	0.024859
B.cells	FERMT3	0.352854	7.235899	3.587432	0.000547	-0.88147	0.036054	0.021362
B.cells	TMEM37	-0.97301	3.751452	-3.58722	0.000547	-0.33158	0.038101	0.023543
B.cells	UGT2B36	-1.28373	2.198922	-3.58599	0.00055	-0.31319	0.039058	0.024659
B.cells	GAMT	-0.96115	4.228598	-3.58309	0.000555	-0.47523	0.037954	0.023452
B.cells	ALDOA	0.55095	8.900702	3.581336	0.000558	-1.17017	0.035253	0.020693
B.cells	SLC22A18	-1.50495	1.143718	-3.57817	0.000564	-0.46945	0.040059	0.025833
B.cells	AADAC	-1.33928	2.701819	-3.56607	0.000587	-0.36342	0.040445	0.025409
B.cells	RUFY3	0.430753	5.26954	3.553033	0.000614	-0.7221	0.040311	0.024296
B.cells	PGLYRP1	0.816597	4.959034	3.54117	0.000638	-0.65912	0.041895	0.025183
B.cells	CREB3L3	-1.42433	1.62088	-3.53818	0.000644	-0.52218	0.044332	0.027858
B.cells	DIPK1A	0.491595	5.446127	3.536263	0.000649	-0.86386	0.041709	0.025124
B.cells	PKM	0.458283	8.60779	3.532047	0.000658	-1.24712	0.039679	0.023214
B.cells	CYB5A	-0.64334	7.336341	-3.53096	0.00066	-1.09643	0.040481	0.024063
B.cells	APOM	-0.96065	4.854579	-3.53017	0.000662	-0.7417	0.042103	0.025816
B.cells	WWOX	0.376285	6.961551	3.529943	0.000662	-1.01687	0.040721	0.024345
B.cells	SORD	-0.77482	4.176195	-3.52758	0.000668	-0.59021	0.042657	0.026445
B.cells	GM16853	-0.91487	2.351019	-3.52218	0.00068	-0.48848	0.044461	0.028131
B.cells	DNAJC15	-0.4058	5.723768	-3.51816	0.000689	-0.88962	0.042469	0.02582
B.cells	CYLD	0.336568	6.329731	3.512613	0.000701	-1.00617	0.042607	0.025666
B.cells	CCL24	-1.93395	3.596362	-3.50894	0.00071	-0.5475	0.044597	0.027958

B.cells	KCNRG	-0.74453	2.320944	-3.50764	0.000713	-0.56036	0.045515	0.029052
B.cells	ZFP106	0.364356	6.288522	3.507021	0.000714	-1.02962	0.042731	0.026034
B.cells	ANGPTL8	-1.47507	1.825356	-3.50198	0.000726	-0.57361	0.046397	0.029838
B.cells	CD274	1.055883	5.805413	3.494622	0.000744	-1.10123	0.044378	0.027148
B.cells	POLR2A	0.302039	6.200996	3.485373	0.000767	-1.03171	0.045222	0.027351
B.cells	P4HA1	0.601766	5.786072	3.476441	0.00079	-1.01191	0.046628	0.028299
B.cells	AQP1	-1.27568	2.964821	-3.46794	0.000812	-0.64592	0.049662	0.031179
B.cells	AGMO	-0.97641	3.650343	-3.46771	0.000813	-0.68033	0.049122	0.0306
B.cells	FARP1	-0.88189	3.619271	-3.458	0.000839	-0.68393	0.050469	0.031175
B.cells	VNN3	-1.40351	1.614791	-3.45332	0.000852	-0.71683	0.052644	0.033331
B.cells	DNAJA1	-0.26196	7.461326	-3.44771	0.000868	-1.32311	0.048592	0.028692
B.cells	GM11808	-0.40811	7.262783	-3.44625	0.000872	-1.32122	0.048745	0.028927
B.cells	ATF4	-0.41804	6.443614	-3.44439	0.000877	-1.19429	0.049429	0.029702
B.cells	CCDC50	0.330103	5.994315	3.440807	0.000887	-1.18886	0.049929	0.030328
B.cells	SEC11C	-0.33048	7.287381	-3.43849	0.000894	-1.3513	0.048919	0.029417
B.cells	BBOX1	-1.68334	0.92351	-3.43823	0.000895	-0.89643	0.054133	0.03513
B.cells	AMBP	-0.82993	4.730423	-3.43613	0.000901	-1.0033	0.05094	0.031764
B.cells	LMO2	0.474505	4.903927	3.435816	0.000902	-1.09715	0.0508	0.031611
B.cells	CREB5	1.536372	2.17534	3.426645	0.000929	-0.75778	0.054395	0.034758
B.cells	MS4A4C	0.898672	3.224383	3.409635	0.000982	-1.13607	0.056248	0.034933
B.cells	IFI206	1.123449	3.940563	3.398302	0.001019	-1.03655	0.05735	0.03513
B.cells	GM20528	-1.61473	-0.01428	-3.3971	0.001023	-1.03122	0.061097	0.039259
B.cells	SH3PXD2B	1.841609	1.543779	3.394366	0.001032	-0.92104	0.059827	0.037794
B.cells	PDK1	0.512018	4.164844	3.376689	0.001092	-1.05772	0.060166	0.036458
B.cells	SLC17A2	-1.57519	1.156237	-3.37665	0.001092	-1.0076	0.063128	0.03964
B.cells	SLC16A2	-1.0062	2.626868	-3.36919	0.001119	-0.91288	0.062856	0.038629
B.cells	AMDHD1	-1.23381	2.26974	-3.36622	0.001129	-0.92466	0.063521	0.03926
B.cells	1300017J0	-0.91271	3.006144	-3.36047	0.00115	-0.96345	0.063557	0.038944
B.cells	PAG1	0.48632	7.038518	3.359448	0.001154	-1.53633	0.059616	0.034897
B.cells	ISG20	0.89753	4.233394	3.355024	0.001171	-1.14752	0.062802	0.038114
B.cells	UPB1	-1.00389	3.0781	-3.35417	0.001174	-0.96505	0.063968	0.039421
B.cells	LGALS8	0.426043	5.110017	3.342979	0.001217	-1.34178	0.063365	0.03815
B.cells	CAR2	0.876988	5.171918	3.342496	0.001219	-1.23545	0.063302	0.038103
B.cells	NAV1	-0.91309	3.595293	-3.34237	0.001219	-1.03844	0.06491	0.039813
B.cells	CYP2C70	-0.97882	4.144913	-3.341	0.001224	-1.20107	0.064344	0.039268
B.cells	FGFR1	-1.96506	1.974297	-3.3393	0.001231	-1.10108	0.066612	0.041893
B.cells	ACVR1B	0.74342	2.543644	3.338454	0.001234	-1.01242	0.066008	0.041316
B.cells	C8G	-1.04372	3.782028	-3.32795	0.001276	-1.15811	0.066623	0.040664
B.cells	FAM45A	0.435525	3.979967	3.321314	0.001304	-1.23292	0.067527	0.040927
B.cells	ABCC2	-1.06005	2.212583	-3.31536	0.001329	-1.05781	0.070471	0.043622
B.cells	CMTM3	-0.76325	3.593966	-3.3116	0.001345	-1.10129	0.069198	0.042309
B.cells	KLHL8	-0.9456	2.402928	-3.31137	0.001346	-1.08373	0.070526	0.043749
B.cells	LDLR	-0.67958	4.577033	-3.30177	0.001387	-1.35388	0.069733	0.041962
B.cells	MAGI3	-0.48596	6.598022	-3.30104	0.00139	-1.64769	0.067535	0.039712
B.cells	TRAPPC6A	0.344416	5.057196	3.299874	0.001396	-1.42869	0.069203	0.041493
B.cells	PPP1R21	0.465646	4.557871	3.290113	0.001439	-1.42001	0.071503	0.042974
B.cells	YPEL1	-0.8483	2.4444	-3.28932	0.001443	-1.1275	0.073953	0.045643

B.cells	QTRT1	-0.58128	4.980734	-3.28349	0.00147	-1.47233	0.072005	0.043106
B.cells	ATP6V1E1	-0.29592	7.59223	-3.28101	0.001481	-1.87132	0.069091	0.040296
B.cells	A330040F1	1.409171	2.508264	3.27951	0.001488	-1.1683	0.074897	0.04661
B.cells	PLG	-0.95279	4.004656	-3.27864	0.001492	-1.33153	0.073131	0.044786
B.cells	XPNPEP3	0.62947	3.250052	3.278225	0.001494	-1.21122	0.074015	0.045749
B.cells	UPP1	2.471092	0.646262	3.27387	0.001515	-1.30135	0.077904	0.049676
B.cells	PFKL	0.429751	5.330071	3.27193	0.001524	-1.5646	0.072431	0.043786
B.cells	GM11290	-0.92669	3.432889	-3.26787	0.001544	-1.2873	0.075295	0.046501
B.cells	CLEC4G	-0.98437	3.468481	-3.26391	0.001563	-1.21513	0.07588	0.046848
B.cells	PCBD1	-1.00552	3.53173	-3.25955	0.001585	-1.32333	0.07628	0.047234
B.cells	TSPAN33	0.986641	2.55857	3.25929	0.001586	-1.23132	0.077474	0.048551
B.cells	SEMA4C	-1.2126	2.202853	-3.25184	0.001624	-1.29101	0.079433	0.049796
B.cells	SLA2	-1.08876	2.746588	-3.24738	0.001647	-1.24292	0.079311	0.049461
B.cells	DDX58	0.54641	4.759373	3.246808	0.00165	-1.56644	0.076808	0.046802
B.cells	APOH	-0.84507	4.983199	-3.24519	0.001658	-1.60299	0.076536	0.046685
B.cells	FGG	-0.63681	6.166651	-3.24438	0.001662	-1.80727	0.075113	0.04521
B.cells	POLG2	-0.41702	5.080463	-3.24297	0.00167	-1.59639	0.076446	0.046745
B.cells	CYP2C68	-1.05818	2.791797	-3.23926	0.001689	-1.28937	0.079889	0.050277
B.cells	ABCA8B	-1.62486	1.118002	-3.23599	0.001706	-1.35041	0.082572	0.052904
B.cells	MTA3	0.3863	6.109959	3.221692	0.001784	-1.85666	0.079191	0.047606
B.cells	LONRF3	-1.2384	1.525709	-3.22137	0.001786	-1.34426	0.085189	0.05412
B.cells	ZFP53	0.435007	4.791451	3.213862	0.001828	-1.65601	0.082122	0.050232
B.cells	F12	-1.46058	1.691224	-3.21277	0.001835	-1.34698	0.086286	0.054925
B.cells	IQCN	-1.43818	1.791223	-3.21263	0.001836	-1.36468	0.086148	0.05478
B.cells	CIITA	0.819761	2.478401	3.20753	0.001865	-1.49029	0.085906	0.054104
B.cells	IGFBP2	-1.18794	4.701389	-3.2075	0.001865	-1.65854	0.082916	0.050859
B.cells	SLCO1B2	-0.88187	4.25024	-3.20227	0.001896	-1.60935	0.084553	0.05199
B.cells	CAV2	-0.72095	2.771749	-3.19851	0.001918	-1.41961	0.087251	0.054612
B.cells	DEPDC7	-1.17568	1.678465	-3.19543	0.001936	-1.37506	0.088961	0.056638
B.cells	CTBS	0.692576	2.934998	3.195419	0.001936	-1.39847	0.087191	0.054693
B.cells	TRIM10	1.614143	0.349121	3.1893	0.001973	-1.44035	0.092267	0.059509
B.cells	FETUB	-1.00805	3.175824	-3.18396	0.002006	-1.46921	0.089279	0.055427
B.cells	SMAP1	0.291141	6.875842	3.181834	0.00202	-2.07397	0.084187	0.050188
B.cells	APOB	-0.86105	4.836413	-3.18165	0.002021	-1.77527	0.08695	0.05312
B.cells	KLHL23	-1.91479	0.171341	-3.17811	0.002043	-1.77365	0.094034	0.060812
B.cells	NUP210L	-0.68842	6.407383	-3.17801	0.002044	-1.97207	0.085132	0.051127
B.cells	GRIA3	-1.22063	3.336555	-3.17451	0.002066	-1.47834	0.089811	0.056029
B.cells	PGRMC1	-0.45941	4.647619	-3.17407	0.002069	-1.68631	0.087956	0.054038
B.cells	SARDH	-1.20205	2.900243	-3.16934	0.002099	-1.45525	0.091431	0.057301
B.cells	METTL7B	-1.35138	2.263028	-3.16768	0.00211	-1.45047	0.0925	0.058558
B.cells	FERMT2	-0.83085	3.451103	-3.16562	0.002123	-1.49714	0.091005	0.056946
B.cells	ZDHHC20	0.248928	6.740107	3.164431	0.002131	-2.08031	0.086377	0.052107
B.cells	MOB3B	-0.41771	4.961752	-3.16003	0.00216	-1.88684	0.0897	0.055266
B.cells	FAM174A	0.43867	6.050716	3.158958	0.002167	-1.97957	0.088165	0.053734
B.cells	PZP	-0.86232	4.39586	-3.15666	0.002183	-1.74821	0.090824	0.056427
B.cells	IGFALS	-1.06924	1.779759	-3.1528	0.002209	-1.48508	0.095482	0.061115
B.cells	DRAM1	1.298768	2.581586	3.150516	0.002224	-1.48878	0.09459	0.059922

B.cells	SERPINA3G	1.798336	3.597185	3.148341	0.002239	-1.62552	0.093359	0.058487
B.cells	MIF	0.475969	7.650894	3.145928	0.002256	-2.26773	0.087888	0.052433
B.cells	UGT3A2	-1.36775	1.174889	-3.14337	0.002274	-1.5576	0.09784	0.06303
B.cells	SH2B2	-0.50656	4.550186	-3.14095	0.002291	-1.81031	0.093046	0.057668
B.cells	GM26511	-0.93289	2.075738	-3.13987	0.002298	-1.52241	0.096791	0.061912
B.cells	VSIG4	-1.08701	4.823941	-3.1315	0.002358	-1.78692	0.094355	0.058291
B.cells	SOD1	-0.39734	5.993562	-3.13078	0.002363	-2.00383	0.092621	0.05652
B.cells	HLX	-0.58329	3.117664	-3.13046	0.002366	-1.6742	0.096954	0.061274
B.cells	SPATA21	-0.64453	3.397572	-3.12179	0.002429	-1.71238	0.098771	0.061933
B.cells	CXCR6	-1.5364	1.255073	-3.11758	0.002461	-1.62809	0.103178	0.066214
B.cells	GM5608	-1.24807	0.923456	-3.11566	0.002475	-1.60419	0.103978	0.066969
B.cells	MORC3	0.324841	5.857789	3.11324	0.002493	-2.09934	0.096491	0.058638
B.cells	PDE6C	-1.655	0.436862	-3.10958	0.002521	-1.74176	0.106022	0.068694
B.cells	PROCA1	-0.71576	2.988273	-3.10637	0.002546	-1.66497	0.101991	0.064364
B.cells	SIPA1L3	0.383533	5.201146	3.105527	0.002553	-2.07933	0.098462	0.060591
B.cells	GCN1	0.378785	4.575371	3.104987	0.002557	-1.88671	0.099445	0.061688
B.cells	GAPDH	0.396679	10.59184	3.103242	0.00257	-2.79892	0.090451	0.052368
B.cells	EMB	0.572599	5.68619	3.103069	0.002572	-2.1673	0.097707	0.060042
B.cells	DNAJC10	0.397236	4.533218	3.102216	0.002578	-1.89592	0.099512	0.062109
B.cells	ELOVL2	-1.15586	2.450205	-3.09941	0.002601	-1.63443	0.103406	0.066139
B.cells	TSTD1	-1.06555	2.589608	-3.09739	0.002617	-1.63274	0.103467	0.066142
B.cells	AMPD1	-0.76835	2.911186	-3.09491	0.002636	-1.71699	0.103371	0.065884
B.cells	CYP7A1	-1.56441	0.739983	-3.09294	0.002652	-1.71474	0.107166	0.070165
B.cells	GRIPAP1	0.313709	5.594976	3.092301	0.002657	-2.11702	0.099178	0.061342
B.cells	SHISA5	0.419158	6.572889	3.08852	0.002688	-2.35085	0.098127	0.060033
B.cells	SEMA4D	0.456804	6.446214	3.087685	0.002695	-2.28884	0.098324	0.060364
B.cells	FABP1	-0.86156	7.428716	-3.08714	0.002699	-2.42821	0.09681	0.058784
B.cells	SERPINA3M	-0.84045	3.688152	-3.08639	0.002705	-1.82002	0.102724	0.065312
B.cells	THEMIS	-1.79507	1.160853	-3.08207	0.002741	-1.72318	0.107996	0.070759
B.cells	ACSM1	-1.78546	1.026687	-3.0809	0.002751	-1.73554	0.108229	0.071178
B.cells	GIMAP1	0.482684	4.06141	3.079996	0.002758	-1.93128	0.103105	0.06551
B.cells	PBX3	-0.39993	5.496226	-3.07842	0.002772	-2.17526	0.100939	0.063073
B.cells	N4BP1	0.434006	5.4056	3.075881	0.002793	-2.15889	0.101432	0.063539
B.cells	PTPN6	0.356359	6.758702	3.075163	0.002799	-2.34221	0.099283	0.061297
B.cells	APOBEC1	0.456757	6.087599	3.07017	0.002842	-2.36238	0.101544	0.063069
B.cells	CSTA2	1.861705	1.96893	3.062944	0.002904	-1.71211	0.110464	0.071515
B.cells	NFKBIE	0.461321	4.879022	3.054493	0.002979	-2.16293	0.107834	0.066991
B.cells	NT5DC1	0.448388	4.5014	3.051527	0.003006	-2.03011	0.108646	0.068073
B.cells	SC5D	-0.53912	4.248539	-3.05079	0.003013	-1.94351	0.109083	0.068669
B.cells	IFIT3	1.454328	3.112471	3.050025	0.00302	-1.79654	0.111076	0.071017
B.cells	DGAT2	-0.58822	3.862389	-3.04839	0.003034	-1.9432	0.109756	0.069691
B.cells	XPO6	0.310022	5.572883	3.047974	0.003038	-2.2206	0.106813	0.06645
B.cells	SNAP23	0.302548	6.222753	3.047745	0.00304	-2.36656	0.10572	0.065259
B.cells	USP25	0.272983	7.051473	3.045634	0.00306	-2.46071	0.104402	0.064087
B.cells	TRIM12C	0.499891	4.726648	3.043473	0.00308	-2.08405	0.108317	0.068611
B.cells	PPFIA4	-0.87977	2.735956	-3.04278	0.003086	-1.77562	0.111807	0.072548
B.cells	MGST1	-0.49778	7.132587	-3.04186	0.003095	-2.54934	0.104268	0.064285

B.cells	BFAR	0.324204	4.85458	3.041558	0.003097	-2.13566	0.108097	0.068539
B.cells	ACOD1	3.280763	2.318035	3.04114	0.003101	-1.77678	0.112556	0.073606
B.cells	GIMAP5	0.628352	3.319876	3.040405	0.003108	-1.96206	0.11077	0.071667
B.cells	CRTC3	0.354056	5.658838	3.035523	0.003154	-2.29413	0.107973	0.06772
B.cells	RBP1	-0.86446	3.578118	-3.03402	0.003168	-1.87519	0.11177	0.071916
B.cells	ANGPTL6	-1.49545	0.97577	-3.03024	0.003204	-1.91967	0.117491	0.077876
B.cells	AOX3	-1.6105	0.998188	-3.0283	0.003223	-1.85323	0.117776	0.078118
B.cells	GPR18	0.480603	4.514903	3.024273	0.003262	-2.20374	0.112358	0.071335
B.cells	BC004004	0.336712	5.147988	3.022636	0.003278	-2.20571	0.111446	0.070343
B.cells	IFIT1BL1	1.934211	0.159661	3.018882	0.003315	-1.90915	0.121689	0.081283
B.cells	TSPO2	1.274578	0.283153	3.016826	0.003336	-1.83199	0.121834	0.081265
B.cells	0610040J0	-0.96792	2.378015	-3.01437	0.00336	-1.83949	0.118328	0.077016
B.cells	CYP2A12	-1.04861	2.85261	-3.00877	0.003417	-1.90903	0.119058	0.076704
B.cells	ATXN7L1	0.320441	6.345264	3.00618	0.003443	-2.46163	0.113169	0.069874
B.cells	EHD1	0.36337	6.1755	2.998883	0.003519	-2.48628	0.115494	0.07109
B.cells	LMBRD1	0.297958	6.356577	2.99828	0.003525	-2.55634	0.115164	0.07079
B.cells	1110008P1	0.361492	5.226277	2.996096	0.003548	-2.3136	0.117664	0.073386
B.cells	ACAT1	-0.48326	6.480379	-2.99321	0.003579	-2.52072	0.116004	0.071285
B.cells	MOSPD1	-0.49015	4.817896	-2.99224	0.003589	-2.21905	0.119101	0.074752
B.cells	SEC14L2	-1.18076	1.70477	-2.98933	0.00362	-1.89106	0.125886	0.082122
B.cells	SPOPL	0.402943	4.42179	2.983861	0.003679	-2.20732	0.122124	0.076827
B.cells	IGF1	-0.79308	5.292109	-2.983	0.003689	-2.29781	0.120447	0.075126
B.cells	MYADM	-0.41792	5.586342	-2.97864	0.003737	-2.49943	0.121094	0.075027
B.cells	RFFL	0.37609	5.915131	2.975709	0.003769	-2.53221	0.121058	0.074741
B.cells	GM19951	0.928277	3.23896	2.974364	0.003784	-2.13379	0.126318	0.080722
B.cells	CYP3A16	-1.41647	0.777697	-2.9741	0.003787	-1.97867	0.131392	0.086472
B.cells	CUL2	0.37234	4.968394	2.972284	0.003808	-2.3501	0.123199	0.07724
B.cells	KLKB1	-1.06972	1.848942	-2.96844	0.003851	-1.93868	0.130344	0.084839
B.cells	STX3	0.94179	2.314188	2.968126	0.003855	-1.96462	0.129377	0.083749
B.cells	SLC25A21	-1.43746	1.867089	-2.96538	0.003886	-1.95201	0.130704	0.085194
B.cells	MEG3	-2.16832	1.040515	-2.96519	0.003888	-2.0271	0.132447	0.087174
B.cells	CCND1	-0.81944	4.140959	-2.96197	0.003925	-2.07683	0.126895	0.080281
B.cells	RBM38	-0.35391	6.898184	-2.95767	0.003975	-2.69676	0.122674	0.074893
B.cells	ABI3	0.628577	4.401261	2.952952	0.004031	-2.29111	0.128844	0.081011
B.cells	TSTD3	-0.83181	1.784277	-2.95256	0.004036	-1.9827	0.134339	0.087174
B.cells	MAN2A1	0.326859	7.183028	2.946059	0.004114	-2.78894	0.125212	0.075753
B.cells	RABGEF1	0.426705	6.01057	2.945022	0.004126	-2.56053	0.127553	0.078416
B.cells	RAPH1	-0.49775	5.055385	-2.94365	0.004143	-2.30654	0.129499	0.080632
B.cells	GM48236	-1.61943	0.1418	-2.94306	0.00415	-2.10563	0.140075	0.092502
B.cells	ITGA8	-1.27837	1.726804	-2.9426	0.004156	-2.00261	0.136558	0.088518
B.cells	UBE2L6	0.795995	4.64931	2.941661	0.004167	-2.31794	0.130337	0.081752
B.cells	GJB2	-1.14252	1.638244	-2.94077	0.004178	-2.00455	0.136752	0.088999
B.cells	SAMHD1	0.472264	7.45997	2.938968	0.0042	-2.89347	0.124987	0.075967
B.cells	EXOC2	0.306941	5.578749	2.934392	0.004257	-2.53563	0.130147	0.080711
B.cells	CTNNA1	-0.34978	6.643473	-2.93186	0.004289	-2.69531	0.128579	0.07867
B.cells	RTL4	-1.80869	0.402884	-2.9297	0.004316	-2.13726	0.142541	0.093987
B.cells	DDX5	-0.17362	9.318404	-2.92603	0.004363	-3.09643	0.124335	0.073701

B.cells	GM15987	0.516657	2.537923	2.925715	0.004367	-2.43711	0.138425	0.08907
B.cells	ACSL3	-0.53587	3.828777	-2.92444	0.004383	-2.28128	0.135604	0.086113
B.cells	SRA1	0.333352	5.239409	2.924369	0.004384	-2.48638	0.132597	0.082797
B.cells	FAM111A	0.363344	5.742953	2.917659	0.004471	-2.58684	0.133792	0.082786
B.cells	AKR1C20	-1.06286	2.839499	-2.91402	0.004518	-2.13368	0.141237	0.090244
B.cells	RETREG1	0.321893	6.437217	2.910756	0.004562	-2.81957	0.133822	0.082166
B.cells	HPN	-0.98081	2.642028	-2.91031	0.004568	-2.08594	0.142143	0.091332
B.cells	TRIM30C	1.159745	1.320225	2.910202	0.004569	-2.07662	0.145182	0.094751
B.cells	GCHFR	-0.87487	3.389908	-2.90821	0.004596	-2.22921	0.140907	0.089817
B.cells	CSNK1G1	0.290984	6.535289	2.903692	0.004657	-2.7653	0.13442	0.082921
B.cells	ANKRD37	0.597217	3.981977	2.90279	0.004669	-2.37699	0.139976	0.089151
B.cells	DERA	0.335575	4.534652	2.902699	0.00467	-2.45129	0.138751	0.08779
B.cells	TTC36	-0.77331	5.028124	-2.90209	0.004678	-2.54412	0.137667	0.086707
B.cells	UBC	-0.32628	8.132317	-2.90195	0.00468	-2.96739	0.131077	0.079541
B.cells	SELL	0.496671	5.472373	2.90189	0.004681	-2.58757	0.136701	0.085655
B.cells	EIF2AK2	0.50542	4.927436	2.898514	0.004727	-2.49394	0.138796	0.087479
B.cells	AGT	-0.9173	4.084746	-2.89739	0.004743	-2.38032	0.140668	0.089731
B.cells	LDHA	0.338917	8.792149	2.896985	0.004748	-3.0953	0.130579	0.078736
B.cells	SULT2A8	-1.7041	0.571296	-2.89297	0.004804	-2.19405	0.150039	0.099764
B.cells	NDRG2	-0.89631	2.964877	-2.891	0.004832	-2.20143	0.144398	0.093635
B.cells	BTG1	0.274267	9.314237	2.88973	0.004849	-3.26459	0.130595	0.078644
B.cells	D430040D2	-1.97689	-0.34621	-2.88788	0.004876	-2.4407	0.152268	0.103149
B.cells	CYP2C23	-1.45466	0.860959	-2.88769	0.004878	-2.19189	0.149343	0.09976
B.cells	SUSD6	0.294646	7.690279	2.887635	0.004879	-2.97728	0.133971	0.082495
B.cells	HFE	-0.75904	3.452689	-2.88746	0.004881	-2.18874	0.143278	0.092835
B.cells	ADGRG3	-1.11889	2.366781	-2.88714	0.004886	-2.13368	0.145784	0.095682
B.cells	IRF2	0.291596	6.689517	2.886097	0.004901	-2.84684	0.136176	0.08491
B.cells	STAT1	0.841797	6.871703	2.883565	0.004937	-2.86803	0.13598	0.084886
B.cells	ARL5A	0.302818	5.831093	2.883065	0.004944	-2.75378	0.138237	0.087407
B.cells	ERBB4	-1.59585	0.909465	-2.88302	0.004945	-2.18106	0.149522	0.100233
B.cells	CEPT1	0.418323	5.374134	2.881662	0.004964	-2.65186	0.139389	0.088713
B.cells	GM4285	0.851645	1.640385	2.880948	0.004975	-2.14813	0.147937	0.098516
B.cells	GM26887	-1.02434	3.10483	-2.87878	0.005006	-2.21698	0.145067	0.094905
B.cells	TMED8	0.541613	2.954093	2.877754	0.005021	-2.27766	0.145489	0.095419
B.cells	MANSC1	-1.74847	0.242039	-2.87597	0.005047	-2.32141	0.152021	0.103197
B.cells	UBE2O	0.468028	4.874388	2.875896	0.005048	-2.6812	0.141174	0.090724
B.cells	USP2	-0.92036	2.711696	-2.87385	0.005078	-2.16775	0.146209	0.096604
B.cells	RGMB	-1.00348	1.845211	-2.87341	0.005084	-2.16213	0.148249	0.099015
B.cells	PML	0.578483	5.273859	2.873163	0.005088	-2.62136	0.140365	0.090021
B.cells	ZUP1	0.386545	5.168406	2.869564	0.005141	-2.6794	0.141727	0.090715
B.cells	HERPUD1	-0.37037	7.853564	-2.86831	0.00516	-3.04671	0.136001	0.08433
B.cells	VAV1	0.290752	6.364742	2.867198	0.005176	-2.82655	0.139344	0.088016
B.cells	LIPC	-0.76769	2.510562	-2.86561	0.0052	-2.26087	0.148474	0.098331
B.cells	TARM1	1.542367	-0.18149	2.860059	0.005284	-2.24725	0.15561	0.106914
B.cells	NEDD9	0.355211	8.003683	2.859947	0.005286	-3.11259	0.136599	0.085143
B.cells	SYK	0.29701	8.336181	2.859232	0.005297	-3.19815	0.135886	0.084407
B.cells	PLXDC2	-0.85462	5.144109	-2.8592	0.005297	-2.55424	0.142914	0.092251

B.cells	GM14221	-1.31202	1.910062	-2.85918	0.005297	-2.19503	0.150474	0.100938
B.cells	RNF145	0.367643	5.159727	2.858249	0.005312	-2.6826	0.142879	0.092362
B.cells	CCDC102A	1.094297	2.048525	2.857696	0.00532	-2.19829	0.150141	0.100794
B.cells	NSF	0.29542	6.770124	2.857692	0.00532	-3.00826	0.139282	0.088382
B.cells	TRAPPC11	0.482177	3.489165	2.856149	0.005344	-2.35692	0.147033	0.097132
B.cells	SIRPB1C	1.65819	1.778958	2.852405	0.005402	-2.21674	0.152384	0.102416
B.cells	CLEC4F	-1.39702	5.458864	-2.85067	0.005429	-2.62583	0.143742	0.092739
B.cells	SERPINA1B	-0.78002	7.994808	-2.85051	0.005431	-3.15553	0.138097	0.086441
B.cells	WASHC4	0.266577	5.792598	2.849876	0.005441	-2.79568	0.142984	0.091999
B.cells	GHR	-0.91856	4.215001	-2.84836	0.005465	-2.54217	0.146906	0.096372
B.cells	PLA2G7	1.609454	4.021195	2.846185	0.005499	-2.29894	0.147942	0.097284
B.cells	TKTL1	-1.06477	1.290296	-2.84438	0.005528	-2.23612	0.154983	0.105337
B.cells	TRIM72	-1.39932	0.692552	-2.83594	0.005663	-2.30203	0.159915	0.108589
B.cells	SMAP2	0.245705	6.821529	2.834569	0.005686	-2.98515	0.145042	0.091785
B.cells	IFI203	0.462798	6.302394	2.834386	0.005689	-2.93276	0.146237	0.093121
B.cells	DPYS	-0.88415	2.829004	-2.83145	0.005737	-2.36221	0.155259	0.103112
B.cells	NCF1	0.436798	5.301832	2.830683	0.005749	-2.75609	0.149267	0.096388
B.cells	CDK5RAP3	0.399225	4.379321	2.830358	0.005755	-2.59608	0.15147	0.098928
B.cells	9030622O2	-1.50008	1.05914	-2.82829	0.005789	-2.28983	0.160063	0.108841
B.cells	OLFR1369-	-2.05336	-0.76388	-2.82756	0.005801	-2.47019	0.164784	0.114616
B.cells	IL4RA	0.429357	4.883706	2.827223	0.005806	-2.73901	0.150583	0.098008
B.cells	LPCAT2	0.576612	4.726381	2.826052	0.005826	-2.57401	0.151124	0.098643
B.cells	TXNDC11	0.313392	5.714713	2.82496	0.005844	-2.84256	0.148901	0.096104
B.cells	AUNIP	-1.02977	1.893508	-2.82088	0.005913	-2.28411	0.159725	0.10764
B.cells	MAP2K4	0.33887	6.321239	2.820125	0.005925	-2.95936	0.148857	0.095273
B.cells	SNTB1	-0.88487	4.264888	-2.81873	0.005949	-2.49401	0.154046	0.101111
B.cells	GM17749	-0.69497	4.634129	-2.81628	0.005991	-2.71814	0.153144	0.100439
B.cells	PLEKHA1	0.448788	4.707996	2.815785	0.005999	-2.78615	0.152964	0.100278
B.cells	OTC	-0.89815	3.438444	-2.81321	0.006043	-2.4795	0.156087	0.10439
B.cells	POU2F1	0.435814	5.39401	2.812898	0.006049	-2.78975	0.151308	0.098928
B.cells	RNF128	-0.87809	2.499728	-2.81174	0.006069	-2.32904	0.158443	0.107498
B.cells	BOK	-1.65656	1.039523	-2.8094	0.006109	-2.42952	0.162191	0.112415
B.cells	BABAM2	0.247515	7.216423	2.808548	0.006124	-3.12435	0.147008	0.094727
B.cells	CLCN3	0.296398	6.389845	2.808152	0.006131	-2.98077	0.14894	0.096985
B.cells	CD63	-0.73137	4.105165	-2.80793	0.006135	-2.50975	0.154438	0.103379
B.cells	6330562C2	-1.21923	1.275201	-2.80763	0.00614	-2.34154	0.16158	0.111876
B.cells	SLC13A3	-1.44495	1.11728	-2.80643	0.006161	-2.32465	0.161989	0.112548
B.cells	ADH1	-0.92213	3.503888	-2.80587	0.006171	-2.50154	0.155924	0.105324
B.cells	PDIA5	-0.87674	2.273787	-2.80578	0.006173	-2.33539	0.159017	0.108991
B.cells	SH3TC1	-0.59075	2.768133	-2.80576	0.006173	-2.43912	0.157765	0.107502
B.cells	HSPA4L	0.498351	4.348791	2.805659	0.006175	-2.58802	0.15384	0.102876
B.cells	UBLCP1	0.342806	4.773952	2.804364	0.006198	-2.73304	0.152804	0.101826
B.cells	SLA	0.397395	5.280678	2.804149	0.006201	-2.86644	0.15158	0.100399
B.cells	A330023F2	0.654757	3.109027	2.803611	0.006211	-2.44237	0.156909	0.106795
B.cells	CKS2	-0.46435	5.959802	-2.80289	0.006224	-2.86717	0.149957	0.09881
B.cells	PTPRC	0.287588	8.915439	2.80283	0.006225	-3.3824	0.14313	0.091007
B.cells	MST1	-1.54974	0.649079	-2.80261	0.006229	-2.36762	0.163211	0.114591

B.cells	DNAH17	-0.88499	3.275665	-2.80027	0.00627	-2.48999	0.156845	0.107048
B.cells	ZBTB46	-0.72702	3.021217	-2.80009	0.006273	-2.42239	0.157483	0.10784
B.cells	PACSIN2	0.260432	5.573303	2.799566	0.006283	-2.92145	0.151218	0.100497
B.cells	GATA4	-1.27507	1.621315	-2.79819	0.006307	-2.36433	0.161058	0.112321
B.cells	LHPP	-0.55515	3.112672	-2.79805	0.00631	-2.47654	0.157263	0.107782
B.cells	TUBGCP4	0.384333	4.42498	2.796445	0.006339	-2.71391	0.154111	0.104128
B.cells	CTTN	-1.10598	1.717261	-2.79633	0.006341	-2.34416	0.160916	0.112275
B.cells	EZH2	-0.39874	6.663574	-2.79309	0.006399	-3.06133	0.149795	0.098439
B.cells	IL10RB	0.343467	5.555085	2.791698	0.006425	-2.84266	0.152728	0.101658
B.cells	GM21188	1.634119	1.578983	2.790117	0.006453	-2.36087	0.163108	0.113978
B.cells	NIPSNAP2	0.305607	4.810103	2.789208	0.00647	-2.76374	0.154988	0.104222
B.cells	SLC8A1	0.80844	6.330185	2.787003	0.006511	-2.82886	0.151928	0.100251
B.cells	MCTP2	0.404297	6.80258	2.785643	0.006536	-3.04501	0.151065	0.099212
B.cells	HEPACAM2	-0.66736	0.779743	-2.7844	0.006559	-2.63095	0.166351	0.117548
B.cells	TEX45	-1.56689	0.222756	-2.78401	0.006566	-2.4522	0.167846	0.119468
B.cells	2010109A1	-0.72028	2.521995	-2.78129	0.006617	-2.4335	0.162637	0.112665
B.cells	ENHO	-0.93418	2.481211	-2.78066	0.006628	-2.40405	0.162743	0.112914
B.cells	TIMD4	-1.02838	3.658253	-2.77989	0.006643	-2.49601	0.159736	0.109429
B.cells	TOB1	-0.37746	5.050855	-2.77884	0.006663	-2.84135	0.156379	0.10538
B.cells	STXBP3	0.325788	5.278015	2.776515	0.006707	-2.93708	0.156196	0.104979
B.cells	IL1A	-1.37224	2.787687	-2.77587	0.006719	-2.3875	0.162512	0.112634
B.cells	DECR1	-0.67601	3.696206	-2.7755	0.006726	-2.56685	0.160174	0.109878
B.cells	CD164L2	-1.15032	1.352514	-2.77373	0.00676	-2.38898	0.166287	0.117516
B.cells	TMEM86B	-0.52458	3.467162	-2.77297	0.006774	-2.56467	0.16076	0.110961
B.cells	RDH7	-0.93235	2.864625	-2.77281	0.006777	-2.49799	0.162312	0.112838
B.cells	HIST1H4N	-1.54127	1.07648	-2.77277	0.006778	-2.46384	0.167025	0.11859
B.cells	FN1	-0.89851	5.580946	-2.77148	0.006803	-3.06843	0.155447	0.104917
B.cells	BSG	0.316552	7.716678	2.77132	0.006806	-3.2966	0.150289	0.098869
B.cells	GM13547	-1.65163	0.514665	-2.77076	0.006816	-2.4439	0.168538	0.120937
B.cells	DUSP18	-1.59186	0.621447	-2.76464	0.006935	-2.48028	0.170835	0.12185
B.cells	S100A4	1.06148	2.87429	2.761114	0.007004	-2.48953	0.166091	0.115194
B.cells	LMLN	-0.8942	1.750464	-2.75961	0.007034	-2.42094	0.169479	0.11916
B.cells	PLBD2	0.385671	4.310294	2.758378	0.007058	-2.77565	0.162793	0.111159
B.cells	STAP1	0.34514	4.891894	2.757972	0.007067	-3.0039	0.161296	0.109374
B.cells	BATF	0.441072	4.183315	2.755179	0.007122	-2.75397	0.163947	0.112248
B.cells	NDNF	-1.36082	-0.47189	-2.75476	0.007131	-2.49264	0.176627	0.127857
B.cells	CD96	1.61833	0.686804	2.752873	0.007169	-2.45802	0.173947	0.124199
B.cells	TMUB1	0.43324	3.138278	2.749857	0.007229	-2.6221	0.168222	0.116368
B.cells	YARS	-0.44402	5.391202	-2.74941	0.007239	-2.941	0.1623	0.109319
B.cells	BC051226	0.608328	2.179737	2.745756	0.007313	-2.52062	0.172076	0.120295
B.cells	ZBTB2	-0.28824	5.709287	-2.74539	0.007321	-3.04556	0.162673	0.109115
B.cells	CCR1	1.087878	2.32125	2.744665	0.007336	-2.48638	0.171696	0.120039
B.cells	SOAT1	0.420163	5.176779	2.739429	0.007444	-2.98794	0.166159	0.111808
B.cells	SQOR	-0.70304	3.496234	-2.73773	0.007479	-2.59462	0.171001	0.117534
B.cells	NDUFA4	-0.29969	8.596899	-2.73673	0.0075	-3.48512	0.157738	0.102154
B.cells	JAK1	0.211948	8.273816	2.734801	0.007541	-3.46169	0.158541	0.103351
B.cells	MCFD2	-0.59065	3.702476	-2.73461	0.007545	-2.64662	0.170439	0.117376

B.cells	OPA1	0.324583	4.835691	2.734551	0.007546	-2.91136	0.167394	0.113731
B.cells	HAO2	-1.49664	1.393032	-2.73447	0.007548	-2.47538	0.176847	0.125162
B.cells	GM12236	-1.44673	1.087964	-2.73325	0.007574	-2.49059	0.177714	0.126408
B.cells	FBXL5	0.396562	6.173273	2.732749	0.007584	-3.19684	0.163882	0.109809
B.cells	MAST4	-0.50388	6.39259	-2.73246	0.00759	-3.18436	0.163315	0.109187
B.cells	KYNU	0.433935	3.853994	2.730106	0.007641	-2.94023	0.170506	0.117633
B.cells	TCEA1	0.205603	7.486226	2.730084	0.007641	-3.38344	0.16097	0.10632
B.cells	ZDHHC21	0.397471	4.271545	2.729105	0.007662	-2.84088	0.169407	0.116429
B.cells	RNF213	0.617606	6.151249	2.728374	0.007678	-3.13089	0.164429	0.11066
B.cells	OMA1	0.526266	3.410326	2.727962	0.007686	-2.68426	0.171747	0.119486
B.cells	PLEKHM3	0.362525	6.068485	2.727201	0.007703	-3.17972	0.164681	0.111085
B.cells	GALNT1	0.232162	6.628673	2.724094	0.00777	-3.247	0.164082	0.109991
B.cells	SGSM3	-0.4629	4.063557	-2.72397	0.007773	-2.84748	0.170895	0.118154
B.cells	BRIP1	-0.50178	4.898207	-2.72165	0.007823	-2.92572	0.169242	0.115885
B.cells	MYCBP2	0.281939	7.768752	2.721337	0.00783	-3.44116	0.16173	0.107061
B.cells	PIKFYVE	0.384774	4.426045	2.720085	0.007857	-2.8938	0.17079	0.117829
B.cells	APOC1	-0.72005	7.949726	-2.71822	0.007898	-3.43969	0.162063	0.107277
B.cells	H2-DMB2	0.44691	3.016521	2.717434	0.007915	-2.95167	0.175301	0.123258
B.cells	PGAP1	0.469066	3.785443	2.713765	0.007997	-2.97849	0.174615	0.121343
B.cells	ARHGEF18	0.346477	5.591269	2.711902	0.008038	-3.16129	0.170205	0.115674
B.cells	PQLC1	-0.31966	4.395337	-2.71076	0.008064	-2.9512	0.173468	0.119806
B.cells	ZBP1	1.032973	3.640152	2.710626	0.008067	-2.77422	0.175566	0.122354
B.cells	GSTT1	-0.99633	2.240293	-2.7092	0.008099	-2.56816	0.179876	0.127559
B.cells	A630001G2	0.329298	5.126842	2.7086	0.008112	-3.06993	0.17179	0.117813
B.cells	ZFP710	-0.34273	6.297621	-2.70673	0.008155	-3.27865	0.169115	0.114399
B.cells	GM2245	1.342307	1.558462	2.706236	0.008166	-2.56791	0.18237	0.130605
B.cells	LRCH3	0.266024	6.497112	2.705423	0.008184	-3.26874	0.168651	0.113948
B.cells	ATG4A	0.432312	4.314693	2.702896	0.008242	-2.9431	0.175377	0.121492
B.cells	DMPK	-0.98921	2.786398	-2.70216	0.008259	-2.57896	0.179701	0.126858
B.cells	BIN2	0.367774	5.165118	2.701602	0.008271	-3.02624	0.173023	0.118815
B.cells	HMGCS2	-0.79639	4.456909	-2.70113	0.008282	-2.94892	0.17498	0.121267
B.cells	CFLAR	0.399135	5.971185	2.700389	0.008299	-3.16678	0.170826	0.116367
B.cells	PKMYT1	-0.70184	3.261877	-2.69834	0.008347	-2.65865	0.178342	0.125954
B.cells	NFE2	0.982749	1.59738	2.698192	0.00835	-2.55427	0.183151	0.131921
B.cells	CD300LF	0.741057	3.385271	2.697987	0.008355	-2.75008	0.177992	0.125543
B.cells	MTFR2	-0.62462	3.615592	-2.69785	0.008358	-2.7845	0.177339	0.124755
B.cells	NPTN	-0.19824	8.043161	-2.6955	0.008413	-3.50436	0.166109	0.110773
B.cells	GBP9	0.666791	3.036901	2.693633	0.008456	-2.74399	0.180432	0.127753
B.cells	CD209F	-2.37457	2.767776	-2.69088	0.008521	-2.59196	0.182263	0.12924
B.cells	TMEM179E	0.248609	5.864754	2.688821	0.008569	-3.19302	0.173868	0.118945
B.cells	ISCA1	0.379208	6.018689	2.688204	0.008584	-3.30168	0.173445	0.118599
B.cells	MAP4K3	0.456937	4.283134	2.687836	0.008593	-2.93483	0.178288	0.124534
B.cells	LRG1	1.133543	2.460703	2.686702	0.00862	-2.63823	0.183546	0.131204
B.cells	TRAF3	0.332795	6.856964	2.686553	0.008623	-3.39349	0.171161	0.116121
B.cells	FXYD1	-0.85264	2.943369	-2.68622	0.008631	-2.69752	0.182136	0.129541
B.cells	NFX1	0.30947	4.900546	2.684217	0.008679	-3.05277	0.177209	0.123005
B.cells	DUSP5	-0.34747	6.834196	-2.67891	0.008807	-3.41357	0.174089	0.117691

B.cells	SLC25A1	0.512868	3.772843	2.678158	0.008826	-2.82708	0.182805	0.128384
B.cells	AZGP1	-0.7134	3.926277	-2.67716	0.00885	-2.92335	0.182471	0.128109
B.cells	TST	-0.82544	3.470042	-2.67665	0.008862	-2.79909	0.183802	0.129882
B.cells	GM32296	1.500765	-0.65404	2.67266	0.00896	-2.7118	0.197399	0.14668
B.cells	CEP295	0.416049	3.949897	2.672273	0.00897	-2.90138	0.183396	0.129137
B.cells	OASL1	1.066059	2.957835	2.671596	0.008987	-2.81176	0.186319	0.132871
B.cells	TRIM26	0.319656	5.330265	2.671507	0.008989	-3.15992	0.179416	0.124381
B.cells	CLCN5	0.401374	5.160349	2.671471	0.00899	-3.11764	0.179901	0.12497
B.cells	ITSN2	0.250253	6.989544	2.670208	0.009021	-3.43356	0.175068	0.119003
B.cells	IL21R	0.433727	5.150614	2.668716	0.009058	-3.22169	0.180663	0.125558
B.cells	MPC2	-0.28798	6.800876	-2.66662	0.00911	-3.42781	0.176563	0.120343
B.cells	RAB3IP	0.400848	4.587906	2.66629	0.009119	-3.06546	0.182864	0.128043
B.cells	PXMP2	-0.75935	4.067436	-2.66442	0.009166	-2.94851	0.184919	0.130242
B.cells	SLC25A10	-0.66932	3.077126	-2.66397	0.009177	-2.71098	0.187861	0.134003
B.cells	HGFAC	-1.10225	1.432763	-2.66254	0.009213	-2.62934	0.193287	0.140624
B.cells	TMEM134	0.282757	6.132006	2.660003	0.009278	-3.3025	0.180093	0.123942
B.cells	IFI213	1.020671	3.327221	2.658254	0.009322	-2.88952	0.188299	0.134386
B.cells	IL11RA1	-0.72864	2.113905	-2.658	0.009329	-2.65913	0.191984	0.139009
B.cells	1700016PC	-0.71127	3.898827	-2.65793	0.009331	-2.98288	0.186592	0.132275
B.cells	REC114	-0.53968	3.19631	-2.65788	0.009332	-2.88952	0.188693	0.134886
B.cells	WDR37	0.291478	5.478401	2.654574	0.009417	-3.22906	0.182519	0.127245
B.cells	AXL	-1.00862	3.708037	-2.6541	0.009429	-2.7679	0.187728	0.133772
B.cells	CSRP2	-0.37536	5.20682	-2.65408	0.009429	-3.17773	0.183307	0.128306
B.cells	ARAP3	-0.81833	3.537048	-2.65401	0.009431	-2.70563	0.18824	0.13441
B.cells	SNRPF	-0.27549	6.76677	-2.65282	0.009462	-3.43536	0.178836	0.123051
B.cells	CD300LB	1.323241	1.973752	2.652777	0.009463	-2.64979	0.192999	0.14061
B.cells	GDA	1.486256	3.902395	2.652113	0.00948	-2.70706	0.187148	0.133454
B.cells	RASA3	0.251758	6.702935	2.65179	0.009489	-3.47485	0.179016	0.123445
B.cells	NFAT5	0.302899	7.501438	2.64963	0.009545	-3.56633	0.177294	0.12112
B.cells	CES2E	-1.13198	1.228112	-2.64895	0.009563	-2.65779	0.195893	0.144331
B.cells	CLDN1	-1.28291	1.493829	-2.64886	0.009565	-2.66138	0.19506	0.143269
B.cells	R3HDM1	0.21994	6.683532	2.645309	0.009659	-3.45056	0.181046	0.124721
B.cells	GRINA	0.469482	6.205395	2.643009	0.00972	-3.32058	0.183261	0.126866
B.cells	AGXT	-0.85899	3.279368	-2.64159	0.009757	-2.86917	0.192201	0.137947
B.cells	ARID5B	0.396305	7.244193	2.640998	0.009773	-3.59174	0.180485	0.123602
B.cells	NGLY1	0.25835	4.849758	2.640746	0.00978	-3.11494	0.187457	0.132144
B.cells	ISG15	0.976167	6.408101	2.634887	0.009937	-3.29217	0.185396	0.127488
B.cells	IDH2	-0.33843	5.667278	-2.63452	0.009947	-3.25904	0.187583	0.130171
B.cells	TCEAL9	-0.36946	5.693961	-2.63311	0.009986	-3.2394	0.187914	0.130389
B.cells	UGT2B5	-1.17616	1.786068	-2.63177	0.010022	-2.70714	0.20038	0.14563
B.cells	ATF3	-0.54304	6.1843	-2.62883	0.010103	-3.49282	0.187802	0.129442
B.cells	EYA3	0.305725	5.473081	2.62816	0.010121	-3.32355	0.18993	0.132106
B.cells	F13B	-1.14398	1.463355	-2.62804	0.010125	-2.70155	0.202467	0.147699
B.cells	SUSD3	0.388205	3.170727	2.625437	0.010197	-3.11361	0.197921	0.141352
B.cells	SLC27A5	-1.17386	1.457834	-2.62446	0.010224	-2.7098	0.203417	0.148355
B.cells	TPST1	0.482087	3.472726	2.62415	0.010232	-3.06227	0.19697	0.140309
B.cells	IGF2	-0.98814	4.193244	-2.62392	0.010239	-3.0868	0.194723	0.137543

B.cells	DHX15	-0.21977	6.794688	-2.62324	0.010258	-3.49919	0.186896	0.128112
B.cells	SMG1	0.194957	7.499087	2.621568	0.010304	-3.64659	0.185367	0.126002
B.cells	KRT8	-1.03548	1.975849	-2.61709	0.010431	-2.74793	0.20451	0.147938
B.cells	ARHGAP31	0.391684	6.152696	2.611883	0.010579	-3.44168	0.19376	0.132825
B.cells	PAQR8	-1.4328	0.223677	-2.61041	0.010621	-2.77603	0.213484	0.157052
B.cells	CRLF3	0.320965	6.899396	2.609774	0.01064	-3.58111	0.191956	0.130546
B.cells	GM15518	-1.49537	0.80065	-2.60808	0.010688	-2.79776	0.21216	0.155074
B.cells	CAMK2D	0.33179	7.214604	2.605873	0.010753	-3.69897	0.192406	0.1301
B.cells	CCL22	-1.77084	-0.37257	-2.60384	0.010812	-2.75497	0.217678	0.161183
B.cells	VTI1A	0.236988	7.233705	2.603764	0.010814	-3.63954	0.192822	0.130448
B.cells	HNRNPDL	-0.18721	7.857159	-2.60273	0.010845	-3.72251	0.191165	0.128432
B.cells	TMEFF1	1.483163	-0.59624	2.601	0.010895	-2.7856	0.219118	0.162875
B.cells	GALNS	0.42838	3.757313	2.599441	0.010941	-3.07149	0.204381	0.144554
B.cells	NECAB3	-1.11685	1.186088	-2.59937	0.010943	-2.77115	0.212955	0.155276
B.cells	IL2RG	0.497622	6.294179	2.599315	0.010945	-3.57034	0.196314	0.134703
B.cells	GPAT3	-0.33476	5.178486	-2.59874	0.010962	-3.38813	0.199814	0.139045
B.cells	KCNH7	-1.54728	-0.13979	-2.59845	0.010971	-2.86097	0.217538	0.161234
B.cells	OGFRL1	0.40753	5.082967	2.59764	0.010995	-3.26818	0.200236	0.139543
B.cells	NES	-1.62157	0.578345	-2.59557	0.011056	-2.81782	0.216027	0.1584
B.cells	CHSY3	-1.03026	1.978293	-2.59334	0.011123	-2.84564	0.212172	0.152919
B.cells	PABPC1L	-0.63322	2.906471	-2.59187	0.011167	-2.9555	0.209313	0.149436
B.cells	FGA	-0.52088	5.727993	-2.59169	0.011173	-3.45883	0.200127	0.138155
B.cells	TUBGCP3	-0.35035	3.88521	-2.58939	0.011242	-3.0854	0.207026	0.145943
B.cells	RARB	-1.40482	1.586463	-2.58808	0.011282	-2.7842	0.215184	0.156023
B.cells	ARHGEF40	-1.30957	0.550928	-2.58694	0.011317	-2.80318	0.219118	0.160777
B.cells	CDKN1C	-0.87506	3.302414	-2.58617	0.01134	-2.93194	0.209779	0.149134
B.cells	GM2000	-0.47061	4.799183	-2.58549	0.011361	-3.25404	0.204893	0.143206
B.cells	AVPI1	-0.61493	2.712104	-2.58449	0.011391	-2.93468	0.212055	0.151976
B.cells	IFIT1	1.569837	2.761439	2.579914	0.011533	-2.84635	0.214178	0.152728
B.cells	AU020206	0.335014	5.320744	2.578842	0.011566	-3.38336	0.205899	0.142368
B.cells	RASGEF1B	0.464021	5.547786	2.577903	0.011595	-3.54204	0.205355	0.141662
B.cells	VAMP4	0.247897	6.154005	2.575803	0.011661	-3.55307	0.203958	0.139753
B.cells	ZADH2	0.448209	3.381757	2.575708	0.011664	-3.03395	0.213141	0.150981
B.cells	RAB20	0.813284	3.301078	2.573872	0.011721	-2.9189	0.213826	0.151687
B.cells	TSPAN15	-0.82464	2.388192	-2.57383	0.011723	-2.83066	0.216966	0.155589
B.cells	MAP3K5	0.287152	7.255072	2.572973	0.01175	-3.7093	0.200977	0.136066
B.cells	CP	-0.58723	4.588111	-2.57143	0.011798	-3.26894	0.209868	0.146894
B.cells	ACSS2	0.586766	3.033515	2.571422	0.011799	-2.99593	0.215129	0.153389
B.cells	HADH	-0.3929	5.394105	-2.56487	0.012008	-3.43916	0.210544	0.145233
B.cells	DOCK10	0.257896	8.80921	2.560963	0.012134	-4.00256	0.200427	0.132973
B.cells	TMEM245	0.35419	4.753996	2.560603	0.012146	-3.33035	0.213685	0.148888
B.cells	NOD1	0.673967	3.387607	2.560348	0.012154	-3.05351	0.218383	0.154733
B.cells	HERC6	0.576214	4.719873	2.55988	0.012169	-3.29401	0.213801	0.149181
B.cells	TIGD2	0.409021	3.85213	2.559675	0.012176	-3.13266	0.216772	0.152868
B.cells	COX4I2	-1.12296	0.956141	-2.55907	0.012195	-2.85309	0.227044	0.165838
B.cells	CALHM2	0.467649	3.088827	2.558916	0.0122	-3.05196	0.219426	0.156294
B.cells	DYNLT1A	-0.43805	4.14434	-2.55854	0.012213	-3.17402	0.215766	0.151877

B.cells	PARP11	0.588836	3.46828	2.556596	0.012276	-3.09922	0.218684	0.155314
B.cells	ATL3	0.262872	5.439187	2.555946	0.012298	-3.46209	0.211938	0.147179
B.cells	PLVAP	-1.22332	1.394862	-2.55563	0.012308	-2.85907	0.226054	0.164778
B.cells	RDH16F2	-1.24951	1.257357	-2.55527	0.01232	-2.85321	0.226553	0.165423
B.cells	TMEM56	-0.95489	2.147941	-2.55401	0.012361	-2.88684	0.223755	0.161654
B.cells	TREM3	1.371735	2.200561	2.55344	0.01238	-2.86432	0.223569	0.161543
B.cells	CARD10	-1.00655	1.098314	-2.55171	0.012438	-2.85856	0.227708	0.166848
B.cells	DNAJB9	-0.33786	5.168187	-2.5517	0.012438	-3.35501	0.213391	0.148988
B.cells	HABP2	-1.34571	0.622763	-2.55147	0.012446	-2.87375	0.229453	0.169067
B.cells	SARDHOS	-1.77604	0.147254	-2.55092	0.012464	-2.92428	0.231212	0.171427
B.cells	S100A11	-0.28933	7.270251	-2.54838	0.012549	-3.8225	0.207504	0.141082
B.cells	ERO1L	0.420086	5.171152	2.547337	0.012583	-3.41096	0.214784	0.149828
B.cells	ASF1A	-0.33635	5.210724	-2.54634	0.012617	-3.44304	0.214804	0.149787
B.cells	SKAP2	0.2182	7.235487	2.544652	0.012674	-3.79007	0.208033	0.141832
B.cells	MBL1	-0.99079	1.455107	-2.54461	0.012675	-2.87786	0.228063	0.166585
B.cells	MALT1	0.466805	7.764534	2.543997	0.012696	-3.97425	0.206305	0.139833
B.cells	IL1R1	1.025124	2.051839	2.543657	0.012707	-2.91988	0.225894	0.163963
B.cells	MAPK6	0.351113	5.88266	2.542065	0.012761	-3.58795	0.212529	0.147719
B.cells	EPS15	0.232164	6.183956	2.541294	0.012788	-3.629	0.211517	0.146622
B.cells	MYO5A	0.386999	5.016572	2.541174	0.012792	-3.46488	0.215467	0.151465
B.cells	TRIM34A	0.607717	3.308515	2.541159	0.012792	-3.15266	0.221404	0.15884
B.cells	GYS2	-1.08724	0.768164	-2.5411	0.012794	-2.885	0.230589	0.170465
B.cells	HMGB2	-0.43358	9.132267	-2.54092	0.0128	-4.01006	0.201918	0.135091
B.cells	TRF	-0.60934	9.658999	-2.53989	0.012835	-4.15583	0.200513	0.133345
B.cells	MIOS	0.383343	3.665658	2.539072	0.012863	-3.19695	0.220584	0.157631
B.cells	UCP2	0.25465	9.571709	2.537139	0.01293	-4.16232	0.201669	0.134078
B.cells	PROK2	1.868875	-0.61756	2.536414	0.012955	-2.93033	0.237199	0.178147
B.cells	TYMS	-0.61102	4.922548	-2.53462	0.013016	-3.33242	0.217828	0.15315
B.cells	PTMA	-0.23901	11.35991	-2.53398	0.013039	-4.40125	0.196899	0.128186
B.cells	THAP1	-0.57255	2.879636	-2.53342	0.013058	-3.015	0.225094	0.162361
B.cells	SULT1D1	-0.90036	2.290136	-2.53257	0.013088	-2.97165	0.227275	0.165211
B.cells	CD36	-0.53109	5.712063	-2.53224	0.013099	-3.61734	0.215225	0.150317
B.cells	ACVR1	-0.59084	3.707524	-2.53103	0.013141	-3.14032	0.222581	0.159254
B.cells	UHMK1	0.320953	4.630018	2.528523	0.013229	-3.33457	0.220108	0.15561
B.cells	NR1I3	-1.24398	1.055611	-2.52826	0.013238	-2.90573	0.233036	0.171907
B.cells	UBAC1	0.349634	4.029628	2.528065	0.013245	-3.25603	0.22222	0.158282
B.cells	STOM	0.61617	3.274711	2.524998	0.013353	-3.08162	0.226351	0.162334
B.cells	SLC2A2	-1.00899	1.627926	-2.52437	0.013375	-2.932	0.232388	0.170063
B.cells	STAT6	0.296518	5.031546	2.52402	0.013388	-3.48051	0.220111	0.154756
B.cells	KYAT3	-0.65531	3.603362	-2.52281	0.013431	-3.19256	0.225341	0.161246
B.cells	BC147527	1.006415	1.109158	2.521207	0.013488	-2.92045	0.234509	0.173322
B.cells	RRP1B	0.416573	4.132089	2.520653	0.013508	-3.38066	0.223452	0.159372
B.cells	PARL	0.310395	4.391466	2.520327	0.013519	-3.40314	0.222532	0.158268
B.cells	SAMSN1	0.332686	7.050554	2.519935	0.013533	-3.72935	0.213352	0.147008
B.cells	GNGT2	0.444989	5.675602	2.51973	0.013541	-3.57263	0.218041	0.152806
B.cells	SERPINA1D	-0.73865	5.558333	-2.51955	0.013547	-3.61064	0.218447	0.153358
B.cells	CDKN2D	-0.2908	6.09877	-2.51904	0.013565	-3.63041	0.216585	0.151108

B.cells	CYP3A44	-1.02329	2.308322	-2.51886	0.013572	-3.01623	0.230048	0.167943
B.cells	LY6A	1.025532	4.339309	2.517743	0.013612	-3.45266	0.223059	0.158939
B.cells	ACVRL1	-1.00039	2.619629	-2.51655	0.013655	-2.94623	0.229656	0.167026
B.cells	ZFP292	0.249326	7.046023	2.515794	0.013682	-3.80581	0.214191	0.147845
B.cells	CWF19L2	0.300866	5.038452	2.512152	0.013815	-3.48587	0.222928	0.157305
B.cells	EGLN1	0.295852	5.631732	2.510941	0.013859	-3.57013	0.221234	0.155037
B.cells	EML4	0.235998	7.138312	2.510252	0.013884	-3.85448	0.216114	0.148825
B.cells	DYM	0.25921	5.781233	2.507866	0.013972	-3.62218	0.221531	0.155152
B.cells	PSME1	0.338881	8.384368	2.507772	0.013975	-4.04927	0.212615	0.144324
B.cells	RGCC	-0.44986	5.613883	-2.50733	0.013992	-3.53772	0.222119	0.156056
B.cells	TUBA1A	-0.39019	5.430541	-2.50624	0.014032	-3.63537	0.222765	0.157054
B.cells	MAPK13	1.164967	0.83712	2.506055	0.014039	-2.94875	0.239705	0.178524
B.cells	MITD1	0.414353	4.433197	2.505393	0.014063	-3.36939	0.226321	0.161643
B.cells	PCNA	-0.35763	6.710032	-2.50528	0.014068	-3.7245	0.218299	0.151721
B.cells	GM26885	-0.86789	2.317843	-2.50446	0.014098	-3.01263	0.234263	0.171688
B.cells	MS4A6C	0.674248	5.165825	2.503566	0.014131	-3.52933	0.224087	0.158827
B.cells	WARS	0.473679	3.723103	2.499895	0.014268	-3.26953	0.231095	0.16619
B.cells	GM16867	0.60077	2.522419	2.498599	0.014317	-3.23598	0.235566	0.171991
B.cells	LRP10	0.254925	6.125795	2.498034	0.014338	-3.7357	0.222445	0.155659
B.cells	ACBD3	0.236778	5.707973	2.498	0.01434	-3.63875	0.223921	0.157481
B.cells	F7	-1.11067	0.719823	-2.49795	0.014342	-2.9672	0.242467	0.180922
B.cells	RNASE6	0.391687	4.491549	2.497298	0.014366	-3.60539	0.22836	0.163034
B.cells	ID1	-0.7449	4.07039	-2.49589	0.01442	-3.16913	0.230402	0.165418
B.cells	B4GALNT1	0.290475	4.892601	2.495411	0.014438	-3.59775	0.227409	0.161758
B.cells	RAB3IL1	-1.007	1.73222	-2.49487	0.014458	-2.97125	0.239176	0.176775
B.cells	CPEB3	-0.54323	4.234811	-2.49283	0.014536	-3.3568	0.230726	0.165294
B.cells	USP40	-0.49303	3.64171	-2.48967	0.014657	-3.3233	0.234033	0.168578
B.cells	SOCS2	-0.64139	3.962092	-2.4895	0.014664	-3.24058	0.232842	0.167082
B.cells	PSMD4	0.19726	6.691408	2.48931	0.014671	-3.84537	0.222977	0.154857
B.cells	TOMM40L	0.708122	1.733558	2.488923	0.014686	-2.99414	0.241277	0.177835
B.cells	SPATS2	-0.96948	2.720159	-2.48825	0.014712	-3.00169	0.237596	0.173205
B.cells	RNF41	0.313704	4.424549	2.486087	0.014795	-3.43598	0.232226	0.165669
B.cells	TRADD	0.415832	3.982911	2.485318	0.014825	-3.34173	0.234016	0.167904
B.cells	MXI1	0.309091	7.063981	2.483651	0.01489	-3.94927	0.223482	0.154477
B.cells	NXN	0.398891	5.325793	2.48322	0.014907	-3.56193	0.229713	0.162239
B.cells	GBP4	0.984147	3.363964	2.480997	0.014994	-3.21614	0.238055	0.171743
B.cells	PRDX4	-0.35547	5.118984	-2.48026	0.015023	-3.53784	0.231631	0.163653
B.cells	PDZRN3	-1.24687	0.932125	-2.47837	0.015098	-3.00745	0.248311	0.184504
B.cells	TSSC4	-0.45083	3.902903	-2.47819	0.015104	-3.28284	0.236794	0.169885
B.cells	CYP2D10	-0.92964	1.738911	-2.47732	0.015139	-3.0316	0.24516	0.180745
B.cells	UBFD1	-0.34023	4.520398	-2.47669	0.015164	-3.46669	0.234517	0.167478
B.cells	RIDA	-0.66567	4.316478	-2.47659	0.015168	-3.44383	0.235279	0.168453
B.cells	GM28192	1.082654	0.064757	2.475775	0.0152	-3.01852	0.252032	0.189751
B.cells	LRRC8B	0.383933	3.358799	2.475043	0.015229	-3.3077	0.239219	0.17337
B.cells	ALDOC	1.135088	0.884667	2.473456	0.015293	-3.01233	0.249295	0.186132
B.cells	ZC3H7A	0.216318	6.883008	2.472138	0.015346	-3.88813	0.226587	0.157822
B.cells	DSCAML1	-1.46335	-0.596	-2.47012	0.015426	-3.07856	0.255271	0.194947

B.cells	POLR2M	-0.24247	5.403452	-2.47008	0.015428	-3.64562	0.231954	0.164996
B.cells	KANSL3	0.278724	4.995066	2.469725	0.015442	-3.61149	0.233462	0.166909
B.cells	BCL2L13	-0.32367	5.133362	-2.46922	0.015463	-3.60518	0.23295	0.166411
B.cells	CASP12	1.380459	-0.47839	2.468901	0.015476	-3.07432	0.25481	0.194579
B.cells	LCP1	0.249766	8.858643	2.468732	0.015483	-4.19963	0.219647	0.150093
B.cells	ARHGAP12	0.320492	5.076663	2.468551	0.01549	-3.6079	0.23316	0.166755
B.cells	DEGS1	-0.29131	6.525466	-2.46842	0.015495	-3.85106	0.22787	0.160163
B.cells	SULT2B1	-0.75506	2.700547	-2.46833	0.015499	-3.1034	0.242151	0.178155
B.cells	CD5L	-0.83619	6.068559	-2.46829	0.0155	-3.66948	0.229523	0.162213
B.cells	INPP5A	0.310102	6.256163	2.467398	0.015537	-3.81028	0.229074	0.161591
B.cells	SYNJ1	-0.2405	6.731012	-2.46663	0.015568	-3.93607	0.227519	0.159626
B.cells	YIPF1	0.252821	5.234712	2.464252	0.015665	-3.63725	0.234044	0.16705
B.cells	DENND2C	-0.75373	2.008648	-2.46386	0.015681	-3.06172	0.24639	0.182751
B.cells	FDX1	-0.41285	4.692703	-2.46305	0.015714	-3.50178	0.236255	0.169736
B.cells	NSRP1	0.284206	5.229533	2.461459	0.015779	-3.62506	0.234728	0.167597
B.cells	TRIM12A	0.357721	5.048891	2.461262	0.015787	-3.58239	0.235402	0.168464
B.cells	SPOP	0.180661	7.366582	2.460448	0.015821	-4.0116	0.227111	0.158034
B.cells	KCNJ2	1.048303	-0.01521	2.459005	0.01588	-3.064	0.25608	0.194496
B.cells	LZTR1	0.553894	2.612289	2.45703	0.015962	-3.1833	0.246008	0.181144
B.cells	ITGA6	-0.53315	4.818835	-2.45703	0.015962	-3.56607	0.23751	0.170355
B.cells	RBMS1	0.210152	7.558573	2.456576	0.015981	-4.02665	0.227434	0.157951
B.cells	TPD52	0.297552	8.000639	2.456201	0.015996	-4.15071	0.225856	0.156042
B.cells	RAB1A	-0.19258	7.08553	-2.45545	0.016027	-3.94322	0.229137	0.160293
B.cells	KANSL1L	-0.42025	7.202302	-2.45502	0.016046	-3.94448	0.228715	0.159853
B.cells	HNRNPA2B	-0.20811	9.287708	-2.45475	0.016057	-4.26797	0.221336	0.150907
B.cells	RAB12	0.270847	5.073594	2.45315	0.016124	-3.62212	0.237227	0.170043
B.cells	PRODH2	-0.98053	2.255274	-2.45064	0.016229	-3.12695	0.24895	0.184493
B.cells	SLC39A4	-1.03297	1.149584	-2.4503	0.016244	-3.0571	0.253397	0.19029
B.cells	1700037H(	-0.4665	3.714085	-2.45004	0.016254	-3.31568	0.24322	0.177239
B.cells	GATA3	-1.10156	1.116393	-2.44989	0.016261	-3.05898	0.253532	0.190517
B.cells	HNRNPA0	-0.19198	7.214278	-2.44878	0.016308	-3.97651	0.230192	0.161047
B.cells	CERKL	0.731624	1.852174	2.447938	0.016343	-3.16828	0.250672	0.187174
B.cells	MANF	-0.23503	6.808848	-2.44766	0.016355	-3.88167	0.23167	0.163086
B.cells	CDC42EP3	-0.33739	5.142958	-2.44727	0.016372	-3.71648	0.237861	0.170855
B.cells	TTC17	0.259397	5.324345	2.447251	0.016373	-3.72321	0.237178	0.169994
B.cells	AMZ1	-0.6426	3.157688	-2.44595	0.016428	-3.33383	0.246009	0.180864
B.cells	POLR2C	0.295826	5.080792	2.444729	0.01648	-3.6339	0.239049	0.171816
B.cells	CAR8	-0.9352	2.375063	-2.44365	0.016526	-3.11525	0.249911	0.185472
B.cells	GVIN1	0.825741	3.037272	2.443235	0.016544	-3.3487	0.247281	0.182175
B.cells	NASP	-0.28088	6.308139	-2.44247	0.016577	-3.81824	0.234868	0.166353
B.cells	PPP2CA	-0.14485	7.762516	-2.44208	0.016594	-4.09087	0.229538	0.159791
B.cells	LPCAT4	-0.64814	3.121289	-2.44115	0.016634	-3.3021	0.247347	0.182095
B.cells	ALYREF	-0.28617	8.216984	-2.43957	0.016702	-4.13801	0.228684	0.158401
B.cells	GOLGA5	0.276935	4.913418	2.43926	0.016715	-3.64529	0.240942	0.173751
B.cells	ATP6AP2	-0.18822	6.938045	-2.4388	0.016735	-3.97096	0.23334	0.164254
B.cells	COL18A1	-0.82993	2.743841	-2.43538	0.016884	-3.21923	0.250885	0.185506
B.cells	SLC38A2	-0.18971	8.351985	-2.43485	0.016907	-4.20771	0.229547	0.158834

B.cells	DNPH1	-1.20813	0.708539	-2.4347	0.016914	-3.10441	0.259197	0.196475
B.cells	CFHR2	-0.79691	2.56401	-2.43458	0.016919	-3.22491	0.251607	0.186599
B.cells	NCKAP1	-0.71445	2.425386	-2.43112	0.017071	-3.13829	0.253899	0.188224
B.cells	CDC45	-0.66128	3.268129	-2.43096	0.017078	-3.27658	0.250505	0.183916
B.cells	A3GALT2	-0.54663	2.675415	-2.42747	0.017232	-3.37978	0.254676	0.18801
B.cells	SLC38A4	-0.74511	3.153421	-2.42726	0.017242	-3.35635	0.25274	0.185538
B.cells	VPS8	0.35207	4.586793	2.426757	0.017264	-3.58829	0.24705	0.178425
B.cells	RSU1	0.255661	5.746299	2.422295	0.017464	-3.78021	0.244858	0.17363
B.cells	SLC17A3	-1.23201	0.779648	-2.4219	0.017481	-3.11159	0.265044	0.19951
B.cells	WNT2	-1.50636	1.077377	-2.42163	0.017494	-3.12248	0.263781	0.197959
B.cells	SPCS1	-0.20241	7.852067	-2.42028	0.017555	-4.13081	0.237374	0.164184
B.cells	SOCS3	0.520735	4.698376	2.419672	0.017582	-3.66875	0.249516	0.179372
B.cells	B930036N1	-0.42087	5.605823	-2.4184	0.01764	-3.76491	0.245948	0.175056
B.cells	HNRNPK	-0.1486	8.860645	-2.41803	0.017656	-4.30345	0.23364	0.159957
B.cells	CTLA2A	0.647174	4.28991	2.417829	0.017666	-3.48736	0.251141	0.181691
B.cells	MTTP	-0.72899	2.566189	-2.41751	0.01768	-3.2116	0.258139	0.190663
B.cells	NFIB	-0.81794	3.880029	-2.4161	0.017744	-3.38276	0.252785	0.184208
B.cells	CBLB	0.252842	7.336459	2.416096	0.017745	-4.19022	0.23931	0.167309
B.cells	TFDP2	0.324709	5.591554	2.415887	0.017754	-3.93216	0.246004	0.175637
B.cells	LCAT	-0.83868	2.143834	-2.41493	0.017798	-3.18086	0.259888	0.193557
B.cells	GM4952	-0.83047	2.667628	-2.41456	0.017815	-3.27218	0.257721	0.190782
B.cells	STAT2	0.512637	5.210285	2.414311	0.017826	-3.73502	0.247496	0.177819
B.cells	BCL2A1B	0.460998	5.877283	2.414245	0.017829	-3.94604	0.244893	0.174556
B.cells	SARAF	-0.34693	5.365255	-2.41407	0.017837	-3.60594	0.246888	0.177117
B.cells	SERPINA10	-1.04525	0.966902	-2.41359	0.017859	-3.12667	0.264835	0.200349
B.cells	TOR4A	0.845277	1.678835	2.412435	0.017912	-3.15858	0.26183	0.196824
B.cells	HIVEP3	0.388302	5.144836	2.412337	0.017917	-3.70793	0.247753	0.178738
B.cells	GBP5	1.183704	2.014589	2.412183	0.017924	-3.21018	0.260426	0.195032
B.cells	BC024386	-1.05201	1.614053	-2.41141	0.01796	-3.14321	0.262236	0.197276
B.cells	H2AFZ	-0.30571	10.35837	-2.41098	0.017979	-4.52135	0.22834	0.154714
B.cells	HRH2	0.957759	1.160498	2.409713	0.018038	-3.1423	0.264149	0.200232
B.cells	CD81	-0.32024	7.604661	-2.40941	0.018052	-4.11843	0.238423	0.167399
B.cells	RAB37	0.685769	2.340912	2.408803	0.01808	-3.20946	0.259203	0.194016
B.cells	GM21762	-1.58453	-1.20491	-2.40839	0.018099	-3.33554	0.274291	0.214216
B.cells	LRP4	-1.07065	2.477171	-2.40817	0.01811	-3.16248	0.258639	0.193482
B.cells	FCNA	-1.08533	4.555419	-2.40803	0.018116	-3.52359	0.250212	0.18262
B.cells	PLK2	0.483916	5.006155	2.407301	0.01815	-3.75595	0.248426	0.180512
B.cells	SLAMF8	1.495512	0.718707	2.407284	0.018151	-3.13884	0.266028	0.203375
B.cells	PDE4B	0.283559	8.527906	2.405018	0.018257	-4.3261	0.23583	0.164225
B.cells	ARHGEF11	0.319539	4.907611	2.404951	0.01826	-3.76937	0.24971	0.181648
B.cells	IGFBP6	1.393194	-0.02144	2.403366	0.018334	-3.1531	0.270739	0.208788
B.cells	EPHA2	-0.6031	2.408212	-2.40319	0.018343	-3.27797	0.260394	0.195159
B.cells	TIMM10	0.4878	3.050855	2.402746	0.018363	-3.36423	0.257735	0.191829
B.cells	AFM	-0.79477	2.294567	-2.40083	0.018453	-3.24392	0.261383	0.196488
B.cells	PER1	0.353226	4.926726	2.400746	0.018458	-3.72546	0.250646	0.182611
B.cells	CLMP	-1.01569	1.140521	-2.40058	0.018465	-3.16288	0.266259	0.202894
B.cells	AI837181	0.422546	3.127875	2.3984	0.018569	-3.36354	0.258837	0.192663

B.cells	SCP2	-0.37243	7.526193	-2.39803	0.018586	-4.14391	0.241396	0.170504
B.cells	CTSS	0.40508	7.501211	2.397358	0.018618	-4.26207	0.241491	0.170693
B.cells	HNRNPH3	0.20466	5.447268	2.397284	0.018622	-3.84136	0.249461	0.180737
B.cells	TCAIM	0.754908	1.789758	2.396458	0.018661	-3.2125	0.264433	0.200144
B.cells	PLCB3	-0.60796	2.612	-2.39643	0.018663	-3.26475	0.260978	0.19562
B.cells	RSAD2	1.138562	4.537164	2.39588	0.018689	-3.4501	0.25315	0.185506
B.cells	FGB	-0.46399	5.797972	-2.39515	0.018724	-3.93437	0.248309	0.179279
B.cells	RAET1E	0.354957	4.113795	2.392746	0.018839	-3.70666	0.255885	0.188498
B.cells	SATB1	0.342086	7.143818	2.392668	0.018843	-4.15243	0.243889	0.173264
B.cells	GM15728	-1.13821	0.500791	-2.39247	0.018852	-3.16695	0.271103	0.208494
B.cells	MIF4GD	0.298547	4.964455	2.390901	0.018928	-3.80812	0.252902	0.184469
B.cells	ST8SIA1	1.349266	0.877835	2.390852	0.018931	-3.16906	0.269951	0.206676
B.cells	ULK2	0.310279	5.457806	2.388922	0.019024	-3.79765	0.251772	0.182332
B.cells	1600012H(	0.468063	2.771016	2.388624	0.019039	-3.34071	0.262771	0.196517
B.cells	ROBO2	-1.55926	0.744725	-2.38815	0.019062	-3.18612	0.27145	0.208001
B.cells	FMO1	-0.97971	2.23787	-2.38738	0.019099	-3.25241	0.265218	0.199709
B.cells	BIRC3	0.267225	7.111789	2.386962	0.019119	-4.15732	0.245454	0.174489
B.cells	GNAT3	-1.29564	-0.04367	-2.38561	0.019186	-3.20155	0.275215	0.213377
B.cells	CNNM2	-0.56885	5.427728	-2.38556	0.019188	-3.71546	0.252184	0.183262
B.cells	KLRD1	-1.23111	3.767425	-2.38515	0.019208	-3.22298	0.258927	0.192108
B.cells	QPCT	-0.68313	2.264701	-2.38498	0.019216	-3.30974	0.265213	0.200332
B.cells	PRKCG	-0.55098	4.448629	-2.38252	0.019337	-3.67206	0.257448	0.189209
B.cells	LBP	-0.61501	3.292366	-2.38196	0.019365	-3.37392	0.262306	0.195535
B.cells	PIGK	0.324492	4.298401	2.37983	0.01947	-3.61934	0.259239	0.190681
B.cells	STX8	0.177308	6.745581	2.37916	0.019503	-4.11233	0.249502	0.178285
B.cells	GPATCH2L	0.254116	5.064269	2.378724	0.019525	-3.79493	0.256234	0.186953
B.cells	EXOC6	0.25435	6.194009	2.37555	0.019683	-3.98212	0.253436	0.181996
B.cells	1700010K2	0.935142	0.709295	2.37504	0.019709	-3.19834	0.276634	0.212058
B.cells	HIF1A	0.27374	6.608469	2.373918	0.019765	-4.07656	0.251963	0.180253
B.cells	KMT5A	0.235731	5.846575	2.373715	0.019775	-3.94106	0.255019	0.184169
B.cells	SULT2A1	-0.78442	3.901678	-2.37346	0.019788	-3.61468	0.263019	0.194502
B.cells	DUS2	-0.48257	3.353616	-2.3717	0.019877	-3.51011	0.26567	0.197905
B.cells	URAH	-0.71357	3.327083	-2.37135	0.019894	-3.50355	0.265782	0.198106
B.cells	SIK1	0.239376	6.868808	2.371179	0.019903	-4.12276	0.251255	0.179506
B.cells	ERCC6L	-0.63081	3.034059	-2.37115	0.019904	-3.41019	0.267028	0.199728
B.cells	MR1	1.237937	0.68263	2.367779	0.020076	-3.22021	0.279337	0.214287
B.cells	ASH1L	0.20519	7.367632	2.366559	0.020138	-4.22133	0.251631	0.178152
B.cells	GM26510	0.383915	4.122817	2.365199	0.020207	-3.68873	0.264904	0.195238
B.cells	SP100	0.303688	7.43248	2.365059	0.020215	-4.24401	0.251374	0.178053
B.cells	ABHD14B	-0.78547	2.125702	-2.36496	0.02022	-3.25323	0.273482	0.2064
B.cells	MICALL2	1.023081	1.195719	2.364596	0.020238	-3.21987	0.277586	0.211868
B.cells	CLEC10A	-1.28077	0.930777	-2.36433	0.020252	-3.21758	0.278768	0.21352
B.cells	CLASP2	0.221973	6.927855	2.362881	0.020327	-4.16193	0.254017	0.181089
B.cells	FPR3	1.399961	-0.4789	2.362452	0.020349	-3.24173	0.285862	0.222669
B.cells	SBNO2	0.25001	5.566821	2.361889	0.020378	-3.95613	0.259624	0.188285
B.cells	IKZF3	0.335127	5.866917	2.36087	0.02043	-4.09442	0.258476	0.186965
B.cells	DCAF8	0.203098	5.699638	2.360683	0.02044	-3.93504	0.259162	0.187868

B.cells	EPB41	0.325302	8.026595	2.360428	0.020453	-4.35937	0.249812	0.176088
B.cells	GM14305	-0.58494	2.260659	-2.35894	0.02053	-3.30491	0.274471	0.207154
B.cells	KBTBD11	1.19587	0.726015	2.35658	0.020653	-3.23633	0.282457	0.216757
B.cells	RUNX2	1.020118	4.240789	2.356438	0.020661	-3.36586	0.267027	0.196582
B.cells	HMGN5	-0.33576	4.876393	-2.35554	0.020708	-3.77537	0.26463	0.193405
B.cells	AKR1C14	-1.0509	0.911524	-2.35485	0.020744	-3.23481	0.281924	0.216136
B.cells	XCR1	-1.23914	0.412393	-2.3546	0.020757	-3.23829	0.284192	0.219226
B.cells	ODF2	0.234846	5.483537	2.354243	0.020776	-3.91893	0.262093	0.190449
B.cells	PTPRF	-1.01525	1.276101	-2.35378	0.0208	-3.24076	0.280299	0.214131
B.cells	ZCCHC7	0.335729	6.744533	2.351833	0.020903	-4.11932	0.256995	0.184361
B.cells	GM36199	-1.28259	-0.13106	-2.35178	0.020905	-3.24886	0.286775	0.223223
B.cells	OTOA	-1.31269	0.757069	-2.35153	0.020919	-3.25902	0.282711	0.2179
B.cells	FAM149A	-1.15997	-0.42534	-2.35093	0.02095	-3.24624	0.288135	0.225245
B.cells	GSDMD	0.415784	4.268046	2.350158	0.020991	-3.74637	0.267285	0.197926
B.cells	HC	-0.84619	2.358321	-2.34992	0.021004	-3.36102	0.275551	0.20876
B.cells	UIMC1	0.227882	5.853468	2.349447	0.021029	-4.01205	0.260642	0.189535
B.cells	LRMDA	0.447236	7.760594	2.349358	0.021034	-4.29784	0.252909	0.179756
B.cells	COLEC11	-0.87997	1.589372	-2.34892	0.021057	-3.26536	0.278963	0.213511
B.cells	GM11837	-1.23325	0.292319	-2.34891	0.021057	-3.24568	0.284829	0.221344
B.cells	DENND4C	0.295235	5.068456	2.348863	0.02106	-3.88123	0.263907	0.193812
B.cells	HK1	0.316069	4.85354	2.345915	0.021217	-3.81394	0.266493	0.195526
B.cells	SLC7A2	1.043148	2.094729	2.344805	0.021276	-3.37135	0.278721	0.211425
B.cells	TAF1C	0.61148	2.170342	2.344695	0.021282	-3.34959	0.278384	0.210981
B.cells	ACOT4	-1.49517	0.046894	-2.34397	0.021321	-3.28554	0.288237	0.223931
B.cells	PTPN11	0.240095	5.063935	2.343508	0.021346	-3.87666	0.266051	0.194887
B.cells	TBC1D8	-0.55338	5.179428	-2.34304	0.021371	-3.59601	0.265586	0.19435
B.cells	MTSS2	-1.28	0.842863	-2.34253	0.021399	-3.2706	0.284627	0.219423
B.cells	ADCK1	0.492437	3.101425	2.342166	0.021418	-3.50528	0.274523	0.206068
B.cells	TRAK1	-0.20454	7.291138	-2.3399	0.021541	-4.24326	0.258055	0.184034
B.cells	MSN	0.205526	8.454473	2.337641	0.021664	-4.46248	0.254236	0.178731
B.cells	PTPN22	0.398537	5.324165	2.337267	0.021684	-3.98122	0.267118	0.19511
B.cells	CPPED1	0.326926	3.886993	2.337251	0.021685	-3.64284	0.273289	0.203074
B.cells	AKR1D1	-0.85604	2.587501	-2.33363	0.021883	-3.41511	0.28126	0.21155
B.cells	GBP8	0.663879	3.65151	2.331261	0.022014	-3.50467	0.277315	0.205926
B.cells	CIART	0.644704	2.328452	2.331145	0.02202	-3.42907	0.283234	0.213662
B.cells	GGTA1	0.331019	5.365487	2.331042	0.022026	-3.98046	0.269861	0.196346
B.cells	1110059E2	0.33079	4.393524	2.330771	0.022041	-3.76437	0.274059	0.201751
B.cells	IARS2	0.338199	4.475268	2.329702	0.0221	-3.78052	0.274143	0.201535
B.cells	PAPSS2	-0.6621	2.513577	-2.32924	0.022126	-3.50368	0.282874	0.213021
B.cells	HEG1	0.311105	5.971346	2.326848	0.022259	-4.08205	0.268933	0.194107
B.cells	PEX16	0.396478	3.474463	2.32616	0.022297	-3.59763	0.279816	0.208185
B.cells	TTC14	0.211234	5.803768	2.325322	0.022344	-4.04267	0.269647	0.19531
B.cells	CAAA0114[illegible]	0.580372	2.131904	2.325215	0.02235	-3.41845	0.28588	0.216335
B.cells	PBX1	-0.39558	5.465012	-2.32499	0.022363	-3.9786	0.271099	0.197212
B.cells	OLFML3	-0.96938	1.284348	-2.32489	0.022368	-3.29107	0.289787	0.221538
B.cells	ZFP456	1.047025	0.791994	2.323923	0.022423	-3.29179	0.292481	0.224891
B.cells	PPP3CC	0.416552	3.993482	2.323403	0.022452	-3.78118	0.277953	0.205735

B.cells	CDC42BPB	-0.64444	3.262023	-2.31702	0.022813	-3.47488	0.285433	0.211574
B.cells	HMOX2	0.196015	6.471899	2.314239	0.022973	-4.1573	0.272635	0.194262
B.cells	MPV17	-0.32304	4.075124	-2.31413	0.022979	-3.7361	0.2832	0.207685
B.cells	SELENBP2	-1.09967	1.116284	-2.3135	0.023015	-3.31641	0.297065	0.22571
B.cells	UBAC2	0.210524	6.679645	2.312049	0.023099	-4.25737	0.272361	0.193625
B.cells	PROCR	1.703578	0.279761	2.311914	0.023106	-3.3139	0.301612	0.231355
B.cells	C8B	-1.06395	0.571894	-2.31152	0.023129	-3.31278	0.3002	0.229667
B.cells	HIST1H2AF	-1.37346	0.617707	-2.31076	0.023173	-3.31837	0.300226	0.22957
B.cells	FGR	0.780657	4.732993	2.310141	0.023209	-3.64091	0.281263	0.204933
B.cells	ST7	0.327751	5.144851	2.30887	0.023283	-3.97492	0.279901	0.202856
B.cells	MOCS1	0.417938	3.367675	2.308618	0.023297	-3.62963	0.287927	0.213143
B.cells	GM26740	0.286027	7.068845	2.305432	0.023483	-4.3025	0.272691	0.193022
B.cells	IRF7	0.794171	6.139097	2.305253	0.023493	-4.02046	0.276727	0.198083
B.cells	GPR132	0.349169	6.00422	2.305131	0.023501	-4.28261	0.277319	0.19883
B.cells	CD28	-0.9802	2.935145	-2.305	0.023508	-3.37339	0.291186	0.216577
B.cells	GOLPH3L	0.338406	4.39604	2.304762	0.023522	-3.87273	0.284486	0.208049
B.cells	PLEK	0.328393	7.520494	2.302928	0.02363	-4.38638	0.271633	0.191263
B.cells	SDF2	0.248129	5.410417	2.302595	0.023649	-4.02377	0.280848	0.202977
B.cells	PRKCB	0.201029	8.511819	2.3022	0.023673	-4.49754	0.267429	0.186296
B.cells	UBE2E3	-0.17647	7.066891	-2.30116	0.023734	-4.32549	0.273614	0.194122
B.cells	SRSF2	-0.21721	7.941182	-2.30108	0.023739	-4.40612	0.26987	0.189455
B.cells	ARL5B	0.334435	5.460574	2.300896	0.02375	-4.05465	0.280656	0.203006
B.cells	PPARD	0.28434	5.613407	2.299212	0.023849	-4.07341	0.280859	0.202556
B.cells	COA5	0.394881	4.468388	2.298122	0.023914	-3.82881	0.286489	0.20945
B.cells	UQCC3	0.337608	4.016511	2.296546	0.024008	-3.7673	0.289116	0.212471
B.cells	GALNT16	-0.85879	0.933714	-2.29651	0.02401	-3.35364	0.303718	0.231491
B.cells	NXPE4	0.627694	2.181788	2.295392	0.024077	-3.5872	0.297802	0.223992
B.cells	2610507B1	0.215391	5.431746	2.295114	0.024094	-4.03103	0.282778	0.20468
B.cells	CEBPZOS	0.343052	4.521965	2.294796	0.024113	-3.81678	0.286893	0.210033
B.cells	FLT4	-0.88547	2.321562	-2.29471	0.024118	-3.37609	0.297137	0.223298
B.cells	NEK7	0.229881	6.476805	2.293136	0.024212	-4.21733	0.278937	0.199293
B.cells	SLC17A5	0.443315	3.572742	2.291127	0.024333	-3.70107	0.292505	0.216665
B.cells	SULT2A2	-0.70352	4.037802	-2.29068	0.02436	-3.81548	0.290346	0.213989
B.cells	CCNYL1	-0.33034	4.635175	-2.29059	0.024366	-3.86695	0.2876	0.21046
B.cells	ARAP2	0.349466	5.924266	2.289942	0.024405	-4.12983	0.281779	0.203268
B.cells	CLEC9A	-1.19078	2.519945	-2.28978	0.024414	-3.36594	0.297461	0.223535
B.cells	SH3BP4	-1.08965	1.369949	-2.28952	0.024431	-3.35191	0.302987	0.230857
B.cells	MTMR6	0.300835	4.718718	2.289267	0.024446	-3.94496	0.287219	0.210382
B.cells	9330111N(	-1.20865	0.392898	-2.289	0.024462	-3.35244	0.307775	0.237345
B.cells	ABCD2	-0.66081	2.633823	-2.28884	0.024471	-3.51537	0.29692	0.223056
B.cells	PTPN18	0.199304	7.11924	2.28759	0.024548	-4.31401	0.277038	0.197196
B.cells	RGS18	-0.58663	3.108266	-2.28725	0.024569	-3.59015	0.295246	0.220496
B.cells	NUDCD3	0.200155	5.998557	2.286138	0.024636	-4.13225	0.282475	0.203685
B.cells	FOXA3	-0.94491	1.425712	-2.28221	0.024877	-3.37924	0.306477	0.232766
B.cells	SRSF3	-0.1812	7.532017	-2.28116	0.024942	-4.40554	0.278497	0.196626
B.cells	GM13483	-0.7807	1.85821	-2.28085	0.024961	-3.4354	0.304768	0.23033
B.cells	SLFN8	0.612405	3.326481	2.279833	0.025024	-3.74201	0.298146	0.221425

B.cells	BASP1	0.710525	5.646818	2.277836	0.025147	-3.842	0.288205	0.208176
B.cells	RGS12	-0.67217	2.240588	-2.27781	0.025149	-3.44157	0.304267	0.228876
B.cells	WWC2	-0.33175	5.107898	-2.27681	0.025212	-3.97158	0.291103	0.21154
B.cells	GSTM7	-0.85704	1.02465	-2.27599	0.025263	-3.37799	0.310846	0.237221
B.cells	CARD9	1.086087	0.997223	2.275398	0.025299	-3.37754	0.310982	0.237526
B.cells	ZFP938	-0.77793	1.450658	-2.27539	0.0253	-3.40422	0.30873	0.234552
B.cells	GMNN	-0.41682	5.558334	-2.27437	0.025364	-4.04243	0.289305	0.2094
B.cells	GM43696	0.528876	2.459243	2.274261	0.025371	-3.56287	0.303936	0.228276
B.cells	RTP4	0.914739	4.174749	2.273968	0.025389	-3.72306	0.295733	0.217638
B.cells	MYCN	-1.38109	-0.18583	-2.27276	0.025465	-3.41354	0.317605	0.246012
B.cells	PRDX5	0.416521	7.635313	2.272515	0.02548	-4.42222	0.280405	0.198013
B.cells	SELENOS	-0.23464	6.113957	-2.27143	0.025548	-4.17371	0.287593	0.206807
B.cells	GM43672	-0.91579	1.700182	-2.27111	0.025568	-3.40724	0.308532	0.23388
B.cells	ASPDH	-1.02694	1.294554	-2.27058	0.025602	-3.40616	0.310544	0.236646
B.cells	4930404N1	1.239543	0.191025	2.270366	0.025616	-3.39379	0.316094	0.244012
B.cells	ADGRG6	1.200079	0.969217	2.268817	0.025714	-3.41668	0.313049	0.23934
B.cells	IDH1	-0.30591	4.88393	-2.26775	0.025782	-3.99331	0.294489	0.21486
B.cells	CHMP3	0.178284	5.884611	2.267448	0.0258	-4.20154	0.289853	0.208986
B.cells	GIMAP6	0.401975	5.672059	2.266396	0.025867	-4.1737	0.291294	0.210495
B.cells	POMT1	-0.75868	2.21731	-2.26512	0.025949	-3.48888	0.308424	0.232125
B.cells	GM42917	-0.77711	1.691044	-2.26359	0.026047	-3.44365	0.3119	0.235966
B.cells	SATB2	-0.71627	1.643339	-2.26296	0.026087	-3.53931	0.312307	0.236425
B.cells	MVB12B	-0.54768	4.074675	-2.26192	0.026154	-3.61654	0.300692	0.221209
B.cells	MGP	1.340368	0.463003	2.261783	0.026163	-3.39949	0.31857	0.244578
B.cells	LIMS1	0.220627	7.288787	2.260612	0.026238	-4.40607	0.286075	0.202439
B.cells	FAM168B	0.178863	6.280422	2.260531	0.026244	-4.26367	0.290668	0.208203
B.cells	TSHZ1	0.334958	5.060403	2.259442	0.026314	-4.08537	0.296353	0.215868
B.cells	ZCCHC2	0.307215	5.422407	2.259022	0.026341	-4.09161	0.294656	0.213784
B.cells	GM44702	-1.46618	0.088947	-2.25866	0.026364	-3.41846	0.320854	0.248084
B.cells	IGLV1	-1.17141	-0.83837	-2.2586	0.026368	-3.40618	0.325584	0.254561
B.cells	UBR4	-0.19679	6.056739	-2.25815	0.026398	-4.21539	0.291709	0.210246
B.cells	ITGAE	-0.85818	2.127038	-2.25811	0.0264	-3.50132	0.310536	0.234544
B.cells	GP9	1.136096	0.659096	2.257312	0.026452	-3.45526	0.318101	0.244588
B.cells	DHX57	0.347859	3.871504	2.256646	0.026495	-3.83254	0.302172	0.223765
B.cells	MIDN	-0.24294	6.064607	-2.25641	0.026511	-4.22242	0.291832	0.210512
B.cells	GM29170	0.669983	1.532706	2.256007	0.026537	-3.45603	0.313675	0.238855
B.cells	PARP16	-1.11593	1.117003	-2.25589	0.026545	-3.40957	0.315772	0.241679
B.cells	IDNK	0.252653	5.52475	2.254565	0.026631	-4.15742	0.295005	0.214146
B.cells	ENPP5	-1.29512	0.877362	-2.25356	0.026697	-3.41565	0.318179	0.243951
B.cells	F630040K0	1.060169	0.611295	2.252799	0.026747	-3.43023	0.319827	0.245922
B.cells	PLCE1	-0.97686	1.279254	-2.25073	0.026883	-3.51338	0.317494	0.242027
B.cells	CERS6	0.334017	7.330325	2.250437	0.026902	-4.42486	0.288363	0.204637
B.cells	BPHL	-0.41514	3.663858	-2.24968	0.026952	-3.78103	0.305621	0.226693
B.cells	ETNK1	0.240513	5.999727	2.24965	0.026954	-4.23538	0.29449	0.212421
B.cells	TRA2B	-0.17442	7.832072	-2.24943	0.026969	-4.51453	0.286092	0.201884
B.cells	2-Mar	0.241443	6.581903	2.248873	0.027005	-4.39947	0.291903	0.209238
B.cells	ARHGAP29	-0.83381	2.520124	-2.24762	0.027088	-3.48681	0.312026	0.234534

B.cells	EID3	1.114041	0.925233	2.247135	0.027121	-3.43266	0.320167	0.245286
B.cells	ATP9B	0.212218	5.858499	2.246572	0.027158	-4.24161	0.29607	0.213941
B.cells	LILR4B	1.172112	3.079256	2.244459	0.027299	-3.49332	0.310471	0.231667
B.cells	HMOX1	-0.71179	4.807778	-2.24443	0.027301	-3.90567	0.302043	0.220786
B.cells	FAM135A	-0.77686	2.016455	-2.24216	0.027453	-3.49954	0.316978	0.239334
B.cells	STRADA	0.315413	4.58715	2.242106	0.027456	-4.01774	0.304244	0.222823
B.cells	NUMA1	0.237902	5.705606	2.241084	0.027525	-4.18503	0.299232	0.216206
B.cells	SNX1	0.196513	5.483558	2.240755	0.027547	-4.18979	0.300287	0.217701
B.cells	DAPK1	-0.49558	4.932787	-2.23973	0.027617	-3.94889	0.302922	0.221275
B.cells	ALDH1L1	-0.63146	3.098676	-2.23969	0.027619	-3.67698	0.311897	0.232864
B.cells	SELENOM	0.772092	2.384919	2.239529	0.02763	-3.55598	0.315473	0.23755
B.cells	GJB1	-0.97521	1.59761	-2.2391	0.027659	-3.47147	0.319473	0.242968
B.cells	HUS1	0.769759	1.653586	2.238889	0.027673	-3.47189	0.319187	0.242695
B.cells	GM13431	-1.19881	0.492651	-2.2383	0.027713	-3.44424	0.325295	0.25082
B.cells	NUPR1	1.166953	2.41246	2.237968	0.027736	-3.56746	0.31544	0.237917
B.cells	PTGS2	1.558508	2.215449	2.23652	0.027834	-3.48172	0.316441	0.239727
B.cells	HGD	-0.81437	2.465599	-2.2363	0.027849	-3.59782	0.315178	0.238112
B.cells	ABCC1	0.386003	4.467713	2.236188	0.027856	-3.96937	0.305277	0.225221
B.cells	HEATR9	1.126915	-0.41964	2.236021	0.027868	-3.44577	0.330108	0.257996
B.cells	NRBF2	0.320297	4.314912	2.236007	0.027869	-3.94338	0.30602	0.226181
B.cells	ABHD2	-0.2506	5.471153	-2.23324	0.028058	-4.15122	0.301762	0.219982
B.cells	NOCT	-0.39134	5.04776	-2.23279	0.028089	-4.06972	0.303796	0.222778
B.cells	LARP4B	0.18898	7.283403	2.232631	0.028099	-4.4524	0.293237	0.209362
B.cells	PANK2	0.25722	5.327592	2.23261	0.028101	-4.11177	0.30245	0.221078
B.cells	NAALADL2	-0.72887	1.964286	-2.23228	0.028123	-3.52025	0.319101	0.242856
B.cells	SOAT2	-0.99027	0.810649	-2.23145	0.02818	-3.45824	0.325406	0.250974
B.cells	METTL23	0.209483	5.675126	2.229634	0.028305	-4.22976	0.301658	0.219788
B.cells	ARMC7	0.419147	3.924467	2.22963	0.028305	-3.81864	0.310165	0.230765
B.cells	CD300C2	-0.71058	4.335062	-2.22954	0.028312	-3.72055	0.308145	0.228151
B.cells	TOR3A	0.481594	4.075925	2.228489	0.028384	-3.97503	0.30972	0.23005
B.cells	SPATA32	-1.20533	0.244996	-2.22799	0.028419	-3.45701	0.329293	0.256002
B.cells	SCAPER	0.253597	6.110801	2.227382	0.028461	-4.29728	0.299877	0.217605
B.cells	SLC22A15	-0.55047	3.077716	-2.22725	0.02847	-3.63706	0.314687	0.23678
B.cells	WDR62	0.428979	3.526858	2.227053	0.028484	-3.87069	0.312441	0.233872
B.cells	HVCN1	0.532744	4.117697	2.225436	0.028596	-3.92577	0.309514	0.230627
B.cells	RGS16	-0.8572	1.351563	-2.22516	0.028615	-3.54685	0.323496	0.249137
B.cells	EBPL	-0.35244	4.469152	-2.22507	0.028622	-3.97332	0.307789	0.228473
B.cells	NKG7	-0.99243	4.013022	-2.22401	0.028695	-3.61702	0.31003	0.231589
B.cells	SLC25A18	-0.49352	4.491338	-2.22394	0.0287	-4.05051	0.30768	0.228526
B.cells	TNFRSF1B	0.413458	4.903674	2.22366	0.02872	-4.00468	0.305671	0.225948
B.cells	CSNK1G3	0.193372	6.624241	2.223652	0.02872	-4.43673	0.297452	0.215379
B.cells	SLC1A5	-0.25122	6.46392	-2.2236	0.028724	-4.42092	0.298207	0.216344
B.cells	TOR1B	-0.37573	3.704769	-2.22311	0.028758	-3.83289	0.311556	0.233788
B.cells	DMGDH	-0.8553	1.667706	-2.2231	0.028759	-3.53441	0.321861	0.247418
B.cells	MNDAL	0.366355	6.786829	2.222985	0.028767	-4.44621	0.296689	0.214595
B.cells	PPARA	-0.75885	2.784821	-2.22183	0.028848	-3.66195	0.316759	0.240308
B.cells	SUB1	-0.18577	8.911934	-2.21999	0.028977	-4.78716	0.288421	0.203129

B.cells	LRRFIP2	0.211972	5.900501	2.219701	0.028997	-4.31416	0.302444	0.220938
B.cells	GM15472	0.854426	1.795978	2.218116	0.029109	-3.55584	0.323621	0.24817
B.cells	IVNS1ABP	-0.25006	6.027762	-2.21799	0.029118	-4.26142	0.302534	0.220604
B.cells	POU2F2	0.364424	5.772063	2.216162	0.029248	-4.38168	0.304487	0.222477
B.cells	SLC38A3	-0.89662	1.479322	-2.21589	0.029267	-3.52464	0.326044	0.250749
B.cells	CDK2AP1	-0.24019	5.884299	-2.21586	0.029269	-4.26142	0.303946	0.221823
B.cells	INSIG2	0.31988	4.405764	2.214547	0.029362	-4.036	0.311869	0.231458
B.cells	TWF1	0.235174	5.464598	2.213136	0.029463	-4.19973	0.307294	0.225199
B.cells	CD163	-1.49635	1.13156	-2.21295	0.029477	-3.49575	0.329281	0.254056
B.cells	SGSH	0.821616	1.453355	2.211799	0.029559	-3.51749	0.328198	0.251987
B.cells	DPP8	0.224841	5.667042	2.210543	0.029649	-4.26337	0.307533	0.224649
B.cells	MYO1E	0.374942	6.198716	2.209394	0.029731	-4.49549	0.304975	0.22164
B.cells	HNRNPM	-0.15638	7.703885	-2.20907	0.029755	-4.59021	0.297813	0.212625
B.cells	SLC10A1	-0.55125	3.248903	-2.20906	0.029756	-3.80816	0.319608	0.240698
B.cells	CHD7	0.249543	6.463817	2.208745	0.029778	-4.45847	0.303699	0.220156
B.cells	BTBD1	0.202376	6.497876	2.208616	0.029787	-4.43159	0.303535	0.219947
B.cells	EEF2K	-0.37399	4.644155	-2.20825	0.029814	-4.00009	0.312586	0.231669
B.cells	RNF114	0.272476	5.645299	2.20328	0.030174	-4.30996	0.311094	0.226895
B.cells	GM11613	0.87607	2.020755	2.201962	0.030271	-3.60287	0.329714	0.251437
B.cells	BRK1	0.19622	6.271039	2.201429	0.03031	-4.37976	0.308152	0.223554
B.cells	SLC35F5	-0.53841	3.11698	-2.20132	0.030318	-3.76435	0.323989	0.244085
B.cells	PSKH1	0.368785	3.273493	2.201129	0.030332	-3.79939	0.323181	0.243024
B.cells	CBFA2T2	0.249635	5.563389	2.201111	0.030333	-4.26238	0.311625	0.228017
B.cells	RTKN2	-1.03505	1.228878	-2.20085	0.030352	-3.50494	0.333923	0.257242
B.cells	ADAM10	0.180748	7.403441	2.199661	0.030439	-4.58578	0.303287	0.216923
B.cells	AGMAT	-0.98639	1.438737	-2.19912	0.030479	-3.55084	0.333589	0.256305
B.cells	GAS2L3	0.436843	4.330262	2.19713	0.030626	-3.97294	0.319472	0.237079
B.cells	RUBCNL	0.39512	3.191015	2.196495	0.030673	-3.98415	0.325322	0.244965
B.cells	NTPCR	0.415147	3.847172	2.195655	0.030735	-3.88025	0.321937	0.240609
B.cells	AU022252	0.562717	2.452615	2.195646	0.030736	-3.66303	0.329181	0.250128
B.cells	NCF4	0.294122	5.316123	2.195179	0.03077	-4.31626	0.314508	0.23102
B.cells	KIF17	-0.97538	1.692096	-2.19507	0.030778	-3.54748	0.333211	0.255541
B.cells	SLC1A4	-0.9541	0.947937	-2.19443	0.030826	-3.52246	0.33721	0.260984
B.cells	RDH9	-1.10959	0.805989	-2.19408	0.030852	-3.51931	0.337979	0.262165
B.cells	UBE2E2	-0.63842	3.465951	-2.19365	0.030884	-3.82454	0.323899	0.243576
B.cells	CD209A	-1.29729	0.102824	-2.1936	0.030888	-3.51811	0.341818	0.267455
B.cells	CD300C	1.147259	0.72621	2.193333	0.030907	-3.51879	0.338412	0.262985
B.cells	PPP1R10	-0.26868	6.277719	-2.19326	0.030913	-4.35595	0.309754	0.225348
B.cells	SMIM12	0.323085	4.0506	2.193055	0.030928	-3.97754	0.320896	0.239838
B.cells	IL1RN	1.36142	2.526967	2.192484	0.030971	-3.61432	0.328818	0.250369
B.cells	MTA1	-0.31052	4.55479	-2.19194	0.031011	-4.04904	0.31836	0.236878
B.cells	BEX4	-1.09115	0.397887	-2.19185	0.031018	-3.51771	0.34023	0.265942
B.cells	BCL2	0.428312	5.016128	2.191535	0.031042	-4.29382	0.316036	0.234043
B.cells	RSRC1	0.210192	6.605429	2.190998	0.031082	-4.46372	0.308303	0.224083
B.cells	PIGYL	0.339651	4.270837	2.189723	0.031177	-4.00749	0.320627	0.23948
B.cells	ANKLE1	-1.04918	0.64315	-2.1892	0.031216	-3.52451	0.33989	0.264931
B.cells	FRYL	0.192041	7.564295	2.188608	0.031261	-4.62707	0.304518	0.218781

B.cells	HNRNPC	-0.14098	7.730209	-2.18704	0.031379	-4.62699	0.303723	0.218008
B.cells	SHC4	-1.13352	0.470109	-2.18671	0.031404	-3.52793	0.340918	0.266838
B.cells	PLTP	-0.7129	5.244881	-2.18541	0.031502	-4.03729	0.315893	0.233933
B.cells	ITIH4	-0.56169	3.988196	-2.18507	0.031528	-4.01343	0.322266	0.242312
B.cells	OLA1	-0.20472	5.835409	-2.18471	0.031555	-4.34371	0.31295	0.230223
B.cells	CEP83OS	0.740661	2.022979	2.184538	0.031569	-3.59653	0.332535	0.256054
B.cells	FAM76B	0.227259	5.401156	2.184101	0.031602	-4.24979	0.315111	0.233181
B.cells	GM20559	0.373242	4.344461	2.18381	0.031624	-4.10697	0.320444	0.240243
B.cells	RFC4	-0.45722	4.603054	-2.1838	0.031625	-4.04462	0.319129	0.23852
B.cells	DRAM2	0.289159	4.847911	2.182739	0.031705	-4.18709	0.31789	0.237105
B.cells	SAA2	1.837935	-1.02347	2.182613	0.031715	-3.53806	0.349083	0.279134
B.cells	GPR35	0.862515	2.516821	2.182512	0.031723	-3.62179	0.329919	0.252996
B.cells	TMBIM1	0.547296	2.851295	2.182163	0.031749	-3.75417	0.328161	0.250766
B.cells	FBXL17	0.20211	7.560342	2.181999	0.031762	-4.6315	0.304537	0.219959
B.cells	TOP1	-0.21922	8.354587	-2.18183	0.031774	-4.71222	0.300753	0.215176
B.cells	DEPTOR	-0.43955	3.469195	-2.18129	0.031816	-3.9467	0.324941	0.246701
B.cells	AUH	0.236708	5.641378	2.180964	0.031841	-4.37865	0.313913	0.23227
B.cells	CHD1	0.177131	6.90203	2.180934	0.031843	-4.53164	0.307716	0.22426
B.cells	ADPRHL2	0.358449	3.241714	2.180425	0.031882	-3.85164	0.326122	0.248349
B.cells	NR3C1	0.252712	7.440275	2.18022	0.031898	-4.59178	0.305114	0.220966
B.cells	S100G	1.070989	0.605317	2.18001	0.031914	-3.58023	0.340178	0.267259
B.cells	MRC2	-0.97193	1.416971	-2.17988	0.031923	-3.55935	0.335778	0.261334
B.cells	RNF180	-1.04742	2.247716	-2.17968	0.031939	-3.56255	0.331341	0.255397
B.cells	GALNT2	0.296809	5.565067	2.179517	0.031951	-4.25858	0.314293	0.23289
B.cells	P2RX4	0.280568	5.534178	2.178571	0.032024	-4.37822	0.314447	0.233376
B.cells	PDLIM2	0.404443	3.759145	2.178405	0.032037	-3.98873	0.323443	0.245243
B.cells	ARFGEF2	0.247831	6.285468	2.17791	0.032075	-4.41309	0.310729	0.228729
B.cells	GM36161	1.218066	0.563057	2.177547	0.032103	-3.54801	0.340409	0.268289
B.cells	ACY3	-0.90235	2.04257	-2.17728	0.032123	-3.60598	0.332431	0.257563
B.cells	GRASP	-0.40096	3.781204	-2.17721	0.032129	-4.04077	0.32333	0.245423
B.cells	SLC35F6	0.41859	2.998461	2.176952	0.032149	-3.86089	0.327391	0.250874
B.cells	DAPK2	-0.68247	2.818088	-2.17685	0.032156	-3.69386	0.328335	0.252136
B.cells	TESK2	0.448249	4.072329	2.176292	0.0322	-4.00703	0.321834	0.243608
B.cells	1600014C1	0.49883	4.275444	2.175961	0.032225	-4.05188	0.320796	0.242289
B.cells	SAA1	1.8245	-0.05604	2.17542	0.032267	-3.54475	0.343813	0.27346
B.cells	ARHGAP21	-0.28954	5.511128	-2.17538	0.03227	-4.26817	0.314562	0.234241
B.cells	B4GALT5	0.297983	5.975954	2.174913	0.032306	-4.43306	0.312254	0.231348
B.cells	PDIA6	-0.23513	6.97381	-2.17455	0.032334	-4.53737	0.307367	0.22517
B.cells	SRD5A3	0.339182	4.511422	2.174541	0.032335	-4.12448	0.319594	0.241147
B.cells	GM26737	-1.15284	0.478605	-2.17296	0.032458	-3.5492	0.341107	0.270275
B.cells	DLG4	-0.72729	3.571009	-2.1729	0.032462	-3.83995	0.324639	0.248021
B.cells	RTL8A	-0.47979	3.632481	-2.17267	0.032481	-3.88252	0.324321	0.247674
B.cells	RPAP2	0.42691	3.02458	2.172491	0.032494	-3.82849	0.32748	0.251957
B.cells	ITIH3	-0.60507	3.636116	-2.17246	0.032496	-3.96487	0.324302	0.247706
B.cells	EWSR1	-0.16171	7.421625	-2.17205	0.032529	-4.61613	0.305457	0.222998
B.cells	ELOC	-0.20662	7.093954	-2.1711	0.032602	-4.5709	0.307466	0.225299
B.cells	TGFBR3	-0.62187	3.337964	-2.17075	0.032629	-3.79609	0.326345	0.250231

B.cells	CD3D	-0.88244	2.678526	-2.16946	0.032731	-3.66586	0.330373	0.255281
B.cells	2810408I1:	-0.96516	1.398496	-2.16869	0.032791	-3.56785	0.337209	0.264763
B.cells	DLAT	0.278478	4.282042	2.168666	0.032793	-4.09343	0.322034	0.244356
B.cells	COL13A1	-1.272	0.62777	-2.1686	0.032798	-3.55997	0.341404	0.270493
B.cells	D2HGDH	0.667542	2.08867	2.167285	0.032901	-3.62975	0.33427	0.260185
B.cells	P4HA3	-1.16523	0.308298	-2.1669	0.032932	-3.56065	0.343975	0.273491
B.cells	CLEC4E	1.50966	2.622943	2.165583	0.033035	-3.62191	0.331987	0.256886
B.cells	TMEM159	0.91738	1.553977	2.165237	0.033063	-3.60799	0.337713	0.264677
B.cells	PARP14	0.485387	5.480462	2.165159	0.033069	-4.33632	0.317225	0.237284
B.cells	FAM20B	0.36026	3.958304	2.164259	0.03314	-4.00501	0.325414	0.247848
B.cells	MARCKS	-0.27009	7.242888	-2.16292	0.033246	-4.63691	0.3094	0.226488
B.cells	PLXND1	-0.40775	3.5086	-2.16289	0.033249	-3.89706	0.328276	0.2513
B.cells	AMDHD2	0.393133	3.693973	2.161831	0.033333	-3.92196	0.327587	0.250272
B.cells	LY6I	1.815454	0.802083	2.161386	0.033368	-3.5854	0.343094	0.271415
B.cells	TBL1X	-0.21272	7.155308	-2.16116	0.033386	-4.58973	0.310092	0.22746
B.cells	SLC45A1	-0.88735	0.608482	-2.16111	0.03339	-3.56852	0.344162	0.272912
B.cells	PRKACB	0.229983	5.548308	2.159061	0.033554	-4.31941	0.319366	0.2386
B.cells	TRAPPC6B	0.181446	6.134079	2.158283	0.033616	-4.43639	0.316559	0.234926
B.cells	IL6ST	0.385937	4.326252	2.158168	0.033625	-4.1837	0.32577	0.247063
B.cells	KCTD17	1.081284	0.958849	2.157787	0.033656	-3.58251	0.3438	0.271405
B.cells	PIM1	-0.24163	9.488973	-2.15633	0.033773	-5.01859	0.30108	0.21444
B.cells	GM26532	-0.34914	5.294849	-2.15468	0.033906	-4.28967	0.322661	0.241343
B.cells	RNASEH1	0.413818	2.84733	2.153609	0.033992	-3.84426	0.335772	0.258585
B.cells	TMEM8	-0.71228	2.000346	-2.15312	0.034032	-3.63828	0.34035	0.26476
B.cells	EEPD1	0.519601	4.881821	2.153037	0.034039	-3.92875	0.325068	0.24436
B.cells	PGAM5	0.320717	3.934831	2.152766	0.034061	-4.05354	0.33	0.250922
B.cells	BBX	0.257947	6.494425	2.152554	0.034078	-4.52072	0.316867	0.233683
B.cells	TMEM104	0.474477	3.273099	2.152205	0.034106	-3.89959	0.333498	0.25575
B.cells	AI182371	-0.74367	2.439191	-2.15167	0.03415	-3.76557	0.33812	0.261886
B.cells	TMEM191(	-0.56074	1.703176	-2.15114	0.034193	-3.66541	0.34227	0.267483
B.cells	GABARAPL	-0.41685	3.974288	-2.1503	0.034261	-3.9595	0.330465	0.251378
B.cells	SEMA6B	-0.93082	1.154578	-2.14962	0.034317	-3.60139	0.345902	0.272053
B.cells	GM29570	-1.00598	0.692445	-2.1491	0.034358	-3.59205	0.348477	0.275817
B.cells	SPARCL1	1.699388	0.3267	2.149	0.034367	-3.58667	0.35053	0.278647
B.cells	APH1C	-0.55268	4.351444	-2.14765	0.034477	-3.93572	0.329464	0.249463
B.cells	MDFIC	0.435003	4.328783	2.147286	0.034507	-4.18035	0.329598	0.249691
B.cells	FKBP2	-0.29366	5.611404	-2.14586	0.034625	-4.32509	0.323783	0.241318
B.cells	ARHGEF3	0.424666	5.916073	2.144713	0.034719	-4.41729	0.322582	0.23963
B.cells	AQP9	-0.73933	2.330322	-2.14419	0.034762	-3.7282	0.341525	0.264968
B.cells	ARL4D	-0.90268	1.705958	-2.14413	0.034766	-3.63763	0.344954	0.269614
B.cells	GRK6	0.239861	5.764162	2.143999	0.034778	-4.38551	0.323359	0.240846
B.cells	F11	-0.96062	0.954836	-2.14088	0.035036	-3.6227	0.351213	0.276285
B.cells	ACP5	0.402136	5.117131	2.140726	0.035049	-4.35336	0.32864	0.246106
B.cells	MSMO1	-0.45384	3.610529	-2.14045	0.035071	-3.88344	0.33661	0.25667
B.cells	ITCH	0.211553	7.100446	2.139975	0.035111	-4.66524	0.318584	0.232997
B.cells	PROX1OS	-1.09311	0.612207	-2.13954	0.035148	-3.6085	0.353335	0.279194
B.cells	PIRB	0.452093	4.941687	2.138854	0.035204	-4.32698	0.32981	0.247759

B.cells	NMRAL1	-0.41686	3.904291	-2.13874	0.035214	-4.05075	0.335295	0.255064
B.cells	MYO9B	0.242448	5.866728	2.137364	0.035329	-4.42155	0.325497	0.241974
B.cells	GPNMB	1.167014	0.898381	2.136978	0.035361	-3.62775	0.352331	0.278027
B.cells	HSD11B1	-0.41008	4.593839	-2.13674	0.035381	-4.16604	0.332137	0.250945
B.cells	COQ3	-0.3119	3.500438	-2.13672	0.035382	-3.99182	0.337968	0.258699
B.cells	MAVS	0.485277	2.932268	2.135787	0.035461	-3.83655	0.341418	0.263025
B.cells	GSTM1	-0.46591	4.059268	-2.13431	0.035585	-4.13853	0.335339	0.255317
B.cells	PHF21A	0.235549	7.069098	2.134112	0.035602	-4.64932	0.319717	0.234796
B.cells	RABEP1	0.182504	6.897474	2.134089	0.035604	-4.64143	0.320584	0.23592
B.cells	GPHN	0.212272	7.486182	2.134072	0.035605	-4.70898	0.31762	0.232085
B.cells	CPT1A	0.21804	5.446894	2.134067	0.035606	-4.40532	0.328027	0.245643
B.cells	CCL6	0.597853	5.306299	2.133854	0.035624	-4.14689	0.328759	0.246648
B.cells	GM550	-1.10666	0.859238	-2.133	0.035696	-3.61228	0.352979	0.279386
B.cells	HMGN2	-0.33783	7.53605	-2.13222	0.035761	-4.64149	0.317409	0.232173
B.cells	TDRD7	0.414087	3.385152	2.131709	0.035805	-4.03256	0.339	0.260723
B.cells	PDE8B	-1.16728	-0.24267	-2.13168	0.035807	-3.62063	0.359285	0.288381
B.cells	GK5	-0.36769	4.404631	-2.13081	0.035881	-4.21793	0.333541	0.25366
B.cells	DQX1	-1.55793	1.215444	-2.1306	0.035898	-3.63988	0.350968	0.277236
B.cells	FKBP15	0.244623	5.239594	2.130366	0.035919	-4.35205	0.329147	0.24792
B.cells	IRAK3	0.446511	4.187779	2.130178	0.035935	-4.32072	0.334693	0.255287
B.cells	SLC22A27	-1.00193	1.124082	-2.12976	0.03597	-3.65046	0.351483	0.278116
B.cells	ASGR2	-0.94994	1.698173	-2.12975	0.035971	-3.68334	0.348264	0.273714
B.cells	SELENOP	-0.3517	8.699951	-2.12971	0.035975	-4.8855	0.311649	0.225257
B.cells	NAMPT	0.369407	6.301782	2.129634	0.035981	-4.52451	0.323655	0.240808
B.cells	DDX43	1.095633	0.153087	2.12936	0.036004	-3.61874	0.357005	0.285771
B.cells	RAP1GDS1	0.216314	6.711436	2.128517	0.036076	-4.61394	0.321947	0.238357
B.cells	ALDH16A1	0.296455	4.358468	2.127558	0.036158	-4.21486	0.334406	0.25472
B.cells	SGIP1	-1.06542	0.175941	-2.12755	0.036158	-3.62204	0.357537	0.286133
B.cells	APOBR	0.850659	2.373103	2.126982	0.036207	-3.6633	0.345188	0.26942
B.cells	TCP11	-0.9485	0.648485	-2.12684	0.036219	-3.62287	0.354859	0.282651
B.cells	TMPRSS6	-0.89827	1.467899	-2.12604	0.036288	-3.69058	0.350612	0.276543
B.cells	CELSR1	1.056633	1.626552	2.125657	0.03632	-3.67441	0.349757	0.275461
B.cells	ECSIT	0.325688	4.015765	2.123494	0.036506	-4.12449	0.337925	0.258459
B.cells	CLEC4A2	-0.71074	3.416946	-2.12309	0.036541	-3.83387	0.341165	0.262882
B.cells	ILRUN	0.241686	5.988088	2.122465	0.036595	-4.51119	0.327508	0.244867
B.cells	DDI2	0.227746	5.943537	2.122429	0.036598	-4.50196	0.327739	0.245172
B.cells	AKAP12	-0.39137	6.169014	-2.12238	0.036602	-4.58966	0.326572	0.243647
B.cells	TLCD2	-0.62869	2.659161	-2.12169	0.036662	-3.80688	0.345603	0.268849
B.cells	ADGB	0.976156	2.335955	2.121092	0.036713	-3.73732	0.347605	0.271548
B.cells	HSD17B7	-0.75761	1.697013	-2.11941	0.036859	-3.66522	0.352289	0.276974
B.cells	SLC40A1	-0.54508	5.835325	-2.11823	0.036961	-4.36802	0.3303	0.247088
B.cells	ZFP652	0.285452	5.654413	2.117985	0.036983	-4.42896	0.331248	0.248408
B.cells	GM3336	-0.9767	1.732452	-2.11778	0.037001	-3.69378	0.352607	0.277143
B.cells	GM15708	-0.43079	3.198583	-2.11678	0.037088	-4.03281	0.344976	0.266389
B.cells	4930594M	1.269991	-0.02762	2.114825	0.037259	-3.64564	0.363668	0.292143
B.cells	CD3G	-0.95317	3.343766	-2.11477	0.037264	-3.81673	0.344553	0.266011
B.cells	CARD19	0.28041	5.613475	2.114629	0.037276	-4.47195	0.332341	0.249746

B.cells	ANKRD24	0.853251	1.054272	2.11426	0.037308	-3.6581	0.357404	0.283711
B.cells	GM33370	0.984699	0.508404	2.114228	0.037311	-3.64779	0.360549	0.288046
B.cells	CCDC141	-0.82171	1.120838	-2.11385	0.037344	-3.69271	0.357023	0.283303
B.cells	TTC7	0.250551	5.873227	2.113434	0.037381	-4.53484	0.330976	0.248358
B.cells	CYP4B1	-1.30328	0.266849	-2.11279	0.037437	-3.64435	0.361951	0.290545
B.cells	CES1D	-0.90587	1.90006	-2.11266	0.037449	-3.73275	0.352593	0.277696
B.cells	SULT1A1	-0.52013	3.923228	-2.11217	0.037493	-4.09828	0.341386	0.26268
B.cells	TRIM30A	0.418759	6.169574	2.111747	0.037529	-4.52974	0.329427	0.246809
B.cells	9130230L2	-0.71327	3.687563	-2.11171	0.037532	-3.97585	0.34267	0.264465
B.cells	DNAL1	-0.64126	1.64713	-2.11158	0.037544	-3.79838	0.354024	0.27994
B.cells	TRAT1	-1.3726	-0.05747	-2.11151	0.03755	-3.64732	0.363843	0.293514
B.cells	BSPRY	0.860295	0.341542	2.111296	0.037569	-3.68687	0.361517	0.290314
B.cells	RNF44	-0.26529	4.833037	-2.11043	0.037646	-4.31003	0.336905	0.25663
B.cells	CD84	0.312192	5.366126	2.108992	0.037773	-4.43143	0.334904	0.253332
B.cells	CCPG1	-0.24803	6.037912	-2.10842	0.037824	-4.51223	0.331358	0.248825
B.cells	SNX20	0.245744	5.514815	2.108222	0.037841	-4.50324	0.334115	0.252475
B.cells	DHX40	-0.31553	7.808142	-2.10803	0.037858	-4.76001	0.322225	0.236935
B.cells	NCEH1	0.407041	4.906119	2.107394	0.037915	-4.21478	0.337599	0.257084
B.cells	SNTB2	0.311873	5.607555	2.106881	0.037961	-4.46584	0.334005	0.252185
B.cells	GPD1	-0.79854	1.749644	-2.10508	0.038121	-3.74014	0.356345	0.281255
B.cells	ACTR6	0.37985	3.522286	2.104821	0.038144	-4.04267	0.346394	0.267783
B.cells	2700081O1	-0.46988	3.137169	-2.10384	0.038232	-3.9386	0.349057	0.270925
B.cells	GALK2	0.249071	4.897213	2.103428	0.038269	-4.35056	0.339479	0.258121
B.cells	PTK2	-0.46446	4.164046	-2.10225	0.038375	-4.05481	0.344102	0.263814
B.cells	METAP1	0.227642	4.718677	2.101967	0.0384	-4.31777	0.341081	0.259905
B.cells	PDIA3	-0.19139	8.546649	-2.10054	0.038528	-4.92583	0.321353	0.233971
B.cells	PRDM1	0.564961	2.929068	2.099243	0.038646	-4.00789	0.351261	0.274017
B.cells	BE692007	0.468859	2.816357	2.099048	0.038664	-4.17461	0.351894	0.274938
B.cells	E230016M	0.620786	2.465953	2.098773	0.038689	-3.8499	0.35387	0.277744
B.cells	B3GALT1	-0.89995	3.129188	-2.09838	0.038724	-3.92247	0.350141	0.272765
B.cells	PID1	-0.37007	5.484883	-2.09805	0.038754	-4.48907	0.337264	0.255593
B.cells	YWHAZ	-0.11552	8.482146	-2.09802	0.038757	-4.9422	0.321679	0.235134
B.cells	AK6	-0.27872	4.761906	-2.09773	0.038783	-4.3132	0.341156	0.260872
B.cells	MXRA7	-0.90681	1.654737	-2.09771	0.038785	-3.70043	0.358493	0.284428
B.cells	CST7	1.001395	2.120473	2.097192	0.038832	-3.71715	0.35583	0.280891
B.cells	PDK3	0.336825	4.945028	2.095968	0.038943	-4.37201	0.340165	0.26002
B.cells	ARRDC4	0.765796	2.427989	2.095787	0.03896	-3.74549	0.354085	0.278887
B.cells	IFT140	0.367705	3.507073	2.095519	0.038984	-4.13407	0.348037	0.27064
B.cells	NTMT1	0.263375	3.588232	2.095457	0.03899	-4.13409	0.347587	0.270048
B.cells	OASL2	1.075819	3.934807	2.095211	0.039012	-4.02234	0.345674	0.267589
B.cells	D430042O(	0.329549	3.792114	2.094919	0.039039	-4.15078	0.34646	0.268691
B.cells	TAP1	0.53595	5.446269	2.094706	0.039058	-4.46931	0.337471	0.256685
B.cells	BAZ2B	-0.1716	8.430417	-2.09441	0.039086	-4.94947	0.321941	0.236245
B.cells	KLHDC10	0.251782	5.434437	2.094099	0.039114	-4.44488	0.337534	0.256859
B.cells	CST3	-0.37546	9.157123	-2.09407	0.039116	-5.09985	0.318288	0.231577
B.cells	GCH1	0.289177	5.663529	2.093697	0.03915	-4.56328	0.336311	0.25523
B.cells	CD40	0.571055	2.17854	2.093457	0.039172	-4.08611	0.3555	0.281241

B.cells	POPDC3	-1.22694	0.894439	-2.09312	0.039203	-3.68433	0.362889	0.291558
B.cells	ATXN3	0.220785	5.054807	2.092736	0.039238	-4.44001	0.339573	0.25978
B.cells	IFNAR2	0.217714	7.128736	2.091901	0.039315	-4.70664	0.328608	0.245588
B.cells	FCHSD2	0.325219	6.823892	2.091744	0.039329	-4.71149	0.330193	0.247681
B.cells	ABCA1	0.383985	5.836161	2.091554	0.039347	-4.57872	0.335392	0.254634
B.cells	AOAH	0.970817	4.784961	2.091473	0.039354	-3.94363	0.341031	0.262205
B.cells	PPIC	-0.67198	2.745716	-2.09137	0.039363	-3.79045	0.352291	0.277517
B.cells	CYBA	0.208303	8.55691	2.090834	0.039413	-4.97225	0.321301	0.236183
B.cells	5430416N(	-0.33431	3.547711	-2.0907	0.039425	-4.10015	0.347812	0.271563
B.cells	FBXO25	0.467357	2.127104	2.090659	0.039429	-3.84343	0.355792	0.28251
B.cells	TBK1	0.24626	5.964636	2.090506	0.039443	-4.57618	0.33471	0.253894
B.cells	FECH	0.387607	5.217188	2.090363	0.039456	-4.49029	0.338699	0.259232
B.cells	RASAL2	-0.41033	5.468995	-2.09014	0.039477	-4.36855	0.337349	0.257421
B.cells	RNF144B	-0.60476	2.562148	-2.08785	0.039688	-3.85005	0.354557	0.280104
B.cells	2210408F2	0.653807	2.856305	2.087689	0.039703	-3.91256	0.352895	0.277857
B.cells	CEP55	-0.65651	3.678611	-2.08768	0.039704	-4.06469	0.348296	0.271572
B.cells	HEATR6	0.262866	4.812778	2.08672	0.039793	-4.34085	0.342573	0.263404
B.cells	SLFN5	0.634546	4.230734	2.085319	0.039923	-4.17114	0.346627	0.268125
B.cells	SLC25A24	-0.48699	3.486183	-2.08328	0.040113	-3.99172	0.352164	0.274382
B.cells	RALGPS2	0.27037	5.773254	2.082591	0.040177	-4.5593	0.339804	0.257559
B.cells	CYP27A1	-0.58601	2.194427	-2.08212	0.040221	-3.89928	0.359726	0.284654
B.cells	TXNIP	0.332685	5.791358	2.081972	0.040235	-4.49987	0.339707	0.257539
B.cells	D730003I1	0.519217	2.591958	2.08173	0.040258	-3.89778	0.357447	0.281597
B.cells	OSBPL1A	-0.48538	3.288159	-2.08122	0.040305	-3.94871	0.353516	0.276329
B.cells	SF3B1	-0.13344	8.256825	-2.08085	0.04034	-4.94411	0.32676	0.240714
B.cells	ALDH3B1	0.406241	3.605843	2.080741	0.040351	-4.10674	0.35173	0.273986
B.cells	LPCAT3	0.268615	5.162712	2.079856	0.040434	-4.43343	0.34358	0.262622
B.cells	ADAR	0.350902	4.311216	2.07881	0.040532	-4.27422	0.348847	0.269295
B.cells	JARID2	0.189237	8.144583	2.078422	0.040569	-5.01433	0.328372	0.242128
B.cells	ATP6V1G2	0.81375	1.237127	2.0774	0.040665	-3.73367	0.366902	0.29385
B.cells	DBNL	0.204516	6.127196	2.077261	0.040679	-4.60308	0.339403	0.256532
B.cells	RECQL5	0.382469	3.722047	2.076504	0.04075	-4.08629	0.352973	0.274484
B.cells	H2-EB2	0.66713	0.547733	2.07546	0.040849	-3.88403	0.371856	0.300309
B.cells	CRBN	0.298144	4.060609	2.075013	0.040891	-4.22607	0.351545	0.272499
B.cells	NREP	-0.92112	1.234795	-2.07489	0.040903	-3.72333	0.367779	0.29481
B.cells	NR5A2	-0.87293	1.145394	-2.07444	0.040946	-3.72119	0.368307	0.295725
B.cells	GM17103	-1.07806	1.028835	-2.07433	0.040956	-3.71124	0.368996	0.296705
B.cells	FCGRT	-0.46129	4.823452	-2.07363	0.041023	-4.30163	0.347589	0.267221
B.cells	CYP2D9	-0.96596	0.530177	-2.07298	0.041084	-3.71294	0.372264	0.301321
B.cells	NHLRC2	0.234521	4.994885	2.07288	0.041094	-4.44882	0.346644	0.266171
B.cells	MASP2	-0.88968	1.894509	-2.07271	0.041111	-3.807	0.364209	0.290203
B.cells	GRCC10	0.187105	6.427807	2.07237	0.041143	-4.65167	0.338876	0.255832
B.cells	KXD1	0.219922	5.765423	2.070947	0.041279	-4.58597	0.343326	0.260897
B.cells	SMOX	0.715067	5.116761	2.070066	0.041363	-4.01387	0.347082	0.265778
B.cells	TTC39B	0.300728	4.653747	2.070055	0.041365	-4.38163	0.349644	0.269226
B.cells	CPN1	-0.78407	1.600653	-2.06809	0.041553	-3.79377	0.368145	0.293788
B.cells	MOSMO	0.278709	4.576833	2.067785	0.041583	-4.35546	0.351069	0.270508

B.cells	H13	-0.1906	6.42662	-2.06769	0.041592	-4.65554	0.340923	0.256967
B.cells	GM13963	1.38934	-0.29578	2.067284	0.041631	-3.71719	0.379526	0.309922
B.cells	GHITM	0.237424	7.4577	2.067006	0.041658	-4.93499	0.335419	0.249891
B.cells	LEFTY1	-1.19055	0.408138	-2.0668	0.041678	-3.71992	0.375255	0.304063
B.cells	FAM120A	0.172126	6.352078	2.066607	0.041696	-4.67082	0.341325	0.257777
B.cells	MRPS34	0.249573	4.826949	2.064835	0.041868	-4.40124	0.350857	0.269474
B.cells	PRRT1	-1.03135	0.466797	-2.06392	0.041957	-3.72144	0.376693	0.304542
B.cells	CD52	0.261204	9.30175	2.063173	0.042029	-5.17901	0.327714	0.238438
B.cells	TOMM34	-0.21194	5.601463	-2.06268	0.042077	-4.54596	0.347545	0.26444
B.cells	WDFY4	-0.25289	7.251372	-2.06093	0.042248	-4.94866	0.339717	0.252906
B.cells	GM41077	-0.91295	0.721818	-2.05693	0.04264	-3.75562	0.379042	0.304723
B.cells	NOC4L	0.321171	3.728892	2.05667	0.042665	-4.20425	0.361244	0.280385
B.cells	WDR44	0.286714	4.70447	2.056441	0.042688	-4.44289	0.35568	0.272933
B.cells	LAIR1	0.905985	4.246709	2.056233	0.042708	-3.95134	0.358278	0.276484
B.cells	CEP250	-0.31181	4.458141	-2.05601	0.042731	-4.34822	0.357075	0.274861
B.cells	MALSU1	0.285287	4.843801	2.0551	0.04282	-4.39982	0.354894	0.272059
B.cells	CHRAC1	0.213266	5.264928	2.055038	0.042826	-4.51622	0.352529	0.268888
B.cells	ABCB7	0.211011	5.334608	2.055025	0.042828	-4.52681	0.35214	0.268367
B.cells	ZFP946	-0.68266	1.39583	-2.05492	0.042838	-3.79655	0.374966	0.299475
B.cells	TLR7	0.577852	2.963787	2.054812	0.042849	-4.05437	0.365679	0.286698
B.cells	SPRYD7	0.603286	2.133927	2.054652	0.042864	-3.86806	0.370561	0.293393
B.cells	ANKIB1	0.210946	5.807453	2.054614	0.042868	-4.61908	0.349511	0.26488
B.cells	HAO1	-0.58637	3.070076	-2.05441	0.042888	-4.07276	0.365059	0.285852
B.cells	TRIM25	0.267138	6.978283	2.052659	0.043062	-4.83757	0.344242	0.256948
B.cells	DYNLL1	-0.17291	7.867224	-2.05126	0.043201	-4.91882	0.340306	0.251085
B.cells	PFKP	0.291674	5.538024	2.049987	0.043328	-4.61019	0.353839	0.268312
B.cells	PROZ	-0.69621	1.915226	-2.04957	0.04337	-3.92403	0.374954	0.296972
B.cells	H2-T22	0.500601	5.391347	2.049008	0.043426	-4.51937	0.354798	0.269818
B.cells	APOL11B	1.848717	-0.04187	2.048546	0.043472	-3.78525	0.386966	0.314035
B.cells	EPHX3	-0.87616	0.657793	-2.04839	0.043487	-3.76507	0.38264	0.308016
B.cells	FHOD1	0.466595	2.907952	2.048258	0.043501	-3.99056	0.369099	0.289371
B.cells	LDHB	0.777077	3.00278	2.047825	0.043544	-3.87633	0.368602	0.288726
B.cells	KRT10	-0.6565	2.100333	-2.04756	0.04357	-3.83816	0.373957	0.296158
B.cells	IFNAR1	0.245773	5.246213	2.047009	0.043626	-4.51341	0.355871	0.271538
B.cells	APCS	0.38601	4.833138	2.046607	0.043666	-4.52376	0.358287	0.274803
B.cells	ARHGAP30	0.16423	7.139253	2.046284	0.043699	-4.83571	0.345452	0.257834
B.cells	PATJ	-0.53078	3.348038	-2.04539	0.043788	-4.22707	0.367334	0.286896
B.cells	PDK4	-0.92313	1.417008	-2.04508	0.04382	-3.78607	0.378851	0.302724
B.cells	PFDN5	0.147922	7.413663	2.044761	0.043852	-4.87237	0.344398	0.256196
B.cells	LACC1	0.50292	3.279516	2.044486	0.04388	-4.09721	0.367735	0.287443
B.cells	TUBB2A	-0.34486	5.517234	-2.0427	0.044061	-4.53773	0.3553	0.270607
B.cells	KRT18	-0.64912	3.557982	-2.04253	0.044077	-4.20239	0.36654	0.285776
B.cells	USB1	0.432394	3.245957	2.042521	0.044078	-4.11377	0.368368	0.288268
B.cells	PATL2	0.684724	2.023827	2.042424	0.044088	-3.90432	0.375632	0.298236
B.cells	P4HTM	-0.59498	2.773581	-2.04242	0.044089	-4.06833	0.371156	0.292082
B.cells	NUDT16	0.471928	2.966991	2.041674	0.044164	-4.03313	0.370338	0.290833
B.cells	LGALS9	0.261806	7.157478	2.041073	0.044225	-4.81754	0.346507	0.258905

B.cells	DYNLL2	-0.34153	5.082989	-2.04097	0.044235	-4.41816	0.358071	0.27432
B.cells	RAD51D	0.405468	2.984907	2.040798	0.044253	-4.08372	0.370232	0.29087
B.cells	GM15417	-0.57192	2.477041	-2.0399	0.044345	-3.93345	0.373418	0.295355
B.cells	CASP4	0.542215	4.029936	2.039731	0.044362	-4.27896	0.364282	0.282865
B.cells	PLD3	0.378663	4.413779	2.039324	0.044403	-4.31914	0.362064	0.279929
B.cells	CD164	-0.24379	6.379209	-2.03924	0.044412	-4.60228	0.350953	0.265034
B.cells	CCDC28B	0.352954	3.504556	2.039056	0.04443	-4.27602	0.367343	0.287151
B.cells	SIDT1	-0.46707	3.376538	-2.03854	0.044483	-4.39943	0.36814	0.288305
B.cells	IGHD	0.404699	3.245617	2.038346	0.044503	-4.21193	0.36891	0.289395
B.cells	ALDOB	-0.56066	4.949636	-2.03808	0.04453	-4.51584	0.359039	0.276047
B.cells	ALDH6A1	-0.6749	2.786139	-2.03757	0.044582	-3.98222	0.371795	0.293307
B.cells	ST6GALNA(	0.387121	3.214358	2.036862	0.044655	-4.23333	0.369554	0.29005
B.cells	TCEA3	-0.62947	2.275566	-2.03661	0.04468	-4.01754	0.375137	0.297812
B.cells	LARP1	0.187866	6.798018	2.03497	0.044849	-4.80288	0.349827	0.263163
B.cells	MS4A4A	0.50397	0.617812	2.033623	0.044987	-4.10459	0.386021	0.313018
B.cells	GM5535	-1.40475	0.151841	-2.03332	0.045019	-3.76641	0.388921	0.317123
B.cells	ISOC1	0.251672	4.993923	2.033311	0.04502	-4.52315	0.359965	0.277171
B.cells	MAN1A	0.248053	8.000333	2.033106	0.045041	-5.04865	0.343258	0.254993
B.cells	RBBP6	-0.16811	7.269431	-2.03271	0.045082	-4.87073	0.347234	0.260356
B.cells	BEND4	0.65461	3.557561	2.032494	0.045104	-3.96455	0.368285	0.288741
B.cells	GIMAP7	0.631219	1.458503	2.032489	0.045105	-4.02925	0.38085	0.306099
B.cells	TCEAL8	-0.40626	3.331023	-2.03242	0.045111	-4.09071	0.369618	0.290569
B.cells	PTDSS2	0.379337	3.06586	2.032422	0.045111	-4.11613	0.371185	0.292721
B.cells	CITED4	-1.26926	-0.2568	-2.03236	0.045118	-3.76968	0.391485	0.321059
B.cells	PTPRG	-0.84709	2.191113	-2.03141	0.045217	-3.91943	0.376968	0.300347
B.cells	ATR	0.394798	3.862209	2.029495	0.045415	-4.29901	0.3677	0.287275
B.cells	SLC9A9	-0.43303	7.120003	-2.0295	0.045415	-4.70904	0.349191	0.262368
B.cells	GM15892	-0.47471	3.443855	-2.02928	0.045437	-4.23819	0.370159	0.290639
B.cells	FAM25C	-0.95251	1.148741	-2.0287	0.045498	-3.82085	0.383997	0.310158
B.cells	DNAL4	0.633194	1.951429	2.028519	0.045516	-3.89361	0.37909	0.303373
B.cells	JUND	-0.23068	10.62781	-2.02797	0.045573	-5.39501	0.330484	0.238444
B.cells	ST3GAL1	0.319909	5.796993	2.027778	0.045593	-4.74581	0.356572	0.272791
B.cells	RBM39	-0.11118	9.586582	-2.02724	0.045649	-5.25085	0.335909	0.245661
B.cells	PTPRD	-0.74729	2.563535	-2.02711	0.045663	-4.00246	0.375397	0.298721
B.cells	RORA	-0.42139	5.020685	-2.02703	0.045672	-4.43311	0.360989	0.278999
B.cells	HMGB1	-0.20913	9.807497	-2.02667	0.045709	-5.22716	0.334749	0.24428
B.cells	BIN3	0.244387	5.271025	2.026492	0.045728	-4.55295	0.359557	0.277191
B.cells	SLC6A13	-0.58756	2.762131	-2.02645	0.045732	-4.00236	0.374208	0.297238
B.cells	IL33	-1.17972	0.25962	-2.02616	0.045762	-3.77588	0.389517	0.318671
B.cells	DIO1	-0.83164	0.977554	-2.02607	0.045772	-3.81116	0.385053	0.312381
B.cells	IMMT	0.18176	6.049157	2.025452	0.045836	-4.70318	0.35528	0.271389
B.cells	ADCK2	0.480551	2.282482	2.025166	0.045866	-3.97722	0.377225	0.301516
B.cells	IQCK	-1.21067	0.158994	-2.02501	0.045883	-3.78002	0.390289	0.31991
B.cells	CCNI	-0.17347	6.783841	-2.02319	0.046074	-4.83405	0.352397	0.266574
B.cells	CDK4	-0.24564	5.844705	-2.02069	0.046337	-4.62882	0.359136	0.274186
B.cells	2310040G2	0.524039	1.291307	2.020547	0.046353	-3.88084	0.386166	0.31122
B.cells	SLC35A3	0.225195	4.775291	2.020309	0.046378	-4.47796	0.36528	0.282472

B.cells	IFI47	0.663596	5.277962	2.020058	0.046404	-4.53134	0.362376	0.278546
B.cells	GM49774	-0.52151	3.174932	-2.01989	0.046422	-4.1678	0.374706	0.295338
B.cells	PPIG	0.145497	6.974522	2.019209	0.046494	-4.86196	0.35308	0.265805
B.cells	COL11A2	-1.08135	0.72136	-2.01857	0.046562	-3.79551	0.390105	0.316555
B.cells	A930037H(	0.548424	3.242306	2.018369	0.046583	-4.19984	0.37468	0.295128
B.cells	AP3D1	0.196639	4.873786	2.018243	0.046597	-4.50802	0.365075	0.282026
B.cells	MAP4K4	0.234352	7.459224	2.017846	0.046639	-4.98898	0.350518	0.262462
B.cells	ARHGAP19	-0.52483	4.256309	-2.01708	0.04672	-4.20963	0.368931	0.287137
B.cells	ZFP69	-0.6031	2.612118	-2.01704	0.046725	-3.98019	0.37873	0.300579
B.cells	GALNT11	0.273433	4.120023	2.016708	0.04676	-4.40758	0.369758	0.288247
B.cells	CLNK	-1.10284	1.404175	-2.0158	0.046857	-3.82396	0.386347	0.311157
B.cells	GM17484	-0.99364	1.264186	-2.0156	0.046878	-3.81823	0.387215	0.312457
B.cells	INAFM1	-0.64003	1.866251	-2.01558	0.04688	-3.91056	0.383498	0.307272
B.cells	B3GAT3	0.319413	4.307364	2.014044	0.047045	-4.36616	0.369719	0.287659
B.cells	PHC3	0.277389	4.499127	2.013949	0.047055	-4.43627	0.368593	0.286129
B.cells	PDE1B	-0.39454	3.114849	-2.01312	0.047143	-4.19703	0.377007	0.297589
B.cells	KIFAP3	-0.40689	2.849059	-2.01312	0.047144	-4.06388	0.37861	0.299798
B.cells	METTL4	0.432851	2.874732	2.012503	0.04721	-4.06307	0.378578	0.299755
B.cells	SNX13	0.280001	5.413359	2.011916	0.047273	-4.58101	0.363589	0.279517
B.cells	WBP11	0.179446	6.346343	2.01182	0.047283	-4.77069	0.358256	0.27235
B.cells	ATP8A1	0.19529	7.601268	2.011634	0.047303	-4.95565	0.351229	0.263
B.cells	GM43331	0.563139	2.057137	2.011487	0.047319	-3.91767	0.383558	0.306927
B.cells	PGGHG	-0.48469	2.820106	-2.01094	0.047378	-4.08738	0.378942	0.30066
B.cells	CD68	-0.47333	4.872198	-2.01078	0.047395	-4.33634	0.366758	0.28397
B.cells	RASSF3	0.237567	6.834864	2.010555	0.04742	-4.91652	0.355533	0.268883
B.cells	C030034I2:	0.401825	2.630863	2.010024	0.047477	-4.14843	0.380157	0.302501
B.cells	MRAS	-1.13935	0.476226	-2.00988	0.047492	-3.80016	0.393509	0.32119
B.cells	PTK6	-1.54336	-0.3416	-2.00813	0.047682	-3.80615	0.400037	0.329129
B.cells	CDH5	-0.61345	4.480665	-2.00702	0.047802	-4.24906	0.371017	0.288218
B.cells	H3F3B	-0.14205	9.934295	-2.00665	0.047842	-5.31162	0.340494	0.247734
B.cells	RIPK1	0.221088	5.973193	2.005946	0.047919	-4.74308	0.362733	0.276742
B.cells	ANXA10	0.943829	0.643116	2.00504	0.048017	-3.82515	0.395461	0.321341
B.cells	URI1	0.21893	5.89939	2.003538	0.048181	-4.72365	0.364656	0.278128
B.cells	PTBP3	0.13732	8.46802	2.002206	0.048327	-5.13403	0.350998	0.259323
B.cells	CD244A	0.607091	3.429777	2.001843	0.048367	-4.0647	0.3802	0.298391
B.cells	MAP3K15	-0.45466	3.326785	-2.00038	0.048527	-4.274	0.381665	0.299692
B.cells	NAGA	-0.39871	4.159018	-2.00028	0.048539	-4.29767	0.376638	0.292858
B.cells	IGFBP4	-0.65855	5.679023	-1.99859	0.048725	-4.57742	0.368038	0.281336
B.cells	ADAP2OS	-0.99232	0.724917	-1.99858	0.048725	-3.8431	0.398304	0.322908
B.cells	MICU2	0.262796	4.939969	1.998502	0.048734	-4.54009	0.372378	0.287186
B.cells	CD47	0.158085	8.628324	1.998251	0.048762	-5.10506	0.351304	0.259214
B.cells	GCAT	-0.58638	2.390839	-1.99753	0.048842	-4.01776	0.387814	0.308662
B.cells	DOP1A	0.337621	4.09244	1.997387	0.048858	-4.36076	0.377429	0.294391
B.cells	FAM220A	0.681547	2.03012	1.997006	0.0489	-4.00411	0.390058	0.311916
B.cells	MRC1	-0.71244	5.240936	-1.99695	0.048906	-4.47242	0.370603	0.285256
B.cells	CAR5A	-1.03201	0.542195	-1.9961	0.049	-3.83354	0.399474	0.32529
B.cells	WDFY1	0.28558	4.332443	1.996007	0.04901	-4.49866	0.37599	0.292749

B.cells	HSD17B13	-0.66822	2.671988	-1.99586	0.049027	-4.08545	0.386075	0.306593
B.cells	EDEM3	-0.29453	5.974164	-1.99576	0.049038	-4.73874	0.366321	0.27967
B.cells	GTDC1	0.221619	6.409058	1.99574	0.04904	-4.82949	0.363809	0.276304
B.cells	GRTP1	-0.84582	0.35364	-1.99569	0.049045	-3.84294	0.400686	0.326998
B.cells	HERC4	0.198151	7.008496	1.994856	0.049138	-4.92297	0.360824	0.271999
B.cells	ITGAX	-0.94004	3.098724	-1.9945	0.049177	-3.86974	0.383975	0.303388
B.cells	FLAD1	0.453383	2.957237	1.994059	0.049227	-4.1442	0.384976	0.304803
B.cells	CDR2	0.547887	2.488604	1.993288	0.049313	-4.10338	0.388288	0.309091
B.cells	TNR	-0.90759	0.036927	-1.99297	0.049349	-3.83398	0.403879	0.331011
B.cells	TBC1D10A	0.326377	3.56507	1.992224	0.049432	-4.3106	0.382087	0.300364
B.cells	TRIO	-0.23394	6.069746	-1.9912	0.049546	-4.77413	0.367453	0.28046
B.cells	FOSL2	0.351485	5.561041	1.991102	0.049557	-4.58907	0.370426	0.28448
B.cells	CASP1	0.393925	3.51676	1.990803	0.04959	-4.3259	0.382661	0.301238
B.cells	TRPC1	-1.22485	-0.17084	-1.99068	0.049604	-3.83469	0.405941	0.333763
B.cells	PCIF1	0.18074	6.021144	1.990437	0.049631	-4.76203	0.367736	0.280973
B.cells	GAB2	0.302614	7.176286	1.989953	0.049686	-4.91162	0.361241	0.272272
B.cells	VPS39	0.38057	3.331542	1.989173	0.049773	-4.19046	0.384383	0.30335
B.cells	APOA5	-0.86179	1.793969	-1.9882	0.049883	-3.95369	0.39413	0.316921
B.cells	FGFR1OP2	0.199134	6.180589	1.988097	0.049894	-4.76838	0.367546	0.280504
B.cells	CD38	0.4163	5.882237	1.98809	0.049895	-4.74432	0.369286	0.282844
B.cells	GFRA1	-0.41301	4.918403	-1.98779	0.049929	-4.61769	0.374984	0.290657
B.cells	ATP11B	0.20863	7.035822	1.986868	0.050033	-4.95096	0.362937	0.274295
B.cells	SPR	-0.38578	3.527522	-1.98682	0.050038	-4.25078	0.383707	0.302436
B.cells	GALC	-0.43547	3.891293	-1.98611	0.050119	-4.2284	0.38185	0.299585
B.cells	SLC26A2	0.350832	3.742645	1.984941	0.050251	-4.34405	0.383515	0.301342
B.cells	CCNA2	-0.51627	5.520186	-1.9837	0.050392	-4.6088	0.373421	0.2871
B.cells	MRTFA	0.224692	7.084199	1.983189	0.05045	-4.99115	0.364297	0.274936
B.cells	NFIL3	0.50381	4.515288	1.982785	0.050496	-4.38731	0.379426	0.295319
B.cells	ANKRD44	0.205034	8.18547	1.982769	0.050497	-5.14159	0.358031	0.266636
B.cells	SNRPE	-0.19125	7.064225	-1.9825	0.050528	-4.93949	0.364412	0.275203
B.cells	MKRN1	0.249885	7.149614	1.982216	0.05056	-5.05078	0.363921	0.274655
B.cells	MGAM	-1.1497	0.078138	-1.98221	0.050561	-3.84422	0.407324	0.334364
B.cells	RGS3	0.723085	2.862627	1.979911	0.050823	-4.01215	0.390639	0.310209
B.cells	HIST1H2AC	-0.91394	1.907677	-1.97972	0.050846	-3.92534	0.396649	0.318614
B.cells	GM50019	-1.1237	-0.06748	-1.97963	0.050855	-3.84307	0.409421	0.336584
B.cells	HPD	-0.56078	4.805638	-1.97962	0.050856	-4.61299	0.378736	0.293932
B.cells	BTLA	0.358741	3.948652	1.979134	0.050912	-4.67748	0.383933	0.301223
B.cells	PHYH	-0.37523	5.40217	-1.97906	0.050921	-4.69083	0.375167	0.289278
B.cells	GRAMD1A	0.34986	4.325204	1.97889	0.05094	-4.41394	0.381639	0.298098
B.cells	5-Mar	0.231625	6.018808	1.978602	0.050973	-4.82011	0.37152	0.284458
B.cells	KIF5A	-1.06493	0.429339	-1.97823	0.051016	-3.85457	0.406165	0.332431
B.cells	SLBP	-0.21612	7.147492	-1.97787	0.051057	-4.93005	0.364951	0.27581
B.cells	ZFP318	0.414888	3.491008	1.977792	0.051066	-4.37683	0.386743	0.30538
B.cells	TUBA1B	-0.34035	7.795232	-1.97731	0.051122	-5.00059	0.361402	0.270997
B.cells	TEK	-0.82926	2.175307	-1.97628	0.05124	-3.94007	0.395729	0.317348
B.cells	SCAMP5	-0.83176	1.229049	-1.97606	0.051266	-3.87361	0.401772	0.325863
B.cells	CYBB	0.380898	7.155496	1.975151	0.05137	-5.03905	0.36613	0.27666

B.cells	MAST3	0.318973	4.169613	1.974374	0.05146	-4.48729	0.384292	0.300923
B.cells	POT1B	0.305049	4.421725	1.97391	0.051514	-4.58854	0.382906	0.29901
B.cells	SEMA4B	-0.32336	5.763505	-1.97306	0.051613	-4.64299	0.375198	0.288329
B.cells	TRAPPC10	0.225596	5.170629	1.972638	0.051661	-4.62653	0.378741	0.293255
B.cells	CRYZ	-0.7871	1.288432	-1.97239	0.05169	-3.89733	0.402937	0.326761
B.cells	ANXA11OS	0.829274	0.812081	1.972334	0.051697	-3.91307	0.406028	0.331136
B.cells	PLGRKT	0.2354	5.796951	1.971767	0.051762	-4.7681	0.375028	0.288381
B.cells	LAPTM4B	-0.53295	3.501356	-1.97138	0.051807	-4.26891	0.388961	0.307563
B.cells	NDUFV3	0.21386	7.098589	1.971029	0.051848	-4.93573	0.367389	0.27839
B.cells	ZFAND4	-0.67722	3.4762	-1.97092	0.051861	-4.17742	0.389117	0.307927
B.cells	STIMATE	0.299098	4.159047	1.970593	0.051899	-4.45524	0.384908	0.302223
B.cells	PRKCZ	0.729096	0.606888	1.970584	0.0519	-3.91262	0.407399	0.333601
B.cells	CTSZ	0.252649	6.598627	1.969837	0.051987	-4.94011	0.370684	0.282644
B.cells	POT1A	0.429484	3.012767	1.968438	0.05215	-4.16568	0.393333	0.313002
B.cells	GM43330	-0.67507	1.526899	-1.96816	0.052183	-3.95519	0.402791	0.326245
B.cells	ZFP839	0.595807	1.537463	1.967933	0.052209	-3.97731	0.402723	0.326174
B.cells	SRSF6	-0.20886	6.210068	-1.96739	0.052273	-4.80299	0.374011	0.286503
B.cells	COLGALT1	-0.21742	5.889717	-1.96717	0.052299	-4.74905	0.375912	0.289152
B.cells	PRDX1	-0.19192	9.719927	-1.96637	0.052393	-5.39089	0.353972	0.26011
B.cells	GM44127	-1.40115	-0.52381	-1.96556	0.052488	-3.8674	0.416512	0.346183
B.cells	MAGED1	-0.7336	2.409871	-1.96528	0.052521	-3.95648	0.397385	0.319143
B.cells	SLC6A6	0.192938	7.287991	1.965258	0.052523	-5.02789	0.367762	0.278624
B.cells	GM19684	0.709446	1.522121	1.96455	0.052607	-3.99631	0.403072	0.327303
B.cells	PSMB10	0.300745	5.706129	1.964505	0.052612	-4.77463	0.377074	0.291332
B.cells	GM27017	-0.38708	3.311249	-1.96433	0.052633	-4.36707	0.391707	0.311435
B.cells	EFNB2	-0.67969	2.469455	-1.96421	0.052647	-4.08208	0.397007	0.318814
B.cells	PLD2	-0.8771	1.042444	-1.96418	0.052651	-3.89148	0.406183	0.331715
B.cells	9130401M	0.268786	4.02313	1.964015	0.05267	-4.4659	0.387289	0.305394
B.cells	FBF1	0.943441	1.539498	1.963528	0.052727	-3.94742	0.40296	0.327408
B.cells	ARHGAP15	0.17344	9.872031	1.963514	0.052729	-5.45439	0.353131	0.259651
B.cells	SPRED2	-0.23888	6.335393	-1.96313	0.052774	-4.80565	0.373336	0.286557
B.cells	ANKRD13D	-0.47317	2.738056	-1.96311	0.052776	-4.1448	0.395307	0.316736
B.cells	SESN1	0.230293	5.939971	1.962766	0.052817	-4.78633	0.375734	0.289798
B.cells	NLRC5	0.513593	5.064856	1.962038	0.052903	-4.66053	0.381197	0.297076
B.cells	VAMP7	-0.28686	4.132998	-1.96195	0.052914	-4.44184	0.386885	0.304883
B.cells	YWHAQ	-0.14991	7.746706	-1.96133	0.052987	-5.0629	0.365645	0.275833
B.cells	BVHT	1.119787	-0.0458	1.960161	0.053126	-3.87522	0.414771	0.34311
B.cells	LNPEP	0.179518	7.335044	1.959202	0.05324	-5.02938	0.369315	0.279665
B.cells	HMMR	-0.6252	4.333361	-1.9586	0.053312	-4.39582	0.387574	0.304293
B.cells	RMDN3	0.327564	3.398108	1.957869	0.053399	-4.28752	0.393783	0.312528
B.cells	1700102P0	-0.99538	0.812589	-1.95759	0.053432	-3.88869	0.410414	0.3359
B.cells	CAVIN2	-0.66096	2.454343	-1.95723	0.053475	-4.03175	0.39983	0.321087
B.cells	E230014E1	-1.04246	-1.38289	-1.95621	0.053597	-3.87819	0.425634	0.357808
B.cells	EIF3L	-0.2061	5.672102	-1.95611	0.053609	-4.75941	0.380343	0.294125
B.cells	JAZF1	-0.51671	3.086872	-1.95357	0.053914	-4.17146	0.398195	0.316954
B.cells	CPQ	-0.34573	5.056734	-1.95334	0.053941	-4.54214	0.385904	0.300081
B.cells	HS1BP3	0.713425	1.087416	1.953134	0.053966	-3.9365	0.411139	0.335152

B.cells	TTF2	0.455498	3.091877	1.952678	0.054021	-4.21756	0.398319	0.317162
B.cells	MYCT1	-0.78454	1.654516	-1.95159	0.054152	-3.94155	0.408312	0.330358
B.cells	ZFR2	0.776467	0.817601	1.95115	0.054206	-3.9236	0.413848	0.338136
B.cells	SLFN4	1.235101	0.48225	1.951006	0.054223	-3.92362	0.416082	0.3413
B.cells	SMTN	-0.72297	2.149503	-1.94958	0.054395	-3.9606	0.406143	0.326371
B.cells	ARHGAP26	0.213183	6.685583	1.949063	0.054458	-5.13532	0.377978	0.287853
B.cells	ELAVL3	-1.08002	1.03829	-1.94891	0.054477	-3.92195	0.413543	0.336866
B.cells	SLC30A1	-0.43477	3.386735	-1.94862	0.054512	-4.32351	0.398308	0.315631
B.cells	ANKRD42	1.282996	0.346625	1.947992	0.054588	-3.88616	0.418449	0.343631
B.cells	EPG5	0.276918	4.420072	1.947758	0.054617	-4.52526	0.392067	0.306951
B.cells	SLC25A38	0.311137	3.517886	1.947312	0.054671	-4.44391	0.397887	0.314926
B.cells	MND1	-0.57896	2.222735	-1.94684	0.054728	-4.07885	0.406359	0.326758
B.cells	NCF2	0.231149	6.576577	1.946394	0.054783	-5.00984	0.379178	0.289586
B.cells	BCDIN3D	0.472426	2.223563	1.946299	0.054794	-4.09274	0.406354	0.326928
B.cells	RNF216	0.159557	6.677461	1.943913	0.055086	-4.99837	0.380243	0.289447
B.cells	SETD7	0.264951	4.9704	1.943657	0.055117	-4.63191	0.390664	0.303579
B.cells	RAB11FIP1	0.494705	4.829945	1.943484	0.055139	-4.47756	0.391536	0.304768
B.cells	STRADB	0.353987	3.238382	1.943172	0.055177	-4.33863	0.401612	0.318726
B.cells	LEPROTL1	0.311653	5.362087	1.941397	0.055395	-4.63563	0.389564	0.301034
B.cells	SNX7	0.889032	1.212304	1.940358	0.055523	-3.92398	0.416623	0.337934
B.cells	GLUD1	-0.16464	7.653063	-1.94033	0.055526	-5.15625	0.376072	0.282483
B.cells	DAZAP1	-0.16586	6.776612	-1.94013	0.055551	-4.95909	0.381308	0.289536
B.cells	NOS2	1.573642	-1.12584	1.939168	0.05567	-3.89807	0.432748	0.360997
B.cells	PGAM1	0.238292	6.892134	1.939098	0.055679	-4.99351	0.380902	0.288857
B.cells	RELB	0.22978	6.088989	1.938605	0.055739	-4.92292	0.385769	0.295526
B.cells	PSME2	0.30891	7.278608	1.938478	0.055755	-5.07632	0.378586	0.285921
B.cells	NEMP2	0.358348	2.854914	1.938394	0.055766	-4.27646	0.406123	0.323403
B.cells	ETFDH	0.232544	4.872939	1.93659	0.055989	-4.65699	0.394487	0.306398
B.cells	FRMD5	-1.00253	2.390592	-1.93574	0.056095	-4.1157	0.410404	0.328641
B.cells	ME1	-1.20582	-0.00982	-1.93571	0.056099	-3.90363	0.426509	0.351307
B.cells	CYP2J5	-0.9416	1.06057	-1.93552	0.056122	-3.95136	0.419239	0.341044
B.cells	IGFBP3	-1.05552	0.690335	-1.93544	0.056133	-3.92761	0.421738	0.34457
B.cells	FAM104A	0.188483	6.153765	1.935324	0.056147	-4.9017	0.386558	0.295983
B.cells	ST6GALNA(	0.345069	3.867281	1.935069	0.056178	-4.4534	0.400847	0.315489
B.cells	TAPBPL	0.327874	4.125165	1.933159	0.056417	-4.51475	0.400083	0.313923
B.cells	SFPQ	-0.14822	8.189863	-1.93307	0.056427	-5.19799	0.375161	0.280355
B.cells	GM35154	-0.82666	1.526848	-1.93291	0.056448	-4.02395	0.417033	0.337556
B.cells	SCAI	0.312685	4.434969	1.932489	0.056501	-4.5902	0.398116	0.311412
B.cells	ATP6V0A2	0.23583	4.619482	1.93226	0.056529	-4.64239	0.39695	0.309928
B.cells	NMT2	0.202942	5.47269	1.93197	0.056565	-4.77845	0.39161	0.302661
B.cells	SH3KBP1	0.161307	7.863843	1.931937	0.05657	-5.17344	0.377091	0.28317
B.cells	NUAK2	0.245267	4.289152	1.931592	0.056613	-4.71625	0.39904	0.312841
B.cells	IL18RAP	1.054411	1.983582	1.93137	0.056641	-3.96976	0.413994	0.333658
B.cells	TRPM2	0.789537	2.540831	1.93128	0.056652	-4.06635	0.410321	0.328535
B.cells	FBXL3	0.209198	5.215741	1.93104	0.056682	-4.73192	0.393209	0.305053
B.cells	9930111J2	0.405511	4.28297	1.930169	0.056792	-4.56442	0.399606	0.313302
B.cells	NICN1	-0.95669	0.820121	-1.92882	0.056961	-3.92639	0.423344	0.345457

B.cells	GM45715	0.759697	1.039938	1.928421	0.057012	-3.95487	0.421947	0.34352
B.cells	PGM1	0.2968	4.592397	1.927229	0.057162	-4.58912	0.398676	0.311403
B.cells	GCDH	-0.56107	3.116618	-1.92715	0.057171	-4.26644	0.408157	0.324463
B.cells	ARNTL	0.298955	5.38742	1.926952	0.057197	-4.78335	0.393676	0.304684
B.cells	CALD1	-0.53748	3.777745	-1.9269	0.057204	-4.38471	0.403878	0.318647
B.cells	FUCA2	-0.27593	4.624115	-1.92652	0.057251	-4.5239	0.398475	0.311349
B.cells	PDE11A	-1.14275	0.072949	-1.92635	0.057274	-3.93414	0.42855	0.353355
B.cells	RAD54B	-0.70936	2.207787	-1.92621	0.057291	-4.07607	0.414127	0.333026
B.cells	IFI208	0.639576	2.936618	1.926206	0.057291	-4.28609	0.409332	0.326343
B.cells	H2-M3	0.403268	4.138363	1.925818	0.057341	-4.5396	0.401565	0.315734
B.cells	CD3E	-0.90298	3.278256	-1.92545	0.057387	-4.14712	0.407106	0.32347
B.cells	GFM2	0.332885	3.590998	1.92543	0.05739	-4.41386	0.405081	0.320667
B.cells	FNDC5	-0.81242	0.474524	-1.92486	0.057462	-4.00481	0.425888	0.34993
B.cells	SCAF1	0.24375	4.690272	1.924488	0.057509	-4.62235	0.398145	0.311303
B.cells	AP5S1	0.536976	2.440536	1.924354	0.057526	-4.12867	0.41268	0.331438
B.cells	LTB	0.386734	3.446775	1.923424	0.057645	-4.53825	0.406103	0.322544
B.cells	URB1	-0.44582	2.176346	-1.92321	0.057673	-4.13323	0.414427	0.33418
B.cells	TCF7L1	-0.66116	3.097343	-1.92319	0.057674	-4.11179	0.408373	0.325727
B.cells	TMEM160	0.199861	5.666014	1.923105	0.057685	-4.83025	0.392028	0.303254
B.cells	FAM20C	-1.05824	1.641237	-1.92288	0.057714	-4.05281	0.417992	0.339265
B.cells	GPR171	-0.37431	4.027921	-1.92191	0.057838	-4.68329	0.402361	0.317851
B.cells	SAT1	-0.21391	8.474503	-1.92182	0.057849	-5.3293	0.375032	0.280884
B.cells	WNT5B	-0.75937	1.562043	-1.92133	0.057912	-3.97818	0.418522	0.340667
B.cells	RBBP7	-0.20977	6.2357	-1.92125	0.057922	-4.89368	0.388508	0.299148
B.cells	STX12	0.207569	5.552332	1.920787	0.057981	-4.81848	0.392735	0.305014
B.cells	GM14029	0.889878	0.297438	1.92073	0.057989	-3.96475	0.427101	0.353017
B.cells	CD2AP	0.237884	6.24029	1.920567	0.058009	-4.95387	0.388479	0.299262
B.cells	PAPLN	-0.81246	0.894565	-1.92038	0.058033	-3.98041	0.423026	0.347265
B.cells	PAPOLA	0.127721	7.055885	1.920328	0.05804	-5.05333	0.383505	0.292558
B.cells	ZCCHC8	0.212805	5.108019	1.920042	0.058077	-4.71941	0.395512	0.308925
B.cells	TNRC18	0.181262	5.931838	1.919826	0.058104	-4.85359	0.39038	0.301921
B.cells	CCDC192	-1.09301	-0.69514	-1.91927	0.058176	-3.9334	0.433896	0.363136
B.cells	KLRB1A	-1.04828	-0.08222	-1.91925	0.058178	-3.95098	0.429716	0.357014
B.cells	WDR86	-0.69239	0.234245	-1.91924	0.058179	-3.97646	0.427535	0.353892
B.cells	E2F8	-0.49668	3.686572	-1.91907	0.058201	-4.31119	0.404554	0.321584
B.cells	SOS1	0.183422	5.916592	1.918736	0.058244	-4.88863	0.390533	0.302302
B.cells	MAGOH	-0.20532	6.296682	-1.91604	0.058591	-4.90595	0.39027	0.299849
B.cells	COL27A1	-0.71283	2.314283	-1.91466	0.05877	-4.18811	0.416596	0.335374
B.cells	RNF157	0.329674	5.375061	1.914248	0.058823	-4.81651	0.396776	0.308128
B.cells	U2AF2	-0.19076	6.245648	-1.91417	0.058832	-4.91031	0.391342	0.300781
B.cells	TUBA1C	-0.26117	7.576145	-1.9138	0.058881	-5.12628	0.383207	0.289955
B.cells	UBE2S	-0.25274	7.624597	-1.91355	0.058914	-5.13128	0.382915	0.28964
B.cells	EVA1A	-0.82287	1.060527	-1.91354	0.058914	-3.98016	0.425046	0.347684
B.cells	SENP7	0.224927	5.356774	1.913192	0.05896	-4.7874	0.396961	0.308582
B.cells	LAMC1	0.308815	4.523339	1.912865	0.059002	-4.67276	0.4023	0.315854
B.cells	IYD	-0.96891	0.499692	-1.91225	0.059082	-3.94927	0.429218	0.353596
B.cells	1700012D1	0.623001	1.843803	1.912115	0.0591	-4.08243	0.420066	0.340673

B.cells	ABHD10	0.291498	3.649409	1.910643	0.059291	-4.42443	0.409103	0.324452
B.cells	IL18R1	0.936592	0.978465	1.910148	0.059356	-3.98405	0.426954	0.349715
B.cells	GBP7	0.595404	4.443583	1.909953	0.059381	-4.56128	0.403964	0.317663
B.cells	LDLRAP1	0.312991	4.099537	1.909736	0.05941	-4.60464	0.406181	0.320771
B.cells	MED12L	-0.41572	3.141983	-1.9093	0.059466	-4.32105	0.412427	0.329558
B.cells	TUBB4B	-0.33057	6.422022	-1.90919	0.059481	-4.93919	0.391488	0.300817
B.cells	TCRG-C2	-1.34772	0.685448	-1.90914	0.059487	-3.98216	0.428966	0.35287
B.cells	KCNMB4	-0.76325	1.763375	-1.90882	0.059529	-4.0678	0.421667	0.342577
B.cells	ITPRIPL1	-0.33794	3.28736	-1.90831	0.059597	-4.38951	0.411745	0.328564
B.cells	FAM129A	-0.35077	5.667894	-1.90795	0.059643	-4.61942	0.39653	0.307604
B.cells	GRPEL2	0.339814	3.176522	1.907381	0.059718	-4.37231	0.412816	0.329914
B.cells	RALGAPB	0.226938	4.920242	1.907124	0.059751	-4.72448	0.401516	0.314378
B.cells	SCAP	-0.32753	4.277309	-1.90636	0.059852	-4.56859	0.405716	0.320316
B.cells	FAM177A	0.617122	1.731422	1.906328	0.059856	-4.087	0.422543	0.343829
B.cells	EPHX2	-0.67667	2.477792	-1.90624	0.059868	-4.19365	0.417527	0.336814
B.cells	GM15283	0.313888	4.261918	1.904889	0.060045	-4.673	0.406346	0.321074
B.cells	CDYL2	0.308408	4.757934	1.90445	0.060103	-4.69547	0.403155	0.31684
B.cells	TRMT61A	0.606766	1.593744	1.90432	0.06012	-4.07667	0.424029	0.346067
B.cells	ARPC5L	0.175441	6.300784	1.904299	0.060123	-4.96691	0.393414	0.303581
B.cells	GBP2	0.899684	4.147241	1.904074	0.060153	-4.53284	0.407088	0.322343
B.cells	UCKL1	0.296269	3.705129	1.904039	0.060157	-4.50999	0.409964	0.326334
B.cells	GM50333	-0.63029	1.191453	-1.90162	0.060477	-4.06638	0.428528	0.350811
B.cells	WDR7	0.272283	5.188411	1.901381	0.060509	-4.78447	0.402056	0.31399
B.cells	IFNG	1.001339	1.241502	1.901366	0.060511	-4.06081	0.428185	0.350433
B.cells	B3GNT5	0.362588	2.546338	1.900432	0.060635	-4.56296	0.419948	0.338216
B.cells	KDM5A	-0.17301	7.014524	-1.89882	0.06085	-5.08898	0.392331	0.299361
B.cells	ZFP518B	0.79215	0.418092	1.898195	0.060933	-3.96755	0.436064	0.359862
B.cells	RPN1	-0.21672	5.957169	-1.89762	0.06101	-4.88252	0.399186	0.308651
B.cells	ZFP768	0.982602	0.794694	1.897528	0.061022	-3.98251	0.433435	0.35634
B.cells	JDP2	-0.5246	5.304027	-1.8973	0.061052	-4.42964	0.403339	0.314419
B.cells	ANKLE2	0.221717	5.177792	1.896998	0.061093	-4.82459	0.404148	0.315592
B.cells	DEFB1	-1.01036	0.86149	-1.89677	0.061124	-4.00638	0.43297	0.355948
B.cells	PVT1	0.38331	5.282726	1.896681	0.061135	-4.84931	0.403475	0.314755
B.cells	STX17	0.313203	4.034327	1.896284	0.061188	-4.5968	0.411678	0.32605
B.cells	FBXW17	0.708444	1.479346	1.894922	0.061371	-4.0597	0.429568	0.350383
B.cells	DSCAM	1.124061	0.029718	1.894753	0.061393	-3.98058	0.43968	0.36489
B.cells	ANG	-0.46492	4.211322	-1.8947	0.061401	-4.6329	0.411231	0.324843
B.cells	VAV3	0.25869	7.363151	1.894348	0.061448	-5.18276	0.391277	0.297671
B.cells	LCT	-1.10963	0.170332	-1.89346	0.061567	-3.96066	0.439364	0.363944
B.cells	UBXN8	0.264339	4.404408	1.892743	0.061664	-4.61492	0.411011	0.323674
B.cells	SOD2	0.413701	6.929524	1.890589	0.061955	-5.12872	0.396173	0.302319
B.cells	WDR48	0.237966	4.298768	1.890372	0.061984	-4.63957	0.413039	0.325343
B.cells	NEK10	-0.75352	1.324395	-1.88994	0.062043	-4.11942	0.433132	0.353473
B.cells	MYO6	-0.51041	3.1857	-1.88983	0.062057	-4.24951	0.420429	0.335676
B.cells	CYP2B9	-0.83949	1.9577	-1.88923	0.062139	-4.14995	0.42876	0.347564
B.cells	IRF9	0.400483	4.515749	1.888828	0.062193	-4.66559	0.411617	0.32373
B.cells	ABCA8A	-1.12175	1.028779	-1.88881	0.062196	-4.01494	0.43519	0.356702

B.cells	FAHD2A	-0.39814	2.845982	-1.88858	0.062226	-4.28042	0.422715	0.339193
B.cells	GTF2IRD1	-0.56935	3.292146	-1.88839	0.062252	-4.23004	0.419715	0.335068
B.cells	ADGRA3	-0.9972	0.459311	-1.88827	0.062269	-3.9668	0.439186	0.362571
B.cells	PAH	-0.64022	3.508909	-1.88807	0.062296	-4.46478	0.418267	0.33317
B.cells	HSPA8	-0.16516	10.08692	-1.88776	0.062338	-5.57504	0.376996	0.277548
B.cells	TMEM265	-0.32761	3.958769	-1.88758	0.062363	-4.52227	0.41528	0.32918
B.cells	ASCC3	0.212295	6.882346	1.887562	0.062365	-5.09901	0.396468	0.303449
B.cells	ACOX1	-0.25108	5.892871	-1.8873	0.062401	-4.92252	0.40272	0.312032
B.cells	TLR12	-0.88445	2.045397	-1.88616	0.062556	-4.07479	0.428374	0.347805
B.cells	NEMF	0.167123	5.95092	1.886122	0.062561	-4.96565	0.402552	0.311992
B.cells	MYL9	0.816454	1.95475	1.886029	0.062574	-4.20987	0.428996	0.348699
B.cells	ACSS1	0.297804	3.946222	1.885974	0.062582	-4.71186	0.415572	0.329906
B.cells	SLC2A6	0.672308	1.781297	1.885758	0.062611	-4.14597	0.430189	0.350507
B.cells	GM9733	1.139132	0.76406	1.884698	0.062756	-4.02657	0.438022	0.360912
B.cells	MAPK9	0.233173	4.751185	1.883624	0.062903	-4.73447	0.411726	0.32326
B.cells	SLC6A19	-0.98248	-0.35949	-1.8811	0.06325	-3.9879	0.448617	0.373904
B.cells	GM38843	-0.89456	0.985485	-1.881	0.063263	-4.02808	0.439026	0.360235
B.cells	CUX2	-0.84684	1.039079	-1.88097	0.063268	-4.01362	0.438649	0.359703
B.cells	GCNT1	0.511107	2.451925	1.8803	0.06336	-4.20329	0.429214	0.346145
B.cells	PSME2B	0.453666	2.815452	1.879675	0.063446	-4.37488	0.426868	0.34281
B.cells	SELENOW	0.201935	7.046449	1.879493	0.063471	-5.16655	0.399133	0.304763
B.cells	GM4869	-0.8139	1.447403	-1.87936	0.063489	-4.0694	0.436313	0.356226
B.cells	ETFB	-0.23556	6.979015	-1.87899	0.063541	-5.09092	0.399658	0.305561
B.cells	MTM1	0.331646	4.433358	1.877869	0.063696	-4.68639	0.416462	0.328252
B.cells	RNASEL	0.492046	3.944163	1.877543	0.063741	-4.43503	0.419717	0.332909
B.cells	ELF2	0.166048	7.39491	1.877359	0.063766	-5.1991	0.397383	0.302465
B.cells	MTFP1	-0.99351	0.572509	-1.8773	0.063774	-3.99506	0.442969	0.365689
B.cells	2010315B0	0.483483	2.024895	1.87729	0.063776	-4.19274	0.432774	0.351225
B.cells	CREB3L1	-0.67375	2.035248	-1.8762	0.063926	-4.10449	0.433467	0.351533
B.cells	FNBP1L	-0.50961	3.079155	-1.87596	0.063961	-4.36722	0.426296	0.341551
B.cells	GM17018	0.343784	3.620053	1.874731	0.064131	-4.47076	0.423521	0.337059
B.cells	C1QA	-0.60314	5.480946	-1.87363	0.064285	-4.79007	0.411927	0.320504
B.cells	RBM4	-0.21932	4.655948	-1.87335	0.064324	-4.71772	0.417375	0.328034
B.cells	G6PDX	0.295034	4.072997	1.87248	0.064445	-4.56293	0.421698	0.333752
B.cells	TRIM30D	0.415724	4.544173	1.872186	0.064486	-4.71698	0.418549	0.329496
B.cells	DBN1	-0.72733	1.388658	-1.87209	0.0645	-4.16806	0.440172	0.359758
B.cells	R3HCC1L	0.207833	5.540599	1.871182	0.064627	-4.92265	0.412563	0.320638
B.cells	XAF1	0.602754	4.033526	1.870546	0.064716	-4.58105	0.422904	0.33467
B.cells	H2-DMA	0.326997	5.184297	1.870104	0.064778	-5.00137	0.415403	0.324276
B.cells	EFCAB14	0.276624	4.713606	1.869676	0.064839	-4.70695	0.418558	0.328651
B.cells	LPCAT1	0.276892	4.107714	1.869543	0.064857	-4.67239	0.42261	0.334267
B.cells	LRIG2	0.277939	4.256808	1.868597	0.06499	-4.66038	0.421787	0.333104
B.cells	IKBKG	0.344744	3.339723	1.868504	0.065003	-4.43	0.427994	0.341717
B.cells	RGP1	0.469994	2.34849	1.868407	0.065017	-4.15736	0.434822	0.351293
B.cells	ABCB11	-0.71554	1.681529	-1.86831	0.065031	-4.13882	0.439488	0.357874
B.cells	DCUN1D1	0.202132	5.787878	1.86748	0.065148	-4.97214	0.412168	0.319423
B.cells	GADD45B	-0.34746	5.322838	-1.86547	0.065432	-4.845	0.416271	0.324258

B.cells	BEX3	-0.27024	4.990762	-1.86546	0.065433	-4.77175	0.418471	0.327269
B.cells	LCA5	0.730099	1.313513	1.865331	0.065452	-4.08511	0.443757	0.362561
B.cells	2210016L2	0.311307	3.97848	1.864643	0.065549	-4.60594	0.42526	0.336797
B.cells	HNMT	-0.98518	-0.38409	-1.86461	0.065553	-3.99729	0.456024	0.380216
B.cells	FCGR4	0.909798	3.388468	1.864547	0.065563	-4.3258	0.429277	0.342387
B.cells	ADAM15	0.643801	2.475369	1.864498	0.06557	-4.20765	0.43558	0.351196
B.cells	DHX30	0.256142	4.509922	1.863751	0.065676	-4.72533	0.421859	0.332034
B.cells	PTPN1	0.204603	7.078745	1.863373	0.065729	-5.18258	0.405038	0.309295
B.cells	NAT8F1	-0.81522	1.619362	-1.8626	0.065839	-4.11386	0.441775	0.360312
B.cells	ZFP110	0.295129	4.039873	1.862019	0.065922	-4.60303	0.425025	0.337113
B.cells	SMCHD1	0.198318	7.994517	1.861979	0.065928	-5.35466	0.399234	0.301979
B.cells	ARHGAP42	-0.68791	1.752056	-1.86193	0.065936	-4.17241	0.440838	0.359277
B.cells	ABHD18	0.437211	3.182254	1.861857	0.065945	-4.39832	0.430874	0.345277
B.cells	ABCG3	0.405146	4.394356	1.861809	0.065952	-4.63773	0.422635	0.333837
B.cells	LARS2	-0.41027	5.571824	-1.8617	0.065967	-4.94691	0.414807	0.323108
B.cells	KIFC1	-0.71271	3.200672	-1.86115	0.066046	-4.30521	0.430747	0.345264
B.cells	THYN1	0.376121	3.271604	1.860602	0.066124	-4.38048	0.43026	0.344841
B.cells	GM17023	1.109314	-0.64235	1.860585	0.066127	-4.03038	0.458051	0.384458
B.cells	SMURF1	0.258036	4.688664	1.860491	0.06614	-4.75483	0.420662	0.331522
B.cells	NXF1	-0.21767	5.180351	-1.86048	0.066142	-4.80796	0.417391	0.327014
B.cells	FAM107B	0.187611	7.858238	1.860215	0.06618	-5.3265	0.400092	0.303605
B.cells	HIST1H2BC	-0.36557	5.260139	-1.86018	0.066184	-4.79066	0.416863	0.326378
B.cells	4930445E1	1.015717	-0.41363	1.858653	0.066403	-4.00602	0.457136	0.382839
B.cells	TANC2	-0.34824	5.521032	-1.85863	0.066407	-4.81281	0.415779	0.324601
B.cells	FAM217B	0.864631	0.616789	1.858461	0.066431	-4.0503	0.449626	0.372125
B.cells	KDM4C	0.195631	6.252217	1.858448	0.066433	-5.03726	0.410993	0.318128
B.cells	EBAG9	0.264914	4.117531	1.85675	0.066677	-4.64971	0.426478	0.338393
B.cells	RAB19	0.432047	2.561986	1.85641	0.066726	-4.42795	0.437201	0.353476
B.cells	GPR84	1.046329	0.260814	1.855873	0.066803	-4.0297	0.453625	0.377002
B.cells	B230369F2	-0.46291	2.457789	-1.8557	0.066828	-4.28595	0.43793	0.354751
B.cells	ELMO1	0.159484	8.719508	1.855681	0.066831	-5.43625	0.396558	0.298011
B.cells	QPRT	-0.61006	2.660001	-1.85536	0.066877	-4.29525	0.436517	0.352856
B.cells	EFNB1	-0.69625	1.912975	-1.85514	0.066909	-4.11609	0.441763	0.360342
B.cells	FAH	-0.52913	3.892683	-1.85495	0.066936	-4.62137	0.428018	0.341033
B.cells	GM36447	-1.08386	0.209364	-1.85453	0.066997	-4.01369	0.454111	0.377974
B.cells	CD59A	-0.47582	2.984691	-1.85433	0.067026	-4.3489	0.434365	0.350001
B.cells	SNX22	-0.89456	-0.37781	-1.85292	0.067229	-4.05571	0.459104	0.384949
B.cells	OSBPL9	0.139856	7.511715	1.852895	0.067233	-5.27069	0.404868	0.3091
B.cells	HERPUD2	-0.18668	5.474289	-1.85287	0.067237	-4.89069	0.41812	0.32714
B.cells	CLOCK	0.219394	5.071637	1.8526	0.067276	-4.8973	0.420813	0.330905
B.cells	L1CAM	-0.43817	3.644556	-1.85106	0.0675	-4.52676	0.431311	0.344644
B.cells	TGFBI	-0.56519	4.412001	-1.85051	0.06758	-4.49334	0.426074	0.337641
B.cells	PRLR	-0.66661	3.152323	-1.8504	0.067595	-4.39119	0.43471	0.349706
B.cells	CDON	-0.58058	1.952332	-1.85025	0.067618	-4.26467	0.443127	0.361652
B.cells	DCLRE1C	0.212487	6.497189	1.850192	0.067626	-5.09343	0.412218	0.318699
B.cells	DARS2	0.630825	2.849125	1.850017	0.067651	-4.26702	0.436819	0.352838
B.cells	BLZF1	-0.33595	3.200504	-1.84992	0.067666	-4.467	0.434376	0.349437

B.cells	TEX9	0.578126	1.820323	1.849498	0.067727	-4.23207	0.444097	0.363191
B.cells	AKR1C13	-0.68521	1.84784	-1.84936	0.067747	-4.1519	0.443901	0.362914
B.cells	H2-Q4	0.370174	4.933329	1.849122	0.067782	-4.84196	0.42259	0.333122
B.cells	HCK	0.266852	5.693071	1.848428	0.067883	-5.11083	0.417713	0.326336
B.cells	SNRNP70	-0.10784	7.511442	-1.84835	0.067895	-5.25952	0.405881	0.310227
B.cells	CD226	0.888548	1.697205	1.848061	0.067937	-4.16875	0.445171	0.364786
B.cells	NFKBIL1	0.28104	3.861655	1.84738	0.068036	-4.57603	0.430045	0.343787
B.cells	ABCB9	-0.3787	3.155352	-1.84735	0.068041	-4.5491	0.434914	0.350611
B.cells	GPD2	0.278988	6.625661	1.846818	0.068119	-5.20591	0.411595	0.31857
B.cells	ABCA13	-1.16958	0.73813	-1.84676	0.068127	-4.07747	0.452069	0.37527
B.cells	CUL1	0.152258	6.562305	1.846742	0.06813	-5.1144	0.412007	0.319132
B.cells	CYP2A22	-0.77935	1.632221	-1.84661	0.068149	-4.16062	0.445634	0.366121
B.cells	CFHR1	-1.08021	0.449486	-1.84635	0.068187	-4.0544	0.454169	0.378456
B.cells	MRPL52	0.180976	7.102833	1.846134	0.068219	-5.23344	0.408505	0.314545
B.cells	S100A16	-0.57237	2.552707	-1.84567	0.068287	-4.22109	0.439317	0.357181
B.cells	STAB2	-0.67314	5.148934	-1.84527	0.068346	-4.66065	0.421594	0.332381
B.cells	CLEC4N	0.648395	4.093598	1.844849	0.068408	-4.5129	0.428725	0.342433
B.cells	HOMEZ	0.700706	1.333922	1.844838	0.068409	-4.09128	0.448047	0.369753
B.cells	MEFV	1.005765	-0.03339	1.844258	0.068495	-4.06008	0.4581	0.38427
B.cells	6030468B1	-1.10828	0.279697	-1.84421	0.068502	-4.04424	0.4558	0.380945
B.cells	SIRT5	-0.63416	1.958147	-1.84389	0.068549	-4.15266	0.443694	0.363676
B.cells	IL4	-1.40196	0.330173	-1.84352	0.068603	-4.05415	0.455431	0.380649
B.cells	FBLIM1	-1.0325	0.928973	-1.84347	0.068611	-4.06691	0.451073	0.37439
B.cells	USP44	-0.95704	-0.6309	-1.84308	0.068668	-4.02545	0.462597	0.391213
B.cells	GYG	0.24708	6.126163	1.842431	0.068764	-5.08481	0.415512	0.324487
B.cells	MASTL	-0.327	3.782	-1.84238	0.068771	-4.58835	0.431268	0.346375
B.cells	TRMT11	0.296673	3.646744	1.841514	0.068899	-4.57485	0.432566	0.348081
B.cells	GM38832	0.792285	0.763984	1.841486	0.068904	-4.09027	0.452978	0.377132
B.cells	FOXN3	0.182925	8.899803	1.8409	0.06899	-5.51936	0.398248	0.300887
B.cells	GM9725	0.58625	2.030059	1.840563	0.06904	-4.26922	0.44407	0.3644
B.cells	ACADS	-0.25945	4.490963	-1.84052	0.069046	-4.7249	0.426976	0.340284
B.cells	CCNG1	0.235337	4.873028	1.840283	0.069082	-4.80074	0.424392	0.33669
B.cells	GRN	-0.25459	7.188808	-1.83968	0.069171	-5.18278	0.409275	0.315748
B.cells	GZMC	1.740524	1.539474	1.839602	0.069183	-4.13218	0.447745	0.369504
B.cells	PTPN3	-0.88084	1.040176	-1.83879	0.069304	-4.11261	0.451891	0.375049
B.cells	NLRC3	0.571017	1.662225	1.837929	0.069431	-4.21484	0.447843	0.368859
B.cells	CYP4A31	-0.91232	0.922941	-1.83783	0.069445	-4.09822	0.453182	0.376541
B.cells	GNS	0.19982	6.931874	1.837186	0.069542	-5.23132	0.411854	0.318726
B.cells	CREB3L2	-0.41963	3.55229	-1.83656	0.069635	-4.49068	0.434538	0.350356
B.cells	ZFP236	0.231293	4.729945	1.836229	0.069685	-4.80747	0.426471	0.339183
B.cells	RCAN1	0.333166	3.708543	1.835963	0.069724	-4.55294	0.433458	0.349063
B.cells	WDR81	-0.30394	3.600126	-1.83595	0.069726	-4.59207	0.434207	0.350117
B.cells	4930595D1	0.60353	1.248821	1.835707	0.069763	-4.23075	0.450838	0.373827
B.cells	SUMO1	-0.09681	7.992553	-1.83564	0.069772	-5.35913	0.405025	0.309862
B.cells	GM32051	0.832167	-0.14913	1.835572	0.069783	-4.14679	0.461074	0.388624
B.cells	CMC1	0.231887	4.601302	1.83557	0.069783	-4.74657	0.427343	0.340541
B.cells	TBC1D7	-0.45008	2.220043	-1.8341	0.070003	-4.2364	0.443953	0.364347

B.cells	STFA3	1.005168	2.751543	1.834059	0.070009	-4.28815	0.440197	0.359
B.cells	MAP2K3	-0.25855	6.01457	-1.83402	0.070016	-5.02081	0.417938	0.327832
B.cells	GM26542	-0.26771	4.802534	-1.83375	0.070056	-4.85251	0.426048	0.339115
B.cells	MICALL1	0.324159	2.97359	1.833705	0.070062	-4.50703	0.438639	0.356823
B.cells	GM43063	-0.76004	0.779437	-1.83369	0.070065	-4.10009	0.454319	0.379266
B.cells	ZMYND11	0.16812	6.466516	1.833561	0.070084	-5.1167	0.41496	0.323768
B.cells	GM34225	-1.08767	-0.06364	-1.83351	0.070092	-4.04557	0.460515	0.388266
B.cells	ITGB3	0.417267	2.991713	1.833089	0.070155	-4.46303	0.438672	0.356838
B.cells	ARRB1	0.304078	3.996665	1.832688	0.070215	-4.57046	0.431842	0.347079
B.cells	TMEM42	0.392034	2.617603	1.831337	0.070418	-4.34315	0.442488	0.361171
B.cells	GM525	-0.70649	0.742971	-1.82969	0.070667	-4.09146	0.457341	0.380971
B.cells	PPFIBP1	-0.39009	3.366596	-1.8294	0.070711	-4.43958	0.438583	0.354273
B.cells	MESD	-0.26382	4.085411	-1.82909	0.070757	-4.61698	0.433641	0.347378
B.cells	UBXN4	0.124663	6.814787	1.828631	0.070827	-5.17363	0.415465	0.322083
B.cells	NFATC1	0.208784	5.77824	1.828231	0.070887	-5.02314	0.42247	0.331667
B.cells	UCP1	-0.96313	-1.00774	-1.82754	0.070992	-4.04411	0.471124	0.400871
B.cells	FRMD4A	-0.55317	3.969412	-1.82732	0.071025	-4.41581	0.43517	0.34912
B.cells	TMEM9B	0.186143	5.558526	1.826416	0.071163	-4.95189	0.424917	0.334304
B.cells	LSAMP	-0.88843	0.929492	-1.82575	0.071263	-4.10449	0.457883	0.380465
B.cells	DBNDD2	-0.42252	2.836435	-1.82513	0.071359	-4.4634	0.444437	0.361051
B.cells	CD209G	-2.04284	1.089148	-1.82492	0.07139	-4.11716	0.457041	0.379071
B.cells	RASSF2	0.28323	4.259468	1.824331	0.07148	-4.77124	0.43479	0.34734
B.cells	COL20A1	-0.61741	1.340253	-1.82373	0.071571	-4.12137	0.455881	0.376949
B.cells	SLC30A9	0.195617	5.265307	1.822748	0.071722	-4.93008	0.428689	0.338333
B.cells	SLC22A23	-0.68724	3.017213	-1.82272	0.071725	-4.37605	0.444303	0.36018
B.cells	DEF6	0.200234	5.457612	1.821932	0.071847	-4.98502	0.427881	0.336726
B.cells	XPA	0.286894	4.138354	1.821283	0.071946	-4.72823	0.437061	0.349611
B.cells	ARNT	0.230077	5.869604	1.820952	0.071997	-5.06992	0.425212	0.333359
B.cells	PSD4	0.265168	4.541706	1.820902	0.072004	-4.78145	0.434266	0.345914
B.cells	LIMD2	0.195863	7.333227	1.820819	0.072017	-5.29778	0.41549	0.320049
B.cells	UBQLN2	-0.26413	4.031371	-1.82036	0.072088	-4.65435	0.438004	0.350991
B.cells	NUDT14	0.238021	4.342372	1.819306	0.07225	-4.75526	0.43642	0.348166
B.cells	0610009L1	0.614668	2.030404	1.818905	0.072311	-4.21539	0.452818	0.371454
B.cells	ZBTB22	0.394707	3.076444	1.818351	0.072397	-4.46769	0.445311	0.360891
B.cells	ACAA2	-0.31604	5.01941	-1.81834	0.072398	-4.89356	0.43175	0.341893
B.cells	GNG7	-1.19595	-0.40318	-1.81803	0.072446	-4.0551	0.470853	0.397653
B.cells	BNIP3L	0.208692	7.263429	1.817964	0.072456	-5.34825	0.416688	0.32131
B.cells	ZCCHC4	0.323728	3.455439	1.817816	0.072479	-4.66591	0.442628	0.357285
B.cells	GDPD5	-0.73984	1.750804	-1.81777	0.072486	-4.16367	0.454849	0.374647
B.cells	TMEM150A	-0.60622	2.327871	-1.81723	0.07257	-4.29084	0.450956	0.368865
B.cells	ARG1	0.57929	3.498182	1.816614	0.072665	-4.6278	0.442956	0.357219
B.cells	PSMG1	-0.33941	3.325411	-1.81597	0.072765	-4.47547	0.444367	0.359108
B.cells	TEX261	0.196184	5.229001	1.815724	0.072803	-4.92398	0.431112	0.34059
B.cells	NCKAP5	-0.96052	1.217214	-1.81555	0.07283	-4.11351	0.459604	0.380929
B.cells	CCDC71	0.420836	2.73695	1.81539	0.072854	-4.40244	0.44856	0.365209
B.cells	DMAC2	0.360298	3.093682	1.81519	0.072885	-4.53959	0.446013	0.361676
B.cells	ALG8	-0.42248	3.226775	-1.81399	0.073071	-4.44789	0.445969	0.360779

B.cells	MYB	-0.31943	5.137938	-1.8136	0.073133	-4.87517	0.432751	0.342188
B.cells	CACNB4	-0.71277	1.615307	-1.81331	0.073177	-4.3048	0.457797	0.377489
B.cells	NR2C1	0.534372	2.276051	1.811525	0.073455	-4.31658	0.454465	0.371491
B.cells	NCOA2	-0.17211	7.684552	-1.81082	0.073565	-5.37924	0.417478	0.319857
B.cells	9230114K1	-0.46523	2.574674	-1.80997	0.073698	-4.27733	0.453105	0.368898
B.cells	SLC36A3	-0.83197	0.101985	-1.80995	0.0737	-4.12385	0.471424	0.395136
B.cells	GM11464	-0.99365	-0.20123	-1.80934	0.073797	-4.07118	0.474027	0.398608
B.cells	SIN3A	0.19548	5.871572	1.809121	0.073831	-5.03343	0.430222	0.336759
B.cells	TNFAIP6	0.917075	1.129558	1.808112	0.073989	-4.1937	0.464002	0.384761
B.cells	MTUS1	-0.52212	3.721745	-1.80793	0.074017	-4.53465	0.445168	0.358001
B.cells	TFEB	0.217375	5.158966	1.807929	0.074018	-4.96701	0.43511	0.343961
B.cells	POFUT1	0.319454	3.458205	1.807695	0.074055	-4.55534	0.447041	0.360687
B.cells	SPG21	-0.16626	6.057126	-1.80768	0.074057	-5.15166	0.42896	0.335515
B.cells	TAX1BP1	0.148516	8.297296	1.807472	0.07409	-5.49584	0.414065	0.315266
B.cells	SNX18	0.256184	6.77742	1.80739	0.074103	-5.09242	0.424102	0.328909
B.cells	GNPTAB	0.276866	4.589659	1.8067	0.074211	-4.81478	0.439062	0.349795
B.cells	STAT5B	0.200676	6.043742	1.806695	0.074212	-5.10258	0.429051	0.335918
B.cells	SPTBN5	-1.00456	-0.00378	-1.80662	0.074224	-4.08577	0.472524	0.397471
B.cells	MFSD6	-0.30342	4.864614	-1.80638	0.074262	-4.95605	0.437148	0.347248
B.cells	MRPS6	-0.21136	6.008603	-1.80625	0.074282	-5.04613	0.42929	0.336398
B.cells	ARRB2	-0.28984	5.195443	-1.80546	0.074406	-4.77833	0.43536	0.344299
B.cells	MPZL1	-0.6959	2.78686	-1.80484	0.074504	-4.3	0.452701	0.368409
B.cells	MAPK8IP3	0.222955	4.521476	1.80441	0.074572	-4.82935	0.440359	0.351237
B.cells	INHBC	-0.93645	0.808252	-1.80415	0.074612	-4.12531	0.467272	0.389494
B.cells	DOCK3	-0.91358	0.039329	-1.80413	0.074617	-4.11713	0.473079	0.397898
B.cells	PAN2	0.335087	3.190864	1.803915	0.07465	-4.49825	0.449791	0.364529
B.cells	CCZ1	0.165095	6.158644	1.803175	0.074767	-5.13408	0.429524	0.335831
B.cells	TRBC2	-0.99105	3.943953	-1.80213	0.074932	-4.42543	0.445346	0.357432
B.cells	GM41496	-0.91337	0.985043	-1.80204	0.074947	-4.13202	0.466914	0.388089
B.cells	PEMT	-0.45678	3.483706	-1.80197	0.074959	-4.61394	0.448623	0.362058
B.cells	LTV1	0.280724	4.232009	1.801439	0.075043	-4.78286	0.443579	0.354752
B.cells	C1QB	-0.49372	6.51237	-1.80098	0.075116	-5.13135	0.427908	0.333034
B.cells	IZUMO4	0.753697	1.453448	1.800862	0.075134	-4.2248	0.463795	0.38342
B.cells	STK39	-0.72889	3.517793	-1.80038	0.075211	-4.34226	0.448973	0.362284
B.cells	OLR1	1.21886	1.333367	1.799981	0.075274	-4.16031	0.465079	0.385167
B.cells	RNF138	0.175489	5.857254	1.799693	0.07532	-5.08114	0.432774	0.339694
B.cells	C79798	-0.48126	1.77973	-1.79934	0.075377	-4.36263	0.461925	0.380736
B.cells	TMEM35B	0.62577	2.207613	1.799085	0.075417	-4.28862	0.458782	0.376351
B.cells	GM15859	0.597026	1.208039	1.798481	0.075513	-4.17499	0.466365	0.387217
B.cells	GM5617	-0.41028	3.238502	-1.79836	0.075532	-4.46204	0.451463	0.365972
B.cells	NDUFS4	0.163998	6.335519	1.798058	0.075581	-5.17377	0.429791	0.33585
B.cells	TSPAN5	0.228286	5.373256	1.797709	0.075637	-5.06883	0.436391	0.345008
B.cells	CKS1B	-0.38994	5.377173	-1.7977	0.075639	-4.92999	0.436364	0.34497
B.cells	ENPP2	-0.63136	2.411456	-1.79693	0.075761	-4.31901	0.457974	0.374919
B.cells	MKLN1OS	-0.95458	0.018205	-1.79654	0.075823	-4.10719	0.476026	0.400913
B.cells	PLEKHM2	0.290771	5.202185	1.796314	0.07586	-4.98628	0.438189	0.347218
B.cells	LIX1	-0.90946	0.723268	-1.79583	0.075938	-4.12209	0.470823	0.393422

B.cells	MCM7	-0.36099	5.384269	-1.79567	0.075963	-4.9472	0.437072	0.345585
B.cells	MID1IP1	-0.349	3.917342	-1.7953	0.076022	-4.65651	0.447504	0.360162
B.cells	CCNB2	-0.47338	5.882225	-1.79486	0.076093	-5.01511	0.433944	0.341123
B.cells	RIPK2	0.284547	4.205503	1.794113	0.076213	-4.87191	0.446125	0.35772
B.cells	CIAO2B	0.243667	4.566233	1.793266	0.07635	-4.87657	0.444142	0.354454
B.cells	TPRA1	-0.41005	2.802051	-1.79273	0.076435	-4.45236	0.457084	0.372523
B.cells	LY96	-0.2807	4.287348	-1.79247	0.076479	-4.80226	0.446417	0.357489
B.cells	FKBP11	-0.49121	1.995525	-1.79186	0.076577	-4.30126	0.46323	0.381212
B.cells	STX5A	0.159225	5.871144	1.791797	0.076587	-5.10431	0.43551	0.342236
B.cells	DDX19B	0.287373	3.529612	1.791478	0.076638	-4.66373	0.452093	0.365302
B.cells	IGFBP7	-0.39016	5.173401	-1.78998	0.076881	-4.86621	0.4416	0.349482
B.cells	NUFIP1	0.227265	4.171093	1.789106	0.077022	-4.82386	0.449104	0.359666
B.cells	XKR5	-0.86046	0.190356	-1.78872	0.077086	-4.10868	0.478629	0.401873
B.cells	KANK2	-0.60937	2.38565	-1.7887	0.077089	-4.27874	0.462078	0.378091
B.cells	STAT3	0.184509	7.950149	1.788582	0.077107	-5.4509	0.42303	0.32385
B.cells	PGAP2	0.234358	5.75264	1.787488	0.077285	-5.07725	0.438269	0.344538
B.cells	GM10974	0.61824	0.824708	1.787372	0.077304	-4.23726	0.474104	0.395195
B.cells	TNIP1	0.285106	4.966974	1.786976	0.077368	-4.98691	0.443765	0.352312
B.cells	CDK19	0.20521	6.390727	1.786832	0.077392	-5.18211	0.433865	0.338649
B.cells	CFAP53	-0.88682	0.830789	-1.78672	0.077411	-4.18493	0.474058	0.395378
B.cells	ALDH1B1	0.580199	1.910302	1.786667	0.077419	-4.24549	0.465926	0.383695
B.cells	TIMD2	-0.7919	0.986598	-1.78658	0.077433	-4.16913	0.472874	0.393676
B.cells	RGS2	-0.21276	6.977171	-1.78623	0.077489	-5.30571	0.429964	0.333352
B.cells	WDR90	-0.61146	1.959845	-1.78586	0.077551	-4.24335	0.465704	0.383424
B.cells	SRSF11	0.105628	7.871425	1.785718	0.077574	-5.4459	0.42398	0.325265
B.cells	NAPSA	0.20216	6.459379	1.785315	0.077639	-5.40228	0.433532	0.338412
B.cells	SP110	0.238217	5.924125	1.785193	0.077659	-5.14026	0.437219	0.343508
B.cells	CSGALNAC	0.241595	4.10884	1.78393	0.077866	-4.78993	0.450002	0.361751
B.cells	EIF4A1	-0.14754	7.526831	-1.78386	0.077878	-5.36272	0.426288	0.328918
B.cells	BRWD3	0.241772	5.109603	1.78361	0.077918	-4.96297	0.442901	0.35183
B.cells	SELENBP1	0.395594	3.795416	1.783461	0.077943	-4.72445	0.452253	0.364999
B.cells	NSMF	0.436013	2.696993	1.783389	0.077955	-4.42398	0.460247	0.376327
B.cells	PPIB	-0.16182	7.605608	-1.78306	0.078009	-5.39157	0.425759	0.328375
B.cells	SRXN1	0.761953	1.094676	1.782901	0.078035	-4.16498	0.472204	0.3936
B.cells	HNRNPR	0.152904	6.29021	1.782789	0.078053	-5.15521	0.434693	0.340639
B.cells	MORN1	-0.68805	1.227517	-1.78262	0.078081	-4.19287	0.4712	0.392179
B.cells	MASP1	-0.7907	1.673868	-1.7826	0.078084	-4.2047	0.467841	0.387344
B.cells	TRAK2	0.222497	5.153104	1.782344	0.078126	-4.98924	0.442595	0.3517
B.cells	CD300LD	-0.67199	3.173108	-1.78223	0.078145	-4.40765	0.456762	0.371665
B.cells	TRP53RKA	0.432627	2.472703	1.781694	0.078233	-4.38419	0.4619	0.379061
B.cells	STAMBP	0.371998	3.374422	1.781628	0.078244	-4.57201	0.455298	0.369681
B.cells	PPARGC1B	0.45532	2.799015	1.78162	0.078245	-4.47008	0.459498	0.375647
B.cells	DHX34	0.469721	2.203437	1.781448	0.078273	-4.37478	0.463893	0.381978
B.cells	GRAP2	0.304273	4.975054	1.781112	0.078328	-5.09965	0.443909	0.353781
B.cells	C130050O	0.750664	1.379067	1.78091	0.078362	-4.19995	0.47012	0.391027
B.cells	FAF2	0.201196	5.155771	1.780325	0.078458	-4.99642	0.442677	0.352163
B.cells	ATXN7	0.197375	6.23066	1.780292	0.078463	-5.17441	0.435201	0.341781

B.cells	DYRK3	-0.52727	2.520243	-1.78008	0.078498	-4.46856	0.461653	0.379091
B.cells	NTAN1	-0.1446	6.261702	-1.77989	0.07853	-5.18707	0.434988	0.341667
B.cells	GM29707	-0.88677	-0.46354	-1.77969	0.078563	-4.10971	0.484278	0.412012
B.cells	TTC23	-0.77277	1.101964	-1.77859	0.078745	-4.17647	0.47284	0.394904
B.cells	GM34466	-1.05692	-1.10689	-1.77845	0.078768	-4.10249	0.48975	0.41993
B.cells	AB124611	0.226838	5.452214	1.778392	0.078777	-5.07902	0.441145	0.349941
B.cells	CD3EAP	0.356966	3.231663	1.778115	0.078823	-4.60176	0.457044	0.372371
B.cells	KCNN4	-0.33056	3.850661	-1.77728	0.078961	-4.92683	0.453127	0.366344
B.cells	ARG2	0.645389	3.174154	1.77636	0.079114	-4.42434	0.458697	0.373651
B.cells	MAP3K11	-0.30645	3.876975	-1.77609	0.079158	-4.70062	0.453616	0.366499
B.cells	KTN1	0.166182	5.826158	1.77574	0.079217	-5.13321	0.439899	0.347257
B.cells	DAND5	-0.56962	3.96611	-1.77444	0.079433	-4.6121	0.454098	0.366097
B.cells	GM12940	-0.27697	4.51886	-1.77309	0.079658	-4.85109	0.450892	0.360969
B.cells	EIF5B	0.142554	7.158051	1.772966	0.079678	-5.3436	0.432434	0.335427
B.cells	WRB	0.688235	1.437969	1.772834	0.0797	-4.20542	0.473621	0.393359
B.cells	OSTC	-0.16333	6.387429	-1.77237	0.079778	-5.19076	0.437729	0.3429
B.cells	1700003F1	-0.62732	2.027827	-1.77235	0.079781	-4.29111	0.469168	0.387133
B.cells	NTNG2	0.594506	3.05713	1.772216	0.079804	-4.45561	0.461514	0.376263
B.cells	CHCHD3	0.126145	6.768694	1.77191	0.079855	-5.28116	0.435165	0.339431
B.cells	GM15543	-0.72016	0.901403	-1.77153	0.079919	-4.18316	0.477825	0.399746
B.cells	SLC8A2	-1.02663	1.394752	-1.7714	0.07994	-4.21537	0.474059	0.394305
B.cells	PPIA	-0.1808	10.92524	-1.77112	0.079987	-5.8788	0.40775	0.302481
B.cells	ZEB2	0.248621	9.36649	1.770853	0.080031	-5.76517	0.41784	0.315979
B.cells	SMIM20	0.215974	4.914811	1.770003	0.080174	-4.95305	0.448286	0.357918
B.cells	PALD1	-0.8279	1.292537	-1.76989	0.080194	-4.19711	0.474961	0.395911
B.cells	NBR1	0.184699	5.455137	1.769845	0.0802	-5.05269	0.444459	0.352614
B.cells	CORO2B	-0.86405	1.197161	-1.7697	0.080224	-4.14312	0.475687	0.396984
B.cells	GM17491	0.56784	1.415352	1.769478	0.080262	-4.20433	0.474026	0.394635
B.cells	TSFM	-0.25715	4.258618	-1.76941	0.080274	-4.78774	0.452986	0.364625
B.cells	DENND1C	0.292409	3.966917	1.769099	0.080326	-4.79804	0.455162	0.367634
B.cells	AKAP10	0.218243	5.860095	1.768713	0.080391	-5.15418	0.441823	0.348881
B.cells	FXYD4	-0.39478	3.001862	-1.76777	0.080549	-4.61168	0.463049	0.378006
B.cells	TMEM189	0.241921	6.387922	1.767365	0.080618	-5.20099	0.438957	0.344204
B.cells	CTSL	-0.23942	6.758061	-1.76656	0.080753	-5.18853	0.43663	0.340843
B.cells	TSC22D4	0.178235	6.684783	1.766315	0.080795	-5.2542	0.437135	0.341663
B.cells	THEMIS2	0.308508	4.692319	1.766257	0.080804	-4.969	0.451156	0.361176
B.cells	UTP6	0.241435	4.42275	1.766177	0.080818	-4.88897	0.453093	0.363938
B.cells	RHOT1	0.178166	5.400436	1.765227	0.080978	-5.05014	0.446563	0.354523
B.cells	NIM1K	-0.47748	2.88905	-1.765	0.081017	-4.45886	0.46477	0.380327
B.cells	DTX3L	0.377255	4.876216	1.764568	0.08109	-4.95372	0.450293	0.360028
B.cells	LMAN2L	0.227853	4.447735	1.764524	0.081097	-4.87289	0.453369	0.364354
B.cells	RNF181	0.277684	4.477136	1.764497	0.081102	-4.85529	0.453158	0.364056
B.cells	RHOB	-0.40339	5.891039	-1.76416	0.08116	-5.08636	0.443104	0.350127
B.cells	OAS2	0.735165	1.571395	1.764067	0.081175	-4.37238	0.474669	0.394879
B.cells	APBB1IP	0.161906	7.638967	1.763762	0.081226	-5.42006	0.431055	0.333685
B.cells	RNF149	0.331544	5.786169	1.763413	0.081286	-5.05816	0.443859	0.35145
B.cells	ACOT1	-0.78617	2.604682	-1.76327	0.081309	-4.36282	0.466905	0.38399

B.cells	RAMP1	-0.33208	4.507186	-1.76312	0.081336	-4.54215	0.452959	0.364194
B.cells	CELF1	0.123553	7.40812	1.762181	0.081495	-5.39209	0.433146	0.336125
B.cells	ANKRD49	0.366672	3.032797	1.761913	0.081541	-4.51395	0.464281	0.379723
B.cells	PLAC8	0.425144	8.2646	1.761828	0.081555	-5.62177	0.427344	0.328262
B.cells	INMT	-0.98885	0.639157	-1.76162	0.081591	-4.17155	0.482418	0.405871
B.cells	HNRNPL	-0.12113	8.426349	-1.76081	0.081728	-5.55732	0.426679	0.326972
B.cells	PPP1R3B	0.534345	2.491892	1.760525	0.081777	-4.33727	0.468771	0.38581
B.cells	SP140	0.237606	6.188606	1.760186	0.081835	-5.24398	0.442005	0.34814
B.cells	PCCA	0.269351	4.599387	1.759934	0.081878	-4.91663	0.453286	0.363904
B.cells	PAQR9	-0.42527	4.029832	-1.75957	0.08194	-4.72974	0.457411	0.369851
B.cells	RUBCN	0.285072	3.888841	1.759525	0.081947	-4.778	0.458439	0.371317
B.cells	CENPO	-0.40924	2.773597	-1.7593	0.081987	-4.46134	0.466666	0.383067
B.cells	HEXIM1	-0.25624	5.48831	-1.75899	0.082039	-5.04752	0.446935	0.355323
B.cells	PROX1	-0.78433	2.15604	-1.75889	0.082056	-4.3242	0.471295	0.38985
B.cells	SLFN1	1.092018	2.335521	1.758591	0.082107	-4.34033	0.469944	0.388015
B.cells	GGPS1	0.16059	5.393996	1.758586	0.082108	-5.09308	0.447604	0.356355
B.cells	TBC1D5	0.182178	7.293343	1.758121	0.082188	-5.38358	0.434358	0.338261
B.cells	FBXO38	0.171568	5.443447	1.75804	0.082202	-5.09963	0.447253	0.356138
B.cells	ART3	-0.8282	0.944132	-1.75791	0.082224	-4.18649	0.480535	0.403676
B.cells	CAST	0.15874	6.266263	1.757673	0.082264	-5.2317	0.441468	0.348216
B.cells	NUP98	0.173489	7.461994	1.757107	0.082361	-5.43812	0.433362	0.336961
B.cells	CHPF	-0.57633	1.242066	-1.75705	0.082372	-4.30902	0.478418	0.400636
B.cells	THEM6	0.34818	3.448768	1.756453	0.082474	-4.70159	0.46218	0.377015
B.cells	SYNC	-0.43768	1.973724	-1.75611	0.082532	-4.38726	0.473309	0.392863
B.cells	ARHGEF15	-1.11148	0.580885	-1.75558	0.082624	-4.14458	0.484264	0.408486
B.cells	PEX14	0.199941	5.405291	1.755336	0.082665	-5.09994	0.448374	0.357232
B.cells	EMILIN1	-0.44669	2.608435	-1.75516	0.082696	-4.38278	0.468788	0.3862
B.cells	WWC1	-0.61965	1.216205	-1.75467	0.08278	-4.25998	0.479375	0.401649
B.cells	SNRNP25	0.295752	3.939991	1.754066	0.082884	-4.77904	0.458959	0.372603
B.cells	HMGA1	0.277708	4.670399	1.75398	0.082899	-4.95505	0.453657	0.365116
B.cells	RAB10	0.164959	7.670572	1.753928	0.082908	-5.47139	0.432625	0.335859
B.cells	TET2	0.179408	6.024646	1.753881	0.082916	-5.22918	0.444017	0.351606
B.cells	CENPK	-0.49285	3.283005	-1.75376	0.082938	-4.50315	0.463789	0.379507
B.cells	RAD52	-0.3764	3.121528	-1.75281	0.0831	-4.57058	0.465485	0.381652
B.cells	CSF3R	0.739133	2.933462	1.752783	0.083106	-4.33038	0.466884	0.383658
B.cells	MAD1L1	-0.19143	5.227133	-1.75255	0.083145	-5.01082	0.450148	0.360066
B.cells	SECISBP2	0.208084	4.786541	1.752196	0.083207	-4.98498	0.453428	0.364581
B.cells	ITGB3BP	-0.31714	3.52337	-1.75168	0.083296	-4.59249	0.462907	0.377795
B.cells	PAQR4	0.808837	1.484926	1.750924	0.083427	-4.28343	0.478677	0.400097
B.cells	MOB4	0.139538	6.832732	1.750784	0.083452	-5.3305	0.439631	0.344851
B.cells	RING1	0.31489	3.585545	1.750075	0.083575	-4.70665	0.463337	0.377763
B.cells	GM26756	1.050896	0.354794	1.749161	0.083734	-4.1778	0.488618	0.413721
B.cells	RBM41	0.273758	3.907296	1.748041	0.083928	-4.80158	0.462398	0.375308
B.cells	AIG1	0.259299	4.903663	1.747896	0.083954	-5.04361	0.45513	0.36507
B.cells	UROC1	-0.71947	1.760508	-1.74708	0.084096	-4.34514	0.478913	0.398672
B.cells	F5	-0.46775	4.094976	-1.74672	0.084159	-4.90973	0.461393	0.373751
B.cells	PCLAF	-0.46362	7.982096	-1.74664	0.084172	-5.45961	0.43386	0.335436

B.cells	CCDC126	0.680571	1.899321	1.746592	0.084181	-4.28239	0.47785	0.397314
B.cells	GNPAT	0.259644	4.15461	1.746251	0.084241	-4.81777	0.461063	0.373263
B.cells	SEC23A	0.238957	4.425257	1.745347	0.084399	-4.90294	0.45927	0.370853
B.cells	4931406CC	-0.39328	3.540099	-1.74519	0.084426	-4.58752	0.465788	0.380162
B.cells	XDH	0.493648	4.807401	1.745177	0.084428	-4.87211	0.456489	0.366985
B.cells	ZDHHC3	0.215776	5.048938	1.745143	0.084434	-4.98674	0.454742	0.364529
B.cells	POLA2	0.316725	4.050563	1.744807	0.084493	-4.7867	0.46212	0.374986
B.cells	VPS33A	0.236535	4.291318	1.743642	0.084697	-4.86964	0.460934	0.372837
B.cells	TRIM35	-0.22272	5.607952	-1.74332	0.084754	-5.19522	0.451398	0.359536
B.cells	DNAH12	-0.89258	1.676699	-1.74317	0.08478	-4.28077	0.480578	0.401098
B.cells	PACS1	0.221457	5.972963	1.743032	0.084805	-5.21347	0.448795	0.355901
B.cells	CDC6	-0.50637	3.269665	-1.74303	0.084806	-4.52945	0.468497	0.383716
B.cells	ESRRA	0.254355	3.981496	1.742378	0.08492	-4.86421	0.463212	0.376484
B.cells	SH3BP5L	-0.47034	2.252113	-1.74187	0.08501	-4.43357	0.476173	0.395211
B.cells	LUC7L2	0.109199	8.527172	1.741749	0.08503	-5.5974	0.431074	0.331891
B.cells	GM26535	0.959341	-0.60061	1.741741	0.085032	-4.16113	0.498422	0.427814
B.cells	UBP1	0.186011	5.448392	1.741731	0.085033	-5.11038	0.452541	0.361589
B.cells	CPSF2	-0.20142	5.649423	-1.74159	0.085058	-5.14754	0.451101	0.359602
B.cells	LRCH1	0.171036	7.432191	1.741331	0.085104	-5.44517	0.438567	0.342288
B.cells	KLRB1B	-0.96144	1.981821	-1.741	0.085162	-4.28209	0.478237	0.398436
B.cells	NECTIN3	-0.78159	1.220532	-1.7409	0.085179	-4.20726	0.484104	0.406992
B.cells	CEP131	0.857698	1.3043	1.740626	0.085228	-4.24206	0.483455	0.406172
B.cells	HORMAD2	-1.02066	1.357831	-1.74057	0.085239	-4.23144	0.48304	0.405598
B.cells	BLNK	-0.2575	7.200732	-1.74003	0.085334	-5.38318	0.44045	0.344961
B.cells	MECOM	1.303548	0.770813	1.739808	0.085373	-4.21604	0.487918	0.4126
B.cells	DHCR24	-0.42578	2.897631	-1.7393	0.085462	-4.57575	0.471842	0.389123
B.cells	PSMB8	0.268401	7.077604	1.739092	0.085499	-5.3932	0.441548	0.346446
B.cells	CLN5	0.254796	4.008222	1.738721	0.085565	-4.81883	0.4637	0.377489
B.cells	KDM5C	-0.23604	6.827805	-1.73825	0.085648	-5.35248	0.443451	0.349171
B.cells	INTS1	-0.36448	3.347025	-1.73812	0.085671	-4.65466	0.468638	0.384704
B.cells	HYOU1	-0.25261	4.380576	-1.73786	0.085717	-4.86143	0.460988	0.37384
B.cells	IFITM1	1.152798	3.489377	1.73779	0.08573	-4.55283	0.467576	0.383243
B.cells	CCPG1OS	-0.60926	1.576044	-1.73731	0.085815	-4.25297	0.482318	0.404319
B.cells	PRADC1	0.338958	3.374688	1.736857	0.085895	-4.64141	0.468649	0.384773
B.cells	PBRM1	0.125949	7.416728	1.736601	0.085941	-5.44342	0.439553	0.343829
B.cells	HNRNPA1	-0.17398	7.807246	-1.73644	0.085969	-5.48413	0.436856	0.340146
B.cells	PRKCE	0.190113	7.233184	1.736303	0.085994	-5.50233	0.440827	0.345688
B.cells	TNFSF11	-1.19974	0.706959	-1.73621	0.086009	-4.21179	0.489087	0.414535
B.cells	BCOR	-0.24165	5.214585	-1.73579	0.086085	-5.02251	0.45532	0.365705
B.cells	ACOT12	-0.8287	0.798615	-1.73546	0.086144	-4.21958	0.488675	0.413574
B.cells	KCP	-0.9133	0.100139	-1.73434	0.086343	-4.19631	0.495008	0.422213
B.cells	NHLRC3	0.392775	3.16365	1.734207	0.086367	-4.60333	0.471305	0.387808
B.cells	GNAQ	0.181224	7.882192	1.733524	0.086489	-5.50576	0.437754	0.340255
B.cells	ISY1	-0.19681	6.241546	-1.73318	0.086549	-5.2596	0.449293	0.35628
B.cells	SNX17	0.185599	5.853791	1.732971	0.086587	-5.18011	0.452059	0.360179
B.cells	FCRLA	-0.31943	5.107141	-1.73279	0.08662	-5.06126	0.457442	0.367787
B.cells	FPR2	1.237505	1.833327	1.732465	0.086678	-4.27864	0.482049	0.402976

B.cells	CLU	-0.62419	5.440247	-1.73214	0.086736	-5.16676	0.45522	0.364698
B.cells	VSTM4	-1.14349	0.147061	-1.73167	0.086819	-4.17541	0.495517	0.422691
B.cells	NOSTRIN	-0.46537	3.426931	-1.73149	0.086852	-4.60949	0.470167	0.385898
B.cells	AMER1	-0.41287	2.080672	-1.73117	0.08691	-4.42835	0.480386	0.400794
B.cells	RAD23A	0.209817	5.807004	1.730728	0.086989	-5.20794	0.452715	0.361403
B.cells	NR2F2	-0.59007	3.13223	-1.73053	0.087024	-4.52935	0.472382	0.389395
B.cells	GM42726	-0.27286	3.69069	-1.73045	0.087038	-4.77921	0.468194	0.38339
B.cells	HLF	-0.86682	1.023669	-1.73043	0.087043	-4.231	0.48859	0.412906
B.cells	YWHAE	-0.10762	8.88218	-1.73017	0.087089	-5.67284	0.431314	0.331916
B.cells	MIS12	-0.38276	3.322859	-1.72855	0.087381	-4.64103	0.472341	0.388241
B.cells	SLCO4A1	-0.48431	3.403213	-1.72822	0.087441	-4.64422	0.471841	0.3876
B.cells	FKBP1B	0.699004	0.793011	1.726864	0.087684	-4.27003	0.493107	0.417252
B.cells	CNPY4	-0.45211	2.433697	-1.72642	0.087764	-4.44093	0.48053	0.398694
B.cells	CSTA3	1.157176	0.791872	1.725702	0.087894	-4.2621	0.493487	0.417492
B.cells	GM48855	0.45755	1.463573	1.725679	0.087899	-4.42503	0.488199	0.409773
B.cells	GM36660	-0.96577	-0.14918	-1.72538	0.087953	-4.17212	0.501005	0.428661
B.cells	MNAT1	0.232205	4.818214	1.725241	0.087978	-5.00102	0.462755	0.373409
B.cells	LCLAT1	0.324213	3.99918	1.725012	0.088019	-4.77614	0.468821	0.382147
B.cells	CHCHD2	-0.12786	8.94643	-1.72494	0.088033	-5.68243	0.433553	0.33299
B.cells	MCM9	0.227485	4.75152	1.724408	0.088129	-5.02129	0.463428	0.374336
B.cells	RBM33	0.161492	5.898903	1.724298	0.088149	-5.22596	0.45507	0.362583
B.cells	ECE1	-0.27032	5.613294	-1.72359	0.088278	-5.19666	0.457592	0.365666
B.cells	ARHGAP39	-0.33234	4.38051	-1.72257	0.088462	-4.9091	0.467396	0.378772
B.cells	MPG	0.266832	3.63806	1.721872	0.08859	-4.78363	0.473419	0.386896
B.cells	SPDL1	-0.48153	2.833783	-1.72085	0.088776	-4.51817	0.479974	0.395978
B.cells	CD200R4	1.037713	1.662182	1.720548	0.088831	-4.26864	0.489052	0.409091
B.cells	CPNE2	-0.56544	3.37335	-1.72051	0.088838	-4.471	0.475859	0.390078
B.cells	PHLDA1	-0.44183	4.035457	-1.7203	0.088876	-4.7961	0.470865	0.383019
B.cells	LY6G5B	-0.67884	1.345565	-1.72023	0.088888	-4.2787	0.491539	0.412775
B.cells	PARN	0.275351	3.875182	1.720037	0.088924	-4.80699	0.472068	0.384818
B.cells	RHOBTB1	-0.4733	3.180961	-1.71987	0.088956	-4.66164	0.477321	0.39234
B.cells	UBL3	0.128026	7.408809	1.717725	0.089347	-5.48682	0.448123	0.349454
B.cells	NR6A1	-0.25113	5.241175	-1.71704	0.089472	-5.13163	0.463992	0.37151
B.cells	DDOST	-0.1832	5.788445	-1.71627	0.089615	-5.16065	0.459984	0.366146
B.cells	FBXO4	0.26188	3.431156	1.716024	0.08966	-4.84114	0.477544	0.390979
B.cells	SLC30A6	0.294571	3.417011	1.716009	0.089663	-4.74827	0.477652	0.391133
B.cells	MAP7D1	-0.19676	5.36793	-1.71587	0.089687	-5.06379	0.46306	0.370514
B.cells	MOB3A	0.231075	4.775701	1.715841	0.089693	-5.02976	0.467434	0.376674
B.cells	PAKAP.1	0.338881	4.53559	1.715423	0.08977	-4.91147	0.469221	0.379434
B.cells	UMAD1	0.206747	5.658219	1.715113	0.089827	-5.17213	0.460934	0.367831
B.cells	TMEM123	0.194383	6.484305	1.715085	0.089833	-5.33012	0.454947	0.359468
B.cells	C1QC	-0.4925	6.384084	-1.71504	0.089841	-5.26353	0.455668	0.360477
B.cells	SP3OS	0.264146	4.205373	1.715031	0.089843	-4.87016	0.471692	0.383018
B.cells	TULP4	-0.18888	5.928036	-1.7145	0.089941	-5.26503	0.459248	0.36524
B.cells	SLC25A45	0.329857	3.260909	1.714291	0.089979	-4.66679	0.479134	0.393406
B.cells	APC	0.175795	6.723147	1.713837	0.090063	-5.35424	0.453726	0.357322
B.cells	TNFSF8	1.071507	-0.51498	1.713158	0.090188	-4.21443	0.509718	0.437282

B.cells	ARMC3	0.5701	1.900651	1.712498	0.09031	-4.40317	0.490577	0.409211
B.cells	ESRRG	-0.74129	1.226258	-1.71229	0.090349	-4.30599	0.495906	0.416999
B.cells	GM37233	-0.93285	0.010375	-1.71227	0.090351	-4.20398	0.505683	0.431319
B.cells	TTC19	0.222363	4.958514	1.712029	0.090397	-5.0934	0.467257	0.375929
B.cells	TNIP2	0.23678	3.816648	1.710718	0.09064	-4.90847	0.476869	0.388734
B.cells	MAP7D3	-0.89841	0.126284	-1.71027	0.090723	-4.22861	0.50588	0.430979
B.cells	TREM1	-0.79752	1.639916	-1.70989	0.090793	-4.32415	0.493741	0.413353
B.cells	IGSF5	-0.77296	1.922281	-1.70952	0.090863	-4.36335	0.491513	0.410296
B.cells	RRAS	0.322147	4.338868	1.709072	0.090946	-4.94296	0.472921	0.383803
B.cells	HSPA14	0.225554	5.034968	1.709037	0.090952	-5.08355	0.467719	0.376441
B.cells	PGM2L1	-0.25664	5.192591	-1.70843	0.091066	-5.1011	0.466551	0.374945
B.cells	ZDHHC17	0.242416	4.015064	1.708423	0.091067	-4.91638	0.475364	0.387439
B.cells	ZFP397	0.240927	3.944609	1.708365	0.091077	-4.84329	0.475898	0.3882
B.cells	TIMELESS	-0.4112	3.491901	-1.70836	0.091079	-4.67881	0.479343	0.393122
B.cells	ATG5	0.155516	5.691584	1.708176	0.091113	-5.18435	0.462874	0.369772
B.cells	MICAL2	-0.73909	1.966497	-1.70817	0.091114	-4.34412	0.491166	0.410161
B.cells	GM31728	0.997882	-0.17439	1.708087	0.091129	-4.21434	0.508332	0.435292
B.cells	AHSA2	0.278955	3.646243	1.707072	0.091319	-4.76714	0.478654	0.391804
B.cells	ZDHHC24	0.770074	1.089708	1.706923	0.091346	-4.27846	0.498623	0.420691
B.cells	NCOA1	0.187557	7.07231	1.706873	0.091356	-5.45242	0.45334	0.356185
B.cells	CEP104	0.310505	3.105243	1.706708	0.091386	-4.67391	0.482801	0.397887
B.cells	D430001F1	1.268912	-0.87048	1.706061	0.091507	-4.18635	0.514877	0.444593
B.cells	VPS4A	0.234172	4.241466	1.705188	0.091671	-4.90258	0.47467	0.386024
B.cells	AMACR	-0.61506	2.326832	-1.70517	0.091674	-4.44859	0.48939	0.407146
B.cells	COL5A3	-0.68713	0.940726	-1.70508	0.09169	-4.38425	0.500377	0.423149
B.cells	LYL1	0.342133	3.172684	1.705062	0.091694	-4.82963	0.482822	0.397688
B.cells	HNRNPF	-0.11809	9.158537	-1.70457	0.091786	-5.75287	0.439438	0.336895
B.cells	ZER1	0.294654	3.262662	1.703948	0.091903	-4.72411	0.482607	0.397231
B.cells	PRELID2	0.613393	2.621255	1.703919	0.091908	-4.50813	0.487575	0.404387
B.cells	GARNL3	-0.56065	2.487215	-1.70234	0.092205	-4.41816	0.489884	0.406698
B.cells	INTS11	0.292153	3.989645	1.702216	0.092229	-4.85173	0.478283	0.390076
B.cells	GM12185	0.375465	3.341306	1.701793	0.092308	-4.76161	0.48345	0.39734
B.cells	TNFRSF25	0.837776	-1.26707	1.701377	0.092387	-4.19917	0.520471	0.451722
B.cells	RFX5	0.485769	1.943489	1.701247	0.092411	-4.46261	0.494477	0.413274
B.cells	DCN	-0.56848	3.138043	-1.70013	0.092621	-4.63532	0.486012	0.400212
B.cells	APOPT1	0.170028	4.910205	1.698646	0.092902	-5.05933	0.473717	0.381468
B.cells	SETX	0.192214	5.905752	1.698097	0.093006	-5.27174	0.466612	0.371095
B.cells	B3GALT5	1.02412	0.779445	1.697526	0.093115	-4.26286	0.506656	0.42829
B.cells	GPR65	0.455051	4.250918	1.69737	0.093144	-4.79258	0.479312	0.388925
B.cells	CCL12	-1.51521	0.036828	-1.69675	0.093262	-4.22666	0.513154	0.437671
B.cells	MRGPRA2A	0.8799	-1.27662	1.69633	0.093342	-4.20655	0.523962	0.453993
B.cells	MPP1	0.196279	6.203718	1.696029	0.093399	-5.36617	0.465089	0.368837
B.cells	BAK1	0.248726	4.936771	1.695893	0.093425	-5.07024	0.474523	0.382092
B.cells	TRAC	-0.64532	2.266621	-1.69575	0.093452	-4.51483	0.495151	0.411564
B.cells	CLK2	0.1899	4.63296	1.695547	0.093491	-5.03617	0.476818	0.385337
B.cells	TSGA10	0.325693	2.968133	1.695381	0.093522	-4.71192	0.489632	0.403611
B.cells	EXT1	-0.22281	7.749502	-1.6952	0.093556	-5.67264	0.453877	0.353367

B.cells	SUZ12	0.155068	6.954117	1.694912	0.093612	-5.41879	0.459673	0.361426
B.cells	FSD2	-1.07783	-0.73763	-1.6945	0.09369	-4.1986	0.519781	0.44766
B.cells	MFSD10	0.261161	4.464143	1.694054	0.093775	-4.97053	0.478385	0.38747
B.cells	SPATA1	-0.26627	3.467697	-1.69391	0.093803	-4.74847	0.486034	0.398404
B.cells	VPS13C	0.256564	4.373385	1.693819	0.09382	-4.99649	0.479075	0.388487
B.cells	CRYBG1	0.26664	4.169377	1.692784	0.094018	-5.03522	0.481433	0.391298
B.cells	PHKB	0.213832	5.588989	1.69187	0.094193	-5.18128	0.471367	0.376547
B.cells	DGKZ	0.209926	6.2774	1.691455	0.094272	-5.33041	0.466339	0.369557
B.cells	S1PR4	-0.30118	4.183137	-1.69135	0.094292	-4.94171	0.482093	0.391768
B.cells	SPRYD3	0.241891	3.699854	1.690909	0.094377	-4.8717	0.486037	0.397293
B.cells	GM10125	-0.48047	2.003063	-1.69021	0.09451	-4.50059	0.499529	0.416674
B.cells	KEL	0.991761	-0.44363	1.690175	0.094518	-4.26708	0.519535	0.445972
B.cells	ANKRD54	-0.3183	3.089479	-1.68981	0.094588	-4.65549	0.490931	0.404351
B.cells	ATP2C1	0.167383	5.85373	1.689733	0.094602	-5.28437	0.469823	0.374406
B.cells	HS3ST3B1	-0.46879	3.380647	-1.68968	0.094612	-4.60898	0.488656	0.401111
B.cells	ATG10	0.206946	5.64086	1.689176	0.094709	-5.22336	0.471638	0.376914
B.cells	GK	-0.28945	4.764608	-1.68898	0.094746	-5.06744	0.478241	0.386298
B.cells	IFT27	-0.34231	3.758551	-1.68879	0.094783	-4.78488	0.485957	0.397315
B.cells	UST	-0.47553	6.028853	-1.688	0.094936	-5.13361	0.46918	0.373287
B.cells	LRRC29	0.434978	1.690453	1.687906	0.094954	-4.42193	0.50274	0.421186
B.cells	PLPP3	-0.59492	4.693864	-1.68739	0.095054	-4.84781	0.479512	0.387578
B.cells	GM14295	-0.59304	1.289141	-1.6867	0.095187	-4.33148	0.506494	0.426434
B.cells	DENND2A	-0.71896	1.374468	-1.68644	0.095237	-4.34638	0.505802	0.425493
B.cells	FAM83F	-0.55344	1.041009	-1.6862	0.095283	-4.5129	0.508514	0.429556
B.cells	SLC25A34	-1.00383	-0.47643	-1.6862	0.095284	-4.21816	0.521052	0.448037
B.cells	GALNT6	0.438783	3.115592	1.686109	0.095301	-4.69715	0.491912	0.405494
B.cells	EHD3	-0.47921	3.517645	-1.68585	0.095351	-4.64346	0.488812	0.401038
B.cells	MIEF1	0.27228	4.21125	1.685498	0.095419	-4.96442	0.483444	0.393486
B.cells	ADIPOR1	0.18303	7.346972	1.685414	0.095435	-5.5591	0.460017	0.36062
B.cells	ACACA	0.226631	5.153719	1.684959	0.095523	-5.15796	0.476471	0.383523
B.cells	LST1	0.342206	5.23223	1.684761	0.095562	-5.02675	0.475878	0.382723
B.cells	GPD1L	-0.23194	5.296236	-1.68436	0.09564	-5.15187	0.475463	0.382174
B.cells	BBC3	-0.41383	2.639565	-1.68426	0.095659	-4.5534	0.496005	0.411552
B.cells	SLC22A1	-0.83855	0.740269	-1.68341	0.095823	-4.29657	0.51157	0.434107
B.cells	CTSO	0.297355	4.26707	1.683172	0.09587	-4.92679	0.483533	0.393667
B.cells	NOP58	-0.22863	5.838554	-1.68301	0.095901	-5.23901	0.47162	0.376884
B.cells	ZFP617	0.494221	2.165547	1.682747	0.095952	-4.51575	0.500018	0.417575
B.cells	ORMDL1	-0.28451	3.609727	-1.6827	0.095963	-4.79205	0.48862	0.401148
B.cells	EPHB4	-1.10318	0.794939	-1.68244	0.096012	-4.25578	0.511121	0.433876
B.cells	BICRAL	0.216906	5.023041	1.682414	0.096017	-5.11367	0.477758	0.385735
B.cells	ELF4	-0.16152	6.785373	-1.6823	0.09604	-5.42545	0.46461	0.367281
B.cells	RFC1	0.220657	5.999025	1.68143	0.096209	-5.25725	0.47099	0.375736
B.cells	SND1	0.14358	7.195863	1.681187	0.096256	-5.50618	0.462165	0.363496
B.cells	EZR	-0.14002	7.933064	-1.68106	0.096281	-5.61509	0.456827	0.35614
B.cells	CMTM8	-0.36432	4.192364	-1.6804	0.09641	-4.89629	0.485127	0.395533
B.cells	9430038I0:	0.196328	4.248537	1.680064	0.096475	-5.0213	0.484729	0.394922
B.cells	TNRC6B	0.1588	8.234075	1.679931	0.096501	-5.66167	0.455112	0.353449

B.cells	ITFG1	0.170283	5.454025	1.67936	0.096613	-5.17132	0.47588	0.382019
B.cells	ABRAXAS2	0.189556	4.960803	1.678985	0.096686	-5.14647	0.479774	0.387473
B.cells	PPP6R2	0.230089	4.368985	1.67876	0.09673	-4.96523	0.484317	0.39393
B.cells	RO60	-0.44171	2.493379	-1.67801	0.096877	-4.49022	0.499559	0.415164
B.cells	SLC7A8	0.720327	2.86439	1.67741	0.096994	-4.55504	0.496886	0.411143
B.cells	APON	0.815997	1.166034	1.677304	0.097015	-4.36188	0.510575	0.431095
B.cells	HACL1	-0.39759	3.523051	-1.67674	0.097126	-4.76278	0.491806	0.403941
B.cells	LIFR	0.383767	3.842745	1.676359	0.097201	-4.93136	0.489307	0.400482
B.cells	MEIS3	-0.82255	0.488054	-1.67627	0.097219	-4.27263	0.516281	0.43967
B.cells	PCP4L1	-0.90738	1.36121	-1.67614	0.097244	-4.33659	0.509098	0.429134
B.cells	TRMT10C	-0.20061	4.996252	-1.67612	0.097248	-5.12565	0.480414	0.387858
B.cells	PNPLA7	0.215458	6.039516	1.675784	0.097314	-5.31579	0.472648	0.376842
B.cells	MDM4	0.156652	5.749077	1.67525	0.097419	-5.27502	0.475027	0.379932
B.cells	SPN	-0.36111	3.586459	-1.67515	0.097439	-4.70946	0.491635	0.403512
B.cells	SH2B3	-0.19545	5.315208	-1.67433	0.097601	-5.20634	0.478725	0.384861
B.cells	C1QTNF12	-0.52026	2.21932	-1.67418	0.097628	-4.49515	0.502925	0.419569
B.cells	INPP5B	0.263838	4.216748	1.674056	0.097654	-4.93106	0.487152	0.396903
B.cells	GM12992	-0.61684	2.041763	-1.67386	0.097693	-4.47652	0.504355	0.421662
B.cells	SIRPB1A	0.827167	-0.18195	1.673295	0.097804	-4.30622	0.523044	0.44873
B.cells	G2E3	0.287823	3.938116	1.673038	0.097855	-4.81068	0.489706	0.40031
B.cells	CCNK	0.191115	4.857081	1.672117	0.098037	-5.13169	0.48329	0.390579
B.cells	PCOLCE2	-0.79236	1.678726	-1.67175	0.09811	-4.41914	0.508584	0.426816
B.cells	GM42477	0.577983	1.273145	1.670935	0.098271	-4.39515	0.512379	0.432029
B.cells	CHP2	-1.34326	-0.09865	-1.67043	0.09837	-4.25954	0.52379	0.449047
B.cells	GM46224	-0.95354	2.389468	-1.67035	0.098386	-4.38747	0.503303	0.419088
B.cells	AATF	0.202209	4.995367	1.670208	0.098415	-5.15148	0.482834	0.389773
B.cells	SLC15A3	0.315441	5.005968	1.670165	0.098424	-5.05643	0.482753	0.389658
B.cells	TAF1D	-0.16248	6.027224	-1.67	0.098456	-5.31441	0.475	0.378786
B.cells	ASNS	-0.60958	1.465562	-1.66926	0.098603	-4.40621	0.511344	0.430293
B.cells	CKLF	0.223834	4.804772	1.668912	0.098672	-5.081	0.484821	0.392235
B.cells	SLC35A4	0.298904	3.561213	1.668791	0.098696	-4.82978	0.49451	0.406076
B.cells	FABP5	-0.32058	7.278075	-1.66859	0.098737	-5.54051	0.466208	0.36616
B.cells	KLF6	-0.24413	7.581942	-1.6684	0.098774	-5.55431	0.46398	0.363095
B.cells	P4HA2	0.924935	0.108055	1.667828	0.098888	-4.25862	0.522842	0.447159
B.cells	L3MBTL3	0.248119	5.067059	1.667553	0.098943	-5.11776	0.483014	0.389579
B.cells	ENY2	-0.17822	5.972518	-1.66755	0.098943	-5.29894	0.47613	0.379881
B.cells	COQ7	-0.25556	4.79596	-1.66704	0.099045	-5.08926	0.485143	0.392736
B.cells	SV2C	0.930989	-0.28271	1.666826	0.099088	-4.2643	0.526187	0.452363
B.cells	PTGR1	-0.37322	3.377249	-1.66652	0.099149	-4.81126	0.496222	0.408713
B.cells	EXOC1	0.259065	4.095264	1.666395	0.099174	-4.94076	0.490579	0.400656
B.cells	SETD5	0.142283	6.628233	1.666174	0.099218	-5.41427	0.471262	0.373473
B.cells	FPR1	0.94811	2.992339	1.665958	0.099261	-4.54817	0.499279	0.413373
B.cells	SLC9A8	0.212742	4.988676	1.665686	0.099315	-5.14717	0.483661	0.39111
B.cells	TRMT1L	0.186487	4.566129	1.66566	0.099321	-5.04497	0.486918	0.395738
B.cells	ZDHHC13	0.249985	3.693777	1.665423	0.099368	-4.90321	0.493725	0.40547
B.cells	MTCH1	0.147042	6.297903	1.664843	0.099484	-5.37158	0.47373	0.377357
B.cells	RNF126	-0.21676	4.767243	-1.66456	0.099541	-5.09924	0.485365	0.39379

B.cells	ANAPC10	0.208551	4.581596	1.664162	0.09962	-5.05546	0.486799	0.395975
B.cells	FBXL7	-0.72268	3.621991	-1.66416	0.099622	-4.6549	0.49429	0.406694
B.cells	GM49041	0.595117	1.334735	1.664094	0.099634	-4.47724	0.512695	0.433405
B.cells	SIK3	-0.1765	10.09847	-1.66403	0.099647	-5.99584	0.446241	0.339616
B.cells	TTLL3	-0.28755	4.027206	-1.66396	0.099661	-4.93265	0.49111	0.402133
B.cells	METRNL	0.61658	3.205494	1.663628	0.099728	-4.57948	0.497584	0.411435
B.cells	IRF8	0.263252	6.49623	1.663599	0.099733	-5.42089	0.472246	0.375421
B.cells	SEC11A	-0.13432	6.406384	-1.66348	0.099757	-5.39872	0.472918	0.376382
B.cells	TAF4	-0.23502	4.075399	-1.66301	0.099852	-4.9258	0.490961	0.401747
B.cells	NUTF2	0.561234	1.951713	1.662786	0.099897	-4.48743	0.507888	0.426251
B.cells	HNRNPH1	-0.17573	6.424984	-1.66262	0.099931	-5.35922	0.472997	0.376407
B.cells	GNMT	0.465544	4.232803	1.661519	0.100151	-5.06017	0.490418	0.400441
B.cells	ENTHD1	-0.82676	0.023444	-1.6615	0.100155	-4.35671	0.524578	0.450156
B.cells	DLEU2	-0.16905	8.40867	-1.66049	0.100358	-5.74937	0.459357	0.356709
B.cells	HEATR5B	0.319333	3.730171	1.66038	0.100381	-4.83687	0.494662	0.406365
B.cells	MXD1	0.216444	6.036212	1.660369	0.100383	-5.30604	0.476874	0.381098
B.cells	LARP7	-0.16316	5.919166	-1.66035	0.100387	-5.28603	0.477759	0.382342
B.cells	1-Mar	-0.68127	1.457886	-1.65984	0.10049	-4.4187	0.513137	0.432928
B.cells	RCN2	-0.23323	5.075901	-1.65975	0.100508	-5.15903	0.484363	0.391471
B.cells	BRCA1	-0.42437	4.143927	-1.65927	0.100604	-4.87034	0.491686	0.401931
B.cells	P2RX1	-0.85939	0.81074	-1.65904	0.10065	-4.32533	0.518591	0.441087
B.cells	GAB3	0.281487	4.746655	1.658776	0.100705	-5.08329	0.486997	0.395449
B.cells	GAPT	-0.61152	1.236779	-1.65832	0.100796	-4.46666	0.515059	0.436259
B.cells	KMT2A	0.185102	6.28827	1.657953	0.100871	-5.38755	0.475239	0.379162
B.cells	MAGI2	0.745893	0.831391	1.657912	0.100879	-4.33416	0.518419	0.441326
B.cells	FYCO1	0.251429	4.137271	1.657762	0.10091	-4.98704	0.491738	0.402602
B.cells	TIMM8B	0.238966	5.242586	1.657737	0.100915	-5.16285	0.483177	0.390406
B.cells	PRDM4	0.318275	3.039382	1.657682	0.100926	-4.73374	0.500418	0.415122
B.cells	TLK1	0.152698	6.854126	1.657218	0.10102	-5.47791	0.471008	0.373428
B.cells	AKIRIN1	0.133324	6.287656	1.657167	0.10103	-5.40326	0.475244	0.379364
B.cells	GM32089	0.812336	-1.09921	1.656826	0.101099	-4.27823	0.534566	0.465938
B.cells	INPPL1	-0.40715	2.471527	-1.65661	0.101143	-4.58344	0.504978	0.422058
B.cells	GLIPR2	0.352486	4.6029	1.656501	0.101165	-5.06324	0.48811	0.397781
B.cells	EIF5	0.13879	8.418516	1.656418	0.101182	-5.77846	0.459541	0.357729
B.cells	GM29966	-0.82832	1.411825	-1.65609	0.101249	-4.41258	0.513616	0.434954
B.cells	EEFSEC	0.275291	4.399413	1.656004	0.101266	-5.08903	0.489692	0.400299
B.cells	EDIL3	-0.64207	1.492552	-1.65529	0.101411	-4.51986	0.512952	0.43423
B.cells	NUDT13	0.41869	2.358115	1.655256	0.101418	-4.60564	0.505894	0.423905
B.cells	F830208F2	0.915178	-0.23725	1.655184	0.101433	-4.28992	0.527398	0.455598
B.cells	PEG3	-0.68402	1.53048	-1.65512	0.101446	-4.43249	0.51264	0.433773
B.cells	CFL1	-0.15669	10.01399	-1.65504	0.101462	-5.97334	0.448187	0.3426
B.cells	H2AFJ	0.146455	7.521162	1.654286	0.101616	-5.54807	0.466547	0.367506
B.cells	HGS	-0.25456	4.094551	-1.65412	0.10165	-4.92522	0.492569	0.404388
B.cells	CENPM	-0.38436	3.916181	-1.65362	0.101751	-4.87536	0.494257	0.406548
B.cells	SERF2	0.107974	9.682679	1.653084	0.101861	-5.91978	0.45153	0.346543
B.cells	PROSCOS	-0.91366	0.287878	-1.65248	0.101984	-4.32206	0.524553	0.450393
B.cells	SCAF11	0.141889	7.16109	1.652222	0.102036	-5.54644	0.470203	0.372151

B.cells	PLS3	-0.63108	2.162516	-1.65145	0.102195	-4.39606	0.509481	0.427973
B.cells	HEMGN	1.073689	0.35427	1.651404	0.102204	-4.31275	0.524467	0.450026
B.cells	PTMS	-0.22928	6.015415	-1.65096	0.102295	-5.28088	0.479406	0.384647
B.cells	NCKAP1L	0.143706	6.137906	1.650579	0.102373	-5.38141	0.478641	0.383397
B.cells	MDK	-0.69757	1.436872	-1.64993	0.102505	-4.4008	0.516212	0.437146
B.cells	ACOT2	0.218043	4.416307	1.649655	0.102562	-5.01803	0.492232	0.402519
B.cells	SLC10A7	0.171872	5.908741	1.64961	0.102571	-5.34205	0.480712	0.386137
B.cells	SSH1	0.254968	3.775324	1.649397	0.102615	-4.9494	0.497284	0.409839
B.cells	CLTA	-0.11801	8.442682	-1.64889	0.102719	-5.74957	0.462155	0.360016
B.cells	CAR9	-0.87214	0.285801	-1.64812	0.102878	-4.28681	0.526751	0.451926
B.cells	OIP5OS1	0.172077	6.02273	1.646018	0.103311	-5.35065	0.482495	0.385816
B.cells	EFCAB9	-0.67873	0.443216	-1.64581	0.103353	-4.32032	0.527404	0.450634
B.cells	RAB3GAP2	0.193169	5.259956	1.645592	0.103398	-5.23823	0.488377	0.394169
B.cells	CNEP1R1	0.211988	4.587392	1.644449	0.103635	-5.07183	0.494542	0.402082
B.cells	HMG20B	0.170112	5.119833	1.644051	0.103717	-5.20853	0.490564	0.39632
B.cells	FIRRE	-0.52321	2.608551	-1.64322	0.103889	-4.57829	0.511162	0.425408
B.cells	MRPS9	0.184005	4.889322	1.642881	0.10396	-5.15271	0.492923	0.3993
B.cells	EXOSC1	0.226434	4.175365	1.642772	0.103982	-5.01068	0.49855	0.407315
B.cells	GOLGA4	0.168352	5.30004	1.64265	0.104008	-5.24312	0.489721	0.394772
B.cells	A930001M	-0.26317	3.967402	-1.64004	0.104549	-5.01174	0.5026	0.410581
B.cells	CHN2	-0.43651	5.358074	-1.6395	0.104663	-5.13439	0.491793	0.395207
B.cells	GM34680	1.057206	0.042973	1.639447	0.104674	-4.28124	0.535368	0.458167
B.cells	CDYL	0.160413	5.659184	1.639135	0.104739	-5.31624	0.489552	0.392015
B.cells	MAPK1IP1	0.42082	2.304416	1.637911	0.104995	-4.56015	0.516638	0.430803
B.cells	FZD6	-0.80513	0.693274	-1.63788	0.105	-4.37511	0.530148	0.450531
B.cells	FGFRL1	-0.96123	0.299565	-1.63787	0.105004	-4.30682	0.533511	0.455484
B.cells	LYPLAL1	-0.80193	1.634017	-1.63786	0.105006	-4.39017	0.522211	0.438908
B.cells	DISP1	0.283871	3.443949	1.637821	0.105013	-4.89194	0.507322	0.41736
B.cells	ZBTB25	0.312381	3.709262	1.637668	0.105045	-4.89918	0.505182	0.414331
B.cells	F2RL1	0.90266	-1.13295	1.636956	0.105194	-4.28103	0.546299	0.47426
B.cells	H2-T24	0.621562	1.828462	1.636281	0.105336	-4.50932	0.521239	0.437161
B.cells	HILPDA	0.456226	4.798635	1.636267	0.105339	-5.04279	0.497124	0.402486
B.cells	NR1H4	-0.70131	1.386536	-1.63621	0.105351	-4.43278	0.524942	0.442572
B.cells	NPEPL1	0.204588	5.049543	1.635579	0.105483	-5.1822	0.49539	0.399927
B.cells	ZFP651	-0.56713	1.578642	-1.63491	0.105624	-4.43904	0.523586	0.440715
B.cells	SEC24B	0.174332	6.179882	1.634876	0.105631	-5.44068	0.486595	0.387749
B.cells	CXCL1	-1.18283	2.815955	-1.63483	0.10564	-4.60043	0.513326	0.42581
B.cells	USP48	0.17787	5.391239	1.634698	0.105668	-5.24133	0.492712	0.396357
B.cells	SREBF2	-0.19038	5.9605	-1.6344	0.10573	-5.39037	0.488288	0.390173
B.cells	PACC1	-0.23721	5.421952	-1.6344	0.105732	-5.28996	0.492472	0.396068
B.cells	GM45606	-0.84851	0.477564	-1.63431	0.10575	-4.34086	0.532916	0.454461
B.cells	9930111J2	0.639417	1.279459	1.634068	0.105801	-4.49327	0.526139	0.444452
B.cells	GBP6	0.932443	1.984096	1.633591	0.105901	-4.45018	0.520408	0.435978
B.cells	SH3GLB1	0.130319	8.546317	1.633338	0.105954	-5.82217	0.468962	0.363286
B.cells	TUBB2B	-0.43062	2.9596	-1.63311	0.106002	-4.71152	0.512356	0.424472
B.cells	SNAP47	-0.42889	2.473639	-1.63293	0.106041	-4.57142	0.516349	0.430291
B.cells	SYNRG	0.183783	5.053855	1.632869	0.106053	-5.23661	0.495556	0.400477

B.cells	HACD1	-0.44618	3.002315	-1.63193	0.106252	-4.65836	0.512462	0.424528
B.cells	ZC3H12D	0.313236	3.479104	1.631831	0.106272	-5.01093	0.508579	0.418976
B.cells	SSR3	-0.16501	6.071068	-1.6318	0.106278	-5.36942	0.488064	0.389814
B.cells	TEC	0.199919	5.751113	1.6315	0.106342	-5.38091	0.490638	0.393358
B.cells	MSH3	0.20829	5.159864	1.630745	0.106502	-5.21711	0.495535	0.400138
B.cells	SLC37A4	0.345249	2.862467	1.630649	0.106522	-4.76036	0.513993	0.426584
B.cells	CMTR1	0.23932	5.166737	1.630602	0.106532	-5.30289	0.495481	0.400084
B.cells	MAST2	-0.22317	5.283184	-1.63026	0.106603	-5.25699	0.49464	0.398876
B.cells	OLFML2B	-0.82121	0.874884	-1.62996	0.106667	-4.37721	0.530689	0.450916
B.cells	SAPCD2	-0.59715	1.790194	-1.62973	0.106717	-4.48591	0.522959	0.439658
B.cells	TIFA	-0.25701	6.262161	-1.62965	0.106734	-5.44933	0.487028	0.388221
B.cells	PPIP5K2	0.207383	4.496011	1.6295	0.106765	-5.11788	0.500862	0.407796
B.cells	WASF2	0.111875	8.064634	1.628728	0.106929	-5.72758	0.473905	0.369502
B.cells	UBE2W	0.113337	6.081677	1.62831	0.107018	-5.4159	0.488994	0.390536
B.cells	GM45716	-0.35925	3.20355	-1.62829	0.107021	-4.7596	0.511879	0.423112
B.cells	TRIM30B	0.473164	1.730639	1.627683	0.107151	-4.69664	0.524263	0.441042
B.cells	ZFP618	-0.94441	0.531603	-1.62756	0.107177	-4.30013	0.534442	0.456052
B.cells	PECR	-0.60254	2.35098	-1.62749	0.107191	-4.59157	0.519085	0.433585
B.cells	TUSC3	-0.214	4.978446	-1.62675	0.107348	-5.19496	0.498331	0.403072
B.cells	PTER	-0.52951	2.433342	-1.62515	0.10769	-4.58736	0.520226	0.433214
B.cells	ADAM17	0.22747	6.061586	1.624917	0.10774	-5.40213	0.49105	0.391671
B.cells	EIPR1	0.189522	4.924643	1.623811	0.107976	-5.19032	0.499981	0.404614
B.cells	UBE2Q2	0.169568	5.51991	1.623647	0.108011	-5.32951	0.495281	0.398018
B.cells	ASB13	0.312808	3.434394	1.623063	0.108136	-4.95626	0.511978	0.42211
B.cells	TSPAN17	-0.5628	1.115501	-1.6229	0.108171	-4.46203	0.531321	0.450237
B.cells	FLT3L	0.415476	2.201527	1.622813	0.10819	-4.68634	0.522158	0.436858
B.cells	PEA15A	-0.3295	3.90143	-1.62271	0.108213	-4.84211	0.508183	0.416679
B.cells	2010110K1	1.087534	-0.03523	1.622573	0.108241	-4.29934	0.541231	0.464948
B.cells	2900060B1	-0.42544	2.614995	-1.62249	0.10826	-4.6106	0.518718	0.431956
B.cells	ZFP961	0.336281	3.308556	1.622442	0.108269	-4.83679	0.513007	0.42372
B.cells	CAPZA2	-0.10067	7.929486	-1.62229	0.108303	-5.71951	0.476783	0.372593
B.cells	NRIP3	-1.04515	0.094317	-1.62227	0.108306	-4.3286	0.540105	0.463339
B.cells	FAM160A2	0.346615	2.604929	1.622235	0.108314	-4.74383	0.518802	0.432116
B.cells	PACS2	-0.25303	4.036916	-1.62201	0.108361	-4.94176	0.507088	0.415312
B.cells	WDR66	-0.53261	1.900032	-1.622	0.108365	-4.53916	0.524684	0.44075
B.cells	ITGB6	1.143301	-0.27325	1.621339	0.108506	-4.29962	0.543307	0.46831
B.cells	MRM1	0.465915	2.067052	1.6209	0.1086	-4.53466	0.523283	0.439057
B.cells	ZBTB10	-0.34156	4.077959	-1.62076	0.10863	-4.90987	0.506756	0.415178
B.cells	ICOS	-0.7679	2.283057	-1.62075	0.108632	-4.45737	0.521478	0.436438
B.cells	AGPAT5	0.170231	5.644905	1.620134	0.108765	-5.29903	0.494301	0.397688
B.cells	RANBP17	-0.79332	1.134101	-1.61967	0.108865	-4.35513	0.531162	0.451026
B.cells	RRAGC	-0.17143	5.819399	-1.61967	0.108866	-5.36295	0.492936	0.395903
B.cells	HCLS1	0.173428	6.691521	1.619581	0.108884	-5.52399	0.486182	0.386442
B.cells	ANK3	-0.82595	1.681131	-1.61953	0.108896	-4.45641	0.526526	0.444263
B.cells	ACOX2	-0.8164	1.030725	-1.61927	0.10895	-4.40109	0.532044	0.452366
B.cells	FABP7	-0.84086	1.989157	-1.61923	0.10896	-4.54518	0.523936	0.440475
B.cells	PPT2	-0.35059	4.199673	-1.61904	0.109	-4.8032	0.505776	0.414237

B.cells	PIR	-0.79986	1.198746	-1.61897	0.109015	-4.35783	0.530612	0.450298
B.cells	ILDR1	-0.47407	1.791756	-1.6189	0.109031	-4.79135	0.525594	0.442937
B.cells	MFSD14B	0.172707	5.841678	1.618662	0.109082	-5.34656	0.492762	0.395802
B.cells	TRIM56	0.258233	3.810038	1.61841	0.109136	-4.95995	0.508923	0.418891
B.cells	ERMARD	-0.32836	3.076068	-1.61817	0.109188	-4.78956	0.514913	0.427573
B.cells	SH2D1B1	0.767031	1.695197	1.618074	0.109209	-4.43777	0.526407	0.44432
B.cells	CITED2	-0.21214	6.149916	-1.61792	0.109241	-5.40098	0.490364	0.392517
B.cells	ZFP954	-0.50725	2.050779	-1.61788	0.10925	-4.58612	0.523419	0.43995
B.cells	CYP4V3	-0.51319	2.78654	-1.6178	0.109269	-4.72835	0.517299	0.431039
B.cells	FKBP5	0.304449	5.30259	1.617594	0.109312	-5.18367	0.496991	0.401887
B.cells	PLEKHA3	0.22817	3.98944	1.617569	0.109318	-5.03279	0.507471	0.41685
B.cells	NPR1	-0.86456	1.263007	-1.61723	0.109392	-4.34916	0.530135	0.449754
B.cells	MVP	0.201174	5.108561	1.617048	0.10943	-5.27184	0.498589	0.404157
B.cells	SELENOI	0.292327	3.422495	1.616521	0.109544	-4.93832	0.512143	0.423806
B.cells	JKAMP	-0.24483	3.680975	-1.6165	0.109549	-4.894	0.510037	0.420769
B.cells	PPT1	-0.18446	6.003515	-1.61649	0.109551	-5.47522	0.491566	0.394425
B.cells	SLC1A2	-0.53111	2.197177	-1.61583	0.109694	-4.54736	0.522485	0.438602
B.cells	EIF2B2	-0.20658	4.936837	-1.61573	0.109717	-5.23901	0.500162	0.406414
B.cells	TRAF6	-0.17123	5.683882	-1.61549	0.109767	-5.3825	0.49427	0.398096
B.cells	CBL	0.153216	7.22396	1.615468	0.109773	-5.57627	0.48238	0.381388
B.cells	REL	0.177916	8.12408	1.615109	0.10985	-5.83212	0.475735	0.372045
B.cells	PQLC2	0.279579	3.590318	1.614669	0.109946	-5.01273	0.51139	0.422193
B.cells	SKINT3	0.734568	0.115469	1.614116	0.110066	-4.39117	0.540885	0.46514
B.cells	ZHX3	-0.27056	4.441922	-1.61404	0.110083	-5.06049	0.50473	0.41242
B.cells	AURKA	-0.40252	3.318106	-1.61325	0.110254	-4.77727	0.514439	0.42583
B.cells	GLIS3	-0.66576	3.068689	-1.61193	0.110541	-4.71868	0.516885	0.429147
B.cells	PTAR1	0.247542	3.690053	1.611654	0.110602	-4.93733	0.511789	0.421911
B.cells	TEX30	-0.19209	5.223272	-1.61129	0.110681	-5.24234	0.499463	0.404419
B.cells	LYZ1	0.896832	-0.68152	1.611136	0.110714	-4.3603	0.548823	0.476599
B.cells	KPNA3	0.162713	6.307968	1.611062	0.110731	-5.46552	0.490954	0.392414
B.cells	HDAC11	-0.87919	0.5008	-1.61082	0.110784	-4.35188	0.53858	0.461274
B.cells	COX7B	-0.15249	7.597144	-1.61048	0.110858	-5.68065	0.481063	0.378695
B.cells	NUP153	0.164731	5.848135	1.610454	0.110863	-5.37619	0.49454	0.39759
B.cells	PANK1	-0.40639	3.430383	-1.61042	0.110871	-4.8169	0.513911	0.425269
B.cells	SYPL	0.172573	5.860238	1.610371	0.110882	-5.4007	0.494445	0.39747
B.cells	CHD1L	0.246456	3.722264	1.610258	0.110906	-4.96293	0.511526	0.421865
B.cells	C9ORF72	0.340323	3.893192	1.610001	0.110962	-5.05063	0.510135	0.41994
B.cells	A	-0.26833	4.78007	-1.60987	0.110991	-5.18633	0.50299	0.4097
B.cells	PRNP	-0.51788	2.012971	-1.60984	0.110997	-4.55627	0.52568	0.442491
B.cells	MDGA1	-0.90726	0.010829	-1.60946	0.11108	-4.34024	0.542837	0.468006
B.cells	THAP7	0.29487	3.607354	1.609284	0.111119	-4.96346	0.512463	0.423554
B.cells	CYSLTR2	0.821332	1.496466	1.609208	0.111136	-4.42224	0.530045	0.449214
B.cells	TMX4	0.266048	4.420261	1.60911	0.111157	-5.10721	0.505875	0.414132
B.cells	POLR2J	0.186867	4.922519	1.609041	0.111173	-5.19978	0.501853	0.408382
B.cells	BPGM	0.318976	4.812429	1.608615	0.111266	-5.32358	0.502954	0.409698
B.cells	TTC21B	0.509886	1.614885	1.608063	0.111387	-4.49175	0.529616	0.447894
B.cells	GM49980	-0.36299	6.157222	-1.60787	0.11143	-5.44818	0.492662	0.394748

B.cells	0610005C1	-0.52576	1.911141	-1.60769	0.111469	-4.57313	0.52711	0.444343
B.cells	PLXNA4OS:	0.8382	0.51532	1.607416	0.111529	-4.36174	0.53904	0.461943
B.cells	ARL6IP4	0.174602	5.733017	1.607286	0.111557	-5.36668	0.495982	0.399608
B.cells	CSF2RB	0.304445	4.838003	1.606225	0.11179	-5.2309	0.503927	0.410017
B.cells	NLRP1A	0.900816	0.449959	1.605958	0.111849	-4.37368	0.540528	0.463374
B.cells	NACC1	0.211617	4.380772	1.60581	0.111882	-5.11951	0.507609	0.415435
B.cells	SLC44A2	0.207625	6.242042	1.605618	0.111924	-5.43005	0.492842	0.39446
B.cells	CTDSP2	-0.18974	4.801724	-1.60519	0.112018	-5.17993	0.504431	0.410803
B.cells	TAGLN2	-0.22408	8.716101	-1.60501	0.112058	-5.83768	0.474187	0.368416
B.cells	CATSPERD	-0.83577	1.329786	-1.60457	0.112155	-4.39042	0.53342	0.452645
B.cells	PSAP	-0.18195	9.249596	-1.60426	0.112223	-5.97989	0.470503	0.363158
B.cells	VPS72	0.209239	4.357985	1.603959	0.11229	-5.1069	0.508292	0.416222
B.cells	BC048403	0.468412	1.255612	1.603848	0.112314	-4.49564	0.534109	0.453736
B.cells	PANX1	0.217982	4.208683	1.603719	0.112343	-5.10846	0.509501	0.417955
B.cells	WDYHV1	0.237603	4.174037	1.603182	0.112461	-5.02237	0.51012	0.418639
B.cells	ST3GAL4	-0.19549	6.423085	-1.60192	0.11274	-5.38872	0.493219	0.393853
B.cells	BRCC3	-0.2022	4.874732	-1.60177	0.112773	-5.20049	0.50547	0.411262
B.cells	EPO	-0.82064	0.415776	-1.60134	0.112869	-4.35492	0.54292	0.465682
B.cells	RTN4RL1	-0.63055	2.381813	-1.60117	0.112907	-4.60289	0.52608	0.440921
B.cells	STXBP1	0.30588	3.786972	1.601054	0.112932	-5.02184	0.514415	0.42405
B.cells	IRAK4	0.213109	4.419459	1.600494	0.113056	-5.15249	0.509623	0.416801
B.cells	CCDC34	-0.35989	4.840796	-1.60021	0.113119	-5.08555	0.506297	0.412019
B.cells	IQGAP2	-0.2176	7.075634	-1.59987	0.113194	-5.39156	0.488696	0.387315
B.cells	FCHO2	0.15694	6.902577	1.59931	0.113319	-5.58139	0.490033	0.389401
B.cells	FARSB	0.189238	5.008108	1.599302	0.113321	-5.24775	0.504954	0.410491
B.cells	FGD3	0.235699	4.660813	1.599247	0.113333	-5.22227	0.507747	0.414489
B.cells	GIMAP1OS	0.510967	0.862202	1.599224	0.113339	-4.60925	0.539505	0.46068
B.cells	STMN1	-0.37088	7.897729	-1.59888	0.113415	-5.6799	0.48253	0.378932
B.cells	ZFP266	0.211413	4.029092	1.598313	0.113541	-5.04509	0.513008	0.422254
B.cells	P4HB	-0.14547	7.267668	-1.59824	0.113558	-5.6263	0.487345	0.385876
B.cells	ST3GAL2	-0.34961	3.085043	-1.59819	0.113569	-4.83097	0.520785	0.433541
B.cells	GLRX2	0.180417	5.111058	1.598096	0.113589	-5.24782	0.504261	0.40977
B.cells	ALPK3	-1.03596	-0.11417	-1.59765	0.113688	-4.33064	0.548441	0.473886
B.cells	CDKN1A	0.319774	5.267608	1.597266	0.113775	-5.15767	0.503437	0.408053
B.cells	TANC1	-0.43164	3.838336	-1.59619	0.114016	-4.84389	0.515703	0.424746
B.cells	LMAN1	-0.25214	4.627609	-1.59618	0.114017	-5.11057	0.509268	0.415516
B.cells	LONP2	0.141961	5.87764	1.595864	0.114088	-5.40129	0.499309	0.401419
B.cells	GM41409	-0.46768	3.031183	-1.59555	0.114158	-4.85335	0.522425	0.434641
B.cells	FBXL22	-0.48229	2.248825	-1.59554	0.11416	-4.67626	0.528997	0.444203
B.cells	NPHP1	-0.86955	0.652168	-1.59523	0.114229	-4.35017	0.542826	0.464413
B.cells	PARP10	0.36451	3.565506	1.594936	0.114295	-4.92327	0.518204	0.428372
B.cells	NTHL1	0.659076	0.544589	1.594679	0.114353	-4.38737	0.543929	0.46606
B.cells	DDX3X	-0.1781	7.320412	-1.59383	0.114543	-5.62534	0.488668	0.386242
B.cells	GPX7	-0.89336	0.610424	-1.59363	0.114587	-4.37266	0.543754	0.465597
B.cells	SNX29	0.216304	6.395771	1.593548	0.114606	-5.51826	0.495855	0.396423
B.cells	SPPL2B	0.482024	2.17345	1.593298	0.114662	-4.62763	0.530302	0.445981
B.cells	GM32401	0.418956	2.445198	1.593169	0.114691	-4.77611	0.528002	0.442623

B.cells	SMLR1	-0.59469	1.822706	-1.59309	0.114709	-4.56297	0.533287	0.450352
B.cells	SLC16A7	-0.34777	3.236151	-1.59292	0.114748	-4.85756	0.521375	0.432991
B.cells	ZC3H4	-0.18087	4.818849	-1.59269	0.114798	-5.18939	0.508432	0.414365
B.cells	PTGS1	-0.43178	3.986721	-1.59234	0.114877	-4.82328	0.515359	0.424185
B.cells	FBXL6	-0.29396	3.399881	-1.59166	0.115031	-4.86727	0.520697	0.431241
B.cells	TMEM241	0.277043	4.310016	1.591044	0.115169	-5.11348	0.513621	0.420621
B.cells	PBX4	0.663718	0.976518	1.590368	0.115321	-4.47215	0.541879	0.46161
B.cells	RBMS2	0.186984	4.872344	1.590324	0.115331	-5.22472	0.509204	0.414201
B.cells	TOMM70A	0.154614	5.752051	1.590269	0.115344	-5.39256	0.502148	0.404189
B.cells	PHF2OS1	-0.61548	1.058613	-1.5901	0.115382	-4.4248	0.541166	0.460682
B.cells	TMEM26	0.506366	2.190341	1.589798	0.11545	-4.7371	0.531552	0.446566
B.cells	GM41790	-0.96191	-0.09939	-1.58944	0.115532	-4.36789	0.551483	0.476081
B.cells	C8A	-0.77762	0.525618	-1.58936	0.115548	-4.39592	0.545971	0.467893
B.cells	SRRT	-0.17803	5.528359	-1.58866	0.115706	-5.35131	0.504424	0.40745
B.cells	CNR2	0.248057	3.101889	1.588613	0.115718	-5.11259	0.524273	0.435919
B.cells	PGLS	-0.14867	7.788311	-1.58741	0.11599	-5.68702	0.487216	0.382925
B.cells	CCDC152	-0.44621	3.284464	-1.58724	0.116029	-4.83557	0.52327	0.434103
B.cells	ZFR	0.13028	6.532381	1.587059	0.116069	-5.54961	0.496967	0.396655
B.cells	RHOD	-0.67072	1.328394	-1.5869	0.116104	-4.44828	0.539896	0.458541
B.cells	POMGNT1	-0.39632	2.312495	-1.5868	0.116127	-4.64598	0.531456	0.446186
B.cells	ASNSD1	0.167051	5.360445	1.586709	0.116149	-5.33996	0.506274	0.409905
B.cells	ATP8B1	-0.69665	0.813793	-1.58592	0.116327	-4.46461	0.544371	0.465416
B.cells	PTPRK	-0.58938	3.178658	-1.58575	0.116366	-4.75448	0.524154	0.435859
B.cells	HPX	-0.31151	5.670685	-1.58568	0.116383	-5.44053	0.503791	0.40665
B.cells	ANAPC2	-0.22952	4.497598	-1.58568	0.116383	-5.13595	0.513259	0.42015
B.cells	TMEM230	-0.19064	4.742854	-1.58551	0.11642	-5.15004	0.511262	0.417302
B.cells	SMARCB1	0.149526	5.410318	1.585492	0.116425	-5.34798	0.505874	0.409619
B.cells	CCDC58	0.228757	4.247902	1.585125	0.116508	-5.12148	0.515301	0.423217
B.cells	CHMP1B	-0.20244	4.698125	-1.58501	0.116536	-5.20119	0.511625	0.418022
B.cells	TRIM11	0.195533	5.06784	1.584904	0.116559	-5.27892	0.50863	0.413741
B.cells	NUDT7	-0.56405	1.74453	-1.58483	0.116574	-4.5702	0.536309	0.453824
B.cells	PNPLA8	0.167948	6.018439	1.58434	0.116687	-5.47168	0.501024	0.403075
B.cells	SLC25A13	-0.23791	4.679744	-1.58394	0.116777	-5.26482	0.511775	0.41846
B.cells	GPLD1	-0.7126	0.934219	-1.58372	0.116827	-4.47451	0.54332	0.464417
B.cells	TACSTD2	0.806596	-0.35025	1.583719	0.116828	-4.40868	0.55465	0.481276
B.cells	LUZP1	-0.19866	5.787075	-1.58372	0.116828	-5.427	0.502862	0.405764
B.cells	EVL	0.194375	6.485099	1.583586	0.116859	-5.56414	0.497338	0.398025
B.cells	DNAJB2	0.378684	2.766414	1.583403	0.1169	-4.75448	0.527615	0.441505
B.cells	GLYAT	-0.57613	2.069154	-1.58308	0.116973	-4.62833	0.533529	0.450247
B.cells	LSP1	-0.1702	8.230432	-1.58278	0.117042	-5.92155	0.483838	0.379312
B.cells	SDCBP	0.143236	7.674968	1.582769	0.117045	-5.71139	0.488087	0.385221
B.cells	AHR	-0.29962	4.719836	-1.58266	0.117069	-5.37778	0.511449	0.418277
B.cells	GM4070	0.583206	2.887167	1.582606	0.117082	-4.81398	0.526599	0.440168
B.cells	UEVLD	0.40197	2.944551	1.582245	0.117164	-4.76794	0.526117	0.439473
B.cells	SLC37A3	0.242286	3.658041	1.582214	0.117171	-5.03791	0.520164	0.430834
B.cells	RECK	-0.76292	1.480341	-1.58188	0.117247	-4.50294	0.538728	0.457745
B.cells	LYVE1	-0.8513	2.42534	-1.58141	0.117356	-4.55792	0.530801	0.446018

B.cells	UOX	0.406959	5.010934	1.581337	0.117372	-5.36502	0.50938	0.415067
B.cells	MAD2L1	-0.34969	4.166093	-1.58102	0.117445	-4.9834	0.516295	0.42503
B.cells	TIAM2	-0.4674	2.628932	-1.58092	0.117468	-4.86462	0.529106	0.443655
B.cells	R3HDM2	0.130025	6.193799	1.580694	0.117519	-5.48632	0.499976	0.401815
B.cells	TMEM219	0.223047	4.624458	1.580375	0.117592	-5.15762	0.512682	0.419871
B.cells	VWCE	-0.84381	0.865529	-1.5802	0.117633	-4.37455	0.544405	0.466251
B.cells	LENG9	0.381573	2.352054	1.579747	0.117736	-4.76733	0.531619	0.447508
B.cells	GOLGA1	0.280244	3.427325	1.579699	0.117747	-4.90438	0.52257	0.43432
B.cells	ATP6V1A	0.17065	6.534998	1.579588	0.117772	-5.54144	0.497413	0.398342
B.cells	AMOTL1	-0.73562	1.696223	-1.5786	0.117999	-4.4409	0.538057	0.456176
B.cells	D11WSU47	0.657279	1.299867	1.577767	0.118191	-4.46976	0.542159	0.461479
B.cells	UTRN	0.193359	7.31422	1.576791	0.118415	-5.63994	0.493451	0.39065
B.cells	RTN2	-0.96941	-0.4318	-1.57598	0.118602	-4.34385	0.558837	0.484972
B.cells	RARG	-0.63068	1.622679	-1.57586	0.11863	-4.52904	0.540703	0.458104
B.cells	LPIN2	0.190285	6.528551	1.575621	0.118685	-5.55464	0.500092	0.399752
B.cells	PLAUR	0.265053	6.837467	1.575355	0.118746	-5.69897	0.497657	0.396434
B.cells	STRBP	-0.15968	7.195368	-1.57498	0.118832	-5.69986	0.494854	0.392667
B.cells	FBXW4	0.276215	4.272711	1.574886	0.118855	-5.10492	0.518306	0.425953
B.cells	IQCH	-1.04242	0.250926	-1.57465	0.118908	-4.40252	0.552735	0.476351
B.cells	SMCR8	-0.32017	3.28105	-1.57451	0.118942	-4.90567	0.526558	0.437918
B.cells	DNAJA4	0.652276	0.616749	1.574184	0.119017	-4.49294	0.549497	0.471713
B.cells	ATP1B1	-0.28808	5.798004	-1.57408	0.11904	-5.35887	0.505907	0.408412
B.cells	PHF11B	0.307412	4.313119	1.573864	0.119091	-5.3211	0.517972	0.425774
B.cells	HDAC3	-0.1981	4.707537	-1.57377	0.119112	-5.17448	0.514735	0.421147
B.cells	ANKS1	0.207891	5.759857	1.573506	0.119173	-5.39694	0.506212	0.409085
B.cells	GAL3ST1	-0.92562	-0.29084	-1.57344	0.11919	-4.35666	0.557572	0.484057
B.cells	PLCB1	-0.39787	4.622522	-1.57333	0.119215	-5.20789	0.515431	0.422267
B.cells	SMARCA4	-0.15673	7.5105	-1.57319	0.119247	-5.73296	0.492401	0.389688
B.cells	TRIM23	0.299023	3.295163	1.572761	0.119346	-4.8727	0.526676	0.438292
B.cells	GM38394	0.657705	0.848639	1.572441	0.11942	-4.48101	0.547714	0.469248
B.cells	MORF4L2	-0.18076	5.404884	-1.57227	0.11946	-5.30486	0.509309	0.413484
B.cells	RNF168	0.273336	4.161361	1.572123	0.119494	-5.06799	0.51947	0.428078
B.cells	EVI5	0.219563	5.166502	1.571975	0.119528	-5.38486	0.511239	0.41631
B.cells	CDK7	0.224343	4.595584	1.571525	0.119633	-5.20755	0.51599	0.423123
B.cells	4933439C1	0.659966	1.200712	1.571469	0.119646	-4.47302	0.544729	0.465046
B.cells	NSDHL	-0.44044	2.406095	-1.5713	0.119685	-4.67947	0.534317	0.449753
B.cells	CAMK2B	-0.39637	3.706454	-1.57099	0.119758	-5.02582	0.523463	0.433853
B.cells	MAOB	-0.69795	1.627366	-1.57028	0.119922	-4.5575	0.541471	0.459851
B.cells	DTX4	0.549443	2.365512	1.570073	0.11997	-4.66269	0.535112	0.450509
B.cells	TFAM	0.222777	4.293459	1.569875	0.120016	-5.15136	0.518909	0.426993
B.cells	TRAF1	0.414318	3.343827	1.569743	0.120047	-5.06594	0.526818	0.438487
B.cells	UROS	0.325668	2.669254	1.569699	0.120057	-4.74757	0.532521	0.446813
B.cells	AKR1E1	-0.42712	2.622552	-1.56944	0.120117	-4.64849	0.532918	0.447522
B.cells	FAM3C	0.188538	5.36557	1.569322	0.120145	-5.27763	0.510148	0.414628
B.cells	PPM1J	0.826429	-0.35731	1.569067	0.120204	-4.37971	0.559071	0.486186
B.cells	ATP5G3	-0.16936	8.015505	-1.56834	0.120375	-5.78504	0.489801	0.38542
B.cells	ASAP2	-0.35553	3.068411	-1.56761	0.120545	-4.95297	0.530303	0.442509

B.cells	CD200	0.526157	1.910379	1.566824	0.120728	-4.73162	0.540829	0.457296
B.cells	4833419F2	0.604548	1.323387	1.56637	0.120834	-4.57863	0.546113	0.465153
B.cells	ERCC3	0.23727	3.695125	1.565931	0.120937	-5.03357	0.525799	0.43546
B.cells	CHCHD6	0.332461	2.968098	1.56581	0.120965	-4.76351	0.531931	0.444359
B.cells	CHMP5	0.159005	5.632148	1.565722	0.120986	-5.40403	0.509862	0.412602
B.cells	ABHD12	0.177393	5.584506	1.565696	0.120992	-5.43547	0.510247	0.413149
B.cells	GGH	0.317569	4.555539	1.565464	0.121047	-5.11192	0.518688	0.425295
B.cells	CD200R2	-0.93074	0.789771	-1.56518	0.121113	-4.46125	0.550848	0.472378
B.cells	RND3	-0.48007	2.943599	-1.56508	0.121137	-4.75694	0.532179	0.444975
B.cells	HIST2H2AA	0.570476	2.225914	1.564736	0.121217	-4.6331	0.538476	0.454175
B.cells	HPS6	0.552899	1.22115	1.564012	0.121387	-4.5471	0.547577	0.467332
B.cells	MTHFD2	-0.24795	5.486209	-1.56391	0.121412	-5.35816	0.511574	0.415041
B.cells	KCTD4	0.422504	2.200261	1.563812	0.121434	-4.79303	0.539058	0.45483
B.cells	CRADD	0.183049	5.224096	1.563333	0.121547	-5.31122	0.513929	0.418239
B.cells	BCL2L1	-0.23623	6.15449	-1.5632	0.121577	-5.58002	0.506408	0.407581
B.cells	ITGA1	-0.45112	3.828562	-1.5629	0.121648	-4.92835	0.525569	0.434966
B.cells	INTS2	-0.29795	4.120259	-1.56266	0.121705	-5.06301	0.523185	0.431449
B.cells	TNNT1	0.647215	1.520318	1.561937	0.121875	-4.46868	0.545791	0.464059
B.cells	CXCR4	-0.22889	6.146916	-1.56187	0.121891	-5.56441	0.507022	0.408024
B.cells	EMC7	-0.13184	6.059195	-1.5613	0.122025	-5.48891	0.507927	0.409233
B.cells	CCNB1	-0.53105	4.032241	-1.56128	0.12203	-4.97647	0.524536	0.43299
B.cells	6330418K0	0.55535	1.506684	1.56103	0.122089	-4.56471	0.546189	0.464615
B.cells	AMT	-0.64512	1.565291	-1.56053	0.122207	-4.56548	0.54592	0.464098
B.cells	HSF5	-0.82494	0.277217	-1.56038	0.122244	-4.41062	0.55732	0.48111
B.cells	CD101	0.775417	0.741284	1.560224	0.122279	-4.43191	0.553182	0.475029
B.cells	PLCL2	0.165574	6.985417	1.559932	0.122349	-5.64245	0.500924	0.399306
B.cells	1700008J0	0.529585	1.842472	1.558863	0.122601	-4.635	0.54453	0.461081
B.cells	RPF2	-0.24353	4.402771	-1.55844	0.122702	-5.20427	0.522967	0.429477
B.cells	GM4707	0.3797	2.866085	1.557992	0.122808	-4.96015	0.536172	0.448322
B.cells	TRIM5	0.29709	3.589282	1.557823	0.122848	-5.04118	0.530022	0.439433
B.cells	FAM167B	-0.58789	2.127548	-1.55694	0.123057	-4.55906	0.543262	0.45784
B.cells	CERK	0.173777	7.028222	1.556729	0.123107	-5.63441	0.502575	0.39957
B.cells	TPCN2	-0.43899	2.597655	-1.55594	0.123296	-4.70558	0.53963	0.452359
B.cells	PCBP1	-0.1288	8.199098	-1.55561	0.123374	-5.8551	0.493767	0.387129
B.cells	LIPA	-0.22693	5.64326	-1.55539	0.123425	-5.38931	0.514099	0.415724
B.cells	DNAJB5	-0.64602	1.175316	-1.55524	0.123462	-4.47409	0.552058	0.470756
B.cells	VPREB2	-0.95728	-0.41404	-1.55521	0.123468	-4.38015	0.566335	0.491994
B.cells	NANOS1	-0.71684	0.634448	-1.5548	0.123567	-4.44926	0.55687	0.478066
B.cells	EGFL7	-0.44393	3.872106	-1.55469	0.123591	-4.88809	0.52877	0.436942
B.cells	INPP5D	0.161689	8.764735	1.554442	0.123652	-5.94509	0.489398	0.381383
B.cells	PNKD	0.224193	4.867546	1.554419	0.123657	-5.26014	0.520465	0.425083
B.cells	A530017D2	0.374214	2.325165	1.554279	0.12369	-4.7635	0.541986	0.456286
B.cells	GTF2H1	-0.16439	5.958143	-1.55414	0.123723	-5.48926	0.511542	0.412443
B.cells	MEA1	0.184174	5.276389	1.554037	0.123748	-5.34224	0.517099	0.4204
B.cells	SPNS1	-0.26995	3.498213	-1.55308	0.123975	-4.99932	0.532034	0.442025
B.cells	TUBB5	-0.23074	9.849372	-1.55303	0.123989	-6.07721	0.481241	0.370407
B.cells	PKD1	0.322051	3.003757	1.55282	0.124039	-4.91014	0.536247	0.448148

B.cells	GM50386	0.548249	0.179027	1.552769	0.124051	-4.55947	0.561069	0.484749
B.cells	HOPX	0.29601	4.034194	1.552364	0.124148	-5.21079	0.527511	0.435535
B.cells	TMA7	0.123636	6.564001	1.552301	0.124163	-5.58744	0.506762	0.405916
B.cells	REPS2	-0.75417	0.905504	-1.55216	0.124198	-4.47145	0.554561	0.475177
B.cells	GM26782	0.280445	2.991571	1.551882	0.124263	-4.86506	0.536352	0.448548
B.cells	SUPT16	-0.12944	7.068147	-1.55154	0.124346	-5.65886	0.502743	0.400537
B.cells	TRMT44	0.465986	1.793941	1.55151	0.124352	-4.67665	0.54672	0.463868
B.cells	THAP2	-0.31934	3.329281	-1.55133	0.124394	-4.94011	0.533469	0.444498
B.cells	BANK1	0.298619	6.09331	1.550946	0.124487	-5.60178	0.510549	0.411642
B.cells	STX2	0.251685	3.698442	1.550843	0.124512	-4.97916	0.530339	0.440021
B.cells	PCBP3	-0.36875	2.616922	-1.55083	0.124515	-4.78657	0.53957	0.453463
B.cells	HTRA3	-0.73969	0.502839	-1.55078	0.124528	-4.44123	0.558158	0.480911
B.cells	GM36862	-0.90531	0.340355	-1.55076	0.124531	-4.38952	0.559616	0.483085
B.cells	SLC7A5	-0.23115	6.118367	-1.5507	0.124546	-5.56239	0.510346	0.41137
B.cells	TMEM106(	-0.34082	3.205819	-1.55043	0.12461	-4.79542	0.534577	0.446214
B.cells	GRAP	0.247504	4.536304	1.550266	0.12465	-5.23372	0.523369	0.43008
B.cells	SOCS4	0.190679	4.684801	1.549975	0.12472	-5.2453	0.5222	0.428443
B.cells	SMPDL3B	0.784145	1.9506	1.549822	0.124756	-4.61709	0.545475	0.462315
B.cells	RAB8A	0.165926	5.454242	1.548694	0.125028	-5.40556	0.51673	0.419843
B.cells	TNFSF12	-0.46192	2.295218	-1.54857	0.125058	-4.67337	0.543389	0.458469
B.cells	SPSB2	0.374736	2.383882	1.548376	0.125104	-4.71663	0.542623	0.457367
B.cells	ADGRE5	-0.15956	7.153842	-1.54793	0.125212	-5.69303	0.503271	0.400565
B.cells	TPM4	0.141764	7.167543	1.547642	0.125281	-5.72466	0.503162	0.400552
B.cells	ENGASE	-0.65951	1.496503	-1.54736	0.125349	-4.57264	0.550651	0.46914
B.cells	LIN54	0.205907	6.14118	1.547308	0.125361	-5.5453	0.511389	0.412261
B.cells	LILRA6	-0.58716	1.213739	-1.54718	0.125393	-4.63688	0.553153	0.472887
B.cells	FUT4	-0.78818	-0.16835	-1.54685	0.125472	-4.42473	0.565609	0.491474
B.cells	4833445I0	-0.67469	0.48418	-1.54676	0.125493	-4.45695	0.559708	0.482653
B.cells	EHMT2	0.192188	4.951712	1.545882	0.125705	-5.32271	0.521726	0.426562
B.cells	CPNE9	-0.66795	1.867904	-1.54582	0.125721	-4.63924	0.548021	0.46478
B.cells	FBXO11	-0.16221	8.797772	-1.54554	0.125789	-6.00478	0.491072	0.383316
B.cells	PLEKHG3	-0.25532	4.378559	-1.54531	0.125844	-5.21335	0.526634	0.433524
B.cells	PDIA4	-0.16797	5.653573	-1.5447	0.12599	-5.41205	0.51649	0.418769
B.cells	SLC25A14	0.362736	2.078186	1.544509	0.126037	-4.74243	0.546756	0.462647
B.cells	KTI12	-0.28434	3.941057	-1.54431	0.126084	-5.10746	0.530738	0.439323
B.cells	METTL2	0.259845	3.27705	1.543994	0.126162	-5.00624	0.536384	0.447605
B.cells	FAM118A	-0.43399	2.069936	-1.54394	0.126174	-4.67756	0.546828	0.462889
B.cells	CC2D1A	0.326133	3.044755	1.54364	0.126247	-4.8983	0.53847	0.450624
B.cells	1700025G(	-0.38021	4.957756	-1.54348	0.126287	-5.08106	0.522319	0.427288
B.cells	TRAFD1	0.264074	5.468862	1.543155	0.126365	-5.43035	0.518231	0.42143
B.cells	ZFP367	-0.2655	5.436634	-1.54251	0.126521	-5.34221	0.518948	0.42201
B.cells	CLEC4A1	0.633221	3.158732	1.542175	0.126603	-4.82002	0.53825	0.449792
B.cells	RNFT1	0.187434	4.495375	1.541584	0.126747	-5.27099	0.526987	0.433563
B.cells	GBF1	0.125311	6.562441	1.541361	0.126801	-5.57894	0.509999	0.409377
B.cells	1810006J0	0.744399	-0.01116	1.541335	0.126807	-4.45049	0.566369	0.49151
B.cells	SERTAD1	-0.21585	5.61417	-1.54126	0.126824	-5.44243	0.517711	0.420336
B.cells	STXBP6	-0.52636	2.953526	-1.54116	0.12685	-4.91513	0.540093	0.452666

B.cells	CHD3	-0.25939	4.533669	-1.54066	0.126971	-5.20977	0.52698	0.433261
B.cells	ATP6V1B2	0.140111	5.98524	1.539874	0.127163	-5.5561	0.515343	0.416442
B.cells	CS	0.135247	6.103857	1.539788	0.127184	-5.54787	0.514377	0.415079
B.cells	CYB5R4	-0.16151	5.869945	-1.53973	0.127197	-5.48674	0.516285	0.417802
B.cells	AGTPBP1	0.201437	5.128653	1.539161	0.127336	-5.42551	0.522771	0.426677
B.cells	STYX	0.183244	4.526153	1.538716	0.127445	-5.2656	0.527933	0.433977
B.cells	GM8797	-0.44842	2.19543	-1.53865	0.127461	-4.66863	0.547924	0.463054
B.cells	SPCS2	-0.12561	7.595587	-1.53843	0.127515	-5.75335	0.502932	0.398507
B.cells	NCOA6	0.146761	5.600455	1.53764	0.127708	-5.47721	0.519554	0.421521
B.cells	RABEPK	0.28794	3.296184	1.537388	0.127769	-4.96524	0.538942	0.44952
B.cells	MPPED2	-0.97109	-0.32287	-1.53735	0.127779	-4.4158	0.571117	0.49708
B.cells	AGTR1A	-0.98993	0.270352	-1.53681	0.127911	-4.43082	0.566039	0.489139
B.cells	MLLT10	0.112103	7.464033	1.536657	0.127949	-5.76771	0.504779	0.40042
B.cells	TPMT	-0.42897	2.097645	-1.53611	0.128081	-4.68789	0.550068	0.465149
B.cells	COG8	0.189295	4.299473	1.534514	0.128474	-5.21573	0.532381	0.438067
B.cells	POU2AF1	0.303729	4.320007	1.534256	0.128538	-5.2942	0.532207	0.437929
B.cells	FBL	-0.16846	6.091074	-1.53416	0.128562	-5.55458	0.51746	0.416916
B.cells	MCEMP1	0.822843	1.650063	1.534085	0.128579	-4.59944	0.555379	0.471765
B.cells	SERINC1	0.13447	6.230989	1.533284	0.128777	-5.60094	0.516541	0.415675
B.cells	CEP85L	0.2593	4.806918	1.533246	0.128786	-5.31269	0.528335	0.432487
B.cells	OSGIN2	0.294763	3.18431	1.533162	0.128807	-5.06004	0.542161	0.452484
B.cells	GM12216	0.294728	4.554087	1.533106	0.12882	-5.23412	0.530462	0.435559
B.cells	1110006O2	-0.77349	0.319918	-1.53265	0.128933	-4.41506	0.567646	0.490062
B.cells	ARCN1	0.155691	5.873425	1.53248	0.128974	-5.51014	0.519509	0.419917
B.cells	GM49067	-0.72741	0.406337	-1.53247	0.128978	-4.43389	0.566858	0.488887
B.cells	ZFP974	0.686347	0.877863	1.532154	0.129055	-4.55407	0.562583	0.48263
B.cells	MFSD5	-0.2375	4.282812	-1.53213	0.12906	-5.20672	0.532792	0.439023
B.cells	SLC12A9	0.240649	4.242634	1.531809	0.12914	-5.27277	0.533272	0.439666
B.cells	NMI	0.230915	5.064624	1.530852	0.129376	-5.39828	0.52686	0.430133
B.cells	GM16337	0.462027	1.967439	1.530764	0.129398	-4.74967	0.553524	0.468855
B.cells	SIGMAR1	-0.27196	3.549056	-1.53074	0.129403	-5.03649	0.539716	0.448674
B.cells	AFG1L	0.297887	3.691074	1.530538	0.129454	-5.12175	0.538505	0.446948
B.cells	EFNA2	-0.89404	1.10216	-1.53025	0.129524	-4.50293	0.561261	0.480354
B.cells	2900097C1	-0.19259	4.909122	-1.52999	0.12959	-5.26513	0.528172	0.432145
B.cells	RYK	-0.52224	2.042505	-1.52989	0.129614	-4.63393	0.552869	0.468027
B.cells	PSMD1	-0.14618	6.546401	-1.5298	0.129637	-5.60811	0.514648	0.412918
B.cells	PRKRIP1	0.211945	3.964552	1.529546	0.129699	-5.14009	0.536233	0.443765
B.cells	CCDC88A	-0.20058	5.372063	-1.52877	0.129892	-5.46148	0.524589	0.426979
B.cells	PIK3CD	0.1905	6.624599	1.528744	0.129898	-5.63381	0.51429	0.412346
B.cells	TBKBP1	0.642295	1.449005	1.52866	0.129919	-4.57105	0.55845	0.47623
B.cells	SLC25A23	-0.4849	2.25448	-1.52856	0.129944	-4.65568	0.551295	0.465704
B.cells	RCL1	0.208843	4.24025	1.528396	0.129984	-5.25148	0.534106	0.44068
B.cells	ARHGEF5	-0.97287	0.327235	-1.52778	0.130137	-4.40365	0.569022	0.491624
B.cells	PRRG2	0.413398	2.322172	1.527198	0.130281	-4.70454	0.551114	0.465399
B.cells	AP2B1	0.134462	6.179239	1.52705	0.130318	-5.55282	0.518315	0.418027
B.cells	ULK4	-0.34381	2.667963	-1.52702	0.130326	-4.87476	0.548076	0.460945
B.cells	UTP25	0.341255	2.617836	1.527013	0.130327	-4.85142	0.548516	0.461588

B.cells	TRMT10A	-0.25303	3.81366	-1.52661	0.130426	-5.10309	0.538151	0.446524
B.cells	COTL1	-0.16316	6.629821	-1.52658	0.130436	-5.64672	0.514635	0.412854
B.cells	SHTN1	-0.46186	3.187667	-1.52618	0.130534	-4.80153	0.543548	0.454535
B.cells	PARVG	-0.19875	5.071901	-1.52606	0.130563	-5.3421	0.527491	0.431357
B.cells	PLAU	-0.92564	0.814983	-1.52599	0.130581	-4.4833	0.564583	0.485591
B.cells	SCARF1	-0.59765	1.587665	-1.52565	0.130666	-4.56904	0.557631	0.475279
B.cells	VEZT	0.219829	4.178132	1.525643	0.130668	-5.16204	0.535037	0.442235
B.cells	PLEKHB2	0.203608	4.419141	1.525582	0.130683	-5.23448	0.53299	0.439278
B.cells	IGHM	-0.32652	8.158942	-1.52517	0.130785	-5.94008	0.502476	0.395968
B.cells	ANXA11	0.162498	5.969579	1.525106	0.130802	-5.56031	0.520141	0.420868
B.cells	GID4	0.207834	4.230623	1.524728	0.130896	-5.18539	0.534893	0.44181
B.cells	GTF2F2	0.147423	5.881638	1.524457	0.130964	-5.53864	0.521098	0.422069
B.cells	MSANTD2	0.177274	4.864755	1.524315	0.130999	-5.3167	0.52957	0.434203
B.cells	ANO6	0.189294	6.75402	1.523713	0.13115	-5.65946	0.514366	0.412042
B.cells	HIST1H2BJ	-0.49185	3.30978	-1.52332	0.131247	-4.90292	0.543482	0.45363
B.cells	S100A6	0.331422	7.580063	1.522864	0.131362	-5.9742	0.507966	0.402915
B.cells	ATG2A	0.188953	5.710857	1.52269	0.131405	-5.5332	0.523193	0.424516
B.cells	GTPBP2	0.214281	4.751688	1.522527	0.131446	-5.28896	0.531216	0.436082
B.cells	FAM149B	0.312991	2.992688	1.522516	0.131449	-4.91898	0.546307	0.457962
B.cells	IKBIP	-0.51061	1.938543	-1.52185	0.131614	-4.61024	0.556094	0.471849
B.cells	INHBA	0.886839	1.050066	1.521159	0.131788	-4.60866	0.564367	0.483933
B.cells	KCTD13	0.380767	2.619439	1.520848	0.131866	-4.75865	0.550364	0.463369
B.cells	ARHGAP33	-0.86926	0.549492	-1.52066	0.131913	-4.434	0.568919	0.490943
B.cells	GM28960	0.768686	-0.82667	1.520591	0.131931	-4.43745	0.58149	0.510055
B.cells	CMPK2	0.430949	3.672891	1.520579	0.131934	-5.07739	0.54119	0.45011
B.cells	HDAC2	-0.19549	5.300553	-1.52046	0.131963	-5.38686	0.527365	0.430226
B.cells	CAPG	0.25505	5.264343	1.520277	0.13201	-5.52915	0.527671	0.430765
B.cells	ZFP623	-0.87253	0.124749	-1.51981	0.132126	-4.42825	0.573037	0.497223
B.cells	CD160	-0.73493	1.916487	-1.51971	0.132153	-4.66732	0.556802	0.473104
B.cells	SURF4	-0.18337	5.782118	-1.51926	0.132265	-5.46639	0.523751	0.424967
B.cells	A530064D(	0.743021	0.81889	1.519142	0.132295	-4.49199	0.566893	0.487889
B.cells	APBA3	0.414421	2.107298	1.51854	0.132446	-4.75474	0.555708	0.470917
B.cells	RAD18	0.282258	4.103468	1.518329	0.132499	-5.15768	0.538285	0.445519
B.cells	AGPAT1	0.29351	3.479015	1.518043	0.132571	-5.03549	0.543666	0.453434
B.cells	ODC1	-0.20927	5.188947	-1.51784	0.132622	-5.38287	0.529079	0.432421
B.cells	2310009A(	0.214933	4.692253	1.51756	0.132693	-5.26084	0.533268	0.438565
B.cells	STK3	-0.18557	5.985237	-1.51748	0.132714	-5.57437	0.522444	0.423058
B.cells	VPS54	0.166953	6.506686	1.517472	0.132715	-5.63181	0.518152	0.416961
B.cells	UPRT	0.61427	0.606501	1.516577	0.132941	-4.53529	0.569811	0.491749
B.cells	FAM185A	0.362963	2.579686	1.516572	0.132942	-4.8726	0.552082	0.465506
B.cells	DMXL1	0.181372	6.604824	1.515743	0.133152	-5.64673	0.517902	0.416494
B.cells	APRT	0.197766	6.416788	1.515715	0.133159	-5.6672	0.519444	0.41868
B.cells	NRIP1	0.205311	6.353871	1.515472	0.13322	-5.5912	0.519961	0.419461
B.cells	MLH1	-0.30689	2.750298	-1.51535	0.133251	-4.82608	0.550613	0.463717
B.cells	PIP4K2A	0.166031	7.239298	1.515065	0.133323	-5.76831	0.51274	0.409297
B.cells	WFDC21	0.743659	2.947804	1.515057	0.133325	-5.14013	0.548879	0.461231
B.cells	WASHC1	0.19954	4.182087	1.515003	0.133339	-5.18458	0.538187	0.445658

B.cells	RASA1	0.160047	6.030636	1.514832	0.133382	-5.55028	0.522627	0.423343
B.cells	SPPL2A	0.192712	6.600738	1.514695	0.133417	-5.69438	0.517936	0.416761
B.cells	MARVELD1	-0.57396	1.253094	-1.51442	0.133487	-4.57138	0.563965	0.483707
B.cells	BAZ1B	0.165822	6.23949	1.514384	0.133495	-5.56693	0.520902	0.421052
B.cells	SLC14A1	0.316527	3.335927	1.514332	0.133509	-5.03494	0.545491	0.456508
B.cells	IL18	-0.31388	3.422293	-1.5135	0.133719	-5.02501	0.545408	0.455627
B.cells	WDFY2	0.215981	5.19857	1.511722	0.134171	-5.41334	0.53139	0.434208
B.cells	ERRFI1	-0.24048	5.004787	-1.51148	0.134232	-5.41493	0.533027	0.436626
B.cells	MCM5	-0.39216	4.910563	-1.51144	0.134242	-5.27068	0.533825	0.437773
B.cells	ITPRID2	0.179581	5.08881	1.511437	0.134243	-5.50256	0.532317	0.435606
B.cells	RUNDC3B	-0.36858	3.848947	-1.51141	0.134249	-5.11733	0.542914	0.450905
B.cells	OSTF1	0.110809	7.480967	1.511207	0.134302	-5.78587	0.512574	0.407641
B.cells	DNTTIP1	0.169611	4.812219	1.510827	0.134398	-5.29745	0.534882	0.439296
B.cells	MAT2A	-0.13759	6.522913	-1.51044	0.134498	-5.63631	0.520646	0.419024
B.cells	TTF1	0.271133	3.466533	1.5099	0.134635	-5.05934	0.546532	0.456417
B.cells	WAPL	0.150565	7.165135	1.509839	0.13465	-5.7392	0.515393	0.411763
B.cells	GLP2R	-0.59277	0.940346	-1.50972	0.134679	-4.59187	0.569067	0.489667
B.cells	LRR1	-0.64416	1.435876	-1.50969	0.134688	-4.53111	0.564563	0.482971
B.cells	FBRSL1	-0.14587	5.766788	-1.50953	0.134728	-5.5193	0.526913	0.428197
B.cells	GM15133	0.472553	1.788244	1.509474	0.134743	-4.73341	0.561386	0.478328
B.cells	TACC1	-0.11194	7.703473	-1.50916	0.134824	-5.84341	0.511166	0.40577
B.cells	CCDC167	-0.2578	4.065969	-1.50892	0.134883	-5.1541	0.541526	0.449038
B.cells	TRAPPC3	0.190995	4.917333	1.50859	0.134969	-5.36495	0.534399	0.438681
B.cells	ABCG2	-0.37807	3.149803	-1.50797	0.135127	-4.82931	0.550064	0.461154
B.cells	BLK	0.28024	4.074052	1.507824	0.135165	-5.20578	0.542022	0.449444
B.cells	NDST2	0.296111	2.934923	1.507566	0.135231	-4.98622	0.551959	0.463945
B.cells	PLEKHA6	-0.43345	1.808761	-1.50745	0.135259	-4.74457	0.561987	0.478706
B.cells	APBB2	-0.47597	4.02877	-1.50722	0.135318	-5.02629	0.542465	0.450036
B.cells	PI4K2B	-0.245	3.903982	-1.50673	0.135444	-5.17409	0.543861	0.451716
B.cells	SPTLC1	0.211946	4.232218	1.506497	0.135504	-5.22187	0.541081	0.447739
B.cells	KLRC1	0.77568	1.444197	1.50624	0.13557	-4.53448	0.565794	0.483905
B.cells	1810020O(	-1.04235	-0.23349	-1.50561	0.135732	-4.43438	0.581736	0.507233
B.cells	SMIM11	-0.18626	4.805042	-1.50444	0.136031	-5.28213	0.537703	0.44148
B.cells	TMEM131	0.143367	6.606387	1.504	0.136145	-5.73279	0.522787	0.420094
B.cells	VPS16	0.173281	4.436967	1.503864	0.13618	-5.30181	0.541078	0.446279
B.cells	VWF	0.773661	1.565048	1.503453	0.136286	-4.69475	0.566703	0.483515
B.cells	ATP6V1C1	0.162399	5.189889	1.50316	0.136361	-5.41706	0.534987	0.43727
B.cells	GADD45A	-0.24578	4.876654	-1.50288	0.136432	-5.43596	0.537749	0.441117
B.cells	CCDC85B	0.235028	3.779227	1.502415	0.136553	-5.09571	0.547256	0.45486
B.cells	METTL22	0.511453	1.382795	1.502409	0.136555	-4.63083	0.568618	0.486211
B.cells	CIRBP	0.157218	7.218243	1.5023	0.136583	-5.75049	0.518219	0.413355
B.cells	POLG	-0.25081	3.808912	-1.50201	0.136659	-5.14697	0.547045	0.45461
B.cells	HNRNPAB	-0.14193	8.05587	-1.50186	0.136696	-5.88447	0.511469	0.40401
B.cells	CDC27	0.159609	6.009717	1.501408	0.136813	-5.56172	0.528257	0.427875
B.cells	GM30054	0.457116	1.953461	1.501022	0.136913	-4.83512	0.563494	0.479006
B.cells	SNHG9	-0.43153	4.309776	-1.50093	0.136936	-5.16123	0.542701	0.448611
B.cells	CLEC4B1	-0.68217	-0.61033	-1.50091	0.136941	-4.51297	0.587099	0.514354

B.cells	RASL11B	-0.79126	0.665224	-1.50091	0.136941	-4.46317	0.575252	0.496464
B.cells	NDUFC2	-0.16107	6.741013	-1.50054	0.137037	-5.68562	0.522183	0.419447
B.cells	TJP2	-0.27019	3.845594	-1.50049	0.137051	-5.19036	0.546725	0.454657
B.cells	B430306N(	-0.38286	2.692804	-1.50017	0.137133	-4.92209	0.556871	0.469567
B.cells	MS4A4B	0.393218	3.207847	1.50015	0.137138	-5.05421	0.552311	0.462887
B.cells	TMEM185E	-0.21686	4.12483	-1.49985	0.137217	-5.25291	0.5443	0.45137
B.cells	EIF2S3X	-0.28375	5.323448	-1.49977	0.137237	-5.41969	0.534033	0.436557
B.cells	E230013L2	0.85388	-0.72305	1.499705	0.137253	-4.42211	0.588118	0.516471
B.cells	TOX	-0.43316	4.351569	-1.49929	0.137362	-5.22096	0.54248	0.448736
B.cells	COL14A1	-0.61696	1.863173	-1.49921	0.137383	-4.66042	0.564454	0.480958
B.cells	PARP3	0.395381	2.362796	1.498635	0.137531	-4.84682	0.560318	0.474596
B.cells	IL2RA	-0.49517	2.557963	-1.4985	0.137564	-4.98708	0.558572	0.472065
B.cells	ICA1	-0.6015	2.463147	-1.49816	0.137654	-4.69767	0.559592	0.47345
B.cells	TM4SF4	-0.55184	2.168937	-1.49793	0.137712	-4.76219	0.562277	0.477426
B.cells	ARHGAP6	-0.36683	3.609197	-1.49773	0.137766	-5.11104	0.54952	0.458725
B.cells	LSM3	-0.20089	5.624923	-1.49723	0.137896	-5.49691	0.532416	0.433843
B.cells	PSMB9	0.298677	6.228284	1.497145	0.137917	-5.60985	0.527353	0.426622
B.cells	OSBPL8	0.154329	7.62098	1.496627	0.138052	-5.86528	0.516062	0.410489
B.cells	SDC2	-0.54122	2.267759	-1.49613	0.138182	-4.73398	0.561842	0.476613
B.cells	TESMIN	-0.67773	0.971475	-1.49608	0.138195	-4.61979	0.57363	0.494099
B.cells	RRNAD1	0.229301	3.384404	1.495996	0.138216	-5.10224	0.551912	0.462049
B.cells	TNNT3	-0.4834	1.772075	-1.49595	0.138227	-4.80703	0.566316	0.483246
B.cells	PBDC1	-0.24555	5.348225	-1.49573	0.138285	-5.45772	0.534941	0.437501
B.cells	ARHGEF37	0.897374	0.616444	1.495694	0.138294	-4.60425	0.576908	0.499045
B.cells	ZFP422	-0.22037	4.395092	-1.49453	0.138597	-5.26631	0.543786	0.449631
B.cells	GTF2B	-0.15668	6.738906	-1.49447	0.138614	-5.70983	0.523955	0.421234
B.cells	RAB4B	0.141448	5.9254	1.494279	0.138663	-5.57181	0.530739	0.430947
B.cells	YWHAH	-0.15139	7.687722	-1.49415	0.138696	-5.83806	0.516171	0.410315
B.cells	RUFY2	0.280235	2.954919	1.494119	0.138705	-5.00355	0.556408	0.468097
B.cells	EME1	-0.46206	2.299791	-1.49355	0.138853	-4.76535	0.562554	0.476892
B.cells	CENPB	0.184838	5.450808	1.493286	0.138923	-5.47061	0.535023	0.436854
B.cells	MMS19	0.168807	4.947005	1.493195	0.138946	-5.40718	0.539317	0.44304
B.cells	GABARAP	0.111939	8.497974	1.492999	0.138997	-6.01045	0.509896	0.401384
B.cells	CACNA1E	-0.42945	4.907691	-1.49283	0.139041	-5.39135	0.539654	0.443597
B.cells	STRIP2	-0.50344	1.016356	-1.49223	0.139199	-4.78894	0.574238	0.494667
B.cells	SLX1B	-0.27575	3.074893	-1.49209	0.139235	-4.9724	0.555632	0.467201
B.cells	MGLL	-0.44487	3.495334	-1.49202	0.139253	-4.91421	0.551918	0.461799
B.cells	HMG20A	0.206083	4.897858	1.490917	0.139543	-5.35062	0.539738	0.444548
B.cells	EIF4EBP1	0.160453	5.454955	1.490833	0.139565	-5.53723	0.534988	0.437707
B.cells	TKFC	-0.38776	3.130684	-1.49075	0.139586	-4.94342	0.555137	0.46696
B.cells	C1S1	-0.50189	1.787052	-1.49073	0.139592	-4.72789	0.567189	0.48474
B.cells	ANKRA2	0.224195	3.792457	1.490605	0.139624	-5.13696	0.549311	0.458439
B.cells	GM15337	-0.54825	2.140023	-1.49055	0.139638	-4.73789	0.563994	0.480006
B.cells	GM17106	0.297697	3.395192	1.49046	0.139662	-5.15023	0.5528	0.463536
B.cells	TPM3-RS7	-0.69911	0.526188	-1.49031	0.139702	-4.50143	0.578774	0.502023
B.cells	ANGPT2	0.792999	0.571482	1.490286	0.139708	-4.47652	0.578353	0.501392
B.cells	STARD9	-0.31726	3.529605	-1.49027	0.139713	-5.05896	0.551617	0.461805

B.cells	PLEKHA5	-0.20416	4.693302	-1.48993	0.139801	-5.44317	0.541495	0.447217
B.cells	GM42047	-0.41283	5.263713	-1.48965	0.139875	-5.31795	0.536613	0.440297
B.cells	UBE2D1	0.143475	5.377113	1.48959	0.139891	-5.5026	0.535648	0.438909
B.cells	CHMP2B	-0.17035	4.813502	-1.4894	0.139942	-5.37553	0.540462	0.445922
B.cells	LY6C2	-0.5821	5.53324	-1.48932	0.139962	-5.31791	0.534324	0.437092
B.cells	RBPMS2	-0.48598	1.246218	-1.48915	0.140005	-4.63378	0.572125	0.492558
B.cells	SSR1	-0.13383	6.554701	-1.48891	0.140069	-5.64751	0.525754	0.425028
B.cells	9330020H(	-0.4077	2.471892	-1.48889	0.140075	-4.78299	0.561009	0.476179
B.cells	NT5C2	0.204474	5.867578	1.488875	0.140079	-5.5666	0.531501	0.433237
B.cells	NFRKB	0.176946	4.478568	1.488799	0.140099	-5.30019	0.543346	0.450318
B.cells	ZFYVE9	-0.49573	2.603777	-1.48772	0.140383	-4.85161	0.560777	0.474876
B.cells	ID3	-0.23102	6.599763	-1.48744	0.140456	-5.65354	0.526312	0.424925
B.cells	SAMM50	0.18133	5.177169	1.487011	0.140569	-5.40791	0.538304	0.442151
B.cells	GM16341	0.52199	0.863635	1.486965	0.140582	-4.68948	0.576669	0.49852
B.cells	PTGR2	0.255211	3.734192	1.486835	0.140616	-5.09959	0.550797	0.460329
B.cells	IL10	-0.55436	2.999738	-1.48678	0.140631	-4.92718	0.557286	0.469834
B.cells	TTYH2	-0.40476	3.075271	-1.48633	0.140749	-4.94022	0.556894	0.468923
B.cells	SLC25A37	0.216058	5.267981	1.485829	0.140881	-5.65295	0.537799	0.441246
B.cells	USP16	0.174922	5.208005	1.485698	0.140916	-5.46854	0.538311	0.441984
B.cells	LCORL	0.156488	6.985276	1.48567	0.140923	-5.75579	0.523381	0.420646
B.cells	RAF1	0.16256	5.986456	1.485614	0.140938	-5.59982	0.53171	0.432508
B.cells	ARMT1	0.244469	3.297769	1.484981	0.141106	-5.04462	0.555236	0.466362
B.cells	MAML1	-0.17384	5.435368	-1.48441	0.141256	-5.50827	0.536678	0.439571
B.cells	ARL6	-0.37703	1.698649	-1.48439	0.141263	-4.7935	0.56961	0.487737
B.cells	PHACTR1	-0.61752	1.519212	-1.48391	0.14139	-4.60763	0.57125	0.490335
B.cells	ARAP1	0.215953	5.049234	1.483751	0.141431	-5.4636	0.539976	0.444499
B.cells	4933412E1	0.477526	1.908564	1.483587	0.141475	-4.79172	0.567699	0.485092
B.cells	PIGA	0.483763	1.918295	1.483587	0.141475	-4.68547	0.56761	0.484961
B.cells	SLX4IP	0.194466	4.853222	1.483266	0.14156	-5.41512	0.541659	0.44698
B.cells	NCOR2	0.16383	4.956649	1.482862	0.141667	-5.48609	0.54077	0.445834
B.cells	SLU7	0.161535	4.955244	1.482815	0.141679	-5.42805	0.540782	0.445851
B.cells	DACH1	-0.64363	1.877933	-1.48279	0.141685	-4.74315	0.567977	0.48571
B.cells	AKT3	0.177457	6.643383	1.48278	0.141689	-5.75264	0.526513	0.425382
B.cells	CHMP2A	0.134539	6.544809	1.482746	0.141698	-5.67934	0.527334	0.42655
B.cells	KLHL3	-0.54198	2.005874	-1.4826	0.141737	-4.64021	0.566815	0.484008
B.cells	GAS7	-0.25509	6.417925	-1.48241	0.141786	-5.7126	0.528393	0.428121
B.cells	PTTG1IP	-0.17047	4.790183	-1.48237	0.141798	-5.36416	0.542201	0.447969
B.cells	SLC4A7	0.181391	5.767425	1.482226	0.141836	-5.61174	0.533861	0.435954
B.cells	LRP5	-0.33623	4.078112	-1.48198	0.141901	-5.03895	0.548375	0.45697
B.cells	SLC29A1	-0.25373	5.215066	-1.48192	0.141918	-5.3122	0.538556	0.442734
B.cells	ALG3	-0.53413	1.77052	-1.48119	0.142111	-4.68735	0.568961	0.487427
B.cells	CLPX	0.161502	5.328169	1.480986	0.142165	-5.49675	0.537597	0.441575
B.cells	TIMM10B	0.155501	6.181583	1.480961	0.142172	-5.61375	0.530378	0.431211
B.cells	ERI3	0.169626	5.177084	1.480643	0.142257	-5.39433	0.538887	0.443606
B.cells	SSB	-0.11718	6.803522	-1.48062	0.142262	-5.73264	0.525189	0.423967
B.cells	DNAJB6	-0.11566	8.310874	-1.48056	0.14228	-5.97729	0.51286	0.406554
B.cells	CACNA1D	0.299468	3.396539	1.480485	0.142299	-5.20117	0.554369	0.466184

B.cells	FTL1-PS1	-0.36294	3.902493	-1.48038	0.142326	-5.14947	0.549917	0.459707
B.cells	DUSP4	-0.48294	0.909125	-1.48031	0.142345	-4.67277	0.576872	0.499654
B.cells	DSTYK	-0.20475	3.925134	-1.47999	0.142431	-5.2293	0.549763	0.459597
B.cells	ZDHHC9	0.231805	4.094804	1.479912	0.142451	-5.27359	0.54828	0.457432
B.cells	LRSAM1	-0.51306	1.610096	-1.47934	0.142603	-4.6573	0.570828	0.49024
B.cells	LRRK1	0.204875	5.612519	1.47907	0.142676	-5.58552	0.535557	0.43866
B.cells	TNFRSF13E	0.197131	4.518479	1.478753	0.14276	-5.38837	0.544938	0.452314
B.cells	FRY	0.179401	6.378637	1.47864	0.14279	-5.7646	0.5291	0.429504
B.cells	GM19585	0.566532	2.331819	1.47839	0.142857	-4.885	0.564272	0.480765
B.cells	CHD8	0.138095	5.733086	1.47836	0.142865	-5.57306	0.534534	0.437343
B.cells	KLF12	-0.36191	3.585919	-1.47833	0.142872	-5.1972	0.553087	0.46428
B.cells	TMEM60	0.221228	4.112089	1.477667	0.14305	-5.20368	0.548974	0.457699
B.cells	ZCCHC24	-0.29721	3.666755	-1.4771	0.143203	-5.10829	0.553225	0.46351
B.cells	NARS2	0.223558	4.271035	1.476451	0.143376	-5.31753	0.547928	0.455961
B.cells	GM614	0.709309	-0.15476	1.476284	0.14342	-4.51521	0.588134	0.515741
B.cells	G6PC3	0.302434	3.106653	1.476242	0.143432	-5.07091	0.558189	0.471053
B.cells	NEMP1	0.426843	3.065406	1.476136	0.14346	-4.90513	0.558556	0.471594
B.cells	SPSB4	0.805991	0.417547	1.476108	0.143468	-4.5094	0.582748	0.507614
B.cells	VDAC3	-0.1273	7.191815	-1.47609	0.143473	-5.83001	0.523152	0.42042
B.cells	RBM28	0.159707	5.311789	1.475916	0.143519	-5.50057	0.538944	0.443088
B.cells	CLASRP	0.291014	3.334689	1.475695	0.143578	-5.01362	0.556208	0.468211
B.cells	DYRK1A	-0.15018	7.419436	-1.47535	0.143671	-5.83635	0.521483	0.418052
B.cells	SQSTM1	-0.16406	7.466775	-1.47475	0.143833	-5.88284	0.5211	0.417683
B.cells	SNF8	0.145314	5.731869	1.474652	0.143858	-5.57712	0.535585	0.43837
B.cells	MORF4L1	-0.07909	8.201561	-1.47453	0.143891	-5.95699	0.515106	0.409247
B.cells	OCIAD2	-0.78989	0.485681	-1.47432	0.143947	-4.51315	0.582347	0.507344
B.cells	ECHDC3	-0.77651	0.833138	-1.47431	0.143949	-4.5664	0.579108	0.502472
B.cells	GBP3	0.4335	3.228626	1.474284	0.143957	-5.08815	0.55733	0.470097
B.cells	CD22	0.353027	2.780412	1.473986	0.144037	-4.97349	0.561458	0.476122
B.cells	KHDC4	0.140786	6.448578	1.473129	0.144268	-5.69205	0.530336	0.430155
B.cells	4931406G(	0.803643	0.347467	1.47262	0.144405	-4.59938	0.584877	0.509889
B.cells	PFKFB4	0.297391	3.15878	1.472223	0.144512	-5.05261	0.559363	0.471774
B.cells	CLUAP1	0.223649	3.885137	1.471558	0.144691	-5.15869	0.55341	0.462465
B.cells	E330009J0	0.353006	2.552592	1.471398	0.144734	-5.06443	0.565301	0.479958
B.cells	CCDC69	-0.28436	3.297575	-1.47102	0.144836	-5.10227	0.558828	0.470329
B.cells	URGCP	0.22468	4.434418	1.46983	0.145157	-5.33865	0.549804	0.456206
B.cells	GM13710	-0.74147	1.36556	-1.46969	0.145197	-4.64953	0.577414	0.496943
B.cells	MRPL54	0.161353	5.906363	1.469328	0.145293	-5.62192	0.537148	0.438149
B.cells	TIMM21	-0.34174	2.602996	-1.4693	0.145302	-4.90116	0.56613	0.480343
B.cells	PDXK	0.211128	4.441778	1.468541	0.145506	-5.36591	0.550115	0.456517
B.cells	C4BP	-0.57607	1.968581	-1.46843	0.145537	-4.7614	0.572254	0.489037
B.cells	TSPAN32	0.333135	2.972272	1.468329	0.145564	-5.06999	0.563146	0.475634
B.cells	KEAP1	0.167284	5.115335	1.468261	0.145582	-5.43529	0.54426	0.448112
B.cells	TLE4	-0.14527	7.814076	-1.46806	0.145638	-5.90844	0.521556	0.415737
B.cells	CAR12	-0.7322	0.013745	-1.4675	0.145788	-4.51863	0.59093	0.516875
B.cells	STFA2L1	0.815896	2.448793	1.466513	0.146056	-4.7512	0.568984	0.483485
B.cells	LPXN	0.178267	5.23201	1.46642	0.146082	-5.60531	0.544314	0.447449

B.cells	GM11508	-0.32383	3.437566	-1.46633	0.146106	-5.15229	0.560073	0.470378
B.cells	FAM76A	0.173373	5.119697	1.466125	0.146162	-5.43637	0.545317	0.448866
B.cells	GPATCH2	0.232669	4.253922	1.465044	0.146456	-5.25362	0.553801	0.460144
B.cells	SCRIB	-0.24034	3.392621	-1.46461	0.146574	-5.11962	0.561719	0.471355
B.cells	UQCRH	0.098386	8.592145	1.464073	0.146721	-6.0647	0.517627	0.408065
B.cells	LCP2	0.432195	5.109024	1.46344	0.146893	-5.07388	0.546896	0.449751
B.cells	EAPP	0.134335	5.602282	1.46342	0.146899	-5.56256	0.542634	0.443617
B.cells	CLCN6	-0.28155	3.649755	-1.46341	0.146902	-5.14754	0.559736	0.468396
B.cells	LGALS1	-0.2706	6.036335	-1.46336	0.146914	-5.6157	0.538917	0.438288
B.cells	OLFR77	-0.54403	1.376904	-1.46325	0.146944	-4.68323	0.580442	0.498961
B.cells	LRP8	-0.62649	2.371033	-1.4628	0.147068	-4.63678	0.571414	0.48544
B.cells	MRVI1	-0.68118	0.497468	-1.46254	0.147139	-4.56686	0.58883	0.511436
B.cells	G3BP2	-0.10818	6.759047	-1.4624	0.147178	-5.75361	0.532919	0.429716
B.cells	POLD1	-0.25361	4.276679	-1.4621	0.147259	-5.25328	0.554308	0.460525
B.cells	DIPK2A	-0.24339	4.112992	-1.46208	0.147266	-5.17795	0.555754	0.462628
B.cells	C130026I2:	0.323341	2.944263	1.462071	0.147267	-5.15231	0.566204	0.477921
B.cells	RNF17	0.958232	-0.02892	1.461784	0.147346	-4.50622	0.593956	0.519237
B.cells	EPAS1	-0.40145	3.448567	-1.4613	0.147478	-4.98667	0.562019	0.471446
B.cells	DOCK5	-0.43311	4.189727	-1.46121	0.147504	-4.95073	0.555424	0.461821
B.cells	GM44710	-0.46684	2.000891	-1.46055	0.147682	-4.8289	0.575676	0.491117
B.cells	LYRM4	-0.20764	4.142278	-1.46015	0.147793	-5.2307	0.556492	0.462893
B.cells	MYBL2	-0.39841	3.080165	-1.46002	0.147829	-4.96349	0.565991	0.476827
B.cells	MRPL43	0.166447	5.608686	1.459768	0.147898	-5.58921	0.543682	0.444564
B.cells	CNST	-0.20726	3.943862	-1.45938	0.148004	-5.20974	0.558252	0.465818
B.cells	KLF11	0.227526	3.555665	1.459278	0.148032	-5.10344	0.561715	0.470886
B.cells	VSIG10L	0.656954	0.298869	1.459264	0.148036	-4.50754	0.591772	0.515509
B.cells	MED21	0.225405	5.21874	1.459176	0.14806	-5.43635	0.547054	0.449597
B.cells	DBR1	-0.2927	3.262038	-1.45834	0.148291	-5.03638	0.565024	0.475179
B.cells	VTI1B	0.123642	5.690918	1.458184	0.148333	-5.59187	0.543621	0.444148
B.cells	SIK2	0.146646	7.126745	1.457869	0.14842	-5.82533	0.531421	0.426744
B.cells	UQCRB	-0.14404	8.047525	-1.45766	0.148477	-5.96285	0.523766	0.415988
B.cells	CD151	0.351077	2.758187	1.457567	0.148503	-4.92852	0.569589	0.481979
B.cells	ANKRD28	0.186667	5.835458	1.457481	0.148526	-5.59104	0.54238	0.442445
B.cells	PLCL1	-0.27919	5.328139	-1.45679	0.148717	-5.56263	0.547283	0.448906
B.cells	SHLD1	0.275894	3.33904	1.456327	0.148844	-5.10627	0.565056	0.474624
B.cells	UXS1	0.208706	4.429155	1.456093	0.148909	-5.35678	0.555332	0.460467
B.cells	GSTA4	-0.59203	1.583658	-1.45609	0.148911	-4.65635	0.581135	0.498399
B.cells	HYAL3	-0.85611	0.035974	-1.4556	0.149045	-4.47799	0.595751	0.520602
B.cells	DSE	-0.45453	3.834005	-1.45557	0.149052	-4.94652	0.560616	0.468439
B.cells	BC029722	0.294153	3.061908	1.455566	0.149054	-5.04876	0.56756	0.478612
B.cells	CDKN1B	0.159605	6.470899	1.455136	0.149173	-5.77181	0.537896	0.435485
B.cells	IQSEC2	0.461759	2.267987	1.453397	0.149654	-4.93061	0.576119	0.49018
B.cells	DUSP8	-0.49449	1.274945	-1.45328	0.149687	-4.75228	0.585353	0.503908
B.cells	DUSP2	-0.2299	5.991936	-1.45326	0.149692	-5.68822	0.54297	0.441928
B.cells	RAB5C	0.138843	6.358629	1.453234	0.149699	-5.69498	0.539828	0.437439
B.cells	GM16638	-0.47738	2.040296	-1.45302	0.149758	-4.71057	0.578221	0.493373
B.cells	UPF3A	0.15869	4.634592	1.452694	0.149849	-5.41141	0.554786	0.459132

B.cells	CEP83	0.154604	5.662564	1.452657	0.149859	-5.5874	0.54581	0.446178
B.cells	ACTR2	0.110861	8.066808	1.452398	0.149931	-6.00303	0.525476	0.417399
B.cells	DSTN	-0.1667	6.886463	-1.45207	0.150021	-5.79706	0.535344	0.431507
B.cells	ING2	0.182958	4.920364	1.452027	0.150034	-5.4014	0.552274	0.455788
B.cells	ABCA6	-0.67986	0.705764	-1.45201	0.150038	-4.68684	0.590722	0.512476
B.cells	2900089D1	-0.36349	2.58395	-1.45192	0.150065	-4.99239	0.573216	0.486413
B.cells	FNDC7	0.695453	-0.36573	1.451891	0.150071	-4.5883	0.600984	0.527952
B.cells	TRAF5	0.183104	5.308971	1.451683	0.150129	-5.57019	0.548912	0.450901
B.cells	XPC	0.264288	3.385386	1.450654	0.150415	-5.0851	0.566449	0.476013
B.cells	ZFP958	0.396958	2.267977	1.450553	0.150443	-4.8765	0.576648	0.491042
B.cells	LRRC43	-0.85995	-0.12202	-1.45052	0.150451	-4.50264	0.599182	0.524752
B.cells	MCPH1	0.197403	4.913551	1.450398	0.150486	-5.38903	0.552841	0.456259
B.cells	OAS3	0.950755	2.402295	1.450146	0.150556	-4.69462	0.575411	0.489414
B.cells	RHD	0.504818	1.667344	1.450007	0.150595	-4.84906	0.582218	0.499575
B.cells	MAPT	-0.48785	1.597756	-1.45	0.150596	-4.72914	0.582867	0.500542
B.cells	ZC3HAV1L	-0.33111	3.053639	-1.44962	0.150702	-5.0697	0.569637	0.480797
B.cells	TPRKB	0.299684	3.157978	1.449228	0.150812	-5.06131	0.56869	0.479667
B.cells	EIF2D	-0.26831	3.5699	-1.44914	0.150836	-5.06525	0.564966	0.474222
B.cells	CCT3	0.139858	5.801241	1.449023	0.150869	-5.61901	0.545286	0.445663
B.cells	LGALS4	-0.49082	2.563012	-1.44896	0.150886	-4.77965	0.574118	0.487723
B.cells	ATP6V0D1	0.100018	7.228201	1.448692	0.150961	-5.86393	0.533218	0.42836
B.cells	GTF3C5	-0.42821	2.258469	-1.44799	0.151158	-4.83326	0.577301	0.492101
B.cells	UBA1	-0.14811	6.00691	-1.44794	0.15117	-5.64808	0.543872	0.443362
B.cells	FOXRED2	-0.57196	1.168266	-1.44791	0.151179	-4.61241	0.587469	0.507264
B.cells	NINJ1	0.245003	5.750418	1.447556	0.151278	-5.55328	0.546141	0.446633
B.cells	ZSWIM1	-0.63837	1.4089	-1.44731	0.151347	-4.66083	0.585266	0.504031
B.cells	YDJC	0.620417	1.21958	1.447093	0.151407	-4.63731	0.587045	0.506771
B.cells	KAT14	0.197631	3.838268	1.446733	0.151508	-5.2362	0.562984	0.471302
B.cells	ERCC1	-0.26268	3.315868	-1.44662	0.15154	-5.10023	0.567691	0.478226
B.cells	LIG1	-0.30872	5.051661	-1.44637	0.151609	-5.37703	0.552227	0.455754
B.cells	ARSA	-0.45884	1.69399	-1.44608	0.151691	-4.72887	0.582599	0.500494
B.cells	HMGXB4	0.209816	4.148587	1.445963	0.151723	-5.28535	0.56021	0.467483
B.cells	ARHGEF4	0.912832	0.759185	1.445845	0.151756	-4.59381	0.591397	0.513715
B.cells	TBC1D20	-0.16759	5.789234	-1.44582	0.151763	-5.62216	0.545806	0.446633
B.cells	GM12166	-0.7144	0.781167	-1.44537	0.15189	-4.55483	0.591189	0.513544
B.cells	HMGB3	-0.27448	4.658182	-1.44469	0.152081	-5.3847	0.555689	0.461365
B.cells	MTUS2	0.892808	0.654472	1.444642	0.152093	-4.53668	0.592392	0.515717
B.cells	MLH3	0.415465	2.01272	1.444616	0.152101	-4.82348	0.579634	0.496619
B.cells	SLC35D2	-0.29735	4.232	-1.44444	0.152149	-5.17797	0.559467	0.46689
B.cells	GM15445	0.514126	0.994582	1.443864	0.152311	-4.67834	0.589168	0.51115
B.cells	BCAP29	0.193391	4.765797	1.443806	0.152328	-5.42303	0.554739	0.460247
B.cells	ASB8	0.242617	3.607063	1.44379	0.152332	-5.17667	0.565062	0.475333
B.cells	JPT1	-0.13487	7.832843	-1.44375	0.152343	-5.96574	0.528471	0.42258
B.cells	C3	-0.30132	6.327835	-1.44362	0.152379	-5.79203	0.541172	0.440743
B.cells	MEX3B	-0.44618	2.233947	-1.44357	0.152394	-4.8066	0.577586	0.493951
B.cells	ZFP467	-0.49936	2.303773	-1.44339	0.152445	-4.74211	0.576942	0.49307
B.cells	AP5Z1	-0.32319	2.539751	-1.44335	0.152454	-4.91622	0.574769	0.489845

B.cells	MTFR1L	0.193479	4.250609	1.443234	0.152488	-5.27857	0.559301	0.467071
B.cells	CDC42	-0.08488	8.943013	-1.44319	0.1525	-6.13543	0.519328	0.409881
B.cells	GM39302	-0.73299	0.880898	-1.44318	0.152505	-4.56282	0.590243	0.512961
B.cells	AMOT	-0.6632	0.863491	-1.44306	0.152537	-4.59879	0.590408	0.513234
B.cells	ADPRH	0.196675	4.93883	1.443013	0.15255	-5.43368	0.553217	0.458231
B.cells	EFL1	0.180587	4.5955	1.442335	0.152741	-5.39986	0.556675	0.462901
B.cells	PPIL2	0.15166	5.124237	1.442246	0.152766	-5.50613	0.55202	0.45618
B.cells	SERPINB9	-0.64791	3.483496	-1.44165	0.152933	-4.84319	0.566722	0.477639
B.cells	RPP14	0.309426	2.846249	1.44164	0.152937	-5.00284	0.572513	0.486183
B.cells	GM28050	-0.72254	0.410517	-1.44163	0.15294	-4.54475	0.595291	0.520238
B.cells	BRWD1	0.13901	6.550877	1.441293	0.153035	-5.76569	0.539951	0.438607
B.cells	PITPNM2	0.272885	3.66119	1.440745	0.153189	-5.18621	0.565589	0.475505
B.cells	CCDC51	0.450886	1.810129	1.44063	0.153221	-4.71303	0.582562	0.50063
B.cells	EMC2	-0.13997	5.762132	-1.44034	0.153304	-5.61357	0.547021	0.448503
B.cells	GM28112	0.779134	0.043725	1.440212	0.153339	-4.57687	0.599309	0.525875
B.cells	BC052040	0.28153	4.165796	1.440046	0.153386	-5.22647	0.561063	0.468946
B.cells	SMIM1	-0.64472	1.759237	-1.43997	0.153407	-4.68734	0.583037	0.501417
B.cells	NCAPD2	-0.3218	4.81058	-1.43966	0.153495	-5.3443	0.555487	0.460604
B.cells	CFAP43	0.270593	3.09234	1.439181	0.15363	-5.0991	0.571214	0.483358
B.cells	BIRC5	-0.4135	5.35755	-1.43879	0.153742	-5.43251	0.551222	0.45394
B.cells	RAG1	-0.51782	1.672599	-1.43817	0.153916	-4.90232	0.584979	0.503185
B.cells	NFKB2	0.218182	5.096214	1.438061	0.153947	-5.55574	0.5539	0.457464
B.cells	LIN9	0.225585	4.133885	1.437644	0.154065	-5.31713	0.56269	0.470086
B.cells	AI467606	-0.2559	4.111903	-1.43746	0.154116	-5.33162	0.562897	0.470432
B.cells	ADAM22	-0.6977	0.796933	-1.43668	0.154338	-4.63555	0.593783	0.516206
B.cells	MARCKSL1	-0.22518	7.792982	-1.43665	0.154346	-5.96247	0.531258	0.4249
B.cells	RBM26	0.119255	6.821937	1.436597	0.154362	-5.80329	0.539456	0.436541
B.cells	LY6G	-0.83239	-0.81776	-1.43654	0.154377	-4.54283	0.609236	0.539869
B.cells	GM10371	0.699581	-0.68835	1.435846	0.154575	-4.54143	0.608525	0.538326
B.cells	GM45894	0.288625	2.290972	1.43575	0.154602	-4.9721	0.580217	0.495577
B.cells	MRPL16	0.21838	3.919331	1.434907	0.154842	-5.2652	0.566032	0.474024
B.cells	ORAI1	0.151394	6.491763	1.434596	0.15493	-5.76563	0.543485	0.441313
B.cells	6430550D2	-0.5518	1.142129	-1.43425	0.155028	-4.69373	0.591819	0.512251
B.cells	TCF19	-0.42343	3.199871	-1.434	0.155099	-4.97694	0.572656	0.483814
B.cells	ATXN1L	0.291906	2.959513	1.433885	0.155132	-5.05371	0.574857	0.48706
B.cells	TNFSF14	0.644812	-0.05669	1.433287	0.155303	-4.53904	0.603323	0.529775
B.cells	STIP1	0.183449	5.419076	1.433209	0.155325	-5.56317	0.552794	0.454945
B.cells	SLCO2A1	-0.79593	2.568296	-1.43316	0.15534	-4.75371	0.57846	0.492511
B.cells	KIF1A	0.70037	-0.59877	1.432996	0.155386	-4.54489	0.608539	0.537802
B.cells	I830077J02	-0.55255	2.758256	-1.43298	0.155389	-4.77425	0.576707	0.489915
B.cells	OSBPL2	0.207049	3.979026	1.432915	0.155409	-5.25049	0.565589	0.47358
B.cells	FBXO10	-0.49963	1.355085	-1.43241	0.155552	-4.67886	0.589802	0.509673
B.cells	TTC39A	-0.45922	2.027911	-1.43229	0.155588	-4.92182	0.58348	0.500243
B.cells	PCDHGC4	-0.78159	0.256002	-1.43193	0.155689	-4.54733	0.600298	0.525644
B.cells	RASA4	0.30119	4.228858	1.43188	0.155704	-5.38582	0.563344	0.470678
B.cells	BUD31	-0.16575	5.474604	-1.43184	0.155714	-5.5638	0.552308	0.454637
B.cells	DLC1	-0.459	4.570858	-1.43179	0.15573	-5.19965	0.560289	0.466252

B.cells	MRPL57	0.155839	5.995401	1.43168	0.155761	-5.64988	0.547769	0.448128
B.cells	LSM11	0.466331	2.171179	1.431651	0.155769	-4.83801	0.582144	0.498453
B.cells	STARD3NL	-0.15352	5.861943	-1.43142	0.155834	-5.67679	0.548928	0.449865
B.cells	INPP5J	0.696715	-1.12022	1.431396	0.155842	-4.53454	0.613511	0.546235
B.cells	NOTCH4	-0.81274	0.210596	-1.43061	0.156068	-4.56656	0.601419	0.526764
B.cells	ZZEF1	0.144713	5.867775	1.430186	0.156188	-5.64207	0.549752	0.45018
B.cells	SRSF5	-0.11992	6.955445	-1.42976	0.156311	-5.81447	0.540521	0.436945
B.cells	AK3	-0.20152	4.328071	-1.42968	0.156333	-5.35347	0.5635	0.470119
B.cells	KDM7A	0.132335	7.129544	1.42953	0.156376	-5.97887	0.539037	0.434907
B.cells	MRPL28	-0.15964	6.016074	-1.4287	0.156614	-5.65962	0.549271	0.44894
B.cells	RBM22	-0.12464	5.603703	-1.42845	0.156686	-5.61191	0.552951	0.454174
B.cells	ACO1	-0.2316	3.712371	-1.42812	0.156781	-5.19465	0.56999	0.478832
B.cells	NOTCH3	-0.74175	1.028947	-1.42753	0.156948	-4.60017	0.595262	0.516176
B.cells	EMCN	-0.5758	1.650943	-1.42751	0.156954	-4.69833	0.589357	0.507331
B.cells	TRIM21	0.410602	2.57157	1.426847	0.157146	-4.96687	0.581154	0.494777
B.cells	SENP1	0.154787	5.013417	1.426785	0.157164	-5.49436	0.558984	0.462291
B.cells	ITGAL	0.223055	6.272091	1.426491	0.157248	-5.62807	0.547975	0.446478
B.cells	EMC1	0.235961	3.622778	1.426414	0.157271	-5.14345	0.571518	0.480651
B.cells	XLR4A	-0.56871	0.802973	-1.42585	0.157434	-4.66354	0.598016	0.520099
B.cells	CAPN5	0.665125	1.546779	1.42583	0.157439	-4.66422	0.590925	0.509461
B.cells	SLC2A1	0.210052	4.676925	1.425531	0.157525	-5.41603	0.562135	0.467132
B.cells	NUDT1	-0.35743	3.00659	-1.4253	0.157591	-5.04154	0.57729	0.489431
B.cells	CRACR2A	0.433836	2.835916	1.425236	0.15761	-4.92679	0.578865	0.491801
B.cells	SPAG5	-0.43876	3.137614	-1.42497	0.157687	-4.97475	0.576084	0.487798
B.cells	SCYL1	0.228093	4.072604	1.424887	0.15771	-5.32175	0.567564	0.475303
B.cells	KLC4	0.295473	2.884186	1.424789	0.157739	-5.063	0.578419	0.491346
B.cells	GM10184	-0.666	1.11236	-1.42476	0.157746	-4.64275	0.595055	0.516157
B.cells	GDF15	-0.82047	1.508239	-1.42297	0.158264	-4.70606	0.593045	0.510934
B.cells	MRPS24	0.134857	6.031221	1.422735	0.158332	-5.69887	0.551888	0.450572
B.cells	DENND6A	0.160656	5.102952	1.422448	0.158415	-5.54788	0.560188	0.462378
B.cells	THAP11	-0.2003	4.457867	-1.42165	0.158646	-5.40762	0.566609	0.471119
B.cells	RNF25	0.266579	3.103588	1.42117	0.158785	-5.09927	0.579292	0.489337
B.cells	MAPKAP1	0.15223	5.764174	1.420483	0.158984	-5.63063	0.55582	0.454615
B.cells	SLC9A3R1	-0.15168	6.690174	-1.42003	0.159115	-5.77693	0.548017	0.44324
B.cells	MAP3K2	0.145722	6.248682	1.419596	0.159241	-5.75282	0.552125	0.448854
B.cells	PRELID1	0.132811	7.529242	1.418981	0.15942	-5.94022	0.541166	0.433384
B.cells	ARHGAP24	0.183521	6.825889	1.418771	0.159481	-5.93082	0.547205	0.442031
B.cells	GM28198	-0.27286	3.85447	-1.41853	0.159551	-5.29254	0.57361	0.480192
B.cells	MAJIN	-0.89483	0.644865	-1.41823	0.159639	-4.57552	0.603822	0.525114
B.cells	CNKSR3	-0.27928	4.754523	-1.41727	0.159918	-5.4352	0.565457	0.468652
B.cells	FCRL6	-0.82771	-0.87747	-1.41721	0.159936	-4.4944	0.618598	0.548071
B.cells	DDB2	0.322274	3.43077	1.417176	0.159946	-5.15349	0.577496	0.486242
B.cells	MAT2B	0.142092	5.679223	1.417166	0.159949	-5.66829	0.557221	0.456742
B.cells	ADGRL4	-0.50929	3.201942	-1.41709	0.159972	-4.89126	0.579608	0.489348
B.cells	IFI27	0.303531	4.998783	1.417062	0.159979	-5.49627	0.563268	0.465477
B.cells	ZYX	0.239584	6.244314	1.41699	0.16	-5.57135	0.552258	0.449612
B.cells	HIP1R	0.173616	5.271328	1.41694	0.160015	-5.61075	0.560837	0.461958

B.cells	PKIB	0.26812	5.453591	1.416853	0.16004	-5.81637	0.559218	0.459656
B.cells	CFP	-0.25659	5.518079	-1.41672	0.16008	-5.61754	0.558646	0.458896
B.cells	ADARB1	-0.4235	3.009129	-1.41668	0.16009	-4.96756	0.581394	0.492088
B.cells	FMN1	-0.41018	3.350464	-1.41651	0.16014	-5.09231	0.578236	0.48748
B.cells	UBE2D2A	-0.07377	8.209532	-1.41643	0.160162	-6.04736	0.535401	0.425808
B.cells	RABL6	0.148219	5.136693	1.416338	0.16019	-5.51694	0.562036	0.463863
B.cells	ENC1	-0.30284	3.181392	-1.41594	0.160306	-5.08362	0.579798	0.4899
B.cells	ADAM32	-0.7432	0.784382	-1.41592	0.160311	-4.66551	0.602471	0.523661
B.cells	POLD3	0.198391	4.695421	1.4158	0.160347	-5.43137	0.565988	0.469704
B.cells	PRKCH	-0.32562	6.999653	-1.41565	0.160392	-5.66068	0.545706	0.440524
B.cells	ZFP39	0.612727	1.023509	1.415444	0.160451	-4.67307	0.600164	0.520231
B.cells	PDGFA	-0.69941	2.114128	-1.41544	0.160453	-4.67248	0.589769	0.504698
B.cells	GM11110	0.381438	1.970897	1.415256	0.160506	-4.85862	0.591136	0.506763
B.cells	PLXNA1	-0.69032	0.899006	-1.41416	0.160828	-4.65219	0.602397	0.522562
B.cells	DCP1B	0.300253	2.830948	1.413624	0.160984	-5.07764	0.584438	0.495447
B.cells	HIST1H2AK	-0.75077	0.954719	-1.41345	0.161033	-4.6177	0.602259	0.521978
B.cells	CNIH1	-0.18137	5.17624	-1.41308	0.161143	-5.50141	0.563135	0.464242
B.cells	TMEM238	-0.33	2.938537	-1.41274	0.161241	-5.13103	0.583554	0.49418
B.cells	NSL1	-0.46178	2.25699	-1.41258	0.161291	-4.79257	0.589944	0.503712
B.cells	H2AFV	-0.17314	8.095101	-1.41254	0.161301	-6.01984	0.537752	0.428167
B.cells	CALCRL	0.263561	5.617508	1.412455	0.161326	-5.59863	0.559208	0.458757
B.cells	MTRF1	0.561003	1.208712	1.412103	0.16143	-4.69522	0.599931	0.518769
B.cells	TRDMT1	0.321955	2.342131	1.412028	0.161452	-4.99302	0.589141	0.50268
B.cells	HEY1	-0.90317	-0.2714	-1.41199	0.161462	-4.5165	0.614364	0.540548
B.cells	TRIM7	0.413158	0.591087	1.411626	0.16157	-4.93264	0.606127	0.52794
B.cells	GM38190	-0.39044	1.823044	-1.41132	0.161659	-4.88162	0.594416	0.51031
B.cells	FAM89A	-0.45344	0.879902	-1.41093	0.161774	-4.76165	0.603717	0.523935
B.cells	MYSM1	0.154762	5.388465	1.410552	0.161885	-5.60471	0.562027	0.462313
B.cells	CSRNP1	-0.19807	6.641086	-1.41024	0.161977	-5.83601	0.551137	0.446518
B.cells	GM20492	-0.41551	1.798274	-1.40988	0.162084	-4.8231	0.595486	0.511055
B.cells	GPATCH11	0.233685	3.800176	1.409703	0.162136	-5.24154	0.576757	0.483433
B.cells	EPM2A	0.639801	1.526051	1.409349	0.16224	-4.66425	0.598297	0.515147
B.cells	FAM126A	0.176773	5.549539	1.408768	0.162411	-5.68446	0.561554	0.460823
B.cells	PRMT9	0.246061	4.074852	1.408247	0.162565	-5.29217	0.575117	0.480378
B.cells	IFI44	1.112718	0.579228	1.408189	0.162582	-4.54468	0.608177	0.529437
B.cells	IGSF9	-0.59337	0.790152	-1.40801	0.162636	-4.63895	0.606138	0.526424
B.cells	PLAA	0.130123	6.069326	1.407519	0.16278	-5.72818	0.557341	0.454633
B.cells	MTERF1A	0.475181	1.405346	1.407341	0.162832	-4.72989	0.600326	0.51768
B.cells	CCDC92	-0.64283	-0.17821	-1.4073	0.162843	-4.61509	0.615786	0.540955
B.cells	CYP4F18	0.222352	3.551567	1.407205	0.162873	-5.46389	0.580082	0.487708
B.cells	DEPDC5	0.170572	4.978079	1.406981	0.162939	-5.52826	0.567123	0.468782
B.cells	CDA	-0.7481	0.894078	-1.40655	0.163067	-4.62005	0.605564	0.525255
B.cells	PLCXD2	0.26273	3.46805	1.406432	0.163101	-5.29243	0.581138	0.488984
B.cells	MCM3AP	-0.19643	3.885875	-1.40603	0.163221	-5.26247	0.577408	0.48342
B.cells	LRATD2	-0.39655	1.700967	-1.40598	0.163236	-4.86427	0.597912	0.513725
B.cells	FMNL2	-0.31293	7.100621	-1.40576	0.1633	-5.73099	0.548768	0.442122
B.cells	DOCK11	0.140463	7.111908	1.405496	0.163378	-5.91276	0.548764	0.442038

B.cells	TBC1D15	-0.13788	6.062519	-1.40489	0.163557	-5.72997	0.558381	0.455334
B.cells	OAT	-0.19249	5.245936	-1.40454	0.163663	-5.57074	0.565847	0.465933
B.cells	NFAM1	0.215347	4.570884	1.40429	0.163736	-5.42546	0.572028	0.474856
B.cells	ANKRD50	-0.28705	2.62264	-1.40412	0.163786	-4.9591	0.590069	0.501417
B.cells	SERHL	-0.26567	3.492794	-1.4035	0.16397	-5.14475	0.582119	0.489739
B.cells	CACNB3	-0.92337	-0.407	-1.4033	0.164029	-4.59211	0.619647	0.545848
B.cells	0610030E2	0.183137	4.591291	1.403248	0.164045	-5.45487	0.572029	0.475035
B.cells	3830403N1	-1.00334	0.704685	-1.40309	0.164093	-4.58509	0.608673	0.529296
B.cells	GM17021	-0.90275	0.125797	-1.40265	0.164224	-4.53003	0.614359	0.53799
B.cells	NEAT1	-0.18973	8.674976	-1.4024	0.164298	-6.17373	0.536283	0.424163
B.cells	PIRA2	-0.32992	3.589246	-1.40219	0.164361	-5.32233	0.581225	0.48872
B.cells	ARF2	-0.23228	4.470722	-1.40215	0.164373	-5.43052	0.573126	0.476894
B.cells	ZCCHC3	0.608566	0.70709	1.401937	0.164435	-4.65059	0.608649	0.529571
B.cells	SCPEP1	0.173243	5.169388	1.401916	0.164441	-5.6668	0.566801	0.467755
B.cells	NPAS2	-0.85673	0.089778	-1.40149	0.16457	-4.55272	0.614715	0.538802
B.cells	FASL	-0.70436	1.037842	-1.40144	0.164584	-4.66359	0.605427	0.524803
B.cells	TSIX	-3.33406	1.906429	-1.40134	0.164614	-4.67146	0.597059	0.512286
B.cells	TMEM65	-0.22242	4.9205	-1.40117	0.164662	-5.47987	0.569045	0.471075
B.cells	TRMT2B	0.254191	3.750232	1.40102	0.164708	-5.22094	0.579736	0.486712
B.cells	S100A10	-0.18595	6.601647	-1.40087	0.164754	-5.93995	0.55409	0.449612
B.cells	ZBTB1	0.17592	5.505188	1.400867	0.164754	-5.64572	0.56379	0.463548
B.cells	GTSF2	-0.91506	0.091247	-1.40081	0.16477	-4.53856	0.614701	0.538873
B.cells	KRT80	0.536488	0.161146	1.400738	0.164792	-4.65147	0.61401	0.537855
B.cells	ROGDI	-0.22851	4.014028	-1.4006	0.164835	-5.35179	0.577305	0.483263
B.cells	VPS26A	-0.12857	6.273847	-1.40035	0.164907	-5.79276	0.557047	0.453873
B.cells	ARL6IP1	0.133378	7.879749	1.399637	0.165121	-5.99157	0.543261	0.434186
B.cells	DDX24	0.129268	6.717234	1.399403	0.165191	-5.83795	0.553313	0.44847
B.cells	RAB8B	0.167572	7.724426	1.399385	0.165196	-6.00868	0.544592	0.436071
B.cells	NOP2	-0.22929	3.635097	-1.3993	0.165222	-5.22296	0.581046	0.488648
B.cells	ABCC5	0.252323	4.301545	1.399258	0.165234	-5.45172	0.574913	0.479668
B.cells	SIPA1L2	0.197202	5.103799	1.399195	0.165253	-5.61286	0.567632	0.469074
B.cells	PAFAH1B3	-0.1962	5.814072	-1.39903	0.165303	-5.69007	0.561275	0.4599
B.cells	RHOV	0.954225	-0.80702	1.398853	0.165355	-4.55988	0.623759	0.552925
B.cells	BDH1	-0.35607	3.282333	-1.39865	0.165415	-5.11596	0.584368	0.493583
B.cells	RHOG	0.164312	7.243088	1.398364	0.165502	-5.92217	0.548818	0.442152
B.cells	NSMCE1	0.204644	4.829662	1.398283	0.165526	-5.43896	0.570189	0.472881
B.cells	SRSF7	-0.1777	6.098296	-1.39754	0.165748	-5.70467	0.559415	0.456753
B.cells	SAMD4B	0.164508	5.014196	1.397226	0.165842	-5.53759	0.569246	0.470806
B.cells	TAF15	0.109728	6.905531	1.396689	0.166003	-5.87996	0.552453	0.446843
B.cells	MID2	-0.88132	0.27499	-1.39642	0.166083	-4.53386	0.614018	0.537474
B.cells	RNF123	0.242553	3.804311	1.396324	0.166113	-5.34047	0.580304	0.487294
B.cells	WDR45	0.275146	2.960786	1.396158	0.166163	-5.09656	0.588163	0.498917
B.cells	SERINC3	-0.09679	9.707046	-1.39607	0.16619	-6.32534	0.528646	0.413523
B.cells	ANP32E	-0.1603	7.305672	-1.39594	0.166228	-5.91844	0.548976	0.442139
B.cells	NBEA	-0.5211	2.602645	-1.39579	0.166273	-4.79657	0.591537	0.504019
B.cells	PROM1	-0.85188	0.403745	-1.39574	0.166287	-4.60723	0.612749	0.535802
B.cells	PLPP1	-0.3266	4.327383	-1.39566	0.166312	-5.32948	0.575493	0.480458

B.cells	SMC3	-0.12837	6.498954	-1.39558	0.166336	-5.80425	0.556013	0.452281
B.cells	HYLS1	-0.28288	3.183514	-1.39535	0.166405	-5.11232	0.586146	0.496015
B.cells	H2-K1	0.293913	8.786019	1.395122	0.166474	-6.17561	0.53642	0.424433
B.cells	SECISBP2L	0.188347	4.964176	1.394997	0.166511	-5.52689	0.569788	0.472075
B.cells	STOML1	0.426266	2.251865	1.394006	0.166809	-4.85935	0.595843	0.509372
B.cells	GAA	-0.31723	3.191942	-1.39129	0.167628	-5.05445	0.589664	0.497252
B.cells	FUS	-0.1021	7.820328	-1.39063	0.167827	-5.99979	0.548104	0.437399
B.cells	RBM17	-0.15489	5.789508	-1.39058	0.167844	-5.65465	0.565969	0.46284
B.cells	RIC8B	0.182704	4.502862	1.390554	0.167851	-5.45893	0.577644	0.479719
B.cells	APPBP2OS	-0.48517	0.985935	-1.39047	0.167876	-4.71317	0.61102	0.529062
B.cells	HERC3	0.215126	4.345366	1.389649	0.168125	-5.42748	0.579778	0.482099
B.cells	SHANK2	-0.53926	1.410698	-1.38942	0.168196	-4.76703	0.607666	0.523198
B.cells	GM15879	-0.74416	0.515986	-1.38862	0.168438	-4.59205	0.617156	0.536784
B.cells	BLOC1S1	0.164588	6.24963	1.388028	0.168616	-5.77048	0.563521	0.457809
B.cells	GIMAP8	0.342883	2.465426	1.387337	0.168826	-5.18373	0.598476	0.508928
B.cells	TBCD	0.178717	4.783335	1.387237	0.168856	-5.51649	0.576773	0.477137
B.cells	MRTFB	0.208154	4.475202	1.38715	0.168883	-5.45464	0.579604	0.481248
B.cells	P2RY10	0.260273	3.930309	1.387126	0.16889	-5.57342	0.584651	0.488602
B.cells	IFI35	0.219594	5.333965	1.387105	0.168897	-5.64272	0.571754	0.469879
B.cells	ZFP41	-0.67127	0.707145	-1.38709	0.168901	-4.6244	0.615573	0.534418
B.cells	MRPL53	0.220251	4.291701	1.387006	0.168927	-5.37104	0.581298	0.483712
B.cells	PARP9	0.271855	5.150171	1.386845	0.168976	-5.60293	0.573424	0.472289
B.cells	AKR1C12	-0.64885	0.756271	-1.38624	0.169159	-4.6157	0.615267	0.533815
B.cells	GM15246	-0.69977	1.046088	-1.38612	0.169196	-4.67348	0.612412	0.529547
B.cells	SERPINA1E	-0.48794	5.030433	-1.38607	0.16921	-5.61678	0.574682	0.473996
B.cells	SMC6	0.131037	7.579287	1.385985	0.169237	-5.97157	0.551975	0.441527
B.cells	BEND5	0.546942	0.115495	1.385768	0.169303	-4.67382	0.621632	0.543468
B.cells	TRAPPC9	0.149347	6.178368	1.385642	0.169342	-5.7487	0.564321	0.45913
B.cells	CCDC77	0.248583	3.291762	1.385525	0.169377	-5.16782	0.590803	0.497548
B.cells	CGRRF1	0.210458	3.907331	1.385137	0.169495	-5.30286	0.585094	0.489295
B.cells	D130040H2	0.35148	2.397794	1.384933	0.169558	-5.01011	0.599358	0.510341
B.cells	WDR19	-0.70888	0.508741	-1.38493	0.169559	-4.61371	0.617779	0.537849
B.cells	VEZF1	-0.12602	6.00178	-1.38472	0.169624	-5.75926	0.565957	0.461693
B.cells	GM43062	-0.37037	1.638658	-1.38464	0.169646	-4.85459	0.606683	0.521318
B.cells	NAB1	0.141145	6.555305	1.384378	0.169727	-5.85489	0.561123	0.454701
B.cells	SORBS3	-0.66573	0.937867	-1.38413	0.169803	-4.6388	0.613741	0.531663
B.cells	CD46	0.40944	2.11028	1.383701	0.169934	-4.92187	0.602603	0.514876
B.cells	ANAPC16	0.131945	5.656735	1.383309	0.170053	-5.68917	0.569747	0.466598
B.cells	TAF2	0.177871	4.171185	1.382586	0.170274	-5.38129	0.583932	0.486584
B.cells	HEBP1	-0.34803	4.67467	-1.38237	0.170339	-5.42887	0.579326	0.479933
B.cells	JAKMIP1	0.270694	3.981458	1.382074	0.170431	-5.33231	0.585889	0.489395
B.cells	ATP6V0C	-0.12204	8.851286	-1.38151	0.170603	-6.20009	0.542859	0.42747
B.cells	IFI27L2A	0.388059	7.373467	1.381285	0.170672	-5.99538	0.555689	0.44548
B.cells	PXYLP1	-0.43103	2.352535	-1.38079	0.170824	-4.85472	0.602168	0.512546
B.cells	SLC16A13	0.829926	-0.01818	1.380604	0.170881	-4.57083	0.625524	0.547488
B.cells	ARMH3	0.153286	5.427651	1.38024	0.170993	-5.67539	0.573617	0.470575
B.cells	CD24A	-0.2053	7.730753	-1.37977	0.171136	-6.18405	0.553405	0.441509

B.cells	GPR157	0.366093	2.137362	1.379617	0.171184	-4.94263	0.6048	0.5159
B.cells	GM36551	0.64706	-0.80034	1.37932	0.171275	-4.53115	0.633993	0.559838
B.cells	TDRKH	-0.55296	1.697145	-1.37902	0.171368	-4.73312	0.60937	0.522437
B.cells	GEMIN8	0.374939	2.284507	1.378482	0.171533	-4.90981	0.604073	0.514218
B.cells	TAPBP	0.210981	6.597076	1.378214	0.171615	-5.8448	0.564145	0.456171
B.cells	DNAJA3	0.235345	3.76841	1.377572	0.171813	-5.30329	0.590544	0.493807
B.cells	ICMT	0.37668	2.429223	1.377237	0.171916	-4.95394	0.6033	0.512664
B.cells	GM16090	-1.05212	-0.96132	-1.3772	0.171927	-4.52533	0.636827	0.563281
B.cells	E230016K2	0.714674	0.143576	1.377055	0.171972	-4.61971	0.625816	0.54628
B.cells	WDR53	0.337031	2.715022	1.376916	0.172015	-5.02877	0.600551	0.508605
B.cells	RTN4	-0.10608	7.592315	-1.37626	0.172218	-6.03251	0.556235	0.444218
B.cells	TRA2A	0.1203	7.075692	1.375913	0.172324	-5.91223	0.560785	0.450801
B.cells	MARS2	-0.28214	2.690445	-1.37578	0.172364	-5.04854	0.601229	0.509377
B.cells	DISC1	-0.34844	3.43956	-1.37513	0.172567	-4.97591	0.594084	0.49906
B.cells	DCAF12	0.137706	6.638146	1.374974	0.172614	-5.92752	0.564673	0.456586
B.cells	GM41556	-0.52453	1.259442	-1.3749	0.172638	-4.75434	0.615157	0.530341
B.cells	LMBRD2	0.233975	4.481892	1.374871	0.172646	-5.40302	0.584306	0.48487
B.cells	CMTM6	0.158804	4.866252	1.374802	0.172667	-5.55319	0.580748	0.479729
B.cells	SIAE	-0.38326	2.612072	-1.37442	0.172784	-4.92063	0.601982	0.511001
B.cells	CCNT2	0.145375	5.37232	1.37436	0.172804	-5.65602	0.576103	0.473215
B.cells	CATSPER2	-0.45648	1.800753	-1.3743	0.172821	-4.85668	0.609845	0.522661
B.cells	BLCAP	0.313149	3.009547	1.374298	0.172823	-5.02823	0.598173	0.505382
B.cells	LRIG3	-0.78664	0.14201	-1.37429	0.172825	-4.60151	0.626292	0.547308
B.cells	CTSC	0.243776	7.322442	1.374204	0.172852	-6.01656	0.558607	0.448209
B.cells	LY6C1	0.975163	-0.32417	1.373804	0.172976	-4.57601	0.631272	0.554578
B.cells	MTO1	0.223476	3.553315	1.373487	0.173074	-5.26839	0.59342	0.498043
B.cells	FAM172A	0.120062	7.557353	1.373035	0.173214	-6.00649	0.557056	0.445657
B.cells	ACAT3	-0.59957	1.453412	-1.37303	0.173215	-4.76222	0.613815	0.52817
B.cells	GM4788	-0.59465	1.237901	-1.37235	0.173425	-4.77606	0.6163	0.531654
B.cells	YIPF4	0.144562	6.515782	1.372107	0.173501	-5.93696	0.566621	0.45908
B.cells	B4GALT7	0.280611	2.869796	1.37203	0.173525	-5.10832	0.600415	0.508117
B.cells	FH1	-0.20198	5.484899	-1.37183	0.173587	-5.66593	0.575944	0.472556
B.cells	TMEM50B	0.252781	4.107732	1.3713	0.173752	-5.42598	0.588682	0.491133
B.cells	QSOX1	-0.2364	4.311358	-1.37128	0.173759	-5.32929	0.586778	0.488358
B.cells	SPP1	0.769186	2.353951	1.371232	0.173773	-4.95337	0.605385	0.515694
B.cells	GM12353	-0.59137	1.278675	-1.37123	0.173774	-4.71659	0.615897	0.531342
B.cells	ENOX2	0.189314	5.852601	1.371229	0.173774	-5.73795	0.572598	0.467847
B.cells	CCDC14	-0.69112	0.809905	-1.37081	0.173903	-4.65564	0.620825	0.538452
B.cells	PRRC1	0.180181	4.219989	1.369478	0.174318	-5.44716	0.588994	0.490273
B.cells	MED11	0.232866	3.312748	1.369343	0.17436	-5.2419	0.597568	0.502811
B.cells	IRF5	0.173108	5.351662	1.369246	0.17439	-5.79123	0.5785	0.475136
B.cells	1700001K1	-0.60117	1.052633	-1.36911	0.174432	-4.75265	0.619566	0.535537
B.cells	SAA3	1.773904	0.738679	1.368878	0.174505	-4.76401	0.622776	0.5403
B.cells	HSF2	-0.19409	4.185277	-1.36827	0.174695	-5.50654	0.589702	0.491158
B.cells	GM40787	0.711807	0.205726	1.36826	0.174698	-4.64716	0.628457	0.548624
B.cells	AARS	-0.18573	5.020707	-1.36807	0.174756	-5.5487	0.581948	0.47998
B.cells	GM43251	1.020542	-0.97845	1.367747	0.174858	-4.54861	0.640539	0.567227

B.cells	OTUD7B	0.180366	5.17042	1.367271	0.175006	-5.55083	0.580928	0.47818
B.cells	SLC37A2	0.340378	3.315046	1.367234	0.175018	-5.13599	0.598331	0.503523
B.cells	TRIM28	-0.17754	5.460856	-1.36695	0.175107	-5.62643	0.578277	0.47439
B.cells	D030028A(	0.315317	2.795422	1.366889	0.175126	-5.17064	0.603333	0.51095
B.cells	TPPP	-0.97554	0.182012	-1.36626	0.175321	-4.58822	0.629653	0.5497
B.cells	KHK	-0.25395	4.3658	-1.36539	0.175594	-5.31132	0.58965	0.489541
B.cells	FNIP2	0.544071	5.343736	1.364919	0.175742	-5.27538	0.58081	0.476536
B.cells	ARF5	0.138965	8.486216	1.364826	0.175771	-6.21173	0.552696	0.436622
B.cells	DUSP11	0.112298	6.33182	1.364391	0.175907	-5.83709	0.572033	0.463773
B.cells	UPF1	-0.13475	5.35091	-1.36426	0.175948	-5.61749	0.580989	0.476674
B.cells	ATG14	-0.22646	3.27883	-1.3637	0.176125	-5.2232	0.600886	0.505054
B.cells	CCNE2	-0.41394	3.75287	-1.3634	0.176218	-5.18177	0.596503	0.498465
B.cells	NIPAL1	-0.60281	1.060638	-1.36272	0.17643	-4.73696	0.623229	0.53752
B.cells	1500015A(	0.379191	1.76661	1.362632	0.176459	-4.87018	0.616218	0.527093
B.cells	REST	0.160334	5.400614	1.362356	0.176546	-5.68392	0.581616	0.476528
B.cells	GM16268	-0.73921	0.706579	-1.36224	0.176584	-4.64475	0.626858	0.543061
B.cells	ST5	-0.44703	2.235439	-1.36195	0.176673	-4.88227	0.611819	0.520633
B.cells	RSF1	0.123995	7.014648	1.361688	0.176756	-5.95209	0.567175	0.455787
B.cells	ARHGEF39	-0.65157	1.960521	-1.36046	0.177142	-4.79589	0.615413	0.525122
B.cells	EPS8	-0.22344	4.963297	-1.36025	0.177209	-5.90305	0.586646	0.48304
B.cells	ADAMTS6	-0.2032	5.940983	-1.36024	0.177213	-5.79273	0.577622	0.470069
B.cells	MN1	-0.94965	0.308399	-1.36022	0.177218	-4.53889	0.631936	0.5498
B.cells	GM10131	-0.70089	1.072727	-1.35985	0.177335	-4.70819	0.624229	0.538405
B.cells	COG3	0.196165	4.292574	1.359789	0.177354	-5.45419	0.592933	0.492283
B.cells	RBM6	0.114034	7.184868	1.359768	0.177361	-5.97587	0.566377	0.454198
B.cells	IER5L	-0.31328	2.89009	-1.35938	0.177484	-5.12909	0.606335	0.512047
B.cells	C9	-0.9229	0.60037	-1.3593	0.177509	-4.67205	0.628979	0.545713
B.cells	GPR183	0.271012	3.488292	1.359244	0.177526	-5.50709	0.600576	0.503625
B.cells	PALM	-0.24343	4.231998	-1.35918	0.177547	-5.41648	0.593504	0.493308
B.cells	GSTM5	-0.62287	1.19189	-1.35884	0.177652	-4.76901	0.623041	0.537014
B.cells	CEBPA	-0.41884	2.906324	-1.35882	0.177659	-5.05687	0.606181	0.512018
B.cells	DYNC2H1	0.237409	3.842041	1.358525	0.177753	-5.34763	0.597203	0.498933
B.cells	MRPS27	0.268865	3.208477	1.358428	0.177784	-5.21114	0.603265	0.507846
B.cells	IMP4	-0.17639	4.46298	-1.35836	0.177805	-5.47209	0.591331	0.490422
B.cells	RAB3GAP1	0.116689	6.044858	1.358168	0.177866	-5.77826	0.576708	0.46934
B.cells	FBXO46	0.351981	2.47185	1.357713	0.17801	-5.02536	0.610754	0.518573
B.cells	CRY1	0.166157	5.502006	1.357413	0.178105	-5.70379	0.58208	0.476633
B.cells	TCOF1	-0.16679	5.717375	-1.3573	0.17814	-5.79062	0.580096	0.473842
B.cells	IL20RB	-0.26281	3.81612	-1.35694	0.178255	-5.27587	0.597977	0.499634
B.cells	METTL9	-0.1764	5.718204	-1.35689	0.17827	-5.62383	0.580178	0.473867
B.cells	PCGF5	0.184229	6.06196	1.356277	0.178465	-5.80837	0.577223	0.469573
B.cells	FRG1	-0.11565	6.660706	-1.35622	0.178483	-5.90148	0.571783	0.461818
B.cells	AHI1	0.304619	3.003332	1.356214	0.178485	-5.0882	0.605981	0.511311
B.cells	MED13L	0.121346	7.196724	1.355527	0.178702	-6.00584	0.567302	0.455142
B.cells	DNAIC1	-0.73341	0.436784	-1.35543	0.178732	-4.63419	0.631782	0.549347
B.cells	OSBPL3	0.433677	3.353205	1.35537	0.178752	-4.95093	0.602966	0.506584
B.cells	PLPP6	0.311494	2.754142	1.355163	0.178818	-5.09919	0.608759	0.515223

B.cells	PRG3	-0.64079	1.171663	-1.35497	0.17888	-4.87602	0.624374	0.538475
B.cells	ATG4B	0.177981	4.800676	1.354906	0.178899	-5.52696	0.589234	0.486769
B.cells	CKAP5	0.19347	6.118962	1.354669	0.178975	-5.75867	0.577122	0.469302
B.cells	HGF	0.539384	2.743326	1.354341	0.179079	-4.97656	0.609124	0.515612
B.cells	RRM1	-0.24826	5.449593	-1.35395	0.179202	-5.60006	0.583681	0.478311
B.cells	MS4A6B	0.304701	4.588359	1.353689	0.179286	-5.6647	0.591822	0.490054
B.cells	KCNQ5	0.176457	7.024096	1.353051	0.179489	-6.02128	0.569905	0.458151
B.cells	RBBP4	-0.10798	7.676556	-1.35259	0.179635	-6.03914	0.564369	0.450029
B.cells	PARVA	-0.58563	1.329681	-1.35221	0.179755	-4.74161	0.624348	0.537084
B.cells	KLHL24	0.149481	6.228518	1.352105	0.17979	-5.82784	0.577484	0.468647
B.cells	CCN1	-0.842	1.098666	-1.35186	0.17987	-4.68071	0.626665	0.540683
B.cells	NEURL1A	-0.87502	-0.39095	-1.3517	0.179919	-4.58309	0.641844	0.563541
B.cells	PEG13	-0.43334	2.114243	-1.35149	0.179986	-4.85863	0.616553	0.525703
B.cells	AASS	-0.65371	1.208483	-1.3513	0.180046	-4.74208	0.625562	0.53918
B.cells	CC2D1B	0.213127	3.659138	1.351196	0.18008	-5.2994	0.60153	0.503678
B.cells	ELOF1	0.164165	5.375272	1.35108	0.180117	-5.64778	0.585341	0.480192
B.cells	CDKN3	-0.39513	4.160534	-1.35082	0.180201	-5.29654	0.596747	0.496751
B.cells	FPGT	0.551463	1.345954	1.35071	0.180235	-4.83029	0.624185	0.537237
B.cells	SPRYD4	-0.4178	1.803638	-1.3506	0.180269	-4.82358	0.619625	0.530472
B.cells	MITF	0.346945	4.369202	1.350429	0.180325	-5.26848	0.594769	0.494008
B.cells	PATL1	0.160004	5.222719	1.350426	0.180326	-5.63234	0.58676	0.482408
B.cells	GM47917	0.50918	0.467794	1.35011	0.180427	-4.76335	0.633217	0.550758
B.cells	GM9949	0.77025	0.483247	1.349751	0.180542	-4.65036	0.633065	0.55065
B.cells	BAIAP2	-0.22558	5.269985	-1.34971	0.180554	-5.6218	0.586485	0.482008
B.cells	IFI30	0.179837	6.746076	1.349278	0.180693	-6.02469	0.57294	0.462657
B.cells	TAZ	0.188846	4.057371	1.349232	0.180707	-5.37303	0.597896	0.498617
B.cells	LRRC51	-0.42941	1.76843	-1.34912	0.180744	-4.87508	0.62015	0.531438
B.cells	PMS1	0.317443	2.650773	1.349053	0.180765	-5.07723	0.611459	0.518568
B.cells	B130034C1	-0.47056	1.038829	-1.34898	0.18079	-4.72368	0.627444	0.542374
B.cells	VIRMA	0.15445	5.71037	1.348545	0.180927	-5.73092	0.582683	0.476454
B.cells	MBD3	0.147992	5.696238	1.348068	0.18108	-5.70252	0.583139	0.476848
B.cells	ARHGAP32	-0.38625	3.193867	-1.34767	0.181209	-4.95246	0.606868	0.511399
B.cells	PPP1R18	-0.13638	6.994866	-1.34765	0.181214	-5.91497	0.571341	0.460053
B.cells	LGALSL	-0.56471	1.281509	-1.34744	0.18128	-4.75912	0.625716	0.539401
B.cells	SKA3	-0.36899	2.738324	-1.34737	0.181305	-5.0073	0.611297	0.518017
B.cells	HSPA4	0.096499	7.667014	1.346723	0.181512	-6.07814	0.565758	0.451796
B.cells	CD86	0.212169	6.31281	1.346605	0.181549	-6.08001	0.577982	0.469221
B.cells	VPS28	0.138214	6.686374	1.346235	0.181668	-5.89505	0.574601	0.464518
B.cells	TMEM140	0.390233	3.514222	1.346152	0.181695	-5.16938	0.60427	0.507452
B.cells	TPX2	-0.35237	5.047457	-1.3461	0.181711	-5.5042	0.58971	0.486272
B.cells	GCSAM	-0.62938	-0.31734	-1.34581	0.181803	-4.70556	0.64266	0.564506
B.cells	AKAP7	0.250237	3.374989	1.345126	0.182025	-5.21498	0.606082	0.509695
B.cells	FARSA	-0.14503	4.90554	-1.34507	0.182042	-5.62237	0.591499	0.488437
B.cells	DHRS9	0.65098	0.196917	1.344965	0.182076	-4.69013	0.63772	0.556771
B.cells	CRIM1	-0.30912	5.191827	-1.34486	0.182111	-5.5317	0.588817	0.48457
B.cells	COG1	0.19007	3.677624	1.344688	0.182166	-5.36471	0.603175	0.505456
B.cells	PKD1L2	0.635532	1.173969	1.344333	0.18228	-4.77761	0.62802	0.541984

B.cells	RBL2	0.184654	4.765126	1.343897	0.18242	-5.55648	0.593205	0.490643
B.cells	BANP	0.17181	4.601582	1.343846	0.182437	-5.49122	0.594748	0.492889
B.cells	AMPD3	0.327818	2.578161	1.343103	0.182676	-5.20195	0.614713	0.521734
B.cells	PIK3R5	0.214578	4.45137	1.342729	0.182797	-5.49184	0.59663	0.495356
B.cells	HPS1	0.254379	3.445228	1.342697	0.182807	-5.28762	0.606264	0.509437
B.cells	AADAT	-0.52627	1.2345	-1.3423	0.182935	-4.79292	0.628078	0.541965
B.cells	CTSB	-0.20824	8.190873	-1.34227	0.182944	-6.09078	0.562367	0.446581
B.cells	ALDH7A1	-0.32366	3.227154	-1.34226	0.182947	-5.16264	0.608377	0.512752
B.cells	HRH4	0.723498	0.27047	1.342151	0.182983	-4.68727	0.637872	0.556687
B.cells	ATP6V0B	0.100616	8.046898	1.341938	0.183052	-6.14706	0.563642	0.448477
B.cells	ARHGAP5	-0.2818	4.809305	-1.34189	0.183067	-5.46354	0.593245	0.4908
B.cells	GM27188	-0.84539	0.619818	-1.34168	0.183137	-4.63584	0.634303	0.551591
B.cells	4931403E2	-0.37256	1.501737	-1.34164	0.183147	-4.91704	0.625393	0.53824
B.cells	GM8251	-0.32003	3.392286	-1.34078	0.183428	-5.31233	0.607318	0.511166
B.cells	GPRIN3	-0.67847	0.638034	-1.34047	0.183527	-4.68943	0.634684	0.551953
B.cells	CDCA3	-0.36457	4.895561	-1.34021	0.18361	-5.49577	0.592962	0.490321
B.cells	BRF2	-0.29676	2.326101	-1.34019	0.183619	-5.06795	0.617746	0.526664
B.cells	A530013C2	0.5216	2.431674	1.339964	0.183691	-4.9337	0.616705	0.525149
B.cells	MORN3	-0.48658	1.323497	-1.33995	0.183695	-4.83342	0.627742	0.54158
B.cells	SNX5	0.118693	7.890887	1.339877	0.183719	-6.09078	0.565532	0.451135
B.cells	RAMP3	0.700342	0.947281	1.339747	0.183761	-4.72696	0.631541	0.547382
B.cells	GM39090	-0.60236	0.497553	-1.3397	0.183777	-4.68052	0.636117	0.554294
B.cells	CREBZF	0.169838	4.715073	1.339212	0.183935	-5.52076	0.595005	0.493101
B.cells	SFT2D2	-0.20213	4.677531	-1.33881	0.184066	-5.50245	0.595551	0.493635
B.cells	A930001A2	-0.81725	-0.71301	-1.33852	0.184157	-4.60156	0.649075	0.573556
B.cells	SF3A2	-0.17678	5.260106	-1.33845	0.184181	-5.64131	0.590068	0.485801
B.cells	PLD1	0.442244	3.080795	1.33839	0.184201	-5.02753	0.610889	0.516187
B.cells	CHRNA9	-0.46754	2.214161	-1.33804	0.184314	-5.02695	0.619474	0.528905
B.cells	COMMD7	0.122928	5.29863	1.338008	0.184325	-5.65852	0.589772	0.4854
B.cells	B230206L0	-0.76233	1.02404	-1.3373	0.184554	-4.76305	0.632003	0.547018
B.cells	CCNL1	-0.11384	7.09056	-1.33691	0.184682	-5.98563	0.573997	0.462183
B.cells	TRIB1	-0.20435	5.765307	-1.33665	0.184768	-5.75603	0.586151	0.479686
B.cells	C430049B0	0.503173	1.844436	1.336383	0.184853	-4.83963	0.623923	0.535102
B.cells	PRMT3	0.208916	4.041123	1.336357	0.184862	-5.45874	0.602422	0.503381
B.cells	ITGB2	0.210382	6.734664	1.336345	0.184865	-5.88987	0.577231	0.467015
B.cells	CCL17	-0.80011	-0.70076	-1.33589	0.185013	-4.70107	0.649975	0.574464
B.cells	DNAJC9	-0.2125	5.617283	-1.33589	0.185014	-5.68558	0.58766	0.481919
B.cells	ODF2L	-0.35769	2.682465	-1.33574	0.185063	-4.969	0.615755	0.523041
B.cells	DGCR2	0.180492	4.849233	1.335547	0.185125	-5.57597	0.594875	0.492522
B.cells	CCNG2	-0.19675	5.189545	-1.33541	0.18517	-5.64302	0.59167	0.487888
B.cells	MPRIP	0.160912	5.481388	1.334563	0.185446	-5.73958	0.589288	0.484195
B.cells	PIPOX	-0.49715	2.011866	-1.33456	0.185446	-4.91423	0.622774	0.533276
B.cells	AMN1	0.243173	3.42505	1.334404	0.185498	-5.26155	0.608873	0.512775
B.cells	SLC31A1	0.161474	5.429507	1.334353	0.185515	-5.71879	0.589773	0.484961
B.cells	NPL	-0.5294	2.535175	-1.33427	0.185541	-4.93211	0.617584	0.525675
B.cells	STAT4	0.247478	5.177658	1.334034	0.185619	-5.74458	0.592218	0.488455
B.cells	SRP68	0.165121	3.983827	1.333316	0.185853	-5.35613	0.60415	0.505285

B.cells	H2AFY	-0.10853	7.697828	-1.33312	0.185916	-6.07954	0.569638	0.455695
B.cells	EVA1B	-0.29386	3.639669	-1.333	0.185956	-5.20601	0.60747	0.510229
B.cells	FAM114A2	-0.14335	5.166561	-1.3328	0.186022	-5.63541	0.592945	0.489048
B.cells	RPA1	0.196743	4.922792	1.331979	0.186291	-5.56723	0.59548	0.492659
B.cells	RSPRY1	-0.13206	5.833379	-1.33182	0.186343	-5.75787	0.586943	0.480402
B.cells	GUCA1A	0.471526	1.4794	1.33167	0.186392	-4.86659	0.629107	0.542323
B.cells	FDPS	-0.25884	4.623809	-1.33166	0.186394	-5.48454	0.598315	0.496898
B.cells	SMG9	0.180236	4.52517	1.331621	0.186408	-5.52252	0.599254	0.498265
B.cells	GM13391	-0.47769	0.765079	-1.33162	0.186409	-4.86847	0.636355	0.553194
B.cells	DGKG	0.436819	2.506906	1.331425	0.186472	-5.00723	0.618882	0.527132
B.cells	ALKBH1	0.150559	4.99535	1.330667	0.18672	-5.67579	0.595455	0.492157
B.cells	NDUFAF4	0.233834	3.867148	1.330184	0.186879	-5.34677	0.606578	0.5081
B.cells	2610037D(	0.16295	5.44268	1.330018	0.186933	-5.71095	0.591588	0.486365
B.cells	MBD2	0.114721	7.044678	1.329827	0.186996	-5.96995	0.576817	0.465205
B.cells	SLC37A1	0.329804	3.202303	1.329461	0.187116	-5.13094	0.613306	0.517845
B.cells	B630019A1	-0.48906	1.596371	-1.32926	0.187182	-4.89664	0.629267	0.541554
B.cells	FARS2	0.10289	6.899126	1.329133	0.187224	-5.98864	0.578363	0.467283
B.cells	PLRG1	-0.18087	4.093452	-1.3289	0.187301	-5.42424	0.604751	0.505348
B.cells	PTH1R	-0.86324	0.264703	-1.32854	0.187419	-4.64987	0.643167	0.562255
B.cells	ORC2	0.185986	4.279461	1.32819	0.187534	-5.47443	0.603376	0.50288
B.cells	SDK1	-0.51545	3.195716	-1.32784	0.187648	-5.04829	0.613999	0.518328
B.cells	MFGE8	-0.43989	2.292559	-1.32777	0.187674	-4.97905	0.622921	0.531515
B.cells	GM49521	0.524395	0.473447	1.327034	0.187914	-4.78533	0.641802	0.559345
B.cells	ZDHHC1	0.450067	1.415954	1.326946	0.187943	-4.80643	0.63217	0.544891
B.cells	ACYP2	0.257423	3.398712	1.326887	0.187963	-5.32049	0.612446	0.515662
B.cells	CYB5R1	-0.25323	3.659051	-1.32671	0.18802	-5.24843	0.609927	0.51197
B.cells	CAMK1	-0.37059	2.966119	-1.32641	0.188121	-5.05055	0.61681	0.52199
B.cells	SVBP	0.168678	5.267752	1.325898	0.188289	-5.65497	0.594625	0.489798
B.cells	GM10863	-0.81753	0.229142	-1.3256	0.188386	-4.63973	0.644454	0.563562
B.cells	RDH11	0.254096	3.215453	1.32545	0.188437	-5.23566	0.61436	0.518679
B.cells	KHSRP	-0.13068	5.998752	-1.32544	0.188441	-5.80219	0.587776	0.480011
B.cells	RAB11FIP3	-0.39311	2.230172	-1.32517	0.18853	-4.92042	0.624106	0.533166
B.cells	USP54	0.272935	2.915478	1.325156	0.188534	-5.17891	0.617308	0.523099
B.cells	SLC20A2	0.229611	4.036306	1.325124	0.188544	-5.39517	0.606376	0.507043
B.cells	CACNA1B	-0.71959	0.056619	-1.32478	0.188659	-4.6723	0.646243	0.566417
B.cells	GEMIN7	0.137515	5.084337	1.324616	0.188712	-5.65761	0.596358	0.492536
B.cells	SYAP1	0.141914	5.053275	1.32459	0.188721	-5.63984	0.596652	0.492962
B.cells	RWDD2B	-0.44463	1.849393	-1.32444	0.188769	-4.9098	0.62792	0.539016
B.cells	EIF1	-0.12574	9.721973	-1.32426	0.18883	-6.40394	0.554311	0.433028
B.cells	XRN1	0.138486	5.924264	1.324253	0.188832	-5.82301	0.58847	0.4813
B.cells	9530052E0	0.463802	1.000649	1.324238	0.188837	-4.8225	0.636519	0.55196
B.cells	I730030J21	0.573556	0.707146	1.323213	0.189176	-4.77299	0.63983	0.556843
B.cells	XKR6	-0.60679	0.862903	-1.32317	0.189192	-4.71952	0.638232	0.55444
B.cells	SHPRH	0.184348	4.541567	1.323134	0.189203	-5.53195	0.601809	0.500517
B.cells	SMIM8	0.192467	4.587829	1.323126	0.189205	-5.50841	0.601367	0.499873
B.cells	4833407H1	0.306612	2.098821	1.322754	0.189328	-5.04875	0.625717	0.535866
B.cells	SCAND1	0.109559	7.018464	1.32265	0.189363	-5.99286	0.578654	0.467297

B.cells	MTHFR	0.231747	3.640415	1.322481	0.189419	-5.28368	0.610503	0.5134
B.cells	TSPOAP1	-0.37616	2.650845	-1.32226	0.189493	-5.0887	0.620219	0.527828
B.cells	LBR	-0.11049	7.219631	-1.32214	0.189531	-5.98175	0.57682	0.464796
B.cells	RCSD1	0.162621	7.091061	1.322069	0.189555	-5.96988	0.577991	0.466462
B.cells	GM11423	-0.54978	0.892029	-1.32203	0.189569	-4.73156	0.637934	0.554275
B.cells	4930438A(	-0.61041	-0.84008	-1.32197	0.189589	-4.63299	0.65576	0.581607
B.cells	SHCBP1	-0.33156	4.69138	-1.32191	0.189607	-5.46876	0.600378	0.498722
B.cells	USP20	0.260589	2.877475	1.321661	0.189691	-5.15209	0.618078	0.524572
B.cells	PODXL	-0.76879	0.550401	-1.32109	0.189879	-4.66518	0.642007	0.559855
B.cells	VCAM1	-0.51562	4.334733	-1.32081	0.189972	-5.34723	0.604452	0.504022
B.cells	TFCP2	0.422619	1.971586	1.320559	0.190057	-4.88668	0.627784	0.538383
B.cells	GM12248	-0.31598	2.619411	-1.32011	0.190207	-5.19155	0.621317	0.528942
B.cells	OSBP	0.155351	5.016429	1.320059	0.190223	-5.66947	0.598044	0.494808
B.cells	4833417C1	0.582899	0.707506	1.319906	0.190274	-4.73357	0.640637	0.557812
B.cells	GRK3	-0.40192	2.91909	-1.31985	0.190292	-5.03735	0.61835	0.524563
B.cells	BOLA3	0.171491	5.736487	1.319739	0.190329	-5.77603	0.591253	0.485002
B.cells	DMWD	-0.52244	2.228423	-1.31961	0.190372	-4.83487	0.625213	0.534789
B.cells	THRB	-0.41218	3.815098	-1.31911	0.190539	-5.26888	0.609814	0.511905
B.cells	RAB40C	0.166929	4.838453	1.318898	0.190609	-5.61151	0.599968	0.4976
B.cells	IFI209	0.194312	5.809713	1.318788	0.190646	-5.88107	0.590796	0.484355
B.cells	UBL7	0.173516	5.130384	1.31876	0.190655	-5.64926	0.597194	0.493606
B.cells	RIMS3	-0.67339	1.085548	-1.3186	0.190709	-4.74493	0.637014	0.552442
B.cells	BC049352	-0.60848	1.083027	-1.3183	0.190807	-4.7702	0.637192	0.552551
B.cells	MMP14	0.45642	3.837765	1.317983	0.190914	-5.36641	0.609787	0.511902
B.cells	ADCY7	0.147117	5.583087	1.317922	0.190934	-5.76929	0.593109	0.487626
B.cells	VIM	-0.20593	8.109275	-1.31779	0.190978	-6.23501	0.569914	0.454513
B.cells	RHOQ	0.18595	5.771108	1.316583	0.191381	-5.80841	0.591731	0.485435
B.cells	CRAMP1L	0.158547	4.984741	1.316397	0.191443	-5.62285	0.599157	0.496309
B.cells	CC2D2A	-0.59428	0.81427	-1.31631	0.191471	-4.80281	0.640408	0.557386
B.cells	LYSMD1	-0.42309	1.591133	-1.3163	0.191477	-4.83333	0.63248	0.545484
B.cells	PTPRB	-0.51393	3.708559	-1.3159	0.191608	-5.18123	0.611442	0.514409
B.cells	B3GNT8	0.395866	2.202747	1.315349	0.191794	-5.08758	0.626318	0.536703
B.cells	2610306M	0.638112	0.680103	1.315311	0.191806	-4.73076	0.641789	0.559901
B.cells	9-Sep	-0.20039	5.640387	-1.31496	0.191923	-5.60432	0.592958	0.48791
B.cells	NFYA	-0.21744	4.152707	-1.3146	0.192046	-5.40772	0.607133	0.508654
B.cells	COG5	0.135989	6.582266	1.314477	0.192086	-5.94445	0.584185	0.475406
B.cells	GM48678	0.280394	3.349644	1.314306	0.192143	-5.33448	0.61495	0.520271
B.cells	4930509H(	0.30857	2.090644	1.314169	0.192189	-5.0553	0.627443	0.538868
B.cells	TOP2A	-0.31222	7.615909	-1.31412	0.192206	-6.06233	0.574733	0.462057
B.cells	NCAPG	-0.40913	3.22832	-1.31403	0.192236	-5.15331	0.616141	0.522082
B.cells	PTPRS	-0.20663	4.390471	-1.31393	0.192268	-5.53446	0.604841	0.505466
B.cells	BRMS1	0.187774	4.236998	1.313929	0.192269	-5.51573	0.606319	0.50763
B.cells	FAF1	0.116926	6.739255	1.313913	0.192275	-5.95875	0.582737	0.473471
B.cells	APP	0.278043	5.938302	1.31384	0.192299	-5.65579	0.590166	0.484148
B.cells	HSPE1-RS1	0.81211	0.18545	1.313693	0.192348	-4.65972	0.646909	0.56816
B.cells	6530409C1	0.426346	1.661527	1.313647	0.192364	-4.98859	0.631768	0.545335
B.cells	GM45442	0.972689	-0.57631	1.313646	0.192364	-4.57776	0.654821	0.580296

B.cells	BIK	-0.6265	1.76969	-1.31362	0.192373	-4.82512	0.630674	0.543698
B.cells	RAB31	-0.18662	4.770711	-1.31347	0.192422	-5.66514	0.601197	0.500197
B.cells	RDH13	0.469083	1.398416	1.313407	0.192444	-4.83854	0.634436	0.549397
B.cells	SNAP29	0.138179	5.5062	1.313361	0.192459	-5.74254	0.59422	0.49006
B.cells	ABCF1	0.119556	6.430861	1.313322	0.192473	-5.91374	0.585585	0.477605
B.cells	RHBDD2	-0.36977	2.284757	-1.31277	0.192657	-4.95188	0.625497	0.53627
B.cells	RRP9	-0.29423	2.59462	-1.3127	0.192683	-5.09917	0.622407	0.531688
B.cells	FAM71A	-0.67266	0.237403	-1.31262	0.192708	-4.71217	0.646369	0.567684
B.cells	3300002A1	0.578548	0.451891	1.312584	0.19272	-4.72045	0.644145	0.564324
B.cells	E130307A1	0.171189	4.490339	1.312335	0.192804	-5.51668	0.603881	0.50445
B.cells	GM28417	0.569044	1.320479	1.312138	0.19287	-4.77434	0.635229	0.551024
B.cells	PCED1A	0.293203	2.508638	1.311874	0.192959	-5.15643	0.623263	0.533194
B.cells	C5AR2	0.594736	1.231827	1.311687	0.193022	-4.80478	0.636133	0.552534
B.cells	MYO1C	0.129633	5.436164	1.31161	0.193048	-5.76806	0.59488	0.491539
B.cells	PCYT1A	0.185944	5.255992	1.311586	0.193055	-5.69173	0.596583	0.494014
B.cells	GM26670	0.552464	0.402458	1.311437	0.193106	-4.68742	0.644657	0.565486
B.cells	VCAN	0.672831	0.76602	1.311223	0.193178	-4.88564	0.640904	0.559808
B.cells	RASSF4	0.187079	4.553252	1.311175	0.193194	-5.73357	0.603278	0.503841
B.cells	ABHD17A	0.114431	6.296745	1.311106	0.193217	-5.86286	0.586828	0.479969
B.cells	LPL	-0.28658	5.132487	-1.3108	0.193321	-5.64463	0.597753	0.495871
B.cells	2700049A(	0.212714	4.236674	1.310631	0.193377	-5.48227	0.606322	0.508432
B.cells	GM16310	-0.57022	1.197906	-1.3105	0.193422	-4.80868	0.636479	0.553335
B.cells	BCLAF1	0.09596	7.155376	1.310317	0.193483	-6.04865	0.578922	0.468896
B.cells	USP46	0.315804	3.281753	1.309947	0.193607	-5.18023	0.615616	0.522345
B.cells	GM26916	-0.46644	0.934105	-1.30973	0.193679	-4.81304	0.639178	0.557579
B.cells	CRYBB3	-0.62497	0.578324	-1.3097	0.193689	-4.7478	0.642838	0.563116
B.cells	CCDC83	-0.90172	0.420205	-1.30943	0.193782	-4.69487	0.644473	0.565646
B.cells	LSM4	0.125653	6.704001	1.308975	0.193935	-5.95196	0.583062	0.474952
B.cells	GM36486	-0.56651	0.805963	-1.30896	0.193942	-4.90172	0.640494	0.559652
B.cells	DIP2C	-0.27044	6.172429	-1.3089	0.193959	-5.74733	0.587983	0.482033
B.cells	4933423P2	-0.38096	1.804113	-1.30887	0.193971	-4.97374	0.630327	0.544342
B.cells	IL3RA	0.260465	3.561284	1.308825	0.193986	-5.27235	0.612879	0.518376
B.cells	ORC1	-0.32888	3.345968	-1.30872	0.194019	-5.19633	0.614986	0.521541
B.cells	B230118H(	-0.32513	2.933488	-1.30869	0.194029	-5.13397	0.619047	0.527561
B.cells	CRP	-0.47237	2.076546	-1.30856	0.194076	-5.02309	0.627584	0.540314
B.cells	TK1	-0.30251	4.882981	-1.30839	0.194134	-5.51416	0.600125	0.499774
B.cells	POLQ	-0.32991	3.166551	-1.30814	0.194218	-5.1359	0.616749	0.524365
B.cells	MRPS7	-0.15862	5.010323	-1.30808	0.194237	-5.65361	0.598913	0.498152
B.cells	SH2D3C	0.208582	4.693449	1.307697	0.194366	-5.6179	0.601935	0.502708
B.cells	GALT	-0.37728	2.389889	-1.30766	0.194379	-5.00117	0.624447	0.535988
B.cells	SMAD4	0.112967	6.23224	1.307652	0.194382	-5.89355	0.587427	0.481626
B.cells	ERCC6L2	0.264438	3.372895	1.307573	0.194408	-5.25677	0.614722	0.521529
B.cells	NEDD4L	0.119423	6.886893	1.306751	0.194686	-6.00629	0.582054	0.47322
B.cells	TGTP2	0.950029	0.773598	1.306245	0.194857	-4.70247	0.641679	0.561235
B.cells	DENND1B	0.136353	7.299486	1.306129	0.194897	-6.0398	0.578375	0.468065
B.cells	TBCK	0.154825	5.26974	1.30601	0.194937	-5.68428	0.597246	0.49529
B.cells	AP3B1	0.101969	7.329007	1.305952	0.194956	-6.07955	0.578106	0.467712

B.cells	CAMSAP2	0.173906	4.496866	1.305825	0.194999	-5.60884	0.604622	0.506114
B.cells	F2R	-0.32585	3.599158	-1.30572	0.195035	-5.18666	0.613323	0.518923
B.cells	TLR4	0.355069	3.122783	1.305167	0.195223	-5.19283	0.618274	0.526015
B.cells	CLTB	-0.21294	4.396264	-1.30516	0.195226	-5.44424	0.605858	0.507699
B.cells	TENT2	0.108758	6.624422	1.304907	0.195311	-5.97679	0.584921	0.477254
B.cells	EIF1B	-0.12937	5.828759	-1.30476	0.195361	-5.78969	0.59233	0.487961
B.cells	LAGE3	0.175599	4.771468	1.30435	0.1955	-5.56612	0.602417	0.502744
B.cells	ZBTB40	0.247238	3.163496	1.304293	0.195519	-5.28133	0.618038	0.525765
B.cells	SEC24D	-0.2443	4.470665	-1.30413	0.195576	-5.33619	0.605304	0.507053
B.cells	ARF4	0.118839	8.479652	1.304072	0.195594	-6.25601	0.568132	0.453537
B.cells	KMT2D	-0.12553	5.945956	-1.30371	0.195716	-5.81971	0.591502	0.486744
B.cells	LZTS2	-0.66807	1.249256	-1.30343	0.195812	-4.72878	0.637472	0.554755
B.cells	ZFYVE27	0.187593	4.063949	1.303376	0.19583	-5.44733	0.609442	0.51299
B.cells	AK7	-0.52001	1.686834	-1.30326	0.195871	-4.91734	0.633018	0.548102
B.cells	GM49336	-0.15517	4.881295	-1.30274	0.196048	-5.62606	0.601814	0.501774
B.cells	GRAMD1B	0.262671	5.536445	1.30271	0.196057	-5.55932	0.595591	0.492705
B.cells	TFDP1	-0.2102	5.887837	-1.30256	0.196108	-5.73495	0.592284	0.487971
B.cells	RAB5B	0.193728	4.382557	1.302299	0.196197	-5.49316	0.606716	0.508923
B.cells	FILIP1	-0.70222	0.904589	-1.30196	0.19631	-4.69617	0.641494	0.560793
B.cells	RNF220	-0.16973	5.723291	-1.30188	0.196339	-5.72341	0.594044	0.490476
B.cells	MRFAP1	-0.1129	6.452488	-1.30162	0.196426	-5.89193	0.587228	0.480654
B.cells	CD247	-0.42226	3.01096	-1.30148	0.196476	-5.0846	0.620229	0.529011
B.cells	DENND2D	0.347008	2.202221	1.301052	0.196621	-5.00387	0.628296	0.541187
B.cells	KLC1	-0.15265	4.702224	-1.301	0.196638	-5.59354	0.603746	0.504819
B.cells	QARS	-0.18297	4.552856	-1.30087	0.196684	-5.57709	0.605181	0.506923
B.cells	ISOC2A	-0.40105	2.013655	-1.30082	0.196702	-4.92428	0.630194	0.544033
B.cells	SLC39A8	-0.46955	2.726982	-1.30081	0.196705	-4.97562	0.623048	0.533346
B.cells	FAM173A	-0.18564	4.68182	-1.30016	0.196926	-5.51406	0.60446	0.505273
B.cells	SPICE1	-0.40087	1.890422	-1.29934	0.197204	-4.95522	0.632654	0.546335
B.cells	STOML3	-0.65921	0.359863	-1.29909	0.197291	-4.69125	0.648376	0.57016
B.cells	ACOXL	0.564256	1.285897	1.298962	0.197334	-4.88254	0.638811	0.555682
B.cells	MGA	0.151472	5.917554	1.298924	0.197347	-5.83958	0.59336	0.488495
B.cells	CEBPB	0.254758	9.155958	1.29816	0.197609	-6.39556	0.564432	0.446623
B.cells	MSRB3	-0.4506	2.604277	-1.29769	0.197768	-4.99096	0.626383	0.536187
B.cells	PTRHD1	-0.19254	4.56189	-1.29748	0.197841	-5.58697	0.607142	0.507778
B.cells	ACTN2	-0.69434	-0.1007	-1.29747	0.197846	-4.70165	0.654144	0.578061
B.cells	ORC4	-0.13745	5.046376	-1.29729	0.197906	-5.67811	0.602511	0.50101
B.cells	ZNRF2	0.133235	5.763077	1.297106	0.197969	-5.80324	0.595726	0.491146
B.cells	TFG	-0.12773	5.864765	-1.29696	0.19802	-5.81987	0.594768	0.48977
B.cells	ALDH8A1	-0.48933	2.197617	-1.29672	0.198102	-5.04488	0.630612	0.542473
B.cells	BIRC2	0.148509	5.673482	1.296385	0.198216	-5.81016	0.596846	0.492562
B.cells	B2M	0.215106	9.94338	1.296063	0.198327	-6.47457	0.55813	0.437399
B.cells	SF3B4	-0.15874	5.494923	-1.29569	0.198454	-5.74121	0.598639	0.495187
B.cells	EXTL2	-0.41887	2.165568	-1.29549	0.198525	-4.98312	0.631239	0.543357
B.cells	NDOR1	0.257547	3.086341	1.29517	0.198633	-5.29626	0.62202	0.529673
B.cells	PSMD9	-0.14043	5.225742	-1.29493	0.198715	-5.70544	0.601201	0.499151
B.cells	STOX2	-0.56474	3.196694	-1.29491	0.198721	-5.08148	0.620926	0.528152

B.cells	NDEL1	0.127454	6.927832	1.294863	0.198739	-6.01589	0.585221	0.476049
B.cells	ZFP607A	0.331302	1.840055	1.294809	0.198757	-5.02298	0.634536	0.54848
B.cells	OXCT1	0.138192	5.719852	1.294628	0.198819	-5.78673	0.596509	0.492414
B.cells	PLP2	-0.1879	5.623922	-1.29445	0.198881	-5.85824	0.597417	0.49381
B.cells	ORC6	-0.19849	5.216442	-1.29438	0.198906	-5.65775	0.601289	0.499477
B.cells	KCTD6	0.277437	2.981269	1.29436	0.198912	-5.21688	0.623064	0.531537
B.cells	KCTD20	-0.15397	4.885027	-1.29429	0.198937	-5.64692	0.604461	0.504124
B.cells	COPG2	0.155226	5.004811	1.293858	0.199084	-5.72099	0.603329	0.502535
B.cells	MCAM	-0.70603	1.173297	-1.29375	0.199121	-4.73941	0.641369	0.559059
B.cells	UBXN2B	0.268357	2.842475	1.293479	0.199215	-5.22395	0.624464	0.533779
B.cells	GM41442	0.676309	-0.19949	1.293388	0.199246	-4.72711	0.655667	0.580861
B.cells	METRN	-0.42042	2.538002	-1.29339	0.199247	-4.96147	0.627508	0.538321
B.cells	AKAP9	0.133317	6.105265	1.293315	0.199271	-5.90485	0.592896	0.48744
B.cells	ITM2C	-0.20242	5.637577	-1.29319	0.199313	-5.64896	0.597304	0.493881
B.cells	HPS3	0.168501	4.626547	1.292812	0.199444	-5.70601	0.607155	0.508186
B.cells	QSOX2	-0.61462	0.948725	-1.29234	0.199606	-4.71167	0.643885	0.563057
B.cells	4930435F1	-0.69025	0.358699	-1.29187	0.19977	-4.74891	0.650014	0.572487
B.cells	PRKCQ	0.404556	3.12892	1.291837	0.19978	-5.13616	0.62181	0.530059
B.cells	WNT4	-0.3947	1.526488	-1.2917	0.199829	-5.00249	0.637948	0.554214
B.cells	PPP1CC	-0.10677	7.584523	-1.29142	0.199923	-6.09979	0.579388	0.468267
B.cells	NSD1	0.102339	7.273059	1.291345	0.19995	-6.08025	0.58224	0.472344
B.cells	ELP1	0.151667	4.553493	1.291316	0.19996	-5.54914	0.607861	0.509495
B.cells	PLAAT3	0.281131	5.19136	1.291213	0.199996	-5.60679	0.601734	0.500527
B.cells	SUOX	-0.43243	1.361958	-1.29117	0.200009	-4.85649	0.639632	0.55682
B.cells	DLGAP4	-0.17583	5.015503	-1.29098	0.200077	-5.7207	0.603416	0.503152
B.cells	SPINT2	-0.19613	4.563442	-1.29073	0.200164	-5.51917	0.607765	0.509631
B.cells	POLRMT	0.351815	1.91437	1.290427	0.200267	-5.05886	0.633998	0.548685
B.cells	SNX32	0.249956	2.812618	1.290413	0.200272	-5.25215	0.624957	0.535148
B.cells	PLA2G4C	0.682494	0.890136	1.290347	0.200295	-4.73344	0.644491	0.564554
B.cells	SEMA4A	0.39419	3.364224	1.290216	0.20034	-5.05548	0.61948	0.527049
B.cells	CLIP2	0.252785	2.886513	1.290214	0.200341	-5.20329	0.62422	0.534101
B.cells	YWHAB	-0.09741	7.88619	-1.29019	0.20035	-6.15779	0.576642	0.464711
B.cells	SERP1	0.108326	7.981742	1.2901	0.20038	-6.20873	0.575775	0.463477
B.cells	GM15345	-0.43802	2.98417	-1.28971	0.200516	-5.20145	0.623264	0.532659
B.cells	CEACAM16	0.529835	1.137316	1.289705	0.200516	-4.83895	0.641956	0.560725
B.cells	2310058D1	0.205934	2.785149	1.289605	0.200551	-5.32232	0.625248	0.535616
B.cells	ZFP513	0.321457	2.534011	1.289334	0.200645	-5.09246	0.627761	0.539431
B.cells	DCAF10	0.144153	5.193751	1.289321	0.200649	-5.71224	0.601727	0.500944
B.cells	DDX3Y	2.735464	2.707432	1.289145	0.20071	-5.24865	0.626049	0.536941
B.cells	MINDY1	0.198814	3.987424	1.288528	0.200924	-5.44965	0.613844	0.518348
B.cells	PPFIA1	0.151437	5.566923	1.288356	0.200984	-5.79215	0.598634	0.496065
B.cells	RHBDD1	0.187757	4.175534	1.288266	0.201015	-5.48378	0.612009	0.515691
B.cells	SIRPB1B	0.728205	1.445504	1.287695	0.201212	-4.8281	0.639605	0.556575
B.cells	ABCB8	0.269734	2.240197	1.287667	0.201222	-5.04974	0.631519	0.544413
B.cells	EAF1	0.211909	4.101569	1.287402	0.201314	-5.46848	0.613148	0.517051
B.cells	CYB561D2	0.236564	3.329402	1.287056	0.201434	-5.29081	0.620737	0.528381
B.cells	EI24	0.155045	4.630195	1.28705	0.201436	-5.59413	0.608015	0.509594

B.cells	NOXRED1	0.544748	0.546984	1.286955	0.201469	-4.8102	0.649005	0.570876
B.cells	CISD2	0.120771	6.732173	1.286645	0.201577	-5.99571	0.588221	0.480721
B.cells	GM4924	-0.69385	0.278075	-1.2865	0.201626	-4.67734	0.651951	0.575279
B.cells	ATXN10	0.129911	6.66392	1.286177	0.201739	-5.95172	0.588855	0.481813
B.cells	SNX10	0.206507	5.029489	1.286122	0.201758	-5.712	0.604297	0.504244
B.cells	LRIF1	0.151643	4.523869	1.286071	0.201776	-5.57418	0.60917	0.511392
B.cells	ADAP1	0.215303	4.152492	1.285653	0.201921	-5.49991	0.612958	0.516795
B.cells	HLTF	0.181962	4.64409	1.285417	0.202003	-5.63853	0.608185	0.509888
B.cells	MLLT3	0.180257	5.728512	1.285157	0.202094	-5.91904	0.59781	0.49483
B.cells	CHD9	0.165167	6.065045	1.284953	0.202165	-5.85622	0.594632	0.49033
B.cells	FAM169B	0.282663	3.62896	1.28467	0.202263	-5.40308	0.61809	0.524867
B.cells	HECTD3	0.268335	2.789878	1.284462	0.202336	-5.16926	0.626419	0.53729
B.cells	L3MBTL1	-0.63082	0.787342	-1.28444	0.202344	-4.75624	0.64683	0.568048
B.cells	TAF1B	0.173337	4.199522	1.284256	0.202407	-5.52523	0.6125	0.516632
B.cells	EPS8L1	-0.92964	0.408966	-1.28407	0.202472	-4.71886	0.650772	0.574158
B.cells	TNNI2	-0.30963	2.310806	-1.2837	0.202601	-5.1616	0.631234	0.544719
B.cells	FOXO4	-0.30148	2.699869	-1.2837	0.202602	-5.18626	0.627321	0.538856
B.cells	TECPR2	0.222477	3.905519	1.283673	0.202611	-5.43678	0.615373	0.521079
B.cells	TRIM37	-0.19802	4.93302	-1.28362	0.202628	-5.56757	0.6054	0.50639
B.cells	RHOH	-0.18939	6.471792	-1.28357	0.202648	-5.9425	0.590819	0.485154
B.cells	HIST1H2AG	-0.58714	2.011327	-1.28323	0.202763	-4.93719	0.634265	0.549434
B.cells	ZFP148	0.106341	6.830997	1.283115	0.202805	-6.00036	0.587475	0.480502
B.cells	DSG2	-0.63569	0.740688	-1.28287	0.202891	-4.76448	0.647314	0.569238
B.cells	DPF2	0.145881	5.127706	1.282659	0.202964	-5.69706	0.603532	0.503937
B.cells	SNX14	-0.19064	4.500746	-1.28255	0.203001	-5.58205	0.609572	0.512829
B.cells	CHEK2	0.249524	3.145946	1.282382	0.203061	-5.24833	0.622869	0.53258
B.cells	FBXW9	-0.31623	2.418265	-1.28228	0.203095	-5.04487	0.63015	0.543522
B.cells	TIFAB	0.453614	2.73524	1.282194	0.203127	-5.06335	0.626966	0.538758
B.cells	STARD8	-0.29498	3.359704	-1.28218	0.20313	-5.23959	0.620749	0.529479
B.cells	MRPL21	-0.15764	5.233418	-1.2821	0.20316	-5.72665	0.60252	0.502611
B.cells	AU040320	-0.19209	4.589216	-1.28194	0.203215	-5.54749	0.608716	0.511737
B.cells	ZDHHC6	0.172897	4.609891	1.281933	0.203217	-5.58839	0.608516	0.511443
B.cells	A630089N(	0.359126	2.672384	1.281725	0.20329	-5.0776	0.627596	0.539795
B.cells	SCYL3	0.190949	3.642548	1.281565	0.203346	-5.41509	0.617956	0.52542
B.cells	GSTP2	0.534373	0.861498	1.281492	0.203371	-4.79722	0.646061	0.567664
B.cells	RRM2	-0.33172	6.880861	-1.28106	0.203522	-5.95014	0.587294	0.480208
B.cells	ZFP428	-0.35403	2.058083	-1.28064	0.20367	-5.06095	0.634298	0.54943
B.cells	ERC2	0.894364	0.862583	1.280571	0.203693	-4.77507	0.646567	0.567992
B.cells	DNASE1L3	-0.24303	6.30437	-1.2803	0.203788	-6.01235	0.592981	0.488242
B.cells	3110056K(	-0.14888	4.723448	-1.27982	0.203958	-5.64867	0.608181	0.510224
B.cells	9530034E1	-0.54957	0.330435	-1.27974	0.203983	-4.77941	0.652412	0.576523
B.cells	4930523C(	0.120627	6.126408	1.279701	0.203998	-5.99012	0.594801	0.490688
B.cells	FNDC3B	-0.16083	6.549271	-1.27905	0.204226	-5.88094	0.591344	0.485153
B.cells	EDARADD	0.253548	3.159788	1.278696	0.204351	-5.35525	0.62422	0.533217
B.cells	CPSF3	0.150593	4.770083	1.278461	0.204433	-5.65316	0.60842	0.509927
B.cells	DEK	-0.13344	7.842	-1.27838	0.20446	-6.15396	0.579566	0.468167
B.cells	HECA	0.1293	6.23169	1.278025	0.204586	-5.93889	0.594484	0.48975

B.cells	SEPHS1	0.161223	4.119783	1.277973	0.204604	-5.47081	0.614744	0.519408
B.cells	GSTP3	-0.34798	3.39573	-1.27797	0.204606	-5.35208	0.621876	0.529981
B.cells	SLC25A47	-0.26059	3.963	-1.2778	0.204666	-5.44487	0.61628	0.521699
B.cells	CTSD	-0.18682	7.133555	-1.27768	0.204707	-5.99613	0.586074	0.477664
B.cells	RNF26	-0.29314	3.515898	-1.2773	0.20484	-5.23549	0.620927	0.528312
B.cells	TANGO2	0.289142	3.93274	1.277132	0.204899	-5.42895	0.616835	0.522256
B.cells	MAPKBP1	-0.32153	3.047991	-1.2769	0.204981	-5.24235	0.625685	0.53529
B.cells	TRMT6	0.176841	4.373913	1.276711	0.205047	-5.54376	0.612644	0.515928
B.cells	MYOF	0.294657	2.65687	1.27652	0.205114	-5.5156	0.629687	0.541182
B.cells	TRAPPC4	0.144424	5.137813	1.276184	0.205233	-5.70976	0.605381	0.505191
B.cells	2610001J0	-0.22206	3.690163	-1.27605	0.205281	-5.38553	0.619479	0.526038
B.cells	IFT122	-0.42578	1.594011	-1.27598	0.205303	-4.85238	0.640577	0.557619
B.cells	ZFP74	0.346132	2.132709	1.275543	0.205458	-5.02828	0.635152	0.549508
B.cells	NFS1	0.167532	4.533914	1.275529	0.205463	-5.61046	0.611289	0.513993
B.cells	ZBTB5	0.328507	2.463409	1.275462	0.205487	-5.10223	0.631802	0.544477
B.cells	GYPC	0.323627	3.080733	1.275089	0.205618	-5.29286	0.625691	0.535218
B.cells	CGAS	-0.45732	2.747045	-1.27508	0.205622	-5.02606	0.629033	0.540209
B.cells	NLRP1B	-0.57432	1.79452	-1.27484	0.205704	-4.84948	0.638779	0.55471
B.cells	HNF4A	-0.65709	0.691681	-1.27458	0.205797	-4.76337	0.650299	0.571972
B.cells	SRPK2	0.139354	7.096447	1.274249	0.205914	-6.03748	0.587436	0.478746
B.cells	TAOK3	0.093739	7.012234	1.273852	0.206054	-6.07949	0.588465	0.480029
B.cells	MZT2	-0.31917	2.206691	-1.27323	0.206273	-4.99891	0.635599	0.54907
B.cells	YIPF5	-0.13876	5.24625	-1.27308	0.206328	-5.70635	0.605554	0.504582
B.cells	MMD	0.23045	4.477186	1.272693	0.206464	-5.5429	0.612996	0.51573
B.cells	JADE2	0.287513	3.270443	1.272595	0.206498	-5.34842	0.62489	0.533345
B.cells	NCBP3	0.138221	5.506298	1.27254	0.206518	-5.77904	0.603062	0.501164
B.cells	ATM	0.189523	4.221236	1.272491	0.206535	-5.52696	0.615497	0.519417
B.cells	PTK7	-0.88041	1.073787	-1.2724	0.206567	-4.6998	0.647238	0.566937
B.cells	MLLT6	0.308731	3.270712	1.271943	0.206729	-5.39766	0.624895	0.533415
B.cells	DDX46	-0.12942	6.040748	-1.27174	0.206801	-5.90166	0.597985	0.493889
B.cells	TMEM69	0.486592	1.585462	1.271668	0.206826	-4.87431	0.641958	0.559088
B.cells	TGFB2	-0.92247	0.46572	-1.2716	0.20685	-4.74979	0.653593	0.576754
B.cells	SFMBT2	-0.6607	0.60235	-1.27135	0.206939	-4.73323	0.652161	0.574593
B.cells	RWDD1	0.117699	6.105394	1.271341	0.206942	-5.91004	0.597374	0.493031
B.cells	STK10	-0.11902	7.315855	-1.27118	0.206997	-6.13807	0.586058	0.476717
B.cells	S1PR5	-0.63214	-0.65106	-1.27115	0.20701	-4.74084	0.665337	0.594969
B.cells	GM1043	-0.45042	1.915295	-1.27104	0.207049	-5.1363	0.638577	0.554093
B.cells	ASXL2	0.124957	6.836454	1.270629	0.207194	-6.03682	0.590771	0.483343
B.cells	GM20234	0.358645	2.059362	1.270468	0.207251	-5.02316	0.637395	0.552146
B.cells	TTC9C	0.141904	4.558959	1.269948	0.207435	-5.62694	0.612484	0.51529
B.cells	RAN	-0.17118	8.400943	-1.26994	0.207438	-6.25449	0.576392	0.463024
B.cells	HOMER3	-0.24834	3.103095	-1.26984	0.207475	-5.21954	0.626852	0.536674
B.cells	PELP1	0.239094	3.358177	1.269694	0.207525	-5.31626	0.624307	0.532909
B.cells	SGPP1	0.191342	5.017924	1.269689	0.207527	-5.63789	0.608035	0.50885
B.cells	TMEM143	-0.48038	1.491704	-1.26957	0.207569	-4.81179	0.643215	0.561319
B.cells	EFHC1	-0.5674	-0.05796	-1.26919	0.207704	-4.77421	0.659675	0.586317
B.cells	INO80DOS	-0.26593	4.442013	-1.26862	0.207907	-5.56163	0.614196	0.517608

B.cells	GM2788	0.67514	0.569462	1.268576	0.207922	-4.71545	0.653409	0.576476
B.cells	NPEPPS	0.127625	7.184287	1.267768	0.208209	-6.11108	0.58863	0.479761
B.cells	MECP2	0.123408	5.847525	1.267428	0.20833	-5.87442	0.601201	0.498093
B.cells	2810006K2	0.382445	2.570884	1.267325	0.208367	-5.11185	0.633374	0.545681
B.cells	CNOT10	-0.14143	5.238486	-1.26727	0.208387	-5.74614	0.607033	0.506636
B.cells	KNG2	-0.42812	2.382731	-1.26717	0.208424	-5.1139	0.635281	0.54856
B.cells	MANBA	0.163889	4.766674	1.267137	0.208434	-5.69804	0.611597	0.513357
B.cells	TRDV4	0.712978	-1.36214	1.266793	0.208556	-4.66446	0.674558	0.608889
B.cells	UGCG	0.156676	6.436724	1.266731	0.208578	-5.99652	0.595729	0.490233
B.cells	MFAP3	0.141044	5.124697	1.266429	0.208686	-5.75712	0.608398	0.508588
B.cells	STARD4	-0.25726	3.526928	-1.26587	0.208886	-5.29537	0.624098	0.531769
B.cells	MCM3	-0.247	5.484871	-1.26582	0.208904	-5.73974	0.604971	0.503563
B.cells	STEAP4	-0.47148	2.205553	-1.26511	0.209156	-5.09035	0.637406	0.551965
B.cells	AQP11	-0.64767	0.397356	-1.26499	0.209199	-4.74852	0.656152	0.580459
B.cells	COX7A2	-0.11317	8.039242	-1.26481	0.209264	-6.21652	0.581048	0.469383
B.cells	PRIM1	-0.28879	4.178781	-1.26435	0.209428	-5.47101	0.617652	0.522809
B.cells	ANKDD1A	-0.60539	2.346094	-1.26434	0.209432	-5.1098	0.635975	0.55016
B.cells	CLDN15	0.506781	0.423974	1.264318	0.209439	-4.80778	0.655871	0.580345
B.cells	NUDT19	0.206515	4.544356	1.264229	0.209471	-5.52519	0.614071	0.517515
B.cells	CARS	-0.23539	4.118134	-1.26422	0.209475	-5.50951	0.618248	0.523692
B.cells	EGFR	-0.61694	2.469637	-1.26416	0.209494	-5.05007	0.63472	0.548272
B.cells	CHD2	0.128253	7.754934	1.264137	0.209503	-6.17178	0.583654	0.473267
B.cells	SRCAP	-0.10486	6.652894	-1.26406	0.209533	-6.00564	0.59389	0.488011
B.cells	MED23	0.196934	3.692708	1.264055	0.209533	-5.40867	0.622451	0.529929
B.cells	GM17745	-0.46976	0.962497	-1.26389	0.209593	-4.91327	0.650226	0.571837
B.cells	TMEM258	-0.1321	6.825258	-1.26376	0.209638	-6.00301	0.592275	0.485789
B.cells	SMN1	0.172767	4.851728	1.263659	0.209674	-5.66985	0.61108	0.513253
B.cells	BCAR3	0.277729	4.496691	1.263319	0.209796	-5.64893	0.614537	0.518455
B.cells	CNTRL	0.134396	5.907012	1.263309	0.2098	-5.86728	0.600938	0.498494
B.cells	ARFRP1	0.170489	4.120918	1.263189	0.209842	-5.49836	0.618221	0.523923
B.cells	DUBR	0.370692	2.378269	1.263148	0.209857	-5.04376	0.635648	0.54996
B.cells	RARRES1	-0.71913	0.889058	-1.26299	0.209912	-4.80499	0.650992	0.573297
B.cells	FAM83D	-0.30699	2.774945	-1.26295	0.209928	-5.19085	0.631631	0.544016
B.cells	TK2	0.240787	3.825305	1.262165	0.210209	-5.41963	0.621262	0.528647
B.cells	MRPS5	-0.17124	4.612666	-1.26216	0.21021	-5.59803	0.613528	0.517188
B.cells	ADGRE4	-0.48742	3.108019	-1.26215	0.210214	-5.1368	0.628407	0.539305
B.cells	PMVK	0.198869	4.327226	1.262085	0.210237	-5.52943	0.616318	0.521313
B.cells	ZFAND6	0.098116	6.978135	1.2619	0.210304	-6.08111	0.590965	0.484282
B.cells	ALCAM	-0.20422	6.646567	-1.26187	0.210313	-6.12083	0.594068	0.488773
B.cells	NSG2	-0.49149	0.587521	-1.26179	0.210344	-4.86644	0.654282	0.578551
B.cells	TMEM273	-0.54784	1.19098	-1.26153	0.210436	-4.80795	0.648097	0.569014
B.cells	NRXN3	-0.62045	1.145579	-1.2613	0.210519	-4.8392	0.64866	0.569801
B.cells	ZFP322A	0.274379	3.041434	1.261075	0.210599	-5.2752	0.62936	0.540702
B.cells	NEURL1B	-0.589	0.43588	-1.26084	0.210683	-4.73388	0.656269	0.581392
B.cells	ETV5	-0.34627	3.199847	-1.26035	0.210861	-5.21268	0.627951	0.538611
B.cells	CELSR2	-0.74843	-0.09555	-1.26034	0.210862	-4.70798	0.661993	0.590268
B.cells	TMEM14A	-0.6618	0.419553	-1.26031	0.210873	-4.74154	0.656534	0.581901

B.cells	RERG	0.763028	0.522461	1.260146	0.210933	-4.71743	0.655468	0.580274
B.cells	GM13212	0.241856	3.205264	1.259891	0.211024	-5.39588	0.628028	0.538661
B.cells	GM26930	0.74886	0.305585	1.258869	0.211391	-4.72296	0.658777	0.584248
B.cells	NME2	0.14417	8.557653	1.258683	0.211458	-6.33021	0.577783	0.46445
B.cells	POLD4	0.136467	5.76477	1.25833	0.211585	-5.88004	0.603812	0.502156
B.cells	BST1	0.267522	3.315218	1.258315	0.211591	-5.56333	0.627787	0.53759
B.cells	DDX60	0.538194	2.866859	1.25821	0.211629	-5.08012	0.632295	0.544371
B.cells	GM1673	-0.37384	2.42109	-1.25767	0.211822	-5.05501	0.636814	0.551485
B.cells	GM15614	-0.31044	2.981808	-1.25766	0.211825	-5.31042	0.631135	0.54295
B.cells	SAP30	-0.17298	5.143228	-1.25752	0.211875	-5.73474	0.609792	0.51129
B.cells	MED31	-0.24615	3.491551	-1.25735	0.211938	-5.27319	0.626024	0.535408
B.cells	ASAH1	0.13672	6.301867	1.257215	0.211987	-5.97714	0.5987	0.49517
B.cells	LEPROT	-0.16057	5.065196	-1.25716	0.212006	-5.68615	0.610547	0.512516
B.cells	EML2	0.310871	2.655573	1.257061	0.212042	-5.22587	0.634432	0.548064
B.cells	VPS13A	0.113989	6.447058	1.257049	0.212046	-5.98522	0.597327	0.493172
B.cells	GOLPH3	-0.10527	5.902166	-1.257	0.212063	-5.90033	0.602499	0.500711
B.cells	PIK3R3	-0.38271	3.43068	-1.25633	0.212304	-5.28186	0.626671	0.536665
B.cells	IFT20	-0.14037	5.442542	-1.25626	0.21233	-5.8043	0.606941	0.507445
B.cells	ATAD3A	0.212331	4.10261	1.256255	0.212333	-5.55122	0.619999	0.526731
B.cells	ANTXR2	-0.28185	7.654982	-1.25624	0.21234	-6.20478	0.586084	0.477134
B.cells	SCAMP3	0.148322	4.819715	1.256157	0.212368	-5.6588	0.61297	0.516346
B.cells	STAM2	0.16894	5.475543	1.255929	0.212451	-5.85208	0.606624	0.50709
B.cells	PABPC4	-0.13641	5.897724	-1.25555	0.212587	-5.90064	0.602579	0.501249
B.cells	SLC35D1	-0.18534	4.114625	-1.25551	0.212602	-5.54597	0.61988	0.52677
B.cells	CAMKMT	0.194808	4.501622	1.255361	0.212655	-5.64049	0.616076	0.521171
B.cells	RPP21	-0.15777	4.873001	-1.25528	0.212686	-5.69342	0.612451	0.515811
B.cells	TBC1D8B	0.224897	3.257326	1.255067	0.212762	-5.36636	0.628406	0.539673
B.cells	PRIM2	0.211875	5.536205	1.254822	0.21285	-5.7817	0.606041	0.506578
B.cells	FRAT2	-0.18553	4.805593	-1.25464	0.212917	-5.71442	0.613108	0.517023
B.cells	OCRL	0.396596	2.602911	1.254596	0.212932	-5.07181	0.635006	0.549708
B.cells	LRAT	-0.70321	-0.03713	-1.25454	0.212953	-4.71873	0.662457	0.591565
B.cells	DNAJC13	0.143132	6.16837	1.254458	0.212982	-5.89465	0.600003	0.497789
B.cells	RPF1	-0.12987	5.149471	-1.25445	0.212986	-5.76577	0.609769	0.512112
B.cells	XRCC3	-0.62944	0.159666	-1.25421	0.213071	-4.70203	0.660459	0.588441
B.cells	HIBADH	0.161179	5.742005	1.253894	0.213186	-5.84107	0.604169	0.503889
B.cells	WRN	0.141058	5.831312	1.253737	0.213242	-5.86161	0.603315	0.502705
B.cells	LILRB4A	0.493926	4.163584	1.25351	0.213325	-5.09901	0.619502	0.526655
B.cells	PAN3	-0.09117	8.767803	-1.25342	0.213359	-6.35697	0.576012	0.463303
B.cells	0610012G(	0.143969	5.039442	1.253362	0.213378	-5.6923	0.610938	0.513986
B.cells	GLIS1	-0.75776	0.394465	-1.25314	0.213458	-4.70877	0.657988	0.584983
B.cells	NVL	0.153798	4.723925	1.252813	0.213577	-5.64063	0.614007	0.518665
B.cells	KIF9	0.371361	1.680578	1.252807	0.21358	-5.06571	0.644553	0.564494
B.cells	LTBR	-0.3341	2.974023	-1.25272	0.21361	-5.09556	0.631359	0.54455
B.cells	EXOC4	0.092778	8.03297	1.252635	0.213642	-6.23071	0.582704	0.473021
B.cells	TPBGL	-0.79517	0.559425	-1.25247	0.213701	-4.69263	0.656247	0.582423
B.cells	IGBP1	0.159914	4.749526	1.252311	0.213759	-5.68996	0.613757	0.518385
B.cells	ZFP820	-0.73496	-0.04486	-1.25228	0.213772	-4.67402	0.662651	0.59231

B.cells	GM7030	0.439735	2.084686	1.251617	0.214011	-5.14329	0.640837	0.558495
B.cells	ELMSAN1	0.161601	6.924696	1.251441	0.214075	-6.10543	0.593381	0.488127
B.cells	TERF2	0.147503	4.800606	1.251221	0.214155	-5.66965	0.613681	0.517965
B.cells	CEP57	0.157455	5.139583	1.251127	0.214188	-5.70728	0.610387	0.5131
B.cells	ADAMTS1	-0.52381	2.937185	-1.25109	0.214201	-5.10978	0.632165	0.545536
B.cells	PAFAH1B2	0.126099	5.489377	1.250817	0.214301	-5.79591	0.60701	0.508223
B.cells	ZFP182	0.209235	4.211679	1.250436	0.21444	-5.56535	0.619453	0.526725
B.cells	CCDC163	-0.40625	2.446867	-1.25036	0.214467	-5.09617	0.637136	0.553243
B.cells	OXLD1	-0.42324	1.813163	-1.25012	0.214554	-4.94738	0.643628	0.563078
B.cells	SUN2	-0.1347	6.122993	-1.25009	0.214567	-5.88979	0.600948	0.499436
B.cells	PRUNE2	0.614194	0.638929	1.249998	0.214599	-4.76535	0.655861	0.581752
B.cells	NAT9	-0.27383	3.244632	-1.24999	0.214601	-5.26552	0.629071	0.5411
B.cells	CDK16	0.215679	3.39772	1.249952	0.214616	-5.32277	0.627537	0.5388
B.cells	SF1	-0.09397	7.215593	-1.24958	0.214752	-6.09791	0.590766	0.484627
B.cells	IL27RA	0.408824	1.535873	1.249554	0.21476	-5.14755	0.646606	0.567614
B.cells	KCND1	-0.7549	-0.19065	-1.24934	0.214838	-4.69275	0.664854	0.595568
B.cells	GM49101	-0.76248	-0.65342	-1.24895	0.214979	-4.68798	0.669801	0.603391
B.cells	XRCC5	0.318419	2.083305	1.248833	0.215023	-5.14765	0.641112	0.559231
B.cells	LY9	0.183312	4.262049	1.248828	0.215024	-5.60121	0.619209	0.526339
B.cells	FBXW7	0.141838	6.448567	1.248175	0.215262	-5.98055	0.598617	0.495526
B.cells	HMGXB3	0.168926	4.408539	1.247775	0.215408	-5.6191	0.618469	0.52458
B.cells	IGKV1-35	-0.65149	-0.53717	-1.24772	0.215429	-4.72164	0.669364	0.601889
B.cells	ZBTB34	0.239245	3.592868	1.24716	0.215632	-5.37418	0.626986	0.536874
B.cells	SPAG7	0.183737	4.55728	1.24701	0.215687	-5.61226	0.617438	0.522743
B.cells	CASR	-0.67897	-0.61039	-1.24652	0.215865	-4.73902	0.670971	0.603668
B.cells	TNS4	0.705396	-0.27421	1.245894	0.216095	-4.72248	0.667976	0.598297
B.cells	DYRK1B	0.341378	1.826196	1.245629	0.216192	-5.02658	0.645961	0.564511
B.cells	TRABD	0.149491	5.363153	1.245471	0.216249	-5.77508	0.610568	0.511613
B.cells	ETFA	-0.14015	6.599323	-1.2453	0.216311	-6.02499	0.598757	0.494356
B.cells	DCAF11	0.1686	4.559305	1.244772	0.216505	-5.64411	0.618557	0.523394
B.cells	NAXD	0.184586	4.128354	1.244759	0.21651	-5.51156	0.622811	0.529711
B.cells	GPX4	0.105428	7.153816	1.244744	0.216515	-6.09835	0.593656	0.486922
B.cells	GM11579	-0.7172	-0.20636	-1.24437	0.216653	-4.74526	0.667663	0.597776
B.cells	PIAS4	0.166982	4.224377	1.244349	0.21666	-5.58268	0.621966	0.528501
B.cells	TFR2	-0.4929	1.213769	-1.24351	0.216967	-4.86805	0.65337	0.575003
B.cells	ANAPC1	0.162699	4.721072	1.243206	0.217079	-5.65581	0.617904	0.521637
B.cells	4933433G1	-0.64637	0.742117	-1.24296	0.217167	-4.82136	0.658448	0.582757
B.cells	PDE7A	0.1414	6.703412	1.242841	0.217212	-6.06015	0.598799	0.493724
B.cells	IARS	-0.16345	5.056202	-1.2428	0.217226	-5.75603	0.614624	0.516911
B.cells	PROS1	-0.36895	2.741429	-1.24257	0.21731	-5.13223	0.637702	0.551393
B.cells	CNTLN	-0.26956	4.34763	-1.24252	0.217328	-5.4307	0.621583	0.527295
B.cells	SPC24	-0.33085	4.768056	-1.24225	0.21743	-5.60345	0.617578	0.521298
B.cells	TXNRD2	0.246981	3.532111	1.242021	0.217513	-5.439	0.629854	0.539613
B.cells	SUMF2	0.290314	2.684259	1.241946	0.217541	-5.20668	0.638434	0.552506
B.cells	GM47200	-0.70883	0.11613	-1.2417	0.21763	-4.71236	0.665366	0.593473
B.cells	SYS1	0.107403	6.417243	1.240904	0.217924	-6.00863	0.602367	0.498252
B.cells	STK4	-0.10653	7.52595	-1.2408	0.217961	-6.18233	0.591916	0.483108

B.cells	PCM1	0.125716	6.494399	1.239907	0.21829	-6.00468	0.602258	0.497573
B.cells	SYBU	-0.72871	0.330557	-1.23985	0.218312	-4.79713	0.664446	0.590655
B.cells	TNFRSF11A	0.474963	2.190262	1.239747	0.218349	-5.0162	0.644928	0.560939
B.cells	SEMA6A	-0.67633	2.821738	-1.23929	0.218517	-5.02406	0.638629	0.551292
B.cells	GM10790	-0.71916	-0.77429	-1.23916	0.218567	-4.6441	0.676386	0.609244
B.cells	GM20274	-0.28999	2.727126	-1.23914	0.218572	-5.21321	0.639595	0.552779
B.cells	S100A9	0.46586	6.814997	1.237739	0.21909	-6.06483	0.600576	0.493798
B.cells	PUS1	0.205327	3.723827	1.237668	0.219116	-5.49279	0.63076	0.538173
B.cells	GRB10	-0.48489	3.090696	-1.23738	0.219223	-5.17887	0.637313	0.547809
B.cells	XPR1	0.134585	6.866995	1.237146	0.219309	-6.0962	0.600313	0.493158
B.cells	AACS	-0.1886	3.861959	-1.23694	0.219385	-5.524	0.629678	0.536297
B.cells	HSPA13	-0.26234	3.082179	-1.23663	0.219498	-5.16291	0.637676	0.548165
B.cells	RASGRP1	0.229478	4.119019	1.236421	0.219577	-5.65952	0.627226	0.532615
B.cells	ZFP446	0.496534	1.164128	1.236262	0.219636	-4.85719	0.657547	0.578251
B.cells	DBF4	-0.19346	5.205532	-1.23588	0.219775	-5.73223	0.61649	0.516924
B.cells	TNFSF9	0.370477	3.435421	1.235845	0.21979	-5.36465	0.634093	0.543039
B.cells	CACNA1A	0.505536	1.424509	1.235843	0.21979	-4.90117	0.654808	0.574274
B.cells	EML5	-0.25661	3.697759	-1.2358	0.219806	-5.50273	0.631448	0.539096
B.cells	SS18L2	-0.24059	3.817204	-1.23558	0.219888	-5.35901	0.63026	0.537398
B.cells	EHBP1L1	-0.1255	6.149657	-1.23542	0.219947	-5.97628	0.607348	0.503654
B.cells	HSPB6	-0.64653	0.40248	-1.23484	0.220162	-4.77158	0.665648	0.591265
B.cells	KRCC1	0.109138	5.9705	1.234718	0.220207	-5.89103	0.609072	0.506439
B.cells	NFIX	-0.24754	3.676603	-1.23448	0.220294	-5.47439	0.631673	0.539936
B.cells	AKAP8L	0.174448	4.8709	1.234341	0.220347	-5.71726	0.619785	0.522349
B.cells	1700020L2	-0.60331	0.390371	-1.23404	0.220457	-4.75565	0.665777	0.591769
B.cells	LDB2	-0.46725	3.160304	-1.23397	0.220483	-5.12133	0.636894	0.547932
B.cells	4931414P1	0.384894	2.099255	1.233825	0.220538	-5.0867	0.647784	0.564364
B.cells	RAB7B	-0.61231	2.804284	-1.23371	0.220581	-4.9503	0.640524	0.553406
B.cells	UAP1	-0.1631	5.112787	-1.23364	0.220608	-5.75506	0.61741	0.518968
B.cells	CLP1	-0.16847	4.608479	-1.23344	0.220681	-5.63317	0.622375	0.526349
B.cells	FTO	0.112784	6.592827	1.233424	0.220687	-6.03262	0.603106	0.498059
B.cells	D130062J1	0.400696	1.642184	1.233215	0.220764	-5.02204	0.652541	0.571749
B.cells	GM13481	-0.70822	0.231781	-1.23311	0.220803	-4.75669	0.667476	0.594605
B.cells	SIAH1B	-0.37461	1.91846	-1.23297	0.220855	-5.02677	0.649661	0.567385
B.cells	A230072E1	0.693419	0.278064	1.232924	0.220872	-4.74667	0.66698	0.593841
B.cells	APOBEC4	-0.59493	0.042895	-1.23291	0.220878	-4.79293	0.669506	0.59773
B.cells	NFYB	-0.1586	5.103926	-1.23288	0.220887	-5.74726	0.617497	0.519257
B.cells	NONO	-0.10736	6.614635	-1.23275	0.220937	-6.00882	0.602898	0.497871
B.cells	PIMREG	0.581345	2.104196	1.232603	0.220991	-4.95673	0.647733	0.564462
B.cells	RBFOX2	-0.46303	2.758111	-1.23254	0.221014	-5.09688	0.640997	0.554289
B.cells	CMC2	-0.2733	4.535117	-1.23243	0.221056	-5.56569	0.623101	0.527545
B.cells	4930481A1	0.408372	1.854963	1.232241	0.221126	-4.98687	0.650367	0.5685
B.cells	CCR9	-0.4655	2.410766	-1.23201	0.22121	-5.01163	0.644628	0.559876
B.cells	RAB2B	0.205495	3.661593	1.231874	0.221263	-5.4819	0.631886	0.540779
B.cells	PREX1	0.149193	6.43388	1.231774	0.221299	-6.00739	0.604683	0.500615
B.cells	VAMP5	0.233217	4.717071	1.231642	0.221349	-5.57681	0.621363	0.525138
B.cells	ANAPC5	0.110695	6.399962	1.231356	0.221455	-5.96893	0.605051	0.501162

B.cells	4-Sep	-0.53023	1.735848	-1.23131	0.221473	-4.90738	0.651675	0.57063
B.cells	ZBTB39	0.374721	1.975209	1.231138	0.221536	-5.09253	0.649208	0.566897
B.cells	UNC50	0.171801	4.105649	1.230787	0.221667	-5.557	0.627721	0.534417
B.cells	EIF4B	-0.11946	6.136378	-1.23056	0.221753	-5.93828	0.607899	0.505165
B.cells	TATDN2	0.138781	5.004884	1.230345	0.221831	-5.76998	0.618968	0.521434
B.cells	DDX20	-0.19384	3.596417	-1.22989	0.222	-5.41102	0.633016	0.542395
B.cells	HELQ	0.281851	2.411841	1.229794	0.222037	-5.16594	0.645099	0.56062
B.cells	LRRC49	-0.50749	1.020554	-1.22978	0.222043	-4.86837	0.659634	0.582726
B.cells	SAT2	-0.86427	-0.24549	-1.22974	0.222057	-4.68966	0.673187	0.603573
B.cells	PPP1R8	-0.18186	4.114233	-1.22947	0.222159	-5.56433	0.627871	0.534747
B.cells	ARID1B	-0.0931	8.198201	-1.22933	0.22221	-6.31751	0.588581	0.47731
B.cells	RFWD3	0.138427	5.6362	1.229253	0.222239	-5.83779	0.612877	0.512595
B.cells	ITPA	-0.16758	4.105721	-1.22869	0.222448	-5.53233	0.628258	0.535141
B.cells	PARVB	0.40652	2.578711	1.228676	0.222454	-5.0853	0.643745	0.558369
B.cells	XRCC2	-0.50402	0.802718	-1.22853	0.222509	-4.84561	0.662322	0.586653
B.cells	TACC3	-0.27502	5.072041	-1.22776	0.222795	-5.70961	0.618785	0.521267
B.cells	OPA3	-0.14658	5.128547	-1.22774	0.222804	-5.78631	0.618231	0.520448
B.cells	UHRF1	-0.28702	4.598357	-1.22771	0.222816	-5.59086	0.623458	0.528187
B.cells	RPN2	-0.13137	6.097946	-1.22769	0.222821	-5.91479	0.608805	0.506589
B.cells	RNF144A	0.232922	3.619246	1.227482	0.2229	-5.41646	0.633249	0.542803
B.cells	CCAR2	-0.23265	3.432111	-1.22738	0.22294	-5.36749	0.635141	0.545685
B.cells	PHB	0.193319	4.493205	1.227296	0.222969	-5.62972	0.624501	0.529826
B.cells	TACC2	-0.33434	3.088667	-1.22712	0.223033	-5.18	0.63863	0.550971
B.cells	CNRIP1	-0.51143	1.648634	-1.22709	0.223047	-4.90682	0.653504	0.573484
B.cells	CAPN3	-0.47733	1.422788	-1.2269	0.223115	-4.92824	0.655873	0.577129
B.cells	ZFP973	-0.57293	0.609416	-1.22683	0.223142	-4.78318	0.664487	0.590321
B.cells	RNF125	0.238021	3.852901	1.226596	0.223231	-5.59633	0.630995	0.539466
B.cells	E2F5	0.241008	2.69996	1.226379	0.223312	-5.29708	0.642784	0.55709
B.cells	NAIP5	0.36978	2.68586	1.226093	0.223419	-5.13484	0.643018	0.557432
B.cells	GM47428	0.623976	0.207827	1.225778	0.223537	-4.81645	0.669066	0.597277
B.cells	MUC13	-0.64965	1.034673	-1.22571	0.223562	-4.82843	0.660242	0.58375
B.cells	GPSM2	-0.3733	2.440784	-1.22547	0.223654	-5.1105	0.645541	0.5615
B.cells	ZBTB42	-0.35843	1.544195	-1.22546	0.223655	-4.98099	0.654871	0.575674
B.cells	MAPK4	-0.70084	0.274036	-1.22516	0.22377	-4.72167	0.668354	0.596505
B.cells	1110038F1	0.169114	4.590808	1.224927	0.223856	-5.68648	0.623792	0.52909
B.cells	HDC	0.651285	3.642871	1.224911	0.223862	-5.27973	0.633274	0.543236
B.cells	CHPT1	0.215691	3.607446	1.224658	0.223957	-5.41416	0.633632	0.543897
B.cells	TMTC2	-0.37251	4.574445	-1.2246	0.223979	-5.62332	0.623955	0.529491
B.cells	ZYG11B	0.133507	5.883084	1.224284	0.224097	-5.9542	0.611133	0.510655
B.cells	ZBED3	-0.22194	3.559356	-1.22423	0.224117	-5.41356	0.634118	0.544779
B.cells	TAF5	0.175725	4.146651	1.223977	0.224213	-5.59472	0.628214	0.536009
B.cells	GM13561	0.322359	1.307424	1.22379	0.224283	-5.1431	0.65736	0.580085
B.cells	TRAPPC8	0.118715	6.225642	1.223498	0.224392	-5.98907	0.607829	0.505964
B.cells	CYP8B1	-0.43015	1.074088	-1.22346	0.224407	-5.02653	0.659825	0.583941
B.cells	CCDC166	-0.61009	0.615517	-1.22338	0.224436	-4.76169	0.664698	0.591427
B.cells	KPNA6	0.173285	3.896998	1.223373	0.224439	-5.5386	0.630716	0.53985
B.cells	MEIS2	-0.49576	2.894046	-1.22327	0.224478	-5.08849	0.640884	0.555131

B.cells	UBE2F	0.12355	6.306137	1.222973	0.22459	-6.02729	0.607055	0.504915
B.cells	GMPR	-0.47214	1.923101	-1.22296	0.224594	-4.97233	0.650909	0.570418
B.cells	UBL5	-0.09444	8.259007	-1.22293	0.224608	-6.29405	0.588643	0.478201
B.cells	TMEM237	0.377879	2.157499	1.222369	0.224817	-5.10434	0.648472	0.56701
B.cells	DNTT	-0.81031	1.449877	-1.22236	0.224821	-4.88266	0.655861	0.578275
B.cells	ATN1	-0.28563	3.221914	-1.2223	0.224843	-5.26938	0.637539	0.550486
B.cells	GINS1	-0.33346	3.197409	-1.2222	0.224879	-5.29947	0.637788	0.550891
B.cells	ZFP329	0.263796	2.511051	1.222034	0.224943	-5.17911	0.644817	0.561607
B.cells	STX18	0.16575	4.637177	1.221906	0.224991	-5.68651	0.623333	0.529377
B.cells	CYP4F16	0.497192	1.441368	1.221881	0.225	-4.9516	0.655951	0.578598
B.cells	MEF2C	0.150484	7.538963	1.221824	0.225022	-6.23907	0.595354	0.488297
B.cells	RBL1	0.210901	4.466968	1.221812	0.225026	-5.59958	0.625022	0.531895
B.cells	MRPS31	-0.19844	3.653241	-1.22161	0.225102	-5.42334	0.63317	0.544112
B.cells	PPIH	-0.15019	5.305306	-1.22142	0.225175	-5.81232	0.616755	0.519735
B.cells	YBX1	-0.11383	8.398771	-1.22118	0.225263	-6.31541	0.587351	0.476938
B.cells	SRFBP1	0.239415	3.231687	1.221107	0.225292	-5.38836	0.63744	0.550744
B.cells	ACTN1	-0.19931	5.757216	-1.22109	0.225298	-5.73505	0.612353	0.513356
B.cells	MAT1A	-0.31637	4.65605	-1.22068	0.225453	-5.7304	0.623403	0.529468
B.cells	EML3	-0.18254	4.139794	-1.22056	0.225499	-5.59928	0.628542	0.537171
B.cells	SLC26A10	-0.62533	0.914242	-1.22018	0.225643	-4.83496	0.662054	0.587732
B.cells	TTLL11	-0.50194	1.608719	-1.21962	0.225853	-4.86317	0.655148	0.576726
B.cells	GPC4	-0.55894	1.1587	-1.2195	0.225898	-4.87198	0.659891	0.584031
B.cells	PAOX	0.343193	2.987177	1.219249	0.225993	-5.14817	0.640979	0.555237
B.cells	RREB1	0.17438	6.814971	1.218819	0.226155	-6.07745	0.60323	0.499161
B.cells	1600002D2	-0.88569	0.257645	-1.21877	0.226172	-4.72337	0.669682	0.599049
B.cells	TFE3	0.178368	4.149746	1.218692	0.226203	-5.58058	0.629265	0.537628
B.cells	ACAP2	0.121445	7.320314	1.218527	0.226265	-6.19688	0.598438	0.492235
B.cells	NAIP2	0.359343	3.006951	1.218478	0.226284	-5.14757	0.640831	0.555056
B.cells	RIOX2	0.175669	3.769866	1.217971	0.226475	-5.45024	0.633366	0.543573
B.cells	USE1	0.151087	5.084893	1.217922	0.226494	-5.74844	0.62026	0.52404
B.cells	CAPNS1	0.101327	7.25812	1.217507	0.226651	-6.15952	0.599494	0.493317
B.cells	BTK	0.133336	6.033608	1.217421	0.226684	-6.01526	0.611206	0.510424
B.cells	LNCPPARA	0.620428	0.60974	1.21729	0.226733	-4.80408	0.666425	0.593639
B.cells	GM26812	-0.82792	0.020142	-1.2169	0.22688	-4.6801	0.672829	0.603534
B.cells	TRIM14	0.219117	4.686216	1.216816	0.226912	-5.68143	0.624464	0.530096
B.cells	NAV2	0.221647	4.841571	1.216805	0.226916	-5.71125	0.622924	0.527809
B.cells	GLTP	0.112876	6.759436	1.216368	0.227082	-6.03857	0.604473	0.500469
B.cells	MSTO1	0.284284	2.681663	1.216332	0.227096	-5.23913	0.644931	0.560627
B.cells	PDCD11	0.168603	4.214015	1.216166	0.227159	-5.619	0.629389	0.5373
B.cells	NCAM1	-0.74723	1.275146	-1.2157	0.227334	-4.90742	0.659818	0.583314
B.cells	PSMD6	-0.15723	5.199485	-1.21566	0.227352	-5.7749	0.61978	0.522982
B.cells	GM11714	0.45593	1.498822	1.215485	0.227417	-4.96007	0.657456	0.579737
B.cells	GPS1	-0.15163	4.905295	-1.21543	0.227439	-5.72271	0.622681	0.527316
B.cells	CENPS	-0.31303	3.529733	-1.21457	0.227764	-5.39431	0.637216	0.548213
B.cells	RHBDF2	0.169532	4.650027	1.213961	0.227995	-5.6406	0.626361	0.531675
B.cells	LTBP4	-0.55453	2.297056	-1.21389	0.228021	-4.98095	0.650303	0.567695
B.cells	WNK1	-0.11577	9.07644	-1.21366	0.22811	-6.44867	0.584166	0.470125

B.cells	RNF40	0.176905	4.021973	1.213103	0.228321	-5.52363	0.63308	0.541309
B.cells	TRIM27	-0.13394	5.675636	-1.21306	0.22834	-5.85947	0.616669	0.516975
B.cells	JUN	-0.32423	7.097589	-1.21247	0.228561	-6.09904	0.60317	0.497075
B.cells	CNP	-0.17105	5.75023	-1.21247	0.228562	-5.92169	0.616157	0.516075
B.cells	LRRC3	-0.53197	0.375073	-1.21241	0.228584	-4.84286	0.671341	0.599327
B.cells	RASGRP3	0.288524	3.586569	1.212237	0.228651	-5.53268	0.637745	0.548216
B.cells	GMCL1	0.142628	4.636774	1.211754	0.228835	-5.70307	0.62738	0.532481
B.cells	ABL2	0.153254	6.21523	1.21174	0.22884	-5.98197	0.611872	0.509588
B.cells	GLUL	-0.16643	6.384395	-1.21161	0.22889	-6.00072	0.610237	0.507262
B.cells	GM20508	-0.58612	0.064606	-1.21146	0.228947	-4.76762	0.674966	0.604808
B.cells	CTNNB1	-0.10018	6.277504	-1.21114	0.229069	-5.98184	0.611325	0.508973
B.cells	GOSR1	0.167074	4.182554	1.211126	0.229075	-5.5932	0.631985	0.53954
B.cells	HPCAL1	-0.15472	6.117607	-1.21088	0.22917	-5.86607	0.612982	0.511352
B.cells	ERCC6	0.20451	3.56729	1.210588	0.22928	-5.55029	0.638361	0.549064
B.cells	GM44148	0.431394	1.90428	1.210239	0.229413	-5.06641	0.655545	0.575128
B.cells	GM27253	-0.52165	1.3478	-1.21015	0.229445	-4.85357	0.661414	0.584094
B.cells	ELP5	-0.15567	4.633093	-1.21013	0.229454	-5.72635	0.627624	0.533079
B.cells	ARC	-0.59832	0.905214	-1.20998	0.229512	-4.85272	0.666126	0.591354
B.cells	HTR7	0.837169	0.754108	1.209889	0.229547	-4.87998	0.667743	0.593852
B.cells	FAM71D	-0.71352	0.095273	-1.20935	0.22975	-4.74534	0.674848	0.605114
B.cells	NCKIPSD	0.216671	2.952621	1.209212	0.229805	-5.3918	0.644651	0.558922
B.cells	PRR33	-0.54537	0.255516	-1.2092	0.229808	-4.85893	0.673112	0.60243
B.cells	HPGDS	-0.271	3.647133	-1.20916	0.229827	-5.50566	0.637549	0.548226
B.cells	RBMX2	-0.26045	3.368821	-1.209	0.229886	-5.32786	0.640384	0.552534
B.cells	BNIP1	0.224782	3.526643	1.20891	0.229921	-5.49273	0.638774	0.550141
B.cells	ALAS1	0.25847	3.683631	1.208865	0.229938	-5.4552	0.637178	0.547743
B.cells	CTDP1	-0.14799	4.660739	-1.20883	0.229949	-5.70421	0.627348	0.533059
B.cells	KAT6B	0.135222	5.844837	1.208462	0.230092	-5.92638	0.615908	0.515826
B.cells	DCAF13	0.143451	4.771338	1.208194	0.230194	-5.74115	0.626545	0.531512
B.cells	TPK1	-0.16922	4.865838	-1.20812	0.230223	-5.75409	0.625605	0.530161
B.cells	ALYREF2	-0.22611	3.243584	-1.2079	0.230306	-5.39331	0.642049	0.554751
B.cells	KDM6A	-0.28243	6.914035	-1.2077	0.230382	-6.07984	0.605771	0.500898
B.cells	NCKAP5L	-0.22715	4.041979	-1.20673	0.230754	-5.54877	0.633993	0.542969
B.cells	RIC8A	0.180296	3.980741	1.206686	0.230772	-5.55965	0.634612	0.543895
B.cells	RFTN2	-0.36038	2.237638	-1.20641	0.230879	-5.17693	0.652512	0.570951
B.cells	ZDHHC2	-0.37288	2.276539	-1.20639	0.230887	-5.16043	0.652106	0.570334
B.cells	ETF1	-0.10428	7.536791	-1.20626	0.230934	-6.20821	0.599851	0.492656
B.cells	FAM241A	0.173128	5.59878	1.20626	0.230935	-5.83174	0.618508	0.519966
B.cells	TRIM33	-0.11467	6.368235	-1.20626	0.230936	-6.03911	0.61102	0.508946
B.cells	EIF1AD	-0.1633	4.923218	-1.20615	0.230977	-5.76596	0.625173	0.529838
B.cells	DNAJC5	0.085512	6.766699	1.205922	0.231065	-6.09316	0.607184	0.503442
B.cells	REEP6	-0.46232	1.791912	-1.20582	0.231104	-5.02521	0.657182	0.578281
B.cells	KCNJ16	-0.84571	-0.02269	-1.20579	0.231115	-4.82632	0.676599	0.608175
B.cells	DYSF	-0.65289	2.592245	-1.20579	0.231116	-5.02874	0.648823	0.565553
B.cells	KPNA1	0.1172	6.778024	1.20551	0.231223	-6.11411	0.607075	0.503382
B.cells	RBM45	-0.24127	3.10947	-1.2054	0.231266	-5.31721	0.643486	0.557551
B.cells	PKD2	-0.53763	0.719693	-1.20532	0.231297	-4.79302	0.668577	0.59586

B.cells	NOL4L	-0.41092	2.680623	-1.20518	0.231349	-5.17949	0.647907	0.564251
B.cells	RNF14	0.124293	5.242687	1.205174	0.231352	-5.83271	0.622011	0.525418
B.cells	TCF25	0.090849	6.939299	1.205134	0.231367	-6.13201	0.605531	0.501177
B.cells	RHBDL3	-0.63642	0.747589	-1.20494	0.231442	-4.81854	0.668336	0.595494
B.cells	NUP62	-0.25835	4.181003	-1.20465	0.231552	-5.51124	0.632797	0.541395
B.cells	LMO7	0.651027	0.761873	1.204393	0.231652	-5.09512	0.668472	0.595532
B.cells	HCST	0.231662	5.375869	1.204174	0.231736	-5.68238	0.621099	0.523867
B.cells	AHRR	-0.62814	-0.36283	-1.20398	0.231811	-4.74589	0.68081	0.614508
B.cells	HHAT	0.455054	1.670909	1.203775	0.23189	-4.97474	0.659009	0.580861
B.cells	CAB39L	0.169336	4.624571	1.203357	0.232051	-5.67917	0.628959	0.535186
B.cells	GM42962	0.528222	-0.60259	1.202871	0.232237	-4.78934	0.684136	0.619092
B.cells	FNBP4	0.112759	6.074991	1.202613	0.232337	-5.99028	0.615124	0.514192
B.cells	GPR68	-0.47948	1.433054	-1.20209	0.232538	-5.07474	0.662568	0.585157
B.cells	ID2	-0.18569	6.76154	-1.20209	0.23254	-6.01243	0.608701	0.504516
B.cells	E2F4	0.138149	5.334947	1.201836	0.232636	-5.81826	0.622602	0.525097
B.cells	STX7	0.106556	6.176182	1.201828	0.232639	-6.01529	0.61436	0.512942
B.cells	MRPL50	-0.17721	4.009842	-1.20148	0.232773	-5.5321	0.636065	0.545024
B.cells	ZBTB14	0.226512	3.004063	1.201256	0.23286	-5.27177	0.646391	0.560622
B.cells	BIRC6	0.072384	7.8393	1.201105	0.232918	-6.29144	0.598686	0.49005
B.cells	VPS37A	0.159883	5.2037	1.201013	0.232954	-5.79758	0.624154	0.527381
B.cells	DCAF5	0.142614	5.52135	1.200605	0.233111	-5.88899	0.621117	0.522857
B.cells	CELF2	0.095995	8.597509	1.200556	0.23313	-6.38925	0.591683	0.47995
B.cells	CFAP77	0.547339	0.931954	1.200491	0.233155	-4.86836	0.668293	0.594083
B.cells	DCAF1	0.170777	5.222364	1.200152	0.233286	-5.7416	0.624243	0.527346
B.cells	GM2396	0.601246	-0.85737	1.200038	0.23333	-4.74165	0.687782	0.62445
B.cells	FAM32A	0.121593	5.312501	1.199568	0.233512	-5.83215	0.623396	0.526187
B.cells	RETREG3	0.129393	5.527566	1.199346	0.233598	-5.91829	0.621274	0.523053
B.cells	LLGL2	0.297999	2.291993	1.199222	0.233646	-5.24526	0.654121	0.572361
B.cells	MGARP	0.731507	-0.49619	1.199154	0.233672	-4.76301	0.684022	0.618449
B.cells	GAREM1	-0.58416	0.757375	-1.19909	0.233699	-4.83511	0.670403	0.597302
B.cells	FAM219A	0.267527	4.171512	1.199066	0.233706	-5.51848	0.6348	0.543199
B.cells	PDE4D	-0.2164	6.428002	-1.199	0.233731	-5.96953	0.61248	0.510125
B.cells	GM26917	-0.19268	5.74366	-1.1986	0.233885	-5.89059	0.619279	0.520063
B.cells	RAD51B	-0.2477	6.152694	-1.19859	0.23389	-5.91906	0.615282	0.514174
B.cells	MRPS23	0.184285	4.411962	1.198345	0.233985	-5.65396	0.632543	0.539706
B.cells	NUDT5	-0.16307	5.148841	-1.19827	0.234012	-5.76755	0.625182	0.528755
B.cells	BBS5	0.540327	0.624034	1.197991	0.234122	-4.91527	0.672081	0.599828
B.cells	CFAP20	-0.13599	5.191326	-1.19793	0.234145	-5.79279	0.62482	0.528274
B.cells	MED30	-0.14876	5.369422	-1.19761	0.234271	-5.79907	0.623173	0.525765
B.cells	ERAP1	0.147281	5.228177	1.19738	0.234359	-5.84895	0.624571	0.527922
B.cells	CERS5	0.129013	5.514163	1.197256	0.234407	-5.92295	0.621744	0.523786
B.cells	PRAM1	-0.23198	3.279629	-1.19719	0.234434	-5.39331	0.644235	0.557421
B.cells	CASP8	0.131124	5.513709	1.197113	0.234463	-5.85694	0.621749	0.52382
B.cells	DEDD	0.150083	4.437275	1.196594	0.234664	-5.7016	0.632778	0.540012
B.cells	RBM15	-0.12613	5.696792	-1.1965	0.234699	-5.90435	0.620253	0.521405
B.cells	MYD88	-0.17993	4.609439	-1.19637	0.234751	-5.707	0.631048	0.537476
B.cells	TMEM167	0.09889	6.804318	1.196248	0.234798	-6.11009	0.609481	0.505664

B.cells	ACAD9	-0.29748	2.542008	-1.19597	0.234907	-5.17507	0.652221	0.569431
B.cells	TBC1D24	0.393552	1.799284	1.195935	0.23492	-5.04835	0.660016	0.581314
B.cells	TTC33	0.205807	3.849347	1.19575	0.234991	-5.52332	0.638803	0.549178
B.cells	GCA	0.578508	1.099128	1.195523	0.235079	-4.9546	0.667601	0.592904
B.cells	NKAIN2	-0.59358	1.350359	-1.19536	0.235142	-4.95041	0.664918	0.588842
B.cells	BRD3	-0.14137	5.712362	-1.19524	0.235188	-5.88604	0.62026	0.521542
B.cells	VPS41	0.13503	5.101022	1.194807	0.235358	-5.82438	0.626432	0.530621
B.cells	IGKV1-135	-0.47801	-0.02384	-1.19471	0.235395	-4.98437	0.679892	0.611894
B.cells	GM12596	-0.2777	2.627238	-1.19458	0.235444	-5.34106	0.651604	0.568439
B.cells	RNF166	0.122958	5.468282	1.194488	0.235482	-5.87847	0.622793	0.525268
B.cells	GRHL1	-0.58171	1.025537	-1.19441	0.235512	-4.85939	0.668529	0.594397
B.cells	GM14636	0.492546	2.274283	1.194067	0.235646	-5.14004	0.655376	0.574227
B.cells	PON3	-0.25977	3.489649	-1.19405	0.235652	-5.46034	0.64278	0.555137
B.cells	ECHS1	-0.17922	5.549982	-1.19362	0.235819	-5.88496	0.622193	0.524348
B.cells	GMFG	0.137803	7.424355	1.193481	0.235874	-6.18801	0.604028	0.497724
B.cells	ZC3H12A	0.16142	5.128189	1.193281	0.235952	-5.86178	0.62637	0.530615
B.cells	MRPL30	0.10447	6.172662	1.193235	0.23597	-6.01732	0.616088	0.515432
B.cells	RBMS3	-0.44718	3.072419	-1.19305	0.23604	-5.22189	0.647202	0.561953
B.cells	KLHL12	0.190576	4.259168	1.192999	0.236061	-5.67293	0.635079	0.54373
B.cells	PSMA7	0.112817	7.683799	1.192939	0.236085	-6.26776	0.601563	0.494306
B.cells	MAP4K1	0.124414	4.801156	1.192639	0.236202	-5.88661	0.629672	0.53577
B.cells	FAM234A	0.246194	4.099085	1.192395	0.236297	-5.5753	0.63674	0.546373
B.cells	BCL9	0.228004	3.399574	1.192088	0.236417	-5.45949	0.643875	0.557269
B.cells	ARMC9	0.28178	2.484131	1.192078	0.236421	-5.17709	0.653355	0.571644
B.cells	GMPR2	0.203017	3.866899	1.191857	0.236507	-5.52193	0.639098	0.550182
B.cells	ACER1	-1.00253	-0.7216	-1.19175	0.236547	-4.68602	0.687702	0.625052
B.cells	CERS4	0.220079	3.651416	1.191529	0.236635	-5.52896	0.641296	0.553561
B.cells	LSR	-0.40877	1.400914	-1.19149	0.236649	-5.17019	0.664781	0.589317
B.cells	CALR	-0.12413	7.35156	-1.19149	0.23665	-6.19996	0.604761	0.49938
B.cells	MAPKAPK2	0.131396	7.207807	1.191271	0.236735	-6.15945	0.606134	0.501406
B.cells	RSRP1	-0.13076	6.653226	-1.19123	0.23675	-6.0627	0.611464	0.509207
B.cells	CLK1	-0.10297	7.55289	-1.19121	0.23676	-6.23701	0.602845	0.496613
B.cells	PYCARD	-0.14957	5.876191	-1.19061	0.236994	-5.97093	0.61936	0.520578
B.cells	SESN2	-0.26594	3.371941	-1.19055	0.237016	-5.34518	0.644507	0.5582
B.cells	CRIP1	-0.15897	8.825299	-1.19046	0.237053	-6.5179	0.591218	0.479634
B.cells	PPP1R12B	0.137252	5.292478	1.190316	0.237108	-5.85792	0.625119	0.529262
B.cells	PTBP2	0.127423	6.156832	1.189941	0.237255	-6.01647	0.616855	0.516782
B.cells	CBX3	-0.12294	8.102288	-1.18977	0.237321	-6.29848	0.598233	0.489584
B.cells	KCTD3	-0.18603	3.747291	-1.18933	0.237494	-5.54924	0.641066	0.552928
B.cells	SFR1	-0.10755	6.44096	-1.18926	0.23752	-6.06244	0.614236	0.512997
B.cells	GM15956	-0.60015	0.27605	-1.18916	0.237559	-4.78951	0.677682	0.609018
B.cells	NUCB1	0.151638	5.383551	1.189092	0.237587	-5.84278	0.624608	0.528374
B.cells	HIC1	-0.54334	2.158311	-1.18866	0.237758	-5.08767	0.657783	0.578162
B.cells	ABCC10	-0.50658	0.65899	-1.18858	0.237787	-4.82248	0.67378	0.602761
B.cells	SMOC1	-0.40669	2.28252	-1.18794	0.238038	-5.27466	0.65702	0.576328
B.cells	GM42567	-0.63814	1.12927	-1.18778	0.2381	-4.89888	0.669285	0.595132
B.cells	FGD2	0.200201	3.69567	1.187397	0.238251	-5.73905	0.642642	0.554254

B.cells	ABHD17B	0.107817	6.542955	1.186866	0.23846	-6.03931	0.614644	0.512145
B.cells	RAB11FIP2	0.226958	3.576284	1.186163	0.238736	-5.50886	0.644659	0.556455
B.cells	TTC41	-0.56512	0.966475	-1.18598	0.238809	-4.84666	0.672134	0.59841
B.cells	COQ10A	0.206881	3.31768	1.185923	0.23883	-5.40083	0.647323	0.56056
B.cells	IFI214	0.422127	1.667368	1.185836	0.238864	-5.15894	0.664624	0.586912
B.cells	UNC5CL	0.559206	0.465408	1.185805	0.238876	-4.96766	0.677562	0.606841
B.cells	PDE6D	0.163316	3.812865	1.185566	0.23897	-5.55477	0.642283	0.553002
B.cells	AK8	-0.35021	2.179586	-1.18547	0.239007	-5.12551	0.659248	0.578731
B.cells	ZFHX3	-0.18182	5.163754	-1.18501	0.23919	-5.76339	0.628636	0.532784
B.cells	DAPK3	0.152842	4.42231	1.18485	0.239252	-5.68056	0.636083	0.543964
B.cells	SARNP	-0.0859	7.797226	-1.18464	0.239336	-6.29208	0.603002	0.495213
B.cells	FEZ2	0.184255	3.693286	1.184374	0.239439	-5.49914	0.643507	0.55519
B.cells	TUBA4A	-0.192	4.513714	-1.18436	0.239443	-5.68608	0.63516	0.542656
B.cells	ZFP46	0.298307	1.77659	1.18432	0.23946	-5.03267	0.663513	0.585594
B.cells	AGXT2	-0.49646	1.612637	-1.18425	0.239487	-5.02986	0.665258	0.588316
B.cells	NR4A2	-0.26353	6.380914	-1.18424	0.239493	-6.06331	0.616631	0.51521
B.cells	LARGE1	-0.24206	5.482959	-1.18421	0.239505	-5.73281	0.625461	0.528263
B.cells	TSPAN7	-0.3429	3.257326	-1.18407	0.239557	-5.21661	0.647996	0.562103
B.cells	ACADM	-0.19006	4.767805	-1.18381	0.239661	-5.76253	0.6327	0.539068
B.cells	BCL6	0.198194	5.992679	1.183698	0.239705	-6.02271	0.620529	0.521023
B.cells	LRRC58	-0.11415	5.945766	-1.18349	0.239787	-5.98385	0.621058	0.521826
B.cells	ALPK1	-0.19876	4.802266	-1.18331	0.239856	-5.85902	0.632461	0.538763
B.cells	HIGD1A	0.12898	6.029655	1.182745	0.240081	-6.00282	0.620584	0.520894
B.cells	CAPN1	0.206779	4.246316	1.182727	0.240088	-5.6313	0.638398	0.547407
B.cells	ISCU	0.115529	7.465509	1.1825	0.240178	-6.28097	0.606689	0.500591
B.cells	CEP44	-0.17747	3.531339	-1.18242	0.240208	-5.4586	0.645732	0.558566
B.cells	AIMP2	-0.26448	3.206699	-1.18224	0.24028	-5.36047	0.649109	0.563689
B.cells	RAP1B	-0.07746	8.543309	-1.18212	0.240327	-6.42049	0.596508	0.485901
B.cells	DGKA	0.169092	5.023185	1.18196	0.240391	-5.90645	0.630634	0.535949
B.cells	SLC38A9	0.154082	5.16902	1.181215	0.240685	-5.87069	0.629563	0.533984
B.cells	B930095G1	-0.72686	0.087994	-1.18118	0.240699	-4.77187	0.682799	0.615027
B.cells	PEX1	0.275343	3.071405	1.181165	0.240705	-5.3724	0.650934	0.566088
B.cells	MCM6	-0.20822	6.155584	-1.18079	0.240852	-5.96363	0.61991	0.519601
B.cells	HEYL	-0.73687	-0.20551	-1.18058	0.240936	-4.7283	0.686154	0.620166
B.cells	ZBTB48	-0.55293	1.123594	-1.18047	0.240978	-4.89044	0.671661	0.597753
B.cells	KLRG1	0.640019	-1.146	1.180444	0.24099	-4.7611	0.696365	0.636626
B.cells	BCAS2	-0.0958	6.380106	-1.18036	0.241025	-6.051	0.617713	0.516452
B.cells	HDLBP	-0.0955	6.655734	-1.18011	0.241122	-6.10348	0.615134	0.512536
B.cells	GKAP1	0.252321	3.541031	1.179901	0.241205	-5.42403	0.646314	0.559005
B.cells	GM8369	0.259364	3.324129	1.179822	0.241236	-5.64234	0.648553	0.562402
B.cells	SIPA1	0.122604	5.681522	1.179475	0.241373	-5.93727	0.62491	0.526729
B.cells	RAB4A	-0.40572	1.578469	-1.17896	0.241576	-5.07343	0.667388	0.590579
B.cells	KIF4	-0.30793	4.394266	-1.17884	0.241625	-5.58879	0.638055	0.546151
B.cells	PCDH15	-0.38716	1.323083	-1.17882	0.241632	-5.0279	0.670125	0.594862
B.cells	WIPF2	0.113081	5.343948	1.178665	0.241694	-5.88919	0.628522	0.531939
B.cells	3110009E1	0.289411	2.175408	1.178434	0.241785	-5.19525	0.661158	0.58106
B.cells	IFITM10	-0.60383	3.144158	-1.17804	0.241941	-5.10847	0.651195	0.565676

B.cells	FAM98B	0.161244	4.474383	1.177976	0.241967	-5.68676	0.637543	0.545117
B.cells	AI839979	0.596109	0.274557	1.177728	0.242066	-4.88688	0.681839	0.612702
B.cells	SLC22A5	0.309857	3.042195	1.177594	0.242119	-5.36756	0.652275	0.567362
B.cells	INAFM2	0.242965	3.225336	1.176986	0.24236	-5.37569	0.650371	0.564724
B.cells	CUL4A	0.129099	5.002429	1.176947	0.242376	-5.8292	0.632235	0.53747
B.cells	BOLL	-0.46529	1.308178	-1.17695	0.242376	-5.04888	0.67062	0.595648
B.cells	HACD2	0.117589	5.732831	1.176747	0.242455	-5.97285	0.624954	0.526687
B.cells	TMEM39B	-0.1836	4.225197	-1.17659	0.242519	-5.66005	0.640094	0.549323
B.cells	PIGF	0.216863	3.388301	1.176569	0.242526	-5.52764	0.648683	0.562268
B.cells	TRP53COR1	-0.57083	1.008827	-1.1765	0.242552	-4.96066	0.673847	0.600752
B.cells	9530062KC	-0.5738	0.793704	-1.17642	0.242585	-4.85261	0.676177	0.604365
B.cells	ANKRD9	0.330153	2.821177	1.176313	0.242627	-5.2477	0.65458	0.571271
B.cells	B3GALT4	0.449803	1.404829	1.17616	0.242688	-5.04825	0.669582	0.594222
B.cells	TACO1	0.285796	3.738306	1.17595	0.242772	-5.51323	0.645075	0.556913
B.cells	CRYZL2	-0.28418	2.609637	-1.17587	0.242802	-5.18213	0.656796	0.574679
B.cells	FFAR2	-0.7189	-0.53401	-1.17586	0.242807	-4.78779	0.690718	0.627122
B.cells	GM47644	0.531404	1.060844	1.175671	0.242883	-4.98561	0.673285	0.600007
B.cells	GRAMD4	0.165234	5.165751	1.175588	0.242916	-5.94156	0.630599	0.53529
B.cells	RELN	-0.3078	3.647841	-1.17538	0.242998	-5.60907	0.646076	0.558478
B.cells	GM14963	0.59851	0.898614	1.17507	0.243122	-4.91756	0.675208	0.602966
B.cells	H2-Q6	0.402459	3.115257	1.174977	0.243159	-5.58157	0.651677	0.566956
B.cells	SETDB2	0.154123	4.580351	1.174879	0.243198	-5.78795	0.636647	0.544341
B.cells	NUTF2-PS1	-0.41721	2.848853	-1.17457	0.243321	-5.25046	0.654638	0.571377
B.cells	CCDC125	0.196245	4.497843	1.174396	0.24339	-5.73477	0.637696	0.545809
B.cells	HAT1	0.140739	6.105162	1.173547	0.243728	-5.98339	0.621656	0.522114
B.cells	PRKAR2A	-0.25508	5.222556	-1.17344	0.243772	-5.81148	0.630411	0.535121
B.cells	FANCI	-0.31367	2.266344	-1.17337	0.2438	-5.12927	0.660809	0.581006
B.cells	CCDC91	-0.32842	2.408137	-1.17331	0.243822	-5.17377	0.659313	0.578719
B.cells	TNFSF10	0.419855	2.307037	1.173128	0.243895	-5.03979	0.66038	0.580349
B.cells	IPO11	0.179697	4.478076	1.173097	0.243907	-5.63964	0.63791	0.546327
B.cells	STPG4	-0.31218	2.37884	-1.17279	0.244029	-5.221	0.659622	0.57927
B.cells	FANCA	0.246349	3.05493	1.172739	0.244051	-5.43622	0.652536	0.568477
B.cells	NPM1	-0.11631	8.143673	-1.17273	0.244055	-6.34975	0.601978	0.493396
B.cells	F9	-0.52108	0.896192	-1.17258	0.244113	-4.93478	0.675474	0.603649
B.cells	SMG7	0.111583	6.385921	1.172564	0.24412	-6.07482	0.618901	0.518145
B.cells	ZBTB11	0.111077	7.088839	1.172316	0.24422	-6.19477	0.612067	0.508147
B.cells	FHL2	-0.74984	0.017257	-1.17212	0.244298	-4.82307	0.685078	0.618615
B.cells	ENKUR	-0.59385	0.339833	-1.17212	0.244299	-4.83287	0.681535	0.6131
B.cells	RYBP	0.138202	5.626574	1.17187	0.244397	-5.9392	0.626385	0.52926
B.cells	IBTK	0.164672	4.642613	1.171495	0.244547	-5.80475	0.636243	0.544156
B.cells	OXR1	-0.12359	6.336267	-1.17129	0.244631	-6.07761	0.619387	0.519212
B.cells	PLA2G12B	-0.58512	1.0508	-1.17122	0.244658	-4.87851	0.673801	0.601477
B.cells	PKIG	0.118364	7.198634	1.171093	0.244708	-6.23205	0.611007	0.506939
B.cells	ROCK2	0.108967	7.468662	1.171023	0.244736	-6.2212	0.608411	0.503143
B.cells	RAD21	-0.1149	7.022909	-1.17074	0.244848	-6.14182	0.612704	0.50948
B.cells	GM14634	-0.44458	1.834434	-1.1701	0.245105	-5.01549	0.665392	0.588799
B.cells	CTNND1	-0.23578	4.294756	-1.17007	0.245117	-5.5583	0.639772	0.549845

B.cells	10-Sep	-0.2567	3.119761	-1.16987	0.245197	-5.31956	0.651862	0.56813
B.cells	MMADHC	-0.15701	4.299266	-1.16983	0.245212	-5.62381	0.639726	0.549784
B.cells	RHOF	-0.22676	3.558194	-1.16981	0.245223	-5.60006	0.64732	0.561241
B.cells	PPID	-0.16377	4.87562	-1.16973	0.245253	-5.74268	0.633893	0.541039
B.cells	TMEM243	0.097196	6.427837	1.169321	0.245417	-6.12114	0.618491	0.518243
B.cells	DIAPH1	0.099868	7.355352	1.16926	0.245441	-6.24603	0.609499	0.505054
B.cells	UBE2J1	0.103772	6.282597	1.169185	0.245472	-6.06641	0.619913	0.520364
B.cells	TSPAN18	-0.53861	1.764014	-1.16914	0.245488	-4.94856	0.666143	0.590107
B.cells	KLRA2	0.613424	2.524963	1.169045	0.245528	-5.08188	0.658083	0.577768
B.cells	FAM126B	0.1941	3.772291	1.168837	0.245611	-5.50399	0.645115	0.55814
B.cells	RASSF1	0.113736	5.940086	1.168823	0.245617	-6.03861	0.623282	0.525454
B.cells	UQCR11	-0.12676	7.224799	-1.16881	0.245621	-6.18135	0.610755	0.506992
B.cells	TMEM80	0.20776	3.276451	1.168716	0.24566	-5.36929	0.650234	0.565932
B.cells	CHST14	0.595438	0.872451	1.168599	0.245706	-4.85137	0.675732	0.605053
B.cells	TOM1	0.165292	5.431971	1.168592	0.245709	-5.88699	0.628321	0.532976
B.cells	PUS3	0.253142	2.37825	1.168586	0.245712	-5.26487	0.659628	0.580251
B.cells	POLR2I	-0.12889	5.116859	-1.16848	0.245754	-5.83453	0.631469	0.537672
B.cells	FBXO9	0.159431	4.752411	1.168464	0.24576	-5.79307	0.635134	0.543155
B.cells	GM42982	0.332211	1.514483	1.168416	0.24578	-5.11268	0.668811	0.594355
B.cells	AGPAT2	-0.23351	4.355109	-1.16838	0.245794	-5.65235	0.639158	0.549196
B.cells	CLIP1	0.148966	5.440971	1.168228	0.245855	-5.86566	0.628231	0.532874
B.cells	MINK1	0.180089	4.171023	1.168022	0.245938	-5.62645	0.641033	0.552121
B.cells	CES2A	-0.5855	0.898465	-1.16794	0.245972	-4.90796	0.67545	0.604748
B.cells	STARD10	-0.2739	4.596544	-1.16771	0.246063	-5.78425	0.636709	0.545636
B.cells	CCDC85C	-0.44641	0.977037	-1.1676	0.246105	-4.94521	0.674599	0.603429
B.cells	PARP4	0.132098	5.335625	1.167592	0.24611	-5.92333	0.629282	0.534524
B.cells	AR	-0.72712	0.218656	-1.16724	0.246253	-4.83552	0.682999	0.616358
B.cells	TCIRG1	0.156575	5.455039	1.167195	0.24627	-5.89083	0.628215	0.532834
B.cells	PRKCSH	0.135595	4.862373	1.166761	0.246444	-5.76125	0.63433	0.541915
B.cells	GALNT10	0.167246	4.614762	1.166623	0.2465	-5.81652	0.636831	0.545687
B.cells	PAXIP1	-0.18237	4.269449	-1.16661	0.246506	-5.65861	0.640337	0.550957
B.cells	ECI2	0.18974	4.557161	1.166255	0.246648	-5.67578	0.637568	0.546722
B.cells	ATP7A	0.123036	5.8288	1.16618	0.246678	-6.01479	0.624833	0.527743
B.cells	RSL1	0.516857	1.039109	1.166046	0.246732	-4.9528	0.674414	0.603038
B.cells	CNOT8	-0.11543	5.284943	-1.16547	0.246965	-5.87178	0.630696	0.536122
B.cells	CLEC1B	-0.37413	3.741016	-1.16427	0.247445	-5.39205	0.647479	0.560131
B.cells	FUCA1	0.115478	5.963141	1.164039	0.24754	-6.07562	0.625079	0.526633
B.cells	CEBPD	0.288266	3.956744	1.163946	0.247577	-5.34009	0.645312	0.556927
B.cells	NDUFA1	-0.11483	6.729501	-1.16375	0.247657	-6.12884	0.617609	0.515609
B.cells	EIF2S3Y	2.112374	1.945318	1.163401	0.247797	-5.29291	0.666663	0.589051
B.cells	CRLF2	0.15924	5.010644	1.163093	0.247921	-5.82782	0.635044	0.540958
B.cells	CALHM6	0.529161	3.048969	1.162716	0.248074	-5.30461	0.655359	0.571423
B.cells	SGF29	0.139248	4.671195	1.162545	0.248143	-5.75828	0.638645	0.546197
B.cells	SAMD8	0.140185	5.289431	1.162436	0.248186	-5.91941	0.632407	0.536877
B.cells	PTGS2OS2	0.597932	-0.48663	1.162282	0.248249	-4.82944	0.693554	0.630409
B.cells	ZBTB16	0.4621	2.808614	1.162243	0.248265	-5.19545	0.657879	0.575258
B.cells	BBS2	-0.53483	0.404044	-1.162	0.248362	-4.87426	0.683828	0.615071

B.cells	PLXNB2	0.214374	3.959065	1.161712	0.248479	-5.55911	0.646188	0.557303
B.cells	GM47071	-0.50987	1.555335	-1.16147	0.248577	-5.02821	0.671593	0.595872
B.cells	CPB2	-0.27245	3.209826	-1.16035	0.24903	-5.44854	0.65491	0.569525
B.cells	GM15247	-0.58085	0.995367	-1.16031	0.249046	-4.96307	0.678529	0.605695
B.cells	PAQR5	-0.77472	0.502008	-1.16004	0.249156	-4.73362	0.683923	0.614149
B.cells	ZMAT2	-0.12157	5.466404	-1.15993	0.2492	-5.91365	0.631822	0.534938
B.cells	GTF2IRD2	0.191125	3.944401	1.159863	0.249227	-5.59014	0.647288	0.558134
B.cells	ATP5D	0.093311	7.898894	1.159642	0.249317	-6.34154	0.608001	0.499976
B.cells	EIF3J2	-0.44448	1.443832	-1.15964	0.249318	-4.96144	0.673667	0.598364
B.cells	TIPIN	-0.23468	5.238466	-1.15947	0.249385	-5.80023	0.63411	0.538469
B.cells	TRDV2-2	0.461744	-1.43025	1.159361	0.249431	-4.75777	0.705303	0.648176
B.cells	LSM14B	0.17164	3.604671	1.159296	0.249457	-5.55415	0.6508	0.563594
B.cells	DNAJB1	-0.1652	6.228259	-1.15909	0.249541	-6.01625	0.624315	0.523903
B.cells	EDA	-0.59691	1.445596	-1.15806	0.249956	-5.01546	0.674323	0.598907
B.cells	PRXL2B	0.291106	2.256934	1.157948	0.250004	-5.21039	0.665621	0.58555
B.cells	FUNDC2	0.125808	7.178207	1.157933	0.25001	-6.23662	0.615561	0.510595
B.cells	NQO2	-0.18266	3.942173	-1.15791	0.250021	-5.55609	0.647959	0.558729
B.cells	UBE2Q1	-0.10287	6.334643	-1.15775	0.250082	-6.07371	0.623819	0.522731
B.cells	GM10501	0.305137	2.138944	1.157717	0.250097	-5.2061	0.666878	0.587493
B.cells	B4GALT6	0.343304	2.991751	1.157267	0.25028	-5.26545	0.658185	0.573919
B.cells	HAVCR1	-0.66671	-0.3843	-1.15705	0.250369	-4.78452	0.694796	0.63041
B.cells	ITPKB	0.132431	7.462345	1.156983	0.250396	-6.25447	0.61313	0.506843
B.cells	SLC39A6	-0.16626	4.11285	-1.15652	0.250584	-5.66446	0.646769	0.556593
B.cells	HSPA1B	0.538522	4.531616	1.156488	0.250597	-5.78664	0.642475	0.550148
B.cells	IL27	0.756175	0.053926	1.1562	0.250714	-4.8148	0.690331	0.623186
B.cells	AW011738	0.416971	2.812185	1.155909	0.250832	-5.18568	0.660646	0.577295
B.cells	RABEP2	0.156891	4.414258	1.155661	0.250934	-5.73078	0.644109	0.552176
B.cells	FMN2	-0.39484	2.359589	-1.15527	0.251091	-5.36033	0.665832	0.584695
B.cells	ZFP62	0.151881	4.580193	1.15486	0.25126	-5.76077	0.642835	0.549834
B.cells	NARS	-0.11912	6.298026	-1.1548	0.251284	-6.05867	0.625562	0.524167
B.cells	DDX54	0.122806	5.820595	1.154706	0.251322	-6.03031	0.630307	0.5312
B.cells	CFAP97	0.192141	3.29046	1.154216	0.251522	-5.4221	0.656537	0.570133
B.cells	RAPGEF1	0.115962	6.775482	1.154089	0.251574	-6.1384	0.621203	0.517442
B.cells	B230217C1	-0.38797	1.478169	-1.15314	0.25196	-5.04132	0.676622	0.600072
B.cells	CAPSL	-0.56887	0.928873	-1.15297	0.252032	-4.99629	0.682608	0.609457
B.cells	UBAP1	0.140976	5.688984	1.152838	0.252084	-5.97558	0.632707	0.533933
B.cells	PLCG2	0.097243	7.02313	1.152632	0.252168	-6.22016	0.619498	0.514465
B.cells	ECHDC2	-0.50217	1.612612	-1.15237	0.252274	-5.01929	0.675167	0.598166
B.cells	ZFP748	0.362458	1.938501	1.152351	0.252283	-5.16041	0.671653	0.592778
B.cells	AP3S2	0.203744	3.43138	1.151866	0.252481	-5.489	0.655824	0.568738
B.cells	GCFC2	0.272174	2.248325	1.151665	0.252564	-5.22556	0.668331	0.587839
B.cells	GATAD2A	-0.10021	7.859185	-1.15155	0.252611	-6.33918	0.611386	0.502867
B.cells	CENPP	-0.22422	5.39603	-1.15155	0.252612	-5.86279	0.635652	0.538559
B.cells	GM45871	0.503535	0.985003	1.151477	0.25264	-4.91435	0.681994	0.608914
B.cells	RASGRP4	0.405072	1.734055	1.151421	0.252663	-5.1055	0.673855	0.59637
B.cells	UBN2	0.110888	7.076175	1.151391	0.252675	-6.23905	0.618979	0.513982
B.cells	TBCEL	0.170103	4.594507	1.151348	0.252693	-5.70621	0.643792	0.550762

B.cells	ITPRIP	0.211228	3.468077	1.151294	0.252715	-5.55017	0.65544	0.56833
B.cells	FBP1	-0.28395	5.143064	-1.15118	0.252763	-5.92733	0.638208	0.542453
B.cells	MEGF8	-0.52986	0.288303	-1.15092	0.252868	-4.85307	0.689799	0.62097
B.cells	PCBP2	-0.07732	8.511828	-1.15074	0.252944	-6.43617	0.605308	0.494024
B.cells	MDP1	0.148562	4.373314	1.150321	0.253114	-5.73236	0.646425	0.554509
B.cells	AMBRA1	0.098115	7.654516	1.150282	0.253129	-6.33804	0.613706	0.506104
B.cells	NAT8L	-0.47233	0.332665	-1.14993	0.253273	-4.97059	0.689636	0.620451
B.cells	AI847159	0.601182	-0.25702	1.149908	0.253283	-4.83515	0.696205	0.630688
B.cells	ARHGAP11	-0.18798	4.784218	-1.14981	0.253324	-5.75493	0.642285	0.548205
B.cells	SELENOT	0.102399	6.182995	1.149529	0.253438	-6.07627	0.628341	0.527313
B.cells	ABHD8	0.318095	2.172201	1.149113	0.253609	-5.1814	0.670049	0.589654
B.cells	STAT5A	-0.1811	3.992851	-1.1488	0.253738	-5.65964	0.650899	0.560689
B.cells	RAI1	-0.1355	6.004853	-1.14879	0.253741	-6.00359	0.630437	0.530147
B.cells	ZFP871	0.146112	5.249328	1.148466	0.253874	-5.88151	0.638031	0.541492
B.cells	KDELR2	-0.12078	5.993878	-1.14846	0.253877	-6.00007	0.630547	0.530383
B.cells	IGSF3	-0.57041	0.826018	-1.14839	0.253904	-4.89216	0.684699	0.612425
B.cells	PEX2	0.20511	3.914925	1.148191	0.253987	-5.59227	0.651733	0.562116
B.cells	GPAA1	0.185298	3.764843	1.148056	0.254042	-5.47037	0.653292	0.564548
B.cells	UBE2K	-0.07978	8.214477	-1.14795	0.254085	-6.41278	0.608858	0.498812
B.cells	EXO1	-0.46365	2.019131	-1.14782	0.254139	-5.0516	0.671758	0.592768
B.cells	SH2D4B	-0.23946	4.439997	-1.14764	0.254215	-5.76722	0.646361	0.554191
B.cells	1190005I0	-0.44005	1.185776	-1.14742	0.254305	-4.93822	0.68093	0.606763
B.cells	CDK13	0.100246	7.414024	1.146901	0.254517	-6.27813	0.61709	0.510368
B.cells	GM45509	-0.40665	1.640462	-1.14667	0.254613	-5.1089	0.676507	0.599412
B.cells	CETN3	-0.13523	5.807387	-1.14639	0.254728	-5.97508	0.633203	0.533856
B.cells	RFX1	-0.18456	3.263376	-1.14615	0.254827	-5.4373	0.659418	0.573071
B.cells	CASD1	0.16456	4.363906	1.146036	0.254872	-5.66804	0.64796	0.555821
B.cells	NEU1	0.161549	4.426111	1.145389	0.255139	-5.7451	0.647634	0.555006
B.cells	BAG2	0.460423	1.435408	1.145349	0.255155	-4.95213	0.679305	0.603163
B.cells	CETN2	-0.1113	5.798667	-1.1453	0.255175	-5.98457	0.633678	0.534233
B.cells	PNISR	0.100453	5.773836	1.145067	0.255272	-6.00182	0.633928	0.534638
B.cells	COA6	0.178115	4.110702	1.145053	0.255277	-5.62113	0.650891	0.559974
B.cells	EOMES	0.720096	-0.77158	1.144709	0.255419	-4.81234	0.703746	0.641436
B.cells	PRORSD1	0.145231	4.586531	1.144653	0.255442	-5.78148	0.646066	0.552682
B.cells	OLFM1	-0.40182	2.469141	-1.1444	0.255548	-5.16431	0.668243	0.586275
B.cells	SERPING1	-0.30469	3.004813	-1.14438	0.255556	-5.3926	0.66255	0.577603
B.cells	NDUFS3	0.120579	6.050235	1.144122	0.255661	-6.05042	0.631239	0.530727
B.cells	BUB1	-0.34425	3.743439	-1.14408	0.25568	-5.46964	0.654792	0.565953
B.cells	HIST1H2BH	-0.69465	0.17295	-1.14373	0.255825	-4.77074	0.693297	0.625079
B.cells	CEP70	0.248159	3.195368	1.143689	0.25584	-5.38555	0.660538	0.574713
B.cells	CHRM3	0.684653	0.831294	1.143655	0.255854	-4.89047	0.686005	0.613757
B.cells	SNX2	0.082001	7.314048	1.143611	0.255872	-6.27621	0.61876	0.512458
B.cells	ABHD6	0.296243	2.548845	1.143456	0.255936	-5.2111	0.667413	0.585143
B.cells	CLSTN1	-0.40992	1.774497	-1.1433	0.256001	-5.00535	0.675756	0.597906
B.cells	UNC13A	-0.48104	1.183593	-1.14274	0.256231	-4.97645	0.682468	0.60803
B.cells	DMTN	-0.53657	0.637339	-1.14261	0.256287	-4.91742	0.688476	0.617416
B.cells	CD8B1	-0.69571	0.660348	-1.1425	0.256331	-4.8758	0.688221	0.617053

B.cells	GM1976	0.295775	2.701657	1.142496	0.256333	-5.23102	0.666085	0.583016
B.cells	ARGLU1	-0.07319	7.597485	-1.14234	0.256397	-6.29903	0.616317	0.508779
B.cells	GM15706	0.522946	1.015787	1.14204	0.256521	-4.87667	0.684436	0.611118
B.cells	KIF11	-0.2992	5.441855	-1.14173	0.256649	-5.88096	0.637779	0.540452
B.cells	IGSF6	0.431343	3.689623	1.141647	0.256683	-5.29609	0.655792	0.567477
B.cells	C1GALT1C1	-0.16591	4.044817	-1.14162	0.256693	-5.7374	0.652093	0.561895
B.cells	SMG6	-0.08315	8.045885	-1.14151	0.256741	-6.39498	0.612077	0.502702
B.cells	TMEM214	-0.14827	4.338367	-1.14143	0.256775	-5.69006	0.649055	0.557379
B.cells	A430035B1	-0.34518	3.071834	-1.14126	0.256845	-5.32632	0.662321	0.577443
B.cells	ATP13A2	0.161716	5.610722	1.141081	0.256917	-5.96974	0.636149	0.538115
B.cells	UBA7	0.257234	3.784743	1.140645	0.257098	-5.50165	0.654986	0.566261
B.cells	MOAP1	-0.4168	0.547771	-1.14053	0.257146	-4.91796	0.689793	0.619614
B.cells	PDZD4	-0.55776	1.698737	-1.14049	0.257162	-4.89291	0.677177	0.600131
B.cells	DUT	-0.225	5.882801	-1.14043	0.257186	-5.9705	0.633519	0.534182
B.cells	MPI	-0.32504	2.225319	-1.14015	0.257301	-5.12963	0.671514	0.5915
B.cells	HSPA1A	0.50962	3.985226	1.140041	0.257348	-5.68246	0.652917	0.563236
B.cells	PIGB	-0.28845	2.703863	-1.14001	0.257362	-5.24881	0.666397	0.58368
B.cells	MTERF4	0.351426	1.913823	1.139693	0.257493	-5.12115	0.675064	0.596756
B.cells	2010310C0	-0.68959	1.298313	-1.13937	0.257626	-4.9017	0.681889	0.607186
B.cells	RNF217	0.444044	2.071962	1.139221	0.257688	-5.19709	0.673492	0.594351
B.cells	CYP2D22	-0.54796	0.864397	-1.13916	0.257713	-4.89345	0.686651	0.614664
B.cells	SGMS1	0.140758	7.102996	1.138635	0.257931	-6.29615	0.622091	0.516933
B.cells	ADRB1	0.750388	0.735849	1.138537	0.257972	-4.87432	0.68846	0.617174
B.cells	RHOU	-0.5413	1.27725	-1.1384	0.258031	-5.04473	0.682513	0.607993
B.cells	MIR22HG	-0.21071	4.089128	-1.13799	0.2582	-5.70351	0.652633	0.562394
B.cells	TMEM120A	-0.19288	3.799392	-1.13792	0.25823	-5.56997	0.65565	0.566947
B.cells	PMF1	0.163169	5.697613	1.137894	0.258239	-5.96509	0.636173	0.537766
B.cells	IZUMO1R	0.566487	0.001693	1.137459	0.25842	-4.97462	0.697077	0.630205
B.cells	MEAK7	-0.56257	0.666682	-1.13687	0.258664	-4.86939	0.689803	0.618978
B.cells	ABCE1	0.149256	5.007871	1.13673	0.258723	-5.85466	0.643606	0.548594
B.cells	GRPEL1	-0.13941	5.877727	-1.13666	0.25875	-6.01622	0.63479	0.535511
B.cells	MED10	-0.14566	4.886924	-1.13635	0.258879	-5.79495	0.644843	0.550572
B.cells	CASTOR1	0.505904	0.626653	1.136292	0.258905	-4.95288	0.690247	0.619833
B.cells	VPS18	0.140986	4.834066	1.136005	0.259024	-5.86612	0.645385	0.551436
B.cells	DDR1	-0.5415	0.137297	-1.13599	0.25903	-4.82572	0.695694	0.628372
B.cells	SCFD1	0.164253	4.929173	1.135893	0.259071	-5.81995	0.644411	0.550016
B.cells	ABITRAM	0.252264	2.584915	1.135857	0.259086	-5.28323	0.668931	0.587095
B.cells	PTCH1	0.296864	3.139748	1.13546	0.259251	-5.38387	0.66303	0.578366
B.cells	SHARPIN	0.142147	4.530571	1.135454	0.259254	-5.78453	0.648505	0.556407
B.cells	MAPKAPK3	0.141886	5.306805	1.135311	0.259313	-6.00277	0.64056	0.544576
B.cells	ETNK2	-0.44238	1.270533	-1.13525	0.259339	-5.04301	0.683153	0.609296
B.cells	DOCK4	-0.16136	6.427831	-1.1352	0.259361	-6.15763	0.629289	0.527842
B.cells	VPS37C	-0.16264	3.950923	-1.13516	0.259377	-5.61136	0.654513	0.565524
B.cells	SLC30A7	0.136142	5.62501	1.13498	0.259451	-5.98266	0.637337	0.539773
B.cells	COL4A4	-0.46585	1.009618	-1.13485	0.259505	-4.97412	0.686017	0.613746
B.cells	TUBE1	-0.45519	1.254723	-1.13477	0.259538	-4.99107	0.683326	0.609606
B.cells	FCRL1	0.247927	2.73778	1.134764	0.259541	-5.55029	0.667299	0.584981

B.cells	MEPCE	-0.1919	4.466254	-1.13411	0.259813	-5.68159	0.649707	0.557775
B.cells	DPH5	0.18903	3.732552	1.133863	0.259917	-5.62285	0.657347	0.569396
B.cells	RINL	0.247801	3.632551	1.133638	0.260011	-5.43934	0.658395	0.571044
B.cells	HTRA2	-0.15918	3.681601	-1.13363	0.260016	-5.58598	0.65788	0.570265
B.cells	SEC22B	-0.12663	5.449712	-1.13346	0.260086	-5.92009	0.639649	0.542896
B.cells	RFTN1	0.133177	6.737622	1.133428	0.260099	-6.20172	0.626743	0.523774
B.cells	AMY1	-0.31369	2.627056	-1.13291	0.260316	-5.32206	0.669456	0.587512
B.cells	VEGFC	-0.50299	1.200834	-1.1325	0.260485	-4.96779	0.684963	0.611431
B.cells	OGG1	-0.21759	3.189389	-1.13223	0.2606	-5.36858	0.663519	0.578623
B.cells	DNASE1L1	0.254576	3.048154	1.132206	0.260609	-5.38809	0.665016	0.580901
B.cells	PGM3	-0.21746	2.89728	-1.13217	0.260626	-5.33574	0.66662	0.583344
B.cells	LCMT2	-0.24937	2.687418	-1.13211	0.260649	-5.31714	0.668858	0.58676
B.cells	RRP8	0.198091	3.730434	1.132054	0.260673	-5.56689	0.657821	0.569976
B.cells	HHEX	-0.1871	4.675113	-1.1319	0.260737	-5.74895	0.648027	0.555184
B.cells	GM17268	0.688002	0.383225	1.131681	0.260829	-4.90743	0.694059	0.625608
B.cells	CHTF8	0.407044	1.341638	1.131596	0.260864	-5.04102	0.683466	0.609173
B.cells	FAU	0.061258	11.4506	1.131471	0.260917	-6.91497	0.582519	0.45988
B.cells	F13A1	-0.64229	2.512117	-1.13114	0.261057	-5.34241	0.670921	0.589928
B.cells	GM9929	-0.37169	1.684976	-1.13107	0.261085	-5.05872	0.679859	0.603687
B.cells	GNAI2	0.072164	9.008515	1.130602	0.26128	-6.52797	0.605508	0.49249
B.cells	ZFP825	0.312054	2.050646	1.130487	0.261329	-5.18935	0.676107	0.597773
B.cells	PON2	-0.12367	6.020273	-1.13028	0.261416	-6.07752	0.634708	0.535269
B.cells	GMIP	0.109434	5.964525	1.130081	0.261499	-6.06299	0.635268	0.536162
B.cells	EDF1	0.09837	7.047067	1.130065	0.261505	-6.21107	0.62449	0.520245
B.cells	SPECC1	0.360235	4.276803	1.129957	0.26155	-5.47444	0.652514	0.562039
B.cells	5730455P1	0.218799	2.914853	1.129828	0.261604	-5.39666	0.666832	0.583803
B.cells	TESPA1	0.297614	2.504605	1.129792	0.26162	-5.36852	0.671216	0.590503
B.cells	LANCL2	0.256029	2.807486	1.129249	0.261847	-5.37424	0.668366	0.58576
B.cells	METTL7A1	-0.27627	2.456994	-1.12916	0.261886	-5.25185	0.672119	0.591506
B.cells	PYGO2	-0.2647	3.374512	-1.12901	0.261948	-5.50346	0.662361	0.576614
B.cells	DOK1	0.177309	3.614618	1.128543	0.262144	-5.62521	0.66018	0.572922
B.cells	TAF5L	0.141475	4.562119	1.128405	0.262202	-5.80155	0.650303	0.558109
B.cells	CRTAM	0.657832	1.176627	1.128192	0.262291	-4.96289	0.686508	0.613265
B.cells	TRP53BP1	0.191763	3.888271	1.127774	0.262467	-5.59604	0.657451	0.568933
B.cells	ADSSL1	-0.17028	4.426295	-1.12777	0.262469	-5.69209	0.651846	0.560476
B.cells	NAA60	0.144963	4.697726	1.127722	0.262488	-5.8048	0.64904	0.556285
B.cells	PHF8	0.121444	5.563796	1.127599	0.26254	-5.99219	0.64018	0.543086
B.cells	CYFIP2	0.120727	6.947339	1.127405	0.262622	-6.27059	0.626381	0.522587
B.cells	NID2	-0.41523	2.19946	-1.12713	0.262737	-5.16654	0.675627	0.596533
B.cells	WDR13	0.231871	2.570105	1.126787	0.262882	-5.29952	0.671781	0.590575
B.cells	CCDC12	-0.09011	6.939585	-1.1266	0.262959	-6.22143	0.626732	0.523008
B.cells	PLPPR1	0.465524	1.149205	1.126524	0.262992	-5.01114	0.687237	0.614489
B.cells	GLRX3	0.11344	6.625536	1.126419	0.263036	-6.16326	0.629849	0.527638
B.cells	NOP53	-0.13155	5.740416	-1.12632	0.263077	-6.02521	0.638732	0.540871
B.cells	CECR2	-0.21353	6.605628	-1.12617	0.26314	-6.20947	0.630064	0.528074
B.cells	SAC3D1	0.215966	3.030557	1.125378	0.263474	-5.40048	0.667545	0.583796
B.cells	SLC22A21	-0.39338	1.348839	-1.12528	0.263517	-5.15167	0.685746	0.611771

B.cells	GSTP1	0.145764	6.928594	1.125094	0.263594	-6.19697	0.627535	0.523789
B.cells	OTUD3	0.292764	2.558441	1.124848	0.263698	-5.34065	0.672769	0.591567
B.cells	KCNAB1	0.475125	1.324987	1.12449	0.263849	-5.09278	0.686428	0.612389
B.cells	SSBP3	-0.13062	5.600863	-1.12424	0.263953	-5.98405	0.641313	0.543723
B.cells	TMEM127	0.145833	4.290896	1.123952	0.264076	-5.70547	0.654955	0.563993
B.cells	ITGB1	0.105664	6.871704	1.123399	0.264309	-6.20614	0.629099	0.525002
B.cells	GM12227	-0.41216	1.113465	-1.12329	0.264355	-5.00267	0.689488	0.616266
B.cells	CDC5L	0.092801	5.768771	1.122886	0.264526	-6.02633	0.640446	0.541537
B.cells	SSU72	0.090047	6.693565	1.122624	0.264637	-6.1736	0.631189	0.527837
B.cells	MRPS22	-0.21773	3.085855	-1.12257	0.264658	-5.42913	0.668406	0.583616
B.cells	1700061N1	-0.84931	-0.1438	-1.12196	0.264918	-4.77769	0.704233	0.638605
B.cells	ITPR1	0.123033	6.992018	1.121951	0.264921	-6.29254	0.628512	0.523638
B.cells	TRIM41	-0.15173	4.185669	-1.12188	0.264951	-5.66707	0.657098	0.566189
B.cells	FLT1	-0.40993	4.243341	-1.12123	0.265226	-5.63579	0.657035	0.565381
B.cells	MAX	0.108884	6.499429	1.120867	0.26538	-6.1289	0.634103	0.531091
B.cells	SCNM1	0.197971	3.621272	1.120641	0.265475	-5.51389	0.663743	0.575445
B.cells	KAT6A	0.110093	6.479855	1.120613	0.265487	-6.15625	0.634299	0.531427
B.cells	ZNHIT2	0.193714	3.310699	1.120507	0.265532	-5.4612	0.667038	0.580494
B.cells	SVIP	-0.3878	1.34626	-1.12002	0.265739	-5.08574	0.688323	0.613265
B.cells	H2-AA	0.285596	8.331767	1.120006	0.265745	-6.73562	0.616047	0.50492
B.cells	HIP1	-0.21227	5.505684	-1.11989	0.265795	-5.82678	0.644157	0.546251
B.cells	GZMB	-0.63922	3.971899	-1.11983	0.265821	-5.30325	0.660047	0.57008
B.cells	HACE1	0.167047	4.490352	1.119789	0.265837	-5.7705	0.654625	0.561912
B.cells	NSMCE3	-0.14285	4.271532	-1.11951	0.265957	-5.69805	0.656907	0.565488
B.cells	DYNC1H1	0.097006	6.255557	1.119198	0.266087	-6.09415	0.636553	0.535189
B.cells	NSUN5	-0.30533	2.124735	-1.11902	0.266162	-5.19299	0.679795	0.600414
B.cells	OXSM	-0.3511	1.788374	-1.11889	0.266218	-5.07164	0.683465	0.6061
B.cells	MANBAL	0.127936	4.531107	1.118831	0.266243	-5.79011	0.654201	0.561577
B.cells	TPST2	0.13183	5.593782	1.118822	0.266247	-5.96321	0.643258	0.545206
B.cells	GM32250	0.617118	-0.2746	1.118544	0.266365	-4.80185	0.706475	0.641889
B.cells	RASL11A	0.636153	0.28073	1.118483	0.266391	-4.84032	0.700195	0.632072
B.cells	AGFG2	-0.1587	4.605897	-1.11848	0.266393	-5.78289	0.653423	0.560444
B.cells	RCC1	0.20049	3.989305	1.118448	0.266405	-5.58516	0.659864	0.570146
B.cells	ALKBH5	-0.10159	7.148219	-1.11803	0.266581	-6.27275	0.627639	0.522318
B.cells	SLC25A11	0.15056	5.162778	1.117821	0.266672	-5.86479	0.64767	0.552069
B.cells	BMT2	0.123196	6.027835	1.117771	0.266693	-6.06971	0.638851	0.538932
B.cells	ZFP831	0.256097	3.063289	1.117593	0.266769	-5.57402	0.669676	0.58533
B.cells	6430548M	0.356304	2.407786	1.117495	0.26681	-5.15186	0.676725	0.596165
B.cells	CACFD1	0.19429	3.174111	1.117409	0.266847	-5.49735	0.668493	0.583628
B.cells	MBOAT7	0.163397	4.278521	1.117357	0.266869	-5.72239	0.656834	0.565982
B.cells	SPINK10	-0.5346	0.791956	-1.11721	0.266931	-4.9082	0.69447	0.623681
B.cells	VIPAS39	0.13864	4.131233	1.117115	0.266972	-5.71573	0.658375	0.568415
B.cells	LDB1	0.165729	4.482765	1.117051	0.266999	-5.76564	0.654704	0.56288
B.cells	PLEKHG2	-0.21469	4.157105	-1.11705	0.266999	-5.66855	0.658104	0.568005
B.cells	SLC18A1	-0.63889	0.497721	-1.11672	0.267141	-4.87925	0.69798	0.62888
B.cells	ARHGAP28	-0.60913	0.598833	-1.11653	0.267219	-4.97301	0.696901	0.627117
B.cells	ZRANB1	0.103094	6.142303	1.115992	0.267449	-6.08302	0.638347	0.537533

B.cells	EDNRB	-0.85976	2.175551	-1.11581	0.267528	-5.09357	0.679938	0.600346
B.cells	GM12743	0.301444	2.382516	1.115739	0.267557	-5.29314	0.677691	0.596923
B.cells	SPATA13	0.163608	5.147731	1.115434	0.267687	-5.94342	0.648531	0.552759
B.cells	UTY	2.24041	2.644423	1.11543	0.267689	-5.5433	0.674905	0.592642
B.cells	GIMAP3	0.337925	2.932139	1.115238	0.267771	-5.71825	0.671813	0.587929
B.cells	ARMCX3	-0.20881	3.573363	-1.11516	0.267804	-5.52055	0.664977	0.577568
B.cells	NUDT9	-0.18414	4.362001	-1.11474	0.267983	-5.75073	0.656948	0.565117
B.cells	ELK4	-0.1189	5.882489	-1.11441	0.268125	-6.04621	0.641285	0.541746
B.cells	TBC1D30	-0.54708	1.06093	-1.11408	0.268265	-5.01871	0.692518	0.619721
B.cells	TLR11	0.459216	-0.67266	1.114062	0.268272	-4.9296	0.711965	0.650271
B.cells	MTIF2	0.146261	4.285566	1.114042	0.26828	-5.75109	0.657747	0.566559
B.cells	TEDC1	-0.40197	1.701406	-1.11396	0.268315	-5.04518	0.685446	0.608798
B.cells	COA3	0.13126	5.812938	1.113864	0.268356	-5.98975	0.641992	0.542987
B.cells	GLIPR1	0.18354	5.059395	1.113484	0.268519	-5.75545	0.649709	0.554691
B.cells	MTRF1L	0.242469	3.1332	1.113439	0.268538	-5.42409	0.669935	0.585241
B.cells	EIF2AK1	0.119079	5.326731	1.113431	0.268542	-5.99372	0.646959	0.550578
B.cells	GM30541	0.355193	0.952964	1.113276	0.268608	-5.14437	0.693719	0.621878
B.cells	GYS1	0.270844	3.38284	1.113248	0.268619	-5.50884	0.667272	0.58126
B.cells	EIF4E	-0.12422	6.950147	-1.11315	0.268659	-6.2703	0.63055	0.526339
B.cells	SBNO1	-0.09699	7.359347	-1.11265	0.268876	-6.28216	0.626492	0.52053
B.cells	GPN3	0.159642	3.982026	1.112557	0.268915	-5.65525	0.660932	0.571862
B.cells	AFDN	0.275225	3.267689	1.112554	0.268916	-5.3253	0.668499	0.583344
B.cells	MIRT1	0.175979	4.950639	1.112468	0.268953	-5.91131	0.650832	0.556671
B.cells	CRYZL1	0.146862	4.570122	1.11245	0.26896	-5.80574	0.654778	0.562601
B.cells	AP2A1	0.168919	4.542647	1.112367	0.268996	-5.77198	0.655064	0.56305
B.cells	ZFP296	-0.25593	2.857765	-1.11174	0.269265	-5.54344	0.673388	0.590373
B.cells	MSL2	0.107342	6.010868	1.111464	0.269382	-6.10199	0.64046	0.540854
B.cells	TMEM71	0.206792	3.858441	1.111452	0.269387	-5.70167	0.662728	0.574253
B.cells	THEM4	-0.34532	2.270582	-1.11136	0.269424	-5.24992	0.679737	0.600211
B.cells	INO80B	0.159848	4.146114	1.110595	0.269754	-5.73574	0.659854	0.569969
B.cells	ZFP81	0.331902	1.873403	1.11052	0.269786	-5.16222	0.68423	0.607193
B.cells	NGRN	0.239944	3.173648	1.110399	0.269838	-5.45086	0.670159	0.585641
B.cells	RAPGEF2	0.159171	7.429198	1.110325	0.26987	-6.27964	0.626416	0.520246
B.cells	CLINT1	-0.08717	8.205941	-1.1103	0.26988	-6.45812	0.618801	0.50912
B.cells	LDLRAD3	0.16484	4.591753	1.110269	0.269893	-5.99175	0.655194	0.563009
B.cells	KRTCAP3	-0.42416	0.893962	-1.11012	0.269958	-5.00068	0.695056	0.624048
B.cells	PER3	-0.44587	1.517982	-1.10967	0.270151	-4.99855	0.688136	0.61334
B.cells	TMEM251	0.149615	4.619848	1.109658	0.270156	-5.80136	0.654902	0.562632
B.cells	ARRDC1	0.155609	4.471227	1.109593	0.270183	-5.83215	0.656451	0.564965
B.cells	AKR1B3	-0.12329	5.402163	-1.10957	0.270193	-5.96497	0.646818	0.550511
B.cells	KLHL26	0.264315	2.727028	1.109513	0.270218	-5.35293	0.674954	0.593086
B.cells	TOPBP1	-0.1599	5.680028	-1.10949	0.270229	-6.0042	0.643976	0.546289
B.cells	PRICKLE1	-0.40599	3.386121	-1.10895	0.270458	-5.31295	0.668316	0.582412
B.cells	ZCCHC18	0.390856	1.04036	1.108698	0.270567	-5.11158	0.693881	0.621764
B.cells	TLR8	0.541178	0.054908	1.108599	0.27061	-4.895	0.704949	0.639036
B.cells	SESTD1	0.509304	2.300083	1.108381	0.270703	-5.10451	0.680022	0.600419
B.cells	SOS2	0.129586	5.600384	1.108297	0.270739	-6.02027	0.645212	0.547728

B.cells	RASA2	0.154057	6.158678	1.108232	0.270767	-6.07442	0.639533	0.539281
B.cells	PPP1R16A	0.255196	2.885752	1.108143	0.270806	-5.34504	0.673687	0.590717
B.cells	ZFP746	0.175054	3.717554	1.107844	0.270934	-5.60095	0.664828	0.57744
B.cells	XLR	-0.47773	2.01867	-1.10781	0.270948	-5.17309	0.68311	0.605395
B.cells	NAIF1	-0.37547	1.251393	-1.10765	0.271019	-5.08438	0.691557	0.618503
B.cells	ZNRD1	-0.12809	5.076686	-1.10752	0.271074	-5.92804	0.650613	0.556077
B.cells	4930430F0	0.394169	1.035161	1.10746	0.271099	-4.98731	0.69396	0.622246
B.cells	PRR13	0.094612	6.510778	1.107251	0.271189	-6.15393	0.636007	0.534313
B.cells	TIA1	0.104291	5.466514	1.107188	0.271216	-5.97112	0.646609	0.550075
B.cells	GM44777	0.501117	0.565153	1.10697	0.271309	-4.94463	0.699295	0.630441
B.cells	DNLZ	0.195133	3.991855	1.106807	0.27138	-5.61938	0.662006	0.573194
B.cells	FZD5	-0.26984	2.536377	-1.10661	0.271466	-5.31384	0.677556	0.597016
B.cells	AW554918	0.125647	5.656196	1.106591	0.271473	-6.01339	0.644735	0.547364
B.cells	SF3B6	-0.08737	7.294759	-1.10571	0.27185	-6.29431	0.628924	0.523219
B.cells	CPNE3	-0.1635	5.367342	-1.10551	0.271936	-5.85664	0.648391	0.552101
B.cells	CENPE	-0.33037	4.9887	-1.10548	0.27195	-5.79903	0.652298	0.557952
B.cells	TRIP11	0.112886	6.069875	1.105337	0.272012	-6.09064	0.641214	0.541425
B.cells	TM2D2	0.12193	5.178235	1.105155	0.272091	-5.95365	0.650339	0.555034
B.cells	SGK3	0.138603	5.592861	1.105117	0.272107	-6.0574	0.646076	0.548671
B.cells	TKT	0.12563	6.932084	1.104818	0.272236	-6.20637	0.632572	0.528726
B.cells	DONSON	0.161108	4.025423	1.104745	0.272267	-5.72332	0.662404	0.573328
B.cells	SYNGR2	0.125379	6.111593	1.104647	0.27231	-6.08703	0.640829	0.540987
B.cells	STK32C	-0.48147	0.553606	-1.1045	0.272374	-4.96263	0.700219	0.631446
B.cells	PILRB1	-0.45796	2.049283	-1.10427	0.272471	-5.1118	0.683629	0.60574
B.cells	GART	0.176922	4.367941	1.104242	0.272484	-5.78685	0.658804	0.567896
B.cells	TINAGL1	-0.49094	2.066409	-1.10405	0.272566	-5.08254	0.683442	0.605522
B.cells	OAS1A	0.492062	2.355225	1.103905	0.27263	-5.2102	0.680292	0.600749
B.cells	TM4SF1	0.489976	2.15291	1.103797	0.272676	-5.14193	0.682497	0.604196
B.cells	OTUD1	-0.2353	3.007254	-1.10346	0.27282	-5.42842	0.673243	0.59019
B.cells	PSMD10	0.210272	3.584601	1.103297	0.272892	-5.53634	0.667072	0.580868
B.cells	STARD7	0.130831	5.358518	1.103043	0.273002	-5.93655	0.648521	0.552984
B.cells	DTNBP1	0.085671	6.683213	1.102891	0.273067	-6.17612	0.635063	0.532997
B.cells	ADAMTS7	-0.5242	0.676356	-1.10286	0.273081	-4.96288	0.69884	0.629948
B.cells	AFAP1	-0.23669	1.955044	-1.10284	0.27309	-5.41633	0.684661	0.607957
B.cells	DDX42	0.103083	5.684077	1.102798	0.273107	-6.01805	0.645182	0.548031
B.cells	CATSPERG1	-0.61679	0.160917	-1.10242	0.273271	-4.89109	0.70465	0.639257
B.cells	ABCC3	-0.45872	2.689429	-1.10233	0.273308	-5.23568	0.676668	0.595895
B.cells	GM13166	0.596078	-0.19353	1.102289	0.273328	-4.84093	0.708678	0.645607
B.cells	RNF7	-0.11774	6.742111	-1.10199	0.273457	-6.17716	0.634472	0.532367
B.cells	COQ8A	0.276685	2.303584	1.101949	0.273474	-5.31931	0.680854	0.602369
B.cells	CLYBL	0.167743	4.489599	1.101928	0.273484	-5.80379	0.657531	0.566821
B.cells	GM49359	0.300855	2.260358	1.101921	0.273486	-5.23768	0.681325	0.603093
B.cells	SSR2	-0.14526	5.189958	-1.1019	0.273495	-5.89208	0.650257	0.555876
B.cells	EML1	-0.55217	1.303195	-1.10138	0.273723	-4.93581	0.692279	0.619504
B.cells	PLEKHB1	-0.43874	0.728002	-1.10104	0.273866	-5.00466	0.698916	0.629607
B.cells	THOC7	-0.09793	6.572603	-1.10058	0.274067	-6.17199	0.637033	0.535213
B.cells	MTMR3	0.088538	7.886466	1.100311	0.274184	-6.43184	0.623966	0.516098

B.cells	MAPRE1	-0.08218	7.148653	-1.10024	0.274214	-6.25884	0.631265	0.526814
B.cells	ZRANB3	-0.25301	3.148069	-1.10003	0.274307	-5.4315	0.672639	0.588964
B.cells	ACE	0.701242	0.341503	1.1	0.274318	-4.97617	0.703557	0.636751
B.cells	PRKD3	0.134958	5.198034	1.099991	0.274322	-5.96456	0.651052	0.5563
B.cells	NUBP1	0.123565	5.473714	1.098605	0.274923	-6.0303	0.649373	0.552563
B.cells	CALML4	-0.47193	1.801954	-1.09859	0.274928	-5.09095	0.6885	0.612004
B.cells	SAP130	0.124243	5.619105	1.098281	0.275064	-6.03696	0.648063	0.550347
B.cells	PPP3CB	0.099337	5.980209	1.097941	0.275211	-6.07988	0.644489	0.544927
B.cells	RNF34	0.150931	4.455799	1.097787	0.275278	-5.75539	0.660267	0.568618
B.cells	TMEM79	-0.52647	0.778123	-1.09776	0.275288	-4.91787	0.700226	0.629857
B.cells	DIAPH2	0.118436	7.666379	1.097357	0.275465	-6.36966	0.627825	0.520124
B.cells	PCF11	-0.1234	6.482932	-1.09715	0.275556	-6.16207	0.639739	0.537582
B.cells	SLC11A1	0.411929	3.505984	1.096403	0.27588	-5.31269	0.671144	0.584318
B.cells	CDC14A	-0.1581	5.803629	-1.09612	0.276001	-6.07796	0.647082	0.548164
B.cells	9330175E1	0.523226	1.002962	1.096	0.276055	-5.05677	0.698555	0.626492
B.cells	RNF170	0.195887	3.319753	1.095973	0.276067	-5.48567	0.67314	0.587406
B.cells	SNRPD2	-0.1072	6.391824	-1.09596	0.276072	-6.12453	0.641086	0.53926
B.cells	WBP1L	0.139758	5.329247	1.095936	0.276083	-5.94449	0.651966	0.555451
B.cells	MAGED2	-0.50915	1.585547	-1.09572	0.276175	-4.98027	0.692148	0.616508
B.cells	HNRNPUL2	-0.09182	6.468104	-1.09551	0.27627	-6.16126	0.64048	0.53823
B.cells	TAP2	0.18517	5.110651	1.095243	0.276385	-5.93819	0.654417	0.559027
B.cells	EZH1	0.199879	3.600438	1.095228	0.276391	-5.62918	0.670325	0.583028
B.cells	GM50232	-0.41283	1.372215	-1.09505	0.276469	-5.12741	0.694679	0.620314
B.cells	TIMM17A	0.118169	5.135955	1.094645	0.276645	-5.92678	0.654483	0.558811
B.cells	HEATR3	0.163611	4.273336	1.094387	0.276758	-5.77816	0.663648	0.572511
B.cells	ARHGEF10	0.47529	2.266878	1.0942	0.276839	-5.13612	0.68529	0.60543
B.cells	GM10053	0.42339	1.424678	1.093649	0.27708	-5.10903	0.694951	0.619853
B.cells	PEPD	-0.12971	5.308275	-1.09361	0.277097	-5.99856	0.653219	0.556389
B.cells	TCF3	-0.11922	6.601305	-1.09296	0.277379	-6.19957	0.64046	0.536994
B.cells	TFB2M	-0.15177	3.953928	-1.09287	0.277419	-5.67426	0.66793	0.578108
B.cells	TNFSF13OS	-0.67609	0.055873	-1.09257	0.277552	-4.88788	0.710943	0.644461
B.cells	FILIP1L	0.151264	5.411773	1.092553	0.277558	-5.98742	0.652657	0.555275
B.cells	GPATCH8	0.087685	7.002079	1.092455	0.277601	-6.28101	0.636441	0.531263
B.cells	OGFOD3	0.21875	3.187381	1.091644	0.277956	-5.47236	0.676886	0.591025
B.cells	MMGT2	0.198746	3.166256	1.091501	0.278018	-5.47411	0.677126	0.591407
B.cells	GM43445	0.299529	1.896237	1.091333	0.278092	-5.25058	0.69105	0.612753
B.cells	SLC15A2	-0.72369	4.597147	-1.0909	0.278279	-5.87015	0.662078	0.5685
B.cells	SEPSECS	-0.22072	3.143326	-1.09056	0.278431	-5.53736	0.677585	0.5921
B.cells	PHF6	0.140165	4.940138	1.090454	0.278476	-5.90749	0.65848	0.563217
B.cells	FBXL18	-0.39004	2.985602	-1.09045	0.278477	-5.3055	0.679293	0.594704
B.cells	CEP78	0.216397	3.001386	1.090423	0.27849	-5.42456	0.679122	0.594443
B.cells	TUT4	0.115369	7.475638	1.090337	0.278527	-6.38948	0.632589	0.524832
B.cells	PHF5A	-0.12015	6.072643	-1.0901	0.278632	-6.11948	0.646764	0.54577
B.cells	NRARP	-0.40579	1.780032	-1.08992	0.278709	-5.15669	0.692513	0.61507
B.cells	UBE2C	-0.30185	6.746366	-1.08985	0.278742	-6.17007	0.63991	0.535711
B.cells	CDK20	0.549444	0.593617	1.089663	0.278823	-4.91192	0.705814	0.63581
B.cells	FAM20A	-0.45884	1.833508	-1.08956	0.278868	-5.12589	0.69192	0.614287

B.cells	RNF146	0.128907	5.30614	1.089459	0.278912	-5.97199	0.654666	0.557729
B.cells	PAF1	-0.13498	4.600235	-1.0894	0.27894	-5.82231	0.662045	0.568828
B.cells	ZFAND2B	0.15458	4.313639	1.089291	0.278986	-5.76946	0.665069	0.57344
B.cells	AVL9	0.117912	6.010486	1.089092	0.279073	-6.1487	0.6474	0.547123
B.cells	D330023K1	-0.36746	2.22594	-1.08897	0.279125	-5.1698	0.687589	0.607922
B.cells	BRAF	0.128729	7.588384	1.088934	0.279142	-6.36658	0.631466	0.523653
B.cells	HIST1H3E	-0.25466	3.610481	-1.08863	0.279278	-5.53041	0.672744	0.585076
B.cells	BUD13	0.167962	3.714639	1.08832	0.279412	-5.63011	0.671812	0.583414
B.cells	UROD	-0.15788	4.467162	-1.08807	0.279523	-5.83758	0.663849	0.571329
B.cells	H3F3A	-0.07836	10.71708	-1.08802	0.279542	-6.88777	0.601591	0.480139
B.cells	UBR5	0.104878	6.888097	1.087809	0.279636	-6.2485	0.638949	0.534157
B.cells	AGAP1	-0.30531	4.225875	-1.08745	0.279795	-5.4192	0.66673	0.575324
B.cells	MPLKIP	0.11896	4.793719	1.086897	0.280037	-5.85597	0.661173	0.566494
B.cells	PKP3	0.242248	2.747004	1.086721	0.280114	-5.59252	0.683095	0.599733
B.cells	HYAL2	-0.41019	1.859326	-1.08662	0.280156	-5.06981	0.69286	0.614772
B.cells	TIMP2	0.315713	4.873204	1.08628	0.280308	-5.63404	0.660433	0.565399
B.cells	AP1S1	0.158325	4.706161	1.086206	0.280341	-5.76531	0.662188	0.568073
B.cells	TOMM22	-0.09781	6.905252	-1.08602	0.280423	-6.25151	0.639519	0.534419
B.cells	KLHDC3	0.126272	4.338451	1.086018	0.280423	-5.7872	0.66607	0.574006
B.cells	ASRGL1	0.233935	3.315534	1.085744	0.280544	-5.56853	0.677162	0.590683
B.cells	PLAG1	0.209089	2.8948	1.08532	0.28073	-5.45414	0.682037	0.597805
B.cells	ELANE	-0.95611	2.340078	-1.08495	0.280895	-5.29498	0.68837	0.607256
B.cells	MGL2	-0.91058	-0.18973	-1.08453	0.281076	-4.91287	0.716898	0.651526
B.cells	STAP2	-0.59914	0.504981	-1.08452	0.281084	-4.8861	0.708937	0.639084
B.cells	HIST1H3I	-0.51774	1.296054	-1.08445	0.281112	-5.00676	0.699995	0.625199
B.cells	CYP7B1	-0.79775	0.01895	-1.0844	0.281135	-4.8337	0.714496	0.647788
B.cells	MEMO1	0.104261	6.363616	1.084065	0.281283	-6.20181	0.645803	0.542988
B.cells	ELMOD2	0.239741	2.807903	1.08406	0.281286	-5.40503	0.683351	0.599479
B.cells	ELDR	-0.48745	3.256101	-1.08333	0.281607	-5.15525	0.678844	0.59231
B.cells	IGTP	0.386686	3.443365	1.083325	0.28161	-5.50566	0.676819	0.589232
B.cells	WASL	0.102816	5.483088	1.083324	0.28161	-5.9899	0.655222	0.556709
B.cells	MAPK3	0.15252	4.874121	1.083063	0.281725	-5.83652	0.661639	0.566276
B.cells	HARS2	0.198428	2.848743	1.082913	0.281792	-5.40883	0.683328	0.59914
B.cells	MID1	-0.43685	5.805162	-1.08288	0.281807	-6.04139	0.651941	0.551816
B.cells	DGCR6	0.207501	3.650268	1.082554	0.28195	-5.50738	0.674851	0.586011
B.cells	MBTPS2	0.169463	3.917498	1.082144	0.282131	-5.68042	0.672172	0.581806
B.cells	RABAC1	0.111183	5.988734	1.082114	0.282145	-6.09809	0.650426	0.549216
B.cells	TNFRSF4	-0.56261	0.30724	-1.08166	0.282345	-4.98154	0.712153	0.643364
B.cells	2310039H(	0.174021	4.246315	1.081622	0.282362	-5.68897	0.668678	0.576619
B.cells	GZMK	0.640307	-1.47202	1.081614	0.282365	-4.78314	0.732619	0.675904
B.cells	GCNT7	-0.21665	2.712006	-1.08148	0.282425	-5.48573	0.685236	0.60182
B.cells	PDE5A	-0.34707	2.569114	-1.08143	0.282449	-5.31476	0.686802	0.604217
B.cells	FBXW11	-0.11909	7.259166	-1.08125	0.282527	-6.33009	0.637513	0.530356
B.cells	TMEM161E	0.135353	4.545354	1.081183	0.282556	-5.83045	0.665506	0.571986
B.cells	TGFBRAP1	0.168522	3.494101	1.080784	0.282732	-5.58237	0.676926	0.589111
B.cells	CLASP1	0.106241	6.373608	1.080742	0.282751	-6.17307	0.646673	0.543737
B.cells	COX6C	-0.08496	8.594079	-1.08037	0.282917	-6.52378	0.624643	0.511263

B.cells	DOCK8	0.11624	7.656272	1.080201	0.282991	-6.38898	0.633928	0.524868
B.cells	CCDC97	-0.19744	3.33761	-1.07988	0.283131	-5.49604	0.678848	0.592007
B.cells	H2AFX	-0.21164	6.482628	-1.07984	0.28315	-6.15063	0.645779	0.542381
B.cells	NAPEPLD	0.457662	1.056502	1.07984	0.28315	-5.02125	0.704074	0.630774
B.cells	CDC42EP2	0.348239	3.08028	1.07974	0.283194	-5.35277	0.681641	0.596292
B.cells	TMEM167E	0.201392	3.755594	1.079343	0.283371	-5.6073	0.674622	0.58528
B.cells	BAG5	0.215648	3.637068	1.078853	0.283588	-5.61553	0.676277	0.587374
B.cells	SETD1B	0.147208	5.026478	1.078579	0.283709	-5.85886	0.661644	0.565252
B.cells	TRAF3IP3	0.13	5.030914	1.078301	0.283832	-5.955	0.661749	0.565242
B.cells	TTPAL	0.14223	4.234118	1.077748	0.284078	-5.78643	0.670492	0.578073
B.cells	CDKL4	-0.43275	1.561956	-1.07753	0.284173	-5.13346	0.699717	0.62275
B.cells	GM16794	0.462327	0.41499	1.0775	0.284188	-4.95387	0.712711	0.642912
B.cells	GM43259	-0.51157	1.452797	-1.07748	0.284196	-5.03327	0.700942	0.624642
B.cells	HSPA9	-0.10537	6.483768	-1.07669	0.284548	-6.17938	0.64765	0.543313
B.cells	CFL2	0.156318	4.631141	1.076402	0.284676	-5.80909	0.66695	0.572211
B.cells	ABTB2	-0.18525	7.412469	-1.07629	0.284728	-6.36679	0.638224	0.529581
B.cells	PKN2	0.104266	6.774243	1.075989	0.284859	-6.26401	0.644684	0.539158
B.cells	SPTLC2	0.093259	6.245992	1.07595	0.284877	-6.15335	0.65009	0.547146
B.cells	KCNJ10	-0.52105	-0.1202	-1.07593	0.284884	-4.86042	0.7196	0.65313
B.cells	ESPN	-0.62957	-0.17599	-1.07586	0.284917	-4.86624	0.720246	0.65417
B.cells	SNX9	0.125321	6.331978	1.075787	0.28495	-6.14967	0.649206	0.545875
B.cells	ANKRD55	-0.48192	0.009583	-1.07549	0.285083	-4.97068	0.718282	0.650988
B.cells	MGRN1	0.134849	5.682428	1.075346	0.285146	-6.07259	0.656082	0.556009
B.cells	CHFR	0.105368	5.641975	1.075233	0.285196	-6.0694	0.656503	0.556651
B.cells	PGD	0.124014	5.716274	1.075048	0.285278	-6.05368	0.655786	0.555514
B.cells	FZD4	-0.46772	1.267832	-1.07432	0.285603	-4.99075	0.704634	0.628845
B.cells	CCDC6	0.153945	4.540905	1.073796	0.285836	-5.85497	0.669167	0.574296
B.cells	SLC38A10	0.124575	5.326136	1.073631	0.28591	-5.97092	0.660911	0.561917
B.cells	MAN2C1OS	0.157989	4.443794	1.073209	0.286098	-5.81436	0.670408	0.575997
B.cells	RNF130	0.102421	7.282781	1.072876	0.286247	-6.30544	0.640934	0.53239
B.cells	PPM1M	-0.14881	4.583696	-1.0728	0.286278	-5.84381	0.668919	0.57394
B.cells	NOSIP	0.116561	5.158105	1.072801	0.28628	-5.96786	0.662844	0.564835
B.cells	HIST1H2BE	-0.3918	2.079102	-1.07254	0.286397	-5.13118	0.696178	0.615484
B.cells	TMEM116	-0.34945	2.038927	-1.07233	0.286492	-5.25087	0.696626	0.616235
B.cells	GM14858	-0.3584	2.296719	-1.07226	0.286522	-5.34476	0.693759	0.611851
B.cells	EGR2	-0.4195	2.237621	-1.07212	0.286582	-5.30172	0.694415	0.612885
B.cells	ERGIC1	0.167019	5.084699	1.072105	0.28659	-5.89631	0.663617	0.566201
B.cells	RWDD3	-0.4812	0.507573	-1.0719	0.286681	-4.95796	0.71394	0.64307
B.cells	HIST2H2BB	-0.63321	-0.261	-1.07187	0.286693	-4.81329	0.722815	0.656935
B.cells	DCAF15	0.196762	3.456621	1.071871	0.286695	-5.59889	0.681028	0.59245
B.cells	LSM5	-0.13459	5.652829	-1.07176	0.286742	-6.02496	0.657664	0.557327
B.cells	FAM129B	-0.32825	3.14002	-1.0716	0.286816	-5.43389	0.684476	0.597737
B.cells	NBEAL1	0.148081	4.937374	1.071303	0.286949	-5.90095	0.665171	0.568697
B.cells	VCPIP1	0.09991	6.167501	1.071292	0.286954	-6.14197	0.652326	0.54955
B.cells	GORASP1	-0.50854	0.733344	-1.07117	0.287007	-4.97931	0.711356	0.639249
B.cells	MLYCD	0.217683	3.124459	1.071121	0.28703	-5.44679	0.684646	0.598147
B.cells	DYNLT1C	0.400288	1.100031	1.070808	0.28717	-5.0592	0.707368	0.632855

B.cells	NDFIP1	0.093185	7.521506	1.070621	0.287254	-6.3691	0.638693	0.529298
B.cells	EXTL3	0.190953	3.56619	1.070554	0.287284	-5.66903	0.680018	0.590908
B.cells	NRP1	-0.31564	4.442319	-1.07043	0.287338	-5.56655	0.670599	0.576672
B.cells	POLM	-0.1772	3.245007	-1.06966	0.287683	-5.59439	0.683775	0.596488
B.cells	LPP	0.133274	7.6885	1.069635	0.287695	-6.39604	0.637262	0.527092
B.cells	EPHA1	-0.62048	0.461905	-1.06955	0.287732	-4.87668	0.714928	0.644475
B.cells	GM10135	0.4971	0.409376	1.069507	0.287752	-4.95807	0.715531	0.645416
B.cells	LIPE	0.148738	3.783042	1.069324	0.287834	-5.78864	0.677935	0.587668
B.cells	DTYMK	-0.17884	5.126752	-1.06931	0.287841	-5.88625	0.663605	0.566092
B.cells	MIA2	0.086967	6.77012	1.069033	0.287965	-6.26943	0.646561	0.540846
B.cells	TSPYL4	0.431668	0.723815	1.069013	0.287974	-4.96067	0.711927	0.63993
B.cells	CNOT6L	0.105183	7.08541	1.068616	0.288152	-6.31246	0.64335	0.53615
B.cells	AP1S3	-0.14718	5.57812	-1.06842	0.28824	-6.14928	0.658871	0.559192
B.cells	USP37	0.118933	6.268931	1.068293	0.288296	-6.14276	0.651704	0.54856
B.cells	XPOT	0.149189	4.512589	1.068261	0.288311	-5.83173	0.67011	0.576066
B.cells	FAM171A1	-0.49541	1.565475	-1.06803	0.288414	-4.99044	0.702383	0.625334
B.cells	RGS7BP	-0.55765	1.883105	-1.06801	0.288423	-5.09974	0.698819	0.619837
B.cells	GLG1	0.103242	6.951102	1.067998	0.288429	-6.2852	0.644716	0.538296
B.cells	GM11998	0.549108	0.318253	1.067985	0.288435	-4.94744	0.71658	0.647379
B.cells	D830050J1	-0.40731	1.312085	-1.06797	0.28844	-5.09203	0.705241	0.629753
B.cells	RNF111	0.104032	6.692893	1.067742	0.288544	-6.24479	0.647453	0.5423
B.cells	CREBL2	0.225344	3.233581	1.067353	0.288718	-5.50968	0.684283	0.597263
B.cells	KRI1	0.136343	4.367396	1.066776	0.288977	-5.79168	0.672467	0.57889
B.cells	TLE1	-0.16568	4.060562	-1.06668	0.28902	-5.68355	0.675758	0.583907
B.cells	PIEZO1	0.131089	5.046187	1.066276	0.289202	-6.02711	0.665303	0.568206
B.cells	ADGRL2	-0.30958	4.208198	-1.06619	0.28924	-5.58058	0.674223	0.581636
B.cells	NUDT8	0.302984	2.208161	1.066137	0.289265	-5.25646	0.696082	0.61492
B.cells	ITPKC	0.356804	1.763821	1.066113	0.289275	-5.21307	0.70105	0.622564
B.cells	PDE4C	-0.45282	3.131277	-1.06586	0.289391	-5.43648	0.685915	0.599371
B.cells	SH3BP2	0.252474	3.288629	1.065832	0.289401	-5.5438	0.684195	0.59676
B.cells	CEP290	0.284511	2.468766	1.065665	0.289477	-5.24848	0.693251	0.610587
B.cells	CRLS1	-0.16802	3.622878	-1.06551	0.289546	-5.61652	0.680616	0.591395
B.cells	DPY19L4	0.149883	4.417697	1.065245	0.289665	-5.78254	0.672059	0.578592
B.cells	ATP2A2	-0.09103	6.456942	-1.06525	0.289666	-6.19517	0.650676	0.546659
B.cells	HBP1	-0.12213	5.788072	-1.06513	0.289717	-6.08983	0.6576	0.556957
B.cells	BMP2	-0.51999	1.613957	-1.06498	0.289783	-4.99334	0.702834	0.625631
B.cells	SLAMF1	0.407406	0.884468	1.064739	0.289893	-5.2005	0.71123	0.638552
B.cells	CHAC2	0.187138	2.893423	1.064115	0.290174	-5.54646	0.688778	0.604242
B.cells	IFFO2	-0.27515	3.364648	-1.06402	0.290217	-5.37423	0.683618	0.596393
B.cells	TMEM132E	-0.55876	0.329919	-1.06399	0.290228	-4.92795	0.71765	0.648888
B.cells	1810062O1	0.322261	2.0989	1.063958	0.290245	-5.24318	0.697581	0.617765
B.cells	RFNG	0.270442	2.228203	1.063906	0.290268	-5.27007	0.696139	0.615548
B.cells	LEKR1	0.534223	1.047991	1.063624	0.290395	-5.00257	0.709424	0.636183
B.cells	PEX11B	0.198458	3.446594	1.063443	0.290477	-5.54621	0.682726	0.595153
B.cells	CISD3	0.241926	3.000972	1.063334	0.290526	-5.49805	0.687596	0.602616
B.cells	ORAI3	0.191297	3.868765	1.063179	0.290596	-5.65615	0.678148	0.588287
B.cells	HSPD1	-0.13232	7.46114	-1.06284	0.290749	-6.36769	0.640624	0.532357

B.cells	GM36738	-0.27353	3.095803	-1.06284	0.290751	-5.35567	0.686557	0.601142
B.cells	FOXP1	0.073525	9.650428	1.062794	0.29077	-6.76126	0.618963	0.500908
B.cells	TSC22D2	0.097527	6.616128	1.062685	0.290819	-6.24258	0.649226	0.545047
B.cells	NAF1	0.169678	3.658508	1.062643	0.290838	-5.64078	0.680424	0.591829
B.cells	DAG1	0.156711	4.867236	1.062553	0.290878	-5.88764	0.667465	0.572275
B.cells	NAIP6	-0.3247	2.310523	-1.06252	0.290893	-5.22604	0.695223	0.614479
B.cells	CEP192	0.130008	5.27108	1.062486	0.290909	-5.96188	0.6632	0.565883
B.cells	TRIM24	0.137962	5.087563	1.062342	0.290974	-5.92911	0.66515	0.568782
B.cells	TMEM144	-0.5017	0.882973	-1.0619	0.291175	-5.01705	0.711404	0.63957
B.cells	TAMM41	0.257572	2.732365	1.061706	0.291261	-5.4136	0.690648	0.607589
B.cells	ARHGEF6	0.128631	5.643537	1.061418	0.291391	-6.03663	0.659387	0.560399
B.cells	TMEM242	-0.14168	4.292008	-1.06127	0.291457	-5.76076	0.673689	0.581886
B.cells	MON2	0.113377	5.7408	1.061242	0.29147	-6.07639	0.658372	0.558883
B.cells	CDC42BPA	-0.266	3.315927	-1.06122	0.291481	-5.41542	0.684245	0.597909
B.cells	CACNA2D1	-0.55507	0.864423	-1.06103	0.291566	-5.05059	0.711616	0.640122
B.cells	USP45	0.161672	4.074097	1.060906	0.291622	-5.79372	0.676029	0.585507
B.cells	1110004F1	0.078972	6.204	1.060717	0.291707	-6.14713	0.653561	0.551907
B.cells	GM6225	0.228897	2.958455	1.060612	0.291755	-5.59915	0.688159	0.604089
B.cells	ACAD10	-0.38693	1.501708	-1.0606	0.29176	-5.11034	0.704381	0.629062
B.cells	SELENOF	-0.13791	5.422799	-1.06054	0.29179	-5.96848	0.661698	0.564074
B.cells	SRF	-0.18272	2.927184	-1.06046	0.291822	-5.45637	0.688503	0.604661
B.cells	COPZ2	-0.32958	2.183281	-1.0603	0.291897	-5.20339	0.696737	0.617343
B.cells	CEP170	0.119194	5.909007	1.060281	0.291905	-6.13098	0.65662	0.556539
B.cells	F8A	0.238324	2.551871	1.0602	0.291941	-5.4537	0.692643	0.611047
B.cells	D130043K2	0.481148	1.054045	1.059847	0.292101	-5.00359	0.70954	0.637154
B.cells	CADM4	0.370187	1.741687	1.059721	0.292158	-5.28815	0.701763	0.625101
B.cells	ING3	-0.12316	4.830612	-1.05966	0.292185	-5.90008	0.668027	0.573621
B.cells	RASIP1	-0.43221	1.808725	-1.05951	0.292254	-5.10266	0.70101	0.623957
B.cells	RIC1	0.130985	6.710291	1.05891	0.292525	-6.3121	0.648429	0.54472
B.cells	SLC23A2	0.140426	5.113974	1.058552	0.292687	-5.99061	0.665028	0.569607
B.cells	PCYOX1L	-0.34916	1.613818	-1.05848	0.292719	-5.15779	0.703202	0.627898
B.cells	ABI2	-0.21363	3.903545	-1.05844	0.29274	-5.61673	0.677949	0.589171
B.cells	VGLL4	-0.10713	6.477378	-1.05838	0.292765	-6.24558	0.65082	0.548454
B.cells	OLFR920	-0.55794	0.137933	-1.05837	0.292768	-4.92382	0.720055	0.65422
B.cells	7-Mar	0.078363	6.926226	1.058308	0.292798	-6.30017	0.646221	0.541663
B.cells	SLC45A4	0.202721	3.337998	1.058234	0.292831	-5.49181	0.684087	0.598562
B.cells	PLOD2	0.559065	1.261605	1.058212	0.292842	-4.96993	0.707182	0.634141
B.cells	A730081D(	0.247613	3.129627	1.058142	0.292873	-5.52365	0.686365	0.602043
B.cells	GM34961	0.480899	0.869528	1.058104	0.29289	-5.03198	0.711643	0.641087
B.cells	AOPEP	0.111701	6.329542	1.057975	0.292949	-6.17624	0.652343	0.550736
B.cells	MAP3K7	0.1089	5.219048	1.057914	0.292977	-5.99855	0.66392	0.568048
B.cells	PPP6C	-0.08041	6.737344	-1.05768	0.293081	-6.26407	0.648152	0.54462
B.cells	MKNK2	-0.1032	6.23257	-1.05767	0.293085	-6.17381	0.653345	0.552327
B.cells	ZFP445	0.123606	4.93802	1.057372	0.293222	-5.91066	0.667067	0.572636
B.cells	ANKHD1	-0.09456	7.647791	-1.05721	0.293294	-6.40326	0.639109	0.531076
B.cells	BATF2	0.604199	0.948583	1.056907	0.293433	-5.00309	0.711161	0.640068
B.cells	STK17B	0.09873	8.129238	1.056624	0.293562	-6.4738	0.634611	0.524177

B.cells	EIF4E3	0.1733	4.420461	1.056008	0.293842	-5.80604	0.673458	0.581304
B.cells	MTHFD1	-0.18495	3.79072	-1.05588	0.293899	-5.66609	0.680242	0.591595
B.cells	GM31462	0.488997	0.152102	1.055549	0.29405	-4.97171	0.721114	0.654714
B.cells	HDAC4	0.161471	5.402881	1.055533	0.294057	-5.97615	0.663113	0.565758
B.cells	OLFR56	0.466618	2.067818	1.055418	0.29411	-5.17697	0.699296	0.620846
B.cells	GPR27	-0.43157	-1.60473	-1.05508	0.294262	-4.78978	0.741832	0.687588
B.cells	SCIMP	-0.25029	3.702449	-1.05492	0.294336	-5.69268	0.681514	0.593369
B.cells	TMEM39A	-0.13851	4.395391	-1.05481	0.294386	-5.84996	0.674038	0.58206
B.cells	ZFP362	0.160859	3.9339	1.05449	0.294532	-5.68723	0.679179	0.589658
B.cells	PISD	0.147126	4.8584	1.054389	0.294578	-5.94768	0.669267	0.574714
B.cells	TRIP4	0.178546	4.852518	1.054163	0.294681	-5.86979	0.669431	0.574856
B.cells	SHMT1	-0.22168	4.012421	-1.05395	0.294777	-5.64243	0.678521	0.588485
B.cells	SNRPB	-0.09558	7.300883	-1.05365	0.294916	-6.34184	0.64409	0.53717
B.cells	IGFBP1	0.378259	4.147259	1.053476	0.294993	-5.8402	0.677088	0.586518
B.cells	RNF167	-0.1363	5.334087	-1.05346	0.295	-6.00485	0.664441	0.567457
B.cells	SCO1	-0.34208	1.930574	-1.05342	0.295021	-5.20342	0.701472	0.623828
B.cells	CTNNBIP1	-0.19088	4.359655	-1.05326	0.295094	-5.66857	0.674836	0.58309
B.cells	GCNT2	-0.30851	4.580489	-1.05285	0.295279	-5.50223	0.672671	0.579629
B.cells	SGPL1	0.124168	5.922758	1.052807	0.295298	-6.13012	0.658498	0.558392
B.cells	4933432I0:	0.544514	0.408312	1.052644	0.295372	-4.96297	0.719091	0.650987
B.cells	ABR	0.226883	6.594873	1.05239	0.295488	-6.01359	0.651676	0.548131
B.cells	TREM2	-0.66959	0.639882	-1.05227	0.295543	-5.03973	0.716541	0.646973
B.cells	ALPK2	-0.31633	1.260876	-1.05208	0.295632	-5.30145	0.709437	0.636013
B.cells	TYROBP	-0.15944	9.095091	-1.05202	0.295656	-6.48743	0.62652	0.511448
B.cells	SUFU	0.122553	5.025085	1.051853	0.295733	-5.97834	0.668117	0.572894
B.cells	TBPL1	-0.11839	4.928003	-1.05163	0.295834	-5.96213	0.669147	0.574445
B.cells	STX16	0.123161	5.756821	1.051606	0.295846	-6.09657	0.660409	0.561341
B.cells	STK38	0.103829	6.531625	1.051371	0.295953	-6.26075	0.652469	0.549362
B.cells	FITM2	-0.39028	0.918829	-1.05111	0.296074	-5.00393	0.713642	0.642258
B.cells	FBXL20	0.16431	5.495646	1.050943	0.296149	-6.08329	0.663432	0.565527
B.cells	GM20732	0.137995	4.58993	1.05069	0.296264	-5.84811	0.673168	0.580113
B.cells	FAM189B	-0.31137	2.569109	-1.05053	0.296336	-5.33529	0.695234	0.61375
B.cells	PRDX2	0.121624	7.774205	1.050373	0.296409	-6.52389	0.640153	0.530991
B.cells	SOX12	-0.5173	0.585357	-1.05	0.296578	-4.91391	0.717983	0.64862
B.cells	COPS9	0.108297	6.68605	1.049822	0.296661	-6.20666	0.651532	0.547414
B.cells	TMUB2	-0.20288	3.181282	-1.04953	0.296796	-5.51428	0.688995	0.603647
B.cells	CNPY3	0.09921	5.300686	1.049074	0.297003	-6.05068	0.666484	0.569187
B.cells	MYLK	-0.41669	1.606214	-1.04882	0.297118	-5.11959	0.707005	0.630875
B.cells	TM2D1	0.094429	5.944576	1.048588	0.297226	-6.1448	0.659799	0.559219
B.cells	TBC1D10B	0.127503	5.147294	1.048427	0.297299	-5.96646	0.66819	0.571836
B.cells	TRPV4	-0.62688	-0.51202	-1.0484	0.297313	-4.83506	0.731431	0.669291
B.cells	ACTG1	-0.11304	11.44474	-1.04835	0.297333	-6.99651	0.605197	0.479952
B.cells	CAR1	0.721303	0.491473	1.048083	0.297457	-4.96756	0.719909	0.651062
B.cells	TSNAX	-0.1446	4.49606	-1.04782	0.297578	-5.84679	0.675277	0.582578
B.cells	TPPP3	0.457401	1.071021	1.047729	0.297619	-5.19048	0.713243	0.640855
B.cells	MAFF	0.236062	3.876198	1.047533	0.297709	-5.68656	0.68197	0.592857
B.cells	GM11523	-0.7853	-0.95948	-1.04747	0.29774	-4.81811	0.736661	0.678139

B.cells	MAML2	0.134044	7.972039	1.047451	0.297747	-6.44481	0.639147	0.528989
B.cells	AA467197	0.693666	0.801131	1.047233	0.297847	-5.02622	0.716438	0.645908
B.cells	RITA1	-0.32313	1.367418	-1.04699	0.29796	-5.15037	0.709988	0.635943
B.cells	2900093K2	-0.25269	2.884005	-1.04683	0.298031	-5.42546	0.692969	0.609735
B.cells	ZC3H14	0.102422	5.656337	1.046666	0.298106	-6.05653	0.663072	0.564499
B.cells	ZFP944	-0.1523	4.397621	-1.04657	0.29815	-5.8477	0.676455	0.584679
B.cells	WDR43	0.114195	6.033494	1.046568	0.298152	-6.16834	0.659123	0.558649
B.cells	PIP4P1	0.086919	5.856707	1.046018	0.298404	-6.14085	0.661401	0.561556
B.cells	ARHGAP22	-0.47663	1.023886	-1.0458	0.298504	-5.17305	0.714472	0.642406
B.cells	N4BP2L1	0.151265	4.595001	1.045596	0.298598	-5.94761	0.674946	0.581753
B.cells	IGIP	-0.51742	0.675003	-1.04543	0.298674	-4.93914	0.718577	0.648851
B.cells	SLC7A6	0.139731	4.274758	1.045325	0.298722	-5.85733	0.6784	0.587073
B.cells	COX17	0.103059	6.919214	1.045204	0.298778	-6.2724	0.650555	0.545438
B.cells	ELK3	-0.13624	5.288608	-1.04484	0.298946	-6.04237	0.66777	0.570829
B.cells	GM38134	0.37665	1.108316	1.044597	0.299057	-5.17477	0.713821	0.641264
B.cells	TCF12	0.102091	8.463403	1.044593	0.299059	-6.5148	0.63512	0.522576
B.cells	EGF	0.468544	0.634522	1.04449	0.299106	-4.94392	0.719269	0.649785
B.cells	TMED5	0.074505	7.227678	1.044008	0.299328	-6.35644	0.64768	0.540933
B.cells	IRGM1	0.277258	4.93997	1.043929	0.299364	-5.92055	0.67156	0.576506
B.cells	SHMT2	-0.18965	4.430904	-1.04373	0.299454	-5.81066	0.677014	0.584735
B.cells	KDM6B	-0.1144	8.152763	-1.04369	0.299473	-6.53111	0.63831	0.527184
B.cells	CPEB2	-0.13547	5.412476	-1.04354	0.299541	-5.9894	0.666544	0.568973
B.cells	DYNLRB1	-0.09725	6.280147	-1.04348	0.299572	-6.18233	0.657447	0.555393
B.cells	SPECC1L	0.13321	5.413581	1.043343	0.299634	-6.13812	0.666532	0.568956
B.cells	IL1R2	0.369622	3.320344	1.04321	0.299695	-5.61334	0.689094	0.603091
B.cells	GM44174	0.567131	-0.56348	1.043137	0.299729	-4.91862	0.733285	0.671817
B.cells	ADORA2B	0.490127	0.37813	1.043131	0.299732	-5.06842	0.722328	0.654491
B.cells	SIN3B	0.090984	6.294475	1.04271	0.299926	-6.17067	0.65752	0.555279
B.cells	ZGRF1	-0.2729	3.956437	-1.04266	0.29995	-5.63083	0.682375	0.592638
B.cells	GM31763	-0.23654	3.257819	-1.0422	0.300162	-5.62901	0.690366	0.604452
B.cells	PHLDB2	-0.3776	2.509693	-1.04198	0.300263	-5.28225	0.698752	0.617207
B.cells	AURKAIP1	0.109506	5.837172	1.041858	0.300318	-6.12276	0.662718	0.562665
B.cells	PSMC3	-0.09998	6.11628	-1.04161	0.300431	-6.15304	0.659893	0.558419
B.cells	ZFP398	0.170425	4.517006	1.041507	0.30048	-5.8489	0.676847	0.583862
B.cells	PTGS2OS	0.667864	-1.09526	1.04109	0.300672	-4.87971	0.740404	0.682566
B.cells	PITPNC1	0.126943	8.799744	1.041078	0.300678	-6.549	0.632731	0.518365
B.cells	PLEC	0.140441	4.805597	1.04084	0.300788	-5.96085	0.673971	0.57939
B.cells	LETMD1	0.218591	2.91201	1.040702	0.300851	-5.46045	0.694605	0.610782
B.cells	STAB1	-0.3687	3.176342	-1.04066	0.300873	-5.39977	0.691681	0.606306
B.cells	WEE1	-0.21951	3.947727	-1.04034	0.301017	-5.61557	0.683369	0.593563
B.cells	HASPIN	0.330944	2.299155	1.040268	0.301052	-5.23728	0.701584	0.621431
B.cells	BCL2A1D	0.254238	4.122114	1.040057	0.301149	-5.93171	0.681534	0.590754
B.cells	N6AMT1	0.269682	2.597729	1.039944	0.301202	-5.34449	0.698304	0.616412
B.cells	RNGTT	0.092756	6.852257	1.039829	0.301255	-6.3046	0.652664	0.547608
B.cells	BGN	-0.34175	2.420901	-1.03934	0.301482	-5.25053	0.700672	0.619712
B.cells	NKIRAS1	0.197968	3.432264	1.039133	0.301576	-5.60096	0.68953	0.602519
B.cells	0610040F0	-0.47903	0.269406	-1.03888	0.301692	-4.92562	0.725466	0.657949

B.cells	FBXO48	0.548256	0.24175	1.038677	0.301787	-4.87278	0.725789	0.658545
B.cells	SOD3	-0.47513	1.286857	-1.03864	0.301805	-5.0828	0.713715	0.639716
B.cells	SLC44A1	0.171846	4.637679	1.038406	0.301913	-5.81201	0.676651	0.582926
B.cells	POP4	-0.17889	4.085123	-1.03815	0.30203	-5.7437	0.682755	0.591971
B.cells	CYB5RL	-0.50902	0.380261	-1.03792	0.302138	-4.90395	0.724551	0.656204
B.cells	BOLA1	0.205228	3.767483	1.037608	0.302282	-5.6104	0.686522	0.597227
B.cells	ERBIN	-0.09812	7.975801	-1.03738	0.302386	-6.46974	0.642292	0.531249
B.cells	MAPK14	0.092609	6.656022	1.037232	0.302456	-6.27042	0.6558	0.551202
B.cells	AP3S1	-0.09464	7.096627	-1.03697	0.302578	-6.33783	0.651253	0.544565
B.cells	ZFP287	-0.48558	0.566909	-1.03695	0.302585	-5.03102	0.722618	0.653053
B.cells	GPRC5C	-0.56835	1.29284	-1.03694	0.302591	-5.04601	0.71425	0.640015
B.cells	ZFAT	-0.2031	3.828322	-1.03673	0.30269	-5.66039	0.685917	0.596472
B.cells	LUC7L3	-0.08369	6.706417	-1.03663	0.302734	-6.26987	0.655304	0.550545
B.cells	4930455G(	-0.40039	1.816178	-1.03639	0.302848	-5.20264	0.708315	0.630952
B.cells	ZFP207	-0.07791	6.934778	-1.03621	0.302931	-6.31536	0.652945	0.547241
B.cells	ZFYVE16	0.218192	3.016629	1.036162	0.302952	-5.49912	0.694852	0.610304
B.cells	SLC35E2	0.147635	3.898481	1.036152	0.302957	-5.73314	0.685151	0.595509
B.cells	ADK	-0.15545	6.968234	-1.03535	0.30333	-6.27753	0.653276	0.546937
B.cells	ADCY10	-0.49998	0.912909	-1.03518	0.30341	-5.01207	0.719389	0.647306
B.cells	F830016B0	-0.71677	0.444299	-1.03506	0.303463	-4.91237	0.724821	0.655785
B.cells	ZFP652OS	-0.54753	-0.171	-1.03489	0.303541	-4.90231	0.732026	0.667132
B.cells	TBC1D22B	0.166872	3.778303	1.03484	0.303566	-5.72314	0.687174	0.59781
B.cells	POLR2B	-0.12012	4.911643	-1.03457	0.303693	-5.94199	0.675051	0.579328
B.cells	ZC3H8	0.270976	2.13952	1.034421	0.303761	-5.29999	0.705567	0.625803
B.cells	CAPZA1	-0.06695	7.715586	-1.03423	0.303848	-6.44123	0.645845	0.535882
B.cells	GM16536	-0.33661	1.530823	-1.03401	0.303951	-5.13078	0.712594	0.636555
B.cells	RNF43	0.372942	1.563577	1.033932	0.303988	-5.29133	0.712221	0.63602
B.cells	CANX	-0.07999	7.35016	-1.03329	0.304287	-6.35783	0.650136	0.541652
B.cells	WASHC2	-0.13635	5.655214	-1.03314	0.304357	-6.08387	0.667815	0.567867
B.cells	RAB43	-0.12347	6.948549	-1.03275	0.304538	-6.33964	0.654554	0.54785
B.cells	RPGRIP1	-0.10907	6.55945	-1.03235	0.304725	-6.20456	0.658715	0.5539
B.cells	USP42	0.167921	3.500066	1.032102	0.30484	-5.61141	0.691494	0.603165
B.cells	ALKBH4	-0.24885	2.574916	-1.03189	0.304938	-5.37508	0.701781	0.61893
B.cells	PNKP	0.151053	4.959191	1.031883	0.304942	-5.94841	0.675627	0.579195
B.cells	CEP135	0.142654	3.853066	1.031844	0.30496	-5.7133	0.687616	0.597328
B.cells	THAP3	0.164041	3.799217	1.031707	0.305023	-5.73241	0.688206	0.598242
B.cells	SDAD1	0.1315	4.473042	1.0316	0.305073	-6.00042	0.680866	0.587196
B.cells	GM16556	-0.34244	1.676001	-1.0312	0.305259	-5.32672	0.711946	0.634857
B.cells	EPN2	-0.49864	1.664749	-1.03119	0.305265	-5.08724	0.712074	0.635055
B.cells	NAA50	-0.09182	6.371464	-1.03085	0.305424	-6.20544	0.660675	0.557248
B.cells	TNFRSF18	-0.25403	2.322619	-1.03081	0.30544	-5.39438	0.704617	0.623719
B.cells	SMCO4	-0.1786	4.10439	-1.03069	0.305497	-5.77474	0.68487	0.593552
B.cells	SNW1	0.083153	6.240739	1.030677	0.305503	-6.20289	0.662043	0.55928
B.cells	IFT52	0.134442	4.249963	1.030328	0.305667	-5.78915	0.683286	0.591252
B.cells	SLC35E3	-0.36176	1.909396	-1.03014	0.305755	-5.18279	0.70929	0.631075
B.cells	NUFIP2	-0.0983	7.215419	-1.03011	0.305769	-6.35133	0.651927	0.544442
B.cells	NRN1	-0.32991	3.109984	-1.03001	0.305813	-5.56961	0.69581	0.610374

B.cells	NUBPL	-0.23544	2.913085	-1.02976	0.30593	-5.40755	0.698	0.613822
B.cells	RALY	-0.10332	6.422986	-1.02974	0.30594	-6.20945	0.660137	0.556688
B.cells	PPIL6	-0.55792	0.219778	-1.02973	0.305944	-4.91013	0.728776	0.661533
B.cells	CAGE1	0.368637	1.94977	1.02957	0.30602	-5.24332	0.708832	0.630494
B.cells	SMIM41	0.482247	0.89661	1.029568	0.306021	-4.99826	0.720897	0.64922
B.cells	S100A13	-0.1142	6.040644	-1.02948	0.306062	-6.1451	0.664142	0.56265
B.cells	MFSD13A	-0.34132	2.008416	-1.02917	0.306205	-5.21319	0.708167	0.629537
B.cells	PBK	-0.32364	3.69023	-1.02914	0.30622	-5.48528	0.689402	0.600763
B.cells	BPNT1	-0.18123	3.339718	-1.02912	0.306229	-5.58581	0.693265	0.606651
B.cells	TSC22D3	0.173036	5.71665	1.028954	0.306308	-6.11741	0.667559	0.567847
B.cells	ILF3	-0.10536	6.541502	-1.02888	0.306342	-6.2387	0.658901	0.554983
B.cells	GLCE	-0.17251	3.79856	-1.02884	0.306359	-5.72982	0.688213	0.599031
B.cells	PITPNB	0.099133	5.507244	1.028793	0.306383	-6.0677	0.669778	0.571209
B.cells	ATP5F1	-0.08459	8.084387	-1.02869	0.306429	-6.4887	0.643063	0.531702
B.cells	IDH3B	0.122028	5.850654	1.02803	0.30674	-6.15659	0.666644	0.566093
B.cells	FNIP1	0.107391	7.760209	1.027844	0.306827	-6.45695	0.646839	0.536867
B.cells	MTDH	0.065056	7.470279	1.027729	0.306881	-6.40553	0.649801	0.541288
B.cells	CTDSPL	-0.30717	3.831098	-1.02761	0.306936	-5.53002	0.688374	0.599079
B.cells	EIF4A3	-0.1075	5.877476	-1.02756	0.306958	-6.1174	0.666361	0.565934
B.cells	ARMH2	-0.53378	0.301427	-1.02734	0.307062	-4.97733	0.728368	0.661015
B.cells	5830408C2	-0.23419	2.989299	-1.02731	0.307075	-5.39615	0.697676	0.613432
B.cells	SLC39A10	-0.16481	3.903206	-1.02709	0.30718	-5.73016	0.687584	0.598088
B.cells	RECQL4	-0.59661	-0.06717	-1.02695	0.307245	-4.82982	0.732696	0.667947
B.cells	KIDINS220	0.117526	5.419693	1.026778	0.307326	-6.02711	0.671213	0.573532
B.cells	MMP9	0.588945	1.381978	1.026729	0.307349	-5.07441	0.715846	0.641733
B.cells	FIS1	0.092193	7.530663	1.026626	0.307397	-6.43253	0.649183	0.540843
B.cells	ZFP358	0.215898	3.044691	1.026547	0.307434	-5.55046	0.69706	0.612811
B.cells	PRIMPOL	0.208463	3.486578	1.026431	0.307489	-5.56662	0.692164	0.605355
B.cells	LMBR1	-0.3633	1.883516	-1.02603	0.307675	-5.12763	0.710202	0.633028
B.cells	GINM1	0.111089	4.990227	1.025955	0.307712	-5.98499	0.675882	0.580609
B.cells	GPR174	0.272013	2.267506	1.025696	0.307833	-5.46034	0.705852	0.626442
B.cells	PDLIM4	-0.4643	2.040162	-1.02569	0.307838	-5.20398	0.708424	0.630415
B.cells	SLC38A6	0.182983	4.233853	1.025678	0.307842	-5.76819	0.684056	0.593089
B.cells	4932438A1	0.134567	7.087671	1.025495	0.307927	-6.30768	0.653811	0.547841
B.cells	LAX1	0.268741	2.319766	1.02538	0.307981	-5.51958	0.705262	0.625594
B.cells	CYSTM1	-0.31783	3.36709	-1.02534	0.307999	-5.63722	0.693565	0.60763
B.cells	MAP4K2	0.154364	5.348887	1.025151	0.308089	-5.97079	0.672096	0.575085
B.cells	KIF18A	-0.2288	3.977118	-1.02504	0.30814	-5.67559	0.686907	0.597521
B.cells	COL5A1	-0.57187	0.493409	-1.02439	0.308445	-4.94602	0.726844	0.658617
B.cells	SDHC	0.12785	5.09505	1.024227	0.308522	-5.94715	0.675386	0.579555
B.cells	GM20712	-0.65747	0.063822	-1.02401	0.308624	-4.9151	0.731931	0.666674
B.cells	SMIM26	-0.17001	3.847983	-1.0235	0.308864	-5.67689	0.68892	0.600399
B.cells	RIT1	0.154838	3.965996	1.023391	0.308915	-5.70963	0.687627	0.598465
B.cells	IMPACT	0.136382	5.540939	1.023239	0.308986	-6.0712	0.670636	0.572801
B.cells	BAD	0.173291	3.912661	1.02319	0.30901	-5.70839	0.688211	0.599379
B.cells	A2ML1	-0.34222	2.471948	-1.02314	0.309034	-5.42245	0.704213	0.623893
B.cells	DNAJB11	-0.09967	6.073023	-1.02305	0.309073	-6.15892	0.665007	0.564394

B.cells	GM14966	0.20786	2.847703	1.022989	0.309104	-5.47531	0.699998	0.617414
B.cells	SOCS5	0.185456	4.029298	1.022954	0.309121	-5.79726	0.686934	0.597449
B.cells	GPS2	-0.08565	5.859455	-1.0228	0.309191	-6.14012	0.66726	0.567827
B.cells	EID2	0.588557	0.075956	1.022662	0.309258	-4.88785	0.731789	0.666967
B.cells	PDLIM1	-0.14917	4.944927	-1.02261	0.30928	-6.0119	0.677007	0.582521
B.cells	GAS2	0.260191	2.456745	1.022341	0.309409	-5.35431	0.704384	0.624334
B.cells	PLK4	-0.24224	3.856529	-1.02233	0.309412	-5.6409	0.688827	0.600486
B.cells	TRIM68	0.431637	0.946883	1.022134	0.309506	-5.00333	0.721624	0.651095
B.cells	SDE2	-0.12155	6.013145	-1.02209	0.309528	-6.17699	0.665638	0.565496
B.cells	S100A8	0.446495	6.633628	1.022075	0.309534	-6.22206	0.659137	0.555811
B.cells	JADE1	0.208408	4.165478	1.021905	0.309614	-5.65741	0.685493	0.595375
B.cells	GTF2A2	0.101536	5.923594	1.021691	0.309715	-6.14596	0.666695	0.566983
B.cells	SLC23A1	0.298844	1.528872	1.021542	0.309785	-5.30039	0.715043	0.640814
B.cells	BAIAP2L1	-0.20145	3.098445	-1.02146	0.309823	-5.60515	0.69732	0.613463
B.cells	HEXA	-0.12475	5.752202	-1.02123	0.309932	-6.15893	0.668615	0.569869
B.cells	NOP56	0.139733	4.591451	1.020913	0.310081	-5.87979	0.681221	0.588723
B.cells	GPSM3	0.111566	6.022347	1.020803	0.310133	-6.17231	0.665934	0.565765
B.cells	PLEKHM1	0.128998	4.771883	1.02058	0.310238	-5.93423	0.679271	0.585861
B.cells	PDE12	-0.14313	3.991564	-1.02055	0.31025	-5.73735	0.687753	0.598726
B.cells	LAMTOR1	0.088425	6.503458	1.020345	0.310349	-6.26533	0.660969	0.55833
B.cells	UBXN2A	0.112093	5.233503	1.02011	0.310459	-6.03565	0.674508	0.578516
B.cells	KRT83	0.593347	-0.26141	1.019788	0.310611	-4.9161	0.736499	0.674185
B.cells	SNHG3	0.158135	5.291632	1.019772	0.310619	-6.01716	0.673959	0.577724
B.cells	PLEKHG1	-0.21856	4.398533	-1.01966	0.310671	-5.76566	0.683587	0.592351
B.cells	EIF3A	-0.07605	7.379667	-1.01918	0.310896	-6.39296	0.652076	0.545332
B.cells	PYCR2	0.206751	3.687049	1.019133	0.31092	-5.58994	0.691383	0.604393
B.cells	GM29666	0.608565	0.244861	1.018862	0.311048	-4.92824	0.730539	0.665178
B.cells	SIPA1L1	0.142499	7.327522	1.018752	0.3111	-6.42873	0.652613	0.546217
B.cells	CHML	-0.30109	1.690018	-1.01864	0.311151	-5.2935	0.713796	0.639003
B.cells	GM20139	0.531899	-0.63311	1.01864	0.311153	-4.87308	0.740826	0.68158
B.cells	HSCB	0.170213	4.143832	1.018555	0.311193	-5.82572	0.686373	0.596839
B.cells	ERLEC1	0.153639	4.341715	1.01855	0.311196	-5.8252	0.684215	0.59356
B.cells	CDIPT	-0.13094	5.093616	-1.01841	0.31126	-5.98358	0.67609	0.581312
B.cells	PRKN	-0.34464	3.428362	-1.01826	0.311332	-5.42024	0.69424	0.608891
B.cells	SOCS7	0.142582	4.207792	1.018217	0.311353	-5.83518	0.685675	0.595825
B.cells	FIGNL1	-0.31696	2.757262	-1.01815	0.311387	-5.36076	0.701715	0.620371
B.cells	PSMG3	0.207933	3.202758	1.017776	0.311561	-5.58684	0.697	0.61287
B.cells	IL1B	0.455009	5.12575	1.017486	0.311698	-5.68151	0.676082	0.581082
B.cells	GM16153	-0.25486	2.536009	-1.01681	0.312017	-5.46782	0.704551	0.624844
B.cells	NTN4	0.628689	0.706687	1.016751	0.312046	-4.91967	0.725501	0.657469
B.cells	EIF5A2	-0.50055	0.577892	-1.01672	0.312063	-5.0096	0.727003	0.659838
B.cells	ATAD2B	0.094252	6.740991	1.016658	0.31209	-6.32919	0.65901	0.555868
B.cells	M1AP	-0.38486	1.320039	-1.01652	0.312157	-5.13616	0.718398	0.646375
B.cells	CYB5B	0.100165	5.866247	1.016314	0.312253	-6.14824	0.668192	0.569572
B.cells	FAM102B	0.17807	4.23575	1.0162	0.312307	-5.78249	0.685711	0.596021
B.cells	CSK	0.097981	6.929767	1.016176	0.312318	-6.33951	0.657049	0.552955
B.cells	A530040E1	0.440276	0.013043	1.016146	0.312333	-5.08224	0.733631	0.670267

B.cells	PERP	-0.44007	1.228461	-1.01604	0.312384	-5.12553	0.719453	0.648022
B.cells	CFDP1	0.102811	5.986865	1.016028	0.312388	-6.15582	0.666917	0.567662
B.cells	GM31718	-0.18557	3.725877	-1.01601	0.312397	-5.72322	0.6913	0.60454
B.cells	FLNA	0.140513	6.097597	1.015935	0.312432	-6.22541	0.665749	0.565915
B.cells	ZFP934	-0.25155	2.990341	-1.01573	0.312528	-5.44413	0.699538	0.617094
B.cells	MRPS35	0.135447	4.266456	1.015402	0.312685	-5.80928	0.685661	0.595707
B.cells	CLIC5	0.445759	0.878367	1.015285	0.31274	-5.1368	0.723806	0.654603
B.cells	HMGCS1	-0.17292	4.453186	-1.01474	0.313	-5.79332	0.683934	0.592789
B.cells	LPAR6	-0.18136	4.523596	-1.01469	0.313023	-5.88491	0.683168	0.591652
B.cells	1700110K1	0.741537	-0.57203	1.014614	0.313058	-4.81371	0.741139	0.681701
B.cells	AMFR	0.091	6.011988	1.01424	0.313236	-6.14573	0.667478	0.56778
B.cells	CPSF6	0.073812	6.68211	1.013983	0.313358	-6.2738	0.660573	0.557408
B.cells	SMO	-0.50958	1.198551	-1.01359	0.313545	-5.01869	0.72113	0.649465
B.cells	FZD7	-0.43471	0.743156	-1.0131	0.313776	-5.0218	0.726814	0.657898
B.cells	PLEKHO1	0.109077	5.499121	1.012553	0.314037	-6.18193	0.67404	0.576495
B.cells	POLR2D	-0.12747	5.184758	-1.01252	0.314053	-6.02327	0.677409	0.581568
B.cells	WIPF1	-0.11097	7.425545	-1.01208	0.314263	-6.43528	0.654124	0.546602
B.cells	RTEL1	-0.29507	2.682882	-1.01195	0.314322	-5.37052	0.705265	0.623757
B.cells	CBX5	0.154544	5.103912	1.011806	0.314392	-5.95785	0.678593	0.583132
B.cells	FGD6	-0.17208	4.002414	-1.01171	0.314437	-5.84397	0.690579	0.601289
B.cells	RBPJ	-0.16943	6.055966	-1.01135	0.314609	-6.21955	0.668582	0.567965
B.cells	DAAM1	-0.14206	4.764899	-1.01123	0.314666	-5.921	0.68241	0.588796
B.cells	E030030I0(	0.215885	2.657085	1.011186	0.314687	-5.44261	0.705716	0.624367
B.cells	OSBPL10	-0.50187	0.973543	-1.01099	0.314778	-4.97873	0.725069	0.654302
B.cells	CYP51	-0.18094	3.916341	-1.01085	0.314846	-5.74702	0.69177	0.602936
B.cells	PABPN1	-0.08222	6.640276	-1.01044	0.315042	-6.28176	0.662803	0.558994
B.cells	MELK	-0.3132	3.016502	-1.01012	0.315196	-5.44974	0.702177	0.618506
B.cells	RCOR1	0.106911	6.85813	1.010047	0.315229	-6.29722	0.660626	0.555798
B.cells	ZSWIM7	0.183204	3.432711	1.009951	0.315275	-5.69552	0.69753	0.611408
B.cells	COMMD8	-0.11331	5.188265	-1.00971	0.31539	-6.03455	0.678322	0.582281
B.cells	ARPC1A	0.092503	6.586291	1.009604	0.31544	-6.29552	0.663469	0.560103
B.cells	PRPF31	0.157494	3.79385	1.009589	0.315447	-5.71967	0.693527	0.605381
B.cells	AAK1	0.113314	6.392459	1.009191	0.315637	-6.25479	0.665781	0.563381
B.cells	PARD3B	0.279759	4.55014	1.009024	0.315717	-5.73262	0.685557	0.593096
B.cells	GM16023	0.279506	1.834877	1.008863	0.315793	-5.29708	0.715946	0.639705
B.cells	RAI14	-0.3421	2.228896	-1.00848	0.315975	-5.32452	0.711464	0.632921
B.cells	SDCCAG8	0.10354	5.914953	1.008354	0.316036	-6.20584	0.670925	0.571212
B.cells	SEC31A	0.087932	5.620105	1.008311	0.316057	-6.12476	0.674066	0.57592
B.cells	RIPOR2	0.13005	7.617108	1.008017	0.316197	-6.43783	0.653127	0.544811
B.cells	GM29994	-0.3365	1.150146	-1.00797	0.316221	-5.15279	0.723862	0.652229
B.cells	ZNFX1	0.211426	4.359029	1.007862	0.316271	-5.8537	0.687698	0.596536
B.cells	IRGM2	0.372165	2.731293	1.007821	0.316291	-5.36058	0.705773	0.624178
B.cells	GM28403	-0.43692	1.458517	-1.00762	0.316389	-5.15175	0.720293	0.646681
B.cells	BCR	-0.12763	5.853797	-1.00723	0.316572	-6.13177	0.671575	0.57229
B.cells	KNTC1	-0.35105	2.818917	-1.00722	0.316577	-5.3066	0.704786	0.622734
B.cells	TMEM177	0.584641	0.336385	1.007132	0.31662	-4.90029	0.73338	0.66725
B.cells	FAM160B2	0.176057	3.264944	1.006842	0.316758	-5.60401	0.699787	0.615213

B.cells	HSPG2	-0.31597	2.741833	-1.00682	0.316769	-5.31042	0.705655	0.624232
B.cells	MYBPC3	-0.38038	1.123244	-1.00668	0.316835	-5.16753	0.724175	0.652983
B.cells	IL1RAP	-0.17816	4.731572	-1.00665	0.316848	-5.92435	0.683637	0.590623
B.cells	CSNK1D	0.070529	6.6346	1.006362	0.316988	-6.30032	0.663331	0.560146
B.cells	GDE1	0.182267	4.883129	1.006359	0.316989	-5.93319	0.681994	0.588136
B.cells	ATP5MPL	-0.08326	8.484004	-1.00635	0.316996	-6.58075	0.644278	0.532042
B.cells	ZFP160	0.214302	2.950859	1.006272	0.317031	-5.50606	0.703303	0.620631
B.cells	TM7SF2	-0.36755	1.754706	-1.00627	0.317032	-5.12471	0.716883	0.641624
B.cells	CYP3A25	-0.39881	1.898523	-1.0061	0.317111	-5.27483	0.715235	0.639068
B.cells	PSTPIP1	0.155253	4.472927	1.00599	0.317166	-5.94798	0.686453	0.594958
B.cells	ENTR1	0.133572	4.880508	1.005805	0.317254	-5.9538	0.682022	0.588333
B.cells	GNG10	0.103632	6.940589	1.005775	0.317268	-6.39072	0.660133	0.555552
B.cells	TBC1D4	-0.25153	4.706501	-1.00559	0.317358	-5.80084	0.68391	0.591229
B.cells	ASS1	0.203284	6.535258	1.005527	0.317387	-6.37848	0.664373	0.561883
B.cells	DGKH	-0.14655	4.909539	-1.00548	0.317411	-6.0641	0.681708	0.587908
B.cells	APOO	-0.18006	3.509146	-1.0052	0.317545	-5.60757	0.697079	0.611346
B.cells	NOL9	0.161783	3.728213	1.005056	0.317613	-5.75221	0.69465	0.607667
B.cells	GM10143	-0.38528	1.245831	-1.00485	0.317709	-5.1073	0.722765	0.65112
B.cells	UBE2N	-0.07451	7.48412	-1.00478	0.317744	-6.43105	0.654508	0.547354
B.cells	LYRM1	0.231531	2.700522	1.004632	0.317816	-5.43244	0.706133	0.625311
B.cells	HOOK2	0.195201	3.558904	1.004595	0.317833	-5.74259	0.696527	0.610554
B.cells	PWWP3A	-0.15949	3.715755	-1.00459	0.317834	-5.70516	0.694788	0.607894
B.cells	ADRB2	-0.23643	4.714467	-1.00419	0.318029	-5.90006	0.683952	0.591317
B.cells	TPM2	0.606814	0.689833	1.004118	0.318062	-4.96893	0.729366	0.661354
B.cells	SPTSSA	-0.10648	6.822761	-1.00402	0.31811	-6.28589	0.661487	0.557625
B.cells	THAP8	0.742527	-0.29985	1.003982	0.318127	-4.85194	0.74106	0.679795
B.cells	IPP	0.248228	2.465742	1.003694	0.318265	-5.38178	0.709084	0.629664
B.cells	DIRAS2	0.343579	0.718948	1.003438	0.318388	-5.2573	0.729264	0.661108
B.cells	INO80D	0.093927	5.995336	1.003392	0.31841	-6.17673	0.670418	0.570852
B.cells	TSPO	0.146604	7.399271	1.003233	0.318486	-6.42985	0.655745	0.548977
B.cells	TLCD1	0.323805	1.624021	1.002444	0.318865	-5.18003	0.719423	0.644972
B.cells	ADGRV1	0.550164	0.711845	1.002403	0.318885	-5.00146	0.730024	0.661541
B.cells	STRN3	0.092673	7.654054	1.002213	0.318976	-6.46445	0.653742	0.545419
B.cells	MIRT2	0.513964	-1.00119	1.001865	0.319143	-4.9015	0.750213	0.693972
B.cells	RELCH	0.088746	6.238997	1.001853	0.319149	-6.24697	0.668511	0.567439
B.cells	HERC1	0.104365	7.056727	1.001851	0.319149	-6.35148	0.659929	0.554667
B.cells	INTS7	0.094262	5.677826	1.001585	0.319277	-6.10279	0.674621	0.57647
B.cells	ASTL	-0.42948	1.424213	-1.00138	0.319377	-5.15494	0.722032	0.648986
B.cells	CSPG5	-0.65333	0.28413	-1.00099	0.319563	-4.93561	0.735401	0.66999
B.cells	ZFP995	-0.24854	2.383748	-1.00078	0.319664	-5.39697	0.711063	0.632074
B.cells	TMCO1	-0.09024	6.265444	-1.0007	0.319705	-6.19819	0.668491	0.567346
B.cells	POLL	-0.3757	1.158885	-1.00069	0.319705	-5.07528	0.725146	0.653966
B.cells	FUOM	0.326176	2.61981	1.00067	0.319717	-5.41945	0.708385	0.627947
B.cells	PAM	-0.1996	4.417759	-1.0006	0.319751	-5.69159	0.688371	0.597315
B.cells	FTL1	-0.10445	11.26205	-1.00017	0.31996	-7.03672	0.618336	0.493848
B.cells	TSPAN12	-0.48744	1.194761	-0.99992	0.320078	-5.07563	0.725072	0.653553
B.cells	ATG2B	0.120323	4.861013	0.999892	0.320091	-6.00362	0.683863	0.590183

B.cells	GM4356	0.467761	0.868166	0.999787	0.320142	-5.08186	0.728881	0.659542
B.cells	FAM72A	-0.48251	1.069392	-0.99953	0.320266	-4.99298	0.726617	0.656006
B.cells	USP11	0.27744	2.611991	0.999449	0.320305	-5.33305	0.708892	0.628481
B.cells	FADD	0.304965	2.421277	0.999195	0.320427	-5.36836	0.711056	0.631925
B.cells	TMEM216	-0.17948	4.069162	-0.99906	0.320492	-5.78225	0.692608	0.603604
B.cells	TXNDC16	0.156308	5.44957	0.998843	0.320597	-6.12327	0.677585	0.58089
B.cells	PKNOX2	-0.69111	-0.82371	-0.99871	0.32066	-4.83809	0.748861	0.691568
B.cells	GM45353	-0.45703	-0.0712	-0.99869	0.32067	-4.93968	0.74005	0.677284
B.cells	ENPP4	-0.33426	2.924155	-0.99864	0.320696	-5.2243	0.705366	0.623225
B.cells	SAYSD1	0.172389	3.323883	0.998541	0.320742	-5.60408	0.700881	0.616355
B.cells	CEBPE	-0.51133	0.2028	-0.99847	0.320777	-5.00026	0.736798	0.672259
B.cells	NANS	-0.14685	4.873876	-0.99816	0.320927	-5.94736	0.683918	0.590468
B.cells	RDH5	-0.32282	2.368316	-0.99811	0.320951	-5.32499	0.711778	0.633107
B.cells	ZBTB49	0.325161	1.051258	0.997938	0.321033	-5.09252	0.727001	0.656798
B.cells	PRPF4	0.174336	3.92555	0.997609	0.321192	-5.70302	0.694426	0.606426
B.cells	MVK	0.276088	2.634356	0.997415	0.321285	-5.35395	0.708876	0.628672
B.cells	PIP4K2B	0.146582	4.178712	0.997395	0.321295	-5.80461	0.691634	0.602228
B.cells	MSH2	0.149409	4.128132	0.997125	0.321425	-5.81045	0.69219	0.603137
B.cells	MED7	0.159459	3.583194	0.997008	0.321481	-5.6868	0.698224	0.612411
B.cells	5430414B1	-0.59312	0.449396	-0.99693	0.321517	-4.92783	0.73413	0.668161
B.cells	LY6G2	-0.53911	0.952461	-0.99689	0.321536	-5.06865	0.728225	0.658914
B.cells	POLR3A	0.198595	3.30507	0.996882	0.321543	-5.66265	0.701327	0.617191
B.cells	GAK	0.084763	6.280821	0.996659	0.32165	-6.2458	0.669047	0.568181
B.cells	PIF1	-0.49697	1.716548	-0.99607	0.321936	-5.12742	0.719502	0.645342
B.cells	PPP1R7	0.122706	4.409711	0.996057	0.321941	-5.83225	0.689232	0.598757
B.cells	GANAB	0.131096	4.730059	0.995977	0.321979	-5.90825	0.685731	0.593443
B.cells	CEP68	0.162629	4.299523	0.995927	0.322003	-5.82864	0.690441	0.600599
B.cells	RCCD1	-0.2693	3.170294	-0.9959	0.322014	-5.47143	0.702974	0.619773
B.cells	GNAS	0.076613	8.931692	0.995684	0.322121	-6.65437	0.641708	0.527958
B.cells	SDHD	-0.1221	5.858408	-0.99566	0.322133	-6.15795	0.673567	0.575107
B.cells	BCO2	-0.38598	0.643324	-0.99556	0.32218	-5.07994	0.731991	0.664902
B.cells	SLC25A30	0.165719	3.550303	0.995374	0.322271	-5.70723	0.698727	0.613316
B.cells	CCNL2	0.079971	6.219089	0.99537	0.322273	-6.25214	0.669732	0.569403
B.cells	A730036I1	-0.67419	-0.20546	-0.99522	0.322344	-4.90074	0.742045	0.680821
B.cells	HRH1	-0.55547	-0.56863	-0.99514	0.322383	-4.84316	0.746325	0.687715
B.cells	ANKS3	-0.1524	4.082767	-0.99457	0.322657	-5.80677	0.693287	0.604488
B.cells	GM29488	-0.56397	0.172443	-0.9943	0.322791	-4.93609	0.738079	0.674013
B.cells	GM16845	0.248851	2.527304	0.994176	0.32285	-5.45519	0.71074	0.631354
B.cells	CD82	-0.12695	5.476033	-0.99417	0.322854	-6.05008	0.678148	0.58161
B.cells	2810454H0	-0.26771	2.514759	-0.99383	0.323018	-5.36882	0.71111	0.631656
B.cells	SPTAN1	-0.10502	6.559592	-0.99324	0.323301	-6.29727	0.667239	0.564568
B.cells	PAWR	-0.47344	0.662659	-0.99299	0.323425	-5.01447	0.732976	0.665307
B.cells	H2-KE6	0.139701	5.147514	0.99289	0.323473	-6.01655	0.682329	0.587311
B.cells	GM17173	-0.53962	0.150476	-0.99276	0.323535	-4.92335	0.739031	0.674914
B.cells	MZB1	-0.19177	5.956787	-0.99255	0.323636	-6.11868	0.673632	0.574326
B.cells	ZFP275	0.321692	1.581356	0.99253	0.323648	-5.17799	0.722255	0.648688
B.cells	ZFP524	0.19484	3.672126	0.992416	0.323703	-5.68923	0.698527	0.612048

B.cells	GM43623	-0.57847	-0.49767	-0.99239	0.323714	-4.91252	0.746746	0.687246
B.cells	BTRC	0.133007	5.211768	0.992232	0.323792	-6.0039	0.681634	0.58642
B.cells	MB21D2	-0.54725	0.883088	-0.992	0.323906	-4.98534	0.730387	0.661562
B.cells	CENPW	-0.22156	4.349764	-0.99197	0.323917	-5.74209	0.691032	0.600763
B.cells	LIN7C	-0.12037	4.956273	-0.99194	0.323936	-5.96792	0.684404	0.590725
B.cells	GOLIM4	-0.18734	5.140893	-0.99161	0.324092	-5.91619	0.682478	0.587805
B.cells	TIMM17B	0.098555	5.161183	0.991614	0.324092	-5.99266	0.682259	0.587473
B.cells	SUDS3	0.11983	5.515338	0.990846	0.324465	-6.10115	0.679072	0.582128
B.cells	TENM3	-0.41721	1.688761	-0.9905	0.324631	-5.19644	0.721771	0.647635
B.cells	PREPL	0.300946	1.734988	0.990455	0.324655	-5.23881	0.721236	0.646816
B.cells	HSDL2	-0.1351	4.684002	-0.99035	0.324706	-5.94194	0.688091	0.59592
B.cells	GM11099	0.688752	-0.70994	0.990233	0.324762	-4.85401	0.749974	0.692358
B.cells	BIN1	0.113799	5.230633	0.990217	0.32477	-6.08828	0.682145	0.586957
B.cells	PCK1	-0.30209	4.182878	-0.99012	0.324815	-5.88125	0.693596	0.604353
B.cells	CDK1	-0.26349	5.506863	-0.98948	0.325129	-5.9753	0.679694	0.582679
B.cells	PCNX3	-0.18025	3.30596	-0.98933	0.325203	-5.62686	0.703933	0.619492
B.cells	ALKBH6	-0.18724	2.755561	-0.98896	0.325381	-5.48327	0.71031	0.629185
B.cells	PCX	-0.29878	2.70013	-0.98886	0.325428	-5.44454	0.710939	0.630166
B.cells	STK35	0.163442	3.447827	0.988801	0.325459	-5.66534	0.702506	0.617215
B.cells	GET4	-0.12295	5.110437	-0.98853	0.325589	-6.00219	0.684273	0.589357
B.cells	CIAO2A	-0.10027	6.679979	-0.98845	0.325628	-6.33935	0.667476	0.564175
B.cells	FBXL15	0.217829	2.696607	0.988051	0.325824	-5.51102	0.711143	0.630519
B.cells	SLC43A2	-0.15367	6.179865	-0.98776	0.325965	-6.24443	0.672828	0.572258
B.cells	VASP	0.117365	6.541669	0.98765	0.326019	-6.2778	0.668989	0.566523
B.cells	SLC7A1	0.148263	5.141375	0.987488	0.326098	-6.13844	0.683992	0.589045
B.cells	IPO9	0.121809	4.771136	0.98724	0.326219	-5.89965	0.688025	0.595234
B.cells	PIN4	0.106592	4.949389	0.987119	0.326278	-6.00497	0.68608	0.592287
B.cells	BCL2A1A	0.534156	2.399522	0.987099	0.326288	-5.24763	0.714528	0.635842
B.cells	MAEA	-0.10456	5.40999	-0.98687	0.326399	-6.07094	0.681083	0.584756
B.cells	1700102H2	-0.48476	0.529845	-0.98682	0.326423	-4.97893	0.736259	0.669772
B.cells	DHDDS	0.143836	4.630539	0.986771	0.326448	-5.89706	0.689563	0.597583
B.cells	ZFP146	0.183957	3.413704	0.986757	0.326454	-5.64813	0.703051	0.618169
B.cells	GM47828	0.364825	0.334612	0.986743	0.326461	-5.16648	0.738571	0.673412
B.cells	GFOD2	-0.32259	2.309358	-0.9867	0.326483	-5.25125	0.715558	0.637455
B.cells	B3GLCT	0.205667	3.311117	0.986607	0.326528	-5.62593	0.704202	0.619946
B.cells	HIST3H2BA	0.336677	0.997637	0.986551	0.326555	-5.15214	0.730751	0.661137
B.cells	KCMF1	0.069108	6.987433	0.985984	0.326832	-6.3798	0.664737	0.55988
B.cells	DFFB	-0.20416	2.981393	-0.98572	0.32696	-5.59971	0.708535	0.626007
B.cells	GM50373	-0.55967	0.358844	-0.98553	0.327051	-4.96845	0.738932	0.673425
B.cells	IKBKB	0.094542	5.778617	0.985422	0.327106	-6.24567	0.67771	0.579186
B.cells	ITGA2	0.408577	0.926385	0.985348	0.327142	-5.14097	0.732229	0.662895
B.cells	EDC4	-0.224	2.984777	-0.98451	0.327549	-5.56561	0.709097	0.626409
B.cells	KNSTRN	-0.33095	3.271497	-0.98444	0.327586	-5.50159	0.705861	0.621432
B.cells	GM31243	0.283015	2.779029	0.984414	0.327598	-5.67324	0.711431	0.630005
B.cells	SERPINA7	-0.52232	0.531403	-0.98428	0.327666	-5.02029	0.737524	0.670619
B.cells	PHF10	-0.127	4.930175	-0.98349	0.328051	-5.9979	0.688003	0.593629
B.cells	GLS2	-0.39253	1.618045	-0.98338	0.328102	-5.23697	0.725326	0.650946

B.cells	2310010J1	0.215903	3.692212	0.983297	0.328145	-5.63839	0.701685	0.614454
B.cells	GM12089	0.395729	0.306607	0.98328	0.328153	-5.04353	0.740749	0.675098
B.cells	GM31522	-0.45326	-1.00368	-0.98307	0.328255	-4.8797	0.756292	0.700186
B.cells	VTA1	0.09582	5.707573	0.983009	0.328285	-6.16099	0.679596	0.581038
B.cells	AIMP1	0.08864	6.38044	0.982811	0.328382	-6.28085	0.672472	0.5703
B.cells	TUBGCP5	0.165607	3.976604	0.982429	0.328569	-5.70485	0.698781	0.609894
B.cells	6430590AC	0.279652	1.647577	0.982353	0.328606	-5.22499	0.725261	0.65074
B.cells	CSNK2A1	0.072956	6.59773	0.982261	0.328652	-6.29765	0.670322	0.567027
B.cells	DHX35	0.251473	2.187953	0.982119	0.328721	-5.4082	0.719014	0.641099
B.cells	CCDC138	-0.20727	4.628632	-0.98204	0.32876	-5.82908	0.691571	0.599015
B.cells	SLC35B3	0.109659	4.482615	0.981771	0.328892	-5.92147	0.693188	0.60159
B.cells	ARMC5	-0.15654	3.383082	-0.9816	0.328978	-5.67165	0.705431	0.620389
B.cells	1110046J0	0.579818	0.031359	0.98126	0.329142	-4.89826	0.744329	0.68115
B.cells	GM42702	-0.27231	2.365724	-0.98087	0.329335	-5.40884	0.716984	0.638514
B.cells	E2F1	-0.19029	4.409532	-0.98084	0.329348	-5.80085	0.693994	0.603214
B.cells	PHETA1	0.479972	0.450779	0.980689	0.329422	-5.0237	0.739329	0.673413
B.cells	FOXR1	0.588256	-0.87975	0.980644	0.329444	-4.81732	0.75509	0.698736
B.cells	AC142100.	-0.51349	0.335219	-0.98064	0.329447	-5.03468	0.740703	0.675577
B.cells	IL1RL1	-0.63401	-0.28284	-0.98055	0.329492	-4.90124	0.7481	0.687305
B.cells	INTS13	-0.14181	4.149388	-0.9805	0.329516	-5.79738	0.696872	0.607658
B.cells	GM47664	0.161258	3.774581	0.980316	0.329605	-5.76765	0.701043	0.614126
B.cells	PCNT	0.119879	5.233516	0.980263	0.329631	-6.08601	0.684971	0.589706
B.cells	FAM117B	0.108576	7.11596	0.980193	0.329666	-6.46119	0.664869	0.55962
B.cells	DGLUCY	0.237956	2.944912	0.980146	0.329689	-5.43588	0.71038	0.628535
B.cells	KLK8	-0.35332	1.605933	-0.98011	0.329707	-5.15269	0.725756	0.652396
B.cells	ATG7	-0.12227	5.786606	-0.97956	0.329974	-6.18838	0.678991	0.580938
B.cells	COLGALT2	-0.71846	-0.12576	-0.97951	0.33	-4.86748	0.746212	0.684762
B.cells	RCHY1	0.100034	5.661194	0.979505	0.330003	-6.15118	0.680341	0.58297
B.cells	SNHG16	-0.28099	2.227913	-0.97936	0.330072	-5.36707	0.718566	0.641439
B.cells	EEF1G	0.096195	7.187877	0.979365	0.330072	-6.39776	0.664115	0.558728
B.cells	ZFP709	0.254462	1.431477	0.979237	0.330135	-5.2703	0.727787	0.655854
B.cells	MLXIPL	-0.65027	0.355448	-0.97892	0.330289	-4.97721	0.740462	0.675851
B.cells	PPIP5K1	0.325365	1.776843	0.97889	0.330305	-5.32662	0.723772	0.649682
B.cells	PIK3R4	0.205818	3.189781	0.978832	0.330334	-5.58845	0.707609	0.624646
B.cells	MED29	-0.14885	3.806911	-0.9787	0.330401	-5.71252	0.700682	0.614053
B.cells	SAMD9L	0.14856	5.184732	0.978453	0.33052	-6.06027	0.685501	0.590986
B.cells	SCD1	0.263752	3.037555	0.978328	0.330582	-5.67375	0.70933	0.627382
B.cells	GAS6	-0.44289	1.545619	-0.97831	0.33059	-5.09968	0.726457	0.653959
B.cells	NT5C3B	-0.17705	3.396774	-0.97799	0.330746	-5.62896	0.705277	0.621275
B.cells	CTNNBL1	0.109406	5.139211	0.97787	0.330807	-6.03621	0.685996	0.591862
B.cells	MMP12	0.672377	-0.05819	0.977857	0.330813	-5.0388	0.745402	0.683892
B.cells	TNPO1	0.087567	7.082293	0.977444	0.331016	-6.40994	0.665223	0.560774
B.cells	SNX30	0.124379	5.755716	0.977262	0.331106	-6.1894	0.679323	0.581959
B.cells	NAGPA	0.165522	3.354165	0.97718	0.331146	-5.68435	0.705757	0.622201
B.cells	DENND5B	0.16892	4.198991	0.977092	0.33119	-5.84385	0.696322	0.607742
B.cells	ABHD11	0.175731	3.541149	0.977065	0.331203	-5.7296	0.703656	0.618971
B.cells	PILRA	-0.34824	3.3927	-0.97702	0.331224	-5.34423	0.705323	0.621534

B.cells	ARHGAP18	-0.15584	6.528672	-0.97693	0.331267	-6.29776	0.671065	0.569598
B.cells	GNL3L	0.11012	4.467102	0.976756	0.331355	-5.90802	0.693359	0.603301
B.cells	HGSNAT	-0.21855	4.642285	-0.9767	0.331383	-5.7749	0.691431	0.600366
B.cells	GNE	-0.1428	4.467546	-0.97666	0.331404	-5.8295	0.693354	0.603294
B.cells	ASB7	0.190562	3.936004	0.976618	0.331423	-5.7621	0.699243	0.612286
B.cells	CCL4	0.441283	7.388292	0.976446	0.331508	-6.04961	0.66202	0.556211
B.cells	IRF2BP2	-0.09018	7.59206	-0.97639	0.331535	-6.45633	0.659897	0.553076
B.cells	TINF2	0.167703	4.054948	0.976119	0.331669	-5.7916	0.69792	0.610401
B.cells	DHX58OS	0.422085	0.831273	0.976098	0.331679	-5.18272	0.734826	0.667657
B.cells	NSFL1C	-0.13785	4.168167	-0.97607	0.331691	-5.78273	0.696664	0.608496
B.cells	TRP53	-0.10395	5.681235	-0.97604	0.33171	-6.1541	0.680125	0.583408
B.cells	GM17276	-0.41039	0.785789	-0.97602	0.331716	-5.2069	0.735363	0.668525
B.cells	SLC27A4	0.208077	2.770367	0.975528	0.33196	-5.42126	0.71261	0.632827
B.cells	ST7L	-0.14567	4.451288	-0.97538	0.332033	-5.88402	0.693774	0.603909
B.cells	HELZ2	0.211569	3.832468	0.975318	0.332064	-5.71881	0.70064	0.614428
B.cells	FBXO5	-0.27019	4.811103	-0.97522	0.332111	-5.86768	0.689818	0.59793
B.cells	SLC22A14	-0.26405	2.744823	-0.97518	0.332132	-5.47492	0.7129	0.633322
B.cells	AIP	0.134946	4.946962	0.975018	0.332211	-5.98618	0.68837	0.595714
B.cells	CFAP45	-0.50878	0.728674	-0.9748	0.332317	-5.04145	0.736357	0.669951
B.cells	IFITM6	0.448193	3.518134	0.974753	0.332342	-5.43184	0.70422	0.619954
B.cells	TMEM165	0.102912	5.311537	0.974448	0.332493	-6.07235	0.684607	0.589899
B.cells	RNF169	0.126472	6.380124	0.974062	0.332683	-6.29411	0.67328	0.572695
B.cells	ZFP653	0.185851	3.131364	0.974041	0.332694	-5.62758	0.70894	0.6269
B.cells	FXYD5	0.16709	6.974009	0.973895	0.332766	-6.2654	0.667016	0.563338
B.cells	GM43260	-0.26371	2.252383	-0.9736	0.332911	-5.37459	0.719077	0.642639
B.cells	ATXN2L	-0.10404	5.47603	-0.97357	0.332926	-6.09845	0.683093	0.587507
B.cells	PRPF8	-0.09166	5.801492	-0.9733	0.333058	-6.17435	0.679664	0.58232
B.cells	CASP6	0.163601	3.628567	0.973053	0.333182	-5.75497	0.703539	0.618694
B.cells	RNF227	-0.33514	1.852006	-0.97275	0.333331	-5.25156	0.723789	0.65013
B.cells	SMYD3	0.115367	6.67093	0.972705	0.333354	-6.33979	0.67038	0.568535
B.cells	SREK1	-0.0763	6.429668	-0.97252	0.333444	-6.29546	0.672941	0.572388
B.cells	GM46560	-0.55537	-0.84515	-0.97244	0.333486	-4.88403	0.755609	0.700713
B.cells	ACBD4	-0.30485	1.764413	-0.97226	0.333575	-5.27314	0.724805	0.651772
B.cells	CBR2	-0.82667	-1.3198	-0.97216	0.333621	-4.83329	0.761376	0.71007
B.cells	POLDIP2	0.15603	4.278307	0.9721	0.333653	-5.82693	0.696298	0.607793
B.cells	NUAK1	-0.25769	3.219187	-0.972	0.333701	-5.61751	0.708146	0.62598
B.cells	THUMPD2	0.267493	1.928752	0.971731	0.333835	-5.27429	0.7229	0.648874
B.cells	GZMM	-0.33002	2.051803	-0.97167	0.333864	-5.29369	0.721478	0.646657
B.cells	USP8	0.099254	5.372996	0.971668	0.333867	-6.12883	0.684296	0.589571
B.cells	1700034P1	-0.46846	1.464772	-0.97164	0.333879	-5.15972	0.728292	0.657297
B.cells	KIFC3	-0.34518	1.625707	-0.97133	0.334036	-5.23325	0.726417	0.654479
B.cells	DNAJC14	0.116768	4.252973	0.971073	0.334161	-5.84459	0.696579	0.608374
B.cells	POLR1E	-0.29252	2.067812	-0.97107	0.334164	-5.32861	0.721293	0.646509
B.cells	DMTF1	0.109162	4.947065	0.971061	0.334167	-6.00856	0.688937	0.596732
B.cells	2510046G1	0.228581	2.620896	0.971054	0.334171	-5.4495	0.714943	0.636641
B.cells	DAB2	-0.41803	4.009728	-0.97094	0.334228	-5.6137	0.699281	0.612529
B.cells	PPA2	0.144554	4.580694	0.970895	0.334249	-5.90295	0.692958	0.602883

B.cells	SAV1	0.118474	4.735258	0.970712	0.33434	-5.99303	0.691258	0.600369
B.cells	ACTR8	0.164133	3.537225	0.970614	0.334389	-5.71835	0.704564	0.620757
B.cells	TAF12	0.116151	5.021001	0.970561	0.334415	-6.01357	0.688128	0.595658
B.cells	TRAM1	-0.07968	7.121393	-0.97039	0.334501	-6.39414	0.665633	0.561911
B.cells	4930477G(	-0.65248	-0.38448	-0.97026	0.334563	-4.90495	0.75025	0.692387
B.cells	CMAH	0.201886	5.561409	0.970123	0.334632	-6.00743	0.682259	0.586875
B.cells	ZFP180	0.166132	3.315586	0.969911	0.334737	-5.66746	0.707063	0.62474
B.cells	UNC45B	0.609816	0.423376	0.969905	0.334739	-4.95525	0.740568	0.677045
B.cells	ACYP1	-0.17427	3.847714	-0.96962	0.33488	-5.71555	0.701092	0.615644
B.cells	TMF1	0.086326	6.243916	0.969179	0.3351	-6.28153	0.674926	0.576084
B.cells	PCED1B	0.175466	4.966857	0.969132	0.335123	-6.00937	0.688725	0.596935
B.cells	HMGN3	-0.21898	3.255903	-0.96906	0.335157	-5.72876	0.707736	0.626074
B.cells	ADAMTS17	-0.62136	0.355692	-0.96879	0.335294	-4.9176	0.741374	0.67875
B.cells	WDR25	0.266244	1.998808	0.968738	0.335318	-5.30517	0.722095	0.648485
B.cells	POLE2	-0.26389	3.014685	-0.96867	0.335352	-5.48831	0.710466	0.630452
B.cells	GM11655	-0.50326	0.316941	-0.96862	0.335375	-4.96245	0.741835	0.679552
B.cells	KCTD1	0.25331	1.719442	0.968615	0.33538	-5.41114	0.725332	0.653584
B.cells	FCF1	-0.0884	6.136138	-0.96842	0.335479	-6.26231	0.676077	0.578068
B.cells	SEC61B	-0.08608	8.272683	-0.96839	0.335492	-6.56925	0.65367	0.544691
B.cells	8030462N1	-0.07782	6.073398	-0.96825	0.335561	-6.22492	0.676749	0.579127
B.cells	HPS5	-0.12672	4.448069	-0.96821	0.335581	-5.93647	0.694426	0.605948
B.cells	ZSCAN21	0.196343	3.394527	0.968139	0.335616	-5.65378	0.706173	0.62402
B.cells	PTK2B	0.10641	7.134222	0.968097	0.335637	-6.43051	0.665498	0.562345
B.cells	TM9SF4	0.134567	4.769644	0.967931	0.335719	-5.97112	0.690885	0.600707
B.cells	ANKRD39	0.141554	3.543857	0.967882	0.335744	-5.73461	0.704494	0.621556
B.cells	EFR3A	0.092141	5.780301	0.967504	0.335931	-6.20309	0.680155	0.584178
B.cells	PAX5	0.168431	5.141192	0.967231	0.336067	-6.13725	0.687086	0.594773
B.cells	DNAJC21	0.109032	5.890572	0.967207	0.336079	-6.18492	0.678972	0.582493
B.cells	TRAF2	0.151404	3.917831	0.96689	0.336237	-5.80532	0.700579	0.615397
B.cells	GSKIP	-0.10415	4.602025	-0.96681	0.336276	-5.96982	0.692995	0.603788
B.cells	PPP2R5D	-0.16258	3.682866	-0.96671	0.336326	-5.72243	0.703206	0.619435
B.cells	IPO8	-0.12413	4.420007	-0.9666	0.336381	-5.89998	0.695003	0.606866
B.cells	GM44899	-0.4193	0.774696	-0.96646	0.336452	-5.05506	0.736685	0.671676
B.cells	VEGFA	-0.19129	4.223513	-0.96636	0.336498	-5.85635	0.697178	0.610277
B.cells	CD44	0.117956	8.587659	0.966361	0.336499	-6.60294	0.650693	0.540465
B.cells	DVL1	-0.26542	2.507773	-0.96594	0.33671	-5.38502	0.716517	0.640189
B.cells	2310011J0.	0.134475	4.446593	0.965798	0.33678	-5.91807	0.694709	0.606643
B.cells	2310009B1	0.123531	4.473004	0.965739	0.336809	-5.91156	0.694417	0.606197
B.cells	KIF24	-0.17893	3.667581	-0.96555	0.336902	-5.64287	0.703377	0.620052
B.cells	CCDC9	0.112253	4.238269	0.965512	0.336922	-5.92821	0.697015	0.610283
B.cells	CPLANE1	-0.14855	4.78519	-0.9654	0.336979	-6.00241	0.690981	0.601089
B.cells	FBXO7	0.142933	4.165071	0.965271	0.337042	-5.88575	0.697827	0.611583
B.cells	AGK	0.201586	3.121713	0.9651	0.337127	-5.54823	0.709526	0.629661
B.cells	SPON1	0.403968	1.404705	0.96508	0.337137	-5.31494	0.729279	0.660455
B.cells	TMSB10	-0.09718	10.73818	-0.96499	0.33718	-7.09723	0.629144	0.509425
B.cells	RELT	0.17271	3.728351	0.96489	0.337232	-5.77034	0.702696	0.619151
B.cells	SDHAF4	0.139446	4.365947	0.964818	0.337268	-5.86046	0.695601	0.60826

B.cells	MRPL23	0.08387	6.069484	0.964806	0.337274	-6.21924	0.677052	0.580086
B.cells	D130020LC	-0.47321	0.782996	-0.96463	0.337362	-5.03731	0.736587	0.672098
B.cells	SHQ1	0.22928	2.60612	0.96459	0.337381	-5.53757	0.715392	0.638887
B.cells	IDS	0.23654	2.698638	0.964203	0.337574	-5.46335	0.7145	0.637274
B.cells	H2-Q7	0.330793	3.889878	0.964182	0.337584	-5.86322	0.701052	0.616497
B.cells	1500011BC	0.147978	4.65787	0.963927	0.337712	-5.99587	0.69254	0.603556
B.cells	EFNA1	-0.55331	0.531324	-0.96364	0.337856	-4.96708	0.73974	0.677019
B.cells	GLB1L	0.247618	2.192734	0.96363	0.33786	-5.34868	0.720301	0.646473
B.cells	FGGY	-0.27113	3.459854	-0.96361	0.337871	-5.53278	0.705873	0.624082
B.cells	LSM12	0.084422	6.360706	0.963495	0.337927	-6.31669	0.674095	0.575678
B.cells	BECN1	0.095294	5.896307	0.96333	0.338009	-6.22627	0.679067	0.583215
B.cells	KMT2C	-0.08411	7.717211	-0.96327	0.33804	-6.52126	0.659816	0.554388
B.cells	ADAP2	0.34222	2.585365	0.963224	0.338062	-5.38118	0.715794	0.639514
B.cells	GM30948	-0.57719	0.467484	-0.96294	0.338206	-5.07756	0.740659	0.678293
B.cells	AGO1	0.163883	3.874896	0.962783	0.338282	-5.79562	0.701371	0.616998
B.cells	MGST2	-0.23037	2.953494	-0.96271	0.338316	-5.55384	0.711751	0.633023
B.cells	PLPBP	0.135062	4.145683	0.962279	0.338534	-5.86889	0.698503	0.612504
B.cells	DPH7	0.366436	1.586984	0.962254	0.338547	-5.20834	0.727632	0.657697
B.cells	FAM83G	-0.44475	0.263823	-0.96221	0.338569	-4.97957	0.743245	0.682339
B.cells	UBN1	-0.07402	6.944041	-0.962	0.338671	-6.37237	0.668278	0.566685
B.cells	PRRG4	0.546096	0.01648	0.961798	0.338774	-4.93244	0.746391	0.687187
B.cells	C5AR1	0.449903	4.179898	0.961674	0.338836	-5.50271	0.6983	0.612122
B.cells	HIST1H3G	-0.46338	1.46486	-0.96106	0.339143	-5.10845	0.729723	0.660372
B.cells	MAP3K8	0.137605	4.64612	0.960985	0.339181	-6.0075	0.693599	0.604439
B.cells	SLC2A8	-0.25096	2.226604	-0.96069	0.339326	-5.36597	0.720906	0.646586
B.cells	TICAM2	0.306244	1.274799	0.960666	0.33934	-5.28325	0.731977	0.663922
B.cells	FKBP1A	-0.08684	7.336878	-0.96057	0.339388	-6.46983	0.6647	0.560873
B.cells	DHCR7	-0.27529	2.184949	-0.96048	0.339435	-5.25493	0.721386	0.647369
B.cells	GGCT	0.224443	3.249782	0.960299	0.339524	-5.66801	0.709278	0.62854
B.cells	BRD9	0.110131	5.185663	0.959916	0.339715	-6.05691	0.68803	0.595726
B.cells	ASAH2	-0.26178	2.945427	-0.95943	0.339956	-5.51255	0.713385	0.634227
B.cells	EIF4H	-0.07595	6.535904	-0.95909	0.340127	-6.31794	0.67402	0.573932
B.cells	WARS2	0.181629	3.512477	0.958822	0.340263	-5.72116	0.707158	0.624435
B.cells	ATF7IP	0.10435	6.540023	0.958677	0.340336	-6.33317	0.673976	0.574028
B.cells	LIPH	0.551674	-0.05189	0.958618	0.340365	-4.9565	0.748694	0.689589
B.cells	FAHD1	-0.33018	1.917334	-0.95857	0.340389	-5.28998	0.725408	0.652886
B.cells	XYLT2	0.269801	1.972195	0.958414	0.340468	-5.30725	0.724772	0.65196
B.cells	PRPF19	-0.11223	5.053951	-0.9581	0.340623	-6.04109	0.690029	0.598485
B.cells	TRP53INP2	0.142006	4.134264	0.95807	0.34064	-5.89838	0.700189	0.614005
B.cells	DOLPP1	0.237736	2.514642	0.958005	0.340672	-5.47189	0.718511	0.642309
B.cells	MARVELD2	0.351285	1.369704	0.957923	0.340714	-5.2664	0.731799	0.663109
B.cells	EIF3B	0.104738	5.890853	0.957917	0.340717	-6.20857	0.680933	0.584724
B.cells	SIRT6	0.228735	2.652878	0.957781	0.340785	-5.50776	0.716942	0.639928
B.cells	PUS7	0.200954	2.95762	0.957609	0.340871	-5.58896	0.713515	0.634574
B.cells	SLC5A3	-0.2248	3.816287	-0.95729	0.341032	-5.75385	0.703848	0.619659
B.cells	JRKL	0.349417	1.537523	0.957209	0.341072	-5.23917	0.729943	0.660223
B.cells	ATIC	0.150273	4.493549	0.957016	0.341169	-5.95093	0.696303	0.608154

B.cells	PITPNM1	0.154055	3.730007	0.95701	0.341172	-5.77873	0.704816	0.621218
B.cells	RPTOR	0.093701	6.294365	0.956972	0.341191	-6.29625	0.6767	0.578419
B.cells	PARD6G	-0.36122	1.981117	-0.95647	0.34144	-5.33924	0.724981	0.652376
B.cells	FHDC1	-0.48567	0.748905	-0.95646	0.341449	-5.03681	0.739442	0.675102
B.cells	CCNDBP1	0.128857	5.692836	0.956418	0.341469	-6.26396	0.683367	0.588368
B.cells	UBALD1	-0.13238	4.983613	-0.95601	0.341672	-6.0037	0.691386	0.600234
B.cells	FBXL14	-0.10992	5.203671	-0.95573	0.341813	-6.04732	0.689122	0.596628
B.cells	SUCO	0.116644	6.354348	0.955629	0.341865	-6.30032	0.676674	0.577837
B.cells	AP5B1	0.369284	1.293299	0.955256	0.342053	-5.16782	0.733728	0.665434
B.cells	C130013H(	0.394405	0.498295	0.955013	0.342175	-5.0114	0.743148	0.680371
B.cells	FANCE	0.205983	2.916052	0.954845	0.34226	-5.55759	0.714924	0.63615
B.cells	RMC1	0.131759	4.652621	0.954782	0.342291	-5.97226	0.695419	0.606136
B.cells	TEX14	-0.15614	5.64791	-0.95458	0.342394	-6.18156	0.684522	0.589571
B.cells	SERGEF	0.144042	4.018452	0.954435	0.342466	-5.89948	0.702469	0.616931
B.cells	TWF2	0.116611	5.240324	0.954421	0.342473	-6.09449	0.68896	0.5963
B.cells	GATM	-0.21459	4.530012	-0.95441	0.342481	-5.79835	0.696776	0.608209
B.cells	CHCHD7	0.131075	4.398636	0.954309	0.342529	-5.88051	0.698233	0.610494
B.cells	ZHX2	0.139402	5.614006	0.954134	0.342617	-6.2034	0.684944	0.590254
B.cells	ENO1B	0.367266	1.327345	0.954006	0.342682	-5.15134	0.733395	0.665053
B.cells	SKIL	-0.10911	7.304614	-0.95375	0.342809	-6.47133	0.666947	0.563196
B.cells	KLHL5	0.113975	4.645979	0.953697	0.342837	-5.97992	0.695619	0.606463
B.cells	4930444A1	0.172266	3.751804	0.953584	0.342894	-5.89248	0.705586	0.621753
B.cells	MPHOSPH(	0.146797	4.258614	0.953423	0.342975	-5.88589	0.699958	0.613086
B.cells	USP38	0.098653	5.889969	0.953273	0.343051	-6.22766	0.682096	0.58588
B.cells	SNAPIN	0.130401	4.083623	0.95307	0.343153	-5.86385	0.702025	0.616095
B.cells	POC1A	-0.21142	3.229083	-0.95283	0.343276	-5.58538	0.71168	0.630941
B.cells	GNB5	0.327865	0.819995	0.952699	0.34334	-5.19357	0.73965	0.674707
B.cells	MYO15	0.424924	1.234713	0.952681	0.343349	-5.14334	0.734746	0.666978
B.cells	SNRPN	-0.71763	-0.6475	-0.95239	0.343498	-4.86593	0.757238	0.702824
B.cells	SH3BP5	0.12134	6.380773	0.952309	0.343536	-6.30469	0.676968	0.578053
B.cells	POFUT2	0.14601	4.257323	0.952281	0.343551	-5.85548	0.700157	0.613256
B.cells	FOCAD	0.235096	2.908895	0.951964	0.34371	-5.51847	0.715505	0.636737
B.cells	NAIP1	-0.56173	-0.47909	-0.95174	0.343822	-4.93807	0.755426	0.699655
B.cells	ASB5	0.409991	0.515697	0.951285	0.344053	-5.09969	0.743459	0.680737
B.cells	INPP5F	0.137859	4.366787	0.951247	0.344072	-6.05156	0.699074	0.611602
B.cells	MACROD1	-0.2682	2.861084	-0.95101	0.344193	-5.47373	0.716051	0.6378
B.cells	PIK3CB	0.249633	4.688415	0.950997	0.344198	-5.65343	0.695509	0.606175
B.cells	GADD45G	-0.22159	4.465474	-0.95089	0.344254	-5.85298	0.697978	0.609991
B.cells	LIG3	0.193933	3.358188	0.950798	0.344299	-5.66829	0.710393	0.629127
B.cells	ZFP385A	-0.15157	3.938147	-0.95072	0.344337	-5.89266	0.703858	0.619096
B.cells	KCTD2	-0.1772	3.355478	-0.95066	0.344368	-5.66049	0.710424	0.629217
B.cells	MATN2	-0.48111	0.980441	-0.95057	0.344414	-5.02098	0.737933	0.672201
B.cells	UCK1	-0.21256	3.265642	-0.95049	0.344455	-5.63516	0.711443	0.630853
B.cells	EFTUD2	0.111727	5.105706	0.950425	0.344487	-6.06309	0.690915	0.599389
B.cells	SUPT3	0.132754	5.480744	0.950365	0.344518	-6.07625	0.686817	0.593178
B.cells	GREB1L	-0.37711	1.947775	-0.95035	0.344526	-5.26821	0.726583	0.654458
B.cells	UFL1	0.140158	3.965196	0.949643	0.344882	-5.78725	0.704022	0.619

B.cells	TGIF1	-0.12657	6.736671	-0.94947	0.34497	-6.43319	0.673747	0.573106
B.cells	SPNS2	-0.33396	1.813184	-0.94942	0.344996	-5.24662	0.728633	0.657285
B.cells	NENF	0.13319	4.583677	0.949277	0.345068	-5.86105	0.69713	0.608576
B.cells	1190007I0	0.182682	3.503332	0.949098	0.345158	-5.63616	0.709222	0.627234
B.cells	FAM167A	0.361975	1.837572	0.948889	0.345264	-5.35473	0.728349	0.65698
B.cells	GM16014	0.512735	-0.09363	0.948844	0.345286	-5.03066	0.751273	0.693184
B.cells	CD55B	-0.56497	0.178504	-0.94881	0.345302	-4.99662	0.747993	0.687968
B.cells	1810030O(	0.119561	4.824335	0.948769	0.345324	-6.01422	0.694469	0.60459
B.cells	2610027KC	0.350801	0.681173	0.948486	0.345468	-5.18441	0.741978	0.678563
B.cells	ITGAD	-0.54971	0.789813	-0.94842	0.3455	-5.0127	0.740685	0.676567
B.cells	NT5C3	0.129577	4.85257	0.948354	0.345534	-6.03641	0.694158	0.604289
B.cells	TIMM9	0.147094	3.882337	0.948145	0.34564	-5.77847	0.704952	0.620868
B.cells	HEATR1	0.140244	4.84445	0.947897	0.345766	-6.039	0.694247	0.604537
B.cells	CXCL9	0.634501	2.080321	0.947858	0.345785	-5.3531	0.725524	0.65287
B.cells	RAB27B	-0.50029	0.615378	-0.94773	0.345852	-5.07914	0.742762	0.680045
B.cells	PSAT1	-0.18805	4.218142	-0.94762	0.345904	-5.89953	0.701194	0.615243
B.cells	PODNL1	-0.48699	-1.02387	-0.94751	0.345962	-4.92188	0.762333	0.711762
B.cells	SCRG1	0.489417	-0.98095	0.947432	0.346001	-4.97112	0.761818	0.710915
B.cells	TMEM135	0.112652	5.7512	0.947426	0.346004	-6.22071	0.684333	0.589571
B.cells	HNRNPUL1	0.071835	7.28107	0.947373	0.346031	-6.4287	0.667982	0.564988
B.cells	ZFP683	0.537015	-1.15907	0.947212	0.346113	-4.95387	0.76399	0.714458
B.cells	RAB33B	-0.1222	4.405753	-0.94714	0.34615	-5.89967	0.699104	0.612125
B.cells	DTD1	0.196001	3.881415	0.946889	0.346276	-5.74297	0.705044	0.621222
B.cells	STARD3	0.156536	4.116736	0.946819	0.346312	-5.84042	0.702408	0.617191
B.cells	LRP1B	-0.32967	1.219155	-0.94662	0.346414	-5.22665	0.735742	0.669067
B.cells	CBS	-0.35732	1.874066	-0.94653	0.346459	-5.32033	0.728062	0.657017
B.cells	MTMR12	0.105237	5.334196	0.946086	0.346683	-6.15435	0.68925	0.596849
B.cells	SETD4	0.172789	2.964232	0.946044	0.346705	-5.64061	0.715741	0.637572
B.cells	FAM220A.1	0.28597	2.200036	0.945773	0.346842	-5.38819	0.724694	0.651379
B.cells	RPAP3	-0.16079	3.841822	-0.9454	0.347029	-5.76345	0.706208	0.622447
B.cells	BAG3	-0.22358	2.967773	-0.945	0.347236	-5.55675	0.716238	0.637947
B.cells	KAT2B	0.108385	6.221283	0.94494	0.347265	-6.31346	0.680143	0.58273
B.cells	ACSL4	0.10867	6.189637	0.944893	0.347289	-6.24834	0.680484	0.58326
B.cells	NEK9	0.123973	5.27603	0.944681	0.347396	-6.1002	0.690405	0.598325
B.cells	ATF7	0.099894	6.314357	0.944414	0.347532	-6.31341	0.679142	0.581388
B.cells	SRC	-0.42745	0.980292	-0.94438	0.34755	-5.09191	0.739386	0.674407
B.cells	NECAP2	0.105123	5.345804	0.944348	0.347565	-6.14649	0.689641	0.597283
B.cells	TAF1	0.108761	5.699019	0.944237	0.347622	-6.16197	0.68579	0.591489
B.cells	UBR3	0.093804	6.575859	0.944149	0.347667	-6.36206	0.67634	0.577249
B.cells	ADAM3	0.423744	-0.17326	0.944095	0.347694	-5.06061	0.753215	0.696458
B.cells	MTHFD1L	0.111842	5.661426	0.943672	0.347909	-6.23492	0.686465	0.592224
B.cells	ZFP790	-0.25045	2.226902	-0.94359	0.34795	-5.40468	0.725048	0.651631
B.cells	PI4KB	0.098802	5.414205	0.943437	0.348028	-6.146	0.689169	0.59636
B.cells	GSTK1	-0.28263	2.449061	-0.94334	0.348076	-5.33493	0.722487	0.647672
B.cells	MON1B	-0.28229	1.881481	-0.94293	0.348286	-5.25029	0.729388	0.65813
B.cells	2310033PC	0.140702	4.144026	0.942778	0.348364	-5.84902	0.703551	0.617981
B.cells	MAGI1	-0.31197	4.373842	-0.94256	0.348472	-5.81191	0.701079	0.614088

B.cells	BCORL1	-0.19586	3.355845	-0.94161	0.348959	-5.72681	0.713409	0.631965
B.cells	CAB39	0.080612	6.974308	0.941255	0.349139	-6.42437	0.67383	0.571503
B.cells	GNPNAT1	-0.13982	4.157449	-0.94089	0.349324	-5.87482	0.704789	0.618188
B.cells	HAGH	-0.13016	5.358255	-0.94083	0.349356	-6.18623	0.691471	0.59787
B.cells	HES6	0.145558	4.105884	0.940624	0.34946	-5.83836	0.705456	0.619162
B.cells	PHYHD1	-0.25754	2.730001	-0.94048	0.349532	-5.44366	0.721125	0.643327
B.cells	MAPK8	0.103575	5.232129	0.940053	0.349752	-6.12866	0.693105	0.600093
B.cells	CNN3	0.171532	4.531127	0.939899	0.34983	-5.94025	0.700865	0.611943
B.cells	PIAS3	-0.17819	2.896492	-0.93989	0.349833	-5.54736	0.719357	0.640424
B.cells	PDCD2L	-0.10287	4.660225	-0.93968	0.34994	-5.99324	0.699428	0.609829
B.cells	PPP2R3A	-0.15817	5.003266	-0.93953	0.350016	-6.10723	0.695628	0.604037
B.cells	SLC29A3	0.168448	3.68183	0.939509	0.350029	-5.73437	0.710402	0.626669
B.cells	VRK2	0.115629	5.691956	0.939508	0.35003	-6.19799	0.688071	0.592565
B.cells	NSUN6	0.169409	3.821373	0.939235	0.350169	-5.78094	0.708933	0.624334
B.cells	CABLES1	-0.28399	4.513404	-0.93916	0.350205	-5.85241	0.701169	0.612423
B.cells	SIDT2	0.120063	5.063847	0.938658	0.350464	-6.06354	0.695287	0.603303
B.cells	2310016D2	0.50784	-0.3578	0.938546	0.350521	-5.01508	0.758239	0.701458
B.cells	SEMA6D	-0.34814	3.713919	-0.93847	0.350558	-5.5614	0.710374	0.626404
B.cells	ASB1	-0.30302	1.712368	-0.93847	0.350561	-5.2498	0.733453	0.662268
B.cells	TBC1D2	-0.3613	1.45751	-0.93822	0.350688	-5.23959	0.736593	0.667119
B.cells	DRAP1	0.079852	6.508223	0.93799	0.350805	-6.32667	0.679723	0.579687
B.cells	LPIN1	0.303576	2.690777	0.93793	0.350836	-5.44588	0.722238	0.644675
B.cells	TFPI	-0.22632	3.468648	-0.93783	0.350889	-5.51892	0.713328	0.630883
B.cells	GSE1	-0.1052	5.664856	-0.93738	0.351114	-6.18525	0.688963	0.593625
B.cells	PPP1R11	0.134794	4.640461	0.937356	0.351129	-5.98112	0.700255	0.610807
B.cells	CCDC62	0.294651	2.858429	0.936995	0.351314	-5.44016	0.720419	0.64201
B.cells	ARHGEF7	0.143737	4.675352	0.936968	0.351327	-5.95438	0.699867	0.610365
B.cells	ZFP420	0.459318	0.275776	0.936902	0.351361	-5.03236	0.75085	0.689853
B.cells	AKR7A5	-0.14217	4.13514	-0.93687	0.351379	-5.89354	0.705905	0.619666
B.cells	ATP2A1	-0.44704	1.448126	-0.93685	0.351387	-5.07191	0.736856	0.667759
B.cells	OTULIN	0.073163	6.57022	0.936765	0.351431	-6.39254	0.679162	0.57909
B.cells	1700066M	-0.31948	1.288003	-0.93641	0.351611	-5.17454	0.739	0.670943
B.cells	ASCC1	0.143013	3.774861	0.936113	0.351765	-5.7821	0.710321	0.626193
B.cells	BACE2	-0.49654	1.120623	-0.93591	0.351867	-5.03023	0.741106	0.674236
B.cells	CCDC186	0.120617	4.760025	0.935872	0.351888	-6.0079	0.699276	0.609319
B.cells	MFAP1A	0.10676	4.832089	0.935607	0.352024	-5.99657	0.698476	0.608099
B.cells	TLL2	-0.5042	0.725481	-0.93559	0.35203	-5.14862	0.74582	0.681679
B.cells	CDC14B	-0.23109	3.454363	-0.93551	0.352073	-5.60166	0.713959	0.631885
B.cells	GM36279	-0.19233	2.959709	-0.93542	0.352119	-5.70158	0.719615	0.640685
B.cells	MS4A3	0.688241	-0.35863	0.935291	0.352186	-4.96608	0.758929	0.702601
B.cells	CDK11B	0.097976	6.82853	0.935229	0.352218	-6.36943	0.676735	0.575318
B.cells	BTBD11	0.304766	4.309098	0.935011	0.352329	-5.68385	0.704408	0.617266
B.cells	MRPS15	0.10013	5.910327	0.934833	0.352421	-6.19508	0.686791	0.590439
B.cells	BC005624	-0.08602	5.761918	-0.93418	0.352757	-6.20512	0.688604	0.593147
B.cells	RPUSD3	-0.4751	0.640562	-0.9341	0.352794	-5.06648	0.747223	0.683997
B.cells	TIMMDC1	0.129326	4.271519	0.934042	0.352826	-5.88253	0.705091	0.618313
B.cells	H2-DMB1	0.207824	4.478206	0.934016	0.352839	-6.23349	0.702777	0.614766

B.cells	CABLES2	-0.15153	3.580958	-0.93384	0.352927	-5.76715	0.712887	0.630361
B.cells	SIAH1A	0.112461	5.079563	0.933678	0.353013	-6.09978	0.696096	0.604692
B.cells	WASHC3	0.120729	4.517912	0.933532	0.353088	-5.91124	0.702334	0.614277
B.cells	NEDD1	0.208762	3.041246	0.93338	0.353166	-5.58411	0.71905	0.6401
B.cells	FAAP100	0.196415	3.097229	0.933378	0.353167	-5.55756	0.718408	0.639104
B.cells	USP4	0.075712	5.660629	0.933298	0.353208	-6.19968	0.68971	0.59512
B.cells	LCMT1	-0.19978	3.124061	-0.93286	0.353433	-5.54994	0.7181	0.638758
B.cells	FNDC3A	0.093454	7.526942	0.932807	0.35346	-6.48892	0.669665	0.565078
B.cells	FMNL3	0.158587	4.258409	0.932737	0.353496	-5.85489	0.705238	0.618908
B.cells	AUTS2	-0.34362	4.397144	-0.9325	0.353618	-5.73058	0.703684	0.616573
B.cells	EME2	0.314791	0.924802	0.932474	0.353631	-5.15157	0.743822	0.67909
B.cells	THAP12	0.116154	4.362104	0.932473	0.353631	-5.92283	0.704076	0.617175
B.cells	TTC25	-0.52791	0.218704	-0.93235	0.353693	-4.98167	0.752304	0.692575
B.cells	NHSL1	-0.36123	1.728678	-0.93233	0.353704	-5.19729	0.734298	0.664125
B.cells	GM30239	0.370145	-0.09316	0.932325	0.353708	-5.08468	0.756085	0.698596
B.cells	OCIAD1	0.086406	5.885315	0.932257	0.353743	-6.21841	0.68726	0.591583
B.cells	ACP1	0.100566	5.923129	0.931988	0.353881	-6.24679	0.686992	0.591079
B.cells	ZFP638	0.087444	6.22747	0.931759	0.353998	-6.30499	0.683691	0.586126
B.cells	PPP4R2	0.090062	6.318519	0.931616	0.354072	-6.32911	0.682707	0.584642
B.cells	EFEMP2	-0.28392	1.944258	-0.9316	0.354082	-5.27913	0.731922	0.660327
B.cells	KANSL2	0.108146	5.119718	0.931483	0.35414	-6.08545	0.695798	0.604484
B.cells	VARS2	-0.39868	1.401859	-0.93131	0.354227	-5.16148	0.738308	0.670381
B.cells	GM33782	0.414528	0.19699	0.931251	0.35426	-5.08366	0.752724	0.693195
B.cells	PUM1	-0.06671	7.56821	-0.931	0.354388	-6.52756	0.66937	0.564715
B.cells	HIC2	-0.23458	2.712677	-0.93087	0.354454	-5.52663	0.722984	0.646459
B.cells	KCNB1	-0.47031	0.988195	-0.93082	0.354481	-5.07509	0.743221	0.678205
B.cells	RYR1	-0.31877	1.831874	-0.93081	0.354488	-5.39551	0.73324	0.662489
B.cells	PIGQ	0.162692	3.519745	0.930432	0.354681	-5.69942	0.713793	0.632253
B.cells	CRIP2	-0.19745	4.565121	-0.9304	0.354696	-5.82336	0.702014	0.614122
B.cells	AKIRIN2	-0.09845	5.756269	-0.93029	0.354756	-6.19423	0.688869	0.594117
B.cells	ZFP672	0.136253	4.004158	0.930273	0.354763	-5.82753	0.708306	0.623814
B.cells	MED28	-0.09078	6.023126	-0.93014	0.354832	-6.23171	0.68598	0.589741
B.cells	ESYT1	0.107949	5.654914	0.929951	0.354929	-6.23138	0.690061	0.595858
B.cells	CRAT	0.213952	3.198294	0.929681	0.355068	-5.59636	0.717575	0.638033
B.cells	SIRT1	-0.10243	5.043074	-0.92963	0.355094	-6.04859	0.696815	0.606099
B.cells	FTH1	0.16574	12.5537	0.929571	0.355124	-7.22396	0.619307	0.491844
B.cells	PIBF1	0.118371	5.059147	0.929263	0.355283	-6.06436	0.696828	0.605929
B.cells	ANKRD61	0.367291	0.799732	0.929042	0.355397	-5.1604	0.745969	0.682268
B.cells	FBXW2	0.078198	6.193608	0.92851	0.355671	-6.31358	0.684924	0.587365
B.cells	ETHE1	0.122257	4.997365	0.928283	0.355788	-6.04653	0.698036	0.607326
B.cells	IFIH1	0.278658	3.912874	0.928198	0.355832	-5.6381	0.710176	0.626001
B.cells	SYT14	0.456412	0.008242	0.928152	0.355856	-5.20215	0.75597	0.697809
B.cells	SHE	-0.49174	0.946591	-0.92793	0.355971	-5.08116	0.744663	0.67992
B.cells	VPS37B	0.125986	7.797142	0.927914	0.355978	-6.61446	0.667808	0.561915
B.cells	GNPTG	-0.16979	3.368723	-0.92761	0.356134	-5.63221	0.716449	0.63571
B.cells	SEC24C	0.105704	4.859773	0.927468	0.356209	-6.0429	0.699648	0.609982
B.cells	ADD1	0.100875	5.795836	0.927372	0.356258	-6.21445	0.689337	0.594333

B.cells	MCCC2	-0.31531	2.436244	-0.92723	0.356329	-5.44703	0.727196	0.652605
B.cells	CHCHD4	0.161124	3.820424	0.927163	0.356366	-5.79294	0.71131	0.62795
B.cells	MED4	-0.12436	4.020834	-0.92693	0.356486	-5.84473	0.709044	0.624555
B.cells	GM43256	-0.50683	0.309245	-0.92661	0.35665	-4.97645	0.752414	0.692533
B.cells	B230208H1	-0.48685	0.861264	-0.92656	0.356679	-5.13445	0.745774	0.681998
B.cells	LIMA1	-0.21004	4.507655	-0.92635	0.356785	-5.98205	0.703574	0.616296
B.cells	ATP2A3	0.117719	5.210591	0.926168	0.35688	-6.1125	0.695763	0.604452
B.cells	BTG2	-0.11774	7.421641	-0.9261	0.356916	-6.50425	0.671852	0.568413
B.cells	CDS2	0.120153	4.708784	0.926035	0.356949	-5.99552	0.701328	0.613017
B.cells	CDK6	0.15947	6.322112	0.925912	0.357012	-6.27127	0.683619	0.586146
B.cells	PDCL	0.160196	3.891934	0.925721	0.357111	-5.81247	0.710501	0.627282
B.cells	UNK	0.146367	4.287183	0.925433	0.35726	-5.93698	0.706045	0.620517
B.cells	CPOX	-0.13032	4.570271	-0.92529	0.357335	-6.01894	0.702874	0.615647
B.cells	SNRPD1	-0.09861	7.17842	-0.92525	0.357353	-6.43177	0.674434	0.572529
B.cells	GM15492	-0.38807	0.742028	-0.9252	0.357379	-5.13239	0.747202	0.684813
B.cells	BMP2K	0.084053	6.936172	0.925153	0.357404	-6.44495	0.677018	0.576403
B.cells	USP36	0.116576	4.970109	0.925122	0.357421	-6.07592	0.698424	0.608832
B.cells	OGFR	0.129632	5.244677	0.925089	0.357438	-6.13074	0.695387	0.604196
B.cells	TJP1	-0.33859	2.024999	-0.92491	0.35753	-5.24315	0.731995	0.660872
B.cells	DALRD3	0.143384	3.729807	0.924696	0.357641	-5.75971	0.712338	0.630316
B.cells	AGO2	0.083435	7.576219	0.924481	0.357752	-6.53842	0.670217	0.566332
B.cells	GM14471	0.470049	0.24931	0.924449	0.357769	-5.00385	0.753139	0.694379
B.cells	GABPA	-0.11556	4.662072	-0.92441	0.357787	-5.99206	0.701849	0.614192
B.cells	SIRT3	0.170764	3.344497	0.92441	0.357789	-5.64117	0.716726	0.637134
B.cells	AHSA1	-0.11703	5.071608	-0.92406	0.35797	-6.0785	0.697299	0.607347
B.cells	KIF3B	0.156793	3.495441	0.924046	0.357977	-5.71624	0.715003	0.634586
B.cells	DDX11	-0.2461	2.934033	-0.92379	0.358109	-5.49642	0.721436	0.644578
B.cells	REEP4	-0.1529	4.356559	-0.92371	0.358152	-5.93777	0.705266	0.619571
B.cells	IKZF4	-0.44431	-0.0706	-0.92327	0.358377	-4.98621	0.757022	0.700815
B.cells	CHURC1	-0.09225	5.66012	-0.92322	0.358405	-6.20154	0.690821	0.597569
B.cells	VPS51	0.145392	3.730024	0.923195	0.358418	-5.8604	0.712335	0.630554
B.cells	TGFA	-0.43429	0.796415	-0.9231	0.35847	-5.15265	0.74655	0.684167
B.cells	GM46218	0.460306	0.58606	0.923062	0.358487	-5.02804	0.749076	0.688175
B.cells	AEN	0.172979	3.664015	0.923052	0.358492	-5.7984	0.713085	0.631731
B.cells	PEX3	-0.14629	3.770542	-0.92272	0.358663	-5.73901	0.711876	0.629987
B.cells	CPED1	-0.30774	2.524781	-0.9227	0.358674	-5.48569	0.726167	0.652206
B.cells	TMEM41A	-0.36257	1.51992	-0.92269	0.358682	-5.18419	0.737939	0.670685
B.cells	SYVN1	-0.17672	4.228615	-0.92266	0.358698	-5.87383	0.706703	0.622006
B.cells	INTS4	0.136511	3.920928	0.922627	0.358713	-5.88855	0.710173	0.627356
B.cells	DCTPP1	-0.15495	5.089369	-0.92238	0.358839	-6.05131	0.697103	0.607365
B.cells	APPL2	0.235934	3.358029	0.922173	0.358948	-5.6107	0.716571	0.637351
B.cells	POU5F1	-0.31689	1.26222	-0.9221	0.358984	-5.25479	0.740993	0.675606
B.cells	LRPAP1	-0.134	4.499631	-0.92175	0.359169	-5.96713	0.703664	0.617521
B.cells	RAB27A	0.151272	4.50985	0.921701	0.359193	-5.8956	0.703549	0.61735
B.cells	RAD51	-0.24976	3.891445	-0.92164	0.359225	-5.72318	0.710506	0.628104
B.cells	BLOC1S6	-0.15822	3.751167	-0.92107	0.359523	-5.72997	0.712095	0.630847
B.cells	NCK2	-0.13563	4.870391	-0.921	0.359554	-6.16407	0.69953	0.611502

B.cells	B9D1	-0.50948	0.371188	-0.92088	0.359621	-4.98754	0.751665	0.69307
B.cells	PDZK1	-0.46694	0.933701	-0.92087	0.359626	-5.13039	0.744907	0.682327
B.cells	MRPS25	-0.13547	4.238863	-0.92079	0.359668	-5.91702	0.706588	0.622428
B.cells	WDR27	0.397709	0.499373	0.920585	0.359772	-5.06365	0.750119	0.690668
B.cells	LGALS3	0.215286	6.142988	0.920484	0.359825	-6.06551	0.685559	0.590319
B.cells	ATP5E	0.069332	8.783189	0.920367	0.359885	-6.7097	0.657613	0.548494
B.cells	BBS7	-0.57416	0.558111	-0.9202	0.35997	-4.97627	0.749412	0.689571
B.cells	ALKBH3	0.173192	3.490416	0.920085	0.360032	-5.71542	0.71506	0.635638
B.cells	PSTK	0.171343	3.061289	0.92007	0.36004	-5.62031	0.719972	0.643273
B.cells	TBC1D23	0.094118	5.852371	0.920025	0.360063	-6.2519	0.68872	0.595192
B.cells	4930513N1	-0.44946	0.475363	-0.91989	0.360132	-5.03466	0.750408	0.691226
B.cells	ITGB7	0.143236	4.620993	0.919763	0.360199	-6.18356	0.702308	0.615956
B.cells	CASC3	-0.10914	5.292913	-0.91974	0.360211	-6.1234	0.694855	0.604541
B.cells	UBR7	0.148133	4.241439	0.919681	0.360242	-5.86359	0.706559	0.622506
B.cells	HEATR5A	-0.12671	5.656048	-0.91945	0.360365	-6.18835	0.690866	0.598586
B.cells	SMIM13	-0.14816	3.919727	-0.91934	0.360418	-5.7997	0.710186	0.628249
B.cells	DDX10	0.092862	5.436521	0.919341	0.360419	-6.18734	0.693274	0.602274
B.cells	BACH1	-0.1256	5.9952	-0.9191	0.360546	-6.33026	0.687165	0.593044
B.cells	SERF1	-0.18528	3.137334	-0.91903	0.36058	-5.54485	0.719099	0.642148
B.cells	TRIM47	-0.24957	2.41312	-0.919	0.360594	-5.41807	0.727465	0.655216
B.cells	KITL	-0.58668	1.956944	-0.91894	0.360629	-5.20268	0.732792	0.663602
B.cells	ATAD1	0.076642	5.731262	0.918911	0.360642	-6.21086	0.690043	0.597438
B.cells	ADHFE1	0.247539	2.690792	0.918792	0.360704	-5.50787	0.724244	0.650243
B.cells	CYSLTR1	-0.24649	2.70023	-0.91862	0.360793	-5.60788	0.724134	0.650172
B.cells	MAFB	0.255461	4.60884	0.918595	0.360807	-5.84745	0.702443	0.61654
B.cells	ZFP472	-0.16965	3.448072	-0.91857	0.360818	-5.7327	0.715543	0.636784
B.cells	PICK1	0.299515	1.538716	0.91846	0.360877	-5.31807	0.737716	0.671514
B.cells	MED12	0.151518	3.781462	0.91843	0.360893	-5.8083	0.711752	0.630903
B.cells	HSPE1	-0.11195	7.179751	-0.91838	0.360917	-6.46026	0.67442	0.573954
B.cells	SLC16A4	-0.29332	1.428027	-0.91837	0.360926	-5.2827	0.739026	0.673585
B.cells	ESS2	0.167751	2.988114	0.918272	0.360975	-5.61317	0.720813	0.645007
B.cells	MIEN1	-0.08487	5.845651	-0.9181	0.361063	-6.2237	0.688846	0.5957
B.cells	SLC46A2	0.422831	-0.09426	0.917944	0.361145	-5.0538	0.757381	0.70278
B.cells	1810013L2	-0.09817	5.901803	-0.91785	0.361192	-6.24638	0.688246	0.594851
B.cells	NIPA2	0.096273	6.042829	0.917627	0.36131	-6.28774	0.68682	0.592605
B.cells	SHF	-0.28634	1.832889	-0.91731	0.361477	-5.28114	0.734612	0.66637
B.cells	CYTH3	-0.13191	4.88921	-0.91692	0.36168	-6.08796	0.699667	0.612076
B.cells	ICE2	0.231763	2.160721	0.916866	0.361707	-5.39629	0.730768	0.660357
B.cells	BCAP31	-0.08848	5.901725	-0.91655	0.361874	-6.2209	0.688523	0.595126
B.cells	ABCA5	0.515343	0.444818	0.916544	0.361875	-5.00641	0.751148	0.692679
B.cells	ASL	-0.14098	4.484774	-0.91643	0.361935	-6.00816	0.704178	0.619101
B.cells	GM15494	-0.49483	0.759331	-0.91641	0.361947	-5.11256	0.747364	0.686696
B.cells	ZFPM2	-0.43256	2.777013	-0.91618	0.362065	-5.37207	0.723605	0.649308
B.cells	TM9SF2	-0.06556	6.683958	-0.91616	0.362076	-6.37686	0.680056	0.5824
B.cells	TSEN2	0.341577	1.266976	0.916129	0.362091	-5.17163	0.741303	0.67715
B.cells	KDM4B	0.143136	4.441359	0.915999	0.362159	-5.98697	0.704664	0.619963
B.cells	VAMP8	0.08463	7.209731	0.915945	0.362187	-6.46146	0.674435	0.573997

B.cells	PLEKHN1	-0.25822	1.876111	-0.91593	0.362193	-5.3938	0.734104	0.665851
B.cells	TMEM163	0.141308	6.026941	0.915725	0.362302	-6.30577	0.687249	0.593261
B.cells	PHC2	0.115008	5.798035	0.915574	0.362381	-6.25858	0.689755	0.597054
B.cells	ZW10	-0.15357	4.014837	-0.91548	0.36243	-5.82055	0.709565	0.627463
B.cells	A530041M	0.237249	2.069945	0.914726	0.362823	-5.40477	0.732608	0.662581
B.cells	RAB13	-0.36044	1.635625	-0.91448	0.362951	-5.235	0.737854	0.670636
B.cells	WDR75	-0.16106	3.575364	-0.91436	0.363012	-5.74218	0.715336	0.635412
B.cells	HCFC1R1	0.108745	5.33224	0.914219	0.363088	-6.14204	0.695658	0.60514
B.cells	GM16675	0.395725	1.196561	0.913587	0.363419	-5.22838	0.743583	0.679186
B.cells	UNC13D	0.17495	3.31115	0.913393	0.36352	-5.7024	0.718857	0.640418
B.cells	AGPS	0.083318	6.813953	0.913284	0.363577	-6.39887	0.679981	0.58092
B.cells	A230059L0	-0.48644	0.47539	-0.91327	0.363587	-5.10921	0.752239	0.692948
B.cells	DENND1A	0.080662	7.497877	0.913176	0.363633	-6.53727	0.672683	0.569964
B.cells	THBS1	0.473385	4.967156	0.913024	0.363713	-5.77164	0.700196	0.611636
B.cells	GLIS2	-0.33887	1.3513	-0.91275	0.363854	-5.2392	0.741827	0.676435
B.cells	COG6	0.20295	3.137898	0.912744	0.36386	-5.56501	0.720931	0.64365
B.cells	MVB12A	0.099305	5.948563	0.912524	0.363975	-6.22404	0.689532	0.595238
B.cells	NCOR1	0.060884	8.098707	0.911962	0.364269	-6.60681	0.666902	0.560886
B.cells	SLC38A7	0.222566	2.59539	0.911579	0.364469	-5.57942	0.727723	0.653791
B.cells	MAP4	0.085349	6.676158	0.911524	0.364498	-6.41656	0.682029	0.583592
B.cells	ARFGEF1	0.093632	6.613928	0.91149	0.364516	-6.37679	0.6827	0.584604
B.cells	GM3448	-0.30681	1.9113	-0.91137	0.364579	-5.29755	0.735729	0.666371
B.cells	LINS1	-0.28361	1.704648	-0.91126	0.364639	-5.22281	0.738167	0.670281
B.cells	FRG2F1	-0.38567	0.702787	-0.91111	0.364712	-5.05584	0.750121	0.689204
B.cells	ACAD11	-0.26228	2.331168	-0.91088	0.364837	-5.33292	0.730803	0.658736
B.cells	CD5	-0.19675	2.693985	-0.9108	0.364877	-5.64758	0.726578	0.652144
B.cells	TXLNG	0.103921	5.107247	0.910696	0.364932	-6.16286	0.699186	0.609809
B.cells	COX7A1	-0.36728	0.986976	-0.91053	0.365019	-5.12333	0.746708	0.683867
B.cells	DVL3	-0.16456	3.300094	-0.9105	0.365036	-5.66561	0.719582	0.641268
B.cells	8-Sep	-0.32981	1.954462	-0.91035	0.365114	-5.1915	0.735221	0.665747
B.cells	PWWP2B	-0.31003	2.082717	-0.9101	0.365244	-5.28355	0.733713	0.663416
B.cells	TOX4	0.079963	5.903687	0.910089	0.36525	-6.26932	0.690414	0.596491
B.cells	2610035D1	-0.18429	4.872984	-0.90977	0.365418	-6.05395	0.701792	0.613877
B.cells	PFKFB3	0.125009	6.273061	0.909701	0.365453	-6.32375	0.68639	0.590409
B.cells	NME7	-0.14258	3.562527	-0.9095	0.365558	-5.73778	0.716577	0.636694
B.cells	ADCY3	-0.33605	3.001893	-0.90947	0.365574	-5.54175	0.723014	0.646704
B.cells	SCFD2	0.104474	5.78946	0.909403	0.36561	-6.22714	0.691664	0.598423
B.cells	IFT74	-0.27161	2.322027	-0.90931	0.365656	-5.4041	0.73091	0.659051
B.cells	CDC42SE1	-0.0905	6.18973	-0.90931	0.36566	-6.29459	0.687295	0.591791
B.cells	ZFP811	-0.68098	-1.02506	-0.90929	0.365668	-4.87106	0.770981	0.723242
B.cells	LTB4R1	0.405587	2.689349	0.909145	0.365745	-5.31588	0.726632	0.652396
B.cells	KCNIP4	-0.57013	0.608323	-0.909	0.365819	-5.11696	0.751259	0.691304
B.cells	PPP1R16B	0.124049	6.504104	0.908861	0.365895	-6.37759	0.683886	0.586744
B.cells	MEF2D	-0.08758	7.288749	-0.90886	0.365897	-6.49752	0.675466	0.574067
B.cells	4921524J1	-0.09111	5.474163	-0.90885	0.365899	-6.17143	0.695129	0.603812
B.cells	GAS8	-0.26836	1.529564	-0.90869	0.365986	-5.27932	0.740293	0.673939
B.cells	MRPS33	-0.07843	6.714474	-0.90805	0.366321	-6.39445	0.682106	0.583664

B.cells	TMEM30A	0.070418	6.581779	0.908002	0.366346	-6.37596	0.683537	0.585829
B.cells	CCSER2	0.122473	4.778998	0.907838	0.366431	-6.09077	0.703352	0.616051
B.cells	PCDH7	0.582909	0.520175	0.90776	0.366473	-5.11032	0.752872	0.693549
B.cells	SEM1	-0.06912	9.100384	-0.90736	0.366685	-6.76928	0.657238	0.546371
B.cells	ARHGAP9	0.12825	5.14536	0.906755	0.367001	-6.05908	0.699949	0.610071
B.cells	MBLAC2	0.211507	2.64789	0.906599	0.367083	-5.55308	0.728347	0.654008
B.cells	COPS4	0.092199	5.581159	0.906255	0.367264	-6.17939	0.695128	0.602835
B.cells	COPS6	0.108378	5.552293	0.906243	0.36727	-6.15882	0.695446	0.603319
B.cells	AIRN	-0.16869	5.663175	-0.90592	0.367441	-6.24638	0.694225	0.601466
B.cells	RESF1	0.102821	6.500817	0.905914	0.367443	-6.37729	0.685082	0.587602
B.cells	ABCC9	0.445255	1.277502	0.905885	0.367459	-5.14557	0.744498	0.679524
B.cells	ICE1	0.151899	4.081492	0.905834	0.367485	-5.8781	0.711885	0.628541
B.cells	LTA4H	-0.14245	5.724021	-0.90583	0.367488	-6.15783	0.693556	0.600448
B.cells	1700028E1	0.31521	1.36693	0.905636	0.36759	-5.18898	0.743431	0.677893
B.cells	3-Sep	-0.37001	-1.00287	-0.90558	0.367617	-5.09055	0.772019	0.723988
B.cells	ATP6V1F	0.073322	6.902317	0.905432	0.367697	-6.43411	0.680751	0.581184
B.cells	DNAJC18	0.149251	3.682567	0.905408	0.36771	-5.83983	0.716422	0.635693
B.cells	COX6A2	-0.5125	2.070766	-0.90533	0.367749	-5.13631	0.735099	0.664874
B.cells	CXCL10	0.378156	4.788598	0.905057	0.367895	-5.853	0.703989	0.616494
B.cells	KIF13B	0.100024	6.510852	0.905038	0.367905	-6.32901	0.685035	0.587631
B.cells	PTPRA	0.072204	6.521638	0.904414	0.368234	-6.37999	0.685416	0.587694
B.cells	DBT	-0.16832	3.826101	-0.90404	0.368429	-5.81445	0.715628	0.633688
B.cells	METTL6	0.131084	4.505421	0.90353	0.3687	-5.98001	0.708234	0.621937
B.cells	GM8113	-0.54383	-0.25687	-0.90351	0.368708	-4.93346	0.764296	0.709979
B.cells	SERPINH1	-0.34803	2.557349	-0.90318	0.368883	-5.35408	0.73074	0.656679
B.cells	DDX39	-0.11625	6.372696	-0.90312	0.368916	-6.33947	0.687731	0.590522
B.cells	ULK1	0.164041	3.637915	0.902904	0.36903	-5.75777	0.71825	0.63727
B.cells	BHMT2	-0.32785	2.060069	-0.90288	0.369041	-5.43309	0.736577	0.665872
B.cells	CAPRIN2	-0.26213	3.181523	-0.90219	0.369405	-5.58038	0.724088	0.645603
B.cells	MTOR	0.106657	4.766611	0.9018	0.369613	-6.0636	0.706325	0.617779
B.cells	DNAJC8	-0.0614	6.901934	-0.90168	0.369678	-6.43586	0.682842	0.582153
B.cells	NUPL2	0.253402	2.367649	0.901541	0.36975	-5.43487	0.733881	0.660482
B.cells	ITGB5	-0.28092	3.058168	-0.90117	0.369946	-5.54503	0.725875	0.648008
B.cells	KIF2C	-0.33473	2.663513	-0.90116	0.369952	-5.3862	0.730464	0.655162
B.cells	USP22	-0.11412	4.389395	-0.90115	0.369958	-5.95447	0.710642	0.62444
B.cells	MAF1	-0.12201	5.342723	-0.90099	0.370043	-6.15394	0.700007	0.60813
B.cells	MSL3	-0.1117	4.998465	-0.90072	0.370186	-6.05694	0.703997	0.614103
B.cells	CPT2	-0.26358	2.829472	-0.9004	0.370351	-5.48843	0.728847	0.652478
B.cells	DAB2IP	-0.33332	2.225574	-0.9003	0.370407	-5.24725	0.735916	0.663555
B.cells	CEP152	0.159857	3.629164	0.90026	0.370428	-5.75079	0.719608	0.638145
B.cells	DLD	-0.1066	4.599821	-0.89983	0.370654	-5.98746	0.708892	0.621298
B.cells	ZFP800	0.098117	5.365723	0.899527	0.370815	-6.18978	0.700361	0.608309
B.cells	PSMB5	0.098401	6.258665	0.899426	0.370869	-6.32854	0.690525	0.593399
B.cells	HIST1H1C	-0.21786	4.106663	-0.89925	0.37096	-5.8049	0.714512	0.630057
B.cells	PSEN1	0.07784	6.297037	0.899224	0.370976	-6.36892	0.690106	0.592785
B.cells	ENDOV	0.28149	2.177707	0.899213	0.370982	-5.37677	0.736845	0.664795
B.cells	SLC25A16	0.150462	3.724214	0.898639	0.371286	-5.7911	0.718926	0.636912

B.cells	SMIM24	0.173234	3.210803	0.898535	0.371341	-5.64487	0.724835	0.646109
B.cells	PEX11A	-0.44869	0.072757	-0.89845	0.371388	-5.07862	0.762197	0.705026
B.cells	HLCS	0.150705	4.15558	0.898326	0.371451	-5.98567	0.714005	0.629404
B.cells	KLHL21	-0.23025	2.689731	-0.89826	0.371484	-5.5846	0.73089	0.655608
B.cells	LRFN1	-0.46786	1.097146	-0.89826	0.371489	-5.11695	0.749762	0.685287
B.cells	WDPCP	-0.20299	3.269533	-0.89822	0.371507	-5.64277	0.724156	0.645117
B.cells	DICER1	0.097859	4.846948	0.898203	0.371517	-6.07132	0.7062	0.617406
B.cells	SERPINB8	-0.4044	-0.3114	-0.89812	0.371563	-5.09674	0.766921	0.712655
B.cells	FLII	0.091474	5.969326	0.897865	0.371696	-6.3049	0.693875	0.598523
B.cells	DPF3	-0.35737	1.667384	-0.89757	0.371854	-5.28436	0.743276	0.674815
B.cells	MTG1	0.263117	2.186733	0.897088	0.372108	-5.44962	0.737321	0.665232
B.cells	SPEN	0.089171	5.922479	0.896872	0.372223	-6.27356	0.694757	0.599578
B.cells	RSL24D1	0.090164	5.674542	0.896828	0.372246	-6.26495	0.697491	0.603731
B.cells	SPACA6	0.38509	1.094819	0.896317	0.372517	-5.14409	0.75033	0.685968
B.cells	G6PC	-0.40369	1.911843	-0.89612	0.372624	-5.36519	0.740571	0.670641
B.cells	BLMH	-0.08616	5.648784	-0.896	0.372685	-6.23132	0.697776	0.604432
B.cells	MLST8	-0.3115	2.044303	-0.89578	0.372802	-5.30777	0.739003	0.668332
B.cells	IL1F9	0.48998	-0.11555	0.895699	0.372846	-5.07223	0.765059	0.709657
B.cells	FAM57B	-0.50595	0.285081	-0.89567	0.37286	-5.02817	0.760147	0.70181
B.cells	UHRF1BP1I	0.099357	5.734451	0.895631	0.372882	-6.24615	0.696829	0.603116
B.cells	CDH22	0.745387	-0.85879	0.895626	0.372885	-4.85657	0.774033	0.724452
B.cells	COBLL1	0.134623	5.069977	0.895564	0.372917	-6.11286	0.704211	0.614393
B.cells	RFX2	-0.19726	3.630115	-0.89542	0.372996	-5.70018	0.720523	0.639553
B.cells	INTS6	-0.12971	6.588587	-0.89532	0.373048	-6.3625	0.687475	0.588991
B.cells	1700094D0	-0.28745	1.476408	-0.89507	0.37318	-5.23092	0.745754	0.679033
B.cells	SNRPA	-0.09672	5.131803	-0.89506	0.373185	-6.11788	0.70352	0.61337
B.cells	SLC16A9	-0.58457	0.530927	-0.89483	0.373307	-5.06977	0.757151	0.697105
B.cells	MCMDC2	-0.17174	3.465667	-0.8948	0.373323	-5.97223	0.722415	0.642486
B.cells	STT3B	0.073456	6.666062	0.894707	0.373373	-6.37456	0.686634	0.587722
B.cells	HIBCH	-0.15076	3.478236	-0.89469	0.373381	-5.70252	0.72227	0.642262
B.cells	TOR1A	0.116478	4.653633	0.894638	0.37341	-5.99795	0.708883	0.62159
B.cells	GABPB1	-0.10336	5.380129	-0.89464	0.373411	-6.14949	0.700754	0.609144
B.cells	IPPK	0.175612	3.400734	0.894616	0.373421	-5.72608	0.723163	0.643648
B.cells	NAA35	-0.10844	4.851078	-0.89443	0.373518	-6.04194	0.706663	0.618258
B.cells	CHERP	-0.10122	5.016827	-0.8943	0.373588	-6.07812	0.704805	0.615468
B.cells	1110051M	-0.18842	3.263469	-0.89409	0.373701	-5.623	0.724748	0.646319
B.cells	SMC1B	-0.61241	-0.03473	-0.89406	0.373718	-4.9582	0.764065	0.708371
B.cells	BMP1	-0.47101	0.911089	-0.89391	0.373797	-5.10138	0.752544	0.690048
B.cells	APBA1	0.16008	4.384633	0.893896	0.373804	-6.08534	0.711921	0.626503
B.cells	EIF4G2	-0.06573	7.71796	-0.8938	0.373855	-6.55763	0.675333	0.571026
B.cells	CAPRIN1	0.064528	7.460311	0.893756	0.373879	-6.53467	0.678081	0.575147
B.cells	SLC35F2	-0.43963	0.357488	-0.8936	0.37396	-5.07947	0.759263	0.700849
B.cells	GM43328	0.234775	2.238918	0.893543	0.373992	-5.51839	0.736706	0.665176
B.cells	PPP2R2D	-0.09103	5.459388	-0.8934	0.374067	-6.18472	0.699886	0.608154
B.cells	CRNKL1	-0.09499	5.293781	-0.89295	0.374306	-6.17692	0.701727	0.611121
B.cells	OXSR1	0.162471	3.496646	0.892925	0.374321	-5.75522	0.72207	0.642478
B.cells	CCDC61	0.194128	2.934126	0.892859	0.374356	-5.57066	0.728579	0.652612

B.cells	NARF	0.11687	4.893244	0.89283	0.374372	-6.09115	0.706202	0.617981
B.cells	ANGPTL4	-0.40117	1.23193	-0.89265	0.374467	-5.13965	0.748695	0.684289
B.cells	TSEN34	-0.15377	4.195681	-0.89262	0.374485	-5.84238	0.714076	0.630147
B.cells	5031425F1	-0.57732	-0.17235	-0.89253	0.374532	-4.94315	0.765771	0.711505
B.cells	IFIT3B	0.518535	1.43036	0.892443	0.374578	-5.2074	0.746317	0.68055
B.cells	PHF1	0.214634	2.695984	0.892372	0.374616	-5.57954	0.731355	0.657028
B.cells	PPP1R15B	-0.10941	5.553021	-0.89178	0.374933	-6.20795	0.699324	0.607095
B.cells	RFC3	-0.13952	4.31335	-0.89147	0.375094	-5.87791	0.713329	0.628493
B.cells	ARHGAP27	-0.24532	2.032169	-0.89144	0.375111	-5.3791	0.73977	0.669675
B.cells	UBE2R2	0.080605	7.144409	0.891187	0.375247	-6.52325	0.682089	0.580875
B.cells	PPP1R9A	-0.2812	3.383193	-0.89116	0.37526	-5.54372	0.724025	0.644992
B.cells	SHLD2	0.150546	3.853936	0.890998	0.375348	-5.85861	0.71863	0.636651
B.cells	ANK2	-0.45656	2.998552	-0.89092	0.375393	-5.46437	0.728498	0.652031
B.cells	RIIAD1	-0.34987	0.993222	-0.8905	0.375613	-5.17493	0.75238	0.689529
B.cells	4833408A1	0.486957	0.127774	0.890227	0.37576	-5.08756	0.762909	0.706377
B.cells	SETD3	0.090456	5.687067	0.890204	0.375772	-6.22613	0.69812	0.605182
B.cells	TMEM51OS	-0.50227	-0.90367	-0.89016	0.375795	-4.91687	0.775432	0.726879
B.cells	NME4	-0.34783	2.364464	-0.89014	0.375804	-5.32133	0.736036	0.663813
B.cells	PACRG	-0.58083	-0.43352	-0.8901	0.375825	-4.92504	0.769827	0.717464
B.cells	DNAJC2	0.08928	5.81945	0.889667	0.376059	-6.25733	0.696866	0.603151
B.cells	NRAP	-0.55967	-1.09404	-0.88966	0.376062	-4.87862	0.777998	0.730961
B.cells	PHACTR2	-0.14283	6.036575	-0.88907	0.376376	-6.15718	0.69471	0.599757
B.cells	DHRS3	-0.21186	4.170119	-0.88906	0.376382	-5.76156	0.715597	0.631745
B.cells	4930526L0	0.412302	-0.8055	0.889039	0.376395	-5.06889	0.774744	0.725429
B.cells	NAA25	0.156447	3.927641	0.88896	0.376437	-5.9014	0.718364	0.636023
B.cells	DAP	-0.12298	6.19848	-0.88881	0.376517	-6.24044	0.692967	0.597099
B.cells	PLA1A	-0.30785	1.841769	-0.88865	0.376602	-5.42639	0.742754	0.67408
B.cells	GM9993	0.349747	1.16551	0.888554	0.376654	-5.22125	0.750846	0.68688
B.cells	GM4013	0.236957	1.984122	0.888242	0.376821	-5.34778	0.741232	0.671497
B.cells	RPP40	-0.34396	1.432828	-0.88816	0.376863	-5.19559	0.747805	0.681866
B.cells	PIGG	0.382596	0.963956	0.887955	0.376974	-5.14146	0.753496	0.690935
B.cells	RARA	-0.10754	5.561492	-0.88782	0.377047	-6.27015	0.700219	0.607987
B.cells	PRPS2	-0.12663	4.382206	-0.88777	0.377073	-5.95347	0.713453	0.62831
B.cells	MMP27	0.519964	-0.20195	0.887233	0.377361	-4.96303	0.768203	0.713906
B.cells	GMPPA	0.172677	3.453598	0.887101	0.377431	-5.73739	0.724532	0.644954
B.cells	CMPK1	0.067968	6.881961	0.886673	0.377661	-6.45945	0.686445	0.586345
B.cells	TENT5C	0.200615	5.86321	0.886588	0.377706	-6.30974	0.697593	0.603213
B.cells	GM15952	-0.35912	1.028007	-0.88646	0.377773	-5.21754	0.753501	0.690099
B.cells	GM47819	0.631003	-0.22199	0.886251	0.377886	-4.96831	0.76889	0.71452
B.cells	NR3C2	-0.36471	1.939637	-0.88548	0.378302	-5.32025	0.743047	0.673117
B.cells	BC005537	-0.0826	7.116434	-0.88545	0.378317	-6.45841	0.684352	0.58281
B.cells	ZFP521	-0.41434	2.100385	-0.88528	0.378408	-5.28732	0.741138	0.670211
B.cells	SYN1	0.456652	0.783746	0.885135	0.378485	-5.13191	0.756942	0.695264
B.cells	GM13822	0.521095	1.17949	0.884861	0.378632	-5.26846	0.752151	0.687722
B.cells	RP9	0.069724	6.834563	0.884856	0.378634	-6.41931	0.687404	0.587588
B.cells	IFI204	0.306732	4.390085	0.884838	0.378644	-5.73956	0.714557	0.628968
B.cells	STAM	0.112547	4.382468	0.884769	0.378681	-5.96081	0.714643	0.629124

B.cells	ZFP688	0.286192	1.695397	0.884764	0.378684	-5.33293	0.745958	0.677956
B.cells	EGFEM1	0.662654	1.229611	0.884634	0.378754	-5.05353	0.751547	0.686812
B.cells	NCK1	0.102568	6.174664	0.884628	0.378757	-6.29697	0.694612	0.598524
B.cells	NDUFB11	-0.07841	7.55899	-0.88424	0.378967	-6.54992	0.679716	0.576052
B.cells	A430033K(	0.421599	0.6406	0.884218	0.378977	-5.14007	0.758821	0.69833
B.cells	UCHL1	-0.34701	0.862756	-0.88415	0.379012	-5.29414	0.75612	0.694034
B.cells	SLMAP	0.089174	6.393027	0.883498	0.379363	-6.36068	0.692872	0.595327
B.cells	PARD3	-0.33984	2.300134	-0.88318	0.379534	-5.39237	0.739592	0.667291
B.cells	ZDHHC16	0.16188	2.888345	0.883153	0.379549	-5.59462	0.732673	0.656474
B.cells	PCGF2	-0.43266	0.79144	-0.88288	0.379694	-5.0846	0.757822	0.695918
B.cells	KCTD18	0.245475	3.044012	0.882795	0.379741	-5.57503	0.730986	0.653682
B.cells	IGKV2-109	-0.39977	-0.87987	-0.88259	0.37985	-5.03462	0.778266	0.729039
B.cells	POLDIP3	-0.08144	6.078814	-0.88253	0.379886	-6.32329	0.696599	0.600793
B.cells	LENG1	0.178905	2.925078	0.88208	0.380125	-5.57982	0.732643	0.655995
B.cells	TMX3	0.094803	5.505888	0.88205	0.380142	-6.18629	0.703169	0.610529
B.cells	9130230N(	-0.51764	0.640779	-0.88196	0.38019	-5.02672	0.759935	0.699055
B.cells	1200007C1	-0.51792	-0.38465	-0.88168	0.380338	-5.03313	0.772589	0.719311
B.cells	BATF3	-0.32177	2.332688	-0.88166	0.380349	-5.39786	0.739633	0.667014
B.cells	PPCS	-0.1896	2.732539	-0.88154	0.380416	-5.54476	0.734921	0.659637
B.cells	CHAF1B	-0.21467	3.382455	-0.8814	0.380493	-5.63147	0.727338	0.647828
B.cells	PSMD14	0.074008	6.618882	0.881344	0.380521	-6.40407	0.690911	0.592017
B.cells	PIK3R6	0.402936	2.596377	0.881246	0.380574	-5.31877	0.736522	0.662194
B.cells	PCBP4	-0.45862	0.472145	-0.88103	0.380689	-5.04249	0.762125	0.70253
B.cells	2410022M	0.298413	1.591485	0.880823	0.380802	-5.24666	0.748613	0.681095
B.cells	GPBP1L1	0.092669	5.913267	0.880672	0.380883	-6.29802	0.698811	0.603964
B.cells	ST6GALNA(	0.417631	0.651842	0.880608	0.380917	-5.09575	0.75998	0.699184
B.cells	GM44659	0.382107	0.682551	0.880495	0.380978	-5.1114	0.759605	0.698595
B.cells	ANAPC11	0.108552	5.923842	0.880408	0.381025	-6.23759	0.698694	0.603814
B.cells	PSTPIP2	0.17109	4.482064	0.880095	0.381194	-6.14567	0.714872	0.628646
B.cells	RNF31	0.190311	3.162894	0.880055	0.381215	-5.65497	0.730051	0.652155
B.cells	REXO1	-0.10818	4.912691	-0.87989	0.381305	-6.1039	0.709997	0.621191
B.cells	ABHD15	-0.28741	2.977881	-0.87983	0.381337	-5.43562	0.73221	0.655558
B.cells	XIST	-3.46956	4.173151	-0.87982	0.381342	-5.56035	0.718393	0.634112
B.cells	DDHD2	-0.12068	4.784447	-0.87967	0.381422	-6.06464	0.711479	0.623461
B.cells	TWNK	-0.21242	2.886068	-0.8794	0.381569	-5.60467	0.733324	0.657363
B.cells	REXO2	-0.10362	6.270041	-0.87928	0.381635	-6.32504	0.694924	0.598334
B.cells	ZFP426	0.194684	2.769149	0.879256	0.381646	-5.56765	0.734695	0.659575
B.cells	ESAM	0.33673	2.065322	0.879145	0.381706	-5.27685	0.743008	0.672673
B.cells	ATP9A	-0.44808	0.837325	-0.87909	0.381735	-5.10361	0.757774	0.696026
B.cells	ZBTB11OS1	0.234132	2.065369	0.878405	0.382105	-5.38797	0.743256	0.672938
B.cells	PPP1R2	0.060871	6.908394	0.878199	0.382216	-6.47172	0.688179	0.58808
B.cells	STIM2	0.103222	6.210089	0.878158	0.382238	-6.33059	0.695817	0.599624
B.cells	DDX6	0.071226	8.314732	0.878156	0.382239	-6.68558	0.673098	0.565505
B.cells	USP39	-0.13918	4.614508	-0.87805	0.382294	-5.99299	0.713646	0.626861
B.cells	ZMYND8	0.091926	6.108671	0.87805	0.382296	-6.26677	0.696934	0.60132
B.cells	UMPS	0.135276	4.099845	0.878047	0.382298	-5.8732	0.719512	0.635906
B.cells	CLEC14A	-0.35908	2.196348	-0.87783	0.382417	-5.31862	0.74181	0.67056

B.cells	E130309D0	-0.13078	3.983364	-0.8776	0.382538	-5.9016	0.721064	0.638109
B.cells	RAD51AP1	-0.29416	3.61991	-0.87729	0.382704	-5.65744	0.725422	0.644712
B.cells	VRK1	0.100065	5.309256	0.877206	0.382752	-6.20056	0.706197	0.615104
B.cells	PIDD1	-0.39135	0.824038	-0.87676	0.382993	-5.11555	0.758951	0.697
B.cells	GM10658	-0.29174	1.776179	-0.8766	0.383077	-5.34352	0.747497	0.678859
B.cells	PSMC3IP	-0.28416	2.412129	-0.87647	0.383152	-5.38188	0.739954	0.666991
B.cells	FN3KRP	-0.32959	1.481688	-0.87614	0.383328	-5.27518	0.751279	0.68464
B.cells	RAB5A	-0.07092	6.913368	-0.87596	0.383427	-6.46827	0.689148	0.588664
B.cells	YPEL3	0.103349	7.069109	0.875695	0.383569	-6.54428	0.687564	0.586156
B.cells	METTL27	-0.50949	0.539053	-0.87562	0.383611	-5.06064	0.762918	0.702962
B.cells	RCN1	0.206179	3.060814	0.875216	0.383828	-5.56667	0.732748	0.655493
B.cells	GM15232	0.310034	1.187425	0.87497	0.383961	-5.3215	0.755041	0.690586
B.cells	RSRC2	0.066395	6.479986	0.874966	0.383963	-6.36727	0.69401	0.596019
B.cells	TCTN2	-0.49279	-0.37598	-0.87482	0.384042	-4.95989	0.774244	0.721271
B.cells	ZSWIM4	-0.13157	4.579223	-0.87444	0.384246	-6.01623	0.71524	0.628535
B.cells	RIOX1	-0.15125	3.521185	-0.87444	0.384247	-5.72812	0.727387	0.647319
B.cells	POU3F1	-0.52832	0.061626	-0.87441	0.384265	-5.05868	0.768814	0.71266
B.cells	4930579G2	-0.22833	2.431609	-0.87437	0.384283	-5.44185	0.74015	0.667242
B.cells	EIF3E	-0.06995	7.095265	-0.87424	0.384357	-6.50725	0.687299	0.586044
B.cells	TLR3	0.386736	1.534056	0.874186	0.384385	-5.30788	0.750858	0.684139
B.cells	SETBP1	0.183278	4.621756	0.873977	0.384498	-6.19767	0.714756	0.627905
B.cells	NISCH	0.088168	6.194239	0.873922	0.384529	-6.30884	0.697154	0.60101
B.cells	ELP6	-0.23471	2.398694	-0.87387	0.384558	-5.49795	0.740539	0.667975
B.cells	SPSB1	-0.37276	1.685361	-0.87378	0.384603	-5.2584	0.74904	0.681356
B.cells	CCNE1	-0.21581	3.530373	-0.87368	0.38466	-5.67223	0.727281	0.647272
B.cells	PMM2	0.100193	4.561806	0.873461	0.384778	-6.02215	0.715438	0.628954
B.cells	ACTR1A	0.076812	6.140647	0.873387	0.384818	-6.33758	0.697745	0.601907
B.cells	ANKS1B	0.374334	1.030517	0.873331	0.384849	-5.20696	0.756944	0.693868
B.cells	CIB1	0.099333	5.732242	0.873324	0.384852	-6.26137	0.702272	0.60879
B.cells	TWSG1	0.191928	3.230079	0.873299	0.384866	-5.69067	0.730772	0.652703
B.cells	SPIDR	0.105517	5.228567	0.87288	0.385093	-6.15928	0.708117	0.617439
B.cells	WTAP	-0.06918	6.931297	-0.8728	0.385135	-6.46845	0.689288	0.588891
B.cells	THUMPD3	0.128817	4.262161	0.872739	0.385169	-5.94733	0.719073	0.634348
B.cells	DGKD	0.080291	7.687633	0.872055	0.38554	-6.63015	0.681457	0.576777
B.cells	HSPA2	-0.18448	3.291574	-0.87202	0.385557	-5.77322	0.730643	0.65186
B.cells	FBXO31	0.170166	3.549697	0.872011	0.385564	-5.75703	0.727642	0.647194
B.cells	GM43329	-0.20627	3.105098	-0.87192	0.385612	-5.65577	0.73282	0.655251
B.cells	SORT1	-0.19412	4.067523	-0.87177	0.385695	-5.76307	0.721704	0.638007
B.cells	IRAK1BP1	-0.54079	0.288762	-0.87154	0.385818	-5.00522	0.76679	0.708761
B.cells	FAM102A	0.149184	4.182226	0.87133	0.385934	-5.94683	0.720517	0.636043
B.cells	GALNT3	-0.43118	1.767982	-0.87127	0.385968	-5.14739	0.748827	0.680238
B.cells	PET100	0.096128	5.786929	0.871134	0.38604	-6.21557	0.702393	0.608296
B.cells	4921511C1	0.220371	2.59393	0.871069	0.386076	-5.54718	0.738999	0.66483
B.cells	DDAH1	-0.25937	2.481818	-0.87078	0.386235	-5.43386	0.740474	0.667012
B.cells	KANK1	-0.3549	1.172928	-0.8707	0.386279	-5.21895	0.756155	0.691774
B.cells	SUGT1	0.080459	5.949959	0.870538	0.386364	-6.29313	0.700735	0.605703
B.cells	TMSB15B1	-0.21855	2.422831	-0.87045	0.38641	-5.52026	0.741185	0.66822

B.cells	IGKV9-124	-0.45865	-1.01492	-0.8702	0.386547	-4.93675	0.782988	0.7352
B.cells	GPM6B	0.312428	2.439081	0.870105	0.386599	-5.38994	0.741024	0.668014
B.cells	BCL3	0.123394	5.236561	0.869941	0.386688	-6.25075	0.708733	0.618052
B.cells	ZFP335	-0.18302	2.842169	-0.86977	0.386782	-5.59582	0.736267	0.66075
B.cells	DGCR8	0.150581	3.779114	0.869748	0.386793	-5.79778	0.725346	0.643746
B.cells	MYO1F	0.217741	5.259228	0.869294	0.38704	-5.79602	0.708478	0.617888
B.cells	ZBTB44	-0.09565	5.853417	-0.86925	0.387063	-6.32128	0.701837	0.60775
B.cells	SNRNP27	0.086591	5.842837	0.869214	0.387084	-6.24397	0.701955	0.60793
B.cells	FAM193A	0.083913	6.704133	0.869178	0.387103	-6.43554	0.692456	0.593526
B.cells	P2RY10B	-0.14005	3.709802	-0.86914	0.387122	-5.96682	0.726147	0.645122
B.cells	CWC27	0.09627	5.662216	0.869072	0.387161	-6.23954	0.703966	0.611024
B.cells	FCER1G	0.19729	8.628675	0.869046	0.387175	-6.54311	0.671777	0.562607
B.cells	SELENOK	-0.07937	7.584969	-0.86893	0.387236	-6.56218	0.682899	0.579214
B.cells	ITGB1BP2	0.424861	0.43994	0.868784	0.387318	-5.04974	0.765186	0.706635
B.cells	CCHCR1	0.366219	1.536464	0.868575	0.387431	-5.19709	0.751868	0.685509
B.cells	PFDN4	-0.11278	5.046958	-0.86842	0.387516	-6.14535	0.710928	0.621751
B.cells	AIFM1	0.118641	4.370662	0.868416	0.387518	-5.96773	0.718609	0.633567
B.cells	ESYT2	0.084373	6.802347	0.86813	0.387673	-6.43954	0.691488	0.592137
B.cells	ARID3B	0.154149	3.836648	0.868037	0.387724	-5.88071	0.724792	0.643099
B.cells	TFEC	-0.35405	2.368464	-0.86793	0.38778	-5.34877	0.741974	0.669917
B.cells	OSTM1	0.103177	4.762239	0.867909	0.387794	-6.08304	0.714199	0.626746
B.cells	MFAP2	-0.54066	0.586159	-0.86743	0.388056	-5.02357	0.763864	0.704019
B.cells	PDAP1	-0.08061	6.715812	-0.86711	0.388231	-6.42044	0.692991	0.593789
B.cells	CKAP4	0.157067	4.030233	0.866848	0.388372	-5.99805	0.72329	0.639937
B.cells	HPSE	0.144037	3.426146	0.866674	0.388467	-6.01365	0.730346	0.650883
B.cells	5730409E0	0.460125	0.23499	0.866223	0.388712	-5.05048	0.768989	0.71139
B.cells	LOCKD	-0.21286	4.472202	-0.86558	0.389061	-5.90203	0.719104	0.632531
B.cells	PARP12	0.275524	3.018486	0.865515	0.389098	-5.47095	0.735952	0.658659
B.cells	ETFRF1	0.213438	3.163487	0.86522	0.38926	-5.61367	0.734355	0.656155
B.cells	SPIC	-0.4913	1.863637	-0.86515	0.389296	-5.30911	0.749771	0.680323
B.cells	SPAST	0.100697	5.207046	0.864995	0.389383	-6.13359	0.710861	0.619954
B.cells	TSKU	-0.52964	-0.3765	-0.86496	0.389404	-4.98685	0.777221	0.724085
B.cells	KLF13	-0.07597	8.183848	-0.86483	0.389471	-6.68032	0.678211	0.5707
B.cells	RBM34	-0.10479	4.465573	-0.86449	0.38966	-5.9926	0.719349	0.633037
B.cells	CHST7	-0.40596	0.725254	-0.86444	0.389688	-5.21166	0.76365	0.702435
B.cells	SUV39H2	-0.2158	2.381659	-0.86443	0.38969	-5.52539	0.743652	0.67083
B.cells	SLIT2	-0.51146	0.983319	-0.86417	0.389833	-5.10164	0.760651	0.697582
B.cells	GRB7	-0.50173	0.183341	-0.864	0.389925	-5.03179	0.770544	0.713307
B.cells	RAB3D	-0.25001	3.11921	-0.8637	0.390091	-5.47826	0.735345	0.657438
B.cells	MTA2	-0.1012	5.782517	-0.86352	0.390191	-6.25724	0.704924	0.610584
B.cells	ZBTB17	-0.13107	3.917458	-0.86314	0.390394	-5.98183	0.726379	0.643226
B.cells	GFER	0.100882	4.406699	0.862531	0.390729	-6.02924	0.72108	0.634691
B.cells	AFF2	0.483217	0.04795	0.862486	0.390754	-5.13378	0.773138	0.716496
B.cells	NCKAP5LO:	-0.52704	0.041134	-0.8624	0.390801	-5.02146	0.773223	0.716667
B.cells	CCDC157	-0.33018	1.103128	-0.8623	0.390856	-5.25014	0.760148	0.695858
B.cells	SMC4	-0.10199	7.287715	-0.86221	0.390905	-6.494	0.688918	0.585871
B.cells	SPA17	-0.35077	0.979282	-0.86213	0.39095	-5.14575	0.76166	0.698342

B.cells	ROPN1L	-0.27074	2.420763	-0.86175	0.391157	-5.48187	0.744537	0.671024
B.cells	PXDN	-0.40543	0.993778	-0.86149	0.391298	-5.09391	0.761748	0.698318
B.cells	GM20275	-0.19814	3.071387	-0.86142	0.391335	-5.69283	0.73684	0.659136
B.cells	ITPK1	0.096012	5.403224	0.861266	0.391421	-6.20305	0.710006	0.617769
B.cells	ARL6IP5	0.079073	7.048458	0.861166	0.391476	-6.52684	0.691764	0.590111
B.cells	LYRM2	0.181002	3.377583	0.861138	0.391491	-5.71764	0.73325	0.653602
B.cells	TMEM234	0.062117	7.040364	0.861011	0.391561	-6.51041	0.691853	0.590244
B.cells	GNB4	-0.23692	2.895468	-0.86096	0.391587	-5.49418	0.738912	0.662432
B.cells	LXN	-0.1448	3.855864	-0.86071	0.391726	-5.82702	0.727805	0.644996
B.cells	PDCD4	0.085476	7.282332	0.860542	0.391818	-6.5684	0.689333	0.586314
B.cells	WDHD1	-0.15871	4.56722	-0.8605	0.391841	-5.99393	0.719614	0.632351
B.cells	AFG3L1	-0.08237	4.862083	-0.86037	0.391914	-6.12002	0.716269	0.627213
B.cells	GM43305	0.177788	7.45195	0.860195	0.392008	-6.66677	0.687565	0.583597
B.cells	DDX1	0.124091	4.964571	0.859898	0.392171	-6.08912	0.715338	0.625459
B.cells	PDE2A	-0.1491	6.050057	-0.85979	0.39223	-6.25384	0.703133	0.606846
B.cells	NCOA3	-0.06849	6.960263	-0.85964	0.392313	-6.49497	0.693125	0.591689
B.cells	TTC8	-0.41655	0.78875	-0.85943	0.39243	-5.09721	0.764803	0.702633
B.cells	KLHL22	0.199773	3.001151	0.859172	0.392569	-5.58497	0.738214	0.660791
B.cells	RAB11A	-0.0663	7.145444	-0.85911	0.392604	-6.51419	0.691219	0.588794
B.cells	PMS2	0.127635	3.954235	0.858982	0.392673	-5.91503	0.727082	0.643496
B.cells	SLC30A4	0.225435	1.941057	0.858909	0.392714	-5.48829	0.750829	0.680564
B.cells	TUBB3	0.374577	0.736309	0.858834	0.392755	-5.14396	0.765469	0.703741
B.cells	INF2	0.174011	3.252642	0.85852	0.392927	-5.83244	0.73542	0.656204
B.cells	PPHLN1	0.113596	4.932629	0.858447	0.392967	-6.14575	0.716017	0.626232
B.cells	FOXO1	0.085636	7.035015	0.858039	0.393191	-6.53058	0.692862	0.590716
B.cells	BACH2OS	0.280602	1.526179	0.857701	0.393377	-5.37281	0.756347	0.688722
B.cells	HSPBAP1	0.154494	3.764205	0.857684	0.393386	-5.96069	0.729781	0.647157
B.cells	DDX50	0.070661	6.369428	0.857467	0.393505	-6.37939	0.700219	0.601952
B.cells	MOB1A	-0.08405	5.970142	-0.85731	0.393593	-6.31582	0.704656	0.608771
B.cells	POLK	-0.15234	3.481233	-0.85708	0.393719	-5.79529	0.733079	0.65251
B.cells	GM42556	0.33919	0.793712	0.856712	0.39392	-5.29898	0.765283	0.703284
B.cells	CTPS	0.160197	3.704287	0.85668	0.393938	-5.81307	0.730478	0.64862
B.cells	GM10802	0.471676	-0.25172	0.856625	0.393968	-5.03005	0.778248	0.724029
B.cells	SLC23A3	-0.45163	1.461516	-0.85657	0.394	-5.23787	0.757131	0.690401
B.cells	DIAPH3	-0.1986	6.026174	-0.85652	0.394027	-6.29838	0.704032	0.60805
B.cells	HDAC8	-0.1073	6.530622	-0.85607	0.394274	-6.4243	0.698436	0.599696
B.cells	GRAMD3	0.111197	6.080829	0.856028	0.394296	-6.45728	0.703423	0.607253
B.cells	ANKRD33B	-0.20123	4.882196	-0.85602	0.394302	-6.19502	0.716918	0.627859
B.cells	SCNN1A	0.46195	0.542515	0.856003	0.39431	-5.13184	0.768376	0.708403
B.cells	PCGF1	-0.31114	1.17851	-0.85596	0.394334	-5.24441	0.760574	0.696017
B.cells	LYRM9	-0.23758	2.696887	-0.85576	0.394441	-5.4718	0.742313	0.667293
B.cells	FCSK	0.380881	0.928399	0.85575	0.394449	-5.15376	0.763631	0.700918
B.cells	PEX6	-0.15075	4.039128	-0.85562	0.394523	-5.9002	0.726593	0.642884
B.cells	ZHX1	0.130323	4.078556	0.855608	0.394527	-5.96546	0.726137	0.642179
B.cells	B4GALT1	-0.07795	7.137524	-0.85559	0.394539	-6.50041	0.691774	0.589759
B.cells	PPP3CA	0.070336	8.812084	0.855524	0.394574	-6.76829	0.67378	0.562916
B.cells	MTERF3	0.130068	4.014483	0.855497	0.394588	-5.93887	0.726878	0.643341

B.cells	PHIP	0.080812	7.276011	0.854755	0.394997	-6.5244	0.690666	0.587822
B.cells	NRF1	0.07093	6.474245	0.85473	0.395011	-6.41145	0.699466	0.601089
B.cells	FDFT1	-0.15628	3.796269	-0.85462	0.395072	-5.79652	0.729833	0.647639
B.cells	PRDX3	-0.10184	5.400912	-0.8543	0.395246	-6.25684	0.711455	0.619347
B.cells	BCL2L14	-0.43292	0.459361	-0.85425	0.395273	-5.30414	0.769851	0.710602
B.cells	MRGPRA2E	0.523967	-0.43033	0.854241	0.39528	-5.05798	0.780942	0.728365
B.cells	BTD	0.235946	2.37707	0.854167	0.395321	-5.49067	0.746551	0.673723
B.cells	NDUFS6	0.101689	5.716521	0.854013	0.395406	-6.24375	0.707905	0.613979
B.cells	1700061G1	-0.34123	1.070717	-0.85373	0.395559	-5.2361	0.762333	0.698787
B.cells	ASB4	0.568943	1.68448	0.853657	0.395602	-5.1862	0.754871	0.687005
B.cells	ADGRG1	-0.3616	1.720983	-0.85363	0.395616	-5.36905	0.754429	0.68632
B.cells	TBXAS1	0.323042	3.468333	0.853501	0.395688	-5.59746	0.733657	0.653831
B.cells	LPGAT1	0.115555	6.245156	0.853442	0.39572	-6.30334	0.702005	0.605199
B.cells	GPAM	-0.19634	3.696477	-0.85341	0.395738	-5.8302	0.730994	0.649715
B.cells	STN1	0.137905	3.886297	0.85326	0.395821	-5.83674	0.728787	0.646367
B.cells	ELF1	0.062105	8.010369	0.853221	0.395843	-6.64938	0.682722	0.576283
B.cells	WDR78	-0.46228	0.561969	-0.85294	0.395995	-5.04334	0.768583	0.70905
B.cells	HEXB	0.096964	5.741415	0.852933	0.396001	-6.31896	0.707625	0.61391
B.cells	CHST12	0.127878	5.763881	0.852917	0.39601	-6.24792	0.707374	0.613526
B.cells	CNPY2	-0.13563	4.719728	-0.85262	0.396174	-5.98248	0.719374	0.631749
B.cells	TRNAU1AP	0.138981	4.126118	0.852228	0.39639	-5.94187	0.726477	0.642368
B.cells	ASAP1	0.08375	7.200045	0.852084	0.396469	-6.57979	0.691968	0.58973
B.cells	ANKRD13A	-0.08463	6.215118	-0.85189	0.396576	-6.33888	0.702831	0.606143
B.cells	IGKC	-0.23313	7.88266	-0.85183	0.396608	-6.73687	0.684575	0.578649
B.cells	ERH	-0.09519	7.372533	-0.85173	0.396664	-6.54981	0.6901	0.586924
B.cells	SP1	0.080099	5.924217	0.851573	0.396752	-6.30775	0.706116	0.611072
B.cells	CBX4	-0.11564	5.468948	-0.85146	0.396816	-6.1908	0.711228	0.618876
B.cells	STXBP5	0.134733	5.383452	0.851248	0.396932	-6.12814	0.712193	0.620439
B.cells	FBH1	0.146469	3.425128	0.851235	0.396938	-5.74298	0.73472	0.655198
B.cells	ADAMDEC1	-0.49131	0.746267	-0.8507	0.397233	-5.10536	0.767183	0.705939
B.cells	KSR1	-0.13817	4.69672	-0.8507	0.397235	-6.07743	0.72027	0.632483
B.cells	11-Sep	-0.12289	6.415184	-0.85047	0.397359	-6.4064	0.700915	0.602936
B.cells	KCTD11	-0.41832	0.546292	-0.85046	0.397364	-5.09282	0.769651	0.70987
B.cells	SINHCAF	-0.10541	4.557551	-0.8504	0.397402	-6.12286	0.721865	0.634938
B.cells	1700047M	-0.476	-0.15234	-0.84966	0.397808	-5.05969	0.778392	0.72412
B.cells	FUNDC1	-0.11024	4.65588	-0.84934	0.397985	-6.04957	0.720785	0.633555
B.cells	OAF	-0.2019	3.151249	-0.84931	0.398003	-5.68073	0.738263	0.66065
B.cells	NOL7	-0.06586	6.906907	-0.84922	0.398053	-6.48867	0.695536	0.595069
B.cells	USP33	0.114255	4.710834	0.849113	0.398112	-6.07333	0.720156	0.632587
B.cells	DDX27	0.102277	4.974515	0.849068	0.398136	-6.14179	0.717146	0.62796
B.cells	RNASET2B	-0.14649	5.139167	-0.84898	0.398183	-6.08964	0.715274	0.625088
B.cells	STAG3	-0.55905	0.607181	-0.84893	0.398213	-5.02511	0.768949	0.709066
B.cells	TBL1XR1	0.080347	6.669065	0.848891	0.398235	-6.4103	0.698154	0.599023
B.cells	AEBP2	0.097145	5.787827	0.848839	0.398264	-6.30124	0.707956	0.613901
B.cells	UBA2	-0.0774	6.457603	-0.84872	0.398329	-6.3922	0.700491	0.60256
B.cells	FGD5	-0.44639	1.663977	-0.84872	0.398332	-5.18144	0.756027	0.688549
B.cells	DTL	-0.18472	4.961318	-0.8487	0.398341	-6.05514	0.717296	0.628191

B.cells	ARMC10	-0.15735	3.275459	-0.84866	0.398361	-5.73116	0.736802	0.65837
B.cells	TSG101	0.09452	5.622255	0.848647	0.39837	-6.22065	0.709815	0.616737
B.cells	LY6D	0.155032	5.594266	0.848435	0.398487	-6.25236	0.710227	0.617257
B.cells	AW549877	0.160757	3.352769	0.847888	0.39879	-5.8154	0.736437	0.657136
B.cells	HECTD2	0.401236	0.291184	0.847738	0.398873	-5.11147	0.773472	0.715525
B.cells	PKN1	0.071049	6.766934	0.847397	0.399062	-6.45215	0.697806	0.597625
B.cells	C330011M	0.385157	0.592367	0.847315	0.399107	-5.0723	0.769938	0.709674
B.cells	PIGW	0.320552	0.765108	0.847139	0.399205	-5.20392	0.767806	0.706329
B.cells	RIOK3	0.076908	7.578172	0.847098	0.399228	-6.63316	0.688932	0.58436
B.cells	PDE7B	0.298774	5.37371	0.846821	0.399381	-6.0114	0.713365	0.621425
B.cells	MREG	0.203924	3.199109	0.846745	0.399423	-6.01047	0.738473	0.660246
B.cells	RHOT2	-0.21583	2.679369	-0.8466	0.399505	-5.53936	0.744626	0.669882
B.cells	WDR18	-0.111	4.82244	-0.84658	0.399513	-6.06813	0.719634	0.631097
B.cells	RFESD	0.214853	2.541257	0.846531	0.399542	-5.59531	0.746271	0.672478
B.cells	SLC16A12	-0.44906	0.427781	-0.84638	0.399628	-5.10118	0.771979	0.713185
B.cells	UVSSA	-0.15049	3.709393	-0.84606	0.399801	-5.80685	0.732492	0.65103
B.cells	NR2C2AP	0.128304	4.44571	0.845863	0.399913	-6.06733	0.723958	0.637887
B.cells	SERPINB2	1.098381	0.991964	0.845727	0.399988	-5.19183	0.765019	0.702251
B.cells	PMM1	0.193608	3.07058	0.84557	0.400075	-5.65436	0.739992	0.662876
B.cells	ENG	-0.23739	4.005662	-0.8455	0.400114	-5.75545	0.729044	0.64587
B.cells	PGLYRP2	-0.26824	2.088786	-0.8455	0.400115	-5.50339	0.751693	0.681249
B.cells	ARIH1	-0.07928	8.728541	-0.84549	0.400121	-6.77926	0.67658	0.566304
B.cells	UBA6	0.0961	5.172539	0.845324	0.400212	-6.21721	0.715648	0.625309
B.cells	PSMA4	-0.07846	6.877207	-0.84522	0.400272	-6.48666	0.696595	0.596325
B.cells	BSN	0.485374	0.764883	0.845187	0.400288	-5.08299	0.767812	0.706872
B.cells	ACADSB	-0.17208	3.270603	-0.84517	0.400298	-5.63732	0.737634	0.659304
B.cells	SFXN5	0.178528	3.363367	0.84471	0.400553	-5.79181	0.736721	0.657736
B.cells	PBLD1	-0.33939	1.75083	-0.84466	0.400578	-5.38331	0.755953	0.687907
B.cells	E430024P1	0.456983	0.53335	0.844489	0.400675	-5.06869	0.770857	0.711603
B.cells	KCNG2	-0.48552	-0.23458	-0.84438	0.400738	-5.02732	0.780431	0.726954
B.cells	MIR17HG	0.254462	2.192534	0.844334	0.400761	-5.43538	0.750627	0.679571
B.cells	LIMK1	-0.26309	2.1198	-0.84431	0.400777	-5.40706	0.751501	0.680952
B.cells	GCSH	-0.131	4.428205	-0.84408	0.400905	-6.02923	0.724334	0.638649
B.cells	MAN2B1	-0.10671	6.851978	-0.84403	0.400928	-6.51339	0.697041	0.596989
B.cells	ASXL1	-0.08292	7.242343	-0.84385	0.40103	-6.50479	0.692759	0.590599
B.cells	GTPBP4	-0.08109	5.956432	-0.84385	0.401031	-6.32968	0.706981	0.612125
B.cells	MPV17L2	0.132351	4.459696	0.843299	0.401337	-6.01167	0.723972	0.638434
B.cells	YY1	-0.05826	7.144963	-0.84328	0.401348	-6.51939	0.693824	0.592446
B.cells	ARMCX6	0.317336	0.977785	0.843267	0.401355	-5.16209	0.765378	0.703357
B.cells	1810009A1	0.202235	2.243882	0.843256	0.401361	-5.47209	0.750011	0.679035
B.cells	GM3604	0.364226	0.595301	0.843244	0.401368	-5.12133	0.770091	0.710868
B.cells	RPP30	-0.13889	3.635635	-0.84311	0.401442	-5.79039	0.733531	0.65331
B.cells	ALG14	0.16546	3.562595	0.843072	0.401463	-5.76884	0.734385	0.654639
B.cells	ZFP189	-0.34161	1.120206	-0.84289	0.401563	-5.18536	0.7637	0.700694
B.cells	ACSL1	0.141479	5.758511	0.842589	0.401732	-6.28659	0.709334	0.615951
B.cells	GSTO1	0.101673	4.594828	0.842574	0.40174	-6.05296	0.722554	0.636232
B.cells	TTC28	-0.17339	4.798912	-0.84245	0.40181	-5.96026	0.720215	0.632691

B.cells	NET1	-0.15929	4.035875	-0.84237	0.401853	-5.92621	0.729006	0.646315
B.cells	4932438H2	-0.45509	-0.27879	-0.84219	0.401952	-5.13676	0.781203	0.728697
B.cells	RPUSD4	-0.19355	2.682051	-0.84154	0.402314	-5.59855	0.745484	0.671344
B.cells	PI4K2A	0.10431	5.639245	0.841482	0.402348	-6.33327	0.711217	0.618301
B.cells	NEK6	-0.17127	3.873937	-0.84131	0.402443	-5.73233	0.731503	0.649486
B.cells	SIRT2	0.083454	5.688086	0.840966	0.402635	-6.29963	0.710952	0.617572
B.cells	IRGC1	0.375876	0.115465	0.840681	0.402794	-5.15273	0.777176	0.721225
B.cells	NF1	-0.08384	6.550388	-0.84067	0.402801	-6.41837	0.701386	0.603023
B.cells	SON	-0.05978	7.67203	-0.84037	0.402965	-6.60623	0.689254	0.584565
B.cells	ZFP770	0.266948	1.760027	0.839765	0.403305	-5.39322	0.757648	0.689218
B.cells	IL13RA1	-0.27001	3.558786	-0.83914	0.403656	-5.58127	0.73671	0.65577
B.cells	WLS	-0.1693	4.263574	-0.83892	0.403774	-5.90609	0.728589	0.643127
B.cells	MAZ	-0.12212	6.241133	-0.83871	0.403893	-6.3693	0.706157	0.60874
B.cells	GM12592	-0.17133	3.124779	-0.83862	0.403943	-5.71098	0.742004	0.663955
B.cells	NDUFS8	0.07978	6.232258	0.838488	0.404018	-6.37595	0.706256	0.608989
B.cells	METTL8	0.222768	2.52734	0.838399	0.404068	-5.53531	0.749117	0.67514
B.cells	PPFIBP2	-0.1676	4.285147	-0.83803	0.404271	-6.17997	0.728575	0.642967
B.cells	UBXN1	0.068658	6.759331	0.837917	0.404337	-6.46019	0.700552	0.60019
B.cells	AP2A2	-0.0874	5.931782	-0.8379	0.404347	-6.28343	0.709781	0.614178
B.cells	SOX6	-0.39408	1.905309	-0.83768	0.404469	-5.41202	0.756886	0.687028
B.cells	DTNB	0.124514	5.293687	0.837287	0.404689	-6.14049	0.717243	0.625308
B.cells	PSMD7	0.099133	5.828246	0.837238	0.404716	-6.28633	0.711191	0.616083
B.cells	GT(ROSA)2	-0.13151	4.594685	-0.83701	0.404845	-6.0378	0.725248	0.637705
B.cells	F10	-0.2391	4.54109	-0.83685	0.404936	-6.17554	0.725866	0.638706
B.cells	WIPI1	0.291845	2.277802	0.836802	0.40496	-5.41415	0.752539	0.680224
B.cells	SCAMP2	0.075864	6.357233	0.836732	0.404999	-6.38879	0.705262	0.607249
B.cells	PARP6	0.182276	2.991541	0.836628	0.405057	-5.69462	0.744006	0.666915
B.cells	LAYN	-0.41794	0.629576	-0.83653	0.405111	-5.14539	0.77268	0.712174
B.cells	GNB1	-0.0489	8.848488	-0.83638	0.405196	-6.80021	0.678114	0.566611
B.cells	APOL7E	0.407891	-0.10248	0.836321	0.405229	-5.09889	0.781824	0.72682
B.cells	SCRN3	-0.3003	1.928035	-0.83629	0.405244	-5.33269	0.756762	0.686974
B.cells	PAFAH1B1	0.054199	8.064884	0.836162	0.405318	-6.68494	0.686527	0.579165
B.cells	MCM10	-0.24311	3.112366	-0.83598	0.40542	-5.53212	0.742583	0.664856
B.cells	HPRT	-0.0901	6.310323	-0.83595	0.405439	-6.36256	0.705795	0.608235
B.cells	TRIB3	-0.40195	1.057747	-0.83547	0.405705	-5.25177	0.767755	0.704142
B.cells	WDR41	0.11733	4.001344	0.835384	0.405753	-5.95527	0.732475	0.648799
B.cells	KLHL42	0.227592	2.445253	0.834603	0.406191	-5.42607	0.751578	0.677859
B.cells	NACC2	-0.30758	1.77434	-0.83421	0.406413	-5.3416	0.759868	0.690723
B.cells	NME3	0.519121	0.405363	0.834147	0.406446	-5.02348	0.776737	0.7175
B.cells	UQCC1	0.132819	3.934429	0.834093	0.406476	-5.89327	0.734105	0.650465
B.cells	SCHIP1	0.361899	1.591487	0.833514	0.406801	-5.22875	0.762441	0.694447
B.cells	SMURF2	0.081368	6.82379	0.833395	0.406868	-6.49451	0.701544	0.600383
B.cells	DDX19A	-0.12603	4.239703	-0.83333	0.406905	-5.94262	0.730877	0.645167
B.cells	THSD4	-0.53328	0.910314	-0.83331	0.406913	-5.20391	0.770813	0.70776
B.cells	TOB2	-0.0867	6.656821	-0.83318	0.406987	-6.50383	0.703415	0.603228
B.cells	KIN	-0.10146	4.642417	-0.83302	0.407079	-6.06783	0.726282	0.638075
B.cells	PHF20	0.077908	6.472356	0.832854	0.407171	-6.41262	0.705527	0.606453

B.cells	CACNA1F	-0.53229	-0.45662	-0.83278	0.40721	-4.98522	0.788011	0.735363
B.cells	DMC1	-0.43541	0.152078	-0.83267	0.407276	-5.02188	0.780346	0.723085
B.cells	SCARB2	0.091014	6.538743	0.832244	0.407514	-6.44896	0.705054	0.605533
B.cells	NUDT21	-0.08487	6.148811	-0.83217	0.407554	-6.36426	0.709415	0.612163
B.cells	TSPAN6	-0.45254	1.103606	-0.83201	0.407644	-5.13931	0.768851	0.704425
B.cells	GM5914	0.166832	3.331997	0.831442	0.407964	-5.73609	0.74231	0.662285
B.cells	SH3BGRL2	0.303695	2.700678	0.831359	0.408011	-5.50435	0.749828	0.674023
B.cells	MKI67	-0.21887	6.498445	-0.8313	0.408043	-6.37978	0.70591	0.606413
B.cells	GM46440	-0.38439	0.637929	-0.83119	0.408107	-5.20181	0.775014	0.713838
B.cells	PAQR7	-0.35438	1.552734	-0.83105	0.408183	-5.23689	0.763726	0.69597
B.cells	LRRC45	0.221082	2.190744	0.830527	0.408478	-5.4761	0.755965	0.68383
B.cells	COL9A3	-0.46146	0.618437	-0.83052	0.408483	-5.11378	0.775257	0.714372
B.cells	1600010M	-0.1251	4.96425	-0.83031	0.408601	-6.1518	0.723278	0.633038
B.cells	PCSK7	0.103527	5.282369	0.830284	0.408614	-6.20396	0.719635	0.627457
B.cells	ZSWIM8	0.09672	4.703109	0.830266	0.408625	-6.11149	0.726284	0.637657
B.cells	CPT1B	-0.26164	1.536488	-0.83012	0.408705	-5.30933	0.763925	0.696419
B.cells	IRF3	0.137645	3.786081	0.83005	0.408746	-5.86568	0.736957	0.654209
B.cells	TSPYL2	0.193922	2.8065	0.82982	0.408875	-5.66509	0.748562	0.672296
B.cells	PNO1	-0.12096	4.446222	-0.82982	0.408877	-6.06146	0.729255	0.642307
B.cells	OVCA2	-0.5112	0.174482	-0.82978	0.4089	-5.01751	0.780806	0.723315
B.cells	AY036118	0.246672	4.212426	0.829763	0.408907	-6.03653	0.731972	0.646501
B.cells	FBXO47	-0.39535	1.069812	-0.82972	0.408929	-5.21172	0.769661	0.705553
B.cells	FBXO22	0.102721	4.616588	0.829322	0.409155	-6.07202	0.727573	0.639387
B.cells	POLB	0.086895	5.332268	0.829179	0.409236	-6.30028	0.719367	0.626824
B.cells	DNAJC25	0.121949	3.856746	0.829053	0.409307	-5.9063	0.736437	0.653106
B.cells	PRSS30	-0.38699	-0.33737	-0.82896	0.409361	-5.03461	0.787589	0.73386
B.cells	EHBP1	-0.1991	3.52414	-0.82886	0.409417	-5.78736	0.74035	0.659217
B.cells	CSTF3	0.080907	6.037918	0.828328	0.409715	-6.34287	0.711681	0.614841
B.cells	8030453O2	-0.46662	-0.75227	-0.82811	0.40984	-4.95066	0.793038	0.742669
B.cells	MCM4	-0.14382	5.68459	-0.82804	0.409877	-6.2401	0.715675	0.620983
B.cells	BCL7B	0.088048	5.531179	0.827966	0.409919	-6.25072	0.717417	0.623641
B.cells	OARD1	0.110186	4.902059	0.827856	0.409981	-6.13454	0.724614	0.63466
B.cells	SPATC1	-0.41086	-1.06407	-0.82781	0.410006	-4.9752	0.796911	0.749132
B.cells	DMAC2L	0.238181	2.257897	0.827656	0.410094	-5.40624	0.755803	0.68313
B.cells	LARS	-0.11834	4.738364	-0.82764	0.410104	-6.07947	0.726501	0.637569
B.cells	PKNOX1	0.12664	4.569323	0.827383	0.410248	-6.04155	0.728563	0.640646
B.cells	GM16576	0.342912	1.513376	0.827304	0.410292	-5.294	0.764978	0.697555
B.cells	MYL6B	-0.35216	0.862198	-0.82639	0.410805	-5.16163	0.773855	0.710573
B.cells	GM48383	0.279564	1.758143	0.826233	0.410896	-5.41168	0.76287	0.693114
B.cells	FAM120C	0.193697	3.050639	0.826043	0.411003	-5.68063	0.747349	0.668697
B.cells	GM15448	0.321439	-0.00747	0.825681	0.411207	-5.1729	0.785146	0.728154
B.cells	RHPN2	-0.31464	0.915486	-0.82552	0.411301	-5.25472	0.773648	0.709731
B.cells	MLF2	0.088165	5.85253	0.825158	0.411503	-6.29588	0.715268	0.618727
B.cells	GRB14	-0.31498	1.939569	-0.82499	0.411599	-5.45861	0.761252	0.689907
B.cells	RAD9B	-0.15645	3.275507	-0.82492	0.411636	-5.80896	0.745174	0.664741
B.cells	KPNB1	-0.08787	5.739818	-0.82486	0.411671	-6.25215	0.716546	0.620671
B.cells	GM16287	-0.41637	-0.44109	-0.82471	0.411757	-5.03602	0.790903	0.737161

B.cells	AU041133	0.286574	1.264215	0.824601	0.411817	-5.26146	0.769532	0.703099
B.cells	GM14296	-0.29461	0.969349	-0.82444	0.411907	-5.20102	0.77318	0.708916
B.cells	INCENP	-0.15695	5.454754	-0.82443	0.411915	-6.15542	0.71979	0.625752
B.cells	5033421B(	0.556591	-0.4422	0.823916	0.412204	-5.0174	0.791225	0.737509
B.cells	GM26936	0.401081	0.478634	0.823712	0.41232	-5.1399	0.779597	0.71897
B.cells	YTHDC1	-0.05934	7.312924	-0.82364	0.41236	-6.56986	0.69922	0.594478
B.cells	PTGES	0.522125	0.477516	0.823453	0.412466	-5.13389	0.779611	0.71901
B.cells	GNL3	0.108668	5.420028	0.823282	0.412563	-6.22481	0.720467	0.626722
B.cells	PRUNE1	0.144435	3.600837	0.823264	0.412573	-5.82396	0.741607	0.659276
B.cells	ITGA9	-0.17589	5.58915	-0.82318	0.41262	-6.23409	0.718538	0.623778
B.cells	1110059G1	-0.12173	3.928256	-0.82316	0.412632	-5.89862	0.737749	0.653302
B.cells	SELPLG	-0.1003	6.845492	-0.82294	0.412757	-6.42691	0.704397	0.602452
B.cells	GALM	-0.27357	2.214948	-0.82278	0.412846	-5.45383	0.7582	0.685355
B.cells	MEIG1	0.530619	-0.47443	0.822521	0.412993	-4.95437	0.791618	0.738627
B.cells	CLEC4D	0.504565	2.949351	0.822315	0.41311	-5.42628	0.749354	0.671658
B.cells	XRCC6	-0.14452	5.144906	-0.82222	0.413165	-6.15521	0.723618	0.631843
B.cells	FIG4	0.089328	4.603641	0.822117	0.413222	-6.11924	0.729865	0.641436
B.cells	HAUS3	-0.11525	4.199727	-0.82188	0.413358	-5.93304	0.734568	0.648753
B.cells	YWHAG	-0.08739	6.203239	-0.82187	0.41336	-6.41177	0.711584	0.613558
B.cells	MEGF11	-0.39924	0.651843	-0.82183	0.413386	-5.17791	0.777432	0.716025
B.cells	GM42829	0.207285	2.205641	0.82182	0.41339	-5.55731	0.758313	0.685764
B.cells	SEMA4G	-0.41004	0.909795	-0.82181	0.413396	-5.1694	0.77422	0.710914
B.cells	USP35	0.468522	0.017078	0.821678	0.41347	-5.11725	0.785402	0.728807
B.cells	FANCL	-0.10919	4.144904	-0.82151	0.413564	-6.02377	0.735209	0.649848
B.cells	2310008N1	-0.34588	0.80481	-0.82148	0.413582	-5.26936	0.775525	0.713104
B.cells	CWC22	0.137403	3.900011	0.821481	0.413582	-5.89179	0.738081	0.654293
B.cells	GUCD1	0.137987	3.805941	0.821297	0.413686	-5.96888	0.739188	0.656029
B.cells	RAMP2	-0.27249	3.013058	-0.82116	0.413761	-5.52938	0.748592	0.670664
B.cells	3110082I1	0.157377	4.199896	0.821148	0.413771	-5.93206	0.734566	0.648875
B.cells	PHLPP2	0.137209	4.283952	0.821113	0.413791	-5.94741	0.733585	0.647359
B.cells	CAML	0.104068	4.427078	0.820982	0.413865	-6.03157	0.731935	0.644832
B.cells	MAN1C1	0.172723	4.292733	0.819611	0.414642	-5.80792	0.734704	0.64781
B.cells	GSTM2	-0.32308	1.633616	-0.81956	0.414671	-5.32482	0.766562	0.697548
B.cells	CYTH1	0.080687	8.072227	0.819317	0.414808	-6.73892	0.692181	0.58318
B.cells	KDR	-0.28881	3.318647	-0.81898	0.414999	-5.59508	0.746453	0.66573
B.cells	SSR4	-0.08098	7.573816	-0.81889	0.415052	-6.60696	0.697754	0.591417
B.cells	ANKRD10	-0.087	5.371393	-0.81886	0.415071	-6.22969	0.722477	0.628825
B.cells	MTRR	0.298115	1.505683	0.818672	0.415175	-5.33464	0.768423	0.70024
B.cells	NSMAF	0.117522	4.903953	0.818229	0.415426	-6.15114	0.727874	0.637165
B.cells	ASTN2	0.384739	1.045238	0.818186	0.415451	-5.20071	0.774116	0.709363
B.cells	USF3	0.103918	4.977969	0.818128	0.415483	-6.20689	0.727019	0.635874
B.cells	UVRAG	0.084962	8.569242	0.817797	0.415672	-6.79217	0.686927	0.575394
B.cells	MYPOPOS	0.330811	1.654931	0.817749	0.415699	-5.34353	0.766588	0.697485
B.cells	KLHL14	0.206776	2.717642	0.81725	0.415982	-5.83246	0.753667	0.677287
B.cells	METTL14	-0.17267	3.09472	-0.81718	0.416025	-5.69802	0.749143	0.670219
B.cells	SESN3	-0.11507	4.83647	-0.8171	0.41607	-6.14222	0.728654	0.638504
B.cells	KLHL32	-0.42431	1.452777	-0.81708	0.41608	-5.20669	0.769074	0.701531

B.cells	VMN2R19	0.499643	-0.79625	0.816957	0.416149	-5.03009	0.797168	0.746726
B.cells	MYBPC2	-0.24094	2.760882	-0.81694	0.41616	-5.6093	0.753146	0.676473
B.cells	KDM3A	0.090839	5.567098	0.816901	0.416181	-6.2896	0.720256	0.625647
B.cells	CCDC122	0.292005	1.315452	0.816816	0.416229	-5.32601	0.770769	0.704213
B.cells	HDDC2	-0.12905	3.817561	-0.81682	0.416229	-5.9024	0.740559	0.656872
B.cells	DET1	-0.24574	2.186379	-0.81673	0.416275	-5.47741	0.760094	0.687374
B.cells	SCAF4	0.078193	6.317786	0.816643	0.416327	-6.41108	0.711746	0.612754
B.cells	CDC20	-0.24985	3.639962	-0.8164	0.416468	-5.74311	0.742658	0.660233
B.cells	GBP2B	1.053383	0.300434	0.816225	0.416565	-5.13439	0.783428	0.724503
B.cells	SELENON	-0.22192	3.694822	-0.81622	0.416571	-5.60405	0.742009	0.65926
B.cells	PTTG1	-0.09781	5.78	-0.81612	0.416622	-6.29857	0.71783	0.622082
B.cells	GM37494	-0.13809	3.825168	-0.81585	0.416779	-5.88223	0.740469	0.656932
B.cells	NDUFS2	-0.08991	6.235293	-0.81574	0.41684	-6.38859	0.712675	0.614302
B.cells	MAIP1	-0.13575	3.588251	-0.81561	0.416912	-5.84316	0.74327	0.661324
B.cells	HAAO	0.152524	3.64437	0.815591	0.416926	-5.94474	0.742605	0.660292
B.cells	ARHGEF26	-0.47381	0.379311	-0.81559	0.416926	-5.07214	0.782436	0.723034
B.cells	CD34	-0.4574	0.953495	-0.81557	0.416939	-5.32625	0.775256	0.711596
B.cells	PAFAH2	0.481331	0.619176	0.815528	0.416962	-5.0568	0.779427	0.718235
B.cells	ANKZF1	0.149287	2.742515	0.815508	0.416973	-5.60784	0.753367	0.677071
B.cells	MYZAP	-0.18611	3.284402	-0.81528	0.417102	-5.73959	0.746879	0.667003
B.cells	SPINDOC	-0.10653	4.427162	-0.81522	0.417135	-6.07157	0.733409	0.646146
B.cells	SNHG12	-0.15918	3.189103	-0.81518	0.417159	-5.7931	0.748016	0.668805
B.cells	CADM1	-0.31462	5.440386	-0.81513	0.417187	-5.95334	0.721704	0.628201
B.cells	PDCD7	0.105517	4.505243	0.814924	0.417305	-6.05628	0.7325	0.644899
B.cells	LRRC40	0.158192	3.249731	0.814694	0.417437	-5.74578	0.747292	0.667832
B.cells	TMEM168	0.10767	4.583494	0.81466	0.417456	-6.09454	0.731589	0.643496
B.cells	NCALD	-0.31703	2.377149	-0.81463	0.417472	-5.32334	0.757779	0.684241
B.cells	CCR3	-0.89702	-0.02075	-0.81445	0.417576	-5.01166	0.787483	0.73138
B.cells	PRMT7	0.195549	3.247895	0.814368	0.417622	-5.76557	0.747314	0.667866
B.cells	COPS7A	0.127864	4.237724	0.814308	0.417656	-5.96219	0.735623	0.649751
B.cells	MIER1	0.071363	6.946828	0.814156	0.417743	-6.52432	0.704706	0.602559
B.cells	UNC119	0.090322	5.47027	0.814016	0.417823	-6.31499	0.721362	0.627909
B.cells	ZFP771	0.112995	4.065583	0.814009	0.417827	-5.96043	0.73764	0.652949
B.cells	MRPL44	0.179602	2.945204	0.813828	0.41793	-5.6506	0.750933	0.673756
B.cells	TLR1	0.283312	1.219121	0.813766	0.417965	-5.47476	0.77196	0.706889
B.cells	MDH1	-0.07587	8.120076	-0.81366	0.418026	-6.71017	0.691795	0.583378
B.cells	AGPAT3	0.102974	5.164345	0.813577	0.418073	-6.24375	0.724871	0.633409
B.cells	ZCCHC10	-0.12378	4.048466	-0.8131	0.418347	-5.98101	0.737956	0.653536
B.cells	TMEM199	0.13346	3.379806	0.813042	0.418378	-5.82649	0.745859	0.665818
B.cells	FCRLS	-0.81609	-1.03209	-0.81303	0.418386	-4.96654	0.80024	0.752635
B.cells	KIF23	-0.16821	5.173452	-0.81298	0.418415	-6.09085	0.724879	0.633393
B.cells	ERCC8	0.180155	2.45611	0.812905	0.418456	-5.58167	0.75694	0.683149
B.cells	H2-D1	0.137995	8.452787	0.812132	0.418897	-6.75534	0.688385	0.578349
B.cells	OGFOD2	0.131248	3.850672	0.811838	0.419065	-5.87441	0.740384	0.657527
B.cells	CEP72	-0.23819	2.208429	-0.81169	0.419151	-5.43077	0.760047	0.68828
B.cells	GM1123	-0.41139	0.265276	-0.81161	0.419196	-5.07178	0.784098	0.726463
B.cells	PARD6A	0.205884	2.688858	0.811497	0.41926	-5.65851	0.754232	0.679145

B.cells	7-Sep	-0.05339	8.277792	-0.81148	0.419271	-6.72465	0.690282	0.581301
B.cells	MRI1	0.114218	3.963231	0.81139	0.419321	-5.95758	0.739058	0.655484
B.cells	MRPS26	0.082155	5.167613	0.81138	0.419326	-6.17321	0.725044	0.633872
B.cells	SMPD5	-0.43077	-0.18019	-0.81136	0.41934	-4.99588	0.789734	0.7355
B.cells	GM21860	-0.57904	-0.14186	-0.81133	0.419356	-5.07904	0.789248	0.734718
B.cells	GM26789	0.401151	0.271556	0.811271	0.419389	-5.16773	0.784019	0.726367
B.cells	RPIA	-0.09832	5.458024	-0.81118	0.419438	-6.23868	0.721712	0.628828
B.cells	ALG12	0.29423	1.395336	0.811147	0.41946	-5.35127	0.770006	0.704086
B.cells	TTBK2	-0.27507	1.314702	-0.81113	0.419468	-5.27387	0.771002	0.705666
B.cells	HIPK2	0.097701	6.550097	0.811061	0.419508	-6.43984	0.709342	0.610012
B.cells	NAAA	-0.16756	3.389339	-0.81102	0.419532	-5.8866	0.745847	0.666125
B.cells	C2CD5	0.109367	4.918041	0.810891	0.419606	-6.12356	0.72794	0.638418
B.cells	DDX49	0.101543	4.208396	0.810573	0.419788	-6.01451	0.736405	0.651265
B.cells	ERLIN2	0.169369	3.099067	0.8104	0.419886	-5.67251	0.749562	0.671724
B.cells	OPRM1	0.205061	2.803916	0.810327	0.419928	-5.7753	0.753102	0.677292
B.cells	XYLB	-0.356	0.922746	-0.80999	0.420119	-5.18899	0.776347	0.71375
B.cells	CDKAL1	0.079768	6.364518	0.809895	0.420175	-6.42387	0.711869	0.613505
B.cells	TUBGCP2	-0.12881	3.994036	-0.80965	0.420317	-5.9076	0.739289	0.655486
B.cells	SERAC1	0.259726	2.177244	0.809539	0.420378	-5.44495	0.761037	0.689518
B.cells	GFRA2	0.27632	2.545769	0.808927	0.420729	-5.46604	0.756984	0.682713
B.cells	WDR74	0.105341	4.042303	0.808864	0.420765	-5.96411	0.73913	0.654868
B.cells	SMARCC1	0.086979	6.606315	0.808701	0.420858	-6.45135	0.709674	0.609753
B.cells	GM9844	-0.16639	2.309102	-0.80865	0.420889	-5.72129	0.759854	0.687222
B.cells	C330013E1	0.411859	0.677864	0.808527	0.420958	-5.13303	0.779976	0.719151
B.cells	ARL8A	0.080558	6.227073	0.808379	0.421042	-6.43601	0.713942	0.616325
B.cells	TAGLN	0.369992	2.304796	0.808283	0.421097	-5.5011	0.759906	0.687463
B.cells	MDC1	0.176271	3.159139	0.808241	0.421121	-5.64807	0.749606	0.671318
B.cells	GM49439	-0.38434	-0.46286	-0.80809	0.421207	-5.1629	0.79444	0.742439
B.cells	CD83	0.156039	5.198788	0.807758	0.421398	-6.51062	0.725846	0.634381
B.cells	5830428M	0.187719	2.548294	0.807727	0.421415	-5.68934	0.757139	0.682945
B.cells	MYC	0.222372	3.533741	0.807522	0.421533	-5.89731	0.745418	0.664547
B.cells	NIN	0.096513	5.920519	0.807406	0.421599	-6.3614	0.717686	0.621829
B.cells	1700027J0	0.268044	2.481407	0.807293	0.421664	-5.65391	0.758051	0.684291
B.cells	EHMT1	0.083991	6.063885	0.806978	0.421845	-6.36788	0.716211	0.619396
B.cells	POLE4	-0.07966	6.652817	-0.80692	0.421881	-6.45597	0.709572	0.609333
B.cells	AIFM2	0.291795	1.347203	0.80674	0.421981	-5.37865	0.77217	0.706328
B.cells	PHF7	0.15232	3.28527	0.806614	0.422053	-5.81208	0.748621	0.669306
B.cells	SLC4A2	-0.1321	3.559435	-0.806	0.422407	-5.85503	0.745842	0.664391
B.cells	MBD5	0.078585	6.903175	0.805878	0.422475	-6.49792	0.707308	0.60536
B.cells	FER	0.217039	3.489418	0.805799	0.422521	-5.62487	0.746675	0.665711
B.cells	PELO	-0.16517	3.125716	-0.80569	0.422584	-5.68478	0.751019	0.672482
B.cells	YEATS2	0.106477	4.376137	0.805134	0.422902	-6.03918	0.73665	0.649643
B.cells	ST3GAL3	-0.09397	5.293649	-0.80484	0.423073	-6.28804	0.726177	0.63337
B.cells	TTK	-0.29915	2.313922	-0.80437	0.423339	-5.39494	0.761836	0.688223
B.cells	SELP	0.47199	1.452771	0.804023	0.42354	-5.27413	0.77266	0.704915
B.cells	ATP5MD	-0.07194	8.035566	-0.80384	0.423643	-6.6932	0.696009	0.586927
B.cells	ZNRF3	-0.12052	5.517512	-0.80364	0.423759	-6.28146	0.724338	0.629605

B.cells	CCDC127	-0.10458	4.15296	-0.80321	0.424009	-5.97663	0.740534	0.654218
B.cells	CBARP	-0.26025	1.923122	-0.8027	0.4243	-5.49042	0.767673	0.696248
B.cells	MFAP4	-0.36704	1.50654	-0.80269	0.424308	-5.31535	0.772811	0.704358
B.cells	COQ9	-0.16867	3.205352	-0.80233	0.424512	-5.69475	0.752275	0.672013
B.cells	TMEM128	-0.09043	5.718829	-0.80219	0.424595	-6.34782	0.722805	0.626661
B.cells	GSDME	0.149605	4.386903	0.802131	0.424628	-5.99642	0.73825	0.650334
B.cells	NPRL2	-0.16324	2.790262	-0.80197	0.424723	-5.6625	0.757276	0.679936
B.cells	EIF4G1	0.069296	6.604378	0.801678	0.424888	-6.47531	0.712748	0.611503
B.cells	6-Sep	-0.1035	5.624977	-0.8016	0.424936	-6.38401	0.723881	0.628421
B.cells	WWP2	0.074359	6.537142	0.801128	0.425205	-6.45372	0.713505	0.612834
B.cells	KLF3	0.090896	6.281206	0.801047	0.425252	-6.48949	0.716399	0.617233
B.cells	NDUFB9	0.076888	7.069533	0.801003	0.425277	-6.5371	0.707531	0.603834
B.cells	USP49	-0.14473	4.534917	-0.80098	0.42529	-6.03711	0.736514	0.647982
B.cells	CD48	-0.09059	6.795574	-0.80096	0.425304	-6.56221	0.710598	0.608457
B.cells	PTS	0.084964	5.573329	0.800898	0.425338	-6.2826	0.724474	0.629522
B.cells	TRP53I11	0.135784	4.565294	0.800867	0.425356	-6.00472	0.736159	0.64744
B.cells	FAM83E	0.328573	1.10583	0.800761	0.425417	-5.23239	0.77797	0.712894
B.cells	DNAJC17	-0.11567	3.568748	-0.80074	0.425428	-5.85292	0.747929	0.665702
B.cells	TMEM248	0.081	5.858866	0.800688	0.425459	-6.32322	0.721204	0.624587
B.cells	ZCCHC17	0.089406	5.162664	0.800627	0.425494	-6.18163	0.729208	0.636812
B.cells	JUP	-0.17809	3.562597	-0.80024	0.425717	-5.74548	0.748281	0.665985
B.cells	CCND2	0.121284	5.400515	0.800055	0.425823	-6.30842	0.726746	0.632841
B.cells	GUK1	0.100983	4.908743	0.800004	0.425853	-6.14086	0.732439	0.641576
B.cells	TOMM20	-0.07676	6.725117	-0.79972	0.426014	-6.46185	0.711739	0.60997
B.cells	KANSL1	-0.07004	8.964116	-0.79971	0.426024	-6.84268	0.687085	0.573121
B.cells	TMEM240	0.491181	0.240106	0.799249	0.426288	-5.05516	0.789399	0.730723
B.cells	TCTN3	0.364762	1.366808	0.799027	0.426416	-5.20376	0.775253	0.708212
B.cells	HABP4	-0.16101	3.054352	-0.79867	0.426621	-5.70792	0.754607	0.6758
B.cells	MICOS10	-0.07336	7.131558	-0.79862	0.426648	-6.52662	0.707324	0.603292
B.cells	NOP16	-0.12372	4.283714	-0.79853	0.426703	-6.03488	0.73997	0.65307
B.cells	ADCY6	-0.38607	0.433361	-0.79845	0.426748	-5.14912	0.786952	0.726873
B.cells	FAM92A	-0.19652	3.301539	-0.79842	0.426768	-5.61498	0.751637	0.671168
B.cells	PBXIP1	0.120562	4.578633	0.798396	0.42678	-6.1448	0.736508	0.647731
B.cells	CDK10	0.210069	2.234353	0.798377	0.426791	-5.49461	0.764559	0.691391
B.cells	SLC39A1	-0.08062	6.161792	-0.79837	0.426793	-6.39814	0.718246	0.619802
B.cells	ADORA3	-0.38401	-0.30137	-0.79833	0.426816	-5.14418	0.796303	0.741848
B.cells	COQ8B	0.189126	3.190292	0.798194	0.426897	-5.68044	0.752972	0.673288
B.cells	FNBP1	-0.08548	8.248905	-0.79804	0.426983	-6.79398	0.694983	0.584872
B.cells	TROAP	-0.33572	1.774645	-0.79801	0.427004	-5.26564	0.770204	0.700348
B.cells	ABHD14A	-0.31275	1.44344	-0.79768	0.427192	-5.25611	0.774453	0.706906
B.cells	PARPBP	-0.35948	2.056928	-0.79758	0.427249	-5.38863	0.766882	0.694963
B.cells	VPS35	-0.07407	6.232281	-0.79753	0.427279	-6.40211	0.717586	0.618722
B.cells	CHRNE	-0.43701	-0.75209	-0.7974	0.427354	-5.00312	0.802097	0.751414
B.cells	LMTK3	0.426688	-0.70138	0.797263	0.427434	-5.04337	0.801486	0.750372
B.cells	TXNDC5	-0.10641	5.179694	-0.79693	0.427627	-6.24047	0.729684	0.637279
B.cells	GM4221	-0.25944	1.846721	-0.79692	0.427633	-5.40582	0.769498	0.699209
B.cells	BLOC1S5	-0.25099	1.955013	-0.79675	0.42773	-5.41878	0.768165	0.697107

B.cells	SDC4	0.131452	6.436865	0.796638	0.427795	-6.43804	0.715297	0.615373
B.cells	ZKSCAN14	0.140859	3.081299	0.796625	0.427803	-5.76488	0.754462	0.675622
B.cells	DCLK2	0.204762	2.72479	0.796545	0.427849	-5.63882	0.758769	0.682394
B.cells	LBH	0.074023	6.308404	0.796502	0.427874	-6.44242	0.716752	0.617631
B.cells	EXT2	-0.13558	3.849599	-0.79618	0.42806	-5.84992	0.745489	0.661425
B.cells	WDR45B	-0.06569	6.330115	-0.79606	0.428132	-6.42524	0.716723	0.617292
B.cells	TRMT2A	0.142913	3.456488	0.795816	0.42827	-5.7798	0.750226	0.66876
B.cells	MTR	0.104457	4.54186	0.795775	0.428294	-6.08228	0.737375	0.648873
B.cells	2300009A(	0.140386	4.080294	0.795381	0.428522	-5.92234	0.742808	0.657404
B.cells	PADI6	-0.46577	0.058128	-0.79536	0.428533	-5.09666	0.792181	0.735184
B.cells	SLC12A7	-0.18904	3.37429	-0.7953	0.428571	-5.70846	0.75121	0.670449
B.cells	ADD3	0.108354	6.047415	0.795202	0.428625	-6.47951	0.719973	0.622397
B.cells	RMI2	-0.17881	3.3602	-0.79519	0.42863	-5.75276	0.751379	0.67074
B.cells	SNX16	0.169172	2.847666	0.79513	0.428667	-5.6974	0.75755	0.680396
B.cells	RGL2	-0.15994	3.300422	-0.79451	0.429025	-5.77486	0.752207	0.67214
B.cells	MPEG1	0.197609	6.081136	0.794499	0.429033	-6.19943	0.719696	0.622076
B.cells	PAICS	-0.0897	6.310596	-0.79445	0.429059	-6.39894	0.717088	0.618114
B.cells	TRIM8	-0.0872	5.616506	-0.79444	0.429065	-6.3103	0.725011	0.630175
B.cells	NXT1	0.12588	4.399059	0.794347	0.42912	-6.0182	0.73916	0.6519
B.cells	CORO1C	0.078251	6.202322	0.794272	0.429164	-6.4145	0.718317	0.620006
B.cells	RINT1	0.130135	3.771156	0.794141	0.42924	-5.85036	0.746584	0.663421
B.cells	SNX24	0.193757	4.299252	0.794078	0.429276	-5.93156	0.740334	0.653741
B.cells	GM26881	-0.42415	0.245715	-0.79402	0.429308	-5.1308	0.789912	0.731736
B.cells	TMC4	-0.24936	1.556285	-0.79374	0.429473	-5.31738	0.773569	0.705635
B.cells	ZFYVE28	-0.29005	0.624372	-0.79371	0.429488	-5.20499	0.785218	0.724144
B.cells	CSAD	-0.14445	3.965874	-0.79359	0.429561	-5.94529	0.744378	0.659922
B.cells	GM9750	-0.37293	0.476616	-0.79347	0.429628	-5.24231	0.787105	0.72718
B.cells	CEP76	0.17725	2.990307	0.79333	0.42971	-5.68774	0.756081	0.678193
B.cells	POLR3K	-0.11973	3.885777	-0.79311	0.429835	-5.90088	0.745449	0.661586
B.cells	BC051537	0.469835	0.044835	0.793023	0.429888	-5.07133	0.792709	0.736151
B.cells	SAP30L	-0.09793	4.309459	-0.79259	0.430141	-6.036	0.740744	0.653929
B.cells	GM50020	0.401937	-0.11132	0.79238	0.43026	-5.06241	0.795028	0.73949
B.cells	ARF3	0.069789	6.29303	0.792303	0.430305	-6.41662	0.7178	0.618872
B.cells	BTF3	-0.05639	8.999494	-0.79211	0.430414	-6.87774	0.687837	0.574034
B.cells	TTC13	0.130707	3.938924	0.792081	0.430433	-5.86727	0.745125	0.660868
B.cells	ARHGAP17	-0.07905	7.245574	-0.79187	0.430553	-6.60393	0.707081	0.602789
B.cells	YAE1D1	-0.1741	3.240113	-0.79187	0.430556	-5.81193	0.75347	0.673891
B.cells	COASY	-0.18105	3.053875	-0.7918	0.430596	-5.68702	0.755713	0.677427
B.cells	IRF4	-0.14574	4.497863	-0.79172	0.430642	-6.04846	0.738528	0.650743
B.cells	ALG10B	0.166792	2.952601	0.791152	0.430973	-5.65626	0.75695	0.679488
B.cells	EID2B	0.217636	1.703903	0.791133	0.430983	-5.40166	0.772216	0.703498
B.cells	SERPINE1	0.356716	0.805177	0.791105	0.431	-5.25461	0.783423	0.721288
B.cells	PGPEP1L	0.369975	-0.29843	0.791024	0.431047	-5.14045	0.797441	0.743769
B.cells	CCT4	-0.06935	6.487167	-0.79087	0.431137	-6.4535	0.715615	0.615853
B.cells	FAM49B	0.052748	9.086243	0.790818	0.431167	-6.91562	0.686915	0.572895
B.cells	DYRK2	0.10349	4.884934	0.790808	0.431172	-6.10767	0.734015	0.643965
B.cells	C77080	-0.78152	0.694235	-0.79078	0.431189	-5.0995	0.784819	0.72358

B.cells	COMMD2	-0.09227	4.927522	-0.79071	0.431228	-6.15408	0.733518	0.643223
B.cells	CACUL1	-0.09155	6.117834	-0.79002	0.431629	-6.40681	0.720368	0.622461
B.cells	CHCHD10	-0.1327	6.141333	-0.78989	0.431707	-6.42151	0.720104	0.62211
B.cells	PMEPA1	0.161421	4.361383	0.789732	0.431797	-6.13744	0.740728	0.653762
B.cells	ZSCAN22	0.348438	1.316109	0.789687	0.431824	-5.22421	0.777639	0.711584
B.cells	FBXO21	-0.13927	3.292952	-0.78951	0.431926	-5.82949	0.753508	0.673566
B.cells	SLC41A3	-0.24654	1.73389	-0.7893	0.432049	-5.39123	0.772624	0.703415
B.cells	ZFP943	0.095342	4.280266	0.788935	0.432261	-6.05382	0.741904	0.655423
B.cells	MAP3K14	0.106031	5.189544	0.788924	0.432267	-6.32257	0.731263	0.639041
B.cells	PTGER4	-0.136	4.681638	-0.7888	0.432341	-6.14986	0.737184	0.648172
B.cells	ABCB6	-0.30658	1.101522	-0.78874	0.432373	-5.17678	0.780549	0.716061
B.cells	HIRIP3	-0.17364	3.169999	-0.78857	0.432475	-5.72829	0.755143	0.676104
B.cells	DSEL	0.359334	0.375304	0.788519	0.432502	-5.09039	0.789702	0.730728
B.cells	PCID2	-0.08836	4.736836	-0.78847	0.432529	-6.16508	0.736538	0.647278
B.cells	B3GNTL1	0.182839	2.673331	0.788061	0.432769	-5.6937	0.761353	0.685637
B.cells	KIF14	0.30739	2.55406	0.788058	0.432771	-5.52138	0.762806	0.687915
B.cells	ULK3	0.220027	2.219052	0.787767	0.43294	-5.50873	0.766973	0.694354
B.cells	CYP2AB1	0.428144	-0.30874	0.787695	0.432982	-5.13703	0.798717	0.744862
B.cells	DNAJC1	0.073754	6.915183	0.787658	0.433003	-6.52862	0.711811	0.609292
B.cells	BORCS6	0.124839	4.007961	0.787514	0.433087	-6.00297	0.745422	0.660685
B.cells	EIF2B4	0.095207	4.102435	0.787387	0.433161	-6.03872	0.744319	0.659012
B.cells	BRCA2	-0.18612	3.555566	-0.78698	0.433399	-5.76765	0.75103	0.669329
B.cells	POSTN	-0.39541	1.260435	-0.78688	0.433459	-5.23353	0.779098	0.713473
B.cells	NFKBIB	0.09587	5.622292	0.786857	0.43347	-6.3523	0.726761	0.631935
B.cells	UPF3B	0.103754	4.979585	0.786574	0.433634	-6.15383	0.734379	0.643392
B.cells	SLC35C1	0.170862	2.749333	0.786186	0.43386	-5.7124	0.761102	0.684664
B.cells	KLHL2	0.093558	5.570335	0.785656	0.434169	-6.33026	0.727692	0.633086
B.cells	SHCBP1L	-0.31126	0.854389	-0.7856	0.434204	-5.26393	0.784547	0.721838
B.cells	RIOK2	-0.10186	4.466641	-0.78555	0.434233	-6.05266	0.740555	0.652841
B.cells	OCEL1	0.135854	3.584489	0.785447	0.434291	-5.85317	0.751027	0.669079
B.cells	GM27216	-0.39618	0.912751	-0.78535	0.43435	-5.22507	0.783813	0.720724
B.cells	ECT2	-0.23129	4.373594	-0.78529	0.434383	-5.89793	0.741651	0.654603
B.cells	TMEM147	0.099929	4.948794	0.785237	0.434414	-6.17416	0.734903	0.644205
B.cells	ZKSCAN6	0.181533	2.942031	0.785235	0.434415	-5.71579	0.758763	0.681209
B.cells	SLC25A12	0.093757	5.092687	0.785184	0.434444	-6.15116	0.733226	0.641639
B.cells	PXN	0.088766	6.266447	0.784873	0.434626	-6.44856	0.719715	0.621129
B.cells	GM43581	0.24781	2.093907	0.784647	0.434758	-5.47229	0.769118	0.697729
B.cells	UTP11	0.101163	4.845019	0.784552	0.434813	-6.08943	0.736115	0.64631
B.cells	MRPS18B	0.122988	3.873974	0.784512	0.434837	-5.9593	0.747572	0.664018
B.cells	TAOK1	0.065422	7.033029	0.784458	0.434868	-6.55957	0.71105	0.608115
B.cells	EMP3	-0.09255	7.421524	-0.78438	0.434911	-6.569	0.706706	0.601585
B.cells	NPM3	-0.11526	5.097579	-0.7843	0.434959	-6.20374	0.733169	0.641801
B.cells	AKR1B10	-0.1424	3.494629	-0.78425	0.434987	-5.90778	0.752104	0.671085
B.cells	LTC4S	0.539862	1.066227	0.784203	0.435017	-5.31666	0.781885	0.717985
B.cells	BEND6	-0.43451	0.170455	-0.78377	0.435269	-5.10185	0.793213	0.736207
B.cells	JHY	-0.5384	-0.37714	-0.7837	0.43531	-5.02926	0.800229	0.747479
B.cells	NOS1AP	-0.30528	3.163666	-0.78366	0.435332	-5.46612	0.756084	0.677437

B.cells	ACER3	0.091114	6.311004	0.783648	0.435341	-6.41102	0.719208	0.620612
B.cells	OLFR1033	-0.50197	0.583769	-0.78357	0.435387	-5.10466	0.787963	0.727831
B.cells	ALOX5AP	0.256492	7.325815	0.783532	0.435409	-6.29156	0.707773	0.603344
B.cells	SLIT1	-0.33017	1.464859	-0.78352	0.435416	-5.31452	0.776904	0.710232
B.cells	ZFP992	-0.1556	3.563813	-0.78321	0.4356	-5.84222	0.751275	0.670079
B.cells	CKAP2	-0.2001	3.667324	-0.78289	0.435782	-5.83121	0.750037	0.668207
B.cells	RDM1	-0.13821	5.028258	-0.78285	0.43581	-6.14164	0.733976	0.643367
B.cells	ZBTB7B	0.215553	2.473173	0.782843	0.435811	-5.64338	0.764468	0.69078
B.cells	HIST1H2BN	-0.35339	1.240034	-0.78259	0.43596	-5.19454	0.779709	0.714968
B.cells	ALAD	-0.15972	4.057214	-0.78256	0.435975	-5.93284	0.745394	0.661094
B.cells	9430015G1	0.246374	1.911053	0.782531	0.435994	-5.37801	0.771372	0.701777
B.cells	SCLT1	0.140998	4.474106	0.782428	0.436054	-6.00086	0.740467	0.653529
B.cells	CARM1	0.098917	4.727042	0.782364	0.436091	-6.09986	0.737496	0.648972
B.cells	NXPE3	0.145676	3.424156	0.782353	0.436098	-5.84391	0.752949	0.672938
B.cells	SPSB3	-0.12123	3.587341	-0.78225	0.436155	-5.84108	0.750993	0.669902
B.cells	AIDA	0.101035	4.437691	0.782082	0.436256	-6.09912	0.740896	0.654263
B.cells	BCL11A	0.13254	5.196907	0.782036	0.436283	-6.27013	0.732015	0.64058
B.cells	CASP2	0.112348	4.01281	0.78192	0.436351	-5.96571	0.745921	0.662074
B.cells	RSPH9	0.29993	1.514419	0.781888	0.436369	-5.39284	0.776288	0.709722
B.cells	9930104L0	0.290169	1.356879	0.781759	0.436444	-5.2681	0.778269	0.712847
B.cells	6720427I0	0.105497	4.894793	0.781604	0.436536	-6.16071	0.735563	0.646084
B.cells	TNFRSF9	0.345975	2.460851	0.781528	0.43658	-5.4238	0.764649	0.691395
B.cells	ROR1	-0.37487	1.374979	-0.78105	0.43686	-5.25518	0.77844	0.712781
B.cells	C6	-0.37082	2.802964	-0.78092	0.436936	-5.5307	0.760876	0.685108
B.cells	TMSB15B2	-0.20587	2.47439	-0.7805	0.437181	-5.58616	0.765078	0.691643
B.cells	ZFP809	0.146293	3.64912	0.780426	0.437225	-5.89938	0.750868	0.669407
B.cells	GM12064	0.255743	0.96957	0.780267	0.437318	-5.41665	0.783739	0.721225
B.cells	HIPK1	0.089549	6.269668	0.780091	0.437421	-6.41397	0.720267	0.622357
B.cells	A130010J1	0.284889	1.72998	0.780036	0.437453	-5.33766	0.774244	0.706167
B.cells	GM36839	0.223685	2.346299	0.779985	0.437483	-5.4767	0.766646	0.69418
B.cells	NINJ2	-0.42169	0.701022	-0.77996	0.437499	-5.19961	0.787124	0.726659
B.cells	GLMP	0.102577	5.839868	0.779532	0.437748	-6.35384	0.725363	0.629973
B.cells	ECM2	-0.51099	0.502848	-0.77934	0.43786	-5.06158	0.789827	0.730818
B.cells	1700123M	0.332354	0.89752	0.779264	0.437905	-5.21312	0.784839	0.72287
B.cells	EAR2	-0.26705	3.55304	-0.7791	0.438001	-5.6994	0.752204	0.671475
B.cells	RHOA	0.041743	9.258828	0.77904	0.438037	-6.91784	0.687315	0.572891
B.cells	BC055324	-0.23068	2.245694	-0.779	0.438063	-5.47042	0.76807	0.696374
B.cells	MCEE	-0.13939	4.194477	-0.77899	0.438068	-5.91946	0.744559	0.659621
B.cells	NCLN	-0.1284	3.839103	-0.77883	0.438158	-5.89172	0.748783	0.666178
B.cells	PDE3B	0.104918	6.955861	0.778785	0.438186	-6.686	0.712675	0.610821
B.cells	GM37065	-0.23409	2.706239	-0.77841	0.438404	-5.68533	0.762653	0.687627
B.cells	CAPN11	-0.49813	1.046105	-0.77835	0.438439	-5.30602	0.783192	0.720118
B.cells	ACY1	-0.30705	1.799123	-0.77755	0.438911	-5.31503	0.774517	0.705571
B.cells	BCAS3OS1	-0.1208	3.793281	-0.77723	0.439099	-5.99059	0.750364	0.667585
B.cells	GM2682	-0.34583	2.755703	-0.77713	0.439155	-5.54648	0.762886	0.687172
B.cells	ZSWIM3	-0.32938	1.503758	-0.77704	0.43921	-5.25954	0.778319	0.711539
B.cells	DENR	-0.07531	6.279481	-0.7769	0.439289	-6.39202	0.721327	0.623009

B.cells	AP3M1	-0.08783	4.881049	-0.77687	0.439306	-6.14184	0.737493	0.647746
B.cells	ZFYVE19	0.146121	3.132938	0.776635	0.439447	-5.76368	0.758437	0.680153
B.cells	0610039K1	-0.46803	0.022539	-0.77647	0.439545	-5.0601	0.797245	0.74163
B.cells	MCL1	-0.07623	8.364811	-0.7758	0.439936	-6.79761	0.698568	0.58819
B.cells	F11R	-0.19403	3.432174	-0.77573	0.439976	-5.78199	0.755318	0.674762
B.cells	AGPAT4	-0.11978	5.023605	-0.77571	0.439992	-6.39144	0.736434	0.645525
B.cells	WDR6	-0.15198	3.277321	-0.77554	0.440092	-5.829	0.757246	0.677741
B.cells	EDC3	0.122768	3.628178	0.775122	0.440336	-5.89112	0.753329	0.671335
B.cells	ZDHHC14	-0.14647	7.022709	-0.77489	0.440471	-6.50088	0.713956	0.610826
B.cells	COL4A1	-0.24754	3.330895	-0.77468	0.440595	-5.67397	0.757057	0.677073
B.cells	WDR76	-0.15245	4.504143	-0.77442	0.440746	-6.01689	0.743045	0.655409
B.cells	NUB1	0.077506	5.534309	0.774307	0.440815	-6.30722	0.730992	0.636936
B.cells	GMFB	-0.08522	5.451593	-0.77427	0.440834	-6.2614	0.731952	0.63841
B.cells	MRS2	0.114732	3.939507	0.774161	0.440901	-5.94648	0.74975	0.665881
B.cells	REEP5	0.064911	7.566136	0.774127	0.440921	-6.63446	0.70789	0.601935
B.cells	RAMAC	-0.07225	5.913686	-0.7741	0.440939	-6.39935	0.726612	0.630272
B.cells	ORMDL2	-0.09306	5.109617	-0.77361	0.441223	-6.21442	0.736203	0.644596
B.cells	SP3	-0.0671	6.891028	-0.7736	0.44123	-6.52413	0.715731	0.613407
B.cells	P2RY6	-0.28194	2.305897	-0.77333	0.441393	-5.4047	0.769842	0.697039
B.cells	1600020E0	-0.09667	6.223144	-0.77319	0.441472	-6.48417	0.723335	0.625088
B.cells	2810403D2	-0.1823	2.774023	-0.77318	0.441481	-5.6446	0.764104	0.688077
B.cells	SH3BGRL3	0.073103	9.061448	0.773154	0.441494	-6.89981	0.691702	0.577618
B.cells	USP24	0.086548	5.459806	0.772978	0.441597	-6.27991	0.732197	0.638574
B.cells	ZFP994	0.170298	2.481603	0.772398	0.441939	-5.61998	0.768232	0.693892
B.cells	TATDN1	0.124959	3.630234	0.772049	0.442144	-5.89851	0.754356	0.672217
B.cells	CTSA	-0.10528	6.259221	-0.77203	0.442157	-6.43339	0.723516	0.624778
B.cells	TIPRL	-0.08287	5.525539	-0.77199	0.442179	-6.28416	0.731971	0.637672
B.cells	IGLC2	-0.33822	3.740399	-0.77171	0.442344	-6.12884	0.75315	0.670288
B.cells	FMC1	0.134611	4.072806	0.771653	0.442377	-5.98292	0.749174	0.664132
B.cells	ARPP21	0.487947	1.234602	0.770979	0.442775	-5.22997	0.784318	0.718885
B.cells	RMI1	0.122692	3.629414	0.770879	0.442834	-5.84105	0.75486	0.672574
B.cells	GM40841	-0.4506	0.40148	-0.77073	0.442924	-5.11859	0.794875	0.735772
B.cells	FBXO30	0.113578	5.032199	0.770443	0.443091	-6.18156	0.738204	0.646983
B.cells	GM26801	-0.33844	0.557742	-0.7704	0.443113	-5.32157	0.792883	0.732725
B.cells	PAK4	-0.18663	2.299468	-0.77039	0.443124	-5.5795	0.771058	0.698103
B.cells	JTB	0.065208	5.842584	0.77032	0.443164	-6.38266	0.728779	0.632549
B.cells	CASC1	-0.25388	1.810453	-0.77031	0.443171	-5.56287	0.777115	0.70766
B.cells	MIPEP	-0.15302	2.843457	-0.77021	0.443229	-5.63838	0.764384	0.687633
B.cells	MXD4	-0.11514	5.790486	-0.77016	0.44326	-6.35296	0.729381	0.633509
B.cells	ACTR3	0.052847	8.876057	0.77006	0.443317	-6.87392	0.694741	0.581391
B.cells	TOR2A	-0.12889	3.670287	-0.76996	0.443374	-5.85449	0.754368	0.672087
B.cells	1600002K0	-0.13803	3.214423	-0.76973	0.443515	-5.79304	0.759952	0.680766
B.cells	NSUN3	0.168357	2.658689	0.769663	0.443551	-5.6121	0.766725	0.691393
B.cells	RAD54L2	0.101962	4.552757	0.769457	0.443673	-6.06102	0.744023	0.656029
B.cells	ABCA9	-0.4244	0.787541	-0.76904	0.443917	-5.23642	0.790469	0.72862
B.cells	PCDH9	0.535921	0.472713	0.768869	0.44402	-5.13681	0.794543	0.735081
B.cells	PLLP	-0.471	-0.20457	-0.7685	0.444237	-5.04964	0.803473	0.749151

B.cells	WBP1	-0.14523	3.813733	-0.76832	0.444342	-5.91458	0.753411	0.670017
B.cells	SNHG10	-0.4595	0.227889	-0.76825	0.444386	-5.07791	0.797905	0.740264
B.cells	PYROXD1	0.155663	2.95488	0.768199	0.444416	-5.74421	0.7638	0.686232
B.cells	CPLX1	-0.61047	-0.57566	-0.7681	0.444473	-4.98168	0.808207	0.756998
B.cells	CCS	-0.12597	4.318203	-0.76787	0.44461	-6.02377	0.747429	0.660861
B.cells	OGFOD1	0.1293	3.611469	0.767841	0.444628	-5.91544	0.755887	0.67399
B.cells	3300005D(	-0.38972	0.756323	-0.76728	0.444956	-5.16371	0.791677	0.729917
B.cells	MYO1B	-0.28569	2.148687	-0.76706	0.445089	-5.48665	0.774254	0.702269
B.cells	NUF2	-0.22551	3.365247	-0.76656	0.445382	-5.68509	0.759355	0.679007
B.cells	CSNK2A2	0.066316	5.948451	0.766525	0.445406	-6.39196	0.728815	0.631895
B.cells	SRPK1	-0.06462	6.11152	-0.76646	0.445444	-6.40885	0.726936	0.629033
B.cells	CHM	0.087147	5.555869	0.766358	0.445504	-6.32325	0.733362	0.638839
B.cells	GM15886	-0.21519	1.881502	-0.76635	0.445507	-5.38338	0.777572	0.707616
B.cells	GLMN	0.174567	2.819036	0.766331	0.44552	-5.67123	0.766003	0.689404
B.cells	EIF3K	0.057348	7.871661	0.766235	0.445577	-6.72206	0.707017	0.598954
B.cells	PPP4R3A	0.05385	6.59174	0.766184	0.445607	-6.49887	0.721436	0.620679
B.cells	WDFY3	-0.13362	5.666809	-0.76614	0.445631	-6.2666	0.732074	0.636869
B.cells	DENND3	0.149006	3.548798	0.765972	0.445733	-5.92644	0.757191	0.675547
B.cells	PSMD3	0.102558	4.642778	0.765871	0.445793	-6.0912	0.744121	0.655329
B.cells	CACNB1	-0.3595	0.846674	-0.76536	0.446098	-5.23183	0.791059	0.728464
B.cells	NT5DC3	0.098298	5.099601	0.764945	0.446341	-6.20527	0.739436	0.647296
B.cells	STUB1	0.084278	5.426852	0.764815	0.446418	-6.2437	0.735627	0.641426
B.cells	ZFP433	-0.37405	0.355661	-0.7644	0.446663	-5.17255	0.797984	0.738726
B.cells	PDP1	0.17325	2.180234	0.764014	0.446893	-5.59734	0.775163	0.702358
B.cells	RECQL	-0.16245	3.363727	-0.76398	0.446913	-5.79511	0.760648	0.679612
B.cells	FXR1	0.068064	6.109299	0.763895	0.446963	-6.41197	0.728183	0.629635
B.cells	MTMR14	-0.0932	5.427711	-0.76378	0.447031	-6.30046	0.736092	0.641727
B.cells	FBXL12OS	-0.29125	0.679534	-0.76367	0.447099	-5.25008	0.794042	0.73239
B.cells	2010013B2	0.167698	3.545259	0.763085	0.447444	-5.83484	0.75861	0.676519
B.cells	TMOD1	0.271721	1.823905	0.763002	0.447493	-5.46263	0.779761	0.709745
B.cells	APAF1	0.09049	5.726771	0.762969	0.447513	-6.35389	0.732761	0.636711
B.cells	NSD2	-0.08646	6.699324	-0.76275	0.447645	-6.49263	0.721573	0.61978
B.cells	UCHL5	0.086921	5.728606	0.762389	0.447857	-6.30309	0.73274	0.636885
B.cells	GM43728	0.380502	-0.48628	0.762355	0.447877	-5.08864	0.809198	0.757045
B.cells	HDDC3	-0.3571	0.928077	-0.7621	0.448032	-5.16604	0.791037	0.72795
B.cells	ANGPTL7	0.514251	-0.05517	0.762029	0.448071	-5.0178	0.803631	0.748096
B.cells	PGGT1B	0.092674	4.818529	0.761953	0.448116	-6.1603	0.743396	0.653309
B.cells	TCTEX1D2	0.166074	3.301638	0.761858	0.448172	-5.73308	0.761563	0.681482
B.cells	GPAT4	0.091891	4.264538	0.761842	0.448182	-6.05682	0.749971	0.663465
B.cells	TIMM50	-0.10995	4.827954	-0.76147	0.448405	-6.11218	0.743284	0.653261
B.cells	MGAT2	-0.07609	5.873385	-0.76145	0.448414	-6.37175	0.731062	0.634521
B.cells	PARK7	-0.07933	6.806683	-0.76133	0.448483	-6.50543	0.72035	0.618253
B.cells	GM47863	0.399419	0.552776	0.761296	0.448506	-5.16398	0.795817	0.735749
B.cells	LY6E	0.095671	9.08245	0.76125	0.448533	-6.92262	0.695002	0.580327
B.cells	2410131K1	0.228594	2.169111	0.760909	0.448736	-5.52207	0.775465	0.703504
B.cells	LGR5	-0.32651	1.073909	-0.76084	0.448778	-5.27448	0.789189	0.725249
B.cells	AAMDC	0.124087	3.299635	0.760758	0.448826	-5.77193	0.761587	0.681735

B.cells	EVI2A	-0.11306	5.251447	-0.76069	0.448863	-6.21044	0.738304	0.645713
B.cells	UBE4A	0.0945	5.03345	0.760662	0.448883	-6.23186	0.740863	0.649643
B.cells	RBM10	-0.09357	4.779494	-0.76061	0.448914	-6.14206	0.743857	0.654253
B.cells	FAM122B	-0.21817	2.015817	-0.7606	0.44892	-5.43104	0.777369	0.706549
B.cells	C130046K2	-0.35185	0.716514	-0.76059	0.448925	-5.20676	0.793728	0.732527
B.cells	GM43112	-0.3446	0.075373	-0.76048	0.448991	-5.17262	0.801945	0.745724
B.cells	GM48099	0.307998	2.773722	0.760435	0.449018	-5.74271	0.768007	0.691851
B.cells	THOC3	-0.11841	4.102819	-0.76039	0.449044	-5.98811	0.751903	0.666731
B.cells	GM33524	0.347341	0.491882	0.76019	0.449164	-5.24493	0.796596	0.737206
B.cells	GM16066	-0.2245	2.012511	-0.76019	0.449166	-5.44372	0.77741	0.706706
B.cells	SMS	-0.07577	6.477089	-0.76016	0.44918	-6.42859	0.724112	0.624132
B.cells	ARF6	-0.07966	6.770128	-0.76005	0.449244	-6.55452	0.720766	0.619089
B.cells	RFK	-0.09542	5.042963	-0.76002	0.449267	-6.25143	0.740751	0.649606
B.cells	IRF1	0.165553	7.149071	0.759845	0.449369	-6.58497	0.716467	0.612657
B.cells	UBAP2L	0.056924	7.113568	0.7598	0.449395	-6.59014	0.716869	0.613268
B.cells	GM42659	-0.10352	4.567867	-0.75938	0.449647	-6.1395	0.746673	0.658432
B.cells	ATP5J2	-0.07611	7.723844	-0.75918	0.449762	-6.67221	0.710378	0.603112
B.cells	PTBP1	-0.07965	6.221744	-0.759	0.449871	-6.39588	0.727424	0.628882
B.cells	TRIT1	0.106813	4.076961	0.758962	0.449894	-6.05688	0.752609	0.667576
B.cells	GANC	0.156115	3.377098	0.758856	0.449957	-5.83206	0.761047	0.680715
B.cells	GM16740	0.199143	2.544974	0.758601	0.450109	-5.5719	0.771278	0.696708
B.cells	SASH3	-0.08346	5.4972	-0.75859	0.450118	-6.31914	0.735871	0.641752
B.cells	POLH	0.126809	4.035992	0.758272	0.450305	-5.96805	0.75321	0.668395
B.cells	WDR92	-0.17206	3.083733	-0.75825	0.450316	-5.76092	0.76473	0.686344
B.cells	SMIM4	0.091334	5.255277	0.75801	0.450461	-6.18918	0.738757	0.646089
B.cells	GRAMD1C	0.214484	2.440736	0.758004	0.450464	-5.5807	0.772624	0.698731
B.cells	EXD2	0.141276	3.156926	0.757981	0.450478	-5.85332	0.763837	0.684947
B.cells	VCP	-0.06068	7.431716	-0.75775	0.450617	-6.62492	0.713879	0.608143
B.cells	IFIT2	0.440963	2.721315	0.757593	0.450709	-5.46122	0.769341	0.693383
B.cells	PIGZ	0.404335	0.017052	0.757473	0.450781	-5.15579	0.803431	0.747514
B.cells	MATK	0.1939	2.36611	0.757222	0.45093	-5.70328	0.773877	0.7004
B.cells	3110040N1	0.142225	3.553549	0.757084	0.451012	-5.9363	0.759374	0.677669
B.cells	NACA	-0.05943	9.080227	-0.75696	0.451084	-6.89943	0.695828	0.581064
B.cells	COQ6	-0.18102	2.285167	-0.75651	0.451357	-5.51592	0.774948	0.702139
B.cells	GM50240	0.275598	1.572659	0.756422	0.451407	-5.33897	0.783837	0.716208
B.cells	TMEM198I	-0.32393	0.786307	-0.75612	0.451584	-5.2272	0.793783	0.732076
B.cells	EIF2B3	0.173478	2.996786	0.756052	0.451628	-5.72757	0.766187	0.688443
B.cells	UBXN6	0.109476	4.786672	0.75601	0.451653	-6.139	0.744657	0.654988
B.cells	MYL12A	-0.0706	7.929477	-0.75597	0.451678	-6.70694	0.708552	0.600118
B.cells	OSBPL7	-0.13446	3.426262	-0.75589	0.451727	-5.83824	0.760956	0.680265
B.cells	VAT1	0.150131	3.491225	0.755783	0.451789	-5.86542	0.760168	0.679069
B.cells	DERL1	-0.05584	6.22546	-0.75567	0.451854	-6.45324	0.727864	0.629322
B.cells	NFKBIZ	0.116246	6.516715	0.755661	0.451861	-6.43621	0.72452	0.62424
B.cells	CRYBA4	0.44704	-0.18755	0.755605	0.451894	-5.07297	0.806302	0.7522
B.cells	GM9917	0.321184	0.773036	0.755598	0.451899	-5.33597	0.793952	0.732407
B.cells	ATP5G2	0.063963	8.844228	0.755519	0.451946	-6.84422	0.698436	0.585084
B.cells	STRAP	-0.08213	6.174275	-0.75514	0.452169	-6.42119	0.728539	0.630354

B.cells	LARP1B	-0.09097	5.599705	-0.75505	0.452227	-6.36156	0.735198	0.640547
B.cells	ZFP975	0.421512	0.810489	0.754924	0.452301	-5.19663	0.793567	0.731872
B.cells	REM2	-0.33425	0.836601	-0.75488	0.452327	-5.33191	0.793235	0.731342
B.cells	NELFCD	-0.10994	4.117826	-0.75488	0.452328	-6.00906	0.752706	0.66756
B.cells	GTF2F1	0.081036	5.030746	0.754778	0.452388	-6.19785	0.741863	0.650819
B.cells	TMED4	-0.14094	3.553347	-0.75404	0.452829	-5.80754	0.759875	0.67837
B.cells	ATP6V1H	-0.0896	6.803114	-0.75391	0.452906	-6.53003	0.721685	0.619694
B.cells	AP4E1	0.165483	3.305374	0.753849	0.452943	-5.7795	0.762886	0.683067
B.cells	NFU1	0.112308	4.549473	0.753791	0.452978	-6.10893	0.747922	0.65982
B.cells	FSHR	-0.30934	0.419984	-0.75371	0.453027	-5.12905	0.798949	0.740114
B.cells	IFRD1	0.094214	8.532379	0.753668	0.453051	-6.84438	0.70229	0.590574
B.cells	RPP38	0.329274	1.226405	0.753645	0.453065	-5.2807	0.788676	0.72372
B.cells	IDH3A	0.106251	4.865883	0.753439	0.453188	-6.13926	0.744218	0.654039
B.cells	ABHD4	0.171289	2.787772	0.753339	0.453247	-5.60846	0.769262	0.692981
B.cells	ELAC2	-0.15431	2.817476	-0.75327	0.453288	-5.721	0.768897	0.692416
B.cells	NAGK	-0.12734	3.956557	-0.75306	0.453414	-5.94487	0.755159	0.670866
B.cells	VPS4B	0.068807	6.231035	0.752748	0.453601	-6.41809	0.728543	0.629731
B.cells	IFIT1BL2	0.508713	-0.46837	0.752489	0.453756	-4.99098	0.810764	0.758768
B.cells	LNX2	0.12532	3.592505	0.752399	0.45381	-5.98504	0.759716	0.677811
B.cells	SDR39U1	0.309718	1.131364	0.752387	0.453817	-5.29368	0.790206	0.725826
B.cells	UQCRC2	0.064826	6.345631	0.752158	0.453954	-6.47015	0.727223	0.627829
B.cells	DNAH7A	-0.45181	-0.11769	-0.752	0.454046	-5.09278	0.806218	0.751473
B.cells	ZFP706	-0.05517	7.871583	-0.752	0.454047	-6.7185	0.709921	0.601719
B.cells	COX16	-0.0613	6.153691	-0.75195	0.454075	-6.42075	0.729435	0.631193
B.cells	MICOS13	0.077075	5.948201	0.751916	0.454099	-6.37705	0.731811	0.634815
B.cells	INTS10	0.142063	3.410048	0.751138	0.454564	-5.80713	0.762363	0.681574
B.cells	PDS5A	0.061329	7.411745	0.751126	0.454572	-6.61318	0.715491	0.609725
B.cells	CANT1	0.123737	3.891787	0.751084	0.454597	-5.92461	0.756533	0.672501
B.cells	MIB1	0.079082	6.189415	0.751021	0.454635	-6.41827	0.729438	0.630874
B.cells	SRGN	0.070681	9.652655	0.750742	0.454801	-7.00052	0.690735	0.572994
B.cells	PRDM15	0.135607	3.462741	0.750671	0.454844	-5.82289	0.761722	0.680749
B.cells	ADAM8	-0.30728	2.602795	-0.7506	0.454889	-5.41869	0.772249	0.697257
B.cells	CENPN	0.220912	2.920239	0.750418	0.454995	-5.68635	0.768344	0.691181
B.cells	DROSHA	0.13353	3.121263	0.75039	0.455012	-5.73841	0.765882	0.687326
B.cells	BAX	0.084435	6.141068	0.750348	0.455038	-6.44602	0.729996	0.631912
B.cells	RHOJ	-0.32275	1.992484	-0.75029	0.455073	-5.34126	0.779823	0.709266
B.cells	MON1A	0.114046	3.876268	0.749981	0.455257	-5.99473	0.756853	0.673166
B.cells	DCTN5	-0.10171	4.598222	-0.74951	0.455542	-6.10657	0.74821	0.659887
B.cells	RAE1	0.097481	4.525674	0.749492	0.45555	-6.10473	0.749073	0.661221
B.cells	ACTR1B	0.118295	4.215968	0.749471	0.455563	-6.02402	0.752771	0.666952
B.cells	GM11457	-0.48269	0.10252	-0.74931	0.455658	-5.08905	0.803967	0.747851
B.cells	GM15788	-0.4433	0.240256	-0.74927	0.455684	-5.09204	0.802189	0.745013
B.cells	MRPL49	-0.14401	3.778216	-0.74926	0.455689	-5.87247	0.758036	0.675222
B.cells	POLR3G	0.232601	2.101279	0.749228	0.455709	-5.51143	0.778604	0.707479
B.cells	DDX23	0.089529	4.71777	0.749176	0.45574	-6.18969	0.746789	0.657813
B.cells	SOX4	-0.18141	5.508952	-0.74879	0.455969	-6.24101	0.737639	0.643593
B.cells	HIST2H3B	-0.37206	1.047683	-0.74875	0.455995	-5.19658	0.792037	0.728645

B.cells	POLR3E	0.108095	3.918387	0.748514	0.456137	-6.00441	0.756518	0.672791
B.cells	GON4L	0.082646	5.15234	0.74851	0.456139	-6.24172	0.741823	0.650073
B.cells	FKBP3	-0.09755	6.130579	-0.7484	0.456207	-6.39227	0.730412	0.632627
B.cells	JPX	-0.17534	3.810956	-0.74835	0.456234	-5.89638	0.757813	0.674827
B.cells	CEP120	0.08202	5.494784	0.748103	0.456384	-6.31158	0.737851	0.643984
B.cells	ANO10	0.180732	3.16746	0.748086	0.456393	-5.72639	0.765675	0.687085
B.cells	GM6034	0.427912	0.50272	0.747848	0.456536	-5.17686	0.799182	0.740023
B.cells	KLHL28	-0.13541	3.44241	-0.74757	0.456703	-5.81287	0.762528	0.682036
B.cells	BTBD19	0.187326	2.725168	0.747558	0.45671	-5.59197	0.771306	0.695783
B.cells	RAC2	0.074978	9.052768	0.747362	0.456828	-6.84487	0.697852	0.583479
B.cells	TMEM68	0.108494	3.807022	0.746684	0.457235	-5.98917	0.758683	0.675434
B.cells	RABGAP1L	-0.08354	7.908574	-0.74657	0.457305	-6.71454	0.710969	0.602598
B.cells	RBM12B2	0.185638	2.316248	0.746331	0.457447	-5.57096	0.77695	0.704165
B.cells	IPCEF1	-0.14705	4.679593	-0.7463	0.457464	-6.18622	0.748223	0.659346
B.cells	PTP4A3	0.083906	5.77385	0.746246	0.457498	-6.35403	0.735345	0.639563
B.cells	ICK	0.232416	2.147459	0.746164	0.457548	-5.45688	0.77905	0.707478
B.cells	VCPKMT	-0.16966	2.877826	-0.74599	0.457649	-5.76261	0.770009	0.693292
B.cells	CD4	-0.45736	1.246276	-0.74591	0.457701	-5.12302	0.790375	0.725475
B.cells	AK2	-0.08681	5.995761	-0.74574	0.457801	-6.36928	0.732766	0.635743
B.cells	CAMK2N1	-0.33959	1.714702	-0.74551	0.457943	-5.41278	0.784465	0.716282
B.cells	PIGT	0.088987	5.640537	0.745429	0.45799	-6.33776	0.7369	0.642164
B.cells	FAM207A	0.101174	4.223482	0.745315	0.458058	-6.0509	0.753669	0.66804
B.cells	TBC1D1	0.086906	7.18844	0.745238	0.458104	-6.57622	0.719084	0.615117
B.cells	SKA1	-0.26678	2.918501	-0.74522	0.458116	-5.59277	0.769509	0.692751
B.cells	MYLPF	-0.28872	2.109786	-0.74517	0.458142	-5.4676	0.77952	0.70851
B.cells	HSPA12B	-0.37016	0.480164	-0.74514	0.458164	-5.12801	0.800152	0.741342
B.cells	GM47689	0.114692	3.369026	0.74495	0.458278	-5.91752	0.764079	0.684181
B.cells	SHB	-0.14769	4.498582	-0.74477	0.458387	-5.99277	0.750529	0.663006
B.cells	PPM1K	-0.15314	3.579795	-0.74466	0.458451	-5.88589	0.761586	0.680189
B.cells	IER3IP1	-0.06141	6.363638	-0.74408	0.458801	-6.45198	0.729112	0.629666
B.cells	RNF115	0.058357	6.978362	0.743775	0.458985	-6.60999	0.72225	0.619084
B.cells	KCNQ1	-0.38807	1.396097	-0.74366	0.459052	-5.24067	0.789335	0.723147
B.cells	SPRTN	0.140058	3.419835	0.743047	0.459423	-5.86431	0.764529	0.683768
B.cells	RANGAP1	-0.09514	5.610379	-0.74295	0.459481	-6.32575	0.738364	0.643348
B.cells	FRS2	0.070264	5.84525	0.742747	0.459604	-6.38563	0.735622	0.639204
B.cells	KDM4D	0.359046	0.328103	0.742635	0.459671	-5.20164	0.803319	0.745245
B.cells	GGA2	0.121364	4.120751	0.742617	0.459682	-6.08246	0.756041	0.670658
B.cells	RAB11FIP4	-0.25755	1.161785	-0.74254	0.459731	-5.3673	0.79264	0.728199
B.cells	AK5	-0.33971	0.348906	-0.74251	0.459749	-5.25949	0.80305	0.744827
B.cells	CLN3	-0.13421	4.768913	-0.74246	0.459775	-6.10733	0.748289	0.658676
B.cells	HIST1H1A	-0.26715	3.773403	-0.74226	0.459896	-5.90836	0.760233	0.677202
B.cells	VPREB3	-0.15794	6.013944	-0.74225	0.459902	-6.4717	0.733659	0.636254
B.cells	ITFG2	-0.12146	3.893716	-0.74207	0.460014	-6.00121	0.758778	0.674939
B.cells	GPANK1	0.129396	3.300008	0.742024	0.46004	-5.85645	0.765992	0.686183
B.cells	ZFP512	0.121854	3.916117	0.741931	0.460095	-5.99541	0.758507	0.674518
B.cells	ALS2CL	-0.44767	0.148166	-0.74179	0.460178	-5.06644	0.805676	0.749057
B.cells	CNDP2	0.102702	5.051865	0.741628	0.460278	-6.22344	0.745019	0.653629

B.cells	CYTH2	-0.12559	3.933028	-0.7415	0.460354	-5.96518	0.758407	0.674331
B.cells	TPR	0.060848	7.524789	0.741113	0.460589	-6.66827	0.71665	0.610336
B.cells	CXCL13	0.681818	-1.21101	0.741079	0.460609	-4.95269	0.823506	0.77817
B.cells	SGO1	-0.25665	3.188246	-0.74089	0.460721	-5.64633	0.767749	0.688739
B.cells	PIANP	-0.31645	-0.06426	-0.74008	0.461212	-5.21655	0.809184	0.754283
B.cells	NFE2L3	-0.22021	2.246069	-0.74003	0.461242	-5.56514	0.77975	0.707413
B.cells	UBOX5	0.256676	1.390449	0.739776	0.461396	-5.36683	0.790505	0.724525
B.cells	ADAM30	-0.28509	0.62147	-0.73971	0.461438	-5.36781	0.800317	0.740193
B.cells	BOLA2	0.091363	5.686348	0.73955	0.461532	-6.33549	0.73819	0.642961
B.cells	SUGCT	-0.21324	2.835527	-0.73955	0.461535	-5.59389	0.772439	0.696057
B.cells	SIL1	0.106679	5.149956	0.739518	0.461551	-6.19621	0.744496	0.652634
B.cells	PLEKHA2	0.078159	7.455833	0.739318	0.461673	-6.63299	0.717832	0.612085
B.cells	4931406P1	0.076681	5.594349	0.739035	0.461844	-6.35775	0.739267	0.644743
B.cells	DLST	0.066556	5.852364	0.738879	0.461937	-6.37608	0.736251	0.640196
B.cells	PEX19	0.119356	3.955615	0.738863	0.461947	-5.94309	0.758765	0.674908
B.cells	VRK3	-0.0846	4.98755	-0.73886	0.461949	-6.23266	0.746418	0.655797
B.cells	PPOX	0.158366	3.108757	0.738727	0.462029	-5.70882	0.769077	0.691048
B.cells	RAB44	0.363627	1.487929	0.738656	0.462072	-5.34418	0.789271	0.722909
B.cells	MVD	0.208923	2.348846	0.738163	0.46237	-5.55489	0.778469	0.705915
B.cells	GM41611	0.378693	0.284798	0.738151	0.462377	-5.18521	0.804656	0.747587
B.cells	GM4117	0.268549	0.660809	0.738098	0.462409	-5.36152	0.799811	0.739836
B.cells	PIAS1	0.057919	7.42613	0.738038	0.462446	-6.65429	0.718168	0.612912
B.cells	SMYD1	0.431037	0.29641	0.737929	0.462512	-5.09571	0.804506	0.747381
B.cells	HERC2	0.082126	5.98752	0.737843	0.462564	-6.39597	0.734678	0.637967
B.cells	RAP1A	0.043237	8.772339	0.737626	0.462695	-6.85861	0.703117	0.590385
B.cells	NCOA4	0.114445	6.199192	0.737548	0.462742	-6.41208	0.73222	0.634234
B.cells	SPACA9	-0.23914	1.758151	-0.73746	0.462793	-5.37964	0.785862	0.717676
B.cells	FHOD3	0.4829	-0.11029	0.737371	0.462849	-5.07175	0.809784	0.755905
B.cells	CYP20A1	0.102233	4.104318	0.737321	0.462879	-6.02371	0.756971	0.672332
B.cells	SLC50A1	0.093237	5.247798	0.737012	0.463067	-6.26953	0.743341	0.65139
B.cells	NEK4	-0.29687	1.202148	-0.73681	0.463189	-5.32058	0.792895	0.72905
B.cells	GM15787	-0.20245	2.641968	-0.7368	0.463195	-5.65748	0.77483	0.700442
B.cells	FAM222B	0.105098	5.370089	0.736693	0.46326	-6.29013	0.7419	0.649241
B.cells	STXBP4	-0.24916	2.23619	-0.73664	0.463289	-5.51582	0.779873	0.708413
B.cells	NT5E	-0.25472	2.431121	-0.73659	0.46332	-5.59021	0.777446	0.704609
B.cells	SPRED1	-0.13314	4.710293	-0.73628	0.463507	-6.12086	0.749712	0.661387
B.cells	GDPD1	-0.309	1.600801	-0.73628	0.463509	-5.36517	0.787845	0.721151
B.cells	GM7072	0.125388	3.842908	0.736057	0.463644	-5.99266	0.760128	0.677605
B.cells	TTPA	-0.28929	1.860466	-0.73595	0.46371	-5.50076	0.784575	0.716057
B.cells	GM50399	-0.48475	0.166375	-0.73576	0.463824	-5.09591	0.806189	0.750586
B.cells	SIMC1	-0.09372	5.702765	-0.73554	0.463955	-6.38062	0.737998	0.643466
B.cells	GM15860	-0.32559	0.373729	-0.73552	0.46397	-5.20376	0.803508	0.746278
B.cells	RTN3	0.071655	8.060675	0.735513	0.463974	-6.76749	0.711027	0.602586
B.cells	ITGA3	-0.307	-0.28405	-0.73535	0.464073	-5.21316	0.81205	0.760048
B.cells	SWI5	-0.08079	7.120072	-0.73516	0.464185	-6.57598	0.721643	0.618643
B.cells	RALGAPA1	0.091573	6.84207	0.735148	0.464195	-6.56449	0.724817	0.62345
B.cells	TPP2	-0.0594	7.082191	-0.73509	0.464227	-6.61247	0.722075	0.619296

B.cells	TLNRD1	-0.12142	4.581796	-0.73496	0.464309	-6.15156	0.751244	0.663952
B.cells	USP47	0.072027	6.472249	0.734648	0.464498	-6.51355	0.729065	0.62994
B.cells	BTG3	-0.08006	5.347109	-0.73451	0.464582	-6.30749	0.742171	0.649985
B.cells	2310015A1	-0.22949	2.091938	-0.73446	0.464611	-5.45764	0.781674	0.711616
B.cells	GNASAS1	0.330344	0.682179	0.734452	0.464617	-5.23835	0.799537	0.740049
B.cells	OFCC1	0.444195	-0.63548	0.73445	0.464618	-5.04617	0.816542	0.767612
B.cells	SNRPD3	-0.0727	6.518599	-0.73425	0.46474	-6.49115	0.728531	0.629153
B.cells	HSP90AA1	-0.07448	7.657631	-0.73393	0.464934	-6.65746	0.715553	0.609577
B.cells	GM43560	-0.28571	0.578606	-0.73393	0.464936	-5.1705	0.800868	0.74228
B.cells	CIAPIN1	0.103873	4.643928	0.733869	0.46497	-6.15648	0.750503	0.662927
B.cells	ZFP277	0.073213	5.309649	0.733847	0.464983	-6.34139	0.742612	0.650753
B.cells	GM15675	0.232925	2.317552	0.733764	0.465034	-5.58585	0.778859	0.707262
B.cells	ERMAP	0.393168	0.529243	0.733603	0.465131	-5.19461	0.801503	0.743316
B.cells	DPH2	-0.34117	0.879573	-0.73357	0.465149	-5.22054	0.797008	0.736119
B.cells	MAOA	-0.38796	0.932781	-0.73354	0.465171	-5.20063	0.796328	0.735037
B.cells	RUSC2	0.348463	0.909936	0.733409	0.465249	-5.30542	0.79662	0.735555
B.cells	SMTNL2	-0.40033	0.4226	-0.73336	0.465282	-5.187	0.802877	0.745599
B.cells	MGAT5	0.081698	6.916886	0.733335	0.465294	-6.54141	0.723962	0.622357
B.cells	MAN2A2	-0.12193	4.595669	-0.73305	0.465468	-6.0482	0.751079	0.663907
B.cells	BRPF3	0.092614	4.451036	0.733027	0.465481	-6.07371	0.752807	0.666585
B.cells	BC030867	-0.28601	2.099983	-0.73301	0.465488	-5.47838	0.781574	0.711648
B.cells	THOP1	-0.23741	2.236401	-0.73298	0.465507	-5.4884	0.77987	0.708954
B.cells	NPLOC4	0.084589	5.537568	0.73292	0.465546	-6.3199	0.739933	0.64673
B.cells	CNBP	-0.05024	8.132464	-0.73291	0.465551	-6.77181	0.710224	0.60166
B.cells	TRIM3	0.167972	2.274801	0.73283	0.465601	-5.66497	0.779391	0.708221
B.cells	MOB1B	-0.06202	6.203813	-0.73267	0.4657	-6.47368	0.732167	0.63486
B.cells	NRXN2	-0.40986	2.095803	-0.73245	0.465834	-5.306	0.781626	0.711767
B.cells	HIST1H1D	0.350186	1.531522	0.732433	0.465841	-5.32747	0.78872	0.72302
B.cells	IMPA1	-0.08364	5.285622	-0.73233	0.465901	-6.26148	0.742895	0.651309
B.cells	AC149090.	-0.17775	5.49949	-0.73205	0.466072	-6.27948	0.74038	0.647508
B.cells	CSE1L	0.081393	5.5229	0.73193	0.466147	-6.3561	0.740105	0.647105
B.cells	NDRG3	0.093791	4.407312	0.731911	0.466159	-6.08658	0.753331	0.667518
B.cells	FAM151B	-0.2791	1.753415	-0.7317	0.466288	-5.40193	0.785921	0.718755
B.cells	ZFP36	0.09873	7.849088	0.731605	0.466344	-6.75394	0.713399	0.606602
B.cells	PLEKHJ1	0.064638	5.969804	0.731586	0.466356	-6.41569	0.734884	0.639181
B.cells	HIST1H3F	-0.45573	0.34671	-0.73144	0.466443	-5.11851	0.803856	0.747432
B.cells	ZCCHC14	-0.34839	1.412865	-0.73132	0.466516	-5.20721	0.790221	0.725633
B.cells	KPTN	-0.16077	4.098478	-0.73127	0.466545	-5.92848	0.757041	0.673402
B.cells	JMJD1C	-0.07687	8.048596	-0.73107	0.466668	-6.74611	0.711162	0.603279
B.cells	ZFP560	-0.11235	4.00302	-0.73097	0.466731	-5.97256	0.758192	0.675193
B.cells	SMIM40	0.357555	0.301755	0.730882	0.466784	-5.19706	0.804437	0.748414
B.cells	TMEM51	0.197403	3.130512	0.73072	0.466882	-5.68502	0.76881	0.691834
B.cells	TMEM11	0.086813	5.070903	0.730427	0.46706	-6.26227	0.745431	0.655584
B.cells	UBAP1L	-0.24198	1.134289	-0.7301	0.467262	-5.34774	0.793758	0.731576
B.cells	LACTB	0.084919	5.139278	0.730042	0.467295	-6.312	0.744622	0.654443
B.cells	MEAF6	0.077394	4.941914	0.730037	0.467297	-6.22874	0.746959	0.658048
B.cells	ELL	0.092716	5.024631	0.729942	0.467355	-6.21581	0.745978	0.656535

B.cells	MIS18BP1	-0.18579	3.964435	-0.72984	0.467415	-5.89824	0.758658	0.676199
B.cells	ALDH4A1	-0.18925	2.912017	-0.72975	0.467472	-5.68617	0.771496	0.69634
B.cells	GM21859	-0.35511	1.780794	-0.72939	0.467688	-5.44988	0.785577	0.71868
B.cells	LRRC4C	0.465627	0.648779	0.729391	0.46769	-5.12153	0.799966	0.741645
B.cells	IER2	-0.10096	8.170209	-0.72939	0.467694	-6.7825	0.709802	0.6016
B.cells	SUPT7L	0.162888	2.767637	0.728889	0.467996	-5.71518	0.773277	0.699406
B.cells	KCNAB2	-0.11507	4.287693	-0.72875	0.468078	-6.10418	0.754766	0.670501
B.cells	ZFP788	-0.18138	2.069958	-0.72866	0.468138	-5.53587	0.781949	0.713199
B.cells	VPS35L	0.07419	5.162292	0.728629	0.468154	-6.26177	0.74435	0.654394
B.cells	4833438C0	-0.17554	2.025244	-0.7286	0.468175	-5.5073	0.782509	0.714086
B.cells	GM5547	0.259948	1.560402	0.728594	0.468175	-5.35458	0.788355	0.723374
B.cells	WHAMM	0.10779	4.147659	0.728518	0.468222	-6.07119	0.756449	0.673141
B.cells	TMEM218	-0.2283	1.874833	-0.72848	0.468248	-5.35613	0.784395	0.717079
B.cells	MRPL38	0.112449	3.863246	0.728451	0.468262	-5.97165	0.759882	0.678499
B.cells	MYL6	-0.06335	9.689609	-0.7283	0.468355	-7.0015	0.693076	0.57693
B.cells	GSN	0.126252	5.844895	0.728253	0.468383	-6.248	0.736339	0.642083
B.cells	USP31	0.130683	3.715776	0.728143	0.46845	-5.9873	0.761669	0.681315
B.cells	SLC5A10	-0.42947	-0.1778	-0.72785	0.468626	-5.08716	0.810663	0.759274
B.cells	GM13205	-0.37666	0.234435	-0.72778	0.468669	-5.11432	0.805308	0.750644
B.cells	6030458C1	0.15334	2.74674	0.727711	0.468713	-5.66659	0.773535	0.700022
B.cells	TUBB6	-0.16701	3.749371	-0.72756	0.468805	-5.96343	0.761261	0.680775
B.cells	GM12655	0.393475	0.353725	0.727452	0.468871	-5.16119	0.803766	0.748225
B.cells	TNFRSF10B	-0.36321	-0.0063	-0.72733	0.468944	-5.16211	0.80843	0.755781
B.cells	CXXC5	-0.09722	5.11182	-0.72729	0.468968	-6.19793	0.744947	0.655526
B.cells	ADIPOR2	0.074032	6.368043	0.727053	0.469114	-6.46546	0.730267	0.632996
B.cells	EIF2S2	-0.06007	7.925804	-0.72696	0.469172	-6.74198	0.712537	0.606134
B.cells	ZFP568	-0.12133	4.08691	-0.72696	0.469173	-6.01654	0.757181	0.674499
B.cells	PIGX	0.085406	5.308726	0.72684	0.469244	-6.27906	0.742623	0.651998
B.cells	ACCS	-0.20997	2.079594	-0.72682	0.469259	-5.50469	0.781829	0.7133
B.cells	SIAH2	-0.08659	4.793136	-0.72678	0.469283	-6.21807	0.748726	0.661442
B.cells	ASTE1	0.152787	2.753642	0.726591	0.469396	-5.73791	0.773449	0.70007
B.cells	AFP	-0.32947	4.371399	-0.72649	0.469456	-6.16797	0.753761	0.669289
B.cells	PRCC	-0.07866	5.107381	-0.72649	0.469459	-6.27806	0.744999	0.655715
B.cells	DPP4	0.100678	5.100888	0.726432	0.469493	-6.34749	0.745076	0.655854
B.cells	SLC36A3OS	-0.28015	1.010121	-0.72642	0.469498	-5.53452	0.79534	0.734901
B.cells	VCL	-0.08807	6.049702	-0.72603	0.469735	-6.46573	0.733955	0.638845
B.cells	DNMT3A	-0.09395	6.044028	-0.726	0.469753	-6.3471	0.734021	0.638954
B.cells	COL25A1	-0.30425	0.981282	-0.72588	0.469829	-5.45129	0.795708	0.735652
B.cells	PLXNA4	-0.52626	2.166702	-0.72588	0.469832	-5.39993	0.78074	0.711809
B.cells	HDAC5	-0.1124	4.378269	-0.72555	0.470031	-6.12682	0.753679	0.669333
B.cells	MEST	-0.23001	3.792937	-0.72552	0.470047	-5.81902	0.760733	0.680327
B.cells	PRPF39	0.083036	5.43876	0.725504	0.470059	-6.31883	0.741093	0.649861
B.cells	HMGCLL1	0.254399	1.17582	0.725499	0.470062	-5.48215	0.793229	0.731692
B.cells	GLCCI1	-0.08791	7.348661	-0.72538	0.470136	-6.61089	0.719046	0.61621
B.cells	MZT1	0.073109	5.278837	0.725372	0.470139	-6.28056	0.742975	0.652761
B.cells	TMEM229A	0.463449	-1.03017	0.725269	0.470202	-4.99702	0.821566	0.777888
B.cells	ACAT2	-0.15498	2.91084	-0.72521	0.470236	-5.69704	0.77151	0.697233

B.cells	CCDC43	-0.13949	3.103767	-0.72515	0.470273	-5.76979	0.769138	0.6935
B.cells	MFSD3	-0.36441	0.642198	-0.72513	0.470289	-5.14965	0.80005	0.742619
B.cells	TRIP6	-0.2797	0.834471	-0.72485	0.470456	-5.23667	0.797585	0.738706
B.cells	GARS	-0.08041	5.511566	-0.72478	0.4705	-6.34484	0.740238	0.648614
B.cells	BRI3BP	0.092237	4.756436	0.72475	0.470519	-6.28129	0.749162	0.662395
B.cells	IL31RA	-0.17244	3.109325	-0.72454	0.470645	-5.74858	0.76907	0.693503
B.cells	DCTN3	-0.07484	5.963361	-0.72454	0.470649	-6.39268	0.734959	0.640541
B.cells	BICD2	-0.11141	4.09822	-0.72452	0.470661	-6.0361	0.757044	0.674679
B.cells	CAPN2	-0.10902	4.14309	-0.72441	0.470724	-6.02637	0.756504	0.673871
B.cells	LMAN1L	-0.26135	1.485183	-0.72438	0.470745	-5.47353	0.789305	0.725603
B.cells	TTC3	0.092263	5.355192	0.724346	0.470765	-6.26265	0.742076	0.651544
B.cells	KBTBD2	0.070449	5.176548	0.724269	0.470813	-6.2746	0.744182	0.654813
B.cells	GINS2	-0.19669	3.493231	-0.72411	0.470913	-5.75825	0.764375	0.686322
B.cells	FAM53B	0.123891	4.652028	0.723938	0.471015	-6.13955	0.750406	0.664548
B.cells	RSF1OS1	0.142699	2.978349	0.72393	0.47102	-5.83376	0.770679	0.696235
B.cells	CHADL	-0.3479	0.647864	-0.72392	0.471026	-5.19308	0.799978	0.742833
B.cells	DAPP1	0.071036	6.482475	0.723872	0.471056	-6.54686	0.728947	0.631531
B.cells	BCL6B	-0.38278	0.649778	-0.72381	0.471095	-5.11206	0.799953	0.742797
B.cells	NAP1L1	-0.08208	7.439515	-0.72375	0.471131	-6.66488	0.718017	0.614933
B.cells	NDUFAF8	0.083051	5.052226	0.723323	0.471391	-6.21125	0.745836	0.657336
B.cells	MPP7	0.158933	7.383215	0.723224	0.471452	-6.58324	0.718833	0.616082
B.cells	EHD4	0.083107	6.076676	0.723205	0.471463	-6.45855	0.733823	0.638908
B.cells	CDCA8	-0.19606	5.109425	-0.72311	0.471519	-6.17044	0.74516	0.65635
B.cells	BC024063	0.337802	0.183577	0.723022	0.471575	-5.16783	0.806166	0.752739
B.cells	AC125149.	0.348054	-0.34803	0.722517	0.471884	-5.29302	0.813468	0.764155
B.cells	GM49417	0.368485	1.078235	0.722447	0.471926	-5.33445	0.79504	0.734488
B.cells	RTRAF	0.068967	7.336334	0.722179	0.47209	-6.66261	0.719771	0.617221
B.cells	RTL5	0.255844	0.491888	0.722158	0.472103	-5.34727	0.802636	0.746693
B.cells	ZFP28	-0.39029	0.2908	-0.72151	0.472499	-5.13069	0.805713	0.751023
B.cells	CNOT7	0.068313	5.288607	0.721413	0.472559	-6.27237	0.743907	0.653568
B.cells	SMIM5	0.454405	0.166279	0.721378	0.472581	-5.06136	0.807326	0.753649
B.cells	FAR1OS	0.185204	2.788806	0.720908	0.472868	-5.85144	0.77439	0.700922
B.cells	SLC49A4	0.117497	6.30055	0.720878	0.472886	-6.48478	0.732348	0.635653
B.cells	BACH2	-0.08902	8.964274	-0.72045	0.473147	-6.9692	0.702349	0.590365
B.cells	SAP25	0.231391	2.354647	0.720304	0.473238	-5.5615	0.779892	0.709633
B.cells	COPB2	-0.06957	5.438302	-0.72005	0.473393	-6.32089	0.742522	0.651331
B.cells	NRD1	0.072842	6.185357	0.72003	0.473406	-6.45813	0.733788	0.637924
B.cells	GPBP1	0.062528	7.723603	0.719709	0.473602	-6.76823	0.716183	0.611244
B.cells	ZBTB37	-0.15941	2.901016	-0.71966	0.473635	-5.71707	0.773114	0.699061
B.cells	CARF	0.212037	2.216581	0.719494	0.473734	-5.529	0.781616	0.712537
B.cells	PLEKHA7	-0.2233	2.084938	-0.71931	0.47385	-5.53447	0.783264	0.715254
B.cells	PSIP1	0.074334	5.994205	0.719147	0.473947	-6.40517	0.736011	0.641537
B.cells	RLF	0.07191	6.699162	0.718993	0.474041	-6.53428	0.727851	0.629102
B.cells	PPIL4	-0.06662	5.46202	-0.71879	0.474163	-6.34472	0.742243	0.65123
B.cells	GM47802	0.401036	0.099495	0.718752	0.474189	-5.13281	0.808607	0.755992
B.cells	ZFP59	0.367383	0.230401	0.718745	0.474193	-5.16782	0.806908	0.75325
B.cells	TMEM164	0.069019	6.945657	0.718612	0.474275	-6.5897	0.725023	0.624944

B.cells	COX14	0.077506	5.656597	0.718578	0.474296	-6.33695	0.739957	0.647784
B.cells	EGR1	-0.17695	6.545199	-0.71857	0.474301	-6.44712	0.729624	0.631954
B.cells	ADAM23	0.242124	3.184267	0.718424	0.47439	-5.72219	0.769627	0.693956
B.cells	ZFP119B	0.249011	1.297642	0.718353	0.474434	-5.3805	0.793203	0.731335
B.cells	COMMD3	0.079615	5.85015	0.718195	0.47453	-6.36227	0.737692	0.644361
B.cells	CCDC47	-0.08223	5.110399	-0.71806	0.474616	-6.22684	0.746395	0.657814
B.cells	KRAS	0.060767	7.278938	0.718045	0.474623	-6.63511	0.72122	0.619275
B.cells	AREL1	0.099842	4.263334	0.718029	0.474632	-6.08038	0.756509	0.67351
B.cells	TMEM208	0.077379	4.89435	0.717907	0.474708	-6.19043	0.748959	0.661846
B.cells	ANXA5	0.093522	6.137389	0.717852	0.474741	-6.41794	0.734345	0.639333
B.cells	LAMP2	0.064049	7.069003	0.717803	0.474771	-6.6327	0.723613	0.622965
B.cells	MKLN1	0.064042	7.159858	0.717753	0.474802	-6.63496	0.722576	0.621395
B.cells	GM826	0.521899	-0.25558	0.717638	0.474872	-5.09598	0.813238	0.76374
B.cells	SAAL1	0.15129	3.298168	0.717548	0.474928	-5.82848	0.76823	0.691954
B.cells	CXCR5	0.22667	2.183855	0.717398	0.475019	-5.66863	0.782026	0.713765
B.cells	PAPSS1	0.096446	4.181725	0.717359	0.475044	-6.06129	0.757492	0.675172
B.cells	ST13	-0.05915	6.987135	-0.71733	0.475064	-6.57981	0.724549	0.624446
B.cells	PNP2	0.394307	0.616927	0.717181	0.475153	-5.17741	0.801913	0.745532
B.cells	TRAM2	-0.09102	4.061754	-0.71714	0.47518	-6.16103	0.758939	0.67743
B.cells	UPP2	0.3096	1.652112	0.717136	0.47518	-5.4067	0.78871	0.724395
B.cells	WDR4	0.138913	2.999114	0.716908	0.475321	-5.76543	0.772025	0.69781
B.cells	YPEL5	0.07639	6.583092	0.716764	0.475409	-6.61409	0.729337	0.631574
B.cells	SARS2	-0.13446	2.675724	-0.71658	0.475519	-5.74299	0.776133	0.704192
B.cells	SERTAD2	-0.09364	6.12155	-0.71635	0.475661	-6.48392	0.734868	0.639818
B.cells	ADSL	-0.11271	4.033205	-0.71567	0.47608	-5.98135	0.760199	0.678364
B.cells	FBXL8	0.225739	1.99347	0.715129	0.476414	-5.4916	0.785427	0.718183
B.cells	CD79B	-0.10382	6.976941	-0.71507	0.476449	-6.65376	0.725603	0.625172
B.cells	GM15726	-0.34546	2.074088	-0.71505	0.476464	-5.51311	0.784414	0.716575
B.cells	GM48293	-0.41675	-0.15995	-0.71493	0.476536	-5.08269	0.813039	0.762511
B.cells	RFXANK	-0.15254	2.879474	-0.71483	0.476596	-5.72697	0.774381	0.70077
B.cells	TAF1A	0.142711	3.171877	0.714659	0.476702	-5.84097	0.770775	0.695115
B.cells	CTIF	-0.17465	2.596638	-0.71462	0.476728	-5.50616	0.777887	0.706332
B.cells	LIPO3	-0.15224	2.915424	-0.71458	0.476753	-5.79086	0.773937	0.70011
B.cells	MCOLN1	0.15141	2.728161	0.714471	0.476818	-5.67673	0.776255	0.703801
B.cells	TAF3	0.076058	5.653075	0.714451	0.47683	-6.36281	0.740957	0.648776
B.cells	CIP2A	0.193586	3.506565	0.714428	0.476845	-5.79494	0.766672	0.688732
B.cells	MRPL9	0.077127	4.787977	0.714282	0.476934	-6.20731	0.751205	0.664633
B.cells	GM27241	-0.18647	2.603068	-0.71414	0.477019	-5.73864	0.777816	0.706349
B.cells	1700037C1	0.157359	3.065274	0.714099	0.477047	-5.7334	0.772096	0.697336
B.cells	MRPL15	0.081357	5.337746	0.713928	0.477152	-6.29287	0.744702	0.654607
B.cells	ZCCHC9	-0.07115	5.404138	-0.71382	0.477216	-6.34416	0.743918	0.653412
B.cells	TCF4	0.096888	7.694523	0.7135	0.477415	-6.59571	0.71747	0.613154
B.cells	RRP7A	0.114016	3.942345	0.713472	0.477433	-6.05302	0.761401	0.680666
B.cells	ARPC3	-0.05209	8.451234	-0.71343	0.477461	-6.82657	0.708978	0.600377
B.cells	ISOC2B	0.200982	2.519105	0.713371	0.477495	-5.60246	0.778885	0.708178
B.cells	AVEN	0.100082	4.926701	0.713316	0.477529	-6.26966	0.749576	0.662284
B.cells	PDHX	0.109111	3.395129	0.713166	0.477621	-5.899	0.768069	0.691185

B.cells	PHOSPHO2	0.102341	3.724911	0.712816	0.477837	-5.96338	0.764042	0.685004
B.cells	SAFB	-0.0715	6.192591	-0.71254	0.478005	-6.4548	0.734685	0.639554
B.cells	0610040B1	0.207164	2.051019	0.712539	0.478008	-5.53393	0.784738	0.717687
B.cells	4930503L1	-0.17872	2.576564	-0.71246	0.478055	-5.59898	0.77817	0.707274
B.cells	CCDC66	0.172302	2.265203	0.712234	0.478195	-5.60311	0.782053	0.713425
B.cells	ANKRD27	0.14924	3.098779	0.712191	0.478222	-5.73277	0.771708	0.697073
B.cells	TMTC3	-0.15531	2.514058	-0.71219	0.478223	-5.61547	0.778948	0.708504
B.cells	TRPC4AP	0.066781	5.651799	0.712115	0.478268	-6.37521	0.741004	0.649256
B.cells	LAT	-0.21988	2.414617	-0.71209	0.478286	-5.48132	0.780187	0.710467
B.cells	PPP2R1A	-0.077	6.00443	-0.712	0.47834	-6.42611	0.736876	0.642922
B.cells	GM28875	-0.10748	4.612323	-0.71196	0.478361	-6.24874	0.753329	0.66834
B.cells	IFNGR1	0.076848	7.03854	0.711944	0.478374	-6.63706	0.724929	0.62469
B.cells	2410018L1	-0.27847	0.727626	-0.71168	0.478539	-5.32006	0.801671	0.744677
B.cells	MS4A8A	-0.39709	0.670009	-0.71162	0.478571	-5.27748	0.802413	0.745874
B.cells	RPE	0.102992	4.554255	0.711102	0.478893	-6.11207	0.754532	0.669609
B.cells	DKC1	0.108076	4.56874	0.710537	0.479241	-6.14401	0.754804	0.669563
B.cells	NHP2	0.09366	5.748278	0.710311	0.47938	-6.38046	0.740917	0.648024
B.cells	DAPL1	-0.36973	-1.31502	-0.71017	0.479465	-5.06556	0.829228	0.788629
B.cells	TEF	-0.13695	3.230001	-0.71	0.479572	-5.82698	0.771182	0.695137
B.cells	GM38973	-0.24881	1.613034	-0.71	0.479575	-5.48856	0.791376	0.72711
B.cells	C330018D2	0.181863	2.325358	0.709694	0.479761	-5.57197	0.782515	0.713003
B.cells	SNRPC	-0.06591	6.245324	-0.70966	0.479785	-6.45932	0.735213	0.639307
B.cells	AZI2	0.064972	5.839646	0.709568	0.479839	-6.41789	0.739949	0.646589
B.cells	TGFBR1	0.091456	6.24383	0.70914	0.480104	-6.4557	0.735488	0.639442
B.cells	DNAH1	-0.40226	-0.10849	-0.70889	0.480259	-5.10209	0.813949	0.763062
B.cells	EXOC8	0.215859	1.951234	0.708859	0.480277	-5.46615	0.787489	0.72063
B.cells	SLCO5A1	-0.24975	-0.20706	-0.70884	0.480288	-5.26629	0.81524	0.765152
B.cells	CLNS1A	-0.0729	5.340846	-0.70876	0.480336	-6.29312	0.746084	0.655789
B.cells	USP19	0.102141	4.704682	0.708614	0.480428	-6.19853	0.753698	0.667538
B.cells	NDUFB10	0.067431	6.731387	0.708469	0.480518	-6.56888	0.729918	0.630961
B.cells	BORA	-0.13866	3.535011	-0.70816	0.480708	-5.86725	0.768097	0.689794
B.cells	LIMK2	0.095137	4.779797	0.708052	0.480776	-6.18611	0.753035	0.666331
B.cells	KIF3C	0.17969	2.145051	0.707773	0.480948	-5.62403	0.785422	0.717113
B.cells	VKORC1L1	-0.08246	5.560377	-0.70776	0.480958	-6.33903	0.743843	0.652093
B.cells	GRIK4	-0.51525	0.238216	-0.70758	0.481067	-5.07112	0.809881	0.75621
B.cells	ARFIP2	-0.18344	2.138474	-0.70743	0.481158	-5.54827	0.785616	0.717396
B.cells	EMP1	0.349542	1.978152	0.706949	0.481457	-5.41271	0.787878	0.72075
B.cells	SHISA8	-0.32984	-0.21439	-0.70689	0.481491	-5.31673	0.81609	0.766009
B.cells	NADK2	0.092315	4.994117	0.706863	0.48151	-6.26561	0.750894	0.662733
B.cells	GAN	0.116914	4.031697	0.706541	0.48171	-6.09492	0.762471	0.680813
B.cells	ZFPM1	-0.11841	4.248218	-0.70648	0.481746	-6.10476	0.759848	0.676722
B.cells	KIF2A	0.065218	6.338703	0.706213	0.481912	-6.51189	0.735065	0.638462
B.cells	ANAPC4	0.087769	4.541254	0.70604	0.482019	-6.13596	0.756316	0.671225
B.cells	PALLD	0.186707	3.882236	0.706024	0.482029	-5.826	0.764288	0.683651
B.cells	5830448L0	-0.29209	0.904406	-0.70591	0.482102	-5.26585	0.801552	0.742729
B.cells	SNN	0.162117	3.649853	0.705856	0.482133	-5.85126	0.767122	0.688137
B.cells	CCL5	0.399412	7.931718	0.705853	0.482135	-6.66208	0.716819	0.610813

B.cells	MED8	0.089027	5.008062	0.705587	0.4823	-6.23176	0.750728	0.662658
B.cells	GGT5	0.388983	0.649699	0.705521	0.482341	-5.17706	0.804836	0.748071
B.cells	TEP1	0.109813	3.914158	0.705509	0.482348	-6.07822	0.763899	0.683157
B.cells	DNAJC7	-0.07001	7.510277	-0.70522	0.482528	-6.70127	0.721593	0.618189
B.cells	DCUN1D4	-0.21244	2.540378	-0.70513	0.482583	-5.61265	0.780825	0.709903
B.cells	NDUFA8	0.088965	5.798653	0.705026	0.482647	-6.36834	0.741375	0.648377
B.cells	UPF2	0.068284	6.075856	0.705006	0.48266	-6.44766	0.738128	0.643392
B.cells	CD302	-0.13033	5.847821	-0.70477	0.482808	-6.42698	0.740798	0.647508
B.cells	6-Mar	0.061426	6.3538	0.704724	0.482834	-6.47978	0.734889	0.63845
B.cells	LRP12	0.274718	2.191669	0.704697	0.482851	-5.49083	0.785191	0.716861
B.cells	SH2B1	0.137035	3.699274	0.704522	0.482959	-5.90439	0.766518	0.687417
B.cells	HSD17B10	-0.09154	5.540491	-0.70451	0.482967	-6.34247	0.744414	0.653072
B.cells	GM15564	0.205041	2.474453	0.704492	0.482978	-5.6756	0.781648	0.711246
B.cells	GM16754	0.390292	0.04601	0.70447	0.482992	-5.12619	0.81268	0.760894
B.cells	ITPKA	0.343998	0.529225	0.70435	0.483066	-5.20151	0.806394	0.75079
B.cells	NOC2L	0.09263	5.059461	0.704152	0.483189	-6.29932	0.750116	0.66191
B.cells	HNRNPH2	0.06064	6.435152	0.703958	0.483309	-6.50338	0.733944	0.637142
B.cells	ENKD1	0.280747	1.3576	0.703945	0.483317	-5.2824	0.795747	0.733834
B.cells	SPTBN4	-0.38548	-0.18469	-0.70391	0.483341	-5.07806	0.815701	0.765945
B.cells	BRPF1	0.092752	4.929241	0.703899	0.483346	-6.24763	0.751668	0.664423
B.cells	ZCRB1	0.060637	6.149583	0.703684	0.483479	-6.43451	0.73737	0.642322
B.cells	ARL13B	-0.11357	3.775076	-0.70345	0.483625	-5.93887	0.765828	0.686237
B.cells	MAPRE2	0.072933	6.802434	0.703138	0.483817	-6.57319	0.730034	0.630873
B.cells	TMEM38B	0.112204	4.174266	0.703114	0.483832	-6.05037	0.761095	0.678763
B.cells	DUSP19	-0.22226	2.048701	-0.70275	0.484061	-5.41896	0.787494	0.720215
B.cells	SACM1L	0.081398	5.504292	0.702654	0.484117	-6.33748	0.745319	0.654217
B.cells	GGA3	0.115482	3.302395	0.702562	0.484174	-5.87142	0.771878	0.695569
B.cells	PLXDC1	-0.18376	3.94302	-0.70255	0.484184	-5.94986	0.764038	0.683278
B.cells	LTBP1	-0.63709	0.730726	-0.70227	0.484356	-5.19317	0.804305	0.747205
B.cells	NDUFA5	0.105508	5.432033	0.702224	0.484384	-6.28192	0.746173	0.655607
B.cells	ZFP335OS	-0.10536	4.456083	-0.70222	0.484389	-6.15593	0.757826	0.673664
B.cells	RCE1	-0.11153	3.757774	-0.70185	0.484617	-5.99059	0.766296	0.686968
B.cells	NBDY	-0.12861	3.659616	-0.70156	0.484797	-5.81609	0.767495	0.688939
B.cells	NRG2	-0.30215	1.685011	-0.70153	0.484817	-5.3869	0.792092	0.727837
B.cells	SMAD5	0.106367	3.702902	0.701472	0.484851	-5.92783	0.766966	0.68811
B.cells	GM42937	-0.36379	0.198102	-0.70146	0.48486	-5.21662	0.811216	0.758529
B.cells	NAA30	0.105688	3.918894	0.701381	0.484908	-5.99208	0.764332	0.683988
B.cells	GTF2H5	-0.06453	6.547345	-0.70136	0.484922	-6.52541	0.733114	0.635727
B.cells	CDH13	-0.42012	2.030566	-0.70106	0.485108	-5.37461	0.787722	0.720914
B.cells	SLC24A1	-0.19042	1.860788	-0.70095	0.485178	-5.62892	0.789866	0.724325
B.cells	ONECUT2	-0.28806	1.559667	-0.70093	0.485189	-5.42971	0.793684	0.730414
B.cells	VAMP1	0.111151	3.997211	0.700876	0.485221	-6.01699	0.763379	0.682539
B.cells	LRRC25	0.196616	3.856093	0.700691	0.485336	-5.86805	0.765096	0.685292
B.cells	GOLGA3	0.105273	4.032459	0.700662	0.485354	-6.00206	0.762951	0.681936
B.cells	EMC8	0.093876	4.721373	0.700661	0.485354	-6.13637	0.754638	0.668984
B.cells	FGFBP3	0.460177	0.028033	0.700404	0.485514	-5.10808	0.813436	0.762258
B.cells	BC065397	0.236633	1.292469	0.7003	0.485579	-5.39157	0.79709	0.736021

B.cells	ETV3	-0.11157	4.950898	-0.7003	0.48558	-6.37733	0.751892	0.664797
B.cells	RAC3	-0.43644	-0.22085	-0.70018	0.485655	-5.09861	0.816699	0.76763
B.cells	ZC3H6	0.138055	3.379852	0.700073	0.48572	-5.87484	0.770925	0.694532
B.cells	NCBP2	-0.09329	4.599992	-0.70007	0.485721	-6.14003	0.756095	0.671342
B.cells	SBK1	-0.12549	3.374185	-0.70001	0.485762	-5.95365	0.770995	0.694641
B.cells	PFAS	-0.15314	4.205445	-0.69983	0.485871	-6.00663	0.760922	0.678773
B.cells	LUM	-0.40126	0.551186	-0.69938	0.486153	-5.17635	0.806897	0.751527
B.cells	GUF1	0.149689	2.589786	0.699243	0.486236	-5.62859	0.780971	0.710186
B.cells	SLC7A6OS	0.087233	4.789888	0.699215	0.486253	-6.17972	0.754069	0.668021
B.cells	MTHFD2L	0.148383	3.033816	0.699063	0.486347	-5.73113	0.775452	0.701527
B.cells	TMCO4	0.115588	4.054604	0.698939	0.486424	-6.09815	0.762937	0.681905
B.cells	RNF141	-0.10245	4.511433	-0.69881	0.486507	-6.0382	0.757413	0.673325
B.cells	ZBTB33	0.136584	2.982117	0.698791	0.486517	-5.76809	0.776093	0.702601
B.cells	HIVEP1	0.080708	6.40877	0.698702	0.486572	-6.52316	0.734968	0.638703
B.cells	GOT2	-0.06873	6.235083	-0.69863	0.486619	-6.51965	0.73699	0.641809
B.cells	TCRG-C4	-0.3548	0.327974	-0.69853	0.486678	-5.22282	0.809795	0.756442
B.cells	TCIM	-0.36596	1.634355	-0.69839	0.486768	-5.30865	0.793	0.729509
B.cells	ITGA5	-0.19961	3.54178	-0.69833	0.486801	-5.74649	0.769195	0.691816
B.cells	LRRC14	0.189224	2.339055	0.698262	0.486846	-5.59643	0.784107	0.715387
B.cells	EPC1	-0.06965	6.811099	-0.69813	0.48693	-6.59375	0.730336	0.631673
B.cells	TM9SF3	-0.04959	7.288714	-0.69795	0.487037	-6.64728	0.724895	0.62333
B.cells	DENND4A	0.078277	10.03042	0.697765	0.487155	-7.12077	0.69434	0.577552
B.cells	PDCD1LG2	0.215451	1.775707	0.697758	0.48716	-5.66388	0.791283	0.726696
B.cells	SEH1L	0.068883	5.334868	0.697511	0.487313	-6.33259	0.747646	0.658227
B.cells	TOPORS	-0.09562	5.749708	-0.69748	0.48733	-6.36216	0.742746	0.650669
B.cells	ABL1	-0.08957	5.653787	-0.69738	0.487394	-6.34896	0.743875	0.652447
B.cells	TMEM9	-0.21259	2.576914	-0.69734	0.48742	-5.5917	0.781207	0.710788
B.cells	RAPGEF5	0.194437	5.011787	0.69714	0.487544	-6.10378	0.751559	0.664261
B.cells	JUNOS	-0.29207	1.820722	-0.69706	0.487597	-5.35814	0.790787	0.725957
B.cells	UBE2G1	0.055306	7.307993	0.696781	0.487768	-6.67251	0.724872	0.623203
B.cells	GPR160	-0.28182	2.407708	-0.6966	0.48788	-5.44225	0.783536	0.714314
B.cells	STT3A	-0.06405	6.169392	-0.6966	0.487882	-6.43833	0.738028	0.64331
B.cells	SERP2	0.261428	0.429349	0.69641	0.487999	-5.30755	0.80886	0.75474
B.cells	ABLIM1	-0.08733	6.121137	-0.69617	0.488146	-6.47271	0.738768	0.644307
B.cells	NR1D2	-0.13106	3.478586	-0.69599	0.488259	-5.88154	0.770438	0.69349
B.cells	KPNA4	0.071361	8.246447	0.695988	0.488262	-6.83941	0.714408	0.607296
B.cells	SLC25A39	0.094625	6.472882	0.695635	0.488482	-6.55095	0.734809	0.638073
B.cells	ANAPC15	0.100691	4.974484	0.695348	0.488661	-6.15613	0.752462	0.665254
B.cells	TALDO1	0.057948	8.310001	0.69534	0.488666	-6.82505	0.713831	0.606265
B.cells	GM5150	-0.31422	2.574091	-0.69533	0.488673	-5.5114	0.78179	0.711212
B.cells	0610010F0	-0.11486	4.458879	-0.69531	0.488684	-6.10413	0.758651	0.674875
B.cells	ST3GAL6	0.120816	5.092875	0.695041	0.488852	-6.11018	0.751135	0.66314
B.cells	PPP1R1C	-0.32767	0.781145	-0.69501	0.488872	-5.40759	0.804658	0.74764
B.cells	KCNG3	0.316405	0.517714	0.694538	0.489166	-5.30947	0.808365	0.753324
B.cells	CDH1	0.338571	1.38751	0.69451	0.489184	-5.28337	0.797163	0.73534
B.cells	CLEC2G	0.284401	0.015976	0.693636	0.489729	-5.37966	0.815697	0.764266
B.cells	MYO7A	0.146813	3.25231	0.693463	0.489837	-5.77618	0.774492	0.698549

B.cells	PHF20L1	0.066885	7.046788	0.693312	0.489931	-6.60386	0.729234	0.628542
B.cells	EIF2B1	-0.11716	3.62049	-0.69331	0.489933	-5.89253	0.769959	0.691446
B.cells	FTCD	-0.3185	1.403237	-0.69313	0.490042	-5.409	0.797733	0.735522
B.cells	ORC3	0.08215	4.958044	0.693117	0.490053	-6.23944	0.75375	0.666261
B.cells	ERMP1	0.104782	3.633634	0.692995	0.490129	-5.9681	0.769814	0.691348
B.cells	BAZ1A	-0.06081	7.694958	-0.69287	0.490205	-6.75506	0.721849	0.617499
B.cells	TMEM126/	0.066173	5.857631	0.692513	0.49043	-6.43374	0.743341	0.650047
B.cells	4930402H2	0.099585	4.453202	0.692388	0.490508	-6.17473	0.760093	0.675998
B.cells	PRR7	0.136361	3.334702	0.692307	0.490559	-5.91764	0.773753	0.69739
B.cells	ATPIF1	-0.07527	7.745006	-0.69183	0.490856	-6.73261	0.721754	0.616982
B.cells	ARL16	-0.16326	2.250651	-0.69183	0.490857	-5.56759	0.787533	0.718931
B.cells	IMPDH2	-0.08718	5.196107	-0.69157	0.491017	-6.27485	0.751485	0.662366
B.cells	PDXDC1	-0.0558	6.616598	-0.69148	0.491077	-6.56015	0.73477	0.636682
B.cells	EP300	-0.06122	6.696726	-0.69146	0.49109	-6.57105	0.733841	0.635264
B.cells	CCL27A	-0.22774	1.684593	-0.69117	0.49127	-5.42265	0.794786	0.730465
B.cells	FASTKD1	0.175665	2.189988	0.691035	0.491354	-5.62902	0.788382	0.720277
B.cells	ANPEP	-0.28994	1.184937	-0.69091	0.491429	-5.50148	0.801175	0.740684
B.cells	RNF2	-0.07061	5.723956	-0.6908	0.491502	-6.37928	0.74525	0.65281
B.cells	GM26724	0.221553	1.517501	0.690794	0.491505	-5.5266	0.796916	0.73387
B.cells	DHFR	-0.15034	4.331798	-0.69071	0.491559	-6.05311	0.761903	0.678644
B.cells	SLC24A3	0.406217	1.280307	0.690689	0.491571	-5.27261	0.799951	0.738763
B.cells	SH3D19	-0.24203	2.244921	-0.69044	0.491725	-5.47488	0.787805	0.719339
B.cells	BROX	-0.07415	4.85097	-0.69036	0.491778	-6.2233	0.755752	0.66905
B.cells	MPO	-0.72726	1.827356	-0.69022	0.491867	-5.42849	0.793087	0.727817
B.cells	RWDD2A	-0.29277	0.48641	-0.69015	0.491907	-5.30192	0.810326	0.755445
B.cells	FIGNL2	0.4526	-0.67701	0.689739	0.492165	-5.0582	0.825649	0.780352
B.cells	PIP5K1A	0.079249	5.595581	0.689694	0.492193	-6.37964	0.747021	0.655466
B.cells	SNX21	-0.16347	2.718125	-0.68961	0.492245	-5.78024	0.78202	0.710171
B.cells	TGM1	-0.37535	0.53017	-0.68944	0.492352	-5.20655	0.809913	0.754783
B.cells	SLC18A2	0.172311	2.251616	0.689322	0.492426	-5.61283	0.787873	0.719549
B.cells	EXOC3L4	0.35851	0.594811	0.689263	0.492463	-5.23934	0.809073	0.753455
B.cells	ZFP131	-0.07671	5.815875	-0.68926	0.492464	-6.38801	0.744418	0.651599
B.cells	BACH2IT1	-0.32659	-0.26225	-0.689	0.49263	-5.11463	0.820447	0.771766
B.cells	VMP1	-0.08963	7.164309	-0.6889	0.492687	-6.62644	0.728852	0.627765
B.cells	CYP4F13	0.165309	2.829707	0.688682	0.492827	-5.6968	0.780794	0.708389
B.cells	TBC1D17	0.114193	3.823308	0.688592	0.492883	-6.01783	0.768519	0.689119
B.cells	SLC17A9	0.198792	2.888147	0.688565	0.4929	-5.70128	0.780066	0.707297
B.cells	PBX2	-0.10194	4.930622	-0.6884	0.493005	-6.20216	0.755128	0.668251
B.cells	ATP10A	0.179444	3.430551	0.688137	0.493168	-5.76351	0.773368	0.69679
B.cells	SLC35C2	0.072877	4.846147	0.688051	0.493222	-6.26144	0.756141	0.669869
B.cells	MERTK	-0.24748	3.774359	-0.68803	0.493234	-5.78859	0.769142	0.690155
B.cells	USP3	-0.06679	6.873279	-0.68801	0.493247	-6.6127	0.732252	0.633111
B.cells	GGCX	-0.22423	1.807957	-0.68781	0.493373	-5.45853	0.793681	0.728958
B.cells	ZFP563	-0.23856	0.800774	-0.68779	0.493388	-5.30209	0.806599	0.749648
B.cells	H2-EB1	0.179117	6.754688	0.687697	0.493444	-6.91302	0.733625	0.635205
B.cells	ALS2	0.093321	4.283789	0.687337	0.49367	-6.10598	0.763027	0.680532
B.cells	WDR91	0.094491	4.750458	0.68726	0.493718	-6.26502	0.757388	0.671748

B.cells	NIPSNAP1	-0.15538	3.134643	-0.68718	0.493766	-5.80358	0.777126	0.702647
B.cells	INPP4B	0.124862	6.480863	0.68714	0.493793	-6.57681	0.7369	0.640154
B.cells	NR4A3	-0.1434	6.012984	-0.68705	0.493848	-6.40003	0.742374	0.648547
B.cells	DOCK1	-0.15338	4.079995	-0.68696	0.493904	-5.86996	0.765506	0.684433
B.cells	FGFR1OP	0.095574	4.601111	0.686795	0.49401	-6.21662	0.759187	0.674602
B.cells	RHOC	0.157706	4.059106	0.686754	0.494035	-5.86796	0.76576	0.68486
B.cells	CHSY1	0.089024	5.079341	0.686445	0.494229	-6.31834	0.753465	0.665747
B.cells	ENTPD7	-0.09564	4.693758	-0.68643	0.494236	-6.17795	0.758093	0.672932
B.cells	MACO1	-0.06661	6.27208	-0.68641	0.494249	-6.49382	0.739359	0.644003
B.cells	CLDND1	-0.08079	4.825073	-0.68631	0.494315	-6.19792	0.756513	0.670504
B.cells	WRNIP1	0.105609	3.743434	0.68607	0.494464	-5.94812	0.769642	0.691056
B.cells	TESK1	-0.11356	3.781202	-0.68604	0.494481	-5.97328	0.769179	0.69033
B.cells	UBASH3B	0.129074	5.794781	0.68581	0.494627	-6.38216	0.744966	0.652734
B.cells	SLF1	0.112024	4.44199	0.685786	0.494642	-6.09925	0.761133	0.677786
B.cells	H2-OA	0.136517	3.19042	0.685738	0.494672	-6.16118	0.776458	0.701815
B.cells	CYB5R3	-0.09102	4.85016	-0.68548	0.494835	-6.19757	0.756212	0.670181
B.cells	DEF8	-0.19726	2.04717	-0.6854	0.494886	-5.48943	0.790772	0.724544
B.cells	RNASEK	0.060948	6.735066	0.685397	0.494887	-6.55334	0.733967	0.635919
B.cells	RASAL1	-0.32235	0.712454	-0.68536	0.494909	-5.34703	0.80787	0.751923
B.cells	HJURP	-0.1077	4.895427	-0.68504	0.495111	-6.11029	0.755804	0.669347
B.cells	N4BP2L2	0.049226	6.786437	0.684912	0.495191	-6.59292	0.733504	0.635027
B.cells	ITGA2B	-0.27063	0.938407	-0.6849	0.495196	-5.31919	0.805091	0.747238
B.cells	INO80	-0.07109	6.666636	-0.68479	0.495268	-6.58484	0.734902	0.637171
B.cells	COPG1	0.084526	5.141092	0.684629	0.495369	-6.29326	0.752915	0.664892
B.cells	MTMR2	-0.0733	5.396075	-0.68448	0.495463	-6.33615	0.749876	0.66019
B.cells	FBXO8	0.084216	4.606605	0.684427	0.495496	-6.17115	0.759334	0.674877
B.cells	NDE1	0.109506	4.58489	0.684053	0.495731	-6.13237	0.759654	0.675349
B.cells	CTDSP1	0.085013	5.3236	0.683851	0.495858	-6.32158	0.750796	0.661678
B.cells	NXPE2	0.193602	1.56591	0.683802	0.495888	-5.72266	0.797149	0.734621
B.cells	MAB21L3	-0.40124	-0.30498	-0.68349	0.496086	-5.21808	0.821458	0.773851
B.cells	PHF11A	0.277217	1.004104	0.683405	0.496138	-5.45739	0.804361	0.746236
B.cells	TMX1	0.080847	5.362	0.683333	0.496183	-6.24732	0.750339	0.661035
B.cells	FLRT3	0.378025	-0.84839	0.683072	0.496347	-5.17095	0.828432	0.785605
B.cells	MMAA	0.22635	1.748976	0.683006	0.496389	-5.49026	0.794815	0.730947
B.cells	NR2F6	-0.142	3.501892	-0.68286	0.496484	-5.86969	0.772863	0.696164
B.cells	UTP20	0.140556	3.652111	0.682846	0.496489	-5.98856	0.771015	0.693259
B.cells	PMEL	0.378257	0.339511	0.682794	0.496522	-5.15546	0.812989	0.760137
B.cells	GATAD2B	0.064083	7.433227	0.682381	0.496782	-6.69221	0.726163	0.62411
B.cells	RBMXL1	0.075311	5.042632	0.682369	0.49679	-6.27342	0.754151	0.66706
B.cells	NDUFA10	0.067478	6.050526	0.682328	0.496816	-6.44847	0.742198	0.648622
B.cells	BBS4	0.205869	2.313596	0.682241	0.49687	-5.48599	0.787666	0.719718
B.cells	DPYSL2	0.078006	6.279623	0.682145	0.49693	-6.51197	0.739512	0.644501
B.cells	GM44284	0.421499	-0.05378	0.68194	0.49706	-5.07374	0.818145	0.768691
B.cells	ATP23	0.261387	1.824257	0.681796	0.49715	-5.52885	0.793858	0.729643
B.cells	GM44699	-0.30501	0.559613	-0.68162	0.497264	-5.21587	0.81012	0.755814
B.cells	PREB	-0.08558	4.736405	-0.68152	0.497322	-6.17833	0.757827	0.672953
B.cells	ZMYM5	0.072143	5.521459	0.681511	0.497329	-6.36279	0.748444	0.658405

B.cells	TMEM43	0.128131	3.43413	0.681501	0.497336	-5.8733	0.773699	0.697793
B.cells	DNASE2A	0.097357	4.427932	0.681472	0.497354	-6.17331	0.761552	0.678757
B.cells	MAST1	-0.38678	1.020113	-0.68144	0.497377	-5.30702	0.804155	0.746241
B.cells	GM44752	0.142721	2.484975	0.681421	0.497386	-5.78283	0.785511	0.716464
B.cells	ZFP219	-0.21278	1.771622	-0.68104	0.497628	-5.404	0.794527	0.730954
B.cells	BSCL2	-0.08463	4.542466	-0.68103	0.497632	-6.15326	0.760167	0.676722
B.cells	SARS	0.06231	5.792679	0.681012	0.497643	-6.42482	0.745234	0.653574
B.cells	CSF2RB2	-0.26559	2.359571	-0.68101	0.497647	-5.46073	0.787088	0.719102
B.cells	CCDC106	-0.33953	0.51131	-0.68098	0.497665	-5.19834	0.810749	0.757006
B.cells	ZMPSTE24	-0.11202	4.942134	-0.68073	0.497824	-6.16316	0.755355	0.669237
B.cells	GIGYF1	0.091625	4.441868	0.680693	0.497845	-6.1419	0.761383	0.678623
B.cells	UBE2M	0.059759	7.20691	0.680673	0.497857	-6.65534	0.728759	0.628344
B.cells	WDR1	-0.07072	7.335798	-0.68063	0.497884	-6.6653	0.727279	0.626093
B.cells	FUBP3	-0.10953	4.08675	-0.68057	0.497924	-6.03267	0.765697	0.685364
B.cells	NTM	-0.3376	0.913874	-0.68042	0.498019	-5.2593	0.805526	0.748628
B.cells	PRRC2B	-0.06589	6.226887	-0.68038	0.498043	-6.48743	0.74013	0.64577
B.cells	CAR13	0.237058	1.31675	0.680374	0.498046	-5.42723	0.800338	0.740312
B.cells	ZFP341	0.192259	1.984672	0.680191	0.498161	-5.49687	0.7919	0.726724
B.cells	SDC3	-0.19612	4.594842	-0.67987	0.498366	-6.02298	0.759664	0.675963
B.cells	1700056E2	0.245107	1.466557	0.679814	0.498398	-5.43572	0.798556	0.737427
B.cells	USP15	0.061089	7.650516	0.679796	0.49841	-6.75636	0.723804	0.620839
B.cells	2200002D(	0.322211	1.337585	0.679616	0.498523	-5.35727	0.800208	0.740137
B.cells	ARID4B	-0.05503	8.259309	-0.67954	0.498569	-6.84012	0.716901	0.61047
B.cells	FRA10AC1	-0.12444	3.408508	-0.6795	0.498594	-5.88501	0.774147	0.698726
B.cells	EIF2S1	-0.06842	6.052768	-0.67937	0.498677	-6.45733	0.742324	0.649157
B.cells	PEX26	0.264958	1.217016	0.679037	0.498888	-5.41618	0.8019	0.742731
B.cells	NEK11	0.344147	0.082898	0.678841	0.499012	-5.18506	0.816636	0.766602
B.cells	HECTD1	0.054778	7.428886	0.678558	0.49919	-6.70581	0.726467	0.625063
B.cells	GM10603	0.336475	0.220408	0.67851	0.499221	-5.24607	0.814833	0.763866
B.cells	XNDC1	0.125079	3.594505	0.678446	0.499261	-5.91602	0.771994	0.695479
B.cells	ZC3H13	0.073547	5.653384	0.678413	0.499282	-6.40293	0.747143	0.65674
B.cells	RALBP1	-0.06419	6.268527	-0.67841	0.499285	-6.48789	0.739902	0.645588
B.cells	IPO13	0.191677	2.264795	0.678196	0.499418	-5.58117	0.788558	0.721748
B.cells	DNAJC30	0.108255	3.959336	0.678162	0.49944	-6.0048	0.767521	0.688511
B.cells	MRM2	-0.25759	2.071278	-0.67813	0.49946	-5.53598	0.791003	0.725643
B.cells	ARID3A	-0.08683	5.513727	-0.6781	0.499477	-6.33831	0.748799	0.659353
B.cells	PDXP	0.285164	1.557173	0.677713	0.499723	-5.38835	0.797678	0.736125
B.cells	LITAF	-0.05549	8.523937	-0.67769	0.499738	-6.88859	0.714176	0.606386
B.cells	NUMB	-0.07084	6.956562	-0.67765	0.499761	-6.60849	0.732027	0.633424
B.cells	MAN1A2	0.059418	6.392544	0.677284	0.499994	-6.51542	0.738748	0.643589
B.cells	EGLN2	0.093766	4.887876	0.676726	0.500346	-6.27448	0.756575	0.671308
B.cells	RFLNB	0.172787	3.463749	0.676606	0.500422	-5.88034	0.773915	0.698474
B.cells	KYAT1	-0.24986	1.503706	-0.67661	0.500422	-5.37933	0.798544	0.737607
B.cells	GM27008	0.255578	1.012976	0.676588	0.500433	-5.33886	0.804851	0.747734
B.cells	PPP2R5E	0.053639	6.820761	0.676411	0.500546	-6.59058	0.733765	0.636167
B.cells	1810046K(	-0.29806	-0.14929	-0.67637	0.500572	-5.30309	0.820019	0.772268
B.cells	GM20300	0.212931	1.343674	0.676125	0.500726	-5.41284	0.800594	0.740905

B.cells	TSPAN2	-0.15768	3.159011	-0.67609	0.500751	-5.68959	0.777685	0.704432
B.cells	CYREN	-0.14892	3.374354	-0.67603	0.500786	-5.80725	0.775019	0.700224
B.cells	FANCC	0.102407	4.74282	0.675914	0.50086	-6.23756	0.75832	0.674082
B.cells	GM43462	0.135098	3.23183	0.675803	0.50093	-5.90146	0.776782	0.703044
B.cells	DPP10	-0.36225	0.411958	-0.67577	0.500951	-5.2701	0.812654	0.760378
B.cells	ZFP35	0.154244	2.543067	0.675693	0.500999	-5.6689	0.785372	0.716674
B.cells	MILR1	-0.10008	4.330073	-0.67556	0.501086	-6.09044	0.763312	0.681908
B.cells	RRAGD	-0.20979	2.299081	-0.67554	0.501098	-5.54527	0.788441	0.721554
B.cells	COX6B2	-0.23404	1.691068	-0.67548	0.501136	-5.46901	0.796151	0.733855
B.cells	4930557K(	-0.23967	1.483636	-0.67526	0.501274	-5.44223	0.798801	0.738099
B.cells	SYTL2	-0.33688	1.257049	-0.67525	0.50128	-5.35164	0.801707	0.742761
B.cells	CXCL16	0.215309	3.468405	0.675199	0.501311	-5.77896	0.773857	0.698459
B.cells	EGLN3	0.192313	3.294296	0.674992	0.501442	-5.86035	0.776009	0.701852
B.cells	TYW5	0.141954	3.139549	0.674973	0.501454	-5.84796	0.777927	0.70488
B.cells	GCKR	-0.29659	1.581851	-0.67493	0.501478	-5.36902	0.797545	0.736086
B.cells	TM6SF1	0.08453	6.252773	0.674899	0.501501	-6.50937	0.740383	0.646376
B.cells	GPX3	-0.33255	1.463214	-0.67485	0.501529	-5.40768	0.799062	0.738558
B.cells	ELAVL1	-0.04382	7.604056	-0.67472	0.501615	-6.73295	0.724781	0.622582
B.cells	MARK4	0.098897	4.541737	0.674616	0.50168	-6.16867	0.760775	0.67802
B.cells	PSMD2	-0.06427	6.149979	-0.67431	0.501874	-6.47926	0.741804	0.648426
B.cells	GM4673	0.157137	2.578427	0.674144	0.501979	-5.65389	0.785217	0.716238
B.cells	CD209D	-0.50621	-0.32218	-0.67383	0.502174	-5.07571	0.82276	0.776414
B.cells	CDK2AP2	0.067833	6.467925	0.673775	0.502212	-6.52464	0.738278	0.642831
B.cells	LRRC59	-0.06881	5.556125	-0.67363	0.502303	-6.36933	0.749011	0.659457
B.cells	LONRF1	0.146691	3.091293	0.673577	0.502338	-5.85825	0.778959	0.706293
B.cells	SNU13	-0.061	6.96176	-0.67346	0.50241	-6.60086	0.732549	0.634184
B.cells	CHDH	-0.36752	1.157054	-0.67331	0.502507	-5.20331	0.803498	0.745385
B.cells	GPALPP1	0.099414	3.737889	0.672898	0.502767	-5.99847	0.771324	0.693797
B.cells	ANKRD23	-0.31131	0.33175	-0.67273	0.502876	-5.1974	0.814597	0.762748
B.cells	MDM1	-0.14029	3.41542	-0.67257	0.502977	-5.94206	0.775417	0.700152
B.cells	VPS13B	-0.05415	8.050399	-0.6723	0.503145	-6.80517	0.720636	0.615519
B.cells	EIF2B5	0.087605	4.703803	0.672231	0.50319	-6.22677	0.759799	0.675659
B.cells	ERAL1	-0.17779	1.918255	-0.67212	0.503262	-5.54233	0.794324	0.730108
B.cells	KCNQ1OT1	-0.0921	6.399833	-0.67195	0.503365	-6.51111	0.739672	0.644533
B.cells	GM16104	0.517383	-0.60575	0.671781	0.503475	-5.00483	0.827095	0.783241
B.cells	VAMP3	0.06826	5.320751	0.671699	0.503527	-6.27772	0.752422	0.664264
B.cells	MKNK1	0.088944	4.508378	0.671678	0.50354	-6.13631	0.762191	0.679465
B.cells	MED16	0.097059	4.12299	0.671563	0.503613	-6.03498	0.766888	0.686798
B.cells	MAP2K3OS	0.406192	-0.12812	0.671284	0.50379	-5.20065	0.82101	0.77303
B.cells	NDUFA7	-0.051	7.909207	-0.67122	0.50383	-6.76085	0.722397	0.618162
B.cells	GOLGB1	0.072239	5.4427	0.670708	0.504155	-6.36777	0.75149	0.662264
B.cells	LSM2	-0.10405	5.695608	-0.6703	0.504416	-6.38567	0.748772	0.65769
B.cells	TMEM107	-0.23258	1.616764	-0.66985	0.5047	-5.36904	0.799397	0.736789
B.cells	CCSER1	0.258793	1.697174	0.669358	0.505011	-5.69712	0.798725	0.735298
B.cells	PRR11	-0.22314	2.847187	-0.66918	0.505123	-5.62332	0.78417	0.712228
B.cells	RBMX	-0.09059	4.578527	-0.66881	0.505358	-6.1197	0.762836	0.678888
B.cells	RBFOX3	0.440889	-0.24977	0.668734	0.505407	-5.07687	0.824082	0.776457

B.cells	ALPL	-0.23313	1.490191	-0.66868	0.505439	-5.51979	0.801378	0.73986
B.cells	SAMD3	-0.35001	0.184815	-0.66856	0.505517	-5.28626	0.818343	0.767178
B.cells	IRAK1	0.072806	5.224747	0.668543	0.505529	-6.31389	0.755047	0.666843
B.cells	CCT7	0.063022	6.427225	0.668387	0.505628	-6.54293	0.740803	0.644888
B.cells	EPB41L2	0.068839	6.975502	0.668291	0.505689	-6.66241	0.734414	0.635125
B.cells	CIT	-0.1876	4.461996	-0.66824	0.505719	-6.05138	0.76425	0.681166
B.cells	PHLDB3	0.1397	2.97973	0.668156	0.505775	-5.81444	0.782513	0.709854
B.cells	ACPP	-0.20978	2.739914	-0.66806	0.505836	-5.74957	0.785515	0.714613
B.cells	ABAT	-0.33255	1.196429	-0.66798	0.505889	-5.31151	0.805161	0.745975
B.cells	GM37768	0.171869	2.087377	0.667839	0.505976	-5.66835	0.793752	0.727779
B.cells	SRM	-0.14292	4.842685	-0.66775	0.506034	-6.18761	0.759641	0.674074
B.cells	GM42067	-0.29613	0.558955	-0.66773	0.506046	-5.19629	0.813439	0.759363
B.cells	CCAR1	0.051103	6.820278	0.667715	0.506055	-6.61049	0.736216	0.637965
B.cells	GPR137	-0.15231	2.506946	-0.66735	0.506284	-5.59036	0.788698	0.719446
B.cells	NR4A1	-0.11679	7.413157	-0.66722	0.50637	-6.75794	0.729623	0.627639
B.cells	NEO1	-0.39791	0.660859	-0.66673	0.506681	-5.17645	0.812455	0.757542
B.cells	TRIB2	0.175501	2.561151	0.666728	0.506682	-5.79877	0.788097	0.718568
B.cells	ZFP503	-0.2867	0.884395	-0.66662	0.506749	-5.28094	0.809545	0.752853
B.cells	NAPRT	-0.32616	0.897988	-0.66662	0.506752	-5.31666	0.809369	0.752569
B.cells	GM45902	-0.22091	1.585805	-0.66655	0.506793	-5.42718	0.800492	0.738322
B.cells	SNHG1	-0.08092	5.230493	-0.66654	0.506802	-6.33887	0.7553	0.667121
B.cells	D330050I1	0.275594	1.118758	0.666185	0.507028	-5.29511	0.806601	0.748041
B.cells	ASH2L	0.095169	4.017187	0.666128	0.507063	-6.02565	0.770094	0.690117
B.cells	LCOR	0.060752	7.538405	0.666088	0.507089	-6.72933	0.728317	0.625692
B.cells	ILF2	-0.07434	5.763945	-0.66594	0.507184	-6.42099	0.749026	0.657398
B.cells	UBE2Z	0.066242	5.437928	0.665926	0.507192	-6.37042	0.752906	0.663392
B.cells	CARD11	0.12638	4.618562	0.665202	0.507653	-6.25171	0.763178	0.678932
B.cells	HPF1	-0.10363	5.467524	-0.66516	0.50768	-6.29105	0.752963	0.663089
B.cells	AW112010	0.227201	7.279978	0.665066	0.507739	-6.61968	0.731688	0.630472
B.cells	PPP4C	0.058269	6.80668	0.664876	0.50786	-6.60086	0.737174	0.638834
B.cells	RAB30	0.153747	2.141089	0.664817	0.507898	-5.96582	0.793932	0.727416
B.cells	TXNL1	-0.06998	6.955558	-0.66481	0.507905	-6.59216	0.735443	0.636192
B.cells	FEN1	-0.12165	4.525584	-0.66476	0.507933	-6.10237	0.764307	0.68072
B.cells	OIT3	-0.24192	2.345496	-0.66452	0.508084	-5.47745	0.791472	0.723365
B.cells	CDK12	-0.06278	6.896517	-0.66414	0.508328	-6.61972	0.736393	0.637399
B.cells	GM42418	-0.14426	11.10924	-0.66412	0.50834	-7.29372	0.689291	0.56692
B.cells	FOXN2	0.060666	6.146142	0.663966	0.50844	-6.50967	0.74518	0.650886
B.cells	TNFAIP8L2	0.107278	3.852483	0.663953	0.508448	-6.06689	0.772814	0.693802
B.cells	NUP214	-0.07308	5.2678	-0.66382	0.50853	-6.34246	0.755646	0.667009
B.cells	CREB3	-0.10132	3.923009	-0.66339	0.508807	-5.97061	0.772226	0.692549
B.cells	CPNE8	0.254393	2.649239	0.663271	0.508883	-5.53279	0.788075	0.717579
B.cells	MLLT1	-0.14491	2.735176	-0.66322	0.508916	-5.66544	0.786994	0.715867
B.cells	SRSF1	-0.07099	6.067856	-0.66305	0.509021	-6.45581	0.746373	0.652479
B.cells	DDX59	0.191912	1.892836	0.663038	0.509031	-5.54191	0.797666	0.732868
B.cells	NID1	-0.22117	2.394724	-0.66286	0.509147	-5.5316	0.791365	0.72277
B.cells	CNOT2	0.05225	7.008824	0.662288	0.509509	-6.64946	0.735641	0.635884
B.cells	GM17655	0.250086	1.183959	0.662222	0.509552	-5.374	0.807092	0.747792

B.cells	NAP1L4	0.055024	6.249563	0.662192	0.509571	-6.49748	0.744521	0.649494
B.cells	ZC3H7B	0.132932	3.353116	0.661996	0.509696	-5.85828	0.779575	0.704115
B.cells	KLRB1F	0.373962	0.424065	0.661862	0.509781	-5.25972	0.816996	0.763878
B.cells	ZFP644	0.061636	6.331418	0.661846	0.509792	-6.53684	0.743557	0.648119
B.cells	DNA2	0.104627	3.583362	0.661663	0.509908	-6.05373	0.776718	0.699716
B.cells	CCDC115	0.089243	4.152653	0.661623	0.509934	-6.11485	0.769708	0.68873
B.cells	MTFR1	0.10894	4.079368	0.661577	0.509963	-6.04046	0.770606	0.690151
B.cells	UBE2G2	0.077548	4.661055	0.661434	0.510054	-6.23675	0.76351	0.679104
B.cells	COPS2	-0.0561	6.020343	-0.66128	0.51015	-6.50155	0.747226	0.653939
B.cells	DGUOK	0.101297	4.460054	0.661238	0.510179	-6.15823	0.765954	0.682989
B.cells	MMGT1	0.166346	2.857236	0.661199	0.510204	-5.66995	0.785768	0.714156
B.cells	PRX	-0.32013	1.104861	-0.66116	0.510227	-5.23005	0.808116	0.749834
B.cells	GM43388	0.241256	0.386691	0.660923	0.51038	-5.56675	0.817496	0.764943
B.cells	DESI2	0.050555	6.03278	0.660885	0.510404	-6.4891	0.747088	0.653762
B.cells	NOL10	0.08082	4.91748	0.6608	0.510459	-6.29256	0.760416	0.674403
B.cells	ACAP1	0.135608	3.923819	0.660736	0.5105	-5.9454	0.772527	0.693348
B.cells	FUT8	-0.07723	5.850748	-0.66054	0.510626	-6.41645	0.749333	0.657192
B.cells	NLN	0.099195	4.446951	0.660269	0.510798	-6.18323	0.766371	0.683452
B.cells	ZFP955B	0.182445	2.201747	0.659907	0.511029	-5.60863	0.79436	0.7277
B.cells	ACTN4	0.054871	6.545001	0.659583	0.511236	-6.55031	0.741347	0.644902
B.cells	PSPH	-0.10999	3.906589	-0.65954	0.511264	-6.0153	0.773038	0.694054
B.cells	CAPN10	-0.15541	2.598593	-0.6595	0.511287	-5.71127	0.789337	0.719779
B.cells	ZMYM4	0.071589	5.733892	0.659389	0.51136	-6.43018	0.750924	0.659644
B.cells	COX4I1	-0.04981	9.086822	-0.65924	0.511454	-6.95099	0.71226	0.600882
B.cells	CHTF18	-0.25472	1.800347	-0.65905	0.511577	-5.42877	0.799479	0.736003
B.cells	USHBP1	-0.32821	1.085061	-0.65904	0.511586	-5.20453	0.808696	0.75078
B.cells	PPARG	-0.25068	2.579221	-0.65897	0.511628	-5.58735	0.789581	0.720234
B.cells	CLEC1A	0.321214	0.972503	0.658936	0.51165	-5.32904	0.810158	0.753132
B.cells	1700112J1	0.350463	0.638697	0.658887	0.511681	-5.22711	0.81451	0.760154
B.cells	PPWD1	0.075038	4.68019	0.658884	0.511682	-6.17165	0.763583	0.679338
B.cells	SCUBE2	-0.44282	-0.26058	-0.65886	0.511696	-5.11198	0.826368	0.779368
B.cells	SPRY2	-0.11523	5.158508	-0.65864	0.511837	-6.35315	0.757916	0.670406
B.cells	ZFP951	0.176126	2.641469	0.658216	0.51211	-5.71877	0.789229	0.719163
B.cells	DNAJC16	0.174794	2.30093	0.657445	0.512603	-5.68731	0.794039	0.726201
B.cells	DDX39B	-0.06467	7.251694	-0.65743	0.51261	-6.65724	0.733987	0.632721
B.cells	GEM	0.111635	5.207345	0.657134	0.512802	-6.36904	0.758114	0.669877
B.cells	RXRB	0.092028	4.393184	0.657088	0.512832	-6.16641	0.767982	0.685233
B.cells	ZBTB38	0.091602	5.068147	0.657034	0.512866	-6.23933	0.75979	0.672478
B.cells	IGF1OS	-0.44962	-1.03209	-0.65701	0.512883	-5.06627	0.837381	0.796739
B.cells	HIPK3	0.073022	5.445686	0.656901	0.512951	-6.38008	0.755253	0.665467
B.cells	HS3ST1	-0.37722	1.500377	-0.65688	0.512967	-5.38556	0.804279	0.742679
B.cells	GM3435	-0.37619	0.205181	-0.65676	0.513039	-5.17277	0.821181	0.76991
B.cells	MYL12B	-0.05227	8.183707	-0.65607	0.513486	-6.84396	0.723811	0.616877
B.cells	HDAC10	-0.20575	1.608346	-0.65599	0.513536	-5.46646	0.803467	0.740775
B.cells	SLC25A25	0.083143	4.951297	0.655778	0.51367	-6.28938	0.761848	0.675076
B.cells	HYPK	-0.06535	5.8151	-0.65558	0.513798	-6.41286	0.751543	0.659099
B.cells	PLSCR2	0.451184	-0.38835	0.655506	0.513844	-5.12038	0.829819	0.783224

B.cells	GM7854	0.356123	0.52802	0.655189	0.514047	-5.22189	0.817804	0.763612
B.cells	MYBBP1A	0.10203	4.85707	0.655179	0.514053	-6.27387	0.763163	0.676992
B.cells	TBC1D10C	0.116275	4.736281	0.654976	0.514183	-6.19591	0.764688	0.679344
B.cells	PIK3C3	0.088583	4.439233	0.654899	0.514233	-6.16933	0.768307	0.685001
B.cells	PLEKHF2	-0.06518	5.470642	-0.65477	0.514319	-6.41963	0.755827	0.665661
B.cells	PPM1L	0.116206	4.617569	0.654701	0.51436	-6.18041	0.766132	0.681667
B.cells	ARHGAP1	0.07604	4.473602	0.654584	0.514435	-6.14411	0.767899	0.684405
B.cells	TRMT5	0.265993	1.213824	0.654197	0.514683	-5.34976	0.809069	0.749413
B.cells	PCBD2	-0.08981	5.24057	-0.65414	0.514717	-6.26727	0.758734	0.67001
B.cells	A630072M	-0.14475	3.151561	-0.65407	0.514762	-5.84948	0.784377	0.71014
B.cells	APBB3	0.21597	1.534528	0.653613	0.515057	-5.4835	0.804921	0.743
B.cells	SGMS2	0.251612	3.643674	0.653593	0.51507	-5.73894	0.778246	0.700667
B.cells	AP1AR	0.094441	5.002368	0.653525	0.515114	-6.22863	0.761608	0.674662
B.cells	SACS	0.180562	2.596254	0.653348	0.515227	-5.79038	0.791363	0.721427
B.cells	GM26982	-0.17634	1.652146	-0.65324	0.515299	-5.49925	0.803406	0.740642
B.cells	SNHG4	-0.11198	3.683196	-0.65318	0.515336	-6.01181	0.777756	0.69997
B.cells	CCDC15	-0.17918	2.810819	-0.65298	0.515464	-5.67426	0.788655	0.717232
B.cells	GM10847	0.323928	0.165978	0.652857	0.515543	-5.20064	0.822795	0.771971
B.cells	NDUFV1	0.080498	5.20278	0.652834	0.515557	-6.30639	0.759189	0.671069
B.cells	MTLN	-0.13541	3.060484	-0.65272	0.515629	-5.75539	0.785517	0.712327
B.cells	ZFP827	0.181473	2.85843	0.652704	0.515641	-5.74483	0.788055	0.716343
B.cells	CEACAM1	0.102266	3.675486	0.652622	0.515693	-6.11512	0.777851	0.700268
B.cells	KLF7	-0.10098	6.159891	-0.65241	0.515829	-6.4226	0.747762	0.653488
B.cells	PHF23	-0.08816	5.240241	-0.65236	0.515862	-6.34403	0.758738	0.670454
B.cells	SMG5	0.067908	5.55464	0.652281	0.515912	-6.38855	0.754964	0.664657
B.cells	POLR1D	0.051967	6.995878	0.652245	0.515936	-6.66275	0.737948	0.638535
B.cells	GM39121	0.369725	-0.94202	0.652175	0.51598	-5.09188	0.837319	0.796263
B.cells	ATP5B	-0.05867	8.182512	-0.65204	0.51607	-6.83529	0.724278	0.61781
B.cells	RNF10	0.063147	6.51711	0.652013	0.516084	-6.62062	0.74355	0.647123
B.cells	SLC25A51	0.075876	5.697758	0.65191	0.516151	-6.4601	0.753254	0.662055
B.cells	1500004A1	-0.14854	2.078931	-0.65186	0.516182	-5.68918	0.797936	0.732211
B.cells	TTC38	-0.17778	2.505389	-0.65174	0.516258	-5.66908	0.792513	0.723606
B.cells	GM16196	-0.23266	1.751417	-0.65173	0.516263	-5.41672	0.80213	0.738937
B.cells	MRPL46	0.123168	3.264758	0.651623	0.516335	-5.8815	0.782961	0.708546
B.cells	SMPDL3A	-0.11927	5.614644	-0.65121	0.516599	-6.39173	0.754247	0.66375
B.cells	SPRY1	-0.35176	1.345776	-0.65121	0.516601	-5.22346	0.80736	0.74746
B.cells	4930426D0	-0.28035	-0.21569	-0.65116	0.51663	-5.3163	0.82786	0.780582
B.cells	NPRL3	0.142467	2.892647	0.651077	0.516685	-5.75885	0.787625	0.715992
B.cells	HIST1H3D	-0.26166	1.963471	-0.65106	0.516698	-5.45966	0.799411	0.734736
B.cells	AKT1S1	-0.111	3.589237	-0.65095	0.516769	-5.92454	0.778921	0.702248
B.cells	AI506816	-0.11547	5.207229	-0.65091	0.516793	-6.33622	0.759135	0.671322
B.cells	CYP4A10	-0.39282	1.117903	-0.65058	0.517007	-5.35443	0.810546	0.752325
B.cells	RNF187	0.071046	6.12757	0.650366	0.517142	-6.44542	0.748367	0.65449
B.cells	EDRF1	0.098861	4.217889	0.650279	0.517198	-6.14451	0.771392	0.690284
B.cells	CDCA2	0.175597	3.79635	0.650255	0.517213	-5.89045	0.776586	0.69843
B.cells	2610021A0	0.164287	2.362444	0.650041	0.517351	-5.65536	0.794582	0.726956
B.cells	SP4	0.087474	5.254382	0.650028	0.51736	-6.31419	0.758812	0.670742

B.cells	WDR59	0.139145	3.072953	0.649812	0.517498	-5.83103	0.785698	0.712747
B.cells	TMCC1	-0.08786	7.946033	-0.64972	0.517559	-6.77803	0.727289	0.62236
B.cells	GM26827	-0.23159	1.344192	-0.64963	0.517615	-5.55779	0.807727	0.747904
B.cells	TARS	-0.09182	4.374393	-0.64943	0.517745	-6.1575	0.769576	0.687446
B.cells	ZSCAN12	-0.23116	1.093811	-0.6494	0.517761	-5.32423	0.810977	0.753125
B.cells	PPP1CB	0.061314	7.803763	0.649256	0.517856	-6.83232	0.728921	0.624862
B.cells	ZFP984	0.092798	3.878402	0.649117	0.517945	-6.06442	0.775675	0.697066
B.cells	UBE2T	-0.13753	4.06115	-0.64885	0.518118	-6.02657	0.773421	0.693605
B.cells	MTREX	0.065331	5.477446	0.648789	0.518156	-6.37267	0.756214	0.666819
B.cells	GM42836	0.296998	-0.40469	0.648744	0.518186	-5.31553	0.830739	0.785317
B.cells	CD300A	-0.18002	4.623283	-0.64872	0.518204	-5.88766	0.766537	0.682887
B.cells	ZEB1	-0.09466	6.848689	-0.64865	0.518243	-6.65456	0.739983	0.641881
B.cells	STFA2	-0.26388	2.469209	-0.64857	0.518299	-5.60944	0.793312	0.725113
B.cells	DCAF4	-0.17616	2.008291	-0.64848	0.518355	-5.53603	0.799182	0.734516
B.cells	MYLIP	0.085154	5.991118	0.648267	0.518492	-6.45063	0.750085	0.657463
B.cells	ZFP52	0.101083	3.859767	0.648255	0.5185	-6.13794	0.775905	0.697653
B.cells	PDPR	0.114243	3.937711	0.648177	0.51855	-6.07383	0.774943	0.696144
B.cells	GM11131	0.288498	0.815809	0.647966	0.518686	-5.3368	0.81471	0.759319
B.cells	TRIM36	-0.14061	3.146179	-0.64768	0.518871	-5.87141	0.785065	0.711702
B.cells	ZFHX2	-0.17859	2.765643	-0.64725	0.519145	-5.72483	0.789986	0.719408
B.cells	TBC1D19	-0.20867	1.749793	-0.64723	0.51916	-5.37411	0.802925	0.74002
B.cells	EHD2	-0.22139	1.82262	-0.64705	0.519278	-5.52963	0.801989	0.738603
B.cells	PLXNC1	-0.10454	5.427157	-0.64704	0.519279	-6.41156	0.757222	0.668114
B.cells	ATP13A1	0.093672	3.809105	0.646854	0.519403	-6.02444	0.776946	0.699019
B.cells	GPR108	-0.09518	4.177602	-0.64684	0.519414	-6.11259	0.772402	0.691891
B.cells	POLR2G	-0.06734	5.51542	-0.64681	0.519428	-6.3792	0.756163	0.666605
B.cells	GTPBP3	0.174414	2.225197	0.646667	0.519523	-5.59694	0.796881	0.73054
B.cells	SLC31A2	0.176719	3.169586	0.646298	0.519761	-5.75961	0.785015	0.711682
B.cells	GM47754	0.337182	0.667894	0.64615	0.519856	-5.24695	0.817086	0.762961
B.cells	LCK	0.12505	3.728178	0.645856	0.520045	-6.0294	0.778055	0.700861
B.cells	PINX1	0.126743	3.022074	0.645788	0.520089	-5.89793	0.786865	0.714792
B.cells	NPC1	0.101924	4.971884	0.645713	0.520138	-6.19764	0.762816	0.67704
B.cells	EOGT	-0.17426	2.532472	-0.64565	0.520181	-5.6475	0.793042	0.724607
B.cells	GM49169	-0.34925	-0.42801	-0.64564	0.520183	-5.18683	0.831607	0.786719
B.cells	GM26802	0.312365	1.069129	0.645634	0.520188	-5.31097	0.811842	0.754692
B.cells	DOP1B	0.108728	4.289272	0.645605	0.520207	-6.10455	0.771136	0.690046
B.cells	ESR1	0.145147	3.611322	0.645563	0.520234	-6.05874	0.779505	0.70321
B.cells	MPHOSPH1	0.082331	4.827879	0.645281	0.520416	-6.25067	0.764732	0.679929
B.cells	PAK1	-0.16441	5.334473	-0.64512	0.520522	-6.24256	0.758664	0.670441
B.cells	FCER2A	0.275423	0.458562	0.645004	0.520595	-5.56248	0.82009	0.767872
B.cells	FOXP4	0.090789	4.962815	0.644742	0.520764	-6.34696	0.76331	0.677503
B.cells	NUP155	-0.07538	5.174039	-0.64461	0.520847	-6.29435	0.760779	0.673556
B.cells	PPP1R9B	0.107509	4.375184	0.644339	0.521024	-6.1014	0.770659	0.688781
B.cells	TIMM23	0.051849	6.942518	0.644126	0.521161	-6.64927	0.740036	0.641337
B.cells	LAMB3	0.11555	4.067884	0.644014	0.521234	-6.19016	0.774549	0.694819
B.cells	TRMT1	0.083005	4.050324	0.643868	0.521328	-6.11476	0.774805	0.695177
B.cells	CEP164	-0.10541	4.052145	-0.6434	0.521633	-6.00152	0.775137	0.695299

B.cells	L2HGDH	-0.22731	1.767651	-0.64329	0.521701	-5.42987	0.803932	0.740924
B.cells	MOCS3	0.253963	1.446277	0.642875	0.521969	-5.39883	0.808138	0.747717
B.cells	STK11IP	0.146698	2.312647	0.642765	0.52204	-5.65054	0.797007	0.729933
B.cells	GM50334	0.420611	-0.42372	0.642699	0.522083	-5.09157	0.832776	0.787583
B.cells	C2CD3	0.075488	4.657214	0.642679	0.522096	-6.20124	0.767768	0.683836
B.cells	ADGRF5	-0.28227	2.598167	-0.64264	0.522119	-5.51324	0.793378	0.724164
B.cells	TBC1D13	0.116193	3.495587	0.642561	0.522172	-5.97948	0.782096	0.706324
B.cells	TGFB3	0.354675	0.567314	0.642464	0.522234	-5.2174	0.819614	0.766234
B.cells	A930015D(	-0.09547	4.760503	-0.64235	0.522307	-6.19516	0.766509	0.681894
B.cells	UFSP2	0.084145	4.93604	0.642246	0.522376	-6.27197	0.764375	0.678611
B.cells	LRRC18	-0.18282	1.588071	-0.64202	0.522524	-5.75905	0.806304	0.744999
B.cells	ZFP54	0.285552	0.794625	0.64196	0.52256	-5.30824	0.816628	0.761627
B.cells	TASOR	0.075054	5.742445	0.641835	0.522641	-6.44382	0.754661	0.663688
B.cells	FAM78B	0.179353	2.115372	0.641785	0.522673	-5.7208	0.799526	0.734189
B.cells	CHP1	-0.0554	7.01438	-0.64178	0.522673	-6.70719	0.739634	0.640607
B.cells	GM6712	0.183136	2.305447	0.64161	0.522786	-5.62856	0.797169	0.730376
B.cells	ZFP429	0.140487	2.448075	0.641319	0.522975	-5.73276	0.795538	0.727613
B.cells	GM49692	0.32906	0.104027	0.640765	0.523332	-5.18005	0.826311	0.776683
B.cells	LYPLA1	0.062048	5.988433	0.640706	0.523371	-6.48735	0.752249	0.659422
B.cells	SMYD2	0.169278	3.028651	0.640706	0.523371	-5.73478	0.78849	0.71605
B.cells	GM10634	-0.27938	0.651485	-0.64036	0.523593	-5.3432	0.819181	0.76504
B.cells	DNHD1	-0.30077	1.01891	-0.64019	0.523707	-5.25197	0.814365	0.757354
B.cells	HK1OS	0.274349	0.741866	0.640144	0.523734	-5.33535	0.817993	0.763223
B.cells	UBQLN1	-0.06137	5.517917	-0.63999	0.523834	-6.40222	0.757975	0.668303
B.cells	EIF3H	-0.05355	7.353901	-0.63995	0.52386	-6.71103	0.736289	0.635025
B.cells	DUS3L	-0.10819	3.300011	-0.63991	0.523883	-5.92399	0.785185	0.710896
B.cells	GM29340	-0.36063	-0.6731	-0.63988	0.523907	-5.08514	0.836675	0.793875
B.cells	MFF	-0.06134	5.906654	-0.63958	0.524097	-6.47946	0.753389	0.661184
B.cells	STARD5	0.102221	4.526318	0.639543	0.524124	-6.10831	0.77007	0.687087
B.cells	ETS1	0.070986	7.418769	0.639433	0.524195	-6.71517	0.735602	0.633928
B.cells	TNFAIP2	0.231124	4.351498	0.639391	0.524222	-5.8367	0.772214	0.690451
B.cells	PRCP	0.071993	5.424232	0.639193	0.52435	-6.42841	0.759258	0.670222
B.cells	PRKD2	0.10032	4.348606	0.638856	0.524568	-6.08736	0.772355	0.690638
B.cells	PSMB6	-0.06828	6.304312	-0.63872	0.524657	-6.52785	0.748765	0.654049
B.cells	ISG20L2	-0.07566	5.083016	-0.63871	0.52466	-6.34445	0.763395	0.676671
B.cells	GM42595	-0.26922	0.917394	-0.6387	0.524672	-5.30335	0.815877	0.759804
B.cells	FOSL1	0.251763	1.662098	0.638658	0.524697	-5.54953	0.806195	0.744259
B.cells	CAMP	-0.23642	4.463959	-0.63856	0.524763	-6.11757	0.770939	0.688472
B.cells	GM43848	-0.14983	2.767038	-0.63835	0.524895	-5.69716	0.792151	0.721844
B.cells	ATRN	0.077674	5.965865	0.638269	0.524949	-6.51623	0.75286	0.660378
B.cells	BLOC1S3	-0.18374	2.381624	-0.63806	0.525083	-5.63925	0.797093	0.729719
B.cells	GALNT12	0.134465	2.991393	0.637845	0.525223	-5.87927	0.789366	0.717495
B.cells	GM21781	0.205383	1.561921	0.637739	0.525292	-5.50395	0.807619	0.746604
B.cells	CSF1	0.286078	2.480595	0.637651	0.525349	-5.64226	0.795833	0.727816
B.cells	DLL4	-0.37101	0.717902	-0.63763	0.525362	-5.17375	0.818625	0.764369
B.cells	ADRM1	0.082165	5.477684	0.637599	0.525382	-6.37255	0.758752	0.669582
B.cells	NAB2	0.122224	3.217989	0.637157	0.525669	-6.03121	0.786846	0.713108

B.cells	CYTH4	0.073331	5.757599	0.636805	0.525897	-6.41533	0.755829	0.664511
B.cells	EVI5L	0.172798	2.262161	0.636725	0.525949	-5.59204	0.799078	0.73243
B.cells	FCGR2B	0.107105	4.881357	0.636664	0.525989	-6.39424	0.766408	0.680957
B.cells	MRNIP	-0.18928	2.538227	-0.63659	0.526035	-5.66515	0.795559	0.72686
B.cells	LYNX1	0.261977	0.898277	0.636467	0.526117	-5.44793	0.816729	0.760798
B.cells	AI987944	0.151992	2.764701	0.636124	0.526339	-5.73843	0.792685	0.72246
B.cells	EFCAB7	-0.29599	0.535523	-0.6361	0.526356	-5.19416	0.821499	0.768641
B.cells	EID1	0.077927	5.155191	0.636056	0.526383	-6.28313	0.763083	0.675969
B.cells	RPUSD1	0.237793	1.47218	0.635958	0.526447	-5.35649	0.809247	0.74892
B.cells	SNRNP48	0.074756	4.916287	0.635907	0.52648	-6.29293	0.765982	0.68049
B.cells	LSM6	-0.07284	6.681324	-0.63587	0.526504	-6.57616	0.744864	0.647859
B.cells	MMRN2	-0.31519	1.228883	-0.63564	0.526651	-5.20357	0.812409	0.754032
B.cells	NRM	-0.10049	5.099836	-0.63536	0.526835	-6.33571	0.763754	0.677155
B.cells	TMEM186	0.200023	2.111676	0.635348	0.526843	-5.56393	0.801003	0.735869
B.cells	PELI2	-0.22898	2.831902	-0.63526	0.526899	-5.55244	0.791835	0.72132
B.cells	ELMO2	0.11335	3.945024	0.635212	0.526931	-6.0039	0.777904	0.699348
B.cells	PGRMC2	-0.09644	3.628957	-0.63493	0.527113	-5.92252	0.78183	0.705583
B.cells	CDC37L1	0.068971	5.167302	0.634878	0.527147	-6.31771	0.762936	0.676007
B.cells	POLR1C	-0.10775	3.945898	-0.63478	0.527211	-6.06165	0.777893	0.699405
B.cells	GPN2	-0.12912	2.713988	-0.63471	0.527255	-5.81081	0.793328	0.723795
B.cells	CD163L1	0.366265	0.60908	0.634675	0.527279	-5.23211	0.820529	0.767405
B.cells	KDM3B	0.056534	6.131762	0.634642	0.5273	-6.53012	0.751365	0.65812
B.cells	1110032A(	0.109609	3.592833	0.634549	0.527361	-5.91897	0.782281	0.706347
B.cells	LEMD3	-0.06622	5.687224	-0.63447	0.527412	-6.46871	0.756672	0.666346
B.cells	5830411N(	0.340611	-1.25694	0.634414	0.527449	-5.11474	0.845242	0.808219
B.cells	FLVCR1	-0.08716	4.670527	-0.63429	0.527531	-6.22546	0.768979	0.685517
B.cells	CASP9	0.123411	2.447605	0.634172	0.527606	-5.77869	0.796712	0.729301
B.cells	COPB1	0.052448	6.220287	0.634137	0.527629	-6.5303	0.750313	0.656606
B.cells	GM26590	-0.19274	1.314817	-0.63381	0.52784	-5.40041	0.811401	0.752677
B.cells	DENND5A	0.063939	6.395889	0.633702	0.527911	-6.55116	0.748333	0.653442
B.cells	KDM1B	-0.12426	3.6758	-0.63362	0.527966	-5.95105	0.781353	0.704856
B.cells	ASB2	-0.24645	2.906427	-0.63361	0.527968	-5.61882	0.791	0.720108
B.cells	RIN2	0.15406	3.687666	0.633425	0.528091	-5.83691	0.781269	0.704669
B.cells	HYKK	-0.29269	0.618386	-0.63331	0.528166	-5.25595	0.820585	0.767459
B.cells	COX5B	-0.06175	8.103976	-0.63314	0.528274	-6.83018	0.728501	0.623162
B.cells	BID	0.103479	3.803317	0.632917	0.528421	-6.07414	0.779831	0.702442
B.cells	RNF11	0.070194	6.207032	0.632771	0.528516	-6.55459	0.750633	0.656981
B.cells	EXOC3L2	0.342512	2.269133	0.632755	0.528527	-5.57874	0.799162	0.733079
B.cells	HP1BP3	0.066442	6.529858	0.632607	0.528623	-6.54679	0.746811	0.651118
B.cells	BOD1L	0.060253	5.740505	0.632549	0.52866	-6.41902	0.756198	0.665595
B.cells	CGGBP1	-0.05735	6.845343	-0.63236	0.528786	-6.62427	0.743098	0.645473
B.cells	SIT1	-0.21053	2.062249	-0.63227	0.528839	-5.58077	0.801811	0.737387
B.cells	NUDT4	0.072769	5.949353	0.632269	0.528843	-6.42978	0.753701	0.661782
B.cells	DCTN1	-0.07426	4.987161	-0.63192	0.529071	-6.25514	0.765287	0.679798
B.cells	DNAJC19	0.062287	5.950115	0.631888	0.529091	-6.47099	0.753692	0.661815
B.cells	DERL2	0.076386	4.925658	0.63188	0.529095	-6.27806	0.766035	0.680963
B.cells	PDE4A	0.170249	3.81993	0.631879	0.529096	-5.88557	0.779625	0.702248

B.cells	ERG	-0.24198	3.921953	-0.63183	0.529128	-5.88915	0.778359	0.700257
B.cells	GTPBP1	0.089726	4.641527	0.631729	0.529194	-6.19798	0.7695	0.686421
B.cells	CCDC112	0.274146	1.151644	0.631693	0.529218	-5.36664	0.813593	0.756403
B.cells	TMEM19	0.093253	3.958743	0.631381	0.52942	-6.04693	0.778078	0.69967
B.cells	PTPN14	0.360573	0.524362	0.630974	0.529685	-5.17203	0.82201	0.769992
B.cells	RBBP9	-0.26735	1.214794	-0.63096	0.529692	-5.36811	0.812952	0.755365
B.cells	KDM5D	0.886927	0.730109	0.630829	0.529779	-5.42884	0.819299	0.765639
B.cells	NDUFS1	-0.05603	5.752161	-0.63077	0.52982	-6.4499	0.756229	0.665809
B.cells	FAM131A	0.321472	0.401139	0.630593	0.529934	-5.30767	0.823638	0.772679
B.cells	TRPM4	0.238992	1.109553	0.630565	0.529952	-5.36463	0.814326	0.757628
B.cells	ALG13	-0.07026	4.514642	-0.63035	0.530091	-6.18338	0.771227	0.689272
B.cells	SAMD10	0.190767	1.732501	0.630296	0.530126	-5.57582	0.806236	0.744743
B.cells	GTF2H3	-0.17727	2.299191	-0.63029	0.530133	-5.60261	0.798958	0.7331
B.cells	GCC1	-0.1832	2.459454	-0.63023	0.530172	-5.62277	0.796913	0.72984
B.cells	JPT2	0.130728	3.513173	0.630164	0.530213	-5.93707	0.783621	0.708767
B.cells	DHRS4	-0.10256	4.374568	-0.63011	0.530247	-6.17096	0.772947	0.691977
B.cells	PINK1	0.091614	5.115722	0.630002	0.530318	-6.32073	0.763899	0.6779
B.cells	VHL	0.090269	3.486608	0.629964	0.530343	-6.01738	0.783953	0.709353
B.cells	TSC1	0.084255	4.710552	0.629775	0.530466	-6.25308	0.768866	0.685644
B.cells	WRAP53	0.142213	2.788872	0.62972	0.530502	-5.68986	0.792768	0.723311
B.cells	GM36975	0.146394	3.276788	0.629585	0.53059	-5.92426	0.786649	0.713576
B.cells	AFF1	0.055691	8.730515	0.629341	0.530749	-6.97892	0.721711	0.613134
B.cells	ZFAND3	0.050087	8.233036	0.628939	0.53101	-6.8828	0.727585	0.621744
B.cells	ANKRD17	-0.05266	7.985576	-0.62876	0.531124	-6.80987	0.730423	0.626042
B.cells	JOSD2	0.093769	4.09679	0.628749	0.531134	-6.04528	0.776796	0.69762
B.cells	PAXBP1	-0.0826	5.312121	-0.6287	0.53117	-6.37499	0.761939	0.67443
B.cells	RNF150	-0.26299	2.909798	-0.62784	0.531728	-5.6699	0.792082	0.721303
B.cells	WDR38	0.248102	0.617809	0.627554	0.531914	-5.28548	0.821691	0.768895
B.cells	ERC1	-0.10084	4.872109	-0.6275	0.531946	-6.20869	0.767713	0.683091
B.cells	CFAP126	-0.27066	1.069649	-0.62741	0.532005	-5.35814	0.815755	0.759334
B.cells	ZBTB45	-0.13483	2.916254	-0.62734	0.532051	-5.75086	0.792	0.721355
B.cells	ZFP36L1	0.068993	7.742233	0.627339	0.532054	-6.77844	0.733642	0.630679
B.cells	NUDT3	0.066322	5.213023	0.627265	0.532102	-6.31678	0.76357	0.676686
B.cells	PTOV1	-0.10073	4.038474	-0.62716	0.53217	-6.01211	0.777957	0.699255
B.cells	DOLK	-0.18267	1.544978	-0.62711	0.532203	-5.42824	0.809562	0.749491
B.cells	USO1	0.064724	5.739311	0.627071	0.532229	-6.44064	0.757225	0.666952
B.cells	PHKA2	0.120964	3.438409	0.626857	0.532368	-5.95716	0.78543	0.711122
B.cells	HSPBP1	0.09472	3.934559	0.626845	0.532376	-6.04671	0.779245	0.701371
B.cells	GM4631	0.28202	0.664941	0.626836	0.532382	-5.21576	0.82107	0.768127
B.cells	NBN	0.119337	3.4476	0.626786	0.532414	-5.91702	0.785315	0.710942
B.cells	DCBLD1	0.167917	3.33755	0.626284	0.532742	-5.81666	0.786694	0.713217
B.cells	GM28529	0.306625	0.724072	0.626251	0.532764	-5.27745	0.820291	0.766978
B.cells	TMEM38A	-0.2223	1.868955	-0.62599	0.532932	-5.43564	0.805373	0.743072
B.cells	FAM199X	0.138606	2.776803	0.625773	0.533076	-5.80469	0.793766	0.724611
B.cells	ZFP112	0.300925	0.391108	0.625717	0.533113	-5.26606	0.824688	0.774291
B.cells	NSUN2	0.075123	5.0541	0.625669	0.533144	-6.32138	0.765498	0.680115
B.cells	SMPD3	-0.35704	-0.20687	-0.62563	0.533167	-5.18924	0.832655	0.787249

B.cells	ZBTB8A	-0.17749	1.978565	-0.62556	0.533214	-5.53523	0.803961	0.740882
B.cells	MSL1	-0.07744	5.419802	-0.62536	0.533343	-6.34858	0.76107	0.673341
B.cells	IQCC	0.226824	1.695654	0.625127	0.533498	-5.45945	0.807611	0.746872
B.cells	CCDC130	-0.20546	2.056293	-0.625	0.533583	-5.54938	0.802962	0.739421
B.cells	NKAP	0.0666	4.989943	0.624971	0.533599	-6.28986	0.766278	0.681458
B.cells	IL16	0.123354	4.60831	0.624892	0.533651	-6.17817	0.770938	0.688737
B.cells	FGD4	-0.20285	4.134435	-0.62487	0.533666	-5.77394	0.77677	0.697882
B.cells	SLC35A2	-0.13537	3.165869	-0.62487	0.533667	-5.84961	0.788851	0.716949
B.cells	LIAS	0.085624	4.310022	0.624856	0.533675	-6.16349	0.774603	0.694479
B.cells	5330417C2	-0.31434	0.541978	-0.62482	0.5337	-5.2921	0.822692	0.771199
B.cells	MS4A1	0.155279	3.936889	0.624807	0.533707	-6.27828	0.779216	0.70173
B.cells	CYCS	-0.07791	7.712668	-0.62465	0.533812	-6.77835	0.733984	0.631729
B.cells	HDGFL2	-0.07677	4.979057	-0.62457	0.533864	-6.24814	0.766411	0.681687
B.cells	GM20721	0.111313	3.72758	0.624184	0.534114	-6.00264	0.781818	0.706007
B.cells	RNF5	0.104572	4.122516	0.624085	0.534178	-6.03195	0.776917	0.698342
B.cells	ISYNA1	0.083594	4.852057	0.62399	0.534241	-6.24925	0.767958	0.684335
B.cells	5031439G(	-0.07304	5.696635	-0.62395	0.534268	-6.48285	0.757738	0.668453
B.cells	ZXDB	-0.12032	3.1887	-0.62372	0.534419	-5.90364	0.788564	0.716869
B.cells	PIK3AP1	0.087042	8.295612	0.623649	0.534464	-6.88723	0.727281	0.621888
B.cells	COL23A1	-0.27674	1.376373	-0.62349	0.534565	-5.39486	0.811753	0.753941
B.cells	MIR142HG	0.05734	8.019491	0.623452	0.534593	-6.81771	0.730447	0.626685
B.cells	TMCO6	0.151677	2.683081	0.623017	0.534877	-5.62867	0.794955	0.727213
B.cells	TM2D3	0.08708	4.227452	0.622963	0.534912	-6.14481	0.775621	0.696645
B.cells	RNF8	-0.07052	4.800789	-0.62292	0.534944	-6.31715	0.768584	0.685615
B.cells	EPB41L3	-0.29332	2.334313	-0.62291	0.534949	-5.56152	0.799399	0.734325
B.cells	BC017158	-0.24075	1.449949	-0.62277	0.535036	-5.42037	0.810796	0.752603
B.cells	TMED7	0.049609	6.308978	0.62276	0.535045	-6.54076	0.750428	0.657423
B.cells	NBEAL2	0.1526	2.635242	0.622745	0.535055	-5.60385	0.795563	0.728204
B.cells	MCTP1	-0.19993	5.535347	-0.62261	0.535144	-6.15956	0.759677	0.671755
B.cells	POLR3F	0.094763	3.882542	0.622491	0.535221	-6.05936	0.779891	0.703407
B.cells	MGMT	0.160635	3.273792	0.622365	0.535304	-5.77022	0.787494	0.715454
B.cells	INTU	-0.40309	0.880465	-0.62219	0.535418	-5.18678	0.818234	0.764723
B.cells	LYAR	0.09711	4.937131	0.622107	0.535472	-6.25128	0.766921	0.683149
B.cells	CBX8	-0.21963	1.617418	-0.62201	0.535537	-5.48832	0.808623	0.749272
B.cells	GM11707	0.274647	0.863582	0.621911	0.535601	-5.31178	0.818456	0.765165
B.cells	HIST1H2BN	-0.24998	2.139918	-0.62189	0.535612	-5.51814	0.801888	0.738499
B.cells	RABGGTB	0.102316	3.732854	0.62179	0.53568	-6.00695	0.781752	0.70649
B.cells	ASPM	-0.20291	3.870548	-0.62157	0.535822	-5.97532	0.78004	0.703851
B.cells	SKI	-0.07952	5.372605	-0.62152	0.535859	-6.34701	0.76164	0.675037
B.cells	ECM1	-0.2085	3.680244	-0.62133	0.535979	-5.97722	0.782408	0.707683
B.cells	S100PBP	0.080365	4.405401	0.621319	0.535989	-6.15824	0.773429	0.693542
B.cells	CTSH	0.089187	5.779307	0.621275	0.536017	-6.51526	0.756746	0.667517
B.cells	PCK2	0.132459	3.244845	0.621204	0.536064	-5.92773	0.787858	0.716333
B.cells	CCDC146	-0.20861	2.867299	-0.62103	0.536179	-5.64332	0.79262	0.723973
B.cells	2310057M	-0.12475	2.710086	-0.62087	0.536279	-5.70678	0.794612	0.727173
B.cells	TRIP10	0.196114	1.355077	0.620767	0.53635	-5.46912	0.81203	0.755091
B.cells	2010007H(	0.212494	0.459338	0.620758	0.536356	-5.42334	0.823785	0.774115

B.cells	ETV1	0.256076	1.377482	0.620725	0.536378	-5.38222	0.811738	0.754637
B.cells	OSCP1	-0.2085	3.25254	-0.62071	0.536386	-5.70446	0.787761	0.716296
B.cells	GM6377	-0.15463	3.091942	-0.62062	0.536447	-5.90473	0.789782	0.719503
B.cells	KRT81	0.346078	-1.53173	0.620614	0.53645	-5.07592	0.850361	0.818079
B.cells	GM48768	0.288078	0.621002	0.620497	0.536527	-5.25943	0.821649	0.770678
B.cells	ETFBKMT	-0.16027	2.261696	-0.62048	0.536539	-5.66119	0.800328	0.736327
B.cells	ATP1A1	0.069549	6.234757	0.62047	0.536544	-6.51441	0.751309	0.659246
B.cells	SLC12A3	-0.21654	1.874484	-0.62034	0.536632	-5.6094	0.805302	0.744297
B.cells	GRHPR	0.119167	4.431109	0.620273	0.536674	-6.18343	0.773113	0.693198
B.cells	SLC12A2	-0.14059	3.544469	-0.62018	0.536737	-5.9872	0.784103	0.710541
B.cells	ACTA2	0.306153	3.320545	0.620123	0.536772	-5.88416	0.786907	0.714995
B.cells	VAC14	0.070024	4.624685	0.619878	0.536933	-6.22999	0.77076	0.689612
B.cells	CPLANE2	-0.32499	0.331465	-0.61976	0.537013	-5.20295	0.825503	0.777119
B.cells	IGSF8	-0.1041	4.266842	-0.61973	0.53703	-6.04687	0.775158	0.696568
B.cells	FXN	-0.09291	4.085717	-0.61969	0.537056	-6.06416	0.777395	0.700089
B.cells	RENBP	-0.09124	4.474184	-0.61916	0.537406	-6.24052	0.772865	0.692704
B.cells	BTF3L4	0.079283	4.269235	0.619134	0.53742	-6.13075	0.775388	0.696669
B.cells	SEC62	0.03824	7.437896	0.619107	0.537438	-6.73836	0.737438	0.637846
B.cells	IRS2	0.096768	5.572327	0.618867	0.537595	-6.46112	0.759636	0.671906
B.cells	SYF2	-0.05161	5.903221	-0.61876	0.537667	-6.47305	0.75567	0.665771
B.cells	LRRCC1	-0.08665	3.748019	-0.61844	0.537877	-6.02115	0.782194	0.707027
B.cells	ABCB1A	-0.29486	2.010725	-0.61796	0.53819	-5.4942	0.804473	0.742176
B.cells	SMAD6	-0.2351	2.733914	-0.61787	0.53825	-5.64313	0.795225	0.727427
B.cells	STAU1	0.064351	6.034578	0.617817	0.538284	-6.50979	0.754562	0.663591
B.cells	KHDRBS3	-0.27683	1.943216	-0.61754	0.538464	-5.39679	0.805343	0.743705
B.cells	FYB	0.179061	6.869674	0.617105	0.538751	-6.1673	0.744665	0.648494
B.cells	CMIP	-0.05989	9.635584	-0.6171	0.538752	-7.11191	0.712968	0.600455
B.cells	PFKM	-0.2465	1.17862	-0.61697	0.53884	-5.34598	0.815269	0.759803
B.cells	SNHG5	0.179053	2.416156	0.616767	0.538973	-5.62094	0.799274	0.734184
B.cells	SCRN2	-0.20102	1.43712	-0.61661	0.539078	-5.39104	0.811897	0.754495
B.cells	TMOD3	0.056965	7.218311	0.616545	0.539119	-6.72024	0.740578	0.642371
B.cells	ZFP592	0.054417	5.89966	0.616537	0.539124	-6.4984	0.756175	0.666391
B.cells	FGF13	0.173261	3.379693	0.616457	0.539177	-6.01618	0.787071	0.714828
B.cells	SLAIN2	0.060909	5.670672	0.616269	0.539301	-6.42177	0.758923	0.670675
B.cells	TEX10	-0.08306	5.266363	-0.61625	0.539313	-6.36673	0.763803	0.678267
B.cells	PPAT	0.103508	4.107428	0.616156	0.539375	-6.08216	0.777999	0.700539
B.cells	WAC	-0.04712	7.414892	-0.61603	0.539455	-6.72906	0.738286	0.638953
B.cells	NQO1	-0.28652	0.792254	-0.61598	0.53949	-5.31265	0.820338	0.76824
B.cells	XYLT1	0.06979	7.355246	0.615943	0.539515	-6.91362	0.738981	0.64002
B.cells	TAF13	-0.08705	4.566883	-0.61583	0.539586	-6.20828	0.772335	0.691711
B.cells	ABRAXAS1	0.167443	2.604808	0.615292	0.539943	-5.67691	0.796867	0.730701
B.cells	DNAJA2	0.045258	7.208069	0.615244	0.539974	-6.71041	0.740698	0.642825
B.cells	GEMIN5	0.106114	3.385628	0.615242	0.539975	-5.99017	0.786997	0.714999
B.cells	GOLGA7	0.056063	5.94	0.615203	0.540001	-6.49977	0.755692	0.665924
B.cells	GM553	0.317482	0.241543	0.614961	0.54016	-5.2448	0.827626	0.780361
B.cells	TFIP11	0.117121	3.322157	0.614921	0.540187	-5.89453	0.787794	0.716318
B.cells	KAT7	0.059825	5.68662	0.614833	0.540244	-6.43842	0.758731	0.670719

B.cells	AGFG1	-0.05727	6.147106	-0.6148	0.540269	-6.53724	0.753219	0.662191
B.cells	ACRBP	-0.22222	1.566441	-0.61463	0.540375	-5.4199	0.810217	0.752227
B.cells	6030443J0	0.283325	0.848488	0.614592	0.540403	-5.25544	0.819598	0.7674
B.cells	ARL4C	-0.11143	5.243589	-0.61449	0.540467	-6.1424	0.76408	0.679117
B.cells	HINT2	-0.09407	4.356845	-0.61433	0.540576	-6.10991	0.774918	0.696149
B.cells	NFKBID	-0.08218	6.350031	-0.61428	0.54061	-6.55539	0.750805	0.658572
B.cells	PPP2R3D	-0.11145	3.751015	-0.61417	0.54068	-6.02715	0.782427	0.707988
B.cells	ZFP518A	0.110814	3.684945	0.614104	0.540724	-5.97363	0.783251	0.709291
B.cells	TRUB1	0.186473	1.825881	0.614103	0.540724	-5.49534	0.806857	0.746935
B.cells	GM48226	0.155511	2.396833	0.614004	0.540789	-5.66973	0.799521	0.735171
B.cells	FLT3	-0.14177	2.584668	-0.61396	0.54082	-5.96512	0.797124	0.73134
B.cells	SUCLA2	-0.06314	5.593683	-0.61395	0.540826	-6.4356	0.759849	0.672587
B.cells	HIGD2A	0.051485	6.065754	0.613948	0.540826	-6.50736	0.754189	0.663805
B.cells	VPS13D	0.070637	6.149191	0.61393	0.540838	-6.49441	0.753194	0.662265
B.cells	CLEC2D	0.142671	4.7558	0.613896	0.54086	-6.21787	0.77002	0.688461
B.cells	AGRN	0.3004	1.254784	0.613859	0.540885	-5.31039	0.814274	0.758888
B.cells	SEC23IP	0.066922	4.499732	0.613398	0.541188	-6.19665	0.773246	0.693433
B.cells	UNKL	-0.10812	3.970057	-0.61332	0.541239	-6.06171	0.779789	0.70375
B.cells	ARMCX4	0.302641	1.19399	0.613257	0.541281	-5.26567	0.815159	0.760253
B.cells	RILPL1	-0.2196	2.31814	-0.61317	0.54134	-5.44954	0.800616	0.736893
B.cells	GPR146	0.123227	3.278936	0.613103	0.541382	-5.86086	0.788425	0.717458
B.cells	ERN1	-0.07813	7.053542	-0.61302	0.541436	-6.64599	0.742589	0.645902
B.cells	ODR4	-0.096	4.042451	-0.61301	0.541444	-6.05839	0.778891	0.70238
B.cells	BORCS7	0.093984	3.716689	0.612951	0.541483	-6.05377	0.782942	0.708796
B.cells	GM32743	-0.33104	-0.48509	-0.61271	0.541643	-5.18679	0.837541	0.796626
B.cells	CENPI	-0.183	2.997583	-0.61253	0.541759	-5.7018	0.792085	0.723169
B.cells	RPRD1A	0.078641	4.057705	0.612527	0.541761	-6.1074	0.778813	0.702147
B.cells	NDUFAF2	-0.089	4.440034	-0.61222	0.541965	-6.14544	0.774286	0.694763
B.cells	GM46652	0.379851	-0.73309	0.611941	0.542148	-5.09316	0.841131	0.802285
B.cells	ZSCAN20	0.309342	1.073033	0.611701	0.542306	-5.29569	0.817311	0.763121
B.cells	NRXN1	-0.25912	2.142242	-0.61138	0.542514	-5.64882	0.803432	0.740897
B.cells	CWF19L1	-0.18146	2.200341	-0.61137	0.542526	-5.58326	0.802685	0.739706
B.cells	PPP1R42	0.324807	0.580161	0.611198	0.542637	-5.27098	0.823801	0.773836
B.cells	SFT2D1	0.052982	5.999884	0.611034	0.542745	-6.49845	0.755587	0.665546
B.cells	RABGGTA	0.129696	2.703471	0.610963	0.542791	-5.74471	0.796256	0.7295
B.cells	GBA2	0.134445	2.547922	0.610935	0.54281	-5.70415	0.798237	0.732663
B.cells	AMZ2	-0.09678	3.901939	-0.61093	0.542815	-5.99014	0.78118	0.705574
B.cells	TRAF3IP2	0.115996	3.229202	0.610922	0.542819	-5.93471	0.789601	0.718908
B.cells	GM43466	-0.24516	1.497294	-0.61075	0.542929	-5.37424	0.811771	0.754381
B.cells	MARK2	0.050255	6.879023	0.610629	0.543012	-6.65654	0.745157	0.649477
B.cells	HBQ1A	0.279289	-1.39639	0.610583	0.543042	-5.19884	0.850151	0.817488
B.cells	HES1	-0.11576	5.161335	-0.61054	0.54307	-6.29646	0.765696	0.681293
B.cells	SLC7A7	0.111251	3.785978	0.610052	0.543392	-6.12482	0.782704	0.707958
B.cells	MYDGF	-0.07698	4.485984	-0.60997	0.543447	-6.1559	0.774033	0.694337
B.cells	RNF6	-0.05973	5.670645	-0.60993	0.543472	-6.45173	0.759614	0.671822
B.cells	ASMT	0.250671	1.052723	0.609861	0.543518	-5.42096	0.81766	0.76393
B.cells	STRN	0.07706	5.530644	0.609561	0.543716	-6.37717	0.761302	0.67446

B.cells	TMEM141	-0.22488	2.239657	-0.60956	0.543718	-5.58711	0.802262	0.739149
B.cells	4930578M	-0.26671	0.63262	-0.60954	0.543733	-5.32147	0.823192	0.772908
B.cells	ECD	0.071025	4.899349	0.609109	0.544014	-6.26795	0.768966	0.686551
B.cells	ZBTB41	0.130039	2.915286	0.60869	0.544291	-5.77374	0.793648	0.725646
B.cells	PGM2	0.095041	4.115633	0.608655	0.544314	-6.0935	0.778606	0.701808
B.cells	NCSTN	0.06992	5.576404	0.608444	0.544453	-6.41547	0.76075	0.673841
B.cells	POLR3C	0.063909	5.018329	0.60838	0.544496	-6.33307	0.767514	0.684392
B.cells	PADI4	-0.29555	1.890862	-0.60818	0.544624	-5.50683	0.806752	0.746617
B.cells	RPRD2	0.064655	5.916624	0.608159	0.544641	-6.4964	0.756661	0.667488
B.cells	SMARCA2	0.062836	6.123463	0.608118	0.544668	-6.53314	0.754187	0.663655
B.cells	ATP5K	-0.0705	7.486519	-0.60808	0.544694	-6.73385	0.738125	0.638944
B.cells	GM20069	-0.33863	-0.52038	-0.60806	0.544703	-5.19086	0.838546	0.798324
B.cells	NCAPG2	-0.12167	5.068495	-0.60796	0.544772	-6.17081	0.766903	0.683437
B.cells	TENT5A	0.091593	5.169086	0.607948	0.54478	-6.35984	0.76568	0.681525
B.cells	GM47448	-0.46458	-0.89373	-0.60792	0.5448	-5.03657	0.843385	0.806631
B.cells	TCERG1	0.052309	6.296507	0.607795	0.544882	-6.56075	0.752125	0.660466
B.cells	ANXA2	0.108616	5.910919	0.607742	0.544917	-6.37923	0.756729	0.667594
B.cells	SLFN3	0.256732	1.4477	0.607483	0.545088	-5.34037	0.812499	0.755931
B.cells	PLSCR4	0.323352	0.494112	0.60745	0.545109	-5.22226	0.825025	0.776224
B.cells	ATF6	-0.05701	6.979377	-0.60742	0.545127	-6.71548	0.744053	0.648079
B.cells	CALM1	-0.04331	9.782329	-0.60733	0.545186	-7.12316	0.71198	0.599482
B.cells	HELLS	-0.13397	4.919254	-0.60727	0.545229	-6.23583	0.768723	0.686371
B.cells	CYP4A14	0.260514	1.833194	0.607254	0.545239	-5.58398	0.807498	0.747915
B.cells	PRR5L	-0.23542	2.756439	-0.60711	0.545334	-5.62962	0.795663	0.728996
B.cells	SUPT20	-0.0679	5.30473	-0.60711	0.545334	-6.35419	0.764034	0.679084
B.cells	LMF2	0.140532	3.103696	0.607016	0.545396	-5.7616	0.791264	0.721997
B.cells	PPBP	0.339747	0.842206	0.60692	0.54546	-5.54327	0.820427	0.768865
B.cells	PTPMT1	0.084851	4.267152	0.606908	0.545467	-6.11462	0.776731	0.699008
B.cells	CDKL2	0.2602	1.163972	0.606894	0.545476	-5.36962	0.816203	0.762022
B.cells	CHST8	-0.47603	-0.80735	-0.60689	0.54548	-5.0501	0.842254	0.80488
B.cells	XRCC1	0.094864	4.144273	0.606744	0.545576	-6.1221	0.778252	0.701454
B.cells	FCGR1	-0.25253	2.432362	-0.60644	0.545777	-5.60671	0.799795	0.735728
B.cells	KLHL25	-0.15038	2.493825	-0.60637	0.545825	-5.64195	0.79901	0.734505
B.cells	MTX3	-0.27824	0.591443	-0.60621	0.545927	-5.32916	0.823737	0.774408
B.cells	TAGAP	0.133574	4.269177	0.606158	0.545963	-6.16914	0.776707	0.699123
B.cells	USF1	-0.08306	4.934416	-0.60613	0.545981	-6.31126	0.768538	0.686306
B.cells	MYH13	-0.36091	-1.61994	-0.60606	0.546027	-4.95754	0.853334	0.823434
B.cells	ICOSL	0.174161	1.327034	0.605911	0.546126	-5.65178	0.814073	0.758815
B.cells	KDSR	0.096205	3.963038	0.605823	0.546184	-6.04075	0.780501	0.705173
B.cells	ASF1B	-0.14156	4.954931	-0.60577	0.546217	-6.21147	0.768288	0.68598
B.cells	C330007PC	-0.07612	5.50907	-0.60562	0.546319	-6.36012	0.761563	0.675519
B.cells	PARP1	0.080062	5.30881	0.605395	0.546468	-6.37564	0.763986	0.679309
B.cells	BAG1	0.051614	6.693589	0.605349	0.546498	-6.62013	0.74742	0.653618
B.cells	PAPOLG	0.125789	3.005898	0.605313	0.546521	-5.86787	0.792501	0.724277
B.cells	HMGA2	-0.50837	1.149583	-0.60531	0.546527	-5.18929	0.816392	0.762642
B.cells	USPL1	0.103672	3.533846	0.605132	0.546641	-5.95852	0.785856	0.713776
B.cells	GM20707	0.158746	1.898927	0.605009	0.546723	-5.54047	0.806649	0.747023

B.cells	GM26787	-0.29579	1.300668	-0.60492	0.546781	-5.34288	0.814417	0.759565
B.cells	JMY	-0.07637	6.282387	-0.6049	0.546796	-6.56577	0.752294	0.661255
B.cells	CHPF2	-0.15117	2.64648	-0.60485	0.546825	-5.75203	0.797063	0.731685
B.cells	CREG1	-0.07756	7.514034	-0.6046	0.546997	-6.73008	0.737892	0.639051
B.cells	ZFP867	0.255861	0.61031	0.604505	0.547056	-5.26329	0.823584	0.774423
B.cells	DUSP10	-0.10268	4.392133	-0.60439	0.547134	-6.24654	0.775281	0.697128
B.cells	TYSND1	0.127791	2.640407	0.604161	0.547284	-5.70409	0.797234	0.73207
B.cells	SAP18	0.051559	7.043708	0.603971	0.547409	-6.6595	0.743386	0.647679
B.cells	GM29114	0.313034	-1.28439	0.603943	0.547428	-5.09474	0.848786	0.816434
B.cells	NLRC4	0.095856	3.885651	0.60394	0.54743	-6.22346	0.781555	0.707199
B.cells	GM12359	-0.18864	1.671224	-0.60391	0.547449	-5.51755	0.809691	0.752147
B.cells	TPM1	0.089729	5.177563	0.603654	0.547619	-6.38201	0.765668	0.682297
B.cells	LIMD1	-0.05261	6.016318	-0.60358	0.547666	-6.57956	0.755557	0.666549
B.cells	ARPC1B	0.049648	8.892807	0.603542	0.547693	-6.98066	0.722062	0.615264
B.cells	MOCS2	0.078418	5.094585	0.603537	0.547697	-6.29922	0.766677	0.683875
B.cells	DNMT3B	0.14891	2.497904	0.603148	0.547955	-5.66828	0.799208	0.73529
B.cells	CTPS2	0.068532	5.066744	0.60309	0.547993	-6.39126	0.767165	0.684564
B.cells	APEX2	0.08894	4.215093	0.602961	0.548078	-6.12393	0.777619	0.700987
B.cells	SMIM19	0.079839	4.75168	0.60292	0.548105	-6.19355	0.771013	0.690595
B.cells	SRSF9	0.051306	6.428408	0.602868	0.54814	-6.57569	0.750794	0.659098
B.cells	AP1M1	0.070994	4.979621	0.602315	0.548506	-6.294	0.76851	0.686354
B.cells	SYDE1	0.298685	0.308678	0.60225	0.548549	-5.1658	0.82805	0.781592
B.cells	GM31323	-0.22895	0.948728	-0.60219	0.548591	-5.37456	0.819584	0.767858
B.cells	GLS	0.060547	7.7257	0.601872	0.548799	-6.78405	0.735849	0.635956
B.cells	RND1	-0.2597	1.145338	-0.60182	0.548831	-5.35746	0.817003	0.763751
B.cells	CAMK1D	-0.06505	8.628354	-0.60179	0.548855	-6.99154	0.725475	0.62018
B.cells	FARP2	-0.1567	2.6131	-0.60167	0.548936	-5.75914	0.798031	0.73323
B.cells	RUNX3	0.092215	5.712717	0.601539	0.54902	-6.52745	0.759626	0.672652
B.cells	SMC5	0.067079	5.613071	0.601519	0.549033	-6.47593	0.760826	0.67452
B.cells	GM47469	-0.30061	0.520329	-0.60139	0.549118	-5.24879	0.82524	0.777243
B.cells	B430010I2:	-0.34117	-0.41456	-0.60137	0.549132	-5.10755	0.837737	0.797674
B.cells	XPO5	0.102951	3.603677	0.601345	0.549149	-5.96681	0.785515	0.713402
B.cells	ATL2	0.064954	5.337211	0.60098	0.549391	-6.3713	0.764334	0.679913
B.cells	CRTC2	-0.10078	4.134514	-0.60095	0.549408	-6.13246	0.77908	0.703062
B.cells	TCF7L2	-0.09837	7.34951	-0.60075	0.549545	-6.71299	0.740397	0.642945
B.cells	RAB11B	0.04674	7.147669	0.600746	0.549546	-6.70677	0.742758	0.646568
B.cells	ADM	-0.25735	1.330377	-0.60047	0.549731	-5.51609	0.814849	0.760243
B.cells	GSAP	0.122595	5.642393	0.600375	0.549792	-6.28049	0.760721	0.674294
B.cells	RSPO3	-0.49042	0.723734	-0.60036	0.5498	-5.20321	0.822818	0.773167
B.cells	AHCY	-0.13315	3.495963	-0.60023	0.549889	-5.93039	0.787154	0.715811
B.cells	ASPSCR1	0.073329	4.685187	0.599871	0.550127	-6.24772	0.77244	0.692519
B.cells	ZFP940	-0.22859	0.734633	-0.59982	0.550161	-5.33863	0.822755	0.772949
B.cells	IRS1	-0.23768	0.821216	-0.59944	0.550413	-5.43882	0.821612	0.771213
B.cells	NDUFB4	0.058966	6.598671	0.59925	0.550539	-6.58743	0.749369	0.656759
B.cells	CLIC4	-0.09064	7.449524	-0.59921	0.550568	-6.79004	0.739373	0.641401
B.cells	FTSJ1	-0.10544	3.078297	-0.5991	0.550638	-5.83326	0.792462	0.724405
B.cells	PTP4A1	0.247115	1.179279	0.599061	0.550664	-5.44679	0.816906	0.763683

B.cells	JAK3	0.159457	2.778845	0.599054	0.550669	-5.75396	0.796259	0.730465
B.cells	SWSAP1	-0.31516	0.278314	-0.59901	0.550699	-5.18943	0.828807	0.783037
B.cells	IFT22	-0.10813	3.654091	-0.5987	0.550902	-6.04524	0.785218	0.713074
B.cells	TDG	0.068938	5.074746	0.598612	0.550962	-6.33329	0.767676	0.685445
B.cells	PSMD13	0.057102	6.203145	0.59844	0.551076	-6.5294	0.754071	0.66429
B.cells	N4BP2	0.077935	5.310122	0.598372	0.551122	-6.38418	0.764815	0.681023
B.cells	ADAMTS14	-0.27231	0.543497	-0.59837	0.551125	-5.36257	0.825283	0.777575
B.cells	2010016I1	-0.31437	-0.03474	-0.59834	0.551146	-5.17266	0.832988	0.790168
B.cells	PMPCA	0.096483	4.024403	0.598237	0.551211	-6.06963	0.780601	0.705866
B.cells	PIGO	0.199949	1.804475	0.598179	0.55125	-5.53691	0.808763	0.750841
B.cells	TNFRSF19	0.249414	0.886333	0.598122	0.551288	-5.42161	0.820754	0.770256
B.cells	GM16552	-0.18836	1.517	-0.59808	0.551317	-5.49038	0.812496	0.756876
B.cells	DHX29	-0.10653	3.415036	-0.59799	0.551376	-5.94525	0.788216	0.71796
B.cells	2700062C0	0.125167	2.646093	0.597983	0.55138	-5.76574	0.79795	0.733485
B.cells	RTCA	-0.08578	4.282973	-0.59784	0.551477	-6.16849	0.777396	0.700858
B.cells	TAPT1	0.078962	5.802801	0.597799	0.551502	-6.43875	0.758865	0.671839
B.cells	FAM129C	-0.11024	3.433215	-0.59775	0.551537	-6.02269	0.787988	0.717672
B.cells	GCLM	-0.07593	6.090034	-0.59737	0.551788	-6.55443	0.755636	0.66658
B.cells	OTUB1	0.051077	5.728978	0.597309	0.551828	-6.44671	0.759968	0.673314
B.cells	RAB3A	-0.15623	2.619056	-0.59713	0.55195	-5.74666	0.7986	0.734191
B.cells	CENPH	-0.18456	3.059161	-0.59691	0.552093	-5.71991	0.793027	0.725257
B.cells	RAB5IF	0.054132	7.307335	0.596634	0.552276	-6.71087	0.741334	0.644422
B.cells	PPP3R1	0.06219	5.5618	0.5965	0.552366	-6.40716	0.762079	0.676548
B.cells	ZFP930	0.158068	2.204594	0.596468	0.552387	-5.67204	0.803929	0.742814
B.cells	RAPGEF6	0.051633	8.049527	0.596381	0.552445	-6.84934	0.732717	0.631321
B.cells	DNMT1	0.09157	5.753517	0.59619	0.552572	-6.39747	0.759767	0.673033
B.cells	HSD17B11	0.077506	4.796644	0.596154	0.552596	-6.30058	0.771388	0.691213
B.cells	CACTIN	-0.09818	3.991663	-0.59613	0.552611	-6.08648	0.781326	0.706885
B.cells	RSPH1	0.257729	1.855808	0.596097	0.552633	-5.58099	0.808428	0.750179
B.cells	ZC3HC1	-0.07986	4.34425	-0.59597	0.552715	-6.19064	0.776955	0.700061
B.cells	TYW3	-0.2491	0.995715	-0.59575	0.552863	-5.33409	0.819648	0.768407
B.cells	SLC16A10	0.09831	7.024829	0.595711	0.55289	-6.68028	0.744646	0.649731
B.cells	APPL1	-0.06992	5.974209	-0.59563	0.552946	-6.51939	0.757116	0.669016
B.cells	1810021B2	-0.38323	0.427969	-0.59562	0.552949	-5.13613	0.827153	0.78062
B.cells	GRIK5	-0.35977	0.167717	-0.59558	0.552974	-5.17156	0.830619	0.786293
B.cells	GM13610	-0.24613	0.396658	-0.59532	0.553151	-5.33534	0.827594	0.781376
B.cells	CSF1R	-0.16732	5.549681	-0.5952	0.553231	-6.28428	0.762248	0.677052
B.cells	TBCA	-0.05426	7.625569	-0.59507	0.553316	-6.7726	0.737647	0.63905
B.cells	KAT5	0.142244	2.921057	0.594974	0.55338	-5.81342	0.7948	0.728463
B.cells	GINS4	-0.10628	3.753755	-0.59497	0.553382	-5.98032	0.784315	0.711778
B.cells	CCL9	-0.17233	2.831707	-0.59495	0.553395	-5.8436	0.795935	0.730276
B.cells	MBTPS1	-0.07076	4.639311	-0.59474	0.553535	-6.25646	0.77339	0.694452
B.cells	GM26749	0.144877	2.222722	0.594506	0.553692	-5.75962	0.80377	0.742755
B.cells	CDKL5	-0.20503	2.066071	-0.59447	0.553714	-5.62577	0.805787	0.745997
B.cells	IP6K1	0.063101	6.598351	0.594472	0.553715	-6.60993	0.749748	0.65759
B.cells	MSI2	0.05401	7.509903	0.594392	0.553768	-6.78627	0.739039	0.641114
B.cells	SLC46A3	-0.13179	3.085031	-0.59421	0.553889	-5.85395	0.792773	0.725197

B.cells	RAB18	0.055731	5.748093	0.594131	0.553942	-6.45281	0.759903	0.673385
B.cells	MYH9	0.047323	7.80338	0.594055	0.553992	-6.81778	0.73563	0.635944
B.cells	PAK1IP1	0.049789	5.901653	0.593871	0.554115	-6.49321	0.758057	0.670576
B.cells	RBBP5	-0.10827	3.457865	-0.5938	0.554162	-5.95082	0.788072	0.717781
B.cells	COL5A2	-0.31869	1.225753	-0.59379	0.554171	-5.31137	0.816706	0.763749
B.cells	HS6ST1	-0.09773	4.156867	-0.59343	0.554405	-6.10027	0.779582	0.70399
B.cells	ATG4D	0.090433	3.954415	0.593181	0.554574	-6.11497	0.782132	0.708032
B.cells	ADAM9	0.071899	5.178783	0.593084	0.554639	-6.43044	0.767058	0.684307
B.cells	ZFP267	-0.18595	1.251689	-0.59301	0.55469	-5.44662	0.816648	0.763308
B.cells	ZFP609	-0.0596	6.067152	-0.59299	0.554699	-6.55303	0.756334	0.66759
B.cells	ANKRD40	0.082433	4.238658	0.592577	0.554977	-6.13236	0.778812	0.702612
B.cells	RNF19A	0.071968	5.375931	0.592558	0.554989	-6.38802	0.764869	0.680721
B.cells	S1PR1	0.113803	4.061514	0.592467	0.55505	-6.19249	0.78101	0.706097
B.cells	TSPAN3	0.091675	4.739337	0.591942	0.5554	-6.27686	0.773009	0.693022
B.cells	KMT5B	0.059036	5.91383	0.591863	0.555453	-6.50831	0.758742	0.670743
B.cells	PRKAR1B	0.377227	-0.37005	0.591297	0.55583	-5.14417	0.839201	0.798882
B.cells	SUPT4A	-0.05824	6.836136	-0.59123	0.555877	-6.68966	0.748086	0.653809
B.cells	MED15	0.063621	5.758733	0.591042	0.556	-6.49128	0.760944	0.673797
B.cells	GAPVD1	0.051047	6.832806	0.590984	0.556039	-6.64712	0.748125	0.653953
B.cells	PIK3CG	-0.07537	4.936727	-0.59075	0.556197	-6.351	0.770929	0.689471
B.cells	A330032B1	0.387715	-0.15579	0.590668	0.55625	-5.15084	0.836313	0.794349
B.cells	RANBP6	0.189308	1.620455	0.590624	0.556279	-5.46198	0.812805	0.756114
B.cells	NFE2L1	0.082913	4.98448	0.590517	0.556351	-6.31161	0.770345	0.688562
B.cells	GZMA	0.415678	4.671597	0.590455	0.556392	-5.97982	0.774183	0.694598
B.cells	EFCAB5	-0.30905	0.504613	-0.59043	0.556412	-5.23971	0.827483	0.779959
B.cells	RBM43	0.127518	2.961078	0.590383	0.55644	-5.84195	0.795565	0.728495
B.cells	SCG5	-0.31256	0.801405	-0.59027	0.556513	-5.26877	0.823557	0.773578
B.cells	RCOR3	0.130358	2.937283	0.590141	0.556601	-5.81421	0.795875	0.728982
B.cells	CNNM4	-0.08774	4.077084	-0.59007	0.556647	-6.11826	0.781545	0.706229
B.cells	TG	0.174395	1.91376	0.589853	0.556793	-5.61016	0.809118	0.750115
B.cells	ZFP511	-0.0823	3.816412	-0.5893	0.557164	-6.06452	0.785323	0.711644
B.cells	BCAM	-0.46435	0.298097	-0.58921	0.557224	-5.17581	0.830799	0.78479
B.cells	MIIP	0.129212	3.057652	0.588945	0.5574	-5.77001	0.795035	0.726965
B.cells	RSAD1	-0.2657	1.298807	-0.58863	0.557609	-5.4381	0.817888	0.763438
B.cells	ATRX	0.049781	7.666179	0.588572	0.557649	-6.78613	0.739154	0.639493
B.cells	PRG4	-0.57532	1.084056	-0.58816	0.557922	-5.46031	0.821011	0.768188
B.cells	NEGR1	-0.38821	-0.13284	-0.58805	0.557998	-5.18329	0.83723	0.794597
B.cells	P2RX7	-0.17732	3.223954	-0.58757	0.558321	-5.75141	0.793758	0.723988
B.cells	EMG1	-0.06491	5.937655	-0.58726	0.558529	-6.49882	0.76037	0.671327
B.cells	ZBED4	0.081729	4.650997	0.587232	0.558545	-6.17859	0.776049	0.69581
B.cells	COX8A	0.045216	9.008145	0.5865	0.559034	-6.98113	0.724918	0.616585
B.cells	GM20536	0.138273	2.340392	0.586485	0.559044	-5.76932	0.805691	0.742318
B.cells	RNASEH2A	0.088064	4.54522	0.585892	0.559441	-6.22577	0.778295	0.698256
B.cells	CARMIL1	0.11287	4.075468	0.585788	0.55951	-6.07873	0.784132	0.707465
B.cells	OVGP1	-0.28574	0.492644	-0.58565	0.559602	-5.19122	0.830369	0.781633
B.cells	E230032D2	0.213831	1.879678	0.585614	0.559627	-5.47876	0.812104	0.752092
B.cells	TMEM173	0.088116	4.250581	0.585454	0.559734	-6.20338	0.782005	0.704119

B.cells	ABCD3	-0.09282	4.193442	-0.58482	0.560161	-6.10365	0.783055	0.705501
B.cells	COMTD1	0.138358	2.92368	0.584781	0.560184	-5.69665	0.799064	0.730876
B.cells	4921509O(	0.367548	0.064578	0.584765	0.560195	-5.17077	0.83652	0.791318
B.cells	HAUS2	0.097889	3.755191	0.584688	0.560246	-6.07471	0.788537	0.714159
B.cells	MPST	-0.10525	3.682692	-0.58421	0.560564	-6.03271	0.789561	0.715714
B.cells	6230400D1	0.154455	2.163361	0.584154	0.560604	-5.66806	0.808948	0.746623
B.cells	FAS	0.137172	3.936884	0.584122	0.560626	-6.02849	0.786371	0.710677
B.cells	PPP6R3	0.048896	7.283025	0.583994	0.560711	-6.72998	0.745756	0.647489
B.cells	SDC1	-0.20821	2.216388	-0.58398	0.560719	-5.71422	0.808262	0.74555
B.cells	CHST10	0.210023	0.375693	0.583876	0.56079	-5.50044	0.832467	0.784703
B.cells	SPARC	-0.14284	5.322477	-0.58373	0.560887	-6.31078	0.769244	0.683854
B.cells	BCKDK	0.078226	4.559456	0.583707	0.560904	-6.16909	0.778621	0.698536
B.cells	DNM2	-0.04776	7.193868	-0.58365	0.560944	-6.72416	0.746806	0.649138
B.cells	MAP3K4	-0.09223	4.074187	-0.58356	0.561004	-6.07058	0.784654	0.708076
B.cells	LZTS1	-0.24007	1.417164	-0.58338	0.561126	-5.40265	0.81875	0.762451
B.cells	SPATA6	0.085784	4.52297	0.583031	0.561356	-6.28466	0.779337	0.699456
B.cells	TMED1	0.150544	2.452472	0.582797	0.561513	-5.65567	0.80549	0.741013
B.cells	METTL26	-0.08896	4.639923	-0.58279	0.561516	-6.2677	0.77789	0.697252
B.cells	IDE	-0.08153	4.161112	-0.58277	0.561533	-6.05171	0.783836	0.706608
B.cells	DZIP3	-0.13986	3.257554	-0.58258	0.561662	-5.83411	0.795243	0.724643
B.cells	KIF15	-0.15012	4.90741	-0.58249	0.561718	-6.22528	0.774632	0.692119
B.cells	INPP5E	-0.14405	2.260693	-0.58236	0.561806	-5.62903	0.808006	0.745048
B.cells	STX4A	-0.06445	4.972734	-0.58232	0.561834	-6.30472	0.773829	0.690903
B.cells	HK3	0.260807	2.677613	0.582125	0.561964	-5.61964	0.802681	0.736511
B.cells	RETNLG	0.24885	3.313557	0.582074	0.561998	-5.89798	0.794574	0.723595
B.cells	KLC3	-0.32381	0.19256	-0.58184	0.562153	-5.20622	0.835335	0.789228
B.cells	CASK	0.077709	5.500078	0.581828	0.562163	-6.47181	0.767462	0.680917
B.cells	CACNA1I	0.209561	-0.17458	0.581421	0.562436	-5.29028	0.840588	0.797457
B.cells	GM36198	-0.14135	2.976425	-0.5813	0.562518	-5.80995	0.799214	0.730643
B.cells	MLEC	0.074471	5.064037	0.580969	0.56274	-6.2636	0.773148	0.689495
B.cells	MAMDC2	0.295223	0.366618	0.580955	0.562749	-5.29029	0.833389	0.785722
B.cells	RCOR2	-0.32322	0.194015	-0.58093	0.562763	-5.21721	0.835703	0.789496
B.cells	BTBD10	-0.06	5.583956	-0.58048	0.563064	-6.41637	0.767064	0.679741
B.cells	YKT6	-0.07555	4.483546	-0.58044	0.563094	-6.18854	0.78058	0.700887
B.cells	TSPAN4	-0.14217	3.356981	-0.58012	0.563307	-5.70696	0.794802	0.723241
B.cells	LFNG	-0.08979	4.413378	-0.5801	0.563322	-6.159	0.781543	0.702294
B.cells	GM5086	0.290414	-0.01663	0.58004	0.563363	-5.17432	0.838927	0.794351
B.cells	CSTF1	0.102105	3.243385	0.579647	0.563627	-5.92584	0.796261	0.725595
B.cells	ZMIZ2	0.073234	4.532649	0.579598	0.56366	-6.28984	0.780079	0.700021
B.cells	DDRGK1	0.064413	5.272107	0.579559	0.563686	-6.35851	0.770973	0.685757
B.cells	GM26520	0.20857	2.362216	0.579517	0.563714	-5.70805	0.807545	0.743601
B.cells	ARID1A	0.049975	6.658177	0.579517	0.563714	-6.61421	0.754238	0.659799
B.cells	SH3D21	-0.18973	1.99564	-0.57932	0.563847	-5.60472	0.812388	0.751298
B.cells	PARP2	0.065448	4.841287	0.578961	0.564088	-6.29523	0.776511	0.694291
B.cells	GM20457	-0.33883	-0.16272	-0.57894	0.5641	-5.12202	0.841186	0.797939
B.cells	SYTL1	-0.23074	1.59739	-0.57878	0.56421	-5.41549	0.817751	0.75997
B.cells	LARP4	0.055764	6.410341	0.578744	0.564233	-6.59374	0.75744	0.664705

B.cells	EGFL6	-0.24858	0.548891	-0.57832	0.564519	-5.40359	0.831941	0.782519
B.cells	XKR8	0.300018	0.500884	0.578131	0.564646	-5.2332	0.832669	0.783627
B.cells	POP5	0.065379	4.525116	0.577764	0.564892	-6.22938	0.781051	0.700786
B.cells	MTF2	-0.06195	5.644163	-0.57728	0.56522	-6.4387	0.767463	0.679365
B.cells	ZMIZ1OS1	0.317617	0.352543	0.577109	0.565332	-5.28509	0.835097	0.787125
B.cells	RIPK3	0.118003	3.285039	0.577078	0.565353	-5.94647	0.796796	0.725515
B.cells	AARSD1	0.103027	3.977143	0.576857	0.565502	-6.03613	0.788057	0.711693
B.cells	EPB41L4B	-0.2048	1.976143	-0.57682	0.565524	-5.56146	0.813635	0.752435
B.cells	PFN2	-0.24591	1.339141	-0.5768	0.565538	-5.41574	0.821979	0.765877
B.cells	CAT	-0.08229	6.72379	-0.57671	0.565602	-6.70641	0.754465	0.659303
B.cells	CYB561A3	-0.07514	5.573777	-0.57669	0.565615	-6.40808	0.76832	0.680753
B.cells	CTSF	0.252895	1.860603	0.576549	0.565709	-5.46613	0.815141	0.754895
B.cells	SLC12A6	-0.06538	8.133974	-0.57647	0.565765	-6.8969	0.737877	0.633988
B.cells	GM14548	-0.1823	1.47249	-0.57634	0.565848	-5.65425	0.820224	0.76312
B.cells	COX20	-0.07198	5.583215	-0.5762	0.565942	-6.41796	0.768205	0.680662
B.cells	CLCN4	0.064813	5.289066	0.576194	0.565948	-6.40401	0.771796	0.68626
B.cells	TICRR	-0.1662	3.009192	-0.57609	0.566017	-5.76441	0.800311	0.731266
B.cells	2900076A(	0.127085	2.621325	0.576071	0.566031	-5.74026	0.805284	0.739199
B.cells	NDUFA13	-0.04976	7.128419	-0.57593	0.566127	-6.69152	0.749698	0.652087
B.cells	PDE6H	0.159834	2.093346	0.575795	0.566217	-5.61571	0.812183	0.750247
B.cells	PHTF2	0.067311	5.998759	0.575685	0.566291	-6.54309	0.763241	0.672979
B.cells	GM27010	-0.1317	2.340877	-0.57554	0.566385	-5.81649	0.808991	0.745184
B.cells	SLC9A3R2	-0.18956	2.701144	-0.57547	0.566433	-5.66679	0.804347	0.737762
B.cells	LAMTOR2	-0.04833	6.577213	-0.57532	0.566539	-6.60159	0.756318	0.662324
B.cells	SNAPC1	0.088752	3.850567	0.575125	0.566667	-6.12422	0.789755	0.714615
B.cells	ANGPT1	-0.24808	0.973826	-0.57495	0.566787	-5.44451	0.826922	0.774214
B.cells	DUSP16	-0.07868	6.833204	-0.57484	0.566856	-6.72798	0.753265	0.657761
B.cells	PDHA1	-0.06956	5.115043	-0.57481	0.566882	-6.35097	0.774037	0.690006
B.cells	AP2S1	0.053007	6.932961	0.574742	0.566925	-6.65034	0.75208	0.65596
B.cells	TMEM154	0.14616	2.583653	0.574681	0.566967	-5.8395	0.805879	0.740378
B.cells	ARFGAP3	-0.10629	3.55174	-0.57455	0.567054	-5.9671	0.793524	0.720703
B.cells	ARAF	0.075612	4.478585	0.574543	0.567059	-6.16953	0.781902	0.702349
B.cells	REV1	-0.06804	5.326262	-0.57428	0.567234	-6.39916	0.771572	0.686081
B.cells	LY75	0.161307	3.332572	0.574205	0.567287	-6.06584	0.796431	0.725238
B.cells	ANO8	-0.27005	0.904446	-0.574	0.567424	-5.31293	0.828078	0.775961
B.cells	ZFP950	0.085101	4.197661	0.573715	0.567617	-6.16602	0.7858	0.708119
B.cells	CMTM7	-0.04994	7.897065	-0.57355	0.56773	-6.84789	0.741167	0.63883
B.cells	MYCL	-0.21367	1.305739	-0.57285	0.568201	-5.54752	0.823457	0.767802
B.cells	APTX	-0.10414	3.052742	-0.57276	0.568262	-5.81111	0.800764	0.731426
B.cells	PPP2R3C	-0.0773	4.333149	-0.57266	0.56833	-6.21282	0.784594	0.705833
B.cells	ZFP229	-0.30246	-0.01049	-0.57259	0.568377	-5.20377	0.841044	0.796433
B.cells	CPEB4	-0.06873	6.51399	-0.57252	0.568421	-6.57936	0.757926	0.664264
B.cells	AC166172.	-0.23332	0.939575	-0.57239	0.56851	-5.3892	0.828307	0.77571
B.cells	DUSP28	0.168742	1.99306	0.572375	0.56852	-5.56531	0.814441	0.753328
B.cells	AMD1	-0.08435	4.581194	-0.57229	0.568581	-6.23322	0.781505	0.700987
B.cells	GM2629	0.238274	0.511245	0.571973	0.568792	-5.31766	0.834231	0.785134
B.cells	ACAD12	-0.26728	0.714351	-0.57157	0.569067	-5.30152	0.831668	0.780819

B.cells	BOP1	-0.08521	4.167475	-0.57149	0.569117	-6.11613	0.787007	0.709325
B.cells	TBC1D14	-0.06231	5.680933	-0.57133	0.569227	-6.4697	0.768319	0.680126
B.cells	TSHZ2	-0.21228	3.393266	-0.57129	0.569251	-5.77279	0.796773	0.724865
B.cells	EXOSC10	-0.07284	4.93082	-0.57107	0.569403	-6.31764	0.777515	0.69461
B.cells	ELN	0.232785	2.40021	0.570964	0.569472	-5.67166	0.809507	0.74532
B.cells	ANAPC7	0.073185	4.359074	0.570844	0.569554	-6.19102	0.784612	0.705811
B.cells	DNAJB14	-0.10854	4.692261	-0.57064	0.56969	-6.06063	0.780467	0.699318
B.cells	SMC2	-0.11878	5.816825	-0.57057	0.569738	-6.41379	0.766666	0.677763
B.cells	CUTA	-0.05516	6.189562	-0.57048	0.569797	-6.52773	0.762156	0.670771
B.cells	BST2	0.112696	6.606914	0.570424	0.569837	-6.49536	0.757143	0.663023
B.cells	ZXDC	-0.08957	4.004625	-0.57037	0.569876	-6.08207	0.789049	0.712868
B.cells	SUMO2	-0.04129	8.656817	-0.57024	0.569964	-6.93838	0.73308	0.626319
B.cells	EPHA4	-0.42805	0.083978	-0.57023	0.569965	-5.07986	0.840133	0.795012
B.cells	HAUS6	0.090143	4.556972	0.5702	0.569988	-6.20416	0.782147	0.702039
B.cells	BCL11B	-0.32699	1.080616	-0.57002	0.570108	-5.39435	0.826794	0.773365
B.cells	MEGF9	0.123274	4.103779	0.569969	0.570145	-6.06223	0.787805	0.710997
B.cells	VAPB	0.075209	5.031604	0.569818	0.570246	-6.3088	0.776272	0.692897
B.cells	PEX7	0.072558	4.45855	0.569781	0.570271	-6.18678	0.783371	0.704039
B.cells	GM29394	0.252211	0.417159	0.569564	0.570418	-5.32077	0.835647	0.78785
B.cells	TMEM87A	-0.06848	5.236916	-0.5691	0.570733	-6.37632	0.773747	0.689144
B.cells	ARMC2	0.303902	-0.1641	0.569035	0.570775	-5.16516	0.843491	0.800856
B.cells	AP4S1	0.064029	4.920297	0.568991	0.570805	-6.32811	0.777645	0.695278
B.cells	KHDRBS1	-0.04003	7.151511	-0.56899	0.570806	-6.71156	0.750659	0.653403
B.cells	GBA	0.09208	4.090254	0.568872	0.570885	-6.07166	0.787974	0.711564
B.cells	PIWIL2	-0.26905	0.700548	-0.56865	0.571037	-5.38415	0.831852	0.781993
B.cells	0610043K1	-0.23981	1.133363	-0.56846	0.57116	-5.50921	0.826095	0.772648
B.cells	LYSMD4	-0.114	3.330154	-0.56835	0.571238	-5.9686	0.797575	0.726862
B.cells	ZFP777	-0.11599	2.809648	-0.56833	0.57125	-5.77751	0.804228	0.73747
B.cells	GM10762	-0.1321	2.549426	-0.56828	0.571283	-5.7559	0.807578	0.742846
B.cells	GM29282	-0.21947	1.712401	-0.56825	0.571308	-5.49243	0.818465	0.760338
B.cells	LDLRAD4	-0.08746	6.109073	-0.56819	0.571345	-6.5434	0.763127	0.672777
B.cells	LMNB2	-0.19104	2.474409	-0.56815	0.571373	-5.587	0.808547	0.744398
B.cells	NHSL2	0.212465	2.487556	0.568114	0.571398	-5.65785	0.808377	0.744126
B.cells	PNRC1	-0.05312	8.470962	-0.56804	0.571444	-6.92273	0.735224	0.629969
B.cells	FAM168A	0.05965	5.93211	0.568015	0.571465	-6.51618	0.765268	0.6761
B.cells	BCAT1	-0.27782	0.852062	-0.56796	0.571504	-5.41689	0.829832	0.778735
B.cells	GLYCTK	-0.25219	0.979545	-0.56753	0.571794	-5.32797	0.828136	0.776065
B.cells	CELF6	-0.3985	-0.70622	-0.56744	0.571853	-5.11921	0.85072	0.813259
B.cells	PSME4	0.056743	7.130098	0.567226	0.571998	-6.73069	0.750913	0.65407
B.cells	MBLAC1	0.310904	-0.19049	0.567128	0.572065	-5.11092	0.843849	0.801784
B.cells	TMEM67	0.181783	1.688091	0.567113	0.572074	-5.58185	0.818784	0.761025
B.cells	LRRC10B	-0.24379	0.595128	-0.56688	0.572229	-5.4651	0.833261	0.784498
B.cells	PRPSAP2	-0.0856	4.045546	-0.56678	0.572301	-6.09345	0.788535	0.71273
B.cells	GM50431	-0.32731	0.122176	-0.56674	0.572328	-5.19313	0.839617	0.794871
B.cells	MS4A6D	0.23632	2.866447	0.566729	0.572334	-5.73489	0.803499	0.736503
B.cells	POLR1B	0.138378	2.442843	0.566695	0.572357	-5.77753	0.808955	0.745237
B.cells	RAB24	0.064733	5.118704	0.566689	0.572362	-6.31858	0.775199	0.691757

B.cells	MFSD14A	-0.04953	6.156841	-0.56664	0.572394	-6.54648	0.76255	0.672059
B.cells	CAPN15	0.072426	4.710463	0.566516	0.572478	-6.30089	0.780241	0.699702
B.cells	UBALD2	-0.06526	7.028455	-0.56645	0.572523	-6.6772	0.752119	0.655974
B.cells	PRMT2	0.180445	1.665423	0.566373	0.572575	-5.51766	0.819081	0.761563
B.cells	KLHL13	-0.37954	0.360663	-0.56634	0.572596	-5.1634	0.836405	0.789674
B.cells	AGBL1	-0.14599	4.663698	-0.56625	0.57266	-6.30344	0.780821	0.700614
B.cells	MYOM1	-0.23214	1.071853	-0.56598	0.572844	-5.418	0.826911	0.774278
B.cells	PLAGL1	-0.24525	1.041672	-0.56595	0.572863	-5.47941	0.827311	0.774929
B.cells	RNF121	0.089574	4.41156	0.565904	0.572893	-6.19578	0.783957	0.705601
B.cells	NUP133	0.092347	3.691763	0.56589	0.572902	-6.00133	0.792991	0.71988
B.cells	PRKACA	-0.08393	4.156404	-0.56588	0.57291	-6.08696	0.787145	0.71063
B.cells	GM35867	-0.31016	0.456051	-0.56574	0.573006	-5.26472	0.835165	0.787699
B.cells	USP5	0.082573	4.34395	0.565279	0.573315	-6.19896	0.784979	0.707043
B.cells	MPHOSPH6	-0.08586	3.799303	-0.56527	0.573321	-6.03929	0.791813	0.717844
B.cells	BUB3	-0.0688	5.956108	-0.56527	0.573322	-6.47011	0.765151	0.676012
B.cells	EXOC3	0.066365	5.06994	0.564966	0.573528	-6.34999	0.775976	0.692941
B.cells	RNMT	0.065595	4.970618	0.564907	0.573568	-6.3574	0.7772	0.69486
B.cells	SPG20	0.153619	2.615271	0.56487	0.573592	-5.59217	0.806912	0.741938
B.cells	YIF1B	-0.07559	4.886916	-0.56467	0.573729	-6.26929	0.778234	0.696527
B.cells	CRACR2B	-0.28921	1.009115	-0.5646	0.573778	-5.31327	0.827931	0.775863
B.cells	SCD2	0.102275	4.726396	0.564476	0.57386	-6.25938	0.780221	0.699667
B.cells	HOOK3	0.054719	6.076111	0.564295	0.573982	-6.53815	0.763699	0.673905
B.cells	DDX31	0.107782	3.220713	0.564269	0.574	-5.91691	0.79915	0.729656
B.cells	UAP1L1	0.09875	3.734573	0.564079	0.574128	-6.00678	0.79263	0.719302
B.cells	UCK2	0.077759	5.87449	0.564049	0.574149	-6.61086	0.76614	0.677704
B.cells	GM11476	0.147768	2.536389	0.56393	0.57423	-5.76485	0.80793	0.743686
B.cells	CRPPA	-0.1612	2.552625	-0.5639	0.57425	-5.71203	0.80772	0.74335
B.cells	ELL2	0.09404	6.734248	0.5639	0.57425	-6.64054	0.755792	0.661675
B.cells	RNASET2A	-0.09638	5.439463	-0.56385	0.574284	-6.33646	0.77144	0.685962
B.cells	FXR2	0.068343	5.267562	0.563608	0.574448	-6.40474	0.773546	0.689363
B.cells	VPREB1	0.262854	2.621584	0.563562	0.574479	-5.76965	0.806831	0.742044
B.cells	FXYD2	0.338082	-0.41192	0.563517	0.574509	-5.1914	0.847054	0.807295
B.cells	PBLD2	-0.23853	0.872415	-0.56334	0.574629	-5.30924	0.829749	0.779035
B.cells	E130102H2	0.232921	1.232168	0.563285	0.574667	-5.44505	0.824975	0.771287
B.cells	FHAD1	-0.2663	0.624492	-0.563	0.574862	-5.40661	0.833058	0.784548
B.cells	TOP3B	0.088607	3.549805	0.562937	0.574903	-5.9707	0.794967	0.723286
B.cells	LMAN2	0.049857	6.112382	0.562871	0.574947	-6.54582	0.76326	0.673502
B.cells	KLRC3	0.253306	-1.20661	0.562859	0.574956	-5.15938	0.857657	0.825473
B.cells	YPEL2	0.102369	4.572346	0.562253	0.575367	-6.2254	0.782133	0.703129
B.cells	RAB23	-0.22539	1.279801	-0.562	0.575536	-5.39218	0.824345	0.770602
B.cells	PPIF	0.11125	3.577224	0.561924	0.57559	-5.93494	0.79462	0.722906
B.cells	GNB2	-0.04566	8.428837	-0.5619	0.575603	-6.91454	0.735878	0.631581
B.cells	ATL1	0.296437	0.498179	0.561837	0.575649	-5.28749	0.834749	0.787516
B.cells	L3MBTL2	0.09109	3.445434	0.561833	0.575651	-5.95761	0.796291	0.725562
B.cells	OPHN1	0.195141	2.997504	0.561804	0.575671	-5.69259	0.802001	0.734661
B.cells	NOTCH2	0.053508	7.16468	0.561793	0.575679	-6.79795	0.750674	0.654198
B.cells	A630023P1	0.287227	0.306091	0.561761	0.5757	-5.32313	0.837329	0.791727

B.cells	TTC4	0.089942	3.541793	0.56173	0.575721	-5.99121	0.795069	0.723619
B.cells	RLIM	0.042952	6.654275	0.561691	0.575747	-6.64688	0.756748	0.66356
B.cells	NAT10	0.111522	3.270157	0.561627	0.575791	-5.93105	0.79852	0.729109
B.cells	TSC22D1	-0.09124	4.872446	-0.56143	0.575925	-6.25933	0.778413	0.697337
B.cells	6330409D2	0.269026	0.333222	0.561346	0.575982	-5.28287	0.836964	0.79119
B.cells	RGS13	-0.32164	-1.05629	-0.56133	0.575995	-5.22194	0.85557	0.822281
B.cells	GM13919	-0.11834	3.111975	-0.5612	0.576082	-5.97759	0.800538	0.73238
B.cells	TCRG-C1	-0.34237	0.880703	-0.56108	0.576163	-5.34757	0.829639	0.779252
B.cells	ATP5A1	-0.04783	7.857813	-0.56105	0.576185	-6.82906	0.742518	0.641746
B.cells	TCTA	-0.16256	2.098287	-0.561	0.576215	-5.52445	0.81361	0.753324
B.cells	4930430E1	0.344245	0.409202	0.560862	0.57631	-5.25173	0.835943	0.789582
B.cells	PDCD1	-0.3601	0.743001	-0.56066	0.57645	-5.34596	0.831474	0.782332
B.cells	CDH24	-0.15302	2.179776	-0.56063	0.576466	-5.64421	0.81255	0.75171
B.cells	ARHGAP35	-0.07335	5.237019	-0.56048	0.576569	-6.35443	0.773921	0.690401
B.cells	DAD1	-0.05154	6.948328	-0.56047	0.576579	-6.68981	0.753242	0.65828
B.cells	A230083N1	-0.318	0.546139	-0.56047	0.576579	-5.21543	0.834107	0.786622
B.cells	KIF18B	0.187907	2.84215	0.560254	0.576723	-5.68172	0.804099	0.73804
B.cells	CSF3	0.458878	-0.096	0.560042	0.576867	-5.17119	0.842921	0.800896
B.cells	CDK9	-0.06138	5.578202	-0.55998	0.57691	-6.44169	0.769893	0.683982
B.cells	LEPR	0.277398	1.823369	0.559744	0.577069	-5.44222	0.817354	0.759346
B.cells	PACSIN1	0.131993	2.953403	0.559578	0.577182	-5.8802	0.80272	0.735853
B.cells	CYBC1	0.084527	4.562008	0.55953	0.577214	-6.24246	0.782412	0.703637
B.cells	GRK4	-0.12049	3.108893	-0.55945	0.577269	-5.87118	0.80073	0.732676
B.cells	LRRC8A	-0.06556	5.427019	-0.55944	0.577279	-6.39773	0.77174	0.686894
B.cells	MOB3C	-0.11293	3.356469	-0.55937	0.577325	-5.88069	0.797574	0.727646
B.cells	ANKFY1	0.05797	5.995175	0.559301	0.57737	-6.54068	0.764824	0.67612
B.cells	SCLY	-0.1079	3.466279	-0.55899	0.57758	-5.88762	0.796269	0.725458
B.cells	FRMD8	-0.08997	4.222806	-0.55892	0.577627	-6.12621	0.786733	0.710342
B.cells	MICU3	0.097752	3.81398	0.558904	0.57764	-6.10648	0.79187	0.718472
B.cells	3110001I2	-0.11739	2.677347	-0.5588	0.577708	-5.78727	0.806358	0.741607
B.cells	ATP6V0A1	0.077777	4.202735	0.558579	0.577861	-6.26827	0.787099	0.710866
B.cells	POGK	-0.15259	2.454489	-0.55842	0.577967	-5.71934	0.809355	0.746329
B.cells	ALMS1	-0.10725	3.824261	-0.55835	0.578017	-6.02904	0.791858	0.718421
B.cells	ZFP729A	-0.11607	3.051831	-0.55828	0.578066	-5.91936	0.80167	0.73403
B.cells	TMEM250-	-0.09069	4.629225	-0.5581	0.578185	-6.16653	0.781849	0.702531
B.cells	GM19605	0.247999	1.260305	0.557978	0.578269	-5.36479	0.825073	0.771611
B.cells	MTMR10	-0.14535	2.897646	-0.55751	0.578589	-5.83459	0.804089	0.737448
B.cells	CEP350	0.060962	6.505585	0.557392	0.578668	-6.60183	0.759305	0.666987
B.cells	ORC5	0.11176	3.474755	0.557093	0.578872	-5.93319	0.79692	0.725796
B.cells	TRDJ1	0.258985	-1.29553	0.556896	0.579005	-5.17364	0.859993	0.828669
B.cells	TMLHE	-0.23476	2.31306	-0.5568	0.579074	-5.51144	0.81185	0.74968
B.cells	PASK	-0.29966	1.295986	-0.55665	0.579173	-5.33675	0.825178	0.771209
B.cells	ZDHHC15	-0.2551	1.125862	-0.55664	0.579183	-5.3474	0.827432	0.774863
B.cells	MTMR9	-0.07877	3.972449	-0.55658	0.579219	-6.07711	0.790636	0.715892
B.cells	PIM3	0.087847	4.641176	0.556358	0.579371	-6.3153	0.78238	0.702774
B.cells	ZDHHC8	0.096157	3.666743	0.556262	0.579437	-6.0316	0.794607	0.722107
B.cells	LBHD1	0.244266	0.16876	0.555926	0.579666	-5.24796	0.840551	0.795969

B.cells	HIST1H4D	0.307887	1.04631	0.555539	0.579929	-5.29127	0.82879	0.776839
B.cells	COPA	0.047571	6.661065	0.55551	0.579949	-6.63251	0.757903	0.664479
B.cells	MPP5	-0.06963	5.379551	-0.55551	0.579949	-6.38891	0.773435	0.688615
B.cells	GABARAPL	-0.06637	7.762001	-0.5555	0.579957	-6.89443	0.744854	0.644425
B.cells	RSPH3A	0.144398	2.558451	0.555471	0.579975	-5.74311	0.808965	0.744865
B.cells	GM15943	-0.31139	0.207344	-0.55522	0.580148	-5.23641	0.840167	0.795226
B.cells	GM37982	-0.11786	2.996043	-0.55507	0.580246	-6.14242	0.803462	0.735988
B.cells	KBTBD7	-0.16183	2.137804	-0.55485	0.580398	-5.59919	0.814557	0.753857
B.cells	BRAP	0.064441	4.924367	0.554769	0.580454	-6.33936	0.779169	0.697611
B.cells	DEAF1	0.100889	3.281829	0.554726	0.580483	-5.91306	0.799806	0.730245
B.cells	MBIP	-0.09683	3.658726	-0.55468	0.580517	-6.05297	0.795015	0.722634
B.cells	BMYC	0.127134	3.380337	0.554572	0.580588	-6.07508	0.798551	0.728282
B.cells	DCAF7	0.055925	5.327488	0.554474	0.580654	-6.39442	0.774201	0.68991
B.cells	GOLM1	-0.1243	3.2957	-0.55443	0.580683	-5.81985	0.79963	0.730057
B.cells	CZIB	0.098205	3.658795	0.554178	0.580856	-5.98706	0.795082	0.722796
B.cells	CCDC171	0.092921	3.57202	0.554159	0.580869	-6.05345	0.796182	0.724543
B.cells	CCNQ	0.110227	3.060749	0.554026	0.58096	-5.87329	0.802732	0.734929
B.cells	TASP1	-0.11106	3.918389	-0.55363	0.58123	-6.06923	0.791991	0.717659
B.cells	KDM2A	-0.03758	7.465818	-0.55358	0.581262	-6.78049	0.748704	0.650174
B.cells	LRRFIP1	0.050744	7.249941	0.553554	0.581281	-6.76203	0.751257	0.654092
B.cells	ATP6V1G1	0.049118	7.177612	0.553183	0.581534	-6.70498	0.752354	0.655432
B.cells	ANKRD46	-0.13024	2.377526	-0.55279	0.581805	-5.56208	0.812187	0.749164
B.cells	NXT2	-0.12295	2.785909	-0.5525	0.581997	-5.70921	0.806904	0.740813
B.cells	GM15441	-0.20306	1.344167	-0.55245	0.582033	-5.40726	0.825735	0.771124
B.cells	SLK	-0.06122	6.477015	-0.55231	0.582128	-6.62031	0.760935	0.668489
B.cells	ADH5	-0.06785	6.03865	-0.552	0.582339	-6.53607	0.76623	0.67671
B.cells	MCRIP2	0.172821	2.015335	0.551923	0.582393	-5.52284	0.816906	0.756885
B.cells	BCL2L2	-0.1948	1.276497	-0.55192	0.582394	-5.39105	0.826631	0.772589
B.cells	ABHD17C	0.080684	4.833665	0.551862	0.582435	-6.25247	0.781011	0.699797
B.cells	HEXIM2	-0.2647	0.998422	-0.55175	0.58251	-5.3019	0.830326	0.778582
B.cells	HTR2B	-0.23127	0.855091	-0.55173	0.582528	-5.36176	0.832237	0.781688
B.cells	BMP6	0.361076	0.450706	0.551604	0.582611	-5.12262	0.837658	0.790516
B.cells	OPTN	0.098244	4.651784	0.551596	0.582617	-6.19532	0.783271	0.703349
B.cells	CENPV	-0.1315	3.40694	-0.55159	0.582621	-6.02683	0.798947	0.728146
B.cells	STX6	-0.06728	5.2207	-0.55142	0.582737	-6.4001	0.776227	0.692344
B.cells	CD74	0.130605	11.27972	0.551409	0.582744	-7.55627	0.70568	0.584942
B.cells	CEP97	-0.11438	2.922474	-0.55135	0.582787	-5.90297	0.805147	0.738081
B.cells	TNFAIP8	0.055398	7.424897	0.55105	0.582989	-6.76031	0.749768	0.651179
B.cells	ZFP991	0.136914	2.80432	0.550938	0.583066	-5.8256	0.806813	0.740556
B.cells	BACE1	-0.1222	2.654059	-0.55077	0.583181	-5.77903	0.808752	0.743688
B.cells	FOXM1	-0.15923	3.10354	-0.55072	0.583216	-5.75954	0.802968	0.734447
B.cells	A930024E0	0.207086	1.108621	0.550581	0.58331	-5.53295	0.829009	0.77637
B.cells	DDX17	0.055549	6.357267	0.55043	0.583413	-6.59341	0.762515	0.670903
B.cells	SHKBP1	-0.08022	4.068341	-0.55041	0.583425	-6.16021	0.790716	0.715046
B.cells	GM49864	0.272668	0.452692	0.55034	0.583474	-5.26876	0.837783	0.790671
B.cells	LAMTOR5	0.060092	5.527925	0.550061	0.583664	-6.45179	0.772594	0.686666
B.cells	GM29243	-0.28264	-0.26495	-0.54992	0.583762	-5.21419	0.847504	0.806695

B.cells	MAPK1IP1l	0.047314	6.060276	0.549783	0.583854	-6.52985	0.766106	0.676593
B.cells	SOX5	0.125843	4.684841	0.549758	0.583872	-6.34935	0.783001	0.703004
B.cells	PRRC2C	0.041819	7.744202	0.549638	0.583954	-6.83429	0.746005	0.645628
B.cells	PHB2	0.052006	6.570723	0.549489	0.584055	-6.61877	0.759946	0.667066
B.cells	ZFP595	-0.13853	1.84184	-0.54944	0.584087	-5.53759	0.819326	0.760894
B.cells	CLPP	-0.07215	4.725598	-0.54941	0.584112	-6.27478	0.782494	0.702228
B.cells	FADS3	-0.24912	0.659123	-0.54934	0.584155	-5.25748	0.83501	0.786316
B.cells	ZFP260	0.069363	4.245881	0.549321	0.58417	-6.1883	0.788485	0.711669
B.cells	MRPL45	0.087727	4.340705	0.549226	0.584235	-6.17164	0.787296	0.709793
B.cells	PCDH17	-0.21666	2.459624	-0.54915	0.584289	-5.61475	0.811268	0.747971
B.cells	GM20470	-0.19472	1.403571	-0.54909	0.584326	-5.51468	0.825098	0.770257
B.cells	GM29417	-0.1879	1.098886	-0.54803	0.585054	-5.45948	0.829572	0.777242
B.cells	RSF1OS2	0.174124	1.663513	0.548019	0.58506	-5.62782	0.822099	0.765142
B.cells	AA386476	0.219422	1.390731	0.54801	0.585066	-5.4212	0.8257	0.770965
B.cells	SSH2	0.06593	8.396317	0.547996	0.585075	-6.91705	0.738776	0.634404
B.cells	CCDC117	0.069349	4.699485	0.54797	0.585093	-6.27434	0.783228	0.703168
B.cells	SPATA2L	0.25599	0.521707	0.54797	0.585094	-5.34196	0.837293	0.789803
B.cells	ERP44	0.041224	6.074446	0.547947	0.585109	-6.55528	0.766335	0.676759
B.cells	GM43813	0.091999	4.535806	0.547665	0.585302	-6.24912	0.785435	0.706446
B.cells	DHX36	-0.0512	5.392248	-0.54744	0.585457	-6.42383	0.774928	0.689907
B.cells	PI4KA	0.056045	6.333121	0.547259	0.585579	-6.62528	0.763466	0.672144
B.cells	ZFP932	0.105725	2.788655	0.547125	0.585671	-5.7613	0.807713	0.741851
B.cells	TCF7	0.184212	2.524319	0.54707	0.585709	-5.80356	0.811132	0.747351
B.cells	ATP8B4	-0.2058	4.781456	-0.54705	0.585719	-5.8192	0.782477	0.701852
B.cells	RAB22A	0.05621	5.60028	0.546592	0.586036	-6.46632	0.772685	0.686062
B.cells	5830432E0	0.204395	1.494203	0.546512	0.586091	-5.48912	0.824944	0.769098
B.cells	LYZ2	0.229608	7.170673	0.546035	0.586417	-6.55504	0.754065	0.656819
B.cells	CHAMP1	0.123861	2.843874	0.545923	0.586494	-5.79358	0.807692	0.740925
B.cells	TSPYL1	-0.07076	4.888609	-0.5455	0.586783	-6.30658	0.782109	0.700025
B.cells	POP1	0.113744	2.708556	0.545291	0.586926	-5.8614	0.809847	0.743825
B.cells	IL23R	0.356843	-0.9447	0.545137	0.587032	-5.10596	0.858502	0.823311
B.cells	SLC35E4	-0.24317	0.657751	-0.54504	0.587098	-5.38998	0.836937	0.787506
B.cells	ITPRIPL2	0.13038	3.897046	0.544758	0.587292	-5.85068	0.794714	0.719788
B.cells	ASCC2	-0.06457	4.605255	-0.54474	0.587306	-6.24297	0.785811	0.705738
B.cells	TRIR	0.052606	6.300182	0.544712	0.587323	-6.57165	0.764974	0.673217
B.cells	FASN	0.124918	3.14635	0.544452	0.587501	-5.89425	0.804373	0.735079
B.cells	NIPAL3	0.117477	3.198166	0.544414	0.587527	-5.91883	0.803709	0.73402
B.cells	FBXO33	-0.0585	5.984659	-0.54427	0.587624	-6.52126	0.768928	0.679239
B.cells	TRPS1	0.068804	7.683996	0.544132	0.58772	-6.92198	0.748603	0.64787
B.cells	TASOR2	0.065235	5.373883	0.543915	0.587869	-6.42636	0.776549	0.690944
B.cells	2-Sep	0.153111	2.22603	0.543796	0.58795	-5.73269	0.816495	0.754236
B.cells	NUP43	-0.12969	2.795528	-0.54365	0.588053	-5.74745	0.809144	0.742427
B.cells	4930590J0	-0.19316	1.547542	-0.54351	0.588146	-5.49145	0.825493	0.768701
B.cells	HRAS	0.068086	4.842094	0.543116	0.588417	-6.27027	0.783496	0.701471
B.cells	CPA6	-0.25838	-0.07229	-0.5429	0.588561	-5.32905	0.847589	0.80427
B.cells	JAG2	0.195086	1.093215	0.542856	0.588595	-5.5171	0.831871	0.77867
B.cells	TET3	-0.05078	6.827605	-0.54275	0.588669	-6.69151	0.759302	0.663883

B.cells	GATD1	0.076323	4.394103	0.542445	0.588876	-6.20392	0.789189	0.710534
B.cells	DCTN2	0.061273	5.367124	0.542441	0.588879	-6.41404	0.777088	0.691546
B.cells	GM49463	-0.32476	-0.05016	-0.54241	0.588898	-5.18236	0.847331	0.803984
B.cells	TOM1L2	0.072474	5.471299	0.542014	0.589172	-6.45377	0.776076	0.689637
B.cells	EIF6	-0.05839	5.835967	-0.54185	0.589286	-6.49914	0.771633	0.682727
B.cells	PPP4R3B	-0.04178	6.842276	-0.54177	0.589341	-6.68767	0.759449	0.663906
B.cells	SEC22C	0.174512	2.04121	0.541653	0.58942	-5.52689	0.819691	0.758848
B.cells	4933411E0	-0.25263	0.555657	-0.54128	0.589679	-5.32268	0.839445	0.790878
B.cells	PLCB4	0.139213	3.876337	0.541255	0.589693	-5.94714	0.796026	0.721123
B.cells	CDC42EP1	-0.32897	0.752796	-0.54108	0.58981	-5.22578	0.836793	0.786585
B.cells	ZSCAN26	-0.07365	4.652438	-0.54104	0.589843	-6.30235	0.786258	0.705734
B.cells	RAP1GAP2	0.105426	4.692548	0.540977	0.589884	-6.3714	0.785757	0.704947
B.cells	TRGV2	0.314479	-0.51519	0.540961	0.589895	-5.26307	0.853978	0.81476
B.cells	SBDS	-0.05105	5.561152	-0.54092	0.589925	-6.46646	0.775	0.688104
B.cells	TOMM5	0.072271	5.458336	0.540884	0.589948	-6.40232	0.776264	0.690077
B.cells	TSR2	0.180116	2.118125	0.540492	0.590217	-5.5817	0.818835	0.75738
B.cells	SNAPC5	-0.07176	4.755447	-0.54039	0.590289	-6.27472	0.785118	0.703832
B.cells	VPS26B	0.114309	3.835261	0.540317	0.590337	-5.85185	0.796695	0.722121
B.cells	GM9967	-0.3424	0.569425	-0.5401	0.590486	-5.23484	0.839416	0.79081
B.cells	DNAJC11	0.072833	4.486406	0.540089	0.590493	-6.22729	0.788483	0.709191
B.cells	PCMTD2	0.097124	3.680281	0.540019	0.590542	-6.0636	0.798664	0.725284
B.cells	CRTAP	-0.13762	3.004923	-0.53997	0.590572	-5.72228	0.807313	0.739042
B.cells	DOCK6	0.190972	1.914885	0.53985	0.590658	-5.50816	0.821503	0.7618
B.cells	LAP3	0.098785	4.901491	0.539826	0.590674	-6.28354	0.783299	0.701052
B.cells	AGA	-0.11825	3.27288	-0.5396	0.590829	-5.88776	0.803972	0.733601
B.cells	DEGS2	0.22316	0.674225	0.539426	0.590949	-5.42813	0.838113	0.788646
B.cells	MCAT	-0.13729	2.553855	-0.53938	0.59098	-5.71481	0.813255	0.748511
B.cells	AURKB	-0.14593	4.237652	-0.5392	0.591102	-6.09484	0.79171	0.714332
B.cells	ICA1L	-0.17761	1.590667	-0.53914	0.591143	-5.65726	0.825885	0.768917
B.cells	CENPC1	0.054148	5.265605	0.539117	0.591161	-6.40473	0.778886	0.69418
B.cells	MRPL42	-0.06916	6.521235	-0.53881	0.59137	-6.60158	0.763566	0.670376
B.cells	FBXO34	-0.05904	5.78838	-0.53868	0.591463	-6.49292	0.772472	0.684195
B.cells	POLR3B	-0.07642	5.512738	-0.53864	0.59149	-6.46188	0.775853	0.689465
B.cells	PTRH2	-0.08053	4.181735	-0.53864	0.591492	-6.15791	0.792429	0.715492
B.cells	NDUFA11	-0.05613	6.910675	-0.5386	0.591515	-6.6936	0.758882	0.663147
B.cells	RAB35	0.065197	4.720448	0.538289	0.59173	-6.27562	0.785862	0.704923
B.cells	MET	-0.20017	1.991608	-0.53819	0.591795	-5.69614	0.820814	0.76054
B.cells	PEX5	0.077176	4.071805	0.53773	0.592114	-6.11825	0.79426	0.717936
B.cells	INSL6	0.193273	1.68935	0.537723	0.592119	-5.57511	0.825056	0.76713
B.cells	MED22	-0.15323	2.384243	-0.53726	0.592436	-5.64469	0.816216	0.752604
B.cells	CDS1	-0.14549	2.788548	-0.53721	0.59247	-5.92557	0.81096	0.744207
B.cells	GM10138	-0.16391	2.471458	-0.53701	0.592606	-5.65274	0.815079	0.750883
B.cells	MAP2K5	0.049697	6.143251	0.536906	0.592681	-6.56782	0.76884	0.677936
B.cells	YTHDF2	-0.04866	6.035232	-0.53687	0.592703	-6.55156	0.770155	0.679991
B.cells	PTPN23	-0.09754	3.647447	-0.53668	0.592837	-6.02975	0.799924	0.726833
B.cells	AHCYL1	0.05332	5.179051	0.536588	0.5929	-6.35946	0.780677	0.696523
B.cells	4930403P2	-0.21547	0.612375	-0.53656	0.592919	-5.32242	0.839721	0.790864

B.cells	WRAP73	0.103128	2.923556	0.536527	0.592942	-5.83389	0.809213	0.741671
B.cells	B3GNT2	0.059523	6.739389	0.536367	0.593052	-6.6162	0.761635	0.666952
B.cells	RGS19	0.069275	5.393744	0.536222	0.593151	-6.44456	0.77803	0.692423
B.cells	SLC25A4	0.06152	7.14787	0.536156	0.593197	-6.75732	0.756739	0.659435
B.cells	MTHFSD	0.144356	2.433778	0.53612	0.593222	-5.67174	0.815578	0.751907
B.cells	TMEM202	0.259512	0.104736	0.535593	0.593585	-5.2532	0.846806	0.802448
B.cells	RBM24	-0.22167	1.008251	-0.53551	0.593644	-5.58924	0.834608	0.782582
B.cells	MRE11A	0.063504	4.349103	0.535391	0.593723	-6.205	0.791233	0.713139
B.cells	C230066G2	-0.29058	0.025289	-0.53539	0.593728	-5.24035	0.847888	0.804236
B.cells	SUPT5	0.055376	6.05341	0.535383	0.593729	-6.53486	0.770123	0.680093
B.cells	CDC23	0.113566	2.927739	0.535318	0.593774	-5.782	0.809359	0.741913
B.cells	KLRK1	-0.26202	3.149025	-0.53518	0.593872	-5.58753	0.806545	0.73736
B.cells	2310022B0	0.119518	2.488564	0.535042	0.593964	-5.664	0.81513	0.751049
B.cells	NUDT22	-0.13239	2.557891	-0.53482	0.59412	-5.6878	0.81432	0.749656
B.cells	FAM192A	0.06256	4.607769	0.534744	0.594169	-6.2493	0.788146	0.708089
B.cells	PRM1	-0.23941	-0.44781	-0.53444	0.594376	-5.30641	0.854562	0.814998
B.cells	MAN2C1	0.087817	3.509599	0.534367	0.594429	-6.00415	0.802068	0.73012
B.cells	USP12	0.062653	6.069178	0.534356	0.594436	-6.56747	0.770111	0.679908
B.cells	KCNIP2	-0.21876	1.248736	-0.53417	0.594565	-5.44536	0.83159	0.777552
B.cells	ABT1	0.094236	3.534654	0.53416	0.594571	-5.93703	0.801748	0.729661
B.cells	PDLIM7	-0.09914	3.638312	-0.53406	0.594644	-6.04129	0.800425	0.727577
B.cells	AP1S2	0.066044	5.081901	0.534002	0.59468	-6.39752	0.782257	0.698937
B.cells	DYNLT1B	0.233646	1.188553	0.533921	0.594736	-5.39804	0.832393	0.778892
B.cells	GTF2E2	-0.05716	5.440806	-0.53357	0.594979	-6.42645	0.777935	0.691988
B.cells	FIP1L1	0.038006	6.639254	0.533453	0.595059	-6.64866	0.763316	0.669314
B.cells	KNOP1	0.064732	4.90183	0.533251	0.595198	-6.32185	0.784617	0.702553
B.cells	ACP6	-0.11146	3.140192	-0.53308	0.595318	-5.88994	0.806931	0.737915
B.cells	EGR3	0.149491	3.533307	0.533055	0.595333	-6.25947	0.801888	0.729887
B.cells	SMG8	-0.12638	2.847118	-0.53304	0.595346	-5.73437	0.810715	0.74396
B.cells	GM44686	-0.09601	3.136727	-0.53294	0.595414	-5.96805	0.806976	0.738054
B.cells	FAAP24	0.192861	2.002599	0.532838	0.595483	-5.63262	0.821734	0.761741
B.cells	TRNT1	-0.06431	4.772449	-0.53269	0.595585	-6.31281	0.786231	0.705249
B.cells	EPC2	0.055225	6.14343	0.53246	0.595743	-6.56773	0.769325	0.678923
B.cells	FAM89B	-0.06583	5.277421	-0.5323	0.595853	-6.42686	0.779953	0.695566
B.cells	VSIR	0.105575	4.468275	0.532192	0.595928	-6.07191	0.790041	0.711424
B.cells	VAMP2	0.064017	4.421644	0.532192	0.595928	-6.21729	0.790627	0.712348
B.cells	ZFP24	0.069659	4.243197	0.532042	0.596032	-6.19562	0.792874	0.715896
B.cells	COA7	0.084078	3.404389	0.532009	0.596054	-5.99446	0.803538	0.732805
B.cells	ADGRL1	-0.12892	2.476047	-0.53198	0.596072	-5.7699	0.815535	0.751977
B.cells	PLOD1	-0.1513	3.410314	-0.53196	0.59609	-5.78959	0.803462	0.732684
B.cells	TMEM63A	0.127645	3.49863	0.531925	0.596112	-5.90299	0.802331	0.730886
B.cells	CHMP4B	0.038677	7.889271	0.531897	0.596131	-6.87747	0.748414	0.646798
B.cells	MCTS2	-0.23007	1.612847	-0.53154	0.596379	-5.44815	0.827127	0.770251
B.cells	PDCL3	-0.07502	4.536557	-0.53142	0.596463	-6.19306	0.789423	0.7101
B.cells	CSNK1E	-0.07198	4.953409	-0.53134	0.596518	-6.35351	0.784212	0.701922
B.cells	VPS26C	-0.08637	4.084075	-0.53106	0.596707	-6.14163	0.795129	0.719115
B.cells	GM16283	0.246491	0.252287	0.531037	0.596725	-5.24094	0.845388	0.799959

B.cells	TSLP	-0.34076	-0.44209	-0.53103	0.596727	-5.13114	0.85488	0.815521
B.cells	MPDU1	0.066446	4.71579	0.530865	0.596844	-6.27534	0.787245	0.706581
B.cells	D930016D(	-0.11543	2.876865	-0.53067	0.59698	-5.80819	0.810737	0.743683
B.cells	CPS1	-0.15687	4.481272	-0.53052	0.597085	-6.30137	0.790316	0.711209
B.cells	MAN2B2	0.103059	3.799809	0.530361	0.597191	-6.04916	0.798933	0.724867
B.cells	RPAP1	0.126357	2.431442	0.530085	0.597381	-5.73883	0.81657	0.753071
B.cells	CCDC90B	-0.10834	3.165156	-0.52999	0.597447	-5.88153	0.807058	0.737894
B.cells	DYNLT1F	-0.08701	5.068561	-0.52987	0.597532	-6.30786	0.782977	0.699842
B.cells	EBI3	-0.08774	4.387447	-0.52967	0.597668	-6.24238	0.791496	0.713262
B.cells	MBNL1	0.058328	8.978349	0.52956	0.597744	-7.00277	0.736121	0.627709
B.cells	RABL2	-0.22183	1.106471	-0.5295	0.597784	-5.33602	0.83408	0.781435
B.cells	CCDC59	0.047177	5.676842	0.529243	0.597963	-6.49236	0.77546	0.688128
B.cells	IQGAP3	0.199736	2.400987	0.529172	0.598012	-5.63266	0.816968	0.753814
B.cells	ALDH9A1	-0.05996	5.005152	-0.52908	0.598075	-6.37273	0.783766	0.701118
B.cells	CDC42EP4	0.143794	2.155265	0.529027	0.598113	-5.66019	0.820185	0.758983
B.cells	CRISPLD2	-0.30385	0.884481	-0.52899	0.598137	-5.34959	0.837055	0.786269
B.cells	BCAR1	0.28941	0.776645	0.528886	0.59821	-5.23281	0.838505	0.788627
B.cells	SMAD7	0.080259	5.164939	0.528778	0.598284	-6.32946	0.78178	0.698006
B.cells	BUB1B	-0.11547	4.016283	-0.5285	0.598474	-6.06971	0.796184	0.720724
B.cells	TSPAN14	0.054336	6.835826	0.528445	0.598515	-6.6783	0.761373	0.666315
B.cells	ADAM33	0.332963	-0.9471	0.52818	0.598698	-5.08491	0.861787	0.827368
B.cells	CEMIP2	0.082763	5.494454	0.528147	0.59872	-6.385	0.777705	0.691718
B.cells	DPP7	-0.17676	1.957266	-0.52813	0.598734	-5.63978	0.822787	0.763268
B.cells	UCHL3	-0.06012	6.235223	-0.528	0.598821	-6.55907	0.768635	0.677627
B.cells	PAIP1	-0.05897	5.907952	-0.52798	0.598836	-6.49669	0.772627	0.683829
B.cells	MLXIP	-0.05443	6.94185	-0.52798	0.598839	-6.72152	0.760099	0.664426
B.cells	NPC2	-0.05545	7.639426	-0.52797	0.598842	-6.83209	0.751782	0.651649
B.cells	MYL4	-0.13371	4.638008	-0.52795	0.598854	-6.24503	0.78835	0.708439
B.cells	ORMDL3	-0.09681	3.803807	-0.52781	0.598952	-6.07068	0.798883	0.725139
B.cells	RAB10OS	0.074943	4.429445	0.527795	0.598964	-6.23009	0.790968	0.71262
B.cells	AGTRAP	0.120992	4.534904	0.527778	0.598975	-6.08302	0.789643	0.710532
B.cells	TMEM94	0.108189	2.610495	0.527739	0.599003	-5.77593	0.814237	0.749623
B.cells	ZBTB24	0.086332	3.32852	0.527453	0.5992	-5.95121	0.80513	0.734888
B.cells	PITRM1	-0.10064	3.560314	-0.52685	0.599619	-5.97251	0.802416	0.730163
B.cells	HIRA	0.062012	5.652742	0.526837	0.599626	-6.47047	0.776171	0.688848
B.cells	UQCRFS1	0.050123	7.164384	0.526827	0.599633	-6.76296	0.757838	0.660461
B.cells	FAM210B	0.08161	3.914295	0.526763	0.599677	-6.23783	0.797904	0.723022
B.cells	CENPJ	0.102283	3.331867	0.52668	0.599735	-5.91779	0.805344	0.734845
B.cells	CTU2	0.095192	3.206943	0.526504	0.599856	-5.98152	0.80698	0.737468
B.cells	GM15327	0.168571	1.294764	0.52645	0.599894	-5.48265	0.832039	0.777755
B.cells	RMND1	0.076249	3.79439	0.525806	0.600339	-6.08521	0.799945	0.725761
B.cells	PRKAR1A	-0.03564	7.625008	-0.52568	0.600425	-6.80915	0.75284	0.652377
B.cells	NIT2	-0.11263	3.588667	-0.5256	0.600481	-5.98352	0.802572	0.729957
B.cells	ATP5J	-0.05072	7.873083	-0.52552	0.600534	-6.84055	0.749905	0.647893
B.cells	AFF4	0.051285	7.842009	0.525297	0.600691	-6.88138	0.750382	0.648519
B.cells	MRPL41	0.085995	4.003451	0.525051	0.600862	-6.06676	0.79751	0.721704
B.cells	DIMT1	-0.09938	3.243055	-0.52487	0.600984	-5.93223	0.807233	0.73717

B.cells	TLN1	0.054247	7.109295	0.52484	0.601008	-6.72627	0.7592	0.661952
B.cells	CDC37	-0.04281	6.605127	-0.52477	0.601055	-6.64545	0.765269	0.671329
B.cells	PTPRM	-0.16892	5.186994	-0.52468	0.601115	-6.15633	0.782649	0.698396
B.cells	BTBD6	0.207081	1.542046	0.524478	0.601259	-5.45141	0.829502	0.772995
B.cells	TMEM129	0.178271	2.084833	0.52447	0.601264	-5.56725	0.822324	0.761419
B.cells	ZC3H10	-0.09833	2.921486	-0.52432	0.60137	-5.89241	0.811451	0.74395
B.cells	CLDN10	0.29828	0.159414	0.524105	0.601517	-5.2968	0.848189	0.803386
B.cells	PPP1CA	0.041753	8.233158	0.524094	0.601525	-6.92589	0.745958	0.641763
B.cells	GM16091	0.146778	2.306149	0.523987	0.601599	-5.70694	0.819499	0.756881
B.cells	SOCS6	0.078354	3.829946	0.523738	0.601771	-6.07056	0.799891	0.725464
B.cells	MYO1D	-0.11654	3.025547	-0.5237	0.601797	-5.91078	0.810216	0.741889
B.cells	THY1	-0.25427	1.573983	-0.52318	0.602158	-5.38747	0.829646	0.772561
B.cells	ATAD5	-0.11388	4.742851	-0.52273	0.602467	-6.23481	0.788755	0.70742
B.cells	SLC35B2	0.071087	4.882082	0.522648	0.602526	-6.31693	0.787013	0.704705
B.cells	TXNRD1	0.071276	5.871273	0.522468	0.602652	-6.45506	0.774763	0.685608
B.cells	ACSBG1	0.247135	-0.5064	0.52246	0.602657	-5.30093	0.857807	0.818644
B.cells	PTPA	-0.05081	5.833043	-0.52232	0.602752	-6.54373	0.775232	0.686379
B.cells	HECW2	0.167197	3.162677	0.522145	0.602875	-5.8116	0.808851	0.739414
B.cells	HAVCR2	0.251835	2.301711	0.521914	0.603036	-5.54756	0.820052	0.757377
B.cells	SUPT6	0.058596	5.808893	0.521734	0.60316	-6.52	0.775529	0.686949
B.cells	9530068E0	0.075491	4.851397	0.521638	0.603227	-6.27093	0.787397	0.705508
B.cells	GH	-0.23315	-0.76502	-0.52162	0.603242	-5.23094	0.861224	0.824716
B.cells	LAMP1	-0.04679	7.757865	-0.52151	0.603315	-6.8764	0.752019	0.650705
B.cells	UNC93B1	0.047749	7.659349	0.521339	0.603434	-6.85908	0.753187	0.652558
B.cells	VIPR1	-0.25833	0.964939	-0.52122	0.603514	-5.32215	0.8378	0.786226
B.cells	2610318N0	0.232205	1.172871	0.521146	0.603568	-5.44605	0.835011	0.781701
B.cells	GOSR2	-0.06115	5.28486	-0.52107	0.603619	-6.43426	0.781998	0.697203
B.cells	ACTR3B	0.206264	-0.01555	0.521036	0.603644	-5.3077	0.851099	0.807931
B.cells	DLGAP5	0.168784	3.248568	0.52097	0.60369	-5.81629	0.807744	0.737869
B.cells	HMGN1	-0.06395	7.26929	-0.52091	0.603729	-6.73743	0.757832	0.659737
B.cells	EXOSC9	-0.0799	4.169312	-0.52091	0.603732	-6.13718	0.795981	0.719202
B.cells	NUP205	0.076188	4.408914	0.520908	0.603733	-6.21325	0.792953	0.714421
B.cells	FCNB	-0.23616	-0.23854	-0.52073	0.603856	-5.28846	0.854157	0.812968
B.cells	RCBTB1	0.080767	3.826201	0.520689	0.603885	-6.1251	0.800341	0.726116
B.cells	ZWINT	-0.06456	5.039001	-0.52057	0.603971	-6.30866	0.785055	0.702039
B.cells	ASB6	-0.12004	2.575022	-0.52048	0.60403	-5.79772	0.816477	0.751869
B.cells	RBIS	0.061409	5.209656	0.520461	0.604043	-6.40481	0.782931	0.698712
B.cells	SLC28A2	0.119321	2.961181	0.520389	0.604093	-6.00885	0.811457	0.743835
B.cells	ICAM1	0.083563	5.898773	0.52033	0.604134	-6.51037	0.774426	0.685434
B.cells	UBE2E1	0.041935	6.229498	0.520306	0.604151	-6.58989	0.770382	0.679152
B.cells	ST6GALNA(	0.102631	5.127632	0.520133	0.604271	-6.41141	0.783951	0.700317
B.cells	NEIL1	-0.12515	3.000738	-0.52011	0.604286	-5.85598	0.810945	0.743027
B.cells	THADA	0.061181	5.881845	0.520089	0.604301	-6.52032	0.774633	0.685772
B.cells	ARRDC3	0.137272	3.403751	0.519831	0.604481	-5.84749	0.805764	0.734784
B.cells	CCDC137	0.109963	2.673176	0.519798	0.604504	-5.79493	0.815215	0.749881
B.cells	ABCB1B	0.139587	3.934965	0.519773	0.604521	-6.0659	0.798973	0.72402
B.cells	FAM3A	0.117669	2.785704	0.519573	0.60466	-5.78513	0.813804	0.747644

B.cells	BCKDHA	0.071051	4.820285	0.519519	0.604697	-6.37835	0.787854	0.706496
B.cells	BC050972	0.317523	-0.27973	0.51917	0.604939	-5.18162	0.854909	0.814183
B.cells	NAT2	0.124621	2.532818	0.51904	0.60503	-5.80513	0.817206	0.753057
B.cells	GM16973	-0.1666	1.894858	-0.51896	0.605084	-5.56248	0.825587	0.766573
B.cells	SLC30A5	0.061358	5.799283	0.518788	0.605205	-6.49015	0.775816	0.687657
B.cells	ZBTB26	0.225371	0.850706	0.518785	0.605207	-5.35483	0.83952	0.789153
B.cells	ITGAV	0.069562	6.550452	0.518752	0.60523	-6.66836	0.766649	0.673425
B.cells	KLC2	0.128566	2.681752	0.518657	0.605295	-5.84312	0.815263	0.74999
B.cells	MTCP1	0.236143	0.737147	0.518611	0.605328	-5.34733	0.841052	0.791646
B.cells	E4F1	0.11672	3.127007	0.518391	0.605481	-5.89942	0.809601	0.740821
B.cells	ZFP956	-0.19645	1.264702	-0.51806	0.605713	-5.40191	0.834151	0.780303
B.cells	SUCLG2	0.076255	4.835404	0.517861	0.605849	-6.30533	0.787945	0.706555
B.cells	MAP2	-0.28938	0.187932	-0.51784	0.605862	-5.21149	0.848694	0.804033
B.cells	AFMID	-0.08053	4.371552	-0.51783	0.605868	-6.25795	0.793775	0.715738
B.cells	TMEM221	-0.32732	-0.26558	-0.51782	0.605876	-5.09443	0.854906	0.814216
B.cells	IFT46	-0.06046	4.502973	-0.51742	0.606156	-6.27989	0.792393	0.713225
B.cells	HINFP	-0.09442	3.494398	-0.51723	0.606289	-5.96885	0.805261	0.733561
B.cells	FIBP	0.065686	4.462532	0.517143	0.606347	-6.23812	0.792945	0.714062
B.cells	NAPA	0.047395	5.92916	0.517076	0.606394	-6.5247	0.774705	0.685494
B.cells	KCTD21	-0.29892	0.182578	-0.51671	0.606647	-5.24548	0.849211	0.804443
B.cells	TNFRSF21	0.122638	4.10783	0.516653	0.606688	-5.95982	0.79753	0.721272
B.cells	3830406C1	0.0709	4.453206	0.516584	0.606736	-6.21286	0.79316	0.714371
B.cells	OLFR543	-0.2858	0.201065	-0.51657	0.606744	-5.20945	0.848959	0.80403
B.cells	ZFP51	0.102046	2.86166	0.516445	0.606833	-5.84371	0.813546	0.746745
B.cells	HDGFL3	-0.2097	1.511028	-0.51636	0.606893	-5.49569	0.831313	0.775324
B.cells	FAM98A	0.086423	3.607656	0.516168	0.607026	-6.02039	0.803984	0.731469
B.cells	RNF219	0.114776	3.193241	0.516085	0.607083	-5.8942	0.809314	0.739966
B.cells	CARMIL2	0.12538	2.704609	0.515994	0.607146	-5.79898	0.815652	0.750136
B.cells	GGACT	0.095644	3.170013	0.515724	0.607334	-5.86709	0.809771	0.740566
B.cells	PFKFB2	-0.12021	2.465839	-0.51563	0.607401	-5.7102	0.818928	0.755237
B.cells	TOGARAM:	-0.05658	5.297825	-0.51532	0.607615	-6.42742	0.7829	0.698086
B.cells	RIF1	-0.06848	5.435862	-0.51517	0.607723	-6.39002	0.781188	0.695438
B.cells	SBF1	0.063587	4.474225	0.514974	0.607856	-6.26434	0.793207	0.714391
B.cells	GLT8D1	0.095879	3.339159	0.514928	0.607888	-5.93113	0.807675	0.73732
B.cells	CNOT4	0.041548	7.523005	0.514796	0.60798	-6.83335	0.755833	0.656259
B.cells	MBP	0.069536	5.172136	0.514757	0.608008	-6.46805	0.784463	0.700645
B.cells	PRELID3B	0.057173	5.574007	0.514744	0.608016	-6.43768	0.779479	0.69285
B.cells	GM20513	0.184273	1.300029	0.514743	0.608017	-5.83875	0.834443	0.780339
B.cells	AMIGO2	-0.29701	0.934211	-0.51453	0.608164	-5.31154	0.839359	0.788328
B.cells	USP7	-0.04411	6.215443	-0.5143	0.608323	-6.57865	0.771607	0.680601
B.cells	KATNBL1	-0.05114	5.963648	-0.51423	0.608374	-6.55679	0.774688	0.685389
B.cells	SRL	0.22488	0.890591	0.513957	0.608564	-5.27552	0.839946	0.789394
B.cells	DYNC1LI1	-0.04468	6.412559	-0.51345	0.608919	-6.64686	0.769205	0.67707
B.cells	GM9530	-0.27312	-0.31965	-0.51344	0.608926	-5.26648	0.856441	0.816484
B.cells	IGLC3	-0.19126	4.04289	-0.51336	0.608981	-6.22526	0.798674	0.723277
B.cells	PSMA5	-0.0641	5.884469	-0.51309	0.609171	-6.51182	0.775659	0.687187
B.cells	NEURL4	0.163288	2.445552	0.512877	0.609317	-5.70962	0.819286	0.756264

B.cells	MEX3C	-0.06145	5.133199	-0.51286	0.609331	-6.33547	0.784953	0.701762
B.cells	GM26759	0.125289	3.123372	0.51277	0.609391	-5.91309	0.810465	0.742163
B.cells	SETDB1	-0.06736	4.832864	-0.51265	0.609478	-6.3218	0.788704	0.707691
B.cells	ZMAT5	0.061545	4.547775	0.512618	0.609497	-6.2677	0.792285	0.713332
B.cells	CNPPD1	-0.0627	5.686802	-0.51256	0.609534	-6.51842	0.778092	0.691072
B.cells	TNFAIP8L1	-0.14259	2.396525	-0.51232	0.609701	-5.64173	0.819928	0.757455
B.cells	ST18	0.284575	0.283819	0.512235	0.609763	-5.39334	0.84817	0.803256
B.cells	PDIK1L	-0.11456	3.065249	-0.51223	0.609767	-5.81004	0.811217	0.743496
B.cells	GM15848	-0.25132	-0.86297	-0.51221	0.60978	-5.24976	0.863703	0.829221
B.cells	SKP1A	-0.04427	6.639256	-0.51208	0.609875	-6.65694	0.766453	0.673088
B.cells	ARMC1	0.059708	4.827556	0.512064	0.609883	-6.30912	0.788771	0.707908
B.cells	2610020CC	-0.0588	4.983149	-0.51186	0.610023	-6.35228	0.786824	0.704909
B.cells	PIGL	-0.09769	2.746364	-0.51178	0.610084	-5.84055	0.815357	0.750196
B.cells	ERF	-0.10921	3.477368	-0.51175	0.610099	-6.03694	0.805902	0.7351
B.cells	ETOHD2	0.116561	2.615429	0.511709	0.610131	-5.74945	0.817065	0.752955
B.cells	CELF4	0.210905	0.6776	0.511689	0.610145	-5.40071	0.842823	0.794624
B.cells	MKKS	0.150568	1.995441	0.511674	0.610155	-5.58275	0.825206	0.76605
B.cells	PIP5K1B	-0.08237	5.568352	-0.51159	0.610215	-6.41074	0.779554	0.693567
B.cells	NAXE	-0.0659	5.283838	-0.51158	0.610223	-6.37581	0.783079	0.699083
B.cells	USP28	0.070984	4.553264	0.511495	0.61028	-6.22439	0.792216	0.713451
B.cells	GTPBP10	-0.12652	2.397542	-0.51139	0.610353	-5.6782	0.819915	0.757598
B.cells	MAP3K3	0.05677	6.795781	0.511307	0.610411	-6.67071	0.76456	0.670334
B.cells	BABAM1	0.055559	5.569892	0.511293	0.61042	-6.46803	0.779535	0.693607
B.cells	GPATCH4	0.100075	3.338867	0.511266	0.610439	-5.9831	0.807683	0.738049
B.cells	INSR	0.071507	5.770425	0.511256	0.610446	-6.5006	0.777062	0.689747
B.cells	TDRP	-0.32712	-0.36439	-0.51114	0.610527	-5.21658	0.857058	0.818111
B.cells	4632427E1	0.073503	4.404143	0.511122	0.61054	-6.23816	0.794097	0.716549
B.cells	TBX2	-0.28047	0.45083	-0.51108	0.61057	-5.24772	0.845897	0.799823
B.cells	GM4258	0.106457	4.929368	0.510943	0.610665	-6.29893	0.787529	0.70618
B.cells	TSN	-0.04142	6.360058	-0.51068	0.61085	-6.60614	0.770022	0.678665
B.cells	ZFP319	0.150362	2.544028	0.510527	0.610955	-5.76882	0.818235	0.75474
B.cells	PRAMEF8	-0.11923	3.188085	-0.51034	0.611086	-5.86493	0.809947	0.741419
B.cells	DHX33	0.087395	3.32737	0.51009	0.611259	-6.02815	0.808207	0.738576
B.cells	GM45370	-0.37265	-1.29914	-0.51008	0.611268	-5.04922	0.870166	0.839855
B.cells	ZFP799	0.208548	0.939029	0.509821	0.611447	-5.37565	0.83974	0.789395
B.cells	TRAPPC12	0.066245	4.201517	0.509595	0.611605	-6.21426	0.797084	0.72095
B.cells	MAMLD1	-0.26336	0.110917	-0.50951	0.611666	-5.29996	0.850983	0.807802
B.cells	MDH2	0.053609	6.490426	0.509374	0.611759	-6.60782	0.768667	0.676519
B.cells	H2-T23	0.09523	6.358381	0.509332	0.611788	-6.59598	0.770274	0.679015
B.cells	GM10101	-0.22202	0.672014	-0.50927	0.611833	-5.34837	0.843347	0.795415
B.cells	FGL1	0.118713	3.523532	0.509232	0.611858	-6.10443	0.805737	0.734801
B.cells	HIST1H2BB	-0.22077	1.116611	-0.50906	0.611978	-5.34944	0.837352	0.785706
B.cells	ADCY4	-0.25423	1.413611	-0.50899	0.612026	-5.45889	0.833374	0.779248
B.cells	MCC	-0.18257	2.092188	-0.50896	0.612046	-5.59625	0.824367	0.764687
B.cells	4930589L2	-0.26181	-0.68311	-0.5089	0.612089	-5.17612	0.861773	0.82596
B.cells	HIST1H2AI	0.223632	2.588783	0.508834	0.612136	-5.75126	0.817848	0.754205
B.cells	KRT222	0.224884	-0.05515	0.508304	0.612506	-5.36707	0.853534	0.81186

B.cells	H2-AB1	0.133765	6.764416	0.508134	0.612625	-6.94273	0.765594	0.671646
B.cells	TTI2	-0.10155	2.749363	-0.50805	0.612682	-5.83937	0.816016	0.751084
B.cells	DHX58	0.142998	2.999964	0.508028	0.612699	-5.92927	0.812757	0.745866
B.cells	AKAP6	-0.36204	0.299684	-0.50793	0.612766	-5.27189	0.84868	0.804044
B.cells	SERTAD4	-0.25666	-0.0023	-0.50777	0.612876	-5.34059	0.852809	0.810872
B.cells	GM7160	0.166437	2.542262	0.507755	0.61289	-5.6852	0.818721	0.755521
B.cells	RTN4IP1	-0.17385	1.927586	-0.50771	0.612922	-5.58493	0.826809	0.768546
B.cells	PREP	-0.06305	5.298922	-0.50761	0.612988	-6.43949	0.783561	0.699742
B.cells	ARL15	0.052137	7.591924	0.507536	0.613042	-6.84741	0.755664	0.656471
B.cells	OS9	0.053747	5.617189	0.507464	0.613093	-6.47549	0.779617	0.693571
B.cells	CDV3	0.048567	7.090195	0.507326	0.613189	-6.72996	0.7617	0.665704
B.cells	URB2	0.118003	2.571455	0.507127	0.613328	-5.80627	0.818379	0.754961
B.cells	DHODH	-0.13856	2.109646	-0.50707	0.613369	-5.6494	0.824444	0.764729
B.cells	DXO	-0.1119	3.302702	-0.507	0.613414	-5.94397	0.80888	0.739792
B.cells	ERP27	-0.16307	1.896721	-0.50693	0.613468	-5.60612	0.827258	0.769296
B.cells	CDC26	0.044855	5.383294	0.50677	0.613578	-6.43009	0.782591	0.698191
B.cells	PRDM11	-0.09937	3.444862	-0.5066	0.613699	-6.04617	0.80709	0.736976
B.cells	PGAP3	-0.22979	1.12543	-0.50659	0.613706	-5.35638	0.837587	0.786077
B.cells	ARHGEF17	-0.34137	-0.37609	-0.50621	0.613973	-5.10482	0.858248	0.819667
B.cells	TRIM44	0.052305	6.053928	0.506175	0.613994	-6.56407	0.77451	0.685456
B.cells	BUD23	0.08688	4.09892	0.505926	0.614168	-6.15801	0.799056	0.723836
B.cells	2610044O1	0.112988	2.24732	0.505783	0.614267	-5.67752	0.823055	0.762129
B.cells	1700017B0	-0.07509	5.285252	-0.50556	0.614422	-6.35896	0.784212	0.700436
B.cells	BRDT	-0.18929	1.567286	-0.50554	0.614435	-5.48985	0.832104	0.776775
B.cells	PADI2	0.150746	2.631853	0.505075	0.614763	-5.79481	0.818311	0.754277
B.cells	NECAP1	0.056744	4.774239	0.505066	0.614769	-6.3709	0.790851	0.710621
B.cells	BMPR1A	-0.22122	2.310682	-0.50486	0.614912	-5.51536	0.822622	0.761102
B.cells	NFATC3	0.047645	7.290076	0.50441	0.615228	-6.83583	0.760252	0.66268
B.cells	SH3YL1	-0.17116	1.030881	-0.50438	0.615248	-5.48237	0.839862	0.788759
B.cells	2500002B1	0.144755	1.915535	0.504278	0.61532	-5.62463	0.828041	0.769668
B.cells	FAM43A	-0.08925	4.098571	-0.50425	0.615338	-6.08991	0.799684	0.724316
B.cells	DTD2	-0.11359	3.491192	-0.50389	0.615592	-5.92228	0.807702	0.736741
B.cells	HELB	0.089344	3.704754	0.503598	0.615796	-6.08737	0.805134	0.732481
B.cells	4931423N1	-0.26664	0.653443	-0.50326	0.616035	-5.28191	0.845637	0.79748
B.cells	LEF1OS1	-0.25452	-0.45613	-0.50301	0.616209	-5.17826	0.860913	0.822516
B.cells	SRP54C	0.159495	1.755516	0.502994	0.616219	-5.59557	0.830888	0.773519
B.cells	MRPL32	0.054943	5.68103	0.502685	0.616436	-6.53746	0.780554	0.693551
B.cells	TNFRSF12A	-0.17241	1.479733	-0.50261	0.616492	-5.55495	0.834605	0.779564
B.cells	H19	-0.16398	6.408472	-0.50254	0.616537	-6.67351	0.771618	0.679675
B.cells	ZFP316	0.257552	0.380007	0.502511	0.616557	-5.30129	0.849463	0.803778
B.cells	UBL4A	-0.09631	4.202976	-0.5024	0.616637	-6.07631	0.79909	0.722764
B.cells	GM4107	-0.29405	0.916265	-0.50236	0.616663	-5.31469	0.84218	0.791962
B.cells	PDPK1	-0.07684	6.462559	-0.50215	0.616809	-6.58819	0.770998	0.678757
B.cells	FOXRED1	-0.13344	2.831724	-0.50212	0.616833	-5.78494	0.816791	0.750942
B.cells	TBRG1	-0.06575	5.618457	-0.50181	0.617049	-6.49444	0.781556	0.694964
B.cells	PURB	0.041802	6.824936	0.50163	0.617175	-6.69381	0.76679	0.672012
B.cells	SNTA1	0.162638	1.865421	0.501599	0.617196	-5.58283	0.829719	0.771474

B.cells	TSPAN13	-0.05736	6.684078	-0.50148	0.617277	-6.65631	0.768512	0.674666
B.cells	POLR2H	-0.06216	4.512761	-0.50118	0.617493	-6.28576	0.795534	0.716829
B.cells	TRMT10B	0.161732	1.681609	0.50104	0.617588	-5.55959	0.8323	0.775606
B.cells	GM45051	-0.1595	1.845863	-0.50102	0.617604	-5.5236	0.830114	0.772073
B.cells	D1ERTD62	0.056903	5.295192	0.500907	0.617682	-6.47638	0.785711	0.701451
B.cells	GM16150	-0.23095	0.174831	-0.50086	0.617717	-5.32505	0.852667	0.808821
B.cells	KMT2E	0.036595	8.273581	0.5007	0.617826	-6.94027	0.749605	0.645692
B.cells	EIF2AK3	0.056766	6.399228	0.500666	0.61785	-6.6659	0.772092	0.68028
B.cells	COPZ1	-0.04413	6.366584	-0.50056	0.617923	-6.63369	0.772495	0.680906
B.cells	PSRC1	-0.23213	0.886949	-0.5001	0.618245	-5.33881	0.843271	0.793127
B.cells	NMT1	0.041202	6.941125	0.500047	0.618284	-6.72175	0.765782	0.670185
B.cells	CRKL	0.056385	5.071994	0.499901	0.618387	-6.37369	0.788779	0.706011
B.cells	LSM8	0.061894	5.264944	0.499859	0.618416	-6.36826	0.786368	0.702236
B.cells	HIST1H2BG	-0.24275	1.128352	-0.49934	0.618777	-5.37827	0.840364	0.787941
B.cells	BTBD8	0.208602	1.572677	0.499292	0.618814	-5.42419	0.8344	0.778288
B.cells	MYO18A	0.066224	4.439945	0.499158	0.618908	-6.26892	0.797106	0.71867
B.cells	TRAPPC13	0.081148	3.808587	0.498372	0.619459	-6.02877	0.805783	0.73165
B.cells	USP6NL	0.05617	5.563085	0.498216	0.619569	-6.51838	0.78368	0.696831
B.cells	ZFP280B	0.172079	1.826966	0.498082	0.619663	-5.53566	0.831777	0.773242
B.cells	ZFP273	-0.22882	0.440289	-0.49743	0.620124	-5.27366	0.850995	0.803793
B.cells	OSM	-0.24347	2.305551	-0.49735	0.620175	-5.5822	0.82593	0.763191
B.cells	SYNPO	0.295607	0.219102	0.496991	0.620429	-5.19564	0.854278	0.808852
B.cells	NIT1	-0.0881	3.750254	-0.49688	0.620506	-6.04161	0.807353	0.73323
B.cells	EIF1A	0.059569	5.711772	0.496594	0.620708	-6.52743	0.782695	0.694332
B.cells	MTERF2	-0.15591	2.099595	-0.49655	0.620735	-5.61761	0.829033	0.767758
B.cells	GM5089	-0.23091	0.395428	-0.49626	0.62094	-5.35321	0.852018	0.805061
B.cells	CLCN7	0.094538	3.608444	0.496188	0.620993	-6.12824	0.809318	0.736266
B.cells	DARS	-0.06155	5.511648	-0.49616	0.621015	-6.45361	0.785201	0.698275
B.cells	PPDPF	0.051337	5.630234	0.496137	0.621029	-6.48736	0.783727	0.695973
B.cells	KIFC5B	0.173298	1.787655	0.495936	0.62117	-5.52831	0.833267	0.774526
B.cells	TSPYL3	-0.23645	0.960124	-0.4958	0.621268	-5.39449	0.844393	0.792634
B.cells	CEP112	-0.22651	1.570821	-0.49577	0.621284	-5.42732	0.836166	0.779297
B.cells	TTC39C	-0.16628	2.230936	-0.49559	0.621411	-5.6873	0.827421	0.765169
B.cells	CTCF	-0.03643	7.101448	-0.49552	0.621462	-6.73973	0.765803	0.668176
B.cells	FAM118B	0.084935	3.464677	0.495404	0.621544	-6.01117	0.81128	0.739393
B.cells	SUMO3	-0.05578	5.724941	-0.49533	0.621595	-6.49077	0.782653	0.694334
B.cells	POLR3D	0.095806	3.27535	0.494955	0.62186	-6.00539	0.813989	0.74346
B.cells	RRP12	0.112168	2.60063	0.494711	0.622031	-5.82644	0.82294	0.757647
B.cells	TAF11	-0.06	4.933052	-0.49445	0.622217	-6.31689	0.793017	0.710068
B.cells	KLHL9	0.070023	4.65586	0.494345	0.622288	-6.23833	0.796517	0.7156
B.cells	EXOC7	0.070406	4.087686	0.494238	0.622364	-6.14947	0.803747	0.727057
B.cells	FOXK2	-0.05719	4.920069	-0.49416	0.622421	-6.35324	0.793181	0.710404
B.cells	PNPO	0.09178	3.744874	0.494115	0.622451	-6.1176	0.808146	0.734027
B.cells	DOT1L	0.065969	5.289755	0.4939	0.622602	-6.44485	0.788644	0.703158
B.cells	FCMR	0.116818	2.105124	0.493795	0.622675	-5.94775	0.829697	0.768363
B.cells	IGHMBP2	0.100794	2.850148	0.493533	0.62286	-5.85959	0.81993	0.752672
B.cells	BARD1	0.123252	3.42386	0.493482	0.622896	-5.93695	0.812458	0.740771

B.cells	GM32031	0.128104	2.845461	0.493397	0.622955	-5.76816	0.819992	0.752789
B.cells	NNT	0.118881	2.482711	0.493341	0.622995	-5.72697	0.824757	0.760431
B.cells	CYB5D2	-0.16697	1.870839	-0.493	0.623237	-5.5837	0.832992	0.773523
B.cells	ERG28	-0.05303	5.118614	-0.49282	0.623365	-6.36201	0.790963	0.706744
B.cells	GM28707	-0.23264	1.154193	-0.49257	0.62354	-5.339	0.842612	0.78922
B.cells	CUL9	-0.13512	2.579779	-0.49256	0.623543	-5.7185	0.8236	0.758554
B.cells	STRN4	0.06236	4.492619	0.492554	0.623549	-6.27783	0.798868	0.719232
B.cells	ABCB10	0.107134	2.977675	0.49241	0.62365	-5.90761	0.818383	0.75027
B.cells	OPLAH	-0.20685	1.500611	-0.49213	0.623847	-5.37453	0.837946	0.781831
B.cells	PLOD3	0.102607	3.277379	0.492114	0.623858	-5.98242	0.814478	0.744137
B.cells	PHKG2	-0.06492	4.528144	-0.49208	0.623879	-6.26334	0.798417	0.718679
B.cells	PNPLA6	0.09739	2.760588	0.491993	0.623944	-5.87301	0.821224	0.754968
B.cells	MRTO4	-0.07941	4.665156	-0.49185	0.624044	-6.30033	0.79668	0.716032
B.cells	ZNHIT3	-0.0857	3.532147	-0.49181	0.624073	-6.02238	0.811176	0.738972
B.cells	NAA15	-0.03838	6.743192	-0.49177	0.6241	-6.69231	0.770877	0.675783
B.cells	NLGN2	-0.31267	0.349554	-0.49167	0.624168	-5.24821	0.853565	0.807334
B.cells	CKM	0.34758	0.1057	0.491554	0.624253	-5.25883	0.856917	0.812837
B.cells	BZW1	-0.03269	7.459579	-0.49155	0.624256	-6.79112	0.762211	0.662455
B.cells	GM13008	-0.19274	1.087607	-0.49145	0.624323	-5.43819	0.843512	0.790972
B.cells	R74862	-0.23767	1.054776	-0.4914	0.624358	-5.39509	0.843956	0.791694
B.cells	IL6	-0.38855	1.106723	-0.49125	0.624469	-5.40244	0.843253	0.790587
B.cells	COX10	0.082953	3.775159	0.490785	0.624795	-6.0681	0.808041	0.734145
B.cells	NMNAT3	0.111256	3.105259	0.490702	0.624853	-5.9495	0.816718	0.747958
B.cells	HOXB4	-0.15658	1.361865	-0.4907	0.624853	-5.58586	0.839811	0.785113
B.cells	SNAPC3	0.066215	4.825524	0.490487	0.625004	-6.35328	0.794653	0.712991
B.cells	ZBTB8OS	0.06181	5.027468	0.490426	0.625047	-6.36438	0.792109	0.708994
B.cells	RRP36	-0.07138	3.775241	-0.49041	0.625056	-6.07169	0.80804	0.734143
B.cells	RASGRP2	0.054675	6.609517	0.490406	0.625061	-6.60185	0.772507	0.678443
B.cells	ZKSCAN1	0.067502	4.068204	0.490342	0.625107	-6.1859	0.804279	0.728181
B.cells	IL15RA	-0.14424	2.38833	-0.49031	0.625127	-5.77322	0.826124	0.763025
B.cells	SMYD4	0.133297	2.820357	0.490041	0.625319	-5.83281	0.820441	0.753939
B.cells	5430405H(	0.087842	3.910079	0.48995	0.625383	-6.09915	0.806307	0.731446
B.cells	KCNK6	0.133866	2.306704	0.489904	0.625415	-5.69711	0.827203	0.764817
B.cells	ACTR5	0.084493	3.477256	0.489898	0.62542	-6.06175	0.811886	0.740312
B.cells	TUBD1	-0.14694	2.095647	-0.48986	0.625443	-5.60465	0.83	0.769318
B.cells	COPRS	-0.21313	0.717795	-0.48986	0.625447	-5.36138	0.848532	0.799348
B.cells	UGDH	-0.07925	4.250685	-0.4897	0.625562	-6.16539	0.802006	0.724591
B.cells	CCDC84	0.114841	2.649641	0.489407	0.625766	-5.80084	0.822874	0.757614
B.cells	SLC52A3	-0.2095	0.451484	-0.48929	0.625847	-5.37929	0.852368	0.805366
B.cells	AC160336.	0.203041	1.371003	0.489203	0.62591	-5.41223	0.839884	0.785055
B.cells	RDH14	0.078644	3.731822	0.489159	0.62594	-6.03333	0.808788	0.735163
B.cells	IGKV1-117	-0.19642	-0.39916	-0.48904	0.626026	-5.32568	0.864129	0.824605
B.cells	TMEM64	-0.07587	5.051385	-0.48859	0.626344	-6.38596	0.792061	0.708728
B.cells	GPR107	0.054449	5.057833	0.488185	0.626627	-6.41707	0.79198	0.708782
B.cells	ZDHHC4	0.065222	4.474275	0.488162	0.626643	-6.2873	0.799357	0.720393
B.cells	ZSCAN18	-0.31079	0.363679	-0.48806	0.626712	-5.20947	0.853645	0.80763
B.cells	KANK3	-0.19831	2.000717	-0.48794	0.626802	-5.54636	0.831528	0.771718

B.cells	TPP1	0.066451	5.393356	0.487875	0.626846	-6.41297	0.787776	0.702194
B.cells	TOP1MT	-0.19276	1.70028	-0.48783	0.62688	-5.46478	0.835537	0.77819
B.cells	5930430L0	-0.25667	0.229599	-0.48781	0.626891	-5.25872	0.855486	0.810641
B.cells	NDUFA6	0.05002	6.56293	0.487762	0.626926	-6.66503	0.773323	0.679698
B.cells	GM11713	-0.14933	2.37115	-0.48762	0.627025	-5.73331	0.826616	0.763843
B.cells	D5ERTD579	0.059307	5.221315	0.487588	0.627048	-6.42349	0.789928	0.705597
B.cells	GGA1	-0.05652	4.730426	-0.48753	0.62709	-6.32245	0.796109	0.715306
B.cells	GM19522	0.202778	0.940675	0.487528	0.627091	-5.3887	0.845773	0.794827
B.cells	MRPL36	0.057872	5.835469	0.487454	0.627143	-6.52788	0.782275	0.693646
B.cells	PUF60	0.053967	5.949591	0.487298	0.627253	-6.55005	0.780863	0.691472
B.cells	TMEM209	0.11315	3.362785	0.487287	0.627261	-5.91874	0.81363	0.7431
B.cells	ATP8B2	0.09082	2.774077	0.487263	0.627278	-5.90481	0.82131	0.755371
B.cells	ZFP983	0.132678	2.38923	0.487213	0.627313	-5.73219	0.826377	0.763506
B.cells	SLC15A4	0.067728	4.804844	0.486997	0.627466	-6.36637	0.795273	0.713965
B.cells	CHST11	-0.0875	6.242747	-0.48662	0.62773	-6.6667	0.777388	0.685977
B.cells	CMBL	-0.18891	2.192141	-0.48661	0.627737	-5.65492	0.829134	0.767838
B.cells	ATG101	-0.0574	5.222366	-0.48658	0.627759	-6.41357	0.790057	0.705744
B.cells	SMIM7	-0.05671	4.932589	-0.48656	0.627774	-6.35595	0.793699	0.711461
B.cells	GM42701	-0.21322	0.844253	-0.48639	0.627891	-5.4136	0.8473	0.797163
B.cells	CNOT3	0.053715	5.46868	0.486015	0.628159	-6.47652	0.787286	0.701014
B.cells	ACOT9	0.062096	4.620859	0.485637	0.628426	-6.32724	0.798103	0.717861
B.cells	TOR1AIP1	0.045392	7.42657	0.48562	0.628438	-6.82884	0.763433	0.663926
B.cells	PLXNA2	-0.20185	2.31233	-0.48517	0.628756	-5.55367	0.828023	0.765585
B.cells	ACOT7	-0.0791	4.076826	-0.48513	0.628782	-6.12003	0.805039	0.728915
B.cells	AGL	0.097811	3.963587	0.484979	0.628891	-6.12336	0.806491	0.73123
B.cells	IRF2BPL	-0.08876	4.091669	-0.48489	0.628955	-6.26075	0.804848	0.728673
B.cells	UBR1	0.062589	5.323458	0.484721	0.629073	-6.45957	0.789249	0.70416
B.cells	CDH23	-0.13594	2.820164	-0.48466	0.629117	-5.74228	0.821331	0.75497
B.cells	MDM2	0.067208	6.41707	0.484305	0.629367	-6.64788	0.775698	0.68316
B.cells	TBC1D16	-0.18986	2.452428	-0.48401	0.629576	-5.57004	0.82617	0.762977
B.cells	ANP32A	-0.0299	7.495527	-0.48389	0.629659	-6.7817	0.762603	0.663054
B.cells	FLOT1	0.07665	4.575358	0.48389	0.62966	-6.21615	0.79868	0.71921
B.cells	PCNP	-0.03937	6.359657	-0.48379	0.62973	-6.63285	0.776403	0.684355
B.cells	TMEM115	0.070824	3.447731	0.483766	0.629748	-6.04222	0.813147	0.74214
B.cells	LLPH	0.041472	6.447819	0.483612	0.629857	-6.66757	0.775321	0.682733
B.cells	CDO1	-0.10962	3.756999	-0.48333	0.630059	-6.10508	0.809149	0.735885
B.cells	TRIM17	0.138542	2.197819	0.483291	0.630084	-5.66403	0.829541	0.768508
B.cells	MED27	0.046692	5.275175	0.48327	0.630099	-6.43706	0.789854	0.705434
B.cells	COX7A2L	-0.04058	6.644119	-0.4832	0.630145	-6.69646	0.772919	0.679054
B.cells	DHRS13	-0.23767	0.959658	-0.48304	0.630258	-5.38997	0.846159	0.795416
B.cells	RAP2A	0.099006	3.974658	0.483042	0.63026	-5.93684	0.806349	0.731441
B.cells	SERINC5	0.074344	5.287482	0.482987	0.630299	-6.39631	0.7897	0.705192
B.cells	CEP89	0.135512	2.805458	0.482918	0.630348	-5.83368	0.821523	0.755634
B.cells	PRPF38A	-0.05823	5.176932	-0.4829	0.630357	-6.41571	0.791086	0.70737
B.cells	CLN6	0.121097	3.482895	0.482789	0.630439	-5.79529	0.812691	0.741572
B.cells	CTDNEP1	0.057968	5.516749	0.482756	0.630462	-6.48517	0.786834	0.700754
B.cells	FMO5	-0.12191	3.247756	-0.48263	0.630552	-5.848	0.815744	0.74644

B.cells	ARMCX2	0.198846	1.41393	0.482492	0.630649	-5.42189	0.840018	0.785494
B.cells	E130317F2	-0.19507	0.77534	-0.48241	0.630709	-5.32029	0.848666	0.799555
B.cells	MIER2	-0.14096	2.002333	-0.48237	0.630737	-5.6392	0.832139	0.772751
B.cells	RAG2	-0.27007	0.168525	-0.48233	0.630762	-5.35124	0.856978	0.81314
B.cells	NDUFS5	-0.0509	6.478554	-0.48233	0.630764	-6.61314	0.774944	0.682239
B.cells	4930557J0	-0.17505	1.834427	-0.48232	0.630771	-5.72644	0.834379	0.776367
B.cells	RHBDD3	0.120883	2.194151	0.4823	0.630785	-5.65782	0.829589	0.76864
B.cells	DTX2	0.069399	4.396914	0.482297	0.630787	-6.2762	0.800949	0.722945
B.cells	PCYT2	0.069132	4.635801	0.482292	0.63079	-6.34325	0.797914	0.718153
B.cells	ARHGDIB	0.055592	8.995183	0.482194	0.63086	-7.05411	0.744828	0.636151
B.cells	ZFP931	0.148942	1.514653	0.482112	0.630918	-5.5417	0.838663	0.783402
B.cells	GM9828	-0.20691	1.076181	-0.48186	0.631097	-5.43091	0.844726	0.793047
B.cells	CABP1	-0.2752	-0.13612	-0.48169	0.631216	-5.23058	0.861403	0.820232
B.cells	4732496CC	-0.1892	0.689806	-0.48147	0.631374	-5.40786	0.850069	0.801687
B.cells	DCSTAMP	0.187452	-1.33737	0.481444	0.63139	-5.27877	0.877959	0.848061
B.cells	SLC4A1AP	0.062815	4.278134	0.481307	0.631488	-6.24055	0.802689	0.725601
B.cells	CD6	0.224878	0.929158	0.481151	0.631598	-5.38577	0.84681	0.796504
B.cells	BLM	-0.11826	3.897628	-0.4811	0.631632	-6.06396	0.807564	0.733391
B.cells	WDR89	-0.11661	2.643388	-0.48108	0.63165	-5.75382	0.823883	0.759438
B.cells	MAD2L2	-0.08891	3.472942	-0.48055	0.632023	-6.02945	0.813185	0.742322
B.cells	CFB	0.216544	4.663824	0.480472	0.632078	-6.38155	0.797916	0.718128
B.cells	GM17435	0.189082	1.133905	0.480455	0.632091	-5.44383	0.844176	0.792221
B.cells	P3H1	0.233857	0.585168	0.48039	0.632136	-5.38298	0.851642	0.804388
B.cells	4931413K1	-0.13273	1.943113	-0.4803	0.6322	-5.67589	0.833302	0.774627
B.cells	KLHDC1	0.130868	2.152219	0.480268	0.632223	-5.68924	0.830518	0.770136
B.cells	TNRC6A	0.045008	6.648204	0.480239	0.632244	-6.68835	0.773216	0.679553
B.cells	EFCAB2	0.137545	2.668273	0.479937	0.632458	-5.76028	0.823771	0.759275
B.cells	CAMTA2	0.07764	3.696678	0.479911	0.632476	-6.11591	0.810365	0.737857
B.cells	CROCC	-0.2109	1.025643	-0.47986	0.63251	-5.351	0.845721	0.794751
B.cells	GM26631	0.176185	1.219353	0.479763	0.63258	-5.49003	0.843098	0.790485
B.cells	TMPO	-0.06205	7.067829	-0.47939	0.632843	-6.73641	0.768264	0.671915
B.cells	PTPN4	0.063238	5.463527	0.479378	0.632853	-6.48815	0.78801	0.702608
B.cells	ACTB	-0.04253	13.9201	-0.47929	0.632915	-7.70029	0.690277	0.555436
B.cells	BCS1L	-0.1889	1.699946	-0.47927	0.632927	-5.5095	0.836721	0.780197
B.cells	PECAM1	0.06993	7.734027	0.479109	0.633044	-6.83262	0.760293	0.659604
B.cells	DCUN1D5	0.035086	6.828224	0.478995	0.633125	-6.71193	0.771247	0.676476
B.cells	IPMK	0.054503	5.867681	0.478864	0.633218	-6.5493	0.783081	0.694815
B.cells	NOMO1	0.087578	3.5373	0.478667	0.633357	-5.99503	0.812698	0.741419
B.cells	CCDC93	0.086821	3.72019	0.478581	0.633418	-6.08946	0.810333	0.737696
B.cells	ULBP1	0.10648	4.251838	0.478219	0.633675	-6.06083	0.803518	0.727022
B.cells	SLC2A3	0.097186	4.519005	0.478193	0.633693	-6.16392	0.800111	0.721646
B.cells	KSR2	-0.19098	2.695294	-0.47813	0.633734	-5.71418	0.823707	0.759223
B.cells	MMAB	-0.20311	1.326097	-0.47804	0.633804	-5.40793	0.841954	0.7887
B.cells	PRMT6	0.10227	2.45108	0.478032	0.633807	-5.79178	0.826927	0.764414
B.cells	RUVBL1	0.060174	4.77265	0.477976	0.633847	-6.32287	0.796893	0.716591
B.cells	ZFP235	0.153476	1.919758	0.477862	0.633928	-5.6035	0.833994	0.775819
B.cells	RNF135	0.145874	2.08421	0.4777	0.634043	-5.60737	0.831802	0.772303

B.cells	MTX1	0.069849	4.556924	0.477672	0.634062	-6.27715	0.799637	0.720954
B.cells	GM527	-0.24294	0.481195	-0.4774	0.634256	-5.36484	0.853588	0.807556
B.cells	SLC9A1	-0.05225	5.416377	-0.47728	0.634342	-6.43997	0.788923	0.70401
B.cells	TTC32	0.078005	4.101346	0.476941	0.634581	-6.10089	0.805578	0.730316
B.cells	KIF13A	0.077585	4.605824	0.476906	0.634605	-6.24566	0.799141	0.720141
B.cells	ZFP598	0.087951	3.44196	0.476801	0.634681	-6.02748	0.814084	0.743865
B.cells	HIST1H3B	0.24617	1.995292	0.476737	0.634725	-5.59542	0.833116	0.774409
B.cells	9430060I0:	-0.19515	1.308677	-0.47672	0.634739	-5.49002	0.842329	0.789331
B.cells	BLOC1S2	-0.06093	4.833886	-0.4767	0.634755	-6.36979	0.79625	0.715597
B.cells	NKRF	-0.10058	2.983842	-0.47663	0.6348	-5.93023	0.820056	0.753415
B.cells	CX3CR1	-0.17253	3.268892	-0.47655	0.634857	-5.80093	0.816334	0.747481
B.cells	LRRC32	-0.18819	1.241998	-0.4761	0.635179	-5.4814	0.843565	0.790983
B.cells	CLSPN	-0.11423	4.411842	-0.47586	0.635348	-6.13783	0.802053	0.72424
B.cells	RAD54L	-0.13039	2.724058	-0.47567	0.635481	-5.75628	0.824003	0.759102
B.cells	ARID4A	0.04718	7.062741	0.475257	0.635776	-6.77476	0.769416	0.672878
B.cells	TOMM7	-0.03867	7.561253	-0.47505	0.635923	-6.80328	0.763413	0.663669
B.cells	DUSP6	-0.08432	4.428378	-0.47476	0.636129	-6.23589	0.80223	0.724225
B.cells	TRIM65	0.09279	3.22435	0.47475	0.636135	-5.99209	0.817762	0.748905
B.cells	FAM13B	0.046328	6.457749	0.474705	0.636168	-6.63784	0.776827	0.684456
B.cells	UBQLN4	-0.0857	3.216072	-0.4746	0.636239	-5.93039	0.81787	0.749094
B.cells	ZFC3H1	-0.04127	6.914629	-0.47455	0.636276	-6.75714	0.77124	0.675807
B.cells	FAM133B	-0.05443	4.911343	-0.47454	0.636282	-6.34522	0.796097	0.714569
B.cells	GM42658	-0.15286	1.544714	-0.47429	0.636462	-5.59864	0.840166	0.784751
B.cells	GAB1	0.100049	4.473058	0.474143	0.636567	-6.42378	0.801836	0.723389
B.cells	ARMCX5	0.105287	2.433168	0.474054	0.63663	-5.79959	0.828342	0.765662
B.cells	BRD8	0.040924	6.236165	0.473718	0.636869	-6.61855	0.779902	0.688788
B.cells	ATP2B4	-0.14933	4.169834	-0.47365	0.636914	-5.84639	0.805896	0.729573
B.cells	GM43378	0.148847	1.243694	0.473397	0.637096	-5.50273	0.844493	0.791548
B.cells	ZWILCH	-0.15376	3.028209	-0.47326	0.637197	-5.77057	0.820725	0.753274
B.cells	TADA2B	0.090589	3.468615	0.473123	0.637292	-5.99717	0.81498	0.744166
B.cells	MRPS11	0.087156	3.950885	0.47298	0.637393	-6.10363	0.808742	0.7343
B.cells	F2RL2	0.270203	0.129948	0.47294	0.637421	-5.25535	0.859728	0.816596
B.cells	ARPC5	0.033476	8.144041	0.472936	0.637424	-6.93443	0.756809	0.6534
B.cells	NEBL	0.203043	1.182115	0.47284	0.637493	-5.65737	0.845327	0.79308
B.cells	ELOVL1	0.06467	4.858115	0.472833	0.637497	-6.3658	0.797158	0.715988
B.cells	CCT6A	-0.05239	5.802009	-0.47228	0.637889	-6.51707	0.785709	0.697598
B.cells	CLDN5	-0.2693	1.006603	-0.47187	0.638181	-5.26563	0.848325	0.797343
B.cells	RAPGEFL1	-0.12793	2.784487	-0.47185	0.638199	-5.8318	0.824523	0.75889
B.cells	TMEM88	0.143099	2.768173	0.471714	0.638293	-5.83181	0.824738	0.759235
B.cells	MRPS30	0.05559	4.726011	0.471692	0.638308	-6.33419	0.799412	0.718974
B.cells	DPM1	-0.04454	5.979025	-0.47153	0.638421	-6.59141	0.783687	0.694345
B.cells	MRPL37	-0.06537	4.2912	-0.47149	0.638452	-6.19368	0.804962	0.727761
B.cells	RICTOR	0.044811	6.409431	0.471168	0.638682	-6.64351	0.778458	0.686125
B.cells	GM15559	0.085041	3.148018	0.471061	0.638758	-5.99756	0.819851	0.75133
B.cells	PDZD8	0.057651	5.970732	0.471056	0.638761	-6.53789	0.78388	0.694556
B.cells	QRFP	0.260558	-0.61178	0.470879	0.638887	-5.2358	0.870678	0.834062
B.cells	4930486L2	0.253093	0.484692	0.470752	0.638977	-5.26088	0.855565	0.809067

B.cells	CDCA7L	-0.12679	3.766654	-0.47072	0.639	-5.90378	0.811805	0.738507
B.cells	FBXW8	0.073457	3.868261	0.470647	0.639052	-6.13289	0.810492	0.736421
B.cells	ZFP874A	-0.12794	2.146516	-0.47052	0.63914	-5.69761	0.833075	0.772555
B.cells	1500009L1	-0.22224	-0.02505	-0.47041	0.639218	-5.24113	0.862603	0.820633
B.cells	SRGAP1	-0.17446	2.559229	-0.47036	0.639256	-5.63162	0.827596	0.763797
B.cells	STON2	-0.14332	3.518101	-0.47013	0.639422	-5.75958	0.815027	0.743797
B.cells	GM14023	-0.19698	1.123999	-0.47009	0.639448	-5.47056	0.846832	0.795003
B.cells	COMMD9	-0.10627	2.646144	-0.47007	0.639462	-5.85793	0.826447	0.762063
B.cells	ARL5C	-0.06811	6.264356	-0.47	0.639509	-6.7133	0.780246	0.689074
B.cells	IL2RB	-0.20861	3.130268	-0.46992	0.639571	-5.68194	0.820083	0.751923
B.cells	4732471J0	-0.14594	1.996323	-0.46939	0.639944	-5.62762	0.835139	0.776183
B.cells	PARP8	0.05361	6.68237	0.469379	0.639954	-6.72905	0.775162	0.681278
B.cells	MBOAT2	-0.29206	0.666194	-0.46938	0.639957	-5.33085	0.853137	0.80541
B.cells	SRMS	0.325882	-0.72874	0.469242	0.640052	-5.08227	0.872308	0.837263
B.cells	GLYR1	0.040765	6.736527	0.46922	0.640067	-6.70965	0.774499	0.680312
B.cells	POLE3	0.079924	4.133596	0.469182	0.640095	-6.15012	0.807134	0.731443
B.cells	GNL1	0.064385	4.299889	0.468909	0.640289	-6.2372	0.805001	0.728117
B.cells	HIST3H2A	0.162766	2.104979	0.468799	0.640367	-5.61399	0.833689	0.773974
B.cells	EIF3M	-0.03333	6.621301	-0.4687	0.640435	-6.69436	0.775911	0.682554
B.cells	TMEM117	0.324044	-0.04691	0.468699	0.640439	-5.20791	0.862968	0.821653
B.cells	FAM110A	0.105388	3.760358	0.468675	0.640456	-5.94247	0.811944	0.739139
B.cells	PDZD11	-0.08028	4.30203	-0.46846	0.640611	-6.14668	0.804974	0.728093
B.cells	ATXN1	0.066753	6.919224	0.468288	0.640731	-6.70979	0.772268	0.676948
B.cells	COMMD10	0.06312	4.694613	0.468057	0.640895	-6.32516	0.799965	0.720291
B.cells	BTBD7	-0.03548	7.004982	-0.46796	0.640963	-6.7568	0.771223	0.67542
B.cells	GM46367	0.148844	2.175914	0.467892	0.641013	-5.70044	0.832743	0.772583
B.cells	3-Mar	-0.08337	6.392222	-0.46775	0.641112	-6.6736	0.778726	0.687084
B.cells	TBL2	0.109121	2.392625	0.467723	0.641134	-5.64214	0.829862	0.76799
B.cells	SPAG9	-0.05674	9.266573	-0.46751	0.641284	-7.13199	0.744266	0.634336
B.cells	AK4	0.21342	1.048143	0.467494	0.641297	-5.45402	0.847923	0.797328
B.cells	6720489N1	-0.18189	0.964398	-0.46741	0.641354	-5.40111	0.849063	0.799185
B.cells	PVRIG	-0.23276	-0.53207	-0.46715	0.641543	-5.18995	0.869674	0.83315
B.cells	PPP1R12C	-0.0539	5.009564	-0.4669	0.641723	-6.36526	0.795974	0.71419
B.cells	SERPINA3F	0.318623	2.19929	0.46666	0.641891	-5.55319	0.832432	0.772283
B.cells	EYA2	-0.19257	1.389743	-0.46662	0.641921	-5.49359	0.843292	0.789861
B.cells	TSPAN31	-0.07836	4.424978	-0.46659	0.641942	-6.18682	0.803401	0.725908
B.cells	PSMG2	-0.08648	3.919974	-0.46643	0.642054	-6.07721	0.809883	0.736184
B.cells	MED18	0.17814	1.412452	0.466382	0.642089	-5.45578	0.842985	0.789362
B.cells	G430095P1	0.195978	0.465518	0.466206	0.642215	-5.3881	0.85589	0.810404
B.cells	PSMB3	-0.04404	7.724991	-0.46615	0.642257	-6.85481	0.762517	0.662193
B.cells	BTBD9	0.042157	8.508871	0.466085	0.642301	-6.99532	0.753171	0.647904
B.cells	UBE4BOS1	-0.20834	0.735049	-0.46605	0.642328	-5.46864	0.852194	0.804361
B.cells	TBC1D9	0.12569	3.953614	0.465914	0.642423	-6.06117	0.809449	0.73558
B.cells	CHORDC1	0.052323	5.082056	0.465877	0.642449	-6.38719	0.795058	0.712853
B.cells	GTPBP8	0.115797	2.317318	0.465852	0.642467	-5.71701	0.830862	0.769864
B.cells	DHDH	0.117952	2.654165	0.465653	0.642609	-5.78213	0.826401	0.762735
B.cells	PLK1	-0.17125	3.704872	-0.46557	0.642669	-5.96283	0.812663	0.74078

B.cells	CLCC1	0.054611	4.329275	0.465438	0.642762	-6.24726	0.804625	0.728059
B.cells	SHPK	0.235673	0.252393	0.465346	0.642828	-5.26295	0.858826	0.815483
B.cells	ALOX5	0.260745	0.791987	0.465298	0.642862	-5.31447	0.851416	0.803355
B.cells	NECTIN1	0.131783	1.073651	0.465233	0.642908	-5.84255	0.847577	0.797094
B.cells	GM11696	-0.14936	1.871722	-0.46514	0.642975	-5.64617	0.836807	0.779613
B.cells	CREBRF	-0.05418	6.98351	-0.46511	0.642994	-6.76143	0.771485	0.676229
B.cells	SLC22A17	0.291288	-0.51359	0.465078	0.643019	-5.12693	0.869427	0.833004
B.cells	RRAGB	0.234387	0.125251	0.464989	0.643082	-5.29807	0.860583	0.818386
B.cells	ZC2HC1A	0.173227	1.741533	0.464949	0.643111	-5.56395	0.838553	0.782466
B.cells	UBE3A	0.039578	6.387077	0.46478	0.643231	-6.63725	0.778789	0.687599
B.cells	PNN	0.042426	6.310373	0.464697	0.643291	-6.61765	0.779734	0.689069
B.cells	ANKMY2	-0.07741	3.602393	-0.4644	0.643503	-5.99808	0.813991	0.743035
B.cells	FEM1C	-0.05145	7.117684	-0.46432	0.643563	-6.80333	0.769853	0.673767
B.cells	DNAJC12	-0.1092	3.069108	-0.4643	0.643575	-5.94201	0.820943	0.754144
B.cells	GM16201	-0.16257	1.818935	-0.46429	0.643578	-5.61797	0.837514	0.780826
B.cells	GM32036	-0.06502	3.651119	-0.46429	0.643579	-6.16392	0.813359	0.742028
B.cells	TRIP13	-0.15037	2.772205	-0.46427	0.643597	-5.77115	0.824844	0.760398
B.cells	1700123O2	-0.05888	4.989927	-0.46419	0.643653	-6.37663	0.796222	0.714885
B.cells	TRP53BP2	0.070093	3.957204	0.464112	0.643708	-6.22172	0.809403	0.735734
B.cells	RFXAP	0.059885	4.179927	0.464094	0.643721	-6.20665	0.806539	0.731187
B.cells	GM11944	-0.08514	4.498486	-0.46395	0.643825	-6.27361	0.802463	0.724732
B.cells	WDSUB1	0.107449	2.44185	0.463919	0.643846	-5.76723	0.829209	0.767418
B.cells	TXNDC9	0.047282	5.512196	0.463794	0.643935	-6.49026	0.789652	0.704612
B.cells	SECTM1A	0.320817	-1.13086	0.463774	0.643949	-5.07786	0.877832	0.847513
B.cells	RRN3	0.060425	4.400393	0.463753	0.643965	-6.25534	0.803715	0.726769
B.cells	THG1L	0.13837	2.021146	0.463689	0.64401	-5.65641	0.834808	0.776532
B.cells	WFS1	0.161134	1.279726	0.463491	0.644152	-5.40099	0.844874	0.792782
B.cells	JCAD	0.172403	1.404554	0.463228	0.644339	-5.43793	0.843309	0.790091
B.cells	ZFP414	-0.09705	3.443642	-0.46312	0.644418	-5.93806	0.816264	0.746567
B.cells	KLHL36	0.147838	2.076404	0.463032	0.644479	-5.65509	0.834285	0.775508
B.cells	RCBTB2	-0.05758	5.187796	-0.46297	0.644525	-6.40896	0.79393	0.711188
B.cells	TLN2	-0.20093	1.298032	-0.46284	0.64462	-5.48051	0.844783	0.79252
B.cells	KLRE1	0.250972	1.626605	0.462624	0.64477	-5.43623	0.840452	0.785366
B.cells	CLUH	0.088398	3.417508	0.462266	0.645026	-6.01858	0.816816	0.74728
B.cells	GM30211	0.120378	4.041214	0.462264	0.645028	-6.22893	0.80874	0.73442
B.cells	ESF1	-0.05013	5.001622	-0.46212	0.645134	-6.38347	0.796486	0.71506
B.cells	BORCS5	-0.06727	4.024358	-0.46206	0.645176	-6.18102	0.808957	0.734792
B.cells	CD59B	-0.29228	0.413	-0.46205	0.645181	-5.24577	0.857057	0.812408
B.cells	LYRM7	-0.18627	1.247217	-0.4619	0.645284	-5.42584	0.845674	0.793826
B.cells	KRR1	0.075055	3.94276	0.461817	0.645347	-6.12191	0.810023	0.736543
B.cells	CCDC191	0.140149	1.993228	0.461676	0.645448	-5.62512	0.835628	0.777596
B.cells	PRPF40B	-0.28498	0.534618	-0.46156	0.645529	-5.23876	0.855399	0.809778
B.cells	MAP1S	0.079547	3.781603	0.461423	0.645629	-6.12955	0.812105	0.739933
B.cells	DOHH	-0.06568	4.347216	-0.4613	0.64572	-6.25791	0.804826	0.728376
B.cells	PITHD1	-0.05981	4.744392	-0.46116	0.645813	-6.37079	0.799761	0.720367
B.cells	PRXL2A	0.094764	4.184431	0.460913	0.645993	-6.41233	0.806913	0.731705
B.cells	BC049715	-0.26923	0.604594	-0.46063	0.646194	-5.30782	0.854438	0.80838

B.cells	PLCD3	-0.19992	1.122441	-0.46062	0.646199	-5.34518	0.847367	0.796835
B.cells	BRIX1	-0.03801	6.144863	-0.46062	0.646203	-6.61046	0.782197	0.692913
B.cells	GM48742	-0.19206	0.618325	-0.46056	0.646242	-5.39105	0.85425	0.808083
B.cells	IFRD2	0.091323	3.30652	0.460416	0.646348	-5.98364	0.818278	0.749897
B.cells	CORO7	0.046972	6.215775	0.460328	0.646411	-6.61636	0.78132	0.691561
B.cells	TXNL4B	0.136151	1.740021	0.460253	0.646464	-5.55799	0.839022	0.783291
B.cells	P3H2	-0.26759	1.190669	-0.46017	0.646522	-5.53204	0.846441	0.795342
B.cells	SERTAD3	0.088662	4.131362	0.460127	0.646555	-6.15821	0.807595	0.732881
B.cells	SNX6	0.038657	6.568189	0.460063	0.646601	-6.6374	0.776978	0.684824
B.cells	DAGLA	-0.30116	-0.78197	-0.45996	0.646675	-5.10542	0.87349	0.840154
B.cells	MFN2	0.074957	3.671067	0.459942	0.646687	-6.02767	0.813536	0.742372
B.cells	ZFP532	0.237016	1.223667	0.459924	0.6467	-5.36993	0.845993	0.794661
B.cells	CCDC18	0.125628	2.722921	0.459842	0.646758	-5.71018	0.825936	0.762235
B.cells	RGL1	-0.0844	5.711711	-0.45972	0.646847	-6.42184	0.787581	0.701445
B.cells	LIN37	-0.07967	3.646925	-0.45945	0.647042	-6.08307	0.813849	0.743031
B.cells	PSPC1	-0.0569	5.702286	-0.4594	0.647074	-6.50466	0.787698	0.701727
B.cells	MRPL19	0.072845	3.923908	0.459365	0.647099	-6.12652	0.810266	0.737342
B.cells	USP34	0.036928	7.703229	0.459328	0.647126	-6.86557	0.763187	0.663711
B.cells	RBM25	0.029814	8.296585	0.459222	0.647202	-6.97158	0.756093	0.652891
B.cells	UNC119B	0.071993	3.551645	0.459202	0.647216	-6.19738	0.815086	0.745087
B.cells	RCC2	-0.04433	6.222377	-0.45903	0.647339	-6.61632	0.781285	0.691722
B.cells	SRGAP3	-0.1382	4.261174	-0.45894	0.6474	-5.95112	0.805976	0.730564
B.cells	9330160F1	0.143228	2.295032	0.458823	0.647487	-5.59227	0.831653	0.771647
B.cells	9530082P2	-0.20319	0.699259	-0.45863	0.647624	-5.29236	0.853191	0.806644
B.cells	GM15411	0.20374	0.212426	0.458446	0.647757	-5.36177	0.859889	0.81768
B.cells	1700084C0	0.104955	2.9115	0.458357	0.64782	-5.92512	0.823501	0.758586
B.cells	ZC3H18	-0.05364	5.270525	-0.45835	0.647823	-6.44072	0.793156	0.710379
B.cells	MNT	0.063515	4.606728	0.458244	0.647902	-6.37294	0.80156	0.723652
B.cells	PRKAA1	0.07141	4.45764	0.458056	0.648036	-6.28303	0.803462	0.726733
B.cells	FAM78A	0.106549	2.648934	0.457956	0.648108	-5.88377	0.826961	0.764281
B.cells	DCUN1D3	0.068473	4.424811	0.457952	0.64811	-6.27	0.803881	0.72743
B.cells	FOXK1	0.05552	4.627409	0.457922	0.648132	-6.34756	0.801296	0.723339
B.cells	LANCL1	0.077284	3.448045	0.457809	0.648213	-5.93109	0.816481	0.747534
B.cells	TMBIM4	0.045779	6.749974	0.457755	0.648251	-6.7129	0.774796	0.68189
B.cells	MIR99AHG	-0.16914	3.430837	-0.45765	0.648327	-5.8766	0.816705	0.747908
B.cells	NAGLU	-0.11411	2.6543	-0.45739	0.64851	-5.77925	0.82689	0.764318
B.cells	TOMT	-0.19335	1.736925	-0.45734	0.648552	-5.51882	0.839113	0.784073
B.cells	GM37401	0.194142	0.875344	0.457182	0.648661	-5.39389	0.850783	0.803086
B.cells	PSMB7	-0.04539	5.624583	-0.45717	0.64867	-6.51834	0.788715	0.703691
B.cells	RAB39	-0.12556	2.115142	-0.45715	0.648684	-5.892	0.834049	0.775892
B.cells	SF3A1	-0.0524	4.851981	-0.45704	0.64876	-6.33976	0.798442	0.719011
B.cells	ETAA1OS	-0.20221	0.543342	-0.45697	0.648814	-5.29917	0.85533	0.810547
B.cells	SHFL	0.159011	1.832835	0.456906	0.648859	-5.5938	0.837826	0.78205
B.cells	SMU1	-0.04439	5.608875	-0.45677	0.648956	-6.50741	0.788945	0.704082
B.cells	GTF2A1	0.055303	5.65525	0.456629	0.649057	-6.51102	0.788403	0.703228
B.cells	9130019O2	-0.26625	0.355077	-0.45648	0.649168	-5.24634	0.858022	0.814996
B.cells	WFDC17	0.176802	5.906168	0.456409	0.649215	-6.57313	0.785298	0.698391

B.cells	PIM2	0.093287	3.150665	0.456247	0.649331	-5.97278	0.820475	0.754066
B.cells	PLA2G6	-0.21028	1.28393	-0.4562	0.649366	-5.39565	0.845341	0.794265
B.cells	FHL1	-0.21976	0.58688	-0.4558	0.649648	-5.32244	0.855128	0.809886
B.cells	FCOR	-0.19262	0.865885	-0.45532	0.649993	-5.40293	0.851442	0.803725
B.cells	NAPG	0.062541	4.542195	0.455191	0.650087	-6.30699	0.802882	0.725635
B.cells	INPP4A	0.057405	6.032154	0.455148	0.650118	-6.52924	0.784128	0.696163
B.cells	DCTN6	0.056371	5.142412	0.455091	0.650159	-6.34002	0.795264	0.713623
B.cells	SMARCD2	-0.04201	5.611886	-0.45494	0.650267	-6.51619	0.789365	0.704368
B.cells	NOM1	0.062778	4.141882	0.454897	0.650298	-6.23568	0.80801	0.733791
B.cells	SWT1	0.051052	5.244266	0.45489	0.650303	-6.45448	0.79398	0.711614
B.cells	DDX55	0.0981	2.934608	0.454777	0.650384	-5.89675	0.823709	0.758891
B.cells	ATG13	0.063124	4.765899	0.454698	0.650441	-6.34551	0.800033	0.721182
B.cells	GLRA1	-0.22142	1.25067	-0.45461	0.650505	-5.52645	0.846203	0.795269
B.cells	CCDC86	-0.05401	5.146452	-0.45452	0.650566	-6.46041	0.795213	0.713582
B.cells	FAM221A	-0.18342	1.467642	-0.45449	0.65059	-5.41068	0.843266	0.790488
B.cells	ZFP407	0.039112	7.039039	0.454313	0.650717	-6.76296	0.771815	0.676976
B.cells	PDCD10	-0.03608	6.713222	-0.45411	0.650866	-6.71048	0.775796	0.68319
B.cells	AIF1	-0.12263	4.896333	-0.45402	0.650928	-6.24468	0.798447	0.718645
B.cells	CC2D2B	0.220513	2.70829	0.453998	0.650943	-5.84671	0.826764	0.763766
B.cells	BRAT1	0.117956	2.183649	0.453868	0.651036	-5.71331	0.833727	0.775063
B.cells	CHD6	0.052303	5.997264	0.453502	0.651299	-6.57729	0.78463	0.697136
B.cells	TMEM97	-0.0891	3.649444	-0.45344	0.651344	-6.01133	0.814442	0.744222
B.cells	MIS18A	0.077527	4.400206	0.453427	0.651353	-6.20827	0.804767	0.728832
B.cells	PDCD2	0.074128	3.817161	0.453391	0.651378	-6.1119	0.812269	0.740756
B.cells	ABHD16A	-0.05888	4.808556	-0.45335	0.651408	-6.32593	0.79956	0.720594
B.cells	MFN1	-0.08045	3.297935	-0.45334	0.651415	-6.00682	0.819019	0.751537
B.cells	INPP1	0.085642	4.03687	0.452933	0.651706	-6.10182	0.809632	0.736281
B.cells	SFSWAP	0.038625	5.786795	0.452884	0.651742	-6.5419	0.787443	0.701273
B.cells	TDP2	-0.0692	4.340054	-0.45276	0.651832	-6.19205	0.805735	0.730108
B.cells	GM37305	-0.17441	1.078554	-0.45273	0.651854	-5.43215	0.848825	0.799452
B.cells	THBS3	0.20821	1.01596	0.452592	0.651951	-5.48285	0.849713	0.80085
B.cells	GM50163	-0.20916	0.458993	-0.45241	0.652081	-5.33676	0.857383	0.813393
B.cells	PDGFB	0.255198	1.062654	0.452356	0.652121	-5.38036	0.849115	0.799901
B.cells	RDH12	0.144393	2.275253	0.45225	0.652197	-5.82301	0.832791	0.773443
B.cells	MEX3A	0.16576	1.577184	0.45204	0.652347	-5.52761	0.842172	0.788669
B.cells	IFT88	-0.13895	1.778503	-0.45188	0.652464	-5.55273	0.839461	0.784268
B.cells	CIC	-0.05864	4.821881	-0.45184	0.652488	-6.36056	0.799687	0.720593
B.cells	PLEKHA8	-0.19255	0.886546	-0.45179	0.652528	-5.31336	0.851549	0.803951
B.cells	ADORA2A	0.099779	3.148747	0.451738	0.652564	-6.1063	0.821275	0.754943
B.cells	TAX1BP3	-0.07171	4.090035	-0.45141	0.652797	-6.21194	0.80925	0.735506
B.cells	RUVBL2	0.08705	3.693884	0.451121	0.653007	-6.05622	0.814544	0.743803
B.cells	ZFP366	-0.20822	1.840557	-0.45102	0.653076	-5.61124	0.839016	0.783174
B.cells	CHIL3	0.340286	2.447415	0.450832	0.653214	-5.74378	0.830936	0.770125
B.cells	TAF7	-0.04959	5.1473	-0.45073	0.653289	-6.40638	0.79596	0.714414
B.cells	ERI2	-0.20233	1.310882	-0.45068	0.653324	-5.36402	0.846192	0.794884
B.cells	A430090L1	0.160823	0.628221	0.45058	0.653395	-5.46717	0.85551	0.810109
B.cells	CENPX	0.049759	6.347296	0.450471	0.653474	-6.61054	0.780971	0.690972

B.cells	SIGLECE	-0.24399	2.293576	-0.45041	0.653515	-5.54694	0.832982	0.773524
B.cells	SLC25A3	-0.03715	7.921066	-0.45011	0.653734	-6.89997	0.76188	0.661404
B.cells	OFD1	-0.07641	3.31561	-0.4501	0.653737	-5.96287	0.819564	0.751872
B.cells	TUBGCP6	-0.11503	2.759296	-0.44994	0.653851	-5.78725	0.826874	0.763615
B.cells	F630028O1	0.221633	1.027185	0.449876	0.653901	-5.33677	0.850121	0.801286
B.cells	OIP5	-0.1446	2.207869	-0.44979	0.653964	-5.60932	0.834194	0.775453
B.cells	A430072P0	0.22682	0.354986	0.449556	0.654131	-5.4024	0.859344	0.816509
B.cells	TCF20	0.046942	7.561094	0.449525	0.654153	-6.82383	0.766212	0.668178
B.cells	PANK4	-0.08241	3.423163	-0.44938	0.654256	-6.03428	0.81816	0.749758
B.cells	CXCR2	-0.24081	-0.00657	-0.44929	0.654319	-5.32934	0.864353	0.824749
B.cells	RNF103	0.067481	4.305165	0.44924	0.654358	-6.19251	0.806748	0.731577
B.cells	CRMP1	-0.2548	-1.07546	-0.44914	0.65443	-5.2013	0.87902	0.849575
B.cells	ZSCAN29	0.07967	3.485733	0.448939	0.654574	-5.99501	0.817344	0.748508
B.cells	YEATS4	0.043661	5.906755	0.448839	0.654646	-6.54284	0.7865	0.699742
B.cells	EVI2	-0.11861	3.35768	-0.44882	0.654657	-5.90247	0.819015	0.751197
B.cells	PDF	0.197551	0.794298	0.448759	0.654703	-5.34798	0.853303	0.806706
B.cells	E2F3	0.073614	5.460693	0.448685	0.654757	-6.38464	0.792078	0.708531
B.cells	MICU1	0.04448	5.891562	0.448683	0.654758	-6.54899	0.786689	0.700082
B.cells	CCT2	-0.04187	6.286032	-0.44826	0.655062	-6.62808	0.78199	0.692528
B.cells	FKTN	0.171672	1.595478	0.448259	0.655063	-5.48383	0.842624	0.789087
B.cells	SIGLEC1	0.299571	0.449661	0.448138	0.65515	-5.27625	0.858277	0.814609
B.cells	DHX38	0.062219	4.560036	0.448031	0.655227	-6.31087	0.803717	0.726636
B.cells	EIF3G	-0.05502	4.982954	-0.44785	0.655355	-6.39589	0.798376	0.718145
B.cells	TUSC2	0.103383	3.017242	0.447688	0.655473	-5.83398	0.823755	0.758597
B.cells	HBS1L	-0.04592	5.694465	-0.44769	0.655474	-6.51969	0.789417	0.704122
B.cells	TUFM	-0.06488	4.731912	-0.44752	0.655592	-6.32991	0.801566	0.723246
B.cells	SLC39A12	-0.35153	0.492802	-0.44734	0.655724	-5.24789	0.857736	0.813742
B.cells	CACYBP	-0.05129	5.736095	-0.44733	0.655734	-6.53157	0.788897	0.703307
B.cells	TRAF4	-0.1048	4.196822	-0.44719	0.655829	-6.1581	0.808414	0.734136
B.cells	A530088E0	0.142529	1.760356	0.447155	0.655857	-5.66132	0.840475	0.785622
B.cells	CTSW	0.200713	2.120663	0.446971	0.655989	-5.58027	0.835642	0.77782
B.cells	ACVR2A	0.079619	4.7385	0.446775	0.65613	-6.33521	0.801482	0.723237
B.cells	9230116N1	-0.17805	0.920334	-0.44655	0.656293	-5.45328	0.851869	0.80433
B.cells	RRAD	0.101828	2.552475	0.446461	0.656356	-6.00267	0.829893	0.768682
B.cells	STIM1	0.057434	7.712751	0.446346	0.656439	-6.86607	0.764643	0.66585
B.cells	F8	-0.15238	3.134673	-0.44634	0.656446	-5.77522	0.822213	0.756349
B.cells	RALGAPA2	0.062511	6.409122	0.446261	0.6565	-6.66985	0.780538	0.69047
B.cells	FAM50A	0.05012	5.186332	0.446079	0.656631	-6.43236	0.795803	0.714398
B.cells	RDH10	0.100817	3.207433	0.446066	0.65664	-5.94174	0.821259	0.754851
B.cells	NEK1	0.100136	3.697931	0.445954	0.65672	-5.96886	0.814862	0.744618
B.cells	CBX2	-0.24963	-0.18673	-0.44576	0.656857	-5.19068	0.867156	0.829519
B.cells	BDP1	0.049042	5.529011	0.445712	0.656895	-6.50764	0.79149	0.707615
B.cells	INVS	0.113017	2.809082	0.445348	0.657157	-5.82291	0.826497	0.763337
B.cells	HP	0.078813	7.365035	0.445335	0.657166	-6.92294	0.768845	0.672419
B.cells	KLHL18	0.066375	4.310869	0.445317	0.657179	-6.24063	0.806949	0.732093
B.cells	LOXL2	-0.18503	1.406795	-0.44529	0.657197	-5.46151	0.845249	0.79369
B.cells	MBOAT1	0.236877	0.682144	0.445239	0.657235	-5.37806	0.855132	0.80984

B.cells	AGBL3	-0.16527	1.72445	-0.44519	0.65727	-5.43536	0.840959	0.786735
B.cells	FLCN	0.068078	4.338996	0.445139	0.657307	-6.27635	0.806588	0.73155
B.cells	DEDD2	-0.07144	4.544003	-0.44512	0.657318	-6.25718	0.803963	0.72739
B.cells	CCL25	0.074651	4.40296	0.444905	0.657475	-6.28074	0.805768	0.73027
B.cells	OTUD5	-0.0437	5.568497	-0.44464	0.657664	-6.51153	0.790995	0.707009
B.cells	GM39556	-0.11847	3.384757	-0.44434	0.657884	-5.91883	0.818939	0.751349
B.cells	PTPN7	0.080151	4.151708	0.444337	0.657885	-6.20374	0.808995	0.735481
B.cells	DANCR	-0.16803	1.545559	-0.44425	0.657949	-5.47079	0.843372	0.790823
B.cells	GM28809	0.237132	-0.12461	0.444231	0.657961	-5.2497	0.86629	0.828369
B.cells	SIRT7	0.045208	4.918849	0.444186	0.657994	-6.38237	0.799189	0.719991
B.cells	GGNBP1	0.20676	1.044976	0.444051	0.658091	-5.39293	0.850167	0.801982
B.cells	TMED10	0.037395	7.769396	0.444044	0.658096	-6.85434	0.763961	0.66512
B.cells	RELA	0.054406	5.114793	0.443824	0.658254	-6.4255	0.796707	0.716216
B.cells	GM42997	-0.22903	0.465543	-0.44373	0.658319	-5.33681	0.858111	0.81509
B.cells	MADD	-0.0553	5.091113	-0.44373	0.658324	-6.43226	0.797007	0.716693
B.cells	SELENOO	0.089621	3.136064	0.443696	0.658346	-5.9422	0.822195	0.756773
B.cells	CEP170B	-0.20379	0.457754	-0.44367	0.658368	-5.29364	0.858219	0.815266
B.cells	VDAC1	0.047534	6.677223	0.443637	0.658389	-6.67712	0.777237	0.685734
B.cells	SERPINA3N	-0.11191	2.557516	-0.44357	0.658436	-5.8463	0.829826	0.769051
B.cells	ARF1	-0.03201	8.02586	-0.44353	0.658466	-6.9097	0.760883	0.660489
B.cells	GM16573	-0.13421	1.655105	-0.44352	0.658476	-5.63242	0.841893	0.788617
B.cells	LGALS2	-0.26444	-0.89826	-0.44323	0.658683	-5.11528	0.876886	0.846644
B.cells	TPCN1	-0.07949	4.187411	-0.44315	0.658741	-6.29083	0.808552	0.735065
B.cells	MXD3	0.175071	1.735683	0.443078	0.658791	-5.5433	0.840825	0.786959
B.cells	GM26901	-0.18602	0.57091	-0.44298	0.658859	-5.31972	0.856678	0.812846
B.cells	AKR1B8	-0.19577	1.592252	-0.44294	0.658892	-5.46985	0.842759	0.790113
B.cells	TDO2	-0.16675	3.218699	-0.44291	0.65891	-6.00187	0.821128	0.755179
B.cells	NUSAP1	-0.10432	5.649629	-0.44269	0.65907	-6.48192	0.79007	0.705837
B.cells	ZFP866	0.092141	2.993086	0.442468	0.659231	-5.93409	0.824168	0.760076
B.cells	LSM1	-0.04686	5.251939	-0.44245	0.659241	-6.43643	0.795068	0.71376
B.cells	KRIT1	0.043061	5.937975	0.442439	0.659252	-6.59031	0.78647	0.700258
B.cells	SRRM1	-0.03096	7.865839	-0.44217	0.659446	-6.90873	0.763016	0.663743
B.cells	KDM1A	0.040725	5.813944	0.442094	0.6595	-6.56397	0.788146	0.702765
B.cells	TMEM18	-0.15152	1.593876	-0.44154	0.659902	-5.52415	0.843211	0.79044
B.cells	EIF3F	-0.03845	7.589953	-0.4415	0.659927	-6.8685	0.766571	0.668957
B.cells	RCN3	-0.14362	1.932131	-0.44144	0.659969	-5.57965	0.838656	0.783043
B.cells	ITGAM	-0.12313	4.619395	-0.44136	0.660031	-6.19513	0.803469	0.726637
B.cells	ARL2BP	0.060653	5.086344	0.441144	0.660186	-6.4052	0.797532	0.717311
B.cells	LSM10	-0.1023	3.07773	-0.44112	0.660202	-5.84239	0.82344	0.758555
B.cells	TUT7	0.036106	7.206797	0.440996	0.660292	-6.77581	0.771216	0.676224
B.cells	RAP1GAP	0.220662	0.58551	0.440943	0.660331	-5.32277	0.856959	0.813029
B.cells	S100A1	-0.08335	4.086611	-0.44083	0.660414	-6.10275	0.810306	0.73764
B.cells	SURF2	0.078808	3.331878	0.440658	0.660536	-6.02103	0.820108	0.753325
B.cells	ACBD5	0.042995	6.231959	0.440589	0.660586	-6.6225	0.783185	0.69492
B.cells	USP21	0.092527	3.140041	0.440549	0.660615	-5.93886	0.822622	0.757385
B.cells	RBM48	-0.08344	3.037834	-0.44047	0.660675	-5.92155	0.823965	0.759544
B.cells	ZFP719	0.117056	2.119349	0.440437	0.660695	-5.63604	0.836147	0.779198

B.cells	TUSC1	0.088367	3.124075	0.440134	0.660914	-5.94878	0.822831	0.757782
B.cells	AU019990	-0.21008	1.211418	-0.44006	0.660969	-5.46779	0.848395	0.799175
B.cells	GM48765	0.176655	0.952243	0.439996	0.661014	-5.44195	0.851929	0.804951
B.cells	CHST2	0.272434	-0.02925	0.439986	0.661021	-5.23071	0.865467	0.827189
B.cells	HTATSF1	-0.05292	4.697996	-0.43989	0.661089	-6.32719	0.802466	0.725304
B.cells	GM17382	0.231715	0.002886	0.439818	0.661142	-5.28508	0.86502	0.826451
B.cells	CBY1	-0.09726	2.846037	-0.43978	0.661169	-5.81026	0.826492	0.763667
B.cells	ZC3H12C	-0.10274	4.431325	-0.43942	0.661432	-6.34248	0.80611	0.730786
B.cells	TXLNA	-0.05316	4.941372	-0.43919	0.661595	-6.37492	0.799631	0.720553
B.cells	CDK2	-0.07849	4.350301	-0.43919	0.661598	-6.18167	0.807178	0.732507
B.cells	GEMIN6	0.11754	2.59595	0.439093	0.661665	-5.75624	0.830072	0.769185
B.cells	COLQ	-0.21121	0.244853	-0.43887	0.661824	-5.28146	0.861956	0.821209
B.cells	HES7	-0.25399	-0.49621	-0.43879	0.661882	-5.15165	0.872257	0.838275
B.cells	CEP85	0.062242	4.470348	0.438789	0.661885	-6.28855	0.80565	0.730177
B.cells	9030404E1	-0.24314	-0.16276	-0.43849	0.662104	-5.28016	0.867818	0.830648
B.cells	FANCM	0.083753	3.67592	0.43819	0.662317	-6.09477	0.816262	0.746709
B.cells	ANK1	0.222105	1.273318	0.438083	0.662394	-5.53172	0.848227	0.798333
B.cells	SPDEF	-0.28955	-0.4296	-0.43782	0.662587	-5.15401	0.871884	0.837003
B.cells	FOPNL	-0.06318	4.468642	-0.43775	0.662637	-6.22455	0.80616	0.73046
B.cells	GM50209	-0.21007	0.629068	-0.43765	0.662706	-5.30253	0.857172	0.812788
B.cells	SNAPC4	0.150823	1.856185	0.43751	0.662809	-5.60378	0.840514	0.785598
B.cells	GM4566	0.147417	1.718905	0.437297	0.662962	-5.64144	0.842384	0.788643
B.cells	ACVR2B	0.167786	1.53111	0.437293	0.662966	-5.52327	0.844922	0.79277
B.cells	ERGIC3	0.048123	5.513051	0.437087	0.663114	-6.47654	0.792968	0.709676
B.cells	YARS2	-0.09261	3.150094	-0.43681	0.663311	-5.94279	0.823338	0.75805
B.cells	CDIP1	0.045623	5.935336	0.436759	0.663351	-6.56501	0.787681	0.701524
B.cells	EHHADH	-0.17208	1.66442	-0.43646	0.663568	-5.52882	0.843127	0.790159
B.cells	GM42722	0.121561	2.335621	0.436446	0.663577	-5.87107	0.834119	0.77555
B.cells	GIN1	-0.05527	3.954097	-0.43639	0.663615	-6.14743	0.812854	0.741409
B.cells	CENPF	-0.13092	5.050794	-0.43619	0.663759	-6.32783	0.798805	0.7192
B.cells	RPAIN	-0.06768	3.771433	-0.43605	0.663866	-6.09872	0.815222	0.745323
B.cells	PLK3	-0.089	4.483011	-0.43598	0.663916	-6.20256	0.806042	0.730706
B.cells	NAA40	0.049749	4.909944	0.435953	0.663934	-6.36202	0.800593	0.722073
B.cells	IIGP1	0.192491	4.709506	0.435728	0.664096	-6.18014	0.803146	0.726181
B.cells	NXF7	-0.26282	-1.34285	-0.43565	0.664154	-5.07307	0.884584	0.859185
B.cells	CTU1	0.195985	0.850388	0.435561	0.664216	-5.40098	0.854202	0.808466
B.cells	VMA21	0.05293	5.176423	0.435522	0.664245	-6.4084	0.797213	0.716803
B.cells	1700096K1	0.115313	2.613489	0.435487	0.66427	-5.75545	0.830422	0.769798
B.cells	SCN1B	0.186542	1.834445	0.435345	0.664373	-5.48536	0.840834	0.78674
B.cells	NFIC	0.063826	4.464453	0.435342	0.664375	-6.24254	0.80628	0.731229
B.cells	GM47230	-0.20283	0.668652	-0.4353	0.664408	-5.38125	0.856698	0.812652
B.cells	MTHFS	-0.09244	5.861718	-0.43494	0.664665	-6.53958	0.7886	0.70339
B.cells	DDC	0.179757	1.455602	0.434748	0.664805	-5.48293	0.845952	0.795196
B.cells	CCDC71L	0.068778	4.455272	0.434662	0.664867	-6.30197	0.806398	0.731536
B.cells	GM12158	0.164804	0.549233	0.434614	0.664901	-5.59154	0.858342	0.815476
B.cells	TMEM131L	0.043575	7.26323	0.434585	0.664923	-6.85908	0.771324	0.676528
B.cells	A1CF	-0.21844	0.569704	-0.43458	0.664929	-5.39446	0.85806	0.815013

B.cells	DTX3	-0.10561	2.572046	-0.43452	0.664966	-5.81577	0.830972	0.770894
B.cells	FBXL4	-0.10141	2.64655	-0.43415	0.66524	-5.8464	0.829984	0.769387
B.cells	SYNJ2BP	0.043888	5.520643	0.434094	0.665278	-6.50823	0.792873	0.710239
B.cells	MIB2	0.102299	2.662247	0.433988	0.665355	-5.84395	0.829776	0.769051
B.cells	DTWD1	0.129848	1.928598	0.433984	0.665357	-5.60146	0.839568	0.784903
B.cells	GM45267	-0.2078	0.245242	-0.43386	0.665445	-5.36444	0.862544	0.822549
B.cells	POLR1A	-0.05816	4.926327	-0.43376	0.665521	-6.45036	0.800385	0.722156
B.cells	SLC41A2	0.12595	2.900939	0.433726	0.665544	-5.87904	0.826618	0.764033
B.cells	ZFP959	-0.07952	3.241052	-0.43366	0.665589	-6.00166	0.822144	0.756837
B.cells	ARHGAP10	0.109899	5.105594	0.433618	0.665622	-6.2302	0.79811	0.718564
B.cells	HPS4	-0.08192	4.16681	-0.43361	0.665625	-6.15187	0.810106	0.73759
B.cells	USP9X	0.042943	7.387216	0.433472	0.665728	-6.84048	0.769817	0.674409
B.cells	RNF13	0.046618	6.065838	0.433246	0.665891	-6.59205	0.786055	0.699699
B.cells	FES	0.086076	4.284767	0.433211	0.665917	-6.08962	0.808588	0.735268
B.cells	TGOLN1	-0.0517	5.839155	-0.43319	0.665932	-6.52453	0.788882	0.704129
B.cells	MPZL3	0.139386	1.727945	0.433167	0.665948	-5.7076	0.842269	0.789473
B.cells	COL4A3BP	0.043894	6.352484	0.433015	0.666058	-6.6741	0.782499	0.694178
B.cells	CLEC16A	0.058269	4.763553	0.43287	0.666163	-6.37024	0.802457	0.725618
B.cells	HMBOX1	0.054793	5.578159	0.432782	0.666227	-6.4827	0.792151	0.709352
B.cells	RIN3	0.049105	5.640444	0.432463	0.666458	-6.56285	0.791369	0.708196
B.cells	ZFP84	-0.07679	3.7569	-0.43231	0.666567	-6.13144	0.815411	0.74632
B.cells	IL6RA	-0.0603	5.504339	-0.43202	0.666779	-6.60316	0.793078	0.710916
B.cells	FAM120B	-0.06514	4.124917	-0.432	0.666792	-6.19958	0.810647	0.738747
B.cells	SYNJ2	-0.11228	2.324633	-0.43159	0.66709	-5.71968	0.834265	0.776775
B.cells	HTT	0.049812	5.362069	0.431326	0.667281	-6.45689	0.794869	0.713927
B.cells	LRRC28	0.107016	3.874144	0.430837	0.667636	-6.0522	0.813889	0.744182
B.cells	DYNC1LI2	-0.05589	4.672528	-0.43081	0.667658	-6.31867	0.803618	0.727826
B.cells	SRBD1	0.067755	4.393343	0.430693	0.66774	-6.22586	0.807192	0.733505
B.cells	GM10552	0.138038	1.54086	0.43068	0.667749	-5.68416	0.844797	0.794082
B.cells	PTGIS	0.321342	-0.12832	0.430629	0.667786	-5.26457	0.86774	0.83176
B.cells	RAPGEF3	-0.20143	1.358645	-0.43056	0.667839	-5.3811	0.847268	0.798114
B.cells	TAF9B	0.191932	0.956005	0.430477	0.667896	-5.37923	0.852756	0.807093
B.cells	GTF3C2	-0.04075	5.474786	-0.43045	0.667913	-6.48504	0.79345	0.711751
B.cells	SAG	-0.09779	3.5306	-0.43042	0.667939	-6.11497	0.818357	0.751331
B.cells	ATF6B	0.062399	4.456866	0.430322	0.668009	-6.28415	0.806377	0.732209
B.cells	RASD1	0.091493	3.88428	0.43032	0.66801	-6.23605	0.813758	0.743972
B.cells	FOXJ3	-0.03932	5.947043	-0.43004	0.668216	-6.58499	0.787535	0.702508
B.cells	4930532G1	0.195342	1.429337	0.429956	0.668274	-5.55803	0.846308	0.796638
B.cells	EEF1AKMT	-0.09364	3.325831	-0.42986	0.668344	-5.99636	0.821033	0.755754
B.cells	MCTS1	0.039271	6.207891	0.42981	0.66838	-6.63998	0.78429	0.6975
B.cells	ATPSCKMT	0.085576	3.937455	0.429774	0.668406	-6.0805	0.813069	0.743012
B.cells	OAZ1	-0.03863	9.534615	-0.42954	0.668577	-7.17466	0.744269	0.635864
B.cells	NEDD8	0.030276	7.48397	0.429505	0.668601	-6.85	0.768644	0.6732
B.cells	PHF3	0.035155	6.980017	0.429443	0.668645	-6.76916	0.774778	0.682714
B.cells	2810002D1	0.123958	1.66458	0.429364	0.668703	-5.54668	0.843125	0.79156
B.cells	RUSC1	0.087666	3.288288	0.429223	0.668805	-6.02161	0.821524	0.756643
B.cells	GM26810	0.241649	0.423092	0.429125	0.668876	-5.29811	0.860083	0.819419

B.cells	AHCYL2	0.048417	6.28947	0.428826	0.669093	-6.71099	0.783279	0.696057
B.cells	CCNT1	0.034365	6.841664	0.4287	0.669184	-6.74014	0.776473	0.685438
B.cells	FAM49A	0.071215	5.390412	0.428202	0.669545	-6.55477	0.794512	0.713877
B.cells	ZFP639	0.064511	4.128657	0.428181	0.669561	-6.19867	0.810598	0.739398
B.cells	ZKSCAN17	0.069592	3.498798	0.428047	0.669658	-6.10741	0.818771	0.752479
B.cells	NSUN4	0.080861	3.189091	0.42778	0.669852	-6.02137	0.822826	0.759033
B.cells	CGNL1	0.198352	1.622928	0.427644	0.66995	-5.50141	0.843687	0.792824
B.cells	SMAD1	0.090764	3.547772	0.427584	0.669994	-5.90066	0.818133	0.751495
B.cells	RTCB	0.048496	5.290693	0.427504	0.670052	-6.48726	0.79577	0.715906
B.cells	GOPC	0.050396	4.545466	0.427456	0.670087	-6.3108	0.805242	0.730913
B.cells	IKZF2	-0.17297	4.781138	-0.42731	0.67019	-6.02627	0.802232	0.726164
B.cells	SNRPG	-0.03822	8.018397	-0.42703	0.670394	-6.92536	0.762201	0.663649
B.cells	GM44987	-0.21103	-0.34886	-0.427	0.670417	-5.23901	0.870824	0.837563
B.cells	GM13012	0.093574	2.775114	0.426666	0.67066	-5.88671	0.828281	0.767985
B.cells	ALKBH7	-0.08677	3.373231	-0.42666	0.670668	-6.03365	0.820412	0.755311
B.cells	SZT2	0.108451	2.318426	0.42642	0.670838	-5.6712	0.834348	0.777831
B.cells	COL4A2	-0.15742	2.620499	-0.4264	0.670854	-5.63363	0.830329	0.771323
B.cells	RTF1	0.032153	6.81408	0.426374	0.670872	-6.74558	0.776811	0.686352
B.cells	OLFR164	-0.20545	-0.16043	-0.42621	0.670991	-5.33528	0.868188	0.833292
B.cells	EMC3	0.043995	5.490353	0.425994	0.671148	-6.48751	0.793254	0.712181
B.cells	GM49961	-0.19265	0.574285	-0.42558	0.671447	-5.41309	0.857997	0.816632
B.cells	RHEBL1	0.15884	1.532009	0.42557	0.671456	-5.53354	0.844917	0.79519
B.cells	CMKLR1	0.220772	2.151939	0.425479	0.671521	-5.52506	0.836573	0.781625
B.cells	METTL15	-0.09251	3.231154	-0.42539	0.671589	-5.88045	0.822274	0.758542
B.cells	FAM234B	0.160356	2.096184	0.425301	0.671651	-5.5211	0.837319	0.782889
B.cells	BBS9	0.072033	4.909527	0.425228	0.671704	-6.39399	0.800598	0.723954
B.cells	HMCN1	-0.25072	1.920186	-0.4252	0.671726	-5.4596	0.839681	0.786746
B.cells	CAMK2A	-0.14399	0.846737	-0.42516	0.671754	-5.43328	0.854252	0.81057
B.cells	JAM3	0.245598	0.244994	0.424728	0.672067	-5.23056	0.862548	0.824316
B.cells	GM12253	-0.28807	-0.86999	-0.42467	0.672107	-5.06732	0.877893	0.850211
B.cells	RBX1	0.036011	7.700144	0.424667	0.672111	-6.86786	0.76603	0.669913
B.cells	GSTCD	-0.08858	3.564196	-0.4246	0.672157	-5.99942	0.817918	0.751665
B.cells	ARL6IP6	-0.04867	5.278997	-0.4246	0.672159	-6.44958	0.795917	0.716629
B.cells	OLFR1259	-0.28202	-1.34501	-0.42444	0.672276	-5.18654	0.884615	0.861563
B.cells	DCP1A	0.049496	4.850131	0.424434	0.67228	-6.40135	0.801354	0.725303
B.cells	PCTP	-0.12242	2.252585	-0.42417	0.67247	-5.66648	0.835227	0.779773
B.cells	RBM3	-0.03726	9.070642	-0.42404	0.672563	-7.1002	0.749703	0.645036
B.cells	ELAC1	0.127699	1.710286	0.42379	0.672748	-5.51779	0.842508	0.791816
B.cells	SP2	0.054273	4.742762	0.423741	0.672784	-6.36399	0.802722	0.727762
B.cells	ZFP874B	0.130516	2.178101	0.423637	0.67286	-5.72335	0.836223	0.781622
B.cells	GTPBP6	-0.09546	2.84005	-0.42363	0.672866	-5.89818	0.827422	0.767363
B.cells	GPIHBP1	-0.10601	4.095185	-0.4236	0.672888	-6.06372	0.81103	0.74103
B.cells	COP1	-0.04362	7.053619	-0.42353	0.672938	-6.78262	0.773879	0.682444
B.cells	HSD17B4	0.050191	5.027211	0.423464	0.672985	-6.40494	0.799104	0.722051
B.cells	NLE1	0.12586	2.257092	0.423453	0.672993	-5.77506	0.835167	0.779913
B.cells	INO80C	-0.07707	3.401246	-0.42343	0.673012	-6.00506	0.820046	0.755486
B.cells	RCAN3	-0.13823	2.117276	-0.42336	0.673061	-5.53654	0.837037	0.782968

B.cells	LAT2	0.055422	4.773693	0.423267	0.673128	-6.50253	0.802327	0.727181
B.cells	ZFP945	-0.08813	3.17689	-0.42322	0.673161	-5.92094	0.822986	0.760228
B.cells	WIZ	0.077131	3.836067	0.422773	0.673487	-6.12118	0.814383	0.74647
B.cells	TMTC1	-0.21054	2.714836	-0.42276	0.673496	-5.57878	0.829079	0.770125
B.cells	NSMCE4A	0.040435	6.083141	0.422491	0.673692	-6.5956	0.78584	0.701265
B.cells	MTAP	0.074412	3.671485	0.422324	0.673814	-6.09093	0.816521	0.750026
B.cells	MFSD2B	0.172139	0.599514	0.422227	0.673884	-5.41716	0.857649	0.816919
B.cells	SSX2IP	0.141978	2.481205	0.421977	0.674066	-5.69391	0.83218	0.77538
B.cells	BLVRA	-0.06003	5.071733	-0.42169	0.674273	-6.33404	0.798539	0.721448
B.cells	GAS2L1	-0.15098	1.765951	-0.42168	0.674283	-5.53647	0.841757	0.790955
B.cells	GM15503	-0.15874	0.81285	-0.42125	0.674593	-5.3782	0.854717	0.812278
B.cells	RNF19B	0.057816	5.758355	0.421224	0.674614	-6.54205	0.789892	0.70787
B.cells	FLYWCH1	0.073547	3.850858	0.42122	0.674617	-6.04967	0.814191	0.746475
B.cells	PSMD5	0.066858	3.835237	0.421003	0.674774	-6.07995	0.814394	0.746803
B.cells	IFT80	0.099733	3.626112	0.421001	0.674776	-6.01542	0.817112	0.751162
B.cells	EEF1B2	-0.03629	8.556525	-0.42095	0.674815	-7.0253	0.755779	0.654833
B.cells	YBX3	0.05928	6.314911	0.420759	0.674952	-6.62983	0.782964	0.697012
B.cells	MAP1LC3B	-0.04472	7.434486	-0.42067	0.675018	-6.87998	0.769243	0.675623
B.cells	HAUS5	-0.09892	2.831524	-0.42056	0.6751	-5.84733	0.827535	0.767979
B.cells	1110020A2	0.210188	0.468534	0.4205	0.67514	-5.29354	0.859455	0.820121
B.cells	UFC1	-0.03548	5.792898	-0.4205	0.675144	-6.5524	0.78946	0.707223
B.cells	TRAF3IP1	0.137741	1.889467	0.420426	0.675194	-5.69979	0.840094	0.788373
B.cells	RET	-0.15156	0.727544	-0.42023	0.675336	-5.57911	0.855888	0.814257
B.cells	FTSJ3	-0.0787	4.016619	-0.42013	0.675407	-6.20298	0.812045	0.743106
B.cells	AREG	0.43877	1.634087	0.420044	0.675472	-5.38959	0.843536	0.794026
B.cells	EMC4	-0.0578	4.771124	-0.41999	0.67551	-6.35572	0.80236	0.727679
B.cells	TRBC1	-0.18291	3.183542	-0.41971	0.675714	-5.71913	0.822898	0.760597
B.cells	RSBN1L	0.037827	7.198782	0.419683	0.675735	-6.79587	0.772108	0.680162
B.cells	FICD	0.183711	0.917558	0.419636	0.675769	-5.40463	0.853282	0.810058
B.cells	C920021L1	0.108416	2.268205	0.419625	0.675777	-5.69838	0.835019	0.780212
B.cells	NOX1	0.185405	1.548335	0.419601	0.675795	-5.49033	0.844696	0.795984
B.cells	ZFP579	0.141327	1.862923	0.41951	0.675861	-5.55885	0.840451	0.789075
B.cells	BRI3	-0.03932	7.328714	-0.41948	0.675881	-6.84573	0.770527	0.677725
B.cells	MRPL11	0.060611	4.26095	0.419364	0.675967	-6.24138	0.808894	0.738152
B.cells	GM16364.1	-0.19052	1.279847	-0.41921	0.676077	-5.50775	0.848338	0.802029
B.cells	TRIAP1	-0.06325	4.111675	-0.41906	0.676191	-6.19563	0.810818	0.741302
B.cells	DUSP22	0.061591	4.486105	0.418991	0.676239	-6.37823	0.806003	0.733632
B.cells	RTN1	0.16684	2.561189	0.418876	0.676322	-5.73471	0.831117	0.774017
B.cells	SERINC4	0.219768	0.365548	0.418863	0.676332	-5.28353	0.860878	0.822719
B.cells	SLC9A5	0.217697	0.362304	0.418588	0.676533	-5.26774	0.860923	0.822854
B.cells	HIST1H4H	-0.20632	0.513275	-0.41853	0.676575	-5.29084	0.858838	0.819412
B.cells	IGF2R	-0.05867	4.985937	-0.41845	0.676636	-6.45668	0.799627	0.723565
B.cells	KANTR	-0.11257	2.529607	-0.41839	0.676678	-5.77136	0.831536	0.774756
B.cells	GM867	0.192666	-0.30876	0.41832	0.676728	-5.42706	0.870262	0.838322
B.cells	HAUS4	-0.0713	4.204699	-0.41828	0.67676	-6.17727	0.809618	0.739473
B.cells	TNKS	-0.04915	6.066905	-0.4182	0.676816	-6.62328	0.786042	0.702113
B.cells	TRAP1	-0.05093	4.381095	-0.41816	0.676845	-6.25357	0.80735	0.735864

B.cells	MRPL13	-0.05562	4.834872	-0.41807	0.676907	-6.3245	0.801548	0.726629
B.cells	MBD4	0.08735	3.212172	0.417995	0.676964	-5.951	0.822523	0.760213
B.cells	AXIN2	-0.20532	-0.02376	-0.41795	0.676998	-5.27494	0.866282	0.831751
B.cells	CTH	-0.1295	3.512521	-0.41792	0.677021	-6.12628	0.818592	0.753886
B.cells	MFSD4A	-0.12431	2.041521	-0.41776	0.677135	-5.72076	0.838052	0.785374
B.cells	CLK4	-0.04113	6.012894	-0.4175	0.677326	-6.59054	0.786714	0.703311
B.cells	GIMAP9	0.082143	3.560572	0.417487	0.677335	-6.12025	0.817966	0.753
B.cells	PFDN1	0.046055	5.109296	0.417407	0.677392	-6.40902	0.798063	0.721263
B.cells	ITM2B	-0.04261	10.00633	-0.41736	0.67743	-7.1944	0.738794	0.629374
B.cells	WDR46	0.081872	3.429733	0.417225	0.677526	-6.06172	0.819673	0.7558
B.cells	SQLE	-0.142	1.97366	-0.41721	0.677533	-5.61935	0.838963	0.78704
B.cells	KIF19A	-0.16317	1.071139	-0.41713	0.677592	-5.42315	0.851182	0.807048
B.cells	LTO1	0.076687	3.526077	0.417134	0.677592	-6.00681	0.818416	0.753791
B.cells	COL1A1	-0.18332	2.255466	-0.41711	0.677607	-5.67308	0.835189	0.78092
B.cells	9330136K2	-0.12923	1.883233	-0.41708	0.677632	-5.70499	0.840178	0.789044
B.cells	SPIN1	0.041389	5.805516	0.417005	0.677685	-6.55725	0.789302	0.707492
B.cells	RALGDS	0.101509	3.822267	0.416894	0.677767	-6.11179	0.814562	0.747712
B.cells	COL1A2	0.150196	2.442649	0.416834	0.67781	-5.74752	0.832693	0.776987
B.cells	GAS5	-0.04733	6.391612	-0.41649	0.678062	-6.66152	0.782014	0.696213
B.cells	MRPL3	0.062152	4.701467	0.416369	0.678149	-6.30511	0.803249	0.729758
B.cells	TXN2	0.040274	6.315861	0.416209	0.678266	-6.64884	0.782952	0.697704
B.cells	MANEA	0.0785	3.728612	0.4162	0.678273	-6.05314	0.815778	0.74981
B.cells	CTSK	-0.21276	0.234472	-0.41606	0.678374	-5.3204	0.862693	0.82633
B.cells	GM43258	-0.1886	0.724157	-0.41601	0.678408	-5.34794	0.855935	0.815185
B.cells	PSMC6	-0.03882	6.141841	-0.41597	0.678437	-6.62221	0.78511	0.701132
B.cells	TIRAP	0.084165	3.005516	0.415927	0.678471	-5.94452	0.82524	0.765095
B.cells	GM42984	-0.20695	0.916614	-0.41591	0.678484	-5.35969	0.853295	0.810844
B.cells	GM16062	0.11159	1.874018	0.415873	0.678511	-5.65287	0.840302	0.789576
B.cells	EIF4EBP2	-0.03132	6.850744	-0.4157	0.678636	-6.73636	0.776361	0.68751
B.cells	ARHGAP23	-0.10103	3.694446	-0.4157	0.678637	-5.915	0.816222	0.750653
B.cells	ALG2	0.081781	3.062397	0.415605	0.678706	-5.85192	0.824491	0.764001
B.cells	KIF20B	-0.11361	4.170808	-0.41541	0.678852	-6.18109	0.810055	0.740892
B.cells	MAP3K1	-0.0364	7.970553	-0.41538	0.678873	-6.97289	0.762775	0.666525
B.cells	DTWD2	-0.09467	2.777791	-0.41518	0.679014	-5.96323	0.828246	0.770208
B.cells	RAB32	0.081488	4.344985	0.415177	0.679018	-6.08637	0.807813	0.737339
B.cells	SMIM10L1	0.053938	4.931338	0.415094	0.679079	-6.35462	0.800321	0.725402
B.cells	ELOVL7	-0.2515	-0.19718	-0.41501	0.679139	-5.19527	0.868702	0.83658
B.cells	GM19325	-0.14777	1.67424	-0.41491	0.679211	-5.68183	0.842994	0.79421
B.cells	CD80	0.098567	3.929604	0.41489	0.679228	-6.21478	0.813171	0.745918
B.cells	EBNA1BP2	0.070509	4.017915	0.414736	0.67934	-6.15876	0.812028	0.744128
B.cells	ZFP605	0.143271	1.633403	0.414705	0.679363	-5.6345	0.843546	0.795175
B.cells	MCM8	-0.15287	1.54016	-0.41445	0.67955	-5.49584	0.844807	0.797268
B.cells	POGLUT3	0.19634	0.979034	0.414393	0.67959	-5.31041	0.852441	0.809797
B.cells	DNAJC27	0.090797	2.442699	0.414359	0.679615	-5.88173	0.832692	0.777511
B.cells	METAP2	0.035301	7.3726	0.414161	0.679759	-6.83583	0.769994	0.677818
B.cells	2700038G2	-0.10003	2.577847	-0.41415	0.679766	-5.78044	0.830895	0.774595
B.cells	NATD1	-0.08952	3.25628	-0.414	0.67988	-6.05036	0.821944	0.760146

B.cells	TVP23B	0.060065	4.042523	0.41367	0.680118	-6.17391	0.81171	0.74378
B.cells	SNIP1	0.063264	3.633076	0.413638	0.680141	-6.06008	0.817021	0.752304
B.cells	GNPDA1	-0.06014	4.395098	-0.41357	0.680192	-6.2908	0.80717	0.736517
B.cells	CYYR1	0.181829	2.282713	0.413514	0.680231	-5.56711	0.834825	0.781105
B.cells	NOP10	-0.04055	7.15867	-0.41346	0.680272	-6.77534	0.772597	0.681982
B.cells	GM2449	-0.17042	1.279792	-0.4133	0.680387	-5.53967	0.848339	0.803231
B.cells	TET1	0.177309	1.135429	0.413246	0.680427	-5.48929	0.850305	0.806477
B.cells	A930014D(	0.221432	-1.05861	0.412946	0.680646	-5.11041	0.880505	0.857132
B.cells	XPO7	0.043169	6.710919	0.412815	0.680742	-6.7376	0.778078	0.690607
B.cells	SYNE3	-0.09748	2.767053	-0.41281	0.680747	-6.04424	0.828388	0.770738
B.cells	PLXNB3	-0.18846	0.164762	-0.41272	0.680808	-5.35553	0.863661	0.828562
B.cells	ZFP689	0.146656	1.407041	0.412703	0.680823	-5.51726	0.846611	0.800466
B.cells	D6WSU163	0.081272	3.1191	0.412589	0.680907	-5.90188	0.823745	0.763249
B.cells	ITK	-0.18106	4.224474	-0.41254	0.680942	-5.92991	0.809363	0.740157
B.cells	STEAP3	0.110869	1.980258	0.412459	0.681001	-5.74791	0.838874	0.787844
B.cells	ZFP566	-0.16111	1.254913	-0.41236	0.681072	-5.48295	0.848678	0.803889
B.cells	MAK16	0.045797	5.072635	0.412241	0.68116	-6.43823	0.798528	0.722886
B.cells	PMPCB	-0.05399	4.974651	-0.41216	0.68122	-6.38832	0.799771	0.72486
B.cells	GSTM4	-0.18057	1.121715	-0.41211	0.681253	-5.443	0.850492	0.80687
B.cells	SETD2	0.038955	6.922415	0.412054	0.681297	-6.75648	0.775483	0.6866
B.cells	PAQR3	0.112575	1.469925	0.412049	0.681301	-5.69681	0.845758	0.7991
B.cells	ARID5A	0.057958	4.681741	0.412042	0.681306	-6.38355	0.8035	0.730795
B.cells	B230307C2	0.071264	3.741811	0.411966	0.681362	-6.07412	0.815607	0.750167
B.cells	SLFN2	0.063296	6.335056	0.411937	0.681382	-6.75404	0.782714	0.69792
B.cells	D330041H(	-0.10387	2.247493	-0.41193	0.681385	-5.76374	0.835295	0.782011
B.cells	SLC25A27	-0.19474	0.260835	-0.41173	0.681536	-5.31434	0.862328	0.826393
B.cells	1810037I1	0.04688	6.482621	0.411718	0.681543	-6.65626	0.78089	0.695059
B.cells	SPTY2D1	-0.04292	5.765398	-0.41165	0.681594	-6.56488	0.789804	0.709077
B.cells	XAB2	0.070104	3.922858	0.411646	0.681595	-6.15744	0.813258	0.746397
B.cells	CSRNP2	0.114555	2.107884	0.411638	0.681601	-5.74989	0.837163	0.785053
B.cells	CTC1	0.079134	3.25694	0.411598	0.681631	-5.91003	0.821935	0.760358
B.cells	DUSP3	0.041712	5.553941	0.411419	0.681761	-6.65158	0.792455	0.713264
B.cells	GM36723	-0.21852	3.324777	-0.41138	0.68179	-5.64029	0.821047	0.758924
B.cells	RPA3	-0.06531	4.97161	-0.41136	0.681803	-6.32193	0.799809	0.724922
B.cells	RUNDC1	0.081665	2.994317	0.411359	0.681805	-5.96114	0.825387	0.765934
B.cells	4732465J0	0.191257	1.227323	0.411328	0.681827	-5.43699	0.849053	0.804506
B.cells	LUC7L	-0.03895	5.622585	-0.41124	0.681895	-6.53793	0.791593	0.711909
B.cells	PPP6R1	-0.03859	5.572544	-0.41113	0.681975	-6.47732	0.792221	0.712906
B.cells	COL15A1	-0.27063	0.332157	-0.41113	0.681975	-5.23479	0.86134	0.824772
B.cells	PES1	0.0469	4.691666	0.411011	0.682059	-6.39294	0.803374	0.730657
B.cells	COQ4	-0.10731	2.66674	-0.41086	0.68217	-5.7157	0.829716	0.773021
B.cells	ZFP458	0.215836	0.876726	0.410858	0.682171	-5.36305	0.853841	0.812459
B.cells	HACD3	-0.04729	4.907611	-0.4106	0.682356	-6.38785	0.800623	0.726359
B.cells	GM22146	-0.13324	2.073446	-0.41053	0.682409	-5.66392	0.837624	0.785975
B.cells	ST8SIA6	-0.16323	1.211567	-0.41052	0.682415	-5.80293	0.849268	0.805032
B.cells	TBC1D12	0.085297	3.768556	0.410494	0.682437	-6.18402	0.815259	0.749782
B.cells	KLRA1	0.213082	0.700337	0.410359	0.682535	-5.57861	0.856262	0.816594

B.cells	VWA8	0.055143	4.975158	0.410319	0.682564	-6.38823	0.799764	0.725062
B.cells	2810001G2	-0.09196	2.479465	-0.41025	0.682617	-5.75095	0.832203	0.777212
B.cells	SERPINB6B	-0.19793	2.936635	-0.41024	0.682625	-5.61502	0.826147	0.767391
B.cells	HIST1H2AE	-0.14054	4.491316	-0.41021	0.682646	-6.26473	0.805936	0.734906
B.cells	B3GNT3	-0.21715	0.711737	-0.4101	0.682726	-5.25144	0.856105	0.816381
B.cells	CBR4	0.105237	2.335594	0.409953	0.682832	-5.69868	0.834119	0.780405
B.cells	TCP1	-0.04483	6.67677	-0.40983	0.682925	-6.71628	0.778498	0.691616
B.cells	GM12979	-0.23121	0.049111	-0.40982	0.682929	-5.21675	0.865268	0.831629
B.cells	BTAF1	0.035376	7.190783	0.409613	0.68308	-6.81226	0.772206	0.681858
B.cells	TOLLIP	0.071546	3.832789	0.409579	0.683106	-6.12844	0.814426	0.748674
B.cells	CASP3	-0.05443	4.814976	-0.40955	0.683127	-6.31652	0.801801	0.728483
B.cells	GM43660	-0.2056	0.127169	-0.40943	0.683218	-5.39446	0.864183	0.829924
B.cells	MMP25	0.21528	0.499533	0.409421	0.683221	-5.33506	0.859028	0.821389
B.cells	MFHAS1	-0.09457	3.729832	-0.40939	0.683245	-6.01499	0.815762	0.750832
B.cells	LHFPL2	-0.149	2.227897	-0.4092	0.683385	-5.57952	0.835557	0.782913
B.cells	SAR1A	-0.04592	5.508767	-0.40917	0.683406	-6.48763	0.793022	0.714595
B.cells	HCAR2	-0.2778	2.377294	-0.40904	0.683499	-5.49312	0.833563	0.779667
B.cells	ASPA	-0.21901	1.375432	-0.40891	0.683592	-5.49088	0.84704	0.801688
B.cells	CCDC114	0.147693	1.271818	0.408913	0.683592	-5.47854	0.848448	0.804
B.cells	MRPL34	0.041436	5.428125	0.408855	0.683635	-6.49843	0.794037	0.716215
B.cells	DDAH2	-0.09461	3.503269	-0.40883	0.683653	-5.95036	0.818713	0.755636
B.cells	SENP6	0.033583	6.681311	0.408792	0.683681	-6.6831	0.778442	0.691657
B.cells	NT5M	0.068163	3.572881	0.408576	0.683839	-6.06445	0.817805	0.754201
B.cells	RLN3	0.19969	-0.84713	0.408419	0.683954	-5.26719	0.87758	0.852776
B.cells	ZFP677	-0.17696	0.788621	-0.40823	0.684093	-5.35333	0.85505	0.814946
B.cells	NUDC	0.044042	5.698731	0.408203	0.684111	-6.51398	0.790638	0.710897
B.cells	CDC16	0.0642	3.893321	0.408181	0.684127	-6.13237	0.813641	0.747539
B.cells	FAM193B	0.062469	4.050857	0.408049	0.684224	-6.17513	0.811603	0.744301
B.cells	IQCB1	-0.04991	4.824078	-0.40802	0.684247	-6.40489	0.801685	0.728468
B.cells	MED24	0.078132	3.187102	0.407947	0.684299	-5.94153	0.822852	0.762435
B.cells	MTMR4	-0.08017	2.648419	-0.40795	0.684299	-5.93889	0.829959	0.773947
B.cells	IGLL1	0.169036	3.916663	0.407929	0.684312	-6.18602	0.813339	0.747117
B.cells	AHDC1	0.04921	4.689967	0.40789	0.68434	-6.31371	0.803395	0.731221
B.cells	LSM7	-0.04146	6.346432	-0.40775	0.684445	-6.65297	0.782573	0.698287
B.cells	MAP2K2	-0.03053	7.299123	-0.40774	0.68445	-6.84188	0.770887	0.680011
B.cells	MOSPD3	-0.05216	4.656046	-0.40771	0.684474	-6.33417	0.803828	0.731935
B.cells	KIF1BP	0.066094	3.865166	0.407706	0.684475	-6.13027	0.814006	0.748227
B.cells	SORBS1	-0.06524	4.747678	-0.40758	0.684567	-6.36254	0.802659	0.730113
B.cells	GM9887	0.138738	1.686181	0.407546	0.684592	-5.59505	0.842833	0.795038
B.cells	CMTR2	0.157837	1.0685	0.407392	0.684705	-5.45477	0.851218	0.808876
B.cells	CCDC174	0.045587	5.082834	0.407362	0.684727	-6.42116	0.798398	0.723412
B.cells	LAD1	-0.29757	-0.99444	-0.40714	0.684891	-5.2082	0.879615	0.856619
B.cells	DDA1	0.051855	5.082621	0.407058	0.68495	-6.40988	0.798401	0.723508
B.cells	MOCOS	-0.21064	1.324581	-0.40691	0.685057	-5.4157	0.84773	0.803254
B.cells	COX15	0.078428	2.891641	0.406901	0.685064	-5.87149	0.826741	0.769005
B.cells	PPM1A	0.033401	6.125745	0.406849	0.685102	-6.60133	0.78531	0.702803
B.cells	NUP88	0.055983	4.421593	0.406807	0.685133	-6.27777	0.80683	0.73695

B.cells	KLF16	-0.08071	3.164065	-0.4068	0.685139	-5.94103	0.823154	0.763199
B.cells	COX19	-0.06416	4.496433	-0.40675	0.685172	-6.28514	0.80587	0.735417
B.cells	TNFAIP1	-0.07978	3.775763	-0.40673	0.685189	-6.09198	0.815166	0.750317
B.cells	ACSL5	0.051332	6.00653	0.406561	0.685313	-6.57313	0.786794	0.705174
B.cells	STYK1	-0.18246	0.538888	-0.40646	0.685388	-5.41881	0.858485	0.821028
B.cells	PEF1	-0.08079	3.347699	-0.40645	0.685391	-5.9748	0.820746	0.759356
B.cells	IMPA2	-0.06255	4.492173	-0.4064	0.685431	-6.26091	0.805925	0.735549
B.cells	EIF3J1	0.032639	7.268033	0.406341	0.685474	-6.83927	0.771265	0.680847
B.cells	CERCAM	0.186359	0.652443	0.406211	0.685569	-5.3207	0.856921	0.818465
B.cells	JAGN1	-0.06812	3.955321	-0.40617	0.685598	-6.18281	0.812838	0.746656
B.cells	FBXO36	-0.19156	1.228268	-0.40612	0.685634	-5.42353	0.84904	0.805514
B.cells	MRPL58	-0.05376	5.199269	-0.40569	0.685947	-6.4235	0.796925	0.721351
B.cells	GM27003	-0.06072	4.204875	-0.40562	0.685999	-6.2198	0.809616	0.741618
B.cells	CD72	-0.08194	4.334961	-0.40561	0.686012	-6.22433	0.807942	0.738937
B.cells	ATG12	0.052341	4.873947	0.405436	0.686136	-6.38474	0.801051	0.727944
B.cells	LMBR1L	-0.05878	4.517308	-0.40515	0.686343	-6.35799	0.805603	0.735241
B.cells	NUS1	0.042643	4.837828	0.405078	0.686398	-6.3761	0.80151	0.72871
B.cells	COX7C	-0.03143	8.585633	-0.40507	0.686403	-7.02452	0.755433	0.656518
B.cells	KCNK13	-0.11348	2.912398	-0.40499	0.68646	-5.99109	0.826467	0.768825
B.cells	FRMD4B	-0.11299	5.267906	-0.40492	0.686517	-6.20303	0.796057	0.720037
B.cells	PTPN13	-0.20283	0.415506	-0.40483	0.686578	-5.38977	0.860188	0.824078
B.cells	CCNO	-0.23383	0.174468	-0.40476	0.686629	-5.26395	0.863526	0.829623
B.cells	UBASH3A	-0.17251	2.009253	-0.40468	0.686689	-5.54052	0.838485	0.788399
B.cells	YIPF3	0.047982	4.904587	0.404485	0.686833	-6.38641	0.800661	0.727373
B.cells	GM12462	-0.19647	-0.49881	-0.40442	0.686883	-5.297	0.872892	0.845273
B.cells	LTF	0.162801	2.942911	0.404415	0.686885	-5.87403	0.826065	0.768189
B.cells	CETN4	-0.18554	-0.08195	-0.40438	0.686908	-5.31509	0.867093	0.835554
B.cells	KIF20A	-0.12509	3.768581	-0.40436	0.686924	-6.05853	0.815259	0.750741
B.cells	KIF5B	0.0281	7.401742	0.404201	0.687041	-6.82702	0.769641	0.678576
B.cells	ARRDC2	-0.1253	1.79539	-0.40414	0.687083	-5.65358	0.84136	0.793181
B.cells	MRPL4	-0.04641	5.176328	-0.40412	0.687099	-6.4616	0.797215	0.721965
B.cells	MTG2	-0.07962	3.031494	-0.40407	0.687133	-5.93001	0.824897	0.76638
B.cells	NRP2	0.132155	3.300945	0.404069	0.687138	-5.97094	0.821359	0.760656
B.cells	4930562C1	-0.21624	0.146658	-0.40401	0.687184	-5.25216	0.863912	0.830372
B.cells	HIF1AN	-0.08149	3.373427	-0.40397	0.687209	-6.02501	0.82041	0.759136
B.cells	PYGL	-0.08111	5.16283	-0.40386	0.68729	-6.35017	0.797385	0.7223
B.cells	KDELR1	0.038205	6.091298	0.403729	0.687387	-6.59465	0.785739	0.703889
B.cells	SEC61A1	-0.04036	5.49084	-0.40359	0.687486	-6.48913	0.793248	0.715813
B.cells	C2CD2	0.089902	3.079676	0.403558	0.687512	-5.90663	0.824263	0.765508
B.cells	EIF3D	-0.04524	5.480464	-0.40341	0.687622	-6.49707	0.793378	0.71603
B.cells	PPP2R2A	0.029236	7.180276	0.403364	0.687654	-6.80745	0.772334	0.682935
B.cells	6530413G1	-0.19533	-0.08741	-0.40335	0.687662	-5.21415	0.867169	0.835943
B.cells	UGGT1	0.043062	5.389379	0.403338	0.687673	-6.48016	0.794525	0.717848
B.cells	IRF2BP1	-0.06061	4.152538	-0.40329	0.687706	-6.1822	0.81029	0.743021
B.cells	FNDC9	0.072402	2.564671	0.403183	0.687787	-5.94473	0.83107	0.776635
B.cells	ZFP143	0.062937	3.815014	0.403176	0.687792	-6.10985	0.814656	0.750081
B.cells	PLEKHG5	-0.17037	1.864551	-0.40304	0.68789	-5.4994	0.840429	0.791943

B.cells	STK16	0.063774	4.37442	0.402798	0.688069	-6.24217	0.807435	0.738575
B.cells	FUT10	0.198893	-0.01434	0.402737	0.688114	-5.30143	0.866151	0.834425
B.cells	GM12971	0.156583	0.967071	0.402625	0.688196	-5.41204	0.852605	0.811994
B.cells	1700094J0	-0.23438	0.092196	-0.40254	0.688258	-5.29961	0.864669	0.831961
B.cells	CENPT	-0.09224	2.566821	-0.40247	0.688307	-5.7636	0.831042	0.776675
B.cells	EPOR	0.298698	-0.66556	0.402471	0.688309	-5.12026	0.875133	0.849636
B.cells	SLC10A3	-0.13512	2.160549	-0.40231	0.688423	-5.63271	0.836458	0.7855
B.cells	GM42699	-0.19112	0.727533	-0.40226	0.688466	-5.44296	0.855888	0.817414
B.cells	CCDC88B	0.092507	3.695402	0.402199	0.688508	-5.87997	0.81621	0.752666
B.cells	TEAD1	-0.18133	1.756692	-0.4021	0.688581	-5.4539	0.841882	0.79437
B.cells	ZFP85	0.210996	0.25725	0.402076	0.688599	-5.27559	0.862378	0.828158
B.cells	ATP5G1	-0.04275	7.643771	-0.40199	0.688665	-6.87934	0.76671	0.674321
B.cells	GM43065	0.19157	0.153169	0.401968	0.688678	-5.39883	0.863821	0.830554
B.cells	EFCC1	-0.20168	0.001369	-0.40191	0.68872	-5.3519	0.865932	0.834061
B.cells	BMX	0.218652	0.02253	0.401464	0.689048	-5.37588	0.865638	0.833623
B.cells	PRDM10	0.052284	5.213796	0.401457	0.689052	-6.46133	0.796741	0.721562
B.cells	PDE8A	0.060073	5.847934	0.401329	0.689146	-6.612	0.788772	0.70894
B.cells	GNG5	0.035679	8.925654	0.401103	0.689312	-7.08306	0.75141	0.650805
B.cells	TMEM59	-0.03622	6.649746	-0.40104	0.689356	-6.69119	0.77883	0.693365
B.cells	LNPK	-0.06445	4.386002	-0.40099	0.689398	-6.24071	0.807287	0.738484
B.cells	SLC39A13	0.086127	2.796524	0.400937	0.689433	-5.90592	0.827998	0.771882
B.cells	FAM53C	-0.06352	3.981286	-0.4009	0.689463	-6.12985	0.812502	0.74685
B.cells	YOD1	0.068585	4.084266	0.400895	0.689465	-6.29236	0.811171	0.744713
B.cells	BICDL1	0.124007	1.878488	0.400842	0.689503	-5.69458	0.840242	0.791843
B.cells	1700030KC	0.144052	1.730931	0.400746	0.689574	-5.60321	0.842229	0.795098
B.cells	RABGAP1	0.042364	5.920481	0.400742	0.689577	-6.60846	0.787866	0.707601
B.cells	DEPDC1A	0.153687	3.001084	0.400682	0.689621	-5.82675	0.825298	0.767502
B.cells	PLAGL2	0.057953	4.367209	0.400639	0.689652	-6.23939	0.807528	0.738871
B.cells	TMEM91	-0.11508	2.459733	-0.40035	0.689861	-5.76747	0.832624	0.77917
B.cells	GM32916	-0.3905	-0.24915	-0.40027	0.689924	-5.23182	0.869594	0.840095
B.cells	SORBS2	-0.19227	1.878636	-0.39998	0.690136	-5.56167	0.840468	0.79196
B.cells	FAM222A	0.099502	2.331884	0.399861	0.690224	-5.77693	0.834395	0.782056
B.cells	KIF1C	0.075596	3.254466	0.399756	0.6903	-5.99674	0.822191	0.762253
B.cells	U2AF1	-0.03183	7.593832	-0.39976	0.690301	-6.86993	0.767522	0.675521
B.cells	TIAL1	0.037998	5.798885	0.399716	0.69033	-6.55895	0.789599	0.710134
B.cells	WDR49	-0.19707	0.34569	-0.39937	0.690584	-5.3467	0.861616	0.826598
B.cells	WDCP	-0.07963	3.079235	-0.39914	0.690754	-6.00299	0.82473	0.766175
B.cells	1600022D1	0.192004	-0.42787	0.399063	0.690809	-5.19868	0.872419	0.844632
B.cells	HIST2H4	0.226628	0.951972	0.399038	0.690827	-5.3034	0.853288	0.812894
B.cells	EXO5	-0.14777	2.006793	-0.39897	0.690879	-5.59113	0.838987	0.78941
B.cells	PGPEP1	0.072137	3.461593	0.398817	0.69099	-6.03542	0.819759	0.758102
B.cells	YTHDF3	0.034205	7.017555	0.398421	0.69128	-6.79774	0.774794	0.686771
B.cells	TSACC	0.155015	1.881856	0.398332	0.691346	-5.64499	0.840711	0.792294
B.cells	INPP5K	0.04498	5.371477	0.39832	0.691355	-6.48602	0.795237	0.718976
B.cells	PEX11G	-0.10205	2.394748	-0.3983	0.691367	-5.74894	0.833841	0.781074
B.cells	IST1	-0.04041	5.536178	-0.39828	0.691382	-6.54232	0.793163	0.715687
B.cells	NEK8	0.105374	2.113602	0.398197	0.691445	-5.75431	0.837599	0.787222

B.cells	CAMKK2	0.040201	5.188179	0.398155	0.691476	-6.54756	0.797553	0.722669
B.cells	SDHAF2	0.051943	4.655098	0.397407	0.692025	-6.3477	0.804825	0.733609
B.cells	MED1	0.033043	5.883036	0.397388	0.692039	-6.59029	0.789298	0.708954
B.cells	NUDCD2	-0.06762	4.534449	-0.39713	0.692229	-6.22696	0.806509	0.736135
B.cells	APEH	0.077469	3.695339	0.396931	0.692375	-6.09841	0.817441	0.75353
B.cells	PDE1A	0.234719	0.441701	0.396429	0.692744	-5.26975	0.861318	0.825067
B.cells	THOC1	-0.04145	5.704802	-0.3964	0.692762	-6.58024	0.791934	0.712705
B.cells	CKB	-0.10227	4.578345	-0.3964	0.692766	-6.1184	0.806218	0.735409
B.cells	HIST1H4M	-0.18732	1.423329	-0.39627	0.692859	-5.4399	0.847858	0.802873
B.cells	BOD1	0.082162	3.243589	0.39618	0.692926	-5.97269	0.823537	0.763243
B.cells	MYO16	-0.2174	-0.30441	-0.39579	0.693212	-5.31398	0.871712	0.842375
B.cells	ACOT11	-0.10345	1.943725	-0.39575	0.693245	-5.718	0.840821	0.791389
B.cells	THOC6	0.058799	4.164901	0.395747	0.693245	-6.26148	0.811537	0.743965
B.cells	MRPS16	0.046194	5.607422	0.39571	0.693272	-6.51044	0.793157	0.714691
B.cells	RAB2A	-0.02284	8.094947	-0.3955	0.693427	-6.95537	0.762604	0.666905
B.cells	CEBPG	-0.04128	5.586056	-0.39544	0.69347	-6.50685	0.793426	0.715162
B.cells	NUP85	-0.07112	4.345133	-0.39539	0.693509	-6.21342	0.809213	0.740332
B.cells	BMPR2	-0.05872	5.519027	-0.39512	0.693707	-6.49825	0.794269	0.716574
B.cells	PRPF18	-0.05367	4.616004	-0.39504	0.693768	-6.3112	0.805736	0.734839
B.cells	FOS	-0.06432	8.489188	-0.39479	0.693949	-7.00436	0.757891	0.659764
B.cells	TSR1	0.07297	3.584651	0.394547	0.694127	-6.11223	0.819071	0.756339
B.cells	GTF3C6	-0.04215	5.226124	-0.39444	0.694204	-6.44478	0.797967	0.722559
B.cells	APOL7C	0.315409	-0.99321	0.394411	0.694227	-5.15091	0.881124	0.858921
B.cells	PLA2G15	0.076476	3.738633	0.394395	0.694239	-6.20823	0.817063	0.753105
B.cells	ACADVL	-0.05594	4.901912	-0.39439	0.694245	-6.39827	0.802084	0.72911
B.cells	FAM98C	-0.06739	3.737769	-0.39438	0.694248	-6.16216	0.817075	0.753123
B.cells	PLSCR3	-0.06356	4.301027	-0.39438	0.694251	-6.26377	0.809781	0.741408
B.cells	PPTC7	0.049791	5.148669	0.394345	0.694276	-6.47111	0.798949	0.724118
B.cells	PPP1R14A	-0.1791	0.601813	-0.39434	0.694281	-5.42285	0.859106	0.82174
B.cells	GM47507	-0.21976	0.732528	-0.39433	0.694286	-5.37987	0.857305	0.818762
B.cells	CCT5	-0.04334	6.571584	-0.39408	0.694473	-6.69739	0.781277	0.696094
B.cells	ATE1	-0.05555	3.945361	-0.39337	0.694991	-6.1573	0.814829	0.749003
B.cells	MIER3	-0.06368	4.078409	-0.39332	0.695032	-6.16099	0.813105	0.746236
B.cells	DCP2	0.049831	5.103401	0.393257	0.695077	-6.4562	0.799966	0.725282
B.cells	BCL7A	0.068588	4.80999	0.393067	0.695217	-6.37415	0.803702	0.731281
B.cells	XPO4	0.042356	5.623363	0.393012	0.695257	-6.55734	0.793396	0.714907
B.cells	PGS1	0.049341	4.740858	0.392951	0.695302	-6.37507	0.804585	0.732689
B.cells	DSCC1	-0.11631	2.435007	-0.39283	0.695389	-5.7226	0.834702	0.781269
B.cells	GM49797	0.049479	4.882769	0.392824	0.695395	-6.38961	0.802773	0.729824
B.cells	PPP1R13L	0.251151	-0.0774	0.392821	0.695398	-5.23159	0.869016	0.837754
B.cells	CFAP36	-0.05726	4.102137	-0.39272	0.695474	-6.17724	0.812798	0.745878
B.cells	SIKE1	0.051377	4.570739	0.392579	0.695575	-6.29341	0.806763	0.736228
B.cells	TPGS1	0.057469	4.393149	0.392455	0.695667	-6.32336	0.809044	0.739876
B.cells	NOP9	0.051142	4.555827	0.392341	0.695751	-6.30925	0.806954	0.736533
B.cells	GM48302	-0.16155	1.63727	-0.39233	0.695757	-5.54049	0.845426	0.798832
B.cells	CENPU	-0.11148	2.562499	-0.39226	0.695811	-5.69182	0.833003	0.778543
B.cells	GIMAP4	0.087519	4.091849	0.392186	0.695865	-6.26203	0.812931	0.746107

B.cells	NUP54	0.047755	4.859149	0.391951	0.696038	-6.39007	0.803159	0.730369
B.cells	8030456M	0.150849	1.090368	0.391844	0.696117	-5.49802	0.852959	0.811133
B.cells	NFYC	-0.02923	6.202043	-0.3918	0.696148	-6.66106	0.786242	0.703597
B.cells	TAF10	0.036356	6.856819	0.391614	0.696286	-6.7456	0.778165	0.690939
B.cells	LEO1	-0.06512	4.138442	-0.3915	0.69637	-6.17937	0.812434	0.745259
B.cells	SLC25A40	0.082159	3.227708	0.391368	0.696467	-5.97194	0.824309	0.764431
B.cells	RSPH10B	-0.24221	0.413611	-0.39136	0.696476	-5.25122	0.862296	0.826631
B.cells	TRMO	0.07784	3.013486	0.391301	0.696516	-5.97535	0.827131	0.769035
B.cells	ZFP369	0.082744	2.919936	0.391176	0.696608	-5.94873	0.828394	0.771067
B.cells	TUFT1	0.085984	2.2113	0.391005	0.696734	-5.82716	0.837895	0.786439
B.cells	PDPN	-0.28882	-0.21403	-0.39048	0.697124	-5.24285	0.871164	0.84129
B.cells	BYSL	-0.05904	3.67185	-0.39045	0.697145	-6.07043	0.818609	0.75516
B.cells	PNPLA2	0.053912	5.509886	0.39022	0.697312	-6.51076	0.795041	0.71757
B.cells	4833418N(	0.162141	1.533706	0.38995	0.697511	-5.48208	0.847059	0.801596
B.cells	TUT1	-0.07929	2.638669	-0.38993	0.697523	-5.79538	0.832216	0.777343
B.cells	TOMM40	0.045988	5.225725	0.389905	0.697545	-6.45674	0.798632	0.723341
B.cells	NANOS3	-0.18589	0.427569	-0.3894	0.697915	-5.28115	0.862225	0.826748
B.cells	GTF2H2	0.054131	3.759481	0.389375	0.697935	-6.16038	0.817467	0.75361
B.cells	MAP3K20	-0.06856	3.798494	-0.38933	0.697969	-6.25705	0.816959	0.752795
B.cells	SLC25A26	0.068207	3.795963	0.389299	0.697991	-6.1743	0.816992	0.752848
B.cells	GLOD4	0.045239	5.050654	0.389227	0.698044	-6.39585	0.800854	0.727033
B.cells	GM49164	0.172573	0.920748	0.389203	0.698062	-5.42219	0.855425	0.815553
B.cells	HDAC9	-0.0663	7.563843	-0.38911	0.698131	-6.86989	0.769644	0.677955
B.cells	PURA	0.037355	5.343404	0.388946	0.698251	-6.47851	0.797142	0.721259
B.cells	IREB2	0.043447	5.493255	0.388582	0.69852	-6.52183	0.79525	0.718346
B.cells	ALKBH8	0.059168	3.835022	0.388504	0.698577	-6.14126	0.816484	0.752283
B.cells	TEAD2	-0.14841	1.154161	-0.38844	0.698623	-5.49205	0.852228	0.810516
B.cells	NEB	0.16191	1.258562	0.388429	0.698632	-5.44847	0.850803	0.808168
B.cells	1700126G(	0.15202	1.104608	0.38833	0.698706	-5.53892	0.852906	0.811684
B.cells	GINS3	-0.11451	2.330816	-0.38832	0.69871	-5.7055	0.836321	0.784483
B.cells	NDUFB2	-0.0417	5.815553	-0.38787	0.699042	-6.56369	0.791199	0.71214
B.cells	IL12B	-0.31151	-0.29714	-0.38786	0.699054	-5.40465	0.87233	0.844092
B.cells	SMARCC2	0.034435	6.020787	0.387855	0.699055	-6.61318	0.788632	0.708082
B.cells	OTULINL	-0.06557	5.347398	-0.38779	0.699102	-6.42017	0.797092	0.721498
B.cells	ZNRD2	0.065395	4.037669	0.387646	0.69921	-6.17363	0.813854	0.748312
B.cells	ING1	0.046633	5.135774	0.387494	0.699322	-6.43118	0.799772	0.725783
B.cells	BMF	0.101913	2.137288	0.387345	0.699432	-5.86939	0.838913	0.788941
B.cells	IKBKE	-0.1659	3.116112	-0.38729	0.699473	-5.78354	0.825895	0.767751
B.cells	ENO3	0.132263	2.077848	0.387237	0.699511	-5.7396	0.839712	0.790255
B.cells	SERPINI1	0.084419	3.178888	0.387222	0.699523	-6.02031	0.825069	0.766419
B.cells	SEMA4F	0.173242	-0.41218	0.38719	0.699546	-5.34045	0.873946	0.846844
B.cells	TECPR1	-0.06716	4.375591	-0.38714	0.699586	-6.2807	0.80949	0.741315
B.cells	TOP3A	-0.07022	3.860539	-0.38712	0.6996	-6.13834	0.816152	0.752024
B.cells	C230037L1	-0.17868	0.378737	-0.38702	0.699672	-5.32218	0.862902	0.82849
B.cells	QRICH1	-0.02965	6.585338	-0.38701	0.699679	-6.72171	0.781621	0.69711
B.cells	MED25	-0.04859	4.496149	-0.38692	0.699742	-6.30272	0.807939	0.738888
B.cells	SNHG6	-0.07907	3.414627	-0.38687	0.699786	-6.02207	0.821972	0.761468

B.cells	ATAT1	0.085859	2.722421	0.386842	0.699803	-5.87965	0.831103	0.776275
B.cells	GM26549	-0.08109	3.018851	-0.38675	0.699868	-5.9256	0.827178	0.769915
B.cells	LRRN3	-0.21778	-0.11923	-0.38645	0.700089	-5.2576	0.869837	0.840122
B.cells	PSMC5	-0.03751	6.00886	-0.38645	0.700089	-6.60997	0.788781	0.708474
B.cells	OTUD4	0.04523	5.091742	0.386319	0.700189	-6.43964	0.800331	0.726797
B.cells	HSDL1	0.049368	4.167902	0.386171	0.700297	-6.214	0.812168	0.745731
B.cells	CIR1	0.041563	5.515333	0.386145	0.700317	-6.57914	0.794972	0.718276
B.cells	MMS22L	-0.07927	4.820836	-0.38614	0.700319	-6.30793	0.803781	0.732299
B.cells	SNRNP35	-0.07713	3.056704	-0.38612	0.700333	-5.96917	0.826679	0.769152
B.cells	FCHSD1	-0.1411	1.15994	-0.38589	0.700504	-5.48957	0.852149	0.810811
B.cells	POLR3H	-0.06744	3.509866	-0.38587	0.700518	-6.07164	0.820725	0.759519
B.cells	GPR137C	0.111378	2.390141	0.385835	0.700546	-5.71824	0.835528	0.783552
B.cells	RP2	0.044672	5.226456	0.385818	0.700558	-6.50441	0.798622	0.724081
B.cells	COPS7B	0.067824	3.34463	0.385573	0.700739	-6.06081	0.82289	0.763089
B.cells	GM15964	0.222189	0.162356	0.38548	0.700808	-5.36912	0.865907	0.833713
B.cells	SLC35G1	0.200709	1.026313	0.385366	0.700892	-5.37927	0.853978	0.813949
B.cells	TFB1M	0.125143	1.706255	0.385333	0.700916	-5.57925	0.844721	0.798714
B.cells	PPP5C	-0.05731	4.346979	-0.38532	0.700926	-6.18415	0.809858	0.74214
B.cells	RAB9	0.042951	4.887523	0.385121	0.701073	-6.40897	0.802931	0.731099
B.cells	TES	0.048053	5.486918	0.384944	0.701203	-6.60734	0.79533	0.719052
B.cells	VARS	-0.04411	5.713285	-0.38494	0.701207	-6.57571	0.792482	0.714535
B.cells	ZFP91	0.031675	6.894237	0.384888	0.701245	-6.76828	0.777817	0.691442
B.cells	SFT2D3	0.077909	2.864525	0.384818	0.701297	-5.85906	0.829219	0.773547
B.cells	RRM2B	0.055833	4.437435	0.384768	0.701333	-6.26661	0.808694	0.740429
B.cells	FASTK	0.076924	3.15048	0.384725	0.701365	-5.92137	0.825443	0.76743
B.cells	CENPL	0.082508	3.393274	0.384721	0.701368	-5.94807	0.822252	0.762263
B.cells	ZFP281	0.056808	4.496464	0.384652	0.701419	-6.28548	0.807935	0.739214
B.cells	ABCF2	0.050582	4.411019	0.384611	0.701449	-6.29023	0.809034	0.740978
B.cells	CDKN2AIPN	0.055267	4.272414	0.384562	0.701486	-6.21816	0.810819	0.743844
B.cells	EAF2	-0.06318	3.647314	-0.38446	0.701561	-6.14309	0.818929	0.756899
B.cells	GM19710	-0.10288	2.595654	-0.38418	0.701764	-5.96484	0.832788	0.779373
B.cells	ATP5L	-0.02938	8.67785	-0.38409	0.701834	-7.05207	0.756273	0.657997
B.cells	9930021J0	0.039189	6.407459	0.383904	0.701971	-6.671	0.783822	0.700933
B.cells	APOC2	-0.0843	4.203003	-0.38369	0.702133	-6.35422	0.811715	0.745349
B.cells	2810405F1	0.170352	0.687122	0.383635	0.70217	-5.41062	0.858639	0.821907
B.cells	ZFPL1	0.064563	3.466605	0.383621	0.70218	-6.01754	0.821291	0.760783
B.cells	HOTAIRM1	-0.2182	0.698917	-0.38359	0.702202	-5.31611	0.858476	0.821638
B.cells	CORO2A	0.057247	5.527432	0.383588	0.702204	-6.47324	0.794819	0.718368
B.cells	NAA10	-0.05418	5.084078	-0.38357	0.702217	-6.43172	0.800429	0.72729
B.cells	TMEM158	0.232164	0.271092	0.383489	0.702278	-5.32062	0.864396	0.831509
B.cells	TMEM53	-0.17828	0.637469	-0.38342	0.702332	-5.39649	0.859323	0.823103
B.cells	IGFLR1	0.1871	0.807622	0.383384	0.702355	-5.43734	0.85698	0.819222
B.cells	PPP1R3E	-0.13849	1.031561	-0.38336	0.702372	-5.39359	0.853906	0.814142
B.cells	KLHDC2	-0.04565	4.860142	-0.38314	0.702539	-6.39704	0.80339	0.731942
B.cells	CBR3	0.183614	0.624626	0.382808	0.702781	-5.44717	0.859772	0.823573
B.cells	TNRC6C	0.03405	7.143837	0.382684	0.702872	-6.81102	0.775003	0.686989
B.cells	OSGEP	-0.04599	4.907558	-0.38268	0.702875	-6.39824	0.802928	0.731119

B.cells	PPP2R1B	-0.05621	4.339805	-0.38245	0.703045	-6.28257	0.810322	0.742894
B.cells	SNX4	0.033428	6.329814	0.382289	0.703165	-6.66602	0.7852	0.702923
B.cells	KIF21B	0.052751	4.865707	0.381968	0.703402	-6.3817	0.803782	0.732316
B.cells	SART1	0.048476	4.872457	0.381922	0.703436	-6.37971	0.803696	0.732184
B.cells	MICAL3	0.074752	3.436889	0.381688	0.703608	-6.08997	0.822387	0.762121
B.cells	SPTBN1	0.045518	6.620496	0.381312	0.703887	-6.73706	0.78209	0.69754
B.cells	PSD	-0.13142	1.633137	-0.3807	0.704341	-5.58235	0.847001	0.801503
B.cells	1-Sep	0.044657	6.037433	0.38053	0.704465	-6.61372	0.789627	0.709053
B.cells	PDCD5	-0.03552	6.418317	-0.3805	0.704488	-6.66988	0.784883	0.701573
B.cells	GSS	0.080358	3.399727	0.38039	0.704568	-6.0081	0.823422	0.76308
B.cells	MAML3	0.054411	6.974808	0.380352	0.704597	-6.80876	0.778013	0.6908
B.cells	SPATA2	-0.06283	4.217056	-0.3803	0.704637	-6.26329	0.812771	0.745941
B.cells	ANKRD16	0.079533	3.026656	0.380141	0.704752	-5.93306	0.828337	0.771131
B.cells	EXOSC3	-0.04486	5.047755	-0.38011	0.704776	-6.42963	0.802112	0.72895
B.cells	CDC73	0.035644	6.174072	0.380031	0.704834	-6.66641	0.787921	0.706447
B.cells	SNRNP200	0.051189	5.038857	0.380009	0.70485	-6.41118	0.802225	0.729139
B.cells	POM121	0.044841	4.883475	0.379719	0.705065	-6.37012	0.804351	0.732312
B.cells	FMNL1	0.036851	6.443857	0.379649	0.705117	-6.7565	0.784706	0.70118
B.cells	MAPK11	0.197558	0.260399	0.379369	0.705323	-5.2774	0.866114	0.832782
B.cells	GM12802	-0.14744	0.658153	-0.37932	0.705362	-5.36724	0.860598	0.823635
B.cells	ZFP354C	0.12239	1.777029	0.379229	0.705427	-5.71493	0.845297	0.798423
B.cells	CLIC1	-0.03675	8.942892	-0.37898	0.70561	-7.09576	0.7545	0.654098
B.cells	PSMA1	-0.03492	6.9166	-0.37892	0.705652	-6.78321	0.778954	0.692064
B.cells	FBXO32	-0.1033	3.711448	-0.37889	0.705681	-6.15627	0.819578	0.75666
B.cells	DEPDC1B	-0.13641	2.954227	-0.37888	0.705687	-5.79693	0.829536	0.772772
B.cells	BCL2L12	-0.06043	4.178233	-0.37874	0.705787	-6.18834	0.813517	0.746929
B.cells	MKS1	0.160959	1.092934	0.378489	0.705975	-5.43067	0.854623	0.813942
B.cells	B3GALNT2	0.065625	3.643954	0.378479	0.705982	-6.1606	0.820468	0.7582
B.cells	MIGA2	0.193813	0.892517	0.378429	0.706019	-5.32142	0.857375	0.818509
B.cells	HGH1	0.118916	1.856316	0.378393	0.706046	-5.59995	0.844232	0.796885
B.cells	CRYL1	0.067114	3.73595	0.378159	0.706219	-6.10993	0.819381	0.756333
B.cells	LATS2	0.037586	5.889771	0.37791	0.706403	-6.6386	0.791824	0.712322
B.cells	CCNJ	-0.13288	2.015982	-0.3778	0.706484	-5.64201	0.842196	0.793428
B.cells	DNM1L	0.040611	5.639142	0.377795	0.706489	-6.55721	0.794974	0.71733
B.cells	VOPP1	0.090115	3.965239	0.377783	0.706497	-5.97341	0.816395	0.751547
B.cells	FYN	-0.05886	7.843798	-0.37764	0.706606	-6.95997	0.767806	0.674653
B.cells	DENND4B	0.05893	3.747946	0.377464	0.706733	-6.186	0.819336	0.756168
B.cells	FAIM	-0.05891	3.865848	-0.37726	0.706886	-6.19531	0.817819	0.753751
B.cells	ATG16L2	0.043625	5.680082	0.37725	0.706892	-6.54644	0.794586	0.716628
B.cells	DHX8	0.043341	5.129659	0.377055	0.707036	-6.46902	0.80159	0.727743
B.cells	SNX15	0.047689	4.880351	0.376926	0.707132	-6.40285	0.804769	0.732837
B.cells	5930403N2	-0.24301	-0.48768	-0.37689	0.707157	-5.16918	0.876883	0.850785
B.cells	GM4876	0.092837	1.628342	0.376824	0.707207	-5.79205	0.847616	0.802236
B.cells	WDR35	-0.2078	0.509893	-0.37664	0.707344	-5.2831	0.86304	0.827618
B.cells	DECR2	-0.10089	2.741577	-0.3764	0.707519	-5.80573	0.832739	0.777851
B.cells	AP2M1	0.038446	6.941455	0.376326	0.707576	-6.76206	0.779005	0.692031
B.cells	MTBP	-0.08047	3.256156	-0.37604	0.707791	-5.98671	0.825927	0.766888

B.cells	MTIF3	-0.07921	3.10156	-0.37603	0.707798	-5.99135	0.827967	0.770194
B.cells	GDI1	0.05157	4.991215	0.375879	0.707908	-6.39173	0.803434	0.730746
B.cells	SNX33	-0.15572	0.759185	-0.37576	0.707999	-5.39923	0.859597	0.822059
B.cells	SLC2A9	-0.09652	2.879571	-0.37543	0.708243	-5.92176	0.830906	0.775062
B.cells	TNKS2	0.029181	7.072389	0.375329	0.708315	-6.79446	0.777397	0.689683
B.cells	PRKAG1	0.035795	5.515312	0.375202	0.708409	-6.50049	0.796781	0.720238
B.cells	GM17494	0.084523	2.389013	0.37518	0.708425	-5.73887	0.837444	0.78571
B.cells	AASDH	0.113342	2.290623	0.375072	0.708505	-5.7666	0.838763	0.787863
B.cells	ATXN7L3	0.051326	4.200833	0.375028	0.708537	-6.24358	0.81359	0.747082
B.cells	COLEC12	-0.14229	2.890318	-0.37497	0.708584	-5.84523	0.830763	0.77483
B.cells	GTF3C4	-0.09534	2.57436	-0.37483	0.708686	-5.77584	0.834967	0.78167
B.cells	LZTFL1	0.05423	5.075487	0.374757	0.708739	-6.48091	0.802359	0.72912
B.cells	AMOTL2	-0.20475	0.684568	-0.37464	0.708822	-5.37489	0.860628	0.823837
B.cells	AP4M1	-0.05597	3.581284	-0.37448	0.708943	-6.08138	0.821657	0.760092
B.cells	ZFP691	0.090039	3.075803	0.374476	0.708947	-5.89739	0.828307	0.770858
B.cells	MAP2K6	-0.07759	3.533157	-0.37447	0.708952	-6.13234	0.822287	0.76111
B.cells	RTL6	0.21883	0.292529	0.374403	0.709001	-5.24749	0.866064	0.832887
B.cells	NDST1	0.070939	4.570663	0.374371	0.709025	-6.19656	0.808819	0.739469
B.cells	SCIN	0.228686	0.090871	0.374354	0.709038	-5.35308	0.868876	0.837562
B.cells	NT5C	0.045533	5.114948	0.374342	0.709046	-6.50438	0.801857	0.728349
B.cells	RPGRIP1L	0.131144	1.613197	0.374294	0.709082	-5.50191	0.847906	0.80289
B.cells	LSM14A	0.028938	6.620649	0.374259	0.709107	-6.72466	0.782963	0.698452
B.cells	GM5165	0.086468	2.638407	0.373946	0.70934	-5.84437	0.834284	0.780373
B.cells	HAP1	0.210038	-0.45019	0.373874	0.709393	-5.20983	0.876645	0.850312
B.cells	TBCC	0.066975	3.579816	0.373753	0.709483	-6.07945	0.821866	0.760287
B.cells	DPYD	-0.11475	3.567148	-0.37365	0.709563	-6.15447	0.822043	0.760585
B.cells	CBFB	0.027163	6.809866	0.373353	0.709779	-6.76008	0.78096	0.695029
B.cells	PQLC3	-0.06391	3.812077	-0.37316	0.709924	-6.21096	0.818991	0.755591
B.cells	ART2B	-0.29058	0.097769	-0.37312	0.709949	-5.2106	0.869151	0.837781
B.cells	CNOT6	0.031692	5.947416	0.372913	0.710105	-6.57965	0.791684	0.712019
B.cells	SIRPA	-0.07454	5.832373	-0.37282	0.710174	-6.36962	0.793128	0.714304
B.cells	SLC20A1	-0.05577	5.219384	-0.37273	0.710242	-6.43695	0.800872	0.726599
B.cells	CDAN1	0.086406	2.943919	0.372688	0.710272	-5.90783	0.830408	0.774104
B.cells	RAD50	0.047181	4.906644	0.372315	0.710549	-6.40821	0.804858	0.73308
B.cells	CPTP	-0.09422	2.248177	-0.37221	0.710629	-5.69665	0.839692	0.789368
B.cells	PPP1R13B	0.046845	5.30235	0.372137	0.710681	-6.50559	0.799819	0.725054
B.cells	SLC1A3	0.291025	-0.29724	0.372127	0.710689	-5.20995	0.87469	0.84721
B.cells	ZFP869	0.063546	4.026546	0.372099	0.710709	-6.18001	0.816199	0.751271
B.cells	SLC48A1	0.04784	5.074284	0.372043	0.710751	-6.3551	0.802718	0.729683
B.cells	MCU	0.046077	6.164566	0.372009	0.710776	-6.62406	0.788968	0.707874
B.cells	MPC1	-0.03542	7.569491	-0.37194	0.710828	-6.90968	0.771657	0.680743
B.cells	PIGN	0.060213	4.40092	0.371888	0.710866	-6.29448	0.811352	0.74352
B.cells	TTC27	0.068202	3.520631	0.371856	0.710889	-6.07345	0.822804	0.761961
B.cells	MAD2L1BP	-0.0533	4.194819	-0.37176	0.710962	-6.27525	0.814019	0.747797
B.cells	TMED2	-0.02975	8.136615	-0.37153	0.711133	-6.94936	0.764846	0.670101
B.cells	APIP	-0.05046	4.220389	-0.37148	0.71117	-6.2328	0.813737	0.747297
B.cells	GM12689	0.211744	-0.48996	0.371415	0.711217	-5.20124	0.877433	0.851848

B.cells	CDC40	0.038238	6.047181	0.371153	0.711412	-6.62935	0.790491	0.710321
B.cells	TLK2	-0.03038	6.825344	-0.37114	0.711422	-6.7648	0.780825	0.695098
B.cells	CDC25B	-0.07241	4.400381	-0.37112	0.711433	-6.34367	0.811417	0.743645
B.cells	RACGAP1	-0.0886	5.205247	-0.37101	0.711516	-6.35023	0.801123	0.727192
B.cells	TENT4B	0.036194	6.368937	0.370853	0.711634	-6.69109	0.786544	0.70401
B.cells	POU5F2	0.110535	1.687999	0.370699	0.711748	-5.67371	0.847438	0.802009
B.cells	GRM8	-0.26324	1.381099	-0.37053	0.711873	-5.27691	0.85168	0.808907
B.cells	ENAH	-0.16496	0.744942	-0.37034	0.712012	-5.49973	0.860498	0.82333
B.cells	TIAM1	0.07278	4.628891	0.370132	0.71217	-6.24973	0.808746	0.739026
B.cells	DOCK2	0.032586	9.34967	0.370106	0.712189	-7.16933	0.750672	0.64803
B.cells	MUTYH	0.180521	0.370874	0.370044	0.712235	-5.25764	0.865697	0.831945
B.cells	TMX2	0.071855	3.46498	0.36987	0.712364	-6.02465	0.823913	0.763394
B.cells	INKA1	0.102965	2.703982	0.36978	0.71243	-5.7248	0.83398	0.779736
B.cells	GM26839	-0.1828	0.656622	-0.36957	0.712584	-5.34901	0.86178	0.825396
B.cells	CHMP1A	0.046243	5.305048	0.369495	0.712643	-6.49536	0.800153	0.725299
B.cells	1700010I1	0.14424	1.215796	0.36915	0.712899	-5.42336	0.854082	0.812767
B.cells	DPAGT1	-0.08119	3.285854	-0.36913	0.712915	-5.9562	0.82627	0.767303
B.cells	CCDC73	-0.06922	3.100981	-0.3691	0.712935	-5.98412	0.828711	0.77126
B.cells	DSN1	-0.09906	2.622227	-0.36909	0.712942	-5.75066	0.83507	0.781601
B.cells	PIGC	-0.08069	2.730331	-0.36901	0.712999	-5.85587	0.833629	0.77927
B.cells	SVIL	0.037241	6.860609	0.368978	0.713026	-6.76908	0.780694	0.694644
B.cells	CARD6	0.092386	3.06005	0.368919	0.71307	-5.94598	0.829252	0.772161
B.cells	SERPINE2	-0.14073	1.29875	-0.36885	0.713119	-5.57741	0.852947	0.81092
B.cells	PLATR25	0.085722	2.729731	0.368672	0.713253	-5.86907	0.833693	0.77929
B.cells	CORO1A	0.037868	8.516071	0.3686	0.713307	-7.0866	0.760649	0.663386
B.cells	LMO4	-0.05211	7.245458	-0.36842	0.713442	-6.8471	0.776045	0.687322
B.cells	NUP37	-0.08104	3.351508	-0.36832	0.713512	-5.97571	0.825488	0.766054
B.cells	EMC10	0.039243	5.502896	0.368179	0.713619	-6.50643	0.797727	0.721557
B.cells	SPATA5	0.045562	5.905372	0.368154	0.713639	-6.61825	0.792656	0.713517
B.cells	TPM3	-0.0218	8.661561	-0.36799	0.713764	-7.04865	0.758938	0.660869
B.cells	POLE	-0.10224	3.301306	-0.36783	0.713876	-5.9681	0.82615	0.767256
B.cells	9930022D1	0.179701	0.319301	0.367698	0.713978	-5.4156	0.866548	0.833609
B.cells	NPHP3	-0.149	1.097931	-0.36733	0.714253	-5.46941	0.855784	0.815822
B.cells	HAUS8	0.051585	4.722045	0.367254	0.714307	-6.32016	0.807674	0.737616
B.cells	GEMIN2	-0.07373	3.260952	-0.36713	0.7144	-5.95298	0.826682	0.768247
B.cells	VBP1	0.039943	4.92592	0.366991	0.714503	-6.4226	0.805063	0.733494
B.cells	CAND2	-0.1576	0.775357	-0.36687	0.71459	-5.3822	0.860225	0.823295
B.cells	GUCY1A1	-0.22279	-0.00341	-0.36667	0.714743	-5.26337	0.871055	0.841369
B.cells	ZFP280C	-0.05233	4.164903	-0.36654	0.714836	-6.26436	0.814861	0.749321
B.cells	GTF2H4	-0.08449	2.307317	-0.36647	0.714889	-5.75942	0.839369	0.789068
B.cells	SLC7A11	0.200318	4.278698	0.366467	0.714892	-5.8836	0.813387	0.746951
B.cells	SORCS2	-0.09043	3.3545	-0.36641	0.714933	-6.10953	0.825449	0.766413
B.cells	1700048O2	-0.26657	-0.24632	-0.36638	0.714954	-5.21251	0.874465	0.847084
B.cells	YIPF6	0.072752	3.457527	0.366258	0.715048	-6.03269	0.824094	0.764237
B.cells	TMEM29	0.04955	4.706351	0.366148	0.715129	-6.32881	0.807876	0.738133
B.cells	1810058I2	0.044578	7.005863	0.366147	0.71513	-6.83158	0.778984	0.69236
B.cells	SLPI	0.122536	4.206306	0.366143	0.715133	-6.16373	0.814324	0.74848

B.cells	ITM2A	0.103462	2.544803	0.365993	0.715244	-5.8799	0.836188	0.783917
B.cells	VPS25	-0.15565	0.881521	-0.36599	0.71525	-5.38132	0.858761	0.821012
B.cells	TNPO3	-0.03312	6.365771	-0.36598	0.715255	-6.69875	0.786902	0.704827
B.cells	ADI1	-0.05829	3.749643	-0.36591	0.715306	-6.08532	0.820267	0.758094
B.cells	MTSS1	0.054175	6.472133	0.365895	0.715317	-6.67576	0.78558	0.702762
B.cells	MAP3K13	-0.18174	0.779749	-0.36582	0.71537	-5.37103	0.860164	0.823358
B.cells	NDFIP2	-0.03637	5.951714	-0.36582	0.715374	-6.59395	0.792075	0.713018
B.cells	WDR11	-0.07507	3.124121	-0.36572	0.715449	-5.91666	0.828494	0.771445
B.cells	LILRA5	-0.16792	1.497471	-0.36559	0.715546	-5.57011	0.850335	0.807194
B.cells	ZFP873	0.131964	0.995152	0.365519	0.715597	-5.45651	0.857211	0.818551
B.cells	AIM2	-0.04908	5.579386	-0.3653	0.715762	-6.60257	0.796802	0.720549
B.cells	HMGCL	0.046473	5.187114	0.365295	0.715763	-6.48982	0.801774	0.728462
B.cells	OSGEPL1	0.087406	2.472736	0.364938	0.71603	-5.73921	0.8374	0.785681
B.cells	PDZD2	0.084215	3.46937	0.364752	0.716168	-6.18825	0.824183	0.764195
B.cells	DGKI	0.306352	0.78	0.364705	0.716203	-5.31967	0.860416	0.82353
B.cells	RBM4B	-0.03955	5.747385	-0.36468	0.71622	-6.59964	0.794877	0.717241
B.cells	SKAP1	-0.10991	4.523681	-0.36418	0.716597	-6.15519	0.81072	0.742279
B.cells	LZIC	0.061446	3.63991	0.364165	0.716605	-6.10998	0.822204	0.760764
B.cells	SLC43A1	-0.14492	1.089863	-0.36375	0.716914	-5.58109	0.856418	0.816765
B.cells	HARS	-0.03975	5.114979	-0.36371	0.716941	-6.43944	0.803141	0.730242
B.cells	CHMP6	-0.07089	3.712475	-0.36371	0.716945	-6.07714	0.821254	0.759315
B.cells	VMAC	0.142583	1.5263	0.363672	0.716971	-5.43966	0.850448	0.80692
B.cells	ACD	0.045221	4.940696	0.363602	0.717023	-6.41138	0.805366	0.7338
B.cells	AAAS	-0.06937	3.794127	-0.36357	0.717044	-6.10746	0.820187	0.757597
B.cells	ENTPD1	-0.05689	6.9129	-0.36353	0.71708	-6.70293	0.780605	0.694605
B.cells	GDI2	-0.02485	8.891579	-0.36319	0.717331	-7.11999	0.756778	0.657436
B.cells	GM19265	-0.17215	0.22098	-0.36295	0.717505	-5.33111	0.86858	0.836815
B.cells	ROBO3	0.233257	-0.8339	0.362871	0.717568	-5.05474	0.883197	0.86169
B.cells	MCM2	-0.08728	4.762793	-0.36266	0.717726	-6.25641	0.807766	0.737622
B.cells	ALG5	-0.05614	4.2129	-0.3626	0.717773	-6.21305	0.814859	0.749011
B.cells	MYO19	0.12982	1.357151	0.362574	0.717788	-5.59741	0.852884	0.810936
B.cells	VSIG10	0.187942	0.47062	0.362457	0.717876	-5.2989	0.865103	0.831155
B.cells	CHEK1	-0.10333	2.722134	-0.36231	0.717984	-5.77763	0.834458	0.780736
B.cells	ZFP764	0.143466	1.074799	0.36228	0.718008	-5.44403	0.856754	0.817324
B.cells	TRIM59	0.066	4.368452	0.362265	0.718019	-6.26496	0.812845	0.745775
B.cells	OGT	0.033757	6.649131	0.362203	0.718065	-6.71226	0.783982	0.699895
B.cells	ZFP82	0.193194	0.121707	0.362165	0.718093	-5.27148	0.869967	0.839268
B.cells	GM13830	-0.22471	0.287554	-0.36212	0.71813	-5.24547	0.867651	0.835436
B.cells	CAMK4	-0.16021	3.194525	-0.36206	0.718175	-5.81125	0.828188	0.770605
B.cells	CCDC28A	-0.09681	2.307492	-0.36194	0.718261	-5.7222	0.840024	0.789869
B.cells	SF3A3	-0.04666	4.984502	-0.36177	0.718388	-6.40916	0.804949	0.73319
B.cells	RASSF8	-0.11654	2.215433	-0.36175	0.718406	-5.75948	0.841266	0.79192
B.cells	ENDOU	-0.16593	0.913658	-0.36148	0.718601	-5.47242	0.859124	0.821166
B.cells	ARHGEF1	0.032312	7.10928	0.361377	0.71868	-6.81923	0.778444	0.691166
B.cells	REPIN1	-0.15702	1.082403	-0.36132	0.718725	-5.40907	0.856801	0.817372
B.cells	SLC39A11	0.06444	4.419343	0.361097	0.718889	-6.21295	0.812383	0.745009
B.cells	UBE2V1	-0.0302	6.983076	-0.361	0.718962	-6.79323	0.780045	0.693718

B.cells	1700120C1	-0.11379	1.482076	-0.36097	0.718981	-5.56634	0.851382	0.808488
B.cells	FAM214B	-0.08506	3.11159	-0.36082	0.719091	-6.02765	0.829529	0.772704
B.cells	TMEM62	0.085418	2.375873	0.360623	0.719241	-5.88795	0.839407	0.788763
B.cells	TMEM87B	0.039919	5.127611	0.360552	0.719294	-6.47907	0.803406	0.730631
B.cells	DCTN4	-0.03172	6.423452	-0.36039	0.719412	-6.6967	0.787134	0.704784
B.cells	THBD	-0.10444	3.145231	-0.36019	0.719562	-6.04084	0.829301	0.772173
B.cells	LIMCH1	0.222757	0.57747	0.359732	0.719905	-5.25484	0.864298	0.829373
B.cells	EEF1E1	-0.05206	4.824221	-0.35951	0.720068	-6.41885	0.807612	0.737022
B.cells	PTCD3	-0.04601	4.845166	-0.35937	0.720178	-6.40944	0.807344	0.736624
B.cells	C1QBP	-0.04878	6.055545	-0.35922	0.720286	-6.60935	0.791997	0.712249
B.cells	SPATA24	0.118726	2.193104	0.358946	0.720491	-5.69671	0.842205	0.793169
B.cells	CCT8	-0.03269	6.762623	-0.35888	0.720544	-6.7622	0.783192	0.698459
B.cells	GM17036	-0.11763	2.038724	-0.35882	0.720583	-5.6874	0.844287	0.796603
B.cells	CAVIN1	0.191005	1.312054	0.358691	0.720681	-5.40925	0.854171	0.812889
B.cells	PPP1R35	0.068754	3.395179	0.35867	0.720697	-6.01125	0.82619	0.767137
B.cells	ZFP654	0.044427	6.120389	0.358657	0.720707	-6.63256	0.791185	0.711094
B.cells	NCAPD3	-0.05896	5.127768	-0.35862	0.720734	-6.37996	0.803729	0.731039
B.cells	SNAI3	0.198278	-0.34486	0.358522	0.720807	-5.25482	0.877208	0.851199
B.cells	KIF21A	-0.17995	0.515306	-0.3585	0.720821	-5.35644	0.865161	0.831119
B.cells	ZFP622	-0.04169	5.377893	-0.35846	0.720853	-6.49552	0.800546	0.725978
B.cells	ARMC6	-0.12584	1.405639	-0.35846	0.720853	-5.49256	0.852891	0.810815
B.cells	AI662270	-0.0446	6.090129	-0.35843	0.720875	-6.64357	0.791564	0.711724
B.cells	DDX56	-0.05464	3.951871	-0.35827	0.720992	-6.18429	0.818894	0.755396
B.cells	GM13402	0.178293	0.307958	0.357962	0.721225	-5.36108	0.868048	0.835938
B.cells	HOMER1	0.049763	6.109837	0.357942	0.72124	-6.62678	0.791317	0.711351
B.cells	GM26632	-0.18293	0.366747	-0.35783	0.721321	-5.31252	0.867228	0.834575
B.cells	LAMA4	-0.22644	1.088771	-0.35782	0.721334	-5.31055	0.857235	0.818006
B.cells	2610002M	0.054374	4.208089	0.357804	0.721342	-6.28849	0.815562	0.750028
B.cells	ATP6AP1	0.0368	5.990589	0.357796	0.721349	-6.62299	0.792812	0.713717
B.cells	PEAR1	0.089515	2.352213	0.357605	0.721491	-5.85912	0.840121	0.789793
B.cells	TCP11L1	0.085216	2.689841	0.357473	0.721589	-5.89758	0.8356	0.78244
B.cells	FEM1B	-0.05098	4.728435	-0.35739	0.721652	-6.36414	0.808897	0.739294
B.cells	TMC7	-0.18475	-0.40026	-0.35735	0.72168	-5.23157	0.878049	0.852592
B.cells	PSMD8	0.034933	6.592353	0.357123	0.721851	-6.75051	0.78537	0.701959
B.cells	TMEM41B	0.051061	4.300772	0.35691	0.722009	-6.22806	0.814431	0.748201
B.cells	SEC61G	0.037398	10.02398	0.356907	0.722012	-7.29009	0.744117	0.638033
B.cells	MSR1	0.091001	3.816756	0.356832	0.722068	-6.05723	0.82073	0.758349
B.cells	BC031181	0.034291	5.703047	0.356752	0.722128	-6.53627	0.796501	0.719556
B.cells	SCMH1	-0.0484	5.849321	-0.35664	0.722211	-6.57415	0.794657	0.716631
B.cells	UBE2B	0.033842	8.25823	0.356491	0.722322	-7.05374	0.765013	0.670153
B.cells	SCYL2	0.046732	4.409687	0.356359	0.722421	-6.29164	0.813022	0.745983
B.cells	FKBPL	0.14107	1.019839	0.356283	0.722477	-5.4644	0.858258	0.819775
B.cells	FBN1	-0.18251	0.898383	-0.35621	0.722531	-5.31634	0.859932	0.82255
B.cells	4930473A(	-0.14489	0.38363	-0.3562	0.722541	-5.39283	0.867069	0.834393
B.cells	NFIA	-0.05986	5.904	-0.35609	0.72262	-6.53717	0.793969	0.715645
B.cells	EDEM2	-0.05768	4.515035	-0.35606	0.722641	-6.24174	0.811661	0.743861
B.cells	NAA20	-0.03625	5.164245	-0.35601	0.722682	-6.46302	0.803335	0.730557

B.cells	ZFP687	0.072199	2.919042	0.355682	0.722926	-5.91288	0.832685	0.777729
B.cells	AMMECR1I	0.050113	4.488762	0.355678	0.722929	-6.334	0.812119	0.744486
B.cells	MTFMT	0.067333	2.527907	0.355351	0.723173	-5.85942	0.838106	0.786359
B.cells	SHROOM2	0.193202	1.506317	0.355178	0.723302	-5.3931	0.851988	0.809056
B.cells	IQCE	0.105521	2.144332	0.354974	0.723454	-5.72651	0.84333	0.794883
B.cells	KIF7	-0.22631	-0.46987	-0.35495	0.723475	-5.16183	0.879444	0.85476
B.cells	RAPGEF4	0.091241	2.563789	0.354784	0.723597	-5.98038	0.837693	0.785723
B.cells	ZFP629	-0.17396	0.926	-0.35463	0.723711	-5.37687	0.859953	0.822328
B.cells	STFA1	0.129969	5.975982	0.354405	0.723879	-6.53122	0.793436	0.71455
B.cells	RILPL2	-0.03879	7.158884	-0.35438	0.723895	-6.84354	0.77874	0.691402
B.cells	FRMD8OS	0.173499	0.736632	0.354159	0.724063	-5.36889	0.86257	0.826674
B.cells	PTPRO	0.129689	2.363394	0.354157	0.724064	-5.72789	0.84038	0.79013
B.cells	PIGV	0.06688	2.921169	0.354122	0.72409	-5.88428	0.832925	0.777966
B.cells	GM43111	-0.1807	-0.1905	-0.35409	0.724111	-5.30444	0.875518	0.848227
B.cells	ZFP574	-0.0671	3.401172	-0.35398	0.724195	-6.038	0.82657	0.767645
B.cells	CD2	-0.0627	3.660392	-0.35393	0.724237	-6.3011	0.823162	0.762127
B.cells	VPS50	0.046397	4.131766	0.353749	0.724369	-6.28406	0.817006	0.752239
B.cells	POLR2L	0.043272	5.414671	0.353706	0.724402	-6.49525	0.800524	0.725855
B.cells	PURG	-0.07637	3.260975	-0.35349	0.724566	-5.95351	0.82842	0.770761
B.cells	GPR141B	0.138554	-0.65516	0.35344	0.7246	-5.3848	0.881953	0.859358
B.cells	CBX6	-0.08437	2.859775	-0.35343	0.724608	-5.82461	0.833741	0.779412
B.cells	TEDC2	-0.16459	0.906107	-0.3534	0.724628	-5.3264	0.860228	0.822915
B.cells	TMEM268	0.078712	3.100152	0.353373	0.72465	-5.87612	0.830548	0.774217
B.cells	4932422M	-0.16047	0.972719	-0.35321	0.724771	-5.3595	0.859309	0.821453
B.cells	NDUFAB1	-0.04541	6.268706	-0.35319	0.724784	-6.62551	0.789769	0.708912
B.cells	2510002D2	0.068669	3.199654	0.352983	0.724941	-6.03606	0.829301	0.772231
B.cells	CDC25A	0.075158	3.793744	0.352661	0.725182	-6.03623	0.821485	0.759667
B.cells	TCP11L2	-0.04858	6.196085	-0.35258	0.725243	-6.71637	0.790744	0.710562
B.cells	HADHB	0.040334	5.576702	0.352142	0.72557	-6.51959	0.798538	0.723059
B.cells	WDR20	0.034173	5.843767	0.352087	0.725611	-6.6082	0.795166	0.717713
B.cells	GM4890	-0.17913	0.373848	-0.35204	0.725646	-5.24323	0.867684	0.835678
B.cells	DNTTIP2	0.036772	5.107858	0.351984	0.725688	-6.4519	0.804498	0.732586
B.cells	UBA3	0.047424	4.076756	0.351905	0.725747	-6.21141	0.817791	0.753953
B.cells	GRSF1	0.034576	5.185543	0.35186	0.72578	-6.48164	0.803507	0.731062
B.cells	JMJD4	-0.11469	1.268434	-0.35182	0.72581	-5.49941	0.855316	0.815249
B.cells	NABP2	-0.04168	4.980112	-0.35172	0.725888	-6.42214	0.806131	0.735285
B.cells	CRYBG2	-0.17432	0.051796	-0.35169	0.725908	-5.32727	0.872187	0.843298
B.cells	PAGR1A	0.188726	0.395669	0.351624	0.725957	-5.30972	0.86738	0.835323
B.cells	GM11772	0.159329	0.302935	0.351397	0.726127	-5.3433	0.868673	0.83759
B.cells	COQ2	-0.04864	4.467496	-0.35138	0.726136	-6.32467	0.812724	0.745999
B.cells	PRKAB2	0.064607	3.392055	0.351329	0.726177	-6.12103	0.82676	0.768685
B.cells	FAM57A	0.151295	1.118395	0.351254	0.726234	-5.47386	0.857376	0.818908
B.cells	ABTB1	-0.06411	4.303875	-0.35123	0.726252	-6.24597	0.814841	0.749473
B.cells	PLPP2	0.086821	1.875681	0.351094	0.726353	-5.8206	0.847035	0.801853
B.cells	HCFC1	-0.04591	4.949877	-0.35098	0.726441	-6.42545	0.806518	0.736167
B.cells	QDPR	0.042007	5.245358	0.350871	0.72652	-6.44029	0.802745	0.730183
B.cells	AP5M1	0.06108	3.584616	0.350548	0.726761	-6.09382	0.824226	0.76484

B.cells	DNMBP	0.071667	3.488772	0.35048	0.726812	-5.9974	0.825487	0.766915
B.cells	HCFC2	0.058709	3.758586	0.350434	0.726846	-6.12099	0.821945	0.761204
B.cells	DMAC1	0.048462	4.611093	0.350285	0.726958	-6.30279	0.81087	0.743387
B.cells	1110002L0	-0.08149	2.8153	-0.3502	0.727019	-5.89099	0.834405	0.781516
B.cells	PSMF1	-0.04723	4.460205	-0.35016	0.727053	-6.33989	0.812818	0.746561
B.cells	QSER1	0.049068	4.323503	0.349837	0.727293	-6.33055	0.814587	0.749538
B.cells	UBXN11	0.096141	1.770334	0.349743	0.727363	-5.68376	0.848465	0.804719
B.cells	FCHO1	0.063746	3.772111	0.349682	0.727409	-6.19642	0.821768	0.761134
B.cells	WDR5	0.048687	4.681603	0.349618	0.727456	-6.33314	0.809962	0.742108
B.cells	MRPS18C	0.040565	5.696786	0.349571	0.727492	-6.53944	0.79702	0.721427
B.cells	RTTN	0.068478	3.609694	0.349473	0.727565	-6.09574	0.823897	0.764583
B.cells	ACOT13	-0.04407	4.43558	-0.3494	0.727621	-6.27583	0.813136	0.747207
B.cells	XXYLT1	0.063268	3.591278	0.349265	0.727721	-6.08045	0.824139	0.764975
B.cells	TMEM119	0.177227	0.305437	0.349249	0.727733	-5.29991	0.868639	0.838145
B.cells	TM7SF3	-0.06205	3.924878	-0.34924	0.727741	-6.06959	0.819771	0.757906
B.cells	NT5DC2	-0.10489	2.432435	-0.34923	0.727744	-5.73402	0.839525	0.79004
B.cells	CEP41	0.125507	1.886422	0.349047	0.727884	-5.50682	0.84689	0.802127
B.cells	SLX4	0.094888	1.820472	0.349002	0.727917	-5.6278	0.847784	0.803599
B.cells	GM34471	0.20344	0.232245	0.348994	0.727924	-5.21274	0.869661	0.83985
B.cells	ZFP758	-0.07941	2.74078	-0.34898	0.727934	-5.84439	0.835399	0.783294
B.cells	GPR141	0.220186	2.440611	0.348976	0.727937	-5.58614	0.839415	0.789861
B.cells	YTHDC2	0.046133	4.693584	0.348884	0.728006	-6.39487	0.809808	0.74187
B.cells	P2RY14	-0.11685	3.467146	-0.34874	0.728113	-5.79127	0.825771	0.767647
B.cells	WDR77	0.064058	3.895337	0.348739	0.728114	-6.07933	0.820156	0.758553
B.cells	USP50	0.06652	3.371827	0.348655	0.728177	-6.04352	0.827027	0.769707
B.cells	GM17529	-0.17169	0.365187	-0.34858	0.728235	-5.36109	0.867805	0.83682
B.cells	COX11	0.05016	3.751078	0.348475	0.728312	-6.16422	0.822051	0.761675
B.cells	ORAI2	-0.04192	5.87495	-0.3483	0.728442	-6.58898	0.794846	0.718016
B.cells	PAIP2	-0.02501	7.620191	-0.34821	0.728513	-6.92365	0.773233	0.683987
B.cells	CASC4	0.07987	2.64714	0.348046	0.728633	-5.92418	0.836782	0.785548
B.cells	1110012L1	0.11267	1.829365	0.347946	0.728708	-5.59794	0.847804	0.803631
B.cells	SERPINB1A	-0.09511	3.600711	-0.34765	0.728929	-6.17534	0.824316	0.765055
B.cells	C130036L2	0.130518	1.354595	0.347562	0.728995	-5.4431	0.854447	0.814406
B.cells	TUG1	0.034972	6.098897	0.347078	0.729358	-6.64307	0.792275	0.713839
B.cells	GM28501	0.14332	0.476037	0.347063	0.729369	-5.42558	0.866605	0.834702
B.cells	SCAMP1	0.103511	2.970331	0.347001	0.729415	-5.78857	0.832672	0.77879
B.cells	ALG1	-0.07023	3.312271	-0.34698	0.729427	-6.03807	0.828141	0.771416
B.cells	EIF4G3	0.026994	7.91677	0.346974	0.729435	-6.96231	0.769864	0.678614
B.cells	RBM8A	-0.03616	5.709751	-0.34694	0.729459	-6.54953	0.797172	0.721615
B.cells	IKZF5	0.056254	3.804869	0.346872	0.729512	-6.13082	0.821665	0.760913
B.cells	SMIM14	0.027312	7.74729	0.34654	0.72976	-6.91805	0.772078	0.681865
B.cells	GM16286	0.034559	5.859685	0.34643	0.729843	-6.58461	0.795442	0.718653
B.cells	NIPSNAP3E	0.040606	5.573981	0.346391	0.729872	-6.53599	0.799051	0.724406
B.cells	GM13986	0.228275	1.376917	0.345819	0.7303	-5.4817	0.854633	0.814292
B.cells	PLBD1	-0.08561	5.389651	-0.34554	0.730506	-6.30969	0.801663	0.728303
B.cells	ZC3H12B	0.181498	1.084598	0.345206	0.73076	-5.31871	0.858648	0.821012
B.cells	CHCHD5	0.07773	3.092559	0.345185	0.730775	-5.93994	0.831501	0.776431

B.cells	ERICH1	-0.06594	3.61294	-0.34506	0.730866	-6.06025	0.824629	0.765269
B.cells	APH1B	-0.09631	2.578014	-0.34472	0.731125	-5.8167	0.838362	0.78771
B.cells	LRRC75A	-0.11038	2.6203	-0.34469	0.731149	-5.77645	0.837795	0.786792
B.cells	ST14	-0.16739	1.167666	-0.34466	0.731168	-5.49078	0.857505	0.819229
B.cells	PPIE	0.043607	4.586932	0.34451	0.731281	-6.34351	0.811945	0.744933
B.cells	GIT2	0.028793	7.020155	0.344502	0.731287	-6.79365	0.781247	0.696142
B.cells	SOX7	0.187223	0.442832	0.344405	0.73136	-5.27491	0.867538	0.835945
B.cells	AI597479	0.112155	1.688645	0.344259	0.731469	-5.55063	0.850375	0.807551
B.cells	PRKDC	0.044202	4.69925	0.344222	0.731497	-6.38811	0.810497	0.742669
B.cells	KLHL11	-0.07714	2.776613	-0.34421	0.731503	-5.93988	0.835706	0.783489
B.cells	U2SURP	-0.02791	6.667671	-0.3442	0.731512	-6.72577	0.785608	0.703064
B.cells	ACKR3	0.197694	0.835269	0.344134	0.731562	-5.5303	0.862089	0.826938
B.cells	CHTOP	-0.04376	5.225925	-0.34399	0.731669	-6.44091	0.803748	0.731881
B.cells	ZFP738	-0.06428	2.843416	-0.34398	0.731681	-5.97037	0.834815	0.782058
B.cells	UFD1	0.036732	5.129038	0.343929	0.731716	-6.45377	0.804984	0.733858
B.cells	IL12RB1	0.193711	0.500116	0.343829	0.731791	-5.35679	0.866741	0.834727
B.cells	PDCD6IP	-0.02291	6.988379	-0.34373	0.731866	-6.80149	0.781639	0.696889
B.cells	ANXA7	0.037083	5.409471	0.343494	0.732042	-6.52794	0.801411	0.728251
B.cells	AMD2	0.187183	0.044662	0.343424	0.732095	-5.18765	0.873107	0.845428
B.cells	PHF13	0.055365	3.504231	0.343418	0.732099	-6.09571	0.826059	0.767915
B.cells	FGL2	0.179455	4.054695	0.343397	0.732115	-5.78058	0.818848	0.756241
B.cells	NIP7	0.039262	4.635807	0.343342	0.732156	-6.35496	0.811314	0.744106
B.cells	CD55	0.084407	4.863884	0.343316	0.732176	-6.31417	0.80838	0.739401
B.cells	NR1H2	0.052557	4.589379	0.343207	0.732257	-6.33063	0.811913	0.745094
B.cells	RC3H1	-0.02989	6.887392	-0.34315	0.732301	-6.78788	0.782886	0.698935
B.cells	UBE2I	-0.02192	7.624242	-0.34304	0.732382	-6.90812	0.77384	0.684777
B.cells	TNS3	0.065118	5.235143	0.342995	0.732416	-6.37311	0.80363	0.73188
B.cells	IGF1R	-0.05049	6.357541	-0.34287	0.732514	-6.62681	0.789469	0.709377
B.cells	ADGRL3	-0.17467	3.47827	-0.34279	0.732573	-5.90717	0.826401	0.768595
B.cells	ACLY	0.030781	6.383195	0.342775	0.732581	-6.66487	0.789149	0.708888
B.cells	SGK1	-0.05167	5.355259	-0.34274	0.732604	-6.5167	0.8021	0.72948
B.cells	TNFRSF26	0.110762	2.617995	0.342547	0.732752	-5.78735	0.837826	0.787258
B.cells	PRC1	-0.09171	5.285692	-0.34247	0.732808	-6.40721	0.802986	0.730925
B.cells	RBM14	-0.05346	3.712533	-0.34245	0.732829	-6.09948	0.823322	0.763644
B.cells	SREK1IP1	0.056635	4.172778	0.342331	0.732914	-6.19684	0.81731	0.753925
B.cells	CSTDC6	0.144416	-1.16323	0.342287	0.732947	-5.2544	0.889924	0.874419
B.cells	CDC34	-0.03672	6.050691	-0.34211	0.733083	-6.62669	0.79338	0.715554
B.cells	CDCA4	-0.05181	4.453152	-0.34193	0.733214	-6.26513	0.813745	0.748194
B.cells	MRPL47	-0.08392	2.974642	-0.34162	0.733447	-5.89356	0.833141	0.779715
B.cells	MALAT1	0.032503	14.72453	0.341538	0.733509	-7.81712	0.692816	0.562476
B.cells	ITGB2L	0.155961	-0.61932	0.341514	0.733527	-5.29504	0.882449	0.86163
B.cells	GPRASP1	0.052881	3.93667	0.341501	0.733537	-6.15024	0.82046	0.759156
B.cells	A430093F1	0.100087	2.363356	0.341489	0.733546	-5.74496	0.841317	0.793126
B.cells	CYP39A1	-0.09901	1.483874	-0.34138	0.733631	-5.8068	0.853244	0.812782
B.cells	SLC39A9	0.041457	4.172579	0.341192	0.733769	-6.24376	0.817385	0.754283
B.cells	KHNYN	0.049492	4.263958	0.341156	0.733795	-6.24213	0.816197	0.752367
B.cells	RANBP10	0.041091	5.698692	0.341137	0.73381	-6.62117	0.797815	0.722908

B.cells	FUBP1	0.026126	6.678668	0.340981	0.733927	-6.74063	0.78554	0.703451
B.cells	KIZ	0.062304	3.359508	0.34097	0.733935	-5.98609	0.82804	0.771546
B.cells	MRM3	0.109917	2.005465	0.340882	0.734001	-5.69669	0.846147	0.801159
B.cells	NANP	0.091072	2.821135	0.340728	0.734117	-5.83474	0.835205	0.783224
B.cells	GRIP1	-0.12043	2.360437	-0.34067	0.734161	-5.84855	0.841376	0.793325
B.cells	LONP1	-0.05842	3.653778	-0.34058	0.73423	-6.10737	0.824185	0.765287
B.cells	MPP6	0.036332	6.194697	0.340468	0.734311	-6.67343	0.791605	0.713026
B.cells	CAAP1	0.043748	4.575271	0.339988	0.734672	-6.34294	0.812457	0.745978
B.cells	FBXO6	0.061441	3.653081	0.339907	0.734732	-6.06732	0.824469	0.765374
B.cells	5033406O(	-0.18472	0.605012	-0.33978	0.734827	-5.31961	0.865667	0.833066
B.cells	RNPC3	0.036904	4.741295	0.339779	0.734829	-6.39703	0.810317	0.742538
B.cells	MIEF2	0.121276	1.112018	0.339451	0.735075	-5.52945	0.85874	0.821473
B.cells	GM13889	-0.16734	1.14195	-0.33936	0.735145	-5.42592	0.858328	0.82081
B.cells	RBM19	0.074531	2.925866	0.339347	0.735153	-5.9104	0.834173	0.781091
B.cells	4833403J1	0.169185	0.509888	0.339303	0.735186	-5.46923	0.867079	0.835347
B.cells	TMEM40	-0.16464	0.497552	-0.33906	0.735369	-5.41011	0.867383	0.835676
B.cells	SLC25A33	0.065374	3.983563	0.338957	0.735446	-6.1188	0.820355	0.758476
B.cells	PIGH	-0.10634	1.827535	-0.33871	0.735628	-5.67122	0.849141	0.805437
B.cells	INTS5	0.068292	2.704825	0.33859	0.735721	-5.88867	0.837311	0.786025
B.cells	METTL1	0.076476	3.431367	0.338297	0.735941	-6.02085	0.827658	0.770399
B.cells	GM42670	-0.15454	0.89839	-0.33819	0.736018	-5.38899	0.861881	0.826615
B.cells	CYTIP	-0.03453	8.087657	-0.33814	0.736057	-7.07391	0.768807	0.676743
B.cells	ZMAT4	-0.20827	0.381713	-0.33812	0.736077	-5.46221	0.869061	0.838561
B.cells	PYGB	-0.04098	4.748164	-0.33809	0.736094	-6.39749	0.810492	0.742674
B.cells	CMYA5	0.204309	0.674059	0.337788	0.736324	-5.35423	0.86499	0.831892
B.cells	MAPK7	-0.07164	3.195275	-0.3376	0.736463	-5.99945	0.83078	0.775624
B.cells	ABCF3	0.051015	3.761251	0.337477	0.736557	-6.16148	0.823318	0.763504
B.cells	CDIPTOS	0.159994	-0.30875	0.337357	0.736647	-5.25486	0.878763	0.854992
B.cells	BET1	0.05339	4.246255	0.33723	0.736743	-6.18519	0.816986	0.753311
B.cells	RAB34	-0.21034	0.235405	-0.33714	0.736809	-5.26258	0.871107	0.842184
B.cells	B230219D2	0.026325	6.252623	0.337114	0.73683	-6.67604	0.79139	0.712387
B.cells	COG4	0.036979	5.336083	0.337061	0.736869	-6.51539	0.802964	0.730799
B.cells	FDXACB1	-0.0789	2.215747	-0.33705	0.736881	-5.77579	0.843882	0.797095
B.cells	CSF2RA	-0.04499	5.196056	-0.33704	0.736884	-6.50399	0.804749	0.733654
B.cells	COMMD1	-0.04352	5.141948	-0.33689	0.737	-6.43603	0.805441	0.73477
B.cells	MYH10	-0.09707	2.573809	-0.33667	0.737164	-5.73004	0.839065	0.789243
B.cells	CKAP2L	-0.07566	4.357364	-0.33662	0.737204	-6.12543	0.815543	0.751032
B.cells	SRP9	-0.02577	7.516452	-0.33659	0.737225	-6.87155	0.775754	0.687802
B.cells	GADD45GI	0.041645	5.128075	0.336535	0.737265	-6.4645	0.805618	0.735109
B.cells	ETV6	-0.03757	8.156803	-0.3364	0.737368	-7.02051	0.767971	0.675716
B.cells	KATNA1	0.044281	4.864725	0.336255	0.737475	-6.3896	0.808993	0.740593
B.cells	2810004N2	-0.05163	4.315225	-0.33625	0.737479	-6.25915	0.81609	0.752009
B.cells	SHROOM3	0.174004	0.981918	0.336215	0.737505	-5.47036	0.860727	0.825062
B.cells	PIK3CA	0.036086	6.128996	0.336167	0.737541	-6.65999	0.79294	0.714979
B.cells	PRF1	-0.175	0.377462	-0.33616	0.737546	-5.40434	0.86912	0.839029
B.cells	GM5544	0.165404	0.129876	0.335912	0.737733	-5.3284	0.872585	0.844857
B.cells	OTUB2	0.110558	1.885457	0.335903	0.73774	-5.66716	0.848354	0.804642

B.cells	CDNF	-0.18526	0.317972	-0.33571	0.737882	-5.27892	0.869952	0.840504
B.cells	G0S2	-0.09813	3.133285	-0.33563	0.737942	-5.87361	0.831602	0.777238
B.cells	MTPN	-0.02519	6.898113	-0.3356	0.737965	-6.7998	0.783358	0.699906
B.cells	EXOSC5	0.039039	5.376885	0.335589	0.737975	-6.49667	0.802444	0.730185
B.cells	ITPR3	-0.0597	4.288295	-0.33553	0.738019	-6.25496	0.816439	0.752652
B.cells	LRP1	0.073625	4.178873	0.33544	0.738088	-6.12646	0.817862	0.754995
B.cells	TRERF1	0.101819	4.396216	0.335411	0.738109	-5.99694	0.815039	0.750446
B.cells	RHAG	0.198449	-0.3642	0.335121	0.738328	-5.37221	0.879614	0.856733
B.cells	SSPN	0.142203	-0.41075	0.335054	0.738378	-5.3915	0.880273	0.85784
B.cells	GOT1	-0.04391	6.081478	-0.33498	0.738436	-6.72067	0.793597	0.71614
B.cells	SAPCD1	0.115948	1.629002	0.334946	0.73846	-5.60854	0.85191	0.810609
B.cells	5330439KC	0.154676	0.538036	0.334775	0.738588	-5.43614	0.866948	0.835622
B.cells	MMP19	-0.14444	1.213067	-0.33477	0.73859	-5.61489	0.857608	0.8201
B.cells	GM44067	-0.10141	1.717425	-0.3345	0.738797	-5.72853	0.850861	0.808701
B.cells	CHUK	0.038939	5.037427	0.334235	0.738994	-6.4613	0.807056	0.73741
B.cells	LYZL4	0.209085	-1.24765	0.334227	0.739	-5.10659	0.892145	0.878153
B.cells	TRIM2	-0.10725	2.325336	-0.33381	0.739315	-5.73634	0.842805	0.795297
B.cells	SDCBP2	-0.13905	1.097863	-0.33346	0.739577	-5.53994	0.859537	0.823052
B.cells	NFATC2IP	0.067195	3.151572	0.333165	0.739798	-6.00965	0.831755	0.777513
B.cells	C1GALT1	-0.03945	6.353776	-0.33313	0.739824	-6.69103	0.790501	0.711214
B.cells	ATXN7L2	-0.08532	2.480154	-0.33308	0.739864	-5.87583	0.840722	0.792205
B.cells	GM48653	-0.20111	-0.16399	-0.33303	0.7399	-5.22334	0.877135	0.852601
B.cells	KLF9	-0.07242	3.693914	-0.33298	0.739935	-6.16051	0.824594	0.765906
B.cells	PELI1	-0.02963	7.676753	-0.33296	0.73995	-6.91814	0.774165	0.685525
B.cells	VAPA	-0.02094	8.028548	-0.33295	0.739958	-6.95931	0.769889	0.678846
B.cells	NOL6	0.082367	2.582305	0.332913	0.739988	-5.79975	0.83935	0.789974
B.cells	LEAP2	-0.10272	3.060255	-0.33278	0.740092	-6.02118	0.832968	0.77954
B.cells	NCS1	0.185769	0.210797	0.33275	0.740111	-5.29735	0.871866	0.843789
B.cells	VDR	0.164242	0.938817	0.332708	0.740142	-5.42203	0.861732	0.826902
B.cells	GALNT4	0.094721	1.622606	0.332476	0.740317	-5.6092	0.852337	0.811377
B.cells	KPNA2	0.056464	4.937072	0.332472	0.74032	-6.4235	0.808449	0.7399
B.cells	G3BP1	0.033179	6.728766	0.332456	0.740332	-6.74226	0.78583	0.703898
B.cells	DCAF17	0.049261	4.054876	0.332418	0.74036	-6.21555	0.819867	0.758298
B.cells	ZFP830	0.056988	3.541014	0.332243	0.740492	-6.08969	0.826606	0.769288
B.cells	SUV39H1	-0.05849	3.622039	-0.3317	0.7409	-6.06133	0.825539	0.76766
B.cells	GM45435	0.175772	0.920103	0.331545	0.741018	-5.43175	0.861991	0.82758
B.cells	TMEM120E	-0.08051	3.044848	-0.33151	0.741041	-6.0912	0.833173	0.780117
B.cells	TXNL4A	-0.04163	4.968748	-0.33137	0.74115	-6.40771	0.808042	0.739484
B.cells	GM17227	-0.06503	2.746669	-0.33123	0.741251	-5.99251	0.837149	0.78667
B.cells	ZFP330	-0.04316	4.432352	-0.3312	0.741279	-6.29624	0.814959	0.75062
B.cells	RMDN2	0.098171	1.873328	0.331198	0.741279	-5.75237	0.848922	0.80601
B.cells	HSH2D	-0.07231	3.210107	-0.33119	0.741281	-6.02283	0.830979	0.776593
B.cells	SART3	-0.03578	5.374984	-0.33117	0.741299	-6.52846	0.80285	0.731178
B.cells	MLKL	0.125267	2.335281	0.331169	0.7413	-5.62685	0.842671	0.795723
B.cells	MFSD8	0.06313	2.979793	0.331068	0.741376	-5.88568	0.834039	0.781585
B.cells	PNPLA1	0.202774	0.231599	0.331056	0.741385	-5.2392	0.871574	0.843623
B.cells	CMTM4	-0.08136	3.141567	-0.331	0.741425	-5.87362	0.831888	0.778079

B.cells	GPR55	0.116414	1.548876	0.330818	0.741565	-5.56403	0.853345	0.813383
B.cells	SCO2	0.070603	2.851352	0.330718	0.74164	-5.94385	0.835751	0.784492
B.cells	ZNHIT1	0.036835	5.422819	0.330662	0.741682	-6.49391	0.802241	0.730307
B.cells	NUCB2	0.042745	4.304904	0.33042	0.741864	-6.28735	0.816612	0.753419
B.cells	ARFIP1	-0.0571	3.797972	-0.33026	0.741981	-6.14356	0.823228	0.764125
B.cells	KLRA9	-0.18705	-0.30711	-0.33023	0.742005	-5.35057	0.879157	0.856479
B.cells	ARHGAP25	0.038823	6.214201	0.330217	0.742017	-6.67557	0.792248	0.714417
B.cells	GM12367	0.200821	0.051988	0.330132	0.742081	-5.28194	0.874094	0.847987
B.cells	ZFP619	0.079564	2.21582	0.330122	0.742089	-5.78389	0.844283	0.79851
B.cells	GM39469	0.073937	2.328802	0.330116	0.742093	-5.79226	0.842758	0.796005
B.cells	TTI1	0.084954	2.712153	0.330027	0.74216	-5.88759	0.837611	0.787575
B.cells	RNF113A1	0.182552	0.42846	0.329972	0.742202	-5.2934	0.868822	0.839184
B.cells	LYPLA2	-0.03941	4.945046	-0.3299	0.742253	-6.41897	0.808346	0.740152
B.cells	PTGES3	-0.02785	7.460041	-0.32983	0.742305	-6.86546	0.776813	0.690113
B.cells	ADSS	0.030282	6.271707	0.329737	0.742379	-6.69714	0.791528	0.713351
B.cells	SLC39A7	-0.0442	4.500813	-0.32968	0.742422	-6.27267	0.814072	0.749412
B.cells	1810055G(	-0.08476	2.268587	-0.32943	0.742607	-5.72329	0.843647	0.797496
B.cells	GM156	0.18761	-1.54566	0.329401	0.742632	-5.18022	0.896692	0.886568
B.cells	ENPP1	0.115212	2.31349	0.329078	0.742875	-5.75051	0.843174	0.796571
B.cells	NDC80	-0.07604	4.625978	-0.32906	0.742889	-6.31491	0.812655	0.746931
B.cells	CD320	0.092168	1.856288	0.32877	0.743107	-5.631	0.849533	0.806802
B.cells	ERI1	0.041325	5.459943	0.328675	0.743179	-6.54133	0.802127	0.729835
B.cells	SNHG8	-0.05125	4.013183	-0.32857	0.743255	-6.19109	0.820785	0.759847
B.cells	GM47350	0.101242	1.355941	0.328315	0.74345	-5.60504	0.856494	0.818162
B.cells	APOLD1	0.168721	1.979983	0.32825	0.743499	-5.47691	0.847976	0.804097
B.cells	ADGRE1	0.095028	4.711233	0.328083	0.743625	-6.33207	0.811894	0.745299
B.cells	TOMM6	0.028314	7.550466	0.327711	0.743905	-6.88509	0.776443	0.688697
B.cells	SDHA	-0.03239	5.691569	-0.32738	0.744158	-6.54017	0.799682	0.725354
B.cells	PLS1	-0.15239	0.910051	-0.32699	0.744452	-5.40541	0.863048	0.82863
B.cells	B230354K1	-0.12757	1.756956	-0.32697	0.744463	-5.56566	0.851411	0.809357
B.cells	DNAJC3	-0.03497	6.725717	-0.32696	0.744469	-6.7276	0.786704	0.704832
B.cells	SH2D1A	0.156643	0.051779	0.326921	0.7445	-5.38523	0.875028	0.848615
B.cells	RRBP1	-0.02698	7.951353	-0.32688	0.744531	-6.95545	0.771645	0.681192
B.cells	FAM136A	0.057732	4.090791	0.326826	0.744572	-6.17131	0.820271	0.758479
B.cells	HOMER2	-0.15071	0.275337	-0.3266	0.744743	-5.44042	0.871889	0.843365
B.cells	INTS3	-0.0503	3.898156	-0.32644	0.744861	-6.15063	0.82279	0.762556
B.cells	FAM204A	-0.03704	4.894011	-0.32644	0.744863	-6.41981	0.809863	0.741708
B.cells	AHCTF1	-0.03837	5.405997	-0.32641	0.744884	-6.51798	0.80331	0.731211
B.cells	UBA5	-0.0389	4.489487	-0.32633	0.744943	-6.28853	0.815085	0.750108
B.cells	4921531C2	-0.11761	1.642005	-0.32632	0.74495	-5.5937	0.85298	0.811947
B.cells	AUP1	0.03324	6.010505	0.326281	0.744983	-6.65043	0.795652	0.719008
B.cells	GM31645	-0.15479	0.726126	-0.32604	0.745167	-5.41974	0.865731	0.832944
B.cells	CCT6B	-0.18691	0.315333	-0.32593	0.745247	-5.2854	0.871472	0.842503
B.cells	ALOX15	-0.39268	-0.82153	-0.32567	0.745444	-5.17549	0.887442	0.86955
B.cells	BC002059	0.075999	2.758564	0.3256	0.745496	-5.861	0.83814	0.787262
B.cells	YLPM1	-0.03568	5.660547	-0.3252	0.745796	-6.57078	0.800517	0.726287
B.cells	PWP2	-0.10602	1.891751	-0.32504	0.745919	-5.6761	0.850045	0.80662

B.cells	BAZ2A	-0.0354	6.97841	-0.32502	0.745936	-6.79093	0.784004	0.700143
B.cells	4732440D(	0.112943	2.03703	0.324983	0.745962	-5.65294	0.848071	0.803368
B.cells	SOCS1	-0.068	5.152296	-0.32481	0.746096	-6.50727	0.807063	0.736676
B.cells	5830418P1	-0.12136	1.463039	-0.32462	0.746235	-5.74219	0.856028	0.816328
B.cells	AA388235	-0.16037	0.991788	-0.32455	0.746285	-5.45346	0.862521	0.827094
B.cells	ZFP608	-0.05335	6.820434	-0.32438	0.746418	-6.76297	0.786144	0.703325
B.cells	QPCTL	-0.0631	3.010203	-0.32386	0.746808	-5.90292	0.835495	0.782133
B.cells	GM47730	-0.16467	-0.65913	-0.32378	0.746866	-5.26433	0.885987	0.866071
B.cells	ICAM2	0.093026	4.282374	0.323719	0.746916	-6.21668	0.818728	0.75493
B.cells	MINDY2	-0.03007	6.610817	-0.32356	0.747034	-6.73828	0.789103	0.707559
B.cells	SNHG20	0.07203	2.355617	0.323439	0.747127	-5.79557	0.844356	0.79655
B.cells	CLEC5A	-0.16589	0.512385	-0.32325	0.747272	-5.39785	0.869716	0.838422
B.cells	HIST1H2AP	0.090923	7.597755	0.323197	0.747309	-6.86468	0.776973	0.688413
B.cells	BBIP1	0.035864	5.888435	0.323014	0.747447	-6.58806	0.798267	0.721994
B.cells	NUBP2	-0.05143	4.451117	-0.32291	0.747528	-6.311	0.816682	0.75147
B.cells	ERO1LB	-0.03079	6.652932	-0.32283	0.747584	-6.82093	0.788671	0.706789
B.cells	PRKX	-0.03318	5.379346	-0.3226	0.74776	-6.5999	0.804733	0.732326
B.cells	4930417O1	-0.13442	0.963159	-0.32254	0.747803	-5.5776	0.863475	0.828056
B.cells	ATP6V0E	-0.02566	7.713213	-0.32246	0.747865	-6.92726	0.775589	0.686269
B.cells	UBE2CBP	0.143013	1.761179	0.322122	0.748121	-5.64346	0.852501	0.809998
B.cells	TARSL2	0.106615	1.415252	0.322002	0.748212	-5.54035	0.857239	0.817843
B.cells	ELP2	-0.0444	4.144774	-0.322	0.748212	-6.2374	0.820672	0.758047
B.cells	ANXA6	0.037466	6.174347	0.321838	0.748335	-6.70387	0.794662	0.716421
B.cells	NFE2L2	0.038344	7.355477	0.32174	0.748409	-6.90604	0.779974	0.69327
B.cells	RBPMS	0.049826	5.842983	0.321735	0.748413	-6.60741	0.798841	0.723084
B.cells	CLMN	-0.15027	0.334512	-0.32171	0.748433	-5.44912	0.872235	0.842815
B.cells	MAP1LC3A	-0.05103	5.476214	-0.32163	0.748492	-6.52758	0.803498	0.730516
B.cells	ZFP282	-0.05138	4.010784	-0.32157	0.748537	-6.25735	0.822424	0.760933
B.cells	FUZ	-0.13686	1.08456	-0.32157	0.748537	-5.47929	0.861796	0.825451
B.cells	SLC19A2	-0.08983	1.834884	-0.32148	0.748607	-5.73296	0.851496	0.808438
B.cells	SHC1	-0.0434	4.384809	-0.32144	0.748635	-6.26961	0.817544	0.753077
B.cells	GMEB2	-0.03239	5.764218	-0.32138	0.748683	-6.57503	0.799839	0.724716
B.cells	CARNMT1	-0.03534	5.353674	-0.32116	0.748846	-6.47471	0.805087	0.733072
B.cells	HSD17B12	-0.03646	6.085483	-0.32108	0.748908	-6.63776	0.795806	0.718294
B.cells	GM20337	0.148436	1.078162	0.321034	0.748942	-5.38551	0.861912	0.825684
B.cells	AP4B1	-0.08845	2.273778	-0.32083	0.749094	-5.7678	0.845564	0.798699
B.cells	HIST2H2BE	0.150646	0.814865	0.320696	0.749198	-5.3623	0.865561	0.831751
B.cells	ZFP760	0.135642	1.032725	0.320675	0.749213	-5.36338	0.86254	0.826732
B.cells	EBP	-0.04801	4.791273	-0.32065	0.749234	-6.37054	0.812306	0.744662
B.cells	ZBTB9	-0.08244	2.511721	-0.32058	0.749287	-5.75268	0.842354	0.793458
B.cells	APPBP2	0.029591	6.248663	0.320501	0.749345	-6.68054	0.793754	0.715107
B.cells	TERF1	0.036047	5.254483	0.320385	0.749432	-6.48131	0.806372	0.735194
B.cells	CALCOCO1	0.056738	3.863697	0.320099	0.749648	-6.16486	0.824399	0.764361
B.cells	SLC25A42	-0.11471	1.940911	-0.32006	0.749676	-5.63026	0.8501	0.806359
B.cells	NELFA	-0.03653	4.630912	-0.31995	0.749758	-6.37225	0.814398	0.74821
B.cells	PIAS2	-0.03609	6.02482	-0.31981	0.749869	-6.64011	0.79659	0.719762
B.cells	KLRA5	-0.17463	2.005641	-0.31971	0.749941	-5.62026	0.84922	0.804979

B.cells	CTTNBP2NL	0.102857	2.52025	0.319657	0.749983	-5.6368	0.842259	0.793542
B.cells	PLA2G12A	-0.05006	4.277535	-0.31964	0.749998	-6.27806	0.818986	0.755672
B.cells	SRPRB	0.046685	4.104103	0.319632	0.750002	-6.21596	0.82125	0.759329
B.cells	4632404H1	0.148212	1.238383	0.319375	0.750196	-5.44209	0.859787	0.822396
B.cells	SLC25A28	0.041185	4.806244	0.319368	0.750201	-6.42193	0.812195	0.744691
B.cells	PABPC1	-0.03193	10.47512	-0.31911	0.750394	-7.3809	0.742923	0.636017
B.cells	NOP14	-0.05099	4.004398	-0.31885	0.750595	-6.23639	0.822893	0.761585
B.cells	MIPOL1	0.045551	4.257052	0.318668	0.75073	-6.24635	0.819602	0.756298
B.cells	GLRX5	-0.03202	6.17176	-0.31826	0.751039	-6.72173	0.795077	0.717107
B.cells	PTPN12	0.042541	6.049828	0.318257	0.751041	-6.57578	0.796613	0.719546
B.cells	CARHSP1	-0.04363	5.430457	-0.31824	0.751054	-6.51942	0.804468	0.732061
B.cells	BCL2L11	-0.0505	7.053365	-0.31798	0.751247	-6.79376	0.784077	0.699771
B.cells	TIMM13	0.030964	6.732513	0.31794	0.75128	-6.75194	0.78806	0.706063
B.cells	CCDC162	0.136725	2.876554	0.31789	0.751318	-5.68169	0.837835	0.786064
B.cells	REEP3	-0.03312	6.727725	-0.31783	0.751367	-6.75036	0.788119	0.706157
B.cells	IL23A	-0.21649	-0.31658	-0.31778	0.751403	-5.23388	0.881839	0.859007
B.cells	UHRF2	-0.02807	6.43989	-0.31771	0.751451	-6.69419	0.791713	0.711866
B.cells	SLC25A22	0.101945	2.495595	0.317519	0.751598	-5.79006	0.84295	0.794502
B.cells	GM20324	0.079342	2.098382	0.317466	0.751639	-5.75669	0.848321	0.803327
B.cells	SLF2	0.033895	6.314028	0.317408	0.751682	-6.63123	0.79329	0.714395
B.cells	SLC33A1	-0.05192	3.794664	-0.31739	0.751696	-6.19481	0.825657	0.766298
B.cells	PRSS57	-0.16581	-0.03051	-0.31734	0.751732	-5.24772	0.87779	0.85227
B.cells	0610010K1	0.037183	5.617735	0.31727	0.751786	-6.51374	0.802084	0.728379
B.cells	TMCO3	-0.05171	3.656165	-0.3172	0.751838	-6.12443	0.82748	0.769258
B.cells	FDX2	0.043479	4.192147	0.317072	0.751936	-6.2675	0.820448	0.757867
B.cells	IBA57	0.082782	1.934328	0.317051	0.751952	-5.71168	0.850551	0.807
B.cells	ZCWPW1	-0.08633	2.765843	-0.31687	0.75209	-5.75696	0.839391	0.788552
B.cells	GM24362	0.142338	0.694716	0.316561	0.752323	-5.38761	0.867885	0.835358
B.cells	PDK2	-0.09175	2.10287	-0.31641	0.752434	-5.74836	0.848563	0.803335
B.cells	TLR6	0.141485	1.070509	0.316042	0.752715	-5.5033	0.862954	0.826826
B.cells	APOL9B	-0.15404	0.574633	-0.31569	0.752982	-5.39865	0.869985	0.838442
B.cells	GNA12	-0.04731	4.652134	-0.31565	0.753013	-6.31151	0.815115	0.748555
B.cells	PLPP5	-0.06119	3.394162	-0.31561	0.753046	-6.02402	0.831603	0.775228
B.cells	CCM2	0.02728	7.391127	0.315392	0.753207	-6.91936	0.78055	0.6936
B.cells	TBP	-0.06722	3.21522	-0.31539	0.753208	-5.98721	0.834001	0.7791
B.cells	GM15832	0.115835	1.685015	0.314721	0.753715	-5.55674	0.854693	0.81317
B.cells	GM34983	0.148711	0.754004	0.314559	0.753838	-5.41074	0.867548	0.834504
B.cells	IFI211	-0.14407	2.960201	-0.31451	0.753878	-5.753	0.837442	0.784863
B.cells	OTUD6B	-0.03889	4.301102	-0.3144	0.753957	-6.26689	0.819738	0.756124
B.cells	MGST3	-0.06371	3.846236	-0.31414	0.754159	-6.24257	0.825694	0.765771
B.cells	CABP4	-0.18091	-0.22452	-0.3141	0.754188	-5.3025	0.881297	0.857511
B.cells	RBKS	-0.06503	3.630745	-0.31395	0.754302	-6.04984	0.828534	0.770442
B.cells	TBL3	0.048135	3.91508	0.313724	0.754469	-6.19348	0.824789	0.764415
B.cells	SLC25A19	0.054763	3.390435	0.313539	0.754609	-6.14291	0.831714	0.775658
B.cells	DTX1	-0.08752	2.069415	-0.3135	0.754641	-5.96024	0.84945	0.804691
B.cells	NDUFAF1	-0.06898	2.81597	-0.31342	0.754697	-5.89742	0.839373	0.788155
B.cells	NDUFA2	-0.02811	7.238115	-0.31334	0.75476	-6.82387	0.782473	0.696872

B.cells	TTYH3	0.041475	5.141527	0.313069	0.754966	-6.4974	0.808865	0.738811
B.cells	B4GALT4	0.112322	1.774117	0.312944	0.75506	-5.52578	0.853475	0.811406
B.cells	TMTC4	-0.12943	1.357794	-0.31252	0.75538	-5.53055	0.859186	0.820985
B.cells	RNF113A2	0.044126	4.271264	0.312472	0.755418	-6.31535	0.820127	0.75709
B.cells	NMNAT1	0.143325	1.326233	0.31236	0.755502	-5.48093	0.859621	0.821737
B.cells	SYT11	0.121217	1.412759	0.312334	0.755522	-5.6043	0.85843	0.819763
B.cells	EEF2KMT	0.058499	3.145957	0.312309	0.755541	-6.02434	0.834963	0.78119
B.cells	CLDN11	0.188014	-0.29485	0.312305	0.755544	-5.26903	0.882295	0.85957
B.cells	RBM15B	-0.0473	4.047089	-0.31215	0.755659	-6.15791	0.823058	0.761843
B.cells	TTLL4	0.061457	3.000367	0.312144	0.755666	-5.97935	0.836906	0.78436
B.cells	CSTDC5	0.114222	5.46156	0.311883	0.755864	-6.45376	0.804769	0.732475
B.cells	STK11	0.0325	5.696969	0.311796	0.755929	-6.56941	0.801772	0.727696
B.cells	ZFP60	0.119901	1.431044	0.311663	0.75603	-5.48681	0.858178	0.819425
B.cells	BNIP2	-0.02772	6.377564	-0.31165	0.756039	-6.70589	0.79318	0.714053
B.cells	TXNDC17	0.036814	6.116505	0.311623	0.756061	-6.62178	0.796463	0.719262
B.cells	D830036C2	-0.15842	0.420218	-0.31161	0.756073	-5.36738	0.87221	0.84276
B.cells	9430091E2	-0.07249	2.439837	-0.31154	0.756121	-5.88403	0.844433	0.796763
B.cells	LMO1	0.148367	1.523387	0.311512	0.756145	-5.47118	0.856909	0.817325
B.cells	SF3B5	0.03223	6.277364	0.311438	0.7562	-6.65216	0.794438	0.716048
B.cells	ARHGAP27	0.11114	0.465307	0.311424	0.756212	-5.52525	0.871579	0.841706
B.cells	DCTD	0.134901	1.018621	0.311396	0.756233	-5.36947	0.863872	0.82887
B.cells	GM16599	-0.03977	4.782001	-0.31133	0.756284	-6.44991	0.813495	0.746488
B.cells	SYNE2	-0.05303	5.373755	-0.31132	0.75629	-6.41613	0.80589	0.73429
B.cells	GM19705	-0.15652	1.868138	-0.31117	0.756407	-5.42536	0.852191	0.809581
B.cells	ANXA1	-0.07755	6.030034	-0.31109	0.756463	-6.49504	0.797554	0.72106
B.cells	ITSN1	0.057875	5.300914	0.310831	0.756661	-6.389	0.806822	0.73587
B.cells	THAP4	-0.0458	4.091332	-0.31081	0.756673	-6.22237	0.822478	0.76107
B.cells	AQR	-0.03217	4.812338	-0.31076	0.756712	-6.40425	0.813103	0.745947
B.cells	UNG	-0.09417	3.369876	-0.31065	0.756795	-5.91189	0.831986	0.776522
B.cells	TEX264	0.039296	4.497588	0.310569	0.75686	-6.30712	0.81718	0.752547
B.cells	METTL3	0.051257	3.319562	0.310489	0.75692	-6.10492	0.832654	0.777634
B.cells	TIMM8A1	0.055448	4.226992	0.310223	0.757122	-6.24822	0.820705	0.758251
B.cells	NRGN	0.09289	3.663682	0.310169	0.757162	-6.12529	0.828099	0.770233
B.cells	SDHB	0.033384	7.274793	0.310146	0.75718	-6.85721	0.782021	0.696568
B.cells	ARL11	0.122556	1.439587	0.310106	0.75721	-5.43464	0.858061	0.819396
B.cells	SERPINB6A	0.051958	5.570318	0.309957	0.757323	-6.4687	0.803383	0.730459
B.cells	SPC25	-0.07906	4.372308	-0.30977	0.757465	-6.15641	0.81881	0.755259
B.cells	RRP15	-0.04882	3.823389	-0.3097	0.757521	-6.19215	0.825995	0.766897
B.cells	CALM3	0.030125	7.10732	0.309643	0.757561	-6.78958	0.784089	0.69991
B.cells	KCTD7	0.143137	0.387211	0.309607	0.757588	-5.31378	0.872673	0.843794
B.cells	DAXX	0.051768	4.352332	0.309595	0.757598	-6.31337	0.81907	0.755704
B.cells	VWA5A	-0.08035	3.406572	-0.30953	0.757645	-5.88375	0.8315	0.775866
B.cells	ATF5	-0.07318	3.267979	-0.30953	0.757647	-6.09117	0.833339	0.778864
B.cells	TBC1D31	-0.05631	4.166685	-0.3095	0.757666	-6.2492	0.821493	0.759631
B.cells	MSRA	-0.03092	6.629496	-0.30947	0.757692	-6.74411	0.790028	0.709298
B.cells	CNOT9	-0.0395	4.541441	-0.30937	0.75777	-6.32377	0.816611	0.751781
B.cells	PCGF3	-0.04303	3.998741	-0.30929	0.757827	-6.24098	0.823691	0.763227

B.cells	ZFP87	0.058213	3.396086	0.309188	0.757906	-6.08662	0.831639	0.776136
B.cells	TFPT	-0.05855	2.965912	-0.30917	0.75792	-5.94242	0.837366	0.785481
B.cells	SPRED3	0.142322	0.719893	0.309114	0.757962	-5.44277	0.868023	0.836089
B.cells	TGFBR2	0.029157	7.292661	0.308951	0.758086	-6.81568	0.7818	0.696372
B.cells	CD79A	0.04598	6.200185	0.308878	0.758141	-6.76034	0.795409	0.717877
B.cells	PYM1	-0.03545	4.692796	-0.3088	0.7582	-6.39736	0.814649	0.748662
B.cells	TMEM204	-0.12591	1.038857	-0.30875	0.758242	-5.37801	0.863591	0.828783
B.cells	LRCH4	0.049573	4.260162	0.308686	0.758287	-6.23045	0.820272	0.757767
B.cells	GM9856	-0.11333	1.246414	-0.3086	0.758349	-5.51277	0.860722	0.824077
B.cells	5430427O1	-0.05705	3.846602	-0.30855	0.758392	-6.21329	0.825689	0.766599
B.cells	C530008M	-0.17148	0.74516	-0.30841	0.758496	-5.33982	0.867671	0.835651
B.cells	NEURL3	0.056832	4.301516	0.308407	0.758498	-6.24676	0.819732	0.756957
B.cells	D030056L2	-0.05841	3.888031	-0.3084	0.758505	-6.16162	0.825145	0.765719
B.cells	QTRT2	-0.13647	1.344368	-0.30832	0.758566	-5.50997	0.859371	0.821882
B.cells	ZFP704	-0.08538	3.792086	-0.30823	0.758632	-6.04159	0.826407	0.767832
B.cells	VIL1	-0.19319	0.018277	-0.30821	0.758646	-5.26878	0.877863	0.852762
B.cells	MTURN	-0.08753	2.270409	-0.30811	0.758725	-5.8093	0.846723	0.801038
B.cells	STRIP1	-0.04615	4.156745	-0.30804	0.75878	-6.24362	0.821623	0.760081
B.cells	HIST1H4C	0.175795	0.309171	0.308032	0.758783	-5.26173	0.873768	0.845904
B.cells	PRKAG2	-0.04019	5.050385	-0.30788	0.7589	-6.47569	0.810085	0.741423
B.cells	CHIL5	0.147158	-0.02332	0.30777	0.758981	-5.30031	0.878516	0.853794
B.cells	ZFP740	0.035194	4.76726	0.307556	0.759143	-6.40248	0.813814	0.747378
B.cells	CAND1	-0.03275	5.132169	-0.3075	0.759186	-6.46293	0.809112	0.739824
B.cells	A430073D2	-0.0928	1.815155	-0.30685	0.759683	-5.70475	0.853279	0.81149
B.cells	HDGF	-0.02893	6.998077	-0.30677	0.759743	-6.79474	0.785779	0.702488
B.cells	FAM122A	0.056513	3.563965	0.306673	0.759813	-6.02686	0.829771	0.77296
B.cells	SPIRE1	0.112286	2.681156	0.306522	0.759928	-5.80598	0.841543	0.792186
B.cells	DHX16	-0.05106	4.087189	-0.30652	0.75993	-6.22798	0.822885	0.76179
B.cells	LIMS2	0.168294	0.424817	0.306506	0.75994	-5.30468	0.872519	0.843455
B.cells	PPCDC	0.055904	3.567947	0.306417	0.760008	-6.07881	0.829718	0.772879
B.cells	GRB2	0.027078	7.898248	0.306265	0.760123	-6.95553	0.774707	0.685129
B.cells	GPC3	0.102427	1.806193	0.306127	0.760228	-5.67367	0.853402	0.811723
B.cells	SAMD4	-0.14066	3.187151	-0.30582	0.760463	-5.82177	0.834772	0.781206
B.cells	GM4951	-0.13017	3.770894	-0.30575	0.760517	-5.98599	0.82704	0.768655
B.cells	SEC16A	0.045049	4.57627	0.305717	0.760539	-6.33369	0.816508	0.75162
B.cells	KIF16B	0.044013	4.843803	0.305531	0.76068	-6.37679	0.813045	0.746046
B.cells	ASNA1	0.037494	4.877847	0.305467	0.760729	-6.44152	0.812606	0.745339
B.cells	MINPP1	0.036492	4.734583	0.305447	0.760744	-6.39331	0.814457	0.748318
B.cells	OSBPL11	0.041032	5.368512	0.305437	0.760751	-6.49556	0.806302	0.735227
B.cells	5330438D1	0.039478	4.842586	0.305433	0.760754	-6.46666	0.813061	0.746071
B.cells	LRRC47	0.039916	4.227098	0.305388	0.760789	-6.25459	0.821055	0.758964
B.cells	UGT1A7C	0.1606	-0.45047	0.305355	0.760813	-5.30722	0.884881	0.864346
B.cells	PIGP	0.049532	4.420664	0.305289	0.760864	-6.21674	0.818531	0.754886
B.cells	PANK3	0.037611	4.591077	0.305086	0.761017	-6.36009	0.816316	0.751353
B.cells	ST6GAL1	0.060145	5.862113	0.304985	0.761094	-6.6068	0.800019	0.725247
B.cells	EMC6	0.031318	5.61708	0.304825	0.761216	-6.57897	0.803131	0.730238
B.cells	CDC7	0.078775	2.873973	0.304737	0.761282	-5.86001	0.838955	0.788176

B.cells	ATP8A2	0.120569	2.725958	0.304654	0.761345	-5.77539	0.840941	0.791466
B.cells	CABYR	0.137058	1.135391	0.304546	0.761427	-5.5111	0.862624	0.827293
B.cells	EMSY	0.032416	5.828738	0.304494	0.761467	-6.61144	0.800442	0.726025
B.cells	CHMP7	-0.07478	2.616172	-0.30448	0.761477	-5.84516	0.842417	0.793933
B.cells	GM48623	0.163725	-0.24558	0.304298	0.761616	-5.28635	0.881973	0.859705
B.cells	MEI4	0.140674	1.201602	0.304234	0.761665	-5.55981	0.861709	0.82587
B.cells	CSNK2B	-0.02847	7.247784	-0.30422	0.761672	-6.85194	0.782689	0.69798
B.cells	SAMD1	-0.04026	4.976188	-0.30406	0.761796	-6.42681	0.811338	0.74355
B.cells	SH3BGRL	0.028647	6.824052	0.304042	0.76181	-6.83647	0.787941	0.706265
B.cells	SLC27A1	0.085124	2.972091	0.303958	0.761874	-5.90528	0.837642	0.78621
B.cells	PAXX	0.055003	3.270058	0.303952	0.761878	-6.01534	0.833669	0.779719
B.cells	SLC25A20	-0.03806	5.597573	-0.30341	0.762293	-6.66326	0.803691	0.730894
B.cells	GPC5	-0.17531	0.598275	-0.30338	0.762316	-5.38431	0.870429	0.839946
B.cells	EPB41L5	-0.06329	3.379051	-0.30319	0.762458	-6.12402	0.832545	0.777476
B.cells	MNS1	-0.0786	2.821224	-0.30314	0.762496	-5.80869	0.839989	0.789646
B.cells	ZFP952	0.079601	2.251647	0.303094	0.76253	-5.76428	0.84767	0.802268
B.cells	5830487J0	0.132081	-0.01733	0.302992	0.762608	-5.36347	0.879086	0.854469
B.cells	DESI1	0.041467	5.729904	0.302794	0.762758	-6.54083	0.802011	0.728298
B.cells	GM49625	-0.10436	2.147374	-0.30269	0.762835	-5.57596	0.849087	0.804662
B.cells	LRMP	-0.0352	6.432878	-0.30267	0.76285	-6.71226	0.793137	0.714194
B.cells	MORC2A	0.046454	4.295558	0.302624	0.762888	-6.28755	0.820483	0.758007
B.cells	F3	-0.2395	0.113979	-0.30192	0.763419	-5.24686	0.877691	0.851545
B.cells	PHAX	0.043587	4.599165	0.301901	0.763436	-6.34166	0.816959	0.7517
B.cells	FKBP9	-0.16275	1.265181	-0.30167	0.76361	-5.38055	0.861619	0.824838
B.cells	MRGBP	-0.05679	3.37422	-0.30164	0.763638	-6.03358	0.833047	0.777852
B.cells	IFITM2	0.043557	7.227901	0.30163	0.763643	-6.80678	0.783651	0.698726
B.cells	MROH1	0.049251	4.199162	0.301401	0.763816	-6.25702	0.822282	0.76024
B.cells	REXO4	0.03971	4.291897	0.301017	0.764108	-6.31162	0.821305	0.758415
B.cells	IQCG	0.209904	0.050135	0.300926	0.764177	-5.21298	0.878962	0.853396
B.cells	SLC19A1	0.12244	1.065999	0.300818	0.764259	-5.48581	0.864745	0.829687
B.cells	EEF1AKMT	0.063511	2.582886	0.300732	0.764325	-5.88016	0.843998	0.795459
B.cells	H1F0	-0.04838	6.001224	-0.30047	0.764525	-6.61155	0.799407	0.723303
B.cells	GM16093	0.084218	2.640752	0.30033	0.76463	-5.82454	0.843297	0.79427
B.cells	PRXL2C	0.03837	4.932963	0.300192	0.764735	-6.4058	0.813062	0.745224
B.cells	PICALM	0.026059	8.404518	0.300044	0.764848	-7.07255	0.769667	0.676588
B.cells	GSK3A	0.032129	5.557032	0.300008	0.764875	-6.53834	0.805052	0.732399
B.cells	SMIM15	-0.0366	4.858356	-0.29998	0.764899	-6.36945	0.814026	0.746799
B.cells	LRRC41	0.038265	4.919545	0.299914	0.764947	-6.43741	0.813235	0.745539
B.cells	GM43936	0.208498	-1.34803	0.299782	0.765047	-5.09327	0.898765	0.887405
B.cells	AV099323	0.139498	1.209882	0.299542	0.76523	-5.45112	0.862833	0.826642
B.cells	TMEM185A	-0.07481	2.540183	-0.29952	0.765243	-5.82128	0.844653	0.796628
B.cells	SLCO4C1	-0.14829	-1.1708	-0.29951	0.765255	-5.2663	0.896171	0.883099
B.cells	CEP95	-0.04175	4.108354	-0.2995	0.765259	-6.21517	0.82379	0.762615
B.cells	FAM53A	0.045326	3.797306	0.299421	0.765321	-6.20965	0.82788	0.769246
B.cells	EPB41L4AC	0.072595	3.25392	0.299203	0.765487	-6.02784	0.835123	0.780996
B.cells	ZBTB21	-0.0477	3.713914	-0.29915	0.76553	-6.19328	0.829021	0.771087
B.cells	TREML4	0.126117	2.758058	0.299029	0.76562	-5.71266	0.841759	0.79189

B.cells	ST3GAL5	-0.04758	6.23691	-0.29866	0.765902	-6.61114	0.79647	0.718875
B.cells	NCAPH	-0.07369	4.175802	-0.29854	0.76599	-6.18361	0.822947	0.761331
B.cells	COX18	0.061104	3.223872	0.298461	0.766051	-5.9762	0.835523	0.78177
B.cells	GM5244	-0.18259	-0.39473	-0.29839	0.766102	-5.26277	0.885407	0.864467
B.cells	ATXN2	-0.03024	6.28335	-0.29817	0.766276	-6.68877	0.795885	0.717996
B.cells	UTP18	-0.03262	5.338906	-0.29816	0.766281	-6.55407	0.80788	0.737125
B.cells	MRPL51	-0.04348	4.486929	-0.29813	0.766304	-6.26842	0.818884	0.754818
B.cells	TIGAR	0.088119	1.851972	0.298122	0.766309	-5.70741	0.854044	0.812232
B.cells	STK24	-0.02495	7.291795	-0.29804	0.766371	-6.88797	0.783308	0.698119
B.cells	NXPH4	-0.188	-0.60095	-0.298	0.766402	-5.21547	0.888248	0.869485
B.cells	SRSF10	-0.02366	6.4448	-0.29786	0.766509	-6.71174	0.793855	0.714786
B.cells	NDUFB8	-0.02731	7.487591	-0.29782	0.76654	-6.89047	0.780894	0.694335
B.cells	AMMECR1	0.043004	4.787165	0.297762	0.766584	-6.39521	0.814987	0.748559
B.cells	ADPGK	0.063562	4.903066	0.297629	0.766684	-6.25306	0.813488	0.746173
B.cells	9230111E0	0.188437	-0.18455	0.297626	0.766687	-5.21042	0.882418	0.859556
B.cells	DIP2A	0.077128	2.079809	0.297549	0.766745	-5.78106	0.850936	0.807176
B.cells	IFI207	0.109352	4.721848	0.297496	0.766786	-6.05804	0.815833	0.749965
B.cells	RNASEH2C	-0.03945	4.998911	-0.29734	0.766904	-6.39413	0.812251	0.744245
B.cells	ST8SIA4	0.037958	7.324778	0.297239	0.766981	-6.88906	0.782901	0.697581
B.cells	GM20404	-0.09944	1.661785	-0.29719	0.767016	-5.62185	0.856649	0.816658
B.cells	RANBP1	-0.03984	7.239938	-0.29708	0.767104	-6.81442	0.783949	0.699263
B.cells	GPR180	0.066571	2.964205	0.296948	0.767202	-5.87113	0.838993	0.78767
B.cells	E130311K1	0.169378	0.244935	0.296845	0.767281	-5.29546	0.876345	0.849525
B.cells	SCARB1	-0.04016	4.995715	-0.29678	0.767332	-6.39094	0.812292	0.744385
B.cells	TIMM29	0.056292	3.296538	0.296753	0.767351	-6.05683	0.834555	0.780436
B.cells	FBXW5	0.053625	3.39907	0.29666	0.767422	-6.04087	0.833192	0.778248
B.cells	ACOX3	0.038249	4.372027	0.296525	0.767525	-6.41495	0.820416	0.75748
B.cells	CDCA7	0.082723	3.246784	0.296308	0.767689	-5.92652	0.835354	0.781674
B.cells	GM37529	0.112796	1.962925	0.29599	0.767932	-5.65941	0.852857	0.810177
B.cells	DPYSL3	-0.17127	0.753878	-0.29582	0.768062	-5.27081	0.869614	0.837873
B.cells	GM48512	-0.11189	1.068935	-0.29568	0.768164	-5.51419	0.865263	0.830616
B.cells	GLDC	0.122477	1.391092	0.295275	0.768476	-5.57352	0.861015	0.823257
B.cells	1110035H1	0.114904	1.216236	0.295253	0.768493	-5.53657	0.863431	0.827267
B.cells	ZFP119A	0.068142	2.125414	0.29508	0.768624	-5.79708	0.850953	0.806692
B.cells	TRAPPC2	0.046318	4.171105	0.295061	0.768639	-6.2514	0.823626	0.762061
B.cells	GM10382	0.15517	0.439288	0.294855	0.768796	-5.32708	0.874367	0.845397
B.cells	BHLHE41	-0.09236	1.739361	-0.29474	0.768882	-5.86839	0.85634	0.815442
B.cells	ATP13A3	0.022847	7.315105	0.294479	0.769082	-6.84375	0.783794	0.698285
B.cells	GLO1	0.041751	5.029475	0.294322	0.769202	-6.46732	0.812659	0.744179
B.cells	PIK3C2A	0.03044	6.854141	0.2943	0.769218	-6.83829	0.789516	0.707319
B.cells	EMILIN2	0.139442	4.009199	0.294261	0.769249	-5.84706	0.825947	0.765617
B.cells	KLRC2	-0.15486	1.332802	-0.29414	0.769342	-5.50831	0.862053	0.8248
B.cells	TAOK2	0.047142	4.127104	0.293958	0.769479	-6.17774	0.824487	0.763144
B.cells	2010320M	-0.08205	2.480423	-0.29384	0.769566	-5.75189	0.846428	0.798957
B.cells	PHF14	-0.02751	6.331947	-0.29372	0.769657	-6.70705	0.796144	0.717751
B.cells	MARCO	0.122202	5.795483	0.293579	0.769768	-6.54141	0.802933	0.728582
B.cells	2010001A1	-0.11323	1.389339	-0.29346	0.769861	-5.46533	0.861339	0.823641

B.cells	POGLUT1	0.0637	2.752407	0.293376	0.769922	-5.93426	0.842757	0.793009
B.cells	RAD17	0.032521	5.023722	0.293151	0.770093	-6.45493	0.81282	0.744467
B.cells	DFFA	-0.06181	2.939835	-0.29303	0.770185	-5.9759	0.840239	0.788922
B.cells	TMEM132E	-0.14003	0.984501	-0.29297	0.770233	-5.52998	0.866949	0.832997
B.cells	TMEM150E	0.138531	1.12138	0.29293	0.770262	-5.50804	0.865047	0.829835
B.cells	FDXR	0.070738	2.625449	0.292914	0.770274	-5.83077	0.844468	0.795852
B.cells	IL10RA	-0.0402	4.971561	-0.2928	0.770361	-6.5512	0.813493	0.745585
B.cells	MRPL35	-0.03547	5.21625	-0.29279	0.770367	-6.50765	0.81034	0.740524
B.cells	E430024I08	0.084991	1.898133	0.292742	0.770405	-5.67403	0.854348	0.812154
B.cells	CLN8	-0.09094	3.202024	-0.29266	0.770471	-5.78488	0.83673	0.783258
B.cells	SUN1	0.04125	3.918531	0.292483	0.770602	-6.23579	0.827229	0.767822
B.cells	ALDH3A2	-0.0472	3.596521	-0.29247	0.770613	-6.11941	0.831483	0.774734
B.cells	STAMBPL1	-0.03743	6.55028	-0.29204	0.770941	-6.68981	0.793665	0.713639
B.cells	UQCR10	-0.03162	6.961957	-0.29184	0.771093	-6.78512	0.788587	0.705511
B.cells	GM42031	-0.08412	4.172175	-0.29177	0.771149	-6.4117	0.82424	0.762498
B.cells	KMT2B	0.04618	3.888591	0.291531	0.771328	-6.18956	0.828041	0.768599
B.cells	DDB1	-0.03563	5.611857	-0.29149	0.771359	-6.53272	0.805679	0.732598
B.cells	NLRX1	0.107143	1.80919	0.291144	0.771623	-5.61903	0.856157	0.81439
B.cells	TYW1	0.04808	3.875123	0.291115	0.771645	-6.19286	0.828373	0.768907
B.cells	2010309G2	0.091478	1.223176	0.290889	0.771818	-5.81426	0.864321	0.827795
B.cells	XIAP	0.023311	6.725324	0.2908	0.771885	-6.77877	0.791836	0.710286
B.cells	ING4	0.047517	4.08954	0.290733	0.771936	-6.21556	0.825633	0.76439
B.cells	ZFP451	0.036524	5.019065	0.290505	0.77211	-6.43703	0.813563	0.744902
B.cells	FHIT	-0.04216	6.167904	-0.29021	0.772334	-6.6448	0.798883	0.721511
B.cells	POU6F1	0.074315	2.610644	0.290148	0.772383	-5.87008	0.845377	0.796605
B.cells	CD7	0.148391	4.199176	0.289991	0.772503	-5.85171	0.824233	0.762203
B.cells	GM10874	0.169013	-0.06869	0.289951	0.772533	-5.22623	0.88248	0.858222
B.cells	MRPL1	0.032184	4.619868	0.289902	0.77257	-6.3782	0.818738	0.753329
B.cells	CTNNA3	0.182439	1.300067	0.28984	0.772617	-5.47459	0.863297	0.826213
B.cells	FBXO44	-0.16545	0.220079	-0.28978	0.772663	-5.30655	0.878393	0.851382
B.cells	SMAGP	0.061164	3.61538	0.289585	0.772811	-6.07957	0.831931	0.7747
B.cells	RBCK1	-0.03121	5.550838	-0.28956	0.772831	-6.56157	0.806729	0.734066
B.cells	1700052K1	0.113476	1.140287	0.289475	0.772896	-5.42778	0.865511	0.829914
B.cells	CR1L	-0.02634	5.693128	-0.28947	0.772899	-6.58455	0.804912	0.731174
B.cells	HTR1F	0.094694	1.429334	0.289411	0.772945	-5.55834	0.86151	0.823274
B.cells	EPRS	0.040137	6.581496	0.289305	0.773026	-6.76336	0.793675	0.713312
B.cells	STARD13	-0.15837	1.070842	-0.28928	0.773042	-5.43646	0.866476	0.831516
B.cells	1110002J0	0.171942	-0.51304	0.288559	0.773594	-5.31263	0.889191	0.869033
B.cells	CASTOR2	-0.07132	3.643855	-0.28842	0.773701	-6.19092	0.831951	0.774222
B.cells	GM43768	0.135406	0.70894	0.288419	0.773701	-5.41604	0.871939	0.840053
B.cells	GM16230	-0.18736	0.238127	-0.28823	0.773843	-5.25721	0.878557	0.851107
B.cells	D830025C0	-0.09124	2.231904	-0.28815	0.773908	-5.69277	0.850917	0.805239
B.cells	GNA11	0.058348	3.954617	0.288129	0.773923	-6.11433	0.827843	0.767555
B.cells	PPM1F	0.09178	2.206568	0.28807	0.773968	-5.62655	0.851262	0.805835
B.cells	SLC36A1	0.053129	2.851189	0.288007	0.774016	-5.97377	0.842537	0.791519
B.cells	GM38604	0.07899	1.796583	0.287957	0.774054	-5.75487	0.856866	0.815077
B.cells	PVR	-0.06109	3.063552	-0.28766	0.774283	-6.07623	0.839817	0.786888

B.cells	CUEDC1	-0.08838	2.113054	-0.28761	0.774317	-5.70511	0.85267	0.807989
B.cells	CHST3	0.05652	4.312271	0.286967	0.774809	-6.34344	0.823712	0.760245
B.cells	GMPS	-0.02767	5.784964	-0.28688	0.774873	-6.59913	0.80468	0.729716
B.cells	WDR83	-0.06483	2.732947	-0.28676	0.774965	-5.88773	0.844735	0.794412
B.cells	SNRPB2	-0.02547	6.282619	-0.28648	0.775178	-6.69013	0.798533	0.719755
B.cells	GM29093	0.089389	1.269702	0.28619	0.775402	-5.52571	0.865002	0.8275
B.cells	NGP	-0.08993	5.022684	-0.28597	0.775573	-6.4282	0.814726	0.745534
B.cells	TXNDC12	-0.04296	3.827411	-0.28595	0.775587	-6.16748	0.830359	0.770756
B.cells	ZFP354B	-0.12737	-0.81695	-0.28583	0.775678	-5.22639	0.894257	0.876964
B.cells	TM4SF5	0.173452	0.261552	0.285772	0.775721	-5.32843	0.879113	0.851135
B.cells	SFXN3	-0.05274	3.370072	-0.28574	0.775742	-6.13679	0.836433	0.780676
B.cells	MRPL48	0.028137	5.282039	0.285717	0.775763	-6.48792	0.81138	0.740215
B.cells	ZFP113	0.133967	1.151185	0.285498	0.77593	-5.40065	0.866669	0.830365
B.cells	ERBB3	-0.14926	1.113728	-0.28533	0.776061	-5.3744	0.86719	0.831275
B.cells	NDUFB6	-0.0368	6.317819	-0.28528	0.776097	-6.68192	0.798197	0.719215
B.cells	CEP63	-0.0398	4.291786	-0.28524	0.776127	-6.35984	0.824265	0.760948
B.cells	RASSF5	0.035197	5.485464	0.28508	0.77625	-6.51117	0.808787	0.736101
B.cells	TTL	-0.082	1.740811	-0.28501	0.776304	-5.68964	0.858519	0.816965
B.cells	ACSF2	0.043932	4.286847	0.284965	0.776338	-6.36068	0.82433	0.761101
B.cells	GNA14	0.216338	-0.0617	0.284881	0.776402	-5.21159	0.883716	0.858909
B.cells	LNCPINT	0.041829	8.328661	0.28485	0.776425	-7.1048	0.773283	0.680108
B.cells	ADAM11	0.161393	-1.14352	0.284554	0.776651	-5.10805	0.899058	0.885107
B.cells	CISH	0.079419	2.887974	0.284369	0.776792	-5.97677	0.843055	0.791385
B.cells	FAM71E1	0.150824	0.837279	0.284224	0.776903	-5.36275	0.87119	0.837812
B.cells	ELK1	-0.08398	1.559348	-0.28412	0.776983	-5.56389	0.861161	0.821171
B.cells	AP1G1	0.030287	6.456316	0.284115	0.776986	-6.71365	0.796583	0.716557
B.cells	CTLA2B	-0.12995	3.353706	-0.28389	0.77716	-5.8015	0.836813	0.781196
B.cells	ZMYND15	-0.18193	0.109664	-0.28374	0.777276	-5.39342	0.881431	0.854932
B.cells	ZFP326	0.030881	5.21559	0.283588	0.777389	-6.49328	0.812392	0.741761
B.cells	SNHG17	-0.08639	1.368538	-0.28356	0.777412	-5.63924	0.863799	0.825558
B.cells	4921516A(	-0.10849	0.76983	-0.28355	0.777419	-5.5164	0.872134	0.839405
B.cells	RMND5A	0.027483	6.766957	0.283368	0.777557	-6.77911	0.792683	0.710423
B.cells	GM3235	0.129745	0.645113	0.283363	0.777561	-5.50184	0.873882	0.842338
B.cells	TTLL12	0.110424	2.217401	0.283349	0.777571	-5.69669	0.852138	0.806325
B.cells	MUL1	0.063292	2.950066	0.283327	0.777588	-5.93466	0.842219	0.790059
B.cells	POLD2	0.068813	3.401936	0.283278	0.777626	-5.98905	0.836169	0.780212
B.cells	PPARGC1A	0.131442	1.419055	0.283002	0.777837	-5.56065	0.863169	0.824492
B.cells	RNF122	-0.08151	2.64878	-0.28297	0.777861	-5.84813	0.84635	0.796794
B.cells	RUNX1	-0.03051	9.201324	-0.28292	0.777903	-7.18842	0.762948	0.663963
B.cells	NUDT18	0.065083	2.627052	0.282741	0.778036	-5.78246	0.846711	0.797303
B.cells	HINT3	-0.04011	4.643869	-0.28242	0.77828	-6.366	0.820071	0.753879
B.cells	ARV1	0.082955	2.101251	0.282359	0.778328	-5.7778	0.854004	0.809146
B.cells	ZFP101	-0.05652	2.916235	-0.28221	0.778445	-5.99603	0.842952	0.791071
B.cells	GM13091	-0.12823	0.904415	-0.28215	0.778486	-5.43449	0.870539	0.836592
B.cells	PRR14	-0.03989	4.524923	-0.28194	0.778649	-6.35327	0.821623	0.756485
B.cells	DR1	0.029336	4.953577	0.281912	0.77867	-6.48949	0.816046	0.747519
B.cells	ATCAYOS	-0.16869	1.220314	-0.28179	0.778764	-5.42947	0.866139	0.829353

B.cells	H2-OB	0.050958	4.007971	0.281711	0.778823	-6.55791	0.828407	0.767497
B.cells	CAMK2G	0.039277	4.893224	0.281589	0.778917	-6.41317	0.816829	0.748852
B.cells	CBX1	-0.02939	6.075378	-0.28151	0.778976	-6.63792	0.801662	0.724638
B.cells	IPO4	0.062868	2.384797	0.281331	0.779114	-5.79952	0.85014	0.803066
B.cells	DCAKD	-0.04934	4.076447	-0.28132	0.779119	-6.20388	0.827505	0.766143
B.cells	GM14410	0.117484	0.850334	0.281261	0.779167	-5.41454	0.871295	0.838069
B.cells	GM49189	0.109573	0.779988	0.281102	0.779289	-5.50147	0.872279	0.839743
B.cells	CUL3	-0.02025	7.198802	-0.28104	0.779337	-6.84591	0.787557	0.702422
B.cells	ME2	0.0368	5.963937	0.280989	0.779375	-6.59247	0.803078	0.727
B.cells	COPE	0.027654	6.316581	0.280962	0.779396	-6.68391	0.798609	0.7199
B.cells	SPIB	-0.06384	4.129765	-0.28089	0.779454	-6.21326	0.826803	0.765103
B.cells	CDC25C	-0.10546	2.030722	-0.2805	0.77975	-5.63326	0.855215	0.811248
B.cells	GALE	0.075954	2.330073	0.280351	0.779863	-5.70161	0.851175	0.804523
B.cells	RALA	-0.02869	5.871704	-0.27995	0.780169	-6.64723	0.804768	0.729103
B.cells	VPS36	0.029663	5.315956	0.279622	0.780421	-6.50207	0.81204	0.740568
B.cells	WDR33	-0.02024	7.186641	-0.27957	0.780458	-6.85984	0.788362	0.702987
B.cells	DHRS7B	0.055877	3.137991	0.279476	0.780532	-6.00413	0.840674	0.786866
B.cells	RARS2	-0.05138	3.441715	-0.27924	0.780715	-6.05226	0.836689	0.780307
B.cells	SNX19	0.052883	2.862011	0.279146	0.780785	-5.92933	0.844464	0.792995
B.cells	DIS3L	0.05876	2.828005	0.279002	0.780895	-5.92033	0.844923	0.793819
B.cells	WDR5B	0.14409	0.670974	0.278885	0.780984	-5.34081	0.874617	0.842895
B.cells	TMEM70	-0.04932	3.930301	-0.27886	0.781002	-6.15836	0.830201	0.769861
B.cells	ZFYVE1	0.039984	4.797159	0.278581	0.781217	-6.41249	0.818834	0.751581
B.cells	ZFP553	0.06352	2.414705	0.278427	0.781335	-5.83352	0.850521	0.803118
B.cells	CCDC17	-0.11871	0.893924	-0.27839	0.781362	-5.5185	0.871492	0.837795
B.cells	BCAT2	0.036744	4.601375	0.278326	0.781412	-6.37279	0.821385	0.755707
B.cells	SLCO3A1	0.100503	4.38978	0.278319	0.781417	-5.87621	0.824153	0.760171
B.cells	IGKV12-46	-0.12619	-0.77013	-0.27814	0.781553	-5.33432	0.894909	0.877441
B.cells	NEFH	-0.16613	-0.09246	-0.27814	0.781556	-5.27312	0.885412	0.861102
B.cells	RC3H2	-0.02674	5.728871	-0.27807	0.781608	-6.59241	0.80682	0.732407
B.cells	ALG6	-0.09233	2.044219	-0.27772	0.781874	-5.67232	0.855576	0.811575
B.cells	LRPPRC	0.034753	5.200579	0.277707	0.781886	-6.50933	0.813606	0.743333
B.cells	CEP128	0.039998	6.336302	0.277557	0.782	-6.66461	0.7991	0.720237
B.cells	RWDD4A	0.04331	3.983066	0.277532	0.782019	-6.22461	0.829504	0.769003
B.cells	TMEM132/	0.127408	0.400445	0.277487	0.782054	-5.38555	0.878425	0.849552
B.cells	RAB6A	0.02481	6.637549	0.277462	0.782073	-6.75743	0.795303	0.714225
B.cells	EPOP	0.102783	1.472765	0.277422	0.782104	-5.56305	0.863441	0.82463
B.cells	INO80E	0.032495	4.165534	0.277148	0.782313	-6.25633	0.827098	0.765161
B.cells	ARHGEF10I	0.080897	2.695098	0.277123	0.782333	-5.87886	0.846718	0.797119
B.cells	LPAR2	-0.06739	2.187833	-0.27709	0.782359	-5.73174	0.853612	0.808444
B.cells	ELOVL5	0.033789	6.195778	0.277039	0.782397	-6.67755	0.800878	0.723113
B.cells	REXO5	0.07977	1.950184	0.276904	0.7825	-5.71248	0.856864	0.813836
B.cells	RAB1B	-0.03143	5.58788	-0.27689	0.782511	-6.56487	0.808625	0.735485
B.cells	NUP210	0.033341	5.041054	0.276837	0.782552	-6.50731	0.815669	0.746774
B.cells	MACROD2	-0.09845	2.251559	-0.27658	0.782747	-5.70624	0.852743	0.807077
B.cells	GCC2	0.035632	5.193926	0.276536	0.782782	-6.45756	0.813692	0.743626
B.cells	NOLC1	0.040404	5.264292	0.276483	0.782822	-6.47343	0.812784	0.74217

B.cells	EED	0.02176	6.114274	0.276479	0.782826	-6.68608	0.801911	0.724814
B.cells	MED14	0.03543	6.321429	0.276391	0.782893	-6.70506	0.799288	0.720663
B.cells	PAM16	-0.03344	4.987535	-0.2762	0.783037	-6.43228	0.81638	0.747929
B.cells	NDRG1	0.076335	2.72107	0.276182	0.783053	-5.97127	0.846386	0.796626
B.cells	TREML2	0.051686	3.901235	0.27566	0.783452	-6.17285	0.830789	0.77096
B.cells	IL17RA	0.03598	5.907729	0.275606	0.783494	-6.66193	0.804735	0.729077
B.cells	ZMYM2	0.030957	5.970893	0.275426	0.783632	-6.62836	0.80393	0.727796
B.cells	ATP11A	-0.08927	2.676915	-0.27527	0.783754	-5.64042	0.847172	0.797677
B.cells	HACD4	0.089984	3.532004	0.27517	0.783828	-5.87608	0.835691	0.778933
B.cells	LRP8OS2	-0.18202	0.163168	-0.27502	0.783942	-5.28092	0.881996	0.855469
B.cells	METTL21A	0.076	1.888583	0.274771	0.784133	-5.63501	0.85792	0.815493
B.cells	A930007I1!	0.056171	4.385631	0.274682	0.784201	-6.43197	0.824409	0.76077
B.cells	SLAMF9	-0.07753	2.637226	-0.2746	0.784264	-6.01812	0.847709	0.7987
B.cells	SSRP1	0.032901	6.322976	0.274579	0.78428	-6.67588	0.799464	0.720825
B.cells	MSS51	0.077232	2.436698	0.27441	0.78441	-5.77087	0.85043	0.803188
B.cells	AHNAK	-0.04918	6.416793	-0.27435	0.784455	-6.86951	0.798279	0.718963
B.cells	PSME3	0.03567	5.282258	0.274335	0.784467	-6.54739	0.812752	0.742035
B.cells	CASP7	-0.05236	4.140272	-0.27428	0.784513	-6.1542	0.827634	0.766004
B.cells	MECR	0.049335	3.581314	0.27427	0.784517	-6.11556	0.835034	0.778014
B.cells	GM11342	0.097376	2.120734	0.274206	0.784566	-5.75254	0.854738	0.810277
B.cells	CRELD1	0.091278	1.843254	0.274203	0.784568	-5.62426	0.858543	0.816552
B.cells	RNPEP	0.038102	5.72187	0.27407	0.78467	-6.61858	0.807107	0.733009
B.cells	CCR2	0.148945	3.092746	0.274021	0.784708	-5.60484	0.841566	0.788663
B.cells	MEN1	-0.06468	2.866329	-0.27393	0.784778	-5.90584	0.844613	0.793689
B.cells	SLC35A1	-0.04603	3.416075	-0.27386	0.784831	-6.03118	0.837236	0.781667
B.cells	PHPT1	-0.04288	4.330971	-0.27385	0.78484	-6.25742	0.825126	0.762015
B.cells	TTLL5	-0.03864	4.471737	-0.27377	0.784899	-6.3103	0.823281	0.759042
B.cells	C030006K1	-0.10905	1.498775	-0.27371	0.784944	-5.51754	0.863292	0.824499
B.cells	TJAP1	0.035311	4.452059	0.273515	0.785095	-6.36936	0.8236	0.759513
B.cells	CHIL1	-0.1351	-0.07877	-0.27345	0.785145	-5.37136	0.885499	0.861495
B.cells	EFCAB11	0.075963	3.410869	0.273008	0.785484	-6.0004	0.837621	0.782007
B.cells	RBSN	-0.05074	2.910659	-0.2728	0.785646	-5.97432	0.844333	0.793011
B.cells	FAM110B	0.160249	0.653098	0.272627	0.785776	-5.3057	0.875412	0.844385
B.cells	NPFF	-0.10998	0.954229	-0.27261	0.785792	-5.46349	0.871191	0.837352
B.cells	BC035044	-0.04896	5.146379	-0.2726	0.785798	-6.54633	0.814813	0.745181
B.cells	EIF4EBP3	0.070552	2.80817	0.272579	0.785812	-5.87744	0.845716	0.795293
B.cells	TGIF2	0.054569	3.122183	0.272397	0.785952	-6.00078	0.84156	0.788436
B.cells	VPS33B	0.053704	2.932531	0.27225	0.786065	-5.97495	0.844125	0.792622
B.cells	GM35853	0.126493	0.184156	0.272149	0.786142	-5.38305	0.882122	0.85556
B.cells	AKAP11	-0.04211	4.648059	-0.27198	0.786268	-6.30567	0.821371	0.755688
B.cells	ZFP280D	0.033239	5.625842	0.271951	0.786294	-6.53223	0.808724	0.735395
B.cells	ACADL	0.030866	6.734458	0.271914	0.786322	-6.7638	0.794662	0.713047
B.cells	WHRN	-0.07327	3.066048	-0.27181	0.786404	-5.90866	0.842338	0.789708
B.cells	AKAP8	-0.02682	5.39229	-0.27166	0.786518	-6.54666	0.811746	0.740196
B.cells	TMEM156	0.045268	3.855526	0.271481	0.786654	-6.22499	0.831815	0.772575
B.cells	GXYLT1	0.031697	5.093219	0.271423	0.786698	-6.50981	0.815607	0.746411
B.cells	ARHGAP27	-0.04858	3.664658	-0.27125	0.786834	-6.12678	0.834348	0.77669

B.cells	PHTF1	-0.0428	4.68503	-0.27115	0.786907	-6.35185	0.820911	0.754941
B.cells	GM43713	-0.08972	2.135076	-0.27102	0.787011	-5.73404	0.854975	0.810453
B.cells	PUS7L	0.075026	1.839535	0.270962	0.787051	-5.80418	0.859029	0.81714
B.cells	CCDC148	0.188216	1.762244	0.270854	0.787134	-5.51288	0.860092	0.818897
B.cells	RNF4	-0.02352	5.964365	-0.27085	0.78714	-6.64858	0.804421	0.728526
B.cells	PPP2CB	0.026715	5.60171	0.270843	0.787143	-6.54824	0.809055	0.735919
B.cells	BAHCC1	-0.13449	1.235974	-0.27075	0.787214	-5.3605	0.867376	0.830963
B.cells	PPIL1	-0.04948	4.052063	-0.27067	0.787274	-6.2277	0.829216	0.768361
B.cells	PRKCD	0.024631	6.897294	0.270367	0.787508	-6.8372	0.792682	0.709907
B.cells	ZFP276	0.061485	2.479933	0.270285	0.787571	-5.86728	0.850316	0.802789
B.cells	MKRN2	0.033548	4.074932	0.270024	0.787771	-6.24139	0.828956	0.768013
B.cells	ZMYM3	-0.07945	2.31007	-0.26997	0.787812	-5.76867	0.852629	0.806687
B.cells	TMCC2	-0.11459	1.686886	-0.26993	0.787841	-5.70934	0.861174	0.820789
B.cells	AI480526	0.083527	2.064128	0.269778	0.78796	-5.68051	0.855989	0.812242
B.cells	ZFP963	-0.0995	1.15161	-0.26976	0.787973	-5.4814	0.868594	0.833107
B.cells	PKD2L2	0.085856	1.170955	0.269671	0.788042	-5.48815	0.868324	0.832659
B.cells	DVL2	0.058743	3.263703	0.269613	0.788086	-6.02776	0.839741	0.785587
B.cells	DDX52	-0.03026	4.930462	-0.2696	0.788099	-6.47458	0.817758	0.749975
B.cells	EPS8L2	-0.13443	0.893203	-0.26958	0.788112	-5.42523	0.872202	0.839109
B.cells	GM15972	0.163558	-0.2921	0.269452	0.788209	-5.25489	0.888973	0.867179
B.cells	OGDH	0.019175	6.625732	0.269365	0.788276	-6.77851	0.79609	0.7154
B.cells	PRPF40A	-0.01914	7.709067	-0.26931	0.788315	-6.9303	0.782599	0.694148
B.cells	UQCRC1	0.026531	6.508803	0.269034	0.78853	-6.7466	0.797692	0.717733
B.cells	PRR16	-0.10932	1.720127	-0.26896	0.788586	-5.68083	0.860855	0.820047
B.cells	ABCB4	-0.06587	3.391454	-0.2688	0.788706	-6.05193	0.838219	0.782799
B.cells	DCAF6	-0.03781	5.908083	-0.26866	0.788815	-6.61595	0.805374	0.729838
B.cells	DPF1	-0.1589	0.292792	-0.26853	0.788916	-5.32577	0.880865	0.853217
B.cells	ECI1	-0.04065	3.920606	-0.26849	0.788949	-6.25147	0.831197	0.771356
B.cells	GM49482	-0.17747	-0.71551	-0.26833	0.789069	-5.15959	0.895092	0.877423
B.cells	SALL2	-0.14499	0.090711	-0.26832	0.789079	-5.29749	0.883731	0.858016
B.cells	SUGP1	-0.03383	4.615277	-0.26774	0.789524	-6.3722	0.822314	0.75664
B.cells	USP1	-0.03408	5.737466	-0.2677	0.789557	-6.57148	0.807801	0.733383
B.cells	SERBP1	-0.02122	8.529561	-0.26768	0.789572	-7.05917	0.772986	0.678543
B.cells	H1FX	-0.11225	2.127581	-0.26763	0.789608	-5.60963	0.855591	0.810899
B.cells	EXOSC7	0.036522	4.629702	0.267302	0.789859	-6.34803	0.822225	0.756488
B.cells	TMEM101	-0.07289	2.322989	-0.26724	0.789904	-5.76184	0.853023	0.806638
B.cells	1810034E1	0.089612	1.895787	0.267226	0.789917	-5.62304	0.858874	0.81628
B.cells	MORN2	0.085333	2.045949	0.266904	0.790165	-5.65357	0.856874	0.812967
B.cells	TGDS	-0.05644	3.1411	-0.26671	0.790312	-5.91578	0.842009	0.788586
B.cells	E430018J2	-0.11695	0.653485	-0.26661	0.790393	-5.41841	0.876214	0.845069
B.cells	AKT1	-0.02958	6.230392	-0.26657	0.790425	-6.68802	0.801676	0.723621
B.cells	WWTR1	0.11926	2.126919	0.266442	0.790519	-5.55194	0.855764	0.811226
B.cells	IL12A	0.081475	2.215059	0.266424	0.790533	-5.87406	0.854559	0.809246
B.cells	TBC1D22A	0.028287	5.871527	0.266197	0.790708	-6.61278	0.806243	0.731006
B.cells	GALK1	-0.04212	5.140655	-0.26614	0.79075	-6.37862	0.815639	0.746035
B.cells	LIN52	0.034876	5.546829	0.266002	0.790857	-6.5515	0.810401	0.737666
B.cells	CD19	-0.05737	3.122136	-0.26597	0.790885	-6.13389	0.842264	0.789171

B.cells	TCEANC	-0.10751	1.730178	-0.2659	0.790933	-5.52366	0.861216	0.820324
B.cells	GM19967	0.089492	1.377066	0.265849	0.790974	-5.58905	0.866102	0.828415
B.cells	GUSB	-0.02906	5.468903	-0.26575	0.791049	-6.57342	0.811403	0.739274
B.cells	CNBD2	-0.04767	3.889219	-0.26568	0.791101	-6.26563	0.832028	0.772516
B.cells	CCDC189	-0.15462	0.183787	-0.26564	0.791133	-5.29388	0.88285	0.856365
B.cells	ZSCAN2	-0.13162	1.067334	-0.26551	0.791238	-5.42772	0.870414	0.835646
B.cells	KREMEN1	0.053495	3.039966	0.265342	0.791363	-5.94902	0.843369	0.791091
B.cells	CATSPERE2	0.098067	1.174644	0.265283	0.791409	-5.45616	0.868917	0.833218
B.cells	TOE1	-0.0657	2.490022	-0.26523	0.791452	-5.82922	0.85081	0.803308
B.cells	ZKSCAN16	0.12849	-0.59074	0.264986	0.791637	-5.29219	0.893822	0.875144
B.cells	ZNHIT6	0.05823	3.231636	0.264903	0.791701	-6.00151	0.840794	0.786964
B.cells	PRKCI	0.059827	3.113961	0.264783	0.791793	-5.97453	0.842374	0.789545
B.cells	LYPD6B	0.157118	0.004013	0.264744	0.791823	-5.34063	0.885405	0.860823
B.cells	C730034F0	0.074747	2.636781	0.2647	0.791857	-5.84783	0.848817	0.800096
B.cells	SPOCK2	-0.12132	0.349422	-0.26469	0.791865	-5.3795	0.880503	0.852609
B.cells	PIK3C2B	0.071813	2.667746	0.264547	0.791974	-5.89757	0.848397	0.799444
B.cells	ZDHHC7	-0.04306	3.834677	-0.26454	0.791981	-6.17756	0.832751	0.773904
B.cells	TJP3	-0.09031	1.707068	-0.26443	0.792061	-5.63212	0.861535	0.821136
B.cells	CYP2C69	-0.11607	1.432054	-0.26439	0.792096	-5.58669	0.865339	0.827445
B.cells	BRD1	0.028344	5.991255	0.264354	0.792123	-6.61953	0.804716	0.728861
B.cells	TNK2	0.056411	3.209858	0.264247	0.792204	-5.99242	0.841097	0.787564
B.cells	2310022A1	0.048488	2.998253	0.264149	0.79228	-6.07496	0.843946	0.792257
B.cells	CEBPZ	0.024122	6.141756	0.26388	0.792487	-6.67545	0.802952	0.725919
B.cells	RXRA	0.064626	3.1994	0.263719	0.79261	-5.88211	0.84144	0.787927
B.cells	SAP18B	0.06397	2.771863	0.263468	0.792803	-5.87589	0.847315	0.797463
B.cells	LACTB2	0.043895	3.94655	0.263395	0.792859	-6.16337	0.83159	0.771846
B.cells	TELO2	-0.07372	2.455163	-0.26318	0.793021	-5.90564	0.851672	0.804562
B.cells	BICD1	0.129029	0.681795	0.263085	0.793097	-5.38255	0.876215	0.845226
B.cells	ADAT2	-0.09938	1.285306	-0.26304	0.793128	-5.56541	0.867772	0.831176
B.cells	PIK3R1	-0.03227	7.694648	-0.26289	0.79325	-6.94432	0.783764	0.695404
B.cells	2410002F2	-0.04297	3.766412	-0.26274	0.793363	-6.14748	0.834133	0.775786
B.cells	DGKE	0.038047	4.880229	0.262606	0.793465	-6.46583	0.819488	0.752107
B.cells	WDR60	0.133515	0.883885	0.262545	0.793512	-5.35435	0.873481	0.840536
B.cells	UBE4B	0.029681	5.48847	0.262456	0.793581	-6.54132	0.811617	0.739487
B.cells	GM16174	0.115895	0.995634	0.262292	0.793706	-5.37038	0.871975	0.837951
B.cells	TMEM220	-0.12601	0.299081	-0.26207	0.793876	-5.38222	0.881839	0.854439
B.cells	ZC3H11A	0.079976	2.01149	0.262039	0.793901	-5.72327	0.857953	0.814747
B.cells	PTPN2	0.02138	6.833794	0.261765	0.794111	-6.8202	0.794709	0.712482
B.cells	ATP1A3	-0.12025	1.709416	-0.26161	0.794234	-5.50474	0.8622	0.821735
B.cells	ZRSR1	0.076863	2.211301	0.261589	0.794246	-5.71405	0.855302	0.810348
B.cells	ANGPTL2	-0.1428	0.84131	-0.26152	0.794299	-5.38085	0.874284	0.841799
B.cells	MEIS1	-0.05494	3.866059	-0.26147	0.794341	-6.15159	0.833009	0.773888
B.cells	SLC35A5	0.03758	3.986264	0.261293	0.794474	-6.19639	0.83148	0.771304
B.cells	SLC22A4	-0.12235	0.816422	-0.26103	0.794675	-5.44467	0.874809	0.842475
B.cells	BTNL9	0.150309	0.689973	0.260957	0.794733	-5.36857	0.876586	0.845448
B.cells	GATD3A	-0.04941	3.544084	-0.26089	0.794787	-6.06124	0.837462	0.780949
B.cells	SURF1	-0.03863	4.36834	-0.26071	0.794925	-6.32417	0.826612	0.763256

B.cells	RBM44	-0.16064	0.135835	-0.26055	0.795043	-5.28091	0.884549	0.858625
B.cells	SSBP4	-0.03374	4.90464	-0.26032	0.795222	-6.36705	0.819691	0.752019
B.cells	MRPS12	0.035477	4.774775	0.26028	0.795253	-6.37089	0.821384	0.754762
B.cells	FLOT2	0.038775	4.443449	0.260211	0.795306	-6.26274	0.82572	0.761782
B.cells	SLC25A44	0.051664	3.239164	0.259972	0.79549	-6.02076	0.841711	0.787776
B.cells	LSMEM1	0.081011	4.06136	0.259959	0.795499	-6.13506	0.830755	0.769955
B.cells	VAV2	-0.0421	4.977829	-0.25993	0.795519	-6.43492	0.818739	0.750561
B.cells	RANBP3	-0.03308	4.303701	-0.25976	0.795652	-6.29492	0.827598	0.764803
B.cells	BLVRB	-0.04364	7.092023	-0.25951	0.795841	-6.9003	0.791832	0.707724
B.cells	KLHL6	0.03503	6.109159	0.259492	0.795859	-6.69047	0.804227	0.727357
B.cells	POP7	0.032821	4.908142	0.259389	0.795938	-6.44392	0.819686	0.752092
B.cells	KAT8	0.042961	3.624929	0.259264	0.796034	-6.16597	0.836591	0.77944
B.cells	ZFYVE21	0.065832	2.542215	0.259233	0.796058	-5.86063	0.851171	0.803277
B.cells	A630052C1	0.136509	0.189023	0.259231	0.79606	-5.33129	0.883888	0.85756
B.cells	PRODH	-0.09258	2.780405	-0.25906	0.796191	-5.76681	0.848002	0.797992
B.cells	CCL2	-0.13176	3.089272	-0.25861	0.796537	-5.87304	0.844055	0.791211
B.cells	LMF1	-0.04842	3.239428	-0.2586	0.796547	-6.09219	0.842036	0.787912
B.cells	CCDC82	0.040109	4.323551	0.25825	0.796814	-6.24337	0.827653	0.764568
B.cells	YME1L1	-0.01864	6.181942	-0.25811	0.796924	-6.67495	0.803608	0.726034
B.cells	4933433G1	-0.08648	1.434512	-0.25809	0.796938	-5.66984	0.866725	0.828575
B.cells	TXNDC15	-0.03332	4.911319	-0.258	0.79701	-6.4712	0.819957	0.752217
B.cells	PTCD2	-0.03361	4.58679	-0.25796	0.797036	-6.38921	0.824196	0.759052
B.cells	RANBP9	0.025234	6.962828	0.257862	0.797113	-6.81274	0.793751	0.710501
B.cells	MATR3	-0.01931	6.50291	-0.25779	0.79717	-6.73638	0.799539	0.719662
B.cells	PITPNA	-0.01814	7.992513	-0.25766	0.797271	-6.99297	0.780972	0.690466
B.cells	SPOUT1	0.066708	2.468742	0.257504	0.797388	-5.77605	0.852497	0.805224
B.cells	MRPS36	-0.03116	5.569029	-0.25739	0.797473	-6.56907	0.811446	0.738659
B.cells	CYB5D1	0.137161	0.775244	0.257169	0.797646	-5.34805	0.875938	0.84407
B.cells	GNB1L	0.052518	3.308245	0.25713	0.797676	-6.02641	0.841146	0.786671
B.cells	TEPSIN	-0.11377	0.762658	-0.2571	0.797697	-5.43507	0.876115	0.844365
B.cells	UBAP2	-0.0254	6.131148	-0.25709	0.797709	-6.6682	0.804254	0.72722
B.cells	CIB2	0.07963	2.152566	0.257032	0.797751	-5.66742	0.856818	0.812371
B.cells	CDK14	-0.09064	5.455475	-0.25696	0.797809	-6.2502	0.812908	0.741045
B.cells	SCAF8	0.021859	6.711338	0.256804	0.797927	-6.79104	0.79691	0.715615
B.cells	APLF	0.079734	2.05909	0.25663	0.798061	-5.70537	0.8581	0.814584
B.cells	AGAP2	-0.05213	2.925575	-0.2566	0.798087	-6.01566	0.846298	0.795188
B.cells	DHX9	-0.03324	6.492589	-0.25656	0.798115	-6.72257	0.799669	0.720044
B.cells	BTBD2	0.048747	3.477347	0.25647	0.798184	-6.06289	0.838881	0.783129
B.cells	ECPAS	-0.02378	6.743157	-0.25644	0.798206	-6.7777	0.796509	0.715073
B.cells	1110065P2	0.042331	3.514697	0.256412	0.798228	-6.0953	0.838381	0.782316
B.cells	BCKDHB	0.048329	3.929893	0.256137	0.79844	-6.17333	0.833	0.773392
B.cells	GM47167	-0.05145	2.927116	-0.25579	0.798705	-6.02374	0.84663	0.795374
B.cells	ABCA3	0.032014	4.193838	0.255609	0.798847	-6.3289	0.829781	0.767927
B.cells	LGR4	-0.08082	2.632615	-0.25549	0.79894	-5.84974	0.85072	0.802028
B.cells	POC5	-0.06284	2.510705	-0.25525	0.799123	-5.80179	0.85245	0.804821
B.cells	NOL12	0.031704	4.012955	0.255151	0.799199	-6.25129	0.832266	0.771873
B.cells	ZFP408	-0.04342	3.716601	-0.25512	0.799223	-6.15113	0.836203	0.778266

B.cells	GM13684	0.047885	3.516914	0.25502	0.7993	-6.20657	0.838874	0.78263
B.cells	MRRF	0.044828	3.454685	0.254856	0.799427	-6.11071	0.839764	0.784022
B.cells	NMNAT2	0.128691	2.111738	0.254583	0.799636	-5.68063	0.857998	0.813865
B.cells	DRG2	-0.04045	3.507943	-0.25455	0.799659	-6.11795	0.839079	0.782861
B.cells	GM20045	0.084137	1.764585	0.254542	0.799668	-5.6792	0.862779	0.821758
B.cells	ZSWIM9	-0.14807	0.544388	-0.25438	0.799793	-5.29882	0.87986	0.850121
B.cells	DDX41	-0.03289	4.356626	-0.25432	0.799841	-6.35284	0.827847	0.764629
B.cells	DNAAF5	0.048869	2.988111	0.254078	0.800025	-6.01625	0.846205	0.794378
B.cells	KCNC3	-0.09882	0.561324	-0.25378	0.800254	-5.52129	0.879733	0.849872
B.cells	GM32569	-0.09214	1.845691	-0.25378	0.800256	-5.85959	0.861801	0.820078
B.cells	GM34084	-0.14628	1.964286	-0.25377	0.800265	-5.55051	0.860167	0.817378
B.cells	KLF10	0.032954	5.248269	0.253719	0.800302	-6.46908	0.816309	0.745995
B.cells	COMMD6	0.036198	4.358964	0.25358	0.800409	-6.30541	0.827946	0.764697
B.cells	GM47601	0.132687	0.02249	0.253418	0.800534	-5.29568	0.887409	0.862745
B.cells	CUL4B	-0.0382	4.524951	-0.25336	0.800579	-6.36863	0.825763	0.76125
B.cells	GM47371	-0.10891	0.907518	-0.2532	0.8007	-5.53579	0.874883	0.841838
B.cells	FIZ1	0.029477	4.553694	0.253057	0.800812	-6.35654	0.825386	0.760672
B.cells	UQCC2	0.030696	6.141014	0.253049	0.800818	-6.6677	0.804866	0.727789
B.cells	THRAP3	-0.01997	8.006361	-0.2529	0.80093	-7.00395	0.781518	0.691019
B.cells	FBXO42	0.029717	5.871653	0.252803	0.801007	-6.62543	0.808306	0.733341
B.cells	CTNS	-0.04573	3.606042	-0.25276	0.801043	-6.18056	0.83793	0.781091
B.cells	ARSK	0.059696	2.705302	0.252728	0.801065	-6.00537	0.85006	0.800926
B.cells	GABBR1	0.11813	2.766527	0.252663	0.801115	-5.72369	0.849229	0.799578
B.cells	OGA	-0.02402	6.76343	-0.25235	0.801353	-6.78442	0.797107	0.715462
B.cells	E230029C0	0.062857	3.508508	0.252254	0.80143	-6.07462	0.839362	0.783323
B.cells	KAT2A	-0.04867	3.199202	-0.25215	0.801512	-6.07189	0.843513	0.790094
B.cells	EXOG	0.081323	1.745846	0.25208	0.801565	-5.62504	0.863337	0.822685
B.cells	0610009B2	-0.0529	3.780562	-0.25204	0.801596	-6.10819	0.835731	0.777415
B.cells	GDF11	-0.07615	1.639394	-0.25155	0.801973	-5.80053	0.864994	0.825225
B.cells	FBXL2	-0.04697	3.231347	-0.25141	0.802077	-6.12851	0.84326	0.789486
B.cells	TPRGL	0.023489	6.927644	0.25137	0.802112	-6.82329	0.795212	0.712292
B.cells	SKIV2L	-0.04107	3.938774	-0.25127	0.802185	-6.19255	0.833806	0.774095
B.cells	PRPF3	0.029204	4.472577	0.251159	0.802274	-6.35539	0.826754	0.762678
B.cells	TMEM183A	0.024383	5.16956	0.251047	0.80236	-6.50263	0.817652	0.748029
B.cells	CAD	-0.09179	1.795668	-0.25099	0.802408	-5.62631	0.862832	0.821655
B.cells	HCCS	-0.0309	4.627385	-0.25089	0.802478	-6.35029	0.824722	0.759424
B.cells	SCML4	0.042647	4.659948	0.250894	0.802479	-6.37622	0.824295	0.758736
B.cells	GM16316	-0.06721	1.803894	-0.25088	0.802485	-5.85127	0.862719	0.821487
B.cells	9530077C0	-0.11462	1.213471	-0.25065	0.80267	-5.46156	0.870959	0.835094
B.cells	GM45669	-0.15602	0.129811	-0.25052	0.802768	-5.26623	0.886244	0.860609
B.cells	ADAM12	-0.1323	0.815122	-0.25048	0.802799	-5.40416	0.876543	0.844387
B.cells	SOX18	-0.15087	0.720608	-0.25046	0.80281	-5.29134	0.877873	0.846607
B.cells	BANF1	0.028455	6.873409	0.250046	0.803132	-6.78539	0.796129	0.713497
B.cells	DPP6	-0.14537	-0.15893	-0.25002	0.803154	-5.32729	0.890592	0.867644
B.cells	IGHJ4	-0.11947	0.337645	-0.24992	0.803229	-5.43591	0.883514	0.855802
B.cells	GM13184	0.062492	2.666577	0.249369	0.803654	-5.95926	0.851526	0.802301
B.cells	SLC25A53	0.043761	3.336328	0.249239	0.803754	-6.08614	0.842501	0.787527

B.cells	TSHZ3	-0.12532	2.187789	-0.24895	0.80398	-5.55723	0.858264	0.813189
B.cells	IFT172	-0.05131	2.410455	-0.24882	0.804073	-5.99653	0.855233	0.808191
B.cells	RER1	-0.02456	6.413933	-0.24874	0.804142	-6.7102	0.802486	0.722976
B.cells	GM3055	0.120445	0.319624	0.248503	0.804322	-5.36419	0.8844	0.85658
B.cells	DHRS1	-0.04449	4.762169	-0.24846	0.804351	-6.37094	0.823792	0.757031
B.cells	MDN1	-0.03688	5.788045	-0.24843	0.804377	-6.63508	0.810494	0.735715
B.cells	GM42941	-0.08099	1.267028	-0.24816	0.804589	-5.62576	0.871082	0.834389
B.cells	MRPL33	-0.02874	6.142277	-0.24811	0.804628	-6.64306	0.805988	0.728555
B.cells	RAD1	-0.06513	2.477895	-0.24801	0.804705	-5.80111	0.85436	0.806814
B.cells	UTP4	0.029764	5.102474	0.24786	0.804818	-6.4694	0.81938	0.750023
B.cells	IER5	-0.03606	6.692584	-0.24777	0.804887	-6.77219	0.799005	0.717566
B.cells	FKBP7	0.084215	1.867273	0.24776	0.804895	-5.68337	0.862746	0.820698
B.cells	NCL	0.029159	7.783001	0.247563	0.805046	-6.94128	0.78538	0.696127
B.cells	DAGLB	-0.03573	4.366228	-0.2474	0.805176	-6.41506	0.82902	0.765593
B.cells	CYFIP1	0.032929	5.64309	0.247393	0.805178	-6.5643	0.812385	0.738857
B.cells	4931428F0	0.118625	0.65018	0.247325	0.80523	-5.35022	0.879746	0.848953
B.cells	RTF2	0.022261	5.957747	0.247316	0.805237	-6.64333	0.808346	0.732418
B.cells	ZNRF1	0.026905	6.496008	0.247278	0.805267	-6.74722	0.801491	0.721535
B.cells	FUT11	-0.04667	3.493603	-0.2469	0.805556	-6.09722	0.840802	0.784548
B.cells	NRG4	-0.09585	2.017195	-0.24669	0.805721	-5.74263	0.860866	0.81744
B.cells	UHRF1BP1	0.097217	1.998967	0.246651	0.80575	-5.61685	0.861117	0.817854
B.cells	CD2BP2	0.03622	4.011388	0.246481	0.805882	-6.22022	0.833896	0.773323
B.cells	ZMYM6	0.073382	2.526746	0.246475	0.805886	-5.80774	0.853879	0.805938
B.cells	METAP1D	-0.04268	3.768583	-0.24642	0.805927	-6.21816	0.837126	0.778567
B.cells	CCDC80	-0.09137	2.683481	-0.24633	0.805998	-5.74252	0.851743	0.802432
B.cells	ZFAND5	-0.01986	7.203792	-0.24623	0.806074	-6.86468	0.792755	0.707561
B.cells	PIGM	0.040513	3.53133	0.24608	0.806191	-6.08992	0.840297	0.783748
B.cells	GM29585	-0.11794	1.247477	-0.24599	0.806259	-5.41246	0.871545	0.835152
B.cells	SSBP2	0.034835	6.284595	0.245822	0.806389	-6.70019	0.80435	0.726002
B.cells	ZRANB2	0.025478	5.45391	0.245768	0.806432	-6.54044	0.815001	0.742988
B.cells	HIKESHI	0.028184	5.228907	0.245729	0.806461	-6.50594	0.817915	0.747657
B.cells	ZCWPW2	0.116609	1.016841	0.245692	0.80649	-5.46527	0.874775	0.840603
B.cells	ZFP346	0.04558	3.658973	0.245445	0.806681	-6.20241	0.838589	0.781086
B.cells	MMRN1	-0.2521	-0.22843	-0.2454	0.806713	-5.20646	0.892451	0.870208
B.cells	MAP2K7	-0.03101	4.294187	-0.24517	0.806896	-6.33487	0.830151	0.767439
B.cells	RNASEH2B	-0.03857	4.594626	-0.24514	0.806918	-6.32451	0.826195	0.761048
B.cells	E2F2	-0.0374	4.99405	-0.24505	0.806987	-6.5725	0.82097	0.752637
B.cells	ANLN	-0.07078	3.747041	-0.24504	0.806992	-6.02097	0.837413	0.779221
B.cells	CCNC	0.035149	4.398502	0.245028	0.807003	-6.3606	0.828775	0.765219
B.cells	CCDC181	-0.08641	1.514839	-0.24488	0.807113	-5.55442	0.867818	0.829184
B.cells	GM26771	-0.13791	-0.2163	-0.24474	0.807224	-5.3276	0.892277	0.870051
B.cells	SNAI1	-0.11476	0.389835	-0.24474	0.807227	-5.32333	0.883624	0.855537
B.cells	TMEM98	-0.1321	0.620631	-0.24472	0.80724	-5.30249	0.880355	0.850072
B.cells	FZR1	-0.03564	4.900739	-0.24432	0.80755	-6.43678	0.822213	0.754713
B.cells	GM43727	-0.13516	-0.59426	-0.2443	0.807563	-5.23089	0.89765	0.879274
B.cells	BPTF	-0.02206	7.356344	-0.24422	0.807629	-6.8825	0.790875	0.704851
B.cells	NEK3	0.095985	1.761696	0.244118	0.807705	-5.64733	0.864421	0.823629

B.cells	ZKSCAN8	-0.11413	0.632724	-0.24408	0.807736	-5.34312	0.880212	0.849882
B.cells	WDR34	-0.12602	1.224098	-0.24394	0.807844	-5.43865	0.8719	0.836107
B.cells	GNAI3	-0.02037	6.443605	-0.24381	0.807945	-6.71778	0.802356	0.723085
B.cells	GNG12	0.026783	6.49518	0.24375	0.807989	-6.72309	0.801702	0.722047
B.cells	ZDBF2	-0.16842	-0.23932	-0.24372	0.808013	-5.27117	0.892636	0.870785
B.cells	4930539J0	-0.11827	0.706555	-0.24368	0.808041	-5.36614	0.879169	0.848219
B.cells	TMEM201	-0.07981	2.102835	-0.24343	0.808236	-5.60109	0.859714	0.815936
B.cells	CLEC2I	0.06002	3.177972	0.24341	0.808251	-6.1201	0.845072	0.791896
B.cells	KDM4A	-0.03356	4.161515	-0.24339	0.808268	-6.32998	0.831932	0.770515
B.cells	CHAF1A	-0.0546	4.186029	-0.24338	0.808272	-6.20786	0.831607	0.769989
B.cells	CSPP1	-0.02793	6.098729	-0.24297	0.808588	-6.66261	0.806849	0.730163
B.cells	GRAMD2	0.110681	0.533275	0.242908	0.80864	-5.38786	0.881733	0.852447
B.cells	SZRD1	-0.02092	5.906997	-0.2428	0.80872	-6.64434	0.809302	0.73411
B.cells	CCDC57	-0.06087	2.546499	-0.2428	0.808725	-5.79669	0.853747	0.806086
B.cells	RANBP2	-0.02923	6.846596	-0.24277	0.808744	-6.78559	0.797366	0.715156
B.cells	2610307P1	-0.06889	4.550996	-0.24257	0.808901	-6.29565	0.826902	0.762422
B.cells	FLNB	-0.05859	5.398208	-0.24247	0.808977	-6.59892	0.815853	0.744667
B.cells	NDUFB7	-0.02513	6.480862	-0.24241	0.809021	-6.72058	0.801987	0.722556
B.cells	SFMBT1	0.028941	6.08199	0.24241	0.809024	-6.60845	0.807063	0.730625
B.cells	PSENEN	0.019684	6.713106	0.242284	0.809121	-6.7657	0.799074	0.7179
B.cells	PAIP2B	-0.03909	3.682629	-0.2419	0.809421	-6.16605	0.838554	0.781211
B.cells	ANKRD22	0.11882	-0.56253	0.241776	0.809514	-5.36942	0.897484	0.879065
B.cells	NDUFAF5	0.056736	2.563951	0.241747	0.809536	-5.88185	0.853657	0.805942
B.cells	GM15489	-0.14511	0.208522	-0.24161	0.809639	-5.28601	0.886499	0.860493
B.cells	ZBTB20	0.026039	8.246328	0.241596	0.809653	-6.9744	0.780104	0.688067
B.cells	SPEF2	0.100017	1.371663	0.24143	0.809781	-5.51403	0.870103	0.833138
B.cells	GPT	0.101689	1.203309	0.241393	0.809809	-5.51382	0.872454	0.837045
B.cells	SLC25A15	0.061074	2.488304	0.241318	0.809867	-5.81504	0.85469	0.807697
B.cells	CD8A	0.147022	1.360615	0.241117	0.810023	-5.45156	0.870257	0.833508
B.cells	GM45820	-0.0761	1.425778	-0.24107	0.810055	-5.60301	0.869348	0.832003
B.cells	CCDC134	0.045265	3.407824	0.240999	0.810114	-6.14157	0.842235	0.78739
B.cells	C1QTNF6	-0.10421	1.904338	-0.24074	0.810317	-5.59607	0.86271	0.821062
B.cells	LYSMD2	-0.09189	1.265146	-0.24066	0.810378	-5.62581	0.87159	0.835788
B.cells	CSRP1	-0.03495	5.881401	-0.24058	0.810436	-6.54023	0.80977	0.735051
B.cells	CFAP298	-0.04309	3.642504	-0.24055	0.810463	-6.1105	0.83909	0.782322
B.cells	CLPTM1	0.02375	5.552635	0.240461	0.81053	-6.56654	0.813999	0.74181
B.cells	WBP4	0.026328	5.270226	0.240388	0.810586	-6.51925	0.817653	0.747666
B.cells	YAP1	0.147814	1.070556	0.240155	0.810766	-5.38065	0.874314	0.840326
B.cells	MICAL1	-0.03926	3.487265	-0.24002	0.81087	-6.18943	0.841169	0.785709
B.cells	MARS	0.052988	3.10364	0.239937	0.810934	-6.0017	0.846331	0.79414
B.cells	ADNP2	0.062245	2.990665	0.23983	0.811017	-5.88828	0.847859	0.796673
B.cells	PIN1	0.028099	5.245371	0.239606	0.81119	-6.47165	0.817975	0.74828
B.cells	KLHL7	0.030867	4.739495	0.239418	0.811335	-6.44529	0.824571	0.758971
B.cells	DPY19L3	-0.0629	2.757574	-0.23937	0.811369	-5.8903	0.85102	0.802014
B.cells	GSTO2	0.121612	0.524993	0.239293	0.811432	-5.40191	0.882003	0.853386
B.cells	PDE1C	0.084918	3.989093	0.23926	0.811458	-6.03261	0.834471	0.77502
B.cells	GM15563	0.078967	1.923249	0.239078	0.811598	-5.75721	0.862449	0.820866

B.cells	MTX2	0.029219	4.838218	0.239056	0.811615	-6.42524	0.823279	0.756916
B.cells	METTL16	0.029686	4.533537	0.23905	0.81162	-6.37938	0.827274	0.763364
B.cells	GID8	-0.02685	5.195402	-0.23897	0.811684	-6.48651	0.818624	0.749427
B.cells	ZFP217	-0.03414	4.011596	-0.23892	0.811721	-6.18636	0.834172	0.774535
B.cells	FCGR3	-0.0753	4.189291	-0.2389	0.811735	-5.99161	0.831816	0.770714
B.cells	SYDE2	-0.13263	-0.10373	-0.23886	0.811765	-5.31119	0.89096	0.86842
B.cells	ELP4	-0.03164	4.963596	-0.23882	0.8118	-6.4498	0.821641	0.754295
B.cells	SLC25A43	-0.12098	-0.21871	-0.23878	0.81183	-5.3275	0.89261	0.871208
B.cells	GSR	0.042908	6.441975	0.238634	0.811941	-6.49996	0.802619	0.723897
B.cells	NFATC2	-0.06107	2.489047	-0.23859	0.811978	-5.92486	0.854679	0.808089
B.cells	CASP8AP2	0.028185	5.182109	0.2385	0.812045	-6.47712	0.818797	0.749739
B.cells	RBM47	-0.0362	6.404832	-0.23842	0.812107	-6.71163	0.803091	0.724646
B.cells	ROMO1	-0.02231	6.595619	-0.23841	0.812113	-6.72135	0.800672	0.720807
B.cells	ALDH1A1	-0.11902	-5.25E-05	-0.23826	0.812228	-5.47512	0.889522	0.865955
B.cells	ATG16L1	0.028466	5.446329	0.238172	0.812299	-6.55924	0.815414	0.744244
B.cells	ECH1	-0.03118	5.784916	-0.23777	0.812612	-6.56469	0.811291	0.737371
B.cells	CPM	0.051467	4.160192	0.237554	0.812777	-6.34255	0.832586	0.771497
B.cells	SCOC	-0.04441	3.752146	-0.23739	0.812903	-6.1305	0.838069	0.780312
B.cells	RUFY1	-0.02477	5.607628	-0.23715	0.813086	-6.58292	0.813831	0.74109
B.cells	ZBTB43	0.044356	3.163113	0.236808	0.813354	-6.03698	0.846252	0.793271
B.cells	9130019P1	-0.13128	0.265967	-0.23677	0.813384	-5.38276	0.886439	0.859796
B.cells	TRMT112	0.021965	7.277188	0.236605	0.81351	-6.86873	0.792834	0.707509
B.cells	GM17251	0.075761	2.365949	0.236085	0.813913	-5.73121	0.857305	0.811308
B.cells	SUMF1	-0.03638	4.240634	-0.23604	0.81395	-6.33237	0.832051	0.770079
B.cells	GDAP2	-0.02923	4.778409	-0.23591	0.81405	-6.44259	0.824968	0.758689
B.cells	GM16618	-0.09319	0.733543	-0.23588	0.814072	-5.47444	0.880021	0.849038
B.cells	XLR4B	-0.07363	1.285179	-0.23586	0.814083	-5.63297	0.872268	0.836126
B.cells	RBM42	-0.02051	6.063078	-0.23585	0.814092	-6.66349	0.808333	0.73204
B.cells	TMEM184E	-0.0346	4.707523	-0.23577	0.814159	-6.47063	0.825898	0.760209
B.cells	MYO1G	0.029303	5.563064	0.235511	0.814357	-6.69467	0.814822	0.742331
B.cells	RMDN1	0.037887	4.528231	0.2355	0.814365	-6.35126	0.828318	0.764028
B.cells	AXDND1	0.090141	1.557778	0.235377	0.81446	-5.70853	0.868557	0.829851
B.cells	CCNH	0.026262	5.363256	0.235084	0.814687	-6.50835	0.817543	0.746543
B.cells	GM11084	0.040079	3.368087	0.235046	0.814716	-6.13672	0.8439	0.789175
B.cells	NMD3	-0.02345	4.999909	-0.2349	0.814828	-6.45392	0.82231	0.754145
B.cells	ENDOD1	-0.04446	2.901638	-0.23456	0.815095	-6.0729	0.850252	0.799568
B.cells	KLRA3	-0.15956	-0.66191	-0.23446	0.815173	-5.30884	0.900113	0.882787
B.cells	SDF4	0.019514	6.506152	0.234336	0.815266	-6.74212	0.802926	0.723272
B.cells	RPA2	0.044805	4.645282	0.234297	0.815296	-6.33168	0.826961	0.761745
B.cells	OAS1G	-0.13887	-0.24881	-0.23428	0.815312	-5.30628	0.894291	0.872775
B.cells	GPN1	-0.04471	3.042468	-0.23424	0.815338	-6.00823	0.848342	0.796497
B.cells	NUDCD1	0.04855	3.226429	0.234106	0.815444	-6.05981	0.845856	0.792474
B.cells	TXK	0.064478	3.287256	0.234066	0.815475	-6.08534	0.845036	0.791134
B.cells	MRPL55	-0.03442	4.367452	-0.23405	0.81549	-6.2844	0.830622	0.767704
B.cells	GM12840	0.100121	3.766389	0.233907	0.815598	-5.91498	0.838606	0.780685
B.cells	MCMBP	0.019917	7.089729	0.233871	0.815626	-6.84008	0.79556	0.711699
B.cells	PSMC2	-0.02761	5.362149	-0.23374	0.815725	-6.51356	0.817616	0.74679

B.cells	KLRA17	0.119538	0.224958	0.233668	0.815783	-5.29925	0.887518	0.8615
B.cells	ZAP70	0.096405	1.982594	0.233475	0.815932	-5.62888	0.862931	0.820496
B.cells	ZKSCAN5	-0.05915	2.402977	-0.23306	0.816251	-5.84191	0.857207	0.811073
B.cells	ACSF3	0.076455	1.59243	0.232998	0.816301	-5.60258	0.868398	0.829559
B.cells	SLC45A3	-0.11532	1.111286	-0.23296	0.81633	-5.46299	0.875121	0.840726
B.cells	ZFP12	0.075446	1.346551	0.232876	0.816396	-5.57191	0.871827	0.835255
B.cells	3300002I0	0.066831	1.970632	0.23281	0.816447	-5.78157	0.863155	0.820891
B.cells	DLGAP1	0.09107	1.398192	0.232484	0.816699	-5.58418	0.871105	0.83414
B.cells	AA465934	-0.08002	1.66043	-0.23248	0.816702	-5.58264	0.867453	0.828083
B.cells	TONSL	-0.08813	1.456718	-0.23242	0.81675	-5.54272	0.870289	0.832785
B.cells	APH1A	0.029581	5.433388	0.232363	0.816793	-6.54611	0.816826	0.745556
B.cells	PTPRJ	0.022058	8.855581	0.232292	0.816848	-7.13955	0.773892	0.677814
B.cells	GDPD3	0.077741	1.793559	0.232204	0.816916	-5.76674	0.865606	0.825024
B.cells	1700029H1	-0.07114	1.58753	-0.23209	0.817002	-5.69425	0.868466	0.829791
B.cells	TAF8	0.044275	3.254648	0.232028	0.817052	-6.07685	0.845627	0.792224
B.cells	TRIM32	0.096337	1.488698	0.231995	0.817078	-5.58517	0.869843	0.832114
B.cells	GM33104	-0.1183	0.440079	-0.23184	0.817196	-5.44253	0.8846	0.85672
B.cells	GM34086	-0.06106	2.658244	-0.2318	0.817231	-5.89921	0.853717	0.805499
B.cells	TPRN	0.066574	2.21301	0.231596	0.817387	-5.79266	0.859815	0.815579
B.cells	EPCAM	-0.06886	2.160039	-0.23156	0.817416	-5.92514	0.860543	0.816781
B.cells	TATDN3	0.058213	2.490952	0.231472	0.817483	-5.84003	0.856002	0.809299
B.cells	TLE3	-0.02891	5.003886	-0.23147	0.817486	-6.45585	0.822412	0.754636
B.cells	4930403D0	0.085641	1.041064	0.231419	0.817524	-5.59034	0.876108	0.842584
B.cells	2610301B2	-0.07372	1.613471	-0.23136	0.817573	-5.60665	0.868106	0.829298
B.cells	CUL7	-0.07865	1.968845	-0.23098	0.817862	-5.63491	0.863269	0.82127
B.cells	LASP1	0.026585	5.455474	0.230899	0.817927	-6.50655	0.816625	0.74533
B.cells	CTLA4	0.075611	2.060232	0.23071	0.818073	-5.92917	0.862008	0.81921
B.cells	EXOSC4	0.043749	3.784732	0.230698	0.818082	-6.1111	0.838599	0.780836
B.cells	GM43113	-0.13114	-0.69248	-0.2305	0.818235	-5.27303	0.900791	0.884329
B.cells	TBC1D32	-0.07198	2.141265	-0.23041	0.818308	-5.77693	0.860891	0.81743
B.cells	SEMA5A	-0.10278	1.106724	-0.2304	0.818316	-5.58107	0.875276	0.841277
B.cells	MSH5	0.076626	2.9838	0.230346	0.818355	-5.80005	0.849378	0.798516
B.cells	NKAPD1	-0.03273	4.086425	-0.22971	0.818848	-6.29083	0.83458	0.774468
B.cells	UBE3C	0.024601	5.784905	0.229466	0.819037	-6.61076	0.812373	0.738749
B.cells	NINL	-0.10266	0.999292	-0.22946	0.819044	-5.44115	0.876786	0.843956
B.cells	PTGER2	0.114489	1.047913	0.229444	0.819054	-5.36511	0.876102	0.842818
B.cells	PRRG1	0.09819	1.915898	0.229363	0.819116	-5.56169	0.864001	0.82273
B.cells	KIRREL3	0.099484	1.272544	0.229338	0.819136	-5.48014	0.872952	0.837574
B.cells	AGO3	0.02546	5.940327	0.229338	0.819136	-6.64062	0.810375	0.73556
B.cells	CTSG	0.234854	-0.2691	0.229115	0.819309	-5.28304	0.894836	0.874227
B.cells	RAVER2	-0.08597	1.423822	-0.2291	0.819322	-5.56501	0.870838	0.834081
B.cells	9830166K0	-0.12562	-1.08122	-0.22909	0.819326	-5.14233	0.906268	0.894137
B.cells	IFT43	-0.08821	1.54627	-0.22905	0.819362	-5.46429	0.869131	0.831247
B.cells	GM33280	0.163642	-0.66423	0.229012	0.819389	-5.16027	0.9004	0.883861
B.cells	KCTD5	0.037921	3.640452	0.228991	0.819405	-6.18831	0.840528	0.784215
B.cells	LLGL1	-0.04625	2.998446	-0.22884	0.819521	-5.99223	0.849179	0.79839
B.cells	PTPRE	-0.03402	5.539856	-0.22883	0.819526	-6.51161	0.815533	0.743865

B.cells	ATP2B1	0.022045	8.327101	0.228822	0.819536	-7.10016	0.780424	0.688333
B.cells	NECTIN4	0.12647	-0.02307	0.228771	0.819575	-5.2799	0.891302	0.868331
B.cells	TRDC	-0.13709	1.265066	-0.2287	0.819631	-5.54343	0.873056	0.837821
B.cells	ZFP658	-0.13554	0.297611	-0.22853	0.819764	-5.24803	0.886719	0.86068
B.cells	VPS11	-0.0318	4.072729	-0.22852	0.819767	-6.26684	0.834762	0.774919
B.cells	CXXC1	-0.03273	4.307295	-0.22847	0.819807	-6.30094	0.831652	0.769888
B.cells	MGME1	0.063109	2.312135	0.228456	0.81982	-5.85489	0.858541	0.81384
B.cells	SGCE	-0.12782	1.156412	-0.22811	0.820087	-5.36955	0.874672	0.840465
B.cells	PLSCR1	0.040055	4.550033	0.228109	0.820089	-6.4459	0.828537	0.764756
B.cells	CPSF1	-0.04303	3.349532	-0.22795	0.820211	-6.05174	0.844526	0.790741
B.cells	GM49662	-0.0603	2.537113	-0.22793	0.820228	-6.24827	0.85555	0.808816
B.cells	RCC1L	-0.05111	3.072184	-0.22788	0.820267	-5.96862	0.848271	0.79687
B.cells	GM38948	0.085307	0.463761	0.227727	0.820385	-5.48575	0.88446	0.856828
B.cells	THTPA	-0.08091	1.360701	-0.22749	0.820566	-5.51935	0.871822	0.835764
B.cells	WIPI2	-0.02538	5.276718	-0.22744	0.82061	-6.52518	0.81904	0.749494
B.cells	ZFP324	0.115725	0.798242	0.227427	0.820617	-5.36553	0.879723	0.848931
B.cells	NADK	-0.02601	6.266885	-0.22723	0.820769	-6.71486	0.806294	0.729115
B.cells	EIF3C	0.019732	7.172674	0.227227	0.820772	-6.88247	0.794838	0.710959
B.cells	MUS81	-0.07025	2.048507	-0.22719	0.820802	-5.68558	0.862272	0.81994
B.cells	PFKFB1	-0.09382	0.961216	-0.22696	0.820979	-5.49852	0.877425	0.845117
B.cells	CALCA	-0.15176	-0.96013	-0.22696	0.82098	-5.28643	0.904648	0.891401
B.cells	DCK	0.02979	5.832657	0.226924	0.821007	-6.62391	0.811854	0.738008
B.cells	PWP1	0.036704	3.903059	0.226843	0.82107	-6.17011	0.837119	0.778777
B.cells	MOGS	0.041441	4.095452	0.226592	0.821264	-6.19718	0.83459	0.774667
B.cells	E330020D1	0.042852	5.11024	0.226502	0.821334	-6.48121	0.821237	0.753144
B.cells	LAMTOR3	0.026216	5.240813	0.226434	0.821387	-6.47459	0.819537	0.750414
B.cells	SLC25A46	0.024134	4.587825	0.226432	0.821388	-6.38465	0.82808	0.764181
B.cells	RFT1	0.057475	2.53189	0.226191	0.821575	-5.90646	0.85572	0.809246
B.cells	DCLRE1B	0.046774	2.770832	0.226176	0.821587	-5.89716	0.85246	0.803888
B.cells	MLX	0.038413	3.767938	0.226016	0.821711	-6.13647	0.839062	0.781925
B.cells	C1D	0.020584	5.843961	0.225828	0.821856	-6.6268	0.811871	0.738059
B.cells	ATP6V1D	0.018307	6.650485	0.225795	0.821882	-6.74454	0.801579	0.721671
B.cells	DBP	0.085049	2.187718	0.225384	0.822201	-5.66859	0.860634	0.817201
B.cells	LAPTM4A	0.018789	7.210016	0.225348	0.822228	-6.8852	0.794629	0.710593
B.cells	PRR5	0.0481	3.479528	0.225292	0.822272	-6.15055	0.843062	0.788355
B.cells	TAT	-0.09142	1.826535	-0.22529	0.822275	-5.68998	0.865623	0.825448
B.cells	NKTR	-0.01858	7.193099	-0.22504	0.822465	-6.8283	0.79492	0.710948
B.cells	RAP2B	0.031009	4.614993	0.224964	0.822527	-6.37792	0.828044	0.76386
B.cells	UNC13B	-0.11989	0.758993	-0.2249	0.822573	-5.34371	0.880652	0.850365
B.cells	DHX37	-0.05304	2.67509	-0.22473	0.82271	-5.87048	0.854056	0.806299
B.cells	GATAD1	0.022886	5.768843	0.224518	0.822872	-6.55592	0.813037	0.739807
B.cells	GM16158	0.093461	0.586451	0.224496	0.82289	-5.59359	0.883112	0.854536
B.cells	PIGS	-0.03182	4.078714	-0.22441	0.822954	-6.25808	0.835152	0.775453
B.cells	DRC7	-0.13998	-0.15036	-0.2244	0.822965	-5.22468	0.893631	0.872194
B.cells	5430431A1	-0.06882	1.423829	-0.22433	0.823021	-5.63661	0.871328	0.834905
B.cells	MSH6	-0.03734	4.38755	-0.22409	0.823202	-6.32491	0.831164	0.768852
B.cells	EEF2	-0.02127	9.105144	-0.22394	0.823318	-7.16901	0.771478	0.674258

B.cells	SOX5OS4	-0.11994	-0.23518	-0.22389	0.823357	-5.3461	0.894965	0.874315
B.cells	GHDC	0.076053	1.628326	0.223821	0.823413	-5.56687	0.868588	0.830228
B.cells	ARFGAP2	0.024401	4.901097	0.22358	0.8236	-6.43706	0.824523	0.758011
B.cells	GM36371	0.087239	0.884673	0.223462	0.823692	-5.39342	0.879149	0.847626
B.cells	ZNRD1AS	0.116637	0.28937	0.223293	0.823822	-5.32245	0.887599	0.861792
B.cells	ENOPH1	0.046011	2.8863	0.223202	0.823894	-6.01187	0.851432	0.801787
B.cells	TMEM223	-0.02874	4.652686	-0.22318	0.823914	-6.38976	0.827809	0.763325
B.cells	TXN1	-0.03155	7.304482	-0.22296	0.824084	-6.81506	0.793812	0.709016
B.cells	GIPC1	0.03454	4.359749	0.222915	0.824116	-6.24744	0.831711	0.76959
B.cells	MRPS14	-0.01995	6.892734	-0.22279	0.824213	-6.8031	0.798987	0.717189
B.cells	PPP4R1	0.026125	5.579566	0.222595	0.824364	-6.57766	0.815759	0.743904
B.cells	MFSD12	-0.04935	2.730413	-0.22257	0.82438	-5.86968	0.853594	0.805297
B.cells	4933421O1	0.060134	2.357702	0.222569	0.824385	-5.84387	0.858694	0.813689
B.cells	B3GALT6	0.088444	1.806345	0.222083	0.824762	-5.58554	0.866558	0.826355
B.cells	CRYBG3	-0.03399	4.474287	-0.22207	0.824773	-6.37964	0.830443	0.767239
B.cells	SUGP2	0.038668	4.059588	0.221756	0.825015	-6.28572	0.836113	0.776258
B.cells	GRK5	-0.03745	5.747033	-0.22164	0.825106	-6.74706	0.814023	0.740635
B.cells	NRAS	0.020777	6.075126	0.221374	0.825311	-6.67038	0.809806	0.733905
B.cells	CENPA	-0.03875	6.681539	-0.22137	0.825318	-6.75676	0.802078	0.721619
B.cells	GM43774	0.061956	2.238278	0.221359	0.825323	-5.83188	0.860787	0.816612
B.cells	RRP1	0.017867	6.518197	0.221183	0.82546	-6.74979	0.804165	0.724959
B.cells	1810024B0	0.054968	2.692562	0.221155	0.825482	-5.86748	0.854574	0.806416
B.cells	GM16272	0.102182	0.363126	0.22088	0.825695	-5.40174	0.887186	0.860425
B.cells	ABCD1	0.026286	4.771292	0.220787	0.825767	-6.45227	0.826845	0.761191
B.cells	P2RX3	0.069404	2.219391	0.220737	0.825806	-5.91747	0.861174	0.817227
B.cells	SAA4	-0.10736	0.640047	-0.22059	0.82592	-5.49077	0.883294	0.853919
B.cells	ZDHHC5	0.022159	5.017726	0.220235	0.826196	-6.46788	0.823792	0.756088
B.cells	PHC1	0.034834	3.5385	0.22009	0.826308	-6.09109	0.843407	0.787896
B.cells	GPR19	0.059829	2.550401	0.219938	0.826426	-5.79777	0.856813	0.809856
B.cells	BAG6	-0.02817	5.438014	-0.21992	0.826441	-6.55094	0.818317	0.747318
B.cells	PRRC2A	-0.02312	5.517966	-0.21982	0.82652	-6.53837	0.817281	0.745657
B.cells	GRK2	0.019621	7.206149	0.219812	0.826524	-6.85031	0.79575	0.711422
B.cells	KLRA4	0.159616	-1.21319	0.219765	0.826561	-5.23062	0.90985	0.899079
B.cells	WWP1	-0.03446	5.227114	-0.21949	0.826774	-6.41211	0.8212	0.751736
B.cells	SLC52A2	0.056746	2.391257	0.219375	0.826864	-5.82657	0.85916	0.813517
B.cells	CREM	-0.03563	6.0952	-0.21914	0.827044	-6.69917	0.810087	0.733886
B.cells	XCL1	-0.12062	2.335401	-0.21905	0.827114	-5.65059	0.860022	0.814844
B.cells	4930484I04	0.063848	1.660784	0.218801	0.827309	-5.74824	0.869357	0.830272
B.cells	PUM3	0.026272	4.640352	0.218582	0.827479	-6.42651	0.828995	0.764258
B.cells	INTS8	-0.02732	4.693172	-0.21837	0.827648	-6.3773	0.8283	0.763238
B.cells	ETS2	-0.03168	5.865691	-0.21836	0.827653	-6.63881	0.813036	0.738726
B.cells	PSMD11	0.013401	7.032517	0.218279	0.827715	-6.85403	0.798174	0.715152
B.cells	ZFP580	-0.06844	2.166288	-0.21792	0.827992	-5.68228	0.862352	0.818942
B.cells	CASS4	-0.06909	3.04899	-0.21781	0.828081	-5.96287	0.850274	0.799114
B.cells	CIPC	-0.05277	2.655218	-0.21779	0.828094	-5.87531	0.855637	0.807919
B.cells	SMC1A	-0.02415	6.748393	-0.21775	0.828125	-6.76093	0.801763	0.720925
B.cells	TIE1	0.091659	1.482911	0.217599	0.828243	-5.48205	0.871838	0.834752

B.cells	MRAP	-0.11992	1.214744	-0.21751	0.828316	-5.44099	0.875594	0.84103
B.cells	SMARCD1	0.028243	4.031896	0.217435	0.82837	-6.26754	0.837057	0.777664
B.cells	ALG11	0.044777	2.610001	0.217388	0.828407	-5.89922	0.856256	0.80905
B.cells	LY86	0.029773	6.704851	0.217326	0.828455	-6.88163	0.802315	0.721908
B.cells	INTS14	0.041329	4.691853	0.217304	0.828472	-6.36391	0.828317	0.763521
B.cells	GM28791	0.036306	3.918458	0.217075	0.82865	-6.25376	0.83857	0.780152
B.cells	4933434E2	-0.02558	5.269667	-0.217	0.828707	-6.52534	0.820753	0.751353
B.cells	COPS3	0.02038	5.92883	0.216975	0.828728	-6.64012	0.812223	0.73769
B.cells	MCRIP1	0.019429	5.919349	0.216953	0.828745	-6.63568	0.812345	0.737884
B.cells	MTMR1	0.029876	4.622377	0.2169	0.828786	-6.35323	0.829232	0.765015
B.cells	UBR2	0.020389	6.362518	0.216793	0.828869	-6.69591	0.806668	0.728836
B.cells	TMBIM6	0.019705	9.142023	0.216564	0.829047	-7.16284	0.772117	0.674632
B.cells	USP30	0.05535	2.252852	0.216498	0.829098	-5.77369	0.861158	0.817217
B.cells	UBE2D3	0.012098	8.966917	0.216339	0.829222	-7.15347	0.774241	0.67793
B.cells	STOML2	0.031496	4.805582	0.216296	0.829256	-6.38394	0.826822	0.761184
B.cells	MEF2B	-0.03856	3.605786	-0.21629	0.829262	-6.14496	0.842757	0.787034
B.cells	SEC14L1	0.031785	4.618739	0.21624	0.829299	-6.34907	0.82928	0.765154
B.cells	AFAP1L1	-0.11189	1.508026	-0.21617	0.829356	-5.43242	0.871488	0.83432
B.cells	EIF1AX	0.023352	6.060349	0.216114	0.829397	-6.66113	0.810534	0.735052
B.cells	GM10851	0.035556	4.366594	0.216113	0.829397	-6.33379	0.832611	0.770543
B.cells	CLPTM1L	0.027689	4.883326	0.216077	0.829426	-6.43544	0.825801	0.759538
B.cells	SASH1	0.04949	5.303699	0.216034	0.829459	-6.36869	0.82031	0.750701
B.cells	5430401H(	-0.12305	-0.47304	-0.216	0.829482	-5.39142	0.89964	0.881499
B.cells	CD1D1	-0.04197	3.932282	-0.2155	0.829875	-6.33814	0.838709	0.780024
B.cells	POC1B	0.027318	4.642223	0.21527	0.830053	-6.36571	0.829324	0.764763
B.cells	AAGAB	0.026918	4.862045	0.215247	0.83007	-6.43397	0.826432	0.760097
B.cells	ABCC4	-0.02677	4.55376	-0.21505	0.830224	-6.45615	0.83049	0.766648
B.cells	SENP3	0.030394	4.324023	0.214981	0.830277	-6.31554	0.83353	0.771567
B.cells	PER2	-0.07972	2.222784	-0.21481	0.830413	-5.6542	0.86194	0.818059
B.cells	TMEM126I	0.047138	2.697386	0.21461	0.830565	-5.88776	0.855425	0.807374
B.cells	ATAD2	0.034487	6.309192	0.214307	0.830801	-6.66766	0.807693	0.730154
B.cells	TMEM138	-0.04538	2.910493	-0.21417	0.830909	-5.91089	0.852519	0.802633
B.cells	POGZ	0.027446	4.431535	0.21403	0.831016	-6.35721	0.832106	0.769367
B.cells	1700109H(	0.042751	3.650709	0.213956	0.831074	-6.15295	0.842513	0.786271
B.cells	RAB6B	-0.08631	1.697022	-0.21392	0.831103	-5.47507	0.869223	0.830181
B.cells	YIF1A	0.030887	4.016242	0.213904	0.831115	-6.18494	0.837622	0.778312
B.cells	GPC6	-0.11894	1.763476	-0.21386	0.831148	-5.62833	0.868299	0.828649
B.cells	DIS3L2	-0.02182	5.906117	-0.21368	0.831292	-6.66024	0.812861	0.738441
B.cells	CASZ1	0.037203	3.474262	0.213571	0.831373	-6.21192	0.844885	0.790163
B.cells	GTF3C3	0.045675	3.102011	0.213549	0.831391	-6.02833	0.849917	0.79839
B.cells	ARL1	-0.02183	5.538643	-0.21351	0.831422	-6.57205	0.817608	0.746036
B.cells	TBRG4	-0.03578	4.075048	-0.21341	0.831499	-6.21811	0.836838	0.777061
B.cells	BAIAP3	-0.11669	-0.32152	-0.21324	0.831629	-5.33288	0.897852	0.878102
B.cells	DAP3	0.024847	5.039126	0.213232	0.831637	-6.48665	0.824112	0.75651
B.cells	KLRB1C	0.116587	1.954564	0.213132	0.831714	-5.5867	0.865647	0.824324
B.cells	UBB	-0.02111	10.85501	-0.21308	0.831756	-7.44984	0.752021	0.643491
B.cells	SMPD1	0.053193	2.643295	0.213007	0.831812	-5.87405	0.856165	0.808685

B.cells	UBE2A	-0.01638	6.786705	-0.21298	0.831836	-6.79946	0.80162	0.720597
B.cells	CNTROB	0.074729	2.044211	0.212897	0.831897	-5.61004	0.864406	0.822272
B.cells	CACNB2	-0.06909	4.420818	-0.21283	0.831948	-6.20321	0.832248	0.76966
B.cells	SH3BP1	-0.0296	5.21446	-0.21282	0.831959	-6.47309	0.821822	0.752842
B.cells	PRDM16	0.126712	0.306557	0.212775	0.831992	-5.32581	0.88883	0.862947
B.cells	1810044D(	0.068556	1.915203	0.212762	0.832002	-5.70768	0.866192	0.825229
B.cells	RASSF7	-0.08692	0.86906	-0.21195	0.832632	-5.47824	0.881129	0.849719
B.cells	CBX7	0.042306	2.651462	0.211901	0.832672	-5.93437	0.856336	0.808644
B.cells	GMEB1	-0.02719	5.025748	-0.21183	0.832725	-6.47233	0.82456	0.756956
B.cells	CDC42BPG	-0.05926	2.57838	-0.21158	0.832919	-5.9061	0.857337	0.810317
B.cells	COIL	0.032467	3.741294	0.211234	0.833191	-6.2445	0.841576	0.784612
B.cells	TSTD2	0.033922	3.84075	0.211216	0.833205	-6.22895	0.840244	0.782444
B.cells	FSCN1	-0.08303	3.378992	-0.21095	0.83341	-6.05109	0.84645	0.792636
B.cells	CTNND2	-0.09848	1.726999	-0.21091	0.833441	-5.57117	0.869093	0.829906
B.cells	MLLT11	0.038249	3.193389	0.210699	0.833607	-6.01698	0.848959	0.796758
B.cells	ADAMTSL1	-0.17549	0.587709	-0.21064	0.833656	-5.33881	0.885118	0.856639
B.cells	GM20743	-0.1088	0.031876	-0.21063	0.833657	-5.39141	0.893058	0.86996
B.cells	CD27	-0.04469	3.318035	-0.21058	0.8337	-6.13216	0.847273	0.794035
B.cells	TSR3	-0.03958	3.569257	-0.2105	0.833765	-6.17332	0.843886	0.788503
B.cells	GM10550	-0.09424	0.590308	-0.21042	0.833824	-5.43987	0.885081	0.856592
B.cells	SCN4A	0.105292	0.410123	0.210406	0.833835	-5.36602	0.887646	0.860888
B.cells	CD69	-0.04386	5.620451	-0.21036	0.833869	-6.60569	0.816818	0.744752
B.cells	NLK	0.025376	5.966033	0.210064	0.834101	-6.66561	0.812359	0.737716
B.cells	DHRS11	-0.03467	4.678666	-0.21002	0.834134	-6.39991	0.829118	0.764637
B.cells	SFI1	-0.02567	5.320089	-0.20996	0.834184	-6.60606	0.820717	0.751104
B.cells	GM37240	0.031279	5.162317	0.209933	0.834203	-6.51374	0.822774	0.754411
B.cells	GM14326	-0.04374	2.868013	-0.20983	0.834286	-6.0313	0.85338	0.804169
B.cells	SSH3	-0.0805	1.522336	-0.20976	0.834336	-5.6238	0.871947	0.834845
B.cells	NUP93	-0.02817	4.586111	-0.20974	0.834355	-6.38716	0.830338	0.766642
B.cells	ZFP26	0.028135	3.995742	0.209385	0.834629	-6.30001	0.838172	0.779445
B.cells	EIF2A	0.01943	5.395584	0.20916	0.834805	-6.56377	0.819735	0.749696
B.cells	C87436	0.032017	3.905756	0.208978	0.834946	-6.26158	0.839374	0.781442
B.cells	RYR2	0.156929	0.059974	0.208945	0.834971	-5.28151	0.892655	0.869617
B.cells	TENT4A	-0.0403	3.460072	-0.20893	0.834984	-6.10464	0.845356	0.791194
B.cells	TULP3	0.067637	1.974251	0.208718	0.835149	-5.6577	0.86566	0.82458
B.cells	ACO2	0.018743	6.116578	0.208559	0.835272	-6.66539	0.810426	0.734826
B.cells	TIMM44	-0.02388	4.84696	-0.20848	0.835337	-6.43512	0.826904	0.761277
B.cells	HMBS	-0.03777	4.451319	-0.20841	0.835391	-6.31942	0.832119	0.769708
B.cells	IWS1	0.020089	5.729671	0.208188	0.835561	-6.62315	0.815406	0.742862
B.cells	DHX32	-0.03386	3.380004	-0.20816	0.83558	-6.13876	0.846436	0.793078
B.cells	NPAT	0.032019	4.485178	0.208096	0.835633	-6.34082	0.831671	0.769059
B.cells	PKDCC	0.086502	1.303453	0.207744	0.835906	-5.50393	0.875011	0.84037
B.cells	NDUFB1-PS	0.015013	8.902498	0.207743	0.835907	-7.14363	0.775611	0.680193
B.cells	LEMD2	-0.02724	4.232797	-0.20773	0.835919	-6.34412	0.835016	0.77461
B.cells	EPN1	-0.02128	6.109989	-0.2077	0.835942	-6.69692	0.81051	0.735169
B.cells	TPGS2	-0.03384	3.805381	-0.20768	0.83596	-6.1845	0.840717	0.78388
B.cells	TECR	-0.02198	6.763011	-0.20763	0.835998	-6.75179	0.802185	0.721926

B.cells	BC003965	-0.03738	3.701969	-0.20755	0.836054	-6.14023	0.842103	0.786153
B.cells	GALNT18	0.10943	2.025829	0.207478	0.836114	-5.60725	0.864946	0.823693
B.cells	GLB1	-0.02568	5.221188	-0.20743	0.836155	-6.49742	0.822006	0.753618
B.cells	HARBI1	0.078756	1.346342	0.20737	0.836198	-5.60872	0.87441	0.839403
B.cells	AGO4	-0.04309	3.167589	-0.20736	0.836204	-6.08055	0.849309	0.797949
B.cells	ZBTB7A	-0.01654	7.04476	-0.20711	0.836403	-6.85505	0.798624	0.716334
B.cells	UFM1	0.022548	5.448609	0.207008	0.836479	-6.55957	0.819046	0.748884
B.cells	DDX47	0.019169	5.550552	0.206999	0.836487	-6.59225	0.817724	0.746761
B.cells	HMCES	-0.03313	4.456587	-0.20692	0.836547	-6.32078	0.832049	0.769854
B.cells	GORASP2	-0.01883	5.534187	-0.2069	0.836563	-6.58057	0.817936	0.747102
B.cells	KCTD14	0.057104	1.055743	0.206804	0.836639	-5.83728	0.878494	0.846257
B.cells	GNA15	0.055679	3.584316	0.20666	0.836751	-5.91015	0.843684	0.788795
B.cells	PUS10	-0.02964	4.485499	-0.20665	0.83676	-6.40244	0.831667	0.769256
B.cells	EP400	0.017568	6.870294	0.206619	0.836783	-6.79367	0.800827	0.719842
B.cells	CCL7	0.156528	1.659903	0.206609	0.83679	-5.60811	0.870028	0.832167
B.cells	FCRL5	-0.09736	-0.65204	-0.20647	0.836899	-5.38487	0.902802	0.887338
B.cells	TTLL1	-0.0666	1.626512	-0.2064	0.836954	-5.62666	0.870493	0.832951
B.cells	MAP4K5	0.032365	4.52083	0.206278	0.837048	-6.40309	0.8312	0.768551
B.cells	EPHX1	-0.05324	3.053436	-0.206	0.837264	-5.98761	0.850857	0.800612
B.cells	SLC35E1	0.030229	4.084727	0.205891	0.837349	-6.23674	0.836986	0.777962
B.cells	IPO7	0.027206	5.656347	0.205811	0.837412	-6.60174	0.816354	0.744659
B.cells	ILVBL	-0.03384	3.757211	-0.20554	0.837624	-6.1966	0.841362	0.78518
B.cells	RIOK1	0.020093	6.007255	0.205494	0.837659	-6.65401	0.811829	0.73753
B.cells	EEF1AKNM	-0.05154	2.318289	-0.20529	0.837817	-5.82305	0.860909	0.817343
B.cells	TRIM39	-0.04113	2.873416	-0.20498	0.838056	-5.91546	0.853306	0.804928
B.cells	GM15446	0.057953	2.166881	0.204898	0.838123	-5.81877	0.862996	0.820902
B.cells	5430427M	-0.09317	1.237849	-0.20466	0.83831	-5.52295	0.875932	0.842491
B.cells	9330159M	0.081133	1.076034	0.2046	0.838355	-5.52588	0.878208	0.846287
B.cells	RSPH3B	0.041457	3.01938	0.204501	0.838432	-5.96412	0.85132	0.801786
B.cells	1700086O(	0.075841	1.546442	0.204353	0.838547	-5.47302	0.871611	0.835309
B.cells	PNP	0.028261	5.924728	0.204313	0.838579	-6.63934	0.812891	0.739498
B.cells	SLC35B1	0.023694	5.751128	0.204286	0.838599	-6.63093	0.815129	0.74308
B.cells	4632428CC	0.113519	0.110114	0.204264	0.838617	-5.26483	0.891935	0.869308
B.cells	PIP4K2C	-0.03025	4.238405	-0.20407	0.83877	-6.3039	0.834942	0.775058
B.cells	CDK5R1	-0.0628	2.111423	-0.20401	0.838815	-5.73073	0.863762	0.822309
B.cells	FOXJ2	-0.03715	3.829076	-0.20399	0.838827	-6.19835	0.8404	0.783938
B.cells	MRPL22	-0.03505	3.858759	-0.20384	0.83895	-6.20848	0.840003	0.783291
B.cells	NMB	-0.06311	1.642171	-0.20359	0.839139	-5.68593	0.870275	0.833107
B.cells	CPEB1	0.101156	0.696287	0.20357	0.839158	-5.35186	0.883576	0.855292
B.cells	TTC7B	0.025663	5.391283	0.203518	0.839198	-6.51859	0.819791	0.750578
B.cells	HDHD2	-0.03192	3.552765	-0.2035	0.839211	-6.13417	0.844108	0.78999
B.cells	ARF4OS	-0.08442	0.845404	-0.20343	0.83927	-5.42309	0.881464	0.851757
B.cells	PFDN6	0.026652	4.874434	0.203382	0.839304	-6.39476	0.826543	0.761456
B.cells	SLC39A3	-0.06258	1.91724	-0.20336	0.839324	-5.70619	0.86645	0.82676
B.cells	LGALS3BP	-0.06632	4.826846	-0.20328	0.83938	-6.12727	0.827168	0.762465
B.cells	CHKA	-0.02302	6.940391	-0.20321	0.839437	-6.82164	0.799941	0.718895
B.cells	UBTD1	0.030907	4.402162	0.203198	0.839447	-6.37741	0.83277	0.771533

B.cells	ZSWIM6	-0.02187	8.155524	-0.20312	0.839508	-7.01739	0.784768	0.694987
B.cells	AZIN1	-0.02199	6.940521	-0.20311	0.839519	-6.85672	0.799939	0.718892
B.cells	CD99L2	-0.04907	2.142314	-0.20303	0.839577	-5.81993	0.863335	0.821603
B.cells	FTX	0.035844	4.311116	0.202969	0.839626	-6.26604	0.833977	0.773491
B.cells	EIF5A	-0.01907	9.061608	-0.20291	0.839673	-7.15539	0.773677	0.677685
B.cells	HIST1H1B	-0.06835	5.277543	-0.20278	0.839775	-6.53292	0.821271	0.752958
B.cells	UTP15	0.030123	3.757308	0.202773	0.839778	-6.19653	0.841361	0.785506
B.cells	PLIN3	-0.03784	3.527331	-0.20254	0.839958	-6.07621	0.84445	0.790558
B.cells	TMEM161/	0.047898	2.853132	0.202534	0.839965	-5.92029	0.853582	0.805528
B.cells	IGKV1-110	-0.08815	0.050225	-0.2025	0.839988	-5.46162	0.892795	0.870781
B.cells	2810013P0	-0.04139	4.123121	-0.20246	0.840024	-6.2055	0.836475	0.777561
B.cells	RBM27	-0.01749	6.399999	-0.20232	0.840133	-6.74507	0.8068	0.729812
B.cells	EIF3I	-0.01942	6.822191	-0.20228	0.840165	-6.82541	0.801435	0.721284
B.cells	CYC1	0.024984	6.36195	0.202239	0.840195	-6.72237	0.807286	0.730585
B.cells	POMT2	0.060138	1.813643	0.202129	0.84028	-5.67247	0.867888	0.829168
B.cells	COG7	0.045706	2.604364	0.202027	0.84036	-5.87843	0.856981	0.811135
B.cells	GM11520	-0.07731	1.043872	-0.20193	0.840433	-5.48476	0.878661	0.847097
B.cells	CD180	-0.02757	5.032672	-0.20192	0.840447	-6.64761	0.824469	0.758129
B.cells	SETD1A	0.026161	4.19796	0.201806	0.840532	-6.29597	0.835479	0.775952
B.cells	PPP2R5A	-0.01454	7.861178	-0.20163	0.840669	-7.00769	0.788412	0.700723
B.cells	GM12905	-0.04679	2.138199	-0.20158	0.840705	-5.77266	0.863392	0.821719
B.cells	2510039O1	0.023509	5.179646	0.201534	0.840744	-6.48762	0.822548	0.755034
B.cells	ZFP169	-0.04924	2.782441	-0.20144	0.840816	-6.06056	0.854547	0.807127
B.cells	CPNE5	-0.10524	0.481176	-0.20143	0.840829	-5.35824	0.886633	0.86044
B.cells	CARNS1	0.074656	2.476067	0.201374	0.840869	-5.70704	0.85874	0.814034
B.cells	GNL2	0.025424	4.675227	0.201362	0.840878	-6.42097	0.829163	0.765711
B.cells	VANGL2	-0.05516	2.225601	-0.20119	0.841013	-5.78478	0.862186	0.819751
B.cells	2610008E1	0.053359	2.466966	0.201103	0.84108	-5.86329	0.858865	0.814305
B.cells	GTF3A	-0.02435	4.569389	-0.20104	0.841133	-6.35927	0.830559	0.768059
B.cells	MIA3	0.019573	5.849323	0.201021	0.841144	-6.63135	0.813862	0.741172
B.cells	FKBP4	-0.02352	5.691682	-0.20101	0.841156	-6.58068	0.815897	0.744433
B.cells	RPRD1B	0.023476	5.480381	0.200962	0.84119	-6.56318	0.818634	0.748838
B.cells	RABL3	0.053682	2.218563	0.200954	0.841196	-5.7789	0.862283	0.819994
B.cells	TAB2	-0.01723	6.780443	-0.20085	0.84128	-6.79563	0.801964	0.722228
B.cells	SLC35B4	0.047424	2.825471	0.20082	0.841301	-5.90287	0.85396	0.806279
B.cells	3010003L2	0.069396	1.349271	0.200754	0.841352	-5.67258	0.874369	0.840062
B.cells	ZFP810	-0.05251	2.914739	-0.20072	0.841379	-5.85505	0.852743	0.804279
B.cells	KDM2B	0.033487	7.133876	0.200716	0.841382	-6.89994	0.797502	0.715157
B.cells	MPV17L	-0.06252	2.006778	-0.2006	0.841475	-5.70676	0.865224	0.824848
B.cells	TMEM263	0.032536	3.98713	0.200513	0.84154	-6.24776	0.838301	0.78066
B.cells	MSRB2	-0.10067	1.023579	-0.20021	0.841773	-5.38824	0.879019	0.847836
B.cells	STK19	0.028795	4.61763	0.200042	0.841907	-6.38644	0.82999	0.767234
B.cells	FAP	0.133472	0.744801	0.199928	0.841996	-5.38668	0.88296	0.854508
B.cells	RERE	0.017741	7.702335	0.199776	0.842114	-6.96635	0.790451	0.704121
B.cells	NOA1	-0.04086	3.137205	-0.19953	0.842309	-6.03266	0.84979	0.799533
B.cells	USF2	-0.02045	6.021725	-0.19945	0.842373	-6.65818	0.811709	0.737846
B.cells	ZFP933	-0.03764	3.490032	-0.19933	0.842464	-6.14833	0.845021	0.791722

B.cells	GTF3C1	0.025133	4.779184	0.199246	0.842528	-6.43626	0.827862	0.763817
B.cells	ESD	0.022638	7.081837	0.199178	0.842581	-6.87237	0.798222	0.716388
B.cells	MYBL1	-0.07221	1.840587	-0.19916	0.842593	-5.60861	0.867585	0.828892
B.cells	SIRT4	-0.11265	0.56797	-0.19914	0.842614	-5.34703	0.88547	0.858727
B.cells	FAM117A	0.027338	5.965212	0.19912	0.842626	-6.7427	0.812436	0.739008
B.cells	F930017D2	0.106092	0.898224	0.198992	0.842726	-5.56605	0.880788	0.850931
B.cells	ALDH18A1	-0.05103	3.070395	-0.19892	0.842781	-5.9897	0.850696	0.801071
B.cells	EARS2	0.062575	1.644083	0.198879	0.842814	-5.61248	0.870319	0.833496
B.cells	ARIH2	0.017713	7.527213	0.198693	0.842959	-6.96464	0.792635	0.70763
B.cells	GSDMC4	-0.07244	0.743558	-0.19867	0.84298	-5.56515	0.882977	0.854639
B.cells	IL18BP	0.076545	3.740937	0.198635	0.843004	-6.05584	0.841649	0.7863
B.cells	SPG7	0.022571	4.467655	0.198033	0.843474	-6.35839	0.831971	0.770663
B.cells	ZFP444	-0.03673	3.63547	-0.19793	0.843551	-6.16375	0.843064	0.788727
B.cells	1300002E1	-0.04592	3.19234	-0.19782	0.843637	-5.92709	0.849042	0.798513
B.cells	ZFP787	0.021392	5.128821	0.197754	0.843692	-6.48421	0.823279	0.75664
B.cells	ANXA4	0.039324	3.74233	0.197687	0.843744	-6.22882	0.84163	0.786428
B.cells	PHF11D	-0.09694	1.34229	-0.19739	0.843973	-5.61228	0.874538	0.84081
B.cells	ZFP40	0.083538	1.347612	0.197321	0.844029	-5.50733	0.874463	0.840703
B.cells	ITIH5	-0.06367	1.943297	-0.19731	0.844035	-5.75216	0.866159	0.826897
B.cells	POLN	-0.05088	3.191009	-0.19706	0.844235	-6.08246	0.84906	0.798806
B.cells	NR2C2	-0.01944	6.925422	-0.19694	0.844324	-6.81776	0.800195	0.719954
B.cells	2210016F1	-0.02203	4.954824	-0.19683	0.844411	-6.48241	0.825556	0.760558
B.cells	FRAT1	-0.03269	3.463579	-0.19661	0.844588	-6.12609	0.845377	0.792812
B.cells	RGS14	0.035761	3.585563	0.196535	0.844643	-6.23789	0.843735	0.790165
B.cells	PHEX	-0.08612	1.372943	-0.19643	0.844728	-5.62073	0.874108	0.840351
B.cells	EMID1	-0.03887	3.280299	-0.19633	0.844806	-6.09177	0.847852	0.796938
B.cells	RMND5B	-0.02569	4.49308	-0.1963	0.844825	-6.35034	0.831634	0.770488
B.cells	CPNE1	-0.0186	6.038278	-0.19617	0.844929	-6.66748	0.811497	0.738045
B.cells	PKD1L3	0.072076	0.837305	0.196161	0.844934	-5.60759	0.88165	0.85295
B.cells	INTS12	-0.02589	4.380987	-0.19606	0.845014	-6.34585	0.833118	0.772895
B.cells	ZFP715	-0.03238	3.662783	-0.19588	0.845156	-6.12929	0.842698	0.788501
B.cells	BRMS1L	0.026568	4.269409	0.195846	0.84518	-6.30905	0.834598	0.7753
B.cells	CCDC180	-0.06535	1.286108	-0.19574	0.845266	-5.73003	0.875326	0.842383
B.cells	SYTL3	-0.07275	2.803793	-0.19556	0.845402	-5.88012	0.854325	0.807587
B.cells	GM6787	0.096542	0.188055	0.195554	0.845408	-5.34186	0.890891	0.868481
B.cells	CSMD3	0.071357	1.302147	0.19554	0.845419	-5.55471	0.875101	0.84202
B.cells	TIPARP	0.023736	7.437182	0.195396	0.845531	-6.9816	0.793761	0.709867
B.cells	GM10521	-0.09373	0.308847	-0.19528	0.845622	-5.32152	0.889163	0.865587
B.cells	NOC3L	-0.04403	3.216026	-0.19523	0.845662	-6.09861	0.848722	0.79839
B.cells	FGF1	0.092497	0.63081	0.195199	0.845685	-5.37039	0.884577	0.857884
B.cells	TCTN1	0.087659	1.315572	0.195184	0.845696	-5.48609	0.874912	0.841719
B.cells	CCL21A	0.296625	0.049265	0.194968	0.845865	-5.28917	0.892881	0.871886
B.cells	CEP162	-0.03666	3.080056	-0.1949	0.845918	-6.02556	0.850565	0.801475
B.cells	RAB7	-0.01487	7.931545	-0.19486	0.84595	-6.98676	0.787603	0.700243
B.cells	GNG4	0.073627	1.462633	0.194825	0.845977	-5.67823	0.872853	0.838365
B.cells	BZW2	0.023016	5.869307	0.194679	0.846091	-6.62362	0.81367	0.741655
B.cells	GFOD1	0.024974	6.621215	0.194543	0.846197	-6.85624	0.804049	0.726305

B.cells	GM48027	0.044764	2.815176	0.194376	0.846327	-5.95228	0.854169	0.807475
B.cells	AFG3L2	-0.02059	4.593409	-0.19434	0.846352	-6.42012	0.830309	0.768494
B.cells	GOLGA2	0.030057	3.814242	0.194181	0.846479	-6.17186	0.840667	0.785389
B.cells	PGP	-0.02706	5.374059	-0.19391	0.84669	-6.50016	0.820082	0.752058
B.cells	SLC8B1	-0.02979	4.721857	-0.19385	0.846736	-6.42275	0.828616	0.765838
B.cells	EMD	-0.0188	6.102062	-0.19385	0.846741	-6.64989	0.810678	0.736968
B.cells	TMEM259	0.02805	4.879001	0.193815	0.846766	-6.47131	0.82655	0.762495
B.cells	ERCC5	-0.03702	2.909402	-0.19379	0.846783	-6.01195	0.852885	0.805463
B.cells	GM31508	-0.09502	0.763062	-0.19376	0.846811	-5.40189	0.882701	0.854985
B.cells	LAPTM5	-0.01568	8.326927	-0.19375	0.846815	-7.04774	0.782719	0.692716
B.cells	GM14798	-0.02867	3.718232	-0.19368	0.84687	-6.2628	0.841953	0.787538
B.cells	ARPP19	-0.01621	6.840626	-0.19362	0.846918	-6.80094	0.801267	0.721991
B.cells	GM31812	0.105506	0.191942	0.193543	0.846978	-5.32082	0.890835	0.868691
B.cells	SEC22A	0.038024	3.176208	0.193452	0.847049	-5.99646	0.849261	0.799543
B.cells	SLC4A9	0.102225	0.122757	0.193375	0.847109	-5.31368	0.891827	0.870361
B.cells	G530011O(	0.078017	1.80368	0.193336	0.84714	-5.71252	0.868097	0.830654
B.cells	MRPL17	0.021929	5.426857	0.193334	0.847141	-6.52744	0.819395	0.750992
B.cells	TMEM170E	0.028126	4.646846	0.193257	0.847201	-6.34407	0.829604	0.767478
B.cells	PDP2	0.029262	3.724878	0.193222	0.847228	-6.23601	0.841864	0.787428
B.cells	CCDC136	-0.11774	0.119291	-0.19318	0.847258	-5.36818	0.891876	0.870445
B.cells	2010009K1	-0.14088	0.112521	-0.19316	0.847274	-5.28845	0.891973	0.870609
B.cells	NCR1	-0.11169	1.170957	-0.19313	0.847296	-5.49687	0.876943	0.845391
B.cells	ZFP780B	-0.03787	3.329247	-0.19307	0.847343	-6.10329	0.84719	0.796145
B.cells	NBAS	0.027577	4.535139	0.193067	0.84735	-6.33598	0.831079	0.769868
B.cells	USP10	-0.02681	4.567936	-0.19304	0.847367	-6.44455	0.830645	0.769166
B.cells	1700019L1	0.09295	0.774577	0.192997	0.847404	-5.47169	0.882538	0.854772
B.cells	PSD3	0.034595	5.794857	0.19285	0.847519	-6.53555	0.81463	0.743374
B.cells	BET1L	0.029349	3.860623	0.192848	0.84752	-6.23004	0.840046	0.784499
B.cells	IL1RL2	-0.12063	0.726101	-0.19273	0.847613	-5.27437	0.883246	0.85595
B.cells	WDR83OS	0.021497	6.074614	0.192473	0.847813	-6.64954	0.811172	0.737641
B.cells	TBC1D9B	0.026354	4.555284	0.192285	0.84796	-6.28935	0.831024	0.769587
B.cells	CDK8	-0.0327	6.559883	-0.19214	0.848071	-6.77014	0.805034	0.727831
B.cells	CSDE1	0.012282	7.576149	0.192108	0.848098	-6.94208	0.792226	0.707529
B.cells	COX6A1	-0.02027	6.768979	-0.19198	0.848194	-6.77476	0.802402	0.723619
B.cells	F730311O2	0.098296	-0.31758	0.19178	0.848354	-5.39161	0.898453	0.881318
B.cells	A630001O1	-0.04473	2.282008	-0.19176	0.84837	-5.95077	0.861754	0.819926
B.cells	ETAA1	0.036028	3.334503	0.191417	0.848638	-6.08888	0.847476	0.796393
B.cells	CMC4	-0.04073	3.064546	-0.1913	0.848729	-5.90566	0.851134	0.802412
B.cells	TAF6	0.034804	3.851886	0.191178	0.848824	-6.18415	0.840517	0.785024
B.cells	SMIM27	0.034291	3.590898	0.191175	0.848827	-6.05429	0.844019	0.790747
B.cells	ALG9	0.032567	3.375018	0.190904	0.849039	-6.1545	0.846929	0.795545
B.cells	PDE10A	-0.08952	2.555984	-0.19076	0.849154	-5.58672	0.858075	0.813956
B.cells	NFKBIA	0.025892	8.130992	0.190534	0.849328	-7.0318	0.785466	0.697045
B.cells	CCNY	0.018442	6.67252	0.190478	0.849371	-6.78213	0.803736	0.72593
B.cells	SEMA7A	-0.05434	2.017527	-0.19044	0.849401	-5.8657	0.865496	0.826341
B.cells	STAU2	0.049697	2.900653	0.190406	0.849427	-5.89827	0.853364	0.806292
B.cells	MRPL14	-0.0263	5.601025	-0.1904	0.849428	-6.56379	0.817481	0.747911

B.cells	GPATCH1	0.038704	2.73622	0.190261	0.849541	-5.95626	0.855608	0.809988
B.cells	EEF1D	-0.01723	7.27018	-0.19025	0.849548	-6.89371	0.796189	0.713952
B.cells	PLCG1	-0.03882	3.409844	-0.1902	0.849589	-6.01026	0.846459	0.794949
B.cells	R3HDM4	-0.02342	6.20323	-0.19018	0.849608	-6.63039	0.809722	0.735476
B.cells	PRMT1	-0.02036	5.803475	-0.19014	0.849632	-6.61544	0.814863	0.743708
B.cells	GJA1	0.11368	1.208053	0.190044	0.849711	-5.36982	0.876791	0.845164
B.cells	GTSE1	-0.10784	1.404909	-0.18998	0.849757	-5.49879	0.874029	0.840569
B.cells	AASDHPPT	-0.03036	3.841771	-0.18962	0.850041	-6.15987	0.840847	0.78551
B.cells	GREM2	0.098272	0.454759	0.189547	0.850098	-5.40575	0.887661	0.863079
B.cells	4930469K1	-0.09306	1.311112	-0.18909	0.850458	-5.5039	0.875842	0.842888
B.cells	HNRNPD	0.013923	7.908504	0.188726	0.85074	-6.98457	0.788711	0.701596
B.cells	4930599N2	-0.05767	1.733523	-0.18859	0.850845	-5.77415	0.86998	0.833149
B.cells	CMSS1	-0.03281	6.448827	-0.18852	0.8509	-6.74918	0.807084	0.730691
B.cells	ZFP626	0.039573	2.562798	0.188498	0.850918	-5.9341	0.858515	0.814157
B.cells	GM43061	-0.09738	0.306464	-0.18849	0.850922	-5.35491	0.890125	0.866843
B.cells	TSPAN9	0.058569	3.290989	0.188457	0.85095	-5.87549	0.848592	0.79783
B.cells	PCSK5	-0.09831	0.558557	-0.18822	0.851139	-5.42214	0.886528	0.860855
B.cells	GLRX	-0.02676	6.214402	-0.18806	0.851264	-6.63591	0.810082	0.735579
B.cells	MED9	0.0353	3.526994	0.188006	0.851303	-6.12354	0.845404	0.792739
B.cells	METTL5	-0.03091	3.739598	-0.18764	0.851591	-6.15099	0.842545	0.788122
B.cells	ZFP516	-0.02715	4.66128	-0.18746	0.851726	-6.44377	0.830279	0.768225
B.cells	FAM114A1	0.059358	2.398626	0.187405	0.851772	-5.80103	0.860771	0.818169
B.cells	ZMYM1	-0.03612	3.329996	-0.18739	0.851783	-6.05278	0.848064	0.797244
B.cells	RAB28	-0.02693	4.828065	-0.18724	0.8519	-6.3623	0.828082	0.764715
B.cells	RDX	0.01455	6.872179	0.187205	0.851929	-6.78653	0.801704	0.722411
B.cells	VDAC2	-0.01676	7.488132	-0.1869	0.85217	-6.91509	0.793951	0.710198
B.cells	UXT	-0.02647	4.452478	-0.18675	0.852283	-6.33219	0.83304	0.772859
B.cells	LETM2	-0.04031	3.131534	-0.18671	0.852314	-5.9762	0.850753	0.801811
B.cells	ZFP526	-0.09058	0.37218	-0.1867	0.852321	-5.35617	0.889186	0.865731
B.cells	D16ERTD47	-0.02824	4.914897	-0.18665	0.852361	-6.43427	0.826941	0.763004
B.cells	WSB2	-0.027	4.493162	-0.18648	0.852497	-6.35702	0.832501	0.772034
B.cells	CABCOCO1	0.088053	0.315412	0.186289	0.852645	-5.34875	0.889997	0.86714
B.cells	THOC5	0.029997	3.564433	0.186079	0.852809	-6.15044	0.8449	0.792279
B.cells	XRN2	-0.01282	7.516217	-0.18591	0.852939	-6.95371	0.793599	0.709772
B.cells	GM28694	-0.09735	-0.23311	-0.18581	0.853016	-5.32981	0.897881	0.880528
B.cells	TANGO6	-0.02811	4.382985	-0.18576	0.853059	-6.32549	0.833961	0.774481
B.cells	MRPL18	0.022476	6.633228	0.185702	0.853104	-6.73543	0.804735	0.727466
B.cells	GM11973	0.040393	3.179185	0.185663	0.853134	-6.12125	0.850107	0.800885
B.cells	MYCBP	-0.02806	4.22905	-0.1854	0.853338	-6.29485	0.836005	0.777879
B.cells	PDE4DIP	0.029455	3.94998	0.185226	0.853476	-6.23585	0.839727	0.783958
B.cells	GM35188	-0.07169	1.73277	-0.18513	0.853552	-5.66821	0.86999	0.833835
B.cells	RALYL	-0.09448	0.59788	-0.1851	0.853575	-5.58392	0.885968	0.860547
B.cells	MBNL2	0.01761	8.406165	0.185095	0.853578	-7.10233	0.78256	0.692502
B.cells	NDUFV2	0.021463	6.136177	0.184917	0.853718	-6.65469	0.811085	0.737684
B.cells	AP1B1	-0.0223	5.190975	-0.18485	0.853773	-6.50103	0.823325	0.757358
B.cells	TIMP3	-0.09813	2.197691	-0.1848	0.85381	-5.6268	0.863541	0.823153
B.cells	ERLIN1	0.022657	4.765624	0.184783	0.853822	-6.53598	0.828904	0.766386

B.cells	BRD7	0.015245	6.307707	0.184686	0.853898	-6.72016	0.808887	0.734179
B.cells	DOK3	0.026876	5.36627	0.184648	0.853928	-6.63047	0.821039	0.753677
B.cells	PMAIP1	-0.03672	4.983149	-0.18464	0.853938	-6.48241	0.826045	0.761759
B.cells	LRBA	0.024074	6.139874	0.184534	0.854017	-6.74788	0.811038	0.737618
B.cells	LDHC	-0.12292	0.610466	-0.18452	0.854031	-5.31845	0.885789	0.860274
B.cells	LATS1	0.021605	4.725566	0.184418	0.854108	-6.42498	0.829432	0.767241
B.cells	ZFP58	0.070712	1.194809	0.184337	0.854171	-5.54747	0.877522	0.846423
B.cells	BTBD3	0.096223	1.337187	0.184308	0.854194	-5.39461	0.875522	0.843081
B.cells	TTC1	-0.01893	4.740195	-0.1843	0.8542	-6.40185	0.829239	0.766929
B.cells	MYEF2	-0.0233	5.328236	-0.18428	0.854213	-6.55493	0.821534	0.754476
B.cells	GM43149	-0.08501	0.774872	-0.18428	0.854214	-5.39818	0.883454	0.856355
B.cells	ENPEP	0.099995	0.401804	0.184264	0.854228	-5.34852	0.888763	0.865273
B.cells	MOV10	-0.03473	3.628623	-0.1842	0.854282	-6.1621	0.844036	0.791048
B.cells	WDR3	0.022763	4.225869	0.184109	0.85435	-6.4022	0.836047	0.778026
B.cells	DIABLO	0.02586	4.259159	0.184044	0.8544	-6.3211	0.835605	0.77731
B.cells	TARS2	-0.02892	3.819829	-0.18381	0.854581	-6.21338	0.841469	0.786883
B.cells	P2RY13	0.109993	1.100765	0.183778	0.854608	-5.3819	0.878847	0.848694
B.cells	DNAJC4	-0.03766	3.061383	-0.18374	0.854638	-5.98759	0.851706	0.803667
B.cells	PIK3IP1	0.046756	2.920799	0.183538	0.854796	-5.97428	0.853619	0.806817
B.cells	SLTM	-0.01527	6.63584	-0.18354	0.854796	-6.79162	0.804702	0.727552
B.cells	PHF12	-0.01855	6.186244	-0.18336	0.854932	-6.69005	0.810443	0.736764
B.cells	SAE1	-0.02007	6.342657	-0.18335	0.854944	-6.70183	0.80844	0.733563
B.cells	CIZ1	0.026625	3.940619	0.183282	0.854997	-6.26776	0.839852	0.784293
B.cells	DLG3	0.085932	0.571754	0.183247	0.855024	-5.39911	0.88634	0.861313
B.cells	MTPAP	-0.02723	4.284578	-0.18321	0.855055	-6.33663	0.835267	0.776826
B.cells	MOB2	-0.01941	5.416589	-0.18319	0.855069	-6.54648	0.820384	0.752732
B.cells	TNF	-0.07068	4.405834	-0.18305	0.85518	-6.14138	0.833658	0.774257
B.cells	PSMD12	-0.01884	5.784758	-0.18299	0.855229	-6.61157	0.815611	0.745101
B.cells	MFSD11	0.031676	3.854162	0.182923	0.855277	-6.23091	0.841009	0.786245
B.cells	HNRNPA3	-0.01581	8.554364	-0.18271	0.855442	-7.06874	0.780796	0.689881
B.cells	PPM1B	0.017075	6.192574	0.18266	0.855483	-6.71881	0.81042	0.736777
B.cells	SREBF1	-0.03204	3.760907	-0.18255	0.855572	-6.13521	0.842335	0.788382
B.cells	IL12RB2	-0.05387	3.672057	-0.18233	0.855738	-5.98015	0.843567	0.790364
B.cells	CAMKK1	-0.08527	0.590902	-0.18204	0.855964	-5.40378	0.886188	0.861119
B.cells	HSPA12A	0.097418	0.150275	0.181987	0.85601	-5.32313	0.892484	0.871728
B.cells	MED19	-0.02722	3.778351	-0.18195	0.85604	-6.14367	0.84214	0.788096
B.cells	A430018G1	-0.06297	1.329295	-0.1819	0.856077	-5.50734	0.875752	0.843659
B.cells	TMA16	-0.03507	3.964843	-0.18179	0.856163	-6.2818	0.839642	0.784027
B.cells	MYNN	-0.02317	4.303762	-0.18168	0.856249	-6.32622	0.835126	0.776666
B.cells	TGFB1	0.01487	8.800362	0.181533	0.856364	-7.14925	0.777835	0.685305
B.cells	HDHD5	-0.04172	2.842941	-0.18152	0.856375	-5.99522	0.854798	0.808907
B.cells	SMARCAD1	-0.02005	4.984115	-0.18145	0.856433	-6.45978	0.826145	0.762153
B.cells	CNTD1	-0.08278	0.620711	-0.18141	0.856459	-5.43892	0.885764	0.860498
B.cells	SLC12A8	0.0888	0.657727	0.181387	0.856479	-5.40371	0.885238	0.859614
B.cells	CLEC4A3	-0.07676	3.297848	-0.18048	0.857186	-5.87648	0.849243	0.799005
B.cells	MRPL40	-0.02647	4.651456	-0.18025	0.857369	-6.37423	0.831245	0.769506
B.cells	NPHS1	0.096156	0.439647	0.180077	0.857504	-5.34828	0.889151	0.865129

B.cells	RAB11FIP4	0.098605	-0.78929	0.179991	0.857572	-5.22276	0.906674	0.895137
B.cells	NIF3L1	0.036393	3.000203	0.179729	0.857777	-5.98083	0.853429	0.805695
B.cells	MARF1	-0.01735	6.147381	-0.17954	0.857921	-6.68958	0.811789	0.738117
B.cells	GM41335	-0.12574	0.493808	-0.17954	0.857926	-5.28007	0.888378	0.863829
B.cells	TADA2A	-0.04251	2.820969	-0.17937	0.858059	-5.87068	0.855875	0.809722
B.cells	ZFP202	0.099569	0.604939	0.179273	0.858134	-5.3726	0.886794	0.861168
B.cells	DNAJB4	-0.02917	3.700143	-0.17924	0.858158	-6.17973	0.843956	0.790154
B.cells	CTBP2	0.039622	3.249836	0.179212	0.858181	-6.04422	0.850036	0.800118
B.cells	GM26674	-0.10862	-1.15959	-0.17906	0.858301	-5.13643	0.911993	0.904372
B.cells	4930522L1	-0.03114	3.684756	-0.17898	0.858362	-6.19615	0.844163	0.790529
B.cells	MRPS17	0.020956	5.224333	0.178894	0.85843	-6.48918	0.823749	0.757399
B.cells	SRPK3	-0.05505	1.292575	-0.17872	0.858567	-5.7289	0.877064	0.844993
B.cells	FASTKD5	-0.07462	0.844458	-0.17869	0.85859	-5.43903	0.88339	0.855579
B.cells	SLIRP	-0.02419	5.558884	-0.17862	0.858646	-6.53495	0.819391	0.750416
B.cells	SNX11	0.044726	2.539196	0.178594	0.858665	-5.81825	0.859736	0.81621
B.cells	HIVEP2	-0.02522	8.386816	-0.17853	0.858712	-7.13438	0.783616	0.69361
B.cells	1810041H1	-0.05725	1.76748	-0.17848	0.858752	-5.78708	0.870416	0.833915
B.cells	SLFN9	0.06475	2.410472	0.178426	0.858796	-5.74448	0.861507	0.819138
B.cells	GM14286	0.09517	0.211974	0.178358	0.85885	-5.26875	0.892409	0.870746
B.cells	CD33	0.085436	2.518088	0.178094	0.859056	-5.58695	0.86014	0.816729
B.cells	GM10130	-0.05416	1.783227	-0.17795	0.859172	-5.70524	0.870311	0.833606
B.cells	ENSA	0.019165	5.388151	0.17785	0.859247	-6.55912	0.82172	0.75406
B.cells	LRRC4	0.03055	3.774431	0.177778	0.859304	-6.30315	0.843069	0.788714
B.cells	MPND	-0.02583	4.813143	-0.1775	0.85952	-6.41642	0.829254	0.766285
B.cells	KBTBD3	0.045316	2.727361	0.1775	0.859522	-5.86438	0.857269	0.812082
B.cells	SPINT1	-0.07161	-1.0651	-0.17742	0.859586	-5.38324	0.910719	0.902306
B.cells	SUPV3L1	0.029747	3.910174	0.177388	0.859609	-6.25748	0.841249	0.78581
B.cells	GM50071	-0.07704	0.514178	-0.17738	0.859616	-5.40149	0.888205	0.863627
B.cells	2610206C1	0.083109	0.40853	0.177072	0.859857	-5.40144	0.889888	0.866195
B.cells	FYTTD1	0.017266	5.267108	0.176874	0.860012	-6.52669	0.823524	0.75675
B.cells	GABPB2	-0.01562	6.502447	-0.17672	0.860135	-6.76526	0.80757	0.731224
B.cells	ZFP993	-0.05476	1.824761	-0.17663	0.860207	-5.75357	0.86997	0.8329
B.cells	AKTIP	0.033842	3.102695	0.176619	0.860212	-6.00539	0.852379	0.803816
B.cells	TRIM13	0.093641	0.46615	0.176317	0.860448	-5.34334	0.889167	0.864975
B.cells	SLC46A1	-0.10359	0.761766	-0.17626	0.860495	-5.41395	0.884956	0.857904
B.cells	BCAS3	0.018017	7.334935	0.175796	0.860856	-6.91374	0.797056	0.714569
B.cells	SLC5A11	-0.10764	-0.01556	-0.1757	0.860935	-5.24124	0.896077	0.876733
B.cells	ZFP763	0.084543	0.433803	0.175591	0.861016	-5.38208	0.889629	0.865869
B.cells	NIPA1	-0.08712	0.13903	-0.17544	0.861138	-5.3882	0.893853	0.872991
B.cells	ZFP65	0.058583	1.662117	0.175427	0.861145	-5.71973	0.872273	0.836827
B.cells	CPLX2	0.043867	3.378009	0.175411	0.861158	-6.15789	0.848677	0.797828
B.cells	RETSAT	-0.05479	1.405172	-0.17535	0.861202	-5.53802	0.875871	0.842838
B.cells	KAZN	-0.10408	0.348768	-0.17532	0.861232	-5.31057	0.890845	0.867948
B.cells	RBM18	-0.02247	4.236008	-0.17498	0.861496	-6.29043	0.837158	0.779142
B.cells	ADCY9	-0.04068	3.136828	-0.17491	0.861549	-6.06164	0.851948	0.803353
B.cells	TADA1	0.022352	4.679062	0.174911	0.861549	-6.37966	0.831281	0.7696
B.cells	ARL8B	-0.01565	5.97123	-0.17475	0.861678	-6.67205	0.814417	0.742451

B.cells	ATPAF1	0.031993	3.553171	0.174678	0.861732	-6.07144	0.84631	0.794147
B.cells	EIF4E2	0.017432	5.989442	0.17461	0.861785	-6.67152	0.814182	0.742074
B.cells	ERP29	0.013655	7.289905	0.174531	0.861847	-6.92834	0.797622	0.715689
B.cells	FBXO45	-0.0303	3.654199	-0.17452	0.861859	-6.12088	0.844949	0.791918
B.cells	ING5	-0.03066	3.456639	-0.17444	0.861919	-6.08973	0.847614	0.796304
B.cells	1700007L1	-0.06742	1.663099	-0.17435	0.861988	-5.52389	0.872259	0.83707
B.cells	TMEM267	-0.05893	1.734822	-0.17435	0.861992	-5.65383	0.871258	0.835403
B.cells	TMC8	-0.03323	3.820781	-0.17416	0.862141	-6.11052	0.842709	0.788338
B.cells	AW146154	-0.05183	2.334177	-0.17407	0.86221	-5.80058	0.862941	0.82166
B.cells	AKNA	0.020212	4.948701	0.174067	0.862211	-6.50272	0.827728	0.764002
B.cells	XPNPEP1	0.022661	4.365592	0.174037	0.862234	-6.36116	0.835434	0.776505
B.cells	ANKRD26	0.049662	2.576265	0.17403	0.86224	-5.82615	0.859609	0.816147
B.cells	SRPR	0.015587	5.794906	0.174017	0.86225	-6.61497	0.816694	0.746213
B.cells	ZFP606	-0.04007	2.487995	-0.17399	0.862274	-5.83773	0.860822	0.81816
B.cells	GM26944	0.073677	0.852005	0.1738	0.86242	-5.44472	0.883731	0.856254
B.cells	IGLV3	-0.09269	-0.70552	-0.17373	0.862473	-5.32467	0.905965	0.894088
B.cells	CPSF7	-0.0166	5.801172	-0.17341	0.862723	-6.62099	0.816774	0.746223
B.cells	D10WSU1(	0.032511	3.652731	0.173399	0.862734	-6.16339	0.845135	0.792214
B.cells	PDLIM5	-0.01867	6.703681	-0.17291	0.86312	-6.84626	0.805386	0.727758
B.cells	CD93	-0.0336	3.89899	-0.1729	0.863123	-6.2279	0.842025	0.786858
B.cells	TIMM22	-0.02304	4.4471	-0.17286	0.863155	-6.33131	0.834713	0.774948
B.cells	SMARCAL1	-0.03527	2.628016	-0.17232	0.863577	-5.90094	0.859538	0.815389
B.cells	FURIN	0.028083	5.290193	0.172227	0.863653	-6.51214	0.823867	0.757168
B.cells	GM31597	0.045242	2.208627	0.172032	0.863806	-5.76772	0.86532	0.824996
B.cells	KARS	-0.02747	4.273346	-0.1719	0.863908	-6.32221	0.837284	0.778937
B.cells	SEL1L	0.019736	5.161227	0.171726	0.864046	-6.50167	0.825555	0.75992
B.cells	SMAD3	-0.02338	6.315623	-0.17165	0.864108	-6.75534	0.810594	0.735885
B.cells	HYAL1	0.058747	1.311575	0.171206	0.864453	-5.55685	0.877839	0.845877
B.cells	PDSS2	-0.02269	5.377267	-0.17112	0.864521	-6.53621	0.82273	0.755421
B.cells	SMNDC1	-0.01445	6.059801	-0.17111	0.864529	-6.68132	0.813882	0.741202
B.cells	GLA	-0.028	4.568639	-0.17106	0.864566	-6.45314	0.833361	0.772626
B.cells	SPAG1	-0.06574	1.4147	-0.17102	0.864599	-5.60549	0.876389	0.843479
B.cells	TMC3	0.122891	-1.08655	0.170952	0.864652	-5.15512	0.91199	0.904131
B.cells	EXOC6B	0.023165	5.502152	0.170932	0.864668	-6.55974	0.821103	0.752818
B.cells	EBF1	0.031076	8.683852	0.170869	0.864717	-7.21796	0.780895	0.689018
B.cells	2310001H1	0.033144	4.065364	0.170866	0.864719	-6.25526	0.840059	0.783546
B.cells	LHFP	0.083361	1.237889	0.170797	0.864774	-5.46374	0.878877	0.847699
B.cells	TMEM222	0.026826	4.289191	0.17068	0.864865	-6.28345	0.837073	0.778745
B.cells	FNTB	0.037596	2.971515	0.170667	0.864875	-5.93107	0.854835	0.807831
B.cells	GM14325	-0.03609	2.324076	-0.17062	0.86491	-5.86524	0.863723	0.822511
B.cells	PPP2R5C	0.01132	7.456887	0.170261	0.865194	-6.91653	0.796117	0.71302
B.cells	KRTCAP2	-0.01665	7.062528	-0.17025	0.8652	-6.81595	0.801085	0.72089
B.cells	MRPS18A	-0.02077	5.09387	-0.17012	0.865302	-6.48322	0.826438	0.761522
B.cells	YBEY	-0.05651	1.366271	-0.17011	0.865313	-5.53711	0.87707	0.844737
B.cells	PDSS1	0.028362	4.315269	0.169997	0.865401	-6.34171	0.836725	0.778219
B.cells	AI504432	-0.0318	2.840905	-0.16981	0.865546	-6.11006	0.85662	0.810897
B.cells	GPR155	-0.03845	3.05644	-0.1698	0.865558	-6.07039	0.853677	0.806052

B.cells	ZRSR2	-0.01645	5.063714	-0.16976	0.865587	-6.49662	0.826834	0.762256
B.cells	ABCA7	-0.0243	3.89815	-0.16963	0.865689	-6.2351	0.842298	0.787435
B.cells	INSYN2B	-0.02555	4.140533	-0.1695	0.865792	-6.36595	0.839055	0.782158
B.cells	WDR12	-0.02663	4.283459	-0.16949	0.8658	-6.32505	0.837149	0.779053
B.cells	GM48696	-0.04312	2.476417	-0.16944	0.865838	-5.91331	0.861622	0.81923
B.cells	ZFP64	-0.02489	5.317866	-0.16941	0.865862	-6.56993	0.823506	0.756942
B.cells	VPS52	0.023298	3.923327	0.169334	0.865921	-6.18592	0.841961	0.786909
B.cells	NEK2	-0.05204	3.092142	-0.1693	0.865948	-5.93451	0.853191	0.805325
B.cells	STAR	-0.07122	1.465058	-0.16912	0.866087	-5.58448	0.875682	0.842604
B.cells	CYP2R1	0.074648	1.009263	0.169109	0.866097	-5.47285	0.882105	0.853343
B.cells	NECAB2	-0.10415	0.442881	-0.16907	0.866124	-5.2839	0.890162	0.866873
B.cells	NDUFAF7	0.024532	4.007139	0.169005	0.866179	-6.26401	0.840838	0.785081
B.cells	NAA38	0.020728	5.467647	0.168764	0.866368	-6.5503	0.821663	0.753849
B.cells	NRDE2	-0.03361	3.371854	-0.16861	0.866487	-6.14983	0.849532	0.799147
B.cells	TMEM260	0.038141	3.273577	0.168453	0.866612	-5.9753	0.850865	0.801358
B.cells	8430429KC	-0.04786	1.679457	-0.16839	0.86666	-5.66846	0.872824	0.8377
B.cells	TNKS1BP1	0.050018	2.080541	0.168209	0.866803	-5.74419	0.867238	0.828442
B.cells	ZEB2OS	-0.02541	4.678362	-0.16805	0.866931	-6.47222	0.832047	0.770672
B.cells	CELA1	0.064812	2.24977	0.168004	0.866964	-5.65572	0.864893	0.824553
B.cells	DNAJB12	0.020727	4.757611	0.167733	0.867176	-6.43987	0.831	0.769039
B.cells	LETM1	-0.02025	4.54276	-0.16773	0.867178	-6.40073	0.833842	0.773652
B.cells	ATPAF2	-0.03726	2.92038	-0.16761	0.867275	-5.93497	0.855674	0.809397
B.cells	CCL8	0.102212	-1.46778	0.167574	0.867301	-5.11309	0.91783	0.914285
B.cells	UTP3	0.019778	5.450096	0.167549	0.867321	-6.55685	0.821916	0.754367
B.cells	GM10563	-0.03054	3.607105	-0.16753	0.867334	-6.21168	0.846352	0.794078
B.cells	GAR1	-0.02673	4.430651	-0.16753	0.867337	-6.36562	0.835329	0.776083
B.cells	MCF2L	-0.08889	0.46165	-0.16725	0.867557	-5.4416	0.890071	0.866721
B.cells	ABLIM2	-0.09703	-0.47962	-0.16715	0.867633	-5.27627	0.903621	0.889665
B.cells	ETL4	-0.05284	2.396305	-0.16708	0.867685	-5.81119	0.862899	0.821367
B.cells	MPHOSPH8	0.019175	4.79616	0.166976	0.86777	-6.42842	0.83052	0.768312
B.cells	MRPL39	-0.02571	3.661118	-0.16696	0.867781	-6.1395	0.845654	0.792973
B.cells	SKA2	-0.04355	3.293834	-0.16693	0.867803	-5.89114	0.85062	0.801119
B.cells	RRAGA	-0.01994	5.020687	-0.16678	0.86792	-6.52604	0.827607	0.763532
B.cells	SNHG15	-0.0301	3.481407	-0.16668	0.868003	-6.2033	0.848125	0.796983
B.cells	SLC39A14	-0.04101	3.169335	-0.16656	0.868098	-5.98647	0.852357	0.803952
B.cells	BMP8A	-0.06037	2.321943	-0.16651	0.868139	-5.74917	0.863972	0.823128
B.cells	DCBLD2	0.061705	1.32551	0.166188	0.868388	-5.49469	0.877965	0.846269
B.cells	B9D2	0.024467	4.930502	0.16615	0.868418	-6.47487	0.828888	0.765513
B.cells	MTF1	0.023793	4.234341	0.166084	0.86847	-6.26533	0.838111	0.780493
B.cells	GM13427	-0.04881	2.062778	-0.16576	0.868722	-5.72219	0.86766	0.829102
B.cells	C3AR1	0.084287	2.290307	0.16574	0.86874	-5.72707	0.864507	0.823873
B.cells	ZFP593	0.034446	3.359126	0.165714	0.86876	-6.10494	0.849877	0.799737
B.cells	ZFAS1	0.025721	5.058651	0.165673	0.868792	-6.51199	0.827204	0.762787
B.cells	IK	0.014304	6.288849	0.165641	0.868818	-6.71705	0.811235	0.737104
B.cells	NEDD4	-0.02443	5.933587	-0.16552	0.868909	-6.58433	0.815821	0.744433
B.cells	EDEM1	-0.01866	6.015715	-0.16537	0.869031	-6.67321	0.814761	0.742756
B.cells	MAU2	0.020099	6.192162	0.165299	0.869086	-6.71792	0.812489	0.739116

B.cells	TMEM86A	0.043366	2.885047	0.165254	0.869121	-5.90762	0.856343	0.810384
B.cells	TBC1D25	-0.04059	2.839206	-0.16495	0.86936	-5.93328	0.857135	0.811527
B.cells	VILL	-0.06481	1.228935	-0.16463	0.869608	-5.55719	0.879554	0.848719
B.cells	DDIT3	-0.02979	4.008692	-0.16454	0.869683	-6.19347	0.841344	0.785576
B.cells	NUP50	-0.0179	5.304963	-0.16439	0.869803	-6.53061	0.824191	0.757753
B.cells	DCPS	0.020864	5.004121	0.164349	0.869831	-6.47266	0.828135	0.764126
B.cells	SPTB	-0.06917	1.120897	-0.16428	0.869887	-5.63018	0.881079	0.851301
B.cells	GM16083	0.066883	1.977218	0.164073	0.870048	-5.58823	0.869074	0.831279
B.cells	NELFE	-0.02289	4.393275	-0.16407	0.870054	-6.2658	0.836212	0.777236
B.cells	GM10785	0.046413	2.451276	0.163924	0.870165	-5.89193	0.862509	0.820447
B.cells	NUDT2	0.032548	3.238769	0.163849	0.870224	-5.9684	0.851731	0.802673
B.cells	SEC24A	0.021981	6.616568	0.163834	0.870236	-6.79454	0.807252	0.730639
B.cells	GTF2E1	0.044194	2.399479	0.163816	0.87025	-5.79189	0.863224	0.821634
B.cells	RAD23B	-0.01262	6.360558	-0.16378	0.870277	-6.7421	0.810526	0.735865
B.cells	MYO10	-0.02932	5.227281	-0.1633	0.870653	-6.47898	0.825496	0.759585
B.cells	ZFAND1	-0.03541	2.727292	-0.1632	0.870736	-5.86377	0.859023	0.814332
B.cells	RNF185	-0.01946	5.117108	-0.16297	0.870911	-6.48471	0.827029	0.762019
B.cells	ESPL1	-0.04619	2.563574	-0.16291	0.870965	-5.8112	0.861356	0.818169
B.cells	TRIP12	-0.01276	7.435654	-0.16265	0.871167	-6.92966	0.797315	0.714458
B.cells	4933407K1	-0.05451	1.39879	-0.16259	0.87121	-5.53378	0.877638	0.845174
B.cells	DNAAF2	-0.03477	2.845924	-0.16236	0.871392	-5.93183	0.857553	0.811875
B.cells	ZFP747	-0.04953	1.605909	-0.16233	0.871414	-5.67906	0.87473	0.840358
B.cells	SMUG1	-0.04008	1.976529	-0.16229	0.87145	-5.78105	0.869555	0.831745
B.cells	HBEGF	0.076495	2.789221	0.162212	0.871509	-5.68594	0.858329	0.813156
B.cells	USP18	-0.05997	2.737474	-0.16212	0.871581	-5.82001	0.859039	0.814341
B.cells	AKAP1	-0.05581	1.799595	-0.16205	0.871633	-5.58707	0.872021	0.835875
B.cells	HMGCR	-0.02387	4.712579	-0.1619	0.871751	-6.35322	0.832429	0.770811
B.cells	MGAT4B	0.029739	3.847759	0.161886	0.871765	-6.15527	0.84396	0.789593
B.cells	POMP	-0.01528	7.268227	-0.16164	0.871958	-6.8852	0.799466	0.717898
B.cells	ZFP971	0.038909	1.911606	0.161521	0.872052	-5.84413	0.870506	0.833321
B.cells	PSMA3	-0.01178	7.755055	-0.16137	0.872173	-6.97033	0.793355	0.708248
B.cells	RRAS2	-0.02323	5.198697	-0.16101	0.872453	-6.50834	0.826074	0.76056
B.cells	AAR2	0.020844	4.021304	0.160962	0.872491	-6.27304	0.841677	0.785915
B.cells	XRCC4	-0.01962	5.185209	-0.16083	0.872594	-6.51483	0.82625	0.760859
B.cells	DPM3	0.016207	6.309203	0.160704	0.872693	-6.68542	0.811668	0.737418
B.cells	ABHD5	-0.03535	3.453436	-0.16055	0.872811	-5.98404	0.849326	0.79846
B.cells	NOB1	-0.02057	4.287446	-0.16048	0.872872	-6.36537	0.83812	0.780144
B.cells	CINP	0.042379	3.163035	0.160473	0.872874	-5.94904	0.853269	0.804943
B.cells	LMTK2	-0.01819	5.616268	-0.1604	0.87293	-6.59586	0.820622	0.751811
B.cells	FANCD2	-0.04511	2.80256	-0.16035	0.87297	-5.79369	0.858193	0.813056
B.cells	PSMA6	0.016424	6.941715	0.16007	0.873191	-6.80461	0.803596	0.724607
B.cells	CIAO1	-0.02578	3.481337	-0.16005	0.873207	-6.09831	0.848948	0.79788
B.cells	ZFP212	0.023495	3.640876	0.159804	0.8734	-6.18602	0.846792	0.794416
B.cells	DAZAP2	0.013035	7.70346	0.159739	0.873451	-6.98536	0.794	0.709469
B.cells	2410006H1	0.023004	7.535004	0.159635	0.873532	-6.96152	0.79611	0.712803
B.cells	SPEF1	0.087781	0.343281	0.159623	0.873542	-5.2882	0.892678	0.870691
B.cells	SEC63	0.012909	7.09953	0.159622	0.873543	-6.8597	0.801597	0.721496

B.cells	ARHGEF12	0.035478	3.963016	0.159621	0.873544	-6.13517	0.842458	0.787328
B.cells	CWC15	-0.01386	6.384372	-0.1596	0.873561	-6.72821	0.810704	0.736004
B.cells	PIK3R2	0.040209	2.306399	0.15941	0.87371	-5.90775	0.865025	0.824471
B.cells	COLEC10	0.075528	0.557157	0.159409	0.87371	-5.38809	0.889616	0.865541
B.cells	GM8066	0.069216	0.498967	0.15934	0.873765	-5.43552	0.890448	0.866941
B.cells	ITGB1BP1	-0.01937	4.649919	-0.15933	0.873775	-6.41853	0.833304	0.772421
B.cells	GM28375	-0.02479	3.206287	-0.15931	0.873788	-6.1452	0.85268	0.804089
B.cells	ZFP846	0.041312	2.431015	0.159218	0.87386	-5.82166	0.863303	0.821655
B.cells	SPATA7	-0.05708	1.309288	-0.15913	0.873926	-5.55483	0.878945	0.847688
B.cells	LOXL3	-0.09317	0.451305	-0.15896	0.874063	-5.35422	0.89113	0.868221
B.cells	TCHP	-0.05166	1.792962	-0.15892	0.874094	-5.62998	0.872161	0.836462
B.cells	TCN2	-0.02991	4.791081	-0.15888	0.874123	-6.366	0.831437	0.769517
B.cells	RFX3	0.016656	6.233922	0.158869	0.874135	-6.72848	0.812635	0.739219
B.cells	RNPEPL1	-0.02321	4.75379	-0.15868	0.874282	-6.43952	0.832	0.770319
B.cells	MCCC1	-0.04203	2.50652	-0.15846	0.874456	-5.87989	0.862369	0.820044
B.cells	COX5A	0.015955	8.546847	0.158452	0.874462	-7.05811	0.783633	0.693142
B.cells	NME6	0.04738	2.576439	0.158354	0.874539	-5.73951	0.86141	0.818451
B.cells	TLR13	0.069215	1.881588	0.158186	0.874671	-5.57209	0.871036	0.834439
B.cells	NLRP12	-0.08607	-0.12085	-0.15816	0.874695	-5.34689	0.899479	0.882167
B.cells	ZMAT1	0.054949	2.090961	0.158078	0.874756	-5.62777	0.868123	0.829614
B.cells	CSTB	0.021144	6.672627	0.157672	0.875075	-6.78611	0.80721	0.73035
B.cells	PRDM9	0.058452	1.146615	0.157625	0.875112	-5.58217	0.881451	0.851746
B.cells	CISD1	-0.02534	5.119405	-0.15757	0.875157	-6.49944	0.827311	0.762626
B.cells	RALB	0.020413	4.247517	0.157534	0.875183	-6.31498	0.838852	0.781362
B.cells	SLC9A7	0.019515	5.115655	0.15713	0.875501	-6.64866	0.82736	0.762705
B.cells	TXNRD3	-0.04928	1.950944	-0.1571	0.875523	-5.6618	0.870165	0.832916
B.cells	CWC25	-0.02307	5.255579	-0.15707	0.87555	-6.5211	0.825525	0.75974
B.cells	2500004C(	-0.06163	1.043374	-0.15704	0.875572	-5.45941	0.882911	0.854193
B.cells	GM10642	0.085326	0.270003	0.156985	0.875614	-5.33929	0.893943	0.872747
B.cells	CDKN2C	-0.04106	3.918847	-0.15666	0.875871	-6.0934	0.843252	0.788599
B.cells	A130014A(	0.04841	1.887317	0.156508	0.875989	-5.69181	0.871052	0.834473
B.cells	POLR2F	0.016561	5.381223	0.156507	0.87599	-6.5367	0.823882	0.757161
B.cells	ZFP982	-0.09515	0.056297	-0.15635	0.876111	-5.3201	0.897018	0.878046
B.cells	PPNR	-0.06571	1.051959	-0.15613	0.876284	-5.54749	0.88279	0.854102
B.cells	GPCPD1	-0.01424	7.329375	-0.1561	0.876307	-6.95828	0.798886	0.717214
B.cells	TAF6L	0.03645	2.800016	0.156082	0.876324	-5.87094	0.858432	0.813586
B.cells	ZFP263	-0.02201	5.167548	-0.15594	0.876434	-6.55995	0.826679	0.761756
B.cells	M6PR	0.015998	6.293265	0.155831	0.876522	-6.72983	0.812066	0.738264
B.cells	FAM214A	-0.02722	5.246692	-0.15581	0.876538	-6.4443	0.825641	0.760092
B.cells	EPB41L1	0.069708	1.011227	0.155698	0.876626	-5.5671	0.883367	0.855141
B.cells	DHRS7	0.039893	4.016547	0.15564	0.876672	-6.09129	0.841941	0.786572
B.cells	TSTA3	0.024055	4.100613	0.155505	0.876777	-6.27127	0.840815	0.784746
B.cells	FAAH	-0.0476	1.451163	-0.15533	0.876917	-5.75818	0.877158	0.844784
B.cells	MEF2A	-0.0147	7.727346	-0.1553	0.876935	-6.94786	0.793891	0.709401
B.cells	SPG11	0.024041	4.094018	0.155291	0.876946	-6.31939	0.840904	0.784908
B.cells	API5	-0.01459	6.03062	-0.15527	0.876959	-6.67983	0.815448	0.743712
B.cells	PRPF4B	0.015141	6.312389	0.154834	0.877305	-6.72443	0.81182	0.737984

B.cells	TRMT12	0.055969	1.30746	0.154706	0.877406	-5.54667	0.87918	0.848291
B.cells	NOL11	-0.0231	4.89374	-0.1546	0.877486	-6.45105	0.83028	0.767793
B.cells	ATG3	-0.01348	6.261249	-0.15434	0.877694	-6.70093	0.812477	0.739176
B.cells	PCGF6	0.032181	2.512206	0.154068	0.877907	-5.90686	0.862389	0.820515
B.cells	ZFP692	0.040908	2.030033	0.153962	0.877991	-5.70624	0.869064	0.831591
B.cells	HK2	-0.02777	5.335441	-0.15392	0.87802	-6.53298	0.82448	0.758521
B.cells	GM43126	-0.07098	0.276018	-0.15389	0.878043	-5.46122	0.893856	0.873131
B.cells	CAMTA1	0.015537	5.835211	0.153815	0.878106	-6.62871	0.817975	0.748055
B.cells	DUSP23	-0.04359	2.373334	-0.15378	0.87813	-5.71624	0.864305	0.823716
B.cells	PHLPP1	-0.0204	7.671226	-0.15376	0.87815	-7.03738	0.794593	0.710782
B.cells	AGAP3	-0.02601	4.127556	-0.15372	0.878182	-6.27778	0.840455	0.784476
B.cells	TMCC3	0.03092	5.475701	0.153699	0.878198	-6.57388	0.822648	0.75558
B.cells	GALNT7	-0.01813	6.060844	-0.15368	0.878209	-6.73063	0.815058	0.743371
B.cells	SLC25A32	0.028205	3.120627	0.15356	0.878306	-6.07039	0.85405	0.806796
B.cells	CNN2	-0.01974	6.538205	-0.15348	0.878371	-6.83572	0.808927	0.733607
B.cells	B3GALT2	-0.06922	0.771125	-0.15347	0.878374	-5.55377	0.886777	0.861286
B.cells	9330151L1	0.053808	0.96571	0.153327	0.87849	-5.57414	0.884012	0.856675
B.cells	GM2A	0.015861	7.22888	0.153096	0.878671	-6.97151	0.800153	0.719672
B.cells	SFN	0.047173	2.056108	0.15295	0.878786	-5.84317	0.868702	0.831149
B.cells	GM28981	0.068443	0.488246	0.152947	0.878789	-5.44853	0.890814	0.868159
B.cells	CYP2C29	0.078154	1.011219	0.152941	0.878793	-5.52341	0.883367	0.85564
B.cells	SS18L1	0.044243	2.325233	0.152871	0.878848	-5.85657	0.86497	0.824957
B.cells	GFPT1	0.022022	5.095805	0.152853	0.878863	-6.58771	0.827621	0.763742
B.cells	ZFP667	-0.03361	2.726732	-0.15271	0.878973	-6.06861	0.859438	0.815825
B.cells	CD300E	-0.11308	0.628868	-0.15268	0.879002	-5.44601	0.888804	0.864811
B.cells	NDUFAF6	-0.03292	2.973661	-0.15265	0.879023	-5.93745	0.856056	0.81024
B.cells	EYA1	0.052632	2.426875	0.152638	0.879032	-5.81253	0.863566	0.822657
B.cells	LRRC20	-0.04645	2.057401	-0.15263	0.879037	-5.71345	0.868684	0.831153
B.cells	MMP13	-0.13663	-1.01302	-0.15263	0.879037	-5.16927	0.912265	0.905118
B.cells	GM1604B	-0.06889	1.489369	-0.1524	0.879215	-5.56061	0.876621	0.844489
B.cells	MCUR1	0.021166	4.462065	0.152374	0.879239	-6.38891	0.835995	0.777475
B.cells	WSB1	-0.01481	6.461454	-0.15226	0.879327	-6.75344	0.809909	0.735397
B.cells	TWISTNB	-0.01585	5.520145	-0.15214	0.879419	-6.59492	0.822069	0.754936
B.cells	RNF215	-0.02906	2.627986	-0.15213	0.879432	-5.95689	0.860795	0.818217
B.cells	FBXO17	-0.08236	0.782245	-0.1521	0.879453	-5.30585	0.886619	0.861292
B.cells	LGMN	-0.02059	7.188159	-0.1519	0.879612	-6.93593	0.800667	0.720732
B.cells	GIT1	-0.02546	3.282244	-0.1519	0.879614	-6.00808	0.851851	0.803517
B.cells	SLC43A3	-0.03408	3.813488	-0.15183	0.879666	-6.04421	0.844668	0.791725
B.cells	FBXL19	0.043482	2.333257	0.151769	0.879715	-5.78989	0.864859	0.825011
B.cells	COL6A1	0.081619	0.367808	0.151573	0.879869	-5.33553	0.892539	0.871368
B.cells	ATMIN	0.033695	2.969649	0.151504	0.879923	-6.04001	0.85611	0.810576
B.cells	CENPQ	-0.02068	5.314475	-0.15148	0.879938	-6.59698	0.824754	0.759364
B.cells	BRD4	-0.00876	8.256086	-0.15146	0.879954	-7.06903	0.787312	0.69966
B.cells	FLI1	0.014908	8.191063	0.151387	0.880015	-7.01304	0.788117	0.700936
B.cells	KBTBD8	-0.07489	0.251382	-0.15127	0.880107	-5.32919	0.89421	0.874249
B.cells	CYP3A13	-0.07284	0.779768	-0.15119	0.880168	-5.44599	0.886654	0.861516
B.cells	BRF1	0.016685	4.828649	0.151062	0.880271	-6.46791	0.831139	0.769792

B.cells	AW209491	-0.06998	1.448036	-0.15102	0.880308	-5.49444	0.877202	0.845722
B.cells	LRRC42	-0.02583	3.272197	-0.15094	0.880369	-6.11803	0.851987	0.803889
B.cells	RACK1	0.010189	8.637453	0.15056	0.880666	-7.105	0.78278	0.692448
B.cells	PRMT5	-0.02828	3.483009	-0.1505	0.880709	-6.09186	0.849316	0.79928
B.cells	SGTA	0.019684	4.959007	0.150388	0.880801	-6.45519	0.829621	0.767118
B.cells	KCTD12B	0.073643	0.963206	0.149764	0.881292	-5.43063	0.884269	0.857474
B.cells	ATP11C	-0.01305	6.869185	-0.14962	0.881405	-6.811	0.80491	0.727585
B.cells	MS4A7	0.067426	1.921581	0.149609	0.881414	-5.79049	0.870792	0.834996
B.cells	GM44649	0.024433	3.949723	0.149466	0.881527	-6.34906	0.843048	0.789198
B.cells	RIMS4	-0.10252	-0.99611	-0.14942	0.881563	-5.18573	0.91225	0.905484
B.cells	STK40	0.016893	5.69472	0.149252	0.881695	-6.6441	0.820003	0.75184
B.cells	MORRBID	0.049153	5.127394	0.149212	0.881726	-6.12149	0.827413	0.763816
B.cells	GM33677	-0.11265	-1.06476	-0.1492	0.881738	-5.16813	0.913237	0.907273
B.cells	CSTDC4	0.080093	3.661016	0.149104	0.881811	-5.9938	0.846934	0.795677
B.cells	BSDC1	0.021477	5.056629	0.149005	0.881889	-6.52988	0.828343	0.765359
B.cells	UGP2	0.019231	5.910415	0.148905	0.881967	-6.65	0.817206	0.747382
B.cells	OSBPL5	0.074551	0.3884	0.148709	0.882122	-5.40616	0.892467	0.871522
B.cells	SMYD5	0.049016	2.056268	0.148562	0.882237	-5.76253	0.868917	0.832096
B.cells	KLHL4	-0.09538	0.018315	-0.14848	0.882303	-5.25581	0.897791	0.880607
B.cells	UBTD2	0.028511	3.37685	0.14832	0.882428	-6.17466	0.850779	0.802142
B.cells	SAP30BP	0.017407	4.838744	0.148313	0.882433	-6.46604	0.831213	0.770158
B.cells	CDC123	-0.01795	5.404958	-0.1483	0.882445	-6.54013	0.823778	0.758113
B.cells	SAR1B	0.013879	5.553451	0.148281	0.882459	-6.60507	0.82184	0.754989
B.cells	ZFP955A	-0.05604	1.472841	-0.14814	0.882566	-5.61913	0.877072	0.845792
B.cells	KCTD10	0.023245	4.043279	0.148064	0.88263	-6.23933	0.841794	0.78747
B.cells	RBM12B1	0.070815	0.935508	0.147988	0.882689	-5.42844	0.884662	0.85858
B.cells	GM20682	0.042086	1.711443	0.147921	0.882742	-5.70108	0.873726	0.840266
B.cells	TSEN15	0.03531	2.776415	0.147819	0.882822	-5.85375	0.858971	0.815771
B.cells	SHANK3	-0.078	1.430051	-0.14772	0.882899	-5.46942	0.877674	0.846889
B.cells	GM38560	-0.07601	0.991541	-0.14768	0.882928	-5.41518	0.883867	0.857271
B.cells	AGBL5	-0.03653	2.244561	-0.14763	0.882973	-5.78495	0.866303	0.827928
B.cells	ZFP646	0.026666	3.903418	0.147541	0.883041	-6.23051	0.84367	0.790586
B.cells	TICAM1	-0.03064	2.819811	-0.14754	0.883044	-6.05854	0.858376	0.814787
B.cells	WDR82	-0.01512	4.82833	-0.14753	0.883048	-6.4474	0.831351	0.770491
B.cells	CDPF1	0.046147	2.220471	0.14747	0.883097	-5.78438	0.866637	0.828483
B.cells	ZPBP	0.050512	1.648971	0.147448	0.883114	-5.61448	0.874601	0.841752
B.cells	MAF	-0.03359	5.654779	-0.14717	0.883336	-6.47125	0.820522	0.753
B.cells	WTIP	-0.05763	1.051984	-0.14708	0.883403	-5.49756	0.88301	0.855872
B.cells	MED17	0.014598	4.867425	0.147056	0.883422	-6.45212	0.830835	0.769688
B.cells	RB1	-0.01489	7.240705	-0.14682	0.883612	-6.907	0.800204	0.720497
B.cells	CAP1	0.012972	6.878742	0.146802	0.883623	-6.8042	0.804788	0.727793
B.cells	PRKAB1	0.030305	3.654114	0.146783	0.883637	-6.06734	0.847027	0.796148
B.cells	SDR42E1	-0.0664	0.45745	-0.14676	0.883653	-5.35538	0.891477	0.870146
B.cells	CPD	-0.02656	4.333844	-0.14673	0.883677	-6.25408	0.837911	0.781228
B.cells	NDUFA9	-0.01824	4.840567	-0.14664	0.883751	-6.43213	0.831189	0.770285
B.cells	BHLHE40	-0.02448	6.323106	-0.1466	0.883785	-6.73958	0.811886	0.739139
B.cells	KMO	0.030723	3.038738	0.146381	0.883954	-6.12973	0.855473	0.809899

B.cells	GM47889	-0.05115	1.560566	-0.14594	0.884305	-5.70647	0.876086	0.843985
B.cells	PKP4	-0.02702	5.958306	-0.14581	0.884401	-6.48917	0.816816	0.746796
B.cells	ZFP949	0.036943	2.594224	0.145713	0.88448	-5.85943	0.861716	0.820099
B.cells	CCDC173	-0.07113	0.767334	-0.14565	0.884527	-5.35803	0.887302	0.862819
B.cells	FMR1	0.015494	5.971489	0.145585	0.88458	-6.63774	0.816645	0.746522
B.cells	BRD2	-0.01273	7.085393	-0.14556	0.884598	-6.86072	0.802392	0.723727
B.cells	IL15	0.041348	3.93993	0.145542	0.884614	-6.18594	0.843417	0.789959
B.cells	RGS9	-0.07876	0.03543	-0.14534	0.884772	-5.3803	0.897882	0.880623
B.cells	SF3B2	0.011411	7.285583	0.14515	0.884923	-6.88125	0.800009	0.719841
B.cells	AP1G2	0.03751	2.809774	0.144976	0.885059	-5.99887	0.858975	0.81537
B.cells	1810059H2	0.058994	1.799204	0.144799	0.885199	-5.75365	0.873035	0.838622
B.cells	CHKB	-0.01867	4.306938	-0.1445	0.885437	-6.33959	0.838795	0.782151
B.cells	TM9SF1	0.018932	5.042685	0.144488	0.885444	-6.46208	0.829045	0.766295
B.cells	H6PD	-0.03293	2.776773	-0.14418	0.885689	-5.8879	0.859504	0.816262
B.cells	PILRB2	-0.05507	2.237136	-0.14411	0.885741	-5.69052	0.866949	0.828608
B.cells	PPME1	-0.01795	4.862972	-0.14405	0.88579	-6.4533	0.831414	0.770231
B.cells	ACBD6	-0.01513	5.436733	-0.14386	0.885941	-6.52896	0.823879	0.758055
B.cells	SPDYA	0.077403	-0.02911	0.143756	0.88602	-5.31356	0.899039	0.882525
B.cells	BMI1	0.027768	3.545427	0.143749	0.886026	-6.06866	0.849027	0.799055
B.cells	ADGRG5	0.082004	0.105614	0.143661	0.886095	-5.37187	0.897094	0.879237
B.cells	GM17178	-0.04808	1.460682	-0.14362	0.886128	-5.59266	0.877793	0.84678
B.cells	UBE2L3	-0.01063	7.560477	-0.14361	0.886133	-6.94133	0.796679	0.714576
B.cells	GM14455	0.049348	1.315653	0.14347	0.886246	-5.58299	0.879836	0.850237
B.cells	SSBP1	-0.01529	5.592236	-0.14344	0.886269	-6.59882	0.82185	0.754859
B.cells	CHID1	0.033623	2.453126	0.143339	0.886349	-5.77763	0.86396	0.823774
B.cells	ZFP141	0.031414	3.367308	0.14331	0.886371	-6.0643	0.851442	0.803088
B.cells	ADCK5	-0.0492	1.510315	-0.14331	0.886374	-5.6192	0.877095	0.845653
B.cells	SLC5A6	-0.06706	0.868812	-0.14271	0.886842	-5.42246	0.886545	0.860966
B.cells	5730480H0	0.030132	2.846707	0.142524	0.88699	-5.94111	0.858913	0.81497
B.cells	SIGIRR	-0.03296	2.175284	-0.14252	0.886996	-5.83257	0.86818	0.830332
B.cells	ZFP707	0.041373	1.958642	0.142411	0.88708	-5.7217	0.871195	0.835394
B.cells	PRICKLE3	0.042098	2.034653	0.142343	0.887133	-5.72316	0.870136	0.833655
B.cells	HIST4H4	0.057065	1.887782	0.14227	0.887191	-5.59983	0.872183	0.837074
B.cells	ATXN7L3B	-0.01354	5.941589	-0.14158	0.887735	-6.63814	0.817709	0.747858
B.cells	SMARCA5	0.011106	7.448521	0.141573	0.887739	-6.92325	0.798471	0.717134
B.cells	CRTC1	0.033684	3.126748	0.14149	0.887805	-5.975	0.855128	0.808812
B.cells	CCR7	-0.02761	4.535564	-0.14114	0.888078	-6.64554	0.836154	0.777757
B.cells	WDR47	0.029122	3.140773	0.141138	0.888082	-6.0256	0.854937	0.808542
B.cells	YTHDF1	-0.01459	5.78074	-0.14094	0.888241	-6.64171	0.819795	0.751266
B.cells	TIGIT	0.074498	-0.09862	0.140926	0.888249	-5.36662	0.900479	0.884718
B.cells	ARL3	-0.02198	4.04145	-0.14082	0.888331	-6.27138	0.842752	0.788568
B.cells	EXOSC2	0.027605	2.945711	0.140773	0.88837	-6.005	0.857603	0.812984
B.cells	CDK5RAP2	0.020783	4.446752	0.140753	0.888385	-6.27047	0.837336	0.779722
B.cells	MAP3K12	0.029911	2.718206	0.140547	0.888548	-5.95027	0.860724	0.818211
B.cells	PHF2	-0.01536	5.076145	-0.14048	0.888604	-6.46213	0.829005	0.766259
B.cells	CSPRS	-0.05902	0.292172	-0.14042	0.888651	-5.42378	0.89484	0.875311
B.cells	PA2G4	-0.01813	6.688076	-0.14021	0.88881	-6.76576	0.808111	0.73265

B.cells	HNRNPU	0.011899	8.26781	0.140203	0.888819	-7.0215	0.788237	0.701133
B.cells	CCSAP	0.038987	2.176115	0.140166	0.888848	-5.75106	0.868215	0.830681
B.cells	METTL25	-0.01715	4.28478	-0.14016	0.888852	-6.36131	0.839496	0.783347
B.cells	GM27201	0.031383	2.321008	0.140136	0.888872	-5.85484	0.866205	0.827341
B.cells	STK25	-0.01817	4.440838	-0.14012	0.888883	-6.35433	0.837415	0.779951
B.cells	B4GAT1	0.051263	1.65015	0.140118	0.888886	-5.57686	0.875555	0.842917
B.cells	CD53	-0.01155	7.920856	-0.1401	0.888903	-6.99234	0.792552	0.707936
B.cells	RAB14	0.009805	7.091867	0.140057	0.888934	-6.85061	0.802975	0.724461
B.cells	TYK2	-0.01994	3.992109	-0.14	0.888977	-6.27786	0.843415	0.789762
B.cells	GPX1	-0.01744	11.17498	-0.13999	0.888983	-7.5566	0.753163	0.646692
B.cells	LRRC8C	-0.01805	6.412612	-0.13962	0.88928	-6.77256	0.811638	0.738325
B.cells	POLI	-0.05065	1.527716	-0.13956	0.889325	-5.54115	0.877275	0.845836
B.cells	MAGT1	-0.01513	5.872067	-0.13954	0.889344	-6.63311	0.81861	0.749534
B.cells	GEN1	-0.0523	2.372171	-0.13934	0.889501	-5.726	0.865498	0.826268
B.cells	PIFO	0.050921	1.246923	0.139324	0.889511	-5.7576	0.881232	0.852524
B.cells	PNLDC1	0.065769	0.924032	0.139148	0.889649	-5.44407	0.885808	0.860242
B.cells	RELL1	0.015331	6.866963	0.139124	0.889669	-6.76034	0.805832	0.729133
B.cells	STAG2	-0.01409	7.464582	-0.13911	0.889678	-6.89086	0.79827	0.717103
B.cells	PCNX	-0.01656	5.518161	-0.1391	0.889687	-6.64614	0.823214	0.757033
B.cells	MSANTD4	-0.02958	2.89703	-0.13865	0.89004	-5.87015	0.85827	0.814439
B.cells	TRAF7	-0.01663	4.585124	-0.13865	0.890045	-6.3951	0.835497	0.777062
B.cells	CHST15	-0.02646	3.54275	-0.13858	0.890098	-6.09971	0.849474	0.799961
B.cells	KCNK5	0.044801	1.47886	0.138439	0.890208	-5.74237	0.877962	0.847247
B.cells	E130215H2	-0.07308	-0.13171	-0.13843	0.890212	-5.33476	0.900959	0.886003
B.cells	POR	0.018285	5.6252	0.138362	0.890269	-6.59968	0.821818	0.75493
B.cells	IL7R	0.030917	4.966863	0.138263	0.890347	-6.44931	0.830445	0.768921
B.cells	NKIRAS2	0.019668	4.042562	0.138217	0.890383	-6.28893	0.842738	0.78897
B.cells	NRG1	-0.05767	2.541088	-0.13813	0.890454	-5.93288	0.863164	0.82264
B.cells	RAB11FIP5	-0.06729	0.808074	-0.13809	0.890482	-5.44676	0.887457	0.863237
B.cells	ANKRD12	-0.01444	7.518738	-0.13809	0.890482	-6.92425	0.797589	0.716206
B.cells	PCMT1	-0.01288	6.63523	-0.13802	0.890541	-6.74934	0.808787	0.734039
B.cells	GGNBP2	0.011207	7.268609	0.137843	0.890678	-6.88151	0.80074	0.72121
B.cells	TRUB2	-0.0187	4.526877	-0.13778	0.890726	-6.39522	0.836271	0.778414
B.cells	PLEKHO2	-0.01495	6.069061	-0.13774	0.890756	-6.73456	0.816061	0.745701
B.cells	DDHD1	0.020987	6.276227	0.137686	0.890802	-6.73223	0.813391	0.741412
B.cells	MCPT8	0.179557	-0.5048	0.137501	0.890947	-5.21094	0.90636	0.89528
B.cells	CLEC12A	0.032494	4.937614	0.137053	0.8913	-6.27967	0.830847	0.769734
B.cells	THUMPD1	0.018198	4.425949	0.136992	0.891349	-6.37207	0.83763	0.780776
B.cells	HAGHL	0.035447	2.911144	0.136972	0.891364	-5.92969	0.858093	0.8144
B.cells	ZGPAT	0.019151	3.668613	0.136941	0.891389	-6.20324	0.847789	0.797421
B.cells	CDK17	-0.01447	6.780519	-0.13691	0.891412	-6.79842	0.806948	0.731252
B.cells	DDX18	-0.01661	4.987355	-0.13685	0.891457	-6.46922	0.830191	0.7687
B.cells	SLC25A36	0.01389	6.285868	0.136846	0.891464	-6.73427	0.813282	0.741406
B.cells	2310061I04	0.022392	3.521012	0.13674	0.891547	-6.14199	0.849785	0.80073
B.cells	ATF1	-0.01054	6.564036	-0.13668	0.891591	-6.76219	0.809713	0.735693
B.cells	ATG4C	0.026184	3.11799	0.136589	0.891667	-6.09857	0.855265	0.809762
B.cells	B020010K1	-0.06341	1.134374	-0.13648	0.891749	-5.50087	0.882841	0.85568

B.cells	DMAP1	0.033392	2.496634	0.136367	0.891841	-5.81648	0.863794	0.823881
B.cells	ZFP775	-0.05765	0.81684	-0.13629	0.891906	-5.47464	0.88735	0.863261
B.cells	CHIC2	0.01005	7.043743	0.135986	0.892141	-6.85333	0.803602	0.726014
B.cells	DNAH8	-0.02771	3.758702	-0.13594	0.892179	-6.21217	0.846573	0.795564
B.cells	ARHGDIA	0.013656	7.188393	0.135796	0.892291	-6.87636	0.801769	0.723159
B.cells	ROCK1	-0.01123	8.134703	-0.13558	0.892464	-7.00908	0.789905	0.704372
B.cells	RPGR	0.044845	1.599467	0.13556	0.892478	-5.60558	0.876284	0.84487
B.cells	VPS53	-0.01468	4.462382	-0.13544	0.892572	-6.37824	0.837145	0.7802
B.cells	ESCO2	-0.03918	3.734708	-0.13517	0.892785	-6.08584	0.846896	0.796163
B.cells	ABCD4	0.029223	2.782602	0.135124	0.892821	-5.88738	0.859857	0.817537
B.cells	RIPOR1	0.023163	3.960811	0.135114	0.892829	-6.17086	0.843852	0.791168
B.cells	CCNF	-0.03718	3.438612	-0.13511	0.892829	-6.05912	0.850902	0.80275
B.cells	MGAT4A	0.018226	5.666978	0.135106	0.892835	-6.55009	0.82129	0.754469
B.cells	MTHFSL	0.01638	5.211119	0.135073	0.892861	-6.5348	0.827248	0.764105
B.cells	PPM1N	0.077788	0.159456	0.13507	0.892863	-5.37284	0.896768	0.87936
B.cells	ARHGEF2	0.014372	5.586467	0.135063	0.892869	-6.59659	0.822339	0.756162
B.cells	ZMIZ1	0.012188	6.978265	0.134989	0.892927	-6.85312	0.804433	0.727442
B.cells	INTS9	-0.01866	4.394064	-0.13482	0.893059	-6.34559	0.838096	0.781725
B.cells	TARDBP	-0.0143	5.771119	-0.13456	0.893266	-6.59727	0.819976	0.752428
B.cells	E2F7	-0.04575	2.983824	-0.13448	0.893327	-5.83795	0.85714	0.813142
B.cells	EFCAB8	0.04788	1.374064	0.134285	0.893483	-5.58799	0.879498	0.850387
B.cells	TDRD3	0.021339	4.059195	0.134266	0.893497	-6.2414	0.842573	0.789188
B.cells	SKP2	0.031767	3.310393	0.134242	0.893516	-5.94143	0.852686	0.805806
B.cells	MAP11	0.032005	2.866952	0.1342	0.89355	-5.93345	0.858741	0.81581
B.cells	TRAIP	0.045333	2.119414	0.134181	0.893564	-5.68551	0.869063	0.832949
B.cells	KALRN	0.045921	2.750236	0.133898	0.893787	-5.81795	0.860343	0.818577
B.cells	BAMBI	-0.02625	3.967331	-0.13371	0.893932	-6.33167	0.843806	0.791366
B.cells	RGS1	0.040336	5.563816	0.13365	0.893983	-6.31891	0.822674	0.756968
B.cells	MMACHC	-0.07277	0.470101	-0.13334	0.894228	-5.345	0.892347	0.872315
B.cells	GPATCH3	-0.02466	3.369567	-0.13329	0.894268	-6.18747	0.851881	0.804764
B.cells	GM16152	-0.03879	2.101073	-0.13319	0.894344	-5.89239	0.869318	0.833688
B.cells	GSG1L	-0.08909	-0.16326	-0.13304	0.894462	-5.24488	0.901478	0.887812
B.cells	THOC2	0.009794	7.106814	0.13302	0.89448	-6.87668	0.802842	0.725266
B.cells	HIST1H2AN	-0.05784	3.254826	-0.13299	0.894507	-6.01452	0.853442	0.807357
B.cells	PHACTR4	0.012845	5.677431	0.132956	0.89453	-6.65037	0.821195	0.754709
B.cells	HADHA	0.013471	5.770414	0.132953	0.894532	-6.62711	0.819986	0.752758
B.cells	OAS1C	0.045382	1.903745	0.132683	0.894746	-5.74887	0.872068	0.838346
B.cells	MED6	0.018663	4.540282	0.132672	0.894755	-6.43054	0.83615	0.779049
B.cells	C530005A1	0.052037	0.742343	0.132447	0.894932	-5.43849	0.888455	0.86591
B.cells	TRP53RKB	0.035096	2.444883	0.1322	0.895127	-5.77519	0.864552	0.825926
B.cells	MBD6	0.023236	3.632499	0.132164	0.895155	-6.13108	0.848318	0.799092
B.cells	EMC9	-0.04316	1.600787	-0.13205	0.895247	-5.62965	0.876309	0.845573
B.cells	FAM171B	0.089482	0.217464	0.132046	0.895248	-5.27741	0.895977	0.878697
B.cells	CCDC107	-0.01824	4.620705	-0.13189	0.895368	-6.36178	0.835081	0.777437
B.cells	GM35769	0.051218	1.096204	0.131776	0.895461	-5.56439	0.883425	0.857517
B.cells	DDIT4	-0.03977	3.744278	-0.13176	0.895474	-6.14652	0.846809	0.796636
B.cells	BICRA	0.01543	5.82128	0.131684	0.895533	-6.62578	0.819325	0.751881

B.cells	AFF3	-0.01897	7.930696	-0.1316	0.895603	-7.02329	0.792484	0.709001
B.cells	LIX1L	-0.05896	0.803407	-0.13156	0.895631	-5.41628	0.887585	0.864521
B.cells	CEACAM10	0.064742	-1.04799	0.131529	0.895656	-5.30344	0.914072	0.910092
B.cells	SET	0.012533	7.946372	0.131522	0.895662	-6.99224	0.792289	0.708692
B.cells	ZFX	0.010498	6.339182	0.131518	0.895664	-6.74446	0.812637	0.741118
B.cells	IMPAD1	0.017784	4.365894	0.131251	0.895875	-6.27567	0.838471	0.783037
B.cells	DIS3	-0.02572	3.258023	-0.13116	0.895943	-6.03785	0.853398	0.807593
B.cells	RNH1	-0.01613	5.988026	-0.13107	0.89602	-6.71732	0.817165	0.748495
B.cells	PTAFR	-0.04688	4.645313	-0.13106	0.896025	-6.16644	0.834755	0.777005
B.cells	ZDHHC23	0.029296	2.221064	0.130694	0.896314	-5.97467	0.867651	0.831337
B.cells	GM46430	-0.03766	1.969969	-0.13068	0.896322	-5.63384	0.871144	0.837161
B.cells	ZFP523	-0.04024	2.19531	-0.13064	0.89636	-5.62935	0.868009	0.831932
B.cells	IER3	-0.03126	6.13903	-0.1306	0.896387	-6.61155	0.815214	0.745454
B.cells	TLR2	0.033529	3.875493	0.130583	0.896402	-6.11546	0.845041	0.793939
B.cells	GM49085	-0.03339	2.025817	-0.13043	0.896519	-5.78848	0.870366	0.835868
B.cells	ACIN1	-0.00907	7.577859	-0.13036	0.896576	-6.95427	0.7969	0.716199
B.cells	FAM219B	0.035575	2.367885	0.130108	0.896776	-5.84477	0.865616	0.82805
B.cells	ZC3H3	0.020109	3.468484	0.130056	0.896818	-6.17379	0.850539	0.803102
B.cells	F730043M1	0.070517	0.260543	0.129896	0.896944	-5.39417	0.895357	0.878041
B.cells	SLC26A11	-0.03091	3.200265	-0.12985	0.89698	-6.07918	0.854185	0.809155
B.cells	ZSCAN25	-0.03714	1.792602	-0.12983	0.897	-5.79394	0.873621	0.841462
B.cells	APEX1	0.016951	5.465947	0.12978	0.897035	-6.56883	0.823952	0.759701
B.cells	PRKCA	0.019943	7.401898	0.129703	0.897096	-6.8086	0.799114	0.71984
B.cells	GM15545	0.046487	1.102359	0.129685	0.89711	-5.48969	0.883338	0.85776
B.cells	PIP4P2	-0.0279	3.706325	-0.12963	0.89715	-6.07223	0.847321	0.79784
B.cells	ISCA2	-0.01794	4.968036	-0.12947	0.897278	-6.44338	0.830487	0.770306
B.cells	BHLHB9	-0.03591	2.203905	-0.12946	0.897287	-5.73677	0.867889	0.831894
B.cells	PALB2	0.036878	2.125531	0.129403	0.897332	-5.75613	0.868978	0.833709
B.cells	ADAL	0.03415	2.279066	0.12938	0.897351	-5.74771	0.866847	0.830157
B.cells	ANGPTL1	0.057741	0.376343	0.12921	0.897485	-5.47488	0.893692	0.875231
B.cells	PP2D1	0.047036	1.690903	0.128841	0.897776	-5.75214	0.875045	0.843874
B.cells	C1RL	0.047711	1.632594	0.128834	0.897782	-5.69572	0.875862	0.845243
B.cells	CIAO3	-0.02256	3.316721	-0.12879	0.897816	-6.11576	0.8526	0.806565
B.cells	WDR26	-0.01037	7.53429	-0.12875	0.897849	-6.94318	0.797448	0.717218
B.cells	C4B	0.05967	2.480976	0.128714	0.897877	-5.88167	0.864053	0.825546
B.cells	8-Mar	-0.03515	3.141103	-0.12869	0.897898	-5.86687	0.854992	0.810535
B.cells	NHEJ1	0.03705	3.867688	0.128561	0.897997	-6.17623	0.845146	0.794375
B.cells	SPCS3	-0.0162	4.704887	-0.12855	0.898008	-6.36508	0.833965	0.776077
B.cells	PRAG1	0.038063	1.923732	0.128539	0.898015	-5.82543	0.871789	0.838517
B.cells	RAB21	0.010719	6.891793	0.12842	0.898108	-6.8334	0.805571	0.730275
B.cells	INIP	0.016115	4.523666	0.128317	0.89819	-6.39259	0.836371	0.780099
B.cells	IGLC1	-0.04954	3.761408	-0.1283	0.898206	-6.44908	0.846578	0.796834
B.cells	CREB1	-0.01144	6.332794	-0.12819	0.898293	-6.74932	0.812731	0.741799
B.cells	PIP5K1C	-0.01532	5.626433	-0.12787	0.89854	-6.61623	0.822023	0.756573
B.cells	GM4129	0.047791	1.28611	0.127709	0.898669	-5.57364	0.880915	0.853691
B.cells	ZFP27	-0.04989	1.016419	-0.12769	0.898688	-5.5041	0.884733	0.860113
B.cells	COL3A1	0.045322	3.678331	0.127607	0.89875	-6.11706	0.847869	0.798759

B.cells	RETNLA	-0.24474	-0.77811	-0.1275	0.898831	-5.31341	0.910413	0.904087
B.cells	ZFP597	0.031367	2.862663	0.1273	0.898992	-5.88865	0.859001	0.817175
B.cells	DOCK9	-0.02528	4.197961	-0.12717	0.899091	-6.32069	0.840911	0.787376
B.cells	GPR34	0.073682	0.588061	0.127134	0.899123	-5.44378	0.890867	0.870533
B.cells	AMIGO1	0.056284	0.613629	0.126984	0.899241	-5.38365	0.890501	0.869933
B.cells	CHCHD1	-0.01429	5.813268	-0.12695	0.899265	-6.63552	0.819621	0.75278
B.cells	GFM1	0.020081	3.89287	0.126923	0.899289	-6.21963	0.845005	0.794119
B.cells	GCLC	0.016859	6.841762	0.126595	0.899548	-6.84899	0.80649	0.731531
B.cells	STX11	-0.02155	4.829435	-0.12604	0.899988	-6.42668	0.832608	0.773742
B.cells	SH3GLB2	0.021845	3.721025	0.125942	0.900063	-6.15302	0.847419	0.797987
B.cells	TEN1	-0.01197	5.852604	-0.12586	0.900125	-6.67143	0.819205	0.752022
B.cells	D230025D1	-0.0175	4.57771	-0.12582	0.900157	-6.40446	0.835945	0.779193
B.cells	POLR2E	0.018333	4.876402	0.125821	0.900159	-6.51881	0.831987	0.772741
B.cells	ZFP668	-0.01909	3.829078	-0.12571	0.900245	-6.22197	0.845962	0.795619
B.cells	BC028528	-0.01659	4.105365	-0.12555	0.900374	-6.46957	0.842249	0.789543
B.cells	SMAD2	0.010707	6.426685	0.125513	0.900402	-6.75497	0.811797	0.740133
B.cells	GM42869	-0.04078	1.342309	-0.12551	0.900406	-5.61312	0.880254	0.852616
B.cells	NDUFB3	0.013928	5.72376	0.125425	0.900472	-6.58784	0.820879	0.75476
B.cells	B3GALNT1	-0.08065	0.351359	-0.12541	0.900482	-5.2859	0.894364	0.876411
B.cells	GM48089	0.065746	1.135961	0.125322	0.900553	-5.43821	0.883171	0.857519
B.cells	GM19557	-0.05548	-0.95554	-0.12529	0.900581	-5.27593	0.913062	0.908768
B.cells	AAMP	-0.0101	5.908148	-0.12517	0.900672	-6.65131	0.818485	0.750896
B.cells	FAR1	0.014313	6.64871	0.125156	0.900684	-6.7895	0.808953	0.735573
B.cells	ATRIP	-0.0303	2.825397	-0.12505	0.900768	-5.98143	0.859612	0.818172
B.cells	PSMB4	-0.01276	6.172049	-0.12502	0.900794	-6.68943	0.815073	0.745399
B.cells	TARBP1	0.031106	2.687423	0.125015	0.900795	-5.91367	0.861508	0.821318
B.cells	CPSF4	0.020055	4.317677	0.124948	0.900848	-6.30496	0.839408	0.784905
B.cells	EXOC5	0.010871	6.376842	0.124604	0.90112	-6.74065	0.812586	0.74121
B.cells	SPNS3	0.029024	3.650358	0.124554	0.901159	-6.17309	0.84853	0.799659
B.cells	ARL4A	-0.0185	4.453783	-0.12425	0.901403	-6.32707	0.837801	0.781975
B.cells	PFN1	0.010047	10.31807	0.124206	0.901434	-7.34535	0.763813	0.664227
B.cells	RHNO1	0.019686	4.345115	0.124154	0.901475	-6.30714	0.83925	0.784344
B.cells	GNG3	-0.04339	1.449153	-0.12411	0.901508	-5.67775	0.878965	0.850145
B.cells	PPP2R5B	-0.04715	1.645269	-0.12359	0.901924	-5.67018	0.876421	0.8456
B.cells	ZFP384	-0.01485	4.90083	-0.12355	0.901949	-6.45901	0.832072	0.772379
B.cells	FHL3	-0.04391	2.081558	-0.12349	0.902001	-5.7046	0.870321	0.835421
B.cells	RETREG2	0.016985	4.884018	0.123445	0.902035	-6.43867	0.832294	0.77275
B.cells	FPGS	0.02797	3.013479	0.1233	0.902149	-5.93189	0.857455	0.814069
B.cells	AKT2	0.014048	5.234185	0.123196	0.902231	-6.50761	0.827681	0.765287
B.cells	ZFYVE26	-0.01621	5.03643	-0.12316	0.902257	-6.48679	0.830283	0.769521
B.cells	SYCE2	-0.0278	4.258668	-0.12303	0.902364	-6.25543	0.840609	0.786364
B.cells	MRPL2	-0.01709	4.862057	-0.12301	0.902382	-6.46327	0.832585	0.773266
B.cells	PHGDH	-0.02674	4.379094	-0.12264	0.902673	-6.36682	0.839155	0.783831
B.cells	SYNCRIP	-0.01051	7.375906	-0.12249	0.902786	-6.91255	0.800261	0.721149
B.cells	ZFP628	-0.0316	2.635685	-0.12239	0.90287	-5.91939	0.862804	0.822864
B.cells	E2F6	-0.03314	2.266229	-0.12235	0.902901	-5.75397	0.867914	0.831384
B.cells	ZMAT3	-0.04068	1.902429	-0.12227	0.902961	-5.80227	0.872981	0.839846

B.cells	PTGES2	0.030727	2.422157	0.122228	0.902996	-5.82835	0.865753	0.827807
B.cells	KCNA2	-0.07725	0.96787	-0.12215	0.903058	-5.44137	0.886154	0.861972
B.cells	TRP53I13	-0.02533	3.021819	-0.1217	0.903411	-5.91751	0.857682	0.814193
B.cells	FASTKD2	-0.02159	2.952537	-0.12169	0.90342	-6.02114	0.858631	0.815764
B.cells	CFAP410	0.027582	2.119698	0.121622	0.903474	-5.80254	0.870135	0.834893
B.cells	NAE1	-0.01838	3.958187	-0.12149	0.903581	-6.23344	0.844974	0.793314
B.cells	ADO	-0.02045	3.708631	-0.12136	0.90368	-6.13999	0.848339	0.798874
B.cells	GM47662	0.06636	-0.79475	0.121318	0.903714	-5.25497	0.911582	0.905369
B.cells	SPATS2L	0.07047	0.9742	0.121261	0.90376	-5.3717	0.886252	0.861994
B.cells	CTDSPL2	-0.01334	5.892856	-0.12053	0.904337	-6.63967	0.81978	0.751863
B.cells	DYNLT3	-0.01479	4.662522	-0.12047	0.904383	-6.40112	0.835937	0.77808
B.cells	NCBP1	-0.0119	5.256901	-0.12038	0.904457	-6.49995	0.828085	0.765315
B.cells	DUSP7	-0.02572	3.559127	-0.12037	0.904462	-6.10452	0.850746	0.802351
B.cells	PHLDB1	0.044997	1.355652	0.120284	0.904531	-5.602	0.881244	0.85309
B.cells	SFXN2	-0.02163	3.18033	-0.11972	0.904974	-6.08343	0.856203	0.811062
B.cells	NR1H3	-0.03957	3.325046	-0.11966	0.905028	-5.99239	0.854229	0.80781
B.cells	XK	0.054294	1.158214	0.119603	0.905069	-5.4449	0.88435	0.857981
B.cells	KDM5B	-0.01922	5.362074	-0.11944	0.905199	-6.57969	0.827049	0.763281
B.cells	FKRP	-0.031	2.332289	-0.11933	0.905282	-5.79385	0.867943	0.830431
B.cells	PTPRCAP	-0.01665	5.807512	-0.1192	0.905387	-6.61631	0.821268	0.753874
B.cells	AGGF1	0.012227	5.384652	0.119009	0.905538	-6.56084	0.826863	0.762802
B.cells	ARVCF	-0.05364	1.228411	-0.11882	0.905688	-5.50132	0.883546	0.856309
B.cells	LSS	0.060735	0.604735	0.118754	0.90574	-5.31965	0.892432	0.871273
B.cells	GM41555	0.065443	0.571556	0.118405	0.906016	-5.35423	0.892908	0.872179
B.cells	TSEN54	-0.02258	2.874509	-0.1184	0.906017	-6.01337	0.860578	0.818096
B.cells	MAP3K9	0.048843	0.308951	0.118386	0.90603	-5.59615	0.896682	0.878561
B.cells	DPM2	0.019175	3.936241	0.118376	0.906038	-6.20928	0.846132	0.794279
B.cells	EPSTI1	0.019899	7.192656	0.118167	0.906203	-6.91943	0.803567	0.725448
B.cells	ABCG1	0.01714	6.591271	0.118148	0.906218	-6.78416	0.811234	0.737695
B.cells	TOM1L1	0.052924	0.714609	0.118121	0.90624	-5.47998	0.890859	0.868729
B.cells	5031434O1	0.056062	0.524887	0.118064	0.906285	-5.35469	0.893577	0.873343
B.cells	ARL10	0.02795	2.295947	0.117886	0.906426	-5.88859	0.868636	0.831472
B.cells	GM43647	-0.07095	-0.94495	-0.11778	0.90651	-5.28257	0.914732	0.909782
B.cells	HECTD4	0.013846	5.548933	0.117678	0.90659	-6.59584	0.824807	0.759512
B.cells	GZF1	0.030335	2.752325	0.116894	0.907209	-5.88562	0.862676	0.821128
B.cells	SSNA1	-0.01258	5.922585	-0.11689	0.907211	-6.62622	0.820257	0.751776
B.cells	GM48960	-0.03912	1.466163	-0.11688	0.907222	-5.6612	0.880611	0.851025
B.cells	ATP5H	-0.00892	7.946365	-0.11686	0.907233	-6.99067	0.794463	0.710595
B.cells	ADAMTS10	-0.02375	3.015513	-0.11663	0.907421	-6.02144	0.859103	0.815181
B.cells	E130308A1	0.016586	4.522919	0.116619	0.907427	-6.37135	0.83872	0.781691
B.cells	LAG3	-0.06018	1.429969	-0.11652	0.907505	-5.4133	0.881173	0.851927
B.cells	KCNK10	-0.05474	0.557275	-0.1163	0.907681	-5.49481	0.893698	0.872876
B.cells	DPH6	0.017974	4.47347	0.116186	0.907768	-6.36742	0.839493	0.782806
B.cells	ZFP90	-0.03514	2.283533	-0.11586	0.908023	-5.79031	0.86946	0.832045
B.cells	SENP2	0.011323	6.656089	0.115773	0.908095	-6.78455	0.811072	0.73671
B.cells	GM16124	-0.03142	2.669782	-0.11563	0.908206	-5.96116	0.864109	0.823202
B.cells	PCCB	0.025613	3.081613	0.115471	0.908334	-5.96423	0.858445	0.813824

B.cells	NRBP1	0.011261	5.827937	0.115366	0.908417	-6.67057	0.821767	0.753931
B.cells	SIVA1	-0.01783	5.470666	-0.11529	0.908475	-6.50902	0.826433	0.761467
B.cells	PQBP1	-0.01545	4.6495	-0.11518	0.908565	-6.39735	0.837276	0.779076
B.cells	TPT1	0.00785	10.10831	0.1151	0.908627	-7.33677	0.768228	0.669271
B.cells	ZFP850	0.040993	1.010206	0.115089	0.908635	-5.51895	0.887375	0.862071
B.cells	TMEM106A	-0.02598	3.645492	-0.11509	0.908637	-6.0945	0.85076	0.801155
B.cells	NR6A1OS	0.034795	2.067272	0.114975	0.908726	-5.85243	0.872488	0.837112
B.cells	TDP1	-0.0252	3.397629	-0.11482	0.90885	-6.03513	0.854153	0.806721
B.cells	SENP5	0.012405	5.992571	0.114759	0.908897	-6.67787	0.819651	0.750502
B.cells	PRPF6	0.011958	5.300623	0.11455	0.909061	-6.53351	0.828689	0.765115
B.cells	ZC3HAV1	0.010197	8.43854	0.114468	0.909126	-7.12603	0.788628	0.701119
B.cells	GM1604A	-0.03018	2.804564	-0.11442	0.909161	-5.98984	0.862275	0.820158
B.cells	TBCB	0.0111	6.070727	0.11437	0.909204	-6.67609	0.818638	0.748885
B.cells	PUSL1	-0.0275	2.317284	-0.1142	0.909338	-5.83686	0.869016	0.831363
B.cells	SNX3	-0.00837	7.730038	-0.11397	0.909518	-6.97933	0.797469	0.715139
B.cells	FAM216A	-0.02884	2.582038	-0.11394	0.909545	-5.82748	0.865346	0.825319
B.cells	VPS29	0.00999	6.521761	0.11385	0.909615	-6.76667	0.812819	0.739602
B.cells	RNF214	-0.01389	4.961908	-0.11381	0.909649	-6.48562	0.833155	0.772429
B.cells	SGO2A	-0.04295	3.063462	-0.11367	0.909759	-5.85247	0.858718	0.814364
B.cells	PRR3	-0.01635	3.987386	-0.1135	0.909891	-6.34731	0.846164	0.793726
B.cells	GSPT1	0.00944	6.707096	0.113488	0.909901	-6.77793	0.810441	0.735848
B.cells	SEC23B	0.012381	5.223375	0.11333	0.910026	-6.50541	0.829705	0.766922
B.cells	PLCB2	-0.02252	3.320212	-0.11319	0.910138	-6.06927	0.855208	0.808695
B.cells	GPC1	-0.06565	0.351919	-0.11316	0.910163	-5.34189	0.896828	0.878253
B.cells	TBXA2R	0.047472	2.36173	0.113077	0.910226	-5.66318	0.868399	0.830556
B.cells	CDKN2AIP	-0.01455	4.567595	-0.11286	0.910399	-6.38962	0.83839	0.78114
B.cells	GM43914	0.051434	0.384486	0.11282	0.910429	-5.43245	0.896359	0.877504
B.cells	ARID2	0.011715	6.543762	0.112743	0.91049	-6.76734	0.812536	0.739325
B.cells	WASHC5	0.016889	4.472587	0.112593	0.910609	-6.36788	0.839658	0.783214
B.cells	A930006K0	0.032576	2.074746	0.112535	0.910654	-5.68975	0.872394	0.837251
B.cells	MMP11	-0.06047	0.128652	-0.11239	0.910771	-5.31651	0.900052	0.883809
B.cells	BAG4	0.015736	4.226706	0.112347	0.910803	-6.30647	0.842948	0.788637
B.cells	PDGFC	-0.06874	1.879122	-0.11218	0.910935	-5.55577	0.875129	0.841863
B.cells	CYB561D1	-0.03774	1.785161	-0.11217	0.910946	-5.69163	0.876447	0.844065
B.cells	TGS1	0.012171	5.214453	0.112102	0.910996	-6.51461	0.829822	0.767247
B.cells	PSMA2	-0.01044	7.21693	-0.1121	0.910999	-6.87266	0.803945	0.725644
B.cells	VPS45	0.01981	3.758422	0.111867	0.911182	-6.16365	0.849255	0.799039
B.cells	4933408B1	-0.03752	1.941966	-0.11177	0.911261	-5.70422	0.874249	0.840452
B.cells	GLE1	0.014539	4.4454	0.111682	0.911329	-6.34416	0.840021	0.783908
B.cells	GM50013	-0.0313	1.85467	-0.11158	0.911409	-5.78347	0.875472	0.842498
B.cells	IMP3	0.011522	5.264975	0.111547	0.911435	-6.55303	0.829157	0.766225
B.cells	DNPEP	0.017625	4.077287	0.111467	0.911498	-6.26135	0.844954	0.791985
B.cells	LDAH	0.015157	4.225642	0.111432	0.911526	-6.32103	0.842962	0.788722
B.cells	LZTS3	-0.0544	0.323606	-0.11135	0.911592	-5.35015	0.897236	0.879112
B.cells	IMPG2	0.06056	0.6613	0.111218	0.911695	-5.41051	0.892383	0.870926
B.cells	RIPPLY3	-0.0769	0.048429	-0.11107	0.91181	-5.22787	0.901214	0.885897
B.cells	MED26	0.017035	4.429093	0.110999	0.911869	-6.37286	0.840239	0.78432

B.cells	SNRNP40	-0.01062	5.982356	-0.111	0.911871	-6.66936	0.819784	0.751127
B.cells	KNL1	0.027792	5.131736	0.110952	0.911906	-6.48925	0.830912	0.769127
B.cells	JMJD8	0.040108	1.564022	0.110949	0.911908	-5.65508	0.879556	0.849392
B.cells	ZFP637	0.031352	2.315939	0.110875	0.911967	-5.78854	0.869035	0.83182
B.cells	JADE3	0.019404	3.471551	0.110798	0.912028	-6.11119	0.853147	0.805503
B.cells	ELOA	-0.01211	5.789735	-0.11071	0.912101	-6.65318	0.822288	0.755165
B.cells	DEXI	-0.0229	2.91114	-0.11063	0.912164	-6.02504	0.860809	0.818162
B.cells	GTF2I	0.012681	5.762327	0.110269	0.912446	-6.56379	0.822727	0.755794
B.cells	IGF2BP3	0.012703	6.5516	0.110151	0.912539	-6.77138	0.812515	0.739367
B.cells	GM17160	0.04924	0.089084	0.110008	0.912652	-5.40696	0.900713	0.884973
B.cells	1810026B0	0.009598	6.570259	0.109867	0.912764	-6.76687	0.812276	0.739016
B.cells	TRP53INP1	0.017272	5.187354	0.109846	0.912781	-6.50354	0.830261	0.768024
B.cells	SENP8	-0.05574	0.568146	-0.10978	0.912832	-5.42324	0.893807	0.873331
B.cells	4930505N2	-0.03486	1.214516	-0.10968	0.91291	-5.6959	0.884584	0.857791
B.cells	SNUPN	0.021943	2.593487	0.109496	0.913057	-5.91474	0.865273	0.825528
B.cells	UNC45A	-0.016	4.303611	-0.10936	0.913164	-6.32876	0.842	0.787181
B.cells	PAK2	0.007618	7.859842	0.109346	0.913176	-6.99866	0.795919	0.712983
B.cells	1700113A1	-0.0301	2.10081	-0.10929	0.913222	-5.8054	0.872116	0.836942
B.cells	KLF5	0.068239	0.36413	0.109257	0.913246	-5.32463	0.896741	0.878319
B.cells	ZFP664	0.016197	4.050718	0.109109	0.913363	-6.26222	0.845395	0.792786
B.cells	FOXD2OS	0.057085	0.311339	0.109044	0.913414	-5.3317	0.897502	0.879649
B.cells	MAFG	0.013394	5.07367	0.109011	0.913441	-6.50731	0.83176	0.770529
B.cells	CARS2	0.015215	3.502896	0.108945	0.913493	-6.11582	0.852805	0.804977
B.cells	DUSP12	0.019763	3.283921	0.108944	0.913494	-6.10121	0.855788	0.809897
B.cells	WDR36	0.013423	4.578146	0.108636	0.913737	-6.4324	0.838424	0.781306
B.cells	ADA	0.039262	2.074866	0.108633	0.91374	-5.68624	0.872573	0.837665
B.cells	MMP8	-0.06678	1.517071	-0.10832	0.913984	-5.54441	0.880494	0.85084
B.cells	UBXN7	0.011602	5.704222	0.108256	0.914038	-6.64108	0.823661	0.757272
B.cells	KIF3A	-0.02733	2.472633	-0.10823	0.914055	-5.8039	0.867132	0.828538
B.cells	DUS4L	0.032578	1.929292	0.108142	0.914128	-5.70037	0.874701	0.841167
B.cells	SLC3A1	-0.04289	0.899213	-0.10798	0.914255	-5.53814	0.889266	0.865603
B.cells	OXNAD1	-0.03303	1.886539	-0.10786	0.914352	-5.63259	0.875305	0.842176
B.cells	N4BP3	-0.02473	2.955418	-0.10786	0.914352	-5.94138	0.860475	0.817509
B.cells	DUS1L	-0.01818	4.064535	-0.10758	0.91457	-6.24391	0.84553	0.792687
B.cells	RB1CC1	-0.01119	6.500368	-0.10742	0.914703	-6.78071	0.813535	0.740723
B.cells	NR1D1	0.050792	1.661874	0.107199	0.914874	-5.57737	0.87875	0.847539
B.cells	HIST1H2AB	0.058851	1.438759	0.106874	0.915131	-5.57892	0.881965	0.852906
B.cells	GM47283	-0.02481	7.990664	-0.10675	0.91523	-7.03324	0.794781	0.710767
B.cells	TCEA2	-0.05116	0.335442	-0.10667	0.915289	-5.3548	0.897719	0.879476
B.cells	NSA2	0.007275	7.622863	0.106559	0.91538	-6.96229	0.799398	0.71808
B.cells	NCOA7	-0.01414	5.299685	-0.10647	0.915449	-6.57375	0.829304	0.766057
B.cells	MRPL12	0.01491	5.090142	0.106468	0.915452	-6.47395	0.832066	0.770539
B.cells	JMJD6	-0.01405	4.999369	-0.10646	0.915456	-6.47302	0.833266	0.772489
B.cells	CNOT11	-0.01612	4.112527	-0.10617	0.915687	-6.2557	0.845177	0.791836
B.cells	ZBTB12	-0.03307	1.665552	-0.10615	0.915702	-5.63645	0.878851	0.847613
B.cells	SHROOM4	-0.06331	0.86592	-0.10587	0.915926	-5.39533	0.89019	0.866724
B.cells	GPR89	0.018566	3.742407	0.105853	0.915939	-6.19049	0.850173	0.800099

B.cells	GM17066	-0.03456	1.747467	-0.10564	0.916105	-5.65063	0.877699	0.845781
B.cells	REEP1	0.056552	0.98372	0.105537	0.916189	-5.35641	0.888509	0.863948
B.cells	MFSD1	0.01229	5.149534	0.105469	0.916242	-6.54403	0.831362	0.769413
B.cells	LAS1L	0.015269	4.172574	0.105343	0.916342	-6.27742	0.84437	0.790645
B.cells	DDX28	-0.03034	1.558733	-0.10533	0.91635	-5.67846	0.880356	0.850275
B.cells	OLFM4	-0.06313	-0.01496	-0.10525	0.91642	-5.42731	0.902876	0.888296
B.cells	MPPE1	-0.01633	4.363196	-0.10523	0.916433	-6.31896	0.841813	0.78649
B.cells	PSMC4	-0.01267	5.46131	-0.10515	0.916494	-6.55322	0.827261	0.762849
B.cells	CSTF2	0.016807	4.139659	0.105138	0.916504	-6.26729	0.844812	0.791439
B.cells	GM15965	-0.02765	2.027609	-0.10494	0.916658	-5.95239	0.873772	0.839401
B.cells	KLRA7	0.078047	0.646446	0.104943	0.916658	-5.49209	0.893332	0.872244
B.cells	GNAL	0.034688	1.371741	0.104876	0.916712	-5.62063	0.882998	0.854855
B.cells	ACACB	0.064439	0.257652	0.104557	0.916964	-5.26329	0.899052	0.881757
B.cells	LRWD1	0.015433	3.781422	0.104435	0.91706	-6.19809	0.849761	0.799472
B.cells	6530402F1	0.053775	-0.17553	0.104311	0.917159	-5.33615	0.905335	0.892426
B.cells	PEBP1	-0.01026	6.440655	-0.10421	0.917236	-6.73912	0.814644	0.742429
B.cells	BLOC1S4	0.016475	3.695064	0.103663	0.917672	-6.16459	0.850931	0.801496
B.cells	UBE2H	-0.00947	8.574917	-0.10359	0.917729	-7.16582	0.787697	0.699694
B.cells	METTL18	0.042217	1.271701	0.103499	0.917801	-5.50345	0.884537	0.857377
B.cells	CRK	-0.01051	6.391053	-0.10338	0.917893	-6.76295	0.815284	0.743544
B.cells	FAM221B	0.052574	0.343919	0.103362	0.91791	-5.36651	0.897807	0.879758
B.cells	EPM2AIP1	0.027064	2.609784	0.10335	0.917919	-5.81693	0.865794	0.826067
B.cells	MRPL10	0.015149	4.602348	0.103249	0.917999	-6.37261	0.838734	0.781509
B.cells	GM36445	0.03605	1.003387	0.103248	0.918	-5.52871	0.888351	0.863792
B.cells	SLC11A2	0.019921	3.918763	0.103187	0.918048	-6.25667	0.847904	0.796545
B.cells	MFSD7A	-0.05184	-0.44923	-0.10316	0.918067	-5.40447	0.909326	0.899363
B.cells	CUX1	0.008652	7.953864	0.102944	0.918241	-7.02628	0.795428	0.711987
B.cells	NDUFS7	-0.00952	6.320866	-0.10294	0.918247	-6.70749	0.816189	0.745099
B.cells	NCOA5	0.015996	4.146435	0.102863	0.918305	-6.30335	0.844837	0.791597
B.cells	DDX21	-0.0116	6.399043	-0.10283	0.918335	-6.76291	0.815181	0.743479
B.cells	2900026A0	-0.02548	3.595436	-0.1026	0.918509	-6.22158	0.852283	0.803842
B.cells	RAC1	-0.00716	7.82414	-0.10252	0.918577	-6.98009	0.797054	0.714574
B.cells	ABRACL	-0.01087	7.550325	-0.1025	0.918593	-6.94807	0.800499	0.72004
B.cells	RFC5	-0.02402	4.185223	-0.10242	0.918655	-6.19926	0.844316	0.790755
B.cells	TNFRSF13C	0.0179	3.92598	0.102417	0.918658	-6.32524	0.847807	0.796482
B.cells	PLA2G4A	0.032087	3.294743	0.102387	0.918682	-5.90023	0.856379	0.810597
B.cells	GM5577	-0.05202	0.20371	-0.10212	0.918892	-5.33976	0.899832	0.883334
B.cells	TMEM109	-0.01934	3.841384	-0.1021	0.918911	-6.20992	0.84895	0.79837
B.cells	SNHG4.1	-0.05116	0.471422	-0.10188	0.91908	-5.50272	0.895969	0.876793
B.cells	PDCD6	-0.01172	6.13963	-0.10188	0.919082	-6.67612	0.818533	0.74889
B.cells	UBE3B	0.012364	4.424846	0.101814	0.919135	-6.38707	0.841104	0.785509
B.cells	STK26	-0.01608	4.048898	-0.10166	0.919259	-6.32186	0.846149	0.793772
B.cells	SMARCE1	0.008404	6.302182	0.101609	0.919297	-6.73817	0.81643	0.745522
B.cells	SPATA48	-0.03055	2.190427	-0.10157	0.919327	-5.80093	0.871618	0.835922
B.cells	IPO5	0.014617	5.437174	0.101397	0.919465	-6.56528	0.827691	0.763738
B.cells	CDH17	0.039959	0.13748	0.101257	0.919576	-5.60461	0.90079	0.885045
B.cells	KIF22	-0.02907	4.043139	-0.10117	0.919641	-6.17335	0.846227	0.793976

B.cells	TMSB4X	-0.01001	12.24836	-0.10104	0.919744	-7.6336	0.743759	0.631974
B.cells	1700006J1	-0.07546	-0.71534	-0.10102	0.91976	-5.19071	0.913054	0.906275
B.cells	MFSD4B4	-0.05955	-0.0204	-0.10093	0.919836	-5.25697	0.903079	0.88898
B.cells	ZFP382	0.03051	2.008207	0.100888	0.919868	-5.8073	0.874163	0.840271
B.cells	PUM2	0.007148	7.324014	0.100885	0.91987	-6.8989	0.80336	0.724713
B.cells	R3HCC1	0.030379	2.353768	0.100722	0.919999	-5.80787	0.869345	0.83228
B.cells	PPP1R37	-0.01323	4.592802	-0.10063	0.92007	-6.39121	0.838861	0.782013
B.cells	GBE1	0.014043	6.483019	0.100553	0.920133	-6.7168	0.814099	0.741924
B.cells	USP32	-0.01148	6.849617	-0.10053	0.920148	-6.83095	0.809397	0.734391
B.cells	HSF1	0.012559	4.997353	0.100507	0.920169	-6.46035	0.833487	0.773255
B.cells	RASGRF2	-0.04071	0.116081	-0.10047	0.9202	-5.48109	0.9011	0.885675
B.cells	CD14	-0.05454	4.260683	-0.10038	0.920269	-5.91866	0.843303	0.789276
B.cells	NUP35	0.020646	3.291207	0.100245	0.920377	-6.05353	0.856459	0.810895
B.cells	TRAPPC5	0.014965	4.379372	0.100038	0.920541	-6.3215	0.841827	0.786741
B.cells	IDUA	-0.0253	2.137056	-0.09982	0.92071	-5.77892	0.872531	0.837398
B.cells	PROX2	0.043928	1.188752	0.099744	0.920774	-5.48086	0.885885	0.859778
B.cells	WDR73	0.019546	2.976485	0.099635	0.92086	-6.01004	0.860905	0.818097
B.cells	LRIG1	-0.05709	0.178544	-0.09957	0.920908	-5.37077	0.900369	0.884254
B.cells	9030025P2	0.032148	1.737859	0.099511	0.920958	-5.706	0.878124	0.846769
B.cells	PLEKHA4	0.034291	2.406918	0.09907	0.921307	-5.74378	0.869034	0.83127
B.cells	GM49602	-0.02879	1.89388	-0.09875	0.921557	-5.73914	0.876255	0.843295
B.cells	1700030J2	0.054063	-0.14389	0.098726	0.921579	-5.41095	0.905381	0.892404
B.cells	FNTA	-0.00922	5.505108	-0.09865	0.92164	-6.59559	0.827263	0.762671
B.cells	EPHB2	-0.0522	1.372664	-0.09862	0.921661	-5.39361	0.883601	0.855601
B.cells	MBD1	0.013648	4.580853	0.098515	0.921746	-6.38632	0.839501	0.78255
B.cells	RAD51C	0.028011	2.245463	0.09837	0.921861	-5.75282	0.871383	0.835132
B.cells	IMMP2L	-0.01606	6.534254	-0.09827	0.921938	-6.78179	0.813937	0.741164
B.cells	FRRS1	0.013706	4.851486	0.098194	0.922	-6.45732	0.83593	0.776718
B.cells	ESCO1	0.010358	5.77786	0.097852	0.922271	-6.64968	0.823788	0.757036
B.cells	BAHD1	-0.02228	2.543391	-0.09776	0.922347	-5.90373	0.867296	0.828385
B.cells	ANP32B	0.010514	8.191649	0.097729	0.922369	-7.02195	0.79299	0.707878
B.cells	CD200R1	0.041489	2.827999	0.097632	0.922446	-5.59211	0.863362	0.82186
B.cells	ENDOG	0.019241	2.943039	0.097595	0.922475	-6.01043	0.861777	0.819234
B.cells	SAFB2	-0.00849	5.993263	-0.09751	0.922541	-6.68247	0.820982	0.75255
B.cells	TADA3	-0.01803	3.403164	-0.09748	0.922568	-6.08761	0.855474	0.808813
B.cells	ANKRD13C	0.010924	5.868682	0.097196	0.922791	-6.66607	0.822616	0.755165
B.cells	IVD	0.013825	4.435995	0.097136	0.922838	-6.38231	0.841533	0.785894
B.cells	ELP3	-0.0225	2.716115	-0.09705	0.922903	-5.87689	0.864919	0.824422
B.cells	CSNK1G2	0.010336	5.468875	0.096869	0.923049	-6.55517	0.827844	0.76365
B.cells	NDUFA12	0.011518	5.362792	0.096772	0.923126	-6.5212	0.829238	0.765944
B.cells	DNAAF3	0.056674	-0.6587	0.096717	0.92317	-5.24421	0.912887	0.905538
B.cells	NAA80	0.024562	2.402845	0.096627	0.923242	-5.78592	0.86926	0.831719
B.cells	TMEM256	-0.01071	6.982152	-0.0966	0.923266	-6.8396	0.808261	0.732187
B.cells	ILKAP	-0.01038	5.651644	-0.09656	0.923294	-6.6005	0.82545	0.759818
B.cells	CREBBP	0.007667	7.888027	0.096526	0.923321	-7.00507	0.7968	0.713957
B.cells	WDTC1	-0.01678	3.851337	-0.09643	0.923396	-6.21525	0.849399	0.798873
B.cells	GM14698	-0.04383	0.738672	-0.09623	0.923554	-5.40905	0.892746	0.871126

B.cells	APMAP	-0.0156	4.080552	-0.09616	0.923608	-6.23595	0.846305	0.793818
B.cells	EEF1A1	0.006974	10.55641	0.096034	0.923711	-7.39557	0.764148	0.662938
B.cells	SGSM2	0.015937	3.786605	0.095981	0.923753	-6.25796	0.850275	0.800382
B.cells	AS3MT	-0.02454	2.69274	-0.09591	0.923806	-5.90653	0.865242	0.825117
B.cells	SMPD4	-0.02134	3.209019	-0.09589	0.923825	-6.01307	0.85814	0.81335
B.cells	SMIM3	0.012654	4.840554	0.095648	0.924017	-6.54181	0.836214	0.777268
B.cells	HOXA7	-0.05846	-0.09629	-0.09555	0.924096	-5.25374	0.904884	0.891644
B.cells	EFHD2	0.011247	6.92246	0.095436	0.924184	-6.77007	0.809094	0.733525
B.cells	TENM2	0.075082	-0.35252	0.095379	0.924229	-5.19401	0.908621	0.898036
B.cells	FBXL12	-0.01408	4.348122	-0.09522	0.924359	-6.33662	0.842784	0.788033
B.cells	LRRC57	-0.02604	2.732576	-0.09515	0.924409	-5.93991	0.864768	0.824269
B.cells	ABI1	0.010075	7.702393	0.095142	0.924417	-6.95616	0.799203	0.717778
B.cells	GDPGP1	0.032354	2.18615	0.094796	0.924691	-5.8251	0.872476	0.836999
B.cells	ALDH2	0.010921	6.861181	0.094658	0.924801	-6.81553	0.80999	0.734903
B.cells	PRDM2	-0.01038	5.970241	-0.09464	0.924813	-6.69562	0.821481	0.75336
B.cells	MYO9A	-0.01438	6.336763	-0.09456	0.924879	-6.71378	0.81673	0.745717
B.cells	GM17477	-0.03903	0.61424	-0.09449	0.924932	-5.47484	0.894735	0.874409
B.cells	COMT	0.011801	5.36738	0.094426	0.924984	-6.52798	0.829366	0.766117
B.cells	YIPF2	-0.02829	1.926928	-0.09395	0.925359	-5.69447	0.876263	0.843177
B.cells	GM15265	0.030694	1.797023	0.093922	0.925384	-5.71973	0.878088	0.846228
B.cells	PDHB	-0.00951	5.703174	-0.09379	0.925491	-6.61267	0.825115	0.759068
B.cells	GM30198	0.032051	1.865066	0.093759	0.925512	-5.87771	0.877132	0.844639
B.cells	FAM8A1	-0.01893	3.231881	-0.09358	0.925654	-5.99664	0.85818	0.813157
B.cells	FAM160B1	-0.01375	4.443367	-0.09357	0.925659	-6.30905	0.84178	0.786197
B.cells	LYN	-0.00874	10.03311	-0.09357	0.925666	-7.36386	0.770741	0.67293
B.cells	ZFP395	-0.01578	4.289929	-0.09331	0.925866	-6.25882	0.843952	0.789577
B.cells	SLC4A4	-0.03234	2.065431	-0.09305	0.926072	-5.78517	0.874478	0.840022
B.cells	FAN1	0.039485	1.159783	0.093046	0.926078	-5.5031	0.887256	0.861436
B.cells	COX6B1	0.00851	7.821955	0.092989	0.926122	-6.97813	0.798098	0.715663
B.cells	TUBG1	-0.02491	3.393021	-0.09282	0.926254	-6.01894	0.856169	0.809626
B.cells	MLF1	-0.048	0.512644	-0.09262	0.926418	-5.34413	0.896561	0.877168
B.cells	GM19696	-0.05643	0.016291	-0.09246	0.926545	-5.28179	0.903741	0.889395
B.cells	POMK	-0.04127	1.068205	-0.09229	0.926677	-5.47276	0.888602	0.863836
B.cells	ARL2	0.020536	3.356926	0.092264	0.926697	-5.99023	0.856662	0.810572
B.cells	GM4316	-0.04396	0.707324	-0.09224	0.926716	-5.45556	0.893763	0.872539
B.cells	PRKAR2B	-0.01856	3.305356	-0.09223	0.926725	-6.15737	0.857366	0.811736
B.cells	FKBP8	0.010451	6.067485	0.092197	0.92675	-6.66428	0.820552	0.751665
B.cells	GM50012	0.02581	1.643382	0.092038	0.926876	-5.71612	0.880481	0.85014
B.cells	PHRF1	-0.00928	5.804424	-0.09197	0.926932	-6.63633	0.824008	0.757192
B.cells	PSMC1	-0.00882	5.889053	-0.09159	0.927227	-6.62596	0.823021	0.755415
B.cells	HIST1H3A	-0.04065	0.953909	-0.09154	0.927267	-5.43961	0.890393	0.866594
B.cells	PTCD1	-0.02062	2.585716	-0.09151	0.927294	-5.90435	0.86743	0.828162
B.cells	SYNE1	0.02007	4.964609	0.091428	0.927359	-6.41201	0.835174	0.77511
B.cells	LSG1	-0.01023	4.928986	-0.09118	0.927553	-6.4824	0.835647	0.775905
B.cells	AKAP13	-0.00772	8.500476	-0.09103	0.927671	-7.09545	0.789804	0.702506
B.cells	SNHG14	-0.02916	1.410261	-0.09092	0.927761	-5.58337	0.883901	0.85574
B.cells	5031425E2	0.011011	5.390925	0.090919	0.927762	-6.57348	0.829544	0.766023

B.cells	1110019D1	0.030119	1.751896	0.090889	0.927786	-5.64999	0.879077	0.847653
B.cells	GM43661	0.028807	2.981343	0.090823	0.927839	-5.91625	0.861966	0.819162
B.cells	COA4	-0.02604	2.161432	-0.09079	0.927868	-5.73857	0.873334	0.838064
B.cells	MCOLN2	0.018548	3.161941	0.09062	0.927999	-6.03392	0.859526	0.815089
B.cells	PROSER1	-0.01377	4.213066	-0.09055	0.928058	-6.32536	0.845251	0.791591
B.cells	FASTKD3	-0.02967	1.993657	-0.0903	0.928255	-5.69151	0.87576	0.84201
B.cells	NUP107	0.011613	5.353646	0.090202	0.928331	-6.52945	0.830109	0.766851
B.cells	0610009E0	0.035225	0.843747	0.090164	0.928361	-5.45039	0.892049	0.869354
B.cells	CEP57L1	-0.02017	3.287731	-0.08991	0.928564	-5.9987	0.857839	0.812311
B.cells	A730063M	0.04275	1.218514	0.089806	0.928645	-5.46706	0.886702	0.860459
B.cells	PSMB1	-0.00782	7.299863	-0.08975	0.928689	-6.90261	0.804947	0.7265
B.cells	NUDT15	0.025556	1.647281	0.089506	0.928882	-5.74008	0.88063	0.85029
B.cells	STX1A	0.036101	1.792697	0.089487	0.928898	-5.60986	0.878582	0.846859
B.cells	S1PR2	-0.03941	1.261742	-0.08947	0.928913	-5.47831	0.886088	0.859449
B.cells	ECSCR	-0.04739	1.207762	-0.08944	0.928932	-5.39853	0.886855	0.860739
B.cells	ACER2	-0.03137	2.516505	-0.08939	0.928974	-5.71561	0.868468	0.829985
B.cells	DHPS	0.011211	4.486343	0.089322	0.929028	-6.38438	0.84162	0.785714
B.cells	NFXL1	0.01858	3.364621	0.089285	0.929057	-6.07927	0.856788	0.810631
B.cells	COG2	-0.01532	3.567744	-0.08905	0.929246	-6.14979	0.854018	0.806083
B.cells	KDM8	-0.02481	1.814343	-0.08903	0.929257	-5.70638	0.878277	0.846373
B.cells	CPSF4L	0.037476	0.499907	0.088765	0.92947	-5.40107	0.896987	0.877928
B.cells	ZFP729B	-0.01408	3.639068	-0.08874	0.929487	-6.19902	0.853048	0.804553
B.cells	TAGAP1	-0.02903	2.214287	-0.08874	0.92949	-5.76195	0.872674	0.837084
B.cells	PPP1R12A	0.007059	8.052992	0.088527	0.929658	-7.02671	0.795453	0.71158
B.cells	HEXDC	0.035307	1.86348	0.088455	0.929715	-5.65852	0.877587	0.845353
B.cells	TNFAIP3	-0.01223	6.763033	-0.08837	0.929781	-6.81476	0.811796	0.737627
B.cells	ZC3H15	-0.00683	7.266033	-0.08835	0.929801	-6.91268	0.805376	0.727379
B.cells	DPY19L1	-0.01408	4.584338	-0.08827	0.929861	-6.3387	0.84031	0.783766
B.cells	MED20	0.012995	4.104119	0.088213	0.929907	-6.25478	0.846753	0.794313
B.cells	PRORP	-0.01293	3.853039	-0.0881	0.93	-6.25892	0.850145	0.799902
B.cells	FBRS	0.010925	4.965395	0.08807	0.93002	-6.49367	0.835239	0.775518
B.cells	NUMBL	-0.03883	1.04252	-0.088	0.930072	-5.48685	0.889208	0.864956
B.cells	TGTP1	0.068369	-0.20272	0.087749	0.930274	-5.27023	0.907242	0.895416
B.cells	DOK2	-0.02604	2.811456	-0.0876	0.930391	-5.77739	0.864449	0.823502
B.cells	TEX2	0.011592	6.723879	0.087512	0.930462	-6.70413	0.812359	0.738554
B.cells	AARS2	-0.04023	0.821033	-0.08749	0.930482	-5.41425	0.89244	0.870379
B.cells	HNRNPLL	-0.02033	4.231786	-0.08736	0.930587	-5.95524	0.845098	0.791642
B.cells	BBOF1	-0.03211	1.302277	-0.08726	0.930662	-5.57623	0.885578	0.858851
B.cells	ASB3	0.010874	4.711015	0.087047	0.930831	-6.41267	0.838683	0.781163
B.cells	ATXN7L1OS	0.034328	1.337166	0.087004	0.930865	-5.57385	0.885083	0.858032
B.cells	ZBTB18	-0.01297	3.894799	-0.08668	0.93112	-6.27765	0.849643	0.799114
B.cells	TEFM	0.038396	1.160891	0.086595	0.931189	-5.5016	0.887588	0.862245
B.cells	PI16	-0.03553	1.135522	-0.08658	0.931198	-5.56504	0.887949	0.862853
B.cells	REX1BD	-0.00928	5.688469	-0.08656	0.931218	-6.577	0.825776	0.760192
B.cells	SETD6	-0.02552	1.423905	-0.08656	0.931219	-5.63764	0.883853	0.855966
B.cells	GM37612	-0.03715	0.985122	-0.08655	0.931226	-5.46333	0.890094	0.866466
B.cells	XPO1	0.011049	5.786847	0.086517	0.931251	-6.61511	0.82449	0.758113

B.cells	BIVM	0.036279	0.882383	0.086354	0.931381	-5.39987	0.891562	0.868943
B.cells	C2CD2L	-0.01836	2.991873	-0.08635	0.931384	-6.0137	0.861963	0.819448
B.cells	PNRC2	0.009612	5.35165	0.085823	0.931802	-6.56651	0.830504	0.767412
B.cells	ANKRD6	0.036576	0.95646	0.085293	0.932221	-5.56196	0.89087	0.867406
B.cells	2610203C2	0.04727	0.354453	0.08526	0.932247	-5.32539	0.899522	0.882029
B.cells	HS2ST1	0.009332	5.689893	0.085228	0.932273	-6.59494	0.826097	0.760381
B.cells	GSTT2	-0.01808	3.554147	-0.08511	0.932368	-6.08916	0.854618	0.806968
B.cells	MAP3K10	0.019398	2.731125	0.085045	0.932418	-5.93385	0.865915	0.825668
B.cells	PPAN	-0.01517	4.072585	-0.08503	0.932431	-6.25326	0.84759	0.795416
B.cells	FAAP20	0.016232	3.120662	0.085022	0.932436	-6.03756	0.860547	0.816769
B.cells	CCDC25	0.011048	4.712121	0.084969	0.932479	-6.40614	0.839013	0.781405
B.cells	HOOK1	-0.02037	2.667358	-0.08494	0.932499	-5.90743	0.866798	0.82717
B.cells	RHEB	-0.00707	7.039284	-0.08476	0.932646	-6.84661	0.80866	0.732421
B.cells	GM19466	-0.03953	0.661272	-0.0846	0.932772	-5.45668	0.895105	0.874637
B.cells	PMP22	-0.03794	1.847479	-0.08455	0.932813	-5.64367	0.878243	0.846278
B.cells	SHLD3	0.021982	2.343634	0.084414	0.932918	-5.8407	0.871299	0.834683
B.cells	ACTR10	-0.007	6.186686	-0.08433	0.932988	-6.69778	0.819629	0.750013
B.cells	SEC61A2	0.013345	3.741527	0.084196	0.933091	-6.20441	0.852074	0.802844
B.cells	CAPZB	0.005666	8.722615	0.084046	0.93321	-7.11637	0.78751	0.698935
B.cells	RGS5	-0.05109	1.107497	-0.08391	0.933319	-5.509	0.888718	0.863962
B.cells	STIL	0.022305	4.156047	0.083873	0.933347	-6.16991	0.846469	0.793726
B.cells	CTSE	-0.01212	5.492809	-0.08385	0.933366	-6.57331	0.828686	0.764732
B.cells	DGKQ	-0.02826	1.470676	-0.0838	0.933402	-5.62121	0.883559	0.855287
B.cells	PRAF2	0.019165	2.133854	0.083627	0.933542	-5.82951	0.874227	0.839705
B.cells	DMXL2	0.029521	2.146865	0.083567	0.933589	-5.8574	0.874045	0.839401
B.cells	NUDT16L1	0.014175	4.234234	0.083549	0.933604	-6.25993	0.845417	0.79204
B.cells	CRIPT	-0.00742	6.218829	-0.08302	0.934025	-6.72984	0.819212	0.749535
B.cells	HSBP1	-0.00987	6.003464	-0.08296	0.934069	-6.63698	0.822009	0.754043
B.cells	GNG11	0.021026	3.831566	0.082473	0.934457	-6.13026	0.850853	0.801076
B.cells	RHOBTB2	0.01783	2.943311	0.082377	0.934533	-6.00897	0.86299	0.82115
B.cells	MIGA1	-0.01675	2.677211	-0.08229	0.9346	-5.90879	0.866666	0.827262
B.cells	TRAPPC2L	0.009114	5.811456	0.082274	0.934615	-6.62443	0.824512	0.758153
B.cells	CLDN34C1	-0.0438	0.809812	-0.08217	0.934696	-5.37095	0.892973	0.871366
B.cells	GM12764	-0.02843	1.453299	-0.08215	0.934711	-5.6789	0.883805	0.855925
B.cells	FOXO3	-0.01005	6.397005	-0.08213	0.93473	-6.74878	0.816907	0.745925
B.cells	GM11755	-0.05155	-0.115	-0.08206	0.934785	-5.28105	0.90634	0.894054
B.cells	CRCP	0.009631	4.449755	0.081999	0.934833	-6.38383	0.842524	0.787504
B.cells	POLR3GL	-0.01362	3.878267	-0.08194	0.934876	-6.13138	0.850221	0.800144
B.cells	SGCB	0.030566	2.212559	0.081787	0.935001	-5.64307	0.873127	0.838146
B.cells	CTR9	-0.01022	4.518969	-0.08151	0.935223	-6.35956	0.841597	0.786071
B.cells	ZFP865	0.01689	3.265059	0.081183	0.93548	-6.05241	0.858571	0.814044
B.cells	COPS5	-0.00894	5.288344	-0.08098	0.935637	-6.53173	0.831377	0.769467
B.cells	WDR24	-0.02665	1.796393	-0.08095	0.935665	-5.63296	0.878961	0.847985
B.cells	NDUFA3	0.007268	8.000282	0.080935	0.935676	-7.00002	0.796504	0.713514
B.cells	TRIM16	-0.02029	2.341778	-0.08089	0.935712	-6.02319	0.871324	0.835223
B.cells	SRR	0.01831	2.599008	0.080876	0.935723	-5.88644	0.867749	0.829269
B.cells	ERCC4	0.017863	2.949475	0.080847	0.935746	-6.03818	0.862906	0.821224

B.cells	NDUFC1	-0.00873	7.367214	-0.08071	0.935851	-6.89341	0.804487	0.726198
B.cells	RBAK	-0.0281	1.336784	-0.08062	0.93593	-5.62818	0.885457	0.858888
B.cells	UTP14A	0.009366	5.238791	0.080594	0.935947	-6.54525	0.832031	0.770529
B.cells	TESC	0.037958	1.561476	0.080557	0.935976	-5.56367	0.882275	0.853541
B.cells	GPSM1	-0.01936	2.672071	-0.08054	0.935989	-5.89797	0.866737	0.827585
B.cells	CCR5	0.020899	4.693422	0.080421	0.936084	-6.17107	0.839267	0.782317
B.cells	GM14302	-0.03486	0.609708	-0.08036	0.936131	-5.41525	0.895846	0.876412
B.cells	SOGA1	-0.01789	3.807296	-0.08034	0.936145	-6.12126	0.851182	0.801853
B.cells	CDK5	-0.01436	3.72259	-0.08033	0.936155	-6.1576	0.852331	0.803746
B.cells	KLRA6	0.04545	-0.71608	0.080237	0.93623	-5.34847	0.914968	0.909255
B.cells	MYG1	-0.01181	3.803891	-0.08023	0.93624	-6.19275	0.851228	0.801929
B.cells	SDHAF3	0.01535	3.044533	0.080192	0.936266	-6.02343	0.861597	0.819055
B.cells	DGAT1	0.015213	7.095267	0.080168	0.936285	-6.85917	0.807946	0.731716
B.cells	SLC36A4	0.017521	3.21113	0.08012	0.936323	-6.13576	0.85931	0.815267
B.cells	RXYLT1	-0.01196	3.864731	-0.08012	0.936326	-6.19811	0.850404	0.800572
B.cells	ZFP61	0.034729	1.174198	0.080093	0.936344	-5.51929	0.887768	0.862777
B.cells	CDT1	0.011033	4.370678	0.080068	0.936364	-6.37173	0.843584	0.789377
B.cells	QK	0.005787	8.142329	0.080046	0.936382	-7.03418	0.794726	0.710699
B.cells	POGLUT2	0.035974	1.00166	0.07968	0.936672	-5.45181	0.890283	0.866985
B.cells	DPP9	-0.01196	4.420233	-0.07965	0.936697	-6.31513	0.842972	0.788346
B.cells	ERCC2	0.021145	1.983619	0.079483	0.936828	-5.78126	0.876385	0.843681
B.cells	C2	0.031021	2.413631	0.079482	0.936829	-5.78078	0.870378	0.833652
B.cells	EPS15L1	-0.00723	6.525246	-0.07939	0.936898	-6.79599	0.815303	0.743531
B.cells	IL5RA	-0.03432	0.975245	-0.07908	0.937148	-5.63796	0.890661	0.867735
B.cells	ANXA3	-0.02428	3.542549	-0.0789	0.937292	-5.97082	0.854833	0.807958
B.cells	SRRD	0.013848	3.465767	0.078854	0.937327	-6.10366	0.85588	0.809688
B.cells	ADPRM	-0.014	3.321315	-0.07874	0.937417	-6.0501	0.857854	0.813003
B.cells	RBM5	-0.00643	6.623423	-0.07871	0.937445	-6.80579	0.814039	0.74161
B.cells	B230217O1	-0.02987	1.757632	-0.07858	0.937543	-5.65104	0.879561	0.849176
B.cells	DDX51	0.029957	1.37149	0.078578	0.937546	-5.55131	0.885019	0.858339
B.cells	PDE3A	-0.05353	0.411945	-0.07841	0.937681	-5.33774	0.898752	0.881538
B.cells	BCL9L	0.01525	3.410778	0.078403	0.937685	-6.07822	0.856631	0.811026
B.cells	NEURL2	-0.03349	0.801683	-0.07824	0.937813	-5.56529	0.893145	0.872048
B.cells	GM21887	-0.02906	2.133944	-0.07822	0.937828	-5.72258	0.87428	0.84035
B.cells	FRMD6	0.017508	3.47737	0.078173	0.937867	-6.14258	0.855722	0.809525
B.cells	PREX2	-0.03299	2.374714	-0.07817	0.937873	-5.66247	0.87092	0.834742
B.cells	TMEM170	0.028349	1.517214	0.078137	0.937896	-5.52469	0.882955	0.854882
B.cells	PPM1D	-0.00913	4.948971	-0.07808	0.937943	-6.45421	0.835919	0.777051
B.cells	TNIK	-0.01631	4.035038	-0.07804	0.937976	-6.34949	0.848154	0.797075
B.cells	TMEM33	-0.00845	5.133185	-0.0779	0.938082	-6.49605	0.833507	0.773082
B.cells	OST4	0.008177	8.049265	0.077633	0.938295	-6.99756	0.796074	0.71281
B.cells	ZFP213	0.022699	2.162406	0.077559	0.938354	-5.71469	0.874029	0.839741
B.cells	SLAMF6	0.014142	4.602908	0.077456	0.938435	-6.3576	0.840677	0.784613
B.cells	SLC3A2	-0.00979	6.772545	-0.07722	0.938622	-6.77641	0.812363	0.738643
B.cells	UTP23	-0.01167	3.577367	-0.07713	0.938692	-6.20624	0.854611	0.807354
B.cells	LRRC61	0.022227	2.011331	0.076779	0.938973	-5.77212	0.876442	0.843385
B.cells	DIP2B	0.007199	7.880088	0.076619	0.939099	-6.9953	0.798468	0.716288

B.cells	TOR1AIP2	0.00655	6.580878	0.076604	0.939112	-6.78505	0.815	0.742667
B.cells	TFAP4	0.021459	2.748617	0.076348	0.939315	-5.86322	0.866291	0.826335
B.cells	CDKL1	0.0327	1.100722	0.075962	0.939621	-5.67188	0.889597	0.865128
B.cells	DLG2	0.038279	2.402232	0.075961	0.939622	-5.53739	0.871249	0.834387
B.cells	ZPR1	0.009707	4.397865	0.075804	0.939746	-6.37062	0.844007	0.789365
B.cells	STAC2	0.027026	1.824947	0.075706	0.939824	-5.78227	0.879385	0.847941
B.cells	CEP19	-0.02129	2.707827	-0.0754	0.940064	-5.8857	0.867209	0.827447
B.cells	CCDC22	-0.01346	3.49155	-0.07518	0.940243	-6.12294	0.85649	0.809623
B.cells	CUL5	0.006816	5.877947	0.075049	0.940345	-6.66948	0.824621	0.757618
B.cells	DPP3	-0.0101	4.728026	-0.07505	0.940348	-6.42604	0.8398	0.782265
B.cells	ELMOD3	-0.02152	3.437093	-0.07458	0.940714	-6.0345	0.8575	0.811005
B.cells	LRRC27	-0.04344	0.302993	-0.07445	0.940824	-5.23082	0.901654	0.884858
B.cells	CABIN1	-0.0082	5.4487	-0.07423	0.940998	-6.61831	0.830628	0.766909
B.cells	SH3PXD2A	-0.01135	5.753145	-0.07392	0.941237	-6.55287	0.826641	0.760449
B.cells	NELFB	0.009324	4.551192	0.073888	0.941266	-6.36975	0.842559	0.786326
B.cells	GM28379	0.038416	0.264238	0.073874	0.941277	-5.39144	0.902323	0.885879
B.cells	HSPH1	-0.0168	4.050366	-0.07376	0.941371	-6.31374	0.849298	0.797379
B.cells	CDCA5	-0.02278	3.028781	-0.07375	0.941377	-5.8896	0.863241	0.820373
B.cells	BRIP1OS	0.008559	5.408086	0.073031	0.941946	-6.55801	0.831194	0.767945
B.cells	ATOX1	-0.00678	8.244709	-0.073	0.94197	-7.09117	0.794781	0.709637
B.cells	ROBO1	-0.03008	2.137687	-0.07298	0.941986	-5.69698	0.875642	0.841115
B.cells	IKZF1	-0.00683	8.378161	-0.07287	0.942075	-7.05972	0.793115	0.707022
B.cells	COQ10B	-0.00737	5.908947	-0.07275	0.942169	-6.68908	0.824624	0.757332
B.cells	NIFK	-0.0098	4.620947	-0.0727	0.942212	-6.42244	0.841646	0.784994
B.cells	TRRAP	0.009602	4.784536	0.072547	0.942331	-6.44448	0.839461	0.781428
B.cells	GM15478	-0.01061	4.735611	-0.07243	0.942423	-6.45175	0.840114	0.782532
B.cells	MBTD1	-0.00717	7.420536	-0.07238	0.942464	-6.95562	0.805162	0.726173
B.cells	DMD	0.025209	2.419953	0.072246	0.942569	-5.74826	0.871699	0.834652
B.cells	PIH1D1	-0.00946	5.06576	-0.07215	0.942645	-6.45811	0.835721	0.775409
B.cells	2410004B1	0.008087	4.71052	0.072017	0.942751	-6.42514	0.840449	0.783155
B.cells	HIST1H4I	0.016547	4.409355	0.071901	0.942843	-6.27119	0.844482	0.789748
B.cells	WDR55	0.013674	2.979495	0.071766	0.94295	-6.02682	0.863942	0.821833
B.cells	MRPS28	0.012676	6.885185	0.071723	0.942984	-6.87718	0.811992	0.737182
B.cells	PJA1	-0.01194	3.699202	-0.07153	0.943136	-6.21108	0.854082	0.805565
B.cells	CNNM3	-0.01446	3.157652	-0.07147	0.943185	-6.02243	0.861489	0.817797
B.cells	SLC16A1	-0.01304	3.905544	-0.07145	0.943205	-6.22816	0.85128	0.800952
B.cells	PHKA1	0.014683	3.411651	0.071435	0.943213	-6.12009	0.858005	0.812037
B.cells	SF3B3	0.006462	6.034328	0.071418	0.943227	-6.68476	0.822988	0.754865
B.cells	IL4I1	-0.01753	2.841673	-0.07128	0.943339	-6.24577	0.865845	0.825031
B.cells	1110038B1	0.011699	4.235292	0.071215	0.943387	-6.30765	0.846823	0.793647
B.cells	2-Mar	-0.00752	5.868752	-0.07121	0.943389	-6.66802	0.825149	0.758365
B.cells	WDR61	0.007991	4.80713	0.071067	0.943505	-6.42913	0.83916	0.781164
B.cells	GM5431	0.035359	0.742737	0.070942	0.943604	-5.55772	0.895437	0.874698
B.cells	GM17056	-0.01864	2.866748	-0.07091	0.943631	-6.10073	0.865498	0.824548
B.cells	NADSYN1	-0.03011	1.092531	-0.07091	0.943633	-5.44712	0.890425	0.866241
B.cells	DRG1	0.005923	6.40915	0.070832	0.943692	-6.74066	0.818122	0.747127
B.cells	GM6710	0.033652	0.761027	0.070633	0.943849	-5.4284	0.895174	0.874285

B.cells	USP14	-0.0066	5.793901	-0.07059	0.943881	-6.65307	0.826127	0.760058
B.cells	EFNA5	0.038668	2.313548	0.070582	0.94389	-5.61683	0.873183	0.837362
B.cells	PRPS1L3	-0.01984	2.045409	-0.07056	0.943904	-5.75855	0.876936	0.843629
B.cells	PATZ1	-0.01049	4.251704	-0.0702	0.944195	-6.30498	0.846602	0.793409
B.cells	PPP1R3F	-0.03123	0.867721	-0.07017	0.944214	-5.43804	0.893643	0.871707
B.cells	PTPN21	-0.0327	0.813665	-0.07014	0.944243	-5.43596	0.894418	0.873017
B.cells	RANGRF	0.031658	1.262196	0.070069	0.944297	-5.50226	0.888006	0.862206
B.cells	POLA1	0.012551	5.968201	0.069921	0.944415	-6.60674	0.82385	0.756387
B.cells	A930029G2	-0.02697	1.230907	-0.06991	0.944421	-5.57416	0.888452	0.862956
B.cells	MEX3D	-0.01632	2.642534	-0.06967	0.944615	-5.99221	0.868603	0.829767
B.cells	KCTD9	0.014276	3.130956	0.069667	0.944616	-5.98214	0.861856	0.818569
B.cells	MIR155HG	0.014724	4.086438	0.069487	0.944759	-6.55832	0.848832	0.79709
B.cells	TRPM7	0.006452	7.284405	0.06926	0.944939	-6.90489	0.806892	0.729231
B.cells	ANKRD11	-0.00565	9.391983	-0.06908	0.945082	-7.2332	0.780593	0.68772
B.cells	FAM13A	0.030315	1.15315	0.068985	0.945157	-5.5929	0.88956	0.864958
B.cells	NSMCE2	0.005345	7.428191	0.068881	0.94524	-6.9163	0.805065	0.726404
B.cells	GM50340	-0.02833	1.533078	-0.06885	0.945263	-5.55239	0.884159	0.855911
B.cells	KLK1B27	-0.0349	-1.42677	-0.06864	0.945429	-5.08699	0.926952	0.929221
B.cells	2510017J1	0.026241	1.414805	0.06828	0.945717	-5.65417	0.885837	0.858822
B.cells	PDRG1	-0.00933	4.496479	-0.06821	0.945774	-6.3827	0.843313	0.788271
B.cells	CCDC32	0.013111	3.31271	0.068176	0.945799	-6.07365	0.85936	0.81468
B.cells	D430020J0	-0.03908	0.440007	-0.06788	0.946035	-5.31008	0.899801	0.882521
B.cells	CLK3	-0.00836	4.807812	-0.06785	0.946056	-6.43342	0.839151	0.781574
B.cells	TBC1D2B	0.016879	4.091379	0.067771	0.946121	-5.94411	0.848765	0.79732
B.cells	SAMD12	0.036997	0.266619	0.067745	0.946142	-5.46902	0.902311	0.88679
B.cells	DST	-0.01735	4.335211	-0.06764	0.946223	-6.11149	0.845478	0.79196
B.cells	ACTL6A	0.007551	5.235041	0.067641	0.946224	-6.52846	0.833479	0.772368
B.cells	DNAH2	0.028049	0.783062	0.067431	0.946391	-5.5146	0.894858	0.874265
B.cells	SNRK	0.009654	4.865356	0.067411	0.946406	-6.41572	0.838384	0.780423
B.cells	CD9	-0.01175	6.033746	-0.06732	0.94648	-6.71823	0.822996	0.755463
B.cells	JAML	0.019329	2.748338	0.067263	0.946524	-5.99274	0.867136	0.827804
B.cells	FAM189A1	-0.02138	2.327494	-0.06719	0.946581	-6.02269	0.872988	0.837551
B.cells	TMEM63B	-0.01113	3.925117	-0.06714	0.946621	-6.32637	0.851014	0.801137
B.cells	ZDHHC12	0.027121	1.360226	0.067028	0.946711	-5.58293	0.886612	0.860414
B.cells	GM49173	-0.05194	-0.87955	-0.06697	0.946755	-5.20472	0.918821	0.915605
B.cells	DCUN1D2	-0.01684	2.743543	-0.06691	0.946804	-5.93954	0.867203	0.827949
B.cells	B130055M	0.019118	2.013314	0.0669	0.946813	-5.78799	0.877387	0.844935
B.cells	PYROXD2	-0.03451	0.182818	-0.0668	0.946888	-5.32247	0.903527	0.889032
B.cells	CCDC88C	0.008314	5.294187	0.066657	0.947006	-6.60331	0.832697	0.771246
B.cells	FANCG	0.020344	2.222876	0.066637	0.947021	-5.67811	0.87445	0.840066
B.cells	TNS1	-0.02321	2.761945	-0.06647	0.947153	-5.74485	0.866948	0.827572
B.cells	RFX7	-0.00718	7.368214	-0.06638	0.947226	-6.91964	0.805826	0.727989
B.cells	GM14321	0.037437	-0.49943	0.066313	0.947278	-5.23386	0.913458	0.906071
B.cells	MRPL20	-0.00677	6.1388	-0.06624	0.947337	-6.71382	0.821629	0.753352
B.cells	UBE2V2	0.006978	5.614121	0.06622	0.947352	-6.61431	0.828484	0.764438
B.cells	AP3M2	-0.01627	2.489505	-0.06617	0.947395	-5.81926	0.87073	0.833891
B.cells	4930549G2	0.013527	2.714781	0.066089	0.947456	-6.00386	0.867601	0.828682

B.cells	MMUT	-0.01469	3.4125	-0.06605	0.94749	-6.08181	0.857994	0.812751
B.cells	SNX27	-0.00636	5.803763	-0.066	0.947529	-6.65555	0.825998	0.760412
B.cells	ZZZ3	0.007528	5.884403	0.065988	0.947536	-6.66603	0.824944	0.758707
B.cells	IGF2BP2	-0.02567	1.457318	-0.06581	0.947675	-5.57429	0.885233	0.858169
B.cells	RNF24	-0.01033	4.233052	-0.06567	0.947788	-6.3313	0.846853	0.794414
B.cells	MFNG	-0.01171	3.090104	-0.06565	0.947805	-6.09796	0.862418	0.820088
B.cells	TNFSF13	-0.0287	1.863945	-0.06557	0.947869	-5.6143	0.879487	0.848538
B.cells	AATK	-0.0393	0.48294	-0.06551	0.947913	-5.30965	0.89918	0.881729
B.cells	SLC25A5	-0.00599	7.608641	-0.06547	0.94795	-6.94946	0.802778	0.723161
B.cells	MED13	-0.0061	7.722854	-0.06547	0.947951	-6.99486	0.801335	0.720866
B.cells	HAUS1	0.013358	3.65784	0.065248	0.948124	-6.11038	0.854645	0.807234
B.cells	ERGIC2	-0.00532	6.017059	-0.06517	0.948185	-6.667	0.823213	0.755922
B.cells	SNX12	-0.00744	4.718214	-0.06517	0.948186	-6.40392	0.840346	0.783757
B.cells	SPHK2	0.012926	3.388566	0.0651	0.948242	-6.06572	0.858321	0.813306
B.cells	FZD1	-0.02907	-0.15494	-0.06499	0.948329	-5.43009	0.908447	0.89748
B.cells	U2AF1L4	-0.0154	3.047054	-0.06479	0.948491	-6.01051	0.86301	0.821071
B.cells	PRPSAP1	-0.00833	4.34081	-0.06478	0.948496	-6.35156	0.845403	0.792034
B.cells	ZFP7	-0.03264	0.286794	-0.06478	0.948497	-5.41206	0.902018	0.886544
B.cells	CDKL3	-0.0177	3.044103	-0.06459	0.948648	-5.94828	0.863051	0.821138
B.cells	EEF1AKMT	0.010268	4.588498	0.064392	0.948804	-6.39211	0.84208	0.786651
B.cells	D17H6S53E	0.009194	4.009289	0.064237	0.948927	-6.26162	0.849875	0.799451
B.cells	TACO1OS	0.022209	1.705089	0.064232	0.94893	-5.65379	0.881727	0.852372
B.cells	SRP19	0.00676	6.048783	0.064205	0.948952	-6.66104	0.8228	0.755325
B.cells	COQ5	0.012323	3.832511	0.064007	0.949109	-6.18089	0.85227	0.803407
B.cells	HELZ	0.00669	6.25998	0.06395	0.949154	-6.71244	0.820055	0.750909
B.cells	4930556J24	-0.01495	2.185085	-0.06393	0.949167	-5.89324	0.874979	0.841086
B.cells	RSL1D1	-0.00703	6.043414	-0.06374	0.949325	-6.6911	0.82287	0.755491
B.cells	DLG1	-0.00564	7.219142	-0.06372	0.949335	-6.89466	0.807723	0.731163
B.cells	CNOT1	0.00521	7.026328	0.063722	0.949335	-6.86795	0.810184	0.735098
B.cells	LAMTOR4	-0.00781	6.205335	-0.0637	0.949355	-6.6712	0.820764	0.752094
B.cells	GM34921	-0.03095	0.841113	-0.06367	0.949374	-5.40547	0.894024	0.873146
B.cells	ZFAND2A	0.011305	3.630572	0.063486	0.949523	-6.08117	0.855017	0.807998
B.cells	CXCL3	-0.05257	-0.07498	-0.06344	0.949559	-5.28054	0.907279	0.895671
B.cells	ARMC8	0.007029	5.019279	0.063275	0.949691	-6.43021	0.836338	0.777415
B.cells	ARPC2	0.003697	9.318766	0.063127	0.949808	-7.23636	0.78149	0.689754
B.cells	EIF4A2	-0.00651	5.530319	-0.06312	0.949816	-6.57125	0.829585	0.766455
B.cells	MRPS10	-0.00925	4.162492	-0.0629	0.949986	-6.27486	0.847805	0.796204
B.cells	RNPS1	-0.00576	6.167896	-0.0629	0.949989	-6.70374	0.82125	0.75297
B.cells	CTNNAL1	0.023569	1.924908	0.062802	0.950066	-5.65557	0.878629	0.847368
B.cells	4930453N2	-0.00842	4.481233	-0.06278	0.950087	-6.37223	0.843517	0.789202
B.cells	SLC16A6	0.011243	4.554893	0.062724	0.950128	-6.40809	0.84253	0.787589
B.cells	EIF4ENIF1	-0.00674	5.556503	-0.06267	0.950172	-6.56651	0.829241	0.765931
B.cells	ZFP808	-0.01989	1.973033	-0.06254	0.950274	-5.63402	0.877952	0.846287
B.cells	MTCH2	0.006282	5.950226	0.062434	0.950358	-6.67644	0.824085	0.757616
B.cells	GM17259	-0.02574	1.365713	-0.06243	0.950364	-5.54037	0.886534	0.860695
B.cells	TERF2IP	-0.01028	3.58313	-0.06235	0.950428	-6.11796	0.855663	0.809219
B.cells	HINT1	-0.00569	7.995864	-0.06231	0.950453	-7.00484	0.797898	0.715679

B.cells	IDH3G	0.008698	5.124044	0.062221	0.950527	-6.50733	0.834948	0.775244
B.cells	ATP5C1	-0.00449	8.358211	-0.06216	0.950574	-7.07924	0.793363	0.708499
B.cells	GM20219	-0.0296	0.646352	-0.06209	0.950633	-5.39075	0.896824	0.878069
B.cells	DEPP1	0.031979	1.043364	0.061952	0.950741	-5.45708	0.89113	0.868439
B.cells	ZFP97	0.022665	1.263623	0.061827	0.95084	-5.57676	0.887988	0.86314
B.cells	MRPL27	-0.00994	4.210901	-0.06161	0.951014	-6.29233	0.847154	0.795203
B.cells	ELOVL6	0.011932	4.451031	0.061487	0.95111	-6.35026	0.843925	0.789906
B.cells	ECHDC1	0.011143	3.820316	0.061482	0.951114	-6.21779	0.852438	0.803895
B.cells	LIG4	-0.02151	1.902715	-0.06146	0.95113	-5.73518	0.878943	0.847943
B.cells	TLE5	0.00583	7.209326	0.061417	0.951166	-6.85683	0.80785	0.731519
B.cells	BICC1	0.037035	0.332453	0.061304	0.951255	-5.34634	0.901359	0.885755
B.cells	SIGLECH	0.047295	0.771994	0.061254	0.951295	-5.24224	0.895019	0.87501
B.cells	1700097N(	0.019251	2.346622	0.061144	0.951382	-5.82999	0.872736	0.837541
B.cells	GFI1	-0.0191	1.55053	-0.06069	0.951743	-5.75264	0.884181	0.856367
B.cells	GM40645	-0.02997	0.751306	-0.06062	0.951802	-5.46329	0.895587	0.875596
B.cells	DCXR	-0.00999	4.317514	-0.06046	0.951929	-6.32766	0.846021	0.792922
B.cells	RAPSN	0.022332	0.715904	0.060285	0.952065	-5.48804	0.896205	0.876493
B.cells	NCAPH2	-0.00709	5.344606	-0.06006	0.952243	-6.50009	0.832421	0.770644
B.cells	DNAJB13	0.018081	2.103334	0.059712	0.95252	-5.74248	0.876533	0.8434
B.cells	BEND3	-0.01599	2.456588	-0.05971	0.952523	-5.88963	0.871596	0.835155
B.cells	WAS	-0.00612	5.349391	-0.05967	0.952555	-6.56479	0.832358	0.770564
B.cells	KMT5C	0.014906	2.877228	0.059613	0.952598	-5.89881	0.865758	0.825472
B.cells	CRY2	0.013439	2.553643	0.059519	0.952673	-5.91236	0.870245	0.832963
B.cells	ZFP512B	-0.01772	2.377698	-0.05948	0.952701	-5.82862	0.872696	0.837049
B.cells	ACP2	-0.01435	3.54465	-0.05944	0.95274	-6.02372	0.856589	0.810319
B.cells	GM20186	0.014718	3.559404	0.059404	0.952764	-6.08212	0.856387	0.809996
B.cells	GM47096	0.020854	1.734536	0.059217	0.952913	-5.5895	0.881793	0.85224
B.cells	PEX10	-0.02534	0.82136	-0.05905	0.953047	-5.45721	0.894854	0.874154
B.cells	ALKBH2	-0.01395	2.187068	-0.05882	0.953225	-5.7797	0.875581	0.841595
B.cells	TTC37	0.011296	3.798842	0.058593	0.953409	-6.25154	0.853399	0.80477
B.cells	KATNB1	-0.0171	2.346793	-0.05856	0.953436	-5.80094	0.873406	0.837954
B.cells	LRRC8D	0.006177	7.366735	0.058303	0.953639	-6.94796	0.80659	0.728758
B.cells	PEX12	0.019156	1.86574	0.058172	0.953743	-5.67059	0.880302	0.849325
B.cells	TMEM203	-0.01276	3.168142	-0.05805	0.953837	-6.01886	0.862187	0.819151
B.cells	KLF8	-0.02813	0.979838	-0.05789	0.953966	-5.53022	0.892961	0.870541
B.cells	RAB29	0.010444	3.635461	0.057706	0.954113	-6.22651	0.855881	0.808569
B.cells	GM30881	0.016261	1.986079	0.057588	0.954207	-5.77299	0.878725	0.846532
B.cells	GSTZ1	-0.0103	4.205829	-0.05755	0.954237	-6.27773	0.848143	0.795835
B.cells	1700021F0	-0.01133	3.209032	-0.05729	0.954444	-6.03002	0.861841	0.81829
B.cells	CTBP1	0.005722	6.500976	0.056788	0.954842	-6.75019	0.818219	0.746722
B.cells	CSTF2T	0.010978	3.017029	0.056648	0.954954	-6.00471	0.86481	0.822802
B.cells	CD37	-0.00612	7.409059	-0.05652	0.955052	-6.90896	0.806601	0.728114
B.cells	IFFO1	0.016165	2.880878	0.05638	0.955167	-5.93516	0.866692	0.825925
B.cells	LMNB1	-0.00727	7.679763	-0.05636	0.955183	-6.93142	0.803167	0.722649
B.cells	ARPC4	-0.00462	7.957802	-0.0562	0.955308	-7.00785	0.799658	0.717078
B.cells	TRIM62	0.016469	1.410517	0.056192	0.955316	-5.66891	0.88732	0.860393
B.cells	ZFP438	0.010946	3.204144	0.055847	0.95559	-6.07005	0.862413	0.818579

B.cells	ENTPD5	-0.00812	3.78751	-0.05568	0.955725	-6.18313	0.854485	0.80544
B.cells	TRIOBP	-0.00672	4.877202	-0.05531	0.956017	-6.40568	0.839855	0.781436
B.cells	SLAMF7	0.008229	5.151332	0.055272	0.956047	-6.71508	0.836208	0.775495
B.cells	IGF2BP1	-0.03773	-0.96106	-0.05513	0.956162	-5.15277	0.921789	0.91911
B.cells	NUCKS1	0.006902	6.576295	0.055106	0.956179	-6.73183	0.817549	0.745331
B.cells	HTATIP2	0.008384	4.480259	0.054944	0.956307	-6.29081	0.84517	0.790118
B.cells	JAM2	0.028124	1.449339	0.054939	0.956311	-5.54651	0.887067	0.859627
B.cells	LENG8	0.007552	4.564084	0.054868	0.956367	-6.39862	0.844044	0.788277
B.cells	YES1	0.011962	4.740029	0.054862	0.956372	-6.42905	0.841687	0.784425
B.cells	C030005K0	0.022676	0.674423	0.054585	0.956592	-5.49376	0.898252	0.878383
B.cells	ZFP386	-0.00846	3.889207	-0.05447	0.956686	-6.28183	0.853242	0.803274
B.cells	NABP1	0.010345	4.743721	0.054463	0.956689	-6.25425	0.841723	0.784393
B.cells	MAPKAPK5	-0.01416	2.128701	-0.05416	0.956926	-5.7282	0.877652	0.843637
B.cells	ANGEL2	0.005779	5.052327	0.053993	0.957062	-6.48521	0.837697	0.777749
B.cells	PF4	0.030353	4.278945	0.053903	0.957134	-6.22477	0.848055	0.794695
B.cells	NDUFAF3	-0.00815	4.160988	-0.05387	0.957157	-6.25428	0.849649	0.797308
B.cells	LPAR1	0.030275	0.763125	0.053829	0.957193	-5.58378	0.897069	0.876324
B.cells	ZFP655	0.007317	4.453763	0.053696	0.957299	-6.37272	0.845701	0.790837
B.cells	ANK	0.011239	3.446389	0.053639	0.957344	-6.24541	0.859375	0.813334
B.cells	TRAJ18	-0.0246	-1.3552	-0.0536	0.957378	-5.23298	0.927862	0.929375
B.cells	ADGRA2	0.027192	0.687625	0.05326	0.957645	-5.51717	0.898292	0.878178
B.cells	ZFP251	0.014354	2.407099	0.05323	0.957669	-5.82417	0.873884	0.8372
B.cells	PPP1R18OS	-0.01939	0.993134	-0.05293	0.957907	-5.42994	0.894053	0.87083
B.cells	STON1	0.027932	1.087662	0.052802	0.958009	-5.4071	0.892699	0.868563
B.cells	DCLRE1A	-0.01725	1.621612	-0.0527	0.958091	-5.66811	0.885091	0.855796
B.cells	SELENOH	-0.00988	5.20549	-0.05265	0.958128	-6.49872	0.835934	0.774589
B.cells	SNAPC2	-0.01132	3.015281	-0.05248	0.958263	-6.04345	0.86564	0.823307
B.cells	RALGPS1	-0.00792	4.92479	-0.05231	0.958401	-6.43312	0.839774	0.780739
B.cells	GM39326	0.019133	1.289251	0.052114	0.958556	-5.62142	0.889932	0.863899
B.cells	YRDC	0.006418	5.035051	0.051819	0.95879	-6.52763	0.838305	0.778445
B.cells	PTEN	0.003929	8.288084	0.051772	0.958827	-7.08973	0.796306	0.71105
B.cells	PYCRL	0.00869	3.541294	0.05176	0.958837	-6.1393	0.858463	0.811495
B.cells	NOL8	0.008368	4.078757	0.051517	0.959029	-6.2981	0.851145	0.799486
B.cells	TENM4	0.025668	1.760128	0.051513	0.959033	-5.53979	0.883243	0.852753
B.cells	ARSB	-0.0124	4.938464	-0.05142	0.959108	-6.36249	0.839591	0.780595
B.cells	KLRI1	0.025844	-0.19768	0.051401	0.959122	-5.41615	0.911438	0.900424
B.cells	JAG1	0.026381	1.3378	0.051349	0.959163	-5.54291	0.889239	0.862839
B.cells	OXA1L	0.006178	4.658758	0.05091	0.959512	-6.44621	0.84333	0.786697
B.cells	GM38115	-0.00719	3.861728	-0.05082	0.959587	-6.38753	0.854091	0.804346
B.cells	ARHGAP45	0.005217	6.710119	0.050731	0.959654	-6.85728	0.81636	0.743027
B.cells	TMEM106E	0.007198	4.565283	0.050551	0.959797	-6.36301	0.844584	0.788747
B.cells	GM3550	-0.01433	1.752212	-0.0505	0.959835	-5.69129	0.883355	0.852958
B.cells	SULF2	0.016247	2.585463	0.050416	0.959905	-5.79812	0.871659	0.833422
B.cells	PRKG1	0.017489	3.504872	0.050304	0.959993	-6.00261	0.858961	0.812394
B.cells	PEX13	0.005791	5.524698	0.050244	0.960041	-6.59419	0.831819	0.767983
B.cells	TMEM14C	0.00512	6.950542	0.05011	0.960148	-6.83401	0.813266	0.7381
B.cells	ZFP1	0.008585	3.874226	0.049862	0.960345	-6.18685	0.853921	0.804098

B.cells	CCNJL	0.018357	1.734552	0.049819	0.960379	-5.61811	0.883605	0.853411
B.cells	GM43707	-0.02677	0.407926	-0.04981	0.960388	-5.32952	0.902608	0.885451
B.cells	PCOLCE	-0.02419	0.911461	-0.04976	0.960422	-5.45079	0.89534	0.873155
B.cells	ZFP942	0.007739	4.309464	0.049674	0.960495	-6.32414	0.848026	0.794443
B.cells	4833439L1	-0.00629	4.862518	-0.04965	0.960516	-6.46657	0.840605	0.782308
B.cells	SLC38A1	0.005432	7.937408	0.049199	0.960872	-7.01741	0.800712	0.718188
B.cells	ADAT1	-0.01612	2.003267	-0.04915	0.960912	-5.69796	0.879812	0.847162
B.cells	OAZ2	0.006215	4.485342	0.049016	0.961017	-6.35894	0.845658	0.790632
B.cells	APLP2	-0.00641	5.805079	-0.04894	0.961074	-6.66432	0.828131	0.76211
B.cells	GM10134	0.023841	0.795093	0.048896	0.961113	-5.51051	0.897014	0.876088
B.cells	BCL10	-0.00435	6.452929	-0.04887	0.96113	-6.78708	0.819685	0.748487
B.cells	MINDY3	0.00519	5.598106	0.048811	0.96118	-6.63732	0.830851	0.766514
B.cells	UBE2J2	0.003921	6.638057	0.048786	0.961199	-6.8181	0.81729	0.744646
B.cells	AGBL2	0.023286	0.26749	0.048738	0.961238	-5.34881	0.904647	0.889027
B.cells	ZC4H2	-0.02416	0.054562	-0.04866	0.961296	-5.31536	0.907748	0.894305
B.cells	PNPT1	0.0074	4.198204	0.048626	0.961327	-6.32045	0.849529	0.796992
B.cells	SRP54A	-0.00785	3.599318	-0.0486	0.961348	-6.13318	0.857669	0.810388
B.cells	GM13708	0.009853	3.088737	0.048574	0.961369	-6.12405	0.864681	0.821983
B.cells	E530011L2	0.023168	1.095538	0.048545	0.961391	-5.49222	0.8927	0.868824
B.cells	FAM91A1	-0.00506	5.007003	-0.04847	0.961453	-6.48169	0.838678	0.779248
B.cells	CSNK1A1	-0.00313	7.684452	-0.04844	0.961476	-6.97149	0.803907	0.723277
B.cells	PRDX6	0.004434	7.110346	0.048396	0.96151	-6.8809	0.811217	0.734928
B.cells	4930581F2	0.010333	3.634004	0.048393	0.961512	-5.96585	0.857195	0.809606
B.cells	SH3RF1	0.008247	4.357213	0.048317	0.961572	-6.33278	0.847383	0.793483
B.cells	MAGOHB	-0.00866	4.560419	-0.04823	0.961638	-6.37919	0.844649	0.789021
B.cells	HAL	0.020367	2.215151	0.048127	0.961723	-5.76258	0.876834	0.84226
B.cells	GMDS	-0.00519	6.245209	-0.04806	0.961778	-6.68257	0.822382	0.7529
B.cells	DACH2	0.024787	0.599965	0.04764	0.962111	-5.44378	0.899938	0.880977
B.cells	PXDC1	0.018192	2.191629	0.047578	0.96216	-5.79915	0.877271	0.842871
B.cells	DNAJC24	-0.00834	3.89302	-0.04754	0.962193	-6.25745	0.85377	0.803903
B.cells	HSP90AB1	-0.0038	9.925498	-0.04747	0.962243	-7.30824	0.776208	0.679658
B.cells	THSD1	0.018046	1.60027	0.047436	0.962272	-5.55254	0.885615	0.856851
B.cells	9830107B1	-0.02241	-0.62676	-0.04722	0.962441	-5.35497	0.917742	0.911575
B.cells	NETO2	0.011699	3.22493	0.047084	0.962552	-6.09838	0.862909	0.819073
B.cells	USP53	-0.00665	4.118641	-0.04707	0.962561	-6.32776	0.850708	0.798952
B.cells	RPH3AL	0.015024	1.299189	0.047012	0.96261	-5.72101	0.889898	0.864135
B.cells	GNA13	-0.00499	7.201206	-0.04674	0.962827	-6.8953	0.810153	0.733252
B.cells	CUEDC2	0.005557	5.245055	0.046695	0.962862	-6.53111	0.835617	0.774292
B.cells	CLTC	-0.00344	7.90636	-0.04655	0.962975	-6.98408	0.8012	0.719028
B.cells	CDADC1	0.008614	4.609962	0.046548	0.962978	-6.33785	0.844087	0.788133
B.cells	SH3GL1	0.006252	4.688824	0.046529	0.962993	-6.36019	0.84303	0.78641
B.cells	HAX1	0.007434	4.020737	0.046467	0.963043	-6.26311	0.852035	0.801178
B.cells	SMPD2	0.013367	2.04317	0.046354	0.963133	-5.78833	0.879357	0.846524
B.cells	TMED9	-0.00387	6.609324	-0.04634	0.963144	-6.77333	0.81776	0.745501
B.cells	A530032D1	-0.01824	0.594014	-0.04621	0.963247	-5.6205	0.900051	0.881328
B.cells	NAA16	0.004828	5.024992	0.046028	0.963392	-6.50878	0.838588	0.779165
B.cells	SRI	0.004728	6.74315	0.045785	0.963585	-6.80154	0.816078	0.742789

B.cells	ZFP142	-0.00837	3.207748	-0.04568	0.963672	-6.06474	0.863194	0.819627
B.cells	DPH3	-0.00468	5.480129	-0.04563	0.963708	-6.5682	0.832554	0.76939
B.cells	IRGQ	0.01354	2.194038	0.045621	0.963715	-5.81053	0.877286	0.843067
B.cells	APOOL	0.007296	3.780654	0.045548	0.963773	-6.2061	0.855347	0.806668
B.cells	AKAP17B	-0.01376	1.696468	-0.04553	0.963786	-5.78114	0.884301	0.854817
B.cells	CDC42SE2	0.003969	7.179375	0.045433	0.963865	-6.87927	0.810482	0.733862
B.cells	GATB	0.010306	3.11047	0.045018	0.964195	-6.02745	0.864624	0.821911
B.cells	MFAP1B	-0.00513	4.945536	-0.04497	0.96423	-6.46317	0.839732	0.780986
B.cells	TOP2B	-0.00506	6.593853	-0.0449	0.964286	-6.78743	0.81809	0.745956
B.cells	PWWP2A	0.005573	5.141602	0.044885	0.964301	-6.51985	0.837123	0.776735
B.cells	TNNI1	-0.03128	-0.907	-0.04482	0.964349	-5.22236	0.921869	0.918953
B.cells	CCL3	0.025463	5.069599	0.044761	0.964399	-5.96079	0.83808	0.778293
B.cells	ZFP157	-0.01164	2.446699	-0.04455	0.964565	-5.8892	0.873923	0.837265
B.cells	2700097O(	0.009513	3.090234	0.044272	0.964787	-6.03633	0.86506	0.822465
B.cells	GORAB	-0.01279	1.673364	-0.04421	0.96484	-5.74399	0.88488	0.855521
B.cells	GPKOW	0.004978	4.488998	0.04417	0.964869	-6.40246	0.846	0.791063
B.cells	CNIH4	0.004782	5.763142	0.043865	0.965111	-6.6236	0.829161	0.76355
B.cells	SH3BGR	-0.03163	-0.59926	-0.04384	0.965127	-5.11286	0.917773	0.911313
B.cells	SGTB	0.021533	0.3418	0.043685	0.965254	-5.3606	0.904111	0.887879
B.cells	GM36756	0.016623	0.926199	0.043565	0.96535	-5.5587	0.895668	0.873604
B.cells	LRP6	0.006207	5.378913	0.043493	0.965407	-6.50023	0.834246	0.771825
B.cells	IFT57	-0.01018	2.891644	-0.04334	0.965527	-5.91822	0.867929	0.827197
B.cells	TNFRSF23	0.01454	1.669914	0.04323	0.965616	-5.69071	0.885053	0.855791
B.cells	PHF11C	0.0112	1.989779	0.043174	0.965661	-5.87659	0.880532	0.848215
B.cells	ATRAID	-0.00567	4.57765	-0.04312	0.965704	-6.37468	0.844926	0.789287
B.cells	MOSPD2	-0.007	4.445481	-0.04308	0.965739	-6.31745	0.846704	0.792205
B.cells	GM30025	-0.01789	1.597885	-0.04283	0.965933	-5.51178	0.886172	0.857532
B.cells	GPR182	0.015332	2.557152	0.042529	0.966173	-5.70409	0.872674	0.835002
B.cells	1110017D1	-0.02624	0.201816	-0.04236	0.966308	-5.33818	0.906247	0.891547
B.cells	HEMK1	-0.01713	1.147753	-0.04209	0.966523	-5.51569	0.892588	0.868476
B.cells	SRRM2	-0.0031	8.62357	-0.04208	0.966528	-7.10726	0.792683	0.70536
B.cells	NCDN	0.00956	2.826122	0.042036	0.966565	-5.9756	0.868933	0.828892
B.cells	FANCF	-0.01562	1.911025	-0.04168	0.966845	-5.62774	0.881739	0.850346
B.cells	RABIF	0.004601	4.708703	0.04148	0.967007	-6.45162	0.843261	0.786703
B.cells	PPA1	-0.00646	4.797054	-0.04143	0.967048	-6.45718	0.842079	0.784787
B.cells	DUSP1	-0.00668	7.412842	-0.04141	0.96706	-6.87775	0.807931	0.729668
B.cells	SRSF4	-0.00372	6.225555	-0.04138	0.967088	-6.70636	0.823223	0.754193
B.cells	MRPS2	0.011378	2.843371	0.041324	0.967131	-5.96542	0.868693	0.828645
B.cells	THRA	-0.00812	3.771843	-0.0413	0.967146	-6.15544	0.855924	0.807511
B.cells	GOLT1B	0.006226	4.631519	0.041186	0.967241	-6.37706	0.844296	0.788436
B.cells	SUCLG1	0.005152	5.422184	0.041072	0.967331	-6.57399	0.833765	0.771295
B.cells	GM48086	-0.01331	1.511775	-0.04104	0.967356	-5.67936	0.887395	0.85995
B.cells	ZFP317	0.008272	2.898111	0.04094	0.967436	-6.03071	0.867934	0.827454
B.cells	RNF20	-0.00459	5.755436	-0.04087	0.967489	-6.66171	0.829373	0.764195
B.cells	EML6	0.014601	2.764743	0.040814	0.967536	-5.91974	0.869785	0.83053
B.cells	HUWE1	-0.004	7.155414	-0.04059	0.967711	-6.88282	0.811218	0.73501
B.cells	UBTF	0.003841	5.879519	0.040577	0.967725	-6.64582	0.827745	0.761591

B.cells	4833420G1	-0.00546	5.494494	-0.04053	0.967763	-6.60561	0.83281	0.769802
B.cells	CCRL2	0.020594	4.741765	0.04052	0.96777	-6.05887	0.842819	0.786104
B.cells	CLEC4A4	-0.01732	-1.00355	-0.04019	0.968036	-5.31021	0.923671	0.922207
B.cells	CLCF1	-0.00941	2.650892	-0.04011	0.968095	-6.04689	0.871368	0.833202
B.cells	FAM124A	0.016128	1.64482	0.039976	0.968203	-5.4964	0.885506	0.85685
B.cells	BCLAF3	-0.00489	4.966172	-0.03993	0.968239	-6.50283	0.83982	0.781226
B.cells	IFI205	0.022183	2.758001	0.039885	0.968275	-5.70533	0.869879	0.830743
B.cells	KLHDC4	0.006682	3.945597	0.039763	0.968372	-6.20517	0.853559	0.803766
B.cells	HSPB11	-0.00897	2.925359	-0.03965	0.968465	-6.00028	0.867557	0.826962
B.cells	SLAIN1	-0.00632	3.6132	-0.03962	0.968487	-6.2228	0.85809	0.811285
B.cells	BORCS8	-0.0054	4.590375	-0.03961	0.968493	-6.38836	0.844848	0.789517
B.cells	PIGU	0.006445	4.215968	0.039605	0.968497	-6.27917	0.849894	0.797788
B.cells	EIF2AK4	-0.00572	4.653928	-0.03955	0.968541	-6.46306	0.843996	0.788121
B.cells	CMAS	-0.00477	5.862643	-0.03945	0.96862	-6.69638	0.827966	0.762042
B.cells	NMRK1	0.006967	3.751312	0.039386	0.968672	-6.1903	0.856204	0.808173
B.cells	ZFP507	0.01205	2.176566	0.039368	0.968686	-5.80416	0.878001	0.844366
B.cells	SEC13	0.004435	5.361757	0.039349	0.968701	-6.53304	0.834565	0.772743
B.cells	DELE1	0.006854	3.426108	0.039334	0.968713	-6.05546	0.860653	0.81552
B.cells	SNX8	0.006893	5.468943	0.039036	0.96895	-6.61427	0.833274	0.770496
B.cells	TNIP3	0.029288	1.037658	0.038932	0.969032	-5.49689	0.894301	0.871604
B.cells	TSC2	-0.00779	3.109391	-0.03887	0.96908	-5.9746	0.865143	0.822815
B.cells	CAAA0111{	-0.00441	5.624186	-0.03876	0.969172	-6.60669	0.831242	0.767195
B.cells	A430005L1	-0.00664	3.764054	-0.03847	0.969403	-6.13609	0.856179	0.808029
B.cells	NUDT6	0.010823	2.183509	0.038417	0.969442	-5.77886	0.878056	0.844364
B.cells	RRS1	0.00589	4.476059	0.038286	0.969546	-6.32255	0.846533	0.792207
B.cells	A530076I1	0.022156	-0.88246	0.038281	0.96955	-5.23276	0.922071	0.919427
B.cells	CCP110	0.011194	2.37539	0.038039	0.969743	-5.74174	0.875366	0.839939
B.cells	ATP1B3	-0.0057	7.281647	-0.03791	0.969843	-6.88228	0.809745	0.732739
B.cells	EXOSC8	0.005554	5.033192	0.037642	0.970058	-6.47378	0.839073	0.780119
B.cells	TRAPPC1	0.00544	5.432947	0.037569	0.970116	-6.57237	0.833768	0.771487
B.cells	MYO3B	0.012307	1.289051	0.037443	0.970216	-5.69339	0.890724	0.865786
B.cells	BC024978	0.011576	1.865186	0.037406	0.970246	-5.78346	0.88254	0.852032
B.cells	GM17354	-0.011	1.393239	-0.0374	0.970248	-5.72164	0.889237	0.863283
B.cells	RNF38	0.003647	6.163439	0.037241	0.970377	-6.72428	0.824176	0.756032
B.cells	CBR1	-0.00662	4.350162	-0.03694	0.970619	-6.31309	0.848229	0.795178
B.cells	CBLL1	0.004265	4.917555	0.036868	0.970673	-6.48893	0.840615	0.782717
B.cells	SLC4A8	-0.01943	0.467759	-0.03684	0.970694	-5.43654	0.90254	0.885833
B.cells	NGDN	-0.00379	5.424919	-0.03684	0.970697	-6.59378	0.833874	0.771738
B.cells	D930030I0	-0.01795	0.408475	-0.03684	0.970697	-5.45601	0.9034	0.887292
B.cells	ZFP703	-0.00629	4.364442	-0.03679	0.970732	-6.40586	0.848036	0.794862
B.cells	SLC2A12	0.011536	1.619007	0.036787	0.970738	-5.74641	0.886026	0.857949
B.cells	RSBN1	-0.00444	5.326631	-0.03675	0.970769	-6.54652	0.835175	0.773853
B.cells	AKR1A1	-0.0034	7.188669	-0.03673	0.970784	-6.87502	0.810934	0.734759
B.cells	TAB1	-0.00809	3.004453	-0.03672	0.970793	-6.01701	0.866613	0.825522
B.cells	AXIN1	0.003143	6.046226	0.036256	0.97116	-6.70162	0.825742	0.758588
B.cells	PEAK1	0.005032	6.584571	0.03623	0.971181	-6.785	0.818741	0.747306
B.cells	PLD4	-0.00516	6.186695	-0.03619	0.971209	-6.8563	0.823909	0.75563

B.cells	TAB3	-0.00609	3.680364	-0.0361	0.971288	-6.17479	0.857359	0.810245
B.cells	SRP72	-0.00297	6.657086	-0.03601	0.971352	-6.81376	0.817803	0.745809
B.cells	BC005561	-0.00514	4.273496	-0.0356	0.971685	-6.31576	0.849301	0.796979
B.cells	NF2	-0.00465	4.781261	-0.03557	0.971703	-6.46389	0.842473	0.785793
B.cells	PRPS1	-0.00649	3.621673	-0.03553	0.97174	-6.1389	0.858161	0.811569
B.cells	GM17231	0.010426	2.734245	0.035428	0.971818	-5.89859	0.870398	0.831858
B.cells	SRGAP2	0.004458	6.60483	0.035369	0.971865	-6.7459	0.818479	0.746895
B.cells	MRPL24	0.003322	5.658173	0.035353	0.971878	-6.61853	0.830833	0.766841
B.cells	WFDC18	0.017174	-0.88323	0.035271	0.971943	-5.26293	0.922122	0.919784
B.cells	TMEM108	-0.00898	5.137851	-0.03525	0.971962	-6.45779	0.837716	0.778031
B.cells	A230072C0	-0.01179	1.545767	-0.03513	0.972059	-5.63375	0.887105	0.859817
B.cells	HKDC1	-0.02098	0.37121	-0.03499	0.972163	-5.4157	0.90398	0.888374
B.cells	DYNC1I2	0.00283	6.441227	0.034888	0.972247	-6.74422	0.820598	0.750359
B.cells	PRR14L	0.003495	6.50182	0.034742	0.972364	-6.75763	0.819813	0.749128
B.cells	BFSP2	0.010176	2.440103	0.034612	0.972467	-5.94212	0.874498	0.838816
B.cells	CXCR3	-0.01953	1.731497	-0.03457	0.9725	-5.46335	0.88447	0.85551
B.cells	IFITM3	-0.00785	7.709419	-0.03456	0.972508	-6.97716	0.804338	0.724389
B.cells	PPM1G	0.00299	6.448565	0.034467	0.972583	-6.77038	0.820503	0.750273
B.cells	PTDSS1	0.00364	5.371518	0.034381	0.972651	-6.59972	0.834617	0.773112
B.cells	TRIM69	0.01309	2.05026	0.034279	0.972732	-5.68184	0.879968	0.847971
B.cells	ARMCX1	0.020644	0.550831	0.034197	0.972797	-5.31037	0.901376	0.884053
B.cells	MAN1B1	-0.00575	4.927787	-0.03417	0.972817	-6.42739	0.840515	0.782725
B.cells	LCN4	0.018542	-1.05402	0.034043	0.97292	-5.23276	0.924606	0.924306
B.cells	PSMB2	0.002857	7.058131	0.034011	0.972945	-6.86244	0.812641	0.737655
B.cells	GM31814	0.015071	-0.06084	0.033925	0.973013	-5.38034	0.91028	0.899205
B.cells	RPP25L	0.005612	3.672149	0.033898	0.973035	-6.20088	0.857471	0.810579
B.cells	CSGALNAC	-0.01238	2.818139	-0.03386	0.973062	-5.82115	0.869232	0.830086
B.cells	GM12703	0.021541	-0.21463	0.033797	0.973115	-5.26108	0.912534	0.903082
B.cells	NPHP4	-0.01642	0.297045	-0.03348	0.973365	-5.40846	0.90507	0.890436
B.cells	AMPD2	-0.00959	2.339378	-0.03347	0.973379	-5.75149	0.875919	0.841313
B.cells	PHYKPL	0.005242	3.470266	0.033455	0.973387	-6.12646	0.860246	0.815256
B.cells	SASS6	-0.00551	4.42508	-0.03341	0.973422	-6.32893	0.847267	0.793881
B.cells	TMEM50A	-0.00285	7.711684	-0.0333	0.973513	-6.96351	0.804334	0.724473
B.cells	THNSL1	0.015367	1.077135	0.03318	0.973606	-5.47036	0.89384	0.871426
B.cells	KCTD12	-0.0042	6.383969	-0.03286	0.973859	-6.66988	0.821428	0.751862
B.cells	REV3L	0.003117	6.713202	0.032844	0.973873	-6.81694	0.817165	0.745002
B.cells	ADNP	0.00312	6.50802	0.032836	0.973879	-6.75349	0.819819	0.74927
B.cells	DNAH10	-0.01963	0.024576	-0.03259	0.974078	-5.26129	0.909179	0.897404
B.cells	DENND6B	0.01318	0.94417	0.032456	0.974181	-5.50463	0.895849	0.874815
B.cells	KLK1	0.027836	-0.20745	0.032089	0.974473	-5.14062	0.912578	0.903243
B.cells	SLC9A6	0.00674	2.81804	0.032088	0.974474	-5.93906	0.869376	0.830415
B.cells	BAP1	0.005803	3.565024	0.032053	0.974502	-6.10774	0.859076	0.813333
B.cells	TMC6	-0.00402	4.632332	-0.03202	0.974532	-6.4094	0.844607	0.78953
B.cells	PPP1R15A	0.004951	7.089434	0.031919	0.974608	-6.89244	0.812372	0.737364
B.cells	MBNL3	0.005279	4.597519	0.031875	0.974644	-6.42036	0.845074	0.790348
B.cells	GM20033	-0.01626	0.595708	-0.03182	0.974687	-5.42376	0.900873	0.883391
B.cells	JUNB	-0.00373	9.343714	-0.0316	0.974859	-7.27144	0.784074	0.692542

B.cells	AGRP	0.011694	1.935634	0.031435	0.974994	-5.74685	0.881728	0.851105
B.cells	P2RY12	0.011255	2.616799	0.031346	0.975064	-5.75647	0.872175	0.835135
B.cells	PFDN2	-0.00356	5.031244	-0.03119	0.975189	-6.491	0.839272	0.780862
B.cells	CHD4	0.00264	7.633169	0.031161	0.975212	-6.95374	0.805437	0.7263
B.cells	TNFRSF14	-0.01793	0.252225	-0.03109	0.975267	-5.32703	0.905858	0.891856
B.cells	ZFP948	0.008392	2.965976	0.031047	0.975302	-6.00304	0.867324	0.827062
B.cells	PHF19	0.009466	1.934693	0.03102	0.975324	-5.64966	0.881741	0.851127
B.cells	ARFGAP1	0.003984	4.620741	0.030967	0.975366	-6.38925	0.844762	0.789837
B.cells	KBTBD4	0.006575	2.78551	0.030917	0.975406	-5.98773	0.869827	0.831225
B.cells	PXMP4	0.005709	3.831766	0.030915	0.975407	-6.16051	0.855433	0.807374
B.cells	MCUB	-0.01253	1.557619	-0.03074	0.975542	-5.70847	0.887086	0.860096
B.cells	PARG	-0.00305	5.560263	-0.03069	0.975589	-6.60469	0.832264	0.769446
B.cells	METTL17	0.006897	2.488504	0.03063	0.975633	-5.92986	0.87397	0.83812
B.cells	SH2D2A	-0.00895	2.764079	-0.03041	0.975811	-5.98863	0.870132	0.83172
B.cells	4930414N(	-0.00688	3.331114	-0.03035	0.975857	-6.07841	0.862292	0.818702
B.cells	ILK	0.003766	5.628263	0.030319	0.975881	-6.58681	0.831368	0.76799
B.cells	SEPHS2	-0.0044	6.39868	-0.03017	0.976002	-6.77749	0.821291	0.751693
B.cells	DPY30	-0.00421	5.414831	-0.03017	0.976003	-6.53013	0.834186	0.772567
B.cells	PCNX4	-0.00888	2.141019	-0.02995	0.976177	-5.71345	0.878932	0.846258
B.cells	TMEM229E	0.005571	4.03146	0.029612	0.976443	-6.13345	0.852888	0.8029
B.cells	RFC2	-0.00402	5.227985	-0.02959	0.976462	-6.51812	0.836823	0.776597
B.cells	CAPN7	0.002977	5.340998	0.029529	0.976509	-6.55429	0.835324	0.774157
B.cells	CERS2	-0.00348	5.262709	-0.02946	0.976562	-6.52466	0.836362	0.775846
B.cells	RARS	0.003558	5.121145	0.029354	0.976649	-6.50026	0.838243	0.77891
B.cells	ACAP3	0.006922	2.238471	0.029254	0.976728	-5.78417	0.877641	0.843968
B.cells	PTPN9	0.003444	5.706865	0.02919	0.976779	-6.63605	0.830493	0.766312
B.cells	SHOC2	0.002739	6.344774	0.028858	0.977043	-6.74196	0.82215	0.752882
B.cells	PPRC1	-0.00423	3.956149	-0.02847	0.977351	-6.29737	0.853911	0.804674
B.cells	UBIAD1	0.006929	1.941616	0.02845	0.977367	-5.74114	0.881819	0.851059
B.cells	PLEKHG6	-0.01225	-0.11425	-0.02843	0.977382	-5.29408	0.911393	0.901075
B.cells	RAD9A	0.008979	2.338849	0.028378	0.977424	-5.80369	0.876234	0.841713
B.cells	GM16541	0.005417	2.915994	0.028292	0.977493	-6.11967	0.86819	0.828308
B.cells	NDUFB5	0.002603	7.037752	0.028243	0.977532	-6.86449	0.813197	0.738523
B.cells	CLPB	0.003935	4.010407	0.02818	0.977582	-6.24938	0.853174	0.803463
B.cells	2610507I0:	-0.01474	0.583661	-0.02813	0.977619	-5.41471	0.901228	0.883788
B.cells	GNPDA2	0.005382	3.100129	0.028103	0.977643	-5.97874	0.865642	0.824076
B.cells	SLC41A1	0.006214	2.387756	0.02799	0.977733	-5.97975	0.875549	0.840569
B.cells	GM34455	0.010883	2.623429	0.027886	0.977816	-5.68744	0.872257	0.835077
B.cells	INTS6L	0.003759	5.229737	0.027791	0.977892	-6.51888	0.8368	0.776656
B.cells	ATF2	-0.00284	6.46092	-0.02778	0.977903	-6.75691	0.820641	0.750485
B.cells	SWAP70	0.004039	5.61218	0.027663	0.977993	-6.6755	0.831741	0.768453
B.cells	DDT	0.003808	5.541533	0.027581	0.978059	-6.61708	0.832672	0.769979
B.cells	AFTPH	-0.00249	6.658529	-0.02754	0.978093	-6.83167	0.818082	0.74641
B.cells	GM10353	0.006162	2.745067	0.0273	0.978282	-5.97496	0.870564	0.832418
B.cells	GM34095	0.014484	-0.39051	0.027292	0.978288	-5.36056	0.915453	0.908187
B.cells	SCCPDH	0.0068	2.465776	0.027222	0.978344	-5.82061	0.874457	0.83892
B.cells	NEPRO	-0.00592	2.709524	-0.02705	0.978485	-6.00208	0.871058	0.833269

B.cells	NOD2	-0.00968	1.576786	-0.02704	0.978491	-5.75348	0.886986	0.859932
B.cells	KLHL20	-0.00588	3.27084	-0.02703	0.978499	-6.10016	0.863288	0.820357
B.cells	PDS5B	0.002684	6.377064	0.02697	0.978545	-6.74705	0.82173	0.752416
B.cells	KLHL15	-0.00354	4.413728	-0.02668	0.978775	-6.37586	0.847808	0.794687
B.cells	CBWD1	0.004524	4.090993	0.026519	0.978903	-6.28215	0.852172	0.801868
B.cells	TMEM175	0.00517	3.375009	0.026488	0.978928	-6.13898	0.861948	0.818006
B.cells	ARL14EP	0.004939	3.524764	0.026363	0.979027	-6.10612	0.859893	0.814605
B.cells	PRR12	0.004708	3.451612	0.026249	0.979118	-6.08129	0.860896	0.816264
B.cells	ZFP773	-0.01397	0.510873	-0.02624	0.979128	-5.4188	0.90238	0.885804
B.cells	HAUS7	0.005693	2.992661	0.025952	0.979354	-5.90737	0.867222	0.826755
B.cells	SBF2	0.003794	5.368073	0.025664	0.979583	-6.56787	0.835056	0.773891
B.cells	ELOB	-0.00213	8.913307	-0.02561	0.979624	-7.15452	0.789628	0.70117
B.cells	ZBTB4	-0.00458	3.655496	-0.0256	0.979631	-6.16525	0.858103	0.811678
B.cells	GM37168	0.010767	0.65738	0.025561	0.979665	-5.4981	0.900259	0.88226
B.cells	GM50218	-0.01373	0.559001	-0.02543	0.979768	-5.39117	0.901683	0.88472
B.cells	NLRP3	-0.01109	4.527197	-0.02527	0.979897	-5.98819	0.84628	0.792329
B.cells	GM43773	0.010179	1.074002	0.025226	0.979932	-5.54641	0.894261	0.872218
B.cells	GM14085	-0.01403	-1.35884	-0.0252	0.97995	-5.23508	0.929637	0.933127
B.cells	RNF139	0.00327	5.218473	0.025173	0.979974	-6.52091	0.83704	0.777239
B.cells	LEF1	0.005328	5.638758	0.025125	0.980012	-6.70287	0.83148	0.7682
B.cells	TRMT61B	0.005987	4.178074	0.024962	0.980141	-6.27918	0.850992	0.800089
B.cells	AC154200.	-0.01007	0.660722	-0.02487	0.980213	-5.44825	0.900211	0.882328
B.cells	UACA	0.009768	2.51718	0.024859	0.980223	-5.64213	0.873834	0.837956
B.cells	TFRC	-0.00303	6.38061	-0.02467	0.980375	-6.67365	0.821773	0.752543
B.cells	MAG	0.015272	0.11897	0.024547	0.980471	-5.2824	0.90808	0.895723
B.cells	GM17092	0.007668	2.1249	0.024482	0.980524	-5.70183	0.879332	0.847169
B.cells	CD177	-0.01249	0.17299	-0.02444	0.980559	-5.45973	0.907292	0.894391
B.cells	SURF6	-0.00401	3.185009	-0.02443	0.980564	-6.06072	0.864565	0.822562
B.cells	GM44751	-0.00818	1.310612	-0.02397	0.980929	-5.67914	0.890874	0.866661
B.cells	ZFP239	-0.01341	-0.06752	-0.0238	0.981062	-5.26165	0.910807	0.900517
B.cells	RASAL3	0.003254	4.10056	0.023789	0.981074	-6.34831	0.852043	0.80199
B.cells	SPI1	-0.0025	6.411888	-0.02374	0.981112	-6.83712	0.821366	0.752021
B.cells	YAF2	-0.00222	6.056106	-0.02372	0.981132	-6.6714	0.826003	0.759509
B.cells	MRPS21	-0.00189	7.034242	-0.0237	0.981142	-6.84694	0.813331	0.739105
B.cells	NFKB1	-0.00311	8.743793	-0.02366	0.981174	-7.15731	0.791732	0.704764
B.cells	4933406I1[illegible]	-0.00436	4.543564	-0.02354	0.981276	-6.54998	0.84606	0.792178
B.cells	ZFP36L2	0.002789	8.376464	0.023468	0.98133	-7.08234	0.796315	0.712007
B.cells	LIPT2	0.009504	1.430834	0.023322	0.981446	-5.51669	0.889159	0.86387
B.cells	MAFK	0.004354	4.524193	0.023286	0.981475	-6.38197	0.846321	0.79264
B.cells	ITPR2	-0.00236	6.912603	-0.02318	0.981559	-6.83127	0.814894	0.74167
B.cells	KIF1B	0.00304	5.385583	0.023143	0.981588	-6.54904	0.834824	0.77388
B.cells	SNRPA1	-0.00337	5.077996	-0.02298	0.981719	-6.49454	0.838908	0.780536
B.cells	STXBP2	0.002775	5.31968	0.022926	0.981761	-6.53493	0.835697	0.775312
B.cells	PSMG4	-0.00267	5.042688	-0.02291	0.981774	-6.49895	0.839378	0.781314
B.cells	CHRNB1	0.010784	0.775917	0.022706	0.981936	-5.40474	0.898548	0.87979
B.cells	PPIL3	-0.0028	4.605985	-0.02249	0.982112	-6.41801	0.845221	0.790897
B.cells	REPS1	-0.00223	5.964436	-0.02243	0.982153	-6.67544	0.827202	0.761545

B.cells	SS18	0.00233	5.946104	0.022417	0.982166	-6.67908	0.827442	0.761933
B.cells	PPP1R14B	0.002396	6.546146	0.022367	0.982206	-6.7569	0.819625	0.749311
B.cells	ZFP120	0.005056	2.536502	0.022365	0.982207	-5.86774	0.873564	0.837782
B.cells	SMDT1	0.001758	7.773748	0.022217	0.982325	-6.98844	0.803903	0.724139
B.cells	P2RY1	0.010193	0.13392	0.022216	0.982325	-5.41345	0.907862	0.895625
B.cells	UQCRQ	-0.00231	7.858453	-0.02221	0.98233	-6.99235	0.802831	0.722434
B.cells	MAPRE3	0.008395	1.502586	0.021894	0.982582	-5.56377	0.888162	0.862268
B.cells	WBP2	0.002524	5.599979	0.021892	0.982583	-6.61516	0.832014	0.769359
B.cells	CYHR1	0.002997	4.48568	0.021864	0.982605	-6.36426	0.846862	0.7936
B.cells	B4GALT3	0.003096	3.874826	0.021784	0.98267	-6.21234	0.855133	0.807221
B.cells	PHKG1	0.009701	1.107052	0.021715	0.982724	-5.54988	0.893811	0.871811
B.cells	WDR70	0.002026	6.1341	0.021498	0.982897	-6.7079	0.825089	0.758014
B.cells	VPS9D1	0.004244	3.05216	0.021361	0.983006	-6.08072	0.866541	0.825913
B.cells	TNPO2	-0.00338	4.122122	-0.02113	0.983191	-6.28517	0.851981	0.801728
B.cells	CCR4	-0.01218	-1.08113	-0.02104	0.983259	-5.22332	0.925689	0.926443
B.cells	A230056P1	0.01034	0.407448	0.020418	0.983756	-5.34892	0.904306	0.889114
B.cells	B3GNT6	-0.01136	-0.72192	-0.02041	0.983759	-5.2431	0.920696	0.917417
B.cells	CXADR	-0.00825	1.645302	-0.02039	0.983782	-5.5661	0.886526	0.859058
B.cells	GPT2	0.007494	1.738624	0.020342	0.983817	-5.65826	0.885202	0.856842
B.cells	COPS8	-0.00212	5.091019	-0.0202	0.983927	-6.49558	0.83913	0.78055
B.cells	TBCCD1	0.004922	2.589728	0.020147	0.983971	-5.90178	0.873232	0.836839
B.cells	TBCE	0.002165	5.119789	0.020143	0.983975	-6.53459	0.838747	0.779941
B.cells	PRPF38B	-0.00137	6.611272	-0.02006	0.984044	-6.80806	0.819167	0.748241
B.cells	DDIAS	0.006137	2.134705	0.019793	0.984253	-5.74109	0.8797	0.847567
B.cells	RAP2C	0.002453	4.944479	0.019742	0.984294	-6.45598	0.841172	0.783835
B.cells	LTN1	0.002255	5.03701	0.019525	0.984466	-6.46728	0.839955	0.781837
B.cells	POLR2K	0.001703	6.77475	0.019458	0.98452	-6.80848	0.817157	0.744932
B.cells	MOK	-0.0122	0.377087	-0.01942	0.984548	-5.28569	0.90486	0.890063
B.cells	ARSG	-0.00519	1.867658	-0.01935	0.984609	-5.70919	0.883486	0.853959
B.cells	ANAPC13	0.001924	5.65737	0.019184	0.984737	-6.59246	0.831749	0.768532
B.cells	TRMT13	-0.00307	3.89491	-0.01893	0.984938	-6.24985	0.855365	0.807214
B.cells	GM16279	-0.00812	1.054801	-0.01885	0.985001	-5.55828	0.895089	0.873558
B.cells	ARHGAP4	0.002281	5.057347	0.01885	0.985004	-6.45572	0.839702	0.781512
B.cells	GM29264	-0.00898	0.505466	-0.01869	0.98513	-5.34908	0.903016	0.886989
B.cells	URM1	0.002567	4.343407	0.018682	0.985137	-6.30109	0.849282	0.7972
B.cells	FANCB	-0.00901	0.677958	-0.01867	0.985146	-5.34937	0.900518	0.88275
B.cells	JOSD1	-0.00268	3.99961	-0.01847	0.985309	-6.27044	0.853996	0.804921
B.cells	PCYOX1	0.002647	4.579765	0.018405	0.985357	-6.3862	0.846151	0.792044
B.cells	GM20342	-0.00354	3.499425	-0.01831	0.985432	-6.14481	0.860829	0.816222
B.cells	TMPRSS3	0.010354	-0.48523	0.018034	0.985652	-5.25041	0.917598	0.911839
B.cells	AKIP1	-0.00406	3.24519	-0.01778	0.985854	-5.99558	0.864373	0.822116
B.cells	ZFP68	0.002599	3.920899	0.017608	0.985991	-6.21217	0.855115	0.806871
B.cells	ABCA2	0.00667	1.527476	0.017326	0.986215	-5.6082	0.888439	0.862455
B.cells	CDK18	-0.01174	-0.22726	-0.01731	0.986228	-5.19575	0.913825	0.905524
B.cells	SIGLECG	-0.00261	4.076853	-0.01726	0.986266	-6.36332	0.852994	0.803412
B.cells	PROSER3	0.006835	1.105594	0.017259	0.986269	-5.51931	0.894468	0.872626
B.cells	STAG1	-0.00125	8.353909	-0.01717	0.986343	-7.06382	0.797186	0.713234

B.cells	RBFA	0.002687	4.826424	0.017135	0.986368	-6.3701	0.842888	0.786825
B.cells	HIST1H3C	-0.00992	1.047316	-0.01713	0.986372	-5.45402	0.895305	0.874042
B.cells	SNED1	-0.00607	1.751149	-0.01702	0.986456	-5.78043	0.885262	0.857112
B.cells	SRP14	0.001225	7.234027	0.016989	0.986484	-6.89018	0.81137	0.735814
B.cells	RBM7	0.001916	5.855126	0.016911	0.986545	-6.65559	0.829247	0.76462
B.cells	IMMP1L	-0.00195	4.995599	-0.01668	0.986726	-6.47906	0.840627	0.783202
B.cells	VEGFB	0.003188	3.802219	0.016671	0.986737	-6.11924	0.856732	0.809656
B.cells	GM29019	-0.00846	0.361473	-0.01656	0.986827	-5.27617	0.905216	0.890986
B.cells	GNG2	0.001851	6.302982	0.016505	0.986869	-6.73966	0.82339	0.755256
B.cells	LRP2BP	0.006126	1.991491	0.016465	0.986901	-5.7734	0.881863	0.851551
B.cells	ATRNL1	-0.00207	6.820202	-0.01618	0.987126	-6.84121	0.816687	0.744518
B.cells	CALU	0.002083	4.640586	0.016164	0.98714	-6.35671	0.845381	0.791098
B.cells	ZFP3	-0.00745	0.562138	-0.01611	0.987181	-5.40885	0.902303	0.886116
B.cells	GM16151	-0.00768	-0.28838	-0.01587	0.987378	-5.34994	0.914724	0.907343
B.cells	TRMU	0.004915	1.548223	0.015814	0.987419	-5.57912	0.888144	0.862227
B.cells	ZFP868	-0.00271	3.676008	-0.01581	0.987424	-6.22398	0.858457	0.812678
B.cells	HDAC7	0.0025	4.31747	0.015725	0.987489	-6.34727	0.849735	0.798302
B.cells	SCPEP1OS	-0.00561	0.502575	-0.01567	0.987535	-5.7135	0.903167	0.887645
B.cells	DIDO1	-0.00176	5.734022	-0.01559	0.987599	-6.63412	0.83084	0.767433
B.cells	5530601H(	0.002812	3.904215	0.015571	0.987612	-6.28225	0.855342	0.807537
B.cells	PPM1E	-0.00333	4.964118	-0.01555	0.987626	-6.44539	0.841047	0.78406
B.cells	GM48796	-0.00727	0.350478	-0.01538	0.987762	-5.36178	0.905376	0.891434
B.cells	ACAD8	0.004138	2.476536	0.015374	0.987768	-5.73805	0.875048	0.840291
B.cells	HDAC1	0.001401	6.039987	0.015091	0.987994	-6.65763	0.826857	0.760954
B.cells	G730013B(	-0.00805	-0.968	-0.01503	0.988043	-5.20995	0.92451	0.924647
B.cells	4930404I0!	0.008413	0.086501	0.015006	0.988061	-5.39193	0.909262	0.897994
B.cells	LYSMD3	0.001893	4.927993	0.014695	0.988308	-6.47287	0.841564	0.784881
B.cells	SLC25A17	-0.00161	5.308731	-0.01469	0.988315	-6.56974	0.836494	0.776605
B.cells	PLIN2	-0.00258	5.831377	-0.0146	0.988386	-6.58783	0.829593	0.765387
B.cells	FAM71F2	0.006123	2.260769	0.01456	0.988416	-5.68205	0.878107	0.845353
B.cells	NUP188	0.002119	4.513741	0.014352	0.988581	-6.34834	0.847121	0.793985
B.cells	ZBTB6	-0.00381	1.997204	-0.01428	0.988639	-5.81258	0.881818	0.851571
B.cells	DOCK7	-0.00297	3.830588	-0.01423	0.988681	-6.11418	0.85638	0.809227
B.cells	ZKSCAN3	-0.00157	4.82207	-0.01421	0.988694	-6.42717	0.842981	0.787199
B.cells	RBBP8	-0.0014	6.609426	-0.01411	0.988774	-6.79987	0.819444	0.748997
B.cells	FFAR1	-0.00626	-0.22509	-0.01403	0.988836	-5.35082	0.91383	0.905806
B.cells	QRSL1	0.002989	3.18623	0.013877	0.98896	-6.04704	0.865222	0.82392
B.cells	LRRC1	-0.00266	3.264954	-0.01386	0.988969	-6.29949	0.864136	0.822118
B.cells	SLC24A5	0.001955	4.423305	0.01364	0.989148	-6.40164	0.84834	0.796066
B.cells	TMEM184(	0.002141	3.390595	0.013617	0.989166	-6.1939	0.862406	0.819279
B.cells	E030042O2	-0.0092	0.180529	-0.01361	0.989172	-5.24421	0.907889	0.895744
B.cells	ZFP410	0.001917	4.138179	0.013536	0.989231	-6.29621	0.852196	0.802437
B.cells	2210408I2	0.003356	3.277757	0.013407	0.989333	-5.94967	0.863959	0.821897
B.cells	KATNAL1	0.006272	0.973387	0.013312	0.989409	-5.42308	0.896403	0.876286
B.cells	GRWD1	-0.00324	2.674334	-0.01322	0.989481	-5.89864	0.872322	0.835814
B.cells	MARK3	0.001103	6.304211	0.013195	0.989502	-6.75605	0.823408	0.755494
B.cells	TMPRSS5	-0.00573	0.722493	-0.01306	0.989606	-5.46141	0.900023	0.882414

B.cells	HDAC6	0.003491	2.206503	0.012626	0.989955	-5.80743	0.878873	0.846878
B.cells	SFXN1	-0.00181	5.121606	-0.01262	0.989959	-6.45262	0.838984	0.780891
B.cells	LRRC75AOS	-0.00619	0.512247	-0.01243	0.990111	-5.39409	0.903067	0.887739
B.cells	PRTN3	0.009673	3.6691	0.012244	0.990258	-5.97462	0.858589	0.813162
B.cells	ABHD13	0.001902	3.557429	0.012192	0.9903	-6.1604	0.860118	0.815693
B.cells	TTC5	0.00142	4.64029	0.012191	0.990301	-6.42963	0.845422	0.791474
B.cells	GM15738	0.005666	0.402397	0.012081	0.990388	-5.38296	0.904661	0.890471
B.cells	FAM210A	0.002017	3.636981	0.011961	0.990483	-6.11614	0.859029	0.813889
B.cells	IAH1	-0.00177	4.521488	-0.01187	0.99056	-6.42718	0.84702	0.794096
B.cells	GSK3B	0.000827	8.105386	0.011848	0.990574	-7.00816	0.800344	0.718694
B.cells	IQGAP1	0.000948	9.247795	0.011772	0.990634	-7.22812	0.786111	0.69621
B.cells	CUTC	-0.00191	3.519969	-0.01175	0.990649	-6.16069	0.860632	0.816543
B.cells	BHMT	0.002769	5.13251	0.01164	0.990739	-6.59014	0.838839	0.780703
B.cells	SYMPK	-0.00162	4.293778	-0.01159	0.990781	-6.33779	0.850092	0.799146
B.cells	YJU2	0.001787	3.454196	0.011577	0.990789	-6.15184	0.861535	0.818039
B.cells	GM15336	-0.00272	1.682133	-0.0115	0.990849	-5.94013	0.88628	0.859369
B.cells	FBXO3	0.001275	5.012921	0.011494	0.990855	-6.46918	0.840433	0.783306
B.cells	BCCIP	0.001057	5.436859	0.01143	0.990906	-6.59325	0.834799	0.774116
B.cells	TPD52L2	-0.00109	5.433659	-0.01123	0.991065	-6.57591	0.834841	0.774208
B.cells	D3ERTD751	-0.00429	1.61523	-0.01116	0.991118	-5.55124	0.88723	0.860994
B.cells	FBXO28	-0.00152	4.523471	-0.01115	0.991125	-6.4417	0.846993	0.794076
B.cells	FRMPD4	0.005461	0.351798	0.010991	0.991256	-5.49045	0.905449	0.891764
B.cells	KLF2	0.002047	8.937753	0.010533	0.99162	-7.15333	0.79022	0.702383
B.cells	TAF4B	-0.00176	5.130162	-0.01006	0.991995	-6.56075	0.8394	0.780981
B.cells	SUSD1	-0.00157	5.183728	-0.01	0.992045	-6.46284	0.838686	0.779821
B.cells	CCDC124	0.000989	5.595782	0.009848	0.992165	-6.59436	0.833262	0.770951
B.cells	TBX21	-0.00427	2.437202	-0.00966	0.992315	-5.68248	0.876298	0.841817
B.cells	IP6K2	-0.0015	3.644965	-0.00955	0.992401	-6.17522	0.859578	0.814028
B.cells	BCL7C	0.000974	5.355918	0.009302	0.992599	-6.53299	0.836588	0.776215
B.cells	SPPL3	-0.00072	7.282604	-0.00925	0.992639	-6.90717	0.81148	0.735676
B.cells	IQSEC1	0.000954	6.277452	0.008902	0.992917	-6.74184	0.824525	0.756594
B.cells	2810402E2	0.002234	2.412627	0.008899	0.99292	-5.80311	0.876795	0.842519
B.cells	ZFP821	0.001364	3.905173	0.008846	0.992962	-6.30162	0.856162	0.808248
B.cells	FSTL1	-0.00325	2.107543	-0.00877	0.993024	-5.75062	0.881084	0.849712
B.cells	BMS1	-0.00114	4.737717	-0.0087	0.993079	-6.44705	0.844899	0.789759
B.cells	THA1	0.00509	0.09883	0.008574	0.993178	-5.28258	0.909955	0.89856
B.cells	GPR137B	-0.00115	5.316315	-0.00818	0.993495	-6.63013	0.837285	0.777244
B.cells	HIST1H1E	0.002946	4.203103	0.008173	0.993497	-6.30323	0.852225	0.801707
B.cells	GM50322	0.004572	0.358739	0.008115	0.993543	-5.33053	0.906258	0.892171
B.cells	CDK5RAP1	-0.00336	2.44604	-0.00805	0.993592	-5.80272	0.876444	0.841903
B.cells	NUP160	0.001147	5.017011	0.007977	0.993653	-6.50121	0.841272	0.783771
B.cells	KCNIP3	-0.00486	0.193763	-0.00777	0.993819	-5.38963	0.908721	0.896338
B.cells	NSD3	0.000652	8.849458	0.007714	0.993863	-7.14775	0.79193	0.704513
B.cells	GATC	0.001818	2.698485	0.007097	0.994354	-5.98636	0.873336	0.836147
B.cells	CXCL2	-0.00332	7.158496	-0.00651	0.994822	-6.68172	0.813951	0.738624
B.cells	MSRB1	0.000831	5.958368	0.006307	0.994982	-6.66471	0.82961	0.763764
B.cells	NECTIN2	-0.00189	2.109704	-0.00606	0.995181	-5.67389	0.882133	0.850136

B.cells	SOWAHC	0.001803	2.917305	0.005914	0.995295	-5.92064	0.870855	0.831282
B.cells	D8ERTD738	-0.00045	7.358725	-0.00578	0.995398	-6.91309	0.811614	0.73463
B.cells	STK38L	0.000799	4.003603	0.005488	0.995634	-6.28815	0.855957	0.806576
B.cells	PPM1H	-0.00148	6.473739	-0.00543	0.995681	-6.24138	0.823062	0.752974
B.cells	A930005H1	-0.00095	3.220496	-0.00539	0.995708	-6.06973	0.866707	0.824346
B.cells	ADAMTS9	-0.00271	2.970839	-0.00527	0.995808	-5.81927	0.870167	0.830092
B.cells	GM17387	-0.00239	0.498836	-0.00525	0.995821	-5.44806	0.905303	0.889137
B.cells	TNFSF13B	0.002352	0.855901	0.005185	0.995875	-5.51774	0.900128	0.880393
B.cells	TGFB1I1	-0.00255	0.90381	-0.00496	0.996053	-5.40494	0.899523	0.879266
B.cells	GLT1D1	0.00236	0.611079	0.004755	0.996217	-5.41418	0.90376	0.88646
B.cells	ANKRD52	0.00071	3.881446	0.004578	0.996358	-6.2368	0.857707	0.809405
B.cells	NEIL3	0.001169	4.375558	0.004511	0.996411	-6.20686	0.850989	0.798349
B.cells	MAP7	-0.00083	3.639692	-0.00447	0.996447	-6.15923	0.861016	0.814869
B.cells	IMPDH1	0.000885	4.326873	0.004426	0.996479	-6.24546	0.851648	0.799432
B.cells	1700056N1	-0.0011	2.033678	-0.00441	0.996488	-5.82423	0.883384	0.8521
B.cells	A530072M	0.002002	0.764735	0.004199	0.996659	-5.46556	0.901563	0.882685
B.cells	COMMD4	0.000482	5.326168	0.004187	0.996669	-6.57659	0.838265	0.777499
B.cells	TRPV2	-0.00051	4.873594	-0.004	0.996821	-6.52067	0.844374	0.787363
B.cells	ACAA1A	-0.00048	5.302507	-0.00381	0.996967	-6.54385	0.838706	0.77807
B.cells	TARBP2	-0.00067	3.242608	-0.00356	0.997164	-6.08656	0.866689	0.824027
B.cells	TMED3	-0.00051	5.491352	-0.00352	0.997197	-6.47039	0.836241	0.774029
B.cells	FEM1A	-0.00066	2.924816	-0.00347	0.997239	-5.99401	0.871095	0.831345
B.cells	ATP5O	0.000349	7.142669	0.003336	0.997346	-6.85782	0.81471	0.73925
B.cells	NME1	0.000414	6.668403	0.003007	0.997608	-6.80115	0.820949	0.749137
B.cells	TCEANC2	0.000385	4.247598	0.002974	0.997634	-6.34471	0.853071	0.801371
B.cells	KLRI2	-0.00131	0.447224	-0.00218	0.998266	-5.42239	0.906936	0.890937
B.cells	CAMSAP1	-0.0004	3.53399	-0.00211	0.998317	-6.14329	0.863224	0.817683
B.cells	SLC12A4	-0.00071	1.745624	-0.00211	0.998322	-5.66915	0.888245	0.859386
B.cells	LRRC63	0.000783	0.544146	0.001868	0.998514	-5.64166	0.905567	0.888613
B.cells	GMPPB	-0.00046	2.629402	-0.00185	0.998532	-5.84244	0.875816	0.838575
B.cells	ADAM19	-0.00031	5.568234	-0.00177	0.998589	-6.69443	0.835796	0.772715
B.cells	HIST2H2AC	-0.00073	2.376405	-0.00165	0.998685	-5.80087	0.879384	0.844528
B.cells	2510009E0	-0.0004	2.702942	-0.00151	0.998799	-5.84324	0.874806	0.836919
B.cells	CD207	0.00093	-0.64958	0.001381	0.998902	-5.24987	0.922998	0.918605
B.cells	JMJD7	0.000342	1.757778	0.001184	0.999058	-5.7223	0.888131	0.859254
B.cells	NIPBL	-8.44E-05	8.615732	-0.00113	0.9991	-7.12357	0.79657	0.709967
B.cells	ZMYND19	-0.00027	3.409984	-0.00112	0.999105	-6.04844	0.86499	0.820673
B.cells	CAP2	0.000507	0.071585	0.00096	0.999236	-5.41023	0.912488	0.900482
B.cells	MCRS1	0.000123	4.318538	0.000899	0.999285	-6.34836	0.852565	0.80019
B.cells	PTP4A2	-4.93E-05	8.699413	-0.00083	0.999341	-7.13252	0.795524	0.70833
B.cells	PJA2	0.000122	4.695864	0.000796	0.999366	-6.39339	0.847466	0.791848
B.cells	ATG9A	0.000112	3.85849	0.000725	0.999423	-6.26153	0.85883	0.81053
B.cells	TNFRSF1A	-8.69E-05	5.171275	-0.00041	0.999674	-6.17077	0.841171	0.781485
B.cells	MGAT1	4.52E-05	4.704416	0.000313	0.999751	-6.39094	0.84743	0.791723
B.cells	NDC1	5.90E-05	3.748037	0.000312	0.999751	-6.13894	0.860423	0.813083
B.cells	GM46620	-7.86E-05	0.683678	-0.00015	0.999884	-5.38834	0.903643	0.885429
B.cells	ACOT8	-2.10E-05	4.129949	-0.00014	0.999885	-6.30829	0.855206	0.804512

B.cells	ENTPD6	-1.82E-06	2.776877	-8.82E-06	0.999993	-5.95744	0.873885	0.835409
