# Supplementary material for: Immune landscape in liver of neonatal mice with phlebotomy-induced anemia
Source: Pediatr Res. 2025 Sep 17;99(4):1602–12. doi: 10.1038/s41390-025-04361-x (PMC12659965; doi:10.1038/s41390-025-04361-x)
Supplement: Supplementary file 8 — Table S8 [file 41390_2025_4361_MOESM8_ESM.pdf]

immunecell	gene	logFC	AveExpr	t	P.Value	B	adj.P.Val.Within	adj.P.Val.Between
T.cells	BNIP3	1.347845	5.086642	8.754251	1.27E-13	20.63048	1.40E-09	2.51E-10
T.cells	GM10076	0.785185	6.671496	7.566621	3.44E-11	15.0945	1.83E-07	3.01E-08
T.cells	TPI1	0.70846	6.690385	7.057968	3.64E-10	12.78487	1.29E-06	2.50E-07
T.cells	ENO1	0.636028	8.066335	6.65239	2.32E-09	10.86012	5.89E-06	1.16E-06
T.cells	SLC16A3	2.583971	3.935418	6.605836	2.86E-09	9.599246	6.46E-06	1.56E-06
T.cells	ALDOA	0.890726	8.900702	6.532852	3.98E-09	10.29311	6.34E-06	1.75E-06
T.cells	CYP2E1	-1.94153	4.291137	-6.50945	4.42E-09	10.37259	7.04E-06	2.15E-06
T.cells	FABP4	1.326324	6.966529	6.480658	5.03E-09	10.33212	6.63E-06	2.21E-06
T.cells	PGK1	0.620528	7.765914	6.286271	1.20E-08	9.241387	1.38E-05	4.50E-06
T.cells	SERPINA6	-3.37977	1.127419	-5.61936	2.20E-07	5.164989	0.000264	6.75E-05
T.cells	LDHA	0.503108	8.792149	5.553402	2.91E-07	6.038909	0.000267	6.84E-05
T.cells	PON1	-1.74259	4.304512	-5.47601	4.03E-07	6.211328	0.000371	9.85E-05
T.cells	LECT2	-2.10254	3.632928	-5.46007	4.31E-07	5.97794	0.000377	0.000105
T.cells	GM11808	-0.49298	7.262783	-5.34247	7.06E-07	5.366446	0.000528	0.000146
T.cells	PKM	0.536542	8.60779	5.257036	1.01E-06	4.856216	0.000644	0.000191
T.cells	UBA52	0.427759	10.38927	5.254913	1.02E-06	4.583627	0.000619	0.000183
T.cells	PFKL	0.537917	5.330071	5.12309	1.75E-06	4.737296	0.001123	0.00032
T.cells	PCMTD1	0.46327	5.924056	5.066046	2.20E-06	4.423031	0.001321	0.00038
T.cells	HSD17B2	-1.74329	2.446494	-5.03296	2.52E-06	4.050839	0.001548	0.000469
T.cells	SERPINF2	-1.58897	3.929119	-4.90771	4.17E-06	4.02406	0.002356	0.00066
T.cells	GM26510	0.642389	4.122817	4.870423	4.85E-06	3.887954	0.002593	0.000742
T.cells	CES1G	-2.60694	0.85155	-4.79181	6.62E-06	2.532774	0.00364	0.001022
T.cells	PDK1	0.592551	4.164844	4.757556	7.58E-06	3.494381	0.0037	0.001028
T.cells	ANKRD37	0.883795	3.981977	4.728709	8.49E-06	3.410014	0.003776	0.001125
T.cells	CORO1B	0.655563	4.252422	4.726111	8.58E-06	3.371453	0.003753	0.001125
T.cells	AKR1C6	-1.66506	4.841164	-4.71974	8.79E-06	3.384704	0.003704	0.001124
T.cells	LPCAT2	0.871717	4.726381	4.697105	9.61E-06	3.294681	0.003713	0.001213
T.cells	SPATA21	-0.86031	3.397572	-4.69506	9.69E-06	3.305459	0.003826	0.001266
T.cells	FABP2	-1.53262	3.257265	-4.69436	9.71E-06	3.211624	0.003838	0.001271
T.cells	APOA1	-1.22373	8.277086	-4.65586	1.13E-05	2.682776	0.003849	0.001245
T.cells	P4HA1	0.719166	5.786072	4.585433	1.48E-05	2.731703	0.00518	0.001659
T.cells	H2-Q10	-1.17051	4.038149	-4.5499	1.70E-05	2.78913	0.005987	0.00193
T.cells	NFKBID	-0.49633	6.350031	-4.50849	1.99E-05	2.308793	0.006459	0.002024
T.cells	TTR	-1.20711	9.202897	-4.44554	2.54E-05	1.776693	0.00747	0.002294
T.cells	CYP3A11	-1.68264	3.352158	-4.38669	3.17E-05	2.177813	0.010352	0.003196
T.cells	DNAJB9	-0.52198	5.168187	-4.35325	3.59E-05	2.004818	0.010828	0.003349
T.cells	GSTA3	-1.65642	4.0016	-4.34907	3.65E-05	2.098546	0.011117	0.003491
T.cells	ARNTL	0.429939	5.38742	4.318906	4.09E-05	1.553407	0.011745	0.00363
T.cells	ATXN7L1	0.453338	6.345264	4.295596	4.46E-05	1.671719	0.012217	0.003737
T.cells	AHSG	-1.0443	9.130873	-4.2111	6.10E-05	0.932245	0.015196	0.004398
T.cells	GM15417	1.600351	2.477041	4.206131	6.21E-05	1.089171	0.017665	0.005352
T.cells	SERPINA3K	2.380387	1.878609	4.172346	7.03E-05	1.330839	0.019119	0.005931
T.cells	SLC44A2	0.431344	6.242042	4.171948	7.04E-05	1.12383	0.017326	0.005252
T.cells	GM45715	1.354631	1.039938	4.169021	7.12E-05	1.174163	0.019483	0.006108

T.cells	GNPTAB	0.487802	4.589659	4.151704	7.58E-05	1.245775	0.018736	0.005771
T.cells	C8B	-1.91165	0.571894	-4.10393	9.02E-05	0.518797	0.023877	0.007274
T.cells	GM16853	-1.01211	2.351019	-4.07539	0.0001	1.162271	0.024898	0.00747
T.cells	LMAN2L	0.40287	4.447735	4.068973	0.000102	0.9737	0.0238	0.007169
T.cells	GM2000	-0.56545	4.799183	-4.05512	0.000108	0.892561	0.024129	0.007358
T.cells	TGIF1	-0.49616	6.736671	-4.05072	0.000109	0.666865	0.023094	0.007055
T.cells	SLC4A1	1.995237	2.002694	4.039417	0.000114	0.802973	0.0257	0.00827
T.cells	CBFA2T3	-0.40447	5.507245	-4.03567	0.000115	0.606227	0.023746	0.007568
T.cells	4931403E2	-1.41547	1.501737	-4.03365	0.000116	0.58927	0.025991	0.008502
T.cells	KNG1	-0.83903	6.039457	-4.03024	0.000118	0.760285	0.023462	0.007546
T.cells	CLDN1	-2.36464	1.493829	-4.01078	0.000126	0.411285	0.027179	0.009013
T.cells	RARRES2	-1.3185	3.7462	-4.00768	0.000128	0.976319	0.025834	0.008521
T.cells	HRG	-1.32967	3.61876	-3.99579	0.000133	0.938586	0.025962	0.008815
T.cells	HBB-BT	1.771301	8.105747	3.993782	0.000134	0.493228	0.023456	0.007807
T.cells	CYP1A2	-1.73527	1.633956	-3.99251	0.000135	0.602419	0.027149	0.00937
T.cells	ID2	-0.7091	6.76154	-3.95118	0.000156	0.61556	0.027547	0.009093
T.cells	ESR1	0.576193	3.611322	3.944789	0.00016	0.526287	0.029762	0.010125
T.cells	AADAC	-1.68245	2.701819	-3.91046	0.00018	0.581004	0.033503	0.011342
T.cells	FAM45A	0.456981	3.979967	3.90797	0.000182	0.564389	0.032551	0.011004
T.cells	HBB-BS	1.628619	11.91128	3.892409	0.000192	-0.37627	0.027852	0.009214
T.cells	TRAPPC6A	0.320734	5.057196	3.888001	0.000195	0.304475	0.03254	0.011284
T.cells	RAB3IP	0.4255	4.587906	3.885769	0.000197	0.288014	0.032887	0.011507
T.cells	UGT2B34	-1.59856	1.748323	-3.88365	0.000198	0.360606	0.035061	0.012512
T.cells	CDK2AP1	-0.29512	5.884299	-3.87349	0.000205	0.056302	0.03261	0.011402
T.cells	APOA2	-0.92199	8.419445	-3.84335	0.000228	-0.21493	0.033716	0.011461
T.cells	E130307A1	0.338851	4.490339	3.782835	0.000282	-0.03137	0.044823	0.014866
T.cells	CES1C	-1.3157	4.023969	-3.77696	0.000287	0.240307	0.045575	0.015286
T.cells	NRROS	0.313069	7.067702	3.749043	0.000316	-0.40905	0.045573	0.015065
T.cells	ITIH2	-1.33424	3.79432	-3.74892	0.000316	0.16311	0.049074	0.016502
T.cells	VAV1	0.305395	6.364742	3.74336	0.000323	-0.3487	0.046559	0.015525
T.cells	HSD17B6	-1.9825	0.767255	-3.73733	0.000329	-0.31353	0.053209	0.018353
T.cells	ALB	-1.06516	9.441682	-3.73109	0.000336	-0.72859	0.044095	0.01466
T.cells	DNAJA1	-0.2207	7.461326	-3.72704	0.000341	-0.56675	0.046159	0.015618
T.cells	SLC35D2	-0.46165	4.232	-3.71495	0.000355	-0.2091	0.050677	0.017595
T.cells	BNIP3L	0.401802	7.263429	3.713198	0.000358	-0.50823	0.047318	0.016231
T.cells	GYPA	2.082879	3.611578	3.70992	0.000362	-0.00505	0.051392	0.018057
T.cells	C130026I2	1.531249	2.944263	3.698497	0.000376	-0.18544	0.053574	0.018881
T.cells	SGSM2	-0.6813	3.786605	-3.68285	0.000397	-0.04168	0.054764	0.019038
T.cells	GPI1	0.296866	7.844302	3.674907	0.000407	-0.76246	0.050708	0.017323
T.cells	EHBP1	-0.75527	3.52414	-3.6662	0.00042	-0.12725	0.056289	0.019934
T.cells	GCSAM	-1.61183	-0.31734	-3.66592	0.00042	-0.40727	0.061367	0.022189
T.cells	HBA-A1	1.620649	10.46682	3.635951	0.000465	-1.00302	0.051454	0.01757
T.cells	MT2	1.728874	2.001416	3.635278	0.000466	-0.26331	0.06232	0.022256
T.cells	HSPA8	-0.24664	10.08692	-3.63408	0.000468	-1.27113	0.0519	0.017804
T.cells	SERPINA1C	-0.97711	7.348457	-3.63234	0.000471	-0.74851	0.05523	0.019293
T.cells	LGALS9	0.314757	7.157478	3.627617	0.000478	-0.96661	0.055732	0.019529
T.cells	UBE2E3	-0.20984	7.066891	-3.62209	0.000487	-0.92657	0.055892	0.019825

T.cells	TMEM38B	0.666118	4.174266	3.617934	0.000494	-0.23565	0.059671	0.021669
T.cells	CYP27A1	-1.59748	2.194427	-3.61574	0.000498	-0.57096	0.062395	0.02295
T.cells	GM50071	-1.40327	0.514178	-3.6124	0.000503	-0.52607	0.064796	0.024281
T.cells	FGF23	2.474141	0.594781	3.611293	0.000505	-0.64531	0.064679	0.024305
T.cells	A1CF	-1.92324	0.569704	-3.59351	0.000536	-0.70803	0.067973	0.025396
T.cells	FAM162A	0.466736	5.640039	3.580662	0.00056	-0.75385	0.062643	0.022699
T.cells	F13B	-1.45004	1.463355	-3.57221	0.000576	-0.49703	0.069678	0.025948
T.cells	SPP2	-1.27998	3.372982	-3.57048	0.000579	-0.35017	0.066746	0.024677
T.cells	MOB3B	-0.53795	4.961752	-3.56786	0.000584	-0.51955	0.064395	0.023771
T.cells	B3GNT2	0.261445	6.739389	3.550617	0.000618	-1.14052	0.064859	0.023443
T.cells	NRG2	1.311736	1.685011	3.544024	0.000632	-0.48313	0.073577	0.027376
T.cells	PROC	-1.1458	2.923674	-3.53124	0.000659	-0.46456	0.073297	0.027207
T.cells	SERPINC1	-0.80236	5.285455	-3.5309	0.00066	-0.72087	0.069492	0.025476
T.cells	SEMA4B	-0.42448	5.763505	-3.5133	0.0007	-0.96325	0.071856	0.026028
T.cells	AGMAT	-1.70503	1.438737	-3.51174	0.000703	-0.72493	0.079219	0.02948
T.cells	FGFR2	-0.31561	5.987704	-3.49894	0.000734	-1.09722	0.073875	0.026742
T.cells	DHX57	0.414181	3.871504	3.488714	0.000759	-0.78898	0.078907	0.029025
T.cells	GM37768	0.715297	2.087377	3.487806	0.000761	-0.60099	0.082144	0.030561
T.cells	ZFP738	0.553203	2.843416	3.481824	0.000776	-0.64606	0.08161	0.030294
T.cells	ICAM1	-0.53005	5.898773	-3.47366	0.000797	-0.97821	0.077077	0.028336
T.cells	HNRNPH3	0.214116	5.447268	3.472355	0.000801	-1.16993	0.077868	0.028791
T.cells	RELN	-0.7657	3.647841	-3.46697	0.000815	-0.82188	0.081098	0.030616
T.cells	CFH	-0.99127	4.930751	-3.46598	0.000817	-0.68746	0.078783	0.029608
T.cells	KEL	2.17094	-0.44363	3.464634	0.000821	-1.13777	0.088911	0.034432
T.cells	AGMO	-1.27877	3.650343	-3.45036	0.00086	-0.7093	0.082298	0.031748
T.cells	MT1	0.783295	6.314344	3.44972	0.000862	-1.17629	0.077489	0.029526
T.cells	RBP4	-0.82116	8.031076	-3.44934	0.000863	-1.41449	0.074531	0.028149
T.cells	WASHC4	0.257503	5.792598	3.448961	0.000864	-1.20316	0.078409	0.02997
T.cells	AKR1C12	-1.54577	0.756271	-3.44708	0.000869	-1.07558	0.087836	0.034616
T.cells	HSD3B7	-0.98609	3.537133	-3.43089	0.000917	-0.74336	0.084981	0.033197
T.cells	CD209F	-3.73472	2.767776	-3.42973	0.00092	-0.91154	0.086468	0.033982
T.cells	CP	-0.83291	4.588111	-3.42861	0.000923	-0.87661	0.082989	0.03236
T.cells	CENPC1	0.249164	5.265605	3.427925	0.000925	-1.25504	0.081728	0.031804
T.cells	RARB	-1.91488	1.586463	-3.4245	0.000936	-0.95472	0.088899	0.035475
T.cells	HBA-A2	1.5235	9.98555	3.419441	0.000951	-1.61394	0.073517	0.028376
T.cells	OSER1	-0.25681	6.59749	-3.41854	0.000954	-1.48384	0.079392	0.031247
T.cells	SERPIND1	-1.1192	2.832849	-3.41416	0.000968	-0.78938	0.08644	0.03504
T.cells	MIF	0.374819	7.650894	3.413769	0.000969	-1.67179	0.077519	0.030668
T.cells	SUOX	-1.13104	1.361958	-3.413	0.000971	-0.93421	0.089349	0.036566
T.cells	GIGYF2	0.311701	5.464029	3.407415	0.000989	-1.26602	0.082308	0.033006
T.cells	SLCO4A1	-0.61586	3.403213	-3.40377	0.001001	-1.02464	0.086565	0.035229
T.cells	ESRRA	0.478546	3.981496	3.401528	0.001008	-0.96569	0.085443	0.03485
T.cells	CD244A	0.977551	3.429777	3.392455	0.001038	-0.84932	0.087551	0.036022
T.cells	NUAK1	-0.57913	3.219187	-3.3911	0.001043	-1.07758	0.087968	0.03632
T.cells	CNNM4	-0.42389	4.077084	-3.39092	0.001043	-1.05491	0.086282	0.035463
T.cells	WNT2	-2.07734	1.077377	-3.3798	0.001081	-1.25984	0.094981	0.039504
T.cells	ODF2	0.241292	5.483537	3.37716	0.00109	-1.4471	0.086105	0.035144

T.cells	CFI	-1.10789	3.743943	-3.37052	0.001114	-0.94592	0.09083	0.037378
T.cells	PSEN2	0.323774	5.03423	3.362654	0.001142	-1.29245	0.089832	0.036633
T.cells	MAN2A1	0.300854	7.183028	3.357112	0.001163	-1.71328	0.086483	0.034926
T.cells	SGCZ	-1.36969	1.627249	-3.35327	0.001177	-1.04416	0.098427	0.041116
T.cells	SERPINA1A	-0.85027	8.012506	-3.35116	0.001185	-1.71472	0.085204	0.034617
T.cells	FXYD1	-1.16051	2.943369	-3.34931	0.001192	-0.96659	0.095554	0.040025
T.cells	APOE	-0.82489	9.241961	-3.3333	0.001255	-1.83412	0.086487	0.034615
T.cells	VWF	2.150938	1.565048	3.331574	0.001262	-1.21163	0.102881	0.04299
T.cells	SLC8A2	-2.25263	1.394752	-3.32418	0.001292	-1.35742	0.104599	0.043743
T.cells	CYP2C37	-1.16969	1.772648	-3.32331	0.001295	-1.08811	0.103714	0.043365
T.cells	SERPINA3N	-1.01586	3.688152	-3.31838	0.001316	-1.08053	0.099987	0.04155
T.cells	PKD1L2	1.821433	1.173969	3.317028	0.001322	-1.34508	0.10581	0.044718
T.cells	PDIA6	-0.28637	6.97381	-3.31286	0.001339	-1.76779	0.093441	0.038423
T.cells	TMEM163	-0.43502	6.026941	-3.30937	0.001354	-1.61876	0.095898	0.039748
T.cells	SWAP70	-0.41286	5.61218	-3.30463	0.001375	-1.58994	0.097625	0.040573
T.cells	DUSP5	-0.40372	6.834196	-3.30016	0.001394	-1.6816	0.095691	0.03949
T.cells	ABAT	-1.6021	1.196429	-3.28793	0.001449	-1.29683	0.111596	0.047415
T.cells	ACBD3	0.243176	5.707973	3.287702	0.00145	-1.68117	0.100801	0.041822
T.cells	SLC17A2	-1.44435	1.156237	-3.27846	0.001493	-1.2867	0.113839	0.048489
T.cells	EEA1	0.286615	6.055281	3.277652	0.001497	-1.71263	0.101931	0.042353
T.cells	MBL2	-1.05449	4.246085	-3.26803	0.001543	-1.28676	0.108096	0.045456
T.cells	KLHL14	-0.60182	2.717642	-3.268	0.001543	-1.41332	0.111887	0.047431
T.cells	ARHGEF10	1.601904	2.266878	3.258927	0.001588	-1.32768	0.11558	0.048972
T.cells	TSTD1	-1.01807	2.589608	-3.25297	0.001618	-1.2248	0.116188	0.049138
T.cells	ERO1L	0.467673	5.171152	3.246207	0.001653	-1.60417	0.111265	0.046325
T.cells	SQOR	-1.16042	3.496234	-3.24429	0.001663	-1.27732	0.115554	0.048699
T.cells	TBL1X	-0.24015	7.155308	-3.24141	0.001678	-2.05524	0.106691	0.044293
T.cells	NIPSNAP2	0.290647	4.810103	3.237932	0.001696	-1.62173	0.113054	0.047643
T.cells	PRKCZ	1.029745	0.606888	3.211614	0.001841	-1.37697	0.134111	0.056656
T.cells	TRMT1L	0.288185	4.566129	3.209364	0.001854	-1.6823	0.122799	0.050971
T.cells	HK1	0.394376	4.85354	3.206896	0.001869	-1.63778	0.122216	0.050674
T.cells	COL6A1	1.646644	0.367808	3.203334	0.001889	-1.65052	0.135237	0.057826
T.cells	CMKLR1	1.864897	2.151939	3.20304	0.001891	-1.50447	0.129923	0.055041
T.cells	BCAR3	-0.59573	4.496691	-3.20102	0.001903	-1.6968	0.123286	0.051745
T.cells	TMEM205	-0.80818	3.71868	-3.19612	0.001932	-1.40637	0.125537	0.053487
T.cells	PGM1	0.453689	4.592397	3.194654	0.001941	-1.61597	0.123085	0.052308
T.cells	ACP6	0.397645	3.140192	3.19272	0.001953	-1.67076	0.127186	0.054707
T.cells	GAMT	-1.05626	4.228598	-3.19179	0.001958	-1.44517	0.124101	0.053163
T.cells	PROCA1	-0.76995	2.988273	-3.19162	0.001959	-1.39323	0.127622	0.055041
T.cells	SELENOW	0.296149	7.046449	3.185861	0.001995	-2.12067	0.117867	0.049689
T.cells	AGBL1	-0.70544	4.663698	-3.18349	0.002009	-1.76049	0.124612	0.053202
T.cells	TESC	-1.67324	1.561476	-3.17778	0.002045	-1.59411	0.134486	0.058527
T.cells	ATP6V1E1	-0.24542	7.59223	-3.17469	0.002065	-2.21547	0.117361	0.04977
T.cells	IGFALS	-1.21439	1.779759	-3.17432	0.002067	-1.50424	0.133828	0.058518
T.cells	ISYNA1	-0.33596	4.852057	-3.17413	0.002068	-1.8355	0.124872	0.053731
T.cells	HSPA5	-0.24463	8.355671	-3.17253	0.002079	-2.31843	0.115346	0.048926
T.cells	PDE6C	-1.57438	0.436862	-3.15709	0.00218	-1.67896	0.143293	0.062993

T.cells	FOSB	-0.70146	6.636378	-3.15664	0.002183	-1.76669	0.124597	0.053014
T.cells	NNT	0.502376	2.482711	3.154095	0.0022	-1.59118	0.137192	0.059775
T.cells	SNCA	1.761486	4.248926	3.152293	0.002212	-1.48676	0.131863	0.057081
T.cells	AMBP	-0.81204	4.730423	-3.14689	0.002249	-1.71292	0.130726	0.05679
T.cells	HSP90AA1	-0.22821	7.657631	-3.14512	0.002262	-2.43575	0.122344	0.052486
T.cells	H1F0	0.341447	6.001224	3.145063	0.002262	-2.23892	0.127023	0.054962
T.cells	OSGIN1	-1.09694	3.869004	-3.1439	0.00227	-1.50826	0.133293	0.05843
T.cells	GM4788	-1.45524	1.237901	-3.14217	0.002282	-1.62201	0.141429	0.063126
T.cells	RTL8A	-0.6474	3.632481	-3.14134	0.002288	-1.58837	0.134007	0.059105
T.cells	RTL8B	-0.70325	3.297356	-3.13738	0.002316	-1.57607	0.135301	0.060151
T.cells	CYBC1	0.361619	4.562008	3.13733	0.002316	-1.93013	0.131494	0.058071
T.cells	DCAF8	0.209171	5.699638	3.13192	0.002355	-2.11819	0.129636	0.056853
T.cells	FKBP9	-1.64499	1.265181	-3.12703	0.00239	-1.74247	0.144693	0.065005
T.cells	CCDC125	0.395311	4.497843	3.121688	0.00243	-1.98888	0.136059	0.060065
T.cells	MTOR	0.266139	4.766611	3.118008	0.002457	-2.08474	0.13608	0.059999
T.cells	KLF4	-0.38819	6.447469	-3.10966	0.002521	-2.21994	0.132471	0.058117
T.cells	HIST1H3D	-0.67614	1.963471	-3.10929	0.002524	-1.70523	0.146583	0.065841
T.cells	PGAM1	0.295799	6.892134	3.109204	0.002524	-2.37769	0.131143	0.057402
T.cells	FETUB	-1.02817	3.175824	-3.10786	0.002535	-1.61932	0.142635	0.063834
T.cells	SIRPB1A	1.766627	-0.18195	3.099584	0.002599	-1.94413	0.156126	0.071139
T.cells	UCP2	0.228064	9.571709	3.098119	0.002611	-2.89236	0.125259	0.054379
T.cells	SRSF10	-0.17449	6.4448	-3.09459	0.002639	-2.42583	0.134468	0.059735
T.cells	ACAA1B	-1.45046	3.318751	-3.09418	0.002642	-1.64992	0.144311	0.065194
T.cells	WDR81	-0.53975	3.600126	-3.09317	0.00265	-1.71314	0.143398	0.064801
T.cells	UGT2B5	-1.4143	1.786068	-3.09147	0.002664	-1.66566	0.149378	0.068275
T.cells	1300017J0	-0.84329	3.006144	-3.09044	0.002672	-1.66041	0.145331	0.066103
T.cells	ZFP329	0.493291	2.511051	3.090308	0.002673	-1.72422	0.146961	0.06702
T.cells	CHIL1	1.571153	-0.07877	3.087536	0.002696	-1.89674	0.156087	0.072389
T.cells	CXCL12	-0.97899	2.838235	-3.08653	0.002704	-1.65013	0.146183	0.066865
T.cells	GM19684	0.900511	1.522121	3.084573	0.00272	-1.65778	0.150771	0.069621
T.cells	COLGALT1	-0.26792	5.889717	-3.08063	0.002753	-2.26047	0.137626	0.062205
T.cells	PIK3R3	-0.74615	3.43068	-3.07072	0.002837	-1.82269	0.148739	0.067909
T.cells	ZDHHC16	0.404487	2.888345	3.068537	0.002856	-1.84699	0.150569	0.069113
T.cells	CCND2	0.400026	5.400515	3.06834	0.002857	-2.21434	0.142267	0.064445
T.cells	LETM1	-0.28673	4.54276	-3.06691	0.00287	-2.12805	0.145052	0.066115
T.cells	JUNB	-0.41127	9.343714	-3.06575	0.00288	-2.55169	0.130102	0.057967
T.cells	LY96	-0.4684	4.287348	-3.06131	0.002919	-1.92657	0.147195	0.067207
T.cells	ADAM22	-1.67077	0.796933	-3.04957	0.003024	-1.95288	0.164029	0.075766
T.cells	GM11290	-0.76215	3.432889	-3.04826	0.003036	-1.89257	0.154583	0.070529
T.cells	GM4258	0.417242	4.929368	3.046991	0.003047	-2.3294	0.149448	0.067763
T.cells	GPR18	0.372123	4.514903	3.042837	0.003085	-2.27291	0.152073	0.069044
T.cells	IDI1	-0.39818	3.90584	-3.03941	0.003117	-2.04351	0.155087	0.070594
T.cells	UGT2B36	-1.11755	2.198922	-3.03787	0.003132	-1.77329	0.161208	0.074214
T.cells	PRELID1	0.220045	7.529242	3.032844	0.003179	-2.68517	0.143146	0.064586
T.cells	ADAMTS14	-1.40772	0.543497	-3.03089	0.003198	-1.89642	0.167588	0.078763
T.cells	SULT2A8	-1.69113	0.571296	-3.0305	0.003202	-1.94236	0.167483	0.078753
T.cells	ZUP1	0.354827	5.168406	3.030231	0.003204	-2.27363	0.151006	0.069303

T.cells	APOBEC1	0.362816	6.087599	3.029612	0.00321	-2.56677	0.147898	0.06766
T.cells	RNF41	0.303608	4.424549	3.028463	0.003221	-2.1742	0.153565	0.071017
T.cells	KLHL26	0.564049	2.727028	3.026415	0.003241	-1.89553	0.159559	0.074707
T.cells	RSRC1	0.226938	6.605429	3.025058	0.003254	-2.5691	0.146174	0.067246
T.cells	EBPL	-0.41476	4.469152	-3.02155	0.003289	-2.10192	0.153411	0.071828
T.cells	TSPO2	1.759709	0.283153	3.021435	0.00329	-2.08356	0.16857	0.080699
T.cells	CYP2A12	-1.09893	2.85261	-3.02126	0.003292	-1.82796	0.159108	0.075137
T.cells	APOF	-0.83415	3.847555	-3.01774	0.003326	-1.92138	0.155579	0.073527
T.cells	2500002B1	0.638513	1.915535	3.016976	0.003334	-1.86152	0.1625	0.077662
T.cells	SLC2A6	1.657883	1.781297	3.016908	0.003335	-2.06957	0.162992	0.077953
T.cells	PDE4DIP	0.467191	3.94998	3.016042	0.003343	-1.98775	0.15522	0.073505
T.cells	METTL7B	-1.38307	2.263028	-3.00856	0.003419	-1.84362	0.1642	0.077972
T.cells	HC	-1.14101	2.358321	-3.00557	0.003449	-1.84925	0.164143	0.078074
T.cells	ATF7IP	0.256164	6.540023	3.005217	0.003453	-2.61463	0.149351	0.069533
T.cells	C8G	-1.03272	3.782028	-3.00298	0.003476	-1.94099	0.159375	0.075403
T.cells	UBE2D2A	-0.11897	8.209532	-2.99917	0.003516	-2.89378	0.145236	0.067137
T.cells	NT5DC1	0.3976	4.5014	2.995306	0.003556	-2.17239	0.159139	0.075021
T.cells	HEMGN	1.686245	0.35427	2.987379	0.003641	-1.98411	0.177866	0.085531
T.cells	APOH	-0.79106	4.983199	-2.98659	0.00365	-2.22017	0.160258	0.075299
T.cells	SERF1	0.398058	3.137334	2.984201	0.003676	-2.10703	0.16725	0.079601
T.cells	NCKAP5L	-0.49131	4.041979	-2.98356	0.003683	-2.14016	0.163872	0.077675
T.cells	PPP1R2	-0.16696	6.908394	-2.98057	0.003715	-2.71373	0.154176	0.072164
T.cells	CAR3	-1.35077	3.476221	-2.97887	0.003734	-2.0168	0.166615	0.079566
T.cells	KDM6B	-0.33628	8.152763	-2.97801	0.003744	-2.70076	0.149886	0.069951
T.cells	IVNS1ABP	-0.2486	6.027762	-2.97697	0.003755	-2.60575	0.157281	0.074318
T.cells	STXBP3	0.304215	5.278015	2.97222	0.003808	-2.44612	0.161608	0.076577
T.cells	SERTAD2	-0.33338	6.12155	-2.96759	0.003861	-2.58816	0.159846	0.075362
T.cells	4930589L2	1.487852	-0.68311	2.963994	0.003902	-2.25718	0.186318	0.091646
T.cells	MAPK1	-0.15096	7.693924	-2.96371	0.003905	-2.94365	0.154251	0.072599
T.cells	SMOX	1.232085	5.116761	2.963712	0.003905	-1.95171	0.163521	0.077999
T.cells	ZFP3	1.085215	0.562138	2.962974	0.003914	-1.9854	0.181189	0.088606
T.cells	PRELID2	0.741516	2.621255	2.953249	0.004028	-2.08789	0.177349	0.08509
T.cells	5830448L0	1.369658	0.904406	2.946711	0.004106	-2.18054	0.186126	0.090103
T.cells	ITGA6	-0.45518	4.818835	-2.94577	0.004117	-2.5489	0.170415	0.080956
T.cells	GM36660	1.510908	-0.14918	2.94494	0.004127	-2.27404	0.190577	0.093069
T.cells	TRAM2	-0.29937	4.061754	-2.94484	0.004128	-2.42943	0.173354	0.082793
T.cells	GC	-0.84183	5.287073	-2.94045	0.004182	-2.38803	0.170029	0.080446
T.cells	ADH1	-1.06278	3.503888	-2.93948	0.004194	-2.06458	0.177016	0.084741
T.cells	NAV1	-0.96018	3.595293	-2.93643	0.004232	-2.02564	0.176815	0.08505
T.cells	ZDHHC12	1.060608	1.360226	2.936079	0.004236	-2.02363	0.185941	0.09054
T.cells	ZFP950	0.368077	4.197661	2.935398	0.004245	-2.34476	0.174428	0.083771
T.cells	MBL1	-1.2174	1.455107	-2.93228	0.004284	-2.07691	0.186568	0.090889
T.cells	CLTB	-0.39266	4.396264	-2.92853	0.004331	-2.33823	0.17589	0.084246
T.cells	HSD3B3	-1.19773	2.014179	-2.92469	0.00438	-2.04417	0.187008	0.090566
T.cells	CDH2	-1.0407	1.947183	-2.92311	0.0044	-2.05351	0.187482	0.090954
T.cells	GBP7	0.842035	4.443583	2.91865	0.004458	-2.31138	0.178901	0.085657
T.cells	FAM217B	1.544369	0.616789	2.911801	0.004548	-2.2907	0.198225	0.096449

T.cells	DNAJB1	-0.30919	6.228259	-2.90812	0.004597	-2.81942	0.17592	0.082996
T.cells	OGG1	-0.41531	3.189389	-2.90173	0.004683	-2.26773	0.191136	0.091297
T.cells	IFT43	-1.05794	1.54627	-2.90071	0.004697	-2.15557	0.198338	0.095788
T.cells	ADAP2OS	1.68197	0.724917	2.89955	0.004713	-2.31457	0.202032	0.098169
T.cells	DYNLT1C	0.845075	1.100031	2.893875	0.004791	-2.11469	0.202952	0.098095
T.cells	BCAS2	-0.17456	6.380106	-2.88722	0.004885	-2.93139	0.180723	0.085569
T.cells	5330439KC	1.48593	0.538036	2.8867	0.004892	-2.28396	0.206168	0.100679
T.cells	CYP2C23	-1.35393	0.860959	-2.88498	0.004917	-2.21945	0.204679	0.100142
T.cells	BCCIP	-0.19569	5.436859	-2.88475	0.00492	-2.81763	0.184622	0.08821
T.cells	INSIG1	-0.33826	5.457849	-2.88323	0.004942	-2.63312	0.184535	0.08832
T.cells	SLBP	-0.20735	7.147492	-2.88016	0.004986	-3.161	0.177608	0.084605
T.cells	DDX5	-0.13561	9.318404	-2.87875	0.005006	-3.33429	0.16907	0.079833
T.cells	GREB1L	-1.18396	1.947775	-2.87697	0.005032	-2.16735	0.19974	0.098242
T.cells	CYP2D26	-1.11393	3.247258	-2.87691	0.005033	-2.19593	0.19398	0.094754
T.cells	ETNK1	0.243049	5.999727	2.876466	0.00504	-2.85564	0.182286	0.087842
T.cells	CES1D	-1.22957	1.90006	-2.87583	0.005049	-2.16205	0.199955	0.098551
T.cells	LIX1L	-1.13366	0.803407	-2.87577	0.00505	-2.21006	0.204944	0.101601
T.cells	GM19951	0.969252	3.23896	2.87413	0.005074	-2.28054	0.194016	0.095193
T.cells	SIGIRR	0.447495	2.175284	2.873706	0.00508	-2.31666	0.198721	0.098077
T.cells	FYCO1	0.438681	4.137271	2.872424	0.005099	-2.37567	0.190124	0.093036
T.cells	CFHR2	-1.05282	2.56401	-2.87117	0.005117	-2.17003	0.196989	0.097395
T.cells	BASP1	1.026476	5.646818	2.871024	0.005119	-2.3006	0.183748	0.0894
T.cells	SLC31A2	0.801029	3.169586	2.870439	0.005128	-2.1984	0.19432	0.095818
T.cells	CYP51	-0.45496	3.916341	-2.87026	0.005131	-2.41836	0.191074	0.093847
T.cells	CHSY3	-1.46807	1.978293	-2.86889	0.005151	-2.1903	0.199603	0.099207
T.cells	YIPF1	0.24323	5.234712	2.867747	0.005168	-2.7162	0.185468	0.090773
T.cells	GYS2	-1.17163	0.768164	-2.86495	0.00521	-2.25499	0.205106	0.103331
T.cells	GM16341	0.839014	0.863635	2.864825	0.005212	-2.18703	0.204667	0.103057
T.cells	2010320M	0.661815	2.480423	2.864781	0.005213	-2.22135	0.19736	0.098528
T.cells	TEK	-1.10274	2.175307	-2.86308	0.005238	-2.1888	0.199051	0.099689
T.cells	ARHGEF17	-1.57896	-0.37609	-2.86038	0.005279	-2.3876	0.210945	0.107467
T.cells	APOM	-0.79596	4.854579	-2.86016	0.005282	-2.52791	0.187519	0.092944
T.cells	ULK2	0.314452	5.457806	2.859472	0.005293	-2.70888	0.184979	0.091449
T.cells	QTRT1	-0.44091	4.980734	-2.85688	0.005333	-2.65814	0.187051	0.093076
T.cells	LRG1	1.425701	2.460703	2.856003	0.005346	-2.20412	0.197993	0.099952
T.cells	P3H1	1.428395	0.585168	2.854114	0.005375	-2.29913	0.206519	0.105544
T.cells	TRA2B	-0.16734	7.832072	-2.85295	0.005393	-3.23317	0.175355	0.086491
T.cells	GM20508	-1.36624	0.064606	-2.85249	0.0054	-2.4029	0.208945	0.107408
T.cells	UBE2W	0.159013	6.081677	2.852264	0.005404	-2.93688	0.182451	0.090877
T.cells	CABP4	1.246721	-0.22452	2.851643	0.005414	-2.24496	0.210303	0.108408
T.cells	PLA2G7	1.965239	4.021195	2.847458	0.005479	-2.22199	0.192858	0.097047
T.cells	KRIT1	0.219763	5.937975	2.840469	0.00559	-2.94715	0.187838	0.093133
T.cells	RAB3GAP2	0.284723	5.259956	2.835776	0.005666	-2.78347	0.191627	0.095655
T.cells	GM31462	1.315515	0.152102	2.833631	0.005701	-2.36552	0.214989	0.110647
T.cells	ERBIN	-0.22965	7.975801	-2.83246	0.00572	-3.19338	0.180194	0.089213
T.cells	RAET1E	0.389551	4.113795	2.831927	0.005729	-2.62967	0.196653	0.099453
T.cells	TMEM245	0.281991	4.753996	2.831701	0.005732	-2.84198	0.193831	0.097725

T.cells	ERO1LB	-0.22429	6.652932	-2.83153	0.005735	-3.10053	0.18568	0.092701
T.cells	TSPYL4	0.926551	0.723815	2.831087	0.005743	-2.27762	0.212249	0.109417
T.cells	EED	-0.17162	6.114274	-2.83026	0.005756	-3.03496	0.187958	0.094271
T.cells	GPATCH1	0.441461	2.73622	2.828866	0.005779	-2.43539	0.203053	0.103817
T.cells	NUP210L	-0.50977	6.407383	-2.82644	0.005819	-2.98734	0.187322	0.094047
T.cells	COPZ2	-0.99382	2.183281	-2.82575	0.005831	-2.29011	0.206067	0.105881
T.cells	CABLES2	-0.37432	3.580958	-2.8249	0.005845	-2.55042	0.199679	0.101985
T.cells	PLEKHB1	-1.03815	0.728002	-2.82281	0.00588	-2.3005	0.213555	0.110786
T.cells	PCBD1	-1.00581	3.53173	-2.81799	0.005962	-2.35741	0.202671	0.103298
T.cells	UTRN	0.272451	7.31422	2.813613	0.006036	-3.18741	0.18783	0.093642
T.cells	VTN	-0.78996	4.267725	-2.8102	0.006095	-2.52252	0.202243	0.102648
T.cells	LAG3	1.607806	1.429969	2.808961	0.006117	-2.49017	0.215594	0.111214
T.cells	GM13561	0.542433	1.307424	2.808738	0.006121	-2.42529	0.216189	0.111619
T.cells	APOC4	-0.77207	5.632488	-2.80649	0.00616	-2.77656	0.196781	0.099262
T.cells	TMEM165	0.234873	5.311537	2.804211	0.0062	-2.91131	0.198916	0.100313
T.cells	CYP2C68	-0.99469	2.791797	-2.80109	0.006256	-2.34657	0.211815	0.10831
T.cells	GJB1	-1.1865	1.59761	-2.7999	0.006277	-2.34045	0.217684	0.112195
T.cells	HNRNPC	-0.14126	7.730209	-2.79883	0.006296	-3.32286	0.189547	0.094715
T.cells	GAPDH	0.272867	10.59184	2.796882	0.006331	-3.77975	0.17772	0.087687
T.cells	EXOC2	0.24845	5.578749	2.796588	0.006336	-2.93636	0.199127	0.100836
T.cells	USP8	0.251716	5.372996	2.793007	0.006401	-2.89681	0.201283	0.10204
T.cells	RAB31	-0.38567	4.770711	-2.7924	0.006412	-2.7493	0.204042	0.103812
T.cells	ZBED4	0.268856	4.650997	2.785188	0.006544	-2.90577	0.208137	0.105432
T.cells	GAN	0.435346	4.031697	2.78432	0.00656	-2.6598	0.211069	0.107388
T.cells	ARRDC4	1.224012	2.427989	2.782136	0.006601	-2.43088	0.219569	0.112774
T.cells	GLUD1	-0.21316	7.653063	-2.77961	0.006648	-3.27973	0.195217	0.097952
T.cells	GIMAP1	0.321136	4.06141	2.779139	0.006657	-2.86607	0.211745	0.108315
T.cells	SLC27A2	-0.83304	3.949769	-2.77804	0.006678	-2.55449	0.212279	0.108747
T.cells	GTF2H1	-0.23752	5.958143	-2.77765	0.006685	-3.08249	0.202859	0.102911
T.cells	OXCT1	-0.21733	5.719852	-2.77694	0.006699	-3.11348	0.203956	0.103667
T.cells	NAP1L1	-0.22463	7.439515	-2.77206	0.006791	-3.4449	0.198209	0.099489
T.cells	ARHGAP23	0.415988	3.694446	2.771287	0.006806	-2.74139	0.21573	0.110582
T.cells	EGR1	-0.72293	6.545199	-2.76977	0.006836	-2.89483	0.202568	0.102433
T.cells	GM26936	0.879003	0.478634	2.767618	0.006877	-2.40585	0.232535	0.121734
T.cells	BOP1	-0.31683	4.167475	-2.76739	0.006882	-2.8077	0.214007	0.109877
T.cells	MAPRE1	-0.15428	7.148653	-2.7588	0.00705	-3.37189	0.204379	0.10264
T.cells	N4BP1	0.374666	5.4056	2.751702	0.007192	-2.94816	0.214603	0.109067
T.cells	ARHGAP29	-0.99809	2.520124	-2.75142	0.007198	-2.43722	0.229043	0.118187
T.cells	MED12L	-0.61854	3.141983	-2.75141	0.007198	-2.50142	0.225856	0.11616
T.cells	ZFP296	-0.44887	2.857765	-2.75124	0.007201	-2.7977	0.227307	0.117082
T.cells	ENPEP	0.781801	0.401804	2.750594	0.007215	-2.47783	0.240212	0.125444
T.cells	FABP1	-0.73604	7.428716	-2.74852	0.007257	-3.26415	0.205523	0.103456
T.cells	MRGPRA2A	1.360971	-1.27662	2.747738	0.007273	-2.67338	0.250059	0.131959
T.cells	FAF2	0.238149	5.155771	2.744131	0.007347	-3.08984	0.217985	0.111041
T.cells	PZP	-0.77236	4.39586	-2.74146	0.007402	-2.73504	0.222437	0.11388
T.cells	DTX1	-0.68972	2.069415	-2.73963	0.00744	-2.58379	0.234414	0.121869
T.cells	DESI1	0.249857	5.729904	2.739189	0.007449	-3.27301	0.21583	0.110149

T.cells	NINJ1	0.369097	5.750418	2.738715	0.007459	-3.10949	0.21573	0.110159
T.cells	LAYN	1.160562	0.629576	2.735263	0.007531	-2.49054	0.242123	0.127759
T.cells	GM4070	1.195773	2.887167	2.734644	0.007544	-2.48749	0.230137	0.120069
T.cells	SH3PXD2A	-0.38037	5.753145	-2.73455	0.007546	-2.98852	0.215716	0.110863
T.cells	RNF169	0.269885	6.380124	2.734294	0.007552	-3.33059	0.212676	0.108954
T.cells	TMEM234	0.158659	7.040364	2.733826	0.007562	-3.3868	0.209519	0.107072
T.cells	CAAA0114[illegible]	0.464749	2.131904	2.733516	0.007568	-2.66109	0.234085	0.122766
T.cells	MORC3	0.231633	5.857789	2.73143	0.007612	-3.19276	0.215896	0.110989
T.cells	4930509H[illegible]	0.478479	2.090644	2.729548	0.007653	-2.61782	0.235061	0.123603
T.cells	KDM4B	-0.33005	4.441359	-2.72954	0.007653	-2.99755	0.222928	0.115778
T.cells	SERPINA7	-1.42446	0.531403	-2.72768	0.007693	-2.57128	0.244078	0.129492
T.cells	GM2629	0.707078	0.511245	2.725815	0.007733	-2.51795	0.244203	0.129984
T.cells	SNAP47	-0.62663	2.473639	-2.7258	0.007733	-2.52543	0.233665	0.123085
T.cells	HMGCS2	-0.88401	4.456909	-2.72431	0.007765	-2.74085	0.223794	0.116818
T.cells	GDA	2.157966	3.902395	2.722597	0.007802	-2.53149	0.226786	0.118969
T.cells	1600002K[illegible]	-0.42359	3.214423	-2.72219	0.007811	-2.68794	0.230332	0.121337
T.cells	OAS1A	1.212743	2.355225	2.720028	0.007858	-2.5098	0.235645	0.124817
T.cells	ZFP683	1.533581	-1.15907	2.716982	0.007925	-2.69953	0.256497	0.138458
T.cells	NRP1	-1.0987	4.442319	-2.71527	0.007963	-2.5297	0.226647	0.118789
T.cells	CLYBL	0.355034	4.489599	2.71307	0.008012	-2.95244	0.227217	0.119108
T.cells	TRIM10	1.679444	0.349121	2.711655	0.008044	-2.63536	0.249763	0.133831
T.cells	PROS1	-1.03527	2.741429	-2.71001	0.008081	-2.53305	0.23717	0.125603
T.cells	DMPK	-1.08782	2.786398	-2.70825	0.00812	-2.53144	0.237486	0.125799
T.cells	PDK3	0.352051	4.945028	2.70659	0.008158	-3.09593	0.226351	0.118789
T.cells	SNTB1	-1.08025	4.264888	-2.7056	0.00818	-2.5883	0.229855	0.121228
T.cells	TIMD2	-1.1788	0.986598	-2.70529	0.008187	-2.57728	0.247467	0.132829
T.cells	CD81	-0.26868	7.604661	-2.70311	0.008237	-3.58402	0.213388	0.110859
T.cells	KYAT3	-0.5947	3.603362	-2.70304	0.008239	-2.8061	0.233602	0.123921
T.cells	ANKS1	0.303541	5.759857	2.69823	0.008349	-3.1778	0.224533	0.117493
T.cells	RTF2	0.19358	5.957747	2.697956	0.008356	-3.29439	0.22353	0.116867
T.cells	PALLD	-0.86837	3.882236	-2.69548	0.008413	-2.59	0.23507	0.124377
T.cells	4833408A1	1.503818	0.127774	2.69493	0.008426	-2.65603	0.255788	0.138201
T.cells	TIGD2	0.326051	3.85213	2.689127	0.008562	-2.94452	0.238036	0.125798
T.cells	SLC22A4	1.462965	0.816422	2.688685	0.008573	-2.69708	0.254871	0.136909
T.cells	NGLY1	0.200941	4.849758	2.68777	0.008594	-3.1278	0.232734	0.122587
T.cells	SNX25	0.219728	5.540177	2.687089	0.008611	-3.31407	0.229129	0.120399
T.cells	GSTM2	-1.1163	1.633616	-2.68332	0.008701	-2.59657	0.252236	0.134931
T.cells	4933421O[illegible]	0.656985	2.357702	2.681151	0.008753	-2.65073	0.249043	0.132837
T.cells	PRKCG	-0.54116	4.448629	-2.67632	0.00887	-3.00885	0.240175	0.126464
T.cells	GM8113	-1.13633	-0.25687	-2.67106	0.009	-2.64364	0.269543	0.14538
T.cells	CYB5A	-0.44983	7.336341	-2.67045	0.009015	-3.46878	0.227127	0.117839
T.cells	PREP	0.218886	5.298922	2.670241	0.00902	-3.43567	0.23785	0.124738
T.cells	AKR1C20	-1.01768	2.839499	-2.66593	0.009128	-2.66515	0.253412	0.134502
T.cells	PROM1	-1.62755	0.403745	-2.66558	0.009136	-2.72257	0.267677	0.143938
T.cells	SLC27A5	-1.18617	1.457834	-2.66476	0.009157	-2.63076	0.261415	0.139983
T.cells	GM31718	-0.58119	3.725877	-2.66275	0.009208	-2.73678	0.249176	0.131935
T.cells	PARP10	0.54995	3.565506	2.659709	0.009285	-2.84738	0.251194	0.133207

T.cells	USP35	1.39742	0.017078	2.659401	0.009293	-2.68642	0.27206	0.147097
T.cells	HEXIM1	-0.33638	5.48831	-2.65848	0.009316	-3.21017	0.240555	0.126486
T.cells	SPOPL	0.298213	4.42179	2.657439	0.009343	-3.09072	0.246544	0.130396
T.cells	ANO6	0.2532	6.75402	2.653213	0.009452	-3.5596	0.235487	0.123064
T.cells	GM17749	-1.12488	4.634129	-2.6531	0.009455	-2.70943	0.247056	0.130546
T.cells	GM31814	1.186671	-0.06084	2.652355	0.009474	-2.74645	0.274591	0.148934
T.cells	NAA50	-0.17622	6.371464	-2.65039	0.009525	-3.52556	0.238259	0.124888
T.cells	PLG	-0.80856	4.004656	-2.64934	0.009553	-2.86942	0.251487	0.133519
T.cells	UGT3A2	-1.11874	1.174889	-2.64781	0.009593	-2.67288	0.268267	0.144784
T.cells	GM15728	-1.2407	0.500791	-2.64732	0.009606	-2.71344	0.272358	0.147596
T.cells	FAM160B2	0.378608	3.264944	2.64414	0.00969	-2.91881	0.257579	0.137503
T.cells	NCKAP1	-0.82098	2.425386	-2.64001	0.009799	-2.67649	0.264858	0.141586
T.cells	RGN	-0.79879	4.650255	-2.63888	0.00983	-3.00365	0.252093	0.133284
T.cells	ZFP367	-0.27662	5.436634	-2.63532	0.009926	-3.53388	0.24933	0.130983
T.cells	CRY1	0.269393	5.502006	2.634416	0.00995	-3.35087	0.248961	0.130772
T.cells	FKBP11	-0.70514	1.995525	-2.63387	0.009965	-2.69601	0.269456	0.144335
T.cells	UHMK1	0.275331	4.630018	2.632458	0.010003	-3.15333	0.254317	0.134442
T.cells	GM15859	0.688441	1.208039	2.629358	0.010088	-2.70399	0.276397	0.148569
T.cells	GULO	-0.94979	1.982596	-2.62723	0.010147	-2.70737	0.27258	0.145723
T.cells	NDEL1	0.245678	6.927832	2.625772	0.010187	-3.47085	0.244192	0.127253
T.cells	FCER2A	1.615019	0.458562	2.619096	0.010374	-2.77203	0.286931	0.153826
T.cells	RNF135	0.648112	2.08421	2.617395	0.010422	-2.77242	0.276639	0.147458
T.cells	S100A11	-0.29892	7.270251	-2.61719	0.010428	-3.54666	0.246057	0.127664
T.cells	ATR	0.39193	3.862209	2.616495	0.010447	-3.16202	0.265774	0.140455
T.cells	MFAP1B	0.225498	4.945536	2.615517	0.010475	-3.35414	0.259351	0.13658
T.cells	C530008M	-1.20453	0.74516	-2.61442	0.010506	-2.74486	0.285091	0.153776
T.cells	ARAP3	-0.94203	3.537048	-2.61431	0.01051	-2.73152	0.267731	0.14229
T.cells	BLOC1S1	0.249919	6.24963	2.610763	0.010611	-3.53957	0.25313	0.132718
T.cells	TUBB2A	-0.40771	5.517234	-2.61073	0.010612	-3.27555	0.257361	0.135452
T.cells	PDZRN3	-1.18873	0.932125	-2.60957	0.010646	-2.75819	0.285614	0.154125
T.cells	CHST11	-0.6161	6.242747	-2.60879	0.010668	-3.18241	0.253375	0.133119
T.cells	ARL6	-0.48246	1.698649	-2.6073	0.010711	-2.88332	0.280914	0.151444
T.cells	NFYC	-0.14792	6.202043	-2.60693	0.010722	-3.61948	0.253769	0.133701
T.cells	UBE2Q1	-0.18965	6.334643	-2.60181	0.010871	-3.56205	0.255048	0.134109
T.cells	SLC40A1	-0.79862	5.835325	-2.60165	0.010876	-3.07868	0.257947	0.136026
T.cells	HIST1H2AC	-0.65787	1.907677	-2.60065	0.010905	-2.93646	0.28185	0.151963
T.cells	NPM1	-0.18718	8.143673	-2.60046	0.010911	-3.96244	0.244801	0.127768
T.cells	FCNB	1.290738	-0.23854	2.599902	0.010928	-2.87607	0.295765	0.161407
T.cells	TAPBPL	0.383777	4.125165	2.59703	0.011013	-3.14707	0.268484	0.143436
T.cells	GZF1	0.531341	2.752325	2.596594	0.011026	-2.92388	0.276925	0.149048
T.cells	CCDC171	0.348242	3.57202	2.595454	0.01106	-3.13555	0.271856	0.145749
T.cells	TET3	-0.19877	6.827605	-2.59538	0.011062	-3.67402	0.252566	0.133121
T.cells	ITGA4	0.248925	7.657286	2.595369	0.011062	-3.85076	0.24786	0.130082
T.cells	SNHG9	-0.50984	4.309776	-2.59443	0.01109	-3.29779	0.267468	0.143021
T.cells	DCUN1D1	0.231225	5.787878	2.591939	0.011165	-3.5239	0.259447	0.137919
T.cells	NOS2	1.962801	-1.12584	2.591737	0.011171	-2.90449	0.303128	0.167152
T.cells	JUP	-0.61076	3.562597	-2.58669	0.011324	-2.88114	0.274581	0.147895

T.cells	SEL1L	0.272561	5.161227	2.583911	0.011409	-3.31074	0.264846	0.142123
T.cells	AU020206	0.2259	5.320744	2.582389	0.011456	-3.6243	0.263892	0.141823
T.cells	SELENBP2	-1.23817	1.116284	-2.58184	0.011473	-2.81284	0.290125	0.159494
T.cells	PTTG1IP	-0.28677	4.790183	-2.58063	0.01151	-3.20906	0.267076	0.144207
T.cells	CDKN1B	0.232287	6.470899	2.580538	0.011513	-3.68741	0.257112	0.137615
T.cells	SLC10A1	-0.66274	3.248903	-2.58053	0.011513	-2.92748	0.27653	0.150528
T.cells	MB21D2	-1.39646	0.883088	-2.58043	0.011517	-2.86682	0.291649	0.160763
T.cells	ASPRV1	1.626851	1.33931	2.580274	0.011521	-2.84689	0.288675	0.158781
T.cells	RBM33	0.191094	5.898903	2.58009	0.011527	-3.59635	0.260463	0.13991
T.cells	SRFBP1	0.358661	3.231687	2.579649	0.011541	-3.18218	0.276638	0.150805
T.cells	COL14A1	-1.08109	1.863173	-2.57898	0.011561	-2.80281	0.285295	0.156746
T.cells	P4HA2	1.3391	0.108055	2.578649	0.011572	-2.89282	0.296765	0.164613
T.cells	LY6I	2.563516	0.802083	2.578294	0.011583	-2.88207	0.29218	0.161532
T.cells	TMEM60	0.298691	4.112089	2.577583	0.011605	-3.24534	0.271198	0.147494
T.cells	CYP2D10	-0.97631	1.738911	-2.57514	0.011682	-2.81119	0.287012	0.158191
T.cells	EPS8L1	-1.62935	0.408966	-2.57474	0.011694	-2.83834	0.295715	0.164226
T.cells	ATN1	-0.532	3.221914	-2.57407	0.011715	-2.95804	0.277586	0.151989
T.cells	ATP2A2	-0.17252	6.456942	-2.57035	0.011833	-3.69664	0.25977	0.139749
T.cells	2010309G2	-0.80293	1.223176	-2.57002	0.011843	-2.86149	0.292329	0.16173
T.cells	TADA1	-0.23488	4.679062	-2.56895	0.011877	-3.45175	0.270649	0.147158
T.cells	DEK	-0.1787	7.842	-2.56738	0.011927	-4.05699	0.252488	0.135048
T.cells	CAST	0.213883	6.266263	2.566409	0.011958	-3.54912	0.261813	0.14139
T.cells	NCOA3	-0.17186	6.960263	-2.56545	0.011989	-3.74396	0.257866	0.138882
T.cells	SBNO1	-0.18248	7.359347	-2.56282	0.012074	-3.80974	0.256827	0.137949
T.cells	DAPK1	-0.64461	4.932787	-2.56121	0.012126	-3.06733	0.271947	0.148071
T.cells	ABCA3	0.257211	4.193838	2.559593	0.012178	-3.34761	0.277107	0.151432
T.cells	ELOVL6	0.30688	4.451031	2.558902	0.012201	-3.5658	0.275503	0.15048
T.cells	PANK2	0.214251	5.327592	2.55672	0.012272	-3.56917	0.27113	0.147458
T.cells	TNFRSF10B	-1.27269	-0.0063	-2.55408	0.012359	-2.9239	0.306902	0.171625
T.cells	F2	-0.7493	4.237274	-2.55246	0.012413	-3.15882	0.278953	0.152862
T.cells	NSRP1	0.225743	5.229533	2.551483	0.012445	-3.54865	0.272769	0.148733
T.cells	RHAG	1.603545	-0.3642	2.551407	0.012448	-2.93071	0.309373	0.173762
T.cells	KDM3A	0.233757	5.567098	2.550855	0.012466	-3.57265	0.270694	0.14744
T.cells	GM10521	0.909153	0.308847	2.550781	0.012468	-2.86201	0.30474	0.17066
T.cells	AQP1	-0.85576	2.964821	-2.54952	0.01251	-2.90066	0.287465	0.158792
T.cells	LTO1	0.38464	3.526077	2.548613	0.012541	-3.14123	0.283976	0.15658
T.cells	6530413G1	-0.96243	-0.08741	-2.54635	0.012617	-2.89373	0.30853	0.173563
T.cells	GPATCH2L	0.232114	5.064269	2.546005	0.012628	-3.45216	0.274743	0.150442
T.cells	PHC3	0.312343	4.499127	2.545733	0.012637	-3.31484	0.278273	0.152841
T.cells	RGS16	-0.75828	1.351563	-2.54501	0.012662	-2.95443	0.298725	0.167004
T.cells	GM47889	-0.82766	1.560566	-2.54246	0.012748	-2.89729	0.298641	0.16649
T.cells	GNPTG	-0.41684	3.368723	-2.54187	0.012768	-3.06645	0.286727	0.158485
T.cells	PTPRJ	-0.22158	8.855581	-2.53748	0.012918	-4.01446	0.25524	0.136734
T.cells	ZNRF1	0.207047	6.496008	2.537446	0.012919	-3.83669	0.269271	0.146015
T.cells	SIRPB1B	1.754242	1.445504	2.536675	0.012946	-2.95975	0.301806	0.16816
T.cells	SLC1A2	-1.05545	2.197177	-2.53579	0.012976	-2.90461	0.296866	0.164942
T.cells	TMEM40	-1.42058	0.497552	-2.53407	0.013036	-2.97823	0.308452	0.17339

T.cells	FGB	-0.48493	5.797972	-2.53356	0.013053	-3.52739	0.27372	0.149658
T.cells	GNB4	0.510779	2.895468	2.533561	0.013053	-3.07391	0.292261	0.162251
T.cells	CCNYL1	-0.36262	4.635175	-2.53154	0.013123	-3.25068	0.28161	0.15507
T.cells	IGKV2-109	-0.96684	-0.87987	-2.53007	0.013175	-2.92294	0.318799	0.181219
T.cells	PBX3	-0.26687	5.496226	-2.52878	0.01322	-3.6636	0.276182	0.151902
T.cells	CNEP1R1	0.240019	4.587392	2.52849	0.01323	-3.50935	0.281915	0.155795
T.cells	SLC38A4	-0.86183	3.153421	-2.5273	0.013272	-3.01121	0.291186	0.162356
T.cells	SAT1	-0.28194	8.474503	-2.52668	0.013294	-3.94002	0.258158	0.1401
T.cells	C1RA	-1.03745	1.359965	-2.52663	0.013295	-2.92603	0.30318	0.170775
T.cells	IFNAR2	0.249472	7.128736	2.526276	0.013308	-3.706	0.26616	0.145545
T.cells	SIRT1	-0.21222	5.043074	-2.52624	0.013309	-3.56714	0.279027	0.154247
T.cells	VPS13C	0.362422	4.373385	2.524894	0.013357	-3.31401	0.283753	0.157446
T.cells	CRYBB3	-0.99634	0.578324	-2.52361	0.013402	-2.91405	0.309525	0.175255
T.cells	SDF2L1	-0.30618	4.888426	-2.5228	0.013431	-3.5462	0.280962	0.15558
T.cells	B020010K1	0.741854	1.134374	2.518559	0.013583	-2.99365	0.308631	0.173548
T.cells	RSPH3B	0.393837	3.01938	2.517565	0.013618	-3.17743	0.296034	0.164941
T.cells	GEM	-0.33218	5.207345	-2.51393	0.01375	-3.67727	0.28365	0.156083
T.cells	IFI27	0.414858	4.998783	2.51252	0.013801	-3.59269	0.28499	0.157404
T.cells	TRIM65	0.42127	3.22435	2.512484	0.013803	-3.20051	0.296637	0.165374
T.cells	SEMA6A	-1.37971	2.821738	-2.51197	0.013821	-2.94441	0.299341	0.167338
T.cells	GM15283	0.368578	4.261918	2.511492	0.013839	-3.42704	0.289774	0.160872
T.cells	STX16	0.231298	5.756821	2.507972	0.013968	-3.7353	0.282234	0.155255
T.cells	GAB2	0.297358	7.176286	2.50289	0.014156	-3.94664	0.276106	0.150409
T.cells	ZFHX3	-0.38157	5.163754	-2.50164	0.014203	-3.38357	0.288976	0.159364
T.cells	SLC37A4	0.493494	2.862467	2.500327	0.014252	-3.11385	0.304381	0.170183
T.cells	ORC1	-0.38095	3.345968	-2.50015	0.014259	-3.40877	0.301082	0.167929
T.cells	MTCH1	0.167462	6.297903	2.499751	0.014274	-3.87177	0.281655	0.154725
T.cells	H2-T24	0.953428	1.828462	2.499411	0.014287	-2.96748	0.311549	0.175251
T.cells	SNRNP70	-0.11082	7.511442	-2.49854	0.014319	-4.06878	0.274016	0.149694
T.cells	2310001H1	0.426117	4.065364	2.49826	0.01433	-3.34202	0.296235	0.164845
T.cells	KCNQ5	0.214626	7.024096	2.49778	0.014348	-4.19812	0.27706	0.151843
T.cells	FNBP1	-0.24089	8.248905	-2.49645	0.014398	-4.0792	0.269919	0.147111
T.cells	SLC2A2	-1.03761	1.627926	-2.4939	0.014495	-2.9732	0.313977	0.177543
T.cells	NTMT1	0.2673	3.588232	2.493309	0.014518	-3.33272	0.300418	0.168243
T.cells	MGARP	1.225164	-0.49619	2.492306	0.014556	-2.99049	0.329306	0.188649
T.cells	GPR146	0.558615	3.278936	2.49218	0.014561	-3.06097	0.30252	0.169862
T.cells	OLR1	2.006165	1.333367	2.492134	0.014563	-3.03797	0.316063	0.179306
T.cells	ZFP595	-0.53491	1.84184	-2.49169	0.01458	-3.01374	0.31247	0.176835
T.cells	TOMM20	-0.17855	6.725117	-2.48965	0.014658	-3.95449	0.280675	0.154711
T.cells	EPB42	1.376323	-0.1647	2.48919	0.014675	-3.06104	0.327831	0.187392
T.cells	MIEN1	-0.1729	5.845651	-2.4846	0.014853	-3.82857	0.289264	0.159553
T.cells	EDEM3	-0.27736	5.974164	-2.48316	0.014909	-3.86118	0.288994	0.159337
T.cells	GORAB	0.560521	1.673364	2.482038	0.014953	-3.09974	0.318807	0.179774
T.cells	GM12940	-0.29399	4.51886	-2.48052	0.015013	-3.58335	0.299446	0.16641
T.cells	FADS2	-0.57449	3.530927	-2.48008	0.01503	-3.30478	0.306198	0.171094
T.cells	TMEM192	0.261615	4.694631	2.479421	0.015056	-3.57046	0.29826	0.165862
T.cells	HNRNPAB	-0.16381	8.05587	-2.47706	0.015149	-4.25912	0.277617	0.151689

T.cells	CFAP298	-0.31171	3.642504	-2.4755	0.015211	-3.46172	0.307475	0.171888
T.cells	AOPEP	0.195664	6.329542	2.472925	0.015314	-3.9441	0.290781	0.160142
T.cells	TUBA1A	-0.33239	5.430541	-2.47227	0.01534	-3.74326	0.296757	0.164327
T.cells	ZDHHC18	0.28182	5.57552	2.468736	0.015482	-3.65892	0.297998	0.164455
T.cells	TSPAN12	-1.19233	1.194761	-2.468	0.015512	-3.03123	0.328981	0.185827
T.cells	PDIA4	-0.21205	5.653573	-2.46724	0.015543	-3.76844	0.297585	0.164352
T.cells	NDUFA4	-0.1994	8.596899	-2.46528	0.015623	-4.32529	0.279315	0.151892
T.cells	GM26511	-0.50299	2.075738	-2.46451	0.015654	-3.25824	0.323765	0.182346
T.cells	BVHT	1.47167	-0.0458	2.463291	0.015704	-3.11626	0.340049	0.193624
T.cells	FRMD8	0.297219	4.222806	2.459645	0.015854	-3.5629	0.31131	0.17272
T.cells	1110046J0[illegible]	1.284982	0.031359	2.458443	0.015903	-3.10599	0.342379	0.194304
T.cells	FOLR2	-1.0927	3.655517	-2.45799	0.015922	-3.09748	0.315578	0.175757
T.cells	SLC25A46	0.203746	4.587825	2.454026	0.016087	-3.65881	0.309965	0.172212
T.cells	POLR2A	0.160979	6.200996	2.453306	0.016117	-3.94652	0.298861	0.164757
T.cells	FOXRED2	-0.78081	1.168266	-2.45314	0.016124	-3.05654	0.334789	0.189558
T.cells	TTF1	0.333141	3.466533	2.453072	0.016127	-3.46073	0.317909	0.177824
T.cells	SLC2A1	0.338831	4.676925	2.452398	0.016155	-3.50405	0.309341	0.17212
T.cells	SLC30A9	0.22294	5.265307	2.452274	0.01616	-3.71021	0.305255	0.169324
T.cells	A230083N[illegible]	-1.20365	0.546139	-2.45213	0.016167	-3.07148	0.339498	0.193105
T.cells	PEMT	-0.61851	3.483706	-2.45039	0.01624	-3.27749	0.318053	0.178331
T.cells	PPIH	-0.20215	5.305306	-2.45031	0.016243	-3.92386	0.305235	0.169514
T.cells	DENND4C	0.255059	5.068456	2.449454	0.016279	-3.7217	0.306873	0.17081
T.cells	ACAT1	-0.32987	6.480379	-2.44729	0.016371	-3.94788	0.297225	0.16461
T.cells	DLGAP4	-0.33724	5.015503	-2.44707	0.01638	-3.56531	0.307241	0.171487
T.cells	MPC1	-0.20551	7.569491	-2.44591	0.016429	-4.08853	0.289979	0.159962
T.cells	DSG2	-1.17301	0.740688	-2.44544	0.01645	-3.08169	0.338302	0.1936
T.cells	PTPRC	0.251041	8.915439	2.443999	0.016511	-4.15039	0.281255	0.154527
T.cells	PLXDC2	-0.60766	5.144109	-2.44287	0.01656	-3.61516	0.306349	0.17182
T.cells	CYP2C70	-0.77894	4.144913	-2.44253	0.016574	-3.36932	0.313343	0.17668
T.cells	AKR1D1	-0.90898	2.587501	-2.44223	0.016587	-3.13307	0.324542	0.184535
T.cells	GM13184	0.698315	2.666577	2.441784	0.016606	-3.13581	0.323964	0.184214
T.cells	TUBB2B	-0.40019	2.9596	-2.44105	0.016638	-3.47946	0.321833	0.182916
T.cells	RBP1	-0.49237	3.578118	-2.4409	0.016645	-3.46172	0.317376	0.179816
T.cells	NUCB2	-0.30002	4.304904	-2.44003	0.016682	-3.46995	0.312213	0.176536
T.cells	GM41611	-1.28088	0.284798	-2.44001	0.016683	-3.11619	0.341781	0.197415
T.cells	CDC42SE1	-0.20118	6.18973	-2.43915	0.01672	-3.90454	0.299187	0.167685
T.cells	MTM1	0.402748	4.433358	2.438937	0.016729	-3.52079	0.311309	0.176116
T.cells	MCEMP1	1.785699	1.650063	2.437586	0.016788	-3.13165	0.33146	0.190677
T.cells	HIST1H2BC	-0.32297	5.260139	-2.4375	0.016792	-3.87436	0.305546	0.172455
T.cells	OAT	-0.24297	5.245936	-2.43696	0.016815	-3.87277	0.305645	0.172706
T.cells	AP4M1	-0.24248	3.581284	-2.435	0.016901	-3.60572	0.317353	0.18137
T.cells	CYP2A22	-1.03067	1.632221	-2.43483	0.016908	-3.08907	0.331593	0.191497
T.cells	OLFR920	1.191912	0.137933	2.434662	0.016915	-3.11132	0.342908	0.199613
T.cells	SULT1A1	-0.69663	3.923228	-2.43457	0.016919	-3.25399	0.314915	0.179674
T.cells	CDK5R1	-0.55911	2.111423	-2.43436	0.016928	-3.22726	0.328038	0.189021
T.cells	CYP3A44	-1.10548	2.308322	-2.43378	0.016954	-3.11445	0.326588	0.188182
T.cells	TTC1	-0.19018	4.740195	-2.43361	0.016961	-3.73311	0.309158	0.175882

T.cells	4931423N1	-1.08017	0.653443	-2.42795	0.017211	-3.10188	0.342903	0.198761
T.cells	CDK10	-0.58171	2.234353	-2.42638	0.017281	-3.1699	0.330932	0.190609
T.cells	WDR18	-0.23281	4.82244	-2.4262	0.017289	-3.78481	0.312169	0.177365
T.cells	NREP	-1.02528	1.234795	-2.42602	0.017297	-3.10619	0.338455	0.19598
T.cells	EAPP	0.175085	5.602282	2.425961	0.017299	-3.89821	0.306713	0.173558
T.cells	ITIH1	-0.93392	2.749811	-2.42411	0.017382	-3.17014	0.328146	0.188359
T.cells	SLCO1B2	-0.70789	4.25024	-2.42318	0.017424	-3.44382	0.317249	0.180839
T.cells	CCNB1IP1	-0.54712	1.863098	-2.42282	0.01744	-3.23251	0.334784	0.193275
T.cells	SEC22A	0.380101	3.176208	2.421003	0.017522	-3.39013	0.326025	0.186905
T.cells	PROZ	-0.96821	1.915226	-2.41955	0.017587	-3.12262	0.335718	0.193814
T.cells	9530068E0	0.297593	4.851397	2.419396	0.017595	-3.65842	0.314212	0.17861
T.cells	SURF2	-0.35479	3.331878	-2.41852	0.017635	-3.4516	0.325388	0.18647
T.cells	SARNP	-0.13292	7.797226	-2.41601	0.017749	-4.31113	0.29455	0.165177
T.cells	ALYREF	-0.19543	8.216984	-2.4159	0.017754	-4.43938	0.291758	0.163259
T.cells	NDC1	-0.2857	3.748037	-2.41589	0.017754	-3.69147	0.322809	0.184886
T.cells	GM45871	0.848621	0.985003	2.41546	0.017774	-3.12784	0.343525	0.199733
T.cells	GRAMD3	-0.25904	6.080829	-2.41316	0.017879	-4.07596	0.307497	0.173913
T.cells	MIPEP	0.352217	2.843457	2.412537	0.017908	-3.39378	0.330821	0.190547
T.cells	IPO9	-0.20662	4.771136	-2.41165	0.017949	-3.82415	0.316741	0.180688
T.cells	ZFP119B	0.666148	1.297642	2.41137	0.017961	-3.17601	0.34253	0.199019
T.cells	CXCR4	-0.27081	6.146916	-2.41052	0.018001	-4.09829	0.307216	0.174059
T.cells	GM36551	1.034457	-0.80034	2.409862	0.018031	-3.2418	0.359281	0.211375
T.cells	MEFV	1.487597	-0.03339	2.409184	0.018063	-3.20206	0.353216	0.207108
T.cells	GCA	-1.34538	1.099128	-2.40853	0.018093	-3.15048	0.344386	0.200901
T.cells	5730522E0	-0.36878	3.610742	-2.40547	0.018235	-3.79857	0.325897	0.188227
T.cells	4833445I0	0.811358	0.48418	2.405237	0.018246	-3.14588	0.349648	0.205386
T.cells	SNX20	0.248106	5.514815	2.405107	0.018253	-3.88126	0.312178	0.178573
T.cells	PRG3	-0.66915	1.171663	-2.40422	0.018294	-3.39518	0.344292	0.201804
T.cells	TERF1	0.187723	5.254483	2.404067	0.018301	-3.98568	0.314021	0.180159
T.cells	RCAN1	-0.41657	3.708543	-2.40262	0.018369	-3.40536	0.325179	0.188375
T.cells	TRAFD1	0.407889	5.468862	2.401934	0.018402	-3.73226	0.312502	0.179606
T.cells	EXOC6	0.211045	6.194009	2.401902	0.018403	-3.99964	0.307416	0.176016
T.cells	DDX58	0.408472	4.759373	2.401412	0.018426	-3.61925	0.317555	0.183312
T.cells	GVIN1	1.033184	3.037272	2.401209	0.018436	-3.23579	0.330138	0.192389
T.cells	VKORC1	-0.40671	4.701765	-2.4007	0.01846	-3.58725	0.317968	0.183758
T.cells	ATXN7	0.229373	6.23066	2.400663	0.018462	-3.97619	0.307161	0.1761
T.cells	C1S1	-0.88486	1.787052	-2.39887	0.018546	-3.15854	0.340596	0.199905
T.cells	SZRD1	-0.1647	5.906997	-2.39786	0.018594	-4.00498	0.31068	0.178378
T.cells	NUDT2	-0.35804	3.238769	-2.39708	0.018631	-3.45962	0.330128	0.192228
T.cells	CD2BP2	0.254397	4.011388	2.395957	0.018685	-3.71274	0.32486	0.188226
T.cells	GALNT3	-0.90915	1.767982	-2.39286	0.018834	-3.17853	0.341983	0.201184
T.cells	ZFP873	0.605016	0.995152	2.39284	0.018835	-3.22052	0.347975	0.205552
T.cells	NUP214	-0.20071	5.2678	-2.39148	0.0189	-3.95481	0.316029	0.182917
T.cells	HYAL1	0.75048	1.311575	2.391126	0.018917	-3.17454	0.34551	0.204201
T.cells	CCL24	-1.6567	3.596362	-2.39094	0.018926	-3.214	0.328186	0.191629
T.cells	SETX	0.207544	5.905752	2.390715	0.018937	-4.06623	0.311501	0.179697
T.cells	APOBEC3	0.204504	6.862598	2.390686	0.018939	-4.27784	0.304824	0.174973

T.cells	FAM20B	0.306372	3.958304	2.390468	0.018949	-3.64819	0.325517	0.189745
T.cells	OTC	-0.82991	3.438444	-2.39024	0.01896	-3.33547	0.329357	0.192558
T.cells	TSPAN17	-0.89233	1.115501	-2.38927	0.019007	-3.17258	0.347088	0.205613
T.cells	CD79B	-0.23595	6.976941	-2.38879	0.019031	-4.39892	0.304082	0.174799
T.cells	9430038I0:	0.225311	4.248537	2.388392	0.01905	-3.75517	0.323441	0.188636
T.cells	PARP3	0.765412	2.362796	2.386795	0.019128	-3.19387	0.338354	0.199161
T.cells	IFITM2	0.304385	7.227901	2.38369	0.01928	-4.10783	0.304552	0.174937
T.cells	DOP1A	0.368934	4.09244	2.383257	0.019301	-3.56117	0.326938	0.190974
T.cells	KLF2	-0.39968	8.937753	-2.38303	0.019312	-4.41991	0.292963	0.166918
T.cells	JUND	-0.21127	10.62781	-2.3826	0.019333	-4.78097	0.281922	0.159318
T.cells	DBI	-0.2229	7.067278	-2.38191	0.019367	-4.25616	0.305734	0.17603
T.cells	HSP90AB1	-0.14158	9.925498	-2.3808	0.019422	-4.72176	0.286851	0.162785
T.cells	H2-Q4	0.364873	4.933329	2.379525	0.019485	-3.92192	0.321225	0.187444
T.cells	CYP3A16	-1.08103	0.777697	-2.37919	0.019502	-3.19261	0.35275	0.210487
T.cells	CASP1	0.502068	3.51676	2.379119	0.019505	-3.42255	0.331664	0.195052
T.cells	MRPL41	-0.28466	4.003451	-2.37766	0.019578	-3.70076	0.328384	0.192877
T.cells	IFI30	-0.37884	6.746076	-2.37754	0.019584	-3.91608	0.308636	0.178698
T.cells	SIAH2	-0.23635	4.793136	-2.37663	0.019629	-3.83315	0.322843	0.189012
T.cells	NFKB1	-0.29356	8.743793	-2.37491	0.019715	-4.35925	0.296061	0.169613
T.cells	GM21188	1.703037	1.578983	2.373869	0.019768	-3.23868	0.348513	0.207333
T.cells	CLU	-0.89607	5.440247	-2.37317	0.019803	-3.78122	0.319547	0.186399
T.cells	IQCN	-0.97554	1.791223	-2.37234	0.019844	-3.21209	0.347173	0.20652
T.cells	GM4285	0.761297	1.640385	2.371158	0.019904	-3.21167	0.348459	0.207621
T.cells	PCBP1	-0.14917	8.199098	-2.37009	0.019958	-4.47416	0.300461	0.173255
T.cells	PREX2	-0.98904	2.374714	-2.37009	0.019958	-3.21977	0.342749	0.203747
T.cells	CPPED1	0.2909	3.886993	2.369638	0.019981	-3.58601	0.331263	0.195437
T.cells	NDRG2	-0.81928	2.964877	-2.36932	0.019997	-3.2857	0.338224	0.200625
T.cells	EI24	0.223101	4.630195	2.367367	0.020097	-3.84151	0.326894	0.192134
T.cells	MTFR1	-0.31357	4.079368	-2.36521	0.020207	-3.6941	0.331875	0.195474
T.cells	CRLF2	0.282467	5.010644	2.365156	0.02021	-3.83193	0.324967	0.190472
T.cells	GATA3	-1.24125	1.116393	-2.364	0.020269	-3.24057	0.355296	0.212505
T.cells	IFNGR2	0.303042	5.782195	2.363191	0.020311	-3.97336	0.320005	0.186873
T.cells	GPHN	0.17843	7.486182	2.362247	0.020359	-4.37628	0.307961	0.178445
T.cells	CIDEB	-0.8308	1.942054	-2.36195	0.020374	-3.23085	0.349051	0.208268
T.cells	R3HCC1L	0.213634	5.540599	2.360425	0.020453	-4.02654	0.322612	0.188706
T.cells	GNL3	-0.23441	5.420028	-2.35915	0.020519	-4.03732	0.323502	0.189684
T.cells	DUS2	-0.34075	3.353616	-2.35872	0.020542	-3.71256	0.338956	0.200995
T.cells	CRLF3	0.231677	6.899396	2.358603	0.020548	-4.26822	0.312848	0.182108
T.cells	REV1	-0.2059	5.326262	-2.35813	0.020572	-4.06157	0.324189	0.190261
T.cells	IGKV1-135	-0.73558	-0.02384	-2.3557	0.020699	-3.2946	0.365774	0.221596
T.cells	POLE4	-0.1724	6.652817	-2.355	0.020736	-4.26791	0.314665	0.184222
T.cells	CEBPA	-0.95586	2.906324	-2.35407	0.020785	-3.24821	0.342461	0.204612
T.cells	RRP1B	0.324822	4.132089	2.353983	0.020789	-3.81012	0.333124	0.197755
T.cells	UPP1	1.920244	0.646262	2.353676	0.020805	-3.26119	0.360317	0.217911
T.cells	MEIS2	-0.97014	2.894046	-2.35245	0.02087	-3.27361	0.342556	0.205033
T.cells	KCNN4	0.397647	3.850661	2.352173	0.020885	-3.79623	0.335246	0.199722
T.cells	EOGT	-0.63468	2.532472	-2.35036	0.02098	-3.31105	0.345357	0.207648

T.cells	ANK1	1.486825	1.273318	2.348787	0.021064	-3.25869	0.355279	0.215397
T.cells	GM50386	0.545846	0.179027	2.348681	0.02107	-3.38256	0.364114	0.222076
T.cells	GOLGA4	0.201788	5.30004	2.348411	0.021084	-3.96503	0.324448	0.192633
T.cells	TET2	0.253828	6.024646	2.348333	0.021088	-3.91933	0.319173	0.188786
T.cells	FBXO17	-0.96611	0.782245	-2.34776	0.021119	-3.24772	0.359219	0.218593
T.cells	RBM12B2	0.461406	2.316248	2.34753	0.021131	-3.42583	0.347043	0.209526
T.cells	SOCS3	0.720778	4.698376	2.347213	0.021148	-3.4744	0.328891	0.196111
T.cells	ZFP869	0.320193	4.026546	2.347203	0.021148	-3.73473	0.333918	0.199813
T.cells	RPA3	-0.23628	4.97161	-2.34717	0.02115	-4.07618	0.326866	0.194625
T.cells	GTDC1	0.232246	6.409058	2.346829	0.021168	-4.10914	0.316407	0.18699
T.cells	AMN1	0.359245	3.42505	2.345478	0.02124	-3.58804	0.33848	0.203626
T.cells	GP9	1.469435	0.659096	2.345457	0.021242	-3.25825	0.360214	0.219903
T.cells	SLC5A3	-0.51398	3.816287	-2.34476	0.021279	-3.57918	0.335506	0.201538
T.cells	WDR74	-0.22667	4.042303	-2.34465	0.021285	-3.83113	0.3338	0.200274
T.cells	SIDT1	-0.81898	3.376538	-2.34461	0.021287	-3.39892	0.338851	0.204019
T.cells	PSEN1	0.176915	6.297037	2.344584	0.021288	-4.1316	0.317211	0.188089
T.cells	SERTAD1	-0.27575	5.61417	-2.34368	0.021337	-4.01533	0.322397	0.191889
T.cells	GM26982	0.663501	1.652146	2.343178	0.021364	-3.2658	0.352535	0.214364
T.cells	BC004004	0.204897	5.147988	2.341839	0.021436	-3.98253	0.326271	0.194761
T.cells	PPP2CA	-0.11203	7.762516	-2.34152	0.021453	-4.41352	0.307514	0.18117
T.cells	P2RY10	-1.1148	3.930309	-2.33964	0.021555	-3.27077	0.335708	0.202109
T.cells	ARL6IP5	0.185128	7.048458	2.338824	0.021599	-4.27307	0.312849	0.185488
T.cells	HBQ1A	1.131623	-1.39639	2.336317	0.021736	-3.3272	0.378388	0.235329
T.cells	FBXO11	-0.21227	8.797772	-2.33603	0.021751	-4.55714	0.300677	0.177349
T.cells	GM29340	-1.1068	-0.6731	-2.3358	0.021764	-3.29951	0.372314	0.23086
T.cells	POLR1A	-0.27006	4.926327	-2.33562	0.021774	-3.95294	0.328241	0.197611
T.cells	IFI203	0.321851	6.302394	2.335017	0.021807	-4.18013	0.318181	0.190307
T.cells	STYX	0.220672	4.526153	2.334488	0.021836	-3.91357	0.331223	0.200115
T.cells	4930599N2	0.863429	1.733523	2.333414	0.021895	-3.27963	0.352741	0.216682
T.cells	DPP4	0.230598	5.100888	2.332831	0.021927	-4.19092	0.326949	0.197468
T.cells	ASGR1	-0.69493	3.372794	-2.33283	0.021927	-3.45008	0.339957	0.207198
T.cells	GM14305	-0.48641	2.260659	-2.33275	0.021932	-3.38434	0.348582	0.213725
T.cells	B230217C1	-0.8492	1.478169	-2.33169	0.02199	-3.2769	0.354772	0.218725
T.cells	SPTAN1	-0.207	6.559592	-2.33165	0.021992	-4.21013	0.316333	0.189884
T.cells	AUTS2	0.532196	4.397144	2.331619	0.021994	-3.89492	0.332189	0.201667
T.cells	AP3D1	0.194518	4.873786	2.331489	0.022001	-3.90584	0.328631	0.199008
T.cells	ALDH1L1	-0.70878	3.098676	-2.33108	0.022024	-3.38615	0.342064	0.209133
T.cells	RDH7	-0.87613	2.864625	-2.33101	0.022028	-3.36871	0.343873	0.210499
T.cells	MRFAP1	-0.15201	6.452488	-2.33033	0.022065	-4.26033	0.317102	0.190623
T.cells	TNFSF14	1.088837	-0.05669	2.329747	0.022098	-3.33978	0.367208	0.228596
T.cells	ADORA2A	0.741496	3.148747	2.329256	0.022125	-3.36237	0.341679	0.20929
T.cells	SPON1	0.957413	1.404705	2.328794	0.022151	-3.31994	0.355358	0.21987
T.cells	VSIG4	-0.94965	4.823941	-2.32864	0.022159	-3.55495	0.329002	0.199972
T.cells	PXDN	-1.01262	0.993778	-2.32752	0.022222	-3.29014	0.359025	0.222752
T.cells	RAMP1	-0.19275	4.507186	-2.32716	0.022242	-4.05968	0.331708	0.202089
T.cells	TRIM33	-0.1704	6.368235	-2.324	0.022418	-4.2797	0.319622	0.192558
T.cells	YWHAZ	-0.10106	8.482146	-2.324	0.022418	-4.58457	0.304664	0.181555

T.cells	COL27A1	-0.66101	2.314283	-2.32358	0.022442	-3.51432	0.35026	0.215575
T.cells	TRIM36	-0.55104	3.146179	-2.32233	0.022512	-3.44247	0.343766	0.211019
T.cells	MYO1C	0.178778	5.436164	2.32199	0.022532	-4.13859	0.326443	0.198014
T.cells	CROT	-0.32044	5.259869	-2.32197	0.022533	-3.93576	0.327747	0.198988
T.cells	LRP10	0.194381	6.125795	2.320786	0.0226	-4.21225	0.321409	0.194469
T.cells	MCTP2	0.253103	6.80258	2.320666	0.022606	-4.30628	0.316521	0.190871
T.cells	GM4952	-0.85433	2.667628	-2.32028	0.022628	-3.37786	0.347515	0.214233
T.cells	MYO1F	0.711569	5.259228	2.31974	0.022659	-3.43625	0.327773	0.19949
T.cells	ZDHHC2	-0.78568	2.276539	-2.3193	0.022684	-3.33602	0.350588	0.216911
T.cells	PML	0.360053	5.273859	2.318583	0.022724	-4.07158	0.327822	0.199758
T.cells	STX17	0.330587	4.034327	2.316301	0.022855	-3.79973	0.338615	0.207333
T.cells	GPAA1	-0.27464	3.764843	-2.31479	0.022941	-3.74678	0.340824	0.209267
T.cells	PCYT2	-0.28865	4.635801	-2.31467	0.022948	-3.91476	0.334188	0.204279
T.cells	MPLKIP	0.184431	4.793719	2.313746	0.023001	-4.05117	0.332997	0.203711
T.cells	4930445E1	1.223373	-0.41363	2.312919	0.023048	-3.37322	0.374396	0.235687
T.cells	GM11508	-0.36367	3.437566	-2.31287	0.023051	-3.8463	0.343349	0.211766
T.cells	CERKL	0.602197	1.852174	2.312843	0.023053	-3.46102	0.35583	0.221313
T.cells	OPA1	0.204165	4.835691	2.312086	0.023096	-4.04865	0.332682	0.203824
T.cells	MAP7	0.284107	3.639692	2.31194	0.023105	-3.89049	0.341787	0.210724
T.cells	CACNA1E	-0.49693	4.907691	-2.31053	0.023186	-4.07497	0.332675	0.203836
T.cells	GM4869	-0.89294	1.447403	-2.31025	0.023202	-3.33061	0.359661	0.224508
T.cells	EPG5	0.26458	4.420072	2.30867	0.023294	-3.89452	0.337051	0.20699
T.cells	GM13431	-1.24001	0.492651	-2.30789	0.02334	-3.35772	0.368211	0.231082
T.cells	2010315BC	0.548782	2.024895	2.307872	0.023341	-3.39685	0.35575	0.22145
T.cells	UCHL3	-0.19108	6.235223	-2.30345	0.023599	-4.31954	0.326622	0.19805
T.cells	GAS2L3	0.26655	4.330262	2.302081	0.02368	-4.11845	0.340998	0.209334
T.cells	BPNT1	-0.40506	3.339718	-2.30199	0.023685	-3.5485	0.348705	0.215194
T.cells	GM26737	-0.89993	0.478605	-2.30194	0.023688	-3.33318	0.371885	0.233009
T.cells	BC024386	-0.98222	1.614053	-2.30079	0.023756	-3.3385	0.362635	0.225963
T.cells	ASB8	0.308821	3.607063	2.300262	0.023787	-3.77363	0.346718	0.213926
T.cells	LGALS8	0.265638	5.110017	2.299697	0.023821	-3.99289	0.335148	0.205352
T.cells	TNFSF12	0.821526	2.295218	2.29949	0.023833	-3.33791	0.35712	0.222123
T.cells	PDLIM4	-1.31321	2.040162	-2.29924	0.023848	-3.33658	0.359176	0.223733
T.cells	LRRFIP2	0.19064	5.900501	2.298518	0.023891	-4.17145	0.329378	0.201199
T.cells	NEMP1	0.474122	3.065406	2.296881	0.023988	-3.65915	0.352002	0.218099
T.cells	EIF4E3	0.303286	4.420461	2.296526	0.024009	-3.92521	0.341404	0.210095
T.cells	HMGCL	-0.2672	5.187114	-2.29395	0.024163	-4.00168	0.336808	0.206422
T.cells	AMPD1	-0.50239	2.911186	-2.29378	0.024174	-3.62196	0.354562	0.219921
T.cells	TMEM56	-0.90133	2.147941	-2.29308	0.024215	-3.36789	0.360708	0.22481
T.cells	GM36279	0.429277	2.959709	2.292896	0.024227	-3.64994	0.354175	0.219825
T.cells	CRPPA	-0.65608	2.552625	-2.29245	0.024253	-3.43412	0.357437	0.22245
T.cells	ATP11B	0.202594	7.035822	2.288879	0.024469	-4.39071	0.32536	0.197334
T.cells	NLRC5	0.488807	5.064856	2.288334	0.024502	-4.08723	0.340203	0.208632
T.cells	SIK3	-0.2195	10.09847	-2.28799	0.024523	-4.79482	0.303507	0.181447
T.cells	IMPDH2	-0.19635	5.196107	-2.28733	0.024563	-4.2404	0.339326	0.208048
T.cells	IFI206	0.685617	3.940563	2.286475	0.024616	-3.84846	0.349096	0.215657
T.cells	CHID1	-0.51933	2.453126	-2.28631	0.024626	-3.42702	0.360999	0.224783

T.cells	ZFP385A	-0.48196	3.938147	-2.28509	0.0247	-3.56523	0.349725	0.215923
T.cells	SRSF2	-0.15807	7.941182	-2.28258	0.024854	-4.63383	0.319808	0.194071
T.cells	TKTL1	-0.88243	1.290296	-2.28213	0.024882	-3.3644	0.371645	0.233643
T.cells	CORO7	0.173814	6.215775	2.282107	0.024883	-4.36605	0.332561	0.203719
T.cells	3-Sep	1.066199	-1.00287	2.281873	0.024898	-3.40332	0.391255	0.249034
T.cells	FCNA	-1.17619	4.555419	-2.28184	0.0249	-3.61517	0.345285	0.213389
T.cells	FOXN3	0.186566	8.899803	2.281514	0.02492	-4.72719	0.312924	0.189177
T.cells	LGMN	-0.314	7.188159	-2.28109	0.024946	-4.29385	0.325316	0.198517
T.cells	PHLDA1	-0.51894	4.035457	-2.27936	0.025053	-3.76527	0.350223	0.217308
T.cells	LAT2	-0.34122	4.773693	-2.27778	0.025151	-3.87816	0.344432	0.213298
T.cells	ARL5A	0.196037	5.831093	2.277311	0.02518	-4.27774	0.336295	0.207249
T.cells	TMEM109	-0.32409	3.841384	-2.27711	0.025193	-3.90694	0.35176	0.219119
T.cells	PROK2	1.760217	-0.61756	2.276996	0.0252	-3.42064	0.388849	0.248026
T.cells	TRAPPC10	0.207814	5.170629	2.276868	0.025208	-4.11742	0.341356	0.211159
T.cells	ERBB4	-1.15909	0.909465	-2.27659	0.025225	-3.3742	0.37576	0.237759
T.cells	TANC2	-0.34069	5.521032	-2.27533	0.025304	-4.09884	0.339134	0.209452
T.cells	RORA	-0.58308	5.020685	-2.27478	0.025338	-3.68415	0.342993	0.212464
T.cells	ATXN7L1O!	0.854889	1.337166	2.273897	0.025393	-3.38865	0.372686	0.235513
T.cells	AKAP10	0.238694	5.860095	2.273572	0.025414	-4.22786	0.336543	0.207739
T.cells	KIF1B	-0.3206	5.385583	-2.27354	0.025416	-3.90414	0.340175	0.210501
T.cells	TMEM179E	0.168254	5.864754	2.272385	0.025488	-4.25069	0.337055	0.208043
T.cells	CCPG1OS	-0.57194	1.576044	-2.27139	0.025551	-3.44873	0.371448	0.234646
T.cells	PLAGL1	-1.19134	1.041672	-2.27123	0.025561	-3.38551	0.375935	0.2382
T.cells	SLC30A5	0.193195	5.799283	2.268689	0.025722	-4.33533	0.338717	0.209245
T.cells	PLEKHG6	0.984113	-0.11425	2.268623	0.025726	-3.43668	0.38698	0.246649
T.cells	CD36	-0.53382	5.712063	-2.26852	0.025733	-4.00621	0.339386	0.209754
T.cells	FCRLA	-0.27007	5.107141	-2.26448	0.02599	-4.34754	0.344898	0.214366
T.cells	TSEN54	-0.34135	2.874509	-2.2644	0.025995	-3.73394	0.362722	0.228127
T.cells	THEMIS2	0.405923	4.692319	2.2641	0.026014	-3.9053	0.348146	0.21697
T.cells	TRIB2	0.404888	2.561151	2.263846	0.02603	-3.84168	0.365291	0.230303
T.cells	GM12655	0.97251	0.353725	2.263292	0.026066	-3.39953	0.383872	0.245018
T.cells	ADAR	0.353793	4.311216	2.263153	0.026075	-3.87243	0.351156	0.219497
T.cells	PPM1M	-0.31351	4.583696	-2.26298	0.026086	-3.8661	0.349001	0.217854
T.cells	YPEL2	0.287587	4.572346	2.262794	0.026098	-4.14896	0.349091	0.217951
T.cells	CALR	-0.19924	7.35156	-2.26265	0.026107	-4.46534	0.32781	0.201774
T.cells	1700003F1	-0.5337	2.027827	-2.26195	0.026152	-3.59434	0.369702	0.234171
T.cells	FGD5	-1.11055	1.663977	-2.26184	0.026159	-3.40043	0.37274	0.236552
T.cells	CAMK2B	-0.37018	3.706454	-2.26151	0.026181	-4.05791	0.355982	0.223528
T.cells	GM16867	0.495445	2.522419	2.261157	0.026203	-3.73217	0.36561	0.231103
T.cells	HERPUD1	-0.20108	7.853564	-2.25912	0.026335	-4.75058	0.325335	0.199779
T.cells	SERPINA1B	-0.56928	7.994808	-2.25668	0.026493	-4.54058	0.32486	0.199499
T.cells	MRTO4	-0.26126	4.665156	-2.25659	0.026499	-4.17371	0.350298	0.218875
T.cells	ARPP21	0.707352	1.234602	2.256525	0.026503	-3.65433	0.378449	0.240792
T.cells	MEAF6	-0.1724	4.941914	-2.25647	0.026507	-4.23625	0.348114	0.217196
T.cells	ARL10	0.481557	2.295947	2.2547	0.026622	-3.58099	0.370354	0.234277
T.cells	ASB3	0.208407	4.711015	2.254586	0.02663	-4.14731	0.350722	0.219053
T.cells	ENTPD5	-0.24922	3.78751	-2.25339	0.026708	-3.92346	0.358298	0.224987

T.cells	PCNA	-0.2115	6.710032	-2.25189	0.026806	-4.5753	0.335388	0.207768
T.cells	ZER1	0.300115	3.262662	2.251817	0.026811	-3.83914	0.362565	0.228695
T.cells	GM29994	-0.74737	1.150146	-2.25144	0.026836	-3.4256	0.380219	0.242661
T.cells	4833419F2	-1.09285	1.323387	-2.25124	0.026849	-3.41914	0.378741	0.241569
T.cells	GLIPR2	0.542457	4.6029	2.250303	0.026911	-3.78304	0.351763	0.22078
T.cells	FCMR	-0.59958	2.105124	-2.24993	0.026935	-3.56676	0.372142	0.236708
T.cells	ILRUN	0.221326	5.988088	2.24993	0.026935	-4.27788	0.340916	0.212462
T.cells	APOC1	-0.58187	7.949726	-2.2496	0.026957	-4.44497	0.326094	0.201198
T.cells	NPTN	-0.14281	8.043161	-2.24956	0.02696	-4.56973	0.325403	0.200675
T.cells	CBFA2T2	0.185308	5.563389	2.2485	0.02703	-4.35061	0.344328	0.215308
T.cells	ELF4	-0.16411	6.785373	-2.24846	0.027033	-4.51211	0.334934	0.208107
T.cells	AZGP1	-0.64566	3.926277	-2.24745	0.0271	-3.74474	0.357774	0.225604
T.cells	CHAF1A	-0.29171	4.186029	-2.24627	0.027178	-4.2186	0.356294	0.224137
T.cells	3830403N1	-1.38938	0.704685	-2.24491	0.027269	-3.42914	0.385937	0.247328
T.cells	A430018G1	0.621303	1.329295	2.244684	0.027284	-3.46758	0.380561	0.243137
T.cells	HMGN1	-0.18884	7.26929	-2.24406	0.027325	-4.67424	0.33292	0.206196
T.cells	FGA	-0.44355	5.727993	-2.24147	0.027499	-4.16018	0.346235	0.215988
T.cells	BEX4	-0.75894	0.397887	-2.24135	0.027507	-3.46205	0.390423	0.250511
T.cells	DHRS9	1.222498	0.196917	2.24002	0.027597	-3.46504	0.393005	0.252423
T.cells	KDM5C	-0.25111	6.827805	-2.23939	0.027639	-4.47259	0.338554	0.20997
T.cells	PARVA	-0.95956	1.329681	-2.23845	0.027703	-3.43972	0.383722	0.244995
T.cells	CD5L	-0.84195	6.068559	-2.23775	0.02775	-3.98689	0.344929	0.21499
T.cells	GLYAT	-0.83787	2.069154	-2.23737	0.027776	-3.48503	0.377514	0.240437
T.cells	TSC22D4	0.171419	6.684783	2.23677	0.027817	-4.50674	0.340255	0.211623
T.cells	SLC25A43	1.076118	-0.21871	2.235787	0.027884	-3.45381	0.398036	0.256612
T.cells	DQX1	-1.35027	1.215444	-2.23477	0.027953	-3.47464	0.385941	0.246921
T.cells	MEG3	-1.51971	1.040515	-2.23261	0.028101	-3.44926	0.388663	0.249081
T.cells	PDE4B	0.22904	8.527906	2.232561	0.028104	-4.72939	0.328182	0.202235
T.cells	MMS19	0.186034	4.947005	2.231919	0.028148	-4.27119	0.356062	0.223584
T.cells	SAMD1	-0.19746	4.976188	-2.22972	0.028299	-4.34355	0.356352	0.224101
T.cells	GM28417	0.730207	1.320479	2.229247	0.028332	-3.50305	0.386958	0.248139
T.cells	SLC30A1	-0.57175	3.386735	-2.22896	0.028352	-3.62371	0.369371	0.234388
T.cells	OTUB2	0.432342	1.885457	2.228526	0.028382	-3.78216	0.382074	0.244505
T.cells	GBP9	0.625079	3.036901	2.228068	0.028413	-3.61734	0.372296	0.236865
T.cells	SULT2A5	-0.92073	2.02078	-2.22761	0.028445	-3.50738	0.380912	0.243816
T.cells	EGLN3	0.484085	3.294296	2.227263	0.028469	-3.88337	0.370142	0.235358
T.cells	AFM	-0.72969	2.294567	-2.22712	0.028479	-3.54326	0.378573	0.242027
T.cells	SYTL2	-1.05516	1.257049	-2.22704	0.028484	-3.46766	0.38751	0.249111
T.cells	THBS3	-1.26838	1.01596	-2.22647	0.028524	-3.46792	0.389614	0.250982
T.cells	KLRC3	0.994761	-1.20661	2.226167	0.028545	-3.50242	0.409515	0.267072
T.cells	MDH1	-0.15554	8.120076	-2.22476	0.028643	-4.79251	0.33233	0.206444
T.cells	FCHSD2	-0.27885	6.823892	-2.22466	0.02865	-4.53484	0.342242	0.21403
T.cells	STX5A	0.166143	5.871144	2.220772	0.028922	-4.33921	0.352625	0.220848
T.cells	LYZ1	1.35803	-0.68152	2.219883	0.028985	-3.50113	0.409109	0.26525
T.cells	SULT2A2	-0.76753	4.037802	-2.21897	0.029049	-3.78736	0.368326	0.232865
T.cells	HDGFL3	1.02242	1.511028	2.218461	0.029085	-3.4733	0.38994	0.249983
T.cells	SLC13A3	-1.17233	1.11728	-2.21707	0.029183	-3.47577	0.394171	0.253033

T.cells	PTDSS2	0.323679	3.06586	2.216735	0.029207	-3.85622	0.377261	0.239689
T.cells	LZTS1	-0.81471	1.417164	-2.21627	0.02924	-3.49872	0.391528	0.251124
T.cells	ERMAP	1.22022	0.529243	2.215071	0.029325	-3.48668	0.399745	0.257667
T.cells	A630001G[illegible]	0.200838	5.126842	2.214703	0.029351	-4.35797	0.360416	0.226842
T.cells	GM12596	-0.40952	2.627238	-2.21451	0.029365	-3.82682	0.381329	0.243202
T.cells	EIF4A3	-0.16683	5.877476	-2.21345	0.029441	-4.46659	0.354348	0.2226
T.cells	FAM129A	-0.49603	5.667894	-2.21333	0.029449	-3.78934	0.356033	0.223903
T.cells	B4GALT7	0.383611	2.869796	2.213162	0.029461	-3.7675	0.379251	0.242065
T.cells	CIRBP	0.17876	7.218243	2.209556	0.02972	-4.62452	0.34638	0.215422
T.cells	PRDM15	0.314283	3.462741	2.207454	0.029871	-3.91019	0.378582	0.239571
T.cells	YWHAQ	-0.12272	7.746706	-2.20648	0.029942	-4.7705	0.343796	0.212965
T.cells	PTPRG	-1.00127	2.191113	-2.20621	0.029962	-3.52577	0.389786	0.248606
T.cells	GATA4	-0.90579	1.621315	-2.20399	0.030123	-3.50078	0.394815	0.253493
T.cells	APCS	0.435779	4.833138	2.203165	0.030183	-4.07118	0.36724	0.232084
T.cells	MYOF	0.740071	2.65687	2.202795	0.03021	-3.65592	0.38572	0.246664
T.cells	IGFBP2	-0.88884	4.701389	-2.20262	0.030222	-3.95936	0.368335	0.233097
T.cells	AGT	-0.76334	4.084746	-2.20162	0.030295	-3.83277	0.3735	0.237517
T.cells	VEZT	0.250072	4.178132	2.201381	0.030313	-4.08406	0.372713	0.236973
T.cells	NFE2L3	0.543585	2.246069	2.201187	0.030327	-3.6845	0.389305	0.25007
T.cells	NUDT5	-0.2014	5.148841	-2.20104	0.030338	-4.39694	0.364629	0.230664
T.cells	GPX3	-1.41049	1.463214	-2.20092	0.030347	-3.50956	0.39622	0.255572
T.cells	CCNE1	-0.29732	3.530373	-2.20065	0.030366	-4.17616	0.378201	0.241317
T.cells	GM9733	1.57991	0.76406	2.200262	0.030395	-3.51921	0.402492	0.260779
T.cells	DDX19B	0.308783	3.529612	2.200091	0.030408	-3.92411	0.378207	0.241499
T.cells	IL2RA	-0.52108	2.557963	-2.19999	0.030415	-3.96115	0.386581	0.248154
T.cells	3-Mar	-0.35097	6.392222	-2.19995	0.030418	-4.42827	0.354514	0.223037
T.cells	CLEC4F	-1.15412	5.458864	-2.19893	0.030493	-3.98448	0.362179	0.229267
T.cells	IFIH1	0.77538	3.912874	2.19873	0.030508	-3.63501	0.375052	0.239391
T.cells	LENG8	0.254228	4.564084	2.198387	0.030533	-4.13843	0.369578	0.235169
T.cells	CCDC61	0.321299	2.934126	2.197077	0.03063	-3.9324	0.383422	0.246472
T.cells	ANKZF1	0.347962	2.742515	2.197056	0.030631	-3.73436	0.385081	0.247789
T.cells	HERC6	0.39752	4.719873	2.197028	0.030633	-4.18057	0.36828	0.234522
T.cells	CTSO	0.368658	4.26707	2.196307	0.030687	-3.94624	0.372066	0.237786
T.cells	ZFP53	0.234928	4.791451	2.195904	0.030717	-4.26371	0.367685	0.234367
T.cells	ANGPTL8	-0.92938	1.825356	-2.19558	0.03074	-3.54564	0.393113	0.254529
T.cells	SCP2	-0.30373	7.526193	-2.19529	0.030762	-4.58571	0.345613	0.217207
T.cells	C4BP	-0.88675	1.968581	-2.19487	0.030793	-3.55299	0.391849	0.253615
T.cells	B3GAT3	0.283909	4.307364	2.193983	0.030859	-4.08845	0.371727	0.237873
T.cells	ALAS2	1.100568	4.928547	2.193662	0.030883	-3.80747	0.366548	0.233859
T.cells	GHR	-0.7381	4.215001	-2.19355	0.030891	-3.92282	0.372503	0.238565
T.cells	ZFP809	0.349503	3.64912	2.190807	0.031096	-3.96656	0.378842	0.24331
T.cells	RARA	-0.29655	5.561492	-2.19058	0.031113	-4.13786	0.362825	0.230803
T.cells	BRI3BP	-0.24829	4.756436	-2.18897	0.031234	-4.23195	0.369488	0.236406
T.cells	VPS39	0.34856	3.331542	2.188408	0.031276	-3.86224	0.381565	0.24616
T.cells	ACSM1	-1.21324	1.026687	-2.18836	0.03128	-3.52426	0.401879	0.262453
T.cells	VEZF1	-0.14955	6.00178	-2.18804	0.031304	-4.54246	0.359228	0.228567
T.cells	MYLIP	0.235056	5.991118	2.188023	0.031305	-4.42067	0.359315	0.228635

T.cells	D1ERTD62.	-0.24957	5.295192	-2.18769	0.03133	-4.1837	0.365016	0.233211
T.cells	PAPSS2	-0.78988	2.513577	-2.18742	0.031351	-3.59867	0.388662	0.252077
T.cells	SORL1	0.587925	5.66068	2.186784	0.031399	-3.87386	0.362011	0.230963
T.cells	MAVS	0.397701	2.932268	2.186643	0.031409	-3.8096	0.385014	0.249187
T.cells	CAMKK1	-1.10347	0.590902	-2.18599	0.031459	-3.53865	0.40583	0.266101
T.cells	GM43112	1.108501	0.075373	2.185653	0.031484	-3.54966	0.41055	0.270041
T.cells	TMEM86B	-0.35107	3.467162	-2.18547	0.031498	-3.90567	0.3804	0.24579
T.cells	COX7A1	0.559838	0.986976	2.184411	0.031578	-3.64545	0.402237	0.263568
T.cells	TMEM38A	0.682456	1.868955	2.184325	0.031585	-3.57972	0.394342	0.257217
T.cells	URI1	0.183591	5.89939	2.184164	0.031597	-4.50242	0.360061	0.229969
T.cells	TRDV4	1.188558	-1.36214	2.183877	0.031619	-3.56767	0.423979	0.281522
T.cells	DNAH17	-0.72431	3.275665	-2.18288	0.031694	-3.75468	0.382555	0.247711
T.cells	RPAIN	-0.25405	3.771433	-2.18236	0.031734	-4.08381	0.378381	0.244362
T.cells	CFP	-0.62239	5.518079	-2.18119	0.031824	-3.84015	0.363836	0.233025
T.cells	BBC3	-0.46254	2.639565	-2.18067	0.031863	-3.72109	0.38826	0.25251
T.cells	RFT1	0.456848	2.53189	2.180142	0.031904	-3.80675	0.389203	0.253315
T.cells	FAM126A	0.272269	5.549539	2.180091	0.031908	-4.26071	0.363577	0.232909
T.cells	COA7	-0.28747	3.404389	-2.18006	0.03191	-3.95221	0.381626	0.24724
T.cells	SHC4	0.685015	0.470109	2.178778	0.032008	-3.60177	0.408497	0.268614
T.cells	TERF2	0.185923	4.800606	2.177994	0.032068	-4.34289	0.370709	0.238376
T.cells	SERINC3	-0.11286	9.707046	-2.17688	0.032154	-5.14551	0.331692	0.208146
T.cells	GM4876	0.582561	1.628342	2.176824	0.032158	-3.61899	0.398189	0.260618
T.cells	FARP1	-0.47409	3.619271	-2.17677	0.032163	-3.81855	0.380729	0.246578
T.cells	A130014A(	0.64377	1.887317	2.176412	0.03219	-3.60292	0.395876	0.258875
T.cells	ARPC2	-0.11477	9.318766	-2.17493	0.032305	-4.91127	0.335474	0.210795
T.cells	UBC	-0.21581	8.132317	-2.17439	0.032347	-4.70125	0.34472	0.218046
T.cells	ID1	-0.69063	4.07039	-2.17375	0.032396	-3.73039	0.378106	0.244421
T.cells	TRDV2-2	0.836588	-1.43025	2.173104	0.032446	-3.58361	0.428065	0.284969
T.cells	AKR1C13	-0.81619	1.84784	-2.17259	0.032486	-3.56913	0.397799	0.260302
T.cells	PPARA	-0.79302	2.784821	-2.17185	0.032544	-3.66092	0.389791	0.253799
T.cells	ATP13A3	0.126016	7.315105	2.171289	0.032588	-4.75691	0.35196	0.223887
T.cells	ETS1	0.186782	7.418769	2.170643	0.032638	-4.75546	0.351314	0.223396
T.cells	STX11	-0.39657	4.829435	-2.16841	0.032813	-4.0277	0.373816	0.240693
T.cells	ELOF1	0.166902	5.375272	2.16833	0.032819	-4.58871	0.369232	0.237076
T.cells	AI182371	-0.8042	2.439191	-2.16667	0.03295	-3.64416	0.395688	0.257844
T.cells	TTC3	-0.21882	5.355192	-2.16528	0.033059	-4.36699	0.370832	0.238113
T.cells	MMAA	-0.6847	1.748976	-2.16487	0.033092	-3.62329	0.402253	0.263361
T.cells	GM20234	0.506274	2.059362	2.164595	0.033113	-3.71472	0.399454	0.26118
T.cells	4930557K(	-0.65318	1.483636	-2.16447	0.033124	-3.64458	0.40466	0.26543
T.cells	KLC1	-0.19897	4.702224	-2.16413	0.03315	-4.28644	0.376346	0.242768
T.cells	PYGM	-0.34947	4.300481	-2.16325	0.03322	-4.20467	0.380107	0.245763
T.cells	GJB2	-0.92107	1.638244	-2.16291	0.033247	-3.57855	0.403607	0.264719
T.cells	CCDC85B	0.290922	3.779227	2.162331	0.033293	-3.99949	0.384745	0.249581
T.cells	ORMDL1	-0.29068	3.609727	-2.16107	0.033393	-4.03165	0.386991	0.251069
T.cells	UBAC2	0.155368	6.679645	2.160115	0.033469	-4.69425	0.361498	0.230861
T.cells	GM29243	1.09992	-0.26495	2.158502	0.033598	-3.58469	0.423573	0.28064
T.cells	MEIS1	0.318391	3.866059	2.15758	0.033672	-4.18402	0.385995	0.250369

T.cells	GDI2	-0.12103	8.891579	-2.15757	0.033673	-4.98806	0.34448	0.217685
T.cells	TRPC1	-1.13848	-0.17084	-2.15661	0.033751	-3.59639	0.422681	0.280461
T.cells	GM32089	1.095591	-1.09921	2.156032	0.033797	-3.60057	0.431559	0.287927
T.cells	PARP6	0.399617	2.991541	2.155988	0.0338	-3.90394	0.393683	0.257002
T.cells	GM28960	1.123013	-0.82667	2.155942	0.033804	-3.60124	0.428935	0.28576
T.cells	PPP1R21	0.25618	4.557871	2.15534	0.033852	-4.27096	0.380013	0.246153
T.cells	PKD2	-0.89884	0.719693	-2.15521	0.033863	-3.57794	0.414322	0.273943
T.cells	E230013L2	1.150827	-0.72305	2.154708	0.033903	-3.61219	0.427941	0.285295
T.cells	PDE4C	-1.05077	3.131277	-2.15462	0.033911	-3.68897	0.392445	0.256324
T.cells	LEF1	0.288808	5.638758	2.153751	0.033981	-4.77557	0.371235	0.239236
T.cells	ZFP143	0.232356	3.815014	2.153264	0.03402	-4.20977	0.38691	0.251718
T.cells	RUFY1	-0.17828	5.607628	-2.15284	0.034055	-4.47632	0.371557	0.239505
T.cells	HMGA1	0.221306	4.670399	2.152078	0.034116	-4.50626	0.379615	0.246018
T.cells	SNX13	0.251328	5.413359	2.151862	0.034134	-4.36119	0.373293	0.241055
T.cells	OFCC1	1.228719	-0.63548	2.151462	0.034166	-3.61013	0.427742	0.285308
T.cells	GM49067	0.776202	0.406337	2.149321	0.034341	-3.59641	0.418041	0.277914
T.cells	ITIH3	-0.63737	3.636116	-2.14883	0.034381	-3.88198	0.388743	0.254154
T.cells	GM43260	-0.43514	2.252383	-2.14875	0.034388	-3.84534	0.401054	0.264142
T.cells	RRM2B	0.213812	4.437435	2.148734	0.034389	-4.36335	0.381775	0.248561
T.cells	AP4E1	0.333528	3.305374	2.148659	0.034395	-4.07877	0.391653	0.25652
T.cells	DDX49	-0.21918	4.208396	-2.14847	0.03441	-4.20937	0.383754	0.250223
T.cells	HNRNPLL	0.52261	4.231786	2.147857	0.03446	-3.74256	0.383552	0.250238
T.cells	2610206C1	0.754103	0.40853	2.147392	0.034499	-3.6246	0.418021	0.278414
T.cells	MRPL21	-0.19414	5.233418	-2.14714	0.03452	-4.48395	0.374969	0.243509
T.cells	SNRPF	-0.15725	6.76677	-2.1471	0.034523	-4.79577	0.36218	0.233334
T.cells	XRCC4	0.213606	5.185209	2.145856	0.034625	-4.38666	0.375976	0.244199
T.cells	DAPK2	-0.85015	2.818088	-2.14552	0.034652	-3.61451	0.396608	0.260918
T.cells	CDKL2	-0.81713	1.163972	-2.14518	0.03468	-3.61905	0.411646	0.273353
T.cells	PTGS1	-0.69309	3.986721	-2.1444	0.034744	-3.71098	0.386624	0.252969
T.cells	CLIP1	0.226974	5.440971	2.143243	0.03484	-4.3841	0.374522	0.243156
T.cells	SEC11C	-0.15312	7.287381	-2.14219	0.034927	-4.8381	0.359187	0.23132
T.cells	MRPL57	0.173705	5.995401	2.142035	0.03494	-4.60038	0.369854	0.239836
T.cells	GM35154	0.874791	1.526848	2.141936	0.034948	-3.642	0.409083	0.271592
T.cells	TRIM14	0.342946	4.686216	2.141844	0.034956	-4.20783	0.380967	0.248752
T.cells	NECAB3	-0.53839	1.186088	-2.14144	0.03499	-3.75893	0.412228	0.274327
T.cells	RMI1	0.253352	3.629414	2.14055	0.035063	-4.11922	0.390165	0.256522
T.cells	ASL	-0.32352	4.484774	-2.14031	0.035083	-4.14068	0.382704	0.25055
T.cells	RALGAPB	0.219115	4.920242	2.140228	0.03509	-4.31177	0.378958	0.247531
T.cells	CLEC5A	1.274778	0.512385	2.139478	0.035153	-3.61666	0.418846	0.279969
T.cells	KLHL24	0.18034	6.228518	2.138637	0.035223	-4.65402	0.368403	0.239112
T.cells	GM10874	-1.13862	-0.06869	-2.13842	0.03524	-3.61755	0.424572	0.28491
T.cells	KLHL22	0.343689	3.001151	2.136525	0.035399	-4.0196	0.396783	0.262347
T.cells	MRC1	-0.92463	5.240936	-2.13582	0.035459	-3.95943	0.377221	0.246656
T.cells	SELL	0.314615	5.472373	2.135487	0.035486	-4.40178	0.375252	0.245168
T.cells	KDR	-0.7585	3.318647	-2.1354	0.035494	-3.75687	0.393954	0.260328
T.cells	CLDN3	-0.90797	1.461002	-2.1352	0.03551	-3.62381	0.410778	0.274153
T.cells	PPIP5K2	0.212266	4.496011	2.13475	0.035548	-4.33549	0.383625	0.252078

T.cells	CD160	-1.29997	1.916487	-2.13387	0.035623	-3.61267	0.406592	0.271046
T.cells	ATG4A	0.289836	4.314693	2.133546	0.03565	-4.25231	0.385199	0.253674
T.cells	AI467606	-0.29603	4.111903	-2.13309	0.035688	-4.275	0.386966	0.255222
T.cells	ZCWPW2	0.906788	1.016841	2.132742	0.035717	-3.62911	0.414898	0.278227
T.cells	PIM1	-0.19962	9.488973	-2.13271	0.03572	-5.1319	0.342588	0.219795
T.cells	LTB	-0.61467	3.446775	-2.13197	0.035783	-3.80235	0.392817	0.260205
T.cells	USP25	0.171991	7.051473	2.131697	0.035806	-4.64723	0.362073	0.235426
T.cells	TTC17	0.19015	5.324345	2.131318	0.035838	-4.45339	0.37651	0.247137
T.cells	PAPOLA	0.110799	7.055885	2.131063	0.03586	-4.77113	0.362037	0.235607
T.cells	CTSH	-0.57593	5.779307	-2.13094	0.03587	-3.88848	0.372655	0.244135
T.cells	ARHGAP10	0.350797	5.105594	2.130918	0.035872	-4.43249	0.378377	0.248757
T.cells	ARHGEF37	1.622198	0.616444	2.130265	0.035927	-3.62271	0.418889	0.281942
T.cells	BIN3	0.230657	5.271025	2.128989	0.036036	-4.34627	0.37796	0.247934
T.cells	PAM	-0.38856	4.417759	-2.1281	0.036111	-4.01831	0.385402	0.254083
T.cells	EFNB1	-0.80221	1.912975	-2.12809	0.036113	-3.63576	0.407787	0.272421
T.cells	GM5547	-0.99863	1.560402	-2.12644	0.036253	-3.62877	0.412239	0.275637
T.cells	TPGS2	-0.24439	3.805381	-2.12488	0.036387	-4.26369	0.392982	0.259557
T.cells	NFX1	0.195462	4.900546	2.124437	0.036425	-4.39643	0.38342	0.251904
T.cells	GCNT1	0.54549	2.451925	2.123751	0.036484	-3.74734	0.405237	0.269917
T.cells	GM16638	-0.49385	2.040296	-2.12351	0.036504	-3.80441	0.409009	0.273061
T.cells	PLA2G12B	-1.04058	1.0508	-2.12318	0.036533	-3.62784	0.418209	0.28076
T.cells	SEMA4D	0.271585	6.446214	2.1218	0.036652	-4.64552	0.370636	0.241952
T.cells	GM29282	1.085019	1.712401	2.121728	0.036658	-3.63127	0.412429	0.27602
T.cells	VSIG10	1.036244	0.47062	2.121418	0.036685	-3.63186	0.424095	0.285869
T.cells	BC147527	0.889706	1.109158	2.121057	0.036716	-3.63677	0.418059	0.280969
T.cells	HMGXB4	0.215201	4.148587	2.120422	0.036771	-4.36197	0.3904	0.258392
T.cells	TGOLN1	-0.24193	5.839155	-2.11989	0.036817	-4.37906	0.375764	0.246662
T.cells	ABCG2	0.399665	3.149803	2.119668	0.036837	-3.94704	0.399296	0.265911
T.cells	EML6	-0.98762	2.764743	-2.11926	0.036872	-3.68076	0.402775	0.268929
T.cells	ADAM10	0.138248	7.403441	2.119131	0.036883	-4.86314	0.362683	0.236373
T.cells	UEVLD	0.349473	2.944551	2.118722	0.036919	-4.1021	0.401147	0.26766
T.cells	MRPL50	-0.23155	4.009842	-2.11836	0.03695	-4.25528	0.391625	0.259961
T.cells	UOX	0.56277	5.010934	2.116003	0.037156	-4.29244	0.384561	0.25351
T.cells	CHCHD7	0.215903	4.398636	2.115533	0.037197	-4.34489	0.389918	0.257991
T.cells	RUFY3	0.204288	5.26954	2.115291	0.037218	-4.50482	0.382319	0.251931
T.cells	NEK7	0.175817	6.476805	2.113833	0.037346	-4.6591	0.372674	0.244078
T.cells	GM45820	-0.66692	1.425778	-2.11366	0.037361	-3.67349	0.417666	0.28098
T.cells	CDC6	-0.31317	3.269665	-2.11334	0.037389	-4.32032	0.400685	0.26706
T.cells	DDX21	-0.1867	6.399043	-2.11252	0.037461	-4.71805	0.373702	0.245058
T.cells	ZYG11B	0.208851	5.883084	2.111798	0.037525	-4.51361	0.378383	0.248785
T.cells	TXNDC11	0.180373	5.714713	2.110023	0.037682	-4.61503	0.38026	0.25054
T.cells	MAPKAPK2	0.192681	7.207807	2.109985	0.037685	-4.76254	0.367617	0.240342
T.cells	4930562C1	-0.93844	0.146658	-2.10978	0.037703	-3.64939	0.431083	0.292618
T.cells	TMX4	0.300028	4.420261	2.109726	0.037708	-4.26087	0.391551	0.259833
T.cells	OGFRL1	0.277088	5.082967	2.108302	0.037834	-4.39236	0.386663	0.255529
T.cells	GCHFR	-0.68521	3.389908	-2.10744	0.03791	-3.89087	0.402165	0.268165
T.cells	RASGRP4	1.031001	1.734055	2.106671	0.037979	-3.65361	0.417746	0.280903

T.cells	GCAT	-0.3994	2.390839	-2.10634	0.038008	-4.03578	0.411618	0.275819
T.cells	PLBD1	-0.86706	5.389651	-2.1046	0.038164	-3.80381	0.385573	0.254298
T.cells	ZC3H6	0.312784	3.379852	2.104239	0.038196	-4.16555	0.403476	0.269064
T.cells	SORT1	0.356598	4.067523	2.104178	0.038202	-4.17306	0.397265	0.263976
T.cells	RAD51AP1	-0.37303	3.61991	-2.1029	0.038317	-4.39156	0.402067	0.267604
T.cells	IKZF4	-0.92844	-0.0706	-2.10257	0.038346	-3.66199	0.436859	0.296646
T.cells	COL20A1	-0.61508	1.340253	-2.10217	0.038382	-3.69026	0.42325	0.285404
T.cells	SYBU	-1.13648	0.330557	-2.10044	0.038538	-3.66609	0.434315	0.294087
T.cells	ALKBH3	0.295177	3.490416	2.099153	0.038654	-4.19247	0.405362	0.269806
T.cells	IL27	1.417323	0.053926	2.097281	0.038824	-3.68177	0.43872	0.29749
T.cells	9030622O2	-1.02035	1.05914	-2.09722	0.038829	-3.68275	0.428934	0.289301
T.cells	EBAG9	0.233828	4.117531	2.097205	0.038831	-4.31767	0.400387	0.26571
T.cells	CPED1	0.64401	2.524781	2.096421	0.038902	-3.86291	0.415172	0.278023
T.cells	TSPAN3	-0.25617	4.739337	-2.09617	0.038924	-4.44431	0.394943	0.261504
T.cells	MID1	-0.59834	5.805162	-2.09588	0.038951	-4.7057	0.385539	0.253868
T.cells	EDNRB	-1.84963	2.175551	-2.09462	0.039066	-3.6805	0.419028	0.281156
T.cells	MRPS26	-0.14896	5.167613	-2.0945	0.039077	-4.59866	0.391679	0.258695
T.cells	NR3C2	-0.95138	1.939637	-2.09383	0.039138	-3.6942	0.42132	0.283098
T.cells	SLC22A27	-0.99753	1.124082	-2.09366	0.039154	-3.68406	0.429115	0.289588
T.cells	FSHR	-0.72909	0.419984	-2.09161	0.039342	-3.67995	0.43765	0.296024
T.cells	LY86	-0.33425	6.704851	-2.09119	0.03938	-4.51953	0.379837	0.248578
T.cells	PTPRD	-0.80678	2.563535	-2.09061	0.039434	-3.79911	0.417274	0.279102
T.cells	2700049A0	0.235876	4.236674	2.089982	0.039491	-4.44565	0.402048	0.266418
T.cells	SKI	-0.29878	5.372605	-2.08949	0.039537	-4.23417	0.391954	0.258322
T.cells	CPEB4	-0.23293	6.51399	-2.08823	0.039653	-4.58319	0.382736	0.250784
T.cells	SLC4A7	0.197174	5.767425	2.087197	0.039749	-4.7103	0.389626	0.256389
T.cells	SPIC	-1.43761	1.863637	-2.08675	0.03979	-3.6867	0.4255	0.28593
T.cells	PUM1	-0.11856	7.56821	-2.08666	0.039798	-4.94344	0.374052	0.243961
T.cells	MMP14	0.559914	3.837765	2.086142	0.039847	-4.25643	0.407114	0.27089
T.cells	CACYBP	-0.1679	5.736095	-2.08502	0.03995	-4.7449	0.390178	0.257199
T.cells	APBB1IP	0.161804	7.638967	2.084158	0.040031	-4.87892	0.373717	0.244156
T.cells	SPOP	0.12389	7.366582	2.083869	0.040058	-4.9142	0.376033	0.24612
T.cells	NRXN2	-0.94693	2.095803	-2.08334	0.040107	-3.75748	0.423583	0.285187
T.cells	IFT172	0.399878	2.410455	2.08268	0.040169	-3.98068	0.420594	0.282828
T.cells	PAPOLG	0.360826	3.005898	2.082302	0.040204	-4.07306	0.414991	0.278281
T.cells	ELAC1	-0.6177	1.710286	-2.08204	0.040229	-3.7212	0.427272	0.288512
T.cells	SP3OS	0.237281	4.205373	2.082006	0.040232	-4.37678	0.403913	0.269165
T.cells	CAR1	1.310069	0.491473	2.081437	0.040285	-3.69611	0.439132	0.298636
T.cells	DSTYK	-0.26466	3.925134	-2.08076	0.040349	-4.19859	0.406476	0.271562
T.cells	SLC41A3	0.793605	1.73389	2.080717	0.040353	-3.69878	0.427046	0.28863
T.cells	HAX1	-0.27469	4.020737	-2.08021	0.040401	-4.27035	0.4056	0.27102
T.cells	EIF2A	-0.14488	5.395584	-2.08008	0.040412	-4.65007	0.393195	0.260896
T.cells	TMEM168	0.23392	4.583494	2.079967	0.040423	-4.38278	0.400479	0.266891
T.cells	TMEM131	0.169325	6.606387	2.079807	0.040438	-4.77988	0.382567	0.252275
T.cells	CTDSPL	-0.35428	3.831098	-2.07903	0.040512	-4.38021	0.40734	0.272847
T.cells	SMLR1	-0.83028	1.822706	-2.07857	0.040555	-3.7311	0.426194	0.288652
T.cells	PODNL1	1.01361	-1.02387	2.078214	0.040589	-3.71236	0.454298	0.312552

T.cells	TTC36	-0.56247	5.028124	-2.07804	0.040605	-4.32327	0.396476	0.264224
T.cells	CDK19	0.168314	6.390727	2.077904	0.040618	-4.86609	0.38444	0.254438
T.cells	AW011738	0.995736	2.812185	2.077893	0.040619	-3.71155	0.416806	0.281088
T.cells	GTPBP2	0.247652	4.751688	2.076413	0.040759	-4.38891	0.399979	0.266765
T.cells	SHANK2	-0.5813	1.410698	-2.07506	0.040887	-3.86411	0.432235	0.293334
T.cells	IFIT1	1.60676	2.761439	2.073618	0.041024	-3.72211	0.420328	0.283004
T.cells	FXYD4	-0.42656	3.001862	-2.0725	0.041131	-4.05037	0.418772	0.281428
T.cells	IFI205	1.448106	2.758001	2.071723	0.041205	-3.71478	0.421364	0.283489
T.cells	PGRMC1	-0.23632	4.647619	-2.07122	0.041253	-4.41476	0.403779	0.269125
T.cells	PEX2	0.313261	3.914925	2.071069	0.041267	-4.2361	0.410515	0.274672
T.cells	PPARGC1A	0.581259	1.419055	2.070573	0.041315	-3.97532	0.434369	0.294491
T.cells	TOP1	-0.16423	8.354587	-2.06949	0.041418	-5.05809	0.371641	0.243042
T.cells	OTUD7B	0.202316	5.17042	2.069327	0.041434	-4.56731	0.39944	0.265568
T.cells	VIPAS39	0.212409	4.131233	2.068848	0.04148	-4.34162	0.408928	0.273583
T.cells	HNRNPL	-0.10779	8.426349	-2.06796	0.041565	-5.14934	0.371037	0.242984
T.cells	HNRNPUL1	0.11236	7.28107	2.067947	0.041567	-4.99621	0.380802	0.250853
T.cells	MRPL4	-0.18759	5.176328	-2.06763	0.041597	-4.58579	0.399387	0.266073
T.cells	LCORL	0.171252	6.985276	2.067526	0.041608	-4.87904	0.383364	0.253012
T.cells	NR1H2	0.255863	4.589379	2.06693	0.041665	-4.42471	0.40472	0.270663
T.cells	STOML1	0.533446	2.251865	2.066847	0.041673	-3.87217	0.426624	0.288865
T.cells	EMILIN1	0.447078	2.608435	2.066039	0.041751	-3.89041	0.423402	0.286269
T.cells	GSAP	0.366738	5.642393	2.065901	0.041765	-4.42548	0.395376	0.263153
T.cells	TWF1	0.191661	5.464598	2.065522	0.041801	-4.51687	0.396971	0.264551
T.cells	KLHDC10	0.191226	5.434437	2.064562	0.041894	-4.63718	0.397479	0.265042
T.cells	AOX3	-0.98288	0.998188	-2.06451	0.041899	-3.73912	0.439281	0.299869
T.cells	KNG2	-0.74256	2.382731	-2.06392	0.041957	-3.82852	0.426034	0.28875
T.cells	DEGS1	-0.23434	6.525466	-2.0631	0.042036	-4.64688	0.388375	0.257601
T.cells	ELL	0.236275	5.024631	2.062286	0.042115	-4.41829	0.401887	0.268834
T.cells	RAB1A	-0.13735	7.08553	-2.06225	0.042119	-4.82957	0.383571	0.253843
T.cells	MED19	-0.21406	3.778351	-2.06095	0.042245	-4.36222	0.413748	0.278579
T.cells	RAD9B	-0.34065	3.275507	-2.0604	0.0423	-4.15586	0.418467	0.282601
T.cells	AKR1B8	0.971521	1.592252	2.060291	0.04231	-3.73164	0.43463	0.29618
T.cells	BCKDHA	0.216876	4.820285	2.059693	0.042368	-4.59619	0.404127	0.270911
T.cells	ALKBH8	0.217204	3.835022	2.05957	0.042381	-4.41687	0.413219	0.278463
T.cells	ENO1B	0.6667	1.327345	2.059565	0.042381	-3.78761	0.437225	0.298579
T.cells	ZBTB40	0.287055	3.163496	2.058485	0.042487	-4.25681	0.419525	0.28408
T.cells	SERPINA1D	-0.59784	5.558333	-2.05848	0.042487	-4.50603	0.39744	0.26576
T.cells	SULT1D1	-0.82263	2.290136	-2.05848	0.042487	-3.80294	0.42786	0.291066
T.cells	ANGPTL7	1.131228	-0.05517	2.057564	0.042577	-3.72888	0.451572	0.311126
T.cells	ANKRD24	0.716063	1.054272	2.056673	0.042665	-3.76166	0.440663	0.302035
T.cells	GM48653	-1.10061	-0.16399	-2.05586	0.042745	-3.73313	0.452874	0.312552
T.cells	HADH	-0.24781	5.394105	-2.05567	0.042764	-4.65336	0.399597	0.267805
T.cells	ANXA7	-0.21007	5.409471	-2.05529	0.042801	-4.48683	0.399458	0.267805
T.cells	HS3ST3B1	-0.72632	3.380647	-2.05519	0.042812	-3.8029	0.418185	0.283368
T.cells	GPX7	-0.9785	0.610424	-2.05465	0.042864	-3.73989	0.445077	0.306081
T.cells	PTAR1	0.226454	3.690053	2.054479	0.042882	-4.3612	0.415277	0.280963
T.cells	CREBZF	0.187422	4.715073	2.054405	0.042889	-4.55822	0.405777	0.27306

T.cells	SLC38A3	-0.82694	1.479322	-2.05399	0.042929	-3.7748	0.436516	0.298964
T.cells	CACNA1B	-0.73674	0.056619	-2.05307	0.043021	-3.80612	0.451263	0.311322
T.cells	PXMP2	-0.53498	4.067436	-2.05165	0.043162	-4.25139	0.413319	0.278928
T.cells	WDR62	0.258122	3.526858	2.050454	0.043281	-4.48336	0.419181	0.283645
T.cells	TNR	-0.67942	0.036927	-2.0501	0.043317	-3.7925	0.453405	0.312668
T.cells	TMCC2	0.907335	1.686886	2.048414	0.043485	-3.81249	0.437522	0.299333
T.cells	REPS2	-0.94044	0.905504	-2.04837	0.043489	-3.75103	0.445272	0.305904
T.cells	PRKD2	0.223817	4.348606	2.048155	0.043511	-4.50044	0.412051	0.278024
T.cells	PSME2B	0.615614	2.815452	2.048019	0.043525	-3.8696	0.426549	0.290148
T.cells	PPDPF	-0.18172	5.630234	-2.04708	0.043619	-4.61299	0.400588	0.268628
T.cells	CYP4F16	0.87193	1.441368	2.046879	0.043639	-3.76529	0.440276	0.301906
T.cells	SNRPE	-0.13836	7.064225	-2.04659	0.043668	-5.04725	0.38779	0.258253
T.cells	TFDP2	0.207334	5.591554	2.045385	0.043789	-4.87048	0.401217	0.269527
T.cells	PARP14	0.425068	5.480462	2.045234	0.043804	-4.54059	0.402226	0.270362
T.cells	DMGDH	-0.80734	1.667706	-2.04514	0.043814	-3.80044	0.438345	0.300622
T.cells	PTPN11	0.169272	5.063935	2.044541	0.043874	-4.58063	0.406032	0.273516
T.cells	ZFP263	0.236368	5.167548	2.044455	0.043883	-4.62554	0.405083	0.272728
T.cells	BIN2	0.347535	5.165118	2.043378	0.043992	-4.19201	0.405322	0.273107
T.cells	YAF2	-0.1391	6.056106	-2.04331	0.043998	-4.83324	0.397234	0.266416
T.cells	POPDC3	-0.76476	0.894439	-2.04313	0.044017	-3.88229	0.446267	0.307599
T.cells	CRISPLD2	-1.04363	0.884481	-2.04125	0.044207	-3.77551	0.447383	0.308237
T.cells	RSAD2	1.366638	4.537164	2.041116	0.044221	-3.87431	0.41205	0.278498
T.cells	MACO1	-0.15288	6.27208	-2.04104	0.044228	-4.83538	0.396197	0.265366
T.cells	FAAH	-0.86447	1.451163	-2.04046	0.044288	-3.76084	0.441945	0.30367
T.cells	AQR	0.163349	4.812338	2.039684	0.044367	-4.57499	0.409759	0.276771
T.cells	MXD1	0.280478	6.036212	2.03967	0.044368	-4.48396	0.398574	0.267499
T.cells	GM6377	-0.85876	3.091942	-2.03864	0.044473	-3.79287	0.426342	0.290589
T.cells	EFCAB8	-0.60057	1.374064	-2.03833	0.044505	-3.85764	0.443149	0.304856
T.cells	TAF1D	-0.14766	6.027224	-2.03814	0.044524	-4.82998	0.398992	0.267872
T.cells	ABCB11	-0.78105	1.681529	-2.03776	0.044563	-3.81255	0.440097	0.302401
T.cells	PPP3CC	0.270933	3.993482	2.036112	0.044732	-4.52404	0.417761	0.284195
T.cells	ZFP746	0.251037	3.717554	2.035932	0.04475	-4.3394	0.42037	0.28646
T.cells	LLGL2	-0.48459	2.291993	-2.03571	0.044773	-3.97177	0.434094	0.298103
T.cells	TOB1	-0.27107	5.050855	-2.03561	0.044783	-4.41481	0.407902	0.276073
T.cells	CD46	0.488869	2.11028	2.0354	0.044805	-3.99166	0.435873	0.299702
T.cells	6430548M	-0.87981	2.407786	-2.03533	0.044811	-3.76416	0.432964	0.297233
T.cells	SARAF	-0.35946	5.365255	-2.03532	0.044813	-4.13161	0.405013	0.273748
T.cells	LENG9	0.511148	2.352054	2.034694	0.044877	-3.93113	0.43357	0.297903
T.cells	I730030J21	0.94302	0.707146	2.034289	0.044919	-3.76589	0.449902	0.311909
T.cells	HIST1H3B	0.535551	1.995292	2.034172	0.044931	-4.25532	0.437065	0.300971
T.cells	TRIM28	-0.17747	5.460856	-2.03279	0.045074	-4.80357	0.405033	0.273777
T.cells	APOB	-0.56005	4.836413	-2.03255	0.045098	-4.39919	0.410792	0.278644
T.cells	GM13822	1.287501	1.17949	2.031138	0.045244	-3.78754	0.447152	0.309013
T.cells	IFI213	0.931675	3.327221	2.029835	0.045379	-4.01691	0.426659	0.291537
T.cells	KCNIP3	1.23215	0.193763	2.029796	0.045383	-3.77008	0.457809	0.318068
T.cells	PDCL	0.268297	3.891934	2.028657	0.045502	-4.40213	0.421517	0.287381
T.cells	HBEGF	-0.96953	2.789221	-2.02844	0.045525	-3.83842	0.432129	0.296412

T.cells	PLEKHM1	0.207046	4.771883	2.027942	0.045576	-4.55213	0.413224	0.280644
T.cells	PPP2R5C	0.109569	7.456887	2.02788	0.045583	-5.00773	0.388858	0.260449
T.cells	NR1I3	-1.01559	1.055611	-2.02748	0.045624	-3.77944	0.449313	0.311378
T.cells	2510039O1	0.18915	5.179646	2.027214	0.045652	-4.62061	0.409434	0.277718
T.cells	KRT83	1.185386	-0.26141	2.025938	0.045786	-3.78114	0.462787	0.323313
T.cells	CCDC166	-0.7414	0.615517	-2.02549	0.045833	-3.81075	0.453775	0.315671
T.cells	POLG2	-0.23512	5.080463	-2.02537	0.045845	-4.51232	0.410353	0.278839
T.cells	NAPSA	0.206564	6.459379	2.024816	0.045903	-4.95422	0.397748	0.268505
T.cells	FAM160A2	0.388542	2.604929	2.024033	0.045985	-4.07291	0.433926	0.299081
T.cells	CRBN	0.242528	4.060609	2.023819	0.046008	-4.38053	0.419916	0.287291
T.cells	GOT1	-0.26512	6.081478	-2.02369	0.046022	-4.67398	0.401165	0.271617
T.cells	GBP5	1.055138	2.014589	2.022676	0.046128	-3.87079	0.439731	0.304525
T.cells	ITPRIPL1	-0.27191	3.28736	-2.02265	0.046131	-4.31105	0.427305	0.293933
T.cells	HFE	-0.62488	3.452689	-2.02252	0.046145	-3.8891	0.425715	0.292583
T.cells	CLUAP1	0.223127	3.885137	2.021766	0.046224	-4.43126	0.421582	0.289246
T.cells	SLC35A1	-0.25942	3.416075	-2.021	0.046305	-4.27761	0.426066	0.293352
T.cells	LDB1	-0.24209	4.482765	-2.0207	0.046336	-4.5017	0.415932	0.284781
T.cells	AB124611	0.23602	5.452214	2.020699	0.046337	-4.61666	0.406918	0.277198
T.cells	RFC4	-0.24765	4.603054	-2.02037	0.046372	-4.73985	0.414804	0.283829
T.cells	BUD31	-0.17687	5.474604	-2.0202	0.046389	-4.74287	0.406712	0.277026
T.cells	CLIC5	0.517797	0.878367	2.020099	0.0464	-4.08151	0.451105	0.314811
T.cells	TM6SF1	0.22831	6.252773	2.019787	0.046433	-4.73749	0.399613	0.271088
T.cells	NASP	-0.15551	6.308139	-2.01954	0.04646	-4.97752	0.399112	0.270734
T.cells	GM13889	-1.03764	1.14195	-2.01944	0.04647	-3.78733	0.448443	0.312612
T.cells	HDAC3	-0.18091	4.707537	-2.01923	0.046492	-4.61728	0.413826	0.283122
T.cells	UBE2A	-0.11685	6.786705	-2.01885	0.046532	-4.99363	0.39481	0.267307
T.cells	GM38973	-0.63095	1.613034	-2.01848	0.046572	-3.92044	0.44372	0.308808
T.cells	ELOVL2	-0.79884	2.450205	-2.01815	0.046607	-3.91441	0.435441	0.301703
T.cells	DHRS11	0.432019	4.678666	2.017919	0.046631	-4.15864	0.414096	0.283586
T.cells	CAMK2D	0.223795	7.214604	2.017627	0.046662	-5.00631	0.391	0.264311
T.cells	5830432E0	0.800342	1.494203	2.017599	0.046665	-3.81507	0.444907	0.309921
T.cells	TRIM56	0.266041	3.810038	2.01707	0.046721	-4.34544	0.422297	0.290725
T.cells	TMEM150A	-0.7132	2.327871	-2.01699	0.04673	-3.87248	0.436642	0.302965
T.cells	SLC24A1	-0.88469	1.860788	-2.01676	0.046755	-3.79116	0.441255	0.306946
T.cells	SPATA6	0.232181	4.52297	2.01615	0.04682	-4.60976	0.415588	0.285182
T.cells	TLE1	-0.32231	4.060562	-2.01602	0.046833	-4.18967	0.41995	0.288889
T.cells	ZFP831	0.384645	3.063289	2.0145	0.046996	-4.31039	0.430653	0.297546
T.cells	ASGR2	-0.86762	1.698173	-2.01383	0.047068	-3.85378	0.444119	0.30933
T.cells	GM34225	-0.7982	-0.06364	-2.01378	0.047073	-3.84285	0.462038	0.324843
T.cells	SPATA13	0.241745	5.147731	2.013172	0.047138	-4.67105	0.411076	0.281219
T.cells	MCM3	-0.24503	5.484871	-2.01287	0.04717	-4.89657	0.407954	0.278634
T.cells	TGTP1	1.442197	-0.20272	2.012025	0.047261	-3.79746	0.463921	0.326762
T.cells	TRADD	0.260746	3.982911	2.011715	0.047295	-4.43937	0.422239	0.290872
T.cells	GLS2	-0.92186	1.618045	-2.01162	0.047305	-3.81823	0.445343	0.31065
T.cells	INTS7	-0.13116	5.677826	-2.00973	0.047508	-4.87234	0.407793	0.277991
T.cells	FAM53B	0.22807	4.652028	2.009119	0.047575	-4.72248	0.417534	0.286196
T.cells	EYA3	0.194034	5.473081	2.008855	0.047603	-4.73268	0.409856	0.279767

T.cells	WDR37	0.184522	5.478401	2.007902	0.047706	-4.69461	0.410371	0.280051
T.cells	CD164L2	-0.75861	1.352514	-2.00713	0.04779	-3.86457	0.450479	0.314403
T.cells	HIBADH	0.18064	5.742005	2.007111	0.047792	-4.88476	0.408021	0.278256
T.cells	ITIH4	-0.54588	3.988196	-2.00617	0.047894	-4.22499	0.42468	0.292423
T.cells	GPLD1	-0.9034	0.934219	-2.00594	0.047919	-3.82297	0.454908	0.31839
T.cells	GM41556	-0.63753	1.259442	-2.0059	0.047924	-3.89243	0.451596	0.315533
T.cells	INHBC	-0.90319	0.808252	-2.00373	0.04816	-3.83304	0.457601	0.320441
T.cells	NSG2	0.609152	0.587521	2.003536	0.048181	-3.91553	0.459874	0.322418
T.cells	FBXL5	0.232405	6.173273	2.003508	0.048185	-4.91859	0.405458	0.276016
T.cells	SLC45A1	-0.66684	0.608482	-2.00239	0.048307	-3.84152	0.459736	0.322669
T.cells	TMEM14A	-0.94498	0.419553	-2.00234	0.048312	-3.81431	0.461689	0.324378
T.cells	ECD	-0.17237	4.899349	-2.00227	0.04832	-4.68807	0.417382	0.286386
T.cells	CRYL1	-0.27007	3.73595	-2.00205	0.048344	-4.41437	0.428492	0.295859
T.cells	FAM89B	0.196741	5.277421	2.000662	0.048496	-4.73247	0.414679	0.283771
T.cells	CDIPTOS	-0.89212	-0.30875	-2.00005	0.048564	-3.81366	0.470252	0.331641
T.cells	CTSF	-0.99326	1.860603	-1.9998	0.048591	-3.82725	0.447899	0.312355
T.cells	ACSL1	0.271308	5.758511	1.999771	0.048595	-4.78634	0.410191	0.280247
T.cells	MGAT5	0.168308	6.916886	1.998892	0.048691	-5.01522	0.400057	0.27166
T.cells	TRBC1	-1.10521	3.183542	-1.99792	0.048799	-3.85227	0.435605	0.301771
T.cells	ABCD1	0.18765	4.771292	1.997876	0.048804	-4.65997	0.420272	0.288726
T.cells	NKG7	-0.95093	4.013022	-1.99751	0.048844	-3.93928	0.42755	0.29504
T.cells	C1QBP	-0.19198	6.055545	-1.99675	0.048928	-4.95028	0.40865	0.278922
T.cells	TTC21B	0.451657	1.614885	1.99625	0.048984	-4.01496	0.451817	0.315728
T.cells	LIMK1	-0.65606	2.1198	-1.99596	0.049016	-3.89055	0.446715	0.311338
T.cells	SNRPG	-0.12612	8.018397	-1.99481	0.049144	-5.29094	0.391679	0.264461
T.cells	PRCP	0.222469	5.424232	1.993695	0.049267	-4.60469	0.416109	0.284698
T.cells	ZMAT3	-0.8416	1.902429	-1.99234	0.049419	-3.86398	0.451545	0.314563
T.cells	HPGDS	-0.70629	3.647133	-1.99177	0.049482	-3.94508	0.434365	0.29984
T.cells	IER5L	-0.42937	2.89009	-1.99106	0.049562	-4.12455	0.442213	0.306444
T.cells	ABCA8A	-1.15795	1.028779	-1.98993	0.049688	-3.83914	0.46194	0.323067
T.cells	POP7	-0.19167	4.908142	-1.98865	0.049832	-4.71022	0.423589	0.29053
T.cells	GUCY1A1	-1.17447	-0.00341	-1.9886	0.049838	-3.83014	0.473114	0.333063
T.cells	FAM241A	0.248527	5.59878	1.988454	0.049854	-4.65338	0.417026	0.285056
T.cells	MRPL53	0.235964	4.291701	1.98824	0.049878	-4.56506	0.429529	0.295646
T.cells	MGAT4B	0.302779	3.847759	1.987851	0.049922	-4.35856	0.433902	0.299446
T.cells	PARK7	-0.15085	6.806683	-1.98698	0.05002	-5.0583	0.406303	0.27605
T.cells	PARP11	0.36652	3.46828	1.986425	0.050083	-4.36252	0.438369	0.303021
T.cells	VTI1B	0.146891	5.690918	1.985908	0.050141	-4.73989	0.41707	0.285018
T.cells	ABCB4	0.404002	3.391454	1.984901	0.050255	-4.29398	0.439965	0.304301
T.cells	HMOX2	0.127455	6.471899	1.984116	0.050344	-4.98304	0.410791	0.279483
T.cells	DCLRE1C	0.215741	6.497189	1.98344	0.050421	-4.79351	0.410866	0.279446
T.cells	GYS1	0.425677	3.38284	1.983094	0.05046	-4.26147	0.440833	0.304775
T.cells	UBA6	0.184639	5.172539	1.981674	0.050622	-4.7434	0.424407	0.290385
T.cells	EMCN	-0.81923	1.650943	-1.98112	0.050685	-3.86711	0.459584	0.320508
T.cells	GRINA	0.429252	6.205395	1.980682	0.050735	-4.47558	0.414697	0.282521
T.cells	UFSP2	0.202186	4.93604	1.980558	0.05075	-4.70294	0.426777	0.292689
T.cells	TNFRSF21	0.351035	4.10783	1.979458	0.050875	-4.3906	0.435206	0.299762

T.cells	DECR2	-0.57896	2.741577	-1.9794	0.050882	-3.97255	0.448823	0.311381
T.cells	SERPINE1	0.895672	0.805177	1.978895	0.05094	-3.86201	0.4688	0.328758
T.cells	EIF4EBP1	0.171886	5.454955	1.978827	0.050948	-4.8175	0.422159	0.28889
T.cells	CHKB	-0.21168	4.306938	-1.97801	0.051042	-4.53775	0.433724	0.298483
T.cells	SORD	-0.33907	4.176195	-1.97753	0.051096	-4.49723	0.435141	0.299687
T.cells	ESF1	-0.15865	5.001622	-1.97638	0.051229	-4.77107	0.427305	0.293304
T.cells	CD320	-0.39896	1.856288	-1.97484	0.051407	-4.09609	0.458713	0.320787
T.cells	SH3YL1	0.566784	1.030881	1.974795	0.051412	-3.95373	0.467302	0.32824
T.cells	STARD3NL	-0.17654	5.861943	-1.97474	0.051418	-4.84939	0.419072	0.286961
T.cells	PDCD7	0.192789	4.505243	1.974699	0.051423	-4.65638	0.432125	0.298007
T.cells	4921516A(	0.897282	0.76983	1.974599	0.051434	-3.85082	0.470049	0.330647
T.cells	TNPO3	-0.14272	6.365771	-1.97428	0.051472	-4.96704	0.41432	0.283117
T.cells	CFLAR	0.254791	5.971185	1.974249	0.051475	-4.77335	0.418037	0.286245
T.cells	PROCR	1.577449	0.279761	1.974048	0.051498	-3.85098	0.475247	0.335474
T.cells	RASSF3	0.215432	6.834864	1.972996	0.05162	-4.95526	0.409941	0.279874
T.cells	RP2	0.201279	5.226456	1.972844	0.051637	-4.7012	0.425139	0.292772
T.cells	DCAF17	0.229151	4.054876	1.972607	0.051665	-4.52343	0.436541	0.302507
T.cells	NDRG1	0.51739	2.72107	1.972464	0.051681	-4.15554	0.44987	0.313972
T.cells	GRN	-0.27868	7.188808	-1.97221	0.051711	-4.81419	0.406666	0.27736
T.cells	ZFYVE1	0.231562	4.797159	1.971986	0.051737	-4.65207	0.429284	0.296519
T.cells	TENT4B	-0.15121	6.368937	-1.97195	0.051741	-4.98161	0.41429	0.283823
T.cells	NEB	-0.63965	1.258562	-1.97142	0.051802	-3.95405	0.464997	0.327357
T.cells	IGF2	-0.78448	4.193244	-1.97078	0.051877	-4.33084	0.435254	0.30188
T.cells	GM15494	-1.10689	0.759331	-1.97031	0.051932	-3.8589	0.47024	0.332302
T.cells	CEACAM16	0.81302	1.137316	1.970212	0.051943	-3.88228	0.466266	0.328831
T.cells	MALSU1	0.220381	4.843801	1.970208	0.051944	-4.65161	0.428905	0.296616
T.cells	FGL1	0.492879	3.523532	1.969339	0.052045	-4.24073	0.442413	0.308188
T.cells	MSANTD2	0.174408	4.864755	1.968445	0.05215	-4.72032	0.429707	0.297277
T.cells	SERPINB8	1.013654	-0.3114	1.968166	0.052182	-3.8594	0.482798	0.343332
T.cells	SF1	-0.11069	7.215593	-1.9678	0.052225	-5.14564	0.407469	0.278562
T.cells	GMFG	0.16561	7.424355	1.966622	0.052363	-5.19467	0.405692	0.277238
T.cells	TENM3	-0.78633	1.688761	-1.9663	0.052401	-3.93539	0.461801	0.325282
T.cells	GBP4	1.003824	3.363964	1.9661	0.052424	-4.01293	0.444706	0.310549
T.cells	PDE3B	0.225325	6.955861	1.965649	0.052477	-5.1391	0.410023	0.281166
T.cells	RAP1B	-0.1184	8.543309	-1.96541	0.052506	-5.18927	0.395523	0.269028
T.cells	LAX1	0.392632	2.319766	1.965371	0.05251	-4.3395	0.455291	0.319908
T.cells	MTHFD1	-0.23647	3.79072	-1.96521	0.052529	-4.59302	0.440446	0.307125
T.cells	TXNIP	0.277691	5.791358	1.964756	0.052582	-4.73308	0.420979	0.290577
T.cells	STAT5B	0.1853	6.043742	1.964502	0.052612	-4.86419	0.418581	0.288607
T.cells	PTPMT1	0.215652	4.267152	1.96433	0.052633	-4.5597	0.435735	0.303259
T.cells	CLK2	0.174263	4.63296	1.963499	0.052731	-4.67721	0.432448	0.30041
T.cells	SKAP1	-0.54747	4.523681	-1.96336	0.052747	-4.35935	0.433517	0.30134
T.cells	MCM6	-0.21825	6.155584	-1.96213	0.052892	-5.10519	0.418653	0.28819
T.cells	RBPMS2	-0.6263	1.246218	-1.96152	0.052965	-3.91642	0.467897	0.330473
T.cells	PDLIM5	-0.21275	6.703681	-1.96127	0.052995	-4.86684	0.41368	0.283959
T.cells	TBL3	-0.22098	3.91508	-1.96023	0.053118	-4.57752	0.441126	0.307309
T.cells	GM39469	0.386709	2.328802	1.960039	0.05314	-4.10942	0.457183	0.321216

T.cells	RASA3	0.178564	6.702935	1.959751	0.053175	-4.90934	0.414177	0.284553
T.cells	ZEB2	0.228271	9.36649	1.959274	0.053231	-5.49626	0.390018	0.264295
T.cells	DDX6	0.128223	8.314732	1.958616	0.05331	-5.28341	0.399739	0.27237
T.cells	RPP25L	0.247447	3.672149	1.958177	0.053362	-4.51216	0.444134	0.31006
T.cells	GM33104	-1.0375	0.440079	-1.95732	0.053464	-3.87863	0.477984	0.339569
T.cells	MOCS1	0.364998	3.367675	1.957194	0.053479	-4.2075	0.447521	0.313035
T.cells	MAP3K8	-0.38185	4.64612	-1.95662	0.053548	-4.26289	0.434846	0.302394
T.cells	OCEL1	0.278832	3.584489	1.955901	0.053634	-4.4068	0.445393	0.311819
T.cells	GM16845	0.362867	2.527304	1.955883	0.053636	-4.29461	0.456132	0.321127
T.cells	ST6GALNA(	0.420954	3.214358	1.95583	0.053642	-4.20204	0.449126	0.315063
T.cells	GUSB	-0.19385	5.468903	-1.95501	0.053741	-4.73643	0.427103	0.296238
T.cells	IL10RA	-0.28195	4.971561	-1.95489	0.053755	-4.59127	0.431932	0.300368
T.cells	IP6K1	0.165581	6.598351	1.953575	0.053913	-5.02622	0.416917	0.287439
T.cells	KBTBD11	1.09588	0.726015	1.953367	0.053938	-3.88518	0.475962	0.338496
T.cells	ACSL3	-0.27299	3.828777	-1.95322	0.053956	-4.56759	0.443857	0.31055
T.cells	KTN1	0.133375	5.826158	1.952951	0.053988	-5.00593	0.424269	0.293817
T.cells	PIK3IP1	0.375084	2.920799	1.952498	0.054043	-4.34864	0.453086	0.318732
T.cells	D830036C2	-1.10276	0.420218	-1.95187	0.054119	-3.88126	0.47929	0.341851
T.cells	4930469K1	0.920104	1.311112	1.95128	0.05419	-3.91516	0.469798	0.333531
T.cells	SMC6	-0.13925	7.579287	-1.95061	0.054271	-5.21862	0.407793	0.280257
T.cells	PLEKHA2	0.148476	7.455833	1.950097	0.054333	-5.24831	0.408936	0.281434
T.cells	TCF21	-1.00523	0.477537	-1.95	0.054345	-3.88677	0.478674	0.341739
T.cells	E2F8	-0.24765	3.686572	-1.94974	0.054376	-4.76047	0.44533	0.312704
T.cells	CLDN5	-1.0112	1.006603	-1.94971	0.054379	-3.88913	0.473023	0.336899
T.cells	MDN1	-0.24426	5.788045	-1.9493	0.054429	-4.86687	0.42468	0.295061
T.cells	GSTT1	-0.79889	2.240293	-1.94898	0.054468	-3.97908	0.460082	0.325764
T.cells	UBFD1	-0.20424	4.520398	-1.94886	0.054483	-4.69064	0.437027	0.305769
T.cells	SSBP4	-0.20652	4.90464	-1.94882	0.054488	-4.64935	0.433249	0.302516
T.cells	ZFAND6	0.124793	6.978135	1.948346	0.054545	-5.08796	0.413389	0.285663
T.cells	GM8797	-0.4473	2.19543	-1.94809	0.054577	-4.11888	0.460547	0.326411
T.cells	SLC25A3	-0.12478	7.921066	-1.94804	0.054583	-5.27329	0.404644	0.278327
T.cells	ZFP799	0.580969	0.939029	1.94625	0.0548	-4.01018	0.474893	0.338811
T.cells	SRP68	0.173798	3.983827	1.945981	0.054833	-4.59207	0.443428	0.311326
T.cells	STAP2	0.990608	0.504981	1.94598	0.054833	-3.89203	0.479542	0.342946
T.cells	BFSP2	0.329805	2.440103	1.945536	0.054888	-4.54158	0.459256	0.325175
T.cells	PRUNE1	0.296066	3.600837	1.944892	0.054966	-4.385	0.447581	0.315093
T.cells	RFXANK	0.332149	2.879474	1.944661	0.054994	-4.29809	0.454919	0.321608
T.cells	RCSD1	0.170126	7.091061	1.944386	0.055028	-5.20185	0.413616	0.286133
T.cells	ZFP292	0.149631	7.046023	1.943952	0.055081	-5.14474	0.414141	0.286492
T.cells	B3GALT1	-0.84819	3.129188	-1.94344	0.055144	-4.11782	0.452671	0.319546
T.cells	RELCH	0.138928	6.238997	1.943057	0.055191	-4.99591	0.422019	0.293192
T.cells	TCF7L1	-0.47673	3.097343	-1.94242	0.055269	-4.22306	0.453165	0.320276
T.cells	STK10	-0.15311	7.315855	-1.9423	0.055284	-5.14579	0.411944	0.284822
T.cells	CDS2	0.194799	4.708784	1.941932	0.055329	-4.72651	0.436982	0.306408
T.cells	GM11099	1.241769	-0.70994	1.941669	0.055361	-3.90133	0.493632	0.356293
T.cells	TSPAN7	-0.54733	3.257326	-1.94092	0.055453	-4.09519	0.451961	0.319253
T.cells	TMEM140	0.634705	3.514222	1.939864	0.055584	-4.1112	0.449374	0.317159

T.cells	TRIM35	-0.20218	5.607952	-1.93984	0.055586	-4.93232	0.428616	0.299203
T.cells	PPIA	-0.14004	10.92524	-1.93969	0.055605	-5.80515	0.379896	0.258098
T.cells	HNRNPA1	-0.13516	7.807246	-1.93962	0.055614	-5.36505	0.407785	0.281478
T.cells	GOLGA2	0.240472	3.814242	1.938087	0.055804	-4.49711	0.447394	0.31491
T.cells	GM15987	-0.571	2.537923	-1.93792	0.055824	-4.1577	0.460452	0.326339
T.cells	TRIM5	0.332955	3.589282	1.937469	0.05588	-4.41204	0.449804	0.317108
T.cells	ANKRD49	0.291143	3.032797	1.936348	0.056019	-4.38011	0.456295	0.32259
T.cells	CUX2	-0.76767	1.039079	-1.93507	0.056179	-3.93586	0.478073	0.341306
T.cells	B630019A1	-0.83153	1.596371	-1.93491	0.056198	-3.93686	0.472123	0.336117
T.cells	SKAP2	0.150369	7.235487	1.934332	0.05627	-5.06886	0.415813	0.287523
T.cells	WDR11	0.313586	3.124121	1.934023	0.056309	-4.33697	0.456313	0.322477
T.cells	IL12RB2	-0.77244	3.672057	-1.9338	0.056337	-4.01007	0.45071	0.31768
T.cells	CRYZL2	-0.40599	2.609637	-1.93346	0.056379	-4.14482	0.461633	0.327349
T.cells	FGG	-0.36377	6.166651	-1.93317	0.056415	-4.87392	0.426003	0.296579
T.cells	ZFP335	-0.32837	2.842169	-1.93263	0.056483	-4.29938	0.459451	0.325486
T.cells	CHD2	0.170205	7.754934	1.931851	0.05658	-5.16002	0.411571	0.284029
T.cells	FAM13B	0.153219	6.457749	1.931255	0.056655	-5.01624	0.424016	0.294601
T.cells	ARID5B	0.272057	7.244193	1.930733	0.056721	-5.07703	0.416527	0.288349
T.cells	LAT	0.373376	2.414617	1.930724	0.056722	-4.29489	0.464553	0.329837
T.cells	PIK3R6	0.684812	2.596377	1.93039	0.056764	-4.11601	0.462673	0.32828
T.cells	CDC42	-0.09541	8.943013	-1.93003	0.056809	-5.3948	0.40083	0.275203
T.cells	NEMP2	0.269001	2.854914	1.929388	0.05689	-4.42343	0.460362	0.326236
T.cells	HNRNPK	-0.08864	8.860645	-1.92826	0.057032	-5.49058	0.402416	0.276346
T.cells	PHF20L1	0.14952	7.046788	1.928028	0.057061	-5.12298	0.419321	0.290733
T.cells	FCGR2B	-0.3023	4.881357	-1.92782	0.057088	-4.74911	0.440383	0.308813
T.cells	SRSF3	-0.11093	7.532017	-1.9274	0.057141	-5.31643	0.414734	0.28687
T.cells	SELENON	-0.65183	3.694822	-1.9272	0.057166	-4.01955	0.45234	0.319281
T.cells	HSPH1	-0.34218	4.050366	-1.92637	0.05727	-4.65575	0.449227	0.316365
T.cells	ZFP120	0.401713	2.536502	1.925875	0.057333	-4.15629	0.465014	0.330112
T.cells	PRRC2A	-0.15214	5.517966	-1.92542	0.057391	-4.92157	0.434887	0.30392
T.cells	UBE2CBP	-0.59923	1.761179	-1.92508	0.057434	-4.20888	0.473372	0.337586
T.cells	U2AF1	-0.11323	7.593832	-1.92464	0.05749	-5.31422	0.415051	0.287086
T.cells	1700012D1	0.453164	1.843803	1.924063	0.057563	-4.18702	0.472699	0.337111
T.cells	1700086O(	0.468939	1.546442	1.923939	0.057579	-4.1067	0.47587	0.339909
T.cells	COPA	-0.13352	6.661065	-1.9233	0.05766	-5.0684	0.42429	0.29496
T.cells	OAF	0.300119	3.151249	1.922823	0.057721	-4.54496	0.459481	0.325331
T.cells	LRMDA	0.344361	7.760594	1.922234	0.057796	-5.11628	0.414249	0.286333
T.cells	NFIB	-0.71388	3.880029	-1.92169	0.057866	-4.20796	0.452494	0.319275
T.cells	CCDC43	-0.26455	3.103767	-1.92043	0.058027	-4.4732	0.460982	0.326577
T.cells	MS4A4B	-0.77674	3.207847	-1.92039	0.058032	-4.03345	0.459902	0.325633
T.cells	LRIG1	-1.09319	0.178544	-1.91991	0.058093	-3.9261	0.49232	0.354316
T.cells	PKMYT1	-0.27946	3.261877	-1.91991	0.058094	-4.62502	0.459342	0.325215
T.cells	DLG4	-0.67322	3.571009	-1.91964	0.058129	-4.1977	0.456151	0.322496
T.cells	DRAM2	0.276662	4.847911	1.91918	0.058187	-4.52389	0.44333	0.311301
T.cells	PTGR2	0.255475	3.734192	1.918638	0.058257	-4.52827	0.45485	0.321245
T.cells	TTC39A	-0.83342	2.027911	-1.91727	0.058433	-3.98077	0.473061	0.337351
T.cells	ZFP651	-0.55221	1.578642	-1.91716	0.058446	-4.05211	0.477865	0.341627

T.cells	YIF1A	-0.21194	4.016242	-1.91695	0.058474	-4.58962	0.452332	0.319262
T.cells	CLCN3	0.171496	6.389845	1.916908	0.058479	-4.99965	0.428699	0.298847
T.cells	DDB2	0.327951	3.43077	1.916637	0.058514	-4.51711	0.458346	0.324528
T.cells	CATSPER2	-0.60418	1.800753	-1.9164	0.058545	-4.05067	0.475485	0.339674
T.cells	1700001K1	-0.86613	1.052633	-1.91601	0.058595	-3.9599	0.483577	0.346955
T.cells	5430405H(	0.280256	3.910079	1.915505	0.05866	-4.5452	0.453445	0.320546
T.cells	SERPINB6A	0.275379	5.570318	1.915282	0.058689	-4.77458	0.436751	0.306143
T.cells	F630040K0	1.042288	0.611295	1.914928	0.058735	-3.93723	0.488393	0.351588
T.cells	ARHGAP27	0.30139	3.664658	1.914646	0.058771	-4.43914	0.455963	0.323008
T.cells	CKM	1.378244	0.1057	1.914504	0.05879	-3.93149	0.493964	0.356647
T.cells	CHKA	-0.18054	6.940391	-1.91387	0.058872	-5.11425	0.423532	0.295132
T.cells	AQP9	-0.76569	2.330322	-1.913	0.058985	-4.00766	0.470015	0.335729
T.cells	TRIM68	-0.68498	0.946883	-1.91293	0.058993	-3.97774	0.484865	0.34889
T.cells	PBXIP1	0.258128	4.578633	1.912797	0.059011	-4.68884	0.446779	0.315397
T.cells	NUP155	-0.15586	5.174039	-1.91251	0.059048	-4.95537	0.440808	0.310366
T.cells	MMP19	-1.07309	1.213067	-1.91243	0.059058	-3.94474	0.481974	0.346562
T.cells	SLC46A3	-0.55118	3.085031	-1.91221	0.059088	-4.07744	0.462093	0.329076
T.cells	ARHGAP17	-0.15971	7.245574	-1.91114	0.059226	-5.18631	0.421245	0.293422
T.cells	SLC30A7	0.205692	5.62501	1.910591	0.059298	-4.83643	0.436991	0.307106
T.cells	GM43063	-0.68824	0.779437	-1.91041	0.059322	-3.99359	0.487423	0.351523
T.cells	POLR2D	-0.1679	5.184758	-1.91012	0.059359	-4.98536	0.441364	0.311098
T.cells	PBDC1	-0.22549	5.348225	-1.90974	0.059408	-4.98164	0.439735	0.309812
T.cells	PTGER4	-0.30979	4.681638	-1.90967	0.059418	-4.64682	0.446411	0.315615
T.cells	DBNDD2	-0.49151	2.836435	-1.9093	0.059467	-4.16929	0.465456	0.332371
T.cells	GM41496	-0.80901	0.985043	-1.90858	0.059561	-3.99563	0.48569	0.350147
T.cells	ARG1	0.70433	3.498182	1.907875	0.059653	-4.27029	0.459379	0.326762
T.cells	FBXL4	0.41153	2.64655	1.906848	0.059788	-4.23543	0.469022	0.335014
T.cells	TRIM69	0.675768	2.05026	1.906194	0.059874	-4.0704	0.475721	0.340852
T.cells	ABHD16A	-0.20401	4.808556	-1.905	0.060031	-4.74631	0.447907	0.316223
T.cells	APON	0.937204	1.166034	1.903887	0.060177	-3.98361	0.486545	0.35021
T.cells	AKR1C14	-0.80576	0.911524	-1.90383	0.060185	-3.96934	0.489334	0.352695
T.cells	PRXL2B	-0.56736	2.256934	-1.90377	0.060192	-4.03554	0.474754	0.33975
T.cells	LSM3	-0.17028	5.624923	-1.90343	0.060238	-5.10664	0.440045	0.309481
T.cells	HSPBAP1	0.391546	3.764205	1.902898	0.060308	-4.38153	0.458952	0.326165
T.cells	IRGM1	0.444025	4.93997	1.902602	0.060347	-4.71926	0.446927	0.31576
T.cells	PLCG1	0.26355	3.409844	1.902496	0.060361	-4.55725	0.462635	0.329535
T.cells	GM26670	0.69734	0.402458	1.902102	0.060413	-3.96636	0.495109	0.358447
T.cells	BST1	0.306571	3.315218	1.901741	0.060461	-4.73975	0.463768	0.330693
T.cells	GM49359	0.439027	2.260358	1.900784	0.060588	-4.20094	0.475345	0.340851
T.cells	FBXL7	-0.81166	3.621991	-1.90072	0.060596	-4.20703	0.460982	0.328181
T.cells	NFATC2	0.829078	2.489047	1.900172	0.06067	-3.97027	0.473159	0.338855
T.cells	ZFYVE28	0.781574	0.624372	1.899746	0.060726	-3.95311	0.493543	0.357125
T.cells	HIC1	-1.25486	2.158311	-1.89944	0.060767	-3.95763	0.476818	0.342322
T.cells	SELENOS	-0.16305	6.113957	-1.89878	0.060855	-4.97635	0.436432	0.306961
T.cells	HARS	-0.15567	5.114979	-1.89832	0.060916	-4.91756	0.446507	0.315769
T.cells	AVIL	-0.53998	2.092681	-1.89737	0.061042	-4.15373	0.478006	0.34381
T.cells	NUP85	-0.21221	4.345133	-1.89728	0.061055	-4.88864	0.454341	0.322925

T.cells	TRP53INP1	0.219762	5.187354	1.89716	0.061071	-4.94122	0.445777	0.315442
T.cells	SRGAP2	0.176293	6.60483	1.897153	0.061072	-5.16846	0.431705	0.303236
T.cells	SNRPD1	-0.13889	7.17842	-1.89621	0.061198	-5.34673	0.426571	0.298754
T.cells	TNFSF10	0.403627	2.307037	1.895991	0.061227	-4.34001	0.476198	0.342159
T.cells	PPP6R2	0.203526	4.368985	1.89577	0.061257	-4.71321	0.454566	0.323081
T.cells	CMTM8	-0.29813	4.192364	-1.89433	0.061451	-4.71583	0.456587	0.325142
T.cells	MSR1	0.64414	3.816756	1.894122	0.061478	-4.1486	0.460474	0.328668
T.cells	CHD9	0.186155	6.065045	1.893711	0.061533	-5.06625	0.437661	0.308869
T.cells	BC005561	0.201904	4.273496	1.893346	0.061582	-4.76384	0.455751	0.324704
T.cells	SLFN2	-0.35589	6.335056	-1.8931	0.061615	-4.7899	0.434994	0.306647
T.cells	COL5A3	-0.53912	0.940726	-1.89276	0.061662	-4.23169	0.491273	0.356407
T.cells	ACSS1	0.226763	3.946222	1.892473	0.0617	-4.84648	0.459131	0.327885
T.cells	B930095G1	-1.06207	0.087994	-1.89217	0.061741	-3.96342	0.500764	0.365066
T.cells	FUT4	-0.89213	-0.16835	-1.89214	0.061745	-3.96914	0.50365	0.367673
T.cells	1700020L2	-0.77912	0.390371	-1.89211	0.06175	-3.98442	0.49738	0.362015
T.cells	NEURL2	0.91255	0.801683	1.89184	0.061786	-3.97979	0.492809	0.35794
T.cells	TMEM62	-0.37263	2.375873	-1.89175	0.061797	-4.34719	0.475673	0.342628
T.cells	LCN2	1.224027	3.398879	1.891514	0.06183	-4.132	0.464835	0.333036
T.cells	MRPS9	0.150865	4.889322	1.891331	0.061854	-4.94054	0.449455	0.319566
T.cells	ZBTB2	-0.14866	5.709287	-1.89024	0.062002	-5.06217	0.441959	0.312851
T.cells	SULT2A1	-0.69115	3.901678	-1.88993	0.062043	-4.39563	0.460393	0.329022
T.cells	FSD2	-1.03989	-0.73763	-1.88963	0.062085	-3.96597	0.511006	0.374401
T.cells	9330175E1	0.564441	1.002962	1.889082	0.062159	-4.25784	0.491708	0.356926
T.cells	UBE2N	-0.10685	7.48412	-1.88857	0.062229	-5.33377	0.424983	0.29811
T.cells	MCCC1	-0.43759	2.50652	-1.88789	0.06232	-4.2738	0.475972	0.342679
T.cells	FOXO3	0.193735	6.397005	1.887148	0.062422	-5.06922	0.436087	0.30774
T.cells	PCGF6	-0.31956	2.512206	-1.88713	0.062423	-4.34912	0.476074	0.342881
T.cells	RUFY2	0.269677	2.954919	1.886742	0.062477	-4.51524	0.471422	0.33883
T.cells	MAST4	-0.25433	6.39259	-1.886	0.062578	-5.20877	0.436197	0.308186
T.cells	ATP13A2	0.207385	5.610722	1.885636	0.062628	-5.0109	0.443984	0.315105
T.cells	CYHR1	0.204591	4.48568	1.885121	0.062698	-4.76082	0.45542	0.32528
T.cells	6330409D2	0.774035	0.333222	1.884549	0.062776	-4.01079	0.500046	0.365287
T.cells	BSPRY	-0.88749	0.341542	-1.88449	0.062784	-3.98401	0.499952	0.365202
T.cells	GM16310	-0.76536	1.197906	-1.88407	0.062842	-4.03265	0.490432	0.356719
T.cells	4833438C0	0.444429	2.025244	1.883873	0.062869	-4.11658	0.481394	0.348701
T.cells	DPF1	-1.05054	0.292792	-1.88383	0.062875	-3.97844	0.500499	0.365896
T.cells	IFI47	0.56727	5.277962	1.883679	0.062895	-4.75952	0.447338	0.318553
T.cells	ABTB1	0.298526	4.303875	1.88362	0.062903	-4.62949	0.457293	0.32731
T.cells	RAP2A	0.3734	3.974658	1.883475	0.062923	-4.32784	0.460705	0.33039
T.cells	GPALPP1	0.225337	3.737889	1.881592	0.063182	-4.61341	0.464148	0.333441
T.cells	ARHGAP21	-0.19082	5.511128	-1.88153	0.06319	-4.97792	0.445923	0.317391
T.cells	SMYD1	-0.99653	0.29641	-1.88128	0.063225	-3.9791	0.501512	0.367081
T.cells	HIST2H4	-0.63141	0.951972	-1.8812	0.063236	-4.15059	0.494187	0.360502
T.cells	UBE2J1	0.133772	6.282597	1.88102	0.06326	-5.11499	0.438205	0.31087
T.cells	WDFY4	-0.1779	7.251372	-1.88002	0.063398	-5.43303	0.429206	0.302939
T.cells	MTSS2	0.930473	0.842863	1.879875	0.063418	-3.98558	0.495994	0.362096
T.cells	NAPG	0.225612	4.542195	1.879501	0.06347	-4.72378	0.456419	0.326769

T.cells	UBL5	-0.10914	8.259007	-1.87886	0.063558	-5.46762	0.41965	0.294885
T.cells	MRS2	0.195241	3.939507	1.878684	0.063583	-4.7631	0.462751	0.332586
T.cells	P4HTM	-0.72694	2.773581	-1.87811	0.063662	-4.11158	0.475077	0.34371
T.cells	CRIM1	-0.51979	5.191827	-1.87805	0.06367	-4.45911	0.449844	0.321344
T.cells	HMGB2	-0.1992	9.132267	-1.87776	0.06371	-5.75812	0.411411	0.288039
T.cells	AVL9	0.17359	6.010486	1.877464	0.063752	-5.05461	0.44159	0.314334
T.cells	DYNLL2	-0.23235	5.082989	-1.87735	0.063768	-4.89019	0.450952	0.322573
T.cells	GM15327	-0.54674	1.294764	-1.87628	0.063916	-4.08571	0.491979	0.358835
T.cells	GM12089	-0.6928	0.306607	-1.87593	0.063965	-4.01465	0.503074	0.368913
T.cells	HEG1	0.207098	5.971346	1.87511	0.064079	-5.05412	0.443281	0.315557
T.cells	VPS8	0.250281	4.586793	1.874013	0.064231	-4.66958	0.458174	0.328388
T.cells	MAP3K9	-0.94309	0.308951	-1.87306	0.064365	-3.99933	0.505218	0.370312
T.cells	BCAP29	-0.20732	4.765797	-1.87187	0.06453	-4.81381	0.457556	0.327522
T.cells	GLRX3	0.136948	6.625536	1.871848	0.064534	-5.27324	0.438704	0.310996
T.cells	LRRK2	0.388062	4.404591	1.871591	0.06457	-4.57795	0.461305	0.330892
T.cells	LSP1	-0.1971	8.230432	-1.8706	0.064708	-5.34787	0.423387	0.297749
T.cells	GM29488	0.80271	0.172443	1.870202	0.064765	-4.03268	0.507837	0.372718
T.cells	HABP4	-0.36685	3.054352	-1.86973	0.064831	-4.31813	0.475977	0.344184
T.cells	SAA1	1.657162	-0.05604	1.869516	0.064861	-3.99263	0.510445	0.375288
T.cells	SPACA6	0.732144	1.094819	1.869328	0.064887	-4.03999	0.497432	0.363529
T.cells	AMDHD1	-0.58547	2.26974	-1.86898	0.064936	-4.21547	0.484463	0.351994
T.cells	ASB13	0.319391	3.434394	1.868974	0.064937	-4.559	0.471916	0.340772
T.cells	SERPINA3G	1.612401	3.597185	1.868845	0.064955	-4.14255	0.470187	0.339245
T.cells	RNF128	-0.72024	2.499728	-1.86854	0.064998	-4.06353	0.48197	0.349795
T.cells	GLCE	-0.25704	3.79856	-1.86805	0.065068	-4.64828	0.46827	0.337439
T.cells	NDUFV3	0.160956	7.098589	1.866015	0.065355	-5.23959	0.436034	0.308443
T.cells	EPN1	-0.16958	6.109989	-1.86593	0.065366	-5.02255	0.445908	0.31705
T.cells	DLC1	-0.66044	4.570858	-1.86537	0.065446	-4.36366	0.461743	0.331151
T.cells	KDELR1	0.135706	6.091298	1.865303	0.065456	-5.11779	0.446138	0.317429
T.cells	EIF3J1	-0.12147	7.268033	-1.86497	0.065502	-5.30592	0.434404	0.307274
T.cells	PHF13	-0.23132	3.504231	-1.86452	0.065566	-4.62966	0.472996	0.341285
T.cells	FERMT3	0.160627	7.235899	1.864424	0.06558	-5.20283	0.434721	0.307635
T.cells	CDC27	0.15044	6.009717	1.863867	0.065659	-5.12605	0.446991	0.318456
T.cells	EHD3	-0.5466	3.517645	-1.86381	0.065667	-4.25629	0.472882	0.341332
T.cells	PARP8	-0.213	6.68237	-1.86332	0.065738	-5.02874	0.440429	0.31273
T.cells	MCM10	-0.30263	3.112366	-1.8621	0.06591	-4.63712	0.478105	0.345905
T.cells	GM10658	-0.48819	1.776179	-1.86164	0.065977	-4.23732	0.492706	0.359113
T.cells	WEE1	-0.23186	3.947727	-1.86124	0.066033	-4.85251	0.469181	0.338132
T.cells	RITA1	-0.48703	1.367418	-1.86086	0.066088	-4.15995	0.497255	0.363467
T.cells	NELFA	0.153023	4.630912	1.860771	0.0661	-4.94482	0.461999	0.331964
T.cells	NFKBIA	-0.20787	8.130992	-1.86064	0.066119	-5.42552	0.426798	0.301213
T.cells	GRB14	-0.78251	1.939569	-1.85998	0.066213	-4.10858	0.490899	0.358008
T.cells	SNRPB	-0.12261	7.300883	-1.85983	0.066234	-5.39394	0.43491	0.308417
T.cells	AP1AR	0.181487	5.002368	1.859428	0.066292	-5.02246	0.458138	0.328937
T.cells	CLUH	-0.28145	3.417508	-1.85939	0.066298	-4.58713	0.474827	0.343773
T.cells	GM5914	0.248284	3.331997	1.859305	0.06631	-4.68532	0.475743	0.344629
T.cells	A930014D(	0.890973	-1.05861	1.85917	0.066329	-4.0058	0.525055	0.389382

T.cells	GPATCH2	0.220526	4.253922	1.858285	0.066456	-4.79083	0.466106	0.336363
T.cells	SLC16A13	-0.98021	-0.01818	-1.85794	0.066505	-4.01088	0.513135	0.378936
T.cells	ANK	0.583127	3.446389	1.857915	0.066509	-4.21826	0.474677	0.344147
T.cells	NSMAF	0.234111	4.903953	1.857855	0.066518	-4.82417	0.459312	0.330469
T.cells	GM50334	-1.09543	-0.42372	-1.85728	0.066601	-4.00642	0.518142	0.383618
T.cells	COLEC11	-0.69962	1.589372	-1.85683	0.066666	-4.07243	0.49539	0.363034
T.cells	GPC1	1.110533	0.351919	1.856571	0.066702	-4.00746	0.50935	0.375821
T.cells	QSOX2	-0.64417	0.948725	-1.85608	0.066774	-4.09065	0.502797	0.369783
T.cells	NXPE3	0.233574	3.424156	1.855501	0.066857	-4.74487	0.475846	0.345414
T.cells	GM12367	1.015526	0.051988	1.853574	0.067135	-4.02087	0.514198	0.380102
T.cells	B230369F2	-0.34016	2.457789	-1.85341	0.067158	-4.42764	0.487141	0.355546
T.cells	SNHG15	-0.32212	3.481407	-1.85316	0.067195	-4.51922	0.476033	0.345702
T.cells	COQ8A	-0.48708	2.303584	-1.85306	0.067209	-4.21017	0.488835	0.357274
T.cells	ABL2	0.18687	6.21523	1.852682	0.067264	-5.13617	0.447516	0.320535
T.cells	ZBTB9	0.355194	2.511721	1.852612	0.067274	-4.37517	0.48655	0.355339
T.cells	MOAP1	0.522823	0.547771	1.852525	0.067287	-4.11602	0.508512	0.375289
T.cells	PRKAR2A	-0.47159	5.222556	-1.85241	0.067303	-4.5876	0.45768	0.329531
T.cells	HGD	-0.70222	2.465599	-1.8518	0.067392	-4.19782	0.487396	0.355976
T.cells	C6	-1.00843	2.802964	-1.85129	0.067466	-4.12493	0.483877	0.352809
T.cells	IRF2	0.153992	6.689517	1.850781	0.06754	-5.22057	0.443201	0.316752
T.cells	SLC25A18	-0.26456	4.491338	-1.85077	0.067541	-5.06324	0.465795	0.336742
T.cells	RESF1	0.17643	6.500817	1.850431	0.067591	-5.16563	0.445151	0.318581
T.cells	SH3D21	-0.60081	1.99564	-1.84961	0.067711	-4.16993	0.493376	0.361425
T.cells	MGST1	-0.27979	7.132587	-1.84873	0.067839	-5.24328	0.439668	0.313485
T.cells	DMXL1	0.184965	6.604824	1.848679	0.067847	-5.16714	0.444957	0.318129
T.cells	LRIG2	0.207432	4.256808	1.848257	0.067908	-4.84488	0.469359	0.339649
T.cells	GPR107	0.17279	5.057833	1.84725	0.068055	-4.93458	0.461457	0.332572
T.cells	AC149090.	-0.40045	5.49949	-1.84696	0.068098	-4.8863	0.456872	0.32864
T.cells	PABPC4	-0.15111	5.897724	-1.84675	0.068128	-5.17301	0.452774	0.325092
T.cells	LIPC	-0.5251	2.510562	-1.84624	0.068203	-4.26541	0.488754	0.357409
T.cells	DHCR24	-0.42632	2.897631	-1.84608	0.068227	-4.36089	0.484512	0.353606
T.cells	MFSD14B	-0.18317	5.841678	-1.84576	0.068273	-4.93068	0.453348	0.325918
T.cells	RDX	-0.1071	6.872179	-1.8456	0.068297	-5.30531	0.442893	0.31675
T.cells	HBQ1B	1.15507	-0.16343	1.845265	0.068347	-4.02222	0.519014	0.385291
T.cells	CYP4A31	-0.8834	0.922941	-1.84437	0.068479	-4.06348	0.506518	0.374201
T.cells	GM14325	0.368636	2.324076	1.844336	0.068483	-4.28496	0.490811	0.359908
T.cells	CD63	-0.59507	4.105165	-1.84426	0.068495	-4.29787	0.471495	0.342507
T.cells	CERS5	0.154457	5.514163	1.844235	0.068498	-5.1007	0.45672	0.329332
T.cells	PTPN7	0.225702	4.151708	1.844022	0.068529	-4.91342	0.471	0.342143
T.cells	1110038F1	0.192026	4.590808	1.842858	0.068701	-4.9302	0.466851	0.338464
T.cells	IDH2	-0.16257	5.667278	-1.8425	0.068754	-5.16351	0.455627	0.328648
T.cells	ST7	0.194846	5.144851	1.841229	0.068942	-5.05648	0.461042	0.333934
T.cells	ACOX1	-0.23204	5.892871	-1.84113	0.068955	-4.9498	0.453308	0.327081
T.cells	PIM2	-0.37503	3.150665	-1.84104	0.06897	-4.39675	0.482272	0.353076
T.cells	NCAM1	-1.32546	1.275146	-1.8406	0.069034	-4.03806	0.503064	0.372128
T.cells	RNASE4	-0.37871	5.17058	-1.84042	0.069062	-4.82697	0.460774	0.333907
T.cells	PPP1R42	-0.9049	0.580161	-1.84036	0.06907	-4.05877	0.510977	0.379388

T.cells	PCGF3	0.197221	3.998741	1.840238	0.069088	-4.79371	0.473134	0.344984
T.cells	EWSR1	-0.10144	7.421625	-1.8402	0.069094	-5.43373	0.437879	0.313629
T.cells	NR4A3	-0.84507	6.012984	-1.84003	0.069119	-4.255	0.452077	0.326178
T.cells	TFB1M	-0.38462	1.706255	-1.83959	0.069185	-4.37799	0.498212	0.367795
T.cells	INPP1	0.29324	4.03687	1.839486	0.0692	-4.63877	0.472727	0.344758
T.cells	GSTP1	0.177056	6.928594	1.839403	0.069212	-5.34164	0.4428	0.318118
T.cells	F3	1.40604	0.113979	1.838734	0.069311	-4.02972	0.516348	0.38482
T.cells	RAP1A	0.090517	8.772339	1.838517	0.069344	-5.52378	0.424662	0.302447
T.cells	STAB2	-0.68024	5.148934	-1.83821	0.069389	-4.56577	0.461	0.33463
T.cells	PPARG	-0.96163	2.579221	-1.83811	0.069404	-4.06818	0.488521	0.359471
T.cells	ADGRE1	-0.79087	4.711233	-1.83807	0.06941	-4.34869	0.465583	0.338774
T.cells	ZFP24	-0.18264	4.243197	-1.83761	0.069478	-4.85449	0.47053	0.34334
T.cells	ISG20L2	-0.16834	5.083016	-1.83742	0.069507	-5.03749	0.461687	0.33549
T.cells	PALD1	-0.87058	1.292537	-1.83729	0.069526	-4.06071	0.502867	0.372817
T.cells	SNHG1	-0.17267	5.230493	-1.83682	0.069596	-5.04939	0.460334	0.334293
T.cells	SEC11A	-0.113	6.406384	-1.83536	0.069814	-5.2586	0.449257	0.323914
T.cells	ITGA8	-0.98915	1.726804	-1.83494	0.069877	-4.0829	0.499303	0.369151
T.cells	FBXO3	0.155853	5.012921	1.834667	0.069918	-4.97858	0.463646	0.336983
T.cells	AGXT	-0.63951	3.279368	-1.83459	0.069929	-4.36611	0.48215	0.353657
T.cells	RNF157	0.238603	5.375061	1.834367	0.069963	-5.11517	0.459865	0.333664
T.cells	TSPAN13	0.151296	6.684078	1.834031	0.070013	-5.33745	0.446492	0.321778
T.cells	DNMBP	-0.36352	3.488772	-1.83357	0.070082	-4.41656	0.480115	0.35175
T.cells	WFDC18	0.87043	-0.88323	1.832848	0.070191	-4.03662	0.53018	0.397462
T.cells	SVBP	0.185265	5.267752	1.832159	0.070294	-4.99292	0.461912	0.335146
T.cells	SBF2	-0.29368	5.368073	-1.83202	0.070315	-4.76043	0.460865	0.334272
T.cells	FKBP4	-0.14591	5.691682	-1.83152	0.07039	-5.22095	0.457719	0.331486
T.cells	SKIL	-0.19773	7.304614	-1.83044	0.070554	-5.22394	0.442062	0.317251
T.cells	GM17023	1.214493	-0.64235	1.829939	0.070629	-4.04104	0.528823	0.395847
T.cells	TOE1	-0.32567	2.490022	-1.82984	0.070643	-4.49246	0.492904	0.362875
T.cells	LCP1	0.176985	8.858643	1.829301	0.070725	-5.44375	0.426815	0.304095
T.cells	MPDU1	0.173038	4.71579	1.82924	0.070735	-4.94794	0.468813	0.34129
T.cells	GM13012	0.263324	2.775114	1.827761	0.070958	-4.66638	0.491052	0.360781
T.cells	GM27241	-0.37287	2.603068	-1.82718	0.071046	-4.53031	0.49305	0.362706
T.cells	CXCL16	0.779332	3.468405	1.827115	0.071056	-4.18381	0.483528	0.354078
T.cells	PHKG2	0.198347	4.528144	1.826517	0.071147	-4.84565	0.472308	0.344033
T.cells	SUSD6	0.175233	7.690279	1.825867	0.071246	-5.2796	0.439684	0.315179
T.cells	ATP5G3	-0.14552	8.015505	-1.82577	0.071261	-5.54525	0.436452	0.31238
T.cells	LUC7L	-0.13078	5.622585	-1.82521	0.071345	-5.15243	0.460767	0.33411
T.cells	KLKB1	-0.7299	1.848942	-1.82512	0.07136	-4.12289	0.50171	0.371104
T.cells	MANSC1	1.011847	0.242039	1.824952	0.071385	-4.05206	0.520149	0.388095
T.cells	DNAJC7	0.135752	7.510277	1.824248	0.071493	-5.48188	0.441482	0.317325
T.cells	NLRP1A	0.831402	0.449959	1.824154	0.071507	-4.11181	0.517728	0.386248
T.cells	TMEM63B	-0.25176	3.925117	-1.82414	0.071509	-4.7902	0.478782	0.350673
T.cells	GM15545	0.474816	1.102359	1.823889	0.071547	-4.21702	0.510201	0.379365
T.cells	NR2C1	-0.50243	2.276051	-1.82355	0.071599	-4.26918	0.496912	0.36737
T.cells	NSD2	-0.13422	6.699324	-1.82327	0.071641	-5.45832	0.449671	0.324928
T.cells	RNF43	-0.99248	1.563577	-1.82317	0.071657	-4.06568	0.50494	0.374849

T.cells	GM28379	0.818797	0.264238	1.822669	0.071734	-4.09244	0.51989	0.388822
T.cells	1700037H(	-0.22975	3.714085	-1.82222	0.071803	-4.78964	0.481067	0.353376
T.cells	B230219D2	0.107136	6.252623	1.821785	0.071869	-5.26982	0.454243	0.329421
T.cells	OXA1L	0.176059	4.658758	1.82172	0.071879	-4.94458	0.470916	0.344382
T.cells	GM44751	0.785556	1.310612	1.821713	0.07188	-4.08249	0.507819	0.377999
T.cells	VCPIP1	0.141443	6.167501	1.821427	0.071924	-5.15311	0.45512	0.33036
T.cells	0610039K1	-0.78425	0.022539	-1.82135	0.071936	-4.11111	0.522715	0.391961
T.cells	POLR1C	-0.22718	3.945898	-1.82058	0.072054	-4.8419	0.479058	0.351728
T.cells	SNX32	0.28486	2.812618	1.819845	0.072167	-4.56694	0.49178	0.363156
T.cells	CTNND1	-0.30386	4.294756	-1.81972	0.072186	-4.70256	0.475612	0.348514
T.cells	MASP1	-0.77299	1.673868	-1.81891	0.07231	-4.12292	0.50505	0.375154
T.cells	A	-0.24732	4.78007	-1.8187	0.072343	-4.92611	0.470896	0.344139
T.cells	OSBPL8	0.152417	7.62098	1.817952	0.072458	-5.43791	0.442009	0.318128
T.cells	INTS3	-0.20828	3.898156	-1.81746	0.072534	-4.78599	0.481037	0.353057
T.cells	DERL1	-0.11929	6.22546	-1.81707	0.072594	-5.12547	0.456386	0.330983
T.cells	FAM57B	0.905531	0.285081	1.81687	0.072625	-4.07849	0.521777	0.390512
T.cells	CTSW	-1.07553	2.120663	-1.81667	0.072657	-4.07721	0.500697	0.371152
T.cells	RPN1	-0.16174	5.957169	-1.81617	0.072734	-5.15374	0.459385	0.333736
T.cells	PRMT9	0.251219	4.074852	1.815817	0.072788	-4.77459	0.479427	0.351696
T.cells	MMADHC	0.188687	4.299266	1.813832	0.073096	-4.83354	0.478741	0.350231
T.cells	NBR1	0.157145	5.455137	1.813464	0.073153	-5.04477	0.466426	0.339189
T.cells	MPP1	0.176841	6.203718	1.813253	0.073186	-5.22235	0.458592	0.33226
T.cells	RBBP7	-0.13911	6.2357	-1.81271	0.07327	-5.31444	0.458347	0.332254
T.cells	SLC39A7	-0.19833	4.500813	-1.81258	0.073291	-4.82554	0.476687	0.348738
T.cells	DUSP2	-0.22558	5.991936	-1.81213	0.073361	-5.24086	0.460883	0.334805
T.cells	WDFY3	-0.30493	5.666809	-1.81207	0.073371	-4.88832	0.464287	0.337856
T.cells	CLPTM1	-0.13839	5.552635	-1.81145	0.073467	-5.12989	0.465825	0.339142
T.cells	MPV17L	0.456414	2.006778	1.811174	0.07351	-4.33027	0.504622	0.374459
T.cells	GM34471	1.027392	0.232245	1.809859	0.073715	-4.06486	0.525878	0.39371
T.cells	UPB1	-0.62374	3.0781	-1.80973	0.073736	-4.24707	0.493283	0.363767
T.cells	GM15448	0.85632	-0.00747	1.809694	0.073741	-4.06582	0.528712	0.396338
T.cells	MAOB	-0.80005	1.627366	-1.80861	0.073911	-4.16333	0.510135	0.37924
T.cells	UBE2V1	-0.12052	6.983076	-1.80853	0.073924	-5.36128	0.452034	0.32675
T.cells	ANKFY1	0.147992	5.995175	1.808082	0.073994	-5.21324	0.462261	0.336046
T.cells	PKDCC	-0.85776	1.303453	-1.80797	0.074011	-4.08431	0.513862	0.382907
T.cells	CDH1	0.904397	1.38751	1.807732	0.074049	-4.09755	0.512892	0.382068
T.cells	LONP2	0.13369	5.87764	1.807546	0.074078	-5.12091	0.463492	0.337218
T.cells	PPP1R18	-0.14444	6.994866	-1.8069	0.074179	-5.3283	0.451913	0.32714
T.cells	2700081O1	-0.24802	3.137169	-1.80688	0.074183	-4.76915	0.493087	0.364225
T.cells	PRR5	-0.29405	3.479528	-1.80679	0.074196	-4.75785	0.489296	0.360772
T.cells	DHRS7	-0.5106	4.016547	-1.80632	0.074271	-4.40421	0.483556	0.355546
T.cells	FTL1-PS1	-0.47306	3.902493	-1.80572	0.074365	-4.50277	0.484803	0.356872
T.cells	NXN	0.214041	5.325793	1.805676	0.074372	-5.1007	0.469463	0.343009
T.cells	IGKV9-124	0.544688	-1.01492	1.805479	0.074403	-4.17687	0.541454	0.409173
T.cells	4933433G1	0.511228	1.434512	1.805281	0.074435	-4.2942	0.512513	0.382341
T.cells	SRSF7	-0.15994	6.098296	-1.80425	0.074597	-5.30938	0.461589	0.336189
T.cells	CACNB2	-0.39592	4.420818	-1.80425	0.074597	-4.91529	0.479432	0.352262

T.cells	RASGRF2	-0.87991	0.116081	-1.80408	0.074623	-4.0759	0.528207	0.397052
T.cells	NCS1	-0.79637	0.210797	-1.80393	0.074648	-4.12557	0.527086	0.396009
T.cells	KLF12	-0.5911	3.585919	-1.80314	0.074773	-4.38011	0.48909	0.360765
T.cells	ATP5H	-0.10522	7.946365	-1.80241	0.074889	-5.54794	0.44343	0.319622
T.cells	ARHGAP1	-0.18493	4.473602	-1.80191	0.074968	-4.79914	0.479698	0.352245
T.cells	ISOC1	0.166178	4.993923	1.8018	0.074985	-5.11156	0.474092	0.347221
T.cells	1110002J0	1.137998	-0.51304	1.801603	0.075017	-4.07586	0.536643	0.404725
T.cells	HMGN2	-0.17991	7.53605	-1.80138	0.075052	-5.60094	0.447576	0.323577
T.cells	PTPRE	0.293599	5.539856	1.801204	0.07508	-4.77623	0.468276	0.342136
T.cells	RNF215	0.340554	2.627986	1.800278	0.075227	-4.38934	0.500781	0.371402
T.cells	CLINT1	-0.11294	8.205941	-1.79897	0.075436	-5.5841	0.441909	0.318546
T.cells	DIPK1A	0.25004	5.446127	1.798808	0.075461	-4.99368	0.470423	0.344061
T.cells	TWISTNB	-0.14527	5.520145	-1.7981	0.075574	-5.16116	0.469636	0.343551
T.cells	ATP5O	-0.1391	7.142669	-1.79796	0.075597	-5.45038	0.452695	0.328388
T.cells	HPD	-0.53448	4.805638	-1.79731	0.0757	-4.79522	0.477283	0.350664
T.cells	NME3	-0.83735	0.405363	-1.79725	0.07571	-4.12951	0.527	0.396312
T.cells	SMAD5	0.201807	3.702902	1.796923	0.075762	-4.79068	0.489316	0.361687
T.cells	CD300LB	1.026691	1.973752	1.796668	0.075803	-4.11384	0.508754	0.379653
T.cells	TNRC18	0.134919	5.931838	1.79623	0.075874	-5.18125	0.465282	0.340241
T.cells	BAIAP2L1	-0.38639	3.098445	-1.79566	0.075965	-4.42984	0.496031	0.368287
T.cells	SF3B4	-0.15582	5.494923	-1.79566	0.075965	-5.21202	0.469904	0.344522
T.cells	HNRNPA2B	-0.10819	9.287708	-1.79507	0.076059	-5.83073	0.431187	0.31024
T.cells	UTP14B	-0.28114	3.081232	-1.79503	0.076067	-4.70147	0.496223	0.368756
T.cells	COX6C	-0.1084	8.594079	-1.79482	0.0761	-5.66382	0.438033	0.316321
T.cells	LIG1	-0.22497	5.051661	-1.79463	0.07613	-5.22433	0.474637	0.349142
T.cells	ARL4C	-0.23742	5.243589	-1.79453	0.076146	-4.95833	0.472582	0.347337
T.cells	PPP5C	-0.19905	4.346979	-1.79373	0.076275	-4.87375	0.482254	0.356468
T.cells	AK8	-0.68082	2.179586	-1.79357	0.076301	-4.14425	0.506403	0.378652
T.cells	TAP1	0.376504	5.446269	1.793465	0.076318	-5.10454	0.470421	0.34578
T.cells	DNASE1L1	0.45205	3.048154	1.79305	0.076385	-4.33582	0.496593	0.369854
T.cells	GM48293	1.033259	-0.15995	1.792556	0.076464	-4.08605	0.533722	0.404529
T.cells	DNAJB2	-0.57039	2.766414	-1.79225	0.076513	-4.19795	0.499756	0.373019
T.cells	LY6G5B	-0.5071	1.345565	-1.7915	0.076635	-4.28463	0.515991	0.388178
T.cells	CRADD	0.165188	5.224096	1.791319	0.076664	-5.05994	0.47279	0.348504
T.cells	KDM5D	2.361719	0.730109	1.791301	0.076667	-4.20134	0.523173	0.39491
T.cells	MTO1	0.2289	3.553315	1.791056	0.076706	-4.75533	0.49097	0.36519
T.cells	SIRPB1C	1.305571	1.778958	1.790759	0.076755	-4.09949	0.510988	0.383782
T.cells	GM44702	-1.02546	0.088947	-1.79036	0.076819	-4.10309	0.530753	0.402457
T.cells	NDUFAF2	-0.18656	4.440034	-1.79019	0.076847	-4.98342	0.481242	0.356671
T.cells	AI837181	0.272155	3.127875	1.790074	0.076865	-4.54005	0.495702	0.369957
T.cells	HINT2	-0.2301	4.356845	-1.79	0.076877	-4.80994	0.482147	0.357517
T.cells	MRPL54	0.147034	5.906363	1.789876	0.076897	-5.28434	0.46555	0.342463
T.cells	FBXL6	-0.26111	3.399881	-1.78952	0.076956	-4.65601	0.492671	0.367335
T.cells	SLC38A2	-0.10807	8.351985	-1.7895	0.076958	-5.64687	0.440447	0.320033
T.cells	SOCS4	0.167866	4.684801	1.789306	0.07699	-5.00675	0.478588	0.35449
T.cells	1700027J0	0.334047	2.481407	1.789155	0.077014	-4.85631	0.502974	0.376964
T.cells	PCBP2	-0.09247	8.511828	-1.78883	0.077067	-5.66271	0.438852	0.318819

T.cells	SCAI	0.234566	4.434969	1.788697	0.077089	-4.92944	0.481297	0.357134
T.cells	ING3	-0.1609	4.830612	-1.78856	0.077112	-5.03083	0.477014	0.353232
T.cells	ACAT2	0.26734	2.91084	1.788451	0.077129	-4.65471	0.498132	0.372635
T.cells	ABCA2	0.613488	1.527476	1.788233	0.077164	-4.23333	0.513885	0.387267
T.cells	FH1	-0.18387	5.484899	-1.78788	0.077221	-5.26222	0.47001	0.346993
T.cells	GNPNAT1	-0.21511	4.157449	-1.78777	0.077239	-4.85628	0.484322	0.36012
T.cells	ECHDC2	-0.71917	1.612612	-1.78741	0.077298	-4.21009	0.512903	0.386637
T.cells	ADORA2B	1.062142	0.37813	1.787389	0.077301	-4.09753	0.527322	0.400127
T.cells	HNRNPM	-0.09515	7.703885	-1.7872	0.077332	-5.56416	0.446972	0.326389
T.cells	A730081D(	0.328005	3.129627	1.787162	0.077338	-4.66032	0.495682	0.37071
T.cells	RALGDS	0.353087	3.822267	1.786919	0.077378	-4.75623	0.488	0.363719
T.cells	BSG	0.174909	7.716678	1.786042	0.077521	-5.45603	0.447243	0.32664
T.cells	FNDC3B	-0.20655	6.549271	-1.78585	0.077551	-5.1002	0.459235	0.337449
T.cells	POLR2B	-0.15067	4.911643	-1.7857	0.077577	-5.11023	0.476569	0.353201
T.cells	APEX1	-0.16554	5.465947	-1.78433	0.0778	-5.24	0.471323	0.348242
T.cells	AAAS	-0.22142	3.794127	-1.78401	0.077853	-4.93385	0.489464	0.364934
T.cells	AKR7A5	-0.23937	4.13514	-1.78391	0.07787	-4.79937	0.485711	0.361487
T.cells	ALG9	0.262553	3.375018	1.783752	0.077895	-4.68835	0.494113	0.369233
T.cells	ZFP710	-0.17817	6.297621	-1.78307	0.078007	-5.35001	0.462535	0.340552
T.cells	AFG3L1	-0.13947	4.862083	-1.78291	0.078032	-5.02247	0.477801	0.354439
T.cells	PXK	0.151356	6.270189	1.782902	0.078034	-5.2886	0.462823	0.340812
T.cells	SF3A2	-0.16946	5.260106	-1.78271	0.078065	-5.17398	0.473522	0.350558
T.cells	CEP164	0.205776	4.052145	1.782627	0.078079	-4.89308	0.486622	0.362547
T.cells	PGLYRP1	0.732001	4.959034	1.782219	0.078146	-4.34661	0.476854	0.353642
T.cells	4930402H2	0.236233	4.453202	1.781919	0.078196	-4.8467	0.482335	0.358716
T.cells	FBXL20	0.258235	5.495646	1.781684	0.078234	-5.04415	0.471103	0.348475
T.cells	CPB2	-0.47939	3.209826	-1.78148	0.078268	-4.40812	0.496058	0.371433
T.cells	RASAL2	-0.26347	5.468995	-1.78095	0.078355	-5.09026	0.471651	0.348968
T.cells	RABEP1	0.125484	6.897474	1.780305	0.078461	-5.3813	0.457007	0.335494
T.cells	PAG1	0.22674	7.038518	1.779906	0.078527	-5.30113	0.455678	0.334357
T.cells	0610040F0	0.895458	0.269406	1.779445	0.078603	-4.10181	0.531053	0.403813
T.cells	XPC	0.255923	3.385386	1.779188	0.078646	-4.69149	0.495111	0.370429
T.cells	SLC50A1	-0.19569	5.247798	-1.77869	0.078728	-5.02263	0.474958	0.351833
T.cells	KRT222	-0.59618	-0.05515	-1.77767	0.078896	-4.25165	0.536043	0.408236
T.cells	RHOB	-0.32045	5.891039	-1.77737	0.078946	-5.17974	0.468877	0.346067
T.cells	NECTIN3	-0.56349	1.220532	-1.77664	0.079068	-4.25584	0.520971	0.394407
T.cells	RASSF8	0.573552	2.215433	1.776523	0.079086	-4.31673	0.509445	0.383696
T.cells	TEC	0.18592	5.751113	1.77636	0.079114	-5.18442	0.470374	0.347787
T.cells	COL18A1	-0.58824	2.743841	-1.77631	0.079121	-4.39362	0.50342	0.378156
T.cells	ARHGAP15	0.13394	9.872031	1.775274	0.079294	-5.83861	0.429099	0.310451
T.cells	NUPR1	1.276001	2.41246	1.774802	0.079372	-4.16394	0.508242	0.382206
T.cells	CLP1	-0.17947	4.608479	-1.77441	0.079437	-5.02343	0.483812	0.359609
T.cells	CDK16	0.242718	3.39772	1.773237	0.079633	-4.68131	0.498099	0.372409
T.cells	CREB3L3	-0.70671	1.62088	-1.77304	0.079665	-4.20161	0.518431	0.391276
T.cells	MSRA	0.131551	6.629496	1.772705	0.079722	-5.42589	0.463087	0.340485
T.cells	ZFP787	0.153	5.128821	1.772317	0.079787	-5.08062	0.479155	0.355133
T.cells	MMP25	1.044045	0.499533	1.771965	0.079846	-4.11359	0.53182	0.404108

T.cells	CENPB	0.159178	5.450808	1.771376	0.079944	-5.22696	0.475679	0.352226
T.cells	F5	-0.74307	4.094976	-1.77137	0.079945	-4.41904	0.490477	0.365774
T.cells	GRTP1	-0.74643	0.35364	-1.77133	0.079952	-4.14432	0.533564	0.405874
T.cells	SRRT	-0.14091	5.528359	-1.77085	0.080032	-5.27181	0.475014	0.351602
T.cells	FRG2F1	-0.63932	0.702787	-1.77049	0.080092	-4.16746	0.529587	0.402202
T.cells	PGGHG	-0.37397	2.820106	-1.77016	0.080148	-4.51279	0.504962	0.379362
T.cells	NRXN3	-0.80165	1.145579	-1.77012	0.080155	-4.19261	0.524348	0.397451
T.cells	AXL	-0.77449	3.708037	-1.7697	0.080225	-4.33868	0.49512	0.370208
T.cells	FKBP3	-0.15754	6.130579	-1.76895	0.080351	-5.39387	0.46912	0.346144
T.cells	DNAJC9	-0.17697	5.617283	-1.76879	0.080377	-5.3673	0.474601	0.351213
T.cells	MPHOSPH8	0.149682	4.79616	1.767902	0.080527	-5.10328	0.483897	0.359592
T.cells	GM43388	-0.74494	0.386691	-1.76787	0.080532	-4.2251	0.534412	0.406506
T.cells	ZFP638	0.131683	6.22747	1.766719	0.080726	-5.31735	0.46936	0.345882
T.cells	PRKCB	0.116328	8.511819	1.76605	0.080839	-5.70696	0.445948	0.324834
T.cells	PIGS	0.214006	4.078714	1.765902	0.080864	-4.82454	0.493029	0.367574
T.cells	TRP53RKA	-0.32832	2.472703	-1.76525	0.080975	-4.51855	0.511628	0.384619
T.cells	CD47	0.109564	8.628324	1.764719	0.081064	-5.65443	0.445388	0.324301
T.cells	GM37168	0.932786	0.65738	1.764337	0.081129	-4.12325	0.533358	0.40489
T.cells	GM26887	-0.79027	3.10483	-1.7641	0.08117	-4.27942	0.50479	0.37839
T.cells	ATG2A	0.235294	5.710857	1.763831	0.081215	-4.97913	0.475946	0.352054
T.cells	A3GALT2	-0.55125	2.675415	-1.76314	0.081332	-4.33726	0.509837	0.38324
T.cells	HPGD	-0.65306	5.673887	-1.76301	0.081354	-4.76706	0.476468	0.352616
T.cells	GM12802	-0.55923	0.658153	-1.76262	0.081421	-4.2123	0.533494	0.405478
T.cells	EARS2	-0.48587	1.644083	-1.76247	0.081447	-4.29398	0.521808	0.394561
T.cells	KANSL3	0.158311	4.995066	1.762412	0.081456	-5.12467	0.483838	0.359437
T.cells	VPREB1	0.425806	2.621584	1.761828	0.081555	-4.93501	0.510803	0.384092
T.cells	E530011L2	0.670386	1.095538	1.760877	0.081717	-4.26205	0.529257	0.40093
T.cells	SAMD4	-0.91134	3.187151	-1.76075	0.081738	-4.30287	0.504923	0.378337
T.cells	DESI2	0.10985	6.03278	1.760414	0.081796	-5.27404	0.473567	0.349609
T.cells	LBR	-0.10148	7.219631	-1.75921	0.082001	-5.5744	0.461641	0.338499
T.cells	HPS5	-0.20633	4.448069	-1.75902	0.082033	-4.89634	0.491519	0.365756
T.cells	GSS	-0.33366	3.399727	-1.75896	0.082044	-4.61559	0.503286	0.376593
T.cells	UBXN4	0.100103	6.814787	1.758721	0.082085	-5.35828	0.465896	0.342491
T.cells	EIF3L	-0.13368	5.672102	-1.75811	0.082189	-5.31102	0.478454	0.353878
T.cells	F7	-0.91003	0.719823	-1.75768	0.082262	-4.15679	0.535148	0.406315
T.cells	SEMA6D	-0.76821	3.713919	-1.75729	0.08233	-4.35051	0.500385	0.373943
T.cells	SH2D4B	-0.29997	4.439997	-1.75706	0.082369	-4.99596	0.492251	0.366536
T.cells	RHD	0.621667	1.667344	1.756448	0.082474	-4.32979	0.52436	0.396166
T.cells	RREB1	0.196283	6.814971	1.756019	0.082548	-5.42682	0.466832	0.343341
T.cells	CDAN1	0.279774	2.943919	1.755614	0.082618	-4.74997	0.509507	0.38251
T.cells	TIA1	0.117378	5.466514	1.755153	0.082697	-5.28803	0.481301	0.35667
T.cells	TNNT3	-0.58112	1.772075	-1.75497	0.082728	-4.34228	0.523126	0.395362
T.cells	LSM14A	-0.10278	6.620649	-1.75488	0.082744	-5.44014	0.468891	0.34549
T.cells	EPS8L2	0.509401	0.893203	1.754746	0.082767	-4.42997	0.533561	0.405267
T.cells	NOL8	-0.21914	4.078757	-1.75467	0.08278	-4.95251	0.496632	0.37091
T.cells	PRDX4	-0.16633	5.118984	-1.75407	0.082884	-5.25728	0.485181	0.360572
T.cells	PDCD2	0.205963	3.817161	1.753438	0.082993	-4.9337	0.499658	0.374013

T.cells	PRIM2	0.192265	5.536205	1.753424	0.082995	-5.35674	0.480625	0.356537
T.cells	ZDHHC20	0.118998	6.740107	1.753386	0.083002	-5.34012	0.467704	0.344794
T.cells	BAHCC1	0.57794	1.235974	1.75331	0.083015	-4.30305	0.529559	0.401863
T.cells	FUOM	0.504996	2.61981	1.752373	0.083177	-4.48256	0.51368	0.387185
T.cells	PARP12	0.472905	3.018486	1.752142	0.083217	-4.45923	0.509087	0.382972
T.cells	CCNE2	-0.28452	3.75287	-1.75203	0.083235	-5.0616	0.500726	0.375286
T.cells	IGFBP1	0.665979	4.147259	1.751958	0.083248	-4.75196	0.496289	0.371191
T.cells	CALM2	-0.09533	8.565355	-1.75126	0.08337	-5.77536	0.449461	0.328452
T.cells	BMPR1A	-0.89697	2.310682	-1.75063	0.083478	-4.18577	0.518124	0.391151
T.cells	FMO5	-0.53526	3.247756	-1.7504	0.083519	-4.33351	0.507301	0.381115
T.cells	EMC7	-0.11681	6.059195	-1.74983	0.083617	-5.30239	0.476264	0.352716
T.cells	VPS41	0.175175	5.101022	1.749461	0.083681	-5.04321	0.486699	0.362399
T.cells	KLF6	-0.25395	7.581942	-1.74901	0.08376	-5.31945	0.460113	0.338427
T.cells	GM30881	0.381067	1.986079	1.748976	0.083766	-4.49925	0.522121	0.395469
T.cells	GM16124	-0.46752	2.669782	-1.74894	0.083772	-4.47537	0.514147	0.38803
T.cells	YBX1	-0.1182	8.398771	-1.7483	0.083884	-5.75207	0.452029	0.331105
T.cells	TAOK1	0.120641	7.033029	1.747926	0.083948	-5.43657	0.466363	0.344057
T.cells	RAB6B	0.580316	1.697022	1.747418	0.084037	-4.26725	0.526354	0.399285
T.cells	SLC35B3	0.163719	4.482615	1.747138	0.084086	-4.97329	0.494351	0.369595
T.cells	HNRNPDL	-0.09311	7.857159	-1.74632	0.084229	-5.67211	0.458042	0.336678
T.cells	FAM76A	0.17266	5.119697	1.746149	0.084258	-5.06456	0.487336	0.363391
T.cells	GSTA4	-0.69601	1.583658	-1.74609	0.084269	-4.22695	0.52778	0.400983
T.cells	PDIK1L	0.261694	3.065249	1.746072	0.084272	-4.75074	0.510468	0.384797
T.cells	JMY	0.208602	6.282387	1.745776	0.084324	-5.19844	0.474726	0.351998
T.cells	F12	-0.78176	1.691224	-1.74522	0.084421	-4.2614	0.526824	0.400223
T.cells	APPBP2OS	0.514037	0.985935	1.744461	0.084554	-4.2795	0.53524	0.408513
T.cells	DPH6	0.202915	4.47347	1.744	0.084635	-5.06043	0.494804	0.370846
T.cells	PCLAF	-0.27343	7.982096	-1.74395	0.084644	-5.86699	0.457022	0.33634
T.cells	DAB2	-0.89507	4.009728	-1.74379	0.084671	-4.4215	0.500012	0.375692
T.cells	HOGA1	-0.58997	2.370342	-1.74323	0.08477	-4.31394	0.518835	0.393437
T.cells	NAMPT	0.291767	6.301782	1.743073	0.084797	-5.16659	0.474763	0.352658
T.cells	ALDOB	-0.50176	4.949636	-1.74306	0.084799	-4.90268	0.489509	0.366187
T.cells	CUL5	-0.12565	5.877947	-1.74305	0.084801	-5.29668	0.47934	0.356844
T.cells	GNAT3	-0.64594	-0.04367	-1.743	0.084811	-4.2591	0.547749	0.420729
T.cells	ILDR1	-0.81405	1.791756	-1.74227	0.084938	-4.24124	0.526144	0.400159
T.cells	GH	0.632399	-0.76502	1.741465	0.08508	-4.2023	0.557519	0.429846
T.cells	NR1D2	-0.30043	3.478586	-1.7413	0.08511	-4.65891	0.50681	0.382112
T.cells	JAG2	-0.47125	1.093215	-1.74125	0.085118	-4.42995	0.534765	0.408318
T.cells	TMEM107	-0.35837	1.616764	-1.73956	0.085416	-4.53048	0.530068	0.403059
T.cells	CDK6	0.203754	6.322112	1.739068	0.085503	-5.47437	0.476668	0.35381
T.cells	2-Mar	0.180803	6.581903	1.738726	0.085564	-5.29426	0.473872	0.351388
T.cells	EMC9	0.647996	1.600787	1.7386	0.085586	-4.21406	0.530258	0.403708
T.cells	ELN	0.675081	2.40021	1.738231	0.085651	-4.40821	0.520806	0.394965
T.cells	SLC25A5	-0.12003	7.608641	-1.73815	0.085665	-5.62509	0.462972	0.341669
T.cells	GM26885	-0.53099	2.317843	-1.73806	0.085681	-4.43085	0.521773	0.395884
T.cells	LPP	0.195394	7.6885	1.737219	0.085831	-5.46769	0.462334	0.3411
T.cells	RIF1	-0.15179	5.435862	-1.73702	0.085866	-5.33963	0.486535	0.363251

T.cells	FADS1	-0.33247	3.059286	-1.73683	0.085899	-4.64078	0.513354	0.388164
T.cells	CXADR	-0.66125	1.645302	-1.73666	0.085931	-4.26019	0.529957	0.403737
T.cells	KEAP1	0.152602	5.115335	1.736237	0.086005	-5.15765	0.490074	0.366697
T.cells	ZKSCAN1	0.185769	4.068204	1.735998	0.086048	-4.97734	0.501804	0.377558
T.cells	TSPAN31	0.245925	4.424978	1.735935	0.086059	-4.89518	0.497778	0.373829
T.cells	NCK1	0.160257	6.174664	1.735746	0.086093	-5.31802	0.478469	0.35606
T.cells	SPATS2	-0.70736	2.720159	-1.7356	0.086119	-4.25691	0.517291	0.392029
T.cells	RNF11	0.184115	6.207032	1.734098	0.086386	-5.21309	0.479351	0.356321
T.cells	HNRNPH1	-0.13471	6.424984	-1.73379	0.086441	-5.42742	0.477049	0.354244
T.cells	5830408C2	-0.28193	2.989299	-1.73255	0.086663	-4.67873	0.516608	0.39018
T.cells	C5AR1	1.02415	4.179898	1.731964	0.086768	-4.32405	0.503058	0.377805
T.cells	MRPL13	-0.15895	4.834872	-1.73191	0.086777	-5.19548	0.495671	0.370981
T.cells	SIAH1A	0.16349	5.079563	1.731358	0.086876	-5.19051	0.493016	0.368608
T.cells	CDC25B	0.209148	4.400381	1.730935	0.086952	-5.2997	0.50064	0.375787
T.cells	CHERP	-0.14376	5.016827	-1.73059	0.087013	-5.19797	0.493716	0.369496
T.cells	ARHGEF5	0.818853	0.327235	1.730548	0.087021	-4.20997	0.548712	0.42097
T.cells	DHX40	-0.21499	7.808142	-1.73045	0.087038	-5.52658	0.463475	0.341871
T.cells	ADTRP	-0.68988	1.835711	-1.73033	0.08706	-4.26912	0.530431	0.403713
T.cells	PRR3	-0.2089	3.987386	-1.72966	0.08718	-4.96672	0.505767	0.380711
T.cells	RDH16F2	-0.83273	1.257357	-1.72916	0.08727	-4.26041	0.537916	0.410958
T.cells	IFT52	0.175223	4.249963	1.729096	0.087282	-4.9913	0.50285	0.37815
T.cells	GM26756	1.122334	0.354794	1.728167	0.087449	-4.16692	0.549434	0.4219
T.cells	PIGO	0.455667	1.804475	1.727929	0.087492	-4.45704	0.531831	0.405359
T.cells	HSPBP1	-0.19007	3.934559	-1.7279	0.087498	-4.98837	0.506911	0.382038
T.cells	FAM110A	0.231461	3.760358	1.727632	0.087546	-4.98976	0.508927	0.383904
T.cells	MNDAL	0.237775	6.786829	1.726296	0.087787	-5.44777	0.476179	0.35324
T.cells	UNG	-0.31692	3.369876	-1.7262	0.087804	-4.89251	0.514415	0.388524
T.cells	CACNB4	-0.73247	1.615307	-1.72544	0.087941	-4.32886	0.535573	0.408126
T.cells	GM10371	0.883485	-0.68835	1.725316	0.087964	-4.16805	0.563983	0.435141
T.cells	UGT1A7C	0.920311	-0.45047	1.724605	0.088093	-4.17433	0.561521	0.432496
T.cells	PIGM	0.225051	3.53133	1.724087	0.088187	-4.81101	0.513729	0.38737
T.cells	TMEM127	0.198376	4.290896	1.723644	0.088267	-4.89507	0.5052	0.379379
T.cells	PITPNM1	-0.27994	3.730007	-1.7229	0.088403	-4.70102	0.512162	0.385475
T.cells	GM43661	0.848647	2.981343	1.72234	0.088504	-4.23747	0.521116	0.393891
T.cells	DDX41	-0.16984	4.356626	-1.72194	0.088578	-5.09429	0.505197	0.379235
T.cells	NINJ2	-0.92426	0.701022	-1.72174	0.088615	-4.21095	0.548541	0.419847
T.cells	RANGAP1	-0.15116	5.610379	-1.72153	0.088651	-5.40114	0.491083	0.366298
T.cells	GM4129	-0.56321	1.28611	-1.72143	0.088669	-4.32568	0.54138	0.413173
T.cells	ADAM33	0.953364	-0.9471	1.720508	0.088838	-4.17115	0.569976	0.439824
T.cells	GM42869	0.510242	1.342309	1.720029	0.088926	-4.32881	0.541571	0.412905
T.cells	EXT1	-0.19889	7.749502	-1.7199	0.088949	-5.64566	0.468611	0.345487
T.cells	GM10382	0.869955	0.439288	1.719592	0.089006	-4.1941	0.552739	0.423439
T.cells	PSD3	-0.251	5.794857	-1.71896	0.089121	-5.19959	0.49028	0.364965
T.cells	GM20712	-0.96403	0.063822	-1.71817	0.089266	-4.20477	0.558474	0.428494
T.cells	TGFA	-0.84814	0.796415	-1.71768	0.089355	-4.222	0.549443	0.42006
T.cells	LRRC59	-0.13054	5.556125	-1.71761	0.089368	-5.32495	0.493551	0.367983
T.cells	LSS	-0.6982	0.604735	-1.71699	0.089482	-4.23744	0.552235	0.422651

T.cells	MTCP1	0.612357	0.737147	1.71671	0.089534	-4.31486	0.550633	0.42118
T.cells	TULP4	-0.17034	5.928036	-1.71635	0.089599	-5.2533	0.489828	0.364685
T.cells	KAZN	-0.92595	0.348768	-1.71621	0.089625	-4.19964	0.555459	0.425958
T.cells	UMAD1	0.156244	5.658219	1.71535	0.089784	-5.32839	0.493236	0.367759
T.cells	CAGE1	0.563682	1.94977	1.715313	0.089791	-4.39588	0.53628	0.407739
T.cells	GOLPH3L	0.197325	4.39604	1.714346	0.089969	-5.10537	0.508062	0.381171
T.cells	TPST1	0.244703	3.472726	1.713942	0.090043	-5.01122	0.518759	0.391162
T.cells	INMT	-0.89979	0.639157	-1.71387	0.090057	-4.22161	0.552909	0.423224
T.cells	GM14634	0.483456	1.834434	1.713797	0.09007	-4.45446	0.538258	0.409399
T.cells	GM43768	0.681623	0.70894	1.71325	0.090171	-4.2788	0.55233	0.422639
T.cells	YOD1	0.244864	4.084266	1.713051	0.090208	-5.01314	0.511916	0.384836
T.cells	PRKAG1	-0.12546	5.515312	-1.71254	0.090302	-5.28054	0.495894	0.370053
T.cells	TNFRSF4	-0.9523	0.30724	-1.71228	0.09035	-4.20858	0.557646	0.427779
T.cells	MYBPC2	-0.30819	2.760882	-1.70995	0.090782	-4.87762	0.529978	0.400657
T.cells	SLC25A16	0.216312	3.724214	1.708121	0.091123	-4.9336	0.519995	0.390588
T.cells	MAGI3	-0.18158	6.598022	-1.70808	0.091131	-5.54383	0.487286	0.360557
T.cells	GM15706	-0.57397	1.015787	-1.70778	0.091186	-4.32365	0.552686	0.421255
T.cells	ESRRG	-0.74561	1.226258	-1.70765	0.091211	-4.26064	0.550079	0.418811
T.cells	UBTD2	-0.31317	3.37685	-1.70735	0.091267	-4.74085	0.524143	0.394634
T.cells	SHLD1	0.323146	3.33904	1.706665	0.091394	-4.64586	0.524723	0.395308
T.cells	NRXN1	-0.47411	2.142242	-1.70638	0.091448	-4.73213	0.539061	0.408844
T.cells	ALKBH2	-0.3201	2.187068	-1.70638	0.091448	-4.55307	0.538517	0.408334
T.cells	CORO2B	-0.6058	1.197161	-1.70607	0.091506	-4.30227	0.550639	0.419797
T.cells	SAMD10	0.434047	1.732501	1.705996	0.09152	-4.49217	0.544053	0.413597
T.cells	APH1C	-0.52476	4.351444	-1.70553	0.091607	-4.4995	0.513103	0.384709
T.cells	EIF4A2	-0.14439	5.530319	-1.70377	0.091936	-5.23434	0.500693	0.37303
T.cells	PTPN6	0.180999	6.758702	1.703575	0.091973	-5.37778	0.486962	0.360587
T.cells	TEX264	-0.21952	4.497588	-1.70355	0.091978	-4.8392	0.512518	0.384015
T.cells	ACOT4	-1.02158	0.046894	-1.70349	0.091989	-4.19867	0.566516	0.434606
T.cells	FIRRE	-0.4059	2.608551	-1.70258	0.092159	-4.61586	0.53555	0.405197
T.cells	DUSP18	1.023983	0.621447	1.702159	0.092239	-4.2009	0.560087	0.428376
T.cells	RNF181	0.208787	4.477136	1.70193	0.092283	-5.08002	0.513512	0.384924
T.cells	DNASE1L3	-0.40499	6.30437	-1.70167	0.092331	-5.08219	0.492725	0.365901
T.cells	MOB3C	-0.37178	3.356469	-1.70155	0.092355	-4.52843	0.526664	0.397238
T.cells	CDK18	0.985099	-0.22726	1.70094	0.092469	-4.19906	0.570925	0.439071
T.cells	IL21R	-0.45978	5.150614	-1.70064	0.092525	-4.66664	0.505823	0.378217
T.cells	BRWD1	0.146206	6.550877	1.700241	0.092601	-5.37175	0.490047	0.363937
T.cells	GNPDA2	0.245197	3.100129	1.700164	0.092615	-4.76826	0.529787	0.40063
T.cells	PPP1R11	-0.21915	4.640461	-1.69996	0.092653	-5.00831	0.511689	0.383864
T.cells	PGPEP1L	-0.90514	-0.29843	-1.69977	0.09269	-4.19722	0.571837	0.440426
T.cells	AKAP12	-0.2055	6.169014	-1.69969	0.092705	-5.66072	0.494303	0.367947
T.cells	ENDOU	0.484185	0.913658	1.699528	0.092736	-4.53136	0.556499	0.425902
T.cells	ATP8A1	0.134157	7.601268	1.699087	0.092819	-5.6045	0.478586	0.353749
T.cells	SLC17A3	-0.81747	0.779648	-1.6989	0.092855	-4.25632	0.558254	0.427813
T.cells	BCL11A	-0.20144	5.196907	-1.69851	0.092928	-5.39386	0.505365	0.378435
T.cells	IRGM2	0.699208	2.731293	1.698486	0.092933	-4.37738	0.534282	0.405316
T.cells	GM17160	-0.70517	0.089084	-1.69797	0.093031	-4.24814	0.567285	0.436331

T.cells	FECH	0.312127	5.217188	1.696619	0.093287	-5.10348	0.505629	0.378962
T.cells	MR1	-0.70429	0.68263	-1.69658	0.093295	-4.28452	0.56002	0.429923
T.cells	NOTCH1	-0.15607	5.292946	-1.69635	0.093337	-5.23018	0.504764	0.378225
T.cells	SF3B6	-0.09945	7.294759	-1.69623	0.09336	-5.66375	0.482397	0.357723
T.cells	PMF1	0.16752	5.697613	1.695813	0.09344	-5.44338	0.500165	0.374083
T.cells	HIST1H2BJ	-0.29308	3.30978	-1.69568	0.093465	-5.08289	0.52788	0.399792
T.cells	SH3KBP1	0.115023	7.863843	1.695501	0.0935	-5.67934	0.476211	0.352191
T.cells	NT5C2	0.178817	5.867578	1.695375	0.093523	-5.39098	0.498245	0.372317
T.cells	THYN1	0.227524	3.271604	1.695366	0.093525	-4.8928	0.528335	0.400217
T.cells	INO80	-0.13688	6.666636	-1.69525	0.093547	-5.53772	0.489312	0.364165
T.cells	CDH23	0.560854	2.820164	1.695091	0.093578	-4.36563	0.533737	0.405375
T.cells	GM33370	0.815528	0.508404	1.694679	0.093656	-4.22706	0.562385	0.432447
T.cells	NUDC	-0.12636	5.698731	-1.69433	0.093722	-5.43607	0.500304	0.374309
T.cells	CDYL2	0.256986	4.757934	1.694216	0.093744	-5.01164	0.51106	0.384243
T.cells	CYP2J5	-0.80333	1.06057	-1.69135	0.094293	-4.27346	0.558432	0.427314
T.cells	COL9A3	-0.91753	0.618437	-1.69041	0.094472	-4.23558	0.564796	0.433018
T.cells	STK35	-0.20422	3.447827	-1.68978	0.094593	-4.95041	0.530338	0.400339
T.cells	GM1976	0.30328	2.701657	1.689581	0.094632	-4.72145	0.539332	0.4088
T.cells	SMYD2	-0.30047	3.028651	-1.6888	0.094782	-4.83349	0.53596	0.405467
T.cells	PWP2	-0.43604	1.891751	-1.68802	0.094932	-4.53541	0.550458	0.418836
T.cells	BHLHE40	-0.56627	6.323106	-1.68702	0.095125	-4.64147	0.498258	0.370562
T.cells	B930036N1	-0.21558	5.605823	-1.68689	0.095151	-5.3665	0.506412	0.378077
T.cells	PRODH2	-0.65803	2.255274	-1.6868	0.095168	-4.44503	0.546186	0.41507
T.cells	MRPL36	0.14805	5.835469	1.686662	0.095194	-5.43222	0.503788	0.375765
T.cells	LIMS2	-0.84636	0.424817	-1.68662	0.095202	-4.24154	0.569122	0.436804
T.cells	ZFP511	-0.17468	3.816412	-1.68583	0.095356	-5.03556	0.527896	0.397718
T.cells	PTPRM	-0.52194	5.186994	-1.68525	0.095467	-4.86077	0.512151	0.383007
T.cells	EVI5	-0.34344	5.166502	-1.68449	0.095615	-4.77357	0.512931	0.383528
T.cells	NEK4	-0.51899	1.202148	-1.68355	0.095796	-4.4382	0.561664	0.428556
T.cells	SLC35E4	0.833543	0.657751	1.682521	0.095996	-4.2523	0.568601	0.435521
T.cells	GM30541	0.660722	0.952964	1.682504	0.096	-4.28685	0.564845	0.431964
T.cells	ST13	-0.10567	6.987135	-1.6824	0.09602	-5.5963	0.49294	0.365209
T.cells	PRDX5	0.288075	7.635313	1.682343	0.096031	-5.49661	0.48575	0.358692
T.cells	ARHGAP12	0.173155	5.076663	1.682324	0.096035	-5.25415	0.514723	0.385174
T.cells	PPP1R15A	-0.25557	7.089434	-1.68207	0.096084	-5.41007	0.491812	0.364282
T.cells	CKAP4	-0.43187	4.030233	-1.68136	0.096223	-4.61734	0.527552	0.396904
T.cells	A630089N(	0.291261	2.672384	1.680682	0.096354	-4.82949	0.544432	0.412498
T.cells	RBM38	-0.14569	6.898184	-1.68003	0.096483	-5.67949	0.494892	0.366839
T.cells	LEPR	-0.90204	1.823369	-1.67982	0.096523	-4.28708	0.554977	0.422486
T.cells	RASL11A	-0.91844	0.28073	-1.6798	0.096526	-4.23252	0.574543	0.440986
T.cells	SOD1	-0.16523	5.993562	-1.67954	0.096577	-5.37117	0.505135	0.376286
T.cells	NUP210	-0.13764	5.041054	-1.6793	0.096625	-5.42547	0.516136	0.386457
T.cells	CTNNA1	-0.16066	6.643473	-1.67897	0.096688	-5.50624	0.497756	0.36974
T.cells	SLC25A21	-0.89475	1.867089	-1.67897	0.09669	-4.37443	0.554431	0.422305
T.cells	KLRD1	-0.85209	3.767425	-1.678	0.096878	-4.39167	0.531673	0.400706
T.cells	4921509O(	1.064315	0.064578	1.677892	0.0969	-4.22311	0.577853	0.444208
T.cells	ZBTB33	0.263544	2.982117	1.677605	0.096956	-4.76797	0.541171	0.409713

T.cells	ST5	-0.42981	2.235439	-1.67719	0.097038	-4.5505	0.550348	0.418507
T.cells	HSPG2	-0.51639	2.741833	-1.67685	0.097104	-4.44678	0.544109	0.4128
T.cells	FAM204A	0.136623	4.894011	1.676569	0.097159	-5.30817	0.518318	0.388859
T.cells	FAM210A	0.214812	3.636981	1.676196	0.097233	-4.92178	0.53324	0.402814
T.cells	GGA3	0.217982	3.302395	1.676169	0.097238	-4.87971	0.537278	0.406582
T.cells	IGF1	-0.49851	5.292109	-1.67553	0.097363	-4.91518	0.513675	0.384778
T.cells	ABCC4	-0.27407	4.55376	-1.67551	0.097368	-4.77732	0.522318	0.392769
T.cells	SMIM3	0.190769	4.840554	1.67547	0.097375	-5.21136	0.518945	0.389646
T.cells	TSFM	-0.16933	4.258618	-1.67545	0.09738	-5.16511	0.525811	0.396009
T.cells	RPAP2	0.270191	3.02458	1.675413	0.097387	-4.76698	0.540653	0.409847
T.cells	TRF	-0.37607	9.658999	-1.67389	0.097687	-5.85397	0.466419	0.341115
T.cells	BBS5	0.633638	0.624034	1.67358	0.097748	-4.37904	0.572076	0.438693
T.cells	TAGLN	0.836977	2.304796	1.673012	0.09786	-4.40853	0.550867	0.418921
T.cells	1700019L1	0.860095	0.774577	1.672939	0.097874	-4.26585	0.570146	0.437141
T.cells	KDM3B	0.116392	6.131762	1.672813	0.097899	-5.48442	0.505272	0.376638
T.cells	RAB27B	-0.97718	0.615378	-1.67258	0.097946	-4.24549	0.572187	0.439223
T.cells	2900060B1	-0.30764	2.614995	-1.67228	0.098005	-4.71562	0.547033	0.415634
T.cells	NTPCR	0.248961	3.847172	1.672267	0.098007	-4.92227	0.532047	0.401626
T.cells	LRCH3	0.137817	6.497112	1.67188	0.098084	-5.46385	0.501227	0.3732
T.cells	TMEM265	-0.21031	3.958769	-1.67168	0.098124	-5.06554	0.530833	0.400596
T.cells	CCDC102A	-0.65772	2.048525	-1.67115	0.098228	-4.35122	0.554503	0.422705
T.cells	PTMA	-0.10577	11.35991	-1.67083	0.098291	-6.42334	0.449157	0.326332
T.cells	ID3	-0.18682	6.599763	-1.66995	0.098466	-5.57955	0.501083	0.372799
T.cells	HIST1H2AB	0.456438	1.438759	1.669418	0.098572	-4.79936	0.563316	0.430559
T.cells	IFI208	0.510187	2.936618	1.668988	0.098657	-4.68568	0.544855	0.413147
T.cells	FKBP5	0.220096	5.30259	1.668433	0.098768	-5.32798	0.516649	0.386882
T.cells	LRRCC1	0.151009	3.748019	1.668389	0.098776	-5.16436	0.535112	0.403992
T.cells	TSHZ2	-0.61582	3.393266	-1.66766	0.098922	-4.56186	0.53995	0.408135
T.cells	PMPCB	-0.16089	4.974651	-1.66673	0.099107	-5.29961	0.521292	0.390885
T.cells	SGSM3	-0.26076	4.063557	-1.66661	0.099131	-4.96781	0.532131	0.400968
T.cells	FBXO7	0.198341	4.165071	1.666366	0.09918	-5.09435	0.530913	0.399882
T.cells	GM32296	0.935664	-0.65404	1.665836	0.099286	-4.23526	0.591679	0.457534
T.cells	REL	-0.17379	8.12408	-1.6657	0.099313	-5.69339	0.485404	0.35842
T.cells	CSTF1	0.208343	3.243385	1.665497	0.099353	-4.97079	0.542068	0.410578
T.cells	MPZL3	0.581338	1.727945	1.665091	0.099434	-4.41963	0.560883	0.428438
T.cells	PPM1K	-0.27416	3.579795	-1.66506	0.099442	-4.9274	0.537971	0.406939
T.cells	DTX3L	0.327788	4.876216	1.664954	0.099462	-5.08848	0.522453	0.392573
T.cells	SH3BP2	0.432433	3.288629	1.664202	0.099613	-4.61763	0.541515	0.410484
T.cells	TBC1D8	-0.41325	5.179428	-1.66369	0.099714	-4.71835	0.518885	0.389439
T.cells	TNFAIP8L1	-0.34821	2.396525	-1.66368	0.099717	-4.63944	0.552509	0.420799
T.cells	ZFP943	0.152811	4.280266	1.663644	0.099724	-5.1669	0.529534	0.39931
T.cells	SCPEP1	-0.25828	5.169388	-1.66363	0.099727	-4.96754	0.519003	0.389548
T.cells	NDUFAF5	-0.2911	2.563951	-1.66341	0.099772	-4.77243	0.55043	0.418878
T.cells	GGPS1	0.127036	5.393996	1.663242	0.099805	-5.31066	0.516373	0.387203
T.cells	AATK	-0.95917	0.48294	-1.66304	0.099847	-4.25026	0.576791	0.443952
T.cells	SPATA2L	0.80432	0.521707	1.662707	0.099912	-4.28048	0.576289	0.443544
T.cells	ADCY7	0.178823	5.583087	1.662589	0.099936	-5.20057	0.51417	0.385337

T.cells	MST1	-0.89774	0.649079	-1.66226	0.100003	-4.28423	0.574644	0.4422
T.cells	ZFP652	0.193752	5.654413	1.661895	0.100076	-5.27991	0.513341	0.384808
T.cells	RAB10	0.136114	7.670572	1.661888	0.100077	-5.64788	0.490424	0.363805
T.cells	TMC8	-0.28671	3.820781	-1.66188	0.100078	-4.80467	0.535054	0.404961
T.cells	GDI1	0.175887	4.991215	1.661582	0.100139	-5.25454	0.521097	0.392075
T.cells	GCKR	-0.70879	1.581851	-1.66149	0.100158	-4.35051	0.562729	0.431087
T.cells	FARSA	-0.13961	4.90554	-1.66119	0.100218	-5.29029	0.522174	0.393052
T.cells	CPN1	-0.63641	1.600653	-1.66035	0.100386	-4.37203	0.563063	0.431162
T.cells	TREM1	0.778168	1.639916	1.660277	0.100401	-4.33807	0.562566	0.430735
T.cells	HPN	-0.60211	2.642028	-1.65938	0.100582	-4.41485	0.550753	0.419056
T.cells	SLC38A9	0.182316	5.16902	1.658121	0.100837	-5.26847	0.521297	0.391101
T.cells	LDHB	0.618614	3.00278	1.657838	0.100894	-4.44513	0.547473	0.415472
T.cells	RASAL1	-0.54205	0.712454	-1.65731	0.101002	-4.53327	0.576505	0.442993
T.cells	D730003I1	0.382267	2.591958	1.657105	0.101043	-4.5812	0.55265	0.420465
T.cells	SPECC1L	0.202814	5.413581	1.657057	0.101053	-5.26398	0.518554	0.388709
T.cells	DEF8	-0.42135	2.04717	-1.65649	0.101168	-4.49196	0.559847	0.427098
T.cells	H6PD	-0.38846	2.776773	-1.6562	0.101226	-4.53562	0.550779	0.418698
T.cells	CCT3	-0.11715	5.801241	-1.65567	0.101334	-5.49543	0.514506	0.385106
T.cells	MORF4L1	-0.06984	8.201561	-1.65566	0.101337	-5.80356	0.487267	0.360216
T.cells	STAT1	0.47936	6.871703	1.655191	0.101431	-5.38972	0.502208	0.37386
T.cells	EEF1A1	-0.09431	10.55641	-1.65473	0.101524	-6.16885	0.461902	0.337586
T.cells	2610203C2	-0.92555	0.354453	-1.65444	0.101584	-4.25559	0.581724	0.448519
T.cells	SLFN8	0.630788	3.326481	1.653548	0.101766	-4.52799	0.54411	0.413297
T.cells	TAPT1	0.150735	5.802801	1.652923	0.101893	-5.52325	0.514511	0.386151
T.cells	PAQR9	-0.48695	4.029832	-1.65286	0.101906	-4.65014	0.535545	0.405694
T.cells	NMNAT1	-0.60788	1.326233	-1.65251	0.101978	-4.43401	0.569168	0.437589
T.cells	ZFP945	0.2578	3.17689	1.65247	0.101986	-4.87129	0.545948	0.415639
T.cells	RBM15	-0.12191	5.696792	-1.65215	0.10205	-5.50506	0.515746	0.387671
T.cells	PRAF2	-0.30203	2.133854	-1.65185	0.102113	-4.67183	0.558925	0.428191
T.cells	GPSM3	0.171782	6.022347	1.651718	0.10214	-5.29028	0.511961	0.384272
T.cells	HOXA7	0.589691	-0.09629	1.651566	0.102171	-4.42568	0.587633	0.455666
T.cells	SLC7A11	1.061178	4.278698	1.651479	0.102188	-4.48972	0.532544	0.403502
T.cells	GM36738	0.412606	3.095803	1.650929	0.102301	-4.56791	0.546947	0.417212
T.cells	FAM131A	0.832196	0.401139	1.650907	0.102306	-4.3131	0.581115	0.449663
T.cells	MN1	0.869263	0.308399	1.650793	0.102329	-4.27468	0.582325	0.45085
T.cells	ALG11	0.288141	2.610001	1.650738	0.10234	-4.70083	0.552966	0.422922
T.cells	RAB27A	0.22273	4.50985	1.65071	0.102346	-5.07003	0.529772	0.401143
T.cells	CHST2	0.976306	-0.02925	1.650158	0.102459	-4.26501	0.586751	0.455312
T.cells	VDAC3	-0.10712	7.191815	-1.65006	0.102479	-5.73021	0.498577	0.372454
T.cells	SERINC4	0.742064	0.365548	1.650016	0.102488	-4.31163	0.581579	0.45035
T.cells	CCNL1	-0.12045	7.09056	-1.64998	0.102495	-5.56831	0.499723	0.373506
T.cells	HSPD1	-0.14603	7.46114	-1.64981	0.102531	-5.80835	0.495543	0.369732
T.cells	BLOC1S4	0.20534	3.695064	1.6496	0.102573	-4.98614	0.539606	0.410681
T.cells	MS4A6C	0.780657	5.165825	1.649482	0.102598	-4.62751	0.521977	0.394243
T.cells	DHFR	-0.21976	4.331798	-1.64863	0.102773	-5.32189	0.531906	0.403732
T.cells	CHMP2A	0.125178	6.544809	1.648608	0.102777	-5.48884	0.50594	0.379609
T.cells	PLSCR3	0.176201	4.301027	1.648551	0.102789	-5.30014	0.532276	0.404089

T.cells	2510002D2	0.260848	3.199654	1.648484	0.102803	-4.88833	0.545668	0.41667
T.cells	PNISR	0.114911	5.773836	1.648255	0.10285	-5.42842	0.514848	0.387944
T.cells	S100A4	1.026912	2.87429	1.64788	0.102927	-4.31694	0.549684	0.4206
T.cells	CDH5	-0.55058	4.480665	-1.64775	0.102953	-4.69592	0.530121	0.402279
T.cells	SEMA7A	-0.50931	2.017527	-1.64775	0.102954	-4.5029	0.56039	0.430815
T.cells	CHRAC1	0.135811	5.264928	1.647213	0.103064	-5.3448	0.520809	0.39373
T.cells	TRMT6	-0.16379	4.373913	-1.6471	0.103087	-5.22383	0.531401	0.403637
T.cells	LSM6	-0.13169	6.681324	-1.64699	0.10311	-5.68068	0.504378	0.378523
T.cells	CDCA5	-0.26161	3.028781	-1.64687	0.103134	-5.10298	0.547773	0.419034
T.cells	RBPMS	0.207695	5.842983	1.646802	0.103149	-5.41698	0.514043	0.387478
T.cells	PMPCA	-0.18946	4.024403	-1.64651	0.103208	-5.14342	0.535619	0.407708
T.cells	ITGA5	0.348838	3.54178	1.646258	0.103261	-4.86363	0.541484	0.413375
T.cells	RNASEH1	-0.24714	2.84733	-1.6461	0.103293	-4.82699	0.550027	0.421469
T.cells	UBE3A	0.11431	6.387077	1.645742	0.103367	-5.50043	0.507854	0.381956
T.cells	CEP95	0.16836	4.108354	1.645476	0.103422	-5.13364	0.534705	0.407086
T.cells	URGCP	0.210262	4.434418	1.64509	0.103502	-5.13676	0.530783	0.40343
T.cells	2210016F1	-0.16005	4.954824	-1.64509	0.103503	-5.20816	0.524579	0.397628
T.cells	SLC22A23	-0.77646	3.017213	-1.64416	0.103695	-4.43505	0.548344	0.420191
T.cells	FASTKD2	-0.21418	2.952537	-1.64396	0.103736	-4.92158	0.549144	0.420986
T.cells	CDKN1A	0.233517	5.267608	1.643901	0.103748	-5.29647	0.521184	0.394722
T.cells	USP40	-0.28867	3.64171	-1.64388	0.103753	-4.94584	0.540678	0.412998
T.cells	RABGAP1L	-0.14735	7.908574	-1.6436	0.10381	-5.76396	0.490967	0.366881
T.cells	CPT1A	0.137413	5.446894	1.642763	0.103984	-5.3712	0.519498	0.39315
T.cells	HUS1	0.36884	1.653586	1.642259	0.104089	-4.63805	0.565898	0.437045
T.cells	H2AFJ	0.115618	7.521162	1.641972	0.104148	-5.67929	0.495659	0.371336
T.cells	PNO1	-0.17356	4.446222	-1.64186	0.104172	-5.28386	0.531381	0.404572
T.cells	GM37240	0.187165	5.162317	1.641681	0.104209	-5.36515	0.522852	0.396643
T.cells	NRP2	-0.81354	3.300945	-1.64164	0.104216	-4.42029	0.545293	0.417734
T.cells	LRP5	-0.23529	4.078112	-1.64157	0.104231	-5.10522	0.535816	0.408796
T.cells	ROBO2	-0.97253	0.744725	-1.6414	0.104266	-4.30601	0.577572	0.44852
T.cells	GM31597	0.360295	2.208627	1.640323	0.104491	-4.61392	0.559155	0.431008
T.cells	SMAP1	0.135571	6.875842	1.640216	0.104513	-5.52922	0.503213	0.378544
T.cells	5330417C2	-0.82076	0.541978	-1.63967	0.104627	-4.31694	0.580496	0.45183
T.cells	FPGT	0.636004	1.345954	1.639563	0.10465	-4.42535	0.570109	0.441861
T.cells	SMPD5	-0.81091	-0.18019	-1.63917	0.104732	-4.2659	0.589973	0.461134
T.cells	RPF2	-0.19603	4.402771	-1.63865	0.104841	-5.22184	0.532168	0.406159
T.cells	PHLDB3	0.216759	2.97973	1.638501	0.104871	-5.05916	0.549529	0.422657
T.cells	SNX24	-0.47487	4.299252	-1.63799	0.104978	-4.7165	0.533413	0.407536
T.cells	ZKSCAN17	0.18982	3.498798	1.637739	0.10503	-5.11316	0.543136	0.416742
T.cells	N4BP3	-0.22958	2.955418	-1.63747	0.105087	-5.01564	0.54983	0.423224
T.cells	ITPKB	0.180243	7.462345	1.637415	0.105098	-5.52546	0.496568	0.373332
T.cells	TCF4	0.158633	7.694523	1.637383	0.105105	-5.74563	0.49396	0.370934
T.cells	GSTO1	-0.17684	4.594828	-1.63719	0.105145	-5.09965	0.529864	0.404455
T.cells	RCC2	-0.11385	6.222377	-1.63706	0.105172	-5.61096	0.510716	0.386552
T.cells	CSF1R	-0.56791	5.549681	-1.63694	0.105197	-4.87173	0.51855	0.39386
T.cells	KAT14	0.161624	3.838268	1.636892	0.105208	-5.1462	0.538993	0.413078
T.cells	RSPO3	-1.27084	0.723734	-1.63683	0.10522	-4.29372	0.578133	0.45048

T.cells	DNAJB13	0.379652	2.103334	1.636242	0.105344	-4.65245	0.560482	0.433833
T.cells	BTBD2	-0.25532	3.477347	-1.63609	0.105376	-4.89822	0.543399	0.417654
T.cells	RDH10	-0.30563	3.207433	-1.63596	0.105402	-4.84162	0.546716	0.420851
T.cells	LAMTOR4	0.16609	6.205335	1.635864	0.105423	-5.43292	0.510913	0.387164
T.cells	ISCA1	0.224111	6.018689	1.635418	0.105517	-5.37158	0.513077	0.389321
T.cells	CPA6	0.813267	-0.07229	1.635414	0.105518	-4.30598	0.588548	0.46119
T.cells	TCTEX1D2	-0.255	3.301638	-1.635	0.105605	-4.93957	0.545556	0.42007
T.cells	ZRSR1	0.385792	2.211301	1.634637	0.105681	-4.6216	0.559121	0.433031
T.cells	HGFAC	-0.64258	1.432763	-1.63463	0.105682	-4.4122	0.568998	0.442506
T.cells	PQLC2	0.326314	3.590318	1.634476	0.105715	-4.76809	0.542016	0.416769
T.cells	VEGFC	-0.61738	1.200834	-1.63413	0.105787	-4.41793	0.571971	0.445424
T.cells	MRPL42	-0.15177	6.521235	-1.63409	0.105795	-5.63548	0.507272	0.384131
T.cells	PHKA2	0.23907	3.438409	1.634058	0.105803	-5.01752	0.543876	0.418553
T.cells	SART3	-0.12759	5.374984	-1.63389	0.105839	-5.47742	0.520603	0.396664
T.cells	SMIM4	0.154232	5.255277	1.633852	0.105846	-5.29435	0.522014	0.397998
T.cells	ALDH4A1	-0.43445	2.912017	-1.63349	0.105922	-4.59801	0.550368	0.424882
T.cells	SNRNP35	0.232772	3.056704	1.633119	0.106	-4.97425	0.548577	0.423327
T.cells	TARS	-0.1708	4.374393	-1.63311	0.106003	-5.24585	0.532509	0.408084
T.cells	TOP3B	0.178096	3.549805	1.632897	0.106047	-5.07753	0.542512	0.417599
T.cells	1700034P1	-0.61136	1.464772	-1.63273	0.106081	-4.5109	0.568589	0.442598
T.cells	MTA1	-0.15638	4.55479	-1.63235	0.106163	-5.30831	0.530515	0.406328
T.cells	HELLS	-0.21613	4.919254	-1.63194	0.106249	-5.49585	0.526355	0.402472
T.cells	MSS51	0.321662	2.436698	1.63167	0.106306	-4.78044	0.55672	0.431323
T.cells	GM7030	0.432505	2.084686	1.631164	0.106413	-4.77448	0.561348	0.435717
T.cells	ZFP277	0.132621	5.309649	1.630979	0.106452	-5.39225	0.52196	0.398394
T.cells	SYNGR2	0.171151	6.111593	1.630622	0.106528	-5.35279	0.512575	0.389697
T.cells	CLCF1	-0.37746	2.650892	-1.63059	0.106535	-4.7173	0.554238	0.429102
T.cells	FAM71F2	0.81064	2.260769	1.62929	0.10681	-4.36537	0.560319	0.434066
T.cells	ZFP931	-0.50944	1.514653	-1.62895	0.106881	-4.41794	0.56993	0.443312
T.cells	MCM7	-0.20328	5.384269	-1.62859	0.106959	-5.56361	0.522452	0.398149
T.cells	LRP12	0.480395	2.191669	1.628288	0.107022	-4.64913	0.561554	0.43519
T.cells	RAB8B	0.196026	7.724426	1.627853	0.107115	-5.55574	0.495761	0.373238
T.cells	KLHL9	0.192177	4.65586	1.627307	0.107231	-5.13955	0.531619	0.406681
T.cells	GPR171	-0.27634	4.027921	-1.62722	0.10725	-5.20283	0.539211	0.413852
T.cells	ARL8B	-0.12542	5.97123	-1.62696	0.107305	-5.42335	0.516074	0.392058
T.cells	ZFP777	0.22726	2.809648	1.6264	0.107424	-4.91818	0.554342	0.428328
T.cells	DNAJC21	0.125581	5.890572	1.626323	0.10744	-5.60427	0.517088	0.393146
T.cells	PTP4A3	0.130292	5.77385	1.626053	0.107498	-5.54924	0.518456	0.394458
T.cells	P2RY12	0.627701	2.616799	1.625831	0.107545	-4.45685	0.556756	0.430823
T.cells	FAM3C	-0.15635	5.36557	-1.62563	0.107588	-5.29195	0.523266	0.399129
T.cells	KAT2B	0.144326	6.221283	1.62554	0.107607	-5.59435	0.513232	0.389751
T.cells	L1CAM	-0.43722	3.644556	-1.6248	0.107764	-4.6998	0.544233	0.418962
T.cells	CEBPD	0.452916	3.956744	1.624635	0.1078	-4.57685	0.540413	0.415354
T.cells	DCAF12	0.151508	6.638146	1.624415	0.107847	-5.522	0.508622	0.385555
T.cells	CMTM3	-0.37568	3.593966	-1.62439	0.107853	-4.66654	0.544855	0.419656
T.cells	MEIS3	0.862858	0.488054	1.624123	0.10791	-4.28785	0.584291	0.457603
T.cells	1-Mar	-0.64537	1.457886	-1.62374	0.107992	-4.42541	0.571702	0.4456

T.cells	E2F1	-0.19076	4.409532	-1.6231	0.108128	-5.35491	0.534917	0.410805
T.cells	NPL	-0.59191	2.535175	-1.62275	0.108203	-4.53673	0.558013	0.432823
T.cells	STARD3	0.232633	4.116736	1.622641	0.108227	-5.03537	0.538465	0.414224
T.cells	E030042O2	0.794983	0.180529	1.622337	0.108292	-4.35562	0.588335	0.462231
T.cells	NEAT1	-0.2716	8.674976	-1.62228	0.108303	-5.53136	0.485662	0.364943
T.cells	RIC8B	0.174737	4.502862	1.622074	0.108348	-5.20201	0.533791	0.409954
T.cells	GUK1	-0.16472	4.908743	-1.62195	0.108375	-5.27249	0.528919	0.405354
T.cells	ZMYND11	0.121038	6.466516	1.621895	0.108387	-5.55786	0.510603	0.388159
T.cells	ERCC5	0.271286	2.909402	1.621729	0.108422	-4.81024	0.553329	0.42862
T.cells	SMIM41	0.391908	0.89661	1.621511	0.108469	-4.69676	0.578957	0.453332
T.cells	ALDH7A1	-0.34609	3.227154	-1.62144	0.108484	-4.78572	0.54938	0.424901
T.cells	CLN6	0.279609	3.482895	1.621301	0.108514	-4.89975	0.546221	0.421901
T.cells	NDUFB4	-0.12278	6.598671	-1.62092	0.108597	-5.61636	0.509183	0.387032
T.cells	STX7	0.129933	6.176182	1.620466	0.108694	-5.42722	0.514077	0.391774
T.cells	GM48302	-0.83732	1.63727	-1.62036	0.108716	-4.32516	0.569519	0.444555
T.cells	ENTPD1	0.233869	6.9129	1.619898	0.108816	-5.44706	0.505571	0.38396
T.cells	SAG	-0.36481	3.5306	-1.61938	0.108927	-4.86232	0.545747	0.421987
T.cells	ADCY10	-0.66779	0.912909	-1.61924	0.108958	-4.4044	0.578865	0.453849
T.cells	CRYBG2	-0.61063	0.051796	-1.61907	0.108995	-4.41411	0.590158	0.464871
T.cells	UIMC1	0.123137	5.853468	1.618992	0.109011	-5.51371	0.517846	0.395598
T.cells	TMEM123	0.153395	6.484305	1.618295	0.109161	-5.54848	0.510503	0.388845
T.cells	EML1	-0.79559	1.303195	-1.61816	0.10919	-4.33874	0.573812	0.44918
T.cells	RFTN2	-0.34365	2.237638	-1.61791	0.109244	-4.82533	0.561879	0.43767
T.cells	CNPY4	-0.27252	2.433697	-1.61785	0.109257	-4.85072	0.559405	0.43529
T.cells	IPCEF1	0.26498	4.679593	1.617734	0.109282	-5.25324	0.531775	0.408918
T.cells	ABCD2	-0.59468	2.633823	-1.61747	0.109338	-4.45596	0.55689	0.432874
T.cells	MAP2	-0.66026	0.187932	-1.61743	0.109347	-4.39809	0.58836	0.463319
T.cells	TSIX	-3.07441	1.906429	-1.61723	0.109391	-4.46131	0.566082	0.441782
T.cells	NAA60	0.165493	4.697726	1.617047	0.109431	-5.26258	0.531557	0.408806
T.cells	HACD2	0.129283	5.732831	1.617022	0.109436	-5.47836	0.519261	0.397194
T.cells	ZKSCAN6	0.346332	2.942031	1.616968	0.109448	-4.75409	0.553037	0.429291
T.cells	GM47730	0.543621	-0.65913	1.615935	0.109671	-4.44166	0.599634	0.474812
T.cells	LNCPINT	-0.3051	8.328661	-1.61589	0.109681	-5.49921	0.489596	0.36979
T.cells	M6PR	0.143699	6.293265	1.615862	0.109687	-5.48735	0.512716	0.391346
T.cells	HAUS3	0.148389	4.199727	1.615734	0.109715	-5.28728	0.537569	0.41484
T.cells	EIF4B	-0.12016	6.136378	-1.61552	0.109761	-5.55082	0.514541	0.393112
T.cells	TMEM191(	-0.34657	1.703176	-1.61522	0.109826	-4.58191	0.568676	0.444825
T.cells	TIPIN	-0.19861	5.238466	-1.61517	0.109836	-5.55165	0.5251	0.403152
T.cells	JADE3	0.198434	3.471551	1.615159	0.109839	-5.08827	0.546475	0.423475
T.cells	GM26740	0.179065	7.068845	1.614762	0.109925	-5.59762	0.503787	0.383201
T.cells	TUBB5	-0.1574	9.849372	-1.61451	0.10998	-6.28571	0.472973	0.354705
T.cells	EPHA4	-1.04113	0.083978	-1.61449	0.109984	-4.29752	0.589733	0.465427
T.cells	WDFY1	0.286118	4.332443	1.614198	0.110048	-4.86692	0.535961	0.413679
T.cells	PRKCD	-0.12576	6.897294	-1.61378	0.110139	-5.63072	0.505749	0.385268
T.cells	PBX1	-0.29345	5.465012	-1.61322	0.110262	-5.1421	0.522417	0.401128
T.cells	XLR	0.764057	2.01867	1.613113	0.110284	-4.4436	0.564655	0.441532
T.cells	MRPS6	-0.14906	6.008603	-1.6129	0.11033	-5.47988	0.516031	0.395174

T.cells	G6PC	-0.78114	1.911843	-1.61286	0.11034	-4.45671	0.566013	0.442896
T.cells	LSM10	-0.32958	3.07773	-1.61282	0.110348	-4.73776	0.551348	0.428764
T.cells	SPRY1	0.682244	1.345776	1.612774	0.110358	-4.41909	0.573263	0.449935
T.cells	RNF213	0.4124	6.151249	1.612123	0.110499	-5.19284	0.51448	0.393816
T.cells	CCNH	0.127428	5.363256	1.611878	0.110553	-5.48636	0.523735	0.402596
T.cells	PCM1	0.121208	6.494399	1.611623	0.110608	-5.66576	0.510498	0.390226
T.cells	ROGDI	-0.33774	4.014028	-1.61128	0.110684	-4.74614	0.539945	0.418264
T.cells	GM4924	0.654124	0.278075	1.610809	0.110786	-4.38749	0.5873	0.464135
T.cells	ZDHHC15	0.409543	1.125862	1.610632	0.110825	-4.70097	0.576229	0.453374
T.cells	4933439C1	0.433983	1.200712	1.610315	0.110894	-4.61768	0.575261	0.452506
T.cells	TRIM21	0.401042	2.57157	1.610307	0.110895	-4.73602	0.557793	0.435585
T.cells	SP140	0.269375	6.188606	1.610098	0.110941	-5.18175	0.514045	0.393895
T.cells	ZCCHC2	0.181743	5.422407	1.609988	0.110965	-5.35075	0.523035	0.402439
T.cells	VIPR1	-0.87924	0.964939	-1.60992	0.110979	-4.31671	0.578316	0.455569
T.cells	BRD3	0.131083	5.712362	1.609908	0.110983	-5.56811	0.519616	0.399203
T.cells	RANBP1	-0.14534	7.239938	-1.6096	0.111051	-5.85488	0.501947	0.382728
T.cells	CCDC167	-0.19936	4.065969	-1.60947	0.111078	-5.1914	0.539313	0.41811
T.cells	R3HDM2	0.106492	6.193799	1.609369	0.111101	-5.54117	0.513985	0.394081
T.cells	SCYL2	0.169871	4.409687	1.608963	0.11119	-5.19584	0.535226	0.414254
T.cells	TCIRG1	0.177308	5.455039	1.608651	0.111258	-5.37433	0.522729	0.402423
T.cells	WLS	0.264307	4.263574	1.608131	0.111372	-5.08734	0.536994	0.416078
T.cells	UBA5	-0.16088	4.489487	-1.60809	0.11138	-5.14707	0.534262	0.41347
T.cells	GM26782	0.22763	2.991571	1.608035	0.111393	-4.8893	0.552625	0.43107
T.cells	KMT5A	0.117002	5.846575	1.60775	0.111455	-5.57763	0.518119	0.398235
T.cells	NAA80	0.328318	2.402845	1.606831	0.111657	-4.69799	0.560002	0.438633
T.cells	POLG	-0.18579	3.808912	-1.60674	0.111678	-5.21991	0.542533	0.421812
T.cells	LPL	-0.46664	5.132487	-1.60645	0.11174	-4.87907	0.526555	0.406611
T.cells	IFT57	0.282969	2.891644	1.606424	0.111747	-4.8633	0.553871	0.432777
T.cells	BAG1	-0.10869	6.693589	-1.60629	0.111776	-5.64561	0.508278	0.389353
T.cells	ZFP654	0.153619	6.120389	1.606136	0.11181	-5.56856	0.514919	0.395668
T.cells	PIGB	-0.34963	2.703863	-1.60535	0.111982	-4.69592	0.556219	0.435436
T.cells	HSD17B13	-0.56583	2.671988	-1.6052	0.112016	-4.6021	0.556618	0.435887
T.cells	WDR49	-0.66184	0.34569	-1.60512	0.112034	-4.40914	0.5865	0.465007
T.cells	AC125149.	1.037846	-0.34803	1.605048	0.112049	-4.3023	0.595692	0.474066
T.cells	SLC39A4	-0.72363	1.149584	-1.6047	0.112125	-4.35783	0.57601	0.454877
T.cells	PIK3CD	0.156541	6.624599	1.604346	0.112204	-5.6446	0.509073	0.390681
T.cells	ZNHIT3	-0.18135	3.532147	-1.60431	0.112212	-5.19641	0.545931	0.425804
T.cells	GM11110	0.408362	1.970897	1.604184	0.11224	-4.568	0.565472	0.444734
T.cells	DYRK1B	0.369989	1.826196	1.604169	0.112243	-4.6151	0.567316	0.446527
T.cells	B4GALT5	0.240816	5.975954	1.604043	0.112271	-5.27292	0.516605	0.397888
T.cells	EEF1B2	-0.10455	8.556525	-1.60384	0.112315	-6.00303	0.487252	0.370312
T.cells	PADI6	-0.71003	0.058128	-1.60376	0.112334	-4.41755	0.590294	0.469079
T.cells	COL4A3BP	0.136686	6.352484	1.603733	0.112339	-5.55967	0.51222	0.393741
T.cells	BAZ2B	-0.10597	8.430417	-1.60362	0.112364	-5.92556	0.488649	0.371653
T.cells	EGLN1	0.138114	5.631732	1.60319	0.112459	-5.52233	0.520863	0.401991
T.cells	GALK2	0.153434	4.897213	1.602532	0.112605	-5.32357	0.530045	0.410592
T.cells	MYCBP2	0.1502	7.768752	1.601705	0.112788	-5.72532	0.497093	0.379442

T.cells	RPIA	-0.13784	5.458024	-1.60167	0.112795	-5.52631	0.523803	0.404653
T.cells	MCRIP1	0.108511	5.919349	1.601448	0.112845	-5.55881	0.518374	0.399579
T.cells	TNFSF11	-0.52465	0.706959	-1.60093	0.112959	-4.77929	0.583353	0.462048
T.cells	VPS33A	0.193053	4.291318	1.600473	0.113061	-5.10025	0.538182	0.41829
T.cells	ADGRL2	-0.34749	4.208198	-1.60044	0.113068	-5.06122	0.539193	0.419259
T.cells	IGHM	-0.23955	8.158942	-1.59995	0.113178	-6.06518	0.493261	0.375784
T.cells	WASHC3	0.162412	4.517912	1.599807	0.113209	-5.21635	0.535648	0.415913
T.cells	RBBP6	-0.10594	7.269431	-1.59952	0.113272	-5.73422	0.503379	0.385375
T.cells	YTHDC2	0.167243	4.693584	1.598955	0.113398	-5.3234	0.533965	0.414259
T.cells	GM17276	0.753279	0.785789	1.597756	0.113665	-4.40092	0.583412	0.462183
T.cells	ECI2	0.200839	4.557161	1.597286	0.11377	-5.23547	0.535904	0.416204
T.cells	HEXDC	0.440938	1.86348	1.597092	0.113813	-4.7174	0.569453	0.448651
T.cells	SRPK3	-0.3895	1.292575	-1.59705	0.113823	-4.68524	0.576809	0.455827
T.cells	ZFP598	-0.22763	3.44196	-1.59704	0.113824	-5.04881	0.549564	0.429379
T.cells	PYGL	0.388819	5.16283	1.597029	0.113827	-4.92283	0.528619	0.409296
T.cells	GM27008	0.431198	1.012976	1.596923	0.113851	-4.59055	0.580443	0.459391
T.cells	CDK4	-0.12933	5.844705	-1.59685	0.113868	-5.64081	0.520529	0.401626
T.cells	GLRX	0.167626	6.214402	1.596315	0.113987	-5.58535	0.516192	0.397559
T.cells	USHBP1	-0.689	1.085061	-1.59624	0.114003	-4.38722	0.579504	0.458546
T.cells	GSDMD	0.286799	4.268046	1.596181	0.114017	-5.02816	0.539415	0.419696
T.cells	NENF	-0.19198	4.583677	-1.59565	0.114136	-5.10829	0.535583	0.416229
T.cells	KIF3B	0.255515	3.495441	1.595334	0.114206	-4.87078	0.548901	0.429113
T.cells	RAPH1	-0.27244	5.055385	-1.59529	0.114216	-5.01756	0.529905	0.41088
T.cells	IFITM6	0.993921	3.518134	1.595189	0.114239	-4.44346	0.548621	0.428843
T.cells	TRAC	-0.73119	2.266621	-1.59518	0.114241	-4.4748	0.564311	0.444036
T.cells	PANK3	0.15733	4.591077	1.59501	0.114279	-5.27214	0.535494	0.416291
T.cells	SSU72	0.102062	6.693565	1.594563	0.114379	-5.65444	0.510853	0.392785
T.cells	PRORSD1	0.155521	4.586531	1.593263	0.11467	-5.32659	0.535982	0.417019
T.cells	RASA4	0.276599	4.228858	1.59309	0.114709	-5.2235	0.540329	0.421192
T.cells	TASOR2	0.154211	5.373883	1.592923	0.114746	-5.42685	0.526528	0.407978
T.cells	MCMDC2	-0.47855	3.465667	-1.59291	0.11475	-4.67434	0.549714	0.430234
T.cells	ITK	-0.82346	4.224474	-1.59285	0.114762	-4.61594	0.540382	0.421243
T.cells	WWC1	-0.54588	1.216205	-1.59284	0.114765	-4.44348	0.578267	0.458007
T.cells	RAB3D	0.448027	3.11921	1.59272	0.114792	-4.60831	0.554025	0.434433
T.cells	HAUS7	-0.22726	2.992661	-1.59217	0.114916	-4.9938	0.555607	0.436135
T.cells	MOB4	0.09519	6.832732	1.592137	0.114923	-5.75538	0.509425	0.39192
T.cells	E2F2	-0.15567	4.99405	-1.59203	0.114948	-5.67789	0.531069	0.412505
T.cells	ACAP2	0.135988	7.320314	1.591995	0.114955	-5.72218	0.503827	0.386637
T.cells	NOXRED1	0.655931	0.546984	1.591528	0.11506	-4.40899	0.587022	0.466856
T.cells	IL13RA1	0.621325	3.558786	1.591301	0.115111	-4.5308	0.548561	0.429455
T.cells	NOL11	-0.17521	4.89374	-1.59116	0.115142	-5.40937	0.532275	0.413798
T.cells	FAM177A	0.435719	1.731422	1.590982	0.115183	-4.59652	0.571609	0.451928
T.cells	GALK1	0.170274	5.140655	1.590903	0.115201	-5.44307	0.529312	0.411035
T.cells	FEN1	-0.18153	4.525584	-1.59044	0.115304	-5.42747	0.53672	0.418207
T.cells	SIN3B	0.108024	6.294475	1.590401	0.115314	-5.59739	0.515674	0.398122
T.cells	NAGLU	0.419364	2.6543	1.590037	0.115396	-4.59613	0.559859	0.440728
T.cells	ZFP112	-0.62157	0.391108	-1.58995	0.115415	-4.45024	0.589079	0.469396

T.cells	GM9967	-0.83321	0.569425	-1.58969	0.115475	-4.39987	0.586726	0.467202
T.cells	FAM220A.:	-0.48182	2.200036	-1.58967	0.11548	-4.57509	0.565614	0.446504
T.cells	BRMS1L	0.155253	4.269409	1.588262	0.115797	-5.30502	0.541038	0.422076
T.cells	WWP1	-0.21367	5.227114	-1.58809	0.115836	-5.24793	0.529457	0.41106
T.cells	COIL	-0.18786	3.741294	-1.58747	0.115977	-5.18691	0.547526	0.428589
T.cells	1700037C1	0.272175	3.065274	1.587357	0.116002	-4.90978	0.555936	0.436737
T.cells	SLU7	-0.14124	4.955244	-1.58722	0.116032	-5.35617	0.532721	0.414378
T.cells	1700030KC	0.413247	1.730931	1.586796	0.116129	-4.70876	0.57289	0.45346
T.cells	MVD	-0.51406	2.348846	-1.58673	0.116144	-4.50466	0.56498	0.44574
T.cells	SLC52A2	-0.36924	2.391257	-1.58656	0.116182	-4.69167	0.564441	0.445242
T.cells	DEPTOR	0.273474	3.469195	1.585708	0.116376	-5.02623	0.550897	0.432349
T.cells	GM28192	0.777626	0.064757	1.585571	0.116407	-4.3314	0.594729	0.475274
T.cells	2610307P1	0.269228	4.550996	1.585558	0.11641	-5.48116	0.537609	0.419537
T.cells	UBE2L6	0.378159	4.64931	1.585524	0.116418	-5.16061	0.536416	0.41839
T.cells	PPP3R1	-0.13354	5.5618	-1.58537	0.116452	-5.44768	0.525465	0.407939
T.cells	ARHGEF18	0.165484	5.591269	1.585273	0.116475	-5.52632	0.525114	0.407634
T.cells	CRACR2B	-0.82849	1.009115	-1.58513	0.116507	-4.37192	0.582259	0.46311
T.cells	ELK1	0.431782	1.559348	1.584714	0.116602	-4.49912	0.575104	0.456209
T.cells	ZFR	0.103164	6.532381	1.584706	0.116604	-5.66689	0.514047	0.397248
T.cells	PTGES	1.106377	0.477516	1.584651	0.116616	-4.33835	0.589249	0.470139
T.cells	HK3	0.865342	2.677613	1.584481	0.116655	-4.45678	0.560813	0.442323
T.cells	NR1H4	-0.67351	1.386536	-1.58418	0.116722	-4.44865	0.577408	0.458578
T.cells	C1QC	-0.49455	6.384084	-1.584	0.116765	-5.28748	0.515835	0.399042
T.cells	NDNF	0.851538	-0.47189	1.583724	0.116827	-4.333	0.602067	0.482902
T.cells	SGTA	-0.15658	4.959007	-1.58343	0.116894	-5.40123	0.532883	0.41537
T.cells	FBXL12	0.165412	4.348122	1.58298	0.116997	-5.3441	0.540541	0.422587
T.cells	TPM3	-0.07555	8.661561	-1.58241	0.117126	-5.97647	0.490488	0.37482
T.cells	PFKFB1	0.600451	0.961216	1.582217	0.117171	-4.45662	0.583685	0.464387
T.cells	OPA3	0.160729	5.128547	1.582081	0.117202	-5.34905	0.531366	0.413555
T.cells	SLC9A7	-0.15143	5.115655	-1.58176	0.117275	-5.57323	0.531528	0.41374
T.cells	CEBPG	0.123501	5.586056	1.581618	0.117308	-5.52774	0.525904	0.408436
T.cells	OS9	0.143477	5.617189	1.581439	0.117349	-5.41757	0.525534	0.408106
T.cells	TSKU	-0.90544	-0.3765	-1.58081	0.117492	-4.33757	0.601962	0.48237
T.cells	EZH1	0.285928	3.600438	1.580526	0.117558	-4.92299	0.550555	0.431902
T.cells	GM27201	-0.28243	2.321008	-1.58007	0.117662	-4.8039	0.566663	0.447783
T.cells	PSMF1	0.164873	4.460205	1.57971	0.117744	-5.32117	0.539979	0.422012
T.cells	PDCD1LG2	-0.90893	1.775707	-1.57954	0.117784	-4.3508	0.573658	0.454756
T.cells	NRAP	0.864034	-1.09404	1.579187	0.117864	-4.32225	0.611801	0.492678
T.cells	ATCAYOS	-0.99537	1.220314	-1.57879	0.117956	-4.37621	0.580865	0.462156
T.cells	PLA2G4C	-0.44539	0.890136	-1.57806	0.118123	-4.70635	0.585189	0.466746
T.cells	HIST1H3A	-0.39854	0.953909	-1.578	0.118136	-4.6995	0.584352	0.46592
T.cells	GM10131	-0.54361	1.072727	-1.57795	0.118148	-4.61217	0.582794	0.464384
T.cells	CD3G	-0.90908	3.343766	-1.57776	0.118191	-4.47388	0.553756	0.435981
T.cells	SLC22A1	-0.79631	0.740269	-1.57776	0.118192	-4.38408	0.587161	0.468709
T.cells	FUBP3	0.187928	4.08675	1.577658	0.118216	-5.22979	0.544551	0.427073
T.cells	SLC35E1	-0.18003	4.084727	-1.57732	0.118294	-5.18518	0.544576	0.427208
T.cells	BATF3	-0.76912	2.332688	-1.57723	0.118313	-4.41271	0.566514	0.448568

T.cells	APOA5	-0.67614	1.793969	-1.57713	0.118338	-4.52308	0.573423	0.455358
T.cells	METTL23	0.11807	5.675126	1.577126	0.118338	-5.52119	0.525351	0.408741
T.cells	BPGM	0.332553	4.812429	1.576989	0.11837	-5.21039	0.535699	0.418736
T.cells	KAT6B	0.140476	5.844837	1.576937	0.118382	-5.53582	0.523338	0.406896
T.cells	R3HDM1	0.099297	6.683532	1.576522	0.118477	-5.7478	0.513649	0.397684
T.cells	GM49085	0.293565	2.025817	1.57634	0.118519	-4.82414	0.570612	0.452788
T.cells	NDRG3	-0.16221	4.407312	-1.57615	0.118563	-5.24309	0.540788	0.42386
T.cells	SARS	0.113133	5.792679	1.575566	0.118697	-5.55966	0.524496	0.408035
T.cells	ZFP36L1	-0.14649	7.742233	-1.57429	0.118991	-5.80207	0.502347	0.386814
T.cells	RAB5C	0.124676	6.358629	1.574048	0.119048	-5.58784	0.518348	0.402095
T.cells	EPS15L1	-0.11214	6.525246	-1.57387	0.11909	-5.70651	0.516396	0.400344
T.cells	UBXN11	0.350537	1.770334	1.573481	0.119179	-4.69863	0.574905	0.457118
T.cells	NT5E	-0.66785	2.431121	-1.57334	0.119211	-4.5056	0.56642	0.448818
T.cells	SEMA5A	-0.55489	1.106724	-1.57296	0.1193	-4.61116	0.583544	0.465756
T.cells	ZBTB11OS1	0.323961	2.065369	1.572913	0.119311	-4.73212	0.571102	0.45351
T.cells	GZMK	0.84966	-1.47202	1.572824	0.119332	-4.33216	0.61826	0.500338
T.cells	FOXK2	-0.1366	4.920069	-1.57279	0.11934	-5.43084	0.535494	0.418911
T.cells	LARP4B	0.105615	7.283403	1.57267	0.119367	-5.77178	0.5076	0.392245
T.cells	ATP5MPL	-0.0985	8.484004	-1.57247	0.119413	-6.02315	0.493962	0.379423
T.cells	PLS3	-0.513	2.162516	-1.57209	0.119501	-4.52719	0.569855	0.452559
T.cells	ARF2	-0.20972	4.470722	-1.57187	0.119554	-5.28252	0.540958	0.424506
T.cells	ZFP65	0.443195	1.662117	1.57161	0.119613	-4.66767	0.576306	0.459071
T.cells	ATP9B	0.129161	5.858499	1.571548	0.119627	-5.4961	0.52425	0.408503
T.cells	NEDD9	0.166426	8.003683	1.571332	0.119678	-5.85949	0.499376	0.384866
T.cells	GM31522	0.685356	-1.00368	1.570978	0.11976	-4.33656	0.611818	0.494512
T.cells	TGFBR2	-0.11614	7.292661	-1.57038	0.1199	-5.77275	0.507494	0.392775
T.cells	MID2	0.868315	0.27499	1.570257	0.119927	-4.35558	0.594541	0.477455
T.cells	HGH1	-0.35523	1.856316	-1.57022	0.119935	-4.72775	0.573794	0.456933
T.cells	RALGPS2	0.146773	5.773254	1.570184	0.119944	-5.66087	0.525262	0.409746
T.cells	KRT81	0.816851	-1.53173	1.570033	0.119979	-4.33655	0.619086	0.501984
T.cells	MIEF1	0.201398	4.21125	1.570023	0.119982	-5.24901	0.544137	0.427957
T.cells	CAP1	0.112903	6.878742	1.569957	0.119997	-5.68934	0.512277	0.397327
T.cells	ZFP991	0.385716	2.80432	1.56981	0.120031	-4.74134	0.561679	0.445079
T.cells	SMU1	-0.11508	5.608875	-1.5685	0.120337	-5.56476	0.528041	0.412052
T.cells	GM48099	0.646233	2.773722	1.568492	0.120339	-4.76036	0.562943	0.445884
T.cells	GM12253	0.912769	-0.86999	1.568201	0.120406	-4.33209	0.610942	0.493479
T.cells	SLC17A5	0.271128	3.572742	1.568024	0.120448	-4.9913	0.552895	0.43624
T.cells	ZFP260	0.147734	4.245881	1.5679	0.120477	-5.31127	0.54456	0.428181
T.cells	UTP18	-0.13491	5.338906	-1.56776	0.12051	-5.5144	0.531275	0.415409
T.cells	RAB3A	-0.28994	2.619056	-1.56728	0.120622	-4.96533	0.565206	0.448176
T.cells	CHST12	0.174605	5.763881	1.56605	0.120909	-5.57975	0.526875	0.411091
T.cells	MRPL10	-0.16863	4.602348	-1.56599	0.120923	-5.36973	0.540894	0.424602
T.cells	SDC2	-0.57419	2.267759	-1.56583	0.12096	-4.55904	0.570132	0.4531
T.cells	P2RY6	0.737528	2.305897	1.565732	0.120984	-4.40543	0.569643	0.45262
T.cells	NMRK1	-0.2337	3.751312	-1.56565	0.121003	-5.06813	0.551387	0.434784
T.cells	RAMP3	0.93186	0.947281	1.565322	0.12108	-4.37003	0.587312	0.470283
T.cells	CAV2	-0.23968	2.771749	-1.56528	0.121089	-5.0926	0.563699	0.447025

T.cells	CYSLTR1	-0.44546	2.70023	-1.56522	0.121105	-4.72608	0.564608	0.447937
T.cells	SMIM20	0.141265	4.914811	1.564616	0.121245	-5.45565	0.537449	0.421496
T.cells	VAPA	-0.078	8.028548	-1.56446	0.121283	-5.91973	0.500857	0.386511
T.cells	PTPRK	-0.59368	3.178658	-1.56413	0.12136	-4.68636	0.55905	0.442509
T.cells	RNF34	0.18079	4.455799	1.563604	0.121483	-5.21397	0.54317	0.427265
T.cells	PIGN	0.201715	4.40092	1.563495	0.121509	-5.28244	0.543844	0.427934
T.cells	TAGLN2	-0.17119	8.716101	-1.56338	0.121537	-6.02886	0.49321	0.379498
T.cells	MYO16	-0.91072	-0.30441	-1.56282	0.121667	-4.36222	0.604573	0.487915
T.cells	EFNA2	-0.91997	1.10216	-1.56281	0.121669	-4.41129	0.585793	0.469229
T.cells	PTGS2OS	1.005046	-1.09526	1.562741	0.121686	-4.34948	0.615373	0.498733
T.cells	SPRYD7	-0.41082	2.133927	-1.56253	0.121735	-4.64179	0.572361	0.455964
T.cells	AMZ1	-0.71363	3.157688	-1.5624	0.121766	-4.4918	0.559315	0.443203
T.cells	LRRC29	0.336782	1.690453	1.562271	0.121797	-4.64085	0.578099	0.461703
T.cells	UXS1	0.176842	4.429155	1.561466	0.121986	-5.31615	0.544126	0.42815
T.cells	LARP1B	-0.15497	5.599705	-1.56108	0.122076	-5.52485	0.530097	0.414603
T.cells	PACS2	0.220777	4.036916	1.560727	0.122161	-5.02004	0.549311	0.433152
T.cells	GPR137C	0.33566	2.390141	1.560339	0.122252	-4.81974	0.570086	0.45369
T.cells	PDLIM1	-0.17009	4.944927	-1.56031	0.122258	-5.5048	0.53816	0.422557
T.cells	1200007C1	0.955577	-0.38465	1.559692	0.122405	-4.35747	0.607058	0.490237
T.cells	RASSF1	0.13592	5.940086	1.559633	0.122419	-5.51534	0.526457	0.41117
T.cells	LRSAM1	0.493067	1.610096	1.559397	0.122475	-4.54152	0.580513	0.463827
T.cells	PCYOX1L	-0.36203	1.613818	-1.559	0.12257	-4.75066	0.580471	0.46398
T.cells	DPM2	0.18827	3.936241	1.558978	0.122574	-5.2117	0.550884	0.434954
T.cells	TPD52	0.183876	8.000639	1.558374	0.122717	-5.79264	0.50286	0.388578
T.cells	SLAMF7	0.160923	5.151332	1.557958	0.122816	-5.76566	0.536468	0.420703
T.cells	DNAJC15	-0.16032	5.723768	-1.55778	0.122858	-5.45352	0.529568	0.414084
T.cells	CYSLTR2	0.842054	1.496466	1.557562	0.12291	-4.41452	0.582546	0.465827
T.cells	ARHGEF3	0.303945	5.916073	1.557442	0.122938	-5.36358	0.527269	0.41194
T.cells	LYL1	0.235401	3.172684	1.557261	0.122981	-5.20752	0.560974	0.444636
T.cells	BRWD3	0.180158	5.109603	1.556679	0.123119	-5.36745	0.537364	0.421511
T.cells	CD74	-0.44524	11.27972	-1.55638	0.12319	-6.20388	0.467244	0.355102
T.cells	SPPL2B	0.405008	2.17345	1.554945	0.123532	-4.70663	0.575322	0.45798
T.cells	TLL2	-0.8255	0.725481	-1.55485	0.123555	-4.45771	0.594354	0.476829
T.cells	PACSIN1	-0.52208	2.953403	-1.55467	0.123596	-4.56073	0.565305	0.448256
T.cells	CEP70	0.220497	3.195368	1.554601	0.123614	-5.13466	0.562231	0.445291
T.cells	PDAP1	-0.10264	6.715812	-1.55412	0.123727	-5.82503	0.519504	0.403834
T.cells	SRP19	-0.11952	6.048783	-1.55339	0.123902	-5.66974	0.52772	0.411637
T.cells	MRPL33	-0.13967	6.142277	-1.55327	0.123931	-5.62243	0.526604	0.410579
T.cells	GM17018	-0.2034	3.620053	-1.55289	0.124021	-5.17947	0.557491	0.440528
T.cells	PRPF19	-0.12354	5.053951	-1.55282	0.124039	-5.574	0.539729	0.423292
T.cells	JAGN1	0.193227	3.955321	1.552647	0.12408	-5.26997	0.55329	0.436465
T.cells	CLK1	-0.11246	7.55289	-1.55244	0.124129	-5.81236	0.51004	0.394894
T.cells	FBXW11	-0.14533	7.259166	-1.55238	0.124143	-5.76856	0.513447	0.398136
T.cells	CAPN15	-0.16279	4.710463	-1.55089	0.124499	-5.36554	0.545233	0.427799
T.cells	PRPS2	-0.15256	4.382206	-1.55073	0.124538	-5.41248	0.549291	0.431731
T.cells	HIST1H2AN	0.345872	3.254826	1.550119	0.124685	-5.38228	0.563887	0.445804
T.cells	TTPAL	-0.15334	4.234118	-1.54971	0.124784	-5.34997	0.551784	0.433901

T.cells	BAP1	-0.19438	3.565024	-1.54891	0.124975	-5.19052	0.56062	0.4424
T.cells	HIF1A	0.151418	6.608469	1.548889	0.124981	-5.67192	0.523349	0.406462
T.cells	ARFIP2	0.308708	2.138474	1.548334	0.125114	-4.80425	0.57905	0.460499
T.cells	PACRG	0.885437	-0.43352	1.5481	0.12517	-4.36987	0.613473	0.494631
T.cells	CTBP2	0.309149	3.249836	1.547993	0.125196	-4.91895	0.564735	0.446551
T.cells	VIRMA	0.134518	5.71037	1.547962	0.125204	-5.60502	0.534208	0.41699
T.cells	FYTTD1	0.117624	5.267108	1.547673	0.125273	-5.50022	0.539678	0.422307
T.cells	MACROD2	-0.49679	2.251559	-1.54675	0.125495	-4.66213	0.578403	0.459521
T.cells	CYTH3	-0.18473	4.88921	-1.54653	0.125549	-5.40237	0.544997	0.427075
T.cells	KLRC2	-0.94838	1.332802	-1.54604	0.125668	-4.4141	0.590479	0.47168
T.cells	F630028O1	0.849445	1.027185	1.54595	0.125689	-4.38175	0.594547	0.475764
T.cells	WDR36	-0.14373	4.578146	-1.54581	0.125722	-5.43474	0.548841	0.431022
T.cells	USO1	0.135193	5.739311	1.545788	0.125728	-5.51309	0.534621	0.417311
T.cells	GM42702	-0.3171	2.365724	-1.54498	0.125923	-4.90659	0.577586	0.458729
T.cells	TNFSF13OS	-0.89745	0.055873	-1.54412	0.126132	-4.41077	0.608668	0.489518
T.cells	RIDA	-0.39476	4.316478	-1.54397	0.126167	-5.13943	0.553021	0.434842
T.cells	4930595D1	-0.48629	1.248821	-1.54396	0.126169	-4.61576	0.592587	0.473576
T.cells	LCAT	-0.53798	2.143834	-1.54389	0.126186	-4.59501	0.58078	0.461944
T.cells	RAI14	0.364785	2.228896	1.543602	0.126257	-4.89919	0.579767	0.460982
T.cells	4930404N1	0.566728	0.191025	1.542746	0.126464	-4.54095	0.607688	0.48819
T.cells	NAT2	0.301561	2.532818	1.542384	0.126552	-4.90054	0.576709	0.45759
T.cells	FCGRT	-0.44168	4.823452	-1.54131	0.126813	-4.89537	0.548359	0.429688
T.cells	NUP62	-0.20521	4.181003	-1.54106	0.126873	-5.40113	0.556374	0.437465
T.cells	D130043K2	-0.55302	1.054045	-1.54087	0.126921	-4.55679	0.596966	0.477255
T.cells	KDELR2	-0.12346	5.993878	-1.5408	0.126936	-5.63698	0.534035	0.41597
T.cells	RECQL	0.217162	3.363727	1.540706	0.12696	-5.21041	0.566728	0.447597
T.cells	GM16268	-0.82286	0.706579	-1.53966	0.127215	-4.40948	0.602525	0.482367
T.cells	IZUMO4	0.615214	1.453448	1.539431	0.127271	-4.55741	0.592501	0.4725
T.cells	IQCB1	-0.1377	4.824078	-1.53933	0.127295	-5.51862	0.549156	0.430222
T.cells	U2AF2	-0.11115	6.245648	-1.53877	0.127433	-5.734	0.531961	0.413692
T.cells	GOT2	-0.11656	6.235083	-1.53871	0.127445	-5.73889	0.532088	0.413813
T.cells	FIGNL2	0.963818	-0.67701	1.538541	0.127488	-4.36971	0.621719	0.501524
T.cells	UCHL5	-0.1248	5.728606	-1.53793	0.127637	-5.6475	0.538512	0.419914
T.cells	SCAND1	0.110073	7.018464	1.537853	0.127656	-5.72333	0.523011	0.405103
T.cells	ABCC9	0.736937	1.277502	1.537246	0.127804	-4.46787	0.595832	0.475509
T.cells	ZFP949	0.300309	2.594224	1.536717	0.127934	-4.89854	0.578804	0.458548
T.cells	GM10974	0.472586	0.824708	1.53639	0.128014	-4.59375	0.60231	0.481768
T.cells	ADGB	0.851587	2.335955	1.536301	0.128036	-4.49143	0.58219	0.462004
T.cells	GM44710	-0.53474	2.000891	-1.53559	0.12821	-4.59639	0.587108	0.46662
T.cells	LDB2	-0.58585	3.160304	-1.53544	0.128247	-4.66047	0.571977	0.451855
T.cells	SERBP1	-0.09011	8.529561	-1.53457	0.128459	-6.11095	0.507083	0.389396
T.cells	UROC1	-0.64158	1.760508	-1.53448	0.128483	-4.56904	0.590917	0.470138
T.cells	MAML3	0.165965	6.974808	1.533631	0.128691	-5.88019	0.525858	0.407028
T.cells	GM26632	-0.67189	0.366747	-1.5335	0.128723	-4.46116	0.610375	0.489217
T.cells	EEF1AKMT4	-0.26161	2.582886	-1.53296	0.128857	-4.91227	0.581095	0.460129
T.cells	RTN4RL1	-0.83274	2.381813	-1.53247	0.128977	-4.4553	0.584048	0.462772
T.cells	GM26771	0.920797	-0.2163	1.531748	0.129155	-4.38713	0.619668	0.497705

T.cells	ATP6V1B2	0.130707	5.98524	1.531353	0.129252	-5.52158	0.538873	0.419055
T.cells	ANP32E	-0.11336	7.305672	-1.53102	0.129336	-6.04226	0.522994	0.40407
T.cells	MACROD1	-0.32777	2.861084	-1.53097	0.129347	-4.94157	0.578265	0.45729
T.cells	ERLIN2	0.273281	3.099067	1.530878	0.12937	-4.92241	0.575172	0.454317
T.cells	SAT2	-0.97802	-0.24549	-1.53081	0.129385	-4.3898	0.620073	0.498569
T.cells	SOX12	-0.46797	0.585357	-1.53036	0.129498	-4.62106	0.608868	0.48729
T.cells	TMEM150I	0.536023	1.12138	1.530179	0.129543	-4.66678	0.601584	0.480098
T.cells	HGS	0.188003	4.094551	1.529877	0.129617	-5.21116	0.562623	0.442056
T.cells	CEP104	0.1985	3.105243	1.529754	0.129648	-5.13051	0.575319	0.454431
T.cells	MCAM	-0.8047	1.173297	-1.5296	0.129687	-4.44412	0.600883	0.479574
T.cells	GM47350	0.342748	1.355941	1.529069	0.129817	-4.76909	0.598672	0.47739
T.cells	ACER2	0.552114	2.516505	1.528962	0.129844	-4.58745	0.583244	0.462272
T.cells	POT1A	0.243427	3.012767	1.528696	0.12991	-5.03133	0.576761	0.455959
T.cells	CD82	0.177065	5.476033	1.528255	0.130019	-5.38024	0.54556	0.425848
T.cells	HIST1H4N	-0.4787	1.07648	-1.52813	0.13005	-4.70516	0.602443	0.481374
T.cells	ZFP874A	0.378695	2.146516	1.528127	0.130051	-4.72515	0.588122	0.467268
T.cells	ZFP236	0.156656	4.729945	1.527896	0.130108	-5.38763	0.55484	0.434917
T.cells	GM3235	0.590605	0.645113	1.527762	0.130141	-4.58196	0.608306	0.487304
T.cells	TST	-0.50392	3.470042	-1.52707	0.130312	-4.85257	0.571375	0.450772
T.cells	GOLGB1	0.134747	5.4427	1.526804	0.130379	-5.52949	0.546534	0.426727
T.cells	RAP1GDS1	0.13374	6.711436	1.526365	0.130489	-5.70569	0.531062	0.411954
T.cells	ZFP951	0.333374	2.641469	1.525889	0.130607	-4.91322	0.582204	0.461555
T.cells	BC028528	-0.19504	4.105365	-1.52587	0.130612	-5.26873	0.563301	0.443131
T.cells	WDR38	0.482061	0.617809	1.525693	0.130656	-4.55583	0.609306	0.48826
T.cells	FAM122A	0.218624	3.563965	1.525639	0.130669	-5.08891	0.570224	0.449859
T.cells	GM47917	-0.59074	0.467794	-1.52561	0.130676	-4.48349	0.611361	0.490298
T.cells	ANKRD6	0.430834	0.95646	1.525399	0.130729	-4.77227	0.604703	0.483728
T.cells	GM43445	0.30857	1.896237	1.524734	0.130895	-4.88516	0.592377	0.471455
T.cells	SAA2	1.264533	-1.02347	1.524683	0.130907	-4.39816	0.63248	0.511284
T.cells	POMK	0.60659	1.068205	1.524321	0.130998	-4.51928	0.603506	0.482569
T.cells	AGK	0.22105	3.121713	1.524223	0.131022	-5.1119	0.576255	0.455794
T.cells	STRN3	0.114923	7.654054	1.524116	0.131049	-5.90387	0.520121	0.401798
T.cells	PTPRO	-0.76991	2.363394	-1.52383	0.13112	-4.48069	0.586275	0.465578
T.cells	MAN1C1	-0.45233	4.292733	-1.52362	0.131172	-4.67564	0.561328	0.441277
T.cells	FAM13A	0.50353	1.15315	1.523193	0.131279	-4.70524	0.602543	0.481684
T.cells	RPH3AL	0.392156	1.299189	1.52315	0.13129	-4.75893	0.600569	0.479733
T.cells	STMN1	-0.21226	7.897729	-1.52285	0.131365	-6.18295	0.517511	0.399368
T.cells	EXOC1	0.188185	4.095264	1.521917	0.131599	-5.26266	0.564341	0.44437
T.cells	A630001O:	-0.38445	2.282008	-1.5218	0.131629	-4.74099	0.587885	0.467364
T.cells	GM42567	-0.84875	1.12927	-1.52141	0.131725	-4.45964	0.603323	0.482747
T.cells	PHIP	0.105017	7.276011	1.521337	0.131744	-5.94051	0.525159	0.406881
T.cells	CYLD	0.127341	6.329731	1.521168	0.131786	-5.64605	0.53654	0.417818
T.cells	PIGW	0.491208	0.765108	1.521054	0.131815	-4.5646	0.608278	0.487839
T.cells	ZFP773	0.782468	0.510873	1.520864	0.131862	-4.45601	0.611758	0.491336
T.cells	MLLT10	0.085123	7.464033	1.520694	0.131905	-5.93876	0.522926	0.405017
T.cells	GM41790	0.99869	-0.09939	1.520608	0.131926	-4.39216	0.620189	0.499869
T.cells	TKFC	-0.34048	3.130684	-1.52055	0.131942	-4.93125	0.576754	0.456962

T.cells	CCZ1	0.107782	6.158644	1.520026	0.132073	-5.72487	0.538757	0.420317
T.cells	VWCE	-0.61795	0.865529	-1.51991	0.132101	-4.51191	0.60706	0.487045
T.cells	2010016I1‹	0.820401	-0.03474	1.519815	0.132126	-4.39617	0.619446	0.499429
T.cells	CYP2B9	-0.656	1.9577	-1.5194	0.132229	-4.64582	0.592581	0.47269
T.cells	B4GALNT1	-0.2563	4.892601	-1.51848	0.132462	-5.01649	0.55531	0.435817
T.cells	FAM124A	-0.60513	1.64482	-1.51836	0.132491	-4.48919	0.597493	0.477057
T.cells	SOAT1	0.306596	5.176779	1.518059	0.132567	-5.0764	0.551862	0.432504
T.cells	WDR5B	0.572422	0.670974	1.517796	0.132634	-4.56353	0.610901	0.490325
T.cells	KBTBD8	-0.57516	0.251382	-1.51755	0.132695	-4.5169	0.616731	0.49621
T.cells	DCK	0.129823	5.832657	1.517275	0.132765	-5.82121	0.543927	0.424905
T.cells	TRDJ1	0.70923	-1.29553	1.516468	0.132969	-4.3989	0.638813	0.518472
T.cells	ATP6V0E	-0.10151	7.713213	-1.5163	0.133011	-5.899	0.521442	0.403686
T.cells	IL2RB	-0.83966	3.130268	-1.5162	0.133036	-4.5366	0.578382	0.458664
T.cells	PBRM1	0.080738	7.416728	1.516054	0.133073	-5.98237	0.524959	0.407118
T.cells	HELB	0.189372	3.704754	1.515929	0.133105	-5.32185	0.570938	0.451461
T.cells	PTPN23	-0.26454	3.647447	-1.51562	0.133183	-5.0097	0.571677	0.452271
T.cells	ASNS	-0.454	1.465562	-1.51554	0.133202	-4.66738	0.600466	0.480581
T.cells	9830107B1	0.769726	-0.62676	1.515368	0.133246	-4.40517	0.629325	0.509325
T.cells	TIMM10B	0.129246	6.181583	1.515175	0.133295	-5.65387	0.539857	0.421528
T.cells	GM16794	0.483224	0.41499	1.515079	0.13332	-4.5949	0.614801	0.494872
T.cells	TIMM50	-0.14546	4.827954	-1.51468	0.133419	-5.55703	0.556847	0.437847
T.cells	IQCG	-0.88703	0.050135	-1.51419	0.133544	-4.43392	0.620319	0.500185
T.cells	ERAP1	0.150339	5.228177	1.513893	0.13362	-5.52704	0.552044	0.433203
T.cells	EML4	0.118766	7.138312	1.513845	0.133632	-5.83342	0.528681	0.410767
T.cells	BMI1	-0.20719	3.545427	-1.51369	0.133672	-5.1943	0.573425	0.454008
T.cells	TIE1	-0.61143	1.482911	-1.51335	0.133757	-4.51141	0.600815	0.48092
T.cells	MRPS33	-0.09827	6.714474	-1.51318	0.133802	-5.84101	0.533899	0.415816
T.cells	KCTD12	-0.18855	6.383969	-1.51272	0.133917	-5.46338	0.538077	0.419817
T.cells	CAR8	-0.56769	2.375063	-1.51262	0.133944	-4.6382	0.589059	0.469412
T.cells	ISG20	0.577614	4.233394	1.511981	0.134105	-4.8281	0.565106	0.445984
T.cells	TBX2	-0.8206	0.45083	-1.51196	0.13411	-4.42846	0.61533	0.495449
T.cells	ANAPC4	0.141643	4.541254	1.511817	0.134147	-5.42768	0.561191	0.442181
T.cells	GM42556	-0.62114	0.793712	-1.51096	0.134366	-4.54378	0.61138	0.491156
T.cells	GM10634	0.850414	0.651485	1.510403	0.134506	-4.41328	0.613339	0.49338
T.cells	A930015D(	0.177893	4.760503	1.510358	0.134518	-5.39968	0.559124	0.4401
T.cells	GM11714	0.584877	1.498822	1.510115	0.13458	-4.5361	0.601776	0.481997
T.cells	1700010I1‹	0.419397	1.215796	1.509901	0.134634	-4.67032	0.605616	0.485898
T.cells	CETN4	-0.48432	-0.08195	-1.50983	0.134652	-4.60443	0.623513	0.503728
T.cells	ZFYVE19	-0.22166	3.132938	-1.50976	0.134669	-5.08953	0.580043	0.460708
T.cells	PADI2	-0.54539	2.631853	-1.50925	0.1348	-4.64412	0.586688	0.467292
T.cells	SPRED2	-0.13228	6.335393	-1.50894	0.134881	-5.76967	0.539612	0.421546
T.cells	TMEM126/	0.111566	5.857631	1.507995	0.135121	-5.69037	0.545479	0.427673
T.cells	ERCC8	-0.23341	2.45611	-1.50794	0.135134	-5.03916	0.589014	0.470139
T.cells	FOCAD	0.294406	2.908895	1.507913	0.135142	-4.98399	0.583038	0.464255
T.cells	UFC1	-0.10064	5.792898	-1.50786	0.135156	-5.63921	0.546279	0.428444
T.cells	NHLRC3	0.346156	3.16365	1.507802	0.13517	-4.84131	0.5797	0.460976
T.cells	EPS8	-0.19721	4.963297	-1.50774	0.135186	-5.80682	0.556624	0.438474

T.cells	NFKBIB	-0.16186	5.622292	-1.50768	0.135203	-5.55513	0.548391	0.430508
T.cells	TEX261	0.125764	5.229001	1.507625	0.135215	-5.55146	0.553291	0.435248
T.cells	EIF1B	-0.10962	5.828759	-1.50733	0.135292	-5.70012	0.545836	0.428041
T.cells	GTF2B	-0.12109	6.738906	-1.50715	0.135336	-5.83865	0.534703	0.417331
T.cells	PPP1R9B	0.246639	4.375184	1.506934	0.135392	-5.12436	0.564069	0.445752
T.cells	ZFP46	-0.30647	1.77659	-1.50654	0.135492	-4.72246	0.59809	0.479389
T.cells	GM43330	-0.50347	1.526899	-1.50652	0.135497	-4.58385	0.601457	0.482728
T.cells	HJURP	0.146522	4.895427	1.506391	0.135532	-5.5824	0.557478	0.439603
T.cells	AMBRA1	0.106468	7.654516	1.506353	0.135541	-5.94565	0.52372	0.407101
T.cells	RNF168	0.170088	4.161361	1.505897	0.135658	-5.45473	0.56683	0.448792
T.cells	GM50240	0.458	1.572659	1.505201	0.135837	-4.65429	0.600872	0.482624
T.cells	KARS	-0.17722	4.273346	-1.50512	0.135858	-5.41672	0.565399	0.447717
T.cells	1700016PC	-0.74388	3.898827	-1.50511	0.135859	-4.64066	0.570199	0.452408
T.cells	ATP13A1	0.180314	3.809105	1.504651	0.135978	-5.20783	0.571354	0.45383
T.cells	VAV2	-0.19057	4.977829	-1.50456	0.136	-5.48841	0.556472	0.439333
T.cells	CCR7	-0.2859	4.535564	-1.50447	0.136024	-5.464	0.562061	0.444804
T.cells	UROD	-0.17902	4.467162	-1.50396	0.136157	-5.39495	0.56293	0.445877
T.cells	IL7R	0.212338	4.966863	1.50388	0.136176	-5.68356	0.55661	0.439744
T.cells	RAP1GAP2	0.730303	4.692548	1.503523	0.136268	-4.59504	0.560071	0.443208
T.cells	CCDC136	0.919816	0.119291	1.503466	0.136283	-4.44625	0.620803	0.503327
T.cells	POSTN	-0.71745	1.260435	-1.50337	0.136306	-4.52338	0.605103	0.487661
T.cells	NIPAL1	-0.7062	1.060638	-1.50331	0.136322	-4.48685	0.607825	0.490397
T.cells	DGCR6	0.190398	3.650268	1.503121	0.136372	-5.25907	0.573406	0.456418
T.cells	PEX6	-0.21122	4.039128	-1.50307	0.136385	-5.26955	0.568396	0.451509
T.cells	UNK	0.175499	4.287183	1.502988	0.136406	-5.43568	0.565222	0.448408
T.cells	GM28791	0.211077	3.918458	1.502809	0.136452	-5.24322	0.569946	0.453041
T.cells	GM50333	0.414908	1.191453	1.502784	0.136458	-4.68835	0.606042	0.488738
T.cells	ZMYND19	-0.22757	3.409984	-1.50259	0.136508	-5.29998	0.576521	0.459498
T.cells	IFI27L2A	0.356493	7.373467	1.502366	0.136566	-5.8782	0.527127	0.411568
T.cells	ELP4	-0.1566	4.963596	-1.50162	0.136759	-5.49584	0.556924	0.440515
T.cells	GM20707	0.364198	1.898927	1.501364	0.136825	-4.67996	0.596772	0.479733
T.cells	MFAP4	0.642212	1.50654	1.501272	0.136848	-4.59684	0.602061	0.484994
T.cells	DCN	-0.53099	3.138043	-1.50122	0.136862	-4.79704	0.580351	0.463481
T.cells	SLC5A11	0.841059	-0.01556	1.501038	0.136909	-4.42568	0.622988	0.505937
T.cells	MINDY3	-0.13937	5.598106	-1.50096	0.136928	-5.55344	0.548991	0.432807
T.cells	APTX	0.214487	3.052742	1.500484	0.137052	-5.01915	0.581574	0.464799
T.cells	SOCS5	-0.25464	4.029298	-1.50011	0.13715	-5.17693	0.568905	0.452425
T.cells	TMEM203	0.285629	3.168142	1.500101	0.137151	-4.98718	0.580063	0.463398
T.cells	SUMO2	-0.07731	8.656817	-1.50008	0.137155	-6.20791	0.512315	0.397744
T.cells	WIPF1	-0.16165	7.425545	-1.49937	0.137339	-5.73967	0.52734	0.411893
T.cells	MTMR6	0.194967	4.718718	1.498624	0.137533	-5.26664	0.560954	0.44448
T.cells	SASH3	-0.12421	5.4972	-1.49852	0.137559	-5.66423	0.551169	0.434986
T.cells	GM12905	0.351126	2.138199	1.497989	0.137698	-4.68932	0.594564	0.477825
T.cells	ACTR2	0.096019	8.066808	1.497814	0.137743	-5.99693	0.519999	0.40515
T.cells	CMAH	-0.24427	5.561409	-1.49781	0.137745	-5.49749	0.550369	0.434416
T.cells	TXNDC17	-0.14162	6.116505	-1.4978	0.137746	-5.67183	0.543499	0.427754
T.cells	DDX28	0.382659	1.558733	1.497438	0.137841	-4.69625	0.602364	0.485758

T.cells	MYBL2	-0.22358	3.080165	-1.49734	0.137868	-5.33603	0.582084	0.465644
T.cells	DDX60	0.693044	2.866859	1.497093	0.137931	-4.65355	0.584888	0.468485
T.cells	GLIS2	-0.43715	1.3513	-1.49678	0.138011	-4.71563	0.605179	0.488725
T.cells	FAM214A	-0.24537	5.246692	-1.49673	0.138024	-5.31899	0.5543	0.438527
T.cells	GOLGA1	0.221688	3.427325	1.496571	0.138066	-5.08042	0.577546	0.461345
T.cells	MDK	-0.59812	1.436872	-1.49653	0.138078	-4.57089	0.604016	0.487627
T.cells	ABCB9	-0.48324	3.155352	-1.4962	0.138162	-4.66443	0.581098	0.46499
T.cells	E130102H2	0.683589	1.232168	1.495883	0.138245	-4.51102	0.606801	0.490576
T.cells	ARHGAP4	-0.1543	5.057347	-1.49585	0.138255	-5.40851	0.556678	0.441053
T.cells	MROH1	-0.24545	4.199162	-1.49578	0.138273	-5.11747	0.567575	0.451722
T.cells	CHAC2	0.237735	2.893423	1.495589	0.138322	-5.01616	0.584538	0.468437
T.cells	B3GNT3	0.654982	0.711737	1.494921	0.138496	-4.49601	0.614482	0.497945
T.cells	SEC23A	0.181561	4.425257	1.494346	0.138646	-5.29576	0.565548	0.449321
T.cells	IMPACT	0.210306	5.540939	1.494144	0.138698	-5.33754	0.551465	0.435584
T.cells	D430042O0	0.20187	3.792114	1.494001	0.138736	-5.19481	0.573689	0.45736
T.cells	GFER	0.137997	4.406699	1.493532	0.138858	-5.42311	0.566078	0.449738
T.cells	C1QTNF12	-0.37907	2.21932	-1.49332	0.138912	-4.81204	0.594709	0.477961
T.cells	CRIP1	0.154263	8.825299	1.492967	0.139006	-6.2586	0.512219	0.397754
T.cells	PRRC2B	-0.10978	6.226887	-1.49282	0.139043	-5.77606	0.543306	0.4276
T.cells	NES	0.880758	0.578345	1.492703	0.139075	-4.46061	0.617097	0.500442
T.cells	OSBPL3	0.471058	3.353205	1.492167	0.139215	-4.70715	0.579893	0.463581
T.cells	COQ10A	0.238248	3.31768	1.491575	0.13937	-4.99581	0.580358	0.464298
T.cells	NKIRAS1	0.241366	3.432264	1.491484	0.139394	-5.10274	0.578861	0.462822
T.cells	GRHPR	0.234212	4.431109	1.491132	0.139486	-5.37419	0.565958	0.450299
T.cells	B230208H1	0.645191	0.861264	1.491095	0.139496	-4.61448	0.613338	0.497302
T.cells	TUBA1C	-0.14332	7.576145	-1.49078	0.13958	-6.05505	0.527074	0.412608
T.cells	ALDH9A1	-0.1291	5.005152	-1.49073	0.139592	-5.57151	0.558666	0.443222
T.cells	RRM2	-0.21982	6.880861	-1.49072	0.139594	-6.10326	0.535447	0.420671
T.cells	TARM1	0.924687	-0.18149	1.490561	0.139636	-4.43609	0.627854	0.511981
T.cells	MYO3B	-0.5815	1.289051	-1.49038	0.139682	-4.54336	0.607473	0.491496
T.cells	IQCK	0.662768	0.158994	1.490291	0.139707	-4.50501	0.62308	0.507167
T.cells	IGSF6	0.656689	3.689623	1.490252	0.139717	-4.68408	0.57551	0.459753
T.cells	SUCO	0.16855	6.354348	1.489632	0.13988	-5.62421	0.542184	0.427129
T.cells	SREBF1	-0.21494	3.760907	-1.48949	0.139916	-5.1646	0.574917	0.459159
T.cells	GIMAP4	0.417836	4.091849	1.489216	0.139989	-4.96824	0.57064	0.455039
T.cells	NANOS1	-0.60488	0.634448	-1.48873	0.140116	-4.53497	0.616826	0.501154
T.cells	TRIM12C	0.217638	4.726648	1.488642	0.14014	-5.34089	0.562517	0.447278
T.cells	H2AFZ	-0.12524	10.35837	-1.48833	0.140223	-6.54801	0.495086	0.382527
T.cells	HTRA3	-0.63271	0.502839	-1.48799	0.140312	-4.51651	0.61865	0.503338
T.cells	TIFAB	0.359645	2.73524	1.487923	0.140329	-5.01711	0.588366	0.473094
T.cells	PHKB	0.153953	5.588989	1.487855	0.140347	-5.57555	0.551657	0.437009
T.cells	SEC22C	0.380354	2.04121	1.487602	0.140414	-4.76524	0.597631	0.482371
T.cells	USP6NL	-0.13112	5.563085	-1.48736	0.140479	-5.68155	0.551981	0.437368
T.cells	SRPK1	-0.09381	6.11152	-1.48728	0.140499	-5.78182	0.545173	0.430741
T.cells	TMEM39B	-0.17052	4.225197	-1.48726	0.140504	-5.44151	0.568924	0.453965
T.cells	GM37305	0.535948	1.078554	1.48723	0.140512	-4.56558	0.610705	0.495486
T.cells	GM17745	0.374983	0.962497	1.487051	0.140559	-4.83871	0.612299	0.497152

T.cells	1810055G(	-0.3876	2.268587	-1.4866	0.140677	-4.68856	0.59481	0.479564
T.cells	KLHL11	-0.27298	2.776613	-1.48632	0.140752	-5.03107	0.588045	0.472836
T.cells	JAKMIP1	0.201306	3.981458	1.486239	0.140773	-5.4222	0.572284	0.457246
T.cells	APLP2	-0.18548	5.805079	-1.48564	0.140933	-5.51859	0.549179	0.434697
T.cells	SELENOM	0.289605	2.384919	1.485356	0.141007	-5.1613	0.593255	0.478213
T.cells	IL10	-0.69305	2.999738	-1.48533	0.141014	-4.6838	0.585096	0.470104
T.cells	KLF5	0.806932	0.36413	1.484897	0.141128	-4.49395	0.620818	0.506011
T.cells	HIST1H1E	0.31097	4.203103	1.48456	0.141217	-5.63804	0.569428	0.454865
T.cells	GM16104	-0.97794	-0.60575	-1.4844	0.141258	-4.42649	0.634461	0.519966
T.cells	SNHG14	-0.42873	1.410261	-1.4844	0.14126	-4.63408	0.606405	0.491662
T.cells	MDM2	0.168827	6.41707	1.484397	0.14126	-5.76086	0.541624	0.42772
T.cells	CCR1	1.022416	2.32125	1.484278	0.141292	-4.44858	0.594106	0.479388
T.cells	SLC35C1	0.291379	2.749333	1.484244	0.141301	-4.96157	0.588406	0.473714
T.cells	CCDC47	0.131945	5.110399	1.483999	0.141366	-5.53706	0.557876	0.443687
T.cells	ANKIB1	0.122021	5.807453	1.483925	0.141385	-5.69186	0.54915	0.435182
T.cells	TFPI	-0.26684	3.468648	-1.4837	0.141446	-5.05584	0.578944	0.464492
T.cells	PSENEN	-0.09703	6.713106	-1.48344	0.141514	-5.80905	0.538004	0.424385
T.cells	RTP4	0.631759	4.174749	1.48339	0.141527	-4.95523	0.569793	0.45546
T.cells	ETNK2	-0.47684	1.270533	-1.48266	0.141721	-4.70277	0.608581	0.494002
T.cells	RNF146	0.145111	5.30614	1.482384	0.141794	-5.55221	0.555658	0.44158
T.cells	HIST2H2AC	0.343503	2.376405	1.482229	0.141835	-5.24425	0.593631	0.479101
T.cells	PLA2G15	0.250513	3.738633	1.482174	0.14185	-5.24601	0.575683	0.461292
T.cells	GM43113	0.849581	-0.69248	1.482105	0.141868	-4.43462	0.635976	0.521757
T.cells	LGALS4	-0.39997	2.563012	-1.48177	0.141957	-4.81808	0.591142	0.476647
T.cells	DYNLL1	-0.09325	7.867224	-1.48162	0.141996	-6.0667	0.524343	0.411223
T.cells	TRAK1	-0.0928	7.291138	-1.48162	0.141996	-6.03066	0.531238	0.41787
T.cells	GSN	0.16194	5.844895	1.480152	0.142387	-5.76862	0.549684	0.435609
T.cells	WWOX	0.127757	6.961551	1.480052	0.142414	-5.85326	0.535959	0.422324
T.cells	INSL6	0.383488	1.68935	1.479977	0.142434	-4.88698	0.60371	0.489088
T.cells	PTK2	-0.32294	4.164046	-1.47985	0.142467	-5.00431	0.570969	0.456551
T.cells	KATNAL1	0.428697	0.973387	1.47974	0.142497	-4.76062	0.613501	0.498936
T.cells	CYFIP1	0.19992	5.64309	1.479561	0.142545	-5.40134	0.552199	0.43817
T.cells	SLC27A1	0.313435	2.972091	1.479551	0.142548	-5.06544	0.586527	0.471987
T.cells	PLK4	-0.20149	3.856529	-1.47888	0.142725	-5.49757	0.575457	0.460674
T.cells	ANKRD33B	0.267306	4.882196	1.478064	0.142944	-5.62784	0.562944	0.447906
T.cells	DBN1	-0.65662	1.388658	-1.47761	0.143065	-4.5924	0.609227	0.493719
T.cells	FKBPL	0.505145	1.019839	1.477476	0.143101	-4.62042	0.614296	0.498821
T.cells	ELANE	1.599246	2.340078	1.477345	0.143136	-4.62515	0.596328	0.480855
T.cells	CACNA1A	0.443721	1.424509	1.477138	0.143192	-4.74948	0.608736	0.493335
T.cells	RIC8A	0.186993	3.980741	1.476966	0.143238	-5.25981	0.574678	0.459539
T.cells	TMEM126I	0.258705	2.697386	1.476645	0.143324	-4.98977	0.591694	0.476329
T.cells	MALT1	0.335039	7.764534	1.475177	0.143717	-5.71398	0.528677	0.414099
T.cells	TSPAN32	0.261376	2.972272	1.475094	0.14374	-5.17593	0.589184	0.473234
T.cells	CUL2	0.155153	4.968394	1.475033	0.143756	-5.50547	0.563231	0.447676
T.cells	WRAP53	0.217266	2.788872	1.474369	0.143934	-5.13062	0.591936	0.475878
T.cells	GM48236	-0.88903	0.1418	-1.47428	0.143958	-4.44925	0.628223	0.512211
T.cells	REV3L	-0.10573	6.713202	-1.47404	0.144023	-5.90609	0.541712	0.426752

T.cells	CAR9	0.588168	0.285801	1.47399	0.144036	-4.56267	0.626198	0.510303
T.cells	SDHAF4	-0.1656	4.365947	-1.47349	0.144172	-5.39001	0.571417	0.455746
T.cells	ALDH6A1	-0.49802	2.786139	-1.47345	0.14418	-4.77284	0.592145	0.476232
T.cells	RASD1	0.232957	3.88428	1.47298	0.144308	-5.44438	0.577968	0.462056
T.cells	IGKV1-35	-0.47138	-0.53717	-1.47194	0.144587	-4.68205	0.638847	0.522842
T.cells	BC050972	0.896222	-0.27973	1.471884	0.144603	-4.44471	0.635174	0.519159
T.cells	CDC37L1	0.121715	5.167302	1.471829	0.144618	-5.60796	0.561873	0.446223
T.cells	DCTN1	-0.14116	4.987161	-1.47177	0.144634	-5.47955	0.564165	0.44847
T.cells	SEPSECS	0.240596	3.143326	1.471459	0.144717	-5.16318	0.58814	0.472111
T.cells	PCBP4	-0.58774	0.472145	-1.47123	0.14478	-4.5767	0.624556	0.508633
T.cells	HMGB1	-0.09639	9.807497	-1.47064	0.144939	-6.50822	0.505763	0.392545
T.cells	GM19522	0.41683	0.940675	1.470553	0.144962	-4.75653	0.618021	0.502417
T.cells	ZBTB34	0.222327	3.592868	1.47042	0.144998	-5.19639	0.582209	0.466734
T.cells	SOS2	0.140616	5.600384	1.470109	0.145082	-5.64838	0.556396	0.441518
T.cells	CES2E	-0.61188	1.228112	-1.46989	0.145142	-4.591	0.614044	0.498739
T.cells	CYP7A1	-0.68051	0.739983	-1.46968	0.145197	-4.5633	0.620813	0.505655
T.cells	SPOCK2	0.605753	0.349422	1.469626	0.145213	-4.53995	0.626278	0.511201
T.cells	SMDT1	0.092192	7.773748	1.469398	0.145274	-6.03486	0.529667	0.415935
T.cells	PPP1R8	-0.15643	4.114233	-1.46903	0.145374	-5.45175	0.5754	0.460636
T.cells	TMEM216	-0.1903	4.069162	-1.46902	0.145378	-5.42085	0.575985	0.461215
T.cells	MRTFA	0.137448	7.084199	1.468855	0.145421	-5.92591	0.538013	0.424139
T.cells	PTPRA	0.090683	6.521638	1.468698	0.145464	-5.86093	0.544914	0.430834
T.cells	FFAR1	0.518899	-0.22509	1.468613	0.145487	-4.56722	0.634397	0.519761
T.cells	ZFP81	0.305709	1.873403	1.468572	0.145498	-4.9382	0.605201	0.490332
T.cells	PPARGC1B	0.321168	2.799015	1.468486	0.145521	-4.96834	0.592721	0.477872
T.cells	DNASE2A	0.225442	4.427932	1.468417	0.14554	-5.18649	0.571338	0.456693
T.cells	GM47507	-0.79473	0.732528	-1.46786	0.145692	-4.5275	0.621145	0.506397
T.cells	SCO2	-0.30173	2.851352	-1.46782	0.145701	-4.9247	0.59224	0.477443
T.cells	SMCO4	-0.1908	4.10439	-1.46747	0.145797	-5.40961	0.575739	0.461313
T.cells	GM38190	0.385394	1.823044	1.467464	0.145798	-4.76561	0.606109	0.49154
T.cells	RNF114	0.162187	5.645299	1.466462	0.14607	-5.60142	0.556878	0.44233
T.cells	AXIN1	-0.0984	6.046226	-1.46611	0.146165	-5.7811	0.552012	0.437435
T.cells	ALDH16A1	0.164078	4.358468	1.465899	0.146223	-5.43695	0.57351	0.458479
T.cells	MRGPRA2E	0.944111	-0.43033	1.464962	0.146478	-4.45814	0.639265	0.524157
T.cells	IQSEC2	0.50826	2.267987	1.464752	0.146536	-4.76612	0.601682	0.486314
T.cells	EDC3	0.174233	3.628178	1.464467	0.146613	-5.30858	0.58352	0.468331
T.cells	HSP90B1	-0.10645	8.976388	-1.46446	0.146615	-6.21024	0.516977	0.403553
T.cells	OPRM1	0.302042	2.803916	1.464391	0.146634	-5.15055	0.594464	0.479202
T.cells	ERBB3	0.672049	1.113728	1.464314	0.146655	-4.55728	0.617502	0.502273
T.cells	DOHH	-0.15785	4.347216	-1.46391	0.146765	-5.47477	0.574127	0.459304
T.cells	TAOK2	0.1723	4.127104	1.46382	0.14679	-5.36727	0.576988	0.462147
T.cells	A430033K0	0.50821	0.6406	1.463761	0.146806	-4.70151	0.624098	0.509198
T.cells	FBXW7	0.130832	6.448567	1.463127	0.146979	-5.8172	0.547936	0.43332
T.cells	POLR1D	-0.09106	6.995878	-1.4624	0.147177	-5.94687	0.541269	0.426893
T.cells	SPR	-0.28491	3.527522	-1.46218	0.147237	-4.99317	0.585423	0.470228
T.cells	PRIM1	-0.1936	4.178781	-1.46203	0.147279	-5.61212	0.576883	0.461781
T.cells	NOP58	-0.13745	5.838554	-1.46175	0.147356	-5.8186	0.555642	0.441057

T.cells	GSTM5	-0.65959	1.19189	-1.46174	0.147358	-4.59266	0.617027	0.501929
T.cells	ALDH2	-0.13685	6.861181	-1.46143	0.147442	-5.85742	0.542924	0.428687
T.cells	DIRAS2	-0.39881	0.718948	-1.46142	0.147446	-4.83863	0.623615	0.508579
T.cells	MCM4	-0.1597	5.68459	-1.46141	0.147448	-5.86702	0.557581	0.442962
T.cells	NCKIPSD	-0.28388	2.952621	-1.4612	0.147505	-4.91687	0.59306	0.477997
T.cells	ZFP235	0.350518	1.919758	1.461162	0.147516	-4.88581	0.607013	0.491927
T.cells	COA4	0.33932	2.161432	1.460955	0.147573	-4.85536	0.603743	0.488683
T.cells	JDP2	-0.45873	5.304027	-1.46053	0.14769	-4.91541	0.562674	0.447988
T.cells	TMEM170I	0.205869	4.646846	1.460333	0.147743	-5.25449	0.571099	0.456339
T.cells	GM17066	-0.35974	1.747467	-1.45999	0.147837	-4.83796	0.609783	0.49484
T.cells	GM13091	0.524464	0.904415	1.459734	0.147907	-4.65831	0.621445	0.506737
T.cells	AFTPH	0.108964	6.658529	1.459601	0.147944	-5.8566	0.545792	0.431812
T.cells	MIRT1	0.191349	4.950639	1.459484	0.147976	-5.54762	0.567297	0.452891
T.cells	NRBP1	0.117551	5.827937	1.459055	0.148094	-5.70495	0.556402	0.442077
T.cells	DNAJB5	-0.52545	1.175316	-1.45848	0.148252	-4.60732	0.618362	0.503476
T.cells	SETD5	0.094736	6.628233	1.458323	0.148295	-5.88406	0.546774	0.432675
T.cells	TCIM	-0.72735	1.634355	-1.45812	0.148351	-4.58905	0.612033	0.497221
T.cells	BLNK	-0.14234	7.200732	-1.45786	0.148423	-6.11651	0.539819	0.425866
T.cells	SDC3	-0.58334	4.594842	-1.45761	0.14849	-4.86068	0.57267	0.457963
T.cells	CD209G	-1.7081	1.089148	-1.45721	0.148601	-4.53119	0.619801	0.505032
T.cells	IL31RA	0.314207	3.109325	1.457102	0.148631	-4.98906	0.592258	0.477457
T.cells	NR2C2AP	0.17792	4.44571	1.45699	0.148662	-5.48116	0.574667	0.46007
T.cells	GM30025	0.479906	1.597885	1.456666	0.148751	-4.6966	0.612911	0.498003
T.cells	PPM1J	-0.72329	-0.35731	-1.45581	0.148987	-4.48798	0.641122	0.526189
T.cells	GM50019	-0.75688	-0.06748	-1.45563	0.149037	-4.49902	0.636971	0.522093
T.cells	FAM220A	0.503647	2.03012	1.455281	0.149133	-4.74293	0.607661	0.4927
T.cells	CFHR1	-0.8247	0.449486	-1.45525	0.14914	-4.52412	0.629629	0.514829
T.cells	AP3B1	0.094317	7.329007	1.454986	0.149214	-5.94831	0.539108	0.425241
T.cells	CCT4	-0.09431	6.487167	-1.45494	0.149227	-5.90289	0.549491	0.435325
T.cells	B2M	0.207552	9.94338	1.454351	0.14939	-6.29687	0.508064	0.395573
T.cells	DDHD2	-0.17821	4.784447	-1.45401	0.149483	-5.41375	0.571095	0.456801
T.cells	P2RX1	-0.71229	0.81074	-1.45392	0.14951	-4.55278	0.624573	0.510241
T.cells	SMIM12	0.159631	4.0506	1.45376	0.149553	-5.40416	0.580639	0.466363
T.cells	ZFP182	0.222463	4.211679	1.453607	0.149596	-5.27999	0.578532	0.464327
T.cells	LBP	-0.45805	3.292366	-1.45353	0.149617	-4.75094	0.590656	0.47636
T.cells	MINDY1	0.233869	3.987424	1.453334	0.149671	-5.09873	0.581468	0.46729
T.cells	NXT1	0.152994	4.399059	1.453327	0.149673	-5.57783	0.576089	0.461967
T.cells	TERF2IP	0.178343	3.58313	1.453238	0.149698	-5.27472	0.586796	0.472578
T.cells	FRMD6	0.230703	3.47737	1.452826	0.149812	-5.34348	0.588338	0.474159
T.cells	SLC25A4	-0.1283	7.14787	-1.45274	0.149837	-6.00113	0.541481	0.428105
T.cells	TMBIM4	0.117396	6.749974	1.451882	0.150074	-5.87225	0.546954	0.433157
T.cells	CD101	0.77218	0.741284	1.451603	0.150151	-4.48875	0.626349	0.51202
T.cells	CSRP2	-0.13412	5.20682	-1.45147	0.150189	-5.79705	0.566394	0.452211
T.cells	GABPB2	-0.09222	6.502447	-1.45143	0.1502	-5.95089	0.550029	0.43619
T.cells	HNRNPF	-0.07471	9.158537	-1.45114	0.15028	-6.32446	0.517968	0.405216
T.cells	SFR1	-0.09866	6.44096	-1.45076	0.150386	-5.89025	0.5511	0.437184
T.cells	OPTN	-0.2166	4.651784	-1.4505	0.150458	-5.38214	0.573936	0.459527

T.cells	GM20721	0.218346	3.72758	1.449922	0.150618	-5.23309	0.586352	0.471771
T.cells	GM46440	0.551965	0.637929	1.449838	0.150642	-4.6636	0.628571	0.514112
T.cells	GM20186	0.31398	3.559404	1.448814	0.150927	-5.10606	0.588859	0.474402
T.cells	GLRX2	0.124631	5.111058	1.448786	0.150935	-5.61555	0.568584	0.454349
T.cells	CEP290	0.262898	2.468766	1.448729	0.150951	-5.02843	0.603511	0.489021
T.cells	1700030J2	0.752206	-0.14389	1.448625	0.15098	-4.54421	0.639993	0.525883
T.cells	NCK2	-0.16889	4.870391	-1.44854	0.151005	-5.66792	0.571686	0.45747
T.cells	YWHAH	-0.10735	7.687722	-1.44823	0.15109	-6.09838	0.536359	0.423047
T.cells	TMEM268	-0.31532	3.100152	-1.44818	0.151103	-4.88869	0.594988	0.480672
T.cells	LMAN1	-0.1681	4.627609	-1.4481	0.151127	-5.50514	0.57483	0.460701
T.cells	METTL1	-0.23705	3.431367	-1.44756	0.151276	-5.26512	0.590784	0.476416
T.cells	PRRG4	0.496288	0.01648	1.447522	0.151287	-4.69119	0.637937	0.523852
T.cells	FSTL1	-0.45055	2.107543	-1.44688	0.151466	-4.85423	0.609172	0.49449
T.cells	RMND1	0.160747	3.79439	1.446556	0.151557	-5.3516	0.586452	0.4719
T.cells	TTBK2	-0.42115	1.314702	-1.44611	0.151683	-4.68915	0.62013	0.505797
T.cells	METTL26	-0.18622	4.639923	-1.44598	0.15172	-5.46875	0.575363	0.461133
T.cells	GM49041	0.703674	1.334735	1.44575	0.151783	-4.53797	0.619851	0.505566
T.cells	ARAF	0.15629	4.478585	1.445615	0.151821	-5.39507	0.577464	0.463287
T.cells	1500004A1	-0.25788	2.078931	-1.44559	0.151828	-5.02773	0.609564	0.495276
T.cells	CATSPERD	-0.59623	1.329786	-1.44537	0.151889	-4.64687	0.61992	0.505776
T.cells	ZC3H7B	0.20377	3.353116	1.445258	0.151921	-5.2887	0.592318	0.478125
T.cells	TLR4	0.405546	3.122783	1.445221	0.151931	-4.89937	0.595402	0.481199
T.cells	ATP1A3	-0.74169	1.709416	-1.44454	0.152123	-4.54035	0.615011	0.500828
T.cells	FAM25C	-0.69533	1.148741	-1.44453	0.152123	-4.58477	0.62281	0.508693
T.cells	KLF16	-0.23511	3.164065	-1.4441	0.152245	-5.10915	0.59531	0.481151
T.cells	ST6GALNA(	-0.24623	5.127632	-1.44406	0.152256	-5.55479	0.569499	0.455562
T.cells	PIBF1	0.141578	5.059147	1.443352	0.152455	-5.61043	0.570842	0.456676
T.cells	CDKL3	0.308037	3.044103	1.443238	0.152487	-5.09181	0.597404	0.48302
T.cells	PATL2	0.423487	2.023827	1.443069	0.152535	-4.83649	0.611288	0.496948
T.cells	OSGIN2	0.227968	3.18431	1.442369	0.152732	-5.26033	0.595595	0.481431
T.cells	MFSD4A	0.275225	2.041521	1.44199	0.152838	-5.12092	0.611123	0.49714
T.cells	GM47664	0.204518	3.774581	1.441687	0.152923	-5.26411	0.587719	0.473786
T.cells	P4HA3	-0.53474	0.308298	-1.44168	0.152925	-4.64878	0.635388	0.521716
T.cells	PNP	0.154279	5.924728	1.441523	0.15297	-5.77515	0.559846	0.44627
T.cells	ATF1	-0.08289	6.564036	-1.44125	0.153048	-5.92027	0.551802	0.438446
T.cells	FCRL1	-0.40031	2.73778	-1.44075	0.153187	-4.88651	0.601618	0.487881
T.cells	NFKB2	-0.23666	5.096214	-1.44065	0.153215	-5.37281	0.570437	0.456886
T.cells	CLCN5	0.191623	5.160349	1.440637	0.153219	-5.50292	0.56961	0.45607
T.cells	GM29966	-0.70771	1.411825	-1.44051	0.153255	-4.63898	0.619838	0.506204
T.cells	PIK3CA	0.122602	6.128996	1.440487	0.153262	-5.80803	0.557264	0.44394
T.cells	GM37233	0.638528	0.010375	1.440122	0.153364	-4.56006	0.639647	0.526376
T.cells	HIST3H2BA	0.297819	0.997637	1.439884	0.153432	-4.95713	0.625633	0.512142
T.cells	MTERF2	0.310126	2.099595	1.439812	0.153452	-5.00153	0.610325	0.496694
T.cells	HMOX1	-0.58045	4.807778	-1.43975	0.153471	-4.99378	0.574167	0.46064
T.cells	SEMA4A	0.563325	3.364224	1.43967	0.153492	-4.63479	0.593184	0.479525
T.cells	DNAJC30	-0.17833	3.959336	-1.4396	0.153513	-5.35784	0.585274	0.471649
T.cells	PELI2	-0.59976	2.831902	-1.43955	0.153526	-4.64216	0.600344	0.48668

T.cells	CYP4F18	0.252765	3.551567	1.4395	0.15354	-5.22163	0.590684	0.477042
T.cells	OSTC	-0.10127	6.387429	-1.43864	0.153782	-5.85954	0.554364	0.441179
T.cells	ADAMTSL1	-1.11973	0.587709	-1.43862	0.153789	-4.53097	0.631817	0.518427
T.cells	PLAUR	0.644875	6.837467	1.438452	0.153836	-4.91506	0.548741	0.435744
T.cells	TMEM259	-0.17789	4.879001	-1.4381	0.153937	-5.52029	0.573607	0.460239
T.cells	TMSB15B2	-0.28308	2.47439	-1.43806	0.153947	-5.06101	0.605581	0.492091
T.cells	0610030E2	0.14503	4.591291	1.438049	0.153951	-5.54141	0.577348	0.46394
T.cells	SIN3A	0.111762	5.871572	1.437871	0.154001	-5.82131	0.560875	0.447779
T.cells	PLA2G4A	0.359226	3.294743	1.437484	0.15411	-5.03924	0.594713	0.481251
T.cells	CAVIN2	-0.52868	2.454343	-1.43728	0.154167	-4.67386	0.606099	0.492713
T.cells	ATP5MD	-0.09982	8.035566	-1.43707	0.154228	-6.12908	0.534283	0.421868
T.cells	INTS14	-0.21812	4.691853	-1.43657	0.154369	-5.48768	0.576637	0.463212
T.cells	4930403P2	-0.51199	0.612375	-1.4363	0.154447	-4.60434	0.632231	0.518953
T.cells	AP4B1	-0.3456	2.273778	-1.4358	0.154589	-4.89215	0.609388	0.495814
T.cells	AGRN	-0.72022	1.254784	-1.43563	0.154635	-4.55269	0.623514	0.510059
T.cells	ARFGEF2	0.140024	6.285468	1.435034	0.154805	-5.73895	0.556874	0.443733
T.cells	VPS54	0.124176	6.506686	1.434877	0.15485	-5.87251	0.554092	0.441085
T.cells	PEX11G	-0.32454	2.394748	-1.43472	0.154894	-4.89087	0.60801	0.494609
T.cells	KCTD20	-0.13638	4.885027	-1.43465	0.154914	-5.5653	0.574798	0.461502
T.cells	KSR2	-0.56671	2.695294	-1.43441	0.154983	-4.80631	0.603923	0.490517
T.cells	BIRC3	-0.14717	7.111789	-1.43422	0.155038	-5.87785	0.54656	0.433851
T.cells	C1QA	-0.52575	5.480946	-1.43409	0.155074	-5.26382	0.567119	0.454031
T.cells	CDK7	0.160841	4.595584	1.433566	0.155223	-5.52449	0.578618	0.465436
T.cells	R74862	-0.50698	1.054776	-1.43313	0.155348	-4.76284	0.626663	0.513675
T.cells	PTH1R	-0.81559	0.264703	-1.43302	0.155378	-4.5483	0.637875	0.525071
T.cells	CSNK2B	-0.10442	7.247784	-1.43295	0.155398	-6.01511	0.544912	0.432372
T.cells	ZKSCAN8	0.491614	0.632724	1.432635	0.155489	-4.66154	0.632629	0.519903
T.cells	SDHAF1	0.193755	4.132974	1.43248	0.155533	-5.39643	0.584694	0.471746
T.cells	MOCS3	-0.44035	1.446277	-1.43236	0.155567	-4.78665	0.621174	0.508397
T.cells	ACTR3B	-0.4586	-0.01555	-1.43229	0.155587	-4.64356	0.641896	0.529462
T.cells	ZPBP	-0.37612	1.648971	-1.43208	0.155647	-4.83491	0.61835	0.505625
T.cells	GM48623	0.492828	-0.24558	1.432075	0.155648	-4.7101	0.645214	0.532941
T.cells	TLR3	-0.82493	1.534056	-1.43205	0.155656	-4.54399	0.61995	0.507243
T.cells	ELAC2	-0.22681	2.817476	-1.43135	0.155855	-5.14927	0.602535	0.489656
T.cells	ABHD5	-0.41923	3.453436	-1.43132	0.155865	-4.7386	0.593959	0.48108
T.cells	SERPINF1	-0.31666	3.83888	-1.43103	0.155947	-5.28572	0.588817	0.476047
T.cells	KLF13	-0.10144	8.183848	-1.43101	0.155952	-6.13685	0.533672	0.421814
T.cells	DGUOK	-0.18142	4.460054	-1.43093	0.155977	-5.44803	0.580618	0.467935
T.cells	MS4A3	1.044925	-0.35863	1.429662	0.156338	-4.49322	0.648385	0.535537
T.cells	MLF1	-0.47015	0.512644	-1.42872	0.156608	-4.7397	0.636727	0.523208
T.cells	PHTF2	0.129277	5.998759	1.427989	0.156818	-5.84029	0.563132	0.44936
T.cells	MFSD3	0.564838	0.642198	1.427898	0.156844	-4.62173	0.635411	0.521613
T.cells	TSEN34	-0.18275	4.195681	-1.42769	0.156904	-5.41417	0.586588	0.472623
T.cells	METTL2	-0.18191	3.27705	-1.42741	0.156983	-5.31456	0.59894	0.484927
T.cells	POLR2J	-0.11808	4.922519	-1.42726	0.157026	-5.67735	0.577102	0.463314
T.cells	SAFB2	-0.09778	5.993263	-1.42662	0.157211	-5.80562	0.563675	0.449929
T.cells	GPR157	-0.34577	2.137362	-1.42616	0.157343	-4.86652	0.61493	0.501051

T.cells	ONECUT2	-0.61829	1.559667	-1.42612	0.157355	-4.65372	0.622972	0.509186
T.cells	ACSF3	-0.3522	1.59243	-1.42584	0.157436	-4.85821	0.622514	0.508815
T.cells	DENND6A	0.126601	5.102952	1.425837	0.157437	-5.66323	0.575142	0.461467
T.cells	SEMA4F	0.713724	-0.41218	1.425749	0.157462	-4.50119	0.651158	0.537973
T.cells	MAP3K5	0.133721	7.255072	1.425164	0.157631	-5.95731	0.547793	0.43489
T.cells	TESK1	-0.19781	3.781202	-1.42478	0.157742	-5.28276	0.592569	0.479234
T.cells	RBMXL1	-0.10827	5.042632	-1.42464	0.157783	-5.75694	0.575927	0.462723
T.cells	SPCS2	-0.08969	7.595587	-1.42438	0.157857	-6.06094	0.54358	0.431049
T.cells	RALYL	-0.66623	0.59788	-1.42385	0.158008	-4.70632	0.636577	0.523844
T.cells	MCM5	-0.22052	4.910563	-1.42374	0.158041	-5.7727	0.577648	0.464631
T.cells	AK3	-0.14802	4.328071	-1.42371	0.158049	-5.53794	0.5853	0.472227
T.cells	GGTA1	0.18077	5.365487	1.423693	0.158055	-5.60055	0.571738	0.458784
T.cells	PLEKHB2	0.197679	4.419141	1.423681	0.158058	-5.24067	0.584097	0.471031
T.cells	GTSF2	-0.7138	0.091247	-1.42339	0.158142	-4.57466	0.643853	0.531338
T.cells	ACCS	-0.32468	2.079594	-1.42331	0.158166	-4.92518	0.61573	0.502814
T.cells	SAP18	0.092092	7.043708	1.42312	0.15822	-5.9991	0.550424	0.43799
T.cells	INPP5J	0.712085	-1.12022	1.422977	0.158262	-4.49558	0.661561	0.549603
T.cells	SATB2	-0.34705	1.643339	-1.42297	0.158265	-5.03225	0.621801	0.509019
T.cells	STAMBPL1	0.12651	6.55028	1.422659	0.158354	-5.97823	0.556611	0.444108
T.cells	GMPPB	-0.31672	2.629402	-1.42252	0.158395	-4.92289	0.608156	0.495319
T.cells	GOPC	0.129205	4.545466	1.422473	0.158408	-5.55346	0.582433	0.469605
T.cells	DLL4	-0.78503	0.717902	-1.42239	0.158432	-4.53048	0.634865	0.522362
T.cells	SDC4	0.169808	6.436865	1.422358	0.158441	-5.95879	0.558043	0.445524
T.cells	GM36161	0.944012	0.563057	1.421584	0.158665	-4.51061	0.637532	0.525041
T.cells	XYLT1	0.140835	7.355246	1.42146	0.158701	-6.12051	0.546942	0.43467
T.cells	SERAC1	0.408908	2.177244	1.421423	0.158711	-4.83131	0.614819	0.502053
T.cells	SREBF2	-0.13961	5.9605	-1.42116	0.158787	-5.76077	0.56458	0.45193
T.cells	MICALL2	0.659307	1.195719	1.420622	0.158944	-4.57179	0.629013	0.516208
T.cells	TRNAU1AP	-0.1836	4.126118	-1.42038	0.159014	-5.42895	0.588839	0.475839
T.cells	CCS	-0.22081	4.318203	-1.42029	0.159041	-5.28189	0.586291	0.473366
T.cells	PDF	-0.4904	0.794298	-1.41961	0.159238	-4.6844	0.635288	0.522406
T.cells	9930111J2	0.623292	1.279459	1.418962	0.159426	-4.63907	0.628506	0.515709
T.cells	THEMIS	-0.87383	1.160853	-1.41887	0.159454	-4.56909	0.630183	0.51747
T.cells	NSUN6	0.207166	3.821373	1.418706	0.1595	-5.32833	0.593541	0.480617
T.cells	CD2AP	0.152845	6.24029	1.418662	0.159513	-5.78848	0.561961	0.449318
T.cells	STK17B	-0.13016	8.129238	-1.41834	0.159607	-5.93928	0.538408	0.426352
T.cells	MYL6B	0.420658	0.862198	1.418081	0.159682	-4.77032	0.634424	0.521983
T.cells	PARVB	0.357461	2.578711	1.417957	0.159718	-4.9818	0.610402	0.497662
T.cells	RAN	-0.12975	8.400943	-1.4179	0.159735	-6.32798	0.535098	0.423208
T.cells	TMX2	-0.19663	3.46498	-1.4178	0.159765	-5.33279	0.598332	0.485541
T.cells	DLEU2	-0.1122	8.40867	-1.41772	0.159789	-6.25071	0.535004	0.423143
T.cells	KDM2B	-0.23212	7.133876	-1.41737	0.15989	-5.83331	0.550803	0.438583
T.cells	GM42941	-0.54378	1.267028	-1.41721	0.159936	-4.61048	0.628799	0.516399
T.cells	TRIM12A	0.174309	5.048891	1.417072	0.159976	-5.54306	0.57742	0.464828
T.cells	ACO2	-0.0951	6.116578	-1.41669	0.160086	-5.87023	0.563664	0.451313
T.cells	PLAC8	0.29947	8.2646	1.416169	0.16024	-6.17639	0.536878	0.425216
T.cells	TNNI2	-0.3823	2.310806	-1.41613	0.160251	-4.82612	0.614234	0.501868

T.cells	2010009K1	-0.79053	0.112521	-1.41606	0.160273	-4.6137	0.645332	0.533482
T.cells	GALNT11	0.184129	4.120023	1.415775	0.160355	-5.32007	0.589687	0.477293
T.cells	2810405F1	0.492892	0.687122	1.415534	0.160425	-4.72028	0.637067	0.525114
T.cells	GNGT2	0.213186	5.675602	1.415498	0.160435	-5.67598	0.569318	0.457074
T.cells	GM14698	-0.48827	0.738672	-1.41539	0.160468	-4.69738	0.63633	0.524363
T.cells	PSMC4	-0.12026	5.46131	-1.41504	0.160569	-5.8016	0.572084	0.459991
T.cells	COL4A2	-0.50343	2.620499	-1.41497	0.160591	-4.76984	0.609966	0.497854
T.cells	CMPK2	0.357855	3.672891	1.414767	0.160649	-5.22425	0.595668	0.483613
T.cells	PIK3CG	0.140997	4.936727	1.414395	0.160758	-5.67023	0.578909	0.467026
T.cells	SULF2	-0.49424	2.585463	-1.41424	0.160803	-4.75409	0.610448	0.49863
T.cells	HK2	-0.27575	5.335441	-1.41359	0.160993	-5.38842	0.573715	0.462087
T.cells	9530062K0	0.581256	0.793704	1.413314	0.161074	-4.63763	0.635544	0.524423
T.cells	CTLA2A	0.447033	4.28991	1.413204	0.161107	-5.03839	0.58743	0.475832
T.cells	WDR7	0.185502	5.188411	1.413175	0.161115	-5.53588	0.575625	0.464078
T.cells	CCL25	0.178017	4.40296	1.413151	0.161122	-5.56858	0.585932	0.474342
T.cells	LRATD2	-0.35085	1.700967	-1.41306	0.161149	-4.86545	0.62272	0.511376
T.cells	SH3GL1	-0.1415	4.688824	-1.41297	0.161174	-5.55955	0.582161	0.470638
T.cells	SETDB1	-0.13819	4.832864	-1.41264	0.161272	-5.64842	0.58027	0.468839
T.cells	SDHAF2	0.132383	4.655098	1.412532	0.161304	-5.65907	0.582605	0.471197
T.cells	GM45267	-0.77145	0.245242	-1.41252	0.161308	-4.52473	0.643414	0.532671
T.cells	POGK	0.277962	2.454489	1.412456	0.161326	-5.12069	0.612251	0.500973
T.cells	ADAP1	-0.20529	4.152492	-1.41242	0.161336	-5.36154	0.589255	0.477854
T.cells	FCRL6	-0.46515	-0.87747	-1.41239	0.161347	-4.70194	0.659804	0.549543
T.cells	CLDND1	-0.14186	4.825073	-1.41123	0.161687	-5.5003	0.581407	0.469416
T.cells	A330040F1	0.601998	2.508264	1.410757	0.161825	-4.90537	0.612925	0.500851
T.cells	MIRT2	0.7177	-1.00119	1.410396	0.161931	-4.50436	0.663232	0.552181
T.cells	RAB5B	0.17891	4.382557	1.410343	0.161947	-5.39425	0.587619	0.47547
T.cells	BTF3	-0.07746	8.999494	-1.40987	0.162087	-6.34698	0.529443	0.418245
T.cells	RILPL1	-0.47217	2.31814	-1.40967	0.162145	-4.72203	0.615817	0.503782
T.cells	TNPO2	0.176974	4.122122	1.409449	0.16221	-5.44416	0.591277	0.479236
T.cells	RNF216	0.093122	6.677461	1.409262	0.162265	-5.96645	0.558077	0.446374
T.cells	NBEAL1	0.169897	4.937374	1.409131	0.162304	-5.51615	0.580489	0.468591
T.cells	ITGA2	0.688605	0.926385	1.409092	0.162315	-4.56809	0.635392	0.523906
T.cells	PRORP	-0.14494	3.853039	-1.40858	0.162466	-5.52732	0.595237	0.48319
T.cells	SRSF5	-0.0974	6.955445	-1.4078	0.162697	-5.92136	0.554953	0.443507
T.cells	NQO2	0.197622	3.942173	1.407651	0.162741	-5.2537	0.59409	0.482334
T.cells	SUZ12	-0.08927	6.954117	-1.40756	0.162769	-6.09845	0.55497	0.443571
T.cells	L3MBTL3	0.156373	5.067059	1.407124	0.162896	-5.67024	0.579186	0.467568
T.cells	RAB5IF	0.099765	7.307335	1.407095	0.162905	-6.03818	0.550545	0.439297
T.cells	MAN1A2	0.097335	6.392544	1.407081	0.162909	-5.88669	0.562075	0.450628
T.cells	SLCO4C1	0.706762	-1.1708	1.406905	0.162961	-4.51051	0.666426	0.556185
T.cells	NRIP3	-0.68216	0.094317	-1.40679	0.162994	-4.66006	0.64781	0.53702
T.cells	RAB11A	-0.08874	7.145444	-1.40664	0.163038	-5.98836	0.552569	0.441396
T.cells	CTNND2	-0.83022	1.726999	-1.40655	0.163067	-4.55785	0.62449	0.513238
T.cells	IQCC	0.410797	1.695654	1.406488	0.163084	-4.83863	0.62493	0.513694
T.cells	GM13205	-0.58217	0.234435	-1.4064	0.163111	-4.613	0.645778	0.534974
T.cells	ORAI2	-0.15053	5.87495	-1.40598	0.163235	-5.68029	0.568914	0.457391

T.cells	EGFR	-0.73399	2.469637	-1.40582	0.163281	-4.7555	0.614373	0.502948
T.cells	SLC9A1	-0.12251	5.416377	-1.40563	0.163337	-5.69281	0.574847	0.463382
T.cells	AAMP	-0.09254	5.908148	-1.40548	0.163383	-5.7733	0.568487	0.457103
T.cells	FGD3	0.228383	4.660813	1.404611	0.16364	-5.30988	0.58548	0.473584
T.cells	HSD17B4	-0.14247	5.027211	-1.40373	0.163902	-5.56927	0.581395	0.469165
T.cells	DDX19A	-0.14558	4.239703	-1.40326	0.164043	-5.57226	0.592146	0.479707
T.cells	TM4SF1	0.661419	2.15291	1.402311	0.164324	-4.70155	0.620985	0.508613
T.cells	CEPT1	0.200577	5.374134	1.402246	0.164343	-5.52933	0.577457	0.465027
T.cells	SAA4	-0.75267	0.640047	-1.40219	0.16436	-4.58043	0.642462	0.530483
T.cells	SMAD1	0.244581	3.547772	1.401927	0.164438	-5.1347	0.601774	0.489352
T.cells	SLC26A2	-0.26024	3.742645	-1.4019	0.164445	-5.1302	0.599134	0.486706
T.cells	EPHX2	-0.50511	2.477792	-1.40181	0.164473	-4.84703	0.61646	0.504159
T.cells	GPR65	-0.66153	4.250918	-1.40172	0.164498	-4.70927	0.5923	0.479896
T.cells	WDR61	0.117546	4.80713	1.401552	0.16455	-5.64275	0.584906	0.472564
T.cells	CBR1	-0.22218	4.350162	-1.40132	0.16462	-5.37122	0.591036	0.478614
T.cells	LEMD3	-0.1258	5.687224	-1.40064	0.164821	-5.73808	0.573956	0.461249
T.cells	FHAD1	0.865386	0.624492	1.400336	0.164912	-4.53182	0.643482	0.531117
T.cells	HOOK2	0.315915	3.558904	1.400057	0.164996	-5.06217	0.602478	0.489523
T.cells	REEP5	-0.09534	7.566136	-1.39987	0.165052	-6.05479	0.550274	0.437929
T.cells	SYDE1	0.452877	0.308678	1.399468	0.165171	-4.7226	0.648178	0.535992
T.cells	TTC39C	0.63568	2.230936	1.399412	0.165188	-4.64219	0.620778	0.508114
T.cells	SKP1A	-0.09392	6.639256	-1.39906	0.165294	-5.95583	0.561956	0.449444
T.cells	TNFAIP6	0.903106	1.129558	1.398661	0.165413	-4.56434	0.636344	0.524032
T.cells	HEMK1	0.498146	1.147753	1.398478	0.165467	-4.70629	0.636084	0.523873
T.cells	PPP1R18OS	-0.36416	0.993134	-1.39843	0.165481	-4.77146	0.638297	0.526129
T.cells	COL4A1	-0.47352	3.330895	-1.39835	0.165506	-4.90921	0.605585	0.493025
T.cells	COMTD1	-0.25451	2.92368	-1.39829	0.165524	-5.0759	0.611169	0.498643
T.cells	RABEPK	0.189249	3.296184	1.398066	0.165591	-5.3421	0.606059	0.49358
T.cells	EID1	-0.14775	5.155191	-1.39779	0.165674	-5.55981	0.581149	0.468791
T.cells	TNFRSF19	0.324077	0.886333	1.397745	0.165687	-5.08687	0.63983	0.527899
T.cells	GALNS	0.192359	3.757313	1.397732	0.165691	-5.35551	0.599789	0.487392
T.cells	SRCAP	-0.08987	6.652894	-1.39729	0.165825	-5.96841	0.561782	0.449809
T.cells	BLOC1S5	0.332944	1.955013	1.397267	0.16583	-4.95674	0.624644	0.512638
T.cells	XCL1	-0.96899	2.335401	-1.39722	0.165843	-4.6098	0.61932	0.507244
T.cells	SPICE1	-0.33001	1.890422	-1.39664	0.166019	-4.95251	0.626018	0.513806
T.cells	GM48855	0.347016	1.463573	1.395511	0.166357	-4.84586	0.632906	0.520316
T.cells	RRP8	-0.17214	3.730434	-1.39516	0.166461	-5.46353	0.601408	0.488569
T.cells	NOB1	-0.13431	4.287446	-1.39515	0.166466	-5.59286	0.593894	0.481053
T.cells	SNAI1	-0.62371	0.389835	-1.39493	0.166533	-4.56447	0.648352	0.53612
T.cells	ADCK2	-0.33037	2.282482	-1.39468	0.166606	-4.85664	0.621355	0.508742
T.cells	IMP4	-0.13562	4.46298	-1.3946	0.166632	-5.5872	0.591545	0.478804
T.cells	SUSD3	-0.25159	3.170727	-1.39451	0.166659	-5.14836	0.609047	0.496337
T.cells	KPNB1	-0.10486	5.739818	-1.39448	0.166667	-5.85862	0.574715	0.462083
T.cells	PCNX	-0.14316	5.518161	-1.39417	0.166761	-5.73224	0.577747	0.465083
T.cells	GM39121	0.762549	-0.94202	1.393427	0.166984	-4.51755	0.668544	0.556837
T.cells	EXOC5	-0.0975	6.376842	-1.39338	0.166997	-5.89365	0.566955	0.454308
T.cells	NUDT8	-0.41803	2.208161	-1.39332	0.167017	-4.77804	0.622909	0.510202

T.cells	ZFP664	0.147025	4.050718	1.392807	0.167171	-5.54078	0.597802	0.48491
T.cells	3300002I08	0.304534	1.970632	1.39238	0.1673	-5.04779	0.626491	0.514007
T.cells	PLSCR1	0.295675	4.550033	1.391792	0.167477	-5.24965	0.5911	0.478613
T.cells	HIVEP1	-0.13331	6.40877	-1.39178	0.167481	-5.88101	0.566766	0.454476
T.cells	DMWD	-0.54081	2.228423	-1.39166	0.167516	-4.69451	0.622867	0.510624
T.cells	ARMC8	0.119108	5.019279	1.391545	0.167551	-5.62604	0.584866	0.472473
T.cells	VPS18	0.163931	4.834066	1.391507	0.167563	-5.49535	0.587319	0.474933
T.cells	KRCC1	0.097962	5.9705	1.391391	0.167598	-5.79934	0.572417	0.460192
T.cells	GM16536	-0.38092	1.530823	-1.39127	0.167635	-4.79798	0.632718	0.520767
T.cells	DYRK2	0.128468	4.884934	1.391211	0.167653	-5.69268	0.586644	0.474379
T.cells	GATD3A	-0.20344	3.544084	-1.39117	0.167666	-5.30157	0.604675	0.492419
T.cells	PALM	0.169858	4.231998	1.390947	0.167732	-5.62432	0.595406	0.483191
T.cells	UTP20	-0.21167	3.652111	-1.39055	0.167852	-5.45586	0.603417	0.491214
T.cells	CLEC2I	0.360926	3.177972	1.390442	0.167885	-5.08506	0.609903	0.49778
T.cells	GM47200	0.658781	0.11613	1.390062	0.168	-4.5976	0.653609	0.542116
T.cells	HEXIM2	-0.45758	0.998422	-1.38872	0.168406	-4.80201	0.64203	0.529572
T.cells	C130050O1	0.66836	1.379067	1.388581	0.168449	-4.5885	0.636563	0.524082
T.cells	TNFSF8	0.830167	-0.51498	1.388466	0.168483	-4.56829	0.664196	0.552397
T.cells	CTDSP2	-0.12721	4.801724	-1.38769	0.168718	-5.6241	0.589942	0.476834
T.cells	MKNK1	-0.17017	4.508378	-1.38733	0.168828	-5.38922	0.59396	0.480804
T.cells	MBNL3	-0.14961	4.597519	-1.38708	0.168905	-5.78111	0.592766	0.479612
T.cells	ILF3	-0.10716	6.541502	-1.38702	0.168923	-5.97684	0.567265	0.454345
T.cells	GM32569	0.314206	1.845691	1.386816	0.168985	-5.30307	0.6307	0.517775
T.cells	DDX51	-0.39445	1.37149	-1.38673	0.169012	-4.85297	0.637461	0.524654
T.cells	RBM39	-0.0608	9.586582	-1.38628	0.169147	-6.42396	0.529401	0.417527
T.cells	PSME2	0.194875	7.278608	1.38615	0.169187	-5.97847	0.557875	0.445232
T.cells	ZBTB22	0.232944	3.076444	1.386037	0.169222	-5.22821	0.613469	0.500488
T.cells	EID3	-0.73959	0.925233	-1.38598	0.169237	-4.6016	0.64389	0.531335
T.cells	CYP8B1	-0.57726	1.074088	-1.38574	0.169311	-4.66497	0.641741	0.529192
T.cells	GM33524	0.702339	0.491882	1.385644	0.169341	-4.58322	0.650184	0.537819
T.cells	THSD4	-0.74695	0.910314	-1.38549	0.169387	-4.72933	0.644106	0.531608
T.cells	4931414P1	0.298129	2.099255	1.385045	0.169524	-5.11274	0.627427	0.514582
T.cells	POLH	0.147134	4.035992	1.384791	0.169601	-5.63708	0.60067	0.48764
T.cells	CRAT	-0.30121	3.198294	-1.38437	0.16973	-5.05689	0.612128	0.499241
T.cells	SPTSSA	-0.10977	6.822761	-1.38433	0.169742	-6.01155	0.563982	0.451331
T.cells	TLCD2	-0.45588	2.659161	-1.38426	0.169763	-4.80194	0.619609	0.506795
T.cells	RAB19	0.303422	2.561986	1.384134	0.169801	-5.09127	0.620966	0.508194
T.cells	CCDC51	0.315348	1.810129	1.383413	0.170021	-4.92517	0.632182	0.519247
T.cells	6230400D1	0.302512	2.163361	1.383125	0.170109	-5.01028	0.627306	0.514329
T.cells	TNFAIP3	-0.19043	6.763033	-1.38283	0.1702	-5.76908	0.565452	0.452616
T.cells	JAK3	0.306414	2.778845	1.382739	0.170228	-5.09056	0.618714	0.505701
T.cells	MIOS	0.155424	3.665658	1.382541	0.170288	-5.46195	0.606493	0.493486
T.cells	RGMB	0.615915	1.845211	1.382048	0.170439	-4.61252	0.632065	0.519361
T.cells	SNRPB2	-0.09189	6.282619	-1.38162	0.170569	-5.96026	0.571829	0.45915
T.cells	MYSM1	0.1197	5.388465	1.381543	0.170593	-5.76057	0.583517	0.470724
T.cells	CTDP1	-0.13258	4.660739	-1.38154	0.170595	-5.60935	0.593194	0.480358
T.cells	ACTR1A	0.096923	6.140647	1.381474	0.170615	-5.88334	0.57367	0.460969

T.cells	GET4	-0.12124	5.110437	-1.38131	0.170665	-5.77233	0.587196	0.474449
T.cells	CCDC192	0.835593	-0.69514	1.381156	0.170712	-4.53794	0.669145	0.557625
T.cells	SSB	-0.08125	6.803522	-1.38034	0.170962	-6.04982	0.565578	0.45289
T.cells	RAC1	-0.07715	7.82414	-1.38032	0.170969	-6.14662	0.552642	0.440205
T.cells	1700007L1	0.373968	1.663099	1.380184	0.17101	-4.89167	0.635171	0.522542
T.cells	ATP7A	0.143131	5.8288	1.379548	0.171205	-5.65711	0.578621	0.465532
T.cells	CPNE9	-0.49234	1.867904	-1.37933	0.171273	-4.90845	0.632713	0.51986
T.cells	A930006K(	0.360447	2.074746	1.379255	0.171295	-4.84219	0.629776	0.516879
T.cells	HIGD2A	0.094728	6.065754	1.378864	0.171415	-5.84026	0.575575	0.462676
T.cells	CSGALNAC	0.281968	2.818139	1.378859	0.171417	-5.34495	0.619375	0.506449
T.cells	9330159M	0.428356	1.076034	1.378614	0.171492	-4.82066	0.644212	0.531693
T.cells	DEF6	0.123384	5.457612	1.377793	0.171744	-5.75688	0.584143	0.471038
T.cells	SCUBE2	-0.87983	-0.26058	-1.37777	0.171752	-4.54861	0.664413	0.55222
T.cells	ZZZ3	-0.12619	5.884403	-1.37724	0.171915	-5.83564	0.5789	0.465661
T.cells	RIPK2	-0.24297	4.205503	-1.37695	0.172003	-5.3047	0.601412	0.487942
T.cells	IAH1	-0.2002	4.521488	-1.37671	0.172077	-5.42229	0.597205	0.483698
T.cells	TRGV2	0.876795	-0.51519	1.375835	0.172348	-4.54859	0.668979	0.556806
T.cells	HAUS6	-0.14841	4.556972	-1.37563	0.172411	-5.69558	0.596839	0.483677
T.cells	SMUG1	0.270839	1.976529	1.375566	0.172431	-5.02089	0.632588	0.519726
T.cells	COL11A2	-0.59917	0.72136	-1.37546	0.172464	-4.69237	0.650686	0.538178
T.cells	MCTS2	-0.54852	1.612847	-1.37546	0.172464	-4.74022	0.637784	0.525011
T.cells	KHSRP	-0.09581	5.998752	-1.37545	0.172466	-5.96163	0.577696	0.464691
T.cells	GM11520	-0.39986	1.043872	-1.37532	0.172508	-4.81228	0.645991	0.533403
T.cells	GRK5	-0.2196	5.747033	-1.37505	0.172592	-5.7828	0.580996	0.468012
T.cells	ZC3H12A	-0.16826	5.128189	-1.37504	0.172592	-5.62209	0.589184	0.476145
T.cells	TMPRSS6	-0.64516	1.467899	-1.37393	0.172938	-4.67718	0.640678	0.527601
T.cells	DUSP23	0.276201	2.373334	1.373901	0.172946	-5.05796	0.627761	0.514485
T.cells	MRPS31	0.155156	3.653241	1.373815	0.172972	-5.46055	0.609913	0.49654
T.cells	NPRL3	0.262858	2.892647	1.372893	0.173258	-5.09427	0.621292	0.507491
T.cells	SV2C	0.699589	-0.28271	1.372511	0.173376	-4.58982	0.667447	0.554502
T.cells	GNAQ	0.121595	7.882192	1.372374	0.173419	-6.10357	0.555175	0.441906
T.cells	YWHAE	-0.06404	8.88218	-1.37218	0.173478	-6.38708	0.542732	0.429815
T.cells	0610040J0	-0.51801	2.378015	-1.37145	0.173706	-4.76154	0.629381	0.515349
T.cells	GM43462	0.223843	3.23183	1.371028	0.173837	-5.30325	0.617514	0.503328
T.cells	TNK2	-0.2047	3.209858	-1.37099	0.173849	-5.3521	0.61782	0.503636
T.cells	SLFN1	1.33099	2.335521	1.370425	0.174024	-4.56539	0.630549	0.516271
T.cells	UBE4B	0.118536	5.48847	1.370071	0.174134	-5.80125	0.587317	0.473094
T.cells	EPSTI1	0.257796	7.192656	1.369907	0.174185	-5.77932	0.565086	0.45126
T.cells	ANP32B	-0.10145	8.191649	-1.36982	0.174212	-6.35042	0.552427	0.438906
T.cells	FBXL18	-0.45587	2.985602	-1.36947	0.17432	-4.96009	0.621461	0.507408
T.cells	FOXJ3	-0.09633	5.947043	-1.36937	0.174352	-5.89612	0.581255	0.467259
T.cells	S100A10	-0.14423	6.601647	-1.36902	0.174461	-6.09767	0.572704	0.458938
T.cells	RHOJ	0.574151	1.992484	1.368699	0.174561	-4.72468	0.635514	0.521932
T.cells	PGD	0.135477	5.716274	1.368555	0.174606	-5.74087	0.584299	0.470605
T.cells	BUD23	-0.16871	4.09892	-1.36847	0.174632	-5.57867	0.60605	0.492301
T.cells	ZFP521	-0.38797	2.100385	-1.36846	0.174637	-5.10636	0.633973	0.52045
T.cells	TM4SF5	0.893692	0.261552	1.367691	0.174875	-4.55437	0.660711	0.548048

T.cells	SAP130	0.123545	5.619105	1.367614	0.174899	-5.81904	0.585584	0.472175
T.cells	DYNC2H1	0.163831	3.842041	1.36757	0.174913	-5.57786	0.609574	0.496121
T.cells	SNX7	-0.54049	1.212304	-1.36756	0.174917	-4.69321	0.646759	0.533766
T.cells	FMNL3	-0.17668	4.258409	-1.36755	0.174918	-5.52178	0.603872	0.490404
T.cells	MSH3	0.121397	5.159864	1.367465	0.174946	-5.83566	0.591698	0.478264
T.cells	IDNK	0.127181	5.52475	1.367095	0.175062	-5.7616	0.586836	0.473506
T.cells	FRYL	0.095101	7.564295	1.367009	0.175088	-6.15368	0.560345	0.447381
T.cells	MBD1	0.144096	4.580853	1.36672	0.175179	-5.63111	0.599491	0.486256
T.cells	RELT	0.24418	3.728351	1.366596	0.175217	-5.22418	0.61114	0.498021
T.cells	NUDT9	-0.19163	4.362001	-1.3665	0.175247	-5.49578	0.602461	0.489356
T.cells	MRPL18	-0.11186	6.633228	-1.36646	0.17526	-6.10014	0.572295	0.459371
T.cells	TESPA1	0.236034	2.504605	1.366379	0.175285	-5.36072	0.628231	0.51532
T.cells	GFRA1	-0.17585	4.918403	-1.36533	0.175612	-5.96666	0.595644	0.482078
T.cells	NEURL3	-0.39486	4.301516	-1.36531	0.17562	-4.92111	0.604002	0.490429
T.cells	PRIMPOL	0.203109	3.486578	1.365174	0.175662	-5.35849	0.615212	0.501717
T.cells	ARF6	-0.11164	6.770128	-1.36484	0.175765	-6.05125	0.571201	0.457983
T.cells	MYCT1	0.515631	1.654516	1.364841	0.175766	-4.71463	0.641125	0.528053
T.cells	ASAP2	-0.2529	3.068411	-1.36439	0.175908	-5.31021	0.62104	0.507875
T.cells	GM12185	0.392866	3.341306	1.364375	0.175913	-4.93716	0.617231	0.504033
T.cells	GM39326	0.318302	1.289251	1.364163	0.175979	-5.06362	0.64641	0.533713
T.cells	ELF2	0.093585	7.39491	1.364162	0.175979	-6.14472	0.56317	0.450317
T.cells	VTA1	0.106598	5.707573	1.363892	0.176064	-5.82632	0.585167	0.472005
T.cells	GM9917	0.584972	0.773036	1.363704	0.176123	-4.70929	0.654014	0.541495
T.cells	GM15614	-0.26664	2.981808	-1.3635	0.176187	-5.28369	0.622315	0.509255
T.cells	NKRF	0.211363	2.983842	1.363198	0.176282	-5.33032	0.622287	0.509333
T.cells	RANBP10	0.140377	5.698692	1.362657	0.176452	-5.80535	0.585285	0.472463
T.cells	ERLEC1	0.164843	4.341715	1.362621	0.176463	-5.529	0.603515	0.490653
T.cells	CTSG	1.34319	-0.2691	1.362448	0.176517	-4.57253	0.669481	0.557867
T.cells	RPGRIP1	-0.0995	6.55945	-1.36186	0.176703	-6.06497	0.573991	0.4616
T.cells	MICU2	0.144888	4.939969	1.361854	0.176704	-5.654	0.595413	0.482888
T.cells	HERPUD2	-0.11681	5.474289	-1.36175	0.176737	-5.69491	0.588264	0.475758
T.cells	UAP1L1	0.219105	3.734573	1.361727	0.176744	-5.24777	0.611842	0.499365
T.cells	GM16599	0.125556	4.782001	1.361677	0.17676	-5.79215	0.597542	0.485016
T.cells	KLRA17	0.72072	0.224958	1.361611	0.17678	-4.55611	0.662107	0.550578
T.cells	UBN2	0.10802	7.076175	1.361252	0.176893	-6.0577	0.567311	0.455196
T.cells	SDHD	-0.12376	5.858408	-1.36094	0.176991	-5.91485	0.583174	0.470973
T.cells	GM15943	-0.74932	0.207344	-1.36086	0.177018	-4.59337	0.662369	0.551149
T.cells	TBC1D8B	0.209076	3.257326	1.360676	0.177075	-5.27097	0.618463	0.506404
T.cells	SMS	-0.09978	6.477089	-1.36067	0.177076	-5.98928	0.575063	0.462991
T.cells	SNHG16	-0.30597	2.227913	-1.36067	0.177076	-5.06502	0.632971	0.521115
T.cells	RING1	-0.21536	3.585545	-1.36067	0.177076	-5.2888	0.613902	0.5018
T.cells	ERN1	-0.16772	7.053542	-1.36026	0.177205	-5.84041	0.567792	0.455733
T.cells	VPS35L	0.114495	5.162292	1.359981	0.177294	-5.6902	0.592627	0.480449
T.cells	FAM122B	-0.27167	2.015817	-1.35997	0.177298	-5.0567	0.636213	0.524412
T.cells	PCYT1A	0.171591	5.255992	1.359428	0.177468	-5.63211	0.591761	0.479412
T.cells	B430306N(	-0.45835	2.692804	-1.3585	0.17776	-4.81442	0.627771	0.515145
T.cells	CD52	0.188461	9.30175	1.358252	0.17784	-6.15035	0.540568	0.428661

T.cells	GPAT3	-0.1253	5.178486	-1.35811	0.177885	-5.92385	0.593526	0.480822
T.cells	GM11084	-0.18848	3.368087	-1.35805	0.177902	-5.36293	0.618289	0.505684
T.cells	CAMSAP2	-0.14834	4.496866	-1.35728	0.178147	-5.62122	0.603041	0.490222
T.cells	PEX13	0.125612	5.524698	1.357262	0.178153	-5.79144	0.589192	0.476394
T.cells	COQ6	0.222216	2.285167	1.356988	0.178239	-5.15499	0.633877	0.521375
T.cells	VMN2R19	0.826895	-0.79625	1.356832	0.178289	-4.55589	0.679275	0.567957
T.cells	ADAM19	-0.21121	5.568234	-1.35651	0.178391	-5.7972	0.588613	0.475984
T.cells	CD37	-0.11162	7.409059	-1.35619	0.178492	-6.15655	0.564578	0.452302
T.cells	LCLAT1	0.211252	3.99918	1.356122	0.178514	-5.37849	0.609855	0.497348
T.cells	FAM234B	-0.36306	2.096184	-1.35606	0.178533	-4.96627	0.636578	0.524389
T.cells	NECTIN2	0.435329	2.109704	1.355865	0.178595	-4.76848	0.636385	0.524227
T.cells	PPIG	0.076055	6.974522	1.355821	0.178609	-6.07825	0.570167	0.457843
T.cells	TMEM219	0.179398	4.624458	1.35582	0.178609	-5.42847	0.601305	0.488799
T.cells	CTR9	0.127778	4.518969	1.355649	0.178664	-5.63012	0.60274	0.490309
T.cells	SLC9A3R2	-0.49331	2.701144	-1.35543	0.178734	-4.82654	0.627967	0.51584
T.cells	BYSL	0.15	3.67185	1.354956	0.178883	-5.4445	0.614376	0.502306
T.cells	GM28875	-0.14918	4.612323	-1.35493	0.178893	-5.79202	0.60147	0.489328
T.cells	LRRC4C	0.672187	0.648779	1.354904	0.1789	-4.6905	0.657626	0.546353
T.cells	CMIP	-0.11263	9.635584	-1.35487	0.178911	-6.42931	0.536751	0.425482
T.cells	ICMT	0.269058	2.429223	1.354421	0.179053	-5.14803	0.632092	0.520136
T.cells	TMCO1	-0.0894	6.265444	-1.35427	0.179102	-5.98149	0.579643	0.467462
T.cells	NLN	0.181785	4.446951	1.353899	0.179219	-5.50377	0.603976	0.491866
T.cells	PTPN12	0.155762	6.049828	1.353834	0.17924	-5.75935	0.582479	0.470438
T.cells	LRRC75A	-0.29372	2.6203	-1.35378	0.179258	-5.21831	0.629378	0.517592
T.cells	EXOSC4	0.182991	3.784732	1.353095	0.179475	-5.46406	0.613203	0.501388
T.cells	IL27RA	-0.48849	1.535873	-1.35297	0.179515	-4.86668	0.645061	0.533761
T.cells	GM5577	0.599957	0.20371	1.352842	0.179556	-4.64604	0.664648	0.553937
T.cells	SNN	-0.19975	3.649853	-1.35267	0.17961	-5.51458	0.615072	0.50339
T.cells	HEYL	0.472072	-0.20551	1.352479	0.179671	-4.81139	0.670773	0.560402
T.cells	TEX45	-0.61388	0.222756	-1.35237	0.179706	-4.68762	0.664364	0.55383
T.cells	KLF3	0.1911	6.281206	1.352261	0.179741	-5.58749	0.57956	0.467956
T.cells	SYNE1	-0.34291	4.964609	-1.35214	0.179778	-5.29171	0.597081	0.485428
T.cells	KHK	-0.25439	4.3658	-1.35187	0.179866	-5.21908	0.605213	0.493715
T.cells	RIC1	0.148334	6.710291	1.351517	0.179978	-5.97088	0.573955	0.46258
T.cells	IMPDH1	0.172009	4.326873	1.351363	0.180027	-5.67762	0.605745	0.494369
T.cells	N4BP2L1	0.221262	4.595001	1.351294	0.180049	-5.37338	0.602088	0.490706
T.cells	ZFP865	0.220367	3.265059	1.351228	0.18007	-5.30421	0.620432	0.509232
T.cells	LSMEM1	0.466057	4.06136	1.351127	0.180102	-5.11001	0.609387	0.498071
T.cells	GNG10	-0.12053	6.940589	-1.35111	0.180108	-6.01947	0.570968	0.459706
T.cells	ANKDD1A	-0.90739	2.346094	-1.35088	0.180182	-4.70288	0.633482	0.522468
T.cells	CDC42EP3	-0.12753	5.142958	-1.34981	0.180522	-5.94947	0.595034	0.483715
T.cells	SLC9A9	-0.33596	7.120003	-1.34975	0.180543	-5.56269	0.568991	0.457811
T.cells	GM29585	-0.46759	1.247477	-1.34967	0.180566	-4.82336	0.649644	0.539084
T.cells	ACSL5	0.133495	6.00653	1.349557	0.180604	-5.8838	0.583523	0.472224
T.cells	RPRD1B	-0.11607	5.480381	-1.34945	0.180636	-5.87304	0.590511	0.479197
T.cells	MTIF3	0.221761	3.10156	1.349437	0.180642	-5.31399	0.623095	0.512
T.cells	NMRAL1	-0.15962	3.904291	-1.34941	0.180649	-5.69624	0.611916	0.500693

T.cells	ADGRA3	-0.54441	0.459311	-1.34918	0.180723	-4.65824	0.661313	0.551093
T.cells	IFFO2	-0.40138	3.364648	-1.34896	0.180796	-4.86694	0.619541	0.508408
T.cells	B3GNT8	0.271367	2.202747	1.348711	0.180874	-5.29007	0.636058	0.525238
T.cells	CTDSPL2	-0.1046	5.892856	-1.34816	0.181052	-5.9851	0.585297	0.474214
T.cells	HAUS8	-0.14679	4.722045	-1.34778	0.181172	-5.63394	0.600999	0.490068
T.cells	PIGP	-0.17774	4.420664	-1.34775	0.181181	-5.47085	0.605104	0.494206
T.cells	GM11696	0.346871	1.871722	1.347678	0.181205	-5.00889	0.640887	0.530555
T.cells	CHMP1B	-0.15711	4.698125	-1.34761	0.181228	-5.51496	0.601324	0.490437
T.cells	PLEKHA3	-0.15822	3.98944	-1.34755	0.181247	-5.47791	0.611024	0.500227
T.cells	GM20513	-0.83957	1.300029	-1.34735	0.181311	-4.66157	0.649177	0.539152
T.cells	RAB4B	-0.10535	5.9254	-1.34728	0.181334	-5.84921	0.584866	0.474049
T.cells	GEMIN8	0.226777	2.284507	1.347123	0.181383	-5.24541	0.634962	0.524658
T.cells	SBNO2	0.137488	5.566821	1.346619	0.181545	-5.67867	0.589871	0.479068
T.cells	CTNNBL1	-0.11505	5.139211	-1.34655	0.181566	-5.76679	0.595603	0.484834
T.cells	GPIHBP1	0.337833	4.095185	1.346009	0.181741	-5.18373	0.610219	0.499345
T.cells	2310033PC	-0.15112	4.144026	-1.34584	0.181794	-5.56263	0.609547	0.498666
T.cells	ALCAM	-0.29105	6.646567	-1.34563	0.181864	-5.62674	0.576058	0.465173
T.cells	FAM172A	0.09599	7.557353	1.344866	0.182108	-6.13135	0.564637	0.453686
T.cells	IMPA1	-0.1126	5.285622	-1.34476	0.182143	-5.82734	0.594444	0.483327
T.cells	CD14	0.830898	4.260683	1.344687	0.182166	-4.89718	0.608371	0.497319
T.cells	TGFBR3	-0.29172	3.337964	-1.3446	0.182194	-5.257	0.62117	0.510317
T.cells	G3BP2	-0.07681	6.759047	-1.34433	0.18228	-6.05589	0.575047	0.463973
T.cells	MAPK3	-0.15301	4.874121	-1.34403	0.182378	-5.62247	0.60025	0.489084
T.cells	ACO1	-0.20974	3.712371	-1.34379	0.182455	-5.23945	0.616278	0.505274
T.cells	ZFP622	-0.12545	5.377893	-1.34345	0.182565	-5.77435	0.593558	0.482501
T.cells	RBBP4	-0.074	7.676556	-1.34342	0.182574	-6.30364	0.563448	0.452592
T.cells	TARSL2	0.378962	1.415252	1.343219	0.182639	-4.83863	0.64907	0.538818
T.cells	ZDHHC4	0.137106	4.474275	1.342733	0.182796	-5.6716	0.606094	0.49504
T.cells	SIAH1B	-0.24457	1.91846	-1.34264	0.182824	-5.21795	0.642037	0.531541
T.cells	GPAM	0.190316	3.696477	1.342228	0.182959	-5.65763	0.61697	0.506099
T.cells	MRPL55	0.143388	4.367452	1.342177	0.182975	-5.6297	0.607697	0.496747
T.cells	ARHGEF12	-0.37363	3.963016	-1.34201	0.183029	-5.00536	0.613271	0.502426
T.cells	TMEM41B	-0.1513	4.300772	-1.34132	0.183253	-5.53408	0.608819	0.498162
T.cells	DGAT2	-0.31815	3.862389	-1.3412	0.18329	-5.09341	0.614874	0.504322
T.cells	ZBP1	0.72252	3.640152	1.340936	0.183376	-4.94894	0.617964	0.507572
T.cells	FAM219A	0.311187	4.171512	1.340554	0.1835	-5.21318	0.610599	0.500255
T.cells	GM47601	0.660492	0.02249	1.340227	0.183606	-4.61133	0.670346	0.561474
T.cells	SNHG8	-0.15559	4.013183	-1.34021	0.183611	-5.54415	0.612785	0.502513
T.cells	TRIM25	0.168432	6.978283	1.340134	0.183636	-5.93126	0.573038	0.462699
T.cells	CACNA1D	0.33322	3.396539	1.340042	0.183666	-5.11308	0.621369	0.511212
T.cells	SLC25A1	0.175223	3.772843	1.340018	0.183673	-5.53779	0.616117	0.505886
T.cells	SLC22A21	0.391758	1.348839	1.339896	0.183713	-4.96488	0.650683	0.541218
T.cells	YTHDF1	-0.10603	5.78074	-1.33948	0.183849	-5.91672	0.58879	0.478603
T.cells	NR2F2	-0.53387	3.13223	-1.33945	0.183856	-4.87339	0.625082	0.515224
T.cells	KMT2A	0.110884	6.28827	1.339401	0.183873	-6.03108	0.582065	0.471906
T.cells	ERG28	-0.11196	5.118614	-1.33922	0.183933	-5.73586	0.597673	0.487593
T.cells	CARMIL2	0.207494	2.704609	1.339126	0.183962	-5.3787	0.631134	0.521477

T.cells	NDST2	0.264781	2.934923	1.33904	0.18399	-5.10957	0.627868	0.518146
T.cells	IRF9	0.291178	4.515749	1.338961	0.184016	-5.38493	0.605871	0.495859
T.cells	PHYKPL	0.156322	3.470266	1.338736	0.184089	-5.43308	0.620381	0.510525
T.cells	2410018L1	0.324117	0.727626	1.338494	0.184167	-5.03661	0.659872	0.551078
T.cells	1700025G(	-0.21536	4.957756	-1.3383	0.18423	-5.64891	0.599893	0.489945
T.cells	LETM2	-0.2735	3.131534	-1.33818	0.18427	-5.10299	0.625137	0.515506
T.cells	PRDX1	-0.10472	9.719927	-1.33808	0.184301	-6.50945	0.538506	0.429185
T.cells	SIGLECG	-0.13414	4.076853	-1.3377	0.184425	-5.77214	0.61218	0.502255
T.cells	IGLL1	0.299862	3.916663	1.337285	0.18456	-5.87304	0.614579	0.504736
T.cells	PLAU	-0.86036	0.814983	-1.33711	0.184615	-4.6171	0.659023	0.550274
T.cells	BBS2	-0.55063	0.404044	-1.33703	0.184643	-4.6979	0.665129	0.556608
T.cells	CCNT1	-0.08684	6.841664	-1.33616	0.184925	-6.04176	0.575639	0.465718
T.cells	NXPE4	0.664622	2.181788	1.336001	0.184977	-4.68221	0.639523	0.530246
T.cells	IFT80	0.201592	3.626112	1.33578	0.185049	-5.55551	0.619046	0.509466
T.cells	PPM1F	-0.29944	2.206568	-1.3355	0.18514	-5.05472	0.639167	0.530117
T.cells	RB1	-0.10193	7.240705	-1.33545	0.185158	-6.20037	0.570457	0.460809
T.cells	STT3A	-0.09695	6.169392	-1.33535	0.18519	-5.91842	0.58447	0.474759
T.cells	9530082P2	-0.43347	0.699259	-1.33487	0.185347	-4.78243	0.661192	0.552991
T.cells	ABCA8B	-0.63137	1.118002	-1.33479	0.185371	-4.72805	0.655004	0.546621
T.cells	VAMP4	0.108976	6.154005	1.334743	0.185388	-5.91171	0.584674	0.475166
T.cells	NUDT16L1	-0.16402	4.234234	-1.33467	0.185413	-5.59567	0.610609	0.50129
T.cells	KPNA6	-0.1654	3.896998	-1.33462	0.185427	-5.42052	0.615275	0.506019
T.cells	MYADM	-0.20449	5.586342	-1.33447	0.185476	-5.63916	0.592233	0.482831
T.cells	STRBP	-0.09345	7.195368	-1.33441	0.185495	-6.29314	0.571043	0.461684
T.cells	HSPE1	-0.12305	7.179751	-1.33402	0.185623	-6.15985	0.571245	0.461974
T.cells	RHBDD1	0.159937	4.175534	1.333913	0.185658	-5.501	0.611419	0.502336
T.cells	2610037D(	0.12427	5.44268	1.33387	0.185672	-5.85902	0.59416	0.484947
T.cells	9430091E2	0.255944	2.439837	1.333283	0.185864	-5.16709	0.635819	0.527465
T.cells	PCED1A	0.258034	2.508638	1.333156	0.185905	-5.15549	0.634835	0.526506
T.cells	INPP5K	0.121879	5.371477	1.333011	0.185953	-5.78523	0.595118	0.486216
T.cells	CNTRL	0.102555	5.907012	1.332848	0.186006	-5.98177	0.587952	0.479057
T.cells	GM49980	-0.2039	6.157222	-1.33277	0.186033	-6.10854	0.584631	0.475749
T.cells	BMF	0.334355	2.137288	1.332428	0.186144	-5.05861	0.640164	0.53217
T.cells	CHIC2	-0.07825	7.043743	-1.33221	0.186215	-6.09667	0.573009	0.464359
T.cells	BZW2	-0.11834	5.869307	-1.33215	0.186234	-5.93439	0.588453	0.47982
T.cells	DRC7	0.425149	-0.15036	1.332103	0.18625	-4.90852	0.673913	0.56728
T.cells	SCN4A	-0.5636	0.410123	-1.33208	0.186257	-4.70845	0.665496	0.558523
T.cells	RPUSD3	0.486403	0.640562	1.332009	0.186281	-4.83463	0.662064	0.554974
T.cells	SLC25A30	-0.20728	3.550303	-1.33174	0.186367	-5.29315	0.620106	0.51197
T.cells	PMS1	0.219585	2.650773	1.331465	0.186459	-5.30408	0.632806	0.525022
T.cells	SF3A1	-0.10734	4.851981	-1.33139	0.186482	-5.80295	0.602148	0.493832
T.cells	PHF14	0.094266	6.331947	1.331363	0.186492	-6.03548	0.582323	0.473904
T.cells	ACBD4	-0.34264	1.764413	-1.3313	0.186513	-4.96882	0.645556	0.538137
T.cells	TSPAN2	0.191327	3.159011	1.330979	0.186618	-5.39958	0.625752	0.5178
T.cells	BCL2L12	-0.14442	4.178233	-1.33083	0.186667	-5.65473	0.611529	0.503409
T.cells	2610301B2	0.277736	1.613471	1.330389	0.186812	-5.08796	0.648219	0.540911
T.cells	PAH	-0.4716	3.508909	-1.33017	0.186884	-5.16973	0.621194	0.513191

T.cells	F13A1	0.497256	2.512117	1.329628	0.187061	-5.37812	0.63569	0.527779
T.cells	EIF2S2	-0.08516	7.925804	-1.32904	0.187254	-6.26705	0.562463	0.454123
T.cells	PIK3R4	0.242608	3.189781	1.328611	0.187395	-5.23251	0.626059	0.51835
T.cells	STAT2	0.267993	5.210285	1.328416	0.187459	-5.60414	0.598142	0.490003
T.cells	CARF	0.309525	2.216581	1.328339	0.187485	-5.06095	0.639934	0.532583
T.cells	RALA	-0.11358	5.871704	-1.3281	0.187565	-5.8813	0.58926	0.481095
T.cells	GALC	-0.35074	3.891293	-1.32792	0.187622	-4.99538	0.616231	0.508391
T.cells	KIZ	0.192794	3.359508	1.32788	0.187636	-5.30888	0.623668	0.515971
T.cells	EFNA1	-0.66885	0.531324	-1.32771	0.187691	-4.65704	0.664635	0.558187
T.cells	TACSTD2	0.78173	-0.35025	1.327525	0.187753	-4.59037	0.677903	0.572108
T.cells	GAB1	0.222935	4.473058	1.32752	0.187754	-5.77989	0.608191	0.500317
T.cells	RNPC3	0.118121	4.741295	1.327405	0.187792	-5.67381	0.604516	0.496611
T.cells	CD86	-0.43699	6.31281	-1.3274	0.187795	-5.30572	0.583406	0.475351
T.cells	SARDH	-0.52257	2.900243	-1.32709	0.187895	-4.94616	0.630157	0.522859
T.cells	ZDHHC6	0.133839	4.609891	1.326952	0.187941	-5.69739	0.606314	0.498564
T.cells	IER2	-0.16433	8.170209	-1.32691	0.187956	-6.17143	0.559353	0.45152
T.cells	APBA3	0.326661	2.107298	1.32636	0.188136	-4.9919	0.641509	0.53462
T.cells	ARIH1	-0.10611	8.728541	-1.32629	0.188161	-6.30615	0.552308	0.444682
T.cells	NR3C1	0.134604	7.440275	1.326277	0.188164	-6.05618	0.568691	0.460913
T.cells	PITPNC1	0.144415	8.799744	1.326139	0.188209	-6.21495	0.551416	0.443868
T.cells	GM4221	0.304647	1.846721	1.326053	0.188238	-5.08784	0.645281	0.538665
T.cells	GGCX	0.366575	1.807957	1.32567	0.188364	-4.92922	0.645844	0.539378
T.cells	PRKAB1	-0.21977	3.654114	-1.32561	0.188384	-5.33611	0.619538	0.512378
T.cells	YTHDC1	-0.07514	7.312924	-1.32543	0.188444	-6.1604	0.570335	0.462767
T.cells	ESAM	-0.49651	2.065322	-1.32542	0.188447	-4.79198	0.642115	0.53554
T.cells	ASAH2	0.279099	2.945427	1.32529	0.18849	-5.2471	0.629516	0.522618
T.cells	ZC3H7A	0.097453	6.883008	1.325276	0.188494	-6.03082	0.57592	0.468363
T.cells	IL1RN	1.103672	2.526967	1.325173	0.188528	-4.68842	0.635478	0.528783
T.cells	NADK2	0.128721	4.994117	1.324847	0.188636	-5.82551	0.601073	0.49375
T.cells	CD38	0.239022	5.882237	1.324808	0.188649	-5.837	0.58912	0.481691
T.cells	SUPT16	-0.07663	7.068147	-1.32469	0.188688	-6.22404	0.573509	0.466069
T.cells	TOMM7	-0.08094	7.561253	-1.32348	0.189087	-6.21602	0.568039	0.460112
T.cells	6430550D2	-0.37582	1.142129	-1.32322	0.189174	-4.95545	0.65663	0.550155
T.cells	GM17036	-0.31422	2.038724	-1.32306	0.189228	-5.12118	0.643526	0.536642
T.cells	PIK3C3	0.144004	4.439233	1.322795	0.189315	-5.62465	0.609628	0.502082
T.cells	DPYS	-0.46245	2.829004	-1.32244	0.189433	-4.99422	0.632178	0.525144
T.cells	SNX8	-0.17458	5.468943	-1.32223	0.189502	-5.92505	0.595604	0.48801
T.cells	GTF2IRD1	-0.24954	3.292146	-1.32217	0.189522	-5.34724	0.625613	0.518494
T.cells	SEC62	-0.06749	7.437896	-1.32211	0.189543	-6.13815	0.56963	0.46198
T.cells	PPP4C	-0.08878	6.80668	-1.32194	0.189599	-6.10478	0.577838	0.4702
T.cells	RNF40	-0.15527	4.021973	-1.3217	0.189679	-5.48526	0.615398	0.508128
T.cells	9-Sep	-0.17362	5.640387	-1.32151	0.189739	-5.62846	0.593299	0.485783
T.cells	FMNL1	-0.10969	6.443857	-1.32151	0.18974	-6.00574	0.582607	0.475037
T.cells	EMILIN2	0.796537	4.009199	1.321449	0.189761	-4.82164	0.615576	0.508351
T.cells	POC1A	-0.17381	3.229083	-1.32096	0.189923	-5.55745	0.626504	0.519704
T.cells	AKAP9	0.109855	6.105265	1.320841	0.189963	-5.96717	0.58709	0.479781
T.cells	DUSP1	-0.21752	7.412842	-1.3208	0.189978	-5.88474	0.569954	0.46263

T.cells	CLEC4G	-0.38701	3.468481	-1.32035	0.190128	-5.04888	0.623131	0.516555
T.cells	PLD2	-0.6234	1.042444	-1.32018	0.190182	-4.64997	0.658102	0.552644
T.cells	NOSIP	0.110376	5.158105	1.320166	0.190187	-5.82066	0.599805	0.492858
T.cells	SMOC1	-0.42404	2.28252	-1.32004	0.19023	-5.08111	0.640005	0.533921
T.cells	CEP170	0.117132	5.909007	1.319991	0.190245	-5.9465	0.589704	0.482663
T.cells	ARL14EP	-0.1908	3.524764	-1.31959	0.19038	-5.40626	0.62234	0.515847
T.cells	ELOVL5	0.136116	6.195778	1.319546	0.190394	-5.90843	0.585889	0.478902
T.cells	TRIM26	0.140863	5.330265	1.319515	0.190404	-5.70547	0.597475	0.490594
T.cells	PHF19	-0.23238	1.934693	-1.31933	0.190465	-5.22588	0.645034	0.539317
T.cells	MAP2K4	0.147598	6.321239	1.319277	0.190483	-5.85643	0.584227	0.477369
T.cells	MGLL	-0.26546	3.495334	-1.31879	0.190646	-5.37958	0.622754	0.516587
T.cells	MED24	0.181329	3.187102	1.318491	0.190745	-5.39103	0.627097	0.521113
T.cells	PEX7	0.126399	4.45855	1.318464	0.190754	-5.66016	0.609362	0.502996
T.cells	NAAA	-0.46832	3.389339	-1.31842	0.190769	-4.84767	0.624244	0.518189
T.cells	LIMS1	0.112053	7.288787	1.318412	0.190771	-6.0546	0.571559	0.464884
T.cells	LZTS2	-0.5656	1.249256	-1.31836	0.190787	-4.7486	0.655051	0.549981
T.cells	ELP5	-0.13551	4.633093	-1.31812	0.190868	-5.69803	0.606964	0.500663
T.cells	PLP2	-0.15157	5.623922	-1.31797	0.190917	-5.94301	0.59352	0.487072
T.cells	EPS15	0.105276	6.183956	1.31761	0.191038	-5.89812	0.586045	0.479639
T.cells	GFRA2	0.239158	2.545769	1.317525	0.191067	-5.41296	0.636224	0.530752
T.cells	CACNB3	0.88499	-0.407	1.317325	0.191134	-4.62302	0.67985	0.576276
T.cells	PSME1	0.156286	8.384368	1.317179	0.191182	-6.25328	0.55753	0.451291
T.cells	ADNP2	0.272955	2.990665	1.317118	0.191203	-5.20158	0.629879	0.524411
T.cells	REXO2	-0.12972	6.270041	-1.31698	0.191247	-5.91616	0.584904	0.478661
T.cells	GM28403	-0.47434	1.458517	-1.31695	0.191259	-4.89177	0.651978	0.547237
T.cells	LRIF1	0.123418	4.523869	1.316783	0.191314	-5.64366	0.608464	0.502535
T.cells	ELOC	-0.09712	7.093954	-1.31671	0.191339	-6.14651	0.574089	0.467834
T.cells	FASTK	0.216201	3.15048	1.316324	0.191468	-5.2481	0.627756	0.522333
T.cells	OLA1	-0.09121	5.835409	-1.3162	0.191509	-5.98302	0.59082	0.484757
T.cells	TBK1	0.13509	5.964636	1.316016	0.191571	-5.86914	0.589095	0.483039
T.cells	FUT10	0.489556	-0.01434	1.315897	0.191611	-4.7786	0.674046	0.570495
T.cells	CPT1B	-0.34848	1.536488	-1.3157	0.191676	-4.89988	0.650983	0.546534
T.cells	RACGAP1	-0.18342	5.205247	-1.31544	0.191763	-5.97831	0.599302	0.493659
T.cells	CETN2	-0.0955	5.798667	-1.31533	0.191799	-5.95113	0.591312	0.485592
T.cells	ZFP961	0.181202	3.308556	1.315272	0.19182	-5.5226	0.625523	0.520449
T.cells	SHPRH	0.138011	4.541567	1.314951	0.191927	-5.70109	0.608516	0.503066
T.cells	PLVAP	-0.5796	1.394862	-1.31448	0.192086	-4.71883	0.653229	0.549231
T.cells	CRELD2	-0.16825	4.759514	-1.31418	0.192187	-5.67384	0.605527	0.5003
T.cells	CDK2	-0.14979	4.350301	-1.31408	0.192219	-5.75425	0.61115	0.506032
T.cells	ZFP944	0.145984	4.397621	1.313944	0.192264	-5.68854	0.610497	0.505365
T.cells	WDR33	-0.07389	7.186641	-1.31379	0.192315	-6.17883	0.573163	0.467611
T.cells	DCTPP1	-0.14403	5.089369	-1.31374	0.192332	-5.92376	0.60103	0.495727
T.cells	RBM10	-0.11536	4.779494	-1.3137	0.192346	-5.79523	0.605254	0.500022
T.cells	BDH1	-0.25405	3.282333	-1.31365	0.192361	-5.3644	0.626056	0.521298
T.cells	CDC25A	-0.17154	3.793744	-1.31323	0.192504	-5.61588	0.618913	0.513987
T.cells	SMAD4	0.095457	6.23224	1.313228	0.192504	-5.94337	0.585725	0.480254
T.cells	IGSF3	-0.41748	0.826018	-1.31313	0.192537	-4.94234	0.661669	0.558248

T.cells	TENT2	0.089662	6.624422	1.312864	0.192626	-6.04961	0.580627	0.475224
T.cells	ZFYVE21	0.287814	2.542215	1.31142	0.193111	-5.1219	0.636711	0.532839
T.cells	CARD10	-0.52523	1.098314	-1.31142	0.193112	-4.71222	0.657727	0.554662
T.cells	USP21	0.211852	3.140041	1.311406	0.193116	-5.34861	0.628193	0.524051
T.cells	TLCD1	-0.33969	1.624021	-1.31113	0.193209	-4.94597	0.650002	0.54662
T.cells	GM16196	-0.30329	1.751417	-1.31108	0.193225	-5.09431	0.648143	0.544688
T.cells	CDA	-0.64616	0.894078	-1.31106	0.193233	-4.70367	0.660751	0.557821
T.cells	COQ5	0.190704	3.832511	1.311037	0.19324	-5.50918	0.618459	0.514051
T.cells	COPS2	-0.08687	6.020343	-1.31091	0.193284	-5.99966	0.588623	0.483702
T.cells	USP24	0.111565	5.459806	1.310839	0.193307	-5.88482	0.596136	0.491352
T.cells	PCYOX1	-0.1621	4.579765	-1.31075	0.193337	-5.58486	0.608113	0.503565
T.cells	RALBP1	0.093831	6.268527	1.310605	0.193386	-6.02861	0.585326	0.480477
T.cells	ZFP715	0.165022	3.662783	1.310562	0.1934	-5.46881	0.620831	0.516633
T.cells	SP4	0.13582	5.254382	1.310253	0.193504	-5.82145	0.598912	0.494405
T.cells	TRIT1	-0.15079	4.076961	-1.31017	0.193531	-5.58612	0.615056	0.510898
T.cells	E130317F2	0.406685	0.77534	1.310161	0.193535	-4.81121	0.662515	0.560028
T.cells	SNX15	-0.15553	4.880351	-1.30999	0.193593	-5.5871	0.603997	0.499614
T.cells	UTP23	0.153396	3.577367	1.309817	0.193651	-5.51361	0.622029	0.518066
T.cells	CDC42BPA	-0.20545	3.315927	-1.30981	0.193652	-5.45113	0.625707	0.521849
T.cells	EIF2S3Y	2.214172	1.945318	1.309582	0.19373	-5.09174	0.645322	0.542137
T.cells	GM48678	-0.25895	3.349644	-1.30958	0.193731	-5.30721	0.625231	0.521368
T.cells	AIFM2	-0.61713	1.347203	-1.30947	0.193767	-4.6836	0.654059	0.551266
T.cells	TMUB1	-0.21781	3.138278	-1.30918	0.193867	-5.14583	0.628247	0.524484
T.cells	NEK9	0.153505	5.27603	1.309123	0.193885	-5.69842	0.598646	0.494212
T.cells	MAT2B	0.106964	5.679223	1.308619	0.194055	-5.89942	0.593564	0.488769
T.cells	ATF6B	0.155135	4.456866	1.308098	0.194231	-5.62395	0.610552	0.505887
T.cells	BMP2	-0.62799	1.613957	-1.30793	0.194288	-4.70527	0.650948	0.547585
T.cells	TGM2	0.521221	4.674438	1.307449	0.19445	-5.06813	0.607559	0.502985
T.cells	CCDC134	0.194054	3.407824	1.307292	0.194503	-5.46011	0.625179	0.521123
T.cells	TREM3	0.790132	2.200561	1.306998	0.194603	-4.74357	0.642415	0.539051
T.cells	SLC36A1	-0.2637	2.851189	-1.30691	0.194633	-5.04285	0.633072	0.529429
T.cells	ZFP532	-0.69389	1.223667	-1.30684	0.194655	-4.68074	0.656683	0.553951
T.cells	SNTA1	-0.41986	1.865421	-1.30652	0.194765	-4.86355	0.647277	0.544311
T.cells	ARL4D	-0.55416	1.705958	-1.30643	0.194794	-4.77176	0.649603	0.546766
T.cells	DPP8	0.103552	5.667042	1.306086	0.194911	-5.91631	0.594077	0.489836
T.cells	PPFIBP2	-0.50359	4.285147	-1.30605	0.194923	-5.01739	0.612924	0.509047
T.cells	ACVR2B	0.325976	1.53111	1.306028	0.194931	-5.10168	0.652161	0.549585
T.cells	SET	-0.08954	7.946372	-1.30576	0.195021	-6.36549	0.564177	0.459776
T.cells	GBP6	0.834729	1.984096	1.305619	0.195069	-4.72406	0.645552	0.542815
T.cells	WFDC21	1.170801	2.947804	1.305555	0.195091	-4.84833	0.631695	0.52847
T.cells	OTUD5	-0.10944	5.568497	-1.30549	0.195114	-5.81466	0.595403	0.491323
T.cells	DOCK5	-0.37182	4.189727	-1.30537	0.195155	-5.08947	0.614246	0.510566
T.cells	ASNA1	-0.12896	4.877847	-1.30528	0.195184	-5.75153	0.604773	0.500921
T.cells	UBXN2A	-0.12412	5.233503	-1.30521	0.195209	-5.74162	0.59993	0.496002
T.cells	PLCB3	-0.28924	2.612	-1.30498	0.195284	-5.07216	0.636552	0.533626
T.cells	KIF21B	0.144609	4.865707	1.304793	0.195349	-5.72197	0.604996	0.501222
T.cells	MTERF3	0.156833	4.014483	1.304668	0.195392	-5.58292	0.616739	0.513249

T.cells	PLAG1	0.180334	2.8948	1.304147	0.195568	-5.39982	0.632839	0.529785
T.cells	RP9	0.075981	6.834563	1.304045	0.195603	-6.15046	0.578935	0.474787
T.cells	OLFR1259	0.839476	-1.34501	1.303844	0.195671	-4.60147	0.696079	0.596099
T.cells	FOPNL	0.134798	4.468642	1.303568	0.195765	-5.69908	0.61093	0.507217
T.cells	MAGI1	-0.36623	4.373842	-1.30187	0.196343	-5.4695	0.613811	0.509252
T.cells	IL17RA	0.134212	5.907729	1.301694	0.196403	-5.98181	0.592891	0.487987
T.cells	ETFA	-0.11047	6.599323	-1.3016	0.196434	-6.11848	0.58368	0.478693
T.cells	FBXL3	-0.12268	5.215741	-1.30144	0.196488	-5.73356	0.602245	0.497534
T.cells	EIF2S3X	-0.18595	5.323448	-1.30117	0.196581	-5.84765	0.600896	0.49616
T.cells	AI506816	-0.15631	5.207229	-1.30059	0.19678	-5.97983	0.602884	0.497785
T.cells	GBP2	0.763714	4.147241	1.299606	0.197114	-5.16013	0.617496	0.513088
T.cells	GPATCH11	0.163911	3.800176	1.299516	0.197145	-5.52805	0.622352	0.518068
T.cells	DHPS	-0.13257	4.486343	-1.29934	0.197207	-5.65354	0.612786	0.508267
T.cells	ELDR	0.355351	3.256101	1.299235	0.197241	-5.23194	0.630035	0.525996
T.cells	MRPL43	0.110961	5.608686	1.299084	0.197293	-5.9734	0.597435	0.492711
T.cells	PFAS	-0.18121	4.205445	-1.29905	0.197304	-5.74305	0.616685	0.512339
T.cells	CTSL	-0.17374	6.758061	-1.29902	0.197313	-5.78761	0.582091	0.477194
T.cells	BBS9	-0.16932	4.909527	-1.29902	0.197313	-5.79812	0.606955	0.502395
T.cells	9130230L2	-0.5732	3.687563	-1.29899	0.197324	-4.95081	0.623935	0.519778
T.cells	NCAPG2	-0.14687	5.068495	-1.29885	0.197372	-5.96538	0.604778	0.500186
T.cells	RAB3GAP1	-0.08891	6.044858	-1.29885	0.197373	-5.96468	0.591568	0.486773
T.cells	CD226	-0.87267	1.697205	-1.29804	0.19765	-4.65694	0.653276	0.549701
T.cells	MIA2	0.094342	6.77012	1.29751	0.197831	-6.00213	0.58278	0.477501
T.cells	RHBDF2	0.159649	4.650027	1.297423	0.197861	-5.55153	0.611414	0.506539
T.cells	MAGT1	0.111953	5.872067	1.297259	0.197917	-5.92437	0.594752	0.489624
T.cells	SLC16A2	-0.37154	2.626868	-1.29715	0.197955	-5.00575	0.639963	0.535944
T.cells	DCUN1D5	-0.06994	6.828224	-1.29704	0.197991	-6.18137	0.582013	0.476795
T.cells	THAP7	0.178945	3.607354	1.296873	0.198049	-5.54787	0.625985	0.521565
T.cells	IGIP	-0.59868	0.675003	-1.29665	0.198126	-4.68729	0.668757	0.565955
T.cells	CCDC77	0.165468	3.291762	1.296318	0.19824	-5.48208	0.630532	0.526325
T.cells	EIF2B3	-0.2341	2.996786	-1.29626	0.198261	-5.31604	0.634738	0.530665
T.cells	ADRB1	0.912629	0.735849	1.296161	0.198293	-4.63959	0.667852	0.56509
T.cells	ZFP946	-0.30815	1.39583	-1.29582	0.198409	-5.02586	0.658224	0.554931
T.cells	5430427M	0.368331	1.237849	1.29483	0.19875	-5.0855	0.66115	0.557734
T.cells	CDC26	0.083763	5.383294	1.294777	0.198768	-5.92532	0.602161	0.496984
T.cells	PRR16	-0.57379	1.720127	-1.29417	0.198975	-4.82777	0.654022	0.550572
T.cells	SLC15A2	-0.88712	4.597147	-1.29405	0.199017	-5.49946	0.612956	0.508238
T.cells	MDM4	0.092472	5.749077	1.294033	0.199024	-5.97326	0.597199	0.492198
T.cells	XRCC6	0.143876	5.144906	1.294028	0.199026	-5.99763	0.605415	0.500547
T.cells	UTP4	-0.126	5.102474	-1.29374	0.199126	-5.77079	0.605996	0.501171
T.cells	ARFGAP1	0.13161	4.620741	1.293734	0.199127	-5.68806	0.612629	0.507937
T.cells	MYH10	0.30527	2.573809	1.293693	0.199141	-5.09008	0.641577	0.53771
T.cells	CBX3	-0.08891	8.102288	-1.29367	0.199149	-6.46057	0.56619	0.461018
T.cells	CCDC58	0.135264	4.247902	1.293548	0.199191	-5.71758	0.61781	0.513286
T.cells	PLOD2	0.497917	1.261605	1.292715	0.199478	-4.87192	0.661392	0.558081
T.cells	LMO1	-0.53993	1.523387	-1.29271	0.19948	-4.82096	0.657512	0.554036
T.cells	COMMD10	0.145753	4.694613	1.292486	0.199556	-5.67868	0.612222	0.507313

T.cells	PHOSPHO2	0.148023	3.724911	1.291712	0.199823	-5.50833	0.626437	0.52134
T.cells	MRPS7	-0.1144	5.010323	-1.29071	0.20017	-5.86538	0.609401	0.50333
T.cells	SNX33	0.427652	0.759185	1.289849	0.200467	-4.84061	0.670948	0.566639
T.cells	MAFF	-0.44217	3.876198	-1.28983	0.200474	-4.97441	0.625487	0.519593
T.cells	9330020H(	-0.26491	2.471892	-1.28967	0.20053	-5.17002	0.645603	0.540304
T.cells	MYPOPOS	0.398981	1.654931	1.289594	0.200555	-5.02225	0.657581	0.552717
T.cells	CAD	-0.29789	1.795668	-1.28951	0.200585	-5.18995	0.655503	0.550559
T.cells	DEFB1	-0.69834	0.86149	-1.28935	0.200641	-4.71925	0.669409	0.565102
T.cells	NFATC3	-0.09166	7.290076	-1.28918	0.200697	-6.30497	0.579005	0.472638
T.cells	A430035B1	-0.27735	3.071834	-1.28908	0.200733	-5.35431	0.636937	0.531563
T.cells	VOPP1	-0.29694	3.965239	-1.28899	0.200764	-5.18908	0.624232	0.518538
T.cells	ATRNL1	-0.1335	6.820202	-1.28885	0.200812	-6.11302	0.585204	0.47897
T.cells	CPSF6	0.067991	6.68211	1.288628	0.200889	-6.16534	0.587037	0.480858
T.cells	TMF1	0.092786	6.243916	1.288533	0.200922	-6.02295	0.592892	0.486787
T.cells	CAMK1D	-0.1426	8.628354	-1.2882	0.201038	-6.21168	0.561859	0.455611
T.cells	NARF	0.136803	4.893244	1.28797	0.201117	-5.76142	0.611523	0.50557
T.cells	TRAP1	-0.11223	4.381095	-1.28783	0.201166	-5.74055	0.618639	0.512864
T.cells	LGALSL	-0.46196	1.281509	-1.28764	0.201231	-4.85151	0.663412	0.559126
T.cells	PIWIL2	-0.62525	0.700548	-1.28704	0.201439	-4.74158	0.672445	0.568446
T.cells	SERF2	0.07306	9.682679	1.287017	0.201448	-6.47462	0.548897	0.442744
T.cells	DLG2	0.426315	2.402232	1.286767	0.201535	-5.05399	0.647206	0.54228
T.cells	RMDN1	0.206804	4.528231	1.286504	0.201626	-5.46948	0.616909	0.511154
T.cells	PCF11	-0.12067	6.482932	-1.28648	0.201634	-6.00626	0.590229	0.48408
T.cells	SLC30A4	0.254966	1.941057	1.286407	0.201659	-5.20616	0.653958	0.549338
T.cells	CST3	-0.22917	9.157123	-1.28606	0.201781	-6.3227	0.555656	0.449516
T.cells	AGPAT5	0.106872	5.644905	1.285699	0.201905	-5.88495	0.601711	0.49576
T.cells	RAD51	-0.19566	3.891445	-1.28544	0.201994	-5.7677	0.626028	0.520674
T.cells	FGFR1OP2	0.10175	6.180589	1.284585	0.202293	-5.99619	0.59446	0.4889
T.cells	MAP3K2	0.114493	6.248682	1.284458	0.202337	-5.95792	0.593545	0.487975
T.cells	FRRS1	0.209033	4.851486	1.284275	0.202401	-5.41029	0.612602	0.507341
T.cells	CNOT6L	0.100044	7.08541	1.284273	0.202402	-6.17989	0.582401	0.476749
T.cells	ENPP5	-0.73757	0.877362	-1.28357	0.202646	-4.66296	0.66998	0.566864
T.cells	TMED8	-0.22147	2.954093	-1.2834	0.202706	-5.25201	0.639401	0.535138
T.cells	SLC8A1	0.467391	6.330185	1.283366	0.202717	-5.38727	0.59245	0.487152
T.cells	FZD6	-0.44333	0.693274	-1.28333	0.202731	-4.95594	0.672754	0.569888
T.cells	FAM192A	0.113287	4.607769	1.283295	0.202742	-5.74518	0.615985	0.511114
T.cells	SLFN4	0.982457	0.48225	1.283214	0.202771	-4.64708	0.675948	0.573281
T.cells	CDC45	-0.18809	3.268129	-1.28306	0.202825	-5.66287	0.634892	0.530612
T.cells	NID1	-0.4576	2.394724	-1.283	0.202844	-4.86506	0.647508	0.543662
T.cells	MAGOH	-0.09554	6.296682	-1.28299	0.202848	-6.12741	0.5929	0.487712
T.cells	OSGEP	0.113501	4.907558	1.282978	0.202853	-5.84825	0.611826	0.506944
T.cells	GM16552	0.341719	1.517	1.28295	0.202863	-4.9638	0.66042	0.557092
T.cells	KCTD18	0.228602	3.044012	1.282915	0.202875	-5.46081	0.638107	0.533931
T.cells	HSDL2	-0.14881	4.684002	-1.28258	0.202991	-5.66485	0.614925	0.510219
T.cells	P2RX3	0.232039	2.219391	1.282276	0.203098	-5.55969	0.650069	0.546537
T.cells	GPR27	0.469914	-1.60473	1.282199	0.203125	-4.60852	0.708288	0.607749
T.cells	BCAR1	-0.58162	0.776645	-1.28215	0.203142	-4.74265	0.671496	0.568921

T.cells	A930001A2	0.745309	-0.71301	1.282017	0.203188	-4.63005	0.694301	0.592983
T.cells	GMIP	0.109728	5.964525	1.281842	0.203249	-5.89301	0.597375	0.492532
T.cells	CHST10	0.616053	0.375693	1.281706	0.203297	-4.69024	0.677566	0.575374
T.cells	SHROOM2	-0.64677	1.506317	-1.2817	0.2033	-4.7521	0.660579	0.557584
T.cells	KHNYN	0.151934	4.263958	1.281507	0.203366	-5.58487	0.620786	0.51641
T.cells	GBA2	0.21935	2.547922	1.281368	0.203415	-5.23294	0.645278	0.541671
T.cells	ZFP82	-0.52443	0.121707	-1.28128	0.203447	-4.77189	0.681437	0.57945
T.cells	RNF6	0.103607	5.670645	1.281216	0.203468	-5.90252	0.60136	0.496604
T.cells	GM50399	-0.64725	0.166375	-1.28115	0.203493	-4.75771	0.680754	0.578798
T.cells	ST8SIA6	0.916087	1.211567	1.280763	0.203627	-4.67183	0.665044	0.562457
T.cells	PA2G4	-0.11354	6.688076	-1.28073	0.203639	-6.22139	0.587734	0.482971
T.cells	5930403N2	-0.65908	-0.48768	-1.28016	0.203837	-4.68223	0.690884	0.589747
T.cells	ZFYVE27	-0.16411	4.063949	-1.28004	0.203879	-5.47806	0.623666	0.519677
T.cells	LRRC3	-0.62056	0.375073	-1.27994	0.203916	-4.66766	0.677651	0.575809
T.cells	INPP4B	-0.34539	6.480863	-1.27989	0.203932	-5.51928	0.590498	0.485861
T.cells	VAPB	0.137795	5.031604	1.279794	0.203965	-5.75264	0.610182	0.505866
T.cells	NXPH4	0.741474	-0.60095	1.279728	0.203989	-4.64018	0.692639	0.591617
T.cells	BORCS7	0.16524	3.716689	1.279655	0.204014	-5.49966	0.628572	0.524744
T.cells	CPEB1	0.549368	0.696287	1.279362	0.204117	-4.74109	0.672849	0.570888
T.cells	USP20	0.233089	2.877475	1.279197	0.204175	-5.17441	0.64064	0.537321
T.cells	GM17251	0.289519	2.365949	1.27899	0.204247	-5.20617	0.648062	0.545061
T.cells	GRK4	-0.2236	3.108893	-1.2789	0.204278	-5.33033	0.637307	0.533948
T.cells	ADAM11	0.627227	-1.14352	1.278808	0.204311	-4.60526	0.701168	0.60089
T.cells	SERTAD4	0.403122	-0.0023	1.278634	0.204372	-5.01836	0.683495	0.58221
T.cells	GSDMC4	0.546138	0.743558	1.278006	0.204593	-4.71881	0.672496	0.570538
T.cells	TRIM16	0.207831	2.341778	1.277872	0.20464	-5.5397	0.648763	0.54577
T.cells	GM43660	-0.63455	0.127169	-1.27761	0.20473	-4.7344	0.681861	0.580498
T.cells	PARP1	-0.11678	5.30881	-1.27758	0.204741	-6.00075	0.606753	0.502593
T.cells	FOXA3	-0.51055	1.425712	-1.27755	0.204753	-4.80525	0.66227	0.559934
T.cells	DACH1	-0.5632	1.877933	-1.27721	0.204871	-4.90193	0.655719	0.552972
T.cells	MTPN	-0.07702	6.898113	-1.2771	0.204911	-6.15039	0.585446	0.480877
T.cells	TENT5A	-0.23665	5.169086	-1.27692	0.204973	-5.44516	0.608832	0.504603
T.cells	RUNX3	-0.20285	5.712717	-1.27661	0.205085	-5.76001	0.601556	0.497057
T.cells	KLRB1F	-0.6801	0.424065	-1.27642	0.20515	-4.74503	0.677727	0.575861
T.cells	LDAH	0.140122	4.225642	1.275724	0.205395	-5.6302	0.622586	0.518396
T.cells	METAP1	0.107913	4.718677	1.275598	0.205439	-5.77326	0.615692	0.511356
T.cells	DAG1	-0.15371	4.867236	-1.27553	0.205463	-5.72911	0.613628	0.509246
T.cells	COX6B1	-0.08997	7.821955	-1.27538	0.205516	-6.31821	0.573919	0.469034
T.cells	ZFP580	0.347378	2.166288	1.274809	0.205717	-4.99968	0.652464	0.549006
T.cells	NEO1	-0.65368	0.660859	-1.2748	0.205721	-4.73713	0.674918	0.572462
T.cells	SLX1B	0.174013	3.074893	1.274474	0.205835	-5.4182	0.639428	0.535357
T.cells	DENND4A	-0.15347	10.03042	-1.27412	0.20596	-6.37265	0.546245	0.441265
T.cells	MCF2L	0.623457	0.46165	1.273976	0.206011	-4.76952	0.678133	0.575918
T.cells	KLHL42	0.23719	2.445253	1.273576	0.206152	-5.22551	0.648562	0.545259
T.cells	LAPTM4B	-0.33353	3.501356	-1.27339	0.206217	-5.25185	0.63331	0.529564
T.cells	RDH14	0.165186	3.731822	1.273307	0.206247	-5.46337	0.630026	0.526208
T.cells	RTN4	-0.08515	7.592315	-1.27317	0.206295	-6.19443	0.57734	0.472654

T.cells	IQSEC1	-0.10689	6.277452	-1.27315	0.206303	-6.06749	0.5948	0.490275
T.cells	TBRG1	-0.14094	5.618457	-1.27263	0.206486	-5.87425	0.603737	0.499601
T.cells	CPSF3	0.116793	4.770083	1.272421	0.20656	-5.78888	0.615429	0.511542
T.cells	OTULINL	-0.27337	5.347398	-1.27233	0.206592	-5.37772	0.60745	0.503386
T.cells	DNAJC14	0.133911	4.252973	1.272254	0.206619	-5.53695	0.622659	0.518959
T.cells	TSPAN15	-0.40539	2.388192	-1.27204	0.206696	-4.88711	0.649396	0.546664
T.cells	OFD1	0.15315	3.31561	1.271825	0.206771	-5.49721	0.635968	0.532812
T.cells	TRMT10C	-0.11194	4.996252	-1.27182	0.206772	-5.88434	0.612292	0.508462
T.cells	XPOT	0.139088	4.512589	1.271693	0.206817	-5.7239	0.61902	0.515365
T.cells	FES	-0.34254	4.284767	-1.27161	0.206847	-5.05393	0.622213	0.518643
T.cells	IKBKB	0.116263	5.778617	1.271429	0.206911	-5.86554	0.601554	0.497537
T.cells	GM3055	-0.50611	0.319624	-1.27132	0.206949	-4.79216	0.680297	0.579121
T.cells	MIB1	0.115058	6.189415	1.271299	0.206957	-5.937	0.595987	0.491879
T.cells	GM5086	0.647114	-0.01663	1.271206	0.20699	-4.6372	0.685446	0.58458
T.cells	UAP1	-0.14106	5.112787	-1.27067	0.207179	-5.76781	0.610681	0.507103
T.cells	MYNN	0.125348	4.303762	1.270608	0.207201	-5.69153	0.621946	0.518657
T.cells	ZCCHC24	0.216192	3.666755	1.270538	0.207226	-5.42456	0.630952	0.527979
T.cells	DDX17	0.103548	6.357267	1.270024	0.207408	-6.04359	0.593726	0.490127
T.cells	MSMO1	-0.21921	3.610529	-1.26995	0.207436	-5.32366	0.631753	0.529074
T.cells	PIEZO1	-0.13492	5.046187	-1.26977	0.207499	-5.82565	0.611601	0.508447
T.cells	G2E3	0.142941	3.938116	1.269628	0.207549	-5.65203	0.6271	0.524392
T.cells	CD27	0.262595	3.318035	1.269107	0.207734	-5.30518	0.635933	0.533866
T.cells	DNAJC18	0.173047	3.682567	1.268902	0.207806	-5.52973	0.630727	0.528552
T.cells	OASL1	0.732846	2.957835	1.268871	0.207817	-4.95176	0.641118	0.539319
T.cells	CACNA1F	0.746353	-0.45662	1.268847	0.207826	-4.64364	0.692237	0.592989
T.cells	SPPL3	-0.07933	7.282604	-1.26833	0.208009	-6.21725	0.581408	0.478372
T.cells	D130062J1	0.394924	1.642184	1.268277	0.208028	-4.93245	0.660388	0.55968
T.cells	SHF	0.275002	1.832889	1.268211	0.208052	-5.13604	0.657562	0.556768
T.cells	RRAGA	-0.12843	5.020687	-1.26804	0.208113	-5.8002	0.611954	0.509517
T.cells	OGFR	0.142313	5.244677	1.267918	0.208156	-5.86662	0.608863	0.50635
T.cells	ZFP524	0.215557	3.672126	1.267897	0.208164	-5.41963	0.630875	0.529007
T.cells	S100A1	0.257497	4.086611	1.267848	0.208181	-5.26222	0.625002	0.522939
T.cells	ADAMDEC1	-0.72011	0.746267	-1.26767	0.208243	-4.70508	0.673815	0.5739
T.cells	BLOC1S6	0.159849	3.751167	1.267538	0.208291	-5.54566	0.629751	0.52798
T.cells	IPO4	-0.23717	2.384797	-1.2673	0.208377	-5.15037	0.649446	0.548512
T.cells	BLVRA	-0.15088	5.071733	-1.26724	0.208397	-5.6989	0.611248	0.508994
T.cells	LUC7L2	-0.06199	8.527172	-1.26688	0.208526	-6.42864	0.565223	0.462486
T.cells	RRP7A	-0.16363	3.942345	-1.2668	0.208554	-5.61532	0.627041	0.525459
T.cells	NUDT3	0.09816	5.213023	1.266576	0.208634	-5.91247	0.609299	0.507314
T.cells	AGTR1A	-0.7498	0.270352	-1.26638	0.208702	-4.70589	0.681049	0.5821
T.cells	CD24A	-0.13171	7.730753	-1.26629	0.208735	-6.5732	0.57553	0.472996
T.cells	BC024978	0.351928	1.865186	1.266263	0.208745	-5.05101	0.657084	0.556872
T.cells	ITGB1	0.108796	6.871704	1.266147	0.208787	-6.01586	0.586848	0.484458
T.cells	CSTDC4	0.80155	3.661016	1.266139	0.208789	-5.02553	0.631034	0.529753
T.cells	TMEM37	-0.40841	3.751452	-1.2661	0.208804	-5.06421	0.629747	0.528422
T.cells	ADAT1	-0.31494	2.003267	-1.26589	0.208876	-5.10944	0.655046	0.554781
T.cells	FBXW9	-0.26112	2.418265	-1.26582	0.208904	-5.11489	0.648957	0.548427

T.cells	ZFP605	0.341078	1.633403	1.26578	0.208917	-5.09914	0.660518	0.56053
T.cells	CTTN	0.476424	1.717261	1.265458	0.209032	-4.82194	0.659274	0.559382
T.cells	ZFP850	0.308727	1.010206	1.265361	0.209067	-5.03318	0.669833	0.570482
T.cells	PDE6H	0.310971	2.093346	1.265335	0.209076	-5.04848	0.65372	0.553563
T.cells	KCNQ1	-0.64326	1.396097	-1.26528	0.209096	-4.75911	0.664051	0.564397
T.cells	BCL3	-0.24114	5.236561	-1.26523	0.209114	-5.45572	0.608974	0.50723
T.cells	CD19	-0.17007	3.122136	-1.26465	0.209319	-5.73174	0.638748	0.5383
T.cells	SLC48A1	0.145195	5.074284	1.264554	0.209354	-5.6424	0.611213	0.509883
T.cells	VPS53	0.115832	4.462382	1.264536	0.209361	-5.65913	0.619722	0.518643
T.cells	CDC37	-0.08107	6.605127	-1.26421	0.209478	-6.11545	0.590403	0.48866
T.cells	SH3D19	-0.35101	2.244921	-1.26419	0.209485	-5.06788	0.651494	0.55171
T.cells	ARMC2	0.620141	-0.1641	1.263868	0.2096	-4.6762	0.687715	0.590006
T.cells	ZFAT	-0.20767	3.828322	-1.26384	0.20961	-5.46717	0.628656	0.528032
T.cells	INTS9	0.128656	4.394064	1.263673	0.209669	-5.75926	0.620679	0.519834
T.cells	TMEM222	0.147728	4.289191	1.263646	0.209679	-5.6935	0.62215	0.521353
T.cells	NAPRT	-0.64716	0.897988	-1.2636	0.209695	-4.74394	0.671523	0.572947
T.cells	GM35867	-0.61367	0.456051	-1.26352	0.209724	-4.76071	0.678218	0.580036
T.cells	LMBR1	-0.35188	1.883516	-1.26322	0.20983	-4.98353	0.656971	0.557571
T.cells	SLFN3	0.338178	1.4477	1.262848	0.209964	-5.0695	0.663462	0.564505
T.cells	AGTRAP	0.235841	4.534904	1.262509	0.210086	-5.53355	0.618876	0.518135
T.cells	LTA4H	-0.14444	5.724021	-1.26225	0.210177	-5.97581	0.602462	0.501421
T.cells	GOLGA5	0.116571	4.913418	1.262122	0.210224	-5.79667	0.613606	0.512876
T.cells	HTR1F	-0.38629	1.429334	-1.26204	0.210254	-4.87446	0.663736	0.565083
T.cells	ZFX	-0.08044	6.339182	-1.26201	0.210266	-6.07354	0.594131	0.492934
T.cells	COL13A1	-0.63484	0.62777	-1.26191	0.210298	-4.7174	0.675794	0.57787
T.cells	RNF13	0.128675	6.065838	1.261692	0.210378	-5.84243	0.597819	0.496823
T.cells	RAF1	0.114242	5.986456	1.261673	0.210385	-5.97254	0.598894	0.497924
T.cells	WHAMM	0.164231	4.147659	1.261496	0.210448	-5.57111	0.624312	0.524092
T.cells	PSMD13	0.087983	6.203145	1.261496	0.210448	-6.11526	0.595964	0.494944
T.cells	1110038B1	-0.13989	4.235292	-1.26127	0.210531	-5.8002	0.623156	0.522893
T.cells	CDH22	0.890738	-0.85879	1.26089	0.210666	-4.61803	0.698902	0.602629
T.cells	IL6	-1.12345	1.106723	-1.26059	0.210774	-4.71018	0.668768	0.570814
T.cells	AHRR	-0.64752	-0.36283	-1.2605	0.210804	-4.65293	0.691183	0.594635
T.cells	ING4	0.160112	4.08954	1.260384	0.210847	-5.61671	0.625322	0.525439
T.cells	HACD3	-0.12506	4.907611	-1.26028	0.210884	-5.72718	0.613874	0.513619
T.cells	FN1	-0.5243	5.580946	-1.26021	0.210909	-5.61036	0.604599	0.504081
T.cells	TMEM88	-0.51563	2.768173	-1.25984	0.211042	-4.88513	0.644234	0.545252
T.cells	PCBD2	-0.12408	5.24057	-1.25983	0.211046	-5.90926	0.609271	0.508994
T.cells	RHOV	0.866994	-0.80702	1.259767	0.211069	-4.64647	0.698092	0.602192
T.cells	SELENBP1	0.262404	3.795416	1.259553	0.211146	-5.43386	0.62955	0.530016
T.cells	CLMP	-0.57013	1.140521	-1.25863	0.211476	-4.78907	0.669197	0.570952
T.cells	DEPDC5	0.11157	4.978079	1.258437	0.211547	-5.90988	0.613801	0.51324
T.cells	PLEKHJ1	-0.09021	5.969804	-1.25796	0.211719	-5.99224	0.600517	0.499457
T.cells	MTIF2	0.134427	4.285566	1.25749	0.211888	-5.67237	0.623949	0.523655
T.cells	TCP1	-0.09751	6.67677	-1.25744	0.211905	-6.24187	0.591101	0.489945
T.cells	SCFD1	0.147538	4.929173	1.257313	0.211951	-5.76876	0.614943	0.514423
T.cells	1700094D(	-0.31894	1.476408	-1.25707	0.212038	-4.98249	0.664715	0.566331

T.cells	MSH2	-0.12365	4.128132	-1.25686	0.212116	-5.8159	0.626171	0.52612
T.cells	CLEC1A	0.748835	0.972503	1.256853	0.212117	-4.68145	0.672284	0.574382
T.cells	SRGAP3	0.338657	4.261174	1.256755	0.212152	-5.30129	0.624293	0.524203
T.cells	HIST1H2AP	0.211324	7.597755	1.256596	0.21221	-6.52726	0.578895	0.477779
T.cells	PPP1R1C	0.72034	0.781145	1.25614	0.212374	-4.74336	0.675245	0.577731
T.cells	PRDX3	-0.11437	5.400912	-1.25586	0.212475	-5.99099	0.608479	0.508156
T.cells	RNF170	0.192632	3.319753	1.255833	0.212485	-5.3429	0.637757	0.538472
T.cells	HMGCS1	-0.17533	4.453186	-1.25564	0.212554	-5.60887	0.621653	0.521818
T.cells	MEX3B	-0.25703	2.233947	-1.25562	0.212563	-5.2733	0.653549	0.555055
T.cells	NRBF2	0.14104	4.314912	1.255616	0.212563	-5.67816	0.623597	0.52383
T.cells	PSMD6	-0.11587	5.199485	-1.25465	0.212913	-5.94099	0.611788	0.511385
T.cells	TMEM67	0.349466	1.688091	1.254479	0.212974	-5.04126	0.662199	0.563887
T.cells	PLXNB3	-0.51215	0.164762	-1.25413	0.213099	-4.78671	0.685243	0.588358
T.cells	KDM6A	-0.23432	6.914035	-1.25409	0.213115	-6.13145	0.588501	0.487643
T.cells	PAXBP1	-0.13276	5.312121	-1.25395	0.213164	-5.89548	0.610232	0.509949
T.cells	GM21860	0.781659	-0.14186	1.253425	0.213356	-4.73869	0.68997	0.593617
T.cells	ANAPC2	-0.13791	4.497598	-1.25302	0.213503	-5.73835	0.621569	0.521938
T.cells	HMBOX1	0.11987	5.578159	1.252951	0.213527	-5.9634	0.606571	0.506471
T.cells	NUP50	-0.09871	5.304963	-1.25292	0.213538	-5.97419	0.610331	0.510338
T.cells	GM46224	0.861024	2.389468	1.252774	0.213591	-4.72873	0.651831	0.553477
T.cells	ALG13	-0.10233	4.514642	-1.25268	0.213627	-5.7995	0.621329	0.521696
T.cells	SPI1	-0.11786	6.411888	-1.25265	0.213635	-6.05827	0.595233	0.494855
T.cells	SLC29A3	0.255836	3.68183	1.25259	0.213658	-5.19727	0.63312	0.53394
T.cells	JRKL	0.412859	1.537523	1.25252	0.213683	-4.94613	0.664444	0.566784
T.cells	3110082I1	-0.17823	4.199896	-1.25238	0.213736	-5.67173	0.625761	0.526365
T.cells	TOGARAM:	-0.11577	5.297825	-1.25217	0.213811	-5.84402	0.610429	0.510546
T.cells	MAMDC2	0.618726	0.366618	1.252052	0.213853	-4.74566	0.682148	0.585623
T.cells	HCAR2	-0.9857	2.377294	-1.25203	0.21386	-4.73985	0.65201	0.553801
T.cells	TK2	0.219638	3.825305	1.251936	0.213895	-5.40525	0.631074	0.531976
T.cells	9530034E1	-0.47864	0.330435	-1.25164	0.214003	-4.80966	0.682701	0.586392
T.cells	BHMT2	-0.48122	2.060069	-1.25144	0.214074	-4.96396	0.656681	0.558898
T.cells	EFHC1	-0.49537	-0.05796	-1.25125	0.214145	-4.79687	0.688673	0.592886
T.cells	GAS2L1	-0.50176	1.765951	-1.25107	0.214211	-4.78975	0.66104	0.563598
T.cells	SLC39A11	0.19237	4.419343	1.250836	0.214294	-5.55187	0.622668	0.52359
T.cells	HSPA4L	0.172679	4.348791	1.250755	0.214324	-5.62991	0.623661	0.524621
T.cells	ATP8B4	0.4088	4.781456	1.250698	0.214344	-5.25058	0.617596	0.51834
T.cells	TK1	-0.18241	4.882981	-1.25067	0.214355	-5.91301	0.61618	0.516877
T.cells	WDR3	-0.12844	4.225869	-1.25064	0.214365	-5.723	0.625394	0.526419
T.cells	UBAP1L	0.318093	1.134289	1.25064	0.214366	-5.01213	0.670493	0.573689
T.cells	MAP4	-0.09391	6.676158	-1.25005	0.214579	-6.15953	0.591974	0.491804
T.cells	CENPS	-0.19247	3.529733	-1.25004	0.214583	-5.71958	0.635611	0.536824
T.cells	PTPN21	0.491169	0.813665	1.249653	0.214724	-4.83517	0.675749	0.579114
T.cells	HILPDA	0.364569	4.798635	1.249577	0.214752	-5.43198	0.617732	0.518359
T.cells	ABCA6	-0.64368	0.705764	-1.24949	0.214783	-4.78994	0.677388	0.580879
T.cells	TTC41	-0.40785	0.966475	-1.24925	0.214872	-4.95303	0.673434	0.576748
T.cells	LAMC1	0.146369	4.523339	1.249169	0.214901	-5.87309	0.621586	0.522462
T.cells	KLHL32	-0.34789	1.452777	-1.24883	0.215025	-5.2105	0.666117	0.56912

T.cells	TBC1D14	-0.11943	5.680933	-1.24881	0.215031	-5.88041	0.605531	0.505953
T.cells	DDOST	-0.10272	5.788445	-1.24875	0.215054	-5.97244	0.60406	0.504442
T.cells	URB1	-0.21686	2.176346	-1.24854	0.215128	-5.22829	0.655419	0.557901
T.cells	PDK2	0.30153	2.10287	1.248201	0.215253	-5.13951	0.656714	0.559224
T.cells	LRRN3	0.471626	-0.11923	1.247892	0.215365	-4.8833	0.690404	0.59494
T.cells	MKRN1	0.168433	7.149614	1.247756	0.215415	-6.02684	0.586033	0.486038
T.cells	GXYLT1	-0.12076	5.093219	-1.24765	0.215455	-5.85247	0.613957	0.514723
T.cells	IL6ST	0.307836	4.326252	1.247266	0.215594	-5.26707	0.624688	0.525984
T.cells	PPARD	0.168282	5.613407	1.247079	0.215662	-5.6674	0.606777	0.507552
T.cells	11-Sep	-0.14357	6.415184	-1.24688	0.215736	-6.10686	0.595865	0.496449
T.cells	ORC5	0.178196	3.474755	1.246856	0.215743	-5.54474	0.636808	0.538798
T.cells	H2-T22	0.25332	5.391347	1.246698	0.215801	-5.85496	0.609832	0.510889
T.cells	ARHGAP30	0.087285	7.139253	1.246332	0.215935	-6.13412	0.58617	0.486729
T.cells	DMTF1	0.109227	4.947065	1.246049	0.216038	-5.83985	0.615988	0.517445
T.cells	TUBA4A	-0.19612	4.513714	-1.24597	0.216065	-5.53623	0.622049	0.523726
T.cells	FAM118B	0.160726	3.464677	1.245936	0.216079	-5.54152	0.636952	0.539243
T.cells	CHST8	0.859284	-0.80735	1.245879	0.2161	-4.64924	0.701127	0.607209
T.cells	MRPL24	0.093192	5.658173	1.245643	0.216187	-5.96488	0.606163	0.507413
T.cells	RELL1	-0.11055	6.866963	-1.24562	0.216194	-6.10219	0.589799	0.490622
T.cells	MFHAS1	-0.36709	3.729832	-1.24558	0.216208	-5.06155	0.633154	0.535411
T.cells	1700120C1	0.2595	1.482076	1.245511	0.216235	-5.17516	0.66603	0.569953
T.cells	KRT8	-0.50865	1.975849	-1.24537	0.216285	-4.92131	0.658675	0.562241
T.cells	CXCL3	1.028609	-0.07498	1.245076	0.216394	-4.65713	0.68989	0.595268
T.cells	GM43581	0.227313	2.093907	1.244762	0.216509	-5.41415	0.657268	0.560558
T.cells	DDR1	0.618553	0.137297	1.244524	0.216596	-4.66011	0.6869	0.592044
T.cells	DRG1	-0.07669	6.40915	-1.24339	0.217011	-6.1641	0.597332	0.497634
T.cells	GM41077	0.488057	0.721818	1.243072	0.217128	-4.85011	0.679145	0.583059
T.cells	WDHD1	-0.13971	4.56722	-1.24293	0.21718	-5.9659	0.622805	0.523944
T.cells	SLC25A37	0.173598	5.267981	1.242753	0.217245	-5.90606	0.613019	0.513874
T.cells	CUL9	-0.29838	2.579779	-1.24267	0.217274	-5.1381	0.651358	0.553852
T.cells	PCGF2	-0.49593	0.79144	-1.24257	0.217313	-4.83335	0.678084	0.582106
T.cells	E230032D2	0.337991	1.879678	1.242275	0.21742	-5.08872	0.661835	0.564915
T.cells	GM20324	-0.24431	2.098382	-1.24214	0.217469	-5.20074	0.658587	0.561537
T.cells	CX3CR1	0.547906	3.268892	1.241341	0.217763	-4.95252	0.641499	0.543791
T.cells	ST8SIA4	0.137878	7.324778	1.24133	0.217767	-6.19338	0.585288	0.485733
T.cells	EPN2	-0.50324	1.664749	-1.24123	0.217805	-4.92744	0.665092	0.568605
T.cells	ZSCAN26	-0.13832	4.652438	-1.24096	0.217905	-5.775	0.621779	0.523343
T.cells	CCR9	0.568862	2.410766	1.240926	0.217916	-4.80078	0.654022	0.557029
T.cells	1700010K2	0.378648	0.709295	1.240892	0.217928	-4.89959	0.679523	0.584004
T.cells	AP3M1	0.110565	4.881049	1.240883	0.217931	-5.80346	0.618575	0.520023
T.cells	IFT27	-0.15946	3.758551	-1.24049	0.218076	-5.71573	0.634454	0.536684
T.cells	FRAT2	0.131055	4.805593	1.240488	0.218077	-5.94378	0.619631	0.521268
T.cells	GAREM1	-0.50303	0.757375	-1.24046	0.218088	-4.83561	0.67879	0.583406
T.cells	DCLK2	0.21685	2.72479	1.240338	0.218132	-5.45445	0.649413	0.552408
T.cells	MELK	-0.20687	3.016502	-1.24027	0.218157	-5.65988	0.645159	0.547979
T.cells	RHOH	-0.12487	6.471792	-1.24003	0.218246	-6.25773	0.596737	0.497843
T.cells	SNU13	-0.07933	6.96176	-1.2399	0.218294	-6.2937	0.59015	0.491141

T.cells	ZFP526	-0.50433	0.37218	-1.23977	0.218341	-4.79848	0.684715	0.590005
T.cells	IRF7	0.574871	6.139097	1.239396	0.218479	-5.4426	0.601249	0.502636
T.cells	PRKAA1	-0.17273	4.45764	-1.23922	0.218545	-5.63623	0.624549	0.52679
T.cells	BRMS1	-0.14699	4.236998	-1.23902	0.218617	-5.67535	0.627669	0.530056
T.cells	ZBTB8OS	-0.11719	5.027468	-1.23886	0.218677	-5.89621	0.61656	0.518557
T.cells	B430010I2:	0.672521	-0.41456	1.238839	0.218684	-4.65629	0.696898	0.603265
T.cells	CRTC1	-0.22672	3.126748	-1.23875	0.218718	-5.39278	0.643588	0.546762
T.cells	BEND3	-0.29437	2.456588	-1.23872	0.218727	-5.18484	0.653377	0.55705
T.cells	SSNA1	0.08983	5.922585	1.238364	0.218859	-6.14882	0.604413	0.506017
T.cells	PRMT1	-0.09501	5.803475	-1.23775	0.219084	-6.09004	0.606128	0.507901
T.cells	GM16066	-0.25745	2.012511	-1.23774	0.21909	-5.20584	0.660261	0.564413
T.cells	EIF5A2	-0.47773	0.577892	-1.23773	0.219093	-4.88811	0.681892	0.587368
T.cells	TMEM156	-0.23967	3.855526	-1.23768	0.219113	-5.28772	0.633404	0.536204
T.cells	WDR91	0.152762	4.750458	1.237375	0.219224	-5.74192	0.620888	0.523079
T.cells	POMT1	-0.2902	2.21731	-1.23637	0.219595	-5.26285	0.658262	0.561568
T.cells	POLR2G	0.096588	5.51542	1.236284	0.219627	-6.0011	0.611053	0.512319
T.cells	ZFP438	0.199617	3.204144	1.236071	0.219706	-5.41634	0.643846	0.546479
T.cells	GPRASP1	0.150377	3.93667	1.235931	0.219758	-5.58807	0.633295	0.535501
T.cells	RRP15	-0.14124	3.823389	-1.23575	0.219826	-5.69409	0.634951	0.537235
T.cells	TIMM23	-0.07641	6.942518	-1.2353	0.219992	-6.24592	0.59186	0.492719
T.cells	WASHC2	-0.14906	5.655214	-1.23524	0.220012	-5.83653	0.609365	0.510716
T.cells	FAM3A	0.251161	2.785704	1.235137	0.220052	-5.19731	0.650148	0.553187
T.cells	SHROOM4	0.722891	0.86592	1.234964	0.220116	-4.74078	0.678835	0.583633
T.cells	POLR3F	0.145059	3.882542	1.23479	0.22018	-5.6455	0.634267	0.536748
T.cells	IFT140	0.183514	3.507073	1.234684	0.220219	-5.52756	0.639662	0.542386
T.cells	SRSF6	-0.09942	6.210068	-1.23444	0.22031	-6.09385	0.601856	0.503192
T.cells	PRAM1	-0.24774	3.279629	-1.23407	0.220446	-5.22385	0.643101	0.546099
T.cells	GTPBP8	0.24429	2.317318	1.234039	0.220458	-5.21286	0.657194	0.560915
T.cells	TICRR	-0.19259	3.009192	-1.23371	0.220579	-5.65553	0.647033	0.55026
T.cells	GM11579	-0.5632	-0.20636	-1.23341	0.220693	-4.82142	0.695522	0.601741
T.cells	4930473A(	0.40845	0.38363	1.233053	0.220824	-4.87957	0.686382	0.592104
T.cells	MPHOSPH:	0.11609	4.827879	1.23301	0.22084	-5.89266	0.621015	0.523263
T.cells	GM11998	-0.50443	0.318253	-1.23274	0.220942	-4.86447	0.68739	0.593179
T.cells	RUNX1	0.109503	9.201324	1.2326	0.220992	-6.55004	0.562421	0.46329
T.cells	PTMS	-0.23878	6.015415	-1.23249	0.221033	-5.45069	0.604559	0.506246
T.cells	PLEKHA6	-0.39681	1.808761	-1.23238	0.221075	-4.91727	0.664758	0.569152
T.cells	HSD17B12	-0.11228	6.085483	-1.23225	0.221122	-6.04222	0.603601	0.50536
T.cells	6430590A(	-0.2711	1.647577	-1.2319	0.221253	-5.09065	0.667171	0.571938
T.cells	NSMCE2	-0.07455	7.428191	-1.23182	0.221282	-6.29747	0.585516	0.486948
T.cells	FAM83G	-0.49076	0.263823	-1.23177	0.2213	-4.76992	0.688229	0.594352
T.cells	GM49189	0.372935	0.779988	1.231692	0.22133	-4.98676	0.680304	0.58591
T.cells	VCAM1	-0.58374	4.334733	-1.2315	0.221403	-5.19044	0.627972	0.530787
T.cells	TCEANC2	0.12745	4.247598	1.231461	0.221416	-5.71028	0.629208	0.532077
T.cells	GFPT1	-0.22407	5.095805	-1.23144	0.221423	-5.52831	0.617266	0.51966
T.cells	ALG12	0.405423	1.395336	1.231417	0.221432	-4.96408	0.670965	0.576021
T.cells	IGKC	-0.26021	7.88266	-1.23137	0.22145	-6.48759	0.579512	0.480899
T.cells	NECAB2	-0.72804	0.442881	-1.23089	0.22163	-4.69131	0.685853	0.591664

T.cells	MCM3AP	0.128049	3.885875	1.230527	0.221764	-5.6487	0.634944	0.537827
T.cells	GM17103	-0.60009	1.028835	-1.23002	0.221953	-4.77896	0.677505	0.582538
T.cells	SLC37A3	-0.22592	3.658041	-1.22989	0.221999	-5.24982	0.638571	0.541466
T.cells	NCLN	-0.15982	3.839103	-1.22948	0.222154	-5.57126	0.636249	0.538857
T.cells	NR4A2	-0.4797	6.380914	-1.22928	0.22223	-5.38073	0.600776	0.502076
T.cells	IGFBP7	-0.25884	5.173401	-1.22909	0.222299	-5.60415	0.617451	0.519311
T.cells	FOXO4	0.250174	2.699869	1.228721	0.222437	-5.27493	0.652981	0.55649
T.cells	GCN1	-0.11785	4.575371	-1.22857	0.222495	-5.75921	0.625932	0.528216
T.cells	TCEA3	-0.4596	2.275566	-1.22856	0.222496	-4.98014	0.659249	0.563123
T.cells	UBXN7	0.101058	5.704222	1.227675	0.222828	-6.04867	0.610916	0.512175
T.cells	GLT1D1	0.63494	0.611079	1.22747	0.222904	-4.72606	0.685288	0.590155
T.cells	CLEC4N	0.556	4.093598	1.227121	0.223034	-5.09408	0.633729	0.535776
T.cells	IKZF1	-0.08622	8.378161	-1.22701	0.223075	-6.48539	0.575147	0.475566
T.cells	MICALL1	0.238469	2.97359	1.226914	0.223112	-5.19652	0.649941	0.552771
T.cells	SLC25A53	0.172362	3.336328	1.226432	0.223292	-5.49562	0.644905	0.547302
T.cells	SCAF1	0.120582	4.690272	1.226376	0.223313	-5.80469	0.625495	0.527072
T.cells	ZCCHC18	0.367211	1.04036	1.226077	0.223425	-5.02783	0.679272	0.583524
T.cells	USP53	-0.15222	4.118641	-1.22552	0.223635	-5.61368	0.634138	0.53587
T.cells	GM20300	0.370093	1.343674	1.225233	0.223741	-4.91061	0.675039	0.579014
T.cells	GM4117	0.426855	0.660809	1.225205	0.223752	-4.90107	0.685472	0.590103
T.cells	TAF13	-0.1495	4.566883	-1.22514	0.223777	-5.6937	0.627751	0.529386
T.cells	IKZF2	-0.57185	4.781138	-1.22493	0.223854	-5.18006	0.624736	0.526294
T.cells	DCP1B	0.221261	2.830948	1.224739	0.223927	-5.35186	0.652839	0.555746
T.cells	NEDD4L	0.084767	6.886893	1.224671	0.223952	-6.26876	0.595667	0.496442
T.cells	AU041133	0.323405	1.264215	1.224449	0.224035	-5.02601	0.676344	0.580645
T.cells	AFF1	0.085254	8.730515	1.224233	0.224117	-6.5548	0.571402	0.471745
T.cells	MTR	0.136586	4.54186	1.223836	0.224266	-5.70781	0.628391	0.530171
T.cells	A930024E0	-0.54151	1.108621	-1.22381	0.224274	-4.82375	0.678921	0.583298
T.cells	MIER2	-0.29701	2.002333	-1.22364	0.224338	-5.12595	0.665442	0.569045
T.cells	CSDE1	-0.06524	7.576149	-1.22344	0.224415	-6.28322	0.586758	0.487295
T.cells	TRIM44	-0.09741	6.053928	-1.22307	0.224551	-6.09482	0.607563	0.508472
T.cells	EGR2	-0.57635	2.237621	-1.22246	0.224784	-4.92307	0.662692	0.565744
T.cells	GM36723	-0.81001	3.324777	-1.22235	0.224823	-4.8229	0.646659	0.548895
T.cells	MRPL44	-0.19424	2.945204	-1.22199	0.224958	-5.44946	0.652221	0.554888
T.cells	GM43061	-0.50147	0.306464	-1.22194	0.224977	-4.84721	0.692086	0.597128
T.cells	PER2	-0.42264	2.222784	-1.2219	0.224995	-4.98208	0.662919	0.566164
T.cells	UBA2	-0.07834	6.457603	-1.22146	0.225158	-6.24406	0.602752	0.50332
T.cells	IQGAP3	-0.2624	2.400987	-1.22128	0.225227	-5.46412	0.660614	0.563515
T.cells	MAPK7	-0.20947	3.195275	-1.22068	0.225452	-5.43441	0.64898	0.551438
T.cells	SGCE	0.495555	1.156412	1.220567	0.225495	-4.92455	0.679451	0.583647
T.cells	RPRD2	0.104298	5.916624	1.220553	0.225501	-6.04593	0.61029	0.511246
T.cells	RBM34	-0.11487	4.465573	-1.22019	0.225637	-5.76365	0.630643	0.532454
T.cells	CYB5RL	-0.4826	0.380261	-1.21988	0.225753	-4.80127	0.691394	0.59664
T.cells	GM10053	0.372519	1.424678	1.219663	0.225836	-5.0733	0.675368	0.579684
T.cells	RBM8A	-0.09132	5.709751	-1.21902	0.22608	-6.08618	0.613153	0.514758
T.cells	CSNK1G3	0.086322	6.624241	1.218903	0.226123	-6.19438	0.60059	0.501818
T.cells	CD80	-0.57053	3.929604	-1.21887	0.226136	-4.92976	0.63832	0.540911

T.cells	WHRN	-0.21375	3.066048	-1.21845	0.226295	-5.52673	0.650873	0.554155
T.cells	HMG20A	0.135096	4.897858	1.218394	0.226315	-5.80762	0.624514	0.526644
T.cells	DDX39B	-0.08479	7.251694	-1.21834	0.226335	-6.364	0.592111	0.493254
T.cells	RTN2	0.711219	-0.4318	1.218234	0.226376	-4.67353	0.704095	0.61082
T.cells	MAP2K1	0.112382	6.311963	1.218205	0.226387	-6.07178	0.604853	0.506374
T.cells	FAM43A	-0.15858	4.098571	-1.21805	0.226447	-5.67661	0.63589	0.538619
T.cells	CNOT6	-0.0756	5.947416	-1.21804	0.226449	-6.11106	0.609865	0.511598
T.cells	ACVRL1	-0.58815	2.619629	-1.218	0.226465	-4.80595	0.657452	0.56125
T.cells	AGAP3	-0.17089	4.127556	-1.2179	0.226502	-5.63879	0.635474	0.538193
T.cells	RAB24	-0.10797	5.118704	-1.21786	0.226517	-5.88342	0.621404	0.523569
T.cells	NDUFS8	-0.08666	6.232258	-1.21752	0.226647	-6.16364	0.605945	0.507609
T.cells	DENND2A	-0.52796	1.374468	-1.21746	0.226667	-4.83475	0.67613	0.581078
T.cells	CDK11B	-0.10855	6.82853	-1.21746	0.226668	-6.11624	0.597817	0.499252
T.cells	SLC22A18	-0.53179	1.143718	-1.21741	0.226688	-4.81097	0.679645	0.584815
T.cells	USF2	-0.09896	6.021725	-1.21722	0.226759	-6.06113	0.60884	0.510683
T.cells	MANBAL	0.106893	4.531107	1.217038	0.226828	-5.82058	0.62971	0.53239
T.cells	S100A9	0.520233	6.814997	1.216948	0.226863	-5.85779	0.598	0.499593
T.cells	STX18	0.13106	4.637177	1.216946	0.226863	-5.80274	0.628203	0.530821
T.cells	DNAJC1	0.096871	6.915183	1.216829	0.226908	-6.15101	0.596644	0.498201
T.cells	CCDC85C	-0.33766	0.977037	-1.21661	0.22699	-5.04038	0.682194	0.587752
T.cells	PIGV	0.188477	2.921169	1.216611	0.22699	-5.3234	0.653002	0.556827
T.cells	ARCN1	0.101077	5.873425	1.216502	0.227031	-6.01731	0.610887	0.512909
T.cells	L2HGDH	-0.30401	1.767651	-1.216	0.227222	-5.14178	0.67018	0.575237
T.cells	CTSS	0.242958	7.501211	1.215959	0.227237	-6.04605	0.588771	0.490415
T.cells	GUCY2C	0.582223	-0.14698	1.215818	0.227291	-4.69198	0.699616	0.606772
T.cells	TOR4A	0.431422	1.678835	1.215534	0.227398	-4.92024	0.67152	0.576843
T.cells	SORBS3	-0.55387	0.937867	-1.21548	0.227419	-4.78003	0.682794	0.588844
T.cells	HP1BP3	0.09207	6.529858	1.215326	0.227477	-6.22261	0.601875	0.504047
T.cells	ATP5L	-0.07471	8.67785	-1.21518	0.227533	-6.49386	0.57326	0.474821
T.cells	WDFY2	0.174487	5.19857	1.215152	0.227543	-5.6934	0.620283	0.523109
T.cells	GSK3B	-0.06616	8.105386	-1.21501	0.227595	-6.40587	0.580757	0.48249
T.cells	LIPT2	0.350713	1.430834	1.214934	0.227626	-5.05661	0.675274	0.580985
T.cells	SPCS1	-0.08106	7.852067	-1.21482	0.227668	-6.34972	0.584105	0.485963
T.cells	IRF4	-0.14291	4.497863	-1.21457	0.227765	-5.95329	0.630183	0.533638
T.cells	HGF	-0.58712	2.743326	-1.21452	0.227784	-4.91437	0.655623	0.56035
T.cells	2610306M	0.4789	0.680103	1.214475	0.2278	-4.89094	0.686757	0.593425
T.cells	5430427O1	-0.19858	3.846602	-1.2143	0.227865	-5.55531	0.63954	0.54349
T.cells	ABR	0.269296	6.594873	1.213683	0.228101	-5.7006	0.601127	0.503734
T.cells	TBC1D20	-0.11526	5.789234	-1.21354	0.228156	-6.00561	0.612191	0.515208
T.cells	DDX23	0.113838	4.71777	1.213456	0.228187	-5.8669	0.627203	0.53082
T.cells	SOD2	0.233041	6.929524	1.21333	0.228235	-6.16763	0.596586	0.499154
T.cells	DNAJC25	-0.16046	3.856746	-1.21333	0.228235	-5.52247	0.639516	0.543732
T.cells	EVI2A	0.257414	5.251447	1.213213	0.22828	-5.346	0.619683	0.523071
T.cells	ZFP422	0.114856	4.395092	1.213148	0.228305	-5.94357	0.631791	0.535712
T.cells	AMIGO2	0.638445	0.934211	1.21297	0.228372	-4.81228	0.683039	0.589898
T.cells	TBCD	0.121488	4.783335	1.212618	0.228506	-5.86683	0.626466	0.530099
T.cells	TUBGCP5	-0.15503	3.976604	-1.21252	0.228544	-5.6121	0.637984	0.542184

T.cells	IRS2	-0.18248	5.572327	-1.21197	0.228754	-5.85417	0.615667	0.518735
T.cells	KLRC1	-0.71371	1.444197	-1.21195	0.22876	-4.7271	0.675735	0.581874
T.cells	STX8	0.076311	6.745581	1.211083	0.229091	-6.18085	0.599879	0.502397
T.cells	CCDC186	0.127638	4.760025	1.21089	0.229165	-5.80325	0.627443	0.531049
T.cells	TGFB1I1	0.54576	0.90381	1.210785	0.229204	-4.82536	0.684387	0.591177
T.cells	GM15708	-0.25023	3.198583	-1.21061	0.229271	-5.32557	0.649948	0.554726
T.cells	CFAP77	0.380872	0.931954	1.21057	0.229287	-5.0325	0.683954	0.590811
T.cells	GM156	0.646661	-1.54566	1.210373	0.229362	-4.67706	0.722994	0.63292
T.cells	VPS37B	-0.19737	7.797142	-1.2103	0.229391	-6.02595	0.585749	0.488132
T.cells	ZC3H12B	0.426877	1.084598	1.210168	0.22944	-4.96682	0.681614	0.588381
T.cells	FBRSL1	-0.09766	5.766788	-1.20997	0.229515	-5.97959	0.613321	0.516546
T.cells	AHCTF1	-0.10758	5.405997	-1.20988	0.22955	-6.00964	0.618347	0.52177
T.cells	DAAM1	0.158967	4.764899	1.209541	0.22968	-5.66324	0.627374	0.531351
T.cells	MMP13	0.969541	-1.01302	1.209408	0.22973	-4.67282	0.714433	0.623965
T.cells	PPP1R13B	0.140206	5.30235	1.209174	0.22982	-5.82511	0.619798	0.523538
T.cells	CSNK1G1	0.096337	6.535289	1.208988	0.229891	-6.17262	0.602744	0.505915
T.cells	SRSF1	-0.08973	6.067856	-1.20887	0.229935	-6.1978	0.609157	0.512569
T.cells	RRAGB	0.450076	0.125251	1.208864	0.229938	-4.91901	0.696448	0.604696
T.cells	GM49169	-0.45944	-0.42801	-1.2087	0.229999	-4.92346	0.705138	0.614127
T.cells	PSMC3	-0.08417	6.11628	-1.20843	0.230105	-6.16957	0.608489	0.511942
T.cells	ARMCX1	0.550585	0.550831	1.208359	0.230131	-4.84614	0.689831	0.597682
T.cells	POLD4	0.098286	5.76477	1.208296	0.230155	-6.10858	0.613349	0.517009
T.cells	SCAMP2	0.091182	6.357233	1.207897	0.230308	-6.08236	0.605179	0.508694
T.cells	AUH	0.11714	5.641378	1.207812	0.230341	-5.96315	0.615064	0.518939
T.cells	XKR6	-0.5555	0.862903	-1.20774	0.230368	-4.77254	0.685016	0.592746
T.cells	DICER1	0.101076	4.846948	1.207666	0.230396	-5.89631	0.626211	0.530584
T.cells	REPS1	0.090859	5.964436	1.207308	0.230533	-6.11583	0.610584	0.514477
T.cells	CEP350	0.103103	6.505585	1.207249	0.230556	-6.1653	0.603149	0.506804
T.cells	DTL	-0.15643	4.961318	-1.2071	0.230613	-6.08371	0.624595	0.529127
T.cells	CEP89	0.213787	2.805458	1.207057	0.23063	-5.56787	0.655731	0.561846
T.cells	USP28	-0.12886	4.553264	-1.20705	0.230634	-5.7831	0.63038	0.535172
T.cells	HAO1	-0.37907	3.070076	-1.20704	0.230638	-5.17375	0.651833	0.557725
T.cells	CD33	-0.67374	2.518088	-1.20693	0.23068	-4.80272	0.659989	0.566411
T.cells	NKAIN2	-0.52603	1.350359	-1.20666	0.230783	-4.95014	0.677556	0.58518
T.cells	HOMER1	0.165031	6.109837	1.206565	0.230818	-5.8782	0.608578	0.512595
T.cells	CERS4	0.160145	3.651416	1.206563	0.230819	-5.71167	0.643345	0.548903
T.cells	SELENOP	-0.17948	8.699951	-1.20639	0.230886	-6.3728	0.573896	0.476964
T.cells	GM27253	-0.34504	1.3478	-1.20621	0.230954	-5.05981	0.677659	0.585251
T.cells	EEF1AKNM	0.257048	2.318289	1.205993	0.231037	-5.21459	0.663104	0.569746
T.cells	GM26930	0.474997	0.305585	1.205231	0.23133	-4.95328	0.694325	0.602752
T.cells	CAMK2N1	-0.575	1.714702	-1.20523	0.231331	-4.90803	0.672695	0.579612
T.cells	GM41442	0.708146	-0.19949	1.204351	0.231668	-4.70193	0.702911	0.611639
T.cells	NFS1	-0.12418	4.533914	-1.20435	0.231668	-5.81681	0.631891	0.53622
T.cells	CCT6A	-0.09557	5.802009	-1.20412	0.231757	-6.12674	0.614116	0.517688
T.cells	TIRAP	0.195126	3.005516	1.203481	0.232003	-5.43692	0.654684	0.559832
T.cells	CD83	-0.63342	5.198788	-1.20288	0.232235	-5.10904	0.623424	0.526876
T.cells	GM49774	-0.23927	3.174932	-1.2027	0.232302	-5.52196	0.652569	0.557408

T.cells	GM48742	-0.43678	0.618325	-1.20257	0.232355	-4.89445	0.691196	0.598492
T.cells	GRPEL2	0.164998	3.176522	1.202394	0.232421	-5.52491	0.652546	0.557415
T.cells	SARS2	-0.15606	2.675724	-1.20239	0.232422	-5.531	0.659951	0.565235
T.cells	MPP5	0.109683	5.379551	1.201965	0.232587	-6.02723	0.621168	0.524382
T.cells	DGCR2	0.122958	4.849233	1.201411	0.2328	-5.89668	0.628784	0.532471
T.cells	EEF1D	-0.08363	7.27018	-1.20101	0.232955	-6.34145	0.595256	0.497896
T.cells	ACY3	-0.42093	2.04257	-1.2009	0.232997	-5.05715	0.669867	0.575962
T.cells	SCAP	-0.15058	4.277309	-1.20069	0.233079	-5.79863	0.63696	0.541282
T.cells	OGDH	-0.06734	6.625732	-1.20066	0.233091	-6.22187	0.604013	0.507026
T.cells	TMEM164	0.09422	6.945657	1.200626	0.233103	-6.21843	0.599651	0.50253
T.cells	GOLGA3	0.14372	4.032459	1.200388	0.233195	-5.64906	0.640491	0.545041
T.cells	GM17529	-0.51293	0.365187	-1.20027	0.233239	-4.84683	0.695593	0.603568
T.cells	CCSER1	-0.67634	1.697174	-1.19995	0.233363	-4.87656	0.675091	0.581743
T.cells	ZMYM2	0.108772	5.970893	1.199872	0.233394	-6.05337	0.613035	0.516518
T.cells	TNRC6B	0.094008	8.234075	1.199851	0.233403	-6.41209	0.582384	0.484986
T.cells	CDIP1	0.100483	5.935336	1.199767	0.233435	-6.05303	0.613529	0.517092
T.cells	MICOS10	-0.08252	7.131558	-1.19968	0.23347	-6.30225	0.59713	0.500169
T.cells	STARD9	-0.2881	3.529605	-1.19967	0.233473	-5.22653	0.647799	0.552923
T.cells	RHOBTB1	-0.3752	3.180961	-1.19957	0.23351	-5.16684	0.652911	0.55835
T.cells	LY6A	0.845053	4.339309	1.19939	0.233581	-5.18146	0.636069	0.540645
T.cells	USP30	0.237108	2.252852	1.199341	0.2336	-5.25641	0.666704	0.572961
T.cells	UBOX5	0.310163	1.390449	1.198827	0.233798	-5.11453	0.680085	0.587019
T.cells	TLE3	-0.1291	5.003886	-1.19876	0.233824	-5.81085	0.626888	0.530876
T.cells	PLEKHM3	0.215312	6.068485	1.19844	0.233948	-5.63511	0.612115	0.515381
T.cells	PRRT1	0.645653	0.466797	1.198326	0.233992	-4.71268	0.694499	0.602287
T.cells	GM17268	0.839558	0.383225	1.198131	0.234068	-4.71453	0.695859	0.603745
T.cells	ZFAND2A	-0.19209	3.630572	-1.19795	0.234139	-5.39249	0.646867	0.551731
T.cells	MFNG	0.17008	3.090104	1.197796	0.234198	-5.51873	0.654798	0.560131
T.cells	ZFP523	-0.32217	2.19531	-1.19766	0.234251	-5.02342	0.668127	0.57432
T.cells	PVR	-0.36399	3.063552	-1.19714	0.234451	-5.04364	0.655286	0.56079
T.cells	RASL11B	-0.47833	0.665224	-1.19709	0.234469	-4.86443	0.691605	0.599455
T.cells	AADAT	-0.48807	1.2345	-1.19682	0.234576	-4.85199	0.68282	0.590225
T.cells	TCF7L2	-0.15361	7.34951	-1.19672	0.234613	-6.30924	0.594772	0.497912
T.cells	RAPSN	-0.43551	0.715904	-1.19658	0.23467	-4.83416	0.690819	0.598896
T.cells	1110020A2	0.411432	0.468534	1.196091	0.234859	-4.96168	0.694665	0.603193
T.cells	RSPH10B	-0.55089	0.413611	-1.19604	0.23488	-4.86606	0.695521	0.60413
T.cells	FBXO38	0.098857	5.443447	1.196006	0.234892	-5.95358	0.621005	0.525257
T.cells	KAT2A	-0.17067	3.199202	-1.19597	0.234907	-5.59039	0.653285	0.559115
T.cells	AK4	-0.55136	1.048143	-1.19588	0.234941	-4.85949	0.685684	0.593578
T.cells	CREB3	-0.16721	3.923009	-1.19566	0.235026	-5.47345	0.642707	0.548064
T.cells	MEGF8	0.543072	0.288303	1.195598	0.23505	-4.75959	0.697479	0.606374
T.cells	RBL1	0.123475	4.466968	1.195548	0.23507	-6.00906	0.634862	0.539884
T.cells	LIMCH1	-0.61336	0.57747	-1.19552	0.235082	-4.80058	0.692969	0.601558
T.cells	ITPA	0.1086	4.105721	1.195263	0.235181	-5.84462	0.640175	0.545398
T.cells	PLAAT3	0.248114	5.19136	1.194735	0.235386	-5.6609	0.624892	0.529288
T.cells	GM13481	-0.5052	0.231781	-1.1947	0.2354	-4.90588	0.698741	0.607568
T.cells	MEPCE	-0.15611	4.466254	-1.19433	0.235542	-5.74873	0.635215	0.540174

T.cells	KLK8	0.311468	1.605933	1.194188	0.235599	-5.10706	0.677508	0.584963
T.cells	GM38115	0.148909	3.861728	1.193994	0.235674	-5.70597	0.643943	0.549418
T.cells	COX19	-0.13772	4.496433	-1.19398	0.23568	-5.85991	0.634782	0.539797
T.cells	DGKA	0.135218	5.023185	1.193923	0.235702	-6.00783	0.627272	0.531943
T.cells	DCTN4	-0.08437	6.423452	-1.19372	0.235779	-6.15889	0.607711	0.511705
T.cells	SLC20A2	0.158977	4.036306	1.193623	0.235818	-5.69142	0.641411	0.546915
T.cells	USP10	-0.12631	4.567936	-1.19354	0.235851	-5.91577	0.633758	0.538898
T.cells	STOX2	-0.51459	3.196694	-1.1934	0.235904	-5.11542	0.653674	0.559899
T.cells	MED13L	0.084693	7.196724	1.193281	0.235952	-6.32518	0.597157	0.500921
T.cells	TIFA	0.129213	6.262161	1.19314	0.236006	-6.30569	0.609935	0.514175
T.cells	PLPP3	-0.3592	4.693864	-1.19291	0.236095	-5.56254	0.632041	0.537212
T.cells	FUT8	0.101053	5.850748	1.192733	0.236165	-6.13285	0.615758	0.520296
T.cells	MFSD7A	0.67561	-0.44923	1.192512	0.236251	-4.71852	0.70971	0.619971
T.cells	3300005D(	-0.52286	0.756323	-1.19214	0.236397	-4.85055	0.691005	0.599756
T.cells	IFITM3	0.299639	7.709419	1.192023	0.236442	-6.06188	0.590632	0.494285
T.cells	ECE1	-0.18199	5.613294	-1.19165	0.236586	-5.86564	0.619428	0.524127
T.cells	IL6RA	-0.28724	5.504339	-1.19156	0.236624	-5.38252	0.620956	0.525733
T.cells	FCHO1	0.13006	3.772111	1.191511	0.236642	-5.94492	0.645737	0.551705
T.cells	FGFR1	0.692457	1.974297	1.191039	0.236826	-4.84543	0.672553	0.580093
T.cells	GM19557	0.526253	-0.95554	1.190967	0.236854	-4.70247	0.718253	0.629269
T.cells	DAD1	-0.08988	6.948328	-1.19096	0.236856	-6.22491	0.601095	0.50511
T.cells	MPHOSPH(	-0.12887	3.799303	-1.19035	0.237095	-5.71959	0.645955	0.551724
T.cells	ANK2	-0.65412	2.998552	-1.18995	0.237252	-5.03856	0.657977	0.56427
T.cells	PLA2G12A	0.1202	4.277535	1.18975	0.23733	-5.94702	0.639265	0.544595
T.cells	YTHDF2	-0.08149	6.035232	-1.18963	0.237375	-6.1569	0.614361	0.518659
T.cells	PI4KA	-0.09714	6.333121	-1.18937	0.23748	-6.18351	0.610232	0.514524
T.cells	ZFP820	-0.47151	-0.04486	-1.1893	0.237504	-4.8775	0.704562	0.6144
T.cells	CHCHD4	0.148877	3.820424	1.189242	0.237529	-5.72494	0.645895	0.551826
T.cells	COMMD4	-0.10571	5.326168	-1.189	0.237623	-6.01968	0.624368	0.529258
T.cells	ABCA7	0.163855	3.89815	1.18888	0.23767	-5.49832	0.644837	0.550739
T.cells	TOMM34	-0.09455	5.601463	-1.1881	0.237977	-6.05002	0.620554	0.525477
T.cells	ITGB7	-0.48623	4.620993	-1.18807	0.237989	-4.97668	0.634461	0.540018
T.cells	SLC43A2	-0.14501	6.179865	-1.18806	0.23799	-6.15526	0.612484	0.517083
T.cells	IARS	-0.11352	5.056202	-1.18765	0.238154	-6.01798	0.628252	0.533597
T.cells	PAICS	-0.09112	6.310596	-1.18764	0.238156	-6.2804	0.610674	0.515284
T.cells	RGS9	-0.47095	0.03543	-1.18743	0.238239	-4.95016	0.703446	0.613616
T.cells	RTEL1	-0.23283	2.682882	-1.18738	0.23826	-5.43498	0.662814	0.570094
T.cells	GM20559	0.293487	4.344461	1.187353	0.238268	-5.27068	0.638436	0.544335
T.cells	APMAP	-0.14957	4.080552	-1.18722	0.238321	-5.6883	0.642252	0.548401
T.cells	NT5C3B	-0.14308	3.396774	-1.18699	0.238412	-5.68095	0.652236	0.559003
T.cells	SPIRE1	-0.52878	2.681156	-1.18698	0.238414	-4.96765	0.66284	0.570244
T.cells	4632404H1	0.472819	1.238383	1.186865	0.23846	-4.92333	0.684707	0.593604
T.cells	PUS10	0.145397	4.485499	1.186836	0.238472	-5.77673	0.636406	0.54235
T.cells	ZFP384	-0.10988	4.90083	-1.18673	0.238513	-5.91704	0.630462	0.536169
T.cells	LILRA6	0.701652	1.213739	1.186524	0.238594	-4.73089	0.685086	0.594155
T.cells	SIAE	0.261706	2.612072	1.18629	0.238686	-5.23403	0.663872	0.57154
T.cells	CHADL	-0.44638	0.647864	-1.18626	0.238697	-4.91999	0.693848	0.603616

T.cells	DLGAP1	0.416637	1.398192	1.186006	0.238797	-4.99266	0.682253	0.591275
T.cells	STFA2	0.570099	2.469209	1.185989	0.238804	-4.95336	0.666011	0.573928
T.cells	NCSTN	0.113489	5.576404	1.185909	0.238836	-5.97281	0.620905	0.526401
T.cells	ZNRD1	-0.09943	5.076686	-1.18546	0.239013	-6.04007	0.628014	0.533906
T.cells	ABCC2	-0.38392	2.212583	-1.18542	0.239026	-5.05552	0.669926	0.5782
T.cells	FAH	-0.36784	3.892683	-1.18542	0.239027	-5.40919	0.645035	0.551796
T.cells	CPSF2	-0.09622	5.649423	-1.18472	0.239304	-6.16925	0.620171	0.525725
T.cells	CCL3	-0.84156	5.069599	-1.18463	0.239337	-5.03703	0.628356	0.534281
T.cells	PITPNA	-0.06941	7.992513	-1.18458	0.239359	-6.39672	0.588107	0.492529
T.cells	G6PDX	0.179477	4.072997	1.184574	0.239361	-5.49029	0.642662	0.549311
T.cells	MIGA2	-0.42971	0.892517	-1.18434	0.239451	-4.96677	0.69037	0.600158
T.cells	2700038G2	-0.20525	2.577847	-1.18418	0.239515	-5.39812	0.664695	0.572752
T.cells	TRIM30D	0.330463	4.544173	1.184172	0.239519	-5.41165	0.635861	0.542254
T.cells	KLRB1A	0.739333	-0.08222	1.183758	0.239682	-4.72478	0.705945	0.616898
T.cells	KCTD1	0.234287	1.719442	1.182817	0.240053	-5.3376	0.678689	0.587266
T.cells	CEP135	-0.11565	3.853066	-1.18281	0.240054	-5.76887	0.64684	0.553426
T.cells	CADM4	0.480694	1.741687	1.182564	0.240152	-4.96637	0.678466	0.587005
T.cells	IGSF5	0.536945	1.922281	1.182068	0.240348	-4.93776	0.676062	0.584219
T.cells	WBP1L	0.142141	5.329247	1.181962	0.24039	-5.79678	0.626055	0.531402
T.cells	NOP2	-0.14381	3.635097	-1.1816	0.240531	-5.65711	0.650643	0.557183
T.cells	MATK	-0.32985	2.36611	-1.18119	0.240695	-5.15374	0.669517	0.577304
T.cells	GM47828	0.285622	0.334612	1.181182	0.240698	-5.22231	0.700791	0.610831
T.cells	RMC1	0.156573	4.652621	1.181174	0.240701	-5.66874	0.635868	0.541716
T.cells	CELF4	0.432403	0.6776	1.181079	0.240739	-4.92772	0.695418	0.605089
T.cells	LYN	-0.0994	10.03311	-1.18074	0.240872	-6.66148	0.562979	0.466456
T.cells	COX7B	-0.08929	7.597144	-1.18049	0.240972	-6.37768	0.594998	0.499216
T.cells	PIR	-0.60359	1.198746	-1.18037	0.241019	-4.76273	0.687478	0.596544
T.cells	KLRA5	-0.85177	2.005641	-1.1802	0.241085	-4.79903	0.675119	0.58335
T.cells	GM15892	-0.1892	3.443855	-1.18002	0.241157	-5.76175	0.653599	0.560473
T.cells	2810402E2	-0.21978	2.412627	-1.17981	0.241241	-5.32002	0.668963	0.576845
T.cells	SGK3	-0.15224	5.592861	-1.17971	0.241281	-5.83797	0.622632	0.528027
T.cells	TFAP4	-0.19853	2.748617	-1.17934	0.241426	-5.57101	0.663921	0.571531
T.cells	MTHFSD	0.241024	2.433778	1.179332	0.24143	-5.30215	0.668645	0.576558
T.cells	SLC39A8	-0.32159	2.726982	-1.17928	0.241451	-5.24315	0.664245	0.571875
T.cells	GPR132	0.278902	6.00422	1.179274	0.241453	-5.63962	0.616863	0.522018
T.cells	RGP1	-0.27712	2.34849	-1.17921	0.241479	-5.03926	0.66993	0.577944
T.cells	B3GALT4	0.433251	1.404829	1.179008	0.241558	-4.99245	0.68434	0.593425
T.cells	ERCC3	-0.14115	3.695125	-1.17889	0.241607	-5.64968	0.649941	0.556869
T.cells	EXOSC9	-0.12055	4.169312	-1.17842	0.241792	-5.87021	0.643077	0.549703
T.cells	HIST1H2AE	0.223882	4.491316	1.178398	0.2418	-6.12542	0.638418	0.544802
T.cells	CSGALNAC	-0.16323	4.10884	-1.17816	0.241895	-5.52088	0.643956	0.550668
T.cells	PROSCOS	-0.67485	0.287878	-1.17812	0.241912	-4.77355	0.701779	0.612391
T.cells	PHF1	-0.37587	2.695984	-1.17802	0.241951	-4.99444	0.664802	0.572773
T.cells	ZFP85	0.441683	0.25725	1.177966	0.241971	-4.93323	0.702261	0.612938
T.cells	S100G	0.646262	0.605317	1.177701	0.242076	-4.85313	0.696888	0.607101
T.cells	USP16	0.11894	5.208005	1.177176	0.242285	-5.90486	0.628243	0.53436
T.cells	TMEM119	0.520752	0.305437	1.177033	0.242341	-4.82073	0.701592	0.61247

T.cells	MPG	0.140387	3.63806	1.176916	0.242388	-5.66667	0.650917	0.558275
T.cells	TSHZ3	-0.68533	2.187789	-1.17633	0.242622	-4.82367	0.672538	0.581424
T.cells	EIF1AX	-0.0896	6.060349	-1.17623	0.242661	-6.22999	0.616246	0.522022
T.cells	DDHD1	0.157973	6.276227	1.176203	0.242671	-6.06962	0.613241	0.518894
T.cells	FAM114A1	0.449027	2.398626	1.176053	0.242731	-4.96055	0.669354	0.578046
T.cells	VASP	0.137214	6.541669	1.17595	0.242772	-5.98511	0.609566	0.515103
T.cells	NUDT16	0.231167	2.966991	1.175841	0.242815	-5.34741	0.66084	0.568995
T.cells	AI839979	0.699215	0.274557	1.175765	0.242845	-4.74128	0.702079	0.61321
T.cells	STAM2	0.137936	5.475543	1.175758	0.242848	-5.96959	0.624454	0.530623
T.cells	PDGFA	-0.47227	2.114128	-1.17573	0.242859	-4.99403	0.673654	0.582656
T.cells	GOLT1B	-0.12438	4.631519	-1.17531	0.243025	-5.93455	0.636482	0.543413
T.cells	GM46430	0.250714	1.969969	1.175244	0.243053	-5.1774	0.675843	0.585219
T.cells	BAZ1A	-0.08125	7.694958	-1.17459	0.243313	-6.43087	0.593839	0.499269
T.cells	ABCB1B	-0.26608	3.934965	-1.17454	0.243334	-5.58431	0.646571	0.554382
T.cells	GLIPR1	0.204301	5.059395	1.174369	0.243401	-5.53417	0.630357	0.537343
T.cells	CD2	-0.1756	3.660392	-1.17436	0.243406	-5.7431	0.650589	0.558681
T.cells	B3GNT5	0.200348	2.546338	1.174272	0.243439	-5.62141	0.667132	0.576269
T.cells	TRMT12	0.33902	1.30746	1.174202	0.243467	-5.04606	0.685985	0.59649
T.cells	EXOC7	0.127002	4.087686	1.174148	0.243489	-5.75151	0.644346	0.552108
T.cells	FBP1	-0.30288	5.143064	-1.17412	0.2435	-5.75114	0.629166	0.536135
T.cells	ASMT	0.431342	1.052723	1.173968	0.243561	-4.98661	0.689922	0.600783
T.cells	TRPC4AP	0.095703	5.651799	1.17393	0.243576	-5.97236	0.621969	0.528626
T.cells	METTL9	0.106149	5.718204	1.173697	0.243668	-6.08854	0.621035	0.527734
T.cells	EIF4EBP3	-0.26115	2.80817	-1.17364	0.243692	-5.3027	0.663209	0.572262
T.cells	UQCRH	0.063312	8.592145	1.173615	0.243701	-6.54673	0.581872	0.487165
T.cells	MORRBID	0.480729	5.127394	1.173436	0.243773	-5.1908	0.629389	0.536541
T.cells	CCND1	-0.41914	4.140959	-1.17336	0.243802	-5.1172	0.643572	0.551478
T.cells	KIF1A	0.602878	-0.59877	1.17333	0.243815	-4.72502	0.715955	0.629129
T.cells	RFX1	-0.15445	3.263376	-1.17324	0.243852	-5.4855	0.65644	0.565113
T.cells	MANF	-0.08573	6.808848	-1.17301	0.243941	-6.24772	0.605888	0.512047
T.cells	TMEM120A	-0.18293	3.799392	-1.17281	0.244021	-5.50402	0.648552	0.556822
T.cells	KLHL23	-0.41732	0.171341	-1.17278	0.244034	-4.98613	0.703706	0.615914
T.cells	TBXAS1	-0.55966	3.468333	-1.17236	0.244203	-5.02496	0.653413	0.562031
T.cells	YIF1B	-0.11303	4.886916	-1.17224	0.24425	-5.95834	0.632819	0.540272
T.cells	TRIM30A	0.228211	6.169574	1.172098	0.244307	-5.92538	0.614724	0.521319
T.cells	F830208F2	0.707214	-0.23725	1.171987	0.244351	-4.71681	0.710181	0.622999
T.cells	GSTK1	-0.28494	2.449061	-1.17198	0.244355	-5.19347	0.668594	0.578197
T.cells	HIF1AN	0.190188	3.373427	1.17191	0.244381	-5.54149	0.654813	0.563518
T.cells	TSPAN9	-0.43016	3.290989	-1.17184	0.244408	-5.0362	0.656032	0.564812
T.cells	RIPOR2	0.119907	7.617108	1.171627	0.244494	-6.37726	0.594959	0.500841
T.cells	KIFC3	0.498886	1.625707	1.171045	0.244727	-4.85144	0.681657	0.591986
T.cells	CBX8	-0.36097	1.617418	-1.17091	0.244782	-5.07102	0.681784	0.592153
T.cells	RBM43	0.192606	2.961078	1.170547	0.244926	-5.50061	0.661626	0.570548
T.cells	HOMER2	0.599403	0.275337	1.17039	0.244989	-4.7891	0.702807	0.614822
T.cells	1600020E0	-0.12673	6.223144	-1.17022	0.245056	-6.13242	0.614627	0.521105
T.cells	RBMS3	-0.44925	3.072419	-1.17001	0.245142	-5.13624	0.659967	0.568876
T.cells	KLRA4	0.82034	-1.21319	1.170004	0.245144	-4.71471	0.726634	0.640784

T.cells	CHN2	-0.30009	5.358074	-1.16991	0.245182	-5.69834	0.626776	0.533815
T.cells	WTIP	0.464145	1.051984	1.169674	0.245276	-4.85386	0.690763	0.601758
T.cells	PREPL	0.280749	1.734988	1.169227	0.245455	-5.16217	0.680251	0.590567
T.cells	MRPL1	0.097088	4.619868	1.168948	0.245567	-5.9193	0.637424	0.54508
T.cells	TM2D1	0.088904	5.944576	1.168699	0.245666	-6.06727	0.618614	0.525423
T.cells	PRTN3	1.042213	3.6691	1.168482	0.245753	-5.14057	0.651253	0.559782
T.cells	MKKS	0.256535	1.995441	1.168406	0.245784	-5.2325	0.676277	0.586461
T.cells	LRBA	-0.12897	6.139874	-1.16837	0.2458	-6.14077	0.615886	0.522574
T.cells	CKAP5	0.109378	6.118962	1.168186	0.245872	-6.28838	0.616177	0.52293
T.cells	CDC73	-0.08708	6.174072	-1.16805	0.245927	-6.17579	0.615409	0.522175
T.cells	MDFIC	0.264159	4.328783	1.167917	0.24598	-5.51748	0.641628	0.549718
T.cells	RYR2	-0.8065	0.059974	-1.16785	0.246006	-4.76791	0.706324	0.618992
T.cells	BRD4	-0.05422	8.256086	-1.16782	0.24602	-6.50546	0.587041	0.492788
T.cells	DMAC1	-0.12762	4.611093	-1.16768	0.246076	-5.81357	0.63755	0.545415
T.cells	CIPC	-0.23075	2.655218	-1.16751	0.246142	-5.34061	0.666308	0.575935
T.cells	ERCC6L	-0.17439	3.034059	-1.16734	0.24621	-5.77135	0.660645	0.569929
T.cells	FBXO22	0.110507	4.616588	1.167342	0.246211	-5.89057	0.637471	0.545372
T.cells	COPZ1	-0.08105	6.366584	-1.16701	0.246343	-6.20572	0.612733	0.519592
T.cells	PSMD9	-0.09617	5.225742	-1.16686	0.246403	-6.01281	0.628755	0.536439
T.cells	DYRK1A	-0.09012	7.419436	-1.16683	0.246415	-6.39573	0.598288	0.50466
T.cells	TSC22D3	0.181957	5.71665	1.166764	0.246443	-5.93239	0.621813	0.529178
T.cells	GM17494	0.185662	2.389013	1.166697	0.24647	-5.37739	0.670314	0.58053
T.cells	SLC9A6	0.195768	2.81804	1.166662	0.246484	-5.39861	0.663868	0.573659
T.cells	PSMB10	0.149084	5.706129	1.16634	0.246613	-6.04583	0.622143	0.529451
T.cells	ATAD5	-0.14853	4.742851	-1.16593	0.246778	-6.10999	0.635979	0.544059
T.cells	ZFP846	0.210753	2.431015	1.165921	0.246782	-5.41877	0.670022	0.58021
T.cells	LY75	0.334366	3.332572	1.165496	0.246953	-5.44951	0.656852	0.566057
T.cells	CD247	-0.40144	3.01096	-1.16484	0.247219	-5.10089	0.662189	0.571392
T.cells	DUSP16	0.131366	6.833204	1.164682	0.247281	-6.29736	0.607403	0.513809
T.cells	NPC2	0.108247	7.639426	1.164268	0.247447	-6.30539	0.596541	0.502525
T.cells	CBX1	0.083163	6.075378	1.163974	0.247566	-6.26621	0.61806	0.525019
T.cells	TNFRSF23	-0.45916	1.669914	-1.16364	0.2477	-4.89964	0.682655	0.593569
T.cells	MDH2	-0.09037	6.490426	-1.16363	0.247705	-6.25207	0.612279	0.519052
T.cells	CD9	0.238018	6.033746	1.163486	0.247763	-5.79056	0.618643	0.525702
T.cells	SNX10	0.188759	5.029489	1.163366	0.247811	-5.74116	0.632856	0.540607
T.cells	MRPS23	-0.13187	4.411962	-1.16292	0.247992	-5.87429	0.641748	0.550093
T.cells	RAB2B	0.158041	3.661593	1.162861	0.248015	-5.6445	0.652709	0.561703
T.cells	GAS5	-0.10196	6.391612	-1.16285	0.24802	-6.22031	0.613651	0.520596
T.cells	ISCA2	0.119182	4.968036	1.162752	0.248059	-5.96871	0.633736	0.5417
T.cells	DIP2C	-0.19208	6.172429	-1.16256	0.248138	-6.02937	0.616704	0.52384
T.cells	CNBP	-0.06153	8.132464	-1.16254	0.248145	-6.5103	0.589906	0.496025
T.cells	PLEKHG1	-0.23843	4.398533	-1.16245	0.24818	-5.55218	0.641942	0.550359
T.cells	TTC14	0.082104	5.803768	1.162417	0.248194	-6.11823	0.621871	0.529244
T.cells	PIP5K1A	0.109419	5.595581	1.162402	0.2482	-6.03177	0.624807	0.53232
T.cells	STON1	-0.52649	1.087662	-1.16217	0.248294	-4.8894	0.691748	0.603451
T.cells	DYSF	-0.63127	2.592245	-1.16198	0.24837	-4.99023	0.668779	0.578783
T.cells	KMO	-0.28025	3.038738	-1.16119	0.248691	-5.27485	0.66256	0.571943

T.cells	GM34680	0.641769	0.042973	1.160688	0.248893	-4.78785	0.708716	0.621851
T.cells	SMAGP	-0.29772	3.61538	-1.16037	0.249024	-5.21323	0.654002	0.563134
T.cells	PCIF1	0.079896	6.021144	1.160296	0.249052	-6.18428	0.619399	0.526656
T.cells	ZBTB18	-0.12963	3.894799	-1.16024	0.249074	-5.79537	0.649892	0.558788
T.cells	HAO2	-0.61382	1.393032	-1.15998	0.249179	-4.937	0.687561	0.599134
T.cells	SDE2	-0.11019	6.013145	-1.15966	0.249309	-6.13684	0.619511	0.526923
T.cells	CD40LG	0.610466	-0.56972	1.15947	0.249386	-4.73877	0.718513	0.632836
T.cells	SIGMAR1	-0.1844	3.549056	-1.15933	0.249445	-5.50636	0.654981	0.564456
T.cells	ANK3	-0.5522	1.681131	-1.15923	0.249483	-4.96759	0.683122	0.594569
T.cells	PPP4R3B	-0.07049	6.842276	-1.15906	0.249553	-6.28865	0.607988	0.515024
T.cells	FUS	-0.06448	7.820328	-1.15905	0.249556	-6.45125	0.594655	0.501191
T.cells	CUEDC1	-0.29195	2.113054	-1.15881	0.249655	-5.2044	0.676517	0.587549
T.cells	ZFP7	0.42346	0.286794	1.158284	0.249867	-5.00681	0.704852	0.618444
T.cells	GARS	-0.09681	5.511566	-1.15814	0.249926	-6.10733	0.626582	0.534837
T.cells	ERP29	-0.07396	7.289905	-1.15793	0.250012	-6.36434	0.601851	0.50901
T.cells	PAXX	-0.17902	3.270058	-1.15787	0.250034	-5.46156	0.659115	0.569278
T.cells	H2-EB1	-0.47347	6.754688	-1.15785	0.250044	-5.93135	0.609195	0.51665
T.cells	CDC23	0.157409	2.927739	1.157748	0.250085	-5.58316	0.66422	0.57473
T.cells	4930526L0	0.625895	-0.8055	1.157632	0.250132	-4.72043	0.722316	0.637575
T.cells	CCDC34	-0.14789	4.840796	-1.15762	0.250136	-6.09969	0.636156	0.544967
T.cells	CBLL1	-0.09924	4.917555	-1.15755	0.250164	-6.01018	0.635054	0.54382
T.cells	ANKRD10	-0.08679	5.371393	-1.15711	0.250343	-6.12811	0.628571	0.537171
T.cells	NME1	-0.11573	6.668403	-1.15711	0.250344	-6.3393	0.610387	0.51812
T.cells	KLHL28	0.169028	3.44241	1.15677	0.250482	-5.53361	0.656558	0.56699
T.cells	GARNL3	-0.35036	2.487215	-1.15658	0.25056	-5.07965	0.670843	0.58232
T.cells	SPINT1	0.519765	-1.0651	1.156572	0.250563	-4.74701	0.726524	0.642693
T.cells	BIRC5	-0.18843	5.35755	-1.15657	0.250564	-6.22317	0.628768	0.537612
T.cells	GNB5	-0.43664	0.819995	-1.1563	0.250672	-4.87469	0.696468	0.610065
T.cells	MFSD14A	-0.08619	6.156841	-1.15573	0.250906	-6.08939	0.617499	0.526027
T.cells	AXIN2	0.386891	-0.02376	1.155542	0.250982	-4.99113	0.709777	0.624706
T.cells	PTPN9	0.115419	5.706865	1.155531	0.250986	-6.02873	0.62382	0.532671
T.cells	STXBP2	0.120072	5.31968	1.155513	0.250994	-5.91627	0.629307	0.538444
T.cells	HEBP1	-0.30534	4.67467	-1.15546	0.251017	-5.55707	0.638549	0.548201
T.cells	NAGPA	-0.17991	3.354165	-1.15541	0.251034	-5.477	0.657866	0.568724
T.cells	CYFIP2	0.090406	6.947339	1.155389	0.251044	-6.43537	0.606542	0.51459
T.cells	PTPN13	-0.59867	0.415506	-1.15536	0.251056	-4.82207	0.70282	0.617135
T.cells	PIF1	0.296647	1.716548	1.15536	0.251056	-5.41809	0.682578	0.595227
T.cells	TMEM229E	0.161575	4.03146	1.155229	0.251109	-5.66167	0.647891	0.558119
T.cells	AAMDC	-0.13731	3.299635	-1.15517	0.251134	-5.59659	0.658675	0.569615
T.cells	HEATR6	0.113648	4.812778	1.154947	0.251224	-5.90869	0.636559	0.546236
T.cells	NR6A1	-0.13109	5.241175	-1.15462	0.251357	-6.04746	0.630425	0.539854
T.cells	A530072M	0.509905	0.764735	1.15459	0.25137	-4.89157	0.697333	0.611441
T.cells	GYG	0.141107	6.126163	1.154561	0.251381	-6.0659	0.617928	0.526715
T.cells	AKR1B3	-0.09263	5.402163	-1.15436	0.251462	-6.11959	0.628134	0.537513
T.cells	BMYC	-0.29432	3.380337	-1.15414	0.251555	-5.38557	0.657478	0.568732
T.cells	CAPN11	-0.63067	1.046105	-1.15366	0.251749	-5.0266	0.692941	0.607134
T.cells	SF3B5	-0.08632	6.277364	-1.15348	0.251824	-6.24725	0.615817	0.524979

T.cells	ITGB1BP2	0.384476	0.43994	1.153471	0.251826	-4.99419	0.702434	0.617542
T.cells	1810034E1	-0.3626	1.895787	-1.15335	0.251876	-5.03217	0.679832	0.593098
T.cells	A530017D2	0.271978	2.325165	1.15326	0.251912	-5.18325	0.673295	0.586067
T.cells	THOC1	-0.09294	5.704802	-1.15276	0.252115	-6.14392	0.623849	0.533698
T.cells	TRDMT1	0.203944	2.342131	1.152709	0.252137	-5.41964	0.673038	0.586056
T.cells	ATP5B	-0.0781	8.182512	-1.1527	0.252139	-6.55374	0.589789	0.498128
T.cells	KCNMB4	-0.29664	1.763375	-1.15264	0.252166	-5.32585	0.68186	0.595563
T.cells	IGFBP6	0.726046	-0.02144	1.152621	0.252173	-4.75607	0.70974	0.625833
T.cells	UHRF1BP1	0.431757	1.998967	1.15262	0.252173	-5.01436	0.678256	0.591675
T.cells	MRPL12	-0.11327	5.090142	-1.15247	0.252234	-6.05862	0.632581	0.542962
T.cells	VPREB2	0.339555	-0.41404	1.152213	0.25234	-5.188	0.716012	0.632825
T.cells	IGKV1-110	-0.34543	0.050225	-1.15217	0.252356	-5.10928	0.708601	0.624725
T.cells	TIMM17A	-0.09611	5.135955	-1.15199	0.252431	-6.00174	0.631926	0.542352
T.cells	IFITM1	0.797044	3.489377	1.151965	0.252441	-5.20282	0.655863	0.567788
T.cells	GM42937	0.602235	0.198102	1.15196	0.252443	-4.81179	0.706255	0.622185
T.cells	INSIG2	0.152265	4.405764	1.151949	0.252447	-5.70934	0.642439	0.553489
T.cells	COPS7A	-0.13592	4.237724	-1.15176	0.252523	-5.80235	0.644881	0.556167
T.cells	COMMD6	-0.13404	4.358964	-1.15167	0.252562	-5.77643	0.643118	0.554311
T.cells	ARHGAP45	0.106977	6.710119	1.151439	0.252656	-6.18862	0.609811	0.519247
T.cells	CMTR2	0.375201	1.0685	1.151398	0.252673	-5.00495	0.692592	0.607503
T.cells	PTP4A2	-0.05698	8.699413	-1.15109	0.2528	-6.54095	0.582909	0.491373
T.cells	ARID3A	-0.11263	5.513727	-1.15097	0.252847	-6.06305	0.626551	0.536956
T.cells	HPX	-0.22349	5.670685	-1.15087	0.252888	-5.92205	0.624331	0.534643
T.cells	SIMC1	-0.10861	5.702765	-1.15079	0.252922	-6.17574	0.623878	0.534173
T.cells	VPS45	0.145805	3.758422	1.150707	0.252955	-5.73725	0.651895	0.563914
T.cells	NACA	-0.06816	9.080227	-1.15057	0.25301	-6.69207	0.57789	0.486316
T.cells	ATXN7L3	-0.12744	4.200833	-1.15043	0.253071	-5.76089	0.645418	0.557139
T.cells	NUDT7	0.375516	1.74453	1.150266	0.253136	-5.06928	0.682149	0.596586
T.cells	MED13	-0.08695	7.722854	-1.15017	0.253174	-6.43263	0.595971	0.505143
T.cells	DCLRE1B	0.189401	2.770832	1.150043	0.253227	-5.40226	0.666572	0.5798
T.cells	SMC3	-0.07467	6.498954	-1.15	0.253246	-6.33953	0.612735	0.522649
T.cells	D330041H(	0.190967	2.247493	1.149883	0.253293	-5.49476	0.674473	0.588301
T.cells	MINPP1	-0.1008	4.734583	-1.14984	0.253309	-5.97893	0.637685	0.548963
T.cells	CAPG	0.25676	5.264343	1.149721	0.253359	-5.63398	0.630095	0.540926
T.cells	SRRD	0.170245	3.465767	1.149682	0.253375	-5.52671	0.656213	0.568697
T.cells	BRCA1	-0.16471	4.143927	-1.14967	0.253381	-5.98411	0.646248	0.558064
T.cells	RBM17	-0.09137	5.789508	-1.14937	0.253504	-6.1694	0.62277	0.533152
T.cells	KLK1B27	0.482885	-1.42677	1.149272	0.253543	-4.7001	0.732561	0.651587
T.cells	FOXD2OS	0.356903	0.311339	1.148952	0.253675	-5.09096	0.704801	0.621059
T.cells	AHDC1	-0.09714	4.689967	-1.14875	0.253756	-5.98097	0.638694	0.549925
T.cells	DHODH	-0.24802	2.109646	-1.14823	0.253972	-5.26727	0.67699	0.591064
T.cells	F11R	-0.3466	3.432174	-1.14792	0.254099	-5.18848	0.657118	0.569856
T.cells	CBR4	0.236745	2.335594	1.147799	0.254148	-5.26227	0.673556	0.587574
T.cells	2900076A(	0.194386	2.621325	1.147709	0.254185	-5.41236	0.669236	0.582935
T.cells	MBOAT7	0.153302	4.278521	1.147582	0.254237	-5.66787	0.644688	0.556689
T.cells	CCT2	-0.07886	6.286032	-1.14758	0.254237	-6.26455	0.616079	0.52643
T.cells	APOBR	0.596631	2.373103	1.147252	0.254372	-4.7801	0.672987	0.58711

T.cells	ZBTB24	0.151774	3.32852	1.147211	0.254389	-5.54654	0.658656	0.571721
T.cells	SAMHD1	0.206421	7.45997	1.146906	0.254515	-6.15002	0.599908	0.509715
T.cells	GM48226	0.213722	2.396833	1.146862	0.254533	-5.4021	0.672628	0.586856
T.cells	CES2A	-0.53652	0.898465	-1.14679	0.254564	-4.90021	0.695675	0.611844
T.cells	DNAJC8	-0.05951	6.901934	-1.14678	0.254565	-6.34246	0.607544	0.517721
T.cells	ZFP626	0.187771	2.562798	1.146388	0.254728	-5.45217	0.670119	0.58431
T.cells	IFI211	-0.73097	2.960201	-1.14633	0.254751	-4.884	0.664147	0.577906
T.cells	WIPF2	0.087683	5.343948	1.146238	0.254789	-6.03439	0.629353	0.540826
T.cells	DYRK3	-0.20745	2.520243	-1.14621	0.2548	-5.65566	0.670761	0.585042
T.cells	RPGRIP1L	0.255244	1.613197	1.146201	0.254805	-5.25807	0.684592	0.599985
T.cells	BANK1	-0.26444	6.09331	-1.14612	0.254837	-5.85255	0.618773	0.52966
T.cells	SLAMF9	-0.34997	2.637226	-1.1459	0.254929	-5.26674	0.668997	0.58325
T.cells	EEF1G	-0.08258	7.187877	-1.1458	0.254971	-6.41505	0.60362	0.513859
T.cells	ETF1	-0.08032	7.536791	-1.14569	0.255015	-6.37245	0.598864	0.508893
T.cells	APOL8	-0.26243	2.828251	-1.14564	0.255035	-5.26943	0.666124	0.580159
T.cells	THG1L	-0.23762	2.021146	-1.14508	0.255264	-5.36725	0.678472	0.593492
T.cells	GM10134	0.665269	0.795093	1.145081	0.255266	-4.79111	0.697428	0.614062
T.cells	ZMIZ1	0.080028	6.978265	1.144842	0.255364	-6.35072	0.606613	0.51707
T.cells	MRPL49	-0.14019	3.778216	-1.14479	0.255386	-5.82638	0.652137	0.565247
T.cells	IRF2BP2	-0.09105	7.59206	-1.14474	0.255406	-6.31484	0.598231	0.50831
T.cells	0610010K1	-0.09677	5.617735	-1.14468	0.255431	-6.12406	0.62559	0.537041
T.cells	UBE2J2	-0.07266	6.638057	-1.14418	0.255636	-6.28895	0.61146	0.522101
T.cells	TMEM273	-0.58105	1.19098	-1.14387	0.255764	-4.77354	0.691428	0.607638
T.cells	WDR48	0.113416	4.298768	1.143859	0.25577	-5.83813	0.644681	0.557318
T.cells	CGAS	0.38465	2.747045	1.143797	0.255795	-5.13015	0.667642	0.581922
T.cells	RBMS1	0.089054	7.558573	1.143713	0.25583	-6.28135	0.598836	0.508982
T.cells	BCL2L14	-0.80856	0.459361	-1.14357	0.25589	-4.77975	0.702879	0.620129
T.cells	CTU2	-0.15589	3.206943	-1.14355	0.255898	-5.64313	0.660759	0.574528
T.cells	RUNX2	0.519702	4.240789	1.143229	0.25603	-5.13593	0.645713	0.55828
T.cells	PECR	-0.38883	2.35098	-1.14297	0.256136	-5.17019	0.673945	0.5885
T.cells	MXI1	0.13281	7.063981	1.142786	0.256213	-6.24193	0.605883	0.516227
T.cells	TLR8	0.613708	0.054908	1.142531	0.256318	-4.75713	0.70963	0.627508
T.cells	H2-K1	0.208586	8.786019	1.142119	0.256488	-6.52117	0.58267	0.492259
T.cells	GM527	0.529603	0.481195	1.141806	0.256618	-4.90886	0.702877	0.620385
T.cells	USP18	0.414478	2.737474	1.14161	0.256699	-5.15772	0.668112	0.582712
T.cells	SNX14	-0.13892	4.500746	-1.14155	0.256724	-5.82851	0.64206	0.554835
T.cells	KCNJ16	-0.88284	-0.02269	-1.14129	0.256831	-4.77337	0.710866	0.629283
T.cells	DHDDS	0.12369	4.630539	1.141213	0.256863	-5.91038	0.640181	0.552946
T.cells	NCALD	-0.48962	2.377149	-1.1412	0.256869	-4.90455	0.673555	0.588729
T.cells	TTYH2	-0.32657	3.075271	-1.14118	0.256877	-5.22442	0.663046	0.577406
T.cells	CDH13	-0.68051	2.030566	-1.14105	0.25693	-4.9509	0.678829	0.594491
T.cells	SNX12	-0.1041	4.718214	-1.14101	0.256946	-5.87498	0.638914	0.551654
T.cells	AGPAT4	-0.20546	5.023605	-1.14099	0.256956	-5.79137	0.63452	0.546984
T.cells	SIPA1L3	0.118797	5.201146	1.140942	0.256975	-6.03651	0.631978	0.544298
T.cells	PRKDC	-0.11212	4.69925	-1.14088	0.257002	-5.95572	0.639188	0.551956
T.cells	ARHGEF7	-0.14785	4.675352	-1.1401	0.257322	-5.80068	0.640115	0.552519
T.cells	GART	-0.13055	4.367941	-1.14	0.257367	-5.95864	0.644575	0.557268

T.cells	PALB2	-0.22944	2.125531	-1.13989	0.257409	-5.36847	0.677997	0.593201
T.cells	PSMB8	0.144511	7.077604	1.139554	0.25755	-6.31703	0.606279	0.516949
T.cells	BEND4	0.263286	3.557561	1.139472	0.257584	-5.41806	0.656506	0.57019
T.cells	RBM12B1	0.420252	0.935508	1.139444	0.257596	-5.00352	0.696415	0.613316
T.cells	UTY	2.606366	2.644423	1.138594	0.257948	-5.30572	0.670902	0.585261
T.cells	CYP2R1	-0.43873	1.009263	-1.13824	0.258096	-4.95926	0.696035	0.612577
T.cells	RRAGD	-0.22504	2.299081	-1.13799	0.258201	-5.45001	0.676139	0.591113
T.cells	CNRIP1	-0.30625	1.648634	-1.13778	0.258285	-5.24072	0.686106	0.6019
T.cells	FBXO28	0.140727	4.523471	1.137778	0.258287	-5.79443	0.643062	0.555635
T.cells	SGPL1	0.144749	5.922758	1.137675	0.25833	-5.8575	0.623036	0.534465
T.cells	ZBTB6	0.254191	1.997204	1.137599	0.258362	-5.27249	0.680748	0.596205
T.cells	DDIAS	-0.24503	2.134705	-1.1374	0.258443	-5.36381	0.678645	0.593969
T.cells	CREB3L1	0.427046	2.035248	1.137162	0.258543	-4.94789	0.680166	0.595733
T.cells	PDPN	0.845409	-0.21403	1.13714	0.258552	-4.75412	0.715402	0.634141
T.cells	TBC1D17	0.154702	3.823308	1.136852	0.258672	-5.70101	0.653308	0.566964
T.cells	NEIL1	-0.17302	3.000738	-1.13682	0.258684	-5.68503	0.665538	0.580098
T.cells	TCF7	-0.3248	2.524319	-1.13654	0.258801	-5.38319	0.672719	0.587955
T.cells	ICA1	-0.36756	2.463147	-1.13642	0.258851	-5.19641	0.673647	0.588983
T.cells	TSC1	0.118934	4.710552	1.136308	0.258898	-5.94145	0.64035	0.553282
T.cells	AP1B1	-0.11946	5.190975	-1.13631	0.258899	-5.90642	0.633435	0.545931
T.cells	ENOX2	0.127715	5.852601	1.136264	0.258917	-6.11481	0.624026	0.535967
T.cells	2300009A(	0.166929	4.080294	1.136253	0.258921	-5.66991	0.64953	0.563074
T.cells	PCDHGC4	-0.44239	0.256002	-1.136	0.259025	-4.97459	0.708028	0.626305
T.cells	PRM1	-0.40182	-0.44781	-1.13585	0.25909	-4.97827	0.719307	0.638702
T.cells	GPX4	0.077088	7.153816	1.135357	0.259294	-6.33163	0.606372	0.517276
T.cells	TUBB6	0.477407	3.749371	1.134486	0.259657	-5.00748	0.655674	0.5689
T.cells	SH3BP1	-0.15365	5.21446	-1.13408	0.259826	-5.7818	0.634521	0.546277
T.cells	PDIA3	-0.0864	8.546649	-1.13397	0.259871	-6.51095	0.58838	0.49792
T.cells	GM14636	0.456274	2.274283	1.133555	0.260046	-5.13377	0.678037	0.593056
T.cells	MFF	0.0886	5.906654	1.133376	0.26012	-6.13567	0.624664	0.536046
T.cells	STRAP	-0.08914	6.174275	-1.13325	0.260173	-6.2738	0.620891	0.532117
T.cells	SMARCAL1	-0.17259	2.628016	-1.13315	0.260216	-5.47398	0.672658	0.587371
T.cells	CD5	-0.29323	2.693985	-1.13313	0.260222	-5.18672	0.67166	0.586294
T.cells	LAMTOR3	0.102313	5.240813	1.132935	0.260304	-5.99881	0.634143	0.546282
T.cells	RSL24D1	-0.08614	5.674542	-1.13289	0.260324	-6.18995	0.627953	0.539734
T.cells	PGGT1B	0.109212	4.818529	1.132854	0.260338	-5.93026	0.640225	0.552756
T.cells	KDM5B	0.167767	5.362074	1.132751	0.260381	-5.92528	0.632407	0.544466
T.cells	ZFP62	0.117364	4.580193	1.13256	0.260461	-5.90358	0.643682	0.556563
T.cells	HACL1	-0.22517	3.523051	-1.13244	0.260511	-5.53553	0.659225	0.573193
T.cells	NELFE	-0.10947	4.393275	-1.13236	0.260545	-5.89527	0.646405	0.559535
T.cells	BCL6	0.166402	5.992679	1.13198	0.260704	-6.0975	0.623674	0.535188
T.cells	GPR141B	0.513252	-0.65516	1.131838	0.260763	-4.77258	0.72439	0.643851
T.cells	PON3	-0.17687	3.489649	-1.13172	0.260813	-5.72835	0.65996	0.573839
T.cells	ANAPC13	0.084319	5.65737	1.130987	0.261119	-6.13873	0.628675	0.540513
T.cells	2210016L2	-0.15429	3.97848	-1.13086	0.261172	-5.73108	0.652983	0.566375
T.cells	GM47754	-0.49608	0.667894	-1.13069	0.261245	-4.93696	0.703502	0.621094
T.cells	4732465J0	-0.55594	1.227323	-1.1306	0.261281	-4.86572	0.694719	0.611555

T.cells	DSCAML1	-0.62448	-0.596	-1.13048	0.261331	-4.77572	0.723721	0.643365
T.cells	ATXN1L	0.196511	2.959513	1.130408	0.261362	-5.4561	0.668162	0.582851
T.cells	4930430E1	0.741965	0.409202	1.130395	0.261367	-4.77612	0.707597	0.625668
T.cells	KIDINS220	0.113939	5.419693	1.13035	0.261386	-5.93075	0.632064	0.544284
T.cells	HSF1	-0.11535	4.997353	-1.13021	0.261443	-5.92382	0.638128	0.550732
T.cells	CRACR2A	0.325936	2.835916	1.130087	0.261496	-5.2221	0.670025	0.584946
T.cells	ARHGAP42	-0.43846	1.752056	-1.12969	0.261663	-5.01105	0.68686	0.603029
T.cells	COP1	-0.0975	7.053619	-1.12901	0.261948	-6.30021	0.609764	0.520482
T.cells	OXSM	-0.2779	1.788374	-1.129	0.261951	-5.15906	0.686748	0.602622
T.cells	NT5DC3	0.129046	5.099601	1.128759	0.262053	-5.87146	0.637446	0.549686
T.cells	GM44649	-0.17622	3.949723	-1.12806	0.262348	-5.6766	0.654671	0.567832
T.cells	FOXM1	-0.17581	3.10354	-1.12805	0.26235	-5.76106	0.667285	0.581362
T.cells	NUP54	-0.10976	4.859149	-1.12788	0.262424	-5.94823	0.641402	0.553723
T.cells	SLC16A7	-0.29563	3.236151	-1.12735	0.262644	-5.19996	0.665676	0.579466
T.cells	A530064D(	0.62648	0.81889	1.127277	0.262675	-4.7757	0.702882	0.619792
T.cells	SNRPD3	-0.08096	6.518599	-1.12684	0.26286	-6.33234	0.618279	0.529091
T.cells	ZDHHC13	0.143247	3.693777	1.12672	0.26291	-5.66618	0.659047	0.572401
T.cells	GM34961	0.496721	0.869528	1.126669	0.262931	-4.91623	0.702301	0.619187
T.cells	PLTP	-0.46086	5.244881	-1.1265	0.263004	-5.38937	0.636394	0.548314
T.cells	ERC2	-0.81403	0.862583	-1.12564	0.263365	-4.83936	0.702786	0.61993
T.cells	SNRPD2	-0.07775	6.391824	-1.12549	0.263427	-6.3204	0.620388	0.531608
T.cells	WDR24	0.276249	1.796393	1.125227	0.263538	-5.21854	0.688192	0.604208
T.cells	HACD1	-0.18931	3.002315	-1.12516	0.263567	-5.62274	0.66976	0.58428
T.cells	AMT	-0.42651	1.565291	-1.12505	0.263614	-5.04968	0.691778	0.608102
T.cells	BTG3	0.099326	5.347109	1.125024	0.263624	-6.04429	0.635229	0.547366
T.cells	AGPAT3	-0.13546	5.164345	-1.125	0.263632	-5.88683	0.63786	0.550158
T.cells	COPE	0.086988	6.316581	1.124856	0.263695	-6.221	0.621446	0.532814
T.cells	GM29170	0.257942	1.532706	1.124793	0.263721	-5.1974	0.692285	0.608717
T.cells	CCL2	-0.76872	3.089272	-1.1247	0.263761	-5.00218	0.668449	0.582969
T.cells	GM14966	0.189429	2.847703	1.124597	0.263804	-5.43764	0.672097	0.586957
T.cells	QSER1	0.107441	4.323503	1.124541	0.263827	-6.03067	0.650093	0.563362
T.cells	KLC2	0.239504	2.681752	1.124343	0.263911	-5.4092	0.674652	0.589738
T.cells	LEFTY1	-0.61202	0.408138	-1.12423	0.263958	-4.76056	0.710031	0.628211
T.cells	NCBP1	-0.07984	5.256901	-1.12397	0.264069	-6.09479	0.636672	0.549028
T.cells	ZDHHC17	0.137695	4.015064	1.123847	0.26412	-5.7387	0.654786	0.56834
T.cells	FSCN1	-0.61038	3.378992	-1.12304	0.264463	-5.11198	0.664369	0.578785
T.cells	DOP1B	-0.20051	4.289272	-1.12294	0.264502	-5.53823	0.650862	0.564308
T.cells	TNFRSF13C	-0.13096	3.92598	-1.12291	0.264517	-5.99253	0.656222	0.570043
T.cells	GMPS	0.080006	5.784964	1.122839	0.264546	-6.20086	0.629226	0.541304
T.cells	TRAPPC2L	-0.09885	5.811456	-1.12279	0.264567	-6.11789	0.628849	0.540905
T.cells	LARP7	-0.07646	5.919166	-1.12278	0.26457	-6.25848	0.627318	0.539285
T.cells	AHCY	0.155671	3.495963	1.122771	0.264575	-5.82974	0.662619	0.576917
T.cells	ITGB1BP1	-0.10867	4.649919	-1.12244	0.264714	-5.92277	0.645664	0.558834
T.cells	RHBDD3	0.237593	2.194151	1.122272	0.264785	-5.23727	0.682427	0.598414
T.cells	TBCA	-0.08337	7.625569	-1.12222	0.264807	-6.41254	0.603606	0.514473
T.cells	BRCC3	-0.10494	4.874732	-1.12214	0.264841	-6.00865	0.642393	0.555424
T.cells	LNPEP	0.086714	7.335044	1.121893	0.264945	-6.34004	0.607702	0.518662

T.cells	GAB3	0.20986	4.746655	1.121396	0.265156	-5.60603	0.644522	0.557515
T.cells	OCIAD2	-0.54002	0.485681	-1.12137	0.265168	-4.86429	0.709431	0.627656
T.cells	RNFT1	-0.12769	4.495375	-1.12103	0.265311	-5.7658	0.648191	0.561516
T.cells	SPDL1	-0.17021	2.833783	-1.12093	0.265351	-5.73641	0.672947	0.588115
T.cells	HERC4	0.095983	7.008496	1.120803	0.265407	-6.27985	0.612363	0.523622
T.cells	PRPF38A	-0.10151	5.176932	-1.12043	0.265566	-6.07936	0.638284	0.551149
T.cells	RGS14	0.249222	3.585563	1.120347	0.2656	-5.36905	0.661639	0.576133
T.cells	ACE	0.854099	0.341503	1.120109	0.265701	-4.79132	0.711729	0.630621
T.cells	SMIM8	-0.11905	4.587829	-1.12002	0.265738	-5.93695	0.646838	0.560383
T.cells	S1PR5	0.613218	-0.65106	1.119984	0.265754	-4.77335	0.727739	0.64823
T.cells	TTC25	-0.48792	0.218704	-1.11998	0.265757	-4.92108	0.713692	0.632774
T.cells	KLRA6	0.676906	-0.71608	1.119969	0.26576	-4.77014	0.728799	0.649399
T.cells	NUBPL	-0.194	2.913085	-1.11996	0.265765	-5.45921	0.671746	0.58712
T.cells	SLC4A9	-0.46082	0.122757	-1.11981	0.265828	-4.92466	0.715243	0.634468
T.cells	KPNA1	0.095605	6.778024	1.11921	0.266082	-6.22823	0.615793	0.52746
T.cells	SAMSN1	0.155723	7.050554	1.119177	0.266096	-6.00871	0.612002	0.523478
T.cells	YIPF4	0.105156	6.515782	1.119131	0.266115	-6.24755	0.619462	0.531333
T.cells	RNF111	0.089856	6.692893	1.119044	0.266153	-6.26731	0.616982	0.528731
T.cells	IWS1	-0.08222	5.729671	-1.11857	0.266354	-6.18039	0.630882	0.543182
T.cells	GABARAP	0.074404	8.497974	1.118474	0.266395	-6.49819	0.592516	0.502897
T.cells	HIST1H3E	0.153397	3.610481	1.118164	0.266526	-5.88779	0.661962	0.576299
T.cells	PCNP	-0.07031	6.359657	-1.11778	0.266687	-6.27078	0.622083	0.533976
T.cells	MS4A6D	0.608723	2.866447	1.117781	0.266689	-5.01254	0.673159	0.588505
T.cells	PHB2	-0.07754	6.570723	-1.11773	0.26671	-6.34235	0.619117	0.530849
T.cells	HIST1H2AI	0.255585	2.588783	1.117449	0.26683	-5.77555	0.677383	0.593186
T.cells	LY6G2	-0.59363	0.952461	-1.1173	0.266893	-4.903	0.702774	0.620863
T.cells	SOS1	0.086538	5.916592	1.117211	0.266931	-6.16064	0.628354	0.540812
T.cells	BCL2A1A	0.824183	2.399522	1.116845	0.267087	-4.84739	0.680275	0.596505
T.cells	THAP4	0.124393	4.091332	1.116823	0.267096	-5.82049	0.654819	0.569077
T.cells	CASP4	0.392193	4.029936	1.116634	0.267176	-5.30948	0.655727	0.57005
T.cells	STEAP3	-0.19724	1.980258	-1.11663	0.267177	-5.51708	0.686725	0.6035
T.cells	CRYBG1	0.192822	4.169377	1.116482	0.267241	-5.62297	0.653666	0.567842
T.cells	CSTA3	0.842838	0.791872	1.116413	0.26727	-4.83422	0.705313	0.623764
T.cells	TIAM1	0.122813	4.628891	1.116307	0.267315	-6.15637	0.646918	0.560625
T.cells	1600014C1	0.238096	4.275444	1.116284	0.267325	-5.66256	0.652103	0.566168
T.cells	LIG4	0.235504	1.902715	1.116096	0.267405	-5.50639	0.687979	0.60483
T.cells	TYROBP	-0.22107	9.095091	-1.11588	0.267496	-6.04696	0.584731	0.495176
T.cells	VPS11	-0.13318	4.072729	-1.1158	0.267532	-5.72519	0.655171	0.569479
T.cells	TIMELESS	-0.14944	3.491901	-1.11538	0.267711	-5.89484	0.664115	0.578832
T.cells	KRT18	-0.38116	3.557982	-1.11512	0.267822	-5.41084	0.663237	0.577815
T.cells	KLHL13	-0.68887	0.360663	-1.115	0.267873	-4.80205	0.712698	0.631549
T.cells	NAPEPLD	0.353631	1.056502	1.114361	0.268144	-5.11495	0.702212	0.619626
T.cells	TRMT10B	0.264686	1.681609	1.114065	0.268271	-5.27375	0.692485	0.609124
T.cells	NDUFAB1	-0.10248	6.268706	-1.11391	0.268338	-6.28903	0.624387	0.53614
T.cells	PLXND1	0.184335	3.5086	1.11387	0.268354	-5.50507	0.664572	0.578959
T.cells	HAL	0.486276	2.215151	1.113549	0.268491	-5.09031	0.684223	0.600369
T.cells	ATXN3	0.095856	5.054807	1.113501	0.268511	-6.03367	0.641771	0.554762

T.cells	HYAL3	-0.56412	0.035974	-1.11339	0.268561	-4.81152	0.718551	0.63785
T.cells	CCHCR1	-0.26609	1.536464	-1.11322	0.268633	-5.34279	0.694749	0.611903
T.cells	DNAL1	-0.37323	1.64713	-1.11319	0.268646	-5.06503	0.693022	0.610025
T.cells	FANCC	-0.12673	4.74282	-1.11306	0.268699	-6.01965	0.646312	0.55974
T.cells	TEX30	-0.09148	5.223272	-1.11278	0.268818	-6.14761	0.639331	0.552424
T.cells	NPEPPS	0.094314	7.184287	1.112683	0.268861	-6.35013	0.611569	0.523073
T.cells	DDX24	0.077689	6.717234	1.112659	0.268871	-6.35379	0.618076	0.529918
T.cells	GLDC	-0.47186	1.391092	-1.11209	0.269114	-4.97773	0.697211	0.61478
T.cells	TPD52L2	0.083081	5.433659	1.112048	0.269132	-6.10533	0.636469	0.549401
T.cells	NRF1	0.069559	6.474245	1.111971	0.269165	-6.3266	0.621655	0.533752
T.cells	PPP1R12B	-0.10055	5.292478	-1.11194	0.269177	-6.07034	0.638504	0.551613
T.cells	BLCAP	0.242842	3.009547	1.111672	0.269293	-5.26912	0.672416	0.587886
T.cells	DCAF5	0.111361	5.52135	1.11145	0.269388	-6.04419	0.635385	0.548276
T.cells	GM36839	0.233189	2.346299	1.111358	0.269427	-5.35786	0.682581	0.598948
T.cells	KMT2C	-0.07835	7.717211	-1.11108	0.269547	-6.46772	0.604657	0.515857
T.cells	GM13008	-0.34005	1.087607	-1.11098	0.269589	-5.11258	0.702295	0.620417
T.cells	HSF5	-0.52165	0.277217	-1.11072	0.269701	-4.877	0.715327	0.634688
T.cells	TEX14	0.160347	5.64791	1.110105	0.269964	-6.0342	0.634053	0.546739
T.cells	GPD1	-0.3844	1.749644	-1.10999	0.270012	-5.10025	0.692334	0.609384
T.cells	UHRF1	-0.14902	4.598357	-1.10997	0.27002	-6.14382	0.649276	0.562951
T.cells	ZFP747	-0.28455	1.605909	-1.10992	0.270042	-5.18883	0.694576	0.611832
T.cells	TACC2	-0.29604	3.088667	-1.10904	0.270421	-5.23024	0.671941	0.587252
T.cells	ZFP646	0.147945	3.903418	1.10898	0.270446	-5.82363	0.659707	0.574087
T.cells	GPCPD1	-0.09215	7.329375	-1.10887	0.270494	-6.33825	0.610513	0.52188
T.cells	AMFR	-0.08777	6.011988	-1.10874	0.270551	-6.05836	0.629006	0.541438
T.cells	ZGPAT	0.134173	3.668613	1.108608	0.270606	-5.64794	0.663212	0.577962
T.cells	RPAP3	-0.12498	3.841822	-1.10838	0.270702	-5.88016	0.660625	0.575209
T.cells	SSR4	-0.09089	7.573816	-1.10829	0.270741	-6.40269	0.607139	0.518449
T.cells	GM11973	0.228265	3.179185	1.108271	0.27075	-5.49681	0.670572	0.585914
T.cells	LPCAT3	0.118759	5.162712	1.107669	0.271009	-5.98903	0.641207	0.554733
T.cells	KPNA2	0.121291	4.937072	1.107645	0.271019	-6.1892	0.644486	0.558229
T.cells	ALG2	-0.16939	3.062397	-1.10762	0.271031	-5.48006	0.672339	0.588128
T.cells	CPSF7	-0.07931	5.801172	-1.10757	0.271051	-6.20115	0.632015	0.544972
T.cells	EIF5A	-0.07753	9.061608	-1.10745	0.271102	-6.74285	0.58698	0.497696
T.cells	SURF6	0.132266	3.185009	1.107422	0.271115	-5.66379	0.670484	0.58614
T.cells	SOX4	0.168202	5.508952	1.107384	0.271132	-6.25619	0.636206	0.549425
T.cells	ACOT11	0.272965	1.943725	1.107376	0.271135	-5.18714	0.68949	0.60673
T.cells	MRPL17	-0.09595	5.426857	-1.10736	0.271141	-6.09235	0.637389	0.550682
T.cells	UBE2S	-0.09709	7.624597	-1.10725	0.271191	-6.6096	0.60644	0.518
T.cells	CTCF	-0.05652	7.101448	-1.10718	0.271219	-6.49981	0.613676	0.525598
T.cells	HAP1	-0.59854	-0.45019	-1.10704	0.271281	-4.77377	0.727567	0.648454
T.cells	TRIM13	0.497936	0.46615	1.106145	0.271665	-4.90643	0.713063	0.632667
T.cells	DMAP1	-0.1964	2.496634	-1.10613	0.271669	-5.43964	0.68125	0.597954
T.cells	GM30211	-0.17796	4.041214	-1.10613	0.271671	-6.08824	0.657936	0.5728
T.cells	LRRC8B	0.112025	3.358799	1.106085	0.27169	-5.87284	0.668144	0.583804
T.cells	FITM2	0.311978	0.918829	1.105961	0.271744	-5.06552	0.705854	0.624787
T.cells	VAMP2	0.117758	4.421644	1.105867	0.271784	-5.76791	0.652309	0.566788

T.cells	A930037H(	0.348625	3.242306	1.105829	0.271801	-5.30354	0.669901	0.5857
T.cells	COX16	-0.07076	6.153691	-1.10569	0.271859	-6.22143	0.627257	0.540106
T.cells	EDARADD	0.140382	3.159788	1.105512	0.271937	-5.85854	0.671196	0.587089
T.cells	SIRT6	0.188911	2.652878	1.104974	0.272169	-5.56049	0.679273	0.595518
T.cells	ETFRF1	-0.24666	3.163487	-1.10475	0.272265	-5.38111	0.671503	0.587246
T.cells	TAF6L	0.180907	2.800016	1.104629	0.272318	-5.52584	0.677025	0.593223
T.cells	POLR3G	-0.24668	2.101279	-1.10442	0.272407	-5.39292	0.687761	0.604865
T.cells	SDF2	0.105302	5.410417	1.104393	0.272419	-5.98407	0.638285	0.551652
T.cells	CENPX	-0.08946	6.347296	-1.10436	0.272435	-6.29932	0.624895	0.537449
T.cells	CECR2	-0.13464	6.605628	-1.10418	0.27251	-6.55542	0.62125	0.533615
T.cells	GM20732	0.116752	4.58993	1.104086	0.272552	-5.88971	0.650233	0.564448
T.cells	RFTN1	-0.10568	6.737622	-1.10364	0.272745	-6.32353	0.619582	0.5319
T.cells	STXBP1	0.145133	3.786972	1.103378	0.272857	-5.90682	0.662327	0.577574
T.cells	SLA2	0.348448	2.746588	1.103325	0.27288	-5.1198	0.678045	0.594571
T.cells	EMP1	0.35546	1.978152	1.103304	0.272889	-5.34989	0.689876	0.607415
T.cells	SLC25A22	0.323098	2.495595	1.103049	0.272999	-5.25914	0.681889	0.598847
T.cells	RASSF4	0.189326	4.553252	1.102941	0.273046	-5.69717	0.650968	0.565558
T.cells	SAA3	1.640715	0.738679	1.102789	0.273111	-4.88152	0.709363	0.628858
T.cells	GCSH	-0.1262	4.428205	-1.10277	0.27312	-5.96566	0.652809	0.567534
T.cells	TMEM161/	-0.2186	2.853132	-1.10268	0.27316	-5.42486	0.67642	0.593009
T.cells	RFK	-0.11449	5.042963	-1.10238	0.273289	-6.0118	0.643845	0.558101
T.cells	OVGP1	-0.39968	0.492644	-1.10236	0.273295	-4.96655	0.713336	0.633397
T.cells	1700084CC	0.21754	2.9115	1.101849	0.273518	-5.48104	0.675586	0.592269
T.cells	H2-D1	0.161947	8.452787	1.101773	0.273551	-6.51077	0.595995	0.507623
T.cells	QK	-0.06425	8.142329	-1.10174	0.273565	-6.53229	0.600209	0.512038
T.cells	HSPA1A	-0.59264	3.985226	-1.10172	0.273575	-5.45334	0.659424	0.574858
T.cells	SEC23B	-0.08919	5.223375	-1.10162	0.273616	-6.09415	0.641236	0.555382
T.cells	RABGAP1	0.088343	5.920481	1.101456	0.273688	-6.24623	0.631204	0.544772
T.cells	ABI3	0.207327	4.401261	1.101216	0.273792	-5.78686	0.653259	0.568428
T.cells	GID4	0.109336	4.230623	1.101181	0.273807	-5.90973	0.655781	0.571151
T.cells	SPNS2	-0.29018	1.813184	-1.1009	0.273928	-5.24697	0.692498	0.610951
T.cells	HIST1H2BB	-0.24257	1.116611	-1.10085	0.273948	-5.38774	0.703425	0.622909
T.cells	SLFN5	-0.49411	4.230734	-1.10078	0.273981	-5.21469	0.65578	0.571251
T.cells	ZFP317	-0.17073	2.898111	-1.10073	0.274002	-5.59241	0.67579	0.592845
T.cells	DDC	-0.29179	1.455602	-1.10056	0.274075	-5.29986	0.698124	0.617116
T.cells	SASH1	0.17651	5.303699	1.1001	0.274275	-6.03147	0.640438	0.554665
T.cells	SUPT6	-0.10507	5.808893	-1.09967	0.274461	-6.10421	0.633361	0.547
T.cells	9930021J0	0.085776	6.407459	1.099629	0.274479	-6.29683	0.624837	0.537982
T.cells	COL5A2	0.506362	1.225753	1.098983	0.274759	-4.98512	0.702892	0.621786
T.cells	AMER1	0.185285	2.080672	1.098277	0.275065	-5.42239	0.689588	0.607298
T.cells	IPPK	0.167445	3.400734	1.098174	0.27511	-5.6594	0.669385	0.585414
T.cells	RRP12	-0.2022	2.60063	-1.09816	0.275117	-5.45919	0.681563	0.598591
T.cells	NRM	-0.11735	5.099836	-1.09815	0.275121	-6.21083	0.644192	0.558372
T.cells	FAF1	0.074387	6.739255	1.097843	0.275254	-6.37026	0.62073	0.533581
T.cells	FAM168A	0.089123	5.93211	1.097656	0.275335	-6.22019	0.632179	0.545748
T.cells	ASPDH	-0.49892	1.294554	-1.09764	0.275341	-4.97203	0.701886	0.620915
T.cells	TRMT44	0.25367	1.793941	1.097549	0.275381	-5.33497	0.69405	0.612403

T.cells	NDUFA6	0.091045	6.56293	1.097372	0.275458	-6.28231	0.623214	0.536348
T.cells	TAX1BP1	0.07604	8.297296	1.097367	0.275461	-6.56235	0.599183	0.511071
T.cells	TAF2	-0.10356	4.171185	-1.09728	0.275499	-5.90569	0.657849	0.573295
T.cells	TRMT1	-0.1049	4.050324	-1.09723	0.275519	-5.90641	0.659646	0.575239
T.cells	PPP1R10	-0.10597	6.277719	-1.09703	0.275609	-6.21257	0.627253	0.540662
T.cells	SIRT4	-0.4782	0.56797	-1.09702	0.275611	-4.98039	0.713432	0.633739
T.cells	JAG1	-0.56501	1.3378	-1.09671	0.275747	-4.9534	0.701403	0.620477
T.cells	ARHGAP26	0.115338	6.685583	1.096537	0.275822	-6.32843	0.621697	0.534751
T.cells	CHCHD3	0.060523	6.768694	1.096194	0.275971	-6.36373	0.620732	0.533632
T.cells	POLRMT	0.215441	1.91437	1.095669	0.276199	-5.45076	0.693062	0.610898
T.cells	KLF10	-0.11552	5.248269	-1.09527	0.276373	-6.00217	0.642932	0.55689
T.cells	TCP11L2	-0.14941	6.196085	-1.09504	0.276472	-6.0831	0.629292	0.542397
T.cells	RNF2	-0.08517	5.723956	-1.09503	0.276476	-6.17833	0.636052	0.549573
T.cells	OAS1C	0.289089	1.903745	1.094995	0.276493	-5.33298	0.693306	0.611217
T.cells	CAPSL	-0.31687	0.928873	-1.09483	0.276565	-5.38354	0.708665	0.628072
T.cells	TIMD4	-0.49234	3.658253	-1.09435	0.276775	-5.19019	0.666439	0.582401
T.cells	ABCB7	0.084788	5.334608	1.094213	0.276834	-6.144	0.641678	0.555888
T.cells	CPSF1	-0.14919	3.349532	-1.09403	0.276915	-5.69888	0.671095	0.587497
T.cells	MCPT8	-1.24977	-0.5048	-1.09402	0.276918	-4.79997	0.73182	0.653912
T.cells	SUPT3	-0.12212	5.480744	-1.09399	0.27693	-6.03865	0.639561	0.553658
T.cells	PRDM9	0.294246	1.146615	1.093922	0.276961	-5.23272	0.705207	0.624611
T.cells	MTMR14	-0.10272	5.427711	-1.0939	0.276971	-6.14235	0.640328	0.554476
T.cells	CELSR2	-0.42707	-0.09555	-1.09372	0.277048	-5.04828	0.725141	0.646564
T.cells	SLC41A1	-0.25452	2.387756	-1.09371	0.277052	-5.28133	0.685796	0.60346
T.cells	NFKBIZ	-0.15959	6.516715	-1.0931	0.277321	-6.06906	0.625213	0.538211
T.cells	FOXR1	-0.54237	-0.87975	-1.09273	0.277481	-4.77237	0.738818	0.6613
T.cells	MZT2	-0.19678	2.206691	-1.09247	0.277597	-5.36694	0.689379	0.607119
T.cells	RWDD2A	-0.41585	0.48641	-1.09224	0.277695	-4.9981	0.716552	0.636899
T.cells	COQ4	-0.18962	2.66674	-1.09221	0.27771	-5.49986	0.682276	0.59945
T.cells	EEF2	-0.07873	9.105144	-1.09215	0.277736	-6.75911	0.589785	0.501112
T.cells	PARP9	0.195339	5.150171	1.092049	0.277779	-5.93751	0.645092	0.559481
T.cells	KLHL3	-0.34628	2.005874	-1.0917	0.27793	-5.08987	0.692732	0.610757
T.cells	PPP1CB	0.085865	7.803763	1.091267	0.27812	-6.51754	0.607834	0.519853
T.cells	GAS6	-0.47444	1.545619	-1.09126	0.278122	-4.95422	0.700129	0.618755
T.cells	UBR4	-0.07825	6.056739	-1.0908	0.278325	-6.17266	0.632592	0.545911
T.cells	TSPAN5	0.11358	5.373256	1.090693	0.278372	-6.14121	0.64245	0.556414
T.cells	CCNG1	0.11657	4.873028	1.090645	0.278393	-5.91833	0.649756	0.564233
T.cells	EVA1A	0.466572	1.060527	1.090484	0.278463	-4.9165	0.708051	0.627402
T.cells	NUDT19	-0.12859	4.544356	-1.09031	0.278539	-5.9218	0.654609	0.569461
T.cells	GM45353	-0.3389	-0.0712	-1.09023	0.278573	-5.03823	0.726262	0.647438
T.cells	RTCB	0.090631	5.290693	1.08974	0.278789	-6.18276	0.644003	0.557919
T.cells	GM20682	0.277342	1.711443	1.089625	0.278839	-5.17726	0.698141	0.616367
T.cells	CCR5	-0.48076	4.693422	-1.08892	0.279149	-5.07081	0.652941	0.567603
T.cells	WDR78	0.404824	0.561969	1.088552	0.27931	-5.03295	0.716607	0.636829
T.cells	PCNX4	-0.26532	2.141019	-1.08853	0.279322	-5.24811	0.691623	0.609489
T.cells	MEN1	-0.17809	2.866329	-1.08849	0.279335	-5.61076	0.68042	0.597317
T.cells	CEACAM10	0.548618	-1.04799	1.088277	0.27943	-4.78928	0.74293	0.665959

T.cells	LCOR	-0.08052	7.538405	-1.08799	0.279555	-6.4555	0.612218	0.524536
T.cells	GM14548	0.60694	1.47249	1.087886	0.279602	-4.81027	0.702101	0.621041
T.cells	SPTA1	0.623569	0.279808	1.087814	0.279634	-4.88962	0.721157	0.64196
T.cells	MTHFD2L	0.183669	3.033816	1.087743	0.279665	-5.50691	0.677857	0.594657
T.cells	SFT2D2	0.131565	4.677531	1.087678	0.279694	-5.89875	0.653176	0.568088
T.cells	IZUMO1R	0.568985	0.001693	1.087643	0.279709	-4.91625	0.725669	0.646968
T.cells	CCDC148	-0.87436	1.762244	-1.08748	0.27978	-4.87578	0.697542	0.616139
T.cells	C130046K2	0.430225	0.716514	1.087368	0.279829	-4.98789	0.714125	0.634304
T.cells	ARFGAP3	0.184578	3.55174	1.087311	0.279855	-5.5352	0.669987	0.586221
T.cells	TOR2A	0.139472	3.670287	1.087008	0.279988	-5.71271	0.668198	0.584439
T.cells	JAM3	-0.42827	0.244994	-1.08685	0.280058	-5.01685	0.721721	0.642882
T.cells	RSPH9	0.230495	1.514419	1.086481	0.280219	-5.54167	0.701439	0.620746
T.cells	IER3IP1	0.070445	6.363638	1.086411	0.28025	-6.26675	0.628735	0.542415
T.cells	SELPLG	0.106877	6.845492	1.086373	0.280267	-6.27648	0.62191	0.535188
T.cells	SLC9A3R1	-0.08861	6.690174	-1.08632	0.28029	-6.33918	0.624102	0.537507
T.cells	POLR2M	-0.07687	5.403452	-1.08593	0.28046	-6.19478	0.642545	0.557272
T.cells	GM43848	0.267643	2.767038	1.085848	0.280498	-5.20717	0.681943	0.599736
T.cells	SF3B2	-0.064	7.285583	-1.08571	0.28056	-6.47679	0.615738	0.528886
T.cells	GM10785	0.263882	2.451276	1.085599	0.280608	-5.38913	0.68681	0.605138
T.cells	USP47	0.085854	6.472249	1.085496	0.280653	-6.29647	0.62719	0.541101
T.cells	ATAD2	-0.11308	6.309192	-1.08547	0.280662	-6.42108	0.62951	0.543568
T.cells	TONSL	-0.25298	1.456718	-1.08528	0.280746	-5.35206	0.70235	0.622195
T.cells	NFKBIE	-0.1438	4.879022	-1.08517	0.280796	-5.99106	0.650209	0.565709
T.cells	GM2396	0.540141	-0.85737	1.084929	0.280902	-4.78665	0.739768	0.663645
T.cells	CD180	-0.1605	5.032672	-1.08491	0.280911	-5.90048	0.647955	0.563417
T.cells	ECSIT	0.116457	4.015765	1.08484	0.280942	-5.92634	0.66301	0.579599
T.cells	SEC23IP	0.090251	4.499732	1.084766	0.280974	-5.95596	0.655804	0.571868
T.cells	ADGRL1	-0.199	2.476047	-1.08463	0.281033	-5.49761	0.686427	0.604995
T.cells	NF1	-0.08638	6.550388	-1.08458	0.281057	-6.29506	0.626081	0.540134
T.cells	WARS	0.163881	3.723103	1.084491	0.281095	-5.73126	0.667403	0.584389
T.cells	CALHM6	0.498237	3.048969	1.084387	0.281141	-5.28296	0.677625	0.595472
T.cells	LMLN	-0.25565	1.750464	-1.08432	0.281171	-5.29627	0.697727	0.617369
T.cells	CHAMP1	-0.17979	2.843874	-1.08428	0.281187	-5.55963	0.680764	0.598879
T.cells	CORO1C	-0.08576	6.202322	-1.0841	0.28127	-6.24874	0.631035	0.545432
T.cells	PPP2R3C	0.110394	4.333149	1.084089	0.281273	-5.95953	0.658276	0.574566
T.cells	SMG6	-0.06175	8.045885	-1.08378	0.281407	-6.58869	0.605284	0.518261
T.cells	LACTB2	0.148132	3.94655	1.083734	0.281429	-5.70896	0.664125	0.580942
T.cells	LRP1	0.306691	4.178873	1.083525	0.281522	-5.29521	0.660652	0.577237
T.cells	BTG1	0.09923	9.314237	1.083479	0.281542	-6.61572	0.588101	0.500368
T.cells	RNASE6	0.25489	4.491549	1.083268	0.281635	-5.53238	0.656035	0.57229
T.cells	ACSS2	0.247478	3.033515	1.083182	0.281673	-5.37631	0.677974	0.59603
T.cells	XRN1	0.089172	5.924264	1.083001	0.281753	-6.22922	0.635173	0.549961
T.cells	SLC6A6	0.102489	7.287991	1.082656	0.281905	-6.26212	0.61586	0.529572
T.cells	KATNA1	-0.11905	4.864725	-1.08256	0.281948	-5.93452	0.650584	0.566532
T.cells	MAST2	-0.09882	5.283184	-1.08242	0.28201	-6.25951	0.644459	0.560007
T.cells	MICU1	0.086349	5.891562	1.082336	0.282046	-6.17052	0.635651	0.550631
T.cells	DDRGK1	0.101524	5.272107	1.082326	0.282051	-6.00208	0.64462	0.56021

T.cells	AMOTL1	-0.39174	1.696223	-1.08186	0.282255	-5.07281	0.699055	0.618956
T.cells	ZFP219	-0.26547	1.771622	-1.08157	0.282384	-5.19424	0.697871	0.617722
T.cells	TICAM2	-0.34636	1.274799	-1.08145	0.282436	-5.08508	0.705709	0.626355
T.cells	ME1	-0.65073	-0.00982	-1.08139	0.282464	-4.80644	0.726352	0.649103
T.cells	DNAJC17	0.116564	3.568748	1.081063	0.282609	-5.76526	0.670188	0.587788
T.cells	ABI2	-0.15813	3.903545	-1.08096	0.282655	-5.78582	0.665145	0.582342
T.cells	FFAR4	0.59936	0.260828	1.080939	0.282664	-4.83736	0.721957	0.64441
T.cells	PDXK	-0.19204	4.441778	-1.08092	0.28267	-5.54336	0.657112	0.573683
T.cells	ZMYM3	0.228483	2.31007	1.080378	0.282912	-5.45204	0.689468	0.60898
T.cells	LTBP1	0.877235	0.730726	1.080298	0.282948	-4.92311	0.714385	0.636359
T.cells	STRADA	0.12371	4.58715	1.080134	0.28302	-5.94106	0.654958	0.571705
T.cells	MANBA	0.124733	4.766674	1.080109	0.283031	-5.91123	0.652307	0.568855
T.cells	ZFP397	0.118422	3.944609	1.080108	0.283031	-5.80467	0.664529	0.582022
T.cells	CCDC28A	0.220169	2.307492	1.080018	0.283072	-5.38256	0.689508	0.609169
T.cells	ODR4	0.138081	4.042451	1.079947	0.283103	-5.75967	0.663063	0.580499
T.cells	TAB2	-0.07695	6.780443	-1.07992	0.283114	-6.30703	0.623253	0.537903
T.cells	NID2	-0.42428	2.19946	-1.07956	0.283275	-5.07852	0.691331	0.611108
T.cells	ARL6IP6	-0.0876	5.278997	-1.07952	0.283291	-6.18901	0.644932	0.560918
T.cells	OPHN1	0.367923	2.997504	1.07899	0.283527	-5.22544	0.679306	0.597871
T.cells	KDM1B	-0.14724	3.6758	-1.07898	0.283529	-5.8161	0.668996	0.586692
T.cells	MVB12B	0.200904	4.074675	1.07876	0.283629	-5.54919	0.663097	0.58031
T.cells	ZXDC	0.155849	4.004625	1.078515	0.283738	-5.64974	0.664227	0.581572
T.cells	TRIM27	-0.08203	5.675636	-1.07842	0.283781	-6.2611	0.639613	0.555152
T.cells	CAMTA1	0.082989	5.835211	1.077652	0.284121	-6.2197	0.637786	0.552915
T.cells	KCTD21	0.562947	0.182578	1.077388	0.284238	-4.88994	0.72442	0.647082
T.cells	PLPP1	-0.23693	4.327383	-1.07729	0.28428	-5.65921	0.659899	0.576655
T.cells	NUTF2	0.245792	1.951713	1.077207	0.284318	-5.4249	0.696198	0.61607
T.cells	PKNOX1	0.117634	4.569323	1.077191	0.284325	-6.02323	0.656304	0.572796
T.cells	CRYZL1	0.117649	4.570122	1.076957	0.284429	-5.91521	0.656292	0.572925
T.cells	LRRC20	0.254069	2.057401	1.076813	0.284493	-5.31683	0.694544	0.614468
T.cells	MYLPF	0.271932	2.109786	1.07661	0.284584	-5.4592	0.693726	0.613697
T.cells	DUSP12	0.153159	3.283921	1.076608	0.284584	-5.67075	0.67562	0.593977
T.cells	TNIP2	0.153764	3.816648	1.076375	0.284688	-5.61993	0.667551	0.585332
T.cells	GM42670	0.399738	0.89839	1.076308	0.284718	-5.02483	0.712876	0.634815
T.cells	MYO5A	-0.14623	5.016572	-1.07612	0.284803	-5.98149	0.649704	0.566167
T.cells	COX10	0.132078	3.775159	1.076114	0.284804	-5.81854	0.668176	0.586073
T.cells	CPNE3	-0.13572	5.367342	-1.07595	0.284878	-5.93128	0.644572	0.560695
T.cells	CEP85L	0.171373	4.806918	1.075944	0.284879	-5.83901	0.65279	0.569512
T.cells	ETFBKMT	0.222686	2.261696	1.075626	0.285021	-5.39738	0.691379	0.611471
T.cells	EGFL6	-0.26674	0.548891	-1.07554	0.28506	-5.3969	0.718513	0.641282
T.cells	GNASAS1	0.291027	0.682179	1.075431	0.285108	-5.2768	0.716367	0.638967
T.cells	PEX5	0.12695	4.071805	1.075288	0.285171	-5.77497	0.663738	0.581518
T.cells	SLC25A24	-0.2668	3.486183	-1.07504	0.285283	-5.3094	0.672566	0.591092
T.cells	SCD2	0.1573	4.726396	1.074932	0.28533	-5.94572	0.653999	0.571051
T.cells	BTBD1	0.080638	6.497876	1.074896	0.285346	-6.31364	0.628309	0.543576
T.cells	IYD	-0.48572	0.499692	-1.07473	0.285421	-4.91483	0.719307	0.642309
T.cells	ZFP35	0.17342	2.543067	1.074486	0.285528	-5.54894	0.687014	0.606937

T.cells	MUS81	-0.26308	2.048507	-1.07432	0.285603	-5.28774	0.694704	0.615346
T.cells	NPM3	-0.10917	5.097579	-1.07417	0.28567	-6.18579	0.648536	0.565333
T.cells	WDR59	0.188644	3.072953	1.073978	0.285755	-5.56064	0.678861	0.598178
T.cells	RPTOR	0.083568	6.294365	1.073934	0.285775	-6.29228	0.631212	0.546889
T.cells	MNAT1	0.106519	4.818214	1.073788	0.28584	-6.06223	0.652643	0.56988
T.cells	GATAD1	-0.08111	5.768843	-1.07345	0.28599	-6.19243	0.638764	0.555102
T.cells	IRAK1	-0.1118	5.224747	-1.07344	0.285997	-5.90208	0.646674	0.563575
T.cells	IQCH	-0.71275	0.250926	-1.07343	0.286	-4.85449	0.723332	0.647183
T.cells	VAMP7	-0.1218	4.132998	-1.07326	0.286074	-5.85231	0.662822	0.581002
T.cells	KIN	-0.09426	4.642417	-1.07324	0.286083	-6.04826	0.655241	0.572821
T.cells	EPB41	0.139543	8.026595	1.073223	0.286092	-6.44252	0.606909	0.52132
T.cells	SIDT2	0.14443	5.063847	1.073144	0.286127	-5.77605	0.64903	0.566172
T.cells	VPS52	-0.11152	3.923327	-1.07249	0.286418	-5.79961	0.666201	0.584717
T.cells	DOK2	-0.33628	2.811456	-1.07238	0.28647	-5.12738	0.683114	0.603136
T.cells	FRMD5	-0.62729	2.390592	-1.07237	0.286471	-5.09428	0.68962	0.610238
T.cells	RAB43	0.1157	6.948549	1.072175	0.286559	-6.28774	0.622147	0.537569
T.cells	GM19696	0.592583	0.016291	1.072073	0.286605	-4.84599	0.727405	0.6519
T.cells	GOLM1	-0.21065	3.2957	-1.07201	0.286631	-5.44094	0.6757	0.595108
T.cells	PPP2CB	0.082772	5.60171	1.071992	0.286641	-6.14477	0.641411	0.558109
T.cells	MOSMO	0.113054	4.576833	1.071582	0.286824	-5.97278	0.656635	0.574306
T.cells	GM5089	0.45086	0.395428	1.071276	0.286961	-4.97387	0.721456	0.645268
T.cells	LPCAT4	-0.31356	3.121289	-1.07124	0.286977	-5.32891	0.678559	0.598188
T.cells	LYRM4	-0.11242	4.142278	-1.07111	0.287036	-5.87957	0.66311	0.581443
T.cells	CLNS1A	-0.07769	5.340846	-1.07105	0.287061	-6.20409	0.645394	0.562397
T.cells	HSPA12A	-0.4872	0.150275	-1.0709	0.287128	-4.92007	0.725434	0.649765
T.cells	TBCCD1	-0.19272	2.589728	-1.07088	0.287138	-5.5356	0.686735	0.607178
T.cells	AI987944	0.232099	2.764701	1.070692	0.287222	-5.37877	0.684094	0.604233
T.cells	SLC16A6	-0.23865	4.554893	-1.07044	0.287334	-5.54401	0.657139	0.574868
T.cells	BRD9	-0.08994	5.185663	-1.07024	0.287425	-6.13749	0.647873	0.564984
T.cells	EIF2B1	-0.12742	3.62049	-1.0699	0.287575	-5.79302	0.671183	0.590288
T.cells	GTF3C2	-0.07531	5.474786	-1.06974	0.287646	-6.1893	0.643652	0.560592
T.cells	INHBA	0.737092	1.050066	1.069529	0.287743	-4.92291	0.711163	0.634033
T.cells	FBXO31	0.167753	3.549697	1.069511	0.28775	-5.66972	0.672255	0.591458
T.cells	PARD3	-0.41395	2.300134	-1.06945	0.287779	-5.13636	0.691452	0.612381
T.cells	KIF7	-0.56608	-0.46987	-1.06939	0.287804	-4.83743	0.735826	0.6614
T.cells	TPPP3	0.697646	1.071021	1.069352	0.287822	-4.83619	0.710828	0.633705
T.cells	FLCN	0.156837	4.338996	1.069203	0.287889	-5.74847	0.660405	0.578685
T.cells	ARMT1	0.120495	3.297769	1.068711	0.288109	-5.78802	0.676483	0.595735
T.cells	TMEM243	0.068901	6.427837	1.067831	0.288504	-6.3694	0.630889	0.546278
T.cells	COQ8B	-0.23656	3.190292	-1.06777	0.288532	-5.40437	0.678771	0.597827
T.cells	GNS	0.109332	6.931874	1.067653	0.288584	-6.26992	0.623727	0.53873
T.cells	AVPI1	-0.35281	2.712104	-1.06745	0.288676	-5.08755	0.686124	0.605948
T.cells	TMEM53	-0.5199	0.637469	-1.06744	0.28868	-4.88985	0.718889	0.641918
T.cells	GCLC	0.102581	6.841762	1.067078	0.288842	-6.49941	0.625202	0.540247
T.cells	ACVR1	-0.17977	3.707524	-1.06687	0.288935	-5.76245	0.671115	0.589598
T.cells	TRIR	-0.07763	6.300182	-1.06682	0.288957	-6.32076	0.632919	0.548551
T.cells	ARHGAP22	0.534176	1.023886	1.066591	0.28906	-5.00518	0.712908	0.635374

T.cells	PILRB2	-0.4891	2.237136	-1.06657	0.289068	-4.9795	0.693724	0.614306
T.cells	PRPF31	-0.11939	3.79385	-1.06642	0.289138	-5.86642	0.669834	0.588295
T.cells	COG8	0.10216	4.299473	1.066068	0.289296	-5.9329	0.662416	0.58016
T.cells	MPRIP	0.095412	5.481388	1.065977	0.289336	-6.23309	0.644957	0.56139
T.cells	MAPRE2	0.099024	6.802434	1.065753	0.289437	-6.25213	0.626045	0.541164
T.cells	SND1	0.077295	7.195863	1.064251	0.290113	-6.3828	0.621326	0.535979
T.cells	TBC1D9B	0.114423	4.555284	1.06425	0.290113	-5.87826	0.659583	0.576865
T.cells	NOSTRIN	0.308123	3.426931	1.063969	0.29024	-5.31521	0.676596	0.59536
T.cells	PPP1R15B	-0.10413	5.553021	-1.06358	0.290416	-6.15116	0.644874	0.561242
T.cells	ADGRE4	-0.53155	3.108019	-1.06355	0.290428	-5.05982	0.681478	0.600768
T.cells	PRKCQ	0.263394	3.12892	1.063486	0.290458	-5.48925	0.681157	0.600435
T.cells	PODXL	0.559499	0.550401	1.063393	0.290499	-4.89392	0.721828	0.645077
T.cells	YEATS4	-0.0752	5.906755	-1.06327	0.290556	-6.28352	0.639734	0.555821
T.cells	TUBGCP3	0.102188	3.88521	1.06318	0.290596	-5.89024	0.669637	0.588017
T.cells	KIT	-0.17086	3.453389	-1.06306	0.290652	-6.02373	0.676192	0.595138
T.cells	NMD3	0.081128	4.999909	1.062613	0.290851	-6.07508	0.65299	0.570152
T.cells	TMEM120I	-0.31494	3.044848	-1.06252	0.290894	-5.32966	0.682449	0.602055
T.cells	ELMOD2	0.206286	2.807903	1.062415	0.290941	-5.44098	0.686102	0.606038
T.cells	PPP1R7	0.088767	4.409711	1.062387	0.290954	-6.02644	0.661755	0.579617
T.cells	RFC2	-0.09966	5.227985	-1.06237	0.290962	-6.21067	0.649632	0.566563
T.cells	ASPSCR1	-0.10391	4.685187	-1.06233	0.290977	-5.97619	0.65765	0.575189
T.cells	PATJ	-0.19182	3.348038	-1.0623	0.290991	-5.89856	0.677801	0.596999
T.cells	MAML2	0.113617	7.972039	1.062249	0.291016	-6.494	0.610485	0.524895
T.cells	UBE2I	-0.05052	7.624242	-1.06221	0.291033	-6.58377	0.61532	0.530007
T.cells	GM43065	-0.60666	0.153169	-1.06197	0.291142	-4.82812	0.728289	0.652637
T.cells	RAD9A	-0.24115	2.338849	-1.06184	0.291201	-5.47348	0.693388	0.614244
T.cells	PNPLA8	0.096484	6.018439	1.061803	0.291217	-6.18573	0.638119	0.554454
T.cells	TLE4	-0.08432	7.814076	-1.06174	0.291247	-6.5188	0.612677	0.527444
T.cells	KBTBD3	0.211929	2.727361	1.061601	0.291308	-5.47031	0.687348	0.607713
T.cells	CSRNP1	-0.12973	6.641086	-1.0616	0.291309	-6.28409	0.629186	0.544988
T.cells	3300002A1	0.379119	0.451891	1.061329	0.291431	-5.02188	0.723581	0.647461
T.cells	MYL12B	-0.07506	8.183707	-1.06111	0.291531	-6.50324	0.60771	0.522171
T.cells	CCDC22	-0.14379	3.49155	-1.06105	0.291559	-5.7375	0.675778	0.595029
T.cells	DSCAM	-0.52446	0.029718	-1.06061	0.291758	-4.98767	0.730838	0.655313
T.cells	HIPK1	0.100406	6.269668	1.060288	0.291901	-6.2361	0.635002	0.550978
T.cells	CSPRS	0.541946	0.292172	1.060223	0.291931	-4.80246	0.726597	0.65065
T.cells	RYK	-0.33611	2.042505	-1.06008	0.291996	-5.13468	0.69858	0.619807
T.cells	CUTA	-0.08066	6.189562	-1.06003	0.292018	-6.25576	0.636155	0.552262
T.cells	GPR182	-0.37635	2.557152	-1.05979	0.292127	-5.12706	0.690534	0.611083
T.cells	TFDP1	-0.10859	5.887837	-1.05978	0.292133	-6.34523	0.640514	0.556981
T.cells	MCM2	-0.15138	4.762793	-1.05964	0.292196	-6.16362	0.657027	0.574705
T.cells	CALCRL	0.155571	5.617508	1.059426	0.292291	-6.14535	0.644531	0.561253
T.cells	H2-DMA	0.256707	5.184297	1.059257	0.292368	-5.73582	0.650916	0.568147
T.cells	UPF3B	0.096297	4.979585	1.058479	0.29272	-6.1563	0.654442	0.571767
T.cells	LITAF	-0.07885	8.523937	-1.05815	0.29287	-6.54074	0.60395	0.518104
T.cells	CARMIL1	-0.15806	4.075468	-1.05779	0.293033	-5.83522	0.667944	0.586474
T.cells	TRIM41	0.121908	4.185669	1.057689	0.293079	-5.77749	0.666285	0.584715

T.cells	PSKH1	0.145957	3.273493	1.057653	0.293095	-5.59223	0.680138	0.599749
T.cells	GRK3	0.423447	2.91909	1.057653	0.293095	-5.02669	0.685593	0.605692
T.cells	COMMD9	0.178239	2.646144	1.057504	0.293163	-5.59655	0.689821	0.610342
T.cells	RBM22	-0.0687	5.603703	-1.05724	0.293283	-6.23746	0.645272	0.562174
T.cells	NDUFAF3	0.108602	4.160988	1.057217	0.293293	-5.97168	0.666656	0.585212
T.cells	TBC1D10B	0.093661	5.147294	1.057207	0.293297	-6.15289	0.651966	0.569363
T.cells	HORMAD2	-0.47873	1.357831	-1.05666	0.293546	-5.11198	0.710108	0.632873
T.cells	ATP5A1	-0.06705	7.857813	-1.05653	0.293604	-6.60926	0.613149	0.528152
T.cells	ZSCAN21	0.153963	3.394527	1.056477	0.293628	-5.76752	0.678285	0.598022
T.cells	SMIM13	-0.12642	3.919727	-1.05647	0.293634	-5.84092	0.670296	0.589345
T.cells	SOCS1	-0.1696	5.152296	-1.05641	0.293657	-6.15634	0.651892	0.569498
T.cells	PDE1B	-0.20822	3.114849	-1.05634	0.293691	-5.4611	0.682575	0.602753
T.cells	ZC3HC1	-0.10237	4.34425	-1.05633	0.293693	-6.02028	0.663904	0.582479
T.cells	HEXA	-0.14444	5.752202	-1.05614	0.29378	-5.90606	0.643108	0.560134
T.cells	BICDL1	0.283819	1.878488	1.056058	0.293819	-5.28353	0.701844	0.623904
T.cells	GM13547	-0.64451	0.514665	-1.05595	0.293866	-4.8763	0.723682	0.648013
T.cells	SAMD8	0.124843	5.289431	1.055867	0.293906	-5.94525	0.649874	0.567422
T.cells	CLIC4	0.133489	7.449524	1.055812	0.293931	-6.47895	0.618853	0.534305
T.cells	ZCCHC7	0.128882	6.744533	1.055746	0.293961	-6.28661	0.62882	0.544919
T.cells	ZC3H4	-0.08577	4.818849	-1.05553	0.29406	-6.08679	0.656901	0.575032
T.cells	PTK6	-0.75801	-0.3416	-1.05541	0.294114	-4.80174	0.737796	0.663817
T.cells	FBXW4	0.191096	4.272711	1.055204	0.294207	-5.62539	0.665054	0.583986
T.cells	GM44127	0.69551	-0.52381	1.055148	0.294233	-4.78976	0.740813	0.667286
T.cells	SBDS	0.080639	5.561152	1.054834	0.294375	-6.1562	0.646018	0.563473
T.cells	CALD1	-0.31381	3.777745	-1.05481	0.294388	-5.44664	0.672578	0.592153
T.cells	CYCS	-0.09957	7.712668	-1.05471	0.294433	-6.58171	0.61529	0.530748
T.cells	ZDHHC14	-0.15593	7.022709	-1.05424	0.294647	-6.37684	0.625201	0.541123
T.cells	PDIA5	-0.40782	2.273787	-1.0541	0.29471	-5.02322	0.695998	0.617623
T.cells	CLCN4	0.101302	5.289066	1.054097	0.294711	-6.06291	0.650225	0.567912
T.cells	MAPKAPK5	0.212081	2.128701	1.053522	0.294973	-5.35058	0.698754	0.6204
T.cells	DSE	-0.41478	3.834005	-1.053	0.295212	-5.13383	0.672484	0.59176
T.cells	MPC2	-0.0985	6.800876	-1.05285	0.295278	-6.32479	0.62885	0.544883
T.cells	TAF4	-0.11076	4.075399	-1.05278	0.295309	-5.88528	0.66883	0.587864
T.cells	MED27	0.078763	5.275175	1.052758	0.29532	-6.14379	0.650945	0.568554
T.cells	GM14963	0.616502	0.898614	1.05259	0.295397	-4.87516	0.718422	0.642183
T.cells	MAPK13	0.603323	0.83712	1.052578	0.295403	-4.84545	0.719414	0.643281
T.cells	GNL1	-0.1212	4.299889	-1.05254	0.29542	-5.86379	0.665449	0.584231
T.cells	MYBPC3	-0.41705	1.123244	-1.05239	0.295487	-5.01302	0.714824	0.638271
T.cells	DOCK2	-0.08175	9.34967	-1.05176	0.295774	-6.69602	0.593693	0.507925
T.cells	IMPAD1	-0.113	4.365894	-1.05171	0.2958	-5.85818	0.664658	0.583504
T.cells	TRIM37	-0.10459	4.93302	-1.05151	0.295889	-6.14034	0.656196	0.574365
T.cells	GM12227	-0.37136	1.113465	-1.0514	0.29594	-5.01678	0.715178	0.638753
T.cells	DDX42	0.074515	5.684077	1.051307	0.295983	-6.23039	0.645146	0.562516
T.cells	2310015A1	-0.3384	2.091938	-1.05121	0.296029	-5.09761	0.699617	0.621655
T.cells	TUBA1B	-0.11532	7.795232	-1.05119	0.296038	-6.698	0.615018	0.530409
T.cells	XRCC1	-0.11147	4.144273	-1.05115	0.296053	-6.02486	0.667992	0.587153
T.cells	B3GALNT1	-0.66873	0.351359	-1.05094	0.29615	-4.82234	0.727518	0.652519

T.cells	AP4S1	0.095325	4.920297	1.050912	0.296163	-6.04307	0.656385	0.574671
T.cells	ASAH1	0.120014	6.301867	1.050505	0.296349	-6.05396	0.636459	0.553192
T.cells	CCNA2	-0.15482	5.520186	-1.04954	0.296789	-6.4116	0.648565	0.5656
T.cells	RPN2	-0.08357	6.097946	-1.0495	0.29681	-6.29034	0.640142	0.556576
T.cells	ZFP553	-0.16548	2.414705	-1.04921	0.296943	-5.583	0.695685	0.616785
T.cells	ZFP518A	0.142752	3.684945	1.049204	0.296944	-5.78244	0.676053	0.595368
T.cells	HSD17B11	0.126839	4.796644	1.048922	0.297073	-5.8967	0.659348	0.577253
T.cells	ACSF2	-0.14022	4.286847	-1.04889	0.297087	-5.8901	0.666984	0.585506
T.cells	ARL6IP4	0.084166	5.733017	1.048289	0.297363	-6.27232	0.646005	0.562629
T.cells	AP3S2	0.133162	3.43138	1.048046	0.297474	-5.79743	0.680591	0.599975
T.cells	C330011M	-0.36633	0.592367	-1.04784	0.29757	-5.0181	0.72548	0.649385
T.cells	RLIM	-0.06575	6.654275	-1.04779	0.297591	-6.34126	0.632775	0.548616
T.cells	CPQ	-0.18834	5.056734	-1.04738	0.29778	-5.71994	0.656241	0.57371
T.cells	NLRP1B	0.514552	1.79452	1.047189	0.297867	-4.9405	0.706333	0.628322
T.cells	ALKBH6	0.170035	2.755561	1.047037	0.297937	-5.46274	0.691218	0.611864
T.cells	CEP57L1	0.149346	3.287731	1.046909	0.297995	-5.79339	0.682978	0.602897
T.cells	ATP5J2	-0.07884	7.723844	-1.04687	0.298013	-6.5841	0.617781	0.532879
T.cells	HIST1H2BN	-0.20645	2.139918	-1.0467	0.298092	-5.7189	0.700866	0.622532
T.cells	SECISBP2L	0.124693	4.964176	1.046698	0.298092	-5.9507	0.657615	0.575481
T.cells	GK5	-0.1433	4.404631	-1.04634	0.298258	-5.97038	0.665981	0.584694
T.cells	CISD3	-0.29674	3.000972	-1.04616	0.298339	-5.23354	0.687407	0.608016
T.cells	GM4316	-0.3228	0.707324	-1.04616	0.29834	-5.24267	0.7238	0.648047
T.cells	SPATC1	0.488768	-1.06407	1.046146	0.298345	-4.80416	0.753111	0.680693
T.cells	HES7	0.567333	-0.49621	1.045823	0.298494	-4.80616	0.743599	0.670086
T.cells	EIF3E	-0.06363	7.095265	-1.04582	0.298494	-6.50147	0.626649	0.542544
T.cells	XAF1	0.306709	4.033526	1.045573	0.298608	-5.7044	0.671584	0.590916
T.cells	NFRKB	0.095506	4.478568	1.045551	0.298618	-5.98359	0.66487	0.583641
T.cells	GM15345	-0.31169	2.98417	-1.04545	0.298666	-5.48206	0.687667	0.608508
T.cells	SMARCA4	0.070342	7.5105	1.0453	0.298734	-6.68663	0.620777	0.536502
T.cells	DNAJA3	0.129687	3.76841	1.045194	0.298782	-5.88019	0.675614	0.595441
T.cells	GPN2	0.174189	2.713988	1.045101	0.298825	-5.562	0.691866	0.613195
T.cells	SLC25A20	-0.1352	5.597573	-1.04491	0.298913	-6.02882	0.648264	0.565939
T.cells	A930029G2	0.347021	1.230907	1.044886	0.298924	-5.13153	0.715339	0.639048
T.cells	FBXL17	0.084806	7.560342	1.044523	0.299091	-6.42935	0.620076	0.535897
T.cells	LBH	0.077816	6.308404	1.04447	0.299115	-6.32163	0.637919	0.554903
T.cells	PLEKHO1	-0.08905	5.499121	-1.04415	0.299263	-6.26657	0.649709	0.567601
T.cells	CDC42SE2	-0.07356	7.179375	-1.04412	0.299278	-6.4377	0.625455	0.541661
T.cells	SCN1B	0.49422	1.834445	1.044041	0.299313	-4.95511	0.705699	0.628537
T.cells	MPZL1	-0.37849	2.78686	-1.04385	0.299402	-5.22122	0.690731	0.612109
T.cells	PDLIM2	-0.23389	3.759145	-1.04358	0.299523	-5.43885	0.675755	0.595771
T.cells	CLEC4B1	0.541255	-0.61033	1.043454	0.299583	-4.83789	0.745501	0.67269
T.cells	GM5544	-0.48476	0.129876	-1.04342	0.299597	-4.90937	0.733238	0.659017
T.cells	ACOT13	-0.11059	4.43558	-1.04337	0.29962	-5.86223	0.665516	0.584659
T.cells	SFT2D3	0.160624	2.864525	1.043314	0.299647	-5.56053	0.689524	0.610788
T.cells	RMDN2	-0.29153	1.873328	-1.04317	0.299713	-5.26058	0.705082	0.627859
T.cells	TBC1D1	-0.09646	7.18844	-1.04312	0.299736	-6.42818	0.625327	0.541526
T.cells	NAPA	-0.08034	5.92916	-1.04308	0.299756	-6.17805	0.643419	0.560847

T.cells	6720489N1	-0.28992	0.964398	-1.04219	0.300167	-5.20074	0.719834	0.644252
T.cells	IGFBP4	-0.276	5.679023	-1.04212	0.300197	-6.07565	0.64725	0.565058
T.cells	SFPQ	-0.06012	8.189863	-1.0421	0.300205	-6.66129	0.611453	0.526912
T.cells	PCMT1	-0.07306	6.63523	-1.04209	0.300212	-6.38904	0.63339	0.550214
T.cells	SRM	-0.15098	4.842685	-1.04202	0.300244	-6.14013	0.659606	0.578385
T.cells	CIR1	0.093009	5.515333	1.041933	0.300283	-6.18779	0.649651	0.567703
T.cells	RAD1	0.173474	2.477895	1.041859	0.300318	-5.62918	0.695747	0.61781
T.cells	ISY1	-0.09941	6.241546	-1.04183	0.300332	-6.25304	0.639062	0.556379
T.cells	SSPN	0.441149	-0.41075	1.041663	0.300408	-4.92489	0.742382	0.669547
T.cells	5-Mar	0.101255	6.018808	1.041515	0.300476	-6.26208	0.642292	0.559954
T.cells	ARPC5L	0.067275	6.300784	1.041453	0.300505	-6.43712	0.638205	0.555582
T.cells	VCAN	0.574655	0.76602	1.041076	0.300679	-5.02832	0.723047	0.648212
T.cells	ASPA	-0.62733	1.375432	-1.04085	0.300785	-4.94343	0.713217	0.637403
T.cells	COLEC12	-0.52201	2.890318	-1.0407	0.300853	-5.09486	0.689314	0.61115
T.cells	GM28529	0.404112	0.724072	1.040651	0.300875	-5.07584	0.723728	0.649084
T.cells	GM16541	0.167371	2.915994	1.040555	0.300919	-5.65696	0.688915	0.61075
T.cells	RHOA	0.044659	9.258828	1.040439	0.300973	-6.788	0.596791	0.511962
T.cells	ARAP1	0.147875	5.049234	1.040348	0.301015	-5.92186	0.656534	0.575639
T.cells	SMG9	0.115674	4.52517	1.040208	0.30108	-5.93523	0.664354	0.584129
T.cells	ABCC1	0.242418	4.467713	1.040089	0.301135	-5.45096	0.665217	0.585098
T.cells	PDE8B	0.533604	-0.24267	1.039718	0.301306	-4.84538	0.739592	0.667138
T.cells	PHF11D	-0.58541	1.34229	-1.03957	0.301373	-4.98049	0.713749	0.638484
T.cells	MRPL46	-0.14034	3.264758	-1.0393	0.301501	-5.77353	0.683521	0.605334
T.cells	ARL5C	-0.10965	6.264356	-1.039	0.301636	-6.48677	0.638732	0.556882
T.cells	CSPG5	-0.51732	0.28413	-1.03889	0.301687	-5.0024	0.730908	0.65768
T.cells	HRH4	0.631597	0.27047	1.038861	0.301702	-4.84237	0.731132	0.657933
T.cells	ZFP704	0.167865	3.792086	1.038844	0.30171	-5.96753	0.67544	0.596572
T.cells	DPH5	0.14149	3.732552	1.038787	0.301736	-5.80437	0.676348	0.597592
T.cells	FASTKD3	-0.23661	1.993657	-1.03876	0.301749	-5.42131	0.703371	0.627217
T.cells	ADRB2	-0.26417	4.714467	-1.03863	0.301809	-5.68806	0.661519	0.581536
T.cells	ARHGEF10I	0.371198	2.695098	1.038335	0.301945	-5.15618	0.692352	0.615307
T.cells	EXT2	-0.16306	3.849599	-1.03821	0.302003	-5.60776	0.674564	0.595864
T.cells	LST1	0.30026	5.23223	1.038016	0.302093	-5.44839	0.653823	0.573366
T.cells	PPFIBP1	-0.20246	3.366596	-1.03797	0.302114	-5.46846	0.681954	0.603927
T.cells	H2-AB1	-0.27506	6.764416	-1.03787	0.30216	-6.41627	0.631539	0.549422
T.cells	SCAMP1	0.337837	2.970331	1.03779	0.302198	-5.18631	0.688072	0.610623
T.cells	GRAMD1B	-0.24182	5.536445	-1.03779	0.302199	-5.62011	0.649341	0.56853
T.cells	TMTC2	-0.37388	4.574445	-1.03768	0.302248	-5.56768	0.663615	0.583962
T.cells	DPYD	-0.34582	3.567148	-1.03767	0.302255	-5.52534	0.678876	0.600566
T.cells	CDS1	-0.33231	2.788548	-1.03764	0.302265	-5.30947	0.690896	0.613719
T.cells	VPS72	-0.10154	4.357985	-1.0376	0.302285	-5.99027	0.666867	0.587492
T.cells	PROX1	-0.46122	2.15604	-1.0374	0.302376	-5.12222	0.70088	0.624662
T.cells	ZFP563	0.286344	0.800774	1.037126	0.302505	-5.13154	0.722723	0.648802
T.cells	KDSR	0.138487	3.963038	1.036828	0.302644	-5.75451	0.673063	0.594228
T.cells	GM42982	0.199167	1.514483	1.036657	0.302723	-5.47527	0.711227	0.636123
T.cells	PRR13	-0.08368	6.510778	-1.0366	0.30275	-6.17338	0.635388	0.553552
T.cells	CLEC4E	0.831573	2.622943	1.036376	0.302853	-4.98867	0.693708	0.616954

T.cells	HABP2	-0.50921	0.622763	-1.03636	0.30286	-4.95129	0.725616	0.652225
T.cells	ZFP629	0.366735	0.926	1.036346	0.302867	-5.1393	0.720693	0.646755
T.cells	DYM	0.089462	5.781233	1.036194	0.302938	-6.17495	0.645969	0.565101
T.cells	IPO5	-0.10248	5.437174	-1.03566	0.303186	-6.29618	0.651016	0.570754
T.cells	GATAD2A	-0.0729	7.859185	-1.03551	0.303253	-6.56156	0.616262	0.53354
T.cells	PHF5A	-0.08166	6.072643	-1.03541	0.3033	-6.39223	0.641723	0.560745
T.cells	ZPR1	-0.10288	4.397865	-1.03533	0.303337	-5.98292	0.666488	0.587509
T.cells	GATB	0.178311	3.11047	1.03528	0.303362	-5.67465	0.68613	0.60894
T.cells	ADPRH	-0.10687	4.93883	-1.0352	0.303396	-6.08033	0.658392	0.578728
T.cells	PHACTR1	0.380205	1.519212	1.035174	0.303411	-5.09061	0.711152	0.636492
T.cells	GSTZ1	-0.14789	4.205829	-1.03517	0.303413	-5.86406	0.669384	0.590658
T.cells	PSMD11	-0.04928	7.032517	-1.03507	0.303459	-6.48295	0.627922	0.545959
T.cells	PJA2	0.130741	4.695864	1.034756	0.303605	-5.94516	0.662204	0.58275
T.cells	TRAT1	0.703537	-0.05747	1.03445	0.303747	-4.8648	0.737094	0.665175
T.cells	GM10642	0.485307	0.270003	1.034371	0.303784	-4.95099	0.731702	0.659187
T.cells	SIVA1	-0.09889	5.470666	-1.03427	0.303831	-6.34499	0.650807	0.570441
T.cells	HPCAL1	0.128492	6.117607	1.033921	0.303993	-5.97805	0.641566	0.560319
T.cells	GM42962	0.485786	-0.60259	1.033221	0.304319	-4.83736	0.746846	0.675757
T.cells	LARS2	0.197003	5.571824	1.03306	0.304393	-6.13911	0.649924	0.569194
T.cells	PIGA	0.244995	1.918295	1.032967	0.304437	-5.34292	0.705762	0.630107
T.cells	DNTTIP2	0.080544	5.107858	1.032937	0.304451	-6.1607	0.656779	0.576595
T.cells	CD200R1	-0.52763	2.827999	-1.03289	0.304472	-4.93337	0.691456	0.614369
T.cells	A530040E1	0.599734	0.013043	1.032614	0.304601	-4.82645	0.736639	0.664492
T.cells	GM50163	-0.34846	0.458993	-1.03261	0.304601	-5.12651	0.729308	0.656317
T.cells	1700048O2	-0.42577	-0.24632	-1.03245	0.304676	-5.16318	0.740958	0.669291
T.cells	NOMO1	-0.15122	3.5373	-1.03227	0.304763	-5.69473	0.680533	0.602539
T.cells	SYMPK	-0.1078	4.293778	-1.03221	0.304791	-5.99007	0.669014	0.589987
T.cells	NCF1	0.359395	5.301832	1.031534	0.305104	-5.20396	0.654465	0.573817
T.cells	DNAJB4	0.144461	3.700143	1.031432	0.305151	-5.71601	0.678574	0.600038
T.cells	XPO5	0.13096	3.603677	1.030768	0.305461	-5.80161	0.680467	0.601882
T.cells	WDR66	-0.21605	1.900032	-1.03056	0.305557	-5.51159	0.707088	0.631122
T.cells	PREB	-0.10534	4.736405	-1.0304	0.305631	-5.97435	0.663287	0.583284
T.cells	C1QB	-0.29629	6.51237	-1.03034	0.305663	-6.03686	0.637168	0.555161
T.cells	AMMECR1	0.103454	4.787165	1.030162	0.305744	-6.15849	0.662527	0.582506
T.cells	DSEL	-0.36557	0.375304	-1.03008	0.305781	-5.00554	0.731727	0.658562
T.cells	IFI35	0.147464	5.333965	1.03001	0.305815	-6.05124	0.654388	0.573736
T.cells	RHNO1	0.105567	4.345115	1.029663	0.305977	-6.10564	0.669175	0.589845
T.cells	TNPO1	0.072494	7.082293	1.029655	0.305981	-6.48303	0.628993	0.546568
T.cells	DRAP1	0.069537	6.508223	1.029481	0.306062	-6.35276	0.637228	0.555373
T.cells	SFT2D1	-0.07893	5.999884	-1.02933	0.306134	-6.16864	0.644604	0.563347
T.cells	PRUNE2	-0.50918	0.638929	-1.0293	0.306148	-4.89321	0.727411	0.653957
T.cells	DUT	-0.12242	5.882801	-1.02927	0.306159	-6.47117	0.646314	0.565191
T.cells	ACADVL	-0.12809	4.901912	-1.02923	0.306181	-5.97131	0.660811	0.580838
T.cells	PIAS4	0.110794	4.224377	1.029009	0.306282	-5.91951	0.671002	0.591935
T.cells	CDK5RAP1	-0.3344	2.44604	-1.02898	0.306295	-5.4329	0.698453	0.621962
T.cells	CCL12	0.929613	0.036828	1.028561	0.306491	-4.84389	0.737516	0.665172
T.cells	FPR3	0.629814	-0.4789	1.028535	0.306504	-4.83349	0.746088	0.67477

T.cells	RNF4	-0.06913	5.964365	-1.02833	0.306601	-6.30024	0.645309	0.564185
T.cells	SLIRP	-0.10046	5.558884	-1.02821	0.306656	-6.2357	0.651256	0.570652
T.cells	PIANP	-0.49703	-0.06426	-1.02815	0.306683	-4.86847	0.739189	0.66725
T.cells	ADIPOR2	0.083263	6.368043	1.027057	0.307195	-6.31684	0.640381	0.558428
T.cells	SLC45A4	-0.1727	3.337998	-1.0266	0.307411	-5.53729	0.686111	0.607811
T.cells	ADM	-0.55511	1.330377	-1.02605	0.307667	-4.96525	0.7179	0.642836
T.cells	G3BP1	-0.07958	6.728766	-1.02605	0.307668	-6.41022	0.635555	0.553172
T.cells	CDC14B	-0.28356	3.454363	-1.02599	0.307696	-5.32472	0.68438	0.605968
T.cells	HECA	0.085916	6.23169	1.025987	0.307696	-6.29768	0.64275	0.560884
T.cells	TPGS1	-0.12828	4.393149	-1.02589	0.307741	-5.92644	0.670034	0.59037
T.cells	CCSER2	0.122777	4.778998	1.025571	0.307892	-5.98502	0.664228	0.584168
T.cells	PDGFC	-0.71354	1.879122	-1.02543	0.30796	-4.94902	0.709107	0.633307
T.cells	THOC7	-0.06599	6.572603	-1.02533	0.308003	-6.45661	0.637815	0.555758
T.cells	MAZ	-0.10218	6.241133	-1.02529	0.308022	-6.4571	0.642621	0.560929
T.cells	GM11655	0.382258	0.316941	1.025244	0.308045	-5.05803	0.734431	0.661439
T.cells	FMR1	-0.07772	5.971489	-1.02485	0.308228	-6.34816	0.646813	0.565296
T.cells	BAIAP2	-0.14252	5.269985	-1.02473	0.308288	-6.06458	0.65716	0.576505
T.cells	LRRC45	-0.20176	2.190744	-1.02404	0.30861	-5.47164	0.704839	0.628406
T.cells	CLEC2D	0.198561	4.7558	1.023896	0.308678	-5.95409	0.665225	0.585194
T.cells	TEPSIN	-0.38938	0.762658	-1.02387	0.308689	-5.05475	0.727832	0.653944
T.cells	PSMB3	-0.07267	7.724991	-1.02384	0.308706	-6.61096	0.621978	0.538792
T.cells	ORC4	-0.08526	5.046376	-1.02367	0.308786	-6.10933	0.660913	0.580605
T.cells	CFAP97	0.131499	3.29046	1.023446	0.308889	-5.688	0.687735	0.609809
T.cells	SMN1	0.100505	4.851728	1.023106	0.309049	-6.14525	0.664135	0.583914
T.cells	TSTA3	0.123337	4.100613	1.022777	0.309204	-5.90244	0.675564	0.596332
T.cells	RINT1	0.120452	3.771156	1.022739	0.309222	-5.85246	0.680606	0.601834
T.cells	CCDC15	0.166973	2.810819	1.022657	0.30926	-5.74943	0.695502	0.618155
T.cells	PLATR25	-0.18121	2.729731	-1.02232	0.309417	-5.58301	0.696774	0.619612
T.cells	CD164	-0.1015	6.379209	-1.02218	0.309484	-6.17407	0.641642	0.559762
T.cells	HMGN5	-0.09492	4.876393	-1.02216	0.309494	-6.21517	0.66383	0.583679
T.cells	ZFP617	0.22349	2.165547	1.022108	0.309518	-5.47206	0.705681	0.629411
T.cells	TIMP2	-0.47128	4.873204	-1.02185	0.309642	-5.20572	0.663878	0.583761
T.cells	E230016M	-0.27816	2.465953	-1.0218	0.309664	-5.33227	0.700925	0.624225
T.cells	SHFL	0.283464	1.832835	1.021746	0.309689	-5.31257	0.710983	0.63532
T.cells	XPA	0.148462	4.138354	1.021515	0.309798	-5.78301	0.674989	0.595947
T.cells	SYNC	-0.18275	1.973724	-1.02151	0.3098	-5.48211	0.708733	0.632938
T.cells	TRIM30C	0.513153	1.320225	1.021011	0.310035	-4.95332	0.71954	0.644783
T.cells	MMACHC	0.409296	0.470101	1.020964	0.310057	-5.07051	0.733408	0.660191
T.cells	ZDBF2	0.658149	-0.23932	1.02069	0.310186	-4.87597	0.74527	0.673413
T.cells	RETSAT	-0.27138	1.405172	-1.02049	0.310279	-5.1402	0.718267	0.643387
T.cells	PRLR	-0.30118	3.152323	-1.02049	0.31028	-5.56714	0.690569	0.612875
T.cells	GM6034	0.496524	0.50272	1.020288	0.310376	-5.01503	0.733056	0.65978
T.cells	MFSD2B	0.402121	0.599514	1.020075	0.310476	-5.00304	0.73156	0.658113
T.cells	IER5	-0.13462	6.692584	-1.01988	0.310567	-6.27077	0.637691	0.555592
T.cells	PNPLA1	-0.53562	0.231599	-1.01915	0.310912	-4.91359	0.738099	0.665329
T.cells	CCDC174	0.086142	5.082834	1.018901	0.31103	-6.15323	0.661709	0.581425
T.cells	GMCL1	0.085386	4.636774	1.018836	0.311061	-6.12838	0.668413	0.588705

T.cells	TXNL4A	-0.09188	4.968748	-1.01867	0.311137	-6.16181	0.663418	0.58329
T.cells	FAM118A	-0.20125	2.069936	-1.01864	0.311151	-5.47842	0.708238	0.632289
T.cells	BMP2K	0.087506	6.936172	1.018587	0.311178	-6.2987	0.634527	0.5522
T.cells	GM15246	-0.37473	1.046088	-1.01855	0.311196	-5.18038	0.724729	0.650541
T.cells	RSPH3A	0.199564	2.558451	1.018504	0.311218	-5.55259	0.700493	0.623773
T.cells	AU022252	0.215369	2.452615	1.018178	0.311372	-5.42068	0.702264	0.625646
T.cells	RNF138	0.08129	5.857254	1.018155	0.311382	-6.23443	0.650313	0.569087
T.cells	NCAPH	-0.14641	4.175802	-1.01786	0.311524	-6.13014	0.67568	0.596464
T.cells	XYLB	-0.31828	0.922746	-1.01752	0.311682	-5.19643	0.727144	0.653151
T.cells	NDUFB5	-0.07107	7.037752	-1.01744	0.31172	-6.51054	0.633421	0.550975
T.cells	TMX1	-0.09171	5.362	-1.0172	0.311832	-6.121	0.657912	0.5774
T.cells	ICE2	0.208055	2.160721	1.017	0.311928	-5.39009	0.707187	0.63129
T.cells	OXLD1	-0.32196	1.813163	-1.0166	0.312117	-5.14238	0.712738	0.637576
T.cells	CDK1	-0.15246	5.506863	-1.01658	0.312129	-6.39441	0.65576	0.575299
T.cells	RBM4	-0.08813	4.655948	-1.01634	0.31224	-6.08376	0.668496	0.58909
T.cells	SLC6A13	-0.32285	2.762131	-1.01621	0.312304	-5.22453	0.697675	0.62097
T.cells	CLEC4A1	0.499009	3.158732	1.016136	0.312337	-5.20015	0.691468	0.614156
T.cells	BBOX1	-0.44983	0.92351	-1.0161	0.312355	-5.02543	0.727132	0.653535
T.cells	FAM126B	0.120295	3.772291	1.016089	0.31236	-5.87182	0.681966	0.603759
T.cells	MMP27	0.576727	-0.20195	1.015799	0.312497	-4.83248	0.745724	0.674324
T.cells	GM20406	0.479192	-0.53168	1.01579	0.312501	-4.88541	0.751252	0.68052
T.cells	FZD7	0.40044	0.743156	1.015785	0.312504	-5.00074	0.730082	0.656863
T.cells	ZFP358	0.159418	3.044691	1.015496	0.31264	-5.72352	0.693248	0.616239
T.cells	SLC7A2	0.536385	2.094729	1.015391	0.31269	-5.13686	0.708238	0.632811
T.cells	ZC3H12C	-0.35723	4.431325	-1.01535	0.312711	-5.45516	0.671896	0.592994
T.cells	HIST1H3C	0.301302	1.047316	1.015081	0.312837	-5.46202	0.725113	0.651639
T.cells	1110032A0	0.135334	3.592833	1.015052	0.312851	-5.75254	0.684733	0.607108
T.cells	INAFM2	0.155988	3.225336	1.015006	0.312873	-5.70681	0.690431	0.613348
T.cells	GM38394	-0.38016	0.848639	-1.01499	0.312882	-5.07881	0.728355	0.655249
T.cells	GSTP3	0.348624	3.39573	1.014956	0.312896	-5.33693	0.687783	0.610448
T.cells	KRT80	0.458213	0.161146	1.014681	0.313027	-4.86539	0.739839	0.667955
T.cells	FNDC3A	0.087972	7.526942	1.014391	0.313164	-6.44031	0.626571	0.544287
T.cells	TRIP11	0.084271	6.069875	1.013935	0.31338	-6.27573	0.6476	0.567003
T.cells	DBF4	-0.10276	5.205532	-1.01363	0.313526	-6.28535	0.660387	0.580955
T.cells	FBXL22	-0.25447	2.248825	-1.01354	0.313568	-5.42611	0.705939	0.630782
T.cells	INTS6	-0.12095	6.588587	-1.01344	0.313618	-6.32938	0.640039	0.559039
T.cells	CDKL4	-0.47992	1.561956	-1.01313	0.313761	-4.99548	0.716931	0.6431
T.cells	EIF5	0.07738	8.418516	1.013125	0.313765	-6.60259	0.614025	0.531383
T.cells	MOCS2	-0.10457	5.094585	-1.01309	0.313784	-6.08642	0.662046	0.5829
T.cells	CD300LF	0.566884	3.385271	1.01303	0.31381	-4.96944	0.688095	0.611315
T.cells	EIF2S1	-0.07437	6.052768	-1.01276	0.31394	-6.37598	0.647851	0.567649
T.cells	SCG5	0.348015	0.801405	1.012617	0.314006	-5.22391	0.729286	0.656972
T.cells	CUL4A	-0.09164	5.002429	-1.01257	0.314027	-6.08022	0.663426	0.58452
T.cells	CDKN3	-0.15712	4.160534	-1.01226	0.314175	-6.16927	0.676164	0.598512
T.cells	TLR2	-0.35983	3.875493	-1.01224	0.314185	-5.25655	0.680528	0.603279
T.cells	CNIH1	-0.09054	5.17624	-1.01197	0.314312	-6.21173	0.660825	0.581941
T.cells	CENPI	-0.15908	2.997583	-1.01193	0.314331	-5.87894	0.694135	0.61832

T.cells	CASD1	0.116174	4.363906	1.011706	0.314439	-5.91132	0.673066	0.595269
T.cells	TRIP13	-0.1823	2.772205	-1.0117	0.314444	-5.82177	0.697668	0.622229
T.cells	SH2B3	-0.10158	5.315208	-1.0116	0.31449	-6.10577	0.658752	0.57972
T.cells	TRAF7	0.092521	4.585124	1.011587	0.314496	-6.05396	0.669712	0.591623
T.cells	PTCD2	0.100108	4.58679	1.011207	0.314677	-6.07513	0.669686	0.591722
T.cells	GM17477	0.338816	0.61424	1.011088	0.314733	-5.12021	0.732357	0.660864
T.cells	GM43696	0.177798	2.459243	1.010996	0.314777	-5.58882	0.702603	0.627831
T.cells	LOXL3	-0.53605	0.451305	-1.01072	0.314908	-4.95318	0.735039	0.663943
T.cells	WIPI1	0.33445	2.277802	1.010698	0.314919	-5.22173	0.705479	0.631093
T.cells	GMEB2	-0.07712	5.764218	-1.01065	0.314942	-6.28123	0.652095	0.572752
T.cells	CNN3	0.1219	4.531127	1.010351	0.315084	-6.16067	0.670529	0.592845
T.cells	FKBP8	-0.0883	6.067485	-1.0099	0.3153	-6.30582	0.647635	0.56817
T.cells	LEF1OS1	0.302107	-0.45613	1.009881	0.315308	-5.16227	0.750145	0.681183
T.cells	ARL3	-0.12976	4.04145	-1.00985	0.315322	-5.86183	0.677984	0.601137
T.cells	SRBD1	0.107343	4.393343	1.009659	0.315414	-6.08497	0.672619	0.595339
T.cells	ZFP386	0.114829	3.889207	1.009562	0.31546	-5.97516	0.680317	0.603788
T.cells	MAFG	0.122484	5.07367	1.009371	0.315551	-5.91578	0.662359	0.584323
T.cells	ISG15	0.452848	6.408101	1.009146	0.315658	-5.81606	0.64266	0.563148
T.cells	PANK4	-0.13198	3.423163	-1.0091	0.315682	-5.85106	0.687507	0.611941
T.cells	ANKRD44	0.092262	8.18547	1.008927	0.315763	-6.5736	0.617281	0.536051
T.cells	NPLOC4	0.09046	5.537568	1.008824	0.315812	-6.21776	0.655447	0.577077
T.cells	CEP170B	-0.3381	0.457754	-1.00882	0.315815	-5.10369	0.734933	0.664657
T.cells	ALG1	0.1712	3.312271	1.008379	0.316024	-5.66658	0.689228	0.614131
T.cells	PRPF40A	-0.0534	7.709067	-1.00834	0.316042	-6.63777	0.623989	0.543383
T.cells	TCOF1	-0.09687	5.717375	-1.00831	0.316055	-6.32428	0.652787	0.574362
T.cells	GFI1	-0.25388	1.55053	-1.00808	0.316165	-5.38431	0.717115	0.645012
T.cells	NOCT	-0.14264	5.04776	-1.00805	0.31618	-6.05926	0.662747	0.585208
T.cells	PLCB1	-0.36458	4.622522	-1.00805	0.316183	-5.48084	0.669146	0.592177
T.cells	MXRA7	-0.38985	1.654737	-1.00801	0.316203	-5.09716	0.715437	0.643146
T.cells	WDTC1	0.125179	3.851337	1.007955	0.316227	-5.93839	0.680899	0.605026
T.cells	SEMA6B	0.418301	1.154578	1.007886	0.316259	-5.01849	0.723525	0.652148
T.cells	PNPT1	0.1141	4.198204	1.007775	0.316312	-5.9996	0.675589	0.599219
T.cells	GRIK4	0.642741	0.238216	1.007714	0.316342	-4.89242	0.738561	0.668977
T.cells	TMSB4X	-0.09249	12.24836	-1.00761	0.316391	-7.09237	0.562808	0.479006
T.cells	GM14455	0.335199	1.315653	1.007561	0.316415	-5.09313	0.720911	0.649254
T.cells	SP110	0.12532	5.924125	1.007372	0.316505	-6.1547	0.64974	0.571158
T.cells	EVI2	0.32749	3.35768	1.007221	0.316577	-5.24941	0.688523	0.613469
T.cells	RCN2	-0.1073	5.075901	-1.00719	0.316592	-6.1583	0.662326	0.58482
T.cells	CCNY	-0.07977	6.67252	-1.00718	0.316595	-6.38176	0.638823	0.559393
T.cells	PPP1R13L	-0.55296	-0.0774	-1.00668	0.316837	-4.92985	0.743806	0.67512
T.cells	UNKL	-0.13903	3.970057	-1.00656	0.316895	-5.87588	0.679077	0.603276
T.cells	1110006O2	0.375376	0.319918	1.006468	0.316937	-5.02861	0.737209	0.667716
T.cells	CALCOCO1	0.19743	3.863697	1.006031	0.317146	-5.53099	0.680709	0.605109
T.cells	TRIP10	0.254491	1.355077	1.006031	0.317146	-5.27918	0.720272	0.648838
T.cells	HIST1H2BH	-0.35294	0.17295	-1.00595	0.317185	-5.15831	0.739643	0.670533
T.cells	PIGF	-0.14565	3.388301	-1.00574	0.317285	-5.82431	0.688048	0.613302
T.cells	4933434E2	-0.0952	5.269667	-1.00535	0.317474	-6.15239	0.659431	0.582043

T.cells	GM34466	0.527935	-1.10689	1.005237	0.317526	-4.81931	0.761152	0.694965
T.cells	FANCB	0.291525	0.677958	1.005167	0.317559	-5.23109	0.73131	0.66143
T.cells	COQ10B	-0.1114	5.908947	-1.00508	0.317599	-5.99553	0.649963	0.571862
T.cells	PTK7	-0.48683	1.073787	-1.00487	0.317704	-5.08785	0.724839	0.65424
T.cells	EEF1AKMT	-0.16767	4.588498	-1.00476	0.317755	-5.75159	0.669661	0.593305
T.cells	F9	0.445612	0.896192	1.0046	0.317831	-5.01207	0.727736	0.657475
T.cells	ARFRP1	-0.10276	4.120918	-1.00459	0.317835	-5.93396	0.676769	0.601078
T.cells	UBL3	0.065844	7.408809	1.004493	0.317882	-6.45265	0.628252	0.548543
T.cells	AKAP17B	0.247345	1.696468	1.004492	0.317883	-5.3983	0.714766	0.643063
T.cells	BC051226	0.183177	2.179737	1.004383	0.317935	-5.49933	0.707038	0.63448
T.cells	LYRM2	0.158887	3.377583	1.004369	0.317942	-5.74645	0.688214	0.613687
T.cells	SNW1	-0.0623	6.240739	-1.00415	0.318044	-6.37496	0.6451	0.566717
T.cells	PKN2	0.077668	6.774243	1.004145	0.318049	-6.44732	0.637353	0.558362
T.cells	HIST1H1B	0.195222	5.277543	1.003688	0.318268	-6.48144	0.659313	0.58227
T.cells	PCOLCE	-0.46481	0.911461	-1.00358	0.318321	-4.99457	0.727486	0.65749
T.cells	NGDN	-0.07923	5.424919	-1.00337	0.318422	-6.24298	0.657119	0.57999
T.cells	GM14221	0.549196	1.910062	1.003354	0.318428	-5.00585	0.711341	0.639584
T.cells	EMID1	-0.12797	3.280299	-1.00325	0.31848	-5.93162	0.689725	0.615674
T.cells	RCC1L	-0.16458	3.072184	-1.00321	0.318497	-5.69743	0.692969	0.619249
T.cells	TEDC1	0.203137	1.701406	1.00313	0.318536	-5.52574	0.714687	0.643326
T.cells	TYSND1	-0.15986	2.640407	-1.00303	0.318585	-5.55499	0.699743	0.626787
T.cells	FDPS	-0.15416	4.623809	-1.00276	0.318713	-5.96948	0.669127	0.59328
T.cells	BBIP1	0.087045	5.888435	1.002743	0.318721	-6.27143	0.650265	0.572756
T.cells	SEC13	-0.0841	5.361757	-1.00255	0.318816	-6.21435	0.658059	0.581231
T.cells	GM11755	0.359187	-0.115	1.002318	0.318925	-5.20686	0.744433	0.676942
T.cells	FLOT1	0.15737	4.575358	1.002176	0.318993	-5.74375	0.669859	0.594228
T.cells	ATE1	-0.10891	3.945361	-1.00206	0.319049	-5.88534	0.679456	0.604807
T.cells	GM30948	0.318789	0.467484	1.002002	0.319077	-5.45118	0.734772	0.666247
T.cells	SEC63	0.061604	7.09953	1.001944	0.319105	-6.53061	0.632672	0.553991
T.cells	ZHX1	0.120241	4.078556	1.001736	0.319205	-5.95151	0.677416	0.602667
T.cells	PRDM4	0.147767	3.039382	1.001382	0.319375	-5.68198	0.693481	0.620483
T.cells	TTC4	0.115263	3.541793	1.001055	0.319532	-5.86261	0.68567	0.611974
T.cells	USP14	-0.07081	5.793901	-1.00103	0.319542	-6.32061	0.651657	0.574786
T.cells	ZFP157	0.194594	2.446699	1.001016	0.319551	-5.5836	0.702801	0.630909
T.cells	TARS2	0.119124	3.819829	1.000876	0.319618	-5.90022	0.681383	0.607296
T.cells	DNMT3A	-0.10357	6.044028	-1.00084	0.319637	-6.21982	0.647979	0.570843
T.cells	HIST1H3G	-0.24367	1.46486	-1.00077	0.31967	-5.53271	0.718497	0.64843
T.cells	VAMP1	0.138552	3.997211	1.000602	0.31975	-5.79109	0.678661	0.604348
T.cells	MAML1	0.092855	5.435368	1.000474	0.319811	-6.20924	0.656964	0.580664
T.cells	URAH	-0.33604	3.327083	-1.00042	0.319839	-5.44993	0.688998	0.615759
T.cells	MAPK6	0.121027	5.88266	1.000277	0.319906	-6.21515	0.65035	0.573568
T.cells	NCKAP5LO:	0.535638	0.041134	1.000175	0.319955	-4.9349	0.741832	0.674752
T.cells	GM17106	0.172918	3.395192	1.000168	0.319959	-5.76094	0.687941	0.614691
T.cells	CEBPZ	-0.07505	6.141756	-0.99997	0.320053	-6.29609	0.646547	0.569492
T.cells	RXRA	-0.27763	3.1994	-0.99983	0.320121	-5.2506	0.690984	0.618149
T.cells	TTC9C	0.083722	4.558959	0.999742	0.320164	-6.09142	0.670108	0.595215
T.cells	NDUFAF7	-0.11729	4.007139	-0.99955	0.320255	-5.88594	0.678509	0.604538

T.cells	BCORL1	0.181517	3.355845	0.999538	0.320262	-5.6933	0.688552	0.615596
T.cells	PSMC2	-0.08613	5.362149	-0.9994	0.32033	-6.24364	0.658053	0.582174
T.cells	CD28	-0.4293	2.935145	-0.99917	0.320441	-5.13397	0.695112	0.622956
T.cells	CMTM7	0.063645	7.897065	0.999144	0.320452	-6.71094	0.621333	0.542592
T.cells	POLR2I	-0.07949	5.116859	-0.9988	0.320615	-6.21916	0.661713	0.586296
T.cells	PDSS1	0.11961	4.315269	0.998702	0.320665	-6.07675	0.673806	0.599526
T.cells	DIS3L	-0.14759	2.828005	-0.99856	0.320734	-5.7045	0.696792	0.624917
T.cells	NSF	0.091316	6.770124	0.998353	0.320833	-6.4306	0.637412	0.560064
T.cells	CRTAM	0.705417	1.176627	0.998297	0.32086	-4.88846	0.723166	0.654406
T.cells	ATP5F1	-0.06239	8.084387	-0.99807	0.32097	-6.67784	0.618698	0.539997
T.cells	PSMB7	-0.07598	5.624583	-0.99792	0.32104	-6.27725	0.654159	0.578329
T.cells	GZMM	-0.272	2.051803	-0.99784	0.321081	-5.35742	0.709076	0.638829
T.cells	CARD9	0.452135	0.997223	0.997707	0.321144	-5.00685	0.726087	0.657847
T.cells	MAMLD1	-0.56506	0.110917	-0.99764	0.321178	-4.88355	0.740673	0.674271
T.cells	NEK1	0.167032	3.697931	0.997515	0.321237	-5.75426	0.68326	0.610321
T.cells	NEMF	0.069599	5.95092	0.997441	0.321273	-6.33186	0.649346	0.573232
T.cells	SYS1	0.068687	6.417243	0.997353	0.321315	-6.38911	0.642527	0.565852
T.cells	RNF113A1	-0.38097	0.42846	-0.99727	0.321355	-5.11989	0.735416	0.668443
T.cells	RNF217	0.508845	2.071962	0.997267	0.321357	-5.04541	0.708754	0.638604
T.cells	NOL7	-0.05606	6.906907	-0.99709	0.32144	-6.55782	0.63544	0.55824
T.cells	TBC1D10A	0.145433	3.56507	0.996979	0.321495	-5.71452	0.68531	0.61272
T.cells	PRKAG2	-0.12438	5.050385	-0.99695	0.321511	-5.96805	0.662708	0.587904
T.cells	SGCB	-0.29187	2.212559	-0.9967	0.321632	-5.31125	0.706515	0.636297
T.cells	CIITA	-0.27378	2.478401	-0.99664	0.321657	-5.5558	0.7023	0.63161
T.cells	OSBPL1A	0.165268	3.288159	0.996631	0.321663	-5.67553	0.689603	0.617541
T.cells	DDX3Y	2.082372	2.707432	0.996528	0.321713	-5.47634	0.698687	0.627655
T.cells	EIF3I	-0.0718	6.822191	-0.99652	0.321715	-6.52221	0.636661	0.559731
T.cells	ZFP467	-0.39525	2.303773	-0.99647	0.32174	-5.03716	0.705066	0.634747
T.cells	CHMP1A	-0.1059	5.305048	-0.99622	0.321861	-6.12155	0.658903	0.58396
T.cells	CD274	0.577332	5.805413	0.995868	0.322032	-5.59583	0.651488	0.57594
T.cells	HSPA9	-0.07442	6.483768	-0.99584	0.322046	-6.41447	0.64156	0.565165
T.cells	HVCN1	0.179267	4.117697	0.995831	0.32205	-5.95578	0.676818	0.603646
T.cells	MGP	0.5434	0.463003	0.995752	0.322088	-4.99841	0.734846	0.668216
T.cells	GM12743	0.221171	2.382516	0.995707	0.32211	-5.48037	0.703818	0.633521
T.cells	PVT1	0.212463	5.282726	0.995665	0.32213	-5.94906	0.659236	0.584399
T.cells	SAMD4B	0.093751	5.014196	0.995648	0.322138	-6.11003	0.66325	0.588784
T.cells	TPBGL	-0.56721	0.559425	-0.99556	0.322179	-4.90303	0.733258	0.666453
T.cells	PSMD5	0.10949	3.835237	0.995528	0.322197	-5.9256	0.681146	0.608432
T.cells	GM12166	-0.43606	0.781167	-0.99549	0.322217	-4.98979	0.729618	0.662361
T.cells	LAPTM4A	-0.07123	7.210016	-0.9953	0.322308	-6.45573	0.63109	0.553928
T.cells	DBNL	0.081499	6.127196	0.994921	0.32249	-6.27912	0.64676	0.570929
T.cells	TRAF3IP1	0.249379	1.889467	0.994899	0.3225	-5.45981	0.71167	0.642392
T.cells	DANCR	-0.24093	1.545559	-0.99456	0.322663	-5.39415	0.717195	0.648693
T.cells	GM44899	-0.37845	0.774696	-0.99455	0.322667	-5.04435	0.729724	0.662737
T.cells	PNPLA6	0.172673	2.760588	0.994297	0.322792	-5.53665	0.697851	0.627236
T.cells	SON	-0.05621	7.67203	-0.99422	0.322829	-6.58036	0.624513	0.547119
T.cells	ADGRG5	0.622444	0.105614	0.994174	0.322851	-4.86921	0.740761	0.675301

T.cells	RWDD1	-0.07034	6.105394	-0.99416	0.322858	-6.36973	0.64708	0.571508
T.cells	TMEM135	0.103488	5.7512	0.994138	0.322868	-6.19481	0.652287	0.577178
T.cells	MAP3K12	0.180041	2.718206	0.994107	0.322884	-5.53723	0.698517	0.628026
T.cells	TUFM	-0.10255	4.731912	-0.9941	0.322889	-6.16176	0.667494	0.5938
T.cells	ASF1A	-0.08979	5.210724	-0.994	0.322934	-6.31138	0.66031	0.585959
T.cells	PAKAP.1	0.148506	4.53559	0.993954	0.322958	-6.00459	0.670461	0.597082
T.cells	DNAJB11	-0.07268	6.073023	-0.9939	0.322985	-6.35664	0.647554	0.572065
T.cells	PEX12	0.249164	1.86574	0.993578	0.32314	-5.36195	0.71205	0.643284
T.cells	DOCK4	-0.15726	6.427831	-0.99353	0.323162	-6.09188	0.642373	0.566584
T.cells	ATF7	0.083156	6.314357	0.993433	0.32321	-6.38407	0.644026	0.56841
T.cells	PHF21A	0.093343	7.069098	0.993337	0.323257	-6.42605	0.633108	0.556591
T.cells	CLOCK	-0.10719	5.071637	-0.99329	0.32328	-6.05998	0.662389	0.588441
T.cells	PLEKHF2	0.074944	5.470642	0.993281	0.323284	-6.30336	0.65644	0.58194
T.cells	CAT	0.120882	6.72379	0.993202	0.323322	-6.4262	0.638081	0.561995
T.cells	TMEM202	0.519392	0.104736	0.992832	0.323501	-4.86974	0.740945	0.675746
T.cells	DVL3	-0.14624	3.300094	-0.99279	0.323522	-5.67372	0.689576	0.618334
T.cells	FBXW8	0.128446	3.868261	0.992676	0.323577	-5.83245	0.680795	0.608641
T.cells	MITD1	0.132269	4.433197	0.992373	0.323724	-5.95541	0.672348	0.599219
T.cells	CTBP1	-0.07291	6.500976	-0.99206	0.323877	-6.4611	0.641751	0.565832
T.cells	PACC1	-0.11513	5.421952	-0.992	0.323906	-6.21668	0.657616	0.583088
T.cells	NMT2	-0.0829	5.47269	-0.99171	0.324047	-6.21795	0.656925	0.582393
T.cells	CFL1	-0.07347	10.01399	-0.99153	0.324133	-6.96979	0.592632	0.513311
T.cells	TMEM11	-0.08939	5.070903	-0.99148	0.324158	-6.19855	0.66292	0.589046
T.cells	TRIM8	-0.09377	5.616506	-0.99144	0.324178	-6.1712	0.654791	0.580177
T.cells	IL2RG	0.183423	6.294179	0.9911	0.324341	-6.25689	0.644947	0.569496
T.cells	NAB1	-0.10209	6.555305	-0.99099	0.324395	-6.19346	0.641145	0.56543
T.cells	AI480526	0.23306	2.064128	0.990937	0.32442	-5.40549	0.709569	0.640749
T.cells	HERC3	0.133328	4.345366	0.990709	0.324531	-5.91089	0.674108	0.601435
T.cells	GM16675	0.375095	1.196561	0.990165	0.324796	-5.18078	0.724108	0.65667
T.cells	RAP2B	0.115581	4.614993	0.989849	0.324949	-5.97308	0.670432	0.597256
T.cells	LRRC41	0.105315	4.919545	0.989815	0.324966	-6.06562	0.665834	0.592214
T.cells	ARSA	0.303071	1.69399	0.989798	0.324974	-5.15357	0.716057	0.64782
T.cells	1810046K0	-0.29691	-0.14929	-0.98957	0.325083	-5.28928	0.746359	0.681931
T.cells	UVSSA	0.154705	3.709393	0.989506	0.325116	-5.69497	0.684324	0.612576
T.cells	ARHGEF6	-0.11574	5.643537	-0.98922	0.325254	-6.03381	0.655222	0.580495
T.cells	RSBN1	0.096008	5.326631	0.988724	0.325496	-6.16694	0.659986	0.585784
T.cells	RBM44	0.401142	0.135835	0.988634	0.32554	-5.13463	0.741838	0.676781
T.cells	FLT4	-0.33896	2.321562	-0.98852	0.325595	-5.198	0.706287	0.636919
T.cells	NIPSNAP3E	-0.08831	5.573981	-0.98847	0.32562	-6.26634	0.656305	0.581815
T.cells	UTP14A	-0.09027	5.238791	-0.98815	0.325776	-6.19006	0.661298	0.58743
T.cells	AURKA	0.137546	3.318106	0.988148	0.325777	-5.96642	0.690607	0.619689
T.cells	GM15952	-0.3475	1.028007	-0.98799	0.325852	-5.15673	0.727132	0.660456
T.cells	KLRA3	0.702515	-0.66191	0.987984	0.325856	-4.86576	0.755217	0.692174
T.cells	GM15503	0.331989	0.81285	0.987921	0.325887	-5.05039	0.730654	0.664415
T.cells	ASB1	0.259123	1.712368	0.987764	0.325964	-5.28213	0.716034	0.648014
T.cells	ZSCAN12	0.251461	1.093811	0.98773	0.325981	-5.2579	0.726058	0.65925
T.cells	PDE4D	0.139749	6.428002	0.987533	0.326076	-6.32273	0.643741	0.568318

T.cells	MON1B	0.235224	1.881481	0.987195	0.326241	-5.30356	0.713316	0.645068
T.cells	SNTB2	0.130452	5.607555	0.987019	0.326327	-6.13495	0.655806	0.581548
T.cells	CABYR	0.449347	1.135391	0.986866	0.326401	-5.04624	0.72538	0.658601
T.cells	HMCES	0.124057	4.456587	0.986711	0.326477	-5.98856	0.673091	0.600484
T.cells	SP3	-0.06377	6.891028	-0.98665	0.326504	-6.51545	0.637024	0.561135
T.cells	RNF214	-0.09775	4.961908	-0.98664	0.326512	-6.10472	0.665451	0.592096
T.cells	IK	0.067054	6.288849	0.98655	0.326556	-6.37087	0.645772	0.57063
T.cells	MBTPS2	0.120927	3.917498	0.986537	0.326562	-5.89857	0.681333	0.609572
T.cells	ZFP619	-0.22533	2.21582	-0.98651	0.326573	-5.33634	0.70797	0.639126
T.cells	1810037I1	0.089708	6.482621	0.986445	0.326607	-6.3666	0.642945	0.567595
T.cells	GAPT	-0.45459	1.236779	-0.98618	0.326735	-5.00271	0.723881	0.656945
T.cells	SIPA1L2	-0.126	5.103799	-0.98573	0.326953	-6.07845	0.663779	0.590099
T.cells	EEF2KMT	0.165391	3.145957	0.985493	0.327071	-5.6049	0.693894	0.623223
T.cells	PYM1	0.08112	4.692796	0.985222	0.327203	-6.17845	0.670234	0.597048
T.cells	THRA	-0.14348	3.771843	-0.98507	0.327276	-5.86267	0.684316	0.612634
T.cells	SLF1	-0.10864	4.44199	-0.98485	0.327387	-6.12238	0.674042	0.601377
T.cells	ASCC3	0.10568	6.882346	0.984822	0.327399	-6.29785	0.637839	0.561885
T.cells	APAF1	0.095275	5.726771	0.984731	0.327443	-6.25019	0.654748	0.580298
T.cells	LAIR1	0.529082	4.246709	0.984402	0.327604	-5.17375	0.67708	0.604793
T.cells	C1QTNF6	0.435915	1.904338	0.984392	0.327609	-5.09698	0.713783	0.645518
T.cells	EYA2	0.28816	1.389743	0.984302	0.327653	-5.34969	0.72209	0.654818
T.cells	IFIT1BL1	0.68106	0.159661	0.983759	0.327918	-4.91266	0.742662	0.677766
T.cells	PGPEP1	-0.14168	3.461593	-0.98366	0.327966	-5.75344	0.68951	0.618339
T.cells	BTRC	0.090719	5.211768	0.983611	0.327991	-6.26344	0.662792	0.588938
T.cells	PNKD	0.105273	4.867546	0.983185	0.328199	-6.13751	0.668271	0.594719
T.cells	MAST3	0.12957	4.169613	0.982971	0.328304	-5.9718	0.678942	0.606477
T.cells	MAD1L1	-0.07482	5.227133	-0.98271	0.328432	-6.26729	0.662913	0.588925
T.cells	HLX	-0.17256	3.117664	-0.98269	0.328441	-5.64199	0.695246	0.624538
T.cells	BCDIN3D	0.177743	2.223563	0.982638	0.328467	-5.50743	0.709389	0.64028
T.cells	C3	-0.22337	6.327835	-0.98215	0.328704	-6.14804	0.646955	0.571322
T.cells	DIAPH2	0.099177	7.666379	0.981852	0.328852	-6.44205	0.627792	0.550525
T.cells	KMT2B	0.120866	3.888591	0.981222	0.329161	-5.89413	0.684327	0.611899
T.cells	KLHL8	-0.2463	2.402928	-0.98085	0.329341	-5.48522	0.70789	0.637853
T.cells	GDF11	-0.22179	1.639394	-0.98015	0.329685	-5.5408	0.720668	0.651773
T.cells	PRKAB2	-0.17919	3.392055	-0.98004	0.329741	-5.61989	0.692781	0.620796
T.cells	RAPGEF4	-0.31169	2.563789	-0.98	0.329758	-5.3055	0.705832	0.635276
T.cells	CTIF	-0.23372	2.596638	-0.97973	0.329892	-5.23297	0.705404	0.634804
T.cells	AKAP13	0.070051	8.500476	0.979539	0.329986	-6.67686	0.617233	0.538669
T.cells	GJA1	-0.51528	1.208053	-0.97929	0.330111	-5.01128	0.727785	0.659939
T.cells	RBMX2	-0.13308	3.368821	-0.97895	0.330275	-5.88253	0.693236	0.621542
T.cells	ENDOD1	0.124101	2.901638	0.978921	0.33029	-5.90884	0.700574	0.629674
T.cells	HIST1H2AK	-0.27193	0.954719	-0.9789	0.3303	-5.44701	0.731938	0.664696
T.cells	2310009A0	0.103531	4.692253	0.978623	0.330437	-6.07938	0.672834	0.59912
T.cells	YTHDF3	0.072835	7.017555	0.978553	0.330471	-6.4291	0.638347	0.561587
T.cells	BLZF1	-0.14483	3.200504	-0.97835	0.330572	-5.70139	0.695871	0.624544
T.cells	LHPP	-0.12851	3.112672	-0.97826	0.330615	-5.87783	0.69725	0.626092
T.cells	GM9725	0.29463	2.030059	0.978137	0.330675	-5.31034	0.714458	0.645296

T.cells	TAF1	-0.081	5.699019	-0.97804	0.330723	-6.34009	0.657693	0.582704
T.cells	RALY	-0.0706	6.422986	-0.97803	0.330727	-6.47155	0.647003	0.571077
T.cells	NAGA	-0.2184	4.159018	-0.97782	0.33083	-5.52751	0.680986	0.608234
T.cells	RRM1	-0.11106	5.449593	-0.97754	0.330967	-6.38078	0.661414	0.586831
T.cells	TMEM35B	0.284977	2.207613	0.977506	0.330986	-5.34688	0.711609	0.642259
T.cells	OIP5OS1	0.079824	6.02273	0.977336	0.33107	-6.33562	0.652893	0.577597
T.cells	2700097O(	0.173721	3.090234	0.97717	0.331151	-5.65236	0.697603	0.626787
T.cells	SSR1	-0.06654	6.554701	-0.97716	0.331156	-6.42599	0.645076	0.569164
T.cells	RMDN3	0.119792	3.398108	0.977131	0.33117	-5.81253	0.692778	0.62144
T.cells	0610005C1	-0.33116	1.911141	-0.97709	0.331189	-5.17898	0.716372	0.647682
T.cells	DDX20	-0.10109	3.596417	-0.97706	0.331204	-5.96296	0.689687	0.61802
T.cells	AGXT2	-0.39492	1.612637	-0.97701	0.331231	-5.16713	0.721197	0.653079
T.cells	BLOC1S2	-0.0922	4.833886	-0.97687	0.331301	-6.19557	0.670685	0.597161
T.cells	HCK	-0.21892	5.693071	-0.97676	0.331351	-5.78137	0.657781	0.583057
T.cells	RGS1	-0.49026	5.563816	-0.97671	0.331378	-5.31133	0.659707	0.585159
T.cells	ST6GALNA(	0.393159	0.651842	0.976316	0.331572	-5.05753	0.737146	0.670904
T.cells	CARNMT1	-0.07879	5.353674	-0.97627	0.331596	-6.24616	0.663042	0.588658
T.cells	XPO4	-0.07877	5.623363	-0.9758	0.331826	-6.33379	0.659095	0.58446
T.cells	RSPH1	0.232281	1.855808	0.975671	0.33189	-5.73392	0.717563	0.649124
T.cells	ENTPD7	-0.10942	4.693758	-0.97555	0.331948	-6.04191	0.673093	0.599844
T.cells	CLNK	-0.55812	1.404175	-0.97555	0.331949	-4.9893	0.724887	0.657329
T.cells	GM36486	0.547216	0.805963	0.975503	0.331972	-4.97403	0.734694	0.668345
T.cells	GM36371	-0.31497	0.884673	-0.97544	0.332002	-5.08002	0.733396	0.666886
T.cells	ZKSCAN16	0.30086	-0.59074	0.97521	0.332117	-5.18302	0.75819	0.694811
T.cells	MYH13	0.471831	-1.61994	0.974985	0.332228	-4.81714	0.775965	0.714968
T.cells	SHQ1	0.189433	2.60612	0.974679	0.332379	-5.60637	0.705961	0.635893
T.cells	NUDCD3	0.063018	5.998557	0.974427	0.332503	-6.37162	0.653925	0.578723
T.cells	CASP7	0.121618	4.140272	0.974413	0.33251	-6.02571	0.681979	0.60945
T.cells	ERG	0.213205	3.921953	0.974285	0.332573	-6.04388	0.685353	0.613172
T.cells	CAB39	0.069767	6.974308	0.973799	0.332813	-6.45711	0.639983	0.563377
T.cells	BRAP	-0.0949	4.924367	-0.97286	0.333278	-6.08446	0.671186	0.596796
T.cells	AKT3	0.086785	6.643383	0.972439	0.333485	-6.54452	0.645795	0.568992
T.cells	NAT8L	0.416416	0.332665	0.972385	0.333512	-5.0228	0.74455	0.678191
T.cells	STARD4	-0.15332	3.526928	-0.97227	0.333571	-5.7324	0.692929	0.620589
T.cells	RAPGEF2	0.124789	7.429198	0.972064	0.333671	-6.41878	0.634413	0.55675
T.cells	CHST15	-0.14574	3.54275	-0.972	0.333701	-5.77449	0.6927	0.620369
T.cells	IRGC1	-0.50676	0.115465	-0.97185	0.333778	-4.89385	0.748237	0.682397
T.cells	GM12592	-0.13509	3.124779	-0.97173	0.333837	-5.84602	0.699288	0.627644
T.cells	TNFRSF11A	0.430234	2.190262	0.971196	0.3341	-5.0857	0.714491	0.644311
T.cells	REST	0.091324	5.400614	0.971172	0.334112	-6.2371	0.664569	0.589245
T.cells	PYCARD	-0.09874	5.876191	-0.97091	0.334244	-6.29909	0.657465	0.581526
T.cells	ACOD1	1.301692	2.318035	0.970791	0.334301	-4.95795	0.712447	0.64212
T.cells	CDKN2C	0.1385	3.918847	0.970639	0.334376	-6.11907	0.6872	0.614196
T.cells	PRSS57	0.355536	-0.03051	0.970417	0.334486	-5.08455	0.751044	0.685595
T.cells	4833417C1	-0.33195	0.707506	-0.97012	0.334635	-5.159	0.738712	0.671716
T.cells	SGO2A	0.180651	3.063462	0.970019	0.334683	-5.96286	0.700585	0.629131
T.cells	GM1673	-0.26703	2.42109	-0.97001	0.334688	-5.31359	0.710796	0.640474

T.cells	LIMK2	0.099577	4.779797	0.969987	0.334699	-6.12025	0.673969	0.599789
T.cells	MAP3K13	0.408136	0.779749	0.969975	0.334705	-5.07832	0.737515	0.670374
T.cells	HSPB6	0.362113	0.40248	0.969872	0.334756	-5.15484	0.743786	0.677437
T.cells	DAPP1	0.090763	6.482475	0.969813	0.334785	-6.27592	0.648503	0.572023
T.cells	CETN3	-0.08345	5.807387	-0.96962	0.33488	-6.35454	0.658555	0.582958
T.cells	GM6710	0.360318	0.761027	0.969155	0.335112	-5.14113	0.738056	0.671016
T.cells	GCH1	0.171073	5.663529	0.969134	0.335122	-5.8842	0.660842	0.585468
T.cells	BEX3	-0.09335	4.990762	-0.96911	0.335133	-6.27811	0.670973	0.596541
T.cells	UCKL1	-0.14418	3.705129	-0.96851	0.335431	-5.71993	0.691225	0.61852
T.cells	TRIM7	-0.3936	0.591087	-0.96834	0.335514	-5.07204	0.741447	0.674493
T.cells	PFKFB4	0.232432	3.15878	0.968065	0.335653	-5.3823	0.700002	0.628167
T.cells	GM50232	-0.27918	1.372215	-0.96786	0.335754	-5.36701	0.728731	0.660252
T.cells	NDUFA3	-0.07004	8.000282	-0.96781	0.335778	-6.63671	0.627402	0.549057
T.cells	FLI1	-0.07436	8.191063	-0.96747	0.335948	-6.68976	0.624895	0.546266
T.cells	LRFN1	-0.39589	1.097146	-0.96723	0.336069	-5.15443	0.733542	0.665595
T.cells	ITPR2	0.078701	6.912603	0.967174	0.336095	-6.47499	0.643324	0.566153
T.cells	PLLP	-0.35975	-0.20457	-0.96702	0.336174	-5.17593	0.75532	0.690114
T.cells	ACAP3	-0.18179	2.238471	-0.96683	0.336265	-5.45635	0.715058	0.644832
T.cells	4930505N2	0.283583	1.214516	0.96668	0.336341	-5.25631	0.731744	0.663459
T.cells	ARFGEF1	0.084314	6.613928	0.96656	0.336401	-6.37438	0.64781	0.570929
T.cells	TMA16	0.136202	3.964843	0.966045	0.336657	-6.03359	0.688108	0.614734
T.cells	SAP18B	-0.17908	2.771863	-0.96601	0.336674	-5.6109	0.706868	0.635503
T.cells	FAR1OS	0.183688	2.788806	0.965691	0.336833	-5.82685	0.706659	0.635303
T.cells	RNF20	-0.08828	5.755436	-0.96537	0.33699	-6.29068	0.660877	0.585069
T.cells	H2AFX	-0.1143	6.482628	-0.96499	0.337181	-6.64893	0.650087	0.573452
T.cells	GM31728	-0.56509	-0.17439	-0.96488	0.337236	-4.89864	0.755313	0.690094
T.cells	TRAF2	0.114619	3.917831	0.964872	0.337241	-5.96025	0.688897	0.615894
T.cells	IMPG2	-0.47493	0.6613	-0.96484	0.337258	-5.03011	0.741288	0.674269
T.cells	RMND5B	-0.1026	4.49308	-0.96455	0.337403	-6.00369	0.680008	0.606205
T.cells	PARL	0.097963	4.391466	0.964528	0.337412	-6.04154	0.681571	0.607927
T.cells	USP33	-0.10742	4.710834	-0.96435	0.337498	-6.04323	0.676671	0.602569
T.cells	CARD19	0.139909	5.613475	0.964305	0.337523	-5.98448	0.663003	0.587615
T.cells	FCF1	-0.06718	6.136138	-0.96424	0.337555	-6.4191	0.655208	0.579127
T.cells	ARRB2	-0.14034	5.195443	-0.96416	0.337593	-5.87922	0.669301	0.594494
T.cells	CDO1	0.219344	3.756999	0.96415	0.337601	-5.66209	0.691402	0.618782
T.cells	PQBP1	-0.09541	4.6495	-0.96413	0.33761	-6.13147	0.67761	0.603604
T.cells	CNKSR3	-0.14951	4.754523	-0.96411	0.337618	-6.07786	0.676004	0.601843
T.cells	SPCS3	-0.09907	4.704887	-0.96362	0.337863	-6.00503	0.67713	0.602826
T.cells	IKZF5	0.117174	3.804869	0.963336	0.338007	-5.90408	0.691041	0.618179
T.cells	CBL	0.078525	7.22396	0.963218	0.338065	-6.43794	0.639616	0.562052
T.cells	CCDC57	0.159123	2.546499	0.963218	0.338065	-5.66367	0.710923	0.640206
T.cells	SLC25A10	-0.1645	3.077126	-0.96312	0.338112	-5.70975	0.702475	0.630825
T.cells	LTBR	0.297307	2.974023	0.962921	0.338214	-5.18036	0.704194	0.632643
T.cells	GM42595	-0.32157	0.917394	-0.96269	0.33833	-5.17386	0.73766	0.66989
T.cells	KRAS	-0.06557	7.278938	-0.96256	0.338392	-6.53262	0.639001	0.561266
T.cells	STK32C	0.381915	0.553606	0.96228	0.338533	-5.06998	0.743779	0.676845
T.cells	SUV39H1	-0.1046	3.622039	-0.96208	0.338636	-6.01694	0.69416	0.621538

T.cells	HCFC1R1	0.10252	5.33224	0.961988	0.33868	-6.09447	0.667859	0.592695
T.cells	SLC23A1	0.199485	1.528872	0.961881	0.338733	-5.55128	0.727663	0.658904
T.cells	PVRIG	0.475178	-0.53207	0.961787	0.33878	-4.87101	0.762104	0.697725
T.cells	SH2B1	-0.13497	3.699274	-0.96177	0.33879	-5.89695	0.692951	0.620319
T.cells	VPS36	0.074027	5.315956	0.961364	0.338991	-6.28554	0.668325	0.593214
T.cells	PECAM1	0.106564	7.734027	0.961298	0.339024	-6.64594	0.632709	0.554626
T.cells	APOOL	0.113145	3.780654	0.961022	0.339162	-5.94307	0.691908	0.619177
T.cells	ACAA2	-0.15893	5.01941	-0.96087	0.339236	-5.98254	0.672821	0.598196
T.cells	TOR1A	0.101835	4.653633	0.960869	0.339239	-6.02582	0.678405	0.604318
T.cells	IL1R1	0.365503	2.051839	0.960758	0.339294	-5.29593	0.719392	0.649716
T.cells	GM38134	0.302445	1.108316	0.960613	0.339367	-5.26608	0.734815	0.667016
T.cells	TOMM6	-0.06429	7.550466	-0.96057	0.33939	-6.61854	0.635349	0.557548
T.cells	LRRC27	-0.39292	0.302993	-0.96041	0.339469	-5.04358	0.748258	0.682162
T.cells	AFAP1	-0.19928	1.955044	-0.96005	0.339649	-5.48407	0.721251	0.651725
T.cells	NUTF2-PS1	-0.20759	2.848853	-0.95985	0.33975	-5.80594	0.706966	0.635785
T.cells	MAPK8	0.083889	5.232129	0.959379	0.339984	-6.22427	0.670282	0.595132
T.cells	PUS7L	0.25739	1.839535	0.959246	0.340051	-5.34545	0.723592	0.654068
T.cells	RAD51C	0.160121	2.245463	0.958396	0.340477	-5.70694	0.71721	0.647029
T.cells	MAIP1	0.118	3.588251	0.95835	0.340499	-5.89661	0.695836	0.62332
T.cells	RBM28	0.076519	5.311789	0.958006	0.340672	-6.2988	0.669271	0.594204
T.cells	VAV3	0.119682	7.363151	0.958003	0.340674	-6.46334	0.638896	0.56122
T.cells	USE1	0.097373	5.084893	0.957926	0.340712	-6.10606	0.672713	0.597992
T.cells	AMMECR1L	0.115642	4.488762	0.957875	0.340738	-5.98609	0.681836	0.608002
T.cells	C77080	-0.53577	0.694235	-0.95776	0.340798	-5.31432	0.742659	0.675733
T.cells	RNF145	0.103226	5.159727	0.957717	0.340817	-6.15996	0.671576	0.596803
T.cells	ATMIN	0.196698	2.969649	0.957704	0.340823	-5.58437	0.705608	0.634313
T.cells	NLRP12	0.504717	-0.12085	0.957577	0.340887	-4.94726	0.756361	0.69125
T.cells	NRIP1	0.118223	6.353871	0.957461	0.340945	-6.24631	0.653674	0.577395
T.cells	GM5244	0.588764	-0.39473	0.957428	0.340962	-4.88417	0.761017	0.696598
T.cells	IVD	-0.10113	4.435995	-0.95727	0.341043	-6.11507	0.682649	0.609062
T.cells	PTPRS	-0.11048	4.390471	-0.95725	0.341053	-6.11332	0.683351	0.609841
T.cells	FRS2	0.071994	5.84525	0.957229	0.341062	-6.31829	0.661243	0.585639
T.cells	BCL2L1	-0.11121	6.15449	-0.95699	0.341184	-6.47438	0.656749	0.580676
T.cells	FTCD	-0.46324	1.403237	-0.95655	0.341402	-5.09732	0.731283	0.662923
T.cells	MLXIPL	-0.55096	0.355448	-0.95625	0.341552	-5.01234	0.748692	0.682583
T.cells	QSOX1	-0.14353	4.311358	-0.95623	0.341564	-5.8149	0.684908	0.611481
T.cells	ELL2	-0.18253	6.734248	-0.95609	0.341633	-6.10347	0.648384	0.57164
T.cells	TRIM11	0.088036	5.06784	0.955437	0.341962	-6.22631	0.673302	0.598988
T.cells	1700061G1	0.274789	1.070717	0.955427	0.341967	-5.32495	0.736767	0.669436
T.cells	THRB	0.253666	3.815098	0.955298	0.342032	-5.70511	0.692623	0.620336
T.cells	CALML4	-0.42068	1.801954	-0.95514	0.342112	-5.11645	0.724757	0.656139
T.cells	SLC36A3	-0.49443	0.101985	-0.95488	0.34224	-4.93225	0.752961	0.687898
T.cells	FAM168B	0.059465	6.280422	0.954764	0.3423	-6.4108	0.655082	0.57928
T.cells	ZNRD1AS	0.388377	0.28937	0.954718	0.342324	-5.07547	0.749803	0.684329
T.cells	GM43256	-0.38952	0.309245	-0.95471	0.34233	-5.07047	0.749469	0.683952
T.cells	EPCAM	0.302205	2.160039	0.954686	0.342339	-5.37205	0.718942	0.649668
T.cells	FHOD1	0.182625	2.907952	0.954673	0.342346	-5.53868	0.706936	0.636293

T.cells	1700096K1	0.235669	2.613489	0.954543	0.342411	-5.38617	0.71164	0.641527
T.cells	STIL	-0.13552	4.156047	-0.95453	0.342418	-6.2223	0.687314	0.614573
T.cells	ZNHIT1	0.089621	5.422819	0.95452	0.342423	-6.15425	0.667919	0.593278
T.cells	AK6	-0.0887	4.761906	-0.95442	0.342474	-6.21576	0.677973	0.604295
T.cells	SLC29A1	-0.10211	5.215066	-0.95441	0.342478	-6.27525	0.671064	0.596719
T.cells	PSPH	-0.11109	3.906589	-0.95425	0.342558	-6.01746	0.691194	0.618931
T.cells	KIF20A	-0.16414	3.768581	-0.95422	0.342575	-6.14682	0.69335	0.621332
T.cells	ZFP101	0.13593	2.916235	0.954076	0.342646	-5.80949	0.706824	0.636304
T.cells	MAP2K7	-0.1003	4.294187	-0.95391	0.342731	-5.98752	0.685236	0.612396
T.cells	LIPE	0.134627	3.783042	0.953753	0.342809	-5.78191	0.693187	0.621206
T.cells	SGMS1	-0.09826	7.102996	-0.95367	0.342852	-6.555	0.643048	0.566366
T.cells	VPS28	-0.08002	6.686374	-0.95348	0.342946	-6.42526	0.64915	0.573018
T.cells	ZFP160	0.14702	2.950859	0.953335	0.34302	-5.74371	0.70632	0.635825
T.cells	ACP5	0.171067	5.117131	0.953301	0.343037	-6.05428	0.672616	0.598622
T.cells	PPP2R5A	0.053793	7.861178	0.953067	0.343155	-6.70438	0.632194	0.554596
T.cells	LUC7L3	-0.05718	6.706417	-0.95282	0.343277	-6.53156	0.649077	0.57273
T.cells	TRAPPC2	-0.11049	4.171105	-0.9521	0.343642	-6.03174	0.687743	0.614734
T.cells	POU3F1	-0.49238	0.061626	-0.95207	0.343656	-5.02991	0.75437	0.689139
T.cells	SCO1	0.244949	1.930574	0.952071	0.343656	-5.39146	0.723361	0.654272
T.cells	NIT2	-0.16332	3.588667	-0.95198	0.343703	-5.78226	0.696842	0.624806
T.cells	GPC4	-0.43223	1.1587	-0.95173	0.34383	-5.04197	0.736069	0.668556
T.cells	PRR33	-0.39953	0.255516	-0.95166	0.343864	-5.05845	0.751145	0.685592
T.cells	IMMT	0.063353	6.049157	0.951572	0.343908	-6.43192	0.659199	0.583552
T.cells	CAPN10	-0.17498	2.598593	-0.95068	0.34436	-5.61068	0.713421	0.642607
T.cells	SLX4	0.178124	1.820472	0.950163	0.34462	-5.49529	0.726437	0.656853
T.cells	CHST7	-0.39003	0.725254	-0.95005	0.344679	-5.16117	0.744536	0.677177
T.cells	GPSM2	0.153992	2.440784	0.949656	0.344876	-5.84326	0.716649	0.645819
T.cells	SACS	0.183507	2.596254	0.949136	0.345139	-5.78852	0.714302	0.643264
T.cells	SUPT7L	-0.15392	2.767637	-0.9491	0.345158	-5.72828	0.71155	0.640205
T.cells	PEA15A	-0.23431	3.90143	-0.94905	0.34518	-5.38676	0.693591	0.620323
T.cells	INTS1	-0.13333	3.347025	-0.94886	0.345277	-5.91406	0.70232	0.629968
T.cells	SORBS1	-0.1201	4.747678	-0.9487	0.345359	-6.11876	0.680464	0.605894
T.cells	PLA2G6	-0.39255	1.28393	-0.94849	0.345467	-5.10318	0.735704	0.667295
T.cells	SLC17A9	0.216469	2.888147	0.948356	0.345533	-5.6102	0.709621	0.638247
T.cells	MYC	-0.26398	3.533741	-0.94833	0.345545	-5.66421	0.699369	0.626884
T.cells	EGLN2	0.103243	4.887876	0.948319	0.345552	-6.18074	0.678311	0.603696
T.cells	TRPM4	-0.31668	1.109553	-0.94805	0.345687	-5.16103	0.738592	0.670681
T.cells	CHD6	0.08288	5.997264	0.947892	0.345768	-6.39372	0.661504	0.585443
T.cells	TMEFF1	0.611252	-0.59624	0.94786	0.345784	-4.89262	0.767397	0.703249
T.cells	MAFB	-0.32806	4.60884	-0.94779	0.345822	-5.51985	0.682601	0.608531
T.cells	2-Mar	0.078454	5.868752	0.947439	0.345998	-6.35164	0.663431	0.587645
T.cells	PCTP	-0.23662	2.252585	-0.94722	0.346106	-5.36739	0.71985	0.649887
T.cells	RBFOX3	0.595073	-0.24977	0.947094	0.346172	-4.87917	0.761464	0.696718
T.cells	ACOXL	-0.43425	1.285897	-0.94679	0.346328	-5.10162	0.735671	0.667771
T.cells	XYLT2	0.236357	1.972195	0.946666	0.346389	-5.32244	0.724407	0.655208
T.cells	PNN	0.062272	6.310373	0.946654	0.346395	-6.49021	0.656832	0.580692
T.cells	SYDE2	0.41313	-0.10373	0.94659	0.346428	-5.06799	0.758976	0.694101

T.cells	B4GALT6	-0.39752	2.991751	-0.9465	0.346471	-5.10586	0.707966	0.636912
T.cells	TIGAR	0.239597	1.851972	0.946339	0.346555	-5.37642	0.726368	0.657513
T.cells	MED7	0.114265	3.583194	0.946266	0.346592	-5.88978	0.698589	0.626625
T.cells	ITCH	0.078722	7.100446	0.946175	0.346638	-6.51171	0.645181	0.568178
T.cells	IST1	-0.07869	5.536178	-0.94616	0.346645	-6.26109	0.668441	0.593468
T.cells	UGP2	0.105054	5.910415	0.94611	0.346671	-6.25432	0.662806	0.587342
T.cells	PNRC2	0.082904	5.35165	0.945889	0.346783	-6.25611	0.671237	0.596633
T.cells	ZNRF3	-0.11103	5.517512	-0.94581	0.346824	-6.28642	0.668724	0.593919
T.cells	CCDC115	0.103608	4.152653	0.945706	0.346876	-5.98219	0.68967	0.616961
T.cells	CNDP2	-0.11615	5.051865	-0.94562	0.346919	-6.06079	0.675802	0.601759
T.cells	GM43813	-0.12594	4.535806	-0.94539	0.347037	-6.07191	0.683728	0.610539
T.cells	C730034F0	-0.2271	2.636781	-0.94527	0.347096	-5.50706	0.713651	0.643714
T.cells	DET1	-0.21498	2.186379	-0.94515	0.347159	-5.51639	0.720924	0.651916
T.cells	TMEM104	0.220622	3.273099	0.945138	0.347164	-5.48079	0.703491	0.632504
T.cells	VILL	-0.34626	1.228935	-0.9448	0.347333	-5.15304	0.736614	0.66959
T.cells	CELF2	0.062562	8.597509	0.94478	0.347346	-6.73534	0.623639	0.545451
T.cells	GAK	0.069421	6.280821	0.944692	0.347391	-6.32726	0.657272	0.581819
T.cells	TSPAN4	-0.17947	3.356981	-0.94463	0.347422	-5.573	0.702162	0.631171
T.cells	IFNAR1	0.1178	5.246213	0.944621	0.347427	-5.95578	0.672839	0.598837
T.cells	FAM129C	0.110512	3.433215	0.944378	0.34755	-6.08752	0.700956	0.629942
T.cells	BCOR	-0.09536	5.214585	-0.94436	0.347561	-6.24772	0.67332	0.599469
T.cells	NUP98	0.080903	7.461994	0.944223	0.347629	-6.54199	0.639914	0.56316
T.cells	SOX5	0.167619	4.684841	0.94422	0.347631	-6.19543	0.68143	0.608428
T.cells	UBE3C	-0.07748	5.784905	-0.94383	0.347827	-6.34547	0.664691	0.590169
T.cells	ALDH18A1	-0.17888	3.070395	-0.94381	0.34784	-5.75872	0.706713	0.636501
T.cells	GMDS	-0.07419	6.245209	-0.9437	0.347896	-6.441	0.657802	0.582676
T.cells	MTSS1	-0.09807	6.472133	-0.94369	0.347899	-6.53604	0.65443	0.579007
T.cells	POLD1	-0.10611	4.276679	-0.94369	0.347899	-6.15648	0.687741	0.6155
T.cells	SLC11A2	0.124451	3.918763	0.943562	0.347965	-6.08338	0.693328	0.621669
T.cells	D17H6S53I	-0.12207	4.009289	-0.94306	0.348218	-5.81991	0.692294	0.620278
T.cells	ASCC2	0.086927	4.605255	0.94278	0.348362	-6.08514	0.683138	0.610098
T.cells	NUDCD2	-0.10233	4.534449	-0.94272	0.348391	-6.19297	0.684231	0.611306
T.cells	TMEM185I	-0.11602	4.12483	-0.94232	0.348597	-5.95297	0.690615	0.618493
T.cells	DCAF10	0.081582	5.193751	0.942207	0.348654	-6.22726	0.674138	0.600381
T.cells	ARHGAP5	0.176916	4.809305	0.942088	0.348715	-5.90247	0.680021	0.606841
T.cells	PIH1D1	0.088532	5.06576	0.942082	0.348718	-6.23607	0.676091	0.602524
T.cells	GM7854	-0.5404	0.52802	-0.94187	0.348825	-4.93645	0.748854	0.683576
T.cells	NKTR	0.06188	7.193099	0.941785	0.348869	-6.52465	0.644305	0.567875
T.cells	EID2	-0.43303	0.075956	-0.94178	0.348872	-5.01401	0.756486	0.692208
T.cells	GM16023	0.243809	1.834877	0.941643	0.348941	-5.3187	0.727187	0.659274
T.cells	SCRN2	-0.2234	1.43712	-0.94161	0.348957	-5.3335	0.73372	0.666603
T.cells	AHCYL2	-0.10623	6.28947	-0.94119	0.34917	-6.25205	0.65778	0.582504
T.cells	DAP	0.091805	6.19848	0.941094	0.349221	-6.42317	0.659137	0.583999
T.cells	ADAM23	0.350063	3.184267	0.940965	0.349286	-5.36714	0.705585	0.635156
T.cells	FTSJ3	-0.1264	4.016619	-0.94091	0.349314	-6.06637	0.692462	0.620616
T.cells	MRPL9	-0.07425	4.787977	-0.94084	0.349348	-6.21616	0.680504	0.607437
T.cells	ZFP740	-0.08684	4.76726	-0.94073	0.349405	-6.11175	0.680822	0.607842

T.cells	MFSD12	0.200138	2.730413	0.940184	0.349685	-5.41923	0.713279	0.64332
T.cells	2010300CC	0.277334	-0.0882	0.940068	0.349744	-5.26376	0.759917	0.695777
T.cells	CASP8	0.079135	5.513709	0.939886	0.349837	-6.27056	0.669904	0.595431
T.cells	SEC61B	-0.06499	8.272683	-0.93953	0.350018	-6.73195	0.629524	0.551489
T.cells	SCAMP5	-0.39154	1.229049	-0.93938	0.350094	-5.0178	0.738131	0.670981
T.cells	KIF19A	0.262345	1.071139	0.93925	0.350161	-5.29527	0.740767	0.673961
T.cells	ABHD10	0.106346	3.649409	0.938529	0.350529	-5.89884	0.699442	0.62753
T.cells	ACTN1	-0.22154	5.757216	-0.93849	0.350548	-5.58477	0.666915	0.591768
T.cells	TCEAL8	-0.15872	3.331023	-0.93845	0.350569	-5.63982	0.704483	0.633114
T.cells	1700006J1	0.637359	-0.71534	0.938025	0.350787	-4.87606	0.771586	0.708633
T.cells	WAS	0.075974	5.349391	0.937954	0.350823	-6.25168	0.673139	0.598695
T.cells	PPT1	0.090493	6.003515	0.937888	0.350857	-6.37613	0.663251	0.5879
T.cells	ATG101	0.082607	5.222366	0.937826	0.350889	-6.27003	0.675075	0.600826
T.cells	BCKDHB	-0.14145	3.929893	-0.93771	0.350948	-5.8833	0.695074	0.622841
T.cells	KANK2	-0.21191	2.38565	-0.93768	0.350961	-5.54331	0.719694	0.650185
T.cells	1500015AC	0.222646	1.76661	0.936999	0.351312	-5.33876	0.73039	0.661802
T.cells	PLCL2	-0.07803	6.985417	-0.93659	0.351519	-6.53232	0.649468	0.572532
T.cells	PNPLA2	0.101225	5.509886	0.936358	0.35164	-6.28099	0.671643	0.596584
T.cells	ARL1	-0.07289	5.538643	-0.9361	0.351774	-6.31597	0.671211	0.596194
T.cells	CPD	0.125322	4.333844	0.935972	0.351837	-6.01694	0.689739	0.616565
T.cells	1700056E2	-0.30576	1.466557	-0.93581	0.351918	-5.24224	0.735768	0.667786
T.cells	NOP16	-0.10472	4.283714	-0.93578	0.351936	-6.1211	0.69052	0.617452
T.cells	RACK1	-0.04874	8.637453	-0.93568	0.351986	-6.8253	0.62569	0.546978
T.cells	TOMM40	-0.08018	5.225725	-0.93551	0.352072	-6.29993	0.675978	0.601485
T.cells	CCDC6	-0.10287	4.540905	-0.93551	0.352074	-6.13545	0.686521	0.613062
T.cells	BPHL	-0.14597	3.663858	-0.93538	0.352142	-5.78626	0.700257	0.628257
T.cells	ITGAX	0.506316	3.098724	0.93526	0.352202	-4.98506	0.709237	0.638259
T.cells	TRAF1	0.481362	3.343827	0.934943	0.352364	-5.16017	0.705531	0.634104
T.cells	GM26827	-0.30144	1.344192	-0.93476	0.352456	-5.34276	0.738078	0.670457
T.cells	SHISA8	-0.46525	-0.21439	-0.93452	0.352582	-5.03508	0.764417	0.700213
T.cells	KLRB1C	0.539344	1.954564	0.934245	0.352722	-5.10303	0.728081	0.659421
T.cells	CYP4A10	0.50178	1.117903	0.93424	0.352725	-5.16592	0.741905	0.674935
T.cells	HECTD2	0.396756	0.291184	0.9342	0.352745	-5.0463	0.755801	0.690624
T.cells	ZC3H15	-0.05648	7.266033	-0.93407	0.35281	-6.6121	0.645768	0.568857
T.cells	RCAN3	0.262403	2.117276	0.933981	0.352857	-5.26216	0.725421	0.656506
T.cells	SPIDR	0.085821	5.228567	0.933382	0.353165	-6.26038	0.67662	0.602293
T.cells	GM17056	0.324627	2.866748	0.933307	0.353203	-5.34451	0.713667	0.643245
T.cells	D5ERTD579	0.091969	5.221315	0.933249	0.353233	-6.20532	0.67673	0.602443
T.cells	CEP295	0.099321	3.949897	0.933116	0.353301	-6.0699	0.69646	0.62419
T.cells	OTUD3	-0.20853	2.558441	-0.9327	0.353517	-5.55808	0.718949	0.648901
T.cells	UBAP1	0.094752	5.688984	0.93259	0.353571	-6.27059	0.669898	0.594787
T.cells	NAA10	-0.09108	5.084078	-0.93247	0.353632	-6.32373	0.679125	0.604895
T.cells	CHD8	-0.06776	5.733086	-0.93226	0.353743	-6.34995	0.66928	0.594096
T.cells	TFB2M	-0.09807	3.953928	-0.93218	0.353782	-5.98552	0.696736	0.624278
T.cells	DCTD	0.256132	1.018621	0.931821	0.353967	-5.31036	0.744438	0.677535
T.cells	TPM1	-0.12228	5.177563	-0.93174	0.354009	-6.14453	0.677832	0.603511
T.cells	MYBL1	-0.19495	1.840587	-0.93173	0.354015	-5.61219	0.730811	0.662233

T.cells	PLEKHN1	0.21954	1.876111	0.931539	0.354111	-5.43581	0.730256	0.661579
T.cells	PUS3	0.158164	2.37825	0.93146	0.354152	-5.60944	0.722051	0.652406
T.cells	ELMO1	0.066104	8.719508	0.931248	0.354261	-6.77671	0.625598	0.546867
T.cells	TTC23	-0.35142	1.101964	-0.93111	0.354333	-5.13086	0.743094	0.676017
T.cells	CDPF1	0.215367	2.220471	0.931082	0.354346	-5.56038	0.724638	0.655323
T.cells	RTL4	-0.51903	0.402884	-0.9308	0.354493	-5.02203	0.755028	0.68932
T.cells	ATP5G2	-0.05818	8.844228	-0.93056	0.354614	-6.88485	0.62406	0.545109
T.cells	GTPBP6	-0.16696	2.84005	-0.9304	0.354697	-5.67721	0.714864	0.644324
T.cells	CFAP53	-0.36405	0.830789	-0.93033	0.354734	-5.24201	0.74791	0.68136
T.cells	SRP14	0.052025	7.234027	0.930227	0.354787	-6.59748	0.64728	0.570174
T.cells	RASGRP3	-0.22458	3.586569	-0.93008	0.354862	-5.7049	0.70296	0.63113
T.cells	MS4A1	-0.29236	3.936889	-0.92967	0.355071	-5.71859	0.697647	0.625053
T.cells	NUP153	-0.0744	5.848135	-0.92962	0.355098	-6.3476	0.668156	0.592666
T.cells	RBX1	0.059546	7.700144	0.929389	0.355218	-6.6917	0.640754	0.562974
T.cells	KIF9	-0.32173	1.680578	-0.92933	0.35525	-5.17261	0.734084	0.665652
T.cells	EIF3B	-0.07434	5.890853	-0.92913	0.355352	-6.42917	0.667627	0.592124
T.cells	COG7	0.168357	2.604364	0.92888	0.35548	-5.59056	0.719049	0.649001
T.cells	MAX	0.075486	6.499429	0.928823	0.35551	-6.38618	0.658492	0.582326
T.cells	KAT7	-0.06816	5.68662	-0.92867	0.35559	-6.36314	0.670719	0.595673
T.cells	IER3	-0.2077	6.13903	-0.92866	0.355593	-6.14931	0.663888	0.588218
T.cells	ACADSB	0.185731	3.270603	0.928373	0.355742	-5.48411	0.708478	0.637228
T.cells	ST8SIA1	-0.58419	0.877835	-0.92828	0.355788	-4.95341	0.747667	0.681064
T.cells	GSR	0.176422	6.441975	0.928097	0.355884	-5.94235	0.659546	0.583488
T.cells	PSMB9	0.141188	6.228284	0.927936	0.355967	-6.43937	0.662784	0.587
T.cells	USP38	0.081093	5.889969	0.92768	0.356099	-6.30506	0.667952	0.59262
T.cells	RETREG1	0.090613	6.437217	0.927621	0.35613	-6.44688	0.659728	0.583663
T.cells	FBXL2	-0.15859	3.231347	-0.92717	0.356363	-5.72226	0.709545	0.638329
T.cells	RAB11FIP3	0.265241	2.230172	0.927095	0.356401	-5.25637	0.725728	0.656382
T.cells	POM121	-0.08069	4.883475	-0.92702	0.356442	-6.20361	0.683573	0.609698
T.cells	E2F4	-0.0759	5.334947	-0.92682	0.356541	-6.32267	0.676702	0.602165
T.cells	GM14858	-0.19775	2.296719	-0.92655	0.356681	-5.77928	0.724877	0.655323
T.cells	TNFRSF26	0.221702	2.617995	0.926397	0.356761	-5.60942	0.719689	0.649542
T.cells	LPIN1	-0.39477	2.690777	-0.92605	0.356939	-5.14711	0.71864	0.648428
T.cells	DAZAP1	-0.05681	6.776612	-0.92603	0.35695	-6.58784	0.655273	0.578722
T.cells	ZBTB46	-0.29058	3.021217	-0.92558	0.357186	-5.30636	0.713643	0.642742
T.cells	GPX1	0.112484	11.17498	0.925471	0.35724	-7.04826	0.593258	0.512297
T.cells	DPY30	-0.08734	5.414831	-0.92506	0.357455	-6.37854	0.67621	0.60134
T.cells	IDH3A	-0.09079	4.865883	-0.92503	0.35747	-6.2379	0.684654	0.610601
T.cells	EMSY	-0.07401	5.828738	-0.92471	0.357635	-6.4015	0.669908	0.594543
T.cells	PARD6A	0.185694	2.688858	0.924668	0.357655	-5.674	0.719122	0.648846
T.cells	TSN	-0.05424	6.360058	-0.9246	0.357692	-6.51764	0.6619	0.585833
T.cells	SLC33A1	0.143205	3.794664	0.92456	0.357711	-5.77494	0.701417	0.629202
T.cells	DNAJB6	-0.05913	8.310874	-0.92441	0.357791	-6.72259	0.633265	0.55487
T.cells	RABGGTB	-0.11812	3.732854	-0.9244	0.357793	-5.91183	0.702396	0.630285
T.cells	ODC1	-0.09978	5.188947	-0.92393	0.35804	-6.21336	0.679932	0.605368
T.cells	FNTB	0.132487	2.971515	0.923759	0.358126	-5.83087	0.71483	0.643915
T.cells	ZCCHC17	-0.07474	5.162664	-0.92369	0.358164	-6.27913	0.680337	0.605822

T.cells	TMEM161E	-0.08998	4.545354	-0.92359	0.358214	-6.11528	0.689894	0.616348
T.cells	STRADB	-0.15321	3.238382	-0.92354	0.35824	-5.69056	0.710544	0.639183
T.cells	ITGAV	0.10423	6.550452	0.923322	0.358353	-6.43608	0.659397	0.582938
T.cells	HRAS	-0.0903	4.842094	-0.92304	0.358501	-6.12649	0.685391	0.611352
T.cells	MOSPD1	-0.11176	4.817896	-0.92286	0.358591	-6.17576	0.685766	0.611832
T.cells	UNC50	-0.11347	4.105649	-0.92256	0.358748	-5.88903	0.696886	0.624165
T.cells	KANK3	0.351993	2.000717	0.922497	0.35878	-5.2364	0.730741	0.66182
T.cells	TLK1	0.06492	6.854126	0.922305	0.358879	-6.55926	0.654886	0.578245
T.cells	GM9993	0.305969	1.16551	0.92225	0.358908	-5.2387	0.744592	0.677419
T.cells	C030034I2	0.162994	2.630863	0.922228	0.35892	-5.63542	0.720449	0.650377
T.cells	NDUFA12	-0.07881	5.362792	-0.9222	0.358932	-6.31775	0.677371	0.602759
T.cells	PLK3	-0.22005	4.483011	-0.92216	0.358956	-5.63352	0.690973	0.617705
T.cells	NCDN	0.168869	2.826122	0.922116	0.358978	-5.66798	0.717288	0.646858
T.cells	AGO2	0.072408	7.576219	0.921939	0.359069	-6.56573	0.644307	0.566771
T.cells	ATG4D	-0.11405	3.954415	-0.92173	0.359178	-5.96327	0.699403	0.626966
T.cells	TLR7	-0.45049	2.963787	-0.92142	0.359341	-5.07879	0.715203	0.64468
T.cells	SEC14L2	-0.36267	1.70477	-0.92131	0.359398	-5.18154	0.735763	0.667716
T.cells	CSTB	0.110103	6.672627	0.921292	0.359405	-6.37816	0.65771	0.581499
T.cells	PITRM1	-0.12153	3.560314	-0.92127	0.359418	-5.93811	0.70565	0.634128
T.cells	CCDC173	0.306888	0.767334	0.92088	0.359619	-5.22042	0.751714	0.685488
T.cells	FANCA	0.132227	3.05493	0.920727	0.359699	-5.93389	0.714046	0.643288
T.cells	ZBTB10	-0.1478	4.077959	-0.92054	0.359796	-5.90059	0.697807	0.62525
T.cells	INKA1	0.151912	2.703982	0.92044	0.359848	-5.76248	0.719764	0.649618
T.cells	LMAN1L	-0.3522	1.485183	-0.92004	0.360054	-5.19472	0.740074	0.672094
T.cells	ABLIM1	0.083844	6.121137	0.919832	0.360163	-6.49517	0.666666	0.590761
T.cells	RO60	-0.21378	2.493379	-0.91925	0.360464	-5.37649	0.723669	0.653726
T.cells	IL1F9	0.557393	-0.11555	0.91907	0.360559	-4.92426	0.767352	0.702903
T.cells	NIFK	-0.09108	4.620947	-0.91904	0.360576	-6.19742	0.689763	0.616173
T.cells	SUPT5	-0.07402	6.05341	-0.91892	0.360639	-6.39201	0.667779	0.592103
T.cells	4930556J2	-0.19272	2.185085	-0.91883	0.360684	-5.49784	0.728709	0.659443
T.cells	MRPS10	0.096075	4.162492	0.918811	0.360694	-6.08236	0.696942	0.624148
T.cells	A130010J1	0.268888	1.72998	0.918734	0.360734	-5.32029	0.736208	0.667893
T.cells	TM7SF2	-0.29034	1.754706	-0.91861	0.360799	-5.22021	0.735798	0.667494
T.cells	4930549G2	0.140915	2.714781	0.918502	0.360855	-5.76541	0.72007	0.649909
T.cells	AP1S3	-0.10032	5.57812	-0.91826	0.360982	-6.41172	0.674999	0.600144
T.cells	MAT1A	-0.25171	4.65605	-0.91822	0.361003	-5.88455	0.689217	0.61575
T.cells	SLC35F5	-0.20491	3.11698	-0.9182	0.361011	-5.58524	0.713574	0.642698
T.cells	ARHGAP6	0.174528	3.609197	0.918187	0.361019	-5.90797	0.705698	0.633956
T.cells	TRAJ18	0.401237	-1.3552	0.918026	0.361103	-4.89414	0.788993	0.72774
T.cells	TCTA	0.229643	2.098287	0.917777	0.361232	-5.31731	0.730178	0.66131
T.cells	NDUFB11	-0.06164	7.55899	-0.91729	0.361488	-6.6817	0.645424	0.568204
T.cells	XIST	-2.85178	4.173151	-0.91729	0.361488	-5.63917	0.696816	0.624367
T.cells	RC3H2	0.070975	5.728871	0.91711	0.36158	-6.31559	0.672742	0.597934
T.cells	YPEL5	0.089738	6.583092	0.916945	0.361666	-6.42393	0.659858	0.583882
T.cells	NIP7	-0.07666	4.635807	-0.91677	0.361755	-6.19262	0.689574	0.616425
T.cells	CEP83	0.071804	5.662564	0.916735	0.361776	-6.39571	0.673752	0.599056
T.cells	TRDC	-0.69349	1.265066	-0.9167	0.361791	-4.97022	0.743987	0.677034

T.cells	PDE12	-0.09267	3.991564	-0.91669	0.361798	-6.03608	0.699678	0.62759
T.cells	SLC39A10	-0.11952	3.903206	-0.91668	0.361802	-5.92167	0.701075	0.629135
T.cells	SLC23A3	-0.45811	1.461516	-0.91667	0.361809	-5.14831	0.740709	0.673348
T.cells	ARL4A	0.10692	4.453783	0.916507	0.361894	-6.04896	0.692415	0.619626
T.cells	HAAO	0.216185	3.64437	0.916477	0.36191	-5.54558	0.705181	0.633748
T.cells	EML5	-0.15799	3.697759	-0.91636	0.361972	-5.90057	0.704332	0.632827
T.cells	ANKRD50	-0.17694	2.62264	-0.91618	0.362065	-5.42933	0.721671	0.652102
T.cells	ARG2	-0.40037	3.174154	-0.91603	0.362141	-5.23413	0.712787	0.642208
T.cells	EIF4ENIF1	-0.07297	5.556503	-0.91572	0.362306	-6.33592	0.675628	0.601082
T.cells	VSIG10L	0.36401	0.298869	0.915617	0.362359	-5.00341	0.760591	0.695734
T.cells	KLHL15	-0.09414	4.413728	-0.91532	0.362512	-6.10209	0.693363	0.620598
T.cells	TRIM72	-0.38578	0.692552	-0.91524	0.362557	-5.11008	0.753964	0.688272
T.cells	MAP4K5	-0.142	4.52083	-0.9152	0.362575	-5.91626	0.691687	0.618762
T.cells	GPR141	0.811572	2.440611	0.914292	0.36305	-4.95827	0.725256	0.655822
T.cells	NCOR2	0.083868	4.956649	0.914089	0.363156	-6.22013	0.685237	0.611535
T.cells	ALDH3A2	0.140473	3.596521	0.913883	0.363264	-5.69939	0.706607	0.635236
T.cells	ARMC3	0.248898	1.900651	0.913664	0.363378	-5.39698	0.734122	0.666006
T.cells	SPINK10	-0.32448	0.791956	-0.91362	0.363402	-5.2058	0.752643	0.686859
T.cells	ABHD6	-0.21925	2.548845	-0.91356	0.363434	-5.39515	0.72349	0.654126
T.cells	SPSB3	-0.10545	3.587341	-0.91352	0.363455	-5.90282	0.706754	0.635497
T.cells	TRIAP1	0.105061	4.111675	0.913351	0.363542	-6.02549	0.698441	0.626308
T.cells	USF1	0.093219	4.934416	0.913245	0.363597	-6.25683	0.685582	0.612127
T.cells	BLVRB	-0.13238	7.092023	-0.91321	0.363618	-6.55252	0.652907	0.57644
T.cells	KLHL4	0.575638	0.018315	0.913144	0.36365	-4.90289	0.76582	0.701806
T.cells	EIF1AD	-0.0881	4.923218	-0.91263	0.363919	-6.28604	0.685755	0.612461
T.cells	FAM171B	0.54972	0.217464	0.912612	0.363929	-4.97336	0.762408	0.698091
T.cells	APBB2	-0.19798	4.02877	-0.91253	0.363973	-5.94943	0.699749	0.627916
T.cells	ATP6V1G2	0.307683	1.237127	0.912523	0.363975	-5.20045	0.745155	0.678614
T.cells	A630023P1	-0.2745	0.306091	-0.91245	0.364013	-5.43451	0.760894	0.696422
T.cells	TMTC1	0.301124	2.714836	0.91222	0.364134	-5.53881	0.720791	0.651441
T.cells	MTAP	-0.11087	3.671485	-0.91221	0.364141	-6.01834	0.705414	0.63433
T.cells	NELFCD	0.091327	4.117826	0.911891	0.364306	-6.13051	0.698344	0.62662
T.cells	UBTD1	0.1501	4.402162	0.911868	0.364318	-5.80953	0.693875	0.62168
T.cells	CMC1	0.0977	4.601302	0.911515	0.364503	-6.01178	0.690761	0.618296
T.cells	EIF2B2	-0.08597	4.936837	-0.91141	0.36456	-6.28663	0.685544	0.612562
T.cells	FERMT2	-0.25158	3.451103	-0.91133	0.364599	-5.44738	0.708929	0.638454
T.cells	FXR1	-0.06355	6.109299	-0.9111	0.364719	-6.41824	0.667603	0.59296
T.cells	MED28	-0.06641	6.023126	-0.91101	0.364768	-6.42282	0.668906	0.59441
T.cells	PYROXD1	0.141274	2.95488	0.911002	0.364771	-5.77098	0.716903	0.647406
T.cells	2610008E1	0.174683	2.466966	0.910972	0.364787	-5.68389	0.724825	0.656253
T.cells	TRMT5	0.262107	1.213824	0.910817	0.364869	-5.36052	0.745545	0.679539
T.cells	DNAAF2	-0.12558	2.845924	-0.91079	0.364884	-5.85226	0.718665	0.649393
T.cells	SERPINA3F	0.570635	2.19929	0.910698	0.364931	-5.26147	0.729206	0.661208
T.cells	EMC4	-0.0995	4.771124	-0.91063	0.364967	-6.14799	0.688116	0.615542
T.cells	ATP2A1	-0.33093	1.448126	-0.91057	0.364998	-5.20566	0.74163	0.67516
T.cells	AP5Z1	-0.18518	2.539751	-0.91048	0.365045	-5.4763	0.723638	0.654977
T.cells	SUDS3	-0.08387	5.515338	-0.91047	0.365051	-6.33015	0.676636	0.60292

T.cells	DOCK10	0.076967	8.80921	0.91044	0.365066	-6.82887	0.627961	0.550112
T.cells	PCMTD2	0.155449	3.680281	0.910289	0.365145	-5.74755	0.705274	0.63454
T.cells	ZFP59	0.369022	0.230401	0.910266	0.365157	-5.1246	0.762187	0.698411
T.cells	RNF220	0.088106	5.723291	0.91026	0.36516	-6.32524	0.67346	0.59946
T.cells	TBX21	-0.5414	2.437202	-0.91024	0.365171	-5.01761	0.725311	0.65687
T.cells	ALDH8A1	-0.36154	2.197617	-0.90996	0.365316	-5.29702	0.729399	0.661322
T.cells	DEGS2	-0.3627	0.674225	-0.90971	0.36545	-5.14222	0.754869	0.689921
T.cells	ABT1	-0.11594	3.534654	-0.90967	0.365471	-5.87404	0.707814	0.637176
T.cells	RFLNB	-0.18901	3.463749	-0.90944	0.36559	-5.78901	0.709056	0.638447
T.cells	FAM72A	0.2586	1.069392	0.908999	0.365822	-5.37325	0.748664	0.682507
T.cells	GM26916	-0.23047	0.934105	-0.90877	0.36594	-5.36609	0.751058	0.685151
T.cells	KIF2A	-0.06111	6.338703	-0.90828	0.366198	-6.54938	0.66522	0.589691
T.cells	RYBP	-0.09811	5.626574	-0.90777	0.366468	-6.18314	0.676388	0.601713
T.cells	PAQR7	-0.42872	1.552734	-0.90767	0.366518	-5.03145	0.741481	0.673963
T.cells	DCSTAMP	0.383711	-1.33737	0.907479	0.36662	-4.92723	0.791121	0.730288
T.cells	GCFC2	0.151989	2.248325	0.907314	0.366707	-5.65828	0.72997	0.661176
T.cells	NDUFS6	-0.07951	5.716521	-0.90725	0.366741	-6.38519	0.675013	0.600365
T.cells	GSTM7	-0.29956	1.02465	-0.90704	0.366851	-5.14481	0.750332	0.684133
T.cells	GLYCTK	-0.34238	0.979545	-0.90697	0.366889	-5.15646	0.751093	0.684991
T.cells	EIF6	0.072879	5.835967	0.906743	0.367007	-6.41296	0.673192	0.598453
T.cells	PSMB4	-0.06999	6.172049	-0.9067	0.36703	-6.45131	0.66809	0.592886
T.cells	DTYMK	-0.09822	5.126752	-0.90648	0.367147	-6.35299	0.684077	0.610443
T.cells	POU2F1	0.102616	5.39401	0.906292	0.367244	-6.32885	0.679956	0.605984
T.cells	PNPO	-0.21191	3.744874	-0.90626	0.367261	-5.52716	0.705763	0.634451
T.cells	GM14410	0.297147	0.850334	0.906233	0.367276	-5.2022	0.753275	0.68763
T.cells	KRI1	0.094683	4.367396	0.906181	0.367303	-6.02877	0.695915	0.623559
T.cells	ZSWIM4	0.142666	4.579223	0.906135	0.367327	-5.83903	0.692594	0.619893
T.cells	RHOU	-0.53933	1.27725	-0.90588	0.367461	-5.0128	0.74613	0.679529
T.cells	ANKRD22	0.483958	-0.56253	0.905854	0.367475	-4.94308	0.777567	0.715138
T.cells	NUP133	-0.09625	3.691763	-0.90574	0.367535	-6.05773	0.70665	0.635415
T.cells	IKBIP	0.25002	1.938543	0.905187	0.367826	-5.31938	0.735358	0.667364
T.cells	9130019P1	0.497887	0.265967	0.90512	0.367862	-5.00679	0.763511	0.699117
T.cells	KANSL1	-0.06262	8.964116	-0.90507	0.36789	-6.87418	0.627343	0.548848
T.cells	HAGHL	0.202727	2.911144	0.904959	0.367947	-5.5816	0.719431	0.649585
T.cells	FAM222A	0.173702	2.331884	0.904921	0.367966	-5.60015	0.728877	0.660159
T.cells	TAPBP	0.136144	6.597076	0.904484	0.368197	-6.28215	0.662211	0.586461
T.cells	FEM1B	-0.09803	4.728435	-0.90417	0.368365	-6.1805	0.690805	0.617926
T.cells	RIPOR1	0.104928	3.960811	0.903915	0.368497	-6.0459	0.702884	0.631336
T.cells	GM20219	-0.37044	0.646352	-0.90378	0.368567	-5.09007	0.757327	0.692289
T.cells	TMEM238	-0.17527	2.938537	-0.90355	0.368688	-5.75331	0.719275	0.649658
T.cells	IGKV1-117	-0.2565	-0.39916	-0.90352	0.368705	-5.26205	0.775291	0.712787
T.cells	SUN1	-0.10219	3.918531	-0.90333	0.368806	-5.9819	0.703555	0.632263
T.cells	RAD18	0.106445	4.103468	0.903195	0.368877	-6.19753	0.700624	0.629027
T.cells	SEC14L1	-0.12889	4.618739	-0.90317	0.368889	-5.89391	0.692519	0.620069
T.cells	AASDHPPT	-0.10786	3.841771	-0.9031	0.368928	-5.9521	0.704774	0.633636
T.cells	GM49625	-0.26535	2.147374	-0.90296	0.369001	-5.29553	0.732205	0.664243
T.cells	A530088E0	-0.2219	1.760356	-0.90277	0.369099	-5.50684	0.738607	0.671456

T.cells	TMEM30A	-0.05993	6.581779	-0.90264	0.369169	-6.42163	0.66244	0.587219
T.cells	GM15788	0.434123	0.240256	0.902601	0.36919	-5.0549	0.764259	0.700523
T.cells	SRGAP1	-0.25869	2.559229	-0.90252	0.369234	-5.48555	0.725447	0.656811
T.cells	1700047M	0.535359	-0.15234	0.902507	0.36924	-4.9235	0.771015	0.708206
T.cells	CD59A	-0.2702	2.984691	-0.9024	0.369298	-5.32753	0.718528	0.649083
T.cells	CHMP3	0.059787	5.884611	0.902384	0.369305	-6.34152	0.672979	0.598749
T.cells	GM4356	-0.34587	0.868166	-0.90231	0.369344	-5.22302	0.753565	0.688424
T.cells	GTF2H5	-0.06123	6.547345	-0.90227	0.369366	-6.55284	0.662957	0.587822
T.cells	KHDC4	0.071552	6.448578	0.902005	0.369505	-6.42867	0.664442	0.589501
T.cells	RAB12	0.080868	5.073594	0.901952	0.369533	-6.18921	0.685437	0.612505
T.cells	ZC3H10	0.15327	2.921486	0.901924	0.369548	-5.62386	0.719552	0.650314
T.cells	RNASEH2C	-0.08651	4.998911	-0.9015	0.369774	-6.24163	0.686821	0.613847
T.cells	ACBD5	0.073767	6.231959	0.901458	0.369794	-6.37692	0.667928	0.593132
T.cells	PAXIP1	-0.09305	4.269449	-0.9013	0.369879	-6.21206	0.698261	0.626504
T.cells	APOL11B	1.05547	-0.04187	0.901189	0.369936	-4.94487	0.769392	0.706312
T.cells	NAT10	-0.14083	3.270157	-0.90098	0.370047	-5.80998	0.714182	0.644244
T.cells	USP48	-0.07621	5.391239	-0.90096	0.370058	-6.27866	0.680783	0.607309
T.cells	TSNAX	-0.09446	4.49606	-0.90031	0.370399	-6.13995	0.695197	0.622929
T.cells	GDPD1	0.297044	1.600801	0.899827	0.370657	-5.34934	0.74207	0.675359
T.cells	GM11464	0.477878	-0.20123	0.899609	0.370772	-4.92072	0.772702	0.710132
T.cells	CDK20	0.401884	0.593617	0.89949	0.370835	-5.06658	0.75905	0.694682
T.cells	BGN	-0.29201	2.420901	-0.89922	0.370978	-5.29079	0.728503	0.660314
T.cells	HAT1	-0.07778	6.105162	-0.89914	0.371021	-6.47093	0.670358	0.595979
T.cells	ALG10B	-0.15244	2.952601	-0.89902	0.371083	-5.66401	0.719831	0.650665
T.cells	GPR89	-0.12234	3.742407	-0.89894	0.371126	-5.93693	0.707126	0.636521
T.cells	SUB1	-0.06208	8.911934	-0.89868	0.371262	-6.87178	0.629024	0.551251
T.cells	COPB1	0.061659	6.220287	0.898419	0.371402	-6.40099	0.668613	0.594216
T.cells	SLC35A3	-0.07841	4.775291	-0.89824	0.3715	-6.17425	0.690826	0.618639
T.cells	SLC16A12	-0.44431	0.427781	-0.89818	0.37153	-5.03682	0.76188	0.698149
T.cells	TMEM68	0.11532	3.807022	0.898069	0.371588	-5.87807	0.706096	0.635588
T.cells	MPPE1	0.139779	4.363196	0.898031	0.371608	-5.82198	0.697287	0.625825
T.cells	MAPKBP1	0.190276	3.047991	0.897838	0.37171	-5.68198	0.718285	0.649227
T.cells	FBXO10	0.284727	1.355085	0.897795	0.371733	-5.19222	0.74618	0.680533
T.cells	SFMBT1	-0.07378	6.08199	-0.89762	0.371828	-6.49244	0.67071	0.596787
T.cells	DAND5	-0.23383	3.96611	-0.89755	0.371862	-5.79995	0.703565	0.633016
T.cells	FAM174A	0.121558	6.050716	0.897498	0.371891	-6.19789	0.671185	0.597326
T.cells	TBCC	-0.11569	3.579816	-0.89745	0.371914	-5.96136	0.709724	0.639865
T.cells	DPYSL2	0.073705	6.279623	0.897431	0.371926	-6.55663	0.667715	0.593538
T.cells	TOLLIP	0.138181	3.832789	0.897373	0.371957	-5.83221	0.705685	0.635385
T.cells	CPLX2	0.133053	3.378009	0.897349	0.37197	-6.17236	0.712962	0.643486
T.cells	ERMARD	0.143133	3.076068	0.89711	0.372097	-5.75121	0.717831	0.648978
T.cells	SRP9	-0.05286	7.516452	-0.89709	0.372107	-6.67296	0.649263	0.57352
T.cells	MRPS36	-0.07914	5.569029	-0.89706	0.372123	-6.40181	0.67854	0.605472
T.cells	E2F3	-0.09193	5.460693	-0.89705	0.372131	-6.43469	0.680205	0.607301
T.cells	ALKBH7	-0.1429	3.373231	-0.89697	0.372172	-5.85635	0.713038	0.643634
T.cells	PLD3	-0.18729	4.413779	-0.89692	0.372195	-5.69963	0.696491	0.625264
T.cells	SLTM	-0.05874	6.63584	-0.89692	0.372196	-6.53179	0.662351	0.587756

T.cells	CHPF2	0.22449	2.64648	0.896738	0.372294	-5.44788	0.724881	0.656858
T.cells	CCNJ	-0.23395	2.015982	-0.89629	0.372531	-5.501	0.735452	0.668639
T.cells	LMNA	-0.26269	4.617164	-0.89622	0.372571	-5.57067	0.693562	0.621985
T.cells	SELP	0.493414	1.452771	0.896139	0.372612	-5.17058	0.744826	0.67925
T.cells	LCP2	0.297915	5.109024	0.896049	0.37266	-5.49937	0.685896	0.613576
T.cells	GM16174	-0.38631	0.995634	-0.89574	0.372822	-5.01775	0.752516	0.688097
T.cells	TNFSF9	-0.25346	3.435421	-0.89567	0.372861	-5.65741	0.71231	0.642964
T.cells	STAG1	-0.04899	8.353909	-0.89531	0.373055	-6.837	0.637286	0.560749
T.cells	YIPF2	-0.23777	1.926928	-0.89529	0.373065	-5.34936	0.736927	0.670583
T.cells	NSUN2	-0.07888	5.0541	-0.89526	0.373079	-6.30945	0.686748	0.614698
T.cells	GM34983	0.325679	0.754004	0.895207	0.373107	-5.22988	0.75661	0.6928
T.cells	ADAM32	-0.42258	0.784382	-0.895	0.373215	-5.1604	0.756094	0.692216
T.cells	ARPC1A	-0.06531	6.586291	-0.89498	0.373227	-6.51515	0.663347	0.589028
T.cells	BICRAL	0.089678	5.023041	0.894911	0.373265	-6.22963	0.68723	0.61523
T.cells	LGALS3	0.333384	6.142988	0.89485	0.373297	-5.55246	0.670039	0.596343
T.cells	GM10550	0.320945	0.590308	0.894766	0.373341	-5.20604	0.759395	0.695957
T.cells	DOT1L	0.095685	5.289755	0.894659	0.373399	-6.27579	0.683099	0.610679
T.cells	ATL1	-0.39259	0.498179	-0.89414	0.373677	-5.14051	0.761293	0.697964
T.cells	SUMF1	0.131251	4.240634	0.894123	0.373684	-5.90802	0.699785	0.628979
T.cells	ANXA4	0.206776	3.74233	0.893935	0.373783	-5.61825	0.70777	0.637803
T.cells	MAPRE3	-0.35163	1.502586	-0.89329	0.374129	-5.11857	0.74475	0.678982
T.cells	ZFP444	0.125822	3.63547	0.893155	0.374199	-5.94963	0.709825	0.639916
T.cells	SLC44A1	-0.14124	4.637679	-0.89312	0.374219	-5.88382	0.693946	0.622326
T.cells	RAD17	-0.07937	5.023722	-0.89308	0.374239	-6.20917	0.687919	0.615693
T.cells	ASXL2	0.069975	6.836454	0.893005	0.374279	-6.55121	0.66027	0.585398
T.cells	LMTK3	-0.46659	-0.70138	-0.89288	0.374345	-4.93168	0.782507	0.721967
T.cells	DCAF7	0.072987	5.327488	0.892743	0.374418	-6.24662	0.683238	0.610577
T.cells	CLN3	-0.18304	4.768913	-0.8926	0.374495	-5.76318	0.691931	0.620171
T.cells	PHKG1	0.319582	1.107052	0.892497	0.374549	-5.29167	0.751441	0.686686
T.cells	NOLC1	-0.09107	5.264292	-0.89178	0.374929	-6.35681	0.684461	0.611995
T.cells	5730480H(	0.131572	2.846707	0.891756	0.374944	-5.80016	0.722848	0.654591
T.cells	MAP1S	0.128211	3.781603	0.891625	0.375014	-5.91153	0.707771	0.637821
T.cells	MEX3D	-0.16996	2.642534	-0.89139	0.375139	-5.70213	0.72618	0.658404
T.cells	CDC42EP4	0.221948	2.155265	0.891247	0.375216	-5.40367	0.73419	0.667404
T.cells	PPP1CC	-0.05091	7.584523	-0.8912	0.37524	-6.78397	0.649427	0.573802
T.cells	TMEM64	-0.10329	5.051385	-0.89117	0.375255	-6.28589	0.687763	0.615728
T.cells	TTLL3	-0.12251	4.027206	-0.89087	0.375414	-5.98438	0.703859	0.633631
T.cells	MTX2	-0.08117	4.838218	-0.89084	0.375432	-6.23715	0.691085	0.619486
T.cells	C3AR1	-0.56054	2.290307	-0.8908	0.375456	-5.12959	0.731962	0.665021
T.cells	TASOR	0.081541	5.742445	0.890762	0.375475	-6.38321	0.677098	0.604109
T.cells	DENR	-0.06449	6.279481	-0.89069	0.375511	-6.47243	0.668918	0.595189
T.cells	ABCF3	0.109856	3.761251	0.890618	0.375551	-5.91717	0.708096	0.638411
T.cells	KBTBD7	0.235173	2.137804	0.890527	0.3756	-5.36849	0.734478	0.667937
T.cells	PCNX3	-0.14104	3.30596	-0.8904	0.375669	-5.73936	0.715404	0.646611
T.cells	GM28809	0.501349	-0.12461	0.890253	0.375746	-4.92278	0.772763	0.711342
T.cells	LPAR1	0.440473	0.763125	0.890238	0.375754	-5.25256	0.757527	0.694025
T.cells	GM16062	0.222693	1.874018	0.890147	0.375803	-5.33806	0.73885	0.672928

T.cells	MDGA1	-0.51789	0.010829	-0.89002	0.375868	-4.93925	0.770426	0.708682
T.cells	RND3	0.322442	2.943599	0.889413	0.376195	-5.28394	0.721774	0.65356
T.cells	CYP7B1	0.639796	0.01895	0.889306	0.376252	-4.92455	0.770816	0.708953
T.cells	CDKN2D	0.070118	6.09877	0.889048	0.37639	-6.54942	0.672181	0.598719
T.cells	EMP3	-0.08342	7.421524	-0.88864	0.376606	-6.59547	0.652336	0.577124
T.cells	PAX5	0.099845	5.141192	0.888566	0.376648	-6.52568	0.6869	0.61496
T.cells	ULK1	0.142365	3.637915	0.888529	0.376667	-5.78121	0.710619	0.641242
T.cells	SNX29	0.107962	6.395771	0.888494	0.376686	-6.3725	0.667676	0.59386
T.cells	HSCB	-0.12919	4.143832	-0.88846	0.376706	-5.97012	0.702552	0.632296
T.cells	VTI1A	0.066784	7.233705	0.888432	0.376719	-6.59206	0.65512	0.580179
T.cells	FAM71D	-0.40328	0.095273	-0.88826	0.376811	-5.09401	0.769619	0.707674
T.cells	MBP	0.11197	5.172136	0.887323	0.377312	-6.16632	0.687089	0.614813
T.cells	ZFP703	-0.15581	4.364442	-0.88721	0.377371	-5.88862	0.699743	0.628794
T.cells	COTL1	-0.08634	6.629821	-0.88715	0.377404	-6.38451	0.664793	0.590377
T.cells	DDAH1	-0.23436	2.481818	-0.88707	0.377447	-5.42618	0.730088	0.662629
T.cells	SELENOI	-0.13814	3.422495	-0.88704	0.377462	-5.83938	0.714776	0.645525
T.cells	ABHD11	0.139825	3.541149	0.886915	0.377531	-5.82708	0.712878	0.643428
T.cells	ADGRE5	-0.0818	7.153842	-0.88655	0.377728	-6.48157	0.657098	0.581924
T.cells	RNF19B	0.11705	5.758355	0.88653	0.377737	-6.16892	0.678196	0.604976
T.cells	SRSF9	-0.05644	6.428408	-0.88621	0.377906	-6.53933	0.668176	0.593861
T.cells	PPCDC	0.131903	3.567947	0.885932	0.378057	-5.83038	0.712886	0.643179
T.cells	SRC	-0.38419	0.980292	-0.88574	0.378158	-5.08696	0.755639	0.691241
T.cells	POR	0.114687	5.6252	0.885724	0.378169	-6.13134	0.680515	0.60745
T.cells	SUMO1	-0.03544	7.992553	-0.8856	0.378236	-6.78662	0.644978	0.568725
T.cells	MAPK1IP1	0.175301	2.304416	0.885545	0.378265	-5.53539	0.733474	0.666322
T.cells	MVK	0.181307	2.634356	0.885411	0.378337	-5.6137	0.728063	0.66026
T.cells	AA467197	0.722115	0.801131	0.885022	0.378545	-4.94893	0.758921	0.694979
T.cells	ZBTB7A	-0.05914	7.04476	-0.88492	0.378601	-6.55004	0.659195	0.58416
T.cells	LXN	0.088732	3.855864	0.884858	0.378633	-6.18993	0.708491	0.63839
T.cells	DGKZ	0.089982	6.2774	0.884602	0.378771	-6.42904	0.670808	0.596814
T.cells	GIGYF1	0.092914	4.441868	0.88454	0.378804	-6.11467	0.699237	0.628105
T.cells	LYSMD4	0.15879	3.330154	0.88445	0.378852	-5.74618	0.717001	0.647886
T.cells	FBXO44	0.380353	0.220079	0.884019	0.379083	-5.14969	0.769286	0.706575
T.cells	ARPC1B	0.065005	8.892807	0.883792	0.379205	-6.77077	0.63256	0.55518
T.cells	STX12	0.082891	5.552332	0.88343	0.3794	-6.2541	0.682553	0.609452
T.cells	HIRA	-0.07299	5.652742	-0.8833	0.379472	-6.45651	0.681021	0.607804
T.cells	SFXN5	-0.1745	3.363367	-0.88282	0.379729	-5.71757	0.71753	0.647899
T.cells	CPLANE1	-0.10516	4.78519	-0.88241	0.379948	-6.21406	0.695151	0.622825
T.cells	CENPV	-0.15317	3.40694	-0.88226	0.380027	-5.98963	0.71715	0.647228
T.cells	CFAP43	-0.17584	3.09234	-0.88178	0.380289	-5.5551	0.722493	0.653067
T.cells	SHANK3	-0.46556	1.430051	-0.88158	0.380396	-5.07561	0.750037	0.683964
T.cells	RGS18	-0.24834	3.108266	-0.8814	0.380493	-5.4528	0.722234	0.652789
T.cells	BLM	0.128365	3.897628	0.881385	0.380499	-6.19013	0.709491	0.638606
T.cells	CCNQ	0.135241	3.060749	0.881342	0.380522	-5.78413	0.723008	0.653655
T.cells	CCDC189	-0.46404	0.183787	-0.88125	0.380571	-5.01371	0.771319	0.708112
T.cells	CWF19L2	0.083886	5.038452	0.881211	0.380593	-6.22694	0.691444	0.618691
T.cells	RFX2	0.13191	3.630115	0.88096	0.380728	-5.96592	0.713921	0.643495

T.cells	OGFOD3	-0.12465	3.187381	-0.88049	0.380979	-5.88424	0.721386	0.651602
T.cells	GM12064	-0.1969	0.96957	-0.8804	0.381027	-5.57038	0.758298	0.69305
T.cells	ZCCHC14	0.386576	1.412865	0.880314	0.381076	-5.08775	0.750784	0.684593
T.cells	DYNC1LI2	-0.08421	4.672528	-0.88007	0.381208	-6.21381	0.697737	0.625321
T.cells	IGLV1	-0.42903	-0.83837	-0.87978	0.381363	-5.0328	0.789883	0.728878
T.cells	MAGED2	-0.30296	1.585547	-0.87961	0.381457	-5.29758	0.748079	0.68154
T.cells	ZBTB20	0.073315	8.246328	0.879528	0.381499	-6.75888	0.643552	0.566241
T.cells	D230025D1	0.096507	4.57771	0.87947	0.38153	-6.15122	0.699296	0.627125
T.cells	LRRC51	-0.23925	1.76843	-0.87942	0.381557	-5.37185	0.745009	0.678116
T.cells	BCAM	0.611977	0.298097	0.879259	0.381644	-5.01997	0.770037	0.706463
T.cells	TRP53BP1	0.117971	3.888271	0.879087	0.381737	-5.95098	0.71028	0.639409
T.cells	ERP27	0.229936	1.896721	0.879058	0.381753	-5.45208	0.742876	0.675833
T.cells	PABPC1L	-0.21561	2.906471	-0.87861	0.381994	-5.56934	0.7262	0.657371
T.cells	ANO10	0.224336	3.16746	0.878586	0.382007	-5.47845	0.721941	0.652622
T.cells	CCDC163	-0.19135	2.446867	-0.87832	0.382153	-5.70856	0.733757	0.665837
T.cells	HRH2	0.311463	1.160498	0.878164	0.382235	-5.23841	0.755295	0.690078
T.cells	4933412E1	0.212818	1.908564	0.878142	0.382247	-5.58361	0.7427	0.675878
T.cells	ANKRD54	-0.10496	3.089479	-0.87813	0.382256	-5.93755	0.723211	0.65404
T.cells	GM867	0.352235	-0.30876	0.87811	0.382264	-5.19451	0.780607	0.71881
T.cells	ENHO	-0.33577	2.481211	-0.87811	0.382267	-5.2564	0.733189	0.665201
T.cells	PSMA2	-0.06134	7.21693	-0.87796	0.382347	-6.66602	0.658815	0.58308
T.cells	ADK	-0.10228	6.968234	-0.87771	0.38248	-6.56381	0.66266	0.587212
T.cells	B230206L0	0.588214	1.02404	0.877472	0.382608	-5.00283	0.757917	0.692918
T.cells	TCEA2	0.373934	0.335442	0.877306	0.382698	-5.059	0.769776	0.706353
T.cells	FNDC9	0.10639	2.564671	0.877086	0.382817	-5.91422	0.732219	0.663929
T.cells	DMAC2L	-0.18926	2.257897	-0.87694	0.382896	-5.51561	0.737294	0.669677
T.cells	PSMA1	-0.06189	6.9166	-0.8769	0.382919	-6.62263	0.663653	0.588239
T.cells	MSRB1	0.102546	5.958368	0.876292	0.383246	-6.28855	0.678559	0.604382
T.cells	P2RY14	0.311035	3.467146	0.876101	0.383349	-5.34943	0.717845	0.64787
T.cells	PHTF1	-0.10327	4.68503	-0.87595	0.383432	-6.18318	0.69838	0.62634
T.cells	SSX2IP	0.176657	2.481205	0.875819	0.383502	-5.77803	0.733972	0.665987
T.cells	GM44686	0.125429	3.136727	0.875711	0.38356	-5.84412	0.723213	0.653963
T.cells	GAS2	0.160608	2.456745	0.875542	0.383651	-5.67038	0.734377	0.666521
T.cells	GNA13	0.083512	7.201206	0.875415	0.38372	-6.51321	0.659724	0.584089
T.cells	RFWD3	-0.06369	5.6362	-0.87536	0.383749	-6.51778	0.683523	0.610114
T.cells	MIR155HG	0.224658	4.086438	0.875337	0.383762	-5.89164	0.707885	0.637012
T.cells	TBCEL	0.120053	4.594507	0.875197	0.383838	-5.94019	0.69981	0.628108
T.cells	BCL7A	0.094239	4.80999	0.875183	0.383846	-6.43192	0.696411	0.624352
T.cells	TAMM41	-0.14773	2.732365	-0.87511	0.383885	-5.80775	0.729832	0.661531
T.cells	DUSP3	0.100618	5.553941	0.875021	0.383934	-6.12367	0.684796	0.611586
T.cells	BMP8A	-0.32946	2.321943	-0.87411	0.384428	-5.29085	0.736768	0.669349
T.cells	9830166K0	0.424378	-1.08122	0.873908	0.384536	-4.90431	0.795244	0.735744
T.cells	PSMD3	0.082841	4.642778	0.873694	0.384652	-6.22738	0.699199	0.627538
T.cells	KPTN	-0.19178	4.098478	-0.87362	0.384694	-5.71543	0.707846	0.637118
T.cells	KHDRBS1	-0.04727	7.151511	-0.87359	0.384709	-6.63946	0.66061	0.585204
T.cells	SYAP1	0.077863	5.053275	0.873454	0.384782	-6.19078	0.692743	0.620407
T.cells	A430073D2	-0.19503	1.815155	-0.87342	0.384799	-5.56434	0.745218	0.678902

T.cells	PIRB	-0.38292	4.941687	-0.87342	0.384803	-5.39026	0.694492	0.622338
T.cells	IP6K2	0.101825	3.644965	0.873404	0.384809	-5.98332	0.715127	0.645211
T.cells	REX1BD	0.069803	5.688469	0.873291	0.38487	-6.37879	0.682863	0.609532
T.cells	SEC61A2	0.108879	3.741527	0.87325	0.384893	-5.95783	0.713571	0.643479
T.cells	UNC13A	-0.32002	1.183593	-0.87324	0.384896	-5.2245	0.755873	0.690923
T.cells	SLFN9	0.22086	2.410472	0.873206	0.384916	-5.6269	0.735301	0.667756
T.cells	ITSN2	0.070143	6.989544	0.873033	0.38501	-6.52222	0.66304	0.587872
T.cells	ANKRD9	-0.18068	2.821177	-0.873	0.38503	-5.70488	0.728531	0.660197
T.cells	CHMP2B	0.083692	4.813502	0.872872	0.385097	-6.14222	0.696507	0.624615
T.cells	STRIP1	-0.10153	4.156745	-0.87287	0.385098	-6.05168	0.706915	0.63614
T.cells	PHF2	0.066522	5.076145	0.872428	0.385338	-6.33625	0.692385	0.620201
T.cells	EXOSC1	0.084886	4.175365	0.872319	0.385397	-6.16397	0.706618	0.63597
T.cells	SLC26A11	0.283779	3.200265	0.872231	0.385445	-5.35366	0.722334	0.653467
T.cells	GM49602	0.209537	1.89388	0.872143	0.385493	-5.4665	0.743899	0.677698
T.cells	PRPF18	0.090462	4.616004	0.872001	0.38557	-6.16522	0.699622	0.628268
T.cells	BCKDK	-0.08136	4.559456	-0.87199	0.385575	-6.20265	0.700516	0.629258
T.cells	RNF227	-0.18672	1.852006	-0.87196	0.385592	-5.64329	0.7446	0.67849
T.cells	CD209A	-0.60113	0.102824	-0.87192	0.385614	-4.96155	0.774432	0.712273
T.cells	ANKRD39	-0.10896	3.543857	-0.87177	0.385695	-5.85994	0.71676	0.647374
T.cells	LMO7	0.635833	0.761873	0.87154	0.38582	-5.09132	0.763066	0.699511
T.cells	RNF121	0.101945	4.41156	0.871486	0.385849	-6.1622	0.70286	0.631992
T.cells	DDX50	0.058085	6.369428	0.871332	0.385933	-6.46382	0.672419	0.598469
T.cells	ATXN2	0.07136	6.28335	0.871255	0.385975	-6.45423	0.673731	0.599913
T.cells	SNHG10	-0.3842	0.227889	-0.87094	0.386147	-5.14063	0.772263	0.710035
T.cells	DUSP28	0.195005	1.99306	0.870888	0.386174	-5.50813	0.742241	0.676072
T.cells	INPP5D	0.072973	8.764735	0.870664	0.386296	-6.86503	0.63687	0.560035
T.cells	PRRG2	0.207238	2.322172	0.870663	0.386296	-5.42089	0.736764	0.670024
T.cells	EPHX3	-0.34777	0.657793	-0.87058	0.386338	-5.12509	0.76485	0.701755
T.cells	ZFP110	0.107796	4.039873	0.870538	0.386364	-6.01803	0.708783	0.638783
T.cells	LDLR	-0.17134	4.577033	-0.87027	0.386512	-5.96774	0.700238	0.629358
T.cells	2310016D2	0.506613	-0.3578	0.8702	0.386548	-4.95	0.782469	0.721926
T.cells	KCNRG	-0.18698	2.320944	-0.86993	0.386693	-5.56977	0.736785	0.670291
T.cells	MED22	-0.19394	2.384243	-0.86974	0.386799	-5.54546	0.735736	0.66922
T.cells	KLF8	-0.34966	0.979838	-0.8696	0.386872	-5.26735	0.75934	0.695905
T.cells	DACH2	0.305047	0.599965	0.869596	0.386876	-5.34692	0.765844	0.703286
T.cells	TMTC3	0.138536	2.514058	0.869558	0.386897	-5.67775	0.733588	0.66685
T.cells	MUL1	-0.17286	2.950066	-0.86945	0.386956	-5.61893	0.726419	0.658849
T.cells	GM30198	-0.32096	1.865066	-0.86943	0.386966	-5.36928	0.744382	0.679033
T.cells	C2CD5	0.079942	4.918041	0.869429	0.386967	-6.33591	0.694864	0.623733
T.cells	CLEC10A	0.553058	0.930777	0.86928	0.387048	-4.97064	0.760177	0.696952
T.cells	PIGQ	0.115127	3.519745	0.869241	0.387069	-5.9044	0.71715	0.648542
T.cells	UBL7	0.092025	5.130384	0.869174	0.387106	-6.22879	0.691536	0.620109
T.cells	RGS7BP	-0.4231	1.883105	-0.86889	0.387262	-5.23269	0.74408	0.678858
T.cells	STUB1	-0.07064	5.426852	-0.86886	0.387277	-6.34486	0.686916	0.615113
T.cells	CAMSAP1	0.121037	3.53399	0.868761	0.38733	-5.96043	0.716919	0.648392
T.cells	GM16152	0.161459	2.101073	0.868718	0.387353	-5.85278	0.74044	0.674762
T.cells	CEP78	0.111925	3.001386	0.868275	0.387594	-5.9208	0.725889	0.658179

T.cells	DNTT	0.276996	1.449877	0.868105	0.387687	-5.70894	0.751684	0.687241
T.cells	ZFP512	0.10429	3.916117	0.868067	0.387708	-6.08595	0.711069	0.641678
T.cells	MFSD1	-0.0941	5.149534	-0.86777	0.387869	-6.13784	0.691667	0.620075
T.cells	4933423P2	-0.26398	1.804113	-0.86769	0.387915	-5.27668	0.745867	0.680585
T.cells	VPS13D	0.085505	6.149191	0.867439	0.38805	-6.34861	0.676201	0.603078
T.cells	9330160F1	-0.17903	2.295032	-0.86742	0.38806	-5.60656	0.737674	0.671366
T.cells	ARMH2	-0.43014	0.301427	-0.86724	0.388159	-5.05408	0.771471	0.709735
T.cells	CCL8	0.451648	-1.46778	0.86717	0.388196	-4.89779	0.802648	0.745455
T.cells	RBM42	0.058747	6.063078	0.86701	0.388283	-6.45106	0.677521	0.604704
T.cells	RGL2	-0.15578	3.300422	-0.86688	0.388352	-5.71758	0.721154	0.653045
T.cells	EPB41L2	0.070771	6.975502	0.866531	0.388545	-6.62775	0.663664	0.589647
T.cells	S100A16	-0.26969	2.552707	-0.86642	0.388606	-5.30132	0.733407	0.666982
T.cells	GRIA3	-0.26332	3.336555	-0.86631	0.388666	-5.75063	0.720567	0.652628
T.cells	8-Sep	-0.28411	1.954462	-0.8663	0.388671	-5.21426	0.743349	0.678205
T.cells	2500004CC	0.264485	1.043374	0.866214	0.388717	-5.26028	0.758729	0.695598
T.cells	TENM4	-0.39974	1.760128	-0.86621	0.388719	-5.19283	0.746605	0.681879
T.cells	PRPSAP2	0.105496	4.045546	0.866162	0.388746	-5.98143	0.709133	0.639893
T.cells	LRPPRC	-0.08356	5.200579	-0.86608	0.388791	-6.31944	0.69087	0.619684
T.cells	IPO7	-0.08556	5.656347	-0.86571	0.388994	-6.42467	0.68403	0.611981
T.cells	SLC4A2	-0.12281	3.559435	-0.86545	0.389135	-5.83564	0.717355	0.64883
T.cells	CYP39A1	-0.19975	1.483874	-0.86524	0.389248	-5.61476	0.751738	0.687528
T.cells	PRKACB	0.069837	5.548308	0.865159	0.389293	-6.36064	0.685898	0.614024
T.cells	ASB5	0.326242	0.515697	0.864788	0.389495	-5.17895	0.768269	0.706435
T.cells	CNOT1	-0.0547	7.026328	-0.86477	0.389506	-6.6381	0.66333	0.589396
T.cells	SCMH1	0.080218	5.849321	0.864723	0.389531	-6.52785	0.681248	0.60903
T.cells	TMEM97	-0.10916	3.649444	-0.86469	0.389548	-6.06037	0.715964	0.647492
T.cells	CYB5D2	0.248161	1.870839	0.864217	0.389807	-5.40097	0.745483	0.680555
T.cells	PANX1	-0.0873	4.208683	-0.86397	0.389943	-6.16198	0.707224	0.637779
T.cells	1190005I0(	0.230606	1.185776	0.863938	0.38996	-5.36757	0.757052	0.693723
T.cells	SPECC1	0.287187	4.276803	0.863916	0.389972	-5.58625	0.706137	0.636571
T.cells	TMEM189	0.106934	6.387922	0.863855	0.390005	-6.34791	0.673219	0.600236
T.cells	NOTCH3	0.419657	1.028947	0.863759	0.390058	-5.08707	0.759724	0.696759
T.cells	TTLL12	-0.2138	2.217401	-0.86345	0.390225	-5.69229	0.73989	0.674287
T.cells	IRF8	-0.12512	6.49623	-0.86319	0.390367	-6.38549	0.671785	0.598667
T.cells	BTD	-0.20337	2.37707	-0.86304	0.390449	-5.5227	0.737274	0.671415
T.cells	FAM219B	0.196283	2.367885	0.863013	0.390465	-5.5695	0.737426	0.671598
T.cells	PAFAH1B1	0.045576	8.064884	0.862903	0.390525	-6.75235	0.648316	0.573157
T.cells	FLT3L	0.284519	2.201527	0.862839	0.39056	-5.25082	0.740193	0.674758
T.cells	DGKE	0.097571	4.880229	0.862116	0.390956	-6.26284	0.697261	0.626531
T.cells	WDR1	-0.07417	7.335798	-0.86204	0.391	-6.60008	0.659563	0.585171
T.cells	CEP85	0.085393	4.470348	0.862019	0.391009	-6.25536	0.703748	0.633764
T.cells	MLH3	-0.21454	2.01272	-0.86174	0.39116	-5.45296	0.743931	0.678682
T.cells	NUP93	-0.07953	4.586111	-0.86135	0.391374	-6.29699	0.701998	0.631966
T.cells	TM2D3	0.099964	4.227452	0.861262	0.391424	-6.04132	0.707707	0.638324
T.cells	STIM2	0.09054	6.210089	0.86121	0.391452	-6.34318	0.676685	0.604046
T.cells	A530076I1	-0.45974	-0.88246	-0.86107	0.391529	-4.94594	0.793868	0.735723
T.cells	MYZAP	-0.12916	3.284402	-0.86063	0.39177	-5.98793	0.722926	0.655336

T.cells	ELOVL1	-0.10999	4.858115	-0.86056	0.39181	-6.06793	0.697697	0.627251
T.cells	SLC35E3	-0.2431	1.909396	-0.86052	0.391832	-5.40858	0.745662	0.680885
T.cells	GM5431	0.476876	0.742737	0.860488	0.391848	-5.08393	0.765469	0.70332
T.cells	MAT2A	-0.06006	6.522913	-0.86046	0.391862	-6.54185	0.671908	0.598842
T.cells	NACC2	0.370207	1.77434	0.860429	0.39188	-5.12138	0.74793	0.683451
T.cells	PRKACA	-0.09222	4.156404	-0.86029	0.391957	-6.0837	0.708843	0.639658
T.cells	KDM5A	-0.06229	7.014524	-0.86027	0.391966	-6.60171	0.664466	0.590734
T.cells	INSYN2B	0.119307	4.140533	0.860097	0.392062	-5.98898	0.709097	0.639956
T.cells	PSIP1	-0.0598	5.994205	-0.86004	0.392093	-6.572	0.679999	0.607772
T.cells	TUT1	0.136955	2.638669	0.86004	0.392093	-5.63848	0.733522	0.667271
T.cells	MYBBP1A	-0.10012	4.85707	-0.85977	0.392241	-6.29287	0.697714	0.62734
T.cells	ANXA5	0.128408	6.137389	0.859733	0.392261	-6.06581	0.677799	0.605372
T.cells	SYNE2	-0.10802	5.373755	-0.85962	0.392324	-6.28481	0.689612	0.618414
T.cells	GDE1	0.131974	4.883129	0.859556	0.392358	-6.11621	0.697303	0.626949
T.cells	NRG1	-0.52443	2.541088	-0.85942	0.392431	-5.19145	0.735136	0.669229
T.cells	TXLNG	0.082408	5.107247	0.859327	0.392484	-6.26593	0.69378	0.623125
T.cells	MOCOS	-0.51738	1.324581	-0.85915	0.392582	-4.99982	0.755531	0.692327
T.cells	ITPK1	-0.07786	5.403224	-0.85902	0.392652	-6.29539	0.689152	0.618057
T.cells	NPR1	-0.39	1.263007	-0.85887	0.392737	-5.11622	0.756577	0.693519
T.cells	AGA	0.160732	3.27288	0.858739	0.392807	-5.71623	0.723114	0.655826
T.cells	ZFP606	0.134106	2.487995	0.858689	0.392834	-5.75591	0.736015	0.670312
T.cells	QTRT2	0.280962	1.344368	0.858615	0.392875	-5.38401	0.755195	0.691967
T.cells	INTS8	-0.07676	4.693172	-0.85859	0.392888	-6.25368	0.700302	0.630415
T.cells	FAM98B	0.081686	4.474383	0.858256	0.393072	-6.24226	0.703944	0.634303
T.cells	MLX	-0.11899	3.767938	-0.85809	0.393161	-5.89027	0.71526	0.646962
T.cells	DNAH8	0.110359	3.758702	0.858063	0.393178	-6.19485	0.715409	0.647128
T.cells	GPD1L	-0.08182	5.296236	-0.85795	0.393237	-6.41788	0.690991	0.620036
T.cells	STXBP6	-0.42437	2.953526	-0.85771	0.393374	-5.27255	0.728652	0.661921
T.cells	OTULIN	-0.05768	6.57022	-0.85754	0.393464	-6.49809	0.671511	0.598571
T.cells	ATP8B1	-0.39181	0.813793	-0.85726	0.393621	-5.09796	0.764616	0.702686
T.cells	FAM210B	0.169308	3.914295	0.857174	0.393666	-5.68302	0.713069	0.644668
T.cells	KCNG3	-0.47795	0.517714	-0.85708	0.393721	-4.99121	0.769714	0.708548
T.cells	GM26839	0.351982	0.656622	0.857037	0.393741	-5.15866	0.767318	0.705831
T.cells	GPANK1	0.114746	3.300008	0.856982	0.393772	-5.90855	0.723018	0.655833
T.cells	SOD3	-0.37706	1.286857	-0.85653	0.394018	-5.1737	0.756796	0.693761
T.cells	TCF25	-0.05296	6.939299	-0.85634	0.394122	-6.57007	0.66615	0.592864
T.cells	GM42701	-0.29642	0.844253	-0.85631	0.394139	-5.29753	0.764358	0.702438
T.cells	CD99L2	0.174569	2.142314	0.856168	0.394219	-5.54966	0.742377	0.677573
T.cells	CBFB	-0.04629	6.809866	-0.85612	0.394243	-6.64506	0.668106	0.595042
T.cells	POU5F1	-0.27499	1.26222	-0.85586	0.394389	-5.26999	0.757215	0.694432
T.cells	ZFP830	0.107965	3.541014	0.855838	0.394401	-5.97196	0.719349	0.651796
T.cells	CIB2	0.307268	2.152566	0.855815	0.394413	-5.16768	0.742206	0.677472
T.cells	HMGB3	-0.09714	4.658182	-0.85533	0.394683	-6.42878	0.701486	0.631962
T.cells	NPEPL1	-0.08331	5.049543	-0.85512	0.394795	-6.25565	0.695309	0.625211
T.cells	BAG6	-0.0826	5.438014	-0.85511	0.394799	-6.37767	0.689229	0.618485
T.cells	GPR137B	-0.18538	5.316315	-0.85496	0.394882	-5.7476	0.691128	0.620663
T.cells	TTL	-0.20195	1.740811	-0.85483	0.394955	-5.46685	0.749167	0.685677

T.cells	E430024I08	0.181831	1.898133	0.85455	0.39511	-5.55194	0.746521	0.682755
T.cells	POT1B	0.111893	4.421725	0.854505	0.395134	-6.16129	0.705242	0.636462
T.cells	ARF1	-0.0486	8.02586	-0.85447	0.395152	-6.76647	0.649985	0.575703
T.cells	MBD2	-0.05561	7.044678	-0.85445	0.395163	-6.6472	0.664609	0.591642
T.cells	LYPLA2	-0.08541	4.945046	-0.85445	0.395165	-6.17589	0.696953	0.627257
T.cells	ERGIC2	-0.05245	6.017059	-0.85439	0.395197	-6.47516	0.680258	0.608813
T.cells	BORCS5	-0.09674	4.024358	-0.85433	0.395233	-6.06077	0.711598	0.643541
T.cells	GIT2	0.054279	7.020155	0.854128	0.395342	-6.64738	0.664978	0.592046
T.cells	VPS37C	0.113979	3.950923	0.853978	0.395425	-5.84047	0.712778	0.644921
T.cells	MLF2	-0.07059	5.85253	-0.85386	0.395489	-6.41141	0.682796	0.611694
T.cells	ANKS1B	-0.36676	1.030517	-0.85386	0.395493	-5.13708	0.761222	0.699517
T.cells	FARS2	0.052945	6.899126	0.853813	0.395516	-6.6068	0.666805	0.594128
T.cells	VCL	0.084874	6.049702	0.853197	0.395856	-6.44917	0.679772	0.608619
T.cells	TCF19	-0.12924	3.199871	-0.85313	0.395893	-6.10577	0.724973	0.658888
T.cells	ORC6	-0.08292	5.216442	-0.85305	0.395938	-6.45178	0.692707	0.622948
T.cells	ZFAND5	-0.0582	7.203792	-0.85298	0.395976	-6.58516	0.662232	0.589418
T.cells	SLX4IP	0.08547	4.853222	0.852608	0.39618	-6.2806	0.698418	0.629406
T.cells	GBA	-0.14122	4.090254	-0.85255	0.396213	-5.75015	0.710557	0.642916
T.cells	IGLC3	-0.50831	4.04289	-0.8524	0.396298	-5.46913	0.711317	0.643828
T.cells	ARHGAP11	0.087424	4.784218	0.852357	0.396319	-6.38524	0.699508	0.6307
T.cells	FUNDC1	-0.08481	4.65588	-0.85231	0.396347	-6.19319	0.701539	0.632965
T.cells	SYK	0.070689	8.336181	0.85215	0.396433	-6.87255	0.645439	0.571348
T.cells	DOCK9	-0.13327	4.197961	-0.85197	0.396532	-6.09472	0.708832	0.641156
T.cells	PDZD4	-0.2843	1.698737	-0.85195	0.396541	-5.32578	0.749894	0.687319
T.cells	ANKHD1	-0.06101	7.647791	-0.85193	0.396553	-6.70481	0.655599	0.582444
T.cells	BUB1B	0.106047	4.016283	0.851884	0.39658	-6.28263	0.711744	0.644406
T.cells	ZBTB26	0.285292	0.850706	0.85173	0.396665	-5.27534	0.76432	0.703708
T.cells	ACKR3	-0.51907	0.835269	-0.85172	0.396671	-5.13972	0.764585	0.70401
T.cells	EEPD1	0.1629	4.881821	0.851694	0.396685	-5.86014	0.697967	0.629066
T.cells	GM14798	-0.10239	3.718232	-0.85146	0.396814	-6.01782	0.716547	0.649784
T.cells	TRP53I13	0.118944	3.021819	0.851131	0.396996	-5.84404	0.727888	0.662629
T.cells	MYO9B	0.078067	5.866728	0.851112	0.397006	-6.37238	0.682593	0.612183
T.cells	FXYD2	0.493153	-0.41192	0.851015	0.39706	-4.98765	0.786273	0.728984
T.cells	SCNM1	-0.1092	3.621272	-0.85093	0.397107	-5.95902	0.718116	0.651702
T.cells	GM20528	-0.43335	-0.01428	-0.85087	0.397137	-5.01392	0.779298	0.721004
T.cells	GM26881	-0.43898	0.245715	-0.8508	0.397177	-5.04207	0.774768	0.715819
T.cells	ATP6V1C1	0.075063	5.189889	0.850727	0.397219	-6.25017	0.693123	0.623859
T.cells	FLT1	-0.36093	4.243341	-0.85058	0.397303	-5.65149	0.708106	0.640516
T.cells	VGLL4	-0.07155	6.477378	-0.85051	0.397341	-6.51178	0.673222	0.601893
T.cells	GOLIM4	-0.13533	5.140893	-0.85048	0.397355	-6.09826	0.693891	0.62471
T.cells	RHOD	-0.31913	1.328394	-0.85048	0.397358	-5.19333	0.756163	0.694614
T.cells	1700123M	-0.26725	0.89752	-0.85021	0.397504	-5.31034	0.763579	0.703068
T.cells	COA3	-0.07588	5.812938	-0.85006	0.397586	-6.40392	0.68348	0.613244
T.cells	PMVK	0.106012	4.327226	0.84998	0.397632	-6.06348	0.706823	0.639179
T.cells	TACC1	-0.05037	7.703473	-0.84996	0.397643	-6.71926	0.654825	0.581843
T.cells	CENPA	-0.09777	6.681539	-0.84943	0.397935	-6.72297	0.670512	0.598815
T.cells	TSPAN33	0.328112	2.55857	0.849366	0.397972	-5.30279	0.735958	0.671633

T.cells	ZFAND1	0.138025	2.727292	0.84919	0.398069	-5.69695	0.733169	0.668488
T.cells	RIN2	0.217498	3.687666	0.849135	0.398099	-5.53859	0.717468	0.650862
T.cells	TMEM177	-0.35558	0.336385	-0.84875	0.398314	-5.15011	0.773734	0.714417
T.cells	8030453O2	0.435933	-0.75227	0.848565	0.398415	-4.92733	0.792842	0.736325
T.cells	MEX3C	0.075742	5.133199	0.84846	0.398473	-6.28569	0.694497	0.625229
T.cells	MRPL32	0.07464	5.68103	0.848334	0.398543	-6.40715	0.685946	0.615827
T.cells	APIP	-0.08116	4.220389	-0.84829	0.398569	-6.1759	0.708968	0.64141
T.cells	FKBP1A	-0.05806	7.336878	-0.84805	0.398702	-6.73292	0.660699	0.588151
T.cells	NHP2	-0.07976	5.748278	-0.84796	0.398751	-6.49344	0.684903	0.614746
T.cells	TRAPPC12	0.086158	4.201517	0.847916	0.398774	-6.11701	0.70927	0.641795
T.cells	PDS5A	-0.04957	7.411745	-0.84776	0.39886	-6.75362	0.659578	0.586938
T.cells	MSN	0.063129	8.454473	0.847701	0.398894	-6.81841	0.644159	0.570158
T.cells	PPM1N	0.532985	0.159456	0.847186	0.399179	-4.96832	0.776811	0.718304
T.cells	THAP3	0.117186	3.799217	0.847136	0.399207	-5.92364	0.715739	0.649181
T.cells	BRIP1OS	0.068639	5.408086	0.847104	0.399224	-6.45505	0.690194	0.620752
T.cells	MMAB	-0.24696	1.326097	-0.84705	0.399257	-5.40913	0.75673	0.695435
T.cells	LAGE3	-0.0801	4.771468	-0.84692	0.399326	-6.27395	0.700198	0.63191
T.cells	MFAP1A	0.070014	4.832089	0.846563	0.399524	-6.27477	0.69924	0.630928
T.cells	PPIB	-0.06394	7.605608	-0.84646	0.399581	-6.66798	0.656685	0.584064
T.cells	BAZ2A	0.071571	6.97841	0.846378	0.399627	-6.62409	0.666089	0.594368
T.cells	SLC31A1	0.090374	5.429507	0.846264	0.39969	-6.25065	0.68986	0.620571
T.cells	PPM1B	0.06232	6.192574	0.846263	0.399691	-6.50209	0.678051	0.607526
T.cells	PIP4K2B	0.106356	4.178712	0.846241	0.399703	-5.97987	0.709635	0.64256
T.cells	PTBP1	-0.06387	6.221744	-0.84623	0.399707	-6.53806	0.677603	0.607033
T.cells	NECAP1	0.08348	4.774239	0.846226	0.399711	-6.16005	0.700155	0.631996
T.cells	DCTN2	0.078227	5.367124	0.846168	0.399743	-6.29025	0.690834	0.621669
T.cells	LIFR	0.283534	3.842745	0.845957	0.39986	-5.486	0.715037	0.648647
T.cells	GM50020	0.365996	-0.11132	0.845772	0.399963	-5.058	0.781541	0.724085
T.cells	KANTR	0.166156	2.529607	0.845652	0.400029	-5.69977	0.736518	0.672919
T.cells	FMO1	-0.35185	2.23787	-0.84538	0.400182	-5.34181	0.741371	0.678509
T.cells	CCAR1	0.049158	6.820278	0.845309	0.40022	-6.63536	0.66848	0.597271
T.cells	HMCN1	-0.33954	1.920186	-0.84528	0.400239	-5.44878	0.74669	0.684539
T.cells	RSL1D1	-0.06898	6.043414	-0.8452	0.400279	-6.52192	0.680344	0.61033
T.cells	GM10603	0.338003	0.220408	0.845191	0.400286	-5.21609	0.77575	0.71764
T.cells	POLR2C	-0.0738	5.080792	-0.84512	0.400326	-6.34432	0.695321	0.626908
T.cells	VCP	-0.05299	7.431716	-0.84508	0.400346	-6.6843	0.65928	0.587203
T.cells	KRTCAP2	-0.06446	7.062528	-0.84477	0.400519	-6.60757	0.66482	0.593355
T.cells	ZDHHC5	-0.06878	5.017726	-0.84473	0.400539	-6.23517	0.696312	0.628133
T.cells	NAIP1	0.447502	-0.47909	0.844647	0.400588	-5.00622	0.788007	0.731852
T.cells	ARHGAP28	-0.41974	0.598833	-0.84464	0.400593	-5.18032	0.769191	0.710282
T.cells	HINT1	-0.05746	7.995864	-0.84455	0.400641	-6.8288	0.650898	0.578176
T.cells	SS18	-0.06622	5.946104	-0.84442	0.400713	-6.48994	0.681844	0.612147
T.cells	ATP5K	-0.07363	7.486519	-0.8443	0.400779	-6.73192	0.658461	0.58647
T.cells	GADD45B	-0.14978	5.322838	-0.84419	0.400841	-6.12048	0.691526	0.622921
T.cells	SPATS2L	-0.46842	0.9742	-0.84416	0.40086	-5.04977	0.762736	0.703034
T.cells	RFXAP	-0.08173	4.179927	-0.84389	0.40101	-6.13017	0.709672	0.64316
T.cells	C920021L1	0.170116	2.268205	0.843873	0.401018	-5.57004	0.740924	0.678297

T.cells	TTC27	0.112675	3.520631	0.843127	0.401433	-5.98848	0.720585	0.655402
T.cells	BC005537	-0.06843	7.116434	-0.84313	0.401433	-6.5197	0.664316	0.592973
T.cells	FOXK1	0.085487	4.627409	0.843001	0.401503	-6.16424	0.702807	0.635533
T.cells	ACTN4	-0.05205	6.545001	-0.84285	0.401588	-6.59469	0.672973	0.602523
T.cells	SYN1	-0.4852	0.783746	-0.84284	0.401595	-5.01363	0.76636	0.707273
T.cells	GRAMD1A	0.130869	4.325204	0.842825	0.401601	-5.95372	0.70762	0.640933
T.cells	FN3KRP	-0.26462	1.481688	-0.8428	0.401615	-5.35373	0.754438	0.693702
T.cells	ACVR1B	-0.17337	2.543644	-0.84229	0.401897	-5.57285	0.737027	0.673733
T.cells	FAM107B	-0.07005	7.858238	-0.84214	0.401982	-6.75313	0.653626	0.581096
T.cells	VIL1	0.471609	0.018277	0.841792	0.402176	-5.03395	0.78024	0.722869
T.cells	GNAL	0.241059	1.371741	0.841784	0.40218	-5.33855	0.756892	0.696202
T.cells	CDK9	0.070272	5.578202	0.841598	0.402284	-6.4011	0.688432	0.619353
T.cells	FBLIM1	0.466837	0.928973	0.841519	0.402327	-5.07171	0.764498	0.704872
T.cells	GM42917	-0.29439	1.691044	-0.84138	0.402406	-5.23157	0.751545	0.690129
T.cells	SRPK2	0.075482	7.096447	0.841227	0.40249	-6.57224	0.665227	0.593772
T.cells	FAM83E	0.290704	1.10583	0.841092	0.402565	-5.24816	0.761558	0.701544
T.cells	ABHD18	-0.17362	3.182254	-0.84071	0.402778	-5.69378	0.727062	0.662295
T.cells	PEX11B	0.118138	3.446594	0.840494	0.402899	-5.90519	0.722844	0.657488
T.cells	COX18	0.127436	3.223872	0.840146	0.403093	-5.85481	0.726592	0.661646
T.cells	TACO1OS	0.243135	1.705089	0.840093	0.403122	-5.3953	0.751868	0.690225
T.cells	GM17491	0.197159	1.415352	0.840011	0.403168	-5.36107	0.756781	0.695826
T.cells	DHX32	0.101987	3.380004	0.839931	0.403212	-5.98766	0.724039	0.65883
T.cells	PDCD10	0.053564	6.713222	0.839189	0.403627	-6.56638	0.672021	0.600626
T.cells	MADD	-0.09248	5.091113	-0.83871	0.403893	-6.19134	0.697148	0.628546
T.cells	NFKBIL1	-0.10695	3.861655	-0.83868	0.403909	-5.91853	0.716778	0.650436
T.cells	RWDD3	0.302907	0.507573	0.838587	0.403963	-5.2132	0.772974	0.714069
T.cells	GM27188	-0.33295	0.619818	-0.83857	0.403974	-5.3054	0.77103	0.711854
T.cells	SH3BGRL3	-0.06805	9.061448	-0.83842	0.404057	-6.886	0.63716	0.562875
T.cells	MAP3K7	-0.06966	5.219048	-0.83831	0.404118	-6.30019	0.695135	0.62644
T.cells	IGKV12-46	0.287848	-0.77013	0.838211	0.404173	-5.20326	0.79543	0.739929
T.cells	PTPN2	-0.0563	6.833794	-0.83815	0.404204	-6.58504	0.670188	0.598904
T.cells	GM37401	-0.31435	0.875344	-0.83811	0.404231	-5.19572	0.76662	0.706906
T.cells	NPRL2	-0.13473	2.790262	-0.83798	0.404304	-5.74529	0.734303	0.670301
T.cells	CCND3	0.07856	7.994145	0.837702	0.404457	-6.91067	0.652786	0.579933
T.cells	ELAVL1	-0.04106	7.604056	-0.83758	0.404524	-6.77583	0.658588	0.586264
T.cells	SMIM27	0.109926	3.590898	0.837582	0.404524	-5.92126	0.721169	0.655559
T.cells	PDZK1	-0.45053	0.933701	-0.83743	0.404609	-5.07145	0.765616	0.705896
T.cells	4833407H1	-0.19178	2.098821	-0.83738	0.404636	-5.44217	0.745824	0.683419
T.cells	OSCP1	-0.19952	3.25254	-0.83727	0.404698	-5.764	0.726693	0.661863
T.cells	GM47662	0.447405	-0.79475	0.837171	0.404754	-4.9491	0.795868	0.740666
T.cells	GLO1	0.09439	5.029475	0.836821	0.404949	-6.27693	0.69812	0.630081
T.cells	GM15848	-0.4057	-0.86297	-0.8368	0.404961	-5.01363	0.797084	0.742217
T.cells	OSTF1	0.052882	7.480967	0.836592	0.405077	-6.59999	0.660429	0.588569
T.cells	BOLA3	0.081804	5.736487	0.836545	0.405104	-6.45326	0.687047	0.617858
T.cells	GM3550	0.14239	1.752212	0.836539	0.405107	-5.72509	0.751662	0.690302
T.cells	MBOAT2	0.40355	0.666194	0.83635	0.405213	-5.23873	0.770228	0.711475
T.cells	GM27017	-0.12377	3.311249	-0.83635	0.405213	-6.02253	0.725732	0.661032

T.cells	ST3GAL5	-0.12375	6.23691	-0.83633	0.405221	-6.27035	0.67931	0.609331
T.cells	REXO4	0.085068	4.291897	0.835886	0.405472	-6.15409	0.70985	0.643397
T.cells	ZFP142	-0.13435	3.207748	-0.83587	0.405481	-5.74881	0.727427	0.663109
T.cells	PPTC7	0.087684	5.148669	0.83579	0.405526	-6.27272	0.696242	0.628283
T.cells	CKS1B	-0.10896	5.377173	-0.83573	0.405558	-6.53986	0.692654	0.624324
T.cells	GRK6	0.0717	5.764162	0.835617	0.405623	-6.41249	0.686617	0.617683
T.cells	NOC4L	-0.0897	3.728892	-0.83545	0.405716	-6.11851	0.718928	0.653737
T.cells	WDR76	-0.09828	4.504143	-0.83535	0.405773	-6.35517	0.706456	0.639791
T.cells	LY6E	0.087933	9.08245	0.835165	0.405876	-6.92654	0.636856	0.563299
T.cells	OTUD6B	0.079832	4.301102	0.834894	0.406028	-6.11248	0.709703	0.643608
T.cells	TMEM115	0.101752	3.447731	0.834749	0.406109	-5.88451	0.723502	0.659137
T.cells	GEMIN2	0.108541	3.260952	0.834671	0.406152	-5.98436	0.726555	0.662606
T.cells	MYO1G	0.102836	5.563064	0.834533	0.40623	-6.27534	0.689748	0.62155
T.cells	BRD1	0.068664	5.991255	0.834508	0.406244	-6.45676	0.683097	0.614184
T.cells	ZFP91	0.05211	6.894237	0.834064	0.406493	-6.6551	0.669271	0.599028
T.cells	GM13684	0.134057	3.516914	0.833991	0.406533	-5.94031	0.722374	0.65808
T.cells	IBTK	0.098616	4.642613	0.833987	0.406536	-6.19221	0.70425	0.637781
T.cells	CD300C2	-0.32389	4.335062	-0.83383	0.406625	-5.4198	0.709159	0.643287
T.cells	GM14295	-0.22938	1.289141	-0.83377	0.406656	-5.32999	0.759527	0.700194
T.cells	BACE2	-0.39708	1.120623	-0.83353	0.406793	-5.09795	0.762408	0.703594
T.cells	POLR3C	0.065582	5.018329	0.833509	0.406804	-6.34344	0.698296	0.631294
T.cells	NPHP3	-0.30501	1.097931	-0.83349	0.406815	-5.22354	0.762797	0.704044
T.cells	2610001J0	0.123407	3.690163	0.833377	0.406878	-5.8757	0.719556	0.65511
T.cells	ARRDC3	0.203109	3.403751	0.833289	0.406927	-5.61785	0.72422	0.660353
T.cells	EFTUD2	-0.07046	5.105706	-0.83319	0.406985	-6.37489	0.696918	0.629849
T.cells	5031434O1	-0.34664	0.524887	-0.83305	0.407062	-5.09891	0.772674	0.71549
T.cells	RNF31	0.176951	3.162894	0.83282	0.407191	-5.60723	0.728163	0.664984
T.cells	REEP6	-0.31782	1.791912	-0.83276	0.407224	-5.27387	0.750991	0.690849
T.cells	MOSPD3	-0.08045	4.656046	-0.83271	0.40725	-6.23683	0.704036	0.637962
T.cells	LANCL2	0.12391	2.807486	0.83255	0.407342	-5.9293	0.734018	0.671707
T.cells	TLK2	-0.05368	6.825344	-0.83214	0.407574	-6.61118	0.670316	0.600744
T.cells	CABLES1	-0.37363	4.513404	-0.83211	0.407589	-5.48398	0.706308	0.640682
T.cells	YWHAG	-0.07681	6.203239	-0.83207	0.407611	-6.43058	0.679828	0.61124
T.cells	RNF144A	0.114091	3.619246	0.832025	0.407636	-6.00231	0.720708	0.656829
T.cells	GM15446	0.182301	2.166881	0.832024	0.407637	-5.64326	0.744682	0.68391
T.cells	CKS2	-0.08979	5.959802	-0.83197	0.407668	-6.56661	0.683584	0.615397
T.cells	MPP7	0.172784	7.383215	0.831969	0.407668	-6.44328	0.661894	0.591488
T.cells	ZFYVE9	-0.34145	2.603777	-0.83196	0.407671	-5.28175	0.737394	0.675651
T.cells	ADAT2	0.212899	1.285306	0.831963	0.407671	-5.52237	0.759592	0.700877
T.cells	ABCA13	0.588681	0.73813	0.831714	0.407811	-5.03204	0.768985	0.711739
T.cells	IRAK4	0.123451	4.419459	0.831564	0.407896	-5.84551	0.707809	0.642492
T.cells	GM20033	-0.41974	0.595708	-0.83151	0.407924	-5.0735	0.771447	0.714602
T.cells	ANGEL2	0.066556	5.052327	0.831349	0.408016	-6.31749	0.69776	0.631322
T.cells	SIPA1	0.068462	5.681522	0.831276	0.408057	-6.39714	0.687902	0.620363
T.cells	HECTD1	0.056395	7.428886	0.83113	0.408139	-6.65037	0.661209	0.590963
T.cells	SLC26A10	0.424027	0.914242	0.830997	0.408214	-5.08475	0.76595	0.708454
T.cells	C130036L2	0.213067	1.354595	0.830937	0.408248	-5.41488	0.758411	0.699841

T.cells	SMYD4	-0.14959	2.820357	-0.83081	0.408317	-5.89516	0.733805	0.671896
T.cells	CAND1	-0.06668	5.132169	-0.83057	0.408456	-6.33368	0.696501	0.630086
T.cells	HMGCLL1	0.172658	1.17582	0.830526	0.408478	-5.76802	0.761463	0.703397
T.cells	NR4A1	-0.20513	7.413157	-0.83048	0.408505	-6.18881	0.661445	0.591318
T.cells	EHBP1L1	0.070195	6.149657	0.830389	0.408556	-6.45684	0.680653	0.612487
T.cells	BUD13	0.093883	3.714639	0.830206	0.408658	-6.05408	0.719159	0.655445
T.cells	BTBD8	0.230436	1.572677	0.830098	0.408719	-5.47512	0.754702	0.695719
T.cells	SLC7A7	0.125696	3.785978	0.829892	0.408835	-6.03931	0.718003	0.65422
T.cells	CEP63	-0.08847	4.291786	-0.82986	0.408851	-6.21172	0.709852	0.645074
T.cells	LMF2	-0.13456	3.103696	-0.82979	0.408893	-5.83212	0.729135	0.66676
T.cells	2610507I0:	-0.41631	0.583661	-0.82966	0.408968	-5.08767	0.771656	0.715144
T.cells	ZFP119A	-0.16681	2.125414	-0.82958	0.409009	-5.54123	0.745378	0.685166
T.cells	RENBP	0.129208	4.474184	0.829564	0.40902	-5.93078	0.706934	0.641821
T.cells	F930017D2	-0.52718	0.898224	-0.82956	0.409021	-5.07646	0.766226	0.708939
T.cells	0610009E0	0.266237	0.843747	0.829517	0.409046	-5.2306	0.767164	0.710043
T.cells	4932438A1	0.091436	7.087671	0.829089	0.409287	-6.54707	0.66657	0.596927
T.cells	PIP4P1	0.053451	5.856707	0.829045	0.409311	-6.47828	0.685413	0.617738
T.cells	CCDC84	-0.1454	2.649641	-0.82868	0.409516	-5.75132	0.737026	0.675532
T.cells	DHX34	-0.19375	2.203437	-0.82868	0.409519	-5.50072	0.744467	0.683968
T.cells	CXCL13	0.659775	-1.21101	0.828494	0.409622	-4.92308	0.803744	0.752107
T.cells	BARD1	-0.11577	3.42386	-0.82845	0.409645	-6.16551	0.724278	0.661249
T.cells	NSMCE3	-0.08018	4.271532	-0.82833	0.409714	-6.12738	0.710567	0.645831
T.cells	EZR	-0.05279	7.933064	-0.82817	0.409807	-6.81972	0.654076	0.583296
T.cells	CAR13	0.171162	1.31675	0.828075	0.409858	-5.67867	0.759503	0.701227
T.cells	OLFR77	0.236883	1.376904	0.82782	0.410001	-5.43222	0.758511	0.70006
T.cells	LY6C2	0.633333	5.53324	0.8278	0.410013	-5.3553	0.69065	0.623608
T.cells	HNRNPA0	-0.04695	7.214278	-0.8277	0.410066	-6.7443	0.664855	0.595102
T.cells	POLDIP2	-0.09536	4.278307	-0.8275	0.410182	-6.16043	0.710609	0.645847
T.cells	KRR1	-0.10485	3.94276	-0.82727	0.410312	-6.02145	0.716128	0.651983
T.cells	GM21762	0.448675	-1.20491	0.826891	0.410525	-4.90598	0.80424	0.752314
T.cells	F730311O2	-0.4339	-0.31758	-0.82676	0.410598	-5.0396	0.788442	0.734073
T.cells	SLC36A4	-0.18759	3.21113	-0.82656	0.41071	-5.67294	0.728408	0.665616
T.cells	SNX19	0.134731	2.862011	0.82643	0.410785	-5.67721	0.734161	0.672139
T.cells	ATAD3A	-0.10334	4.10261	-0.82627	0.410874	-6.16299	0.713906	0.649351
T.cells	OTUD1	-0.12227	3.007254	-0.82623	0.410898	-5.91174	0.731763	0.66945
T.cells	TTC7	-0.08501	5.873227	-0.82594	0.41106	-6.39356	0.685975	0.618212
T.cells	TPM4	0.058699	7.167543	0.825925	0.41107	-6.7057	0.666158	0.596332
T.cells	RPE	-0.09533	4.554255	-0.82562	0.411239	-6.13429	0.706772	0.641415
T.cells	PDP1	0.150054	2.180234	0.825617	0.411243	-5.64718	0.745635	0.685216
T.cells	ZFP770	0.186963	1.760027	0.825552	0.41128	-5.59687	0.752718	0.693268
T.cells	PCDH9	0.370944	0.472713	0.825209	0.411474	-5.3425	0.77504	0.718606
T.cells	RABGGTA	-0.16663	2.703471	-0.8251	0.411533	-5.54525	0.737129	0.675405
T.cells	AP2S1	0.057907	6.932961	0.824969	0.411609	-6.63301	0.669967	0.600393
T.cells	TMLHE	0.240124	2.31306	0.824579	0.41183	-5.56415	0.743932	0.682903
T.cells	ZFP768	0.326711	0.794694	0.824478	0.411887	-5.25245	0.769764	0.712363
T.cells	9230114K1	-0.1327	2.574674	-0.82426	0.412008	-5.73819	0.739666	0.678049
T.cells	MMP8	0.627422	1.517071	0.823949	0.412186	-5.03202	0.75751	0.698449

T.cells	UBE2K	-0.04675	8.214477	-0.82387	0.412232	-6.81822	0.651133	0.579703
T.cells	HIST1H2AG	-0.19832	2.011327	-0.82378	0.412279	-5.82604	0.749136	0.688972
T.cells	KLRB1B	-0.51707	1.981821	-0.82358	0.412392	-5.10361	0.749634	0.689563
T.cells	CCDC191	0.19223	1.993228	0.823562	0.412405	-5.54269	0.749441	0.689345
T.cells	LMNB1	-0.06953	7.679763	-0.82356	0.412406	-6.90134	0.65908	0.588439
T.cells	RAD21	-0.05589	7.022909	-0.82325	0.41258	-6.73513	0.669147	0.59937
T.cells	PABPN1	0.04907	6.640276	0.822931	0.412762	-6.64559	0.675166	0.605904
T.cells	MTFR2	-0.12246	3.615592	-0.82273	0.412876	-6.14529	0.723034	0.6592
T.cells	ATP2B4	0.172122	4.169834	0.82244	0.413039	-5.90548	0.714219	0.649208
T.cells	IDH1	-0.11985	4.88393	-0.82208	0.413243	-5.94412	0.703031	0.636451
T.cells	CCDC18	0.1254	2.722921	0.82185	0.413373	-5.92097	0.738272	0.675969
T.cells	IPO11	0.085572	4.478076	0.821742	0.413434	-6.23931	0.709616	0.64377
T.cells	CREG1	-0.10005	7.514034	-0.82161	0.413507	-6.51554	0.66251	0.591646
T.cells	PHYH	-0.14495	5.40217	-0.82146	0.413592	-6.2182	0.695	0.6275
T.cells	SUN2	-0.06691	6.122993	-0.82135	0.413655	-6.48832	0.683757	0.615049
T.cells	PHF8	0.07166	5.563796	0.821135	0.413778	-6.37979	0.692555	0.6247
T.cells	XRN2	-0.04594	7.516217	-0.82103	0.413839	-6.72816	0.662598	0.591679
T.cells	HTT	-0.0725	5.362069	-0.82084	0.413943	-6.36644	0.695721	0.628327
T.cells	RET	0.257228	0.727544	0.820807	0.413964	-5.38081	0.77231	0.714793
T.cells	TMEM258	-0.06425	6.825258	-0.82024	0.414283	-6.63058	0.673393	0.603469
T.cells	ETFDH	0.081255	4.872939	0.820217	0.414298	-6.21465	0.703808	0.637178
T.cells	ARHGAP25	0.07951	6.214201	0.81996	0.414444	-6.47327	0.682838	0.613896
T.cells	GMPR2	0.109377	3.866899	0.819926	0.414463	-6.00395	0.720045	0.655355
T.cells	RAB9	0.070209	4.887523	0.819367	0.41478	-6.29675	0.703713	0.637224
T.cells	GTPBP4	-0.06081	5.956432	-0.81916	0.414897	-6.49126	0.686905	0.618586
T.cells	LRRC32	0.36992	1.241998	0.819143	0.414907	-5.10887	0.763968	0.705291
T.cells	EXTL2	-0.24089	2.165568	-0.81906	0.414953	-5.41831	0.748265	0.687415
T.cells	PPAT	-0.09205	4.107428	-0.81877	0.415118	-6.21629	0.716221	0.651345
T.cells	GM553	-0.42261	0.241543	-0.81876	0.415124	-5.05231	0.78132	0.725257
T.cells	COPG2	0.080412	5.004811	0.818398	0.41533	-6.29522	0.70185	0.635378
T.cells	MCL1	-0.07247	8.364811	-0.81828	0.415397	-6.7542	0.650414	0.578644
T.cells	LRRC18	0.195061	1.588071	0.817864	0.415634	-5.69579	0.758049	0.698782
T.cells	HSPA1B	-0.35712	4.531616	-0.81782	0.415656	-6.0653	0.709394	0.643839
T.cells	1700052K1	0.230917	1.140287	0.817811	0.415663	-5.34984	0.765716	0.707532
T.cells	CAMK1	-0.27365	2.966119	-0.81774	0.415707	-5.30621	0.734897	0.67251
T.cells	STIMATE	0.105398	4.159047	0.817718	0.415716	-6.03518	0.715387	0.650551
T.cells	INPP5B	0.104521	4.216748	0.817595	0.415787	-6.04279	0.714456	0.649569
T.cells	RDH11	0.120885	3.215453	0.817485	0.415849	-5.88475	0.730779	0.667971
T.cells	CMTR1	0.11751	5.166737	0.817429	0.415881	-6.18297	0.699285	0.63266
T.cells	PHF3	0.053548	6.980017	0.817307	0.41595	-6.63576	0.671165	0.601515
T.cells	SMYD5	-0.19959	2.056268	-0.8173	0.415954	-5.63107	0.750108	0.68986
T.cells	PAN2	0.111089	3.190864	0.817236	0.41599	-5.88485	0.731184	0.668429
T.cells	CTPS2	0.074096	5.066744	0.817106	0.416065	-6.35021	0.700868	0.634424
T.cells	H2-T23	0.146483	6.358381	0.817027	0.416109	-6.32374	0.680683	0.612015
T.cells	SPATA1	-0.10459	3.467697	-0.81703	0.416109	-5.87084	0.726634	0.663299
T.cells	IQGAP2	-0.07965	7.075634	-0.81682	0.416226	-6.52977	0.669713	0.599916
T.cells	COL25A1	0.220878	0.981282	0.816633	0.416333	-5.66872	0.768455	0.710837

T.cells	D830025C(	-0.25559	2.231904	-0.81663	0.416335	-5.34567	0.747149	0.686552
T.cells	ATF3	-0.24361	6.1843	-0.81658	0.416363	-6.01885	0.683371	0.615046
T.cells	CENPL	0.100517	3.393274	0.816543	0.416384	-6.1152	0.727855	0.664758
T.cells	DNAJC11	-0.08193	4.486406	-0.81649	0.416415	-6.21879	0.710119	0.644851
T.cells	SLC22A17	-0.45867	-0.51359	-0.81642	0.416452	-4.96492	0.794655	0.741016
T.cells	TOPBP1	-0.08091	5.680028	-0.81638	0.416475	-6.53632	0.691215	0.623784
T.cells	PFN2	-0.21634	1.339141	-0.81637	0.416481	-5.57209	0.762302	0.703863
T.cells	DFFB	-0.12571	2.981393	-0.81599	0.416702	-5.91356	0.734856	0.672535
T.cells	RAB35	0.078249	4.720448	0.815938	0.416728	-6.2067	0.706578	0.640763
T.cells	MTCH2	-0.06063	5.950226	-0.8158	0.41681	-6.53362	0.687228	0.619196
T.cells	SEC61A1	-0.06835	5.49084	-0.81551	0.416971	-6.30238	0.694572	0.627211
T.cells	CHTF18	-0.17881	1.800347	-0.81491	0.417316	-5.71657	0.755224	0.695304
T.cells	GRIP1	0.187164	2.360437	0.814785	0.417385	-5.88193	0.745766	0.68456
T.cells	GPC5	0.346367	0.598275	0.814775	0.41739	-5.29567	0.775898	0.718919
T.cells	CD55B	-0.38238	0.178504	-0.81452	0.417537	-5.17466	0.783239	0.727347
T.cells	TICAM1	0.188997	2.819811	0.814444	0.417579	-5.55167	0.738091	0.675868
T.cells	PSTK	0.118871	3.061289	0.814401	0.417604	-5.82599	0.734086	0.671343
T.cells	SCNN1A	-0.46094	0.542515	-0.81438	0.417616	-5.05151	0.77687	0.720033
T.cells	PPP1R3F	0.312373	0.867721	0.814188	0.417725	-5.19311	0.771302	0.713606
T.cells	OSTM1	0.088139	4.762239	0.814028	0.417816	-6.10804	0.706553	0.640409
T.cells	LAPTM5	-0.05693	8.326927	-0.81393	0.417873	-6.76711	0.651753	0.579954
T.cells	ZCCHC10	0.087584	4.048466	0.813735	0.417983	-6.20345	0.718114	0.653393
T.cells	BHMT	0.196173	5.13251	0.813565	0.41808	-6.14018	0.70079	0.63397
T.cells	SLC25A47	-0.14507	3.963	-0.81346	0.418139	-5.93027	0.719549	0.654954
T.cells	DERA	0.070744	4.534652	0.812855	0.418484	-6.24732	0.71077	0.644766
T.cells	GM15832	0.31234	1.685015	0.812742	0.418549	-5.19291	0.757917	0.69801
T.cells	NIPBL	0.046278	8.615732	0.812659	0.418596	-6.94818	0.648018	0.575572
T.cells	POFUT1	-0.11172	3.458205	-0.81251	0.418684	-5.90734	0.728249	0.664479
T.cells	METTL16	0.074273	4.533537	0.81244	0.418722	-6.26946	0.710787	0.64491
T.cells	PDCD1	0.429727	0.743001	0.811587	0.419208	-5.2622	0.774531	0.71689
T.cells	NSD3	-0.05684	8.849458	-0.81156	0.419222	-6.89149	0.644923	0.572128
T.cells	GM38843	0.460099	0.985485	0.811494	0.419261	-5.00798	0.770325	0.712078
T.cells	LMF1	0.139105	3.239428	0.811492	0.419263	-5.78492	0.73223	0.668821
T.cells	ZBTB7B	-0.38926	2.473173	-0.81143	0.419299	-5.09247	0.744981	0.683231
T.cells	RASGEF1B	-0.25558	5.547786	-0.81138	0.419327	-5.76711	0.695038	0.627199
T.cells	FAM221A	-0.18164	1.467642	-0.81105	0.419512	-5.61209	0.762025	0.702687
T.cells	ST6GALNA(	0.135272	3.867281	0.810969	0.419561	-5.8742	0.721934	0.657334
T.cells	GM29114	0.397059	-1.28439	0.81083	0.419641	-4.95712	0.810526	0.758515
T.cells	PPIL1	-0.0937	4.052063	-0.8104	0.419887	-6.2742	0.71893	0.654144
T.cells	LSM7	-0.059	6.346432	-0.81037	0.419902	-6.62577	0.682589	0.613679
T.cells	CD69	-0.1227	5.620451	-0.81025	0.419974	-6.50399	0.693897	0.626248
T.cells	HIST1H1A	0.15622	3.773403	0.810125	0.420044	-6.34547	0.723465	0.6593
T.cells	5830487J0	0.217053	-0.01733	0.810037	0.420094	-5.42268	0.787855	0.732608
T.cells	CISD2	-0.06045	6.732173	-0.81002	0.420105	-6.60222	0.676651	0.607211
T.cells	EGR3	-0.58536	3.533307	-0.80998	0.420127	-5.16144	0.727394	0.663775
T.cells	KIF24	0.089889	3.667581	0.809741	0.420263	-6.1502	0.725195	0.661407
T.cells	ESS2	0.112153	2.988114	0.809627	0.420328	-5.85138	0.73639	0.674099

T.cells	FGFR1OP	0.086318	4.601111	0.809511	0.420394	-6.27394	0.710072	0.644551
T.cells	VPS16	0.069885	4.436967	0.809325	0.420501	-6.24731	0.712709	0.647575
T.cells	ENPP4	0.276824	2.924155	0.80931	0.42051	-5.26839	0.737452	0.675423
T.cells	DDIT4	0.170795	3.744278	0.809251	0.420543	-6.06977	0.723941	0.660225
T.cells	KRT10	0.156024	2.100333	0.809132	0.420612	-5.70665	0.751259	0.691151
T.cells	ZBTB49	0.218539	1.051258	0.809127	0.420614	-5.29264	0.769188	0.7116
T.cells	TBC1D25	0.154735	2.839206	0.80904	0.420664	-5.76359	0.738865	0.677136
T.cells	E2F7	-0.14042	2.983824	-0.80898	0.4207	-6.05697	0.736461	0.674444
T.cells	BC003965	0.107135	3.701969	0.80897	0.420704	-6.0281	0.724632	0.661101
T.cells	SERPING1	-0.23077	3.004813	-0.80893	0.420728	-5.53883	0.736113	0.674051
T.cells	EAF2	-0.09499	3.647314	-0.80872	0.420844	-6.10887	0.725537	0.662107
T.cells	9930022D1	0.408761	0.319301	0.808695	0.420861	-5.0966	0.781942	0.72629
T.cells	RAB20	-0.35229	3.301078	-0.80841	0.421024	-5.24241	0.731239	0.66861
T.cells	TMEM79	0.295727	0.778123	0.80833	0.42107	-5.25298	0.773948	0.717235
T.cells	DIP2B	-0.06276	7.880088	-0.80824	0.421121	-6.74788	0.659293	0.588648
T.cells	CXXC5	-0.0714	5.11182	-0.8081	0.421202	-6.42851	0.701949	0.635842
T.cells	HIST3H2A	0.213224	2.104979	0.808038	0.421238	-5.55639	0.751207	0.691348
T.cells	CNOT4	-0.05242	7.523005	-0.80801	0.421253	-6.74336	0.664654	0.594574
T.cells	SNF8	-0.06252	5.731869	-0.80774	0.421406	-6.44672	0.692321	0.625035
T.cells	PNPLA7	0.105215	6.039516	0.807345	0.421635	-6.24534	0.687654	0.619772
T.cells	VWA8	0.08065	4.975158	0.807215	0.421709	-6.32765	0.704409	0.638412
T.cells	MAJIN	-0.52429	0.644865	-0.8072	0.421719	-5.01411	0.776584	0.720131
T.cells	ERF	-0.15239	3.477368	-0.8071	0.421774	-5.85778	0.728637	0.665615
T.cells	PRPF3	0.069339	4.472577	0.806799	0.421947	-6.24907	0.712453	0.647513
T.cells	DNPH1	-0.20361	0.708539	-0.80667	0.422019	-5.53839	0.775475	0.719012
T.cells	SYNJ2	0.162223	2.324633	0.806639	0.422039	-5.6326	0.747809	0.687433
T.cells	AP1G1	0.071993	6.456316	0.806633	0.422042	-6.49317	0.681196	0.612758
T.cells	CACNA2D1	0.251102	0.864423	0.806594	0.422065	-5.58575	0.772766	0.715907
T.cells	CCDC138	0.117099	4.628632	0.806337	0.422212	-6.23039	0.710031	0.644839
T.cells	TMEM39A	-0.0873	4.395391	-0.80625	0.422263	-6.15114	0.713781	0.649042
T.cells	GRAMD2	0.377755	0.533275	0.806067	0.422367	-5.05378	0.778624	0.722679
T.cells	LRRC25	-0.36182	3.856093	-0.80594	0.422439	-5.29068	0.722524	0.658947
T.cells	FAP	0.553744	0.744801	0.805921	0.422451	-5.04636	0.774936	0.718499
T.cells	IL1A	-0.42952	2.787687	-0.80581	0.422516	-5.24495	0.74014	0.678836
T.cells	CCT8	-0.05479	6.762623	-0.80564	0.422612	-6.67921	0.676566	0.607733
T.cells	CALM1	-0.0465	9.782329	-0.80561	0.422626	-7.0801	0.631754	0.558755
T.cells	MTHFD2	-0.08964	5.486209	-0.80508	0.422931	-6.46701	0.696673	0.629906
T.cells	AA386476	0.228822	1.390731	0.804844	0.423069	-5.45724	0.764075	0.70602
T.cells	GMNN	-0.08995	5.558334	-0.80481	0.423089	-6.58206	0.695538	0.628732
T.cells	RYR1	0.370436	1.831874	0.804781	0.423105	-5.1714	0.756534	0.69744
T.cells	GBF1	0.050766	6.562441	0.804645	0.423183	-6.56365	0.679908	0.611443
T.cells	PSMG3	0.117592	3.202758	0.804559	0.423232	-5.97766	0.733537	0.671386
T.cells	DPY19L1	0.103969	4.584338	0.804546	0.42324	-6.11921	0.711021	0.64606
T.cells	AEBP2	0.077949	5.787827	0.804243	0.423414	-6.38886	0.69194	0.624814
T.cells	COX6B2	0.201725	1.691068	0.80423	0.423421	-5.56169	0.758937	0.700262
T.cells	CHORDC1	-0.06564	5.082056	-0.80421	0.423434	-6.37525	0.703073	0.637213
T.cells	PLD4	-0.14112	6.186695	-0.8038	0.423668	-6.22113	0.685996	0.617939

T.cells	BDP1	0.071217	5.529011	0.802627	0.424343	-6.38629	0.697215	0.629813
T.cells	CCRL2	0.463764	4.741765	0.802426	0.424458	-5.53682	0.709734	0.643882
T.cells	PLXNA4	-0.68377	2.166702	-0.80241	0.424469	-5.14153	0.752168	0.691712
T.cells	ZFP335OS	-0.0904	4.456083	-0.80237	0.424492	-6.24862	0.714329	0.649023
T.cells	TMEM132/	0.341011	0.400445	0.802157	0.424613	-5.13007	0.782725	0.726574
T.cells	TBC1D10C	0.107465	4.736281	0.801232	0.425145	-6.25225	0.710385	0.644301
T.cells	MRPL28	-0.06299	6.016074	-0.80104	0.425255	-6.57814	0.69012	0.621806
T.cells	INO80C	-0.10557	3.401246	-0.80093	0.425322	-5.97929	0.732118	0.668754
T.cells	IL4	-0.61599	0.330173	-0.80088	0.425346	-5.05356	0.784476	0.72836
T.cells	UHRF1BP1I	0.07695	5.734451	0.800742	0.425428	-6.35468	0.694532	0.626755
T.cells	GM20139	0.403533	-0.63311	0.800662	0.425474	-4.97243	0.801596	0.748149
T.cells	CAR5A	0.370476	0.542195	0.800641	0.425486	-5.15294	0.780753	0.724143
T.cells	THSD1	-0.22318	1.60027	-0.80052	0.425557	-5.43702	0.762416	0.703246
T.cells	SLC23A2	0.095951	5.113974	0.800399	0.425625	-6.21403	0.704347	0.637781
T.cells	SPNS3	0.120674	3.650358	0.800104	0.425795	-6.1767	0.728016	0.66443
T.cells	TAF5L	0.076777	4.562119	0.800063	0.425819	-6.25819	0.713186	0.647785
T.cells	IL23R	0.488336	-0.9447	0.799861	0.425935	-4.9592	0.807206	0.754949
T.cells	ZFP281	0.098361	4.496464	0.799789	0.425977	-6.11157	0.714244	0.649015
T.cells	CANX	-0.04827	7.35016	-0.79967	0.426043	-6.70335	0.669577	0.599448
T.cells	ADGRL3	-0.49941	3.47827	-0.79966	0.42605	-5.38876	0.730847	0.66767
T.cells	CSTA2	0.559241	1.96893	0.799658	0.426052	-5.17792	0.756121	0.696299
T.cells	EXOC8	-0.20006	1.951234	-0.79952	0.426131	-5.47435	0.756422	0.696679
T.cells	NDE1	0.079962	4.58489	0.799452	0.426171	-6.39332	0.712819	0.647492
T.cells	STIP1	-0.07324	5.419076	-0.79934	0.426235	-6.46923	0.699505	0.632671
T.cells	PSMD2	-0.05857	6.149979	-0.79934	0.426238	-6.53438	0.688032	0.619927
T.cells	CBWD1	0.099324	4.090993	0.799153	0.426343	-6.1654	0.720813	0.65653
T.cells	VPS13A	0.054631	6.447058	0.798987	0.426439	-6.61501	0.683419	0.614851
T.cells	FRMD8OS	0.391927	0.736632	0.798966	0.426451	-5.082	0.777353	0.720729
T.cells	TOR3A	0.168697	4.075925	0.798875	0.426504	-5.94459	0.721058	0.656858
T.cells	REEP3	-0.06723	6.727725	-0.79867	0.426619	-6.58131	0.679088	0.610113
T.cells	CDIPT	-0.08343	5.093616	-0.79865	0.426631	-6.26996	0.704671	0.638511
T.cells	LHFPL2	-0.32087	2.227897	-0.79862	0.42665	-5.20706	0.751728	0.691515
T.cells	SH2D3C	0.104994	4.693449	0.798354	0.426804	-6.21645	0.711073	0.645664
T.cells	RUVBL1	-0.06822	4.77265	-0.79834	0.426813	-6.38751	0.709801	0.644242
T.cells	PLIN2	-0.13679	5.831377	-0.79826	0.426857	-6.19111	0.693011	0.625552
T.cells	MRPS34	0.076336	4.826949	0.798145	0.426925	-6.25754	0.708931	0.643322
T.cells	GM44067	0.159552	1.717425	0.79804	0.426986	-5.75711	0.760411	0.701463
T.cells	ANKRD52	0.094239	3.881446	0.797912	0.42706	-6.07888	0.72423	0.660517
T.cells	MMUT	-0.15143	3.4125	-0.79779	0.427131	-5.82101	0.731932	0.669234
T.cells	YES1	-0.14783	4.740029	-0.79767	0.427198	-6.16512	0.710325	0.644959
T.cells	AKAP11	-0.08993	4.648059	-0.79759	0.427243	-6.2264	0.711802	0.646622
T.cells	AVEN	-0.0877	4.926701	-0.79756	0.427264	-6.33318	0.707335	0.641626
T.cells	2410022M	0.229927	1.591485	0.797377	0.427368	-5.3448	0.762603	0.70408
T.cells	CKAP2L	0.095196	4.357364	0.797173	0.427486	-6.37014	0.716525	0.651972
T.cells	CDC42BPB	-0.28757	3.262023	-0.79713	0.427509	-5.30685	0.734454	0.672153
T.cells	E130311K1	0.426392	0.244935	0.797079	0.427541	-5.05185	0.786015	0.730954
T.cells	CSNK1E	-0.08438	4.953409	-0.79691	0.427639	-6.30857	0.706999	0.641306

T.cells	PUF60	0.064762	5.949591	0.79664	0.427794	-6.54474	0.691303	0.623778
T.cells	ERRFI1	-0.14243	5.004787	-0.79638	0.427942	-5.99781	0.706234	0.640459
T.cells	SIT1	-0.15659	2.062249	-0.79624	0.428027	-5.8428	0.754692	0.695143
T.cells	ITSN1	-0.18311	5.300914	-0.79604	0.428143	-5.81879	0.701522	0.635236
T.cells	SPAST	0.067707	5.207046	0.795979	0.428176	-6.38403	0.703013	0.636904
T.cells	PLAA	0.058227	6.069326	0.795943	0.428197	-6.50752	0.689432	0.621804
T.cells	VAMP8	0.061482	7.209731	0.795901	0.428221	-6.62783	0.671851	0.602397
T.cells	HDAC7	0.084875	4.31747	0.795857	0.428247	-6.31295	0.717286	0.652915
T.cells	LYZ2	0.396448	7.170673	0.795851	0.42825	-6.11495	0.672446	0.603059
T.cells	1700097N(	0.147434	2.346622	0.795704	0.428335	-5.91715	0.749914	0.689762
T.cells	ZFP709	0.171663	1.431477	0.79533	0.428552	-5.47604	0.765608	0.70767
T.cells	ARPP19	-0.05133	6.840626	-0.79532	0.428558	-6.64897	0.677614	0.608778
T.cells	2810403D2	0.13635	2.774023	0.795277	0.428582	-5.81431	0.742825	0.68174
T.cells	ALPK3	-0.4084	-0.11417	-0.79494	0.428778	-5.11047	0.792878	0.738844
T.cells	MARK2	0.052577	6.879023	0.79438	0.429101	-6.63076	0.677428	0.60841
T.cells	MRPL34	0.059476	5.428125	0.794211	0.429199	-6.45359	0.700047	0.633515
T.cells	ZSCAN25	-0.2037	1.792602	-0.79418	0.429215	-5.50863	0.75987	0.700957
T.cells	APOO	-0.09067	3.509146	-0.79417	0.429221	-6.10488	0.731054	0.668276
T.cells	ANXA11	0.077998	5.969579	0.794171	0.429222	-6.35945	0.691524	0.624037
T.cells	GM43774	0.198981	2.238278	0.793687	0.429503	-5.55984	0.752575	0.692382
T.cells	ZFP874B	0.195438	2.178101	0.793652	0.429523	-5.61317	0.753595	0.693558
T.cells	TXN2	-0.05936	6.315861	-0.79356	0.429578	-6.56319	0.686386	0.618131
T.cells	VDR	0.380934	0.938817	0.793156	0.42981	-5.145	0.775188	0.718089
T.cells	PSTPIP1	0.139552	4.472927	0.79303	0.429883	-5.9028	0.71589	0.650874
T.cells	EIF3M	-0.04221	6.621301	-0.79277	0.430037	-6.66374	0.681986	0.613152
T.cells	OSBPL5	-0.33144	0.3884	-0.79268	0.430084	-5.20909	0.784901	0.729258
T.cells	CCNJL	0.197948	1.734552	0.792641	0.430109	-5.57492	0.761523	0.702492
T.cells	SLC46A2	0.297088	-0.09426	0.791969	0.430498	-5.20516	0.794013	0.73929
T.cells	RAB23	-0.21569	1.279801	-0.79185	0.430566	-5.4808	0.769901	0.711646
T.cells	RAMP2	-0.24941	3.013058	-0.79177	0.430611	-5.49816	0.74045	0.678197
T.cells	TMEM87A	-0.07702	5.236916	-0.7915	0.43077	-6.32305	0.704241	0.637524
T.cells	PET100	-0.0678	5.786929	-0.7914	0.430827	-6.4167	0.695534	0.627871
T.cells	2010310CC	0.45837	1.298313	0.791397	0.43083	-5.12519	0.769629	0.711393
T.cells	DPH7	0.207009	1.586984	0.79125	0.430916	-5.55783	0.764689	0.705789
T.cells	DDX43	-0.35711	0.153087	-0.791	0.431062	-5.143	0.789844	0.734508
T.cells	YPEL3	0.079844	7.069109	0.79084	0.431154	-6.65642	0.675778	0.605969
T.cells	SNHG3	0.086758	5.291632	0.790632	0.431274	-6.44647	0.703525	0.636743
T.cells	CDYL	0.060444	5.659184	0.790361	0.431432	-6.45181	0.697701	0.630369
T.cells	LEPROT	0.08545	5.065196	0.79031	0.431462	-6.20294	0.707135	0.64088
T.cells	EIF3H	-0.05009	7.353901	-0.79028	0.431477	-6.76611	0.671429	0.601319
T.cells	GM37612	0.236592	0.985122	0.789999	0.431642	-5.39926	0.775233	0.718019
T.cells	AK2	0.067773	5.995761	0.789982	0.431652	-6.52294	0.692407	0.624588
T.cells	HSDL1	0.075546	4.167902	0.78983	0.431741	-6.15145	0.721615	0.657272
T.cells	ZFP180	0.099954	3.315586	0.789742	0.431792	-5.99559	0.735625	0.673069
T.cells	FARP2	-0.21505	2.6131	-0.78955	0.431905	-5.49862	0.747363	0.686414
T.cells	DUSP4	-0.28304	0.909125	-0.78937	0.432006	-5.19338	0.776557	0.719723
T.cells	NME4	-0.15815	2.364464	-0.78937	0.432008	-5.88709	0.751558	0.69119

T.cells	SLC7A5	-0.09166	6.118367	-0.78936	0.432013	-6.5694	0.690488	0.62262
T.cells	DHX15	-0.0498	6.794688	-0.78935	0.43202	-6.6654	0.679993	0.611006
T.cells	CRYBG3	0.103385	4.474287	0.789311	0.432042	-6.13884	0.71664	0.65178
T.cells	1810059H2	0.181653	1.799204	0.789017	0.432213	-5.88979	0.761178	0.702251
T.cells	IFIT2	0.332397	2.721315	0.788697	0.432399	-5.62	0.745543	0.684544
T.cells	GM3604	0.24396	0.595301	0.788345	0.432604	-5.32206	0.782048	0.726309
T.cells	WDR20	-0.06127	5.843767	-0.78834	0.432606	-6.46777	0.694793	0.627644
T.cells	ZFP68	0.081552	3.920899	0.788341	0.432606	-6.1411	0.72565	0.662158
T.cells	RIMS3	-0.33801	1.085548	-0.78831	0.432623	-5.24796	0.773487	0.716486
T.cells	ARL2BP	-0.07879	5.086344	-0.78829	0.432634	-6.39438	0.706797	0.64102
T.cells	GM32916	-0.5652	-0.24915	-0.7881	0.432748	-5.18962	0.796998	0.743632
T.cells	HHEX	-0.10293	4.675113	-0.78804	0.43278	-6.1866	0.713396	0.648486
T.cells	NDUFC2	-0.06154	6.741013	-0.78802	0.432793	-6.68792	0.680821	0.61224
T.cells	FZD5	-0.19099	2.536377	-0.78795	0.432832	-5.49934	0.748655	0.688253
T.cells	MYO1E	-0.11003	6.198716	-0.78787	0.432883	-6.64263	0.689233	0.621554
T.cells	FGF1	0.323168	0.63081	0.787705	0.432976	-5.15884	0.781425	0.725689
T.cells	MEF2D	-0.05997	7.288749	-0.78765	0.43301	-6.71981	0.672422	0.602989
T.cells	VKORC1L1	0.069983	5.560377	0.787593	0.433041	-6.43347	0.699262	0.632727
T.cells	PAIP2B	-0.09727	3.682629	-0.78754	0.433073	-6.0411	0.729561	0.666695
T.cells	TNS1	-0.25539	2.761945	-0.78747	0.433111	-5.43237	0.744861	0.684003
T.cells	ZC3HAV1L	-0.19244	3.053639	-0.78715	0.4333	-5.60456	0.740161	0.678677
T.cells	GM29019	0.310905	0.361473	0.787072	0.433344	-5.13924	0.786352	0.731455
T.cells	SDHA	-0.05996	5.691569	-0.78675	0.433534	-6.43077	0.697492	0.630736
T.cells	NOA1	-0.11782	3.137205	-0.78666	0.433582	-5.94158	0.73891	0.677234
T.cells	RBM47	-0.14701	6.404832	-0.78644	0.433714	-6.12094	0.686322	0.61841
T.cells	BCL9	0.119432	3.399574	0.786374	0.433751	-5.9516	0.734552	0.672382
T.cells	ARRDC2	-0.22971	1.79539	-0.78631	0.433791	-5.40346	0.761574	0.703062
T.cells	TRIM47	0.226105	2.41312	0.786136	0.43389	-5.37823	0.751061	0.69111
T.cells	GM17092	-0.1563	2.1249	-0.78597	0.433986	-5.72196	0.755949	0.696688
T.cells	MCM8	-0.17249	1.54016	-0.78595	0.433996	-5.64483	0.765957	0.708111
T.cells	GM43672	-0.3017	1.700182	-0.7859	0.43403	-5.26178	0.763206	0.704999
T.cells	RHEB	-0.05115	7.039284	-0.78565	0.434174	-6.67433	0.676528	0.607718
T.cells	POC1B	-0.07234	4.642223	-0.78559	0.434207	-6.28562	0.714236	0.649658
T.cells	CCDC107	0.077275	4.620705	0.785549	0.434232	-6.2869	0.714584	0.650047
T.cells	4930453N2	-0.0764	4.481233	-0.7855	0.434263	-6.27189	0.716838	0.652586
T.cells	PES1	-0.07181	4.691666	-0.78528	0.434389	-6.27698	0.71354	0.648833
T.cells	EFCAB14	0.098678	4.713606	0.784944	0.434584	-6.13722	0.713402	0.648571
T.cells	3110009E1	-0.14087	2.175408	-0.78476	0.43469	-5.72651	0.755497	0.696113
T.cells	RNF17	-0.46982	-0.02892	-0.78456	0.434806	-5.06958	0.79394	0.740114
T.cells	ATP6V0A1	0.147925	4.202735	0.784417	0.434892	-5.72922	0.721871	0.65797
T.cells	DNAH7A	0.417974	-0.11769	0.784262	0.434983	-5.07792	0.795609	0.741962
T.cells	TSTD3	-0.20741	1.784277	-0.78377	0.43527	-5.43679	0.762484	0.703931
T.cells	PRNP	0.213789	2.012971	0.783606	0.435365	-5.47318	0.758572	0.699492
T.cells	CARD6	-0.16717	3.06005	-0.78355	0.435401	-5.76204	0.740895	0.679414
T.cells	STK16	0.091887	4.37442	0.783496	0.43543	-6.20058	0.719249	0.655011
T.cells	VPS29	-0.05503	6.521761	-0.78346	0.435451	-6.5747	0.685154	0.617008
T.cells	STAC2	-0.24719	1.824947	-0.78337	0.435504	-5.51275	0.761787	0.703204

T.cells	PTPA	-0.05819	5.833043	-0.78336	0.43551	-6.52759	0.69592	0.628986
T.cells	CD177	0.453953	0.17299	0.783191	0.435608	-5.0434	0.790641	0.736313
T.cells	PTGS2OS2	0.433005	-0.48663	0.783081	0.435672	-4.99999	0.802416	0.749962
T.cells	ASPH	0.246243	4.664011	0.782885	0.435787	-5.59817	0.714696	0.649924
T.cells	ZFP579	-0.19216	1.862923	-0.7826	0.435955	-5.53636	0.761463	0.702697
T.cells	MOSPD2	0.123805	4.445481	0.782336	0.436107	-5.93803	0.718514	0.654079
T.cells	DDX3X	-0.07424	7.320412	-0.78219	0.43619	-6.62208	0.673258	0.60378
T.cells	AGTPBP1	0.090139	5.128653	0.782073	0.436261	-6.2959	0.707508	0.641799
T.cells	NDUFB9	0.057069	7.069533	0.782042	0.436279	-6.71011	0.677098	0.608039
T.cells	ACTR8	-0.10328	3.537225	-0.78184	0.436397	-6.00232	0.733484	0.670897
T.cells	SLC15A3	0.172297	5.005968	0.781638	0.436516	-5.85472	0.709647	0.644069
T.cells	DYNLRB1	-0.05565	6.280147	-0.78117	0.436789	-6.57055	0.689727	0.621838
T.cells	HYOU1	-0.09526	4.380576	-0.78115	0.436798	-6.07549	0.719999	0.655611
T.cells	CHD1	0.04932	6.90203	0.780331	0.43728	-6.67931	0.680556	0.611471
T.cells	TMEM208	-0.06568	4.89435	-0.78031	0.437294	-6.27367	0.712189	0.646622
T.cells	LCMT2	-0.12472	2.687418	-0.7803	0.4373	-5.81716	0.748549	0.687582
T.cells	ZFP707	-0.17846	1.958642	-0.78008	0.437426	-5.56327	0.76093	0.701689
T.cells	COX5B	-0.05838	8.103976	-0.78008	0.437427	-6.87613	0.662259	0.591377
T.cells	SAE1	-0.0569	6.342657	-0.77987	0.437551	-6.70311	0.689253	0.621113
T.cells	CORO2A	0.096478	5.527432	0.779748	0.437622	-6.3175	0.702087	0.635413
T.cells	TARBP2	0.110948	3.242608	0.779683	0.43766	-5.94739	0.739263	0.67716
T.cells	IGHJ4	0.267192	0.337645	0.779566	0.437728	-5.3797	0.789172	0.734158
T.cells	NDUFA7	0.046939	7.909207	0.779509	0.437761	-6.79998	0.66521	0.594708
T.cells	NBN	-0.10143	3.4476	-0.77934	0.437862	-6.06504	0.735914	0.673371
T.cells	PPP2R1A	-0.06372	6.00443	-0.77902	0.438046	-6.5388	0.694649	0.627203
T.cells	TRAPPC4	-0.07205	5.137813	-0.77885	0.438149	-6.294	0.7084	0.642524
T.cells	ABHD17C	-0.08623	4.833665	-0.77878	0.43819	-6.26299	0.713287	0.647997
T.cells	1110065P2	-0.1145	3.514697	-0.77878	0.438192	-5.83777	0.734845	0.672229
T.cells	ZFP414	-0.12067	3.443642	-0.77855	0.438324	-5.92601	0.736023	0.673634
T.cells	SEC24C	0.074658	4.859773	0.778448	0.438383	-6.22417	0.712866	0.647619
T.cells	CXCL2	-0.4566	7.158496	-0.77842	0.438403	-6.13569	0.676726	0.607479
T.cells	PPBP	0.570349	0.842206	0.778377	0.438425	-5.14246	0.780396	0.724208
T.cells	TMED2	-0.04964	8.136615	-0.77809	0.438593	-6.82823	0.66188	0.591249
T.cells	IGHMBP2	0.120327	2.850148	0.778011	0.43864	-5.8394	0.745936	0.685012
T.cells	H2-Q6	0.398325	3.115257	0.777998	0.438647	-5.48958	0.741493	0.679978
T.cells	MYB	-0.08449	5.137938	-0.7779	0.438708	-6.53115	0.708398	0.642798
T.cells	RBIS	0.068997	5.209656	0.777863	0.438726	-6.40706	0.707251	0.641529
T.cells	EML3	-0.0936	4.139794	-0.77736	0.439023	-6.12611	0.724735	0.661177
T.cells	TBC1D32	0.183522	2.141265	0.777278	0.439069	-5.67059	0.758127	0.698982
T.cells	XK	-0.22554	1.158214	-0.77713	0.439158	-5.4652	0.775071	0.71838
T.cells	ATP6V1F	0.052496	6.902317	0.777103	0.439172	-6.60773	0.680837	0.612264
T.cells	BAD	-0.10769	3.912661	-0.77695	0.439264	-6.00349	0.728459	0.665442
T.cells	CACFD1	0.108284	3.174111	0.776921	0.439279	-5.90365	0.740695	0.67926
T.cells	HHAT	-0.24539	1.670909	-0.77691	0.439288	-5.394	0.766191	0.708259
T.cells	TCEANC	0.207135	1.730178	0.776506	0.439523	-5.54428	0.765454	0.707273
T.cells	TSACC	0.244864	1.881856	0.77641	0.439579	-5.5272	0.762847	0.704299
T.cells	TCEAL9	-0.07539	5.693961	-0.77628	0.439655	-6.49376	0.699999	0.633453

T.cells	ZFP719	0.167165	2.119349	0.775949	0.43985	-5.5342	0.759026	0.699819
T.cells	MRPL30	0.05182	6.172662	0.775351	0.440201	-6.576	0.693113	0.62533
T.cells	SESN1	0.067657	5.939971	0.77517	0.440307	-6.54227	0.696838	0.629481
T.cells	0610009B2	-0.12167	3.780562	-0.77493	0.440447	-6.0159	0.731743	0.668522
T.cells	ERCC2	-0.16646	1.983619	-0.77489	0.440471	-5.59674	0.761976	0.70279
T.cells	RAVER2	0.299142	1.423822	0.774485	0.44071	-5.21823	0.771934	0.714015
T.cells	PIGU	0.093139	4.215968	0.774213	0.44087	-6.16817	0.72496	0.660776
T.cells	GRAP	0.097974	4.536304	0.774082	0.440947	-6.22015	0.719734	0.654911
T.cells	CXCR3	-0.50839	1.731497	-0.77397	0.441013	-5.03576	0.766703	0.708062
T.cells	ACADS	-0.08829	4.490963	-0.77393	0.441035	-6.15603	0.720472	0.655738
T.cells	USP50	-0.11633	3.371827	-0.7738	0.441114	-5.93111	0.7389	0.676503
T.cells	STOML2	0.078485	4.805582	0.773725	0.441157	-6.35291	0.715369	0.650063
T.cells	SDCBP2	-0.27925	1.097863	-0.77363	0.441215	-5.35275	0.7777	0.720702
T.cells	CSF3	0.637305	-0.096	0.773421	0.441336	-4.99132	0.798918	0.745135
T.cells	SLC5A10	-0.39348	-0.1778	-0.77203	0.442157	-5.0834	0.801755	0.747268
T.cells	GM15559	0.106541	3.148018	0.771503	0.442466	-5.96292	0.744218	0.681522
T.cells	MIEF2	0.239947	1.112018	0.771466	0.442488	-5.34141	0.779108	0.721217
T.cells	UBE4A	0.077799	5.03345	0.771304	0.442583	-6.31565	0.71321	0.646719
T.cells	RDH13	0.23696	1.398416	0.771027	0.442747	-5.35252	0.774111	0.71562
T.cells	ALS2	-0.09662	4.283789	-0.7709	0.442821	-6.0147	0.725392	0.660428
T.cells	GM13710	-0.4533	1.36556	-0.77064	0.442977	-5.12159	0.774682	0.716403
T.cells	PLA1A	-0.33752	1.841769	-0.7706	0.442997	-5.23485	0.766432	0.707003
T.cells	TMEM181/	-0.07873	4.58903	-0.77056	0.443024	-6.19129	0.720408	0.655004
T.cells	EDRF1	0.088199	4.217889	0.77049	0.443064	-6.22449	0.726472	0.661817
T.cells	FOXRED1	-0.15066	2.831724	-0.77028	0.443187	-5.80025	0.749542	0.687844
T.cells	EIF3C	0.051992	7.172674	0.770269	0.443193	-6.74865	0.679497	0.609555
T.cells	P2RY10B	0.115954	3.709802	0.770247	0.443207	-6.00234	0.73485	0.671242
T.cells	GGNBP2	-0.05008	7.268609	-0.77024	0.443209	-6.7034	0.678021	0.607929
T.cells	PPIL4	-0.05606	5.46202	-0.77024	0.443213	-6.43178	0.706332	0.639287
T.cells	NSUN5	-0.14672	2.124735	-0.77005	0.443325	-5.7061	0.761569	0.701572
T.cells	FAM83D	-0.13752	2.774945	-0.76975	0.443501	-5.83363	0.750501	0.68914
T.cells	PURB	-0.05149	6.824936	-0.76969	0.443535	-6.62858	0.684873	0.615684
T.cells	TCAIM	0.230402	1.789758	0.769683	0.44354	-5.40332	0.76733	0.708297
T.cells	PRDM1	0.303779	2.929068	0.769506	0.443644	-5.35187	0.7479	0.686321
T.cells	ABHD2	-0.0725	5.471153	-0.76933	0.443748	-6.32123	0.706187	0.639503
T.cells	PRR14L	-0.0577	6.50182	-0.76931	0.443762	-6.64075	0.689904	0.621414
T.cells	NSL1	0.131819	2.25699	0.7693	0.443766	-5.8814	0.759306	0.699343
T.cells	LONRF3	-0.23511	1.525709	-0.76929	0.443774	-5.41754	0.771899	0.713707
T.cells	NDUFAF6	-0.11518	2.973661	-0.76912	0.443869	-5.90046	0.7472	0.685641
T.cells	NDFIP1	0.058988	7.521506	0.769008	0.443938	-6.65691	0.674197	0.604133
T.cells	SCLY	0.121921	3.466279	0.768846	0.444033	-5.83157	0.739006	0.676381
T.cells	TTK	0.148967	2.313922	0.768677	0.444134	-5.89792	0.758505	0.698479
T.cells	KBTBD4	-0.11853	2.78551	-0.76835	0.444328	-5.89998	0.750531	0.689464
T.cells	YIPF3	0.077264	4.904587	0.768303	0.444354	-6.22638	0.715488	0.649983
T.cells	PDZD2	0.135412	3.46937	0.76826	0.44438	-6.11502	0.739051	0.676478
T.cells	CLTC	0.048745	7.90636	0.7682	0.444415	-6.72908	0.668471	0.597878
T.cells	KCTD3	-0.08662	3.747291	-0.76803	0.444515	-6.14876	0.734462	0.671347

T.cells	CNNM2	-0.18244	5.427728	-0.7678	0.44465	-6.03647	0.707105	0.640762
T.cells	ARL6IP1	0.05165	7.879749	0.767775	0.444667	-6.90014	0.668902	0.598494
T.cells	OSBPL9	0.046835	7.511715	0.767391	0.444894	-6.76193	0.674509	0.60482
T.cells	LRRC61	0.199489	2.011331	0.767366	0.444908	-5.50102	0.763757	0.704884
T.cells	CRK	0.063808	6.391053	0.767303	0.444946	-6.56116	0.691857	0.624008
T.cells	TRPM2	-0.39571	2.540831	-0.76723	0.444991	-5.17773	0.754709	0.694602
T.cells	1300002E1	-0.1258	3.19234	-0.76699	0.445129	-5.88291	0.743712	0.682175
T.cells	CTNNBIP1	0.110108	4.359655	0.76694	0.44516	-6.03164	0.72438	0.660369
T.cells	GM49961	-0.29168	0.574285	-0.76689	0.445191	-5.28863	0.788822	0.733702
T.cells	PRKX	-0.07531	5.379346	-0.76671	0.445295	-6.29447	0.707879	0.641982
T.cells	GINS2	-0.11233	3.493231	-0.76662	0.44535	-6.20172	0.738683	0.676588
T.cells	TXNL1	-0.05892	6.955558	-0.76661	0.445355	-6.71747	0.683066	0.61442
T.cells	CCDC152	-0.19995	3.284464	-0.76637	0.445495	-5.70361	0.742169	0.680535
T.cells	CRTAP	-0.15041	3.004923	-0.76623	0.445578	-5.71696	0.74686	0.685848
T.cells	RCBTB2	0.073534	5.187796	0.766198	0.4456	-6.36667	0.710952	0.64542
T.cells	VSTM4	0.472819	0.147061	0.766172	0.445615	-5.04044	0.796419	0.742546
T.cells	HIBCH	0.100931	3.478236	0.766153	0.445626	-5.92732	0.738933	0.676875
T.cells	1700061N1	-0.46514	-0.1438	-0.766	0.445719	-5.08623	0.801629	0.748564
T.cells	IFT88	-0.18015	1.778503	-0.76584	0.445811	-5.51292	0.767768	0.70969
T.cells	MAP4K3	0.115988	4.283134	0.765754	0.445862	-6.04894	0.725633	0.661922
T.cells	MRM3	-0.18507	2.005465	-0.76543	0.446053	-5.6351	0.763858	0.705377
T.cells	COL1A1	-0.33904	2.255466	-0.76535	0.446101	-5.38528	0.759573	0.700492
T.cells	ITGAD	0.421222	0.789813	0.765212	0.446183	-5.09459	0.785015	0.729649
T.cells	SMARCA2	-0.08142	6.123463	-0.76514	0.446225	-6.28704	0.696061	0.629065
T.cells	JOSD1	-0.09339	3.99961	-0.76485	0.4464	-6.04212	0.730292	0.667423
T.cells	MIS18A	-0.08139	4.400206	-0.76483	0.44641	-6.37085	0.723717	0.660023
T.cells	2900093K2	-0.1435	2.884005	-0.76482	0.446418	-5.78918	0.748897	0.688468
T.cells	ADAL	0.145595	2.279066	0.764726	0.446471	-5.67597	0.759169	0.70015
T.cells	CASZ1	0.116052	3.474262	0.764696	0.446489	-5.97336	0.738999	0.677264
T.cells	TBC1D24	-0.23978	1.799284	-0.76438	0.446677	-5.36916	0.767409	0.709684
T.cells	COG3	0.089715	4.292574	0.764254	0.446751	-6.15463	0.725478	0.662197
T.cells	TMEM51O:	0.395323	-0.90367	0.764218	0.446772	-4.97459	0.815388	0.765087
T.cells	WDR46	-0.10805	3.429733	-0.76411	0.446839	-6.06639	0.739742	0.67832
T.cells	ZFP692	-0.14878	2.030033	-0.76358	0.447151	-5.62397	0.763436	0.705428
T.cells	PDCD5	-0.0541	6.418317	-0.76339	0.447261	-6.61111	0.69143	0.624405
T.cells	GM48796	-0.2297	0.350478	-0.76333	0.447301	-5.38629	0.792794	0.73922
T.cells	SENP1	0.05907	5.013417	0.763091	0.447441	-6.41935	0.71376	0.64943
T.cells	GRAMD4	0.075792	5.165751	0.763082	0.447446	-6.56296	0.711306	0.646681
T.cells	KATNBL1	0.058256	5.963648	0.763055	0.447462	-6.54697	0.698583	0.632486
T.cells	ANKRD11	0.049816	9.391983	0.762937	0.447532	-7.05406	0.646326	0.574979
T.cells	WNT4	-0.26179	1.526488	-0.76281	0.447609	-5.30363	0.772131	0.715718
T.cells	FOSL2	-0.25912	5.561041	-0.76273	0.447654	-5.51085	0.704976	0.639738
T.cells	CYP3A25	-0.31037	1.898523	-0.76271	0.447669	-5.35316	0.765698	0.708388
T.cells	4930539J0.	0.285808	0.706555	0.762668	0.447692	-5.26283	0.786484	0.732269
T.cells	TGFBI	-0.30735	4.412001	-0.76234	0.447888	-5.48158	0.723524	0.660676
T.cells	CCNI	-0.05263	6.783841	-0.76226	0.447932	-6.64565	0.685729	0.618471
T.cells	CNR2	0.196466	3.101889	0.761957	0.448113	-5.4887	0.745229	0.685281

T.cells	PLPP6	0.176718	2.754142	0.761916	0.448138	-5.57541	0.751092	0.691942
T.cells	GM17354	-0.20246	1.393239	-0.76187	0.448167	-5.45008	0.774447	0.718627
T.cells	TSPO	0.094667	7.399271	0.761669	0.448285	-6.68909	0.676231	0.608079
T.cells	SF3B1	-0.03769	8.256825	-0.76161	0.448319	-6.91986	0.663203	0.593751
T.cells	NOC2L	-0.07629	5.059461	-0.76144	0.448418	-6.41235	0.713017	0.649064
T.cells	FILIP1L	0.087428	5.411773	0.761433	0.448425	-6.36031	0.70736	0.642729
T.cells	COL1A2	-0.27736	2.442649	-0.76141	0.448437	-5.45417	0.756379	0.698096
T.cells	GM7072	0.108404	3.842908	0.761405	0.448441	-6.03657	0.732879	0.671422
T.cells	SAR1B	-0.05806	5.553451	-0.76134	0.448482	-6.41795	0.705097	0.640205
T.cells	KIF3C	0.171913	2.145051	0.761327	0.448488	-5.5908	0.761463	0.703903
T.cells	SMO	-0.25858	1.198551	-0.76105	0.44865	-5.41816	0.777842	0.722723
T.cells	FEZ2	0.113009	3.693286	0.761013	0.448674	-5.83091	0.735357	0.67428
T.cells	TOMM40L	0.168178	1.733558	0.760886	0.44875	-5.48133	0.768544	0.712075
T.cells	GGH	0.145501	4.555539	0.760767	0.448821	-5.95914	0.721183	0.658317
T.cells	C9	0.474978	0.60037	0.760431	0.44902	-5.15995	0.788361	0.734934
T.cells	FAM222B	-0.10367	5.370089	-0.76031	0.449091	-6.20659	0.708027	0.64363
T.cells	KLK1	-0.56191	-0.20745	-0.76012	0.449205	-4.97302	0.802773	0.751674
T.cells	PTGES3	0.047131	7.460041	0.759996	0.449279	-6.8241	0.6753	0.607339
T.cells	CSF3R	0.354897	2.933462	0.759934	0.449316	-5.25202	0.748063	0.688905
T.cells	GHITM	0.078309	7.4577	0.759802	0.449394	-6.73432	0.675335	0.607422
T.cells	PURA	0.061731	5.343404	0.759695	0.449458	-6.3203	0.708455	0.644282
T.cells	BCAT2	-0.08575	4.601375	-0.75965	0.449485	-6.16903	0.720436	0.657734
T.cells	GM28050	-0.29684	0.410517	-0.75956	0.449537	-5.22269	0.791726	0.739086
T.cells	GPS1	-0.07085	4.905295	-0.75952	0.449562	-6.33281	0.715506	0.652252
T.cells	GLTP	-0.05537	6.759436	-0.75946	0.449596	-6.60917	0.686108	0.619442
T.cells	ADCY9	-0.15624	3.136828	-0.75943	0.449613	-5.80901	0.744643	0.685171
T.cells	KCNH7	0.411151	-0.13979	0.759346	0.449666	-5.03843	0.801557	0.750523
T.cells	2900089D1	-0.23057	2.58395	-0.75926	0.449717	-5.43969	0.753976	0.695875
T.cells	DNAAF3	-0.41791	-0.6587	-0.75921	0.449749	-5.0054	0.810928	0.761463
T.cells	PDK4	-0.32527	1.417008	-0.75912	0.4498	-5.23159	0.774033	0.718836
T.cells	AOAH	-0.4402	4.784961	-0.75908	0.449824	-5.39683	0.717454	0.654548
T.cells	TAF4B	-0.13118	5.130162	-0.75864	0.450088	-6.17574	0.711879	0.648343
T.cells	ZBTB37	0.152751	2.901016	0.75852	0.450157	-5.67362	0.74861	0.689842
T.cells	ENDOV	-0.21511	2.177707	-0.75848	0.450181	-5.46939	0.760903	0.70386
T.cells	CISH	0.289792	2.887974	0.75845	0.450199	-5.37318	0.74883	0.690092
T.cells	GAA	-0.17425	3.191942	-0.75845	0.450201	-5.58029	0.743718	0.68428
T.cells	QPRT	-0.25924	2.660001	-0.75842	0.450218	-5.46775	0.752686	0.694482
T.cells	AMOTL2	-0.42314	0.684568	-0.75838	0.450243	-5.10371	0.786872	0.733682
T.cells	LONP1	0.092319	3.653778	0.758374	0.450244	-6.10298	0.736013	0.675543
T.cells	UCK2	-0.0906	5.87449	-0.75835	0.450259	-6.51219	0.699994	0.635046
T.cells	ADARB1	-0.16847	3.009129	-0.75832	0.450276	-5.79302	0.746789	0.68777
T.cells	SSRP1	-0.06077	6.322976	-0.7582	0.450347	-6.71143	0.692924	0.627166
T.cells	UTP6	0.078864	4.42275	0.75815	0.450377	-6.26804	0.723349	0.661238
T.cells	OGFOD1	0.105859	3.611469	0.758038	0.450444	-5.99189	0.736716	0.676339
T.cells	FHL3	0.216255	2.081558	0.758011	0.45046	-5.54376	0.762551	0.705745
T.cells	2-Sep	0.190897	2.22603	0.757527	0.450749	-5.60133	0.760193	0.703015
T.cells	MZT1	0.055098	5.278837	0.757481	0.450776	-6.46408	0.709598	0.645759

T.cells	VMP1	-0.0844	7.164309	-0.75705	0.451031	-6.61756	0.679946	0.612868
T.cells	TMEM230	-0.06831	4.742854	-0.75697	0.45108	-6.25296	0.718247	0.655626
T.cells	DCUN1D2	-0.15579	2.743543	-0.75681	0.451177	-5.75154	0.751386	0.693196
T.cells	MPHOSPH9	0.08043	4.258614	0.756804	0.451179	-6.29642	0.726146	0.664578
T.cells	ZFP84	-0.10351	3.7569	-0.75679	0.451189	-6.08474	0.734415	0.673926
T.cells	NDUFS2	-0.06324	6.235293	-0.75659	0.451306	-6.59451	0.694407	0.629033
T.cells	TMEM218	-0.15581	1.874833	-0.75653	0.451344	-5.59495	0.766223	0.710196
T.cells	LRP8	-0.22132	2.371033	-0.75628	0.451489	-5.51794	0.757715	0.70049
T.cells	GRAP2	0.183806	4.975054	0.756275	0.451494	-5.91914	0.714488	0.651524
T.cells	G730013B(	0.376828	-0.968	0.75603	0.451641	-4.98259	0.816687	0.768594
T.cells	5430401H(	-0.46315	-0.47304	-0.75599	0.451662	-5.05378	0.807687	0.758118
T.cells	SNRNP27	-0.05527	5.842837	-0.75599	0.451664	-6.52604	0.700603	0.636009
T.cells	NCF2	0.094169	6.576577	0.755857	0.451744	-6.42193	0.689061	0.623149
T.cells	SMC2	-0.09613	5.816825	-0.75563	0.451877	-6.66352	0.701016	0.636523
T.cells	NT5DC2	-0.11977	2.432435	-0.75552	0.451944	-6.00679	0.756669	0.699394
T.cells	CYSTM1	-0.33023	3.36709	-0.75547	0.451973	-5.46	0.740901	0.681443
T.cells	TPPP	-0.49945	0.182012	-0.75542	0.452004	-5.04739	0.795917	0.744525
T.cells	SLC12A3	0.157922	1.874484	0.755413	0.452009	-5.88358	0.766229	0.710329
T.cells	TCEA1	0.046418	7.486226	0.755224	0.452122	-6.77261	0.675002	0.607715
T.cells	TAX1BP3	-0.094	4.090035	-0.75519	0.452141	-6.15795	0.728915	0.667993
T.cells	GM40787	-0.34469	0.205726	-0.75505	0.452223	-5.17525	0.795493	0.744227
T.cells	CCNG2	-0.07808	5.189545	-0.755	0.452253	-6.46926	0.711032	0.647929
T.cells	SECTM1A	0.458504	-1.13086	0.754744	0.452408	-4.96721	0.819668	0.772408
T.cells	E130308A1	0.073379	4.522919	0.754612	0.452487	-6.35654	0.721825	0.660149
T.cells	HEPACAM2	-0.49456	0.779743	-0.75455	0.452522	-5.0487	0.785313	0.73258
T.cells	CAPN7	-0.05877	5.340998	-0.75426	0.452696	-6.41378	0.708602	0.645409
T.cells	BIRC2	0.070369	5.673482	0.754237	0.452711	-6.44524	0.703293	0.639462
T.cells	PPP6C	-0.04465	6.737344	-0.75417	0.452749	-6.6705	0.686557	0.62081
T.cells	ZFP668	0.084303	3.829078	0.754169	0.452752	-6.13723	0.73322	0.673163
T.cells	PI16	0.382442	1.135522	0.75414	0.452769	-5.08725	0.779064	0.725527
T.cells	SRP72	-0.05017	6.657086	-0.75406	0.452815	-6.62922	0.687806	0.622218
T.cells	BCAS3	0.068138	7.334935	0.753906	0.452909	-6.65735	0.677321	0.610587
T.cells	GM43251	0.523398	-0.97845	0.753891	0.452918	-4.98163	0.816878	0.769391
T.cells	SP2	0.073404	4.742762	0.753831	0.452953	-6.32828	0.718249	0.656279
T.cells	ZKSCAN5	-0.15903	2.402977	-0.75365	0.453062	-5.66649	0.757171	0.700431
T.cells	ADHFE1	-0.20316	2.690792	-0.75364	0.453069	-5.53624	0.752279	0.694847
T.cells	PAK4	-0.14003	2.299468	-0.75356	0.453116	-5.72657	0.758937	0.702451
T.cells	CROCC	0.216412	1.025643	0.75353	0.453134	-5.45813	0.780989	0.727768
T.cells	IMMP2L	-0.10294	6.534254	-0.75338	0.453224	-6.56835	0.689722	0.624355
T.cells	FBXW5	-0.11152	3.39907	-0.7533	0.453272	-5.89151	0.740367	0.681294
T.cells	TSR2	0.183935	2.118125	0.753224	0.453316	-5.62838	0.762041	0.706015
T.cells	ABCD3	0.094268	4.193442	0.752872	0.453527	-6.11205	0.727449	0.666458
T.cells	COPRS	-0.30407	0.717795	-0.75256	0.453712	-5.19727	0.786868	0.734153
T.cells	PRXL2A	-0.21712	4.184431	-0.75231	0.453865	-5.8095	0.727931	0.666758
T.cells	HIPK3	-0.07006	5.445686	-0.75158	0.4543	-6.37497	0.70799	0.64383
T.cells	CAMP	-0.31063	4.463959	-0.75153	0.454327	-5.83127	0.723875	0.661683
T.cells	ADCY4	-0.41336	1.413611	-0.75143	0.454388	-5.16954	0.775376	0.720342

T.cells	DNAJC5	0.043811	6.766699	0.751284	0.454477	-6.61323	0.68717	0.620695
T.cells	OAS1G	0.464847	-0.24881	0.750925	0.454692	-5.01034	0.804935	0.754642
T.cells	NIF3L1	-0.11285	3.000203	-0.75089	0.454711	-5.89922	0.748257	0.689507
T.cells	NEU1	-0.09476	4.426111	-0.75082	0.454753	-6.10657	0.72457	0.662689
T.cells	PGAP2	0.087701	5.75264	0.750781	0.454778	-6.35964	0.703166	0.638681
T.cells	RRBP1	-0.05353	7.951353	-0.75065	0.454855	-6.75938	0.668995	0.600789
T.cells	L3MBTL1	-0.29651	0.787342	-0.75053	0.454931	-5.27167	0.786444	0.733378
T.cells	ZZEF1	-0.06168	5.867775	-0.75048	0.454955	-6.4604	0.701336	0.636718
T.cells	NCR1	-0.51415	1.170957	-0.75009	0.455194	-5.07429	0.779995	0.725769
T.cells	RUNDC3B	0.126763	3.848947	0.749765	0.455386	-6.1806	0.734558	0.673712
T.cells	TXNDC16	0.100562	5.44957	0.7494	0.455606	-6.37703	0.708711	0.644455
T.cells	MRVI1	-0.26256	0.497468	-0.74904	0.455823	-5.33384	0.792419	0.739792
T.cells	MSRB2	0.275282	1.023579	0.749035	0.455825	-5.32804	0.783115	0.729061
T.cells	CAAP1	0.071704	4.575271	0.749027	0.455829	-6.30035	0.722901	0.660479
T.cells	ANAPC16	0.054872	5.656735	0.748722	0.456012	-6.49863	0.705625	0.640954
T.cells	A230056P1	-0.28167	0.407448	-0.74834	0.456243	-5.26137	0.79441	0.741804
T.cells	CORO1A	-0.064	8.516071	-0.74833	0.456247	-6.94338	0.661502	0.591985
T.cells	H3F3B	-0.03921	9.934295	-0.74814	0.456359	-7.1985	0.640594	0.569137
T.cells	DGKH	-0.12114	4.909539	-0.74765	0.456652	-6.07504	0.718033	0.654716
T.cells	RAPGEF3	0.226949	1.358645	0.747469	0.456764	-5.48033	0.777861	0.722769
T.cells	CSF2RA	0.097156	5.196056	0.747456	0.456772	-6.20007	0.713398	0.64958
T.cells	C1GALT1C1	0.103766	4.044817	0.747446	0.456778	-6.03457	0.732194	0.670735
T.cells	FNBP4	0.052956	6.074991	0.747437	0.456783	-6.5908	0.699354	0.633878
T.cells	CHAF1B	-0.10433	3.382455	-0.747	0.457043	-6.14935	0.743218	0.683419
T.cells	MARCKSL1	-0.08925	7.792982	-0.74698	0.457059	-6.86254	0.672652	0.604458
T.cells	GM49164	0.202288	0.920748	0.746852	0.457135	-5.56146	0.785551	0.731855
T.cells	TRAPPC1	-0.08071	5.432947	-0.74685	0.457135	-6.47166	0.709587	0.64552
T.cells	CCL4	-0.45931	7.388292	-0.74681	0.45716	-5.8435	0.678853	0.611328
T.cells	PRRC2C	0.044485	7.744202	0.746637	0.457263	-6.83626	0.673396	0.605316
T.cells	HTATIP2	-0.10772	4.480259	-0.74652	0.457336	-5.96624	0.725031	0.662971
T.cells	ZFPM1	-0.07992	4.248218	-0.74647	0.457362	-6.39961	0.72884	0.667267
T.cells	CHRNA9	0.200702	2.214161	0.746472	0.457363	-5.72402	0.763038	0.70612
T.cells	NR6A1OS	0.147517	2.067272	0.745584	0.457897	-5.88339	0.766196	0.709278
T.cells	GM41555	-0.26576	0.571556	-0.74546	0.457972	-5.38533	0.792387	0.739405
T.cells	GBP8	0.260846	3.65151	0.745181	0.458139	-5.46536	0.739331	0.678765
T.cells	CLIP2	0.171716	2.886513	0.74518	0.458139	-5.48049	0.752193	0.693368
T.cells	NSUN3	-0.13902	2.658689	-0.74516	0.458149	-5.66981	0.756063	0.697776
T.cells	EIF2B4	0.068432	4.102435	0.745092	0.458192	-6.22514	0.731846	0.670329
T.cells	S100A8	0.42028	6.633628	0.744953	0.458276	-6.1062	0.691133	0.624745
T.cells	TSPAN6	0.222744	1.103606	0.744751	0.458397	-5.58559	0.782977	0.728668
T.cells	KIF1C	0.133601	3.254466	0.744746	0.4584	-5.75252	0.745981	0.686365
T.cells	TMEM80	0.100594	3.276451	0.744673	0.458444	-5.89968	0.745611	0.685955
T.cells	DSCC1	-0.1203	2.435007	-0.74465	0.458456	-5.98688	0.759881	0.702202
T.cells	CD55	-0.15145	4.863884	-0.74418	0.458742	-6.17314	0.719619	0.656503
T.cells	CZIB	-0.10485	3.658795	-0.74406	0.458815	-5.97692	0.739468	0.678918
T.cells	GID8	0.063192	5.195402	0.743964	0.458871	-6.40325	0.714247	0.650513
T.cells	NICN1	-0.27018	0.820121	-0.74395	0.458877	-5.30295	0.788253	0.734705

T.cells	DUSP19	-0.16344	2.048701	-0.74358	0.4591	-5.60553	0.766896	0.710174
T.cells	TRIM34A	0.202205	3.308515	0.743496	0.459153	-5.67403	0.745441	0.685785
T.cells	ARID1B	-0.04222	8.198201	-0.74345	0.45918	-6.9659	0.667376	0.598528
T.cells	IL18	-0.16973	3.422293	-0.74334	0.459249	-5.65685	0.743531	0.683683
T.cells	SCLT1	-0.09363	4.474106	-0.74322	0.459319	-6.26436	0.72609	0.663958
T.cells	FFAR2	0.458632	-0.53401	0.743194	0.459335	-5.02629	0.812674	0.763132
T.cells	CEP55	-0.12947	3.678611	-0.74279	0.459579	-6.29808	0.739396	0.678944
T.cells	MED30	-0.06241	5.369422	-0.74275	0.459605	-6.50058	0.711691	0.647728
T.cells	RGS10	-0.07888	5.509055	-0.74271	0.459624	-6.35062	0.709447	0.645215
T.cells	SRSF11	-0.03402	7.871425	-0.74228	0.459883	-6.88178	0.672764	0.604191
T.cells	GM2449	-0.30257	1.279792	-0.74212	0.459983	-5.29251	0.780818	0.725836
T.cells	CNOT11	0.082843	4.112527	0.741612	0.460288	-6.17806	0.73285	0.670946
T.cells	MRPS22	0.094666	3.085855	0.741595	0.460298	-6.04852	0.750019	0.690393
T.cells	OLFML3	-0.27696	1.284348	-0.74146	0.460377	-5.25445	0.781074	0.725884
T.cells	CACNB1	0.226594	0.846674	0.741333	0.460456	-5.50087	0.788815	0.73483
T.cells	E2F5	-0.11269	2.69996	-0.74115	0.460569	-5.87718	0.756693	0.698045
T.cells	CEP128	0.072327	6.336302	0.740725	0.460823	-6.70124	0.697289	0.631034
T.cells	BTBD19	-0.16845	2.725168	-0.74065	0.460871	-5.5821	0.756545	0.697766
T.cells	RAB18	-0.05689	5.748093	-0.74052	0.460946	-6.44021	0.706636	0.641517
T.cells	B130055M	-0.14744	2.013314	-0.74043	0.461002	-5.7591	0.768768	0.711827
T.cells	ABHD17B	-0.05156	6.542955	-0.73996	0.461283	-6.61276	0.694049	0.6277
T.cells	ANKRD40	0.079437	4.238658	0.739833	0.461361	-6.17586	0.731174	0.669313
T.cells	GUCA1A	0.23653	1.4794	0.73983	0.461363	-5.39326	0.778069	0.722709
T.cells	CDT1	-0.07034	4.370678	-0.73981	0.461374	-6.35544	0.728998	0.666862
T.cells	NANP	-0.13498	2.821135	-0.73975	0.461409	-5.87798	0.754928	0.696277
T.cells	MAP2K3	0.090294	6.01457	0.73965	0.461472	-6.42111	0.702402	0.637086
T.cells	METRNL	-0.39209	3.205494	-0.73951	0.461558	-5.20752	0.748417	0.688929
T.cells	UNC45B	0.419814	0.423376	0.739491	0.461568	-5.09814	0.796742	0.744324
T.cells	UBE2E2	-0.21357	3.465951	-0.73944	0.461599	-5.70791	0.744035	0.683976
T.cells	6030468B1	-0.45064	0.279697	-0.73909	0.461809	-5.06237	0.799499	0.747421
T.cells	4833403J1	0.330381	0.509888	0.738942	0.4619	-5.28302	0.795381	0.742735
T.cells	EHMT1	0.056874	6.063885	0.738939	0.461901	-6.60441	0.701781	0.636406
T.cells	CD93	0.094995	3.89899	0.738479	0.46218	-6.26372	0.737048	0.676076
T.cells	RBAK	-0.2224	1.336784	-0.73839	0.462235	-5.41744	0.780828	0.726026
T.cells	ETL4	-0.24999	2.396305	-0.73835	0.462255	-5.4186	0.762439	0.704952
T.cells	SLC16A1	-0.08265	3.905544	-0.73827	0.462305	-6.32577	0.736938	0.675963
T.cells	UCP1	0.368966	-1.00774	0.738229	0.462331	-4.99589	0.822978	0.774842
T.cells	TKT	-0.0643	6.932084	-0.73814	0.462386	-6.69105	0.688187	0.621372
T.cells	TDRD7	-0.16804	3.385152	-0.7378	0.462589	-5.66353	0.74564	0.685898
T.cells	CCNB2	-0.11328	5.882225	-0.73778	0.462603	-6.73448	0.704744	0.63984
T.cells	PTCD3	-0.06812	4.845166	-0.73771	0.462645	-6.41201	0.721466	0.658583
T.cells	ZFP930	0.149453	2.204594	0.73764	0.462686	-5.72311	0.765736	0.708797
T.cells	4933408B1	0.165555	1.941966	0.737193	0.462957	-5.71753	0.770275	0.714056
T.cells	THTPA	0.224468	1.360701	0.737135	0.462992	-5.34177	0.780408	0.725711
T.cells	TULP3	0.16457	1.974251	0.736931	0.463115	-5.65809	0.769716	0.713534
T.cells	EIF3A	-0.04191	7.379667	-0.73678	0.463206	-6.79482	0.681241	0.613904
T.cells	MSL3	-0.06335	4.998465	-0.73672	0.463246	-6.43348	0.718971	0.655985

T.cells	SLPI	0.272489	4.206306	0.736426	0.463421	-5.76725	0.731953	0.670713
T.cells	GPRC5C	-0.44847	1.29284	-0.73633	0.463477	-5.11205	0.781599	0.72735
T.cells	STARD5	0.092719	4.526318	0.736125	0.463603	-6.16275	0.726682	0.664822
T.cells	ZFP810	0.135583	2.914739	0.736064	0.46364	-5.83659	0.753588	0.695333
T.cells	MARK3	-0.05027	6.304211	-0.73569	0.463868	-6.57332	0.698044	0.632781
T.cells	SUCLG1	-0.07367	5.422184	-0.73553	0.463961	-6.41575	0.712116	0.648512
T.cells	FLRT3	0.436521	-0.84839	0.735512	0.463974	-5.02345	0.820048	0.772015
T.cells	MAF	0.165053	5.654779	0.735488	0.463989	-6.12248	0.70838	0.644327
T.cells	GFOD2	0.172676	2.309358	0.735473	0.463998	-5.63054	0.763933	0.707198
T.cells	UBL4A	-0.09383	4.202976	-0.73542	0.464028	-6.21669	0.732008	0.670904
T.cells	KTI12	-0.09492	3.941057	-0.7354	0.464043	-6.2219	0.736348	0.675813
T.cells	EVL	0.075104	6.485099	0.735118	0.464213	-6.59865	0.695191	0.629676
T.cells	BRI3	0.059735	7.328714	0.73506	0.464248	-6.69268	0.682028	0.615061
T.cells	CFL2	0.081197	4.631141	0.735035	0.464263	-6.26383	0.724964	0.663033
T.cells	DFFA	0.115444	2.939835	0.735005	0.464282	-5.92804	0.753162	0.694988
T.cells	KIF14	0.15123	2.55406	0.734662	0.464489	-6.0392	0.759736	0.702526
T.cells	ALAS1	0.14314	3.683631	0.734511	0.464581	-5.8761	0.740638	0.680791
T.cells	SGF29	-0.06598	4.671195	-0.7345	0.46459	-6.32199	0.724308	0.662332
T.cells	SLC43A1	0.19143	1.089863	0.734413	0.46464	-5.66817	0.785171	0.731718
T.cells	5330438D1	0.069677	4.842586	0.734328	0.464692	-6.43694	0.721508	0.659193
T.cells	PHF23	-0.08164	5.240241	-0.73431	0.464701	-6.36723	0.715052	0.651937
T.cells	CYBA	-0.07092	8.55691	-0.73411	0.464824	-6.76372	0.663287	0.594471
T.cells	KCNK5	-0.18176	1.47886	-0.73403	0.46487	-5.64058	0.778338	0.72392
T.cells	RUSC2	0.407571	0.909936	0.734025	0.464876	-5.10995	0.788351	0.735454
T.cells	AC166172.	-0.26826	0.939575	-0.73402	0.464881	-5.29597	0.787826	0.734849
T.cells	KLHL6	0.068776	6.109159	0.733842	0.464987	-6.70337	0.701133	0.636428
T.cells	RLN3	0.375556	-0.84713	0.733695	0.465075	-5.02146	0.820025	0.772241
T.cells	PPID	-0.07029	4.87562	-0.73361	0.465129	-6.40402	0.72097	0.65868
T.cells	GM4707	0.157453	2.866085	0.733574	0.465149	-5.86621	0.754415	0.696568
T.cells	TBXA2R	0.192943	2.36173	0.733437	0.465233	-5.73762	0.763033	0.70645
T.cells	ATL3	0.061174	5.439187	0.733159	0.465401	-6.41487	0.711843	0.648498
T.cells	RUVBL2	0.093802	3.693884	0.733096	0.465439	-6.1775	0.740466	0.680782
T.cells	GM29417	-0.16056	1.098886	-0.733	0.465499	-5.6398	0.785012	0.731728
T.cells	PRR14	0.080787	4.524923	0.732966	0.465518	-6.26013	0.726705	0.665216
T.cells	MEF2B	0.120279	3.605786	0.732809	0.465614	-5.87978	0.741939	0.682477
T.cells	TBC1D30	-0.35608	1.06093	-0.7328	0.465621	-5.23966	0.785682	0.732525
T.cells	9130230N(	0.256897	0.640779	0.732764	0.465641	-5.42243	0.793129	0.741124
T.cells	ENY2	-0.05729	5.972518	-0.73271	0.465674	-6.58929	0.703305	0.638961
T.cells	PRPS1	-0.08997	3.621673	-0.73266	0.465705	-6.1501	0.741673	0.682175
T.cells	ANAPC10	-0.06952	4.581596	-0.73243	0.465846	-6.29149	0.725775	0.664201
T.cells	2010109A1	-0.16783	2.521995	-0.73215	0.466016	-5.6492	0.760285	0.703439
T.cells	PRKRIP1	-0.07784	3.964552	-0.73172	0.466273	-6.14922	0.735958	0.675869
T.cells	FCRLS	0.675623	-1.03209	0.73166	0.466311	-4.99321	0.823426	0.77654
T.cells	TMEM87B	0.072316	5.127611	0.731566	0.466368	-6.27885	0.716875	0.654345
T.cells	PROX2	0.251408	1.188752	0.73145	0.466438	-5.36565	0.783429	0.730155
T.cells	8030456M	0.274491	1.090368	0.731328	0.466512	-5.26538	0.785163	0.732165
T.cells	MICAL2	-0.35126	1.966497	-0.73118	0.466602	-5.19764	0.76985	0.714552

T.cells	TMEM18	0.204989	1.593876	0.731163	0.466613	-5.44829	0.776328	0.721992
T.cells	HIPK2	0.075556	6.550097	0.731017	0.466702	-6.53977	0.694168	0.628974
T.cells	ZFP513	-0.16826	2.534011	-0.73101	0.466706	-5.58072	0.760079	0.703363
T.cells	GRWD1	-0.11788	2.674334	-0.73097	0.466731	-5.92918	0.757681	0.700623
T.cells	WFDC17	0.369821	5.906168	0.730959	0.466737	-6.04434	0.704362	0.640352
T.cells	PPP1R37	0.07154	4.592802	0.730804	0.466831	-6.30909	0.725592	0.664243
T.cells	HIST2H2BB	-0.23026	-0.261	-0.73072	0.466881	-5.40505	0.809332	0.760207
T.cells	RFC5	-0.10025	4.185223	-0.73043	0.467059	-6.3506	0.732301	0.671949
T.cells	PLXNA1	0.284328	0.899006	0.730184	0.467208	-5.34443	0.788544	0.736373
T.cells	HSPA12B	-0.22983	0.480164	-0.73013	0.467239	-5.39463	0.795993	0.744989
T.cells	RBM7	0.067586	5.855126	0.730051	0.467289	-6.47791	0.705176	0.641529
T.cells	ACOX2	-0.34323	1.030725	-0.73003	0.4673	-5.22839	0.786215	0.733684
T.cells	ABHD14B	-0.23811	2.125702	-0.73003	0.467304	-5.34764	0.767097	0.711691
T.cells	PATZ1	0.075991	4.251704	0.729896	0.467383	-6.28593	0.731203	0.670814
T.cells	SOCS7	0.08466	4.207792	0.729857	0.467407	-6.15152	0.731928	0.671635
T.cells	ZFP954	-0.18903	2.050779	-0.72983	0.467426	-5.58703	0.768391	0.713175
T.cells	ZFP398	-0.0872	4.517006	-0.72966	0.467525	-6.31123	0.726835	0.665957
T.cells	SERTAD3	0.107354	4.131362	0.729652	0.467532	-6.15135	0.733192	0.673144
T.cells	CARM1	-0.07229	4.727042	-0.72961	0.467555	-6.26432	0.723395	0.662079
T.cells	GLRX5	0.060236	6.17176	0.729589	0.46757	-6.58986	0.70014	0.635978
T.cells	AGO4	0.151277	3.167589	0.729576	0.467578	-5.74389	0.749306	0.691449
T.cells	2410002F2	-0.09484	3.766412	-0.72938	0.467699	-6.03299	0.739256	0.680061
T.cells	GM50373	-0.33585	0.358844	-0.72934	0.467724	-5.20737	0.798162	0.74764
T.cells	UBXN6	0.090021	4.786672	0.729328	0.467729	-6.20275	0.722421	0.66102
T.cells	TIAL1	-0.0516	5.798885	-0.72915	0.467837	-6.5523	0.706074	0.6427
T.cells	HIVEP2	-0.08426	8.386816	-0.72915	0.467837	-6.97042	0.665853	0.59803
T.cells	GM2682	-0.42259	2.755703	-0.72913	0.467849	-5.26956	0.756293	0.699511
T.cells	PYGB	0.071853	4.748164	0.729087	0.467875	-6.28257	0.723049	0.661783
T.cells	ATF4	-0.07053	6.443614	-0.72885	0.468022	-6.5883	0.695844	0.631387
T.cells	KCNA2	-0.49521	0.96787	-0.72857	0.46819	-5.09892	0.787326	0.735383
T.cells	BMPR2	0.09332	5.519027	0.728382	0.468304	-6.34699	0.710558	0.647935
T.cells	TMPO	-0.06015	7.067829	-0.72829	0.468361	-6.91813	0.686073	0.62059
T.cells	TOR1AIP1	-0.05769	7.42657	-0.72825	0.468385	-6.73315	0.680517	0.614424
T.cells	RPA2	-0.08439	4.645282	-0.72817	0.468432	-6.47418	0.724732	0.663914
T.cells	FANCM	0.089223	3.67592	0.727985	0.468547	-6.24702	0.740766	0.682111
T.cells	PMS2	-0.07483	3.954235	-0.7278	0.468661	-6.25987	0.736129	0.676892
T.cells	COA5	0.112588	4.468388	0.727644	0.468754	-6.07429	0.727634	0.667283
T.cells	D2HGDH	-0.18315	2.08867	-0.72755	0.468809	-5.50424	0.767737	0.712964
T.cells	RTN3	0.058004	8.060675	0.72736	0.468927	-6.85909	0.670799	0.603856
T.cells	SMIM14	0.045443	7.74729	0.727322	0.46895	-6.83105	0.675585	0.609146
T.cells	COPS8	-0.05921	5.091019	-0.72719	0.469033	-6.37061	0.717469	0.655906
T.cells	PARN	0.091958	3.875182	0.727175	0.46904	-6.08011	0.737444	0.678485
T.cells	TRMT10A	-0.08518	3.81366	-0.7271	0.469088	-6.18686	0.738468	0.679648
T.cells	AHNAK	-0.22256	6.416793	-0.72709	0.469094	-6.0142	0.696267	0.632138
T.cells	1-Mar	0.452296	4.477654	0.72704	0.469122	-5.3368	0.727481	0.667202
T.cells	LARGE1	-0.11874	5.482959	-0.727	0.469146	-6.2651	0.711138	0.648788
T.cells	SMARCE1	-0.0444	6.302182	-0.72697	0.469164	-6.66685	0.698076	0.634158

T.cells	EXOSC8	-0.07115	5.033192	-0.72689	0.469212	-6.50313	0.718407	0.657007
T.cells	DHRS3	0.141528	4.170119	0.726824	0.469254	-5.96989	0.732551	0.672996
T.cells	MAPK9	0.073524	4.751185	0.726713	0.469322	-6.27495	0.723	0.662215
T.cells	MGA	0.068972	5.917554	0.726644	0.469364	-6.49947	0.70418	0.641056
T.cells	LPGAT1	-0.07985	6.245156	-0.72656	0.469415	-6.4953	0.698978	0.635242
T.cells	GM11772	-0.36382	0.302935	-0.72646	0.469476	-5.0398	0.799164	0.749469
T.cells	LRP1B	0.210436	1.219155	0.726197	0.469636	-5.44529	0.782894	0.730713
T.cells	TFG	0.051856	5.864765	0.726177	0.469648	-6.59054	0.705022	0.642113
T.cells	MREG	-0.16542	3.199109	-0.72611	0.469687	-6.04761	0.748774	0.691582
T.cells	SH2D1B1	0.430999	1.695197	0.726069	0.469714	-5.07659	0.774562	0.721127
T.cells	PPM1E	0.099367	4.964118	0.725415	0.470113	-6.50989	0.719678	0.658506
T.cells	ANAPC7	-0.06786	4.359074	-0.72533	0.470165	-6.28709	0.729582	0.669691
T.cells	LRRC57	-0.17064	2.732576	-0.72533	0.470167	-5.73082	0.756843	0.700699
T.cells	6-Mar	-0.05144	6.3538	-0.72512	0.470294	-6.55592	0.697404	0.633515
T.cells	GM16283	-0.26644	0.252287	-0.72511	0.470296	-5.23111	0.800237	0.750702
T.cells	CACNA1I	0.194799	-0.17458	0.725064	0.470327	-5.35149	0.807932	0.759656
T.cells	LSM14B	0.083505	3.604671	0.725019	0.470355	-6.0771	0.742111	0.683929
T.cells	SLA	0.148964	5.280678	0.725003	0.470365	-5.88078	0.714546	0.652754
T.cells	B3GNTL1	0.160901	2.673331	0.724948	0.470398	-5.67625	0.757854	0.701886
T.cells	VAT1	0.152935	3.491225	0.724531	0.470653	-5.72737	0.744308	0.686173
T.cells	KCTD12B	0.33955	0.963206	0.724253	0.470822	-5.17675	0.787883	0.736241
T.cells	PDCL3	0.08116	4.536557	0.724034	0.470956	-6.18723	0.726953	0.666688
T.cells	GM10762	0.119007	2.549426	0.723983	0.470987	-5.87442	0.760274	0.704592
T.cells	CCDC88B	0.223421	3.695402	0.72396	0.471001	-5.36056	0.740888	0.682481
T.cells	PRKCH	0.131631	6.999653	0.723857	0.471065	-6.50483	0.687549	0.622503
T.cells	ZFC3H1	-0.0527	6.914629	-0.72379	0.471105	-6.6715	0.688874	0.623982
T.cells	IFI204	0.328121	4.390085	0.723787	0.471108	-5.52123	0.729361	0.669415
T.cells	BCL2	0.201974	5.016128	0.723582	0.471233	-5.90718	0.719138	0.657927
T.cells	TUFT1	-0.13269	2.2113	-0.72355	0.471251	-5.71519	0.766104	0.711341
T.cells	PHF11C	-0.18077	1.989779	-0.72318	0.471476	-5.56701	0.770193	0.715873
T.cells	4930426D(	-0.28036	-0.21569	-0.72291	0.471644	-5.26803	0.809329	0.761161
T.cells	DUS1L	-0.08514	4.064535	-0.72289	0.471654	-6.23249	0.735043	0.675782
T.cells	GM27216	-0.33958	0.912751	-0.72283	0.471692	-5.23266	0.789099	0.737722
T.cells	TRIB1	-0.13235	5.765307	-0.72247	0.47191	-6.09218	0.707425	0.644691
T.cells	GNG5	0.053246	8.925654	0.722459	0.471919	-6.97609	0.658514	0.590403
T.cells	RNF180	0.314537	2.247716	0.722402	0.471954	-5.30848	0.765875	0.711004
T.cells	5530601H(	-0.09894	3.904215	-0.72202	0.47219	-6.18391	0.738077	0.679135
T.cells	ASB4	-0.47332	1.68448	-0.7217	0.472383	-5.20076	0.776114	0.722454
T.cells	ZFP983	0.151296	2.38923	0.721611	0.472438	-5.73331	0.763905	0.708445
T.cells	CENPM	-0.09433	3.916181	-0.72148	0.47252	-6.36989	0.738077	0.679015
T.cells	ELOVL7	-0.40896	-0.19718	-0.72137	0.472587	-5.03736	0.809618	0.761289
T.cells	DYNC1LI1	-0.05204	6.412559	-0.72075	0.472964	-6.59156	0.697577	0.633733
T.cells	YARS	-0.08023	5.391202	-0.72074	0.472974	-6.52449	0.713888	0.652013
T.cells	MRPS27	-0.09987	3.208477	-0.72067	0.473014	-6.06696	0.749953	0.692876
T.cells	TIAM2	-0.18668	2.628932	-0.72047	0.473135	-5.81047	0.75981	0.704208
T.cells	H2-AA	-0.23567	8.331767	-0.72045	0.473147	-6.74283	0.667875	0.600846
T.cells	GM47096	0.194771	1.734536	0.720314	0.473232	-5.48939	0.775259	0.721974

T.cells	RAPGEF5	0.135696	5.011787	0.720199	0.473302	-6.35657	0.720038	0.659057
T.cells	MMP12	0.573564	-0.05819	0.720182	0.473312	-5.04099	0.8071	0.758842
T.cells	ZFP607A	-0.14681	1.840055	-0.72013	0.473344	-5.6466	0.773421	0.719903
T.cells	IFRD2	-0.09538	3.30652	-0.71991	0.473478	-6.11015	0.748297	0.691166
T.cells	TSG101	0.059999	5.622255	0.719847	0.473518	-6.49211	0.710167	0.648033
T.cells	CAMTA2	0.127879	3.696678	0.719781	0.473559	-5.78385	0.741741	0.683752
T.cells	GM10863	0.385324	0.229142	0.719658	0.473634	-5.12609	0.801917	0.752948
T.cells	MPI	-0.13691	2.225319	-0.71965	0.473639	-5.73379	0.766746	0.712323
T.cells	H2-OB	-0.10708	4.007971	-0.71964	0.473645	-6.42057	0.73655	0.677856
T.cells	4921511C1	0.168351	2.59393	0.71957	0.473687	-5.62778	0.760409	0.705058
T.cells	PAPSS1	0.080919	4.181725	0.719565	0.473691	-6.12176	0.733666	0.674585
T.cells	2510017J1	-0.2159	1.414805	-0.7194	0.47379	-5.53943	0.780908	0.728672
T.cells	HES6	0.073226	4.105884	0.7189	0.474098	-6.30876	0.735303	0.676237
T.cells	TGFB2	-0.47409	0.46572	-0.71885	0.474131	-5.17447	0.798084	0.748297
T.cells	TANK	0.089774	6.451404	0.718426	0.474389	-6.44784	0.697606	0.633628
T.cells	RHOT1	0.05927	5.400436	0.718243	0.474501	-6.39856	0.714468	0.652476
T.cells	UNC13B	-0.37814	0.758993	-0.71761	0.474888	-5.09127	0.793646	0.742538
T.cells	IFITM10	-0.44615	3.144158	-0.71741	0.475011	-5.25346	0.752184	0.694951
T.cells	ITPRIP	0.131243	3.468077	0.717407	0.475014	-5.83588	0.746711	0.688716
T.cells	ING5	0.09498	3.456639	0.717313	0.475072	-6.01661	0.746904	0.688935
T.cells	CD200	0.379743	1.910379	0.717209	0.475136	-5.16242	0.773374	0.719213
T.cells	ZFP280B	0.157213	1.826966	0.717073	0.475219	-5.71338	0.774827	0.720883
T.cells	TAF1A	0.111976	3.171877	0.717002	0.475263	-5.96268	0.751714	0.69442
T.cells	GM13986	0.534272	1.376917	0.717002	0.475263	-5.14113	0.782706	0.729962
T.cells	ATG12	0.083971	4.873947	0.71685	0.475356	-6.19707	0.723427	0.662332
T.cells	NUDT18	0.139514	2.627052	0.71649	0.475577	-5.63015	0.761197	0.705146
T.cells	MECR	-0.09792	3.581314	-0.71645	0.475604	-6.05642	0.745001	0.686683
T.cells	TOM1L1	-0.26969	0.714609	-0.71624	0.475731	-5.31729	0.794644	0.743703
T.cells	ARMCX5	0.133033	2.433168	0.716044	0.475851	-5.72346	0.764528	0.709159
T.cells	TRIM17	0.152875	2.197819	0.715996	0.475881	-5.69627	0.768589	0.713831
T.cells	CDK5	-0.10752	3.72259	-0.71597	0.475896	-5.97039	0.74263	0.684184
T.cells	UROS	-0.11776	2.669254	-0.71595	0.475911	-5.74932	0.760474	0.704537
T.cells	ADAM12	-0.31656	0.815122	-0.7158	0.476004	-5.26799	0.792899	0.741872
T.cells	SLC37A1	0.121265	3.202303	0.715408	0.476242	-5.9629	0.751542	0.694375
T.cells	POLR2H	-0.06302	4.512761	-0.71529	0.476314	-6.37912	0.729644	0.669511
T.cells	DENND2D	0.240067	2.202221	0.715142	0.476405	-5.25849	0.768663	0.714026
T.cells	NLRX1	-0.24977	1.80919	-0.71505	0.476463	-5.37967	0.77549	0.721872
T.cells	PTCH1	0.124616	3.139748	0.715038	0.47647	-6.02066	0.752602	0.695646
T.cells	PPP3CA	-0.04795	8.812084	-0.71485	0.476587	-6.96751	0.66194	0.594101
T.cells	MLLT3	0.082309	5.728512	0.714675	0.476693	-6.56284	0.709864	0.647367
T.cells	FAM189B	0.161717	2.569109	0.714656	0.476705	-5.74747	0.762341	0.70687
T.cells	ZFP729B	-0.09512	3.639068	-0.71456	0.476762	-6.01413	0.744176	0.686157
T.cells	ACOT7	-0.07881	4.076826	-0.71434	0.4769	-6.25174	0.736862	0.677921
T.cells	FLII	0.058418	5.969326	0.714089	0.477053	-6.55506	0.706006	0.643185
T.cells	CD72	-0.09197	4.334961	-0.71401	0.477101	-6.38037	0.73258	0.673139
T.cells	1700112J1	-0.38164	0.638697	-0.71399	0.477114	-5.11449	0.796155	0.746014
T.cells	IRAK2	-0.24471	6.796286	-0.71399	0.477117	-5.72255	0.692909	0.628558

T.cells	WWTR1	-0.31229	2.126919	-0.71398	0.47712	-5.29054	0.769967	0.71579
T.cells	PDE1C	-0.23741	3.989093	-0.71394	0.477143	-5.77233	0.738322	0.67965
T.cells	RNF8	0.064247	4.800789	0.713878	0.477183	-6.36981	0.724912	0.664476
T.cells	CEP72	-0.12641	2.208429	-0.71368	0.477306	-5.84655	0.76858	0.714248
T.cells	0610043K1	-0.34936	1.133363	-0.71364	0.477329	-5.22184	0.787386	0.735914
T.cells	GLCCI1	-0.0633	7.348661	-0.71329	0.477544	-6.81493	0.684447	0.619103
T.cells	GM44284	-0.40591	-0.05378	-0.71296	0.477747	-5.03854	0.808804	0.760821
T.cells	SLC25A14	-0.13872	2.078186	-0.71286	0.477809	-5.66217	0.77099	0.717104
T.cells	D330023K1	0.211065	2.22594	0.712786	0.477855	-5.48604	0.76843	0.71418
T.cells	TGM1	0.394404	0.53017	0.712705	0.477905	-5.10397	0.798281	0.748666
T.cells	BECN1	-0.05831	5.896307	-0.71264	0.477945	-6.50826	0.707337	0.644852
T.cells	DTNB	-0.07733	5.293687	-0.71257	0.477988	-6.4311	0.717046	0.655762
T.cells	RHOQ	-0.09758	5.771108	-0.71234	0.478129	-6.32979	0.709344	0.647103
T.cells	ECT2	0.115312	4.373594	0.712325	0.478139	-6.41631	0.73211	0.672776
T.cells	TTC32	-0.07554	4.101346	-0.71231	0.478149	-6.27102	0.736624	0.677893
T.cells	ZFP868	-0.09611	3.676008	-0.71228	0.478169	-6.09719	0.743728	0.685966
T.cells	LRRC49	-0.19286	1.020554	-0.71221	0.47821	-5.53959	0.78954	0.738539
T.cells	ASB7	0.108827	3.936004	0.712089	0.478285	-6.10148	0.739381	0.68102
T.cells	PIRA2	-0.35827	3.589246	-0.71186	0.478428	-5.26237	0.745188	0.687682
T.cells	LCMT1	-0.11386	3.124061	-0.71173	0.478506	-5.88381	0.753046	0.696677
T.cells	CD68	-0.2111	4.872198	-0.71171	0.478515	-5.69748	0.723913	0.663592
T.cells	GAR1	-0.07834	4.430651	-0.71158	0.478597	-6.38006	0.731171	0.671807
T.cells	MRPS25	-0.07775	4.238863	-0.71157	0.478606	-6.25968	0.734344	0.675403
T.cells	THOC6	0.076545	4.164901	0.711405	0.478706	-6.29203	0.735625	0.676835
T.cells	CFDP1	0.054528	5.986865	0.71103	0.478937	-6.57592	0.706188	0.643428
T.cells	PROX1OS	-0.34515	0.612207	-0.71079	0.479087	-5.18732	0.79729	0.747363
T.cells	APOL9B	0.359148	0.574633	0.710307	0.479383	-5.12687	0.798347	0.748331
T.cells	POLR3K	-0.07368	3.885777	-0.71014	0.479486	-6.21016	0.741047	0.682534
T.cells	CHP1	-0.05921	7.01438	-0.71006	0.479534	-6.58344	0.690426	0.62563
T.cells	TELO2	-0.14438	2.455163	-0.70974	0.479733	-5.89057	0.765444	0.710426
T.cells	LILRA5	0.366571	1.497471	0.709661	0.479782	-5.2275	0.782115	0.729604
T.cells	TMEM250-	-0.08435	4.629225	-0.70957	0.479836	-6.2641	0.728817	0.668749
T.cells	NSUN4	-0.11791	3.189091	-0.70953	0.479866	-5.88188	0.752892	0.696098
T.cells	SRA1	0.067249	5.239409	0.709007	0.480186	-6.34273	0.719095	0.657608
T.cells	FNDC7	0.396367	-0.36573	0.708906	0.480248	-5.05747	0.815805	0.768528
T.cells	3110056K0	-0.0661	4.723448	-0.70887	0.480271	-6.30584	0.72753	0.667134
T.cells	KCNK10	0.237924	0.557275	0.708694	0.480379	-5.46925	0.799094	0.749145
T.cells	TMEM176A	-0.14075	4.388779	-0.70868	0.480386	-5.92177	0.733051	0.673428
T.cells	TRMT61B	0.144444	4.178074	0.708556	0.480464	-6.08156	0.736547	0.677426
T.cells	PHRF1	-0.05455	5.804424	-0.7085	0.480498	-6.54551	0.709964	0.647454
T.cells	WDR86	-0.29839	0.234245	-0.70808	0.480759	-5.09181	0.805177	0.756113
T.cells	TXN1	0.07211	7.304482	0.708027	0.480791	-6.80244	0.686478	0.621104
T.cells	MAP3K1	-0.05189	7.970553	-0.70744	0.481155	-6.87251	0.676607	0.609906
T.cells	KYNU	-0.21322	3.853994	-0.70723	0.481282	-5.52865	0.742675	0.684038
T.cells	ZFP839	0.214267	1.537463	0.707133	0.481343	-5.34805	0.782454	0.729572
T.cells	LAD1	-0.49939	-0.99444	-0.70711	0.48136	-5.03403	0.828173	0.782697
T.cells	IL3RA	0.115478	3.561284	0.706843	0.481523	-5.92516	0.747658	0.689699

T.cells	ZFP677	-0.24834	0.788621	-0.70681	0.481541	-5.29011	0.795795	0.744978
T.cells	CSTF2T	0.107845	3.017029	0.706549	0.481705	-5.88489	0.756889	0.70031
T.cells	GM19325	0.259969	1.67424	0.706541	0.48171	-5.41049	0.780117	0.726957
T.cells	IPO8	0.067071	4.420007	0.70635	0.481828	-6.29319	0.733308	0.673494
T.cells	GM10802	-0.39483	-0.25172	-0.70628	0.481873	-5.03741	0.814583	0.766899
T.cells	GM10184	-0.19821	1.11236	-0.70621	0.481913	-5.60245	0.790031	0.738397
T.cells	MECP2	0.05276	5.847525	0.7061	0.481982	-6.56713	0.710021	0.647253
T.cells	2810408I1:	-0.19281	1.398496	-0.70606	0.48201	-5.5869	0.784968	0.732549
T.cells	VCPKMT	-0.12848	2.877826	-0.70588	0.482119	-5.891	0.759267	0.703079
T.cells	RBL2	0.08375	4.765126	0.705616	0.482282	-6.22445	0.727613	0.667155
T.cells	ENTR1	0.077249	4.880508	0.705581	0.482304	-6.28182	0.725718	0.665015
T.cells	PAN3	-0.04131	8.767803	-0.70555	0.482326	-6.9926	0.664539	0.596813
T.cells	NPC1	0.130543	4.971884	0.705415	0.482406	-5.89986	0.724221	0.663375
T.cells	ACACB	-0.29779	0.257652	-0.70496	0.482687	-5.26757	0.805334	0.756438
T.cells	PTAFR	0.480151	4.645313	0.704851	0.482756	-5.27889	0.729585	0.669552
T.cells	AAR2	-0.07163	4.021304	-0.70474	0.482823	-6.16665	0.739938	0.681283
T.cells	KLHL20	0.121652	3.27084	0.704723	0.482835	-5.97295	0.752571	0.695662
T.cells	LOCKD	0.096577	4.472202	0.704629	0.482893	-6.47909	0.732444	0.672786
T.cells	UBE2M	0.046642	7.20691	0.704313	0.483089	-6.80519	0.688494	0.623495
T.cells	ADGRF5	-0.28558	2.598167	-0.70431	0.483089	-5.44795	0.764065	0.708835
T.cells	EHD1	0.080878	6.1755	0.7043	0.483097	-6.45139	0.70477	0.641657
T.cells	MMS22L	-0.08737	4.820836	-0.70419	0.483163	-6.51658	0.726698	0.666322
T.cells	GSKIP	0.063764	4.602025	0.703934	0.483323	-6.24197	0.730299	0.670503
T.cells	2010110K1	-0.26484	-0.03523	-0.70385	0.483374	-5.41935	0.81064	0.762789
T.cells	RELB	-0.07399	6.088989	-0.7038	0.483407	-6.51863	0.706151	0.643343
T.cells	GADD45A	-0.1143	4.876654	-0.70356	0.483553	-6.19254	0.725782	0.665502
T.cells	MBNL1	0.0653	8.978349	0.703551	0.483561	-6.95249	0.661369	0.593698
T.cells	GUF1	0.105829	2.589786	0.703326	0.4837	-5.84212	0.764209	0.709246
T.cells	MTERF1A	-0.19456	1.405346	-0.70312	0.483829	-5.43622	0.784847	0.733108
T.cells	CUX1	0.044476	7.953864	0.703082	0.483852	-6.95313	0.67693	0.610974
T.cells	RANBP2	0.067849	6.846596	0.702965	0.483924	-6.67443	0.694139	0.630112
T.cells	MND1	-0.12199	2.222735	-0.70284	0.484001	-5.9333	0.77055	0.716708
T.cells	LSM5	-0.05895	5.652829	-0.70272	0.484079	-6.63075	0.713156	0.651489
T.cells	NHLRC2	0.064966	4.994885	0.702624	0.484136	-6.36657	0.723845	0.663562
T.cells	H2-OA	-0.21484	3.19042	-0.70257	0.48417	-5.64562	0.753937	0.69774
T.cells	INTS6L	0.081849	5.229737	0.702537	0.48419	-6.31029	0.720012	0.659237
T.cells	WBP2	0.067776	5.599979	0.70249	0.484219	-6.43386	0.714009	0.652477
T.cells	NEDD8	-0.03823	7.48397	-0.70247	0.484234	-6.83921	0.684183	0.619137
T.cells	RBM3	-0.04582	9.070642	-0.70231	0.484329	-7.12604	0.659984	0.592461
T.cells	TCRG-C2	-0.56266	0.685448	-0.7023	0.484338	-5.09313	0.797641	0.748146
T.cells	TATDN2	0.072042	5.004884	0.70226	0.484362	-6.25099	0.723681	0.66345
T.cells	4833418N(	-0.26201	1.533706	-0.70217	0.484415	-5.33171	0.782586	0.730734
T.cells	JHY	0.446791	-0.37714	0.702077	0.484475	-5.05062	0.816875	0.770625
T.cells	MEIG1	-0.39667	-0.47443	-0.70207	0.484482	-5.02981	0.818657	0.772707
T.cells	BST2	0.130384	6.606914	0.701936	0.484563	-6.36188	0.697918	0.634586
T.cells	QRFP	-0.35033	-0.61178	-0.70185	0.484615	-5.12427	0.821179	0.775663
T.cells	STK25	0.069396	4.440838	0.701848	0.484618	-6.27472	0.732963	0.674031

T.cells	DPP6	-0.42057	-0.15893	-0.70185	0.484619	-5.06319	0.812891	0.765981
T.cells	PEBP1	-0.04962	6.440655	-0.70128	0.484969	-6.71345	0.700659	0.63763
T.cells	FAM83F	-0.34105	1.041009	-0.7012	0.485018	-5.17049	0.79142	0.741017
T.cells	ANKRD13C	-0.06535	5.868682	-0.70107	0.485103	-6.49871	0.70979	0.647881
T.cells	ITPR1	0.070295	6.992018	0.701063	0.485105	-6.64925	0.691962	0.627933
T.cells	MAD2L1	-0.0886	4.166093	-0.70101	0.485138	-6.36624	0.737638	0.679329
T.cells	1110059G1	-0.07592	3.928256	-0.70098	0.485156	-6.17708	0.741608	0.683841
T.cells	DUSP8	-0.22257	1.274945	-0.70093	0.485187	-5.43093	0.787272	0.736216
T.cells	POLR2K	-0.04594	6.77475	-0.7008	0.485271	-6.7367	0.695377	0.631795
T.cells	ATAD2B	0.052203	6.740991	0.700773	0.485285	-6.70165	0.695909	0.632398
T.cells	GM31763	-0.12833	3.257819	-0.70056	0.485416	-6.00869	0.752931	0.696813
T.cells	PIAS3	0.102231	2.896492	0.700443	0.48549	-5.86722	0.759088	0.703854
T.cells	SHPK	-0.35064	0.252393	-0.70043	0.485497	-5.08733	0.805578	0.757526
T.cells	HSPE1-RS1	0.327948	0.18545	0.700047	0.485736	-5.24702	0.807046	0.759023
T.cells	PDCD6IP	-0.03941	6.988379	-0.69997	0.485786	-6.67033	0.692262	0.628154
T.cells	LIMD1	-0.04869	6.016318	-0.6998	0.485887	-6.62696	0.707671	0.645402
T.cells	INPP5E	-0.14346	2.260693	-0.69937	0.486158	-5.62485	0.770281	0.716659
T.cells	CLTA	-0.03991	8.442682	-0.69927	0.486218	-6.96107	0.6698	0.60342
T.cells	COPS5	-0.05716	5.288344	-0.69915	0.486292	-6.47577	0.719422	0.658843
T.cells	2310008N1	-0.17782	0.80481	-0.69901	0.486379	-5.68047	0.795908	0.746403
T.cells	UST	-0.1332	6.028853	-0.69896	0.486411	-6.5505	0.70747	0.645438
T.cells	MFSD13A	-0.17704	2.008416	-0.69894	0.486424	-5.60035	0.774666	0.72185
T.cells	ECSCR	0.319281	1.207762	0.698913	0.486441	-5.22983	0.788737	0.738101
T.cells	FARSB	-0.06055	5.008108	-0.69874	0.486546	-6.44545	0.723994	0.664097
T.cells	STFA3	0.47183	2.751543	0.698588	0.486643	-5.31561	0.761814	0.707168
T.cells	RFFL	0.093373	5.915131	0.69856	0.486661	-6.32964	0.709293	0.647562
T.cells	ZFP800	0.061848	5.365723	0.698412	0.486752	-6.4405	0.718164	0.657568
T.cells	EIF2D	0.088689	3.5699	0.698367	0.486781	-6.07212	0.74789	0.691274
T.cells	EGFEM1	0.492461	1.229611	0.698348	0.486793	-5.13277	0.78835	0.737762
T.cells	RBBP8	-0.05451	6.609426	-0.69814	0.486922	-6.68262	0.698231	0.63523
T.cells	GAPVD1	0.048909	6.832806	0.698133	0.486926	-6.67269	0.694707	0.631292
T.cells	GM43328	-0.19063	2.238918	-0.69802	0.486994	-5.52962	0.770658	0.717396
T.cells	LIN9	0.071501	4.133885	0.697907	0.487067	-6.38067	0.738433	0.680552
T.cells	ZKSCAN14	0.100232	3.081299	0.697881	0.487083	-5.92275	0.756175	0.700782
T.cells	2310011J0	-0.07887	4.446593	-0.69779	0.487141	-6.2265	0.733237	0.674654
T.cells	FBRS	-0.0822	4.965395	-0.69751	0.487312	-6.19749	0.724693	0.665033
T.cells	MEGF9	-0.10816	4.103779	-0.6975	0.487318	-6.19294	0.738935	0.681176
T.cells	NQO1	0.326493	0.792254	0.697399	0.487383	-5.18028	0.796132	0.746931
T.cells	TIMM8A1	-0.08615	4.226992	-0.69738	0.487395	-6.304	0.736882	0.678884
T.cells	TPR	0.044054	7.524789	0.697251	0.487475	-6.84628	0.683895	0.619359
T.cells	TNFRSF1B	0.199557	4.903674	0.697146	0.487541	-5.62282	0.725705	0.666263
T.cells	GALNT16	-0.19951	0.933714	-0.69703	0.487614	-5.46951	0.793607	0.744079
T.cells	SHMT1	-0.09733	4.012421	-0.69694	0.487669	-6.22415	0.74046	0.683034
T.cells	NDC80	0.095271	4.625978	0.696884	0.487704	-6.52941	0.730273	0.671481
T.cells	KMT5B	0.057166	5.91383	0.69651	0.487937	-6.52117	0.709314	0.647938
T.cells	GM15472	0.356742	1.795978	0.696379	0.488018	-5.18034	0.778377	0.726621
T.cells	TLR12	-0.34817	2.045397	-0.69634	0.488041	-5.24655	0.774022	0.721611

T.cells	AREL1	0.074343	4.263334	0.696092	0.488197	-6.23978	0.736278	0.678508
T.cells	CEP192	0.060467	5.27108	0.695924	0.488302	-6.50079	0.719703	0.659738
T.cells	NT5C	-0.07021	5.114948	-0.69587	0.488337	-6.40576	0.722248	0.662612
T.cells	UBR1	-0.06916	5.323458	-0.69572	0.488429	-6.48088	0.718851	0.65882
T.cells	CAPZB	-0.03849	8.722615	-0.69551	0.488563	-6.9605	0.665557	0.599344
T.cells	GM28981	-0.3366	0.488246	-0.69545	0.488599	-5.12127	0.801583	0.753661
T.cells	TIMP3	-0.35774	2.197691	-0.69539	0.488635	-5.36176	0.771374	0.718696
T.cells	FIZ1	0.060178	4.553694	0.695376	0.488644	-6.32813	0.731466	0.673093
T.cells	TNNI1	0.457319	-0.907	0.695348	0.488661	-5.01603	0.827041	0.783407
T.cells	SLF2	-0.05581	6.314028	-0.69533	0.488671	-6.60985	0.702918	0.640901
T.cells	GM11713	0.212839	2.37115	0.695299	0.488691	-5.52157	0.768368	0.715242
T.cells	SMPD3	-0.38336	-0.20687	-0.69515	0.488786	-5.10003	0.814175	0.768422
T.cells	SLC25A13	-0.08986	4.679744	-0.69496	0.4889	-6.28401	0.729386	0.670811
T.cells	SNX11	-0.13896	2.539196	-0.69488	0.488953	-5.70911	0.765466	0.712015
T.cells	SALL2	-0.33393	0.090711	-0.69485	0.488974	-5.14106	0.808762	0.762152
T.cells	RTL5	0.229151	0.491888	0.694749	0.489035	-5.33064	0.801517	0.753768
T.cells	RAE1	0.064836	4.525674	0.694642	0.489101	-6.35419	0.731929	0.673842
T.cells	SCPEP1OS	0.333023	0.502575	0.694607	0.489123	-5.17961	0.801325	0.753631
T.cells	WDR83OS	-0.05785	6.074614	-0.69455	0.489156	-6.60483	0.706738	0.645427
T.cells	FAM169B	-0.151	3.62896	-0.69445	0.489223	-5.86762	0.746895	0.690921
T.cells	LYZL4	0.378504	-1.24765	0.693995	0.489505	-4.99576	0.833368	0.791399
T.cells	DIAPH1	0.054086	7.355352	0.693955	0.48953	-6.66323	0.686528	0.623052
T.cells	FAR1	0.05997	6.64871	0.693935	0.489542	-6.72138	0.69761	0.635436
T.cells	LAMTOR5	0.057261	5.527925	0.693855	0.489592	-6.52644	0.715534	0.655581
T.cells	COL15A1	-0.46137	0.332157	-0.69383	0.489608	-5.03728	0.804395	0.757512
T.cells	WNK1	-0.05573	9.07644	-0.6938	0.489628	-6.99948	0.660231	0.59393
T.cells	GM10851	-0.07673	4.366594	-0.69358	0.489763	-6.39503	0.734563	0.677212
T.cells	TAF11	-0.06032	4.933052	-0.69355	0.489781	-6.41364	0.725223	0.666623
T.cells	TGDS	0.113322	3.1411	0.693524	0.489799	-5.85565	0.755156	0.700714
T.cells	EBP	-0.07969	4.791273	-0.69343	0.489859	-6.33081	0.72755	0.669259
T.cells	ASF1B	-0.0928	4.954931	-0.69328	0.489953	-6.58732	0.724907	0.666252
T.cells	ZFP866	0.106333	2.993086	0.693109	0.490058	-5.97745	0.757784	0.703713
T.cells	GNB1	-0.0317	8.848488	-0.69277	0.490269	-7.04049	0.663946	0.598032
T.cells	MTREX	-0.05249	5.477446	-0.69255	0.490408	-6.5102	0.71677	0.656955
T.cells	CAB39L	0.074413	4.624571	0.691985	0.49076	-6.30014	0.730875	0.672923
T.cells	AFG1L	0.118967	3.691074	0.691883	0.490824	-6.0246	0.746441	0.690691
T.cells	PI4K2B	0.086659	3.903982	0.691583	0.491011	-6.17472	0.742864	0.686624
T.cells	CARNS1	0.17978	2.476067	0.691503	0.491061	-5.72135	0.767163	0.714453
T.cells	SMC4	-0.05295	7.287715	-0.69149	0.491072	-6.94335	0.688127	0.624898
T.cells	USP4	-0.04682	5.660629	-0.69148	0.491073	-6.45903	0.713956	0.653855
T.cells	ZDHHC21	0.079369	4.271545	0.691427	0.491109	-6.22833	0.736726	0.679645
T.cells	ILF2	-0.05333	5.763945	-0.69141	0.491117	-6.65506	0.712289	0.651986
T.cells	GEMIN7	0.055899	5.084337	0.691142	0.491287	-6.4137	0.723321	0.664537
T.cells	EBF1	0.082821	8.683852	0.691062	0.491337	-7.29478	0.666672	0.601182
T.cells	DIABLO	0.073034	4.259159	0.691061	0.491338	-6.26889	0.736932	0.679981
T.cells	MAPK1IP1I	-0.04627	6.060276	-0.69101	0.49137	-6.57749	0.707528	0.646726
T.cells	FAM8A1	-0.10868	3.231881	-0.69086	0.491463	-5.90164	0.754211	0.699702

T.cells	CHUK	-0.06908	5.037427	-0.69084	0.491476	-6.31896	0.724088	0.665407
T.cells	NPFF	0.239346	0.954229	0.690806	0.491497	-5.32396	0.793871	0.745447
T.cells	HEATR5A	-0.08055	5.656048	-0.69053	0.491669	-6.42168	0.714183	0.654176
T.cells	ZFP942	0.081786	4.309464	0.690316	0.491804	-6.24641	0.736329	0.679211
T.cells	PPP6R1	0.048769	5.572544	0.69018	0.491889	-6.49172	0.715607	0.655784
T.cells	CSNK2A1	-0.03921	6.59773	-0.69014	0.491917	-6.69137	0.699192	0.637331
T.cells	TRP53BP2	0.093903	3.957204	0.689826	0.49211	-6.08534	0.742362	0.68604
T.cells	DRG2	-0.07819	3.507943	-0.68976	0.49215	-6.13233	0.749924	0.694685
T.cells	TRAF5	0.067023	5.308971	0.689459	0.49234	-6.51218	0.720156	0.660838
T.cells	RASA2	0.079926	6.158678	0.689417	0.492366	-6.51211	0.706443	0.645388
T.cells	MVB12A	0.060672	5.948563	0.689314	0.492431	-6.4968	0.709811	0.649196
T.cells	ZWINT	-0.06202	5.039001	-0.6891	0.492562	-6.40928	0.724661	0.665952
T.cells	ADAM15	0.244851	2.475369	0.688818	0.492741	-5.42544	0.767986	0.71534
T.cells	DOCK3	0.38859	0.039329	0.688427	0.492986	-5.07113	0.811489	0.765597
T.cells	CLCN6	0.107645	3.649755	0.687879	0.49333	-6.03049	0.748555	0.692552
T.cells	B230217O1	0.183136	1.757632	0.687784	0.493389	-5.65257	0.781146	0.729978
T.cells	KCNIP4	-0.41286	0.608323	-0.68768	0.493454	-5.21098	0.801578	0.753665
T.cells	4632427E1	0.074458	4.404143	0.687596	0.493507	-6.29865	0.735917	0.678188
T.cells	9030025P2	0.177989	1.737859	0.687373	0.493647	-5.59043	0.781494	0.73048
T.cells	ATXN1	-0.09821	6.919224	-0.68729	0.493701	-6.47003	0.695215	0.632389
T.cells	COX7A2	-0.0458	8.039242	-0.68724	0.493731	-6.95472	0.677781	0.612978
T.cells	JTB	-0.04927	5.842584	-0.68711	0.493811	-6.49377	0.712372	0.651631
T.cells	1110008P1	0.078445	5.226277	0.687009	0.493875	-6.25506	0.722372	0.662909
T.cells	CCR2	-0.47294	3.092746	-0.6869	0.493942	-5.16238	0.758015	0.703501
T.cells	PAQR8	-0.289	0.223677	-0.6869	0.493946	-5.29572	0.808526	0.761899
T.cells	IL18RAP	0.37663	1.983582	0.686706	0.494065	-5.2334	0.77727	0.72559
T.cells	ITGA9	-0.12184	5.58915	-0.68645	0.494225	-6.40577	0.716682	0.656365
T.cells	TPCN2	-0.20141	2.597655	-0.68596	0.494533	-5.45723	0.766815	0.713464
T.cells	WRB	-0.19847	1.437969	-0.68588	0.494581	-5.46169	0.787086	0.736844
T.cells	TUBE1	0.154519	1.254723	0.685876	0.494586	-5.73281	0.790334	0.740605
T.cells	HNRNPD	-0.03628	7.908504	-0.68587	0.49459	-6.98402	0.680059	0.615421
T.cells	PRKRA	0.104285	3.39958	0.685855	0.494599	-5.8787	0.753082	0.697722
T.cells	SERPINB6B	0.369298	2.936635	0.685277	0.494962	-5.29924	0.761366	0.706839
T.cells	FDX1	-0.08855	4.692703	-0.68525	0.494981	-6.2941	0.731789	0.673125
T.cells	TUSC3	-0.07007	4.978446	-0.68504	0.495108	-6.38554	0.727089	0.667801
T.cells	ATPIF1	-0.05731	7.745006	-0.68501	0.495127	-6.88481	0.682939	0.618299
T.cells	RABL2	-0.17575	1.106471	-0.68492	0.495189	-5.56419	0.793381	0.743758
T.cells	SLC39A14	0.17287	3.169335	0.684816	0.495251	-5.65273	0.757394	0.70232
T.cells	GM11837	-0.38026	0.292319	-0.68449	0.495455	-5.14227	0.808239	0.760882
T.cells	DLD	-0.06184	4.599821	-0.68432	0.495564	-6.3057	0.733602	0.675023
T.cells	CDCA8	-0.1057	5.109425	-0.68422	0.495628	-6.63495	0.725202	0.665528
T.cells	ZMPSTE24	-0.0821	4.942134	-0.68407	0.495722	-6.35619	0.72796	0.668664
T.cells	BE692007	0.167387	2.816357	0.683995	0.495767	-5.85101	0.76373	0.709438
T.cells	SUCLG2	-0.08223	4.835404	-0.68367	0.49597	-6.29328	0.72976	0.670782
T.cells	LGALS2	0.369726	-0.89826	0.683573	0.496033	-5.01187	0.830218	0.786688
T.cells	ZSWIM6	-0.06953	8.155524	-0.6835	0.496081	-6.74802	0.676902	0.611574
T.cells	RBM15B	-0.07243	4.047089	-0.68342	0.496131	-6.21159	0.742868	0.68568

T.cells	FGF13	0.1247	3.379693	0.68337	0.49616	-6.27852	0.754138	0.698535
T.cells	AW549877	-0.11689	3.352769	-0.68328	0.496218	-5.92631	0.754596	0.699058
T.cells	ZFPL1	0.088323	3.466605	0.683062	0.496354	-6.00514	0.752661	0.696848
T.cells	BORCS8	0.079895	4.590375	0.682967	0.496413	-6.1977	0.733811	0.675389
T.cells	GM43259	-0.23379	1.452797	-0.6828	0.49652	-5.5009	0.787573	0.73699
T.cells	CYP4A14	0.311765	1.833194	0.682716	0.496571	-5.39275	0.780866	0.729238
T.cells	PHYHD1	-0.16791	2.730001	-0.68241	0.496767	-5.63086	0.765261	0.711406
T.cells	TAGAP1	0.152417	2.214287	0.682305	0.49683	-5.79195	0.774199	0.721706
T.cells	2810001G2	0.116656	2.479465	0.682116	0.496949	-5.75441	0.769591	0.716406
T.cells	PLEKHA7	-0.16527	2.084938	-0.68201	0.497015	-5.67609	0.776456	0.724351
T.cells	MRPL39	-0.0751	3.661118	-0.68198	0.497036	-6.13899	0.749367	0.693276
T.cells	POLE2	-0.1094	3.014685	-0.68189	0.49709	-6.09591	0.760369	0.70587
T.cells	PPFIA1	0.062951	5.566923	0.681851	0.497116	-6.50337	0.71779	0.657465
T.cells	HIST2H2BE	-0.20682	0.814865	-0.68183	0.49713	-5.47053	0.798941	0.750387
T.cells	BACH1	0.090234	5.9952	0.681765	0.497169	-6.4385	0.710868	0.649687
T.cells	CHST14	-0.28681	0.872451	-0.68175	0.497179	-5.2687	0.797908	0.749208
T.cells	PI4K2A	-0.08088	5.639245	-0.6816	0.497276	-6.3855	0.716616	0.656226
T.cells	MNS1	0.091469	2.821224	0.681372	0.497417	-6.14098	0.76369	0.709814
T.cells	DIP2A	-0.16596	2.079809	-0.68089	0.497718	-5.56291	0.776545	0.724721
T.cells	FBXO9	0.079966	4.752411	0.680891	0.49772	-6.26858	0.73113	0.672781
T.cells	EIF1	-0.05315	9.721973	-0.68084	0.497753	-7.12685	0.653244	0.585876
T.cells	LRRC43	0.34423	-0.12202	0.680785	0.497786	-5.14491	0.815909	0.770432
T.cells	TMEM141	-0.29965	2.239657	-0.68061	0.497896	-5.30744	0.773757	0.721507
T.cells	NET1	-0.08705	4.035875	-0.68054	0.497941	-6.31205	0.743056	0.686335
T.cells	ADAMTS17	-0.36826	0.355692	-0.68027	0.498108	-5.13051	0.807216	0.760371
T.cells	GM11423	0.255651	0.892029	0.680271	0.498111	-5.24582	0.797558	0.74913
T.cells	GM17655	0.200744	1.183959	0.6802	0.498155	-5.47246	0.792345	0.743079
T.cells	ZSCAN18	-0.32341	0.363679	-0.6802	0.498156	-5.2506	0.807072	0.760203
T.cells	IL18BP	-0.30309	3.740937	-0.68016	0.498183	-5.61947	0.748018	0.692082
T.cells	GM11131	0.249172	0.815809	0.680079	0.498231	-5.38319	0.798924	0.750777
T.cells	ANO8	-0.24652	0.904446	-0.68001	0.498276	-5.37229	0.797335	0.748975
T.cells	4-Sep	0.299136	1.735848	0.680007	0.498277	-5.25227	0.782577	0.73187
T.cells	CCL5	-0.4746	7.931718	-0.6799	0.498343	-6.36288	0.680348	0.615998
T.cells	AARSD1	0.078767	3.977143	0.679868	0.498364	-6.27875	0.744042	0.687686
T.cells	NDUFB10	-0.04982	6.731387	-0.67983	0.498386	-6.73424	0.699116	0.636928
T.cells	MRPL52	0.054573	7.102833	0.679525	0.498581	-6.76156	0.693256	0.630439
T.cells	JAZF1	-0.1625	3.086872	-0.67947	0.498615	-5.69815	0.759133	0.705007
T.cells	RHOBTB2	-0.14218	2.943311	-0.6794	0.498657	-5.72049	0.761592	0.707846
T.cells	ACOT12	-0.31089	0.798615	-0.67933	0.4987	-5.1922	0.799232	0.751333
T.cells	KCNJ10	0.290718	-0.1202	0.679241	0.49876	-5.12999	0.815876	0.770789
T.cells	GM15337	-0.23502	2.140023	-0.67923	0.498766	-5.47882	0.775494	0.723881
T.cells	FAM76B	0.050788	5.401156	0.678808	0.499032	-6.53417	0.720488	0.661211
T.cells	JMJD1C	-0.05973	8.048596	-0.67858	0.499175	-6.8465	0.678548	0.614375
T.cells	PPAN	-0.084	4.072585	-0.67855	0.499195	-6.27411	0.742443	0.686282
T.cells	ANKS3	-0.0901	4.082767	-0.67837	0.49931	-6.091	0.742272	0.686115
T.cells	GM17021	-0.39422	0.125797	-0.67821	0.49941	-5.06628	0.811391	0.765943
T.cells	ZDHHC3	-0.06497	5.048938	-0.67807	0.499497	-6.37481	0.726248	0.667941

T.cells	VAMP5	0.099248	4.717071	0.678063	0.499502	-6.22625	0.731715	0.674141
T.cells	ZMYM6	0.160295	2.526746	0.677977	0.499557	-5.7278	0.768774	0.716509
T.cells	GALNT7	-0.06407	6.060844	-0.67781	0.499663	-6.62	0.709814	0.649418
T.cells	ADAM3	0.322734	-0.17326	0.677703	0.49973	-5.11459	0.816849	0.772364
T.cells	NR1H3	0.28519	3.325046	0.677644	0.499767	-5.46996	0.755069	0.700807
T.cells	OSBPL2	0.089163	3.979026	0.67758	0.499807	-5.99619	0.744012	0.688184
T.cells	OSBP	-0.06563	5.016429	-0.6773	0.499983	-6.37542	0.726782	0.668725
T.cells	PLEKHO2	-0.07304	6.069061	-0.67727	0.500001	-6.41109	0.709682	0.649411
T.cells	HIST1H4H	-0.19053	0.513275	-0.67718	0.500061	-5.53115	0.804369	0.75799
T.cells	DIO1	-0.25736	0.977554	-0.67706	0.500137	-5.28302	0.796029	0.748334
T.cells	TLN2	-0.27092	1.298032	-0.67692	0.500225	-5.34925	0.790319	0.741777
T.cells	SPDEF	-0.41536	-0.4296	-0.67681	0.500295	-5.03435	0.821554	0.778253
T.cells	SOCS6	-0.07773	3.829946	-0.67675	0.500333	-6.12487	0.746519	0.691371
T.cells	TMEM59	-0.04984	6.649746	-0.67674	0.500338	-6.63234	0.700412	0.639174
T.cells	CHEK1	-0.10646	2.722134	-0.67673	0.500346	-6.07004	0.765399	0.713014
T.cells	YWHAB	-0.03914	7.88619	-0.67667	0.500381	-6.90672	0.681053	0.617568
T.cells	ZDHHC23	0.173306	2.221064	0.676342	0.500589	-5.54697	0.774083	0.723053
T.cells	CD3EAP	0.109053	3.231663	0.676297	0.500618	-5.97215	0.756661	0.70301
T.cells	PPP1R9A	-0.1727	3.383193	-0.67625	0.500649	-5.78948	0.75408	0.700052
T.cells	FKRP	0.149517	2.332289	0.676199	0.50068	-5.6322	0.772147	0.72082
T.cells	RAB29	-0.1091	3.635461	-0.67617	0.5007	-6.0075	0.749802	0.695155
T.cells	PGP	-0.06429	5.374059	-0.67609	0.500748	-6.55665	0.720929	0.662313
T.cells	ATL2	-0.05263	5.337211	-0.67606	0.500766	-6.53335	0.72153	0.662993
T.cells	GM6712	0.155655	2.305447	0.676039	0.50078	-5.69754	0.772614	0.721358
T.cells	TACC3	-0.09232	5.072041	-0.67592	0.500858	-6.5934	0.725869	0.667932
T.cells	GM26590	-0.15437	1.314817	-0.67587	0.500887	-5.5197	0.790021	0.74153
T.cells	SLC35B4	0.116876	2.825471	0.675779	0.500945	-5.8889	0.763619	0.711043
T.cells	CSNK2A2	-0.04471	5.948451	-0.67544	0.50116	-6.60812	0.711655	0.651867
T.cells	HSD17B10	-0.06915	5.540491	-0.67516	0.501338	-6.48384	0.718254	0.659313
T.cells	ZFP995	0.121994	2.383748	0.675123	0.501359	-5.83372	0.771289	0.719857
T.cells	TUBGCP4	0.071633	4.42498	0.675055	0.501402	-6.31154	0.736593	0.680109
T.cells	ENO3	-0.26484	2.077848	-0.67495	0.501471	-5.38656	0.776618	0.726006
T.cells	GM2A	-0.06897	7.22888	-0.67493	0.501481	-6.65693	0.691312	0.629039
T.cells	ETV1	-0.26095	1.377482	-0.67477	0.501584	-5.31382	0.788946	0.740365
T.cells	CDH24	0.140583	2.179776	0.674686	0.501636	-5.71836	0.774838	0.724042
T.cells	DNAJC19	0.051284	5.950115	0.674621	0.501677	-6.58661	0.711628	0.651949
T.cells	MYO10	-0.09825	5.227281	-0.67453	0.501732	-6.36042	0.72336	0.665237
T.cells	ATP5J	-0.04847	7.873083	-0.67453	0.501734	-6.93069	0.681287	0.618
T.cells	CCDC157	0.221449	1.103128	0.67435	0.501848	-5.4068	0.793824	0.746182
T.cells	LETMD1	-0.10917	2.91201	-0.67431	0.501875	-5.89005	0.762167	0.709582
T.cells	ZFP382	0.140854	2.008207	0.674249	0.501912	-5.83361	0.777835	0.727657
T.cells	PRPF4B	0.049864	6.312389	0.67414	0.501981	-6.67305	0.705816	0.645537
T.cells	NMI	0.083765	5.064624	0.674073	0.502024	-6.39729	0.726024	0.668357
T.cells	EPB41L4AC	-0.14098	3.25392	-0.67387	0.502151	-5.88578	0.756318	0.702962
T.cells	YDJC	0.198163	1.21958	0.673862	0.502157	-5.54023	0.791752	0.743875
T.cells	RAB11FIP4	-0.34939	-0.78929	-0.67331	0.502504	-5.03316	0.828334	0.786761
T.cells	RNF167	-0.06878	5.334087	-0.67329	0.502516	-6.4468	0.7217	0.663609

T.cells	RUNDC1	-0.1267	2.994317	-0.67322	0.502563	-5.77086	0.760845	0.708264
T.cells	MYL9	0.329121	1.95475	0.673171	0.502594	-5.40934	0.778863	0.729055
T.cells	TRAPPC6B	0.045667	6.134079	0.673146	0.50261	-6.57042	0.708755	0.649004
T.cells	GALNT2	0.075389	5.565067	0.673144	0.502611	-6.40594	0.71794	0.659368
T.cells	RDH9	-0.33898	0.805989	-0.67298	0.502717	-5.19157	0.799297	0.752713
T.cells	DAB2IP	-0.16137	2.225574	-0.67273	0.502874	-5.67702	0.774334	0.723647
T.cells	DDX54	-0.05447	5.820595	-0.67261	0.502953	-6.62052	0.714005	0.654802
T.cells	UBR2	-0.05075	6.362518	-0.67209	0.503276	-6.59868	0.705647	0.645167
T.cells	ERCC6L2	-0.10036	3.372895	-0.67195	0.503366	-6.02939	0.754966	0.701197
T.cells	PAFAH1B2	0.052753	5.489377	0.671901	0.503399	-6.47446	0.71973	0.661098
T.cells	CCT5	-0.05456	6.571584	-0.67165	0.503557	-6.73671	0.70242	0.641539
T.cells	TMEM241	-0.09577	4.310016	-0.67157	0.503608	-6.19832	0.739282	0.683232
T.cells	GM46560	-0.21918	-0.84515	-0.67117	0.503862	-5.3822	0.830141	0.788568
T.cells	ZFP763	0.222085	0.433803	0.671067	0.503928	-5.41547	0.806686	0.761137
T.cells	OSBPL7	-0.09957	3.426262	-0.67088	0.504047	-5.95938	0.754172	0.700397
T.cells	POLA1	-0.07644	5.968201	-0.67085	0.504062	-6.72069	0.712082	0.65255
T.cells	KALRN	0.158349	2.750236	0.670755	0.504125	-5.85352	0.765751	0.713773
T.cells	TEX9	0.128556	1.820323	0.670675	0.504176	-5.89489	0.781948	0.7325
T.cells	EIF4H	-0.04006	6.535904	-0.67052	0.504276	-6.7164	0.702988	0.642462
T.cells	ZFP324	-0.26715	0.798242	-0.67045	0.504316	-5.30397	0.800115	0.753662
T.cells	MFAP3	0.059564	5.124697	0.670376	0.504365	-6.43167	0.725799	0.668228
T.cells	EYA1	0.141235	2.426875	0.670335	0.504391	-5.96188	0.771348	0.720329
T.cells	TWF2	-0.07322	5.240324	-0.67029	0.50442	-6.31975	0.723904	0.66608
T.cells	MAPKAPK3	0.102184	5.306805	0.670147	0.504511	-6.11408	0.722816	0.664872
T.cells	FAAP24	0.157166	2.002599	0.670087	0.504549	-5.8445	0.778748	0.728906
T.cells	DDB1	-0.05985	5.611857	-0.67007	0.504561	-6.54822	0.717847	0.659252
T.cells	MTMR12	-0.06276	5.334196	-0.66988	0.504682	-6.43081	0.722369	0.664388
T.cells	ITPR3	-0.08423	4.288295	-0.66986	0.504692	-6.31576	0.739644	0.684017
T.cells	NOTCH2	-0.07263	7.16468	-0.6698	0.50473	-6.49229	0.693044	0.631388
T.cells	FAM189A1	0.132109	2.327494	0.669564	0.504881	-6.15437	0.773205	0.722461
T.cells	SPEF2	0.17535	1.371663	0.66926	0.505074	-5.63707	0.790059	0.742057
T.cells	GCDH	-0.16086	3.116618	-0.66918	0.505126	-5.85069	0.759632	0.706909
T.cells	PKD1	0.151966	3.003757	0.669148	0.505145	-5.64782	0.761567	0.709137
T.cells	LSM11	0.155983	2.171179	0.66904	0.505213	-5.75432	0.77598	0.725817
T.cells	TAB1	0.108521	3.004453	0.668992	0.505244	-5.98462	0.761555	0.709202
T.cells	ZFP971	-0.17487	1.911606	-0.6686	0.505491	-5.47963	0.78078	0.731243
T.cells	ITGB2L	-0.32222	-0.61932	-0.66852	0.505541	-5.05587	0.826416	0.784486
T.cells	RBM45	-0.10034	3.10947	-0.66833	0.505666	-5.95139	0.760013	0.707289
T.cells	WFS1	-0.21456	1.279726	-0.6683	0.50568	-5.27772	0.791963	0.744207
T.cells	GM16287	0.342861	-0.44109	0.668188	0.505754	-5.0414	0.823146	0.780633
T.cells	MTFMT	0.106544	2.527907	0.668086	0.505819	-5.76274	0.770046	0.718839
T.cells	ABCB8	0.114355	2.240197	0.667927	0.50592	-5.67893	0.775104	0.724667
T.cells	GM15543	-0.18601	0.901403	-0.66752	0.50618	-5.4916	0.799093	0.752246
T.cells	UBE2V2	-0.05301	5.614121	-0.66731	0.506311	-6.56116	0.718645	0.659859
T.cells	FAM20A	0.301892	1.833508	0.667119	0.506434	-5.30342	0.782625	0.733114
T.cells	PLEKHA8	0.249825	0.886546	0.667107	0.506441	-5.228	0.799459	0.752662
T.cells	CKB	-0.23122	4.578345	-0.66692	0.506563	-5.60603	0.73575	0.679268

T.cells	LIN54	0.070763	6.14118	0.666673	0.506717	-6.61208	0.710326	0.650419
T.cells	KLC4	-0.12724	2.884186	-0.66633	0.506933	-5.77893	0.764667	0.712181
T.cells	EIF4EBP2	0.038238	6.850744	0.666332	0.506934	-6.75761	0.699111	0.637733
T.cells	NDUFB3	0.053277	5.72376	0.666064	0.507105	-6.58582	0.717179	0.658113
T.cells	FAAP100	-0.13052	3.097229	-0.66601	0.507141	-5.71988	0.761005	0.708045
T.cells	SRL	-0.19401	0.890591	-0.66595	0.507178	-5.4308	0.799738	0.752851
T.cells	TPRKB	-0.11548	3.157978	-0.66592	0.507193	-5.89982	0.759964	0.706856
T.cells	AKT1	-0.0549	6.230392	-0.66563	0.50738	-6.68314	0.709094	0.649062
T.cells	TUBGCP6	-0.11984	2.759296	-0.66557	0.50742	-5.89156	0.766921	0.714905
T.cells	NDOR1	0.099736	3.086341	0.665414	0.507518	-6.04086	0.76129	0.708432
T.cells	MRRF	-0.09203	3.454685	-0.6649	0.507847	-6.04795	0.754994	0.701315
T.cells	TMSB15B1	-0.13089	2.422831	-0.66484	0.507885	-5.78326	0.772754	0.721739
T.cells	RNF14	0.059426	5.242687	0.664745	0.507944	-6.35646	0.725119	0.667265
T.cells	DAPL1	0.334631	-1.31502	0.66471	0.507966	-5.03859	0.840366	0.800667
T.cells	USP44	-0.20571	-0.6309	-0.66451	0.508093	-5.3784	0.8276	0.785706
T.cells	WRNIP1	-0.07466	3.743434	-0.66451	0.508096	-6.15326	0.750093	0.695771
T.cells	MTURN	0.128727	2.270409	0.664271	0.508245	-5.89357	0.77541	0.724941
T.cells	SLCO2A1	-0.35554	2.568296	-0.66423	0.508272	-5.39727	0.770227	0.718977
T.cells	HSPA2	-0.09429	3.291574	-0.66408	0.508368	-6.19706	0.757776	0.70469
T.cells	FANCI	0.100664	2.266344	0.664007	0.508414	-5.96142	0.775481	0.725104
T.cells	TOX4	0.050547	5.903687	0.664003	0.508416	-6.4899	0.714358	0.655294
T.cells	GMFB	0.058986	5.451593	0.663975	0.508434	-6.43759	0.721701	0.663595
T.cells	GM17227	0.104263	2.746669	0.663924	0.508467	-5.93676	0.767139	0.715482
T.cells	CCL21A	0.902802	0.049265	0.663891	0.508487	-5.13448	0.815083	0.771183
T.cells	SNX5	-0.04774	7.890887	-0.66381	0.508536	-6.85517	0.682906	0.620038
T.cells	UBE2H	0.054718	8.574917	0.663812	0.508538	-6.94024	0.672387	0.608348
T.cells	GM43149	0.258005	0.774872	0.663694	0.508613	-5.27618	0.801922	0.755858
T.cells	AKIRIN2	-0.05271	5.756269	-0.66364	0.508648	-6.57353	0.716744	0.658065
T.cells	TRBC2	-0.39305	3.943953	-0.66345	0.508769	-5.55074	0.746787	0.692137
T.cells	AASS	0.312651	1.208483	0.6632	0.508928	-5.25436	0.79438	0.746907
T.cells	JPT2	0.095977	3.513173	0.66307	0.509011	-6.14987	0.754242	0.70057
T.cells	PDS5B	-0.04645	6.377064	-0.66279	0.50919	-6.7621	0.707128	0.646951
T.cells	VPS50	0.074256	4.131766	0.662463	0.509398	-6.17238	0.744081	0.688834
T.cells	WAPL	0.064431	7.165135	0.662252	0.509532	-6.5587	0.694735	0.633137
T.cells	ARHGAP39	-0.11359	4.38051	-0.66223	0.509547	-6.12844	0.739914	0.684173
T.cells	PDE3A	0.390457	0.411945	0.661973	0.50971	-5.192	0.809059	0.764104
T.cells	ZFP934	-0.10271	2.990341	-0.66192	0.509745	-6.04922	0.763486	0.711285
T.cells	DMD	0.205683	2.419953	0.661681	0.509897	-5.55341	0.773358	0.722729
T.cells	SRPR	-0.04794	5.794906	-0.66157	0.509965	-6.50921	0.716631	0.657965
T.cells	HARS2	0.092838	2.848743	0.661438	0.510051	-5.8936	0.765925	0.714234
T.cells	KNSTRN	-0.12242	3.271497	-0.66138	0.51009	-6.2043	0.758663	0.705883
T.cells	PABPC1	-0.05349	10.47512	-0.66105	0.510302	-7.31092	0.644431	0.577749
T.cells	DUSP7	0.112772	3.559127	0.660983	0.510342	-6.00636	0.753758	0.700367
T.cells	TMEM8	-0.21527	2.000346	-0.66096	0.510356	-5.35404	0.780696	0.731404
T.cells	GNA14	-0.42078	-0.0617	-0.66094	0.510367	-5.12252	0.817699	0.774523
T.cells	SPC25	-0.09053	4.372308	-0.66088	0.51041	-6.48159	0.740051	0.684693
T.cells	GALE	-0.13548	2.330073	-0.66081	0.510455	-5.68421	0.774924	0.724752

T.cells	PEX3	-0.08054	3.770542	-0.66071	0.510513	-6.07885	0.750172	0.696312
T.cells	POU5F2	0.176716	1.687999	0.660679	0.510536	-5.53415	0.7862	0.737849
T.cells	GM29264	0.270448	0.505466	0.660625	0.51057	-5.21102	0.807363	0.762509
T.cells	CLMN	-0.32918	0.334512	-0.66043	0.510696	-5.17743	0.810466	0.766195
T.cells	ZMAT4	0.432379	0.381713	0.660428	0.510696	-5.19548	0.809608	0.765192
T.cells	XNDC1	-0.07937	3.594505	-0.6604	0.510713	-6.22651	0.753157	0.699821
T.cells	FCGR4	0.404535	3.388468	0.660368	0.510735	-5.42818	0.756664	0.703853
T.cells	BIN1	0.058059	5.230633	0.659974	0.510986	-6.50635	0.725986	0.668867
T.cells	AA388235	-0.28225	0.991788	-0.65996	0.510997	-5.33847	0.798762	0.752581
T.cells	RECQL4	0.221281	-0.06717	0.659891	0.511039	-5.35614	0.817968	0.775046
T.cells	LYRM1	0.138106	2.700522	0.659663	0.511185	-5.68343	0.768693	0.717741
T.cells	1700113A1	-0.17156	2.10081	-0.6596	0.511225	-5.56154	0.779142	0.729812
T.cells	A330023F2	0.232461	3.109027	0.659531	0.511269	-5.36039	0.761651	0.70963
T.cells	DRAM1	0.274533	2.581586	0.659398	0.511354	-5.43819	0.770783	0.720133
T.cells	YJU2	-0.07904	3.454196	-0.65924	0.511458	-6.07358	0.75583	0.702876
T.cells	SERPINB1A	-0.12968	3.600711	-0.659	0.511611	-6.22978	0.753385	0.700053
T.cells	PSD	-0.22586	1.633137	-0.65892	0.511659	-5.36759	0.787519	0.739432
T.cells	CCDC12	-0.04417	6.939585	-0.65876	0.511764	-6.69697	0.698604	0.637966
T.cells	PF4	0.425671	4.278945	0.658735	0.511778	-5.78193	0.741941	0.687015
T.cells	TCN2	-0.1459	4.791081	-0.65836	0.512018	-5.90399	0.733408	0.677357
T.cells	EME1	-0.11945	2.299791	-0.65819	0.512127	-5.95534	0.775796	0.72603
T.cells	MOB1A	0.05718	5.970142	0.658157	0.512147	-6.47211	0.714111	0.655527
T.cells	B4GALT1	-0.04975	7.137524	-0.65807	0.512202	-6.72082	0.695478	0.634566
T.cells	FAM221B	-0.31001	0.343919	-0.65803	0.512229	-5.17258	0.810654	0.766581
T.cells	ZFP212	0.070172	3.640876	0.658024	0.512232	-6.18653	0.752702	0.699441
T.cells	FMN2	-0.13401	2.359589	-0.65796	0.512275	-6.16309	0.774752	0.724857
T.cells	GM9750	-0.26894	0.476616	-0.65794	0.512289	-5.34555	0.808244	0.763807
T.cells	MAP1LC3A	-0.09331	5.476214	-0.65765	0.512473	-6.36313	0.722136	0.664732
T.cells	TSLP	-0.39678	-0.44209	-0.65744	0.512604	-5.04418	0.825063	0.783695
T.cells	SLC24A3	-0.40444	1.280307	-0.65743	0.51261	-5.17497	0.793789	0.7471
T.cells	DDX39	-0.05783	6.372696	-0.65735	0.512665	-6.79233	0.707633	0.648422
T.cells	UBA1	-0.05326	6.00691	-0.65729	0.512699	-6.58187	0.713517	0.655081
T.cells	KCTD14	-0.21348	1.055743	-0.65724	0.512736	-5.42062	0.797804	0.751856
T.cells	AIRN	0.100895	5.663175	0.657198	0.512761	-6.51605	0.719089	0.661413
T.cells	ABHD13	0.079853	3.557429	0.657029	0.512869	-6.08063	0.754121	0.701359
T.cells	ESCO2	0.096097	3.734708	0.657009	0.512882	-6.43351	0.751111	0.697907
T.cells	ANKRD55	-0.33713	0.009583	-0.65665	0.513113	-5.09242	0.817017	0.77414
T.cells	BOK	0.222556	1.039523	0.656283	0.513347	-5.48579	0.798611	0.752463
T.cells	GM26812	-0.32825	0.020142	-0.65593	0.513573	-5.17472	0.817249	0.774213
T.cells	SAP30	0.060616	5.143228	0.655666	0.513741	-6.57481	0.728206	0.6715
T.cells	0610010F0	-0.08782	4.458879	-0.65531	0.513968	-6.22234	0.739555	0.684538
T.cells	CUTC	0.079961	3.519969	0.655287	0.513985	-6.11808	0.755393	0.702663
T.cells	GM49173	0.464153	-0.87955	0.655251	0.514007	-5.03958	0.833885	0.794024
T.cells	DTX3	0.137028	2.572046	0.655181	0.514053	-5.73644	0.771704	0.721441
T.cells	QRICH1	-0.03984	6.585338	-0.65516	0.514063	-6.698	0.704826	0.645189
T.cells	DPP10	0.367924	0.411958	0.655152	0.514071	-5.16494	0.810099	0.766077
T.cells	CSF2RB	0.238307	4.838003	0.655133	0.514083	-5.56578	0.733247	0.677352

T.cells	FAM78A	-0.11359	2.648934	-0.65469	0.514365	-5.9468	0.770369	0.720054
T.cells	IPMK	0.058533	5.867681	0.654678	0.514375	-6.57572	0.716371	0.658351
T.cells	E230016K2	0.367852	0.143576	0.654579	0.514438	-5.0636	0.81499	0.771972
T.cells	9930104L0	0.174057	1.356879	0.654461	0.514514	-5.5265	0.793091	0.746391
T.cells	RANBP3	-0.06245	4.303701	-0.65446	0.514514	-6.28701	0.742151	0.687649
T.cells	HIST2H3B	-0.18502	1.047683	-0.6544	0.514552	-5.61859	0.79862	0.752831
T.cells	ZC2HC1A	-0.21485	1.741533	-0.65439	0.514559	-5.4806	0.786263	0.738454
T.cells	JMJD6	0.067646	4.999369	0.654349	0.514585	-6.39428	0.730578	0.67448
T.cells	SOX6	0.307272	1.905309	0.654198	0.514683	-5.44096	0.783372	0.735147
T.cells	CSTDC6	0.279581	-1.16323	0.65412	0.514732	-5.0544	0.839195	0.80054
T.cells	NRD1	0.055362	6.185357	0.654029	0.514791	-6.56648	0.711239	0.652632
T.cells	SLC25A26	-0.09527	3.795963	-0.65395	0.514839	-6.10768	0.750705	0.697531
T.cells	NUFIP2	-0.05178	7.215419	-0.65366	0.51503	-6.73732	0.694901	0.63435
T.cells	CYTH4	0.11088	5.757599	0.653508	0.515125	-5.92463	0.718227	0.660625
T.cells	PLEKHG2	-0.09608	4.157105	-0.65343	0.515177	-6.21279	0.744682	0.690736
T.cells	MLXIP	-0.05263	6.94185	-0.65336	0.515222	-6.77901	0.699223	0.639236
T.cells	CDCA3	-0.09985	4.895561	-0.65331	0.51525	-6.61013	0.732364	0.67671
T.cells	TMEM132I	-0.33135	0.329919	-0.65321	0.515316	-5.15152	0.811668	0.768363
T.cells	SVIL	0.057366	6.860609	0.653052	0.515417	-6.64923	0.700512	0.640747
T.cells	CPNE2	-0.16103	3.37335	-0.65252	0.515758	-5.77107	0.757967	0.706187
T.cells	CD84	0.098472	5.366126	0.652492	0.515776	-6.24401	0.724615	0.668082
T.cells	RC3H1	-0.0459	6.887392	-0.65247	0.515792	-6.73296	0.700087	0.640377
T.cells	MRPS18A	-0.05551	5.09387	-0.65235	0.515869	-6.52871	0.729089	0.673192
T.cells	CASC1	-0.19771	1.810453	-0.65205	0.516062	-5.61371	0.78512	0.737689
T.cells	TRMU	0.152187	1.548223	0.652001	0.516092	-5.5388	0.789763	0.743085
T.cells	CYTH1	0.054554	8.072227	0.652	0.516093	-6.91063	0.681529	0.619709
T.cells	SURF1	-0.08377	4.36834	-0.6519	0.516155	-6.17702	0.741139	0.687017
T.cells	EVA1B	-0.1493	3.639669	-0.6519	0.516159	-5.70549	0.753428	0.701092
T.cells	SH3GLB1	0.045723	8.546317	0.651838	0.516196	-6.94498	0.674235	0.611594
T.cells	BCL2L13	-0.06576	5.133362	-0.65163	0.516328	-6.45543	0.728438	0.672635
T.cells	TRABD	-0.06161	5.363153	-0.65154	0.516387	-6.44843	0.724663	0.668372
T.cells	FIGNL1	-0.11267	2.757262	-0.65144	0.516453	-6.07945	0.768565	0.718664
T.cells	GM19705	-0.35133	1.868138	-0.65129	0.516549	-5.156	0.784102	0.736686
T.cells	SAPCD1	0.131008	1.629002	0.651148	0.51664	-5.84967	0.78833	0.741599
T.cells	CHMP7	0.120924	2.616172	0.651147	0.516641	-5.84159	0.771011	0.72152
T.cells	GM32250	-0.35256	-0.2746	-0.65109	0.516679	-5.04679	0.822743	0.781859
T.cells	IARS2	0.075091	4.475268	0.651083	0.516681	-6.2689	0.739351	0.685141
T.cells	FBXO5	-0.09632	4.811103	-0.65104	0.516707	-6.62334	0.733763	0.678766
T.cells	RICTOR	0.050074	6.409431	0.650983	0.516746	-6.64011	0.707707	0.649224
T.cells	GM47167	0.104412	2.927116	0.650882	0.516811	-5.95786	0.76563	0.715305
T.cells	RAD54L	-0.09656	2.724058	-0.6508	0.516866	-6.12884	0.76914	0.719405
T.cells	PCGF5	0.06442	6.06196	0.650778	0.516878	-6.70441	0.713297	0.655583
T.cells	NCF4	0.111766	5.316123	0.650628	0.516974	-6.08891	0.725476	0.669394
T.cells	GM16337	0.197455	1.967439	0.650251	0.517216	-5.5179	0.782634	0.734885
T.cells	ARGLU1	0.030951	7.597485	0.650099	0.517314	-6.91812	0.689156	0.628386
T.cells	PDLIM7	-0.10336	3.638312	-0.65004	0.517354	-6.01395	0.753723	0.701605
T.cells	STAU1	-0.05778	6.034578	-0.64994	0.517415	-6.53344	0.713996	0.656332

T.cells	GM28375	0.081968	3.206287	0.649779	0.51752	-6.04455	0.761102	0.710126
T.cells	RIPPLY3	0.386489	0.048429	0.649719	0.517558	-5.10363	0.817102	0.775281
T.cells	TRIM24	0.062357	5.087563	0.649663	0.517594	-6.45864	0.729455	0.673932
T.cells	DHX37	0.108673	2.67509	0.649115	0.517947	-5.90775	0.770385	0.7208
T.cells	TRIM30B	0.357644	1.730639	0.649105	0.517953	-5.12135	0.786935	0.739981
T.cells	DLAT	0.063196	4.282042	0.648767	0.518171	-6.28848	0.742966	0.689391
T.cells	SLC35B1	-0.06175	5.751128	-0.64857	0.518299	-6.52486	0.718701	0.661815
T.cells	AGAP2	0.126813	2.925575	0.648392	0.518412	-5.77735	0.76605	0.716024
T.cells	MGAM	-0.36372	0.078138	-0.64838	0.518419	-5.13752	0.816684	0.774988
T.cells	4930435F1	0.374692	0.358699	0.648296	0.518474	-5.11765	0.811561	0.768978
T.cells	IGLC2	0.500704	3.740399	0.648265	0.518493	-5.58513	0.752105	0.699975
T.cells	TNFRSF14	-0.35421	0.252225	-0.64826	0.518494	-5.12379	0.813502	0.771254
T.cells	ZFP982	0.379117	0.056297	0.648164	0.518558	-5.11638	0.817084	0.775464
T.cells	HCST	0.179962	5.375869	0.647998	0.518665	-5.73335	0.724827	0.668858
T.cells	NUCB1	0.071984	5.383551	0.647967	0.518685	-6.35359	0.724702	0.668715
T.cells	DYNLT3	-0.06744	4.662522	-0.64795	0.518695	-6.2519	0.736609	0.682275
T.cells	YLPM1	0.052947	5.660547	0.647799	0.518794	-6.59225	0.720175	0.663595
T.cells	LIN52	-0.06082	5.546829	-0.64775	0.518828	-6.59443	0.72203	0.665699
T.cells	ATXN2L	-0.05235	5.47603	-0.64766	0.518884	-6.52018	0.723187	0.667015
T.cells	BLOC1S3	0.183015	2.381624	0.647477	0.519001	-5.55088	0.775492	0.727072
T.cells	BTBD10	0.04624	5.583956	0.647471	0.519005	-6.62645	0.721424	0.665076
T.cells	THBD	0.136777	3.145231	0.647237	0.519156	-6.09332	0.762267	0.711851
T.cells	ZFP354C	0.135717	1.777029	0.646977	0.519323	-5.87537	0.786114	0.739591
T.cells	MCPH1	0.058171	4.913551	0.64687	0.519392	-6.49901	0.732443	0.677852
T.cells	FAM110B	0.341961	0.653098	0.646684	0.519512	-5.16644	0.806218	0.763165
T.cells	IGF1R	-0.07731	6.357541	-0.64666	0.519527	-6.57043	0.708903	0.651172
T.cells	TMEM214	0.066634	4.338367	0.646591	0.519571	-6.23224	0.742022	0.688851
T.cells	PDE1A	0.356036	0.441701	0.64632	0.519746	-5.14017	0.810051	0.767747
T.cells	UBXN8	0.074447	4.404408	0.646219	0.519812	-6.20642	0.740916	0.687658
T.cells	MAP2K5	0.045874	6.143251	0.646163	0.519847	-6.65262	0.712351	0.655162
T.cells	LANCL1	0.077174	3.448045	0.64616	0.51985	-6.04974	0.757081	0.706208
T.cells	MS4A7	0.384234	1.921581	0.646146	0.519858	-5.28723	0.783562	0.736839
T.cells	PXN	0.069771	6.266447	0.646056	0.519917	-6.48097	0.710367	0.652945
T.cells	HEATR3	-0.08231	4.273336	-0.64594	0.519994	-6.20203	0.743112	0.690281
T.cells	RNF219	0.096748	3.193241	0.645875	0.520033	-6.08656	0.761442	0.711367
T.cells	SLC52A3	0.256747	0.451484	0.645783	0.520093	-5.27502	0.809874	0.767736
T.cells	RBMS2	0.058787	4.872344	0.645779	0.520095	-6.38734	0.733125	0.678915
T.cells	ATAD1	0.03901	5.731262	0.645736	0.520123	-6.62651	0.719024	0.662877
T.cells	NOM1	0.067812	4.141882	0.64568	0.520159	-6.28667	0.745321	0.692873
T.cells	MRPS5	-0.06093	4.612666	-0.64565	0.520176	-6.41051	0.737439	0.683852
T.cells	NAA25	-0.09447	3.927641	-0.64556	0.520237	-6.15024	0.748934	0.697046
T.cells	DDX31	0.091413	3.220713	0.645421	0.520326	-6.04406	0.760971	0.710984
T.cells	RNASEL	0.155047	3.944163	0.645317	0.520393	-5.86968	0.748655	0.696862
T.cells	SPARCL1	0.451317	0.3267	0.645245	0.52044	-5.2003	0.812144	0.770597
T.cells	CD302	-0.13834	5.847821	-0.64521	0.520463	-6.21276	0.71713	0.660893
T.cells	ITGA2B	0.247417	0.938407	0.645109	0.520527	-5.27695	0.80107	0.757656
T.cells	KPNA4	-0.05957	8.246447	-0.64477	0.520745	-6.86437	0.679307	0.61832

T.cells	AIG1	0.092477	4.903663	0.644763	0.52075	-6.24748	0.732734	0.67862
T.cells	MTX1	-0.06998	4.556924	-0.64438	0.520995	-6.36339	0.738704	0.685306
T.cells	TTC19	0.073796	4.958514	0.644286	0.521058	-6.32671	0.732032	0.677705
T.cells	MVP	0.075354	5.108561	0.644227	0.521096	-6.29274	0.729553	0.674885
T.cells	CERS6	0.100542	7.330325	0.644102	0.521177	-6.52063	0.693765	0.634456
T.cells	ATRX	-0.04134	7.666179	-0.6439	0.52131	-6.91247	0.688502	0.628607
T.cells	IFFO1	0.141749	2.880878	0.643878	0.521321	-5.87989	0.76717	0.718172
T.cells	CWC25	0.089183	5.255579	0.643714	0.521427	-6.27499	0.727132	0.67232
T.cells	POU6F1	0.16927	2.610644	0.643713	0.521428	-5.59164	0.771854	0.723677
T.cells	MRE11A	-0.0532	4.349103	-0.64339	0.521635	-6.38167	0.74238	0.689575
T.cells	PDE11A	0.380077	0.072949	0.642754	0.522047	-5.15023	0.817628	0.776798
T.cells	DDX18	-0.05793	4.987355	-0.64263	0.522126	-6.45357	0.731982	0.67758
T.cells	GPR84	0.381951	0.260814	0.642505	0.522208	-5.09251	0.81419	0.772782
T.cells	ACTA2	0.337293	3.320545	0.642406	0.522272	-5.71073	0.760049	0.709773
T.cells	PRKD3	-0.06094	5.198034	-0.64239	0.522285	-6.49619	0.728504	0.673633
T.cells	PGLYRP2	-0.23774	2.088786	-0.64238	0.522288	-5.41438	0.781432	0.734513
T.cells	GM12158	0.153286	0.549233	0.642329	0.522322	-5.80013	0.808939	0.766629
T.cells	DKC1	-0.06819	4.56874	-0.64224	0.522378	-6.42743	0.738938	0.685585
T.cells	HKDC1	-0.3973	0.37121	-0.64221	0.522397	-5.14534	0.812177	0.770485
T.cells	CENPN	0.100237	2.920239	0.641702	0.522727	-6.24683	0.767102	0.717965
T.cells	NUP35	-0.09203	3.291207	-0.64165	0.522762	-6.09513	0.760715	0.710602
T.cells	COX5A	-0.04837	8.546847	-0.64159	0.522802	-7.03889	0.675419	0.613908
T.cells	FHDC1	0.289358	0.748905	0.641537	0.522834	-5.24275	0.805494	0.762681
T.cells	STYK1	-0.34823	0.538888	-0.64117	0.523071	-5.09848	0.8093	0.767263
T.cells	H2-EB2	0.23946	0.547733	0.641155	0.523081	-5.41117	0.809139	0.767074
T.cells	CDKAL1	0.048396	6.364518	0.640973	0.523198	-6.69506	0.70968	0.652541
T.cells	SHB	-0.1029	4.498582	-0.64097	0.523203	-6.15573	0.740268	0.687335
T.cells	SPAG9	-0.07572	9.266573	-0.64082	0.523298	-6.94954	0.664468	0.601922
T.cells	POLR3B	-0.07455	5.512738	-0.64072	0.523362	-6.48496	0.723493	0.668214
T.cells	ACOT9	-0.07284	4.620859	-0.64067	0.523396	-6.24513	0.738227	0.685013
T.cells	RUBCNL	0.119527	3.191015	0.640659	0.523401	-5.93329	0.762435	0.712823
T.cells	TGFB3	0.316121	0.567314	0.64064	0.523414	-5.21732	0.808784	0.766764
T.cells	ROCK2	0.045069	7.468662	0.640347	0.523603	-6.86124	0.692144	0.632862
T.cells	DAXX	0.085319	4.352332	0.64027	0.523653	-6.26883	0.742717	0.690263
T.cells	GM8066	0.214553	0.498967	0.640223	0.523683	-5.39104	0.810025	0.768366
T.cells	PEX11A	0.357313	0.072757	0.640191	0.523704	-5.08372	0.817806	0.777516
T.cells	CASP2	0.06268	4.01281	0.640102	0.523762	-6.3228	0.748432	0.696878
T.cells	WDR26	0.046219	7.53429	0.639897	0.523894	-6.74183	0.691115	0.631856
T.cells	ODF2L	0.10936	2.682465	0.63967	0.524041	-5.89314	0.771222	0.723262
T.cells	6030458C1	-0.10274	2.74674	-0.63966	0.524045	-5.86713	0.770106	0.72197
T.cells	ERCC6	-0.10259	3.56729	-0.63962	0.524071	-6.00122	0.755993	0.705674
T.cells	A230072E1	-0.25889	0.278064	-0.63954	0.524127	-5.33425	0.814049	0.773243
T.cells	CXCL9	0.60773	2.080321	0.639502	0.52415	-5.20413	0.781747	0.735476
T.cells	VPS37A	0.078836	5.2037	0.63943	0.524196	-6.26763	0.728566	0.674251
T.cells	CPEB3	0.139542	4.234811	0.63937	0.524235	-5.97884	0.744691	0.692705
T.cells	HIST1H2BE	0.131512	2.079102	0.639122	0.524396	-5.86312	0.781833	0.735608
T.cells	GPR19	-0.10798	2.550401	-0.63883	0.524585	-5.96269	0.773582	0.726076

T.cells	PDHX	0.071281	3.395129	0.638651	0.524701	-6.13857	0.758996	0.709236
T.cells	CTSC	0.112673	7.322442	0.638571	0.524753	-6.64225	0.694499	0.635808
T.cells	METRN	-0.11629	2.538002	-0.63846	0.524823	-5.94377	0.773798	0.726391
T.cells	STOML3	-0.26069	0.359863	-0.63807	0.525079	-5.26282	0.812623	0.77189
T.cells	TOP2A	-0.09185	7.615909	-0.63792	0.525173	-7.09897	0.689894	0.630828
T.cells	MED10	-0.0621	4.886924	-0.6379	0.525185	-6.38298	0.733862	0.680621
T.cells	ZFP319	0.148053	2.544028	0.637864	0.525211	-5.8137	0.773693	0.726477
T.cells	PTPRB	-0.24667	3.708559	-0.63762	0.525372	-5.70809	0.75365	0.703379
T.cells	MRPL37	-0.06125	4.2912	-0.63747	0.525466	-6.3479	0.743804	0.692098
T.cells	SMCHD1	0.053356	7.994517	0.63746	0.525473	-6.9843	0.683994	0.624302
T.cells	PUSL1	-0.11491	2.317284	-0.63746	0.525475	-5.81361	0.777653	0.731174
T.cells	CRP	0.243284	2.076546	0.637442	0.525484	-5.47166	0.781878	0.736085
T.cells	GM43111	0.347652	-0.1905	0.637438	0.525487	-5.07316	0.822713	0.78393
T.cells	TAF3	-0.05037	5.653075	-0.63734	0.525549	-6.59471	0.721258	0.666371
T.cells	B4GAT1	0.200867	1.65015	0.637337	0.525552	-5.43789	0.789412	0.744886
T.cells	DNAH10	-0.33584	0.024576	-0.63732	0.525565	-5.12431	0.818757	0.77929
T.cells	MTFR1L	-0.07224	4.250609	-0.63722	0.525627	-6.13742	0.744486	0.692907
T.cells	TUG1	0.051286	6.098897	0.637191	0.525647	-6.61021	0.714018	0.658155
T.cells	TES	0.072471	5.486918	0.637092	0.525711	-6.4592	0.723974	0.669464
T.cells	MUC13	0.271624	1.034673	0.637048	0.52574	-5.35579	0.800407	0.757741
T.cells	TM4SF4	-0.23534	2.168937	-0.63683	0.52588	-5.49049	0.780363	0.734304
T.cells	APRT	-0.07011	6.416788	-0.6364	0.52616	-6.65271	0.709208	0.652526
T.cells	2310061I0	-0.08419	3.521012	-0.63639	0.526169	-6.04986	0.757175	0.707305
T.cells	TMED10	0.044168	7.769396	0.636049	0.526387	-6.82912	0.687879	0.628521
T.cells	SLC3A1	0.206036	0.899213	0.635933	0.526463	-5.44296	0.803292	0.760939
T.cells	LRP2BP	0.178186	1.991491	0.635837	0.526525	-5.73964	0.783811	0.738187
T.cells	ZC3H13	0.054827	5.653384	0.635767	0.526571	-6.54337	0.721655	0.666668
T.cells	HECTD3	-0.11626	2.789878	-0.63567	0.526633	-5.76671	0.769849	0.722006
T.cells	4930438A0	-0.29271	-0.84008	-0.63563	0.526662	-5.06274	0.835234	0.798602
T.cells	SLC25A11	-0.06919	5.162778	-0.63557	0.526697	-6.37482	0.729706	0.675875
T.cells	GTF2F1	0.050038	5.030746	0.63516	0.526965	-6.47017	0.731893	0.678528
T.cells	GTF2I	0.055748	5.762327	0.635124	0.526988	-6.52311	0.719884	0.664849
T.cells	IREB2	0.055283	5.493255	0.634939	0.527108	-6.53047	0.724279	0.6699
T.cells	IGTP	0.248884	3.443365	0.634749	0.527231	-5.68229	0.758599	0.709255
T.cells	DDX27	-0.0553	4.974515	-0.63462	0.527313	-6.5029	0.732824	0.679675
T.cells	ST7L	0.087614	4.451288	0.634476	0.527409	-6.11735	0.741538	0.689672
T.cells	ULBP1	-0.08022	4.251838	-0.6344	0.527456	-6.41127	0.744885	0.693512
T.cells	TEF	-0.1065	3.230001	-0.6344	0.527459	-5.8989	0.762257	0.713517
T.cells	METTL25	0.060371	4.28478	0.633853	0.527813	-6.30641	0.744331	0.692995
T.cells	LYPLAL1	-0.23477	1.634017	-0.63378	0.52786	-5.47294	0.790144	0.746029
T.cells	PITHD1	-0.06561	4.744392	-0.63377	0.527866	-6.38621	0.736644	0.684185
T.cells	SLC25A23	-0.15364	2.25448	-0.63375	0.527881	-5.64432	0.779192	0.73327
T.cells	TNFRSF12A	0.218571	1.479733	0.633746	0.527883	-5.39063	0.79289	0.749235
T.cells	GM44987	-0.2619	-0.34886	-0.63354	0.528016	-5.20065	0.826103	0.788298
T.cells	CDC34	-0.05059	6.050691	-0.63335	0.528139	-6.66415	0.715201	0.659818
T.cells	BAHD1	0.118245	2.543391	0.633276	0.528188	-5.79826	0.774141	0.727477
T.cells	ATXN7L2	-0.11043	2.480154	-0.63325	0.528207	-5.99023	0.775244	0.728757

T.cells	FCGR1	0.327526	2.432362	0.633236	0.528214	-5.31847	0.776078	0.729727
T.cells	RETNLG	0.343984	3.313557	0.633172	0.528256	-5.55184	0.760822	0.712056
T.cells	CBS	-0.26302	1.874066	-0.63316	0.528263	-5.36917	0.78589	0.741144
T.cells	N4BP2L2	-0.03595	6.786437	-0.63311	0.528298	-6.7471	0.703384	0.64644
T.cells	WDR12	0.072013	4.283459	0.633103	0.528301	-6.34525	0.744354	0.693092
T.cells	LIN37	0.085275	3.646925	0.633006	0.528363	-6.12335	0.755124	0.705502
T.cells	CHEK2	-0.08052	3.145946	-0.63296	0.528392	-6.12672	0.763702	0.71541
T.cells	ATG16L2	-0.05584	5.680082	-0.63295	0.528401	-6.57229	0.721224	0.666686
T.cells	BRK1	0.045345	6.271039	0.632778	0.528512	-6.63489	0.711642	0.655811
T.cells	ANG	-0.15704	4.211322	-0.63271	0.528553	-6.04368	0.745567	0.694516
T.cells	PEX10	0.211301	0.82136	0.632631	0.528608	-5.40054	0.804704	0.763194
T.cells	UBE2E1	-0.03882	6.229498	-0.63251	0.528684	-6.68171	0.712312	0.656596
T.cells	CLEC2G	-0.31412	0.015976	-0.6325	0.528695	-5.18214	0.819376	0.780467
T.cells	ALOX5	0.398209	0.791987	0.632173	0.528905	-5.08211	0.805453	0.7639
T.cells	OLFR1369-	0.374184	-0.76388	0.632005	0.529015	-5.15351	0.834111	0.797607
T.cells	DTX2	0.069533	4.396914	0.631692	0.529218	-6.34383	0.742903	0.691114
T.cells	ADRM1	-0.05951	5.477684	-0.63156	0.529304	-6.57144	0.72499	0.670679
T.cells	ACER1	-0.46253	-0.7216	-0.63138	0.529423	-5.05014	0.833553	0.796859
T.cells	XCR1	-0.39088	0.412393	-0.63137	0.529428	-5.13711	0.812636	0.772177
T.cells	GM20743	0.233002	0.031876	0.631252	0.529504	-5.42154	0.819616	0.780432
T.cells	AMD1	-0.07278	4.581194	-0.63078	0.529813	-6.32557	0.740138	0.687872
T.cells	NMB	-0.17507	1.642171	-0.63069	0.529869	-5.52304	0.790825	0.746493
T.cells	RCOR1	0.052027	6.85813	0.630574	0.529946	-6.7392	0.702976	0.645635
T.cells	PLPP5	-0.11785	3.394162	-0.63052	0.52998	-5.8106	0.760235	0.711021
T.cells	TMCO6	0.115434	2.683081	0.630229	0.53017	-5.77766	0.772696	0.725315
T.cells	SYNE3	0.107012	2.767053	0.629735	0.530492	-6.1396	0.77143	0.72375
T.cells	SIRT2	0.057485	5.688086	0.629542	0.530618	-6.4236	0.722198	0.667277
T.cells	COA6	-0.07487	4.110702	-0.62947	0.530664	-6.2107	0.748406	0.697261
T.cells	GM35853	0.330489	0.184156	0.629311	0.530768	-5.089	0.817542	0.777709
T.cells	2210408F2	-0.29088	2.856305	-0.62931	0.530771	-5.29548	0.76988	0.722046
T.cells	PTPN3	0.306888	1.040176	0.6293	0.530775	-5.25531	0.801985	0.759439
T.cells	CTDSP1	-0.06172	5.3236	-0.62924	0.530812	-6.47719	0.728176	0.674126
T.cells	PTRHD1	-0.07487	4.56189	-0.62921	0.530836	-6.31672	0.74082	0.688574
T.cells	TFIP11	0.107927	3.322157	0.629097	0.530908	-5.87768	0.761846	0.712748
T.cells	ZFP628	-0.12753	2.635685	-0.62889	0.531045	-5.85786	0.773825	0.726567
T.cells	AXDND1	-0.31369	1.557778	-0.6286	0.531229	-5.24999	0.792951	0.748699
T.cells	PANK1	-0.11479	3.430383	-0.62855	0.531263	-6.04425	0.760212	0.710716
T.cells	ZFP623	0.287791	0.124749	0.628186	0.531501	-5.2218	0.819053	0.779182
T.cells	CD200R2	-0.43447	0.789771	-0.62812	0.531546	-5.11361	0.806923	0.764945
T.cells	GNG12	-0.06178	6.49518	-0.62807	0.531576	-6.55344	0.709489	0.652684
T.cells	ARHGEF15	-0.34754	0.580885	-0.6275	0.531946	-5.15421	0.810999	0.769684
T.cells	TM9SF4	-0.07362	4.769644	-0.62744	0.53199	-6.29083	0.737989	0.685067
T.cells	GINS4	0.074033	3.753755	0.62732	0.532066	-6.2481	0.755112	0.70474
T.cells	RDH5	-0.18983	2.368316	-0.62714	0.532183	-5.55787	0.779061	0.732522
T.cells	TRMT112	-0.04334	7.277188	-0.62684	0.532377	-6.87931	0.697272	0.639049
T.cells	PQLC3	0.093513	3.812077	0.626841	0.532379	-6.1108	0.754119	0.703766
T.cells	TFEC	-0.29561	2.368464	-0.62684	0.532381	-5.30002	0.779058	0.732607

T.cells	FANCE	0.111131	2.916052	0.626789	0.532413	-5.87791	0.769509	0.721534
T.cells	LMO2	0.099905	4.903927	0.62655	0.532569	-6.14656	0.735753	0.682741
T.cells	PKIG	-0.04362	7.198634	-0.62647	0.532618	-6.9737	0.698514	0.640488
T.cells	OSGEPL1	0.108513	2.472736	0.626445	0.532637	-5.80927	0.777232	0.730531
T.cells	RAC3	0.356244	-0.22085	0.62615	0.53283	-5.12059	0.825712	0.787432
T.cells	SLC45A3	-0.26629	1.111286	-0.62604	0.532902	-5.35116	0.801398	0.758855
T.cells	WASF2	0.033348	8.064634	0.625945	0.532964	-6.98211	0.68493	0.625408
T.cells	BC052040	0.08153	4.165796	0.625929	0.532974	-6.313	0.748123	0.697111
T.cells	9930111J2	0.165329	4.28297	0.625884	0.533004	-5.83925	0.746146	0.694849
T.cells	HMGA2	-0.28257	1.149583	-0.62588	0.533004	-5.58152	0.800709	0.758125
T.cells	1810021B2	0.342661	0.427969	0.625883	0.533004	-5.15463	0.813786	0.773474
T.cells	ATP5G1	-0.05139	7.643771	-0.62575	0.533092	-6.90781	0.6915	0.632804
T.cells	NOX1	0.218811	1.548335	0.625744	0.533095	-5.49675	0.793565	0.749791
T.cells	WAC	-0.03928	7.414892	-0.62557	0.533206	-6.80599	0.695156	0.636852
T.cells	PPRC1	-0.07894	3.956149	-0.62516	0.533473	-6.15399	0.751884	0.701354
T.cells	1810009A1	0.109146	2.243882	0.625154	0.53348	-5.80133	0.781467	0.735574
T.cells	IMMP1L	0.054831	4.995599	0.625024	0.533565	-6.45694	0.734438	0.681351
T.cells	SMARCC1	-0.04667	6.606315	-0.62493	0.533625	-6.82975	0.708153	0.651472
T.cells	GLYR1	0.042955	6.736527	0.624745	0.533748	-6.7435	0.706067	0.649129
T.cells	TRAK2	0.066248	5.153104	0.62472	0.533764	-6.38949	0.731828	0.678386
T.cells	FPR1	0.468968	2.992339	0.624697	0.533779	-5.30265	0.768405	0.720435
T.cells	SULT2B1	-0.23545	2.700547	-0.62453	0.533889	-5.36378	0.773537	0.726317
T.cells	WIPI2	0.053368	5.276718	0.624158	0.534131	-6.50723	0.730085	0.676221
T.cells	RRP1	-0.03774	6.518197	-0.62375	0.534401	-6.75306	0.709921	0.653405
T.cells	CSF2RB2	0.299062	2.359571	0.623683	0.534442	-5.2402	0.779823	0.733612
T.cells	USP37	-0.05025	6.268931	-0.62359	0.534504	-6.72049	0.71394	0.657979
T.cells	ALOX15	-0.69303	-0.82153	-0.62342	0.534613	-5.05434	0.837549	0.801372
T.cells	DDI2	0.055452	5.943537	0.623418	0.534615	-6.56854	0.719218	0.663967
T.cells	LMBR1L	-0.07281	4.517308	-0.62338	0.53464	-6.3476	0.742788	0.690861
T.cells	GLB1L	0.13875	2.192734	0.623372	0.534645	-5.61999	0.782757	0.737024
T.cells	POGLUT3	-0.23901	0.979034	-0.62311	0.534815	-5.29688	0.804566	0.762435
T.cells	CYREN	0.117181	3.374354	0.622826	0.535002	-5.88079	0.762515	0.713383
T.cells	MTHFR	0.129465	3.640415	0.622712	0.535077	-5.67095	0.757965	0.708121
T.cells	ASRGL1	0.086719	3.315534	0.622282	0.535358	-6.2537	0.763738	0.714633
T.cells	GM38832	0.306003	0.763984	0.622207	0.535407	-5.14169	0.808865	0.767196
T.cells	GM34455	0.263798	2.623429	0.622126	0.53546	-5.35321	0.775743	0.72858
T.cells	HDDC3	0.204594	0.928077	0.62209	0.535484	-5.4317	0.805891	0.763744
T.cells	VPS9D1	0.118742	3.05216	0.621665	0.535762	-5.82736	0.768454	0.720062
T.cells	FASN	-0.11379	3.14635	-0.62164	0.535779	-5.95593	0.766825	0.71818
T.cells	CLSPN	-0.0811	4.411842	-0.6216	0.535801	-6.54014	0.74524	0.693326
T.cells	PELP1	-0.08156	3.358177	-0.62139	0.535942	-6.13112	0.763277	0.714051
T.cells	NR2F6	-0.1261	3.501892	-0.62115	0.536098	-5.82526	0.760934	0.711259
T.cells	ASNSD1	-0.05141	5.360445	-0.62085	0.536298	-6.50365	0.729691	0.675464
T.cells	SUSD1	0.063605	5.183728	0.620814	0.536319	-6.58115	0.732613	0.678795
T.cells	HMGXB3	0.070289	4.408539	0.620618	0.536448	-6.25087	0.745558	0.693617
T.cells	SNHG17	-0.17516	1.368538	-0.62061	0.53645	-5.47215	0.798412	0.754824
T.cells	RTCA	-0.06327	4.282973	-0.62042	0.536577	-6.37519	0.747675	0.696058

T.cells	GRK2	-0.04082	7.206149	-0.62007	0.536807	-6.87096	0.699827	0.641694
T.cells	CNTD1	0.222174	0.620711	0.620063	0.536811	-5.3942	0.811935	0.770728
T.cells	DISC1	-0.1943	3.43956	-0.61978	0.536999	-5.38813	0.762041	0.712793
T.cells	TGIF2	-0.0981	3.122183	-0.61973	0.537031	-5.96684	0.767513	0.719115
T.cells	DOK3	0.063907	5.36627	0.619718	0.537037	-6.68359	0.729595	0.675572
T.cells	CLPB	-0.06601	4.010407	-0.61959	0.537122	-6.2133	0.75229	0.701576
T.cells	ANKRD17	-0.04214	7.985576	-0.61953	0.537159	-6.90902	0.687566	0.628094
T.cells	ALKBH4	-0.11305	2.574916	-0.61931	0.537302	-5.83947	0.777035	0.730245
T.cells	GM26549	-0.13402	3.018851	-0.6191	0.537442	-5.68315	0.769303	0.721337
T.cells	CCT6B	-0.31056	0.315333	-0.61902	0.537492	-5.19226	0.817517	0.777674
T.cells	TAOK3	0.040967	7.012234	0.618993	0.537513	-6.68534	0.70291	0.645485
T.cells	ZFP667	0.117933	2.726732	0.618949	0.537542	-5.92488	0.774383	0.727264
T.cells	ARID4B	-0.04117	8.259309	-0.61889	0.537579	-6.96175	0.683308	0.623483
T.cells	DENND6B	0.187787	0.94417	0.61866	0.537731	-5.4877	0.80606	0.764321
T.cells	TCTN1	0.206826	1.315572	0.618527	0.537818	-5.48818	0.799363	0.756515
T.cells	SH3GLB2	-0.08236	3.721025	-0.61852	0.537822	-6.11902	0.757219	0.707559
T.cells	PDCD11	-0.06572	4.214015	-0.61826	0.537993	-6.29734	0.74884	0.698
T.cells	IFT20	-0.05638	5.442542	-0.61814	0.538071	-6.47831	0.728338	0.674547
T.cells	LAMTOR1	0.043879	6.503458	0.618132	0.538077	-6.67624	0.711058	0.654914
T.cells	PSME3	0.064229	5.282258	0.618005	0.538161	-6.50345	0.730983	0.677569
T.cells	WWP2	0.046029	6.537142	0.618004	0.538162	-6.68746	0.710516	0.654305
T.cells	MDP1	0.064941	4.373314	0.617923	0.538215	-6.27955	0.746152	0.69497
T.cells	SPAG1	0.175979	1.4147	0.617799	0.538296	-5.60924	0.797584	0.754622
T.cells	SUV39H2	-0.10252	2.381659	-0.61775	0.538327	-5.96541	0.780423	0.734614
T.cells	ZFP113	0.191548	1.151185	0.617748	0.538329	-5.51976	0.802321	0.760177
T.cells	CCDC88C	-0.05858	5.294187	-0.6175	0.538489	-6.57331	0.730786	0.677514
T.cells	CDCA7	-0.09566	3.246784	-0.61749	0.5385	-6.23592	0.76536	0.717245
T.cells	RNF38	-0.04956	6.163439	-0.61738	0.538568	-6.61907	0.716554	0.661321
T.cells	SH3BGRL	0.048854	6.824052	0.617352	0.538589	-6.75243	0.705913	0.649276
T.cells	CREBRF	-0.06	6.98351	-0.61723	0.538668	-6.74572	0.703367	0.646432
T.cells	EIF3F	-0.04147	7.589953	-0.61718	0.538704	-6.91983	0.693764	0.635611
T.cells	IGLC1	0.399333	3.761408	0.616931	0.538866	-5.67992	0.756529	0.70714
T.cells	PIAS2	-0.05366	6.02482	-0.61675	0.538988	-6.64585	0.718806	0.664027
T.cells	THAP2	0.094553	3.329281	0.616696	0.53902	-6.06633	0.763938	0.715793
T.cells	GM10847	0.219905	0.165978	0.616557	0.539111	-5.38441	0.82026	0.781609
T.cells	CNIH4	0.051705	5.763142	0.616435	0.539192	-6.58161	0.723075	0.668941
T.cells	EMC10	-0.05173	5.502896	-0.61635	0.539249	-6.50805	0.727344	0.673805
T.cells	CCDC130	0.160753	2.056293	0.616199	0.539347	-5.657	0.786159	0.741678
T.cells	DEAF1	-0.07893	3.281829	-0.61613	0.53939	-6.10168	0.764756	0.716825
T.cells	GTF2F2	-0.04741	5.881638	-0.61611	0.539402	-6.60467	0.721139	0.666787
T.cells	BCLAF3	0.053181	4.966172	0.616059	0.539439	-6.54713	0.736225	0.684026
T.cells	FBL	-0.05103	6.091074	-0.61594	0.539518	-6.68837	0.717729	0.662945
T.cells	PSTPIP2	0.232925	4.482064	0.615712	0.539666	-5.61232	0.744321	0.693374
T.cells	GOSR1	-0.06915	4.182554	-0.61571	0.539669	-6.20782	0.749372	0.699179
T.cells	LCK	0.087632	3.728178	0.615673	0.539692	-6.23352	0.757096	0.708077
T.cells	LYSMD1	-0.1516	1.591133	-0.61566	0.5397	-5.54192	0.794427	0.751443
T.cells	ZFP608	0.075973	6.820434	0.615597	0.539742	-6.79769	0.705971	0.649711

T.cells	MED8	0.06013	5.008062	0.615574	0.539757	-6.42857	0.735528	0.683335
T.cells	DHX38	0.071513	4.560036	0.615434	0.539849	-6.28594	0.743012	0.691991
T.cells	ZMYM5	0.052375	5.521459	0.615367	0.539893	-6.50728	0.727039	0.673729
T.cells	LSM2	-0.0642	5.695608	-0.6152	0.540006	-6.69883	0.724181	0.670471
T.cells	YEATS2	0.058853	4.376137	0.615116	0.540058	-6.37391	0.746104	0.695572
T.cells	2900097C1	0.063536	4.909122	0.615087	0.540077	-6.29695	0.737174	0.685342
T.cells	B4GALT3	0.071986	3.874826	0.615012	0.540126	-6.10575	0.754595	0.705385
T.cells	RBFOX2	-0.24788	2.758111	-0.61501	0.54013	-5.45754	0.773835	0.727648
T.cells	ZBTB14	0.077386	3.004063	0.6147	0.540332	-6.03881	0.769676	0.722747
T.cells	GM49336	-0.05587	4.881295	-0.61468	0.540346	-6.42153	0.73775	0.685955
T.cells	DDX1	0.060318	4.964571	0.61455	0.54043	-6.53121	0.736387	0.684395
T.cells	GM17231	-0.17567	2.734245	-0.61429	0.540603	-5.64703	0.774394	0.728303
T.cells	FAM207A	-0.06514	4.223482	-0.61426	0.54062	-6.26592	0.748818	0.698739
T.cells	JCAD	-0.18251	1.404554	-0.61425	0.54063	-5.4594	0.797913	0.755734
T.cells	NTM	-0.27989	0.913874	-0.61392	0.540843	-5.27281	0.806964	0.766108
T.cells	ANKRD23	-0.24362	0.33175	-0.61383	0.540903	-5.25694	0.817577	0.778598
T.cells	RNF44	-0.06585	4.833037	-0.6134	0.541184	-6.31004	0.738895	0.687029
T.cells	CNOT3	-0.0514	5.46868	-0.61338	0.541202	-6.56695	0.728353	0.674978
T.cells	WDR77	-0.07695	3.895337	-0.61306	0.541408	-6.21119	0.754708	0.705256
T.cells	NABP1	-0.13707	4.743721	-0.61302	0.541434	-5.81419	0.740388	0.688802
T.cells	CERS2	-0.06113	5.262709	-0.61291	0.541508	-6.39483	0.731753	0.67895
T.cells	FBXO30	0.081833	5.032199	0.612841	0.541555	-6.27442	0.735576	0.683321
T.cells	HDAC4	0.08537	5.402881	0.612803	0.54158	-6.31918	0.729438	0.676306
T.cells	ZFP808	0.117399	1.973033	0.61271	0.541641	-5.81573	0.788115	0.744057
T.cells	GM49864	0.22045	0.452692	0.6126	0.541713	-5.39905	0.815501	0.776178
T.cells	GM42418	-0.11865	11.10924	-0.61257	0.541735	-7.32781	0.640844	0.577052
T.cells	10-Sep	-0.11194	3.119761	-0.61245	0.541809	-5.84782	0.768025	0.720715
T.cells	LENG1	0.098916	2.925078	0.612448	0.541814	-5.87335	0.771402	0.724629
T.cells	TRMT2B	-0.08004	3.750232	-0.61235	0.541878	-6.18199	0.757183	0.70821
T.cells	PLAGL2	0.069732	4.367209	0.612183	0.541988	-6.25529	0.746711	0.696185
T.cells	GM13391	-0.21576	0.765079	-0.61208	0.542055	-5.40189	0.809803	0.769583
T.cells	PDE8A	0.086437	5.847934	0.612067	0.542064	-6.45175	0.72213	0.668105
T.cells	SLK	0.059334	6.477015	0.611962	0.542133	-6.60559	0.711923	0.656525
T.cells	GNMT	0.186865	4.232803	0.611747	0.542275	-6.02218	0.749088	0.698905
T.cells	RIOK3	0.05035	7.578172	0.611482	0.54245	-6.78868	0.694611	0.636825
T.cells	GM26535	0.336629	-0.60061	0.610791	0.542905	-5.06991	0.835373	0.799626
T.cells	NATD1	0.10049	3.25628	0.610739	0.54294	-6.10434	0.766022	0.718366
T.cells	TUBB3	-0.25307	0.736309	-0.61068	0.542978	-5.24684	0.810705	0.770511
T.cells	PEX14	0.054132	5.405291	0.610514	0.543088	-6.55258	0.729738	0.676681
T.cells	SF3A3	-0.05432	4.984502	-0.61051	0.543092	-6.54111	0.736714	0.684655
T.cells	USP15	0.044691	7.650516	0.610489	0.543104	-6.89144	0.693559	0.63568
T.cells	FKTN	-0.19426	1.595478	-0.61041	0.543158	-5.46849	0.795207	0.752374
T.cells	SERPINE2	-0.2581	1.29875	-0.61033	0.543208	-5.29954	0.800528	0.758605
T.cells	SMAD2	0.041014	6.426685	0.610123	0.543345	-6.70008	0.713064	0.657777
T.cells	PIN1	-0.0492	5.245371	-0.61004	0.543402	-6.57091	0.732382	0.679777
T.cells	ARMC7	0.133285	3.924467	0.609873	0.54351	-5.72993	0.754564	0.705291
T.cells	GM49521	0.198256	0.473447	0.609716	0.543614	-5.43725	0.815502	0.776332

T.cells	CARS	0.079877	4.118134	0.609609	0.543685	-6.33056	0.751273	0.701509
T.cells	GM20342	0.084979	3.499425	0.609586	0.5437	-6.16916	0.761834	0.713693
T.cells	GM20470	-0.19479	1.403571	-0.60958	0.543704	-5.47575	0.798645	0.75653
T.cells	GM49692	0.196878	0.104027	0.609334	0.543866	-5.46639	0.822287	0.784371
T.cells	GM45606	-0.25775	0.477564	-0.60923	0.543936	-5.32385	0.815426	0.77633
T.cells	SLC12A2	-0.13222	3.544469	-0.6092	0.543954	-5.92798	0.761061	0.712889
T.cells	VRK3	0.053386	4.98755	0.608767	0.54424	-6.46804	0.736663	0.684979
T.cells	SETD3	0.049177	5.687067	0.608743	0.544256	-6.54762	0.725102	0.671768
T.cells	PHF7	0.086403	3.28527	0.608727	0.544266	-6.10876	0.765522	0.718214
T.cells	GM35769	-0.23074	1.096204	-0.60865	0.544316	-5.34075	0.804179	0.76329
T.cells	MBD6	-0.08801	3.632499	-0.6086	0.544347	-6.03821	0.759551	0.711308
T.cells	ITFG2	0.070285	3.893716	0.608362	0.544507	-6.31453	0.755088	0.706156
T.cells	CHP2	-0.49005	-0.09865	-0.60832	0.544537	-5.09669	0.826032	0.789039
T.cells	INTS12	0.063058	4.380987	0.608246	0.544584	-6.30718	0.746827	0.696643
T.cells	TRAPPC3	-0.06134	4.917333	-0.60818	0.54463	-6.40254	0.737833	0.686319
T.cells	ERI3	0.050816	5.177084	0.608155	0.544644	-6.47728	0.733514	0.681374
T.cells	NEURL4	0.143948	2.445552	0.608139	0.544655	-5.82554	0.780143	0.73519
T.cells	KLRA7	0.55854	0.646446	0.608102	0.544679	-5.10683	0.812342	0.772886
T.cells	DDX52	-0.05274	4.930462	-0.608	0.544743	-6.46837	0.737614	0.686068
T.cells	1810024B0	-0.11892	2.692562	-0.60787	0.544832	-5.83231	0.775815	0.730156
T.cells	ESCO1	0.053105	5.77786	0.607773	0.544896	-6.5477	0.723614	0.670072
T.cells	5033421B0	0.396955	-0.4422	0.607721	0.544931	-5.08045	0.832415	0.796595
T.cells	DMTN	-0.3013	0.637339	-0.60767	0.544963	-5.17851	0.812508	0.773081
T.cells	NXT2	0.106692	2.785909	0.607657	0.544973	-5.7909	0.774185	0.728262
T.cells	CRMP1	0.346006	-1.07546	0.607522	0.545062	-5.06261	0.844299	0.810769
T.cells	NINL	0.179325	0.999292	0.607424	0.545126	-5.55684	0.805932	0.765409
T.cells	BBOF1	-0.17201	1.302277	-0.60735	0.545179	-5.54348	0.800465	0.759025
T.cells	JPX	-0.09919	3.810956	-0.60727	0.545228	-6.22404	0.756499	0.707876
T.cells	PRPF39	0.056239	5.43876	0.607224	0.545259	-6.4924	0.729186	0.676523
T.cells	TAF1B	0.060808	4.199522	0.607106	0.545337	-6.32322	0.749901	0.700302
T.cells	INPPL1	0.119668	2.471527	0.606969	0.545427	-5.75923	0.779694	0.73481
T.cells	FBXO42	0.0549	5.871653	0.6069	0.545473	-6.61985	0.722087	0.668472
T.cells	GM16573	0.190196	1.655105	0.606812	0.545531	-5.43006	0.794149	0.751741
T.cells	ZFP346	0.083178	3.658973	0.606433	0.545781	-6.23439	0.759292	0.711044
T.cells	KCTD13	0.126421	2.619439	0.606304	0.545867	-5.70287	0.777293	0.731962
T.cells	SLC10A7	-0.04705	5.908741	-0.60624	0.545909	-6.67096	0.721659	0.667928
T.cells	RBM27	-0.04142	6.399999	-0.6062	0.545933	-6.70234	0.713678	0.658855
T.cells	MLST8	-0.16099	2.044303	-0.60592	0.546119	-5.63464	0.787594	0.743836
T.cells	STAT5A	-0.08924	3.992851	-0.6056	0.546333	-6.0771	0.753831	0.704642
T.cells	SAP30L	-0.05791	4.309459	-0.60557	0.546351	-6.30083	0.748462	0.698459
T.cells	GM6787	-0.22628	0.188055	-0.60551	0.546393	-5.33616	0.821208	0.783274
T.cells	ADCY6	-0.26769	0.433361	-0.60544	0.546439	-5.24051	0.816702	0.777965
T.cells	MED4	-0.05798	4.020834	-0.60507	0.546685	-6.2976	0.753599	0.704187
T.cells	PRKCSH	0.053582	4.862373	0.604924	0.546779	-6.40002	0.739412	0.687912
T.cells	RBM26	0.038447	6.821937	0.604866	0.546817	-6.79152	0.707342	0.651403
T.cells	NFIA	0.082912	5.904	0.604707	0.546922	-6.507	0.722248	0.668284
T.cells	TSPYL2	0.114901	2.8065	0.604564	0.547017	-5.90347	0.774613	0.72853

T.cells	5031439G(	0.057519	5.696635	0.604447	0.547094	-6.58643	0.725696	0.672256
T.cells	TMC6	0.06646	4.632332	0.604341	0.547164	-6.24792	0.743373	0.692501
T.cells	GM48383	0.160072	1.758143	0.604117	0.547313	-5.66887	0.793247	0.750278
T.cells	4930444A1	-0.10986	3.751804	-0.60376	0.547547	-6.04238	0.758419	0.709921
T.cells	GM13963	0.397039	-0.29578	0.603757	0.547551	-5.06885	0.830685	0.794452
T.cells	LCA5	0.162827	1.313513	0.603738	0.547564	-5.61333	0.801223	0.759734
T.cells	TBC1D7	0.100517	2.220043	0.603539	0.547696	-5.81003	0.785054	0.740827
T.cells	PBK	-0.0927	3.69023	-0.60339	0.547796	-6.38468	0.759473	0.711191
T.cells	RTF1	-0.03502	6.81408	-0.60329	0.547859	-6.80096	0.707683	0.652005
T.cells	KBTBD2	-0.0473	5.176548	-0.60309	0.547995	-6.4529	0.734403	0.682474
T.cells	LCN4	-0.28425	-1.05402	-0.60306	0.548014	-5.12122	0.844907	0.81152
T.cells	DLG1	0.04288	7.219142	0.60295	0.548086	-6.81872	0.701217	0.644748
T.cells	FAM136A	-0.0705	4.090791	-0.60294	0.548092	-6.33392	0.752639	0.703417
T.cells	UQCRC2	-0.03978	6.345631	-0.60284	0.548161	-6.72166	0.715232	0.660618
T.cells	MTMR1	-0.07381	4.622377	-0.60278	0.548195	-6.20207	0.743658	0.693093
T.cells	GM21859	0.402807	1.780794	0.602755	0.548215	-5.18788	0.792851	0.750112
T.cells	CIB1	-0.05519	5.732242	-0.60248	0.548398	-6.55959	0.725231	0.672119
T.cells	TADA2A	-0.09749	2.820969	-0.60245	0.548418	-5.96294	0.774503	0.728849
T.cells	CDKN1C	-0.14474	3.302414	-0.6024	0.548451	-6.02252	0.766145	0.719152
T.cells	ZMIZ1OS1	-0.29399	0.352543	-0.60228	0.548532	-5.2778	0.818698	0.780628
T.cells	PAWR	-0.25813	0.662659	-0.60223	0.548562	-5.26067	0.813021	0.773954
T.cells	TBRG4	0.075525	4.075048	0.602091	0.548655	-6.2332	0.752907	0.703929
T.cells	2310022B(	-0.12746	2.488564	-0.60209	0.548657	-5.57682	0.780323	0.735699
T.cells	DVL2	0.101741	3.263703	0.601917	0.54877	-6.02088	0.766878	0.72005
T.cells	RASSF2	-0.08822	4.259468	-0.60176	0.548876	-6.14393	0.749895	0.700381
T.cells	MAPK11	-0.25307	0.260399	-0.60162	0.548964	-5.26532	0.820551	0.782772
T.cells	PPIL3	-0.05564	4.605985	-0.60104	0.549354	-6.41858	0.744443	0.693886
T.cells	BBX	0.060437	6.494425	0.601015	0.549368	-6.65515	0.713314	0.658349
T.cells	ORMDL2	-0.05407	5.109617	-0.60051	0.549705	-6.50309	0.736133	0.684351
T.cells	COX7A2L	-0.0396	6.644119	-0.6005	0.54971	-6.75947	0.71101	0.65573
T.cells	CTC1	0.074607	3.25694	0.600059	0.550002	-6.12215	0.767575	0.720588
T.cells	N6AMT1	-0.1227	2.597729	-0.60004	0.550014	-5.78806	0.779061	0.733925
T.cells	PSMD4	0.038152	6.691408	0.600034	0.550018	-6.764	0.710249	0.654887
T.cells	BPTF	-0.0424	7.356344	-0.59999	0.550047	-6.86248	0.699626	0.642876
T.cells	FIS1	0.044006	7.530663	0.599963	0.550065	-6.86535	0.696867	0.639764
T.cells	GM29394	0.217862	0.417159	0.599906	0.550103	-5.37236	0.818199	0.779793
T.cells	PSMA4	-0.04263	6.877207	-0.59984	0.550147	-6.81432	0.707265	0.65152
T.cells	IGF2R	0.074851	4.985937	0.599701	0.550239	-6.36813	0.738194	0.686767
T.cells	ERC1	-0.07507	4.872109	-0.59969	0.550249	-6.35721	0.740096	0.68895
T.cells	DUS3L	-0.07917	3.300011	-0.59963	0.550286	-6.07416	0.76683	0.719787
T.cells	SPEF1	0.227846	0.343281	0.599574	0.550324	-5.35038	0.819557	0.781475
T.cells	SLC25A45	0.110639	3.260909	0.599414	0.55043	-5.80958	0.767561	0.720637
T.cells	ARMC6	-0.14434	1.405639	-0.59927	0.550525	-5.6153	0.800315	0.758858
T.cells	RNPS1	-0.0384	6.167896	-0.59919	0.550578	-6.7664	0.718789	0.664699
T.cells	SNX4	0.043469	6.329814	0.599024	0.550689	-6.63184	0.716215	0.661719
T.cells	ZFP955B	0.112672	2.201747	0.59891	0.550764	-5.89003	0.786188	0.742353
T.cells	TOP1MT	0.167101	1.70028	0.598582	0.550982	-5.61047	0.795222	0.75292

T.cells	LIPA	-0.09442	5.64326	-0.5985	0.551035	-6.20964	0.727548	0.674694
T.cells	ETFB	-0.05932	6.979015	-0.59839	0.55111	-6.76156	0.705873	0.650105
T.cells	MGAT1	0.065039	4.704416	0.598232	0.551214	-6.38077	0.743155	0.692631
T.cells	CTDNEP1	-0.05644	5.516749	-0.59805	0.551339	-6.54806	0.729633	0.677161
T.cells	LRP6	-0.06718	5.378913	-0.59802	0.551357	-6.44714	0.731911	0.679766
T.cells	CTU1	0.214398	0.850388	0.597896	0.551438	-5.42128	0.810554	0.771057
T.cells	EIF1A	-0.06174	5.711772	-0.59788	0.551446	-6.50787	0.726421	0.673521
T.cells	MTPAP	-0.06989	4.284578	-0.59788	0.551447	-6.30011	0.750235	0.70082
T.cells	MYO6	-0.18127	3.1857	-0.59749	0.551708	-5.51512	0.769071	0.722685
T.cells	ZDHHC1	0.168127	1.415954	0.597482	0.551713	-5.46473	0.800326	0.759142
T.cells	ZFP52	0.087234	3.859767	0.597226	0.551883	-6.12277	0.757468	0.709278
T.cells	GLT8D1	0.082254	3.339159	0.596998	0.552035	-6.0638	0.766415	0.719628
T.cells	PRC1	0.09378	5.285692	0.596953	0.552064	-6.70002	0.733459	0.681675
T.cells	CAMKMT	0.081915	4.501622	0.596951	0.552066	-6.24318	0.746571	0.696718
T.cells	DOCK1	-0.15623	4.079995	-0.59685	0.552132	-5.6763	0.753713	0.704948
T.cells	MARS2	-0.08273	2.690445	-0.59684	0.552139	-6.01988	0.777702	0.732739
T.cells	KLRA1	0.230431	0.700337	0.596806	0.552162	-5.65319	0.813294	0.774415
T.cells	LYST	0.064625	6.729745	0.596806	0.552162	-6.56903	0.709875	0.654818
T.cells	SLC49A4	0.125243	6.30055	0.596769	0.552187	-6.20942	0.716809	0.662689
T.cells	HNRNPH2	0.040189	6.435152	0.596567	0.552321	-6.70281	0.714668	0.660293
T.cells	SNAPC3	0.06065	4.825524	0.59652	0.552352	-6.46186	0.74117	0.690559
T.cells	STN1	-0.08105	3.886297	-0.59631	0.552491	-6.07259	0.757156	0.708909
T.cells	6330562C2	-0.23851	1.275201	-0.59572	0.552883	-5.29171	0.803356	0.762463
T.cells	MEI4	0.153885	1.201602	0.595682	0.55291	-5.85754	0.804686	0.764024
T.cells	P2RY13	-0.35351	1.100765	-0.59565	0.552929	-5.17701	0.806511	0.766168
T.cells	CEP19	-0.13925	2.707827	-0.59537	0.553118	-5.79089	0.778041	0.732763
T.cells	RALGPS1	-0.06443	4.92479	-0.59527	0.55318	-6.47556	0.74008	0.688924
T.cells	GM29570	-0.21561	0.692445	-0.59477	0.553515	-5.40315	0.814425	0.775025
T.cells	ZFP267	0.148049	1.251689	0.594411	0.553755	-5.544	0.80426	0.763077
T.cells	TJAP1	-0.05657	4.452059	-0.59434	0.553802	-6.41806	0.748314	0.698099
T.cells	EAR2	0.265027	3.55304	0.594331	0.553808	-5.48688	0.763653	0.715788
T.cells	CFAP45	0.29499	0.728674	0.59425	0.553863	-5.237	0.813763	0.774294
T.cells	FAM98C	0.087361	3.737769	0.594133	0.55394	-6.10112	0.760477	0.712143
T.cells	DUSP22	-0.07205	4.486105	-0.5941	0.55396	-6.36737	0.747739	0.69747
T.cells	MITF	0.176411	4.369202	0.594051	0.553995	-5.73109	0.749715	0.699743
T.cells	CDC16	0.066249	3.893321	0.594048	0.553997	-6.26254	0.757813	0.709073
T.cells	CNOT2	0.035129	7.008824	0.593627	0.554277	-6.86423	0.706353	0.650222
T.cells	CMTM6	-0.06594	4.866252	-0.59355	0.554331	-6.23094	0.741447	0.690203
T.cells	2310010J1	-0.11091	3.692212	-0.59353	0.55434	-5.97624	0.761364	0.713131
T.cells	ZSWIM1	0.1858	1.4089	0.593523	0.554347	-5.59269	0.801534	0.759888
T.cells	RAB44	0.264221	1.487929	0.593332	0.554474	-5.41705	0.800112	0.758267
T.cells	TRAF3IP3	0.057361	5.030914	0.593328	0.554476	-6.45726	0.738693	0.687088
T.cells	GM10138	-0.13524	2.471458	-0.59309	0.554632	-5.78305	0.782653	0.737858
T.cells	GTF3C3	-0.09397	3.102011	-0.59297	0.554712	-6.05712	0.771615	0.725028
T.cells	WSB1	-0.04814	6.461454	-0.59297	0.554713	-6.65596	0.715213	0.660301
T.cells	COPS4	-0.04555	5.581159	-0.59268	0.55491	-6.57434	0.729736	0.676828
T.cells	MICOS13	-0.04793	5.948201	-0.59254	0.555	-6.60381	0.723701	0.669964

T.cells	CEBPB	0.164861	9.155958	0.592522	0.555013	-6.60157	0.672929	0.612746
T.cells	SCAF8	-0.03992	6.711338	-0.59219	0.555234	-6.76695	0.7115	0.655985
T.cells	2610044O1	0.096862	2.24732	0.591623	0.555613	-5.81159	0.787252	0.742801
T.cells	TMEM229A	0.341247	-1.03017	0.591498	0.555696	-5.05105	0.847341	0.813596
T.cells	AFF4	0.052603	7.842009	0.591493	0.5557	-6.82434	0.693744	0.635707
T.cells	CHTOP	-0.05451	5.225925	-0.59142	0.555751	-6.53036	0.736089	0.683745
T.cells	DST	0.118564	4.335211	0.591329	0.555809	-6.07108	0.751054	0.700928
T.cells	TMCC3	-0.19223	5.475701	-0.59129	0.555836	-5.84674	0.731943	0.67903
T.cells	KSR1	-0.07038	4.69672	-0.59111	0.555955	-6.452	0.744998	0.693992
T.cells	ARMCX4	-0.21432	1.19399	-0.59097	0.55605	-5.42958	0.806178	0.765051
T.cells	VPS26B	0.09128	3.835261	0.590811	0.556155	-6.01184	0.75963	0.710903
T.cells	ACRBP	-0.17716	1.566441	-0.59056	0.556323	-5.49312	0.799458	0.75725
T.cells	CRY2	0.116094	2.553643	0.590556	0.556325	-5.77322	0.781896	0.736746
T.cells	C2CD2L	-0.09572	2.991873	-0.59032	0.556479	-5.99839	0.774215	0.727876
T.cells	ZFP771	-0.05769	4.065583	-0.59019	0.556567	-6.34136	0.755692	0.706449
T.cells	GM36862	-0.32103	0.340355	-0.59015	0.556597	-5.12316	0.821776	0.783546
T.cells	MIR17HG	-0.12437	2.192534	-0.59011	0.556621	-5.81936	0.788278	0.74424
T.cells	BC065397	0.168459	1.292469	0.589894	0.556766	-5.49934	0.804396	0.763181
T.cells	TMEM63A	-0.14812	3.49863	-0.58988	0.556777	-5.72686	0.76542	0.717764
T.cells	MAGOHB	-0.07602	4.560419	-0.58976	0.556854	-6.42091	0.747295	0.696903
T.cells	4931428F0	-0.20054	0.65018	-0.58975	0.556865	-5.41431	0.816083	0.776965
T.cells	VAC14	-0.05307	4.624685	-0.58971	0.55689	-6.35805	0.746211	0.695663
T.cells	ARID1A	0.038425	6.658177	0.589621	0.556948	-6.77242	0.712662	0.657395
T.cells	DMAC2	-0.09117	3.093682	-0.58954	0.557	-6.05336	0.77244	0.726007
T.cells	SERPINA11	-0.20684	1.813558	-0.58952	0.557016	-5.45643	0.795028	0.752329
T.cells	SIRT3	0.082063	3.344497	0.589128	0.557278	-6.04012	0.768354	0.7211
T.cells	EDEM1	0.058401	6.015715	0.5889	0.55743	-6.52972	0.723405	0.669505
T.cells	SSR2	-0.05808	5.189958	-0.58887	0.557449	-6.48884	0.737045	0.685076
T.cells	CSPP1	0.050422	6.098729	0.588679	0.557578	-6.69731	0.722126	0.667985
T.cells	GM42997	0.238893	0.465543	0.588474	0.557715	-5.37416	0.819954	0.781372
T.cells	PYROXD2	-0.30021	0.182818	-0.58834	0.557807	-5.12106	0.825171	0.787614
T.cells	ROBO3	0.213266	-0.8339	0.588278	0.557846	-5.28979	0.844183	0.81016
T.cells	TMED1	0.114569	2.452472	0.588071	0.557984	-5.8045	0.784141	0.739524
T.cells	EPB41L5	-0.08034	3.379051	-0.58806	0.557993	-6.29823	0.76794	0.720705
T.cells	ZWILCH	0.107918	3.028209	0.587909	0.558092	-6.13981	0.774037	0.727844
T.cells	CCL6	-0.23394	5.306299	-0.58785	0.55813	-5.71702	0.735237	0.683153
T.cells	CAMK4	-0.317	3.194525	-0.58776	0.558195	-5.44274	0.771141	0.724533
T.cells	NLRC4	-0.08462	3.885651	-0.58754	0.558337	-6.21716	0.759214	0.710778
T.cells	THADA	0.056721	5.881845	0.587426	0.558415	-6.56548	0.725726	0.672359
T.cells	SNED1	-0.20227	1.751149	-0.5874	0.558432	-5.55392	0.796613	0.754262
T.cells	8030462N1	-0.0356	6.073398	-0.58732	0.558484	-6.68351	0.722587	0.668783
T.cells	LRRC8A	-0.05162	5.427019	-0.58727	0.558522	-6.55187	0.733232	0.680939
T.cells	PRPF8	-0.04258	5.801492	-0.5872	0.558567	-6.6166	0.727047	0.673877
T.cells	STX2	-0.08202	3.698442	-0.58712	0.558621	-5.99002	0.762428	0.714541
T.cells	CREB3L2	-0.15171	3.55229	-0.58696	0.558727	-5.6951	0.764997	0.717513
T.cells	TMED7	0.038312	6.308978	0.586759	0.558861	-6.64617	0.718878	0.66459
T.cells	PGM2L1	-0.06258	5.192591	-0.58642	0.559086	-6.57514	0.737385	0.685696

T.cells	DAGLB	-0.08168	4.366228	-0.58635	0.559135	-6.20134	0.751282	0.701685
T.cells	HSD17B7	-0.14377	1.697013	-0.58609	0.559307	-5.61045	0.79786	0.755837
T.cells	CASP9	0.111891	2.447605	0.585945	0.559405	-5.72048	0.784498	0.740226
T.cells	TMEM251	-0.06638	4.619848	-0.58593	0.559413	-6.29275	0.746991	0.696807
T.cells	DENND5B	-0.06362	4.198991	-0.58592	0.559422	-6.48116	0.754124	0.705018
T.cells	INSR	0.063969	5.770425	0.585698	0.559571	-6.59118	0.72781	0.674894
T.cells	SMAP2	-0.04329	6.821529	-0.58569	0.559578	-6.70103	0.710694	0.65543
T.cells	CXXC1	-0.06062	4.307295	-0.5856	0.559637	-6.36283	0.752283	0.702973
T.cells	ATP1A1	-0.06069	6.234757	-0.58557	0.559658	-6.50068	0.720202	0.666257
T.cells	AKT1S1	-0.0819	3.589237	-0.58552	0.559689	-6.043	0.764573	0.717194
T.cells	RNF7	-0.0433	6.742111	-0.58521	0.559898	-6.85895	0.71204	0.657023
T.cells	4930557J0	0.137057	1.834427	0.585208	0.559899	-5.97308	0.795472	0.753178
T.cells	PLOD3	0.113984	3.277379	0.585068	0.559992	-5.89185	0.77004	0.723596
T.cells	2610002M	0.063579	4.208089	0.585042	0.560009	-6.39818	0.754039	0.705094
T.cells	RBBP9	0.207897	1.214794	0.584657	0.560268	-5.44231	0.806825	0.766455
T.cells	FIG4	0.050175	4.603641	0.584602	0.560304	-6.40178	0.747515	0.697525
T.cells	MKLN1	-0.04323	7.159858	-0.58442	0.560424	-6.79445	0.705502	0.649669
T.cells	OAZ2	-0.06033	4.485342	-0.5844	0.560438	-6.28147	0.749515	0.699889
T.cells	PFDN5	-0.03421	7.413663	-0.58434	0.560481	-6.84383	0.701454	0.645102
T.cells	NRN1	-0.20762	3.109984	-0.58423	0.560555	-5.71265	0.773147	0.727209
T.cells	SWI5	-0.04623	7.120072	-0.58408	0.560654	-6.88713	0.706187	0.650409
T.cells	GM48027	0.11927	2.815176	0.583945	0.560745	-5.81495	0.778377	0.733238
T.cells	CLPX	-0.05122	5.328169	-0.58383	0.560821	-6.50382	0.735461	0.683737
T.cells	CTSA	0.07538	6.259221	0.583737	0.560884	-6.50201	0.720129	0.66625
T.cells	FAHD2A	-0.10493	2.845982	-0.58332	0.561165	-5.80307	0.778043	0.732763
T.cells	CD207	0.379481	-0.64958	0.583302	0.561175	-5.07357	0.841594	0.807492
T.cells	RIOX2	-0.0626	3.769866	-0.5831	0.561311	-6.17012	0.762004	0.714259
T.cells	CDC123	-0.05088	5.404958	-0.58303	0.561355	-6.59166	0.734373	0.68249
T.cells	EP400	-0.03858	6.870294	-0.583	0.561379	-6.7807	0.710413	0.655208
T.cells	TMEM223	-0.05447	4.652686	-0.58281	0.561505	-6.44826	0.746967	0.697005
T.cells	GM46218	-0.23349	0.58606	-0.58267	0.5616	-5.30848	0.818603	0.78051
T.cells	HSF2	-0.06315	4.185277	-0.58264	0.561619	-6.40259	0.754893	0.706173
T.cells	KLHDC2	0.05045	4.860142	0.582619	0.561633	-6.50243	0.743473	0.693031
T.cells	TEX2	-0.07532	6.723879	-0.58248	0.561728	-6.45162	0.712809	0.657988
T.cells	RHPN2	-0.16939	0.915486	-0.58226	0.561875	-5.53104	0.812728	0.773471
T.cells	CSNK1G2	0.045598	5.468875	0.58187	0.562135	-6.58305	0.733704	0.681509
T.cells	LONRF1	-0.13927	3.091293	-0.58162	0.562302	-5.72232	0.774224	0.728179
T.cells	PCOLCE2	-0.34032	1.678726	-0.58154	0.562356	-5.22957	0.79924	0.757373
T.cells	LRIG3	-0.32193	0.14201	-0.58137	0.562468	-5.13889	0.827301	0.790511
T.cells	MED12	0.083316	3.781462	0.581352	0.562482	-6.07801	0.762268	0.714443
T.cells	MED16	-0.06291	4.12299	-0.58106	0.562675	-6.25801	0.756415	0.707796
T.cells	CTNNA3	0.322281	1.300067	0.580979	0.562733	-5.3774	0.806072	0.765612
T.cells	CALU	-0.05987	4.640586	-0.5809	0.562783	-6.29766	0.747625	0.697672
T.cells	NSA2	-0.03126	7.622863	-0.58076	0.562879	-6.92198	0.698821	0.642157
T.cells	MFSD5	-0.07783	4.282812	-0.58069	0.562926	-6.20775	0.753691	0.704756
T.cells	IRS1	-0.17425	0.821216	-0.58062	0.562971	-5.58822	0.814789	0.776003
T.cells	BACH2	-0.04984	8.964274	-0.58033	0.563169	-7.30925	0.677867	0.618705

T.cells	LOXL2	0.201639	1.406795	0.580299	0.563189	-5.45547	0.804141	0.763522
T.cells	XAB2	0.074544	3.922858	0.580154	0.563286	-6.23732	0.75984	0.71194
T.cells	CTH	0.174806	3.512521	0.580133	0.563301	-5.96708	0.766906	0.720117
T.cells	BAMBI	0.11412	3.967331	0.580129	0.563303	-6.06547	0.759077	0.71106
T.cells	GLE1	-0.05394	4.4454	-0.58004	0.563365	-6.40121	0.750928	0.701672
T.cells	SPP1	0.283254	2.353951	0.579943	0.563428	-5.67788	0.78719	0.74375
T.cells	E130215H2	0.200098	-0.13171	0.579823	0.563508	-5.45196	0.832393	0.796926
T.cells	PHF2OS1	-0.15857	1.058613	-0.5798	0.563526	-5.50857	0.810456	0.771018
T.cells	NUMA1	0.045407	5.705606	0.579791	0.56353	-6.63013	0.729839	0.677511
T.cells	SPC24	-0.08911	4.768056	-0.57968	0.563602	-6.61368	0.745475	0.695448
T.cells	POLK	0.078047	3.481233	0.579602	0.563657	-6.14231	0.767447	0.720809
T.cells	ACAD12	-0.22071	0.714351	-0.57946	0.563752	-5.36907	0.816757	0.778456
T.cells	ENKUR	-0.31364	0.339833	-0.57939	0.5638	-5.11782	0.82365	0.786638
T.cells	PRKN	-0.19661	3.428362	-0.57888	0.564143	-5.64185	0.76875	0.722181
T.cells	GM11613	0.261892	2.020755	0.578656	0.564293	-5.38901	0.793566	0.751097
T.cells	CWF19L1	-0.11545	2.200341	-0.57859	0.564338	-5.85581	0.790365	0.747405
T.cells	ZFP287	0.256937	0.566909	0.578524	0.564381	-5.29191	0.819919	0.782114
T.cells	COMMD2	0.049488	4.927522	0.578389	0.564472	-6.48827	0.743246	0.692796
T.cells	GRASP	-0.12822	3.781204	-0.57816	0.564629	-5.85111	0.762774	0.715317
T.cells	SESN2	-0.12436	3.371941	-0.5781	0.564663	-5.78585	0.769848	0.723511
T.cells	ATG4C	0.118943	3.11799	0.577994	0.564738	-5.77674	0.774268	0.72868
T.cells	PPP1R12A	-0.03773	8.052992	-0.57778	0.564883	-6.94883	0.692491	0.635233
T.cells	CASS4	0.20811	3.04899	0.577733	0.564913	-5.61128	0.775473	0.730174
T.cells	ABCE1	-0.05663	5.007871	-0.57765	0.564972	-6.49788	0.741934	0.691431
T.cells	SERPINA1E	-0.20009	5.030433	-0.5775	0.565067	-6.2988	0.741556	0.690997
T.cells	HDHD2	-0.0706	3.552765	-0.57746	0.565096	-6.12194	0.766715	0.720011
T.cells	CAML	0.056828	4.427078	0.577446	0.565106	-6.33254	0.751734	0.702701
T.cells	SLC25A40	0.086786	3.227708	0.577071	0.565358	-6.09309	0.772608	0.726673
T.cells	COL4A4	-0.18574	1.009618	-0.57658	0.565688	-5.48192	0.812174	0.772961
T.cells	TAF5	-0.06022	4.146651	-0.57657	0.565697	-6.35696	0.75678	0.70838
T.cells	SFN	0.218366	2.056108	0.57642	0.565796	-5.42582	0.793291	0.750839
T.cells	GM34086	-0.22188	2.658244	-0.57642	0.565797	-5.34765	0.78261	0.738371
T.cells	PSAP	-0.06347	9.249596	-0.57639	0.565815	-7.00605	0.674173	0.614612
T.cells	INPP5A	0.059305	6.256163	0.57636	0.565836	-6.66525	0.721535	0.668009
T.cells	ACP1	-0.04663	5.923129	-0.57629	0.565881	-6.68331	0.726994	0.674232
T.cells	HADHA	-0.04564	5.770414	-0.57624	0.565915	-6.60115	0.729511	0.677106
T.cells	LSR	-0.26782	1.400914	-0.57602	0.566067	-5.31869	0.805122	0.764735
T.cells	DROSHA	0.072507	3.121263	0.575984	0.566089	-6.06556	0.774542	0.72902
T.cells	GM5535	-0.32416	0.151841	-0.57526	0.566577	-5.20023	0.828476	0.791942
T.cells	4930430F0	-0.15169	1.035161	-0.57525	0.566584	-5.51532	0.812214	0.772752
T.cells	MAP3K15	-0.12562	3.326785	-0.57519	0.566626	-5.93653	0.771389	0.72509
T.cells	NAE1	-0.06676	3.958187	-0.57512	0.566672	-6.22452	0.76048	0.712478
T.cells	RBM6	0.037221	7.184868	0.574593	0.567026	-6.86967	0.70731	0.651376
T.cells	VPS25	-0.19032	0.881521	-0.57442	0.567142	-5.408	0.815503	0.776301
T.cells	TBC1D16	-0.21802	2.452428	-0.57423	0.567272	-5.4595	0.787278	0.743215
T.cells	STX6	-0.05794	5.2207	-0.57389	0.567502	-6.46151	0.739608	0.688205
T.cells	KLHDC4	-0.06904	3.945597	-0.57388	0.567504	-6.25751	0.76122	0.713071

T.cells	GM3435	-0.26023	0.205181	-0.57381	0.567553	-5.3014	0.828056	0.791244
T.cells	EXOSC3	-0.04731	5.047755	-0.57374	0.567602	-6.57766	0.742505	0.691558
T.cells	TUBD1	-0.13128	2.095647	-0.57368	0.567639	-5.70252	0.793626	0.750767
T.cells	PNLDC1	-0.18217	0.924032	-0.57364	0.56767	-5.54781	0.814805	0.775618
T.cells	IFIT1BL2	0.349347	-0.46837	0.573518	0.56775	-5.06801	0.840669	0.80619
T.cells	1-Sep	-0.05082	6.037433	-0.57312	0.568017	-6.68624	0.72634	0.67298
T.cells	SH3BGR	-0.34074	-0.59926	-0.57302	0.568087	-5.05346	0.843437	0.809437
T.cells	FAIM	0.070646	3.865848	0.572819	0.568221	-6.22213	0.76289	0.715032
T.cells	ITFG1	0.046615	5.454025	0.572716	0.56829	-6.48771	0.736004	0.684148
T.cells	RALGAPA1	0.060658	6.84207	0.572641	0.568341	-6.71797	0.713237	0.658214
T.cells	GM15336	0.145439	1.682133	0.572606	0.568365	-5.62205	0.801356	0.759856
T.cells	SUGT1	-0.04221	5.949959	-0.57244	0.568478	-6.61606	0.72785	0.674777
T.cells	RNF185	-0.06014	5.117108	-0.57218	0.568651	-6.3495	0.741821	0.690662
T.cells	GM10125	-0.13044	2.003063	-0.57209	0.568712	-5.73548	0.795795	0.753205
T.cells	SLC25A15	-0.11509	2.488304	-0.57197	0.568792	-5.7951	0.787168	0.743157
T.cells	DCAF4	0.122308	2.008291	0.571771	0.568928	-5.6884	0.795774	0.753231
T.cells	MIB2	-0.13255	2.662247	-0.57164	0.569015	-5.69134	0.784145	0.739666
T.cells	SLC41A2	-0.27613	2.900939	-0.5716	0.569041	-5.28595	0.77994	0.734771
T.cells	MRPL20	-0.04309	6.1388	-0.57144	0.569155	-6.74043	0.724939	0.671464
T.cells	NDUFA11	-0.04451	6.910675	-0.57106	0.569408	-6.85027	0.712376	0.657359
T.cells	GM20069	0.32965	-0.52038	0.570842	0.569555	-5.10568	0.842252	0.808337
T.cells	MAB21L3	-0.37006	-0.30498	-0.57081	0.569577	-5.12454	0.838198	0.803523
T.cells	RAI1	0.048055	6.004853	0.570573	0.569736	-6.71282	0.72714	0.674224
T.cells	SNRNP40	-0.038	5.982356	-0.57054	0.569758	-6.75882	0.72751	0.674647
T.cells	KLHL12	-0.07228	4.259168	-0.57047	0.569805	-6.32064	0.756409	0.707805
T.cells	SRGN	0.046823	9.652655	0.570452	0.569818	-7.14322	0.669397	0.609143
T.cells	OGT	0.042859	6.649131	0.57038	0.569867	-6.71579	0.716609	0.662269
T.cells	H2-KE6	-0.07324	5.147514	-0.57028	0.569934	-6.3191	0.741379	0.690589
T.cells	7-Sep	-0.02831	8.277792	-0.57023	0.56997	-7.05779	0.690629	0.632959
T.cells	TPP2	-0.03717	7.082191	-0.57018	0.570003	-6.81963	0.709612	0.654387
T.cells	BTNL9	-0.32835	0.689973	-0.57003	0.570106	-5.18296	0.819702	0.781802
T.cells	KIF18B	0.097178	2.84215	0.569863	0.570216	-6.20071	0.780974	0.736432
T.cells	GPBP1L1	0.048396	5.913267	0.569669	0.570347	-6.6197	0.728649	0.67613
T.cells	F2R	-0.16895	3.599158	-0.56967	0.570347	-5.57773	0.76776	0.721119
T.cells	GM15972	-0.3037	-0.2921	-0.56964	0.570367	-5.17111	0.837956	0.803486
T.cells	OLFR543	-0.19836	0.201065	-0.56957	0.570412	-5.45578	0.828744	0.792591
T.cells	ZFP90	-0.14826	2.283533	-0.56949	0.570465	-5.68237	0.79086	0.748069
T.cells	SPHK2	-0.10405	3.388566	-0.56948	0.570477	-5.88434	0.771415	0.725423
T.cells	AFAP1L1	0.180734	1.508026	0.569472	0.57048	-5.62905	0.804777	0.764373
T.cells	GMPPA	-0.10534	3.453598	-0.56923	0.57064	-5.89991	0.770284	0.72417
T.cells	TYMS	-0.08145	4.922548	-0.56916	0.57069	-6.61475	0.745158	0.695178
T.cells	XBP1	-0.05442	6.135195	-0.56905	0.570763	-6.50134	0.724998	0.672148
T.cells	GGA2	0.069038	4.120751	0.568881	0.570879	-6.37161	0.758776	0.710961
T.cells	A630052C1	0.213895	0.189023	0.568631	0.571048	-5.40145	0.828967	0.793179
T.cells	RNF123	0.096109	3.804311	0.568488	0.571145	-6.04209	0.764215	0.717334
T.cells	IPO13	0.125983	2.264795	0.56846	0.571164	-5.75958	0.791194	0.748725
T.cells	DPH2	-0.18644	0.879573	-0.56845	0.571168	-5.49869	0.816221	0.778114

T.cells	CLPTM1L	0.06114	4.883326	0.568393	0.571209	-6.35894	0.745819	0.696109
T.cells	BTBD6	0.169611	1.542046	0.568364	0.571229	-5.57063	0.804162	0.763922
T.cells	MDM1	0.073761	3.41542	0.568354	0.571236	-6.35027	0.770948	0.725139
T.cells	AP3S1	0.043095	7.096627	0.568314	0.571262	-6.78202	0.70938	0.65452
T.cells	MRPL45	-0.06595	4.340705	-0.56807	0.571424	-6.35973	0.755143	0.706727
T.cells	PP2D1	0.168672	1.690903	0.567779	0.571624	-5.69462	0.801794	0.760798
T.cells	PFN1	-0.03795	10.31807	-0.56733	0.57193	-7.26732	0.659644	0.598538
T.cells	FKBP1B	-0.2544	0.793011	-0.5672	0.572018	-5.21289	0.818179	0.78019
T.cells	EXOSC5	-0.04798	5.376885	-0.56717	0.572035	-6.59389	0.737878	0.686787
T.cells	AKAP7	-0.10442	3.374989	-0.5671	0.572085	-5.82362	0.772	0.726144
T.cells	GM2245	0.318859	1.558462	0.567037	0.572126	-5.11933	0.804229	0.763772
T.cells	KANSL1L	-0.07296	7.202302	-0.56703	0.572128	-6.86905	0.708003	0.652757
T.cells	UBE4BOS1	0.183674	0.735049	0.567033	0.572128	-5.59971	0.819244	0.781447
T.cells	SSR3	0.046512	6.071068	0.566664	0.572379	-6.60867	0.726578	0.673666
T.cells	CBR3	-0.27867	0.624626	-0.56658	0.572436	-5.26634	0.821501	0.783899
T.cells	ABCG3	0.161963	4.394356	0.566373	0.572575	-5.78047	0.754651	0.705887
T.cells	MRPL2	0.056542	4.862057	0.566283	0.572636	-6.52421	0.74672	0.696756
T.cells	GSTM1	-0.11428	4.059268	-0.56623	0.57267	-6.11983	0.760382	0.712501
T.cells	ZNHIT6	-0.08767	3.231636	-0.56611	0.57275	-6.10494	0.774711	0.729105
T.cells	EDA	0.215102	1.445596	0.566041	0.5728	-5.62036	0.806492	0.766251
T.cells	ARV1	0.134496	2.101251	0.565844	0.572933	-5.75519	0.794688	0.752457
T.cells	TEAD1	-0.20798	1.756692	-0.56571	0.573027	-5.46039	0.800871	0.75975
T.cells	GM37982	-0.13919	2.996043	-0.56567	0.573053	-5.89051	0.778836	0.734015
T.cells	ZFP60	-0.15851	1.431044	-0.56566	0.573061	-5.56115	0.806756	0.766664
T.cells	FAS	0.162644	3.936884	0.565604	0.573096	-5.74572	0.762485	0.715055
T.cells	MBD5	0.044312	6.903175	0.565264	0.573326	-6.76509	0.713079	0.65842
T.cells	TMC7	-0.19619	-0.40026	-0.56517	0.573387	-5.37079	0.840675	0.80673
T.cells	LAS1L	-0.05815	4.172574	-0.56498	0.57352	-6.34765	0.758508	0.710469
T.cells	STK39	-0.26513	3.517793	-0.56496	0.573535	-5.43678	0.769798	0.723535
T.cells	GM8369	-0.11748	3.324129	-0.56495	0.573537	-6.10686	0.773167	0.727445
T.cells	ARFIP1	0.081058	3.797972	0.564865	0.573596	-6.11103	0.764948	0.71792
T.cells	TPM2	0.292866	0.689833	0.564713	0.573699	-5.26972	0.820375	0.782768
T.cells	RERE	0.03849	7.702335	0.564619	0.573763	-6.9762	0.700276	0.644019
T.cells	EFNB2	-0.17304	2.469455	-0.5646	0.573776	-5.68508	0.7882	0.744998
T.cells	MCMBP	-0.03584	7.089729	-0.56451	0.573837	-6.88619	0.71007	0.655107
T.cells	CD53	0.044207	7.920856	0.564143	0.574085	-6.78063	0.696814	0.640177
T.cells	RARG	-0.26323	1.622679	-0.56412	0.574103	-5.25038	0.803361	0.762822
T.cells	CXCR2	0.335111	-0.00657	0.563762	0.574343	-5.12812	0.833291	0.798183
T.cells	SLC27A4	-0.12724	2.770367	-0.56367	0.574408	-5.56429	0.782877	0.738959
T.cells	MGAT4A	-0.0559	5.666978	-0.56356	0.574482	-6.59161	0.733319	0.68174
T.cells	TMEM184(	0.068125	3.390595	0.56349	0.574527	-6.18225	0.772009	0.726353
T.cells	VPS13B	-0.03525	8.050399	-0.56306	0.574819	-7.00965	0.694769	0.638096
T.cells	COG5	0.047648	6.582266	0.562952	0.574892	-6.72689	0.718282	0.664732
T.cells	GRB10	-0.20168	3.090696	-0.56246	0.575225	-5.71824	0.777247	0.732729
T.cells	ITPKC	0.186392	1.763821	0.562408	0.575261	-5.49261	0.800816	0.760274
T.cells	COPS6	-0.04996	5.552293	-0.56237	0.575289	-6.58721	0.735224	0.684206
T.cells	ALAD	-0.08655	4.057214	-0.56232	0.575318	-6.23756	0.760486	0.713284

T.cells	TBC1D2	-0.22497	1.45751	-0.56224	0.575375	-5.36284	0.80635	0.766775
T.cells	ZFP867	0.170103	0.61031	0.562216	0.575392	-5.45936	0.82184	0.785036
T.cells	CTBS	0.115866	2.934998	0.562137	0.575445	-5.71863	0.779978	0.73594
T.cells	RFNG	-0.11476	2.228203	-0.56212	0.575456	-5.73533	0.792493	0.750551
T.cells	NAALADL2	-0.17766	1.964286	-0.56205	0.575502	-5.4805	0.797213	0.756079
T.cells	MRPS18B	-0.06603	3.873974	-0.56204	0.575508	-6.27242	0.763637	0.71696
T.cells	SDC1	-0.13994	2.216388	-0.562	0.575539	-5.95141	0.792703	0.750798
T.cells	F8A	-0.10455	2.551871	-0.56194	0.575581	-5.90346	0.786739	0.743825
T.cells	AGAP1	0.114308	4.225875	0.561932	0.575584	-6.02049	0.757596	0.709972
T.cells	GM29707	0.276744	-0.46354	0.561679	0.575756	-5.08431	0.841868	0.808817
T.cells	S1PR2	0.222777	1.261742	0.561597	0.575811	-5.34264	0.809905	0.771005
T.cells	CCDC9	-0.05704	4.238269	-0.56122	0.576069	-6.24958	0.757384	0.70978
T.cells	ZFP871	0.056677	5.249328	0.561172	0.5761	-6.48834	0.740279	0.690081
T.cells	SLC8B1	-0.12295	4.721857	-0.56114	0.576124	-5.84278	0.749157	0.70029
T.cells	IGLV3	-0.27743	-0.70552	-0.56108	0.576161	-5.18701	0.846442	0.814324
T.cells	RECK	-0.19917	1.480341	-0.56103	0.576195	-5.59828	0.805936	0.766377
T.cells	CCDC180	0.297512	1.286108	0.561005	0.576213	-5.1515	0.809462	0.770525
T.cells	TRP53	-0.04506	5.681235	-0.56093	0.576263	-6.63396	0.733082	0.681847
T.cells	ZBTB41	-0.08555	2.915286	-0.56089	0.576291	-6.00749	0.780325	0.73644
T.cells	BLK	0.067999	4.074052	0.560449	0.576591	-6.45256	0.760197	0.713119
T.cells	STXBP5	0.081584	5.383452	0.560432	0.576602	-6.30075	0.738037	0.687597
T.cells	PLCE1	-0.32329	1.279254	-0.56039	0.57663	-5.28259	0.809586	0.770774
T.cells	TRPV2	0.069397	4.873594	0.560207	0.576755	-6.27505	0.746593	0.697473
T.cells	SERPINB9	0.254001	3.483496	0.560148	0.576795	-5.5348	0.770394	0.725003
T.cells	TMEM19	0.069733	3.958743	0.56003	0.576875	-6.14261	0.762178	0.715524
T.cells	5031425F1	-0.33174	-0.17235	-0.56	0.576893	-5.10087	0.836393	0.802594
T.cells	ATP9A	0.272754	0.837325	0.559884	0.576974	-5.23875	0.817663	0.780417
T.cells	NANS	-0.0599	4.873876	-0.55986	0.576989	-6.4807	0.746588	0.697533
T.cells	ATP1B1	-0.06806	5.798004	-0.55966	0.577127	-6.70481	0.731148	0.679836
T.cells	BET1	0.066181	4.246255	0.559507	0.57723	-6.25896	0.757248	0.709848
T.cells	RBM41	0.075053	3.907296	0.55942	0.57729	-6.15284	0.763063	0.716577
T.cells	GIN1	0.050946	3.954097	0.559286	0.57738	-6.29538	0.762258	0.715651
T.cells	C79798	0.119131	1.77973	0.55928	0.577385	-5.79129	0.800529	0.760274
T.cells	CNP	-0.05387	5.75023	-0.55928	0.577388	-6.75489	0.731939	0.680747
T.cells	SLC25A34	0.317421	-0.47643	0.559253	0.577403	-5.07947	0.84211	0.809442
T.cells	ATP8B2	-0.09422	2.774077	-0.5591	0.577504	-5.85723	0.782811	0.739565
T.cells	DYNC1I2	-0.03658	6.441227	-0.55891	0.577639	-6.68872	0.72058	0.667799
T.cells	GM45442	0.322695	-0.57631	0.558693	0.577784	-5.14165	0.843996	0.811809
T.cells	NOL12	0.052294	4.012955	0.558676	0.577795	-6.29413	0.761246	0.714595
T.cells	GM29093	-0.12025	1.269702	-0.55867	0.577797	-5.64662	0.80976	0.771253
T.cells	DNAJC12	-0.10883	3.069108	-0.55867	0.5778	-5.96577	0.777625	0.733613
T.cells	CHPF	-0.17651	1.242066	-0.55863	0.577829	-5.45478	0.810263	0.771863
T.cells	SCCPDH	0.098699	2.465776	0.558558	0.577875	-5.88855	0.788266	0.746073
T.cells	RNF149	0.120963	5.786169	0.558495	0.577918	-6.17221	0.731344	0.680232
T.cells	TTI1	0.104382	2.712153	0.558465	0.577938	-5.98352	0.783904	0.740994
T.cells	P2RY1	-0.2431	0.13392	-0.55846	0.577944	-5.24149	0.83067	0.796034
T.cells	RCOR3	0.109759	2.937283	0.558365	0.578006	-5.83422	0.779938	0.736373

T.cells	PBX4	0.142101	0.976518	0.558358	0.578011	-5.7342	0.815111	0.777629
T.cells	NOP53	0.052305	5.740416	0.558313	0.578042	-6.60167	0.732102	0.681103
T.cells	KIF15	-0.08538	4.90741	-0.55831	0.578047	-6.63702	0.746022	0.697083
T.cells	STARD10	-0.12528	4.596544	-0.5582	0.578119	-6.23112	0.751281	0.70316
T.cells	PHEX	-0.18404	1.372943	-0.55814	0.578159	-5.64793	0.807884	0.769133
T.cells	NAT8F1	0.226375	1.619362	0.557904	0.57832	-5.43291	0.803421	0.763984
T.cells	TBC1D5	0.049794	7.293343	0.557825	0.578374	-6.78399	0.706801	0.652421
T.cells	TXNDC5	-0.06371	5.179694	-0.55772	0.578442	-6.44161	0.741445	0.692004
T.cells	OGA	-0.04284	6.76343	-0.55772	0.578442	-6.76036	0.71534	0.662155
T.cells	PTPN18	0.039639	7.11924	0.557462	0.57862	-6.78923	0.709596	0.655687
T.cells	FNIP1	-0.05191	7.760209	-0.55743	0.578645	-6.82942	0.699358	0.644095
T.cells	KLRI2	-0.36686	0.447224	-0.55736	0.578688	-5.14708	0.824853	0.789429
T.cells	D10WSU1(	-0.08288	3.652731	-0.55736	0.578689	-6.14012	0.767459	0.722118
T.cells	GALNT4	0.135584	1.622606	0.557181	0.578812	-5.558	0.803365	0.764163
T.cells	GM15726	-0.2266	2.074088	-0.55691	0.578999	-5.65516	0.795248	0.754668
T.cells	ISOC2A	-0.14396	2.013655	-0.55682	0.579056	-5.5881	0.79633	0.755948
T.cells	GEN1	0.121279	2.372171	0.556799	0.579072	-5.98572	0.789931	0.748449
T.cells	GON4L	0.04665	5.15234	0.556784	0.579082	-6.52341	0.741906	0.692725
T.cells	ACSBG1	0.290161	-0.5064	0.556671	0.579159	-5.13569	0.842678	0.810782
T.cells	ENPP1	-0.21664	2.31349	-0.55653	0.579253	-5.46926	0.790975	0.749725
T.cells	PWWP2A	-0.0499	5.141602	-0.55645	0.579307	-6.57497	0.742086	0.693004
T.cells	PROSER3	-0.15079	1.105594	-0.55644	0.579316	-5.61077	0.812753	0.775352
T.cells	SLC38A6	0.095058	4.233853	0.556366	0.579366	-6.0608	0.757462	0.710788
T.cells	CSAD	-0.08588	3.965874	-0.55614	0.579517	-6.14911	0.762167	0.716165
T.cells	TNFRSF18	-0.17348	2.322619	-0.55582	0.579736	-5.44096	0.791091	0.749732
T.cells	PFKP	0.076988	5.538024	0.555768	0.579773	-6.41326	0.735722	0.685571
T.cells	TTPA	-0.22051	1.860466	-0.55565	0.579853	-5.50062	0.799378	0.759459
T.cells	ZBTB5	0.097518	2.463409	0.555509	0.579949	-5.955	0.788624	0.746832
T.cells	STK24	-0.03689	7.291795	-0.55535	0.58006	-6.89439	0.707109	0.652975
T.cells	OGFOD2	-0.07223	3.850672	-0.55533	0.580071	-6.14232	0.764346	0.718621
T.cells	TJP3	0.133739	1.707068	0.5552	0.58016	-5.7325	0.80216	0.762777
T.cells	ATP23	0.161362	1.824257	0.555133	0.580205	-5.74452	0.800049	0.760326
T.cells	4930486L2	-0.30093	0.484692	-0.55472	0.58049	-5.14146	0.824551	0.789294
T.cells	YKT6	0.05631	4.483546	0.55471	0.580494	-6.33317	0.753558	0.706225
T.cells	CLK3	-0.05402	4.807812	-0.55459	0.580575	-6.40262	0.748058	0.699913
T.cells	SLC2A9	0.135457	2.879571	0.554353	0.580736	-5.78556	0.781324	0.738595
T.cells	FANCL	-0.05395	4.144904	-0.55422	0.58083	-6.39454	0.759342	0.713083
T.cells	ZNFX1	0.121048	4.359029	0.554084	0.58092	-6.05149	0.75568	0.70885
T.cells	PTCD1	0.101952	2.585716	0.553923	0.58103	-5.84553	0.786512	0.744706
T.cells	OMA1	0.103251	3.410326	0.553906	0.581041	-5.89742	0.772032	0.727816
T.cells	ABCF1	-0.03871	6.430861	-0.55379	0.581123	-6.76158	0.721091	0.669135
T.cells	USP54	-0.08985	2.915478	-0.55353	0.581294	-5.97583	0.780692	0.73797
T.cells	SLIT1	-0.14525	1.464859	-0.55343	0.581368	-5.76014	0.806599	0.768355
T.cells	GM17387	-0.21725	0.498836	-0.55342	0.581374	-5.34738	0.824289	0.789254
T.cells	CC2D2A	-0.2669	0.81427	-0.5534	0.581386	-5.26681	0.818474	0.78237
T.cells	TOM1	-0.07543	5.431971	-0.55316	0.581551	-6.33234	0.737577	0.688072
T.cells	SAR1A	-0.04908	5.508767	-0.5531	0.58159	-6.53777	0.736297	0.686603

T.cells	ZFP984	0.073894	3.878402	0.55291	0.58172	-6.07657	0.763923	0.718525
T.cells	WDR43	0.047406	6.033494	0.55282	0.581782	-6.68388	0.727607	0.676685
T.cells	MASP2	-0.21843	1.894509	-0.55276	0.58182	-5.51864	0.798844	0.759294
T.cells	TCTN2	-0.25072	-0.37598	-0.55274	0.581834	-5.19742	0.840617	0.808716
T.cells	PTTG1	-0.05822	5.78	-0.55266	0.58189	-6.49186	0.731793	0.681486
T.cells	BCR	0.049231	5.853797	0.552615	0.581921	-6.71612	0.730572	0.680101
T.cells	EPB41L1	-0.27182	1.011227	-0.55242	0.582057	-5.28102	0.814862	0.77829
T.cells	ASTE1	0.091163	2.753642	0.552394	0.582072	-5.96265	0.783543	0.741488
T.cells	ZFP456	-0.2151	0.791994	-0.55227	0.58216	-5.37273	0.818884	0.783048
T.cells	RBM24	0.139464	1.008251	0.552151	0.582238	-5.92426	0.814917	0.778369
T.cells	ZMAT2	0.042864	5.466404	0.552088	0.582281	-6.60755	0.737003	0.687578
T.cells	TNRC6C	0.038087	7.143837	0.552086	0.582282	-6.87115	0.709537	0.656206
T.cells	HIKESHI	-0.04452	5.228907	-0.55191	0.5824	-6.61844	0.740972	0.692139
T.cells	8-Mar	0.161755	3.141103	0.551857	0.582438	-5.57027	0.776732	0.733552
T.cells	ZDHHC9	0.096061	4.094804	0.551828	0.582458	-5.97609	0.760201	0.714339
T.cells	BHLHE41	-0.15705	1.739361	-0.55177	0.582497	-5.75075	0.801636	0.762714
T.cells	ZFP146	0.073677	3.413704	0.551627	0.582595	-6.17667	0.771973	0.728009
T.cells	GM37494	0.077749	3.825168	0.551367	0.582773	-6.12241	0.764842	0.719772
T.cells	ZHX3	0.068363	4.441922	0.551035	0.582999	-6.34912	0.754267	0.707564
T.cells	DGKD	0.0383	7.687633	0.551002	0.583022	-7.02394	0.700842	0.646434
T.cells	HLCS	-0.11135	4.15558	-0.55095	0.583061	-5.94641	0.759159	0.713226
T.cells	WIZ	-0.0721	3.836067	-0.55087	0.58311	-6.27012	0.764654	0.719609
T.cells	SLC1A4	-0.18267	0.947937	-0.55085	0.583124	-5.48334	0.816021	0.779794
T.cells	MRPL58	-0.05344	5.199269	-0.55083	0.583137	-6.54856	0.741468	0.692817
T.cells	DHDH	-0.13389	2.654165	-0.55073	0.583209	-5.67683	0.785301	0.743686
T.cells	GLP2R	0.188665	0.940346	0.5506	0.583296	-5.4517	0.81616	0.780008
T.cells	PIK3CB	0.140117	4.688415	0.550575	0.583313	-5.86318	0.750079	0.702784
T.cells	GM49662	-0.37407	2.537113	-0.55045	0.583402	-5.21195	0.787374	0.746169
T.cells	ERMP1	0.071434	3.633634	0.550297	0.583503	-6.09076	0.768154	0.723749
T.cells	GM12353	0.197836	1.278675	0.55018	0.583583	-5.43	0.809981	0.772796
T.cells	ALG8	-0.08498	3.226775	-0.55004	0.583676	-6.11201	0.775234	0.732088
T.cells	TDRKH	-0.13385	1.697145	-0.55002	0.583691	-5.7415	0.802398	0.763905
T.cells	GM10130	0.135822	1.783227	0.549941	0.583746	-5.6666	0.800846	0.762087
T.cells	USP31	-0.0793	3.715776	-0.54982	0.583828	-6.19823	0.766732	0.722225
T.cells	AP1G2	0.122115	2.809774	0.549816	0.583832	-5.90146	0.782553	0.740663
T.cells	KMT2D	0.041825	5.945956	0.549776	0.583859	-6.62667	0.72905	0.67875
T.cells	PTGS2	0.466759	2.215449	0.549565	0.584003	-5.23592	0.793096	0.753031
T.cells	MKRN2	0.05348	4.074932	0.549465	0.584071	-6.25516	0.760543	0.715063
T.cells	CREB1	-0.03922	6.332794	-0.5493	0.584184	-6.71485	0.722694	0.6715
T.cells	NEK10	0.169061	1.324395	0.549249	0.58422	-5.67883	0.809149	0.771917
T.cells	GM29666	0.256625	0.244861	0.549232	0.584231	-5.31011	0.828999	0.795412
T.cells	NHSL1	0.143638	1.728678	0.54914	0.584293	-5.6814	0.801829	0.763328
T.cells	RSL1	0.201394	1.039109	0.549124	0.584304	-5.46233	0.814352	0.778097
T.cells	CLN8	0.203113	3.202024	0.548951	0.584423	-5.43817	0.775666	0.732736
T.cells	RAB21	-0.03776	6.891793	-0.5489	0.584456	-6.76985	0.713601	0.661232
T.cells	GAS8	-0.12963	1.529564	-0.54887	0.584481	-5.60461	0.805427	0.767647
T.cells	9030404E1	-0.2075	-0.16276	-0.54877	0.584545	-5.44697	0.83661	0.804651

T.cells	GDPGP1	-0.1512	2.18615	-0.54875	0.58456	-5.78062	0.793619	0.753822
T.cells	PEF1	0.081211	3.347699	0.548524	0.584714	-6.08033	0.773123	0.729996
T.cells	COX17	0.042308	6.919214	0.548446	0.584768	-6.79492	0.713158	0.660942
T.cells	NEK8	-0.12918	2.113602	-0.54842	0.584786	-5.69116	0.794916	0.755541
T.cells	PDHA1	-0.05095	5.115043	-0.54831	0.584859	-6.51253	0.742882	0.695024
T.cells	BACE1	-0.09747	2.654059	-0.54823	0.584918	-5.87933	0.785303	0.744323
T.cells	COMMD7	0.039007	5.29863	0.547863	0.585167	-6.52572	0.739805	0.691578
T.cells	NADSYN1	0.178031	1.092531	0.547804	0.585207	-5.44809	0.813375	0.777447
T.cells	RHOT2	-0.10325	2.679369	-0.54775	0.585242	-5.90493	0.784855	0.743901
T.cells	CYP2D9	0.237337	0.530177	0.547696	0.585281	-5.2627	0.82371	0.789692
T.cells	GM33677	-0.37066	-1.06476	-0.54764	0.585321	-5.06924	0.853679	0.825418
T.cells	SPTY2D1	-0.05662	5.765398	-0.54751	0.585411	-6.39734	0.732035	0.682736
T.cells	1810026B0	-0.03795	6.570259	-0.54739	0.585489	-6.73929	0.718818	0.66767
T.cells	SEC31A	0.039383	5.620105	0.547182	0.585632	-6.55529	0.734445	0.685632
T.cells	VARS2	0.164808	1.401859	0.546983	0.585769	-5.60233	0.807742	0.771053
T.cells	ATP6AP2	-0.03576	6.938045	-0.54696	0.585782	-6.75052	0.712853	0.660963
T.cells	ENAH	-0.29262	0.744942	-0.54685	0.585857	-5.20236	0.819749	0.785276
T.cells	GM3336	-0.27424	1.732452	-0.54681	0.585888	-5.31807	0.801761	0.764021
T.cells	FAM20C	-0.36942	1.641237	-0.54652	0.586088	-5.27534	0.803407	0.765961
T.cells	GSTM4	0.202936	1.121715	0.546431	0.586146	-5.43024	0.812842	0.777116
T.cells	GM33280	0.351305	-0.66423	0.546378	0.586182	-5.06979	0.846061	0.816623
T.cells	LPIN2	0.051847	6.528551	0.546366	0.586191	-6.75353	0.719497	0.668581
T.cells	PPP2R5E	0.032766	6.820761	0.546154	0.586336	-6.83305	0.71475	0.663223
T.cells	LTB4R1	0.30569	2.689349	0.545964	0.586466	-5.27612	0.784679	0.744116
T.cells	TMEM167	0.034464	6.804318	0.545884	0.586521	-6.82466	0.715017	0.663589
T.cells	H2AFY	-0.03102	7.697828	-0.54581	0.586574	-7.05583	0.70068	0.64731
T.cells	GLUL	0.060111	6.384395	0.545715	0.586636	-6.68366	0.72185	0.671446
T.cells	SHMT2	-0.06837	4.430904	-0.54559	0.586719	-6.44574	0.754454	0.709001
T.cells	PLCL1	-0.07939	5.328139	-0.54554	0.586753	-6.61465	0.739311	0.691511
T.cells	SCHIP1	-0.22234	1.591487	-0.54548	0.586798	-5.35699	0.804306	0.767278
T.cells	MFSD10	-0.07133	4.464143	-0.54545	0.586815	-6.25902	0.753888	0.708348
T.cells	BAK1	0.062846	4.936771	0.545401	0.586851	-6.44451	0.745881	0.6991
T.cells	GIT1	-0.07325	3.282244	-0.5454	0.586852	-5.97842	0.774265	0.732047
T.cells	E130309D0	-0.05703	3.983364	-0.54533	0.586901	-6.36284	0.762116	0.717902
T.cells	TNFSF13B	-0.22553	0.855901	-0.54531	0.586911	-5.37445	0.817709	0.783136
T.cells	SHCBP1L	-0.16742	0.854389	-0.54521	0.58698	-5.51648	0.817737	0.783204
T.cells	CSTF2	-0.05765	4.139659	-0.5452	0.586991	-6.41733	0.759432	0.714818
T.cells	MASTL	0.064662	3.782	0.545172	0.587008	-6.33788	0.765587	0.72198
T.cells	SNX9	-0.05265	6.331978	-0.54495	0.587157	-6.6473	0.722736	0.672563
T.cells	DHRS1	-0.09156	4.762169	-0.54482	0.58725	-6.20708	0.74886	0.702661
T.cells	STK38L	0.062374	4.003603	0.544806	0.587259	-6.26494	0.761798	0.717656
T.cells	PTPN1	0.055671	7.078745	0.544629	0.58738	-6.69709	0.710613	0.65881
T.cells	GM6225	-0.15927	2.958455	-0.54463	0.58738	-5.61502	0.779967	0.738874
T.cells	EPHX1	-0.14641	3.053436	-0.54455	0.587437	-5.72738	0.7783	0.736955
T.cells	GM42726	-0.07333	3.69069	-0.54432	0.587591	-6.10626	0.767197	0.724029
T.cells	SLC14A1	0.099984	3.335927	0.544292	0.58761	-5.9975	0.77336	0.731213
T.cells	SNAPIN	0.063748	4.083623	0.544249	0.58764	-6.20126	0.760424	0.716155

T.cells	TRPS1	0.06851	7.683996	0.544121	0.587728	-6.81375	0.700945	0.64787
T.cells	DAPK3	-0.06244	4.42231	-0.54403	0.587789	-6.2222	0.754649	0.709459
T.cells	A730063M	-0.18713	1.218514	-0.54389	0.587888	-5.51941	0.811171	0.775638
T.cells	GM44752	0.107158	2.484975	0.543652	0.588049	-5.81406	0.788516	0.748868
T.cells	GPM6B	-0.17041	2.439081	-0.54351	0.588146	-5.61715	0.789341	0.74988
T.cells	SLC25A19	-0.09119	3.390435	-0.54344	0.588192	-5.97825	0.772601	0.730315
T.cells	SHCBP1	-0.07701	4.69138	-0.54321	0.588355	-6.61563	0.750325	0.70442
T.cells	HDAC2	-0.0479	5.300553	-0.54314	0.5884	-6.62757	0.740065	0.692575
T.cells	POLL	0.140718	1.158885	0.543056	0.588458	-5.58488	0.812485	0.777167
T.cells	UTP11	-0.05106	4.845019	-0.54286	0.588593	-6.44231	0.747812	0.7015
T.cells	OTUB1	0.035546	5.728978	0.542214	0.589035	-6.62695	0.733479	0.684657
T.cells	GM48768	0.197393	0.621002	0.541571	0.589476	-5.39712	0.823428	0.789404
T.cells	ZFP39	-0.17029	1.023509	-0.54142	0.589583	-5.56051	0.81602	0.780667
T.cells	RIPK1	-0.05639	5.973193	-0.54131	0.589655	-6.50103	0.729838	0.680234
T.cells	CHIL5	0.269958	-0.02332	0.541305	0.589659	-5.11577	0.835415	0.803692
T.cells	TPCN1	-0.0982	4.187411	-0.54125	0.589698	-6.12351	0.759902	0.714907
T.cells	CYP2D22	-0.22227	0.864397	-0.54097	0.589891	-5.33327	0.819047	0.78415
T.cells	1700123O2	-0.05348	4.989927	-0.54081	0.589999	-6.47061	0.746347	0.699137
T.cells	PRMT3	0.065152	4.041123	0.540778	0.59002	-6.32309	0.762514	0.71785
T.cells	SPATA7	0.133695	1.309288	0.540677	0.59009	-5.66022	0.810903	0.774559
T.cells	ASB6	-0.09956	2.575022	-0.54055	0.590179	-5.88998	0.788139	0.747805
T.cells	TAGAP	-0.20359	4.269177	-0.54046	0.590239	-5.60522	0.758598	0.713393
T.cells	TMEM106E	0.058365	4.565283	0.540245	0.590387	-6.37851	0.753542	0.707616
T.cells	ZFYVE16	0.122441	3.016629	0.540051	0.59052	-5.69751	0.780337	0.738802
T.cells	GM48765	-0.16013	0.952243	-0.53998	0.590569	-5.55801	0.817433	0.782446
T.cells	BTG2	-0.0599	7.421641	-0.53992	0.59061	-6.80832	0.70637	0.653544
T.cells	HADHB	-0.04985	5.576702	-0.5399	0.590625	-6.53629	0.73651	0.687988
T.cells	E430024P1	-0.22977	0.53335	-0.53981	0.590684	-5.32853	0.825156	0.791612
T.cells	R3HCC1	0.109219	2.353768	0.53981	0.590685	-5.93922	0.792075	0.752559
T.cells	IFRD1	0.066083	8.532379	0.539693	0.590765	-6.89068	0.688792	0.633692
T.cells	EPM2A	0.236242	1.526051	0.539658	0.59079	-5.3075	0.806962	0.77012
T.cells	B9D1	-0.17539	0.371188	-0.53955	0.590862	-5.52847	0.82817	0.795241
T.cells	MED14	0.056974	6.321429	0.539385	0.590977	-6.67692	0.724264	0.673994
T.cells	GATD1	-0.06188	4.394103	-0.53905	0.591205	-6.29245	0.756733	0.711254
T.cells	NAA15	-0.03228	6.743192	-0.53865	0.591485	-6.81691	0.717609	0.666247
T.cells	IL15RA	-0.14379	2.38833	-0.53862	0.591502	-5.73513	0.791783	0.752101
T.cells	ZBTB4	0.115115	3.655496	0.538618	0.591504	-5.75478	0.769493	0.726048
T.cells	CLEC9A	-0.34441	2.519945	-0.53856	0.591544	-5.18397	0.78944	0.749353
T.cells	TMEM69	0.144471	1.585462	0.538345	0.591692	-5.64834	0.806215	0.769166
T.cells	A430093F1	0.189806	2.363356	0.538263	0.591748	-5.4116	0.792228	0.752709
T.cells	ZFP217	0.056537	4.011596	0.538217	0.59178	-6.25939	0.763334	0.71897
T.cells	SH3BP5L	0.116961	2.252113	0.53808	0.591874	-5.7656	0.794215	0.755075
T.cells	SHLD3	-0.10072	2.343634	-0.53806	0.59189	-5.90772	0.79258	0.753157
T.cells	TMEM71	0.091222	3.858441	0.537931	0.591976	-6.14016	0.765977	0.72213
T.cells	MRAP	-0.19651	1.214744	-0.53788	0.592009	-5.58659	0.81296	0.777249
T.cells	RALGAPA2	0.065098	6.409122	0.537827	0.592048	-6.65694	0.72306	0.672685
T.cells	PDXDC1	0.035793	6.616598	0.537671	0.592155	-6.73178	0.719718	0.668846

T.cells	SMIM40	-0.29615	0.301755	-0.53747	0.59229	-5.13051	0.829943	0.797397
T.cells	ZFP36	-0.07096	7.849088	-0.53738	0.592357	-6.78615	0.699968	0.646373
T.cells	HCLS1	0.050539	6.691521	0.5372	0.592479	-6.68706	0.718644	0.667593
T.cells	C1GALT1	0.044463	6.353776	0.53697	0.592637	-6.84348	0.724204	0.673974
T.cells	H2-DMB1	0.248775	4.478206	0.536913	0.592676	-5.71871	0.755583	0.710102
T.cells	DUSP10	-0.09063	4.392133	-0.53685	0.592717	-6.18101	0.757053	0.711819
T.cells	VMAC	-0.16071	1.5263	-0.5366	0.592888	-5.50159	0.807694	0.771028
T.cells	LRRC8C	0.056906	6.412612	0.536293	0.593103	-6.72179	0.723513	0.673057
T.cells	GM21781	0.142256	1.561921	0.536147	0.593204	-5.6267	0.807211	0.770343
T.cells	SMC5	0.044039	5.613071	0.535957	0.593334	-6.70132	0.736725	0.688307
T.cells	THAP1	-0.0848	2.879636	-0.53581	0.593434	-6.00902	0.783623	0.742812
T.cells	D030028A(	-0.15991	2.795422	-0.5357	0.593511	-5.58278	0.785111	0.744588
T.cells	CD300LG	-0.22724	1.259921	-0.5354	0.593716	-5.31771	0.812708	0.777129
T.cells	SERINC5	0.056601	5.287482	0.53538	0.593731	-6.62632	0.74217	0.694751
T.cells	TNFRSF9	-0.27319	2.460851	-0.53538	0.593733	-5.35748	0.791049	0.751611
T.cells	ETHE1	0.052971	4.997365	0.535324	0.59377	-6.49651	0.747053	0.700385
T.cells	D16ERTD47	-0.07476	4.914897	-0.53513	0.593902	-6.27769	0.748447	0.702011
T.cells	FNIP2	-0.27465	5.343736	-0.53506	0.593953	-5.69119	0.741226	0.69368
T.cells	TBPL1	-0.04645	4.928003	-0.53505	0.593958	-6.50687	0.748225	0.701755
T.cells	BEND5	-0.15639	0.115495	-0.53498	0.594004	-5.49641	0.833856	0.802247
T.cells	SRRM2	-0.0308	8.62357	-0.5349	0.594064	-7.0959	0.688134	0.633183
T.cells	CAVIN1	0.267444	1.312054	0.53474	0.594172	-5.30458	0.811757	0.776076
T.cells	RASAL3	0.060423	4.10056	0.534735	0.594175	-6.28313	0.762341	0.718157
T.cells	TMSB10	-0.04415	10.73818	-0.53471	0.594191	-7.50511	0.655844	0.597039
T.cells	XPR1	0.050452	6.866995	0.534473	0.594356	-6.74031	0.716104	0.664973
T.cells	GM34921	0.185234	0.841113	0.534374	0.594424	-5.46995	0.820389	0.786355
T.cells	DDIT3	0.078046	4.008692	0.534334	0.594451	-6.16471	0.763923	0.720049
T.cells	WASHC5	-0.07083	4.472587	-0.53429	0.594485	-6.24366	0.755964	0.710811
T.cells	RRAGC	-0.04935	5.819399	-0.53425	0.594507	-6.52871	0.733294	0.684663
T.cells	FASTKD5	0.167118	0.844458	0.534086	0.594622	-5.4752	0.820328	0.786354
T.cells	NUS1	0.045572	4.837828	0.534067	0.594635	-6.41014	0.749752	0.703685
T.cells	TMEM134	0.045885	6.132006	0.533896	0.594753	-6.64569	0.728185	0.678799
T.cells	GTF2H3	-0.10312	2.299191	-0.53371	0.594879	-5.89144	0.794056	0.755213
T.cells	VRK1	0.045463	5.309256	0.533639	0.59493	-6.61423	0.741919	0.694527
T.cells	SRF	-0.07927	2.927184	-0.53351	0.595016	-5.88337	0.782928	0.742135
T.cells	SNHG5	-0.10698	2.416156	-0.53333	0.595146	-5.8975	0.792074	0.752835
T.cells	TSGA10	0.086665	2.968133	0.532783	0.595521	-5.97912	0.782486	0.741568
T.cells	ZFP958	0.118622	2.267977	0.53276	0.595536	-5.79213	0.794922	0.756151
T.cells	4930484I0	-0.13469	1.660784	-0.53274	0.595547	-5.66686	0.805855	0.769025
T.cells	AKAP6	-0.32895	0.299684	-0.53255	0.595683	-5.28382	0.830869	0.798729
T.cells	NRDE2	0.078126	3.371854	0.532463	0.595741	-6.21061	0.775398	0.733377
T.cells	RRAS2	0.05853	5.198697	0.532215	0.595912	-6.53554	0.744065	0.697104
T.cells	AHI1	0.087225	3.003332	0.532028	0.596041	-6.00004	0.781866	0.741028
T.cells	TRMT61A	-0.13271	1.593744	-0.53162	0.596321	-5.64088	0.807071	0.77066
T.cells	BC024063	0.186881	0.183577	0.531565	0.596361	-5.39544	0.833035	0.801446
T.cells	SOX18	-0.30668	0.720608	-0.53155	0.596372	-5.15758	0.823058	0.789585
T.cells	ZFP213	-0.10907	2.162406	-0.53155	0.596373	-5.80526	0.796813	0.758572

T.cells	LRRC40	-0.07318	3.249731	-0.53154	0.59638	-6.14488	0.777535	0.735972
T.cells	TMEM185/	0.111006	2.540183	0.531526	0.596387	-5.79089	0.790066	0.750644
T.cells	THEM6	-0.08164	3.448768	-0.53135	0.596508	-6.1659	0.774054	0.731943
T.cells	4933407K1	-0.1377	1.39879	-0.53124	0.596587	-5.54396	0.810615	0.774885
T.cells	ARHGEF39	-0.12634	1.960521	-0.53123	0.596594	-5.99732	0.800441	0.76288
T.cells	EAF1	0.069113	4.101569	0.531153	0.596645	-6.24855	0.762737	0.718765
T.cells	GM13919	0.072081	3.111975	0.531061	0.596708	-6.30592	0.779954	0.738835
T.cells	DNAJA2	-0.03083	7.208069	-0.53104	0.59672	-6.88034	0.710976	0.659217
T.cells	C2CD2	0.118238	3.079676	0.531009	0.596744	-5.74707	0.780522	0.739499
T.cells	NFYB	-0.04578	5.103926	-0.53099	0.596757	-6.63305	0.74566	0.698987
T.cells	LAMA4	0.281831	1.088771	0.530943	0.59679	-5.29622	0.816282	0.781589
T.cells	MOV10	0.090767	3.628623	0.530794	0.596892	-6.02713	0.770957	0.728289
T.cells	GM16201	-0.2464	1.818935	-0.53065	0.596995	-5.26724	0.803033	0.765889
T.cells	CALHM2	-0.12018	3.088827	-0.53052	0.59708	-5.64137	0.780398	0.739311
T.cells	PAM16	-0.04943	4.987535	-0.53051	0.59709	-6.47576	0.747661	0.701255
T.cells	PI4KB	0.043573	5.414205	0.530328	0.597214	-6.57238	0.740534	0.693028
T.cells	PEX16	-0.07734	3.474463	-0.53005	0.597408	-6.02935	0.773696	0.731533
T.cells	ARL16	0.093747	2.250651	0.530047	0.597408	-5.81527	0.795325	0.756867
T.cells	SMIM19	0.057904	4.75168	0.529976	0.597457	-6.30997	0.751708	0.706022
T.cells	RAB2A	0.025945	8.094947	0.529806	0.597574	-6.94391	0.6969	0.643306
T.cells	SERPINA3N	0.147628	2.557516	0.529708	0.597642	-5.61614	0.789849	0.750522
T.cells	SETD1A	-0.05212	4.19796	-0.52959	0.597725	-6.33503	0.761167	0.717043
T.cells	CHM	-0.053	5.555869	-0.52957	0.597739	-6.48877	0.738165	0.690446
T.cells	ZFR2	0.193512	0.817601	0.529399	0.597855	-5.34433	0.821363	0.787731
T.cells	SNX18	0.089115	6.77742	0.529375	0.597872	-6.2719	0.718032	0.667363
T.cells	GREM2	-0.25132	0.454759	-0.52923	0.597974	-5.28657	0.82808	0.795708
T.cells	RABIF	-0.04751	4.708703	-0.5292	0.597994	-6.40957	0.752438	0.706928
T.cells	DHX58OS	0.2181	0.831273	0.529072	0.598081	-5.42063	0.821111	0.787432
T.cells	GM614	0.248958	-0.15476	0.528879	0.598215	-5.19246	0.839475	0.809285
T.cells	AARS	-0.05163	5.020707	-0.52882	0.598258	-6.53555	0.747151	0.700814
T.cells	DAZAP2	-0.03613	7.70346	-0.52881	0.598265	-6.92146	0.703116	0.65038
T.cells	GRIPAP1	0.046063	5.594976	0.528779	0.598284	-6.52405	0.737512	0.689699
T.cells	GNG4	0.280805	1.462633	0.528559	0.598436	-5.20928	0.809649	0.773768
T.cells	PLOD1	0.165895	3.410314	0.528448	0.598512	-5.60196	0.774913	0.732982
T.cells	SHTN1	0.21197	3.187667	0.528232	0.598662	-5.40293	0.778812	0.737589
T.cells	TM7SF3	0.077669	3.924878	0.528149	0.598719	-6.0654	0.76597	0.722611
T.cells	CFAP20	-0.04325	5.191326	-0.52812	0.598739	-6.56672	0.744368	0.69758
T.cells	OST4	-0.04402	8.049265	-0.52808	0.598766	-6.98005	0.69771	0.644244
T.cells	WASHC1	-0.05661	4.182087	-0.52793	0.598871	-6.24385	0.761548	0.717488
T.cells	F730043M	-0.32508	0.260543	-0.52743	0.599215	-5.11855	0.831812	0.80031
T.cells	ARSK	0.111217	2.705302	0.5274	0.599237	-5.90312	0.787336	0.747741
T.cells	GM17178	0.132227	1.460682	0.527296	0.599309	-5.63023	0.809697	0.774071
T.cells	SATB1	0.069154	7.143818	0.527275	0.599323	-6.7642	0.712195	0.660839
T.cells	PRKCI	-0.09245	3.113961	-0.52727	0.599325	-6.00656	0.780119	0.739288
T.cells	INTS13	-0.05669	4.149388	-0.52717	0.599398	-6.3041	0.76211	0.71829
T.cells	ITGA1	-0.20232	3.828562	-0.52709	0.599454	-5.59004	0.767648	0.724732
T.cells	HTRA2	-0.05341	3.681601	-0.52708	0.599459	-6.27416	0.770198	0.727703

T.cells	EEFSEC	0.065481	4.399413	0.52696	0.599541	-6.4544	0.75782	0.713308
T.cells	TIMM8B	0.059115	5.242586	0.526881	0.599596	-6.49708	0.743518	0.69676
T.cells	KCP	-0.28304	0.100139	-0.52687	0.599602	-5.16491	0.83481	0.803882
T.cells	DNAJC2	-0.03766	5.81945	-0.52667	0.599739	-6.73292	0.733879	0.685683
T.cells	MGAT2	0.041068	5.873385	0.526623	0.599774	-6.65336	0.732984	0.684663
T.cells	QPCT	-0.17244	2.264701	-0.52652	0.599848	-5.52918	0.795185	0.757053
T.cells	EXOSC7	-0.04988	4.629702	-0.5265	0.599862	-6.4925	0.753888	0.708837
T.cells	TLR13	-0.28811	1.881588	-0.52621	0.600063	-5.20181	0.80207	0.765248
T.cells	SAMD3	0.32516	0.184815	0.52617	0.600088	-5.15172	0.833226	0.802183
T.cells	TBC1D15	0.042485	6.062519	0.526165	0.600091	-6.64462	0.729853	0.681195
T.cells	ABCC3	-0.24194	2.689429	-0.52611	0.600132	-5.47895	0.787617	0.748248
T.cells	BCAP31	-0.03905	5.901725	-0.52573	0.60039	-6.64501	0.732556	0.68443
T.cells	WDR19	0.211578	0.508741	0.525662	0.600439	-5.35326	0.827241	0.795218
T.cells	SH3RF1	0.06504	4.357213	0.52564	0.600455	-6.40013	0.758585	0.714509
T.cells	TPP1	0.066909	5.393356	0.525593	0.600487	-6.34935	0.741029	0.694193
T.cells	PKNOX2	-0.31982	-0.82371	-0.52557	0.600506	-5.09779	0.852316	0.82517
T.cells	LHFP	0.208728	1.237889	0.525389	0.600628	-5.43464	0.813877	0.779323
T.cells	RILPL2	-0.06012	7.158884	-0.5253	0.600693	-6.61254	0.712053	0.660969
T.cells	TMEM65	-0.06835	4.9205	-0.52486	0.600994	-6.38312	0.749196	0.703544
T.cells	ZFP780B	0.075972	3.329247	0.524833	0.601013	-6.16933	0.776596	0.735404
T.cells	SLC16A10	0.088679	7.024829	0.524672	0.601124	-6.58463	0.714352	0.663561
T.cells	ERGIC1	-0.06847	5.084699	-0.52461	0.601165	-6.38573	0.74642	0.700416
T.cells	PPP2R2D	0.038734	5.459388	0.524552	0.601208	-6.64683	0.740123	0.693168
T.cells	MMD	0.08556	4.477186	0.524494	0.601247	-6.18554	0.756737	0.712385
T.cells	ETAA1	-0.06333	3.334503	-0.52445	0.601279	-6.28957	0.776504	0.73541
T.cells	PLCG2	0.035496	7.02313	0.524071	0.601541	-6.84751	0.7144	0.663745
T.cells	POMT2	-0.12321	1.813643	-0.52404	0.60156	-5.67419	0.803583	0.767302
T.cells	MXD4	-0.06524	5.790486	-0.52389	0.601668	-6.57849	0.734622	0.686931
T.cells	NOL6	0.103087	2.582305	0.523855	0.60169	-5.82865	0.789801	0.75109
T.cells	TJP1	-0.19503	2.024999	-0.52375	0.60176	-5.38996	0.799771	0.762821
T.cells	DPY19L3	0.103166	2.757574	0.523742	0.601768	-5.94973	0.786689	0.747437
T.cells	MAP7D1	0.054002	5.36793	0.523737	0.601772	-6.35248	0.741678	0.69506
T.cells	GM26542	0.058837	4.802534	0.523473	0.601955	-6.49126	0.751256	0.706156
T.cells	POLI	-0.14339	1.527716	-0.52335	0.602038	-5.57256	0.808807	0.773531
T.cells	STEAP4	0.146746	2.205553	0.52318	0.602158	-5.85151	0.796569	0.759114
T.cells	GM15247	0.212391	0.995367	0.523102	0.602212	-5.49696	0.81854	0.785083
T.cells	CERK	0.043006	7.028222	0.523088	0.602222	-6.88261	0.714355	0.663762
T.cells	GM1123	-0.26038	0.265276	-0.52303	0.602264	-5.15363	0.832063	0.801163
T.cells	1700056N1	-0.10516	2.033678	-0.52301	0.602278	-5.79236	0.799656	0.762751
T.cells	ATG5	0.038095	5.691584	0.522851	0.602386	-6.58111	0.736354	0.688979
T.cells	D6WSU163	0.068627	3.1191	0.52247	0.60265	-6.12727	0.780462	0.740195
T.cells	POLE	-0.08132	3.301306	-0.52246	0.602659	-6.3468	0.777262	0.736452
T.cells	UNC13D	0.101933	3.31115	0.522313	0.602759	-5.84758	0.77709	0.736294
T.cells	PEAK1	0.064244	6.584571	0.522235	0.602813	-6.65727	0.721677	0.672199
T.cells	SLC20A1	-0.06464	5.219384	-0.52218	0.602853	-6.4449	0.74432	0.698274
T.cells	GM16316	-0.09324	1.803894	-0.52205	0.602944	-6.02379	0.803918	0.767915
T.cells	TMEM183A	0.039822	5.16956	0.521981	0.602989	-6.52399	0.745159	0.699281

T.cells	TFRC	-0.04606	6.38061	-0.52191	0.603035	-6.75284	0.725018	0.676096
T.cells	CYB561D2	0.071764	3.329402	0.521884	0.603056	-6.11388	0.77677	0.736031
T.cells	STRN4	0.051218	4.492619	0.521699	0.603185	-6.39979	0.756646	0.712589
T.cells	FOS	-0.10125	8.489188	-0.52164	0.603228	-6.66081	0.691172	0.637563
T.cells	ECHS1	0.06274	5.549982	0.521261	0.603488	-6.55678	0.738776	0.692058
T.cells	GM42477	-0.17965	1.273145	-0.52122	0.603516	-5.38571	0.813567	0.779523
T.cells	ZRANB1	0.036944	6.142303	0.521194	0.603535	-6.7127	0.728941	0.680754
T.cells	NEFH	0.214143	-0.09246	0.521163	0.603556	-5.39612	0.838887	0.809655
T.cells	GM9844	-0.09362	2.309102	-0.52111	0.603593	-5.92322	0.794832	0.757397
T.cells	PIGT	-0.04787	5.640537	-0.52072	0.603863	-6.62315	0.737265	0.690353
T.cells	RNF103	-0.06611	4.305165	-0.52072	0.603865	-6.21111	0.759855	0.7165
T.cells	MMGT1	-0.09138	2.857236	-0.52062	0.603936	-5.97419	0.785081	0.745983
T.cells	WDR90	-0.12267	1.959845	-0.52056	0.603977	-5.72817	0.801103	0.764842
T.cells	SAMM50	0.04662	5.177169	0.520308	0.604149	-6.54317	0.745031	0.699344
T.cells	1110002L0	-0.09687	2.8153	-0.52031	0.604149	-5.92231	0.785823	0.74686
T.cells	MSH5	0.102929	2.9838	0.520289	0.604163	-6.07507	0.782845	0.743368
T.cells	CKAP2	-0.07927	3.667324	-0.52025	0.60419	-6.38238	0.770873	0.729363
T.cells	CCDC50	0.043981	5.994315	0.520232	0.604202	-6.60005	0.731386	0.683614
T.cells	NDUFS7	0.036332	6.320866	0.520218	0.604212	-6.74267	0.726	0.677427
T.cells	LEMD2	0.061454	4.232797	0.520138	0.604267	-6.22363	0.761098	0.717982
T.cells	HEATR9	0.277213	-0.41964	0.519901	0.604432	-5.12637	0.845195	0.817153
T.cells	USP32	-0.05321	6.849617	-0.51971	0.604563	-6.69675	0.717518	0.667618
T.cells	VSIR	-0.16454	4.468275	-0.51955	0.604674	-5.54923	0.757232	0.713445
T.cells	ACBD6	0.040965	5.436733	0.519299	0.60485	-6.5759	0.740837	0.694525
T.cells	ZCCHC4	0.07095	3.455439	0.51926	0.604877	-6.25882	0.77474	0.733904
T.cells	HIGD1A	0.044519	6.029655	0.51926	0.604877	-6.68472	0.730966	0.683154
T.cells	NDUFA2	0.035285	7.238115	0.519151	0.604953	-6.90055	0.711228	0.660558
T.cells	ASB2	-0.15754	2.906427	-0.51894	0.6051	-5.80033	0.784387	0.745317
T.cells	TMEM106(	-0.07675	3.205819	-0.51867	0.605288	-6.04275	0.779112	0.739135
T.cells	FAM135A	0.172127	2.016455	0.518648	0.605302	-5.50554	0.800263	0.763996
T.cells	BANF1	-0.04066	6.873409	-0.51863	0.605316	-6.93831	0.717131	0.667398
T.cells	YAE1D1	0.086161	3.240113	0.518561	0.605363	-6.14122	0.77851	0.73845
T.cells	SNHG4	-0.06122	3.683196	-0.5185	0.605404	-6.33743	0.77077	0.729418
T.cells	RAG2	0.161619	0.168525	0.51841	0.605467	-5.73552	0.83418	0.804313
T.cells	CPT2	0.120842	2.829472	0.518328	0.605524	-5.84307	0.785748	0.746979
T.cells	FAM71E1	0.214845	0.837279	0.518183	0.605625	-5.39077	0.821756	0.789592
T.cells	LASP1	0.055127	5.455474	0.518141	0.605654	-6.3665	0.740523	0.694406
T.cells	POGLUT1	0.110324	2.752407	0.518117	0.605671	-5.76211	0.787113	0.748663
T.cells	CEMIP2	-0.07655	5.494454	-0.51805	0.605717	-6.3369	0.739871	0.693673
T.cells	IRGQ	0.128711	2.194038	0.517887	0.605831	-5.74523	0.797072	0.760443
T.cells	TRMO	-0.08672	3.013486	-0.51785	0.605859	-5.97528	0.782497	0.743307
T.cells	KLRI1	0.295918	-0.19768	0.517677	0.605977	-5.13464	0.841057	0.812703
T.cells	FABP5	0.086681	7.278075	0.517582	0.606043	-6.80245	0.710584	0.660151
T.cells	SEPHS1	0.042252	4.119783	0.517419	0.606156	-6.43972	0.763214	0.720873
T.cells	PRR7	-0.10238	3.334702	-0.51733	0.606219	-5.90852	0.776852	0.73681
T.cells	ABHD8	0.093567	2.172201	0.517321	0.606224	-5.94383	0.797463	0.761026
T.cells	GPS2	-0.03269	5.859455	-0.5173	0.606236	-6.68963	0.733787	0.686807

T.cells	LTF	0.249144	2.942911	0.517049	0.606413	-5.56391	0.783796	0.744908
T.cells	COX15	0.072504	2.891641	0.517048	0.606414	-6.01388	0.784702	0.745971
T.cells	GM26724	0.14889	1.517501	0.516743	0.606626	-5.61608	0.809513	0.775161
T.cells	CDKL1	-0.23288	1.100722	-0.51668	0.606673	-5.42189	0.81713	0.784191
T.cells	SPSB2	0.109461	2.383882	0.516449	0.60683	-5.69941	0.793925	0.756735
T.cells	LSM8	-0.04225	5.264944	-0.51638	0.60688	-6.65421	0.743957	0.69843
T.cells	PEPD	-0.06261	5.308275	-0.51634	0.606905	-6.3238	0.743229	0.697589
T.cells	ANGPT1	-0.18795	0.973826	-0.51597	0.607162	-5.51921	0.819756	0.787112
T.cells	SLC25A12	-0.05072	5.092687	-0.51583	0.607263	-6.40142	0.747134	0.701935
T.cells	SEPHS2	0.060598	6.39868	0.515561	0.607448	-6.74704	0.725427	0.676942
T.cells	RPP14	-0.08217	2.846249	-0.51525	0.607666	-6.02072	0.786039	0.747398
T.cells	XRCC3	0.163122	0.159666	0.515241	0.607671	-5.46047	0.83497	0.805338
T.cells	DDX10	-0.0407	5.436521	-0.51516	0.607724	-6.6028	0.741395	0.695426
T.cells	NOP10	-0.03673	7.15867	-0.51512	0.607755	-6.93103	0.713043	0.662886
T.cells	AGO3	-0.04714	5.940327	-0.51494	0.607882	-6.61284	0.732993	0.68582
T.cells	SURF4	0.051511	5.782118	0.514891	0.607914	-6.53831	0.735622	0.688847
T.cells	GM1604A	0.123476	2.804564	0.514873	0.607927	-5.85338	0.786777	0.748369
T.cells	USP34	-0.0325	7.703229	-0.51484	0.607947	-6.96539	0.704293	0.652975
T.cells	RIT1	0.072323	3.965996	0.514608	0.608111	-6.0491	0.76644	0.724582
T.cells	FIP1L1	0.027771	6.639254	0.514475	0.608203	-6.82028	0.721485	0.672618
T.cells	GM26532	0.10409	5.294849	0.514383	0.608268	-6.07445	0.743774	0.698271
T.cells	MFAP2	-0.26952	0.586159	-0.51437	0.608275	-5.25357	0.827019	0.795985
T.cells	EPRS	-0.05716	6.581496	-0.51432	0.608315	-6.77982	0.722429	0.673701
T.cells	GM20337	0.210179	1.078162	0.514264	0.608351	-5.32703	0.817934	0.785181
T.cells	ACOX3	0.074634	4.372027	0.514209	0.608389	-6.10529	0.759447	0.716442
T.cells	NAGK	0.086316	3.956557	0.514085	0.608475	-5.99058	0.766627	0.72483
T.cells	GM45716	-0.08412	3.20355	-0.51379	0.60868	-6.04754	0.779908	0.740296
T.cells	POLR2E	-0.0594	4.876402	-0.51362	0.608797	-6.51085	0.75101	0.706653
T.cells	MPPED2	0.267729	-0.32287	0.513576	0.608829	-5.26591	0.844238	0.816557
T.cells	KCTD9	-0.06983	3.130956	-0.51324	0.609064	-6.13804	0.781186	0.741905
T.cells	RER1	-0.03944	6.413933	-0.51322	0.609078	-6.74497	0.725337	0.677111
T.cells	UQCC1	0.062205	3.934429	0.513145	0.60913	-6.25503	0.767157	0.725503
T.cells	THRAP3	-0.03106	8.006361	-0.51303	0.609208	-7.05506	0.699623	0.64779
T.cells	SPG20	0.11042	2.615271	0.512831	0.609349	-5.72381	0.790315	0.752709
T.cells	TNS3	0.077266	5.235143	0.512765	0.609395	-6.41573	0.744945	0.699797
T.cells	PTBP2	0.042635	6.156832	0.512703	0.609437	-6.72274	0.729572	0.682072
T.cells	TRPV4	-0.28269	-0.51202	-0.51259	0.609516	-5.11516	0.847822	0.821069
T.cells	NSMCE4A	-0.03415	6.083141	-0.51243	0.609627	-6.79836	0.73079	0.683546
T.cells	BTBD11	-0.14476	4.309098	-0.51216	0.609814	-6.00333	0.760696	0.718199
T.cells	LLGL1	0.078804	2.998446	0.51214	0.60983	-6.04155	0.783522	0.744879
T.cells	DARS	-0.04774	5.511648	-0.51212	0.609847	-6.61413	0.740301	0.694553
T.cells	CCDC181	-0.128	1.514839	-0.5121	0.609857	-5.66508	0.810127	0.776253
T.cells	PRKG1	-0.16046	3.504872	-0.5119	0.609994	-5.87014	0.774628	0.734519
T.cells	NPAT	-0.05493	4.485178	-0.5118	0.610069	-6.47313	0.757677	0.714752
T.cells	ORMDL3	0.06805	3.803807	0.511676	0.610153	-6.27807	0.769422	0.728466
T.cells	AHR	-0.19371	4.719836	-0.51161	0.610197	-5.71112	0.753672	0.710146
T.cells	FGFRL1	-0.22645	0.299565	-0.51159	0.610214	-5.36151	0.83254	0.803031

T.cells	MYL12A	-0.03962	7.929477	-0.51152	0.610264	-6.93441	0.700844	0.649449
T.cells	CMYA5	-0.28873	0.674059	-0.5114	0.610343	-5.26118	0.825573	0.794771
T.cells	FOXN2	-0.03682	6.146142	-0.5113	0.610416	-6.72136	0.729748	0.682568
T.cells	GM28501	0.180375	0.476037	0.511014	0.610615	-5.40136	0.82925	0.799282
T.cells	ACER3	-0.06595	6.311004	-0.511	0.610626	-6.37306	0.72703	0.679523
T.cells	PSMA3	-0.02947	7.755055	-0.51096	0.610651	-6.97386	0.703623	0.652757
T.cells	MSI2	0.034396	7.509903	0.51093	0.610674	-7.01693	0.707545	0.657227
T.cells	CCM2	-0.03413	7.391127	-0.51093	0.610676	-6.98199	0.709453	0.659403
T.cells	ADAMTS9	-0.2681	2.970839	-0.51078	0.610779	-5.58066	0.78401	0.745757
T.cells	MRPL51	-0.05039	4.486929	-0.51063	0.610882	-6.45286	0.757647	0.714995
T.cells	ITGAM	0.170593	4.619395	0.510627	0.610885	-5.88579	0.755384	0.712363
T.cells	PID1	-0.13262	5.484883	-0.5106	0.6109	-6.01122	0.74075	0.695404
T.cells	APEH	0.082347	3.695339	0.5106	0.610904	-6.12191	0.771307	0.730923
T.cells	ZBTB39	0.136549	1.975209	0.510488	0.610982	-5.67549	0.801797	0.766783
T.cells	CLCN7	0.098035	3.608444	0.510291	0.611119	-6.00573	0.772894	0.732797
T.cells	ARHGEF11	0.060446	4.907611	0.510133	0.611229	-6.41501	0.750552	0.706788
T.cells	ZFP956	0.141215	1.264702	0.510127	0.611234	-5.57334	0.814772	0.782172
T.cells	TRIO	-0.05153	6.069746	-0.50984	0.611437	-6.61275	0.731168	0.684385
T.cells	OPLAH	0.178747	1.500611	0.509828	0.611442	-5.42143	0.81056	0.777173
T.cells	CIZ1	0.057524	3.940619	0.509358	0.61177	-6.29062	0.767541	0.726291
T.cells	ZFAND4	-0.12932	3.4762	-0.50922	0.611864	-6.02927	0.775629	0.735769
T.cells	NMNAT2	-0.31259	2.111738	-0.50901	0.612014	-5.33857	0.79984	0.764271
T.cells	2900026A(	0.081709	3.595436	0.508998	0.612022	-6.41224	0.773546	0.733369
T.cells	ETV3	-0.08969	4.950898	-0.50896	0.612046	-6.28314	0.75023	0.706214
T.cells	PPP4R3A	0.026901	6.59174	0.508595	0.612303	-6.83048	0.722916	0.674787
T.cells	MTF2	0.038078	5.644163	0.508579	0.612314	-6.7175	0.73859	0.692826
T.cells	RAB32	-0.11299	4.344985	-0.50855	0.612331	-5.82321	0.760599	0.718343
T.cells	FDXACB1	-0.08888	2.215747	-0.50844	0.612412	-5.87131	0.798	0.762222
T.cells	TSEN2	-0.15048	1.266976	-0.50839	0.612449	-5.51524	0.81521	0.782593
T.cells	PPP2R5D	-0.05911	3.682866	-0.50835	0.612477	-6.29205	0.772051	0.731738
T.cells	ORAI3	0.079405	3.868765	0.508043	0.612688	-6.10187	0.768917	0.72805
T.cells	PRDM10	-0.05193	5.213796	-0.50804	0.612691	-6.53905	0.745908	0.701295
T.cells	DDX46	-0.03907	6.040748	-0.50772	0.612913	-6.73491	0.732268	0.685422
T.cells	APPL1	-0.04875	5.974209	-0.50757	0.613016	-6.65304	0.733413	0.686709
T.cells	MSTO1	0.089643	2.681663	0.507238	0.613251	-5.96187	0.790167	0.752637
T.cells	GM42659	-0.05986	4.567867	-0.50707	0.613365	-6.3254	0.757254	0.714171
T.cells	GOSR2	-0.05156	5.28486	-0.50701	0.613407	-6.4703	0.745082	0.700081
T.cells	PPME1	0.051371	4.862972	0.506865	0.613512	-6.4275	0.752222	0.708365
T.cells	TENT5C	-0.17381	5.86321	-0.50683	0.613537	-6.05963	0.735398	0.688911
T.cells	STON2	-0.18654	3.518101	-0.50682	0.613541	-5.48012	0.775411	0.735384
T.cells	GM16272	0.197576	0.363126	0.506169	0.613998	-5.3609	0.832927	0.803023
T.cells	TYW1	-0.06199	3.875123	-0.50612	0.614035	-6.28753	0.769638	0.728327
T.cells	MRC2	-0.20158	1.416971	-0.50594	0.614159	-5.42311	0.813457	0.7799
T.cells	HINFP	0.068045	3.494398	0.505852	0.61422	-6.15618	0.776285	0.736125
T.cells	TAF10	-0.0361	6.856819	-0.50581	0.614246	-6.84182	0.719464	0.670358
T.cells	ADGRV1	-0.2834	0.711845	-0.50544	0.614508	-5.20369	0.826674	0.795512
T.cells	PHB	0.058787	4.493205	0.505342	0.614576	-6.42127	0.759194	0.71612

T.cells	ST14	-0.19985	1.167666	-0.5051	0.614747	-5.50813	0.818255	0.785615
T.cells	HLF	-0.21889	1.023669	-0.50509	0.614755	-5.3626	0.820906	0.788764
T.cells	PNP2	0.194821	0.616927	0.505015	0.614805	-5.47161	0.828437	0.797726
T.cells	DPH3	0.039687	5.480129	0.504985	0.614826	-6.5918	0.742446	0.696794
T.cells	UPP2	0.228099	1.652112	0.504713	0.615016	-5.38959	0.809555	0.775193
T.cells	GM47230	-0.19284	0.668652	-0.50412	0.615433	-5.42128	0.827911	0.796838
T.cells	DR1	0.042931	4.953577	0.504115	0.615435	-6.4879	0.751733	0.70731
T.cells	DPM1	-0.03865	5.979025	-0.50402	0.615503	-6.66888	0.734499	0.687438
T.cells	TBC1D2B	0.113663	4.091379	0.503984	0.615526	-5.81346	0.766516	0.724536
T.cells	JAML	-0.24235	2.748338	-0.50386	0.615616	-5.33304	0.790085	0.752135
T.cells	NUDT22	0.099608	2.557891	0.503808	0.615649	-5.80302	0.793481	0.75616
T.cells	MIR99AHG	0.121204	3.430837	0.503685	0.615735	-6.15252	0.778025	0.738061
T.cells	DPP9	-0.05555	4.420233	-0.50341	0.615931	-6.38218	0.760846	0.718116
T.cells	METTL8	0.099549	2.52734	0.50338	0.615949	-5.90985	0.794027	0.756955
T.cells	GM43728	0.257715	-0.48628	0.503271	0.616025	-5.11874	0.84963	0.823109
T.cells	ZFP994	0.095828	2.481603	0.50325	0.61604	-5.81	0.794846	0.757971
T.cells	IKBKG	0.083694	3.339723	0.502982	0.616228	-5.93823	0.779625	0.740176
T.cells	PRADC1	-0.06671	3.374688	-0.50296	0.61624	-6.19752	0.779011	0.739462
T.cells	E2F6	-0.0876	2.266229	-0.50287	0.616305	-5.94377	0.798709	0.762678
T.cells	RRS1	-0.05476	4.476059	-0.5028	0.616353	-6.42398	0.759887	0.717206
T.cells	BC051537	0.275276	0.044835	0.502755	0.616386	-5.19071	0.839578	0.811245
T.cells	GM12979	-0.26247	0.049111	-0.5027	0.616425	-5.15995	0.839498	0.811155
T.cells	GNB2	-0.03183	8.428837	-0.50253	0.616542	-7.08042	0.694825	0.642508
T.cells	PSMG4	0.046113	5.042688	0.50244	0.616607	-6.49811	0.750221	0.706057
T.cells	GM16230	0.251839	0.238127	0.502431	0.616613	-5.30021	0.835947	0.80699
T.cells	CIAPIN1	-0.05526	4.643928	-0.50231	0.616701	-6.43127	0.757011	0.713972
T.cells	AP2B1	0.035939	6.179239	0.502283	0.616717	-6.66596	0.731178	0.684097
T.cells	PRRC1	0.055719	4.219989	0.50206	0.616874	-6.28872	0.764404	0.722485
T.cells	CENPU	-0.08336	2.562499	-0.50178	0.617069	-6.03531	0.793676	0.756663
T.cells	CHD7	0.082014	6.463817	0.501627	0.617177	-6.23423	0.726742	0.678802
T.cells	C230066G2	0.230605	0.025289	0.501579	0.61721	-5.29321	0.840247	0.811934
T.cells	S100A13	-0.04692	6.040644	-0.50113	0.617525	-6.59955	0.733962	0.686995
T.cells	AP3M2	0.119081	2.489505	0.500993	0.617621	-5.63509	0.795231	0.758377
T.cells	CD300E	0.435225	0.628868	0.500983	0.617628	-5.15162	0.8292	0.798628
T.cells	SLC16A9	0.346576	0.530927	0.5009	0.617687	-5.16616	0.831025	0.800846
T.cells	OSM	0.311149	2.305551	0.500817	0.617745	-5.29401	0.798532	0.762336
T.cells	CENPJ	0.06359	3.331867	0.500726	0.617808	-6.22575	0.78028	0.740908
T.cells	DBP	-0.13833	2.187718	-0.50041	0.618029	-5.75387	0.80085	0.764903
T.cells	SMIM5	-0.29453	0.166279	-0.49982	0.618444	-5.1294	0.838422	0.809218
T.cells	BC048403	-0.14589	1.255612	-0.49974	0.618496	-5.40333	0.818169	0.785131
T.cells	SPG21	-0.03825	6.057126	-0.49971	0.618522	-6.69655	0.734189	0.686943
T.cells	RSF1OS1	-0.07882	2.978349	-0.49964	0.618571	-6.06775	0.787059	0.748412
T.cells	GM10143	0.120348	1.245831	0.49949	0.618675	-5.73291	0.818396	0.785373
T.cells	GPN3	-0.05385	3.982026	-0.49934	0.618782	-6.29822	0.769537	0.727821
T.cells	FHOD3	0.181632	-0.11029	0.498791	0.619165	-5.5525	0.844025	0.815675
T.cells	BC055324	-0.08537	2.245694	-0.49874	0.619198	-6.03008	0.800522	0.764017
T.cells	GM20536	-0.12697	2.340392	-0.49862	0.619288	-5.64282	0.798817	0.762074

T.cells	ZFP28	-0.2256	0.2908	-0.4986	0.619303	-5.28426	0.836469	0.806737
T.cells	TMEM138	-0.06698	2.910493	-0.49834	0.619482	-6.13575	0.788626	0.750179
T.cells	A2ML1	-0.18869	2.471948	-0.49833	0.619488	-5.5829	0.796455	0.759386
T.cells	CD151	0.107292	2.758187	0.498255	0.619542	-5.79538	0.791337	0.753409
T.cells	HLTF	-0.06163	4.64409	-0.49823	0.619561	-6.34405	0.758376	0.71489
T.cells	SUCLA2	-0.03886	5.593683	-0.49816	0.619607	-6.65905	0.74227	0.696252
T.cells	USF3	-0.05361	4.977969	-0.49765	0.619966	-6.46456	0.752711	0.708493
T.cells	PSMG2	-0.06161	3.919974	-0.49745	0.620104	-6.34113	0.770913	0.729669
T.cells	SDR42E1	0.192859	0.45745	0.497425	0.620124	-5.29649	0.833386	0.80341
T.cells	PPWD1	-0.03826	4.68019	-0.49738	0.620159	-6.53447	0.757793	0.714391
T.cells	NSMCE1	0.05025	4.829662	0.497288	0.62022	-6.51798	0.755238	0.711435
T.cells	PFKFB2	0.105776	2.465839	0.49713	0.620332	-5.69894	0.796601	0.759861
T.cells	UBAC1	0.049672	4.029628	0.497122	0.620337	-6.34435	0.769008	0.727508
T.cells	ST3GAL2	-0.09094	3.085043	-0.4971	0.620351	-5.97432	0.785567	0.746891
T.cells	CD40	-0.17491	2.17854	-0.49699	0.620428	-5.69197	0.80177	0.765993
T.cells	GM20274	-0.10608	2.727126	-0.49695	0.620458	-5.79335	0.791928	0.754395
T.cells	SCAMP3	-0.04804	4.819715	-0.4969	0.620493	-6.39165	0.755408	0.711718
T.cells	D430040D2	0.309592	-0.34621	0.496745	0.620602	-5.08148	0.848538	0.821727
T.cells	OSBPL10	0.20044	0.973543	0.496676	0.62065	-5.35967	0.823787	0.792167
T.cells	NAA20	-0.03892	5.164245	-0.49667	0.620654	-6.54853	0.749548	0.704997
T.cells	APLF	-0.11546	2.05909	-0.49664	0.620675	-5.78093	0.803928	0.768623
T.cells	TMEM42	-0.10063	2.617603	-0.49657	0.620728	-5.70947	0.793884	0.756777
T.cells	CD163L1	-0.31257	0.60908	-0.49622	0.620971	-5.15059	0.830782	0.800299
T.cells	GLIS1	0.196409	0.394465	0.496129	0.621035	-5.44844	0.834793	0.805082
T.cells	LRCH1	0.037911	7.432191	0.49599	0.621132	-6.95132	0.712227	0.661929
T.cells	ZFP963	0.151886	1.15161	0.495934	0.621172	-5.46713	0.820724	0.788339
T.cells	RNASET2B	-0.07259	5.139167	-0.4956	0.62141	-6.37142	0.750284	0.705683
T.cells	CXCL10	0.22769	4.788598	0.495546	0.621444	-5.93365	0.756253	0.712615
T.cells	AP5S1	0.123622	2.440536	0.495519	0.621464	-5.67852	0.797386	0.760733
T.cells	TAF15	0.028707	6.905531	0.495159	0.621716	-6.91033	0.720959	0.671951
T.cells	RASSF7	0.145013	0.86906	0.495131	0.621736	-5.57973	0.826154	0.794847
T.cells	F2RL1	0.267468	-1.13295	0.495059	0.621787	-5.10339	0.864067	0.840284
T.cells	TSPAN18	-0.20247	1.764014	-0.49493	0.621879	-5.42719	0.809705	0.775384
T.cells	APPL2	0.124539	3.358029	0.494878	0.621914	-5.80763	0.781156	0.741773
T.cells	CLEC14A	-0.20705	2.196348	-0.4948	0.621966	-5.43729	0.801868	0.766173
T.cells	ZW10	-0.05517	4.014837	-0.49465	0.622076	-6.39117	0.769667	0.728412
T.cells	IDUA	0.125833	2.137056	0.494645	0.622078	-5.56899	0.802939	0.767478
T.cells	ARMCX3	0.076821	3.573363	0.494509	0.622173	-6.06643	0.777406	0.737457
T.cells	UBE2D1	-0.04034	5.377113	-0.49423	0.622366	-6.51977	0.746402	0.701422
T.cells	TRAF3	-0.0523	6.856964	-0.49422	0.622377	-6.78384	0.721813	0.673111
T.cells	PDHB	0.036376	5.703174	0.494183	0.622402	-6.6984	0.740917	0.695083
T.cells	ARHGAP32	-0.09166	3.193867	-0.494	0.622529	-5.95637	0.784141	0.745399
T.cells	JADE1	-0.06771	4.165478	-0.49395	0.622564	-6.31325	0.767141	0.725514
T.cells	EPHA1	0.252068	0.461905	0.493865	0.622626	-5.23087	0.833833	0.804218
T.cells	MCTS1	0.036316	6.207891	0.493589	0.622821	-6.71203	0.732597	0.685513
T.cells	ADA	0.125952	2.074866	0.493582	0.622825	-5.79062	0.804234	0.769077
T.cells	SYTL1	-0.15841	1.59739	-0.49345	0.622917	-5.54125	0.812919	0.779374

T.cells	CHCHD1	0.04021	5.813268	0.493386	0.622963	-6.72862	0.739172	0.693103
T.cells	PPA2	0.05298	4.580694	0.493171	0.623114	-6.45044	0.760094	0.717335
T.cells	FZR1	0.049725	4.900739	0.493145	0.623133	-6.59607	0.754618	0.710973
T.cells	MRNIP	-0.10404	2.538227	-0.49305	0.623197	-5.95102	0.795928	0.759288
T.cells	BMT2	-0.04764	6.027835	-0.49279	0.623386	-6.58388	0.735692	0.689092
T.cells	ZFP691	0.108515	3.075803	0.492753	0.623409	-5.82532	0.786419	0.748125
T.cells	MRPL48	-0.03627	5.282039	-0.49268	0.623459	-6.5817	0.74821	0.703582
T.cells	CSRNP2	0.123252	2.107884	0.492414	0.623648	-5.70935	0.803904	0.768642
T.cells	ZFP51	0.074123	2.86166	0.492111	0.62386	-6.00236	0.790499	0.752794
T.cells	NDUFA9	0.044651	4.840567	0.49208	0.623882	-6.52024	0.755978	0.712455
T.cells	CATSPERE2	0.153587	1.174644	0.491325	0.624414	-5.42798	0.821577	0.789096
T.cells	CELSR1	-0.16948	1.626552	-0.49094	0.624685	-5.70599	0.813275	0.779358
T.cells	P3H2	-0.37522	1.190669	-0.49094	0.624686	-5.21534	0.821282	0.788856
T.cells	TGFBRAP1	-0.06559	3.494101	-0.49081	0.624776	-6.06467	0.779782	0.739909
T.cells	NEURL1B	0.145096	0.43588	0.490716	0.624843	-5.53474	0.835318	0.805569
T.cells	SEC61G	-0.04283	10.02398	-0.49064	0.624895	-7.30187	0.672569	0.616954
T.cells	CENPH	-0.08181	3.059161	-0.49062	0.624909	-6.24736	0.787466	0.748918
T.cells	SERPINI1	-0.08522	3.178888	-0.49059	0.624935	-6.08412	0.785344	0.746428
T.cells	GM50431	0.260066	0.122176	0.49044	0.625037	-5.204	0.841216	0.812615
T.cells	ACTR10	-0.03124	6.186686	-0.49036	0.625093	-6.7309	0.733752	0.686477
T.cells	STAG3	-0.19096	0.607181	-0.49025	0.625172	-5.53042	0.832113	0.801757
T.cells	GM12216	-0.08697	4.554087	-0.49014	0.62525	-6.22303	0.761348	0.718419
T.cells	SNAPC4	-0.13179	1.856185	-0.49005	0.625315	-5.70033	0.809086	0.774429
T.cells	DHX58	0.139963	2.999964	0.489965	0.625372	-5.81916	0.788517	0.750207
T.cells	TFR2	-0.17232	1.213769	-0.48984	0.625459	-5.42306	0.820855	0.78841
T.cells	HDAC9	-0.0723	7.563843	-0.48982	0.625472	-6.85524	0.71121	0.660707
T.cells	FAM89A	-0.16818	0.879902	-0.4897	0.625557	-5.37659	0.827034	0.795805
T.cells	ARL5B	0.067164	5.460574	0.489583	0.625641	-6.4333	0.745908	0.700592
T.cells	ATP6V1H	-0.05073	6.803114	-0.4895	0.625699	-6.71653	0.72358	0.674889
T.cells	H1FX	0.102784	2.127581	0.4895	0.6257	-6.05958	0.804161	0.768683
T.cells	TRIP6	0.139607	0.834471	0.489382	0.625783	-5.50818	0.827878	0.796822
T.cells	MRPS30	0.041565	4.726011	0.489217	0.625899	-6.53416	0.758397	0.715098
T.cells	CCDC117	-0.04561	4.699485	-0.48921	0.625905	-6.49109	0.758851	0.715626
T.cells	PLEKHG5	-0.16592	1.864551	-0.48916	0.625937	-5.55591	0.808934	0.774359
T.cells	C030006K1	0.149118	1.498775	0.489116	0.62597	-5.57661	0.815615	0.782272
T.cells	SLC35F6	0.113348	2.998461	0.488879	0.626138	-5.6807	0.788615	0.750367
T.cells	FBXO34	0.043097	5.78838	0.488738	0.626237	-6.62027	0.740463	0.694334
T.cells	BZW1	-0.02423	7.459579	-0.48873	0.626244	-6.97094	0.712958	0.66277
T.cells	PUM3	-0.04607	4.640352	-0.48856	0.626365	-6.45798	0.759934	0.716884
T.cells	TOM1L2	0.057812	5.471299	0.488552	0.626369	-6.48941	0.745794	0.70049
T.cells	COPG1	-0.0485	5.141092	-0.48834	0.62652	-6.50824	0.751435	0.707096
T.cells	RPUSD1	0.121157	1.47218	0.488103	0.626685	-5.68001	0.816232	0.78314
T.cells	ZFP507	0.1082	2.176566	0.488095	0.626691	-5.88546	0.803403	0.767954
T.cells	SNAPC5	0.051092	4.755447	0.487981	0.626771	-6.42169	0.758013	0.714776
T.cells	EP300	0.034981	6.696726	0.487949	0.626794	-6.78662	0.725441	0.677171
T.cells	GCNT2	0.196674	4.580489	0.487908	0.626823	-5.52741	0.761015	0.718266
T.cells	ESPL1	-0.06984	2.563574	-0.4877	0.626973	-6.24631	0.796467	0.759763

T.cells	CREBBP	-0.0315	7.888027	-0.48744	0.627154	-6.9826	0.706141	0.655126
T.cells	SORBS2	0.159357	1.878636	0.487354	0.627214	-5.76628	0.80884	0.774418
T.cells	TRAPPC13	-0.05776	3.808587	-0.4872	0.627324	-6.22845	0.774425	0.733945
T.cells	APH1A	0.052032	5.433388	0.487092	0.627399	-6.5158	0.746516	0.701544
T.cells	CCDC83	-0.33815	0.420205	-0.48703	0.627442	-5.18737	0.835779	0.806549
T.cells	GM4566	0.139842	1.718905	0.486969	0.627485	-5.66175	0.811751	0.777974
T.cells	NLGN2	0.219456	0.349554	0.486962	0.62749	-5.42911	0.837105	0.80814
T.cells	UFM1	0.040665	5.448609	0.486553	0.627779	-6.61173	0.746259	0.701313
T.cells	H2-M3	0.090136	4.138363	0.486341	0.627929	-6.20932	0.768683	0.72738
T.cells	SKINT3	0.260799	0.115469	0.48628	0.627972	-5.15193	0.841511	0.813477
T.cells	BAZ1B	0.036781	6.23949	0.486246	0.627996	-6.8293	0.733023	0.68608
T.cells	ZFP74	0.101954	2.132709	0.486178	0.628044	-5.78556	0.804229	0.769188
T.cells	MTRF1	-0.14639	1.208712	-0.48615	0.628066	-5.5765	0.821113	0.789211
T.cells	ETAA1OS	0.15813	0.543342	0.486114	0.628089	-5.44109	0.833473	0.803944
T.cells	ASS1	0.086151	6.535258	0.486066	0.628123	-6.78121	0.72813	0.680504
T.cells	PPM1G	-0.03108	6.448565	-0.48599	0.628173	-6.84197	0.729561	0.682148
T.cells	PAQR3	-0.14455	1.469925	-0.48596	0.628197	-5.49514	0.816307	0.783514
T.cells	TTLL4	-0.07655	3.000367	-0.48594	0.628214	-6.01576	0.788668	0.750868
T.cells	PRKAR1A	-0.02562	7.625008	-0.48583	0.628287	-6.96559	0.710367	0.66017
T.cells	KLHDC3	-0.04628	4.338451	-0.48581	0.628306	-6.32934	0.765218	0.723422
T.cells	BC049352	0.225276	1.083027	0.485595	0.628456	-5.30333	0.823435	0.792101
T.cells	6530402F1	0.24359	-0.17553	0.48555	0.628487	-5.18637	0.847018	0.82027
T.cells	COMMD3	0.039777	5.85015	0.485509	0.628516	-6.68615	0.739511	0.693717
T.cells	SYT14	0.270755	0.008242	0.485448	0.628559	-5.28221	0.843537	0.816137
T.cells	GM38604	0.115937	1.796583	0.485035	0.628851	-5.70644	0.810566	0.776658
T.cells	ZFP276	0.103875	2.479933	0.485	0.628876	-5.7431	0.798197	0.762039
T.cells	GM33782	0.243288	0.19699	0.484757	0.629048	-5.17056	0.84026	0.812059
T.cells	GPNMB	0.309267	0.898381	0.484756	0.629048	-5.20655	0.827139	0.796396
T.cells	MID1IP1	-0.0692	3.917342	-0.48461	0.629154	-6.26321	0.77279	0.732283
T.cells	ZBTB17	-0.05969	3.917458	-0.48451	0.629219	-6.32291	0.772788	0.732306
T.cells	DCAF11	-0.05741	4.559305	-0.48437	0.629323	-6.3036	0.76167	0.719393
T.cells	SLC2A3	0.082007	4.519005	0.483875	0.629671	-6.22656	0.762364	0.720339
T.cells	USP19	-0.05691	4.704682	-0.48376	0.629752	-6.41715	0.759173	0.716625
T.cells	KLF9	-0.10667	3.693914	-0.48374	0.629763	-5.98029	0.776696	0.737073
T.cells	MRPS18C	-0.041	5.696786	-0.48373	0.62977	-6.68086	0.742334	0.697103
T.cells	EPHB2	0.14986	1.372664	0.483577	0.629881	-5.65189	0.818373	0.786291
T.cells	USP3	0.037272	6.873279	0.48357	0.629886	-6.84759	0.722822	0.674694
T.cells	ELF1	0.027842	8.010369	0.483524	0.629919	-7.0156	0.704424	0.653674
T.cells	SDHC	-0.04523	5.09505	-0.4835	0.629938	-6.51698	0.752505	0.708936
T.cells	RAB3IL1	-0.19357	1.73222	-0.48311	0.630209	-5.34978	0.811784	0.778521
T.cells	EFCAB5	0.178038	0.504613	0.482956	0.630321	-5.47369	0.834482	0.805537
T.cells	SIL1	0.055571	5.149956	0.482949	0.630326	-6.45984	0.751572	0.707902
T.cells	SPRYD3	-0.07407	3.699854	-0.48274	0.630473	-5.95389	0.776592	0.737108
T.cells	PGAP1	0.129578	3.785443	0.482626	0.630554	-5.82728	0.775094	0.735354
T.cells	FICD	-0.184	0.917558	-0.4826	0.630569	-5.40058	0.826782	0.796402
T.cells	LNPK	0.05436	4.386002	0.482396	0.630717	-6.43952	0.764657	0.723164
T.cells	BRIX1	-0.0285	6.144863	-0.48238	0.630726	-6.81761	0.734845	0.688606

T.cells	AFF3	0.048197	7.930696	0.482325	0.630767	-7.16638	0.705699	0.655211
T.cells	C5AR2	-0.24179	1.231827	-0.48232	0.630771	-5.26676	0.820967	0.789479
T.cells	LRRC63	0.270755	0.544146	0.482309	0.630779	-5.21889	0.833742	0.804705
T.cells	COL3A1	0.158159	3.678331	0.482108	0.630921	-5.98085	0.776969	0.737647
T.cells	TMEM242	0.045989	4.292008	0.481883	0.63108	-6.40955	0.766282	0.725193
T.cells	CPSF4L	-0.14933	0.499907	-0.48175	0.631175	-5.46609	0.83457	0.805879
T.cells	POLDIP3	0.0343	6.078814	0.481742	0.631179	-6.73766	0.735944	0.690032
T.cells	RGS5	-0.30312	1.107497	-0.48171	0.631205	-5.29861	0.823263	0.792393
T.cells	TXNDC12	0.055772	3.827411	0.481675	0.631227	-6.23536	0.77436	0.734664
T.cells	CDH17	-0.26166	0.13748	-0.48162	0.631269	-5.1731	0.841383	0.814028
T.cells	SH2D2A	-0.13627	2.764079	-0.48161	0.631273	-5.8108	0.793148	0.756726
T.cells	CRIPT	0.034785	6.218829	0.481603	0.631278	-6.71623	0.733615	0.687347
T.cells	THOC2	-0.02783	7.106814	-0.48153	0.63133	-6.89655	0.719006	0.670581
T.cells	CLEC1B	0.191608	3.741016	0.481521	0.631336	-5.6267	0.775871	0.736451
T.cells	PINX1	0.069885	3.022074	0.481065	0.631658	-6.16448	0.788551	0.751479
T.cells	4930417O1	0.279479	0.963159	0.480848	0.631813	-5.17584	0.825936	0.795852
T.cells	EPAS1	-0.13209	3.448567	-0.48082	0.63183	-5.77332	0.781006	0.742708
T.cells	GABARAPL	-0.05203	7.762001	-0.48082	0.631831	-6.91876	0.708404	0.658674
T.cells	ANKRD28	-0.04353	5.835458	-0.48055	0.632022	-6.74858	0.740009	0.695074
T.cells	SERPINH1	0.188384	2.557349	0.480551	0.632023	-5.50483	0.79685	0.761474
T.cells	SIK2	0.041195	7.126745	0.480457	0.632089	-6.80627	0.718682	0.670538
T.cells	NUAK2	0.076433	4.289152	0.480431	0.632108	-6.08358	0.766332	0.725658
T.cells	DDX59	0.10025	1.892836	0.480374	0.632148	-5.82825	0.808857	0.775685
T.cells	EIF4G1	0.031724	6.604378	0.480175	0.632289	-6.83325	0.727238	0.680395
T.cells	4930404I0	-0.2708	0.086501	-0.47978	0.632572	-5.2108	0.842345	0.815749
T.cells	C4B	-0.26274	2.480976	-0.47972	0.632612	-5.53483	0.798221	0.763253
T.cells	ETV5	-0.11169	3.199847	-0.47971	0.632616	-5.89539	0.785398	0.748141
T.cells	GM49417	-0.2314	1.078235	-0.47969	0.632629	-5.41265	0.823804	0.79361
T.cells	NBEAL2	-0.1226	2.635242	-0.47964	0.632665	-5.54034	0.795453	0.760014
T.cells	PRDM16	-0.23775	0.306557	-0.47948	0.632784	-5.30557	0.838198	0.810914
T.cells	TBKBP1	-0.24098	1.449005	-0.47913	0.633029	-5.24043	0.81697	0.785675
T.cells	LMAN2	0.033754	6.112382	0.479125	0.633033	-6.70263	0.735385	0.690061
T.cells	TNFSF13	0.257156	1.863945	0.478874	0.633211	-5.26229	0.809383	0.776707
T.cells	TPRGL	-0.03773	6.927644	-0.47886	0.633223	-6.79655	0.721932	0.674611
T.cells	1600022D1	-0.20469	-0.42787	-0.47873	0.63331	-5.17583	0.852111	0.827764
T.cells	ATOX1	-0.03913	8.244709	-0.47869	0.633337	-6.99334	0.700688	0.650349
T.cells	WDR70	-0.03372	6.1341	-0.47856	0.633435	-6.76852	0.735024	0.689763
T.cells	KNTC1	-0.08713	2.818917	-0.47852	0.633461	-6.15904	0.792169	0.756444
T.cells	COG6	0.077957	3.137898	0.478009	0.633824	-6.03207	0.786495	0.749948
T.cells	RAD23A	0.047141	5.807004	0.477932	0.633878	-6.65127	0.740485	0.69625
T.cells	WDR44	0.061168	4.70447	0.477668	0.634065	-6.34237	0.759177	0.717999
T.cells	GM17435	-0.18464	1.133905	-0.47755	0.63415	-5.36777	0.822775	0.792977
T.cells	PHF12	-0.03625	6.186244	-0.47749	0.634191	-6.76424	0.734157	0.68901
T.cells	PRRG1	-0.16526	1.915898	-0.47744	0.63423	-5.56922	0.808438	0.775975
T.cells	SHOC2	0.036861	6.344774	0.47737	0.634277	-6.71998	0.731526	0.686014
T.cells	GM47428	-0.25759	0.207827	-0.47733	0.634306	-5.20488	0.840056	0.813718
T.cells	GM26789	0.212465	0.271556	0.477165	0.634422	-5.33714	0.838856	0.812314

T.cells	RRP9	-0.08267	2.59462	-0.47712	0.634453	-5.92378	0.796181	0.761529
T.cells	SRXN1	0.216254	1.094676	0.476843	0.63465	-5.23434	0.8235	0.794039
T.cells	TSPYL1	-0.05824	4.888609	-0.47674	0.634723	-6.2893	0.756024	0.714507
T.cells	HSPA4	0.027057	7.667014	0.476734	0.634727	-6.98273	0.709932	0.661325
T.cells	NGRN	0.076903	3.173648	0.476562	0.63485	-6.09479	0.785862	0.749465
T.cells	WDR75	-0.0604	3.575364	-0.47653	0.634874	-6.24018	0.778776	0.741134
T.cells	DDAH2	0.066439	3.503269	0.476481	0.634907	-6.29575	0.780043	0.742623
T.cells	ACOT8	-0.05879	4.129949	-0.47645	0.63493	-6.25098	0.769091	0.729791
T.cells	DNA2	0.045206	3.583362	0.476447	0.634932	-6.54134	0.778635	0.740978
T.cells	ZC3H12D	0.08152	3.479104	0.476318	0.635023	-6.18273	0.780468	0.743178
T.cells	GM20404	-0.12248	1.661785	-0.47621	0.635099	-5.69095	0.813071	0.781751
T.cells	4732471J0	-0.13448	1.996323	-0.47597	0.635269	-5.60921	0.806976	0.774586
T.cells	GM16754	0.244631	0.04601	0.475932	0.635297	-5.20227	0.84311	0.817711
T.cells	ZAP70	0.145748	1.982594	0.475881	0.635333	-5.71479	0.807225	0.774882
T.cells	AZI2	-0.03685	5.839646	-0.47585	0.635357	-6.60814	0.739939	0.696016
T.cells	NAA30	-0.05874	3.918894	-0.47584	0.635362	-6.20275	0.772763	0.734245
T.cells	P4HB	-0.03578	7.267668	-0.47583	0.635367	-6.84976	0.71639	0.668888
T.cells	ANGPTL4	0.161228	1.23193	0.475793	0.635395	-5.51475	0.820965	0.791229
T.cells	WDR82	0.037808	4.82833	0.475726	0.635443	-6.48351	0.757055	0.71591
T.cells	RAB11FIP2	0.079244	3.576284	0.475241	0.635787	-6.09859	0.77876	0.74146
T.cells	DZIP3	-0.07621	3.257554	-0.47495	0.63599	-6.16076	0.784377	0.748173
T.cells	ADAMTS6	-0.06201	5.940983	-0.47487	0.636053	-6.61327	0.738244	0.694337
T.cells	SFI1	0.047543	5.320089	0.474836	0.636075	-6.60046	0.748686	0.706446
T.cells	CCDC126	0.165825	1.899321	0.47463	0.636221	-5.48555	0.808739	0.777037
T.cells	ZFP458	0.19247	0.876726	0.474395	0.636388	-5.45605	0.827541	0.799449
T.cells	BRD8	-0.0288	6.236165	-0.47432	0.636444	-6.85392	0.733328	0.688704
T.cells	ALKBH5	-0.03383	7.148219	-0.47431	0.636451	-6.90871	0.718332	0.671437
T.cells	CIART	-0.15135	2.328452	-0.47416	0.636556	-5.5577	0.800967	0.767869
T.cells	PPP1R14A	-0.23647	0.601813	-0.47402	0.636655	-5.23519	0.832664	0.805641
T.cells	CLASRP	-0.07203	3.334689	-0.47396	0.636694	-6.0714	0.783014	0.746698
T.cells	ATG14	-0.06089	3.27883	-0.47385	0.636771	-6.13575	0.784001	0.74789
T.cells	KYAT1	0.128182	1.503706	0.473851	0.636774	-5.64984	0.815966	0.785738
T.cells	SPG7	-0.04159	4.467655	-0.47382	0.636793	-6.41073	0.763249	0.72355
T.cells	SDK1	-0.10351	3.195716	-0.47376	0.636841	-6.18704	0.785471	0.749622
T.cells	NECAP2	0.043209	5.345804	0.473724	0.636865	-6.55934	0.748251	0.706075
T.cells	KIF21A	-0.22518	0.515306	-0.47372	0.63687	-5.27281	0.834282	0.807612
T.cells	MTFP1	-0.17336	0.572509	-0.47368	0.636893	-5.46446	0.833211	0.80633
T.cells	ACTR5	-0.06742	3.477256	-0.47366	0.636908	-6.14402	0.780501	0.743772
T.cells	SENP2	-0.03812	6.656089	-0.47361	0.636942	-6.75938	0.726387	0.680792
T.cells	FRMPD4	-0.25812	0.351798	-0.47357	0.636977	-5.23828	0.837348	0.811298
T.cells	PIGC	0.098065	2.730331	0.473429	0.637074	-5.76055	0.793751	0.759481
T.cells	PPCS	0.087179	2.732539	0.473179	0.637251	-5.84032	0.793712	0.759536
T.cells	HDAC11	-0.18964	0.5008	-0.47291	0.637445	-5.42754	0.834553	0.808232
T.cells	3010003L2	0.113415	1.349271	0.47289	0.637457	-5.81504	0.818803	0.789405
T.cells	LSM12	-0.03276	6.360706	-0.47272	0.637581	-6.78562	0.731263	0.6867
T.cells	PARVG	-0.05053	5.071901	-0.47259	0.637673	-6.44845	0.752899	0.711836
T.cells	BAG3	-0.16028	2.967773	-0.47241	0.637799	-5.45494	0.789517	0.754822

T.cells	GALNT6	0.200386	3.115592	0.472367	0.637829	-5.3967	0.786891	0.75173
T.cells	LIPH	-0.23864	-0.05189	-0.47235	0.637844	-5.23349	0.844962	0.820903
T.cells	DIS3	-0.06021	3.258023	-0.47218	0.63796	-6.24061	0.784369	0.748832
T.cells	RCC1	-0.05089	3.989305	-0.47203	0.638065	-6.46011	0.771536	0.733829
T.cells	SP100	0.065928	7.43248	0.471964	0.638116	-6.65491	0.713718	0.66679
T.cells	INTS4	0.05699	3.920928	0.471835	0.638207	-6.28007	0.772728	0.735268
T.cells	FANCD2	-0.07238	2.80256	-0.47183	0.638208	-6.1611	0.792461	0.758509
T.cells	MUTYH	-0.14719	0.370874	-0.47174	0.638278	-5.48495	0.83699	0.811545
T.cells	CMC4	0.075001	3.064546	0.471679	0.638318	-5.98386	0.787797	0.753001
T.cells	CYP2C69	0.213118	1.432054	0.471607	0.638369	-5.40202	0.817281	0.787972
T.cells	GM47689	0.052573	3.369026	0.471546	0.638412	-6.2784	0.782408	0.746669
T.cells	GM12764	-0.15712	1.453299	-0.47135	0.638551	-5.51758	0.816891	0.787545
T.cells	GM43936	0.280081	-1.34803	0.471347	0.638554	-5.07652	0.86984	0.851211
T.cells	CPNE5	-0.14665	0.481176	-0.47121	0.638653	-5.61661	0.834921	0.809146
T.cells	ZC3H8	0.089028	2.13952	0.471111	0.638722	-5.87967	0.80438	0.772705
T.cells	HOMEZ	0.138138	1.333922	0.471109	0.638724	-5.52431	0.819085	0.790204
T.cells	MESD	0.047187	4.085411	0.47107	0.638751	-6.34443	0.769865	0.731984
T.cells	ANGPT2	-0.16599	0.571482	-0.47096	0.638828	-5.4735	0.833231	0.807119
T.cells	RNGTT	-0.03313	6.852257	-0.47083	0.638918	-6.85323	0.723166	0.677718
T.cells	TGS1	0.039653	5.214453	0.470804	0.63894	-6.54841	0.750477	0.709336
T.cells	TMEM204	-0.17158	1.038857	-0.47073	0.638992	-5.32555	0.824533	0.796709
T.cells	URM1	0.049816	4.343407	0.470705	0.639011	-6.33543	0.765393	0.726746
T.cells	MRGBP	0.06278	3.37422	0.470649	0.63905	-6.19035	0.782316	0.746616
T.cells	TEX10	-0.04945	5.266363	-0.4706	0.639083	-6.57731	0.749596	0.708311
T.cells	CDCA2	0.06952	3.79635	0.470316	0.639287	-6.45667	0.774903	0.737971
T.cells	NT5M	0.056389	3.572881	0.469956	0.639544	-6.24477	0.778819	0.742701
T.cells	CDCA7L	0.073215	3.766654	0.469951	0.639547	-6.31678	0.775422	0.738707
T.cells	ZNRF2	0.043153	5.763077	0.469875	0.639601	-6.52027	0.741222	0.698808
T.cells	HOXB4	0.135747	1.361865	0.46987	0.639605	-5.58254	0.818571	0.789845
T.cells	GM39090	-0.1475	0.497553	-0.46983	0.639632	-5.50502	0.834614	0.809062
T.cells	SLC18A1	0.242887	0.497721	0.469677	0.639742	-5.27512	0.834611	0.809126
T.cells	MOGS	-0.05867	4.095452	-0.46949	0.639875	-6.37248	0.76969	0.732153
T.cells	TLN1	0.041675	7.109295	0.469453	0.639902	-6.79177	0.718966	0.673229
T.cells	PCCA	-0.06017	4.599387	-0.46939	0.639944	-6.36824	0.760981	0.721954
T.cells	MON2	0.041667	5.7408	0.469366	0.639964	-6.59342	0.741595	0.699373
T.cells	DTWD2	-0.09302	2.777791	-0.46916	0.640112	-5.96503	0.792903	0.759566
T.cells	GM26787	-0.18594	1.300668	-0.46902	0.64021	-5.48307	0.819698	0.791449
T.cells	CD7	-0.31672	4.199176	-0.46889	0.640302	-5.44803	0.76789	0.730161
T.cells	SPRED3	-0.19204	0.719893	-0.46871	0.64043	-5.39318	0.83046	0.804346
T.cells	AC154200.	0.150034	0.660722	0.468681	0.640451	-5.45556	0.831564	0.805669
T.cells	FAM173A	0.049665	4.68182	0.468632	0.640486	-6.40557	0.759565	0.720428
T.cells	ADSSL1	0.052431	4.426295	0.468474	0.640599	-6.38223	0.763962	0.725574
T.cells	TRERF1	-0.13974	4.396216	-0.46837	0.640674	-5.8566	0.764481	0.726207
T.cells	PAPLN	-0.17052	0.894565	-0.46832	0.640711	-5.44466	0.827209	0.800491
T.cells	ACP2	-0.12385	3.54465	-0.46829	0.640733	-5.73345	0.779315	0.743624
T.cells	ART2B	-0.34979	0.097769	-0.46802	0.64092	-5.13181	0.842132	0.818503
T.cells	RASGRP2	0.038185	6.609517	0.468004	0.640933	-6.80826	0.727154	0.682897

T.cells	KLHL7	-0.0601	4.739495	-0.468	0.640938	-6.25942	0.758576	0.719392
T.cells	ATF6	-0.03767	6.979377	-0.46796	0.640963	-6.82978	0.721086	0.675901
T.cells	SLC12A4	0.13814	1.745624	0.467935	0.640983	-5.58901	0.81154	0.781882
T.cells	CHD3	0.060522	4.533669	0.467871	0.641028	-6.39692	0.762112	0.723533
T.cells	SIK1	0.044657	6.868808	0.467772	0.641099	-6.68143	0.722895	0.678028
T.cells	HS3ST1	0.309767	1.500377	0.467718	0.641137	-5.27478	0.816027	0.78728
T.cells	PIM3	0.064763	4.641176	0.467654	0.641182	-6.37707	0.760263	0.721441
T.cells	CEACAM1	-0.07344	3.675486	-0.46762	0.641209	-6.10532	0.777019	0.741107
T.cells	CMBL	0.188902	2.192141	0.467456	0.641323	-5.53	0.803428	0.772373
T.cells	PSMD7	0.039716	5.828246	0.467414	0.641353	-6.73941	0.74013	0.698038
T.cells	GM550	-0.13272	0.859238	-0.46739	0.641373	-5.65469	0.827866	0.801535
T.cells	FDFT1	-0.0626	3.796269	-0.46734	0.641408	-6.23109	0.774904	0.738691
T.cells	DNAJC24	-0.05947	3.89302	-0.46732	0.641419	-6.35247	0.773215	0.736705
T.cells	2310039H(	0.06172	4.246315	0.467319	0.641421	-6.22933	0.767073	0.729496
T.cells	DUSP6	0.066408	4.428378	0.467202	0.641505	-6.34493	0.763926	0.725835
T.cells	9530052E0	-0.15498	1.000649	-0.46707	0.6416	-5.43225	0.825241	0.798446
T.cells	MAPK14	-0.03333	6.656022	-0.46678	0.641804	-6.80415	0.726388	0.682192
T.cells	FMN1	-0.16867	3.350464	-0.46678	0.641805	-5.63128	0.782736	0.747944
T.cells	SETD4	0.077581	2.964232	0.466651	0.641897	-5.94459	0.78958	0.756025
T.cells	FAM114A2	-0.03902	5.166561	-0.46642	0.642064	-6.53198	0.75129	0.711075
T.cells	B230307C2	0.066424	3.741811	0.466397	0.642078	-6.12788	0.775857	0.739843
T.cells	FBN1	-0.19365	0.898383	-0.46638	0.642089	-5.3545	0.827139	0.800718
T.cells	ARF5	0.041747	8.486216	0.466343	0.642117	-7.04387	0.696858	0.648311
T.cells	DPAGT1	-0.07534	3.285854	-0.4663	0.64215	-6.10111	0.783877	0.74929
T.cells	CADM1	-0.21779	5.440386	-0.46618	0.642231	-5.88323	0.746652	0.705677
T.cells	GNB1L	0.060005	3.308245	0.466133	0.642266	-6.28569	0.783481	0.748826
T.cells	ZDHHC7	-0.06034	3.834677	-0.46586	0.642463	-6.2162	0.774233	0.73804
T.cells	EIF4G2	-0.02603	7.71796	-0.46581	0.6425	-7.00724	0.709112	0.662433
T.cells	CIAO1	0.059194	3.481337	0.465789	0.642512	-6.12333	0.780429	0.745356
T.cells	RNMT	0.041935	4.970618	0.465556	0.642678	-6.54504	0.754625	0.715136
T.cells	NDUFS4	0.033809	6.335519	0.465509	0.642712	-6.76229	0.73168	0.688497
T.cells	TUBG1	-0.07552	3.393021	-0.46544	0.642761	-6.29052	0.781985	0.747279
T.cells	GNPDA1	-0.06978	4.395098	-0.4652	0.642929	-6.12966	0.7645	0.726754
T.cells	CIAO3	0.065535	3.316721	0.465199	0.642933	-6.1183	0.783331	0.748897
T.cells	ZFP131	0.042545	5.815875	0.465002	0.643073	-6.59686	0.740337	0.698587
T.cells	DCBLD2	-0.12123	1.32551	-0.46493	0.643125	-5.61696	0.81924	0.791566
T.cells	CITED2	-0.04929	6.149916	-0.46485	0.643179	-6.67469	0.734761	0.692141
T.cells	MAN2B2	0.09761	3.799809	0.464733	0.643265	-5.90092	0.774843	0.738951
T.cells	GLB1	0.046141	5.221188	0.464705	0.643285	-6.51655	0.750362	0.710284
T.cells	FXN	0.043578	4.085717	0.464653	0.643322	-6.48994	0.769859	0.733107
T.cells	BUB3	-0.03758	5.956108	-0.46465	0.643326	-6.81188	0.737991	0.695911
T.cells	HMG20B	0.038522	5.119833	0.464599	0.643361	-6.52471	0.752084	0.712304
T.cells	CHCHD2	-0.02611	8.94643	-0.46451	0.643422	-7.20704	0.689614	0.640335
T.cells	NBDY	0.061521	3.659616	0.464486	0.643441	-6.14768	0.777297	0.741853
T.cells	LTC4S	0.320993	1.066227	0.464454	0.643464	-5.39347	0.824026	0.797332
T.cells	ZFP827	-0.0883	2.85843	-0.46441	0.643496	-6.09309	0.791464	0.758577
T.cells	HIST1H1C	0.071681	4.106663	0.464229	0.643625	-6.4155	0.769495	0.73268

T.cells	PGS1	0.052978	4.740858	0.464157	0.643676	-6.32738	0.758553	0.719859
T.cells	GM16973	-0.11818	1.894858	-0.46414	0.64369	-5.6902	0.80882	0.77918
T.cells	PDPR	0.066257	3.937711	0.464112	0.643708	-6.26683	0.772435	0.736133
T.cells	GPR137	0.104785	2.506946	0.464066	0.643741	-5.60507	0.797755	0.76603
T.cells	EXOG	0.103507	1.745846	0.464063	0.643743	-5.78354	0.811536	0.782414
T.cells	PHLPP1	0.053327	7.671226	0.464002	0.643787	-6.97081	0.709864	0.663465
T.cells	CLDN15	-0.18054	0.423974	-0.46377	0.643951	-5.31257	0.835993	0.811737
T.cells	LFNG	0.066903	4.413378	0.463709	0.643996	-6.17665	0.764185	0.726529
T.cells	GM5617	0.103115	3.238502	0.463609	0.644067	-5.77986	0.784714	0.750687
T.cells	TINF2	-0.05803	4.054948	-0.4634	0.644216	-6.34413	0.770394	0.733898
T.cells	ARC	-0.17302	0.905214	-0.46339	0.644225	-5.50175	0.827012	0.801084
T.cells	CCDC71	0.079807	2.73695	0.463346	0.644255	-5.99075	0.793633	0.761313
T.cells	TLR6	-0.2219	1.070509	-0.46334	0.64426	-5.31014	0.823947	0.797414
T.cells	LRRC14	-0.09545	2.339055	-0.46312	0.644419	-5.8678	0.800776	0.769865
T.cells	RNF122	0.12695	2.64878	0.463086	0.644441	-5.72752	0.795211	0.763261
T.cells	GM44148	0.114589	1.90428	0.463005	0.644498	-5.85249	0.808649	0.779228
T.cells	NECTIN4	-0.23983	-0.02307	-0.46298	0.644518	-5.18381	0.844417	0.822084
T.cells	RIIAD1	-0.14857	0.993222	-0.46296	0.644532	-5.49204	0.825379	0.79921
T.cells	NUBP1	-0.03885	5.473714	-0.46278	0.644657	-6.69528	0.74609	0.705598
T.cells	TMEM50A	-0.0324	7.711684	-0.46256	0.644816	-6.94155	0.709213	0.663055
T.cells	MMP9	0.323112	1.381978	0.462504	0.644856	-5.19319	0.818201	0.790773
T.cells	PLXNB2	-0.0677	3.959065	-0.46248	0.644876	-6.21851	0.772063	0.736082
T.cells	SENP8	-0.2049	0.568146	-0.46246	0.644885	-5.35832	0.833293	0.808864
T.cells	JMJD4	-0.11335	1.268434	-0.46236	0.644956	-5.60615	0.820292	0.793286
T.cells	DCAF1	0.04542	5.222364	0.462354	0.644964	-6.59166	0.750342	0.710651
T.cells	KCTD10	0.062445	4.043279	0.462308	0.644996	-6.18121	0.770597	0.734369
T.cells	GT(ROSA)2	0.055631	4.594685	0.461814	0.645349	-6.42355	0.761285	0.723298
T.cells	2810013PC	0.077318	4.123121	0.461802	0.645358	-6.20421	0.769435	0.732855
T.cells	ELOA	0.040476	5.789735	0.461756	0.645391	-6.65893	0.740992	0.699632
T.cells	RCCD1	-0.07951	3.170294	-0.46145	0.645611	-6.15217	0.786187	0.752617
T.cells	GM45435	0.19125	0.920103	0.461261	0.645744	-5.49695	0.827014	0.801219
T.cells	DNAL4	-0.11024	1.951429	-0.46121	0.645783	-5.73943	0.808064	0.778581
T.cells	TCHP	-0.10904	1.792962	-0.46119	0.645792	-5.7266	0.81095	0.782019
T.cells	LARP1	0.033915	6.798018	0.461031	0.645908	-6.83102	0.7243	0.680347
T.cells	SMARCD1	0.040611	4.031896	0.461009	0.645924	-6.45293	0.771055	0.734809
T.cells	GM15965	-0.15934	2.027609	-0.46099	0.645939	-5.51612	0.806681	0.776946
T.cells	HACE1	0.051461	4.490352	0.460922	0.645987	-6.44916	0.763115	0.725501
T.cells	PDRG1	-0.04669	4.496479	-0.46085	0.646035	-6.4622	0.763009	0.725385
T.cells	DNMT1	-0.04445	5.753517	-0.46053	0.646269	-6.81331	0.741679	0.700514
T.cells	NRARP	-0.11767	1.780032	-0.46051	0.646281	-5.82513	0.811236	0.782415
T.cells	KLRG1	0.249096	-1.146	0.460404	0.646356	-5.12624	0.866265	0.848625
T.cells	MRM2	-0.13204	2.071278	-0.46035	0.646392	-5.79828	0.805939	0.776111
T.cells	SSH3	-0.13717	1.522336	-0.46018	0.646515	-5.67051	0.81595	0.788045
T.cells	IKZF3	-0.04969	5.866917	-0.46016	0.646534	-6.84503	0.739778	0.69831
T.cells	COX14	-0.04117	5.656597	-0.46013	0.646555	-6.55935	0.743307	0.702414
T.cells	TMUB2	0.070417	3.181282	0.459447	0.647041	-6.04735	0.786424	0.752721
T.cells	DLG3	0.13405	0.571754	0.459382	0.647087	-5.62542	0.833965	0.809375

T.cells	GPC6	-0.24887	1.763476	-0.45913	0.647267	-5.48089	0.811933	0.783107
T.cells	NVL	-0.04087	4.723925	-0.45901	0.647351	-6.5046	0.759516	0.721189
T.cells	RAB11FIP1	0.148678	4.829945	0.459011	0.647353	-5.846	0.757699	0.719064
T.cells	IRF5	-0.07469	5.351662	-0.45887	0.647457	-6.25394	0.748815	0.708721
T.cells	GLOD4	0.039984	5.050654	0.458785	0.647514	-6.53689	0.753929	0.714686
T.cells	RFC1	-0.04205	5.999025	-0.45874	0.647547	-6.77734	0.737929	0.696062
T.cells	MED11	-0.06974	3.312748	-0.45873	0.647551	-6.03386	0.784096	0.750107
T.cells	HAVCR2	-0.27226	2.301711	-0.45873	0.647555	-5.31333	0.80216	0.771503
T.cells	COLQ	0.1298	0.244853	0.458541	0.647689	-5.61418	0.840189	0.8169
T.cells	ITPRID2	-0.06405	5.08881	-0.45808	0.648019	-6.25015	0.753659	0.714071
T.cells	MAFK	-0.08406	4.524193	-0.45787	0.648167	-6.18765	0.76343	0.725444
T.cells	CEP76	0.062051	2.990307	0.457744	0.648259	-6.25151	0.790341	0.757121
T.cells	GCC1	-0.12886	2.459454	-0.45759	0.648373	-5.63645	0.799904	0.768398
T.cells	PWWP2B	-0.12074	2.082717	-0.45726	0.648606	-5.67995	0.806847	0.776582
T.cells	GATC	0.095101	2.698485	0.457245	0.648616	-5.9759	0.795739	0.763396
T.cells	PHLDB2	-0.17493	2.509693	-0.45704	0.648762	-5.53438	0.799224	0.767439
T.cells	PIGX	-0.0435	5.308726	-0.45694	0.648838	-6.53142	0.750314	0.709944
T.cells	INTS11	-0.05474	3.989645	-0.45678	0.648948	-6.37402	0.773058	0.736545
T.cells	PINK1	0.056948	5.115722	0.456533	0.649126	-6.45244	0.75375	0.7139
T.cells	SLC19A2	-0.10288	1.834884	-0.45642	0.649209	-5.77581	0.811632	0.782173
T.cells	IL5RA	0.133297	0.975245	0.456338	0.649266	-5.79835	0.827465	0.801084
T.cells	NDUFB7	0.035324	6.480862	0.456123	0.64942	-6.82166	0.730824	0.687346
T.cells	RTN4IP1	0.10338	1.927586	0.455938	0.649552	-5.87582	0.809941	0.78026
T.cells	HPSE	0.079209	3.426146	0.455834	0.649627	-6.12727	0.78306	0.74841
T.cells	DCUN1D4	-0.11219	2.540378	-0.4558	0.64965	-5.82688	0.798847	0.767086
T.cells	GM10790	0.235266	-0.77429	0.455708	0.649717	-5.09804	0.86057	0.841074
T.cells	MTMR9	-0.04685	3.972449	-0.45561	0.649785	-6.32473	0.773469	0.737144
T.cells	FAHD1	0.148068	1.917334	0.455578	0.64981	-5.56008	0.810128	0.780516
T.cells	ADPRHL2	-0.0583	3.241714	-0.4555	0.649866	-6.09652	0.786323	0.752287
T.cells	CIAO2A	-0.0343	6.679979	-0.45531	0.650003	-6.90327	0.727535	0.683682
T.cells	NGP	0.164517	5.022684	0.455247	0.650047	-6.123	0.755337	0.715977
T.cells	PAGR1A	-0.16825	0.395669	-0.4552	0.650082	-5.48219	0.8383	0.814323
T.cells	CD96	-0.29532	0.686804	-0.45518	0.650098	-5.21332	0.832841	0.807769
T.cells	SPNS1	0.065414	3.498213	0.455121	0.650138	-6.12076	0.781788	0.747042
T.cells	KCTD2	0.068272	3.355478	0.454959	0.650254	-6.09054	0.784362	0.750028
T.cells	1810006J0	-0.23614	-0.01116	-0.45487	0.65032	-5.17453	0.846041	0.823598
T.cells	RAB11FIP5	-0.18132	0.808074	-0.45474	0.650407	-5.43882	0.830658	0.805108
T.cells	BTLA	-0.14574	3.948652	-0.45446	0.650612	-5.82389	0.77399	0.737783
T.cells	VRK2	0.047491	5.691956	0.454389	0.650662	-6.58089	0.744093	0.702844
T.cells	MYCBP	-0.0478	4.22905	-0.45436	0.650685	-6.45304	0.769107	0.732059
T.cells	RAD52	-0.07771	3.121528	-0.45433	0.650707	-6.01378	0.788564	0.754975
T.cells	UBE2B	0.038482	8.25823	0.4542	0.650798	-7.01415	0.70208	0.654433
T.cells	UQCC2	0.040995	6.141014	0.453963	0.650968	-6.7758	0.736603	0.694184
T.cells	DIPK2A	0.059689	4.112992	0.453691	0.651163	-6.21934	0.771159	0.734624
T.cells	AIDA	-0.04771	4.437691	-0.45338	0.651383	-6.38671	0.765526	0.728072
T.cells	GM47371	0.162375	0.907518	0.4533	0.651444	-5.53293	0.828875	0.803185
T.cells	EPC1	0.034948	6.811099	0.453296	0.651446	-6.87652	0.725509	0.681519

T.cells	CWC15	-0.02971	6.384372	-0.45326	0.651469	-6.80428	0.732555	0.68967
T.cells	TBC1D22B	0.05635	3.778303	0.453215	0.651504	-6.28209	0.777005	0.741566
T.cells	ESYT2	-0.03571	6.802347	-0.45319	0.651521	-6.80704	0.725653	0.681702
T.cells	A230072C(	-0.12961	1.545767	-0.4531	0.651587	-5.58295	0.817073	0.789101
T.cells	DUBR	0.139772	2.378269	0.453039	0.65163	-5.53014	0.801913	0.771049
T.cells	ZFP395	0.055575	4.289929	0.452908	0.651724	-6.36291	0.768084	0.731113
T.cells	FBH1	0.068765	3.425128	0.452881	0.651744	-6.00183	0.78322	0.748924
T.cells	ZFP973	-0.17355	0.609416	-0.45286	0.651758	-5.36261	0.83444	0.809908
T.cells	GM27010	0.08147	2.340877	0.45284	0.651773	-5.99438	0.802589	0.771858
T.cells	BEND6	0.253886	0.170455	0.45267	0.651896	-5.17202	0.842698	0.819869
T.cells	1700029H1	-0.19301	1.58753	-0.45266	0.651902	-5.26484	0.816307	0.788224
T.cells	PAK2	-0.02683	7.859842	-0.45207	0.652329	-6.94862	0.708852	0.662156
T.cells	NR5A2	-0.18134	1.145394	-0.4518	0.652521	-5.3299	0.825067	0.79819
T.cells	UBE2G1	-0.02858	7.307993	-0.45163	0.652639	-6.9246	0.717969	0.672405
T.cells	ATM	-0.04911	4.221236	-0.45121	0.652942	-6.40597	0.770179	0.732921
T.cells	TMC4	-0.11401	1.556285	-0.45094	0.653137	-5.59103	0.817997	0.789403
T.cells	KIFC1	0.083499	3.200672	0.450789	0.653245	-6.37812	0.788317	0.754172
T.cells	GPR174	0.132355	2.267506	0.450596	0.653384	-5.66329	0.805097	0.774029
T.cells	RNF26	0.060184	3.515898	0.450559	0.653411	-6.31397	0.782767	0.747611
T.cells	ZC3H11A	0.112991	2.01149	0.450372	0.653544	-5.73565	0.809794	0.779624
T.cells	IMPA2	0.046945	4.492173	0.450198	0.65367	-6.51055	0.765751	0.72765
T.cells	LYPD6B	0.287377	0.004013	0.450158	0.653698	-5.15445	0.847139	0.824361
T.cells	CEBPZOS	-0.0529	4.521965	-0.45013	0.653722	-6.34221	0.765236	0.727047
T.cells	EIF3G	-0.04125	4.982954	-0.44959	0.654107	-6.56941	0.757674	0.717899
T.cells	SRRM1	-0.02399	7.865839	-0.44934	0.654287	-7.06176	0.709832	0.662539
T.cells	NCOA2	-0.03524	7.684552	-0.44927	0.654335	-6.97015	0.712757	0.66589
T.cells	GPT	0.163061	1.203309	0.449134	0.654434	-5.48139	0.825089	0.79751
T.cells	NSDHL	0.099833	2.406095	0.4491	0.654458	-5.81264	0.803069	0.771304
T.cells	GNA12	0.05806	4.652134	0.4488	0.654674	-6.29413	0.763403	0.724615
T.cells	PHPT1	-0.05007	4.330971	-0.44867	0.654769	-6.41346	0.76896	0.731172
T.cells	MAP7D3	0.200629	0.126284	0.448386	0.654972	-5.32781	0.845278	0.821911
T.cells	METAP1D	0.058295	3.768583	0.448376	0.654979	-6.3167	0.778783	0.742764
T.cells	NUP88	-0.04663	4.421593	-0.44829	0.655039	-6.42163	0.767388	0.729424
T.cells	PHC2	-0.04846	5.798035	-0.44818	0.65512	-6.60955	0.743878	0.702001
T.cells	FGL2	-0.3059	4.054695	-0.44818	0.655123	-5.4153	0.773771	0.736911
T.cells	2010001A1	0.13517	1.389339	0.448168	0.655128	-5.52681	0.821648	0.793621
T.cells	VPS4B	-0.03429	6.231035	-0.44815	0.655138	-6.66254	0.736624	0.693589
T.cells	ZFP566	0.123454	1.254913	0.447924	0.655304	-5.67181	0.824133	0.796615
T.cells	METTL27	0.179556	0.539053	0.44783	0.655371	-5.48959	0.837488	0.812634
T.cells	ZFP141	0.071606	3.367308	0.447636	0.655511	-6.16924	0.785864	0.751244
T.cells	U2AF1L4	-0.07587	3.047054	-0.44763	0.655516	-6.12925	0.791558	0.757968
T.cells	ELOB	-0.02888	8.913307	-0.44742	0.655663	-7.19457	0.693154	0.643781
T.cells	UBE2G2	-0.04576	4.661055	-0.44742	0.655668	-6.37567	0.763249	0.724674
T.cells	ATP1B3	0.050032	7.281647	0.44737	0.655702	-6.96272	0.719298	0.673687
T.cells	2610507B1	-0.03261	5.431746	-0.44733	0.65573	-6.60372	0.750068	0.709291
T.cells	ARHGDIA	-0.0355	7.188393	-0.44715	0.65586	-6.90697	0.72082	0.675471
T.cells	ABCF2	-0.04222	4.411019	-0.44703	0.655947	-6.47611	0.767571	0.729798

T.cells	PRDM2	-0.04275	5.970241	-0.44702	0.655957	-6.6232	0.740985	0.698797
T.cells	UBR5	0.035645	6.888097	0.446978	0.655984	-6.81154	0.725743	0.681166
T.cells	CAPN3	0.176378	1.422788	0.446966	0.655993	-5.38508	0.82103	0.793057
T.cells	MED17	0.03449	4.867425	0.446954	0.656001	-6.4991	0.759698	0.720585
T.cells	BACH2OS	-0.1219	1.526179	-0.44678	0.656126	-5.67258	0.819195	0.790848
T.cells	BCL2L11	0.062625	7.053365	0.446389	0.656408	-6.75499	0.723326	0.678184
T.cells	FTSJ1	0.053271	3.078297	0.446033	0.656664	-6.16793	0.7914	0.757568
T.cells	ALMS1	-0.05073	3.824261	-0.44591	0.656756	-6.47945	0.778198	0.742005
T.cells	MS4A6B	-0.1809	4.588359	-0.44577	0.65685	-5.79706	0.764889	0.72639
T.cells	FURIN	-0.08097	5.290193	-0.44574	0.656875	-6.21396	0.752852	0.712335
T.cells	ATP2A3	0.040828	5.210591	0.445495	0.657051	-6.64615	0.754209	0.713974
T.cells	H13	-0.0324	6.42662	-0.44545	0.657085	-6.75975	0.733739	0.690209
T.cells	GM15492	-0.15624	0.742028	-0.44544	0.657087	-5.46074	0.834102	0.808474
T.cells	9430015G1	0.092055	1.911053	0.44518	0.657278	-5.83397	0.812475	0.782664
T.cells	GPR35	-0.22282	2.516821	-0.44514	0.657305	-5.33027	0.801474	0.769599
T.cells	ADAMTS10	0.068045	3.015513	0.445012	0.657399	-6.09635	0.792521	0.759002
T.cells	ACSL4	-0.04196	6.189637	-0.44496	0.657434	-6.67568	0.737687	0.694823
T.cells	MED1	-0.02854	5.883036	-0.44481	0.657541	-6.71365	0.742824	0.700795
T.cells	POLE3	-0.0495	4.133596	-0.44476	0.657577	-6.46295	0.772784	0.735778
T.cells	GANAB	-0.04367	4.730059	-0.44476	0.65758	-6.47053	0.762444	0.723663
T.cells	SCRN3	-0.1192	1.928035	-0.44465	0.657662	-5.7102	0.812165	0.782386
T.cells	PTP4A1	-0.18185	1.179279	-0.44455	0.657735	-5.42994	0.825952	0.798844
T.cells	GM19605	0.13709	1.260305	0.444544	0.657735	-5.62494	0.824449	0.797048
T.cells	ME2	0.049599	5.963937	0.44445	0.657803	-6.57914	0.741465	0.699296
T.cells	CDC25C	-0.08376	2.030722	-0.44445	0.657804	-6.10791	0.810291	0.780187
T.cells	LGALS1	0.061061	6.036335	0.444392	0.657845	-6.75079	0.740251	0.697888
T.cells	PHGDH	-0.06205	4.379094	-0.44397	0.65815	-6.60026	0.768788	0.730995
T.cells	ZEB2OS	0.047608	4.678362	0.443839	0.658243	-6.61349	0.763634	0.724971
T.cells	ZHX2	-0.06023	5.614006	-0.44375	0.658309	-6.51695	0.747651	0.706335
T.cells	PSMA7	0.032702	7.683799	0.443496	0.658491	-7.01098	0.71353	0.666807
T.cells	TRAIP	-0.0822	2.119414	-0.443	0.658847	-6.04884	0.809364	0.77861
T.cells	DEPP1	-0.2267	1.043364	-0.44286	0.658946	-5.29563	0.829183	0.802268
T.cells	REEP1	-0.18572	0.98372	-0.44273	0.659045	-5.38477	0.830294	0.803673
T.cells	UBN1	0.026485	6.944041	0.442613	0.659127	-6.87094	0.725806	0.680878
T.cells	MTUS1	-0.15796	3.721745	-0.4426	0.659139	-5.67997	0.780664	0.744779
T.cells	M1AP	0.13077	1.320039	0.442463	0.659235	-5.61271	0.824045	0.796271
T.cells	RBCK1	0.036376	5.550838	0.442381	0.659294	-6.6603	0.749065	0.707872
T.cells	MNT	-0.0587	4.606728	-0.44215	0.659461	-6.31166	0.765223	0.726784
T.cells	FTL1	-0.04347	11.26205	-0.44198	0.659581	-7.38634	0.658	0.603966
T.cells	A530032D1	0.261368	0.594014	0.441968	0.659592	-5.13886	0.83759	0.81264
T.cells	MAN2A2	-0.05318	4.595669	-0.44187	0.659661	-6.41814	0.765414	0.727102
T.cells	CD6	-0.23694	0.929158	-0.44168	0.659796	-5.21022	0.831312	0.805176
T.cells	MMRN2	-0.18776	1.228883	-0.44166	0.659811	-5.31674	0.825734	0.798499
T.cells	LNCPPARA	0.176308	0.60974	0.441635	0.659832	-5.42103	0.837295	0.812351
T.cells	EMD	0.031625	6.102062	0.441559	0.659886	-6.75818	0.73978	0.697247
T.cells	PIK3C2B	-0.07776	2.667746	-0.44156	0.659887	-6.13826	0.799434	0.767187
T.cells	TMEM147	0.045037	4.948794	0.441481	0.659943	-6.4863	0.759331	0.719998

T.cells	DCAF13	0.040081	4.771338	0.4414	0.660001	-6.51004	0.762382	0.72357
T.cells	SLC25A51	-0.04025	5.697758	-0.44126	0.660102	-6.70228	0.746579	0.705172
T.cells	BAG5	-0.06887	3.637068	-0.44117	0.660164	-6.20873	0.782157	0.74684
T.cells	GM4631	0.151827	0.664941	0.441092	0.660223	-5.41524	0.836258	0.811185
T.cells	GM16153	-0.08094	2.536009	-0.44094	0.660335	-6.04625	0.80181	0.770127
T.cells	GM14296	0.134761	0.969349	0.440839	0.660406	-5.43837	0.830562	0.804444
T.cells	TIMM44	0.037485	4.84696	0.440768	0.660457	-6.54049	0.76108	0.722197
T.cells	GM31243	-0.18335	2.779029	-0.44075	0.660472	-5.60383	0.797433	0.764979
T.cells	UCHL1	0.101942	0.862756	0.440686	0.660516	-5.92508	0.832553	0.806851
T.cells	EFL1	-0.04248	4.5955	-0.44065	0.66054	-6.46015	0.765417	0.727291
T.cells	IDH3G	0.044376	5.124044	0.439994	0.661015	-6.62187	0.756758	0.716752
T.cells	USP12	0.045525	6.069178	0.439943	0.661052	-6.6243	0.740751	0.698138
T.cells	A630072M	0.077701	3.151561	0.439827	0.661136	-6.0664	0.791226	0.757203
T.cells	RAB14	-0.02475	7.091867	-0.43936	0.661471	-6.86668	0.723799	0.678613
T.cells	AKIRIN1	0.028639	6.287656	0.439346	0.661483	-6.75142	0.737105	0.693978
T.cells	GM11476	0.156596	2.536389	0.43927	0.661537	-5.45498	0.802268	0.77038
T.cells	PSPC1	0.040534	5.702286	0.439094	0.661665	-6.69073	0.746936	0.705478
T.cells	POGLUT2	-0.14276	1.00166	-0.43899	0.661737	-5.55143	0.830441	0.804066
T.cells	FLNA	-0.06355	6.097597	-0.43875	0.661914	-6.48452	0.740284	0.697838
T.cells	ZFAND3	-0.02946	8.233036	-0.43867	0.661972	-7.04089	0.705306	0.65759
T.cells	CAND2	0.120215	0.775357	0.438427	0.662146	-5.63847	0.834672	0.809316
T.cells	CTPS	-0.05342	3.704287	-0.43839	0.66217	-6.37639	0.781425	0.746023
T.cells	RHOC	-0.11652	4.059106	-0.43837	0.662188	-5.76159	0.775193	0.7387
T.cells	CYB5D1	-0.18282	0.775244	-0.43835	0.6622	-5.40112	0.834674	0.809336
T.cells	STT3B	-0.0284	6.666062	-0.43835	0.662203	-6.79799	0.730815	0.686988
T.cells	STK26	0.051635	4.048898	0.437987	0.662464	-6.40745	0.775372	0.739023
T.cells	CSE1L	-0.0367	5.5229	-0.43799	0.662464	-6.6926	0.749973	0.709312
T.cells	MRAS	0.254445	0.476226	0.437944	0.662495	-5.16017	0.840294	0.81621
T.cells	NFIL3	-0.161	4.515288	-0.43789	0.662537	-5.71828	0.76725	0.729491
T.cells	EIF4E2	-0.03395	5.989442	-0.43773	0.66265	-6.72813	0.742098	0.700179
T.cells	SMIM26	-0.05848	3.847983	-0.43769	0.662677	-6.18806	0.778896	0.743195
T.cells	GALNT18	-0.2409	2.025829	-0.43767	0.662696	-5.41207	0.811541	0.781844
T.cells	GK	0.063288	4.764608	0.437615	0.662733	-6.41219	0.76294	0.72449
T.cells	4930477G(	0.263729	-0.38448	0.437561	0.662772	-5.1768	0.856667	0.836026
T.cells	DHX35	0.093856	2.187953	0.437422	0.662872	-5.82261	0.808585	0.778411
T.cells	E230029C(	0.112518	3.508508	0.437418	0.662875	-5.87753	0.784883	0.750334
T.cells	SNRNP200	0.043323	5.038857	0.437407	0.662883	-6.57751	0.758226	0.719043
T.cells	KIF5A	-0.23262	0.429339	-0.43736	0.662919	-5.23289	0.841179	0.817395
T.cells	NXF7	0.22826	-1.34285	0.437355	0.66292	-5.09214	0.87524	0.858576
T.cells	CAPZA1	-0.02275	7.715586	-0.43666	0.66342	-6.98114	0.713973	0.667649
T.cells	BCL2A1D	0.317746	4.122114	0.436438	0.663583	-5.29011	0.774458	0.737942
T.cells	RLF	0.036131	6.699162	0.436292	0.663688	-6.77082	0.730613	0.686904
T.cells	EDC4	-0.07391	2.984777	-0.43605	0.663862	-6.10487	0.794581	0.761818
T.cells	NADK	-0.04645	6.266885	-0.43599	0.663909	-6.60473	0.737801	0.695303
T.cells	4930481A1	0.112397	1.854963	0.435946	0.663938	-5.69214	0.815051	0.786135
T.cells	MRPL3	-0.047	4.701467	-0.4358	0.664043	-6.50271	0.764391	0.726335
T.cells	DXO	-0.07022	3.302702	-0.43553	0.664241	-6.16608	0.788907	0.755179

T.cells	COX4I1	-0.02573	9.086822	-0.43552	0.664249	-7.20531	0.692093	0.64288
T.cells	DEPDC1B	0.079264	2.954227	0.43532	0.664391	-6.31111	0.795128	0.762625
T.cells	SFXN3	0.103489	3.370072	0.435228	0.664458	-5.74411	0.78771	0.75388
T.cells	H2AFV	-0.03408	8.095101	-0.4352	0.664476	-7.24669	0.707852	0.660968
T.cells	AKAP1	-0.10031	1.799595	-0.43506	0.664577	-5.76793	0.816067	0.78757
T.cells	IKBKE	0.177976	3.116112	0.434998	0.664624	-5.70445	0.792233	0.759254
T.cells	SCRG1	0.231314	-0.98095	0.434966	0.664647	-5.14271	0.868594	0.850774
T.cells	RIOX1	-0.05415	3.521185	-0.43491	0.664686	-6.20769	0.78503	0.750745
T.cells	RNF187	0.034288	6.12757	0.434908	0.664689	-6.77048	0.740132	0.698203
T.cells	MIER3	0.049682	4.078409	0.434859	0.664724	-6.37095	0.775222	0.739206
T.cells	TMX3	0.038129	5.505888	0.434536	0.664958	-6.56423	0.750617	0.710449
T.cells	ILVBL	0.051434	3.757211	0.434496	0.664987	-6.33351	0.780862	0.745888
T.cells	MBTD1	-0.03626	7.420536	-0.43449	0.664988	-6.9216	0.718766	0.673572
T.cells	CREM	-0.08063	6.0952	-0.43449	0.664988	-6.38853	0.740674	0.698888
T.cells	DTX4	-0.19194	2.365512	-0.43445	0.665022	-5.39407	0.805741	0.775351
T.cells	PLEKHM2	-0.07358	5.202185	-0.43429	0.665134	-6.30835	0.755789	0.716542
T.cells	EFR3A	0.034812	5.780301	0.434164	0.665227	-6.63011	0.745971	0.705123
T.cells	COPB2	0.032285	5.438302	0.434051	0.665309	-6.62908	0.751765	0.711869
T.cells	USP7	0.028659	6.215443	0.434021	0.66533	-6.76213	0.738661	0.696632
T.cells	BC002059	0.079777	2.758564	0.433772	0.665511	-5.9378	0.798641	0.767065
T.cells	EIF4G3	-0.02774	7.91677	-0.43371	0.665553	-7.01006	0.710722	0.664471
T.cells	D330050I1	0.138373	1.118758	0.43371	0.665555	-5.52253	0.828652	0.802845
T.cells	GTF2H4	0.070393	2.307317	0.433675	0.665581	-5.95241	0.806798	0.776753
T.cells	FAM102A	0.112937	4.182226	0.433623	0.665618	-5.72658	0.773408	0.737273
T.cells	CASR	0.250866	-0.61039	0.433252	0.665887	-5.14472	0.86142	0.842433
T.cells	SMTNL2	0.136035	0.4226	0.433208	0.665919	-5.69603	0.841704	0.818629
T.cells	DGKI	-0.38551	0.78	-0.4332	0.665927	-5.17392	0.83498	0.810544
T.cells	AP2A1	0.050368	4.542647	0.432897	0.666144	-6.43182	0.767138	0.730094
T.cells	NUP205	-0.04379	4.408914	-0.43281	0.666205	-6.50207	0.769459	0.732822
T.cells	ZFP27	-0.1231	1.016419	-0.4328	0.666211	-5.60336	0.830559	0.805338
T.cells	BNIP2	0.030594	6.377564	0.432698	0.666288	-6.77516	0.735954	0.693747
T.cells	ZFP706	-0.02228	7.871583	-0.43264	0.666327	-7.14588	0.711451	0.665486
T.cells	TRAPPC9	0.038736	6.178368	0.432551	0.666394	-6.67198	0.739281	0.697636
T.cells	CD48	-0.03863	6.795574	-0.43243	0.66648	-6.90947	0.729019	0.685784
T.cells	ZFP1	-0.0538	3.874226	-0.43235	0.666538	-6.30009	0.778803	0.743887
T.cells	MYEF2	0.036314	5.328236	0.432329	0.666555	-6.76924	0.753638	0.714379
T.cells	GM15956	-0.18603	0.27605	-0.43217	0.666671	-5.29818	0.844476	0.822155
T.cells	CEP162	0.058353	3.080056	0.432157	0.66668	-6.1607	0.792877	0.760511
T.cells	ARL8A	0.039112	6.227073	0.432122	0.666704	-6.65069	0.738466	0.696725
T.cells	LEKR1	-0.15316	1.047991	-0.43212	0.666705	-5.59013	0.82997	0.804717
T.cells	COQ3	0.050977	3.500438	0.432103	0.666718	-6.14171	0.785398	0.751667
T.cells	DNAJC10	-0.04197	4.533218	-0.43207	0.666741	-6.44408	0.767302	0.73038
T.cells	POU2AF1	0.053591	4.320007	0.431917	0.666853	-6.69326	0.771005	0.734764
T.cells	ASPM	0.079074	3.870548	0.431899	0.666866	-6.52053	0.778867	0.744014
T.cells	MSANTD4	-0.07505	2.89703	-0.43182	0.666927	-5.87587	0.796154	0.764445
T.cells	SCIMP	0.147602	3.702449	0.431776	0.666955	-5.69592	0.781827	0.747526
T.cells	PDZD8	0.040071	5.970732	0.431487	0.667165	-6.71531	0.742854	0.701888

T.cells	GM36756	0.119119	0.926199	0.431474	0.667174	-5.65939	0.832344	0.807643
T.cells	PER1	0.081133	4.926726	0.431375	0.667246	-6.045	0.760606	0.722622
T.cells	SAP30BP	0.038408	4.838744	0.431176	0.66739	-6.54352	0.762205	0.724439
T.cells	DOCK11	-0.03532	7.111908	-0.43096	0.667545	-6.88134	0.724079	0.680039
T.cells	KIF3A	0.072525	2.472633	0.430733	0.667711	-5.99205	0.804213	0.773774
T.cells	CALCA	0.294761	-0.96013	0.430549	0.667844	-5.13968	0.868719	0.851171
T.cells	FAM71A	-0.24661	0.237403	-0.43022	0.668079	-5.16274	0.845746	0.823394
T.cells	GMEB1	-0.04049	5.025748	-0.43017	0.668118	-6.59547	0.759293	0.720751
T.cells	SLC37A2	0.094585	3.315046	0.430156	0.668129	-5.93812	0.789189	0.7559
T.cells	SLC25A42	-0.13803	1.940911	-0.43013	0.668146	-5.59067	0.813995	0.785357
T.cells	F830016B0	0.289177	0.444299	0.430044	0.66821	-5.19309	0.841829	0.818707
T.cells	TATDN3	-0.08946	2.490952	-0.42995	0.668276	-5.84836	0.80398	0.773468
T.cells	OAZ1	0.03073	9.534615	0.429247	0.668788	-7.28876	0.685928	0.635899
T.cells	9330136K2	-0.13109	1.883233	-0.42921	0.668814	-5.61277	0.815532	0.786799
T.cells	MTUS2	-0.22141	0.654472	-0.42892	0.669027	-5.32272	0.838362	0.814194
T.cells	RAP2C	-0.04531	4.944479	-0.42889	0.66905	-6.41263	0.761137	0.722615
T.cells	KAT8	-0.05369	3.624929	-0.42875	0.669146	-6.26911	0.784156	0.74965
T.cells	LDLRAP1	0.06372	4.099537	0.428706	0.66918	-6.25206	0.775803	0.739813
T.cells	EML2	-0.11331	2.655573	-0.42866	0.669214	-5.7155	0.801477	0.770144
T.cells	USP45	-0.06582	4.074097	-0.42865	0.669222	-6.13566	0.776248	0.740337
T.cells	SNRK	-0.05862	4.865356	-0.42813	0.6696	-6.26136	0.762786	0.724389
T.cells	OCRL	-0.11716	2.602911	-0.42798	0.669707	-5.73058	0.802731	0.771471
T.cells	TEFM	0.150464	1.160891	0.427898	0.669766	-5.52543	0.829195	0.803062
T.cells	NOL9	-0.05259	3.728213	-0.4279	0.669766	-6.30226	0.782625	0.747723
T.cells	ABCB1A	-0.2709	2.010725	-0.4277	0.669909	-5.26271	0.813503	0.784323
T.cells	CPTP	-0.08219	2.248177	-0.42755	0.670017	-5.8221	0.809167	0.779161
T.cells	TECR	-0.0351	6.763011	-0.42749	0.670061	-6.82076	0.730727	0.687154
T.cells	TNKS1BP1	0.10807	2.080541	0.427474	0.670073	-5.71718	0.812226	0.782802
T.cells	RBM5	-0.02854	6.623423	-0.42744	0.670095	-6.80424	0.733041	0.689829
T.cells	GM525	0.110956	0.742971	0.427348	0.670165	-5.60455	0.837014	0.812481
T.cells	NMT1	-0.02777	6.941125	-0.42707	0.670364	-6.87719	0.727855	0.683844
T.cells	KCNK6	0.135782	2.306704	0.427069	0.670368	-5.49342	0.808181	0.777996
T.cells	STK4	-0.02832	7.52595	-0.42685	0.670523	-6.99865	0.71828	0.672845
T.cells	BMX	0.270638	0.02253	0.426731	0.670612	-5.1706	0.850748	0.829034
T.cells	JUN	-0.088	7.097589	-0.42661	0.670697	-6.88107	0.72529	0.680932
T.cells	STRIP2	-0.18964	1.016356	-0.42642	0.670842	-5.38487	0.831985	0.806499
T.cells	USP39	0.047043	4.614508	0.42631	0.670918	-6.52266	0.767208	0.729695
T.cells	ZFP764	0.128973	1.074799	0.426306	0.670921	-5.55241	0.830894	0.805197
T.cells	TBC1D9	-0.12737	3.953614	-0.4262	0.671001	-5.87577	0.778743	0.743243
T.cells	ARL11	0.134166	1.439587	0.42618	0.671012	-5.49215	0.824111	0.797077
T.cells	PPP2R2A	-0.02447	7.180276	-0.42616	0.671029	-6.91589	0.723932	0.67939
T.cells	YBX3	-0.04452	6.314911	-0.4261	0.671068	-6.81028	0.738264	0.695979
T.cells	GM8251	-0.07361	3.392286	-0.42586	0.671245	-6.28637	0.788727	0.755073
T.cells	GRSF1	-0.03189	5.185543	-0.42576	0.671317	-6.60792	0.757429	0.71833
T.cells	ORC3	0.036987	4.958044	0.425748	0.671326	-6.57915	0.761334	0.722894
T.cells	ASXL1	-0.03206	7.242343	-0.42556	0.67146	-6.9193	0.722986	0.678353
T.cells	5930430L0	0.131443	0.229599	0.425527	0.671486	-5.62329	0.846891	0.82449

T.cells	AURKB	-0.06421	4.237652	-0.42541	0.671569	-6.60483	0.773843	0.737589
T.cells	TCF3	-0.03059	6.601305	-0.42535	0.671618	-7.00127	0.733564	0.690616
T.cells	JAK1	-0.02944	8.273816	-0.4252	0.671722	-6.97332	0.706289	0.659231
T.cells	DSTN	-0.04267	6.886463	-0.42488	0.67196	-6.76841	0.728863	0.685259
T.cells	POLR3D	0.068267	3.27535	0.424668	0.672111	-6.10486	0.790852	0.757725
T.cells	1810062O1	0.088361	2.0989	0.424625	0.672142	-5.91161	0.812088	0.782938
T.cells	SIKE1	0.044386	4.570739	0.424616	0.672148	-6.38828	0.768068	0.730899
T.cells	CWC27	0.035336	5.662216	0.424571	0.672181	-6.69289	0.749349	0.709038
T.cells	PLCD3	0.118982	1.122441	0.424351	0.672341	-5.62764	0.830113	0.80458
T.cells	IL10RB	0.055908	5.555085	0.424139	0.672495	-6.27239	0.751167	0.711308
T.cells	CBX4	-0.04241	5.468948	-0.42407	0.672544	-6.64807	0.752632	0.71305
T.cells	1110004F1	-0.02304	6.204	-0.42399	0.672604	-6.8128	0.740217	0.698634
T.cells	PPP3CB	0.028238	5.980209	0.423909	0.672662	-6.77608	0.743976	0.703042
T.cells	TMEM98	-0.12411	0.620631	-0.42376	0.672772	-5.6629	0.83952	0.816165
T.cells	CPOX	0.049694	4.570271	0.423663	0.672841	-6.41795	0.768076	0.731289
T.cells	4930513N1	-0.14443	0.475363	-0.42337	0.673056	-5.49462	0.842261	0.819518
T.cells	ZFP652OS	-0.2138	-0.171	-0.42328	0.673116	-5.19461	0.854558	0.834353
T.cells	CHRM3	-0.27915	0.831294	-0.42321	0.673171	-5.1911	0.835559	0.811457
T.cells	E330020D1	-0.05863	5.11024	-0.42298	0.673339	-6.61143	0.758761	0.720453
T.cells	UACA	0.091529	2.51718	0.422968	0.673346	-5.99987	0.804478	0.774366
T.cells	PSMB6	0.033464	6.304312	0.422967	0.673346	-6.82645	0.738538	0.696896
T.cells	CCDC90B	-0.06529	3.165156	-0.42285	0.673429	-6.08722	0.792819	0.760537
T.cells	TRAF3IP2	0.058182	3.229202	0.422843	0.673437	-6.22673	0.791675	0.759183
T.cells	SLC7A6OS	0.042184	4.789888	0.422823	0.673451	-6.43875	0.764274	0.726912
T.cells	RBSN	0.06687	2.910659	0.422598	0.673615	-5.96975	0.797379	0.765984
T.cells	COPS9	0.033464	6.68605	0.422552	0.673648	-6.81276	0.73218	0.689579
T.cells	JARID2	0.03544	8.144583	0.42251	0.673678	-7.01236	0.708364	0.662157
T.cells	PIFO	-0.18923	1.246923	-0.42247	0.67371	-5.41519	0.827795	0.802274
T.cells	PBLD1	-0.16773	1.75083	-0.42247	0.673711	-5.5354	0.818471	0.791117
T.cells	TAF6	-0.05078	3.851886	-0.42231	0.673826	-6.4057	0.780635	0.74627
T.cells	UQCRB	-0.03279	8.047525	-0.42209	0.673982	-7.05267	0.709926	0.664081
T.cells	ELP6	-0.07322	2.398694	-0.42203	0.674028	-6.06884	0.806627	0.777161
T.cells	DTWD1	0.088099	1.928598	0.421976	0.674067	-5.82617	0.815205	0.787387
T.cells	PAFAH2	0.213718	0.619176	0.421806	0.674191	-5.27198	0.839547	0.816564
T.cells	ZFP54	0.133328	0.794625	0.421752	0.67423	-5.59693	0.836247	0.812595
T.cells	NCOA5	-0.04886	4.146435	-0.42171	0.674259	-6.39643	0.775462	0.740272
T.cells	NDUFB1-PS	0.023092	8.902498	0.42167	0.67429	-7.23879	0.69628	0.648479
T.cells	NUP160	-0.0414	5.017011	-0.42153	0.674391	-6.67578	0.760362	0.722591
T.cells	SIGLECE	0.297981	2.293576	0.421303	0.674557	-5.25393	0.808538	0.779528
T.cells	SCOC	0.06279	3.752146	0.421221	0.674616	-6.18023	0.782393	0.748528
T.cells	AKNA	-0.04259	4.948701	-0.42115	0.67467	-6.45396	0.761536	0.724008
T.cells	APOL7C	0.311674	-0.99321	0.421137	0.674677	-5.11929	0.870437	0.854022
T.cells	SERP1	0.02876	7.981742	0.421135	0.674678	-7.03948	0.710986	0.665386
T.cells	CDC14A	-0.04724	5.803629	-0.42112	0.674692	-6.71927	0.746955	0.706979
T.cells	TGTP2	0.270886	0.773598	0.421115	0.674693	-5.31284	0.836642	0.813164
T.cells	TRAPPC11	0.058429	3.489165	0.421046	0.674743	-6.09216	0.787048	0.754027
T.cells	SESN3	-0.04431	4.83647	-0.42084	0.674896	-6.55637	0.76347	0.7263

T.cells	CD1D1	-0.06813	3.932282	-0.42073	0.67497	-6.34289	0.77922	0.744812
T.cells	NUDT21	-0.02999	6.148811	-0.4207	0.674993	-6.86109	0.741142	0.700243
T.cells	GM47283	-0.07161	7.990664	-0.42065	0.675032	-7.14637	0.710842	0.665245
T.cells	ZFP518B	-0.12594	0.418092	-0.42064	0.675038	-5.46497	0.843344	0.821262
T.cells	UPF3A	0.035896	4.634592	0.420568	0.675091	-6.48186	0.766961	0.730393
T.cells	RABL3	0.083359	2.218563	0.420394	0.675217	-5.88422	0.809972	0.781192
T.cells	LATS1	-0.03997	4.725566	-0.42023	0.675339	-6.44693	0.76551	0.728566
T.cells	GM12248	-0.10121	2.619411	-0.41998	0.67552	-5.81747	0.802891	0.772584
T.cells	PLSCR2	0.2929	-0.38835	0.419623	0.675779	-5.12819	0.859189	0.839963
T.cells	GM30054	0.114782	1.953461	0.419533	0.675845	-5.78362	0.815186	0.787092
T.cells	5830428M	-0.09113	2.548294	-0.4195	0.675867	-5.87187	0.804345	0.774179
T.cells	NCL	-0.03575	7.783001	-0.41936	0.675968	-7.09198	0.714615	0.669212
T.cells	REXO1	-0.03935	4.912691	-0.41918	0.676104	-6.55064	0.762674	0.724936
T.cells	AGO1	0.053771	3.874896	0.419002	0.676231	-6.32179	0.780759	0.74622
T.cells	TMEM170	0.119623	1.517214	0.418989	0.67624	-5.55812	0.82334	0.796797
T.cells	DONSON	-0.04627	4.025423	-0.41876	0.676407	-6.38947	0.778218	0.74313
T.cells	LAMP2	0.036276	7.069003	0.418671	0.676472	-6.71903	0.726447	0.682775
T.cells	GPR160	-0.20475	2.407708	-0.41854	0.676566	-5.33078	0.807151	0.777387
T.cells	FASTKD1	-0.08449	2.189988	-0.41822	0.676801	-5.87533	0.811316	0.782108
T.cells	CASTOR1	-0.21892	0.626653	-0.41804	0.67693	-5.20893	0.840403	0.816861
T.cells	MYLK	0.129115	1.606214	0.417925	0.677015	-5.64005	0.822119	0.794936
T.cells	EXOSC2	0.055483	2.945711	0.417845	0.677074	-6.19297	0.797703	0.76587
T.cells	TNFAIP1	0.074261	3.775763	0.417694	0.677184	-6.07308	0.782959	0.748422
T.cells	RCHY1	-0.0338	5.661194	-0.41759	0.677256	-6.65563	0.750311	0.710176
T.cells	GM15738	0.156079	0.402397	0.417406	0.677393	-5.40165	0.844789	0.822143
T.cells	METTL7A1	0.101517	2.456994	0.417247	0.677509	-5.66122	0.806721	0.776533
T.cells	ZYX	0.080529	6.244314	0.417054	0.67765	-6.22997	0.740678	0.698873
T.cells	GM31645	0.171643	0.726126	0.416823	0.677818	-5.41475	0.838944	0.814955
T.cells	SERPINA10	-0.17339	0.966902	-0.41653	0.678033	-5.3615	0.834518	0.809639
T.cells	CNOT8	-0.03167	5.284943	-0.41649	0.678064	-6.60088	0.75713	0.717997
T.cells	ANGPTL2	0.173526	0.84131	0.416339	0.678171	-5.46368	0.836874	0.812471
T.cells	BRAF	0.044347	7.588384	0.416248	0.678237	-6.86058	0.718649	0.673421
T.cells	CANT1	-0.05963	3.891787	-0.41624	0.678243	-6.1384	0.781336	0.746412
T.cells	CPNE8	-0.17597	2.649239	-0.41603	0.678398	-5.47233	0.803634	0.772711
T.cells	CCL27A	0.108596	1.684593	0.415384	0.678867	-5.65941	0.821669	0.793949
T.cells	USP49	-0.05369	4.534917	-0.41527	0.67895	-6.48098	0.770544	0.733438
T.cells	RAD51D	0.07691	2.984907	0.415014	0.679137	-5.89702	0.797967	0.765783
T.cells	CCDC88A	-0.04303	5.372063	-0.41497	0.679166	-6.64074	0.756103	0.716555
T.cells	AIMP2	-0.06193	3.206699	-0.41493	0.679201	-6.21493	0.793988	0.761072
T.cells	CYP2C29	-0.21772	1.011219	-0.41485	0.679254	-5.3472	0.834198	0.809004
T.cells	SDR39U1	-0.12165	1.131364	-0.41481	0.679289	-5.65439	0.83195	0.806312
T.cells	GANC	-0.07396	3.377098	-0.41479	0.679297	-6.05334	0.790944	0.757499
T.cells	SPRY2	-0.05806	5.158508	-0.41461	0.679434	-6.58933	0.759762	0.720887
T.cells	CHFR	0.032696	5.641975	0.41427	0.67968	-6.65618	0.751501	0.711275
T.cells	ZBTB25	0.056223	3.709262	0.414236	0.679704	-6.3126	0.78504	0.750591
T.cells	FAM98A	0.051651	3.607656	0.41421	0.679724	-6.23918	0.786842	0.752716
T.cells	SDF4	-0.02823	6.506152	-0.41413	0.679781	-6.76951	0.736944	0.694366

T.cells	DDX11	-0.07241	2.934033	-0.41385	0.679985	-6.10019	0.798882	0.767044
T.cells	FAN1	-0.15223	1.159783	-0.41384	0.679995	-5.45827	0.831419	0.805833
T.cells	TPRN	0.099713	2.21301	0.41382	0.680008	-5.7953	0.81196	0.782589
T.cells	PRKCE	0.03646	7.233184	0.413772	0.680043	-7.00555	0.724903	0.680552
T.cells	2610035D1	-0.09203	4.872984	-0.41372	0.680081	-6.176	0.764681	0.726779
T.cells	RFX7	0.035164	7.368214	0.413611	0.680161	-6.96259	0.722688	0.67801
T.cells	UBAP2	0.030293	6.131148	0.413532	0.680218	-6.79891	0.743228	0.701767
T.cells	MEF2C	0.038283	7.538963	0.413428	0.680294	-7.0658	0.719895	0.6748
T.cells	TMCO4	0.054129	4.054604	0.413392	0.680321	-6.36928	0.778947	0.743536
T.cells	OLFR164	-0.2212	-0.16043	-0.41339	0.680322	-5.17932	0.856417	0.835967
T.cells	2810454H(	-0.0804	2.514759	-0.4132	0.680457	-5.93801	0.806463	0.776138
T.cells	TSC22D1	-0.05589	4.872446	-0.4132	0.680458	-6.3983	0.764691	0.726849
T.cells	TMEM154	-0.12466	2.583653	-0.41308	0.680545	-5.65678	0.805213	0.77467
T.cells	KCNJ2	-0.16378	-0.01521	-0.41301	0.680596	-5.24953	0.853634	0.832685
T.cells	CARHSP1	-0.04111	5.430457	-0.41276	0.680781	-6.68742	0.755128	0.715678
T.cells	SKA3	-0.06362	2.738324	-0.4126	0.680898	-6.19211	0.802438	0.771378
T.cells	MYO7A	0.066127	3.25231	0.412448	0.681009	-6.07793	0.793197	0.760456
T.cells	A330032B1	-0.23086	-0.15579	-0.41234	0.681086	-5.25105	0.856355	0.836004
T.cells	DOCK6	-0.1296	1.914885	-0.4121	0.681264	-5.5653	0.81745	0.789277
T.cells	CEP83OS	0.095318	2.022979	0.412068	0.681287	-5.82589	0.815465	0.786909
T.cells	CEP112	0.152175	1.570821	0.411997	0.681339	-5.54278	0.8238	0.796862
T.cells	GM45669	-0.23882	0.129811	-0.41188	0.681423	-5.19577	0.850889	0.829404
T.cells	TTYH3	0.043635	5.141527	0.411828	0.681462	-6.56945	0.760078	0.721501
T.cells	AP2A2	-0.03613	5.931782	-0.41179	0.681487	-6.60498	0.746612	0.7058
T.cells	NFAT5	0.041467	7.501438	0.411782	0.681496	-6.87864	0.720531	0.675623
T.cells	TACO1	-0.07098	3.738306	-0.41177	0.681506	-6.31625	0.784551	0.75024
T.cells	GM15964	0.257543	0.162356	0.411753	0.681517	-5.21402	0.850269	0.828655
T.cells	CARD11	-0.08319	4.618562	-0.41161	0.681623	-6.2163	0.769155	0.732082
T.cells	POLQ	-0.05728	3.166551	-0.41133	0.681827	-6.30893	0.794932	0.762272
T.cells	RAB5A	0.026276	6.913368	0.411094	0.681999	-6.88719	0.730417	0.686791
T.cells	AGPAT1	0.068655	3.479015	0.411036	0.682041	-6.08155	0.789388	0.75573
T.cells	SUFU	-0.03601	5.025085	-0.411	0.682066	-6.60665	0.762308	0.723906
T.cells	YY1	-0.02216	7.144963	-0.4109	0.682138	-6.922	0.726596	0.682426
T.cells	AI504432	0.052072	2.840905	0.410708	0.68228	-6.30732	0.800912	0.769316
T.cells	MIS18BP1	0.057135	3.964435	0.410295	0.682582	-6.54691	0.780893	0.745754
T.cells	AW209491	0.147696	1.448036	0.410013	0.682788	-5.54825	0.826431	0.799873
T.cells	GM37065	0.07192	2.706239	0.40993	0.682849	-6.2953	0.803362	0.772401
T.cells	RNASEH2B	-0.04119	4.594626	-0.40989	0.682876	-6.59786	0.76986	0.732869
T.cells	HEXB	0.040924	5.741415	0.409536	0.683137	-6.61128	0.750157	0.709934
T.cells	FBXO8	0.039702	4.606605	0.409511	0.683155	-6.43984	0.769652	0.732721
T.cells	SETD1B	-0.04714	5.026478	-0.40951	0.683159	-6.4008	0.762384	0.724206
T.cells	SNAPC1	-0.06047	3.850567	-0.40944	0.683204	-6.13695	0.782902	0.748313
T.cells	GRIK5	-0.19496	0.167717	-0.40939	0.683241	-5.33655	0.850531	0.82899
T.cells	PARPBP	0.095099	2.056928	0.409273	0.683329	-6.09942	0.815192	0.786628
T.cells	CYBB	-0.12376	7.155496	-0.40925	0.683345	-6.31521	0.726515	0.682561
T.cells	TMEM29	-0.0414	4.706351	-0.40918	0.683397	-6.48487	0.767919	0.730738
T.cells	CEP57	-0.03554	5.139583	-0.40906	0.683486	-6.64211	0.760437	0.721978

T.cells	MOB3A	-0.05289	4.775701	-0.40905	0.683496	-6.30525	0.766717	0.729329
T.cells	CCDC69	-0.0528	3.297575	-0.409	0.683531	-6.33031	0.792728	0.75996
T.cells	CD4	-0.25374	1.246276	-0.40894	0.683573	-5.18432	0.830187	0.80457
T.cells	POLR3A	0.064264	3.30507	0.408772	0.683695	-6.17626	0.792594	0.759815
T.cells	CCDC124	-0.02963	5.595782	-0.40876	0.683701	-6.71996	0.752633	0.712879
T.cells	WDR45B	-0.02712	6.330115	-0.40862	0.683809	-6.75968	0.740228	0.69847
T.cells	FBF1	0.167982	1.539498	0.408507	0.683889	-5.55268	0.824734	0.798096
T.cells	GM20457	-0.19765	-0.16272	-0.40848	0.683907	-5.2381	0.856855	0.836731
T.cells	ZBTB43	-0.06688	3.163113	-0.4084	0.683966	-6.00353	0.795135	0.762871
T.cells	GZMC	-0.47769	1.539474	-0.4084	0.683969	-5.23947	0.824734	0.798106
T.cells	TNFRSF13E	0.040004	4.518479	0.408387	0.683977	-6.56153	0.771185	0.73463
T.cells	DPM3	-0.03172	6.309203	-0.40829	0.684051	-6.76026	0.740578	0.698916
T.cells	DCBLD1	-0.07616	3.33755	-0.40821	0.684105	-6.15874	0.792014	0.759191
T.cells	ZFP362	0.047462	3.9339	0.408132	0.684164	-6.29516	0.781431	0.746695
T.cells	PRPF40B	0.203497	0.534618	0.40784	0.684377	-5.33181	0.843737	0.820802
T.cells	FDX2	0.043156	4.192147	0.40757	0.684574	-6.37566	0.777199	0.741491
T.cells	TIMM21	0.065037	2.602996	0.407402	0.684698	-6.06672	0.805616	0.775096
T.cells	ZFP932	-0.05682	2.788655	-0.40719	0.68485	-6.02079	0.802256	0.771173
T.cells	CCNB1	-0.0711	4.032241	-0.40711	0.684913	-6.59076	0.780071	0.744963
T.cells	PBX2	-0.04677	4.930622	-0.40704	0.684961	-6.48996	0.764403	0.726576
T.cells	MINK1	0.060803	4.171023	0.406878	0.685081	-6.12802	0.777631	0.742108
T.cells	SLC39A1	-0.03412	6.161792	-0.40672	0.685197	-6.70285	0.743409	0.702098
T.cells	1700028E1	-0.11191	1.36693	-0.40655	0.685318	-5.57024	0.828335	0.802347
T.cells	TMEM50B	-0.07332	4.107732	-0.40654	0.685327	-6.16867	0.778743	0.743453
T.cells	GM5165	-0.07094	2.638407	-0.4064	0.685427	-5.98801	0.804975	0.774489
T.cells	8430429K0	-0.09473	1.679457	-0.40635	0.685471	-5.6815	0.822536	0.795419
T.cells	SUGP2	0.055044	4.059588	0.4063	0.685504	-6.36328	0.77959	0.744466
T.cells	RNF150	0.179489	2.909798	0.40627	0.685526	-5.6273	0.800069	0.768671
T.cells	TM2D2	0.039845	5.178235	0.406265	0.68553	-6.47703	0.760136	0.721641
T.cells	GPKOW	0.036866	4.488998	0.405955	0.685756	-6.42176	0.772196	0.735697
T.cells	ALG5	0.05098	4.2129	0.405831	0.685848	-6.28879	0.777026	0.74142
T.cells	E4F1	0.070622	3.127007	0.405825	0.685851	-6.08458	0.796295	0.764168
T.cells	POC5	-0.06727	2.510705	-0.40569	0.685948	-6.01606	0.80746	0.777395
T.cells	ANKRD42	0.210791	0.346625	0.405399	0.686164	-5.3044	0.847746	0.825616
T.cells	PSMD14	0.025669	6.618882	0.405387	0.686172	-6.87486	0.735942	0.693425
T.cells	PIDD1	-0.10633	0.824038	-0.40523	0.686286	-5.71521	0.838711	0.814766
T.cells	TGFB1	0.025931	8.800362	0.404856	0.686561	-7.21504	0.700416	0.652607
T.cells	SLAMF6	-0.04761	4.602908	-0.40484	0.686573	-6.59825	0.77028	0.733491
T.cells	APOC2	0.131878	4.203003	0.40483	0.68658	-5.86413	0.777268	0.741702
T.cells	SNX17	0.033835	5.853791	0.40463	0.686727	-6.69873	0.7488	0.708403
T.cells	4930579G2	-0.05948	2.431609	-0.40456	0.686781	-6.14453	0.808938	0.779193
T.cells	HPS3	0.053412	4.626547	0.404543	0.686791	-6.3215	0.769869	0.733025
T.cells	A730036I1	0.263688	-0.20546	0.404525	0.686803	-5.15561	0.858305	0.838422
T.cells	GRPEL1	-0.03722	5.877727	-0.40444	0.68687	-6.74368	0.748395	0.707931
T.cells	TFE3	0.058744	4.149746	0.40437	0.686917	-6.11904	0.778203	0.742819
T.cells	SNX22	-0.22124	-0.37781	-0.40436	0.686928	-5.19344	0.861626	0.842441
T.cells	ANKRD26	0.076683	2.576265	0.404323	0.686951	-6.03766	0.806308	0.776081

T.cells	UBQLN1	-0.03089	5.517917	-0.40381	0.687329	-6.63446	0.754549	0.715198
T.cells	POLB	-0.0359	5.332268	-0.40341	0.687618	-6.60877	0.757724	0.71899
T.cells	I830077J02	-0.18211	2.758256	-0.40338	0.687643	-5.38365	0.803049	0.772389
T.cells	PCSK5	-0.21282	0.558557	-0.40308	0.687865	-5.27442	0.843766	0.821204
T.cells	DOK1	0.056352	3.614618	0.403062	0.687876	-6.16458	0.787696	0.754312
T.cells	INPP4A	-0.05342	6.032154	-0.40285	0.688028	-6.39163	0.745821	0.705263
T.cells	STAR	0.133882	1.465058	0.402799	0.688068	-5.63202	0.826761	0.800835
T.cells	TFPT	0.060503	2.965912	0.402742	0.68811	-6.0213	0.799301	0.7681
T.cells	FANCF	-0.10577	1.911025	-0.40265	0.68818	-5.77023	0.818512	0.790969
T.cells	TRMT2A	-0.0492	3.456488	-0.40243	0.688339	-6.26325	0.79051	0.757687
T.cells	KIF13B	0.034932	6.510852	0.402406	0.688356	-6.78092	0.737781	0.695927
T.cells	KIFAP3	0.058532	2.849059	0.402367	0.688385	-6.04905	0.801408	0.770601
T.cells	AP5B1	-0.14337	1.293299	-0.40233	0.688415	-5.4633	0.829959	0.804667
T.cells	ANAPC1	0.037905	4.721072	0.402201	0.688507	-6.53959	0.768265	0.731482
T.cells	AI662270	0.038616	6.090129	0.402199	0.688508	-6.77046	0.744843	0.704126
T.cells	IPP	0.065706	2.465742	0.402178	0.688524	-6.05086	0.808357	0.778862
T.cells	IFIT3B	0.242559	1.43036	0.402168	0.688531	-5.35701	0.827406	0.801608
T.cells	MGST3	0.062911	3.846236	0.402126	0.688562	-6.35285	0.783591	0.749513
T.cells	RAB28	0.045936	4.828065	0.402111	0.688573	-6.41362	0.766409	0.729306
T.cells	GSE1	0.034283	5.664856	0.401824	0.688783	-6.69723	0.752045	0.712514
T.cells	NCAPD2	-0.05174	4.81058	-0.4018	0.688798	-6.69729	0.766712	0.729663
T.cells	MRPL15	-0.03301	5.337746	-0.40172	0.688862	-6.67411	0.75763	0.719045
T.cells	ZRANB3	-0.05603	3.148069	-0.40161	0.688941	-6.2934	0.796026	0.764265
T.cells	ADCK1	0.082734	3.101425	0.401586	0.688958	-5.87995	0.796864	0.765258
T.cells	SFXN2	0.084467	3.18033	0.401549	0.688985	-5.76728	0.795448	0.76358
T.cells	CASP6	-0.05842	3.628567	-0.40154	0.688992	-6.1638	0.787448	0.754115
T.cells	SOGA1	-0.06364	3.807296	-0.40151	0.68901	-6.26291	0.78428	0.750373
T.cells	ATP6V0D1	-0.02558	7.228201	-0.40148	0.689034	-6.78869	0.725886	0.68221
T.cells	ACTL6A	-0.031	5.235041	-0.4014	0.689091	-6.70099	0.759392	0.721135
T.cells	BMS1	0.038514	4.737717	0.401288	0.689177	-6.564	0.767976	0.731198
T.cells	CEP44	0.042988	3.531339	0.401269	0.68919	-6.26324	0.789177	0.756167
T.cells	GM16576	-0.12012	1.513376	-0.40116	0.689272	-5.7054	0.825864	0.799864
T.cells	TWSG1	-0.06835	3.230079	-0.40116	0.689272	-6.11009	0.794556	0.762574
T.cells	METTL3	-0.05352	3.319562	-0.40083	0.689516	-6.16779	0.793068	0.760731
T.cells	EXOC6B	-0.06045	5.502152	-0.40068	0.689623	-6.27988	0.754926	0.71589
T.cells	ZRSR2	0.030974	5.063714	0.400569	0.689704	-6.541	0.762447	0.724682
T.cells	CDKN2AIP	-0.03778	4.567595	-0.40056	0.689707	-6.51071	0.771043	0.734761
T.cells	BCL9L	0.060393	3.410778	0.400551	0.689717	-6.13801	0.791438	0.758803
T.cells	TECPR1	0.063918	4.375591	0.40032	0.689886	-6.2461	0.774504	0.738728
T.cells	METTL22	-0.1136	1.382795	-0.40019	0.689979	-5.59557	0.828527	0.802919
T.cells	NDUFV1	-0.03637	5.20278	-0.40001	0.690113	-6.62478	0.760162	0.721991
T.cells	ZFP106	0.037071	6.288522	0.399754	0.690302	-6.69308	0.741716	0.700519
T.cells	JMJD8	-0.1222	1.564022	-0.39973	0.690321	-5.63641	0.825159	0.798945
T.cells	KCTD4	-0.08972	2.200261	-0.39965	0.690376	-5.9178	0.813434	0.78496
T.cells	LGR4	-0.09978	2.632615	-0.39963	0.69039	-5.91899	0.805554	0.775585
T.cells	ABRACL	0.03419	7.550325	0.399616	0.690403	-6.97385	0.720809	0.676369
T.cells	GM28198	-0.05512	3.85447	-0.39931	0.690626	-6.39557	0.783668	0.749774

T.cells	INO80DOS	-0.05834	4.442013	-0.39916	0.690741	-6.50555	0.773343	0.737655
T.cells	GNG3	-0.0999	1.449153	-0.3991	0.690782	-5.81006	0.827292	0.801718
T.cells	GLS	0.034878	7.7257	0.398986	0.690865	-6.91209	0.717948	0.673249
T.cells	CDC5L	0.02456	5.768771	0.398985	0.690867	-6.74788	0.750493	0.710944
T.cells	UBLCP1	-0.03882	4.773952	-0.39894	0.690897	-6.47756	0.767565	0.730909
T.cells	EMC3	0.031803	5.490353	0.398876	0.690947	-6.64093	0.755235	0.716476
T.cells	SRMS	0.247225	-0.72874	0.398815	0.690991	-5.10308	0.868714	0.851712
T.cells	SNX21	0.094062	2.718125	0.398734	0.691051	-5.82968	0.804004	0.773988
T.cells	EXD2	-0.05726	3.156926	-0.39872	0.691063	-6.19295	0.796094	0.764597
T.cells	LZTS3	-0.15159	0.323606	-0.39846	0.691253	-5.39866	0.848616	0.827347
T.cells	DHRS13	0.158036	0.959658	0.398279	0.691385	-5.50507	0.836662	0.812862
T.cells	MCUB	0.236584	1.557619	0.39811	0.691509	-5.24052	0.825503	0.799484
T.cells	GM7160	0.119097	2.542262	0.398083	0.691529	-5.71088	0.807415	0.777893
T.cells	BLMH	-0.02788	5.648784	-0.39793	0.691641	-6.74967	0.752785	0.713509
T.cells	MTLN	-0.07019	3.060484	-0.39773	0.691785	-5.92615	0.79818	0.766839
T.cells	ROBO1	0.171449	2.137687	0.397525	0.691938	-5.49221	0.815	0.786839
T.cells	1810041H1	-0.13411	1.76748	-0.39743	0.692008	-5.58439	0.821816	0.79498
T.cells	GPATCH3	-0.06071	3.369567	-0.39738	0.692043	-6.19399	0.792696	0.760333
T.cells	SNRPC	-0.02677	6.245324	-0.3972	0.692176	-6.83513	0.742887	0.701879
T.cells	PRSS30	0.187214	-0.33737	0.397028	0.692304	-5.14884	0.86165	0.842902
T.cells	SLAIN1	0.042462	3.6132	0.39702	0.69231	-6.42366	0.788419	0.755255
T.cells	MCM9	-0.03803	4.75152	-0.39647	0.692712	-6.58721	0.768528	0.731873
T.cells	AMY1	-0.09706	2.627056	-0.39646	0.692718	-5.85557	0.806256	0.776447
T.cells	1700066M	0.103027	1.288003	0.396349	0.692802	-5.61752	0.830913	0.805898
T.cells	NDUFV2	-0.03382	6.136177	-0.39632	0.692825	-6.78455	0.744834	0.704196
T.cells	TM9SF1	-0.03936	5.042685	-0.3963	0.69284	-6.55447	0.763487	0.725964
T.cells	MPND	0.041543	4.813143	0.396289	0.692847	-6.56372	0.767458	0.730618
T.cells	BAG4	-0.04237	4.226706	-0.39621	0.692902	-6.38726	0.777692	0.742643
T.cells	SWT1	0.036894	5.244266	0.396127	0.692966	-6.55053	0.760016	0.721901
T.cells	CLEC4D	0.336141	2.949351	0.395969	0.693082	-5.29884	0.800474	0.769532
T.cells	BRPF3	-0.03826	4.451036	-0.39588	0.693148	-6.40911	0.773811	0.738035
T.cells	MIIP	0.058997	3.057652	0.395765	0.693232	-6.11526	0.79854	0.767246
T.cells	BC005624	-0.02892	5.761918	-0.39563	0.693334	-6.67444	0.751268	0.711655
T.cells	AREG	0.422749	1.634087	0.395482	0.69344	-5.29162	0.824614	0.798277
T.cells	CELA1	-0.17922	2.24977	-0.39513	0.693701	-5.36988	0.813272	0.784828
T.cells	ATP11C	0.026672	6.869185	0.395074	0.69374	-6.88548	0.732695	0.690155
T.cells	BTK	0.032468	6.033608	0.394942	0.693837	-6.8416	0.746692	0.706418
T.cells	C2CD3	0.032402	4.657214	0.394821	0.693926	-6.56625	0.770298	0.734032
T.cells	TDRD3	-0.05201	4.059195	-0.39481	0.693933	-6.2454	0.78077	0.746354
T.cells	RAB10OS	0.047106	4.429445	0.394541	0.694132	-6.34811	0.774271	0.738763
T.cells	PCK1	-0.12783	4.182878	-0.39445	0.694197	-6.11648	0.778593	0.743851
T.cells	TMOD1	-0.12898	1.823905	-0.39442	0.694222	-5.63004	0.821102	0.794305
T.cells	RAMAC	-0.02953	5.913686	-0.39441	0.694225	-6.72476	0.748721	0.708859
T.cells	SMPD1	-0.07568	2.643295	-0.39402	0.694513	-5.96046	0.806098	0.776523
T.cells	PPP4R1	-0.03362	5.579566	-0.39394	0.694573	-6.72677	0.754403	0.715587
T.cells	ICOSL	0.204887	1.327034	0.393934	0.694578	-5.17655	0.830326	0.805468
T.cells	CASC3	0.036403	5.292913	0.393881	0.694617	-6.59706	0.759309	0.721339

T.cells	TMEM143	0.100082	1.491704	0.393842	0.694646	-5.65709	0.827258	0.801817
T.cells	EHHADH	-0.14137	1.66442	-0.3938	0.694676	-5.52744	0.824052	0.797977
T.cells	OIP5	-0.06831	2.207869	-0.39377	0.6947	-6.09256	0.814039	0.78601
T.cells	TRAPPC8	0.031149	6.225642	0.393763	0.694704	-6.74109	0.743453	0.702854
T.cells	HELQ	-0.06019	2.411841	-0.39363	0.694802	-6.0624	0.81031	0.781607
T.cells	SH3BP4	0.172861	1.369949	0.393549	0.694861	-5.35825	0.829525	0.804607
T.cells	CRAMP1L	0.034374	4.984741	0.393456	0.69493	-6.59646	0.764618	0.727643
T.cells	MAN2C1OS	0.041232	4.443794	0.393368	0.694994	-6.52057	0.77402	0.738684
T.cells	POLR1B	-0.07299	2.442843	-0.39333	0.695023	-6.00545	0.809745	0.78098
T.cells	SLCO5A1	-0.12632	-0.20706	-0.39321	0.695111	-5.39427	0.859409	0.840695
T.cells	ARVCF	0.099504	1.228411	0.393188	0.695127	-5.84854	0.832168	0.807848
T.cells	ENOPH1	0.059752	2.8863	0.393042	0.695234	-6.14255	0.801698	0.771499
T.cells	SELENOK	-0.03155	7.584969	-0.39297	0.695284	-6.87679	0.720903	0.676914
T.cells	FAM49B	-0.02289	9.086243	-0.39292	0.695322	-7.17322	0.696753	0.649218
T.cells	SLC16A4	-0.09601	1.428027	-0.39252	0.695617	-5.72246	0.828443	0.803491
T.cells	SLC25A36	-0.03379	6.285868	-0.39244	0.695678	-6.71702	0.74244	0.701899
T.cells	RAG1	0.087366	1.672599	0.392432	0.695683	-6.1224	0.823901	0.798048
T.cells	RNH1	-0.04265	5.988026	-0.39232	0.695762	-6.65955	0.747463	0.707744
T.cells	KIF2C	-0.07275	2.663513	-0.3923	0.695784	-6.22538	0.805731	0.776358
T.cells	FBXO47	-0.12093	1.069812	-0.39221	0.695844	-5.71263	0.835138	0.811529
T.cells	UQCRC1	-0.02902	6.508803	-0.39216	0.695885	-6.87731	0.738701	0.697556
T.cells	ACIN1	-0.02097	7.577859	-0.39195	0.696039	-7.03371	0.721019	0.67713
T.cells	FAM53A	-0.04722	3.797306	-0.39183	0.696127	-6.28863	0.785398	0.752312
T.cells	4930578M	0.150164	0.63262	0.391583	0.696309	-5.42739	0.843376	0.821573
T.cells	SUMF2	0.069072	2.684259	0.39151	0.696362	-6.00962	0.805355	0.776034
T.cells	MCOLN1	-0.07551	2.728161	-0.39116	0.69662	-5.82526	0.804559	0.775191
T.cells	CLDN11	-0.2422	-0.29485	-0.39112	0.69665	-5.1483	0.861101	0.843113
T.cells	GNAI3	0.02567	6.443605	0.391111	0.696656	-6.79714	0.739793	0.699029
T.cells	XPNPEP1	0.041918	4.365592	0.391086	0.696675	-6.37948	0.775388	0.740667
T.cells	CDC7	-0.05997	2.873973	-0.39101	0.696728	-6.2165	0.80192	0.772077
T.cells	BIK	-0.16725	1.76969	-0.39099	0.696742	-5.4936	0.822104	0.796155
T.cells	AKAP8	0.02892	5.39229	0.390924	0.696794	-6.67936	0.757605	0.719836
T.cells	GM45902	-0.09962	1.585805	-0.39087	0.696835	-5.68513	0.82551	0.800263
T.cells	ZFYVE26	-0.04064	5.03643	-0.39083	0.69686	-6.5378	0.763725	0.727006
T.cells	CFAP36	-0.03994	4.102137	-0.39071	0.696955	-6.41139	0.780014	0.74617
T.cells	DISP1	-0.05413	3.443949	-0.39061	0.697024	-6.20147	0.791684	0.759965
T.cells	FAM171A1	0.137615	1.565475	0.390591	0.697039	-5.53144	0.825887	0.800725
T.cells	ADPGK	-0.08532	4.903066	-0.39052	0.697094	-6.09138	0.76603	0.729718
T.cells	ERLIN1	-0.04346	4.765624	-0.39049	0.697111	-6.461	0.768413	0.732516
T.cells	4930403D(	0.142393	1.041064	0.390463	0.697134	-5.46388	0.835677	0.812479
T.cells	NPHS1	0.16638	0.439647	0.390405	0.697176	-5.38777	0.847036	0.826164
T.cells	TMEM221	-0.22429	-0.26558	-0.39016	0.697353	-5.12274	0.860598	0.842589
T.cells	ATPAF1	0.048639	3.553171	0.390149	0.697365	-6.27184	0.789792	0.757744
T.cells	PLS1	-0.1636	0.910051	-0.38986	0.697577	-5.37985	0.838352	0.815608
T.cells	PRKAR1B	0.207911	-0.37005	0.389788	0.69763	-5.26579	0.862771	0.845114
T.cells	AQP11	0.182038	0.397356	0.389641	0.697739	-5.30586	0.848102	0.827355
T.cells	MFGE8	-0.09754	2.292559	-0.38899	0.698219	-5.85859	0.813219	0.785161

T.cells	HSH2D	0.078451	3.210107	0.38869	0.69844	-5.97013	0.796756	0.765476
T.cells	SNHG12	-0.06032	3.189103	-0.38853	0.698561	-6.149	0.797192	0.765927
T.cells	GM32401	-0.10475	2.445198	-0.38838	0.698668	-5.75914	0.810709	0.781974
T.cells	GM43258	-0.13104	0.724157	-0.38824	0.698773	-5.5044	0.842734	0.820343
T.cells	ARID5A	0.043669	4.681741	0.388091	0.698882	-6.4999	0.77091	0.734878
T.cells	NAA40	-0.03174	4.909944	-0.38792	0.699006	-6.5955	0.767005	0.730318
T.cells	THOC5	-0.04711	3.564433	-0.38782	0.699083	-6.2566	0.790672	0.758208
T.cells	UNC5CL	0.183302	0.465408	0.38771	0.699163	-5.41168	0.847767	0.826454
T.cells	ARMC1	-0.03305	4.827556	-0.38763	0.699224	-6.5588	0.768445	0.732038
T.cells	FILIP1	-0.18085	0.904589	-0.38741	0.699387	-5.32402	0.839564	0.816439
T.cells	GM50218	0.117982	0.559001	0.386873	0.69978	-5.7152	0.846344	0.824386
T.cells	COLEC10	-0.17352	0.557157	-0.38669	0.699912	-5.29269	0.846379	0.824467
T.cells	ALG6	-0.08609	2.044219	-0.38665	0.699942	-5.89941	0.818564	0.791107
T.cells	MARK4	-0.04766	4.541737	-0.38648	0.700068	-6.37598	0.773748	0.738011
T.cells	PSMD8	0.029035	6.592353	0.386464	0.700081	-6.88812	0.738678	0.697058
T.cells	BRPF1	0.03828	4.929241	0.386358	0.70016	-6.58586	0.767003	0.730092
T.cells	CSTF3	-0.0296	6.037918	-0.3863	0.700204	-6.73612	0.74801	0.707901
T.cells	SLC7A8	0.190664	2.86439	0.386145	0.700317	-5.54522	0.803587	0.773278
T.cells	BOLA2	0.035694	5.686348	0.386124	0.700332	-6.70555	0.753984	0.714864
T.cells	PKP3	0.093783	2.747004	0.38596	0.700453	-5.89996	0.805715	0.775813
T.cells	MRM1	0.101283	2.067052	0.385913	0.700488	-5.61087	0.818144	0.790623
T.cells	SECISBP2	0.038558	4.786541	0.385823	0.700555	-6.45891	0.769481	0.733004
T.cells	GTF3C5	-0.07889	2.258469	-0.38582	0.700559	-5.94812	0.814627	0.786426
T.cells	GINS1	-0.05717	3.197409	-0.38578	0.700584	-6.40739	0.797579	0.766152
T.cells	PRMT7	0.069146	3.247895	0.385721	0.70063	-6.18104	0.796672	0.765101
T.cells	H3F3A	-0.02105	10.71708	-0.38545	0.700828	-7.56821	0.672736	0.621587
T.cells	A530013C2	0.210133	2.431674	0.385404	0.700863	-5.30042	0.811567	0.782765
T.cells	RCBTB1	-0.05028	3.826201	-0.38502	0.701149	-6.24106	0.786698	0.753054
T.cells	CYB5R4	-0.03365	5.869945	-0.38485	0.701272	-6.64371	0.751198	0.711462
T.cells	DNLZ	0.049369	3.991855	0.384721	0.701368	-6.33463	0.783769	0.74967
T.cells	BC031181	0.030092	5.703047	0.384648	0.701422	-6.61655	0.754041	0.714795
T.cells	ZFP609	0.030267	6.067152	0.38462	0.701443	-6.78348	0.747853	0.707589
T.cells	DNAJC13	-0.03663	6.16837	-0.38454	0.701504	-6.66305	0.746142	0.7056
T.cells	AASDH	-0.0872	2.290623	-0.3844	0.701605	-5.92228	0.814406	0.786013
T.cells	MDC1	0.056757	3.159139	0.384333	0.701654	-6.14581	0.798629	0.767251
T.cells	PRKAR2B	0.070673	3.305356	0.384235	0.701727	-6.06778	0.796001	0.764136
T.cells	SMPD2	0.090388	2.04317	0.384161	0.701781	-5.80279	0.818954	0.79144
T.cells	ZFP251	0.079567	2.407099	0.38394	0.701945	-5.89431	0.812371	0.783486
T.cells	CNBD2	-0.05729	3.889219	-0.38386	0.702004	-6.29618	0.785681	0.751841
T.cells	CD200R4	-0.26267	1.662182	-0.38353	0.70225	-5.24529	0.82627	0.799967
T.cells	NOTCH4	-0.20451	0.210596	-0.38325	0.702451	-5.27398	0.853648	0.832918
T.cells	SLC4A4	-0.14601	2.065431	-0.38322	0.702476	-5.54204	0.81881	0.791101
T.cells	NAF1	0.044591	3.658508	0.38319	0.702499	-6.30807	0.789943	0.756821
T.cells	STAG2	-0.02885	7.464582	-0.38318	0.702507	-7.00857	0.724783	0.680735
T.cells	SPTBN4	0.170853	-0.18469	0.383025	0.70262	-5.27382	0.861247	0.842203
T.cells	CBR2	0.286301	-1.3198	0.383005	0.702635	-5.11207	0.883412	0.86913
T.cells	DENND1B	0.033736	7.299486	0.382858	0.702744	-6.86736	0.727541	0.683976

T.cells	NCOA4	0.057809	6.199192	0.382765	0.702812	-6.52428	0.745904	0.705262
T.cells	ELMO2	0.055642	3.945024	0.382516	0.702997	-6.21517	0.785023	0.751068
T.cells	ZNHIT2	0.053051	3.310699	0.382367	0.703107	-6.08809	0.796383	0.764509
T.cells	TDRP	0.248307	-0.36439	0.382154	0.703264	-5.16935	0.864992	0.846849
T.cells	SLC38A7	0.094242	2.59539	0.38208	0.703319	-5.78808	0.809353	0.780043
T.cells	BSDC1	-0.05056	5.056629	-0.38205	0.703342	-6.44114	0.765632	0.728407
T.cells	TMTC4	-0.12684	1.357794	-0.38179	0.703537	-5.60093	0.832273	0.807462
T.cells	PLRG1	-0.03612	4.093452	-0.38178	0.703539	-6.4488	0.782537	0.7483
T.cells	CIP2A	-0.0565	3.506565	-0.38144	0.703795	-6.43857	0.793133	0.760675
T.cells	LPXN	-0.05229	5.23201	-0.3814	0.703825	-6.36155	0.762824	0.725013
T.cells	HSBP1	0.034808	6.003464	0.38122	0.703954	-6.71699	0.749652	0.709652
T.cells	UBE2O	0.057721	4.874388	0.381176	0.703987	-6.49119	0.769041	0.73232
T.cells	C330007P(	0.033841	5.50907	0.380796	0.704268	-6.69825	0.758118	0.71956
T.cells	GM16014	0.206994	-0.09363	0.380761	0.704294	-5.2429	0.860076	0.840879
T.cells	IFNGR1	-0.03959	7.03854	-0.38072	0.704323	-6.74224	0.732315	0.689564
T.cells	2310022A1	0.054118	2.998253	0.3807	0.704339	-6.17399	0.802336	0.771646
T.cells	KLRE1	-0.2318	1.626605	-0.38057	0.704432	-5.28461	0.827495	0.801649
T.cells	MAP3K4	0.042176	4.074187	0.380467	0.704512	-6.39837	0.7831	0.748884
T.cells	NFU1	0.045752	4.549473	0.380419	0.704547	-6.41391	0.774741	0.739047
T.cells	BC049715	0.181378	0.604594	0.380056	0.704815	-5.40535	0.846712	0.824839
T.cells	ALDH1B1	0.084472	1.910302	0.379972	0.704878	-5.79772	0.822232	0.795469
T.cells	CEP250	0.040326	4.458141	0.37993	0.704908	-6.54157	0.776341	0.741034
T.cells	CCNC	-0.0439	4.398502	-0.37977	0.705024	-6.41849	0.777387	0.742265
T.cells	A930005H1	0.052553	3.220496	0.379762	0.705032	-6.12507	0.798327	0.767004
T.cells	NPHP4	-0.13162	0.297045	-0.37973	0.705056	-5.54106	0.852575	0.831935
T.cells	2010007H(	0.082165	0.459338	0.379597	0.705155	-5.8333	0.849476	0.828195
T.cells	ARMH3	0.035674	5.427651	0.379418	0.705287	-6.59181	0.759515	0.721301
T.cells	SAC3D1	-0.04814	3.030557	-0.37924	0.705419	-6.21332	0.801752	0.771068
T.cells	RSF1	0.027545	7.014648	0.379209	0.705442	-6.90937	0.732712	0.690126
T.cells	GNAI2	0.020031	9.008515	0.379207	0.705444	-7.16884	0.700304	0.652875
T.cells	LRRK1	0.047404	5.612519	0.379184	0.705461	-6.57813	0.756346	0.717598
T.cells	CDC40	0.031474	6.047181	0.379108	0.705517	-6.72706	0.748944	0.708978
T.cells	COMMD1	-0.03577	5.141948	-0.37907	0.705543	-6.61746	0.764438	0.72708
T.cells	CD8B1	-0.24596	0.660348	-0.37903	0.705575	-5.20917	0.845653	0.823606
T.cells	NAA16	0.028514	5.024992	0.378773	0.705765	-6.65251	0.766461	0.729525
T.cells	PLPBP	0.04859	4.145683	0.378747	0.705783	-6.22678	0.781838	0.747606
T.cells	IL1RL2	0.219769	0.726101	0.378639	0.705864	-5.20681	0.844406	0.82225
T.cells	COASY	-0.06772	3.053875	-0.37847	0.705991	-6.03858	0.801331	0.770752
T.cells	RAB34	0.2088	0.235405	0.378304	0.706111	-5.25784	0.853754	0.83359
T.cells	MICU3	-0.06117	3.81398	-0.37829	0.706124	-6.1551	0.787713	0.754648
T.cells	MCU	0.037919	6.164566	0.378284	0.706126	-6.71816	0.746956	0.706848
T.cells	MAK16	-0.03191	5.072635	-0.37828	0.70613	-6.60969	0.765637	0.728666
T.cells	SLC12A6	0.031887	8.133974	0.378091	0.706269	-7.17312	0.714347	0.66918
T.cells	TBL2	0.065311	2.392625	0.378073	0.706282	-5.8453	0.813356	0.785135
T.cells	SLC7A1	-0.04631	5.141375	-0.37795	0.706375	-6.63723	0.764448	0.72732
T.cells	ERP44	-0.02331	6.074446	-0.37792	0.706399	-6.71713	0.748482	0.708669
T.cells	TRIM23	0.05449	3.295163	0.377734	0.706533	-6.12184	0.796984	0.765694

T.cells	ATRAID	0.04335	4.57765	0.377499	0.706707	-6.32698	0.774248	0.7389
T.cells	IDE	-0.03491	4.161112	-0.37742	0.706765	-6.43729	0.781565	0.747533
T.cells	LYAR	-0.03868	4.937131	-0.37711	0.706997	-6.67133	0.767985	0.731634
T.cells	TNS4	-0.22004	-0.27421	-0.37705	0.707042	-5.15826	0.863564	0.845732
T.cells	FBXO32	-0.09591	3.711448	-0.37692	0.70714	-6.11433	0.789537	0.757074
T.cells	MARVELD1	-0.16288	1.253094	-0.37681	0.707218	-5.28686	0.834471	0.810646
T.cells	CYC1	-0.03378	6.36195	-0.37675	0.707261	-6.87642	0.743626	0.703237
T.cells	GM12703	0.182535	-0.21463	0.376522	0.707431	-5.32718	0.862411	0.84444
T.cells	ACADM	-0.05489	4.767805	-0.37651	0.707437	-6.3617	0.770929	0.735202
T.cells	MACF1	0.033636	8.000182	0.376472	0.707468	-6.86007	0.716519	0.671935
T.cells	1110035H1	0.122734	1.216236	0.376469	0.70747	-5.56684	0.835162	0.811548
T.cells	N4BP2	-0.03537	5.310122	-0.37643	0.707502	-6.70007	0.761537	0.724185
T.cells	TMEM209	0.057236	3.362785	0.376425	0.707503	-6.25906	0.79577	0.764523
T.cells	GM43914	0.198802	0.384486	0.376416	0.707509	-5.18497	0.850904	0.830515
T.cells	PTPN4	0.037046	5.463527	0.37622	0.707655	-6.70757	0.758899	0.721144
T.cells	SMC1B	0.208313	-0.03473	0.376139	0.707715	-5.27136	0.858941	0.840334
T.cells	GM16279	0.122221	1.054801	0.376096	0.707746	-5.64117	0.838196	0.815296
T.cells	LMBRD2	0.047141	4.481892	0.376021	0.707802	-6.43532	0.775925	0.741167
T.cells	RCL1	-0.04126	4.24025	-0.37593	0.707868	-6.41747	0.78017	0.746183
T.cells	SHARPIN	0.03859	4.530571	0.375871	0.707913	-6.42747	0.775072	0.740177
T.cells	TCP11L1	-0.09017	2.689841	-0.37587	0.707916	-5.78121	0.80793	0.779081
T.cells	PACSIN2	0.028686	5.573303	0.375486	0.708198	-6.666	0.75708	0.719076
T.cells	AS3MT	-0.08091	2.69274	-0.37547	0.708208	-5.89819	0.807945	0.779101
T.cells	CCNK	0.032775	4.857081	0.375293	0.708341	-6.57421	0.76944	0.733558
T.cells	TRA2A	0.025523	7.075692	0.375275	0.708355	-6.91451	0.731759	0.68962
T.cells	CDCA4	-0.03587	4.453152	-0.3752	0.708414	-6.59134	0.776493	0.741853
T.cells	PLXNC1	0.095115	5.427157	0.375143	0.708453	-6.00531	0.759587	0.722008
T.cells	DNAJB12	0.035783	4.757611	0.374948	0.708597	-6.52573	0.771171	0.735592
T.cells	FLNB	0.101623	5.398208	0.374845	0.708673	-6.33521	0.760084	0.72259
T.cells	NFAM1	0.088356	4.570884	0.37483	0.708685	-5.80158	0.774431	0.739426
T.cells	DCP1A	0.034491	4.850131	0.37467	0.708803	-6.54102	0.76956	0.733709
T.cells	BABAM2	0.027846	7.216423	0.374661	0.70881	-6.89163	0.729429	0.686932
T.cells	GNAS	-0.02344	8.931692	-0.37466	0.708811	-7.17192	0.701586	0.654912
T.cells	ZSCAN2	0.129304	1.067334	0.374552	0.70889	-5.60641	0.838042	0.815131
T.cells	2610020C0	0.031427	4.983149	0.374316	0.709066	-6.6079	0.767337	0.731031
T.cells	NCEH1	0.081935	4.906119	0.374271	0.709099	-6.04012	0.768674	0.7326
T.cells	ZFP426	0.060605	2.769149	0.374148	0.70919	-6.04988	0.80667	0.77752
T.cells	GUCD1	0.066201	3.805941	0.373865	0.7094	-6.0703	0.788189	0.75545
T.cells	1700126G0	-0.1278	1.104608	-0.37306	0.709999	-5.56091	0.838238	0.8147
T.cells	OSBPL11	0.043072	5.368512	0.372814	0.710179	-6.50931	0.76154	0.723588
T.cells	ARRB1	0.06474	3.996665	0.372691	0.71027	-5.94623	0.785536	0.751756
T.cells	TINAGL1	-0.16637	2.066409	-0.37231	0.710555	-5.45423	0.820547	0.793368
T.cells	ALG3	0.097034	1.77052	0.372228	0.710614	-5.77374	0.826026	0.799921
T.cells	TFEB	-0.03347	5.158966	-0.372	0.710783	-6.66167	0.76526	0.727972
T.cells	CPLANE2	0.147201	0.331465	0.371677	0.711023	-5.43104	0.853162	0.832601
T.cells	TMEM158	-0.17816	0.271092	-0.37167	0.711025	-5.41875	0.854318	0.833998
T.cells	ARL2	0.059343	3.356926	0.371665	0.711031	-6.13498	0.797038	0.76544

T.cells	TEP1	0.056998	3.914158	0.371647	0.711045	-6.15873	0.787082	0.753662
T.cells	GM16083	-0.11525	1.977218	-0.3716	0.71108	-5.67837	0.822195	0.795387
T.cells	AK5	-0.16896	0.348906	-0.3716	0.71108	-5.34725	0.852828	0.832198
T.cells	SLC19A1	0.102011	1.065999	0.371577	0.711097	-5.70686	0.83921	0.815788
T.cells	PHACTR4	-0.02636	5.677431	-0.37156	0.711112	-6.78264	0.75634	0.717568
T.cells	SNUPN	-0.05973	2.593487	-0.3712	0.711376	-5.95388	0.810951	0.781981
T.cells	NACC1	-0.03806	4.380772	-0.37119	0.711387	-6.43272	0.778915	0.74404
T.cells	SEM1	-0.02267	9.100384	-0.37118	0.711394	-7.21903	0.699937	0.652444
T.cells	TUBB4B	-0.04207	6.422022	-0.37106	0.711482	-6.95644	0.743795	0.702956
T.cells	HES1	-0.04829	5.161335	-0.37088	0.711612	-6.66914	0.765363	0.728082
T.cells	COX7C	-0.02254	8.585633	-0.37081	0.711663	-7.16725	0.708227	0.661887
T.cells	PRDX6	-0.02899	7.110346	-0.3706	0.711821	-6.85735	0.732342	0.689695
T.cells	UNC119	-0.05704	5.47027	-0.37045	0.711936	-6.14697	0.760048	0.721917
T.cells	RUSC1	-0.093	3.288288	-0.37041	0.711961	-5.74813	0.798436	0.767122
T.cells	GIMAP1OS	0.121931	0.862202	0.370267	0.712069	-5.5696	0.843232	0.820653
T.cells	GM15265	0.090236	1.797023	0.370238	0.712091	-5.82334	0.825703	0.799609
T.cells	NIPAL3	-0.08149	3.198166	-0.37023	0.712094	-5.87063	0.800059	0.769048
T.cells	TTC28	0.055341	4.798912	0.369971	0.712289	-6.43835	0.771692	0.735565
T.cells	DCTN5	-0.03758	4.598222	-0.36984	0.712386	-6.48553	0.775199	0.739692
T.cells	5430416N(	-0.0422	3.547711	-0.36983	0.71239	-6.30999	0.7938	0.761639
T.cells	GM10552	0.159666	1.54086	0.3698	0.712416	-5.35926	0.830495	0.805366
T.cells	CASP3	0.035428	4.814976	0.369727	0.71247	-6.5398	0.771412	0.735247
T.cells	CCDC137	-0.06018	2.673176	-0.36963	0.712542	-5.9955	0.809602	0.780404
T.cells	ADGRG1	0.087108	1.720983	0.36955	0.712602	-6.06688	0.827139	0.801344
T.cells	XPO6	0.030198	5.572883	0.369377	0.71273	-6.63799	0.75833	0.719939
T.cells	RAC2	0.028657	9.052768	0.369235	0.712836	-7.19834	0.700807	0.653495
T.cells	GM40645	0.214882	0.751306	0.369195	0.712865	-5.19883	0.845383	0.823323
T.cells	SLC38A10	0.032676	5.326136	0.369091	0.712942	-6.62437	0.762573	0.724941
T.cells	DHCR7	-0.07883	2.184949	-0.36905	0.712972	-5.76325	0.818575	0.791164
T.cells	ARHGAP9	-0.05353	5.14536	-0.36878	0.713175	-6.26071	0.765819	0.72862
T.cells	ATP6AP1	0.030868	5.990589	0.36871	0.713225	-6.70978	0.751317	0.711675
T.cells	ZFP420	-0.1438	0.275776	-0.36827	0.713555	-5.41778	0.854826	0.83446
T.cells	RASGRP1	0.066928	4.119019	0.368256	0.713563	-6.24899	0.784	0.74989
T.cells	SKIV2L	0.047739	3.938774	0.368097	0.713681	-6.27055	0.787249	0.753669
T.cells	TBC1D19	0.074303	1.749793	0.367962	0.713781	-5.80679	0.827069	0.801051
T.cells	TWNK	0.066995	2.886068	0.367857	0.713859	-6.06331	0.806183	0.776163
T.cells	THEM4	0.086435	2.270582	0.3676	0.71405	-5.90464	0.817435	0.789622
T.cells	GTPBP1	-0.03914	4.641527	-0.36757	0.714071	-6.49726	0.774878	0.739209
T.cells	DNAAF5	0.049255	2.988111	0.367494	0.714129	-6.21793	0.804332	0.774022
T.cells	BOLL	-0.09963	1.308178	-0.36743	0.714176	-5.83619	0.835321	0.811046
T.cells	RPA1	-0.03578	4.922792	-0.36741	0.71419	-6.66268	0.769969	0.733455
T.cells	ATAT1	-0.06533	2.722421	-0.36707	0.714444	-5.9866	0.809337	0.779873
T.cells	NFYA	-0.04118	4.152707	-0.36694	0.714539	-6.44053	0.783649	0.749453
T.cells	HECW2	0.137403	3.162677	0.366922	0.714554	-5.63302	0.801347	0.770395
T.cells	ST6GAL1	-0.05242	5.862113	-0.36675	0.714684	-6.79135	0.754013	0.7147
T.cells	RDH12	-0.08814	2.275253	-0.36659	0.7148	-6.01429	0.817635	0.789772
T.cells	SPRYD4	0.083854	1.803638	0.366455	0.714901	-5.72294	0.826357	0.800196

T.cells	A430005L1	-0.04854	3.764054	-0.36642	0.714928	-6.21685	0.790658	0.757728
T.cells	SHE	-0.19001	0.946591	-0.36623	0.715065	-5.3062	0.842432	0.819503
T.cells	PCGF1	-0.1163	1.17851	-0.36607	0.715187	-5.51942	0.838055	0.814266
T.cells	YIPF6	0.061279	3.457527	0.366059	0.715195	-6.08718	0.796147	0.764262
T.cells	PERP	0.164981	1.228461	0.36604	0.71521	-5.38655	0.837115	0.813136
T.cells	SAAL1	0.048895	3.298168	0.365906	0.715309	-6.33814	0.799046	0.767679
T.cells	PIP4K2C	-0.04499	4.238405	-0.36565	0.7155	-6.32915	0.782347	0.747926
T.cells	ZFP408	0.049168	3.716601	0.365628	0.715516	-6.23472	0.791615	0.758874
T.cells	STARD13	-0.19031	1.070842	-0.36551	0.7156	-5.36501	0.840215	0.816832
T.cells	ELP2	-0.03759	4.144774	-0.36542	0.715673	-6.39646	0.784022	0.749904
T.cells	GM14085	0.193727	-1.35884	0.365313	0.71575	-5.14212	0.887248	0.873807
T.cells	TGFBR1	0.03923	6.24383	0.365089	0.715917	-6.70697	0.747794	0.707384
T.cells	MRPS16	-0.03222	5.607422	-0.36465	0.716247	-6.68421	0.758917	0.720041
T.cells	NOL10	0.037953	4.91748	0.364548	0.716319	-6.48865	0.770852	0.734061
T.cells	CD3E	-0.20561	3.278256	-0.36424	0.71655	-5.50063	0.800068	0.768451
T.cells	GCLM	0.040418	6.090034	0.363994	0.716732	-6.68714	0.750826	0.710646
T.cells	FLT3	-0.08936	2.584668	-0.36399	0.716733	-5.91839	0.81267	0.783476
T.cells	SMCR8	-0.07671	3.28105	-0.36394	0.716774	-5.87437	0.800018	0.768451
T.cells	CTSZ	0.047761	6.598627	0.363887	0.716811	-6.63741	0.742229	0.700662
T.cells	ERGIC3	-0.03154	5.513051	-0.36369	0.716961	-6.62824	0.760739	0.722194
T.cells	GM15563	0.096451	1.923249	0.363562	0.717053	-5.81836	0.824909	0.79807
T.cells	SEC24D	-0.07021	4.470665	-0.36329	0.717256	-5.94954	0.778875	0.743524
T.cells	PTS	-0.03	5.573329	-0.3632	0.717321	-6.66855	0.759703	0.72105
T.cells	GM12462	0.114553	-0.49881	0.363157	0.717355	-5.56827	0.870999	0.853671
T.cells	FAM234A	0.09846	4.099085	0.363095	0.717401	-5.83537	0.785437	0.751258
T.cells	B3GALT5	0.246362	0.779445	0.363009	0.717464	-5.18933	0.846387	0.823922
T.cells	PRF1	-0.20503	0.377462	-0.36296	0.717497	-5.24701	0.854057	0.833188
T.cells	ABCA5	0.169217	0.444818	0.362839	0.717591	-5.33654	0.852768	0.831656
T.cells	CD209D	0.26112	-0.32218	0.36281	0.717613	-5.14511	0.867559	0.849549
T.cells	RRNAD1	0.047483	3.384404	0.362736	0.717668	-6.14997	0.798204	0.766436
T.cells	GBP3	-0.11538	3.228626	-0.36266	0.717725	-5.87871	0.801012	0.7698
T.cells	NKAP	0.027775	4.989943	0.362455	0.717877	-6.62911	0.76979	0.732983
T.cells	DYNLT1B	0.122542	1.188553	0.362451	0.71788	-5.58527	0.838646	0.814744
T.cells	FMC1	0.047577	4.072806	0.362421	0.717902	-6.37341	0.785903	0.751937
T.cells	CUL4B	-0.04121	4.524951	-0.36198	0.718228	-6.49888	0.778044	0.742605
T.cells	TRP53INP2	0.060607	4.134264	0.361907	0.718285	-6.0564	0.784938	0.750731
T.cells	MBOAT1	-0.17664	0.682144	-0.36187	0.718312	-5.38402	0.848372	0.826379
T.cells	HMBS	-0.04458	4.451319	-0.36183	0.718344	-6.54159	0.779339	0.744146
T.cells	ITGAE	-0.11485	2.127038	-0.3618	0.718365	-5.75554	0.821265	0.793861
T.cells	UTP15	0.040662	3.757308	0.361393	0.718668	-6.31114	0.791855	0.758766
T.cells	ALPK1	0.058606	4.802266	0.361355	0.718696	-6.40595	0.77339	0.737014
T.cells	CDC42EP1	-0.20018	0.752796	-0.36101	0.718952	-5.27313	0.847472	0.825024
T.cells	CCDC112	0.13608	1.151644	0.36086	0.719065	-5.50396	0.839966	0.815979
T.cells	DPP7	0.140788	1.957266	0.360649	0.719222	-5.47674	0.82496	0.797993
T.cells	UTP25	0.063094	2.617836	0.360534	0.719307	-5.99509	0.812784	0.783495
T.cells	CHSY1	-0.03716	5.079341	-0.36044	0.719376	-6.60036	0.768872	0.731648
T.cells	DLGAP5	0.061447	3.248568	0.360403	0.719405	-6.38595	0.801315	0.769907

T.cells	SPIB	0.051703	4.129765	0.360094	0.719636	-6.52389	0.785627	0.751311
T.cells	STK11	-0.02951	5.696969	-0.35999	0.719712	-6.67774	0.758289	0.719249
T.cells	GM42836	0.162571	-0.40469	0.359966	0.719731	-5.35788	0.869977	0.852248
T.cells	PYCR2	0.053067	3.687049	0.359786	0.719865	-6.21879	0.793515	0.760657
T.cells	SOX7	-0.18478	0.442832	-0.35962	0.719986	-5.22892	0.853603	0.832517
T.cells	0610012G(	0.034253	5.039442	0.359616	0.719992	-6.46865	0.769648	0.732609
T.cells	RAB11FIP4	-0.17447	1.161785	-0.35958	0.720021	-5.19941	0.839935	0.816058
T.cells	ZFP560	0.039053	4.00302	0.359461	0.720108	-6.39416	0.787878	0.754064
T.cells	TRP53RKB	-0.07027	2.444883	-0.35946	0.720111	-5.89949	0.816043	0.787474
T.cells	GOLPH3	0.02513	5.902166	0.359234	0.720277	-6.68096	0.75481	0.715297
T.cells	MTERF4	-0.07843	1.913823	-0.35914	0.720344	-5.8516	0.825892	0.799287
T.cells	CYP4V3	0.141534	2.78654	0.35902	0.720436	-5.58603	0.809825	0.78016
T.cells	GM43727	0.192397	-0.59426	0.358844	0.720568	-5.14874	0.873717	0.857029
T.cells	ZFP472	0.048974	3.448072	0.358843	0.720568	-6.25652	0.797839	0.765972
T.cells	BOD1L	0.025797	5.740505	0.358736	0.720648	-6.71812	0.757576	0.718639
T.cells	TDP1	-0.04734	3.397629	-0.35868	0.720687	-6.35766	0.798747	0.767083
T.cells	DNAJC3	0.031631	6.725717	0.358475	0.720843	-6.79459	0.740865	0.699241
T.cells	CRELD1	-0.10455	1.843254	-0.35843	0.720873	-5.61662	0.827204	0.801012
T.cells	DDA1	0.034633	5.082621	0.358414	0.720888	-6.58746	0.768932	0.731974
T.cells	SMYD3	0.035393	6.67093	0.358253	0.721008	-6.81416	0.741785	0.700326
T.cells	DNAIC1	-0.18158	0.436784	-0.3581	0.721121	-5.26131	0.853757	0.832954
T.cells	EMC2	-0.02758	5.762132	-0.35799	0.721205	-6.68538	0.757205	0.718271
T.cells	SPINT2	-0.04013	4.563442	-0.35794	0.721245	-6.44954	0.778006	0.742645
T.cells	NUCKS1	-0.02976	6.576295	-0.35792	0.721254	-6.96199	0.743377	0.702173
T.cells	MAP4K2	0.03832	5.348887	0.357859	0.721302	-6.64631	0.764317	0.726583
T.cells	ZFP688	0.08716	1.695397	0.357725	0.721402	-5.77587	0.829959	0.804331
T.cells	TSR3	-0.05326	3.569257	-0.35772	0.721402	-6.24784	0.795662	0.763482
T.cells	SLC39A9	0.03448	4.172579	0.357597	0.721497	-6.35188	0.78493	0.750786
T.cells	INO80E	-0.03058	4.165534	-0.35742	0.721628	-6.42682	0.785098	0.750963
T.cells	EIF3K	-0.02116	7.871661	-0.35724	0.721761	-7.0709	0.721932	0.677383
T.cells	TMEM116	0.09247	2.038927	0.356963	0.72197	-5.82007	0.823644	0.796799
T.cells	ZFAS1	0.044668	5.058651	0.356839	0.722062	-6.5621	0.769417	0.732581
T.cells	MPEG1	0.129645	6.081136	0.356808	0.722086	-5.97507	0.751826	0.712021
T.cells	GM14286	-0.17064	0.211974	-0.35678	0.722107	-5.22973	0.85815	0.838287
T.cells	ANXA2	0.108822	5.910919	0.35675	0.722129	-5.87913	0.754728	0.715403
T.cells	MARF1	0.026493	6.147381	0.356729	0.722145	-6.78967	0.750699	0.710709
T.cells	SLC22A15	-0.07719	3.077716	-0.35668	0.72218	-5.84782	0.804601	0.774106
T.cells	LDLRAD4	0.04377	6.109073	0.35648	0.722331	-6.75139	0.751351	0.711467
T.cells	MAP3K14	0.048808	5.189544	0.35647	0.722338	-6.43841	0.767144	0.729916
T.cells	ATIC	-0.03915	4.493549	-0.3564	0.722392	-6.57436	0.779305	0.744238
T.cells	STAB1	-0.13854	3.176342	-0.3562	0.722538	-5.6583	0.802814	0.77207
T.cells	EGFL7	-0.0664	3.872106	-0.35615	0.722579	-6.26082	0.790315	0.757275
T.cells	ATP11A	0.144452	2.676915	0.355954	0.722723	-5.3384	0.811899	0.782946
T.cells	UQCR11	-0.02916	7.224799	-0.35587	0.722789	-6.96118	0.732599	0.689884
T.cells	GM46367	-0.09204	2.175914	-0.35579	0.722848	-5.82739	0.821109	0.793971
T.cells	TMEM101	0.070297	2.322989	0.355739	0.722883	-5.92754	0.818395	0.790731
T.cells	NDFIP2	0.029955	5.951714	0.355703	0.722911	-6.64892	0.754031	0.714773

T.cells	SERINC1	0.030409	6.230989	0.355657	0.722945	-6.60861	0.74928	0.709237
T.cells	MAG	-0.20027	0.11897	-0.35558	0.723005	-5.2247	0.859941	0.840684
T.cells	KLHL5	0.037814	4.645979	0.355448	0.723101	-6.36608	0.776653	0.74132
T.cells	OIT3	0.123688	2.345496	0.355326	0.723192	-5.55858	0.818034	0.79037
T.cells	NEK11	0.173728	0.082898	0.355142	0.723329	-5.25917	0.8607	0.841682
T.cells	LYVE1	0.197233	2.42534	0.35513	0.723338	-5.47385	0.816572	0.788645
T.cells	RABGEF1	0.055143	6.01057	0.35489	0.723517	-6.43357	0.753093	0.713815
T.cells	TMEM160	-0.03157	5.666014	-0.35487	0.723535	-6.60874	0.758987	0.720705
T.cells	PRG4	-0.38355	1.084056	-0.35468	0.723672	-5.38019	0.841589	0.818725
T.cells	ZCCHC8	-0.02869	5.108019	-0.35465	0.723699	-6.63972	0.768625	0.732018
T.cells	AI847159	0.17191	-0.25702	0.3546	0.723734	-5.27369	0.867294	0.849789
T.cells	HTR7	0.289105	0.754108	0.354555	0.723767	-5.22438	0.847847	0.826273
T.cells	IGSF8	0.039421	4.266842	0.354416	0.723871	-6.46122	0.783405	0.749371
T.cells	CENPF	0.060027	5.050794	0.354254	0.723992	-6.80895	0.769653	0.733194
T.cells	ZMYND15	0.25925	0.109664	0.354091	0.724114	-5.19131	0.860232	0.841191
T.cells	ATF5	-0.07807	3.267979	-0.35408	0.724124	-6.03539	0.801261	0.770495
T.cells	GDF15	-0.24208	1.508239	-0.35405	0.724148	-5.29218	0.83364	0.809141
T.cells	CLK4	-0.02732	6.012894	-0.35393	0.724237	-6.74328	0.7531	0.713816
T.cells	SFXN1	-0.03501	5.121606	-0.35373	0.724386	-6.64362	0.768437	0.731794
T.cells	EPM2AIP1	-0.07452	2.609784	-0.35365	0.72444	-5.85872	0.813249	0.784804
T.cells	USP9X	0.030284	7.387216	0.353652	0.724442	-6.90369	0.730015	0.687083
T.cells	PYCRL	-0.04162	3.541294	-0.35346	0.724582	-6.31157	0.796431	0.764772
T.cells	MRPS15	-0.02895	5.910327	-0.35328	0.724717	-6.71688	0.754987	0.715997
T.cells	HPS6	-0.12488	1.22115	-0.35318	0.724794	-5.45269	0.839205	0.815809
T.cells	MED25	-0.03519	4.496149	-0.353	0.724929	-6.42909	0.779516	0.74486
T.cells	AKAP8L	0.041761	4.8709	0.353	0.724929	-6.46387	0.772944	0.737129
T.cells	LMNB2	0.06776	2.474409	0.352847	0.725043	-6.08834	0.815879	0.788023
T.cells	ZSWIM8	-0.04265	4.703109	-0.35282	0.725063	-6.24298	0.77588	0.740635
T.cells	BICC1	-0.19228	0.332453	-0.35263	0.725208	-5.29052	0.856147	0.836425
T.cells	NME6	-0.07147	2.576439	-0.35254	0.725273	-5.94611	0.814036	0.785899
T.cells	4632428CC	0.13815	0.110114	0.352467	0.725327	-5.41423	0.860427	0.841649
T.cells	RCOR2	-0.11414	0.194015	-0.35241	0.725368	-5.6534	0.858809	0.839699
T.cells	UBE2F	0.031812	6.306137	0.352254	0.725486	-6.70408	0.748328	0.708487
T.cells	RFX3	0.028133	6.233922	0.352026	0.725657	-6.83506	0.749657	0.709975
T.cells	LAP3	-0.05104	4.901491	-0.35153	0.726026	-6.48078	0.772914	0.736984
T.cells	KLRA2	-0.24197	2.524963	-0.35122	0.726257	-5.31672	0.815593	0.787505
T.cells	PAIP1	-0.02987	5.907952	-0.3512	0.726277	-6.72991	0.755622	0.71674
T.cells	CHRNB1	0.126779	0.775917	0.35112	0.726334	-5.48465	0.848306	0.826737
T.cells	SLC25A33	0.073515	3.983563	0.350834	0.726547	-5.92961	0.789367	0.756286
T.cells	AHSA1	0.032103	5.071608	0.35037	0.726895	-6.6437	0.770475	0.733907
T.cells	FXYD5	0.058473	6.974009	0.350305	0.726943	-6.58997	0.738004	0.696058
T.cells	ZBTB8A	-0.07389	1.978565	-0.35007	0.727118	-5.81988	0.826258	0.799945
T.cells	PTER	-0.10179	2.433342	-0.34982	0.727304	-5.72168	0.817889	0.790015
T.cells	ZGRF1	-0.05442	3.956437	-0.34979	0.727326	-6.48631	0.790285	0.757235
T.cells	SYNJ1	-0.03854	6.731012	-0.34974	0.727368	-6.58578	0.742226	0.700951
T.cells	GTF2A2	0.024646	5.923594	0.349516	0.727533	-6.82768	0.75598	0.716886
T.cells	NDUFB6	-0.03341	6.317819	-0.34942	0.727609	-6.84493	0.749264	0.709061

T.cells	TCP11	-0.14454	0.648485	-0.34929	0.727704	-5.3037	0.85144	0.830199
T.cells	SLC39A13	0.070292	2.796524	0.349221	0.727754	-5.87925	0.811291	0.78208
T.cells	DEPDC7	-0.14192	1.678465	-0.34919	0.727776	-5.48017	0.831965	0.806778
T.cells	PNRC1	-0.02811	8.470962	-0.34891	0.727988	-7.05854	0.71367	0.667815
T.cells	CMSS1	-0.04599	6.448827	-0.34876	0.728098	-6.86823	0.747153	0.706502
T.cells	WDR6	-0.04801	3.277321	-0.34872	0.728132	-6.28827	0.802665	0.771715
T.cells	OTOA	-0.15727	0.757069	-0.34868	0.728162	-5.34916	0.849492	0.827749
T.cells	FBXL12OS	-0.09065	0.679534	-0.34836	0.728396	-5.67342	0.851075	0.829633
T.cells	NEBL	0.202291	1.182115	0.348333	0.728418	-5.35409	0.841524	0.818146
T.cells	RAB22A	0.02891	5.60028	0.348051	0.728629	-6.65247	0.761734	0.723564
T.cells	BCS1L	0.097763	1.699946	0.347991	0.728674	-5.75503	0.831786	0.806528
T.cells	PAK1IP1	0.022931	5.901653	0.347972	0.728688	-6.73329	0.756558	0.717531
T.cells	RAD51B	0.04692	6.152694	0.34789	0.72875	-6.87573	0.752271	0.712558
T.cells	EIF5B	0.022681	7.158051	0.347866	0.728767	-6.92378	0.735333	0.692899
T.cells	RSBN1L	-0.02465	7.198782	-0.34765	0.72893	-6.94833	0.734655	0.692137
T.cells	MBIP	-0.04549	3.658726	-0.34759	0.728977	-6.31118	0.795888	0.763852
T.cells	UPF1	0.025573	5.35091	0.347205	0.729262	-6.64582	0.766043	0.728835
T.cells	UQCC3	0.041182	4.016511	0.347156	0.729299	-6.30134	0.789488	0.756436
T.cells	DPY19L4	0.037383	4.417697	0.347136	0.729314	-6.42518	0.78237	0.748035
T.cells	PTPN14	-0.15942	0.524362	-0.34712	0.729326	-5.35986	0.854044	0.833576
T.cells	HCFC2	0.04292	3.758586	0.346825	0.729547	-6.30404	0.794097	0.761894
T.cells	SRR	-0.0643	2.599008	-0.34682	0.729552	-5.91278	0.815127	0.786876
T.cells	MLYCD	-0.07158	3.124459	-0.34682	0.729552	-5.81137	0.805534	0.775458
T.cells	MAPK4	0.152175	0.274036	0.3468	0.729565	-5.35167	0.858854	0.839396
T.cells	KCNC3	-0.11195	0.561324	-0.34679	0.729574	-5.55974	0.853336	0.832727
T.cells	PAF1	0.03633	4.600235	0.346578	0.729732	-6.39528	0.779151	0.744298
T.cells	ZFP503	-0.15648	0.884395	-0.34636	0.729894	-5.28869	0.84717	0.825357
T.cells	OXNAD1	-0.07563	1.886539	-0.34611	0.730079	-5.783	0.828303	0.802749
T.cells	TTC7B	0.035363	5.391283	0.346101	0.730089	-6.57455	0.765344	0.728158
T.cells	GM20492	-0.0988	1.798274	-0.34606	0.730119	-5.58352	0.829949	0.804737
T.cells	FAM216A	0.052876	2.582038	0.346022	0.730148	-6.14301	0.815438	0.7874
T.cells	RABEP2	-0.03457	4.414258	-0.34598	0.730179	-6.51124	0.78243	0.748275
T.cells	FAM185A	0.065421	2.579686	0.345846	0.73028	-5.99927	0.815481	0.787511
T.cells	ZFP568	0.042965	4.08691	0.345781	0.730329	-6.39081	0.788235	0.755167
T.cells	MYL4	-0.05157	4.638008	-0.34576	0.730346	-6.72989	0.778486	0.743671
T.cells	ABTB2	-0.04916	7.412469	-0.34576	0.730348	-6.96028	0.731105	0.688392
T.cells	CPNE1	-0.02594	6.038278	-0.34575	0.730354	-6.74581	0.754222	0.715237
T.cells	DCP2	0.034032	5.103401	0.345569	0.730487	-6.60926	0.770409	0.73413
T.cells	MPV17L2	0.042412	4.459696	0.345291	0.730696	-6.42845	0.781806	0.747428
T.cells	TOP2B	-0.02847	6.593853	-0.34525	0.73073	-6.92561	0.744963	0.704325
T.cells	GM15411	-0.09641	0.212426	-0.34511	0.73083	-5.69713	0.860239	0.841141
T.cells	EFEMP2	-0.0894	1.944258	-0.34506	0.730872	-5.65373	0.827418	0.801629
T.cells	BTBD3	-0.15014	1.337187	-0.34481	0.731054	-5.41414	0.838892	0.815339
T.cells	ITGB2	-0.06607	6.734664	-0.34475	0.731101	-6.40888	0.742683	0.701643
T.cells	GM30239	0.096342	-0.09316	0.344387	0.731373	-5.58917	0.866383	0.848475
T.cells	METTL6	-0.03771	4.505421	-0.3443	0.731436	-6.48401	0.781208	0.746675
T.cells	BICRA	0.028117	5.82128	0.344253	0.731473	-6.81908	0.758311	0.719825

T.cells	CUL1	-0.02176	6.562305	-0.34415	0.731547	-6.87562	0.745696	0.705131
T.cells	BUB1	-0.05412	3.743439	-0.34412	0.731576	-6.55326	0.794762	0.762697
T.cells	SH3PXD2B	0.215035	1.543779	0.344048	0.731627	-5.30497	0.835126	0.810826
T.cells	APOLD1	0.121911	1.979983	0.343786	0.731823	-5.69414	0.827055	0.801099
T.cells	TCERG1	-0.02152	6.296507	-0.34367	0.731912	-6.88776	0.750272	0.710461
T.cells	STAMBP	-0.06055	3.374422	-0.34366	0.73192	-6.06049	0.801484	0.770667
T.cells	STX4A	0.03347	4.972734	0.343577	0.73198	-6.42718	0.77308	0.737141
T.cells	EIF4E	-0.03159	6.950147	-0.34341	0.732105	-6.90577	0.739301	0.697674
T.cells	HIST1H4D	-0.09885	1.04631	-0.34322	0.732245	-5.83442	0.844741	0.822265
T.cells	RTN1	0.192765	2.561189	0.343045	0.732379	-5.37114	0.816516	0.788413
T.cells	NTHL1	-0.10362	0.544589	-0.34294	0.73246	-5.51199	0.854398	0.833874
T.cells	ABHD15	-0.09063	2.977881	-0.34264	0.732684	-5.82393	0.809056	0.779392
T.cells	GM41409	-0.08493	3.031183	-0.34251	0.732778	-6.04726	0.808085	0.778261
T.cells	GM16740	0.065679	2.544974	0.342287	0.732947	-5.9678	0.816984	0.788858
T.cells	ZFP760	0.096237	1.032725	0.342212	0.733003	-5.60147	0.845248	0.822744
T.cells	CXCL1	0.272687	2.815955	0.342183	0.733025	-5.5744	0.812013	0.78296
T.cells	WDR60	-0.10595	0.883885	-0.34182	0.733301	-5.65221	0.848078	0.826332
T.cells	NTAN1	-0.02239	6.261702	-0.34181	0.733302	-6.78088	0.751213	0.711499
T.cells	PRX	0.108949	1.104861	0.341729	0.733366	-5.6121	0.843879	0.821291
T.cells	TBC1D4	0.07261	4.706501	0.341723	0.73337	-6.28281	0.778106	0.742997
T.cells	RPAP1	0.059058	2.431442	0.341701	0.733386	-6.0081	0.819075	0.791556
T.cells	ZEB1	0.040947	6.848689	0.341669	0.733411	-6.88859	0.741292	0.699995
T.cells	SGK1	0.041251	5.355259	0.341626	0.733443	-6.62326	0.766779	0.729725
T.cells	NUF2	-0.05258	3.365247	-0.34139	0.733622	-6.41612	0.802081	0.771348
T.cells	EMC8	-0.03087	4.721373	-0.34137	0.733637	-6.59229	0.777902	0.742776
T.cells	FAM151B	-0.1036	1.753415	-0.34119	0.733768	-5.687	0.831797	0.806793
T.cells	ADAM30	0.186836	0.62147	0.340909	0.733981	-5.19156	0.853229	0.832571
T.cells	RALB	0.053426	4.247517	0.340876	0.734006	-5.98077	0.786344	0.752711
T.cells	ATPSCKMT	-0.05139	3.937455	-0.34085	0.734028	-6.27144	0.791867	0.759235
T.cells	BSCL2	-0.03553	4.542466	-0.34081	0.734055	-6.38366	0.781124	0.746557
T.cells	CTLA2B	-0.19256	3.353706	-0.34067	0.734157	-5.50096	0.80238	0.771704
T.cells	FGR	-0.21637	4.732993	-0.34042	0.73435	-5.41737	0.777785	0.74264
T.cells	DLST	0.024104	5.852364	0.340358	0.734394	-6.71398	0.758346	0.719858
T.cells	CELF1	0.018654	7.40812	0.340278	0.734454	-6.99358	0.732088	0.689361
T.cells	LATS2	-0.03377	5.889771	-0.34015	0.734547	-6.54236	0.757704	0.719154
T.cells	DSN1	0.052407	2.622227	0.340146	0.734554	-6.17229	0.815715	0.787624
T.cells	BRD2	-0.02359	7.085393	-0.34014	0.734562	-6.93101	0.737463	0.69561
T.cells	FHIT	-0.03506	6.167904	-0.33993	0.734716	-6.85509	0.752959	0.713624
T.cells	RBM19	0.055684	2.925866	0.339881	0.734752	-6.07878	0.810166	0.781019
T.cells	ECI1	0.043404	3.920606	0.339828	0.734792	-6.28469	0.792195	0.759696
T.cells	POMGNT1	0.062987	2.312495	0.339715	0.734877	-5.89861	0.821435	0.794467
T.cells	L3MBTL2	0.038253	3.445434	0.339589	0.734972	-6.29359	0.800732	0.769828
T.cells	LY6D	-0.04647	5.594266	-0.33956	0.734992	-6.72363	0.762797	0.725135
T.cells	APOPT1	-0.02588	4.910205	-0.3394	0.735113	-6.55815	0.774694	0.739119
T.cells	UBTF	-0.02395	5.879519	-0.3393	0.735186	-6.76645	0.757897	0.719444
T.cells	MSH6	-0.03753	4.38755	-0.33927	0.735209	-6.58161	0.783895	0.749961
T.cells	PSMA6	0.024622	6.941715	0.339009	0.735407	-6.98949	0.740012	0.698516

T.cells	RNPEPL1	-0.03985	4.75379	-0.33867	0.735658	-6.49686	0.777763	0.742458
T.cells	CMPK1	-0.02015	6.881961	-0.33853	0.735766	-6.93157	0.741236	0.699749
T.cells	ZFP975	0.138581	0.810489	0.338314	0.735929	-5.5442	0.850059	0.828636
T.cells	CTSK	-0.16696	0.234472	-0.33829	0.735944	-5.28436	0.861118	0.841996
T.cells	STX3	0.125585	2.314188	0.338243	0.735982	-5.55897	0.821803	0.794718
T.cells	LTN1	0.028375	5.03701	0.338138	0.736061	-6.61224	0.772855	0.736726
T.cells	IQGAP1	-0.02337	9.247795	-0.33802	0.736146	-7.20977	0.702524	0.655285
T.cells	CCDC14	-0.11266	0.809905	-0.33784	0.736282	-5.61267	0.85013	0.828754
T.cells	TXNL4B	-0.07657	1.740021	-0.33773	0.736367	-5.73597	0.832547	0.807627
T.cells	EFCAB11	-0.05203	3.410869	-0.33771	0.736385	-6.43959	0.801804	0.770953
T.cells	THUMPD1	0.033012	4.425949	0.337523	0.736522	-6.51289	0.783705	0.749508
T.cells	SEC16A	0.044155	4.57627	0.337309	0.736684	-6.32834	0.781049	0.746423
T.cells	ZFP266	-0.03283	4.029092	-0.33715	0.736799	-6.40469	0.790759	0.757878
T.cells	RAB40C	0.0337	4.838453	0.337033	0.736891	-6.52469	0.776436	0.740994
T.cells	CCR3	-0.34643	-0.02075	-0.33701	0.736906	-5.18316	0.866187	0.848193
T.cells	PCED1B	-0.04964	4.966857	-0.33668	0.737159	-6.50423	0.774186	0.738393
T.cells	CHD1L	-0.03907	3.722264	-0.33668	0.73716	-6.30445	0.796253	0.764422
T.cells	XKR5	-0.09808	0.190356	-0.33652	0.737274	-5.58951	0.862097	0.843313
T.cells	TMEM176B	-0.05389	5.475623	-0.33649	0.737301	-6.46742	0.765332	0.728044
T.cells	ZFP512B	0.079644	2.377698	0.336346	0.737407	-5.88421	0.82075	0.793662
T.cells	NDUFB8	-0.0233	7.487591	-0.33633	0.737421	-7.04355	0.731243	0.688443
T.cells	ST3GAL6	0.040701	5.092875	0.33626	0.737472	-6.53272	0.771984	0.735904
T.cells	ZFP451	0.031746	5.019065	0.336256	0.737474	-6.59194	0.773273	0.737418
T.cells	LRRC10B	0.090013	0.595128	0.336193	0.737522	-5.87767	0.854304	0.833973
T.cells	NDUFA5	-0.03789	5.432033	-0.33612	0.73758	-6.63972	0.766087	0.728988
T.cells	JOSD2	-0.03937	4.09679	-0.33611	0.737581	-6.30552	0.789551	0.756595
T.cells	MEST	-0.11055	3.792937	-0.33595	0.737703	-5.8756	0.794984	0.763059
T.cells	TREM2	0.26412	0.639882	0.335945	0.737708	-5.208	0.853446	0.832979
T.cells	PIK3R2	0.0709	2.306399	0.335757	0.73785	-5.92615	0.822068	0.795322
T.cells	SAYSD1	-0.05735	3.323883	-0.3357	0.737892	-5.92885	0.803439	0.773127
T.cells	PPP1R35	0.044187	3.395179	0.335648	0.737932	-6.26913	0.802149	0.771595
T.cells	NONO	-0.02038	6.614635	-0.33558	0.737986	-6.94848	0.745854	0.705452
T.cells	GM15441	0.099143	1.344167	0.335418	0.738104	-5.57812	0.840108	0.816941
T.cells	CCDC71L	0.046427	4.455272	0.335264	0.73822	-6.35869	0.783286	0.749291
T.cells	RWDD4A	-0.03973	3.983066	-0.33513	0.738319	-6.36323	0.791687	0.759221
T.cells	C1RL	0.15223	1.632594	0.335069	0.738367	-5.38535	0.834737	0.81052
T.cells	BRAT1	0.077393	2.183649	0.334976	0.738436	-5.76011	0.824453	0.798197
T.cells	PIGL	0.050066	2.746364	0.334717	0.738631	-6.03329	0.814211	0.785881
T.cells	CYB5B	0.024433	5.866247	0.334233	0.738996	-6.78592	0.75913	0.720589
T.cells	4732440D0	-0.09588	2.03703	-0.33396	0.739203	-5.71891	0.827665	0.801665
T.cells	SPATA32	0.198172	0.244996	0.333942	0.739214	-5.20633	0.861664	0.842589
T.cells	ICK	0.077247	2.147459	0.333871	0.739267	-5.82191	0.825611	0.799214
T.cells	AGBL5	-0.05829	2.244561	-0.33379	0.739327	-5.95952	0.823809	0.797086
T.cells	CCDC28B	0.050656	3.504556	0.333744	0.739363	-6.21725	0.800751	0.76965
T.cells	ARSB	0.068259	4.938464	0.333592	0.739478	-6.35834	0.77529	0.739618
T.cells	FTX	0.039325	4.311116	0.333491	0.739553	-6.51375	0.786361	0.752684
T.cells	LILRB4A	-0.16279	4.163584	-0.33313	0.739821	-5.48029	0.7891	0.755929

T.cells	SREK1	-0.02014	6.429668	-0.33308	0.739864	-6.85473	0.749686	0.709735
T.cells	CABCOCO1	0.107635	0.315412	0.332819	0.740059	-5.53366	0.860488	0.841342
T.cells	NFE2L2	0.03735	7.355477	0.332807	0.740068	-6.84945	0.734123	0.691692
T.cells	TMEM220	0.153606	0.299081	0.332788	0.740082	-5.30885	0.860803	0.841724
T.cells	GM49463	-0.16924	-0.05016	-0.33279	0.740083	-5.28882	0.86757	0.849923
T.cells	PLEK	-0.06871	7.520494	-0.33263	0.740202	-6.43559	0.731429	0.688522
T.cells	AGGF1	-0.02725	5.384652	-0.33249	0.740305	-6.62332	0.767693	0.730738
T.cells	XDH	-0.20006	4.807401	-0.33241	0.74037	-5.49004	0.777776	0.742579
T.cells	PSMC1	0.023085	5.889053	0.332142	0.740568	-6.78433	0.758983	0.720625
T.cells	ACTR6	-0.04406	3.522286	-0.33213	0.740579	-6.25586	0.800671	0.769701
T.cells	KIF17	-0.10177	1.692096	-0.33211	0.740593	-5.7622	0.834362	0.809897
T.cells	GM42658	0.091165	1.544714	0.332019	0.740661	-5.69138	0.837131	0.813229
T.cells	NDUFA10	-0.02455	6.050526	-0.33195	0.740709	-6.79911	0.756214	0.717398
T.cells	CASK	-0.0459	5.500078	-0.33178	0.740844	-6.43958	0.765758	0.728538
T.cells	ARMC9	-0.05971	2.484131	-0.33164	0.740945	-5.92397	0.819697	0.792372
T.cells	LZTR1	0.060846	2.612289	0.331543	0.741019	-5.95566	0.817335	0.789552
T.cells	COPS3	0.022579	5.92883	0.331492	0.741057	-6.81359	0.758368	0.71994
T.cells	BC030867	0.069416	2.099983	0.331331	0.741178	-6.14091	0.826872	0.800947
T.cells	CGGBP1	0.023247	6.845343	0.331052	0.741389	-6.90956	0.742981	0.701852
T.cells	KLHDC1	-0.08433	2.152219	-0.33096	0.741457	-5.70314	0.826055	0.799794
T.cells	GM43773	-0.12401	1.074002	-0.33072	0.741635	-5.46619	0.846376	0.824231
T.cells	G430095P1	-0.15325	0.465518	-0.33067	0.74168	-5.2688	0.858018	0.838279
T.cells	C430049B0	0.091366	1.844436	0.33026	0.741985	-5.74001	0.831846	0.806799
T.cells	TTI2	0.050239	2.749363	0.330229	0.742008	-6.0591	0.815073	0.786748
T.cells	PTOV1	0.037431	4.038474	0.330227	0.742009	-6.37618	0.791722	0.759027
T.cells	D3ERTD751	0.079863	1.61523	0.330187	0.74204	-5.81111	0.836144	0.811955
T.cells	CCDC127	-0.03303	4.15296	-0.33011	0.742099	-6.38242	0.789678	0.756612
T.cells	PLBD2	0.047885	4.310294	0.330039	0.742151	-6.15365	0.786878	0.753317
T.cells	DUS4L	-0.0716	1.929292	-0.32999	0.742184	-5.84988	0.830259	0.80491
T.cells	TPST2	-0.04276	5.593782	-0.32997	0.742206	-6.32247	0.764381	0.726876
T.cells	HS2ST1	-0.0281	5.689893	-0.32983	0.742308	-6.67929	0.762754	0.724974
T.cells	TFCP2	-0.08457	1.971586	-0.3295	0.742554	-5.69988	0.829677	0.804105
T.cells	NUBP2	-0.03857	4.451117	-0.32944	0.742602	-6.49795	0.784575	0.750506
T.cells	PGAP3	0.109443	1.12543	0.329144	0.742825	-5.58266	0.845784	0.823281
T.cells	TJP2	-0.04237	3.845594	-0.32901	0.742924	-6.43692	0.795569	0.76334
T.cells	EID2B	0.070921	1.703903	0.32874	0.743129	-5.74756	0.834996	0.810227
T.cells	ZFP993	-0.10675	1.824761	-0.3287	0.743159	-5.5779	0.83273	0.807524
T.cells	KANSL2	0.027887	5.119718	0.32849	0.743318	-6.65472	0.773191	0.736817
T.cells	RERG	-0.18225	0.522461	-0.32828	0.743476	-5.26654	0.857596	0.837326
T.cells	GM17484	-0.13349	1.264186	-0.32806	0.743639	-5.49314	0.84343	0.820305
T.cells	CSMD3	-0.10899	1.302147	-0.32797	0.743713	-5.50775	0.842711	0.819441
T.cells	TAF9B	-0.12016	0.956005	-0.32796	0.743715	-5.47908	0.84929	0.827359
T.cells	MAD2L2	0.045296	3.472942	0.327946	0.743728	-6.25999	0.802519	0.771434
T.cells	MYL6	-0.02427	9.689609	-0.32782	0.743822	-7.26111	0.697128	0.648883
T.cells	FLAD1	0.064619	2.957237	0.327755	0.743872	-5.96286	0.811903	0.78263
T.cells	NAB2	-0.09188	3.217989	-0.3277	0.743915	-5.71707	0.807145	0.776973
T.cells	9330151L1	-0.10491	0.96571	-0.32728	0.744229	-5.51822	0.849259	0.827287

T.cells	TMCC1	0.034604	7.946033	0.327279	0.74423	-7.06692	0.725415	0.681241
T.cells	IL23A	0.207262	-0.31658	0.32718	0.744305	-5.20246	0.874041	0.857309
T.cells	PRMT5	-0.04106	3.483009	-0.3271	0.744368	-6.32891	0.802482	0.771418
T.cells	SVIP	0.107038	1.34626	0.326927	0.744496	-5.53998	0.842029	0.818648
T.cells	NFIC	0.050355	4.464453	0.326809	0.744585	-6.12622	0.784903	0.750631
T.cells	NAIP6	-0.13667	2.310523	-0.32679	0.7446	-5.32972	0.823964	0.796995
T.cells	DNAJA4	0.166147	0.616749	0.326771	0.744613	-5.24417	0.855938	0.835408
T.cells	ZDHHC24	-0.12435	1.089708	-0.32658	0.744758	-5.47668	0.846981	0.824505
T.cells	PFDN6	0.029273	4.874434	0.326239	0.745014	-6.62188	0.777849	0.742129
T.cells	CGRRF1	0.039902	3.907331	0.326227	0.745024	-6.27303	0.795024	0.762379
T.cells	HAVCR1	0.193419	-0.3843	0.326165	0.74507	-5.14717	0.875574	0.858985
T.cells	C230037L1	0.141604	0.378737	0.326014	0.745184	-5.29931	0.860774	0.841021
T.cells	FOXP1	0.017483	9.650428	0.325663	0.745449	-7.41243	0.698113	0.649837
T.cells	COX4I2	0.10624	0.956141	0.32554	0.745542	-5.54413	0.84973	0.827751
T.cells	GM48960	0.080008	1.466163	0.325127	0.745853	-5.79304	0.840048	0.816212
T.cells	GM13402	0.124233	0.307958	0.324973	0.745969	-5.49286	0.862182	0.842905
T.cells	NAT9	0.056972	3.244632	0.324931	0.746001	-6.05363	0.807081	0.776839
T.cells	SLC25A32	-0.04998	3.120627	-0.32486	0.746058	-6.09123	0.80934	0.779524
T.cells	GM26801	0.09169	0.557742	0.324844	0.746067	-5.82342	0.857364	0.837088
T.cells	RABAC1	-0.03084	5.988734	-0.32477	0.74612	-6.59059	0.758571	0.719735
T.cells	GM47863	0.180743	0.552776	0.324695	0.746179	-5.22495	0.857459	0.837234
T.cells	MPST	-0.04558	3.682692	-0.32468	0.746187	-6.27733	0.799147	0.767455
T.cells	PGAM5	0.034897	3.934831	0.324651	0.746212	-6.40698	0.794614	0.762088
T.cells	NCAPD3	-0.03301	5.127768	-0.32465	0.746216	-6.75105	0.773489	0.737197
T.cells	STK40	-0.03451	5.69472	-0.3246	0.746253	-6.55274	0.763635	0.725652
T.cells	PAFAH1B3	0.029773	5.814072	0.324591	0.746257	-6.90723	0.761576	0.723245
T.cells	PDE4A	-0.0877	3.81993	-0.32419	0.746557	-5.91604	0.796677	0.764642
T.cells	2410004B1	0.028776	4.71052	0.323919	0.746764	-6.49364	0.780817	0.745983
T.cells	LSM4	0.022367	6.704001	0.323904	0.746776	-6.96057	0.746385	0.705709
T.cells	H19	-0.10313	6.408472	-0.32387	0.746802	-6.65919	0.751397	0.71154
T.cells	PTGR1	-0.04328	3.377249	-0.32386	0.74681	-6.46636	0.804672	0.774186
T.cells	SMPDL3A	-0.07841	5.614644	-0.32384	0.746825	-6.16876	0.765019	0.72744
T.cells	BRIP1	-0.0374	4.898207	-0.32384	0.746826	-6.72198	0.777513	0.742096
T.cells	ABCC5	0.057454	4.301545	0.323793	0.746859	-6.25852	0.788063	0.754525
T.cells	NEDD1	0.054242	3.041246	0.323604	0.747002	-6.11089	0.81079	0.781476
T.cells	GABPB1	0.02672	5.380129	0.323369	0.747179	-6.70996	0.769088	0.732252
T.cells	C330013E1	0.143405	0.677864	0.323348	0.747195	-5.37315	0.855055	0.834578
T.cells	PDE2A	0.036729	6.050057	0.323316	0.74722	-6.87256	0.757519	0.718721
T.cells	HAUS5	0.04427	2.831524	0.323271	0.747253	-6.28627	0.81463	0.786081
T.cells	RAB7B	0.185481	2.804284	0.323175	0.747326	-5.35435	0.81513	0.786684
T.cells	RBFA	-0.0407	4.826424	-0.32312	0.747367	-6.42135	0.778775	0.743644
T.cells	CBX5	-0.03163	5.103912	-0.32308	0.747398	-6.7332	0.773906	0.737933
T.cells	PHETA1	-0.13738	0.450779	-0.32299	0.747465	-5.39144	0.859424	0.839894
T.cells	TOMM70A	0.023825	5.752051	0.322982	0.747472	-6.7397	0.762645	0.724741
T.cells	SLC10A3	0.101055	2.160549	0.322786	0.74762	-5.62063	0.827117	0.800923
T.cells	UGCG	0.037034	6.436724	0.32255	0.747798	-6.69999	0.751068	0.711082
T.cells	UFL1	0.038928	3.965196	0.322502	0.747834	-6.25689	0.79423	0.761739

T.cells	CDK2AP2	0.027388	6.467925	0.322347	0.747951	-6.75399	0.750566	0.710491
T.cells	SYPL	0.027767	5.860238	0.322169	0.748085	-6.72353	0.760963	0.722679
T.cells	INO80D	0.0229	5.995336	0.322057	0.74817	-6.78237	0.75864	0.719974
T.cells	CYP4F13	0.082961	2.829707	0.322032	0.748189	-5.70303	0.814859	0.786295
T.cells	RPRD1A	-0.03124	4.057705	-0.32193	0.748266	-6.40082	0.792603	0.759904
T.cells	PCDH15	-0.09672	1.323083	-0.32191	0.748284	-5.57084	0.842956	0.81996
T.cells	GZMB	-0.22007	3.971899	-0.32177	0.748385	-5.57435	0.794153	0.761747
T.cells	FRMD4A	0.089306	3.969412	0.321703	0.748437	-5.89005	0.794197	0.761799
T.cells	SRP54A	-0.0422	3.599318	-0.32138	0.748677	-6.1741	0.801012	0.769733
T.cells	NBEA	-0.11667	2.602645	-0.32099	0.748979	-5.53197	0.819209	0.791427
T.cells	KCNG2	-0.18868	-0.23458	-0.32088	0.749058	-5.16256	0.873125	0.856338
T.cells	ELP3	0.051026	2.716115	0.320823	0.749101	-6.07892	0.817118	0.788933
T.cells	1700102PC	0.145247	0.812589	0.320817	0.749106	-5.35974	0.852856	0.831803
T.cells	FUNDC2	0.026429	7.178207	0.320682	0.749208	-7.01255	0.738739	0.696786
T.cells	ARF4	-0.02576	8.479652	-0.32066	0.749222	-7.0627	0.717249	0.671999
T.cells	RNF19A	0.031826	5.375931	0.320511	0.749337	-6.59687	0.769507	0.732695
T.cells	TBC1D13	-0.05325	3.495587	-0.32042	0.749404	-6.10148	0.802888	0.772084
T.cells	CASP8AP2	-0.02614	5.182109	-0.3204	0.74942	-6.70652	0.772886	0.736676
T.cells	PRPF38B	0.017023	6.611272	0.320401	0.74942	-6.89548	0.74829	0.70794
T.cells	U2SURP	-0.01935	6.667671	-0.32039	0.749427	-6.91251	0.747335	0.70683
T.cells	VAMP3	0.026037	5.320751	0.319986	0.749734	-6.55591	0.770653	0.733978
T.cells	BSN	-0.11336	0.764883	-0.31997	0.749749	-5.6297	0.853976	0.833147
T.cells	RIOK2	0.031877	4.466641	0.319615	0.750015	-6.44889	0.785722	0.751705
T.cells	RNASEH2A	-0.03425	4.54522	-0.3196	0.750026	-6.56323	0.784328	0.750065
T.cells	TTLL11	0.075373	1.608719	0.319376	0.750195	-5.82388	0.837991	0.813975
T.cells	WRN	0.027009	5.831312	0.319331	0.750229	-6.75439	0.761851	0.723739
T.cells	HPRT	-0.0239	6.310323	-0.31929	0.75026	-6.9062	0.753636	0.714152
T.cells	FBXL19	-0.06858	2.333257	-0.31919	0.750338	-5.91312	0.824442	0.797743
T.cells	PITPNM2	0.041843	3.66119	0.319182	0.750341	-6.37531	0.800137	0.768809
T.cells	UBAP2L	0.019175	7.113568	0.319158	0.750359	-6.93315	0.740046	0.69836
T.cells	PRICKLE1	-0.10961	3.386121	-0.31886	0.750583	-5.78955	0.80528	0.774803
T.cells	ENPP2	-0.11357	2.411456	-0.31817	0.751104	-5.60549	0.82335	0.796243
T.cells	ZFP12	0.085668	1.346551	0.318073	0.75118	-5.61265	0.84331	0.8202
T.cells	COG2	-0.04685	3.567744	-0.31803	0.751213	-6.14436	0.802174	0.771067
T.cells	SOCS2	0.051472	3.962092	0.317905	0.751306	-6.4108	0.795068	0.762674
T.cells	CAPN2	0.050111	4.14309	0.317875	0.751329	-6.07954	0.791827	0.758842
T.cells	ZFP788	-0.06032	2.069958	-0.31783	0.751363	-5.87565	0.829703	0.803897
T.cells	DYNLT1A	-0.03536	4.14434	-0.31762	0.751522	-6.50369	0.791804	0.758871
T.cells	CAPZA2	-0.01618	7.929486	-0.31753	0.751587	-7.05261	0.726796	0.682981
T.cells	PLEC	0.046841	4.805597	0.317533	0.751588	-6.24656	0.780067	0.745035
T.cells	WDR83	0.050819	2.732947	0.317461	0.751643	-6.09627	0.817411	0.789274
T.cells	IRAK3	0.070577	4.187779	0.317208	0.751833	-6.29023	0.791028	0.75796
T.cells	SYNCRIP	-0.02028	7.375906	-0.31718	0.751858	-7.04874	0.735978	0.693586
T.cells	SACM1L	0.03081	5.504292	0.317074	0.751935	-6.59284	0.767842	0.730683
T.cells	SUPV3L1	0.043602	3.910174	0.316971	0.752013	-6.30534	0.796	0.763839
T.cells	PHAX	0.034233	4.599165	0.31693	0.752044	-6.50904	0.783714	0.749329
T.cells	DHRS7B	-0.04816	3.137991	-0.3169	0.752066	-6.14583	0.809984	0.78043

T.cells	EIF4A1	-0.0207	7.526831	-0.3169	0.752067	-6.99934	0.733464	0.690678
T.cells	ZFP825	0.062631	2.050646	0.316802	0.75214	-5.92462	0.830063	0.804411
T.cells	GM12992	-0.093	2.041763	-0.31661	0.752284	-5.77924	0.830229	0.804633
T.cells	HIST2H2AA	0.075938	2.225914	0.316472	0.75239	-5.95809	0.826796	0.800588
T.cells	TNIP1	0.05242	4.966974	0.316321	0.752504	-6.343	0.777227	0.741824
T.cells	DEXI	0.056904	2.91114	0.316216	0.752584	-6.00788	0.814136	0.785535
T.cells	MLH1	-0.04417	2.750298	-0.31618	0.752607	-6.07839	0.817091	0.789071
T.cells	BOLA1	0.042564	3.767483	0.316134	0.752646	-6.33803	0.798567	0.767063
T.cells	AMACR	-0.09905	2.326832	-0.31609	0.752678	-5.72791	0.82492	0.798438
T.cells	ARSG	-0.09046	1.867658	-0.3158	0.752902	-5.50959	0.833487	0.808836
T.cells	ACAD10	-0.09664	1.501708	-0.31566	0.753004	-5.60881	0.840374	0.817123
T.cells	ERI2	0.082514	1.310882	0.315649	0.753013	-5.77402	0.843986	0.821468
T.cells	TXNDC15	-0.03713	4.911319	-0.31553	0.7531	-6.43476	0.778205	0.743175
T.cells	PDE6D	-0.03266	3.812865	-0.31542	0.753186	-6.35053	0.79775	0.76625
T.cells	ZFP433	0.119072	0.355661	0.315391	0.753208	-5.48686	0.862283	0.843558
T.cells	COX6A1	-0.02509	6.768979	-0.31531	0.753273	-6.91073	0.746172	0.705753
T.cells	ARFGAP2	0.026453	4.901097	0.315276	0.753295	-6.55857	0.778385	0.743429
T.cells	MAPKAP1	0.027163	5.764174	0.315268	0.753301	-6.67517	0.763341	0.725779
T.cells	PRICKLE3	0.083082	2.034653	0.315248	0.753316	-5.68878	0.830362	0.805154
T.cells	RARS2	-0.04245	3.441715	-0.31524	0.753321	-6.23153	0.804457	0.774249
T.cells	CLEC4A2	-0.13871	3.416946	-0.31515	0.753394	-5.49686	0.804906	0.774797
T.cells	ATP6V0C	-0.02469	8.851286	-0.31509	0.753435	-7.10559	0.711747	0.666043
T.cells	RNF130	0.026434	7.282781	0.31498	0.753519	-6.84311	0.737536	0.695797
T.cells	ATP6V1G1	0.022646	7.177612	0.314758	0.753687	-6.90394	0.739296	0.697903
T.cells	CYB561D1	-0.07896	1.785161	-0.31472	0.753715	-5.81414	0.835037	0.810909
T.cells	GM36447	-0.128	0.209364	-0.31468	0.753744	-5.43454	0.865119	0.847196
T.cells	ZFP326	-0.02541	5.21559	-0.31462	0.753789	-6.66587	0.772873	0.737085
T.cells	VIM	0.042472	8.109275	0.314422	0.753941	-7.08099	0.723878	0.680069
T.cells	ARHGAP33	-0.10506	0.549492	-0.31438	0.753971	-5.61371	0.85859	0.83927
T.cells	PELI1	0.023188	7.676753	0.314203	0.754107	-6.99704	0.73108	0.688317
T.cells	TMEM131L	0.022499	7.26323	0.313751	0.754449	-7.12415	0.738183	0.696385
T.cells	FAM117A	0.036158	5.965212	0.313725	0.754469	-6.77392	0.760208	0.72201
T.cells	LTBP4	0.103274	2.297056	0.313304	0.754787	-5.76986	0.826019	0.799613
T.cells	TANGO6	0.035577	4.382985	0.313076	0.75496	-6.4623	0.78807	0.754578
T.cells	BMP6	-0.19632	0.450706	-0.31306	0.754973	-5.1491	0.861015	0.841771
T.cells	AA465934	0.067709	1.66043	0.313048	0.754981	-5.87679	0.837934	0.813951
T.cells	MEAK7	-0.13992	0.666682	-0.31304	0.754991	-5.33479	0.856852	0.836738
T.cells	DBR1	0.042095	3.262038	0.312798	0.755171	-6.26519	0.808339	0.778577
T.cells	GM16091	0.060117	2.306149	0.312695	0.755249	-6.03234	0.825934	0.79958
T.cells	NARS2	0.040112	4.271035	0.312564	0.755348	-6.37739	0.790145	0.757079
T.cells	MTBP	-0.04212	3.256156	-0.31256	0.75535	-6.3415	0.808446	0.778768
T.cells	MAPT	-0.08224	1.597756	-0.31246	0.755426	-5.7245	0.839208	0.815544
T.cells	PIGG	-0.10815	0.963956	-0.31224	0.755592	-5.51485	0.851352	0.83005
T.cells	EIF3J2	0.093219	1.443832	0.312092	0.755706	-5.60594	0.842271	0.819056
T.cells	VNN3	-0.14473	1.614791	-0.31182	0.755912	-5.40454	0.839144	0.815233
T.cells	XPO1	-0.02751	5.786847	-0.31167	0.756025	-6.82243	0.763772	0.725894
T.cells	HBS1L	0.024534	5.694465	0.311473	0.756174	-6.71893	0.76537	0.727763

T.cells	EIF2AK1	0.028418	5.326731	0.31146	0.756184	-6.60119	0.771763	0.735252
T.cells	RNF115	0.020746	6.978362	0.311442	0.756198	-6.88699	0.743441	0.70221
T.cells	PXMP4	0.045042	3.831766	0.311361	0.756259	-6.23917	0.79827	0.766496
T.cells	SCAF4	0.024415	6.317786	0.311316	0.756294	-6.77892	0.754649	0.715243
T.cells	GM26749	0.059327	2.222722	0.311155	0.756415	-6.00283	0.827803	0.801638
T.cells	WNT5B	0.086913	1.562043	0.310886	0.756619	-5.66403	0.840263	0.816546
T.cells	NIT1	0.046059	3.750254	0.310807	0.756679	-6.17691	0.799856	0.768329
T.cells	SYTL3	-0.13578	2.803793	-0.31067	0.756782	-5.60676	0.817106	0.788849
T.cells	IL1B	-0.19194	5.12575	-0.31061	0.756826	-5.72732	0.775391	0.739502
T.cells	SHKBP1	0.03766	4.068341	0.310601	0.756835	-6.32903	0.794135	0.761593
T.cells	AFMID	0.051427	4.371552	0.310412	0.756978	-6.17174	0.788717	0.755197
T.cells	GDPD5	0.122111	1.750804	0.310411	0.756979	-5.45609	0.836704	0.812315
T.cells	ANAPC11	-0.02947	5.923842	-0.31033	0.757039	-6.71831	0.761519	0.723265
T.cells	DHX8	-0.02797	5.129659	-0.31024	0.75711	-6.6186	0.775323	0.739435
T.cells	USP2	-0.07151	2.711696	-0.30978	0.757454	-5.88577	0.819099	0.790979
T.cells	POLR3GL	-0.04018	3.878267	-0.30939	0.757753	-6.21574	0.798078	0.765787
T.cells	GPN1	-0.04358	3.042468	-0.30927	0.757841	-6.1688	0.813282	0.78381
T.cells	LRAT	-0.15895	-0.03713	-0.30888	0.758142	-5.26408	0.871777	0.85387
T.cells	THAP8	-0.18988	-0.29985	-0.3088	0.758199	-5.22578	0.876926	0.860138
T.cells	AMPD3	0.0946	2.578161	0.308717	0.758263	-5.69708	0.822025	0.79407
T.cells	ABHD17A	-0.02024	6.296745	-0.30867	0.758301	-6.83383	0.755813	0.716004
T.cells	TMEM106/	0.077464	3.645492	0.308339	0.75855	-5.8493	0.802573	0.770924
T.cells	KATNB1	0.058259	2.346793	0.308189	0.758664	-6.05664	0.826407	0.799339
T.cells	SP1	-0.02178	5.924217	-0.30816	0.758687	-6.78965	0.762296	0.723586
T.cells	TOP3A	-0.04119	3.860539	-0.30814	0.758702	-6.34969	0.798688	0.766373
T.cells	CCL22	0.259933	-0.37257	0.308094	0.758736	-5.20326	0.87845	0.86206
T.cells	CHMP5	0.026074	5.632148	0.307859	0.758914	-6.62411	0.767459	0.729511
T.cells	MED26	-0.03617	4.429093	-0.30761	0.759102	-6.4811	0.788664	0.75444
T.cells	AEN	0.044093	3.664015	0.307529	0.759164	-6.31259	0.802401	0.770685
T.cells	GPR180	-0.05534	2.964205	-0.30747	0.75921	-5.96653	0.815162	0.785839
T.cells	GM13166	0.146791	-0.19353	0.307423	0.759244	-5.27381	0.875112	0.857926
T.cells	GM5150	0.175318	2.574091	0.307225	0.759395	-5.39349	0.82238	0.794485
T.cells	MED21	0.034229	5.21874	0.307208	0.759407	-6.63724	0.774738	0.738114
T.cells	FYB	-0.1078	6.869674	-0.30711	0.75948	-6.01684	0.746323	0.70497
T.cells	SUMO3	-0.02498	5.724941	-0.30693	0.759615	-6.77329	0.765919	0.727834
T.cells	GDPD3	-0.09448	1.793559	-0.30693	0.759619	-5.72165	0.836954	0.811977
T.cells	RNF25	0.051535	3.103588	0.306768	0.759741	-6.00574	0.812683	0.782926
T.cells	TMEM91	-0.05867	2.459733	-0.30654	0.759915	-6.07084	0.824609	0.797079
T.cells	ABCD4	0.053637	2.782602	0.306502	0.759943	-5.94285	0.818635	0.789953
T.cells	SNAI3	0.172154	-0.34486	0.306299	0.760097	-5.17541	0.878225	0.86169
T.cells	CNPY3	-0.0242	5.300686	-0.30611	0.760238	-6.61123	0.773406	0.736509
T.cells	ABCA9	0.20752	0.787541	0.306099	0.760249	-5.20077	0.856202	0.835028
T.cells	QARS	-0.03159	4.552856	-0.30607	0.76027	-6.54658	0.786588	0.752004
T.cells	CYB5R3	-0.03854	4.85016	-0.30601	0.760318	-6.32329	0.781322	0.745805
T.cells	HOMER3	-0.0384	3.103095	-0.30591	0.76039	-6.24473	0.812744	0.782987
T.cells	CNOT10	-0.02553	5.238486	-0.30587	0.760426	-6.66147	0.774494	0.737823
T.cells	LMTK2	-0.02704	5.616268	-0.3056	0.760631	-6.69486	0.76791	0.730173

T.cells	PIP5K1B	0.034242	5.568352	0.305594	0.760632	-6.73837	0.768743	0.731148
T.cells	MAF1	-0.03159	5.342723	-0.30538	0.760797	-6.66092	0.772676	0.735755
T.cells	CYTH2	0.040971	3.933028	0.305371	0.760802	-6.30142	0.797678	0.765202
T.cells	SLCO3A1	-0.09403	4.38978	-0.30529	0.760862	-5.91088	0.789496	0.755535
T.cells	SLC39A12	0.232142	0.492802	0.305221	0.760916	-5.22979	0.86189	0.842031
T.cells	LIG3	-0.05109	3.358188	-0.30515	0.760972	-6.13545	0.808089	0.777583
T.cells	RIOK1	-0.02377	6.007255	-0.3051	0.761011	-6.74072	0.761147	0.722306
T.cells	GLIS3	-0.13183	3.068689	-0.30502	0.761067	-5.73245	0.81338	0.78389
T.cells	RPP40	-0.07754	1.432828	-0.30477	0.761261	-5.75864	0.843949	0.820402
T.cells	CACTIN	0.036735	3.991663	0.30476	0.761265	-6.40614	0.796683	0.76404
T.cells	EOMES	0.194316	-0.77158	0.304423	0.761521	-5.18463	0.886732	0.872289
T.cells	PPP1R3B	0.106937	2.491892	0.304203	0.761688	-5.44532	0.824082	0.796771
T.cells	GTF2E1	0.065059	2.399479	0.304108	0.76176	-5.86969	0.825799	0.798825
T.cells	AY036118	0.086665	4.212426	0.304022	0.761826	-6.25876	0.792726	0.759528
T.cells	FLOT2	0.039336	4.443449	0.303937	0.76189	-6.27705	0.788601	0.754668
T.cells	WDR53	-0.05514	2.715022	-0.30391	0.761907	-6.03809	0.819951	0.791854
T.cells	POP5	-0.02627	4.525116	-0.30389	0.761923	-6.49953	0.787148	0.752954
T.cells	EIPR1	-0.02647	4.924643	-0.30387	0.761938	-6.60241	0.780074	0.744632
T.cells	AI413582	0.056919	4.68032	0.303829	0.761972	-6.04987	0.784393	0.749721
T.cells	SLC9A5	0.108203	0.362304	0.30365	0.762108	-5.51189	0.864485	0.845439
T.cells	PHACTR2	0.035547	6.036575	0.303578	0.762163	-6.70038	0.760701	0.722011
T.cells	CHPT1	0.043909	3.607446	0.303571	0.762168	-6.16605	0.803622	0.772523
T.cells	UBASH3A	0.061431	2.009253	0.303547	0.762187	-6.14986	0.833083	0.807647
T.cells	SGTB	-0.14069	0.3418	-0.30323	0.762427	-5.2846	0.864972	0.845971
T.cells	GM31508	0.117029	0.763062	0.303025	0.762583	-5.48148	0.856833	0.836155
T.cells	HYLS1	-0.03976	3.183514	-0.30299	0.762612	-6.2932	0.811425	0.781739
T.cells	WDR92	0.048842	3.083733	0.302972	0.762623	-6.2075	0.813252	0.783912
T.cells	CBX6	-0.04466	2.859775	-0.30295	0.762636	-6.1864	0.817366	0.788812
T.cells	KDM2A	-0.01648	7.465818	-0.30276	0.762784	-6.99407	0.736541	0.693947
T.cells	ARF3	-0.02287	6.29303	-0.3027	0.76283	-6.72215	0.756376	0.716977
T.cells	ATG13	0.032966	4.765899	0.302698	0.762831	-6.52822	0.782958	0.748117
T.cells	ASTL	0.12018	1.424213	0.302505	0.762978	-5.5185	0.844259	0.821041
T.cells	TYW5	-0.0476	3.139549	-0.30223	0.763185	-6.18889	0.812284	0.782855
T.cells	EPOR	0.201282	-0.66556	0.302189	0.763218	-5.14113	0.884781	0.87015
T.cells	ALPK2	-0.11937	1.260876	-0.30214	0.763255	-5.43249	0.847364	0.824839
T.cells	ARHGAP31	-0.04513	6.152696	-0.30213	0.763262	-6.56175	0.758833	0.719872
T.cells	ANXA3	-0.12628	3.542549	-0.30207	0.763306	-5.55427	0.804937	0.77413
T.cells	CLASP1	0.022676	6.373608	0.301633	0.76364	-6.8592	0.755259	0.715543
T.cells	RBBP5	0.038555	3.457865	0.301606	0.763661	-6.30804	0.806701	0.776044
T.cells	BC017158	-0.08697	1.449949	-0.30145	0.763779	-5.70584	0.844059	0.820647
T.cells	PKN1	-0.02105	6.766934	-0.30133	0.763871	-6.83238	0.748627	0.707833
T.cells	GGACT	0.044863	3.170013	0.301232	0.763945	-6.04946	0.812029	0.78241
T.cells	DIDO1	0.025799	5.734022	0.301014	0.76411	-6.74973	0.766439	0.728569
T.cells	NABP2	-0.02786	4.980112	-0.30085	0.764236	-6.56868	0.779673	0.744078
T.cells	CCDC93	-0.05403	3.72019	-0.3005	0.764498	-6.05708	0.80218	0.770722
T.cells	TRP53I11	0.032847	4.565294	0.300361	0.764607	-6.5899	0.787021	0.752844
T.cells	PSME4	-0.0242	7.130098	-0.30026	0.764683	-6.94778	0.742646	0.700987

T.cells	JPT1	-0.02085	7.832843	-0.30025	0.764695	-7.13532	0.730906	0.687409
T.cells	ALKBH1	0.027915	4.99535	0.300184	0.764741	-6.58037	0.77941	0.743931
T.cells	KLC3	-0.13646	0.19256	-0.30011	0.764796	-5.37158	0.868431	0.850247
T.cells	FAM199X	0.058997	2.776803	0.300096	0.764808	-5.95265	0.819423	0.791324
T.cells	THA1	-0.17433	0.09883	-0.30008	0.764818	-5.18315	0.870258	0.852461
T.cells	ST3GAL1	-0.04628	5.796993	-0.29986	0.764988	-6.61507	0.765408	0.727547
T.cells	TRIM32	0.092441	1.488698	0.299839	0.765004	-5.73115	0.843522	0.820208
T.cells	ABCC10	0.110235	0.65899	0.299819	0.765019	-5.37465	0.85939	0.83934
T.cells	KCMF1	-0.01715	6.987433	-0.29952	0.76525	-6.90614	0.745157	0.703875
T.cells	ABCB10	-0.06005	2.977675	-0.29947	0.765286	-5.95038	0.81584	0.786992
T.cells	2410006H1	0.035229	7.535004	0.29932	0.765398	-7.01746	0.735965	0.693228
T.cells	IL1RL1	-0.18779	-0.28284	-0.2993	0.765417	-5.16363	0.87786	0.861622
T.cells	PCCB	-0.05531	3.081613	-0.29921	0.765483	-5.99238	0.813931	0.784749
T.cells	LRRC28	-0.05942	3.874144	-0.29905	0.765602	-6.20336	0.799518	0.767676
T.cells	MAP2K2	-0.01795	7.299123	-0.29899	0.765646	-6.9869	0.739917	0.697861
T.cells	PFKFB3	0.029317	6.273061	0.298816	0.765782	-6.91055	0.757318	0.718112
T.cells	CEBPE	0.170818	0.2028	0.298647	0.76591	-5.19797	0.868362	0.850252
T.cells	RELA	-0.03116	5.114793	-0.29862	0.76593	-6.52509	0.777426	0.741688
T.cells	FAM104A	0.02345	6.153765	0.298575	0.765965	-6.78581	0.759367	0.720541
T.cells	SNHG6	0.046782	3.414627	0.298375	0.766117	-6.19554	0.807849	0.777631
T.cells	POMP	-0.0227	7.268227	-0.29837	0.766124	-6.95635	0.740435	0.698529
T.cells	MAP11	0.069829	2.866952	0.298365	0.766125	-5.81278	0.817883	0.789572
T.cells	SDCCAG8	0.026974	5.914953	0.298111	0.766318	-6.67794	0.763604	0.725398
T.cells	KLHL18	0.040439	4.310869	0.298015	0.766391	-6.27512	0.791804	0.758526
T.cells	TARDBP	-0.02255	5.771119	-0.29764	0.766679	-6.78362	0.766258	0.728356
T.cells	GM11944	-0.03798	4.498486	-0.29762	0.766695	-6.57937	0.788623	0.754624
T.cells	UBE2T	0.038366	4.06115	0.297472	0.766804	-6.55983	0.79649	0.763886
T.cells	DHX29	-0.04143	3.415036	-0.29711	0.767083	-6.19999	0.808404	0.777825
T.cells	ZFP687	0.046586	2.919042	0.296957	0.767196	-6.06635	0.817538	0.788661
T.cells	RANBP9	0.023853	6.962828	0.296685	0.767403	-6.88397	0.746271	0.704745
T.cells	SAMD9L	-0.05067	5.184732	-0.29635	0.767655	-6.26243	0.776965	0.740618
T.cells	MTHFD1L	-0.02552	5.661426	-0.29628	0.767708	-6.83496	0.768634	0.730862
T.cells	R3HDM4	0.029924	6.20323	0.296192	0.767778	-6.68796	0.759267	0.719937
T.cells	GRB7	0.107597	0.183341	0.296167	0.767797	-5.57222	0.8696	0.851182
T.cells	MCCC2	0.086883	2.436244	0.296013	0.767914	-5.83396	0.826672	0.799583
T.cells	GM9929	0.075648	1.684976	0.295897	0.768002	-5.69919	0.840765	0.816474
T.cells	GPR68	0.160637	1.433054	0.295849	0.768039	-5.33126	0.84554	0.822211
T.cells	TMEM26	-0.09321	2.190341	-0.29582	0.768059	-5.76919	0.831261	0.805083
T.cells	SENP6	0.019083	6.681311	0.295725	0.768134	-6.849	0.751091	0.710513
T.cells	DERL2	-0.02842	4.925658	-0.29551	0.768296	-6.5441	0.781528	0.746138
T.cells	MTMR2	0.023042	5.396075	0.29546	0.768335	-6.72449	0.773261	0.73643
T.cells	MPV17	0.033027	4.075124	0.295401	0.768381	-6.33237	0.796687	0.764015
T.cells	ST3GAL3	0.029233	5.293649	0.295309	0.76845	-6.61571	0.775054	0.738543
T.cells	BIVM	0.094991	0.882383	0.295255	0.768491	-5.49192	0.856065	0.834976
T.cells	GM1043	-0.0859	1.915295	-0.29518	0.768552	-5.9201	0.836421	0.811382
T.cells	NME7	0.032236	3.562527	0.294896	0.768765	-6.33007	0.805955	0.775087
T.cells	CLSTN1	-0.08878	1.774497	-0.29482	0.768824	-5.58123	0.839074	0.814611

T.cells	TOMM22	-0.0198	6.905252	-0.29477	0.768857	-6.97182	0.74729	0.70623
T.cells	ANXA6	-0.03467	6.174347	-0.29472	0.768896	-6.59008	0.759763	0.720769
T.cells	ZBTB11	0.022241	7.088839	0.294613	0.768981	-6.94614	0.744187	0.702683
T.cells	MMP11	-0.09171	0.128652	-0.29461	0.768985	-5.64324	0.870667	0.852801
T.cells	TUSC1	0.050465	3.124075	0.294488	0.769076	-6.04197	0.813962	0.784692
T.cells	SPAG7	0.033355	4.55728	0.294472	0.769088	-6.47186	0.78806	0.754009
T.cells	C87436	-0.03595	3.905756	-0.29427	0.769239	-6.34979	0.799738	0.767825
T.cells	COLGALT2	-0.19061	-0.12576	-0.29419	0.769304	-5.18407	0.875647	0.858892
T.cells	TRAF6	-0.02336	5.683882	-0.29413	0.769345	-6.79115	0.768243	0.730755
T.cells	GM14326	-0.06155	2.868013	-0.29404	0.769419	-5.90103	0.818672	0.790354
T.cells	YRDC	-0.02828	5.035051	-0.294	0.769447	-6.62265	0.779598	0.744097
T.cells	S100PBP	0.029972	4.405401	0.293927	0.769502	-6.42538	0.790768	0.757259
T.cells	DENND2C	-0.06816	2.008648	-0.2938	0.769602	-5.79141	0.834697	0.809518
T.cells	GM39302	-0.10974	0.880898	-0.29368	0.769689	-5.51134	0.856144	0.83531
T.cells	SS18L1	-0.07099	2.325233	-0.29344	0.769875	-5.88777	0.828913	0.802487
T.cells	CCN1	0.170999	1.098666	0.293201	0.770055	-5.3446	0.852128	0.830345
T.cells	ZMYND8	0.023594	6.108671	0.293041	0.770178	-6.75249	0.761082	0.722316
T.cells	ACOT2	0.031554	4.416307	0.292969	0.770232	-6.37687	0.79077	0.757162
T.cells	ARHGEF2	0.025291	5.586467	0.292945	0.77025	-6.65682	0.770129	0.732894
T.cells	MKLN1OS	-0.14749	0.018205	-0.29293	0.77026	-5.27441	0.873042	0.855649
T.cells	SEC22B	0.024719	5.449712	0.292555	0.770548	-6.67233	0.772521	0.735782
T.cells	ITGB5	-0.10337	3.058168	-0.29244	0.770637	-5.66825	0.81538	0.786427
T.cells	SETD2	0.021821	6.922415	0.292299	0.770743	-6.91981	0.74719	0.70625
T.cells	TMEM144	-0.11477	0.882973	-0.29224	0.77079	-5.51136	0.856273	0.835484
T.cells	SIPA1L1	0.036779	7.327522	0.292145	0.77086	-6.89474	0.740361	0.69834
T.cells	UQCRFS1	-0.02143	7.164384	-0.29204	0.77094	-7.00362	0.743104	0.701542
T.cells	SMG5	-0.02267	5.55464	-0.29194	0.771018	-6.73336	0.77069	0.733697
T.cells	MZB1	-0.03479	5.956787	-0.29193	0.771024	-6.9272	0.763709	0.725529
T.cells	EXO5	0.074183	2.006793	0.291855	0.771081	-5.8602	0.834914	0.809837
T.cells	HRH1	0.148177	-0.56863	0.291761	0.771153	-5.1458	0.884604	0.86989
T.cells	MAP4K1	-0.02366	4.801156	-0.29173	0.771176	-6.68017	0.783931	0.749277
T.cells	GM44777	0.102068	0.565153	0.291714	0.771189	-5.5313	0.862404	0.842965
T.cells	PTGES2	0.056823	2.422157	0.291643	0.771243	-5.92588	0.827146	0.800584
T.cells	TMEM199	0.04243	3.379806	0.291623	0.771258	-6.11004	0.80949	0.779543
T.cells	MRPS28	0.043307	6.885185	0.291452	0.771388	-6.89644	0.747828	0.707083
T.cells	WBP11	0.019193	6.346343	0.291369	0.771451	-6.88883	0.757011	0.717771
T.cells	SKP2	0.038918	3.310393	0.291335	0.771477	-6.34601	0.810765	0.781068
T.cells	SETBP1	-0.15352	4.621756	-0.29105	0.771692	-5.56396	0.787202	0.753092
T.cells	DVL1	-0.06517	2.507773	-0.29105	0.771698	-5.85723	0.825645	0.798723
T.cells	IGHD	0.040971	3.245617	0.290881	0.771824	-6.35632	0.812067	0.782525
T.cells	ECH1	-0.03202	5.784916	-0.29078	0.771897	-6.61081	0.766802	0.729128
T.cells	DHRS4	-0.03751	4.374568	-0.2907	0.771964	-6.43911	0.791642	0.758349
T.cells	C9ORF72	-0.08036	3.893192	-0.29057	0.772061	-5.89566	0.800292	0.768617
T.cells	ARIH2	0.023835	7.527213	0.290408	0.772184	-6.91151	0.737129	0.694683
T.cells	PMP22	-0.1459	1.847479	-0.29038	0.772209	-5.41901	0.83804	0.81365
T.cells	GCNT7	-0.05358	2.712006	-0.29019	0.77235	-5.97007	0.82189	0.794362
T.cells	SQSTM1	-0.03093	7.466775	-0.29015	0.772382	-6.84723	0.73814	0.695888

T.cells	GM42067	-0.08298	0.558955	-0.28992	0.772556	-5.60773	0.862655	0.843399
T.cells	POP4	-0.03444	4.085123	-0.28988	0.772589	-6.47545	0.796833	0.764626
T.cells	KDM4C	-0.02617	6.252217	-0.28985	0.772606	-6.72345	0.758735	0.71986
T.cells	PRPS1L3	-0.05767	2.045409	-0.28984	0.772614	-5.93511	0.834317	0.809272
T.cells	POLD3	0.027259	4.695421	0.289742	0.772692	-6.64374	0.785926	0.751757
T.cells	LAMP1	0.023381	7.757865	0.289703	0.772722	-6.96278	0.733283	0.690322
T.cells	ZFP658	-0.15718	0.297611	-0.28939	0.772964	-5.21781	0.867921	0.849566
T.cells	F11	-0.13329	0.954836	-0.28929	0.773039	-5.36806	0.855217	0.834201
T.cells	AGBL3	0.075849	1.72445	0.289165	0.773132	-5.68528	0.840571	0.816568
T.cells	MSL1	-0.02717	5.419802	-0.289	0.773256	-6.65931	0.773354	0.736814
T.cells	AGFG1	-0.02225	6.147106	-0.28899	0.773268	-6.75948	0.760731	0.722045
T.cells	ACTG1	-0.02581	11.44474	-0.28867	0.773511	-7.57861	0.674539	0.623166
T.cells	D430001F1	0.190847	-0.87048	0.288535	0.773613	-5.15187	0.89099	0.877688
T.cells	ZFP189	0.087705	1.120206	0.288497	0.773642	-5.5939	0.852094	0.830511
T.cells	PPM1H	-0.09931	6.473739	-0.28849	0.773646	-5.875	0.755152	0.71557
T.cells	LYRM7	-0.08115	1.247217	-0.28847	0.773663	-5.6935	0.849666	0.827585
T.cells	CENPE	-0.04957	4.9887	-0.28837	0.773735	-6.78573	0.780955	0.745776
T.cells	SMIM10L1	0.029981	4.931338	0.288302	0.773791	-6.50502	0.781968	0.746967
T.cells	SC5D	0.043817	4.248539	0.287816	0.774161	-6.23865	0.794213	0.761346
T.cells	BATF2	0.169894	0.948583	0.287786	0.774184	-5.31591	0.855482	0.834529
T.cells	LRWD1	-0.02961	3.781422	-0.28778	0.774186	-6.40302	0.802631	0.771309
T.cells	GM38948	0.07441	0.463761	0.287709	0.774243	-5.69359	0.864843	0.845859
T.cells	TTC5	0.024568	4.64029	0.287551	0.774364	-6.58081	0.787217	0.753141
T.cells	MBLAC2	-0.0646	2.64789	-0.28747	0.774423	-5.82943	0.823404	0.796079
T.cells	PARP4	0.029869	5.335625	0.287458	0.774434	-6.52586	0.774942	0.738702
T.cells	IGBP1	-0.02985	4.749526	-0.28742	0.774462	-6.50681	0.785277	0.750864
T.cells	APPBP2	0.021577	6.248663	0.287326	0.774535	-6.78228	0.759097	0.720206
T.cells	TLR1	0.1649	1.219121	0.287258	0.774587	-5.23466	0.850299	0.828423
T.cells	MATR3	-0.01603	6.50291	-0.28685	0.7749	-6.90556	0.754894	0.715275
T.cells	6030443J0	0.106453	0.848488	0.286797	0.774939	-5.45827	0.857582	0.837137
T.cells	FGGY	0.067546	3.459854	0.286771	0.774958	-5.99094	0.808639	0.778507
T.cells	MRPL47	-0.04759	2.974642	-0.28667	0.775034	-6.17252	0.817538	0.789107
T.cells	CRYZ	-0.09177	1.288432	-0.28646	0.775192	-5.55882	0.849216	0.827059
T.cells	FBXO4	0.052194	3.431156	0.286361	0.775271	-5.93588	0.809226	0.779216
T.cells	CLDN10	-0.09749	0.159414	-0.28623	0.775371	-5.7133	0.871014	0.853414
T.cells	SEMA4C	-0.11508	2.202853	-0.28614	0.775443	-5.35006	0.831932	0.806311
T.cells	TANGO2	-0.05346	3.93274	-0.28613	0.775445	-6.24949	0.800122	0.768424
T.cells	GM12689	-0.15481	-0.48996	-0.28552	0.77591	-5.17753	0.884179	0.869072
T.cells	PEAR1	-0.05321	2.352213	-0.28522	0.776143	-6.04958	0.829511	0.803143
T.cells	LRR1	-0.05912	1.435876	-0.28522	0.776143	-5.97275	0.846786	0.823867
T.cells	GM43560	-0.09046	0.578606	-0.28521	0.776151	-5.42605	0.863248	0.843723
T.cells	2810004N2	-0.03165	4.315225	-0.28514	0.776208	-6.4795	0.793598	0.76047
T.cells	IMP3	-0.02214	5.264975	-0.28493	0.776363	-6.68678	0.776749	0.740656
T.cells	GM50322	0.097335	0.358739	0.284851	0.776424	-5.61491	0.867517	0.848944
T.cells	1700110K1	0.186014	-0.57203	0.284837	0.776435	-5.13647	0.885805	0.871155
T.cells	SMG7	-0.02158	6.385921	-0.28477	0.776488	-6.82761	0.757295	0.717899
T.cells	ANKRA2	0.036609	3.792457	0.284694	0.776545	-6.20336	0.803018	0.771663

T.cells	MIA3	-0.02358	5.849323	-0.28464	0.776584	-6.65832	0.76655	0.728725
T.cells	ZFP322A	-0.04961	3.041434	-0.28456	0.776644	-6.08959	0.816733	0.787979
T.cells	ARHGEF26	-0.14564	0.379311	-0.28426	0.776874	-5.30759	0.867225	0.848535
T.cells	TLNRD1	0.033303	4.581796	0.284252	0.776882	-6.60173	0.788933	0.754946
T.cells	FBXL14	-0.02314	5.203671	-0.28408	0.777014	-6.68348	0.777985	0.741988
T.cells	STARD8	-0.05492	3.359704	-0.28377	0.777251	-6.0321	0.811146	0.781086
T.cells	PPM1A	0.018734	6.125745	0.283695	0.777308	-6.74615	0.762005	0.723201
T.cells	TBP	-0.04125	3.21522	-0.28365	0.777342	-6.25215	0.813792	0.784232
T.cells	UNC93B1	-0.02132	7.659349	-0.2836	0.777381	-7.03803	0.735981	0.692976
T.cells	CDON	-0.06558	1.952332	-0.28341	0.777524	-5.94997	0.837316	0.81229
T.cells	0610040B1	-0.06768	2.051019	-0.28336	0.777567	-5.80355	0.835459	0.810064
T.cells	ATRN	-0.03107	5.965865	-0.28323	0.777664	-6.66918	0.764837	0.726468
T.cells	MFSD11	-0.04749	3.854162	-0.2831	0.777761	-6.12987	0.802248	0.770474
T.cells	SIRT7	-0.02366	4.918849	-0.28294	0.777882	-6.52116	0.78324	0.748016
T.cells	GM13830	-0.1344	0.287554	-0.2824	0.778299	-5.38132	0.869545	0.850967
T.cells	GM26759	-0.0673	3.123372	-0.28237	0.778316	-5.91739	0.815828	0.786466
T.cells	BHLHB9	-0.04912	2.203905	-0.28225	0.778413	-6.03284	0.832899	0.806889
T.cells	2510046G1	-0.05104	2.620896	-0.28211	0.778516	-5.98974	0.825117	0.797611
T.cells	VDAC1	-0.02201	6.677223	-0.28193	0.778654	-6.93629	0.752871	0.712487
T.cells	SCARF1	-0.10353	1.587665	-0.28189	0.77869	-5.48484	0.844525	0.820916
T.cells	PLPPR1	0.102708	1.149205	0.281778	0.778772	-5.52992	0.852888	0.830993
T.cells	TPX2	-0.04146	5.047457	-0.28165	0.778867	-6.79958	0.781154	0.745613
T.cells	A230059L0	0.131136	0.47539	0.281457	0.779018	-5.43679	0.865889	0.846812
T.cells	TMBIM1	0.070065	2.851295	0.280964	0.779395	-5.79813	0.820845	0.792798
T.cells	AIMP1	0.019045	6.38044	0.280665	0.779623	-6.88973	0.757948	0.718717
T.cells	CATSPERG1	-0.16057	0.160917	-0.28064	0.779644	-5.23816	0.872018	0.854465
T.cells	DGLUCY	0.084187	2.944912	0.280359	0.779857	-5.55542	0.819116	0.790881
T.cells	ITM2C	0.048762	5.637577	0.280287	0.779912	-6.21559	0.7708	0.733785
T.cells	NTN4	0.153923	0.706687	0.280209	0.779972	-5.28914	0.861405	0.841688
T.cells	TNKS	-0.02599	6.066905	-0.28001	0.780121	-6.79195	0.763348	0.725139
T.cells	ARHGAP27	0.070878	2.032169	0.279956	0.780165	-5.67588	0.836124	0.811323
T.cells	TBCK	0.027442	5.26974	0.279951	0.780169	-6.55627	0.777239	0.741425
T.cells	TRMT13	0.035476	3.89491	0.279878	0.780225	-6.34045	0.801755	0.770382
T.cells	ARL15	0.02315	7.591924	0.279654	0.780396	-7.03853	0.737423	0.695101
T.cells	METTL14	0.043523	3.09472	0.279577	0.780455	-6.15928	0.816355	0.787799
T.cells	2310058D1	0.039365	2.785149	0.27952	0.780499	-6.08908	0.82207	0.794613
T.cells	LIX1	0.11112	0.723268	0.279278	0.780684	-5.46836	0.861085	0.841571
T.cells	CASTOR2	-0.0586	3.643855	-0.27911	0.780812	-6.24158	0.806309	0.775936
T.cells	TUBGCP2	0.030387	3.994036	0.279001	0.780896	-6.44405	0.799964	0.768476
T.cells	FDXR	-0.05206	2.625449	-0.27882	0.781036	-5.96762	0.825032	0.798349
T.cells	0610009L1	-0.10152	2.030404	-0.27877	0.781072	-5.46429	0.836157	0.811698
T.cells	GM13708	0.065414	3.088737	0.278624	0.781184	-5.84485	0.816465	0.78818
T.cells	PEX19	0.035348	3.955615	0.278552	0.781239	-6.29253	0.800658	0.769394
T.cells	D130020L0	0.102199	0.782996	0.278517	0.781266	-5.52737	0.859931	0.840359
T.cells	DENND1C	-0.06082	3.966917	-0.2785	0.781279	-5.8917	0.800453	0.769156
T.cells	INTS10	-0.03645	3.410048	-0.27849	0.78129	-6.28298	0.810573	0.781171
T.cells	FOXO1	-0.02123	7.035015	-0.27845	0.781315	-7.01288	0.746792	0.706201

T.cells	ADGRL4	0.062892	3.201942	0.27839	0.781363	-6.09548	0.814385	0.785721
T.cells	CDK12	-0.02133	6.896517	-0.27834	0.781402	-6.89598	0.74914	0.708929
T.cells	ANKRD46	0.047385	2.377526	0.278264	0.78146	-5.85493	0.829651	0.803973
T.cells	HTATSF1	-0.02418	4.697996	-0.27818	0.781524	-6.59065	0.787348	0.753717
T.cells	KIF13A	-0.0372	4.605824	-0.27817	0.781529	-6.39921	0.788989	0.755653
T.cells	DNHD1	-0.10745	1.01891	-0.27807	0.781607	-5.45339	0.855388	0.834951
T.cells	VPS33B	0.04254	2.932531	0.278062	0.781614	-6.10617	0.819344	0.79169
T.cells	C530005A1	0.093951	0.742343	0.27794	0.781707	-5.44367	0.860716	0.841419
T.cells	OAS3	0.198754	2.402295	0.277928	0.781717	-5.40398	0.829188	0.80348
T.cells	1500009L1	0.076149	-0.02505	0.277889	0.781746	-5.65167	0.875662	0.859537
T.cells	9130401M	0.031676	4.02313	0.277641	0.781936	-6.34057	0.799439	0.768124
T.cells	RTRAF	-0.02025	7.336334	-0.2776	0.78197	-7.03893	0.741709	0.700455
T.cells	ECM1	-0.10526	3.680244	-0.27741	0.782115	-5.90377	0.805648	0.775523
T.cells	IGF2BP3	-0.02417	6.5516	-0.27727	0.782222	-6.90339	0.755016	0.715985
T.cells	CCDC82	0.030023	4.323551	0.277176	0.782292	-6.47784	0.794035	0.761803
T.cells	CLDN34C1	-0.0863	0.809812	-0.27717	0.782295	-5.6986	0.859414	0.840007
T.cells	SCML4	-0.035	4.659948	-0.27688	0.782517	-6.54755	0.788025	0.754732
T.cells	PITPNB	0.020324	5.507244	0.276873	0.782524	-6.70753	0.773075	0.737138
T.cells	ABL1	0.026981	5.653787	0.276857	0.782536	-6.71124	0.770517	0.734136
T.cells	ZMYM4	0.022595	5.733892	0.276799	0.782581	-6.77674	0.769122	0.73251
T.cells	MRPL19	0.031641	3.923908	0.276762	0.782609	-6.3982	0.801231	0.770375
T.cells	PKP4	0.036514	5.958306	0.27674	0.782626	-6.68345	0.765226	0.727963
T.cells	CAMK2G	0.029138	4.893224	0.276689	0.782665	-6.5782	0.783882	0.749877
T.cells	NECTIN1	0.138834	1.073651	0.276603	0.78273	-5.31897	0.854337	0.83394
T.cells	5730409E0	0.144874	0.23499	0.276548	0.782773	-5.23922	0.870571	0.853591
T.cells	ZMIZ2	-0.03139	4.532649	-0.27653	0.782783	-6.41131	0.790294	0.757442
T.cells	KIF18A	-0.03643	3.977118	-0.27651	0.7828	-6.5452	0.800269	0.769246
T.cells	MLEC	0.024355	5.064037	0.276306	0.782958	-6.64723	0.780862	0.746338
T.cells	OXR1	0.021706	6.336267	0.276283	0.782976	-6.89787	0.758707	0.720365
T.cells	TNRC6A	-0.02018	6.648204	-0.27625	0.783001	-6.89935	0.753366	0.714137
T.cells	SLC6A19	-0.10023	-0.35949	-0.27622	0.783022	-5.51531	0.882249	0.86781
T.cells	BRF1	-0.02335	4.828649	-0.27622	0.783023	-6.59201	0.785027	0.751245
T.cells	ADO	-0.03188	3.708631	-0.27621	0.783029	-6.3649	0.805132	0.775034
T.cells	NUP37	0.034745	3.351508	0.276126	0.783096	-6.43136	0.811643	0.782775
T.cells	CCDC17	0.110656	0.893924	0.276015	0.78318	-5.47563	0.857792	0.838175
T.cells	GPSM1	-0.04401	2.672071	-0.276	0.783194	-6.14433	0.824166	0.797751
T.cells	AP1S2	0.030955	5.081901	0.275815	0.783334	-6.48474	0.780547	0.746032
T.cells	ADAM8	0.123726	2.602795	0.275783	0.783358	-5.46094	0.825453	0.799321
T.cells	CXCR5	0.082563	2.183855	0.275595	0.783503	-5.81827	0.833275	0.808691
T.cells	HBP1	-0.02635	5.788072	-0.27541	0.783645	-6.67895	0.76818	0.73151
T.cells	PTPN22	-0.083	5.324165	-0.27528	0.783743	-5.91856	0.776283	0.741019
T.cells	SLC35B2	0.029279	4.882082	0.275194	0.783809	-6.54307	0.78408	0.750196
T.cells	CYP2AB1	-0.15635	-0.30874	-0.27511	0.783873	-5.23563	0.881247	0.866709
T.cells	TECPR2	-0.03954	3.905519	-0.27506	0.783915	-6.26441	0.801563	0.770929
T.cells	SLC9A8	0.029676	4.988676	0.274991	0.783964	-6.51358	0.782193	0.748053
T.cells	RAB1B	0.024562	5.58788	0.274896	0.784037	-6.68893	0.771667	0.735696
T.cells	CHML	0.063571	1.690018	0.274731	0.784164	-5.84924	0.842584	0.820015

T.cells	AMIGO1	-0.07491	0.613629	-0.27472	0.784174	-5.6717	0.863207	0.84491
T.cells	PDE7B	-0.10877	5.37371	-0.27468	0.784201	-6.1049	0.775414	0.740128
T.cells	GM50013	0.060961	1.85467	0.274295	0.784498	-5.86329	0.839469	0.816293
T.cells	CCPG1	0.028486	6.037912	0.274293	0.784499	-6.64339	0.763849	0.726587
T.cells	TTC13	0.036164	3.938924	0.27428	0.784509	-6.23957	0.800959	0.770309
T.cells	PPP2R3D	0.038033	3.751015	0.274045	0.78469	-6.3223	0.804363	0.774348
T.cells	GPR34	-0.18647	0.588061	-0.27404	0.784694	-5.21087	0.863702	0.845533
T.cells	RBKS	-0.05429	3.630745	-0.27383	0.784857	-5.99527	0.806548	0.777012
T.cells	4930581F2	0.049126	3.634004	0.273797	0.784879	-5.98791	0.806489	0.776941
T.cells	ZMYM1	0.035465	3.329996	0.273794	0.784882	-6.30538	0.812037	0.783542
T.cells	KCNQ1OT1	0.028053	6.399833	0.273655	0.784988	-6.87295	0.757616	0.719401
T.cells	ATG16L1	-0.02712	5.446329	-0.27359	0.785035	-6.62217	0.774141	0.738763
T.cells	ARID4A	0.022729	7.062741	0.273193	0.785342	-6.91272	0.746323	0.70638
T.cells	COQ9	-0.04934	3.205352	-0.27299	0.7855	-5.99928	0.814322	0.786492
T.cells	ELMSAN1	-0.04141	6.924696	-0.27296	0.78552	-6.57428	0.748661	0.709135
T.cells	AV099323	-0.08051	1.209882	-0.27292	0.785553	-5.75426	0.851726	0.831366
T.cells	DNAJC4	0.053264	3.061383	0.272844	0.78561	-5.90784	0.816969	0.789677
T.cells	EZH2	-0.02462	6.663574	-0.27261	0.785786	-7.09695	0.753104	0.71436
T.cells	ZCRB1	0.017639	6.149583	0.272254	0.786062	-6.81263	0.76192	0.72473
T.cells	EMG1	-0.02138	5.937655	-0.27223	0.786081	-6.85808	0.765584	0.729019
T.cells	RGS13	-0.17605	-1.05629	-0.27213	0.786159	-5.14761	0.89612	0.885508
T.cells	G530011O(	-0.07749	1.80368	-0.2721	0.78618	-5.90968	0.840433	0.817921
T.cells	PGM2	0.038037	4.115633	0.271982	0.78627	-6.23456	0.797771	0.766972
T.cells	NDUFAF1	-0.04455	2.81597	-0.27196	0.786283	-6.07951	0.821499	0.795217
T.cells	STRN	0.027568	5.530644	0.271635	0.786536	-6.62845	0.772666	0.73735
T.cells	CENPK	-0.04068	3.283005	-0.27163	0.786536	-6.37916	0.812898	0.784955
T.cells	ZBTB48	0.098754	1.123594	0.271589	0.786571	-5.55207	0.853379	0.83355
T.cells	PICK1	0.073213	1.538716	0.271522	0.786622	-5.73032	0.845455	0.824001
T.cells	SLC35C2	-0.02753	4.846147	-0.27135	0.786755	-6.38982	0.784717	0.751563
T.cells	NR2C2	0.019962	6.925422	0.271273	0.786814	-6.9788	0.748649	0.709292
T.cells	DOLPP1	0.048864	2.514642	0.271269	0.786817	-6.05821	0.827093	0.801943
T.cells	FRA10AC1	0.035185	3.408508	0.271141	0.786915	-6.2923	0.810601	0.782246
T.cells	VPS35	-0.01961	6.232281	-0.27113	0.786923	-6.816	0.760495	0.723112
T.cells	PPIL6	0.117615	0.219778	0.270948	0.787063	-5.37151	0.870868	0.854749
T.cells	4931406P1	0.023152	5.594349	0.270931	0.787076	-6.67099	0.771554	0.736069
T.cells	DHX9	-0.0256	6.492589	-0.27079	0.787186	-6.9177	0.756026	0.71789
T.cells	SERHL	-0.03958	3.492794	-0.27066	0.787286	-6.18091	0.809061	0.780414
T.cells	SNX6	0.018007	6.568189	0.270635	0.787302	-6.82454	0.754732	0.716381
T.cells	ZFP169	0.061534	2.782441	0.270474	0.787426	-6.00544	0.82212	0.796053
T.cells	ADAMTS1	-0.1083	2.937185	-0.27032	0.78754	-5.73038	0.819258	0.792639
T.cells	MRPL35	0.023402	5.21625	0.270321	0.787543	-6.73243	0.778179	0.743921
T.cells	PRDX2	0.026763	7.774205	0.27027	0.787582	-7.08025	0.734381	0.692792
T.cells	GM26674	-0.13581	-1.15959	-0.27026	0.787592	-5.17251	0.898193	0.888163
T.cells	WDR13	0.054125	2.570105	0.270254	0.787595	-5.77559	0.826061	0.800778
T.cells	GSTCD	0.037931	3.564196	0.270204	0.787633	-6.32155	0.80776	0.778942
T.cells	GATAD2B	-0.01981	7.433227	-0.26992	0.787852	-7.02008	0.740081	0.699486
T.cells	PPM1D	-0.02191	4.948971	-0.26988	0.787885	-6.66721	0.782895	0.749586

T.cells	IFIT3	0.170047	3.112471	0.269775	0.787962	-5.58051	0.816029	0.7889
T.cells	NIM1K	-0.06296	2.88905	-0.26971	0.788016	-5.88574	0.820147	0.793819
T.cells	ESD	0.024883	7.081837	0.2697	0.788019	-6.94986	0.746	0.706374
T.cells	CRTC2	-0.03927	4.134514	-0.26969	0.788024	-6.29996	0.797431	0.766777
T.cells	SMPD4	-0.03769	3.209019	-0.26965	0.788059	-6.31829	0.814255	0.786783
T.cells	S1PR1	0.046159	4.061514	0.269504	0.78817	-6.32983	0.798746	0.768337
T.cells	PRR11	0.044483	2.847187	0.269458	0.788205	-6.3536	0.820921	0.794744
T.cells	ARPC3	0.016089	8.451234	0.269199	0.788403	-7.131	0.723185	0.679959
T.cells	TMED3	-0.03463	5.491352	-0.26911	0.78847	-6.43104	0.773353	0.738355
T.cells	MCTP1	-0.05329	5.535347	-0.26902	0.78854	-6.67489	0.772584	0.737451
T.cells	DIMT1	-0.03744	3.243055	-0.26899	0.788564	-6.21418	0.81363	0.786038
T.cells	4931413K1	-0.06534	1.943113	-0.26892	0.788616	-5.76197	0.837801	0.81498
T.cells	ALYREF2	0.037887	3.243584	0.268848	0.788673	-6.21553	0.81362	0.786027
T.cells	SLC25A39	0.031645	6.472882	0.268816	0.788697	-6.7864	0.756363	0.718451
T.cells	GM2788	0.083045	0.569462	0.268805	0.788706	-5.66591	0.864063	0.846688
T.cells	INTU	0.123907	0.880465	0.268792	0.788716	-5.49065	0.858051	0.839406
T.cells	QPCTL	-0.04231	3.010203	-0.26875	0.788745	-6.0115	0.817912	0.791147
T.cells	MAU2	0.026065	6.192162	0.268642	0.788831	-6.80616	0.761186	0.724088
T.cells	CDKL5	0.064446	2.066071	0.2686	0.788863	-5.98416	0.835487	0.812198
T.cells	RPP38	-0.09666	1.226405	-0.2685	0.788939	-5.55887	0.85141	0.831377
T.cells	GM12359	-0.06709	1.671224	-0.26842	0.789002	-5.74549	0.84294	0.821163
T.cells	SPTBN5	-0.14995	-0.00378	-0.26833	0.789071	-5.20903	0.875244	0.860269
T.cells	1110059E2	0.027978	4.393524	0.268329	0.789071	-6.50202	0.792781	0.761269
T.cells	CKLF	-0.03008	4.804772	-0.26826	0.789123	-6.46028	0.785451	0.752604
T.cells	GORASP2	0.019848	5.534187	0.268238	0.789141	-6.65797	0.772604	0.737475
T.cells	GLMN	0.045487	2.819036	0.268225	0.789151	-6.08388	0.821442	0.795366
T.cells	MEMO1	-0.01957	6.363616	-0.26819	0.78918	-6.90946	0.758237	0.720647
T.cells	HS6ST1	-0.04214	4.156867	-0.26818	0.789183	-6.17257	0.797029	0.766308
T.cells	HIST1H4M	-0.0741	1.423329	-0.26814	0.789212	-5.84759	0.84765	0.826848
T.cells	PIKFYVE	-0.0375	4.426045	-0.26813	0.78922	-6.24785	0.792199	0.760588
T.cells	KCNIP2	-0.09939	1.248736	-0.26797	0.789348	-5.52538	0.850983	0.830911
T.cells	PUM2	0.014839	7.324014	0.267849	0.78944	-6.99926	0.741916	0.701673
T.cells	METAP2	0.01755	7.3726	0.267818	0.789463	-7.0337	0.741099	0.700725
T.cells	ROCK1	-0.01834	8.134703	-0.26774	0.789526	-7.0578	0.728399	0.686025
T.cells	MTRR	0.075101	1.505683	0.267683	0.789567	-5.72754	0.846083	0.825028
T.cells	EEF2K	-0.02876	4.644155	-0.26754	0.789679	-6.61996	0.788346	0.756102
T.cells	TIMM17B	-0.01867	5.161183	-0.26738	0.789799	-6.67469	0.779189	0.745361
T.cells	CLIC1	-0.01938	8.942892	-0.26735	0.789821	-7.30828	0.715194	0.67089
T.cells	FAM78B	-0.07755	2.115372	-0.2672	0.789941	-5.74205	0.834655	0.811318
T.cells	GM34084	-0.17777	1.964286	-0.26707	0.790036	-5.33771	0.837498	0.814786
T.cells	4930503L1	-0.04501	2.576564	-0.26697	0.790116	-6.06434	0.826035	0.801047
T.cells	RNF139	0.02783	5.218473	0.26686	0.790199	-6.58975	0.778229	0.744273
T.cells	EGF	0.087748	0.634522	0.266825	0.790225	-5.50785	0.8629	0.845487
T.cells	STXBP4	0.05811	2.23619	0.266691	0.790328	-6.03117	0.832423	0.808696
T.cells	ATP8A2	0.09562	2.725958	0.266571	0.79042	-5.77154	0.823316	0.797763
T.cells	EIF2AK4	0.030653	4.653928	0.266403	0.790549	-6.54103	0.788332	0.756156
T.cells	VPS4A	-0.02718	4.241466	-0.26616	0.790732	-6.4433	0.795776	0.764945

T.cells	2310040G2	0.070153	1.291307	0.266133	0.790757	-5.50619	0.850456	0.830365
T.cells	NPAS2	-0.16171	0.089778	-0.26574	0.791059	-5.18199	0.873961	0.858571
T.cells	FUT11	-0.04235	3.493603	-0.26562	0.79115	-6.14662	0.809574	0.781091
T.cells	STAM	0.02519	4.382468	0.26546	0.791273	-6.48287	0.793497	0.762061
T.cells	FCRL5	0.146168	-0.65204	0.265424	0.791301	-5.17466	0.888628	0.876521
T.cells	PARP16	-0.10071	1.117003	-0.26533	0.79137	-5.49635	0.854062	0.83454
T.cells	ACAT3	-0.10565	1.453412	-0.26525	0.791432	-5.52124	0.84763	0.826796
T.cells	GMPR	0.060951	1.923101	0.264994	0.791631	-5.96056	0.83886	0.816134
T.cells	4932438H2	-0.1639	-0.27879	-0.26479	0.791787	-5.20885	0.881448	0.867604
T.cells	ITM2A	-0.07359	2.544803	-0.26472	0.791841	-5.82075	0.827276	0.802146
T.cells	PRPF6	0.021361	5.300623	0.264293	0.792169	-6.64494	0.777609	0.743028
T.cells	S1PR4	-0.03273	4.183137	-0.26424	0.792208	-6.52772	0.797493	0.766518
T.cells	GM47644	0.121154	1.060844	0.264131	0.792294	-5.44458	0.855587	0.836104
T.cells	4933411E0	0.108731	0.555657	0.264026	0.792374	-5.42398	0.865346	0.847919
T.cells	FBXO46	-0.05604	2.47185	-0.26397	0.792414	-5.9231	0.828864	0.803954
T.cells	PDCD4	0.022755	7.282332	0.263517	0.792765	-6.92919	0.743753	0.703353
T.cells	OLFR1033	-0.15492	0.583769	-0.26296	0.793193	-5.29499	0.865418	0.847533
T.cells	DENND3	0.050074	3.548798	0.26294	0.793209	-6.08874	0.809568	0.780468
T.cells	CAPNS1	0.01881	7.25812	0.262535	0.79352	-6.89665	0.74443	0.703929
T.cells	TDG	0.021708	5.074746	0.262495	0.79355	-6.68953	0.782148	0.748045
T.cells	PIK3AP1	0.028643	8.295612	0.262474	0.793567	-7.1791	0.727116	0.6839
T.cells	ZCCHC9	-0.02037	5.404138	-0.26243	0.793603	-6.69614	0.776345	0.741226
T.cells	TIMM13	-0.01912	6.732513	-0.26241	0.793613	-6.9424	0.75335	0.714314
T.cells	XIAP	0.016986	6.725324	0.262405	0.79362	-6.88562	0.753472	0.714457
T.cells	LCT	-0.10138	0.170332	-0.26205	0.793896	-5.48189	0.873708	0.857507
T.cells	EXOC3	0.02554	5.06994	0.261831	0.794061	-6.54433	0.782462	0.748327
T.cells	IRF3	-0.03428	3.786081	-0.26175	0.794122	-6.28307	0.805481	0.775571
T.cells	ARHGAP27	-0.08962	0.465307	-0.26171	0.794153	-5.47692	0.867975	0.850603
T.cells	TMEM33	-0.02282	5.133185	-0.26162	0.794226	-6.56743	0.781344	0.747067
T.cells	E030030I06	-0.04359	2.657085	-0.26155	0.794279	-6.01319	0.826244	0.800364
T.cells	COMT	0.026174	5.36738	0.261343	0.794436	-6.60425	0.777276	0.742212
T.cells	OXSR1	-0.03667	3.496646	-0.26119	0.794553	-6.24475	0.810818	0.781903
T.cells	ADD1	-0.02362	5.795836	-0.26117	0.794572	-6.67579	0.769779	0.733433
T.cells	SPTLC2	-0.01972	6.245992	-0.26094	0.794745	-6.71576	0.761976	0.724365
T.cells	LARP4	-0.02052	6.410341	-0.26094	0.794745	-6.81504	0.759146	0.721058
T.cells	PROSER1	-0.03066	4.213066	-0.26091	0.794772	-6.43595	0.797816	0.766543
T.cells	GM36199	0.147118	-0.13106	0.26074	0.7949	-5.22363	0.879727	0.864956
T.cells	OCIAD1	0.018904	5.885315	0.260712	0.794921	-6.74452	0.768222	0.731693
T.cells	RETREG3	-0.02381	5.527566	-0.26055	0.795047	-6.64487	0.774465	0.739059
T.cells	CENPW	0.033264	4.349764	0.260545	0.795049	-6.6178	0.795357	0.763694
T.cells	TPK1	0.02955	4.865838	0.260137	0.795363	-6.52897	0.786291	0.752897
T.cells	CTSB	-0.04015	8.190873	-0.26005	0.795429	-6.84338	0.729255	0.686338
T.cells	RPGR	0.057097	1.599467	0.260044	0.795435	-5.84834	0.846372	0.824526
T.cells	PRAG1	0.081978	1.923732	0.259894	0.79555	-5.64362	0.840222	0.817133
T.cells	WARS2	0.046355	3.512477	0.259818	0.795608	-6.07972	0.810686	0.781808
T.cells	NDUFA1	0.020215	6.729501	0.259768	0.795647	-6.88884	0.753824	0.714859
T.cells	BCL10	-0.01903	6.452929	-0.25963	0.795749	-6.83555	0.758561	0.720428

T.cells	ICE1	-0.03867	4.081492	-0.25957	0.795797	-6.24838	0.800344	0.769596
T.cells	METTL17	0.045647	2.488504	0.259422	0.795913	-6.02293	0.82961	0.804475
T.cells	MCOLN2	0.039844	3.161941	0.259312	0.795997	-6.17012	0.817118	0.789547
T.cells	TMEM267	-0.06922	1.734822	-0.2593	0.796004	-5.74618	0.8438	0.821515
T.cells	PTK2B	-0.02479	7.134222	-0.25892	0.7963	-6.88792	0.746942	0.706926
T.cells	ATP5C1	-0.01444	8.358211	-0.25888	0.796333	-7.19218	0.726491	0.68325
T.cells	RARRES1	-0.12402	0.889058	-0.25885	0.79635	-5.48286	0.85999	0.841088
T.cells	ALDH3B1	0.065011	3.605843	0.258845	0.796356	-5.77298	0.80898	0.779889
T.cells	ZFP955A	0.06689	1.472841	0.258802	0.796389	-5.83805	0.848784	0.827557
T.cells	CCDC122	-0.09811	1.315452	-0.25873	0.796447	-5.44397	0.851792	0.831185
T.cells	TXK	-0.1109	3.287256	-0.25851	0.796618	-5.57279	0.814813	0.786899
T.cells	KCTD7	0.093817	0.387211	0.258496	0.796625	-5.42665	0.869731	0.852963
T.cells	NEPRO	-0.04497	2.709524	-0.25843	0.796673	-6.07987	0.825491	0.799675
T.cells	D030056L2	-0.03254	3.888031	-0.2584	0.796698	-6.46035	0.803846	0.773877
T.cells	GIMAP8	-0.09477	2.465426	-0.25821	0.796842	-5.60183	0.830043	0.805139
T.cells	GM43623	0.149827	-0.49767	0.258195	0.796856	-5.17897	0.887157	0.874214
T.cells	ARF4OS	-0.08783	0.845404	-0.25812	0.796913	-5.48224	0.860835	0.842243
T.cells	SPINDOC	0.025526	4.427162	0.258013	0.796997	-6.52506	0.794136	0.762419
T.cells	5031425E2	0.025212	5.390925	0.257833	0.797135	-6.64304	0.777088	0.742285
T.cells	ZMAT5	-0.02348	4.547775	-0.25763	0.797288	-6.52217	0.792038	0.759961
T.cells	CERCAM	0.075648	0.652443	0.257537	0.797363	-5.63712	0.864654	0.846916
T.cells	SMIM24	-0.0406	3.210803	-0.25737	0.797488	-6.07866	0.816298	0.788772
T.cells	MXD3	0.054096	1.735683	0.257346	0.797509	-6.07312	0.843865	0.821806
T.cells	EFHD2	0.028744	6.92246	0.257345	0.79751	-6.68242	0.750608	0.711344
T.cells	TG	0.105147	1.91376	0.257284	0.797557	-5.36761	0.840492	0.817747
T.cells	1810013L2	-0.02337	5.901803	-0.25707	0.79772	-6.67892	0.768217	0.731879
T.cells	H2-DMB2	-0.0743	3.016521	-0.25702	0.79776	-5.772	0.819942	0.793082
T.cells	SMURF2	0.020171	6.82379	0.256869	0.797877	-6.91049	0.75237	0.713357
T.cells	UBASH3B	0.063087	5.794781	0.25665	0.798046	-6.21241	0.770105	0.734152
T.cells	INPP5F	0.031753	4.366787	0.256619	0.798069	-6.50746	0.795369	0.763926
T.cells	MEF2A	-0.02124	7.727346	-0.25662	0.798071	-6.9471	0.737116	0.695699
T.cells	MFSD4B4	0.120808	-0.0204	0.256453	0.798197	-5.31938	0.877958	0.86309
T.cells	RAPGEF6	-0.01812	8.049527	-0.25604	0.798515	-7.08174	0.731987	0.689622
T.cells	FRY	0.028841	6.378637	0.256	0.798546	-6.77264	0.760242	0.722466
T.cells	ACTR1B	-0.03247	4.215968	-0.2559	0.798624	-6.36696	0.798342	0.767333
T.cells	SNAP23	-0.02743	6.222753	-0.25539	0.799017	-6.55054	0.76293	0.725794
T.cells	RBM18	0.026972	4.236008	0.255378	0.799024	-6.35253	0.797981	0.767069
T.cells	MSL2	0.020676	6.010868	0.255342	0.799052	-6.74249	0.766598	0.730106
T.cells	CCDC80	-0.10112	2.683481	-0.25517	0.799182	-5.58208	0.826414	0.800962
T.cells	CYP3A13	0.119802	0.779768	0.254809	0.799462	-5.35743	0.862561	0.844556
T.cells	BCLAF1	-0.0145	7.155376	-0.25478	0.799485	-7.00466	0.746981	0.707274
T.cells	HPF1	-0.02593	5.467524	-0.25475	0.799507	-6.78377	0.77608	0.741302
T.cells	DGAT1	-0.0477	7.095267	-0.25463	0.799604	-6.73039	0.748	0.708458
T.cells	RHBDD2	0.058609	2.284757	0.254566	0.799649	-5.77353	0.833867	0.809959
T.cells	TMEM184E	-0.03609	4.707523	-0.25448	0.799715	-6.38874	0.789529	0.757153
T.cells	BAG2	0.066659	1.435408	0.254434	0.799751	-5.74805	0.84995	0.829311
T.cells	GSTP2	0.10937	0.861498	0.25439	0.799785	-5.33621	0.86098	0.842667

T.cells	PPA1	-0.02878	4.797054	-0.25433	0.799829	-6.62914	0.787933	0.755312
T.cells	MIDN	-0.02368	6.064607	-0.25425	0.799896	-6.68507	0.765666	0.729143
T.cells	SCRIB	0.029864	3.392621	0.254218	0.799917	-6.32301	0.813312	0.785451
T.cells	GM9530	-0.13633	-0.31965	-0.25418	0.799946	-5.25353	0.884094	0.870815
T.cells	P2RX7	-0.10491	3.223954	-0.25363	0.800371	-5.50318	0.816411	0.789238
T.cells	POLR2F	-0.02021	5.381223	-0.25362	0.800378	-6.68375	0.777597	0.743242
T.cells	CNOT7	-0.01922	5.288607	-0.25361	0.800386	-6.60434	0.779227	0.745161
T.cells	PRR5L	-0.11753	2.756439	-0.25353	0.800444	-5.51364	0.825057	0.799577
T.cells	URB2	0.044624	2.571455	0.253529	0.800448	-6.05186	0.828502	0.803702
T.cells	TMEM186	0.057217	2.111676	0.253369	0.800571	-5.91755	0.837121	0.814122
T.cells	MBNL2	0.022209	8.406165	0.253282	0.800638	-7.05552	0.726087	0.683297
T.cells	PRPF4	-0.03139	3.92555	-0.25313	0.800754	-6.37249	0.803593	0.774087
T.cells	GPATCH8	0.015792	7.002079	0.253024	0.800837	-6.97871	0.749581	0.710556
T.cells	PTEN	0.015189	8.288084	0.252775	0.801029	-7.17796	0.728035	0.685639
T.cells	ST18	0.121051	0.283819	0.252652	0.801124	-5.50594	0.872214	0.856697
T.cells	KREMEN1	-0.04503	3.039966	-0.25258	0.801177	-5.96079	0.819803	0.793542
T.cells	HIVEP3	0.039638	5.144836	0.252559	0.801196	-6.37393	0.781764	0.748388
T.cells	RNASET2A	-0.03689	5.439463	-0.25244	0.801289	-6.50403	0.776573	0.742323
T.cells	LZIC	-0.02988	3.63991	-0.25229	0.801405	-6.37495	0.808789	0.780464
T.cells	KIF5B	0.013712	7.401742	0.252257	0.801428	-7.00806	0.742821	0.702866
T.cells	ANXA10	0.101191	0.643116	0.252133	0.801524	-5.44905	0.865211	0.848282
T.cells	VBP1	-0.02087	4.92592	-0.25212	0.80153	-6.61435	0.785642	0.75302
T.cells	NUP107	-0.02356	5.353646	-0.25212	0.801537	-6.70499	0.778082	0.744107
T.cells	PSD4	0.034418	4.541706	0.2519	0.801703	-6.3007	0.792492	0.761157
T.cells	LRRC4	-0.03934	3.774431	-0.25189	0.801713	-6.27006	0.806338	0.777587
T.cells	FEM1C	0.025333	7.117684	0.251715	0.801845	-6.86215	0.74762	0.708509
T.cells	AP2M1	-0.02036	6.941455	-0.25171	0.801846	-6.91974	0.750611	0.711993
T.cells	RNF144B	-0.07819	2.562148	-0.25166	0.801889	-5.61416	0.828676	0.804301
T.cells	STFA2L1	0.160103	2.448793	0.251454	0.802047	-5.4669	0.830793	0.806861
T.cells	TMBIM6	0.019499	9.142023	0.251402	0.802087	-7.20048	0.714055	0.669723
T.cells	MED20	-0.03007	4.104119	-0.25123	0.802222	-6.31851	0.800361	0.770541
T.cells	HINT3	-0.03219	4.643869	-0.2512	0.80224	-6.36373	0.790665	0.75905
T.cells	ZFP318	-0.05857	3.491008	-0.25115	0.802283	-5.99897	0.81151	0.783802
T.cells	ANKMY2	-0.03217	3.602393	-0.25112	0.802303	-6.21429	0.809473	0.781381
T.cells	CIT	-0.04046	4.461996	-0.25103	0.802375	-6.66818	0.79392	0.762909
T.cells	PSMD10	-0.03703	3.584601	-0.25102	0.802378	-6.22433	0.809798	0.781769
T.cells	REC114	-0.05921	3.19631	-0.25088	0.802492	-5.89552	0.81692	0.790282
T.cells	NEGR1	-0.16886	-0.13284	-0.2508	0.802551	-5.19541	0.8804	0.866904
T.cells	SUPT20	-0.02244	5.30473	-0.25077	0.802571	-6.61227	0.778943	0.745251
T.cells	CNOT9	-0.02172	4.541441	-0.25073	0.802607	-6.60797	0.792496	0.761257
T.cells	MKNK2	-0.0191	6.23257	-0.2507	0.80263	-6.82838	0.762761	0.726247
T.cells	NME2	-0.02229	8.557653	-0.25066	0.802656	-7.22639	0.723594	0.680735
T.cells	ZFP592	-0.017	5.89966	-0.25063	0.802679	-6.80337	0.76853	0.733009
T.cells	NETO2	0.042575	3.22493	0.250588	0.802714	-6.32479	0.816393	0.789667
T.cells	HOOK3	0.020967	6.076111	0.25056	0.802735	-6.69847	0.765467	0.729417
T.cells	RBPJ	0.040756	6.055966	0.250502	0.80278	-6.61221	0.765816	0.729826
T.cells	NCOA6	0.019736	5.600455	0.250455	0.802817	-6.68246	0.77375	0.739141

T.cells	PSMC6	-0.0173	6.141841	-0.25009	0.803102	-6.86052	0.76452	0.728178
T.cells	1700102H2	-0.0725	0.529845	-0.25001	0.80316	-5.68337	0.86763	0.851214
T.cells	MLKL	0.069464	2.335281	0.249543	0.80352	-5.8212	0.833427	0.809727
T.cells	SETD7	-0.02908	4.9704	-0.24927	0.803734	-6.46667	0.785427	0.752508
T.cells	AURKAIP1	0.020243	5.837172	0.249112	0.803852	-6.77016	0.770181	0.734626
T.cells	GM11342	0.061442	2.120734	0.249016	0.803926	-5.9895	0.837563	0.814704
T.cells	CTNNB1	0.016265	6.277504	0.249011	0.80393	-6.79647	0.762543	0.7257
T.cells	DECR1	-0.05155	3.696206	-0.24879	0.8041	-6.13198	0.808353	0.779792
T.cells	EXOC4	0.014929	8.03297	0.248766	0.804118	-7.07584	0.732798	0.691116
T.cells	EPO	0.106718	0.415776	0.248736	0.804142	-5.36968	0.870272	0.854261
T.cells	GM28112	0.138589	0.043725	0.248677	0.804188	-5.27226	0.877565	0.863133
T.cells	ZFP366	0.157287	1.840557	0.248437	0.804373	-5.31833	0.842978	0.82114
T.cells	RETNLA	0.492425	-0.77811	0.248235	0.804528	-5.18956	0.894091	0.883079
T.cells	SMG1	0.01547	7.499087	0.247955	0.804744	-6.99343	0.742025	0.70159
T.cells	PIGYL	0.026961	4.270837	0.247781	0.804878	-6.49033	0.79826	0.767613
T.cells	HDGF	-0.01612	6.998077	-0.24763	0.804998	-7.06597	0.7505	0.711504
T.cells	GM43713	-0.06126	2.135076	-0.2476	0.805016	-5.90179	0.837631	0.814627
T.cells	TMEM9	-0.04812	2.576914	-0.24752	0.805082	-6.10053	0.829341	0.804678
T.cells	QDPR	-0.02229	5.245358	-0.24749	0.805103	-6.63849	0.780875	0.747084
T.cells	TCF12	-0.01872	8.463403	-0.24737	0.805192	-7.16948	0.725967	0.68307
T.cells	MAP3K3	0.021458	6.795781	0.247177	0.805344	-6.90155	0.753948	0.715592
T.cells	ZFP618	-0.11949	0.531603	-0.24708	0.80542	-5.31057	0.868364	0.851849
T.cells	MARVELD2	0.096521	1.369704	0.247078	0.805421	-5.49551	0.852173	0.832246
T.cells	WDR45	0.040868	2.960786	0.246757	0.805668	-6.04155	0.8222	0.796266
T.cells	ARHGEF40	-0.10039	0.550928	-0.24674	0.805678	-5.48119	0.867987	0.851433
T.cells	FAM92A	-0.03716	3.301539	-0.24673	0.805687	-6.2416	0.815909	0.788752
T.cells	ABLIM2	-0.14458	-0.47962	-0.24655	0.805832	-5.17555	0.888275	0.876136
T.cells	NDUFS1	-0.01655	5.752161	-0.24651	0.805858	-6.78075	0.771975	0.736741
T.cells	RBM4B	-0.02017	5.747385	-0.24646	0.805896	-6.80128	0.772058	0.736839
T.cells	ZBED3	0.028292	3.559356	0.246361	0.805974	-6.43121	0.81118	0.783114
T.cells	NFATC2IP	-0.03225	3.151572	-0.24633	0.805999	-6.33893	0.818672	0.79205
T.cells	GM45370	0.155713	-1.29914	0.246316	0.806009	-5.13606	0.904717	0.896267
T.cells	2810006K2	-0.05098	2.570884	-0.24602	0.806235	-6.06724	0.82952	0.804976
T.cells	GPR183	0.107918	3.488292	0.245856	0.806363	-5.54143	0.812547	0.784764
T.cells	FAM129B	-0.05461	3.14002	-0.2458	0.806409	-6.14146	0.818952	0.792415
T.cells	CCT7	0.017354	6.427225	0.245713	0.806474	-6.90849	0.76033	0.723125
T.cells	SETD6	0.056393	1.423905	0.245701	0.806483	-5.73526	0.851204	0.831149
T.cells	SELENOO	0.040867	3.136064	0.245677	0.806502	-6.08336	0.819025	0.792503
T.cells	BORA	0.030416	3.535011	0.245557	0.806594	-6.41019	0.811712	0.783785
T.cells	DNM2	-0.01704	7.193868	-0.24504	0.806995	-6.91299	0.747406	0.707926
T.cells	WDR47	-0.04222	3.140773	-0.24493	0.807081	-6.06899	0.819123	0.79252
T.cells	TPM3-RS7	-0.09148	0.526188	-0.24477	0.8072	-5.44222	0.868736	0.852304
T.cells	CLPP	-0.02422	4.725598	-0.24473	0.807231	-6.55887	0.790345	0.758356
T.cells	NOP9	-0.02315	4.555827	-0.24467	0.80728	-6.55722	0.793382	0.76195
T.cells	GTF2A1	-0.02418	5.65525	-0.24465	0.807295	-6.67288	0.773907	0.738982
T.cells	FNBP1L	0.049155	3.079155	0.244598	0.807335	-6.156	0.820261	0.793916
T.cells	1810020O0	0.161632	-0.23349	0.244575	0.807352	-5.19942	0.883661	0.870469

T.cells	AZIN1	-0.02114	6.940521	-0.24446	0.807444	-6.95754	0.75171	0.713037
T.cells	FAM120C	0.04359	3.050639	0.244425	0.807468	-6.12671	0.820789	0.794588
T.cells	MIF4GD	-0.02756	4.964455	-0.24424	0.807611	-6.50979	0.78616	0.753384
T.cells	MED31	-0.03318	3.491551	-0.24381	0.807945	-6.22882	0.813007	0.785016
T.cells	TMEM175	0.038773	3.375009	0.243516	0.80817	-6.19685	0.815241	0.787566
T.cells	MAP2K3OS	-0.15235	-0.12812	-0.24345	0.808224	-5.24556	0.882045	0.868103
T.cells	LPAR6	0.039481	4.523596	0.243386	0.80827	-6.34281	0.794381	0.762788
T.cells	PIK3R5	0.042836	4.45137	0.243315	0.808325	-6.13646	0.795678	0.764358
T.cells	ATXN7L3B	0.018129	5.941589	0.243129	0.808469	-6.75166	0.769386	0.733328
T.cells	TENT4A	-0.03496	3.460072	-0.24291	0.808636	-6.2689	0.813775	0.785831
T.cells	GADD45GII	0.022067	5.128075	0.242833	0.808697	-6.68286	0.783696	0.750204
T.cells	LPAR2	0.043434	2.187833	0.242744	0.808766	-5.9167	0.837436	0.814177
T.cells	IL18R1	-0.12291	0.978465	-0.2427	0.808804	-5.33559	0.860518	0.842026
T.cells	PRR12	-0.03237	3.451612	-0.24256	0.808908	-6.22709	0.81393	0.786112
T.cells	ZFP229	0.099249	-0.01049	0.242545	0.808919	-5.41951	0.879826	0.865521
T.cells	MTMR3	0.016298	7.886466	0.242277	0.809127	-7.07078	0.736324	0.694831
T.cells	UCK1	-0.03762	3.265642	-0.24218	0.809199	-6.27715	0.817447	0.790232
T.cells	RAD54B	-0.04963	2.207787	-0.24214	0.809229	-6.12471	0.837159	0.813831
T.cells	LYPLA1	0.01834	5.988433	0.241999	0.809341	-6.79187	0.768684	0.732618
T.cells	KLF7	-0.03418	6.159891	-0.24196	0.809373	-6.58284	0.765707	0.729129
T.cells	CDK8	-0.03222	6.559883	-0.24182	0.809477	-6.88908	0.758832	0.72109
T.cells	UBA7	0.053427	3.784743	0.241733	0.809547	-6.00729	0.807965	0.779027
T.cells	ZFP974	-0.09185	0.877863	-0.24149	0.809732	-5.5677	0.862691	0.844647
T.cells	RNF113A2	-0.02705	4.271264	-0.24144	0.809774	-6.44922	0.799225	0.768578
T.cells	MRPS35	-0.0231	4.266456	-0.24106	0.81007	-6.49344	0.79942	0.768789
T.cells	GNE	-0.02642	4.467546	-0.24101	0.810104	-6.43386	0.795798	0.764505
T.cells	FAM49A	-0.03975	5.390412	-0.24084	0.810234	-6.51311	0.779372	0.745139
T.cells	BBS4	0.059752	2.313596	0.240745	0.81031	-5.74147	0.835402	0.811771
T.cells	NCOR1	0.012675	8.098707	0.240666	0.810371	-7.11568	0.732993	0.691048
T.cells	GM43647	0.145173	-0.94495	0.240555	0.810457	-5.18327	0.898793	0.888669
T.cells	AATF	-0.02417	4.995367	-0.24044	0.810544	-6.5519	0.786364	0.753409
T.cells	TMEM117	0.1622	-0.04691	0.240377	0.810594	-5.20474	0.880896	0.866809
T.cells	HAGH	-0.03725	5.358255	-0.24028	0.810667	-6.37306	0.779939	0.745836
T.cells	4833420G1	0.02449	5.494494	0.240275	0.810673	-6.74119	0.77754	0.743012
T.cells	CITED4	0.139754	-0.2568	0.240189	0.81074	-5.31188	0.885049	0.871871
T.cells	ADAM9	-0.02746	5.178783	-0.24019	0.810741	-6.52215	0.783111	0.749572
T.cells	VDAC2	-0.0169	7.488132	-0.24	0.810886	-7.03094	0.743254	0.702897
T.cells	HOTAIRM1	0.131858	0.698917	0.239947	0.810927	-5.29123	0.866327	0.849046
T.cells	ALOX5AP	0.071294	7.325815	0.239219	0.81149	-6.54746	0.74622	0.706271
T.cells	GSDME	-0.03396	4.386903	-0.23918	0.811523	-6.43643	0.797532	0.766486
T.cells	MCUR1	0.032212	4.462065	0.239139	0.811552	-6.29192	0.79618	0.764884
T.cells	PRMT2	-0.07696	1.665423	-0.23911	0.811572	-5.5283	0.847975	0.826771
T.cells	ANLN	0.036422	3.747041	0.239004	0.811655	-6.52914	0.809134	0.780259
T.cells	MYO1D	0.049088	3.025547	0.238994	0.811663	-5.98285	0.822406	0.796082
T.cells	CEP41	-0.05585	1.886422	-0.23898	0.811671	-5.81485	0.843771	0.821707
T.cells	PPIP5K1	0.057974	1.776843	0.238821	0.811797	-5.91899	0.84591	0.824232
T.cells	4930522L1	-0.03009	3.684756	-0.23855	0.812009	-6.385	0.810331	0.781649

T.cells	AKTIP	0.037811	3.102695	0.238449	0.812084	-6.06452	0.821037	0.794412
T.cells	BID	-0.03361	3.803317	-0.23834	0.812169	-6.27444	0.808166	0.779074
T.cells	PXDC1	-0.11286	2.191629	-0.2383	0.812197	-5.4884	0.838058	0.814801
T.cells	GPR108	-0.0306	4.177602	-0.23827	0.812225	-6.31068	0.801368	0.771
T.cells	HPS1	-0.0412	3.445228	-0.23826	0.81223	-6.1008	0.814721	0.786876
T.cells	CC2D2B	-0.15871	2.70829	-0.23786	0.81254	-5.53231	0.828488	0.803286
T.cells	NUMBL	0.101973	1.04252	0.237612	0.812732	-5.39655	0.860118	0.84138
T.cells	PPOX	-0.04011	3.108757	-0.23752	0.812805	-6.05689	0.821046	0.794405
T.cells	GM39556	-0.04729	3.384757	-0.23743	0.812874	-6.15443	0.815953	0.788328
T.cells	JUNOS	-0.08849	1.820722	-0.23734	0.812939	-5.54627	0.845205	0.823384
T.cells	ZFP637	-0.04677	2.315939	-0.23687	0.813308	-5.99505	0.835839	0.812188
T.cells	5830411N(	0.12454	-1.25694	0.236838	0.81333	-5.17783	0.905609	0.896888
T.cells	MORN1	0.077667	1.227517	0.236781	0.813374	-5.53645	0.856551	0.837137
T.cells	JCHAIN	0.294151	-0.4625	0.236466	0.813618	-5.21937	0.889649	0.877469
T.cells	KAT5	-0.0483	2.921057	-0.23616	0.813852	-5.99428	0.824526	0.798857
T.cells	ALDOC	-0.11294	0.884667	-0.2361	0.813902	-5.34721	0.863173	0.845409
T.cells	PHF6	0.021749	4.940138	0.236097	0.813904	-6.67892	0.7878	0.755223
T.cells	SRI	-0.02014	6.74315	-0.23607	0.813921	-6.85116	0.756306	0.718249
T.cells	TUSC2	0.04417	3.017242	0.235978	0.813995	-5.99194	0.822741	0.796784
T.cells	KIF4	-0.03392	4.394266	-0.23585	0.814095	-6.70512	0.797575	0.766853
T.cells	YIPF5	-0.01997	5.24625	-0.23576	0.814167	-6.60831	0.782367	0.748893
T.cells	FCOR	0.124033	0.865885	0.235647	0.814252	-5.21092	0.863537	0.845956
T.cells	RASIP1	-0.10053	1.808725	-0.23562	0.814269	-5.43242	0.845433	0.824079
T.cells	WTAP	0.014005	6.931297	0.2356	0.814288	-6.99247	0.753088	0.714586
T.cells	IGSF9	-0.07911	0.790152	-0.23555	0.814328	-5.5197	0.865007	0.847738
T.cells	MANEA	-0.03449	3.728612	-0.23555	0.814329	-6.2435	0.809649	0.781224
T.cells	GM10563	-0.04329	3.607105	-0.23553	0.814345	-6.08213	0.811872	0.78387
T.cells	B3GALNT2	-0.03157	3.643954	-0.23548	0.814377	-6.34174	0.811197	0.783067
T.cells	GM16364.:	-0.10505	1.279847	-0.23545	0.8144	-5.4799	0.855545	0.836283
T.cells	TCRG-C4	-0.13458	0.327974	-0.23542	0.814424	-5.22301	0.874025	0.85869
T.cells	GM14023	0.092506	1.123999	0.235343	0.814487	-5.50026	0.858546	0.839915
T.cells	SYCE2	-0.03327	4.258668	-0.23531	0.814509	-6.58135	0.800021	0.769786
T.cells	C030005K(	0.084531	0.674423	0.235169	0.814622	-5.49755	0.867257	0.850547
T.cells	GM42031	-0.04937	4.172175	-0.23512	0.81466	-6.62552	0.801585	0.771711
T.cells	UNC119B	0.03109	3.551645	0.235097	0.814677	-6.32806	0.812888	0.785155
T.cells	GEMIN6	-0.03844	2.59595	-0.23507	0.814699	-6.17566	0.830586	0.806312
T.cells	TOPORS	0.024303	5.749708	0.235063	0.814703	-6.74146	0.773509	0.738553
T.cells	FAM117B	-0.02883	7.11596	-0.23506	0.814707	-6.69926	0.749943	0.710989
T.cells	ABRAXAS2	0.021698	4.960803	0.234753	0.814943	-6.57665	0.78756	0.754997
T.cells	TSTD2	-0.03168	3.84075	-0.2347	0.814982	-6.28199	0.807735	0.778922
T.cells	LGR5	0.056669	1.073909	0.234584	0.815074	-5.9056	0.859664	0.841245
T.cells	MGME1	-0.05174	2.312135	-0.2345	0.815136	-5.95665	0.836059	0.812799
T.cells	JKAMP	-0.03053	3.680975	-0.23401	0.815514	-6.14318	0.810727	0.782513
T.cells	SGIP1	-0.10223	0.175941	-0.234	0.815524	-5.38295	0.877234	0.862598
T.cells	SOWAHC	-0.092	2.917305	-0.23392	0.815587	-5.56581	0.824806	0.79935
T.cells	ZBTB42	-0.05883	1.544195	-0.23387	0.815623	-5.64847	0.850694	0.830462
T.cells	PAOX	-0.04686	2.987177	-0.23387	0.81563	-6.01467	0.823509	0.797797

T.cells	MAP4K4	0.023436	7.459224	0.233858	0.815636	-6.9907	0.74432	0.704416
T.cells	MCC	-0.08952	2.092188	-0.23377	0.8157	-5.53298	0.840274	0.817934
T.cells	AGBL2	0.083933	0.26749	0.233456	0.815947	-5.46455	0.875557	0.860542
T.cells	GTF3A	-0.01973	4.569389	-0.23344	0.815961	-6.59283	0.79474	0.763518
T.cells	GM40841	-0.0805	0.40148	-0.23329	0.816075	-5.67491	0.872929	0.857413
T.cells	CUEDC2	-0.02092	5.245055	-0.23326	0.8161	-6.67134	0.782697	0.749349
T.cells	RCE1	0.028355	3.757774	0.232963	0.816329	-6.35084	0.809592	0.78107
T.cells	XLR4A	0.074769	0.802973	0.232792	0.816461	-5.5821	0.865331	0.847965
T.cells	TMED9	-0.01512	6.609324	-0.23236	0.816794	-6.8824	0.759348	0.721542
T.cells	KCTD17	-0.1143	0.958849	-0.23209	0.817006	-5.36815	0.862645	0.844414
T.cells	GRAMD1C	-0.06183	2.440736	-0.23183	0.817205	-5.77198	0.834374	0.810387
T.cells	FGD2	-0.08363	3.69567	-0.23181	0.81722	-5.58753	0.811106	0.782581
T.cells	HELZ	-0.01845	6.25998	-0.23177	0.817254	-6.84521	0.765434	0.728674
T.cells	GM48089	0.088671	1.135961	0.231743	0.817273	-5.59172	0.859221	0.840338
T.cells	SMARCB1	-0.01543	5.410318	-0.23171	0.817302	-6.78106	0.780293	0.746136
T.cells	NCOA7	-0.02717	5.299685	-0.23164	0.817353	-6.56343	0.782247	0.748447
T.cells	CD59B	0.116495	0.413	0.231354	0.817574	-5.3972	0.873334	0.857422
T.cells	4930594M	0.110111	-0.02762	0.231344	0.817582	-5.34478	0.882006	0.867973
T.cells	GM16150	0.073434	0.174831	0.230996	0.817852	-5.62264	0.878012	0.86324
T.cells	SNX16	0.040462	2.847666	0.230884	0.817938	-6.04686	0.826813	0.80144
T.cells	CST7	0.075681	2.120473	0.230877	0.817944	-5.78427	0.840462	0.817814
T.cells	CIC	-0.0232	4.821881	-0.23081	0.817994	-6.57349	0.79079	0.758599
T.cells	D11WSU47	0.071634	1.299867	0.230739	0.81805	-5.64109	0.856115	0.836709
T.cells	PRCC	0.019691	5.107381	0.230717	0.818068	-6.63425	0.785704	0.752611
T.cells	PPP1R12C	0.021671	5.009564	0.230693	0.818087	-6.53693	0.787443	0.754663
T.cells	GM13427	0.046698	2.062778	0.230651	0.818119	-5.94921	0.841554	0.819152
T.cells	LEO1	-0.02752	4.138442	-0.23002	0.818604	-6.43734	0.803392	0.773323
T.cells	AKR1B10	0.033723	3.494629	0.229888	0.81871	-6.2755	0.815147	0.787305
T.cells	RNF126	-0.02139	4.767243	-0.2298	0.818777	-6.63571	0.792063	0.759905
T.cells	GM49797	0.022866	4.882769	0.229578	0.81895	-6.55818	0.789998	0.757534
T.cells	FHL2	0.150302	0.017257	0.229497	0.819013	-5.23934	0.881449	0.867286
T.cells	ZFP87	0.03599	3.396086	0.229463	0.819039	-6.17321	0.816961	0.789543
T.cells	F2RL2	0.12972	0.129948	0.229162	0.819272	-5.24438	0.879225	0.8646
T.cells	SMARCC2	-0.01534	6.020787	-0.22915	0.819281	-6.82823	0.769925	0.733932
T.cells	GM4013	-0.0603	1.984122	-0.22907	0.819342	-5.59006	0.84336	0.821191
T.cells	ITGB3	0.060297	2.991713	0.228728	0.819608	-5.7698	0.824442	0.798545
T.cells	NPHP1	-0.07002	0.652168	-0.22872	0.819616	-5.6771	0.868984	0.852205
T.cells	ZFP748	0.057321	1.938501	0.228485	0.819797	-5.84019	0.844226	0.822336
T.cells	GM28694	0.083118	-0.23311	0.228403	0.81986	-5.52543	0.886409	0.873474
T.cells	ARMCX2	-0.07495	1.41393	-0.22825	0.819976	-5.5954	0.854243	0.834468
T.cells	TRIM62	0.058725	1.410517	0.228174	0.820038	-5.66146	0.854308	0.834551
T.cells	CBARP	-0.11023	1.923122	-0.22816	0.820049	-5.32845	0.844518	0.822744
T.cells	AW554918	-0.01938	5.656196	-0.2281	0.820096	-6.76507	0.776303	0.741564
T.cells	PIP4K2A	-0.02179	7.239298	-0.22807	0.820122	-6.89989	0.748965	0.709591
T.cells	6530409C1	0.07446	1.661527	0.228047	0.820136	-5.63946	0.849501	0.828749
T.cells	TRIM39	-0.03856	2.873416	-0.228	0.820173	-5.96404	0.826642	0.801285
T.cells	NSD1	0.01429	7.273059	0.227873	0.820271	-7.00068	0.748392	0.708928

T.cells	NEURL1A	0.143786	-0.39095	0.227837	0.820299	-5.16694	0.889549	0.877362
T.cells	RAPGEFL1	-0.03808	2.784487	-0.22784	0.8203	-6.26315	0.8283	0.803273
T.cells	HAUS2	0.029016	3.755191	0.227831	0.820304	-6.37007	0.81037	0.781868
T.cells	RFESD	0.050794	2.541257	0.227787	0.820338	-5.92144	0.83285	0.808727
T.cells	FCGR3	-0.09209	4.189291	-0.22759	0.820491	-5.6622	0.80247	0.772516
T.cells	TTF2	0.031186	3.091877	0.227585	0.820494	-6.36097	0.822583	0.796473
T.cells	C1D	0.015369	5.843961	0.227544	0.820526	-6.8098	0.773012	0.737742
T.cells	ZC3HAV1	0.018206	8.43854	0.227491	0.820567	-7.10019	0.728865	0.686344
T.cells	CLEC4A4	0.100387	-1.00355	0.227329	0.820693	-5.19091	0.901833	0.892452
T.cells	IL12A	0.042823	2.215059	0.227314	0.820705	-6.23365	0.838989	0.816152
T.cells	TPRA1	0.04886	2.802051	0.227278	0.820733	-5.86592	0.827972	0.802935
T.cells	LUM	-0.11912	0.551186	-0.22721	0.820784	-5.33187	0.870956	0.854775
T.cells	GM10353	-0.04029	2.745067	-0.22697	0.820974	-6.07005	0.829095	0.804229
T.cells	ADGRA2	0.120709	0.687625	0.226944	0.820991	-5.37087	0.868355	0.851564
T.cells	GIMAP3	0.128775	2.932139	0.226715	0.821169	-5.60935	0.825608	0.800112
T.cells	PKD2L2	0.057778	1.170955	0.226674	0.821201	-5.58965	0.858981	0.840267
T.cells	THNSL1	-0.09002	1.077135	-0.22667	0.821201	-5.4825	0.860793	0.84246
T.cells	2810002D1	-0.04825	1.66458	-0.22625	0.821526	-5.77328	0.849766	0.828976
T.cells	GPAT4	-0.02155	4.264538	-0.22613	0.821624	-6.43365	0.801439	0.771168
T.cells	ACYP1	-0.02869	3.847714	-0.22588	0.821817	-6.36838	0.809134	0.780215
T.cells	ZRANB2	0.017006	5.45391	0.225326	0.822246	-6.74559	0.780578	0.746197
T.cells	LSM1	0.01832	5.251939	0.225318	0.822252	-6.65604	0.784151	0.750405
T.cells	MIGA1	0.032806	2.677211	0.22491	0.822568	-6.09413	0.831264	0.80621
T.cells	SIRPA	-0.055	5.832373	-0.22476	0.822687	-6.10905	0.774109	0.738468
T.cells	GM35188	-0.07497	1.73277	-0.22464	0.822775	-5.69193	0.849122	0.827706
T.cells	FBXO6	-0.04422	3.653081	-0.22461	0.822802	-5.91161	0.813178	0.784655
T.cells	BCAT1	0.058581	0.852062	0.224531	0.822862	-5.99729	0.866093	0.848249
T.cells	HIST1H2BN	0.051741	1.240034	0.224455	0.822922	-5.91648	0.858579	0.839162
T.cells	CDV3	-0.01566	7.090195	-0.22442	0.822951	-7.03422	0.752369	0.713092
T.cells	FAU	-0.00988	11.4506	-0.22414	0.823164	-7.62244	0.681531	0.631675
T.cells	LAMTOR2	0.015177	6.577213	0.223895	0.823356	-6.84116	0.761379	0.723403
T.cells	ZFP775	-0.07078	0.81684	-0.22377	0.823449	-5.6115	0.867023	0.84917
T.cells	ANXA11OS	-0.10819	0.812081	-0.22373	0.823487	-5.31851	0.867115	0.849312
T.cells	AFG3L2	0.019814	4.593409	0.223657	0.823541	-6.47794	0.796324	0.764531
T.cells	HDGFL2	-0.01872	4.979057	-0.22355	0.82362	-6.68013	0.789422	0.756377
T.cells	SLC35D1	-0.02746	4.114625	-0.22339	0.823745	-6.35317	0.80504	0.774806
T.cells	TROAP	-0.04764	1.774645	-0.22323	0.82387	-6.0246	0.84868	0.826961
T.cells	AR	0.149194	0.218656	0.223089	0.823981	-5.21663	0.878903	0.863495
T.cells	SHISA5	0.040527	6.572889	0.222743	0.824249	-6.45677	0.761778	0.723604
T.cells	FGD6	0.038104	4.002414	0.222646	0.824324	-6.20304	0.807368	0.777293
T.cells	SLC35G1	-0.08144	1.026313	-0.22252	0.824424	-5.62139	0.863354	0.844367
T.cells	POFUT2	0.033908	4.257323	0.222362	0.824545	-6.1683	0.802811	0.771844
T.cells	XPNPEP3	0.033682	3.250052	0.222245	0.824635	-6.12946	0.821282	0.793807
T.cells	LRP4	0.093918	2.477171	0.222152	0.824708	-5.56843	0.835709	0.811077
T.cells	GM14029	-0.10065	0.297438	-0.22194	0.824876	-5.37321	0.877698	0.861808
T.cells	SLC18A2	-0.04508	2.251616	-0.22166	0.825093	-5.89473	0.839992	0.816304
T.cells	CNTLN	-0.02835	4.34763	-0.22163	0.825111	-6.54924	0.801222	0.770059

T.cells	OVCA2	0.107194	0.174482	0.221592	0.825143	-5.35374	0.880121	0.864802
T.cells	UBQLN4	-0.0296	3.216072	-0.22155	0.825174	-6.19271	0.82194	0.794695
T.cells	TRRAP	0.023938	4.784536	0.221508	0.825207	-6.50985	0.793353	0.760751
T.cells	PDSS2	-0.02236	5.377267	-0.22147	0.825237	-6.68215	0.782794	0.748302
T.cells	DDX56	-0.02381	3.951871	-0.22117	0.82547	-6.45038	0.808465	0.778648
T.cells	NIN	0.022366	5.920519	0.221071	0.825547	-6.7128	0.773284	0.737172
T.cells	FAM91A1	0.020264	5.007003	0.22096	0.825633	-6.47457	0.789427	0.756175
T.cells	CEP68	-0.02831	4.299523	-0.22093	0.825659	-6.41241	0.802145	0.771216
T.cells	B3GLCT	-0.0371	3.311117	-0.2209	0.825676	-6.16489	0.820235	0.792725
T.cells	TNKS2	-0.01383	7.072389	-0.22085	0.825722	-6.942	0.753372	0.713936
T.cells	CHMP6	-0.03057	3.712475	-0.22059	0.825924	-6.33621	0.812859	0.783998
T.cells	THUMPD2	-0.04397	1.928752	-0.22056	0.825944	-5.86365	0.846188	0.823881
T.cells	TXLNA	-0.01966	4.941372	-0.22055	0.825952	-6.63432	0.790613	0.757634
T.cells	ATP10A	-0.03048	3.430551	-0.22034	0.826112	-6.4635	0.818132	0.790239
T.cells	DHX33	-0.02892	3.32737	-0.21986	0.826486	-6.27503	0.820319	0.792539
T.cells	RXYLT1	0.028318	3.864731	0.219721	0.826595	-6.20999	0.810437	0.78078
T.cells	PLSCR4	-0.10518	0.494112	-0.2197	0.826611	-5.34371	0.874301	0.857493
T.cells	SLC35E2	0.027819	3.898481	0.219501	0.826766	-6.23291	0.809901	0.78006
T.cells	BAIAP3	-0.12527	-0.32152	-0.21925	0.826963	-5.21557	0.890571	0.877245
T.cells	PLXNA4OS1	0.092586	0.51532	0.219228	0.826978	-5.43506	0.874017	0.857111
T.cells	1810030O0	-0.02353	4.824335	-0.21917	0.827022	-6.47775	0.793184	0.760317
T.cells	RHEBL1	-0.06053	1.532009	-0.21903	0.827129	-5.75727	0.854321	0.833249
T.cells	GM47819	0.146635	-0.22199	0.218939	0.827202	-5.20316	0.888627	0.874841
T.cells	SMIM7	0.021136	4.932589	0.218811	0.827302	-6.52151	0.791303	0.758029
T.cells	TASP1	0.034965	3.918389	0.218724	0.827369	-6.30392	0.809637	0.779736
T.cells	RCN1	0.039912	3.060814	0.218431	0.827597	-6.03425	0.825603	0.798692
T.cells	6720427I0	0.021936	4.894793	0.21814	0.827823	-6.61459	0.792276	0.759046
T.cells	TTC38	-0.05768	2.505389	-0.21737	0.828425	-5.76601	0.836689	0.811417
T.cells	MTRF1L	0.030905	3.1332	0.21717	0.828576	-6.28444	0.825018	0.797388
T.cells	STOM	-0.06141	3.274711	-0.21704	0.828678	-5.73851	0.82242	0.794254
T.cells	A930001M	-0.03397	3.967402	-0.21683	0.828838	-6.19807	0.809694	0.77909
T.cells	FKBP7	-0.05748	1.867273	-0.21682	0.828847	-5.81134	0.848929	0.825963
T.cells	EHD2	-0.04463	1.82262	-0.21668	0.828954	-6.06005	0.849819	0.826995
T.cells	KIF1BP	-0.02844	3.865166	-0.21648	0.829116	-6.28102	0.811623	0.781369
T.cells	STK19	0.024491	4.61763	0.216445	0.829139	-6.50703	0.797951	0.765174
T.cells	NUDT4	0.018004	5.949353	0.216159	0.829362	-6.81149	0.774281	0.737327
T.cells	UHRF2	-0.01428	6.43989	-0.21614	0.829376	-6.8958	0.765731	0.727326
T.cells	MFSD6	0.029712	4.864614	0.216029	0.829463	-6.60421	0.79351	0.759934
T.cells	2700062C0	0.032932	2.646093	0.21594	0.829532	-6.10272	0.834238	0.808332
T.cells	HEATR1	0.025993	4.84445	0.215937	0.829534	-6.5482	0.793872	0.760361
T.cells	PKD1L3	-0.06376	0.837305	-0.21588	0.829578	-5.69611	0.868871	0.850014
T.cells	PLXDC1	0.036326	3.94302	0.215821	0.829624	-6.48258	0.810198	0.779682
T.cells	APBB3	-0.0683	1.534528	-0.2156	0.829799	-5.59093	0.855421	0.833792
T.cells	COX20	0.020549	5.583215	0.215569	0.829821	-6.71349	0.780772	0.744962
T.cells	SNRPA	0.016549	5.131803	0.215383	0.829965	-6.71267	0.788794	0.75441
T.cells	PJA1	-0.03549	3.699202	-0.21537	0.829976	-6.09468	0.814731	0.785102
T.cells	IGFLR1	-0.09256	0.807622	-0.21472	0.830481	-5.48865	0.869968	0.85089

T.cells	ZCCHC3	0.05473	0.70709	0.214518	0.830637	-5.79963	0.871934	0.8533
T.cells	NLRP3	-0.11564	4.527197	-0.2144	0.830731	-5.67524	0.800059	0.767292
T.cells	GTF3C1	-0.02089	4.779184	-0.21429	0.830811	-6.57409	0.795517	0.761928
T.cells	TMEM201	-0.04675	2.102835	-0.21416	0.830913	-5.8699	0.845006	0.820867
T.cells	MRPL11	0.02178	4.26095	0.214044	0.831006	-6.53609	0.804884	0.773029
T.cells	HOPX	-0.05112	4.034194	-0.21368	0.831285	-6.02991	0.809015	0.777943
T.cells	TMA7	-0.01337	6.564001	-0.21363	0.831327	-6.87706	0.764036	0.72504
T.cells	TMEM173	-0.03152	4.250581	-0.21346	0.831456	-6.25749	0.805072	0.773273
T.cells	PLD1	-0.09299	3.080795	-0.21326	0.831611	-5.48649	0.826599	0.798889
T.cells	GLRA1	0.111371	1.25067	0.213117	0.831726	-5.44237	0.861354	0.84061
T.cells	TMEM14C	0.01666	6.950542	0.213092	0.831746	-6.96495	0.757375	0.717317
T.cells	ECHDC3	-0.10616	0.833138	-0.21304	0.831785	-5.39051	0.86947	0.850418
T.cells	ASH2L	0.022062	4.017187	0.212986	0.831828	-6.42032	0.809325	0.778354
T.cells	GM42047	-0.03626	5.263713	-0.21292	0.831877	-6.73899	0.786853	0.751808
T.cells	HSD11B1	-0.05482	4.593839	-0.21288	0.831909	-5.93732	0.798855	0.76596
T.cells	MOB1B	-0.01474	6.203813	-0.21279	0.831982	-6.80391	0.770293	0.732388
T.cells	MYCL	-0.08463	1.305739	-0.21269	0.832055	-5.52025	0.860289	0.839328
T.cells	TM9SF3	0.012335	7.288714	0.212679	0.832067	-6.95629	0.751592	0.710599
T.cells	DDX55	-0.03426	2.934608	-0.21267	0.832072	-6.14198	0.829327	0.802161
T.cells	HYPK	0.016202	5.8151	0.212655	0.832086	-6.75617	0.7771	0.740357
T.cells	HDAC10	-0.04899	1.608346	-0.21264	0.832101	-5.8119	0.854457	0.832298
T.cells	SASS6	-0.02339	4.42508	-0.21263	0.832107	-6.59209	0.801906	0.769569
T.cells	MSRB3	-0.06688	2.604277	-0.21256	0.832162	-5.71592	0.835521	0.809566
T.cells	AIFM1	0.021427	4.370662	0.212456	0.83224	-6.49838	0.802892	0.770735
T.cells	SEH1L	0.015433	5.334868	0.212357	0.832317	-6.74071	0.785588	0.750335
T.cells	FZD4	0.088134	1.267832	0.212224	0.832421	-5.39652	0.861022	0.840277
T.cells	PCK2	0.029085	3.244845	0.212223	0.832422	-6.40757	0.823549	0.795329
T.cells	DCAF15	-0.02816	3.456621	-0.21202	0.832578	-6.33039	0.819625	0.790728
T.cells	GM15879	0.07998	0.515986	0.211894	0.832677	-5.50807	0.875681	0.858092
T.cells	UVRAG	0.021021	8.569242	0.211892	0.832679	-7.0374	0.730072	0.685839
T.cells	SPAG5	-0.03378	3.137614	-0.21177	0.832777	-6.41469	0.825542	0.797808
T.cells	RSF1OS2	-0.05661	1.663513	-0.2117	0.832828	-5.79753	0.853398	0.831195
T.cells	ZFP148	0.013631	6.830997	0.211622	0.832889	-6.92994	0.759429	0.71986
T.cells	UBXN1	0.01342	6.759331	0.211581	0.832921	-6.92813	0.760663	0.721298
T.cells	ZFP275	-0.06134	1.581356	-0.21113	0.833269	-5.58442	0.854979	0.833214
T.cells	YBEY	-0.05622	1.366271	-0.21099	0.833379	-5.62217	0.859123	0.838263
T.cells	API5	-0.01547	6.03062	-0.21096	0.833405	-6.80215	0.773322	0.736236
T.cells	MCAT	0.035683	2.553855	0.210924	0.833432	-6.10471	0.836474	0.811042
T.cells	UBE2D3	-0.00981	8.966917	-0.21086	0.83348	-7.22456	0.723512	0.678468
T.cells	POLR3H	-0.02573	3.509866	-0.21064	0.833653	-6.35759	0.818645	0.789794
T.cells	NDUFB2	-0.01704	5.815553	-0.21046	0.833791	-6.78417	0.777095	0.740708
T.cells	B9D2	0.022552	4.930502	0.210435	0.833813	-6.66681	0.792805	0.759186
T.cells	MYOM1	-0.09117	1.071853	-0.21043	0.833815	-5.398	0.864825	0.845211
T.cells	CYYR1	-0.08233	2.282713	-0.21031	0.833908	-5.62556	0.841596	0.817262
T.cells	FXR2	-0.01985	5.267562	-0.21015	0.834033	-6.674	0.786787	0.752188
T.cells	LLPH	0.014069	6.447819	0.210041	0.834119	-6.89409	0.766052	0.727886
T.cells	FRAT1	0.027172	3.463579	0.2099	0.834228	-6.25442	0.8195	0.790939

T.cells	NSMF	-0.04805	2.696993	-0.20985	0.834271	-5.81666	0.833782	0.807998
T.cells	BCL11B	0.145561	1.080616	0.209693	0.83439	-5.27256	0.864655	0.845168
T.cells	ITGB3BP	0.023643	3.52337	0.209605	0.834458	-6.353	0.818396	0.789699
T.cells	4932422M	-0.07541	0.972719	-0.20958	0.834475	-5.48984	0.866752	0.847743
T.cells	C8A	-0.09537	0.525618	-0.20954	0.834505	-5.35115	0.875496	0.858349
T.cells	KIF20B	-0.03391	4.170808	-0.20949	0.83455	-6.64426	0.806527	0.775628
T.cells	NEDD4	-0.02203	5.933587	-0.20948	0.834554	-6.79791	0.775022	0.738483
T.cells	GM44659	-0.08135	0.682551	-0.20943	0.834597	-5.40738	0.872418	0.85467
T.cells	PASK	-0.06065	1.295986	-0.20923	0.834752	-5.89139	0.860481	0.840271
T.cells	GM16093	-0.05171	2.640752	-0.20919	0.834779	-5.85758	0.834839	0.809437
T.cells	USP36	-0.02308	4.970109	-0.20911	0.834842	-6.494	0.792096	0.758629
T.cells	MERTK	-0.09291	3.774359	-0.20908	0.834867	-5.69468	0.813776	0.784305
T.cells	WDPCP	0.029827	3.269533	0.209042	0.834897	-6.33583	0.823094	0.7954
T.cells	GM28707	-0.07909	1.154193	-0.20884	0.835055	-5.50926	0.863227	0.84359
T.cells	CFAP126	-0.07746	1.069649	-0.20884	0.835055	-5.53883	0.864868	0.845574
T.cells	HARBI1	0.067596	1.346342	0.208779	0.835101	-5.6486	0.859508	0.839098
T.cells	TIPRL	-0.0167	5.525539	-0.20877	0.835106	-6.7345	0.782211	0.746988
T.cells	GM31812	-0.09261	0.191942	-0.20871	0.835151	-5.39044	0.882074	0.866438
T.cells	GM42699	-0.07567	0.727533	-0.20846	0.835346	-5.62608	0.871544	0.853696
T.cells	2410131K1	0.058374	2.169111	0.208434	0.83537	-5.72784	0.843757	0.82017
T.cells	TADA3	0.031161	3.403164	0.208429	0.835373	-6.16594	0.820624	0.792493
T.cells	TESMIN	-0.08922	0.971475	-0.20824	0.835523	-5.51192	0.866863	0.847986
T.cells	TALDO1	-0.01492	8.310001	-0.20814	0.835598	-7.08232	0.73446	0.691383
T.cells	EIF3D	0.01719	5.480464	0.207641	0.835987	-6.74309	0.783367	0.748168
T.cells	SF3B3	-0.0139	6.034328	-0.20758	0.836037	-6.85053	0.773611	0.736731
T.cells	ARHGAP18	0.027775	6.528672	0.207366	0.836201	-6.81048	0.765085	0.726678
T.cells	SLC39A3	0.059985	1.91724	0.20714	0.836377	-5.65867	0.849134	0.826213
T.cells	ATPAF2	0.031482	2.92038	0.206926	0.836544	-6.17905	0.830175	0.803481
T.cells	IGF1OS	0.133148	-1.03209	0.206915	0.836552	-5.17009	0.907226	0.896712
T.cells	MTDH	0.010422	7.470279	0.206858	0.836597	-7.02367	0.74903	0.707906
T.cells	STX1A	0.056509	1.792697	0.206758	0.836674	-5.84081	0.851525	0.829101
T.cells	FZD1	0.107746	-0.15494	0.206504	0.836872	-5.21111	0.889622	0.875187
T.cells	GM17173	-0.10369	0.150476	-0.20648	0.836892	-5.25488	0.883552	0.867801
T.cells	IFI44	0.141578	0.579228	0.206437	0.836924	-5.28917	0.875094	0.857532
T.cells	BMP1	0.091924	0.911089	0.205999	0.837265	-5.45247	0.868646	0.849748
T.cells	WDR41	-0.03175	4.001344	-0.2059	0.837342	-6.07606	0.810264	0.77977
T.cells	SMPDL3B	-0.12423	1.9506	-0.20581	0.837413	-5.41164	0.848586	0.82555
T.cells	ARNT	-0.02332	5.869604	-0.20575	0.837456	-6.66459	0.776765	0.740253
T.cells	UBIAD1	0.036458	1.941616	0.205675	0.837517	-5.90845	0.848757	0.825794
T.cells	CENPT	-0.03196	2.566821	-0.2053	0.837812	-6.07356	0.836897	0.811713
T.cells	SCYL3	-0.02593	3.642548	-0.20517	0.837911	-6.23338	0.816851	0.787817
T.cells	RNF141	-0.01999	4.511433	-0.2051	0.837962	-6.54199	0.800986	0.769021
T.cells	GM16286	-0.01552	5.859685	-0.20489	0.838133	-6.78258	0.776939	0.740727
T.cells	CNST	-0.01934	3.943862	-0.20487	0.838141	-6.49863	0.811316	0.781305
T.cells	BROX	-0.01873	4.85097	-0.20482	0.838186	-6.48352	0.794865	0.761846
T.cells	GM42722	0.071445	2.335621	0.20468	0.838293	-5.63371	0.841265	0.817169
T.cells	PPHLN1	0.021221	4.932629	0.204551	0.838393	-6.61516	0.7934	0.760206

T.cells	CCNO	0.121008	0.174468	0.20444	0.83848	-5.20995	0.883124	0.867756
T.cells	DNAJC16	0.056238	2.30093	0.204418	0.838497	-5.73482	0.841922	0.81797
T.cells	MCEE	0.031754	4.194477	0.204406	0.838506	-6.21952	0.80674	0.77599
T.cells	EVI5L	0.044136	2.262161	0.204346	0.838553	-5.86277	0.842657	0.818853
T.cells	EXTL3	0.033233	3.56619	0.204317	0.838575	-6.18165	0.818259	0.789679
T.cells	GPC3	-0.07069	1.806193	-0.20425	0.838628	-5.56831	0.851347	0.829302
T.cells	ELAVL3	-0.08081	1.03829	-0.20417	0.838694	-5.64165	0.866168	0.847192
T.cells	CAR2	0.034012	5.171918	0.204161	0.838697	-6.71703	0.78912	0.755163
T.cells	AAGAB	0.02188	4.862045	0.204154	0.838703	-6.47597	0.794666	0.761706
T.cells	CNTROB	0.041733	2.044211	0.204098	0.838746	-5.99937	0.846801	0.823847
T.cells	AGRP	-0.04876	1.935634	-0.20394	0.838869	-6.03114	0.848872	0.826338
T.cells	NHSL2	-0.095	2.487556	-0.20376	0.839009	-5.49504	0.838392	0.813752
T.cells	ZFP597	0.037308	2.862663	0.203627	0.839113	-6.04758	0.831339	0.805306
T.cells	SCIN	-0.08729	0.090871	-0.20359	0.83914	-5.59334	0.884781	0.86979
T.cells	PPP1R16B	-0.02069	6.504104	-0.20358	0.839153	-6.94655	0.765687	0.72768
T.cells	TMEM260	0.040185	3.273577	0.203546	0.839176	-5.99106	0.823676	0.796151
T.cells	THUMPD3	-0.02329	4.262161	-0.20351	0.839204	-6.43521	0.805508	0.774545
T.cells	ABCB6	0.055712	1.101522	0.203399	0.83929	-5.61263	0.864938	0.845718
T.cells	GCC2	0.020353	5.193926	0.203375	0.83931	-6.61118	0.788727	0.754713
T.cells	FKBP2	-0.02006	5.611404	-0.2033	0.839369	-6.73754	0.781316	0.745992
T.cells	COMMD8	-0.01869	5.188265	-0.20314	0.839491	-6.59914	0.788828	0.754832
T.cells	FEM1A	0.029989	2.924816	0.203141	0.839492	-6.10588	0.830176	0.803914
T.cells	MAEA	-0.01627	5.40999	-0.20288	0.839694	-6.69263	0.784883	0.750187
T.cells	PFDN4	0.01891	5.046958	0.202864	0.839708	-6.7106	0.791352	0.757807
T.cells	A430072P0	0.073213	0.354986	0.20279	0.839765	-5.65905	0.879555	0.863436
T.cells	BICD2	0.023374	4.09822	0.20278	0.839773	-6.44116	0.808494	0.778088
T.cells	PKIB	-0.05708	5.453591	-0.20261	0.839902	-6.28694	0.784121	0.749285
T.cells	RGS3	-0.07868	2.862627	-0.20251	0.839985	-5.58532	0.831351	0.805315
T.cells	ATP6V1D	-0.01328	6.650485	-0.20249	0.84	-6.84696	0.763163	0.724728
T.cells	CAP2	-0.09428	0.071585	-0.20207	0.840329	-5.40139	0.885438	0.870279
T.cells	CRTC3	-0.02112	5.658838	-0.20191	0.84045	-6.62494	0.78072	0.745028
T.cells	CLASP2	0.015796	6.927855	0.20188	0.840474	-6.91783	0.758608	0.719165
T.cells	MIR22HG	-0.07635	4.089128	-0.20174	0.840586	-5.54888	0.808911	0.778306
T.cells	MECOM	-0.1476	0.770813	-0.2017	0.840615	-5.35037	0.871656	0.853547
T.cells	PER3	0.06393	1.517982	0.201562	0.840722	-5.60636	0.857148	0.83601
T.cells	SPATA5	-0.0204	5.905372	-0.20133	0.840901	-6.75046	0.776377	0.739942
T.cells	CTNS	0.03965	3.606042	0.201242	0.840972	-6.003	0.817777	0.788866
T.cells	GM32051	-0.09909	-0.14913	-0.20123	0.84098	-5.40288	0.88983	0.875654
T.cells	PURG	-0.03381	3.260975	-0.20117	0.841031	-6.12384	0.824165	0.796478
T.cells	LRPAP1	0.025511	4.499631	0.201032	0.841135	-6.38448	0.801448	0.769552
T.cells	RMND5A	-0.01573	6.766957	-0.20078	0.841335	-6.89764	0.761378	0.722499
T.cells	PPP1CA	0.011923	8.233158	0.200733	0.841368	-7.2222	0.736481	0.693607
T.cells	RAD54L2	0.019302	4.552757	0.20066	0.841425	-6.53211	0.800487	0.768438
T.cells	NISCH	-0.0162	6.194239	-0.20063	0.841448	-6.77477	0.771318	0.734112
T.cells	CTTNBP2NI	0.071719	2.52025	0.200616	0.841459	-5.47698	0.838036	0.813153
T.cells	DARS2	0.044313	2.849125	0.2006	0.841472	-6.09412	0.831851	0.805748
T.cells	ELMOD3	-0.04894	3.437093	-0.20032	0.84169	-6.0679	0.821041	0.792751

T.cells	TAP2	0.029873	5.110651	0.20017	0.841808	-6.51904	0.790612	0.756742
T.cells	GM11707	0.101412	0.863582	0.200017	0.841926	-5.25996	0.870011	0.851676
T.cells	SLC4A8	0.090846	0.467759	0.199895	0.842022	-5.44946	0.877775	0.861116
T.cells	B130034C1	0.048965	1.038829	0.199795	0.842099	-5.66489	0.866593	0.847569
T.cells	FAM149A	-0.10813	-0.42534	-0.19968	0.84219	-5.21421	0.895527	0.882729
T.cells	PAK1	0.064564	5.334473	0.199648	0.842214	-6.08876	0.786621	0.7521
T.cells	TMED5	-0.01219	7.227678	-0.19957	0.842277	-6.92063	0.753616	0.713485
T.cells	MCRS1	0.019077	4.318538	0.199536	0.842301	-6.5659	0.804888	0.773674
T.cells	GPBP1	-0.01452	7.723603	-0.19935	0.842444	-7.06944	0.745189	0.703704
T.cells	4930590J0	0.057456	1.547542	0.199276	0.842505	-5.68773	0.856744	0.835672
T.cells	ABHD12	-0.0198	5.584506	-0.19915	0.842605	-6.63444	0.782185	0.746882
T.cells	PEX26	-0.07065	1.217016	-0.19913	0.842618	-5.58513	0.863132	0.843383
T.cells	GFM2	0.028204	3.590998	0.1991	0.842642	-6.23286	0.818213	0.7895
T.cells	LMBRD1	-0.01949	6.356577	-0.19892	0.842783	-6.71589	0.768678	0.731045
T.cells	DYNC1H1	-0.01456	6.255557	-0.19886	0.842831	-6.74856	0.770438	0.733114
T.cells	HIST1H3I	-0.05008	1.296054	-0.19869	0.84296	-5.95061	0.861666	0.841615
T.cells	GGNBP1	0.080071	1.044976	0.198475	0.843129	-5.48348	0.86654	0.847547
T.cells	SNAPC2	-0.03193	3.015281	-0.19847	0.843133	-6.19678	0.828965	0.802366
T.cells	METTL21A	0.053764	1.888583	0.198464	0.843138	-5.57048	0.850263	0.827906
T.cells	ATF2	-0.01575	6.46092	-0.19837	0.843215	-6.87498	0.766886	0.728989
T.cells	GM47071	-0.06625	1.555335	-0.19824	0.84331	-5.7401	0.856688	0.835641
T.cells	REEP4	0.021867	4.356559	0.197662	0.843763	-6.61771	0.804477	0.773107
T.cells	COX8A	0.011567	9.008145	0.197646	0.843776	-7.29515	0.724028	0.679219
T.cells	NBAS	0.021611	4.535139	0.197597	0.843814	-6.48439	0.801239	0.769294
T.cells	DNMT3B	-0.03399	2.497904	-0.19738	0.843985	-6.04805	0.838911	0.814226
T.cells	FUCA1	-0.01921	5.963141	-0.19697	0.844305	-6.63212	0.775783	0.739502
T.cells	NR1D1	-0.06127	1.661874	-0.19694	0.844324	-5.83842	0.854842	0.833529
T.cells	PISD	-0.02433	4.8584	-0.19693	0.844334	-6.48242	0.795409	0.7626
T.cells	AKIP1	0.028094	3.24519	0.196901	0.844357	-6.31806	0.824905	0.797621
T.cells	HERC2	-0.0178	5.98752	-0.19689	0.844367	-6.73971	0.775355	0.739
T.cells	2510009E0	0.059777	2.702942	0.196658	0.844546	-5.60109	0.835047	0.809764
T.cells	HPS4	-0.03783	4.16681	-0.19665	0.844551	-6.09856	0.807931	0.777441
T.cells	TRIP12	0.013344	7.435654	0.196462	0.844699	-6.95364	0.750332	0.709874
T.cells	ANPEP	0.118155	1.184937	0.196372	0.84477	-5.30527	0.864055	0.844753
T.cells	G6PC3	-0.0372	3.106653	-0.19628	0.844842	-6.05335	0.827485	0.800798
T.cells	TNF	0.105656	4.405834	0.1962	0.844904	-5.72453	0.803583	0.772361
T.cells	SBF1	0.019622	4.474225	0.196127	0.84496	-6.46993	0.802343	0.770892
T.cells	REPIN1	-0.05617	1.082403	-0.19612	0.844969	-5.70753	0.866047	0.847163
T.cells	SCD1	-0.04802	3.037555	-0.1961	0.844979	-6.09769	0.828775	0.802339
T.cells	VARS	-0.01575	5.713285	-0.1961	0.84498	-6.86884	0.780181	0.744753
T.cells	PSMD12	0.015119	5.784758	0.196091	0.844989	-6.78225	0.778921	0.743273
T.cells	B3GNT6	-0.10333	-0.72192	-0.19599	0.845066	-5.18693	0.901808	0.89067
T.cells	RECQL5	-0.02873	3.722047	-0.19593	0.845115	-6.20074	0.816082	0.787202
T.cells	ARL13B	-0.02751	3.775076	-0.19578	0.845232	-6.18324	0.815106	0.786041
T.cells	5033406O(	0.080949	0.605012	0.195756	0.845251	-5.479	0.87538	0.858472
T.cells	USP11	0.035109	2.611991	0.19568	0.84531	-6.07103	0.836759	0.811895
T.cells	SPN	0.025872	3.586459	0.195397	0.84553	-6.36485	0.818725	0.79019

T.cells	LACTB	0.020545	5.139278	0.195103	0.84576	-6.54282	0.790526	0.756808
T.cells	CHCHD6	-0.02498	2.968098	-0.19505	0.845801	-6.24706	0.830231	0.803953
T.cells	CCNL2	0.012551	6.219089	0.194898	0.84592	-6.83084	0.771448	0.734417
T.cells	PPNR	0.073272	1.051959	0.194784	0.846009	-5.5405	0.866804	0.847986
T.cells	H2-Q7	0.088225	3.889878	0.194745	0.846039	-5.92499	0.813152	0.783652
T.cells	TYK2	0.030563	3.992109	0.194718	0.846061	-6.06709	0.811278	0.781437
T.cells	INO80B	-0.02139	4.146114	-0.19466	0.846103	-6.46945	0.808463	0.778101
T.cells	CRLS1	-0.02383	3.622878	-0.19462	0.846136	-6.27751	0.818065	0.789515
T.cells	IL16	0.02721	4.60831	0.194411	0.8463	-6.57568	0.800068	0.768169
T.cells	ICAM2	-0.04021	4.282374	-0.19437	0.846332	-6.45804	0.805979	0.775171
T.cells	KCNK13	-0.04683	2.912398	-0.19437	0.846334	-6.087	0.831274	0.805294
T.cells	HSPA14	0.01847	5.034968	0.194199	0.846465	-6.69552	0.792407	0.759149
T.cells	GM14471	0.073069	0.24931	0.194063	0.846572	-5.46999	0.882579	0.867265
T.cells	SLC28A2	-0.04266	2.961181	-0.19403	0.846595	-6.05313	0.830376	0.804299
T.cells	MYO15	-0.0683	1.234713	-0.19394	0.846667	-5.61811	0.863269	0.843851
T.cells	ZFP938	-0.05114	1.450658	-0.19375	0.846817	-5.70721	0.85909	0.838807
T.cells	GM42984	0.06435	0.916614	0.193608	0.846927	-5.66056	0.86946	0.851368
T.cells	HIST1H2AF	0.056791	0.617707	0.193388	0.847098	-5.86071	0.875314	0.858483
T.cells	E230014E1	0.096273	-1.38289	0.193365	0.847116	-5.1625	0.915434	0.907469
T.cells	TNNT1	-0.05675	1.520318	-0.19328	0.847181	-5.65414	0.857746	0.837223
T.cells	ANKRD27	0.034912	3.098779	0.193094	0.847329	-5.9863	0.827805	0.801267
T.cells	CLCC1	0.017561	4.329275	0.193065	0.847351	-6.47233	0.805142	0.774293
T.cells	PCX	-0.04535	2.70013	-0.19281	0.847551	-6.01017	0.835274	0.810244
T.cells	ZFP811	-0.12395	-1.02506	-0.19277	0.847584	-5.16288	0.908137	0.898565
T.cells	RAB13	-0.0677	1.635625	-0.19268	0.847648	-5.58406	0.855526	0.8346
T.cells	CAMKK2	-0.0154	5.188179	-0.19268	0.847654	-6.72631	0.789667	0.756049
T.cells	ENDOG	0.029907	2.943039	0.192639	0.847683	-6.11588	0.830716	0.804803
T.cells	LAMB3	-0.03211	4.067884	-0.19262	0.847698	-6.33637	0.809907	0.780007
T.cells	1700021F0	0.027133	3.209032	0.192558	0.847747	-6.22867	0.825751	0.798883
T.cells	SELENOT	-0.01415	6.182995	-0.19253	0.847765	-6.78947	0.772093	0.735412
T.cells	SFSWAP	-0.01254	5.786795	-0.1925	0.84779	-6.77889	0.779048	0.743571
T.cells	ZCWPW1	-0.0343	2.765843	-0.19239	0.84788	-6.0748	0.834056	0.80883
T.cells	GALM	0.078076	2.214948	0.192204	0.848023	-5.47289	0.844491	0.82139
T.cells	GYPC	-0.03707	3.080733	-0.19217	0.848049	-6.19948	0.828184	0.801845
T.cells	ZFP330	-0.01896	4.432352	-0.19152	0.848559	-6.48877	0.803725	0.772396
T.cells	PEX1	0.034724	3.071405	0.191354	0.848687	-6.1449	0.828841	0.802254
T.cells	SMC1A	-0.01433	6.748393	-0.19123	0.848784	-7.04246	0.762773	0.724208
T.cells	CYB561A3	0.019034	5.573777	0.191141	0.848853	-6.70715	0.783327	0.748289
T.cells	ZSCAN22	-0.05858	1.316109	-0.19096	0.848998	-5.68447	0.862334	0.842423
T.cells	ADCK5	0.054494	1.510315	0.190607	0.849271	-5.67312	0.85873	0.83804
T.cells	LEAP2	-0.06487	3.060255	-0.19058	0.849293	-5.85934	0.829288	0.802698
T.cells	SKA1	0.033871	2.918501	0.190349	0.849472	-6.38862	0.83202	0.805892
T.cells	KNL1	0.029142	5.131736	0.190248	0.849551	-6.84946	0.79148	0.757747
T.cells	SPRED1	-0.02754	4.710293	-0.1902	0.849589	-6.47435	0.799054	0.76669
T.cells	LYSMD2	0.074779	1.265146	0.189928	0.849801	-5.51164	0.863699	0.843844
T.cells	DNPEP	-0.02354	4.077287	-0.18958	0.850073	-6.37403	0.810839	0.780378
T.cells	AGL	0.035944	3.963587	0.189533	0.850109	-6.17024	0.812922	0.782852

T.cells	GSTO2	0.093112	0.524993	0.189454	0.850171	-5.34725	0.878333	0.861417
T.cells	LY6C1	-0.12518	-0.32417	-0.18936	0.850248	-5.23247	0.895221	0.881997
T.cells	C130013H(	0.060169	0.498295	0.189104	0.850445	-5.47744	0.878993	0.862135
T.cells	UBB	-0.01481	10.85501	-0.18885	0.850647	-7.57403	0.695487	0.646389
T.cells	THBS1	0.131375	4.967156	0.188736	0.850732	-5.77487	0.794861	0.761463
T.cells	ZFP97	0.055081	1.263623	0.188701	0.85076	-5.6622	0.864055	0.844102
T.cells	NDUFS5	-0.01428	6.478554	-0.18848	0.85093	-6.92671	0.76814	0.730086
T.cells	B230354K1	0.052427	1.756956	0.188478	0.850934	-5.79677	0.854526	0.832607
T.cells	NHEJ1	-0.04229	3.867688	-0.18836	0.851029	-6.29797	0.814844	0.785128
T.cells	RTKN2	-0.04517	1.228878	-0.18835	0.851035	-5.91343	0.86473	0.844917
T.cells	RAB33B	-0.02058	4.405753	-0.18827	0.851093	-6.37038	0.805006	0.773491
T.cells	GLG1	-0.01456	6.951102	-0.18823	0.851131	-6.94228	0.759961	0.720593
T.cells	WDCP	-0.02695	3.079235	-0.18812	0.851217	-6.27025	0.829476	0.802648
T.cells	RASA1	0.016245	6.030636	0.187909	0.851379	-6.7459	0.776021	0.739381
T.cells	PHF11B	-0.06653	4.313119	-0.18779	0.851471	-5.8364	0.806745	0.775601
T.cells	RAB8A	0.01549	5.454242	0.187784	0.851476	-6.70712	0.786207	0.751349
T.cells	ETS2	0.025878	5.865691	0.187688	0.851551	-6.58344	0.778926	0.742803
T.cells	RB1CC1	0.016215	6.500368	0.187539	0.851668	-6.84093	0.767854	0.729854
T.cells	SOAT2	-0.09609	0.810649	-0.18713	0.851986	-5.26683	0.873249	0.855143
T.cells	MYO19	-0.05043	1.357151	-0.18703	0.852063	-5.79217	0.8626	0.842303
T.cells	GM20045	-0.05229	1.764585	-0.18693	0.85214	-5.74034	0.85474	0.832834
T.cells	ZFP282	-0.02172	4.010784	-0.18658	0.85242	-6.48832	0.812758	0.78247
T.cells	ELK3	0.018402	5.288608	0.18637	0.852581	-6.7251	0.789673	0.755125
T.cells	DNAH12	-0.08308	1.676699	-0.18633	0.852609	-5.58201	0.856685	0.83496
T.cells	STAT4	0.032883	5.177658	0.186213	0.852704	-6.53954	0.791677	0.757488
T.cells	MED18	0.044033	1.412452	0.18577	0.853051	-5.85688	0.862089	0.841197
T.cells	GM14302	-0.05766	0.609708	-0.1855	0.853262	-5.58706	0.877875	0.860226
T.cells	GM15133	0.060022	1.788244	0.185451	0.8533	-5.61298	0.854936	0.832529
T.cells	USB1	0.034916	3.245957	0.185323	0.8534	-6.04957	0.82733	0.799465
T.cells	MARCO	0.093431	5.795483	0.185032	0.853628	-6.26852	0.781034	0.744701
T.cells	CYTIP	-0.01753	8.087657	-0.18503	0.853632	-7.07575	0.741503	0.698667
T.cells	HAUS4	-0.0198	4.204699	-0.18499	0.853657	-6.58825	0.809627	0.778422
T.cells	SH3TC1	0.058139	2.768133	0.184942	0.853698	-5.54477	0.836286	0.810185
T.cells	ERCC4	-0.03393	2.949475	-0.18487	0.853755	-6.09316	0.832877	0.806108
T.cells	CC2D1A	-0.03248	3.044755	-0.18481	0.8538	-6.06735	0.83109	0.803985
T.cells	USP46	0.035146	3.281753	0.184532	0.854019	-6.08484	0.826765	0.798701
T.cells	PPP2R5B	-0.06454	1.645269	-0.18449	0.85405	-5.63807	0.857796	0.835893
T.cells	RAB39	-0.06627	2.115142	-0.18439	0.854126	-5.62749	0.848781	0.825044
T.cells	CEP152	0.020416	3.629164	0.183954	0.854471	-6.46799	0.820396	0.791067
T.cells	PDE5A	-0.03429	2.569114	-0.1839	0.854516	-6.24385	0.840232	0.814746
T.cells	SUGCT	0.046118	2.835527	0.183852	0.854551	-5.85486	0.835205	0.80873
T.cells	ZBTB38	0.022829	5.068147	0.183773	0.854613	-6.43684	0.794166	0.76001
T.cells	SPSB1	0.078382	1.685361	0.183562	0.854778	-5.47859	0.857109	0.835018
T.cells	4732496C(	-0.06344	0.689806	-0.1835	0.85483	-5.50378	0.876496	0.858438
T.cells	ZADH2	-0.02801	3.381757	-0.18349	0.854836	-6.10573	0.824986	0.796535
T.cells	7-Mar	-0.01128	6.926226	-0.18348	0.854843	-6.91155	0.761462	0.721694
T.cells	BTF3L4	0.017027	4.269235	0.183459	0.854858	-6.50414	0.80863	0.777102

T.cells	PHLDB1	-0.0744	1.355652	-0.18326	0.855015	-5.42959	0.863504	0.84276
T.cells	STAU2	-0.03143	2.900653	-0.18325	0.855021	-6.18868	0.833998	0.807322
T.cells	ACOT1	0.08113	2.604682	0.183053	0.855176	-5.64224	0.839585	0.814053
T.cells	DOCK8	-0.01654	7.656272	-0.18298	0.855232	-7.0133	0.748986	0.707275
T.cells	GM19710	0.04371	2.595654	0.182789	0.855382	-6.01888	0.839756	0.814322
T.cells	ATG3	0.013183	6.261249	0.182755	0.855408	-6.77255	0.773041	0.735342
T.cells	STPG4	0.041284	2.37884	0.18267	0.855476	-5.88577	0.843866	0.819284
T.cells	FUCA2	0.022944	4.624115	0.182611	0.855522	-6.30085	0.8022	0.769666
T.cells	MPO	0.200251	1.827356	0.18212	0.855905	-5.49083	0.854403	0.832032
T.cells	MGL2	0.167501	-0.18973	0.182103	0.855919	-5.21336	0.893985	0.879982
T.cells	CYB5R1	0.028584	3.659051	0.182059	0.855953	-6.14631	0.819868	0.790706
T.cells	ROMO1	-0.013	6.595619	-0.18199	0.856005	-6.88752	0.76721	0.728646
T.cells	FAM50A	0.016385	5.186332	0.181923	0.856059	-6.6198	0.792072	0.757808
T.cells	TRIM2	-0.03558	2.325336	-0.18185	0.856119	-6.12877	0.844883	0.820619
T.cells	SERGEF	-0.02313	4.018452	-0.18172	0.856221	-6.3828	0.813246	0.782858
T.cells	NKAPD1	-0.02012	4.086425	-0.18171	0.856229	-6.43522	0.811999	0.781378
T.cells	SCAF11	-0.0116	7.16109	-0.1817	0.85623	-7.05674	0.757443	0.717278
T.cells	EPB41L3	-0.09431	2.334313	-0.18165	0.856271	-5.53797	0.844712	0.820414
T.cells	GM15478	0.02026	4.735611	0.181647	0.856275	-6.59337	0.800182	0.767385
T.cells	HUWE1	-0.01415	7.155414	-0.18158	0.856328	-6.98598	0.75754	0.717406
T.cells	TMEM248	-0.01436	5.858866	-0.18137	0.856494	-6.75355	0.780199	0.743816
T.cells	LDLRAD3	0.023504	4.591753	0.181239	0.856595	-6.60437	0.802901	0.770547
T.cells	ORC2	0.017432	4.279461	0.180927	0.856839	-6.56757	0.808711	0.777324
T.cells	FAAP20	-0.0377	3.120662	-0.18051	0.857162	-5.83618	0.830131	0.802954
T.cells	G0S2	-0.07144	3.133285	-0.18016	0.857443	-5.53789	0.829895	0.802717
T.cells	GM16090	-0.12354	-0.96132	-0.18015	0.857445	-5.1588	0.90984	0.899416
T.cells	TMEM132E	0.051756	0.984501	0.1801	0.857486	-5.90632	0.871004	0.852137
T.cells	RNPEP	-0.02118	5.72187	-0.18	0.857562	-6.69195	0.782771	0.746913
T.cells	FAM32A	0.014657	5.312501	0.179892	0.857649	-6.62577	0.790053	0.755474
T.cells	FBXO33	0.016969	5.984659	0.179826	0.857701	-6.66858	0.77813	0.741468
T.cells	MIPOL1	-0.01831	4.257052	-0.17977	0.857747	-6.49903	0.809121	0.778001
T.cells	KDM7A	0.019672	7.129544	0.179554	0.857913	-6.69685	0.758212	0.718208
T.cells	TMEM159	0.067925	1.553977	0.179315	0.8581	-5.58805	0.85993	0.838758
T.cells	FCER1G	0.047594	8.628675	0.179297	0.858115	-6.63715	0.732861	0.688859
T.cells	4921531C2	-0.05222	1.642005	-0.17928	0.858124	-5.71445	0.85823	0.836709
T.cells	KCTD11	-0.07552	0.546292	-0.17921	0.858183	-5.40094	0.879615	0.862571
T.cells	EIF2AK3	0.015877	6.399228	0.179197	0.858193	-6.90635	0.770862	0.732959
T.cells	IFT46	0.017247	4.502973	0.1791	0.858269	-6.47357	0.80464	0.772694
T.cells	HNRNPA3	0.011089	8.554364	0.179062	0.858299	-7.28913	0.734098	0.690285
T.cells	AGPS	0.012862	6.813953	0.179058	0.858302	-6.92173	0.763654	0.724546
T.cells	UBA3	-0.01809	4.076756	-0.17896	0.858376	-6.42469	0.812421	0.781951
T.cells	GRCC10	0.013086	6.427807	0.178802	0.858503	-6.82179	0.770363	0.732465
T.cells	LYSMD3	-0.01995	4.927993	-0.17876	0.858535	-6.48635	0.79695	0.76371
T.cells	KRTCAP3	0.067442	0.893962	0.178729	0.85856	-5.42499	0.872777	0.854403
T.cells	MAN2C1	0.022541	3.509599	0.178659	0.858615	-6.27094	0.822884	0.794468
T.cells	PLGRKT	-0.01851	5.796951	-0.1785	0.858735	-6.67126	0.781443	0.74546
T.cells	CTLA4	-0.07156	2.060232	-0.17848	0.858753	-5.65304	0.850194	0.827155

T.cells	SPPL2A	0.02063	6.600738	0.178457	0.858773	-6.79284	0.767352	0.728964
T.cells	MOK	-0.08745	0.377087	-0.1784	0.858817	-5.37689	0.882961	0.866758
T.cells	ZBTB1	0.017052	5.505188	0.178141	0.85902	-6.74914	0.786618	0.751573
T.cells	COL23A1	0.053118	1.376373	0.178127	0.859031	-5.79975	0.86337	0.843066
T.cells	SNHG20	0.033246	2.355617	0.17801	0.859123	-5.88674	0.844561	0.820443
T.cells	CRCP	0.015421	4.449755	0.177989	0.859139	-6.55764	0.805607	0.774002
T.cells	ROR1	-0.08317	1.374979	-0.17798	0.859145	-5.42836	0.863397	0.843119
T.cells	FIBP	-0.01735	4.462532	-0.17797	0.859155	-6.50054	0.805375	0.773726
T.cells	CHMP4B	0.011316	7.889271	0.177886	0.859219	-7.02324	0.745263	0.703343
T.cells	HDAC8	0.015546	6.530622	0.177837	0.859258	-7.01716	0.768571	0.730453
T.cells	PLCXD2	0.037383	3.46805	0.1775	0.859521	-6.00703	0.823838	0.795518
T.cells	THOP1	-0.03167	2.236401	-0.17734	0.859644	-6.16479	0.847035	0.823278
T.cells	EMB	-0.04521	5.68619	-0.17728	0.859691	-6.27687	0.783593	0.747921
T.cells	SPTBN1	-0.01627	6.620496	-0.1772	0.859755	-6.94105	0.767193	0.728725
T.cells	CCAR2	0.023208	3.432111	0.177067	0.859861	-6.3137	0.824555	0.796362
T.cells	GM10501	-0.03548	2.138944	-0.17687	0.860018	-5.95292	0.849012	0.825578
T.cells	NEK3	0.046201	1.761696	0.176413	0.860373	-5.92576	0.856424	0.834395
T.cells	KLHL21	0.042208	2.689731	0.176315	0.86045	-5.90756	0.838721	0.813137
T.cells	ATG7	-0.02076	5.786606	-0.17631	0.860452	-6.59357	0.782083	0.745984
T.cells	MTHFSL	-0.02071	5.211119	-0.17627	0.860483	-6.44819	0.792329	0.758033
T.cells	GM22146	0.039036	2.073446	0.175965	0.860724	-5.97122	0.850605	0.827223
T.cells	E330009J0	-0.04606	2.552592	-0.1755	0.861084	-5.88573	0.841584	0.816354
T.cells	ALS2CL	-0.08006	0.148166	-0.17546	0.861117	-5.38766	0.888308	0.87277
T.cells	TMEM256	-0.01473	6.982152	-0.1754	0.86117	-6.99131	0.761437	0.721666
T.cells	SLC22A5	-0.04184	3.042195	-0.17517	0.861349	-5.99262	0.832352	0.805396
T.cells	NUFIP1	0.018022	4.171093	0.175141	0.861369	-6.44638	0.811425	0.780494
T.cells	TNFAIP8	-0.01372	7.424897	-0.17512	0.861387	-7.03092	0.753834	0.712909
T.cells	ZC4H2	0.061695	0.054562	0.174885	0.86157	-5.48618	0.890175	0.87521
T.cells	RNF10	0.015006	6.51711	0.174834	0.86161	-6.82445	0.769501	0.731228
T.cells	P2RX4	-0.02576	5.534178	-0.17482	0.861617	-6.42046	0.786813	0.751515
T.cells	TMEM240	-0.08344	0.240106	-0.17469	0.861722	-5.39335	0.886478	0.870762
T.cells	IRAK1BP1	0.066073	0.288762	0.174689	0.861723	-5.58965	0.885512	0.869587
T.cells	DAGLA	-0.10327	-0.78197	-0.17463	0.861773	-5.16034	0.907014	0.895806
T.cells	KIRREL3	0.073122	1.272544	0.174574	0.861814	-5.42493	0.866169	0.846148
T.cells	METTL5	0.021107	3.739598	0.174403	0.861947	-6.34071	0.819366	0.790067
T.cells	SOX5OS4	-0.07296	-0.23518	-0.17424	0.862076	-5.46043	0.895974	0.882404
T.cells	TXNRD2	0.030247	3.532111	0.174228	0.862085	-6.18605	0.823209	0.794685
T.cells	GNL3L	0.015024	4.467102	0.173955	0.862298	-6.50821	0.80602	0.77432
T.cells	ERICH1	0.02846	3.61294	0.173885	0.862353	-6.14236	0.82171	0.792966
T.cells	PSMC5	-0.01261	6.00886	-0.17379	0.862428	-6.84375	0.778407	0.741814
T.cells	SGMS2	-0.09675	3.643674	-0.17375	0.862455	-5.49658	0.821141	0.7923
T.cells	FBXO36	-0.05824	1.228268	-0.17368	0.862511	-5.67576	0.867031	0.847387
T.cells	POLD2	0.025161	3.401936	0.173673	0.86252	-6.39964	0.82563	0.797682
T.cells	VEGFB	-0.02282	3.802219	-0.17358	0.862592	-6.35582	0.818209	0.788884
T.cells	MFN1	0.026808	3.297935	0.173573	0.862598	-6.11507	0.827568	0.800033
T.cells	GM26631	0.048826	1.219353	0.173523	0.862637	-5.7111	0.867204	0.847654
T.cells	ARMCX6	0.06012	0.977785	0.173235	0.862863	-5.41704	0.871984	0.853417

T.cells	GM36198	-0.03381	2.976425	-0.17314	0.862934	-6.0547	0.833645	0.807297
T.cells	EFCAB2	-0.03475	2.668273	-0.1731	0.862966	-6.08015	0.839453	0.814247
T.cells	HNRNPUL2	-0.01124	6.468104	-0.17308	0.862987	-6.88125	0.77041	0.732525
T.cells	MRPL27	-0.01969	4.210901	-0.17267	0.863303	-6.5063	0.810964	0.780128
T.cells	REXO5	0.034344	1.950184	0.172523	0.863421	-5.9688	0.853354	0.83085
T.cells	KDM4A	0.021201	4.161515	0.172111	0.863743	-6.34355	0.811869	0.781357
T.cells	USPL1	0.021891	3.533846	0.172092	0.863758	-6.29726	0.823449	0.795131
T.cells	TATDN1	-0.02353	3.630234	-0.17203	0.86381	-6.22451	0.821661	0.793005
T.cells	CSNK1A1	-0.00898	7.684452	-0.17198	0.863849	-7.05565	0.749657	0.708372
T.cells	HIST1H2BG	0.045765	1.128352	0.171671	0.864089	-5.90041	0.869266	0.850144
T.cells	VPS26A	-0.01367	6.273847	-0.17164	0.864109	-6.77427	0.774008	0.736741
T.cells	ARID2	0.013425	6.543762	0.171638	0.864114	-6.92749	0.769291	0.731226
T.cells	TAF8	-0.02491	3.254648	-0.17155	0.864184	-6.24113	0.82865	0.801337
T.cells	SRD5A3	-0.02673	4.511422	-0.17153	0.864201	-6.26379	0.805479	0.773792
T.cells	RAB7	0.011431	7.931545	0.171489	0.864231	-7.01248	0.745467	0.703517
T.cells	CPS1	0.051659	4.481272	0.171441	0.864269	-6.2968	0.806028	0.774442
T.cells	SNRPA1	-0.01703	5.077996	-0.17132	0.864363	-6.75213	0.795233	0.761701
T.cells	LEPROTL1	0.02642	5.362087	0.171233	0.864432	-6.36937	0.790142	0.755718
T.cells	TNIK	-0.04048	4.035038	-0.17119	0.864462	-6.11048	0.81419	0.784168
T.cells	ANXA1	0.031083	6.030034	0.171135	0.864509	-6.72791	0.778291	0.741816
T.cells	SYF2	-0.01128	5.903221	-0.17113	0.864513	-6.76851	0.780528	0.74444
T.cells	SINHCAF	-0.01449	4.557551	-0.17095	0.864656	-6.72556	0.804707	0.772878
T.cells	ZFP940	-0.04471	0.734633	-0.17073	0.864827	-5.70288	0.877106	0.85966
T.cells	GM27003	-0.01854	4.204875	-0.17045	0.865042	-6.48621	0.811185	0.780584
T.cells	MED15	0.01544	5.758733	0.170413	0.865075	-6.70813	0.783191	0.747538
T.cells	MCRIP2	0.042119	2.015335	0.170294	0.865168	-5.75413	0.85222	0.829613
T.cells	DEPDC1A	-0.03357	3.001084	-0.17023	0.865216	-6.4143	0.833513	0.807177
T.cells	PTGER2	0.073537	1.047913	0.17018	0.865258	-5.39867	0.870957	0.852229
T.cells	BANP	0.015412	4.601582	0.170102	0.865319	-6.57995	0.80395	0.77203
T.cells	PTGIS	-0.12296	-0.12832	-0.17008	0.865334	-5.26399	0.894249	0.880536
T.cells	GM11457	0.085711	0.10252	0.170027	0.865377	-5.39419	0.889633	0.874925
T.cells	MIER1	-0.01172	6.946828	-0.16996	0.865426	-6.9649	0.762403	0.723249
T.cells	SMARCD2	0.011219	5.611886	0.169399	0.86587	-6.82029	0.785945	0.750771
T.cells	PSMC3IP	-0.03027	2.412129	-0.16934	0.865917	-6.163	0.844802	0.820701
T.cells	NARS	-0.01365	6.298026	-0.16925	0.865984	-6.8346	0.773835	0.736575
T.cells	ZSCAN20	-0.067	1.073033	-0.16909	0.866111	-5.54706	0.870629	0.851833
T.cells	ZKSCAN3	0.014992	4.82207	0.169066	0.866131	-6.51846	0.800106	0.767472
T.cells	NDUFAF8	0.014468	5.052226	0.169044	0.866148	-6.63119	0.795955	0.762571
T.cells	ECHDC1	0.025012	3.820316	0.168972	0.866205	-6.29135	0.81841	0.789174
T.cells	STK11IP	-0.03023	2.312647	-0.16892	0.866242	-5.94837	0.846697	0.822981
T.cells	CREBL2	-0.03674	3.233581	-0.16887	0.866284	-5.90264	0.829312	0.802171
T.cells	4930414N0	-0.02831	3.331114	-0.16883	0.866312	-6.24539	0.827491	0.800015
T.cells	ITPRIPL2	0.047846	3.897046	0.168652	0.866456	-5.71759	0.817018	0.787551
T.cells	ADAM17	-0.02112	6.061586	-0.16857	0.86652	-6.68101	0.77801	0.741497
T.cells	MET	-0.08647	1.991608	-0.16853	0.866554	-5.43838	0.85286	0.830427
T.cells	GNG11	0.050327	3.831566	0.168367	0.866679	-5.86402	0.818276	0.789023
T.cells	PARG	-0.01295	5.560263	-0.16815	0.866849	-6.73066	0.786948	0.751981

T.cells	GNA11	-0.02273	3.954617	-0.16808	0.866904	-6.42094	0.816022	0.786355
T.cells	LRRC1	-0.02551	3.264954	-0.168	0.866966	-6.4029	0.828815	0.801593
T.cells	ACPP	0.058304	2.739914	0.167978	0.866984	-5.75042	0.83868	0.81339
T.cells	GIMAP7	-0.08169	1.458503	-0.16774	0.867172	-5.41792	0.863309	0.842964
T.cells	GZMA	-0.16325	4.671597	-0.16763	0.86726	-5.72743	0.803005	0.770895
T.cells	PLCB4	-0.03596	3.876337	-0.1675	0.867358	-6.138	0.817554	0.788151
T.cells	ADAMTS7	0.050648	0.676356	0.167376	0.867456	-5.74627	0.87861	0.861541
T.cells	TTLL1	0.036156	1.626512	0.167288	0.867526	-5.89211	0.860054	0.839118
T.cells	SELENOH	-0.02038	5.20549	-0.16726	0.867546	-6.79911	0.793374	0.759587
T.cells	UFD1	0.013548	5.129038	0.166914	0.867819	-6.65627	0.794747	0.761225
T.cells	PPP2R1B	-0.01965	4.339805	-0.16679	0.867918	-6.44514	0.809045	0.778142
T.cells	NXF1	-0.01453	5.180351	-0.16674	0.867958	-6.6487	0.793825	0.76015
T.cells	GM5608	-0.08375	0.923456	-0.16672	0.86797	-5.28811	0.873749	0.855712
T.cells	IFT22	-0.01909	3.654091	-0.16652	0.868129	-6.53662	0.821664	0.79318
T.cells	NAXE	-0.01605	5.283838	-0.16641	0.868214	-6.6639	0.79197	0.758019
T.cells	CDK14	-0.08317	5.455475	-0.1664	0.868224	-5.83277	0.788902	0.754406
T.cells	FLYWCH1	0.019663	3.850858	0.166352	0.86826	-6.38823	0.818024	0.788868
T.cells	GALT	-0.0407	2.389889	-0.166	0.868535	-5.80825	0.845409	0.821594
T.cells	CHURC1	0.012652	5.66012	0.165901	0.868613	-6.76352	0.785259	0.75012
T.cells	YARS2	-0.02456	3.150094	-0.16583	0.868669	-6.25459	0.831055	0.804402
T.cells	E430018J2:	-0.05581	0.653485	-0.16583	0.868672	-5.56287	0.879061	0.862216
T.cells	CHAC1	-0.06308	0.269965	-0.1658	0.868695	-5.54661	0.886658	0.871447
T.cells	BC029722	-0.02859	3.061908	-0.16573	0.868746	-6.07818	0.832708	0.806379
T.cells	STK3	0.016371	5.985237	0.165732	0.868746	-6.79158	0.779503	0.743361
T.cells	COL5A1	-0.08269	0.493409	-0.16569	0.868778	-5.34632	0.882224	0.866058
T.cells	KDM4D	-0.07565	0.328103	-0.1656	0.868851	-5.37024	0.885502	0.870042
T.cells	GPR155	0.028031	3.05644	0.165592	0.868856	-6.24055	0.832811	0.806501
T.cells	HIST1H1D	0.04225	1.531522	0.165134	0.869215	-6.00809	0.861893	0.841519
T.cells	SMIM11	-0.01478	4.805042	-0.16508	0.869261	-6.57206	0.800588	0.768294
T.cells	PMM1	-0.02425	3.07058	-0.16504	0.869292	-6.29391	0.832546	0.806308
T.cells	A930007I1!	0.034727	4.385631	0.164931	0.869375	-6.31694	0.808209	0.777343
T.cells	TBC1D12	0.035501	3.768556	0.164893	0.869404	-6.11004	0.819545	0.79081
T.cells	HEATR5B	0.026226	3.730171	0.164773	0.869499	-6.20636	0.820255	0.791686
T.cells	C2	0.06157	2.413631	0.164662	0.869586	-5.70767	0.844957	0.821223
T.cells	TESK2	0.024601	4.072329	0.1646	0.869635	-6.44068	0.813946	0.784183
T.cells	SEMA4G	-0.08209	0.909795	-0.16458	0.869651	-5.3462	0.874017	0.856278
T.cells	RNASEK	0.012779	6.735066	0.164399	0.869792	-6.79442	0.76638	0.728176
T.cells	DHX16	0.020079	4.087189	0.164359	0.869824	-6.45126	0.813673	0.783882
T.cells	ABITRAM	-0.02599	2.584915	-0.16431	0.869859	-6.07978	0.841705	0.817345
T.cells	SIRT5	-0.03997	1.958147	-0.16426	0.8699	-5.81302	0.853663	0.83172
T.cells	RND1	0.083079	1.145338	0.164184	0.869961	-5.28278	0.869405	0.850723
T.cells	PYGO2	-0.03094	3.374512	-0.16405	0.870068	-6.19878	0.826861	0.79959
T.cells	B3GALT6	-0.04645	1.806345	-0.16394	0.870154	-5.79691	0.856583	0.835292
T.cells	SNIP1	0.017169	3.633076	0.163845	0.870227	-6.3845	0.822053	0.793914
T.cells	ETV6	-0.01568	8.156803	-0.16364	0.870387	-7.10301	0.742069	0.700022
T.cells	DPP3	-0.01666	4.728026	-0.16345	0.870537	-6.57666	0.801982	0.770183
T.cells	ANGPTL6	0.062804	0.97577	0.163386	0.870587	-5.4781	0.872723	0.854931

T.cells	RAB11B	-0.00985	7.147669	-0.16338	0.870593	-7.01371	0.759248	0.720025
T.cells	HDDC2	0.017913	3.817561	0.163373	0.870597	-6.45642	0.818639	0.789968
T.cells	ZFP593	-0.02504	3.359126	-0.16328	0.870671	-6.28813	0.827148	0.800128
T.cells	GM43331	-0.04531	2.057137	-0.16319	0.870739	-5.58557	0.851764	0.829651
T.cells	AIF1	-0.05886	4.896333	-0.1631	0.870813	-5.94806	0.798938	0.76667
T.cells	HSPA13	0.02679	3.082179	0.163023	0.870873	-5.98848	0.832328	0.806413
T.cells	NOD1	0.067936	3.387607	0.162809	0.871041	-5.54849	0.826617	0.799652
T.cells	FAM167A	-0.06174	1.837572	-0.16275	0.871084	-5.65546	0.855982	0.834887
T.cells	DCUN1D3	0.020825	4.424811	0.162745	0.871091	-6.40079	0.807494	0.776903
T.cells	RGS2	-0.01738	6.977171	-0.16263	0.871179	-6.85519	0.762187	0.723635
T.cells	TARBP1	0.02487	2.687423	0.162485	0.871294	-6.26155	0.839764	0.815435
T.cells	SGO1	0.029337	3.188246	0.162372	0.871383	-6.40236	0.830341	0.804179
T.cells	SLC24A5	0.015191	4.423305	0.162346	0.871404	-6.69488	0.807521	0.777007
T.cells	CCDC92	0.070257	-0.17821	0.162283	0.871453	-5.35136	0.895612	0.883046
T.cells	TM9SF2	-0.00949	6.683958	-0.16225	0.871475	-6.86781	0.767267	0.729612
T.cells	B230118H(	0.028371	2.933488	0.162233	0.871492	-6.14357	0.835122	0.8099
T.cells	SLC1A5	-0.01271	6.46392	-0.16215	0.871561	-7.06117	0.771101	0.734098
T.cells	PPIL2	0.013297	5.124237	0.162021	0.871659	-6.64224	0.794833	0.76202
T.cells	SSBP3	0.013958	5.600863	0.162008	0.871669	-6.77675	0.786312	0.751975
T.cells	AKT2	-0.01475	5.234185	-0.1619	0.87175	-6.61109	0.79286	0.759699
T.cells	1500011B(	-0.02027	4.65787	-0.16177	0.871856	-6.52606	0.803254	0.772012
T.cells	PARD6G	-0.03586	1.981117	-0.16163	0.871964	-6.13088	0.853222	0.831705
T.cells	PIN4	0.01345	4.949389	0.161614	0.871979	-6.61466	0.79798	0.765772
T.cells	1110012L1	0.044496	1.829365	0.161588	0.871999	-5.68282	0.85614	0.835221
T.cells	MYCN	0.058001	-0.18583	0.161373	0.872168	-5.56041	0.895853	0.883318
T.cells	AMDHD2	-0.02501	3.693973	-0.16129	0.872229	-6.1422	0.821005	0.793018
T.cells	GABPA	-0.01497	4.662072	-0.16119	0.87231	-6.577	0.803264	0.771952
T.cells	GM43305	0.023601	7.45195	0.16093	0.872516	-7.2516	0.754135	0.714329
T.cells	NUP188	0.016966	4.513741	0.160899	0.87254	-6.55299	0.805987	0.775216
T.cells	MEA1	-0.01514	5.276389	-0.16085	0.872582	-6.62257	0.792215	0.75893
T.cells	NFIX	0.027636	3.676603	0.160616	0.872762	-6.23314	0.821363	0.793515
T.cells	MRPS14	-0.01051	6.892734	-0.16052	0.872839	-7.01236	0.763755	0.725563
T.cells	GM43329	0.039093	3.105098	0.160495	0.872857	-5.87081	0.832016	0.806241
T.cells	CD22	0.028368	2.780412	0.160403	0.872929	-6.15899	0.838125	0.813557
T.cells	SLC25A44	-0.0283	3.239164	-0.16027	0.873034	-6.04523	0.829505	0.803239
T.cells	DGKQ	-0.04935	1.470676	-0.16024	0.873055	-5.59302	0.863194	0.843732
T.cells	PTDSS1	-0.01415	5.371518	-0.16023	0.873068	-6.65385	0.790513	0.756952
T.cells	ZFP672	-0.01928	4.004158	-0.16009	0.873176	-6.31354	0.815314	0.786355
T.cells	ATP6V0B	0.010847	8.046898	0.160049	0.873207	-7.01629	0.744027	0.702656
T.cells	PHLPP2	-0.02313	4.283952	-0.15975	0.873441	-6.29143	0.81027	0.780326
T.cells	NFXL1	-0.03126	3.364621	-0.15974	0.873448	-6.0217	0.827253	0.800549
T.cells	FNDC5	-0.08162	0.474524	-0.15939	0.873728	-5.31822	0.88296	0.867529
T.cells	TNFRSF1A	0.04307	5.171275	0.159339	0.873765	-5.82333	0.794314	0.761291
T.cells	ST3GAL4	0.015708	6.423085	0.15929	0.873804	-6.73172	0.772131	0.735216
T.cells	BFAR	0.014887	4.85458	0.159155	0.87391	-6.48011	0.800051	0.768073
T.cells	ADGRG3	0.033747	2.366781	0.158815	0.874177	-6.02941	0.846387	0.823222
T.cells	PIGH	0.035378	1.827535	0.158762	0.874219	-5.92079	0.85672	0.835658

T.cells	RETREG2	-0.0171	4.884018	-0.15851	0.874418	-6.56047	0.79973	0.767544
T.cells	SYT11	0.06237	1.412759	0.158382	0.874517	-5.52613	0.864813	0.845365
T.cells	MGST2	-0.02758	2.953494	-0.15829	0.874593	-6.17236	0.835341	0.809912
T.cells	ATG4B	-0.0167	4.800676	-0.15816	0.874693	-6.51142	0.801238	0.769345
T.cells	USP22	0.015238	4.389395	0.15812	0.874723	-6.49408	0.808716	0.77822
T.cells	GM36445	0.039844	1.003387	0.158074	0.874759	-5.70699	0.872804	0.855074
T.cells	MTTP	-0.03723	2.566189	-0.15795	0.874853	-5.92856	0.84266	0.818739
T.cells	SKA2	0.021528	3.293834	0.157751	0.875013	-6.40174	0.828957	0.802379
T.cells	ZC3H3	-0.02085	3.468484	-0.15772	0.87504	-6.21115	0.825699	0.798489
T.cells	PADI4	-0.0596	1.890862	-0.1577	0.875049	-5.78391	0.855566	0.834309
T.cells	OTUD4	-0.01363	5.091742	-0.15757	0.875154	-6.68682	0.795984	0.763213
T.cells	MAN1A	0.017011	8.000333	0.157464	0.875239	-7.07233	0.745239	0.703851
T.cells	CNNM3	-0.02345	3.157652	-0.15745	0.875246	-6.19639	0.831506	0.805425
T.cells	TMEM9B	0.013777	5.558526	0.157085	0.875536	-6.59911	0.787627	0.753359
T.cells	1700094J0	0.064615	0.092196	0.157035	0.875575	-5.54497	0.890835	0.877055
T.cells	TRNT1	0.014154	4.772449	0.156958	0.875636	-6.61478	0.801749	0.770036
T.cells	A430090L1	-0.04752	0.628221	-0.15679	0.875771	-5.58682	0.880187	0.864115
T.cells	SPACA9	0.063413	1.758151	0.156779	0.875776	-5.33208	0.858124	0.837408
T.cells	MOB2	-0.01275	5.416589	-0.15678	0.87578	-6.69934	0.790159	0.756356
T.cells	9230111E0	0.094302	-0.18455	0.156774	0.87578	-5.1897	0.896378	0.883832
T.cells	FAM120A	-0.01049	6.352078	-0.15666	0.875871	-6.84215	0.773608	0.736949
T.cells	LNX2	0.02604	3.592505	0.156564	0.875945	-6.14058	0.823393	0.795815
T.cells	DALRD3	0.018505	3.729807	0.156545	0.87596	-6.31145	0.820847	0.79278
T.cells	D830050J1	-0.05761	1.312085	-0.15646	0.876023	-5.50832	0.866772	0.847933
T.cells	IFT74	0.029185	2.322027	0.156219	0.876216	-6.10089	0.847422	0.824475
T.cells	ELP1	0.013566	4.553493	0.156067	0.876336	-6.53047	0.805879	0.774838
T.cells	GM41335	-0.05239	0.493808	-0.1558	0.876548	-5.8042	0.883025	0.867518
T.cells	SLC12A7	0.026295	3.37429	0.155691	0.876632	-6.21357	0.827623	0.800757
T.cells	CSF1	-0.06123	2.480595	-0.15556	0.876735	-5.83451	0.844458	0.820925
T.cells	METTL18	0.043439	1.271701	0.15555	0.876743	-5.72793	0.867735	0.848984
T.cells	SAFB	-0.01165	6.192591	-0.15551	0.876773	-6.87173	0.776564	0.740348
T.cells	MAOA	-0.05571	0.932781	-0.15539	0.876865	-5.6416	0.874367	0.85704
T.cells	PRAMEF8	0.030029	3.188085	0.155285	0.876951	-6.04789	0.831105	0.804949
T.cells	TRAM1	-0.00948	7.121393	-0.15527	0.87696	-7.03164	0.760396	0.721467
T.cells	ARMC5	0.019254	3.383082	0.155225	0.876998	-6.2541	0.827459	0.800611
T.cells	ACADL	0.013304	6.734458	0.154876	0.877272	-6.95207	0.767142	0.729364
T.cells	TMPRSS3	-0.04836	-0.48523	-0.15484	0.877299	-5.64226	0.902678	0.891548
T.cells	CDK5RAP3	0.018574	4.379321	0.154817	0.877318	-6.38869	0.809117	0.778788
T.cells	SNRPN	-0.10604	-0.6475	-0.15478	0.877344	-5.15921	0.905964	0.895569
T.cells	ATRIP	-0.02769	2.825397	-0.15455	0.877531	-6.15576	0.83805	0.813308
T.cells	PBLD2	-0.05747	0.872415	-0.15444	0.877615	-5.44257	0.875683	0.858723
T.cells	CCSAP	-0.02652	2.176115	-0.15441	0.877636	-6.09116	0.850392	0.828175
T.cells	ARHGEF4	-0.09476	0.759185	-0.1541	0.877884	-5.36667	0.877984	0.861479
T.cells	GALNT1	-0.01061	6.628673	-0.15397	0.877982	-6.89139	0.769111	0.731688
T.cells	SREK1IP1	-0.01867	4.172778	-0.15395	0.877997	-6.43313	0.813034	0.783465
T.cells	FBXO25	-0.03241	2.127104	-0.15395	0.877998	-5.74482	0.851401	0.829349
T.cells	MIS12	0.021999	3.322859	0.153743	0.878163	-6.34173	0.828783	0.802243

T.cells	MKS1	-0.0552	1.092934	-0.15368	0.878216	-5.52728	0.871437	0.853562
T.cells	KNOP1	0.013536	4.90183	0.153666	0.878223	-6.65318	0.799763	0.767749
T.cells	STFA1	-0.07057	5.975982	-0.1532	0.878592	-6.24934	0.780794	0.745208
T.cells	CWC22	-0.01909	3.900011	-0.15316	0.878621	-6.38161	0.818299	0.789544
T.cells	ARPC5	-0.00923	8.144041	-0.15291	0.878815	-7.07965	0.74346	0.701604
T.cells	SETDB2	-0.01615	4.580351	-0.15268	0.878995	-6.55185	0.806036	0.774808
T.cells	CLEC4A3	0.078465	3.297848	0.15259	0.879069	-5.60825	0.829707	0.802973
T.cells	TXNRD1	0.017896	5.871273	0.152325	0.879278	-6.61397	0.782868	0.747566
T.cells	GM44174	-0.08662	-0.56348	-0.15219	0.879382	-5.21485	0.904939	0.894069
T.cells	ITGB6	0.101837	-0.27325	0.15203	0.87951	-5.2042	0.899075	0.886923
T.cells	PHC1	0.017063	3.5385	0.151982	0.879547	-6.32396	0.825235	0.79777
T.cells	NCKAP5	0.055534	1.217214	0.15188	0.879627	-5.65633	0.869505	0.851008
T.cells	FADS3	0.050274	0.659123	0.151814	0.879679	-5.53298	0.880471	0.864304
T.cells	TAB3	-0.02306	3.680364	-0.15179	0.879702	-6.15532	0.822598	0.794664
T.cells	SSBP1	0.01196	5.592236	0.151773	0.879712	-6.78293	0.787827	0.753477
T.cells	CUL3	-0.00867	7.198802	-0.15169	0.879776	-6.99105	0.759681	0.720533
T.cells	TENM2	0.102554	-0.35252	0.151598	0.879849	-5.21578	0.900673	0.888962
T.cells	GM45894	-0.03425	2.290972	-0.15149	0.879936	-5.70874	0.84876	0.826024
T.cells	HDHD5	-0.0261	2.842941	-0.15145	0.879966	-6.16548	0.838275	0.813439
T.cells	DENND4B	-0.02556	3.747946	-0.15137	0.88003	-6.06062	0.821345	0.79322
T.cells	UBE2L3	0.008714	7.560477	0.150688	0.880565	-7.0728	0.753873	0.713509
T.cells	LUZP1	-0.0146	5.787075	-0.15046	0.880745	-6.78708	0.784869	0.749625
T.cells	PEG3	-0.05901	1.53048	-0.15034	0.880838	-5.55449	0.863984	0.843897
T.cells	CCDC59	0.010333	5.676842	0.150141	0.880995	-6.76304	0.786923	0.751997
T.cells	NMNAT3	0.030349	3.105259	0.150043	0.881073	-6.05437	0.833978	0.807799
T.cells	ZFP516	0.026995	4.66128	0.149871	0.881207	-6.12971	0.805261	0.773622
T.cells	LDHC	0.082891	0.610466	0.148965	0.881921	-5.37472	0.882724	0.866109
T.cells	2610027K0	-0.05445	0.681173	-0.14882	0.882036	-5.47644	0.881324	0.864453
T.cells	VPS26C	0.017453	4.084075	0.148768	0.882076	-6.47514	0.816333	0.786413
T.cells	PCDH17	0.060979	2.459624	0.148725	0.88211	-5.60735	0.846783	0.822774
T.cells	CASC4	0.021408	2.64714	0.148711	0.882121	-6.30735	0.843215	0.818494
T.cells	HIP1	-0.02543	5.505684	-0.14844	0.882334	-6.41747	0.790527	0.755972
T.cells	SHC1	0.015935	4.384809	0.148299	0.882445	-6.42079	0.810809	0.779952
T.cells	CCDC146	-0.05242	2.867299	-0.14822	0.88251	-5.66267	0.839043	0.813617
T.cells	KITL	0.099575	1.956944	0.147877	0.882776	-5.41827	0.856417	0.834574
T.cells	IDH3B	-0.01337	5.850654	-0.14773	0.882896	-6.82386	0.784381	0.748871
T.cells	DNAH2	-0.06129	0.783062	-0.14771	0.882907	-5.41584	0.879311	0.86225
T.cells	GFOD1	0.015794	6.621215	0.147686	0.882927	-6.93261	0.770816	0.732976
T.cells	SPOUT1	-0.0272	2.468742	-0.14748	0.883092	-6.00596	0.846609	0.822782
T.cells	POLR2L	-0.01336	5.414671	-0.14745	0.88311	-6.73026	0.792156	0.758016
T.cells	MTG1	-0.03416	2.186733	-0.14732	0.883213	-5.92123	0.852	0.82929
T.cells	KLRK1	-0.1036	3.149025	-0.14711	0.88338	-5.30948	0.833733	0.807395
T.cells	PLK2	-0.02829	5.006155	-0.14705	0.883426	-6.45476	0.799506	0.766721
T.cells	PWP1	-0.0176	3.903059	-0.14705	0.883429	-6.36582	0.819675	0.790629
T.cells	GM50340	0.037501	1.533078	0.147015	0.883455	-5.86937	0.864619	0.844506
T.cells	GM4890	0.048417	0.373848	0.14701	0.883459	-5.53952	0.887423	0.872144
T.cells	LRCH4	0.018977	4.260162	0.14694	0.883514	-6.36733	0.813094	0.782832

T.cells	PGLS	-0.0094	7.788311	-0.14691	0.883538	-7.18574	0.750692	0.709598
T.cells	HNRNPU	0.008948	8.26781	0.146826	0.883603	-7.23671	0.742569	0.700192
T.cells	PCDH7	-0.10035	0.520175	-0.14667	0.883725	-5.30144	0.884515	0.868632
T.cells	GM11523	-0.10202	-0.95948	-0.14662	0.883768	-5.17172	0.914334	0.905032
T.cells	CDC20	0.02389	3.639962	0.14656	0.883813	-6.5491	0.824555	0.796462
T.cells	SQLE	0.03693	1.97366	0.146508	0.883854	-5.85676	0.856095	0.834249
T.cells	CCDC66	0.025354	2.265203	0.146493	0.883866	-6.03339	0.850497	0.827513
T.cells	SLC11A1	0.067174	3.505984	0.146473	0.883882	-5.57947	0.82705	0.799437
T.cells	AW112010	0.050305	7.279978	0.146408	0.883933	-6.71171	0.759395	0.71971
T.cells	RANGRF	-0.03848	1.262196	-0.14613	0.88415	-5.87181	0.870042	0.850973
T.cells	4931406CC	-0.02932	3.540099	-0.14586	0.884363	-6.00146	0.826681	0.798789
T.cells	SMURF1	0.016949	4.688664	0.145753	0.884448	-6.47742	0.805534	0.773665
T.cells	RANBP6	-0.03731	1.620455	-0.14563	0.884545	-5.70296	0.863237	0.842607
T.cells	GTF3C6	-0.01166	5.226124	-0.14532	0.884792	-6.68088	0.795987	0.762259
T.cells	SENP3	0.016383	4.324023	0.144952	0.885078	-6.43499	0.812523	0.781725
T.cells	PDGFB	-0.09605	1.062654	-0.14486	0.885151	-5.24322	0.874454	0.855961
T.cells	PWWP3A	0.015672	3.715755	0.144834	0.885171	-6.44242	0.823755	0.79508
T.cells	GM26520	-0.05097	2.362216	-0.14468	0.885292	-5.77028	0.849269	0.825616
T.cells	OLFR56	-0.06631	2.067818	-0.14465	0.885313	-5.53241	0.854915	0.832414
T.cells	GGA1	0.013926	4.730426	0.144461	0.885465	-6.4984	0.805144	0.772987
T.cells	ZBTB16	0.077222	2.808614	0.144406	0.885508	-5.44765	0.840822	0.815462
T.cells	XKR8	-0.06604	0.500884	-0.14418	0.885683	-5.3663	0.88561	0.869565
T.cells	NRGN	-0.0227	3.663682	-0.14412	0.885734	-6.67622	0.824777	0.796368
T.cells	TNIP3	0.129085	1.037658	0.14395	0.885867	-5.26111	0.875003	0.856734
T.cells	ATP6V0A2	0.01648	4.619482	0.143936	0.885879	-6.38625	0.807172	0.775491
T.cells	MBTPS1	-0.01331	4.639311	-0.14386	0.885942	-6.55473	0.806811	0.775072
T.cells	PHF11A	0.086289	1.004104	0.143804	0.885982	-5.24577	0.875662	0.857551
T.cells	PPP6R3	-0.00946	7.283025	-0.14375	0.886025	-7.0164	0.759954	0.720095
T.cells	FAM149B	-0.02444	2.992688	-0.14313	0.886513	-6.06455	0.837723	0.811559
T.cells	ZFP821	-0.01561	3.905173	-0.14302	0.886601	-6.51764	0.820661	0.791211
T.cells	PPP2R3A	-0.0199	5.003266	-0.14297	0.886637	-6.60733	0.800559	0.767392
T.cells	SLC7A6	-0.01501	4.274758	-0.14283	0.886746	-6.50529	0.813865	0.783117
T.cells	GFM1	-0.01604	3.89287	-0.14276	0.886806	-6.43918	0.820911	0.791507
T.cells	TDO2	-0.05851	3.218699	-0.14266	0.886885	-5.89931	0.833494	0.806527
T.cells	GM20275	0.03098	3.071387	0.1421	0.887324	-5.97974	0.836291	0.810011
T.cells	ANKRD61	0.048143	0.799732	0.142048	0.887365	-5.55269	0.880137	0.862831
T.cells	TFAM	-0.01418	4.293459	-0.14195	0.887439	-6.56436	0.81355	0.782938
T.cells	LSG1	-0.01219	4.928986	-0.14189	0.887492	-6.62666	0.801954	0.769225
T.cells	MYH9	0.009409	7.80338	0.141885	0.887494	-7.05351	0.751421	0.71007
T.cells	GM42829	0.042703	2.205641	0.141862	0.887512	-5.6014	0.852757	0.829791
T.cells	RCN3	-0.04323	1.932131	-0.14168	0.887655	-5.61432	0.858021	0.836137
T.cells	PRXL2C	0.015596	4.932963	0.141517	0.887783	-6.47415	0.801882	0.769156
T.cells	KDM1A	-0.00986	5.813944	-0.14149	0.887807	-6.81387	0.786065	0.750521
T.cells	PLK1	-0.027	3.704872	-0.14134	0.887924	-6.58051	0.82443	0.795904
T.cells	DOCK7	-0.02267	3.830588	-0.14131	0.887946	-6.2489	0.822095	0.793124
T.cells	SNX1	-0.00962	5.483558	-0.14103	0.888167	-6.74019	0.791962	0.757507
T.cells	ATP5E	-0.00847	8.783189	-0.14091	0.888259	-7.23464	0.734896	0.691033

T.cells	ADAP2	-0.06736	2.585365	-0.14087	0.888293	-5.44091	0.845498	0.821164
T.cells	ACAD8	-0.03216	2.476536	-0.14076	0.888382	-5.77442	0.847572	0.823663
T.cells	RANBP17	0.040898	1.134101	0.140707	0.888422	-5.73506	0.873552	0.855009
T.cells	AP1M1	0.011833	4.979621	0.140658	0.88846	-6.68232	0.801037	0.768275
T.cells	CTSD	0.020032	7.133555	0.140625	0.888486	-6.70789	0.762921	0.723563
T.cells	EFCC1	-0.0783	0.001369	-0.14034	0.888714	-5.24139	0.896043	0.882417
T.cells	CHCHD5	-0.02683	3.092559	-0.14031	0.888733	-6.03413	0.835892	0.809774
T.cells	BET1L	-0.01917	3.860623	-0.14011	0.888895	-6.23156	0.821538	0.79265
T.cells	FOXJ2	-0.03085	3.829076	-0.14008	0.888919	-5.92147	0.822123	0.793346
T.cells	SFMBT2	0.055517	0.60235	0.140017	0.888965	-5.48916	0.884045	0.867826
T.cells	GM19585	0.081631	2.331819	0.139954	0.889015	-5.41925	0.850338	0.8271
T.cells	UBALD1	-0.01569	4.983613	-0.13977	0.889157	-6.53667	0.800965	0.768292
T.cells	ANTXR2	-0.03897	7.654982	-0.13968	0.88923	-6.65183	0.753955	0.713228
T.cells	SPG11	0.019746	4.094018	0.13967	0.889239	-6.29956	0.817222	0.787555
T.cells	TMED4	-0.02012	3.553347	-0.13966	0.889247	-6.2102	0.827252	0.799495
T.cells	WBP1	0.018477	3.813733	0.139602	0.889292	-6.42541	0.822407	0.793723
T.cells	BRF2	0.024696	2.326101	0.139499	0.889373	-5.99394	0.850448	0.827289
T.cells	DPF2	0.011971	5.127706	0.139497	0.889375	-6.65823	0.79836	0.76524
T.cells	SMIM1	0.063853	1.759237	0.139462	0.889402	-5.45415	0.861364	0.840441
T.cells	LIN7C	0.013019	4.956273	0.139369	0.889476	-6.58575	0.80146	0.768936
T.cells	GM31323	0.040431	0.948728	0.139229	0.889586	-5.65532	0.877197	0.859649
T.cells	1700008J0	0.031729	1.842472	0.139174	0.889629	-5.96955	0.859753	0.838563
T.cells	PCP4L1	0.04181	1.36121	0.139033	0.88974	-5.91884	0.869106	0.849876
T.cells	ECM2	0.054141	0.502848	0.139015	0.889755	-5.61567	0.886021	0.870384
T.cells	ZFP354B	0.038575	-0.81695	0.138984	0.889779	-5.53478	0.91262	0.902848
T.cells	TRAF4	-0.02234	4.196822	-0.13892	0.889827	-6.50163	0.815327	0.785434
T.cells	CACUL1	-0.01358	6.117834	-0.1388	0.889924	-6.76217	0.780677	0.744576
T.cells	CCDC106	-0.05751	0.51131	-0.13867	0.890024	-5.43275	0.885853	0.870279
T.cells	IL33	0.05052	0.25962	0.138657	0.890036	-5.54735	0.890869	0.876381
T.cells	ANKRD13A	-0.01045	6.215118	-0.1385	0.890162	-6.84836	0.778959	0.74263
T.cells	EBI3	0.041534	4.387447	0.138459	0.890193	-5.70597	0.811825	0.781395
T.cells	FBXO48	-0.04387	0.24175	-0.13839	0.890248	-5.58736	0.891226	0.87687
T.cells	NLRC3	-0.02927	1.662225	-0.13827	0.890344	-5.94444	0.863245	0.842972
T.cells	SLMAP	-0.0113	6.393027	-0.13823	0.890375	-6.83495	0.775828	0.738996
T.cells	DTD1	0.020732	3.881415	0.137996	0.890557	-6.36506	0.821152	0.792526
T.cells	AIP	-0.01507	4.946962	-0.13793	0.890607	-6.5637	0.801628	0.769364
T.cells	GM26944	0.053658	0.852005	0.137744	0.890756	-5.4257	0.879104	0.862185
T.cells	MRPL14	0.014683	5.601025	0.137594	0.890874	-6.71426	0.78986	0.755484
T.cells	FKBP15	0.013796	5.239594	0.137552	0.890907	-6.55523	0.796343	0.763124
T.cells	OLFML2B	-0.04555	0.874884	-0.13754	0.890921	-5.72175	0.878653	0.86164
T.cells	PACS1	-0.01592	5.972963	-0.1375	0.89095	-6.67118	0.783241	0.747704
T.cells	IL1RAP	-0.02281	4.731572	-0.13747	0.890973	-6.36356	0.805539	0.774007
T.cells	NAA35	0.012745	4.851078	0.137459	0.890981	-6.57633	0.803367	0.771436
T.cells	FAM167B	-0.04099	2.127548	-0.13717	0.891204	-5.70385	0.854326	0.832288
T.cells	AMZ2	0.017002	3.901939	0.137103	0.891261	-6.28749	0.820839	0.792226
T.cells	FBXO45	0.015495	3.654199	0.13702	0.891327	-6.43057	0.82544	0.797729
T.cells	STAT6	0.015583	5.031546	0.136925	0.891401	-6.43376	0.800162	0.767738

T.cells	MKI67	-0.02182	6.498445	-0.13684	0.891466	-7.0977	0.774041	0.737032
T.cells	CDC42BPG	-0.04503	2.57838	-0.13681	0.891489	-5.66866	0.845699	0.822008
T.cells	RAD23B	-0.00828	6.360558	-0.13645	0.891777	-6.86939	0.77663	0.739874
T.cells	SMARCAD1	0.010972	4.984115	0.136384	0.891828	-6.6666	0.801194	0.768777
T.cells	ZFP61	-0.04103	1.174198	-0.13614	0.892023	-5.73193	0.873146	0.854776
T.cells	NDUFA8	-0.01313	5.798653	-0.13584	0.892257	-6.75567	0.786693	0.751673
T.cells	MBD3	0.010716	5.696238	0.135762	0.892318	-6.79914	0.788518	0.753836
T.cells	GM1604B	-0.04582	1.489369	-0.13576	0.892321	-5.71915	0.866999	0.847449
T.cells	GEMIN5	-0.01726	3.385628	-0.13573	0.892343	-6.34843	0.830763	0.803923
T.cells	DBT	-0.02001	3.826101	-0.13549	0.892529	-6.31445	0.82255	0.79414
T.cells	CDADC1	0.02016	4.609962	0.135484	0.892537	-6.43143	0.808121	0.777001
T.cells	ENC1	-0.02517	3.181392	-0.13547	0.892547	-6.04545	0.834597	0.808516
T.cells	ARRDC1	-0.01684	4.471227	-0.13536	0.892632	-6.43178	0.810669	0.780007
T.cells	HYAL2	-0.04421	1.859326	-0.13515	0.892804	-5.5972	0.859914	0.8388
T.cells	NRAS	-0.0102	6.075126	-0.13495	0.892961	-6.78726	0.781874	0.745964
T.cells	SNX3	0.007991	7.730038	0.134922	0.892981	-7.07783	0.753099	0.712378
T.cells	TPT1	0.007491	10.10831	0.134882	0.893012	-7.42564	0.713502	0.666781
T.cells	TSC2	-0.02065	3.109391	-0.13447	0.893337	-6.11079	0.836127	0.810306
T.cells	CLEC16A	0.015107	4.763553	0.134357	0.893426	-6.51857	0.80549	0.773865
T.cells	RASSF5	0.014382	5.485464	0.134322	0.893453	-6.57078	0.792452	0.758467
T.cells	GTF2IRD2	0.020112	3.944401	0.134276	0.89349	-6.2137	0.820528	0.791713
T.cells	YAP1	-0.07233	1.070556	-0.13422	0.893531	-5.41224	0.875379	0.857589
T.cells	ZFP202	0.071236	0.604939	0.13422	0.893533	-5.32511	0.884578	0.868753
T.cells	4933432I0[illegible]	-0.0785	0.408312	-0.13391	0.893776	-5.22151	0.888532	0.873631
T.cells	FBXO21	0.017668	3.292952	0.133805	0.893861	-6.28923	0.832715	0.806337
T.cells	GTF2E2	-0.01048	5.440806	-0.13378	0.89388	-6.75449	0.79329	0.759543
T.cells	LIMD2	-0.01128	7.333227	-0.13365	0.893983	-7.04697	0.760014	0.720584
T.cells	NCBP3	0.010639	5.506298	0.133641	0.89399	-6.75994	0.792116	0.758182
T.cells	CDK13	0.009497	7.414024	0.133579	0.894039	-7.01536	0.758623	0.718997
T.cells	IGF2BP2	-0.05216	1.457318	-0.13352	0.894085	-5.47138	0.867846	0.848646
T.cells	IFT122	-0.02694	1.594011	-0.13341	0.894171	-5.88339	0.865196	0.845482
T.cells	PFDN1	-0.01088	5.109296	-0.13317	0.894364	-6.66946	0.799321	0.766741
T.cells	NDST1	-0.02324	4.570663	-0.13309	0.894426	-6.2255	0.80911	0.778325
T.cells	TMEM86A	0.041357	2.885047	0.132969	0.89452	-5.65523	0.840471	0.815707
T.cells	VMA21	-0.0122	5.176423	-0.13287	0.8946	-6.6573	0.798109	0.765335
T.cells	AUP1	-0.01132	6.010505	-0.13274	0.894699	-6.75754	0.783192	0.747791
T.cells	GGT5	0.077532	0.649699	0.13274	0.894701	-5.23859	0.883801	0.868057
T.cells	GM13610	0.042187	0.396658	0.132694	0.894737	-5.60297	0.888834	0.874195
T.cells	CEP131	-0.0406	1.3043	-0.13194	0.895333	-5.80183	0.871306	0.852573
T.cells	GM14321	0.070874	-0.49943	0.131709	0.895514	-5.18283	0.907287	0.896355
T.cells	GM15564	-0.04226	2.474453	-0.1316	0.895603	-5.70658	0.848671	0.825284
T.cells	HTR2B	-0.04374	0.855091	-0.13156	0.895631	-5.58246	0.880142	0.86328
T.cells	UBE2Z	-0.01067	5.437928	-0.13156	0.895633	-6.67051	0.79377	0.759949
T.cells	GM17259	0.04163	1.365713	0.131458	0.895712	-5.66454	0.870104	0.851139
T.cells	NAXD	-0.01536	4.128354	-0.13138	0.895769	-6.37166	0.817609	0.788216
T.cells	UBE2C	-0.0218	6.746366	-0.1313	0.895836	-7.14436	0.770606	0.732825
T.cells	ZFP207	0.007537	6.934778	0.131077	0.896012	-6.99341	0.767323	0.729024

T.cells	PLXNA2	-0.04583	2.31233	-0.13104	0.89604	-5.70381	0.851774	0.82912
T.cells	FGD4	-0.04483	4.134435	-0.1307	0.896307	-5.7945	0.817497	0.788237
T.cells	MAN2B1	-0.01729	6.851978	-0.13061	0.89638	-6.70241	0.768764	0.730803
T.cells	CCP110	-0.02174	2.37539	-0.13013	0.896758	-6.156	0.850566	0.827877
T.cells	CCNDBP1	-0.018	5.692836	-0.13012	0.896767	-6.57139	0.789206	0.754863
T.cells	MTG2	-0.01997	3.031494	-0.12998	0.896879	-6.12779	0.838089	0.81294
T.cells	MTA3	0.014312	6.109959	0.129938	0.896911	-6.71171	0.781792	0.746185
T.cells	KCTD5	-0.01635	3.640452	-0.12993	0.89692	-6.36122	0.82666	0.799286
T.cells	RAD50	0.01172	4.906644	0.129859	0.896973	-6.6898	0.803362	0.771623
T.cells	PPFIA4	-0.0452	2.735956	-0.12979	0.897028	-5.57157	0.843688	0.819675
T.cells	4931406G(	0.083738	0.347467	0.129733	0.897072	-5.27544	0.890226	0.875933
T.cells	PSMA5	-0.01214	5.884469	-0.1297	0.897102	-6.80675	0.785792	0.750901
T.cells	WDR35	-0.04298	0.509893	-0.12965	0.897136	-5.66084	0.886988	0.871992
T.cells	INTS5	0.018753	2.704825	0.129613	0.897167	-6.14106	0.84428	0.820385
T.cells	NOP14	0.015587	4.004398	0.129607	0.897172	-6.44425	0.8199	0.791248
T.cells	SNX30	0.012604	5.755716	0.129383	0.897349	-6.80526	0.788084	0.753597
T.cells	GM826	-0.0933	-0.25558	-0.12933	0.897393	-5.20776	0.902344	0.890717
T.cells	CTSE	-0.0137	5.492809	-0.12928	0.89743	-6.76252	0.792785	0.759133
T.cells	OASL2	-0.10082	3.934807	-0.12925	0.897457	-5.58148	0.821188	0.792783
T.cells	TCTN3	-0.04786	1.366808	-0.12919	0.897498	-5.54206	0.870083	0.85148
T.cells	CMAS	0.014185	5.862643	0.129162	0.897523	-6.67095	0.78618	0.751357
T.cells	POLA2	0.0155	4.050563	0.129146	0.897535	-6.52669	0.819046	0.790232
T.cells	SPTB	-0.0547	1.120897	-0.1291	0.897571	-5.53147	0.874903	0.857318
T.cells	KLHL25	0.023752	2.493825	0.129054	0.897608	-5.97411	0.848301	0.825229
T.cells	FABP7	-0.07512	1.989157	-0.12883	0.897782	-5.50292	0.857992	0.836906
T.cells	GIPC1	-0.01454	4.359749	-0.12882	0.897794	-6.46089	0.813349	0.783488
T.cells	PIGZ	-0.07575	0.017052	-0.12879	0.897813	-5.21985	0.896847	0.884035
T.cells	ABHD14A	0.032805	1.44344	0.128757	0.897842	-5.79874	0.868585	0.849698
T.cells	SLC25A38	-0.01975	3.517886	-0.12851	0.898038	-6.17179	0.829062	0.802151
T.cells	PQLC1	-0.01692	4.395337	-0.12804	0.89841	-6.19688	0.812843	0.78291
T.cells	TSR1	-0.01856	3.584651	-0.12779	0.898602	-6.30283	0.827852	0.800827
T.cells	ZSWIM3	0.04203	1.503758	0.127727	0.898655	-5.62806	0.867566	0.848543
T.cells	MAP3K20	-0.01604	3.798494	-0.12764	0.898722	-6.54125	0.823868	0.796087
T.cells	CAAA0111{	0.011973	5.624186	0.127585	0.898767	-6.67679	0.790577	0.756643
T.cells	ZC3H14	0.009265	5.656337	0.12745	0.898874	-6.76963	0.790002	0.756018
T.cells	GM46652	0.074546	-0.73309	0.127286	0.899003	-5.18393	0.912212	0.903018
T.cells	NAIP2	-0.05498	3.006951	-0.12723	0.899045	-5.39338	0.838705	0.81391
T.cells	ADI1	0.015066	3.749643	0.127195	0.899075	-6.31781	0.824776	0.797262
T.cells	CD300A	0.046886	4.623283	0.127093	0.899155	-5.71334	0.808669	0.778124
T.cells	MYD88	0.016786	4.609439	0.126871	0.899331	-6.41178	0.808922	0.778436
T.cells	PTBP3	-0.00705	8.46802	-0.12686	0.899337	-7.19108	0.741233	0.699157
T.cells	SDAD1	-0.01366	4.473042	-0.12676	0.899419	-6.56959	0.811418	0.781398
T.cells	VPREB3	0.01659	6.013944	0.126715	0.899453	-7.10599	0.783635	0.748586
T.cells	ESPN	-0.07622	-0.17599	-0.12662	0.899529	-5.19739	0.9009	0.889212
T.cells	TMC3	-0.08199	-1.08655	-0.12653	0.899597	-5.15303	0.919456	0.91195
T.cells	CCR4	-0.06895	-1.08113	-0.12651	0.899612	-5.18041	0.919345	0.911813
T.cells	SLC3A2	0.014886	6.772545	0.126512	0.899614	-6.73329	0.770289	0.732942

T.cells	SLC36A3OS	-0.06822	1.010121	-0.1265	0.899623	-5.38427	0.877242	0.860405
T.cells	KCND1	0.071846	-0.19065	0.126497	0.899626	-5.22791	0.901196	0.889573
T.cells	GM24362	-0.0478	0.694716	-0.12643	0.899681	-5.471	0.883477	0.867984
T.cells	CD44	0.015185	8.587659	0.126338	0.899751	-6.96388	0.739223	0.69685
T.cells	RSU1	-0.01172	5.746299	-0.12634	0.899752	-6.60653	0.788396	0.754202
T.cells	ERAL1	0.025939	1.918255	0.126145	0.899904	-5.86599	0.859537	0.83901
T.cells	AHCYL1	0.009465	5.179051	0.126127	0.899918	-6.65768	0.798593	0.766234
T.cells	GM50209	-0.04814	0.629068	-0.12599	0.90003	-5.46514	0.884813	0.869627
T.cells	SAMD12	-0.05347	0.266619	-0.12592	0.900077	-5.5807	0.892038	0.878422
T.cells	GBE1	-0.01465	6.483019	-0.12574	0.900222	-6.78063	0.775448	0.738949
T.cells	UTP3	0.010814	5.450096	0.125633	0.900308	-6.76192	0.793801	0.76052
T.cells	POGZ	-0.01212	4.431535	-0.12543	0.900468	-6.53687	0.812292	0.782407
T.cells	APBA1	-0.03048	4.384633	-0.12535	0.90053	-6.06603	0.813153	0.78343
T.cells	F8	0.030346	3.134673	0.125335	0.900543	-6.07578	0.836411	0.811163
T.cells	UBQLN2	0.013107	4.031371	0.1252	0.900649	-6.43503	0.819664	0.791171
T.cells	BBS7	-0.0551	0.558111	-0.12505	0.900769	-5.47696	0.886314	0.871394
T.cells	TMPRSS5	-0.04167	0.722493	-0.12503	0.900785	-5.5914	0.88305	0.867425
T.cells	EEF1E1	-0.01326	4.824221	-0.12483	0.900944	-6.66698	0.805118	0.773926
T.cells	ADORA3	-0.06714	-0.30137	-0.12478	0.900981	-5.22358	0.903561	0.892461
T.cells	CSK	-0.00919	6.929767	-0.12432	0.901342	-6.99638	0.767657	0.729888
T.cells	TBCE	0.010428	5.119789	0.124227	0.901418	-6.65822	0.799758	0.76761
T.cells	CENPP	-0.0149	5.39603	-0.12407	0.901538	-6.87028	0.794778	0.761729
T.cells	AKR1A1	-0.00932	7.188669	-0.12404	0.901563	-6.96214	0.763166	0.724647
T.cells	KCNAB1	0.045225	1.324987	0.123997	0.901599	-5.67286	0.871181	0.853085
T.cells	GSG1L	0.065532	-0.16326	0.123987	0.901607	-5.33814	0.900769	0.889079
T.cells	1110017D1	-0.07731	0.201816	-0.12388	0.901689	-5.24208	0.893426	0.88012
T.cells	ISOC2B	0.028412	2.519105	0.123822	0.901738	-5.91499	0.848091	0.825247
T.cells	CCDC91	0.028472	2.408137	0.123786	0.901766	-5.87099	0.850212	0.827813
T.cells	GM4107	-0.07522	0.916265	-0.12374	0.901799	-5.29329	0.879216	0.862863
T.cells	ZFP64	-0.01565	5.317866	-0.12366	0.901868	-6.61145	0.796184	0.763437
T.cells	ARID3B	-0.02502	3.836648	-0.12356	0.901946	-6.00535	0.823274	0.795601
T.cells	GORASP1	0.051249	0.733344	0.123505	0.901987	-5.46207	0.882834	0.867316
T.cells	GM12236	0.065882	1.087964	0.123452	0.902029	-5.38234	0.875832	0.858814
T.cells	EFCAB7	0.03486	0.535523	0.123405	0.902066	-5.66934	0.886763	0.872094
T.cells	DPYSL3	0.062589	0.753878	0.123324	0.902131	-5.31222	0.882428	0.866847
T.cells	TSEN15	-0.0176	2.776415	-0.12326	0.902182	-6.23533	0.84319	0.819455
T.cells	SESTD1	-0.04286	2.300083	-0.12323	0.902207	-5.78597	0.852283	0.830396
T.cells	CARS2	0.013784	3.502896	0.123103	0.902305	-6.21695	0.829519	0.80308
T.cells	TBC1D31	0.015265	4.166685	0.122638	0.902672	-6.54756	0.817448	0.788479
T.cells	GM15860	0.035431	0.373729	0.122458	0.902814	-5.68307	0.890297	0.876253
T.cells	ZFP959	-0.0164	3.241052	-0.12233	0.902917	-6.27578	0.834697	0.809148
T.cells	GPT2	-0.0402	1.738624	-0.12228	0.902952	-5.6519	0.863417	0.843679
T.cells	ERH	0.00955	7.372533	0.122162	0.903048	-7.14593	0.760255	0.721267
T.cells	KLHL36	0.034457	2.076404	0.121987	0.903186	-5.75391	0.856881	0.835839
T.cells	D930016D(	0.020785	2.876865	0.121836	0.903305	-6.05519	0.841576	0.817466
T.cells	DDT	0.013255	5.541533	0.121728	0.903391	-6.73192	0.792442	0.759046
T.cells	GM26802	-0.0698	1.069129	-0.12162	0.90348	-5.25654	0.876507	0.859632

T.cells	MAP3K10	0.023439	2.731125	0.121594	0.903497	-5.98133	0.844344	0.820816
T.cells	ZFP992	-0.02442	3.563813	-0.1215	0.903569	-6.0112	0.828644	0.802066
T.cells	RGL1	0.016803	5.711711	0.12143	0.903626	-6.68505	0.789397	0.75554
T.cells	ZFP280C	-0.01284	4.164903	-0.12136	0.90368	-6.50378	0.817481	0.788794
T.cells	TXNRD3	0.025077	1.950944	0.121203	0.903806	-5.94132	0.859303	0.838922
T.cells	GM49439	0.044494	-0.46286	0.12117	0.903831	-5.54416	0.90715	0.897089
T.cells	ORAI1	-0.01378	6.491763	-0.12115	0.903851	-6.5879	0.775581	0.739331
T.cells	YME1L1	-0.00676	6.181942	-0.12105	0.903923	-6.8426	0.781041	0.745735
T.cells	NF2	0.012191	4.781261	0.121045	0.90393	-6.60064	0.80618	0.77539
T.cells	LIMA1	-0.02562	4.507655	-0.12091	0.904036	-6.35035	0.811178	0.781335
T.cells	PAIP2	0.006546	7.620191	0.120894	0.904049	-7.14465	0.755998	0.716482
T.cells	PRODH	0.030932	2.780405	0.120773	0.904145	-5.9939	0.843429	0.819828
T.cells	PON2	0.010011	6.020273	0.120531	0.904336	-6.86219	0.783972	0.749201
T.cells	GM43062	-0.02567	1.638658	-0.12042	0.904423	-5.80517	0.865435	0.846371
T.cells	TLR11	-0.05737	-0.67266	-0.12032	0.904504	-5.23124	0.911501	0.902486
T.cells	PIK3C2A	0.010378	6.854141	0.120184	0.90461	-6.94057	0.769307	0.732105
T.cells	XLR4B	-0.03143	1.285179	-0.12017	0.90462	-5.7073	0.872338	0.854821
T.cells	ZXDB	-0.02086	3.1887	-0.11989	0.904839	-6.07343	0.835755	0.810845
T.cells	ZFP428	-0.01954	2.058083	-0.11989	0.904843	-6.19204	0.857309	0.83675
T.cells	D8ERTD738	-0.00738	7.358725	-0.11985	0.90487	-7.02555	0.760559	0.72198
T.cells	CBLB	0.011174	7.336459	0.119547	0.905113	-7.01797	0.760943	0.722459
T.cells	PSAT1	-0.01454	4.218142	-0.11953	0.905126	-6.69802	0.816569	0.787979
T.cells	PSMB1	-0.00784	7.299863	-0.11951	0.90514	-7.06884	0.761574	0.723195
T.cells	NLE1	-0.0286	2.257092	-0.11948	0.905168	-5.94692	0.853478	0.832169
T.cells	MAST1	0.077234	1.020113	0.119422	0.905212	-5.30215	0.877548	0.861279
T.cells	CELF6	0.080544	-0.70622	0.11929	0.905317	-5.17907	0.912186	0.903553
T.cells	GABBR1	-0.05123	2.766527	-0.11902	0.905531	-5.71856	0.843744	0.820461
T.cells	GM19466	-0.04538	0.661272	-0.11887	0.905652	-5.52747	0.884647	0.869907
T.cells	ARHGAP19	-0.01688	4.256309	-0.11882	0.905684	-6.59399	0.815866	0.787143
T.cells	AP1S1	0.011713	4.706161	0.11882	0.905688	-6.60367	0.807618	0.777347
T.cells	SUGP1	-0.01186	4.615277	-0.11878	0.905721	-6.51946	0.809278	0.779316
T.cells	TMEM167B	0.019591	3.755594	0.118751	0.905742	-6.1694	0.825138	0.79819
T.cells	CD300C	0.061966	0.72621	0.118748	0.905744	-5.22948	0.883359	0.868339
T.cells	ACY1	-0.03619	1.799123	-0.11837	0.906042	-5.69505	0.862317	0.842932
T.cells	NIPA2	0.010392	6.042829	0.118331	0.906074	-6.76161	0.783572	0.749046
T.cells	SYNPO	-0.06581	0.219102	-0.11824	0.906146	-5.24903	0.893467	0.880763
T.cells	GM12840	-0.05978	3.766389	-0.11823	0.906156	-5.67907	0.824937	0.798051
T.cells	GM36975	0.025908	3.276788	0.118183	0.90619	-6.05604	0.834097	0.808998
T.cells	KDM8	0.022833	1.814343	0.117883	0.906428	-5.9317	0.862022	0.842681
T.cells	PATL1	0.011437	5.222719	0.117848	0.906455	-6.60306	0.798244	0.766439
T.cells	PAQR5	-0.06506	0.502008	-0.11783	0.90647	-5.26097	0.887815	0.87398
T.cells	ASAP1	0.0097	7.200045	0.117763	0.906523	-6.99768	0.7633	0.72539
T.cells	GRHL1	0.042715	1.025537	0.117644	0.906617	-5.58825	0.877441	0.861366
T.cells	AMOT	-0.04231	0.863491	-0.11761	0.906647	-5.55304	0.88064	0.865252
T.cells	NUDCD1	0.016815	3.226429	0.117488	0.90674	-6.32632	0.835044	0.810254
T.cells	GM47469	0.040489	0.520329	0.117259	0.906921	-5.60231	0.88745	0.873631
T.cells	NCOA1	0.0108	7.07231	0.117192	0.906973	-6.94163	0.765513	0.728073

T.cells	ADCY3	-0.03142	3.001893	-0.11709	0.907053	-6.11692	0.839281	0.815447
T.cells	UBE2Q2	0.010767	5.51991	0.117005	0.907121	-6.62571	0.792898	0.760252
T.cells	GSPT1	0.007358	6.707096	0.116979	0.907142	-6.94155	0.771874	0.735535
T.cells	THOC3	0.012241	4.102819	0.11689	0.907212	-6.53798	0.818698	0.790843
T.cells	KIFC5B	0.023452	1.787655	0.116745	0.907327	-6.03269	0.86254	0.843499
T.cells	CCL7	-0.0951	1.659903	-0.11663	0.907415	-5.4622	0.865021	0.8465
T.cells	PMAIP1	0.030174	4.983149	0.116483	0.907534	-6.12713	0.802579	0.771755
T.cells	TRUB1	0.026769	1.825881	0.116382	0.907614	-5.80417	0.861799	0.842626
T.cells	PRKCA	-0.01342	7.401898	-0.11634	0.907647	-6.98263	0.759815	0.721509
T.cells	ZFP40	0.036982	1.347612	0.116321	0.907662	-5.66732	0.871115	0.853915
T.cells	IL12RB1	0.071303	0.500116	0.116265	0.907706	-5.2473	0.887852	0.874263
T.cells	TLE5	-0.00924	7.209326	-0.11622	0.907743	-6.91458	0.763139	0.725397
T.cells	SLC1A3	-0.08254	-0.29724	-0.11611	0.907825	-5.24317	0.903869	0.893831
T.cells	PCBP3	-0.01692	2.616922	-0.11598	0.907933	-6.24464	0.846591	0.824365
T.cells	RPP21	-0.0113	4.873001	-0.11591	0.90799	-6.59517	0.80458	0.774199
T.cells	IBA57	0.023622	1.934328	0.115853	0.908032	-5.87332	0.859699	0.840163
T.cells	TEAD2	-0.0356	1.154161	-0.11577	0.908098	-5.64084	0.87491	0.858587
T.cells	SPRTN	-0.01761	3.419835	-0.11574	0.908122	-6.21924	0.831411	0.806174
T.cells	SPA17	-0.02976	0.979282	-0.11551	0.908299	-5.74291	0.878353	0.862812
T.cells	PSMG1	0.015249	3.325411	0.115418	0.908376	-6.23116	0.833183	0.808323
T.cells	RINL	0.025705	3.632551	0.115277	0.908487	-5.91755	0.827432	0.801443
T.cells	APP	0.031436	5.938302	0.115194	0.908553	-6.1385	0.785428	0.751618
T.cells	NRG4	-0.05052	2.017195	-0.11517	0.908573	-5.55758	0.858098	0.838278
T.cells	ZFP410	-0.01197	4.138179	-0.11501	0.908696	-6.47894	0.818045	0.790244
T.cells	CCDC32	-0.01622	3.31271	-0.115	0.908708	-6.2574	0.833422	0.808608
T.cells	SERPINB2	-0.1773	0.991964	-0.11499	0.908711	-5.26198	0.878103	0.862508
T.cells	MCFD2	-0.02178	3.702476	-0.11489	0.908792	-6.09458	0.826128	0.799895
T.cells	NUMB	-0.00976	6.956562	-0.11474	0.908908	-6.92266	0.767523	0.730629
T.cells	MICAL3	-0.01571	3.436889	-0.11464	0.908992	-6.38907	0.831091	0.805846
T.cells	SPIN1	0.009057	5.805516	0.114614	0.909011	-6.7497	0.787791	0.754428
T.cells	GM47802	0.057336	0.099495	0.114562	0.909052	-5.32479	0.895867	0.88419
T.cells	NTNG2	0.042069	3.05713	0.11432	0.909243	-5.70576	0.838237	0.814416
T.cells	FAM160B1	0.012243	4.443367	0.114252	0.909297	-6.50381	0.812427	0.783607
T.cells	RHOG	-0.01169	7.243088	-0.11419	0.90935	-6.90753	0.762555	0.72485
T.cells	AC142100.	0.065594	0.335219	0.114055	0.909452	-5.26043	0.891143	0.878482
T.cells	TIGIT	-0.06299	-0.09862	-0.11403	0.909472	-5.24962	0.899854	0.889134
T.cells	NUDT14	0.011843	4.342372	0.113967	0.909522	-6.45532	0.814282	0.785866
T.cells	NAP1L4	-0.00699	6.249563	-0.11375	0.909698	-6.89442	0.779913	0.745246
T.cells	PILRA	0.046851	3.3927	0.113568	0.909838	-5.54471	0.83192	0.80697
T.cells	TANC1	0.022118	3.838336	0.113564	0.909841	-6.24139	0.823599	0.797024
T.cells	RAB6A	0.008586	6.637549	0.113373	0.909992	-6.84131	0.773091	0.737294
T.cells	GSK3A	0.009132	5.557032	0.113171	0.910151	-6.74335	0.792232	0.759878
T.cells	GM45509	-0.02733	1.640462	-0.11308	0.910225	-5.93554	0.8654	0.84738
T.cells	TMEM94	-0.01843	2.610495	-0.11287	0.910388	-6.00653	0.846714	0.824863
T.cells	MIR142HG	0.008151	8.019491	0.112856	0.9104	-7.13299	0.749246	0.70957
T.cells	ZFP26	-0.0116	3.995742	-0.1128	0.910446	-6.46453	0.820679	0.793674
T.cells	NT5C3	-0.01338	4.85257	-0.11274	0.910492	-6.49905	0.804951	0.774968

T.cells	SPATA24	0.023159	2.193104	0.112559	0.910635	-6.08453	0.854708	0.8345
T.cells	WDR4	-0.01566	2.999114	-0.11256	0.910638	-6.21	0.839334	0.815999
T.cells	CXCR6	-0.06707	1.255073	-0.11249	0.91069	-5.26551	0.872928	0.856555
T.cells	MICAL1	-0.01497	3.487265	-0.11236	0.910797	-6.2996	0.830148	0.805035
T.cells	WASL	0.008333	5.483088	0.112168	0.910944	-6.6908	0.793558	0.76153
T.cells	HECTD4	0.010233	5.548933	0.112131	0.910974	-6.73473	0.792377	0.760135
T.cells	HMMR	0.018985	4.333361	0.112112	0.910989	-6.72075	0.814448	0.786299
T.cells	TSHZ1	-0.01408	5.060403	-0.11206	0.911028	-6.58706	0.801179	0.770551
T.cells	CBY1	0.015161	2.846037	0.111917	0.911143	-6.25134	0.842234	0.819584
T.cells	TMEM51	0.043225	3.130512	0.111812	0.911226	-5.50945	0.836852	0.813123
T.cells	MPP6	0.009276	6.194697	0.111697	0.911317	-6.86041	0.780883	0.746646
T.cells	MTHFS	0.028893	5.861718	0.111607	0.911388	-6.32376	0.78679	0.7536
T.cells	CR1L	0.008152	5.693128	0.11152	0.911457	-6.72365	0.789797	0.757146
T.cells	ZFPM2	0.049964	2.777013	0.111446	0.911516	-5.69161	0.843544	0.821166
T.cells	MEGF11	-0.05034	0.651843	-0.11134	0.911601	-5.41812	0.884834	0.871136
T.cells	PPT2	-0.02097	4.199673	-0.11131	0.911621	-6.05352	0.81691	0.789292
T.cells	CCDC114	0.027937	1.271818	0.111302	0.911629	-5.7651	0.8726	0.856261
T.cells	MLLT1	-0.01664	2.735176	-0.11129	0.911639	-6.11329	0.84434	0.822122
T.cells	JAM2	0.053754	1.449339	0.111062	0.911819	-5.49405	0.869126	0.852091
T.cells	ACAA1A	-0.01314	5.302507	-0.11099	0.911877	-6.49221	0.796806	0.765479
T.cells	ARHGEF1	-0.00797	7.10928	-0.11095	0.911905	-6.98225	0.764872	0.727927
T.cells	STIM1	-0.01113	7.712751	-0.11093	0.911924	-7.0934	0.754478	0.715802
T.cells	ABHD4	-0.02442	2.787772	-0.11068	0.91212	-5.7901	0.84334	0.820975
T.cells	5730455P1	0.015176	2.914853	0.110637	0.912155	-6.21255	0.840929	0.818077
T.cells	PLIN3	0.016883	3.527331	0.110602	0.912182	-6.17431	0.829398	0.804254
T.cells	MRPS24	-0.00802	6.031221	-0.1106	0.912182	-6.84174	0.783778	0.750105
T.cells	HIP1R	-0.01078	5.271328	-0.11051	0.912252	-6.70208	0.797368	0.766181
T.cells	CCDC62	0.030081	2.858429	0.11049	0.912271	-5.86791	0.841998	0.819402
T.cells	HEY1	0.064614	-0.2714	0.110475	0.912283	-5.21097	0.903345	0.893851
T.cells	PTRH2	-0.01327	4.181735	-0.11033	0.9124	-6.39421	0.817248	0.789793
T.cells	FUZ	-0.03978	1.08456	-0.11028	0.912437	-5.5621	0.876287	0.860844
T.cells	HK1OS	0.046942	0.741866	0.110179	0.912517	-5.41504	0.883063	0.869078
T.cells	NAIF1	-0.03267	1.251393	-0.11003	0.912637	-5.63193	0.873036	0.856863
T.cells	POU2F2	-0.01655	5.772063	-0.10978	0.912833	-6.73377	0.78842	0.755633
T.cells	TMOD3	0.008737	7.218311	0.109541	0.913022	-6.93729	0.763014	0.725813
T.cells	CHTF8	0.029586	1.341638	0.109457	0.913088	-5.74322	0.871267	0.854766
T.cells	PIP5K1C	0.011045	5.626433	0.109456	0.913089	-6.67576	0.791021	0.758703
T.cells	LIPO3	-0.01743	2.915424	-0.10945	0.913092	-6.19699	0.840952	0.818168
T.cells	CEP120	-0.00928	5.494784	-0.1093	0.913208	-6.72266	0.79338	0.761511
T.cells	KLF11	0.012142	3.555665	0.109279	0.913229	-6.32672	0.828901	0.803746
T.cells	ACTB	-0.00772	13.9201	-0.10926	0.913246	-7.92428	0.655142	0.602511
T.cells	TOMT	-0.04136	1.736925	-0.10919	0.913298	-5.60205	0.863559	0.845466
T.cells	INF2	-0.02454	3.252642	-0.10914	0.913339	-5.97614	0.834585	0.810591
T.cells	EXOSC10	0.010915	4.93082	0.109006	0.913445	-6.59825	0.80359	0.773599
T.cells	XPO7	-0.00859	6.710919	-0.10884	0.913573	-6.98293	0.771871	0.736246
T.cells	GPD2	-0.02089	6.625661	-0.10881	0.913597	-6.52656	0.773363	0.737996
T.cells	HCFC1	0.010132	4.949877	0.108632	0.91374	-6.70077	0.803257	0.773262

T.cells	CHDH	0.054982	1.157054	0.10862	0.91375	-5.34999	0.874933	0.859311
T.cells	GPRIN3	-0.04343	0.638034	-0.10844	0.913891	-5.50604	0.885226	0.871846
T.cells	APH1B	0.029624	2.578014	0.108388	0.913934	-5.78281	0.847446	0.826102
T.cells	MORC2A	-0.01338	4.295558	-0.10819	0.914094	-6.42616	0.815281	0.787574
T.cells	LRMP	0.00854	6.432878	0.108162	0.914113	-7.02291	0.776814	0.742078
T.cells	CPEB2	0.018	5.412476	0.107996	0.914243	-6.16704	0.794986	0.76347
T.cells	JMJD7	0.022559	1.757778	0.107942	0.914286	-5.90865	0.863292	0.845196
T.cells	LGALS3BP	0.036538	4.826846	0.107633	0.914531	-5.99637	0.805676	0.776044
T.cells	CD8A	-0.07538	1.360615	-0.10762	0.914541	-5.27266	0.871139	0.854604
T.cells	WDYHV1	-0.01119	4.174037	-0.10752	0.91462	-6.48396	0.81765	0.790277
T.cells	SS18L2	-0.01516	3.817204	-0.10741	0.914707	-6.27909	0.824273	0.798171
T.cells	SNX2	0.005627	7.314048	0.107122	0.914935	-7.11676	0.761665	0.724196
T.cells	NDUFC1	0.008646	7.367214	0.107112	0.914943	-7.06827	0.760747	0.723125
T.cells	ZFP758	-0.01858	2.74078	-0.10692	0.915097	-6.042	0.844646	0.822573
T.cells	CRYBA4	-0.06316	-0.18755	-0.10683	0.915165	-5.20233	0.902091	0.892315
T.cells	MLLT6	-0.03204	3.270712	-0.10672	0.915254	-5.86127	0.83462	0.810566
T.cells	NUSAP1	0.014863	5.649629	0.106686	0.91528	-6.98004	0.790962	0.75863
T.cells	GPATCH4	0.014527	3.338867	0.106402	0.915505	-6.34024	0.833386	0.80904
T.cells	AC160336.	-0.03788	1.371003	-0.10632	0.915569	-5.54206	0.871133	0.854581
T.cells	4930455G(	-0.05727	1.816178	-0.10629	0.915594	-5.3061	0.862458	0.844071
T.cells	SRSF4	-0.00668	6.225555	-0.1062	0.915667	-6.94147	0.780764	0.746599
T.cells	C330018D2	0.020278	2.325358	0.10616	0.915696	-5.9644	0.852633	0.832205
T.cells	TBL1XR1	-0.00751	6.669065	-0.10607	0.915769	-6.93742	0.772961	0.737462
T.cells	ACTR3	0.006277	8.876057	0.105828	0.915959	-7.19751	0.735234	0.693561
T.cells	DEDD2	-0.01545	4.544003	-0.10573	0.916038	-6.32946	0.811041	0.78247
T.cells	ITIH5	-0.025	1.943297	-0.10565	0.916102	-5.94523	0.860012	0.841188
T.cells	EDIL3	-0.05278	1.492552	-0.10564	0.916106	-5.42599	0.868773	0.851795
T.cells	GAL3ST1	-0.05903	-0.29084	-0.10559	0.916151	-5.21441	0.90425	0.895066
T.cells	2310009B1	-0.01027	4.473004	-0.10503	0.916589	-6.51262	0.812667	0.784168
T.cells	INVS	-0.02008	2.809082	-0.1046	0.916931	-6.066	0.843936	0.821397
T.cells	ICOS	-0.05616	2.283057	-0.10459	0.916938	-5.37989	0.853992	0.833503
T.cells	CAMK2A	0.032001	0.846737	0.103991	0.917412	-5.49048	0.882145	0.867558
T.cells	DCAKD	0.013273	4.076447	0.103759	0.917596	-6.43285	0.820277	0.793033
T.cells	EBNA1BP2	-0.01304	4.017915	-0.10369	0.917647	-6.41903	0.821361	0.794326
T.cells	WDR73	-0.01588	2.976485	-0.10369	0.917652	-6.13643	0.84088	0.817683
T.cells	TRUB2	0.010923	4.526877	0.103665	0.91767	-6.53521	0.811976	0.783152
T.cells	1700017B(	0.011073	5.285252	0.103602	0.91772	-6.69116	0.798179	0.766791
T.cells	3830406C1	-0.01267	4.453206	-0.10344	0.917847	-6.33858	0.813329	0.78476
T.cells	POP1	0.017134	2.708556	0.103379	0.917896	-6.10746	0.845972	0.823801
T.cells	NEIL3	0.014229	4.375558	0.103339	0.917928	-6.70742	0.814756	0.786458
T.cells	FCHO2	-0.00888	6.902577	-0.10324	0.918003	-6.85435	0.769487	0.733031
T.cells	9430060I0:	0.035214	1.308677	0.10304	0.918165	-5.6426	0.873039	0.85658
T.cells	TOB2	-0.01022	6.656821	-0.10293	0.918255	-6.77838	0.773783	0.738163
T.cells	STARD7	-0.00884	5.358518	-0.10268	0.918449	-6.73323	0.796857	0.765344
T.cells	GM26917	0.013326	5.74366	0.102539	0.918561	-6.69015	0.789946	0.757181
T.cells	IRF1	-0.01901	7.149071	-0.1025	0.91859	-6.91452	0.7652	0.728128
T.cells	9330111N(	0.051127	0.392898	0.102474	0.918612	-5.35796	0.891176	0.878695

T.cells	FLVCR1	0.012318	4.670527	0.102396	0.918674	-6.43692	0.809346	0.780144
T.cells	ENSA	0.008842	5.388151	0.102363	0.918701	-6.67523	0.796324	0.764713
T.cells	NOL4L	0.027469	2.680623	0.102356	0.918706	-5.95975	0.846504	0.824565
T.cells	GM15232	0.042302	1.187425	0.102302	0.918749	-5.42911	0.875421	0.859516
T.cells	NUDT13	0.023034	2.358115	0.102263	0.918779	-5.85705	0.852674	0.831995
T.cells	MARCKS	-0.0103	7.242888	-0.10207	0.918929	-7.01248	0.763575	0.726238
T.cells	EMC1	-0.013	3.622778	-0.10201	0.918983	-6.24684	0.828717	0.803242
T.cells	ANKLE2	-0.00954	5.177792	-0.10195	0.91903	-6.67487	0.80012	0.769215
T.cells	MTMR10	0.027538	2.897646	0.101903	0.919064	-5.83813	0.842376	0.819613
T.cells	GTF2H2	-0.01001	3.759481	-0.10179	0.919157	-6.45469	0.826165	0.800192
T.cells	RIPK3	0.016372	3.285039	0.10172	0.91921	-6.22166	0.835053	0.810827
T.cells	BTBD7	-0.00615	7.004982	-0.10172	0.919213	-6.97194	0.767703	0.731064
T.cells	HELZ2	-0.02593	3.832468	-0.10156	0.919337	-5.91095	0.824806	0.798593
T.cells	RMI2	-0.01333	3.3602	-0.10151	0.919378	-6.45718	0.833639	0.809176
T.cells	4933433G1	0.052583	0.742117	0.101498	0.919385	-5.36407	0.88422	0.870274
T.cells	SLC15A4	-0.01219	4.804844	-0.10147	0.919405	-6.51059	0.806893	0.777286
T.cells	DCXR	-0.0167	4.317514	-0.10136	0.919497	-6.21748	0.815842	0.787919
T.cells	VWA5A	-0.02492	3.406572	-0.10122	0.919603	-5.86791	0.832815	0.808167
T.cells	TIPARP	0.011807	7.437182	0.101052	0.919738	-6.92154	0.760313	0.722421
T.cells	ALPL	-0.01981	1.490191	-0.10045	0.920215	-6.14013	0.869782	0.85251
T.cells	NUPL2	0.022124	2.367649	0.100383	0.920268	-5.92227	0.852782	0.831964
T.cells	TRIOBP	0.009305	4.877202	0.100151	0.920451	-6.55393	0.80585	0.775877
T.cells	TBC1D22A	0.009621	5.871527	0.100147	0.920454	-6.63051	0.787932	0.754694
T.cells	AACS	0.013676	3.861959	0.100132	0.920466	-6.25524	0.824538	0.798117
T.cells	ITGA3	0.043445	-0.28405	0.09998	0.920587	-5.30975	0.905114	0.895641
T.cells	WDR55	0.015276	2.979495	0.099965	0.920598	-6.1259	0.84111	0.817995
T.cells	DAP3	0.008501	5.039126	0.099877	0.920668	-6.70104	0.802906	0.77243
T.cells	HMGN3	0.014428	3.255903	0.099819	0.920714	-6.49923	0.835887	0.811738
T.cells	KCNAB2	0.013206	4.287693	0.099763	0.920758	-6.42022	0.816653	0.788772
T.cells	WDR25	0.022916	1.998808	0.099756	0.920764	-5.78269	0.85989	0.840636
T.cells	RFC3	-0.00969	4.31335	-0.09969	0.920812	-6.6125	0.81618	0.788217
T.cells	WDR5	-0.00991	4.681603	-0.09959	0.920895	-6.6059	0.809428	0.780181
T.cells	LILR4B	-0.06617	3.079256	-0.09927	0.921152	-5.37619	0.839378	0.815832
T.cells	TTC39B	0.017396	4.653747	0.098908	0.921435	-6.14468	0.81008	0.780935
T.cells	ZFP790	-0.02034	2.226902	-0.09873	0.921575	-5.93774	0.855647	0.835522
T.cells	TDP2	0.0102	4.340054	0.09871	0.921592	-6.55794	0.81584	0.787813
T.cells	CYP4B1	0.062799	0.266849	0.098593	0.921685	-5.25058	0.894171	0.882289
T.cells	CCDC86	0.009657	5.146452	0.098312	0.921907	-6.63323	0.80111	0.770362
T.cells	SLC35A5	-0.0136	3.986264	-0.0983	0.921919	-6.15297	0.822382	0.795648
T.cells	ZSWIM7	-0.01731	3.432711	-0.09828	0.921934	-6.1018	0.832716	0.808
T.cells	SERP2	-0.03407	0.429349	-0.09817	0.922019	-5.47966	0.890918	0.878373
T.cells	ABRAXAS1	0.017256	2.604808	0.098149	0.922036	-6.1514	0.848398	0.826839
T.cells	ILKAP	-0.00762	5.651644	-0.09803	0.922127	-6.80741	0.792009	0.759641
T.cells	TOMM5	-0.00911	5.458336	-0.09784	0.922278	-6.78552	0.79548	0.7638
T.cells	DNAH1	-0.03287	-0.10849	-0.09776	0.922344	-5.62345	0.901728	0.891726
T.cells	PRPSAP1	-0.00954	4.34081	-0.09765	0.922431	-6.50502	0.815826	0.787979
T.cells	AFF2	0.060684	0.04795	0.097629	0.922448	-5.2658	0.898571	0.887872

T.cells	FUBP1	-0.00555	6.678668	-0.09744	0.922597	-6.97437	0.773808	0.738326
T.cells	INCENP	0.010943	5.454754	0.097439	0.922599	-6.89305	0.795544	0.763918
T.cells	MTX3	0.034886	0.591443	0.097371	0.922652	-5.59275	0.887683	0.874576
T.cells	EIF2B5	-0.00919	4.703803	-0.09736	0.922661	-6.64927	0.809164	0.780057
T.cells	PMEPA1	-0.03492	4.361383	-0.09729	0.92272	-5.79714	0.815447	0.787528
T.cells	GM15787	-0.02309	2.641968	-0.09728	0.922728	-5.93757	0.847688	0.826124
T.cells	ADGRG6	-0.06145	0.969217	-0.09721	0.922779	-5.3523	0.880186	0.865447
T.cells	AP5M1	-0.01456	3.584616	-0.09713	0.922839	-6.18406	0.829868	0.804739
T.cells	ASH1L	0.006855	7.367632	0.097023	0.922928	-7.02686	0.76182	0.724301
T.cells	NFATC1	0.011069	5.77824	0.096801	0.923104	-6.53713	0.789744	0.75713
T.cells	TBC1D23	0.009184	5.852371	0.09663	0.923239	-6.62708	0.788421	0.755583
T.cells	CREB5	0.045256	2.17534	0.09663	0.923239	-5.62747	0.85664	0.836998
T.cells	RRN3	0.00957	4.400393	0.096215	0.923568	-6.50858	0.814729	0.78678
T.cells	AI597479	0.024387	1.688645	0.096098	0.92366	-5.71836	0.866071	0.848454
T.cells	GTSE1	-0.02743	1.404909	-0.09605	0.923702	-6.02548	0.871613	0.855185
T.cells	GM9949	-0.05228	0.483247	-0.09604	0.923709	-5.31011	0.889841	0.877363
T.cells	RPP30	0.010234	3.635635	0.095709	0.923969	-6.43471	0.828914	0.803779
T.cells	PIAS1	-0.00589	7.42613	-0.09555	0.924095	-7.04853	0.76081	0.723294
T.cells	SHROOM3	0.044359	0.981918	0.095526	0.924113	-5.5102	0.879935	0.865347
T.cells	ZFP644	0.006705	6.331418	0.095504	0.92413	-6.91729	0.779917	0.745675
T.cells	MED29	0.011302	3.806911	0.095426	0.924192	-6.30051	0.825717	0.799987
T.cells	EPB41L4B	-0.01921	1.976143	-0.09534	0.92426	-6.12673	0.860489	0.841792
T.cells	FOSL1	-0.05727	1.662098	-0.09531	0.92428	-5.2853	0.866588	0.849174
T.cells	HIST1H4C	0.034368	0.309171	0.095264	0.924321	-5.57382	0.893323	0.881697
T.cells	SLC46A1	-0.04945	0.761766	-0.09526	0.924323	-5.43141	0.884296	0.870685
T.cells	AUNIP	-0.01711	1.893508	-0.09518	0.924388	-6.12888	0.86209	0.843729
T.cells	NCAPH2	0.007518	5.344606	0.094959	0.924562	-6.77836	0.797529	0.766509
T.cells	KHDRBS3	0.029594	1.943216	0.094711	0.924759	-5.7754	0.861126	0.842684
T.cells	LRRFIP1	0.007665	7.249941	0.094624	0.924827	-6.92074	0.763855	0.726993
T.cells	CD300LD	-0.04611	3.173108	-0.09445	0.924964	-5.52378	0.837604	0.814367
T.cells	3110001I2:	-0.01684	2.677347	-0.09432	0.925068	-5.97556	0.847013	0.825719
T.cells	SSH2	-0.00889	8.396317	-0.09383	0.925457	-7.18723	0.744248	0.704244
T.cells	ASCC1	-0.01148	3.774861	-0.09381	0.925475	-6.30527	0.826314	0.800928
T.cells	LSAMP	-0.03928	0.929492	-0.0938	0.925482	-5.48928	0.880972	0.866877
T.cells	PPP1R3E	0.030481	1.031561	0.093678	0.925577	-5.44351	0.878955	0.86446
T.cells	IRF2BP1	-0.0099	4.152538	-0.09366	0.925594	-6.48882	0.819301	0.792588
T.cells	SCARB1	0.009522	4.995715	0.093424	0.925778	-6.59618	0.803844	0.774215
T.cells	OARD1	-0.00917	4.902059	-0.09335	0.925836	-6.65423	0.805547	0.776237
T.cells	RSRC2	-0.00547	6.479986	-0.09335	0.925841	-6.88669	0.777298	0.742874
T.cells	SENP7	-0.00896	5.356774	-0.09333	0.925856	-6.64798	0.797309	0.766472
T.cells	SPATA2	-0.0129	4.217056	-0.09303	0.926092	-6.38979	0.818108	0.791211
T.cells	ZFP933	0.014611	3.490032	0.093022	0.926097	-6.23115	0.83164	0.807384
T.cells	LTV1	-0.01195	4.232009	-0.09277	0.926294	-6.41537	0.817832	0.790942
T.cells	LRRC58	-0.00728	5.945766	-0.09275	0.926311	-6.762	0.786756	0.754092
T.cells	PNKP	-0.01256	4.959191	-0.09239	0.926597	-6.42804	0.804508	0.775189
T.cells	CS	0.006283	6.103857	0.092363	0.926618	-6.8496	0.783946	0.750874
T.cells	MAN1B1	-0.01413	4.927787	-0.09226	0.926703	-6.40511	0.805079	0.775895

T.cells	PXYLP1	0.019368	2.352535	0.092116	0.926814	-5.95723	0.853231	0.833542
T.cells	SGSH	-0.03689	1.453355	-0.092	0.926907	-5.42195	0.870664	0.854645
T.cells	FADD	0.022037	2.421277	0.091876	0.927004	-5.94118	0.851911	0.831958
T.cells	NUP43	0.013571	2.795528	0.091677	0.927162	-6.2248	0.844761	0.823343
T.cells	GNG2	0.010357	6.302982	0.09164	0.927191	-6.61603	0.78042	0.74676
T.cells	1600012H(	0.016985	2.771016	0.091535	0.927274	-5.88289	0.845228	0.823904
T.cells	QRSL1	-0.01307	3.18623	-0.09151	0.927292	-6.3283	0.837356	0.814442
T.cells	1110019D1	0.024756	1.751896	0.091402	0.92738	-5.72816	0.86484	0.847588
T.cells	LARS	0.009802	4.738364	0.091396	0.927384	-6.58133	0.808533	0.780007
T.cells	CTNNAL1	-0.02119	1.924908	-0.09137	0.927404	-5.98902	0.861481	0.843521
T.cells	GM34095	0.028941	-0.39051	0.091371	0.927404	-5.69595	0.907444	0.899547
T.cells	ICA1L	-0.03836	1.590667	-0.09134	0.92743	-5.47603	0.867981	0.851395
T.cells	SENP5	0.007856	5.992571	0.091332	0.927436	-6.79362	0.785923	0.753241
T.cells	SYVN1	0.01238	4.228615	0.09129	0.927469	-6.50885	0.817895	0.791165
T.cells	NFE2	0.037161	1.59738	0.091206	0.927535	-5.43058	0.86785	0.851259
T.cells	SCARB2	0.010126	6.538743	0.0911	0.927619	-6.66133	0.776264	0.741917
T.cells	GM19265	-0.03044	0.22098	-0.09109	0.927627	-5.5764	0.895092	0.884463
T.cells	TXNDC9	-0.00715	5.512196	-0.09097	0.927719	-6.73664	0.794512	0.763442
T.cells	PFKM	0.027726	1.17862	0.090872	0.9278	-5.63719	0.876056	0.861267
T.cells	BCL2A1B	0.044978	5.877283	0.090856	0.927812	-5.73809	0.787976	0.755721
T.cells	EPC2	0.007271	6.14343	0.090588	0.928025	-6.84553	0.783244	0.750173
T.cells	ZFP36L2	0.009706	8.376464	0.090586	0.928026	-7.0686	0.744584	0.704964
T.cells	ADIPOR1	-0.0094	7.346972	-0.09057	0.928039	-6.88858	0.762177	0.725455
T.cells	SLC25A27	0.031171	0.260835	0.090558	0.928049	-5.54689	0.894292	0.883539
T.cells	CNN2	-0.01301	6.538205	-0.09051	0.928084	-6.6216	0.776274	0.741974
T.cells	PDPK1	-0.01124	6.462559	-0.09045	0.928134	-6.80288	0.777605	0.743538
T.cells	DIAPH3	-0.01348	6.026174	-0.09037	0.928195	-6.98135	0.785325	0.752626
T.cells	ELK4	-0.00706	5.882489	-0.09033	0.928225	-6.85104	0.787883	0.755643
T.cells	XRCC2	0.025332	0.802718	0.090278	0.928271	-5.69518	0.883483	0.870344
T.cells	GALNT10	-0.01205	4.614762	-0.09025	0.928296	-6.4378	0.810793	0.782789
T.cells	ANGPTL1	0.033048	0.376343	0.090201	0.928331	-5.54992	0.891978	0.880711
T.cells	FTO	-0.00669	6.592827	-0.09018	0.928346	-6.87577	0.775314	0.740847
T.cells	SLC35A2	0.017727	3.165869	0.090084	0.928424	-6.01515	0.837741	0.815017
T.cells	CSNK1D	0.005276	6.6346	0.089656	0.928763	-6.85972	0.774581	0.740086
T.cells	GM15445	-0.03371	0.994582	-0.08962	0.928793	-5.42664	0.879685	0.865841
T.cells	ZFP239	0.034112	-0.06752	0.089343	0.929012	-5.47466	0.9009	0.891767
T.cells	ZFP574	-0.01181	3.401172	-0.08934	0.929012	-6.3314	0.833308	0.809807
T.cells	PMEL	-0.03768	0.339511	-0.08932	0.929027	-5.4609	0.892715	0.881749
T.cells	ATXN10	0.007	6.66392	0.089261	0.929077	-6.90356	0.774066	0.739496
T.cells	TYW3	0.026648	0.995715	0.088965	0.929311	-5.6681	0.879663	0.86585
T.cells	COG1	0.010816	3.677624	0.08883	0.929418	-6.26581	0.828129	0.803683
T.cells	EHMT2	0.008352	4.951712	0.08856	0.929632	-6.64322	0.804644	0.775744
T.cells	MFSD8	0.012175	2.979793	0.088536	0.929651	-6.13479	0.841261	0.819509
T.cells	CCNF	0.013258	3.438612	0.088407	0.929753	-6.54056	0.832605	0.80914
T.cells	KPNA3	0.006566	6.307968	0.088372	0.929781	-6.92843	0.780332	0.747032
T.cells	GM21887	-0.02147	2.133944	-0.08813	0.929969	-6.00541	0.857439	0.839068
T.cells	IL4I1	-0.02532	2.841673	-0.08807	0.930024	-5.98739	0.843884	0.822737

T.cells	COPS7B	0.010963	3.34463	0.088017	0.930062	-6.35155	0.834372	0.811322
T.cells	CFB	-0.05358	4.663824	-0.08795	0.930113	-6.09102	0.809895	0.782076
T.cells	HMGCR	-0.01023	4.712579	-0.08791	0.930148	-6.48243	0.809004	0.781016
T.cells	RAB37	-0.02035	2.340912	-0.08776	0.930263	-5.85476	0.853454	0.834326
T.cells	SARDHOS	-0.05038	0.147254	-0.08776	0.930264	-5.35309	0.896573	0.886762
T.cells	BAX	0.008281	6.141068	0.087732	0.930288	-6.78332	0.783286	0.750589
T.cells	HSPB11	-0.0169	2.925359	-0.08764	0.930361	-6.04115	0.842294	0.820897
T.cells	CRIP2	-0.0156	4.565121	-0.08755	0.930431	-6.29822	0.811703	0.78429
T.cells	SELENOF	0.00882	5.422799	0.087404	0.930548	-6.65512	0.79612	0.765824
T.cells	WSB2	0.01262	4.493162	0.08731	0.930623	-6.26196	0.813024	0.785903
T.cells	TAF12	0.007489	5.021001	0.087295	0.930634	-6.68758	0.803385	0.774439
T.cells	ZBTB44	-0.00768	5.853417	-0.08719	0.930716	-6.80008	0.788402	0.756709
T.cells	GIMAP5	0.017929	3.319876	0.08705	0.930828	-6.1215	0.834837	0.812002
T.cells	RGS12	-0.01449	2.240588	-0.08694	0.930915	-6.13032	0.855383	0.836751
T.cells	DNAJC27	-0.0153	2.442699	-0.0868	0.931029	-6.05691	0.851501	0.832061
T.cells	6330418KC	0.027215	1.506684	0.08666	0.931138	-5.62908	0.869621	0.853999
T.cells	SAP25	0.023675	2.354647	0.086606	0.931181	-5.83966	0.85319	0.834101
T.cells	NOP56	0.008453	4.591451	0.086603	0.931183	-6.60491	0.811221	0.783767
T.cells	COG4	0.00729	5.336083	0.086586	0.931196	-6.71652	0.797682	0.767687
T.cells	GTPBP10	0.015587	2.397542	0.08657	0.931209	-5.98358	0.852367	0.833107
T.cells	IFI214	-0.02305	1.667368	-0.0865	0.931267	-5.95961	0.866485	0.850193
T.cells	GM16151	-0.03038	-0.28838	-0.08639	0.931351	-5.51393	0.90537	0.897649
T.cells	TIMM9	0.009857	3.882337	0.086368	0.931369	-6.3962	0.824313	0.799392
T.cells	NEK6	0.018633	3.873937	0.086198	0.931504	-5.90375	0.824469	0.799624
T.cells	GINS3	-0.01423	2.330816	-0.08609	0.931593	-6.2065	0.853648	0.834707
T.cells	ZFP273	0.027463	0.440289	0.086058	0.931615	-5.57727	0.890699	0.879732
T.cells	UBP1	-0.00757	5.448392	-0.08602	0.931643	-6.66559	0.795659	0.765339
T.cells	GLMP	-0.01042	5.839868	-0.08597	0.931681	-6.62022	0.788644	0.757043
T.cells	DNTTIP1	-0.00741	4.812219	-0.08597	0.931684	-6.54923	0.807184	0.779015
T.cells	SPEN	-0.00687	5.922479	-0.08593	0.931717	-6.77159	0.787171	0.755304
T.cells	NCKAP1L	-0.00646	6.137906	-0.08577	0.931843	-6.74596	0.783342	0.750807
T.cells	ATP2B1	0.007836	8.327101	0.085735	0.931871	-7.06223	0.745418	0.706421
T.cells	CSTDC5	0.036603	5.46156	0.085631	0.931953	-6.23786	0.795422	0.765109
T.cells	SPATA48	-0.01818	2.190427	-0.0856	0.931978	-6.01949	0.85635	0.838042
T.cells	ZFP58	0.025517	1.194809	0.085268	0.932241	-5.67621	0.875738	0.861681
T.cells	MTA2	0.007148	5.782517	0.084902	0.932531	-6.83662	0.789668	0.75848
T.cells	ADD3	-0.01058	6.047415	-0.08468	0.932706	-6.76424	0.784948	0.752927
T.cells	HS1BP3	0.029666	1.087416	0.084646	0.932734	-5.46326	0.877853	0.864344
T.cells	DNAJB14	-0.01624	4.692261	-0.08462	0.932753	-6.09815	0.809375	0.781876
T.cells	ALDH1A1	-0.0505	-5.25E-05	-0.08457	0.932793	-5.26547	0.899539	0.890843
T.cells	TTC37	0.012634	3.798842	0.084557	0.932805	-6.38641	0.825867	0.801564
T.cells	AGFG2	0.008051	4.605897	0.08452	0.932834	-6.66898	0.810956	0.783758
T.cells	SMTN	0.0177	2.149503	0.084359	0.932962	-5.97729	0.857139	0.839201
T.cells	LACC1	0.024988	3.279516	0.084321	0.932992	-5.76548	0.835597	0.813232
T.cells	PARD3B	0.015802	4.55014	0.084293	0.933014	-6.49069	0.811978	0.784976
T.cells	GM48086	0.022009	1.511775	0.084264	0.933037	-5.76928	0.869522	0.854212
T.cells	UPRT	-0.02879	0.606501	-0.0841	0.933165	-5.53211	0.887383	0.875985

T.cells	D930030I0	-0.04253	0.408475	-0.08409	0.933172	-5.33351	0.891335	0.880815
T.cells	ITGAL	-0.01864	6.272091	-0.08398	0.933265	-6.1731	0.780966	0.748299
T.cells	ENG	-0.01604	4.005662	-0.0839	0.933325	-6.35459	0.822022	0.797056
T.cells	HCCS	-0.00763	4.627385	-0.0839	0.933329	-6.56286	0.810562	0.78338
T.cells	GRM8	0.057659	1.381099	0.08384	0.933373	-5.26643	0.872079	0.857419
T.cells	GHDC	0.026656	1.628326	0.08384	0.933373	-5.54529	0.867246	0.851548
T.cells	ZSWIM9	-0.03373	0.544388	-0.08376	0.933437	-5.52697	0.888621	0.877592
T.cells	CCDC162	-0.02862	2.876554	-0.08373	0.933461	-5.82929	0.843221	0.822516
T.cells	ZMAT1	0.022631	2.090961	0.083711	0.933476	-5.75822	0.858269	0.840685
T.cells	GM17382	-0.03777	0.002886	-0.08364	0.933529	-5.39102	0.89948	0.890918
T.cells	RHOF	-0.02344	3.558194	-0.08346	0.933676	-5.80292	0.830363	0.807087
T.cells	CGNL1	-0.03782	1.622928	-0.0834	0.933721	-5.50056	0.867352	0.85174
T.cells	ULK3	-0.02198	2.219052	-0.08328	0.93382	-5.73818	0.855798	0.837756
T.cells	EPHB4	0.048187	0.794939	0.083195	0.933884	-5.35406	0.883638	0.871592
T.cells	ZFP429	-0.01689	2.448075	-0.08315	0.933923	-5.88689	0.851397	0.832453
T.cells	KIF11	-0.01266	5.441855	-0.08314	0.933931	-6.94061	0.795777	0.765905
T.cells	HASPIN	0.014503	2.299155	0.083132	0.933934	-6.17411	0.854257	0.835908
T.cells	PICALM	0.006806	8.404518	0.083047	0.934002	-7.05903	0.74411	0.705294
T.cells	ING2	0.007568	4.920364	0.083024	0.93402	-6.63001	0.805214	0.777127
T.cells	PDZD11	-0.00935	4.30203	-0.08297	0.93406	-6.54767	0.81654	0.790619
T.cells	ZFP316	-0.03541	0.380007	-0.08289	0.934125	-5.45627	0.891905	0.881724
T.cells	RARS	-0.00754	5.121145	-0.08286	0.934147	-6.66724	0.801568	0.772798
T.cells	TIMM29	-0.01235	3.296538	-0.08283	0.934177	-6.21763	0.835277	0.813059
T.cells	JADE2	-0.02013	3.270443	-0.08273	0.934254	-5.94086	0.835768	0.81365
T.cells	GM15886	0.016947	1.881502	0.082704	0.934274	-5.82032	0.862323	0.845698
T.cells	PIGK	0.009313	4.298401	0.082627	0.934335	-6.37183	0.816607	0.790702
T.cells	CHRNE	-0.04217	-0.75209	-0.08251	0.934428	-5.20039	0.914841	0.909894
T.cells	NDUFS3	0.00659	6.050235	0.082212	0.934664	-6.85217	0.78503	0.753154
T.cells	6-Sep	-0.00794	5.624977	-0.08202	0.934812	-6.87235	0.792621	0.762155
T.cells	IL12B	0.078203	-0.29714	0.081956	0.934867	-5.24218	0.905701	0.89861
T.cells	SAPCD2	-0.01443	1.790194	-0.08178	0.935005	-6.21371	0.864241	0.848047
T.cells	AMD2	0.032397	0.044662	0.081777	0.935009	-5.3836	0.898789	0.890179
T.cells	RAPGEF1	-0.0067	6.775482	-0.08171	0.935061	-6.88434	0.772243	0.738201
T.cells	3110040N1	0.012085	3.553549	0.081584	0.935162	-6.33019	0.83059	0.807473
T.cells	BRCA2	0.011625	3.555566	0.08119	0.935475	-6.43353	0.830552	0.80749
T.cells	TRIP4	0.009827	4.852518	0.081062	0.935576	-6.62621	0.806586	0.77886
T.cells	CPLX1	0.056227	-0.57566	0.08081	0.935776	-5.19917	0.911369	0.905722
T.cells	ATP6V1A	0.007553	6.534998	0.080792	0.93579	-6.79662	0.776461	0.743218
T.cells	TIMMDC1	-0.00839	4.271519	-0.08075	0.935827	-6.47278	0.817241	0.791559
T.cells	TMCO3	-0.01093	3.656165	-0.08066	0.935895	-6.23814	0.82867	0.805233
T.cells	ASTN2	0.03085	1.045238	0.08058	0.935958	-5.55499	0.878833	0.865873
T.cells	GM15489	0.031576	0.208522	0.080514	0.936011	-5.55905	0.895493	0.886228
T.cells	UGGT1	-0.00694	5.389379	-0.08051	0.936015	-6.65541	0.796856	0.767305
T.cells	BTAF1	-0.00615	7.190783	-0.0805	0.936022	-6.90147	0.765009	0.729773
T.cells	4921524J1	-0.0058	5.474163	-0.08046	0.936052	-6.78881	0.795329	0.765496
T.cells	SPTLC1	0.009755	4.232218	0.0804	0.936101	-6.34859	0.817966	0.792425
T.cells	SLC25A25	-0.00792	4.951297	-0.08017	0.93628	-6.63107	0.804787	0.776721

T.cells	NAV2	0.011367	4.841571	0.080165	0.936287	-6.56546	0.806785	0.779097
T.cells	BATF	0.018391	4.183315	0.080095	0.936343	-5.86065	0.81887	0.793505
T.cells	LYRM9	-0.01448	2.696887	-0.07999	0.936425	-6.09801	0.846783	0.827014
T.cells	AU040320	0.010121	4.589216	0.079951	0.936457	-6.39613	0.811398	0.78459
T.cells	MINDY2	0.005554	6.610817	0.079687	0.936666	-6.95968	0.775129	0.741704
T.cells	RGCC	-0.01101	5.613883	-0.07965	0.936693	-6.6858	0.79282	0.762579
T.cells	BCO2	-0.03109	0.643324	-0.07948	0.936827	-5.39309	0.8868	0.875695
T.cells	ETOHD2	-0.01786	2.615429	-0.07947	0.936841	-5.77679	0.848338	0.828987
T.cells	XRCC5	-0.01403	2.083305	-0.07931	0.936968	-6.1007	0.858561	0.841389
T.cells	TVP23B	-0.01014	4.042523	-0.07922	0.93704	-6.26654	0.821477	0.796768
T.cells	GM26810	-0.03717	0.423092	-0.07917	0.937074	-5.42343	0.891193	0.881129
T.cells	MEX3A	0.018444	1.577184	0.078898	0.937292	-5.91835	0.86839	0.853353
T.cells	EXOC3L4	-0.04448	0.594811	-0.0789	0.937294	-5.28003	0.887766	0.876964
T.cells	KIF16B	-0.00964	4.843803	-0.07882	0.937354	-6.46878	0.806745	0.779234
T.cells	SMARCA5	-0.00451	7.448521	-0.07866	0.93748	-7.12775	0.760552	0.724784
T.cells	RAB4A	0.025779	1.578469	0.078434	0.93766	-5.63023	0.868365	0.853428
T.cells	ZFAND2B	0.009461	4.313639	0.078382	0.937702	-6.3912	0.816464	0.7909
T.cells	NFE2L1	0.009693	4.98448	0.078311	0.937758	-6.49272	0.804184	0.776268
T.cells	IIGP1	0.036087	4.709506	0.078181	0.937861	-6.11866	0.809196	0.782233
T.cells	CFAP410	0.016393	2.119698	0.07816	0.937877	-5.77431	0.857858	0.840683
T.cells	SDHAF3	-0.01057	3.044533	-0.07798	0.93802	-6.24399	0.840177	0.819356
T.cells	PDE10A	-0.03324	2.555984	-0.07797	0.938026	-5.58482	0.849475	0.830567
T.cells	OLFM1	-0.03775	2.469141	-0.07781	0.938158	-5.38152	0.851137	0.832576
T.cells	NEK2	0.012521	3.092142	0.07778	0.938179	-6.45586	0.839276	0.818271
T.cells	WDSUB1	0.016706	2.44185	0.077726	0.938222	-5.82887	0.85166	0.833208
T.cells	TRPM7	-0.00586	7.284405	-0.07767	0.938264	-7.00131	0.763387	0.728141
T.cells	SYNJ2BP	0.006055	5.520643	0.077654	0.938279	-6.74248	0.794494	0.764787
T.cells	GTPBP3	-0.01534	2.225197	-0.0776	0.938325	-5.96753	0.855824	0.838269
T.cells	ZFP952	-0.0158	2.251647	-0.0775	0.938403	-5.93242	0.855315	0.837663
T.cells	MRPS17	0.006499	5.224333	0.077391	0.938487	-6.7174	0.799835	0.771169
T.cells	CCL9	0.031304	2.831707	0.077363	0.93851	-5.60204	0.844215	0.82428
T.cells	CDR2	-0.02162	2.488604	-0.07728	0.938576	-5.80581	0.850764	0.832184
T.cells	IFI209	-0.00841	5.809713	-0.07727	0.938585	-6.92365	0.789315	0.758711
T.cells	TSPOAP1	-0.01606	2.650845	-0.07727	0.938585	-6.08426	0.847662	0.828437
T.cells	KANK1	-0.02842	1.172928	-0.07723	0.938614	-5.53093	0.876316	0.863185
T.cells	ARAP2	-0.01033	5.924266	-0.07718	0.938651	-6.678	0.787272	0.756295
T.cells	CD34	-0.05196	0.953495	-0.07704	0.938768	-5.36744	0.880686	0.86849
T.cells	WBP4	-0.00649	5.270226	-0.07689	0.938884	-6.68949	0.799077	0.770255
T.cells	MRI1	-0.00875	3.963231	-0.0768	0.938959	-6.36908	0.823021	0.798808
T.cells	CCNT2	0.006795	5.37232	0.076711	0.939026	-6.64296	0.797234	0.768081
T.cells	TSPAN14	-0.00596	6.835826	-0.07645	0.939235	-6.97544	0.771368	0.737445
T.cells	CRKL	0.006514	5.071994	0.076187	0.939443	-6.65451	0.802879	0.774618
T.cells	DMC1	-0.02257	0.152078	-0.07606	0.939539	-5.6514	0.896945	0.888213
T.cells	GIMAP9	-0.01067	3.560572	-0.07598	0.939607	-6.41864	0.830753	0.807927
T.cells	NUB1	0.006534	5.534309	0.075937	0.939641	-6.63796	0.79453	0.764736
T.cells	MRPL16	0.008529	3.919331	0.075644	0.939874	-6.4347	0.824119	0.79995
T.cells	BCAS3OS1	0.011551	3.793281	0.075613	0.939898	-6.18909	0.826466	0.802761

T.cells	RPF1	0.005837	5.149471	0.07557	0.939932	-6.7116	0.801535	0.773025
T.cells	FBXW17	-0.02657	1.479346	-0.07526	0.940174	-5.54317	0.870834	0.856224
T.cells	BTBD9	-0.00586	8.508871	-0.07491	0.940456	-7.1328	0.743061	0.70414
T.cells	HIST1H4I	0.010122	4.409355	0.074868	0.940489	-6.67926	0.815345	0.789263
T.cells	GKAP1	-0.0125	3.541031	-0.07467	0.940645	-6.20944	0.831501	0.808627
T.cells	ACACA	-0.00804	5.153719	-0.07457	0.940728	-6.64455	0.801766	0.773164
T.cells	CPSF4	0.008213	4.317677	0.074524	0.940762	-6.56557	0.817054	0.791372
T.cells	LMO4	-0.00782	7.245458	-0.07447	0.940803	-7.09516	0.764684	0.729438
T.cells	KMT2E	-0.00434	8.273581	-0.07416	0.941053	-7.1716	0.747198	0.708915
T.cells	GM16158	-0.03185	0.586451	-0.07397	0.941203	-5.48801	0.888855	0.877989
T.cells	POLR3E	-0.00814	3.918387	-0.07393	0.941236	-6.42727	0.824637	0.800299
T.cells	PCSK7	-0.0076	5.282369	-0.07375	0.941375	-6.61711	0.799616	0.770496
T.cells	AIM2	0.009749	5.579386	0.073745	0.94138	-6.57048	0.794263	0.764151
T.cells	FPR2	-0.06329	1.833327	-0.07364	0.941467	-5.30138	0.864313	0.848128
T.cells	RRP36	0.008193	3.775241	0.073405	0.941649	-6.33193	0.827406	0.803583
T.cells	LIAS	0.007416	4.310022	0.07332	0.941717	-6.53731	0.817479	0.791716
T.cells	1810044D(	-0.0183	1.915203	-0.07323	0.941787	-5.85329	0.862815	0.846267
T.cells	UGDH	0.0105	4.250685	0.073022	0.941953	-6.28423	0.818656	0.793091
T.cells	PCNT	0.006419	5.233516	0.072922	0.942033	-6.76878	0.800683	0.771698
T.cells	AU019990	-0.04402	1.211418	-0.07274	0.942173	-5.26843	0.876731	0.863077
T.cells	BICD1	-0.02472	0.681795	-0.07258	0.942308	-5.61901	0.887236	0.875883
T.cells	PREX1	0.007505	6.43388	0.072541	0.942335	-6.7987	0.779296	0.746357
T.cells	VANGL2	0.012352	2.225601	0.072377	0.942465	-6.13782	0.857029	0.839142
T.cells	PAQR4	0.031809	1.484926	0.071991	0.942771	-5.53862	0.871535	0.856682
T.cells	AARS2	-0.02413	0.821033	-0.07197	0.942791	-5.58421	0.884631	0.872631
T.cells	USP5	0.007663	4.34395	0.071952	0.942803	-6.55025	0.817163	0.791187
T.cells	CMTM4	0.01112	3.141567	0.071578	0.943099	-6.25006	0.839833	0.818163
T.cells	FAM214B	0.027047	3.11159	0.071433	0.943214	-5.55378	0.840438	0.818846
T.cells	ZFP407	0.004839	7.039039	0.070943	0.943604	-6.98156	0.769247	0.734136
T.cells	JAK2	0.008826	6.674709	0.070848	0.943679	-6.70737	0.775623	0.741624
T.cells	PHF10	0.007051	4.930175	0.07077	0.943741	-6.61314	0.806852	0.77854
T.cells	SLC35F2	-0.02555	0.357488	-0.07074	0.943762	-5.56076	0.894368	0.884118
T.cells	DCPS	0.00645	5.004121	0.070522	0.943938	-6.69379	0.805505	0.776955
T.cells	RNF166	0.007027	5.468282	0.070489	0.943964	-6.49825	0.797095	0.766985
T.cells	HDAC6	-0.01484	2.206503	-0.0703	0.944117	-5.94956	0.857971	0.839867
T.cells	4933406I1!	0.011019	4.543564	0.070192	0.944199	-6.59829	0.813931	0.78699
T.cells	CCL17	0.049888	-0.70076	0.070109	0.944265	-5.20337	0.915833	0.910492
T.cells	ZFP341	-0.01535	1.984672	-0.07006	0.944301	-5.83025	0.862265	0.845064
T.cells	TRIM3	0.017108	2.274801	0.070021	0.944335	-5.75162	0.856653	0.838274
T.cells	LY9	-0.00898	4.262049	-0.06993	0.94441	-6.37732	0.819122	0.793181
T.cells	ARHGAP35	-0.00634	5.237019	-0.06987	0.944452	-6.75492	0.801275	0.771954
T.cells	WDR89	0.012674	2.643388	0.06972	0.944574	-6.02142	0.849574	0.829747
T.cells	AFP	-0.03333	4.371399	-0.06972	0.944574	-6.26363	0.817102	0.790792
T.cells	IL11RA1	-0.01958	2.113905	-0.06957	0.944692	-5.59829	0.859761	0.842056
T.cells	BCL6B	-0.02921	0.649778	-0.0695	0.944752	-5.40196	0.888522	0.877024
T.cells	ANAPC15	0.00687	4.974484	0.069459	0.944782	-6.65279	0.806044	0.777627
T.cells	ILK	-0.00655	5.628263	-0.06934	0.944878	-6.74233	0.794216	0.763628

T.cells	UQCR10	0.00567	6.961957	0.069236	0.944958	-6.98994	0.770592	0.735807
T.cells	FAM193B	0.007425	4.050857	0.069186	0.944998	-6.48853	0.823036	0.797935
T.cells	BORCS6	0.010064	4.007961	0.068997	0.945148	-6.27353	0.823901	0.798935
T.cells	GABARAPL	-0.01133	3.974288	-0.06859	0.945471	-6.17241	0.824737	0.799753
T.cells	MAP3K11	0.009696	3.876975	0.068509	0.945535	-6.28323	0.826551	0.801927
T.cells	COQ2	0.007103	4.467496	0.068336	0.945673	-6.55186	0.81566	0.788901
T.cells	CAPN5	0.026417	1.546779	0.067952	0.945977	-5.57523	0.871364	0.855769
T.cells	1700109H(	-0.0104	3.650709	-0.06781	0.946088	-6.31793	0.831074	0.807156
T.cells	BOD1	0.010108	3.243589	0.06756	0.946288	-6.24877	0.838797	0.81641
T.cells	TMEM237	-0.01855	2.157499	-0.06752	0.946318	-5.78519	0.859569	0.841469
T.cells	RBM48	0.009226	3.037834	0.067411	0.946407	-6.21281	0.842696	0.821145
T.cells	TPMT	-0.01656	2.097645	-0.06734	0.946459	-5.75657	0.860728	0.842921
T.cells	APEX2	0.007053	4.215093	0.06727	0.946519	-6.52074	0.820612	0.794697
T.cells	UBE2R2	0.005027	7.144409	0.066925	0.946793	-6.98846	0.767994	0.732492
T.cells	SSH1	0.011388	3.775324	0.066785	0.946903	-6.05013	0.828798	0.804533
T.cells	ANKRD16	0.010815	3.026656	0.066675	0.946991	-6.13304	0.842908	0.821491
T.cells	RGS19	-0.00749	5.393744	-0.06661	0.947039	-6.60959	0.799046	0.769113
T.cells	TRIM59	0.007732	4.368452	0.066554	0.947087	-6.65555	0.817776	0.791383
T.cells	SH2D1A	-0.03672	0.051779	-0.06654	0.947099	-5.2316	0.901204	0.892298
T.cells	4930532G1	-0.02944	1.429337	-0.06641	0.9472	-5.56081	0.873762	0.858818
T.cells	POLR1E	-0.01526	2.067812	-0.06641	0.947204	-5.89776	0.861306	0.843709
T.cells	SNHG4.1	-0.02577	0.471422	-0.06635	0.947246	-5.62795	0.892762	0.881978
T.cells	CLEC12A	-0.02376	4.937614	-0.06635	0.947251	-5.79136	0.807328	0.778955
T.cells	SLIT2	-0.02789	0.983319	-0.06616	0.947403	-5.58874	0.882575	0.869546
T.cells	CNPPD1	-0.00711	5.686802	-0.06603	0.947507	-6.66054	0.793779	0.762892
T.cells	WRAP73	0.008931	2.923556	0.065932	0.947581	-6.1919	0.844882	0.823883
T.cells	SART1	-0.0062	4.872457	-0.06589	0.947613	-6.63056	0.80853	0.780386
T.cells	YPEL1	-0.0115	2.4444	-0.06587	0.947631	-6.05303	0.854049	0.834938
T.cells	HDLBP	0.004464	6.655734	0.065451	0.947962	-6.84814	0.776716	0.7426
T.cells	GRB2	0.005173	7.898248	0.06535	0.948043	-6.99737	0.755143	0.717355
T.cells	HERC1	-0.0055	7.056727	-0.06535	0.948044	-6.94678	0.769691	0.734356
T.cells	ZC3H18	-0.00569	5.270525	-0.06511	0.948236	-6.7176	0.801492	0.771798
T.cells	NIPSNAP1	0.011076	3.134643	0.065074	0.948262	-6.18819	0.841086	0.819073
T.cells	CABIN1	-0.00559	5.4487	-0.06499	0.948325	-6.75312	0.798269	0.767979
T.cells	USP42	-0.00775	3.500066	-0.06488	0.948418	-6.30739	0.834185	0.810786
T.cells	ATG10	0.006821	5.64086	0.064826	0.948459	-6.65397	0.794807	0.763881
T.cells	PHKA1	-0.0091	3.411651	-0.06471	0.948555	-6.37608	0.835871	0.812784
T.cells	TREML4	-0.03738	2.758058	-0.06436	0.948832	-5.38191	0.848299	0.827763
T.cells	SGPP1	0.008309	5.017924	0.064298	0.948878	-6.46183	0.806125	0.777305
T.cells	NUDT15	-0.01511	1.647281	-0.064	0.949119	-5.8111	0.869773	0.853757
T.cells	TMEM43	-0.00966	3.43413	-0.06396	0.94915	-6.19442	0.835471	0.812364
T.cells	ANKRD13D	-0.01085	2.738056	-0.06394	0.949162	-6.13417	0.848681	0.82825
T.cells	TOR1B	-0.00973	3.704769	-0.06389	0.949202	-6.14304	0.830387	0.806276
T.cells	ZBTB45	-0.00897	2.916254	-0.06386	0.949228	-6.22911	0.845281	0.824176
T.cells	LRP8OS2	-0.03705	0.163168	-0.0638	0.949276	-5.32143	0.899247	0.889729
T.cells	TRMT11	0.007951	3.646744	0.063724	0.949334	-6.32126	0.831474	0.807615
T.cells	XXYLT1	-0.00836	3.591278	-0.06362	0.949414	-6.3368	0.832515	0.808867

T.cells	UBXN2B	-0.00981	2.842475	-0.06359	0.949441	-6.18364	0.846687	0.825903
T.cells	GTF3C4	0.011079	2.57436	0.063319	0.949655	-6.08238	0.851816	0.832152
T.cells	GSTT2	0.008724	3.554147	0.063252	0.949709	-6.39693	0.833213	0.809775
T.cells	MRPS11	-0.00801	3.950885	-0.06324	0.949721	-6.46272	0.825788	0.800883
T.cells	GINM1	0.00546	4.990227	0.063231	0.949725	-6.61078	0.806629	0.778044
T.cells	EEF1AKMT:	0.009705	3.325831	0.063115	0.949817	-6.30803	0.837514	0.81496
T.cells	ATP5D	0.004029	7.898894	0.063103	0.949827	-7.12575	0.755211	0.717548
T.cells	CDK17	-0.00514	6.780519	-0.06289	0.949999	-6.94767	0.774684	0.740237
T.cells	EPOP	-0.01832	1.472765	-0.0624	0.950384	-5.69962	0.873479	0.858138
T.cells	GM38560	-0.02822	0.991541	-0.06233	0.950442	-5.43424	0.882974	0.86969
T.cells	EXOC3L2	0.031145	2.269133	0.062194	0.950549	-5.72713	0.857972	0.839348
T.cells	ANP32A	-0.0028	7.495527	-0.06214	0.950595	-7.09635	0.762402	0.725715
T.cells	MRPL23	-0.00395	6.069484	-0.0621	0.950625	-6.87403	0.787436	0.755104
T.cells	BRD7	0.003892	6.307707	0.062026	0.950682	-6.89405	0.7832	0.750113
T.cells	STAP1	-0.00763	4.891894	-0.06192	0.95077	-6.52938	0.80869	0.780271
T.cells	MS4A4C	-0.04542	3.224383	-0.06185	0.950824	-5.48114	0.839707	0.817338
T.cells	UPF2	0.004616	6.075856	0.061788	0.950871	-6.83679	0.787322	0.754971
T.cells	EFNA5	-0.02602	2.313548	-0.06167	0.950963	-5.74828	0.857123	0.838312
T.cells	GM3448	0.017495	1.9113	0.061526	0.951079	-5.71363	0.864916	0.847741
T.cells	RNF24	0.009185	4.233052	0.0615	0.9511	-6.27191	0.820823	0.794713
T.cells	TOX	-0.0143	4.351569	-0.0611	0.951415	-6.3983	0.818703	0.792096
T.cells	2610021A(	0.011583	2.362444	0.061047	0.951459	-6.01044	0.856257	0.837173
T.cells	NLK	-0.00624	5.966033	-0.06104	0.951463	-6.73865	0.78936	0.757282
T.cells	ECPAS	-0.00407	6.743157	-0.06102	0.951478	-7.02183	0.775592	0.741074
T.cells	MTF1	-0.00702	4.234341	-0.0606	0.951815	-6.37323	0.820993	0.79476
T.cells	NSFL1C	0.006087	4.168167	0.060561	0.951846	-6.46198	0.82222	0.796225
T.cells	FAM120B	0.00792	4.124917	0.060501	0.951893	-6.31576	0.823023	0.797184
T.cells	GM10135	0.023971	0.409376	0.060479	0.951911	-5.45803	0.894809	0.883949
T.cells	AKR1E1	-0.01219	2.622552	-0.06023	0.952105	-5.92014	0.851443	0.831257
T.cells	UNC45A	0.00781	4.303611	0.060207	0.952127	-6.33942	0.81977	0.793267
T.cells	PPIC	-0.01504	2.745716	-0.05997	0.952315	-5.83341	0.84913	0.828466
T.cells	ERI1	0.005326	5.459943	0.059893	0.952376	-6.82236	0.798668	0.768186
T.cells	CHD4	0.003844	7.633169	0.059813	0.952439	-7.11408	0.760309	0.723088
T.cells	PSMD1	-0.00426	6.546401	-0.05969	0.952534	-6.93464	0.779269	0.745298
T.cells	DELE1	0.007149	3.426108	0.059645	0.952573	-6.30393	0.836208	0.812948
T.cells	NCBP2	-0.00567	4.599992	-0.05959	0.952617	-6.59625	0.814344	0.786837
T.cells	LRRC42	-0.00815	3.272197	-0.05912	0.952993	-6.22611	0.839382	0.816562
T.cells	MRTFB	0.007202	4.475202	0.058991	0.953092	-6.479	0.816925	0.789684
T.cells	PARP2	0.00462	4.841287	0.058601	0.953402	-6.70069	0.810389	0.781753
T.cells	REM2	0.016404	0.836601	0.058137	0.953771	-5.87877	0.886898	0.873973
T.cells	GM43126	-0.02805	0.276018	-0.05793	0.953939	-5.33973	0.898126	0.887723
T.cells	TSPYL3	-0.02301	0.960124	-0.05786	0.953989	-5.55436	0.884442	0.871017
T.cells	AHSA2	-0.00726	3.646243	-0.05782	0.954019	-6.29423	0.832552	0.808327
T.cells	TUT4	-0.0049	7.475638	-0.05779	0.954048	-7.08596	0.763475	0.726591
T.cells	SBK1	0.006641	3.374185	0.057773	0.95406	-6.50966	0.837676	0.81447
T.cells	TNFAIP8L2	-0.0125	3.852483	-0.05764	0.954162	-5.89308	0.828687	0.8037
T.cells	2210408I2:	-0.0098	3.277757	-0.05764	0.954165	-6.2076	0.839499	0.816658

T.cells	5430431A1	0.015077	1.423829	0.057563	0.954227	-5.69694	0.875276	0.859867
T.cells	ADNP	-0.00411	6.50802	-0.05754	0.954242	-6.92163	0.780401	0.746428
T.cells	KXD1	0.00505	5.765423	0.057517	0.954263	-6.74318	0.79363	0.762023
T.cells	SMAD3	0.006635	6.315623	0.057269	0.95446	-6.81604	0.783808	0.7505
T.cells	D430020J0	-0.01867	0.440007	-0.05703	0.954648	-5.68475	0.894828	0.883838
T.cells	MYO1B	-0.02278	2.148687	-0.05699	0.954679	-5.5897	0.861123	0.842856
T.cells	ZFHX2	-0.01397	2.765643	-0.05688	0.95477	-5.92248	0.849244	0.82854
T.cells	IGF2BP1	0.033988	-0.96106	0.056829	0.954809	-5.16712	0.923365	0.918896
T.cells	1190007I0	0.007153	3.503332	0.056589	0.955	-6.35832	0.83524	0.811744
T.cells	DUSP11	-0.00377	6.33182	-0.05654	0.955042	-6.84976	0.783521	0.75028
T.cells	HIST4H4	0.015339	1.887782	0.056435	0.955122	-5.85406	0.866192	0.849054
T.cells	SRPRB	0.006038	4.104103	0.056416	0.955137	-6.45566	0.823994	0.798284
T.cells	UQCRQ	0.004672	7.858453	0.056411	0.955141	-7.1031	0.756875	0.719064
T.cells	GNPAT	-0.00647	4.15461	-0.0564	0.955154	-6.42018	0.823055	0.797163
T.cells	PSMB5	-0.00463	6.258665	-0.05631	0.955225	-6.89873	0.78482	0.75181
T.cells	VEGFA	0.008358	4.223513	0.05616	0.955341	-6.47612	0.821776	0.795635
T.cells	IFNG	0.037232	1.241502	0.056102	0.955387	-5.33024	0.878869	0.864443
T.cells	MRPL38	-0.00652	3.863246	-0.05603	0.955441	-6.39145	0.828486	0.803654
T.cells	IL15	-0.01827	3.93993	-0.05601	0.955461	-5.95588	0.827053	0.80194
T.cells	RNF125	0.01224	3.852901	0.05564	0.955754	-6.09581	0.828679	0.803976
T.cells	RHBDL3	-0.02095	0.747589	-0.0556	0.955783	-5.58554	0.888672	0.876487
T.cells	CENPQ	-0.00551	5.314475	-0.05534	0.955989	-6.80587	0.801767	0.77198
T.cells	SLAMF1	-0.02478	0.884468	-0.05524	0.956076	-5.47179	0.885946	0.873225
T.cells	MAP1LC3B	0.005142	7.434486	0.0552	0.956104	-6.98554	0.764188	0.727736
T.cells	HIRIP3	0.007154	3.169999	0.055123	0.956165	-6.42058	0.841541	0.819463
T.cells	GM15518	0.021147	0.80065	0.054991	0.95627	-5.56726	0.887614	0.875261
T.cells	HDAC1	-0.00383	6.039987	-0.05496	0.956294	-6.82781	0.788714	0.756543
T.cells	PIPOX	-0.01509	2.011866	-0.0549	0.956345	-5.88068	0.863778	0.84629
T.cells	ANKLE1	0.013507	0.64315	0.054857	0.956376	-5.7906	0.890758	0.879098
T.cells	LRRC8D	0.00542	7.366735	0.054848	0.956383	-6.90474	0.765362	0.72911
T.cells	D130040H2	0.01211	2.397794	0.054772	0.956444	-5.91913	0.856308	0.837275
T.cells	CPM	-0.00758	4.160192	-0.05468	0.956519	-6.69448	0.822952	0.79722
T.cells	ARHGAP24	-0.00564	6.825889	-0.05467	0.956524	-7.03777	0.774802	0.740199
T.cells	CABP1	-0.01957	-0.13612	-0.05466	0.95653	-5.6093	0.906464	0.898357
T.cells	MAGED1	-0.01591	2.409871	-0.05416	0.956927	-5.72462	0.856075	0.837066
T.cells	EPHA2	-0.00889	2.408212	-0.05407	0.956999	-6.17668	0.856107	0.837137
T.cells	FAM111A	-0.00473	5.742953	-0.05406	0.95701	-6.85625	0.794034	0.762938
T.cells	PHF20	0.003972	6.472356	0.054026	0.957036	-6.88169	0.781031	0.747601
T.cells	PILRB1	0.027848	2.049283	0.053994	0.957062	-5.37068	0.863051	0.845534
T.cells	ROPN1L	-0.01631	2.420763	-0.05394	0.957102	-5.75418	0.855865	0.836863
T.cells	TMEM41A	0.014325	1.51992	0.053909	0.957129	-5.79234	0.873387	0.85809
T.cells	MORN2	0.010062	2.045949	0.053834	0.957189	-6.074	0.863116	0.845633
T.cells	SCYL1	0.006967	4.072604	0.053834	0.957189	-6.39762	0.82458	0.799272
T.cells	KLRA9	0.032543	-0.30711	0.053731	0.957271	-5.255	0.909944	0.902748
T.cells	KMT5C	-0.00944	2.877228	-0.05372	0.957276	-6.12399	0.847111	0.826306
T.cells	CRNKL1	0.00442	5.293781	0.053559	0.957407	-6.70709	0.802144	0.772567
T.cells	MRPS2	0.010206	2.843371	0.053537	0.957425	-6.20589	0.847759	0.827095

T.cells	FAM193A	-0.00406	6.704133	-0.05333	0.957588	-6.93291	0.777016	0.74282
T.cells	INIP	-0.00467	4.523666	-0.05315	0.957734	-6.63673	0.816365	0.789332
T.cells	MARS	-0.00818	3.10364	-0.05287	0.957954	-6.25906	0.84299	0.821206
T.cells	GALNT12	-0.00854	2.991393	-0.05283	0.957988	-6.21726	0.845125	0.823782
T.cells	SZT2	0.010114	2.318426	0.052697	0.958092	-5.90632	0.858032	0.839375
T.cells	PDCD6	-0.00496	6.13963	-0.05268	0.958102	-6.76558	0.787115	0.754691
T.cells	RTL6	0.02105	0.292529	0.052622	0.958152	-5.51254	0.897996	0.888004
T.cells	5430414B1	0.021097	0.449396	0.052458	0.958282	-5.52919	0.894859	0.884173
T.cells	TADA2B	0.007256	3.468615	0.052339	0.958377	-6.30413	0.836099	0.813033
T.cells	GATM	0.015263	4.530012	0.052231	0.958462	-5.88285	0.816308	0.789387
T.cells	VPS51	-0.00666	3.730024	-0.05182	0.958791	-6.36004	0.831183	0.807244
T.cells	MLLT11	0.00747	3.193389	0.051803	0.958803	-6.14771	0.841303	0.819389
T.cells	BC035044	0.006455	5.146379	0.051707	0.958879	-6.82832	0.805017	0.776044
T.cells	SLC22A14	0.011067	2.744823	0.051637	0.958935	-6.03047	0.84985	0.8297
T.cells	TIMM22	0.004884	4.4471	0.051594	0.958969	-6.5677	0.817838	0.791312
T.cells	ABCG1	0.006486	6.591271	0.051506	0.959038	-6.81729	0.779122	0.745475
T.cells	ZBTB21	0.00712	3.713914	0.051377	0.959141	-6.26119	0.831486	0.807661
T.cells	DOLK	0.012822	1.544978	0.051346	0.959166	-5.63397	0.873109	0.857885
T.cells	CUL7	0.01287	1.968845	0.051293	0.959207	-5.82984	0.864826	0.847836
T.cells	GPR55	-0.02603	1.548876	-0.05127	0.959223	-5.22676	0.873033	0.857792
T.cells	CBX2	-0.01584	-0.18673	-0.05121	0.959277	-5.65517	0.907715	0.900151
T.cells	STAT3	0.005457	7.950149	0.051149	0.959322	-6.88023	0.755487	0.717818
T.cells	GLA	-0.00965	4.568639	-0.05104	0.959408	-6.21667	0.815596	0.788673
T.cells	ACAD11	0.012627	2.331168	0.050961	0.959472	-5.73653	0.857803	0.839337
T.cells	PLCB2	0.010794	3.320212	0.050806	0.959594	-5.9062	0.838901	0.816559
T.cells	ZFP653	0.007146	3.131364	0.050791	0.959606	-6.26172	0.84248	0.820861
T.cells	STK38	0.004066	6.531625	0.050753	0.959637	-6.90269	0.780175	0.746729
T.cells	FOXP4	0.004997	4.962815	0.050223	0.960058	-6.78121	0.808597	0.780097
T.cells	CLN5	-0.00773	4.008222	-0.05018	0.960089	-6.10982	0.82622	0.801105
T.cells	FBXW2	0.003333	6.193608	0.050019	0.96022	-6.85933	0.786396	0.753819
T.cells	MGMT	0.010484	3.273792	0.049993	0.960241	-6.03981	0.840022	0.817647
T.cells	ZFP369	-0.00822	2.919936	-0.04994	0.96028	-6.15054	0.846748	0.825736
T.cells	ACAD9	0.00904	2.542008	0.049761	0.960425	-6.01682	0.854051	0.834463
T.cells	PFDN2	0.004345	5.031244	0.049064	0.960979	-6.62749	0.807559	0.778707
T.cells	TMEM108	-0.00769	5.137851	-0.04904	0.961001	-6.82253	0.805616	0.776399
T.cells	TMEM70	-0.00656	3.930301	-0.049	0.961026	-6.35334	0.827891	0.802941
T.cells	UBR7	-0.00518	4.241439	-0.04886	0.961143	-6.56924	0.822097	0.796018
T.cells	ANAPC5	-0.00301	6.399962	-0.04881	0.961181	-6.9782	0.782936	0.749592
T.cells	GM49101	0.029139	-0.65342	0.048769	0.961214	-5.19585	0.917759	0.912035
T.cells	KIF23	0.006167	5.173452	0.048677	0.961286	-6.78365	0.804968	0.775645
T.cells	GM13212	0.010886	3.205264	0.048628	0.961325	-5.90294	0.841541	0.819323
T.cells	ZFP280D	0.004157	5.625842	0.048619	0.961333	-6.80764	0.796774	0.765938
T.cells	SLAMF8	0.035638	0.718707	0.048611	0.961339	-5.27727	0.889957	0.877966
T.cells	MMGT2	-0.00688	3.166256	-0.04842	0.961491	-6.16505	0.842348	0.820212
T.cells	AAK1	-0.00442	6.392459	-0.04822	0.961647	-6.8465	0.78313	0.749786
T.cells	SEC24A	0.005554	6.616568	0.047929	0.961881	-6.83942	0.779166	0.745179
T.cells	NAA38	0.004244	5.467647	0.047899	0.961905	-6.77278	0.799693	0.769401

T.cells	PLEKHG3	0.005809	4.378559	0.04784	0.961952	-6.55867	0.819621	0.793098
T.cells	RABL6	-0.0038	5.136693	-0.0478	0.961985	-6.65326	0.8057	0.776537
T.cells	MON1A	-0.006	3.876268	-0.04765	0.962103	-6.33976	0.828967	0.804267
T.cells	NDUFAF4	0.006146	3.867148	0.04761	0.962134	-6.38372	0.829137	0.804482
T.cells	MGRN1	0.005369	5.682428	0.047559	0.962175	-6.63426	0.795818	0.764839
T.cells	ACVR2A	-0.00695	4.7385	-0.04741	0.962297	-6.51145	0.812984	0.785214
T.cells	SLC35A4	-0.00623	3.561213	-0.04737	0.962322	-6.34128	0.83488	0.811381
T.cells	PMM2	-0.0046	4.561806	-0.04736	0.962335	-6.44516	0.816235	0.789099
T.cells	FRMD4B	-0.014	5.267906	-0.04732	0.962369	-6.11649	0.803314	0.773751
T.cells	CALM3	-0.00325	7.10732	-0.04721	0.962451	-7.06788	0.770557	0.735156
T.cells	MORF4L2	0.004181	5.404884	0.047048	0.962581	-6.65053	0.800855	0.770852
T.cells	B4GALT4	0.012667	1.774117	0.047	0.96262	-5.71876	0.869197	0.852803
T.cells	FAM57A	0.01783	1.118395	0.046884	0.962712	-5.53477	0.88212	0.868487
T.cells	ITPKA	0.016261	0.529225	0.046768	0.962803	-5.58927	0.893875	0.882816
T.cells	HACD4	0.018267	3.532004	0.046686	0.962869	-5.64545	0.835482	0.812107
T.cells	AMPD2	0.009516	2.339378	0.046573	0.962959	-5.95762	0.858253	0.839535
T.cells	TRIB3	0.016575	1.057747	0.046332	0.96315	-5.70974	0.883378	0.87003
T.cells	ZNRD2	0.005355	4.037669	0.046178	0.963272	-6.49941	0.826047	0.800848
T.cells	GM16556	-0.01398	1.676001	-0.04616	0.963287	-5.79383	0.871189	0.855228
T.cells	NOS1AP	-0.0151	3.163666	-0.04611	0.963328	-5.79773	0.842494	0.820575
T.cells	MYO18A	-0.00562	4.439945	-0.04607	0.963359	-6.36066	0.818579	0.791944
T.cells	MED6	-0.00497	4.540282	-0.0458	0.963569	-6.58198	0.816727	0.789769
T.cells	LINS1	-0.01116	1.704648	-0.0458	0.963573	-5.69663	0.870629	0.854598
T.cells	COX6A2	0.018757	2.070766	0.045781	0.963588	-5.65658	0.863488	0.845943
T.cells	KLHL2	-0.00487	5.570335	-0.04562	0.963712	-6.63041	0.79793	0.76746
T.cells	HNMT	0.023014	-0.38409	0.045221	0.964033	-5.18939	0.912417	0.905701
T.cells	COX11	-0.00485	3.751078	-0.04511	0.964119	-6.39507	0.831408	0.807389
T.cells	SWSAP1	0.018798	0.278314	0.044973	0.96423	-5.4198	0.898969	0.889227
T.cells	HIC2	-0.00897	2.712677	-0.04497	0.964236	-6.04441	0.8511	0.831057
T.cells	SLC12A9	-0.0071	4.242634	-0.04492	0.964269	-6.2957	0.822235	0.796418
T.cells	ZSCAN29	-0.00666	3.485733	-0.04479	0.964376	-6.15125	0.836399	0.813373
T.cells	NCAPG	-0.00742	3.22832	-0.04475	0.964405	-6.45511	0.841268	0.81922
T.cells	ADPRM	-0.00626	3.321315	-0.04473	0.964421	-6.17165	0.839506	0.817103
T.cells	FAM102B	-0.00869	4.23575	-0.04473	0.964423	-6.07489	0.822363	0.796571
T.cells	ZFP689	-0.01058	1.407041	-0.04472	0.964431	-5.85277	0.876473	0.861781
T.cells	MS4A8A	0.028252	0.670009	0.044683	0.964461	-5.29936	0.891103	0.879619
T.cells	ART3	-0.01865	0.944132	-0.04431	0.964755	-5.42655	0.88566	0.873034
T.cells	FCSK	0.015657	0.928399	0.044135	0.964896	-5.55039	0.885973	0.873426
T.cells	SCFD2	-0.00415	5.78946	-0.04409	0.964928	-6.71902	0.794006	0.762955
T.cells	GM15675	-0.01262	2.317552	-0.04401	0.964995	-5.8226	0.858729	0.84037
T.cells	CHCHD10	-0.00493	6.141333	-0.04396	0.965035	-7.0182	0.787708	0.755529
T.cells	GM12971	0.013869	0.967071	0.043839	0.965131	-5.55734	0.885204	0.872541
T.cells	AW146154	-0.01061	2.334177	-0.04383	0.96514	-5.89945	0.858408	0.840007
T.cells	METTL4	0.006695	2.874732	0.043355	0.965517	-6.12564	0.848021	0.827587
T.cells	DCAF6	-0.00524	5.908083	-0.04333	0.965534	-6.68926	0.791878	0.760581
T.cells	CCDC141	0.01328	1.120838	0.043313	0.96555	-5.69512	0.882152	0.868943
T.cells	COBLL1	0.004693	5.069977	0.043201	0.965639	-6.71414	0.807032	0.778543

T.cells	ENKD1	-0.00934	1.3576	-0.04307	0.965742	-5.93034	0.877471	0.863261
T.cells	FHL1	0.018079	0.58688	0.043055	0.965754	-5.42574	0.892792	0.881943
T.cells	UBR3	-0.00343	6.575859	-0.04301	0.965788	-6.90868	0.779996	0.746599
T.cells	TEDC2	0.012205	0.906107	0.042911	0.965869	-5.69585	0.886416	0.874175
T.cells	SMAD6	0.019828	2.733914	0.042884	0.96589	-5.5454	0.850716	0.830867
T.cells	USP1	0.003578	5.737466	0.042863	0.965907	-6.91047	0.794941	0.764232
T.cells	9130019O2	0.018457	0.355077	0.042852	0.965916	-5.47814	0.897447	0.887655
T.cells	THAP12	0.004039	4.362104	0.042809	0.96595	-6.50619	0.820042	0.794061
T.cells	GM9887	-0.01208	1.686181	-0.04276	0.965991	-5.733	0.871014	0.85543
T.cells	DHX36	-0.00303	5.392248	-0.04269	0.966047	-6.73212	0.801172	0.771621
T.cells	NDUFA13	0.002925	7.128419	0.04258	0.966132	-6.97085	0.770291	0.735238
T.cells	SH3BP5	0.004523	6.380773	0.042527	0.966174	-6.82021	0.783449	0.750702
T.cells	4833439L1	0.004166	4.862518	0.042335	0.966327	-6.60261	0.81083	0.78317
T.cells	PRMT6	0.00733	2.45108	0.042214	0.966423	-5.98646	0.856157	0.837573
T.cells	PEG13	-0.0128	2.114243	-0.04216	0.966463	-5.61158	0.862673	0.845456
T.cells	CD79A	0.004153	6.200185	0.04215	0.966474	-7.10348	0.786665	0.75458
T.cells	PPP4R2	-0.00335	6.318519	-0.04172	0.96682	-6.83048	0.78478	0.752186
T.cells	DMXL2	0.022799	2.146865	0.041586	0.966922	-5.3815	0.862309	0.844788
T.cells	ARMC10	-0.0053	3.275459	-0.04146	0.967023	-6.32094	0.840666	0.818725
T.cells	GM13483	-0.01101	1.85821	-0.0414	0.967071	-5.8335	0.867929	0.851656
T.cells	WDR34	0.013408	1.224098	0.0413	0.96715	-5.7671	0.880394	0.866805
T.cells	GM50012	-0.01347	1.643382	-0.04119	0.967233	-5.48804	0.872146	0.856783
T.cells	NIPA1	0.02087	0.13903	0.040997	0.96739	-5.29248	0.902147	0.893381
T.cells	GDAP2	-0.00468	4.778409	-0.04081	0.967536	-6.43331	0.812673	0.785267
T.cells	PPP1R14B	0.003131	6.546146	0.040719	0.967611	-6.972	0.780815	0.747561
T.cells	MAGI2	-0.01513	0.831391	-0.0406	0.967707	-5.51583	0.88824	0.87642
T.cells	RRAS	0.010644	4.338868	0.040549	0.967747	-5.92745	0.820782	0.79497
T.cells	ZFP639	0.004477	4.128657	0.04045	0.967826	-6.4766	0.824687	0.799635
T.cells	ATG9A	0.006299	3.85849	0.040415	0.967853	-6.14707	0.829731	0.805671
T.cells	HNRNPR	-0.00242	6.29021	-0.04037	0.967892	-6.94998	0.785354	0.752936
T.cells	APC	-0.00328	6.723147	-0.04031	0.967933	-6.91245	0.777691	0.743915
T.cells	EHD4	0.003927	6.076676	0.04011	0.968096	-6.76346	0.789232	0.757425
T.cells	IQCE	0.011299	2.144332	0.039887	0.968273	-5.7246	0.862581	0.845043
T.cells	9530077C0	0.009747	1.213471	0.039764	0.968371	-5.94053	0.880848	0.867223
T.cells	RDM1	-0.00471	5.028258	-0.03954	0.968547	-6.71717	0.808365	0.779951
T.cells	SLC43A3	0.005249	3.813488	0.039413	0.96865	-6.4477	0.830852	0.806776
T.cells	DYNLT1F	-0.0044	5.068561	-0.03935	0.968701	-6.71142	0.807633	0.779076
T.cells	SNX27	-0.00303	5.803763	-0.03914	0.968865	-6.76101	0.794315	0.763303
T.cells	WWC2	0.004024	5.107898	0.039118	0.968885	-6.6847	0.806915	0.778242
T.cells	LYNX1	0.014708	0.898277	0.038912	0.969048	-5.60091	0.887204	0.874985
T.cells	GM9828	0.01308	1.076181	0.038901	0.969057	-5.64226	0.883667	0.870671
T.cells	ZFP69	0.009486	2.612118	0.038746	0.96918	-5.88965	0.853661	0.834284
T.cells	TRAPPC5	0.004918	4.379372	0.038703	0.969214	-6.37376	0.820307	0.794268
T.cells	ALG14	-0.00578	3.562595	-0.03849	0.969383	-6.25653	0.835568	0.812572
T.cells	TSSC4	0.004631	3.902903	0.038458	0.969409	-6.43365	0.829178	0.804912
T.cells	ATP2C1	0.00333	5.85373	0.038342	0.969502	-6.70008	0.793417	0.762394
T.cells	SCAPER	0.00402	6.110801	0.038273	0.969557	-6.66781	0.788815	0.756961

T.cells	HIST1H3F	0.013311	0.34671	0.038081	0.969709	-5.71358	0.898256	0.888638
T.cells	GM43378	0.010112	1.243694	0.038062	0.969724	-5.64794	0.880347	0.866777
T.cells	LPCAT1	0.004513	4.107714	0.038006	0.969769	-6.45696	0.825354	0.800414
T.cells	POLM	-0.00427	3.245007	-0.03793	0.96983	-6.40557	0.841573	0.81988
T.cells	LRRC75AOS	0.014313	0.512247	0.037866	0.96988	-5.5266	0.894926	0.884597
T.cells	TBCB	-0.00288	6.070727	-0.03773	0.969989	-6.80498	0.78953	0.757907
T.cells	ACAP1	0.005479	3.923819	0.037689	0.970021	-6.37816	0.828786	0.804585
T.cells	THAP11	-0.00369	4.457867	-0.03764	0.970061	-6.63365	0.818854	0.792714
T.cells	TAF1C	0.007855	2.170342	0.037597	0.970094	-5.89345	0.862193	0.844812
T.cells	TSC22D2	0.00283	6.616128	0.037067	0.970515	-6.87197	0.78012	0.746564
T.cells	SHLD2	0.005146	3.853936	0.036835	0.9707	-6.31667	0.830487	0.806237
T.cells	UBE3B	-0.00362	4.424846	-0.03596	0.971397	-6.46439	0.820325	0.793628
T.cells	GNL2	-0.00358	4.675227	-0.03595	0.971406	-6.5612	0.815699	0.788116
T.cells	WDR27	-0.01296	0.499373	-0.03573	0.971582	-5.45429	0.896178	0.885169
T.cells	SYNRG	0.00344	5.053855	0.035688	0.971611	-6.57708	0.808798	0.779852
T.cells	FMNL2	0.006849	7.100621	0.03557	0.971706	-6.73489	0.772199	0.736657
T.cells	EXO1	-0.00769	2.019131	-0.03548	0.971781	-6.09528	0.866112	0.848567
T.cells	SLC30A6	0.004721	3.417011	0.035324	0.971901	-6.27672	0.839306	0.816205
T.cells	RPUSD4	-0.00585	2.682051	-0.03519	0.972007	-6.12926	0.853353	0.833066
T.cells	ULK4	0.006565	2.667963	0.034913	0.972228	-6.02393	0.853753	0.833452
T.cells	CISD1	0.003921	5.119405	0.034694	0.972402	-6.74599	0.807891	0.778522
T.cells	RSAD1	-0.01417	1.298807	-0.03448	0.972574	-5.57138	0.88061	0.86587
T.cells	LRRC47	0.003318	4.227098	0.034426	0.972615	-6.48521	0.824398	0.798139
T.cells	5830418P1	0.008132	1.463039	0.034229	0.972771	-6.10667	0.877441	0.861931
T.cells	ZDHHC8	0.004254	3.666743	0.034114	0.972863	-6.38625	0.834976	0.810688
T.cells	SNRNP25	0.003982	3.939991	0.033899	0.973034	-6.49181	0.829925	0.804559
T.cells	4930523C0	0.00244	6.126408	0.033788	0.973123	-6.95137	0.789908	0.757074
T.cells	EIF2AK2	-0.00492	4.927436	-0.0337	0.973196	-6.50917	0.811622	0.782781
T.cells	AFDN	0.009431	3.267689	0.033547	0.973314	-5.6812	0.842607	0.81985
T.cells	CMC2	-0.00441	4.535117	-0.03294	0.973797	-6.68226	0.818849	0.791575
T.cells	SPARC	0.007908	5.322477	0.03254	0.974115	-6.37426	0.804407	0.77448
T.cells	RSRP1	0.00288	6.653226	0.032476	0.974166	-6.86322	0.780541	0.746327
T.cells	GNG7	-0.01661	-0.40318	-0.03238	0.974244	-5.27366	0.915072	0.908161
T.cells	CYP20A1	-0.0038	4.104318	-0.03229	0.974312	-6.33684	0.826853	0.801217
T.cells	ATG2B	0.003309	4.861013	0.032254	0.974342	-6.53933	0.812842	0.784517
T.cells	9230116N1	-0.00981	0.920334	-0.03221	0.974377	-5.67345	0.888308	0.875401
T.cells	PPIF	0.004335	3.577224	0.032064	0.974493	-6.34298	0.836746	0.813057
T.cells	PRDM11	0.004766	3.444862	0.032035	0.974516	-6.32659	0.839247	0.816058
T.cells	SMG8	0.005365	2.847118	0.031394	0.975026	-6.06591	0.850631	0.829801
T.cells	HGSNAT	-0.00755	4.642285	-0.03131	0.975094	-6.05618	0.816869	0.789363
T.cells	NOD2	-0.01656	1.576786	-0.0312	0.975179	-5.32637	0.875302	0.859638
T.cells	SLC25A28	0.003363	4.806244	0.031169	0.975205	-6.53493	0.813848	0.785769
T.cells	RAP1GAP	-0.01406	0.58551	-0.03116	0.975212	-5.39307	0.89501	0.883641
T.cells	TRP53COR1	0.011256	1.008827	0.031137	0.975231	-5.70939	0.886544	0.873313
T.cells	MYDGF	0.003166	4.485984	0.031086	0.975271	-6.43557	0.819758	0.792805
T.cells	PPIE	-0.00278	4.586932	-0.031	0.97534	-6.62902	0.817891	0.790581
T.cells	TTC33	-0.00416	3.849347	-0.03087	0.975445	-6.33777	0.831625	0.806979

T.cells	SORCS2	0.004716	3.3545	0.03074	0.975546	-6.52467	0.840959	0.818169
T.cells	MYG1	-0.00314	3.803891	-0.03073	0.975551	-6.43925	0.832478	0.808001
T.cells	RSPRY1	0.002396	5.833379	0.030713	0.975567	-6.75432	0.795164	0.763617
T.cells	RRAD	-0.0105	2.552475	-0.03065	0.975621	-5.62038	0.856295	0.83663
T.cells	PIMREG	-0.00733	2.104196	-0.0306	0.975658	-6.13672	0.86498	0.847125
T.cells	B3GALT2	0.011318	0.771125	0.030585	0.975669	-5.63659	0.891289	0.879098
T.cells	HNF4A	0.014201	0.691681	0.03056	0.975689	-5.41153	0.89288	0.88104
T.cells	RUBCN	0.003878	3.888841	0.030553	0.975695	-6.38663	0.830884	0.806093
T.cells	ISCU	-0.00245	7.465509	-0.03029	0.975904	-7.04868	0.766303	0.729697
T.cells	ZFP41	-0.0087	0.707145	-0.03024	0.975944	-5.7335	0.89257	0.880661
T.cells	CDC42EP2	-0.01099	3.08028	-0.03009	0.976061	-5.6781	0.846174	0.824436
T.cells	MRPL40	-0.00308	4.651456	-0.02995	0.976172	-6.62723	0.816699	0.789162
T.cells	ACTN2	0.014121	-0.1007	0.029894	0.976219	-5.36979	0.908891	0.900635
T.cells	PDP2	-0.00376	3.724878	-0.02976	0.976324	-6.31783	0.833963	0.80978
T.cells	ING1	0.002709	5.135774	0.029747	0.976336	-6.66693	0.807809	0.778593
T.cells	EFCAB9	-0.00882	0.443216	-0.02969	0.976385	-5.56886	0.897872	0.88714
T.cells	SPDYA	0.013901	-0.02911	0.029683	0.976387	-5.34119	0.907434	0.898847
T.cells	ITM2B	-0.00272	10.00633	-0.02968	0.976387	-7.276	0.723345	0.679898
T.cells	INTS2	0.003917	4.120259	0.029522	0.976515	-6.47552	0.826555	0.800918
T.cells	THY1	0.012353	1.573983	0.029333	0.976666	-5.52771	0.875357	0.859705
T.cells	KCTD6	0.006182	2.981269	0.029258	0.976725	-5.91247	0.848064	0.82671
T.cells	TTLL5	-0.00286	4.471737	-0.02926	0.976725	-6.59414	0.820022	0.79312
T.cells	MILR1	0.003511	4.330073	0.029242	0.976738	-6.40656	0.822649	0.796254
T.cells	NAIP5	0.012518	2.68586	0.029203	0.976768	-5.41976	0.853727	0.833532
T.cells	NOC3L	0.005079	3.216026	0.029202	0.97677	-6.25675	0.843589	0.821328
T.cells	IDS	0.008081	2.698638	0.029156	0.976806	-5.68193	0.853481	0.833236
T.cells	TCF20	-0.00229	7.561094	-0.02914	0.976819	-7.09363	0.764644	0.727758
T.cells	EDF1	0.002011	7.047067	0.029137	0.976821	-6.96172	0.773607	0.738246
T.cells	ENGASE	0.011773	1.496503	0.029079	0.976867	-5.55388	0.876883	0.861566
T.cells	GM32743	-0.00939	-0.48509	-0.02888	0.977029	-5.65952	0.916752	0.910363
T.cells	GM4951	0.013424	3.770894	0.028838	0.977059	-5.84128	0.833098	0.808808
T.cells	BABAM1	-0.00227	5.569892	-0.02877	0.977115	-6.80187	0.799918	0.769301
T.cells	MED23	0.003362	3.692708	0.028763	0.977119	-6.34231	0.834569	0.81057
T.cells	SLC2A8	0.005796	2.226604	0.028499	0.977329	-5.91234	0.8626	0.844341
T.cells	GNA15	-0.0058	3.584316	-0.02835	0.977451	-6.08141	0.836612	0.813047
T.cells	PRELID3B	-0.00226	5.574007	-0.02828	0.977505	-6.78035	0.799844	0.76924
T.cells	1110051M	0.004247	3.263469	0.028205	0.977562	-6.21241	0.842687	0.820339
T.cells	DNM1L	0.002333	5.639142	0.028089	0.977654	-6.76991	0.798666	0.767846
T.cells	INAFM1	-0.00862	1.866251	-0.02801	0.977718	-5.61708	0.869622	0.852847
T.cells	TIMM10	-0.00448	3.050855	-0.02799	0.977734	-6.14269	0.846735	0.825206
T.cells	1600010M	0.003081	4.96425	0.027987	0.977736	-6.68728	0.810947	0.78241
T.cells	SNAP29	0.002206	5.5062	0.027956	0.97776	-6.76104	0.801071	0.770693
T.cells	GOLGA7	0.002037	5.94	0.027922	0.977787	-6.79468	0.793248	0.761441
T.cells	TREML2	0.003932	3.901235	0.027891	0.977812	-6.37514	0.830651	0.805908
T.cells	TTC8	-0.01124	0.78875	-0.02771	0.977955	-5.44539	0.891	0.878795
T.cells	EDEM2	0.003853	4.515035	0.027572	0.978065	-6.33646	0.819309	0.792301
T.cells	RBMX	-0.0025	4.578527	-0.02732	0.978268	-6.60863	0.81818	0.791011

T.cells	SSBP2	-0.00287	6.284595	-0.02728	0.9783	-6.9108	0.787213	0.754318
T.cells	BCL7B	-0.00222	5.531179	-0.02723	0.97834	-6.73725	0.80075	0.770317
T.cells	PUS7	-0.00415	2.95762	-0.02707	0.978464	-6.21389	0.848698	0.827538
T.cells	ARPC4	-0.00175	7.957802	-0.02679	0.978691	-7.12374	0.758076	0.720026
T.cells	PSMB2	0.001717	7.058131	0.026624	0.97882	-7.01231	0.773735	0.738293
T.cells	KCNB1	-0.01082	0.988195	-0.02648	0.978933	-5.50999	0.887324	0.874138
T.cells	ENTHD1	-0.01535	0.023444	-0.0264	0.978996	-5.29128	0.906743	0.897871
T.cells	GM43707	0.011156	0.407926	0.026231	0.979133	-5.42534	0.898958	0.888339
T.cells	LY6G	0.015903	-0.81776	0.026207	0.979151	-5.22019	0.923991	0.919068
T.cells	FBXL8	0.006055	1.99347	0.026041	0.979284	-5.89701	0.867498	0.850053
T.cells	RFX5	0.006397	1.943489	0.025959	0.979348	-5.79079	0.868474	0.851235
T.cells	FER	0.006726	3.489418	0.025868	0.979421	-5.85642	0.838754	0.815422
T.cells	GM4673	-0.0049	2.578427	-0.02583	0.979455	-5.95062	0.856151	0.83636
T.cells	BIRC6	-0.00124	7.8393	-0.02573	0.979528	-7.12162	0.760151	0.722441
T.cells	RTTN	0.003326	3.609694	0.025637	0.979605	-6.44315	0.836481	0.812713
T.cells	GM19967	0.007785	1.377066	0.025621	0.979617	-5.61013	0.879606	0.864777
T.cells	HOOK1	0.004108	2.667358	0.02542	0.979777	-6.17574	0.854503	0.834364
T.cells	ZFP446	-0.00746	1.164128	-0.02512	0.980014	-5.65783	0.883892	0.870045
T.cells	KAT6A	0.00195	6.479855	0.025028	0.980089	-6.89539	0.783997	0.750422
T.cells	1600002D2	-0.01706	0.257645	-0.025	0.980108	-5.23898	0.902061	0.892229
T.cells	FNTA	0.001816	5.505108	0.025002	0.980109	-6.7367	0.801485	0.771066
T.cells	ACLY	-0.00181	6.383195	-0.02492	0.980172	-6.82037	0.785715	0.752463
T.cells	GM10101	-0.00824	0.672014	-0.02487	0.980214	-5.591	0.893714	0.882051
T.cells	LBHD1	-0.00802	0.16876	-0.02479	0.98028	-5.52817	0.903861	0.894486
T.cells	MS4A4A	-0.01051	0.617812	-0.02465	0.98039	-5.4448	0.894834	0.883403
T.cells	DPF3	0.007254	1.667384	0.024546	0.980472	-5.80418	0.87399	0.858014
T.cells	FCHSD1	0.008261	1.15994	0.024278	0.980686	-5.51819	0.884141	0.87021
T.cells	RNF5	0.002948	4.122516	0.02403	0.980883	-6.41226	0.82718	0.801437
T.cells	SAV1	-0.00239	4.735258	-0.02376	0.9811	-6.5341	0.815932	0.787954
T.cells	CDK5RAP2	0.002397	4.446752	0.023658	0.981178	-6.53577	0.821268	0.794312
T.cells	CIAO2B	-0.00253	4.566233	-0.02356	0.981257	-6.55269	0.819054	0.791678
T.cells	TMEM129	0.005055	2.084833	0.023474	0.981325	-6.00459	0.866183	0.848269
T.cells	ABI1	0.002041	7.702393	0.0233	0.981464	-7.03574	0.762925	0.725522
T.cells	TAT	0.0098	1.826535	0.023293	0.981469	-5.58121	0.871231	0.854421
T.cells	MORN3	0.009217	1.323497	0.023084	0.981635	-5.43792	0.881222	0.866476
T.cells	AGPAT2	0.004874	4.355109	0.022964	0.98173	-6.14794	0.823064	0.796402
T.cells	APOL7E	0.011799	-0.10248	0.022844	0.981827	-5.24696	0.909921	0.901489
T.cells	DGKG	0.009884	2.506906	0.022627	0.981999	-5.46737	0.858169	0.838462
T.cells	MFN2	0.002997	3.671067	0.022576	0.98204	-6.21694	0.835946	0.811743
T.cells	IL1R2	0.015672	3.320344	0.022347	0.982222	-5.42119	0.842616	0.81971
T.cells	GGCT	0.005792	3.249782	0.022343	0.982224	-5.90484	0.843957	0.821321
T.cells	PLEKHA1	0.003684	4.707996	0.022221	0.982322	-6.39471	0.816654	0.788657
T.cells	AK7	0.007393	1.686834	0.022005	0.982493	-5.71925	0.874264	0.857864
T.cells	SNRNP48	0.001905	4.916287	0.021955	0.982533	-6.6784	0.812873	0.784143
T.cells	LZTFL1	-0.00272	5.075487	-0.02174	0.982706	-6.58162	0.809973	0.780687
T.cells	DGCR8	-0.00235	3.779114	-0.02162	0.982803	-6.50079	0.834037	0.809374
T.cells	CC2D1B	-0.00268	3.659138	-0.02153	0.982873	-6.29389	0.836298	0.812081

T.cells	TUT7	-0.00156	7.206797	-0.02143	0.982955	-6.87622	0.771823	0.735712
T.cells	FASL	-0.01267	1.037842	-0.02139	0.982983	-5.25762	0.88713	0.873489
T.cells	CAPN1	-0.00435	4.246316	-0.02137	0.982999	-6.05461	0.825289	0.798917
T.cells	HYKK	0.008166	0.618386	0.021212	0.983124	-5.47571	0.895571	0.883746
T.cells	FPGS	-0.0036	3.013479	-0.02102	0.983274	-6.09571	0.848629	0.826849
T.cells	COQ7	0.002197	4.79596	0.020979	0.98331	-6.69958	0.815172	0.786846
T.cells	MAPK8IP3	-0.00212	4.521476	-0.0209	0.983373	-6.48209	0.820242	0.79291
T.cells	DTNBP1	-0.00126	6.683213	-0.02078	0.983466	-6.9084	0.781113	0.746619
T.cells	BCL2L2	0.006433	1.276497	0.020452	0.983729	-5.52181	0.882484	0.867908
T.cells	2310057M	0.00297	2.710086	0.020378	0.983787	-6.08098	0.854476	0.833996
T.cells	ZFP948	-0.00523	2.965976	-0.02031	0.983846	-5.94144	0.849564	0.828089
T.cells	SIGLECH	-0.01506	0.771994	-0.02027	0.983874	-5.18997	0.89254	0.880169
T.cells	MED9	-0.00255	3.526994	-0.02011	0.983999	-6.41057	0.838887	0.815302
T.cells	RWDD2B	-0.00492	1.849393	-0.02009	0.984013	-5.87173	0.871191	0.854253
T.cells	MALAT1	0.001515	14.72453	0.019905	0.984164	-7.98577	0.650557	0.597265
T.cells	TAF7	0.001642	5.1473	0.01984	0.984215	-6.67471	0.808749	0.779395
T.cells	CAR12	0.007157	0.013745	0.0197	0.984327	-5.52441	0.907853	0.899023
T.cells	ZFP445	-0.00177	4.93802	-0.01964	0.984378	-6.60193	0.812584	0.783976
T.cells	MMRN1	-0.01033	-0.22843	-0.01955	0.984445	-5.6753	0.912794	0.905103
T.cells	TMEM198B	0.007277	0.786307	0.019387	0.984576	-5.47978	0.892253	0.880009
T.cells	SLC4A1AP	-0.00201	4.278134	-0.01936	0.9846	-6.46481	0.824787	0.798569
T.cells	CEP97	0.002636	2.922474	0.019198	0.984726	-6.33439	0.850397	0.829319
T.cells	DCTN3	0.001523	5.963361	0.019083	0.984818	-6.79529	0.793957	0.762045
T.cells	HDC	0.01178	3.642871	0.019015	0.984872	-5.7397	0.836697	0.812896
T.cells	MRPL22	-0.00238	3.858759	-0.01895	0.984923	-6.42428	0.832632	0.808026
T.cells	ADSL	-0.00208	4.033205	-0.0189	0.984962	-6.46655	0.82936	0.804113
T.cells	TNFAIP2	-0.00939	4.351498	-0.01888	0.984978	-5.63611	0.823422	0.79702
T.cells	ZBTB12	-0.00371	1.665552	-0.0188	0.985045	-5.95262	0.8748	0.858857
T.cells	PDXP	0.004766	1.557173	0.018742	0.985089	-5.93942	0.876934	0.861448
T.cells	SDCBP	0.001476	7.674968	0.018713	0.985112	-6.97244	0.763758	0.726587
T.cells	DENND5A	-0.00163	6.395889	-0.01852	0.985265	-6.77633	0.786221	0.752957
T.cells	TNFRSF25	-0.00863	-1.26707	-0.01843	0.985335	-5.18602	0.934266	0.93177
T.cells	IFI207	-0.00871	4.721848	-0.01833	0.985414	-5.75673	0.816563	0.788918
T.cells	A530041M	0.003716	2.069945	0.018272	0.985463	-5.90567	0.866879	0.849345
T.cells	MBD4	-0.00244	3.212172	-0.01827	0.985465	-6.35256	0.844863	0.822785
T.cells	PDE7A	-0.0016	6.703412	-0.01793	0.985735	-6.9531	0.780914	0.746636
T.cells	ERCC1	0.002332	3.315868	0.017525	0.986057	-6.28213	0.843053	0.820585
T.cells	DEDD	-0.00175	4.437275	-0.01744	0.986126	-6.50913	0.821987	0.79539
T.cells	GM47448	0.01162	-0.89373	0.017409	0.986149	-5.17785	0.926674	0.922416
T.cells	PIP4P2	-0.00294	3.706325	-0.01735	0.986193	-6.19919	0.835661	0.81174
T.cells	SUPT4A	-0.00134	6.836136	-0.01731	0.986227	-6.99145	0.77857	0.744007
T.cells	DCLRE1A	-0.00576	1.621612	-0.01713	0.98637	-5.54704	0.875834	0.860204
T.cells	TMEM128	0.001521	5.718829	0.017096	0.986399	-6.78799	0.798516	0.767517
T.cells	CENPO	0.002528	2.773597	0.017019	0.98646	-6.19569	0.853419	0.833085
T.cells	TMEM263	0.001829	3.98713	0.017014	0.986464	-6.55718	0.830383	0.805423
T.cells	TEN1	-0.00128	5.852604	-0.01701	0.986468	-6.79952	0.796103	0.764664
T.cells	FRG1	0.001044	6.660706	0.016913	0.986544	-7.01871	0.78167	0.747662

T.cells	SMNDC1	0.001105	6.059801	0.016827	0.986612	-6.83182	0.792379	0.7603
T.cells	IL20RB	0.002545	3.81612	0.016576	0.986812	-6.27014	0.8337	0.809389
T.cells	PGM3	0.002462	2.89728	0.015935	0.987322	-6.03225	0.851484	0.83048
T.cells	2610318N(	0.003997	1.172871	0.015909	0.987343	-5.97726	0.885168	0.871267
T.cells	CSRP1	0.001484	5.881401	0.015547	0.987631	-6.87039	0.796101	0.76429
T.cells	RBM25	-0.00078	8.296585	-0.01542	0.987734	-7.20712	0.753696	0.714622
T.cells	PTPRCAP	0.001414	5.807512	0.015332	0.987801	-6.92107	0.797433	0.765895
T.cells	EME2	0.00444	0.924802	0.01531	0.987819	-5.55229	0.890232	0.877333
T.cells	PSRC1	-0.00373	0.886949	-0.0151	0.987984	-5.89586	0.890989	0.878256
T.cells	MATN2	0.006413	0.980441	0.015045	0.98803	-5.42728	0.889121	0.875978
T.cells	GBP2B	0.021215	0.300434	0.015016	0.988054	-5.24663	0.902795	0.892678
T.cells	MYO9A	-0.00201	6.336763	-0.01491	0.988136	-6.72676	0.787937	0.754693
T.cells	ZFP655	0.001739	4.453763	0.014898	0.988147	-6.41418	0.822214	0.795311
T.cells	MTMR4	0.002537	2.648419	0.014817	0.988211	-6.0408	0.856384	0.836287
T.cells	SRP54C	0.004162	1.755516	0.014721	0.988288	-5.71033	0.87377	0.857312
T.cells	TET1	0.004281	1.135429	0.014531	0.988439	-5.79504	0.886081	0.872214
T.cells	CDNF	0.006192	0.317972	0.014411	0.988535	-5.43035	0.902491	0.892243
T.cells	SLC39A6	-0.00165	4.11285	-0.01428	0.988641	-6.41506	0.828615	0.802894
T.cells	SPSB4	-0.00473	0.417547	-0.01415	0.988742	-5.6757	0.900477	0.889793
T.cells	BNIP1	-0.00223	3.526643	-0.01412	0.988764	-6.24354	0.839647	0.816113
T.cells	POLN	-0.00238	3.191009	-0.01409	0.988793	-6.39865	0.846026	0.823771
T.cells	IL4RA	-0.00323	4.883706	-0.01401	0.988857	-6.01181	0.814313	0.785861
T.cells	MRPS21	0.000831	7.034242	0.013739	0.989069	-7.01024	0.7757	0.740254
T.cells	CAPRIN2	0.002932	3.181523	0.01364	0.989148	-6.14084	0.846284	0.824059
T.cells	SLAIN2	0.001066	5.670672	0.013433	0.989313	-6.7141	0.800024	0.768934
T.cells	FBXL15	0.002529	2.696607	0.013236	0.989469	-5.9979	0.855581	0.835297
T.cells	F10	0.005226	4.54109	0.013184	0.989511	-6.09446	0.820715	0.793503
T.cells	CAPRIN1	-0.00073	7.460311	-0.01308	0.989597	-7.07539	0.768244	0.731567
T.cells	OLFM4	0.008417	-0.01496	0.013074	0.989598	-5.28765	0.909336	0.900666
T.cells	FAM53C	0.001683	3.981286	0.013045	0.989621	-6.29601	0.831154	0.805965
T.cells	DTD2	0.002133	3.491192	0.012984	0.98967	-6.2369	0.840395	0.817033
T.cells	CD163	0.01042	1.13156	0.012966	0.989684	-5.28336	0.886238	0.872443
T.cells	UBALD2	0.001022	7.028455	0.012527	0.990033	-7.06675	0.776008	0.740525
T.cells	KIF22	0.001922	4.043139	0.012392	0.990141	-6.6675	0.830215	0.804731
T.cells	APOBEC4	-0.00419	0.042895	-0.01226	0.990245	-5.59559	0.908399	0.899402
T.cells	S100A6	0.006208	7.580063	0.012088	0.990382	-6.17067	0.766364	0.729279
T.cells	CINP	0.002086	3.163035	0.012079	0.99039	-6.25689	0.846861	0.824694
T.cells	VHL	0.001701	3.486608	0.01188	0.990548	-6.10184	0.840705	0.817302
T.cells	SLC12A8	-0.00545	0.657727	-0.01174	0.990659	-5.36938	0.895956	0.88418
T.cells	GM48512	0.003893	1.068935	0.011602	0.99077	-5.55733	0.887721	0.874139
T.cells	GM9856	0.003279	1.246414	0.011589	0.99078	-5.68168	0.884189	0.869839
T.cells	SEC24B	-0.00106	6.179882	-0.01156	0.990803	-6.78674	0.791067	0.758266
T.cells	DCTN6	0.00113	5.142412	0.011495	0.990854	-6.55679	0.809853	0.780488
T.cells	CBX7	-0.00186	2.651462	-0.01147	0.990878	-6.04359	0.856679	0.836515
T.cells	1810058I2	0.001309	7.005863	0.011342	0.990976	-6.83915	0.776405	0.741038
T.cells	PLPP2	-0.00258	1.875681	-0.01125	0.991048	-5.84471	0.871769	0.854783
T.cells	PLEKHA5	-0.00127	4.693302	-0.01125	0.991053	-6.60929	0.818115	0.790331

T.cells	NXPE2	0.005177	1.56591	0.01106	0.9912	-5.40212	0.877872	0.862187
T.cells	TCRG-C1	0.00724	0.880703	0.011047	0.991211	-5.33107	0.891492	0.878761
T.cells	PCID2	-0.00093	4.736836	-0.01086	0.99136	-6.62018	0.817381	0.789423
T.cells	2010013B2	-0.00213	3.545259	-0.01063	0.991546	-6.09524	0.839716	0.816134
T.cells	PUS1	0.001408	3.723827	0.010601	0.991566	-6.33612	0.836341	0.812088
T.cells	TAZ	0.0012	4.057371	0.01049	0.991654	-6.33552	0.830082	0.804585
T.cells	DIS3L2	0.00085	5.906117	0.010289	0.991814	-6.78047	0.796133	0.764251
T.cells	ADH5	-0.00095	6.03865	-0.01027	0.991828	-6.8546	0.793748	0.761436
T.cells	IRF2BPL	0.00167	4.091669	0.010022	0.992026	-6.35711	0.829534	0.803891
T.cells	METTL15	-0.00141	3.231154	-0.00998	0.992062	-6.27116	0.845793	0.823375
T.cells	CCDC25	0.000863	4.712121	0.009544	0.992407	-6.65398	0.818085	0.790204
T.cells	GM32036	0.001137	3.651119	0.009478	0.992459	-6.30888	0.837917	0.813911
T.cells	CHIL3	-0.00951	2.447415	-0.00943	0.992501	-5.483	0.860958	0.841649
T.cells	RIMS4	-0.00588	-0.99611	-0.00937	0.992541	-5.17678	0.930147	0.926115
T.cells	HAUS1	0.00119	3.65784	0.00937	0.992545	-6.46364	0.83779	0.813784
T.cells	CDKN2AIPI	0.000938	4.272414	0.009267	0.992627	-6.52054	0.82625	0.799985
T.cells	SIGLEC1	0.006189	0.449661	0.008998	0.992841	-5.24805	0.900498	0.889747
T.cells	BCL7C	0.000686	5.355918	0.008924	0.9929	-6.72538	0.806266	0.776257
T.cells	SLC25A17	-0.00072	5.308731	-0.00892	0.992903	-6.75368	0.807127	0.777278
T.cells	PLEKHA4	0.002937	2.406918	0.008902	0.992917	-5.6895	0.861743	0.842652
T.cells	CASP12	0.004822	-0.47839	0.008766	0.993025	-5.21228	0.919429	0.912985
T.cells	PPP1R16A	0.001371	2.885752	0.008608	0.993152	-6.15834	0.8525	0.831577
T.cells	GM32031	0.001914	2.845461	0.008506	0.993232	-5.90894	0.853274	0.832547
T.cells	IGFBP3	-0.00459	0.690335	-0.00849	0.993248	-5.33759	0.895648	0.883953
T.cells	FAM133B	0.000708	4.911343	0.008444	0.993282	-6.65159	0.81441	0.786046
T.cells	ESYT1	-0.00072	5.654914	-0.00827	0.993423	-6.82388	0.800858	0.769951
T.cells	EMC6	-0.00065	5.61708	-0.00822	0.993462	-6.77626	0.801544	0.770762
T.cells	DENND1A	-0.00057	7.497877	-0.008	0.993635	-7.05274	0.768154	0.731466
T.cells	NANOS3	-0.00246	0.427569	-0.00797	0.993658	-5.60482	0.901018	0.890478
T.cells	CNPY2	0.00092	4.719728	0.007691	0.993881	-6.50391	0.818018	0.790339
T.cells	NELFB	-0.00071	4.551192	-0.0076	0.993951	-6.55497	0.821139	0.794059
T.cells	ACYP2	0.001291	3.398712	0.007511	0.994024	-6.17028	0.842777	0.819954
T.cells	DDX47	0.000528	5.550552	0.007509	0.994026	-6.77288	0.802798	0.772282
T.cells	GM37529	0.002637	1.962925	0.007468	0.994058	-5.66574	0.870476	0.853364
T.cells	SMAD7	-0.00092	5.164939	-0.0074	0.994109	-6.55342	0.809829	0.780633
T.cells	BACH2IT1	-0.0023	-0.26225	-0.0072	0.994271	-5.52096	0.915152	0.907832
T.cells	SLC38A1	-0.00066	7.937408	-0.00708	0.99437	-7.07077	0.760628	0.722689
T.cells	PDCD2L	0.000609	4.660225	0.006835	0.994562	-6.51569	0.819258	0.791768
T.cells	HP	-0.00132	7.365035	-0.00679	0.994599	-6.81944	0.77061	0.734336
T.cells	SH3BGRL2	0.002329	2.700678	0.006727	0.994648	-5.76805	0.856285	0.836144
T.cells	PTPRF	0.00249	1.276101	0.006508	0.994822	-5.39368	0.884263	0.869989
T.cells	RIN3	-0.00062	5.640444	-0.00639	0.994913	-6.69407	0.801398	0.770551
T.cells	ARHGDIB	-0.00055	8.995183	-0.00593	0.995282	-7.27271	0.742734	0.701905
T.cells	MRPS12	-0.00058	4.774775	-0.00592	0.995288	-6.61757	0.817262	0.789401
T.cells	OAS2	-0.00344	1.571395	-0.00588	0.995323	-5.38123	0.878454	0.862985
T.cells	GADD45G	0.00113	4.465474	0.005599	0.995545	-6.32127	0.822992	0.796273
T.cells	PIK3R1	-0.0006	7.694648	-0.00552	0.995611	-6.9929	0.764985	0.727813

T.cells	GM43466	-0.00176	1.497294	-0.00545	0.995665	-5.6211	0.879919	0.864813
T.cells	FGFBP3	-0.00313	0.028033	-0.00544	0.995671	-5.30817	0.909426	0.900833
T.cells	FANCG	-0.00102	2.222876	-0.00523	0.995837	-5.98441	0.865675	0.847548
T.cells	ENTPD6	0.001014	2.776877	0.005001	0.996021	-5.86689	0.854943	0.834653
T.cells	UMPS	0.000499	4.099845	0.004949	0.996062	-6.54824	0.829814	0.804485
T.cells	TOR1AIP2	-0.00035	6.580878	-0.00488	0.996115	-6.86276	0.784542	0.750808
T.cells	GM44699	0.001542	0.559613	0.004778	0.996198	-5.58095	0.898647	0.887728
T.cells	GM49482	-0.00257	-0.71551	-0.00473	0.99624	-5.24416	0.924702	0.919701
T.cells	CCDC97	-0.00063	3.33761	-0.00464	0.99631	-6.22931	0.844207	0.821748
T.cells	GAS7	0.000616	6.417925	0.004544	0.996385	-6.9204	0.787442	0.754222
T.cells	SLC5A6	-0.00184	0.868812	-0.00448	0.996439	-5.44542	0.892431	0.880137
T.cells	MAD2L1BP	-0.00045	4.194819	-0.00441	0.996489	-6.55699	0.828037	0.802376
T.cells	SLC2A12	0.001166	1.619007	0.004333	0.996553	-5.78251	0.877514	0.86198
T.cells	RXRB	0.000478	4.393184	0.004327	0.996558	-6.4986	0.824337	0.797958
T.cells	GM46620	0.001537	0.683678	0.004277	0.996597	-5.63105	0.896148	0.884675
T.cells	MAP2K6	0.000739	3.533157	0.004105	0.996734	-6.22569	0.840493	0.817289
T.cells	HDAC5	0.000481	4.378269	0.004083	0.996752	-6.54214	0.824614	0.798289
T.cells	MBLAC1	-0.00158	-0.19049	-0.00406	0.99677	-5.40334	0.913891	0.906405
T.cells	ZFP729A	0.000673	3.051831	0.003953	0.996855	-6.15079	0.849663	0.828315
T.cells	PPM1L	0.000545	4.617569	0.003904	0.996894	-6.51476	0.820169	0.793007
T.cells	ACD	-0.00034	4.940696	-0.00375	0.997013	-6.66611	0.814203	0.785953
T.cells	PELO	-0.00056	3.125716	-0.00367	0.997077	-6.15984	0.848249	0.826708
T.cells	FYN	0.000594	7.843798	0.003673	0.997078	-6.88221	0.762401	0.72496
T.cells	CD3D	-0.00154	2.678526	-0.0035	0.997218	-5.57819	0.856839	0.83706
T.cells	DHX30	0.000361	4.509922	0.00343	0.997271	-6.54902	0.822166	0.795469
T.cells	PGRMC2	0.000368	3.628957	0.003356	0.99733	-6.31975	0.838679	0.815215
T.cells	SMIM15	-0.00031	4.858356	-0.00334	0.99734	-6.58049	0.81572	0.78779
T.cells	ABCA1	0.000818	5.836161	0.00316	0.997486	-6.25842	0.797881	0.766677
T.cells	FTH1	0.000494	12.5537	0.003096	0.997537	-7.65615	0.684973	0.636041
T.cells	GM48696	0.000592	2.476417	0.003012	0.997603	-6.06324	0.860748	0.841849
T.cells	GM45051	-0.00073	1.845863	-0.00284	0.997737	-5.73016	0.873049	0.856775
T.cells	NUDT1	-0.00049	3.00659	-0.00279	0.997777	-6.23411	0.85053	0.829558
T.cells	RBM14	0.00029	3.712533	0.002676	0.997871	-6.39765	0.837099	0.813467
T.cells	SH2B2	0.000518	4.550186	0.002661	0.997882	-6.11648	0.821419	0.794723
T.cells	ANKRD12	0.000226	7.518738	0.002646	0.997895	-7.02363	0.768043	0.731688
T.cells	NUDT6	0.000544	2.183509	0.00226	0.998202	-5.81919	0.866645	0.848947
T.cells	UXT	-0.00021	4.452478	-0.00205	0.998372	-6.57548	0.823506	0.797046
T.cells	NKIRAS2	-0.00019	4.042562	-0.00167	0.998674	-6.37988	0.831327	0.806271
T.cells	SDHB	-0.00013	7.274793	-0.00161	0.998717	-7.04798	0.77271	0.736898
T.cells	CHST3	0.000161	4.312271	0.001294	0.99897	-6.72446	0.826328	0.800278
T.cells	RAB30	0.000503	2.141089	0.001279	0.998982	-5.58825	0.867778	0.850112
T.cells	GM26901	0.00037	0.57091	0.001261	0.998997	-5.60315	0.898946	0.888032
T.cells	ADSS	6.86E-05	6.271707	0.000988	0.999214	-6.9146	0.79063	0.757841
T.cells	BRDT	0.000234	1.567286	0.000896	0.999287	-5.82238	0.879176	0.863839
T.cells	CCDC73	0.000117	3.100981	0.000729	0.99942	-6.09145	0.849392	0.827797
T.cells	GM16618	0.000119	0.733543	0.000401	0.999681	-5.64605	0.89597	0.884159
T.cells	GIMAP6	5.36E-05	5.672059	0.000376	0.999701	-6.75957	0.801587	0.770665

T.cells	2200002D(	3.65E-05	1.337585	0.000106	0.999915	-5.74122	0.884021	0.869499
