# Supplementary material for: Immune landscape in liver of neonatal mice with phlebotomy-induced anemia
Source: Pediatr Res. 2025 Sep 17;99(4):1602–12. doi: 10.1038/s41390-025-04361-x (PMC12659965; doi:10.1038/s41390-025-04361-x)
Supplement: Supplementary file 9 — Table S9 [file 41390_2025_4361_MOESM9_ESM.pdf]

immunecell	gene	logFC	AveExpr	t	P.Value	B	adj.P.Val. Within	adj.P.Val. Between
NK.cells	CYP2E1	-2.28151	4.291137	-8.2577	1.34E-12	18.09425	1.47E-08	1.87E-09
NK.cells	GM10076	1.002377	6.671496	8.129161	2.45E-12	17.72097	1.41E-08	3.02E-09
NK.cells	ENO1	0.861107	8.066335	7.997815	4.56E-12	17.01064	1.72E-08	5.01E-09
NK.cells	TPI1	0.902467	6.690385	7.412008	7.08E-11	14.43983	2.04E-07	5.80E-08
NK.cells	PGK1	0.813619	7.765914	6.854056	9.27E-10	11.81891	2.10E-06	5.45E-07
NK.cells	MT1	1.830149	6.314344	6.809303	1.14E-09	11.81361	2.20E-06	6.80E-07
NK.cells	BNIP3	1.38142	5.086642	6.743567	1.53E-09	11.54558	2.55E-06	9.03E-07
NK.cells	SLC16A3	1.192412	3.935418	6.716953	1.73E-09	11.42814	2.60E-06	1.05E-06
NK.cells	ALDOA	0.943897	8.900702	6.671881	2.12E-09	10.87054	2.63E-06	1.07E-06
NK.cells	FABP4	1.39065	6.966529	6.559852	3.52E-09	10.65327	4.04E-06	1.69E-06
NK.cells	EPHA2	-2.58898	2.408212	-6.06825	3.15E-08	6.605945	3.51E-05	1.23E-05
NK.cells	UBA52	0.538774	10.38927	5.894202	6.73E-08	7.263316	5.70E-05	1.85E-05
NK.cells	BLNK	-1.08423	7.200732	-5.89206	6.79E-08	7.877664	5.97E-05	2.03E-05
NK.cells	MT2	2.469259	2.001416	5.8041	9.94E-08	7.069261	8.78E-05	3.32E-05
NK.cells	SERPINA6	-3.51713	1.127419	-5.72865	1.38E-07	5.775934	0.000115	4.51E-05
NK.cells	LECT2	-1.80517	3.632928	-5.66178	1.83E-07	6.962025	0.000138	5.36E-05
NK.cells	PKM	0.64319	8.60779	5.515675	3.41E-07	5.946969	0.000225	7.67E-05
NK.cells	P4HA1	0.85876	5.786072	5.473517	4.08E-07	6.116648	0.000264	9.52E-05
NK.cells	KNG1	-1.08252	6.039457	-5.24954	1.04E-06	5.199147	0.000636	0.00021
NK.cells	PROC	-1.55225	2.923674	-5.14296	1.61E-06	4.866719	0.000981	0.000319
NK.cells	MIF	0.713054	7.650894	5.113745	1.81E-06	4.457441	0.000981	0.000308
NK.cells	HBB-BT	1.925049	8.105747	5.066805	2.20E-06	4.234863	0.001126	0.000358
NK.cells	GPI1	0.430832	7.844302	4.912417	4.10E-06	3.628501	0.002017	0.000586
NK.cells	LILRB4A	0.910541	4.163584	4.884671	4.58E-06	3.911384	0.002282	0.000709
NK.cells	GAPDH	0.553586	10.59184	4.826633	5.77E-06	2.93349	0.002508	0.000705
NK.cells	SERPINF2	-1.35115	3.929119	-4.78557	6.79E-06	3.634069	0.003092	0.000957
NK.cells	CAR3	-2.3885	3.476221	-4.77951	6.95E-06	3.596812	0.003113	0.000987
NK.cells	PGAM1	0.556466	6.892134	4.754391	7.67E-06	3.197227	0.003148	0.00096
NK.cells	FABP2	-1.40006	3.257265	-4.74211	8.05E-06	3.463146	0.003348	0.001105
NK.cells	CYP1A2	-2.05434	1.633956	-4.73511	8.28E-06	2.952338	0.003431	0.00118
NK.cells	MBL2	-1.35891	4.246085	-4.72484	8.62E-06	3.383489	0.003324	0.001129
NK.cells	AKR1C6	-1.48082	4.841164	-4.71638	8.91E-06	3.305196	0.003299	0.001136
NK.cells	LDHA	0.493218	8.792149	4.690685	9.85E-06	2.671421	0.003334	0.001102
NK.cells	GSTA3	-1.55563	4.0016	-4.60185	1.39E-05	2.962719	0.004913	0.001652
NK.cells	H2-Q10	-1.04072	4.038149	-4.5538	1.68E-05	2.788018	0.005742	0.001918
NK.cells	SERPINA3K	2.272388	1.878609	4.535714	1.80E-05	2.662713	0.006167	0.002123
NK.cells	PON1	-1.19855	4.304512	-4.5292	1.84E-05	2.667429	0.005946	0.002014
NK.cells	2010007H(	-2.17465	0.459338	-4.51985	1.91E-05	1.517284	0.006359	0.002297
NK.cells	HSD17B2	-1.3732	2.446494	-4.46466	2.36E-05	2.406615	0.007425	0.002596
NK.cells	HBB-BS	1.742586	11.91128	4.421135	2.78E-05	1.335434	0.007412	0.002261
NK.cells	RASGRP4	1.804612	1.734055	4.392473	3.10E-05	1.834417	0.009386	0.003288
NK.cells	GFRA1	-1.17552	4.918403	-4.37046	3.37E-05	2.141685	0.009489	0.003218
NK.cells	SLC49A4	0.789352	6.30055	4.360387	3.50E-05	1.924129	0.009429	0.003184
NK.cells	CD79A	-0.77429	6.200185	-4.34021	3.77E-05	1.859348	0.009954	0.003367

NK.cells	VTN	-1.14469	4.267725	-4.33312	3.88E-05	1.986842	0.010289	0.003608
NK.cells	GYPA	1.94843	3.611578	4.315523	4.14E-05	1.973279	0.010806	0.003838
NK.cells	APOA1	-1.15244	8.277086	-4.3095	4.23E-05	1.354173	0.010076	0.003422
NK.cells	RARRES2	-1.28873	3.7462	-4.30539	4.30E-05	1.939713	0.010784	0.003918
NK.cells	SDHAF1	0.625283	4.132974	4.296043	4.45E-05	1.819625	0.010875	0.003972
NK.cells	HPS4	-0.60971	4.16681	-4.28087	4.71E-05	1.72044	0.011271	0.004158
NK.cells	IL1R2	2.207097	3.320344	4.26759	4.95E-05	1.725746	0.011758	0.004431
NK.cells	HSPBAP1	0.894523	3.764205	4.250701	5.27E-05	1.758383	0.012196	0.00459
NK.cells	SLC17A2	-1.93109	1.156237	-4.20421	6.25E-05	1.201134	0.013979	0.005577
NK.cells	CFI	-1.23595	3.743943	-4.20308	6.28E-05	1.582686	0.013444	0.005199
NK.cells	HBA-A1	1.692012	10.46682	4.196692	6.43E-05	0.70364	0.012156	0.004372
NK.cells	GM11808	-0.44174	7.262783	-4.19464	6.48E-05	1.052451	0.012752	0.004811
NK.cells	UGT3A2	-1.74666	1.174889	-4.19367	6.50E-05	1.255106	0.013975	0.005709
NK.cells	CORO1B	0.613626	4.252422	4.192876	6.52E-05	1.458156	0.013341	0.005246
NK.cells	ITIH2	-1.35769	3.79432	-4.19014	6.59E-05	1.538646	0.013434	0.005342
NK.cells	STAT5B	0.396561	6.043742	4.181672	6.79E-05	1.137129	0.013124	0.005153
NK.cells	SERPINC1	-0.92263	5.285455	-4.1782	6.88E-05	1.306253	0.013274	0.005315
NK.cells	HBA-A2	1.668928	9.98555	4.163699	7.26E-05	0.644621	0.012404	0.00483
NK.cells	VAMP4	0.378366	6.154005	4.161972	7.30E-05	1.112993	0.013136	0.005393
NK.cells	CBFA2T3	-0.56941	5.507245	-4.16108	7.33E-05	1.205945	0.013264	0.005504
NK.cells	SORD	-0.94799	4.176195	-4.16016	7.35E-05	1.444343	0.013532	0.005726
NK.cells	SLC9A8	0.507033	4.988676	4.141326	7.88E-05	1.225778	0.014046	0.005874
NK.cells	IGSF9	-2.18728	0.790152	-4.13831	7.96E-05	0.39901	0.014963	0.006635
NK.cells	TSTD1	-1.4124	2.589608	-4.12652	8.31E-05	1.312628	0.014678	0.006498
NK.cells	CECR2	-0.92895	6.605628	-4.11796	8.57E-05	1.059094	0.013818	0.005939
NK.cells	HSD17B6	-2.12329	0.767255	-4.11782	8.58E-05	0.869112	0.015087	0.006987
NK.cells	RAB43	-0.44692	6.948549	-4.1177	8.58E-05	0.824115	0.013747	0.005883
NK.cells	ROGDI	-0.97354	4.014028	-4.11638	8.62E-05	1.318077	0.014367	0.006392
NK.cells	TIMD2	-1.86727	0.986598	-4.11036	8.81E-05	0.828681	0.015159	0.007065
NK.cells	GM40645	2.132098	0.751306	4.101142	9.11E-05	0.724689	0.015518	0.007299
NK.cells	TTR	-1.13285	9.202897	-4.08368	9.71E-05	0.430587	0.014367	0.006045
NK.cells	ENPEP	-2.441	0.401804	-4.07305	0.000101	0.284971	0.016817	0.007933
NK.cells	CFH	-0.90045	4.930751	-4.06446	0.000104	1.038251	0.015992	0.007132
NK.cells	GM13205	-2.08604	0.234435	-4.03427	0.000116	0.28776	0.01889	0.008794
NK.cells	SNCA	1.651178	4.248926	3.994761	0.000134	0.907074	0.020225	0.008668
NK.cells	PRELID1	0.336043	7.529242	3.98491	0.000138	0.305379	0.019645	0.008086
NK.cells	TMEM205	-0.96953	3.71868	-3.98212	0.00014	0.88639	0.020801	0.009054
NK.cells	ANGPTL8	-1.63715	1.825356	-3.97696	0.000142	0.775423	0.021534	0.0097
NK.cells	REXO2	0.416545	6.270041	3.971532	0.000145	0.443168	0.020287	0.00871
NK.cells	ALAS2	1.496334	4.928547	3.95991	0.000151	0.685623	0.021316	0.009324
NK.cells	CYP2D10	-1.49459	1.738911	-3.94709	0.000158	0.657179	0.023131	0.010587
NK.cells	CES1G	-2.09006	0.85155	-3.93489	0.000165	0.378726	0.024194	0.011226
NK.cells	AKAP12	-0.85682	6.169014	-3.92292	0.000173	0.439473	0.023031	0.009977
NK.cells	APOF	-0.98685	3.847555	-3.91102	0.00018	0.611037	0.024316	0.01098
NK.cells	AHSG	-0.9987	9.130873	-3.91098	0.00018	-0.1536	0.022465	0.009478
NK.cells	MZB1	-0.93021	5.956787	-3.8982	0.000188	0.49806	0.024369	0.010705
NK.cells	WDFY4	-0.77398	7.251372	-3.89358	0.000191	0.386161	0.024024	0.010463

NK.cells	GULO	-1.41236	1.982596	-3.88262	0.000199	0.509603	0.026732	0.012451
NK.cells	CD79B	-0.65502	6.976941	-3.87704	0.000203	0.179984	0.025017	0.01099
NK.cells	FCRLA	-0.99851	5.107141	-3.85934	0.000216	0.469021	0.026859	0.012122
NK.cells	LILR4B	0.999179	3.079256	3.85869	0.000216	0.402994	0.027691	0.012838
NK.cells	CYP2C37	-1.35086	1.772648	-3.85048	0.000223	0.390911	0.028514	0.013512
NK.cells	HRG	-1.15266	3.61876	-3.84999	0.000223	0.437461	0.027732	0.01285
NK.cells	GSTO1	0.428764	4.594828	3.846309	0.000226	0.281931	0.027398	0.012658
NK.cells	UBR4	-0.3107	6.056739	-3.83935	0.000231	0.059011	0.027185	0.012396
NK.cells	CES1C	-1.18145	4.023969	-3.83628	0.000234	0.350052	0.028046	0.013221
NK.cells	PTGR1	-1.06998	3.377249	-3.82246	0.000245	0.39712	0.029422	0.013921
NK.cells	C1RA	-1.53359	1.359965	-3.81763	0.00025	0.106918	0.030542	0.014896
NK.cells	APOE	-0.94035	9.241961	-3.80918	0.000257	-0.42654	0.027669	0.01225
NK.cells	GIGYF2	0.40221	5.464029	3.795293	0.00027	-0.01122	0.030428	0.014058
NK.cells	SLC27A2	-1.05713	3.949769	-3.78927	0.000275	0.20862	0.031481	0.014863
NK.cells	PFKL	0.46362	5.330071	3.778891	0.000285	-0.04642	0.031625	0.014678
NK.cells	GSTP1	0.436567	6.928594	3.776489	0.000288	-0.29705	0.030876	0.014113
NK.cells	APOH	-0.95532	4.983199	-3.77056	0.000294	-0.00884	0.032147	0.015161
NK.cells	CYP3A11	-1.27711	3.352158	-3.76446	0.0003	0.204507	0.033337	0.016085
NK.cells	SLC38A4	-1.18158	3.153421	-3.7589	0.000306	0.191576	0.033774	0.016418
NK.cells	VPREB3	-0.93016	6.013944	-3.74591	0.00032	-0.12014	0.033525	0.015628
NK.cells	NR4A2	-0.55193	6.380914	-3.74284	0.000323	-0.60832	0.033393	0.015532
NK.cells	SLFN1	1.078893	2.335521	3.73952	0.000327	-0.03074	0.035577	0.01749
NK.cells	SERPINA1C	-1.00402	7.348457	-3.69613	0.000379	-0.61366	0.037936	0.016745
NK.cells	C8G	-1.13186	3.782028	-3.68391	0.000395	-0.09734	0.041198	0.019014
NK.cells	CXCL12	-1.06702	2.838235	-3.68251	0.000397	-0.02204	0.041788	0.019549
NK.cells	OSER1	-0.36924	6.59749	-3.66933	0.000415	-0.5311	0.040616	0.018153
NK.cells	CD59B	-2.38177	0.413	-3.66921	0.000415	-0.61823	0.044578	0.02156
NK.cells	ADTRP	-1.45971	1.835711	-3.65857	0.000431	-0.1988	0.044498	0.021261
NK.cells	IL7R	0.723523	4.966863	3.658436	0.000431	-0.38657	0.042449	0.019487
NK.cells	TAF7	-0.3734	5.1473	-3.65263	0.000439	-0.40458	0.042816	0.019622
NK.cells	FAM162A	0.549649	5.640039	3.642394	0.000455	-0.51519	0.043636	0.019817
NK.cells	ERO1L	0.509519	5.171152	3.631942	0.000471	-0.53396	0.044302	0.020499
NK.cells	SULT2A5	-1.57269	2.02078	-3.63138	0.000472	-0.2224	0.046453	0.022392
NK.cells	UGT2B34	-1.4239	1.748323	-3.63047	0.000473	-0.23542	0.046644	0.02259
NK.cells	ALDH2	-0.51874	6.861181	-3.63043	0.000474	-0.49679	0.043193	0.019594
NK.cells	RHAG	2.258093	-0.3642	3.599846	0.000525	-0.65602	0.052946	0.02568
NK.cells	IL6RA	0.536222	5.504339	3.575223	0.00057	-0.68068	0.052212	0.023046
NK.cells	KCNQ5	0.419692	7.024096	3.567624	0.000584	-1.04501	0.051939	0.022452
NK.cells	CAPG	0.660576	5.264343	3.562871	0.000594	-0.67415	0.053645	0.023807
NK.cells	FADS2	-1.12532	3.530927	-3.56122	0.000597	-0.38569	0.055062	0.025074
NK.cells	GC	-0.95096	5.287073	-3.54812	0.000624	-0.76701	0.055589	0.024566
NK.cells	MIDN	-0.34585	6.064607	-3.54529	0.000629	-0.89988	0.055046	0.024156
NK.cells	RPP38	1.541021	1.226405	3.540477	0.00064	-0.59413	0.059516	0.02799
NK.cells	ZFP738	0.655817	2.843416	3.536885	0.000647	-0.46569	0.058082	0.026976
NK.cells	MGST1	-0.61633	7.132587	-3.53593	0.000649	-1.02371	0.054458	0.023986
NK.cells	FFAR2	1.963048	-0.53401	3.534766	0.000652	-0.77918	0.061119	0.029729
NK.cells	ZC3HAV1L	-0.58993	3.053639	-3.52437	0.000675	-0.65262	0.059383	0.027513

NK.cells	GM4129	-1.37727	1.28611	-3.52273	0.000678	-0.62242	0.060987	0.028943
NK.cells	ITGB3	0.657203	2.991713	3.519011	0.000687	-0.59168	0.059744	0.027825
NK.cells	CRLF3	0.395687	6.899396	3.514572	0.000697	-1.13713	0.056764	0.02517
NK.cells	UFSP2	0.452815	4.93604	3.49005	0.000755	-0.83746	0.06293	0.028111
NK.cells	RNASE4	-0.71315	5.17058	-3.48613	0.000765	-0.92216	0.062942	0.028127
NK.cells	NR4A3	-0.49727	6.012984	-3.48406	0.00077	-1.28449	0.062151	0.02757
NK.cells	ITGB2L	-1.52869	-0.61932	-3.48189	0.000776	-0.93676	0.068681	0.033353
NK.cells	CCDC80	0.959165	2.683481	3.480457	0.00078	-0.63306	0.065342	0.030527
NK.cells	SGCZ	-1.38891	1.627249	-3.44937	0.000863	-0.74323	0.072447	0.03364
NK.cells	AI182371	-1.20633	2.439191	-3.44701	0.00087	-0.69818	0.071565	0.033034
NK.cells	DSCAM	1.235879	0.029718	3.446359	0.000872	-0.70064	0.074215	0.035374
NK.cells	RELN	-1.1827	3.647841	-3.44548	0.000874	-0.72631	0.070274	0.032045
NK.cells	NOD2	1.587942	1.576786	3.429042	0.000922	-0.87372	0.075979	0.035157
NK.cells	SLAMF6	0.571629	4.602908	3.403939	0.001	-1.06466	0.077931	0.034067
NK.cells	RGN	-0.94515	4.650255	-3.40309	0.001003	-1.08225	0.077875	0.034073
NK.cells	MED14	-0.3604	6.321429	-3.39958	0.001014	-1.47109	0.076313	0.032781
NK.cells	SPOPL	0.425047	4.42179	3.391392	0.001042	-1.09997	0.080104	0.035113
NK.cells	GAMT	-0.90617	4.228598	-3.383	0.00107	-1.05877	0.08201	0.035961
NK.cells	GM19951	0.995905	3.23896	3.364728	0.001135	-1.07366	0.087282	0.03837
NK.cells	FGA	-0.65099	5.727993	-3.36429	0.001136	-1.39325	0.084076	0.035832
NK.cells	FADS1	-0.85171	3.059286	-3.35295	0.001178	-0.96142	0.090186	0.039537
NK.cells	MDFIC	0.612678	4.328783	3.34918	0.001193	-1.25847	0.088994	0.038513
NK.cells	MGARP	2.054301	-0.49619	3.34591	0.001205	-1.35373	0.096115	0.044272
NK.cells	DOK3	-0.80669	5.36627	-3.34228	0.001219	-1.07316	0.088465	0.037897
NK.cells	APOB	-0.88139	4.836413	-3.33347	0.001254	-1.33812	0.090616	0.039131
NK.cells	APOC4	-0.84434	5.632488	-3.33163	0.001262	-1.4725	0.089539	0.03839
NK.cells	GM2A	0.390621	7.22888	3.3315	0.001262	-1.61234	0.087421	0.036721
NK.cells	PDIK1L	0.672753	3.065249	3.326892	0.001281	-1.064	0.093324	0.041529
NK.cells	CCND3	0.402054	7.994145	3.32582	0.001285	-1.8654	0.086668	0.036266
NK.cells	PZP	-0.8801	4.39586	-3.32457	0.00129	-1.27594	0.091474	0.040218
NK.cells	GYS2	-1.39546	0.768164	-3.32231	0.0013	-1.19172	0.096614	0.044647
NK.cells	CFLAR	0.467631	5.971185	3.321247	0.001304	-1.50353	0.089335	0.03872
NK.cells	DCLRE1B	0.628515	2.770832	3.310989	0.001347	-1.0997	0.096279	0.043341
NK.cells	HSD3B3	-1.33297	2.014179	-3.30902	0.001356	-1.08008	0.097417	0.044468
NK.cells	ASGR1	-0.88136	3.372794	-3.30722	0.001363	-1.1662	0.095443	0.042982
NK.cells	IFNGR2	0.486593	5.782195	3.299514	0.001397	-1.52138	0.093782	0.040664
NK.cells	RBP4	-0.7989	8.031076	-3.29256	0.001428	-1.94705	0.092164	0.038791
NK.cells	ITIH1	-1.16153	2.749811	-3.28593	0.001458	-1.1522	0.101304	0.045627
NK.cells	PIK3R5	0.383975	4.45137	3.280842	0.001482	-1.60142	0.099775	0.043986
NK.cells	BSG	0.353994	7.716678	3.275096	0.001509	-1.94146	0.095346	0.040689
NK.cells	LPCAT2	0.794338	4.726381	3.27474	0.001511	-1.25764	0.099719	0.044256
NK.cells	SIGLECG	-0.73668	4.076853	-3.27438	0.001513	-1.19492	0.100698	0.045099
NK.cells	RITA1	-1.10145	1.367418	-3.26965	0.001535	-1.24976	0.105882	0.049092
NK.cells	MRPL54	0.342837	5.906363	3.2601	0.001582	-1.70607	0.101347	0.044151
NK.cells	RASA3	0.298038	6.702935	3.252477	0.001621	-1.87209	0.101889	0.043875
NK.cells	RHPN2	-1.2161	0.915486	-3.24945	0.001636	-1.29552	0.111159	0.051904
NK.cells	PHACTR4	-0.30422	5.677431	-3.24939	0.001636	-1.70667	0.103468	0.045464

NK.cells	PLG	-0.91585	4.004656	-3.24459	0.001661	-1.43022	0.107131	0.048001
NK.cells	APOA2	-0.78841	8.419445	-3.24226	0.001673	-2.15332	0.100464	0.042677
NK.cells	ALB	-0.94767	9.441682	-3.23864	0.001692	-2.30499	0.099538	0.041843
NK.cells	TIMD4	-1.63677	3.658253	-3.23546	0.001709	-1.27396	0.108835	0.04935
NK.cells	FETUB	-1.00805	3.175824	-3.23442	0.001715	-1.32802	0.109628	0.050146
NK.cells	NGLY1	0.270929	4.849758	3.230235	0.001737	-1.66242	0.107743	0.048368
NK.cells	SPP2	-1.03881	3.372982	-3.22446	0.001769	-1.38278	0.111584	0.051048
NK.cells	NEAT1	-0.4328	8.674976	-3.22191	0.001783	-2.20304	0.103339	0.044325
NK.cells	EFNA2	-1.92333	1.10216	-3.21212	0.001838	-1.47371	0.118544	0.055875
NK.cells	DNAJA1	-0.21756	7.461326	-3.20968	0.001852	-2.10697	0.107734	0.047003
NK.cells	PRELID2	0.92636	2.621255	3.208683	0.001858	-1.39153	0.115859	0.053807
NK.cells	PHC3	0.393632	4.499127	3.207776	0.001863	-1.67202	0.11263	0.051134
NK.cells	SLC27A5	-1.41395	1.457834	-3.20222	0.001896	-1.39235	0.119359	0.05619
NK.cells	BAIAP2	-0.43858	5.269985	-3.20034	0.001907	-1.86288	0.112791	0.050719
NK.cells	SERPINA1C	-1.39172	0.966902	-3.19365	0.001947	-1.4784	0.122254	0.058004
NK.cells	GM15494	-1.65281	0.759331	-3.18735	0.001985	-1.48946	0.124431	0.059044
NK.cells	HSD3B7	-0.76567	3.537133	-3.18547	0.001997	-1.47386	0.11943	0.054833
NK.cells	GCHFR	-0.98134	3.389908	-3.18305	0.002012	-1.48165	0.120001	0.055169
NK.cells	PRODH2	-1.2606	2.255274	-3.18106	0.002024	-1.4109	0.122222	0.057114
NK.cells	PIPOX	-1.21585	2.011866	-3.1781	0.002043	-1.42641	0.122684	0.05778
NK.cells	ARHGAP21	-0.39575	5.511128	-3.1773	0.002048	-1.85206	0.116391	0.052486
NK.cells	TSC22D2	-0.26907	6.616128	-3.17631	0.002054	-2.08178	0.114478	0.05099
NK.cells	PPFIA4	-1.4247	2.735956	-3.17075	0.00209	-1.55424	0.12286	0.057383
NK.cells	SGMS2	1.230825	3.643674	3.168265	0.002106	-1.46617	0.12154	0.056283
NK.cells	APOM	-0.83238	4.854579	-3.16283	0.002142	-1.82344	0.120787	0.055171
NK.cells	CYP2C70	-0.91053	4.144913	-3.16064	0.002156	-1.70725	0.122325	0.056459
NK.cells	FURIN	0.424641	5.290193	3.157798	0.002175	-1.98986	0.120403	0.054991
NK.cells	LCN2	1.789557	3.398879	3.156642	0.002183	-1.49846	0.123877	0.058014
NK.cells	HIST1H2AC	-1.38257	1.907677	-3.15561	0.00219	-1.49161	0.126692	0.060537
NK.cells	ANKRD37	0.679923	3.981977	3.151713	0.002216	-1.66562	0.123696	0.05754
NK.cells	RNF216	-0.28269	6.677461	-3.14728	0.002247	-2.05638	0.119181	0.053773
NK.cells	F2	-0.86602	4.237274	-3.14508	0.002262	-1.76064	0.123628	0.057729
NK.cells	CEPT1	0.430683	5.374134	3.144925	0.002263	-1.96599	0.121534	0.055938
NK.cells	HEXIM1	-0.42231	5.48831	-3.14464	0.002265	-1.95831	0.121326	0.055789
NK.cells	CEP290	0.92287	2.468766	3.141672	0.002286	-1.51863	0.127544	0.061013
NK.cells	SIK3	-0.3133	10.09847	-3.13482	0.002334	-2.70944	0.115646	0.049996
NK.cells	ZEB2OS	-1.03048	4.678362	-3.1322	0.002353	-1.53537	0.125861	0.058493
NK.cells	ANKZF1	0.538615	2.742515	3.129482	0.002373	-1.58279	0.13008	0.062062
NK.cells	BPGM	0.64381	4.812429	3.126635	0.002393	-1.94236	0.126539	0.058951
NK.cells	AGXT	-1.00863	3.279368	-3.1253	0.002403	-1.6433	0.129492	0.061666
NK.cells	SNX13	0.411578	5.413359	3.122829	0.002421	-1.98652	0.125402	0.05845
NK.cells	GM8369	0.639311	3.324129	3.121774	0.002429	-1.83135	0.129404	0.062059
NK.cells	BCL2	0.476637	5.016128	3.120969	0.002435	-2.21784	0.126153	0.059267
NK.cells	ZFP106	0.341363	6.288522	3.119634	0.002445	-2.10164	0.123766	0.057361
NK.cells	FFAR4	1.53756	0.260828	3.116737	0.002467	-1.68364	0.135921	0.068136
NK.cells	GM28875	-0.44014	4.612323	-3.11582	0.002474	-1.98169	0.127294	0.060439
NK.cells	PAX5	-0.70242	5.141192	-3.10912	0.002525	-1.8366	0.12834	0.060269

NK.cells	IL27	1.983041	0.053926	3.107556	0.002537	-1.80448	0.138627	0.069624
NK.cells	PDE7B	0.982618	5.37371	3.105713	0.002551	-1.93516	0.128121	0.060301
NK.cells	RBP1	-0.87293	3.578118	-3.10114	0.002587	-1.6925	0.132541	0.063903
NK.cells	CHAC1	-1.86075	0.269965	-3.09999	0.002596	-1.90798	0.13932	0.070244
NK.cells	EHD1	0.367549	6.1755	3.099252	0.002602	-2.20715	0.127468	0.059637
NK.cells	SERPINF1	-0.88101	3.83888	-3.09336	0.002649	-1.75231	0.133632	0.064372
NK.cells	TSPO2	1.529314	0.283153	3.090521	0.002672	-1.73151	0.140991	0.0713
NK.cells	URI1	0.305192	5.89939	3.089168	0.002683	-2.23387	0.129557	0.061099
NK.cells	FAM241A	0.397364	5.59878	3.089049	0.002684	-2.12982	0.130143	0.061618
NK.cells	CD300LD	-1.61685	3.173108	-3.08839	0.002689	-1.68136	0.134978	0.066
NK.cells	THAP3	0.415106	3.799217	3.082127	0.002741	-1.95542	0.135408	0.065753
NK.cells	TST	-0.94654	3.470042	-3.08151	0.002746	-1.7682	0.136081	0.066435
NK.cells	CDK5R1	-1.07759	2.111423	-3.07749	0.002779	-1.68445	0.140021	0.069408
NK.cells	AMBP	-0.72059	4.730423	-3.07424	0.002807	-2.04227	0.135387	0.064969
NK.cells	TMEM86B	-0.55379	3.467162	-3.06581	0.002879	-1.82937	0.140973	0.068267
NK.cells	DNAJB6	-0.21227	8.310874	-3.0614	0.002918	-2.68152	0.131485	0.060085
NK.cells	GPR35	1.424031	2.516821	3.061273	0.002919	-1.7445	0.143435	0.070601
NK.cells	ZFP651	-1.06553	1.578642	-3.05952	0.002934	-1.74325	0.145479	0.072654
NK.cells	1500011B(	0.424153	4.65787	3.059166	0.002938	-2.12787	0.138887	0.066743
NK.cells	TINF2	-0.44991	4.054948	-3.0579	0.002949	-2.01765	0.140152	0.068005
NK.cells	SERPINA1A	-0.78989	8.012506	-3.05625	0.002963	-2.62774	0.132073	0.061135
NK.cells	4632428C(	-1.77959	0.110114	-3.05554	0.00297	-2.05233	0.14874	0.076309
NK.cells	PSEN2	0.293046	5.03423	3.043034	0.003084	-2.27171	0.142544	0.068038
NK.cells	NRROS	0.341113	7.067702	3.042413	0.003089	-2.34895	0.138261	0.064333
NK.cells	VSIG4	-1.28977	4.823941	-3.03678	0.003142	-1.90433	0.143996	0.069159
NK.cells	TMEM150I	1.564999	1.12138	3.034279	0.003166	-1.85477	0.152255	0.077001
NK.cells	HHEX	-0.44239	4.675113	-3.03411	0.003167	-2.1522	0.144319	0.069754
NK.cells	SLC11A1	-1.43056	3.505984	-3.03371	0.003171	-1.78474	0.146879	0.072105
NK.cells	KCNQ1	-1.6566	1.396097	-3.03364	0.003172	-1.88327	0.151625	0.076462
NK.cells	TOB1	-0.37416	5.050855	-3.02689	0.003237	-2.24738	0.145516	0.070028
NK.cells	PPBP	1.910828	0.842206	3.026451	0.003241	-1.81406	0.155039	0.078726
NK.cells	LRIF1	0.326232	4.523869	3.024668	0.003258	-2.15448	0.146899	0.071303
NK.cells	PEMT	-0.77561	3.483706	-3.02028	0.003301	-1.94659	0.149772	0.073959
NK.cells	AADAC	-1.10499	2.701819	-3.02003	0.003304	-1.85295	0.151547	0.075602
NK.cells	CASZ1	-0.60311	3.474262	-3.01887	0.003315	-1.92937	0.149794	0.074128
NK.cells	CROT	-0.40071	5.259869	-3.01753	0.003328	-2.34256	0.145825	0.070718
NK.cells	SGSM3	-0.49185	4.063557	-3.01715	0.003332	-2.10669	0.148471	0.073157
NK.cells	ELOVL2	-1.0882	2.450205	-3.01187	0.003385	-1.85947	0.153422	0.077171
NK.cells	PGAP1	0.582899	3.785443	3.011816	0.003386	-2.10817	0.150368	0.074357
NK.cells	CIDEB	-0.98718	1.942054	-3.00987	0.003406	-1.83878	0.154931	0.078523
NK.cells	SLC38A2	-0.21905	8.351985	-3.00849	0.00342	-2.80251	0.140775	0.065817
NK.cells	CREB3L3	-1.20762	1.62088	-3.00705	0.003434	-1.86069	0.155852	0.079658
NK.cells	SERPIND1	-0.92565	2.832849	-3.00338	0.003472	-1.92071	0.153778	0.077384
NK.cells	OAF	-0.78169	3.151249	-3.00297	0.003476	-1.89707	0.153042	0.076738
NK.cells	SAMHD1	0.474518	7.45997	2.994367	0.003566	-2.73319	0.14664	0.069215
NK.cells	KCTD13	0.637001	2.619439	2.988414	0.00363	-1.94179	0.159936	0.08016
NK.cells	HMGCS2	-0.85728	4.456909	-2.98377	0.00368	-2.22732	0.157166	0.076754

NK.cells	NIPSNAP2	0.30299	4.810103	2.97712	0.003754	-2.31397	0.158343	0.076881
NK.cells	GSTP2	1.257957	0.861498	2.97705	0.003754	-1.98464	0.168047	0.085806
NK.cells	FGF23	2.005454	0.594781	2.973013	0.003799	-2.00678	0.170147	0.087113
NK.cells	HDDC2	0.419681	3.817561	2.961658	0.003929	-2.22191	0.167015	0.081063
NK.cells	SRCAP	-0.23122	6.652894	-2.95536	0.004003	-2.66832	0.162478	0.075745
NK.cells	GM15448	1.387898	-0.00747	2.952422	0.004037	-2.17521	0.180383	0.091633
NK.cells	ITGAV	0.334036	6.550452	2.950966	0.004055	-2.78555	0.163425	0.07657
NK.cells	APRT	0.346708	6.416788	2.949185	0.004076	-2.72887	0.163753	0.077114
NK.cells	SLC16A7	-0.85906	3.236151	-2.94818	0.004088	-1.99387	0.17177	0.084356
NK.cells	AKT3	0.353736	6.643383	2.947089	0.004101	-2.6735	0.163198	0.076805
NK.cells	AKR1C20	-1.04828	2.839499	-2.9468	0.004105	-2.05152	0.172799	0.085384
NK.cells	1300017J0	-0.77441	3.006144	-2.94498	0.004127	-2.07499	0.172696	0.085256
NK.cells	CHMP2A	0.241477	6.544809	2.943427	0.004146	-2.7076	0.163805	0.077389
NK.cells	FAM220A	0.907887	2.03012	2.942539	0.004157	-2.03138	0.175312	0.087774
NK.cells	KIT	0.771467	3.453389	2.935466	0.004244	-2.21071	0.174498	0.085525
NK.cells	MRPL57	0.291682	5.995401	2.93404	0.004262	-2.63015	0.167957	0.079822
NK.cells	UPB1	-0.92035	3.0781	-2.93335	0.00427	-2.05088	0.175487	0.086696
NK.cells	DNAJA3	0.506145	3.76841	2.929703	0.004316	-2.21996	0.174946	0.085591
NK.cells	HBQ1B	1.625166	-0.16343	2.926077	0.004362	-2.1248	0.186984	0.095945
NK.cells	FYCO1	0.383121	4.137271	2.916719	0.004483	-2.429	0.179177	0.086687
NK.cells	PTPRJ	-0.27471	8.855581	-2.91588	0.004494	-3.07052	0.166942	0.07612
NK.cells	RAP2B	0.37354	4.614993	2.914399	0.004514	-2.4534	0.177894	0.085875
NK.cells	PI16	1.289265	1.135522	2.91392	0.00452	-2.1045	0.187467	0.09463
NK.cells	OTC	-0.91846	3.438444	-2.91023	0.004569	-2.2279	0.182426	0.089331
NK.cells	S100G	1.386342	0.605317	2.90515	0.004637	-2.0918	0.192592	0.09755
NK.cells	CSPRS	1.475945	0.292172	2.902748	0.004669	-2.29819	0.194219	0.098782
NK.cells	CYP2D26	-0.98789	3.247258	-2.90163	0.004685	-2.22891	0.185753	0.091156
NK.cells	NDUFV3	0.27561	7.098589	2.899952	0.004708	-2.91893	0.175598	0.082183
NK.cells	KDM6B	-0.29928	8.152763	-2.89772	0.004738	-3.08036	0.173409	0.080064
NK.cells	MMADHC	0.354805	4.299266	2.891751	0.004821	-2.43571	0.184747	0.090136
NK.cells	SYNPO	-1.64876	0.219102	-2.89068	0.004836	-2.28089	0.196466	0.101133
NK.cells	2310008N1	-1.67589	0.80481	-2.88959	0.004852	-2.304	0.194736	0.099654
NK.cells	FZD7	-1.21161	0.743156	-2.88947	0.004853	-2.1779	0.194918	0.099825
NK.cells	NUDT4	-0.28562	5.949353	-2.88941	0.004854	-2.77536	0.180224	0.086365
NK.cells	F13B	-1.20178	1.463355	-2.88706	0.004887	-2.13758	0.192811	0.098116
NK.cells	TIMM9	0.419136	3.882337	2.886791	0.004891	-2.37833	0.185909	0.091739
NK.cells	INHBC	-1.43591	0.808252	-2.88674	0.004892	-2.17838	0.194726	0.099926
NK.cells	AQP1	-1.01397	2.964821	-2.88589	0.004904	-2.14751	0.188495	0.094222
NK.cells	CPLX2	-1.02105	3.378009	-2.88399	0.004931	-2.1629	0.187771	0.093504
NK.cells	COA4	0.785766	2.161432	2.878667	0.005008	-2.16145	0.193053	0.097432
NK.cells	BE692007	0.687606	2.816357	2.877868	0.005019	-2.35358	0.191156	0.095785
NK.cells	SELENON	-0.48564	3.694822	-2.87728	0.005028	-2.47006	0.188644	0.093562
NK.cells	BC048403	0.799707	1.255612	2.872733	0.005094	-2.16427	0.195708	0.100731
NK.cells	MGP	1.674446	0.463003	2.872288	0.005101	-2.22939	0.198064	0.103063
NK.cells	CNNM2	-0.45607	5.427728	-2.87221	0.005102	-2.86772	0.183793	0.089772
NK.cells	A430005L1	0.415226	3.764054	2.872069	0.005104	-2.43781	0.188448	0.094045
NK.cells	PPP2CA	-0.15936	7.762516	-2.87071	0.005124	-3.07262	0.177472	0.084318

NK.cells	MBL1	-1.07947	1.455107	-2.87034	0.005129	-2.17034	0.19512	0.100497
NK.cells	ACP1	0.288602	5.923129	2.870037	0.005134	-2.79849	0.182432	0.088748
NK.cells	WWOX	0.280667	6.961551	2.868907	0.005151	-2.96275	0.179653	0.08636
NK.cells	ATF4	-0.33065	6.443614	-2.86433	0.005219	-2.8529	0.182907	0.088333
NK.cells	ABCC2	-0.90809	2.212583	-2.86265	0.005245	-2.20234	0.195081	0.099649
NK.cells	KLHL6	0.37877	6.109159	2.861535	0.005262	-2.81792	0.183976	0.089565
NK.cells	NME2	0.300787	8.557653	2.860906	0.005271	-3.24456	0.177354	0.083794
NK.cells	MARCKS	-0.54551	7.242888	-2.85598	0.005346	-2.62671	0.182878	0.087495
NK.cells	MRPS10	0.39484	4.162492	2.855007	0.005361	-2.502	0.191531	0.095441
NK.cells	AHI1	0.591734	3.003332	2.853769	0.005381	-2.30683	0.195017	0.09876
NK.cells	ZFP53	0.341918	4.791451	2.849881	0.005441	-2.71771	0.190896	0.094599
NK.cells	BTK	-0.57967	6.033608	-2.84978	0.005443	-2.38629	0.187369	0.091391
NK.cells	BRD2	-0.22983	7.085393	-2.84718	0.005484	-3.04287	0.18497	0.089166
NK.cells	9130401M	0.348849	4.02313	2.846721	0.005491	-2.58608	0.193672	0.097208
NK.cells	GM38832	-1.45873	0.763984	-2.84563	0.005508	-2.34949	0.203465	0.10658
NK.cells	PDK1	0.379413	4.164844	2.843695	0.005539	-2.58543	0.193806	0.097338
NK.cells	GM2000	-0.46731	4.799183	-2.83799	0.00563	-2.69161	0.19401	0.096604
NK.cells	LRG1	1.284787	2.460703	2.837619	0.005636	-2.25898	0.20096	0.103148
NK.cells	POLD3	0.371306	4.695421	2.83696	0.005647	-2.65337	0.194313	0.097021
NK.cells	MS4A6B	0.462771	4.588359	2.835134	0.005676	-3.00955	0.195081	0.097569
NK.cells	DNAJB9	-0.35442	5.168187	-2.83346	0.005704	-2.68974	0.193666	0.096249
NK.cells	OTUD5	-0.26228	5.568497	-2.83149	0.005736	-2.82981	0.192505	0.095542
NK.cells	CES2A	-1.34393	0.898465	-2.82941	0.00577	-2.2834	0.206529	0.109193
NK.cells	CEACAM1	-0.84506	3.675486	-2.82893	0.005778	-2.26742	0.198062	0.101139
NK.cells	WNK1	-0.25262	9.07644	-2.82879	0.00578	-3.37118	0.182656	0.087022
NK.cells	GSTA4	-1.09721	1.583658	-2.82863	0.005783	-2.2678	0.204404	0.107221
NK.cells	HMGXB4	0.378401	4.148587	2.823696	0.005865	-2.60843	0.198885	0.100621
NK.cells	UBC	-0.29264	8.132317	-2.82054	0.005918	-3.23236	0.18851	0.090534
NK.cells	GALK2	0.302793	4.897213	2.818064	0.00596	-2.78728	0.198663	0.099446
NK.cells	TSPAN15	-0.87726	2.388192	-2.81718	0.005975	-2.29312	0.206308	0.106808
NK.cells	CAR1	1.82221	0.491473	2.815853	0.005998	-2.33402	0.212323	0.112754
NK.cells	GM15327	-0.93988	1.294764	-2.81518	0.00601	-2.30241	0.209764	0.110371
NK.cells	GPR171	-0.53429	4.027921	-2.81161	0.006071	-2.73413	0.202612	0.103039
NK.cells	PRDM1	0.614847	2.929068	2.810943	0.006083	-2.5609	0.205992	0.106359
NK.cells	SLC22A18	-1.21848	1.143718	-2.80892	0.006118	-2.33157	0.212245	0.112102
NK.cells	KLF4	-0.45867	6.447469	-2.80661	0.006158	-2.87609	0.196712	0.09703
NK.cells	TMEM192	0.320103	4.694631	2.80529	0.006181	-2.79076	0.20216	0.101932
NK.cells	CLEC4G	-0.84398	3.468481	-2.80132	0.006251	-2.39192	0.207616	0.106259
NK.cells	PPP1R14B	0.284345	6.546146	2.799907	0.006277	-3.03657	0.198235	0.097761
NK.cells	ATG4A	0.40851	4.314693	2.799516	0.006284	-2.75445	0.204989	0.104081
NK.cells	FAM149A	-1.50094	-0.42534	-2.79746	0.00632	-2.46372	0.220863	0.11898
NK.cells	ACAA1B	-1.13854	3.318751	-2.79636	0.00634	-2.49626	0.208816	0.107384
NK.cells	9130230N(	-1.63386	0.640779	-2.79508	0.006363	-2.45908	0.217463	0.116028
NK.cells	MXD1	0.29498	6.036212	2.794159	0.00638	-3.10056	0.200497	0.100042
NK.cells	FAF2	0.299263	5.155771	2.793432	0.006393	-2.86116	0.203165	0.102639
NK.cells	GM20492	-0.94474	1.798274	-2.78707	0.006509	-2.36136	0.216421	0.113727
NK.cells	NRGN	0.605995	3.663682	2.787068	0.006509	-2.78714	0.210423	0.107976

NK.cells	ARL5A	0.275312	5.831093	2.784947	0.006549	-3.03853	0.203855	0.102039
NK.cells	METTL7B	-1.15199	2.263028	-2.78487	0.00655	-2.40149	0.215095	0.112692
NK.cells	MICU2	0.324948	4.939969	2.783195	0.006581	-2.88166	0.207033	0.104962
NK.cells	WWC2	-0.49035	5.107898	-2.78164	0.00661	-2.63929	0.206872	0.104777
NK.cells	CES2E	-1.17347	1.228112	-2.78012	0.006639	-2.37839	0.219503	0.117048
NK.cells	CYP3A44	-1.13145	2.308322	-2.77947	0.006651	-2.4157	0.215956	0.113686
NK.cells	EBF1	-0.58935	8.683852	-2.77837	0.006671	-3.34093	0.196337	0.095284
NK.cells	SPTY2D1	-0.2641	5.765398	-2.77707	0.006696	-3.03216	0.205326	0.103535
NK.cells	VWF	1.518384	1.565048	2.77012	0.006829	-2.39591	0.222473	0.117565
NK.cells	KLHL14	-0.98659	2.717642	-2.76701	0.006889	-2.43644	0.219892	0.114416
NK.cells	TOP3B	0.410882	3.549805	2.766244	0.006904	-2.61422	0.217153	0.111979
NK.cells	IL1R1	1.029645	2.051839	2.761339	0.007	-2.44924	0.224493	0.117828
NK.cells	GPAT4	0.315872	4.264538	2.76061	0.007014	-2.78187	0.217136	0.110941
NK.cells	CDH2	-0.91995	1.947183	-2.75729	0.00708	-2.43532	0.226371	0.118853
NK.cells	MALSU1	0.345517	4.843801	2.755629	0.007113	-2.90676	0.217164	0.110083
NK.cells	NAV1	-0.81837	3.595293	-2.75068	0.007213	-2.53249	0.223636	0.114787
NK.cells	GCNT2	-0.35756	4.580489	-2.74974	0.007232	-3.17012	0.220346	0.11179
NK.cells	SMAD1	0.485833	3.547772	2.747037	0.007287	-2.64921	0.223796	0.115553
NK.cells	IGF1R	0.425421	6.357541	2.746833	0.007291	-3.01737	0.214545	0.106884
NK.cells	RNFT1	0.313756	4.495375	2.746489	0.007298	-2.89294	0.220628	0.112648
NK.cells	GM29994	-0.90882	1.150146	-2.74639	0.0073	-2.44888	0.232032	0.123633
NK.cells	CCR5	0.399938	4.693422	2.743074	0.007368	-3.11607	0.221218	0.112692
NK.cells	CES1D	-1.13252	1.90006	-2.74232	0.007384	-2.46345	0.230721	0.121947
NK.cells	TRAF1	0.459874	3.343827	2.741672	0.007397	-3.07918	0.225756	0.117216
NK.cells	CLEC5A	1.577414	0.512385	2.739143	0.00745	-2.553	0.236221	0.127353
NK.cells	CACNA1E	-1.20918	4.907691	-2.73894	0.007454	-2.61028	0.221085	0.112743
NK.cells	EHD4	-0.29473	6.076676	-2.73569	0.007522	-3.17467	0.218224	0.109715
NK.cells	SPATA21	-0.61551	3.397572	-2.73554	0.007525	-2.63841	0.22719	0.118225
NK.cells	PRSS30	-1.21374	-0.33737	-2.73279	0.007583	-2.57672	0.241609	0.131605
NK.cells	LAIR1	0.99567	4.246709	2.727905	0.007688	-2.62823	0.227833	0.116737
NK.cells	ACY3	-1.1207	2.04257	-2.72729	0.007701	-2.49153	0.235522	0.124255
NK.cells	SPHK2	-0.47228	3.388566	-2.72389	0.007774	-2.72985	0.232415	0.120399
NK.cells	UBXN4	0.174406	6.814787	2.719669	0.007866	-3.29817	0.22282	0.110339
NK.cells	EPS8L1	-1.80491	0.408966	-2.71712	0.007922	-2.54775	0.24651	0.132565
NK.cells	TNFRSF1B	0.332647	4.903674	2.714348	0.007984	-3.22054	0.231539	0.117514
NK.cells	GM38560	-1.26927	0.991541	-2.71354	0.008002	-2.53308	0.245593	0.131173
NK.cells	GP9	1.469533	0.659096	2.710218	0.008076	-2.52802	0.24851	0.133055
NK.cells	AKR1D1	-0.95532	2.587501	-2.70665	0.008156	-2.60694	0.243196	0.126844
NK.cells	FAM83D	-0.55634	2.774945	-2.70389	0.008219	-2.70986	0.243782	0.1267
NK.cells	SLC19A2	-0.75746	1.834884	-2.70054	0.008296	-2.57228	0.248667	0.130558
NK.cells	CP	-0.61185	4.588111	-2.7001	0.008306	-2.94659	0.238568	0.120977
NK.cells	GM14636	1.16896	2.274283	2.69427	0.008441	-2.57781	0.249906	0.130306
NK.cells	GLUL	-0.35981	6.384395	-2.69318	0.008467	-3.25544	0.234929	0.116493
NK.cells	HIVEP3	0.39225	5.144836	2.692484	0.008483	-3.1126	0.23934	0.120727
NK.cells	METTL21A	0.628097	1.888583	2.691733	0.008501	-2.60616	0.251364	0.132311
NK.cells	TIGD2	0.394334	3.85213	2.691581	0.008504	-2.86458	0.244037	0.125283
NK.cells	ANAPC2	-0.33812	4.497598	-2.68936	0.008557	-3.03593	0.242589	0.123499

NK.cells	CCR1	1.416025	2.32125	2.687991	0.008589	-2.57777	0.250953	0.131501
NK.cells	INTS6	-0.36023	6.588587	-2.68637	0.008628	-3.34757	0.235358	0.117031
NK.cells	ARHGEF40	-1.28834	0.550928	-2.68629	0.00863	-2.59951	0.257746	0.138444
NK.cells	TJP2	-0.36025	3.845594	-2.68542	0.00865	-3.10644	0.245257	0.126442
NK.cells	CFP	-0.75292	5.518079	-2.68467	0.008668	-2.81136	0.239168	0.120797
NK.cells	RAD21	-0.23545	7.022909	-2.68069	0.008764	-3.44847	0.235856	0.116733
NK.cells	CASP12	1.499122	-0.47839	2.677148	0.00885	-2.72099	0.266022	0.144792
NK.cells	APOA5	-1.13071	1.793969	-2.67545	0.008892	-2.61152	0.257653	0.136395
NK.cells	KEL	1.675435	-0.44363	2.672919	0.008954	-2.6789	0.267744	0.145799
NK.cells	LY6D	-0.63334	5.594266	-2.67188	0.008979	-2.97053	0.24459	0.123451
NK.cells	LY6A	1.028049	4.339309	2.66923	0.009045	-3.11788	0.25049	0.128283
NK.cells	WDR59	0.571287	3.072953	2.667542	0.009087	-2.76012	0.255904	0.133261
NK.cells	CD19	-0.82087	3.122136	-2.66648	0.009114	-2.66792	0.255869	0.133336
NK.cells	SLC2A2	-1.02001	1.627926	-2.66521	0.009146	-2.63434	0.261833	0.139221
NK.cells	TNFAIP6	1.544992	1.129558	2.664616	0.009161	-2.63916	0.26381	0.1413
NK.cells	LZTFL1	0.399695	5.075487	2.66262	0.009211	-3.12246	0.249215	0.127071
NK.cells	PAK1	-0.48845	5.334473	-2.66164	0.009236	-3.34753	0.248247	0.126381
NK.cells	SPAG9	-0.30525	9.266573	-2.6612	0.009247	-3.84503	0.234055	0.113399
NK.cells	GSTT1	-0.96668	2.240293	-2.65859	0.009314	-2.67851	0.261354	0.138434
NK.cells	CCNYL1	-0.33781	4.635175	-2.65764	0.009338	-3.11478	0.252183	0.129596
NK.cells	ATF3	-0.78095	6.1843	-2.65538	0.009396	-3.10227	0.247353	0.124558
NK.cells	CYP2C23	-1.28067	0.860959	-2.65428	0.009424	-2.66599	0.26819	0.144781
NK.cells	TRERF1	-0.37869	4.396216	-2.65304	0.009456	-3.38118	0.254562	0.131413
NK.cells	ERF	-0.52213	3.477368	-2.65068	0.009518	-2.93605	0.258719	0.135313
NK.cells	ABHD18	0.52851	3.182254	2.650531	0.009522	-2.88763	0.259872	0.136451
NK.cells	FBXO33	-0.25312	5.984659	-2.64971	0.009543	-3.39027	0.249154	0.126344
NK.cells	STPG4	-0.73097	2.37884	-2.64843	0.009577	-2.70405	0.26337	0.139869
NK.cells	SLC30A9	0.262311	5.265307	2.645861	0.009644	-3.25598	0.252828	0.129672
NK.cells	NFKBIZ	0.372147	6.516715	2.644473	0.009681	-3.42409	0.248124	0.125511
NK.cells	AFF1	-0.22588	8.730515	-2.64447	0.009681	-3.80424	0.240039	0.118011
NK.cells	NUDT14	0.336901	4.342372	2.643558	0.009705	-3.05386	0.256359	0.133547
NK.cells	CLDN1	-1.34048	1.493829	-2.64342	0.009709	-2.66934	0.267599	0.144592
NK.cells	FPR3	1.532103	-0.4789	2.640921	0.009775	-2.74957	0.275995	0.153219
NK.cells	TSSC4	0.454349	3.902903	2.639731	0.009807	-2.93239	0.258345	0.135886
NK.cells	SSPN	1.390956	-0.41075	2.638633	0.009836	-2.80107	0.275711	0.153442
NK.cells	PKDCC	-1.22228	1.303453	-2.63826	0.009846	-2.70535	0.268666	0.146351
NK.cells	MCTP1	0.828658	5.535347	2.637525	0.009866	-3.06585	0.252083	0.130213
NK.cells	RALGAPB	0.287453	4.920242	2.63675	0.009887	-3.23267	0.254423	0.132597
NK.cells	MRPS35	0.318264	4.266456	2.636027	0.009907	-3.09887	0.256936	0.135247
NK.cells	BCDIN3D	0.606861	2.223563	2.635994	0.009907	-2.73995	0.264963	0.143157
NK.cells	GM47802	-1.45169	0.099495	-2.63579	0.009913	-2.75232	0.273593	0.15192
NK.cells	ZNHIT1	0.278841	5.422819	2.626594	0.010165	-3.27852	0.2582	0.132546
NK.cells	HC	-0.92428	2.358321	-2.62602	0.01018	-2.76855	0.270384	0.144436
NK.cells	AMPD3	1.024633	2.578161	2.623572	0.010248	-2.71272	0.270708	0.14398
NK.cells	HSPA5	-0.21882	8.355671	-2.62026	0.010341	-3.79239	0.24992	0.123318
NK.cells	RABGAP1	0.268708	5.920481	2.618132	0.010401	-3.40658	0.259611	0.132405
NK.cells	ZGRF1	-0.51736	3.956437	-2.6181	0.010402	-3.15524	0.267387	0.139846

NK.cells	STRIP2	-1.09249	1.016356	-2.6139	0.010521	-2.73186	0.282108	0.152722
NK.cells	DENND5B	-0.58766	4.198991	-2.61173	0.010583	-2.90392	0.269907	0.140274
NK.cells	9030622O2	-1.33453	1.05914	-2.60976	0.01064	-2.74313	0.283895	0.153581
NK.cells	SERPINA11	-0.94141	1.813558	-2.60681	0.010725	-2.75622	0.282327	0.15107
NK.cells	TNIP1	0.370379	4.966974	2.605958	0.01075	-3.37918	0.269289	0.138501
NK.cells	0610005C1	-0.85364	1.911141	-2.60408	0.010805	-2.77098	0.282594	0.151228
NK.cells	ATG101	-0.29806	5.222366	-2.60355	0.01082	-3.30353	0.268855	0.138012
NK.cells	SYNE2	-0.40979	5.373755	-2.60236	0.010855	-3.28989	0.268244	0.137668
NK.cells	RASAL2	-0.56637	5.468995	-2.60191	0.010868	-3.1522	0.267861	0.137368
NK.cells	IQCH	-1.73591	0.250926	-2.60046	0.010911	-2.80362	0.289764	0.159143
NK.cells	TMEM165	0.249863	5.311537	2.600159	0.01092	-3.37475	0.268495	0.138249
NK.cells	APCS	0.479118	4.833138	2.599694	0.010934	-3.32422	0.270431	0.140236
NK.cells	GTPBP8	0.620667	2.317318	2.59816	0.010979	-2.82866	0.281448	0.150646
NK.cells	IFNAR1	0.273031	5.246213	2.59667	0.011023	-3.41642	0.269577	0.139053
NK.cells	KCTD12	-0.3184	6.383969	-2.59626	0.011036	-3.45419	0.265013	0.134734
NK.cells	SAP18	0.200603	7.043708	2.59529	0.011065	-3.6654	0.262547	0.13233
NK.cells	PIK3R6	0.714425	2.596377	2.590638	0.011204	-2.99999	0.283206	0.151022
NK.cells	KEAP1	-0.25311	5.115335	-2.58979	0.01123	-3.40406	0.272673	0.140961
NK.cells	PROZ	-0.89307	1.915226	-2.58969	0.011233	-2.82631	0.286129	0.154104
NK.cells	CLDN3	-1.08714	1.461002	-2.58708	0.011312	-2.78656	0.289019	0.156749
NK.cells	CLTC	-0.17839	7.90636	-2.58698	0.011315	-3.79327	0.262337	0.131022
NK.cells	CLYBL	0.394268	4.489599	2.585872	0.011349	-3.20829	0.27626	0.144383
NK.cells	KLRA9	-0.67676	-0.30711	-2.58479	0.011382	-2.98057	0.296978	0.165227
NK.cells	GM49980	-0.55483	6.157222	-2.58453	0.01139	-3.53261	0.269427	0.13808
NK.cells	SLCO5A1	-1.12808	-0.20706	-2.58338	0.011426	-2.8719	0.296688	0.1651
NK.cells	NUP155	-0.27465	5.174039	-2.58248	0.011453	-3.38161	0.27358	0.142404
NK.cells	GM4952	-0.89198	2.667628	-2.58194	0.01147	-2.89296	0.284094	0.152761
NK.cells	N4BP1	0.358358	5.4056	2.581006	0.011499	-3.42378	0.27263	0.141745
NK.cells	GM3336	-1.21956	1.732452	-2.58056	0.011512	-2.79988	0.288128	0.157012
NK.cells	MBOAT7	0.341184	4.278521	2.579366	0.01155	-3.24942	0.277371	0.1465
NK.cells	LBP	-0.70382	3.292366	-2.57889	0.011564	-2.90281	0.281518	0.150637
NK.cells	CYP2A12	-0.85229	2.85261	-2.57746	0.011609	-2.96778	0.283388	0.152755
NK.cells	METTL9	0.293968	5.718204	2.577462	0.011609	-3.44775	0.271435	0.141045
NK.cells	MAP3K1	-0.21594	7.970553	-2.5761	0.011651	-3.86621	0.262866	0.132795
NK.cells	SFMBT2	1.092876	0.60235	2.57486	0.01169	-2.81174	0.294042	0.163316
NK.cells	MFSD12	0.711561	2.730413	2.574131	0.011713	-2.82783	0.284754	0.154081
NK.cells	TMEM38B	0.352281	4.174266	2.573306	0.011739	-3.27435	0.278695	0.148169
NK.cells	OLA1	0.225841	5.835409	2.571781	0.011787	-3.51919	0.272081	0.141826
NK.cells	HSPB6	1.268745	0.40248	2.571479	0.011797	-2.83871	0.295276	0.165027
NK.cells	GM36862	-1.37987	0.340355	-2.56967	0.011854	-2.87245	0.296403	0.165849
NK.cells	ITIH4	-0.64568	3.988196	-2.56813	0.011903	-3.17754	0.281148	0.150236
NK.cells	GM13561	0.788011	1.307424	2.567014	0.011939	-2.84273	0.29304	0.162069
NK.cells	FGG	-0.48188	6.166651	-2.5653	0.011994	-3.61752	0.273098	0.142014
NK.cells	SMG9	0.321565	4.52517	2.563329	0.012057	-3.3137	0.280841	0.149168
NK.cells	RNASEH1	0.483265	2.84733	2.557212	0.012256	-2.99472	0.290918	0.15788
NK.cells	9530062K0	1.283554	0.793704	2.556942	0.012265	-2.87806	0.300069	0.167228
NK.cells	LRP4	-1.27347	2.477171	-2.55614	0.012291	-2.84901	0.292545	0.159789

NK.cells	CPEB2	-0.28028	5.412476	-2.5555	0.012312	-3.53235	0.279907	0.147363
NK.cells	SERTAD2	-0.30454	6.12155	-2.55529	0.012319	-3.65966	0.276944	0.144483
NK.cells	PRORSD1	0.338317	4.586531	2.555223	0.012321	-3.26819	0.283402	0.150791
NK.cells	ATP9B	0.220682	5.858499	2.553235	0.012387	-3.61117	0.27832	0.145959
NK.cells	ABCC1	0.372103	4.467713	2.553	0.012395	-3.38814	0.284196	0.151747
NK.cells	MIRT2	1.325384	-1.00119	2.552322	0.012417	-2.95277	0.308609	0.17684
NK.cells	STAP1	0.33558	4.891894	2.550553	0.012476	-3.44506	0.28239	0.1503
NK.cells	COPS7A	0.368412	4.237724	2.55034	0.012483	-3.26759	0.285181	0.153106
NK.cells	PEX11A	1.243863	0.072757	2.549841	0.0125	-2.87939	0.303661	0.172021
NK.cells	PAQR9	-0.6668	4.029832	-2.54839	0.012548	-3.09693	0.286074	0.1544
NK.cells	TLCD2	-0.79407	2.659161	-2.54679	0.012602	-2.91622	0.292039	0.160759
NK.cells	BFAR	0.257463	4.85458	2.546535	0.01261	-3.4108	0.282548	0.151279
NK.cells	MAP2K1	0.261428	6.311963	2.546235	0.01262	-3.66566	0.276434	0.145264
NK.cells	SAMSN1	0.265023	7.050554	2.545969	0.012629	-3.86436	0.27339	0.142349
NK.cells	CALM2	-0.1798	8.565355	-2.54572	0.012638	-3.98025	0.267265	0.136484
NK.cells	HOGA1	-0.79011	2.370342	-2.54554	0.012644	-2.92647	0.293313	0.162182
NK.cells	FOSB	-0.42777	6.636378	-2.54378	0.012703	-3.65148	0.275641	0.144314
NK.cells	LLPH	0.208778	6.447819	2.543372	0.012717	-3.68519	0.276422	0.145171
NK.cells	TMEM132I	-1.66293	0.984501	-2.54233	0.012752	-2.94068	0.300374	0.169198
NK.cells	ZSWIM6	-0.24107	8.155524	-2.53995	0.012834	-4.00978	0.270893	0.13904
NK.cells	HSPA1A	1.002834	3.985226	2.538309	0.01289	-3.35094	0.289098	0.156444
NK.cells	CCND1	-0.86394	4.140959	-2.53733	0.012923	-2.97687	0.288637	0.155937
NK.cells	TTC36	-0.65369	5.028124	-2.5359	0.012972	-3.45849	0.285361	0.152447
NK.cells	NT5C	0.27787	5.114948	2.535186	0.012997	-3.56728	0.285001	0.152269
NK.cells	GDE1	0.437022	4.883129	2.532899	0.013076	-3.39931	0.287204	0.153648
NK.cells	CD300LF	1.179314	3.385271	2.531987	0.013108	-2.90317	0.293915	0.160437
NK.cells	CD47	0.185328	8.628324	2.531263	0.013133	-4.01034	0.271703	0.138822
NK.cells	FAM111A	0.294378	5.742953	2.529341	0.0132	-3.59197	0.284236	0.150822
NK.cells	NXPE3	0.422522	3.424156	2.529161	0.013207	-3.2209	0.294319	0.160878
NK.cells	ARMC3	0.79818	1.900651	2.525913	0.013321	-2.94485	0.3032	0.16841
NK.cells	RBM44	1.330058	0.135835	2.524615	0.013367	-2.92511	0.311884	0.177192
NK.cells	P4HA3	-1.25275	0.308298	-2.52066	0.013508	-2.94881	0.313555	0.17725
NK.cells	SMIM4	0.277214	5.255277	2.52023	0.013523	-3.50098	0.291037	0.154513
NK.cells	ABT1	-0.38776	3.534654	-2.51878	0.013575	-3.21603	0.299268	0.16231
NK.cells	FXYD1	-0.77837	2.943369	-2.5166	0.013653	-3.08423	0.303136	0.165529
NK.cells	RAB3IL1	-1.13506	1.73222	-2.5157	0.013686	-2.93941	0.308729	0.171427
NK.cells	ZFP26	0.318695	3.995742	2.515214	0.013703	-3.35909	0.298379	0.161089
NK.cells	ASPH	0.781579	4.664011	2.513271	0.013774	-3.09248	0.296378	0.158655
NK.cells	SOCS7	-0.33303	4.207792	-2.51166	0.013832	-3.37942	0.298652	0.161063
NK.cells	CIRBP	0.238953	7.218243	2.510737	0.013866	-3.88483	0.285458	0.148358
NK.cells	FBRSL1	-0.22285	5.766788	-2.51061	0.013871	-3.66524	0.291738	0.154505
NK.cells	QTRT1	-0.44042	4.980734	-2.51024	0.013885	-3.47426	0.295202	0.158038
NK.cells	EPB41L3	-1.34095	2.334313	-2.50847	0.01395	-2.95121	0.308079	0.170579
NK.cells	RACK1	0.154427	8.637453	2.5069	0.014007	-4.13271	0.280617	0.143508
NK.cells	SEMA4B	-0.48119	5.763505	-2.50627	0.014031	-3.37585	0.292957	0.155605
NK.cells	UOX	0.621504	5.010934	2.505959	0.014042	-3.58984	0.296287	0.158975
NK.cells	OSGIN1	-0.42612	3.869004	-2.50482	0.014084	-3.46208	0.30149	0.164361

NK.cells	GM16853	-0.6758	2.351019	-2.50452	0.014096	-3.03681	0.308462	0.17153
NK.cells	P2RX4	0.339116	5.534178	2.501862	0.014195	-3.6026	0.29535	0.157666
NK.cells	RHOU	-1.32334	1.27725	-2.50081	0.014234	-2.95924	0.314897	0.177745
NK.cells	ATR	0.43738	3.862209	2.500534	0.014244	-3.38032	0.302866	0.165489
NK.cells	ADH1	-0.79416	3.503888	-2.50007	0.014262	-3.22942	0.304504	0.167192
NK.cells	0610009E0	-1.0378	0.843747	-2.49946	0.014285	-2.97898	0.316964	0.180112
NK.cells	FKBP11	-0.76565	1.995525	-2.4979	0.014343	-2.97335	0.312235	0.17462
NK.cells	SHISA8	-1.55993	-0.21439	-2.49475	0.014463	-3.03386	0.324467	0.186599
NK.cells	TICAM2	1.181462	1.274799	2.493954	0.014493	-3.00293	0.317253	0.179294
NK.cells	PRDX4	-0.37803	5.118984	-2.49341	0.014514	-3.44098	0.299409	0.161225
NK.cells	BMP8A	-0.86601	2.321943	-2.4933	0.014518	-3.03426	0.312282	0.174275
NK.cells	SULT2A8	-1.37948	0.571296	-2.49086	0.014611	-2.98469	0.321108	0.183411
NK.cells	SNIP1	-0.34383	3.633076	-2.49078	0.014614	-3.30708	0.306622	0.168444
NK.cells	CCDC58	0.339816	4.247902	2.489857	0.01465	-3.42603	0.303798	0.165757
NK.cells	NDUFA4	-0.25191	8.596899	-2.48976	0.014654	-4.13796	0.284622	0.146857
NK.cells	CSNK1G1	0.228998	6.535289	2.488898	0.014687	-3.8509	0.293541	0.15559
NK.cells	FMNL1	-0.21437	6.443857	-2.48877	0.014692	-3.92443	0.293943	0.156018
NK.cells	SLC30A6	0.42081	3.417011	2.488022	0.014721	-3.2541	0.307652	0.169936
NK.cells	PTOV1	-0.46451	4.038474	-2.48742	0.014744	-3.18633	0.304788	0.167157
NK.cells	RTL8B	-0.45113	3.297356	-2.48654	0.014778	-3.36325	0.308388	0.170855
NK.cells	IGF1	-0.66714	5.292109	-2.48438	0.014862	-3.50725	0.300008	0.162103
NK.cells	KLRA1	-0.88446	0.700337	-2.48272	0.014927	-3.15808	0.32149	0.184573
NK.cells	HGFAC	-0.96747	1.432763	-2.48224	0.014945	-3.00814	0.317956	0.180934
NK.cells	DUSP5	-0.28546	6.834196	-2.48199	0.014955	-3.9743	0.293147	0.155723
NK.cells	SLC4A7	0.290273	5.767425	2.481844	0.014961	-3.74634	0.297875	0.160457
NK.cells	CTDSP2	-0.27584	4.801724	-2.48168	0.014967	-3.52972	0.302227	0.16483
NK.cells	RABEPK	0.421497	3.296184	2.480026	0.015032	-3.28361	0.309963	0.172217
NK.cells	AGT	-0.75136	4.084746	-2.47669	0.015164	-3.41211	0.307495	0.169522
NK.cells	GRIP1	-1.10266	2.360437	-2.4763	0.015179	-3.03212	0.315584	0.177916
NK.cells	SDK1	-0.8681	3.195716	-2.47605	0.015189	-3.1475	0.311637	0.173879
NK.cells	SAA1	2.240688	-0.05604	2.475954	0.015193	-3.04408	0.327304	0.190348
NK.cells	SLC4A1	1.121988	2.002694	2.474914	0.015235	-3.04138	0.317618	0.180067
NK.cells	TRAPPC6A	0.237914	5.057196	2.474206	0.015263	-3.62327	0.303361	0.165516
NK.cells	NREP	-1.08838	1.234795	-2.47361	0.015287	-3.01332	0.321336	0.184305
NK.cells	KLHDC10	0.292746	5.434437	2.472718	0.015322	-3.62809	0.30184	0.164194
NK.cells	HTRA3	-0.68021	0.502839	-2.47135	0.015377	-3.11494	0.325735	0.188725
NK.cells	UBXN2B	0.474986	2.842475	2.469942	0.015434	-3.24157	0.314579	0.177195
NK.cells	DDX3X	-0.25614	7.320412	-2.4699	0.015436	-3.97347	0.294115	0.156429
NK.cells	MMP8	1.679959	1.517071	2.468383	0.015497	-3.02497	0.32145	0.184115
NK.cells	SPIB	-0.79268	4.129765	-2.468	0.015512	-3.14764	0.309043	0.171259
NK.cells	ACSS2	0.459497	3.033515	2.465463	0.015615	-3.35166	0.315742	0.177292
NK.cells	GFOD1	-0.30607	6.621215	-2.46397	0.015676	-3.94533	0.299534	0.160734
NK.cells	TMEM150A	-0.84488	2.327871	-2.46332	0.015702	-3.07621	0.319506	0.181267
NK.cells	RDH7	-0.80871	2.864625	-2.46151	0.015777	-3.2105	0.316932	0.178996
NK.cells	AGMO	-0.78318	3.650343	-2.46137	0.015783	-3.17038	0.313204	0.175148
NK.cells	CHKB	-0.29184	4.306938	-2.46091	0.015802	-3.52797	0.310125	0.172039
NK.cells	GPR84	1.491639	0.260814	2.46089	0.015802	-3.08218	0.329628	0.192525

NK.cells	H2AFV	-0.27184	8.095101	-2.46009	0.015835	-4.14827	0.292997	0.154946
NK.cells	GM10131	-1.19162	1.072727	-2.45933	0.015867	-3.03959	0.325612	0.188619
NK.cells	BAZ1A	-0.20767	7.694958	-2.45868	0.015894	-4.07591	0.294756	0.156968
NK.cells	ZC3H12B	1.168034	1.084598	2.458671	0.015894	-3.04557	0.325554	0.188667
NK.cells	CD81	-0.34868	7.604661	-2.4565	0.015984	-3.92652	0.296346	0.157762
NK.cells	RAB3IP	0.335665	4.587906	2.452235	0.016162	-3.62446	0.312708	0.172547
NK.cells	SCAND1	0.19507	7.018464	2.451976	0.016173	-3.96492	0.301507	0.16129
NK.cells	GM20186	0.480526	3.559404	2.451176	0.016207	-3.44823	0.317728	0.17776
NK.cells	KLKB1	-0.88995	1.848942	-2.45046	0.016237	-3.08241	0.326097	0.186627
NK.cells	CCDC85B	0.319543	3.779227	2.446335	0.016412	-3.49089	0.319413	0.177677
NK.cells	SLCO1B2	-0.65652	4.25024	-2.44591	0.01643	-3.52604	0.317158	0.175448
NK.cells	ALG9	0.428528	3.375018	2.445368	0.016453	-3.33885	0.321363	0.179939
NK.cells	SELL	0.384032	5.472373	2.442743	0.016565	-3.76373	0.313007	0.170257
NK.cells	MRPL16	0.348197	3.919331	2.441668	0.016611	-3.4785	0.320777	0.177935
NK.cells	MAD2L1BF	-0.31483	4.194819	-2.44042	0.016665	-3.58477	0.319968	0.17693
NK.cells	SNN	-0.80962	3.649853	-2.43865	0.016742	-3.12761	0.323566	0.179999
NK.cells	MTUS1	-0.80105	3.721745	-2.43638	0.01684	-3.22996	0.324596	0.180257
NK.cells	SEC14L2	-0.9183	1.70477	-2.4354	0.016883	-3.11178	0.334933	0.190945
NK.cells	SERHL	0.387948	3.492794	2.433284	0.016975	-3.43688	0.326472	0.18216
NK.cells	GM26510	0.376638	4.122817	2.432949	0.01699	-3.55707	0.323391	0.179105
NK.cells	UBAC1	0.305672	4.029628	2.432749	0.016999	-3.52577	0.323845	0.17962
NK.cells	CLCN7	0.507153	3.608444	2.432213	0.017023	-3.37852	0.325904	0.181857
NK.cells	TMEM273	0.581768	1.19098	2.431282	0.017064	-3.26822	0.337998	0.194761
NK.cells	B4GALT7	0.432411	2.869796	2.43118	0.017068	-3.35415	0.329549	0.185905
NK.cells	CYP2J5	-1.15689	1.06057	-2.42954	0.01714	-3.09639	0.339493	0.195998
NK.cells	ZFP36L2	-0.23319	8.376464	-2.42901	0.017164	-4.39594	0.304149	0.160035
NK.cells	IER2	-0.29629	8.170209	-2.42676	0.017264	-4.28347	0.306331	0.161486
NK.cells	CYP4F18	-0.92901	3.551567	-2.42612	0.017293	-3.11189	0.328314	0.183751
NK.cells	SLC25A18	-0.71614	4.491338	-2.4256	0.017315	-3.37992	0.323705	0.179091
NK.cells	R3HCC1L	0.255655	5.540599	2.423501	0.01741	-3.82741	0.319778	0.174351
NK.cells	LIMK2	0.302075	4.779797	2.421924	0.01748	-3.67699	0.323453	0.178503
NK.cells	TRUB1	0.673611	1.825881	2.421203	0.017513	-3.16069	0.338164	0.194019
NK.cells	TUBA1C	-0.29715	7.576145	-2.42086	0.017529	-4.15809	0.310168	0.165372
NK.cells	SRXN1	-0.87544	1.094676	-2.42074	0.017534	-3.12225	0.341915	0.198059
NK.cells	SLC12A3	-1.1794	1.874484	-2.42058	0.017541	-3.11327	0.337917	0.193844
NK.cells	MAMLD1	1.330536	0.110917	2.419614	0.017585	-3.13707	0.347359	0.203777
NK.cells	LGR5	-1.02663	1.073909	-2.41828	0.017645	-3.11844	0.342992	0.198579
NK.cells	1500004A1	0.504125	2.078931	2.417595	0.017676	-3.2822	0.337869	0.193254
NK.cells	TNFAIP8	0.22655	7.424897	2.417032	0.017702	-4.12222	0.311786	0.16669
NK.cells	5033406O(	1.369586	0.605012	2.415384	0.017777	-3.15256	0.346398	0.201852
NK.cells	RAB8A	0.230033	5.454242	2.414558	0.017815	-3.86147	0.322189	0.176545
NK.cells	ISOC1	0.27539	4.993923	2.412135	0.017926	-3.78302	0.325739	0.179515
NK.cells	IRS1	-1.13662	0.821216	-2.4114	0.01796	-3.13265	0.34687	0.20167
NK.cells	USP28	0.301703	4.553264	2.41119	0.01797	-3.6566	0.327903	0.181787
NK.cells	AZGP1	-0.62596	3.926277	-2.40621	0.018201	-3.54771	0.334758	0.186339
NK.cells	CCL24	-1.58171	3.596362	-2.40461	0.018276	-3.24147	0.337004	0.188544
NK.cells	PRCP	0.349237	5.424232	2.404342	0.018288	-3.61225	0.327869	0.179271

NK.cells	5730522E0	-0.86892	3.610742	-2.40333	0.018336	-3.25297	0.337289	0.188738
NK.cells	MAK16	0.248767	5.072635	2.401463	0.018424	-3.79767	0.331028	0.181718
NK.cells	MS4A4A	-1.19426	0.617812	-2.39975	0.018505	-3.15868	0.354005	0.206162
NK.cells	FOLR2	-1.00802	3.655517	-2.39856	0.018561	-3.2854	0.338158	0.189837
NK.cells	WASHC4	0.22151	5.792598	2.398277	0.018575	-3.87651	0.327469	0.178905
NK.cells	APBB1IP	0.203856	7.638967	2.397777	0.018598	-4.20996	0.318532	0.169982
NK.cells	ZDHHC13	0.39246	3.693777	2.397604	0.018607	-3.44412	0.337963	0.189732
NK.cells	FAM13A	-1.36474	1.15315	-2.39713	0.018629	-3.16981	0.351156	0.203705
NK.cells	INSIG1	-0.32179	5.457849	-2.39662	0.018653	-3.77141	0.329118	0.180723
NK.cells	CYSLTR1	0.631916	2.70023	2.396547	0.018657	-3.36076	0.343058	0.19514
NK.cells	DDX5	-0.13145	9.318404	-2.3953	0.018717	-4.46108	0.310638	0.162502
NK.cells	H2AFJ	0.191055	7.521162	2.39465	0.018748	-4.19051	0.319094	0.171019
NK.cells	PPARG	0.685148	2.579221	2.392352	0.018858	-3.38678	0.343684	0.196829
NK.cells	RASD1	-0.57576	3.88428	-2.39184	0.018883	-3.50039	0.336996	0.189983
NK.cells	SLC44A2	0.299619	6.242042	2.391815	0.018884	-3.94169	0.325268	0.177914
NK.cells	HIF1A	0.25037	6.608469	2.391105	0.018918	-4.10403	0.323486	0.176216
NK.cells	LGALS3	0.398731	6.142988	2.390893	0.018929	-4.02465	0.325752	0.178514
NK.cells	DNAL1	-0.76645	1.64713	-2.39087	0.01893	-3.22344	0.348548	0.202305
NK.cells	ANKRD13C	-0.24297	5.868682	-2.39068	0.018939	-3.98386	0.327095	0.179883
NK.cells	GM44284	-1.37118	-0.05378	-2.39051	0.018947	-3.20818	0.357614	0.212133
NK.cells	SLPI	1.16675	4.206306	2.389708	0.018986	-3.28539	0.335367	0.18859
NK.cells	PSMG4	0.269721	5.042688	2.388747	0.019033	-3.77383	0.331177	0.18447
NK.cells	SERPINA1E	-0.61866	7.994808	-2.38829	0.019055	-4.31051	0.31684	0.169977
NK.cells	SPRED3	1.177642	0.719893	2.387907	0.019073	-3.17785	0.35346	0.208248
NK.cells	SVBP	0.274365	5.267752	2.387721	0.019082	-3.85065	0.330059	0.183537
NK.cells	GM10847	1.140513	0.165978	2.387435	0.019096	-3.18272	0.356428	0.211537
NK.cells	DENND6A	0.262617	5.102952	2.386019	0.019166	-3.80818	0.331588	0.184811
NK.cells	FEZ2	0.349695	3.693286	2.384283	0.019251	-3.53381	0.339679	0.1928
NK.cells	CALHM6	0.752316	3.048969	2.383513	0.019288	-3.57845	0.34299	0.196552
NK.cells	SLC35C2	0.264092	4.846147	2.383153	0.019306	-3.71644	0.33384	0.187037
NK.cells	PFDN1	0.237106	5.109296	2.382583	0.019334	-3.84448	0.332523	0.185761
NK.cells	GM12248	-0.55277	2.619411	-2.38172	0.019377	-3.40403	0.345388	0.199264
NK.cells	MRPL23	0.194864	6.069484	2.381246	0.0194	-3.97715	0.327926	0.181059
NK.cells	CAPSL	-1.51046	0.928873	-2.3771	0.019606	-3.20989	0.357076	0.210304
NK.cells	MICAL2	-0.54257	1.966497	-2.37648	0.019637	-3.51751	0.35153	0.204513
NK.cells	P2RX3	-0.94916	2.219391	-2.37648	0.019637	-3.24367	0.350192	0.20308
NK.cells	UNC93B1	-0.28987	7.659349	-2.37182	0.019871	-3.97081	0.326077	0.175536
NK.cells	AATF	0.265132	4.995367	2.371023	0.019911	-3.86231	0.339367	0.189159
NK.cells	TANK	0.287999	6.451404	2.370174	0.019954	-4.0983	0.332031	0.181892
NK.cells	SLC7A11	1.360188	4.278698	2.37012	0.019957	-3.34206	0.343043	0.193234
NK.cells	A	-0.37123	4.78007	-2.36676	0.020127	-3.7992	0.342062	0.191423
NK.cells	WDR86	-0.77531	0.234245	-2.36594	0.02017	-3.22335	0.366315	0.21743
NK.cells	EPHX2	-0.80206	2.477792	-2.3654	0.020197	-3.34274	0.354123	0.204382
NK.cells	MS4A6C	0.606872	5.165825	2.365363	0.020199	-3.86119	0.340085	0.18965
NK.cells	FBLIM1	-1.4093	0.928973	-2.36507	0.020214	-3.22541	0.362493	0.213373
NK.cells	KDM2A	-0.15035	7.465818	-2.36489	0.020223	-4.25587	0.328555	0.177905
NK.cells	UNC50	0.305601	4.105649	2.364024	0.020268	-3.68646	0.345813	0.195552

NK.cells	SULT2A1	-0.78319	3.901678	-2.36235	0.020354	-3.61692	0.34785	0.197145
NK.cells	0610012G(	0.250749	5.039442	2.360532	0.020448	-3.84093	0.34251	0.191357
NK.cells	GLRX2	0.242004	5.111058	2.359481	0.020502	-3.88627	0.342141	0.191264
NK.cells	TTF1	0.401895	3.466533	2.359373	0.020508	-3.56229	0.350709	0.200233
NK.cells	HMGB2	-0.34719	9.132267	-2.35886	0.020534	-4.5479	0.322145	0.171099
NK.cells	SPRY2	-0.32419	5.158508	-2.35827	0.020565	-4.08853	0.341898	0.191151
NK.cells	DAB2	-1.09704	4.009728	-2.35817	0.02057	-3.41979	0.347854	0.197363
NK.cells	CACNA1I	-1.03791	-0.17458	-2.35758	0.020601	-3.2743	0.370508	0.221955
NK.cells	MEIS1	0.550874	3.866059	2.357239	0.020619	-3.48442	0.348606	0.198461
NK.cells	IGF2BP3	0.260645	6.5516	2.352674	0.020858	-4.11203	0.338234	0.185124
NK.cells	ARHGAP15	0.192019	9.872031	2.349287	0.021037	-4.70991	0.32398	0.169513
NK.cells	PSME2B	0.545815	2.815452	2.348956	0.021055	-3.4859	0.360134	0.206354
NK.cells	RASSF3	0.288147	6.834864	2.347691	0.021122	-4.1603	0.339389	0.184724
NK.cells	CDC42EP3	-0.30589	5.142958	-2.34719	0.021149	-3.96728	0.348114	0.193699
NK.cells	LIPC	-0.71414	2.510562	-2.34687	0.021166	-3.35903	0.36218	0.208421
NK.cells	HIST1H2BC	-0.45292	5.260139	-2.34527	0.021252	-3.82673	0.348078	0.193557
NK.cells	ENO3	0.640599	2.077848	2.345108	0.02126	-3.46419	0.365154	0.211524
NK.cells	GM11707	0.725609	0.863582	2.343885	0.021326	-3.33429	0.372524	0.218935
NK.cells	EPB42	1.157373	-0.1647	2.343346	0.021355	-3.25768	0.378353	0.225387
NK.cells	HKDC1	-1.27829	0.37121	-2.34233	0.021409	-3.25814	0.375317	0.222316
NK.cells	SLFN2	0.33556	6.335056	2.342232	0.021415	-4.22028	0.34309	0.18833
NK.cells	CHERP	-0.24259	5.016827	-2.34139	0.02146	-3.90096	0.350207	0.195605
NK.cells	GALNT6	0.396258	3.115592	2.339376	0.021569	-3.806	0.361697	0.20686
NK.cells	ATP13A1	0.319176	3.809105	2.338571	0.021613	-3.67584	0.358172	0.203057
NK.cells	CDC27	0.229416	6.009717	2.335849	0.021762	-4.08444	0.347964	0.191708
NK.cells	CXXC5	-0.28982	5.11182	-2.33547	0.021782	-3.89019	0.352686	0.196624
NK.cells	CTSG	2.289818	-0.2691	2.33528	0.021793	-3.2871	0.382477	0.228369
NK.cells	UBLCP1	0.283607	4.773952	2.334633	0.021828	-3.82217	0.354572	0.198687
NK.cells	ESYT2	-0.18812	6.802347	-2.33302	0.021917	-4.32714	0.344697	0.188312
NK.cells	NUPR1	1.412595	2.41246	2.332669	0.021936	-3.31252	0.36821	0.212825
NK.cells	XPO6	0.219424	5.572883	2.331599	0.021995	-4.03138	0.351233	0.195173
NK.cells	2510046G1	0.513689	2.620896	2.331446	0.022004	-3.45002	0.367181	0.211885
NK.cells	OASL1	1.24261	2.957835	2.330852	0.022036	-3.38708	0.365369	0.209955
NK.cells	TRMT1L	0.236934	4.566129	2.32975	0.022097	-3.85914	0.357134	0.201024
NK.cells	TSPOAP1	-0.62344	2.650845	-2.32837	0.022174	-3.4601	0.368351	0.212447
NK.cells	SMC3	-0.19308	6.498954	-2.32488	0.022369	-4.21935	0.349743	0.191616
NK.cells	SLC17A5	0.437402	3.572742	2.324805	0.022373	-3.63238	0.365462	0.207896
NK.cells	LAMC1	0.360099	4.523339	2.324022	0.022417	-3.87019	0.360273	0.202707
NK.cells	TRMO	0.459112	3.013486	2.323815	0.022429	-3.53493	0.368552	0.211422
NK.cells	PILRB1	-1.22614	2.049283	-2.32328	0.022459	-3.29185	0.373947	0.217181
NK.cells	TNFRSF4	0.724503	0.30724	2.322713	0.022491	-3.44865	0.383934	0.228178
NK.cells	SEMA4C	-0.52996	2.202853	-2.32171	0.022547	-3.48899	0.37328	0.21674
NK.cells	FAM76A	0.250943	5.119697	2.321222	0.022575	-3.95863	0.357248	0.199954
NK.cells	JAGN1	0.372158	3.955321	2.320919	0.022592	-3.73129	0.363557	0.206559
NK.cells	SLC22A23	-0.96706	3.017213	-2.31909	0.022696	-3.39697	0.369925	0.212527
NK.cells	ANKRD11	-0.16995	9.391983	-2.31798	0.022759	-4.69301	0.336361	0.178316
NK.cells	FBXO10	-0.83096	1.355085	-2.31783	0.022768	-3.30617	0.379494	0.223051

NK.cells	LCLAT1	0.410793	3.99918	2.317224	0.022802	-3.69454	0.364735	0.207265
NK.cells	MSRB1	0.311856	5.958368	2.315539	0.022898	-4.03306	0.3548	0.196665
NK.cells	IGFBP2	-0.81626	4.701389	-2.31543	0.022904	-3.90785	0.361561	0.203703
NK.cells	CEP95	0.30374	4.108354	2.31364	0.023007	-3.75516	0.365949	0.207649
NK.cells	CCDC191	0.64193	1.993228	2.312287	0.023085	-3.40671	0.37822	0.2206
NK.cells	SLFN4	1.561666	0.48225	2.312127	0.023094	-3.31201	0.386943	0.230059
NK.cells	PLA2G12A	0.400797	4.277535	2.309996	0.023217	-3.69271	0.366836	0.207559
NK.cells	MANBAL	0.254212	4.531107	2.309526	0.023244	-3.86614	0.36544	0.206246
NK.cells	WSB1	-0.21802	6.461454	-2.30876	0.023289	-4.18703	0.355001	0.195555
NK.cells	FGB	-0.43101	5.797972	-2.30818	0.023322	-4.13644	0.358551	0.199334
NK.cells	RNMT	0.252796	4.970618	2.30792	0.023338	-3.98635	0.363034	0.204022
NK.cells	FAM71A	-1.32273	0.237403	-2.30716	0.023382	-3.34036	0.389871	0.233018
NK.cells	MRPL39	0.331167	3.661118	2.306863	0.023399	-3.69246	0.370254	0.211955
NK.cells	JOSD1	-0.30573	3.99961	-2.30619	0.023439	-3.80512	0.368373	0.210193
NK.cells	GM13547	-1.28761	0.514665	-2.30584	0.023459	-3.32267	0.388242	0.231632
NK.cells	KDM2B	-0.31282	7.133876	-2.30442	0.023542	-4.50996	0.352224	0.192867
NK.cells	SULT1D1	-0.82241	2.290136	-2.30365	0.023588	-3.43507	0.379056	0.220992
NK.cells	VPS13C	0.308305	4.373385	2.301942	0.023688	-3.93866	0.367906	0.209091
NK.cells	HPGD	-0.78747	5.673887	-2.30147	0.023716	-3.88629	0.360787	0.201771
NK.cells	GHR	-0.73164	4.215001	-2.30125	0.023729	-3.81449	0.368783	0.210163
NK.cells	SMAP1	0.189026	6.875842	2.300947	0.023747	-4.37223	0.354342	0.195163
NK.cells	ZFP992	0.422601	3.563813	2.299397	0.023838	-3.7014	0.373144	0.214448
NK.cells	CDK11B	-0.20851	6.82853	-2.29845	0.023895	-4.35256	0.355287	0.196073
NK.cells	GM35154	-1.31447	1.526848	-2.29832	0.023902	-3.33835	0.384776	0.227257
NK.cells	RHOQ	-0.28367	5.771108	-2.29808	0.023917	-4.21102	0.360966	0.20199
NK.cells	1600022D1	1.138966	-0.42787	2.294826	0.024111	-3.39156	0.398339	0.241016
NK.cells	NT5DC1	0.291287	4.5014	2.294585	0.024125	-3.96442	0.369809	0.210199
NK.cells	MRPL34	0.219026	5.428125	2.294213	0.024148	-4.07674	0.364696	0.204993
NK.cells	KDM5C	-0.29758	6.827805	-2.29394	0.024164	-4.30199	0.357118	0.197206
NK.cells	TOB2	-0.21913	6.656821	-2.29296	0.024223	-4.35237	0.358364	0.198328
NK.cells	MED8	0.272859	5.008062	2.29156	0.024307	-3.98725	0.367341	0.20804
NK.cells	WDR37	0.23873	5.478401	2.291209	0.024328	-4.08618	0.364755	0.205471
NK.cells	DLST	0.195284	5.852364	2.29083	0.024351	-4.13962	0.362713	0.203448
NK.cells	MAN1B1	-0.3344	4.927787	-2.28974	0.024417	-4.0207	0.367784	0.209048
NK.cells	SRA1	0.234197	5.239409	2.289553	0.024428	-4.07677	0.366067	0.207295
NK.cells	PTMS	-0.24267	6.015415	-2.28931	0.024443	-4.30982	0.361827	0.202922
NK.cells	TSC22D4	0.212183	6.684783	2.2892	0.02445	-4.29907	0.358213	0.199176
NK.cells	SERPINA3N	-0.60143	3.688152	-2.28875	0.024477	-3.74209	0.374705	0.216638
NK.cells	TM9SF2	-0.15421	6.683958	-2.28769	0.024542	-4.28762	0.358218	0.1996
NK.cells	ISG20	0.564796	4.233394	2.287404	0.024559	-3.88457	0.371644	0.213699
NK.cells	PNPLA1	-1.47495	0.231599	-2.28735	0.024562	-3.39937	0.394753	0.23885
NK.cells	TIMM10B	0.225843	6.181583	2.286888	0.02459	-4.19597	0.360926	0.202537
NK.cells	CARD10	-0.68185	1.098314	-2.28381	0.024778	-3.43719	0.392105	0.234247
NK.cells	PICK1	0.682661	1.538716	2.283057	0.024825	-3.45026	0.389511	0.231762
NK.cells	ZFP329	0.465238	2.511051	2.282576	0.024854	-3.53267	0.383842	0.225736
NK.cells	SUN2	-0.21591	6.122993	-2.28228	0.024872	-4.23905	0.36355	0.204246
NK.cells	VCL	-0.27819	6.049702	-2.28109	0.024946	-4.18025	0.364568	0.20491

NK.cells	GM43661	-0.53884	2.981343	-2.27881	0.025087	-3.73366	0.38326	0.223975
NK.cells	ZCCHC4	0.405078	3.455439	2.27841	0.025112	-3.77258	0.380534	0.221179
NK.cells	MRPL53	0.359612	4.291701	2.278002	0.025137	-3.82248	0.375775	0.216148
NK.cells	SEC22A	0.397822	3.176208	2.277057	0.025196	-3.68269	0.382521	0.223213
NK.cells	2610035D1	-0.42085	4.872984	-2.27658	0.025225	-4.03364	0.37288	0.21294
NK.cells	HIBADH	0.29572	5.742005	2.274964	0.025327	-4.08556	0.3689	0.208196
NK.cells	1810030O(	-0.24623	4.824335	-2.27463	0.025347	-4.08693	0.374019	0.213668
NK.cells	CUL4A	0.246376	5.002429	2.273188	0.025438	-4.01494	0.373805	0.212832
NK.cells	FAM129C	-0.5429	3.433215	-2.27277	0.025464	-3.5702	0.382733	0.222471
NK.cells	PELI1	0.18685	7.676753	2.272217	0.025499	-4.50978	0.35916	0.197814
NK.cells	GM13212	0.444513	3.205264	2.271116	0.025568	-3.70357	0.384142	0.224292
NK.cells	BBOX1	-1.05158	0.92351	-2.27068	0.025596	-3.38878	0.397587	0.239099
NK.cells	PIGX	0.235173	5.308726	2.270546	0.025604	-4.14906	0.372179	0.21164
NK.cells	SHLD1	0.367391	3.33904	2.270167	0.025628	-3.80533	0.383369	0.223608
NK.cells	ACP5	0.397851	5.117131	2.268824	0.025713	-4.12684	0.373314	0.213257
NK.cells	PRKCI	-0.51788	3.113961	-2.26865	0.025724	-3.6096	0.384734	0.225485
NK.cells	AARS	-0.28578	5.020707	-2.2686	0.025727	-4.03805	0.373855	0.21383
NK.cells	ZBTB40	0.410071	3.163496	2.266856	0.025838	-3.71019	0.385628	0.225615
NK.cells	DYSF	-1.35464	2.592245	-2.2662	0.02588	-3.41242	0.388991	0.229401
NK.cells	NLRP1A	1.260679	0.449959	2.265828	0.025904	-3.39324	0.401768	0.243573
NK.cells	RINL	0.314697	3.632551	2.264569	0.025984	-4.00221	0.383664	0.223338
NK.cells	TMEM37	-0.50843	3.751452	-2.26353	0.026051	-3.79907	0.383079	0.222823
NK.cells	LTB4R1	0.634834	2.689349	2.263222	0.02607	-3.70171	0.389255	0.22963
NK.cells	PRUNE2	1.073393	0.638929	2.262972	0.026086	-3.39765	0.401482	0.243116
NK.cells	BICD1	1.0887	0.681795	2.259973	0.02628	-3.40254	0.403213	0.243579
NK.cells	PRKCG	-0.8527	4.448629	-2.25878	0.026357	-3.56997	0.380964	0.219721
NK.cells	TSPYL1	-0.28256	4.888609	-2.25855	0.026372	-4.01755	0.378452	0.217108
NK.cells	BUD13	0.32669	3.714639	2.258255	0.026391	-3.82801	0.385193	0.224406
NK.cells	DEK	-0.21285	7.842	-2.25742	0.026445	-4.55551	0.362058	0.200262
NK.cells	MRPL52	0.205007	7.102833	2.256535	0.026503	-4.47738	0.366086	0.204514
NK.cells	RAB27A	0.278044	4.50985	2.256444	0.026508	-4.11096	0.380613	0.219824
NK.cells	ACAT1	-0.34352	6.480379	-2.2564	0.026511	-4.33909	0.369517	0.208089
NK.cells	NCOA6	0.2049	5.600455	2.255429	0.026575	-4.20102	0.374427	0.213454
NK.cells	GM13919	-0.7845	3.111975	-2.25506	0.026598	-3.45861	0.388704	0.228902
NK.cells	UPP2	0.96351	1.652112	2.254887	0.02661	-3.45017	0.397352	0.238446
NK.cells	TGFB1I1	-1.1714	0.90381	-2.2545	0.026635	-3.41301	0.401864	0.243523
NK.cells	KLRA7	-0.70524	0.646446	-2.25424	0.026652	-3.85796	0.403429	0.245368
NK.cells	GSTM1	-0.52726	4.059268	-2.25407	0.026663	-3.82072	0.383201	0.223196
NK.cells	IKBKG	0.378591	3.339723	2.252912	0.026739	-3.75635	0.38801	0.227916
NK.cells	BRAF	-0.24051	7.588384	-2.25005	0.026928	-4.56193	0.36578	0.203205
NK.cells	PACC1	-0.31873	5.421952	-2.24992	0.026936	-4.1528	0.377855	0.215838
NK.cells	SRF	0.337005	2.927184	2.249495	0.026964	-3.73371	0.392303	0.231401
NK.cells	2-Sep	0.597305	2.22603	2.248963	0.026999	-3.60128	0.396514	0.236176
NK.cells	PLEKHA5	-0.24385	4.693302	-2.24707	0.027125	-4.28529	0.383278	0.220879
NK.cells	NOP10	0.202031	7.15867	2.246181	0.027184	-4.46467	0.369359	0.206335
NK.cells	E130307A1	0.276755	4.490339	2.245137	0.027254	-3.97614	0.384449	0.222591
NK.cells	S1PR5	-1.10787	-0.65106	-2.2451	0.027256	-3.42929	0.415438	0.256787

NK.cells	CDK7	0.309182	4.595584	2.244973	0.027265	-4.0045	0.383841	0.221953
NK.cells	PTER	-0.73479	2.433342	-2.24475	0.027279	-3.52486	0.396541	0.235772
NK.cells	PIGC	0.429033	2.730331	2.24247	0.027432	-3.69468	0.396116	0.234499
NK.cells	ST7	0.297566	5.144851	2.242393	0.027437	-4.15352	0.381984	0.219277
NK.cells	IGKV2-109	-1.21356	-0.87987	-2.23983	0.027609	-3.48924	0.420204	0.260077
NK.cells	SARNP	-0.15258	7.797226	-2.23957	0.027627	-4.57979	0.368764	0.204328
NK.cells	CSTDC4	1.251991	3.661016	2.238189	0.027721	-3.6496	0.392811	0.229709
NK.cells	FCOR	1.09829	0.865885	2.238159	0.027723	-3.44237	0.409715	0.248275
NK.cells	SH3PXD2A	-0.31856	5.753145	-2.23582	0.027881	-4.19094	0.382383	0.217347
NK.cells	KRT80	-0.94279	0.161146	-2.23475	0.027954	-3.44725	0.416577	0.254257
NK.cells	SLC25A1	0.405668	3.772843	2.230452	0.028249	-3.83766	0.397783	0.231456
NK.cells	SMUG1	0.52886	1.976529	2.230391	0.028253	-3.58219	0.408694	0.243324
NK.cells	NEBL	-0.66662	1.182115	-2.22959	0.028308	-3.75704	0.413842	0.249055
NK.cells	FAM217B	1.084055	0.616789	2.229214	0.028334	-3.4549	0.417388	0.253037
NK.cells	HSPG2	-0.69602	2.741833	-2.22669	0.028509	-3.56	0.405998	0.239177
NK.cells	PLEKHA1	0.348069	4.707996	2.226456	0.028525	-4.15119	0.39416	0.226486
NK.cells	GTF2H1	-0.22706	5.958143	-2.22538	0.0286	-4.28748	0.3871	0.219108
NK.cells	PKIG	-0.27587	7.198634	-2.2249	0.028633	-4.28933	0.379969	0.211782
NK.cells	NEURL2	1.022513	0.801683	2.224328	0.028673	-3.47092	0.418347	0.25313
NK.cells	ERCC6L2	0.417698	3.372895	2.224259	0.028678	-3.79965	0.402439	0.235666
NK.cells	EGFL7	-0.62142	3.872106	-2.22297	0.028768	-3.73293	0.400214	0.232734
NK.cells	MYO9B	0.222496	5.866728	2.222255	0.028818	-4.33769	0.388624	0.220405
NK.cells	ANAPC4	0.248922	4.541254	2.22092	0.028912	-4.08973	0.396535	0.229144
NK.cells	UBA5	0.242913	4.489487	2.220905	0.028913	-4.04028	0.396843	0.229474
NK.cells	CNTD1	-1.02267	0.620711	-2.21987	0.028986	-3.47155	0.420667	0.255836
NK.cells	FOSL2	-0.25465	5.561041	-2.21958	0.029006	-4.50616	0.390506	0.223061
NK.cells	HMGB1	-0.20917	9.807497	-2.21913	0.029038	-4.89959	0.366467	0.198287
NK.cells	METTL1	0.485615	3.431367	2.218466	0.029085	-3.78743	0.403212	0.236982
NK.cells	COL27A1	-1.23636	2.314283	-2.21846	0.029085	-3.47999	0.410055	0.244462
NK.cells	IL1RN	1.764217	2.526967	2.217277	0.029169	-3.48474	0.408742	0.243309
NK.cells	FAM168A	-0.20781	5.93211	-2.21717	0.029176	-4.37715	0.388337	0.221309
NK.cells	CEP295	0.318377	3.949897	2.217004	0.029188	-3.95747	0.400077	0.233864
NK.cells	TM6SF1	-0.22777	6.252773	-2.21695	0.029192	-4.51255	0.386473	0.219342
NK.cells	SERINC3	-0.15783	9.707046	-2.21529	0.02931	-4.84091	0.367815	0.199533
NK.cells	GPALPP1	0.296181	3.737889	2.215116	0.029322	-3.9587	0.40223	0.235671
NK.cells	UQCC2	0.239124	6.141014	2.212994	0.029473	-4.40938	0.389197	0.221002
NK.cells	TMCO6	0.467962	2.683081	2.21287	0.029482	-3.68049	0.409969	0.24333
NK.cells	ATXN7L1	0.21733	6.345264	2.212044	0.029541	-4.42842	0.388343	0.219895
NK.cells	LRRC29	0.564616	1.690453	2.209966	0.02969	-3.5483	0.418138	0.251172
NK.cells	PAFAH1B3	-0.38231	5.814072	-2.20816	0.029821	-4.08568	0.393976	0.224255
NK.cells	IRF8	-0.32589	6.49623	-2.20774	0.029851	-4.45443	0.389965	0.220091
NK.cells	CFAP20	-0.22917	5.191326	-2.20752	0.029867	-4.20906	0.397677	0.228302
NK.cells	CTSH	-0.51505	5.779307	-2.20601	0.029976	-3.9106	0.395178	0.225024
NK.cells	LRRC63	1.250176	0.544146	2.205474	0.030015	-3.49488	0.42766	0.260597
NK.cells	SWSAP1	1.076149	0.278314	2.204787	0.030065	-3.49606	0.42961	0.262828
NK.cells	MBLAC1	-1.21008	-0.19049	-2.204	0.030122	-3.53194	0.433	0.266574
NK.cells	CD164	-0.23567	6.379209	-2.20251	0.030231	-4.35284	0.393189	0.222501

NK.cells	ZFP709	0.545284	1.431477	2.20171	0.030289	-3.59119	0.423916	0.255699
NK.cells	TEX264	0.247822	4.497588	2.200871	0.030351	-4.11951	0.405052	0.234884
NK.cells	TMSB15B1	-0.48082	2.422831	-2.20051	0.030377	-3.74782	0.417903	0.248935
NK.cells	SLC18A1	-1.33801	0.497721	-2.19957	0.030446	-3.51069	0.430707	0.262874
NK.cells	MRC1	-0.9779	5.240936	-2.19857	0.03052	-3.88072	0.401456	0.230741
NK.cells	USP2	-0.58495	2.711696	-2.19812	0.030553	-3.68853	0.417026	0.247682
NK.cells	RHD	0.799795	1.667344	2.197722	0.030582	-3.61002	0.423644	0.25511
NK.cells	IL1A	-1.26913	2.787687	-2.19284	0.030944	-3.53453	0.419812	0.248503
NK.cells	MAP2K3	-0.27023	6.01457	-2.19279	0.030948	-4.43437	0.399929	0.227159
NK.cells	FSD2	-1.23236	-0.73763	-2.1927	0.030954	-3.53751	0.442752	0.274103
NK.cells	UPP1	1.667979	0.646262	2.19264	0.030959	-3.51785	0.433593	0.263776
NK.cells	2310022B0	0.473217	2.488564	2.189206	0.031216	-3.65851	0.424167	0.251685
NK.cells	ENDOG	0.387626	2.943039	2.188744	0.031251	-3.8546	0.421272	0.248755
NK.cells	KDSR	0.304227	3.963038	2.188328	0.031282	-4.04099	0.414852	0.241872
NK.cells	TKTL1	-0.78723	1.290296	-2.1872	0.031367	-3.55668	0.431901	0.260798
NK.cells	GM26737	-1.04442	0.478605	-2.18697	0.031384	-3.52562	0.437226	0.266745
NK.cells	TRAF3	0.266597	6.856964	2.186947	0.031386	-4.5247	0.397211	0.223373
NK.cells	SERPINA3F	1.246717	2.19929	2.186678	0.031407	-3.65547	0.42602	0.254319
NK.cells	KLRG1	0.740718	-1.146	2.186552	0.031416	-3.58638	0.447987	0.279085
NK.cells	KANK2	-0.80054	2.38565	-2.1856	0.031488	-3.57011	0.424902	0.253261
NK.cells	CWC25	-0.27227	5.255579	-2.18477	0.031551	-4.35713	0.406943	0.233965
NK.cells	CEBPD	0.529212	3.956744	2.184215	0.031593	-3.777	0.414966	0.242746
NK.cells	MDN1	-0.30069	5.788045	-2.18377	0.031627	-4.4011	0.403702	0.23078
NK.cells	PYGM	-0.44431	4.300481	-2.18326	0.031665	-4.0442	0.412826	0.24071
NK.cells	1600014C1	0.404326	4.275444	2.183204	0.03167	-4.19618	0.412982	0.240891
NK.cells	SIK1	-0.20686	6.868808	-2.18276	0.031704	-4.56347	0.397212	0.224133
NK.cells	ADD1	0.21461	5.795836	2.182735	0.031706	-4.40289	0.403655	0.230929
NK.cells	PINX1	0.423041	3.022074	2.181971	0.031764	-3.8627	0.420847	0.249577
NK.cells	ERO1LB	-0.22417	6.652932	-2.18192	0.031767	-4.46055	0.398499	0.225585
NK.cells	ZFP467	0.644716	2.303773	2.181166	0.031825	-3.65893	0.425495	0.254831
NK.cells	TOR1AIP2	-0.16636	6.580878	-2.18082	0.031852	-4.55856	0.398994	0.226274
NK.cells	TSPAN33	0.850471	2.55857	2.1805	0.031876	-3.61581	0.423864	0.253105
NK.cells	SULT2A2	-0.65851	4.037802	-2.17954	0.03195	-4.03787	0.414747	0.243005
NK.cells	6720489N1	-0.83837	0.964398	-2.17921	0.031975	-3.54852	0.434408	0.264747
NK.cells	ALG12	0.741061	1.395336	2.178925	0.031997	-3.61318	0.431593	0.261729
NK.cells	4632404H1	0.964693	1.238383	2.176491	0.032184	-3.55751	0.434679	0.263586
NK.cells	CD22	-0.69219	2.780412	-2.1756	0.032253	-3.63415	0.425136	0.252712
NK.cells	BTF3L4	0.272915	4.269235	2.173699	0.0324	-4.06205	0.417139	0.243079
NK.cells	MRE11A	0.237457	4.349103	2.173066	0.032449	-4.13879	0.416638	0.242695
NK.cells	BCL2A1A	1.373505	2.399522	2.172824	0.032468	-3.56949	0.429049	0.256265
NK.cells	TRMT61B	-0.41656	4.178074	-2.17208	0.032526	-4.20386	0.417998	0.244071
NK.cells	ZFP991	0.53675	2.80432	2.171639	0.03256	-3.78552	0.426733	0.253704
NK.cells	CTSZ	0.330765	6.598627	2.171063	0.032605	-4.36118	0.403201	0.228427
NK.cells	KRCC1	0.170292	5.9705	2.169275	0.032745	-4.44916	0.408106	0.232973
NK.cells	GDF15	-1.34951	1.508239	-2.16905	0.032763	-3.56734	0.436451	0.263847
NK.cells	NCSTN	0.241431	5.576404	2.168553	0.032802	-4.33107	0.410578	0.235707
NK.cells	BC003965	0.3601	3.701969	2.165422	0.033048	-3.99617	0.422703	0.249322

NK.cells	ID1	-0.76782	4.07039	-2.1648	0.033097	-3.73501	0.420366	0.246909
NK.cells	B230208H1	-1.20999	0.861264	-2.16469	0.033106	-3.56439	0.441196	0.269997
NK.cells	PRDX5	0.372747	7.635313	2.164413	0.033128	-4.68492	0.398469	0.223676
NK.cells	LIMD2	0.213564	7.333227	2.164182	0.033146	-4.70029	0.400275	0.225622
NK.cells	MARS2	-0.44081	2.690445	-2.16398	0.033162	-3.77439	0.429191	0.256753
NK.cells	MPLKIP	0.225534	4.793719	2.163562	0.033195	-4.20788	0.415818	0.242312
NK.cells	RTN2	1.279742	-0.4318	2.162843	0.033252	-3.58072	0.449897	0.280382
NK.cells	GBP5	0.840884	2.014589	2.162841	0.033252	-3.82558	0.433586	0.261964
NK.cells	GM47863	-0.98903	0.552776	-2.16244	0.033284	-3.57873	0.443256	0.273006
NK.cells	1700016P0	-0.43678	3.898827	-2.16235	0.033291	-4.2949	0.421452	0.248752
NK.cells	SNRNP27	0.199481	5.842837	2.161891	0.033328	-4.40267	0.409318	0.23574
NK.cells	YWHAQ	-0.15114	7.746706	-2.1607	0.033422	-4.73058	0.397806	0.223875
NK.cells	TLR4	0.825153	3.122783	2.159015	0.033557	-3.64235	0.426405	0.255265
NK.cells	SENP1	0.223127	5.013417	2.158239	0.033619	-4.26681	0.414448	0.242372
NK.cells	DDIT4	0.595774	3.744278	2.157549	0.033675	-4.01409	0.422433	0.251219
NK.cells	USP49	-0.38081	4.534917	-2.15696	0.033722	-4.12647	0.417439	0.245987
NK.cells	F13A1	1.761112	2.512117	2.156762	0.033738	-3.61839	0.430346	0.260261
NK.cells	TLR1	0.846449	1.219121	2.15673	0.033741	-3.64094	0.43882	0.269787
NK.cells	COL18A1	-0.7127	2.743841	-2.15656	0.033754	-3.78637	0.428846	0.25865
NK.cells	BATF	0.279473	4.183315	2.156333	0.033772	-4.2553	0.419652	0.248565
NK.cells	MED10	0.253066	4.886924	2.156242	0.03378	-4.24992	0.415236	0.243777
NK.cells	ABCC4	0.261622	4.55376	2.156091	0.033792	-4.24269	0.417321	0.246068
NK.cells	MOB1B	-0.17258	6.203813	-2.15581	0.033815	-4.51074	0.407107	0.235025
NK.cells	CCNA2	-0.46254	5.520186	-2.15538	0.033849	-4.42425	0.411305	0.239644
NK.cells	TARM1	1.306343	-0.18149	2.155097	0.033872	-3.57914	0.448199	0.280889
NK.cells	CDC25C	-0.63275	2.030722	-2.15463	0.03391	-3.7643	0.43348	0.264292
NK.cells	ROBO2	-1.36776	0.744725	-2.15412	0.033951	-3.57847	0.441973	0.27406
NK.cells	IGBP1	0.253552	4.749526	2.154032	0.033958	-4.27586	0.416095	0.245172
NK.cells	SLC25A16	0.323863	3.724214	2.153542	0.033998	-4.05775	0.422561	0.252352
NK.cells	FOXO1	-0.19486	7.035015	-2.15334	0.034014	-4.7047	0.402067	0.230136
NK.cells	IGLC3	-1.26921	4.04289	-2.1531	0.034033	-3.78339	0.42054	0.250134
NK.cells	MAOB	-0.94595	1.627366	-2.15276	0.034061	-3.636	0.436125	0.267556
NK.cells	APOC1	-0.55206	7.949726	-2.15255	0.034078	-4.7397	0.396599	0.224405
NK.cells	FABP1	-0.5716	7.428716	-2.15211	0.034114	-4.72356	0.399703	0.227834
NK.cells	NDUFS7	0.183214	6.320866	2.151669	0.03415	-4.51782	0.406393	0.235067
NK.cells	TCEAL8	-0.52604	3.331023	-2.15134	0.034176	-3.72786	0.42507	0.255552
NK.cells	INPP5J	1.098032	-1.12022	2.151324	0.034178	-3.59412	0.454511	0.289188
NK.cells	PPP1R16A	0.455747	2.885752	2.151047	0.0342	-3.84018	0.42793	0.258843
NK.cells	WDFY3	-0.59264	5.666809	-2.14834	0.034421	-3.93134	0.411585	0.240284
NK.cells	BIRC3	0.237954	7.111789	2.148081	0.034442	-4.65588	0.402763	0.230903
NK.cells	ANPEP	-1.10395	1.184937	-2.14749	0.03449	-3.61162	0.440312	0.272474
NK.cells	IL18R1	0.502668	0.978465	2.14742	0.034496	-3.95802	0.441687	0.274042
NK.cells	CYP2C68	-0.67301	2.791797	-2.14741	0.034497	-3.84583	0.429772	0.260569
NK.cells	ZCCHC24	-0.51181	3.666755	-2.14725	0.03451	-3.84249	0.424147	0.254334
NK.cells	KLHL23	-1.33528	0.171341	-2.14425	0.034757	-3.61919	0.449476	0.281335
NK.cells	LRCH3	0.192059	6.497112	2.14346	0.034822	-4.60544	0.408648	0.236073
NK.cells	TNS4	1.205276	-0.27421	2.14338	0.034829	-3.59892	0.452511	0.284994

NK.cells	RAG2	-1.4552	0.168525	-2.14331	0.034835	-3.60255	0.449495	0.281537
NK.cells	ZFP516	0.418043	4.66128	2.142633	0.03489	-3.98926	0.420315	0.248748
NK.cells	FCGRT	-0.53793	4.823452	-2.14174	0.034965	-4.04819	0.41945	0.247846
NK.cells	SEMA4D	0.283757	6.446214	2.14162	0.034974	-4.65602	0.409355	0.236924
NK.cells	ETNK2	-0.86988	1.270533	-2.14052	0.035066	-3.63167	0.443211	0.273915
NK.cells	CMTM8	-0.5418	4.192364	-2.13839	0.035243	-3.94141	0.425838	0.253082
NK.cells	FGFR1OP2	0.199017	6.180589	2.136233	0.035424	-4.52176	0.414252	0.240214
NK.cells	ORC1	-0.42484	3.345968	-2.13604	0.03544	-4.0439	0.432282	0.259967
NK.cells	HNRNPL	-0.13668	8.426349	-2.13572	0.035467	-4.89972	0.400555	0.225726
NK.cells	COQ2	0.284846	4.467496	2.135305	0.035502	-4.21152	0.425048	0.252151
NK.cells	CDKN1A	-0.3245	5.267608	-2.1353	0.035502	-4.42976	0.419968	0.246596
NK.cells	JUNB	-0.22017	9.343714	-2.13296	0.035699	-5.14598	0.396646	0.220636
NK.cells	PALB2	0.583548	2.125531	2.132809	0.035712	-3.75039	0.44202	0.269766
NK.cells	ITIH3	-0.58072	3.636116	-2.13218	0.035765	-4.04238	0.432285	0.258798
NK.cells	CFHR1	-1.24356	0.449486	-2.13118	0.03585	-3.61639	0.453806	0.28309
NK.cells	RSRC1	0.191273	6.605429	2.130695	0.035891	-4.62581	0.413649	0.238612
NK.cells	ARF4OS	-0.84379	0.845404	-2.13014	0.035938	-3.6271	0.451101	0.280158
NK.cells	SETBP1	0.357726	4.621756	2.129588	0.035985	-4.56178	0.426152	0.252338
NK.cells	SHCBP1	-0.43838	4.69138	-2.12926	0.036013	-4.2998	0.425707	0.251929
NK.cells	HERPUD1	-0.27759	7.853564	-2.12919	0.036019	-4.74028	0.40599	0.230717
NK.cells	GALK1	0.318699	5.140655	2.128994	0.036035	-4.26485	0.422842	0.248836
NK.cells	4833445I0	-1.03992	0.48418	-2.12687	0.036216	-3.62353	0.455237	0.283944
NK.cells	MVP	0.257038	5.108561	2.126596	0.03624	-4.3673	0.424603	0.2497
NK.cells	GRAMD4	0.297508	5.165751	2.125861	0.036303	-4.41488	0.424554	0.249549
NK.cells	PCBD1	-0.63626	3.53173	-2.12369	0.036489	-4.04197	0.436917	0.261929
NK.cells	RGS2	-0.22719	6.977171	-2.12301	0.036548	-4.75225	0.415136	0.238103
NK.cells	REV3L	-0.1847	6.713202	-2.12127	0.036698	-4.68213	0.417906	0.24032
NK.cells	CLEC4F	-1.16391	5.458864	-2.12018	0.036792	-4.17774	0.425844	0.249199
NK.cells	C1QA	-0.83968	5.480946	-2.11981	0.036824	-4.12896	0.425703	0.24916
NK.cells	SCNN1A	1.231312	0.542515	2.119499	0.036851	-3.63344	0.458572	0.285991
NK.cells	RCOR2	-1.24869	0.194015	-2.11893	0.036901	-3.63791	0.460992	0.288909
NK.cells	TMPO	-0.24073	7.067829	-2.11809	0.036974	-4.76226	0.415691	0.238801
NK.cells	UBE2O	0.380993	4.874388	2.117999	0.036982	-4.32066	0.429599	0.253862
NK.cells	LPCAT4	-0.35823	3.121289	-2.11739	0.037035	-4.30846	0.441081	0.266715
NK.cells	MAML3	0.334315	6.974808	2.117273	0.037045	-4.57736	0.416271	0.239586
NK.cells	ASGR2	-0.89132	1.698173	-2.11727	0.037045	-3.71842	0.450644	0.277483
NK.cells	PROCA1	-0.44074	2.988273	-2.11674	0.037091	-3.98545	0.441965	0.267952
NK.cells	SNHG17	0.604922	1.368538	2.11652	0.037111	-3.74312	0.45289	0.280348
NK.cells	ARHGAP12	0.268323	5.076663	2.115921	0.037163	-4.36048	0.428296	0.253061
NK.cells	MSN	0.175455	8.454473	2.115789	0.037175	-4.96447	0.407156	0.230379
NK.cells	WDR13	-0.46638	2.570105	-2.11561	0.037191	-3.79648	0.444759	0.271451
NK.cells	SPATA6	0.269298	4.52297	2.112993	0.03742	-4.40905	0.434112	0.258015
NK.cells	MIOS	0.316235	3.665658	2.112105	0.037498	-4.09037	0.439804	0.264577
NK.cells	CANX	0.153309	7.35016	2.112085	0.0375	-4.74842	0.416136	0.238785
NK.cells	RNF149	0.330391	5.786169	2.111695	0.037534	-4.56113	0.426008	0.249458
NK.cells	PDZRN3	-1.0147	0.932125	-2.10936	0.03774	-3.6604	0.45991	0.286476
NK.cells	CISD2	0.181024	6.732173	2.108961	0.037776	-4.70778	0.421477	0.243878

NK.cells	TM4SF1	0.93214	2.15291	2.108482	0.037818	-3.73565	0.451517	0.27723
NK.cells	PDCD10	0.15453	6.713222	2.107733	0.037885	-4.70005	0.421597	0.244361
NK.cells	EPS8	-0.78719	4.963297	-2.10737	0.037917	-3.94153	0.432815	0.256675
NK.cells	MPST	-0.35824	3.682692	-2.10724	0.037929	-4.12403	0.441231	0.265998
NK.cells	TACO1	0.450461	3.738306	2.106854	0.037963	-4.16421	0.440862	0.265655
NK.cells	ISCA1	0.26876	6.018689	2.106131	0.038027	-4.67738	0.426011	0.249401
NK.cells	AGXT2	-0.87744	1.612637	-2.10538	0.038095	-3.72505	0.455211	0.282199
NK.cells	FBXL5	0.328811	6.173273	2.105346	0.038097	-4.52429	0.425024	0.248565
NK.cells	PAPSS2	-0.62989	2.513577	-2.10465	0.03816	-3.89943	0.449069	0.275489
NK.cells	DUSP18	-1.12959	0.621447	-2.10396	0.038222	-3.66106	0.462072	0.29055
NK.cells	HACD4	0.753618	3.532004	2.103917	0.038225	-3.81937	0.442233	0.267964
NK.cells	UBE2S	-0.24998	7.624597	-2.1039	0.038227	-4.84194	0.415882	0.23911
NK.cells	MLH1	0.375023	2.750298	2.103858	0.038231	-3.93591	0.44747	0.273856
NK.cells	SELENBP2	-0.9624	1.116284	-2.10339	0.038272	-3.69171	0.458633	0.286749
NK.cells	CD209F	-1.92305	2.767776	-2.10322	0.038288	-3.75825	0.447352	0.273906
NK.cells	CKS1B	-0.38824	5.377173	-2.10238	0.038363	-4.48886	0.430563	0.255018
NK.cells	RRP1B	0.322679	4.132089	2.101665	0.038427	-4.31875	0.438696	0.264302
NK.cells	MEF2D	-0.19396	7.288749	-2.10139	0.038452	-4.769	0.418396	0.242094
NK.cells	CCNB1IP1	-0.6859	1.863098	-2.1008	0.038505	-3.73182	0.453948	0.281721
NK.cells	EEF1B2	0.163682	8.556525	2.100757	0.038509	-5.02441	0.410537	0.233846
NK.cells	COLEC11	-0.79239	1.589372	-2.09947	0.038626	-3.71076	0.456133	0.284404
NK.cells	RFXANK	0.413503	2.879474	2.099306	0.03864	-3.9737	0.447347	0.274395
NK.cells	PTGES	1.263223	0.477516	2.098536	0.03871	-3.67414	0.463852	0.293618
NK.cells	MS4A6D	0.872688	2.866447	2.09754	0.0388	-3.86805	0.447435	0.275062
NK.cells	NR1I3	-0.96931	1.055611	-2.09749	0.038805	-3.69848	0.459821	0.289265
NK.cells	IL31RA	0.462034	3.109325	2.097189	0.038832	-3.99771	0.445801	0.273271
NK.cells	CDIPTOS	0.980645	-0.30875	2.096618	0.038884	-3.67112	0.469393	0.300735
NK.cells	LIX1	-1.06737	0.723268	-2.09592	0.038947	-3.67141	0.462134	0.292419
NK.cells	RBBP8	-0.18853	6.609426	-2.09573	0.038965	-4.72919	0.42296	0.248247
NK.cells	ZBTB25	0.331956	3.709262	2.095689	0.038969	-4.23408	0.441793	0.26912
NK.cells	GZMA	0.548269	4.671597	2.095637	0.038973	-4.94073	0.435444	0.262007
NK.cells	IP6K1	0.205504	6.598351	2.095616	0.038975	-4.69709	0.42303	0.248323
NK.cells	BANP	0.252777	4.601582	2.095108	0.039021	-4.29445	0.43601	0.26269
NK.cells	SHQ1	0.483815	2.60612	2.094498	0.039077	-3.95314	0.449475	0.27781
NK.cells	MVK	-0.42944	2.634356	-2.09414	0.03911	-4.01835	0.449284	0.277677
NK.cells	NRAP	1.197306	-1.09404	2.09356	0.039163	-3.6958	0.475414	0.307974
NK.cells	CAMK2B	-0.5012	3.706454	-2.0915	0.039352	-4.15701	0.443324	0.270196
NK.cells	NCK1	0.221486	6.174664	2.091419	0.039359	-4.62031	0.427184	0.252254
NK.cells	EEF1G	0.188132	7.187877	2.091342	0.039366	-4.79895	0.420746	0.245239
NK.cells	ACTR2	0.150522	8.066808	2.088699	0.03961	-4.94332	0.417427	0.239987
NK.cells	FBXO46	0.511353	2.47185	2.087662	0.039706	-3.91412	0.454689	0.280844
NK.cells	ARHGAP18	-0.28025	6.528672	-2.08705	0.039762	-4.78817	0.427995	0.251056
NK.cells	DMPK	-0.88786	2.786398	-2.08538	0.039917	-3.76056	0.453322	0.279121
NK.cells	XLR	-1.0132	2.01867	-2.08471	0.03998	-3.74706	0.458598	0.285352
NK.cells	AMBRA1	0.165794	7.654516	2.084572	0.039992	-4.91253	0.421361	0.243948
NK.cells	TNFRSF13C	-0.48259	3.92598	-2.08435	0.040013	-4.01508	0.44561	0.270697
NK.cells	APH1B	-0.72449	2.578014	-2.08379	0.040065	-3.77302	0.454747	0.281211

NK.cells	UBAP2L	0.150632	7.113568	2.083743	0.04007	-4.76752	0.424787	0.247859
NK.cells	MYO7A	-0.59985	3.25231	-2.08294	0.040145	-3.79771	0.450152	0.276282
NK.cells	ARF4	-0.16657	8.479652	-2.08282	0.040156	-5.07714	0.416194	0.238869
NK.cells	F630028O1	1.11706	1.027185	2.082786	0.040159	-3.69024	0.465509	0.293915
NK.cells	KNG2	-0.69284	2.382731	-2.08254	0.040182	-3.88313	0.456088	0.283057
NK.cells	MCTP2	0.266685	6.80258	2.081115	0.040316	-4.7378	0.427791	0.250601
NK.cells	TTC14	0.174051	5.803768	2.079961	0.040424	-4.60402	0.434759	0.257976
NK.cells	3830406C1	0.243555	4.453206	2.07949	0.040468	-4.38957	0.443669	0.267963
NK.cells	RDH9	-1.01517	0.805989	-2.07936	0.04048	-3.71342	0.468734	0.296628
NK.cells	KMT2C	-0.17175	7.717211	-2.07902	0.040512	-4.89743	0.422469	0.24487
NK.cells	4931403E2	-0.51536	1.501737	-2.07715	0.040689	-3.86649	0.465367	0.291798
NK.cells	FBXL7	-0.86523	3.621991	-2.07682	0.04072	-3.96812	0.450732	0.275175
NK.cells	WNT2	-1.2369	1.077377	-2.07586	0.040811	-3.70481	0.468594	0.295738
NK.cells	AFF2	-1.26823	0.04795	-2.07581	0.040816	-3.70143	0.475935	0.30432
NK.cells	ABL1	-0.23096	5.653787	-2.07367	0.041019	-4.61378	0.438562	0.261114
NK.cells	G430095P1	0.593301	0.465518	2.073602	0.041026	-3.86753	0.474203	0.301652
NK.cells	DDAH2	-0.55045	3.503269	-2.07348	0.041037	-3.92222	0.452972	0.277278
NK.cells	IGFALS	-0.70476	1.779759	-2.07047	0.041324	-3.80608	0.466314	0.291554
NK.cells	CNPY3	0.215714	5.300686	2.069351	0.041432	-4.40951	0.442244	0.264607
NK.cells	SF3B1	-0.12374	8.256825	-2.06935	0.041433	-5.01006	0.423081	0.243704
NK.cells	5830448L0	0.698929	0.904406	2.069007	0.041465	-3.78619	0.472514	0.299183
NK.cells	EIF2S1	0.191472	6.052768	2.068383	0.041525	-4.66159	0.437279	0.259475
NK.cells	COL14A1	-0.84236	1.863173	-2.06748	0.041612	-3.79117	0.465728	0.291777
NK.cells	ZFP292	0.188163	7.046023	2.065552	0.041798	-4.83463	0.430816	0.253156
NK.cells	TMED10	0.167246	7.769396	2.06518	0.041834	-4.87942	0.426176	0.248258
NK.cells	FOCAD	0.42344	2.908895	2.064774	0.041874	-4.09665	0.458445	0.284268
NK.cells	IBTK	0.281537	4.642613	2.064687	0.041882	-4.42658	0.446638	0.270892
NK.cells	PCYT2	-0.27766	4.635801	-2.06426	0.041923	-4.43915	0.446684	0.271067
NK.cells	TRIM56	0.301336	3.810038	2.06387	0.041961	-4.2456	0.452266	0.277515
NK.cells	P2RY10B	0.36526	3.709802	2.062657	0.042079	-4.20215	0.452949	0.278739
NK.cells	DAPL1	0.686971	-1.31502	2.062574	0.042087	-3.78195	0.488418	0.320526
NK.cells	PLA2G12B	-1.00486	1.0508	-2.06255	0.04209	-3.734	0.471471	0.300162
NK.cells	9130019P1	-0.66448	0.265967	-2.06234	0.04211	-3.92712	0.477091	0.306873
NK.cells	SLC38A3	-0.79528	1.479322	-2.0619	0.042153	-3.80857	0.468432	0.296811
NK.cells	HSD17B13	-0.69258	2.671988	-2.0614	0.042202	-3.94845	0.460084	0.287249
NK.cells	CAPN7	-0.1944	5.340998	-2.06124	0.042217	-4.5363	0.441976	0.266685
NK.cells	ATP11B	0.187933	7.035822	2.060853	0.042255	-4.91803	0.430882	0.254428
NK.cells	TLL2	-1.25028	0.725481	-2.06037	0.042302	-3.73066	0.473792	0.303364
NK.cells	LCP2	0.279782	5.109024	2.060116	0.042327	-4.74995	0.443519	0.268622
NK.cells	ZFP524	0.343584	3.672126	2.060113	0.042327	-4.27088	0.453206	0.279584
NK.cells	PRKCQ	0.373062	3.12892	2.059903	0.042348	-4.37128	0.456928	0.283913
NK.cells	MED17	0.191492	4.867425	2.059758	0.042362	-4.45413	0.445132	0.27055
NK.cells	OTUB1	0.166278	5.728978	2.059575	0.04238	-4.59514	0.439409	0.264162
NK.cells	SPATC1	1.013529	-1.06407	2.059202	0.042417	-3.73832	0.486709	0.319289
NK.cells	TREM1	1.135238	1.639916	2.05895	0.042441	-3.73396	0.467299	0.296313
NK.cells	ACSM1	-1.13606	1.026687	-2.0589	0.042446	-3.75367	0.471643	0.301406
NK.cells	SKIL	-0.21806	7.304614	-2.05886	0.04245	-4.90071	0.429151	0.253096

NK.cells	PPOX	-0.43124	3.108757	-2.05794	0.042541	-4.01048	0.457066	0.284752
NK.cells	NR1H4	-0.84935	1.386536	-2.05782	0.042552	-3.78925	0.469088	0.298779
NK.cells	PLPP3	-0.76565	4.693864	-2.05754	0.04258	-4.13979	0.446294	0.27264
NK.cells	CTDSPL	-0.59009	3.831098	-2.05736	0.042598	-4.0726	0.452123	0.279313
NK.cells	DPM2	0.319628	3.936241	2.0573	0.042603	-4.22241	0.451408	0.278498
NK.cells	SPRED2	-0.21898	6.335393	-2.0572	0.042613	-4.72646	0.435429	0.260536
NK.cells	SORBS2	-1.04344	1.878636	-2.05715	0.042618	-3.78092	0.465619	0.294956
NK.cells	FADS3	-1.01076	0.659123	-2.05635	0.042697	-3.73062	0.474267	0.305435
NK.cells	TACC1	-0.14152	7.703473	-2.05609	0.042722	-4.9483	0.426597	0.251117
NK.cells	MRPS24	0.176643	6.031221	2.055788	0.042752	-4.70767	0.43742	0.263084
NK.cells	RRM2	-0.45669	6.880861	-2.05539	0.042791	-4.8229	0.431884	0.257051
NK.cells	ITGA1	-0.30027	3.828562	-2.05529	0.042802	-4.66455	0.45214	0.279854
NK.cells	STXBP6	-0.67045	2.953526	-2.05524	0.042806	-4.08141	0.458136	0.286755
NK.cells	GM29966	-1.01488	1.411825	-2.05455	0.042875	-3.7907	0.468909	0.299342
NK.cells	ATP13A2	0.368148	5.610722	2.054482	0.042881	-4.33571	0.44019	0.266335
NK.cells	NDUFS4	0.171566	6.335519	2.054375	0.042892	-4.76012	0.435429	0.261015
NK.cells	STK35	-0.32761	3.447827	-2.05262	0.043066	-4.15854	0.455461	0.283484
NK.cells	G730013B(	1.072856	-0.968	2.052452	0.043082	-3.74759	0.486829	0.320516
NK.cells	ITPA	0.258582	4.105721	2.0523	0.043098	-4.30201	0.450974	0.278424
NK.cells	SLIT1	-1.05065	1.464859	-2.05179	0.043148	-3.74363	0.469278	0.299797
NK.cells	SLC8A2	-1.3367	1.394752	-2.05179	0.043148	-3.74629	0.469775	0.300381
NK.cells	ARHGEF3	0.350577	5.916073	2.05136	0.043191	-4.71541	0.438925	0.265076
NK.cells	TRIP10	-0.67674	1.355077	-2.04866	0.04346	-3.80115	0.472627	0.302058
NK.cells	SRSF2	-0.17894	7.941182	-2.04767	0.043559	-4.98004	0.428639	0.251677
NK.cells	SUB1	-0.15987	8.911934	-2.04735	0.043592	-5.18216	0.422466	0.245091
NK.cells	ABHD6	0.540835	2.548845	2.046941	0.043633	-3.90068	0.464826	0.292731
NK.cells	ISY1	-0.19361	6.241546	-2.04492	0.043836	-4.8425	0.440732	0.264694
NK.cells	ACOX3	-0.30905	4.372027	-2.04445	0.043883	-4.28737	0.453282	0.278835
NK.cells	SLC9A9	-0.30878	7.120003	-2.04434	0.043894	-4.98282	0.434967	0.258312
NK.cells	EIF3B	0.204532	5.890853	2.043586	0.043971	-4.69146	0.443057	0.267632
NK.cells	CATSPERD	-0.95472	1.329786	-2.04307	0.044023	-3.78091	0.474539	0.303917
NK.cells	NCAPD2	-0.37743	4.81058	-2.04286	0.044044	-4.49375	0.450303	0.275914
NK.cells	KITL	-1.48875	1.956944	-2.04252	0.044079	-3.76519	0.470071	0.298791
NK.cells	CAR5A	-1.01289	0.542195	-2.04238	0.044093	-3.76403	0.480214	0.310771
NK.cells	KIF7	1.138764	-0.46987	2.041506	0.044181	-3.75288	0.487611	0.320029
NK.cells	KLRA17	-0.9733	0.224958	-2.04143	0.044189	-3.75454	0.48252	0.313925
NK.cells	IGFBP3	-1.08199	0.690335	-2.04137	0.044195	-3.76472	0.479141	0.309896
NK.cells	GM12689	1.186261	-0.48996	2.041304	0.044202	-3.76087	0.48776	0.320216
NK.cells	ESRRA	0.300379	3.981496	2.041056	0.044227	-4.30527	0.455952	0.282839
NK.cells	FGR	0.43919	4.732993	2.040113	0.044323	-4.53605	0.451418	0.277332
NK.cells	CBR1	0.339372	4.350162	2.038652	0.044472	-4.36825	0.454884	0.28059
NK.cells	SNX27	-0.19014	5.803763	-2.03837	0.044501	-4.64495	0.445058	0.269505
NK.cells	SIVA1	0.273932	5.470666	2.038167	0.044521	-4.61156	0.447289	0.272055
NK.cells	RNF150	0.998367	2.909798	2.036834	0.044658	-3.87298	0.465878	0.292518
NK.cells	SPECC1L	0.253679	5.413581	2.035455	0.044799	-4.66088	0.449696	0.273373
NK.cells	PRTN3	1.479173	3.6691	2.0344	0.044907	-4.10246	0.462132	0.28725
NK.cells	DENND1A	0.168091	7.497877	2.034251	0.044923	-4.96658	0.436319	0.258209

NK.cells	ZFP800	0.204675	5.365723	2.033442	0.045006	-4.635	0.450944	0.274317
NK.cells	PLK3	-0.30533	4.483011	-2.03219	0.045135	-4.56876	0.457883	0.281478
NK.cells	ZDHHC17	0.291087	4.015064	2.03131	0.045226	-4.31919	0.461657	0.285489
NK.cells	CLEC4D	1.435325	2.949351	2.030757	0.045284	-3.83395	0.469319	0.294282
NK.cells	BC024063	-0.89844	0.183577	-2.02968	0.045395	-3.77833	0.490103	0.318158
NK.cells	CTNND2	0.716145	1.726999	2.029296	0.045435	-3.96236	0.478833	0.304855
NK.cells	GM4788	-0.87428	1.237901	-2.02751	0.045622	-3.83319	0.483947	0.309805
NK.cells	S100A4	-0.33294	2.87429	-2.02643	0.045734	-4.70624	0.472365	0.296312
NK.cells	XPO5	0.316535	3.603677	2.026251	0.045753	-4.25821	0.467205	0.290382
NK.cells	DPYS	-0.65344	2.829004	-2.02586	0.045793	-4.05544	0.472687	0.296709
NK.cells	EGLN1	0.2062	5.631732	2.025827	0.045797	-4.71166	0.453176	0.274444
NK.cells	IYD	-0.96827	0.499692	-2.0249	0.045894	-3.78544	0.49021	0.316907
NK.cells	ALYREF	-0.21622	8.216984	-2.02432	0.045955	-5.08367	0.436614	0.25579
NK.cells	AOX3	-0.99175	0.998188	-2.02406	0.045982	-3.82085	0.486648	0.312743
NK.cells	RBM34	-0.22503	4.465573	-2.02361	0.04603	-4.44708	0.46197	0.284147
NK.cells	FAM207A	0.256998	4.223482	2.021768	0.046224	-4.40114	0.465212	0.286535
NK.cells	SLC40A1	-0.60566	5.835325	-2.02088	0.046318	-4.40257	0.454622	0.274257
NK.cells	DENND4C	0.214654	5.068456	2.019781	0.046434	-4.68507	0.460652	0.280176
NK.cells	SH3D19	-0.73518	2.244921	-2.01789	0.046635	-3.87714	0.481825	0.303456
NK.cells	ACTA2	1.011364	3.320545	2.01724	0.046703	-4.13453	0.474079	0.294689
NK.cells	BOLA2	0.224322	5.686348	2.016554	0.046776	-4.7057	0.457513	0.275991
NK.cells	SIL1	0.273141	5.149956	2.016548	0.046777	-4.53722	0.461213	0.280143
NK.cells	WDR90	-0.48008	1.959845	-2.01615	0.04682	-4.0598	0.4839	0.306262
NK.cells	STON2	0.450767	3.518101	2.016145	0.04682	-4.2155	0.472671	0.293271
NK.cells	NEK2	-0.52246	3.092142	-2.01614	0.046821	-4.17982	0.475713	0.296767
NK.cells	C330007P(	-0.24127	5.50907	-2.01559	0.04688	-4.59602	0.458924	0.277651
NK.cells	FAM53A	0.274675	3.797306	2.013965	0.047053	-4.38826	0.472038	0.291774
NK.cells	EGR1	-0.45153	6.545199	-2.01378	0.047073	-4.79262	0.452951	0.270297
NK.cells	SPOUT1	0.483871	2.468742	2.013215	0.047133	-4.01386	0.481697	0.302986
NK.cells	2500002B1	0.569044	1.915535	2.012941	0.047163	-3.93856	0.485731	0.30773
NK.cells	MTMR6	0.243853	4.718718	2.012226	0.04724	-4.55229	0.465806	0.28488
NK.cells	MRPL36	0.222835	5.835469	2.011696	0.047297	-4.71945	0.458058	0.276251
NK.cells	OSTF1	0.135603	7.480967	2.011631	0.047304	-4.99004	0.446899	0.263882
NK.cells	EXOC3	0.217931	5.06994	2.01134	0.047335	-4.60181	0.463354	0.282319
NK.cells	DIPK1A	0.290712	5.446127	2.010739	0.0474	-4.654	0.46099	0.279469
NK.cells	DNAH1	-1.28322	-0.10849	-2.0095	0.047533	-3.81071	0.50215	0.326619
NK.cells	GM15283	0.301368	4.261918	2.009204	0.047565	-4.54098	0.47013	0.289293
NK.cells	GEM	-0.2903	5.207345	-2.00844	0.047648	-4.75156	0.463709	0.281969
NK.cells	SLC22A15	-0.30673	3.077716	-2.00827	0.047666	-4.39287	0.478804	0.29924
NK.cells	PHKB	0.2389	5.588989	2.007531	0.047747	-4.65593	0.461458	0.279334
NK.cells	CPN1	-0.74813	1.600653	-2.00716	0.047787	-3.89466	0.490018	0.312242
NK.cells	PSMD4	0.14712	6.691408	2.006277	0.047883	-4.92063	0.454434	0.271208
NK.cells	ASTL	-0.76944	1.424213	-2.00581	0.047934	-3.91445	0.492022	0.314136
NK.cells	SMIM20	0.231662	4.914811	2.004871	0.048036	-4.55861	0.467004	0.28541
NK.cells	KIF4	-0.41444	4.394266	-2.0047	0.048055	-4.52458	0.470672	0.289577
NK.cells	RNF157	0.299032	5.375061	2.004554	0.04807	-4.7571	0.463786	0.281785
NK.cells	M1AP	0.814675	1.320039	2.002742	0.048268	-3.84757	0.494607	0.316216

NK.cells	JPT1	-0.1711	7.832843	-2.0015	0.048405	-5.09543	0.449051	0.264084
NK.cells	GM14221	-1.09137	1.910062	-2.00115	0.048443	-3.84593	0.490844	0.311519
NK.cells	H2AFX	-0.34656	6.482628	-2.00072	0.048491	-4.86896	0.458224	0.274406
NK.cells	MRPS9	0.212061	4.889322	2.000305	0.048536	-4.56027	0.469314	0.286965
NK.cells	PHB	0.285712	4.493205	2.000156	0.048552	-4.50602	0.472117	0.290166
NK.cells	RBM38	-0.23255	6.898184	-2.00002	0.048567	-4.91435	0.455379	0.271403
NK.cells	TKT	0.214003	6.932084	1.999154	0.048662	-4.89262	0.455668	0.271471
NK.cells	RARB	-1.09831	1.586463	-1.99787	0.048805	-3.86447	0.494849	0.315477
NK.cells	NCEH1	0.344254	4.906119	1.996629	0.048941	-4.50324	0.471372	0.287992
NK.cells	ULBP1	-0.36039	4.251838	-1.99651	0.048955	-4.43826	0.476031	0.293285
NK.cells	DAND5	-0.60124	3.96611	-1.99618	0.048992	-4.25626	0.478082	0.29575
NK.cells	TRIM30C	0.869594	1.320225	1.99561	0.049054	-3.87922	0.497761	0.318368
NK.cells	USP46	0.462866	3.281753	1.995142	0.049106	-4.15093	0.483277	0.30164
NK.cells	GM16201	0.416954	1.818935	1.994875	0.049136	-4.28914	0.494049	0.314262
NK.cells	SERPINA3G	0.828535	3.597185	1.994202	0.049211	-4.52193	0.48133	0.299373
NK.cells	IFITM6	1.108856	3.518134	1.993472	0.049292	-3.94185	0.482309	0.30026
NK.cells	GM13684	0.304752	3.516914	1.992614	0.049388	-4.50151	0.482863	0.300687
NK.cells	HGD	-0.72059	2.465599	-1.99153	0.049509	-4.04227	0.491359	0.309982
NK.cells	RUFY1	-0.21175	5.607628	-1.99116	0.04955	-4.64618	0.468674	0.284092
NK.cells	GM21859	-1.05799	1.780794	-1.99083	0.049587	-3.90227	0.496458	0.316141
NK.cells	KLHL20	0.399887	3.27084	1.989452	0.049742	-4.28331	0.486325	0.303793
NK.cells	ZFP330	0.24011	4.432352	1.989305	0.049759	-4.49645	0.477899	0.29413
NK.cells	MASP1	-0.87341	1.673868	-1.98763	0.049947	-3.88211	0.498352	0.318204
NK.cells	MTFP1	-1.04299	0.572509	-1.98743	0.049969	-3.83394	0.506704	0.328183
NK.cells	PURA	0.173357	5.343404	1.987387	0.049974	-4.70386	0.471571	0.287398
NK.cells	RTP4	0.593121	4.174749	1.985938	0.050138	-4.51182	0.479927	0.297305
NK.cells	WARS	0.349275	3.723103	1.985514	0.050186	-4.36497	0.4832	0.301247
NK.cells	FAHD1	-0.72676	1.917334	-1.98478	0.050269	-3.92949	0.496525	0.3171
NK.cells	RAB1A	-0.14849	7.08553	-1.98462	0.050287	-4.9533	0.459409	0.274644
NK.cells	FYB	0.263285	6.869674	1.984527	0.050298	-5.16434	0.460897	0.2763
NK.cells	IDH1	-0.31894	4.88393	-1.98449	0.050301	-4.38629	0.474837	0.292006
NK.cells	SLC25A20	-0.23626	5.597573	-1.98376	0.050385	-4.81594	0.469775	0.286481
NK.cells	BLK	-0.4829	4.074052	-1.9837	0.050392	-4.18904	0.480655	0.298894
NK.cells	CD72	-0.47848	4.334961	-1.983	0.050471	-4.26057	0.478772	0.296805
NK.cells	C030005K0	-0.8894	0.674423	-1.98277	0.050497	-3.85323	0.505925	0.328612
NK.cells	DNAJC9	-0.28129	5.617283	-1.98174	0.050615	-4.73811	0.469636	0.286811
NK.cells	CYFIP2	-0.19486	6.947339	-1.98165	0.050625	-5.03648	0.460361	0.276385
NK.cells	TCAIM	0.559465	1.789758	1.981401	0.050653	-4.03463	0.497482	0.319117
NK.cells	FECH	0.380582	5.217188	1.981277	0.050667	-4.64591	0.472466	0.290151
NK.cells	GM10603	0.929054	0.220408	1.981034	0.050695	-3.86884	0.509406	0.333545
NK.cells	GAB3	0.286234	4.746655	1.980922	0.050708	-4.64783	0.475818	0.294131
NK.cells	ZYX	-0.23477	6.244314	-1.98065	0.050739	-4.95992	0.465238	0.282219
NK.cells	ALDH4A1	-0.47663	2.912017	-1.98051	0.050755	-4.1654	0.489137	0.30965
NK.cells	GPR27	0.793474	-1.60473	1.980451	0.050762	-3.85349	0.523256	0.351025
NK.cells	SPIN1	0.189741	5.805516	1.980399	0.050768	-4.74543	0.468311	0.285689
NK.cells	RAB7B	-1.19444	2.804284	-1.97995	0.050819	-3.86148	0.489931	0.310706
NK.cells	UGT2B36	-0.68099	2.198922	-1.97968	0.05085	-4.03372	0.494422	0.316045

NK.cells	CLK3	-0.23177	4.807812	-1.97944	0.050878	-4.56996	0.475381	0.293977
NK.cells	SMIM12	0.25982	4.0506	1.979087	0.050918	-4.46727	0.480824	0.300369
NK.cells	TMEM135	0.210802	5.7512	1.97908	0.050919	-4.83742	0.468693	0.28648
NK.cells	4930578M	-0.94079	0.63262	-1.9785	0.050986	-3.85488	0.506245	0.330496
NK.cells	PSD3	-0.4103	5.794857	-1.9784	0.050997	-4.50599	0.468386	0.286309
NK.cells	VAMP5	0.371529	4.717071	1.978196	0.05102	-4.42581	0.47603	0.295063
NK.cells	MYBPC2	-0.78117	2.760882	-1.97612	0.051259	-3.94831	0.491593	0.312253
NK.cells	VAV1	0.189906	6.364742	1.975938	0.05128	-4.86651	0.465668	0.282509
NK.cells	RAB13	-0.88156	1.635625	-1.97575	0.051301	-3.88112	0.500003	0.322338
NK.cells	ATF7IP	0.203697	6.540023	1.975254	0.051359	-4.91073	0.464446	0.281412
NK.cells	GM15832	0.60183	1.685015	1.975237	0.051361	-4.0117	0.499631	0.322122
NK.cells	CD2AP	0.262586	6.24029	1.97331	0.051583	-4.76567	0.467315	0.284466
NK.cells	NLRP12	1.16902	-0.12085	1.973304	0.051584	-3.85079	0.514293	0.33952
NK.cells	DNASE1L3	-0.50108	6.30437	-1.9733	0.051585	-4.62857	0.466866	0.283959
NK.cells	MORC3	0.198702	5.857789	1.973104	0.051607	-4.78692	0.470004	0.287573
NK.cells	VPS16	0.212918	4.436967	1.972086	0.051725	-4.56358	0.480268	0.299486
NK.cells	0610030E2	0.254687	4.591291	1.971823	0.051756	-4.52822	0.479155	0.298223
NK.cells	DTWD1	0.560997	1.928598	1.971638	0.051777	-3.99616	0.498757	0.32122
NK.cells	IL21R	-0.26197	5.150614	-1.9716	0.051782	-4.89703	0.475144	0.293679
NK.cells	PYCRL	0.307034	3.541294	1.970584	0.0519	-4.34741	0.487515	0.307465
NK.cells	NAP1L4	0.148749	6.249563	1.969153	0.052067	-4.89028	0.469214	0.285728
NK.cells	0610009B2	0.399597	3.780562	1.967983	0.052204	-4.31763	0.487308	0.306434
NK.cells	RASGRP1	0.319274	4.119019	1.96754	0.052255	-4.6382	0.484833	0.303677
NK.cells	CRYBG1	0.240069	4.169377	1.967334	0.05228	-4.76583	0.484466	0.303251
NK.cells	ACSS1	0.371021	3.946222	1.9671	0.052307	-4.39165	0.486095	0.305227
NK.cells	GPATCH2L	0.203248	5.064269	1.966904	0.05233	-4.64103	0.477992	0.295963
NK.cells	RPGRIP1L	0.625989	1.613197	1.966426	0.052386	-3.95911	0.503485	0.325935
NK.cells	GM553	-1.04094	0.241543	-1.96551	0.052494	-3.86866	0.514017	0.338909
NK.cells	UBAC2	0.160574	6.679645	1.965367	0.052511	-5.041	0.466544	0.283351
NK.cells	STMN1	-0.39206	7.897729	-1.96515	0.052536	-5.16952	0.458112	0.273957
NK.cells	ASAP2	-0.37302	3.068411	-1.96509	0.052543	-4.42404	0.492561	0.313376
NK.cells	ARRDC1	0.254443	4.471227	1.965024	0.052551	-4.62119	0.482271	0.301377
NK.cells	STFA2	0.9348	2.469209	1.964306	0.052636	-3.97101	0.497125	0.318817
NK.cells	EXOSC4	0.329062	3.784732	1.964249	0.052642	-4.39573	0.487373	0.307358
NK.cells	SLC25A45	0.336238	3.260909	1.963137	0.052773	-4.31612	0.49173	0.312162
NK.cells	GM42658	0.627986	1.544714	1.963109	0.052777	-3.99682	0.504615	0.327423
NK.cells	PQLC3	-0.32256	3.812077	-1.96274	0.052821	-4.45275	0.487698	0.307483
NK.cells	RTL4	-1.20383	0.402884	-1.96233	0.052869	-3.87356	0.513479	0.338185
NK.cells	CHRM3	0.953623	0.831294	1.961977	0.052911	-3.94222	0.510168	0.334203
NK.cells	GM12236	-0.93972	1.087964	-1.96166	0.052948	-3.93283	0.508196	0.331887
NK.cells	AI839979	1.065223	0.274557	1.96069	0.053063	-3.88	0.515171	0.339856
NK.cells	ARHGAP24	-0.39897	6.825889	-1.96037	0.053101	-4.60145	0.4668	0.283357
NK.cells	NCOA3	-0.14722	6.960263	-1.95923	0.053236	-5.01551	0.466354	0.282598
NK.cells	ZNFX1	0.401543	4.359029	1.959188	0.053242	-4.50551	0.484923	0.303822
NK.cells	CD300LG	-0.81337	1.259921	-1.95853	0.053321	-3.92803	0.508101	0.331452
NK.cells	RARG	0.48326	1.622679	1.958462	0.053328	-4.23008	0.505327	0.328127
NK.cells	SERPINA1D	-0.54579	5.558333	-1.95819	0.05336	-4.81548	0.476263	0.2942

NK.cells	TMX4	0.282109	4.420261	1.955884	0.053636	-4.61965	0.486608	0.304649
NK.cells	YARS	-0.29241	5.391202	-1.95547	0.053685	-4.72319	0.479634	0.296697
NK.cells	ACSL4	0.206691	6.189637	1.954814	0.053764	-4.88308	0.473952	0.290353
NK.cells	MBD2	0.147412	7.044678	1.954764	0.05377	-5.07931	0.467917	0.283523
NK.cells	RAB32	-0.48256	4.344985	-1.95424	0.053833	-4.17223	0.487462	0.305792
NK.cells	ABCA8A	-1.15441	1.028779	-1.95372	0.053896	-3.91011	0.512474	0.335592
NK.cells	NXF1	-0.21295	5.180351	-1.95353	0.053918	-4.68047	0.481414	0.299013
NK.cells	SLC25A37	0.357766	5.267981	1.952798	0.054007	-4.64443	0.481206	0.298481
NK.cells	RSBN1L	-0.15414	7.198782	-1.95177	0.05413	-5.11998	0.467813	0.283108
NK.cells	LCAT	-0.6421	2.143834	-1.95115	0.054206	-4.06415	0.50474	0.325899
NK.cells	AS3MT	-0.62396	2.69274	-1.951	0.054224	-4.01764	0.500581	0.320962
NK.cells	FAM234A	0.332505	4.099085	1.95099	0.054225	-4.58964	0.490093	0.308644
NK.cells	ZFP617	0.558298	2.165547	1.950648	0.054266	-4.08618	0.504575	0.325828
NK.cells	ADAM19	0.326969	5.568234	1.950437	0.054292	-4.86287	0.479391	0.296451
NK.cells	POFUT2	0.276167	4.257323	1.949986	0.054346	-4.52396	0.489052	0.307643
NK.cells	DYM	0.186824	5.781233	1.94853	0.054523	-4.83974	0.479174	0.295292
NK.cells	GM11772	-0.93302	0.302935	-1.94733	0.054669	-3.89132	0.521144	0.344368
NK.cells	PCGF5	0.232514	6.06196	1.946609	0.054757	-4.96395	0.477857	0.293706
NK.cells	ZNRF1	0.196701	6.496008	1.9466	0.054758	-4.97189	0.474757	0.290178
NK.cells	PECR	-0.72708	2.35098	-1.94652	0.054767	-4.06844	0.505286	0.325662
NK.cells	KLF5	-0.90997	0.36413	-1.94582	0.054853	-3.95532	0.520871	0.344366
NK.cells	CBR3	-1.11457	0.624626	-1.94567	0.054871	-3.89585	0.518827	0.341977
NK.cells	GTF2B	-0.18758	6.738906	-1.94512	0.054938	-5.01632	0.473441	0.288646
NK.cells	PPP1R10	-0.22305	6.277719	-1.94426	0.055044	-4.88244	0.477065	0.292686
NK.cells	C8A	-0.90817	0.525618	-1.94413	0.05506	-3.91371	0.520214	0.343477
NK.cells	CACNA1F	1.171943	-0.45662	1.943796	0.0551	-3.89174	0.52799	0.352974
NK.cells	LARP7	-0.17134	5.919166	-1.9426	0.055247	-4.87555	0.480558	0.295994
NK.cells	TG	0.515946	1.91376	1.941209	0.055418	-4.13384	0.510556	0.331409
NK.cells	CCDC34	-0.35457	4.840796	-1.94109	0.055433	-4.67542	0.488545	0.305488
NK.cells	MCEMP1	1.232809	1.650063	1.940711	0.055479	-3.93029	0.512591	0.333848
NK.cells	NR2C2AP	0.295127	4.44571	1.940648	0.055487	-4.57868	0.491455	0.308866
NK.cells	UBE2CBP	0.773826	1.761179	1.940571	0.055497	-4.08248	0.511732	0.332818
NK.cells	MRPL58	0.216299	5.199269	1.9402	0.055542	-4.81681	0.485921	0.302504
NK.cells	LPL	-0.47335	5.132487	-1.94004	0.055562	-4.54653	0.486409	0.303137
NK.cells	WDR62	0.34119	3.526858	1.939588	0.055618	-4.47832	0.498297	0.317098
NK.cells	SQLE	0.686228	1.97366	1.939256	0.055659	-4.01286	0.510095	0.331233
NK.cells	SLC41A2	-0.39009	2.900939	-1.9389	0.055703	-4.49554	0.503016	0.322877
NK.cells	EPB41	0.225469	8.026595	1.938787	0.055717	-5.36395	0.465759	0.279945
NK.cells	AVIL	-0.52053	2.092681	-1.93803	0.055811	-4.1764	0.5093	0.330359
NK.cells	C8B	-0.89452	0.571894	-1.93801	0.055813	-3.91709	0.521122	0.344619
NK.cells	ZFP719	0.511278	2.119349	1.936751	0.055969	-4.05433	0.509197	0.330725
NK.cells	CAR8	-0.69703	2.375063	-1.93588	0.056077	-4.08302	0.507238	0.328759
NK.cells	CHID1	0.410798	2.453126	1.935804	0.056087	-4.15183	0.506642	0.328061
NK.cells	HERC4	0.180404	7.008496	1.935623	0.056109	-5.07114	0.473116	0.289002
NK.cells	GPAT3	-0.29922	5.178486	-1.93562	0.05611	-4.6104	0.486284	0.304108
NK.cells	SPATS2	-0.44232	2.720159	-1.93544	0.056132	-4.35139	0.504607	0.325667
NK.cells	CCNB2	-0.42559	5.882225	-1.93529	0.056151	-4.90989	0.481173	0.298229

NK.cells	2300009A(	0.298216	4.080294	1.935267	0.056154	-4.53911	0.494379	0.313571
NK.cells	METTL8	0.485423	2.52734	1.934604	0.056236	-4.17872	0.50638	0.327669
NK.cells	DNTTIP1	0.19633	4.812219	1.934042	0.056306	-4.67088	0.489262	0.307677
NK.cells	HTRA2	0.265883	3.681601	1.934002	0.056311	-4.42493	0.497653	0.317513
NK.cells	UCHL1	-1.0386	0.862756	-1.93317	0.056415	-3.91588	0.519556	0.343737
NK.cells	PLP2	-0.27067	5.623922	-1.93308	0.056427	-4.87289	0.483613	0.301113
NK.cells	HSPA1B	0.793086	4.531616	1.93265	0.05648	-4.72866	0.491615	0.310537
NK.cells	TIMMDC1	0.248697	4.271519	1.932419	0.056509	-4.56013	0.493542	0.31286
NK.cells	SUSD3	0.298049	3.170727	1.931725	0.056596	-4.51776	0.501951	0.322719
NK.cells	PWP1	0.286268	3.903059	1.931611	0.056611	-4.47546	0.496448	0.316221
NK.cells	STK11	-0.18176	5.696969	-1.93077	0.056716	-4.87512	0.483792	0.301041
NK.cells	ACTN1	-0.23704	5.757216	-1.9298	0.056838	-4.90145	0.484036	0.300815
NK.cells	CD59A	-0.53484	2.984691	-1.92946	0.05688	-4.15266	0.504654	0.325027
NK.cells	GNS	0.25804	6.931874	1.92858	0.056992	-4.84756	0.475901	0.291517
NK.cells	CAPRIN1	0.132627	7.460311	1.928007	0.057064	-5.1315	0.47215	0.287406
NK.cells	FARSB	0.216087	5.008108	1.927837	0.057085	-4.72101	0.489837	0.307725
NK.cells	RSAD2	0.840759	4.537164	1.92776	0.057095	-4.42228	0.493317	0.311786
NK.cells	ACVR2A	0.319909	4.7385	1.927529	0.057124	-4.68146	0.491826	0.310043
NK.cells	TEP1	0.354233	3.914158	1.926806	0.057216	-4.38384	0.498397	0.317486
NK.cells	ZFYVE16	0.402747	3.016629	1.925917	0.057328	-4.26728	0.505803	0.325732
NK.cells	ZFHX3	-0.34654	5.163754	-1.92409	0.05756	-4.50223	0.490921	0.307364
NK.cells	SARDH	-0.7388	2.900243	-1.92368	0.057612	-4.16203	0.507926	0.327446
NK.cells	GBF1	-0.14175	6.562441	-1.92334	0.057655	-5.00961	0.480724	0.295763
NK.cells	RTKN2	-0.82715	1.228878	-1.92297	0.057702	-3.96932	0.520888	0.343145
NK.cells	SRMS	-1.27015	-0.72874	-1.9227	0.057737	-3.92619	0.536518	0.362413
NK.cells	CASP1	0.447986	3.51676	1.922671	0.057741	-4.30229	0.503232	0.322086
NK.cells	SUMF1	0.293179	4.240634	1.922524	0.057759	-4.5711	0.49778	0.315725
NK.cells	APH1C	-0.3214	4.351444	-1.92137	0.057907	-4.69802	0.497859	0.315232
NK.cells	BCR	-0.24543	5.853797	-1.92074	0.057987	-4.80639	0.486794	0.302465
NK.cells	MRAS	-1.03649	0.476226	-1.92068	0.057995	-3.93307	0.527852	0.351269
NK.cells	ZSCAN29	0.322257	3.485733	1.919942	0.058089	-4.38267	0.504696	0.323195
NK.cells	TENT5A	0.260733	5.169086	1.919611	0.058132	-4.82648	0.49208	0.308464
NK.cells	PCNA	-0.2439	6.710032	-1.91946	0.058151	-5.03362	0.480833	0.295553
NK.cells	LNX2	-0.28414	3.592505	-1.91821	0.058312	-4.58199	0.50487	0.322621
NK.cells	MPHOSPH8	-0.20153	4.79616	-1.91793	0.058348	-4.72279	0.495812	0.312073
NK.cells	APLP2	-0.21121	5.805079	-1.91712	0.058452	-5.01229	0.488538	0.303727
NK.cells	KLF3	0.196125	6.281206	1.916997	0.058468	-5.08477	0.485061	0.299753
NK.cells	HAO2	-0.97687	1.393032	-1.91676	0.058499	-4.03258	0.52208	0.343435
NK.cells	NFIB	-0.64014	3.880029	-1.91646	0.058537	-4.358	0.502878	0.320561
NK.cells	GM11837	-1.05216	0.292319	-1.91532	0.058684	-3.95222	0.531406	0.354551
NK.cells	SRGAP3	-0.30358	4.261174	-1.9152	0.0587	-4.86185	0.500547	0.317524
NK.cells	P2RX7	0.333996	3.223954	1.914466	0.058795	-4.64516	0.508422	0.327045
NK.cells	ZFP207	0.136926	6.934778	1.914394	0.058804	-5.07712	0.480855	0.294977
NK.cells	CCDC50	0.178464	5.994315	1.914344	0.05881	-4.936	0.487683	0.302817
NK.cells	LNCPINT	-0.24654	8.328661	-1.91337	0.058937	-5.44576	0.471607	0.284058
NK.cells	OLFM1	-0.37994	2.469141	-1.91206	0.059107	-4.52111	0.516096	0.334803
NK.cells	ARHGEF37	1.248295	0.616444	1.911728	0.05915	-3.97707	0.53073	0.352617

NK.cells	TMEM179I	0.164315	5.864754	1.911291	0.059207	-4.9112	0.490437	0.304859
NK.cells	IKBKB	0.176117	5.778617	1.911053	0.059238	-4.95754	0.491071	0.305675
NK.cells	FMN2	-0.87238	2.359589	-1.91036	0.059328	-4.09368	0.517164	0.336428
NK.cells	PIN4	0.187011	4.949389	1.909846	0.059395	-4.78404	0.497394	0.313256
NK.cells	IGLL1	-0.94493	3.916663	-1.9095	0.05944	-4.36226	0.505178	0.322485
NK.cells	GM42869	-0.62718	1.342309	-1.90943	0.05945	-4.03419	0.52516	0.346429
NK.cells	RIDA	-0.48977	4.316478	-1.90929	0.059468	-4.61714	0.502149	0.31896
NK.cells	PIGN	0.296525	4.40092	1.908265	0.059602	-4.62541	0.502284	0.318538
NK.cells	ZFP64	-0.25518	5.317866	-1.90768	0.059678	-4.87844	0.495694	0.31071
NK.cells	FNDC9	-0.32898	2.564671	-1.90699	0.059769	-4.33702	0.516661	0.33572
NK.cells	CC2D2B	-1.33253	2.70829	-1.90661	0.059819	-4.0407	0.515544	0.334541
NK.cells	UQCRQ	0.184744	7.858453	1.906441	0.059841	-5.24952	0.477174	0.289907
NK.cells	ZFP101	-0.3472	2.916235	-1.9064	0.059847	-4.38972	0.513931	0.332676
NK.cells	PACSIN2	0.16102	5.573303	1.905885	0.059914	-4.95017	0.494004	0.309212
NK.cells	MAP1S	0.318151	3.781603	1.904975	0.060034	-4.50939	0.507959	0.325392
NK.cells	SCIMP	-0.59703	3.702449	-1.90479	0.060059	-4.26829	0.508565	0.326125
NK.cells	TMC7	-1.11517	-0.40026	-1.90412	0.060146	-3.94981	0.541029	0.365775
NK.cells	CEACAM1(	0.984265	-1.04799	1.903847	0.060183	-3.94581	0.546287	0.372404
NK.cells	SRSF5	-0.15036	6.955445	-1.9038	0.060189	-5.08264	0.484318	0.298102
NK.cells	CYP20A1	0.249002	4.104318	1.903543	0.060223	-4.58283	0.505499	0.322803
NK.cells	PEBP1	0.175011	6.440655	1.903072	0.060285	-5.01534	0.488231	0.302706
NK.cells	FN1	-0.66846	5.580946	-1.90223	0.060397	-4.87309	0.495139	0.310347
NK.cells	ABCA13	-1.25445	0.73813	-1.90102	0.060556	-3.978	0.533629	0.355524
NK.cells	RFNG	-0.44931	2.228203	-1.90059	0.060614	-4.19342	0.521901	0.341156
NK.cells	TMCC3	-0.34859	5.475701	-1.89873	0.060861	-4.89376	0.497758	0.312465
NK.cells	PLEKHA3	-0.24459	3.98944	-1.898	0.060959	-4.6238	0.509002	0.325883
NK.cells	MFF	0.172577	5.906654	1.897913	0.06097	-4.95151	0.494548	0.308955
NK.cells	ZBTB16	-0.32577	2.808614	-1.89685	0.061113	-4.84169	0.518133	0.337193
NK.cells	NR1D2	-0.30521	3.478586	-1.8965	0.06116	-4.52536	0.512931	0.331038
NK.cells	CDKN1C	-0.58142	3.302414	-1.89634	0.06118	-4.36672	0.514293	0.332736
NK.cells	CAV2	-0.59515	2.771749	-1.89592	0.061237	-4.10794	0.51842	0.337765
NK.cells	BTBD9	-0.15975	8.508871	-1.89589	0.061241	-5.37913	0.475648	0.287914
NK.cells	RABL6	0.185859	5.136693	1.895679	0.061269	-4.79275	0.500299	0.316249
NK.cells	TTYH2	-0.5087	3.075271	-1.89568	0.061269	-4.29932	0.516055	0.334925
NK.cells	FCNA	-1.00012	4.555419	-1.89527	0.061325	-4.39542	0.504688	0.321561
NK.cells	KCNB1	-1.02941	0.988195	-1.89524	0.061328	-3.96935	0.532553	0.355103
NK.cells	CBX4	-0.23259	5.468948	-1.89524	0.061328	-4.87435	0.497808	0.313486
NK.cells	PLCE1	-0.62874	1.279254	-1.89508	0.061349	-4.30994	0.530219	0.352254
NK.cells	ARPC4	0.146111	7.957802	1.893802	0.061521	-5.2828	0.479582	0.292981
NK.cells	GM41496	-0.9248	0.985043	-1.89379	0.061522	-4.00141	0.532579	0.355716
NK.cells	AKAP10	0.228196	5.860095	1.893528	0.061558	-4.93354	0.494894	0.310674
NK.cells	TTC39A	-0.65919	2.027911	-1.89343	0.061572	-4.16389	0.524265	0.345638
NK.cells	ARF2	-0.33715	4.470722	-1.89339	0.061577	-4.57557	0.505331	0.322927
NK.cells	KCTD20	-0.2197	4.885027	-1.89331	0.061587	-4.73182	0.502194	0.319224
NK.cells	MAP3K20	-0.61511	3.798494	-1.89324	0.061596	-4.14151	0.510467	0.329026
NK.cells	PAQR8	0.880092	0.223677	1.892609	0.061682	-4.01376	0.538885	0.363598
NK.cells	TRIO	-0.19721	6.069746	-1.89249	0.061698	-5.04614	0.493476	0.309063

NK.cells	EYA3	0.203195	5.473081	1.892015	0.061762	-4.93378	0.498088	0.314289
NK.cells	MAP3K15	-0.47283	3.326785	-1.89146	0.061837	-4.37586	0.514699	0.333704
NK.cells	OMA1	0.328412	3.410326	1.89035	0.061987	-4.49726	0.514943	0.333488
NK.cells	SEC62	-0.10828	7.437896	-1.88872	0.062207	-5.22581	0.485857	0.298455
NK.cells	FRMD8	0.24474	4.222806	1.888652	0.062217	-4.73928	0.509873	0.326432
NK.cells	LRP8OS2	1.144963	0.163168	1.887659	0.062352	-3.98165	0.542546	0.365813
NK.cells	MERTK	-0.8605	3.774359	-1.88752	0.062371	-4.17596	0.513794	0.330874
NK.cells	BRD4	-0.10197	8.256086	-1.88706	0.062433	-5.36916	0.480386	0.292249
NK.cells	DNPEP	0.281335	4.077287	1.886472	0.062514	-4.59482	0.511458	0.328537
NK.cells	DCTN3	0.17713	5.963361	1.885649	0.062626	-4.98456	0.497168	0.312026
NK.cells	TUBB2B	-0.60395	2.9596	-1.88559	0.062634	-4.16483	0.520136	0.339252
NK.cells	LY6I	1.762066	0.802083	1.885362	0.062665	-3.99609	0.537337	0.36027
NK.cells	HSP90AB1	0.139167	9.925498	1.885052	0.062707	-5.60904	0.468564	0.279598
NK.cells	OLFR164	-1.15528	-0.16043	-1.88453	0.062778	-3.97137	0.545205	0.370326
NK.cells	ZBTB46	-0.33502	3.021217	-1.88453	0.062779	-4.60146	0.519653	0.33899
NK.cells	GJB1	-0.78281	1.59761	-1.88452	0.06278	-4.08767	0.530925	0.352679
NK.cells	NCF1	0.314822	5.301832	1.884185	0.062826	-4.73538	0.502129	0.31823
NK.cells	GMPPA	0.33178	3.453598	1.884076	0.062841	-4.50071	0.516281	0.33503
NK.cells	NRP1	0.446517	4.442319	1.883853	0.062871	-4.59975	0.508657	0.326021
NK.cells	RAB12	0.19481	5.073594	1.882311	0.063083	-4.81729	0.505207	0.32098
NK.cells	SLC46A1	-1.16964	0.761766	-1.88185	0.063146	-3.99085	0.539284	0.362093
NK.cells	CISH	0.360492	2.887974	1.880865	0.063282	-4.65089	0.522715	0.341736
NK.cells	PPP1R9A	-0.66774	3.383193	-1.88052	0.063329	-4.19573	0.518831	0.337232
NK.cells	CAMK2N1	0.68773	1.714702	1.880434	0.063341	-4.23085	0.532042	0.35327
NK.cells	CHRNE	-0.70554	-0.75209	-1.87995	0.063407	-4.04408	0.552232	0.378459
NK.cells	GTF3A	0.199738	4.569389	1.879701	0.063442	-4.77141	0.509654	0.326479
NK.cells	GSDMD	0.310372	4.268046	1.879588	0.063458	-4.71375	0.511968	0.329262
NK.cells	ALKBH1	0.201119	4.99535	1.878652	0.063587	-4.85785	0.506585	0.322953
NK.cells	SMYD1	1.029927	0.29641	1.878503	0.063608	-3.98934	0.543751	0.368095
NK.cells	DSTN	-0.22995	6.886463	-1.87837	0.063626	-5.02493	0.492413	0.306481
NK.cells	HEY1	-1.01817	-0.2714	-1.87792	0.063689	-3.99108	0.548435	0.37412
NK.cells	CD209G	-2.21523	1.089148	-1.87787	0.063695	-4.01465	0.537282	0.360251
NK.cells	AK2	0.211234	5.995761	1.877434	0.063756	-4.93491	0.499172	0.314465
NK.cells	SP3	-0.15293	6.891028	-1.87656	0.063877	-5.13203	0.493124	0.307022
NK.cells	PDE6C	-0.95623	0.436862	-1.87505	0.064087	-4.01017	0.544839	0.368086
NK.cells	PPP2R5E	-0.13597	6.820761	-1.87429	0.064193	-5.1581	0.49541	0.308461
NK.cells	CLIP1	0.193516	5.440971	1.873654	0.064281	-5.0032	0.505771	0.320861
NK.cells	COG7	0.431306	2.604364	1.873513	0.064301	-4.25934	0.527821	0.347246
NK.cells	ACOX1	-0.24011	5.892871	-1.87337	0.064321	-4.95879	0.502351	0.31693
NK.cells	DOLK	-0.5622	1.544978	-1.87305	0.064365	-4.07352	0.536325	0.357813
NK.cells	AGAP1	0.456371	4.225875	1.872645	0.064422	-4.43612	0.51521	0.332304
NK.cells	SYNC	-0.44166	1.973724	-1.87179	0.064542	-4.22083	0.533116	0.353981
NK.cells	KLF8	-0.86035	0.979838	-1.87138	0.0646	-4.07841	0.54117	0.364015
NK.cells	G6PDX	0.279008	4.072997	1.871236	0.06462	-4.59025	0.516523	0.334009
NK.cells	ARSG	0.449155	1.867658	1.870826	0.064677	-4.21376	0.533969	0.355374
NK.cells	DIRAS2	-0.9972	0.718948	-1.87081	0.064679	-3.99735	0.543305	0.3669
NK.cells	C130036L2	0.627971	1.354595	1.870685	0.064697	-4.08449	0.538118	0.360494

NK.cells	POLR2L	0.204503	5.414671	1.869971	0.064797	-4.9379	0.506657	0.322276
NK.cells	SCYL3	0.260774	3.642548	1.868271	0.065036	-4.57758	0.521911	0.338877
NK.cells	MFSD14A	-0.15334	6.156841	-1.86759	0.065132	-5.03723	0.502887	0.316103
NK.cells	COL13A1	-1.0306	0.62777	-1.86736	0.065165	-4.0039	0.546535	0.368762
NK.cells	CLNS1A	0.173675	5.340846	1.867046	0.065209	-4.91726	0.509088	0.323557
NK.cells	GALNS	0.296009	3.757313	1.863747	0.065676	-4.58873	0.524742	0.339063
NK.cells	SAA2	1.631267	-1.02347	1.861668	0.065972	-4.00164	0.565649	0.388132
NK.cells	BRIX1	-0.14331	6.144863	-1.86157	0.065986	-5.0639	0.507788	0.318025
NK.cells	GID8	0.195315	5.195402	1.861425	0.066007	-4.87317	0.515077	0.326548
NK.cells	PCED1B	0.298937	4.966857	1.860925	0.066078	-4.87955	0.517065	0.328822
NK.cells	ITSN1	-0.31966	5.300914	-1.85977	0.066243	-4.81398	0.515234	0.326077
NK.cells	GRCC10	0.147191	6.427807	1.859632	0.066263	-5.15171	0.506594	0.316063
NK.cells	GLYAT	-0.66404	2.069154	-1.85908	0.066343	-4.20702	0.541206	0.357025
NK.cells	CST7	0.357476	2.120473	1.857569	0.066559	-4.73326	0.542171	0.357241
NK.cells	SLC25A19	0.312816	3.390435	1.856808	0.066668	-4.56093	0.531896	0.345297
NK.cells	PCBP3	-0.45361	2.616922	-1.85655	0.066706	-4.28034	0.538129	0.352893
NK.cells	DEPDC1A	-0.53641	3.001084	-1.85649	0.066714	-4.51381	0.535024	0.349165
NK.cells	GM11508	-0.41532	3.437566	-1.85636	0.066733	-4.51353	0.531519	0.344983
NK.cells	SELENOW	0.184584	7.046449	1.855972	0.066789	-5.2587	0.503476	0.312117
NK.cells	1810020O(	1.20991	-0.23349	1.855103	0.066914	-4.01511	0.561781	0.382478
NK.cells	CLIP2	-0.28094	2.886513	-1.85478	0.06696	-4.56832	0.535948	0.350756
NK.cells	IGLV3	-1.18977	-0.70552	-1.85422	0.067041	-4.01075	0.565802	0.387827
NK.cells	NAPSA	0.256173	6.459379	1.853968	0.067078	-5.06813	0.507925	0.317905
NK.cells	HIST1H3G	-0.75906	1.46486	-1.85335	0.067167	-4.13566	0.547559	0.365515
NK.cells	CLOCK	0.211935	5.071637	1.852173	0.067338	-4.90644	0.518615	0.330955
NK.cells	GM47889	-0.66777	1.560566	-1.85216	0.06734	-4.169	0.546769	0.364917
NK.cells	TPRN	-0.44253	2.21301	-1.85213	0.067345	-4.3752	0.541416	0.358357
NK.cells	MXD4	-0.24011	5.790486	-1.85208	0.067352	-5.08355	0.513047	0.324414
NK.cells	CDC26	0.157996	5.383294	1.851886	0.06738	-4.90067	0.516193	0.328201
NK.cells	RAB5IF	0.158046	7.307335	1.851532	0.067431	-5.23986	0.501513	0.311202
NK.cells	NUPL2	0.486751	2.367649	1.851184	0.067482	-4.28739	0.540155	0.357107
NK.cells	0610040J0	-0.66994	2.378015	-1.85113	0.06749	-4.17802	0.540071	0.357004
NK.cells	MEFV	1.121839	-0.03339	1.851095	0.067495	-4.02347	0.560085	0.381742
NK.cells	SKINT3	0.939239	0.115469	1.851064	0.067499	-4.03048	0.558827	0.380168
NK.cells	ITPKB	-0.17163	7.462345	-1.85105	0.067501	-5.41538	0.500351	0.309894
NK.cells	DCUN1D1	0.193986	5.787878	1.850899	0.067523	-5.00819	0.513067	0.324772
NK.cells	GM43062	-0.52711	1.638658	-1.85015	0.067633	-4.15053	0.546275	0.364838
NK.cells	GM50020	0.914613	-0.11132	1.849776	0.067687	-4.02329	0.560898	0.383157
NK.cells	LENG9	0.452528	2.352054	1.849565	0.067717	-4.32093	0.54043	0.357861
NK.cells	ADAM17	0.251376	6.061586	1.849533	0.067722	-5.03052	0.511104	0.322757
NK.cells	TSPAN17	-0.69464	1.115501	-1.84926	0.067761	-4.0876	0.550604	0.370435
NK.cells	PIGK	0.220074	4.298401	1.848847	0.067822	-4.75823	0.524952	0.339177
NK.cells	NRXN3	-0.87453	1.145579	-1.84828	0.067905	-4.09519	0.550806	0.37035
NK.cells	TUBA1B	-0.27715	7.795232	-1.84793	0.067956	-5.34674	0.498461	0.307897
NK.cells	ABCC3	-0.88196	2.689429	-1.84666	0.068142	-4.16322	0.539191	0.355503
NK.cells	LIPO3	0.445174	2.915424	1.846462	0.068171	-4.33101	0.537358	0.353361
NK.cells	RAG1	-0.92051	1.672599	-1.84553	0.068307	-4.10957	0.548099	0.366077

NK.cells	GM10134	1.024894	0.795093	1.845278	0.068345	-4.04871	0.555405	0.375174
NK.cells	1810026B(	0.144763	6.570259	1.844758	0.068421	-5.18421	0.509175	0.319652
NK.cells	C030034I2	0.366318	2.630863	1.844341	0.068482	-4.42591	0.540238	0.35685
NK.cells	CYP2B9	-0.7788	1.9577	-1.84404	0.068527	-4.2385	0.545748	0.363678
NK.cells	SRSF7	-0.21295	6.098296	-1.84382	0.068559	-5.07265	0.512792	0.324103
NK.cells	CAMK4	0.318415	3.194525	1.843648	0.068584	-5.0906	0.53567	0.351499
NK.cells	IL4	-0.82568	0.330173	-1.84357	0.068596	-4.28852	0.559318	0.38063
NK.cells	LRP10	0.175406	6.125795	1.843211	0.068649	-5.12143	0.512645	0.324133
NK.cells	LAD1	-1.21277	-0.99444	-1.84187	0.068847	-4.03946	0.571518	0.395826
NK.cells	SSU72	0.136656	6.693565	1.841433	0.068911	-5.18682	0.50906	0.31979
NK.cells	FLT4	-0.69959	2.321562	-1.84091	0.068988	-4.15522	0.543642	0.361341
NK.cells	MSTO1	0.382903	2.681663	1.840899	0.06899	-4.43322	0.5407	0.35774
NK.cells	WBP4	0.188232	5.270226	1.840705	0.069019	-4.94085	0.520048	0.332974
NK.cells	GPR182	-0.69694	2.557152	-1.84053	0.069045	-4.17002	0.541716	0.359098
NK.cells	RCC1	-0.30407	3.989305	-1.84025	0.069087	-4.62431	0.530158	0.345075
NK.cells	PAG1	-0.21381	7.038518	-1.84013	0.069105	-5.36924	0.506435	0.316991
NK.cells	ZDHHC5	-0.17324	5.017726	-1.83975	0.06916	-4.88806	0.52211	0.335422
NK.cells	RHOA	0.093737	9.258828	1.838814	0.0693	-5.57724	0.490358	0.298379
NK.cells	RUFY2	0.318106	2.954919	1.838797	0.069302	-4.51525	0.538982	0.355605
NK.cells	UACA	-0.87269	2.51718	-1.83767	0.069469	-4.08586	0.54351	0.360218
NK.cells	DNAJC19	0.164302	5.950115	1.837105	0.069554	-5.08885	0.51619	0.327558
NK.cells	MBTPS2	0.28152	3.917498	1.836938	0.069579	-4.63204	0.532199	0.346693
NK.cells	GM26549	-0.32931	3.018851	-1.83676	0.069606	-4.52728	0.539447	0.35553
NK.cells	GZMC	0.643166	1.539474	1.83645	0.069652	-4.83698	0.551623	0.370546
NK.cells	A3GALT2	-0.51966	2.675415	-1.83574	0.069757	-4.34234	0.542734	0.35929
NK.cells	ERCC6	0.302501	3.56729	1.833944	0.070027	-4.64942	0.536218	0.350944
NK.cells	MTFR2	-0.37913	3.615592	-1.83388	0.070036	-4.63896	0.535828	0.350497
NK.cells	MIPOL1	0.235668	4.257052	1.833858	0.070039	-4.75407	0.530682	0.344295
NK.cells	CRCP	0.212024	4.449755	1.833794	0.070049	-4.75706	0.529146	0.342461
NK.cells	GDA	1.097969	3.902395	1.833628	0.070074	-4.2469	0.53352	0.347724
NK.cells	GM14455	0.607931	1.315653	1.833108	0.070152	-4.16044	0.554752	0.373862
NK.cells	DOP1A	0.278081	4.09244	1.83289	0.070185	-4.72648	0.532021	0.346135
NK.cells	NAPRT	-0.85612	0.897988	-1.83272	0.07021	-4.12036	0.55826	0.378315
NK.cells	NR1H3	0.679675	3.325046	1.832341	0.070267	-4.3353	0.538297	0.35375
NK.cells	AIFM1	0.230958	4.370662	1.831666	0.070369	-4.77091	0.530324	0.343849
NK.cells	CALCA	-0.7381	-0.96013	-1.83119	0.07044	-4.22551	0.574929	0.398948
NK.cells	ADI1	-0.27927	3.749643	-1.83043	0.070555	-4.57716	0.535585	0.350254
NK.cells	LARP1B	-0.20737	5.599705	-1.83036	0.070565	-5.04331	0.520896	0.332685
NK.cells	PRIMPOL	0.338252	3.486578	1.830059	0.070611	-4.54961	0.53771	0.352929
NK.cells	LMF1	0.326653	3.239428	1.829943	0.070628	-4.56001	0.539715	0.355376
NK.cells	POGLUT1	0.389395	2.752407	1.829021	0.070768	-4.41754	0.54415	0.360505
NK.cells	IL12A	-0.83328	2.215059	-1.82896	0.070777	-4.12957	0.548575	0.365948
NK.cells	AXL	-0.60801	3.708037	-1.82817	0.070896	-4.49787	0.536941	0.351367
NK.cells	FAM169B	0.354141	3.62896	1.826763	0.07111	-4.69372	0.53886	0.352661
NK.cells	FHOD3	1.21515	-0.11029	1.826434	0.07116	-4.04673	0.570163	0.391388
NK.cells	DYRK1B	0.505083	1.826196	1.826014	0.071224	-4.22417	0.553888	0.371021
NK.cells	CTSS	0.4039	7.501211	1.825432	0.071312	-5.13263	0.508917	0.317012

NK.cells	GM50399	-1.13491	0.166375	-1.82356	0.071598	-4.06361	0.569677	0.389548
NK.cells	CYP51	-0.30879	3.916341	-1.82306	0.071673	-4.67962	0.538361	0.351148
NK.cells	GM47428	1.071585	0.207827	1.822716	0.071727	-4.05804	0.56932	0.389431
NK.cells	NCF2	0.269213	6.576577	1.822547	0.071752	-5.00521	0.517275	0.326225
NK.cells	KNSTRN	-0.48996	3.271497	-1.82233	0.071786	-4.57145	0.543612	0.357715
NK.cells	WDR89	0.380576	2.643388	1.822026	0.071832	-4.43423	0.54878	0.364161
NK.cells	NOL12	0.218542	4.012955	1.821956	0.071843	-4.70126	0.537579	0.350544
NK.cells	UNKL	-0.27348	3.970057	-1.82194	0.071845	-4.77558	0.537926	0.350963
NK.cells	AMZ1	-0.7488	3.157688	-1.82089	0.072006	-4.22524	0.545426	0.359498
NK.cells	TMEM191(	-0.40678	1.703176	-1.82006	0.072134	-4.30218	0.557842	0.374535
NK.cells	UBFD1	-0.24213	4.520398	-1.81942	0.072232	-4.78767	0.534669	0.346417
NK.cells	IFI27L2A	0.513786	7.373467	1.818785	0.07233	-5.28476	0.512264	0.320169
NK.cells	DEDD2	-0.27084	4.544003	-1.81878	0.07233	-4.80542	0.534479	0.346408
NK.cells	GM50431	-0.82308	0.122176	-1.81849	0.072376	-4.12755	0.571314	0.391752
NK.cells	ZFP593	0.343105	3.359126	1.818418	0.072386	-4.61434	0.544093	0.358044
NK.cells	ANKRD40	0.238937	4.238658	1.818199	0.07242	-4.74486	0.536939	0.349488
NK.cells	TRADD	0.270276	3.982911	1.818152	0.072427	-4.74619	0.539008	0.351985
NK.cells	HNRNPH1	-0.17782	6.424984	-1.81737	0.072547	-5.14875	0.519595	0.329083
NK.cells	GUK1	0.201712	4.908743	1.817175	0.072578	-4.95025	0.531557	0.343303
NK.cells	DDX18	0.199752	4.987355	1.816835	0.072631	-4.94505	0.530929	0.342649
NK.cells	CASD1	0.243943	4.363906	1.816515	0.07268	-4.75815	0.535928	0.348769
NK.cells	CEBPZOS	0.237903	4.521965	1.816467	0.072688	-4.82767	0.534656	0.347254
NK.cells	TOMM34	-0.1681	5.601463	-1.81644	0.072692	-5.07436	0.526055	0.336974
NK.cells	RAB44	0.88444	1.487929	1.816243	0.072722	-4.1683	0.559656	0.377931
NK.cells	GM32569	-0.92685	1.845691	-1.81531	0.072867	-4.16657	0.557406	0.374396
NK.cells	NSD3	-0.13192	8.849458	-1.8146	0.072977	-5.64562	0.501949	0.308304
NK.cells	SMARCD1	0.219295	4.031896	1.81456	0.072983	-4.75252	0.539543	0.352548
NK.cells	P2RY6	-0.81959	2.305897	-1.81368	0.07312	-4.1232	0.554185	0.370222
NK.cells	TTC41	-0.86701	0.966475	-1.81361	0.07313	-4.09546	0.565496	0.384292
NK.cells	9930111J2	0.382278	4.28297	1.813147	0.073202	-4.74586	0.53813	0.350571
NK.cells	TMEM42	0.341223	2.617603	1.81077	0.073573	-4.44984	0.553877	0.368325
NK.cells	CD84	0.23618	5.366126	1.810605	0.073599	-5.08094	0.531442	0.34121
NK.cells	NECTIN4	-1.08144	-0.02307	-1.8105	0.073615	-4.06526	0.576392	0.396378
NK.cells	ITGAX	-0.26896	3.098724	-1.81012	0.073675	-4.98413	0.549991	0.363532
NK.cells	HECW2	-0.63548	3.162677	-1.80976	0.073731	-4.3447	0.549544	0.362912
NK.cells	PRKDC	0.207817	4.69925	1.809346	0.073796	-4.93987	0.536999	0.347836
NK.cells	CDKN3	-0.41758	4.160534	-1.80895	0.073858	-4.79676	0.541367	0.35326
NK.cells	TRF	-0.41199	9.658999	-1.80888	0.073869	-5.69859	0.498563	0.303161
NK.cells	CTSO	0.314469	4.26707	1.808446	0.073937	-4.71243	0.540664	0.352454
NK.cells	A430093F1	0.414906	2.363356	1.80798	0.07401	-4.47777	0.556596	0.37179
NK.cells	9330159M	-0.86598	1.076034	-1.80726	0.074123	-4.09687	0.568031	0.385496
NK.cells	SLC16A10	-0.22202	7.024829	-1.80682	0.074193	-5.51434	0.519583	0.326867
NK.cells	ETS1	0.175018	7.418769	1.806126	0.074301	-5.42214	0.516966	0.32348
NK.cells	A930005H1	0.286309	3.220496	1.805763	0.074359	-4.60379	0.550695	0.363709
NK.cells	GSTK1	-0.49264	2.449061	-1.805	0.074478	-4.32875	0.557689	0.371864
NK.cells	SNX20	0.209599	5.514815	1.803704	0.074684	-5.05022	0.533471	0.341695
NK.cells	CABP4	-1.15461	-0.22452	-1.80361	0.074698	-4.07555	0.581653	0.40084

NK.cells	RCOR3	-0.37664	2.937283	-1.80311	0.074777	-4.49063	0.554715	0.367335
NK.cells	SLC39A14	0.383765	3.169335	1.802884	0.074813	-4.57835	0.55278	0.365047
NK.cells	MKI67	-0.39815	6.498445	-1.80262	0.074856	-5.27522	0.525805	0.332767
NK.cells	MLLT11	0.302316	3.193389	1.800519	0.075189	-4.56096	0.554702	0.365499
NK.cells	ARHGEF18	0.214859	5.591269	1.799961	0.075278	-5.13218	0.535246	0.342136
NK.cells	RBMS1	0.135677	7.558573	1.799779	0.075307	-5.45494	0.519692	0.323949
NK.cells	MED27	0.159206	5.275175	1.797898	0.075607	-5.03697	0.53928	0.345918
NK.cells	SEL1L	0.192435	5.161227	1.797896	0.075607	-4.98798	0.540204	0.347018
NK.cells	GSR	0.236076	6.441975	1.797588	0.075656	-5.22708	0.52992	0.334935
NK.cells	CAR13	-0.85056	1.31675	-1.79732	0.075698	-4.10683	0.572399	0.386405
NK.cells	RNF144B	-0.53432	2.562148	-1.79654	0.075825	-4.31549	0.562311	0.373552
NK.cells	PIWIL2	0.859484	0.700548	1.796276	0.075866	-4.15971	0.578324	0.393541
NK.cells	FBXL20	0.262126	5.495646	1.795993	0.075912	-5.09527	0.538035	0.344495
NK.cells	GOLGB1	0.184941	5.4427	1.795572	0.075979	-5.03733	0.538616	0.345036
NK.cells	PPIL6	-1.00113	0.219778	-1.79467	0.076123	-4.09161	0.583458	0.399352
NK.cells	TRA2B	-0.12782	7.832072	-1.79376	0.076269	-5.45387	0.521026	0.323508
NK.cells	SEC24C	0.187283	4.859773	1.792715	0.076439	-4.96382	0.545019	0.351789
NK.cells	CHIL1	0.930546	-0.07877	1.792613	0.076455	-4.10949	0.587132	0.403592
NK.cells	KATNAL1	0.784522	0.973387	1.792286	0.076508	-4.15276	0.577877	0.392085
NK.cells	CYB5A	-0.31167	7.336341	-1.79219	0.076524	-5.37599	0.525143	0.328463
NK.cells	CYP4V3	0.512163	2.78654	1.792123	0.076534	-4.5167	0.56229	0.372827
NK.cells	SPI1	-0.36515	6.411888	-1.79163	0.076614	-4.64308	0.532706	0.337122
NK.cells	DISC1	-0.23786	3.43956	-1.78965	0.076934	-4.88331	0.557965	0.366923
NK.cells	ALDOB	-0.47144	4.949636	-1.78954	0.076953	-4.98404	0.545435	0.351826
NK.cells	NMNAT2	-0.39499	2.111738	-1.78952	0.076955	-4.91586	0.56924	0.380736
NK.cells	GM15848	-1.05987	-0.86297	-1.78927	0.076995	-4.08983	0.595387	0.413592
NK.cells	IFFO2	-0.33	3.364648	-1.78908	0.077026	-4.67222	0.558595	0.36783
NK.cells	MFSD6	-0.24695	4.864614	-1.78875	0.07708	-5.14487	0.546132	0.352888
NK.cells	ALDH7A1	-0.38616	3.227154	-1.78866	0.077095	-4.58343	0.559753	0.369352
NK.cells	FARS2	0.130759	6.899126	1.788559	0.077111	-5.32247	0.529711	0.333464
NK.cells	4930557J0	-0.59237	1.834427	-1.78755	0.077275	-4.42732	0.572496	0.384229
NK.cells	PTK2	-0.41366	4.164046	-1.78691	0.077379	-4.56334	0.552963	0.360275
NK.cells	RAB5B	0.256296	4.382557	1.786617	0.077427	-4.79091	0.551149	0.35819
NK.cells	POLR2B	-0.18992	4.911643	-1.78652	0.077442	-4.97704	0.546783	0.352982
NK.cells	MIR155HG	0.38289	4.086438	1.785378	0.077629	-4.9748	0.554236	0.361495
NK.cells	2700097O(	0.35195	3.090234	1.784721	0.077736	-4.59261	0.56261	0.371906
NK.cells	TENT4B	-0.16149	6.368937	-1.78469	0.077741	-5.24644	0.535558	0.339466
NK.cells	BCL2A1D	0.325809	4.122114	1.784463	0.077779	-5.16488	0.553939	0.361465
NK.cells	USP3	0.160772	6.873279	1.783982	0.077857	-5.33245	0.531524	0.334955
NK.cells	CASS4	0.684447	3.04899	1.783869	0.077876	-4.35384	0.56296	0.372581
NK.cells	COQ10A	0.274724	3.31768	1.78379	0.077889	-4.65286	0.560686	0.369818
NK.cells	FILIP1L	0.254211	5.411773	1.783721	0.0779	-4.97592	0.543305	0.348882
NK.cells	LARP4B	0.143669	7.283403	1.783324	0.077965	-5.33828	0.528377	0.331224
NK.cells	TMEM204	-0.77149	1.038857	-1.78289	0.078037	-4.12461	0.580402	0.394211
NK.cells	FAM49A	0.283838	5.390412	1.782665	0.078073	-5.11085	0.543591	0.349289
NK.cells	TADA1	-0.21187	4.679062	-1.7825	0.078101	-4.90234	0.549431	0.356322
NK.cells	BOK	-1.00337	1.039523	-1.78071	0.078394	-4.12162	0.58223	0.394777

NK.cells	TMEM40	0.943576	0.497552	1.779762	0.078551	-4.13408	0.587837	0.401133
NK.cells	PTPRD	-0.64398	2.563535	-1.77943	0.078605	-4.41874	0.569858	0.378872
NK.cells	SORBS3	-0.82854	0.937867	-1.77913	0.078655	-4.15078	0.584026	0.39652
NK.cells	PIK3R3	-0.46571	3.43068	-1.77758	0.078912	-4.64849	0.56399	0.370577
NK.cells	ATP1B3	0.241706	7.281647	1.777002	0.079007	-5.40191	0.532627	0.333095
NK.cells	PRKD2	0.25554	4.348606	1.776029	0.079168	-4.82732	0.556999	0.36172
NK.cells	RBM33	0.155963	5.898903	1.775993	0.079175	-5.1757	0.544177	0.346443
NK.cells	TPGS2	-0.2526	3.805381	-1.77575	0.079214	-4.79671	0.56157	0.367351
NK.cells	MYL9	0.781801	1.95475	1.774893	0.079357	-4.36266	0.577446	0.387102
NK.cells	TATDN2	-0.17426	5.004884	-1.77479	0.079374	-5.08622	0.551531	0.355604
NK.cells	CCDC9	0.202078	4.238269	1.774763	0.079379	-4.8596	0.557924	0.363274
NK.cells	HAAO	-0.39519	3.64437	-1.77435	0.079447	-4.59449	0.562932	0.369392
NK.cells	PCLAF	-0.41004	7.982096	-1.77414	0.079482	-5.52227	0.527456	0.327479
NK.cells	6-Sep	-0.19791	5.624977	-1.77413	0.079485	-5.27702	0.546419	0.349688
NK.cells	STX11	0.251918	4.829435	1.77319	0.079641	-5.05809	0.55375	0.357861
NK.cells	CD1D1	-0.3888	3.932282	-1.77191	0.079854	-4.72272	0.561795	0.36732
NK.cells	ATP1B1	-0.30835	5.798004	-1.77189	0.079858	-5.06419	0.546263	0.348729
NK.cells	NGRN	0.367997	3.173648	1.771331	0.079952	-4.57598	0.568247	0.375274
NK.cells	ZFP35	0.405595	2.543067	1.771279	0.07996	-4.42388	0.573671	0.381919
NK.cells	PPP6C	-0.12618	6.737344	-1.77085	0.080031	-5.31757	0.538621	0.339965
NK.cells	BTG1	0.161808	9.314237	1.770732	0.080052	-5.74159	0.51826	0.316455
NK.cells	FBXO5	-0.40835	4.811103	-1.76981	0.080207	-4.96438	0.55442	0.358994
NK.cells	AGTR1A	-1.07639	0.270352	-1.76938	0.080278	-4.14818	0.593683	0.407387
NK.cells	JAG2	-0.67942	1.093215	-1.76924	0.080303	-4.2383	0.586353	0.398222
NK.cells	HAO1	-0.5049	3.070076	-1.76922	0.080305	-4.55213	0.569135	0.376924
NK.cells	NDRG2	-0.51887	2.964877	-1.76915	0.080318	-4.53907	0.570037	0.378029
NK.cells	MCMDC2	0.516415	3.465667	1.769022	0.080339	-4.49175	0.565754	0.372798
NK.cells	GM38948	0.675568	0.463761	1.769	0.080342	-4.1895	0.591952	0.405246
NK.cells	RWDD3	0.822288	0.507573	1.768552	0.080418	-4.15248	0.59156	0.40496
NK.cells	TMEM71	0.27668	3.858441	1.767896	0.080528	-4.93091	0.56242	0.369102
NK.cells	QK	-0.128	8.142329	-1.7678	0.080544	-5.47064	0.527411	0.327609
NK.cells	UHRF1BP1	0.185421	5.734451	1.767298	0.080629	-5.14591	0.546784	0.350533
NK.cells	BTBD10	-0.17048	5.583956	-1.76685	0.080705	-5.1019	0.548021	0.352134
NK.cells	ZFPM1	-0.27158	4.248218	-1.76678	0.080716	-4.89924	0.559131	0.365475
NK.cells	JAK3	0.354732	2.778845	1.766684	0.080732	-4.68198	0.571637	0.380729
NK.cells	GM20275	0.346138	3.071387	1.766608	0.080745	-4.69057	0.569123	0.37768
NK.cells	ANGPT1	0.675063	0.973826	1.766587	0.080749	-4.30425	0.587411	0.400361
NK.cells	KIF15	-0.38757	4.90741	-1.76634	0.080791	-5.00708	0.553618	0.358994
NK.cells	BIRC5	-0.40147	5.35755	-1.76522	0.08098	-5.12324	0.55085	0.354947
NK.cells	OCIAD2	-0.8814	0.485681	-1.76457	0.08109	-4.16575	0.592928	0.406787
NK.cells	PTCH1	0.425115	3.139748	1.764557	0.081092	-4.58375	0.569664	0.377855
NK.cells	TWSG1	-0.40103	3.230079	-1.7634	0.081288	-4.54746	0.569229	0.377367
NK.cells	RMND1	0.273986	3.79439	1.763268	0.08131	-4.65703	0.564413	0.371488
NK.cells	LPCAT1	0.258123	4.107714	1.762758	0.081397	-4.84349	0.561759	0.368389
NK.cells	PRF1	0.47419	0.377462	1.76267	0.081412	-4.56392	0.594252	0.408651
NK.cells	SOCS4	0.207553	4.684801	1.762142	0.081502	-4.92645	0.556905	0.36261
NK.cells	UBL3	0.12766	7.408809	1.76197	0.081531	-5.41524	0.534606	0.336174

NK.cells	UGT2B5	-0.76785	1.786068	-1.76186	0.081549	-4.31863	0.581752	0.39312
NK.cells	SH3BP2	0.418523	3.288629	1.761683	0.08158	-4.60031	0.568727	0.377046
NK.cells	KRT18	-0.55726	3.557982	-1.76117	0.081667	-4.682	0.566425	0.374312
NK.cells	GM48089	-0.94536	1.135961	-1.76116	0.081669	-4.17317	0.587486	0.400392
NK.cells	UNC13A	0.765475	1.183593	1.760549	0.081773	-4.20676	0.587064	0.4001
NK.cells	FAM3A	0.371025	2.785704	1.760044	0.081859	-4.51844	0.573052	0.382727
NK.cells	FMNL3	-0.2854	4.258409	-1.76001	0.081865	-4.81119	0.560487	0.367359
NK.cells	GM48099	0.662521	2.773722	1.75995	0.081875	-4.60621	0.573155	0.38287
NK.cells	RSPH3B	0.310146	3.01938	1.759772	0.081905	-4.63153	0.571038	0.380314
NK.cells	HIC1	-0.4557	2.158311	-1.75966	0.081925	-4.74908	0.578496	0.389583
NK.cells	CD300LB	0.987684	1.973752	1.759584	0.081938	-4.19867	0.580108	0.391623
NK.cells	C230066G1	0.902562	0.025289	1.75876	0.082078	-4.16603	0.598105	0.413685
NK.cells	THOC1	-0.16966	5.704802	-1.75804	0.082202	-5.19906	0.549579	0.353556
NK.cells	SLC16A13	1.045308	-0.01818	1.757296	0.082329	-4.13808	0.599271	0.41482
NK.cells	COL25A1	-0.97155	0.981282	-1.7569	0.082397	-4.18846	0.590295	0.403589
NK.cells	SCRG1	-0.94314	-0.98095	-1.75645	0.082474	-4.13877	0.608029	0.426388
NK.cells	HMGN2	-0.24947	7.53605	-1.75641	0.082481	-5.42259	0.53489	0.336469
NK.cells	VWCE	-0.88012	0.865529	-1.75606	0.082541	-4.1638	0.591327	0.40515
NK.cells	CYTH2	-0.27262	3.933028	-1.75589	0.08257	-4.81132	0.564611	0.37206
NK.cells	PIK3C2B	-0.60174	2.667746	-1.75589	0.08257	-4.33507	0.575471	0.385393
NK.cells	EDIL3	-0.65804	1.492552	-1.75567	0.082607	-4.37518	0.585759	0.398255
NK.cells	CNDP2	0.220849	5.051865	1.75449	0.082811	-5.06356	0.556244	0.361058
NK.cells	ITGA2B	0.646971	0.938407	1.754162	0.082868	-4.25333	0.591852	0.405021
NK.cells	WDR75	0.282948	3.575364	1.753899	0.082913	-4.73711	0.568787	0.376416
NK.cells	PCF11	-0.18596	6.482932	-1.75356	0.082971	-5.28257	0.544505	0.347252
NK.cells	RPP25L	0.278068	3.672149	1.753326	0.083012	-4.75091	0.56799	0.375538
NK.cells	CCNT1	-0.12828	6.841664	-1.7527	0.08312	-5.38558	0.541985	0.344153
NK.cells	DHPS	0.205527	4.486343	1.751965	0.083247	-4.94342	0.562028	0.367704
NK.cells	INPP5K	-0.19411	5.371477	-1.75148	0.083331	-5.06399	0.554645	0.358925
NK.cells	BEND5	0.698209	0.115495	1.751382	0.083348	-4.19853	0.600348	0.415462
NK.cells	MFSD14B	-0.1747	5.841678	-1.75054	0.083493	-5.17161	0.551097	0.354542
NK.cells	NAV2	-0.2746	4.841571	-1.75053	0.083496	-5.06371	0.559437	0.364552
NK.cells	PAK2	0.111547	7.859842	1.750035	0.083582	-5.55646	0.534865	0.335396
NK.cells	PPNR	-0.98358	1.051959	-1.74974	0.083633	-4.15544	0.592503	0.405478
NK.cells	RCN1	0.324687	3.060814	1.749547	0.083666	-4.67705	0.574825	0.383556
NK.cells	HOMER3	-0.36657	3.103095	-1.74796	0.083943	-4.48507	0.575765	0.383828
NK.cells	LAMTOR4	0.194792	6.205335	1.747485	0.084025	-5.24583	0.549533	0.352205
NK.cells	CCT6A	0.178565	5.802009	1.747426	0.084035	-5.18941	0.552869	0.356184
NK.cells	OGG1	-0.3212	3.189389	-1.74735	0.084048	-4.56597	0.575018	0.383076
NK.cells	ARPP21	1.020175	1.234602	1.747095	0.084093	-4.24701	0.592215	0.404542
NK.cells	SATB1	0.214531	7.143818	1.745332	0.084401	-5.55201	0.543465	0.343822
NK.cells	DDIAS	-0.49259	2.134705	-1.74512	0.084438	-4.42666	0.585962	0.395322
NK.cells	UST	-0.35292	6.028853	-1.74441	0.084562	-5.32713	0.552798	0.355035
NK.cells	PRKAR2A	-0.30667	5.222556	-1.74416	0.084607	-5.18176	0.55953	0.363193
NK.cells	SRFBP1	0.329797	3.231687	1.744007	0.084633	-4.67148	0.576537	0.383887
NK.cells	USP5	0.240715	4.34395	1.743889	0.084654	-4.91684	0.566967	0.372197
NK.cells	EFTUD2	0.186178	5.105706	1.743372	0.084745	-5.09509	0.560799	0.364599

NK.cells	LRRK1	-0.22049	5.612519	-1.7429	0.084828	-5.23643	0.556779	0.359543
NK.cells	COMTD1	0.345139	2.92368	1.742374	0.08492	-4.58065	0.580061	0.387731
NK.cells	GRAMD2	-0.87844	0.533275	-1.74167	0.085043	-4.1682	0.60189	0.41459
NK.cells	PUM3	0.1982	4.640352	1.74119	0.085129	-5.00667	0.565879	0.369953
NK.cells	CNOT7	-0.1546	5.288607	-1.74104	0.085155	-5.09016	0.560394	0.363407
NK.cells	TOMM5	0.208863	5.458336	1.740665	0.085221	-5.1477	0.559091	0.361829
NK.cells	HDAC11	-0.96231	0.5008	-1.74004	0.085331	-4.17662	0.602874	0.415646
NK.cells	GM12940	-0.25182	4.51886	-1.73968	0.085396	-4.95211	0.567565	0.371783
NK.cells	PRPF19	-0.18611	5.053951	-1.73911	0.085495	-5.0827	0.56336	0.366525
NK.cells	SERF1	0.340331	3.137334	1.73807	0.08568	-4.54528	0.580768	0.386961
NK.cells	TUBB2A	-0.26216	5.517234	-1.73755	0.085772	-5.16759	0.560641	0.362254
NK.cells	DGKI	-1.39467	0.78	-1.7366	0.08594	-4.1998	0.602936	0.413562
NK.cells	ZFP532	-0.95459	1.223667	-1.73611	0.086027	-4.1992	0.598912	0.408627
NK.cells	FGL1	0.403414	3.523532	1.735807	0.086082	-4.77129	0.578506	0.383331
NK.cells	IQCN	-0.75927	1.791223	-1.73572	0.086098	-4.29479	0.593805	0.402257
NK.cells	TESPA1	0.376789	2.504605	1.735266	0.086178	-4.70056	0.587453	0.394467
NK.cells	D6WSU163	0.373344	3.1191	1.73524	0.086183	-4.49685	0.58204	0.387783
NK.cells	PROX1OS	-0.8571	0.612207	-1.73499	0.086227	-4.2266	0.604465	0.41586
NK.cells	AMPD1	-0.43391	2.911186	-1.73447	0.08632	-4.61536	0.583997	0.390438
NK.cells	NCAPH2	-0.17981	5.344606	-1.73403	0.086398	-5.13638	0.563008	0.36498
NK.cells	MCM9	0.217546	4.75152	1.733863	0.086428	-5.02833	0.568047	0.371139
NK.cells	SLC13A3	-0.84073	1.11728	-1.73381	0.086438	-4.2853	0.60001	0.410621
NK.cells	FADD	0.46342	2.421277	1.733126	0.08656	-4.53853	0.588826	0.396265
NK.cells	NDRG1	0.478843	2.72107	1.731632	0.086827	-4.58661	0.587333	0.393512
NK.cells	NR2F2	-0.59808	3.13223	-1.73163	0.086827	-4.50038	0.583707	0.389036
NK.cells	NSDHL	-0.45996	2.406095	-1.731	0.086941	-4.46367	0.590571	0.397225
NK.cells	MTAP	0.26801	3.671485	1.73047	0.087035	-4.84025	0.579673	0.383595
NK.cells	WDR45B	-0.13296	6.330115	-1.72945	0.087218	-5.31666	0.556975	0.356791
NK.cells	B130034C1	0.584715	1.038829	1.729413	0.087225	-4.25062	0.603138	0.413388
NK.cells	TPPP	-1.25893	0.182012	-1.72929	0.087247	-4.16965	0.610991	0.423367
NK.cells	LEMD3	-0.17013	5.687224	-1.72877	0.087341	-5.23778	0.562373	0.363453
NK.cells	PLOD3	0.38204	3.277379	1.72869	0.087355	-4.621	0.583123	0.388696
NK.cells	TMEM181A	0.203244	4.58903	1.727509	0.087568	-4.98647	0.571727	0.375226
NK.cells	TRIM36	-0.3085	3.146179	-1.72741	0.087586	-4.80732	0.584276	0.390624
NK.cells	SLC16A2	-0.4586	2.626868	-1.7272	0.087624	-4.54101	0.588865	0.396419
NK.cells	CD9	-0.27096	6.033746	-1.72666	0.087721	-5.33621	0.559456	0.360668
NK.cells	EXOC1	0.253531	4.095264	1.72618	0.087808	-4.87246	0.575988	0.38076
NK.cells	GPR55	0.397744	1.548876	1.726115	0.08782	-4.58693	0.598514	0.408717
NK.cells	TOP1	-0.16559	8.354587	-1.72513	0.087998	-5.58975	0.540346	0.338446
NK.cells	PJA1	-0.236	3.699202	-1.72486	0.088047	-4.94389	0.579431	0.385357
NK.cells	RFFL	0.250783	5.915131	1.72477	0.088063	-5.12433	0.560453	0.362304
NK.cells	LDAH	0.22084	4.225642	1.724763	0.088065	-4.92579	0.57486	0.379752
NK.cells	KNL1	-0.37438	5.131736	-1.72473	0.08807	-5.15825	0.567084	0.370293
NK.cells	DHX35	-0.4241	2.187953	-1.72465	0.088086	-4.5065	0.592773	0.401921
NK.cells	ACTR6	0.292169	3.522286	1.724225	0.088162	-4.74651	0.580976	0.387383
NK.cells	DHCR24	-0.4418	2.897631	-1.72393	0.088216	-4.54702	0.586467	0.394291
NK.cells	TRPM7	-0.14868	7.284405	-1.7239	0.088221	-5.49389	0.549067	0.348967

NK.cells	PHF13	-0.24496	3.504231	-1.72359	0.088277	-4.82686	0.581134	0.387777
NK.cells	TEX30	-0.19274	5.223272	-1.72354	0.088286	-5.0985	0.566304	0.369658
NK.cells	FKBP5	0.271363	5.30259	1.723345	0.088322	-5.15536	0.56563	0.368843
NK.cells	GM30211	-0.59078	4.041214	-1.72315	0.088358	-4.65151	0.576457	0.382144
NK.cells	TMA7	0.122874	6.564001	1.723105	0.088365	-5.3979	0.555025	0.356232
NK.cells	MED11	0.279664	3.312748	1.722468	0.088481	-4.74724	0.582812	0.390224
NK.cells	FTCD	-0.80979	1.403237	-1.72194	0.088578	-4.31286	0.59983	0.411707
NK.cells	CDH1	0.676916	1.38751	1.721893	0.088586	-4.3556	0.599973	0.411887
NK.cells	GM50334	-1.17218	-0.42372	-1.72167	0.088627	-4.1702	0.616608	0.43316
NK.cells	DCAF15	0.271987	3.456621	1.721566	0.088645	-4.84858	0.581551	0.388924
NK.cells	TMEM237	0.439513	2.157499	1.72083	0.08878	-4.56545	0.593046	0.403497
NK.cells	CS	0.144019	6.103857	1.720815	0.088782	-5.28427	0.558868	0.361527
NK.cells	PWWP2B	-0.40746	2.082717	-1.7207	0.088803	-4.58277	0.593715	0.404336
NK.cells	SYNGR2	0.187464	6.111593	1.720571	0.088827	-5.2555	0.558803	0.361449
NK.cells	NPL	-0.74963	2.535175	-1.72052	0.088836	-4.34334	0.589679	0.399281
NK.cells	ZRSR2	-0.15883	5.063714	-1.72036	0.088866	-5.09244	0.567663	0.372207
NK.cells	ILDR1	0.643341	1.791756	1.720129	0.088907	-4.44171	0.596325	0.407736
NK.cells	CD5L	-0.67456	6.068559	-1.72	0.088931	-4.99116	0.559164	0.362028
NK.cells	3830403N1	-1.1495	0.704685	-1.71969	0.088987	-4.22056	0.606188	0.42033
NK.cells	VPREB1	-0.96297	2.621584	-1.71965	0.088996	-4.41237	0.588912	0.39852
NK.cells	RELL1	-0.18472	6.866963	-1.71964	0.088998	-5.31883	0.552511	0.354104
NK.cells	ZFP746	0.25038	3.717554	1.719325	0.089054	-4.83074	0.579327	0.386576
NK.cells	HELQ	0.380015	2.411841	1.717996	0.089298	-4.52084	0.591573	0.401495
NK.cells	SLC25A40	0.275492	3.227708	1.717953	0.089306	-4.86191	0.584346	0.392495
NK.cells	PGM1	0.231443	4.592397	1.717928	0.08931	-5.04764	0.572469	0.377866
NK.cells	IQGAP3	-0.62301	2.400987	-1.71745	0.089397	-4.44731	0.591927	0.401847
NK.cells	SNHG3	0.248878	5.291632	1.717007	0.089479	-5.13265	0.566926	0.370991
NK.cells	SH2B3	-0.22057	5.315208	-1.71676	0.089524	-5.02635	0.566725	0.370813
NK.cells	IGKC	-0.55109	7.88266	-1.71517	0.089818	-5.46673	0.546823	0.345738
NK.cells	AFM	-0.55077	2.294567	-1.71427	0.089982	-4.48433	0.595318	0.404124
NK.cells	A630001G2	0.219515	5.126842	1.714138	0.090007	-5.05783	0.570476	0.373526
NK.cells	INO80DOS	-0.3389	4.442013	-1.71359	0.090108	-4.95698	0.576488	0.380843
NK.cells	ERMAP	-1.01812	0.529243	-1.71341	0.090141	-4.20422	0.611504	0.424733
NK.cells	5031425F1	0.835808	-0.17235	1.713259	0.090169	-4.24789	0.618018	0.433106
NK.cells	GM26887	-0.51257	3.10483	-1.71282	0.090251	-4.69391	0.588419	0.395598
NK.cells	SETD5	0.136145	6.628233	1.712233	0.090359	-5.38301	0.558448	0.358809
NK.cells	EXOSC1	0.225644	4.175365	1.711603	0.090476	-4.91827	0.579737	0.384473
NK.cells	SUSD6	0.164625	7.690279	1.711427	0.090508	-5.55848	0.549955	0.348677
NK.cells	FAHD2A	-0.31984	2.845982	-1.71008	0.090759	-4.64247	0.592083	0.399642
NK.cells	SRSF3	-0.127	7.532017	-1.70943	0.090878	-5.53815	0.551844	0.351002
NK.cells	MID1IP1	-0.33981	3.917342	-1.70921	0.09092	-4.75785	0.582609	0.388255
NK.cells	MRPL24	0.146846	5.658173	1.708919	0.090974	-5.25222	0.567564	0.37002
NK.cells	CRP	-0.61612	2.076546	-1.70841	0.091069	-4.46682	0.598989	0.408908
NK.cells	CPT1B	-0.51861	1.536488	-1.70827	0.091095	-4.35873	0.603888	0.415094
NK.cells	CD24A	-0.35786	7.730753	-1.70814	0.091119	-5.33406	0.550205	0.349366
NK.cells	CIP2A	-0.38294	3.506565	-1.70809	0.091129	-4.80302	0.586222	0.392961
NK.cells	GM50019	-1.01103	-0.06748	-1.70801	0.091144	-4.19786	0.618689	0.434023

NK.cells	METTL15	0.32318	3.231154	1.707868	0.09117	-4.69883	0.588658	0.396065
NK.cells	CDA	-0.87685	0.894078	-1.70781	0.091181	-4.2533	0.60977	0.422677
NK.cells	C130046K2	-0.79271	0.716514	-1.70775	0.091191	-4.2522	0.611407	0.424775
NK.cells	DIAPH2	0.175961	7.666379	1.707463	0.091245	-5.55185	0.550735	0.350204
NK.cells	SFPQ	-0.12147	8.189863	-1.70726	0.091284	-5.64359	0.54644	0.345155
NK.cells	ITGAD	-0.70924	0.789813	-1.70698	0.091336	-4.36263	0.610758	0.42421
NK.cells	RAD9B	-0.30101	3.275507	-1.70609	0.091503	-4.7945	0.58905	0.396223
NK.cells	TBK1	0.198124	5.964636	1.705826	0.091551	-5.26408	0.565714	0.367699
NK.cells	TARSL2	-0.526	1.415252	-1.70527	0.091655	-4.36537	0.606063	0.417597
NK.cells	IGF2R	0.205961	4.985937	1.705096	0.091688	-5.24188	0.574336	0.378115
NK.cells	UBXN8	0.212851	4.404408	1.704354	0.091827	-5.01204	0.57995	0.384637
NK.cells	CD200R1	0.303528	2.827999	1.70268	0.092141	-4.98022	0.595299	0.402731
NK.cells	NHLRC3	0.348507	3.16365	1.702662	0.092145	-4.71338	0.592297	0.398988
NK.cells	CCDC138	-0.33215	4.628632	-1.70229	0.092215	-4.95161	0.579518	0.383171
NK.cells	B230219D2	0.125818	6.252623	1.701646	0.092336	-5.34021	0.565796	0.36643
NK.cells	MAN2A1	0.203276	7.183028	1.701565	0.092351	-5.41246	0.557962	0.3571
NK.cells	GRN	-0.3076	7.188808	-1.70114	0.092431	-5.14406	0.558098	0.357197
NK.cells	RBMXL1	0.18159	5.042632	1.700257	0.092598	-5.10384	0.576527	0.379545
NK.cells	AHCTF1	-0.18772	5.405997	-1.70019	0.092611	-5.19528	0.573389	0.375724
NK.cells	AI662270	0.199351	6.090129	1.700019	0.092643	-5.3231	0.567532	0.368674
NK.cells	DCTN6	0.185047	5.142412	1.699792	0.092686	-5.13561	0.575664	0.378592
NK.cells	CEP85L	0.293685	4.806918	1.699554	0.092731	-5.013	0.578573	0.382189
NK.cells	CASP4	0.544415	4.029936	1.699448	0.092751	-4.69835	0.585372	0.39059
NK.cells	GJB2	-0.55061	1.638244	-1.69919	0.0928	-4.5005	0.60685	0.417583
NK.cells	ZFP362	-0.23872	3.9339	-1.69845	0.092939	-4.89386	0.586791	0.391977
NK.cells	SMIM26	0.266515	3.847983	1.698125	0.093001	-4.83417	0.587633	0.392973
NK.cells	9430038I0	0.209314	4.248537	1.697188	0.093179	-4.92376	0.584912	0.389056
NK.cells	ABCA3	0.215313	4.193838	1.696057	0.093394	-4.93786	0.58627	0.390063
NK.cells	OIT3	-0.65017	2.345496	-1.69569	0.093464	-4.36788	0.602816	0.410662
NK.cells	2010016I1	0.58773	-0.03474	1.69568	0.093465	-4.38316	0.624862	0.438737
NK.cells	MAMDC2	-0.58731	0.366618	-1.69476	0.093641	-4.45426	0.62127	0.434152
NK.cells	DNAH8	-0.29876	3.758702	-1.69461	0.09367	-4.95249	0.590295	0.39508
NK.cells	REPS1	0.150479	5.964436	1.69444	0.093702	-5.35267	0.571049	0.37155
NK.cells	ZFP982	0.968556	0.056297	1.69434	0.093721	-4.24424	0.624188	0.437909
NK.cells	METRNL	-0.31126	3.205494	-1.69408	0.093769	-5.09182	0.595232	0.401277
NK.cells	ZFP366	-1.0706	1.840557	-1.69399	0.093787	-4.26716	0.607603	0.416814
NK.cells	TOP2A	-0.34569	7.615909	-1.6937	0.093843	-5.62773	0.557129	0.355007
NK.cells	TRPM2	0.809669	2.540831	1.691787	0.094208	-4.34093	0.603011	0.409915
NK.cells	LRRC43	-0.92398	-0.12202	-1.69176	0.094214	-4.23829	0.627734	0.441397
NK.cells	ATP11A	0.272666	2.676915	1.690934	0.094372	-4.92769	0.602472	0.408763
NK.cells	LBR	-0.1281	7.219631	-1.69034	0.094486	-5.51102	0.562922	0.360365
NK.cells	MAPK6	0.222975	5.88266	1.690247	0.094504	-5.31197	0.574318	0.374031
NK.cells	EXOC3L2	-1.14568	2.269133	-1.68903	0.094737	-4.33821	0.607503	0.414056
NK.cells	ZFP523	0.44501	2.19531	1.688737	0.094794	-4.47599	0.60818	0.414998
NK.cells	AGTPBP1	0.209794	5.128653	1.688582	0.094824	-5.21811	0.581914	0.382533
NK.cells	AMACR	-0.58851	2.326832	-1.68636	0.095253	-4.4817	0.609405	0.414418
NK.cells	AP3S2	0.281664	3.43138	1.686022	0.095318	-4.80264	0.599445	0.40198

NK.cells	2310033P(	0.228155	4.144026	1.685621	0.095395	-4.97967	0.593142	0.3942
NK.cells	B4GALT5	0.231317	5.975954	1.68536	0.095446	-5.31061	0.57704	0.374666
NK.cells	EID1	0.174288	5.155191	1.684879	0.095539	-5.2356	0.584194	0.383508
NK.cells	NARF	0.209551	4.893244	1.684629	0.095587	-5.12717	0.586498	0.386399
NK.cells	CCN1	-1.04216	1.098666	-1.68446	0.09562	-4.27475	0.620995	0.429483
NK.cells	XK	0.810756	1.158214	1.684409	0.09563	-4.26677	0.620437	0.428778
NK.cells	CMTM7	-0.15194	7.897065	-1.68334	0.095838	-5.55438	0.561598	0.355762
NK.cells	FAH	-0.45966	3.892683	-1.68251	0.095998	-4.92399	0.596616	0.398055
NK.cells	SGK3	0.253426	5.592861	1.682071	0.096084	-5.08266	0.581563	0.37978
NK.cells	CHRAC1	0.161447	5.264928	1.681721	0.096152	-5.22967	0.584434	0.383401
NK.cells	CDCA8	-0.37331	5.109425	-1.68124	0.096246	-5.21714	0.585801	0.385212
NK.cells	SLC2A1	0.220762	4.676925	1.680419	0.096406	-5.11837	0.589621	0.390234
NK.cells	COL4A3BP	0.154566	6.352484	1.680087	0.096471	-5.44957	0.574973	0.372454
NK.cells	BCL7A	-0.32134	4.80999	-1.67993	0.096502	-4.97553	0.588442	0.388798
NK.cells	MEPCE	-0.24845	4.466254	-1.67945	0.096595	-5.04571	0.591491	0.392654
NK.cells	ZFP414	0.291787	3.443642	1.678909	0.096701	-4.8496	0.600662	0.404186
NK.cells	SLC16A6	-0.25394	4.554893	-1.67882	0.096718	-5.17682	0.590703	0.391897
NK.cells	FRMD5	-0.42374	2.390592	-1.67851	0.096779	-5.09262	0.610266	0.416292
NK.cells	CASK	0.243192	5.500078	1.678332	0.096814	-5.16237	0.582374	0.381817
NK.cells	ASS1	0.327238	6.535258	1.6781	0.096859	-5.50497	0.573399	0.37099
NK.cells	PRPF39	0.181159	5.43876	1.678076	0.096864	-5.23531	0.58291	0.38249
NK.cells	TSPAN14	0.169965	6.835826	1.678025	0.096874	-5.42302	0.570822	0.367898
NK.cells	PHF2OS1	0.60461	1.058613	1.677729	0.096932	-4.32961	0.622651	0.432215
NK.cells	ABCG2	0.354318	3.149803	1.67756	0.096965	-4.69503	0.603325	0.407806
NK.cells	NOS2	1.354799	-1.12584	1.677226	0.09703	-4.23023	0.643406	0.459446
NK.cells	IRF2BPL	-0.24291	4.091669	-1.67721	0.097033	-5.20684	0.594833	0.397441
NK.cells	DHRS1	-0.29504	4.762169	-1.67695	0.097084	-5.03291	0.588866	0.390187
NK.cells	LMNB1	-0.19438	7.679763	-1.67671	0.097131	-5.62578	0.563654	0.359803
NK.cells	LPXN	0.179367	5.23201	1.676634	0.097147	-5.38263	0.584723	0.385217
NK.cells	PCGF1	0.585931	1.17851	1.676409	0.097191	-4.37376	0.621525	0.431267
NK.cells	SLC31A2	0.478945	3.169586	1.676401	0.097192	-4.59931	0.603146	0.408039
NK.cells	PIGZ	0.874099	0.017052	1.676233	0.097225	-4.2776	0.63252	0.445456
NK.cells	THRAP3	-0.1216	8.006361	-1.6759	0.097291	-5.70666	0.560907	0.356764
NK.cells	ARHGAP11	-0.25117	4.784218	-1.67587	0.097297	-5.09653	0.58867	0.390245
NK.cells	IRF4	-0.33626	4.497863	-1.67565	0.09734	-4.90149	0.59121	0.393381
NK.cells	PXK	0.172041	6.270189	1.675566	0.097356	-5.37104	0.575683	0.374442
NK.cells	ZMAT5	0.18434	4.547775	1.675518	0.097366	-5.09406	0.590766	0.392834
NK.cells	1700034P1	-0.91605	1.464772	-1.67472	0.097524	-4.30457	0.619534	0.428237
NK.cells	SIGLECH	-1.21583	0.771994	-1.67403	0.097659	-4.2409	0.626593	0.436807
NK.cells	SMARCAD:	0.174905	4.984115	1.672073	0.098045	-5.14415	0.589415	0.3892
NK.cells	MTERF3	0.242057	4.014483	1.671943	0.098071	-4.97146	0.598071	0.39989
NK.cells	PAH	-0.55485	3.508909	-1.67173	0.098113	-4.82023	0.602638	0.405633
NK.cells	RBAK	-0.57697	1.336784	-1.6717	0.098119	-4.40886	0.622694	0.430899
NK.cells	SYT14	0.914824	0.008242	1.671522	0.098155	-4.32035	0.63531	0.447195
NK.cells	ASPDH	-0.73573	1.294554	-1.67125	0.098208	-4.382	0.623091	0.431615
NK.cells	COBLL1	-0.23613	5.069977	-1.67113	0.098231	-5.18202	0.588655	0.388619
NK.cells	TSPAN2	-0.50192	3.159011	-1.67019	0.098419	-4.43269	0.606426	0.410212

NK.cells	DLC1	-0.56296	4.570858	-1.67014	0.098429	-4.78858	0.593679	0.394406
NK.cells	RECQL5	0.313788	3.722047	1.669879	0.09848	-4.75288	0.601316	0.403898
NK.cells	PDK4	-0.73919	1.417008	-1.66872	0.098711	-4.35323	0.623385	0.431029
NK.cells	IGFBP7	-0.35049	5.173401	-1.66864	0.098727	-5.07658	0.589106	0.388256
NK.cells	DDIT3	-0.30096	4.008692	-1.66841	0.098772	-4.85855	0.599511	0.401072
NK.cells	GOLGA4	0.154644	5.30004	1.668225	0.098809	-5.27244	0.587986	0.38698
NK.cells	FBXW11	-0.1737	7.259166	-1.66777	0.0989	-5.57012	0.570966	0.366493
NK.cells	GM15543	-0.71808	0.901403	-1.66772	0.09891	-4.29447	0.628258	0.437416
NK.cells	KDM6A	-0.3594	6.914035	-1.6671	0.099033	-5.48945	0.573925	0.370234
NK.cells	ABCC9	0.814012	1.277502	1.66694	0.099065	-4.33206	0.624702	0.433166
NK.cells	PARL	0.197303	4.391466	1.666768	0.099099	-5.08808	0.596074	0.39727
NK.cells	GM26944	0.711034	0.852005	1.666579	0.099137	-4.31151	0.628727	0.438432
NK.cells	COPG2	0.193878	5.004811	1.666467	0.09916	-5.19337	0.590603	0.390637
NK.cells	TXNRD2	0.321214	3.532111	1.666139	0.099225	-4.86126	0.603924	0.4071
NK.cells	ST3GAL3	0.195108	5.293649	1.665489	0.099355	-5.22588	0.588181	0.387804
NK.cells	SLC25A4	0.177011	7.14787	1.665262	0.0994	-5.58366	0.572049	0.368341
NK.cells	SMLR1	-0.59512	1.822706	-1.66494	0.099465	-4.47256	0.619727	0.427384
NK.cells	SLC1A5	-0.17855	6.46392	-1.66456	0.09954	-5.48697	0.577942	0.375622
NK.cells	CD274	0.472377	5.805413	1.664335	0.099586	-5.48561	0.583679	0.382675
NK.cells	BC024386	-0.70271	1.614053	-1.66374	0.099704	-4.4086	0.621681	0.430054
NK.cells	H2AFZ	-0.18974	10.35837	-1.66354	0.099746	-6.0636	0.545268	0.337182
NK.cells	GM26724	0.499801	1.517501	1.663384	0.099776	-4.53232	0.622587	0.431245
NK.cells	HECTD1	0.122204	7.428886	1.663236	0.099806	-5.63812	0.569647	0.365878
NK.cells	ABI3	0.326862	4.401261	1.662997	0.099854	-5.08188	0.596122	0.39812
NK.cells	CYP27A1	-0.69736	2.194427	-1.66278	0.099897	-4.34807	0.616263	0.423384
NK.cells	D1ERTD62[illegible]	0.21776	5.295192	1.661984	0.100058	-5.09626	0.588167	0.388676
NK.cells	RAB33B	-0.20446	4.405753	-1.66193	0.100068	-5.02455	0.596082	0.39842
NK.cells	CBX2	-0.83124	-0.18673	-1.66145	0.100165	-4.27566	0.638813	0.452789
NK.cells	LGALS4	-0.5181	2.563012	-1.66096	0.100265	-4.52752	0.612849	0.419556
NK.cells	GM16066	-0.47131	2.012511	-1.66082	0.100291	-4.49068	0.617956	0.426086
NK.cells	AHDC1	-0.17987	4.689967	-1.66051	0.100355	-5.13541	0.59354	0.395613
NK.cells	ADGRE4	-0.76108	3.108019	-1.6602	0.100416	-4.47627	0.607837	0.413511
NK.cells	B4GALT6	0.513379	2.991751	1.660149	0.100427	-4.57162	0.608903	0.41485
NK.cells	CDC23	0.31354	2.927739	1.659903	0.100477	-4.71296	0.60949	0.41559
NK.cells	URAH	-0.49087	3.327083	-1.65987	0.100483	-4.81438	0.605836	0.410998
NK.cells	XBP1	-0.1723	6.135195	-1.65969	0.10052	-5.31876	0.580798	0.380094
NK.cells	AADAT	-0.62443	1.2345	-1.65968	0.100522	-4.40073	0.625251	0.435624
NK.cells	HIRA	-0.18207	5.652742	-1.65967	0.100525	-5.30374	0.585018	0.385234
NK.cells	ZFR2	0.689431	0.817601	1.659004	0.100658	-4.31991	0.629197	0.441003
NK.cells	KCTD14	0.78389	1.055743	1.658647	0.100731	-4.29514	0.62694	0.438294
NK.cells	STYX	0.184926	4.526153	1.658332	0.100794	-5.11946	0.595003	0.398113
NK.cells	KCNN4	0.271712	3.850661	1.658283	0.100804	-5.21518	0.60108	0.405682
NK.cells	CRYBA4	0.98498	-0.18755	1.658122	0.100837	-4.25911	0.638821	0.453916
NK.cells	NEDD1	0.334701	3.041246	1.658095	0.100842	-4.77262	0.608449	0.414966
NK.cells	GM34471	-0.81677	0.232245	-1.6579	0.100882	-4.29409	0.634783	0.448729
NK.cells	ABCB9	-0.23761	3.155352	-1.65789	0.100884	-5.15292	0.607404	0.413712
NK.cells	COL9A3	-0.81152	0.618437	-1.65755	0.100953	-4.34396	0.631092	0.444046

NK.cells	ODF2	0.154336	5.483537	1.657142	0.101035	-5.28385	0.586506	0.387953
NK.cells	NTN4	1.016536	0.706687	1.65702	0.10106	-4.2714	0.630252	0.443085
NK.cells	CMKLR1	0.85162	2.151939	1.65701	0.101062	-4.41268	0.616658	0.425642
NK.cells	FAM185A	0.36336	2.579686	1.656912	0.101082	-4.71678	0.612695	0.420652
NK.cells	HACL1	-0.36694	3.523051	-1.65597	0.101274	-4.83005	0.604495	0.410196
NK.cells	CD101	0.460205	0.741284	1.655871	0.101293	-4.62511	0.630385	0.443225
NK.cells	SYVN1	-0.32147	4.228615	-1.65581	0.101306	-4.93556	0.598111	0.402264
NK.cells	STT3A	-0.13881	6.169392	-1.65517	0.101435	-5.39962	0.581379	0.381242
NK.cells	BET1L	0.234532	3.860623	1.654555	0.101561	-4.97557	0.602347	0.406822
NK.cells	CFAP43	0.344372	3.09234	1.653893	0.101696	-4.68864	0.609862	0.415919
NK.cells	GRSF1	0.156283	5.185543	1.652915	0.101895	-5.22277	0.591131	0.392845
NK.cells	BLOC1S1	0.17895	6.24963	1.65269	0.101941	-5.44217	0.581764	0.381497
NK.cells	GIMAP1	0.216156	4.06141	1.652463	0.101987	-5.16494	0.601205	0.405533
NK.cells	EZR	-0.12734	7.933064	-1.65225	0.10203	-5.71277	0.567279	0.364234
NK.cells	YIPF2	0.455365	1.926928	1.651983	0.102085	-4.53144	0.620848	0.430662
NK.cells	ARVCF	-0.79414	1.228411	-1.65193	0.102097	-4.3323	0.627425	0.439105
NK.cells	MTERF1A	0.513735	1.405346	1.651793	0.102124	-4.4454	0.625752	0.436951
NK.cells	CHD1	0.124638	6.90203	1.651783	0.102126	-5.55069	0.576103	0.374976
NK.cells	FCHO1	-0.27166	3.772111	-1.65141	0.102203	-5.01556	0.603983	0.409231
NK.cells	AHSA1	0.190312	5.071608	1.650709	0.102346	-5.23562	0.592721	0.394919
NK.cells	FHAD1	0.757292	0.624492	1.650013	0.102489	-4.39559	0.633788	0.447124
NK.cells	PITHD1	0.209388	4.744392	1.649976	0.102496	-5.14863	0.595643	0.398717
NK.cells	TBX21	-0.29574	2.437202	-1.64995	0.102501	-5.18553	0.616691	0.425155
NK.cells	GM47664	0.248513	3.774581	1.649369	0.102621	-4.98417	0.604394	0.409848
NK.cells	LACTB2	0.248924	3.94655	1.648778	0.102742	-4.96234	0.602832	0.408058
NK.cells	PPFIBP1	-0.31395	3.366596	-1.64872	0.102754	-4.79802	0.608117	0.414706
NK.cells	FAM160A2	0.339799	2.604929	1.648623	0.102774	-4.7126	0.615134	0.423606
NK.cells	ZBTB44	-0.16745	5.853417	-1.64843	0.102813	-5.41507	0.585804	0.387
NK.cells	CDC25A	-0.33169	3.793744	-1.64817	0.102868	-4.83778	0.60422	0.409932
NK.cells	CHURC1	-0.1487	5.66012	-1.64811	0.102879	-5.36625	0.587506	0.389178
NK.cells	BUD31	-0.18071	5.474604	-1.64811	0.102879	-5.26937	0.589144	0.391193
NK.cells	UAP1	-0.195	5.112787	-1.64762	0.102979	-5.25845	0.592645	0.395289
NK.cells	NFX1	0.173427	4.900546	1.647165	0.103074	-5.2107	0.594598	0.397763
NK.cells	ECHDC3	-0.85709	0.833138	-1.64693	0.103123	-4.34423	0.632168	0.445409
NK.cells	ERI3	0.176194	5.177084	1.646843	0.10314	-5.18441	0.592132	0.394717
NK.cells	AC149090.	-0.35919	5.49949	-1.64624	0.103265	-5.2982	0.589637	0.391451
NK.cells	IGKV1-110	-0.90939	0.050225	-1.64588	0.103339	-4.27492	0.640083	0.455577
NK.cells	METTL5	0.251373	3.739598	1.645726	0.103371	-4.92073	0.605445	0.41117
NK.cells	BTG3	0.160015	5.347109	1.64522	0.103475	-5.33521	0.590988	0.393309
NK.cells	LAMB3	-0.2531	4.067884	-1.64515	0.10349	-5.20024	0.602462	0.407567
NK.cells	PSME2	0.231986	7.278608	1.645063	0.103508	-5.67807	0.574116	0.372718
NK.cells	RAD52	-0.33658	3.121528	-1.64483	0.103556	-4.75741	0.611105	0.418584
NK.cells	MRPS27	0.312452	3.208477	1.643493	0.103833	-4.82347	0.611638	0.418215
NK.cells	GHITM	0.187927	7.4577	1.643156	0.103903	-5.67361	0.573936	0.371726
NK.cells	TSG101	0.169249	5.622255	1.642767	0.103983	-5.3074	0.590119	0.391235
NK.cells	POLR1B	0.379421	2.442843	1.642322	0.104075	-4.70251	0.619056	0.427558
NK.cells	NUS1	0.160401	4.837828	1.642047	0.104133	-5.19229	0.597138	0.400124

NK.cells	CRIP1	-0.19888	8.825299	-1.64202	0.104139	-5.96028	0.562508	0.358094
NK.cells	ZBTB24	0.260091	3.32852	1.64161	0.104223	-4.81744	0.611052	0.417414
NK.cells	TMEM60	0.236327	4.112089	1.640481	0.104458	-4.97472	0.604592	0.408755
NK.cells	SQOR	-0.41775	3.496234	-1.6404	0.104475	-4.73558	0.610221	0.41583
NK.cells	GM15337	-0.47811	2.140023	-1.63993	0.104572	-4.67748	0.622818	0.43205
NK.cells	STX18	0.210009	4.637177	1.639566	0.104649	-5.14115	0.599837	0.403202
NK.cells	TTC8	0.667628	0.78875	1.639541	0.104654	-4.39184	0.635645	0.448754
NK.cells	ELMSAN1	-0.19914	6.924696	-1.63877	0.104815	-5.59275	0.579594	0.378582
NK.cells	PSKH1	0.243697	3.273493	1.638726	0.104824	-4.86495	0.612271	0.419085
NK.cells	TMED7	-0.12181	6.308978	-1.63863	0.104844	-5.45686	0.584968	0.385167
NK.cells	ZC3H12D	0.354816	3.479104	1.638399	0.104892	-4.8753	0.610379	0.41687
NK.cells	GM30948	-1.17376	0.467484	-1.6383	0.104914	-4.29325	0.638736	0.453297
NK.cells	CMTR2	0.617815	1.0685	1.638171	0.10494	-4.40018	0.632967	0.445793
NK.cells	PPP2R2D	-0.15379	5.459388	-1.63805	0.104965	-5.32083	0.592473	0.394575
NK.cells	THA1	0.781401	0.09883	1.637808	0.105016	-4.35026	0.642302	0.458029
NK.cells	PF4	0.972774	4.278945	1.637638	0.105052	-4.87521	0.603077	0.40782
NK.cells	AP2A1	0.229861	4.542647	1.637064	0.105172	-5.12696	0.600966	0.405058
NK.cells	ACAT3	-0.66754	1.453412	-1.63692	0.105201	-4.45111	0.62959	0.441409
NK.cells	SECISBP2	0.177959	4.786541	1.635875	0.105421	-5.23085	0.599522	0.402763
NK.cells	GSE1	-0.16723	5.664856	-1.63544	0.105513	-5.37463	0.591664	0.393176
NK.cells	TMEM132I	-0.98223	0.329919	-1.63535	0.105532	-4.28276	0.641165	0.456083
NK.cells	ZFYVE28	-0.4975	0.624372	-1.63531	0.10554	-4.44902	0.638321	0.452369
NK.cells	KLHDC1	0.344975	2.152219	1.635087	0.105586	-4.75681	0.623775	0.433698
NK.cells	HERPUD2	-0.14898	5.474289	-1.63472	0.105663	-5.32512	0.593397	0.395442
NK.cells	SAYSD1	0.251226	3.323883	1.634511	0.105707	-4.89365	0.612899	0.419867
NK.cells	ZFP608	-0.40719	6.820434	-1.6343	0.105752	-5.09041	0.581536	0.380943
NK.cells	SCN1B	0.480297	1.834445	1.634092	0.105795	-4.6543	0.626811	0.437649
NK.cells	UBN1	-0.11432	6.944041	-1.63355	0.105909	-5.58544	0.580806	0.379774
NK.cells	IER3	0.399871	6.13903	1.632287	0.106176	-5.27086	0.589056	0.388825
NK.cells	TLE5	-0.12831	7.209326	-1.63163	0.106314	-5.70449	0.580083	0.377668
NK.cells	CTSL	-0.22732	6.758061	-1.63088	0.106472	-5.36059	0.584017	0.382652
NK.cells	CCDC146	-0.32421	2.867299	-1.63038	0.106578	-5.01302	0.619182	0.426619
NK.cells	DGAT1	-0.2241	7.095267	-1.63038	0.106579	-5.84097	0.581074	0.379252
NK.cells	NPTN	-0.10963	8.043161	-1.63033	0.106589	-5.75471	0.572889	0.369377
NK.cells	RAB5A	-0.12132	6.913368	-1.63005	0.106648	-5.59796	0.58266	0.381178
NK.cells	GM31462	0.74704	0.152102	1.62979	0.106704	-4.35038	0.645071	0.460127
NK.cells	STX5A	-0.13172	5.871144	-1.62975	0.106713	-5.43725	0.591837	0.39246
NK.cells	JAK1	0.111279	8.273816	1.629729	0.106717	-5.85043	0.570917	0.36707
NK.cells	KLC4	-0.32866	2.884186	-1.62954	0.106757	-4.77537	0.619025	0.426504
NK.cells	2310022A1	0.290981	2.998253	1.628639	0.106948	-4.8535	0.618775	0.425488
NK.cells	QSER1	-0.26203	4.323503	-1.62806	0.10707	-4.96319	0.60696	0.410184
NK.cells	NENF	0.208304	4.583677	1.627058	0.107284	-5.08362	0.604934	0.4075
NK.cells	GSTM5	-0.77645	1.19189	-1.62698	0.107299	-4.40055	0.636645	0.447819
NK.cells	PDCD1	0.507563	0.743001	1.626907	0.107316	-4.85186	0.640973	0.453438
NK.cells	SOAT1	0.240228	5.176779	1.626499	0.107403	-5.27497	0.599566	0.400967
NK.cells	4933423P2	-0.48881	1.804113	-1.62611	0.107486	-4.52445	0.630792	0.440531
NK.cells	SWT1	0.166706	5.244266	1.62608	0.107492	-5.32682	0.598958	0.400317

NK.cells	AURKB	-0.37675	4.237652	-1.62606	0.107496	-5.08354	0.608089	0.411694
NK.cells	GADD45B	-0.26418	5.322838	-1.62566	0.107581	-5.34915	0.598252	0.399604
NK.cells	DNAH17	-0.48188	3.275665	-1.62558	0.107598	-4.87273	0.616957	0.423039
NK.cells	MASP2	-0.66865	1.894509	-1.62539	0.10764	-4.52788	0.629932	0.439698
NK.cells	FERMT3	0.143799	7.235899	1.624974	0.107728	-5.66664	0.581485	0.379056
NK.cells	CSRP1	-0.20648	5.881401	-1.62478	0.10777	-5.38103	0.593413	0.393669
NK.cells	NOP53	-0.16841	5.740416	-1.62418	0.107898	-5.44783	0.595015	0.395375
NK.cells	ZCCHC18	0.48283	1.04036	1.623772	0.107985	-4.59363	0.638641	0.450791
NK.cells	GPRC5C	-0.8678	1.29284	-1.62301	0.108148	-4.40308	0.636213	0.448024
NK.cells	HIST2H3B	-0.6761	1.047683	-1.62274	0.108206	-4.43524	0.638571	0.451096
NK.cells	GM43445	0.437002	1.896237	1.622287	0.108303	-4.59906	0.630448	0.440716
NK.cells	ELAVL1	-0.09781	7.604056	-1.62193	0.10838	-5.70892	0.578625	0.376053
NK.cells	ADAMDEC	0.907727	0.746267	1.621666	0.108436	-4.35517	0.641483	0.455199
NK.cells	GM16196	-0.54134	1.751417	-1.62151	0.10847	-4.49322	0.631827	0.442691
NK.cells	PXDN	-0.74771	0.993778	-1.62139	0.108496	-4.34922	0.639091	0.452123
NK.cells	GM46560	-0.98955	-0.84515	-1.62138	0.108497	-4.27755	0.657091	0.475797
NK.cells	LSM7	0.150796	6.346432	1.621033	0.108572	-5.50545	0.58963	0.389713
NK.cells	NR4A1	-0.26068	7.413157	-1.62074	0.108635	-5.74061	0.580281	0.378365
NK.cells	C130026I2	0.543891	2.944263	1.620431	0.108701	-4.58165	0.620568	0.428652
NK.cells	MAB21L3	-1.0283	-0.30498	-1.62024	0.108743	-4.3246	0.651749	0.469225
NK.cells	SORL1	0.21869	5.66068	1.620068	0.108779	-5.57695	0.595728	0.397527
NK.cells	PELP1	0.279964	3.358177	1.619766	0.108844	-4.89305	0.616711	0.423956
NK.cells	LEF1	0.294017	5.638758	1.619458	0.10891	-5.53986	0.595924	0.397936
NK.cells	ORAI3	0.275205	3.868765	1.619333	0.108937	-4.97523	0.61199	0.418034
NK.cells	FAM83G	-0.74753	0.263823	-1.61923	0.108959	-4.32401	0.646173	0.462103
NK.cells	TET2	0.157215	6.024646	1.61917	0.108973	-5.47422	0.592483	0.393688
NK.cells	IDUA	0.397078	2.137056	1.618727	0.109068	-4.60583	0.628163	0.438765
NK.cells	CHMP6	0.27309	3.712475	1.618348	0.10915	-4.99786	0.613431	0.42003
NK.cells	NT5C3	0.200284	4.85257	1.618316	0.109157	-5.27199	0.603002	0.406917
NK.cells	ALOX5AP	0.548405	7.325815	1.618229	0.109175	-5.2589	0.58104	0.379869
NK.cells	SETD7	0.197779	4.9704	1.618131	0.109197	-5.26076	0.601936	0.405614
NK.cells	CDS2	0.18843	4.708784	1.618126	0.109198	-5.22139	0.604307	0.408579
NK.cells	IER5L	-0.28876	2.89009	-1.61808	0.109207	-4.96352	0.621074	0.429798
NK.cells	NFKBIE	0.248508	4.879022	1.617667	0.109297	-5.23751	0.602978	0.406654
NK.cells	PUS7L	0.439466	1.839535	1.616355	0.10958	-4.6411	0.632434	0.44297
NK.cells	POR	0.191202	5.6252	1.61555	0.109755	-5.41212	0.597412	0.398747
NK.cells	TENT5C	0.374213	5.86321	1.615288	0.109812	-5.37289	0.595281	0.396199
NK.cells	PEG3	-0.64096	1.53048	-1.61478	0.109921	-4.49742	0.635389	0.447057
NK.cells	EPM2A	0.640729	1.526051	1.614716	0.109935	-4.46599	0.635432	0.44712
NK.cells	PCP4L1	-0.89133	1.36121	-1.61442	0.110001	-4.39882	0.637014	0.449207
NK.cells	2210408F2	0.453935	2.856305	1.614414	0.110001	-4.76364	0.622815	0.430915
NK.cells	BUB1	-0.37624	3.743439	-1.61424	0.110039	-5.05821	0.614551	0.420517
NK.cells	CTSC	0.279853	7.322442	1.614059	0.110078	-5.6641	0.582402	0.380639
NK.cells	RSPH9	-0.73641	1.514419	-1.61358	0.110183	-4.4138	0.635543	0.447668
NK.cells	TMBIM4	0.163621	6.749974	1.613467	0.110207	-5.49817	0.587418	0.387
NK.cells	KIF11	-0.3488	5.441855	-1.61343	0.110215	-5.40835	0.599059	0.401354
NK.cells	MAP2	0.863892	0.187932	1.613223	0.11026	-4.3339	0.648397	0.46454

NK.cells	PBX3	-0.18976	5.496226	-1.61315	0.110277	-5.4434	0.59857	0.400806
NK.cells	RBM22	-0.12899	5.603703	-1.61309	0.110288	-5.39855	0.597605	0.399609
NK.cells	CAAA0114	0.372743	2.131904	1.612925	0.110325	-4.69885	0.629652	0.440194
NK.cells	TMEM220	-0.80135	0.299081	-1.61268	0.110378	-4.34955	0.647323	0.463237
NK.cells	EIF2AK3	-0.17473	6.399228	-1.61195	0.110538	-5.5311	0.590851	0.39115
NK.cells	OSGEPL1	0.347774	2.472736	1.611209	0.110699	-4.68076	0.626783	0.436605
NK.cells	MAPKAPK2	0.168606	7.207807	1.610917	0.110762	-5.6106	0.583736	0.382753
NK.cells	WDR55	0.288723	2.979495	1.610765	0.110796	-4.83966	0.622015	0.430562
NK.cells	GRK4	-0.32554	3.108893	-1.61015	0.110929	-4.83431	0.620804	0.429152
NK.cells	CERS6	0.220222	7.330325	1.609394	0.111095	-5.66559	0.582666	0.381805
NK.cells	ZFP296	-0.47691	2.857765	-1.60908	0.111163	-4.68514	0.623157	0.432537
NK.cells	TMEM131l	-0.15875	7.26323	-1.60877	0.111232	-5.66648	0.583251	0.382623
NK.cells	WHRN	-0.45526	3.066048	-1.60848	0.111296	-4.6865	0.621205	0.430144
NK.cells	GYS1	0.384007	3.38284	1.608204	0.111356	-4.8703	0.618249	0.426403
NK.cells	SCPEP1OS	0.864226	0.502575	1.608125	0.111373	-4.34959	0.645692	0.461951
NK.cells	LAMTOR2	0.123293	6.577213	1.608056	0.111388	-5.558	0.589277	0.390123
NK.cells	SLC24A1	-0.35863	1.860788	-1.60759	0.111491	-4.80473	0.632592	0.444969
NK.cells	DUSP28	0.438988	1.99306	1.607574	0.111494	-4.59829	0.631332	0.443335
NK.cells	NME1	0.201105	6.668403	1.60728	0.111559	-5.59771	0.588472	0.389335
NK.cells	MYB	-0.26498	5.137938	-1.60719	0.111578	-5.24989	0.602143	0.406338
NK.cells	B3GAT3	0.224556	4.307364	1.607095	0.111599	-5.15076	0.609707	0.415874
NK.cells	SPC24	-0.34904	4.768056	-1.60671	0.111683	-5.26861	0.605499	0.4107
NK.cells	IDNK	0.161166	5.52475	1.606597	0.111709	-5.42817	0.598655	0.402152
NK.cells	TSN	0.118698	6.360058	1.606219	0.111792	-5.53286	0.591198	0.393006
NK.cells	SYDE1	-0.76676	0.308678	-1.60617	0.111802	-4.337	0.647585	0.465081
NK.cells	UBL5	-0.11468	8.259007	-1.60598	0.111844	-5.81438	0.574626	0.372883
NK.cells	YAE1D1	-0.38854	3.240113	-1.60574	0.111897	-4.79175	0.619579	0.428835
NK.cells	MLF2	0.155592	5.85253	1.605734	0.111898	-5.43846	0.595717	0.398758
NK.cells	VKORC1	-0.26612	4.701765	-1.60567	0.111913	-5.17331	0.606102	0.41174
NK.cells	FAM174A	0.204122	6.050716	1.605314	0.111991	-5.47829	0.593948	0.39672
NK.cells	ZFP867	0.655829	0.61031	1.605276	0.111999	-4.38511	0.644642	0.461558
NK.cells	GM45902	-0.48339	1.585805	-1.60515	0.112026	-4.54755	0.635222	0.44926
NK.cells	BIRC2	-0.16203	5.673482	-1.60506	0.112046	-5.47129	0.59732	0.400977
NK.cells	PRRC2C	0.110561	7.744202	1.604967	0.112067	-5.78462	0.579068	0.378527
NK.cells	DUSP22	-0.28625	4.486105	-1.60472	0.112122	-5.01634	0.608071	0.41456
NK.cells	GM17749	-0.56409	4.634129	-1.60461	0.112146	-4.87878	0.606719	0.412879
NK.cells	MAPK9	0.179596	4.751185	1.604454	0.11218	-5.2677	0.605652	0.41159
NK.cells	NR3C1	-0.15574	7.440275	-1.60427	0.11222	-5.78691	0.581707	0.38191
NK.cells	XPNPEP1	0.198768	4.365592	1.604248	0.112226	-5.15223	0.609173	0.41604
NK.cells	AC125149.	-1.03552	-0.34803	-1.60399	0.112284	-4.32122	0.654044	0.474395
NK.cells	BSCL2	-0.18064	4.542466	-1.60378	0.112328	-5.20141	0.607558	0.41409
NK.cells	2210016L2	0.241788	3.97848	1.60331	0.112433	-5.10067	0.613024	0.420854
NK.cells	PVT1	0.293273	5.282726	1.602494	0.112613	-5.4124	0.601586	0.406218
NK.cells	RNF170	0.254465	3.319753	1.602455	0.112622	-4.9233	0.61961	0.429031
NK.cells	GM26670	-0.65797	0.402458	-1.60146	0.112842	-4.36209	0.648449	0.465854
NK.cells	ZUP1	0.206087	5.168406	1.60104	0.112935	-5.3479	0.603617	0.408053
NK.cells	TIMM29	-0.29226	3.296538	-1.60065	0.113023	-4.89676	0.620852	0.430033

NK.cells	TMEM56	-0.58464	2.147941	-1.60054	0.113046	-4.58468	0.63169	0.443994
NK.cells	PYGB	-0.17013	4.748164	-1.60015	0.113133	-5.30661	0.60744	0.413102
NK.cells	YKT6	0.189517	4.483546	1.60004	0.113157	-5.17813	0.609862	0.416227
NK.cells	POLN	-0.36685	3.191009	-1.59985	0.1132	-4.96781	0.621839	0.431546
NK.cells	GM27010	0.40741	2.340877	1.599767	0.113218	-4.67963	0.629856	0.441903
NK.cells	SNX12	-0.16926	4.718214	-1.59944	0.11329	-5.21187	0.607825	0.413781
NK.cells	PHYH	-0.29693	5.40217	-1.59879	0.113436	-5.33762	0.601668	0.406232
NK.cells	ZKSCAN3	0.168269	4.82207	1.598721	0.11345	-5.20895	0.606935	0.412864
NK.cells	H3F3B	-0.10563	9.934295	-1.59833	0.113538	-6.06869	0.562222	0.358259
NK.cells	ARHGAP32	0.490617	3.193867	1.598005	0.11361	-4.56174	0.621985	0.43223
NK.cells	GNA11	-0.30187	3.954617	-1.59795	0.113622	-4.9764	0.614903	0.423176
NK.cells	IGHM	-0.37027	8.158942	-1.59795	0.113622	-5.72745	0.57732	0.376437
NK.cells	E230029C0	0.473484	3.508508	1.597733	0.11367	-4.7373	0.619046	0.428485
NK.cells	BRI3	0.153555	7.328714	1.597205	0.113788	-5.62532	0.584536	0.385328
NK.cells	TCF7L2	-0.38642	7.34951	-1.59711	0.11381	-5.2421	0.584354	0.385123
NK.cells	DOK1	0.251816	3.614618	1.597095	0.113813	-4.99218	0.618058	0.427321
NK.cells	MTFR1L	0.190336	4.250609	1.596781	0.113883	-5.13881	0.612274	0.419883
NK.cells	MITD1	0.22532	4.433197	1.596195	0.114014	-5.22868	0.611021	0.417771
NK.cells	TRAIP	-0.47225	2.119414	-1.59531	0.114212	-4.63887	0.633501	0.445858
NK.cells	IL1RAP	-0.27256	4.731572	-1.59498	0.114285	-5.19469	0.609186	0.414672
NK.cells	SMIM10L1	0.197969	4.931338	1.594583	0.114374	-5.22266	0.607561	0.412536
NK.cells	4833407H1	0.384246	2.098821	1.59429	0.11444	-4.64418	0.634042	0.446492
NK.cells	SGCE	-0.94291	1.156412	-1.59411	0.11448	-4.35702	0.64312	0.458443
NK.cells	IL12RB1	0.615296	0.500116	1.593841	0.114541	-4.57768	0.649559	0.466949
NK.cells	SORBS1	-0.21112	4.747678	-1.59363	0.114589	-5.36184	0.609289	0.41499
NK.cells	1700029H1	-0.57882	1.58753	-1.59251	0.11484	-4.47375	0.639066	0.453447
NK.cells	STX3	0.547737	2.314188	1.592441	0.114855	-4.61679	0.632101	0.444385
NK.cells	NUAK2	-0.40938	4.289152	-1.59236	0.114872	-4.67229	0.613581	0.420629
NK.cells	UROC1	-0.63984	1.760508	-1.5919	0.114977	-4.56932	0.6374	0.451339
NK.cells	DNAJC10	0.193163	4.533218	1.591805	0.114998	-5.17647	0.611333	0.417831
NK.cells	HMMR	-0.38463	4.333361	-1.59175	0.11501	-5.23213	0.613173	0.420162
NK.cells	ENOPH1	0.32049	2.8863	1.591728	0.115015	-4.84295	0.626675	0.437427
NK.cells	ADGRG1	-0.79709	1.720983	-1.59168	0.115026	-4.46279	0.63778	0.451852
NK.cells	ANKRD52	0.227695	3.881446	1.591505	0.115065	-5.07096	0.617355	0.425518
NK.cells	TM7SF2	-0.58137	1.754706	-1.59107	0.115163	-4.4695	0.637462	0.451605
NK.cells	CCNL1	-0.12954	7.09056	-1.59074	0.115237	-5.64145	0.588321	0.389319
NK.cells	RBM6	0.124946	7.184868	1.590557	0.115279	-5.68217	0.587491	0.388334
NK.cells	PRKAR2B	0.416624	3.305356	1.590549	0.115281	-4.72425	0.622738	0.43262
NK.cells	NDUFA8	0.182254	5.798653	1.590359	0.115323	-5.44311	0.599828	0.403656
NK.cells	4930402H2	0.211357	4.453202	1.589933	0.11542	-5.2325	0.612313	0.419259
NK.cells	ATP6V1C1	0.164369	5.189889	1.589188	0.115588	-5.30599	0.605809	0.411035
NK.cells	GM4117	-0.65996	0.660809	-1.58916	0.115595	-4.43008	0.64858	0.466227
NK.cells	VPS53	0.159582	4.462382	1.58881	0.115673	-5.2102	0.612469	0.419683
NK.cells	IGFBP1	0.559435	4.147259	1.588633	0.115713	-5.15444	0.615379	0.42342
NK.cells	PROSER3	-0.65482	1.105594	-1.58852	0.115739	-4.43033	0.64424	0.460832
NK.cells	L3MBTL1	-0.79685	0.787342	-1.58837	0.115774	-4.39008	0.647342	0.46497
NK.cells	TMEM107	-0.50266	1.616764	-1.58739	0.115995	-4.52768	0.640231	0.454683

NK.cells	CADM1	-0.67508	5.440386	-1.58662	0.11617	-4.95922	0.604814	0.409018
NK.cells	TMA16	0.303957	3.964843	1.586482	0.1162	-5.06907	0.618378	0.426169
NK.cells	LSAMP	-0.74694	0.929492	-1.58633	0.116235	-4.43546	0.647324	0.463756
NK.cells	TMEM127	0.18599	4.290896	1.586101	0.116287	-5.17374	0.615353	0.422437
NK.cells	FBXO7	0.221767	4.165071	1.585919	0.116328	-5.15356	0.616518	0.423999
NK.cells	AKR1E1	-0.36291	2.622552	-1.58489	0.116562	-4.73226	0.631691	0.442878
NK.cells	CTNNBIP1	-0.31825	4.359655	-1.58485	0.116571	-4.89411	0.615386	0.421979
NK.cells	F830208F2	0.995186	-0.23725	1.58468	0.116609	-4.33415	0.659548	0.479551
NK.cells	PGRMC1	-0.2094	4.647619	-1.5842	0.116718	-5.20732	0.612883	0.418807
NK.cells	RUFY3	0.20213	5.26954	1.584097	0.116742	-5.26768	0.607183	0.411618
NK.cells	VGLL4	-0.14063	6.477378	-1.58329	0.116926	-5.66846	0.596896	0.398215
NK.cells	AAR2	0.193299	4.021304	1.583009	0.11699	-5.109	0.619326	0.426449
NK.cells	PPIL1	0.267013	4.052063	1.582701	0.117061	-5.10812	0.61904	0.426125
NK.cells	ERLIN1	-0.27732	4.765624	-1.58266	0.11707	-4.98599	0.612433	0.417745
NK.cells	DAPK1	-0.30804	4.932787	-1.58213	0.117192	-5.22201	0.611169	0.415815
NK.cells	TRAM1	-0.12346	7.121393	-1.58177	0.117273	-5.65323	0.591428	0.39127
NK.cells	BHMT2	-0.57465	2.060069	-1.58128	0.117385	-4.65375	0.638179	0.450596
NK.cells	TTC33	0.254668	3.849347	1.581201	0.117403	-5.05098	0.621208	0.428715
NK.cells	PRPF38B	0.101795	6.611272	1.580554	0.117551	-5.61228	0.595966	0.397172
NK.cells	SMC2	-0.276	5.816825	-1.58011	0.117653	-5.49565	0.603109	0.406269
NK.cells	PMM2	0.162818	4.561806	1.579757	0.117734	-5.26041	0.614587	0.42082
NK.cells	CHCHD6	0.329893	2.968098	1.579752	0.117735	-4.73715	0.629505	0.439905
NK.cells	DTD1	0.285321	3.881415	1.579553	0.11778	-5.08069	0.620908	0.428883
NK.cells	ANP32E	-0.16682	7.305672	-1.5794	0.117815	-5.70841	0.589798	0.389939
NK.cells	RYK	0.472406	2.042505	1.579311	0.117836	-4.63698	0.638348	0.451519
NK.cells	PRRC2B	-0.1356	6.226887	-1.57904	0.117897	-5.58294	0.59941	0.402008
NK.cells	BGN	-0.51905	2.420901	-1.57896	0.117917	-4.60625	0.634717	0.446968
NK.cells	PAN3	-0.10794	8.767803	-1.57886	0.117939	-5.94024	0.577042	0.374606
NK.cells	ZDHHC20	0.111689	6.740107	1.578788	0.117956	-5.66729	0.594816	0.396354
NK.cells	EIF2B3	0.33952	2.996786	1.578536	0.118014	-4.86346	0.629233	0.439973
NK.cells	GCH1	0.199884	5.663529	1.578522	0.118017	-5.52835	0.604499	0.408498
NK.cells	RPP14	0.313127	2.846249	1.57836	0.118054	-4.85518	0.630662	0.441904
NK.cells	GM19705	0.512474	1.868138	1.578143	0.118104	-4.69158	0.640029	0.454179
NK.cells	TEF	-0.30109	3.230001	-1.57771	0.118204	-4.88073	0.62703	0.437363
NK.cells	EFCAB9	-0.58723	0.443216	-1.57764	0.118219	-4.43846	0.653944	0.472608
NK.cells	PITPNC1	-0.15001	8.799744	-1.57747	0.11826	-6.07675	0.57677	0.374602
NK.cells	VPS37B	-0.17425	7.797142	-1.57719	0.118323	-5.9904	0.585538	0.385295
NK.cells	TUBB4B	-0.24338	6.422022	-1.57641	0.118504	-5.58309	0.598372	0.400639
NK.cells	EIF2AK1	-0.16852	5.326731	-1.57601	0.118595	-5.36546	0.608491	0.413219
NK.cells	KSR1	-0.21914	4.69672	-1.57546	0.118723	-5.34641	0.614605	0.420745
NK.cells	CYB561D1	0.52318	1.785161	1.575002	0.118828	-4.59043	0.642151	0.456474
NK.cells	TSPAN7	-0.44877	3.257326	-1.57483	0.118868	-4.71951	0.62806	0.438161
NK.cells	TAF1B	0.196504	4.199522	1.574405	0.118966	-5.17617	0.619217	0.426903
NK.cells	PHB2	0.137636	6.570723	1.574318	0.118986	-5.60122	0.597557	0.399636
NK.cells	PDRG1	0.194252	4.496479	1.573051	0.119279	-5.2562	0.616458	0.423848
NK.cells	1110046J0	0.835829	0.031359	1.571986	0.119526	-4.37641	0.659377	0.480215
NK.cells	UBE2Q1	-0.13337	6.334643	-1.57182	0.119563	-5.5421	0.599676	0.403041

NK.cells	ZFP12	-0.45716	1.346551	-1.57173	0.119585	-4.58175	0.646413	0.463085
NK.cells	GM45606	0.780965	0.477564	1.571604	0.119615	-4.42735	0.654948	0.47443
NK.cells	TMEM8	-0.45224	2.000346	-1.57123	0.119701	-4.62489	0.64007	0.454877
NK.cells	CNOT11	0.212597	4.112527	1.571209	0.119706	-5.15428	0.620028	0.42892
NK.cells	TEX45	-0.86051	0.222756	-1.57118	0.119714	-4.3937	0.657473	0.477919
NK.cells	GRK6	0.16281	5.764162	1.571142	0.119722	-5.44965	0.60483	0.409664
NK.cells	CCDC43	0.272202	3.103767	1.571111	0.119729	-4.92572	0.629515	0.441161
NK.cells	CNPY2	-0.22984	4.719728	-1.57109	0.119734	-5.19732	0.614393	0.421761
NK.cells	GM19466	-0.68366	0.661272	-1.57106	0.119741	-4.47158	0.653134	0.472165
NK.cells	MRPS30	0.171844	4.726011	1.570647	0.119837	-5.28949	0.614335	0.421868
NK.cells	RACGAP1	-0.30042	5.205247	-1.57061	0.119844	-5.40233	0.609927	0.416277
NK.cells	ADGRA2	-0.86014	0.687625	-1.57058	0.119853	-4.43903	0.652874	0.472016
NK.cells	DUSP6	-0.29165	4.428378	-1.57056	0.119858	-5.0916	0.61709	0.425378
NK.cells	ALDH16A1	-0.22821	4.358468	-1.5704	0.119894	-5.12764	0.617739	0.426207
NK.cells	CMIP	-0.14183	9.635584	-1.57039	0.119897	-6.12484	0.570792	0.367987
NK.cells	CSTB	-0.19398	6.672627	-1.57037	0.119901	-5.63848	0.596645	0.399614
NK.cells	TFB1M	0.458085	1.706255	1.569838	0.120025	-4.6203	0.643281	0.458874
NK.cells	IL2RA	0.483035	2.557963	1.569634	0.120072	-4.9235	0.635073	0.448237
NK.cells	TIGIT	0.411525	-0.09862	1.56912	0.120192	-4.81781	0.661249	0.482703
NK.cells	CPB2	-0.36553	3.209826	-1.56903	0.120213	-4.91034	0.629059	0.440316
NK.cells	LSM14B	0.214053	3.604671	1.568685	0.120293	-5.07314	0.625481	0.435655
NK.cells	PIN1	0.168588	5.245371	1.567624	0.120541	-5.35888	0.610713	0.416492
NK.cells	ARHGEF17	-1.00679	-0.37609	-1.56758	0.120552	-4.34672	0.664703	0.486955
NK.cells	C430049B(	-0.61665	1.844436	-1.56757	0.120554	-4.51273	0.64279	0.457832
NK.cells	ZFP938	0.477919	1.450658	1.567397	0.120594	-4.59241	0.64662	0.46293
NK.cells	PRLR	-0.56094	3.152323	-1.5669	0.120711	-4.81987	0.630583	0.441727
NK.cells	GM34983	-0.88549	0.754004	-1.56612	0.120893	-4.375	0.654047	0.47257
NK.cells	NUP210L	-0.34749	6.407383	-1.56588	0.120949	-5.48476	0.600699	0.403792
NK.cells	FRYL	0.121907	7.564295	1.565018	0.121151	-5.84512	0.590378	0.3913
NK.cells	GCLM	0.200971	6.090034	1.564699	0.121226	-5.49418	0.603565	0.407879
NK.cells	CHST11	-0.22988	6.242747	-1.56453	0.121265	-5.74971	0.602184	0.406202
NK.cells	GTF2F2	0.142188	5.881638	1.564384	0.1213	-5.5138	0.605455	0.410335
NK.cells	TIMM8B	0.218264	5.242586	1.56414	0.121357	-5.31435	0.611292	0.417805
NK.cells	AC154200.	0.60537	0.660722	1.563679	0.121465	-4.46183	0.654968	0.474796
NK.cells	PSMC1	0.138942	5.889053	1.563557	0.121494	-5.48375	0.605388	0.410567
NK.cells	PLXND1	-0.33971	3.5086	-1.56341	0.121528	-4.79137	0.627441	0.438695
NK.cells	ITGA4	0.169484	7.657286	1.563399	0.121531	-5.86674	0.589557	0.390846
NK.cells	PDGFC	-1.12493	1.879122	-1.56335	0.121543	-4.43088	0.643037	0.459036
NK.cells	GDPD3	0.623944	1.793559	1.563207	0.121576	-4.55494	0.643867	0.460171
NK.cells	HPS3	0.212697	4.626547	1.563061	0.121611	-5.26978	0.616977	0.425339
NK.cells	FOXJ3	-0.13063	5.947043	-1.56271	0.121693	-5.53923	0.604861	0.410055
NK.cells	APOBEC3	0.173837	6.862598	1.562668	0.121703	-5.67914	0.596614	0.399736
NK.cells	TK1	-0.31447	4.882981	-1.56258	0.121724	-5.26785	0.614603	0.422385
NK.cells	BAMBI	-0.2729	3.967331	-1.56251	0.12174	-5.24338	0.623124	0.43329
NK.cells	MKNK2	-0.13901	6.23257	-1.56247	0.121749	-5.59162	0.602276	0.406809
NK.cells	CDK5	0.251195	3.72259	1.562211	0.121811	-5.06685	0.625423	0.436358
NK.cells	EHBP1	-0.3794	3.52414	-1.56176	0.121916	-4.92853	0.627294	0.438961

NK.cells	FAM49B	0.100451	9.086243	1.561612	0.121952	-6.02993	0.5771	0.376065
NK.cells	OTULINL	0.181037	5.347398	1.561549	0.121967	-5.63838	0.61033	0.417326
NK.cells	EHMT1	0.151599	6.063885	1.561402	0.122001	-5.53565	0.603802	0.409135
NK.cells	CDK2AP2	0.142544	6.467925	1.560879	0.122125	-5.62098	0.600193	0.404738
NK.cells	UQCRFS1	0.139397	7.164384	1.560574	0.122197	-5.73574	0.593962	0.397119
NK.cells	LIMA1	-0.58417	4.507655	-1.56016	0.122295	-4.77621	0.618121	0.427787
NK.cells	UBE2E3	-0.1168	7.066891	-1.56011	0.122306	-5.69638	0.59483	0.398368
NK.cells	EID2B	-0.42518	1.703903	-1.55989	0.122359	-4.5971	0.64478	0.462534
NK.cells	CREB5	0.839989	2.17534	1.559835	0.122371	-4.54232	0.640213	0.456538
NK.cells	KCNRG	-0.36397	2.320944	-1.55967	0.122412	-4.80149	0.638809	0.454743
NK.cells	3300002I0	0.403636	1.970632	1.559609	0.122425	-4.7595	0.642192	0.459194
NK.cells	CMAS	0.180531	5.862643	1.559362	0.122483	-5.5192	0.6057	0.412064
NK.cells	HSPA2	-0.33047	3.291574	-1.55877	0.122624	-4.95951	0.629625	0.442867
NK.cells	NDUFS3	0.153259	6.050235	1.558641	0.122654	-5.54607	0.604051	0.410144
NK.cells	MOSMO	0.177593	4.576833	1.558277	0.12274	-5.36214	0.617567	0.427452
NK.cells	RNF114	0.182789	5.645299	1.558166	0.122766	-5.49488	0.607733	0.414943
NK.cells	GPD1	-0.57829	1.749644	-1.55806	0.122792	-4.59408	0.644428	0.462458
NK.cells	PHLDB3	-0.33667	2.97973	-1.55799	0.122808	-4.83931	0.632589	0.446907
NK.cells	ZCCHC8	-0.15941	5.108019	-1.55765	0.122888	-5.37208	0.612656	0.421294
NK.cells	TRMT112	0.13151	7.277188	1.55756	0.12291	-5.76626	0.593044	0.396605
NK.cells	PXMP2	-0.42101	4.067436	-1.55733	0.122966	-5.13446	0.622316	0.433695
NK.cells	GBP7	0.369551	4.443583	1.556943	0.123057	-5.36534	0.618805	0.429267
NK.cells	PTGS1	-0.35605	3.986721	-1.55626	0.123218	-4.9958	0.623072	0.435057
NK.cells	P2RX1	-0.78322	0.81074	-1.55603	0.123274	-4.45884	0.653623	0.475348
NK.cells	IER5	-0.20898	6.692584	-1.55594	0.123295	-5.66285	0.598261	0.403629
NK.cells	PPP1R16B	-0.18384	6.504104	-1.55571	0.123351	-5.71041	0.599954	0.405817
NK.cells	PLPP1	-0.39192	4.327383	-1.55567	0.12336	-5.0187	0.619887	0.43117
NK.cells	MAGOH	-0.14944	6.296682	-1.55556	0.123384	-5.58833	0.601822	0.408192
NK.cells	GM4070	0.40349	2.887167	1.555487	0.123403	-5.12882	0.633472	0.448847
NK.cells	HEATR5A	0.199731	5.656048	1.555377	0.123429	-5.46395	0.607635	0.415576
NK.cells	RDH10	-0.33599	3.207433	-1.55445	0.123649	-4.91542	0.631019	0.445185
NK.cells	VTI1A	0.134815	7.233705	1.55445	0.123649	-5.73793	0.593992	0.397984
NK.cells	PDP2	-0.22324	3.724878	-1.55375	0.123816	-5.11823	0.626287	0.439106
NK.cells	FRRS1	0.205833	4.851486	1.553532	0.123868	-5.312	0.615765	0.425568
NK.cells	UHMK1	0.178	4.630018	1.553446	0.123889	-5.26336	0.617818	0.42821
NK.cells	H3F3A	-0.11794	10.71708	-1.55328	0.123929	-6.20358	0.564041	0.36151
NK.cells	NAGPA	0.260561	3.354165	1.552914	0.124016	-4.98152	0.629792	0.4438
NK.cells	1810021B2	-0.94214	0.427969	-1.55267	0.124075	-4.39135	0.658205	0.481442
NK.cells	FCGR3	0.281659	4.189291	1.552615	0.124087	-5.34967	0.621926	0.433632
NK.cells	PSMD7	0.176071	5.828246	1.55249	0.124117	-5.46847	0.606798	0.414317
NK.cells	ZER1	0.238808	3.262662	1.552355	0.12415	-5.0319	0.63066	0.444987
NK.cells	ADAM3	0.64141	-0.17326	1.552095	0.124212	-4.47942	0.664263	0.489655
NK.cells	GPR180	0.305208	2.964205	1.551674	0.124313	-4.88167	0.633595	0.448996
NK.cells	CLSPN	-0.30263	4.411842	-1.55149	0.124357	-5.25514	0.61994	0.431285
NK.cells	TAX1BP1	0.122088	8.297296	1.551254	0.124413	-5.93209	0.584854	0.387089
NK.cells	ANO10	0.349068	3.16746	1.551192	0.124428	-4.90218	0.631658	0.446504
NK.cells	GPNMB	0.572818	0.898381	1.550623	0.124564	-4.73586	0.654086	0.475701

NK.cells	PBDC1	-0.24387	5.348225	-1.54995	0.124725	-5.39412	0.612225	0.420603
NK.cells	CCR2	0.250744	3.092746	1.548915	0.124974	-5.58326	0.634262	0.448252
NK.cells	TCF21	-0.87636	0.477537	-1.54863	0.125042	-4.4073	0.659779	0.482213
NK.cells	SRRT	-0.15786	5.528359	-1.54851	0.125072	-5.4774	0.611451	0.419019
NK.cells	FOXM1	-0.37221	3.10354	-1.54834	0.125114	-4.9205	0.634159	0.448302
NK.cells	LRRC4	0.251956	3.774431	1.547717	0.125263	-5.18794	0.628062	0.440205
NK.cells	R3HDM2	0.121957	6.193799	1.547502	0.125314	-5.55343	0.605641	0.411633
NK.cells	NANOS1	-0.57467	0.634448	-1.5473	0.125364	-4.5551	0.658506	0.480512
NK.cells	ACAA2	-0.24121	5.01941	-1.5468	0.125484	-5.40839	0.616413	0.425456
NK.cells	YTHDC2	-0.19326	4.693584	-1.5467	0.125508	-5.31957	0.619439	0.42936
NK.cells	D130043K2	-0.67844	1.054045	-1.5465	0.125556	-4.49035	0.654349	0.475157
NK.cells	ZFP810	0.371612	2.914739	1.546345	0.125593	-4.81985	0.636244	0.451296
NK.cells	GM16541	0.29394	2.915994	1.546267	0.125612	-4.96925	0.636232	0.45132
NK.cells	HSD17B11	-0.24845	4.796644	-1.54616	0.125638	-5.09744	0.61848	0.428325
NK.cells	CLINT1	0.117425	8.205941	1.545697	0.12575	-5.8967	0.587741	0.389646
NK.cells	SIGMAR1	0.254349	3.549056	1.54566	0.125759	-5.05917	0.630278	0.443577
NK.cells	NUDT16	0.313337	2.966991	1.544859	0.125952	-4.92744	0.636541	0.45119
NK.cells	MMP25	-0.76658	0.499533	-1.54394	0.126175	-4.46634	0.661568	0.483536
NK.cells	PRMT3	0.224856	4.041123	1.543414	0.126302	-5.21442	0.627414	0.438352
NK.cells	ZFP943	0.184992	4.280266	1.543001	0.126402	-5.1901	0.625162	0.435685
NK.cells	SLC36A1	-0.28269	2.851189	-1.54281	0.126449	-4.88123	0.638755	0.453391
NK.cells	CD300C2	-0.63056	4.335062	-1.54263	0.126493	-4.69546	0.624647	0.435107
NK.cells	MOB3A	0.187788	4.775701	1.542527	0.126517	-5.37016	0.620522	0.429847
NK.cells	TPM4	-0.12357	7.167543	-1.54245	0.126536	-5.83801	0.598649	0.402174
NK.cells	ZSCAN18	0.930751	0.363679	1.542103	0.12662	-4.41552	0.663285	0.486131
NK.cells	MYPOPOS	0.551535	1.654931	1.541564	0.126751	-4.66443	0.650484	0.469214
NK.cells	RBM18	-0.1825	4.236008	-1.54156	0.126753	-5.20904	0.625679	0.436704
NK.cells	KCNJ2	0.735525	-0.01521	1.541346	0.126804	-4.38521	0.667092	0.491565
NK.cells	1110032A(	0.267253	3.592833	1.541085	0.126868	-4.96057	0.631763	0.444685
NK.cells	YAF2	-0.13112	6.056106	-1.54107	0.126871	-5.57235	0.608804	0.415214
NK.cells	PTGS2	1.043457	2.215449	1.540769	0.126945	-4.5869	0.645117	0.462094
NK.cells	DCAKD	0.268593	4.076447	1.540125	0.127101	-5.08619	0.627762	0.439087
NK.cells	DCTD	0.573499	1.018621	1.539588	0.127232	-4.52053	0.657366	0.478213
NK.cells	CRKL	-0.15883	5.071994	-1.53954	0.127243	-5.40054	0.618437	0.427223
NK.cells	MYO1F	0.181452	5.259228	1.539081	0.127356	-5.70171	0.6167	0.425172
NK.cells	ALS2	-0.19076	4.283789	-1.53898	0.12738	-5.25136	0.625807	0.436875
NK.cells	HPN	-0.45812	2.642028	-1.53836	0.127531	-4.77065	0.641468	0.457425
NK.cells	GPATCH11	0.228742	3.800176	1.538047	0.127608	-5.13558	0.630377	0.443064
NK.cells	APOL8	-0.33061	2.828251	-1.53794	0.127635	-4.90792	0.63967	0.455219
NK.cells	FIRRE	-0.50083	2.608551	-1.53761	0.127715	-4.69451	0.641791	0.458124
NK.cells	GM34086	-0.26556	2.658244	-1.53733	0.127783	-5.16161	0.641311	0.457569
NK.cells	KIF20B	-0.35303	4.170808	-1.5373	0.12779	-5.26244	0.626872	0.43871
NK.cells	HTATIP2	0.191975	4.480259	1.53715	0.127828	-5.31188	0.623961	0.43499
NK.cells	CLP1	-0.19069	4.608479	-1.53712	0.127835	-5.29373	0.62276	0.433449
NK.cells	ACP6	-0.32273	3.140192	-1.537	0.127864	-4.89085	0.636672	0.451593
NK.cells	LYRM2	0.316387	3.377583	1.536939	0.127879	-4.95139	0.6344	0.448621
NK.cells	KLK8	0.28393	1.605933	1.536901	0.127889	-5.02877	0.651567	0.471295

NK.cells	FMNL2	-0.48193	7.100621	-1.53673	0.127931	-5.20218	0.5999	0.404492
NK.cells	RIN3	-0.14784	5.640444	-1.53584	0.128149	-5.63137	0.613972	0.42159
NK.cells	8430429K(	0.423516	1.679457	1.535238	0.128296	-4.64354	0.652167	0.470953
NK.cells	VEGFA	0.287144	4.223513	1.534692	0.12843	-5.20185	0.627814	0.438945
NK.cells	CREBBP	-0.11419	7.888027	-1.53456	0.128462	-5.88314	0.594233	0.39641
NK.cells	TSHZ1	-0.18724	5.060403	-1.53436	0.128511	-5.55148	0.619966	0.428954
NK.cells	BVHT	0.948308	-0.0458	1.534141	0.128566	-4.40281	0.669552	0.494511
NK.cells	JAM3	-0.78663	0.244994	-1.53403	0.128593	-4.4489	0.666618	0.490543
NK.cells	TTC13	0.226866	3.938924	1.533044	0.128836	-5.1207	0.630861	0.443087
NK.cells	DEF8	0.371399	2.04717	1.532807	0.128894	-4.7603	0.649096	0.467073
NK.cells	SSBP2	0.196586	6.284595	1.532787	0.128899	-5.64572	0.609018	0.415114
NK.cells	KDM5D	2.083333	0.730109	1.532598	0.128946	-4.59368	0.662123	0.484512
NK.cells	CD93	-0.345	3.89899	-1.53231	0.129016	-4.96695	0.63124	0.443701
NK.cells	CLEC16A	-0.19169	4.763553	-1.53231	0.129018	-5.36365	0.623086	0.433151
NK.cells	SEMA7A	0.581847	2.017527	1.532194	0.129045	-4.58957	0.649386	0.467565
NK.cells	ZFP212	-0.20022	3.640876	-1.53203	0.129085	-5.1564	0.633697	0.446989
NK.cells	VPS39	0.274663	3.331542	1.531725	0.129161	-4.96397	0.636769	0.450976
NK.cells	PALM	-0.35189	4.231998	-1.53075	0.129401	-4.94308	0.629109	0.440227
NK.cells	ATP10A	0.243865	3.430551	1.530366	0.129496	-5.29673	0.636952	0.450229
NK.cells	CMC1	0.15837	4.601302	1.530155	0.129548	-5.39525	0.625831	0.435803
NK.cells	HAGHL	0.35941	2.911144	1.529079	0.129815	-4.90855	0.642828	0.457185
NK.cells	FAM222A	-0.35143	2.331884	-1.52902	0.12983	-4.82104	0.648464	0.46464
NK.cells	BOD1L	0.133851	5.740505	1.527938	0.130098	-5.52815	0.616813	0.422852
NK.cells	GM36447	-0.82408	0.209364	-1.52726	0.130265	-4.43216	0.670397	0.493539
NK.cells	8030453O2	-0.85071	-0.75227	-1.5271	0.130305	-4.38102	0.680208	0.506883
NK.cells	NEK4	0.592195	1.202148	1.527103	0.130305	-4.57031	0.660424	0.480117
NK.cells	LITAF	-0.11989	8.523937	-1.52695	0.130344	-5.97907	0.59163	0.391651
NK.cells	CUX2	-0.66875	1.039079	-1.52685	0.130368	-4.49259	0.662051	0.482343
NK.cells	MPP7	0.258501	7.383215	1.526573	0.130437	-5.92244	0.60181	0.404286
NK.cells	TMEM70	0.246007	3.930301	1.526461	0.130465	-5.14031	0.633823	0.445077
NK.cells	GM27188	-0.94116	0.619818	-1.52646	0.130466	-4.43445	0.666255	0.487993
NK.cells	RBM39	-0.07808	9.586582	-1.52542	0.130724	-6.15949	0.582825	0.380463
NK.cells	IFI213	0.471648	3.327221	1.525374	0.130735	-5.23363	0.640158	0.452869
NK.cells	SLC37A4	0.295208	2.862467	1.525364	0.130738	-4.96673	0.644655	0.458763
NK.cells	ITCH	0.142525	7.100446	1.524508	0.130951	-5.78118	0.605235	0.407988
NK.cells	AW209491	0.644187	1.448036	1.524252	0.131015	-4.57104	0.658928	0.47751
NK.cells	FAM43A	-0.39471	4.098571	-1.52414	0.131043	-4.77093	0.633133	0.443603
NK.cells	CDC6	-0.37541	3.269665	-1.52406	0.131062	-4.96852	0.641081	0.453955
NK.cells	MAGED1	-0.5494	2.409871	-1.52396	0.131088	-4.61376	0.649439	0.464944
NK.cells	CSRP2	-0.25114	5.20682	-1.52375	0.131141	-5.23262	0.622673	0.430167
NK.cells	CHSY3	-0.63345	1.978293	-1.52358	0.131183	-4.73119	0.653679	0.470627
NK.cells	KLHL12	0.219989	4.259168	1.52304	0.131318	-5.311	0.631765	0.441884
NK.cells	CFAP53	-0.80218	0.830789	-1.52295	0.131339	-4.501	0.665262	0.486115
NK.cells	UNC119	-0.14833	5.47027	-1.52237	0.131484	-5.60546	0.62037	0.427527
NK.cells	ZFP120	0.27925	2.536502	1.522016	0.131574	-4.94813	0.648364	0.464068
NK.cells	CXCR3	-0.32628	1.731497	-1.52191	0.131599	-5.20706	0.656282	0.474573
NK.cells	GM27017	-0.31282	3.311249	-1.5218	0.131628	-5.03125	0.640841	0.454173

NK.cells	PLEKHA2	-0.14852	7.455833	-1.52176	0.131637	-5.81441	0.602175	0.404683
NK.cells	ARHGAP45	-0.13888	6.710119	-1.52176	0.131639	-5.80645	0.608939	0.413171
NK.cells	ANXA1	-0.27442	6.030034	-1.52153	0.131695	-5.69442	0.615195	0.421141
NK.cells	ANAPC16	0.139482	5.656735	1.521188	0.131781	-5.50832	0.618659	0.42572
NK.cells	PPP1R3B	0.276962	2.491892	1.521104	0.131802	-5.05645	0.648825	0.464951
NK.cells	SPEF1	-0.82177	0.343281	-1.52043	0.131971	-4.4272	0.670345	0.493828
NK.cells	TRAPPC3	0.182249	4.917333	1.519412	0.132227	-5.37585	0.625702	0.435323
NK.cells	GSDMC4	-0.60619	0.743558	-1.51935	0.132241	-4.52901	0.666306	0.488911
NK.cells	SND1	0.12079	7.195863	1.519218	0.132276	-5.80315	0.604675	0.408566
NK.cells	CDS1	-0.8624	2.788548	-1.51919	0.132284	-4.47332	0.646069	0.461893
NK.cells	SIDT1	0.289486	3.376538	1.51869	0.132409	-5.42263	0.640373	0.454574
NK.cells	THUMPD3	0.212918	4.262161	1.518531	0.132449	-5.22811	0.631895	0.443513
NK.cells	ARL10	0.345081	2.295947	1.518519	0.132452	-4.8438	0.650883	0.468453
NK.cells	NPHS1	0.73676	0.439647	1.51823	0.132524	-4.49115	0.66937	0.493351
NK.cells	GOSR1	0.194663	4.182554	1.517684	0.132662	-5.23562	0.632652	0.444774
NK.cells	ABRACL	0.146164	7.550325	1.517478	0.132714	-5.88364	0.601475	0.405022
NK.cells	ADAP2	0.619	2.585365	1.51741	0.132731	-4.64862	0.64805	0.46505
NK.cells	HOOK2	-0.26421	3.558904	-1.51732	0.132754	-5.20042	0.638617	0.452668
NK.cells	HS1BP3	0.536598	1.087416	1.517183	0.132788	-4.56166	0.662857	0.484906
NK.cells	PLD4	-0.46681	6.186695	-1.51689	0.132861	-4.99367	0.613891	0.420924
NK.cells	THYN1	0.250519	3.271604	1.516715	0.132906	-5.06051	0.641385	0.456609
NK.cells	CDKN2AIP	-0.17387	4.567595	-1.51617	0.133045	-5.36439	0.629	0.44069
NK.cells	MORRBID	-0.26004	5.127394	-1.5159	0.133113	-5.61146	0.62373	0.433932
NK.cells	GCDH	-0.42246	3.116618	-1.5157	0.133164	-4.93023	0.642884	0.458964
NK.cells	BC005537	-0.13121	7.116434	-1.51543	0.133231	-5.76275	0.605395	0.41065
NK.cells	HADH	-0.21546	5.394105	-1.51536	0.133249	-5.50745	0.621237	0.43082
NK.cells	VASP	0.15818	6.541669	1.515358	0.133249	-5.73113	0.610632	0.417273
NK.cells	UBR2	-0.12402	6.362518	-1.51535	0.13325	-5.69862	0.612274	0.41936
NK.cells	FAM167B	-0.59528	2.127548	-1.51501	0.133338	-4.57612	0.652538	0.471875
NK.cells	SLCO4A1	-0.46562	3.403213	-1.51493	0.133358	-4.87004	0.640116	0.455453
NK.cells	TXNDC9	0.144008	5.512196	1.514866	0.133373	-5.51463	0.620136	0.429492
NK.cells	PPM1M	-0.21028	4.583696	-1.51486	0.133375	-5.26827	0.628847	0.440739
NK.cells	HMOX2	0.123573	6.471899	1.514597	0.133441	-5.63231	0.611271	0.41828
NK.cells	4930426D0	-0.83284	-0.21569	-1.51454	0.133457	-4.42907	0.676029	0.503806
NK.cells	WFDC21	1.297354	2.947804	1.514493	0.133468	-4.68219	0.644521	0.461429
NK.cells	CLIC4	-0.25748	7.449524	-1.51442	0.133487	-5.69737	0.602383	0.407095
NK.cells	IL20RB	-0.26604	3.81612	-1.51397	0.133601	-5.12568	0.636149	0.450558
NK.cells	RBM4	-0.16315	4.655948	-1.51396	0.133602	-5.35041	0.628165	0.440148
NK.cells	CENPM	-0.29873	3.916181	-1.51376	0.133655	-5.20568	0.635192	0.449341
NK.cells	SRSF6	-0.15011	6.210068	-1.51327	0.133776	-5.60382	0.613675	0.421669
NK.cells	ARHGAP22	-0.95724	1.023886	-1.5131	0.13382	-4.46776	0.663493	0.487159
NK.cells	ADSSL1	-0.30153	4.426295	-1.51288	0.133878	-4.94453	0.630337	0.44324
NK.cells	MICALL2	-0.7351	1.195719	-1.51287	0.133879	-4.48509	0.661774	0.484914
NK.cells	L1CAM	-0.32487	3.644556	-1.51265	0.133936	-5.12107	0.637794	0.453122
NK.cells	PLAA	0.128116	6.069326	1.512263	0.134033	-5.63875	0.614972	0.423686
NK.cells	NHP2	0.184049	5.748278	1.512116	0.134071	-5.56067	0.617942	0.427524
NK.cells	MEG3	-1.00533	1.040515	-1.51191	0.134122	-4.53397	0.663326	0.487401

NK.cells	REM2	-0.80264	0.836601	-1.51184	0.13414	-4.49307	0.665371	0.49017
NK.cells	DBI	-0.16989	7.067278	-1.51177	0.134159	-5.79025	0.605841	0.412177
NK.cells	RNF17	0.912531	-0.02892	1.511567	0.13421	-4.47216	0.674124	0.502226
NK.cells	GK	-0.29683	4.764608	-1.51117	0.134311	-5.19345	0.627364	0.439676
NK.cells	PTPN4	-0.182	5.463527	-1.51064	0.134446	-5.54551	0.62098	0.431491
NK.cells	ASB8	0.238626	3.607063	1.510531	0.134474	-5.11536	0.638556	0.454433
NK.cells	ARHGEF26	-0.89123	0.379311	-1.51027	0.13454	-4.4415	0.670403	0.497134
NK.cells	GID4	0.187651	4.230623	1.510178	0.134564	-5.26715	0.632593	0.446699
NK.cells	REEP6	-0.55776	1.791912	-1.50998	0.134614	-4.67267	0.656262	0.47818
NK.cells	RILPL2	-0.17754	7.158884	-1.50971	0.134682	-5.68633	0.605392	0.411864
NK.cells	VPS72	0.18504	4.357985	1.509573	0.134718	-5.28069	0.631383	0.445325
NK.cells	CYP8B1	-0.55281	1.074088	-1.50892	0.134884	-4.64148	0.663965	0.488002
NK.cells	ULK4	0.332291	2.667963	1.508367	0.135026	-4.8981	0.648622	0.467181
NK.cells	RNF180	-0.80358	2.247716	-1.50791	0.135143	-4.51453	0.653053	0.472872
NK.cells	SLC9A7	-0.21663	5.115655	-1.50732	0.135295	-5.3845	0.625782	0.436624
NK.cells	SAMD12	-0.88802	0.266619	-1.50714	0.135338	-4.489	0.67321	0.49966
NK.cells	HIST1H2BE	-0.53002	1.116611	-1.50694	0.13539	-4.62882	0.664627	0.488041
NK.cells	PI4K2A	-0.18364	5.639245	-1.50681	0.135423	-5.56062	0.62088	0.430372
NK.cells	4921509O(	0.964079	0.064578	1.506473	0.13551	-4.43305	0.675347	0.502749
NK.cells	COPE	0.135764	6.316581	1.50614	0.135596	-5.66693	0.614674	0.422564
NK.cells	SCP2	-0.2192	7.526193	-1.50613	0.135598	-5.85069	0.603635	0.408572
NK.cells	SESTD1	0.429429	2.300083	1.505651	0.135721	-4.9888	0.653269	0.47276
NK.cells	SRP54C	0.45272	1.755516	1.505453	0.135772	-4.72009	0.658655	0.480058
NK.cells	THAP4	-0.21248	4.091332	-1.50479	0.135943	-5.19459	0.636435	0.450235
NK.cells	ANKS3	-0.22772	4.082767	-1.50409	0.136123	-5.18471	0.636887	0.450631
NK.cells	RBM28	0.148515	5.311789	1.504065	0.136129	-5.52245	0.625229	0.435475
NK.cells	SLA	0.184596	5.280678	1.503424	0.136294	-5.60811	0.625521	0.436033
NK.cells	1700056N1	0.378402	2.033678	1.503315	0.136321	-4.77051	0.656849	0.477312
NK.cells	AMD1	-0.18481	4.581194	-1.50287	0.136436	-5.44157	0.632131	0.44476
NK.cells	IL33	-0.88278	0.25962	-1.50274	0.13647	-4.42803	0.674672	0.501552
NK.cells	PRDM9	0.585949	1.146615	1.502419	0.136552	-4.57749	0.665698	0.489412
NK.cells	S100A13	-0.14003	6.040644	-1.50231	0.13658	-5.70272	0.618426	0.42713
NK.cells	6330409D2	0.667628	0.333222	1.502278	0.136589	-4.53005	0.673922	0.500623
NK.cells	PNPO	0.26025	3.744874	1.502076	0.136641	-5.19177	0.640133	0.455421
NK.cells	CCDC115	0.188067	4.152653	1.50207	0.136642	-5.28233	0.636217	0.450281
NK.cells	CD300A	0.523254	4.623283	1.502002	0.13666	-4.81851	0.631731	0.444421
NK.cells	SGSM2	-0.21192	3.786605	-1.50122	0.136861	-5.30108	0.640422	0.455192
NK.cells	CXCL13	1.322297	-1.21101	1.500945	0.136933	-4.39841	0.690461	0.52299
NK.cells	MSMO1	-0.28707	3.610529	-1.50073	0.136989	-5.01145	0.642222	0.457516
NK.cells	AGMAT	-0.64914	1.438737	-1.50029	0.137103	-4.64513	0.663886	0.486229
NK.cells	ETFBKMT	0.376332	2.261696	1.499677	0.13726	-4.81337	0.656195	0.475385
NK.cells	MAP3K5	-0.159	7.255072	-1.49798	0.137702	-5.81932	0.610475	0.414399
NK.cells	TNFRSF9	0.266807	2.460851	1.49769	0.137776	-5.438	0.656175	0.473633
NK.cells	GM47350	0.463838	1.355941	1.497138	0.137919	-4.65125	0.667594	0.488566
NK.cells	SLAMF1	0.456962	0.884468	1.496751	0.138019	-4.85681	0.672362	0.495105
NK.cells	FUBP1	0.107094	6.678668	1.496669	0.138041	-5.73545	0.616226	0.421393
NK.cells	DCP1B	-0.28073	2.830948	-1.49631	0.138135	-5.0483	0.652912	0.469174

NK.cells	POU2AF1	-0.37408	4.320007	-1.49576	0.138277	-5.11808	0.63844	0.450205
NK.cells	GM43936	0.988117	-1.34803	1.495625	0.138312	-4.40177	0.695104	0.526994
NK.cells	COX10	0.222029	3.775159	1.49533	0.138389	-5.20698	0.643694	0.457133
NK.cells	DBF4	-0.19334	5.205532	-1.49532	0.138392	-5.54511	0.629999	0.439289
NK.cells	HGS	0.214565	4.094551	1.495232	0.138415	-5.20143	0.640609	0.453088
NK.cells	TAPT1	0.191086	5.802801	1.494896	0.138502	-5.52698	0.624375	0.432188
NK.cells	POU5F1	0.523159	1.26222	1.494868	0.13851	-4.62872	0.668539	0.490388
NK.cells	RB1CC1	-0.14183	6.500368	-1.49459	0.138583	-5.75076	0.617876	0.42402
NK.cells	TIMM10	0.317865	3.050855	1.494297	0.138659	-4.91669	0.650752	0.466845
NK.cells	CCDC32	-0.27441	3.31271	-1.49421	0.138682	-5.03685	0.648191	0.463456
NK.cells	DDX3Y	2.876151	2.707432	1.493805	0.138787	-5.04316	0.654128	0.471464
NK.cells	TTLL3	-0.26614	4.027206	-1.49368	0.138818	-5.13825	0.641258	0.454502
NK.cells	PRDM4	0.278046	3.039382	1.493134	0.138962	-4.97806	0.650865	0.467227
NK.cells	PRR33	-0.7209	0.255516	-1.49304	0.138986	-4.50407	0.678777	0.504833
NK.cells	KXD1	0.144934	5.765423	1.492863	0.139033	-5.63569	0.624725	0.433129
NK.cells	UROS	0.296317	2.669254	1.49283	0.139042	-4.8691	0.654504	0.47211
NK.cells	RSPO3	-1.12786	0.723734	-1.49253	0.139119	-4.50442	0.673995	0.498533
NK.cells	SLC39A9	-0.17671	4.172579	-1.49253	0.13912	-5.22599	0.639857	0.452939
NK.cells	ITGA8	-0.71213	1.726804	-1.49234	0.13917	-4.65229	0.66387	0.484983
NK.cells	DYNLL2	-0.20071	5.082989	-1.49201	0.139256	-5.51908	0.63116	0.441835
NK.cells	ANGEL2	0.150455	5.052327	1.49196	0.139269	-5.43254	0.631451	0.442224
NK.cells	MGME1	0.411102	2.312135	1.491886	0.139289	-4.83668	0.658036	0.477277
NK.cells	TBC1D13	0.254968	3.495587	1.491788	0.139314	-5.14211	0.646409	0.461887
NK.cells	SLC35D2	-0.2528	4.232	-1.49147	0.139397	-5.26253	0.639286	0.452634
NK.cells	EPC2	-0.14348	6.14343	-1.49116	0.139479	-5.66236	0.621192	0.429334
NK.cells	SRPK1	0.110171	6.11152	1.491112	0.139491	-5.70902	0.621489	0.429718
NK.cells	PSMA6	0.144284	6.941715	1.491022	0.139515	-5.75252	0.613802	0.419942
NK.cells	DGKH	0.221438	4.909539	1.490517	0.139648	-5.43348	0.632938	0.444404
NK.cells	WAS	0.138427	5.349391	1.490365	0.139687	-5.54941	0.62877	0.438995
NK.cells	SOD1	-0.16379	5.993562	-1.4903	0.139704	-5.64071	0.622719	0.431192
NK.cells	ZDHHC21	0.19143	4.271545	1.489936	0.1398	-5.35937	0.639081	0.452499
NK.cells	MKNK1	-0.20597	4.508378	-1.48983	0.139828	-5.22025	0.636809	0.449591
NK.cells	LIN7C	-0.16657	4.956273	-1.48958	0.139893	-5.42224	0.632536	0.444082
NK.cells	GM10642	0.773651	0.270003	1.48945	0.139928	-4.49684	0.678815	0.505955
NK.cells	ZBTB2	-0.15427	5.709287	-1.48884	0.140088	-5.53947	0.625776	0.43515
NK.cells	GM13431	-0.85092	0.492651	-1.48874	0.140114	-4.45065	0.676917	0.503131
NK.cells	ELOVL6	-0.24851	4.451031	-1.48813	0.140275	-5.39393	0.638029	0.450979
NK.cells	ANXA11	0.142988	5.969579	1.488075	0.140289	-5.68918	0.623641	0.432322
NK.cells	HMGXB3	0.186585	4.408539	1.487789	0.140365	-5.35034	0.638498	0.451612
NK.cells	FNTA	-0.13007	5.505108	-1.48736	0.140478	-5.55489	0.628064	0.438075
NK.cells	IL6ST	0.262584	4.326252	1.487269	0.140502	-5.33055	0.639289	0.45269
NK.cells	AMDHD1	-0.50497	2.26974	-1.48703	0.140565	-4.78906	0.659394	0.479413
NK.cells	PRXL2B	-0.39055	2.256934	-1.48701	0.14057	-4.7665	0.659521	0.479583
NK.cells	D430040D[illegible]	-0.88534	-0.34621	-1.4868	0.140624	-4.45122	0.685951	0.51561
NK.cells	ARHGEF12	0.338236	3.963016	1.486402	0.14073	-5.05251	0.642948	0.457481
NK.cells	TICRR	-0.36881	3.009192	-1.48623	0.140775	-4.99673	0.652246	0.469786
NK.cells	MRPS16	0.15656	5.607422	1.486067	0.140819	-5.57901	0.627252	0.437082

NK.cells	GCKR	-0.62321	1.581851	-1.48555	0.140955	-4.63589	0.666823	0.488944
NK.cells	VNN3	-0.58826	1.614791	-1.48534	0.141009	-4.68667	0.666496	0.488563
NK.cells	CDH22	1.161015	-0.85879	1.483983	0.14137	-4.41356	0.692636	0.523769
NK.cells	WBP1	-0.2567	3.813733	-1.48384	0.141407	-5.20111	0.645496	0.460046
NK.cells	NOP16	0.226152	4.283714	1.483704	0.141444	-5.27242	0.640949	0.454073
NK.cells	CYP7A1	-0.7072	0.739983	-1.48335	0.141536	-4.577	0.676108	0.501111
NK.cells	COG5	0.141475	6.582266	1.482952	0.141643	-5.768	0.619204	0.426082
NK.cells	RREB1	0.211819	6.814971	1.482852	0.14167	-5.70567	0.617048	0.423354
NK.cells	CHIL3	1.28402	2.447415	1.481923	0.141916	-4.71763	0.658917	0.478182
NK.cells	CSNK2A1	0.101699	6.59773	1.48184	0.141938	-5.74577	0.619061	0.426015
NK.cells	PDE6H	0.389461	2.093346	1.481831	0.141941	-4.7978	0.662443	0.482914
NK.cells	LAYN	-0.82316	0.629576	-1.48177	0.141957	-4.48675	0.677236	0.502962
NK.cells	GLRA1	-0.67237	1.25067	-1.48174	0.141965	-4.69308	0.670917	0.494359
NK.cells	TMEM64	0.20168	5.051385	1.481726	0.141969	-5.53152	0.633595	0.444756
NK.cells	PHEX	-0.69783	1.372943	-1.4816	0.142001	-4.62041	0.66968	0.492682
NK.cells	DDX54	0.155053	5.820595	1.481493	0.142031	-5.60112	0.626319	0.43536
NK.cells	LMTK2	-0.15445	5.616268	-1.48068	0.142248	-5.58881	0.628242	0.438215
NK.cells	CHST7	-0.85175	0.725254	-1.47951	0.142559	-4.49732	0.676258	0.502411
NK.cells	GNA14	1.077993	-0.0617	1.479508	0.142559	-4.45564	0.684343	0.513508
NK.cells	INPP1	-0.19221	4.03687	-1.47927	0.142622	-5.47051	0.643333	0.458245
NK.cells	TSPAN6	0.803547	1.103606	1.479087	0.142671	-4.52876	0.672407	0.497288
NK.cells	RILPL1	-0.52981	2.31814	-1.4789	0.14272	-4.65014	0.660202	0.480819
NK.cells	OSBPL1A	0.326636	3.288159	1.478871	0.142729	-4.94407	0.650623	0.468018
NK.cells	PRXL2A	0.384495	4.184431	1.478771	0.142756	-5.21149	0.641906	0.456539
NK.cells	HFE	-0.47602	3.452689	-1.47867	0.142782	-4.73928	0.649014	0.465952
NK.cells	SMPD1	0.326197	2.643295	1.478444	0.142843	-4.96364	0.656974	0.476618
NK.cells	CRYBB3	-0.56566	0.578324	-1.47838	0.142859	-4.66735	0.67776	0.504773
NK.cells	SKA1	-0.39617	2.918501	-1.47811	0.142933	-5.06057	0.654256	0.47305
NK.cells	ING5	0.239336	3.456639	1.478102	0.142934	-5.11562	0.648975	0.46602
NK.cells	DDX42	0.125389	5.684077	1.477938	0.142978	-5.6143	0.627603	0.438041
NK.cells	TASOR2	0.162086	5.373883	1.477844	0.143003	-5.56359	0.630533	0.441879
NK.cells	PPP1R11	0.194543	4.640461	1.477832	0.143006	-5.40795	0.63752	0.450994
NK.cells	PPP5C	-0.2005	4.346979	-1.47761	0.143066	-5.26524	0.640339	0.454717
NK.cells	MAX	0.133589	6.499429	1.477566	0.143078	-5.72016	0.619974	0.428282
NK.cells	NR2F6	-0.27741	3.501892	-1.47748	0.143101	-5.12365	0.648533	0.465554
NK.cells	SLC25A14	0.334397	2.078186	1.477473	0.143102	-4.84246	0.662594	0.484361
NK.cells	ZFP267	-0.45485	1.251689	-1.47714	0.143191	-4.65362	0.670906	0.495709
NK.cells	BIN2	0.153963	5.165118	1.476909	0.143253	-5.65214	0.632513	0.444638
NK.cells	TNNT1	0.504403	1.520318	1.476837	0.143272	-4.66923	0.668193	0.492101
NK.cells	AGK	0.280915	3.121713	1.476573	0.143343	-5.02264	0.652256	0.470739
NK.cells	ATP2C1	0.144199	5.85373	1.476432	0.143381	-5.60065	0.626007	0.436307
NK.cells	SLC27A1	0.503451	2.972091	1.476207	0.143441	-4.76676	0.653728	0.47282
NK.cells	DPP10	-0.84662	0.411958	-1.47619	0.143445	-4.52262	0.679465	0.507692
NK.cells	ZNRD1	-0.16384	5.076686	-1.47611	0.143467	-5.48055	0.633354	0.44592
NK.cells	ADCY10	-0.73822	0.912909	-1.47608	0.143474	-4.534	0.674346	0.500678
NK.cells	AHSA2	-0.21577	3.646243	-1.47555	0.143617	-5.18767	0.647348	0.464248
NK.cells	ZMAT4	0.525501	0.381713	1.475499	0.143631	-4.96016	0.680009	0.508384

NK.cells	KAT14	0.1961	3.838268	1.475209	0.143709	-5.1985	0.645577	0.461926
NK.cells	DCAF8	0.119969	5.699638	1.474594	0.143874	-5.58055	0.627767	0.438763
NK.cells	GFRA2	-0.67182	2.545769	-1.47426	0.143964	-4.58791	0.658266	0.479112
NK.cells	CYP4A14	0.622178	1.833194	1.473883	0.144065	-4.76863	0.665376	0.488853
NK.cells	CTU1	0.615923	0.850388	1.473748	0.144101	-4.60819	0.675316	0.502433
NK.cells	EEF2KMT	0.24807	3.145957	1.473723	0.144108	-5.1129	0.652341	0.471368
NK.cells	P2RY10	-0.22813	3.930309	-1.47372	0.144109	-5.61118	0.644684	0.461191
NK.cells	NRD1	0.135654	6.185357	1.473261	0.144232	-5.71326	0.623209	0.433291
NK.cells	VPS37C	0.196449	3.950923	1.473236	0.144239	-5.2322	0.644484	0.461104
NK.cells	SLC12A2	0.320667	3.544469	1.473065	0.144285	-5.14906	0.648438	0.466414
NK.cells	TRIM23	0.254392	3.295163	1.472968	0.144311	-5.07332	0.650877	0.469694
NK.cells	SULF2	-0.22631	2.585463	-1.47294	0.144319	-5.42087	0.657872	0.47907
NK.cells	ADK	0.193724	6.968234	1.472821	0.14435	-5.84277	0.615938	0.424079
NK.cells	PTPRB	-0.56365	3.708559	-1.47228	0.144497	-4.97463	0.646868	0.464583
NK.cells	PPCDC	0.253502	3.567947	1.472159	0.144529	-5.12084	0.648239	0.466454
NK.cells	ILF3	-0.14402	6.541502	-1.47211	0.144541	-5.71515	0.619917	0.429397
NK.cells	TSKU	0.920794	-0.3765	1.471999	0.144572	-4.4545	0.687977	0.520507
NK.cells	HOMER1	-0.18774	6.109837	-1.47152	0.1447	-5.68335	0.623989	0.434692
NK.cells	PHACTR1	-0.70106	1.519212	-1.4715	0.144706	-4.54042	0.668613	0.493923
NK.cells	SLC29A1	-0.22322	5.215066	-1.4713	0.14476	-5.41521	0.632426	0.445728
NK.cells	OAS1C	0.443344	1.903745	1.471164	0.144797	-4.85703	0.664747	0.488838
NK.cells	LRRC8B	-0.19084	3.358799	-1.4708	0.144895	-5.27197	0.65036	0.469647
NK.cells	SF1	-0.10278	7.215593	-1.47074	0.144911	-5.84979	0.613761	0.421868
NK.cells	NIPA1	0.771225	0.13903	1.470453	0.144989	-4.5045	0.682836	0.513768
NK.cells	SLC43A1	0.638704	1.089863	1.470238	0.145047	-4.62714	0.673117	0.500466
NK.cells	ADCY7	0.147818	5.583087	1.470044	0.1451	-5.63844	0.629089	0.441749
NK.cells	DMGDH	-0.55374	1.667706	-1.46947	0.145255	-4.72558	0.667743	0.492932
NK.cells	VCAN	0.885766	0.76602	1.469216	0.145324	-4.58711	0.676899	0.505528
NK.cells	NADK	0.162667	6.266885	1.468869	0.145418	-5.68507	0.623112	0.4339
NK.cells	BLOC1S3	0.404519	2.381624	1.468759	0.145447	-4.85663	0.660601	0.483452
NK.cells	TMEM185I	-0.22102	4.12483	-1.46863	0.145482	-5.23853	0.643486	0.460562
NK.cells	GM1123	-0.6387	0.265276	-1.46847	0.145526	-4.55611	0.682036	0.512736
NK.cells	ACBD3	0.133495	5.707973	1.468181	0.145604	-5.59136	0.628465	0.4408
NK.cells	SERTAD1	-0.18615	5.61417	-1.46792	0.145676	-5.61944	0.629429	0.442061
NK.cells	TUBB5	-0.19214	9.849372	-1.46758	0.145766	-6.30374	0.590932	0.393112
NK.cells	HSF1	-0.16545	4.997353	-1.46691	0.145949	-5.48803	0.635604	0.4503
NK.cells	GNAQ	0.172006	7.882192	1.466671	0.146014	-5.78583	0.608699	0.415594
NK.cells	PLCG2	-0.12927	7.02313	-1.46647	0.146069	-5.75018	0.616577	0.425684
NK.cells	TCEA3	-0.47825	2.275566	-1.46619	0.146143	-4.86084	0.662182	0.485831
NK.cells	LYZL4	0.877424	-1.24765	1.465913	0.14622	-4.43519	0.698118	0.535762
NK.cells	GM42047	-0.3608	5.263713	-1.4659	0.146223	-5.43779	0.633066	0.44707
NK.cells	I830077J02	0.651228	2.758256	1.465876	0.14623	-4.62909	0.657383	0.479357
NK.cells	JARID2	-0.12195	8.144583	-1.46573	0.14627	-6.13049	0.606315	0.412685
NK.cells	RTL8A	-0.24099	3.632481	-1.46567	0.146286	-5.30592	0.648785	0.467946
NK.cells	MCRIP2	-0.37213	2.015335	-1.46396	0.146751	-4.845	0.666318	0.490061
NK.cells	RRAGD	0.510861	2.299081	1.463156	0.146971	-4.69412	0.663474	0.486369
NK.cells	GRB7	-0.76377	0.183341	-1.46311	0.146982	-4.53646	0.684997	0.515818

NK.cells	IER3IP1	0.109632	6.363638	1.463029	0.147005	-5.74129	0.624137	0.434346
NK.cells	VMAC	0.4998	1.5263	1.462985	0.147018	-4.66907	0.671251	0.496935
NK.cells	DYNLT1B	0.6374	1.188553	1.462848	0.147055	-4.60871	0.67468	0.501662
NK.cells	TRAP1	-0.16431	4.381095	-1.46282	0.147063	-5.35382	0.642998	0.459034
NK.cells	ZFP36L1	-0.14982	7.742233	-1.46272	0.147089	-5.9603	0.611381	0.41803
NK.cells	FOXA3	-0.57529	1.425712	-1.46263	0.147115	-4.65606	0.67227	0.498411
NK.cells	SCD2	-0.26508	4.726396	-1.46245	0.147163	-5.35226	0.639668	0.454727
NK.cells	RBKS	0.253394	3.630745	1.462148	0.147246	-5.21552	0.650428	0.468879
NK.cells	MARS	-0.31366	3.10364	-1.46193	0.147306	-5.0257	0.655612	0.475855
NK.cells	HECTD2	0.498069	0.291184	1.461401	0.147451	-4.71853	0.684021	0.514656
NK.cells	GAR1	0.214044	4.430651	1.461323	0.147472	-5.39807	0.642649	0.458734
NK.cells	RHOT2	-0.33013	2.679369	-1.46122	0.1475	-4.96437	0.659816	0.481644
NK.cells	WDR48	0.169029	4.298768	1.461162	0.147516	-5.37704	0.643925	0.460422
NK.cells	LRATD2	-0.44208	1.700967	-1.46074	0.147631	-4.72879	0.6699	0.495129
NK.cells	ZNHIT2	0.228526	3.310699	1.459605	0.147943	-5.11347	0.654474	0.473989
NK.cells	TBC1D2	0.517093	1.45751	1.459584	0.147948	-4.73616	0.673013	0.499058
NK.cells	THOC5	0.222876	3.564433	1.459343	0.148015	-5.1746	0.651978	0.470763
NK.cells	RAD50	0.16882	4.906644	1.459277	0.148033	-5.49903	0.638949	0.453508
NK.cells	B3GALT5	-0.40351	0.779445	-1.45924	0.148044	-5.07808	0.679935	0.508675
NK.cells	MAGI3	-0.20478	6.598022	-1.45809	0.14836	-5.74562	0.623822	0.433071
NK.cells	SBNO1	-0.11902	7.359347	-1.45788	0.148416	-5.88546	0.616747	0.42399
NK.cells	AMN1	0.267253	3.42505	1.457878	0.148417	-5.07299	0.65428	0.473064
NK.cells	SLC35B4	0.286396	2.825471	1.457269	0.148585	-5.0656	0.66053	0.4812
NK.cells	IL10	-0.56281	2.999738	-1.45688	0.148692	-4.91966	0.658798	0.478962
NK.cells	ELANE	1.449047	2.340078	1.45682	0.148709	-4.76042	0.66538	0.487832
NK.cells	STT3B	0.113091	6.666062	1.456676	0.148748	-5.75533	0.623483	0.432493
NK.cells	KLRI1	-0.36096	-0.19768	-1.45664	0.148759	-5.02351	0.691357	0.523474
NK.cells	CAPN11	-0.98665	1.046105	-1.4563	0.148853	-4.61414	0.678679	0.505882
NK.cells	CASC4	0.308989	2.64714	1.455979	0.14894	-5.03271	0.662635	0.483935
NK.cells	PARP10	0.285827	3.565506	1.455332	0.149119	-5.23364	0.654079	0.472089
NK.cells	BCOR	-0.18455	5.214585	-1.45496	0.149221	-5.51589	0.638265	0.451025
NK.cells	IFITM2	0.207519	7.227901	1.454735	0.149284	-5.79397	0.619315	0.426447
NK.cells	SUFU	-0.15872	5.025085	-1.45432	0.149399	-5.5259	0.640203	0.453608
NK.cells	SPATA2	-0.23089	4.217056	-1.45427	0.149411	-5.32347	0.648027	0.463951
NK.cells	AP3S1	0.118576	7.096627	1.454016	0.149483	-5.91209	0.620684	0.428322
NK.cells	KIF5A	-0.73164	0.429339	-1.45372	0.149564	-4.56806	0.686231	0.515767
NK.cells	GM42659	-0.19348	4.567867	-1.45358	0.149603	-5.39314	0.644741	0.459746
NK.cells	PDE4B	0.166942	8.527906	1.453137	0.149726	-6.09329	0.607714	0.411853
NK.cells	PHKA2	0.289427	3.438409	1.452939	0.149781	-5.09072	0.655931	0.474608
NK.cells	KPNA1	-0.12653	6.778024	-1.45283	0.14981	-5.87402	0.623837	0.432533
NK.cells	CYHR1	0.178476	4.48568	1.452683	0.149852	-5.41828	0.645677	0.461039
NK.cells	GTF3C2	-0.12945	5.474786	-1.45232	0.149954	-5.57612	0.63629	0.448658
NK.cells	TEX261	0.142764	5.229001	1.451841	0.150085	-5.55955	0.638643	0.451893
NK.cells	ZFP639	0.201683	4.128657	1.45171	0.150122	-5.32144	0.649294	0.465962
NK.cells	FZD4	-0.57599	1.267832	-1.45159	0.150154	-4.63795	0.677894	0.504585
NK.cells	FGFBP3	0.818151	0.028033	1.451459	0.150191	-4.55598	0.690702	0.52226
NK.cells	ABCE1	0.175843	5.007871	1.451393	0.15021	-5.51154	0.640768	0.454723

NK.cells	MGLL	-0.41347	3.495334	-1.45116	0.150275	-4.98583	0.655562	0.474401
NK.cells	TXN2	0.131067	6.315861	1.450765	0.150384	-5.73974	0.628393	0.438709
NK.cells	BBS7	0.86145	0.558111	1.450736	0.150392	-4.51519	0.685285	0.514922
NK.cells	MST1	-0.75243	0.649079	-1.4501	0.150569	-4.59676	0.684722	0.513855
NK.cells	HSDL1	0.192153	4.167902	1.449876	0.150631	-5.2178	0.649355	0.466043
NK.cells	TFEC	0.598417	2.368464	1.449852	0.150638	-4.73	0.66719	0.48997
NK.cells	GM525	-0.70848	0.742971	-1.44907	0.150856	-4.48298	0.684495	0.513002
NK.cells	GNG5	0.124549	8.925654	1.448752	0.150944	-6.12991	0.605385	0.408579
NK.cells	USP8	0.138118	5.372996	1.448542	0.151003	-5.60709	0.638465	0.451113
NK.cells	CNOT6	-0.11151	5.947416	-1.44838	0.151048	-5.68795	0.632984	0.443984
NK.cells	SLC30A1	-0.30303	3.386735	-1.4482	0.151098	-5.14745	0.657823	0.476855
NK.cells	H2-T24	0.553698	1.828462	1.448033	0.151145	-4.74126	0.673452	0.498019
NK.cells	PECAM1	0.199105	7.734027	1.447555	0.151278	-5.94311	0.616377	0.422638
NK.cells	AEBP2	0.139314	5.787827	1.447484	0.151298	-5.7698	0.634618	0.446168
NK.cells	CDKAL1	0.13113	6.364518	1.447349	0.151336	-5.77322	0.629151	0.439105
NK.cells	NRARP	-0.40513	1.780032	-1.44654	0.151561	-4.95039	0.674757	0.499097
NK.cells	ASCC1	0.197249	3.774861	1.446406	0.151599	-5.28052	0.65478	0.472237
NK.cells	ZKSCAN1	0.189547	4.068204	1.446031	0.151704	-5.31446	0.651983	0.468522
NK.cells	TDG	0.156335	5.074746	1.445945	0.151728	-5.52371	0.642191	0.455584
NK.cells	LARP1	0.124326	6.798018	1.445714	0.151793	-5.83691	0.625841	0.434274
NK.cells	ZCCHC7	0.186933	6.744533	1.444377	0.152168	-5.83853	0.627219	0.435361
NK.cells	CEP128	-0.19649	6.336302	-1.44432	0.152183	-5.70691	0.631069	0.440337
NK.cells	ZFP445	0.156195	4.93802	1.443958	0.152285	-5.49088	0.644455	0.458022
NK.cells	HPX	-0.2733	5.670685	-1.44343	0.152432	-5.66059	0.637403	0.44896
NK.cells	KLHL15	-0.18196	4.413728	-1.44328	0.152476	-5.38948	0.649554	0.464955
NK.cells	PDCD2L	-0.16083	4.660225	-1.44327	0.152478	-5.37257	0.647151	0.461776
NK.cells	ARL4C	-0.16938	5.243589	-1.44267	0.152648	-5.70219	0.641503	0.454508
NK.cells	TUT7	-0.10967	7.206797	-1.44265	0.152654	-5.89133	0.62289	0.430334
NK.cells	GM10863	0.804483	0.229142	1.442546	0.152682	-4.54929	0.691843	0.522562
NK.cells	COA6	-0.20264	4.110702	-1.44227	0.152758	-5.32241	0.652521	0.469228
NK.cells	CALHM2	-0.22821	3.088827	-1.44219	0.152781	-5.19203	0.662635	0.482777
NK.cells	LTA4H	-0.19145	5.724021	-1.44218	0.152784	-5.68044	0.636892	0.448633
NK.cells	TLK2	-0.11015	6.825344	-1.44199	0.152839	-5.84875	0.626459	0.435167
NK.cells	AP5B1	0.52408	1.293299	1.441963	0.152846	-4.69522	0.680817	0.507587
NK.cells	FAM25C	-0.66893	1.148741	-1.44163	0.152941	-4.65488	0.682304	0.509679
NK.cells	ZBTB39	0.407024	1.975209	1.44113	0.15308	-4.87758	0.67385	0.498208
NK.cells	MAPKAPK3	0.160229	5.306805	1.440717	0.153197	-5.70619	0.640895	0.454215
NK.cells	DDX23	0.166538	4.71777	1.440517	0.153253	-5.46095	0.646592	0.461725
NK.cells	GBP3	0.381745	3.228626	1.440336	0.153304	-5.20294	0.661242	0.481274
NK.cells	HAUS1	0.259026	3.65784	1.440309	0.153312	-5.21673	0.656983	0.47556
NK.cells	PRAG1	0.361733	1.923732	1.440169	0.153351	-4.99357	0.674373	0.499129
NK.cells	SNAPC5	0.172669	4.755447	1.439925	0.15342	-5.48044	0.646226	0.461312
NK.cells	WDR25	0.374078	1.998808	1.439876	0.153434	-4.82242	0.67361	0.498088
NK.cells	SMNDC1	-0.11252	6.059801	-1.4397	0.153483	-5.72544	0.633691	0.44486
NK.cells	ATXN7	-0.13923	6.23066	-1.4397	0.153484	-5.81233	0.632069	0.442749
NK.cells	HERC6	0.265862	4.719873	1.439575	0.153519	-5.55425	0.646571	0.461769
NK.cells	ANKRD42	-0.88707	0.346625	-1.43936	0.153579	-4.5229	0.690616	0.521611

NK.cells	ELMOD3	-0.43683	3.437093	-1.43922	0.153618	-4.99809	0.659169	0.478743
NK.cells	CFHR2	-0.46413	2.56401	-1.43897	0.153691	-4.94266	0.667895	0.490589
NK.cells	SMAGP	-0.32855	3.61538	-1.43884	0.153726	-5.04764	0.657403	0.476496
NK.cells	RNASEH2A	-0.19592	4.54522	-1.43866	0.153778	-5.45617	0.648271	0.464396
NK.cells	RDH14	0.217143	3.731822	1.438597	0.153795	-5.19411	0.656252	0.47503
NK.cells	ATM	0.190303	4.221236	1.438338	0.153869	-5.39646	0.651437	0.468727
NK.cells	5430401H(	0.935988	-0.47304	1.43677	0.154313	-4.51579	0.699221	0.534714
NK.cells	JUND	-0.15747	10.62781	-1.43674	0.154321	-6.40405	0.591875	0.392678
NK.cells	ELP3	0.297894	2.716115	1.436615	0.154357	-4.99768	0.666366	0.489395
NK.cells	ALDH3B1	0.446323	3.605843	1.436421	0.154412	-4.77675	0.657497	0.477454
NK.cells	PPM1E	-0.38303	4.964118	-1.43581	0.154584	-5.21842	0.644202	0.46005
NK.cells	GBP4	0.424694	3.363964	1.435709	0.154614	-5.3187	0.659896	0.481003
NK.cells	CAB39L	0.190907	4.624571	1.435697	0.154617	-5.41265	0.647498	0.464419
NK.cells	RIPOR1	-0.24605	3.960811	-1.43474	0.15489	-5.17223	0.653994	0.47352
NK.cells	USP7	-0.11722	6.215443	-1.43473	0.154892	-5.7188	0.632214	0.444712
NK.cells	TBC1D5	0.141086	7.293343	1.434593	0.154931	-5.91221	0.622083	0.431574
NK.cells	PNISR	0.118426	5.773836	1.434564	0.154939	-5.67134	0.636416	0.450222
NK.cells	ZADH2	0.206388	3.381757	1.434559	0.154941	-5.27179	0.659719	0.481222
NK.cells	GM15564	0.392096	2.474453	1.434475	0.154964	-4.98429	0.668798	0.493523
NK.cells	RBBP4	-0.10368	7.676556	-1.43431	0.155012	-5.99976	0.618525	0.427074
NK.cells	SNX10	0.276117	5.029489	1.434271	0.155022	-5.32633	0.64357	0.459745
NK.cells	ASPRV1	0.803662	1.33931	1.434153	0.155056	-4.64599	0.680345	0.509479
NK.cells	IVNS1ABP	-0.15426	6.027762	-1.43412	0.155064	-5.65067	0.633996	0.447171
NK.cells	PPARA	-0.47064	2.784821	-1.43407	0.155081	-4.97031	0.665677	0.489418
NK.cells	FAM102B	0.221323	4.23575	1.434001	0.155099	-5.38364	0.651295	0.470065
NK.cells	GLUD1	-0.1136	7.653063	-1.43393	0.15512	-6.02818	0.618743	0.427407
NK.cells	GYG	0.161927	6.126163	1.433797	0.155157	-5.87371	0.633061	0.445989
NK.cells	IPO11	0.197889	4.478076	1.433753	0.15517	-5.38558	0.648926	0.466929
NK.cells	ALKBH6	0.250234	2.755561	1.433698	0.155186	-5.02998	0.66597	0.489857
NK.cells	GATAD2A	-0.11183	7.859185	-1.4337	0.155186	-6.06258	0.616838	0.424984
NK.cells	KCTD17	0.587348	0.958849	1.433634	0.155204	-4.7244	0.684262	0.514981
NK.cells	INVS	0.339049	2.809082	1.433579	0.15522	-4.99881	0.665434	0.489161
NK.cells	IFI209	0.211183	5.809713	1.433026	0.155377	-5.70332	0.636496	0.450023
NK.cells	PCBD2	-0.16901	5.24057	-1.43202	0.155663	-5.59524	0.642377	0.457539
NK.cells	CDC14A	-0.1963	5.803629	-1.43187	0.155708	-5.68766	0.636969	0.450489
NK.cells	ZFP367	-0.21764	5.436634	-1.43165	0.15577	-5.59418	0.640488	0.455154
NK.cells	ARMCX6	0.547268	0.977785	1.431565	0.155794	-4.59576	0.684967	0.515291
NK.cells	PEX1	0.333464	3.071405	1.431509	0.15581	-5.04631	0.663682	0.486168
NK.cells	GGT5	-0.62159	0.649699	-1.43122	0.155894	-4.67968	0.688368	0.520118
NK.cells	BANK1	-0.40661	6.09331	-1.43115	0.155914	-5.38565	0.634207	0.447044
NK.cells	GSTCD	0.269527	3.564196	1.431024	0.155948	-5.18091	0.658775	0.4797
NK.cells	TRMT5	0.531029	1.213824	1.430919	0.155979	-4.70707	0.682531	0.512155
NK.cells	CWF19L2	0.163881	5.038452	1.430665	0.156051	-5.51515	0.64433	0.460579
NK.cells	GM34095	0.906715	-0.39051	1.430481	0.156104	-4.48833	0.699268	0.535653
NK.cells	PPP1CC	-0.10514	7.584523	-1.4302	0.156184	-6.00439	0.620193	0.429171
NK.cells	GM20682	-0.41065	1.711443	-1.43008	0.156219	-4.77587	0.677426	0.505429
NK.cells	AMOTL2	-0.8117	0.684568	-1.43	0.156242	-4.57349	0.688006	0.520058

NK.cells	ANKFY1	0.14951	5.995175	1.428471	0.15668	-5.65824	0.636497	0.44925
NK.cells	RBFOX2	-0.52078	2.758111	-1.42845	0.156685	-4.86621	0.668245	0.491607
NK.cells	TMEM203	0.285497	3.168142	1.427874	0.156851	-5.11914	0.66437	0.486241
NK.cells	YWHAZ	-0.07433	8.482146	-1.42787	0.156851	-6.11743	0.613451	0.419384
NK.cells	JUNOS	-0.58148	1.820722	-1.42753	0.15695	-4.69812	0.678103	0.504942
NK.cells	TARBP2	0.250095	3.242608	1.427409	0.156984	-5.15714	0.663727	0.485392
NK.cells	CYB5RL	-0.65363	0.380261	-1.42689	0.157133	-4.55917	0.693419	0.525836
NK.cells	PROK2	1.123145	-0.61756	1.426409	0.157272	-4.51601	0.704329	0.54076
NK.cells	COPS9	0.131173	6.68605	1.425978	0.157396	-5.83631	0.631193	0.44157
NK.cells	APOC2	0.339797	4.203003	1.42584	0.157436	-5.33933	0.65517	0.473174
NK.cells	2010013B2	-0.45188	3.545259	-1.42413	0.15793	-4.83734	0.66325	0.482539
NK.cells	HPSE	0.332433	3.426146	1.423989	0.15797	-5.07528	0.66444	0.484198
NK.cells	NIP7	0.154921	4.635807	1.423978	0.157972	-5.44895	0.652458	0.468167
NK.cells	HDAC1	-0.11894	6.039987	-1.42379	0.158027	-5.73784	0.638843	0.450249
NK.cells	ODC1	0.173927	5.188947	1.423374	0.158147	-5.60677	0.647114	0.461109
NK.cells	KCNK5	0.408033	1.47886	1.42336	0.158151	-4.91066	0.68429	0.51125
NK.cells	H2-DMB1	-0.8884	4.478206	-1.42305	0.158242	-4.77167	0.654211	0.470417
NK.cells	LAT2	-0.16753	4.773693	-1.42283	0.158305	-5.67495	0.651348	0.466624
NK.cells	VAMP1	-0.19115	3.997211	-1.4224	0.158429	-5.40829	0.659113	0.476834
NK.cells	GM41442	-0.85625	-0.19949	-1.42235	0.158443	-4.52144	0.702171	0.535836
NK.cells	METTL23	0.122009	5.675126	1.421956	0.158557	-5.70156	0.642937	0.455277
NK.cells	TMEM221	0.861589	-0.26558	1.42177	0.158611	-4.49333	0.703131	0.537178
NK.cells	1200007C1	0.797683	-0.38465	1.421466	0.158699	-4.56554	0.70448	0.539081
NK.cells	ACVR1	-0.33436	3.707524	-1.42127	0.158756	-5.12304	0.662313	0.48118
NK.cells	VDAC1	0.142229	6.677223	1.421137	0.158795	-5.81998	0.633419	0.443002
NK.cells	GM16618	-0.55343	0.733543	-1.42078	0.158897	-4.65006	0.692889	0.522786
NK.cells	MASTL	-0.22708	3.782	-1.4204	0.159008	-5.30665	0.662004	0.480419
NK.cells	COX17	0.128792	6.919214	1.420129	0.159087	-5.87508	0.631633	0.440398
NK.cells	DOCK7	0.369226	3.830588	1.419918	0.159148	-4.9544	0.661643	0.479993
NK.cells	ZDHHC24	0.575525	1.089708	1.419674	0.159219	-4.68876	0.689615	0.518118
NK.cells	NF1	-0.13227	6.550388	-1.41868	0.159509	-5.81718	0.635649	0.445456
NK.cells	COG3	0.188433	4.292574	1.418622	0.159525	-5.42831	0.657569	0.474363
NK.cells	PIR	-0.60699	1.198746	-1.41857	0.15954	-4.65771	0.688946	0.517003
NK.cells	GM48293	0.860856	-0.15995	1.418374	0.159597	-4.5073	0.703225	0.536921
NK.cells	HES7	-0.76472	-0.49621	-1.41836	0.159601	-4.51977	0.706807	0.541953
NK.cells	SAMD4	-0.67029	3.187151	-1.41811	0.159674	-4.89988	0.668671	0.489309
NK.cells	GAS2	-0.27843	2.456745	-1.41762	0.159816	-5.10368	0.676261	0.499417
NK.cells	CDCA3	-0.31528	4.895561	-1.41758	0.159829	-5.55663	0.651889	0.466666
NK.cells	TMEM176I	-0.2164	5.475623	-1.41722	0.159934	-5.66931	0.646437	0.459338
NK.cells	RASL11A	-0.65072	0.28073	-1.4169	0.160027	-4.64281	0.699217	0.530758
NK.cells	CETN4	0.579932	-0.08195	1.41655	0.160128	-4.63121	0.70308	0.536275
NK.cells	MRC2	-0.59626	1.416971	-1.4165	0.160142	-4.7045	0.687348	0.51441
NK.cells	A230072C(	0.451351	1.545767	1.416049	0.160275	-4.77562	0.686033	0.512662
NK.cells	POT1B	0.216685	4.421725	1.415942	0.160306	-5.47732	0.65695	0.473278
NK.cells	FKBP9	-0.73535	1.265181	-1.41583	0.160339	-4.61693	0.688944	0.516724
NK.cells	GM12802	-0.52656	0.658153	-1.41573	0.160367	-4.62543	0.695285	0.525526
NK.cells	SNX30	-0.21917	5.755716	-1.41522	0.160517	-5.46861	0.644293	0.456103

NK.cells	INCENP	-0.2228	5.454754	-1.41488	0.160617	-5.667	0.647243	0.46015
NK.cells	OSBPL10	0.680125	0.973543	1.414623	0.160691	-4.59648	0.692424	0.521378
NK.cells	ZC3H12C	0.615848	4.431325	1.414485	0.160732	-4.90915	0.657274	0.473643
NK.cells	ATP1A3	0.64363	1.709416	1.41401	0.160871	-4.67968	0.684778	0.510976
NK.cells	CHMP1A	0.157345	5.305048	1.413905	0.160901	-5.67044	0.6487	0.462338
NK.cells	HILPDA	0.285964	4.798635	1.413757	0.160945	-5.64171	0.653654	0.468997
NK.cells	GPKOW	-0.14828	4.488998	-1.41359	0.160993	-5.47435	0.656704	0.473105
NK.cells	BBX	0.159897	6.494425	1.41355	0.161005	-5.81944	0.637225	0.447415
NK.cells	CRY1	0.165268	5.502006	1.413496	0.161021	-5.64785	0.646784	0.459949
NK.cells	DCXR	-0.23213	4.317514	-1.41326	0.16109	-5.34456	0.658455	0.475467
NK.cells	HAGH	-0.19322	5.358255	-1.41281	0.161224	-5.63029	0.648553	0.461964
NK.cells	TBCD	0.167673	4.783335	1.41229	0.161375	-5.53529	0.654363	0.469586
NK.cells	RASA1	0.133004	6.030636	1.412138	0.161419	-5.78218	0.642221	0.453636
NK.cells	FBXL6	-0.22143	3.399881	-1.41205	0.161446	-5.23829	0.668124	0.488104
NK.cells	ILVBL	0.210685	3.757211	1.411899	0.16149	-5.32484	0.66454	0.483322
NK.cells	GNGT2	0.274794	5.675602	1.411621	0.161571	-5.52264	0.645757	0.45831
NK.cells	GM5244	-0.71923	-0.39473	-1.41122	0.16169	-4.61714	0.707876	0.542728
NK.cells	1700006J1	0.973126	-0.71534	1.410725	0.161835	-4.51791	0.711575	0.547764
NK.cells	IMP3	0.136767	5.264975	1.410641	0.161859	-5.61989	0.650243	0.463886
NK.cells	RNF40	-0.18622	4.021973	-1.4104	0.161931	-5.3433	0.662573	0.480245
NK.cells	MRPL43	0.158447	5.608686	1.410119	0.162013	-5.6484	0.647002	0.459581
NK.cells	LMBRD1	0.143724	6.356577	1.409987	0.162052	-5.80235	0.639783	0.450149
NK.cells	CDH5	-0.49423	4.480665	-1.4094	0.162223	-5.06362	0.658533	0.474491
NK.cells	TRMT12	0.516542	1.30746	1.408871	0.162381	-4.68793	0.69121	0.518491
NK.cells	WHAMM	-0.18783	4.147659	-1.40868	0.162437	-5.44144	0.662257	0.479194
NK.cells	ABRAXAS1	0.34984	2.604808	1.408385	0.162524	-4.99333	0.677821	0.500372
NK.cells	PRKCA	-0.18492	7.401898	-1.40773	0.162718	-5.96595	0.63069	0.43803
NK.cells	UNK	0.219281	4.287183	1.407663	0.162737	-5.38475	0.660868	0.477725
NK.cells	RRP7A	0.211154	3.942345	1.407493	0.162787	-5.37797	0.664305	0.482369
NK.cells	EEF1A1	0.090964	10.55641	1.407339	0.162833	-6.50381	0.601679	0.40131
NK.cells	DERA	0.159568	4.534652	1.4073	0.162844	-5.43831	0.658414	0.474522
NK.cells	TMC3	1.017677	-1.08655	1.406671	0.16303	-4.48701	0.716546	0.55493
NK.cells	TMEM14C	0.134206	6.950542	1.406616	0.163047	-5.89899	0.634968	0.443797
NK.cells	ATRNL1	0.204729	6.820202	1.40648	0.163087	-5.71452	0.636209	0.445418
NK.cells	GM33677	1.034739	-1.06476	1.406403	0.16311	-4.49887	0.716328	0.554604
NK.cells	LSM5	-0.15478	5.652829	-1.40604	0.163216	-5.72242	0.647446	0.460217
NK.cells	ADPRHL2	0.211279	3.241714	1.405751	0.163303	-5.18632	0.671348	0.49226
NK.cells	ROR1	-0.56448	1.374979	-1.40535	0.163421	-4.77939	0.690508	0.518625
NK.cells	PLXNA4OS	-0.78556	0.51532	-1.40508	0.163502	-4.55746	0.699526	0.531204
NK.cells	COMMD10	0.169675	4.694613	1.404837	0.163574	-5.55047	0.656832	0.473009
NK.cells	ATL3	0.131356	5.439187	1.404645	0.16363	-5.68593	0.649526	0.463432
NK.cells	UPF1	-0.1224	5.35091	-1.40443	0.163694	-5.65797	0.650387	0.464613
NK.cells	BEX3	-0.16856	4.990762	-1.40394	0.163841	-5.6461	0.653916	0.46956
NK.cells	UCKL1	0.200077	3.705129	1.403812	0.163878	-5.34591	0.666681	0.486742
NK.cells	GM15965	0.429398	2.027609	1.40339	0.164003	-4.90157	0.683744	0.510143
NK.cells	ARID1B	-0.10521	8.198201	-1.40306	0.164101	-6.07743	0.623221	0.429681
NK.cells	TNNI1	-0.67554	-0.907	-1.40305	0.164104	-4.67291	0.71472	0.553503

NK.cells	MTHFD1L	0.150486	5.661426	1.403048	0.164105	-5.7784	0.647362	0.461121
NK.cells	SULT1A1	-0.34326	3.923228	-1.40271	0.164205	-5.24849	0.664497	0.484074
NK.cells	ZBTB5	0.34978	2.463409	1.402692	0.16421	-4.95283	0.679267	0.504141
NK.cells	IL16	0.259333	4.60831	1.402536	0.164257	-5.45127	0.657685	0.475002
NK.cells	YIPF3	-0.15182	4.904587	-1.4022	0.164356	-5.56913	0.654763	0.47115
NK.cells	DPH5	0.217608	3.732552	1.401886	0.16445	-5.33951	0.666406	0.486842
NK.cells	GM30881	0.438495	1.986079	1.401809	0.164473	-4.79554	0.684172	0.5111
NK.cells	C1QB	-0.43481	6.51237	-1.40118	0.16466	-5.60909	0.639151	0.450653
NK.cells	SSBP4	0.146634	4.90464	1.401159	0.164667	-5.62147	0.654762	0.471283
NK.cells	OPLAH	0.516995	1.500611	1.401117	0.164679	-4.72513	0.6892	0.518097
NK.cells	ALDH8A1	-0.51983	2.197617	-1.40095	0.16473	-4.91172	0.681993	0.508242
NK.cells	AFAP1	-0.38354	1.955044	-1.40086	0.164755	-4.86057	0.684492	0.511682
NK.cells	FASTK	0.252265	3.15048	1.400712	0.1648	-5.13744	0.672271	0.494976
NK.cells	ACAD8	0.315242	2.476536	1.400202	0.164952	-4.97344	0.679132	0.50447
NK.cells	RNF19A	0.154387	5.375931	1.400191	0.164956	-5.66825	0.650143	0.465363
NK.cells	DEFB1	-0.71768	0.86149	-1.40013	0.164975	-4.67666	0.695879	0.527633
NK.cells	GOLGA5	0.149765	4.913418	1.399645	0.165118	-5.56344	0.654676	0.471563
NK.cells	CENPE	-0.34391	4.9887	-1.39963	0.165122	-5.60212	0.653936	0.470576
NK.cells	PID1	-0.29001	5.484883	-1.39955	0.165148	-5.48536	0.64908	0.46412
NK.cells	LRFN1	-0.69565	1.097146	-1.39944	0.165181	-4.66027	0.693409	0.524383
NK.cells	MRPL40	0.188543	4.651456	1.399421	0.165186	-5.4944	0.657259	0.475018
NK.cells	RO60	-0.33238	2.493379	-1.39934	0.165209	-4.92714	0.67896	0.504419
NK.cells	IKZF1	0.121316	8.378161	1.399339	0.16521	-6.14255	0.621547	0.428209
NK.cells	NUSAP1	-0.27823	5.649629	-1.39892	0.165335	-5.75641	0.647477	0.462041
NK.cells	FBXO42	0.153585	5.871653	1.398847	0.165357	-5.73563	0.645323	0.459221
NK.cells	KLF11	-0.2196	3.555665	-1.3988	0.165371	-5.15718	0.668183	0.489816
NK.cells	BATF3	0.515465	2.332688	1.39873	0.165392	-4.84478	0.680606	0.506795
NK.cells	NMRAL1	-0.31199	3.904291	-1.39872	0.165395	-5.13674	0.664686	0.485113
NK.cells	LAMP1	0.131632	7.757865	1.398635	0.165421	-5.97087	0.627339	0.435766
NK.cells	DCAF12	0.139721	6.638146	1.39843	0.165482	-5.8736	0.637947	0.449653
NK.cells	TIMM13	0.127502	6.732513	1.398254	0.165535	-5.87573	0.637045	0.448482
NK.cells	CPNE9	-0.70543	1.867904	-1.39811	0.165578	-4.71277	0.685392	0.513583
NK.cells	ARSB	-0.21601	4.938464	-1.39738	0.165795	-5.811	0.65443	0.471799
NK.cells	GM3448	-0.47815	1.9113	-1.39726	0.165833	-4.77782	0.684944	0.513261
NK.cells	ASXL1	-0.12556	7.242343	-1.39722	0.165845	-5.95594	0.632199	0.442488
NK.cells	GM27008	0.46028	1.012976	1.397115	0.165876	-4.77847	0.69429	0.526281
NK.cells	GM16124	0.374485	2.669782	1.397083	0.165885	-5.04509	0.677157	0.502602
NK.cells	POSTN	-0.6977	1.260435	-1.39689	0.165942	-4.67033	0.691702	0.522785
NK.cells	MTFMT	-0.2459	2.527907	-1.39686	0.165953	-5.05175	0.678607	0.504695
NK.cells	WBP1L	0.164404	5.329247	1.39669	0.166003	-5.6316	0.650599	0.46689
NK.cells	CWC22	0.215907	3.900011	1.396552	0.166044	-5.3291	0.664729	0.485912
NK.cells	TCF4	-0.21645	7.694523	-1.39649	0.166064	-5.73354	0.627934	0.437211
NK.cells	SRRD	-0.19581	3.465767	-1.39642	0.166083	-5.3558	0.669087	0.491853
NK.cells	BC031181	0.123662	5.703047	1.396178	0.166157	-5.69605	0.646958	0.462253
NK.cells	MBLAC2	0.274669	2.64789	1.395871	0.166249	-5.15139	0.677381	0.503385
NK.cells	AK7	-0.55673	1.686834	-1.3958	0.166269	-4.79161	0.687267	0.517019
NK.cells	ZFP944	-0.20597	4.397621	-1.39542	0.166384	-5.40022	0.659772	0.479535

NK.cells	BAHD1	0.307378	2.543391	1.395327	0.166412	-5.01251	0.678448	0.504929
NK.cells	PMF1	-0.19096	5.697613	-1.39496	0.166522	-5.66784	0.647011	0.462534
NK.cells	LMBR1	-0.42868	1.883516	-1.39491	0.166538	-4.82281	0.685231	0.514326
NK.cells	EFNA5	-0.81716	2.313548	-1.3948	0.16657	-4.71509	0.680803	0.508267
NK.cells	RARS2	0.249469	3.441715	1.39448	0.166667	-5.16905	0.66933	0.492609
NK.cells	LPIN2	0.168197	6.528551	1.394438	0.16668	-5.79358	0.638996	0.452046
NK.cells	SHROOM4	-0.80711	0.86592	-1.39405	0.166797	-4.62744	0.695833	0.529354
NK.cells	RAB28	-0.19855	4.828065	-1.3937	0.166903	-5.42145	0.655516	0.474226
NK.cells	SMOX	0.20953	5.116761	1.39359	0.166935	-5.82221	0.652679	0.470489
NK.cells	SCAPER	0.143471	6.110801	1.393358	0.167004	-5.81665	0.643012	0.457685
NK.cells	PCDH17	-0.59367	2.459624	-1.39325	0.167038	-4.80621	0.679305	0.506666
NK.cells	BYSL	-0.18034	3.67185	-1.39319	0.167055	-5.33263	0.667015	0.489863
NK.cells	TSPAN31	0.238787	4.424978	1.393157	0.167065	-5.31421	0.6595	0.479701
NK.cells	MYZAP	-0.43876	3.284402	-1.39315	0.167066	-4.86498	0.670917	0.495173
NK.cells	PEX16	0.21804	3.474463	1.392767	0.167183	-5.25309	0.669	0.492768
NK.cells	AU020206	0.155522	5.320744	1.392526	0.167256	-5.72852	0.650682	0.468205
NK.cells	UBALD1	-0.17848	4.983613	-1.3925	0.167264	-5.55477	0.653986	0.47262
NK.cells	A1CF	-0.69126	0.569704	-1.39179	0.167478	-4.66604	0.698952	0.534637
NK.cells	EXOSC10	-0.16117	4.93082	-1.39167	0.167513	-5.59914	0.654505	0.473633
NK.cells	CDK10	-0.33554	2.234353	-1.39157	0.167543	-4.95476	0.681616	0.510545
NK.cells	ECT2	-0.3392	4.373594	-1.39117	0.167663	-5.41496	0.66001	0.48118
NK.cells	NUDT8	0.36835	2.208161	1.390888	0.16775	-4.93195	0.681885	0.511197
NK.cells	GM16794	0.548977	0.41499	1.390873	0.167755	-4.67438	0.700586	0.537315
NK.cells	FAM20A	-0.4988	1.833508	-1.39019	0.16796	-4.89619	0.685748	0.516831
NK.cells	GART	0.208938	4.367941	1.390188	0.167962	-5.47832	0.660066	0.481644
NK.cells	SLAMF9	-0.81046	2.637226	-1.39013	0.167979	-4.62229	0.67749	0.505408
NK.cells	CALU	-0.15648	4.640586	-1.38998	0.168023	-5.52087	0.657366	0.478073
NK.cells	TMCO4	0.202562	4.054604	1.389956	0.168032	-5.49356	0.663184	0.485935
NK.cells	EFCAB11	-0.3249	3.410869	-1.38991	0.168047	-5.25259	0.669641	0.494729
NK.cells	GM12596	-0.2719	2.627238	-1.38989	0.168052	-5.2314	0.677591	0.505634
NK.cells	MRPS18A	0.162409	5.09387	1.389605	0.168138	-5.57045	0.652903	0.472158
NK.cells	CLEC4A3	-0.75209	3.297848	-1.38936	0.168213	-4.80334	0.670781	0.49648
NK.cells	PXYLP1	-0.33125	2.352535	-1.38905	0.168305	-5.02914	0.680403	0.509883
NK.cells	AV099323	-0.61492	1.209882	-1.38877	0.168391	-4.70144	0.69223	0.52647
NK.cells	SLC3A2	-0.15045	6.772545	-1.38874	0.168401	-5.9551	0.636663	0.450973
NK.cells	RBPJ	-0.19918	6.055966	-1.38841	0.168501	-5.90109	0.643541	0.460166
NK.cells	PTPMT1	0.180796	4.267152	1.387911	0.168652	-5.40453	0.661068	0.483857
NK.cells	CSF2RB2	0.710289	2.359571	1.387503	0.168776	-4.64069	0.68033	0.510429
NK.cells	0610039K1	0.936342	0.022539	1.387468	0.168786	-4.52137	0.704751	0.544672
NK.cells	NAPG	0.175574	4.542195	1.387274	0.168845	-5.5248	0.658339	0.480351
NK.cells	SEC61A2	0.209575	3.741527	1.387227	0.16886	-5.30411	0.666316	0.491176
NK.cells	GPR18	0.195077	4.514903	1.387199	0.168868	-5.65317	0.658609	0.480716
NK.cells	2310040G2	0.445451	1.291307	1.38718	0.168874	-4.66037	0.69138	0.525815
NK.cells	TMEM156	0.229155	3.855526	1.386901	0.168959	-5.31906	0.665174	0.489621
NK.cells	ABCD1	-0.17802	4.771292	-1.38688	0.168965	-5.44378	0.656076	0.477297
NK.cells	TRIM26	-0.15162	5.330265	-1.38682	0.168984	-5.67816	0.650589	0.469927
NK.cells	NCAM1	-0.78503	1.275146	-1.38663	0.169042	-4.75596	0.691549	0.52606

NK.cells	STK10	-0.1172	7.315855	-1.38661	0.169046	-6.06256	0.631503	0.444663
NK.cells	CNRIP1	-0.54742	1.648634	-1.38659	0.169052	-4.74906	0.687663	0.520628
NK.cells	GM12064	-0.70148	0.96957	-1.38655	0.169065	-4.57913	0.694745	0.530545
NK.cells	UBIAD1	0.335683	1.941616	1.386463	0.169092	-4.86409	0.684631	0.516434
NK.cells	TMEM251	0.184116	4.619848	1.386058	0.169215	-5.45855	0.657571	0.479445
NK.cells	PSMA7	0.120739	7.683799	1.385815	0.169289	-6.07854	0.628035	0.44028
NK.cells	CARHSP1	-0.18809	5.430457	-1.38557	0.169363	-5.6136	0.649611	0.468793
NK.cells	APOBEC4	-0.68964	0.042895	-1.38549	0.169388	-4.60819	0.704535	0.544567
NK.cells	GABPB1	-0.14079	5.380129	-1.3852	0.169478	-5.70145	0.650102	0.469609
NK.cells	ARHGAP30	0.104794	7.139253	1.38493	0.169559	-5.96581	0.633175	0.447245
NK.cells	HOMER2	-0.62507	0.275337	-1.38483	0.169589	-4.68732	0.702065	0.541389
NK.cells	SGSH	-0.44176	1.453355	-1.38471	0.169626	-4.84372	0.689692	0.523986
NK.cells	DNAJC2	0.128903	5.81945	1.384683	0.169634	-5.7536	0.645829	0.46405
NK.cells	CD200R2	0.332817	0.789771	1.384615	0.169655	-5.3445	0.696633	0.533761
NK.cells	TUBGCP4	0.176802	4.42498	1.384293	0.169753	-5.5074	0.6595	0.482487
NK.cells	CYP4A31	-0.65535	0.922941	-1.38416	0.169794	-4.7057	0.695234	0.531883
NK.cells	GSTM4	-0.58965	1.121715	-1.38395	0.169856	-4.71217	0.693152	0.529102
NK.cells	TBC1D8B	-0.21962	3.257326	-1.3837	0.169935	-5.2904	0.671191	0.498708
NK.cells	4930404N1	-0.73894	0.191025	-1.38355	0.16998	-4.58845	0.70296	0.543122
NK.cells	TMTC1	-0.59203	2.714836	-1.38349	0.169997	-4.90571	0.676698	0.506346
NK.cells	SLC30A7	0.159615	5.62501	1.383044	0.170134	-5.69069	0.647716	0.467109
NK.cells	TYMS	-0.27154	4.922548	-1.38303	0.170138	-5.61439	0.654586	0.476334
NK.cells	GM17259	0.561181	1.365713	1.383014	0.170143	-4.72488	0.690604	0.525879
NK.cells	ZFP654	0.153852	6.120389	1.382761	0.170221	-5.83334	0.642919	0.460787
NK.cells	NAIP6	-0.31127	2.310523	-1.38233	0.170354	-5.09572	0.680834	0.512587
NK.cells	STOML1	0.429821	2.251865	1.382278	0.170368	-4.84406	0.681436	0.513424
NK.cells	ASB1	-0.4029	1.712368	-1.38211	0.170421	-4.85471	0.687002	0.521276
NK.cells	GGPS1	0.116943	5.393996	1.381698	0.170546	-5.71251	0.649967	0.470652
NK.cells	LRRC8A	-0.1493	5.427019	-1.38159	0.170579	-5.66965	0.649645	0.470219
NK.cells	CHN2	-0.22129	5.358074	-1.38153	0.170598	-5.96894	0.650318	0.471123
NK.cells	COA3	0.14788	5.812938	1.381382	0.170643	-5.73158	0.645892	0.46524
NK.cells	FSTL1	-0.59521	2.107543	-1.38129	0.17067	-4.77194	0.68292	0.515798
NK.cells	BCL11A	-0.35635	5.196907	-1.38126	0.17068	-5.22996	0.651894	0.473314
NK.cells	RHOV	0.928202	-0.80702	1.38052	0.170907	-4.57455	0.714358	0.559634
NK.cells	ATG7	-0.15886	5.786606	-1.3802	0.171006	-5.77904	0.646964	0.465896
NK.cells	PLXNC1	-0.20202	5.427157	-1.37973	0.171149	-5.75324	0.650785	0.47075
NK.cells	COPRS	-0.58207	0.717795	-1.37956	0.171202	-4.68191	0.698617	0.53666
NK.cells	KIF20A	-0.34253	3.768581	-1.3792	0.171312	-5.40298	0.667258	0.493088
NK.cells	TSPAN13	0.157451	6.684078	1.379162	0.171324	-5.81978	0.63867	0.454648
NK.cells	RGS10	0.151573	5.509055	1.378396	0.171559	-5.74107	0.65033	0.470124
NK.cells	F7	-0.62266	0.719823	-1.37828	0.171593	-4.72579	0.698965	0.537207
NK.cells	SMAD6	0.590031	2.733914	1.378211	0.171616	-4.84715	0.678052	0.507958
NK.cells	HNF4A	-0.68941	0.691681	-1.37817	0.171628	-4.65626	0.699263	0.537627
NK.cells	SSR4	0.125118	7.573816	1.378001	0.171681	-6.05915	0.630509	0.44398
NK.cells	CRYBG3	0.250105	4.474287	1.377599	0.171804	-5.30255	0.660782	0.4842
NK.cells	RALB	-0.23862	4.247517	-1.37735	0.171882	-5.1309	0.663062	0.487336
NK.cells	AC166172.	0.560465	0.939575	1.377143	0.171945	-4.71412	0.696951	0.534338

NK.cells	MAFF	-0.23587	3.876198	-1.37704	0.171977	-5.53213	0.666777	0.492435
NK.cells	COX8A	0.095456	9.008145	1.376203	0.172235	-6.29106	0.618129	0.427059
NK.cells	MAML2	-0.1547	7.972039	-1.37597	0.172305	-6.15429	0.627829	0.439687
NK.cells	AA467197	0.543032	0.801131	1.375379	0.172489	-5.041	0.699813	0.537011
NK.cells	PLAC8	0.402333	8.2646	1.375073	0.172583	-6.00155	0.625557	0.436336
NK.cells	RCC1L	0.288711	3.072184	1.374887	0.172641	-5.1397	0.676258	0.504271
NK.cells	HGF	-0.56529	2.743326	-1.37433	0.172813	-4.90895	0.679617	0.509139
NK.cells	SOD2	0.28635	6.929524	1.374142	0.172871	-5.96994	0.638182	0.453145
NK.cells	SLC25A5	0.116582	7.608641	1.373995	0.172917	-6.0639	0.631725	0.444716
NK.cells	9330111N(	0.637118	0.392898	1.373961	0.172927	-4.70669	0.704146	0.543587
NK.cells	HTR7	1.083825	0.754108	1.373899	0.172946	-4.62435	0.700316	0.538168
NK.cells	YOD1	0.238568	4.084266	1.373648	0.173024	-5.41764	0.666034	0.490676
NK.cells	TIMM17A	0.137215	5.135955	1.373535	0.173059	-5.64141	0.655587	0.476553
NK.cells	CD40	-0.7573	2.17854	-1.37314	0.17318	-4.70535	0.685427	0.517625
NK.cells	GNG12	0.164197	6.49518	1.37309	0.173197	-5.71703	0.642349	0.459032
NK.cells	LYRM9	0.296977	2.696887	1.372979	0.173231	-5.12835	0.680093	0.510251
NK.cells	ST5	-0.44515	2.235439	-1.37292	0.173249	-4.85988	0.684839	0.516853
NK.cells	ZBTB4	-0.22528	3.655496	-1.37274	0.173305	-5.31729	0.670345	0.496928
NK.cells	ANAPC11	0.149056	5.923842	1.372602	0.173348	-5.79208	0.647878	0.466583
NK.cells	RGS19	0.170434	5.393744	1.372322	0.173435	-5.67594	0.653053	0.473604
NK.cells	CD164L2	-0.5785	1.352514	-1.37224	0.17346	-4.74621	0.694019	0.529988
NK.cells	CEP55	-0.35648	3.678611	-1.37202	0.173527	-5.35988	0.670112	0.49681
NK.cells	CTDSP1	-0.15915	5.3236	-1.37186	0.173577	-5.67152	0.653742	0.474671
NK.cells	IGF2	-0.48473	4.193244	-1.37161	0.173655	-5.45466	0.664943	0.489952
NK.cells	PITPNB	0.1206	5.507244	1.371603	0.173658	-5.72917	0.651941	0.472353
NK.cells	CISD3	0.253228	3.000972	1.371313	0.173748	-5.30558	0.677119	0.506495
NK.cells	TBC1D10A	0.196227	3.56507	1.371105	0.173812	-5.38826	0.671426	0.498659
NK.cells	E130309D(	-0.19583	3.983364	-1.37087	0.173885	-5.38396	0.66728	0.49295
NK.cells	4930526L0	-0.79306	-0.8055	-1.37018	0.174099	-4.54264	0.717912	0.563517
NK.cells	UBE4BOS1	-0.55678	0.735049	-1.36996	0.174169	-4.80521	0.701456	0.540047
NK.cells	SOCS6	-0.2021	3.829946	-1.36965	0.174266	-5.29272	0.669606	0.495625
NK.cells	FRMPD4	0.832468	0.351798	1.369498	0.174312	-4.59768	0.705657	0.545947
NK.cells	ZFP652OS	-0.68961	-0.171	-1.36899	0.17447	-4.60957	0.711666	0.554077
NK.cells	RDH16F2	-0.64896	1.257357	-1.36862	0.174587	-4.78158	0.696727	0.532765
NK.cells	FERMT2	-0.35538	3.451103	-1.36733	0.174988	-5.09274	0.674749	0.501621
NK.cells	MEN1	-0.32111	2.866329	-1.3673	0.174996	-5.05366	0.680718	0.509849
NK.cells	LGALSL	-0.49068	1.281509	-1.36721	0.175025	-4.81913	0.697179	0.532818
NK.cells	MED20	-0.18723	4.104119	-1.36646	0.17526	-5.40078	0.668151	0.492881
NK.cells	MIS18A	-0.19282	4.400206	-1.36627	0.175318	-5.55885	0.665182	0.488864
NK.cells	GPR183	0.240481	3.488292	1.366236	0.17533	-5.5905	0.674371	0.501439
NK.cells	AIMP2	0.272058	3.206699	1.366147	0.175358	-5.21913	0.677237	0.505414
NK.cells	MAP11	0.29138	2.866952	1.366107	0.17537	-5.14337	0.680711	0.510235
NK.cells	ABCB4	-0.36589	3.391454	-1.36601	0.175401	-5.10438	0.675355	0.502843
NK.cells	NIPSNAP1	-0.28396	3.134643	-1.36601	0.175402	-5.19817	0.677972	0.506449
NK.cells	RWDD1	0.112639	6.105394	1.36596	0.175416	-5.8752	0.648362	0.466256
NK.cells	FAU	0.068086	11.4506	1.365851	0.17545	-6.68194	0.598623	0.401838
NK.cells	TIRAP	0.320755	3.005516	1.365699	0.175498	-5.00559	0.679292	0.508313

NK.cells	SLC36A3	-0.65062	0.101985	-1.36555	0.175545	-4.65794	0.709705	0.551126
NK.cells	FZD1	-0.68674	-0.15494	-1.36522	0.175647	-4.60751	0.712655	0.555147
NK.cells	CEP250	-0.18617	4.458141	-1.36492	0.175741	-5.54482	0.664895	0.488431
NK.cells	VPS50	0.180416	4.131766	1.36387	0.176071	-5.41214	0.668166	0.493172
NK.cells	NCAPG2	-0.22739	5.068495	-1.36385	0.176076	-5.59493	0.658823	0.480478
NK.cells	CHAF1A	-0.26107	4.186029	-1.36375	0.176109	-5.46222	0.667621	0.492462
NK.cells	CAMK2D	0.179662	7.214604	1.363447	0.176203	-5.99771	0.637953	0.45267
NK.cells	GM16014	0.831167	-0.09363	1.36342	0.176212	-4.5873	0.712119	0.554715
NK.cells	LRMDA	0.216817	7.760594	1.363388	0.176222	-6.21074	0.63276	0.445843
NK.cells	RMDN3	0.211848	3.398108	1.363365	0.176229	-5.24654	0.675585	0.503411
NK.cells	C1QC	-0.44825	6.384084	-1.36302	0.176338	-5.63602	0.645942	0.463395
NK.cells	SLC46A3	-0.3206	3.085031	-1.36294	0.176363	-5.07685	0.678777	0.508005
NK.cells	FXYD4	-0.30445	3.001862	-1.36293	0.176365	-5.18066	0.679628	0.509182
NK.cells	ST6GAL1	0.265942	5.862113	1.362672	0.176447	-5.72505	0.651019	0.470318
NK.cells	CPNE2	-0.38356	3.37335	-1.36215	0.17661	-5.06776	0.675836	0.504299
NK.cells	TRAPPC2L	0.140853	5.811456	1.361799	0.176721	-5.75723	0.651514	0.471313
NK.cells	NUCB2	-0.31867	4.304904	-1.36174	0.17674	-4.92217	0.666429	0.491501
NK.cells	LSM4	0.118351	6.704001	1.361565	0.176795	-5.95488	0.642851	0.459776
NK.cells	A430033K(	0.58026	0.6406	1.361446	0.176832	-4.75165	0.704266	0.544315
NK.cells	PDGFB	-0.3995	1.062654	-1.361	0.176971	-5.15315	0.699793	0.538192
NK.cells	EIF4EBP1	0.170147	5.454955	1.360783	0.177041	-5.55234	0.65501	0.476348
NK.cells	MAVS	0.278786	2.932268	1.360666	0.177078	-5.12753	0.680341	0.511005
NK.cells	PCCA	0.186452	4.599387	1.360577	0.177106	-5.59253	0.663484	0.4879
NK.cells	ANK1	0.669278	1.273318	1.360419	0.177156	-4.81441	0.697572	0.535249
NK.cells	CARNS1	-0.47479	2.476067	-1.36025	0.17721	-4.92252	0.685033	0.517659
NK.cells	GM34921	-0.6741	0.841113	-1.35999	0.177289	-4.6434	0.702137	0.541814
NK.cells	VILL	-0.56091	1.228935	-1.35958	0.177419	-4.73311	0.69804	0.536185
NK.cells	LYN	-0.12884	10.03311	-1.35954	0.177433	-6.40724	0.611651	0.419694
NK.cells	ZFP809	0.233092	3.64912	1.359511	0.177442	-5.36407	0.673037	0.501297
NK.cells	SNHG6	0.263752	3.414627	1.359208	0.177538	-5.23907	0.675417	0.504658
NK.cells	PDE2A	-0.21548	6.050057	-1.35919	0.177542	-5.77284	0.649186	0.468961
NK.cells	LONRF3	-0.49389	1.525709	-1.35872	0.17769	-4.80645	0.694921	0.532084
NK.cells	ABI2	-0.21011	3.903545	-1.35869	0.177701	-5.48928	0.670464	0.498043
NK.cells	IFI208	0.334391	2.936618	1.358564	0.177741	-5.38457	0.680296	0.511663
NK.cells	GTF2IRD2	0.202018	3.944401	1.358554	0.177744	-5.421	0.670052	0.497514
NK.cells	PTGIS	0.961229	-0.12832	1.358536	0.17775	-4.6373	0.712492	0.557151
NK.cells	JUP	-0.25778	3.562597	-1.35841	0.17779	-5.29402	0.673914	0.502866
NK.cells	4930453N2	0.161464	4.481233	1.35835	0.177809	-5.56575	0.664664	0.490173
NK.cells	CCZ1	-0.11936	6.158644	-1.35806	0.177901	-5.83072	0.648129	0.467889
NK.cells	GIMAP5	0.227197	3.319876	1.358024	0.177912	-5.51263	0.676381	0.506363
NK.cells	CHCHD4	0.243396	3.820424	1.357888	0.177955	-5.2729	0.671304	0.499415
NK.cells	4921524J1	-0.12524	5.474163	-1.35761	0.178041	-5.72947	0.654821	0.477003
NK.cells	ICAM1	-0.19345	5.898773	-1.3576	0.178046	-5.82823	0.650661	0.471397
NK.cells	POLI	0.439551	1.527716	1.357517	0.178072	-4.82233	0.6949	0.532366
NK.cells	IMMT	0.113611	6.049157	1.357393	0.178111	-5.83454	0.649195	0.469428
NK.cells	CABCOCO1	0.580649	0.315412	1.357145	0.17819	-4.67795	0.707733	0.550701
NK.cells	BRIP1	-0.22197	4.898207	-1.35697	0.178245	-5.61722	0.660511	0.484809

NK.cells	B430010I2	0.775739	-0.41456	1.356881	0.178273	-4.56275	0.71558	0.561977
NK.cells	SLC4A2	-0.2093	3.559435	-1.3566	0.178361	-5.31834	0.673946	0.503304
NK.cells	MPG	0.192313	3.63806	1.356396	0.178427	-5.37133	0.673149	0.50231
NK.cells	TBL1X	-0.12467	7.155308	-1.35635	0.178442	-5.99728	0.638519	0.455459
NK.cells	DLG1	-0.11316	7.219142	-1.35609	0.178525	-6.01824	0.637909	0.454705
NK.cells	UTY	2.673035	2.644423	1.355945	0.17857	-5.25589	0.683298	0.516485
NK.cells	ABCB7	0.129617	5.334608	1.355914	0.17858	-5.7135	0.656195	0.479228
NK.cells	RHOJ	0.575325	1.992484	1.355532	0.178701	-4.78658	0.690163	0.52608
NK.cells	LRP6	0.183798	5.378913	1.355467	0.178722	-5.6266	0.65587	0.478789
NK.cells	ZBP1	0.369781	3.640152	1.354828	0.178924	-5.592	0.673557	0.502752
NK.cells	FAM210B	0.199334	3.914295	1.354659	0.178978	-5.50621	0.670784	0.499008
NK.cells	TMED5	-0.08975	7.227678	-1.35465	0.178981	-6.03733	0.638235	0.455038
NK.cells	KAZN	-0.77689	0.348768	-1.35446	0.179042	-4.62443	0.707828	0.551094
NK.cells	MCUB	0.490163	1.557619	1.354218	0.179118	-4.95988	0.69503	0.532895
NK.cells	SIAE	-0.33534	2.612072	-1.35415	0.17914	-5.0171	0.684068	0.517498
NK.cells	TRIM34A	0.322189	3.308515	1.352992	0.179508	-5.268	0.677904	0.508085
NK.cells	FARP1	-0.21357	3.619271	-1.35298	0.17951	-5.51399	0.67474	0.50371
NK.cells	STOX2	-0.59966	3.196694	-1.35247	0.179673	-4.97985	0.679417	0.509845
NK.cells	YPEL1	-0.31996	2.4444	-1.35206	0.179805	-5.01303	0.687161	0.520726
NK.cells	C130050O:	-0.6531	1.379067	-1.35203	0.179816	-4.66657	0.69829	0.536384
NK.cells	SART3	-0.13759	5.374984	-1.35197	0.179832	-5.71814	0.65752	0.479967
NK.cells	TNFAIP8L2	0.205952	3.852483	1.351616	0.179946	-5.42925	0.672835	0.500882
NK.cells	KCNK6	0.475824	2.306704	1.351545	0.179969	-4.78031	0.688684	0.522897
NK.cells	GM48226	0.346074	2.396833	1.351255	0.180061	-4.97822	0.687889	0.521678
NK.cells	HIC2	-0.30912	2.712677	-1.35091	0.180172	-5.14137	0.684831	0.517166
NK.cells	MAP4K3	0.221295	4.283134	1.350418	0.180328	-5.45987	0.669022	0.495193
NK.cells	LXN	-0.21861	3.855864	-1.3504	0.180336	-5.32187	0.673336	0.501117
NK.cells	ZFP703	-0.21884	4.364442	-1.35021	0.180396	-5.54535	0.668209	0.494145
NK.cells	EBPL	-0.19585	4.469152	-1.35004	0.180449	-5.50552	0.667158	0.492789
NK.cells	MAPK1IP1	0.332439	2.304416	1.349566	0.180601	-4.95581	0.689625	0.523475
NK.cells	CERKL	0.3964	1.852174	1.348892	0.180816	-5.0149	0.694446	0.53028
NK.cells	CALML4	-0.5964	1.801954	-1.3488	0.180846	-4.79128	0.694972	0.531085
NK.cells	GPR107	0.148499	5.057833	1.348323	0.180999	-5.63212	0.661729	0.485318
NK.cells	ARL14EP	0.232735	3.524764	1.348271	0.181015	-5.29086	0.677163	0.506485
NK.cells	VSIG10L	-0.52481	0.298869	-1.34812	0.181063	-4.68239	0.710918	0.554052
NK.cells	NIPSNAP3I	0.151099	5.573981	1.34787	0.181144	-5.7215	0.656619	0.478581
NK.cells	C9ORF72	0.248992	3.893192	1.347853	0.181149	-5.52067	0.673419	0.501506
NK.cells	MREG	-0.55134	3.199109	-1.34784	0.181154	-5.0025	0.680492	0.511287
NK.cells	MRPL27	0.199881	4.210901	1.347817	0.181161	-5.46929	0.670208	0.497091
NK.cells	MYO1G	0.159052	5.563064	1.347732	0.181188	-5.85278	0.656727	0.478737
NK.cells	PLEKHA8	-0.49425	0.886546	-1.34728	0.181331	-4.73977	0.704978	0.545388
NK.cells	NDUFV1	0.151586	5.20278	1.346247	0.181664	-5.69116	0.66061	0.484106
NK.cells	KCNJ10	-0.69188	-0.1202	-1.34622	0.181672	-4.58268	0.71578	0.561337
NK.cells	GNG4	-0.44026	1.462633	-1.34608	0.18172	-4.97752	0.698878	0.537272
NK.cells	WDR12	0.201349	4.283459	1.346038	0.181732	-5.48223	0.669802	0.496725
NK.cells	SLC7A7	-0.41149	3.785978	-1.34589	0.181778	-4.97173	0.674834	0.503661
NK.cells	LZTR1	0.261166	2.612289	1.345849	0.181792	-5.18304	0.686867	0.52038

NK.cells	MAPK14	-0.11021	6.656022	-1.3458	0.18181	-5.96836	0.646361	0.464985
NK.cells	KIDINS220	0.13586	5.419693	1.345515	0.1819	-5.75362	0.658462	0.481383
NK.cells	INTS14	-0.23186	4.691853	-1.34548	0.18191	-5.57451	0.665702	0.491237
NK.cells	PDK3	0.200879	4.945028	1.345381	0.181943	-5.64986	0.663173	0.487843
NK.cells	PARD6A	-0.31638	2.688858	-1.34526	0.181983	-5.16388	0.686075	0.519491
NK.cells	CYP2D22	-0.5975	0.864397	-1.34498	0.182072	-4.71844	0.705216	0.546546
NK.cells	TMEM167I	-0.2362	3.755594	-1.34474	0.182148	-5.33255	0.675142	0.504357
NK.cells	1110051M	0.273032	3.263469	1.344579	0.182201	-5.16106	0.680162	0.511376
NK.cells	RTN4RL1	-0.3141	2.381813	-1.34456	0.182206	-5.34866	0.689257	0.524074
NK.cells	GBP8	0.263573	3.65151	1.34433	0.182281	-5.46571	0.676201	0.505898
NK.cells	2010320M	0.395093	2.480423	1.344317	0.182285	-4.91377	0.688234	0.522653
NK.cells	KPNB1	0.130243	5.739818	1.343541	0.182535	-5.76766	0.655773	0.47765
NK.cells	ANKIB1	0.125713	5.807453	1.343425	0.182572	-5.84638	0.655108	0.476765
NK.cells	SOX4	-0.33581	5.508952	-1.34331	0.182608	-5.58083	0.65805	0.480809
NK.cells	ZFP512B	0.364302	2.377698	1.343216	0.18264	-5.05832	0.689792	0.524592
NK.cells	GPLD1	-0.60775	0.934219	-1.34253	0.182862	-4.74935	0.70525	0.546354
NK.cells	IMPG2	-0.66169	0.6613	-1.34248	0.182876	-4.73034	0.708161	0.550529
NK.cells	F2RL1	0.501625	-1.13295	1.342474	0.182879	-4.77955	0.727463	0.578627
NK.cells	KCTD1	0.344763	1.719442	1.341442	0.183213	-5.01197	0.697186	0.535167
NK.cells	MMACHC	0.704726	0.470101	1.341276	0.183266	-4.65725	0.710456	0.554179
NK.cells	IGSF6	0.570339	3.689623	1.341075	0.183331	-4.95686	0.676794	0.506782
NK.cells	PLEKHB2	0.166077	4.419141	1.340715	0.183448	-5.53405	0.669407	0.496774
NK.cells	MED31	-0.25139	3.491551	-1.34057	0.183495	-5.1873	0.678815	0.509812
NK.cells	EIF3M	0.086804	6.621301	1.340497	0.183518	-5.98154	0.647636	0.467306
NK.cells	NDUFC1	0.135039	7.367214	1.340399	0.18355	-6.05082	0.64044	0.45771
NK.cells	POLH	-0.2074	4.035992	-1.3402	0.183615	-5.42872	0.673275	0.502195
NK.cells	ANKRD46	0.285868	2.377526	1.340085	0.183652	-4.95418	0.690302	0.525911
NK.cells	MGST3	0.326128	3.846236	1.339523	0.183834	-5.23875	0.6752	0.505083
NK.cells	DHX30	0.173705	4.509922	1.339512	0.183837	-5.5575	0.668494	0.495839
NK.cells	CD180	-0.44196	5.032672	-1.33942	0.183868	-4.98296	0.663262	0.488694
NK.cells	GM34084	0.821412	1.964286	1.339312	0.183902	-4.75341	0.694616	0.532302
NK.cells	MAD2L1	-0.2432	4.166093	-1.33909	0.183974	-5.47191	0.671959	0.500695
NK.cells	DYNLL1	-0.10526	7.867224	-1.33896	0.184017	-6.13312	0.635666	0.451688
NK.cells	NFKBIA	0.167668	8.130992	1.33895	0.184019	-6.2147	0.633163	0.448385
NK.cells	CLN6	0.230604	3.482895	1.338934	0.184024	-5.37298	0.678903	0.510321
NK.cells	1110004F1	0.090546	6.204	1.338886	0.18404	-5.88826	0.651701	0.473096
NK.cells	NLK	0.138578	5.966033	1.338825	0.18406	-5.92853	0.654032	0.476241
NK.cells	ANAPC10	0.15877	4.581596	1.338807	0.184066	-5.5514	0.667774	0.494955
NK.cells	AQP9	-0.41146	2.330322	-1.33845	0.184181	-5.03335	0.691016	0.527069
NK.cells	EDC3	0.202149	3.628178	1.338013	0.184323	-5.34241	0.677954	0.508504
NK.cells	TGFBRAP1	0.201342	3.494101	1.337804	0.184391	-5.29639	0.679368	0.510462
NK.cells	POLA2	0.218575	4.050563	1.337204	0.184586	-5.45368	0.674125	0.502896
NK.cells	SDHC	0.150582	5.09505	1.337014	0.184648	-5.6614	0.663622	0.488526
NK.cells	PTPRS	-0.23786	4.390471	-1.33656	0.184796	-5.3702	0.670687	0.498417
NK.cells	AHCYL1	-0.12303	5.179051	-1.33653	0.184804	-5.67562	0.662785	0.487599
NK.cells	KLHDC2	0.133317	4.860142	1.336368	0.184858	-5.7405	0.665969	0.492034
NK.cells	ANKRD27	0.246226	3.098779	1.33606	0.184958	-5.2231	0.683853	0.516859

NK.cells	PTPRE	0.165656	5.539856	1.335962	0.18499	-5.81114	0.659204	0.482945
NK.cells	NEDD4	-0.17131	5.933587	-1.33567	0.185086	-5.83108	0.65532	0.477819
NK.cells	CDC42BPA	-0.36323	3.315927	-1.33557	0.185119	-5.02518	0.68162	0.513997
NK.cells	ORAI1	-0.12053	6.491763	-1.3352	0.185239	-6.0271	0.649856	0.470614
NK.cells	ZKSCAN5	-0.32185	2.402977	-1.33517	0.185247	-5.06643	0.69106	0.527338
NK.cells	ULK2	0.15699	5.457806	1.335116	0.185266	-5.73102	0.660016	0.484359
NK.cells	ANKLE2	0.15698	5.177792	1.334994	0.185306	-5.66884	0.662798	0.488188
NK.cells	ADGRA3	-0.63162	0.459311	-1.33498	0.185309	-4.6399	0.711624	0.55665
NK.cells	MTHFR	-0.17291	3.640415	-1.33486	0.185351	-5.51721	0.678298	0.509568
NK.cells	CXCL9	-1.23472	2.080321	-1.33448	0.185473	-4.7271	0.694626	0.532315
NK.cells	ADPRM	0.210986	3.321315	1.33421	0.185561	-5.29021	0.681758	0.514329
NK.cells	EMC7	-0.10376	6.059195	-1.33418	0.185572	-5.86349	0.654271	0.47659
NK.cells	ACOD1	0.924838	2.318035	1.333915	0.185658	-5.13491	0.692251	0.528981
NK.cells	FBXO6	0.199842	3.653081	1.333618	0.185755	-5.41931	0.678617	0.509832
NK.cells	RBL2	0.151969	4.765126	1.333289	0.185862	-5.72228	0.667546	0.494414
NK.cells	G2E3	0.198731	3.938116	1.332981	0.185963	-5.3959	0.676008	0.506009
NK.cells	DPH6	0.191329	4.47347	1.332853	0.186005	-5.55617	0.670586	0.498563
NK.cells	CD320	-0.34774	1.856288	-1.33259	0.186091	-4.92613	0.697659	0.536303
NK.cells	AK4	0.595225	1.048143	1.332097	0.186252	-4.79047	0.706501	0.548713
NK.cells	RTRAF	0.1179	7.336334	1.332014	0.186279	-6.09278	0.642754	0.460644
NK.cells	ETS2	0.19191	5.865691	1.331339	0.1865	-5.77162	0.657236	0.480096
NK.cells	CDK20	0.671339	0.593617	1.331311	0.186509	-4.66927	0.711534	0.555931
NK.cells	ATP6V1A	0.143884	6.534998	1.331262	0.186525	-5.86892	0.650671	0.471248
NK.cells	EGF	-0.58037	0.634522	-1.33119	0.186549	-4.67853	0.711095	0.555371
NK.cells	NADK2	-0.16986	4.994117	-1.33086	0.186657	-5.6426	0.666037	0.4921
NK.cells	SLC5A10	-0.78753	-0.1778	-1.33072	0.186701	-4.61297	0.720027	0.568255
NK.cells	SECISBP2L	0.159929	4.964176	1.329959	0.186953	-5.69791	0.666337	0.49291
NK.cells	KIF2C	-0.3791	2.663513	-1.32996	0.186954	-5.14603	0.68981	0.525502
NK.cells	GM16310	-0.54189	1.197906	-1.32937	0.187146	-4.81302	0.705226	0.547559
NK.cells	NSF	0.143828	6.770124	1.329346	0.187154	-5.97339	0.648522	0.468913
NK.cells	CGNL1	-0.66005	1.622928	-1.32917	0.187212	-4.76258	0.700718	0.541189
NK.cells	RESF1	0.141071	6.500817	1.329149	0.187218	-5.95233	0.651145	0.472494
NK.cells	NAPEPLD	0.562918	1.056502	1.329066	0.187246	-4.75632	0.706732	0.54978
NK.cells	TMEM201	0.381495	2.102835	1.328873	0.187309	-4.93185	0.695665	0.534131
NK.cells	SAP130	0.141677	5.619105	1.32879	0.187336	-5.79601	0.659815	0.484395
NK.cells	SLX4	0.328405	1.820472	1.328486	0.187437	-4.95065	0.698633	0.538472
NK.cells	NRXN1	-0.67579	2.142242	-1.32839	0.187467	-4.86292	0.695251	0.533676
NK.cells	VPS33A	0.170527	4.291318	1.328227	0.187522	-5.50535	0.673112	0.502712
NK.cells	PARN	0.1935	3.875182	1.328067	0.187574	-5.43418	0.677338	0.508601
NK.cells	TTC9C	-0.14038	4.558959	-1.32777	0.187673	-5.5824	0.670408	0.49903
NK.cells	TCEAL9	-0.17028	5.693961	-1.32726	0.187839	-5.74046	0.659074	0.483681
NK.cells	EZH2	-0.18061	6.663574	-1.32715	0.187876	-5.94407	0.649558	0.470858
NK.cells	GTF3C3	0.260349	3.102011	1.326935	0.187947	-5.26965	0.685268	0.519932
NK.cells	MSS51	0.350787	2.436698	1.326791	0.187994	-5.03208	0.692172	0.529672
NK.cells	NHLRC2	0.144986	4.994885	1.32645	0.188107	-5.67114	0.66603	0.493332
NK.cells	FUT11	0.222239	3.493603	1.326366	0.188134	-5.37174	0.68124	0.514395
NK.cells	LY6C2	-0.31213	5.53324	-1.32623	0.188179	-5.99727	0.660666	0.48607

NK.cells	STARD10	-0.3026	4.596544	-1.32589	0.18829	-5.59516	0.670029	0.499037
NK.cells	GM19696	0.738372	0.016291	1.325694	0.188356	-4.65722	0.71792	0.566901
NK.cells	SYF2	-0.10204	5.903221	-1.32564	0.188374	-5.85022	0.657008	0.481282
NK.cells	4930445E1	0.732562	-0.41363	1.325541	0.188407	-4.6	0.722598	0.573712
NK.cells	CCL9	-0.42596	2.831707	-1.32532	0.18848	-5.07805	0.688064	0.524267
NK.cells	CD2BP2	0.1738	4.011388	1.324976	0.188593	-5.48354	0.675952	0.507444
NK.cells	KANSL1	-0.10717	8.964116	-1.32488	0.188625	-6.34285	0.627584	0.442095
NK.cells	GM45669	-0.77908	0.129811	-1.32485	0.188636	-4.64565	0.71669	0.565268
NK.cells	ELP2	0.163143	4.144774	1.324679	0.188691	-5.51981	0.674597	0.505587
NK.cells	KIF17	-0.56976	1.692096	-1.32463	0.188708	-4.8327	0.699987	0.541288
NK.cells	MRAP	-0.74861	1.214744	-1.32452	0.188744	-4.71157	0.705046	0.548569
NK.cells	PMVK	-0.21414	4.327226	-1.32424	0.188835	-5.40601	0.672748	0.503182
NK.cells	GM5617	0.280256	3.238502	1.324061	0.188896	-5.18337	0.683861	0.518775
NK.cells	JDP2	-0.1927	5.304027	-1.32398	0.188921	-5.86368	0.662944	0.489826
NK.cells	C1S1	-0.44114	1.787052	-1.32389	0.188952	-4.92434	0.698985	0.54024
NK.cells	GM31243	-0.62899	2.779029	-1.3234	0.189116	-4.88141	0.688611	0.525624
NK.cells	CDK1	-0.28233	5.506863	-1.32337	0.189123	-5.78589	0.660928	0.487194
NK.cells	AMER1	-0.31476	2.080672	-1.32333	0.189137	-4.96571	0.695897	0.535933
NK.cells	PDE1B	-0.29667	3.114849	-1.32304	0.189232	-5.13157	0.685136	0.520818
NK.cells	APON	0.602169	1.166034	1.322887	0.189284	-4.83382	0.705565	0.549919
NK.cells	ZFP90	0.398212	2.283533	1.322805	0.189311	-4.98686	0.693772	0.53312
NK.cells	GM24362	-0.57013	0.694716	-1.32271	0.189344	-4.72868	0.710602	0.557189
NK.cells	ZFP688	0.411159	1.695397	1.322555	0.189394	-4.91698	0.699952	0.541937
NK.cells	GM17036	-0.39931	2.038724	-1.32232	0.189474	-5.00171	0.696337	0.536881
NK.cells	EIF5A	0.116488	9.061608	1.322055	0.18956	-6.34484	0.626672	0.441571
NK.cells	SPTAN1	-0.1287	6.559592	-1.32203	0.18957	-5.97855	0.650572	0.473415
NK.cells	GM15247	0.671753	0.995367	1.321879	0.189618	-4.77018	0.707384	0.552722
NK.cells	CD163L1	0.490145	0.60908	1.321745	0.189663	-4.9632	0.711521	0.558714
NK.cells	GM13166	-0.70581	-0.19353	-1.3215	0.189744	-4.63629	0.720199	0.571348
NK.cells	KAT2B	0.144754	6.221283	1.321211	0.18984	-5.91425	0.65388	0.478105
NK.cells	SIRPB1A	0.738544	-0.18195	1.32116	0.189857	-4.65362	0.720073	0.571321
NK.cells	SDAD1	0.16653	4.473042	1.320925	0.189935	-5.65557	0.671275	0.502035
NK.cells	RARRES1	-0.81984	0.889058	-1.32082	0.18997	-4.68477	0.70852	0.554714
NK.cells	SGMS1	0.180112	7.102996	1.320277	0.19015	-5.95599	0.645296	0.466881
NK.cells	LDLRAD3	-0.37583	4.591753	-1.32025	0.19016	-5.13464	0.670078	0.50069
NK.cells	GM2682	0.241034	2.755703	1.320234	0.190165	-5.79556	0.688853	0.526938
NK.cells	WDR6	-0.23261	3.277321	-1.32021	0.190174	-5.35838	0.683462	0.519346
NK.cells	HSPA8	-0.10541	10.08692	-1.3202	0.190175	-6.53056	0.617163	0.429686
NK.cells	RETSAT	0.408765	1.405172	1.320051	0.190225	-4.79852	0.703023	0.547104
NK.cells	HBS1L	-0.12457	5.694465	-1.31965	0.190358	-5.81771	0.659069	0.485613
NK.cells	COG2	0.208558	3.567744	1.319354	0.190457	-5.37364	0.68048	0.515366
NK.cells	ZCRB1	0.104849	6.149583	1.319243	0.190494	-5.87587	0.654584	0.47967
NK.cells	CTCF	-0.09033	7.101448	-1.31919	0.190512	-6.04504	0.645311	0.467141
NK.cells	GM8113	-0.84961	-0.25687	-1.31909	0.190545	-4.57348	0.720888	0.573249
NK.cells	ZFP287	0.454717	0.566909	1.31909	0.190545	-4.9427	0.711975	0.560291
NK.cells	GM42984	0.649328	0.916614	1.319073	0.190551	-4.71671	0.708226	0.554875
NK.cells	RWDD2B	0.418951	1.849393	1.318965	0.190587	-4.93786	0.698328	0.540721

NK.cells	FAM124A	-0.54185	1.64482	-1.31896	0.19059	-4.74541	0.700486	0.543804
NK.cells	TRIM12A	0.176606	5.048891	1.318587	0.190713	-5.67671	0.665658	0.49477
NK.cells	ADAMTSL1	-1.0078	0.587709	-1.31847	0.190751	-4.70794	0.711931	0.560164
NK.cells	SERPINB6A	0.199796	5.570318	1.318188	0.190846	-5.73636	0.660509	0.487745
NK.cells	MDM2	0.176347	6.41707	1.317954	0.190924	-5.91728	0.65217	0.476434
NK.cells	STIM2	0.139166	6.210089	1.317928	0.190932	-5.93513	0.654198	0.479187
NK.cells	LRSAM1	-0.46752	1.610096	-1.31724	0.191161	-4.81963	0.701237	0.544885
NK.cells	PALLD	-0.36514	3.882236	-1.31706	0.191221	-5.18027	0.677637	0.511561
NK.cells	4933432I0	0.644354	0.408312	1.317027	0.191233	-4.72949	0.714072	0.563437
NK.cells	HNRNPA0	-0.09485	7.214278	-1.31697	0.191253	-6.07741	0.644574	0.466243
NK.cells	GBP6	0.480349	1.984096	1.316776	0.191317	-5.16777	0.697292	0.539331
NK.cells	CCNQ	0.230485	3.060749	1.316703	0.191341	-5.29027	0.68607	0.52342
NK.cells	ADAT2	0.464904	1.285306	1.316614	0.191371	-4.87442	0.704681	0.549952
NK.cells	SLC39A11	0.223851	4.419343	1.31638	0.191449	-5.46009	0.672261	0.504183
NK.cells	CYBA	0.126293	8.55691	1.316024	0.191568	-6.26595	0.631915	0.449412
NK.cells	THBS3	-0.61486	1.01596	-1.31597	0.191588	-4.80263	0.707727	0.554373
NK.cells	DHX38	0.172528	4.560036	1.315793	0.191645	-5.59579	0.670935	0.50242
NK.cells	DTD2	0.265619	3.491192	1.315354	0.191792	-5.29857	0.682016	0.517806
NK.cells	SEC23B	-0.12845	5.223375	-1.3152	0.191845	-5.74662	0.66448	0.493486
NK.cells	GM48796	-0.53297	0.350478	-1.31477	0.191987	-4.7527	0.715094	0.565352
NK.cells	USP33	0.183097	4.710834	1.31477	0.191987	-5.52864	0.669617	0.500792
NK.cells	DIAPH3	-0.25369	6.026174	-1.31467	0.19202	-5.96284	0.65652	0.482788
NK.cells	CYB561D2	0.220052	3.329402	1.314609	0.192041	-5.31664	0.683679	0.520443
NK.cells	VPS8	0.178379	4.586793	1.313557	0.192394	-5.5869	0.671819	0.502711
NK.cells	ALDH1L1	-0.36225	3.098676	-1.31345	0.192431	-5.17507	0.687032	0.524025
NK.cells	ZFP638	0.113139	6.22747	1.31329	0.192483	-5.94236	0.65547	0.480317
NK.cells	USP54	-0.25302	2.915478	-1.3131	0.192549	-5.22888	0.688962	0.526761
NK.cells	SLC38A1	-0.13561	7.937408	-1.31265	0.192698	-6.20303	0.638952	0.458217
NK.cells	FAM126A	-0.15829	5.549539	-1.31239	0.192786	-5.826	0.66223	0.489792
NK.cells	AGAP3	-0.18859	4.127556	-1.31233	0.192807	-5.58341	0.676534	0.50957
NK.cells	UTP15	0.182414	3.757308	1.312033	0.192905	-5.42533	0.680314	0.514971
NK.cells	5031425E2	0.148303	5.390925	1.311834	0.192972	-5.77372	0.663809	0.492114
NK.cells	SPRYD3	0.164227	3.699854	1.311608	0.193048	-5.50994	0.680902	0.515881
NK.cells	MRPL41	0.187115	4.003451	1.311607	0.193049	-5.47579	0.677799	0.51154
NK.cells	CLCF1	-0.26871	2.650892	-1.31151	0.193081	-5.3293	0.691744	0.531216
NK.cells	THUMPD2	0.319099	1.928752	1.311302	0.193151	-5.00398	0.699315	0.542009
NK.cells	IQGAP2	-0.12883	7.075634	-1.31123	0.193174	-6.08926	0.647247	0.469674
NK.cells	PRRG4	-0.72541	0.01648	-1.31114	0.193205	-4.63906	0.719793	0.57158
NK.cells	MFSD4A	0.393175	2.041521	1.311053	0.193235	-4.99293	0.698127	0.540312
NK.cells	MTM1	0.261803	4.433358	1.310071	0.193566	-5.41395	0.673648	0.505869
NK.cells	NRM	-0.20056	5.099836	-1.30987	0.193632	-5.70064	0.666934	0.49657
NK.cells	EIF2S3Y	2.336027	1.945318	1.309859	0.193637	-5.11976	0.699367	0.542128
NK.cells	ETV5	-0.36008	3.199847	-1.30983	0.193646	-5.13252	0.686269	0.523537
NK.cells	CPTP	-0.30362	2.248177	-1.30982	0.193651	-5.03297	0.69618	0.537582
NK.cells	CDK6	0.204992	6.322112	1.309663	0.193703	-5.91794	0.654809	0.479956
NK.cells	PNRC2	0.143964	5.35165	1.309412	0.193787	-5.72186	0.664416	0.493146
NK.cells	DEPP1	0.642499	1.043364	1.309351	0.193808	-4.76863	0.708948	0.55594

NK.cells	DYNC1LI2	-0.15821	4.672528	-1.30903	0.193916	-5.61734	0.67123	0.502588
NK.cells	KRT81	0.73091	-1.53173	1.309016	0.193921	-4.59716	0.736577	0.597144
NK.cells	SMURF1	0.168295	4.688664	1.308865	0.193972	-5.63828	0.671067	0.502362
NK.cells	BCAR3	-0.36718	4.496691	-1.30875	0.19401	-5.3475	0.673007	0.505102
NK.cells	MED9	0.231418	3.526994	1.308687	0.194032	-5.35292	0.682897	0.51892
NK.cells	RAB30	-0.51842	2.141089	-1.30836	0.194142	-4.90369	0.697459	0.539397
NK.cells	EID2	0.624378	0.075956	1.308225	0.194188	-4.71856	0.719538	0.571343
NK.cells	ZXDB	-0.23339	3.1887	-1.3075	0.194433	-5.32307	0.687076	0.52422
NK.cells	TM2D2	-0.13369	5.178235	-1.30744	0.194454	-5.76398	0.66682	0.49598
NK.cells	AP4B1	0.329071	2.273778	1.307159	0.194548	-5.08645	0.696748	0.53788
NK.cells	MSH2	0.169251	4.128132	1.306611	0.194733	-5.57822	0.677935	0.511072
NK.cells	ADO	0.217902	3.708631	1.306506	0.194769	-5.31768	0.682227	0.517106
NK.cells	RUNX3	-0.15263	5.712717	-1.30614	0.194892	-6.10409	0.662201	0.489206
NK.cells	HIGD1A	0.123885	6.029655	1.305983	0.194946	-5.9628	0.659059	0.484929
NK.cells	PGLYRP1	0.182564	4.959034	1.305362	0.195156	-6.04847	0.670266	0.499748
NK.cells	ARHGAP29	-0.45998	2.520124	-1.30502	0.195273	-4.94424	0.695378	0.534991
NK.cells	PLPP5	-0.20187	3.394162	-1.30498	0.195285	-5.44132	0.686282	0.522139
NK.cells	UHRF1	-0.28084	4.598357	-1.30383	0.195676	-5.55043	0.674613	0.505352
NK.cells	MRTO4	0.197073	4.665156	1.303739	0.195707	-5.62824	0.673936	0.504413
NK.cells	NANP	0.323998	2.821135	1.303711	0.195717	-5.14099	0.6929	0.530975
NK.cells	DPYSL2	0.127997	6.279623	1.303709	0.195717	-6.02702	0.657795	0.482243
NK.cells	NCF4	0.190224	5.316123	1.303591	0.195757	-5.70667	0.667376	0.495391
NK.cells	2010109A1	-0.31459	2.521995	-1.30276	0.196041	-5.14223	0.696445	0.535823
NK.cells	TMEM183A	-0.11959	5.16956	-1.30262	0.196087	-5.73116	0.669245	0.497804
NK.cells	PTPRO	0.645295	2.363394	1.302453	0.196144	-4.78978	0.698112	0.538305
NK.cells	RIN2	0.357952	3.687666	1.302446	0.196147	-5.10471	0.684325	0.518833
NK.cells	ANKDD1A	0.790893	2.346094	1.302197	0.196231	-4.87253	0.698294	0.538651
NK.cells	FAM3C	0.148822	5.36557	1.302149	0.196247	-5.67087	0.667278	0.495248
NK.cells	DROSHA	0.197573	3.121263	1.301795	0.196368	-5.32294	0.690185	0.527338
NK.cells	NR3C2	-0.54475	1.939637	-1.30157	0.196446	-4.89139	0.702586	0.544993
NK.cells	DCAF1	0.168349	5.222364	1.301541	0.196455	-5.68463	0.668714	0.497423
NK.cells	P4HA2	0.67955	0.108055	1.301128	0.196595	-4.67893	0.722279	0.573605
NK.cells	E2F3	-0.19441	5.460693	-1.30098	0.196645	-5.72012	0.666325	0.494271
NK.cells	DCAF7	-0.11806	5.327488	-1.30075	0.196723	-5.79254	0.667659	0.496108
NK.cells	D030028A0	-0.2412	2.795422	-1.30032	0.19687	-5.42372	0.693581	0.532478
NK.cells	MED13	-0.11833	7.722854	-1.30018	0.196917	-6.15596	0.64411	0.464257
NK.cells	PTPN22	-0.14874	5.324165	-1.30012	0.19694	-6.1425	0.667693	0.496334
NK.cells	F8A	0.267841	2.551871	1.300043	0.196965	-5.24222	0.696131	0.536181
NK.cells	SCG5	0.60069	0.801405	1.299638	0.197103	-4.79428	0.714756	0.563121
NK.cells	RMI1	0.191686	3.629414	1.29962	0.19711	-5.36535	0.684925	0.520536
NK.cells	ARHGEF10	-0.48354	2.695098	-1.29952	0.197145	-4.92488	0.69463	0.534245
NK.cells	LYNX1	-0.69198	0.898277	-1.29946	0.197163	-4.71781	0.713712	0.561608
NK.cells	HAVCR2	0.602578	2.301711	1.299421	0.197178	-4.93315	0.698761	0.540123
NK.cells	AZI2	0.107987	5.839646	1.299369	0.197195	-5.90444	0.662545	0.489474
NK.cells	SIRT2	0.120744	5.688086	1.299314	0.197214	-5.8571	0.664054	0.491544
NK.cells	PSMC3	-0.11322	6.11628	-1.29925	0.197236	-5.9262	0.659801	0.485741
NK.cells	RBM24	-0.68634	1.008251	-1.29888	0.197361	-4.78413	0.712772	0.559993

NK.cells	CEP57L1	0.25447	3.287731	1.298517	0.197486	-5.30361	0.688712	0.525716
NK.cells	BCAS3OS1	-0.18929	3.793281	-1.29847	0.197503	-5.50461	0.68349	0.518379
NK.cells	DIP2B	0.113436	7.880088	1.297388	0.197873	-6.20782	0.642834	0.462978
NK.cells	TBCEL	0.172249	4.594507	1.29727	0.197913	-5.60956	0.675303	0.507352
NK.cells	PANX1	0.155835	4.208683	1.297047	0.19799	-5.62543	0.679232	0.512866
NK.cells	SLCO3A1	0.204785	4.38978	1.297021	0.197998	-5.85549	0.677385	0.510287
NK.cells	CYB5D1	0.653192	0.775244	1.29694	0.198026	-4.72716	0.715303	0.564267
NK.cells	GM4566	-0.38526	1.718905	-1.29659	0.198145	-5.05074	0.705189	0.549797
NK.cells	LSM1	0.123329	5.251939	1.296412	0.198207	-5.78695	0.668664	0.498377
NK.cells	METAP1	0.133047	4.718677	1.296348	0.198229	-5.6484	0.674043	0.505831
NK.cells	CATSPERG1	0.774683	0.160917	1.29623	0.19827	-4.66603	0.721969	0.574183
NK.cells	FAM98A	0.198967	3.607656	1.296086	0.198319	-5.40016	0.685402	0.521743
NK.cells	PLCB4	0.258035	3.876337	1.295919	0.198376	-5.49523	0.682636	0.517913
NK.cells	RPGR	-0.43889	1.599467	-1.29586	0.198397	-4.8507	0.706461	0.551799
NK.cells	THEM6	0.253941	3.448768	1.295543	0.198505	-5.35131	0.687044	0.524299
NK.cells	RIOX2	0.170963	3.769866	1.29545	0.198537	-5.41455	0.683731	0.519639
NK.cells	1600020E0	-0.15509	6.223144	-1.29528	0.198594	-5.96938	0.658988	0.485386
NK.cells	RNF144A	0.22747	3.619246	1.295271	0.198599	-5.38174	0.685283	0.521877
NK.cells	IKBKE	0.323875	3.116112	1.295205	0.198621	-5.52522	0.690494	0.529235
NK.cells	FNBP1	-0.12455	8.248905	-1.29518	0.19863	-6.35927	0.639298	0.458773
NK.cells	PDHA1	0.144135	5.115043	1.295126	0.198649	-5.74755	0.670041	0.500594
NK.cells	A430035B1	-0.48284	3.071834	-1.29511	0.198653	-4.9697	0.690954	0.529887
NK.cells	SREK1	-0.09754	6.429668	-1.29496	0.198704	-5.95102	0.65695	0.482697
NK.cells	RASGRP3	-0.46403	3.586569	-1.2947	0.198796	-5.06419	0.68562	0.522511
NK.cells	CYP2AB1	0.75745	-0.30874	1.2946	0.198829	-4.67182	0.727109	0.582289
NK.cells	ZSCAN26	0.162743	4.652438	1.294112	0.198997	-5.67906	0.674715	0.507457
NK.cells	PSMD10	0.227365	3.584601	1.293999	0.199036	-5.36934	0.68564	0.522766
NK.cells	ARHGEF39	-0.55972	1.960521	-1.29387	0.19908	-4.92405	0.702624	0.54696
NK.cells	ANO6	0.15699	6.75402	1.293757	0.199119	-5.97426	0.653764	0.478649
NK.cells	GM39090	-0.5644	0.497553	-1.29348	0.199213	-4.73107	0.718308	0.569729
NK.cells	FANCI	0.304918	2.266344	1.293451	0.199224	-5.07428	0.699392	0.542403
NK.cells	EXOSC5	0.14153	5.376885	1.293328	0.199266	-5.7655	0.667411	0.497426
NK.cells	ZFP623	-0.71319	0.124749	-1.29332	0.199271	-4.67064	0.722363	0.575658
NK.cells	RUNX1	-0.12607	9.201324	-1.29323	0.199301	-6.46993	0.630269	0.447237
NK.cells	NRBF2	0.170898	4.314912	1.293075	0.199354	-5.56292	0.678148	0.512488
NK.cells	MGAT4B	0.20522	3.847759	1.292355	0.199602	-5.47598	0.682961	0.519444
NK.cells	CKLF	0.16473	4.804772	1.292321	0.199613	-5.64071	0.673202	0.505789
NK.cells	CHST8	-0.97597	-0.80735	-1.29225	0.199637	-4.60806	0.732642	0.591218
NK.cells	RAE1	0.15376	4.525674	1.292121	0.199682	-5.62654	0.676033	0.509803
NK.cells	HPD	-0.3524	4.805638	-1.29204	0.199709	-5.7029	0.673194	0.50585
NK.cells	ANP32A	0.072256	7.495527	1.291876	0.199767	-6.14364	0.646573	0.469391
NK.cells	SMAD3	-0.15531	6.315623	-1.29164	0.199849	-6.04155	0.658105	0.485065
NK.cells	FAM45A	0.163259	3.979967	1.291619	0.199855	-5.51077	0.681604	0.517653
NK.cells	EGR3	-0.42307	3.533307	-1.29156	0.199874	-5.42112	0.686201	0.524146
NK.cells	DHX57	0.199709	3.871504	1.291245	0.199985	-5.41082	0.68272	0.519259
NK.cells	CCDC88C	0.143026	5.294187	1.291233	0.199989	-5.87485	0.668273	0.499096
NK.cells	ARFIP1	0.199601	3.797972	1.29059	0.200211	-5.47403	0.684041	0.520651

NK.cells	HMCES	-0.17905	4.456587	-1.28992	0.200443	-5.643	0.677892	0.511266
NK.cells	SEC23A	0.161869	4.425257	1.28937	0.200632	-5.62169	0.678611	0.511777
NK.cells	TRAPPC13	-0.19296	3.808587	-1.28896	0.200773	-5.40619	0.684935	0.520803
NK.cells	OSBPL9	0.092794	7.511715	1.288931	0.200784	-6.15524	0.647903	0.469789
NK.cells	FBXW8	0.178585	3.868261	1.288779	0.200837	-5.52516	0.684321	0.520021
NK.cells	CST3	-0.26455	9.157123	-1.28875	0.200846	-6.26714	0.632164	0.448835
NK.cells	PRC1	-0.28942	5.285692	-1.28837	0.200978	-5.79574	0.670144	0.499964
NK.cells	RLN3	0.350541	-0.84713	1.287973	0.201116	-5.05184	0.735339	0.593032
NK.cells	DLGAP4	-0.17082	5.015503	-1.28746	0.201295	-5.72602	0.673331	0.504045
NK.cells	CTU2	0.21396	3.206943	1.287257	0.201364	-5.37671	0.691901	0.530137
NK.cells	LAMTOR3	0.139626	5.240813	1.287239	0.201371	-5.72614	0.671056	0.500959
NK.cells	BOLA3	0.147979	5.736487	1.287037	0.201441	-5.91079	0.66608	0.494138
NK.cells	ZHX2	0.173814	5.614006	1.286987	0.201458	-5.88898	0.667306	0.495826
NK.cells	GM27216	-0.63444	0.912751	-1.28611	0.201761	-4.79415	0.716856	0.565424
NK.cells	MAML1	-0.1409	5.435368	-1.28605	0.201785	-5.81454	0.669661	0.498578
NK.cells	STX16	0.143012	5.756821	1.286005	0.201799	-5.85382	0.666437	0.494136
NK.cells	BTRC	-0.16217	5.211768	-1.2858	0.201869	-5.71207	0.671958	0.501767
NK.cells	RNF10	-0.12034	6.51711	-1.28528	0.202052	-6.0229	0.659207	0.484069
NK.cells	CLASP2	-0.10701	6.927855	-1.28517	0.202089	-6.14411	0.655161	0.47858
NK.cells	GM2629	0.616231	0.511245	1.285061	0.202127	-4.68896	0.721571	0.572176
NK.cells	TRPM4	0.383472	1.109553	1.284486	0.202327	-4.98186	0.715488	0.562986
NK.cells	DNMBP	-0.21886	3.488772	-1.2844	0.202356	-5.41273	0.690283	0.526943
NK.cells	MBD1	0.169133	4.580853	1.2839	0.202531	-5.61155	0.679432	0.511317
NK.cells	EXTL2	-0.42207	2.165568	-1.28268	0.202955	-4.9663	0.705412	0.547228
NK.cells	GM43328	0.316093	2.238918	1.282638	0.202971	-5.15988	0.704632	0.546124
NK.cells	1810037I1	0.132537	6.482621	1.282498	0.20302	-5.99738	0.661074	0.485315
NK.cells	DHRS7	-0.2666	4.016547	-1.28248	0.203026	-5.48417	0.686018	0.519804
NK.cells	ECSIT	0.17972	4.015765	1.282309	0.203086	-5.5329	0.686026	0.519893
NK.cells	OXLD1	-0.40551	1.813163	-1.28224	0.20311	-4.94231	0.70917	0.552752
NK.cells	AFF4	0.116427	7.842009	1.282025	0.203185	-6.24889	0.647784	0.467422
NK.cells	POP5	0.123643	4.525116	1.281786	0.203269	-5.71879	0.680826	0.51267
NK.cells	FAM122A	0.223078	3.563965	1.281389	0.203408	-5.32008	0.690742	0.526574
NK.cells	HMGCL	0.16922	5.187114	1.281294	0.203441	-5.69067	0.674086	0.503312
NK.cells	NFATC3	0.115617	7.290076	1.281223	0.203466	-6.16801	0.653157	0.474713
NK.cells	SLC10A3	-0.38658	2.160549	-1.28108	0.203516	-5.03456	0.705501	0.547629
NK.cells	CSTA2	0.851551	1.96893	1.281002	0.203543	-4.85569	0.707542	0.550594
NK.cells	SIPA1L1	-0.15142	7.327522	-1.28067	0.20366	-6.27142	0.652791	0.474481
NK.cells	STX7	0.119654	6.176182	1.280507	0.203716	-5.84498	0.664152	0.48996
NK.cells	FOXN3	0.121254	8.899803	1.280406	0.203752	-6.39721	0.637624	0.454217
NK.cells	ZFP277	0.120749	5.309649	1.280086	0.203863	-5.83125	0.672847	0.501953
NK.cells	TNFAIP3	0.141519	6.763033	1.280082	0.203865	-6.20192	0.658334	0.482047
NK.cells	WDR46	0.234723	3.429733	1.279766	0.203975	-5.39998	0.692139	0.528931
NK.cells	GBA	-0.18518	4.090254	-1.27932	0.204131	-5.51894	0.685293	0.519489
NK.cells	BCAR1	-0.65428	0.776645	-1.27924	0.20416	-4.72897	0.720386	0.569635
NK.cells	IL17RA	0.161967	5.907729	1.279232	0.204163	-5.89733	0.666833	0.493859
NK.cells	NECAP2	-0.14505	5.345804	-1.27917	0.204183	-5.74091	0.672481	0.501646
NK.cells	CLUAP1	0.179116	3.885137	1.279124	0.2042	-5.46087	0.687411	0.52249

NK.cells	POLDIP2	0.192924	4.278307	1.279019	0.204237	-5.52825	0.683357	0.516816
NK.cells	DSE	0.265697	3.834005	1.27878	0.204321	-5.50292	0.68794	0.523282
NK.cells	PHF11B	0.226603	4.313119	1.278779	0.204321	-5.79668	0.683	0.51635
NK.cells	NID2	-0.44892	2.19946	-1.27799	0.204597	-5.02148	0.705702	0.547914
NK.cells	MRPL45	0.199258	4.340705	1.277457	0.204785	-5.52631	0.683311	0.516322
NK.cells	DQX1	-1.03116	1.215444	-1.27743	0.204795	-4.75603	0.716254	0.563216
NK.cells	RANBP17	-0.53975	1.134101	-1.27737	0.204816	-4.81412	0.717134	0.56449
NK.cells	DNM1L	0.124159	5.639142	1.277326	0.204831	-5.89704	0.67011	0.497992
NK.cells	RBBP6	-0.09935	7.269431	-1.27727	0.204852	-6.11773	0.653929	0.475895
NK.cells	PSMB8	0.171548	7.077604	1.277036	0.204933	-6.18337	0.65581	0.478488
NK.cells	KDM8	-0.32497	1.814343	-1.27697	0.204957	-5.03045	0.709812	0.553974
NK.cells	GLRX3	0.119289	6.625536	1.276743	0.205036	-6.03404	0.660337	0.48458
NK.cells	LYRM7	0.471513	1.247217	1.27654	0.205108	-4.8704	0.716036	0.562813
NK.cells	ARMCX5	0.255792	2.433168	1.27599	0.205301	-5.21294	0.70381	0.54476
NK.cells	1110059E2	0.179906	4.393524	1.275689	0.205407	-5.53975	0.683439	0.515981
NK.cells	CSTF2T	0.241925	3.017029	1.275365	0.205521	-5.23693	0.697746	0.536156
NK.cells	SNHG9	-0.34034	4.309776	-1.27512	0.205607	-5.50435	0.684301	0.517221
NK.cells	GM15859	0.407773	1.208039	1.274899	0.205685	-4.84958	0.717038	0.563826
NK.cells	UBE2E2	-0.33429	3.465951	-1.27476	0.205736	-5.36376	0.693045	0.529512
NK.cells	A530076I1	0.744053	-0.88246	1.274718	0.205749	-4.635	0.740038	0.597509
NK.cells	USP50	0.215904	3.371827	1.274626	0.205781	-5.41115	0.694027	0.5309
NK.cells	B430306N(	-0.50337	2.692804	-1.2746	0.205789	-4.86342	0.701162	0.541025
NK.cells	SIT1	0.395652	2.062249	1.274086	0.205972	-5.06311	0.70829	0.550825
NK.cells	TBC1D2B	0.180282	4.091379	1.273642	0.206129	-5.6702	0.687287	0.52082
NK.cells	PTPRA	0.096254	6.521638	1.273347	0.206233	-6.0128	0.662655	0.486882
NK.cells	IFI206	0.336687	3.940563	1.27333	0.206239	-5.7485	0.688848	0.523154
NK.cells	GM29570	-0.59816	0.692445	-1.27256	0.206512	-4.7609	0.723624	0.572975
NK.cells	FBXL3	0.126649	5.215741	1.272423	0.206559	-5.80225	0.675965	0.505234
NK.cells	ASB3	0.142482	4.711015	1.272247	0.206621	-5.70558	0.681111	0.512384
NK.cells	FAM78A	0.258498	2.648934	1.272241	0.206623	-5.31749	0.702581	0.542647
NK.cells	COX16	0.094937	6.153691	1.272191	0.206641	-5.9636	0.666515	0.492212
NK.cells	DUSP4	-0.35626	0.909125	-1.27219	0.206643	-5.01166	0.72126	0.569537
NK.cells	PLA2G7	0.846516	4.021195	1.27174	0.206801	-5.03674	0.688344	0.522452
NK.cells	EGFR	-0.61934	2.469637	-1.2716	0.20685	-5.02393	0.704616	0.545511
NK.cells	BST2	0.232472	6.606914	1.271364	0.206934	-5.9103	0.662126	0.486195
NK.cells	ACSF2	-0.18959	4.286847	-1.27127	0.206965	-5.6203	0.685599	0.518634
NK.cells	GM26771	0.527125	-0.2163	1.271263	0.206969	-4.88623	0.733763	0.587799
NK.cells	DENND6B	-0.51398	0.94417	-1.27096	0.207075	-4.81495	0.721186	0.569293
NK.cells	EIF2S3X	-0.21841	5.323448	-1.27051	0.207237	-5.79409	0.675287	0.504184
NK.cells	ARID5B	0.171497	7.244193	1.270085	0.207387	-6.23252	0.656113	0.478173
NK.cells	SPATA48	0.362594	2.190427	1.270077	0.207389	-5.09092	0.707887	0.550394
NK.cells	EMB	0.164774	5.68619	1.270076	0.20739	-6.24088	0.671619	0.49937
NK.cells	CARD9	0.606255	0.997223	1.269643	0.207544	-4.78087	0.720744	0.569084
NK.cells	R3HDM1	0.098355	6.683532	1.269581	0.207566	-6.06017	0.661647	0.485807
NK.cells	PBX4	0.44163	0.976518	1.269573	0.207568	-4.96254	0.72097	0.569412
NK.cells	1700061N1	-0.99136	-0.1438	-1.26945	0.20761	-4.63079	0.73327	0.587485
NK.cells	CMAH	0.244656	5.561409	1.269085	0.207741	-5.73	0.672878	0.501551

NK.cells	FTH1	0.229819	12.5537	1.268875	0.207816	-6.81213	0.606203	0.412975
NK.cells	ATP5B	0.106551	8.182512	1.268867	0.207819	-6.29039	0.646966	0.466322
NK.cells	TMEM186	0.386029	2.111676	1.268839	0.207829	-5.02846	0.708728	0.552163
NK.cells	TMTC2	-0.34075	4.574445	-1.26875	0.207861	-5.66267	0.68293	0.515628
NK.cells	FAM210A	0.192766	3.636981	1.268461	0.207963	-5.41881	0.692723	0.529397
NK.cells	ZFP280D	0.144665	5.625842	1.268345	0.208004	-5.82029	0.67232	0.500953
NK.cells	ASB5	0.524607	0.515697	1.268029	0.208116	-4.8195	0.726194	0.57753
NK.cells	MNDAL	0.192009	6.786829	1.267948	0.208145	-6.11128	0.660797	0.48507
NK.cells	SRD5A3	0.18078	4.511422	1.267638	0.208256	-5.66484	0.683931	0.516858
NK.cells	TMEM19	0.170041	3.958743	1.267286	0.208381	-5.53043	0.689863	0.52501
NK.cells	SNF8	0.116253	5.731869	1.266353	0.208713	-5.89876	0.672608	0.500063
NK.cells	RGS18	-0.29509	3.108266	-1.26602	0.208832	-5.30752	0.699672	0.537983
NK.cells	IL23R	0.589026	-0.9447	1.265778	0.208918	-4.79697	0.743802	0.602118
NK.cells	GM20342	-0.23278	3.499425	-1.2657	0.208944	-5.40115	0.695563	0.532216
NK.cells	LARGE1	0.203777	5.482959	1.265661	0.20896	-5.83792	0.675126	0.503636
NK.cells	ERLEC1	0.174491	4.341715	1.265437	0.209039	-5.61284	0.686804	0.519995
NK.cells	RSRP1	-0.12548	6.653226	-1.26535	0.209071	-6.04736	0.663377	0.487586
NK.cells	PQLC2	-0.25273	3.590318	-1.26507	0.209171	-5.34165	0.694612	0.531118
NK.cells	EP400	-0.09893	6.870294	-1.26506	0.209173	-6.07929	0.661223	0.48477
NK.cells	DERL1	-0.09013	6.22546	-1.26481	0.209263	-5.95734	0.66775	0.49369
NK.cells	FASTKD3	-0.39179	1.993657	-1.26444	0.209396	-5.00816	0.711891	0.55556
NK.cells	GM4285	0.359324	1.640385	1.264	0.209552	-5.00952	0.715976	0.561092
NK.cells	MOV10	0.203432	3.628623	1.263782	0.20963	-5.53237	0.694843	0.531
NK.cells	ZC3H8	0.31444	2.13952	1.263702	0.209659	-5.08241	0.710607	0.553481
NK.cells	VEZF1	-0.10953	6.00178	-1.26356	0.209709	-5.93852	0.670499	0.497107
NK.cells	E4F1	0.263122	3.127007	1.263397	0.209768	-5.31898	0.700116	0.53855
NK.cells	FGD4	-0.48506	4.134435	-1.26295	0.209929	-5.05591	0.689851	0.52382
NK.cells	ADHFE1	0.341611	2.690792	1.262844	0.209966	-5.13754	0.705009	0.545326
NK.cells	PROX1	-0.57453	2.15604	-1.26252	0.210082	-4.94501	0.710912	0.553592
NK.cells	NPR1	-0.65853	1.263007	-1.26213	0.21022	-4.74573	0.720586	0.567696
NK.cells	RPAP3	0.19616	3.841822	1.262097	0.210233	-5.48782	0.69312	0.528405
NK.cells	MRPS17	0.136114	5.224333	1.261896	0.210305	-5.77886	0.678862	0.508515
NK.cells	CACNB4	-0.53395	1.615307	-1.26185	0.210322	-4.98546	0.716766	0.56223
NK.cells	GM42567	-0.56876	1.12927	-1.26163	0.210401	-4.92174	0.722117	0.56999
NK.cells	FCRL1	0.444232	2.73778	1.261387	0.210487	-4.99247	0.704907	0.545062
NK.cells	NAB2	-0.21252	3.217989	-1.26104	0.210613	-5.55699	0.70005	0.538071
NK.cells	PTP4A2	-0.06878	8.699413	-1.26035	0.210858	-6.40162	0.645362	0.46216
NK.cells	MAPK1	-0.08049	7.693924	-1.26003	0.210975	-6.22501	0.655233	0.475354
NK.cells	MZT1	0.118538	5.278837	1.259944	0.211005	-5.80266	0.679392	0.508452
NK.cells	PSMD14	0.097404	6.618882	1.259781	0.211064	-6.06044	0.665871	0.489842
NK.cells	PLA1A	-0.45319	1.841769	-1.25921	0.21127	-5.01808	0.715972	0.559691
NK.cells	PUS1	0.197592	3.723827	1.25898	0.211352	-5.49481	0.696034	0.531259
NK.cells	NDST1	-0.21933	4.570663	-1.25846	0.211538	-5.55296	0.687544	0.519087
NK.cells	FAM8A1	0.231451	3.231881	1.258385	0.211565	-5.31516	0.701534	0.538833
NK.cells	PRMT5	0.219101	3.483009	1.257932	0.211728	-5.39344	0.699234	0.535264
NK.cells	MMS19	0.133498	4.947005	1.257434	0.211908	-5.75639	0.684398	0.514117
NK.cells	ZBED3	-0.20814	3.559356	-1.25718	0.212	-5.43316	0.698943	0.534454

NK.cells	SIMC1	-0.14786	5.702765	-1.25702	0.212058	-5.92256	0.676783	0.503498
NK.cells	NXPE4	0.549772	2.181788	1.256779	0.212144	-4.89437	0.713696	0.555425
NK.cells	CD3E	-0.21849	3.278256	-1.25625	0.212335	-5.97132	0.702443	0.538955
NK.cells	PTPN13	0.400646	0.415506	1.255548	0.212588	-5.1072	0.733872	0.583824
NK.cells	FAS	0.217521	3.936884	1.255541	0.212591	-5.69266	0.695929	0.529371
NK.cells	AFG1L	0.222446	3.691074	1.255415	0.212636	-5.54939	0.698508	0.53306
NK.cells	TRAK1	-0.11032	7.291138	-1.25485	0.212841	-6.09059	0.662066	0.482423
NK.cells	SOX7	-0.67322	0.442832	-1.25482	0.212851	-4.71199	0.733912	0.583679
NK.cells	UBE2F	0.127069	6.306137	1.253623	0.213284	-5.96249	0.672934	0.496159
NK.cells	LRRC8D	-0.1284	7.366735	-1.25353	0.213318	-6.17768	0.662324	0.481724
NK.cells	OAS1A	0.527461	2.355225	1.253441	0.21335	-5.0769	0.714125	0.553832
NK.cells	SNRPD1	-0.12221	7.17842	-1.25284	0.213567	-6.1415	0.664452	0.484407
NK.cells	1110038F1	0.160201	4.590808	1.252629	0.213644	-5.70134	0.69076	0.520591
NK.cells	PQBP1	-0.16189	4.6495	-1.25238	0.213735	-5.65022	0.69015	0.519819
NK.cells	VPS54	0.128673	6.506686	1.252341	0.213748	-6.03218	0.671174	0.493625
NK.cells	ST8SIA4	-0.14217	7.324778	-1.25228	0.213769	-6.2451	0.662998	0.482522
NK.cells	TGFBR1	0.154955	6.24383	1.252234	0.213787	-5.94676	0.673825	0.49727
NK.cells	ZFP771	0.16492	4.065583	1.251937	0.213895	-5.54914	0.696237	0.52843
NK.cells	CAPN10	0.27543	2.598593	1.251851	0.213926	-5.22204	0.711787	0.550464
NK.cells	RNASET2A	-0.18001	5.439463	-1.25177	0.213956	-5.86595	0.682008	0.508638
NK.cells	PRKCZ	0.395446	0.606888	1.25157	0.214028	-4.96168	0.733545	0.581908
NK.cells	SNHG5	-0.29813	2.416156	-1.25134	0.214112	-5.22181	0.71388	0.553447
NK.cells	KANSL3	0.130287	4.995066	1.251164	0.214175	-5.81402	0.686721	0.515168
NK.cells	C3	-0.26243	6.327835	-1.25097	0.214246	-6.01686	0.67316	0.496435
NK.cells	ENHO	-0.4113	2.481211	-1.25055	0.214397	-5.09054	0.713388	0.552666
NK.cells	STAT3	0.112201	7.950149	1.250324	0.21448	-6.36211	0.657136	0.474669
NK.cells	ID2	0.141767	6.76154	1.250272	0.214499	-6.23068	0.668934	0.490636
NK.cells	GM27241	-0.30488	2.603068	-1.25018	0.214531	-5.28239	0.712079	0.550844
NK.cells	TNFSF13	-0.40351	1.863945	-1.25005	0.214581	-5.14566	0.720058	0.562283
NK.cells	TPX2	-0.25988	5.047457	-1.24971	0.214704	-5.8178	0.68657	0.514742
NK.cells	HMGN5	-0.15886	4.876393	-1.2494	0.214815	-5.74586	0.688346	0.517263
NK.cells	ZC3H10	0.247026	2.921486	1.249267	0.214865	-5.22009	0.708894	0.546237
NK.cells	LRRC59	-0.12022	5.556125	-1.24907	0.214936	-5.85921	0.681354	0.507673
NK.cells	LSM3	-0.15285	5.624923	-1.24906	0.214939	-5.8919	0.68065	0.506701
NK.cells	MRGPRA2/	0.65615	-1.27662	1.248825	0.215026	-4.65861	0.755081	0.613904
NK.cells	ADCY6	-0.50982	0.433361	-1.24865	0.215089	-4.86637	0.736117	0.585512
NK.cells	CSGALNAC	0.500786	2.818139	1.248466	0.215156	-5.01733	0.71014	0.548072
NK.cells	2310061I0	0.18789	3.521012	1.248132	0.215278	-5.45793	0.702781	0.537625
NK.cells	VPREB2	-0.82178	-0.41404	-1.24805	0.215309	-4.65621	0.74576	0.599755
NK.cells	EED	-0.09172	6.114274	-1.24788	0.215371	-5.99645	0.675931	0.500242
NK.cells	NDUFA7	0.088233	7.909207	1.247586	0.215477	-6.25806	0.658057	0.475948
NK.cells	RAPGEFL1	-0.45719	2.784487	-1.24751	0.215506	-4.95759	0.710695	0.54895
NK.cells	PTPRF	-0.49927	1.276101	-1.24717	0.215628	-4.79717	0.727152	0.572604
NK.cells	PHKG2	-0.15203	4.528144	-1.2471	0.215654	-5.67485	0.692389	0.523117
NK.cells	LRRC45	-0.30948	2.190744	-1.24668	0.215809	-5.11944	0.717327	0.558482
NK.cells	GATA4	-0.56727	1.621315	-1.24667	0.215812	-4.8037	0.723513	0.567394
NK.cells	RAB1B	-0.12801	5.58788	-1.246	0.216055	-5.91016	0.681679	0.508296

NK.cells	GM13391	-0.58661	0.765079	-1.24571	0.216162	-4.77663	0.733025	0.581383
NK.cells	XRCC5	0.273149	2.083305	1.245531	0.216227	-5.25533	0.718591	0.560506
NK.cells	ASPM	-0.33271	3.870548	-1.24549	0.216244	-5.57737	0.699503	0.533319
NK.cells	ZFP704	-0.3196	3.792086	-1.24532	0.216303	-5.3944	0.70033	0.534539
NK.cells	TLR11	0.580484	-0.67266	1.24519	0.216352	-4.72579	0.749118	0.605191
NK.cells	TM2D3	0.168072	4.227452	1.245087	0.21639	-5.5906	0.695758	0.52816
NK.cells	MKKS	0.341939	1.995441	1.244854	0.216475	-5.04207	0.719543	0.562095
NK.cells	SRPK2	-0.12012	7.096447	-1.2448	0.216496	-6.17129	0.666431	0.4877
NK.cells	AGPAT5	0.118839	5.644905	1.244723	0.216523	-5.88755	0.681095	0.507816
NK.cells	HEATR5B	0.21363	3.730171	1.244429	0.21663	-5.48523	0.700983	0.535813
NK.cells	KDR	-0.4497	3.318647	-1.24405	0.21677	-5.14848	0.705339	0.542153
NK.cells	ABCD2	-0.40246	2.633823	-1.24404	0.216773	-5.06348	0.712652	0.552581
NK.cells	ZFP282	-0.18374	4.010784	-1.24389	0.216829	-5.60682	0.698029	0.531846
NK.cells	PCDH9	-0.88654	0.472713	-1.24384	0.216846	-4.73686	0.736268	0.586829
NK.cells	SLAIN1	-0.171	3.6132	-1.24381	0.216858	-5.59128	0.702218	0.537764
NK.cells	IL2RG	0.206836	6.294179	1.243582	0.216941	-6.16911	0.674492	0.499176
NK.cells	GM16283	0.566013	0.252287	1.2435	0.216971	-4.74569	0.738723	0.590564
NK.cells	HOXB4	0.49407	1.361865	1.243347	0.217027	-4.84041	0.726453	0.572694
NK.cells	OFD1	0.195332	3.31561	1.243222	0.217073	-5.37228	0.705371	0.542446
NK.cells	UTP14B	-0.28673	3.081232	-1.24315	0.217101	-5.23406	0.707865	0.546035
NK.cells	MSL1	0.147835	5.419802	1.243086	0.217123	-5.79758	0.683401	0.511631
NK.cells	AKR1B8	0.574774	1.592252	1.242631	0.217289	-4.84966	0.724093	0.569296
NK.cells	CD247	0.191127	3.01096	1.242612	0.217296	-5.79716	0.708772	0.547274
NK.cells	4930562C1	-0.6216	0.146658	-1.24243	0.217364	-4.7468	0.740067	0.592707
NK.cells	UBASH3A	0.312923	2.009253	1.242234	0.217435	-5.33734	0.719553	0.562891
NK.cells	RABGGTB	0.188655	3.732854	1.242161	0.217462	-5.50205	0.70111	0.536574
NK.cells	MS4A4C	0.40823	3.224383	1.241813	0.21759	-5.5517	0.706723	0.544328
NK.cells	NPFF	-0.47395	0.954229	-1.24127	0.217788	-4.86986	0.731788	0.579943
NK.cells	UFM1	0.130357	5.448609	1.241126	0.217842	-5.83395	0.683902	0.511841
NK.cells	BTBD8	0.495624	1.572677	1.240766	0.217974	-4.89138	0.725092	0.570161
NK.cells	NAMPT	0.225466	6.301782	1.240244	0.218167	-5.89924	0.675296	0.500095
NK.cells	CD27	-0.20307	3.318035	-1.24009	0.218223	-5.63037	0.706266	0.543486
NK.cells	DDX1	0.163728	4.964571	1.239883	0.2183	-5.76051	0.688989	0.519223
NK.cells	GM43848	0.273424	2.767038	1.239711	0.218363	-5.20579	0.712152	0.552013
NK.cells	NCKIPSD	-0.27833	2.952621	-1.23958	0.218411	-5.14558	0.710164	0.549189
NK.cells	GM5431	-0.80409	0.742737	-1.23956	0.218419	-4.73479	0.73423	0.583991
NK.cells	BPNT1	-0.18229	3.339718	-1.2395	0.218439	-5.49829	0.706036	0.543331
NK.cells	FASL	-0.24839	1.037842	-1.23946	0.218455	-5.5483	0.730967	0.57927
NK.cells	GM47371	-0.58749	0.907518	-1.23933	0.218502	-4.82803	0.732406	0.581401
NK.cells	CYFIP1	0.170288	5.64309	1.239117	0.218582	-5.78481	0.682003	0.509688
NK.cells	GM14548	0.650962	1.47249	1.238993	0.218627	-4.75307	0.726188	0.572426
NK.cells	CTNND1	-0.32297	4.294756	-1.23899	0.21863	-5.23231	0.695961	0.529204
NK.cells	UPF3A	0.126661	4.634592	1.238634	0.21876	-5.72792	0.692641	0.52434
NK.cells	ALG8	-0.27551	3.226775	-1.23795	0.219013	-5.27446	0.707965	0.545605
NK.cells	VPS26C	0.217621	4.084075	1.237901	0.21903	-5.44428	0.698889	0.532768
NK.cells	2610203C2	-0.70681	0.354453	-1.23768	0.219111	-4.71252	0.739383	0.591023
NK.cells	GM21887	0.426802	2.133944	1.237296	0.219253	-5.07574	0.72007	0.562583

NK.cells	MRPS15	0.118193	5.910327	1.236858	0.219415	-5.95893	0.680625	0.506607
NK.cells	DNAH7A	-0.72775	-0.11769	-1.23661	0.219506	-4.72549	0.745356	0.599144
NK.cells	ARHGAP19	-0.26365	4.256309	-1.23609	0.219698	-5.55115	0.69779	0.530709
NK.cells	TRPV4	0.715546	-0.51202	1.236064	0.219709	-4.68478	0.74981	0.605923
NK.cells	SIAH1A	0.14541	5.079563	1.235914	0.219764	-5.77795	0.689208	0.518733
NK.cells	SNX5	0.109411	7.890887	1.23591	0.219766	-6.19855	0.660764	0.479683
NK.cells	CKAP2	-0.26662	3.667324	-1.23575	0.219824	-5.5257	0.704001	0.539573
NK.cells	EXOC6	0.123032	6.194009	1.235718	0.219837	-5.99806	0.677774	0.502935
NK.cells	SMARCA4	-0.12617	7.5105	-1.23538	0.219961	-6.14895	0.664733	0.484948
NK.cells	WDR73	0.23865	2.976485	1.23518	0.220036	-5.28644	0.711629	0.550252
NK.cells	TMEM159	0.388131	1.553977	1.234659	0.220229	-5.1466	0.727146	0.572668
NK.cells	COL5A3	-0.64684	0.940726	-1.23439	0.220329	-4.79766	0.733907	0.582662
NK.cells	SLBP	-0.12059	7.147492	-1.23419	0.220401	-6.17287	0.668493	0.49033
NK.cells	MCM7	-0.22214	5.384269	-1.23396	0.220486	-5.84765	0.686406	0.515042
NK.cells	PSRC1	-0.5564	0.886949	-1.23376	0.220561	-4.82213	0.734503	0.583703
NK.cells	1700113A1	0.33428	2.10081	1.233581	0.220628	-5.10838	0.721174	0.564361
NK.cells	GM50340	0.472896	1.533078	1.233455	0.220675	-4.93506	0.727376	0.573365
NK.cells	FCRL6	-0.67289	-0.87747	-1.23344	0.220681	-4.67832	0.754342	0.613046
NK.cells	KRR1	0.185905	3.94276	1.233252	0.22075	-5.53794	0.701441	0.53629
NK.cells	FLOT2	0.150471	4.443449	1.233022	0.220836	-5.72142	0.696179	0.528893
NK.cells	ALG10B	-0.23396	2.952601	-1.23254	0.221013	-5.30772	0.711976	0.551298
NK.cells	COX7C	0.088848	8.585633	1.232496	0.221031	-6.39787	0.654265	0.471294
NK.cells	FAM189A1	-0.56312	2.327494	-1.23249	0.221032	-4.99799	0.718714	0.560968
NK.cells	NDUFAF6	0.233047	2.973661	1.232455	0.221047	-5.32868	0.71175	0.550976
NK.cells	HTR1F	0.380366	1.429334	1.232444	0.22105	-4.9603	0.728515	0.575152
NK.cells	CLU	-0.43586	5.440247	-1.23206	0.221192	-5.87847	0.685829	0.514607
NK.cells	CHCHD3	0.080816	6.768694	1.232033	0.221203	-6.12576	0.672297	0.495922
NK.cells	SHCBP1L	0.532976	0.854389	1.231975	0.221225	-4.81525	0.734864	0.584633
NK.cells	TDRD3	0.17209	4.059195	1.231797	0.221291	-5.61265	0.700214	0.534829
NK.cells	DDB2	0.220728	3.43077	1.231699	0.221327	-5.57707	0.706867	0.544279
NK.cells	FYTTD1	-0.11259	5.267108	-1.23152	0.221394	-5.83579	0.687614	0.517188
NK.cells	1700112J1	-0.64713	0.638697	-1.23131	0.221473	-4.78726	0.73726	0.588293
NK.cells	CACNA2D1	0.803245	0.864423	1.230905	0.221623	-4.74554	0.734752	0.584777
NK.cells	ZFP946	0.428881	1.39583	1.230801	0.221662	-4.88905	0.728883	0.576257
NK.cells	DOCK1	-0.30613	4.079995	-1.23042	0.221805	-5.26004	0.699994	0.534985
NK.cells	SUGP1	-0.14038	4.615277	-1.23031	0.221845	-5.74087	0.694382	0.527124
NK.cells	GBP9	0.260402	3.036901	1.230247	0.221868	-5.48046	0.711072	0.55081
NK.cells	EIF2B1	-0.19549	3.62049	-1.23007	0.221932	-5.43298	0.704851	0.542032
NK.cells	LIMD1	-0.10842	6.016318	-1.23004	0.221944	-5.97727	0.679924	0.507061
NK.cells	AIG1	0.159273	4.903663	1.229932	0.221985	-5.89317	0.691379	0.523014
NK.cells	HYAL1	-0.40959	1.311575	-1.2299	0.221996	-4.92313	0.729811	0.57798
NK.cells	ZFP619	-0.30674	2.21582	-1.22967	0.222083	-5.09332	0.719925	0.563702
NK.cells	POFUT1	0.196336	3.458205	1.229612	0.222105	-5.44332	0.706575	0.544589
NK.cells	TADA2B	-0.22777	3.468615	-1.22946	0.22216	-5.39096	0.706465	0.544442
NK.cells	3300002A1	0.52048	0.451891	1.229438	0.222169	-4.81336	0.739343	0.592072
NK.cells	RBBP5	0.201521	3.457865	1.229376	0.222192	-5.47118	0.706579	0.544616
NK.cells	TAB1	0.258343	3.004453	1.229288	0.222226	-5.32374	0.71142	0.551553

NK.cells	SNHG4.1	0.633602	0.471422	1.228855	0.222387	-4.8411	0.739126	0.591984
NK.cells	TWISTNB	0.119447	5.520145	1.228486	0.222525	-5.90514	0.685008	0.514504
NK.cells	CTNNA1	-0.14085	6.643473	-1.22834	0.222581	-6.05628	0.673561	0.498707
NK.cells	RBM25	0.074895	8.296585	1.228303	0.222593	-6.36311	0.657099	0.47628
NK.cells	RECQL4	-0.60926	-0.06717	-1.22806	0.222684	-4.72872	0.745163	0.601193
NK.cells	GM14321	-0.64207	-0.49943	-1.22801	0.222702	-4.71313	0.750046	0.608461
NK.cells	PPP2R5D	0.186025	3.682866	1.227703	0.222817	-5.52049	0.704191	0.541819
NK.cells	BBS5	0.623941	0.624034	1.227229	0.222994	-4.79841	0.737425	0.590044
NK.cells	GM42937	0.586186	0.198102	1.227122	0.223034	-4.84208	0.742184	0.597064
NK.cells	ATP6AP2	-0.09747	6.938045	-1.22711	0.22304	-6.04941	0.670595	0.494986
NK.cells	1700025G(	-0.16673	4.957756	-1.22688	0.223126	-6.0346	0.690819	0.523074
NK.cells	MBD4	0.245975	3.212172	1.226863	0.223131	-5.3364	0.709199	0.549115
NK.cells	ENPP2	-0.41976	2.411456	-1.22682	0.223147	-5.0854	0.717806	0.561484
NK.cells	ATP6V1D	0.093496	6.650485	1.226755	0.223172	-6.06076	0.67349	0.498995
NK.cells	CYB5B	0.115711	5.866247	1.226745	0.223175	-5.93475	0.681459	0.51001
NK.cells	HRAS	0.133756	4.842094	1.226722	0.223184	-5.78834	0.692021	0.524761
NK.cells	MORN3	-0.34615	1.323497	-1.22663	0.223219	-5.12832	0.729681	0.578778
NK.cells	GM47200	-0.60567	0.11613	-1.22644	0.223289	-4.78722	0.74314	0.598549
NK.cells	DDAH1	-0.34497	2.481818	-1.22606	0.223431	-5.12202	0.717349	0.560604
NK.cells	F2R	-0.2	3.599158	-1.22585	0.22351	-5.63821	0.705446	0.543518
NK.cells	TMEM184I	-0.17081	4.707523	-1.22526	0.223733	-5.78695	0.694261	0.527288
NK.cells	GPANK1	0.207814	3.300008	1.225029	0.223818	-5.40634	0.709119	0.548416
NK.cells	GRHPR	0.226773	4.431109	1.224969	0.22384	-5.65236	0.697152	0.531439
NK.cells	GPR146	0.234776	3.278936	1.224568	0.223991	-5.34928	0.709595	0.548945
NK.cells	UPF2	0.11105	6.075856	1.224377	0.224063	-5.99323	0.680382	0.507922
NK.cells	EMID1	-0.20939	3.280299	-1.22427	0.224104	-5.50365	0.70958	0.549026
NK.cells	ARRB2	0.145517	5.195443	1.224135	0.224153	-5.87821	0.689433	0.520578
NK.cells	ETV1	-0.63156	1.377482	-1.22357	0.224364	-4.76247	0.730354	0.579039
NK.cells	THBS1	0.662987	4.967156	1.223448	0.224411	-5.39325	0.691922	0.524006
NK.cells	LRIG1	0.506282	0.178544	1.223233	0.224492	-4.97152	0.743693	0.598669
NK.cells	PDE4A	-0.21318	3.81993	-1.22306	0.224556	-5.73988	0.703962	0.541099
NK.cells	URB2	-0.26524	2.571455	-1.22299	0.224584	-5.26762	0.717322	0.560225
NK.cells	MYOM1	-0.56433	1.071853	-1.22291	0.224612	-4.82699	0.73373	0.584074
NK.cells	5330417C2	-0.5985	0.541978	-1.22279	0.224659	-4.84084	0.739623	0.59281
NK.cells	RASSF2	0.182446	4.259468	1.222785	0.22466	-5.66071	0.699322	0.534589
NK.cells	VIPR1	0.430706	0.964939	1.222503	0.224766	-5.03671	0.73507	0.586087
NK.cells	PPP1R12A	-0.08786	8.052992	-1.2222	0.22488	-6.36449	0.660841	0.481244
NK.cells	MAP2K4	0.137038	6.321239	1.221691	0.225072	-6.06074	0.678203	0.505152
NK.cells	TNIP3	0.703065	1.037658	1.221664	0.225082	-5.04064	0.734329	0.585149
NK.cells	RANBP10	0.131404	5.698692	1.221659	0.225084	-6.02726	0.684567	0.513983
NK.cells	MAN2B2	-0.20737	3.799809	-1.22139	0.225185	-5.56824	0.704386	0.541979
NK.cells	ALOX5	0.584767	0.791987	1.221308	0.225216	-4.88327	0.737057	0.589289
NK.cells	DLD	-0.13692	4.599821	-1.22097	0.225344	-5.68486	0.695961	0.530215
NK.cells	GK5	-0.20311	4.404631	-1.2209	0.22537	-5.67776	0.698006	0.533136
NK.cells	ASB2	0.284536	2.906427	1.220803	0.225406	-5.54114	0.713925	0.555884
NK.cells	A430018G1	-0.41015	1.329295	-1.22075	0.225426	-4.89526	0.731104	0.580803
NK.cells	IFT80	0.241064	3.626112	1.22074	0.22543	-5.5309	0.70623	0.544863

NK.cells	TSIX	-2.56445	1.906429	-1.22012	0.225663	-4.93094	0.724804	0.571759
NK.cells	SERPINA3N	-0.31186	2.557516	-1.22004	0.225695	-5.21189	0.717724	0.561543
NK.cells	NACC2	0.451091	1.77434	1.2195	0.225898	-4.97464	0.726249	0.574123
NK.cells	CACNA1A	0.418982	1.424509	1.219108	0.226046	-5.02298	0.730092	0.579945
NK.cells	MMRN1	1.246354	-0.22843	1.219044	0.22607	-4.68606	0.748541	0.607185
NK.cells	NDC1	-0.21808	3.748037	-1.21904	0.226073	-5.4538	0.704971	0.543651
NK.cells	OS9	0.111436	5.617189	1.218722	0.226192	-5.98884	0.68544	0.516105
NK.cells	ATG4C	0.37023	3.11799	1.218671	0.226211	-5.00465	0.71169	0.553289
NK.cells	KIFC3	-0.33217	1.625707	-1.21857	0.226248	-5.18071	0.727879	0.576766
NK.cells	GM35853	0.622049	0.184156	1.218533	0.226263	-4.75105	0.743891	0.60033
NK.cells	LY6G2	-0.64557	0.952461	-1.21819	0.226392	-4.86299	0.735311	0.587804
NK.cells	TRAF3IP3	0.131737	5.030914	1.218054	0.226444	-5.87792	0.691502	0.52482
NK.cells	CTLA4	0.281137	2.060232	1.218019	0.226457	-5.54635	0.723125	0.57005
NK.cells	CCDC122	0.473686	1.315452	1.217974	0.226474	-4.8975	0.731294	0.581975
NK.cells	CUEDC1	-0.30926	2.113054	-1.21783	0.226529	-5.20584	0.722549	0.569257
NK.cells	4930590J0	0.406836	1.547542	1.217798	0.226541	-5.00002	0.728738	0.578289
NK.cells	ZBTB42	0.291424	1.544195	1.217549	0.226635	-5.12114	0.728775	0.578343
NK.cells	UBE4B	-0.12583	5.48847	-1.21715	0.226785	-5.90854	0.686766	0.518364
NK.cells	GM29243	-0.35421	-0.26495	-1.21714	0.22679	-5.11833	0.748954	0.608299
NK.cells	GM43126	-0.48437	0.276018	-1.21711	0.226802	-4.93694	0.742859	0.599235
NK.cells	GM50232	-0.44122	1.372215	-1.21697	0.226855	-5.01406	0.730668	0.581291
NK.cells	CEBPB	-0.20683	9.155958	-1.21684	0.226905	-6.61383	0.650074	0.468083
NK.cells	DHRS3	-0.22878	4.170119	-1.21679	0.226924	-5.62536	0.700508	0.537767
NK.cells	EIF6	0.121123	5.835967	1.216757	0.226935	-5.96684	0.683193	0.513397
NK.cells	ARG2	0.513777	3.174154	1.21672	0.226949	-5.04605	0.711088	0.552881
NK.cells	NINJ2	0.619896	0.701022	1.216575	0.227004	-4.86416	0.738107	0.592279
NK.cells	URGCP	0.16453	4.434418	1.216526	0.227022	-5.75745	0.697729	0.533874
NK.cells	ACSF3	-0.36291	1.59243	-1.21635	0.227088	-5.03016	0.728269	0.577864
NK.cells	GNPTG	-0.18715	3.368723	-1.21609	0.227187	-5.47181	0.709157	0.550133
NK.cells	DNAJC5	0.080473	6.766699	1.21565	0.227354	-6.12392	0.674186	0.500654
NK.cells	PRDX3	0.13761	5.400912	1.21529	0.227491	-5.91545	0.688202	0.520143
NK.cells	ARC	-0.5656	0.905214	-1.21529	0.227491	-4.86914	0.736406	0.589437
NK.cells	APPL1	-0.12648	5.974209	-1.21472	0.227706	-6.05846	0.682736	0.512114
NK.cells	PTGS2OS2	0.675427	-0.48663	1.214602	0.227752	-4.72981	0.752525	0.612944
NK.cells	GAS6	-0.54368	1.545619	-1.21438	0.227836	-4.87517	0.729866	0.579379
NK.cells	GM26885	-0.39762	2.317843	-1.21372	0.228086	-5.13782	0.721677	0.567415
NK.cells	ASCC3	0.123543	6.882346	1.21372	0.228087	-6.18745	0.673822	0.499719
NK.cells	ST3GAL4	0.124448	6.423085	1.213696	0.228096	-6.06359	0.678476	0.506149
NK.cells	MEIG1	-0.77579	-0.47443	-1.21355	0.228153	-4.67222	0.752742	0.613232
NK.cells	LIMS1	0.113013	7.288787	1.213225	0.228275	-6.18237	0.669918	0.494229
NK.cells	GM29417	-0.45542	1.098886	-1.21284	0.228423	-4.87768	0.73556	0.587241
NK.cells	RAB4B	0.106507	5.9254	1.212651	0.228494	-5.99115	0.684026	0.513496
NK.cells	METTL22	0.373286	1.382795	1.212431	0.228577	-5.02212	0.732425	0.582783
NK.cells	TMEM69	0.428605	1.585462	1.212122	0.228695	-4.97416	0.730189	0.579594
NK.cells	PVR	0.231777	3.063552	1.212043	0.228725	-5.48592	0.7141	0.556253
NK.cells	C4BP	-0.46194	1.968581	-1.21194	0.228765	-5.06858	0.725982	0.573454
NK.cells	GLS	-0.11103	7.7257	-1.2119	0.228778	-6.30214	0.66582	0.488552

NK.cells	NDUFB9	0.10596	7.069533	1.211698	0.228856	-6.19487	0.672449	0.497633
NK.cells	OCIAD1	0.101118	5.885315	1.211545	0.228915	-6.00121	0.684494	0.51439
NK.cells	VSTM4	-0.82069	0.147061	-1.21122	0.22904	-4.71627	0.746311	0.603556
NK.cells	GM10785	0.329737	2.451276	1.211211	0.229042	-5.2345	0.720809	0.566123
NK.cells	SPDYA	-0.52524	-0.02911	-1.21097	0.229134	-4.86625	0.748342	0.606659
NK.cells	MEF2A	-0.10515	7.727346	-1.21087	0.229172	-6.29062	0.665926	0.488943
NK.cells	GLYR1	-0.10089	6.736527	-1.21055	0.229296	-6.10914	0.676006	0.5027
NK.cells	RCAN1	-0.23897	3.708543	-1.21045	0.229332	-5.35151	0.70746	0.546971
NK.cells	SH3TC1	-0.28109	2.768133	-1.21027	0.229401	-5.22843	0.717579	0.561476
NK.cells	GPS2	0.094408	5.859455	1.209478	0.229703	-5.97914	0.685515	0.515415
NK.cells	HK1OS	0.452232	0.741866	1.209294	0.229774	-4.96845	0.740421	0.594349
NK.cells	CLK4	-0.11256	6.012894	-1.20922	0.229801	-5.98581	0.683939	0.513283
NK.cells	ABHD10	0.167983	3.649409	1.20919	0.229813	-5.51236	0.708668	0.54819
NK.cells	EYA1	0.441966	2.426875	1.208685	0.230007	-5.08818	0.72226	0.567305
NK.cells	PHF8	-0.12	5.563796	-1.20843	0.230106	-5.95355	0.689088	0.519878
NK.cells	UFD1	0.117372	5.129038	1.208103	0.230229	-5.86226	0.693796	0.526291
NK.cells	TJP1	0.432922	2.024999	1.207797	0.230346	-4.97853	0.727165	0.573883
NK.cells	F12	-0.52306	1.691224	-1.2075	0.230458	-5.00966	0.730958	0.579374
NK.cells	CEP97	0.216132	2.922474	1.207388	0.230503	-5.46512	0.717516	0.559935
NK.cells	CPPED1	0.153649	3.886993	1.206754	0.230746	-5.57625	0.707296	0.545316
NK.cells	GM42836	-0.73525	-0.40469	-1.20668	0.230775	-4.71703	0.754589	0.614402
NK.cells	SLC3A1	-0.38342	0.899213	-1.2066	0.230804	-5.06389	0.739875	0.592561
NK.cells	GM28707	-0.54303	1.154193	-1.20654	0.230828	-4.85849	0.737033	0.588379
NK.cells	GNG10	-0.11926	6.940589	-1.20628	0.230928	-6.1875	0.675598	0.5009
NK.cells	CREB1	-0.09753	6.332794	-1.20614	0.230983	-6.10883	0.68178	0.509448
NK.cells	ATF2	-0.11368	6.46092	-1.20601	0.23103	-6.09171	0.680472	0.507697
NK.cells	RNASEL	0.244161	3.944163	1.205732	0.231138	-5.6117	0.706688	0.544674
NK.cells	EZH1	0.196887	3.600438	1.205673	0.23116	-5.58603	0.710353	0.54991
NK.cells	HAT1	0.13404	6.105162	1.205635	0.231175	-5.99467	0.684112	0.512873
NK.cells	HBQ1A	0.590706	-1.39639	1.205473	0.231237	-4.73343	0.765589	0.631821
NK.cells	BORA	-0.218	3.535011	-1.20537	0.231276	-5.46297	0.711053	0.550996
NK.cells	CEP41	-0.34481	1.886422	-1.2051	0.231382	-5.08409	0.728937	0.576852
NK.cells	5830411N(	-0.4014	-1.25694	-1.2049	0.231456	-4.99579	0.764098	0.629564
NK.cells	RC3H2	0.111318	5.728871	1.204863	0.231471	-5.92963	0.687986	0.518464
NK.cells	RMC1	0.155543	4.652621	1.204623	0.231563	-5.74893	0.699198	0.534334
NK.cells	ASF1B	-0.23023	4.954931	-1.20417	0.231738	-5.84997	0.696029	0.530042
NK.cells	ARHGEF7	-0.17006	4.675352	-1.20412	0.231755	-5.73079	0.69896	0.534183
NK.cells	CIPC	0.219542	2.655218	1.204052	0.231783	-5.44766	0.720537	0.565083
NK.cells	CC2D2A	0.607585	0.81427	1.203802	0.231879	-4.8368	0.740824	0.594853
NK.cells	WRAP73	-0.21359	2.923556	-1.20361	0.231953	-5.33694	0.71763	0.56106
NK.cells	MCOLN2	-0.25253	3.161941	-1.20357	0.23197	-5.30915	0.715058	0.557354
NK.cells	6230400D1	0.30754	2.163361	1.203564	0.231971	-5.16868	0.725899	0.573052
NK.cells	DEDD	0.142539	4.437275	1.203401	0.232034	-5.7237	0.701466	0.537983
NK.cells	WDR91	-0.16863	4.750458	-1.20316	0.232125	-5.74622	0.698171	0.533388
NK.cells	DAAM1	0.1676	4.764899	1.202945	0.232209	-5.69899	0.698019	0.533279
NK.cells	ARAP1	0.187294	5.049234	1.202783	0.232271	-5.74207	0.695043	0.529075
NK.cells	GM15246	-0.61824	1.046088	-1.20245	0.232399	-4.84115	0.738237	0.591432

NK.cells	RAB11FIP5	0.627847	0.808074	1.202451	0.232399	-4.80571	0.740894	0.595355
NK.cells	HNRNPH3	0.097022	5.447268	1.202446	0.232401	-5.92244	0.6909	0.523273
NK.cells	MRPS28	0.224958	6.885185	1.202395	0.232421	-6.08227	0.676159	0.502739
NK.cells	HDAC6	-0.31641	2.206503	-1.20238	0.232427	-5.16644	0.725427	0.57266
NK.cells	USP47	0.1078	6.472249	1.202183	0.232503	-6.12872	0.680406	0.508596
NK.cells	GCSAM	-0.37676	-0.31734	-1.20168	0.232697	-5.11462	0.754087	0.614566
NK.cells	1700056E2	0.397456	1.466557	1.201025	0.232949	-5.05084	0.734658	0.585169
NK.cells	5730480H(	0.251236	2.846707	1.200793	0.233039	-5.26347	0.719624	0.563254
NK.cells	RNF167	-0.14253	5.334087	-1.20055	0.233134	-5.90094	0.693303	0.52559
NK.cells	TENM3	-0.49274	1.688761	-1.20029	0.233235	-4.99701	0.732541	0.581777
NK.cells	USP14	0.104303	5.793901	1.200073	0.233317	-5.99098	0.688726	0.519024
NK.cells	IGLV1	-0.77894	-0.83837	-1.19944	0.23356	-4.68793	0.761684	0.62432
NK.cells	MMP13	1.041767	-1.01302	1.199313	0.233611	-4.68417	0.763638	0.627349
NK.cells	TAF4	-0.15306	4.075399	-1.19912	0.233684	-5.62754	0.707283	0.544654
NK.cells	OSM	0.529183	2.305551	1.199	0.233732	-5.11258	0.726388	0.572146
NK.cells	GM15952	-0.49686	1.028007	-1.19866	0.233863	-4.90891	0.740733	0.592969
NK.cells	SLC6A13	-0.31486	2.762131	-1.1985	0.233926	-5.26729	0.721613	0.565063
NK.cells	THRA	-0.23037	3.771843	-1.19837	0.233976	-5.46533	0.710724	0.549408
NK.cells	FANCE	0.235638	2.916052	1.197659	0.234251	-5.37076	0.720455	0.562921
NK.cells	MEX3A	-0.39915	1.577184	-1.19749	0.234318	-5.0427	0.735144	0.584333
NK.cells	SEMA4F	0.38127	-0.41218	1.197482	0.234319	-5.03277	0.757559	0.61752
NK.cells	KDM1A	0.101434	5.813944	1.197149	0.234449	-6.01011	0.68994	0.519459
NK.cells	FOXP1	0.083146	9.650428	1.196666	0.234636	-6.58192	0.651811	0.467063
NK.cells	SLC25A22	0.355475	2.495595	1.195733	0.234998	-5.22203	0.726509	0.570258
NK.cells	OXSR1	0.194815	3.496646	1.195401	0.235127	-5.53081	0.715637	0.55471
NK.cells	RBM4B	-0.11883	5.747385	-1.1953	0.235168	-6.0202	0.691829	0.521033
NK.cells	GM45267	0.428946	0.245242	1.195195	0.235207	-5.03444	0.7516	0.607196
NK.cells	ZFP84	-0.1993	3.7569	-1.19501	0.235278	-5.56822	0.712839	0.550755
NK.cells	CD69	0.230315	5.620451	1.194912	0.235317	-5.9751	0.693148	0.522972
NK.cells	CDH24	-0.30657	2.179776	-1.19463	0.235426	-5.16763	0.729976	0.575557
NK.cells	QSOX2	0.504202	0.948725	1.194615	0.235432	-4.86053	0.743659	0.595591
NK.cells	TECPR2	0.214687	3.905519	1.19443	0.235504	-5.48675	0.711246	0.548643
NK.cells	SPCS3	0.133688	4.704887	1.194307	0.235552	-5.76584	0.702747	0.5366
NK.cells	TIMM17B	0.10775	5.161183	1.194271	0.235566	-5.84469	0.697945	0.529827
NK.cells	PEX3	0.169741	3.770542	1.194106	0.235631	-5.54086	0.712708	0.550814
NK.cells	ACSL3	-0.2251	3.828777	-1.19395	0.235691	-5.51461	0.712087	0.549922
NK.cells	SMIM41	-0.60294	0.89661	-1.19349	0.235871	-4.7944	0.744308	0.59686
NK.cells	GM36445	-0.38698	1.003387	-1.19341	0.2359	-4.96181	0.743109	0.595106
NK.cells	ASPA	-0.71667	1.375432	-1.19319	0.235989	-4.863	0.738949	0.589055
NK.cells	POLR2M	0.107563	5.403452	1.1931	0.236022	-5.92665	0.69547	0.526629
NK.cells	ZFP354B	-0.60427	-0.81695	-1.19291	0.236096	-4.67219	0.763824	0.626136
NK.cells	GM15563	0.374828	1.923249	1.192655	0.236196	-5.156	0.732868	0.580437
NK.cells	RNF181	0.178439	4.477136	1.192633	0.236204	-5.68737	0.705218	0.540625
NK.cells	MPRIP	-0.13394	5.481388	-1.19249	0.236261	-5.95853	0.694656	0.52572
NK.cells	TFIP11	-0.17915	3.322157	-1.19241	0.236292	-5.58999	0.717581	0.558315
NK.cells	SFT2D3	0.226166	2.864525	1.192395	0.236297	-5.29911	0.722544	0.565469
NK.cells	DTX3L	0.220323	4.876216	1.191668	0.23658	-5.88228	0.701472	0.534993

NK.cells	NRG2	0.55976	1.685011	1.191625	0.236597	-4.90994	0.736001	0.584674
NK.cells	RRN3	0.142351	4.400393	1.191522	0.236637	-5.72706	0.706508	0.542141
NK.cells	LDB2	-0.3935	3.160304	-1.19122	0.236755	-5.25942	0.719816	0.561205
NK.cells	SFMBT1	-0.1334	6.08199	-1.1911	0.236801	-5.97523	0.688887	0.51739
NK.cells	6430548M	-0.39923	2.407786	-1.19096	0.236856	-5.02088	0.728024	0.573155
NK.cells	GM17018	0.174121	3.620053	1.190867	0.236893	-5.63597	0.714851	0.554177
NK.cells	GM17268	-0.79993	0.383225	-1.19048	0.237044	-4.78294	0.750605	0.606615
NK.cells	FCSK	0.500982	0.928399	1.19029	0.237119	-4.89252	0.744452	0.597578
NK.cells	SLFN8	0.275554	3.326481	1.190176	0.237163	-5.61879	0.718017	0.559061
NK.cells	ARG1	0.347094	3.498182	1.190126	0.237183	-5.57536	0.716164	0.556416
NK.cells	EML3	-0.16311	4.139794	-1.19006	0.237208	-5.6923	0.709282	0.546593
NK.cells	DDHD2	-0.1403	4.784447	-1.18994	0.237256	-5.87158	0.70244	0.53691
NK.cells	UBE2M	0.09746	7.20691	1.189863	0.237285	-6.22685	0.677369	0.501894
NK.cells	CTLA2A	0.194981	4.28991	1.189355	0.237484	-5.99444	0.708078	0.54464
NK.cells	TANC2	-0.21525	5.521032	-1.189	0.237621	-5.80234	0.695099	0.526394
NK.cells	HIST2H4	-0.60165	0.951972	-1.18893	0.237649	-4.86177	0.744603	0.597763
NK.cells	POLR2E	0.158375	4.876402	1.188852	0.237681	-5.894	0.701862	0.535945
NK.cells	ADORA2A	0.208641	3.148747	1.188695	0.237743	-5.7135	0.720344	0.562446
NK.cells	MIEF2	0.41875	1.112018	1.188616	0.237774	-4.96274	0.742806	0.595224
NK.cells	CDKN1B	0.119413	6.470899	1.188238	0.237922	-6.17801	0.685498	0.512864
NK.cells	ONECUT2	-0.48106	1.559667	-1.188	0.238016	-5.01857	0.738059	0.588028
NK.cells	BCL6	-0.18063	5.992679	-1.18787	0.238068	-6.08668	0.690433	0.519812
NK.cells	ORMDL1	-0.18233	3.609727	-1.1878	0.238092	-5.52483	0.715607	0.555463
NK.cells	TSC22D3	0.198165	5.71665	1.187512	0.238206	-5.92835	0.693457	0.523933
NK.cells	YBX3	0.14937	6.314911	1.186951	0.238426	-6.10635	0.687616	0.515389
NK.cells	CCDC71	0.255828	2.73695	1.186785	0.238492	-5.30489	0.725616	0.569425
NK.cells	BTBD6	0.372779	1.542046	1.186497	0.238605	-5.11392	0.738807	0.588759
NK.cells	E230016M	0.352507	2.465953	1.186429	0.238631	-5.16785	0.728585	0.573828
NK.cells	USP4	0.090162	5.660629	1.186296	0.238684	-5.97501	0.6944	0.525046
NK.cells	ST6GALNA	0.182043	5.127632	1.186291	0.238686	-6.07096	0.69998	0.532895
NK.cells	BPHL	-0.22351	3.663858	-1.18546	0.23901	-5.44177	0.71579	0.555321
NK.cells	PDS5A	-0.08995	7.411745	-1.18545	0.239017	-6.24386	0.676628	0.500302
NK.cells	EEF1AKMT	0.251905	2.582886	1.185434	0.239022	-5.24146	0.727538	0.572276
NK.cells	RORA	0.169763	5.020685	1.185344	0.239057	-6.0541	0.701333	0.534768
NK.cells	RABEP2	-0.15635	4.414258	-1.18513	0.239142	-5.70061	0.707755	0.543992
NK.cells	GM11973	-0.22177	3.179185	-1.1851	0.239154	-5.57916	0.721032	0.56303
NK.cells	APOL11B	1.225099	-0.04187	1.185037	0.239179	-4.84011	0.756931	0.615771
NK.cells	M6PR	0.112657	6.293265	1.184714	0.239306	-6.09728	0.68812	0.516386
NK.cells	4632427E1	0.181917	4.404143	1.184599	0.239351	-5.59873	0.707921	0.544271
NK.cells	MAP7D1	-0.11864	5.36793	-1.18453	0.239376	-5.91175	0.697742	0.529869
NK.cells	EXTL3	0.202134	3.56619	1.183881	0.239633	-5.56351	0.717336	0.557397
NK.cells	PMEPA1	-0.20289	4.361383	-1.18386	0.23964	-5.90154	0.708805	0.545197
NK.cells	TMEM123	0.125943	6.484305	1.183537	0.239769	-6.11731	0.686765	0.514035
NK.cells	PROSER1	-0.1708	4.213066	-1.18297	0.239993	-5.67389	0.710908	0.547872
NK.cells	RASA2	0.148642	6.158678	1.182966	0.239994	-6.06367	0.690432	0.518985
NK.cells	LRP8	-0.38486	2.371033	-1.1822	0.240298	-5.12991	0.731022	0.576963
NK.cells	HSD17B7	-0.37333	1.697013	-1.18209	0.240339	-5.01083	0.738489	0.587889

NK.cells	GABPA	-0.13887	4.662072	-1.18199	0.240379	-5.76304	0.70624	0.541364
NK.cells	MBOAT1	0.333857	0.682144	1.181857	0.240431	-5.34332	0.749886	0.604786
NK.cells	SLC25A39	0.150154	6.472882	1.181634	0.24052	-6.14183	0.687299	0.51486
NK.cells	USP38	-0.10779	5.889969	-1.18155	0.240555	-6.0857	0.693335	0.523288
NK.cells	THAP11	-0.16508	4.457867	-1.18146	0.240588	-5.6933	0.708411	0.54461
NK.cells	MDC1	0.235836	3.159139	1.18143	0.2406	-5.35578	0.722393	0.564655
NK.cells	GNB1L	0.212248	3.308245	1.180931	0.240797	-5.48712	0.720772	0.562453
NK.cells	PSMC3IP	0.33299	2.412129	1.18089	0.240813	-5.19177	0.730569	0.576651
NK.cells	GM26749	-0.26255	2.222722	-1.18061	0.240924	-5.33164	0.732658	0.579712
NK.cells	CHAC2	0.210281	2.893423	1.180394	0.241009	-5.3896	0.72529	0.569081
NK.cells	MPI	-0.33636	2.225319	-1.1802	0.241084	-5.06961	0.732629	0.579798
NK.cells	HIST1H4N	-0.56431	1.07648	-1.18014	0.24111	-4.88963	0.745436	0.598611
NK.cells	NOP9	0.1408	4.555827	1.180125	0.241116	-5.76511	0.707369	0.543393
NK.cells	ASXL2	0.110195	6.836454	1.180092	0.241129	-6.17176	0.683564	0.509964
NK.cells	ARPC1B	0.088577	8.892807	1.179718	0.241277	-6.53449	0.662858	0.481688
NK.cells	IL18RAP	0.259384	1.983582	1.179512	0.241359	-5.66209	0.735304	0.583821
NK.cells	FUCA2	-0.14331	4.624115	-1.17946	0.24138	-5.7554	0.706643	0.542466
NK.cells	MAD2L2	-0.19959	3.472942	-1.1794	0.241403	-5.5324	0.718987	0.560128
NK.cells	SLC22A1	-0.56671	0.740269	-1.17932	0.241436	-4.88361	0.749228	0.604345
NK.cells	GBP2	0.429284	4.147241	1.179301	0.241442	-5.81145	0.711729	0.549715
NK.cells	MON2	-0.11753	5.7408	-1.17862	0.241711	-5.9896	0.694889	0.526094
NK.cells	CNIH4	0.123168	5.763142	1.178526	0.241749	-5.95617	0.694656	0.525824
NK.cells	EPHX3	-0.47367	0.657793	-1.17851	0.241756	-4.92054	0.750162	0.606065
NK.cells	STXBP3	0.132305	5.278015	1.178242	0.241862	-5.93857	0.699734	0.533033
NK.cells	XYLB	-0.50713	0.922746	-1.17797	0.241968	-4.88588	0.747167	0.601715
NK.cells	CCNB1	-0.3205	4.032241	-1.17779	0.24204	-5.65219	0.712961	0.551919
NK.cells	N4BP3	-0.26295	2.955418	-1.1777	0.242075	-5.29919	0.724613	0.568708
NK.cells	MTHFSL	0.126251	5.211119	1.177661	0.242092	-5.956	0.700437	0.534109
NK.cells	SLC25A28	-0.15088	4.806244	-1.1775	0.242157	-5.77526	0.704711	0.540221
NK.cells	PFN2	-0.53009	1.339141	-1.17745	0.242175	-4.91281	0.742487	0.594923
NK.cells	GPBP1L1	0.114327	5.913267	1.177229	0.242264	-6.05099	0.693093	0.523962
NK.cells	USP40	-0.24496	3.64171	-1.17717	0.242286	-5.49816	0.717163	0.558173
NK.cells	EPRS	-0.13977	6.581496	-1.17703	0.242343	-6.24557	0.686181	0.514354
NK.cells	ZC3H4	-0.12422	4.818849	-1.17686	0.24241	-5.78912	0.704578	0.540235
NK.cells	KHSRP	-0.10793	5.998752	-1.1768	0.242432	-6.02942	0.692204	0.52278
NK.cells	TSFM	0.154469	4.258618	1.176484	0.242559	-5.68995	0.710537	0.548834
NK.cells	PLCL2	0.115449	6.985417	1.176474	0.242564	-6.20782	0.68204	0.508712
NK.cells	PHF19	-0.30847	1.934693	-1.1764	0.242592	-5.13614	0.735846	0.585503
NK.cells	RBM42	-0.09332	6.063078	-1.1762	0.242673	-6.07181	0.691536	0.521982
NK.cells	BMYC	0.311793	3.380337	1.176109	0.242708	-5.40477	0.71999	0.562456
NK.cells	UBE2C	-0.27205	6.746366	-1.17596	0.242766	-6.2247	0.684488	0.512176
NK.cells	CMTM6	0.131511	4.866252	1.17586	0.242807	-5.80585	0.704076	0.539699
NK.cells	NAT8F1	-0.47131	1.619362	-1.17578	0.242841	-5.04514	0.739354	0.590724
NK.cells	PCIF1	0.099238	6.021144	1.175414	0.242985	-6.05483	0.691972	0.522773
NK.cells	UQCC3	0.15775	4.016511	1.175321	0.243022	-5.65523	0.71313	0.552796
NK.cells	SIN3A	0.120149	5.871572	1.175078	0.243118	-5.97819	0.693526	0.525058
NK.cells	SHOC2	-0.10272	6.344774	-1.17486	0.243207	-6.11432	0.688621	0.518248

NK.cells	PSMB5	0.119046	6.258665	1.174724	0.243259	-6.09712	0.689511	0.519562
NK.cells	WAC	-0.0819	7.414892	-1.1746	0.243309	-6.29906	0.677668	0.503177
NK.cells	DRAM1	0.391843	2.581586	1.174293	0.243431	-5.26784	0.728705	0.575704
NK.cells	HIST2H2AA	0.409655	2.225914	1.174292	0.243431	-5.12069	0.732622	0.581427
NK.cells	TLN2	-0.39298	1.298032	-1.17424	0.243453	-5.15431	0.742947	0.596627
NK.cells	AMY1	-0.32257	2.627056	-1.17416	0.243482	-5.26356	0.728206	0.575009
NK.cells	CYREN	0.263468	3.374354	1.174011	0.243543	-5.33513	0.720055	0.563223
NK.cells	MAP3K3	-0.11905	6.795781	-1.17386	0.243604	-6.18176	0.683981	0.512084
NK.cells	SETDB2	0.149864	4.580351	1.173631	0.243695	-5.79103	0.707108	0.544723
NK.cells	DRAP1	0.090046	6.508223	1.173273	0.243838	-6.15814	0.686935	0.516306
NK.cells	CYP2A22	-0.45829	1.632221	-1.17325	0.243847	-5.0733	0.739211	0.59134
NK.cells	SEC31A	0.095247	5.620105	1.173125	0.243897	-5.993	0.696149	0.529231
NK.cells	CYBB	0.451254	7.155496	1.173118	0.243899	-5.58502	0.680305	0.507086
NK.cells	PDCD6	0.119631	6.13963	1.173112	0.243902	-6.09326	0.690743	0.521631
NK.cells	PHF20L1	0.102151	7.046788	1.172715	0.24406	-6.23626	0.681414	0.508692
NK.cells	SAMM50	0.129348	5.177169	1.172672	0.244077	-5.88403	0.700795	0.535871
NK.cells	VIPAS39	0.140284	4.131233	1.172632	0.244093	-5.66508	0.7119	0.551703
NK.cells	ADGRL2	-0.36478	4.208198	-1.17251	0.244141	-5.38455	0.711076	0.55053
NK.cells	C1QBP	0.149218	6.055545	1.172497	0.244147	-6.03279	0.691615	0.522932
NK.cells	DNAJA4	0.306088	0.616749	1.172248	0.244247	-5.28874	0.750627	0.608404
NK.cells	FRY	0.152954	6.378637	1.172093	0.244309	-6.06761	0.688271	0.518294
NK.cells	GM45353	-0.52283	-0.0712	-1.17195	0.244367	-4.79629	0.758466	0.620135
NK.cells	ATP5G2	0.089701	8.844228	1.171879	0.244394	-6.51993	0.663339	0.483919
NK.cells	DST	0.267709	4.335211	1.171866	0.244399	-5.53947	0.709719	0.548634
NK.cells	CGAS	0.244818	2.747045	1.171704	0.244464	-5.58057	0.726891	0.573525
NK.cells	SFXN1	0.152508	5.121606	1.171323	0.244616	-5.84968	0.70138	0.537057
NK.cells	ZDHHC3	0.128378	5.048938	1.171087	0.24471	-5.88279	0.702146	0.538204
NK.cells	SIKE1	-0.14293	4.570739	-1.17078	0.244835	-5.74262	0.70721	0.54547
NK.cells	SLC9A3R2	-0.30214	2.701144	-1.17077	0.244838	-5.37481	0.727394	0.574601
NK.cells	PNKD	0.15169	4.867546	1.170573	0.244916	-5.84198	0.704062	0.541055
NK.cells	ERGIC3	0.11784	5.513051	1.170497	0.244946	-5.95857	0.697269	0.531457
NK.cells	TMEM53	-0.5612	0.637469	-1.17045	0.244965	-4.85963	0.750392	0.608671
NK.cells	CYP2D9	-0.53484	0.530177	-1.17035	0.245006	-4.8589	0.751608	0.610487
NK.cells	SPN	0.156144	3.586459	1.170314	0.24502	-5.7916	0.71776	0.560755
NK.cells	ARHGAP23	-0.2902	3.694446	-1.17029	0.245028	-5.31295	0.716594	0.559072
NK.cells	GM14295	-0.36418	1.289141	-1.17013	0.245095	-5.01048	0.74306	0.597791
NK.cells	FDX2	0.148752	4.192147	1.169841	0.245209	-5.69879	0.71126	0.551425
NK.cells	AFG3L2	0.11164	4.593409	1.169795	0.245227	-5.84455	0.706982	0.545301
NK.cells	KIF1A	0.609953	-0.59877	1.169689	0.24527	-4.76121	0.764549	0.629973
NK.cells	CRTC3	0.120529	5.658838	1.16944	0.245369	-6.07002	0.695796	0.529437
NK.cells	JAML	0.302935	2.748338	1.169116	0.245499	-5.41915	0.726931	0.574178
NK.cells	KIF3B	0.187759	3.495441	1.169082	0.245513	-5.49887	0.718797	0.562377
NK.cells	ABHD2	-0.11429	5.471153	-1.16895	0.245564	-5.99032	0.697759	0.532314
NK.cells	EWSR1	-0.08117	7.421625	-1.16891	0.245581	-6.28184	0.67765	0.504191
NK.cells	GM11110	0.24167	1.970897	1.168444	0.245768	-5.34073	0.735808	0.586861
NK.cells	OGFOD1	0.186099	3.611469	1.168359	0.245803	-5.57105	0.717844	0.560681
NK.cells	ZFP369	-0.23545	2.919936	-1.16809	0.245911	-5.39672	0.725391	0.571678

NK.cells	WDR27	-0.47718	0.499373	-1.16803	0.245933	-4.87529	0.752363	0.61147
NK.cells	TAF1C	0.281315	2.170342	1.167854	0.246005	-5.21666	0.73367	0.583743
NK.cells	1600012H(	0.228219	2.771016	1.167569	0.24612	-5.28511	0.727158	0.574079
NK.cells	CAPZA2	-0.0705	7.929486	-1.16707	0.24632	-6.34405	0.67296	0.497442
NK.cells	CDC5L	0.089564	5.768771	1.166993	0.246351	-6.02387	0.695103	0.528313
NK.cells	MTMR4	-0.25991	2.648419	-1.16639	0.246593	-5.24761	0.728502	0.576475
NK.cells	HIP1	0.15313	5.505684	1.166364	0.246604	-6.10259	0.697854	0.532407
NK.cells	ICA1L	-0.34282	1.590667	-1.16636	0.246604	-5.2427	0.740213	0.593682
NK.cells	CRYZL1	0.148518	4.570122	1.165586	0.246917	-5.75665	0.707732	0.546839
NK.cells	LNCPPARA	0.565087	0.60974	1.165441	0.246975	-4.86749	0.751253	0.610578
NK.cells	CENPF	-0.28034	5.050794	-1.16501	0.24715	-5.94403	0.702638	0.53979
NK.cells	HNRNPC	-0.06946	7.730209	-1.16477	0.247244	-6.33803	0.674969	0.50109
NK.cells	ERH	0.120697	7.372533	1.164746	0.247255	-6.2867	0.678592	0.506112
NK.cells	SMCO4	0.176861	4.10439	1.164643	0.247296	-5.73226	0.712706	0.554384
NK.cells	ECE1	-0.17601	5.613294	-1.16461	0.24731	-5.9915	0.696727	0.531582
NK.cells	HVCN1	0.263271	4.117697	1.164593	0.247317	-5.64837	0.712564	0.554182
NK.cells	PIK3C2A	0.121483	6.854141	1.164496	0.247356	-6.17529	0.683881	0.513547
NK.cells	CLK1	-0.09127	7.55289	-1.16443	0.247383	-6.33935	0.676762	0.503685
NK.cells	MRPS18B	0.186791	3.873974	1.164232	0.247462	-5.55855	0.715181	0.558064
NK.cells	NPHP4	-0.51625	0.297045	-1.16422	0.247467	-4.9091	0.754809	0.616415
NK.cells	GM42829	0.271183	2.205641	1.164199	0.247475	-5.28939	0.73338	0.584581
NK.cells	NUDT6	0.300032	2.183509	1.163778	0.247645	-5.21482	0.733625	0.585154
NK.cells	SRP72	0.088572	6.657086	1.163747	0.247657	-6.19514	0.685903	0.51665
NK.cells	HEATR1	0.167555	4.84445	1.163421	0.247789	-5.81875	0.70482	0.543399
NK.cells	SERPINB8	0.58723	-0.3114	1.163408	0.247794	-4.79805	0.761778	0.627154
NK.cells	CDH17	-0.38181	0.13748	-1.16339	0.247801	-5.1387	0.75663	0.61939
NK.cells	CDKL4	-0.40876	1.561956	-1.16335	0.247817	-5.13116	0.740534	0.595362
NK.cells	KLF12	-0.24613	3.585919	-1.16323	0.247867	-5.70591	0.718289	0.562842
NK.cells	PIM2	0.220488	3.150665	1.163206	0.247876	-5.47383	0.723011	0.5697
NK.cells	SDHAF2	0.150186	4.655098	1.163128	0.247907	-5.75189	0.706829	0.546339
NK.cells	GM5608	-0.53938	0.923456	-1.16308	0.247925	-4.88036	0.747704	0.606094
NK.cells	AGL	0.238611	3.963587	1.162944	0.247981	-5.54601	0.714218	0.557001
NK.cells	UBR7	0.165927	4.241439	1.162848	0.24802	-5.70845	0.711239	0.55271
NK.cells	IQCK	-0.63756	0.158994	-1.16276	0.248056	-4.78062	0.756384	0.619211
NK.cells	TACC3	-0.22426	5.072041	-1.16271	0.248078	-5.90309	0.702414	0.540136
NK.cells	CCR7	0.223097	4.535564	1.162513	0.248156	-5.98242	0.7081	0.548264
NK.cells	GPAM	-0.31214	3.696477	-1.16245	0.248182	-5.37289	0.717094	0.561219
NK.cells	NOXRED1	0.488424	0.546984	1.162261	0.248257	-4.9254	0.751965	0.612582
NK.cells	FABP7	-0.68066	1.989157	-1.16185	0.248422	-4.99864	0.735778	0.588643
NK.cells	ZFP691	0.289968	3.075803	1.161832	0.248431	-5.27768	0.723827	0.571118
NK.cells	ANKRD17	-0.09037	7.985576	-1.16149	0.248567	-6.38373	0.672395	0.498236
NK.cells	B930036N1	-0.19086	5.605823	-1.16149	0.248567	-5.93875	0.696805	0.53236
NK.cells	SMAP2	0.096429	6.821529	1.161485	0.248571	-6.17538	0.684215	0.514643
NK.cells	1810059H2	-0.5987	1.799204	-1.16146	0.248582	-4.96185	0.737889	0.591846
NK.cells	MID1	-0.46823	5.805162	-1.1613	0.248644	-5.93155	0.694724	0.529504
NK.cells	SUV39H2	-0.27208	2.381659	-1.16101	0.248761	-5.28034	0.731437	0.582562
NK.cells	CCL12	1.046068	0.036828	1.160872	0.248819	-4.78371	0.757781	0.621877

NK.cells	RANGRF	0.490526	1.262196	1.16072	0.24888	-4.96626	0.743891	0.601146
NK.cells	DIABLO	0.155204	4.259159	1.160716	0.248882	-5.70512	0.711049	0.553065
NK.cells	NID1	-0.42225	2.394724	-1.16068	0.248895	-5.04097	0.731293	0.582516
NK.cells	FAM120B	0.156877	4.124917	1.160528	0.248958	-5.71571	0.712495	0.555158
NK.cells	TMEM245	0.147533	4.753996	1.160151	0.249111	-5.84401	0.706052	0.545611
NK.cells	TMEM164	0.105719	6.945657	1.159684	0.2493	-6.20608	0.683445	0.513419
NK.cells	ANKRD49	0.235046	3.032797	1.159644	0.249316	-5.32413	0.724827	0.5725
NK.cells	D030056L2	0.200904	3.888031	1.159407	0.249412	-5.6184	0.71557	0.559125
NK.cells	EGR2	-0.41866	2.237621	-1.15906	0.249554	-5.25815	0.733581	0.585445
NK.cells	KBTBD8	-0.5451	0.251382	-1.15904	0.24956	-4.83161	0.7559	0.618664
NK.cells	DIO1	-0.46123	0.977554	-1.15896	0.249592	-4.94455	0.747658	0.606352
NK.cells	ERCC6L	-0.28476	3.034059	-1.15886	0.249635	-5.40776	0.724829	0.572673
NK.cells	GPC4	-0.52022	1.1587	-1.15874	0.249681	-4.92887	0.745617	0.603392
NK.cells	KLHL21	-0.25948	2.689731	-1.1586	0.249738	-5.42978	0.728599	0.578333
NK.cells	ZFYVE9	-0.44169	2.603777	-1.15812	0.249934	-5.12171	0.729942	0.579923
NK.cells	6530413G1	0.488855	-0.08741	1.157611	0.250141	-4.83442	0.760569	0.625002
NK.cells	2010315B0	0.280503	2.024895	1.157521	0.250177	-5.19117	0.736703	0.589411
NK.cells	OTUD6B	-0.1308	4.301102	-1.15725	0.250285	-5.72529	0.711879	0.553383
NK.cells	GM5089	0.527624	0.395428	1.157105	0.250346	-4.90125	0.755044	0.616907
NK.cells	PIAS3	-0.20195	2.896492	-1.15708	0.250356	-5.38435	0.72709	0.575481
NK.cells	PTPN7	-0.1588	4.151708	-1.15638	0.25064	-5.88391	0.714078	0.556035
NK.cells	SNRPD3	0.105182	6.518599	1.156171	0.250726	-6.15511	0.689142	0.520623
NK.cells	SNX25	0.121911	5.540177	1.156105	0.250752	-5.95501	0.699333	0.53503
NK.cells	POLR2D	-0.13626	5.184758	-1.1559	0.250836	-5.91007	0.703075	0.540406
NK.cells	ELN	0.466841	2.40021	1.15583	0.250864	-5.19684	0.733162	0.583985
NK.cells	ARL6IP4	0.116037	5.733017	1.15565	0.250938	-6.02475	0.697349	0.532271
NK.cells	6030458C1	0.210247	2.74674	1.155252	0.2511	-5.39662	0.729402	0.578432
NK.cells	UMAD1	0.134197	5.658219	1.154993	0.251205	-5.96249	0.698151	0.533503
NK.cells	ATP5E	0.078881	8.783189	1.154829	0.251272	-6.53991	0.666235	0.489127
NK.cells	MRPS33	0.093229	6.714474	1.1547	0.251325	-6.18445	0.687178	0.518144
NK.cells	TMEM87B	0.124261	5.127611	1.154542	0.251389	-5.89105	0.703736	0.541564
NK.cells	SSH1	0.163261	3.775324	1.154392	0.25145	-5.66886	0.718192	0.562403
NK.cells	FAM172A	0.093624	7.557353	1.154327	0.251477	-6.32742	0.678558	0.506229
NK.cells	ARCN1	0.110637	5.873425	1.15425	0.251508	-6.03709	0.6959	0.530577
NK.cells	RNF113A2	0.158823	4.271264	1.154057	0.251587	-5.71498	0.712853	0.554803
NK.cells	STK38	0.10755	6.531625	1.15405	0.25159	-6.18105	0.689064	0.520966
NK.cells	ODF2L	0.287547	2.682465	1.153659	0.251749	-5.20994	0.730109	0.580133
NK.cells	GM29019	0.645278	0.361473	1.153645	0.251755	-4.72817	0.756126	0.618789
NK.cells	FAM149B	0.214409	2.992688	1.153494	0.251816	-5.4446	0.726705	0.575241
NK.cells	MRPL2	0.154005	4.862057	1.153461	0.25183	-5.83284	0.70655	0.546078
NK.cells	ZC3H6	0.213096	3.379852	1.153438	0.251839	-5.51055	0.72248	0.569078
NK.cells	BUB1B	-0.21648	4.016283	-1.15269	0.252145	-5.68085	0.715731	0.559383
NK.cells	TMEM260	0.21405	3.273577	1.152624	0.252171	-5.51571	0.723777	0.571066
NK.cells	CENPP	-0.1991	5.39603	-1.15249	0.252228	-5.95188	0.701041	0.538358
NK.cells	MAP3K10	0.238625	2.731125	1.152471	0.252234	-5.37771	0.729715	0.579806
NK.cells	6430590A0	0.299323	1.647577	1.152286	0.25231	-5.11587	0.741733	0.597609
NK.cells	NECAB2	-0.56044	0.442881	-1.15214	0.25237	-4.91368	0.755343	0.617954

NK.cells	GM15892	-0.26181	3.443855	-1.15207	0.252396	-5.55232	0.721924	0.568504
NK.cells	GM14471	0.556272	0.24931	1.151957	0.252444	-4.83645	0.757554	0.621302
NK.cells	PDLIM7	-0.21002	3.638312	-1.15188	0.252475	-5.53499	0.719814	0.565472
NK.cells	NSRP1	0.123784	5.229533	1.151729	0.252537	-5.91632	0.702796	0.541037
NK.cells	VPS51	0.177788	3.730024	1.151711	0.252545	-5.62712	0.718821	0.5641
NK.cells	RBM27	-0.09128	6.399999	-1.15144	0.252654	-6.16866	0.690595	0.523749
NK.cells	COQ8A	-0.29836	2.303584	-1.15125	0.252734	-5.22154	0.734472	0.587042
NK.cells	CPEB1	-0.45023	0.696287	-1.15124	0.252737	-4.9792	0.752499	0.61386
NK.cells	NFKBIB	0.134271	5.622292	1.150808	0.252914	-6.02276	0.699007	0.535368
NK.cells	GM4013	0.291792	1.984122	1.150678	0.252967	-5.12949	0.738341	0.592459
NK.cells	SLC44A1	-0.2082	4.637679	-1.15038	0.25309	-5.58784	0.709598	0.550419
NK.cells	CSGALNAC	0.152821	4.10884	1.150233	0.253149	-5.67134	0.715264	0.558609
NK.cells	APOBR	0.192846	2.373103	1.149932	0.253273	-5.70829	0.73439	0.586249
NK.cells	CREM	-0.15855	6.0952	-1.14956	0.253426	-6.20455	0.694665	0.528591
NK.cells	SNRNP70	-0.0615	7.511442	-1.14935	0.253511	-6.34169	0.680146	0.508198
NK.cells	PDE8A	-0.16262	5.847934	-1.14913	0.253601	-6.06929	0.697376	0.532317
NK.cells	NFATC2	-0.1813	2.489047	-1.14901	0.253652	-5.74573	0.733513	0.584551
NK.cells	DOCK4	-0.16994	6.427831	-1.14882	0.253729	-6.07922	0.691383	0.523941
NK.cells	TUBA4A	-0.16493	4.513714	-1.14856	0.253836	-5.81674	0.711583	0.552692
NK.cells	STAG2	-0.10683	7.464582	-1.14838	0.253911	-6.30412	0.680767	0.509134
NK.cells	BLOC1S4	0.175216	3.695064	1.148347	0.253923	-5.55052	0.720401	0.565466
NK.cells	ZMYND8	0.109466	6.108671	1.14775	0.254168	-6.06386	0.694834	0.529012
NK.cells	ANK	-0.18431	3.446389	-1.14774	0.254171	-5.83475	0.723199	0.569707
NK.cells	ARF5	0.108853	8.486216	1.147478	0.25428	-6.52618	0.670535	0.495183
NK.cells	TOMT	-0.51288	1.736925	-1.14737	0.254324	-4.98054	0.742071	0.597565
NK.cells	ANGPTL6	-0.52378	0.97577	-1.14733	0.254341	-4.88753	0.750643	0.610349
NK.cells	NIN	0.119092	5.920519	1.147282	0.25436	-6.14654	0.696798	0.531902
NK.cells	MRPS25	-0.15651	4.238863	-1.14713	0.254421	-5.73737	0.714626	0.557458
NK.cells	SLC17A3	-0.55777	0.779648	-1.1471	0.254435	-4.9229	0.752868	0.61376
NK.cells	STAP2	-0.66537	0.504981	-1.14662	0.254634	-4.79367	0.756091	0.618645
NK.cells	RING1	0.190685	3.585545	1.146427	0.254712	-5.57931	0.721775	0.567917
NK.cells	GCA	0.571515	1.099128	1.146006	0.254885	-4.96626	0.74934	0.608671
NK.cells	CLDN5	-0.61652	1.006603	-1.14589	0.254932	-4.85882	0.750387	0.610298
NK.cells	LAG3	0.294071	1.429969	1.145845	0.254951	-5.39572	0.745608	0.603161
NK.cells	ESF1	0.115416	5.001622	1.145754	0.254989	-5.89144	0.706567	0.546107
NK.cells	ATXN3	0.12072	5.054807	1.145654	0.25503	-5.88917	0.706002	0.545299
NK.cells	NDUFAF4	0.187621	3.867148	1.145653	0.25503	-5.60605	0.718723	0.563628
NK.cells	GEMIN5	0.181113	3.385628	1.145522	0.255084	-5.59876	0.723951	0.571262
NK.cells	WRN	0.11995	5.831312	1.145442	0.255117	-6.03349	0.697818	0.533694
NK.cells	CARNMT1	-0.11492	5.353674	-1.14542	0.255124	-5.94518	0.70284	0.54084
NK.cells	D830050J1	0.476411	1.312085	1.145123	0.255249	-4.94278	0.747029	0.605305
NK.cells	PITPNM2	-0.18227	3.66119	-1.14506	0.255276	-5.68559	0.721044	0.567023
NK.cells	GRIK4	-0.75925	0.238216	-1.14473	0.255409	-4.80855	0.759455	0.623706
NK.cells	GM14963	0.582814	0.898614	1.144462	0.255521	-4.93084	0.751955	0.612413
NK.cells	ZNRF2	0.104288	5.763077	1.144274	0.255599	-6.07657	0.698853	0.53495
NK.cells	AARSD1	0.173495	3.977143	1.143863	0.255768	-5.69433	0.717863	0.562329
NK.cells	A530072M	0.495378	0.764735	1.14386	0.255769	-4.97093	0.753476	0.614876

NK.cells	GM21781	0.379324	1.561921	1.143756	0.255812	-5.0633	0.744466	0.601422
NK.cells	POGLUT3	0.449067	0.979034	1.143729	0.255823	-4.96092	0.751043	0.611243
NK.cells	KLHL8	-0.32893	2.402928	-1.14358	0.255885	-5.28477	0.735084	0.587523
NK.cells	NAA60	0.141957	4.697726	1.143493	0.255921	-5.7965	0.710126	0.551177
NK.cells	SNX32	0.227535	2.812618	1.143146	0.256064	-5.37477	0.73056	0.580915
NK.cells	ABCB11	-0.42374	1.681529	-1.14313	0.25607	-5.08894	0.743124	0.599477
NK.cells	ZFP821	0.209701	3.905173	1.142874	0.256176	-5.48684	0.718641	0.563667
NK.cells	TMEM63A	-0.23566	3.49863	-1.14244	0.256355	-5.53149	0.723051	0.570238
NK.cells	SSB	-0.08271	6.803522	-1.1424	0.256372	-6.22939	0.688033	0.520125
NK.cells	GM13012	0.261575	2.775114	1.142343	0.256396	-5.30171	0.730973	0.581869
NK.cells	XAF1	0.308549	4.033526	1.142152	0.256474	-5.7613	0.717254	0.561884
NK.cells	ACO1	-0.16766	3.712371	-1.14213	0.256484	-5.61118	0.720729	0.566929
NK.cells	SH2D1A	0.243999	0.051779	1.142097	0.256497	-5.50657	0.761632	0.627656
NK.cells	LUZP1	-0.12989	5.787075	-1.1421	0.256497	-6.0833	0.698601	0.535114
NK.cells	GM13889	-0.57015	1.14195	-1.14186	0.256595	-4.90937	0.749309	0.609068
NK.cells	LUM	-0.63285	0.551186	-1.14163	0.256692	-4.86115	0.756094	0.61922
NK.cells	CSRNP2	-0.2923	2.107884	-1.1414	0.256785	-5.27779	0.738542	0.593065
NK.cells	EHBP1L1	-0.11998	6.149657	-1.14137	0.2568	-6.02863	0.694981	0.529978
NK.cells	MAPRE1	-0.07829	7.148653	-1.14117	0.256879	-6.27207	0.684704	0.51551
NK.cells	HERC1	-0.1068	7.056727	-1.14083	0.257022	-6.28426	0.685673	0.516899
NK.cells	GM49692	0.520931	0.104027	1.140593	0.25712	-4.88808	0.761303	0.627283
NK.cells	KCNIP3	0.461859	0.193763	1.140402	0.257199	-5.15301	0.760272	0.625764
NK.cells	COMMD7	0.09421	5.29863	1.140395	0.257202	-5.97434	0.703995	0.54294
NK.cells	CRIM1	-0.18348	5.191827	-1.14025	0.257263	-6.09426	0.705125	0.544601
NK.cells	CBX3	-0.10493	8.102288	-1.1402	0.257282	-6.44464	0.675028	0.502242
NK.cells	COQ7	-0.18888	4.79596	-1.14014	0.257307	-5.70533	0.709332	0.550667
NK.cells	TEC	-0.13334	5.751113	-1.13983	0.257436	-6.06475	0.699415	0.536269
NK.cells	PMS2	0.147264	3.954235	1.13907	0.257751	-5.75099	0.719083	0.564096
NK.cells	BIK	-0.44122	1.76969	-1.1389	0.257823	-5.14128	0.743141	0.5995
NK.cells	PDZD4	-0.56029	1.698737	-1.13884	0.257844	-4.87585	0.743937	0.600704
NK.cells	CSE1L	-0.12163	5.5229	-1.1388	0.257863	-5.9964	0.702327	0.540078
NK.cells	GATB	0.255045	3.11047	1.138347	0.258051	-5.41204	0.72841	0.577777
NK.cells	ADARB1	-0.3254	3.009129	-1.13833	0.258056	-5.31036	0.729523	0.579408
NK.cells	KDM5A	-0.09577	7.014524	-1.13803	0.258181	-6.26612	0.686918	0.518407
NK.cells	TBC1D14	-0.11996	5.680933	-1.13763	0.258348	-6.00127	0.700793	0.5381
NK.cells	DPYSL3	-0.62128	0.753878	-1.13751	0.2584	-4.83041	0.754761	0.61718
NK.cells	SURF6	0.176175	3.185009	1.137261	0.258502	-5.47644	0.727593	0.57697
NK.cells	JADE2	-0.20554	3.270443	-1.13722	0.258518	-5.68334	0.726657	0.575613
NK.cells	IFIT1BL2	0.675726	-0.46837	1.137161	0.258544	-4.78586	0.768823	0.638631
NK.cells	CD38	0.249559	5.882237	1.137019	0.258603	-5.95982	0.698679	0.535312
NK.cells	ZFP382	0.312385	2.008207	1.136743	0.258717	-5.27837	0.740611	0.59635
NK.cells	ABHD14B	-0.34989	2.125702	-1.13674	0.258719	-5.1289	0.7393	0.594405
NK.cells	ZDHHC18	0.11895	5.57552	1.136624	0.258767	-6.11385	0.701903	0.540056
NK.cells	COPG1	-0.12667	5.141092	-1.13651	0.258814	-5.9637	0.706497	0.54666
NK.cells	DCAF11	-0.14243	4.559305	-1.13646	0.258833	-5.81833	0.712701	0.555597
NK.cells	EIF2B4	-0.12821	4.102435	-1.13627	0.258915	-5.7544	0.717615	0.562727
NK.cells	KIF2A	0.093602	6.338703	1.136197	0.258945	-6.20713	0.693911	0.52879

NK.cells	RCL1	0.148134	4.24025	1.136113	0.258979	-5.77496	0.716129	0.560611
NK.cells	NAALADL2	-0.35573	1.964286	-1.13596	0.259042	-5.1285	0.741102	0.597278
NK.cells	ALG2	-0.21046	3.062397	-1.13583	0.259098	-5.3602	0.728938	0.57935
NK.cells	ETV6	0.130932	8.156803	1.135801	0.259109	-6.37846	0.675275	0.502756
NK.cells	ABCC5	0.192247	4.301545	1.135113	0.259396	-5.81036	0.715788	0.560032
NK.cells	SUPT5	-0.10845	6.05341	-1.13509	0.259406	-6.09294	0.697198	0.533374
NK.cells	OSBPL8	0.111817	7.62098	1.135008	0.25944	-6.353	0.681013	0.510598
NK.cells	RAB2A	-0.0596	8.094947	-1.13495	0.259465	-6.46079	0.676201	0.503907
NK.cells	NBN	0.193389	3.4476	1.13465	0.259589	-5.56786	0.725224	0.573592
NK.cells	ANXA5	0.131155	6.137389	1.134369	0.259706	-6.12899	0.696504	0.532343
NK.cells	HNRNPF	-0.07334	9.158537	-1.13426	0.25975	-6.59131	0.665718	0.489459
NK.cells	XPO1	0.130548	5.786847	1.134209	0.259773	-6.06021	0.700177	0.537624
NK.cells	GM9967	-0.72442	0.569425	-1.1337	0.259984	-4.82971	0.757532	0.621798
NK.cells	IFIT1BL1	0.597359	0.159661	1.133698	0.259986	-5.01219	0.762235	0.628912
NK.cells	FBXO22	0.129986	4.616588	1.133671	0.259997	-5.8303	0.712716	0.555615
NK.cells	TPMT	-0.31053	2.097645	-1.13316	0.260209	-5.16154	0.740368	0.596098
NK.cells	A530040E1	0.676462	0.013043	1.132967	0.260291	-4.77799	0.764031	0.631762
NK.cells	IVD	-0.15785	4.435995	-1.13294	0.260301	-5.75695	0.714753	0.558659
NK.cells	GM11755	-0.77289	-0.115	-1.13277	0.260374	-4.75219	0.76551	0.634041
NK.cells	BMT2	-0.1174	6.027835	-1.13275	0.260381	-6.08826	0.697866	0.534467
NK.cells	CRTAM	0.302118	1.176627	1.132722	0.260393	-5.56673	0.750726	0.611717
NK.cells	GPHN	0.108944	7.486182	1.132242	0.260594	-6.30335	0.682949	0.513393
NK.cells	YTHDC1	-0.07471	7.312924	-1.1322	0.260613	-6.33238	0.684722	0.515875
NK.cells	GM47828	-0.55462	0.334612	-1.13193	0.260726	-4.84033	0.760517	0.62641
NK.cells	DHRS7B	0.215541	3.137991	1.13189	0.260741	-5.45483	0.729032	0.579451
NK.cells	ZFAND5	-0.08795	7.203792	-1.13185	0.260758	-6.27015	0.685842	0.517444
NK.cells	RNF20	0.118007	5.755436	1.131441	0.260929	-6.07736	0.701197	0.538837
NK.cells	FITM2	-0.40925	0.918829	-1.13045	0.261345	-4.93311	0.754734	0.616875
NK.cells	TDP2	-0.15597	4.340054	-1.13031	0.261403	-5.744	0.716812	0.560902
NK.cells	PSTPIP1	0.140121	4.472927	1.130295	0.261409	-6.00354	0.715381	0.558832
NK.cells	GM49864	-0.59926	0.452692	-1.13022	0.26144	-4.82849	0.760064	0.624934
NK.cells	C5AR2	0.623101	1.231827	1.130173	0.26146	-4.85371	0.751177	0.611581
NK.cells	ANXA6	-0.11136	6.174347	-1.1299	0.261572	-6.26072	0.697334	0.533137
NK.cells	WDR3	0.136958	4.225869	1.129809	0.261613	-5.78972	0.718044	0.562888
NK.cells	ARID4B	-0.08477	8.259309	-1.12959	0.261705	-6.48551	0.675898	0.503162
NK.cells	TTC27	0.192041	3.520631	1.129474	0.261753	-5.57467	0.725705	0.574153
NK.cells	RAD18	0.17759	4.103468	1.129374	0.261795	-5.78207	0.719367	0.564949
NK.cells	RRAD	-0.21732	2.552475	-1.12924	0.261851	-5.62074	0.736365	0.58993
NK.cells	UBE2A	-0.08425	6.786705	-1.1291	0.26191	-6.19478	0.690961	0.524364
NK.cells	ACLY	-0.09503	6.383195	-1.12899	0.261957	-6.13092	0.695153	0.530287
NK.cells	PHRF1	-0.10433	5.804424	-1.12866	0.262093	-6.0723	0.701215	0.539041
NK.cells	RAB3D	0.22891	3.11921	1.128538	0.262146	-5.52107	0.730104	0.580875
NK.cells	GM14023	-0.47996	1.123999	-1.12854	0.262147	-4.99139	0.7524	0.614011
NK.cells	FGFR2	-0.13352	5.987704	-1.12853	0.262148	-6.01422	0.699289	0.536297
NK.cells	2500004C0	0.433083	1.043374	1.128323	0.262236	-4.94049	0.753395	0.615502
NK.cells	KCTD18	0.274292	3.044012	1.128006	0.262369	-5.43963	0.731212	0.582351
NK.cells	SNAI1	-0.4989	0.389835	-1.1274	0.262622	-4.86994	0.761506	0.627172

NK.cells	H1F0	0.165976	6.001224	1.127278	0.262675	-6.09094	0.699809	0.536576
NK.cells	HIVEP2	-0.14773	8.386816	-1.12703	0.262779	-6.56554	0.67525	0.502202
NK.cells	NAA16	0.105753	5.024992	1.126993	0.262795	-5.96832	0.710142	0.551475
NK.cells	XCL1	-0.23357	2.335401	-1.12694	0.262817	-5.94664	0.739477	0.594366
NK.cells	ATG13	-0.13942	4.765899	-1.12668	0.262926	-5.91104	0.713042	0.55559
NK.cells	UBTD1	0.20344	4.402162	1.126443	0.263026	-5.59575	0.717063	0.56133
NK.cells	SPINK10	-0.5071	0.791956	-1.12603	0.2632	-4.92204	0.757478	0.620881
NK.cells	PNP2	0.628528	0.616927	1.125765	0.263312	-4.85586	0.759486	0.624095
NK.cells	TOPBP1	-0.14589	5.680028	-1.12545	0.263444	-6.06447	0.703734	0.542257
NK.cells	HAUS3	-0.1358	4.199727	-1.12519	0.263552	-5.76434	0.719562	0.565131
NK.cells	SERINC4	-0.56393	0.365548	-1.12515	0.26357	-4.8692	0.762374	0.62871
NK.cells	COASY	0.241752	3.053875	1.124936	0.263661	-5.41959	0.73208	0.583443
NK.cells	RNF215	-0.20752	2.627986	-1.12494	0.263661	-5.38194	0.736793	0.590396
NK.cells	SMG1	0.078868	7.499087	1.124601	0.263802	-6.36625	0.684804	0.515675
NK.cells	YY1	-0.07181	7.144963	-1.12448	0.263853	-6.29794	0.688445	0.520823
NK.cells	ZFP825	0.291195	2.050646	1.124446	0.263868	-5.22641	0.743233	0.60016
NK.cells	CDYL2	0.166502	4.757934	1.124142	0.263996	-5.8897	0.713548	0.556658
NK.cells	SLC25A26	-0.18913	3.795963	-1.1241	0.264015	-5.65114	0.723947	0.571792
NK.cells	RCSD1	0.132822	7.091061	1.124051	0.264034	-6.2225	0.689001	0.521686
NK.cells	PDCD6IP	-0.06592	6.988379	-1.12372	0.264172	-6.33359	0.690061	0.523232
NK.cells	TBCB	0.098155	6.070727	1.123476	0.264277	-6.13604	0.69962	0.536839
NK.cells	EPHA1	-0.63581	0.461905	-1.12343	0.264296	-4.83162	0.761266	0.627487
NK.cells	CCDC134	-0.23581	3.407824	-1.12335	0.26433	-5.44182	0.728189	0.578167
NK.cells	GM49439	0.69399	-0.46286	1.123269	0.264364	-4.7633	0.771974	0.64384
NK.cells	SP3OS	0.172324	4.205373	1.123226	0.264382	-5.65112	0.719501	0.565515
NK.cells	SLC25A21	-0.54784	1.867089	-1.12304	0.26446	-5.16628	0.745293	0.603644
NK.cells	PTPN11	0.105513	5.063935	1.122939	0.264503	-5.9499	0.710275	0.552269
NK.cells	KNTC1	-0.31269	2.818917	-1.1229	0.264519	-5.35999	0.734676	0.587876
NK.cells	B020010K1	0.46973	1.134374	1.122889	0.264524	-5.03289	0.753577	0.616064
NK.cells	CEP19	0.282768	2.707827	1.122559	0.264664	-5.37925	0.736081	0.589786
NK.cells	BORCS7	0.179699	3.716689	1.122456	0.264707	-5.56528	0.724982	0.57354
NK.cells	R3HDM4	-0.12446	6.20323	-1.12204	0.264883	-6.1036	0.69843	0.535258
NK.cells	PTGES2	0.280635	2.422157	1.12192	0.264934	-5.24007	0.739292	0.594664
NK.cells	ZCCHC9	-0.10386	5.404138	-1.12185	0.264965	-6.02285	0.706856	0.547306
NK.cells	CYP4A10	0.665219	1.117903	1.121799	0.264986	-4.99554	0.753979	0.61662
NK.cells	ATP23	-0.41241	1.824257	-1.12171	0.265021	-5.18526	0.745987	0.604634
NK.cells	KIFAP3	0.233018	2.849059	1.121305	0.265194	-5.29292	0.73481	0.587749
NK.cells	TXNIP	0.160482	5.791358	1.12121	0.265235	-6.06196	0.703006	0.541534
NK.cells	OPA1	0.124267	4.835691	1.120612	0.265488	-5.87395	0.713687	0.556318
NK.cells	IGF2BP2	0.40941	1.457318	1.120315	0.265614	-5.06373	0.751123	0.611281
NK.cells	PPHLN1	0.143346	4.932629	1.119959	0.265765	-5.90046	0.713059	0.55503
NK.cells	LIPH	0.62411	-0.05189	1.119824	0.265822	-4.83345	0.768686	0.637551
NK.cells	DNAJC24	0.182705	3.89302	1.119262	0.26606	-5.69576	0.724771	0.571569
NK.cells	CHUK	-0.12137	5.037427	-1.11913	0.266117	-5.94556	0.712406	0.553659
NK.cells	CNKSR3	-0.22508	4.754523	-1.11888	0.266221	-5.75785	0.715557	0.558089
NK.cells	TRIM35	-0.13686	5.607952	-1.11866	0.266315	-6.08137	0.706534	0.545006
NK.cells	RTN4	-0.08423	7.592315	-1.11835	0.266446	-6.34731	0.68601	0.515766

NK.cells	TEFM	0.477605	1.160891	1.11712	0.26697	-4.98786	0.756867	0.617369
NK.cells	OXSM	0.30835	1.788374	1.116964	0.267036	-5.15075	0.749736	0.606729
NK.cells	MRPL50	-0.16268	4.009842	-1.11677	0.267117	-5.61444	0.725062	0.570369
NK.cells	PDE10A	-0.34649	2.555984	-1.11647	0.267246	-5.36936	0.74111	0.593915
NK.cells	SUCLG1	0.133848	5.422184	1.11633	0.267306	-5.98017	0.709832	0.548379
NK.cells	S100A16	-0.33721	2.552707	-1.11613	0.267391	-5.17509	0.741146	0.593996
NK.cells	RAB6B	0.33413	1.697022	1.115852	0.267509	-5.17089	0.75077	0.608387
NK.cells	PEX7	0.133755	4.45855	1.115727	0.267562	-5.7725	0.720185	0.563418
NK.cells	TUBG1	-0.26142	3.393021	-1.11571	0.267572	-5.53221	0.731824	0.580382
NK.cells	TESK1	-0.16376	3.781202	-1.11565	0.267596	-5.68866	0.72756	0.574182
NK.cells	ZFP59	-0.56938	0.230401	-1.11556	0.267632	-4.86725	0.767575	0.633755
NK.cells	PSMB10	0.149275	5.706129	1.115497	0.26766	-6.14406	0.706812	0.544224
NK.cells	GAS2L1	0.37735	1.765951	1.115402	0.267701	-5.09481	0.74999	0.607298
NK.cells	OLFR543	-0.53125	0.201065	-1.1153	0.267743	-4.90815	0.767915	0.634293
NK.cells	ZFP148	0.087007	6.830997	1.115015	0.267866	-6.23267	0.694989	0.527513
NK.cells	E230016K2	-0.56653	0.143576	-1.11495	0.267894	-4.87096	0.768582	0.635391
NK.cells	KLRB1A	0.285077	-0.08222	1.114839	0.267941	-5.48021	0.771208	0.639433
NK.cells	NPHP3	0.453075	1.097931	1.114789	0.267962	-4.98204	0.757587	0.618824
NK.cells	NDUFA9	0.126867	4.840567	1.114018	0.268291	-5.89499	0.716667	0.557873
NK.cells	KHDRBS3	-0.48034	1.943216	-1.11397	0.26831	-5.03855	0.74862	0.604719
NK.cells	HCK	-0.3737	5.693071	-1.11345	0.268535	-5.36366	0.70787	0.544991
NK.cells	FAM136A	0.184436	4.090791	1.113405	0.268552	-5.66072	0.72512	0.569851
NK.cells	GM15972	-0.63631	-0.2921	-1.11314	0.268668	-4.8365	0.774737	0.643799
NK.cells	RRM1	-0.17477	5.449593	-1.11302	0.268716	-6.01296	0.71053	0.548853
NK.cells	5031439G(	-0.17617	5.696635	-1.11268	0.268862	-5.75295	0.7079	0.545192
NK.cells	INPP4A	-0.12081	6.032154	-1.11256	0.268915	-6.14146	0.704345	0.540138
NK.cells	CNOT3	0.113664	5.46868	1.112394	0.268984	-6.03493	0.710327	0.54872
NK.cells	TSPAN12	-0.52984	1.194761	-1.11237	0.268996	-4.97551	0.757536	0.618005
NK.cells	SCFD1	0.145586	4.929173	1.112353	0.269002	-5.91641	0.716106	0.557024
NK.cells	GM49164	0.463164	0.920748	1.11209	0.269114	-4.99492	0.760822	0.622867
NK.cells	A730063M	0.510016	1.218514	1.111262	0.269468	-4.96599	0.758157	0.618046
NK.cells	CAD	0.337114	1.795668	1.111053	0.269558	-5.23525	0.751585	0.608279
NK.cells	MED4	0.136423	4.020834	1.111045	0.269561	-5.7227	0.726809	0.571767
NK.cells	RAB11FIP2	0.194305	3.576284	1.110757	0.269685	-5.65042	0.731785	0.579057
NK.cells	ERRFI1	-0.14682	5.004787	-1.11049	0.269798	-6.07875	0.716231	0.556527
NK.cells	XRN2	-0.07097	7.516217	-1.11041	0.269832	-6.40668	0.689757	0.518958
NK.cells	HPCAL1	0.104417	6.117607	1.110227	0.269912	-6.25249	0.704365	0.539604
NK.cells	TIMM23	0.083084	6.942518	1.110087	0.269971	-6.29073	0.695708	0.527385
NK.cells	CD14	0.671772	4.260683	1.110038	0.269992	-5.23806	0.724288	0.568267
NK.cells	IFI203	0.164166	6.302394	1.109715	0.270131	-6.22956	0.702415	0.536885
NK.cells	TAF3	0.109813	5.653075	1.109671	0.27015	-6.05425	0.709292	0.546679
NK.cells	TUBB3	0.465899	0.736309	1.109576	0.270191	-4.95055	0.763797	0.626807
NK.cells	MEF2B	-0.19331	3.605786	-1.1092	0.270352	-5.57438	0.73146	0.578863
NK.cells	ZFP706	-0.08039	7.871583	-1.10917	0.270364	-6.37567	0.686099	0.514048
NK.cells	NCAPD3	-0.14949	5.127768	-1.10916	0.27037	-6.00636	0.714909	0.554851
NK.cells	GM15964	0.690965	0.162356	1.109123	0.270385	-4.86817	0.770445	0.637012
NK.cells	REXO1	-0.12721	4.912691	-1.10893	0.270466	-5.9234	0.717278	0.558183

NK.cells	MRPL12	0.141491	5.090142	1.108346	0.270719	-5.94112	0.715875	0.555564
NK.cells	PIBF1	0.133764	5.059147	1.107836	0.270937	-5.91366	0.716468	0.556274
NK.cells	S100A9	0.451878	6.814997	1.107647	0.271019	-6.1122	0.697839	0.529793
NK.cells	TRRAP	-0.1367	4.784536	-1.10749	0.271086	-5.88863	0.719431	0.560618
NK.cells	CRIP2	-0.23685	4.565121	-1.10715	0.271231	-5.61969	0.721808	0.564053
NK.cells	MLX	0.169366	3.767938	1.107096	0.271255	-5.64562	0.730515	0.576722
NK.cells	ZFP446	0.412461	1.164128	1.106969	0.27131	-5.01657	0.759752	0.620039
NK.cells	XYLT2	0.252917	1.972195	1.106134	0.271669	-5.31906	0.750547	0.606739
NK.cells	UFC1	0.085765	5.792898	1.105836	0.271798	-6.09344	0.708618	0.545559
NK.cells	MAFK	-0.20558	4.524193	-1.10583	0.271798	-5.76729	0.722252	0.565173
NK.cells	ROMO1	0.094158	6.595619	1.105812	0.271808	-6.21117	0.700137	0.533502
NK.cells	MDM4	0.097905	5.749077	1.105798	0.271814	-6.07321	0.709084	0.546225
NK.cells	ETV3	-0.16657	4.950898	-1.10573	0.271844	-6.04669	0.717635	0.558499
NK.cells	ARL6IP1	-0.09449	7.879749	-1.10572	0.271845	-6.43632	0.686803	0.514768
NK.cells	ATP6V0B	0.085454	8.046898	1.105637	0.271883	-6.37782	0.685088	0.512379
NK.cells	KTI12	0.195817	3.941057	1.105603	0.271898	-5.6748	0.728614	0.574421
NK.cells	TFG	0.10368	5.864765	1.105533	0.271928	-6.06771	0.707854	0.544508
NK.cells	OSGIN2	0.220279	3.18431	1.105415	0.271979	-5.54052	0.736962	0.586708
NK.cells	PROX2	0.477049	1.188752	1.105324	0.272018	-4.9723	0.75947	0.620184
NK.cells	CRACR2A	0.227377	2.835916	1.105193	0.272074	-5.61729	0.740839	0.592423
NK.cells	NEIL1	-0.2633	3.000738	-1.10517	0.272085	-5.39586	0.739002	0.589712
NK.cells	PER1	0.143859	4.926726	1.105143	0.272096	-5.9755	0.717895	0.558934
NK.cells	TMEM126I	0.228068	2.697386	1.104685	0.272293	-5.37227	0.742387	0.594919
NK.cells	CDK2AP1	-0.10781	5.884299	-1.10459	0.272333	-6.11044	0.707646	0.544421
NK.cells	FAM72A	-0.47815	1.069392	-1.10443	0.272401	-4.95731	0.760839	0.622464
NK.cells	LGR4	-0.38833	2.632615	-1.10437	0.272428	-5.20793	0.743112	0.595992
NK.cells	AKAP7	0.211494	3.374989	1.104255	0.272479	-5.44491	0.734849	0.583809
NK.cells	TGDS	0.195499	3.1411	1.104247	0.272482	-5.51209	0.737441	0.587621
NK.cells	GM38973	-0.38322	1.613034	-1.10401	0.272584	-5.1569	0.754624	0.613229
NK.cells	DGLUCY	0.204048	2.944912	1.103902	0.272631	-5.53187	0.739624	0.590976
NK.cells	CSF3R	0.521283	2.933462	1.103888	0.272637	-5.03752	0.739751	0.591164
NK.cells	IFIT1	0.439915	2.761439	1.103635	0.272746	-5.56101	0.741778	0.594151
NK.cells	DMAC1	0.138555	4.611093	1.103516	0.272798	-5.83689	0.721413	0.564394
NK.cells	PIAS4	0.146553	4.224377	1.103133	0.272963	-5.72844	0.725897	0.570693
NK.cells	GM20508	-0.51663	0.064606	-1.10289	0.27307	-4.86791	0.773007	0.640738
NK.cells	B4GALNT1	0.102584	4.892601	1.102703	0.273149	-6.17432	0.718812	0.56028
NK.cells	SIGLECE	0.665024	2.293576	1.102022	0.273443	-5.00827	0.747717	0.602537
NK.cells	LPCAT3	0.137305	5.162712	1.101964	0.273468	-5.93601	0.716119	0.556298
NK.cells	SLC22A17	0.6551	-0.51359	1.101872	0.273508	-4.78771	0.78008	0.651397
NK.cells	PTDSS2	0.200701	3.06586	1.101699	0.273583	-5.46561	0.739067	0.589755
NK.cells	NMNAT3	0.236747	3.105259	1.101648	0.273605	-5.51281	0.738628	0.589109
NK.cells	HIST1H2BJ	-0.28193	3.30978	-1.10162	0.273615	-5.60164	0.736357	0.585766
NK.cells	RNF41	0.14244	4.424549	1.101526	0.273658	-5.72851	0.724108	0.567935
NK.cells	SLX1B	-0.20161	3.074893	-1.10124	0.273782	-5.43169	0.739138	0.589795
NK.cells	DHX36	0.093457	5.392248	1.100766	0.273987	-6.04411	0.713932	0.553075
NK.cells	RAP1B	-0.06656	8.543309	-1.10063	0.274047	-6.56684	0.681015	0.506646
NK.cells	GM43329	-0.23938	3.105098	-1.10051	0.274096	-5.49532	0.738918	0.589466

NK.cells	USP22	0.138509	4.389395	1.100461	0.274119	-5.74326	0.724773	0.568783
NK.cells	GM16151	-0.57162	-0.28838	-1.10036	0.274161	-4.82514	0.777733	0.647832
NK.cells	ESYT1	-0.11232	5.654914	-1.10002	0.274308	-6.16138	0.711123	0.549269
NK.cells	TXNDC11	0.115497	5.714713	1.099842	0.274387	-6.06967	0.710485	0.548379
NK.cells	ST8SIA6	0.377838	1.211567	1.099714	0.274442	-5.42404	0.760316	0.621618
NK.cells	POLR2A	0.087328	6.200996	1.099575	0.274502	-6.15747	0.705319	0.541103
NK.cells	TCN2	-0.21214	4.791081	-1.0992	0.274667	-5.74206	0.72041	0.562967
NK.cells	SLC39A6	0.136851	4.11285	1.099166	0.27468	-5.83798	0.727794	0.573711
NK.cells	NVL	0.126506	4.723925	1.099002	0.274751	-5.85278	0.721137	0.564126
NK.cells	ZFP984	0.158772	3.878402	1.098901	0.274795	-5.65149	0.730365	0.57758
NK.cells	PYCR2	0.196178	3.687049	1.09889	0.274799	-5.60232	0.732472	0.580664
NK.cells	SDC2	-0.3885	2.267759	-1.09878	0.274847	-5.19046	0.7483	0.604108
NK.cells	POMT1	-0.35354	2.21731	-1.09867	0.274896	-5.22324	0.748869	0.604977
NK.cells	SNED1	-0.50403	1.751149	-1.09865	0.274903	-5.02295	0.754152	0.612869
NK.cells	ABHD5	-0.18194	3.453436	-1.09861	0.274919	-5.65975	0.735052	0.584528
NK.cells	BRD3	-0.1289	5.712362	-1.09829	0.27506	-5.978	0.710625	0.54892
NK.cells	STK17B	-0.09407	8.129238	-1.09823	0.275085	-6.49013	0.685354	0.513214
NK.cells	ZFP512	0.151314	3.916117	1.097955	0.275205	-5.81932	0.730227	0.577156
NK.cells	TRMT2A	0.193336	3.456488	1.097668	0.27533	-5.50333	0.735467	0.584684
NK.cells	HMGA1	0.193393	4.670399	1.097506	0.2754	-5.73183	0.722183	0.565314
NK.cells	FPR2	0.954884	1.833327	1.097031	0.275607	-4.88857	0.753941	0.611845
NK.cells	UBA3	0.138563	4.076756	1.097024	0.27561	-5.72533	0.728889	0.574826
NK.cells	TLE4	-0.09957	7.814076	-1.09675	0.275727	-6.43674	0.689142	0.518142
NK.cells	KAT2A	0.192085	3.199202	1.096747	0.27573	-5.59306	0.73858	0.589169
NK.cells	SMCHD1	-0.10294	7.994517	-1.09658	0.275803	-6.56165	0.687314	0.515596
NK.cells	ACOT4	-0.68264	0.046894	-1.09634	0.275905	-4.82962	0.774594	0.64329
NK.cells	RBCK1	0.106101	5.550838	1.096289	0.27593	-6.09003	0.712962	0.551987
NK.cells	PNRC1	-0.09158	8.470962	-1.096	0.276056	-6.57143	0.68261	0.508938
NK.cells	PER2	-0.40181	2.222784	-1.09568	0.276193	-5.13183	0.749957	0.605682
NK.cells	TMEM161I	0.132135	4.545354	1.09551	0.276269	-5.82663	0.724224	0.567827
NK.cells	DNAJC11	0.141381	4.486406	1.095218	0.276396	-5.80262	0.724943	0.568827
NK.cells	A930001A:	-0.45965	-0.71301	-1.09516	0.276419	-5.02668	0.784065	0.65727
NK.cells	GM16536	-0.3566	1.530823	-1.09436	0.276768	-5.05667	0.758543	0.617903
NK.cells	SAA3	1.66897	0.738679	1.094155	0.276859	-4.92672	0.767666	0.63171
NK.cells	LRAT	-0.60131	-0.03713	-1.09411	0.27688	-4.85894	0.776712	0.645463
NK.cells	IDH2	-0.14571	5.667278	-1.0941	0.276882	-5.92206	0.712758	0.550773
NK.cells	TNFRSF26	0.300433	2.617995	1.093925	0.276959	-5.40739	0.746256	0.599576
NK.cells	SLC41A1	-0.23794	2.387756	-1.0934	0.277187	-5.41298	0.749299	0.603623
NK.cells	GM29114	-0.37827	-1.28439	-1.09299	0.277368	-5.01813	0.792052	0.668613
NK.cells	CYP2C29	-0.54379	1.011219	-1.09274	0.277476	-5.02693	0.765483	0.62749
NK.cells	AKIRIN2	-0.10518	5.756269	-1.09243	0.27761	-6.10012	0.712895	0.550012
NK.cells	EEA1	0.107856	6.055281	1.091978	0.27781	-6.14566	0.710058	0.545559
NK.cells	SPRED1	-0.18415	4.710293	-1.09168	0.27794	-5.84642	0.724725	0.566492
NK.cells	MINDY3	0.106843	5.598106	1.091256	0.278125	-6.10579	0.715308	0.552774
NK.cells	FAM120A	0.08381	6.352078	1.091123	0.278183	-6.21522	0.707261	0.54135
NK.cells	GM10125	-0.25844	2.003063	-1.09087	0.278292	-5.37824	0.755069	0.611065
NK.cells	IFNAR2	0.106044	7.128736	1.09076	0.278342	-6.2927	0.699077	0.529869

NK.cells	NADSYN1	0.414787	1.092531	1.090726	0.278357	-5.01682	0.765512	0.626782
NK.cells	CC2D1B	0.152033	3.659138	1.090386	0.278506	-5.69225	0.736464	0.583662
NK.cells	CASR	-0.6449	-0.61039	-1.09033	0.278529	-4.82189	0.785455	0.657187
NK.cells	DCBLD1	-0.36773	3.33755	-1.09026	0.278561	-5.22802	0.740038	0.588916
NK.cells	VPS18	-0.14656	4.834066	-1.09015	0.278608	-5.81633	0.723564	0.564918
NK.cells	RASAL1	-0.58164	0.712454	-1.09002	0.278664	-4.94066	0.769917	0.633547
NK.cells	SLC39A7	0.134667	4.500813	1.089982	0.278683	-5.80094	0.727198	0.570204
NK.cells	UBQLN4	0.178989	3.216072	1.089514	0.278888	-5.53663	0.741581	0.591114
NK.cells	HYOU1	0.143125	4.380576	1.089077	0.27908	-5.80825	0.728699	0.572522
NK.cells	DCTN4	-0.08611	6.423452	-1.089	0.279115	-6.25346	0.706684	0.540893
NK.cells	PANK1	-0.26841	3.430383	-1.08899	0.279116	-5.47245	0.739192	0.587891
NK.cells	ZMYM3	-0.3094	2.31007	-1.08865	0.279266	-5.2684	0.751774	0.606631
NK.cells	HSBP1	0.121069	6.003464	1.088617	0.279281	-6.09616	0.71115	0.547371
NK.cells	IFI214	0.375941	1.667368	1.088591	0.279293	-5.2633	0.759095	0.617568
NK.cells	SELENOH	-0.17688	5.20549	-1.08848	0.279341	-6.03187	0.71972	0.559668
NK.cells	SCYL1	-0.16211	4.072604	-1.08844	0.27936	-5.77139	0.732082	0.5776
NK.cells	IL4I1	-0.33085	2.841673	-1.08843	0.279365	-5.46149	0.745775	0.597727
NK.cells	FLAD1	0.235183	2.957237	1.08807	0.279522	-5.45124	0.744733	0.595848
NK.cells	IL10RB	0.117473	5.555085	1.087885	0.279602	-6.0575	0.716248	0.554353
NK.cells	NARS2	0.152475	4.271035	1.087184	0.27991	-5.8468	0.730376	0.574661
NK.cells	CHCHD1	0.112846	5.813268	1.086947	0.280015	-6.11397	0.713647	0.550622
NK.cells	MYBBP1A	0.159035	4.85707	1.086774	0.280091	-5.94104	0.723969	0.565529
NK.cells	RFTN2	-0.35102	2.237638	-1.08652	0.280201	-5.21843	0.753085	0.60839
NK.cells	KCNH7	-0.631	-0.13979	-1.08651	0.280207	-4.82738	0.780596	0.649929
NK.cells	RRP8	0.177749	3.730434	1.086336	0.280283	-5.65365	0.73634	0.58367
NK.cells	OGDH	0.074133	6.625732	1.086067	0.280402	-6.24567	0.705003	0.538591
NK.cells	TMEM9	-0.30895	2.576914	-1.08587	0.280486	-5.31128	0.749244	0.602908
NK.cells	MCMBP	0.086916	7.089729	1.085787	0.280525	-6.31748	0.700119	0.531772
NK.cells	PAM16	0.119838	4.987535	1.085612	0.280602	-5.95604	0.722551	0.563899
NK.cells	XCR1	-0.58109	0.412393	-1.08556	0.280623	-4.98358	0.774113	0.640436
NK.cells	NOC3L	0.228023	3.216026	1.085383	0.280703	-5.59491	0.742065	0.592486
NK.cells	PPAT	0.159341	4.107428	1.0853	0.280739	-5.79831	0.732175	0.577969
NK.cells	LAMP2	0.102769	7.069003	1.084581	0.281056	-6.22176	0.700336	0.532392
NK.cells	IL11RA1	-0.29801	2.113905	-1.08436	0.281155	-5.17363	0.754491	0.611162
NK.cells	PARP11	0.248348	3.46828	1.084165	0.281239	-5.55563	0.739251	0.588565
NK.cells	UTP11	0.122934	4.845019	1.084163	0.28124	-5.91472	0.7241	0.566437
NK.cells	MIER1	0.088482	6.946828	1.084149	0.281246	-6.31578	0.701619	0.534243
NK.cells	HDGF	-0.09401	6.998077	-1.0841	0.281267	-6.31545	0.701081	0.533482
NK.cells	TEX2	0.107706	6.723879	1.08407	0.281281	-6.38218	0.703967	0.537569
NK.cells	CLCN6	-0.19698	3.649755	-1.08396	0.281331	-5.63546	0.737235	0.585668
NK.cells	AKR1C14	-0.45898	0.911524	-1.0837	0.281446	-4.98608	0.768302	0.632121
NK.cells	SHFL	0.34682	1.832835	1.083628	0.281476	-5.20995	0.757696	0.616156
NK.cells	TSPYL2	0.244042	2.8065	1.083579	0.281498	-5.44741	0.746656	0.5997
NK.cells	MITF	0.292209	4.369202	1.083298	0.281622	-5.51055	0.729298	0.574243
NK.cells	PRR13	0.083135	6.510778	1.083285	0.281627	-6.25983	0.706219	0.541006
NK.cells	ZBTB10	-0.21054	4.077959	-1.08323	0.28165	-5.6481	0.7325	0.578933
NK.cells	COPB1	0.082186	6.220287	1.083149	0.281687	-6.20855	0.709302	0.545441

NK.cells	4930455G(	0.423125	1.816178	1.083115	0.281702	-5.13132	0.757886	0.616561
NK.cells	IFT52	0.133174	4.249963	1.083085	0.281715	-5.76721	0.730607	0.576196
NK.cells	TOMM70A	0.098995	5.752051	1.083083	0.281717	-6.08772	0.714303	0.552598
NK.cells	PUM1	-0.07211	7.56821	-1.08296	0.281772	-6.4232	0.695121	0.52537
NK.cells	CHTOP	-0.12834	5.225925	-1.08288	0.281805	-5.96707	0.719968	0.560787
NK.cells	GPATCH1	0.205968	2.73622	1.082594	0.281932	-5.45864	0.747447	0.601106
NK.cells	ATP5A1	-0.08592	7.857813	-1.08255	0.281952	-6.44832	0.692116	0.52124
NK.cells	FOSL1	0.428829	1.662098	1.08246	0.281991	-5.18264	0.759649	0.61936
NK.cells	IFNGR1	0.090912	7.03854	1.082239	0.282089	-6.52918	0.700656	0.533372
NK.cells	B3GNT6	0.611088	-0.72192	1.082068	0.282165	-4.79749	0.787493	0.661888
NK.cells	YPEL2	0.20165	4.572346	1.082042	0.282176	-5.76356	0.727074	0.571321
NK.cells	MYO5A	0.188954	5.016572	1.081872	0.282252	-5.82107	0.722236	0.564336
NK.cells	EI24	0.11537	4.630195	1.081764	0.282299	-5.92101	0.726442	0.57049
NK.cells	TAGAP1	0.3694	2.214287	1.081706	0.282325	-5.1808	0.75335	0.61016
NK.cells	FMO1	-0.4422	2.23787	-1.0814	0.28246	-5.22408	0.753082	0.609809
NK.cells	PWWP2A	0.127887	5.141602	1.081178	0.282558	-5.96853	0.72088	0.56257
NK.cells	FANCM	0.186435	3.67592	1.081135	0.282577	-5.69633	0.736944	0.586022
NK.cells	ZFP839	-0.36192	1.537463	-1.08104	0.282618	-5.05375	0.761079	0.621952
NK.cells	LACC1	0.35997	3.279516	1.080937	0.282665	-5.21573	0.741355	0.592544
NK.cells	RLIM	0.078396	6.654275	1.080936	0.282665	-6.25142	0.704702	0.539413
NK.cells	UBE2R2	0.092227	7.144409	1.080868	0.282695	-6.37038	0.699546	0.532131
NK.cells	GM16740	-0.26149	2.544974	-1.08076	0.282745	-5.34719	0.749604	0.60488
NK.cells	GM39326	0.387082	1.289251	1.080655	0.28279	-5.11117	0.763934	0.626373
NK.cells	FOPNL	0.134944	4.468642	1.080444	0.282883	-5.86274	0.728208	0.573476
NK.cells	NUP210	-0.11493	5.041054	-1.08039	0.282909	-6.06583	0.72197	0.564414
NK.cells	CABLES1	-0.22312	4.513404	-1.0801	0.283034	-6.05199	0.727764	0.57288
NK.cells	SAC3D1	0.184394	3.030557	1.079833	0.283154	-5.52829	0.744187	0.597086
NK.cells	BRI3BP	0.147306	4.756436	1.079593	0.28326	-5.87696	0.72511	0.569137
NK.cells	RGS5	0.661526	1.107497	1.079443	0.283326	-4.99935	0.76608	0.629953
NK.cells	FUNDC1	0.128343	4.65588	1.079285	0.283396	-5.8927	0.726207	0.57082
NK.cells	6330418K(	0.370975	1.506684	1.079208	0.283431	-5.11976	0.76148	0.623103
NK.cells	AGBL2	0.516907	0.26749	1.079084	0.283485	-4.87897	0.775857	0.644988
NK.cells	ICE1	0.165818	4.081492	1.078994	0.283525	-5.76997	0.732506	0.580105
NK.cells	BAP1	-0.18117	3.565024	-1.07898	0.283529	-5.5971	0.738222	0.588504
NK.cells	PPP3CB	0.090598	5.980209	1.078966	0.283538	-6.14802	0.711906	0.550238
NK.cells	ATRIP	0.242622	2.825397	1.078426	0.283777	-5.48377	0.746819	0.600987
NK.cells	ATP8B1	-0.48819	0.813793	-1.07819	0.283883	-4.98978	0.769823	0.635592
NK.cells	CMBL	-0.41221	2.192141	-1.07814	0.283903	-5.22789	0.75398	0.611705
NK.cells	PSENEN	0.081957	6.713106	1.078133	0.283907	-6.25855	0.704435	0.53938
NK.cells	PRKD3	0.117774	5.198034	1.077736	0.284083	-6.0753	0.720924	0.562768
NK.cells	GGA3	-0.18659	3.302395	-1.07736	0.284249	-5.48465	0.741902	0.593332
NK.cells	SPPL2A	0.131989	6.600738	1.07732	0.284268	-6.26227	0.706031	0.54128
NK.cells	RGS7BP	-0.68231	1.883105	-1.07721	0.284316	-4.94359	0.757941	0.617246
NK.cells	GNL1	0.129262	4.299889	1.076768	0.284513	-5.88292	0.731197	0.577327
NK.cells	PLS3	-0.40031	2.162516	-1.07608	0.28482	-5.05678	0.755736	0.612975
NK.cells	LRRC27	0.45865	0.302993	1.075898	0.2849	-4.9791	0.777242	0.645524
NK.cells	D430001F1	-0.78699	-0.87048	-1.07583	0.284931	-4.77507	0.791145	0.666909

NK.cells	TRAF5	-0.1287	5.308971	-1.07555	0.285053	-6.05994	0.720937	0.561756
NK.cells	SLC39A4	-0.34313	1.149584	-1.07498	0.28531	-5.20744	0.767763	0.630792
NK.cells	AEN	0.194542	3.664015	1.074885	0.285351	-5.6616	0.739211	0.588196
NK.cells	DLL4	-0.6964	0.717902	-1.07484	0.285372	-4.81524	0.772782	0.638426
NK.cells	UMPS	0.153083	4.099845	1.074681	0.285442	-5.73752	0.734379	0.581123
NK.cells	IL10RA	0.172399	4.971561	1.07442	0.285558	-5.85189	0.724817	0.567277
NK.cells	ITPR2	-0.10022	6.912603	-1.07441	0.285564	-6.31942	0.704012	0.537435
NK.cells	SCRN3	-0.34692	1.928035	-1.0744	0.285567	-5.19548	0.758799	0.617411
NK.cells	GPATCH3	0.201852	3.369567	1.07418	0.285665	-5.60298	0.742557	0.593189
NK.cells	IL3RA	-0.1619	3.561284	-1.07402	0.285738	-5.76749	0.740417	0.590101
NK.cells	FAM104A	0.100683	6.153765	1.073811	0.28583	-6.17139	0.712126	0.549079
NK.cells	PLEKHF2	0.112251	5.470642	1.073795	0.285836	-5.99163	0.719465	0.559622
NK.cells	PDE1A	-0.5991	0.441701	-1.07324	0.286085	-4.9051	0.776584	0.643805
NK.cells	E330020D1	-0.20398	5.11024	-1.07306	0.286166	-5.91231	0.723892	0.565478
NK.cells	PRDM10	-0.12237	5.213796	-1.07231	0.286497	-6.07799	0.72345	0.564128
NK.cells	NNT	-0.27641	2.482711	-1.07202	0.286628	-5.23488	0.753848	0.608721
NK.cells	FIBP	0.126744	4.462532	1.071998	0.286639	-5.85238	0.731711	0.576093
NK.cells	SUPT4A	-0.0992	6.836136	-1.07134	0.286931	-6.32966	0.706528	0.539415
NK.cells	LOXL3	0.653067	0.451305	1.071297	0.286952	-4.87526	0.77778	0.644267
NK.cells	NTAN1	-0.07958	6.261702	-1.0712	0.286997	-6.23283	0.712638	0.548116
NK.cells	GM45894	-0.2184	2.290972	-1.07087	0.287141	-5.36067	0.75657	0.612245
NK.cells	PPP1R21	0.151859	4.557871	1.070742	0.2872	-5.86211	0.731185	0.57482
NK.cells	MAPKAP1	0.104782	5.764174	1.070624	0.287253	-6.1192	0.718055	0.555837
NK.cells	PPP1R13L	-0.71058	-0.0774	-1.07049	0.287312	-4.82611	0.7841	0.653887
NK.cells	PGS1	0.122569	4.740858	1.070439	0.287335	-5.95339	0.729177	0.5719
NK.cells	SH3BP5	0.147987	6.380773	1.070097	0.287488	-6.0835	0.71146	0.5466
NK.cells	ZGPAT	-0.12652	3.668613	-1.06998	0.287542	-5.79214	0.741049	0.589498
NK.cells	GM19684	0.302256	1.522121	1.069837	0.287605	-5.35581	0.765411	0.625764
NK.cells	PSMG1	0.183676	3.325411	1.069777	0.287632	-5.52695	0.744888	0.595155
NK.cells	STARD13	-0.56567	1.070842	-1.06956	0.287731	-5.00299	0.770641	0.633667
NK.cells	SAT1	-0.12604	8.474503	-1.06955	0.287732	-6.49529	0.689508	0.515688
NK.cells	DCUN1D3	0.136281	4.424811	1.069463	0.287772	-5.94224	0.732667	0.577227
NK.cells	MAF1	-0.13557	5.342723	-1.06932	0.287835	-6.03047	0.722632	0.562699
NK.cells	ZFP189	-0.41649	1.120206	-1.06906	0.287951	-5.0156	0.770217	0.632922
NK.cells	IFT22	-0.25709	3.654091	-1.06883	0.288057	-5.39037	0.741398	0.589857
NK.cells	CDKN2AIPI	-0.13608	4.272414	-1.06857	0.288173	-5.82557	0.734533	0.579839
NK.cells	FBXO34	-0.10565	5.78838	-1.06854	0.288187	-6.14823	0.717992	0.555879
NK.cells	WASL	-0.09329	5.483088	-1.06848	0.288214	-6.06149	0.72129	0.560664
NK.cells	CCDC181	0.322384	1.514839	1.068162	0.288355	-5.1859	0.7659	0.626215
NK.cells	PTTG1IP	-0.11578	4.790183	-1.06789	0.288478	-5.9072	0.729077	0.571737
NK.cells	GM37240	-0.14908	5.162317	-1.0677	0.288563	-6.01272	0.725012	0.565932
NK.cells	KIF13A	0.180527	4.605824	1.06762	0.288598	-5.74184	0.7311	0.574772
NK.cells	CCNG2	-0.14898	5.189545	-1.06758	0.288616	-5.98397	0.724715	0.565529
NK.cells	STFA2L1	0.663443	2.448793	1.067257	0.288761	-5.09553	0.755341	0.610461
NK.cells	REL	-0.11811	8.12408	-1.06719	0.288791	-6.52569	0.693635	0.521289
NK.cells	SLC39A1	0.105102	6.161792	1.066878	0.288932	-6.15062	0.714322	0.550661
NK.cells	USP19	-0.14293	4.704682	-1.06686	0.28894	-5.93338	0.730123	0.573458

NK.cells	RRP9	0.211455	2.59462	1.0668	0.288966	-5.45254	0.753683	0.608154
NK.cells	KLRA6	-0.3254	-0.71608	-1.06643	0.289131	-5.33557	0.792538	0.666843
NK.cells	RAB19	-0.18586	2.561986	-1.06601	0.289323	-5.7413	0.75428	0.608903
NK.cells	PPA1	0.14944	4.797054	1.065999	0.289327	-5.97869	0.729329	0.572183
NK.cells	D730003I1	0.253629	2.591958	1.065816	0.289409	-5.35723	0.753939	0.60844
NK.cells	8-Mar	0.231777	3.141103	1.065753	0.289437	-5.52395	0.747728	0.599228
NK.cells	GM50013	0.275441	1.85467	1.065688	0.289466	-5.31776	0.762366	0.621095
NK.cells	CARF	0.300019	2.216581	1.065649	0.289484	-5.26607	0.758217	0.614885
NK.cells	CEP83OS	0.33153	2.022979	1.065203	0.289685	-5.20642	0.76054	0.618462
NK.cells	GM42067	-0.44109	0.558955	-1.06516	0.289702	-4.95084	0.777528	0.64415
NK.cells	ERBB4	-0.56327	0.909465	-1.06512	0.289721	-5.00714	0.773425	0.637921
NK.cells	AFAP1L1	-0.54772	1.508026	-1.06501	0.289771	-4.97306	0.76647	0.627464
NK.cells	SH3GLB2	0.157763	3.721025	1.064819	0.289857	-5.73769	0.741396	0.590079
NK.cells	GNG11	0.275284	3.831566	1.064437	0.290029	-5.5551	0.740445	0.588501
NK.cells	OSTM1	0.114904	4.762239	1.063871	0.290284	-5.96562	0.730367	0.573639
NK.cells	LRRC1	-0.2077	3.264954	-1.06386	0.290291	-5.71073	0.747006	0.598049
NK.cells	SURF4	0.115839	5.782118	1.063556	0.290426	-6.11404	0.719265	0.557674
NK.cells	NOD1	0.197299	3.387607	1.063494	0.290454	-5.84175	0.745628	0.596129
NK.cells	NOA1	0.204834	3.137205	1.063126	0.29062	-5.54661	0.748445	0.600385
NK.cells	CHP1	-0.08474	7.01438	-1.0631	0.29063	-6.40408	0.706097	0.538961
NK.cells	NDUFB5	0.090454	7.037752	1.063071	0.290645	-6.35662	0.70585	0.538621
NK.cells	CDR2	0.264144	2.488604	1.062981	0.290685	-5.46695	0.755794	0.611371
NK.cells	PMP22	-0.52674	1.847479	-1.06291	0.290719	-5.05802	0.763135	0.622377
NK.cells	SH2D4B	-0.24041	4.439997	-1.06288	0.29073	-5.75882	0.733914	0.579066
NK.cells	DNAJC7	-0.10653	7.510277	-1.06268	0.29082	-6.35319	0.700891	0.531648
NK.cells	ACBD5	-0.09523	6.231959	-1.06246	0.29092	-6.21827	0.714444	0.550946
NK.cells	DSG2	-0.46335	0.740688	-1.0624	0.290949	-5.0414	0.776005	0.641884
NK.cells	LHPP	-0.25731	3.112672	-1.06232	0.290983	-5.32776	0.748739	0.600922
NK.cells	NHSL2	-0.45644	2.487556	-1.0618	0.29122	-5.16018	0.756229	0.611709
NK.cells	TSPAN18	-0.4854	1.764014	-1.06138	0.291407	-5.03725	0.764523	0.624283
NK.cells	PJA2	0.147422	4.695864	1.061064	0.291551	-5.91205	0.731505	0.575414
NK.cells	H6PD	-0.22103	2.776773	-1.06092	0.291618	-5.4313	0.75294	0.60703
NK.cells	ACACB	-0.60842	0.257652	-1.06091	0.291618	-4.87496	0.782104	0.651052
NK.cells	CNTLN	-0.17371	4.34763	-1.06076	0.291686	-5.86813	0.735345	0.581154
NK.cells	GARNL3	-0.28401	2.487215	-1.06067	0.291729	-5.34371	0.756232	0.61206
NK.cells	MEIS3	0.405582	0.488054	1.060615	0.291754	-5.07411	0.779388	0.647028
NK.cells	DFFB	-0.20225	2.981393	-1.0606	0.291759	-5.57867	0.750623	0.603703
NK.cells	CHMP3	0.08041	5.884611	1.060488	0.291811	-6.14335	0.718561	0.55688
NK.cells	ABCA6	-0.56833	0.705764	-1.06043	0.291837	-4.99684	0.77683	0.643195
NK.cells	ZFP955B	-0.26251	2.201747	-1.06034	0.291878	-5.36505	0.759494	0.617026
NK.cells	NUP93	0.131054	4.586111	1.060149	0.291964	-5.91395	0.732775	0.577418
NK.cells	SMCR8	-0.2256	3.28105	-1.0598	0.292124	-5.4794	0.747379	0.598899
NK.cells	C2CD2L	-0.23478	2.991873	-1.05965	0.29219	-5.42814	0.750641	0.60374
NK.cells	FBXO48	-0.48499	0.24175	-1.05961	0.292209	-4.92243	0.782435	0.651698
NK.cells	PIGYL	0.154982	4.270837	1.059541	0.29224	-5.77266	0.736329	0.582649
NK.cells	ACD	0.121548	4.940696	1.059053	0.292461	-5.97877	0.729215	0.572142
NK.cells	SPTA1	0.595022	0.279808	1.058807	0.292572	-4.98048	0.782268	0.651481

NK.cells	SERTAD4	-0.68723	-0.0023	-1.05864	0.292646	-4.82876	0.785608	0.65664
NK.cells	CDK9	0.111874	5.578202	1.058636	0.292649	-6.05515	0.722266	0.562245
NK.cells	XKR6	0.323137	0.862903	1.057985	0.292944	-5.25075	0.775411	0.641205
NK.cells	AREL1	0.150509	4.263334	1.057966	0.292953	-5.74258	0.736678	0.583344
NK.cells	DYNC1I2	-0.07902	6.441227	-1.05771	0.293067	-6.24582	0.712974	0.549128
NK.cells	WDR36	0.120177	4.578146	1.057595	0.293121	-5.94601	0.733199	0.578374
NK.cells	NUF2	-0.2444	3.365247	-1.05754	0.293148	-5.62897	0.746702	0.598253
NK.cells	TMEM30A	0.072594	6.581779	1.057356	0.293229	-6.32531	0.711473	0.547021
NK.cells	SLC9A5	-0.48539	0.362304	-1.05718	0.293308	-4.96264	0.781294	0.650416
NK.cells	YWHAE	-0.06331	8.88218	-1.05695	0.293412	-6.60687	0.687403	0.513264
NK.cells	PHLDB1	-0.37202	1.355652	-1.05677	0.293497	-5.14811	0.769666	0.632911
NK.cells	CCDC136	-0.3105	0.119291	-1.05676	0.2935	-5.47524	0.784166	0.655021
NK.cells	DNASE2A	-0.17025	4.427932	-1.05674	0.29351	-5.71941	0.734857	0.581067
NK.cells	GLCCI1	-0.12362	7.348661	-1.05652	0.293608	-6.32817	0.703346	0.53569
NK.cells	GINS2	0.243717	3.493231	1.056318	0.293701	-5.60274	0.745264	0.59644
NK.cells	GM8251	-0.24804	3.392286	-1.05624	0.293737	-5.62883	0.746398	0.598146
NK.cells	GM15559	0.179456	3.148018	1.056168	0.293769	-5.60313	0.749148	0.602238
NK.cells	GM26802	0.447769	1.069129	1.056166	0.29377	-5.09484	0.773001	0.638075
NK.cells	CYP4F13	0.236466	2.829707	1.056077	0.29381	-5.43665	0.752749	0.607616
NK.cells	HCFC2	0.166294	3.758586	1.055908	0.293887	-5.67737	0.742294	0.592125
NK.cells	GM12166	0.482546	0.781167	1.055887	0.293897	-4.9694	0.776368	0.643235
NK.cells	LOCKD	-0.21072	4.472202	-1.05581	0.29393	-5.89778	0.734368	0.580479
NK.cells	GTF2H2	-0.1372	3.759481	-1.0558	0.293934	-5.73426	0.742284	0.59211
NK.cells	NRXN2	-0.55733	2.095803	-1.05564	0.294008	-5.08441	0.761121	0.620258
NK.cells	NEU1	0.160617	4.426111	1.055638	0.29401	-5.74634	0.734877	0.581318
NK.cells	GOLPH3L	0.148454	4.39604	1.054999	0.2943	-5.88196	0.735498	0.582004
NK.cells	DIDO1	-0.11172	5.734022	-1.05468	0.294446	-6.12312	0.720862	0.560804
NK.cells	PAPSS1	-0.12444	4.181725	-1.05467	0.294448	-5.8705	0.737873	0.58557
NK.cells	GM11713	-0.21771	2.37115	-1.05463	0.29447	-5.65492	0.758266	0.615827
NK.cells	SIK2	-0.0984	7.126745	-1.05461	0.294477	-6.372	0.705965	0.539477
NK.cells	GM16973	0.316451	1.894858	1.05457	0.294495	-5.22097	0.763731	0.624038
NK.cells	MYO1B	-0.3459	2.148687	-1.05439	0.294577	-5.33379	0.760865	0.619724
NK.cells	UBE2V2	0.103794	5.614121	1.054089	0.294714	-6.10917	0.722394	0.562796
NK.cells	SLC20A2	0.175179	4.036306	1.053787	0.294852	-5.71926	0.73992	0.588175
NK.cells	KLHL5	0.126761	4.645979	1.053501	0.294982	-5.85214	0.733229	0.578432
NK.cells	ERAL1	0.263355	1.918255	1.053419	0.295019	-5.27087	0.763971	0.624042
NK.cells	MOAP1	0.377736	0.547771	1.053274	0.295086	-4.99191	0.779935	0.648295
NK.cells	EOGT	-0.2509	2.532472	-1.0531	0.295165	-5.41365	0.75693	0.613554
NK.cells	NUMBL	0.336676	1.04252	1.053055	0.295185	-5.24675	0.774131	0.6395
NK.cells	HIST1H2AK	-0.46132	0.954719	-1.05261	0.295389	-5.08802	0.775499	0.641183
NK.cells	ADNP2	0.259929	2.990665	1.052499	0.295439	-5.41954	0.752052	0.605916
NK.cells	AC142100.	-0.62826	0.335219	-1.05108	0.296084	-4.8829	0.784331	0.652752
NK.cells	LRRC57	-0.29522	2.732576	-1.05095	0.296145	-5.38282	0.756472	0.610733
NK.cells	TMEM160	0.097263	5.666014	1.050558	0.296325	-6.17269	0.723863	0.56306
NK.cells	SLC7A5	-0.14631	6.118367	-1.05039	0.2964	-6.24017	0.718966	0.55604
NK.cells	ERGIC2	-0.08184	6.017059	-1.05032	0.296434	-6.14459	0.720059	0.557619
NK.cells	SF3B5	0.098681	6.277364	1.049912	0.29662	-6.22378	0.717253	0.553673

NK.cells	PCDH15	0.344459	1.323083	1.049788	0.296676	-5.13505	0.772765	0.635535
NK.cells	PIKFYVE	0.164966	4.426045	1.049721	0.296707	-5.72202	0.737474	0.58301
NK.cells	IPO5	0.14165	5.437174	1.04971	0.296713	-6.06663	0.726354	0.566828
NK.cells	DDX31	0.191671	3.220713	1.049649	0.29674	-5.56242	0.750973	0.602929
NK.cells	HSPE1	0.121115	7.179751	1.049605	0.296761	-6.36151	0.707619	0.540036
NK.cells	NACC1	0.127032	4.380772	1.049366	0.29687	-5.8861	0.737976	0.583895
NK.cells	SAMD9L	0.144415	5.184732	1.04934	0.296881	-6.06002	0.729113	0.570972
NK.cells	SLC45A4	-0.17513	3.337998	-1.04924	0.296928	-5.60126	0.749647	0.60114
NK.cells	STAU2	-0.28384	2.900653	-1.0491	0.29699	-5.3501	0.754601	0.608507
NK.cells	NME6	0.330168	2.576439	1.04902	0.297028	-5.19325	0.758297	0.614023
NK.cells	TMEM88	-0.30283	2.768173	-1.04852	0.297258	-5.44266	0.756393	0.610949
NK.cells	TROAP	-0.3551	1.774645	-1.04851	0.297263	-5.21247	0.767807	0.628064
NK.cells	CCNT2	0.106954	5.37232	1.048125	0.297438	-6.05182	0.72756	0.5684
NK.cells	MDH2	0.100139	6.490426	1.048035	0.297479	-6.26221	0.715455	0.550998
NK.cells	PIK3R1	-0.11128	7.694648	-1.04767	0.297645	-6.54965	0.702802	0.532994
NK.cells	RPRD1A	-0.12735	4.057705	-1.04764	0.297661	-5.78494	0.742223	0.589801
NK.cells	MTG2	0.181126	3.031494	1.047203	0.29786	-5.58295	0.754126	0.607074
NK.cells	PREX1	-0.10475	6.43388	-1.04682	0.298036	-6.3764	0.716805	0.552367
NK.cells	SLC2A9	0.243408	2.879571	1.046399	0.298229	-5.5385	0.756446	0.610012
NK.cells	GYPC	0.238126	3.080733	1.046085	0.298373	-5.61857	0.754157	0.606668
NK.cells	POLR3GL	0.153857	3.878267	1.046043	0.298392	-5.69894	0.745157	0.593352
NK.cells	ADCY9	0.310509	3.136828	1.046019	0.298404	-5.32258	0.75352	0.605722
NK.cells	CBARP	-0.22269	1.923122	-1.04559	0.298601	-5.62897	0.767638	0.626649
NK.cells	EDEM2	0.156062	4.515035	1.045419	0.298679	-5.82546	0.738254	0.583128
NK.cells	ZFP62	0.137221	4.580193	1.045266	0.29875	-5.85574	0.737531	0.582103
NK.cells	1190005I0	0.364759	1.185776	1.045169	0.298794	-5.07907	0.776224	0.639783
NK.cells	TIGAR	0.286582	1.851972	1.045168	0.298794	-5.29429	0.768462	0.628046
NK.cells	1110006O2	-0.49403	0.319918	-1.04482	0.298955	-4.90531	0.786592	0.655423
NK.cells	CCRL2	0.284334	4.741765	1.044697	0.299011	-6.10074	0.735887	0.579618
NK.cells	GM48653	-0.6449	-0.16399	-1.04463	0.29904	-4.83647	0.792363	0.664323
NK.cells	MACF1	0.087806	8.000182	1.044442	0.299128	-6.53468	0.70086	0.529376
NK.cells	PARD3B	-0.30053	4.55014	-1.044	0.29933	-5.64066	0.738419	0.582796
NK.cells	INMT	-0.54904	0.639157	-1.0435	0.299561	-4.99033	0.783528	0.649764
NK.cells	B3GALT4	0.384479	1.404829	1.043498	0.299562	-5.18664	0.774525	0.636089
NK.cells	CPLANE1	-0.13861	4.78519	-1.04307	0.29976	-6.04237	0.736085	0.578997
NK.cells	ZFP518A	0.179251	3.684945	1.043051	0.299768	-5.65452	0.748367	0.597003
NK.cells	GIMAP7	0.22097	1.458503	1.043033	0.299777	-5.7367	0.773902	0.635141
NK.cells	LARP4	-0.09601	6.410341	-1.04251	0.300019	-6.24101	0.718344	0.553533
NK.cells	BAIAP3	-0.35818	-0.32152	-1.04248	0.300033	-5.23341	0.794983	0.667508
NK.cells	MXD3	-0.32385	1.735683	-1.0419	0.300297	-5.32372	0.770673	0.630677
NK.cells	RAB31	-0.21908	4.770711	-1.04176	0.300364	-5.58805	0.736245	0.579671
NK.cells	TNFRSF14	-0.55243	0.252225	-1.04155	0.300461	-4.94325	0.788122	0.657375
NK.cells	FBXO32	-0.25393	3.711448	-1.04151	0.30048	-5.7821	0.748068	0.597101
NK.cells	FBXO4	0.159885	3.431156	1.041491	0.300488	-5.69782	0.751231	0.601777
NK.cells	PLRG1	0.131024	4.093452	1.041325	0.300564	-5.80128	0.743781	0.590857
NK.cells	SPRTN	-0.18212	3.419835	-1.04131	0.300569	-5.65533	0.751359	0.602038
NK.cells	PDE4C	-0.50907	3.131277	-1.0413	0.300578	-5.31997	0.754631	0.606892

NK.cells	ATIC	0.151496	4.493549	1.041256	0.300596	-5.91739	0.739319	0.584322
NK.cells	GM16230	-0.60321	0.238127	-1.04117	0.300635	-4.93193	0.78829	0.657764
NK.cells	ARMC7	-0.13807	3.924467	-1.04109	0.300671	-5.93415	0.745674	0.593707
NK.cells	BRK1	0.08601	6.271039	1.041062	0.300685	-6.24359	0.719846	0.556163
NK.cells	DDX28	0.262021	1.558733	1.041003	0.300713	-5.28827	0.772732	0.634106
NK.cells	CSAD	-0.18578	3.965874	-1.04094	0.300743	-5.72124	0.74521	0.593069
NK.cells	GM26982	-0.23618	1.652146	-1.04077	0.300819	-5.32665	0.771679	0.63256
NK.cells	FDPS	-0.19583	4.623809	-1.04031	0.301033	-5.82432	0.738188	0.582503
NK.cells	PARP14	0.209425	5.480462	1.040255	0.301058	-6.14889	0.728748	0.568777
NK.cells	ARIH1	-0.09071	8.728541	-1.03984	0.301252	-6.64345	0.694379	0.519746
NK.cells	TNFSF13OS	0.636346	0.055873	1.039621	0.301351	-4.92335	0.791066	0.661692
NK.cells	LYPLA1	0.093435	5.988433	1.03935	0.301476	-6.20438	0.723455	0.561136
NK.cells	FLRT3	0.61424	-0.84839	1.039295	0.301501	-4.87942	0.801949	0.678613
NK.cells	RRAGC	0.108225	5.819399	1.039167	0.301561	-6.10584	0.725293	0.563783
NK.cells	MRRF	0.169602	3.454685	1.039146	0.30157	-5.66933	0.751538	0.602143
NK.cells	APEX1	0.135169	5.465947	1.039095	0.301594	-6.02458	0.729151	0.569358
NK.cells	F630040K0	0.429159	0.611295	1.038416	0.301908	-5.15473	0.785031	0.651998
NK.cells	SERINC1	0.086229	6.230989	1.03835	0.301938	-6.25237	0.721354	0.557634
NK.cells	CD33	0.588125	2.518088	1.0381	0.302054	-4.9985	0.762835	0.618353
NK.cells	SLC36A3OS	0.372606	1.010121	1.037935	0.302131	-5.32338	0.780383	0.644825
NK.cells	COL1A1	-0.43993	2.255466	-1.03786	0.302165	-5.27357	0.76586	0.622886
NK.cells	CCDC171	0.168172	3.57202	1.037699	0.30224	-5.69103	0.750821	0.600485
NK.cells	A330023F2	0.162494	3.109027	1.037625	0.302274	-5.8545	0.756073	0.608271
NK.cells	ERCC5	0.197117	2.909402	1.037374	0.30239	-5.5024	0.758357	0.611709
NK.cells	SLC25A47	-0.21062	3.963	-1.03735	0.302401	-5.6923	0.746423	0.594035
NK.cells	TNKS2	-0.07143	7.072389	-1.03699	0.302567	-6.42413	0.712504	0.544933
NK.cells	SUZ12	-0.08728	6.954117	-1.03697	0.302579	-6.35439	0.713768	0.54673
NK.cells	ZFP622	-0.11343	5.377893	-1.03624	0.302914	-6.06423	0.731422	0.571482
NK.cells	PRKCH	-0.1326	6.999653	-1.03618	0.302942	-6.66485	0.713845	0.546254
NK.cells	CDK12	0.092173	6.896517	1.035923	0.303063	-6.34087	0.715087	0.547946
NK.cells	HNRNPA2B	-0.08129	9.287708	-1.03558	0.303221	-6.71403	0.690161	0.512749
NK.cells	B3GNT5	-0.28601	2.546338	-1.03548	0.303271	-5.41589	0.763611	0.618572
NK.cells	PRKAR1A	-0.06275	7.625008	-1.03527	0.303367	-6.49907	0.707599	0.537029
NK.cells	MYLIP	-0.13667	5.991118	-1.03508	0.303453	-6.10013	0.725137	0.562019
NK.cells	KIF23	-0.18776	5.173452	-1.035	0.30349	-5.99192	0.734093	0.574973
NK.cells	RBBP7	-0.10051	6.2357	-1.03471	0.303627	-6.2533	0.722544	0.558308
NK.cells	ZFP41	0.428562	0.707145	1.034677	0.303641	-5.04588	0.785245	0.651136
NK.cells	REEP4	0.152466	4.356559	1.034204	0.303861	-5.91835	0.743436	0.588411
NK.cells	ATG4D	0.146393	3.954415	1.034196	0.303865	-5.81302	0.747946	0.595035
NK.cells	GM48765	0.390109	0.952243	1.034091	0.303914	-5.09755	0.782565	0.646872
NK.cells	E030042O2	0.642102	0.180529	1.033843	0.304029	-4.88603	0.791754	0.660973
NK.cells	LSM8	0.113729	5.264944	1.033484	0.304196	-6.05574	0.733371	0.573996
NK.cells	CD300C	-0.58819	0.72621	-1.03342	0.304226	-4.84869	0.785256	0.651272
NK.cells	PSMG2	0.166289	3.919974	1.033305	0.304279	-5.77589	0.74835	0.595926
NK.cells	SERINC5	-0.18641	5.287482	-1.03328	0.304289	-5.81247	0.733122	0.573667
NK.cells	LEPROT	-0.12287	5.065196	-1.03327	0.304294	-5.97948	0.735574	0.577228
NK.cells	LRCH1	0.096605	7.432191	1.033045	0.3044	-6.43064	0.710023	0.540464

NK.cells	GNL3	0.12956	5.420028	1.032754	0.304536	-6.05862	0.731946	0.571667
NK.cells	TENM4	-0.61073	1.760128	-1.03248	0.304664	-4.95566	0.773565	0.633015
NK.cells	ZFHX2	-0.24596	2.765643	-1.03225	0.304772	-5.52972	0.761938	0.615615
NK.cells	TCEA1	0.074633	7.486226	1.032158	0.304813	-6.45355	0.709782	0.539814
NK.cells	SMPDL3B	0.529081	1.9506	1.032068	0.304855	-5.17294	0.771357	0.629731
NK.cells	CCNDBP1	0.136483	5.692836	1.031813	0.304974	-6.18347	0.72914	0.567512
NK.cells	ATP2A3	-0.12109	5.210591	-1.03178	0.304987	-6.0636	0.734439	0.575183
NK.cells	SAP30BP	-0.11354	4.838744	-1.03161	0.30507	-5.99176	0.738602	0.581249
NK.cells	KRT8	-0.38917	1.975849	-1.03072	0.305482	-5.25982	0.771889	0.629762
NK.cells	GLTP	-0.08486	6.759436	-1.03042	0.305622	-6.33595	0.718319	0.551335
NK.cells	PHF23	0.145071	5.240241	1.030323	0.305669	-5.96813	0.73488	0.575176
NK.cells	TUFM	0.137454	4.731912	1.030072	0.305786	-5.96375	0.740514	0.583417
NK.cells	CNR2	0.206105	3.101889	1.03007	0.305787	-5.56402	0.758901	0.610494
NK.cells	PSME4	-0.09966	7.130098	-1.03006	0.305793	-6.37159	0.714342	0.545737
NK.cells	A930014D(	0.540094	-1.05861	1.029846	0.305891	-4.80456	0.807973	0.68538
NK.cells	EPC1	0.095578	6.811099	1.029671	0.305973	-6.35572	0.717764	0.550709
NK.cells	TRMT11	0.149926	3.646744	1.029471	0.306066	-5.74581	0.7527	0.601423
NK.cells	RNASEH2B	0.143275	4.594626	1.029465	0.306069	-5.92921	0.742044	0.585763
NK.cells	SNAPIN	0.130237	4.083623	1.029452	0.306076	-5.84041	0.747769	0.594154
NK.cells	GM43305	-0.21393	7.45195	-1.02936	0.306118	-6.47774	0.710907	0.541023
NK.cells	PARP16	-0.45119	1.117003	-1.02881	0.306374	-5.03862	0.782383	0.645417
NK.cells	CIT	-0.23117	4.461996	-1.0286	0.306472	-5.94115	0.743932	0.588162
NK.cells	POMT2	-0.2916	1.813643	-1.02857	0.306487	-5.23692	0.774203	0.633139
NK.cells	GM29394	0.43613	0.417159	1.028468	0.306535	-5.03121	0.790692	0.658236
NK.cells	ACOX2	-0.49903	1.030725	-1.0283	0.306612	-5.05185	0.783439	0.647205
NK.cells	INPP5A	0.13357	6.256163	1.028127	0.306695	-6.16495	0.724237	0.559689
NK.cells	ATP11C	-0.08598	6.869185	-1.0274	0.307037	-6.31389	0.717632	0.550603
NK.cells	PBLD2	-0.4303	0.872415	-1.0273	0.307081	-4.99836	0.785386	0.650617
NK.cells	GM43727	-0.54118	-0.59426	-1.02723	0.307116	-4.87806	0.802978	0.67768
NK.cells	GBP2B	1.463663	0.300434	1.027199	0.307129	-4.90428	0.792198	0.661064
NK.cells	RMND5A	-0.09168	6.766957	-1.02714	0.307159	-6.35171	0.718732	0.552241
NK.cells	MRPS12	0.126329	4.774775	1.02699	0.307227	-5.96183	0.740546	0.583798
NK.cells	PIF1	-0.44339	1.716548	-1.02682	0.307306	-5.1897	0.775446	0.635759
NK.cells	LYPLAL1	-0.48496	1.634017	-1.02677	0.307331	-5.07208	0.776412	0.637242
NK.cells	CTBS	0.214637	2.934998	1.026698	0.307363	-5.44107	0.761334	0.614611
NK.cells	ZFP811	-0.72339	-1.02506	-1.02647	0.307473	-4.81116	0.80815	0.686191
NK.cells	VPS45	0.172248	3.758422	1.026378	0.307513	-5.68913	0.751953	0.600805
NK.cells	PAFAH1B2	0.095937	5.489377	1.026324	0.307539	-6.09405	0.732639	0.57255
NK.cells	PGLS	-0.09639	7.788311	-1.02632	0.30754	-6.37952	0.707824	0.537055
NK.cells	AGPAT4	0.179387	5.023605	1.026241	0.307577	-6.02488	0.737782	0.580022
NK.cells	CD80	0.355627	3.929604	1.026079	0.307653	-5.42476	0.750052	0.598002
NK.cells	CASP9	0.22756	2.447605	1.025855	0.307759	-5.34057	0.767025	0.623234
NK.cells	EXT2	-0.13993	3.849599	-1.02567	0.307845	-5.83168	0.751	0.599466
NK.cells	QPCT	-0.1916	2.264701	-1.0256	0.307878	-5.7465	0.769143	0.626503
NK.cells	PEX2	0.171832	3.914925	1.025507	0.307921	-5.75613	0.750262	0.598431
NK.cells	5830428M	0.230619	2.548294	1.024972	0.308173	-5.50841	0.766331	0.621764
NK.cells	MTPAP	0.144926	4.284578	1.024824	0.308242	-5.86405	0.746575	0.592477

NK.cells	RAF1	0.107605	5.986456	1.024176	0.308546	-6.18085	0.728301	0.565362
NK.cells	RPAIN	-0.15794	3.771433	-1.02357	0.308833	-5.66819	0.753351	0.601645
NK.cells	MCEE	-0.17953	4.194477	-1.02341	0.308906	-5.70258	0.748571	0.594671
NK.cells	NSD2	-0.10465	6.699324	-1.02335	0.308934	-6.33828	0.72094	0.554618
NK.cells	MLXIP	0.097379	6.94185	1.023282	0.308966	-6.39379	0.718325	0.550886
NK.cells	ERG28	0.097647	5.118614	1.023094	0.309054	-6.05846	0.738244	0.579592
NK.cells	SLC38A6	0.184976	4.233853	1.023028	0.309086	-5.73498	0.748128	0.594044
NK.cells	GPD1L	-0.14759	5.296236	-1.02283	0.309181	-5.95058	0.736355	0.576801
NK.cells	S100A6	-0.17645	7.580063	-1.02222	0.309464	-6.80669	0.712076	0.541359
NK.cells	9330020H(	0.259093	2.471892	1.022069	0.309537	-5.35336	0.768894	0.624071
NK.cells	MTMR1	0.124992	4.622377	1.021884	0.309624	-5.95503	0.744462	0.587852
NK.cells	DCAF6	0.135091	5.908083	1.021484	0.309812	-6.17172	0.730449	0.567306
NK.cells	ZDHHC14	-0.24344	7.022709	-1.02142	0.309844	-6.03306	0.718344	0.549993
NK.cells	FRG2F1	-0.3791	0.702787	-1.02128	0.30991	-5.04039	0.790004	0.655794
NK.cells	ADAMTS7	-0.5557	0.676356	-1.02106	0.31001	-4.93277	0.790413	0.6564
NK.cells	CCNO	0.476285	0.174468	1.020826	0.310122	-5.04448	0.796554	0.665699
NK.cells	INAFM2	-0.23787	3.225336	-1.02045	0.310298	-5.40966	0.760851	0.611631
NK.cells	RCOR1	-0.09889	6.85813	-1.02033	0.310357	-6.34834	0.72044	0.552846
NK.cells	4921531C2	-0.36708	1.642005	-1.02018	0.310425	-5.16472	0.779226	0.639271
NK.cells	ANG	-0.25175	4.211322	-1.02007	0.310478	-5.82228	0.749644	0.595224
NK.cells	SNAP29	0.113137	5.5062	1.019942	0.310539	-6.00207	0.735198	0.574223
NK.cells	GM29170	0.293942	1.532706	1.019924	0.310547	-5.18809	0.780512	0.641356
NK.cells	SH3GLB1	0.081566	8.546317	1.019635	0.310683	-6.59606	0.702575	0.527758
NK.cells	CHKA	-0.1091	6.940391	-1.01945	0.310769	-6.35675	0.71966	0.551931
NK.cells	PDCD1LG2	0.210687	1.775707	1.019012	0.310978	-5.74393	0.777773	0.637443
NK.cells	CPNE8	-0.40397	2.649239	-1.01897	0.310997	-5.21779	0.767598	0.622152
NK.cells	NEB	0.367053	1.258562	1.018741	0.311106	-5.16204	0.783865	0.646711
NK.cells	SMARCA5	-0.07436	7.448521	-1.01863	0.311159	-6.4624	0.714205	0.54438
NK.cells	ELMO1	0.080528	8.719508	1.018482	0.311228	-6.67289	0.700759	0.525481
NK.cells	BRAP	-0.10826	4.924367	-1.01845	0.311242	-6.04235	0.741762	0.584045
NK.cells	SOGA1	0.313502	3.807296	1.018393	0.31127	-5.3242	0.754328	0.602504
NK.cells	BMPR1A	-0.31332	2.310682	-1.01835	0.311291	-5.43997	0.771524	0.628121
NK.cells	AASDH	0.286877	2.290623	1.018282	0.311322	-5.39238	0.771758	0.628471
NK.cells	PLK1	-0.28938	3.704872	-1.01823	0.311346	-5.78587	0.755491	0.604224
NK.cells	RHBDL3	-0.47756	0.747589	-1.01752	0.311681	-5.04527	0.790424	0.656337
NK.cells	CEBPZ	-0.08823	6.141756	-1.01749	0.311695	-6.22407	0.72878	0.564875
NK.cells	STAC2	0.487062	1.824947	1.017424	0.311728	-5.07014	0.777679	0.636998
NK.cells	GALNT2	-0.10902	5.565067	-1.01709	0.311884	-6.24771	0.735334	0.574174
NK.cells	GM15860	-0.48778	0.373729	-1.01641	0.312207	-4.93857	0.795672	0.66359
NK.cells	CD160	-0.22702	1.916487	-1.01633	0.312247	-5.88631	0.777361	0.635747
NK.cells	SSX2IP	-0.29546	2.481205	-1.01625	0.312282	-5.40628	0.770771	0.625842
NK.cells	EPHB2	-0.51766	1.372664	-1.01608	0.312362	-4.99152	0.783768	0.64543
NK.cells	PAXX	0.164755	3.270058	1.015858	0.312469	-5.64467	0.761669	0.612292
NK.cells	CEP350	0.100031	6.505585	1.015598	0.312592	-6.3225	0.725523	0.559592
NK.cells	ADIPOR2	0.095747	6.368043	1.015338	0.312715	-6.26742	0.727021	0.561867
NK.cells	NUDT3	-0.09909	5.213023	-1.01533	0.312718	-6.05662	0.739733	0.580229
NK.cells	B930095G1	-0.40411	0.087994	-1.01525	0.312757	-5.18342	0.799116	0.669133

NK.cells	GM13402	-0.46288	0.307958	-1.01518	0.312791	-5.03802	0.796466	0.665097
NK.cells	6720427I0	-0.12793	4.894793	-1.0151	0.312828	-5.99696	0.743279	0.585495
NK.cells	CHMP1B	-0.11996	4.698125	-1.01479	0.312975	-5.96624	0.745479	0.588753
NK.cells	NXPH4	-0.65489	-0.60095	-1.01466	0.313039	-4.84691	0.807478	0.682228
NK.cells	GSN	-0.16771	5.844895	-1.01465	0.31304	-6.02481	0.732749	0.570284
NK.cells	GRPEL2	0.180318	3.176522	1.014605	0.313063	-5.5697	0.762743	0.614272
NK.cells	SPEN	-0.0952	5.922479	-1.01453	0.3131	-6.19445	0.731896	0.569075
NK.cells	2310039H0	0.149585	4.246315	1.014271	0.313221	-5.81449	0.7507	0.596385
NK.cells	2310001H1	0.178424	4.065364	1.013689	0.313497	-5.8384	0.753259	0.59969
NK.cells	NAGA	-0.16134	4.159018	-1.013	0.313824	-5.88469	0.752662	0.5983
NK.cells	ARL6IP6	0.113734	5.278997	1.012885	0.313879	-6.02921	0.740097	0.579977
NK.cells	KIF18B	-0.28187	2.84215	-1.01284	0.313901	-5.49318	0.767731	0.620701
NK.cells	ARHGAP39	0.191831	4.38051	1.012752	0.313942	-5.83285	0.750159	0.594701
NK.cells	NEMF	-0.0841	5.95092	-1.0126	0.314014	-6.23318	0.73269	0.569285
NK.cells	PCDHGC4	-0.51703	0.256002	-1.01225	0.31418	-4.96522	0.798553	0.667147
NK.cells	TMEM11	0.112749	5.070903	1.012124	0.31424	-6.05939	0.742674	0.583609
NK.cells	4932422M	-0.35114	0.972719	-1.01189	0.31435	-5.16756	0.790002	0.654141
NK.cells	BRPF3	-0.12352	4.451036	-1.0116	0.314492	-5.84603	0.749667	0.593835
NK.cells	KPNA6	0.134791	3.896998	1.011585	0.314497	-5.7995	0.75594	0.603064
NK.cells	AI597479	0.290041	1.688645	1.011559	0.314509	-5.23567	0.781514	0.641283
NK.cells	GM49662	-0.58555	2.537113	-1.01128	0.31464	-5.09629	0.771639	0.626441
NK.cells	ENOX2	0.134396	5.852601	1.011165	0.314697	-6.17018	0.734104	0.571233
NK.cells	LOXL2	-0.45935	1.406795	-1.01099	0.314778	-5.03035	0.784904	0.646473
NK.cells	2610507B1	0.086891	5.431746	1.01098	0.314785	-6.12325	0.738756	0.577982
NK.cells	ING4	-0.16329	4.08954	-1.01022	0.315146	-5.74917	0.754316	0.600265
NK.cells	NR6A1	-0.1567	5.241175	-1.0101	0.315203	-5.98578	0.74137	0.58137
NK.cells	MAU2	-0.11107	6.192162	-1.00993	0.315284	-6.29918	0.730863	0.566201
NK.cells	PLVAP	-0.4673	1.394862	-1.00991	0.315295	-5.0284	0.785571	0.64706
NK.cells	NKAIN2	-0.45705	1.350359	-1.00988	0.315311	-5.17479	0.786098	0.64786
NK.cells	TFDP2	0.133354	5.591554	1.009706	0.315392	-6.23737	0.737507	0.57578
NK.cells	EBNA1BP2	0.155122	4.017915	1.009459	0.315509	-5.81984	0.755157	0.601701
NK.cells	RAB6A	-0.0848	6.637549	-1.00945	0.315511	-6.32619	0.726025	0.559381
NK.cells	ZFP729B	-0.15648	3.639068	-1.00871	0.315867	-5.70941	0.759532	0.608458
NK.cells	HMBS	0.168272	4.451319	1.008531	0.315952	-5.89918	0.750307	0.594961
NK.cells	ADSS	0.085439	6.271707	1.00851	0.315962	-6.30092	0.730074	0.56556
NK.cells	CUL2	0.109256	4.968394	1.008506	0.315964	-6.12648	0.744498	0.586458
NK.cells	HYAL3	-0.58171	0.035974	-1.00806	0.316178	-4.87178	0.801944	0.672724
NK.cells	IDI1	-0.17219	3.90584	-1.00799	0.316212	-5.68617	0.756489	0.604109
NK.cells	ATP2A2	-0.07909	6.456942	-1.0079	0.316253	-6.31235	0.728049	0.562693
NK.cells	CPNE5	-0.52419	0.481176	-1.00788	0.31626	-4.959	0.79657	0.664461
NK.cells	MDK	-0.37599	1.436872	-1.00788	0.316263	-5.19432	0.785162	0.647053
NK.cells	1600002K0	0.169859	3.214423	1.007832	0.316286	-5.62518	0.764403	0.615845
NK.cells	CCDC88A	-0.1975	5.372063	-1.00767	0.316361	-5.70266	0.739998	0.579964
NK.cells	AIM2	0.124347	5.579386	1.00748	0.316454	-6.22456	0.737698	0.576653
NK.cells	BCL7B	0.099486	5.531179	1.00738	0.316501	-6.13595	0.738232	0.577441
NK.cells	CIAO2B	0.12576	4.566233	1.0073	0.31654	-5.99205	0.749012	0.593177
NK.cells	GM47644	0.463847	1.060844	1.007252	0.316563	-5.11128	0.78963	0.653928

NK.cells	DPP8	0.097057	5.667042	1.00702	0.316673	-6.15251	0.736728	0.575374
NK.cells	SH3KBP1	0.077822	7.863843	1.00699	0.316688	-6.55242	0.712869	0.541249
NK.cells	CYP2R1	-0.45045	1.009263	-1.00689	0.316734	-5.03537	0.790244	0.655034
NK.cells	ABCC10	0.391866	0.65899	1.006823	0.316767	-5.02215	0.794434	0.661439
NK.cells	D130020L0	0.463381	0.782996	1.006722	0.316816	-5.02916	0.792948	0.659185
NK.cells	B4GALT1	-0.08086	7.137524	-1.00663	0.316857	-6.45984	0.720662	0.552358
NK.cells	DSCAML1	-0.61064	-0.596	-1.00641	0.316965	-4.86844	0.809689	0.684903
NK.cells	PARP6	0.206495	2.991541	1.006258	0.317038	-5.57796	0.767022	0.619929
NK.cells	IRS2	-0.15492	5.572327	-1.0062	0.317066	-6.14115	0.737823	0.576963
NK.cells	STAB1	-0.36587	3.176342	-1.0054	0.317449	-5.38892	0.765141	0.616964
NK.cells	KPNA2	0.154873	4.937072	1.005349	0.317472	-6.05093	0.74514	0.587458
NK.cells	WASHC1	0.119281	4.182087	1.005238	0.317525	-5.8747	0.753646	0.59998
NK.cells	CHST3	-0.25656	4.312271	-1.00509	0.317596	-5.62676	0.752172	0.597856
NK.cells	LY6G	0.614597	-0.81776	1.005019	0.31763	-4.90129	0.812673	0.689505
NK.cells	UTP3	0.11026	5.450096	1.004904	0.317685	-6.119	0.73942	0.579243
NK.cells	SHC4	0.451766	0.470109	1.004847	0.317713	-5.01174	0.797014	0.665302
NK.cells	PRRG2	0.287341	2.322172	1.004727	0.31777	-5.23116	0.775051	0.631926
NK.cells	SOCS3	-0.17765	4.698376	-1.00466	0.317801	-6.13403	0.747818	0.591493
NK.cells	GLG1	0.090265	6.951102	1.004475	0.317891	-6.39704	0.722959	0.555577
NK.cells	1810046K0	-0.5869	-0.14929	-1.00447	0.317893	-4.88304	0.804505	0.676888
NK.cells	PEF1	-0.18367	3.347699	-1.00424	0.318003	-5.62641	0.763169	0.614233
NK.cells	RHOD	-0.32933	1.328394	-1.00423	0.318008	-5.24005	0.786755	0.649713
NK.cells	GM550	-0.45416	0.859238	-1.00413	0.318055	-5.01864	0.792345	0.658249
NK.cells	CD7	-0.18933	4.199176	-1.00401	0.318115	-6.32989	0.753452	0.599878
NK.cells	NUDT22	0.218058	2.557891	1.003623	0.318299	-5.44642	0.772597	0.628077
NK.cells	SOAT2	-0.27472	0.810649	-1.00324	0.318485	-5.4217	0.793378	0.659434
NK.cells	PIK3R4	0.205566	3.189781	1.003235	0.318485	-5.56618	0.765421	0.617227
NK.cells	KDM4D	0.315706	0.328103	1.002973	0.318611	-5.31751	0.799293	0.668429
NK.cells	PFDN2	-0.10691	5.031244	-1.00277	0.31871	-6.03819	0.744615	0.586589
NK.cells	NUP205	0.138117	4.408914	1.002675	0.318754	-5.88868	0.751613	0.59684
NK.cells	KLF9	-0.19948	3.693914	-1.00262	0.318782	-5.78799	0.759741	0.608848
NK.cells	LENG1	0.182876	2.925078	1.002296	0.318936	-5.5449	0.768669	0.622105
NK.cells	ILKAP	-0.09886	5.651644	-1.00194	0.319107	-6.16766	0.737788	0.576778
NK.cells	CCHCR1	-0.35679	1.536464	-1.00177	0.319189	-5.19811	0.78493	0.646826
NK.cells	B3GALT1	-0.42276	3.129188	-1.00174	0.319202	-5.46781	0.76631	0.618801
NK.cells	ZFP281	0.134732	4.496464	1.001582	0.319279	-5.933	0.750704	0.595744
NK.cells	GM13091	0.474256	0.904415	1.001439	0.319348	-5.00786	0.792452	0.658427
NK.cells	D830036C2	-0.58332	0.420218	-1.00139	0.319369	-4.90572	0.798266	0.667341
NK.cells	LYPD6B	0.386815	0.004013	1.00138	0.319376	-5.26022	0.8033	0.675096
NK.cells	CUTA	-0.08545	6.189562	-1.00054	0.319782	-6.28996	0.731857	0.568571
NK.cells	RYR2	-0.68183	0.059974	-1.00044	0.319829	-4.96108	0.802621	0.674293
NK.cells	CD3G	-0.1651	3.343766	-1.00044	0.31983	-6.27832	0.763838	0.615474
NK.cells	NAA38	0.111812	5.467647	1.00025	0.319919	-6.13767	0.739829	0.580234
NK.cells	ZFP934	0.230995	2.990341	1.00017	0.319958	-5.49752	0.767914	0.621654
NK.cells	RAPH1	-0.12057	5.055385	-1.00009	0.319994	-6.1689	0.744424	0.586945
NK.cells	ENDOU	0.55074	0.913658	1.000044	0.320018	-4.96588	0.792342	0.658617
NK.cells	RER1	-0.08859	6.413933	-0.9998	0.320137	-6.32642	0.729399	0.565239

NK.cells	TYROBP	-0.11171	9.095091	-0.99935	0.320353	-6.81698	0.70072	0.524721
NK.cells	ZFP112	-0.53352	0.391108	-0.99911	0.320466	-4.93906	0.798617	0.668601
NK.cells	SMPD2	-0.29908	2.04317	-0.99904	0.320501	-5.28276	0.778954	0.638604
NK.cells	ADPGK	0.215766	4.903066	0.999014	0.320514	-5.77732	0.74613	0.589762
NK.cells	VPS28	0.089783	6.686374	0.998942	0.320549	-6.40079	0.726426	0.561228
NK.cells	RHBDD3	0.248141	2.194151	0.998738	0.320647	-5.29768	0.777183	0.635973
NK.cells	POMK	-0.43222	1.068205	-0.99861	0.320707	-5.06645	0.790496	0.656238
NK.cells	ZKSCAN16	-0.56256	-0.59074	-0.99819	0.32091	-4.84266	0.810551	0.68734
NK.cells	CPM	0.208302	4.160192	0.998134	0.320938	-5.89532	0.75451	0.602316
NK.cells	PPP1R8	-0.13181	4.114233	-0.9981	0.320954	-5.89551	0.755032	0.603087
NK.cells	HSPB11	0.196858	2.925359	0.998001	0.321002	-5.63101	0.768666	0.623437
NK.cells	FMC1	0.161155	4.072806	0.997802	0.321098	-5.83788	0.755502	0.603908
NK.cells	TMEM214	0.12359	4.338367	0.997745	0.321126	-5.85956	0.752491	0.599468
NK.cells	TRIM14	0.154084	4.686216	0.997742	0.321127	-6.03914	0.748566	0.593692
NK.cells	SELENBP1	0.204252	3.795416	0.997712	0.321142	-5.81451	0.758662	0.608596
NK.cells	MXI1	0.114601	7.063981	0.997588	0.321202	-6.49363	0.722327	0.555691
NK.cells	SETX	0.103667	5.905752	0.997579	0.321206	-6.25007	0.73498	0.573901
NK.cells	IFRD2	0.189028	3.30652	0.99745	0.321268	-5.62892	0.764266	0.61702
NK.cells	SEC24D	-0.12299	4.470665	-0.99739	0.321296	-6.04057	0.750995	0.597354
NK.cells	SLC25A13	-0.14164	4.679744	-0.99736	0.321313	-6.02768	0.748638	0.593897
NK.cells	NDUFB11	-0.08028	7.55899	-0.9973	0.32134	-6.51477	0.716994	0.54816
NK.cells	MMGT2	0.154009	3.166256	0.997277	0.321351	-5.69305	0.765882	0.619459
NK.cells	THEMIS	0.227543	1.160853	0.996999	0.321486	-5.84115	0.789391	0.655087
NK.cells	TMEM250-	0.152778	4.629225	0.996962	0.321504	-5.86639	0.749207	0.594838
NK.cells	TNRC6B	-0.08284	8.234075	-0.99677	0.321597	-6.66225	0.70979	0.538114
NK.cells	SLK	-0.09067	6.477015	-0.99657	0.321692	-6.44784	0.728709	0.565123
NK.cells	GM20559	0.159575	4.344461	0.996423	0.321764	-5.9414	0.752422	0.599709
NK.cells	4930532G1	-0.36009	1.429337	-0.99638	0.321786	-5.36625	0.7862	0.650384
NK.cells	KIF5B	-0.06425	7.401742	-0.99636	0.321795	-6.46345	0.718683	0.550795
NK.cells	FBP1	-0.23917	5.143064	-0.99627	0.321836	-6.09202	0.743444	0.586567
NK.cells	LRRK2	-0.34	4.404591	-0.99617	0.321884	-5.4038	0.751742	0.598801
NK.cells	FTSJ1	-0.16251	3.078297	-0.99616	0.321893	-5.5796	0.766897	0.621319
NK.cells	RETREG1	0.104214	6.437217	0.996008	0.321965	-6.40105	0.729144	0.565898
NK.cells	SPARCL1	0.750828	0.3267	0.995839	0.322046	-4.98222	0.799395	0.67075
NK.cells	ZC3H7B	0.179214	3.353116	0.995756	0.322086	-5.67174	0.76373	0.616628
NK.cells	DAP3	0.110017	5.039126	0.995738	0.322095	-6.0483	0.744606	0.588359
NK.cells	AGPAT3	-0.11067	5.164345	-0.9956	0.322162	-6.18301	0.743216	0.586332
NK.cells	ZFP747	-0.28884	1.605909	-0.99543	0.322242	-5.26852	0.784145	0.647392
NK.cells	RAP1GDS1	0.091645	6.711436	0.995156	0.322376	-6.401	0.726187	0.56167
NK.cells	CHD6	-0.10564	5.997264	-0.99509	0.322406	-6.24532	0.734005	0.572949
NK.cells	POC1A	-0.20196	3.229083	-0.99506	0.322425	-5.60376	0.765193	0.618846
NK.cells	EXOSC2	0.180603	2.945711	0.994703	0.322595	-5.59234	0.768722	0.623855
NK.cells	RASSF4	-0.32006	4.553252	-0.99448	0.322705	-5.32855	0.750457	0.596655
NK.cells	HAUS8	-0.12392	4.722045	-0.99405	0.322912	-5.99284	0.748884	0.593973
NK.cells	AIMP1	0.081848	6.38044	0.993922	0.322973	-6.33416	0.730468	0.56719
NK.cells	EIF4E3	0.141959	4.420461	0.993372	0.32324	-5.97538	0.75265	0.599184
NK.cells	9930111J2	0.38208	1.279459	0.99334	0.323255	-5.22108	0.78912	0.653892

NK.cells	TMEM141	0.372392	2.239657	0.992821	0.323507	-5.27565	0.777905	0.636829
NK.cells	PTPRC	0.086386	8.915439	0.992424	0.323699	-6.8103	0.703737	0.528933
NK.cells	ZFP3	0.439389	0.562138	0.992348	0.323736	-5.0233	0.797845	0.667327
NK.cells	NTMT1	0.114109	3.588232	0.992344	0.323738	-5.79744	0.762261	0.613479
NK.cells	RCBTB1	0.135175	3.826201	0.992127	0.323843	-5.8778	0.759536	0.609547
NK.cells	CD302	-0.19677	5.847821	-0.99201	0.323899	-6.09434	0.736808	0.576215
NK.cells	7-Sep	-0.05855	8.277792	-0.9919	0.323951	-6.64506	0.710473	0.538569
NK.cells	TRAPPC6B	0.075711	6.134079	0.991884	0.323961	-6.27879	0.73365	0.571689
NK.cells	TRIM17	0.31548	2.197819	0.991806	0.323999	-5.22035	0.778396	0.637911
NK.cells	OARD1	0.124736	4.902059	0.991755	0.324023	-6.01868	0.747347	0.591689
NK.cells	MAP4K1	0.104022	4.801156	0.991665	0.324067	-6.07191	0.748481	0.59339
NK.cells	ZFAT	-0.18495	3.828322	-0.99162	0.324091	-5.7262	0.759512	0.609679
NK.cells	SLAMF8	0.725706	0.718707	0.991452	0.32417	-4.96283	0.795961	0.664861
NK.cells	STAB2	-0.33296	5.148934	-0.99128	0.324256	-5.86866	0.74458	0.587854
NK.cells	APP	0.233255	5.938302	0.991088	0.324347	-5.89302	0.735808	0.575167
NK.cells	DEPDC7	-0.41189	1.678465	-0.9908	0.324487	-5.16291	0.784516	0.647746
NK.cells	KNOP1	0.113706	4.90183	0.990698	0.324537	-6.00971	0.74735	0.59219
NK.cells	PRELID3B	0.101009	5.574007	0.990693	0.324539	-6.14313	0.739842	0.581211
NK.cells	PIK3IP1	0.241292	2.920799	0.990594	0.324587	-5.54459	0.769961	0.625798
NK.cells	TUBB6	0.194665	3.749371	0.990274	0.324743	-5.86713	0.760415	0.611651
NK.cells	NDNF	-0.30856	-0.47189	-0.99027	0.324745	-5.30241	0.810404	0.687787
NK.cells	F730311O2	0.630781	-0.31758	0.989774	0.324986	-4.87033	0.808517	0.685046
NK.cells	10-Sep	0.262725	3.119761	0.989625	0.325058	-5.31821	0.767657	0.622709
NK.cells	LGALS2	0.56865	-0.89826	0.989613	0.325064	-4.84972	0.815643	0.696242
NK.cells	MSH6	0.15866	4.38755	0.989489	0.325124	-5.88451	0.75315	0.601122
NK.cells	CAPZB	-0.06089	8.722615	-0.98939	0.325172	-6.696	0.705766	0.532789
NK.cells	NAIF1	-0.3652	1.251393	-0.98925	0.325241	-5.11067	0.789587	0.655949
NK.cells	GNE	0.146449	4.467546	0.989151	0.325289	-5.78246	0.752244	0.599841
NK.cells	PHAX	0.127359	4.599165	0.989118	0.325305	-5.99298	0.750757	0.597664
NK.cells	NAF1	0.155846	3.658508	0.988691	0.325513	-5.7021	0.761456	0.613626
NK.cells	GOLT1B	-0.13395	4.631519	-0.98863	0.32554	-5.98025	0.750392	0.597252
NK.cells	AI467606	0.179016	4.111903	0.988603	0.325555	-5.81632	0.756278	0.605968
NK.cells	GM44752	-0.20777	2.484975	-0.98852	0.325597	-5.51582	0.775034	0.634031
NK.cells	SLC35E1	-0.12187	4.084727	-0.98832	0.325693	-5.92413	0.756588	0.606502
NK.cells	KANK3	0.393638	2.000717	0.988146	0.325778	-5.21662	0.780713	0.642818
NK.cells	GMEB2	-0.09422	5.764218	-0.98791	0.325894	-6.17291	0.737733	0.578919
NK.cells	PPARGC1B	0.21419	2.799015	0.987888	0.325903	-5.6036	0.771375	0.628725
NK.cells	SLC12A8	0.464221	0.657727	0.987868	0.325913	-5.03195	0.796694	0.667284
NK.cells	TBC1D22B	0.138677	3.778303	0.987735	0.325978	-5.87487	0.760084	0.611824
NK.cells	PRPS1	-0.1666	3.621673	-0.98766	0.326015	-5.72183	0.761878	0.614497
NK.cells	ZDHHC6	0.12784	4.609891	0.987614	0.326037	-5.92692	0.750636	0.597825
NK.cells	FCRLS	-0.92542	-1.03209	-0.9876	0.326046	-4.8785	0.817213	0.699313
NK.cells	SURF2	0.165171	3.331878	0.987473	0.326105	-5.68564	0.765209	0.619473
NK.cells	NR1H2	-0.14098	4.589379	-0.98747	0.326107	-5.95433	0.750868	0.598167
NK.cells	STAG3	-0.63124	0.607181	-0.98711	0.326284	-4.93175	0.797302	0.668318
NK.cells	MRPL11	0.135889	4.26095	0.98708	0.326297	-5.87492	0.754585	0.603752
NK.cells	GM12089	-0.46277	0.306607	-0.98704	0.326318	-4.92993	0.800929	0.673936

NK.cells	NIT1	0.163637	3.750254	0.986938	0.326366	-5.7309	0.760405	0.612407
NK.cells	GM26827	-0.27783	1.344192	-0.98686	0.326404	-5.48643	0.788482	0.654791
NK.cells	PDPN	0.739395	-0.21403	0.986685	0.326489	-4.9097	0.807308	0.683745
NK.cells	APPBP2OS	-0.31732	0.985935	-0.98649	0.326585	-5.15711	0.792889	0.661361
NK.cells	EPG5	0.136677	4.420072	0.986211	0.326721	-5.87178	0.753075	0.601262
NK.cells	VPS33B	0.187152	2.932531	0.985882	0.326881	-5.5621	0.770249	0.62681
NK.cells	RCAN3	0.237464	2.117276	0.985845	0.3269	-5.41564	0.779771	0.641192
NK.cells	ANKRD16	0.188588	3.026656	0.985346	0.327143	-5.59977	0.769581	0.625295
NK.cells	CBY1	-0.19572	2.846037	-0.9852	0.327213	-5.52003	0.771693	0.628541
NK.cells	CARM1	-0.12144	4.727042	-0.98485	0.327383	-5.92522	0.750402	0.596624
NK.cells	NDUFA5	0.128529	5.432033	0.98459	0.327512	-6.15925	0.742563	0.58514
NK.cells	INPP5B	0.129324	4.216748	0.984534	0.327539	-5.95447	0.75625	0.605297
NK.cells	SYNCRIP	-0.077	7.375906	-0.98415	0.327725	-6.50374	0.72133	0.554362
NK.cells	THUMPD1	0.11784	4.425949	0.984109	0.327748	-5.98261	0.753976	0.601834
NK.cells	RASAL3	-0.11346	4.10056	-0.98406	0.327773	-6.01445	0.757675	0.607334
NK.cells	CUL4B	-0.13909	4.524951	-0.98346	0.328065	-5.96391	0.753163	0.600376
NK.cells	FASTKD2	0.172147	2.952537	0.983401	0.328094	-5.55918	0.771199	0.627232
NK.cells	UBE2K	-0.06532	8.214477	-0.98339	0.328097	-6.60637	0.712629	0.541773
NK.cells	RAB3GAP1	0.077223	6.044858	0.983137	0.328223	-6.2712	0.736169	0.575559
NK.cells	TIMM44	0.107121	4.84696	0.982896	0.328341	-6.00591	0.74953	0.595165
NK.cells	GUCY2C	0.400468	-0.14698	0.982833	0.328372	-5.01924	0.808122	0.68383
NK.cells	CITED2	-0.10979	6.149916	-0.9828	0.328388	-6.3383	0.73501	0.573972
NK.cells	ATXN7L1O	0.350922	1.337166	0.982738	0.328418	-5.21635	0.790215	0.656225
NK.cells	DAZAP1	-0.07692	6.776612	-0.98248	0.328544	-6.38163	0.728136	0.564144
NK.cells	RASIP1	-0.4135	1.808725	-0.98242	0.328573	-5.12885	0.784614	0.647806
NK.cells	PPIE	0.112799	4.586932	0.982339	0.328614	-5.99839	0.752465	0.599643
NK.cells	MPP5	0.121514	5.379551	0.982166	0.328698	-6.05463	0.743557	0.586606
NK.cells	LRRC25	0.395215	3.856093	0.982076	0.328742	-5.30918	0.760782	0.612035
NK.cells	PSMD13	0.085563	6.203145	0.981847	0.328855	-6.29352	0.734423	0.573413
NK.cells	BLCAP	0.196928	3.009547	0.981718	0.328918	-5.54365	0.77054	0.626795
NK.cells	MEX3B	-0.24651	2.233947	-0.98171	0.328922	-5.45929	0.779599	0.640464
NK.cells	GM39469	0.212009	2.328802	0.981237	0.329153	-5.40778	0.778804	0.639037
NK.cells	PSAT1	-0.17059	4.218142	-0.98116	0.329189	-5.96942	0.75696	0.606338
NK.cells	AK8	-0.28011	2.179586	-0.98105	0.329246	-5.33207	0.780558	0.641767
NK.cells	GCLC	-0.12978	6.841762	-0.98078	0.329379	-6.36011	0.727747	0.563721
NK.cells	MYO6	0.162599	3.1857	0.980668	0.329432	-5.85348	0.768839	0.624114
NK.cells	CEP192	0.10631	5.27108	0.980638	0.329447	-6.13585	0.745099	0.588919
NK.cells	TSGA10	0.21931	2.968133	0.979775	0.329871	-5.45159	0.772206	0.628263
NK.cells	NCBP2	-0.11963	4.599992	-0.9794	0.330055	-5.97003	0.753666	0.600468
NK.cells	SERF2	0.057353	9.682679	0.979243	0.330132	-6.87729	0.698431	0.521302
NK.cells	H2-OA	-0.24464	3.19042	-0.97918	0.330163	-5.68791	0.769821	0.624585
NK.cells	MBD6	-0.17179	3.632499	-0.97909	0.330208	-5.65838	0.764715	0.616955
NK.cells	HTR2B	-0.39199	0.855091	-0.97875	0.330375	-5.1031	0.797552	0.666574
NK.cells	ABHD4	0.21076	2.787772	0.97871	0.330394	-5.45307	0.774641	0.631715
NK.cells	NCAPH	-0.20655	4.175802	-0.97849	0.330503	-5.88654	0.758622	0.60782
NK.cells	TMEFF1	0.62363	-0.59624	0.978453	0.33052	-4.92572	0.815228	0.694029
NK.cells	2610021A0	0.227427	2.362444	0.978004	0.330741	-5.45895	0.779756	0.639411

NK.cells	TCOF1	-0.11636	5.717375	-0.97792	0.330783	-6.23174	0.741379	0.582411
NK.cells	GOLPH3	-0.07428	5.902166	-0.97788	0.330802	-6.27684	0.739326	0.579422
NK.cells	SNX17	0.096685	5.853791	0.977821	0.330831	-6.22855	0.739863	0.580203
NK.cells	MINDY1	-0.16473	3.987424	-0.97768	0.330902	-5.68334	0.760923	0.611158
NK.cells	MRPS36	0.109613	5.569029	0.976999	0.331236	-6.1799	0.743603	0.584999
NK.cells	TAF4B	-0.14966	5.130162	-0.97678	0.331343	-6.1919	0.74852	0.592267
NK.cells	UTP14A	0.110247	5.238791	0.976568	0.331447	-6.08219	0.747299	0.590486
NK.cells	LRP1	-0.25787	4.178873	-0.97657	0.331449	-5.53216	0.7593	0.608169
NK.cells	RRS1	0.14264	4.476059	0.976419	0.331521	-5.88391	0.755914	0.603185
NK.cells	FAM204A	0.10324	4.894011	0.976366	0.331547	-6.04759	0.75118	0.596218
NK.cells	NLRC3	0.207197	1.662225	0.976254	0.331602	-5.58174	0.788638	0.652336
NK.cells	SFXN5	0.226421	3.363367	0.97613	0.331663	-5.57092	0.768676	0.622263
NK.cells	MYH9	0.070135	7.80338	0.976016	0.331719	-6.59598	0.719114	0.54994
NK.cells	TOP3A	-0.17095	3.860539	-0.97573	0.331859	-5.76007	0.763121	0.613871
NK.cells	FLII	0.091558	5.969326	0.975329	0.332058	-6.28842	0.739613	0.578944
NK.cells	TMEM263	-0.14491	3.98713	-0.97516	0.332142	-5.86479	0.761972	0.611803
NK.cells	LEKR1	0.480795	1.047991	0.974962	0.332239	-5.07239	0.796481	0.663973
NK.cells	COX5A	0.090712	8.546847	0.974701	0.332368	-6.64916	0.711612	0.538973
NK.cells	SERPINB2	1.234933	0.991964	0.97444	0.332497	-5.09364	0.797155	0.66522
NK.cells	TGFB1	-0.07743	8.800362	-0.9744	0.332517	-6.69616	0.708923	0.535306
NK.cells	AXIN1	-0.07725	6.046226	-0.97439	0.332523	-6.29432	0.73876	0.577955
NK.cells	TUBA1A	-0.13649	5.430541	-0.97437	0.332531	-6.2603	0.745619	0.587947
NK.cells	TTC7	0.104759	5.873227	0.974226	0.332602	-6.30514	0.74068	0.580779
NK.cells	HIST1H3B	0.395608	1.995292	0.974177	0.332626	-5.45136	0.78518	0.646969
NK.cells	MYO1D	-0.25905	3.025547	-0.97372	0.33285	-5.41353	0.773454	0.628882
NK.cells	FAM57B	0.496141	0.285081	0.973562	0.33293	-5.02068	0.806118	0.678758
NK.cells	BTAF1	-0.07935	7.190783	-0.9732	0.33311	-6.45637	0.726564	0.560269
NK.cells	PGGT1B	0.111655	4.818529	0.972939	0.333238	-6.01275	0.752888	0.598557
NK.cells	NKAPD1	-0.13339	4.086425	-0.97269	0.333359	-5.86832	0.761222	0.610949
NK.cells	CTDSPL2	0.097135	5.892856	0.972683	0.333365	-6.25437	0.740839	0.580985
NK.cells	IFNG	-0.24908	1.241502	-0.97263	0.333388	-5.8014	0.794563	0.661263
NK.cells	WASHC3	0.108378	4.517912	0.972571	0.33342	-5.99231	0.756298	0.603669
NK.cells	POLR2J	0.106759	4.922519	0.97249	0.33346	-6.02414	0.751713	0.596916
NK.cells	RNH1	0.114855	5.988026	0.972319	0.333545	-6.27247	0.739782	0.579496
NK.cells	ALPL	-0.42409	1.490191	-0.9723	0.333554	-5.10711	0.791587	0.656759
NK.cells	ADAR	0.153372	4.311216	0.972064	0.333671	-5.92041	0.758653	0.607237
NK.cells	AIP	0.117634	4.946962	0.97173	0.333836	-6.09341	0.751437	0.596606
NK.cells	ACVR2B	0.460214	1.53111	0.971713	0.333845	-5.02825	0.791098	0.656086
NK.cells	AGFG2	0.129291	4.605897	0.971699	0.333851	-5.96893	0.755299	0.602297
NK.cells	HERC2	-0.09941	5.98752	-0.97169	0.333856	-6.25611	0.739787	0.57957
NK.cells	SNX29	0.13594	6.395771	0.971574	0.333913	-6.24492	0.735271	0.573037
NK.cells	CSPG5	-0.58864	0.28413	-0.97152	0.333939	-4.98773	0.80613	0.67923
NK.cells	AHNAK	-0.14798	6.416793	-0.97106	0.334166	-6.57245	0.735061	0.572808
NK.cells	NOMO1	0.1638	3.5373	0.971049	0.334173	-5.70516	0.767561	0.620614
NK.cells	KCTD9	0.16578	3.130956	0.971017	0.334189	-5.68109	0.772271	0.627671
NK.cells	ATRAID	0.121691	4.57765	0.970988	0.334203	-5.93671	0.755641	0.602903
NK.cells	CCDC71L	0.153095	4.455272	0.970573	0.334409	-5.91313	0.757089	0.605114

NK.cells	UHRF1BP1	-0.34594	1.998967	-0.97034	0.334526	-5.28788	0.785617	0.648037
NK.cells	SARAF	-0.09241	5.365255	-0.97031	0.334538	-6.28744	0.746807	0.590088
NK.cells	CCDC25	-0.11431	4.712121	-0.97027	0.334558	-6.01871	0.754171	0.600916
NK.cells	STIM1	-0.11111	7.712751	-0.97014	0.334624	-6.58641	0.720984	0.552758
NK.cells	FBXO3	0.101683	5.012921	0.970047	0.334669	-6.03716	0.75077	0.595905
NK.cells	PNKP	0.132011	4.959191	0.97004	0.334673	-6.05451	0.751376	0.596797
NK.cells	WDCP	0.189415	3.079235	0.969353	0.335013	-5.62709	0.77325	0.629083
NK.cells	ZFP236	0.118387	4.729945	0.969264	0.335058	-5.98264	0.754281	0.600874
NK.cells	TMEM238	0.2008	2.938537	0.969254	0.335063	-5.71521	0.77489	0.631591
NK.cells	GM43149	0.403991	0.774872	0.969129	0.335125	-5.0835	0.800592	0.670758
NK.cells	XPNPEP3	0.186535	3.250052	0.968981	0.335198	-5.56602	0.771263	0.626197
NK.cells	RGCC	-0.13588	5.613883	-0.96838	0.335496	-6.29578	0.744332	0.586534
NK.cells	BASP1	0.228179	5.646818	0.967889	0.33574	-6.09235	0.743964	0.586215
NK.cells	SCAF1	0.113644	4.690272	0.967872	0.335749	-5.99654	0.754731	0.602035
NK.cells	B3GALT6	0.352019	1.806345	0.967822	0.335774	-5.24909	0.788228	0.652353
NK.cells	PRPF40B	-0.57033	0.534618	-0.96771	0.33583	-4.97366	0.803501	0.675878
NK.cells	CREB3	-0.14343	3.923009	-0.96758	0.335894	-5.73644	0.76349	0.615159
NK.cells	MYCT1	-0.38748	1.654516	-0.96755	0.33591	-5.11912	0.790035	0.655228
NK.cells	CASC1	-0.39084	1.810453	-0.96744	0.335962	-5.22046	0.788179	0.652418
NK.cells	PANK2	0.099835	5.327592	0.967421	0.335972	-6.1301	0.747538	0.591598
NK.cells	EEF2K	-0.16565	4.644155	-0.9673	0.336034	-5.83269	0.755254	0.603049
NK.cells	SACS	0.225855	2.596254	0.967254	0.336056	-5.67961	0.778897	0.638426
NK.cells	PBXIP1	0.129987	4.578633	0.96725	0.336057	-6.0865	0.755998	0.604161
NK.cells	AURKAIP1	0.093013	5.837172	0.967167	0.336099	-6.25834	0.741841	0.58336
NK.cells	SCARF1	-0.42741	1.587665	-0.96716	0.336105	-5.05553	0.790832	0.656585
NK.cells	SMTNL2	-0.60332	0.4226	-0.96712	0.336123	-4.93056	0.804861	0.678191
NK.cells	LIN54	0.108403	6.14118	0.966813	0.336275	-6.40179	0.738465	0.578462
NK.cells	KLF2	0.184153	8.937753	0.966665	0.336348	-6.68098	0.708195	0.535151
NK.cells	CRPPA	0.349911	2.552625	0.966612	0.336374	-5.21526	0.779409	0.639234
NK.cells	AMD2	-0.43737	0.044662	-0.96646	0.336453	-4.98531	0.809468	0.685429
NK.cells	CRTC2	-0.15865	4.134514	-0.96635	0.336506	-5.85073	0.761064	0.611791
NK.cells	TUSC2	0.202531	3.017242	0.966332	0.336514	-5.55425	0.773972	0.631111
NK.cells	GNMT	0.268671	4.232803	0.966262	0.336549	-5.92337	0.75994	0.610123
NK.cells	DMC1	-0.49893	0.152078	-0.96618	0.336591	-4.92503	0.808156	0.683427
NK.cells	NFAM1	0.303059	4.570884	0.965943	0.336707	-5.27181	0.756205	0.60447
NK.cells	MRPL28	0.101397	6.016074	0.965353	0.337001	-6.23784	0.740475	0.580844
NK.cells	STX8	0.068155	6.745581	0.965111	0.337121	-6.42018	0.732542	0.569242
NK.cells	GM46218	-0.46419	0.58606	-0.96486	0.337245	-5.00382	0.803762	0.67568
NK.cells	PRX	-0.41068	1.104861	-0.96479	0.33728	-5.10185	0.797492	0.666013
NK.cells	FBXO38	0.088768	5.443447	0.964307	0.337522	-6.18062	0.747129	0.590404
NK.cells	TMEM87A	-0.10706	5.236916	-0.96418	0.337588	-6.13164	0.749449	0.593808
NK.cells	GM42031	-0.29809	4.172175	-0.96399	0.33768	-5.90726	0.76154	0.611771
NK.cells	GPR132	0.127495	6.00422	0.963956	0.337697	-6.50904	0.740866	0.581352
NK.cells	LRCH4	0.140869	4.260162	0.963781	0.337784	-5.89063	0.760533	0.610335
NK.cells	TAF10	0.079816	6.856819	0.963781	0.337784	-6.4476	0.731456	0.567771
NK.cells	TRIM3	0.261628	2.274801	0.963724	0.337813	-5.31907	0.783613	0.644999
NK.cells	ZC3H3	0.142382	3.468484	0.963616	0.337867	-5.75315	0.769647	0.623932

NK.cells	TREML4	0.512871	2.758058	0.963515	0.337917	-5.13332	0.777926	0.6364
NK.cells	SMOC1	-0.41147	2.28252	-0.96337	0.33799	-5.29231	0.783522	0.644924
NK.cells	ACAD11	-0.25539	2.331168	-0.96311	0.338119	-5.33558	0.782947	0.644052
NK.cells	RNF141	-0.15194	4.511433	-0.96296	0.338194	-5.73417	0.757665	0.606146
NK.cells	CHSY1	-0.10241	5.079341	-0.96292	0.338213	-6.26171	0.751226	0.596638
NK.cells	FTL1-PS1	-0.26369	3.902493	-0.96291	0.338221	-5.62793	0.764636	0.616507
NK.cells	SEPHS2	0.130382	6.39868	0.962818	0.338265	-6.36633	0.736496	0.575136
NK.cells	3110040N1	0.168151	3.553549	0.962594	0.338377	-5.81023	0.768771	0.622584
NK.cells	DCAF4	0.232999	2.008291	0.962321	0.338513	-5.38288	0.786949	0.650031
NK.cells	CAMKK2	0.1044	5.188179	0.962279	0.338534	-6.07499	0.750171	0.594984
NK.cells	DGKZ	0.106639	6.2774	0.961959	0.338694	-6.36071	0.73814	0.577315
NK.cells	TCTN3	-0.3763	1.366808	-0.9619	0.338722	-5.17825	0.794744	0.661872
NK.cells	GFI1	-0.26335	1.55053	-0.96143	0.338956	-5.43027	0.792672	0.658695
NK.cells	UBE2N	-0.06649	7.48412	-0.96126	0.339044	-6.51708	0.725031	0.558499
NK.cells	DYNLT1C	0.350659	1.100031	0.961223	0.339062	-5.15325	0.798079	0.667074
NK.cells	ASNSD1	0.096101	5.360445	0.96117	0.339088	-6.14049	0.748489	0.592514
NK.cells	PRR5L	0.293299	2.756439	0.960981	0.339183	-5.56086	0.778391	0.637077
NK.cells	TNKS1BP1	0.249471	2.080541	0.96097	0.339188	-5.43068	0.786361	0.649166
NK.cells	9930104L0	0.334818	1.356879	0.960793	0.339277	-5.16378	0.794991	0.662389
NK.cells	S100A8	0.480065	6.633628	0.960771	0.339287	-6.12739	0.734327	0.571931
NK.cells	MRPL47	0.227579	2.974642	0.960583	0.339382	-5.52524	0.775836	0.633286
NK.cells	IFT20	0.097307	5.442542	0.960365	0.33949	-6.19522	0.747566	0.591322
NK.cells	TRIB2	0.229089	2.561151	0.960327	0.339509	-5.63896	0.780685	0.640697
NK.cells	GM10501	-0.27309	2.138944	-0.96026	0.339541	-5.31309	0.785669	0.648266
NK.cells	CCL17	-0.66181	-0.70076	-0.95985	0.339746	-4.94441	0.820423	0.701673
NK.cells	INSYN2B	-0.13062	4.140533	-0.95963	0.339856	-6.00282	0.76276	0.613415
NK.cells	BCCIP	0.082932	5.436859	0.959244	0.340052	-6.17977	0.748096	0.591765
NK.cells	ACER3	0.107703	6.311004	0.959232	0.340058	-6.26927	0.738347	0.577537
NK.cells	DUBR	0.216859	2.378269	0.959094	0.340127	-5.5492	0.783327	0.644343
NK.cells	DARS	0.110718	5.511648	0.959078	0.340135	-6.16675	0.747256	0.590534
NK.cells	KIF3A	-0.22512	2.472633	-0.95891	0.340221	-5.42784	0.782265	0.642728
NK.cells	OLFM4	0.620467	-0.01496	0.958694	0.340327	-4.99125	0.812189	0.688836
NK.cells	KCNAB1	0.435567	1.324987	0.95859	0.340379	-5.16971	0.795924	0.663725
NK.cells	D3ERTD751	0.305814	1.61523	0.958281	0.340534	-5.28085	0.792446	0.658536
NK.cells	EGLN3	0.253662	3.294296	0.957948	0.340701	-5.6841	0.772644	0.628551
NK.cells	TRAPPC4	0.100256	5.137813	0.957449	0.340952	-6.1072	0.751515	0.597278
NK.cells	MARVELD2	-0.30374	1.369704	-0.9574	0.340975	-5.36377	0.795387	0.663294
NK.cells	BBC3	0.207817	2.639565	0.95719	0.341081	-5.5102	0.780302	0.640303
NK.cells	DECR1	-0.20988	3.696206	-0.95713	0.341113	-5.7505	0.767984	0.621772
NK.cells	NAE1	0.125832	3.958187	0.957101	0.341126	-5.90476	0.764962	0.617255
NK.cells	CENPN	-0.24378	2.920239	-0.9567	0.341328	-5.62748	0.777009	0.635533
NK.cells	CLTB	-0.12005	4.396264	-0.95657	0.341395	-6.04748	0.759938	0.609964
NK.cells	BAX	0.099527	6.141068	0.956474	0.341441	-6.32381	0.740282	0.581057
NK.cells	CAMK2A	0.352775	0.846737	0.956332	0.341512	-5.04766	0.80169	0.673319
NK.cells	TBC1D9	-0.47264	3.953614	-0.95633	0.341512	-5.19074	0.765014	0.617587
NK.cells	TSPAN5	0.108978	5.373256	0.956312	0.341522	-6.27506	0.748862	0.593656
NK.cells	GM13184	-0.33415	2.666577	-0.95589	0.341733	-5.25695	0.779985	0.640245

NK.cells	LTBR	-0.26409	2.974023	-0.95581	0.341776	-5.34327	0.77638	0.634825
NK.cells	GGCX	0.291877	1.807957	0.955708	0.341826	-5.29546	0.790146	0.655751
NK.cells	RNF146	0.105474	5.30614	0.955666	0.341847	-6.14877	0.749617	0.59494
NK.cells	MRPL22	0.155056	3.858759	0.955171	0.342096	-5.81752	0.766107	0.61964
NK.cells	GRK3	0.305393	2.91909	0.954948	0.342208	-5.35387	0.777023	0.636096
NK.cells	1110008P1	0.104755	5.226277	0.954874	0.342245	-6.16296	0.750517	0.596533
NK.cells	SUMO3	-0.10297	5.724941	-0.9548	0.342283	-6.17942	0.744919	0.588308
NK.cells	VCPKMT	0.173638	2.877826	0.954794	0.342285	-5.77545	0.777506	0.636827
NK.cells	HES6	0.144135	4.105884	0.954736	0.342314	-5.82499	0.763264	0.615432
NK.cells	TNKS	-0.10699	6.066905	-0.95466	0.342354	-6.28023	0.741106	0.582734
NK.cells	CHD7	0.092187	6.463817	0.954609	0.342378	-6.50756	0.736707	0.57633
NK.cells	ECM2	-0.58236	0.502848	-0.95459	0.342386	-4.97511	0.805863	0.680285
NK.cells	GM26535	-0.54735	-0.60061	-0.95459	0.342387	-4.90603	0.819408	0.701438
NK.cells	PHTF2	-0.10624	5.998759	-0.95444	0.342463	-6.27967	0.741864	0.58384
NK.cells	SAP30L	-0.10614	4.309459	-0.95397	0.342698	-5.97434	0.76093	0.612129
NK.cells	SLC28A2	0.188897	2.961181	0.953918	0.342726	-5.81464	0.77653	0.63554
NK.cells	TBX2	-0.51594	0.45083	-0.95391	0.34273	-4.98754	0.806496	0.681471
NK.cells	SBK1	-0.19779	3.374185	-0.95375	0.342808	-5.59654	0.771715	0.628315
NK.cells	PCK1	-0.27918	4.182878	-0.95374	0.342816	-5.93093	0.762381	0.614331
NK.cells	BRCA2	0.19514	3.555566	0.953717	0.342827	-5.73891	0.769611	0.625151
NK.cells	GTF2IRD1	-0.23508	3.292146	-0.95367	0.342849	-5.58537	0.772669	0.629751
NK.cells	FAM214A	-0.15143	5.246692	-0.9535	0.342934	-6.07709	0.750287	0.596452
NK.cells	MAPK8IP3	-0.10543	4.521476	-0.95346	0.342955	-6.03862	0.758508	0.608617
NK.cells	NRIP1	0.103155	6.353871	0.953265	0.343055	-6.46558	0.737923	0.578361
NK.cells	MED25	0.108338	4.496149	0.953239	0.343068	-6.00302	0.758797	0.609061
NK.cells	SEMA6D	0.288318	3.713919	0.953191	0.343092	-5.70933	0.767779	0.622467
NK.cells	1110020A2	0.4148	0.468534	0.952999	0.343189	-5.0799	0.80628	0.681246
NK.cells	DENND2A	0.409158	1.374468	0.952962	0.343207	-5.13659	0.79533	0.664316
NK.cells	GRASP	-0.17971	3.781204	-0.95287	0.343252	-5.80858	0.767002	0.621315
NK.cells	IRGC1	0.494048	0.115465	0.952362	0.34351	-4.96031	0.810591	0.688104
NK.cells	KMT5A	0.087327	5.846575	0.952307	0.343537	-6.26109	0.74356	0.586714
NK.cells	FBXO47	-0.46797	1.069812	-0.95219	0.343595	-5.08106	0.798995	0.670113
NK.cells	CCDC61	0.185793	2.934126	0.952184	0.3436	-5.59296	0.776847	0.636264
NK.cells	CUEDC2	0.107421	5.245055	0.952184	0.3436	-6.11276	0.750305	0.596628
NK.cells	B230118H(	-0.30986	2.933488	-0.95217	0.343606	-5.2618	0.776854	0.636275
NK.cells	TMPRSS6	-0.28932	1.467899	-0.95195	0.343718	-5.48694	0.79421	0.662769
NK.cells	PRKCSH	0.097573	4.862373	0.951855	0.343765	-6.07853	0.754632	0.603075
NK.cells	DLG3	0.426393	0.571754	0.951638	0.343875	-5.0599	0.805025	0.679602
NK.cells	SAAL1	0.178436	3.298168	0.95162	0.343884	-5.72662	0.772599	0.629994
NK.cells	CWC27	0.099814	5.662216	0.951245	0.344073	-6.19398	0.74562	0.589938
NK.cells	ADCK2	0.193986	2.282482	0.951201	0.344095	-5.52113	0.784513	0.648121
NK.cells	MRPL44	0.192773	2.945204	0.951196	0.344098	-5.59018	0.776717	0.636283
NK.cells	SELENOP	-0.15399	8.699951	-0.9512	0.344098	-6.64958	0.712462	0.542106
NK.cells	GM28403	0.413922	1.458517	0.951182	0.344105	-5.17784	0.794322	0.663138
NK.cells	KLC1	0.104722	4.702224	0.951173	0.344109	-6.0124	0.75645	0.605918
NK.cells	WDR49	0.434786	0.34569	0.950932	0.344231	-5.08024	0.807913	0.68402
NK.cells	NFIC	-0.12797	4.464453	-0.95071	0.344343	-5.95164	0.759371	0.610093

NK.cells	MOGS	0.151928	4.095452	0.9506	0.344399	-5.88758	0.763597	0.616402
NK.cells	RHOBTB2	0.192296	2.943311	0.950129	0.344637	-5.62241	0.776956	0.636664
NK.cells	2210408I2	0.221715	3.277757	0.950113	0.344645	-5.5657	0.773053	0.630769
NK.cells	TRIM28	-0.11523	5.460856	-0.94995	0.344726	-6.13907	0.748087	0.593636
NK.cells	5430405H(	-0.15657	3.910079	-0.94986	0.344774	-5.83432	0.76573	0.619865
NK.cells	GPN1	-0.16808	3.042468	-0.94936	0.345027	-5.65152	0.775797	0.635212
NK.cells	FUT8	-0.09865	5.850748	-0.94899	0.345211	-6.27712	0.743721	0.587557
NK.cells	CMC2	-0.16811	4.535117	-0.9489	0.345256	-6.05292	0.758565	0.609477
NK.cells	ACOT9	0.117199	4.620859	0.948793	0.345312	-6.01068	0.757588	0.608024
NK.cells	MAGOHB	0.140955	4.560419	0.948792	0.345313	-6.09712	0.758276	0.609048
NK.cells	CAMK1	-0.2477	2.966119	-0.94879	0.345315	-5.48067	0.776689	0.63668
NK.cells	KLHL42	0.236107	2.445253	0.948492	0.345464	-5.3892	0.782809	0.646095
NK.cells	1700048O2	-0.69635	-0.24632	-0.94849	0.345468	-4.91058	0.815261	0.69626
NK.cells	CD83	0.27553	5.198788	0.948269	0.345577	-5.9696	0.751037	0.598493
NK.cells	DNAL4	0.307354	1.951429	0.948265	0.34558	-5.24718	0.788659	0.655093
NK.cells	TCF3	-0.10376	6.601305	-0.94824	0.345594	-6.31769	0.735396	0.575574
NK.cells	EIF5	0.077449	8.418516	0.948173	0.345626	-6.68691	0.715664	0.547214
NK.cells	FABP5	-0.1734	7.278075	-0.94803	0.345696	-6.48874	0.727978	0.564898
NK.cells	ZFP60	-0.33856	1.431044	-0.94803	0.345698	-5.18265	0.794874	0.664716
NK.cells	A230056P1	0.444169	0.407448	0.947983	0.345722	-5.03084	0.80725	0.68392
NK.cells	FKBP4	0.100843	5.691682	0.947942	0.345743	-6.22473	0.745499	0.590433
NK.cells	GM1043	-0.28695	1.915295	-0.94794	0.345743	-5.58212	0.789089	0.655859
NK.cells	NRDE2	-0.18045	3.371854	-0.94783	0.345799	-5.75049	0.771958	0.629831
NK.cells	IWS1	-0.08497	5.729671	-0.94729	0.346073	-6.24718	0.745208	0.589925
NK.cells	CELF1	-0.06173	7.40812	-0.94723	0.346101	-6.52042	0.726693	0.563013
NK.cells	DDX59	0.262424	1.892836	0.947149	0.346144	-5.35084	0.789498	0.656453
NK.cells	AP3M1	0.103178	4.881049	0.947132	0.346153	-6.00976	0.754767	0.604079
NK.cells	2610307P1	0.280818	4.550996	0.94699	0.346225	-5.8234	0.758521	0.609697
NK.cells	PHYKPL	-0.14224	3.470266	-0.94695	0.346244	-5.71265	0.770954	0.628325
NK.cells	MSH3	0.116432	5.159864	0.946712	0.346366	-6.08395	0.751738	0.599586
NK.cells	SQSTM1	-0.09583	7.466775	-0.94632	0.346566	-6.58072	0.726424	0.562393
NK.cells	GM31718	0.213204	3.725877	0.946015	0.346719	-5.57516	0.768385	0.624201
NK.cells	SHTN1	-0.33463	3.187667	-0.946	0.346725	-5.3067	0.774636	0.63362
NK.cells	EML1	-0.35406	1.303195	-0.94598	0.346739	-5.26001	0.796957	0.667709
NK.cells	NAIP1	0.581055	-0.47909	0.945512	0.346974	-4.9061	0.819102	0.701841
NK.cells	2510039O1	-0.09865	5.179646	-0.94536	0.347052	-6.15087	0.752173	0.599693
NK.cells	NASP	-0.09842	6.308139	-0.94521	0.347129	-6.3214	0.739543	0.581225
NK.cells	ACER2	0.269428	2.516505	0.945105	0.347181	-5.4703	0.782929	0.645975
NK.cells	FBXW9	-0.21952	2.418265	-0.94489	0.347288	-5.41384	0.784165	0.647811
NK.cells	EPN2	-0.49517	1.664749	-0.94449	0.347495	-5.0845	0.793125	0.661675
NK.cells	GSDME	-0.16313	4.386903	-0.94447	0.347501	-5.92446	0.761264	0.613416
NK.cells	PLEKHA6	-0.2764	1.808761	-0.9442	0.347642	-5.32867	0.791404	0.659125
NK.cells	XIST	-3.77513	4.173151	-0.94412	0.347683	-5.47192	0.763716	0.617192
NK.cells	GOLM1	0.15512	3.2957	0.94371	0.347889	-5.80473	0.773868	0.632536
NK.cells	KANSL1L	-0.17511	7.202302	-0.94364	0.347925	-6.31322	0.729769	0.567356
NK.cells	NME7	0.146678	3.562527	0.943455	0.348019	-5.69232	0.770765	0.627905
NK.cells	SCOC	0.171772	3.752146	0.943333	0.348081	-5.73138	0.768568	0.624611

NK.cells	NSD1	0.06994	7.273059	0.94331	0.348093	-6.50471	0.728997	0.566293
NK.cells	ARL3	0.150579	4.04145	0.943178	0.34816	-5.80439	0.76523	0.61967
NK.cells	FIS1	0.079518	7.530663	0.943077	0.348211	-6.5513	0.726191	0.562357
NK.cells	GM12353	-0.37603	1.278675	-0.94296	0.348272	-5.16725	0.797758	0.66925
NK.cells	FUT10	0.524608	-0.01434	0.942889	0.348307	-4.92843	0.813485	0.693718
NK.cells	CBS	-0.35746	1.874066	-0.94281	0.348347	-5.29965	0.790625	0.658266
NK.cells	GNAS	-0.06623	8.931692	-0.94274	0.348381	-6.77056	0.71114	0.540876
NK.cells	SIGLEC1	-0.67317	0.449661	-0.94255	0.34848	-4.95136	0.807804	0.684895
NK.cells	TMEM108	-0.24956	5.137851	-0.94219	0.348663	-5.96157	0.752721	0.601316
NK.cells	ALPK3	-0.5161	-0.11417	-0.94213	0.348694	-5.0257	0.814713	0.69587
NK.cells	ZFP950	0.127034	4.197661	0.942096	0.348711	-5.96075	0.763434	0.617262
NK.cells	SLC4A1AP	0.112954	4.278134	0.942047	0.348735	-5.9773	0.762511	0.615881
NK.cells	TMEM161/	0.20549	2.853132	0.941905	0.348808	-5.5726	0.779043	0.640794
NK.cells	POU2F1	0.131218	5.39401	0.941858	0.348832	-6.18092	0.74983	0.597043
NK.cells	SFT2D2	0.119346	4.677531	0.941786	0.348868	-6.0834	0.757945	0.609071
NK.cells	DNAJC12	0.214214	3.069108	0.9413	0.349116	-5.63066	0.776513	0.637129
NK.cells	RPF1	-0.09169	5.149471	-0.94126	0.349137	-6.13596	0.752589	0.601288
NK.cells	SAMD8	0.110372	5.289431	0.94121	0.349162	-6.15008	0.751009	0.59895
NK.cells	DDX52	-0.09932	4.930462	-0.94065	0.349447	-6.10942	0.75507	0.605105
NK.cells	GUCD1	-0.17243	3.805941	-0.94054	0.349504	-5.76241	0.767947	0.624353
NK.cells	ZMYND11	0.081286	6.466516	0.940505	0.349521	-6.35681	0.73786	0.57978
NK.cells	GM41556	-0.34828	1.259442	-0.94042	0.349565	-5.18514	0.79799	0.670176
NK.cells	RABIF	-0.09435	4.708703	-0.94039	0.349578	-6.09031	0.75759	0.608859
NK.cells	USP31	-0.16437	3.715776	-0.94039	0.349581	-5.81743	0.768989	0.625922
NK.cells	RAB11FIP4	0.519558	-0.78929	0.940353	0.349598	-4.89598	0.823066	0.709392
NK.cells	ILF2	-0.097	5.763945	-0.94025	0.349652	-6.25564	0.745677	0.591232
NK.cells	AKAP1	-0.33026	1.799595	-0.94009	0.349732	-5.17991	0.791514	0.660224
NK.cells	SCML4	0.134654	4.659948	0.939934	0.349812	-6.11406	0.758146	0.609753
NK.cells	FAM89B	0.110193	5.277421	0.939796	0.349883	-6.1585	0.751144	0.599397
NK.cells	ZFP646	0.161669	3.903418	0.939785	0.349888	-5.83588	0.766821	0.622767
NK.cells	GM17021	-0.54769	0.125797	-0.93971	0.349926	-4.96201	0.811765	0.691778
NK.cells	MCRIP1	0.077163	5.919349	0.939684	0.34994	-6.28176	0.74394	0.588822
NK.cells	LCA5	0.376017	1.313513	0.939345	0.350113	-5.13895	0.797339	0.669412
NK.cells	KATNA1	-0.11231	4.864725	-0.93926	0.350155	-6.08072	0.755816	0.606463
NK.cells	SPATA32	-0.42504	0.244996	-0.93919	0.350195	-5.15656	0.810305	0.689602
NK.cells	AKAP8L	-0.12172	4.8709	-0.93914	0.350215	-6.08841	0.755746	0.606359
NK.cells	HINT3	0.115457	4.643869	0.939116	0.35023	-6.08706	0.758329	0.610203
NK.cells	FAM222B	0.119828	5.370089	0.938555	0.350516	-6.20676	0.7501	0.598181
NK.cells	TFR2	-0.35531	1.213769	-0.93855	0.350518	-5.16236	0.79854	0.671509
NK.cells	GM16158	0.350453	0.586451	0.938521	0.350534	-5.29595	0.806137	0.683313
NK.cells	9230111E0	0.505022	-0.18455	0.938513	0.350538	-5.00217	0.815579	0.698095
NK.cells	CCDC77	0.162882	3.291762	0.938276	0.350659	-5.66515	0.773914	0.633917
NK.cells	LRPPRC	0.110796	5.200579	0.938239	0.350678	-6.14518	0.752012	0.601136
NK.cells	ATP2B4	-0.14098	4.169834	-0.93817	0.350715	-6.20668	0.763754	0.618638
NK.cells	FAM83F	-0.35056	1.041009	-0.93812	0.35074	-5.2699	0.800625	0.674891
NK.cells	HDAC3	0.106421	4.707537	0.938037	0.350781	-6.01873	0.757604	0.609476
NK.cells	DHDH	0.236053	2.654165	0.938021	0.350789	-5.45838	0.781382	0.645318

NK.cells	HDDC3	0.390015	0.928077	0.937817	0.350893	-5.11042	0.802084	0.677063
NK.cells	GM33280	0.661982	-0.66423	0.93723	0.351193	-4.8679	0.822057	0.707915
NK.cells	CIAO1	-0.13688	3.481337	-0.93719	0.351213	-5.75677	0.772219	0.630913
NK.cells	NSUN2	-0.10297	5.0541	-0.93669	0.351471	-6.14337	0.754425	0.6041
NK.cells	ANO8	-0.38627	0.904446	-0.93653	0.351551	-5.1637	0.803083	0.678101
NK.cells	CLK2	0.09741	4.63296	0.936417	0.351609	-6.04623	0.759215	0.611328
NK.cells	4833408A1	0.500895	0.127774	0.936404	0.351616	-5.0518	0.812556	0.692914
NK.cells	IQCB1	-0.11393	4.824078	-0.93633	0.351652	-6.03696	0.757037	0.608084
NK.cells	2410022M	-0.29218	1.591485	-0.93593	0.351859	-5.24689	0.794919	0.665471
NK.cells	TMX3	-0.09236	5.505888	-0.93587	0.351889	-6.1963	0.749436	0.596797
NK.cells	RHBDD1	0.129357	4.175534	0.935855	0.351897	-5.88714	0.76457	0.619314
NK.cells	TNNI2	-0.399	2.310806	-0.93564	0.352006	-5.05452	0.786445	0.652296
NK.cells	URB1	0.211871	2.176346	0.935397	0.352132	-5.39463	0.788179	0.654801
NK.cells	ESRRG	-0.40284	1.226258	-0.93493	0.352372	-5.15352	0.79974	0.672565
NK.cells	HAUS4	0.138765	4.204699	0.934923	0.352375	-5.94498	0.764645	0.619092
NK.cells	RAB11FIP1	0.145271	4.829945	0.934829	0.352423	-6.30659	0.75749	0.608468
NK.cells	IMPACT	-0.10758	5.540939	-0.93461	0.352536	-6.27403	0.749443	0.596638
NK.cells	FNIP2	0.276593	5.343736	0.934362	0.352662	-6.00219	0.751666	0.600006
NK.cells	ALKBH5	0.079506	7.148219	0.93436	0.352663	-6.49889	0.731596	0.570608
NK.cells	NMRK1	0.162633	3.751312	0.934309	0.352689	-5.76153	0.769878	0.627235
NK.cells	ADAM32	-0.54005	0.784382	-0.93389	0.352903	-5.052	0.805092	0.681313
NK.cells	ZDHHC16	0.158441	2.888345	0.933582	0.353062	-5.59766	0.779947	0.642768
NK.cells	TAOK1	0.074866	7.033029	0.933563	0.353072	-6.4462	0.73286	0.572733
NK.cells	CBX7	0.17327	2.651462	0.933277	0.353219	-5.58829	0.782736	0.647109
NK.cells	ZFP318	0.194231	3.491008	0.933104	0.353307	-5.8005	0.772901	0.632213
NK.cells	RABL3	0.250537	2.218563	0.93289	0.353417	-5.37384	0.78786	0.655051
NK.cells	NAT2	0.228919	2.532818	0.932805	0.353461	-5.48336	0.784137	0.649351
NK.cells	DHDDS	0.129397	4.630539	0.932791	0.353468	-5.97048	0.759764	0.61253
NK.cells	NAIP5	-0.19889	2.68586	-0.93277	0.353481	-5.70713	0.78233	0.646592
NK.cells	MAST1	-0.51468	1.020113	-0.93261	0.353562	-5.13895	0.802232	0.677288
NK.cells	4933412E1	0.292108	1.908564	0.932604	0.353564	-5.3934	0.791551	0.660769
NK.cells	CCNE2	-0.23082	3.75287	-0.93254	0.353599	-5.81202	0.76986	0.62773
NK.cells	COX7A2L	0.073658	6.644119	0.932512	0.353611	-6.41602	0.737143	0.579185
NK.cells	FLI1	-0.0849	8.191063	-0.93228	0.353731	-6.64291	0.720269	0.554806
NK.cells	ID3	-0.16318	6.599763	-0.93227	0.353734	-6.17142	0.737633	0.579932
NK.cells	PIK3C3	-0.11529	4.439233	-0.93225	0.353744	-6.01585	0.761953	0.615885
NK.cells	FYN	-0.12972	7.843798	-0.93223	0.353758	-6.68469	0.724019	0.560205
NK.cells	KRT222	-0.50248	-0.05515	-0.93167	0.354045	-4.96871	0.815364	0.698039
NK.cells	SEC16A	0.126487	4.57627	0.931651	0.354054	-6.0091	0.760384	0.613699
NK.cells	MCOLN1	0.188846	2.728161	0.931468	0.354148	-5.52704	0.781832	0.646087
NK.cells	RAPGEF3	0.449844	1.358645	0.931433	0.354166	-5.06072	0.798144	0.671164
NK.cells	FRG1	-0.07468	6.660706	-0.9314	0.354182	-6.41518	0.73696	0.579099
NK.cells	KIF18A	-0.18603	3.977118	-0.93114	0.354319	-5.84318	0.767267	0.624065
NK.cells	INPP4B	0.14229	6.480863	0.931107	0.354334	-6.52618	0.738949	0.582048
NK.cells	ACOT12	-0.42828	0.798615	-0.93102	0.35438	-5.0871	0.804919	0.681764
NK.cells	COL4A2	-0.33704	2.620499	-0.93099	0.354396	-5.32161	0.783101	0.648099
NK.cells	GM34961	0.397391	0.869528	0.930914	0.354433	-5.16487	0.804058	0.680445

NK.cells	PCK2	-0.23063	3.244845	-0.93088	0.354451	-5.54099	0.775771	0.636958
NK.cells	D330050I1	0.360054	1.118758	0.930565	0.354613	-5.14932	0.801221	0.675844
NK.cells	TMEM98	-0.51547	0.620631	-0.93043	0.354684	-4.96086	0.807269	0.685326
NK.cells	E2F7	-0.26533	2.983824	-0.93032	0.354736	-5.57826	0.779004	0.641773
NK.cells	GM26740	0.102129	7.068845	0.93005	0.354878	-6.54921	0.732634	0.572826
NK.cells	PATL2	0.27319	2.023827	0.929909	0.35495	-5.45823	0.790357	0.65922
NK.cells	MYO3B	0.226954	1.289051	0.929892	0.354959	-5.53702	0.799165	0.672823
NK.cells	SLC25A53	0.156961	3.336328	0.929706	0.355055	-5.71476	0.774879	0.635587
NK.cells	PCSK5	-0.47586	0.558557	-0.92961	0.355103	-5.07956	0.808026	0.686615
NK.cells	CHM	0.101912	5.555869	0.929498	0.355162	-6.19788	0.749446	0.59756
NK.cells	2610318N(	-0.35377	1.172871	-0.92947	0.355175	-5.29396	0.800567	0.675062
NK.cells	CCDC82	0.121466	4.323551	0.929356	0.355235	-5.99554	0.763453	0.618465
NK.cells	FAM221A	-0.34401	1.467642	-0.92907	0.355381	-5.17961	0.797198	0.669708
NK.cells	ZFP626	0.193865	2.562798	0.92864	0.355604	-5.5287	0.784349	0.649781
NK.cells	PEAK1	-0.10542	6.584571	-0.92862	0.355617	-6.53015	0.738334	0.580966
NK.cells	SEC11A	-0.06785	6.406384	-0.92852	0.355667	-6.40288	0.740309	0.583896
NK.cells	MUL1	0.195104	2.950066	0.92828	0.35579	-5.5985	0.779906	0.642936
NK.cells	RARS	0.101769	5.121145	0.928081	0.355892	-6.14889	0.754845	0.605344
NK.cells	CLEC4E	0.677041	2.622943	0.928039	0.355914	-5.20638	0.78376	0.648929
NK.cells	HEG1	0.135054	5.971346	0.927835	0.356019	-6.16498	0.745357	0.591246
NK.cells	HADHB	0.094935	5.576702	0.92735	0.35627	-6.24081	0.75006	0.59797
NK.cells	LETM1	0.102488	4.54276	0.927268	0.356312	-6.05336	0.761803	0.615444
NK.cells	GM20337	0.410875	1.078162	0.927053	0.356423	-5.08906	0.80262	0.677768
NK.cells	SLC24A5	-0.12321	4.423305	-0.92691	0.356498	-6.02865	0.763172	0.617605
NK.cells	TPRA1	0.183734	2.802051	0.926777	0.356565	-5.6401	0.782025	0.646166
NK.cells	METAP2	0.074455	7.3726	0.926735	0.356587	-6.52696	0.730135	0.568973
NK.cells	NDOR1	0.165898	3.086341	0.926714	0.356598	-5.75686	0.778683	0.641076
NK.cells	EMSY	-0.0903	5.828738	-0.92645	0.356735	-6.29392	0.747275	0.594015
NK.cells	EIF3D	0.094679	5.480464	0.926396	0.356762	-6.21949	0.751192	0.599802
NK.cells	BC049715	0.486693	0.604594	0.926298	0.356813	-5.10436	0.80843	0.686997
NK.cells	ADH5	0.104975	6.03865	0.92603	0.356951	-6.31679	0.744979	0.590735
NK.cells	ZFP119A	-0.19565	2.125414	-0.92581	0.357066	-5.49671	0.790147	0.658825
NK.cells	ZFP61	-0.37119	1.174198	-0.92547	0.357243	-5.19787	0.801566	0.676641
NK.cells	LYSMD3	-0.10725	4.927993	-0.9253	0.357331	-6.12938	0.757507	0.609546
NK.cells	H2-EB2	0.423576	0.547733	0.925255	0.357352	-5.13349	0.809182	0.688518
NK.cells	KCNQ1OT1	0.118934	6.399833	0.925254	0.357352	-6.34883	0.740954	0.585074
NK.cells	GM16364.:	-0.31027	1.279847	-0.92512	0.357423	-5.43815	0.80029	0.674657
NK.cells	MON1A	-0.13049	3.876268	-0.92505	0.357458	-5.89967	0.769582	0.627655
NK.cells	ILK	-0.10087	5.628263	-0.92502	0.357475	-6.26652	0.749581	0.597778
NK.cells	GNASAS1	0.3818	0.682179	0.924952	0.357509	-5.15108	0.807541	0.68599
NK.cells	RBM7	0.098754	5.855126	0.924804	0.357585	-6.26554	0.747033	0.594102
NK.cells	THOP1	0.287334	2.236401	0.924762	0.357607	-5.35887	0.788826	0.657056
NK.cells	PSTK	0.147108	3.061289	0.92453	0.357727	-5.73629	0.779118	0.642174
NK.cells	POLK	0.143528	3.481233	0.924416	0.357786	-5.8398	0.774207	0.634714
NK.cells	KDELR2	0.089041	5.993878	0.924273	0.35786	-6.28907	0.745514	0.591863
NK.cells	C920021L1	-0.24393	2.268205	-0.92393	0.358039	-5.35114	0.788485	0.656627
NK.cells	MALT1	0.148354	7.764534	0.923915	0.358046	-6.79153	0.725996	0.563482

NK.cells	SKA3	-0.20882	2.738324	-0.9239	0.358055	-5.5915	0.782918	0.648097
NK.cells	USP25	0.074721	7.051473	0.923829	0.35809	-6.5228	0.733787	0.574778
NK.cells	MTUS2	-0.51978	0.654472	-0.92368	0.358169	-5.0557	0.807916	0.686759
NK.cells	TOMM20	-0.08135	6.725117	-0.92352	0.358249	-6.41548	0.737384	0.580059
NK.cells	SLC35G1	0.384287	1.026313	0.923307	0.35836	-5.23604	0.803394	0.679792
NK.cells	IFIT2	0.395129	2.721315	0.923264	0.358383	-5.57705	0.783118	0.648502
NK.cells	CIB2	-0.33944	2.152566	-0.92325	0.358391	-5.16448	0.78986	0.658841
NK.cells	EAPP	0.07866	5.602282	0.922701	0.358674	-6.23986	0.749996	0.598669
NK.cells	LYAR	0.129753	4.937131	0.922621	0.358716	-6.09977	0.757526	0.609858
NK.cells	POLR3H	0.151136	3.509866	0.922309	0.358877	-5.77093	0.773963	0.634664
NK.cells	UBE2Z	-0.08622	5.437928	-0.92224	0.358914	-6.20548	0.751849	0.6015
NK.cells	MTIF2	0.113633	4.285566	0.922202	0.358933	-5.9767	0.764982	0.621109
NK.cells	LRRC20	0.257524	2.057401	0.922069	0.359002	-5.39108	0.791087	0.660857
NK.cells	ATXN1L	0.180824	2.959513	0.921865	0.359108	-5.62935	0.780404	0.644505
NK.cells	TNFRSF25	0.307107	-1.26707	0.921699	0.359194	-5.15794	0.831505	0.724849
NK.cells	UQCR11	0.094154	7.224799	0.921498	0.359298	-6.47044	0.73197	0.572529
NK.cells	KLRB1F	-0.26196	0.424065	-0.92137	0.359365	-5.62577	0.810826	0.691867
NK.cells	H2-Q6	-0.24314	3.115257	-0.92128	0.359412	-6.06013	0.778575	0.64203
NK.cells	SPDEF	-0.54911	-0.4296	-0.92096	0.359576	-4.96249	0.82135	0.708601
NK.cells	GM47754	0.542507	0.667894	0.920822	0.359649	-4.97954	0.807847	0.687413
NK.cells	GM48696	-0.21683	2.476417	-0.92077	0.359674	-5.57606	0.786105	0.653714
NK.cells	LY96	-0.18348	4.287348	-0.92071	0.359707	-5.68843	0.764962	0.621598
NK.cells	RBM12B1	0.417174	0.935508	0.920646	0.359741	-5.11063	0.80459	0.682333
NK.cells	FLT1	-0.35155	4.243341	-0.92051	0.359811	-5.76904	0.765468	0.622379
NK.cells	COLGALT2	0.625684	-0.12576	0.920474	0.35983	-4.93466	0.817588	0.702758
NK.cells	GM15232	-0.25586	1.187425	-0.92045	0.359842	-5.46515	0.801537	0.677594
NK.cells	NLRP1B	-0.36663	1.79452	-0.92038	0.35988	-5.25237	0.794229	0.666257
NK.cells	GM15788	-0.54113	0.240256	-0.92031	0.359916	-4.97216	0.81308	0.695655
NK.cells	ABCA9	-0.60433	0.787541	-0.92008	0.360032	-4.99782	0.806389	0.685249
NK.cells	SPTLC1	0.11742	4.232218	0.919873	0.360142	-5.97439	0.765596	0.622658
NK.cells	BC017158	0.330378	1.449949	0.919836	0.360161	-5.25923	0.798368	0.672763
NK.cells	XRCC3	-0.42841	0.159666	-0.91981	0.360176	-5.00425	0.81407	0.69731
NK.cells	UTP6	0.117309	4.42275	0.919797	0.360182	-6.02407	0.763406	0.619365
NK.cells	ZBTB37	0.191119	2.901016	0.919764	0.360199	-5.5976	0.781092	0.646157
NK.cells	TMX1	-0.09792	5.362	-0.91964	0.360265	-6.13871	0.752706	0.603387
NK.cells	MAGED2	-0.35568	1.585547	-0.91936	0.360409	-5.24459	0.796736	0.670393
NK.cells	AP5M1	0.144322	3.584616	0.919194	0.360495	-5.81991	0.773093	0.634205
NK.cells	NEIL3	-0.19441	4.375558	-0.91912	0.360531	-5.96579	0.763947	0.620396
NK.cells	RAI1	-0.10137	6.004853	-0.9191	0.360543	-6.27765	0.745478	0.592884
NK.cells	FASTKD5	0.367534	0.844458	0.91901	0.360591	-5.11661	0.805696	0.684416
NK.cells	DIS3	0.162763	3.258023	0.918972	0.360611	-5.71406	0.776903	0.639998
NK.cells	PARP9	0.166672	5.150171	0.918472	0.360871	-6.17959	0.75533	0.607318
NK.cells	GM4258	0.178995	4.929368	0.918401	0.360907	-6.05349	0.75784	0.611063
NK.cells	SETD3	0.083826	5.687067	0.918396	0.36091	-6.28069	0.749265	0.598308
NK.cells	LMTK3	-0.33481	-0.70138	-0.91815	0.36104	-5.20773	0.825004	0.714626
NK.cells	SLC15A3	0.284782	5.005968	0.918126	0.361051	-5.51665	0.756994	0.609781
NK.cells	VAMP3	-0.08462	5.320751	-0.91793	0.361151	-6.15513	0.75349	0.604536

NK.cells	PRR14	0.120685	4.524923	0.917819	0.361211	-6.02593	0.762554	0.618116
NK.cells	RIT1	0.128034	3.965996	0.917612	0.361318	-5.8603	0.769084	0.627877
NK.cells	MOSPD3	-0.11019	4.656046	-0.91731	0.361476	-6.0397	0.761257	0.616031
NK.cells	MVB12A	-0.09065	5.948563	-0.917	0.361638	-6.28883	0.746622	0.594288
NK.cells	GM17227	0.17947	2.746669	0.916932	0.361673	-5.62529	0.78345	0.649683
NK.cells	FGFR1OP	-0.14487	4.601111	-0.91688	0.361702	-5.89174	0.761886	0.617005
NK.cells	RFX2	-0.22642	3.630115	-0.91683	0.361726	-5.55693	0.773096	0.633914
NK.cells	MRPL10	0.122313	4.602348	0.916566	0.361863	-6.03332	0.761872	0.617049
NK.cells	ABCB10	-0.18181	2.977675	-0.91636	0.361971	-5.68528	0.780728	0.645635
NK.cells	FDFT1	-0.1496	3.796269	-0.91615	0.362078	-5.82579	0.771165	0.631152
NK.cells	ARHGEF11	0.152389	4.907611	0.916149	0.362081	-5.89118	0.758384	0.611926
NK.cells	JAG1	-0.27865	1.3378	-0.91564	0.362347	-5.59678	0.800271	0.675944
NK.cells	KLRA5	-0.19166	2.005641	-0.91558	0.362376	-6.13405	0.79225	0.663511
NK.cells	FANCD2	-0.22056	2.80256	-0.91535	0.3625	-5.53285	0.782791	0.649056
NK.cells	MELK	-0.22551	3.016502	-0.91531	0.362517	-5.71803	0.780272	0.645205
NK.cells	USP9X	-0.08301	7.387216	-0.91521	0.362573	-6.57887	0.730695	0.571304
NK.cells	FLCN	-0.13907	4.338996	-0.91516	0.362599	-5.94636	0.764894	0.621902
NK.cells	PLGRKT	0.097631	5.796951	0.914938	0.362713	-6.33064	0.748323	0.597251
NK.cells	MINK1	0.120327	4.171023	0.914895	0.362735	-6.00725	0.766829	0.624911
NK.cells	TNFRSF13E	-0.15572	4.518479	-0.91489	0.362737	-5.78893	0.762833	0.618896
NK.cells	AP5S1	-0.23741	2.440536	-0.91445	0.362969	-5.44998	0.787073	0.655794
NK.cells	ZW10	-0.13336	4.014837	-0.91438	0.363002	-5.92905	0.768633	0.627688
NK.cells	ATAD2	-0.12681	6.309192	-0.91418	0.363108	-6.39661	0.742594	0.588912
NK.cells	CARD6	0.211164	3.06005	0.913788	0.363313	-5.66493	0.77976	0.64479
NK.cells	MMP14	0.404645	3.837765	0.91366	0.36338	-5.55228	0.770684	0.631066
NK.cells	SENP2	-0.08322	6.656089	-0.91354	0.363444	-6.42129	0.738742	0.583462
NK.cells	GIT1	-0.13972	3.282244	-0.91334	0.363549	-5.68354	0.777155	0.640933
NK.cells	HSPE1-RS1	0.539393	0.18545	0.91333	0.363553	-5.01129	0.814314	0.698548
NK.cells	NDUFS6	0.099101	5.716521	0.913323	0.363557	-6.2578	0.749227	0.598942
NK.cells	SLC9A3R1	-0.08722	6.690174	-0.91322	0.363612	-6.4426	0.738365	0.582925
NK.cells	IGF1OS	0.626185	-1.03209	0.913131	0.363657	-4.90773	0.829351	0.722552
NK.cells	ACAD10	0.279133	1.501708	0.912931	0.363762	-5.35666	0.798294	0.673561
NK.cells	ITGB1	0.075392	6.871704	0.912772	0.363845	-6.52075	0.736359	0.58011
NK.cells	EPSTI1	0.126633	7.192656	0.912731	0.363866	-6.67715	0.732827	0.57495
NK.cells	HDAC4	0.118069	5.402881	0.912379	0.36405	-6.25137	0.752763	0.604416
NK.cells	VOPP1	0.137128	3.965239	0.91217	0.36416	-6.05289	0.769207	0.629185
NK.cells	9030025P2	-0.28371	1.737859	-0.91208	0.364207	-5.34727	0.795456	0.669464
NK.cells	TLK1	0.077362	6.854126	0.911846	0.364329	-6.47213	0.736553	0.580661
NK.cells	CDK5RAP1	-0.33986	2.44604	-0.91168	0.364415	-5.49632	0.787007	0.656503
NK.cells	SOX18	0.529106	0.720608	0.911679	0.364417	-5.00399	0.807761	0.688754
NK.cells	SYS1	0.075307	6.417243	0.911595	0.364461	-6.37862	0.741392	0.587814
NK.cells	PDIA4	-0.0925	5.653573	-0.91158	0.364469	-6.21205	0.749935	0.600445
NK.cells	THG1L	0.274628	2.021146	0.911559	0.36448	-5.34887	0.792065	0.664315
NK.cells	RHOG	0.099003	7.243088	0.911515	0.364503	-6.52875	0.732274	0.574454
NK.cells	TBC1D23	0.091474	5.852371	0.911381	0.364573	-6.25373	0.747701	0.597152
NK.cells	DAB2IP	-0.27837	2.225574	-0.9112	0.364666	-5.37194	0.789627	0.660645
NK.cells	NIPAL1	-0.30544	1.060638	-0.91099	0.36478	-5.36297	0.803625	0.682416

NK.cells	ERC1	-0.14246	4.872109	-0.91089	0.364829	-5.99752	0.758789	0.613767
NK.cells	CALD1	-0.2491	3.777745	-0.91075	0.364902	-5.73471	0.77138	0.632791
NK.cells	GLIPR1	-0.10513	5.059395	-0.9106	0.36498	-6.27244	0.756657	0.610622
NK.cells	USP32	-0.09969	6.849617	-0.91059	0.364988	-6.43992	0.736603	0.580934
NK.cells	ELAVL3	-0.48686	1.03829	-0.9105	0.365036	-5.13264	0.803896	0.682893
NK.cells	RALY	-0.08237	6.422986	-0.91039	0.36509	-6.40007	0.741328	0.587895
NK.cells	SART1	0.104532	4.872457	0.910258	0.365162	-6.1166	0.758785	0.613829
NK.cells	DDX24	0.077452	6.717234	0.910252	0.365164	-6.44926	0.738065	0.583099
NK.cells	HNRNPA3	0.071368	8.554364	0.910247	0.365167	-6.74397	0.718052	0.554033
NK.cells	ETF1	-0.07432	7.536791	-0.9102	0.365191	-6.54937	0.729061	0.569955
NK.cells	ALDH1B1	-0.31144	1.910302	-0.91012	0.365236	-5.20339	0.79339	0.666604
NK.cells	IRGM1	0.221199	4.93997	0.909977	0.365308	-6.109	0.758015	0.612733
NK.cells	CDIP1	-0.09002	5.935336	-0.90989	0.365353	-6.27815	0.74677	0.595986
NK.cells	HIST1H2AF	-0.43799	0.617707	-0.90987	0.365365	-5.16763	0.809016	0.691005
NK.cells	STX2	-0.13498	3.698442	-0.90984	0.365381	-5.78418	0.772301	0.634279
NK.cells	RANBP6	-0.27112	1.620455	-0.90946	0.365582	-5.29022	0.796866	0.672032
NK.cells	ATG12	0.105894	4.873947	0.909397	0.365613	-6.13433	0.758768	0.613872
NK.cells	GM20234	0.202092	2.059362	0.909347	0.365639	-5.55941	0.791608	0.663882
NK.cells	HIGD2A	0.070827	6.065754	0.909269	0.36568	-6.33216	0.745311	0.593838
NK.cells	HERC3	0.121015	4.345366	0.909257	0.365687	-6.0812	0.764821	0.622972
NK.cells	ARV1	0.249668	2.101251	0.909139	0.365749	-5.47104	0.791109	0.663154
NK.cells	ANK3	-0.4126	1.681131	-0.90914	0.365749	-5.29154	0.796137	0.670945
NK.cells	MBNL3	-0.13303	4.597519	-0.90911	0.365762	-6.11009	0.761927	0.618656
NK.cells	PLIN3	0.190338	3.527331	0.909021	0.36581	-5.57565	0.774293	0.637416
NK.cells	CD209D	-0.69313	-0.32218	-0.9089	0.365874	-4.91837	0.820583	0.709415
NK.cells	SZRD1	-0.0754	5.906997	-0.90872	0.365966	-6.2769	0.747088	0.596649
NK.cells	LRRC41	0.109308	4.919545	0.908687	0.365986	-6.10813	0.758248	0.613282
NK.cells	ZFP868	-0.14597	3.676008	-0.90842	0.366125	-5.86131	0.772721	0.634993
NK.cells	PPT1	-0.11109	6.003515	-0.90821	0.366236	-6.25813	0.746255	0.595257
NK.cells	DNAAF3	0.532855	-0.6587	0.907628	0.366542	-4.93074	0.825388	0.71664
NK.cells	DDT	0.115466	5.541533	0.907623	0.366545	-6.26509	0.751764	0.603163
NK.cells	BATF2	-0.5353	0.948583	-0.90755	0.366582	-5.08482	0.805592	0.685411
NK.cells	L3MBTL3	0.129723	5.067059	0.90742	0.366652	-6.07862	0.75715	0.611242
NK.cells	TYW5	-0.17748	3.139549	-0.907	0.366872	-5.71618	0.779524	0.645064
NK.cells	9530082P2	-0.34899	0.699259	-0.90687	0.36694	-5.12816	0.808744	0.690376
NK.cells	TCF25	0.063317	6.939299	0.906859	0.366946	-6.49352	0.736272	0.580345
NK.cells	SMIM3	0.129141	4.840554	0.906843	0.366955	-6.04598	0.759828	0.615266
NK.cells	EXOC2	0.085923	5.578749	0.906563	0.367102	-6.27858	0.751599	0.602777
NK.cells	DUSP16	0.146678	6.833204	0.90646	0.367156	-6.29535	0.73759	0.58212
NK.cells	PLEKHM3	0.141486	6.068485	0.90611	0.36734	-6.15102	0.746168	0.594717
NK.cells	MRS2	0.12334	3.939507	0.906024	0.367386	-5.89547	0.770422	0.63103
NK.cells	S100A11	-0.08799	7.270251	-0.90591	0.367446	-6.68214	0.732849	0.575149
NK.cells	DNAH12	-0.36469	1.676699	-0.9059	0.367449	-5.39282	0.797139	0.672027
NK.cells	NDUFAF1	-0.19084	2.81597	-0.9055	0.367659	-5.57495	0.783877	0.651169
NK.cells	CBL	0.07854	7.22396	0.905309	0.367762	-6.51733	0.733683	0.576038
NK.cells	DCLRE1C	-0.1098	6.497189	-0.90523	0.367805	-6.28449	0.741716	0.587847
NK.cells	FKBP3	0.102853	6.130579	0.905	0.367925	-6.37337	0.745831	0.593894

NK.cells	PTMA	-0.07997	11.35991	-0.90496	0.367946	-7.15512	0.689804	0.513503
NK.cells	CASP8AP2	0.094914	5.182109	0.904836	0.368011	-6.19283	0.756529	0.609858
NK.cells	MTBP	-0.17454	3.256156	-0.90466	0.368105	-5.73614	0.778792	0.643533
NK.cells	REC114	-0.18432	3.19631	-0.90457	0.368153	-5.75389	0.779494	0.644662
NK.cells	TUSC1	-0.20361	3.124075	-0.90357	0.36868	-5.52831	0.780909	0.646313
NK.cells	2310009A(	0.119612	4.692253	0.903557	0.368685	-6.01853	0.762699	0.618711
NK.cells	EIF2D	0.141037	3.5699	0.903291	0.368826	-5.8023	0.775685	0.638428
NK.cells	SAA4	0.453452	0.640047	0.903289	0.368827	-5.1264	0.810717	0.692612
NK.cells	HIST1H1C	-0.20757	4.106663	-0.90327	0.368838	-5.82712	0.769445	0.628961
NK.cells	ACADM	-0.13908	4.767805	-0.90315	0.368898	-6.0635	0.761833	0.617501
NK.cells	SIRPB1B	0.621196	1.445504	0.903139	0.368906	-5.03029	0.800921	0.677296
NK.cells	SMARCB1	0.079952	5.410318	0.903086	0.368934	-6.2113	0.754515	0.606564
NK.cells	RHOH	0.131745	6.471792	0.902351	0.369322	-6.33819	0.743123	0.58904
NK.cells	NCKAP5	-0.41597	1.217214	-0.90232	0.369338	-5.19446	0.804258	0.68178
NK.cells	FDXR	0.188719	2.625449	0.902167	0.369419	-5.59697	0.787359	0.655681
NK.cells	PTGES3	0.070565	7.460041	0.9021	0.369455	-6.56456	0.732202	0.573134
NK.cells	MRPL32	0.092628	5.68103	0.901915	0.369553	-6.29617	0.751991	0.602234
NK.cells	MRPL3	0.126479	4.701467	0.901843	0.36959	-6.02763	0.763138	0.6189
NK.cells	CEP120	-0.0894	5.494784	-0.90173	0.369648	-6.25988	0.754097	0.605381
NK.cells	TMEM80	-0.1449	3.276451	-0.90154	0.369751	-5.69748	0.779757	0.643994
NK.cells	IGFBP4	-0.26553	5.679023	-0.90128	0.36989	-6.19263	0.752245	0.602401
NK.cells	NCL	0.097019	7.783001	0.900916	0.370081	-6.63044	0.72894	0.568213
NK.cells	IGKV1-117	-0.46542	-0.39916	-0.90087	0.370106	-4.92318	0.824433	0.713453
NK.cells	UNG	0.243424	3.369876	0.90085	0.370116	-5.65605	0.778866	0.64251
NK.cells	NELFB	-0.09986	4.551192	-0.90075	0.37017	-6.07522	0.765145	0.621782
NK.cells	PI4KA	0.086887	6.333121	0.900379	0.370365	-6.40519	0.745207	0.59185
NK.cells	GLO1	0.116471	5.029475	0.900081	0.370522	-6.17549	0.760032	0.613844
NK.cells	NUFIP2	-0.07856	7.215419	-0.90004	0.370542	-6.53849	0.735515	0.577603
NK.cells	IFITM1	0.446663	3.489377	0.899616	0.370768	-5.81335	0.778042	0.640903
NK.cells	MALAT1	-0.07819	14.72453	-0.89958	0.37079	-7.53459	0.657988	0.468997
NK.cells	TNPO3	-0.07678	6.365771	-0.89945	0.370857	-6.39596	0.745131	0.591632
NK.cells	MEA1	0.095636	5.276389	0.899326	0.370922	-6.2029	0.757413	0.609862
NK.cells	GPN3	-0.10989	3.982026	-0.8988	0.371202	-5.98949	0.772294	0.632332
NK.cells	E2F5	0.182241	2.69996	0.898733	0.371236	-5.57341	0.787347	0.655297
NK.cells	INPPL1	-0.25481	2.471527	-0.89873	0.371236	-5.32193	0.790062	0.659474
NK.cells	ITGA9	-0.26596	5.58915	-0.89868	0.371264	-5.83669	0.753864	0.604667
NK.cells	CENPV	-0.20289	3.40694	-0.89861	0.371299	-5.81177	0.779008	0.64257
NK.cells	CLEC4B1	0.456085	-0.61033	0.898574	0.37132	-5.00332	0.827681	0.718455
NK.cells	SBF1	0.096232	4.474225	0.898469	0.371376	-6.11128	0.766598	0.623815
NK.cells	PCMTD1	0.101669	5.924056	0.898406	0.371409	-6.29625	0.750084	0.599137
NK.cells	TSC22D1	-0.14419	4.872446	-0.89793	0.371663	-6.00655	0.762412	0.617054
NK.cells	HNRNPM	-0.05961	7.703885	-0.89741	0.371938	-6.60681	0.731128	0.570466
NK.cells	GM28960	-0.26113	-0.82667	-0.8972	0.372049	-5.39352	0.831283	0.723295
NK.cells	1810013L2	0.090349	5.901803	0.897168	0.372066	-6.29897	0.75114	0.599888
NK.cells	TOE1	0.20168	2.490022	0.896799	0.372262	-5.55155	0.79097	0.659687
NK.cells	PDLIM4	0.302915	2.040162	0.896623	0.372355	-5.53812	0.796402	0.668094
NK.cells	2010110K1	-0.52854	-0.03523	-0.89648	0.372433	-5.02621	0.821743	0.707789

NK.cells	RFX5	0.247025	1.943489	0.896334	0.372508	-5.40422	0.797564	0.669977
NK.cells	FGGY	-0.26515	3.459854	-0.89613	0.372615	-5.52518	0.779547	0.642373
NK.cells	MYCBP2	0.085015	7.768752	0.895985	0.372694	-6.66823	0.730723	0.569795
NK.cells	NISCH	0.084576	6.194239	0.895585	0.372907	-6.32972	0.748163	0.595457
NK.cells	SINHCAF	0.105774	4.557551	0.895449	0.372978	-6.1064	0.766779	0.623255
NK.cells	DNAJC25	-0.12101	3.856746	-0.89543	0.372989	-5.90688	0.774904	0.635532
NK.cells	IGKV1-135	-0.49457	-0.02384	-0.89538	0.373017	-5.01109	0.821601	0.707924
NK.cells	4933433G1	-0.28884	1.434512	-0.89531	0.373051	-5.35115	0.80371	0.679824
NK.cells	TMF1	0.075106	6.243916	0.895302	0.373057	-6.38349	0.747606	0.59467
NK.cells	SLC7A2	0.427116	2.094729	0.895184	0.37312	-5.38749	0.795747	0.667461
NK.cells	HRH2	0.306194	1.160498	0.895178	0.373123	-5.32298	0.80704	0.685021
NK.cells	YWHAB	-0.06166	7.88619	-0.89451	0.373477	-6.65009	0.729872	0.568148
NK.cells	PIRB	0.329085	4.941687	0.894252	0.373615	-5.59993	0.762816	0.616777
NK.cells	KLHL2	0.100415	5.570335	0.894144	0.373673	-6.27283	0.755647	0.606122
NK.cells	POLR1E	0.254105	2.067812	0.894109	0.373691	-5.42095	0.796541	0.668176
NK.cells	TFPT	0.161992	2.965912	0.894097	0.373697	-5.6273	0.785832	0.651692
NK.cells	GM13830	-0.52564	0.287554	-0.89393	0.373785	-5.01014	0.81823	0.702101
NK.cells	DAP	-0.10488	6.19848	-0.89366	0.373929	-6.37184	0.748558	0.595744
NK.cells	GM27253	-0.33899	1.3478	-0.89352	0.374003	-5.20032	0.805238	0.681859
NK.cells	TMEM170	0.313665	1.517214	0.893441	0.374047	-5.19368	0.803183	0.678657
NK.cells	1700084C0	0.184142	2.9115	0.893383	0.374078	-5.71897	0.786477	0.652847
NK.cells	B3GALNT1	-0.60976	0.351359	-0.89337	0.374085	-4.94113	0.817442	0.700995
NK.cells	EIF4G2	-0.06095	7.71796	-0.89323	0.374161	-6.60715	0.731711	0.571151
NK.cells	GMFB	-0.09202	5.451593	-0.89315	0.374203	-6.20343	0.756996	0.608369
NK.cells	CIITA	-0.4547	2.478401	-0.89307	0.374246	-5.20724	0.791626	0.660887
NK.cells	TCF20	-0.08532	7.561094	-0.89259	0.374501	-6.58987	0.733768	0.57379
NK.cells	GTF3C6	0.086736	5.226124	0.892381	0.374611	-6.19652	0.759912	0.612353
NK.cells	UBE2E1	-0.06861	6.229498	-0.89238	0.374612	-6.3508	0.748554	0.595484
NK.cells	PLXNA2	-0.36836	2.31233	-0.89211	0.374757	-5.29169	0.794144	0.664249
NK.cells	LYST	0.085366	6.729745	0.891957	0.374837	-6.55897	0.743151	0.587458
NK.cells	CCS	0.123583	4.318203	0.891417	0.375125	-6.06022	0.770859	0.628408
NK.cells	PEX12	0.281922	1.86574	0.891259	0.375209	-5.33088	0.799864	0.672799
NK.cells	GGCT	0.177651	3.249782	0.89123	0.375225	-5.85276	0.783353	0.647391
NK.cells	MLEC	0.101605	5.064037	0.891191	0.375245	-6.12817	0.762265	0.615511
NK.cells	GM11457	-0.56843	0.10252	-0.89105	0.375323	-4.99148	0.821464	0.706612
NK.cells	UBE2D1	0.079252	5.377113	0.890771	0.37547	-6.25042	0.758867	0.610284
NK.cells	GIMAP6	0.132236	5.672059	0.890613	0.375554	-6.41225	0.755513	0.60534
NK.cells	ARRDC2	-0.27611	1.79539	-0.89043	0.375651	-5.34926	0.8009	0.674347
NK.cells	CYP7B1	0.629071	0.01895	0.890422	0.375656	-4.98429	0.822666	0.708458
NK.cells	TNF	0.244712	4.405834	0.890272	0.375736	-6.07246	0.770056	0.627158
NK.cells	BC051537	-0.53145	0.044835	-0.89011	0.375823	-4.98896	0.822426	0.708005
NK.cells	SLC25A10	-0.18643	3.077126	-0.8899	0.375935	-5.66894	0.785754	0.650863
NK.cells	MEAK7	-0.3882	0.666682	-0.88949	0.376156	-5.13649	0.815086	0.69626
NK.cells	VIRMA	0.090488	5.71037	0.889447	0.376177	-6.32459	0.755476	0.605118
NK.cells	INTS11	0.134831	3.989645	0.889301	0.376255	-5.94835	0.775295	0.634859
NK.cells	GM43331	0.227744	2.057137	0.889062	0.376382	-5.38842	0.798327	0.669997
NK.cells	4930469K1	0.280145	1.311112	0.88893	0.376453	-5.52471	0.807374	0.684036

NK.cells	HCFC1	-0.10948	4.949877	-0.88859	0.376634	-6.13111	0.764558	0.618244
NK.cells	ZMYM1	-0.15146	3.329996	-0.8884	0.376737	-5.78265	0.783426	0.64678
NK.cells	CLPTM1	0.081024	5.552635	0.88818	0.376854	-6.25797	0.757677	0.608018
NK.cells	CAAP1	-0.10747	4.575271	-0.88817	0.376857	-6.04807	0.768885	0.624789
NK.cells	RPP30	-0.13553	3.635635	-0.88801	0.376945	-5.80037	0.77983	0.641446
NK.cells	GM16552	0.285626	1.517	0.887976	0.376963	-5.26401	0.805129	0.680398
NK.cells	CHD1L	0.120343	3.722264	0.887609	0.377159	-5.91175	0.778814	0.640014
NK.cells	ZBTB21	0.135802	3.713914	0.887548	0.377192	-5.87016	0.778911	0.64017
NK.cells	GM2245	0.200185	1.558462	0.887293	0.377328	-5.72437	0.804625	0.679843
NK.cells	CD200R4	0.223995	1.662182	0.887285	0.377333	-5.88419	0.803368	0.677886
NK.cells	CCT7	0.076343	6.427225	0.887237	0.377359	-6.43907	0.7478	0.593701
NK.cells	NKAP	0.086637	4.989943	0.886975	0.377499	-6.15498	0.764107	0.617982
NK.cells	BRCA1	-0.18865	4.143927	-0.88666	0.377667	-5.95277	0.773888	0.632787
NK.cells	SLC16A9	-0.64446	0.530927	-0.88659	0.377705	-4.99182	0.817201	0.699665
NK.cells	EPOP	0.283447	1.472765	0.886583	0.377709	-5.35161	0.805666	0.681595
NK.cells	DYNLT1A	0.151577	4.14434	0.886282	0.37787	-5.84215	0.773883	0.63285
NK.cells	ENY2	-0.08719	5.972518	-0.88608	0.377978	-6.32771	0.752917	0.601514
NK.cells	LRRC58	0.080596	5.945766	0.886068	0.377985	-6.31894	0.753219	0.601962
NK.cells	RMI2	0.175695	3.3602	0.88605	0.377994	-5.77218	0.78307	0.646885
NK.cells	KCNG2	0.546909	-0.23458	0.885815	0.37812	-4.94408	0.826704	0.714936
NK.cells	GMPR	-0.39292	1.923101	-0.88578	0.378141	-5.15794	0.800212	0.67335
NK.cells	AREG	0.376154	1.634087	0.885773	0.378143	-5.87841	0.803708	0.678781
NK.cells	PTPRK	-0.31316	3.178658	-0.88568	0.378194	-5.57311	0.785213	0.650258
NK.cells	1700028E1	0.274699	1.36693	0.885632	0.378218	-5.27299	0.806954	0.683878
NK.cells	EFCAB2	0.246104	2.668273	0.885491	0.378294	-5.50858	0.791274	0.659613
NK.cells	PRPS1L3	0.221327	2.045409	0.885273	0.378411	-5.48299	0.798738	0.671237
NK.cells	SAMD10	0.234531	1.732501	0.885206	0.378446	-5.49226	0.802516	0.677132
NK.cells	NUCKS1	-0.09958	6.576295	-0.88499	0.378564	-6.42462	0.74613	0.591821
NK.cells	BECN1	0.081844	5.896307	0.884688	0.378724	-6.33862	0.753779	0.60318
NK.cells	3300005D(	0.537019	0.756323	0.88457	0.378788	-4.95901	0.814425	0.695902
NK.cells	GM28529	0.406245	0.724072	0.884538	0.378805	-5.14522	0.814821	0.696526
NK.cells	GCC1	0.225204	2.459454	0.884173	0.379001	-5.53893	0.793768	0.663905
NK.cells	NDUFA3	0.072324	8.000282	0.884147	0.379015	-6.68018	0.73039	0.56902
NK.cells	LMLN	0.287277	1.750464	0.884145	0.379016	-5.28549	0.802299	0.677123
NK.cells	TRMT44	0.260903	1.793941	0.88412	0.379029	-5.39774	0.801773	0.676305
NK.cells	ATG9A	-0.12301	3.85849	-0.88411	0.379037	-5.96073	0.777219	0.638559
NK.cells	CPSF3	0.097548	4.770083	0.88406	0.379062	-6.10659	0.766636	0.622574
NK.cells	DNAJC18	0.130178	3.682567	0.883864	0.379167	-5.94054	0.779279	0.641812
NK.cells	PHACTR2	-0.1254	6.036575	-0.88376	0.379225	-6.25754	0.752194	0.601132
NK.cells	TXNDC17	0.096336	6.116505	0.883718	0.379245	-6.33687	0.751293	0.599809
NK.cells	CAPN3	-0.28584	1.422788	-0.88354	0.379339	-5.36477	0.806274	0.683598
NK.cells	NDUFAB1	0.103264	6.268706	0.883338	0.379449	-6.36895	0.749579	0.597446
NK.cells	SHROOM3	-0.44319	0.981918	-0.88328	0.379482	-5.19214	0.811656	0.692152
NK.cells	ARL13B	-0.13093	3.775076	-0.88325	0.379496	-5.85765	0.778195	0.640435
NK.cells	EGFEM1	-0.62533	1.229611	-0.8831	0.37958	-5.07504	0.808628	0.687497
NK.cells	RAPGEF1	0.081862	6.775482	0.882938	0.379664	-6.46508	0.743906	0.589184
NK.cells	FASN	-0.1672	3.14635	-0.88288	0.379698	-5.81784	0.785596	0.651815

NK.cells	GM7160	0.24445	2.542262	0.882794	0.379742	-5.57061	0.792778	0.66287
NK.cells	HYKK	-0.39155	0.618386	-0.88279	0.379742	-5.11369	0.816122	0.69928
NK.cells	APOL9B	-0.36211	0.574633	-0.88259	0.379851	-5.2282	0.816667	0.700203
NK.cells	CENPK	-0.20171	3.283005	-0.8825	0.379899	-5.72099	0.783986	0.649418
NK.cells	NCBP3	0.088768	5.506298	0.882369	0.37997	-6.2402	0.75821	0.610468
NK.cells	ZFP652	0.10431	5.654413	0.882307	0.380003	-6.31847	0.756525	0.607962
NK.cells	LMO4	0.12351	7.245458	0.882177	0.380073	-6.51231	0.738704	0.58165
NK.cells	RLF	0.082746	6.699162	0.881628	0.380368	-6.44278	0.74512	0.590683
NK.cells	SLC11A2	-0.14789	3.918763	-0.88158	0.380397	-5.9934	0.776892	0.63822
NK.cells	FOXJ2	-0.14289	3.829076	-0.88149	0.380444	-5.92802	0.777941	0.639815
NK.cells	TMEM143	0.290524	1.491704	0.881351	0.380517	-5.24047	0.805846	0.682807
NK.cells	BRWD3	0.114729	5.109603	0.881009	0.380702	-6.1404	0.763317	0.617525
NK.cells	UBE3A	0.068297	6.387077	0.880818	0.380804	-6.42654	0.748821	0.596006
NK.cells	WARS2	0.160901	3.512477	0.880782	0.380824	-5.80015	0.781873	0.645669
NK.cells	GATAD1	0.082399	5.768843	0.880691	0.380873	-6.25641	0.755798	0.606398
NK.cells	BOLA1	0.158595	3.767483	0.880085	0.381199	-5.81712	0.779407	0.641274
NK.cells	TRIP12	0.065212	7.435654	0.879893	0.381303	-6.58773	0.73765	0.579056
NK.cells	CSTF2	0.131266	4.139659	0.87985	0.381326	-5.93787	0.775055	0.634703
NK.cells	H2-AB1	-0.46394	6.764416	-0.87954	0.381492	-5.98511	0.745203	0.590086
NK.cells	CETN2	-0.07635	5.798667	-0.87952	0.381506	-6.33416	0.756074	0.606166
NK.cells	MS4A3	0.620703	-0.35863	0.879232	0.381659	-5.01446	0.829753	0.719467
NK.cells	GM42702	-0.25641	2.365724	-0.87906	0.381752	-5.43238	0.796385	0.667077
NK.cells	GM12185	0.182778	3.341306	0.878444	0.382084	-5.79557	0.785131	0.649473
NK.cells	MARK3	-0.06974	6.304211	-0.87837	0.382125	-6.39874	0.750939	0.598055
NK.cells	TNFSF8	0.33515	-0.51498	0.878276	0.382175	-5.39006	0.832163	0.722934
NK.cells	NOM1	0.113334	4.141882	0.878164	0.382235	-6.00035	0.775728	0.635163
NK.cells	B4GALT3	-0.12278	3.874826	-0.87813	0.382253	-5.8315	0.778851	0.639901
NK.cells	CTNNB1	-0.06735	6.277504	-0.87786	0.382401	-6.37302	0.751403	0.598548
NK.cells	N4BP2L2	0.058453	6.786437	0.877608	0.382535	-6.4934	0.745827	0.590208
NK.cells	GM31812	-0.37726	0.191942	-0.87718	0.382764	-5.18184	0.82372	0.709181
NK.cells	SYCE2	0.176007	4.258668	0.877082	0.382819	-5.97754	0.77474	0.633359
NK.cells	PIAS1	-0.06523	7.42613	-0.87707	0.382824	-6.57074	0.738777	0.579901
NK.cells	CLCN5	-0.13636	5.160349	-0.87707	0.382826	-6.07394	0.764311	0.617659
NK.cells	GLCE	-0.13926	3.79856	-0.87668	0.383038	-5.89655	0.78032	0.64169
NK.cells	ZFP426	0.177995	2.769149	0.876625	0.383066	-5.62429	0.79251	0.660363
NK.cells	7-Mar	0.059787	6.926226	0.876528	0.383118	-6.53008	0.744518	0.588231
NK.cells	GCNT1	0.196911	2.451925	0.876345	0.383217	-5.58557	0.796378	0.666314
NK.cells	PRDX6	0.069126	7.110346	0.876214	0.383288	-6.62192	0.742545	0.585348
NK.cells	PPM1H	0.104776	6.473739	0.875833	0.383494	-6.643	0.749784	0.595942
NK.cells	PLEC	0.10155	4.805597	0.875672	0.383581	-6.23748	0.768796	0.624282
NK.cells	TNFRSF19	-0.42024	0.886333	-0.87559	0.383624	-5.12362	0.815553	0.696225
NK.cells	ISYNA1	0.10491	4.852057	0.875515	0.383666	-6.1474	0.768259	0.623541
NK.cells	DEF6	0.082091	5.457612	0.875425	0.383715	-6.35342	0.761303	0.613141
NK.cells	EIF4E2	0.079313	5.989442	0.87538	0.383739	-6.37679	0.75525	0.604142
NK.cells	DTWD2	0.183833	2.777791	0.875102	0.38389	-5.73293	0.792625	0.660695
NK.cells	KREMEN1	0.148783	3.039966	0.874955	0.383969	-5.75244	0.789501	0.655914
NK.cells	GM45871	0.34144	0.985003	0.874899	0.384	-5.20904	0.814347	0.69451

NK.cells	TPK1	0.114495	4.865838	0.874784	0.384062	-6.13345	0.768107	0.623458
NK.cells	HIBCH	0.130585	3.478236	0.874657	0.38413	-5.80743	0.784308	0.648018
NK.cells	LRP12	0.344116	2.191669	0.874654	0.384132	-5.34252	0.799658	0.671631
NK.cells	RAB24	0.096785	5.118704	0.874136	0.384413	-6.10691	0.765624	0.619218
NK.cells	NOP14	0.12646	4.004398	0.873796	0.384597	-5.98532	0.778775	0.638789
NK.cells	SUPT3	0.111752	5.480744	0.873562	0.384723	-6.20546	0.761685	0.613068
NK.cells	SKP1A	-0.0696	6.639256	-0.87329	0.38487	-6.46341	0.748561	0.593609
NK.cells	GSTM2	-0.35188	1.633616	-0.8732	0.384921	-5.24857	0.807093	0.682336
NK.cells	TUBGCP6	-0.20673	2.759296	-0.87289	0.385089	-5.57234	0.793512	0.661364
NK.cells	GLIS1	-0.48629	0.394465	-0.8728	0.385138	-5.0493	0.82233	0.70634
NK.cells	XRCC4	0.10127	5.185209	0.872735	0.385171	-6.17604	0.765073	0.618256
NK.cells	CRYZ	-0.33421	1.288432	-0.87257	0.385264	-5.19513	0.811307	0.689098
NK.cells	FSHR	0.327817	0.419984	0.872546	0.385274	-5.09544	0.822013	0.705929
NK.cells	CKAP2L	-0.15725	4.357364	-0.87242	0.385342	-5.99689	0.774651	0.632736
NK.cells	CDC37	0.063425	6.605127	0.872418	0.385343	-6.49263	0.748944	0.594354
NK.cells	FAM129A	0.098784	5.667894	0.872278	0.385419	-6.44246	0.759547	0.610086
NK.cells	BCAT1	0.44679	0.852062	0.872272	0.385423	-5.17604	0.816668	0.69755
NK.cells	RRNAD1	0.125547	3.384404	0.872261	0.385429	-5.83983	0.786076	0.650124
NK.cells	FZD5	-0.18702	2.536377	-0.8717	0.385734	-5.61886	0.796573	0.665819
NK.cells	PPP6R3	-0.06754	7.283025	-0.87156	0.385811	-6.57302	0.741741	0.583437
NK.cells	D330023K1	0.299862	2.22594	0.871474	0.385855	-5.32329	0.800309	0.671619
NK.cells	ENPP1	-0.24554	2.31349	-0.87143	0.385878	-5.61958	0.799254	0.669986
NK.cells	RASGRP2	0.092563	6.609517	0.871011	0.386107	-6.36721	0.749582	0.594523
NK.cells	6-Mar	-0.06911	6.3538	-0.8707	0.386277	-6.41718	0.752511	0.598905
NK.cells	TNRC18	-0.07713	5.931838	-0.87062	0.386322	-6.31093	0.75729	0.605992
NK.cells	PRG3	-0.52884	1.171663	-0.8706	0.386328	-5.14218	0.813538	0.691809
NK.cells	ARL5C	0.138817	6.264356	0.870485	0.386393	-6.32837	0.753521	0.600456
NK.cells	DAPK3	-0.10374	4.42231	-0.87	0.386656	-6.04507	0.774714	0.63221
NK.cells	FAM219B	0.211834	2.367885	0.869981	0.386667	-5.58168	0.799051	0.669408
NK.cells	POLR1A	-0.11871	4.926327	-0.86997	0.386673	-6.12907	0.768867	0.623406
NK.cells	8030456M	-0.27999	1.090368	-0.86958	0.386884	-5.35984	0.814597	0.693771
NK.cells	SPOP	0.063916	7.366582	0.869367	0.387001	-6.54151	0.741233	0.582647
NK.cells	FNDC5	-0.39044	0.474524	-0.86881	0.387301	-5.18618	0.822205	0.705957
NK.cells	LDLRAD4	0.114734	6.109073	0.868514	0.387464	-6.45449	0.755334	0.603635
NK.cells	KLRD1	-0.13617	3.767425	-0.86837	0.387543	-6.42776	0.782384	0.644295
NK.cells	TRAF3IP2	0.148369	3.229202	0.868349	0.387554	-5.82672	0.788749	0.654018
NK.cells	5930403N2	0.569676	-0.48768	0.868326	0.387567	-4.92653	0.834243	0.725175
NK.cells	GM39121	0.502122	-0.94202	0.868322	0.387569	-4.9462	0.839991	0.734369
NK.cells	FOXD2OS	0.396288	0.311339	0.868322	0.387569	-5.11783	0.824234	0.709274
NK.cells	PLTP	-0.29959	5.244881	-0.86831	0.387574	-5.86773	0.765196	0.618334
NK.cells	GVIN1	0.243757	3.037272	0.868109	0.387685	-5.91688	0.791032	0.657565
NK.cells	HSCB	0.129776	4.143832	0.868065	0.387709	-6.04526	0.777966	0.637624
NK.cells	PNO1	0.12002	4.446222	0.868058	0.387713	-6.05618	0.774436	0.63228
NK.cells	CCDC127	-0.10331	4.15296	-0.86804	0.387724	-5.98519	0.777859	0.637462
NK.cells	CHEK2	0.166391	3.145946	0.86796	0.387766	-5.65781	0.789738	0.655584
NK.cells	GM16152	0.264099	2.101073	0.867943	0.387775	-5.48951	0.802271	0.674917
NK.cells	PIMREG	-0.31339	2.104196	-0.86791	0.387793	-5.48863	0.802234	0.674858

NK.cells	CENPA	-0.13501	6.681539	-0.86772	0.387895	-6.49751	0.748878	0.594192
NK.cells	MAGI2	-0.3575	0.831391	-0.86772	0.387897	-5.18874	0.817787	0.699225
NK.cells	UGP2	-0.10414	5.910415	-0.86754	0.387995	-6.33479	0.757589	0.607101
NK.cells	NAGK	0.150285	3.956557	0.867455	0.388041	-5.8095	0.78016	0.641037
NK.cells	APEH	-0.14505	3.695339	-0.86745	0.388046	-5.9329	0.783233	0.645714
NK.cells	PSME1	0.105601	8.384368	0.86714	0.388213	-6.80199	0.730038	0.566827
NK.cells	DTX3	-0.17401	2.572046	-0.86708	0.388245	-5.70177	0.7966	0.666393
NK.cells	MGAM	-0.51414	0.078138	-0.86705	0.388262	-5.03261	0.827146	0.714216
NK.cells	RBM8A	0.083781	5.709751	0.866965	0.388308	-6.28639	0.759878	0.610685
NK.cells	ISCU	0.084337	7.465509	0.866566	0.388525	-6.58056	0.74043	0.581658
NK.cells	1700008J0	-0.30793	1.842472	-0.86598	0.388844	-5.32222	0.806172	0.680431
NK.cells	TCRG-C1	-0.22085	0.880703	-0.86574	0.388976	-5.88771	0.817956	0.698926
NK.cells	GM31814	0.346632	-0.06084	0.865731	0.388981	-5.13609	0.829669	0.717437
NK.cells	DZIP3	0.203199	3.257554	0.865607	0.389048	-5.63821	0.789162	0.654279
NK.cells	VPS52	-0.11133	3.923327	-0.86558	0.389066	-5.87725	0.781293	0.642269
NK.cells	TCF19	0.197102	3.199871	0.865236	0.389251	-5.79433	0.789913	0.655488
NK.cells	GM49041	-0.36001	1.334735	-0.86513	0.389308	-5.22884	0.812438	0.690407
NK.cells	PDS5B	-0.08001	6.377064	-0.8651	0.389324	-6.42252	0.753082	0.600028
NK.cells	PDCD5	0.073988	6.418317	0.864995	0.389383	-6.43116	0.752616	0.599395
NK.cells	PIAS2	0.091677	6.02482	0.864921	0.389423	-6.35659	0.757071	0.606015
NK.cells	ENAH	0.396187	0.744942	0.8647	0.389543	-5.19734	0.819797	0.701954
NK.cells	TTC19	-0.10461	4.958514	-0.86459	0.389602	-6.19563	0.769379	0.624378
NK.cells	BAG2	-0.3227	1.435408	-0.86448	0.389663	-5.22428	0.811299	0.688712
NK.cells	PBK	-0.22889	3.69023	-0.86388	0.389991	-5.76458	0.784502	0.647049
NK.cells	THADA	0.086452	5.881845	0.863874	0.389995	-6.41809	0.759083	0.608753
NK.cells	GM43462	-0.16297	3.23183	-0.86384	0.390012	-5.79311	0.789935	0.655356
NK.cells	TRIM12C	0.127939	4.726648	0.86349	0.390205	-6.12471	0.772621	0.628803
NK.cells	IFT27	-0.16507	3.758551	-0.86327	0.390326	-5.81097	0.783993	0.646077
NK.cells	TSC1	-0.10857	4.710552	-0.86321	0.390355	-6.11736	0.772848	0.629182
NK.cells	TEX10	0.109939	5.266363	0.862911	0.390521	-6.21714	0.76655	0.619594
NK.cells	PLEKHB1	-0.34299	0.728002	-0.86265	0.390661	-5.18441	0.820779	0.703061
NK.cells	A530013C2	0.289067	2.431674	0.862569	0.390708	-5.50974	0.799954	0.670548
NK.cells	CDKN2D	-0.09651	6.09877	-0.86245	0.390774	-6.31655	0.757032	0.605533
NK.cells	GM42701	-0.3943	0.844253	-0.86239	0.390807	-5.15047	0.81934	0.700863
NK.cells	CTSB	-0.13167	8.190873	-0.86238	0.390812	-6.60463	0.733681	0.571286
NK.cells	KCTD10	0.120244	4.043279	0.862014	0.391012	-5.97718	0.781011	0.641343
NK.cells	BTBD7	-0.06173	7.004982	-0.86192	0.391061	-6.52722	0.747044	0.590614
NK.cells	INTS9	0.111775	4.394064	0.861645	0.391214	-6.02935	0.776986	0.635225
NK.cells	CR1L	0.071819	5.693128	0.861608	0.391234	-6.31953	0.761969	0.612682
NK.cells	IFI205	0.550096	2.758001	0.861319	0.391392	-5.24362	0.796446	0.664954
NK.cells	MPHOSPH	0.103794	4.827879	0.861238	0.391436	-6.12174	0.772021	0.627744
NK.cells	TSACC	0.329102	1.881856	0.861072	0.391528	-5.37718	0.807035	0.681368
NK.cells	SYNE1	-0.15789	4.964609	-0.86093	0.391604	-6.20124	0.770436	0.625362
NK.cells	AACS	-0.12422	3.861959	-0.86082	0.391665	-5.93183	0.783317	0.644866
NK.cells	SLC35F6	0.183071	2.998461	0.860815	0.391668	-5.60313	0.793566	0.660549
NK.cells	TCTEX1D2	0.276697	3.301638	0.860625	0.391772	-5.30537	0.79003	0.655022
NK.cells	GMFG	0.092021	7.424355	0.860135	0.392041	-6.57671	0.742964	0.584046

NK.cells	LRP1B	0.288774	1.219155	0.860042	0.392092	-5.30378	0.815619	0.694131
NK.cells	LDLRAP1	0.130736	4.099537	0.859371	0.39246	-6.04349	0.781148	0.640845
NK.cells	AFDN	0.139078	3.267689	0.859319	0.392489	-5.92041	0.79099	0.655861
NK.cells	COG8	0.10204	4.299473	0.859073	0.392624	-6.00703	0.778803	0.637342
NK.cells	GM13963	-0.59113	-0.29578	-0.85907	0.392628	-4.96162	0.834674	0.724187
NK.cells	LY6C1	0.413121	-0.32417	0.858958	0.392686	-5.25154	0.835033	0.724758
NK.cells	ARID1A	-0.06843	6.658177	-0.85891	0.39271	-6.46769	0.751704	0.596835
NK.cells	GM49417	0.37581	1.078235	0.858871	0.392734	-5.34254	0.817535	0.697089
NK.cells	JRKL	0.324236	1.537523	0.858647	0.392857	-5.27126	0.811888	0.688263
NK.cells	RNF14	0.079907	5.242687	0.858559	0.392906	-6.25763	0.76784	0.620844
NK.cells	RPIA	-0.09992	5.458024	-0.85844	0.392973	-6.21469	0.765361	0.617138
NK.cells	PRKCD	0.09068	6.897294	0.858194	0.393106	-6.33708	0.749016	0.592948
NK.cells	ZFP180	0.14553	3.315586	0.858191	0.393107	-5.75092	0.79042	0.655116
NK.cells	ZFP420	0.380104	0.275776	0.858166	0.393121	-5.14093	0.827499	0.71288
NK.cells	PSMB6	0.084998	6.304312	0.858065	0.393177	-6.41504	0.755703	0.602856
NK.cells	POP4	-0.12294	4.085123	-0.85805	0.393185	-6.05953	0.781318	0.641275
NK.cells	SLFN9	0.239802	2.410472	0.857983	0.393222	-5.62905	0.801271	0.671885
NK.cells	EXT1	0.115785	7.749502	0.857603	0.393431	-6.67354	0.739783	0.579241
NK.cells	HAX1	0.135564	4.020737	0.857491	0.393492	-5.8946	0.782354	0.64262
NK.cells	NAA25	0.148891	3.927641	0.857176	0.393665	-5.91688	0.783496	0.644428
NK.cells	KCNAB2	0.111449	4.287693	0.857172	0.393667	-6.16678	0.779264	0.638002
NK.cells	NOTCH2	-0.08031	7.16468	-0.85709	0.393711	-6.58045	0.746331	0.588918
NK.cells	PURB	-0.06525	6.824936	-0.85658	0.393991	-6.51756	0.750549	0.594678
NK.cells	TMEM119	-0.47321	0.305437	-0.85618	0.394213	-4.99202	0.828194	0.713053
NK.cells	G6PC	-0.37755	1.911843	-0.85601	0.394308	-5.39336	0.808358	0.681956
NK.cells	MS4A8A	0.466475	0.670009	0.855993	0.394316	-5.18181	0.823648	0.705899
NK.cells	ERLIN2	-0.17545	3.099067	-0.85573	0.394462	-5.63159	0.794171	0.659876
NK.cells	KANTR	0.217417	2.529607	0.855477	0.3946	-5.54162	0.801014	0.670485
NK.cells	CCDC174	0.088292	5.082834	0.855443	0.394618	-6.19646	0.770822	0.624502
NK.cells	FRA10AC1	0.146545	3.408508	0.855384	0.394651	-5.79424	0.790479	0.654302
NK.cells	ZFP526	0.37648	0.37218	0.855114	0.3948	-5.1292	0.827577	0.712069
NK.cells	LPGAT1	-0.11955	6.245156	-0.85509	0.394811	-6.24633	0.757547	0.604743
NK.cells	OXR1	-0.08377	6.336267	-0.85471	0.395021	-6.44134	0.756708	0.603403
NK.cells	CCDC85C	-0.23854	0.977037	-0.85467	0.395044	-5.44209	0.820266	0.700431
NK.cells	PHLDB2	-0.28234	2.509693	-0.85417	0.395321	-5.5004	0.801951	0.671242
NK.cells	IGKV9-124	-0.48412	-1.01492	-0.85383	0.395506	-4.89514	0.845775	0.740372
NK.cells	USP1	-0.09692	5.737466	-0.85375	0.395553	-6.33355	0.764007	0.613702
NK.cells	H2-KE6	0.106069	5.147514	0.853282	0.395809	-6.23802	0.770805	0.624061
NK.cells	NLN	0.121828	4.446951	0.853162	0.395875	-6.065	0.778963	0.636409
NK.cells	GM16599	-0.11053	4.782001	-0.85313	0.39589	-6.10169	0.77505	0.630504
NK.cells	ASB4	-0.56719	1.68448	-0.8531	0.395911	-5.17005	0.812056	0.687251
NK.cells	COX7A1	0.32234	0.986976	0.852918	0.396009	-5.19436	0.820648	0.700729
NK.cells	GOLGA3	-0.11676	4.032459	-0.85279	0.396079	-5.95152	0.783835	0.643861
NK.cells	RFX7	-0.08525	7.368214	-0.85278	0.396086	-6.61235	0.745556	0.586762
NK.cells	CCDC12	-0.06309	6.939585	-0.85277	0.396093	-6.54129	0.750358	0.593805
NK.cells	E430018J2	0.357932	0.653485	0.852666	0.396149	-5.16185	0.82479	0.707372
NK.cells	TSC2	0.155526	3.109391	0.85257	0.396202	-5.69432	0.794802	0.660707

NK.cells	GM43774	-0.23749	2.238278	-0.85257	0.396202	-5.50057	0.805303	0.676912
NK.cells	TDO2	-0.30681	3.218699	-0.85233	0.396332	-5.7665	0.793513	0.658797
NK.cells	BIN3	0.103193	5.271025	0.852275	0.396364	-6.15387	0.769394	0.622218
NK.cells	BICRAL	0.109687	5.023041	0.852126	0.396447	-6.11011	0.772266	0.626559
NK.cells	GLIS3	0.287921	3.068689	0.852078	0.396473	-5.73901	0.795308	0.661604
NK.cells	RAD23B	0.061383	6.360558	0.851896	0.396574	-6.43552	0.756962	0.603694
NK.cells	CENPT	-0.18346	2.566821	-0.85167	0.3967	-5.53559	0.801393	0.670927
NK.cells	DNM2	-0.06408	7.193868	-0.85157	0.396755	-6.59041	0.747569	0.58985
NK.cells	NFKB2	-0.12124	5.096214	-0.85156	0.396759	-6.24824	0.771464	0.625322
NK.cells	BCKDHA	0.120215	4.820285	0.851355	0.396872	-6.09967	0.774763	0.630208
NK.cells	BARD1	-0.17509	3.42386	-0.85107	0.39703	-5.86378	0.791394	0.655276
NK.cells	SHKBP1	0.113171	4.068341	0.850797	0.397181	-6.01825	0.783888	0.643644
NK.cells	GM38134	0.294186	1.108316	0.850711	0.397228	-5.34989	0.819644	0.698875
NK.cells	ABCG1	-0.11868	6.591271	-0.85054	0.397325	-6.44478	0.754802	0.599987
NK.cells	FAM102A	-0.11399	4.182226	-0.84993	0.397662	-6.17394	0.783012	0.641784
NK.cells	COPZ2	-0.23827	2.183281	-0.84988	0.397685	-5.44748	0.806941	0.678489
NK.cells	VHL	-0.11565	3.486608	-0.84981	0.397726	-5.88503	0.791251	0.654368
NK.cells	ST3GAL1	0.143931	5.796993	0.849281	0.398019	-6.29749	0.76427	0.613744
NK.cells	POP7	0.105187	4.908142	0.849253	0.398034	-6.11868	0.77454	0.629122
NK.cells	AY036118	0.235883	4.212426	0.848933	0.398211	-6.06123	0.782684	0.641425
NK.cells	NUP50	-0.08395	5.304963	-0.84889	0.398236	-6.25721	0.769936	0.62221
NK.cells	TTC39B	0.098071	4.653747	0.848831	0.398268	-6.28677	0.777507	0.633593
NK.cells	CSMD3	0.279305	1.302147	0.848635	0.398376	-5.32141	0.817765	0.695508
NK.cells	GM5577	-0.43074	0.20371	-0.84849	0.398456	-5.04866	0.831439	0.717092
NK.cells	NAT9	-0.18685	3.244632	-0.84843	0.39849	-5.63325	0.794167	0.658977
NK.cells	RAB37	0.154808	2.340912	0.848267	0.39858	-5.82009	0.805054	0.675845
NK.cells	FAM199X	0.175344	2.776803	0.848266	0.39858	-5.6896	0.799783	0.6677
NK.cells	DOCK11	0.077611	7.111908	0.848126	0.398658	-6.59383	0.74935	0.591845
NK.cells	OCRL	0.218671	2.602911	0.848099	0.398673	-5.61388	0.801881	0.670989
NK.cells	NGDN	-0.07999	5.424919	-0.84807	0.398689	-6.298	0.768551	0.620305
NK.cells	UBE2G2	0.098733	4.661055	0.84784	0.398817	-6.08121	0.777422	0.633638
NK.cells	MOCS3	-0.32762	1.446277	-0.84783	0.398824	-5.26451	0.815989	0.692918
NK.cells	GPR137B	0.175721	5.316315	0.847546	0.398979	-5.87696	0.769805	0.622251
NK.cells	TMEM134	0.082554	6.132006	0.847436	0.39904	-6.3946	0.760438	0.608282
NK.cells	OSBPL5	-0.35041	0.3884	-0.84741	0.399052	-5.24583	0.829123	0.713663
NK.cells	MAP4	0.074137	6.676158	0.847215	0.399163	-6.51427	0.754258	0.599208
NK.cells	OSBPL2	-0.11529	3.979026	-0.84721	0.399164	-5.92278	0.785436	0.645932
NK.cells	TBRG4	0.132453	4.075048	0.84701	0.399276	-5.93432	0.784303	0.644244
NK.cells	MIA2	0.064638	6.77012	0.847005	0.399279	-6.51788	0.753197	0.597675
NK.cells	RGMB	0.265979	1.845211	0.846681	0.399459	-5.40576	0.811093	0.685603
NK.cells	2810403D2	-0.19599	2.774023	-0.8466	0.399502	-5.58301	0.799816	0.66812
NK.cells	MTSS2	-0.43979	0.842863	-0.84648	0.399573	-5.07199	0.823453	0.704991
NK.cells	RPA1	0.106761	4.922792	0.846473	0.399574	-6.20876	0.774369	0.629357
NK.cells	IQSEC1	-0.09	6.277452	-0.84645	0.39959	-6.36929	0.758781	0.606064
NK.cells	SERPINE1	0.377861	0.805177	0.846375	0.399629	-5.20503	0.823922	0.705749
NK.cells	PUS3	0.170653	2.37825	0.846298	0.399672	-5.60619	0.804601	0.675565
NK.cells	PPM1N	-0.47173	0.159456	-0.84613	0.399763	-5.09973	0.831995	0.718555

NK.cells	SLF1	0.126563	4.44199	0.846056	0.399806	-6.03946	0.779986	0.637889
NK.cells	RHNO1	0.124566	4.345115	0.846004	0.399834	-6.01215	0.781123	0.639634
NK.cells	CDK16	0.138982	3.39772	0.845173	0.400296	-5.74437	0.792864	0.656976
NK.cells	GM26782	0.13809	2.991571	0.845125	0.400322	-5.72279	0.797728	0.664442
NK.cells	PRPF31	0.12307	3.79385	0.845075	0.40035	-5.90714	0.788151	0.649786
NK.cells	SERP2	0.244469	0.429349	0.845013	0.400385	-5.39607	0.82916	0.713512
NK.cells	KLF7	0.11998	6.159891	0.844936	0.400427	-6.33957	0.760624	0.608388
NK.cells	CHCHD10	-0.14972	6.141333	-0.84466	0.40058	-6.30042	0.761002	0.60879
NK.cells	WTAP	0.063368	6.931297	0.844494	0.400673	-6.52165	0.752094	0.595571
NK.cells	PTBP3	-0.05453	8.46802	-0.84392	0.400992	-6.78071	0.735463	0.570791
NK.cells	ZFP846	-0.2153	2.431015	-0.8435	0.401226	-5.49774	0.805392	0.675463
NK.cells	DPY30	-0.10399	5.414831	-0.84342	0.401272	-6.27601	0.770034	0.621654
NK.cells	HELLS	0.167293	4.919254	0.843413	0.401274	-6.14597	0.775788	0.630291
NK.cells	FNTB	0.161459	2.971515	0.843085	0.401456	-5.71417	0.798859	0.665495
NK.cells	COMMD9	-0.20076	2.646144	-0.84288	0.401571	-5.5594	0.802785	0.67159
NK.cells	GM16675	0.352236	1.196561	0.842862	0.40158	-5.29039	0.820526	0.699209
NK.cells	1700097N(	-0.22417	2.346622	-0.84267	0.401684	-5.62997	0.806417	0.677287
NK.cells	NSUN4	0.143907	3.189091	0.842614	0.401718	-5.83959	0.796245	0.661618
NK.cells	ZBTB49	0.248167	1.051258	0.842599	0.401727	-5.27023	0.822328	0.702138
NK.cells	BMP1	-0.43042	0.911089	-0.84246	0.401805	-5.14159	0.824069	0.704948
NK.cells	ALG1	0.16288	3.312271	0.842413	0.40183	-5.77517	0.79477	0.65944
NK.cells	HIST1H2BN	-0.31438	1.240034	-0.84214	0.40198	-5.27787	0.819988	0.698606
NK.cells	F3	0.684772	0.113979	0.842133	0.401986	-4.985	0.834047	0.720798
NK.cells	TTPA	-0.3275	1.860466	-0.84212	0.401993	-5.41302	0.812349	0.686662
NK.cells	AFMID	0.132352	4.371552	0.842011	0.402054	-6.00829	0.782202	0.640392
NK.cells	MTF2	-0.08322	5.644163	-0.84142	0.402383	-6.3084	0.76789	0.618231
NK.cells	1810062O1	0.239347	2.0989	0.841007	0.402613	-5.46597	0.810122	0.682444
NK.cells	4930435F1	-0.40008	0.358699	-0.84094	0.40265	-5.20179	0.831677	0.716253
NK.cells	D230025D1	0.107012	4.57771	0.840926	0.402658	-6.1263	0.780444	0.636961
NK.cells	OLR1	0.627477	1.333367	0.840636	0.40282	-5.11851	0.819685	0.697244
NK.cells	JTB	0.067634	5.842584	0.840554	0.402865	-6.35163	0.7659	0.615066
NK.cells	SERTAD3	0.165819	4.131362	0.840318	0.402997	-5.87253	0.785921	0.645124
NK.cells	TMUB2	-0.15424	3.181282	-0.84022	0.403052	-5.72509	0.79724	0.662408
NK.cells	ANKRD12	-0.08092	7.518738	-0.83998	0.403184	-6.6377	0.746969	0.587132
NK.cells	ATG10	0.096762	5.64086	0.839908	0.403226	-6.30433	0.768294	0.618647
NK.cells	CYB5R4	0.082993	5.869945	0.839898	0.403231	-6.33423	0.765657	0.614713
NK.cells	GNAL	0.239024	1.371741	0.839709	0.403336	-5.41736	0.819361	0.696716
NK.cells	TGM1	0.443912	0.53017	0.839327	0.40355	-5.11725	0.829835	0.713334
NK.cells	GM20219	0.402221	0.646352	0.839265	0.403584	-5.08915	0.828381	0.711055
NK.cells	PTPRG	-0.35062	2.191113	-0.83916	0.403641	-5.41139	0.809297	0.681189
NK.cells	VTA1	0.077746	5.707573	0.83908	0.403687	-6.31494	0.767595	0.617721
NK.cells	RFTN1	-0.09434	6.737622	-0.83901	0.403728	-6.50724	0.755827	0.600285
NK.cells	DANCR	0.289351	1.545559	0.83872	0.403888	-5.30553	0.817215	0.693712
NK.cells	SNX16	0.170854	2.847666	0.838703	0.403898	-5.68028	0.801329	0.669043
NK.cells	DIP2A	0.204993	2.079809	0.83853	0.403995	-5.52714	0.810657	0.683573
NK.cells	SMPD3	-0.55915	-0.20687	-0.83845	0.404041	-4.95818	0.839124	0.728425
NK.cells	WDHD1	-0.13797	4.56722	-0.8384	0.404066	-6.09697	0.780857	0.637883

NK.cells	NPLOC4	-0.08916	5.537568	-0.83823	0.404162	-6.2931	0.769556	0.620912
NK.cells	PHF21A	0.084596	7.069098	0.838198	0.40418	-6.58371	0.752083	0.594994
NK.cells	VEGFB	0.149858	3.802219	0.837968	0.404308	-5.82112	0.789894	0.65169
NK.cells	ACRBP	-0.31318	1.566441	-0.83778	0.404411	-5.23478	0.816957	0.693529
NK.cells	AVEN	0.103667	4.926701	0.837755	0.404427	-6.27192	0.77665	0.631631
NK.cells	DR1	-0.08268	4.953577	-0.83771	0.404453	-6.20959	0.776336	0.631159
NK.cells	ADAM15	0.284654	2.475369	0.837611	0.404508	-5.43486	0.805837	0.676223
NK.cells	ZMYM6	0.237373	2.526746	0.837599	0.404515	-5.53303	0.805213	0.675258
NK.cells	GZMM	-0.25744	2.051803	-0.8372	0.404737	-5.4647	0.811313	0.684365
NK.cells	APBB2	-0.26735	4.02877	-0.83701	0.404845	-5.73532	0.787593	0.64783
NK.cells	ARF6	-0.08235	6.770128	-0.83668	0.40503	-6.53067	0.755934	0.600331
NK.cells	GTPBP2	0.108651	4.751688	0.836674	0.405032	-6.1249	0.779184	0.635032
NK.cells	1700001K1	-0.3406	1.052633	-0.83624	0.405272	-5.28924	0.823902	0.703915
NK.cells	TUG1	0.080907	6.098897	0.836226	0.405282	-6.37107	0.763644	0.611712
NK.cells	CTBP2	-0.19422	3.249836	-0.83607	0.405367	-5.65888	0.797057	0.66228
NK.cells	SELENOF	0.100036	5.422799	0.83602	0.405397	-6.23303	0.771432	0.623444
NK.cells	NUP188	0.115307	4.513741	0.835769	0.405538	-6.04758	0.782041	0.639493
NK.cells	9930022D1	0.335848	0.319301	0.835667	0.405595	-5.31084	0.833074	0.718669
NK.cells	MGMT	0.211107	3.273792	0.835298	0.405802	-5.6209	0.796769	0.662078
NK.cells	TTC3	0.09475	5.355192	0.835165	0.405876	-6.26529	0.772216	0.624877
NK.cells	GM17066	-0.26906	1.747467	-0.83504	0.405949	-5.33853	0.81531	0.690946
NK.cells	ZFP566	0.300548	1.254913	0.83502	0.405957	-5.30072	0.821391	0.70047
NK.cells	DHX37	0.188431	2.67509	0.834998	0.40597	-5.58645	0.803987	0.673351
NK.cells	C2CD2	0.15806	3.079676	0.834703	0.406135	-5.77953	0.799102	0.665939
NK.cells	WNT4	-0.26629	1.526488	-0.83446	0.40627	-5.38248	0.818032	0.69545
NK.cells	CKS2	-0.12724	5.959802	-0.83446	0.40627	-6.31287	0.765239	0.614739
NK.cells	CCDC83	-0.5089	0.420205	-0.83441	0.406301	-5.16044	0.831806	0.717173
NK.cells	MEIS2	0.322283	2.894046	0.834301	0.40636	-5.46863	0.801339	0.669546
NK.cells	PIGU	0.132501	4.215968	0.834155	0.406441	-5.94063	0.785551	0.645363
NK.cells	ST8SIA1	0.245004	0.877835	0.834132	0.406455	-5.67854	0.826078	0.708149
NK.cells	SYPL	0.097756	5.860238	0.834019	0.406518	-6.21635	0.766383	0.616523
NK.cells	ARL8B	-0.07404	5.97123	-0.83401	0.406525	-6.3265	0.765108	0.614621
NK.cells	ZFP605	0.269985	1.633403	0.833904	0.406582	-5.43248	0.816714	0.693481
NK.cells	DERL2	0.093874	4.925658	0.833899	0.406585	-6.17706	0.777214	0.632781
NK.cells	TTLL11	0.312044	1.608719	0.833773	0.406656	-5.24301	0.817018	0.694003
NK.cells	PCBP1	-0.06539	8.199098	-0.83369	0.406703	-6.73437	0.739999	0.577753
NK.cells	HIF1AN	-0.14809	3.373427	-0.83368	0.406708	-5.86499	0.795575	0.660817
NK.cells	NUBP1	0.085325	5.473714	0.833364	0.406885	-6.3491	0.771055	0.623379
NK.cells	TBCC	0.151486	3.579816	0.833042	0.407066	-5.7763	0.793548	0.657229
NK.cells	GM42595	-0.34865	0.917394	-0.83268	0.407267	-5.1714	0.826145	0.707881
NK.cells	TOGARAM	-0.08614	5.297825	-0.83244	0.407406	-6.24965	0.773405	0.626765
NK.cells	STIMATE	0.120086	4.159047	0.832405	0.407423	-6.00035	0.786757	0.646952
NK.cells	RAB34	-0.49978	0.235405	-0.83237	0.407441	-5.04992	0.834696	0.721524
NK.cells	CDK2	-0.14289	4.350301	-0.83229	0.407486	-5.95308	0.784497	0.643541
NK.cells	GM48302	-0.31517	1.63727	-0.8321	0.407596	-5.35197	0.817219	0.694053
NK.cells	GM43256	-0.43449	0.309245	-0.8321	0.407596	-5.05288	0.833765	0.720137
NK.cells	TAF6	0.143688	3.851886	0.832017	0.407641	-5.89301	0.790401	0.65259

NK.cells	SLC22A27	-0.37228	1.124082	-0.83168	0.407829	-5.26865	0.823652	0.704149
NK.cells	PLCB1	0.221712	4.622522	0.8316	0.407875	-5.99578	0.781369	0.638854
NK.cells	4930539J0	-0.39659	0.706555	-0.8316	0.407878	-5.10779	0.828859	0.712363
NK.cells	PRPSAP2	0.123881	4.045546	0.831245	0.408074	-5.90325	0.788431	0.649285
NK.cells	CLASRP	0.153615	3.334689	0.831128	0.40814	-5.74786	0.796912	0.662305
NK.cells	UBXN6	0.108707	4.786672	0.830787	0.408332	-6.15405	0.779934	0.636116
NK.cells	ARRDC3	-0.18171	3.403751	-0.83061	0.40843	-5.79812	0.796343	0.661141
NK.cells	TRP53RKB	-0.22036	2.444883	-0.83054	0.40847	-5.44735	0.807929	0.679014
NK.cells	KDM7A	0.077029	7.129544	0.830348	0.408579	-6.62058	0.753085	0.596005
NK.cells	4930579G2	-0.18766	2.431609	-0.8297	0.408943	-5.57907	0.80873	0.679343
NK.cells	BAG5	0.158888	3.637068	0.829615	0.408991	-5.85969	0.794178	0.656951
NK.cells	IK	-0.06633	6.288849	-0.82927	0.409184	-6.43938	0.763166	0.610333
NK.cells	CHMP7	0.182471	2.616172	0.829216	0.409216	-5.64323	0.806493	0.676044
NK.cells	TKFC	-0.21509	3.130684	-0.8292	0.409227	-5.63859	0.800266	0.666436
NK.cells	9830107B1	0.424659	-0.62676	0.829053	0.409307	-5.02066	0.846947	0.739771
NK.cells	WWP1	0.112102	5.227114	0.829021	0.409325	-6.218	0.775425	0.628662
NK.cells	KLRC3	0.227052	-1.20661	0.828761	0.409472	-5.35992	0.854315	0.751827
NK.cells	TPT1	0.050929	10.10831	0.828376	0.409688	-7.07723	0.721164	0.549
NK.cells	CBX6	-0.18252	2.859775	-0.82812	0.409832	-5.61196	0.803929	0.671768
NK.cells	D930030I0	-0.41108	0.408475	-0.82811	0.409837	-5.14201	0.834211	0.719132
NK.cells	P3H1	-0.37137	0.585168	-0.82787	0.409974	-5.23549	0.831988	0.715624
NK.cells	EMILIN1	0.19107	2.608435	0.827611	0.410119	-5.51378	0.806979	0.676503
NK.cells	INTS13	-0.11166	4.149388	-0.82748	0.410192	-5.97267	0.788473	0.648173
NK.cells	LFNG	-0.09455	4.413378	-0.82739	0.410241	-6.25639	0.785348	0.643433
NK.cells	ASH1L	0.069943	7.367632	0.827186	0.410359	-6.59969	0.751295	0.592721
NK.cells	SNX21	-0.20825	2.718125	-0.82711	0.410399	-5.60531	0.805647	0.674623
NK.cells	USP53	0.109424	4.118641	0.827046	0.410438	-6.03369	0.788837	0.648854
NK.cells	ZFP597	-0.20345	2.862663	-0.827	0.410466	-5.55667	0.803894	0.671933
NK.cells	ST6GALNA	0.391818	0.651842	0.826946	0.410494	-5.11589	0.831151	0.714528
NK.cells	ENKD1	-0.30023	1.3576	-0.82686	0.410545	-5.25527	0.822345	0.700671
NK.cells	TRAPPC11	0.126763	3.489165	0.826588	0.410696	-5.83763	0.796345	0.660486
NK.cells	GM49173	-0.63607	-0.87955	-0.8262	0.410917	-4.95403	0.850603	0.745776
NK.cells	SEMA6B	0.347914	1.154578	0.826179	0.410927	-5.2074	0.824868	0.704842
NK.cells	RND3	-0.3082	2.943599	-0.82609	0.410977	-5.37979	0.802915	0.670689
NK.cells	CDCA7L	-0.1622	3.766654	-0.82607	0.410987	-5.95469	0.793026	0.655503
NK.cells	EPS8L2	-0.45587	0.893203	-0.82587	0.411099	-5.08579	0.828128	0.710109
NK.cells	RELT	0.132966	3.728351	0.8257	0.411197	-5.96606	0.793483	0.656331
NK.cells	FCGR2B	-0.22098	4.881357	-0.82563	0.411235	-5.79337	0.779843	0.635601
NK.cells	CLCN3	0.080343	6.389845	0.825581	0.411264	-6.46252	0.762382	0.60946
NK.cells	USP18	0.299061	2.737474	0.825571	0.411269	-5.51424	0.805412	0.674678
NK.cells	LILRA6	0.471163	1.213739	0.825317	0.411413	-5.07049	0.824132	0.70393
NK.cells	THY1	0.168232	1.573983	0.825262	0.411444	-5.94455	0.819665	0.696954
NK.cells	CDKL2	0.302278	1.163972	0.825129	0.411519	-5.33991	0.824751	0.704941
NK.cells	PGAP2	0.102173	5.75264	0.82508	0.411547	-6.33107	0.769705	0.62045
NK.cells	MSH5	-0.30327	2.9838	-0.825	0.411594	-5.40554	0.802429	0.670158
NK.cells	AA386476	0.319283	1.390731	0.824989	0.411598	-5.26514	0.821934	0.700513
NK.cells	DCBLD2	-0.27492	1.32551	-0.82483	0.411687	-5.28224	0.822743	0.701794

NK.cells	GPR89	0.126748	3.742407	0.824762	0.411726	-5.96773	0.793315	0.656222
NK.cells	DCTPP1	0.126272	5.089369	0.824703	0.41176	-6.19987	0.777409	0.632098
NK.cells	LAMTOR1	0.067779	6.503458	0.824429	0.411914	-6.47095	0.761085	0.607746
NK.cells	CD5	0.135124	2.693985	0.824336	0.411967	-5.95109	0.80594	0.675773
NK.cells	PABPN1	0.062539	6.640276	0.824196	0.412046	-6.49449	0.759525	0.605536
NK.cells	SLC8A1	0.281416	6.330185	0.824151	0.412072	-5.9932	0.763065	0.610802
NK.cells	MRPS11	-0.139	3.950885	-0.82391	0.412207	-5.94285	0.790831	0.652738
NK.cells	TXLNG	0.086621	5.107247	0.823709	0.412321	-6.29182	0.777201	0.632123
NK.cells	MED12L	0.164326	3.141983	0.823638	0.412361	-5.75579	0.800519	0.667667
NK.cells	FNDC3A	0.077666	7.526942	0.823582	0.412393	-6.61894	0.749505	0.590947
NK.cells	GLT1D1	0.411637	0.611079	0.823574	0.412398	-5.11689	0.831663	0.716335
NK.cells	GM47662	-0.29631	-0.79475	-0.82353	0.412422	-5.28288	0.849514	0.74484
NK.cells	PLK4	-0.16136	3.856529	-0.82351	0.412434	-5.9705	0.791954	0.65453
NK.cells	RNF220	0.097464	5.723291	0.823414	0.412488	-6.2898	0.770044	0.6214
NK.cells	2010310CC	-0.47243	1.298313	-0.82321	0.412602	-5.17298	0.823175	0.702875
NK.cells	COIL	-0.1183	3.741294	-0.82288	0.412789	-5.97655	0.793653	0.65683
NK.cells	CUL5	-0.0703	5.877947	-0.82266	0.412912	-6.37163	0.76868	0.618974
NK.cells	PPID	-0.10382	4.87562	-0.82243	0.413043	-6.15437	0.780461	0.636577
NK.cells	RIMS4	-0.55826	-0.99611	-0.8222	0.413173	-4.94811	0.852731	0.74945
NK.cells	ITPKA	-0.40581	0.529225	-0.82213	0.413212	-5.10479	0.833353	0.718397
NK.cells	NUFIP1	0.09946	4.171093	0.821711	0.413452	-6.05878	0.78912	0.649311
NK.cells	PTP4A1	-0.28791	1.179279	-0.82163	0.413497	-5.42092	0.825507	0.705694
NK.cells	ATG5	0.070129	5.691584	0.821331	0.413667	-6.31381	0.771295	0.622481
NK.cells	GALNT7	-0.0903	6.060844	-0.82118	0.413753	-6.45158	0.767033	0.616114
NK.cells	TUBGCP5	0.130029	3.976604	0.821177	0.413754	-5.87906	0.791432	0.652921
NK.cells	RFT1	0.210833	2.53189	0.820873	0.413926	-5.58293	0.808838	0.679819
NK.cells	SELENOI	0.132984	3.422495	0.820631	0.414063	-5.93969	0.79806	0.663275
NK.cells	CCDC186	0.09985	4.760025	0.820493	0.414142	-6.13778	0.782163	0.639069
NK.cells	1700030J2	0.455649	-0.14389	0.820368	0.414212	-5.1147	0.842165	0.732498
NK.cells	GM50071	0.342174	0.514178	0.82033	0.414234	-5.15205	0.833836	0.719251
NK.cells	MYL4	-0.21476	4.638008	-0.82009	0.41437	-5.99677	0.783599	0.641378
NK.cells	PLAGL1	-0.29765	1.041672	-0.81993	0.414463	-5.44971	0.827223	0.708925
NK.cells	PLLP	-0.50624	-0.20457	-0.81991	0.41447	-4.99971	0.842938	0.733883
NK.cells	R74862	-0.46804	1.054776	-0.81979	0.41454	-5.08034	0.827059	0.708762
NK.cells	ARHGEF6	-0.08517	5.643537	-0.81953	0.414689	-6.38509	0.771851	0.623902
NK.cells	GCSH	0.118981	4.428205	0.819505	0.414702	-6.08878	0.786074	0.645372
NK.cells	ZC3H15	-0.05875	7.266033	-0.81948	0.414718	-6.63134	0.753302	0.596359
NK.cells	KCNJ16	-0.67955	-0.02269	-0.81937	0.414781	-5.01434	0.840625	0.730447
NK.cells	AKAP9	0.078492	6.105265	0.819346	0.414792	-6.4445	0.766522	0.615989
NK.cells	ERCC4	-0.16106	2.949475	-0.81929	0.414827	-5.80596	0.803765	0.672565
NK.cells	GTPBP6	-0.17738	2.84005	-0.81909	0.414938	-5.67937	0.805091	0.674639
NK.cells	PSMA5	0.094724	5.884469	0.81899	0.414994	-6.36085	0.769065	0.61984
NK.cells	TRIM47	-0.24863	2.41312	-0.81873	0.415141	-5.38287	0.810287	0.682787
NK.cells	CBFB	-0.05497	6.809866	-0.81864	0.415195	-6.54999	0.758467	0.604146
NK.cells	FBXO11	-0.08639	8.797772	-0.81859	0.415219	-6.82384	0.736243	0.571647
NK.cells	TADA2A	0.175195	2.820969	0.818433	0.415311	-5.64171	0.805323	0.67519
NK.cells	CCSER2	-0.099	4.778998	-0.81832	0.415376	-6.2474	0.78194	0.639385

NK.cells	NUP133	-0.12054	3.691763	-0.81823	0.415425	-5.90538	0.794832	0.659031
NK.cells	MOK	0.523891	0.377087	0.818226	0.415428	-5.00647	0.835564	0.72265
NK.cells	SLC25A23	-0.23872	2.25448	-0.81818	0.415455	-5.43879	0.812227	0.685916
NK.cells	AA465934	-0.25139	1.66043	-0.8181	0.4155	-5.38499	0.819536	0.697366
NK.cells	RBM41	0.118299	3.907296	0.818046	0.41553	-5.99228	0.792258	0.655121
NK.cells	GM48742	-0.33448	0.618325	-0.81787	0.415632	-5.19635	0.832526	0.717865
NK.cells	HLCS	0.131765	4.15558	0.817862	0.415635	-6.06751	0.789304	0.650614
NK.cells	BRMS1	0.110776	4.236998	0.817807	0.415666	-6.05455	0.788338	0.649141
NK.cells	QRICH1	-0.05884	6.585338	-0.81754	0.415819	-6.50036	0.761165	0.608154
NK.cells	ZFP40	0.303456	1.347612	0.817354	0.415923	-5.32228	0.823566	0.703584
NK.cells	ZFP46	0.196916	1.77659	0.817323	0.415941	-5.37048	0.818254	0.695245
NK.cells	DESI2	0.058557	6.03278	0.816889	0.416188	-6.41381	0.767592	0.617588
NK.cells	GM44899	0.300761	0.774696	0.816507	0.416405	-5.2666	0.830819	0.714972
NK.cells	SPINT1	0.364228	-1.0651	0.816377	0.416479	-5.04521	0.854117	0.752433
NK.cells	TAP2	-0.12128	5.110651	-0.81625	0.41655	-6.30007	0.778293	0.633784
NK.cells	CYP3A16	-0.3773	0.777697	-0.81619	0.416584	-5.19509	0.830782	0.714965
NK.cells	ETL4	-0.27787	2.396305	-0.81619	0.416584	-5.43336	0.810743	0.683513
NK.cells	HK1	0.08988	4.85354	0.816128	0.41662	-6.28784	0.781305	0.638336
NK.cells	HEXIM2	-0.35052	0.998422	-0.81613	0.41662	-5.21663	0.828018	0.710595
NK.cells	DENND1B	-0.07792	7.299486	-0.81609	0.416642	-6.61637	0.753157	0.596334
NK.cells	H2-OB	-0.22825	4.007971	-0.81604	0.416672	-5.8294	0.791302	0.653565
NK.cells	SRBD1	0.109345	4.393343	0.815855	0.416775	-6.12076	0.786729	0.646624
NK.cells	COPS5	-0.08277	5.288344	-0.81575	0.416836	-6.26428	0.776218	0.630712
NK.cells	ACVR1B	0.176485	2.543644	0.815726	0.416849	-5.62745	0.808944	0.680778
NK.cells	CUL3	-0.05393	7.198802	-0.81561	0.416915	-6.61364	0.754293	0.598085
NK.cells	COX7B	0.070752	7.597144	0.815378	0.417047	-6.691	0.749888	0.591526
NK.cells	DXO	-0.15802	3.302702	-0.8153	0.417092	-5.84627	0.799832	0.66666
NK.cells	SPIRE1	0.309715	2.681156	0.814955	0.417288	-5.48898	0.807356	0.67848
NK.cells	G3BP1	-0.07506	6.728766	-0.81469	0.417438	-6.52087	0.759704	0.606188
NK.cells	MRPL38	0.13069	3.863246	0.814603	0.417488	-5.84794	0.793112	0.656527
NK.cells	FIG4	0.085925	4.603641	0.81447	0.417564	-6.12949	0.784329	0.643135
NK.cells	TMC8	-0.14049	3.820781	-0.81442	0.417595	-5.87685	0.793619	0.657303
NK.cells	LMO7	0.560195	0.761873	0.814413	0.417597	-5.23627	0.831069	0.71566
NK.cells	DCAF5	-0.08669	5.52135	-0.8144	0.417603	-6.35281	0.773589	0.626912
NK.cells	HNRNPAB	-0.07058	8.05587	-0.81431	0.417657	-6.73067	0.74476	0.584233
NK.cells	CDCA5	-0.20496	3.028781	-0.81426	0.417685	-5.74421	0.803139	0.671992
NK.cells	PFAS	0.159469	4.205445	0.813711	0.417997	-6.00356	0.789359	0.650545
NK.cells	NDFIP2	-0.07114	5.951714	-0.81369	0.418006	-6.433	0.76892	0.619675
NK.cells	SLC43A2	0.126271	6.179865	0.813503	0.418115	-6.37709	0.766293	0.615751
NK.cells	NFYA	0.136327	4.152707	0.813497	0.418119	-5.88387	0.789986	0.6515
NK.cells	DACH1	0.391804	1.877933	0.813348	0.418204	-5.3219	0.817559	0.694114
NK.cells	ZFP202	-0.45108	0.604939	-0.81303	0.418387	-5.10483	0.833599	0.719217
NK.cells	4930505N2	-0.26423	1.214516	-0.81295	0.418428	-5.36588	0.825964	0.707141
NK.cells	TULP4	-0.08502	5.928036	-0.81273	0.418558	-6.40345	0.769516	0.620277
NK.cells	PIGH	-0.26313	1.827535	-0.81216	0.418879	-5.37904	0.818959	0.695451
NK.cells	CAPZA1	-0.04914	7.715586	-0.81207	0.418932	-6.68657	0.749611	0.590344
NK.cells	CHCHD2	0.056022	8.94643	0.811735	0.419124	-6.86901	0.735982	0.570586

NK.cells	ITGAL	-0.09436	6.272091	-0.81168	0.419153	-6.56678	0.766033	0.614677
NK.cells	PIK3CD	0.094822	6.624599	0.811311	0.419366	-6.49611	0.761995	0.608677
NK.cells	4833438C(	0.182057	2.025244	0.811114	0.419478	-5.48852	0.81656	0.691865
NK.cells	GPCPD1	0.076434	7.329375	0.811109	0.419481	-6.58499	0.753992	0.59691
NK.cells	SYMPK	0.104817	4.293778	0.810864	0.419621	-6.0701	0.789135	0.649609
NK.cells	SYK	-0.10434	8.336181	-0.81083	0.41964	-6.56043	0.742721	0.580471
NK.cells	GM15956	0.389459	0.27605	0.810524	0.419816	-5.09719	0.838402	0.726518
NK.cells	KBTBD11	0.26884	0.726015	0.810523	0.419816	-5.57209	0.832725	0.717497
NK.cells	ATCAYOS	-0.39086	1.220314	-0.81048	0.419838	-5.33609	0.826535	0.707716
NK.cells	CCL7	0.660762	1.659903	0.810429	0.41987	-5.26105	0.821072	0.699154
NK.cells	ZFP324	-0.36014	0.798242	-0.81035	0.419916	-5.20191	0.831817	0.716119
NK.cells	RSPH1	-0.40591	1.855808	-0.81021	0.419996	-5.30466	0.818649	0.695413
NK.cells	PHIP	-0.07235	7.276011	-0.8102	0.42	-6.59256	0.754595	0.598039
NK.cells	BMPR2	-0.11249	5.519027	-0.81012	0.420047	-6.29263	0.774739	0.628029
NK.cells	3110009E1	-0.19334	2.175408	-0.80982	0.420216	-5.53314	0.814714	0.689388
NK.cells	CNN3	-0.16874	4.531127	-0.80954	0.420376	-5.90187	0.786323	0.645804
NK.cells	TMEM241	0.127201	4.310016	0.809486	0.420409	-6.09008	0.788942	0.649795
NK.cells	OXNAD1	0.222609	1.886539	0.80934	0.420492	-5.41588	0.81827	0.695195
NK.cells	PHC1	0.125664	3.5385	0.809221	0.42056	-5.79464	0.798154	0.664032
NK.cells	TRMT10C	-0.08903	4.996252	-0.80918	0.420584	-6.22662	0.780846	0.637635
NK.cells	MBNL1	-0.07561	8.978349	-0.80916	0.420595	-6.9537	0.735631	0.570734
NK.cells	PXN	0.081582	6.266447	0.808915	0.420736	-6.51714	0.766098	0.61555
NK.cells	RNF34	-0.09862	4.455799	-0.80879	0.420806	-6.11312	0.787215	0.647374
NK.cells	DGCR2	0.101596	4.849233	0.808765	0.420822	-6.17135	0.782573	0.640323
NK.cells	SATB2	-0.3837	1.643339	-0.80869	0.420864	-5.19548	0.821277	0.700061
NK.cells	MZT2	-0.18615	2.206691	-0.80857	0.420931	-5.48141	0.814329	0.689212
NK.cells	TPP2	-0.05963	7.082191	-0.8081	0.421201	-6.62107	0.756788	0.601901
NK.cells	DNHD1	0.372785	1.01891	0.808033	0.42124	-5.1485	0.829051	0.712536
NK.cells	YIPF4	0.083429	6.515782	0.807944	0.421291	-6.5385	0.763239	0.611492
NK.cells	ZFP580	0.223504	2.166288	0.807938	0.421295	-5.49625	0.814826	0.690183
NK.cells	TMUB1	0.125294	3.138278	0.807522	0.421533	-5.78302	0.802978	0.6719
NK.cells	ZMIZ1OS1	0.378829	0.352543	0.807502	0.421545	-5.25431	0.837434	0.725996
NK.cells	NSUN3	0.166277	2.658689	0.807479	0.421558	-5.61548	0.808801	0.680925
NK.cells	GM43112	0.311408	0.075373	0.807422	0.421591	-5.24188	0.840947	0.731603
NK.cells	ING1	0.087191	5.135774	0.807407	0.421599	-6.26454	0.779211	0.635564
NK.cells	PHPT1	0.115521	4.330971	0.807329	0.421643	-6.03826	0.788694	0.649964
NK.cells	GM50333	-0.26253	1.191453	-0.80729	0.421664	-5.33546	0.826895	0.709275
NK.cells	TMEM116	-0.27802	2.038927	-0.80729	0.421666	-5.40898	0.816392	0.692763
NK.cells	PRMT2	0.203818	1.665423	0.80706	0.421798	-5.54566	0.821003	0.70002
NK.cells	TMEM43	0.133565	3.43413	0.806916	0.42188	-5.87886	0.799409	0.66645
NK.cells	TACC2	-0.17975	3.088667	-0.80681	0.421939	-5.73111	0.803578	0.672939
NK.cells	GM33524	-0.48164	0.491882	-0.80676	0.421968	-5.01677	0.835674	0.723311
NK.cells	GPR174	0.169901	2.267506	0.80676	0.42197	-5.83499	0.813583	0.688486
NK.cells	KLRC1	-0.14703	1.444197	-0.80626	0.422258	-6.07914	0.823748	0.704574
NK.cells	TIMM22	-0.10045	4.4471	-0.80621	0.422283	-6.06877	0.787318	0.648108
NK.cells	GABARAPL	-0.11875	3.974288	-0.80621	0.422286	-6.05227	0.792936	0.656694
NK.cells	NDE1	-0.11191	4.58489	-0.80609	0.422354	-6.15586	0.785688	0.645661

NK.cells	NAIP2	0.14589	3.006951	0.806045	0.42238	-5.94941	0.804568	0.674664
NK.cells	GATC	0.219077	2.698485	0.805937	0.422441	-5.60432	0.808316	0.680517
NK.cells	POLR2F	0.076779	5.381223	0.805831	0.422503	-6.30554	0.776343	0.631562
NK.cells	BAHCC1	-0.25247	1.235974	-0.80578	0.422532	-5.46715	0.82634	0.708764
NK.cells	WDR4	0.146659	2.999114	0.80567	0.422595	-5.73539	0.804663	0.674855
NK.cells	PLOD2	0.338058	1.261605	0.805578	0.422648	-5.31434	0.82602	0.70826
NK.cells	PKP4	0.105709	5.958306	0.805544	0.422667	-6.49121	0.769647	0.621496
NK.cells	ANKRD13A	0.073433	6.215118	0.805541	0.422669	-6.43616	0.766688	0.617068
NK.cells	KLHL13	-0.52031	0.360663	-0.80546	0.422718	-5.0474	0.837331	0.726251
NK.cells	CENPO	-0.17229	2.773597	-0.80516	0.422888	-5.62994	0.807489	0.679271
NK.cells	ATG14	-0.12524	3.27883	-0.80514	0.422896	-5.83465	0.801368	0.669789
NK.cells	SLC7A6	-0.09938	4.274758	-0.80465	0.423181	-6.12563	0.789797	0.651652
NK.cells	CAMK2G	0.093673	4.893224	0.804443	0.423299	-6.28419	0.782488	0.640552
NK.cells	EYA2	-0.17852	1.389743	-0.80428	0.423395	-5.82244	0.824881	0.706151
NK.cells	4833419F2	-0.34836	1.323387	-0.80413	0.423478	-5.25602	0.825707	0.707454
NK.cells	2810001G2	0.146901	2.479465	0.804073	0.423512	-5.68928	0.811437	0.685078
NK.cells	AVL9	0.081011	6.010486	0.804039	0.423531	-6.46068	0.76947	0.620953
NK.cells	AGAP2	0.1352	2.925575	0.804011	0.423547	-5.89074	0.806001	0.676629
NK.cells	TNFRSF21	0.154013	4.10783	0.803858	0.423635	-5.95474	0.791789	0.654741
NK.cells	CLDND1	-0.08451	4.825073	-0.80341	0.42389	-6.19647	0.783296	0.642002
NK.cells	4930556J2	-0.1966	2.185085	-0.80322	0.423999	-5.53256	0.815052	0.691036
NK.cells	4930589L2	0.426794	-0.68311	0.803218	0.424003	-4.9974	0.851117	0.748333
NK.cells	EML2	-0.1665	2.655573	-0.80319	0.424017	-5.79997	0.809293	0.682054
NK.cells	GM11579	-0.2512	-0.20636	-0.80303	0.424108	-5.55256	0.845008	0.738562
NK.cells	ABCG3	0.144965	4.394356	0.802909	0.424181	-6.13899	0.788385	0.64992
NK.cells	COPS3	-0.07083	5.92883	-0.8029	0.424186	-6.38053	0.77042	0.62274
NK.cells	MAN1C1	-0.14404	4.292733	-0.8029	0.424187	-5.94767	0.78959	0.651761
NK.cells	CBFA2T2	0.082007	5.563389	0.802734	0.424281	-6.36168	0.774657	0.629173
NK.cells	DSEL	-0.34038	0.375304	-0.8025	0.424416	-5.11286	0.837616	0.726987
NK.cells	LRRC10B	-0.46252	0.595128	-0.8023	0.42453	-5.0918	0.83484	0.722591
NK.cells	FAM129B	0.249573	3.14002	0.802214	0.42458	-5.63398	0.803408	0.673296
NK.cells	TNFSF11	-0.33604	0.706959	-0.80214	0.424623	-5.53265	0.833432	0.720416
NK.cells	ZBED4	0.110071	4.650997	0.802004	0.424701	-6.02241	0.785348	0.645622
NK.cells	SWI5	-0.08161	7.120072	-0.80198	0.424713	-6.57584	0.756784	0.602734
NK.cells	FBXO21	-0.12471	3.292952	-0.8019	0.42476	-5.91391	0.80156	0.670492
NK.cells	NUDT1	0.193557	3.00659	0.801783	0.424828	-5.70218	0.805024	0.675892
NK.cells	DNAJB4	0.121138	3.700143	0.801746	0.424849	-5.93062	0.796661	0.662994
NK.cells	EEF2	0.068896	9.105144	0.801706	0.424872	-6.94661	0.734652	0.570391
NK.cells	GM12971	0.289588	0.967071	0.801468	0.425009	-5.24779	0.830304	0.715414
NK.cells	TNFRSF23	-0.25533	1.669914	-0.80109	0.425227	-5.42808	0.821797	0.701745
NK.cells	THOC7	-0.06471	6.572603	-0.80088	0.425347	-6.52339	0.763378	0.61225
NK.cells	LYRM4	0.102045	4.142278	0.800729	0.425435	-6.02567	0.791753	0.65516
NK.cells	PEX14	0.087271	5.405291	0.800672	0.425468	-6.30451	0.776865	0.632525
NK.cells	TLE3	-0.09315	5.003886	-0.80044	0.425602	-6.21071	0.781564	0.639723
NK.cells	GM15728	0.402728	0.500791	0.800431	0.425607	-5.11448	0.836426	0.725078
NK.cells	HIST1H2BN	-0.2486	2.139918	-0.80025	0.425711	-5.60376	0.815993	0.692871
NK.cells	MRTFA	0.08378	7.084199	0.799954	0.425882	-6.65118	0.757548	0.603887

NK.cells	TSR1	0.137422	3.584651	0.799908	0.425908	-5.92406	0.798425	0.665679
NK.cells	JOSD2	0.107776	4.09679	0.799902	0.425912	-5.99899	0.792295	0.656258
NK.cells	ARL6IP5	0.063387	7.048458	0.799897	0.425914	-6.6977	0.757954	0.604488
NK.cells	PMS1	0.166509	2.650773	0.799846	0.425944	-5.71128	0.809734	0.683203
NK.cells	OVGP1	-0.35712	0.492644	-0.79975	0.425999	-5.1192	0.836528	0.725455
NK.cells	NAA80	0.19545	2.402845	0.799583	0.426095	-5.50964	0.812822	0.687966
NK.cells	GNPTAB	-0.09905	4.589659	-0.79938	0.426211	-6.25719	0.786542	0.647443
NK.cells	LSM2	0.114312	5.695608	0.79918	0.426328	-6.34725	0.773583	0.627848
NK.cells	PARP1	0.104076	5.30881	0.799145	0.426348	-6.23199	0.778088	0.634646
NK.cells	LYSMD2	0.25371	1.265146	0.799085	0.426383	-5.50166	0.826933	0.710216
NK.cells	CXCL3	0.571296	-0.07498	0.798851	0.426518	-5.12212	0.84388	0.737202
NK.cells	CCDC189	-0.42533	0.183787	-0.79882	0.426538	-5.1177	0.840588	0.731927
NK.cells	ACCS	-0.1869	2.079594	-0.79857	0.426682	-5.61549	0.81703	0.694373
NK.cells	ZFP438	-0.14707	3.204144	-0.79842	0.426764	-5.80107	0.80333	0.672989
NK.cells	UBR1	0.094732	5.323458	0.797966	0.427028	-6.31836	0.778491	0.634495
NK.cells	1500009L1	0.273038	-0.02505	0.797679	0.427194	-5.29455	0.843831	0.736332
NK.cells	MEAF6	-0.0777	4.941914	-0.79757	0.427255	-6.23533	0.782975	0.641354
NK.cells	STUB1	0.080162	5.426852	0.797471	0.427314	-6.28317	0.777292	0.632768
NK.cells	MKS1	0.328262	1.092934	0.797353	0.427383	-5.2296	0.829706	0.713902
NK.cells	2010009K1	0.582798	0.112521	0.797309	0.427408	-5.0431	0.842079	0.733612
NK.cells	XXYLT1	0.146534	3.591278	0.797159	0.427494	-5.79693	0.799042	0.666023
NK.cells	GTF2E1	0.199184	2.399479	0.797086	0.427537	-5.55718	0.813516	0.688477
NK.cells	FAM89A	-0.26141	0.879902	-0.7969	0.427645	-5.31448	0.832378	0.718249
NK.cells	SENP7	0.08594	5.356774	0.796871	0.427661	-6.31054	0.778111	0.634145
NK.cells	SAR1B	0.070879	5.553451	0.796811	0.427695	-6.3267	0.775816	0.630682
NK.cells	9030404E1	0.532029	-0.16276	0.796223	0.428035	-4.97514	0.845876	0.739463
NK.cells	CCT8	0.067373	6.762623	0.796211	0.428042	-6.55859	0.762131	0.609902
NK.cells	MED29	0.11343	3.806911	0.796209	0.428043	-5.90538	0.796724	0.662209
NK.cells	GM48383	0.220346	1.758143	0.796063	0.428128	-5.58412	0.8217	0.701048
NK.cells	HCFC1R1	0.088341	5.33224	0.795987	0.428172	-6.28414	0.778662	0.634688
NK.cells	NR2C2	-0.07227	6.925422	-0.79584	0.428256	-6.57899	0.760306	0.60719
NK.cells	BOLL	-0.27818	1.308178	-0.79562	0.428383	-5.46081	0.827361	0.709994
NK.cells	CEP83	-0.08041	5.662564	-0.79559	0.428404	-6.33575	0.77487	0.628983
NK.cells	WDR92	0.164703	3.083733	0.795258	0.428593	-5.79421	0.805725	0.675897
NK.cells	JAK2	0.081063	6.674709	0.794953	0.428769	-6.67949	0.7634	0.611709
NK.cells	2900097C1	0.093279	4.909122	0.794932	0.428782	-6.13482	0.783899	0.642529
NK.cells	TMEM117	-0.56551	-0.04691	-0.79489	0.428808	-5.01892	0.844689	0.737499
NK.cells	KPTN	-0.16308	4.098478	-0.79474	0.428895	-5.92279	0.793512	0.65732
NK.cells	PSAP	-0.09875	9.249596	-0.7947	0.428916	-6.82424	0.734561	0.569525
NK.cells	HIPK1	0.082482	6.269668	0.794356	0.429115	-6.47634	0.768208	0.618819
NK.cells	TPR	0.058942	7.524789	0.794319	0.429137	-6.70252	0.753897	0.597573
NK.cells	E2F2	-0.13685	4.99405	-0.79406	0.429284	-6.14859	0.783121	0.641218
NK.cells	TMC4	0.232349	1.556285	0.793866	0.429399	-5.35362	0.824725	0.705635
NK.cells	TXN1	0.10023	7.304482	0.793773	0.429453	-6.6134	0.756446	0.601321
NK.cells	PTBP1	-0.07465	6.221744	-0.79374	0.429471	-6.45166	0.76882	0.619722
NK.cells	GIN1	-0.09391	3.954097	-0.79369	0.429502	-5.97486	0.795462	0.660106
NK.cells	GM12359	-0.23136	1.671224	-0.79297	0.429916	-5.43559	0.823738	0.70364

NK.cells	CNNM3	-0.14189	3.157652	-0.79279	0.430023	-5.81572	0.805489	0.675214
NK.cells	CASC3	-0.08937	5.292913	-0.79276	0.430037	-6.2554	0.780031	0.636253
NK.cells	FBXW17	0.257224	1.479346	0.792728	0.430059	-5.41441	0.826126	0.707475
NK.cells	CHD2	0.07129	7.754934	0.792571	0.43015	-6.74577	0.751767	0.59412
NK.cells	RNF145	0.092195	5.159727	0.792532	0.430172	-6.31408	0.781593	0.638637
NK.cells	KLHL22	0.168544	3.001151	0.79249	0.430196	-5.69374	0.807389	0.678184
NK.cells	CDCA7	0.204859	3.246784	0.792202	0.430363	-5.71856	0.804527	0.673673
NK.cells	CCT4	0.062857	6.487167	0.792046	0.430454	-6.5257	0.76629	0.61561
NK.cells	MACROD2	0.259573	2.251559	0.791999	0.430481	-5.5057	0.816682	0.692647
NK.cells	TRIM24	0.096244	5.087563	0.79186	0.430562	-6.21399	0.782557	0.640114
NK.cells	ARHGEF1	0.063529	7.10928	0.79172	0.430643	-6.6537	0.75918	0.605112
NK.cells	SLC27A4	-0.15932	2.770367	-0.79163	0.430698	-5.6	0.810321	0.68281
NK.cells	AQR	0.073727	4.812338	0.79158	0.430724	-6.22143	0.7858	0.645104
NK.cells	TTC7B	-0.08445	5.391283	-0.79074	0.431213	-6.36739	0.779471	0.634972
NK.cells	TRIP6	0.317312	0.834471	0.790706	0.431232	-5.14959	0.834838	0.720786
NK.cells	STK26	-0.10764	4.048898	-0.79065	0.431266	-6.14075	0.795358	0.659157
NK.cells	GM32743	-0.51042	-0.48509	-0.79025	0.431498	-4.9661	0.851643	0.747836
NK.cells	NIPAL3	0.14542	3.198166	0.790187	0.431533	-5.88655	0.805608	0.675108
NK.cells	GLOD4	0.083237	5.050654	0.790171	0.431542	-6.23397	0.783469	0.641181
NK.cells	DAZAP2	-0.06295	7.70346	-0.79008	0.431596	-6.685	0.752917	0.595578
NK.cells	RNGTT	0.063165	6.852257	0.789786	0.431766	-6.6137	0.762574	0.609959
NK.cells	TMEM67	0.28217	1.688091	0.789595	0.431877	-5.33638	0.824153	0.704276
NK.cells	HLF	0.305719	1.023669	0.789584	0.431883	-5.36515	0.832457	0.7174
NK.cells	JMY	-0.08814	6.282387	-0.78949	0.43194	-6.53396	0.769116	0.619783
NK.cells	GM47507	-0.40605	0.732528	-0.78933	0.432033	-5.22801	0.836123	0.723262
NK.cells	QPRT	-0.23481	2.660001	-0.78896	0.432248	-5.64296	0.812165	0.68565
NK.cells	ANKS1B	-0.33265	1.030517	-0.78891	0.432274	-5.25975	0.832371	0.717424
NK.cells	KHK	-0.13718	4.3658	-0.78887	0.432296	-5.94511	0.791576	0.653864
NK.cells	HIST3H2BA	0.275454	0.997637	0.788571	0.432472	-5.26561	0.832784	0.7182
NK.cells	HSPH1	0.157955	4.050366	0.788482	0.432524	-6.11167	0.79534	0.659766
NK.cells	DNPH1	0.38191	0.708539	0.788308	0.432625	-5.11079	0.836426	0.724029
NK.cells	CHPF	-0.1999	1.242066	-0.78798	0.432816	-5.57816	0.829718	0.71347
NK.cells	RERG	0.469199	0.522461	0.787964	0.432825	-5.08236	0.83878	0.727875
NK.cells	VMA21	0.087759	5.176423	0.787827	0.432905	-6.2561	0.78199	0.639498
NK.cells	F830016B0	0.522135	0.444299	0.787823	0.432907	-5.09765	0.83977	0.729456
NK.cells	RGL1	-0.16027	5.711711	-0.78756	0.43306	-6.09712	0.77573	0.630081
NK.cells	NFAT5	-0.08565	7.501438	-0.78753	0.433077	-6.66501	0.755196	0.599457
NK.cells	RBBP9	-0.31764	1.214794	-0.78737	0.433172	-5.292	0.830059	0.714131
NK.cells	MIEN1	-0.0666	5.845651	-0.78723	0.433254	-6.39545	0.774172	0.627889
NK.cells	AASS	-0.37809	1.208483	-0.78722	0.433256	-5.23205	0.830138	0.714358
NK.cells	SLC39A10	-0.11803	3.903206	-0.78706	0.433349	-5.98015	0.797103	0.66286
NK.cells	APPL2	0.152148	3.358029	0.786844	0.433477	-5.95572	0.803671	0.673046
NK.cells	TRNAU1AP	0.125933	4.126118	0.786827	0.433487	-5.98774	0.794434	0.658811
NK.cells	CCR3	0.750007	-0.02075	0.786822	0.43349	-5.10437	0.845689	0.739315
NK.cells	NEO1	-0.42222	0.660859	-0.78681	0.433497	-5.16415	0.837029	0.725457
NK.cells	FTSJ3	-0.13813	4.016619	-0.78651	0.433674	-6.02518	0.795744	0.660898
NK.cells	SLC4A9	0.393181	0.122757	0.786426	0.433721	-5.10904	0.843858	0.736488

NK.cells	ZBTB17	-0.11997	3.917458	-0.78623	0.433837	-6.01497	0.796932	0.662767
NK.cells	MRGPRA2I	0.497195	-0.43033	0.786214	0.433844	-5.06489	0.850938	0.747898
NK.cells	S100PBP	0.095188	4.405401	0.785523	0.434247	-6.08376	0.791104	0.653964
NK.cells	GAREM1	-0.35011	0.757375	-0.78544	0.434295	-5.21116	0.83581	0.723797
NK.cells	NOL9	0.116291	3.728213	0.785418	0.434308	-5.99694	0.799205	0.666409
NK.cells	KCTD7	0.328127	0.387211	0.785078	0.434506	-5.16178	0.840494	0.731405
NK.cells	CLASP1	0.074731	6.373608	0.784924	0.434596	-6.45227	0.768065	0.619279
NK.cells	LRBA	-0.09236	6.139874	-0.78475	0.434696	-6.5349	0.770762	0.623393
NK.cells	PELI2	-0.2694	2.831902	-0.78469	0.434733	-5.47657	0.810065	0.683554
NK.cells	MMD	0.105142	4.477186	0.784517	0.434833	-6.29355	0.790251	0.652962
NK.cells	MAP4K4	0.082325	7.459224	0.784439	0.434879	-6.75227	0.755674	0.600944
NK.cells	CD207	0.536083	-0.64958	0.784369	0.43492	-4.99979	0.853762	0.752983
NK.cells	GM17173	0.303464	0.150476	0.784342	0.434936	-5.28637	0.843505	0.736455
NK.cells	FBXL18	-0.26256	2.985602	-0.78415	0.435047	-5.60536	0.808191	0.680678
NK.cells	GRAP2	0.103336	4.975054	0.784146	0.43505	-6.57394	0.784359	0.644013
NK.cells	GM46430	0.205992	1.969969	0.784041	0.435112	-5.42503	0.820657	0.700245
NK.cells	IGKV1-35	-0.48424	-0.53717	-0.78399	0.435139	-4.96341	0.852313	0.750738
NK.cells	SLC38A7	0.209758	2.59539	0.783988	0.435143	-5.57735	0.812956	0.688171
NK.cells	BIRC6	0.043102	7.8393	0.783852	0.435222	-6.76769	0.751388	0.594745
NK.cells	KLRE1	-0.15194	1.626605	-0.78384	0.435229	-6.15733	0.824918	0.707002
NK.cells	MOB1A	0.072714	5.970142	0.783715	0.435302	-6.40464	0.772727	0.62653
NK.cells	OIP5	0.219127	2.207869	0.783708	0.435306	-5.52152	0.817719	0.695689
NK.cells	NEFH	0.347495	-0.09246	0.783691	0.435316	-5.26077	0.846606	0.741599
NK.cells	POLR2H	0.091844	4.512761	0.783683	0.43532	-6.13815	0.789828	0.652473
NK.cells	CX3CR1	0.248309	3.268892	0.783654	0.435337	-5.71722	0.80475	0.675456
NK.cells	TMTC3	0.210127	2.514058	0.783478	0.43544	-5.37065	0.813954	0.689816
NK.cells	PLEKHJ1	-0.06295	5.969804	-0.78341	0.43548	-6.44496	0.772731	0.62659
NK.cells	RTTN	0.129903	3.609694	0.783284	0.435553	-5.9921	0.800632	0.669175
NK.cells	AI847159	-0.26395	-0.25702	-0.78328	0.435554	-5.42457	0.848713	0.745098
NK.cells	BDP1	0.080568	5.529011	0.783197	0.435604	-6.34357	0.77786	0.634407
NK.cells	SLC52A3	0.249719	0.451484	0.782993	0.435723	-5.43633	0.839679	0.730702
NK.cells	BNIP3L	0.085129	7.263429	0.782992	0.435724	-6.69711	0.757892	0.604552
NK.cells	RABAC1	0.071746	5.988734	0.782913	0.43577	-6.46019	0.772512	0.626391
NK.cells	ZFP456	0.32853	0.791994	0.782856	0.435803	-5.25366	0.835373	0.723825
NK.cells	AGBL1	-0.26423	4.663698	-0.78268	0.435907	-5.88054	0.788038	0.650005
NK.cells	GM156	-0.29805	-1.54566	-0.78221	0.43618	-5.25463	0.86481	0.772552
NK.cells	AMT	-0.32121	1.565291	-0.78219	0.436191	-5.33929	0.825681	0.708657
NK.cells	OPA3	0.092196	5.128547	0.78218	0.436199	-6.21229	0.782553	0.641771
NK.cells	GPN2	0.155226	2.713988	0.78216	0.43621	-5.71167	0.811505	0.68638
NK.cells	CCND2	0.100702	5.400515	0.782035	0.436283	-6.45713	0.779363	0.636959
NK.cells	NFIA	-0.13143	5.904	-0.78181	0.436413	-6.27319	0.773494	0.62812
NK.cells	CACTIN	-0.12396	3.991663	-0.7818	0.436418	-5.97449	0.796043	0.662465
NK.cells	CMTR1	0.120437	5.166737	0.781775	0.436435	-6.22953	0.782104	0.641152
NK.cells	LY6G5B	-0.27084	1.345565	-0.78171	0.436472	-5.33499	0.828423	0.713081
NK.cells	GRTP1	-0.34941	0.35364	-0.78168	0.436488	-5.14204	0.840921	0.733001
NK.cells	GM36756	0.273433	0.926199	0.781471	0.436613	-5.34511	0.833683	0.721569
NK.cells	IFT57	0.18832	2.891644	0.781362	0.436677	-5.58853	0.809336	0.683259

NK.cells	GM13481	-0.41831	0.231781	-0.78133	0.436695	-5.14056	0.84247	0.735683
NK.cells	HDHD5	0.145856	2.842941	0.78105	0.436859	-5.88972	0.80993	0.684273
NK.cells	PRIM1	-0.1499	4.178781	-0.78101	0.436882	-6.08732	0.793805	0.659305
NK.cells	CATSPER2	0.245837	1.800753	0.780889	0.436954	-5.44663	0.822754	0.704476
NK.cells	RNASET2B	-0.11345	5.139167	-0.78083	0.436989	-6.2856	0.782428	0.642012
NK.cells	A730081D(	-0.17389	3.129627	-0.78078	0.437019	-5.78739	0.80644	0.678981
NK.cells	E430024P1	-0.39753	0.53335	-0.78069	0.437072	-5.13333	0.838642	0.72986
NK.cells	IL15RA	0.196429	2.38833	0.780661	0.437087	-5.71882	0.815497	0.693187
NK.cells	GM20274	-0.178	2.727126	-0.78043	0.437225	-5.64403	0.811344	0.686802
NK.cells	ITGB7	0.091523	4.620993	0.780422	0.437227	-6.55274	0.788544	0.651542
NK.cells	SSR1	0.064078	6.554701	0.780317	0.437288	-6.5005	0.765982	0.617412
NK.cells	MOB4	0.058188	6.832732	0.780306	0.437295	-6.57394	0.762797	0.612652
NK.cells	TEX14	-0.12121	5.64791	-0.78023	0.437339	-6.35622	0.776473	0.633215
NK.cells	NDUFA2	0.063784	7.238115	0.780082	0.437426	-6.63615	0.758179	0.605802
NK.cells	HAUS2	-0.12215	3.755191	-0.78007	0.437435	-5.98494	0.79888	0.667482
NK.cells	SMU1	-0.06919	5.608875	-0.77993	0.437513	-6.37409	0.776928	0.633926
NK.cells	GM43623	-0.44035	-0.49767	-0.77991	0.437528	-5.05735	0.851805	0.751241
NK.cells	GM49189	-0.2895	0.779988	-0.77986	0.437555	-5.29395	0.835525	0.725075
NK.cells	EMC8	-0.09427	4.721373	-0.77948	0.437776	-6.15333	0.787435	0.649898
NK.cells	STARD3NL	0.072822	5.861943	0.779435	0.437805	-6.49403	0.774061	0.629586
NK.cells	GPR108	-0.10505	4.177602	-0.77943	0.43781	-6.08009	0.7939	0.65981
NK.cells	LACTB	-0.08967	5.139278	-0.77933	0.437865	-6.26145	0.782506	0.642398
NK.cells	WDR81	0.147586	3.600126	0.778968	0.438078	-5.75411	0.800866	0.670623
NK.cells	LENG8	0.100691	4.564084	0.778854	0.438146	-6.13856	0.789336	0.652869
NK.cells	SPATA13	0.094226	5.147731	0.778753	0.438204	-6.4189	0.782443	0.642363
NK.cells	TRP53I11	-0.1289	4.565294	-0.77863	0.438277	-6.02052	0.789322	0.652908
NK.cells	TM4SF4	-0.28123	2.168937	-0.7786	0.438294	-5.49452	0.81832	0.697927
NK.cells	ABTB2	-0.12322	7.412469	-0.77853	0.438337	-6.69855	0.756315	0.603181
NK.cells	GCN1	0.088735	4.575371	0.778338	0.438448	-6.11952	0.789203	0.652784
NK.cells	SUMO1	-0.03792	7.992553	-0.77828	0.438479	-6.76838	0.749779	0.593559
NK.cells	BOD1	0.14594	3.243589	0.778271	0.438487	-5.82858	0.805177	0.677447
NK.cells	ARPC3	0.052021	8.451234	0.778165	0.438549	-6.84069	0.744655	0.586064
NK.cells	TMEM219	0.102775	4.624458	0.777818	0.438753	-6.10938	0.788867	0.652086
NK.cells	ARAP2	0.104993	5.924266	0.777602	0.438879	-6.45854	0.773624	0.629016
NK.cells	MRPL1	-0.08095	4.619868	-0.77751	0.438932	-6.16029	0.788929	0.652323
NK.cells	N4BP2L1	-0.11904	4.595001	-0.77747	0.438956	-6.10809	0.789224	0.652788
NK.cells	GM34466	-0.44865	-1.10689	-0.77731	0.439051	-4.95395	0.859934	0.764949
NK.cells	ITGB6	-0.48878	-0.27325	-0.77724	0.439091	-5.09102	0.849326	0.74747
NK.cells	LY75	-0.15912	3.332572	-0.77693	0.439274	-6.21059	0.804387	0.676271
NK.cells	POGK	0.196918	2.454489	0.776924	0.439277	-5.62833	0.815098	0.692995
NK.cells	TGM2	0.283928	4.674438	0.776879	0.439304	-5.73156	0.788311	0.651483
NK.cells	GATAD2B	-0.06961	7.433227	-0.77652	0.439512	-6.64484	0.756444	0.603471
NK.cells	RNF31	0.152643	3.162894	0.776454	0.439553	-5.80023	0.806544	0.679645
NK.cells	ACAA1A	0.088807	5.302507	0.776429	0.439568	-6.31997	0.781002	0.640353
NK.cells	POM121	-0.08324	4.883475	-0.77626	0.439665	-6.21372	0.785935	0.647909
NK.cells	TEK	-0.3045	2.175307	-0.77622	0.43969	-5.38787	0.818636	0.698634
NK.cells	CREB3L1	-0.17079	2.035248	-0.77591	0.439871	-5.75969	0.82058	0.701458

NK.cells	FKBP7	-0.26447	1.867273	-0.77569	0.440003	-5.41701	0.822661	0.704782
NK.cells	MRPS31	0.118315	3.653241	0.775668	0.440015	-5.88292	0.800819	0.670624
NK.cells	NIPA2	0.076648	6.042829	0.775403	0.440171	-6.44029	0.772572	0.627583
NK.cells	FAM83E	-0.27042	1.10583	-0.77527	0.440251	-5.35624	0.832166	0.720065
NK.cells	SYN1	-0.42686	0.783746	-0.77526	0.440256	-5.1426	0.836221	0.726537
NK.cells	SLC25A15	-0.20356	2.488304	-0.77509	0.440353	-5.51062	0.814994	0.692966
NK.cells	RAP1GAP	-0.37457	0.58551	-0.77503	0.440391	-5.16599	0.838727	0.730617
NK.cells	GPIHBP1	-0.19283	4.095185	-0.77501	0.440402	-5.8432	0.795512	0.662681
NK.cells	LGALS3BP	0.154008	4.826846	0.774225	0.440863	-6.30631	0.787261	0.649635
NK.cells	UCHL5	-0.08034	5.728606	-0.77404	0.440971	-6.36107	0.776671	0.633529
NK.cells	FLOT1	0.111788	4.575358	0.773993	0.440999	-6.10226	0.790243	0.654199
NK.cells	PPP2R3C	-0.10136	4.333149	-0.77396	0.441022	-6.08517	0.793126	0.658625
NK.cells	CHP2	-0.66496	-0.09865	-0.7739	0.441054	-5.02844	0.847927	0.744985
NK.cells	ACOT11	-0.14849	1.943725	-0.77388	0.441064	-5.76814	0.822186	0.703897
NK.cells	ZFP974	-0.38201	0.877863	-0.77372	0.441162	-5.20114	0.835515	0.725107
NK.cells	PRRT1	-0.20765	0.466797	-0.77367	0.441187	-5.60941	0.840716	0.733432
NK.cells	CENPJ	0.132278	3.331867	0.773376	0.441363	-5.85911	0.805366	0.677427
NK.cells	SH3GL1	0.094295	4.688824	0.772944	0.441617	-6.13724	0.789428	0.652429
NK.cells	KIF1BP	0.119244	3.865166	0.772793	0.441706	-5.94458	0.799294	0.667559
NK.cells	GM44659	0.402223	0.682551	0.772591	0.441825	-5.04462	0.838574	0.729391
NK.cells	AU040320	0.104898	4.589216	0.77258	0.441831	-6.1242	0.790636	0.654295
NK.cells	ELAC1	0.189982	1.710286	0.772234	0.442035	-5.48245	0.825715	0.709015
NK.cells	GBE1	-0.09772	6.483019	-0.77211	0.442107	-6.49738	0.768516	0.620876
NK.cells	DEAF1	-0.11938	3.281829	-0.77202	0.44216	-5.9027	0.806388	0.678766
NK.cells	SNAP23	0.081509	6.222753	0.772009	0.442168	-6.39108	0.771521	0.625413
NK.cells	PLXNB3	0.397353	0.164762	0.771973	0.442189	-5.08278	0.845203	0.7402
NK.cells	PTCD2	0.095414	4.58679	0.771861	0.442255	-6.15583	0.790709	0.654606
NK.cells	ZFP521	-0.39755	2.100385	-0.77175	0.442322	-5.27437	0.820874	0.701578
NK.cells	IRF2BP2	0.068702	7.59206	0.771515	0.442459	-6.64533	0.755861	0.602219
NK.cells	ZFYVE26	-0.10635	5.03643	-0.77149	0.442476	-6.13212	0.785391	0.646626
NK.cells	WRB	0.27489	1.437969	0.771295	0.442589	-5.31009	0.829121	0.714758
NK.cells	KLHL24	0.082842	6.228518	0.771291	0.442591	-6.43791	0.771461	0.625555
NK.cells	GM48086	0.233076	1.511775	0.770997	0.442764	-5.45261	0.828398	0.713369
NK.cells	BZW1	-0.04696	7.459579	-0.77079	0.442888	-6.67702	0.757642	0.604587
NK.cells	GTPBP1	-0.09362	4.641527	-0.7704	0.443116	-6.22861	0.790646	0.654058
NK.cells	TANGO2	0.161976	3.93274	0.770058	0.443318	-5.97225	0.799193	0.667156
NK.cells	PPDPF	-0.07363	5.630234	-0.76995	0.443385	-6.36816	0.779061	0.636404
NK.cells	HIST1H4I	0.160468	4.409355	0.769928	0.443395	-6.05551	0.793484	0.658408
NK.cells	SLC19A1	0.285763	1.065999	0.769881	0.443423	-5.32447	0.834477	0.722597
NK.cells	CYBC1	0.11524	4.562008	0.769205	0.443822	-6.09	0.792135	0.655945
NK.cells	SNX33	-0.2923	0.759185	-0.7691	0.443884	-5.2588	0.838849	0.729177
NK.cells	SREBF1	-0.11969	3.760907	-0.76908	0.443893	-5.95535	0.801738	0.670787
NK.cells	IFT43	-0.25644	1.54627	-0.76891	0.443997	-5.33005	0.828944	0.713482
NK.cells	PRNP	0.242744	2.012971	0.768858	0.444026	-5.43515	0.82313	0.704285
NK.cells	RRP12	0.171556	2.60063	0.768743	0.444095	-5.6557	0.815871	0.692895
NK.cells	TMEM94	-0.14867	2.610495	-0.76864	0.444153	-5.65964	0.815749	0.692755
NK.cells	PPIP5K2	0.092132	4.496011	0.767849	0.444623	-6.14797	0.793058	0.6576

NK.cells	DMAP1	0.174454	2.496634	0.767657	0.444736	-5.59673	0.81729	0.695309
NK.cells	GATA3	-0.15709	1.116393	-0.7676	0.444772	-5.94039	0.834482	0.722552
NK.cells	GALNT10	0.097086	4.614762	0.767534	0.444809	-6.29034	0.791643	0.655572
NK.cells	SARS	0.063738	5.792679	0.767328	0.444931	-6.43275	0.77776	0.634491
NK.cells	CPLANE2	-0.37831	0.331465	-0.76724	0.444981	-5.14769	0.844429	0.738583
NK.cells	GEN1	0.254773	2.372171	0.767123	0.445052	-5.55186	0.818825	0.697918
NK.cells	PSMB7	-0.07186	5.624583	-0.76706	0.445091	-6.36268	0.779724	0.637519
NK.cells	RNF25	0.143042	3.103588	0.767024	0.445111	-5.75573	0.809851	0.683862
NK.cells	SIRPB1C	-0.50825	1.778958	-0.76699	0.44513	-5.19379	0.826182	0.709525
NK.cells	GM16337	-0.17611	1.967439	-0.76698	0.445139	-5.76694	0.823836	0.705817
NK.cells	ANLN	-0.1804	3.747041	-0.76667	0.445319	-5.93646	0.802043	0.671826
NK.cells	RNF26	-0.14803	3.515898	-0.76663	0.445345	-5.86074	0.804838	0.676161
NK.cells	SLC16A12	-0.40716	0.427781	-0.76662	0.445349	-5.13429	0.843202	0.736776
NK.cells	CC2D1A	0.140004	3.044755	0.766537	0.445399	-5.84896	0.810569	0.685089
NK.cells	UCP2	0.076022	9.571709	0.766481	0.445432	-6.9814	0.735003	0.571285
NK.cells	CEP170B	-0.29716	0.457754	-0.7664	0.44548	-5.24269	0.84282	0.736169
NK.cells	ROCK1	-0.05881	8.134703	-0.76628	0.445548	-6.77475	0.750945	0.594586
NK.cells	SRPK3	-0.33382	1.292575	-0.76628	0.445553	-5.18486	0.832266	0.719294
NK.cells	TET1	0.286228	1.135429	0.766224	0.445584	-5.40085	0.834242	0.722442
NK.cells	ARAP3	-0.12553	3.537048	-0.76611	0.445651	-6.12434	0.804582	0.675768
NK.cells	YLPM1	-0.0773	5.660547	-0.766	0.445719	-6.38953	0.779304	0.636981
NK.cells	4933408B1	0.282024	1.941966	0.765818	0.445824	-5.34979	0.824153	0.706472
NK.cells	ATG16L2	-0.08334	5.680082	-0.76574	0.445868	-6.36121	0.779075	0.636674
NK.cells	GM19585	-0.14636	2.331819	-0.7657	0.445894	-6.2474	0.819323	0.698883
NK.cells	SPSB4	-0.42506	0.417547	-0.76542	0.446063	-5.10021	0.843429	0.737196
NK.cells	NOL7	-0.05562	6.906907	-0.76539	0.446078	-6.59512	0.76496	0.615428
NK.cells	MYBL2	-0.19571	3.080165	-0.76485	0.446397	-5.71789	0.810279	0.684721
NK.cells	1700019L1	0.327613	0.774577	0.76485	0.446398	-5.31274	0.838946	0.730047
NK.cells	RAD51	-0.16903	3.891445	-0.76479	0.446431	-6.01732	0.800443	0.669436
NK.cells	ANAPC1	0.090452	4.721072	0.764609	0.446541	-6.19589	0.790518	0.654224
NK.cells	CASP8	0.073222	5.513709	0.764558	0.44657	-6.37879	0.781161	0.639945
NK.cells	APOPT1	0.070454	4.910205	0.764493	0.446609	-6.22795	0.788274	0.650803
NK.cells	ATP6V0D1	0.051189	7.228201	0.764385	0.446673	-6.61588	0.761334	0.610164
NK.cells	ZFP963	0.269725	1.15161	0.764316	0.446714	-5.27958	0.834185	0.722606
NK.cells	SH2D1B1	0.174249	1.695197	0.764174	0.446798	-5.90905	0.827372	0.711835
NK.cells	DNAJC15	0.071956	5.723768	0.764145	0.446816	-6.52761	0.778701	0.636358
NK.cells	GM4221	-0.33766	1.846721	-0.76409	0.446846	-5.17267	0.825483	0.708855
NK.cells	GADD45G	-0.17951	4.465474	-0.76371	0.447073	-5.97646	0.793562	0.659197
NK.cells	DCUN1D4	0.202665	2.540378	0.763657	0.447105	-5.66287	0.816895	0.695473
NK.cells	RHOF	-0.12412	3.558194	-0.76364	0.447113	-6.12226	0.804468	0.676055
NK.cells	TSR3	0.126166	3.569257	0.763625	0.447124	-6.01111	0.804334	0.675847
NK.cells	GM11655	-0.34748	0.316941	-0.76354	0.447171	-5.1725	0.844763	0.739816
NK.cells	ESCO1	-0.07385	5.77786	-0.76285	0.447586	-6.42848	0.778535	0.635866
NK.cells	IGLC2	-0.42925	3.740399	-0.76282	0.447602	-5.87517	0.802745	0.672975
NK.cells	FCHSD1	-0.24587	1.15994	-0.76259	0.447741	-5.38398	0.834579	0.723151
NK.cells	QRSL1	-0.15998	3.18623	-0.76255	0.447764	-5.77127	0.80947	0.68354
NK.cells	SPSB1	0.276003	1.685361	0.762532	0.447772	-5.45072	0.827989	0.712676

NK.cells	KIF21A	-0.37218	0.515306	-0.76208	0.448041	-5.17517	0.842976	0.736343
NK.cells	BC004004	0.080205	5.147988	0.76167	0.448284	-6.24397	0.786153	0.64741
NK.cells	GM15503	0.217395	0.81285	0.761623	0.448312	-5.40916	0.839198	0.730399
NK.cells	DDX10	0.070465	5.436521	0.76155	0.448355	-6.3788	0.782754	0.642274
NK.cells	MRPS22	0.137744	3.085855	0.761391	0.44845	-5.79063	0.810921	0.685718
NK.cells	GPR141	0.364073	2.440611	0.761001	0.448681	-5.59768	0.818843	0.698194
NK.cells	BICRA	0.088378	5.82128	0.760882	0.448752	-6.33154	0.778246	0.635513
NK.cells	CFDP1	0.072824	5.986865	0.760864	0.448763	-6.40908	0.776314	0.63259
NK.cells	IMP4	0.090526	4.46298	0.760744	0.448834	-6.14485	0.794288	0.660016
NK.cells	LAGE3	0.091615	4.771468	0.76068	0.448872	-6.19384	0.790613	0.654399
NK.cells	IDH3G	0.100057	5.124044	0.760631	0.448901	-6.2584	0.786436	0.648007
NK.cells	CTSE	-0.10324	5.492809	-0.76057	0.448939	-6.32752	0.782092	0.641388
NK.cells	NAT10	0.140451	3.270157	0.760391	0.449044	-5.8492	0.808673	0.682361
NK.cells	PALD1	0.331322	1.292537	0.760381	0.44905	-5.28202	0.833144	0.720938
NK.cells	SNAP47	0.151219	2.473639	0.760084	0.449227	-5.73142	0.818436	0.697728
NK.cells	DEPDC1B	-0.20414	2.954227	-0.76007	0.449234	-5.83772	0.81253	0.688463
NK.cells	NOL8	-0.11525	4.078757	-0.7597	0.449456	-6.05921	0.798891	0.667368
NK.cells	POLR2C	0.084654	5.080792	0.759595	0.449517	-6.26099	0.786947	0.649041
NK.cells	SASH3	0.079287	5.4972	0.759521	0.449562	-6.345	0.782041	0.641591
NK.cells	FKBPL	0.300581	1.019839	0.75948	0.449586	-5.25487	0.83658	0.726706
NK.cells	1700096K1	0.183684	2.613489	0.759462	0.449596	-5.63424	0.816713	0.695213
NK.cells	0610043K1	0.344735	1.133363	0.759222	0.44974	-5.31804	0.835148	0.724511
NK.cells	CAR2	0.113622	5.171918	0.759165	0.449773	-6.64339	0.785871	0.647522
NK.cells	FCHSD2	-0.11478	6.823892	-0.75915	0.44978	-6.56185	0.766632	0.618415
NK.cells	ZFP788	0.170817	2.069958	0.759009	0.449866	-5.58076	0.823431	0.705912
NK.cells	NDUFB2	0.074866	5.815553	0.758903	0.449929	-6.41238	0.778313	0.636043
NK.cells	BRF2	0.162888	2.326101	0.758599	0.45011	-5.63663	0.820258	0.700962
NK.cells	JAKMIP1	0.126088	3.981458	0.758511	0.450163	-6.10532	0.800062	0.66941
NK.cells	MAGT1	0.077067	5.872067	0.758505	0.450166	-6.39677	0.777653	0.635089
NK.cells	A130014A(	0.216028	1.887317	0.758273	0.450304	-5.49278	0.825702	0.709566
NK.cells	CACNB2	-0.30042	4.420818	-0.75824	0.450326	-5.73883	0.794792	0.661273
NK.cells	REXO5	0.167219	1.950184	0.758185	0.450357	-5.6986	0.82492	0.708327
NK.cells	BRF1	-0.07743	4.828649	-0.75801	0.450458	-6.24747	0.789934	0.653808
NK.cells	SARS2	-0.13718	2.675724	-0.75799	0.450475	-5.72412	0.815947	0.694179
NK.cells	EVA1B	-0.12865	3.639669	-0.75797	0.450487	-5.99273	0.804187	0.675811
NK.cells	SLC35A4	0.125461	3.561213	0.757862	0.450549	-5.92739	0.805138	0.677312
NK.cells	GM7854	0.411661	0.52802	0.757805	0.450583	-5.14657	0.842814	0.736913
NK.cells	IQCE	0.201672	2.144332	0.757676	0.45066	-5.5959	0.822508	0.704596
NK.cells	ING2	-0.08739	4.920364	-0.75762	0.450693	-6.20666	0.788846	0.65222
NK.cells	ABLIM1	0.083597	6.121137	0.757554	0.450733	-6.52939	0.774752	0.630787
NK.cells	FUOM	0.240658	2.61981	0.757543	0.450739	-5.61776	0.816635	0.695355
NK.cells	ICOS	0.152663	2.283057	0.757219	0.450932	-6.07146	0.82079	0.70202
NK.cells	TGIF2	-0.14086	3.122183	-0.75712	0.45099	-5.80823	0.810478	0.685853
NK.cells	CLEC4N	0.331213	4.093598	0.757099	0.451004	-5.62353	0.798713	0.66756
NK.cells	CENPB	0.085336	5.450808	0.757085	0.451012	-6.34535	0.782586	0.642809
NK.cells	PSEN1	0.061613	6.297037	0.757054	0.45103	-6.5213	0.772711	0.627845
NK.cells	CAMTA1	0.07058	5.835211	0.75693	0.451104	-6.41	0.778083	0.635987

NK.cells	MIF4GD	0.092576	4.964455	0.756899	0.451122	-6.26397	0.788324	0.651604
NK.cells	TFRC	0.093634	6.38061	0.756667	0.451261	-6.35886	0.771743	0.626487
NK.cells	FES	-0.1323	4.284767	-0.75666	0.451264	-6.00054	0.79642	0.664129
NK.cells	NCKAP5LO	0.415135	0.041134	0.756606	0.451298	-5.142	0.849034	0.747303
NK.cells	CRYL1	-0.12903	3.73595	-0.75609	0.451606	-5.91625	0.80322	0.674427
NK.cells	CNOT1	-0.05733	7.026328	-0.75605	0.45163	-6.63426	0.764498	0.615395
NK.cells	SLC35E4	-0.35537	0.657751	-0.75597	0.451679	-5.21579	0.841371	0.734695
NK.cells	PPP1R3F	0.283882	0.867721	0.755929	0.451701	-5.31484	0.838708	0.730422
NK.cells	INPP5D	-0.0758	8.764735	-0.75586	0.451742	-6.86807	0.744875	0.58633
NK.cells	GM45715	0.262518	1.039938	0.755621	0.451885	-5.31213	0.836572	0.726997
NK.cells	TRIT1	-0.10773	4.076961	-0.75542	0.452006	-6.02297	0.799148	0.668112
NK.cells	MDP1	0.087311	4.373314	0.755369	0.452035	-6.18039	0.795594	0.662648
NK.cells	TGFA	-0.24747	0.796415	-0.75529	0.452084	-5.54659	0.839653	0.732013
NK.cells	AAGAB	-0.08958	4.862045	-0.75526	0.452097	-6.203	0.78977	0.65375
NK.cells	SPNS2	-0.30692	1.813184	-0.75489	0.452319	-5.25849	0.826906	0.711761
NK.cells	GPR68	-0.17364	1.433054	-0.75462	0.452482	-5.93697	0.831661	0.719403
NK.cells	FAM135A	-0.26832	2.016455	-0.75447	0.45257	-5.38944	0.824375	0.707879
NK.cells	PTPN9	-0.07883	5.706865	-0.7544	0.452613	-6.44845	0.779847	0.638838
NK.cells	PHYHD1	-0.19306	2.730001	-0.75429	0.452681	-5.62635	0.815556	0.694057
NK.cells	ZFP85	0.367946	0.25725	0.754237	0.452711	-5.1497	0.846554	0.74343
NK.cells	SRSF9	-0.06226	6.428408	-0.7542	0.452734	-6.47898	0.771451	0.626172
NK.cells	SLC39A12	0.597036	0.492802	0.753833	0.452953	-5.08397	0.843548	0.738619
NK.cells	TBCE	0.076908	5.119789	0.753753	0.453	-6.28033	0.786753	0.649427
NK.cells	GLMN	0.151489	2.819036	0.753728	0.453015	-5.77082	0.814463	0.692372
NK.cells	RET	-0.24138	0.727544	-0.75371	0.453025	-5.48365	0.840564	0.73382
NK.cells	PEX5	-0.1003	4.071805	-0.75361	0.453086	-6.03057	0.799246	0.668652
NK.cells	LONP1	0.119955	3.653778	0.753587	0.4531	-5.93607	0.804289	0.676476
NK.cells	HIST1H4H	0.330725	0.513275	0.753521	0.453139	-5.20032	0.843287	0.738199
NK.cells	NEMP2	0.118104	2.854914	0.753487	0.453159	-5.88857	0.814023	0.691687
NK.cells	DUSP7	-0.15114	3.559127	-0.7533	0.453273	-5.87439	0.805436	0.678266
NK.cells	CNIH1	-0.08944	5.17624	-0.75305	0.453419	-6.24779	0.786086	0.648471
NK.cells	GM7030	-0.20864	2.084686	-0.75301	0.453443	-5.79402	0.823527	0.706729
NK.cells	SERPINB9	-0.1284	3.483496	-0.75293	0.453494	-6.40622	0.806353	0.679757
NK.cells	WDFY2	0.123643	5.19857	0.752888	0.453517	-6.09221	0.785822	0.648068
NK.cells	LBHD1	-0.33605	0.16876	-0.75289	0.453518	-5.11789	0.847686	0.745367
NK.cells	SEC61B	0.06102	8.272683	0.752464	0.453771	-6.81995	0.750666	0.595
NK.cells	GNB5	-0.19782	0.819995	-0.75211	0.453981	-5.54387	0.839641	0.732163
NK.cells	GM17655	0.25171	1.183959	0.752009	0.454043	-5.3959	0.835042	0.724797
NK.cells	TTBK2	0.244345	1.314702	0.751915	0.454099	-5.32722	0.833396	0.722168
NK.cells	NMI	0.099783	5.064624	0.751867	0.454128	-6.36455	0.78764	0.650625
NK.cells	GM4356	0.312476	0.868166	0.751812	0.454161	-5.33613	0.839031	0.731195
NK.cells	AP4E1	0.151359	3.305374	0.751676	0.454243	-5.83733	0.808759	0.68334
NK.cells	SFR1	-0.06311	6.44096	-0.75168	0.454243	-6.52964	0.771536	0.626223
NK.cells	CTDP1	-0.08689	4.660739	-0.75166	0.454254	-6.1714	0.792435	0.658042
NK.cells	ZFP422	0.110879	4.395092	0.751443	0.454382	-6.04708	0.795713	0.663008
NK.cells	RSF1OS2	-0.23022	1.663513	-0.75092	0.454695	-5.51703	0.829583	0.715609
NK.cells	COX20	-0.08178	5.583215	-0.75037	0.455022	-6.41225	0.782211	0.641803

NK.cells	ACACA	0.093962	5.153719	0.750355	0.455033	-6.30863	0.787272	0.649523
NK.cells	GM28050	0.360562	0.410517	0.750325	0.455051	-5.14373	0.845586	0.741126
NK.cells	GM11099	0.395948	-0.70994	0.749954	0.455273	-5.16189	0.860021	0.764668
NK.cells	RRAGB	0.314721	0.125251	0.749943	0.45528	-5.27581	0.849237	0.747155
NK.cells	TXNRD3	0.233955	1.950944	0.749825	0.455351	-5.40593	0.826156	0.71024
NK.cells	AP3D1	0.070664	4.873786	0.749801	0.455365	-6.20461	0.790591	0.654764
NK.cells	SELP	-0.43914	1.452771	-0.74967	0.455444	-5.29233	0.832387	0.720174
NK.cells	AI480526	0.186301	2.064128	0.749638	0.455463	-5.62847	0.824747	0.708042
NK.cells	AFF3	0.107206	7.930696	0.749552	0.455514	-6.73383	0.755173	0.601414
NK.cells	COX6A2	-0.41483	2.070766	-0.74945	0.455575	-5.24141	0.824665	0.70797
NK.cells	DVL3	0.125782	3.300094	0.749435	0.455585	-5.83757	0.80953	0.68417
NK.cells	RUNX2	0.149191	4.240789	0.74905	0.455815	-6.31275	0.798247	0.666658
NK.cells	SAP25	-0.23494	2.354647	-0.74892	0.455894	-5.54101	0.821242	0.702616
NK.cells	2810004N1	0.104501	4.315225	0.748909	0.4559	-6.10804	0.797354	0.665313
NK.cells	TBCA	0.06133	7.625569	0.748897	0.455907	-6.75064	0.75872	0.60674
NK.cells	NEURL1B	-0.28781	0.43588	-0.74872	0.456014	-5.2569	0.84544	0.741132
NK.cells	CCDC173	0.330105	0.767334	0.748606	0.456082	-5.1989	0.84122	0.734391
NK.cells	LSP1	-0.07682	8.230432	-0.74848	0.456159	-6.90382	0.751971	0.59669
NK.cells	PSMG3	0.152236	3.202758	0.747951	0.456475	-5.79353	0.81135	0.686479
NK.cells	CYP3A25	-0.2922	1.898523	-0.74766	0.456649	-5.46907	0.8275	0.711931
NK.cells	CAST	0.058454	6.266263	0.74749	0.456751	-6.5848	0.774882	0.63051
NK.cells	TAF13	-0.09722	4.566883	-0.74709	0.456994	-6.17592	0.794909	0.661205
NK.cells	IL23A	0.513272	-0.31658	0.74695	0.457076	-5.04217	0.855638	0.757416
NK.cells	MCTS1	0.062364	6.207891	0.746938	0.457083	-6.51832	0.77556	0.63173
NK.cells	HNRNPK	-0.0431	8.860645	-0.74669	0.457232	-6.90615	0.745372	0.5868
NK.cells	SPECC1	0.216432	4.276803	0.746655	0.457252	-5.88067	0.798384	0.666659
NK.cells	WFDC17	0.358799	5.906168	0.746644	0.45726	-6.16678	0.779079	0.637095
NK.cells	ADCY3	-0.23364	3.001893	-0.74664	0.457263	-5.79545	0.813853	0.690737
NK.cells	POC5	0.168103	2.510705	0.746208	0.457522	-5.63087	0.819898	0.700367
NK.cells	GM47283	0.16632	7.990664	0.746204	0.457524	-6.77672	0.755126	0.601288
NK.cells	NOP56	0.092505	4.591451	0.746178	0.457539	-6.18712	0.794616	0.660966
NK.cells	NFU1	0.109538	4.549473	0.745744	0.4578	-6.10434	0.795117	0.661863
NK.cells	IFI207	0.328504	4.721848	0.745704	0.457824	-5.64682	0.79306	0.658716
NK.cells	SAT2	-0.51905	-0.24549	-0.74556	0.45791	-5.02441	0.854719	0.756348
NK.cells	FAAP20	0.119203	3.120662	0.745536	0.457925	-5.91733	0.812398	0.688814
NK.cells	GDF11	-0.189	1.639394	-0.74552	0.457936	-5.7085	0.83074	0.717783
NK.cells	USB1	0.14634	3.245957	0.745436	0.457986	-5.84438	0.810866	0.686425
NK.cells	SCFD2	0.075183	5.78946	0.745362	0.45803	-6.46342	0.780444	0.639519
NK.cells	WBP11	0.062151	6.346343	0.745284	0.458077	-6.50906	0.773952	0.629703
NK.cells	ANKHD1	-0.06321	7.647791	-0.74519	0.458132	-6.70995	0.759009	0.607302
NK.cells	PDSS1	0.119588	4.315269	0.744896	0.45831	-6.09716	0.797923	0.666398
NK.cells	SP140	0.110941	6.188606	0.744838	0.458345	-6.36657	0.775785	0.632534
NK.cells	TMEM140	0.165043	3.514222	0.744787	0.458375	-5.98659	0.807598	0.681422
NK.cells	CENPQ	-0.09362	5.314475	-0.74461	0.458482	-6.38961	0.78603	0.64815
NK.cells	UTRN	0.075092	7.31422	0.744585	0.458497	-6.75784	0.762808	0.613062
NK.cells	ZFP871	-0.08648	5.249328	-0.74453	0.458533	-6.31982	0.786799	0.649366
NK.cells	TOR1AIP1	-0.06589	7.42657	-0.74443	0.45859	-6.69904	0.761526	0.61122

NK.cells	MCM4	0.110799	5.68459	0.744383	0.458618	-6.43069	0.781674	0.641621
NK.cells	TIMM8A1	0.127103	4.226992	0.74418	0.458741	-6.04463	0.798983	0.668247
NK.cells	SLC10A1	-0.17156	3.248903	-0.74413	0.45877	-5.86982	0.810831	0.686686
NK.cells	ACPP	0.142473	2.739914	0.743963	0.458871	-6.13746	0.817071	0.696524
NK.cells	KLHL7	-0.09038	4.739495	-0.74391	0.458904	-6.23411	0.792849	0.65885
NK.cells	ZC3H11A	0.217015	2.01149	0.743277	0.459285	-5.5199	0.826091	0.711046
NK.cells	URM1	0.098274	4.343407	0.743266	0.459291	-6.04465	0.797585	0.666378
NK.cells	6430550D2	-0.29191	1.142129	-0.74324	0.459304	-5.30083	0.836997	0.728437
NK.cells	GM13483	0.239815	1.85821	0.74323	0.459313	-5.48887	0.828003	0.714083
NK.cells	ELAC2	-0.13836	2.817476	-0.74314	0.459366	-5.79234	0.816117	0.695307
NK.cells	FUS	-0.05032	7.820328	-0.74308	0.459406	-6.73746	0.757052	0.604931
NK.cells	DNAJC1	0.061733	6.915183	0.743035	0.45943	-6.65543	0.767381	0.62037
NK.cells	MGAT5	0.072217	6.916886	0.742987	0.459459	-6.64371	0.767361	0.620368
NK.cells	2510009E0	0.213387	2.702942	0.742817	0.459562	-5.49624	0.817526	0.697627
NK.cells	IL2RB	-0.11957	3.130268	-0.7428	0.45957	-6.45632	0.81228	0.689383
NK.cells	ZFP110	0.108779	4.039873	0.742774	0.459588	-6.04288	0.801235	0.672151
NK.cells	TMEM132A	-0.40524	0.400445	-0.74275	0.459605	-5.0591	0.846421	0.74373
NK.cells	LMO1	-0.23377	1.523387	-0.74243	0.459797	-5.60044	0.832195	0.720918
NK.cells	RARA	-0.08949	5.561492	-0.74242	0.459803	-6.36732	0.78312	0.644302
NK.cells	NUP214	-0.07567	5.2678	-0.74238	0.459826	-6.33832	0.786581	0.649591
NK.cells	DCAF10	-0.07607	5.193751	-0.74238	0.459828	-6.3155	0.787456	0.650932
NK.cells	ABCB1A	0.137904	2.010725	0.742353	0.459841	-6.25811	0.826101	0.711218
NK.cells	XKR5	-0.31857	0.190356	-0.74202	0.46004	-5.16826	0.849249	0.748133
NK.cells	SLC23A3	-0.34368	1.461516	-0.74191	0.460111	-5.39965	0.833109	0.722182
NK.cells	TRDJ1	-0.20634	-1.29553	-0.74188	0.460124	-5.44919	0.868184	0.779615
NK.cells	CALR	-0.07177	7.35156	-0.74151	0.460348	-6.67832	0.762506	0.613116
NK.cells	PLA2G4C	-0.34334	0.890136	-0.7415	0.460357	-5.2568	0.840323	0.733878
NK.cells	GTF2H5	-0.06345	6.547345	-0.74147	0.460371	-6.54317	0.771749	0.626997
NK.cells	RAD54L2	0.083907	4.552757	0.741358	0.460441	-6.20881	0.795208	0.662847
NK.cells	CAMSAP1	0.130117	3.53399	0.741224	0.460522	-5.92545	0.80749	0.681997
NK.cells	PGM2L1	-0.09979	5.192591	-0.74121	0.460531	-6.33259	0.787599	0.651228
NK.cells	CSNK1G3	0.059941	6.624241	0.741017	0.460647	-6.61432	0.77086	0.625877
NK.cells	ZFP444	0.130978	3.63547	0.740916	0.460707	-5.93948	0.806257	0.680211
NK.cells	PGM3	-0.12698	2.89728	-0.74083	0.46076	-5.75729	0.815269	0.694383
NK.cells	ZFYVE27	0.100591	4.063949	0.740787	0.460785	-6.03235	0.801075	0.672216
NK.cells	THAP2	0.140924	3.329281	0.740329	0.461062	-5.8652	0.810306	0.686273
NK.cells	FKBP15	0.080866	5.239594	0.74026	0.461103	-6.29671	0.787358	0.650761
NK.cells	SERPING1	-0.18955	3.004813	-0.73989	0.461328	-5.76342	0.814554	0.692692
NK.cells	E2F1	-0.13007	4.409532	-0.7397	0.461442	-6.07084	0.797591	0.666183
NK.cells	TIMP2	-0.14615	4.873204	-0.73958	0.461514	-6.28274	0.792051	0.657682
NK.cells	NELFA	0.080465	4.630912	0.739388	0.46163	-6.17594	0.79494	0.662154
NK.cells	RIOK1	0.062747	6.007255	0.739372	0.46164	-6.5177	0.778677	0.637262
NK.cells	2200002D0	0.364345	1.337585	0.73921	0.461738	-5.27148	0.835424	0.725756
NK.cells	STAMBPL1	-0.09435	6.55028	-0.739	0.461864	-6.43995	0.772513	0.627817
NK.cells	GREM2	0.373226	0.454759	0.738654	0.462073	-5.19869	0.846964	0.743958
NK.cells	ASB7	-0.13986	3.936004	-0.73854	0.46214	-5.96333	0.803662	0.675356
NK.cells	GAL3ST1	-0.43277	-0.29084	-0.73837	0.462242	-5.04793	0.856556	0.759591

NK.cells	MTSS1	-0.1232	6.472133	-0.73826	0.462313	-6.3302	0.773623	0.62935
NK.cells	LDB1	-0.09343	4.482765	-0.7382	0.462347	-6.23588	0.79708	0.665198
NK.cells	MOB2	-0.07016	5.416589	-0.73812	0.462398	-6.37495	0.785973	0.648156
NK.cells	GM15738	0.322925	0.402397	0.737911	0.462522	-5.19674	0.847634	0.745185
NK.cells	ZFP507	0.208219	2.176566	0.737911	0.462522	-5.6039	0.825243	0.709339
NK.cells	MRPS26	0.066945	5.167613	0.737664	0.462672	-6.31089	0.788918	0.652698
NK.cells	FAM117B	-0.07496	7.11596	-0.73762	0.462696	-6.72828	0.766195	0.618237
NK.cells	ELP5	0.088892	4.633093	0.737502	0.46277	-6.21346	0.79528	0.662507
NK.cells	NUDT21	-0.06878	6.148811	-0.7374	0.462832	-6.50061	0.777383	0.635133
NK.cells	SRI	0.067713	6.74315	0.737359	0.462856	-6.62243	0.770487	0.62471
NK.cells	ADAM10	-0.05931	7.403441	-0.73702	0.463059	-6.67	0.763031	0.613449
NK.cells	TMCO3	-0.11286	3.656165	-0.73698	0.463084	-5.94589	0.807187	0.680913
NK.cells	SEMA5A	0.407094	1.106724	0.736807	0.46319	-5.18509	0.838808	0.730985
NK.cells	SLC45A1	-0.31563	0.608482	-0.73679	0.463201	-5.13626	0.84514	0.741172
NK.cells	CSF2RB	0.279593	4.838003	0.736465	0.463397	-5.55372	0.793142	0.659005
NK.cells	CELF4	0.333127	0.6776	0.736399	0.463437	-5.19424	0.844446	0.739877
NK.cells	BIVM	0.290429	0.882383	0.736144	0.463592	-5.21133	0.841957	0.735735
NK.cells	UCHL3	-0.0788	6.235223	-0.73593	0.463722	-6.4642	0.776786	0.633977
NK.cells	ADGB	-0.41625	2.335955	-0.73565	0.463889	-5.28392	0.823697	0.706674
NK.cells	SPP1	0.436038	2.353951	0.73556	0.463946	-5.49289	0.823474	0.706321
NK.cells	SKA2	-0.15226	3.293834	-0.73556	0.463946	-5.83865	0.811895	0.688093
NK.cells	OPHN1	-0.28503	2.997504	-0.73555	0.463948	-5.47299	0.815527	0.693789
NK.cells	FANCF	0.216611	1.911025	0.735518	0.463971	-5.57245	0.828991	0.715074
NK.cells	PROCR	0.586907	0.279761	0.73518	0.464176	-5.09991	0.849736	0.748315
NK.cells	GCFC2	0.158174	2.248325	0.735149	0.464194	-5.64608	0.824867	0.708482
NK.cells	PYM1	0.076857	4.692796	0.734779	0.464419	-6.24436	0.795064	0.661903
NK.cells	MTA3	-0.07703	6.109959	-0.73477	0.464422	-6.58707	0.778323	0.636297
NK.cells	RNASEH2C	-0.08173	4.998911	-0.73458	0.46454	-6.3184	0.791415	0.656317
NK.cells	ALYREF2	0.144143	3.243584	0.73453	0.464569	-5.76313	0.81259	0.689179
NK.cells	METTL17	0.151683	2.488504	0.734419	0.464637	-5.73238	0.821886	0.703814
NK.cells	SLFN3	0.254683	1.4477	0.734271	0.464726	-5.40518	0.834887	0.724502
NK.cells	GPT2	-0.28828	1.738624	-0.73421	0.464762	-5.34716	0.831231	0.718669
NK.cells	EMC6	0.0729	5.61708	0.734203	0.464768	-6.3668	0.784101	0.645146
NK.cells	GLP2R	-0.28683	0.940346	-0.73414	0.464803	-5.31575	0.841303	0.734802
NK.cells	WIPI2	-0.07614	5.276718	-0.73412	0.46482	-6.33117	0.788119	0.651307
NK.cells	SASS6	-0.11306	4.42508	-0.73388	0.464963	-6.10812	0.798283	0.666978
NK.cells	KIF9	-0.29157	1.680578	-0.73382	0.464999	-5.27294	0.831972	0.719898
NK.cells	RNF121	0.107974	4.41156	0.733656	0.465099	-6.13488	0.798445	0.667236
NK.cells	SNRK	-0.09175	4.865356	-0.73351	0.465185	-6.26003	0.793017	0.658882
NK.cells	CCT2	0.063545	6.286032	0.733236	0.465354	-6.50965	0.776283	0.633384
NK.cells	PPP4R3A	-0.04705	6.59174	-0.73306	0.465461	-6.58795	0.772732	0.628019
NK.cells	POLA1	-0.11526	5.968201	-0.73291	0.465553	-6.44759	0.779992	0.639022
NK.cells	BPTF	-0.06156	7.356344	-0.73288	0.46557	-6.69086	0.763932	0.614798
NK.cells	BRAT1	-0.19601	2.183649	-0.73274	0.465658	-5.51488	0.825684	0.710018
NK.cells	NEK11	0.381134	0.082898	0.73251	0.465795	-5.12009	0.85228	0.752704
NK.cells	PDCD4	0.063186	7.282332	0.73241	0.465855	-6.78418	0.764779	0.616092
NK.cells	TK2	-0.12896	3.825305	-0.73237	0.465879	-5.97694	0.805518	0.678331

NK.cells	LCMT1	0.12467	3.124061	0.732364	0.465883	-5.89402	0.814066	0.691698
NK.cells	SLC48A1	0.091623	5.074284	0.732096	0.466046	-6.16184	0.790531	0.655218
NK.cells	SLC50A1	0.082447	5.247798	0.732046	0.466076	-6.36153	0.788474	0.652061
NK.cells	SAPCD2	-0.21047	1.790194	-0.73202	0.46609	-5.59244	0.830598	0.717912
NK.cells	PAQR7	0.232887	1.552734	0.731983	0.466115	-5.52174	0.833578	0.722667
NK.cells	OAT	-0.09904	5.245936	-0.73198	0.466115	-6.26027	0.788496	0.652095
NK.cells	PPP2R5C	-0.04413	7.456887	-0.73188	0.466175	-6.73504	0.762783	0.613186
NK.cells	MAST2	-0.09672	5.283184	-0.73179	0.466229	-6.29939	0.788055	0.651477
NK.cells	4931423N1	-0.35115	0.653443	-0.73179	0.466234	-5.22248	0.844968	0.741016
NK.cells	MYO18A	-0.09843	4.439945	-0.73155	0.466378	-6.09142	0.798215	0.666992
NK.cells	RAB39	0.351122	2.115142	0.731461	0.466432	-5.24833	0.826652	0.711593
NK.cells	TRAK2	-0.08548	5.153104	-0.73119	0.466598	-6.30938	0.789872	0.653919
NK.cells	HOOK1	-0.15568	2.667358	-0.73099	0.466719	-5.74489	0.820055	0.700759
NK.cells	MECR	0.122279	3.581314	0.730756	0.46686	-5.93873	0.808844	0.683209
NK.cells	CLEC4A2	-0.31316	3.416946	-0.73058	0.466965	-5.40299	0.810848	0.686414
NK.cells	1700094D0	-0.23076	1.476408	-0.73049	0.467024	-5.33931	0.834913	0.724531
NK.cells	CYTH1	0.074715	8.072227	0.730471	0.467033	-6.74523	0.756132	0.60303
NK.cells	D330041H0	-0.1652	2.247493	-0.73042	0.467065	-5.66965	0.825261	0.709171
NK.cells	SLC25A17	0.07601	5.308731	0.730188	0.467206	-6.33034	0.788106	0.651362
NK.cells	EDF1	0.057087	7.047067	0.730171	0.467216	-6.66014	0.767823	0.62059
NK.cells	MROH1	0.10966	4.199162	0.730075	0.467274	-6.09785	0.80136	0.671796
NK.cells	WDR41	0.097978	4.001344	0.73001	0.467314	-6.05453	0.803748	0.675505
NK.cells	ZFP358	0.157822	3.044691	0.729889	0.467388	-5.74892	0.815418	0.69373
NK.cells	TASP1	0.120469	3.918389	0.729724	0.467488	-6.12101	0.80482	0.677122
NK.cells	F11R	-0.19769	3.432174	-0.72955	0.467595	-5.71902	0.810733	0.686391
NK.cells	GM16286	0.066906	5.859685	0.729425	0.46767	-6.43782	0.781682	0.641565
NK.cells	GM15675	-0.2655	2.317552	-0.72878	0.468061	-5.44988	0.824461	0.708268
NK.cells	ZFP949	-0.14778	2.594224	-0.72873	0.468095	-5.80124	0.82103	0.702861
NK.cells	EPHB4	-0.46386	0.794939	-0.72863	0.468156	-5.14341	0.843617	0.738924
NK.cells	NDUFS8	-0.06656	6.232258	-0.72853	0.468215	-6.47632	0.777325	0.635188
NK.cells	GATD1	0.092905	4.394103	0.728391	0.468299	-6.15841	0.799083	0.668547
NK.cells	METTL14	-0.1463	3.09472	-0.72826	0.468382	-5.78611	0.814863	0.693166
NK.cells	DLG2	0.336376	2.402232	0.728153	0.468444	-5.3619	0.823409	0.706674
NK.cells	TMPRSS3	-0.43105	-0.48523	-0.7281	0.468474	-5.02132	0.860087	0.765689
NK.cells	STON1	0.368672	1.087662	0.7281	0.468476	-5.20706	0.839898	0.732975
NK.cells	OST4	0.06975	8.049265	0.727883	0.468608	-6.80423	0.756458	0.603946
NK.cells	PSMD8	0.067988	6.592353	0.727767	0.468679	-6.57076	0.77314	0.628961
NK.cells	2810013P0	-0.13283	4.123121	-0.72776	0.468682	-6.05213	0.802346	0.673718
NK.cells	ZBTB41	-0.14292	2.915286	-0.72776	0.468683	-5.75248	0.817068	0.696747
NK.cells	GM4876	0.15227	1.628342	0.727692	0.468725	-5.73599	0.833074	0.722134
NK.cells	LPIN1	-0.15062	2.690777	-0.72763	0.468761	-5.99358	0.819837	0.701122
NK.cells	GAPVD1	-0.05879	6.832806	-0.72758	0.468792	-6.59841	0.770359	0.624776
NK.cells	TOR1B	0.108622	3.704769	0.727506	0.468838	-5.99192	0.807413	0.68164
NK.cells	DHX58OS	0.339258	0.831273	0.727212	0.469018	-5.28699	0.843155	0.738417
NK.cells	GABPB2	0.060562	6.502447	0.727187	0.469033	-6.551	0.774182	0.630632
NK.cells	COL4A1	0.225573	3.330895	0.727179	0.469037	-5.71146	0.81197	0.68884
NK.cells	TERF2	0.079004	4.800606	0.727133	0.469065	-6.22888	0.794215	0.661231

NK.cells	HUS1	0.221774	1.653586	0.727082	0.469097	-5.44336	0.832757	0.721736
NK.cells	CLEC1A	0.410395	0.972503	0.726861	0.469231	-5.1627	0.841481	0.735589
NK.cells	MCUR1	0.097792	4.462065	0.726592	0.469395	-6.14753	0.79851	0.667566
NK.cells	SH3BGR	0.505637	-0.59926	0.726388	0.469519	-4.94746	0.861834	0.768475
NK.cells	CD34	0.483204	0.953495	0.726239	0.469611	-5.23403	0.841858	0.736126
NK.cells	TAF1	-0.07324	5.699019	-0.72621	0.46963	-6.4464	0.783809	0.645083
NK.cells	RTL5	-0.35909	0.491888	-0.72619	0.469642	-5.08483	0.847745	0.745625
NK.cells	GM9844	0.124712	2.309102	0.725964	0.469778	-5.92524	0.824915	0.708961
NK.cells	ITPRIP	-0.15693	3.468077	-0.72585	0.469847	-5.75794	0.810638	0.686501
NK.cells	GM15726	0.354802	2.074088	0.725767	0.469899	-5.47479	0.827843	0.713649
NK.cells	CEP131	0.318278	1.3043	0.725622	0.469987	-5.34603	0.837549	0.729085
NK.cells	ADCK5	0.222558	1.510315	0.7254	0.470123	-5.46888	0.835045	0.724922
NK.cells	WDR43	0.072872	6.033494	0.725315	0.470174	-6.50551	0.7801	0.63919
NK.cells	AP1AR	0.102421	5.002368	0.725184	0.470254	-6.178	0.792293	0.657805
NK.cells	MAD1L1	-0.07114	5.227133	-0.72506	0.470327	-6.32375	0.789632	0.653708
NK.cells	PPP3CC	0.108808	3.993482	0.724903	0.470426	-6.23851	0.804466	0.676578
NK.cells	CD151	0.185185	2.758187	0.724693	0.470554	-5.59285	0.819591	0.70029
NK.cells	KRIT1	0.065125	5.937975	0.724528	0.470655	-6.48021	0.781324	0.640994
NK.cells	ZFP276	0.152306	2.479933	0.724442	0.470707	-5.70439	0.823035	0.705776
NK.cells	GM15489	0.403162	0.208522	0.724327	0.470777	-5.14192	0.851731	0.751809
NK.cells	PDSS2	0.088527	5.377267	0.724298	0.470795	-6.34596	0.787928	0.651157
NK.cells	PUM2	-0.04803	7.324014	-0.7242	0.470857	-6.68246	0.76526	0.616847
NK.cells	CNOT6L	-0.06362	7.08541	-0.72404	0.470955	-6.70565	0.767999	0.621019
NK.cells	ITPRIPL1	-0.11276	3.28736	-0.724	0.470975	-5.94903	0.813085	0.690274
NK.cells	CITED4	-0.27999	-0.2568	-0.72344	0.471319	-5.51535	0.858241	0.761867
NK.cells	TCF7	0.145544	2.524319	0.723058	0.471553	-6.15572	0.823259	0.705336
NK.cells	ZFP993	-0.23697	1.824761	-0.7228	0.471711	-5.46098	0.832149	0.719268
NK.cells	GRAMD1A	0.102609	4.325204	0.722471	0.471912	-6.21526	0.801574	0.671063
NK.cells	SPATS2L	0.394049	0.9742	0.722134	0.472118	-5.19666	0.843087	0.736757
NK.cells	EMC2	-0.0635	5.762132	-0.72205	0.472171	-6.44716	0.784455	0.644902
NK.cells	ST13	0.055611	6.987135	0.721984	0.47221	-6.61872	0.770177	0.623281
NK.cells	IRF5	-0.13306	5.351662	-0.72193	0.472245	-6.06787	0.789304	0.652325
NK.cells	OTUB2	-0.27527	1.885457	-0.72186	0.472285	-5.34206	0.831575	0.718377
NK.cells	CXCR6	0.143225	1.255073	0.721856	0.472288	-6.07047	0.839521	0.731075
NK.cells	HBEGF	-0.29709	2.789221	-0.72162	0.47243	-5.55853	0.820454	0.700606
NK.cells	DDX39B	-0.06491	7.251694	-0.72092	0.472858	-6.67606	0.767678	0.619039
NK.cells	PHF20	0.064484	6.472356	0.720923	0.472859	-6.51798	0.776695	0.632616
NK.cells	CEP162	-0.12616	3.080056	-0.72087	0.472893	-5.8393	0.817319	0.695269
NK.cells	1110002J0	-0.4592	-0.51304	-0.72058	0.473071	-5.08063	0.862856	0.7684
NK.cells	SPC25	-0.1443	4.372308	-0.7204	0.473181	-6.15817	0.801581	0.670883
NK.cells	EIF3F	-0.05731	7.589953	-0.72036	0.473204	-6.77124	0.763804	0.613407
NK.cells	2610037D(	0.090382	5.44268	0.720318	0.47323	-6.27547	0.788791	0.651194
NK.cells	WDR76	-0.12368	4.504143	-0.72009	0.473372	-6.16269	0.799993	0.668492
NK.cells	GM48623	-0.45694	-0.24558	-0.71997	0.473444	-5.00265	0.859376	0.762858
NK.cells	OTUD1	-0.15008	3.007254	-0.71996	0.47345	-5.75516	0.818219	0.696929
NK.cells	MAEA	0.070837	5.40999	0.719953	0.473453	-6.34215	0.789178	0.651845
NK.cells	ZSCAN22	-0.30367	1.316109	-0.71984	0.473521	-5.28323	0.839349	0.730517

NK.cells	SDF2	0.069819	5.410417	0.719734	0.473587	-6.39809	0.789173	0.651901
NK.cells	ZC3H18	-0.07714	5.270525	-0.71957	0.473686	-6.34951	0.790833	0.654445
NK.cells	MORC2A	-0.10545	4.295558	-0.71955	0.473701	-6.09964	0.802506	0.672454
NK.cells	SEMA4A	-0.09671	3.364224	-0.71937	0.473813	-6.3202	0.813909	0.690241
NK.cells	EXOSC3	0.077702	5.047755	0.718998	0.474038	-6.30699	0.79375	0.658701
NK.cells	GPT	-0.27626	1.203309	-0.71891	0.474094	-5.37066	0.841061	0.733095
NK.cells	SEC22C	-0.18873	2.04121	-0.71885	0.474129	-5.58049	0.830498	0.716245
NK.cells	CPEB3	-0.16499	4.234811	-0.71817	0.474544	-5.96219	0.803519	0.673999
NK.cells	DMAC2L	0.191088	2.257897	0.717418	0.475007	-5.52697	0.827798	0.712297
NK.cells	RRBP1	-0.05744	7.951353	-0.71733	0.475061	-6.75654	0.759949	0.60791
NK.cells	FLT3	0.297117	2.584668	0.717319	0.475068	-5.36435	0.823731	0.705852
NK.cells	DNTT	-0.49105	1.449877	-0.7173	0.475079	-5.24973	0.837947	0.728478
NK.cells	RND1	0.293215	1.145338	0.717285	0.475089	-5.31557	0.841807	0.734669
NK.cells	ARGLU1	-0.04329	7.597485	-0.71724	0.475118	-6.72519	0.763983	0.613927
NK.cells	OXCT1	0.068149	5.719852	0.717175	0.475156	-6.44542	0.785788	0.646871
NK.cells	2700081O1	-0.17187	3.137169	-0.71712	0.475189	-5.65424	0.816903	0.695086
NK.cells	GM17023	0.472741	-0.64235	0.717066	0.475223	-5.10281	0.864844	0.772046
NK.cells	HPS1	0.123254	3.445228	0.71706	0.475227	-5.96932	0.813123	0.689154
NK.cells	2510002D2	0.12522	3.199654	0.717038	0.475241	-5.92663	0.816135	0.693882
NK.cells	STAT1	0.180834	6.871703	0.717029	0.475246	-6.70212	0.772331	0.626459
NK.cells	RGS13	0.491553	-1.05629	0.716839	0.475363	-5.01341	0.87016	0.781011
NK.cells	SGF29	0.07731	4.671195	0.716821	0.475374	-6.24319	0.798264	0.666086
NK.cells	MIR99AHG	0.289729	3.430837	0.716791	0.475393	-5.63033	0.813299	0.689493
NK.cells	NAA35	-0.08099	4.851078	-0.71673	0.475427	-6.22551	0.796108	0.66279
NK.cells	INTS3	-0.10117	3.898156	-0.7167	0.475445	-6.01271	0.807598	0.680606
NK.cells	TMEM170I	-0.10705	4.646846	-0.71657	0.475526	-6.06206	0.798556	0.666605
NK.cells	HLTF	0.091228	4.64409	0.716501	0.47557	-6.27125	0.798589	0.666656
NK.cells	AW146154	0.195817	2.334177	0.716326	0.475678	-5.63141	0.826847	0.710944
NK.cells	DET1	0.198981	2.186379	0.716281	0.475705	-5.595	0.828691	0.713883
NK.cells	UEVLD	0.160693	2.944551	0.715979	0.475891	-5.77734	0.819277	0.699158
NK.cells	NUP107	-0.08551	5.353646	-0.71597	0.475898	-6.32881	0.79012	0.653815
NK.cells	EDEM1	0.076295	6.015715	0.715814	0.475993	-6.46358	0.782307	0.641939
NK.cells	ZKSCAN6	0.183716	2.942031	0.715725	0.476047	-5.64816	0.819308	0.699278
NK.cells	IFI30	-0.12547	6.746076	-0.71572	0.476051	-6.37796	0.773786	0.629018
NK.cells	DCTN5	0.091306	4.598222	0.715695	0.476065	-6.16586	0.79914	0.667778
NK.cells	ZRANB2	0.069355	5.45391	0.715637	0.476102	-6.35813	0.788932	0.652065
NK.cells	UTP18	-0.07475	5.338906	-0.71547	0.476201	-6.3651	0.790295	0.654251
NK.cells	CD82	0.075994	5.476033	0.715377	0.476261	-6.43873	0.78867	0.651791
NK.cells	EEF1AKMT	0.095306	4.588498	0.715337	0.476285	-6.29222	0.799257	0.668096
NK.cells	ZBTB11OS1	0.187256	2.065369	0.715056	0.476458	-5.49882	0.830389	0.716749
NK.cells	SLC22A21	-0.25381	1.348839	-0.71487	0.476574	-5.45532	0.839423	0.731238
NK.cells	CSRNP1	-0.09569	6.641086	-0.71483	0.476599	-6.5866	0.775187	0.631105
NK.cells	PIP5K1A	0.074277	5.595581	0.714647	0.47671	-6.4356	0.787512	0.649767
NK.cells	ZFP54	0.287373	0.794625	0.714467	0.47682	-5.32539	0.846551	0.742664
NK.cells	SPATA24	0.203115	2.193104	0.714366	0.476883	-5.63324	0.828877	0.714397
NK.cells	RASL11B	-0.30156	0.665224	-0.71432	0.476911	-5.26736	0.848206	0.745386
NK.cells	EPS15	0.062996	6.183956	0.714159	0.47701	-6.49177	0.780608	0.639343

NK.cells	5430416N(	-0.10314	3.547711	-0.71408	0.477057	-5.98716	0.812153	0.688037
NK.cells	GM12979	0.352441	0.049111	0.71393	0.477151	-5.16141	0.856203	0.758325
NK.cells	GM12367	0.370002	0.051988	0.713743	0.477266	-5.22222	0.856251	0.758333
NK.cells	DUS2	0.142811	3.353616	0.713602	0.477353	-5.88471	0.814657	0.691877
NK.cells	BC002059	0.150408	2.758564	0.71352	0.477403	-5.74164	0.821991	0.703458
NK.cells	SLCO2A1	-0.38418	2.568296	-0.71338	0.477491	-5.43501	0.824351	0.707209
NK.cells	PTPN6	0.083755	6.758702	0.713306	0.477535	-6.53922	0.774031	0.629352
NK.cells	GM12227	-0.23056	1.113465	-0.71314	0.477638	-5.40336	0.8427	0.736473
NK.cells	NPRL3	0.139082	2.892647	0.712964	0.477746	-5.80082	0.820412	0.701008
NK.cells	THBD	0.279375	3.145231	0.712823	0.477833	-5.5656	0.817296	0.696143
NK.cells	GPSM3	0.067792	6.022347	0.71278	0.477859	-6.54732	0.782701	0.642571
NK.cells	NUTF2-PS1	-0.22097	2.848853	-0.71263	0.477953	-5.75318	0.820953	0.701937
NK.cells	REST	0.081606	5.400614	0.712462	0.478055	-6.34615	0.790039	0.653813
NK.cells	DNAJC4	0.131682	3.061383	0.712375	0.478108	-5.83482	0.818329	0.697799
NK.cells	GM26812	-0.43927	0.020142	-0.71236	0.478119	-5.06526	0.856746	0.759342
NK.cells	KIRREL3	-0.28438	1.272544	-0.71213	0.478262	-5.3332	0.840699	0.733386
NK.cells	SLC35F5	-0.16228	3.11698	-0.71208	0.478289	-5.80486	0.817645	0.69672
NK.cells	COX6A1	0.06855	6.768979	0.71192	0.478389	-6.60649	0.773987	0.629395
NK.cells	CLDN10	0.425281	0.159414	0.71192	0.478389	-5.15094	0.854946	0.756439
NK.cells	QDPR	0.076617	5.245358	0.711768	0.478482	-6.33	0.791928	0.656734
NK.cells	RAP1GAP2	0.144603	4.692548	0.711349	0.47874	-6.20123	0.798724	0.666974
NK.cells	PPIL3	0.081914	4.605985	0.711299	0.478771	-6.22119	0.799764	0.668588
NK.cells	A530017D:	0.180209	2.325165	0.711255	0.478799	-5.57196	0.827702	0.712407
NK.cells	BRIP1OS	0.079076	5.408086	0.710998	0.478957	-6.33714	0.790334	0.653881
NK.cells	TM7SF3	-0.11279	3.924878	-0.71075	0.479111	-5.96772	0.808186	0.681606
NK.cells	CSNK1E	-0.09827	4.953409	-0.71075	0.479111	-6.19986	0.795783	0.662368
NK.cells	HIST1H2AF	0.169691	7.597755	0.710412	0.479319	-6.8016	0.765045	0.615477
NK.cells	EARS2	-0.21523	1.644083	-0.71023	0.479429	-5.41791	0.836661	0.726423
NK.cells	2610044O:	-0.14518	2.24732	-0.71014	0.479489	-5.61928	0.829084	0.714367
NK.cells	METTL26	-0.09562	4.639923	-0.70991	0.479629	-6.28354	0.799752	0.66842
NK.cells	MMP12	0.562098	-0.05819	0.709508	0.479876	-5.10628	0.858439	0.761842
NK.cells	GINM1	0.071237	4.990227	0.709395	0.479946	-6.30279	0.795553	0.662062
NK.cells	2610020C(	-0.07145	4.983149	-0.70937	0.479958	-6.33662	0.795638	0.662192
NK.cells	CCDC107	0.088085	4.620705	0.709321	0.479992	-6.20313	0.799983	0.668915
NK.cells	VAV2	-0.14086	4.977829	-0.7091	0.48013	-6.00163	0.795701	0.662383
NK.cells	NFATC1	-0.07018	5.77824	-0.70905	0.480156	-6.5206	0.786194	0.647787
NK.cells	GSTT2	-0.14007	3.554147	-0.70893	0.480232	-5.89575	0.812922	0.689204
NK.cells	AIF1	-0.24151	4.896333	-0.7088	0.480313	-5.85743	0.796676	0.663944
NK.cells	VPS13A	0.060213	6.447058	0.708788	0.480321	-6.55608	0.778346	0.63589
NK.cells	4833418N(	0.2777	1.533706	0.708515	0.480489	-5.35896	0.838055	0.729153
NK.cells	ZFP933	-0.12595	3.490032	-0.70819	0.480689	-5.95832	0.813707	0.690652
NK.cells	CEP112	-0.27312	1.570821	-0.70799	0.480813	-5.43436	0.837586	0.728556
NK.cells	OTULIN	0.052414	6.57022	0.707848	0.480902	-6.60689	0.77691	0.633985
NK.cells	REXO4	0.087121	4.291897	0.707811	0.480924	-6.15316	0.803948	0.675512
NK.cells	BSPRY	-0.19192	0.341542	-0.70771	0.480989	-5.66713	0.853272	0.753982
NK.cells	FAM126B	0.11052	3.772291	0.707675	0.481009	-5.95176	0.810257	0.685405
NK.cells	ORC5	-0.12104	3.474755	-0.70764	0.48103	-5.96749	0.813894	0.691124

NK.cells	BICC1	0.393859	0.332453	0.707599	0.481055	-5.19136	0.853389	0.754228
NK.cells	CSF1R	0.242896	5.549681	0.707599	0.481056	-5.99755	0.788896	0.65235
NK.cells	WFS1	-0.22672	1.279726	-0.70755	0.481084	-5.32469	0.841272	0.734662
NK.cells	MAN2B1	-0.08953	6.851978	-0.70746	0.481144	-6.59297	0.773636	0.629195
NK.cells	A230083N1	-0.35299	0.546139	-0.70735	0.481209	-5.21599	0.85064	0.749883
NK.cells	AU022252	0.179645	2.452615	0.707192	0.481307	-5.58625	0.826523	0.711242
NK.cells	CACNB1	-0.3733	0.846674	-0.70718	0.481317	-5.16408	0.846789	0.74371
NK.cells	ITSN2	0.061976	6.989544	0.707143	0.481337	-6.65395	0.772043	0.626866
NK.cells	TMCC2	0.371663	1.686886	0.707069	0.481383	-5.32012	0.836121	0.726576
NK.cells	EBI3	0.188806	4.387447	0.706947	0.481459	-5.66754	0.802794	0.67402
NK.cells	SNW1	0.05315	6.240739	0.706851	0.481518	-6.52522	0.780758	0.640126
NK.cells	PWWP3A	-0.09721	3.715755	-0.70677	0.481566	-6.06193	0.810947	0.686747
NK.cells	EXOSC7	0.086966	4.629702	0.706744	0.481584	-6.21474	0.799875	0.669497
NK.cells	BCL2L2	0.254134	1.276497	0.706592	0.481678	-5.2747	0.841313	0.735037
NK.cells	NTNG2	0.14105	3.05713	0.706534	0.481714	-6.12508	0.819029	0.699529
NK.cells	CCDC22	-0.12475	3.49155	-0.70644	0.481769	-5.87502	0.813689	0.691125
NK.cells	FXYD5	0.082769	6.974009	0.706315	0.48185	-6.83849	0.772223	0.627269
NK.cells	ZNHIT3	0.115394	3.532147	0.706293	0.481863	-5.93563	0.813191	0.690344
NK.cells	SPTSSA	-0.066	6.822761	-0.70604	0.482021	-6.63748	0.774079	0.629916
NK.cells	LCT	0.368387	0.170332	0.705851	0.482137	-5.1459	0.855596	0.758024
NK.cells	APOO	0.119625	3.509146	0.705664	0.482252	-5.87925	0.813583	0.69089
NK.cells	TNS3	-0.22537	5.235143	-0.70559	0.4823	-5.67726	0.792738	0.658482
NK.cells	2610001J0	-0.12134	3.690163	-0.70556	0.482317	-5.91318	0.811369	0.687419
NK.cells	DHX8	-0.07579	5.129659	-0.70529	0.482481	-6.33455	0.793995	0.660536
NK.cells	RGL2	-0.14183	3.300422	-0.70523	0.482518	-5.81634	0.816143	0.695051
NK.cells	MINDY2	0.064556	6.610817	0.70517	0.482558	-6.53291	0.776542	0.633863
NK.cells	GRM8	0.45661	1.381099	0.705019	0.482652	-5.1843	0.8401	0.733178
NK.cells	PKD1	0.126303	3.003757	0.705012	0.482656	-5.92177	0.819798	0.700831
NK.cells	GM28192	0.289663	0.064757	0.704906	0.482722	-5.2275	0.856962	0.760499
NK.cells	TBRG1	-0.08119	5.618457	-0.70488	0.482736	-6.47445	0.788188	0.651656
NK.cells	TRAPPC10	0.073483	5.170629	0.704622	0.482897	-6.32945	0.79361	0.659831
NK.cells	ITPR1	0.074483	6.992018	0.704463	0.482996	-6.68175	0.772219	0.627207
NK.cells	SERPINA7	-0.35093	0.531403	-0.70446	0.483	-5.23858	0.851054	0.75071
NK.cells	KMT2A	0.072169	6.28827	0.704304	0.483095	-6.54989	0.780452	0.639646
NK.cells	PPIG	-0.04526	6.974522	-0.70405	0.483251	-6.69547	0.772607	0.627604
NK.cells	4930473A0	-0.29164	0.38363	-0.70385	0.483378	-5.2091	0.853232	0.753971
NK.cells	STYK1	0.164946	0.538888	0.703768	0.483426	-5.81322	0.851234	0.750768
NK.cells	MTDH	0.042633	7.470279	0.70302	0.483891	-6.70203	0.767504	0.619263
NK.cells	SGO2A	-0.20231	3.063462	-0.703	0.483906	-5.85322	0.820017	0.700088
NK.cells	LDLR	0.170089	4.577033	0.702876	0.483979	-5.98402	0.801562	0.671244
NK.cells	TNFAIP8L1	-0.13362	2.396525	-0.70267	0.48411	-5.85527	0.828334	0.71332
NK.cells	BLM	0.14077	3.897628	0.702514	0.484204	-6.10726	0.809818	0.684142
NK.cells	PLD3	-0.10674	4.413779	-0.7025	0.484212	-6.23284	0.803555	0.674383
NK.cells	FAM133B	-0.07415	4.911343	-0.70223	0.484383	-6.26925	0.797568	0.66518
NK.cells	PKMYT1	-0.15082	3.261877	-0.702	0.484524	-5.8744	0.817605	0.696514
NK.cells	SERBP1	0.050406	8.529561	0.701974	0.484539	-6.92139	0.755473	0.601519
NK.cells	FBXW2	0.054373	6.193608	0.701895	0.484589	-6.55368	0.782361	0.64193

NK.cells	PMM1	0.162987	3.07058	0.701878	0.484599	-5.72352	0.819964	0.700231
NK.cells	MCM10	0.182494	3.112366	0.701775	0.484663	-5.70458	0.819448	0.699443
NK.cells	RUBCN	0.129564	3.888841	0.701569	0.484791	-5.87496	0.809925	0.684558
NK.cells	ATP2B1	0.06493	8.327101	0.701402	0.484894	-6.89453	0.757763	0.604998
NK.cells	DIS3L2	0.066162	5.906117	0.701375	0.484911	-6.49185	0.785742	0.647176
NK.cells	ADIPOR1	0.075207	7.346972	0.701318	0.484946	-6.70195	0.768955	0.621732
NK.cells	TPD52	0.098578	8.000639	0.701309	0.484952	-6.66253	0.76147	0.610523
NK.cells	1700012D1	-0.22441	1.843803	-0.70102	0.485129	-5.45969	0.835266	0.724667
NK.cells	AFG3L1	0.062231	4.862083	0.700998	0.485145	-6.29076	0.798158	0.666295
NK.cells	EEF1E1	0.097102	4.824221	0.700925	0.48519	-6.25899	0.798613	0.666997
NK.cells	CBLL1	-0.07594	4.917555	-0.70061	0.485386	-6.28621	0.797493	0.66534
NK.cells	RBSN	0.122306	2.910659	0.70057	0.485411	-5.81189	0.821942	0.703576
NK.cells	GM15492	-0.28195	0.742028	-0.70052	0.48544	-5.29727	0.849268	0.747297
NK.cells	GM16341	0.21208	0.863635	0.700516	0.485445	-5.54734	0.84771	0.744777
NK.cells	RENBP	0.133581	4.474184	0.700465	0.485476	-5.91911	0.802826	0.673619
NK.cells	GM46224	0.423024	2.389468	0.700126	0.485687	-5.30303	0.82843	0.713934
NK.cells	FBL	0.072265	6.091074	0.700035	0.485743	-6.50412	0.783572	0.644043
NK.cells	ZFAND2B	0.094392	4.313639	0.699911	0.485821	-6.13246	0.804774	0.676716
NK.cells	MAPT	-0.21878	1.597756	-0.69971	0.485947	-5.47457	0.838379	0.729895
NK.cells	NPC1	0.097492	4.971884	0.699632	0.485994	-6.25798	0.79685	0.664526
NK.cells	SVIL	-0.06654	6.860609	-0.69947	0.486093	-6.62652	0.774584	0.630536
NK.cells	ABHD8	0.14877	2.172201	0.699271	0.486218	-5.77035	0.831148	0.718479
NK.cells	RRAGA	0.081533	5.020687	0.69913	0.486306	-6.29798	0.796266	0.663771
NK.cells	CRLS1	0.105724	3.622878	0.698953	0.486416	-5.94172	0.813181	0.690148
NK.cells	IRAK3	0.269755	4.187779	0.698888	0.486456	-5.67164	0.806299	0.679389
NK.cells	CSNK2A2	0.056255	5.948451	0.698792	0.486516	-6.48686	0.785251	0.646919
NK.cells	GRB2	0.061836	7.898248	0.698725	0.486558	-6.73251	0.762644	0.61276
NK.cells	CAPN15	-0.08087	4.710463	-0.69856	0.486663	-6.29042	0.799987	0.669648
NK.cells	ATRN	-0.07899	5.965865	-0.69836	0.486783	-6.52342	0.785045	0.646672
NK.cells	KATNBL1	0.063045	5.963648	0.698334	0.486801	-6.51619	0.785072	0.646712
NK.cells	STK39	0.129435	3.517793	0.698289	0.486829	-6.28649	0.814469	0.692306
NK.cells	ATP8A2	0.158349	2.725958	0.698197	0.486886	-6.08258	0.82424	0.707744
NK.cells	RALGPS2	0.094965	5.773254	0.698116	0.486937	-6.35351	0.787318	0.650191
NK.cells	ARHGAP6	-0.20923	3.609197	-0.69794	0.487043	-5.73863	0.813349	0.690563
NK.cells	NSUN5	0.176323	2.124735	0.69788	0.487083	-5.58329	0.831743	0.719678
NK.cells	RTEL1	-0.18165	2.682882	-0.69788	0.487085	-5.69593	0.824775	0.708593
NK.cells	AHR	0.131457	4.719836	0.697844	0.487106	-6.34313	0.799874	0.669542
NK.cells	MAG	-0.33197	0.11897	-0.69779	0.487139	-5.27134	0.857304	0.760921
NK.cells	SP1	-0.06286	5.924217	-0.69776	0.487158	-6.4431	0.785536	0.647464
NK.cells	BCLAF3	-0.08284	4.966172	-0.69775	0.487166	-6.2696	0.796918	0.664966
NK.cells	FAM220A.1	0.187066	2.200036	0.697573	0.487275	-5.65765	0.83087	0.718229
NK.cells	CAMKK1	-0.31062	0.590902	-0.69662	0.487869	-5.23765	0.851623	0.751308
NK.cells	PLEKHG1	0.149169	4.398533	0.696562	0.487904	-6.0108	0.804131	0.675833
NK.cells	YPEL5	0.077667	6.583092	0.696504	0.487941	-6.55907	0.778184	0.635977
NK.cells	SZT2	-0.16427	2.318426	-0.69594	0.488291	-5.57657	0.829714	0.716238
NK.cells	GM10563	-0.10738	3.607105	-0.69591	0.488312	-6.12512	0.813763	0.691014
NK.cells	HMGB3	-0.11784	4.658182	-0.6959	0.488316	-6.24824	0.800998	0.671091

NK.cells	HDAC2	0.082733	5.300553	0.695897	0.488318	-6.32829	0.793304	0.659196
NK.cells	SAP30	-0.09413	5.143228	-0.69581	0.488373	-6.26284	0.795181	0.662101
NK.cells	CCL2	-0.44907	3.089272	-0.69578	0.488393	-5.45174	0.820133	0.701061
NK.cells	CKAP5	-0.08794	6.118962	-0.69568	0.488451	-6.51683	0.783619	0.644391
NK.cells	AKR7A5	-0.10452	4.13514	-0.69565	0.488471	-6.07031	0.807323	0.680981
NK.cells	SRM	0.136155	4.842685	0.695647	0.488474	-6.2308	0.79878	0.667699
NK.cells	HPS6	-0.20241	1.22115	-0.69556	0.48853	-5.53265	0.843559	0.738473
NK.cells	KRI1	0.083731	4.367396	0.695546	0.488538	-6.14268	0.804508	0.676592
NK.cells	TNFRSF10E	0.269046	-0.0063	0.695535	0.488544	-5.35335	0.859338	0.764078
NK.cells	HS6ST1	0.124191	4.156867	0.695512	0.488559	-5.9363	0.807059	0.680569
NK.cells	GZMB	0.13272	3.971899	0.695425	0.488613	-6.67908	0.809308	0.684082
NK.cells	NUAK1	-0.26571	3.219187	-0.69502	0.488864	-5.48945	0.818547	0.698642
NK.cells	GALNT11	0.091564	4.120023	0.694946	0.488911	-6.12464	0.807524	0.681349
NK.cells	2210016F1	0.071069	4.954824	0.694911	0.488934	-6.32633	0.797452	0.665707
NK.cells	GM43728	-0.34261	-0.48628	-0.69469	0.489071	-5.11743	0.865611	0.774543
NK.cells	ZMYND15	0.513972	0.109664	0.694596	0.48913	-5.11145	0.857853	0.761896
NK.cells	SIDT2	-0.07808	5.063847	-0.69452	0.489176	-6.35984	0.796147	0.663848
NK.cells	UBE2L6	0.25302	4.64931	0.694452	0.48922	-5.82608	0.801122	0.671574
NK.cells	ERICH1	-0.12891	3.61294	-0.69444	0.48923	-5.88952	0.813709	0.691229
NK.cells	P3H2	-0.43553	1.190669	-0.69438	0.489262	-5.33228	0.843966	0.739433
NK.cells	NSUN6	0.107214	3.821373	0.694323	0.4893	-6.09707	0.81116	0.687297
NK.cells	FGL2	-0.13638	4.054695	-0.69416	0.489403	-6.51153	0.808318	0.682912
NK.cells	SERPINE2	-0.23593	1.29875	-0.69411	0.489432	-5.5004	0.84259	0.737328
NK.cells	ZFAND2A	0.09182	3.630572	0.69376	0.489651	-6.10844	0.813745	0.691165
NK.cells	AATK	0.39163	0.48294	0.693445	0.489848	-5.15075	0.853331	0.754452
NK.cells	NDUFA10	0.061466	6.050526	0.693089	0.49007	-6.52313	0.784717	0.646308
NK.cells	RSPRY1	-0.06388	5.833379	-0.6929	0.490187	-6.43903	0.787278	0.65033
NK.cells	E230032D2	0.231876	1.879678	0.692794	0.490254	-5.46983	0.835533	0.726029
NK.cells	STAMBP	0.132808	3.374422	0.692751	0.490281	-5.89056	0.816924	0.696468
NK.cells	ZFP971	-0.1673	1.911606	-0.6927	0.490312	-5.60929	0.835131	0.725392
NK.cells	LRRC40	-0.12284	3.249731	-0.69238	0.490511	-5.89375	0.818459	0.699023
NK.cells	ZBTB12	0.210056	1.665552	0.692307	0.490559	-5.43058	0.838236	0.730529
NK.cells	PCOLCE	-0.32242	0.911461	-0.69226	0.490591	-5.26711	0.847828	0.746006
NK.cells	RAB11B	0.050572	7.147669	0.692231	0.490607	-6.68505	0.771917	0.627166
NK.cells	PRR3	-0.09503	3.987386	-0.69222	0.490611	-6.14143	0.809422	0.684844
NK.cells	METTL6	-0.09827	4.505421	-0.69222	0.490611	-6.14117	0.80314	0.67504
NK.cells	GM48236	-0.29699	0.1418	-0.69184	0.490852	-5.35546	0.857738	0.762315
NK.cells	CCDC163	0.201682	2.446867	0.691825	0.49086	-5.66362	0.828419	0.715019
NK.cells	SOX12	0.334825	0.585357	0.691722	0.490925	-5.15091	0.852012	0.752994
NK.cells	MAJIN	0.407752	0.644865	0.691714	0.490929	-5.20384	0.851247	0.75175
NK.cells	TNFSF12	0.126413	2.295218	0.691614	0.490992	-5.95189	0.830314	0.718055
NK.cells	NT5M	0.110807	3.572881	0.691525	0.491048	-5.92886	0.814487	0.692981
NK.cells	PPM1L	-0.11803	4.617569	-0.69152	0.49105	-6.1695	0.801787	0.673121
NK.cells	CNPY4	0.17093	2.433697	0.691473	0.49108	-5.59308	0.828583	0.715295
NK.cells	KATNB1	-0.16402	2.346793	-0.69146	0.491089	-5.74578	0.829669	0.717026
NK.cells	GM43696	-0.1597	2.459243	-0.69094	0.491413	-5.67946	0.828651	0.714904
NK.cells	USP20	-0.11606	2.877475	-0.69081	0.491494	-5.86885	0.823445	0.706635

NK.cells	GM16272	0.309065	0.363126	0.690685	0.491573	-5.23319	0.855275	0.757825
NK.cells	STAT4	-0.10299	5.177658	-0.69048	0.491698	-6.57237	0.795438	0.662956
NK.cells	EXOC6B	0.081472	5.502152	0.690386	0.49176	-6.44876	0.791571	0.657023
NK.cells	TMEM65	0.106346	4.9205	0.69032	0.491801	-6.20713	0.798517	0.667756
NK.cells	DDRGK1	0.067578	5.272107	0.690316	0.491804	-6.38807	0.79431	0.661251
NK.cells	GM10138	-0.22433	2.471458	-0.69023	0.491858	-5.49152	0.828498	0.714897
NK.cells	RAB40C	-0.07878	4.838453	-0.69008	0.491952	-6.30257	0.799545	0.669325
NK.cells	HIPK2	0.076549	6.550097	0.68974	0.492164	-6.58585	0.779415	0.638277
NK.cells	TADA3	-0.1151	3.403164	-0.68971	0.492181	-5.93682	0.817153	0.696714
NK.cells	NDUFAF8	0.070938	5.052226	0.689399	0.492378	-6.31649	0.797343	0.665579
NK.cells	TMEM50B	0.146068	4.107732	0.688953	0.492657	-5.94935	0.809093	0.683513
NK.cells	8030462N1	-0.05117	6.073398	-0.68879	0.492757	-6.50609	0.785595	0.647185
NK.cells	ZFYVE21	-0.19148	2.542215	-0.68869	0.49282	-5.56886	0.828439	0.714076
NK.cells	A530032D1	0.410902	0.594014	0.688507	0.492936	-5.08944	0.853231	0.753881
NK.cells	A230072E1	0.348357	0.278064	0.688271	0.493084	-5.20594	0.857351	0.760608
NK.cells	TOR3A	0.177828	4.075925	0.688148	0.493161	-5.91752	0.809644	0.684386
NK.cells	PCBP4	-0.28899	0.472145	-0.68807	0.493211	-5.2994	0.854842	0.756521
NK.cells	POU2F2	0.145021	5.772063	0.687956	0.493282	-6.26412	0.78927	0.652822
NK.cells	ATP6V1G2	-0.23493	1.237127	-0.68793	0.493299	-5.41551	0.845028	0.740615
NK.cells	ANXA10	0.305699	0.643116	0.687594	0.493509	-5.26775	0.852638	0.752966
NK.cells	DNLZ	-0.11169	3.991855	-0.68759	0.493512	-6.0347	0.810669	0.686016
NK.cells	1190007I0	0.121924	3.503332	0.687538	0.493544	-5.89213	0.816651	0.695429
NK.cells	KYNU	0.175752	3.853994	0.68725	0.493724	-5.76569	0.812352	0.688722
NK.cells	TM2D1	0.057609	5.944576	0.687239	0.493731	-6.49981	0.787229	0.649783
NK.cells	GFER	0.078027	4.406699	0.687224	0.49374	-6.16623	0.805626	0.678207
NK.cells	EP300	-0.0573	6.696726	-0.68718	0.49377	-6.60806	0.778399	0.636318
NK.cells	IDH3B	0.075549	5.850654	0.686612	0.494124	-6.49503	0.78846	0.651597
NK.cells	CMTM4	0.147144	3.141567	0.686596	0.494134	-5.80569	0.821237	0.702633
NK.cells	2810405F1	0.300964	0.687122	0.686581	0.494144	-5.26631	0.852203	0.752251
NK.cells	IPCEF1	0.100107	4.679593	0.686358	0.494284	-6.49643	0.802451	0.673217
NK.cells	GM49602	0.163475	1.89388	0.686256	0.494347	-5.68508	0.836826	0.727483
NK.cells	NUP62	-0.1337	4.181003	-0.68611	0.494441	-6.04887	0.808489	0.68269
NK.cells	DIP2C	-0.14876	6.172429	-0.68592	0.494558	-6.28493	0.784663	0.645893
NK.cells	RAP2A	-0.08911	3.974658	-0.68591	0.494565	-6.23133	0.811003	0.686645
NK.cells	FSCN1	-0.27276	3.378992	-0.68587	0.494591	-5.81484	0.818307	0.698122
NK.cells	TMEM63B	-0.12219	3.925117	-0.68564	0.494735	-6.0061	0.811608	0.687623
NK.cells	C2	-0.28207	2.413631	-0.68563	0.494739	-5.50187	0.830294	0.717157
NK.cells	ENDOD1	0.113089	2.901638	0.685402	0.494884	-5.9804	0.824211	0.707528
NK.cells	TMEM216	-0.1201	4.069162	-0.68538	0.494898	-6.03747	0.809851	0.68491
NK.cells	MTOR	0.082801	4.766611	0.685298	0.494949	-6.18709	0.801402	0.671741
NK.cells	CHST2	-0.29757	-0.02925	-0.68524	0.494985	-5.36913	0.861472	0.767566
NK.cells	CD37	-0.07183	7.409059	-0.68522	0.494998	-6.68369	0.770256	0.624099
NK.cells	TOMM40	0.076824	5.225725	0.685107	0.495069	-6.32474	0.795893	0.66321
NK.cells	EIF4H	0.05051	6.535904	0.684984	0.495146	-6.58639	0.780398	0.639465
NK.cells	DCPS	0.079541	5.004121	0.684971	0.495154	-6.31672	0.798547	0.667326
NK.cells	GM5914	0.132563	3.331997	0.68459	0.495393	-5.85159	0.818886	0.69923
NK.cells	SRP68	0.076937	3.983827	0.684555	0.495415	-6.05163	0.810891	0.686661

NK.cells	BACH2	-0.08258	8.964274	-0.68452	0.495435	-6.99313	0.752554	0.597771
NK.cells	RP2	0.090949	5.226456	0.684477	0.495464	-6.18884	0.795884	0.663315
NK.cells	WDR38	0.261986	0.617809	0.684471	0.495468	-5.26013	0.853095	0.754037
NK.cells	AP4S1	0.071034	4.920297	0.684263	0.495599	-6.31075	0.799655	0.669042
NK.cells	VPS36	-0.06579	5.315956	-0.68375	0.49592	-6.38018	0.795155	0.661849
NK.cells	MED7	0.136376	3.583194	0.683575	0.496031	-5.71284	0.816144	0.694587
NK.cells	CORO1A	0.06425	8.516071	0.683468	0.496098	-6.98375	0.757933	0.605469
NK.cells	CAPN2	-0.07538	4.14309	-0.68338	0.496155	-6.32892	0.809297	0.68385
NK.cells	A330040F1	0.25721	2.508264	0.683352	0.496171	-5.78931	0.829465	0.715677
NK.cells	IL18BP	-0.30674	3.740937	-0.6831	0.496328	-5.68935	0.814208	0.691548
NK.cells	3-Mar	-0.11617	6.392222	-0.68306	0.496357	-6.57162	0.782415	0.642344
NK.cells	PEX6	-0.11333	4.039128	-0.68296	0.49642	-6.0617	0.810563	0.685832
NK.cells	LLGL2	0.146814	2.291993	0.682876	0.496471	-5.80492	0.832174	0.719994
NK.cells	RUVBL2	0.119209	3.693884	0.682808	0.496514	-6.00015	0.814785	0.692454
NK.cells	SPG7	-0.06995	4.467655	-0.68276	0.496544	-6.22751	0.805356	0.6777
NK.cells	ATP8B4	-0.09487	4.781456	-0.68274	0.496559	-6.66963	0.801566	0.671806
NK.cells	CCDC47	0.071274	5.110399	0.682128	0.496941	-6.31594	0.797712	0.665827
NK.cells	C2CD5	0.0835	4.918041	0.68197	0.497041	-6.28698	0.800021	0.66944
NK.cells	HIST3H2A	0.272318	2.104979	0.681945	0.497057	-5.36752	0.834626	0.723947
NK.cells	MIR142HG	-0.05846	8.019491	-0.68163	0.497257	-6.82392	0.763674	0.614148
NK.cells	CSTF1	0.117081	3.243385	0.681441	0.497374	-5.86424	0.820431	0.701506
NK.cells	SGTB	0.277376	0.3418	0.681406	0.497396	-5.30055	0.85713	0.760432
NK.cells	SLC26A11	-0.12402	3.200265	-0.68135	0.497432	-6.09378	0.820964	0.702376
NK.cells	DEGS2	0.239939	0.674225	0.681309	0.497456	-5.46454	0.852839	0.753478
NK.cells	TTI2	0.134108	2.749363	0.681155	0.497554	-5.7333	0.826559	0.711344
NK.cells	MED26	-0.09465	4.429093	-0.68108	0.497603	-6.22281	0.805923	0.678862
NK.cells	BTG2	-0.08201	7.421641	-0.68103	0.497634	-6.72866	0.770536	0.624597
NK.cells	PRAM1	-0.15783	3.279629	-0.68076	0.4978	-5.66707	0.819983	0.700933
NK.cells	NACA	0.048922	9.080227	0.680589	0.49791	-7.01684	0.751667	0.596427
NK.cells	CDC40	0.065176	6.047181	0.680529	0.497948	-6.51463	0.786572	0.648962
NK.cells	TMEM128	0.071036	5.718829	0.680501	0.497965	-6.48754	0.790457	0.654921
NK.cells	COA7	0.098474	3.404389	0.680492	0.497971	-5.96694	0.818445	0.698504
NK.cells	RNF219	0.136285	3.193241	0.680482	0.497977	-5.87429	0.821051	0.70262
NK.cells	CIAPIN1	0.083669	4.643928	0.680438	0.498005	-6.30508	0.803324	0.674843
NK.cells	PTK2B	0.067744	7.134222	0.680258	0.498118	-6.73864	0.773859	0.629689
NK.cells	GTSE1	-0.31507	1.404909	-0.68008	0.498229	-5.46174	0.843485	0.738589
NK.cells	CCNF	-0.16379	3.438612	-0.67991	0.498335	-5.9474	0.818023	0.69807
NK.cells	IPP	0.154239	2.465742	0.679758	0.498433	-5.68689	0.830099	0.717223
NK.cells	GPBP1	0.056949	7.723603	0.679745	0.498442	-6.80714	0.767061	0.619577
NK.cells	TECPR1	0.09147	4.375591	0.67959	0.49854	-6.34884	0.806571	0.68016
NK.cells	KAT6B	0.070939	5.844837	0.679489	0.498603	-6.46071	0.788964	0.65291
NK.cells	ASCC2	0.073849	4.605255	0.679378	0.498673	-6.22132	0.803791	0.675852
NK.cells	MRM2	0.285001	2.071278	0.679347	0.498693	-5.43047	0.83505	0.725224
NK.cells	CAPRIN2	-0.18276	3.181523	-0.67934	0.498696	-5.79291	0.821196	0.70317
NK.cells	PLAU	-0.37676	0.814983	-0.67923	0.498768	-5.28006	0.851028	0.751035
NK.cells	EPO	0.32983	0.415776	0.679206	0.498782	-5.18373	0.856173	0.759415
NK.cells	SPINT2	-0.08607	4.563442	-0.67917	0.498804	-6.25222	0.804296	0.676695

NK.cells	1700123O2	0.081706	4.989927	0.679138	0.498824	-6.28257	0.799157	0.668708
NK.cells	RAB3GAP2	-0.07017	5.259956	-0.6791	0.498851	-6.43176	0.795922	0.6637
NK.cells	WNT5B	-0.2003	1.562043	-0.67887	0.498994	-5.52972	0.841596	0.735742
NK.cells	PRRC2A	-0.06562	5.517966	-0.67878	0.499051	-6.39193	0.792946	0.659077
NK.cells	RNF227	-0.21674	1.852006	-0.6782	0.499415	-5.50911	0.838382	0.73008
NK.cells	1700021F0	0.132205	3.209032	0.678132	0.499459	-5.80293	0.82141	0.703038
NK.cells	SSR2	0.081168	5.189958	0.677902	0.499604	-6.33023	0.797422	0.665324
NK.cells	MATK	0.190955	2.36611	0.677661	0.499756	-5.64841	0.832155	0.719753
NK.cells	XRN1	0.063726	5.924264	0.677554	0.499824	-6.53877	0.78879	0.651908
NK.cells	SMIM27	0.103204	3.590898	0.677444	0.499893	-5.9814	0.816944	0.695719
NK.cells	INO80	-0.06772	6.666636	-0.67736	0.499945	-6.60215	0.780057	0.638663
NK.cells	TFCP2	0.18884	1.971586	0.677241	0.500021	-5.54574	0.837134	0.727855
NK.cells	GM14286	-0.3249	0.211974	-0.6771	0.500112	-5.16314	0.859702	0.764414
NK.cells	THAP7	0.116411	3.607354	0.676849	0.500269	-6.01576	0.816938	0.695658
NK.cells	WRAP53	0.138681	2.788872	0.676504	0.500487	-5.72569	0.827137	0.711724
NK.cells	BBIP1	-0.07042	5.888435	-0.6765	0.500487	-6.4513	0.789469	0.652895
NK.cells	SMS	-0.06112	6.477089	-0.67646	0.500513	-6.55979	0.782529	0.642283
NK.cells	GM13708	0.13067	3.088737	0.676342	0.500589	-5.92726	0.823423	0.705811
NK.cells	THRB	-0.22986	3.815098	-0.67617	0.500699	-5.77661	0.814506	0.691688
NK.cells	ITGB3BP	0.110469	3.52337	0.67609	0.500748	-5.8839	0.81809	0.697326
NK.cells	PGLYRP2	0.150715	2.088786	0.67553	0.501102	-5.91964	0.836033	0.725786
NK.cells	SIRT4	-0.34353	0.56797	-0.67547	0.501142	-5.24112	0.855439	0.757115
NK.cells	MRPL14	0.087079	5.601025	0.675355	0.501212	-6.39842	0.792998	0.658203
NK.cells	CCNI	-0.05506	6.783841	-0.67521	0.501303	-6.63116	0.779053	0.636881
NK.cells	ZFP27	0.244772	1.016419	0.675101	0.501373	-5.35699	0.849668	0.747743
NK.cells	PANK3	-0.07938	4.591077	-0.67509	0.501378	-6.20342	0.805121	0.676972
NK.cells	GM11131	0.245245	0.815809	0.675082	0.501385	-5.46575	0.852244	0.751922
NK.cells	OLFML3	-0.23992	1.284348	-0.67504	0.501413	-5.40295	0.846239	0.742198
NK.cells	ANK2	-0.38868	2.998552	-0.67497	0.501459	-5.49725	0.824649	0.707651
NK.cells	EPHX1	0.167422	3.053436	0.674954	0.501466	-5.80629	0.823967	0.706571
NK.cells	HIST1H4M	-0.28626	1.423329	-0.67473	0.501607	-5.37949	0.844512	0.739429
NK.cells	SOWAHC	-0.30118	2.917305	-0.6747	0.501628	-5.36088	0.825703	0.70934
NK.cells	GNB2	-0.05135	8.428837	-0.67452	0.501741	-6.88446	0.760218	0.608515
NK.cells	PGAM5	0.091474	3.934831	0.674329	0.501862	-6.09668	0.813281	0.689671
NK.cells	EDEM3	-0.09963	5.974164	-0.67425	0.501914	-6.42593	0.788738	0.651618
NK.cells	ZBTB33	0.128769	2.982117	0.674142	0.50198	-5.78207	0.82503	0.70824
NK.cells	HTATSF1	0.073422	4.697996	0.673917	0.502122	-6.27055	0.80412	0.675254
NK.cells	TPM2	0.384982	0.689833	0.673527	0.502369	-5.20913	0.854481	0.755079
NK.cells	HAUS5	0.137733	2.831524	0.673237	0.502553	-5.82184	0.8274	0.711479
NK.cells	FOXK1	-0.07751	4.627409	-0.67303	0.50268	-6.25329	0.805337	0.676814
NK.cells	TMOD3	0.059964	7.218311	0.673023	0.502688	-6.69493	0.77463	0.629714
NK.cells	GM50012	-0.154	1.643382	-0.67299	0.50271	-5.67107	0.842354	0.735401
NK.cells	UGT1A7C	0.381494	-0.45047	0.672888	0.502773	-5.09899	0.869409	0.779445
NK.cells	GPAA1	0.089272	3.764843	0.672809	0.502824	-6.04237	0.815854	0.693306
NK.cells	GARS	0.067838	5.511566	0.672615	0.502946	-6.4492	0.794798	0.66046
NK.cells	CTH	0.208318	3.512521	0.672189	0.503216	-5.96826	0.819051	0.698438
NK.cells	PGGHG	-0.16435	2.820106	-0.67219	0.503217	-5.70291	0.827636	0.712024

NK.cells	GM43063	-0.28235	0.779437	-0.67217	0.50323	-5.2619	0.853504	0.753584
NK.cells	ATP7A	0.072641	5.8288	0.671944	0.503372	-6.41408	0.791025	0.654862
NK.cells	UBL7	0.084818	5.130384	0.671936	0.503377	-6.29376	0.799364	0.66772
NK.cells	KCTD2	-0.11001	3.355478	-0.67183	0.503442	-5.97362	0.82099	0.701605
NK.cells	CD74	-0.22935	11.27972	-0.67142	0.503706	-7.09287	0.729166	0.562835
NK.cells	STARD8	-0.20152	3.359704	-0.67139	0.503724	-5.54155	0.820937	0.701596
NK.cells	PHTF1	-0.09565	4.68503	-0.67138	0.503728	-6.24126	0.804731	0.676187
NK.cells	STRBP	-0.06816	7.195368	-0.67137	0.503737	-6.67364	0.774984	0.630543
NK.cells	GXYLT1	0.072604	5.093219	0.671345	0.503751	-6.37373	0.79981	0.668546
NK.cells	GNPDA2	-0.10673	3.100129	-0.67133	0.503763	-5.91461	0.824153	0.706683
NK.cells	NDUFAF2	0.087454	4.440034	0.67116	0.503868	-6.18928	0.807701	0.68084
NK.cells	SMIM14	0.0546	7.74729	0.670941	0.504007	-6.69663	0.768608	0.621019
NK.cells	SLC5A6	-0.30595	0.868812	-0.6709	0.504031	-5.25005	0.852353	0.752016
NK.cells	CEP44	-0.09267	3.531339	-0.6709	0.504033	-5.96485	0.818819	0.698352
NK.cells	UBE2W	-0.04613	6.081677	-0.67069	0.504168	-6.48094	0.788109	0.650587
NK.cells	GMNN	-0.10609	5.558334	-0.6706	0.504223	-6.42634	0.794323	0.660156
NK.cells	RPUSD3	-0.3158	0.640562	-0.67026	0.504436	-5.27634	0.855382	0.756993
NK.cells	GM44987	-0.38028	-0.34886	-0.67026	0.50444	-5.03252	0.868261	0.77807
NK.cells	ERAP1	0.072348	5.228177	0.670195	0.50448	-6.43424	0.798271	0.666317
NK.cells	KMT2E	-0.04627	8.273581	-0.66997	0.504622	-6.87235	0.762659	0.612171
NK.cells	ZFP280C	-0.08654	4.164903	-0.66993	0.504651	-6.18257	0.811132	0.68637
NK.cells	EEF1AKMT	0.133838	3.325831	0.669874	0.504684	-5.92714	0.821439	0.702587
NK.cells	DHRS11	-0.08761	4.678666	-0.66979	0.50474	-6.4025	0.804889	0.676635
NK.cells	CPSF4L	0.290146	0.499907	0.669752	0.504761	-5.1863	0.8572	0.76003
NK.cells	CDKN2C	-0.13003	3.918847	-0.66918	0.505124	-6.14948	0.814541	0.691303
NK.cells	PIP4K2B	0.091257	4.178712	0.669037	0.505215	-6.1168	0.811362	0.686388
NK.cells	SETD4	0.119744	2.964232	0.668963	0.505262	-5.84861	0.826331	0.710004
NK.cells	ZFP7	-0.30691	0.286794	-0.66893	0.505281	-5.28335	0.860387	0.764867
NK.cells	RHOB	-0.15319	5.891039	-0.6686	0.505495	-6.35681	0.790892	0.654587
NK.cells	ARID3B	0.117431	3.836648	0.668422	0.505605	-6.01537	0.815689	0.693114
NK.cells	PSD4	0.082747	4.541706	0.668391	0.505625	-6.29349	0.807085	0.679644
NK.cells	SPAST	-0.07226	5.207046	-0.66837	0.50564	-6.32888	0.799055	0.667171
NK.cells	9530052E0	-0.23923	1.000649	-0.66822	0.505736	-5.34498	0.851359	0.750006
NK.cells	B630019A1	0.189207	1.596371	0.668018	0.505862	-5.68529	0.843747	0.737721
NK.cells	PPP2R5B	0.224935	1.645269	0.667997	0.505875	-5.56273	0.843125	0.736722
NK.cells	EIF1B	0.060857	5.828759	0.667509	0.506185	-6.46401	0.791877	0.655882
NK.cells	VAPB	0.079636	5.031604	0.667491	0.506197	-6.32282	0.801412	0.670601
NK.cells	AI413582	0.084603	4.68032	0.667456	0.506219	-6.3374	0.805654	0.677194
NK.cells	SEC61G	0.066423	10.02398	0.667374	0.506271	-7.15655	0.743719	0.58364
NK.cells	EIF3C	0.05403	7.172674	0.667033	0.506488	-6.72986	0.776136	0.631894
NK.cells	PACS2	0.084575	4.036916	0.666948	0.506542	-6.15668	0.813544	0.689579
NK.cells	CNST	-0.07843	3.943862	-0.66689	0.506578	-6.17324	0.814683	0.691373
NK.cells	TBC1D31	0.113071	4.166685	0.66657	0.506782	-6.12211	0.811957	0.687173
NK.cells	ARFGEF1	0.063678	6.613928	0.666456	0.506855	-6.6149	0.78266	0.641933
NK.cells	GAS7	0.107676	6.417925	0.666348	0.506924	-6.61244	0.784963	0.645464
NK.cells	CACYBP	0.070216	5.736095	0.666293	0.506959	-6.46865	0.793034	0.657834
NK.cells	EXD2	0.121239	3.156926	0.666288	0.506962	-5.9148	0.824391	0.706797

NK.cells	CHMP4B	0.045092	7.889271	0.66626	0.50698	-6.8431	0.767856	0.619561
NK.cells	ACTR1B	-0.09028	4.215968	-0.66625	0.506985	-6.20326	0.811355	0.68627
NK.cells	RGS12	-0.17853	2.240588	-0.66598	0.507159	-5.57062	0.836029	0.725126
NK.cells	GM43813	0.102783	4.535806	0.665762	0.507297	-6.23597	0.807743	0.680345
NK.cells	STARD9	-0.18399	3.529605	-0.66559	0.507406	-5.64101	0.820071	0.699746
NK.cells	RFC5	0.130926	4.185223	0.66544	0.507501	-6.13272	0.812021	0.687094
NK.cells	CNPPD1	-0.07592	5.686802	-0.66531	0.507584	-6.47259	0.793904	0.659019
NK.cells	NXT2	-0.14675	2.785909	-0.66527	0.507612	-5.63116	0.829308	0.714447
NK.cells	SNAPC4	0.220429	1.856185	0.664917	0.507834	-5.5034	0.841012	0.733201
NK.cells	GM15545	0.222098	1.102359	0.66488	0.507858	-5.34608	0.850631	0.748724
NK.cells	DENND4A	-0.06521	10.03042	-0.66479	0.507913	-7.2517	0.743965	0.584027
NK.cells	IKZF3	0.084313	5.866917	0.664793	0.507913	-6.65865	0.791761	0.655769
NK.cells	MCC	-0.25087	2.092188	-0.66477	0.507931	-5.4502	0.838024	0.728406
NK.cells	CRBN	0.082439	4.060609	0.664514	0.508091	-6.17783	0.813544	0.689656
NK.cells	NMB	-0.16149	1.642171	-0.66446	0.508126	-5.69675	0.843731	0.737649
NK.cells	GM17382	0.385093	0.002886	0.664456	0.508128	-5.1049	0.86487	0.772012
NK.cells	MANBA	0.081631	4.766674	0.664302	0.508226	-6.29177	0.804953	0.67628
NK.cells	CCL25	0.105073	4.40296	0.664149	0.508323	-6.19399	0.809366	0.68322
NK.cells	ARL5B	-0.08516	5.460574	-0.66408	0.508368	-6.48593	0.796605	0.663403
NK.cells	MYCBP	0.09789	4.22905	0.663946	0.508453	-6.09738	0.811486	0.686568
NK.cells	MKRN2	0.075401	4.074932	0.663781	0.508557	-6.12383	0.813369	0.689535
NK.cells	CEP135	0.086557	3.853066	0.663768	0.508566	-6.04567	0.816089	0.693807
NK.cells	USE1	0.074182	5.084893	0.663509	0.508731	-6.33426	0.801113	0.670499
NK.cells	CDV3	0.060202	7.090195	0.663501	0.508736	-6.66683	0.777372	0.634092
NK.cells	CCL3	-0.15072	5.069599	-0.66341	0.508795	-6.60715	0.801297	0.670794
NK.cells	SLC35C1	0.132082	2.749333	0.663353	0.50883	-5.8539	0.829765	0.715562
NK.cells	GM31522	0.308398	-1.00368	0.663267	0.508885	-5.04809	0.878067	0.794199
NK.cells	LLGL1	0.121467	2.998446	0.663196	0.508931	-5.87591	0.826657	0.710643
NK.cells	DLGAP5	-0.16893	3.248568	-0.66299	0.509064	-5.93541	0.823654	0.705748
NK.cells	PIM3	0.089817	4.641176	0.662789	0.50919	-6.37535	0.806668	0.678984
NK.cells	RPN2	-0.06402	6.097946	-0.6626	0.509313	-6.51557	0.789298	0.652095
NK.cells	SET	0.057477	7.946372	0.661975	0.509709	-6.84877	0.768238	0.619603
NK.cells	USF3	0.084534	4.977969	0.66173	0.509865	-6.29081	0.803328	0.673049
NK.cells	MRPS2	0.171898	2.843371	0.661516	0.510002	-5.78953	0.829548	0.714337
NK.cells	FAM13B	-0.06055	6.457749	-0.66143	0.510058	-6.57048	0.785682	0.64597
NK.cells	ACADSB	0.110587	3.270603	0.661364	0.510099	-5.9261	0.824227	0.7059
NK.cells	THEMIS2	0.203695	4.692319	0.661117	0.510256	-5.71105	0.806784	0.678619
NK.cells	SHB	-0.11584	4.498582	-0.66111	0.510258	-6.14381	0.809137	0.682289
NK.cells	GM47230	-0.36692	0.668652	-0.66099	0.510336	-5.13494	0.857208	0.758977
NK.cells	GM36371	-0.2365	0.884673	-0.66093	0.510377	-5.28269	0.854417	0.754437
NK.cells	MPHOSPH	0.089886	3.799303	0.660883	0.510406	-6.06332	0.817693	0.695701
NK.cells	SLC25A25	-0.07537	4.951297	-0.66051	0.510641	-6.36416	0.80365	0.673856
NK.cells	CDCA2	-0.14435	3.79635	-0.66002	0.510959	-6.05886	0.81773	0.696097
NK.cells	RINT1	0.098317	3.771156	0.659705	0.511158	-6.00982	0.81804	0.696675
NK.cells	ZFP667	0.147193	2.726732	0.659649	0.511193	-5.83553	0.831007	0.717218
NK.cells	GRAMD1B	0.090594	5.536445	0.659601	0.511224	-6.52055	0.796618	0.663267
NK.cells	INPP5F	0.109116	4.366787	0.659579	0.511238	-6.13036	0.810743	0.68522

NK.cells	OGT	-0.05548	6.649131	-0.65942	0.51134	-6.63694	0.783431	0.643048
NK.cells	IBA57	-0.15991	1.934328	-0.65941	0.511344	-5.60198	0.840993	0.733212
NK.cells	GADD45GI	0.076448	5.128075	0.659353	0.511383	-6.34466	0.801519	0.670878
NK.cells	GMPS	0.057483	5.784964	0.6593	0.511417	-6.49848	0.793652	0.65872
NK.cells	FBXL4	-0.1607	2.64655	-0.65927	0.511439	-5.67003	0.832012	0.718848
NK.cells	ARPP19	0.049971	6.840626	0.659133	0.511523	-6.67606	0.781186	0.639676
NK.cells	ACOT7	-0.07133	4.076826	-0.65908	0.511557	-6.39361	0.814286	0.690837
NK.cells	MRNIP	0.160577	2.538227	0.659067	0.511566	-5.8087	0.833371	0.721055
NK.cells	RNF185	-0.07689	5.117108	-0.65896	0.511637	-6.28136	0.801651	0.671118
NK.cells	IFT88	0.197492	1.778503	0.658921	0.511659	-5.45147	0.842971	0.73645
NK.cells	NUDT18	0.126971	2.627052	0.658548	0.511898	-5.75547	0.832256	0.719341
NK.cells	PLD2	-0.259	1.042444	-0.65853	0.511909	-5.34761	0.852385	0.751738
NK.cells	RAD17	-0.0699	5.023722	-0.65852	0.511915	-6.30183	0.802776	0.672927
NK.cells	ZFR	0.049703	6.532381	0.658479	0.511942	-6.63135	0.784804	0.645247
NK.cells	STFA3	0.369951	2.751543	0.658343	0.512029	-5.56418	0.830697	0.716919
NK.cells	YDJC	0.244778	1.21958	0.658283	0.512067	-5.42657	0.850109	0.748135
NK.cells	EFR3A	0.055075	5.780301	0.658219	0.512108	-6.55344	0.793707	0.659001
NK.cells	GORAB	0.187039	1.673364	0.658152	0.512151	-5.54204	0.844309	0.738786
NK.cells	PDXK	0.093172	4.441778	0.658098	0.512185	-6.19249	0.809829	0.684022
NK.cells	SPON1	0.218624	1.404705	0.658078	0.512198	-5.72055	0.847738	0.744323
NK.cells	TRDV2-2	0.174108	-1.43025	0.657841	0.51235	-5.3679	0.884336	0.805329
NK.cells	ATXN2	-0.06569	6.28335	-0.65783	0.512354	-6.50847	0.78774	0.649907
NK.cells	PHC2	0.079489	5.798035	0.657804	0.512373	-6.47223	0.793496	0.658752
NK.cells	GM3550	-0.16253	1.752212	-0.6574	0.512633	-5.59579	0.843621	0.737375
NK.cells	SLC17A9	0.164901	2.888147	0.65711	0.512817	-5.8258	0.829385	0.714542
NK.cells	WDR78	0.307107	0.561969	0.657102	0.512822	-5.24511	0.859	0.762271
NK.cells	SPAG5	-0.15841	3.137614	-0.65692	0.512936	-5.91599	0.826317	0.709622
NK.cells	TNFRSF18	0.095457	2.322619	0.656748	0.513049	-6.15506	0.836527	0.725919
NK.cells	ASAP1	0.061413	7.200045	0.656742	0.513053	-6.7508	0.777404	0.633768
NK.cells	TGFBR2	0.056492	7.292661	0.656256	0.513364	-6.71429	0.776573	0.632304
NK.cells	RAPGEF6	0.052228	8.049527	0.655996	0.51353	-6.87004	0.767828	0.619206
NK.cells	MTLN	0.119702	3.060484	0.655795	0.513659	-5.84766	0.827541	0.71155
NK.cells	RBIS	0.071627	5.209656	0.655697	0.513722	-6.37677	0.801216	0.670259
NK.cells	ALAS1	0.140394	3.683631	0.655672	0.513738	-5.92329	0.819813	0.699344
NK.cells	VAC14	0.073544	4.624685	0.65542	0.513899	-6.18997	0.80829	0.681368
NK.cells	CEP152	0.10933	3.629164	0.655356	0.51394	-5.96822	0.820485	0.700526
NK.cells	5830408C2	-0.13371	2.989299	-0.6553	0.513978	-5.76333	0.828429	0.713115
NK.cells	THNSL1	-0.29519	1.077135	-0.65524	0.514014	-5.2938	0.852662	0.752054
NK.cells	RHOT1	-0.06103	5.400436	-0.6552	0.514043	-6.40938	0.798924	0.666828
NK.cells	A730036I1	-0.40111	-0.20546	-0.65519	0.514049	-5.15988	0.869336	0.779318
NK.cells	GM1604A	0.221502	2.804564	0.654749	0.514329	-5.54925	0.830737	0.716939
NK.cells	KIFC1	-0.18825	3.200672	-0.65467	0.514377	-5.93237	0.825796	0.709086
NK.cells	MYO19	-0.24539	1.357151	-0.65467	0.514381	-5.40713	0.849066	0.746386
NK.cells	ZFP874B	0.175043	2.178101	0.654614	0.514416	-5.71243	0.838618	0.729545
NK.cells	DRG2	-0.09892	3.507943	-0.65451	0.514485	-5.96625	0.821984	0.703049
NK.cells	YME1L1	-0.04333	6.181942	-0.6545	0.514488	-6.55566	0.789608	0.652616
NK.cells	COX6B1	0.05526	7.821955	0.654309	0.514611	-6.82283	0.770446	0.623515

NK.cells	MRPS6	0.070854	6.008603	0.654298	0.514619	-6.47723	0.791664	0.655798
NK.cells	SYTL2	0.153553	1.257049	0.654293	0.514621	-6.00809	0.85035	0.748493
NK.cells	POLB	-0.06735	5.332268	-0.65422	0.51467	-6.42129	0.799742	0.668282
NK.cells	AKIP1	0.131334	3.24519	0.654151	0.514712	-5.88889	0.825242	0.708276
NK.cells	PFKP	0.078497	5.538024	0.653978	0.514823	-6.58117	0.797302	0.664512
NK.cells	YAP1	0.383713	1.070556	0.653753	0.514968	-5.24771	0.852776	0.752609
NK.cells	TXK	0.101388	3.287256	0.65371	0.514995	-6.43262	0.824748	0.707647
NK.cells	RAPGEF2	0.08357	7.429198	0.653613	0.515057	-6.76711	0.775014	0.630587
NK.cells	GM42962	0.309336	-0.60259	0.653409	0.515188	-5.11836	0.874599	0.788433
NK.cells	RSF1	-0.05528	7.014648	-0.65332	0.515245	-6.70452	0.77984	0.637942
NK.cells	PGPEP1L	-0.27907	-0.29843	-0.65317	0.515341	-5.26836	0.870588	0.781879
NK.cells	B230206L0	-0.35342	1.02404	-0.65314	0.51536	-5.35515	0.853374	0.753693
NK.cells	IMPA2	0.07632	4.492173	0.653139	0.515361	-6.37495	0.80993	0.684385
NK.cells	KDM5B	0.095052	5.362074	0.652987	0.515459	-6.44882	0.799412	0.668069
NK.cells	PDE5A	-0.19829	2.569114	-0.65296	0.515478	-5.68371	0.833719	0.72207
NK.cells	ZFP472	-0.10864	3.448072	-0.65278	0.515591	-6.00695	0.822773	0.704662
NK.cells	A630052C1	0.332747	0.189023	0.652612	0.515699	-5.19487	0.864222	0.771538
NK.cells	SKI	-0.07316	5.372605	-0.65261	0.515701	-6.43157	0.799304	0.667956
NK.cells	MRPL37	-0.0845	4.2912	-0.65245	0.515806	-6.13563	0.812401	0.688384
NK.cells	HINFP	-0.10703	3.494398	-0.65241	0.515831	-5.96947	0.8222	0.703821
NK.cells	PLCXD2	0.095497	3.46805	0.652164	0.515987	-6.2471	0.822667	0.704442
NK.cells	PRKRA	-0.1091	3.39958	-0.65204	0.516068	-5.89358	0.823536	0.705792
NK.cells	ANXA4	0.127535	3.74233	0.651801	0.51622	-6.03315	0.819432	0.699101
NK.cells	ABHD12	0.068043	5.584506	0.651446	0.516448	-6.48169	0.797081	0.66428
NK.cells	DMXL2	0.33561	2.146865	0.6513	0.516542	-5.32567	0.839395	0.730998
NK.cells	SENP8	0.305831	0.568146	0.651157	0.516634	-5.29465	0.859629	0.763782
NK.cells	GM43259	-0.31326	1.452797	-0.65104	0.516708	-5.31462	0.848228	0.74524
NK.cells	AXIN2	-0.22503	-0.02376	-0.651	0.516734	-5.42712	0.867348	0.776436
NK.cells	INTS1	-0.124	3.347025	-0.65099	0.516739	-5.9415	0.824353	0.706997
NK.cells	A330032B1	-0.40027	-0.15579	-0.6509	0.516802	-5.14977	0.869079	0.779286
NK.cells	RNF13	-0.06976	6.065838	-0.65067	0.516947	-6.45406	0.791344	0.655566
NK.cells	9230116N1	0.252691	0.920334	0.6506	0.516992	-5.35844	0.855071	0.756465
NK.cells	SLC1A3	0.531985	-0.29724	0.650597	0.516994	-5.05994	0.870939	0.782471
NK.cells	STAG1	-0.04396	8.353909	-0.65053	0.517034	-6.90452	0.76469	0.615115
NK.cells	ADCK1	0.119331	3.101425	0.650501	0.517056	-5.94547	0.827407	0.711956
NK.cells	MPV17L	0.191844	2.006778	0.650393	0.517125	-5.55959	0.841171	0.734022
NK.cells	DCTN2	0.067737	5.367124	0.649943	0.517414	-6.40461	0.79995	0.668643
NK.cells	MTHFD2	0.094242	5.486209	0.649919	0.51743	-6.40702	0.798521	0.66643
NK.cells	TRIM65	-0.12952	3.22435	-0.64966	0.517595	-5.8561	0.826164	0.709805
NK.cells	SUV39H1	-0.10357	3.622039	-0.64958	0.517645	-5.98243	0.821233	0.701993
NK.cells	ST6GALNA	-0.12883	3.214358	-0.64953	0.517677	-5.93069	0.826289	0.710002
NK.cells	ITGAM	0.180331	4.619395	0.649424	0.517748	-6.06513	0.809004	0.682773
NK.cells	PRPF4	0.100342	3.92555	0.649374	0.51778	-6.07119	0.81749	0.696088
NK.cells	RASSF5	0.068706	5.485464	0.648961	0.518046	-6.46853	0.79885	0.66661
NK.cells	NLRP3	0.298669	4.527197	0.64868	0.518227	-5.72327	0.810611	0.684725
NK.cells	MORN2	0.205516	2.045949	0.648432	0.518386	-5.48351	0.841549	0.733758
NK.cells	NEDD9	0.074251	8.003683	0.648392	0.518412	-6.88188	0.769505	0.62164

NK.cells	MCPH1	0.078101	4.913551	0.648129	0.518581	-6.29916	0.806045	0.677489
NK.cells	GM15265	-0.20102	1.797023	-0.64811	0.518593	-5.55729	0.844768	0.738865
NK.cells	1700010I1	0.237601	1.215796	0.648002	0.518663	-5.33459	0.852208	0.750928
NK.cells	CASP6	0.106419	3.628567	0.647801	0.518792	-6.00711	0.821774	0.702213
NK.cells	GM3604	0.243324	0.595301	0.647766	0.518815	-5.32567	0.860227	0.764004
NK.cells	STXBP4	0.216708	2.23619	0.647705	0.518854	-5.55934	0.839193	0.729947
NK.cells	UGGT1	-0.06306	5.389379	-0.64731	0.519105	-6.41822	0.80053	0.668746
NK.cells	MFNG	-0.09921	3.090104	-0.64724	0.519156	-6.01801	0.828697	0.712943
NK.cells	RBX1	0.051396	7.700144	0.647141	0.519217	-6.78859	0.773274	0.627117
NK.cells	ZSCAN2	0.269612	1.067334	0.647055	0.519273	-5.40022	0.85436	0.754271
NK.cells	AK3	-0.08551	4.328071	-0.64662	0.519556	-6.17644	0.813512	0.689049
NK.cells	ST3GAL2	-0.14537	3.085043	-0.64658	0.519578	-5.78401	0.828874	0.713302
NK.cells	C030006K1	0.234001	1.498775	0.646541	0.519604	-5.42757	0.848933	0.745474
NK.cells	CHMP5	0.061845	5.632148	0.646505	0.519627	-6.43117	0.797727	0.664503
NK.cells	AMMECR1	-0.08627	4.488762	-0.64642	0.519685	-6.21911	0.811548	0.686017
NK.cells	2310016D2	-0.17923	-0.3578	-0.64623	0.519804	-5.72494	0.873153	0.784994
NK.cells	CYB5D2	0.20301	1.870839	0.645876	0.520033	-5.54798	0.844526	0.738019
NK.cells	BSDC1	0.09	5.056629	0.645324	0.520389	-6.34262	0.80524	0.675612
NK.cells	ZFP74	0.156161	2.132709	0.645315	0.520394	-5.65275	0.84147	0.732881
NK.cells	GM48512	-0.21598	1.068935	-0.64522	0.520456	-5.45221	0.855082	0.754874
NK.cells	4933434E2	0.072483	5.269667	0.645191	0.520474	-6.36528	0.802667	0.671617
NK.cells	EXOG	0.190055	1.745846	0.644984	0.520608	-5.52395	0.846433	0.740894
NK.cells	JPT2	0.129996	3.513173	0.644943	0.520634	-5.93138	0.824188	0.705359
NK.cells	USP24	0.06604	5.459806	0.644697	0.520793	-6.43174	0.800479	0.668194
NK.cells	GPX1	0.0919	11.17498	0.64466	0.520816	-7.19821	0.734948	0.569965
NK.cells	SLC25A43	-0.31897	-0.21871	-0.6444	0.520982	-5.18549	0.87198	0.782611
NK.cells	NFRKB	0.068424	4.478568	0.644334	0.521027	-6.25603	0.812368	0.686802
NK.cells	ANKRD55	0.259092	0.009583	0.644258	0.521076	-5.33784	0.868978	0.77769
NK.cells	LSG1	-0.06845	4.928986	-0.64408	0.521193	-6.3103	0.806887	0.678332
NK.cells	PRAMEF8	-0.13065	3.188085	-0.644	0.52124	-5.89901	0.828298	0.712041
NK.cells	ZPBP	-0.18417	1.648971	-0.64388	0.521323	-5.57307	0.847737	0.743201
NK.cells	INKA1	-0.15718	2.703982	-0.64381	0.521364	-5.69172	0.83436	0.721746
NK.cells	TSPAN9	-0.14832	3.290989	-0.64363	0.521483	-5.95029	0.827015	0.710119
NK.cells	SAFB	-0.05958	6.192591	-0.64354	0.521539	-6.55538	0.791724	0.65505
NK.cells	BOP1	-0.0871	4.167475	-0.64351	0.521557	-6.14198	0.816178	0.693041
NK.cells	GM41790	-0.30014	-0.09939	-0.6434	0.521627	-5.35519	0.870409	0.780357
NK.cells	LSM11	0.180386	2.171179	0.643315	0.521685	-5.6835	0.841087	0.732655
NK.cells	SAMD4B	0.074157	5.014196	0.643109	0.521818	-6.28363	0.805854	0.67699
NK.cells	NRIP3	-0.35392	0.094317	-0.64311	0.52182	-5.27725	0.867866	0.776248
NK.cells	MACO1	-0.06007	6.27208	-0.64304	0.521862	-6.53498	0.790781	0.653713
NK.cells	RNASEK	0.052979	6.735066	0.642977	0.521903	-6.63152	0.785311	0.64536
NK.cells	ZDHHC4	0.078057	4.474275	0.642644	0.522118	-6.26928	0.812649	0.687328
NK.cells	PLEKHG6	-0.22249	-0.11425	-0.6422	0.522406	-5.27603	0.871107	0.780939
NK.cells	USP44	0.333521	-0.6309	0.642095	0.522473	-5.07937	0.877934	0.792301
NK.cells	DPM1	-0.05728	5.979025	-0.64194	0.522575	-6.52848	0.794723	0.65933
NK.cells	SNHG20	-0.12928	2.355617	-0.64193	0.52258	-5.72067	0.839236	0.729289
NK.cells	ABCA8B	-0.31019	1.118002	-0.64188	0.522614	-5.34169	0.855049	0.754817

NK.cells	TMEM39A	-0.07841	4.395391	-0.64169	0.522735	-6.23673	0.813936	0.689088
NK.cells	EFHC1	-0.32171	-0.05796	-0.64128	0.523002	-5.15846	0.870584	0.780081
NK.cells	GM15336	0.153791	1.682133	0.641193	0.523056	-5.71305	0.848014	0.743275
NK.cells	CCAR1	0.046325	6.820278	0.641181	0.523063	-6.65966	0.784958	0.644242
NK.cells	PILRA	0.263296	3.3927	0.641147	0.523086	-5.44934	0.826433	0.708741
NK.cells	STX17	0.1055	4.034327	0.64099	0.523187	-6.10014	0.818531	0.696275
NK.cells	HNRNPH2	0.052686	6.435152	0.640896	0.523248	-6.56661	0.789541	0.651259
NK.cells	TRIM72	-0.30728	0.692552	-0.64053	0.523486	-5.25526	0.860946	0.764111
NK.cells	MTHFS	-0.11764	5.861718	-0.64049	0.523509	-6.54828	0.796483	0.661767
NK.cells	TFE3	0.094839	4.149746	0.640456	0.523533	-6.06536	0.817236	0.694081
NK.cells	TSTD2	0.096087	3.84075	0.640142	0.523736	-6.09184	0.821238	0.700209
NK.cells	TSHZ3	0.191682	2.187789	0.640053	0.523794	-5.83795	0.841942	0.733176
NK.cells	ADORA2B	0.337036	0.37813	0.639852	0.523923	-5.24892	0.865283	0.771053
NK.cells	SMG7	-0.05359	6.385921	-0.63981	0.523951	-6.65239	0.790463	0.652383
NK.cells	KIFC5B	0.185381	1.787655	0.639685	0.524031	-5.59428	0.847091	0.741485
NK.cells	TBPL1	-0.06915	4.928003	-0.63958	0.524102	-6.31849	0.807975	0.679446
NK.cells	SDR39U1	0.216507	1.131364	0.639435	0.524193	-5.50024	0.855551	0.755134
NK.cells	PLOD1	0.143702	3.410314	0.639339	0.524255	-5.92391	0.826658	0.7088
NK.cells	SS18	0.056844	5.946104	0.639061	0.524435	-6.60441	0.795889	0.660554
NK.cells	ZFP658	0.347308	0.297611	0.638979	0.524489	-5.15715	0.866547	0.772936
NK.cells	ANKRD50	-0.11062	2.62264	-0.6387	0.52467	-5.80438	0.836859	0.724628
NK.cells	ALKBH4	-0.13203	2.574916	-0.63848	0.524814	-5.79471	0.837581	0.725646
NK.cells	GM20732	0.085113	4.58993	0.63822	0.52498	-6.14876	0.812624	0.686166
NK.cells	FBRS	-0.07753	4.965395	-0.63817	0.525012	-6.29245	0.808051	0.679062
NK.cells	OAS3	0.277981	2.402295	0.638096	0.525061	-5.75944	0.839831	0.72929
NK.cells	ARL11	-0.20414	1.439587	-0.63774	0.525293	-5.45361	0.852378	0.749147
NK.cells	ZFP710	-0.09875	6.297621	-0.63751	0.525443	-6.36777	0.792421	0.654498
NK.cells	GM49067	-0.30256	0.406337	-0.63736	0.525536	-5.16179	0.865924	0.771075
NK.cells	GAA	-0.13582	3.191942	-0.63728	0.525592	-5.80587	0.830299	0.713625
NK.cells	KLRA3	-0.24227	-0.66191	-0.63706	0.525734	-5.6167	0.880011	0.794277
NK.cells	NIT2	0.129172	3.588667	0.636987	0.525779	-5.94948	0.825355	0.705791
NK.cells	SMG5	0.061968	5.55464	0.636966	0.525793	-6.43984	0.801318	0.668203
NK.cells	SDCCAG8	0.063537	5.914953	0.636634	0.526008	-6.51124	0.797094	0.661646
NK.cells	RNF44	-0.07645	4.833037	-0.63662	0.526017	-6.27279	0.81015	0.681878
NK.cells	CCP110	0.164258	2.37539	0.63655	0.526063	-5.65147	0.840681	0.730185
NK.cells	CXCR4	0.098934	6.146916	0.636276	0.52624	-6.48082	0.794483	0.657502
NK.cells	SLIRP	0.079627	5.558884	0.636162	0.526314	-6.40274	0.801525	0.668382
NK.cells	EIF3G	0.072758	4.982954	0.635856	0.526513	-6.33999	0.808488	0.679228
NK.cells	PIGG	-0.25216	0.963956	-0.63583	0.526529	-5.3455	0.858941	0.759571
NK.cells	CRMP1	0.242038	-1.07546	0.635765	0.526572	-5.40321	0.885682	0.803796
NK.cells	GM30198	-0.33495	1.865066	-0.63576	0.526578	-5.30916	0.847342	0.740786
NK.cells	ATAD3A	0.102264	4.10261	0.635361	0.526834	-6.14222	0.819268	0.696217
NK.cells	EMC9	-0.16542	1.600787	-0.63536	0.526836	-5.63245	0.850735	0.746395
NK.cells	AGBL3	-0.22649	1.72445	-0.63505	0.527034	-5.36008	0.849149	0.743929
NK.cells	NUP37	0.119941	3.351508	0.635004	0.527066	-5.96704	0.828581	0.71103
NK.cells	ACSBG1	0.185157	-0.5064	0.634856	0.527162	-5.62253	0.878237	0.791514
NK.cells	ELOA	0.066455	5.789735	0.634728	0.527245	-6.47715	0.79876	0.664411

NK.cells	HACD1	-0.17872	3.002315	-0.63444	0.527434	-5.64614	0.83295	0.718052
NK.cells	ZFP873	0.221355	0.995152	0.634371	0.527477	-5.34964	0.858545	0.759274
NK.cells	GM12462	0.374421	-0.49881	0.634255	0.527552	-5.05598	0.878136	0.791465
NK.cells	ZFP560	0.088967	4.00302	0.634168	0.527608	-6.09929	0.820496	0.698412
NK.cells	D11WSU47	0.256945	1.299867	0.634073	0.527671	-5.34107	0.854606	0.752979
NK.cells	FCMR	-0.21009	2.105124	-0.63395	0.527753	-5.54774	0.844289	0.736322
NK.cells	MSANTD4	-0.12044	2.89703	-0.63382	0.527834	-5.77369	0.834272	0.720281
NK.cells	SEC24B	0.064482	6.179882	0.633808	0.527842	-6.5756	0.794098	0.657381
NK.cells	NFE2L1	-0.08541	4.98448	-0.63374	0.527886	-6.30086	0.808477	0.679627
NK.cells	ARHGAP27	0.116687	3.664658	0.633687	0.527921	-5.91902	0.824684	0.705062
NK.cells	GTPBP3	0.158389	2.225197	0.633592	0.527983	-5.64922	0.842762	0.733883
NK.cells	DPYD	-0.19382	3.567148	-0.63359	0.527984	-6.00743	0.825896	0.70699
NK.cells	GDI2	0.042804	8.891579	0.633478	0.528057	-6.97245	0.762515	0.609623
NK.cells	ACIN1	-0.04098	7.577859	-0.63337	0.528125	-6.80887	0.777638	0.632354
NK.cells	AA388235	0.284454	0.991788	0.633337	0.528149	-5.38453	0.858588	0.759541
NK.cells	UTP25	0.128039	2.617836	0.633226	0.528221	-5.81417	0.837789	0.725972
NK.cells	ATP5L	0.044991	8.67785	0.633118	0.528291	-6.97336	0.764953	0.61329
NK.cells	GM26810	0.33021	0.423092	0.633052	0.528333	-5.24966	0.865992	0.771633
NK.cells	GM16867	0.18882	2.522419	0.632924	0.528417	-5.72917	0.838994	0.727928
NK.cells	GIT2	0.04972	7.020155	0.632752	0.528529	-6.68915	0.784158	0.642276
NK.cells	MEF2C	-0.10182	7.538963	-0.63269	0.528568	-6.49301	0.778091	0.633074
NK.cells	RNASE6	0.23692	4.491549	0.632646	0.528597	-5.56163	0.81449	0.689133
NK.cells	PLEKHA7	-0.17939	2.084938	-0.63235	0.528791	-5.64484	0.844546	0.736917
NK.cells	RASSF1	-0.05629	5.940086	-0.6323	0.528823	-6.57793	0.79696	0.661948
NK.cells	FBXL19	-0.17316	2.333257	-0.63215	0.528918	-5.6343	0.84139	0.731867
NK.cells	NPAT	0.087059	4.485178	0.632076	0.528968	-6.24874	0.814568	0.689331
NK.cells	KCTD6	0.13727	2.981269	0.632037	0.528994	-5.78106	0.833214	0.718794
NK.cells	TM9SF3	-0.04134	7.288714	-0.63202	0.529007	-6.74775	0.781011	0.637575
NK.cells	CWF19L1	0.1747	2.200341	0.631988	0.529025	-5.61164	0.843078	0.734582
NK.cells	EIF2B5	0.076716	4.703803	0.631958	0.529045	-6.29679	0.811895	0.685167
NK.cells	ACAD9	0.141917	2.542008	0.631837	0.529124	-5.71573	0.838763	0.727667
NK.cells	ZFP511	0.078476	3.816412	0.631626	0.529261	-6.12677	0.822896	0.702407
NK.cells	DGCR8	0.100007	3.779114	0.631483	0.529354	-6.0299	0.823358	0.703176
NK.cells	SESN1	0.066947	5.939971	0.631359	0.529435	-6.53843	0.797051	0.662125
NK.cells	MLLT10	0.043512	7.464033	0.631094	0.529607	-6.76678	0.779051	0.634759
NK.cells	TIPARP	0.078775	7.437182	0.630838	0.529774	-6.74944	0.779365	0.635297
NK.cells	TRABD	0.069357	5.363153	0.630814	0.529789	-6.4007	0.803982	0.673059
NK.cells	GM30239	-0.31475	-0.09316	-0.63067	0.529883	-5.09886	0.872869	0.783354
NK.cells	ITGA2	-0.15011	0.926385	-0.63063	0.529909	-5.84804	0.859533	0.76149
NK.cells	SLC25A24	-0.09905	3.486183	-0.6306	0.529928	-6.16465	0.826996	0.709172
NK.cells	IZUMO1R	-0.20176	0.001693	-0.63032	0.530111	-5.70668	0.871619	0.781422
NK.cells	KLRI2	0.157304	0.447224	0.630275	0.53014	-5.99695	0.865774	0.771817
NK.cells	NANOS3	0.261189	0.427569	0.630001	0.530319	-5.26979	0.866031	0.772313
NK.cells	HEXB	0.073646	5.741415	0.62994	0.530358	-6.43054	0.799429	0.666198
NK.cells	NOS1AP	0.187175	3.163666	0.629927	0.530367	-5.78279	0.831022	0.715756
NK.cells	GATD3A	0.105318	3.544084	0.629792	0.530455	-5.9969	0.826276	0.708271
NK.cells	BEND6	-0.30975	0.170455	-0.62966	0.53054	-5.25504	0.8694	0.777926

NK.cells	CSTDC5	0.245654	5.46156	0.62962	0.530567	-6.22073	0.802795	0.671474
NK.cells	MANEA	0.124362	3.728612	0.629356	0.530739	-5.87906	0.823984	0.704731
NK.cells	CCDC102A	0.129972	2.048525	0.629183	0.530852	-5.99709	0.845105	0.738451
NK.cells	FEM1B	-0.08512	4.728435	-0.62916	0.530869	-6.25927	0.811686	0.685389
NK.cells	TMEM167	0.048168	6.804318	0.629131	0.530886	-6.67278	0.786787	0.646901
NK.cells	TAX1BP3	-0.10129	4.090035	-0.62911	0.5309	-6.08153	0.819515	0.697678
NK.cells	RUSC2	0.339256	0.909936	0.629035	0.530948	-5.24914	0.859747	0.76223
NK.cells	ZFP52	0.085671	3.859767	0.628782	0.531113	-6.25564	0.822359	0.702237
NK.cells	ADAP2OS	0.344968	0.724917	0.628714	0.531157	-5.20877	0.862151	0.766214
NK.cells	ADAM23	0.219737	3.184267	0.628631	0.531211	-5.72318	0.830764	0.715587
NK.cells	CNNM4	-0.08916	4.077084	-0.6286	0.53123	-6.11645	0.819675	0.698027
NK.cells	NIPBL	0.045071	8.615732	0.628517	0.531286	-6.93993	0.765749	0.615192
NK.cells	SF3A1	-0.06625	4.851981	-0.62852	0.531287	-6.30533	0.81018	0.683143
NK.cells	ULK3	0.163596	2.219052	0.628478	0.531311	-5.6516	0.842935	0.735073
NK.cells	STK19	-0.08116	4.61763	-0.6283	0.531426	-6.29249	0.813039	0.687633
NK.cells	UQCR10	0.062419	6.961957	0.628298	0.531429	-6.69133	0.784931	0.644187
NK.cells	IFITM3	0.151119	7.709419	0.628295	0.53143	-6.69915	0.776196	0.630922
NK.cells	LIX1L	0.280533	0.803407	0.627985	0.531633	-5.24613	0.861197	0.764722
NK.cells	GRK5	-0.09147	5.747033	-0.6279	0.531691	-6.69623	0.799424	0.666516
NK.cells	TEAD2	0.253825	1.154161	0.627874	0.531705	-5.32275	0.85665	0.757376
NK.cells	SLC15A2	-0.38742	4.597147	-0.62781	0.531745	-6.30488	0.813352	0.688226
NK.cells	DDX27	0.068925	4.974515	0.627638	0.531859	-6.36775	0.808802	0.681125
NK.cells	ANKS1	-0.07842	5.759857	-0.62755	0.531915	-6.44409	0.799319	0.666416
NK.cells	KAT8	0.099911	3.624929	0.62728	0.532092	-6.02166	0.825464	0.707271
NK.cells	BCL2L14	0.383166	0.459361	0.627265	0.532102	-5.283	0.865818	0.772339
NK.cells	UIMC1	-0.05562	5.853468	-0.62696	0.532301	-6.54359	0.798335	0.664886
NK.cells	GM16091	0.150182	2.306149	0.626778	0.53242	-5.77001	0.842092	0.733879
NK.cells	GNB4	-0.13108	2.895468	-0.62666	0.532496	-5.88081	0.834646	0.721991
NK.cells	MCAM	-0.30832	1.173297	-0.62658	0.53255	-5.3133	0.856604	0.757403
NK.cells	PSMD12	0.059774	5.784758	0.626495	0.532604	-6.48358	0.799159	0.666202
NK.cells	CERS2	-0.06847	5.262709	-0.62644	0.53264	-6.38342	0.805447	0.675955
NK.cells	LSR	-0.21818	1.400914	-0.62641	0.532659	-5.60419	0.853667	0.752633
NK.cells	TLR12	-0.24247	2.045397	-0.62636	0.532691	-5.56521	0.845409	0.739292
NK.cells	CEBPA	-0.21496	2.906324	-0.62609	0.532869	-5.61226	0.834681	0.721899
NK.cells	STK16	-0.09251	4.37442	-0.62563	0.533171	-6.17162	0.816466	0.693106
NK.cells	ASRGL1	-0.12208	3.315534	-0.62556	0.533212	-6.01432	0.82958	0.713836
NK.cells	TCF12	-0.05514	8.463403	-0.62547	0.533275	-6.94036	0.767916	0.618579
NK.cells	U2AF1	-0.0463	7.593832	-0.62522	0.53344	-6.80125	0.777967	0.633807
NK.cells	RNF19B	0.08433	5.758355	0.625169	0.53347	-6.42869	0.799666	0.667035
NK.cells	SMIM24	0.108521	3.210803	0.625157	0.533478	-5.91603	0.830889	0.716053
NK.cells	MVB12B	-0.09141	4.074675	-0.62507	0.533538	-6.23135	0.820155	0.699044
NK.cells	TCEA2	0.264831	0.335442	0.625036	0.533557	-5.2777	0.867713	0.775634
NK.cells	PRICKLE1	0.168937	3.386121	0.62503	0.533561	-5.95676	0.828699	0.712569
NK.cells	DMAC2	0.122172	3.093682	0.625005	0.533577	-5.89559	0.832356	0.71839
NK.cells	IFI204	0.233687	4.390085	0.624837	0.533687	-5.83344	0.816274	0.692933
NK.cells	ALDOC	0.296752	0.884667	0.624739	0.533751	-5.28506	0.860548	0.763912
NK.cells	TXNL4A	-0.07153	4.968748	-0.62465	0.533807	-6.3351	0.809204	0.681876

NK.cells	ZFP217	-0.0834	4.011596	-0.6246	0.533844	-6.06523	0.820934	0.700288
NK.cells	PPP3R1	-0.06005	5.5618	-0.62403	0.534216	-6.44418	0.802363	0.670938
NK.cells	TRIM7	-0.28102	0.591087	-0.62376	0.534394	-5.28717	0.864731	0.770541
NK.cells	NUB1	0.056838	5.534309	0.623578	0.53451	-6.4781	0.802694	0.671575
NK.cells	CDC25B	-0.10811	4.400381	-0.62346	0.534585	-6.31012	0.816488	0.693113
NK.cells	MTCH2	0.058885	5.950226	0.623435	0.534604	-6.52261	0.797699	0.663843
NK.cells	RALA	-0.06082	5.871704	-0.62341	0.534623	-6.51653	0.79864	0.665296
NK.cells	CPNE1	-0.05555	6.038278	-0.62332	0.534677	-6.52963	0.796646	0.662218
NK.cells	FTL1	0.067595	11.26205	0.622865	0.534977	-7.27971	0.736853	0.572806
NK.cells	GM28694	0.373854	-0.23311	0.622855	0.534983	-5.08311	0.875562	0.788613
NK.cells	GOT2	-0.05797	6.235083	-0.62273	0.535063	-6.59898	0.794298	0.658777
NK.cells	HSPA4L	0.096358	4.348791	0.622694	0.535089	-6.17739	0.817121	0.694296
NK.cells	BEND3	-0.16445	2.456588	-0.62267	0.535104	-5.70014	0.840735	0.731841
NK.cells	SYAP1	0.061773	5.053275	0.622596	0.535153	-6.35751	0.808514	0.680833
NK.cells	GZF1	0.171568	2.752325	0.622519	0.535203	-5.63515	0.836996	0.725879
NK.cells	RIOX1	-0.09687	3.521185	-0.62251	0.535211	-5.98352	0.82736	0.710516
NK.cells	EIF4EBP3	0.148825	2.80817	0.622343	0.535318	-5.77984	0.836292	0.724782
NK.cells	EVA1A	-0.25768	1.060527	-0.62225	0.535379	-5.32775	0.858625	0.760886
NK.cells	DDX17	-0.05894	6.357267	-0.62212	0.535462	-6.58897	0.792843	0.656663
NK.cells	RWDD2A	-0.27035	0.48641	-0.62206	0.5355	-5.30122	0.866099	0.77317
NK.cells	GABARAPL	0.070301	7.762001	0.621879	0.535622	-6.8852	0.776336	0.631527
NK.cells	CD8B1	0.178418	0.660348	0.621811	0.535666	-5.85859	0.863827	0.769497
NK.cells	4930522L1	-0.1024	3.684756	-0.62181	0.53567	-6.05257	0.825325	0.707433
NK.cells	DALRD3	-0.09183	3.729807	-0.62176	0.535699	-6.00699	0.824766	0.706564
NK.cells	ELP4	0.074337	4.963596	0.621682	0.535751	-6.36251	0.809605	0.682755
NK.cells	ZC3H12A	0.080013	5.128189	0.621594	0.535808	-6.39463	0.807605	0.679637
NK.cells	E230013L2	-0.35521	-0.72305	-0.62153	0.535847	-5.06393	0.882068	0.799697
NK.cells	ITM2A	0.127563	2.544803	0.621313	0.535992	-6.03512	0.83965	0.730396
NK.cells	PRR5	0.123275	3.479528	0.621294	0.536005	-6.03403	0.827911	0.711648
NK.cells	LZTS2	-0.31334	1.249256	-0.62109	0.536137	-5.24892	0.856317	0.757262
NK.cells	CDK13	-0.05033	7.414024	-0.62087	0.536281	-6.78905	0.780622	0.63793
NK.cells	MYADM	0.117711	5.586342	0.620577	0.536475	-6.20036	0.802463	0.671255
NK.cells	ZFP143	0.093488	3.815014	0.620504	0.536522	-6.01203	0.824115	0.705184
NK.cells	UBE2V1	-0.04831	6.983076	-0.62035	0.536623	-6.71199	0.785876	0.645654
NK.cells	YWHAG	0.065009	6.203239	0.620207	0.536717	-6.54531	0.795154	0.65984
NK.cells	XPOT	0.080729	4.512589	0.620084	0.536797	-6.24384	0.815619	0.691663
NK.cells	COL20A1	0.198352	1.340253	0.619965	0.536876	-5.35805	0.855548	0.75552
NK.cells	GPC6	0.334413	1.763476	0.619832	0.536963	-5.48271	0.850103	0.746784
NK.cells	GM15987	-0.25651	2.537923	-0.61966	0.537077	-5.47269	0.840235	0.730899
NK.cells	OLFR1259	0.262201	-1.34501	0.61958	0.537128	-5.42811	0.890554	0.814125
NK.cells	COA5	0.096607	4.468388	0.619399	0.537246	-6.22263	0.816168	0.692744
NK.cells	ABL2	0.076362	6.21523	0.619092	0.537447	-6.51677	0.795036	0.65993
NK.cells	KPNA4	0.059073	8.246447	0.619	0.537508	-6.93612	0.771218	0.623688
NK.cells	TMEM101	0.158154	2.322989	0.618566	0.537792	-5.65389	0.842961	0.735622
NK.cells	COPA	-0.04871	6.661065	-0.61844	0.537876	-6.65324	0.789739	0.651954
NK.cells	GUSB	0.07107	5.468903	0.618325	0.537951	-6.34585	0.80399	0.674024
NK.cells	GM13986	-0.49564	1.376917	-0.61825	0.538002	-5.20338	0.855074	0.755318

NK.cells	PPP6R2	0.079026	4.368985	0.618146	0.538068	-6.17895	0.817389	0.695011
NK.cells	EVI5L	-0.1541	2.262161	-0.61803	0.538147	-5.65507	0.843734	0.736977
NK.cells	MFSD1	0.074188	5.149534	0.618023	0.538149	-6.30738	0.807856	0.680077
NK.cells	GPRASP1	0.087105	3.93667	0.618005	0.538161	-6.08716	0.822722	0.70343
NK.cells	LYVE1	-0.32986	2.42534	-0.61781	0.538289	-5.46913	0.841661	0.733714
NK.cells	GM12655	0.304884	0.353725	0.617724	0.538345	-5.26859	0.868383	0.777205
NK.cells	LGALS9	-0.07681	7.157478	-0.61769	0.538369	-6.66832	0.783887	0.643188
NK.cells	TRAF3IP1	0.179473	1.889467	0.617374	0.538575	-5.68982	0.848489	0.744848
NK.cells	IQCG	0.376664	0.050135	0.617334	0.538601	-5.17987	0.872374	0.78391
NK.cells	RASA4	0.17693	4.228858	0.617314	0.538615	-5.87824	0.819113	0.697935
NK.cells	EGLN2	-0.08481	4.887876	-0.61727	0.538645	-6.29611	0.811038	0.685269
NK.cells	GSG1L	0.422108	-0.16326	0.617211	0.538682	-5.08119	0.875191	0.788599
NK.cells	FBH1	-0.10068	3.425128	-0.61713	0.538734	-5.93317	0.82908	0.713756
NK.cells	MARK4	0.086084	4.541737	0.617113	0.538746	-6.22858	0.815269	0.691915
NK.cells	BTD	0.158723	2.37707	0.617067	0.538776	-5.69194	0.842274	0.734873
NK.cells	WIPI1	0.217543	2.277802	0.617067	0.538777	-5.52721	0.843535	0.736905
NK.cells	NCLN	0.094009	3.839103	0.616965	0.538844	-6.05128	0.82393	0.705613
NK.cells	KCTD12B	0.302387	0.963206	0.616661	0.539043	-5.26659	0.86043	0.764482
NK.cells	NUP54	-0.07185	4.859149	-0.61654	0.539121	-6.29729	0.811388	0.685978
NK.cells	TANGO6	0.086644	4.382985	0.616533	0.539127	-6.20329	0.817217	0.695131
NK.cells	PRPF18	0.075307	4.616004	0.616491	0.539155	-6.27967	0.814359	0.690641
NK.cells	QRFP	-0.24797	-0.61178	-0.61642	0.539204	-5.39648	0.881142	0.798672
NK.cells	TNPO1	0.053424	7.082293	0.616363	0.539239	-6.7035	0.78477	0.644819
NK.cells	H2-T22	0.123331	5.391347	0.616332	0.539259	-6.53352	0.804927	0.675901
NK.cells	OVCA2	-0.34068	0.174482	-0.61632	0.539269	-5.18995	0.870737	0.781438
NK.cells	PIP4P1	0.05272	5.856707	0.616008	0.539472	-6.44021	0.799401	0.66728
NK.cells	CENPH	0.160684	3.059161	0.615988	0.539485	-5.84088	0.833743	0.721317
NK.cells	2410018L1	0.203399	0.727626	0.615925	0.539527	-5.49276	0.863579	0.769626
NK.cells	ITFG1	-0.05781	5.454025	-0.61568	0.539686	-6.40929	0.804383	0.674864
NK.cells	GM11696	0.199419	1.871722	0.615366	0.539894	-5.54299	0.849022	0.74575
NK.cells	ACTL6A	0.063797	5.235041	0.615227	0.539985	-6.39089	0.80711	0.679123
NK.cells	SNRPB	-0.05107	7.300883	-0.61519	0.540008	-6.76767	0.782488	0.641167
NK.cells	OASL2	0.316131	3.934807	0.615187	0.540012	-5.8684	0.823042	0.704154
NK.cells	TRIP13	-0.17088	2.772205	-0.61492	0.540188	-5.81158	0.837644	0.727363
NK.cells	NUDT15	0.175962	1.647281	0.614911	0.540193	-5.54852	0.851971	0.750475
NK.cells	GM43260	-0.16847	2.252383	-0.61436	0.540556	-5.60254	0.844457	0.73813
NK.cells	FAM214B	-0.17643	3.11159	-0.6139	0.540856	-5.70515	0.833594	0.720698
NK.cells	OSBPL7	-0.0968	3.426262	-0.61384	0.5409	-6.02091	0.829654	0.714419
NK.cells	ATRX	-0.0478	7.666179	-0.61375	0.540954	-6.82773	0.778492	0.634905
NK.cells	3110082I1	0.122277	4.199896	0.613531	0.541101	-6.01542	0.820051	0.699245
NK.cells	STOM	0.18912	3.274711	0.613419	0.541174	-5.59088	0.831549	0.717483
NK.cells	SKP2	0.128921	3.310393	0.613384	0.541197	-5.85837	0.831103	0.716771
NK.cells	USF2	0.057107	6.021725	0.613204	0.541315	-6.56098	0.797912	0.664715
NK.cells	MILR1	-0.09607	4.330073	-0.61308	0.5414	-6.05217	0.818447	0.696774
NK.cells	GJA1	-0.3451	1.208053	-0.61304	0.541424	-5.26011	0.857864	0.759993
NK.cells	PIM1	-0.06546	9.488973	-0.61283	0.541563	-7.13341	0.757578	0.603606
NK.cells	SLC35E3	-0.21837	1.909396	-0.61283	0.541563	-5.45166	0.848835	0.745353

NK.cells	ZFP952	-0.14768	2.251647	-0.6128	0.541582	-5.68785	0.844466	0.738294
NK.cells	BTLA	0.129559	3.948652	0.612497	0.541781	-6.21826	0.823156	0.704281
NK.cells	RHEB	-0.04654	7.039284	-0.61244	0.54182	-6.72972	0.785833	0.646218
NK.cells	RDH11	0.114385	3.215453	0.612353	0.541876	-5.89012	0.832292	0.718828
NK.cells	GM11342	0.271479	2.120734	0.612176	0.541992	-5.40629	0.846135	0.741088
NK.cells	GM47917	0.212969	0.467794	0.612016	0.542098	-5.41982	0.867504	0.775963
NK.cells	UXS1	0.080476	4.429155	0.611965	0.542131	-6.26146	0.817229	0.695043
NK.cells	TLNRD1	-0.1067	4.581796	-0.61193	0.542155	-6.15662	0.815355	0.692102
NK.cells	GM16845	0.14678	2.527304	0.611854	0.542204	-5.75689	0.840965	0.732847
NK.cells	SCAF4	-0.05452	6.317786	-0.61177	0.542257	-6.60192	0.794377	0.659492
NK.cells	SMYD3	0.064696	6.67093	0.611763	0.542265	-6.71153	0.790182	0.653039
NK.cells	RALGPS1	-0.092	4.92479	-0.61146	0.542461	-6.23186	0.811163	0.685649
NK.cells	CSDE1	-0.03745	7.576149	-0.61126	0.542593	-6.79102	0.779541	0.636962
NK.cells	DHODH	-0.15987	2.109646	-0.6112	0.542638	-5.61251	0.846276	0.741635
NK.cells	SNX18	0.066068	6.77742	0.611186	0.542644	-6.74549	0.788922	0.651283
NK.cells	D430020J0	-0.32018	0.440007	-0.61116	0.54266	-5.21729	0.867868	0.776854
NK.cells	ZMAT3	0.168142	1.902429	0.611047	0.542737	-5.78526	0.848924	0.745933
NK.cells	RSRC2	0.042577	6.479986	0.610983	0.542779	-6.62062	0.792447	0.656709
NK.cells	ZFP354C	-0.20098	1.777029	-0.61094	0.542808	-5.57153	0.850531	0.748538
NK.cells	PGPEP1	0.126871	3.461593	0.610859	0.54286	-5.78176	0.829213	0.714274
NK.cells	WEE1	-0.12404	3.947727	-0.61084	0.542874	-5.96705	0.823168	0.704675
NK.cells	TMEM259	0.082076	4.879001	0.610782	0.542911	-6.35342	0.811721	0.686643
NK.cells	ZFP940	0.215946	0.734633	0.610613	0.543022	-5.37224	0.864016	0.770564
NK.cells	SH2D2A	0.100332	2.764079	0.610596	0.543034	-6.34129	0.83797	0.728297
NK.cells	BFSP2	-0.22631	2.440103	-0.61054	0.543073	-5.54017	0.842071	0.734891
NK.cells	H2-Q7	-0.15664	3.889878	-0.61051	0.543087	-6.39001	0.823885	0.705838
NK.cells	PSMF1	0.076077	4.460205	0.610454	0.543127	-6.26932	0.816847	0.694726
NK.cells	TNFSF13B	-0.31125	0.855901	-0.6103	0.543226	-5.25042	0.862435	0.767996
NK.cells	CAVIN1	0.317847	1.312054	0.6103	0.543229	-5.30575	0.856518	0.758324
NK.cells	GT(ROSA)2	0.093176	4.594685	0.610236	0.543271	-6.20133	0.815197	0.692169
NK.cells	MEGF9	-0.13068	4.103779	-0.61008	0.54337	-6.02465	0.821238	0.70172
NK.cells	TTPAL	-0.08056	4.234118	-0.60988	0.543504	-6.14538	0.819629	0.699238
NK.cells	AI837181	0.097606	3.127875	0.609804	0.543556	-5.89914	0.83339	0.721101
NK.cells	4930438A(	0.289729	-0.84008	0.609689	0.543632	-5.11074	0.884815	0.805146
NK.cells	PLAGL2	-0.08318	4.367209	-0.60956	0.543716	-6.16806	0.81799	0.696662
NK.cells	PABPC1	-0.05629	10.47512	-0.60942	0.543808	-7.30018	0.746528	0.587774
NK.cells	TBC1D19	0.187125	1.749793	0.609401	0.543821	-5.41357	0.850881	0.749296
NK.cells	FMO5	-0.14606	3.247756	-0.60935	0.543858	-5.8077	0.831887	0.718743
NK.cells	DNMT3A	0.069163	6.044028	0.609326	0.543871	-6.52761	0.797645	0.664919
NK.cells	FAM160B2	0.105426	3.264944	0.609259	0.543915	-5.89626	0.831672	0.718436
NK.cells	SNX8	-0.14766	5.468943	-0.60905	0.544055	-6.10064	0.80456	0.67579
NK.cells	MAGI1	-0.17106	4.373842	-0.60894	0.544128	-6.19475	0.817909	0.696742
NK.cells	SEC11C	-0.05657	7.287381	-0.60869	0.54429	-6.72649	0.782918	0.642496
NK.cells	HIST1H2BE	-0.20368	2.079102	-0.6086	0.544351	-5.52574	0.846666	0.74272
NK.cells	CRACR2B	-0.32435	1.009115	-0.60858	0.544365	-5.24348	0.860443	0.765139
NK.cells	FBXO31	-0.1005	3.549697	-0.60836	0.54451	-6.07724	0.828114	0.712949
NK.cells	MAN1A2	0.049413	6.392544	0.608211	0.544607	-6.6102	0.793486	0.658702

NK.cells	CCDC6	-0.07546	4.540905	-0.60817	0.544635	-6.35284	0.815857	0.693549
NK.cells	TRIM25	0.088065	6.978283	0.607993	0.544751	-6.58276	0.786551	0.648048
NK.cells	MINPP1	0.068743	4.734583	0.607908	0.544807	-6.28624	0.813485	0.689819
NK.cells	GM43647	0.221783	-0.94495	0.607762	0.544903	-5.51537	0.886218	0.807778
NK.cells	SHROOM2	0.311102	1.506317	0.607755	0.544908	-5.32023	0.854012	0.754641
NK.cells	HSH2D	-0.1199	3.210107	-0.60773	0.544925	-5.96991	0.832359	0.719718
NK.cells	GM32250	0.244852	-0.2746	0.607678	0.544959	-5.33712	0.877286	0.7929
NK.cells	F930017D2	0.395286	0.898224	0.60767	0.544964	-5.2481	0.861884	0.7675
NK.cells	MSRB3	-0.20207	2.604277	-0.60752	0.545064	-5.5978	0.83999	0.732009
NK.cells	PTCD1	0.130681	2.585716	0.607421	0.545129	-5.75449	0.840225	0.732387
NK.cells	IFIH1	0.135208	3.912874	0.607093	0.545345	-6.12961	0.8236	0.705912
NK.cells	TPM1	-0.08379	5.177563	-0.60677	0.545556	-6.4274	0.808088	0.681559
NK.cells	2-Mar	-0.0594	5.868752	-0.60676	0.545564	-6.53664	0.799746	0.668569
NK.cells	BCAS3	0.058097	7.334935	0.6067	0.545605	-6.78738	0.782361	0.641838
NK.cells	MFAP4	0.26062	1.50654	0.606533	0.545715	-5.46604	0.854009	0.754884
NK.cells	UBXN1	0.046101	6.759331	0.606524	0.545721	-6.67532	0.789136	0.652224
NK.cells	BICDL1	-0.12951	1.878488	-0.60651	0.545732	-5.91277	0.849231	0.74712
NK.cells	ANXA3	0.218582	3.542549	0.606456	0.545766	-5.63553	0.828203	0.713351
NK.cells	MADD	0.064669	5.091113	0.606178	0.54595	-6.47509	0.809138	0.683307
NK.cells	MICOS13	0.056545	5.948201	0.605945	0.546104	-6.5424	0.798793	0.667257
NK.cells	MARK2	0.046535	6.879023	0.605914	0.546124	-6.70271	0.787722	0.650188
NK.cells	SNX11	0.149905	2.539196	0.605684	0.546276	-5.65161	0.840814	0.733708
NK.cells	ARL4D	-0.19052	1.705958	-0.60559	0.54634	-5.64972	0.851444	0.750925
NK.cells	KDM3B	-0.0515	6.131762	-0.60555	0.546368	-6.56874	0.796596	0.663908
NK.cells	GM26632	-0.2558	0.366747	-0.60542	0.546451	-5.34295	0.868828	0.779405
NK.cells	KCNC3	-0.25911	0.561324	-0.60542	0.546453	-5.29761	0.866279	0.775206
NK.cells	GM11998	0.289574	0.318253	0.605334	0.546508	-5.29388	0.869465	0.780455
NK.cells	SLC35A3	-0.06307	4.775291	-0.60529	0.546539	-6.30508	0.812987	0.689479
NK.cells	HIST1H3I	-0.23823	1.296054	-0.60516	0.546624	-5.4821	0.856725	0.759601
NK.cells	ICA1	-0.20966	2.463147	-0.60512	0.546652	-5.63382	0.841778	0.735357
NK.cells	EPAS1	0.132875	3.448567	0.605091	0.546669	-5.99853	0.829375	0.715481
NK.cells	TAF1A	0.112335	3.171877	0.604883	0.546806	-5.95439	0.832838	0.721064
NK.cells	PPIC	0.167484	2.745716	0.604803	0.546859	-5.65645	0.838202	0.729682
NK.cells	CHMP2B	-0.06759	4.813502	-0.6048	0.546859	-6.27417	0.812521	0.688876
NK.cells	CHCHD5	-0.12702	3.092559	-0.60476	0.546888	-5.86412	0.833833	0.722683
NK.cells	ECSCR	0.29598	1.207762	0.604756	0.54689	-5.2947	0.857867	0.761568
NK.cells	DBP	-0.20791	2.187718	-0.60463	0.546973	-5.58581	0.845281	0.741149
NK.cells	SLC35A5	0.080947	3.986264	0.604479	0.547073	-6.09616	0.822691	0.705039
NK.cells	CAR9	0.321646	0.285801	0.604362	0.547151	-5.15392	0.869891	0.781463
NK.cells	KIN	-0.06869	4.642417	-0.60431	0.547188	-6.27109	0.814613	0.692326
NK.cells	DPF1	-0.35821	0.292792	-0.60402	0.547376	-5.19102	0.869799	0.781491
NK.cells	NES	-0.33638	0.578345	-0.60399	0.547395	-5.26386	0.866057	0.775319
NK.cells	SNRNP40	0.052378	5.982356	0.603988	0.547398	-6.56638	0.798383	0.667102
NK.cells	EXOC5	-0.04927	6.376842	-0.60399	0.547398	-6.60797	0.793673	0.659816
NK.cells	GOT1	-0.07845	6.081478	-0.60373	0.547569	-6.57897	0.797342	0.665341
NK.cells	ELF1	-0.03944	8.010369	-0.60363	0.547635	-6.9126	0.774634	0.630558
NK.cells	PDF	0.24955	0.794298	0.603499	0.547722	-5.3099	0.863424	0.770788

NK.cells	TCRG-C2	-0.17815	0.685448	-0.60304	0.548025	-6.05968	0.865204	0.773285
NK.cells	GTF2H3	0.160027	2.299191	0.602924	0.548102	-5.64682	0.844395	0.73937
NK.cells	TOLLIP	-0.10249	3.832789	-0.60284	0.548156	-6.03546	0.825114	0.708486
NK.cells	H2-T23	-0.09925	6.358381	-0.60268	0.548266	-6.6444	0.794402	0.660433
NK.cells	ZFP831	-0.1184	3.063289	-0.60256	0.548342	-6.12093	0.834736	0.723933
NK.cells	ENC1	0.118434	3.181392	0.602525	0.548367	-5.91061	0.833253	0.721565
NK.cells	DYRK1A	-0.05932	7.419436	-0.60196	0.548745	-6.73899	0.782098	0.64139
NK.cells	SCMH1	-0.0809	5.849321	-0.60192	0.548771	-6.44234	0.800722	0.670047
NK.cells	CXCR5	0.234909	2.183855	0.601807	0.548843	-5.53168	0.846115	0.742019
NK.cells	DIMT1	0.104715	3.243055	0.601775	0.548864	-5.94616	0.832719	0.720476
NK.cells	HIST4H4	0.222271	1.887782	0.601769	0.548868	-5.49785	0.849901	0.748153
NK.cells	GM27201	-0.12878	2.321008	-0.60164	0.548955	-5.71371	0.844396	0.73921
NK.cells	BMP6	-0.36963	0.450706	-0.60152	0.549032	-5.12668	0.868577	0.778677
NK.cells	C2CD3	0.065423	4.657214	0.60138	0.549126	-6.2761	0.815264	0.692774
NK.cells	RAMP2	0.193324	3.013058	0.601278	0.549193	-5.6792	0.835686	0.725238
NK.cells	YBX1	0.052183	8.398771	0.600968	0.549399	-6.9221	0.770989	0.624369
NK.cells	CDIPT	0.069568	5.093616	0.60074	0.54955	-6.39238	0.810157	0.684619
NK.cells	PAQR3	0.159716	1.469925	0.60058	0.549656	-5.61124	0.855589	0.757281
NK.cells	TPST2	0.057119	5.593782	0.6005	0.549709	-6.5861	0.804094	0.675188
NK.cells	SELENOK	-0.05039	7.584969	-0.60043	0.549756	-6.81747	0.780449	0.638797
NK.cells	TAF8	0.106624	3.254648	0.600342	0.549814	-5.97339	0.832879	0.720633
NK.cells	PDE8B	0.302932	-0.24267	0.600247	0.549877	-5.17726	0.878	0.79419
NK.cells	XKR8	-0.28714	0.500884	-0.60001	0.550036	-5.26407	0.868194	0.777968
NK.cells	MDGA1	0.308078	0.010829	0.599985	0.550051	-5.24165	0.874644	0.788623
NK.cells	LEAP2	0.18113	3.060255	0.599973	0.550059	-5.89924	0.835321	0.724542
NK.cells	OGFOD2	0.085417	3.850672	0.599858	0.550135	-6.11529	0.82544	0.708793
NK.cells	4933433G1	0.322621	0.742117	0.599809	0.550168	-5.26284	0.865038	0.772788
NK.cells	SLC2A6	0.204161	1.781297	0.59915	0.550605	-5.57484	0.851992	0.751007
NK.cells	MAP2K2	-0.0419	7.299123	-0.59912	0.550625	-6.78839	0.784175	0.644102
NK.cells	QARS	-0.07556	4.552856	-0.5991	0.55064	-6.3064	0.817164	0.695278
NK.cells	USP10	-0.07927	4.567936	-0.59876	0.550862	-6.30814	0.817062	0.695152
NK.cells	GM20743	0.295827	0.031876	0.598732	0.550882	-5.2785	0.87488	0.788604
NK.cells	SPR	0.095881	3.527522	0.598701	0.550903	-6.09249	0.829952	0.71559
NK.cells	ECHDC2	-0.24585	1.612612	-0.5983	0.551167	-5.46658	0.854544	0.754842
NK.cells	DTX4	0.159722	2.365512	0.598155	0.551265	-5.83132	0.844897	0.739229
NK.cells	BRDT	-0.21558	1.567286	-0.59755	0.551666	-5.45543	0.855128	0.755954
NK.cells	GTF2I	0.059467	5.762327	0.597544	0.551672	-6.5097	0.802809	0.672667
NK.cells	TRIM62	0.142978	1.410517	0.597109	0.551961	-5.66494	0.857153	0.75928
NK.cells	2610008E1	0.146554	2.466966	0.59699	0.55204	-5.76351	0.843606	0.737303
NK.cells	ZFP319	-0.17019	2.544028	-0.59699	0.55204	-5.73085	0.842627	0.735724
NK.cells	COMMD3	0.061075	5.85015	0.596961	0.552059	-6.49486	0.801752	0.671046
NK.cells	GPSM1	-0.12852	2.672071	-0.59693	0.552079	-5.8118	0.841003	0.733109
NK.cells	SNRPA	0.060915	5.131803	0.596909	0.552094	-6.36759	0.810446	0.684602
NK.cells	PPP3CA	-0.04748	8.812084	-0.59672	0.55222	-6.96352	0.766976	0.617967
NK.cells	TMOD1	0.213023	1.823905	0.596718	0.552221	-5.5407	0.851825	0.750635
NK.cells	TPST1	0.129664	3.472726	0.596642	0.552271	-5.94401	0.830922	0.717017
NK.cells	FHIT	0.08073	6.167904	0.59655	0.552333	-6.55466	0.797939	0.665227

NK.cells	YIPF5	-0.05996	5.24625	-0.59649	0.552372	-6.37902	0.809054	0.682518
NK.cells	POLR3C	0.057853	5.018329	0.596392	0.552437	-6.39314	0.811829	0.686906
NK.cells	MLYCD	0.102603	3.124459	0.596378	0.552447	-5.95012	0.835291	0.724085
NK.cells	ECH1	-0.07105	5.784916	-0.59601	0.552689	-6.48539	0.802537	0.672519
NK.cells	GM43251	0.440716	-0.97845	0.595953	0.552729	-5.071	0.88861	0.811664
NK.cells	TNR	0.31211	0.036927	0.595819	0.552818	-5.13471	0.875113	0.789139
NK.cells	IL1F9	0.359777	-0.11555	0.595713	0.552889	-5.16256	0.877131	0.792485
NK.cells	SLC25A30	0.09179	3.550303	0.595689	0.552905	-6.06239	0.829952	0.715716
NK.cells	CLN8	0.149051	3.202024	0.595685	0.552908	-5.83375	0.834315	0.722686
NK.cells	EVL	0.063062	6.485099	0.595675	0.552914	-6.74313	0.794152	0.659567
NK.cells	FAP	0.361369	0.744801	0.595658	0.552926	-5.31287	0.865808	0.773782
NK.cells	SMC5	0.058355	5.613071	0.595545	0.553	-6.58155	0.80461	0.675798
NK.cells	PYROXD1	0.115914	2.95488	0.59533	0.553143	-5.86953	0.837427	0.727744
NK.cells	ARFGAP1	0.07037	4.620741	0.59532	0.55315	-6.27333	0.816694	0.694775
NK.cells	ELDR	-0.21644	3.256101	-0.59505	0.553328	-5.68181	0.833636	0.721704
NK.cells	LIN9	-0.08861	4.133885	-0.59498	0.553378	-6.16982	0.822695	0.704287
NK.cells	CCDC28A	0.13181	2.307492	0.594941	0.553402	-5.76602	0.845636	0.741002
NK.cells	TRMT2B	0.096224	3.750232	0.594931	0.553409	-6.07322	0.827458	0.711848
NK.cells	MGRN1	0.072012	5.682428	0.594873	0.553448	-6.47804	0.803773	0.674571
NK.cells	ABCD3	-0.0915	4.193442	-0.59479	0.553501	-6.10364	0.821959	0.703123
NK.cells	PCYOX1	-0.09174	4.579765	-0.59475	0.55353	-6.12166	0.817198	0.695604
NK.cells	ITGA3	-0.20228	-0.28405	-0.59454	0.553668	-5.43156	0.879367	0.796319
NK.cells	1110012L1	0.163542	1.829365	0.594492	0.553701	-5.61332	0.851755	0.750923
NK.cells	ALG5	0.080102	4.2129	0.594489	0.553703	-6.21401	0.821718	0.702744
NK.cells	PROSCOS	-0.31656	0.287878	-0.59414	0.553937	-5.28006	0.872062	0.783829
NK.cells	TLR2	0.237751	3.875493	0.593824	0.554146	-5.53158	0.826244	0.709487
NK.cells	HOOK3	0.053541	6.076111	0.593734	0.554206	-6.56774	0.799371	0.667327
NK.cells	1810009A1	0.129369	2.243882	0.593729	0.554209	-5.69874	0.8468	0.742436
NK.cells	IQCC	0.18934	1.695654	0.593456	0.554391	-5.56423	0.854004	0.753879
NK.cells	GM45435	0.295632	0.920103	0.593269	0.554516	-5.35946	0.864144	0.770337
NK.cells	SLFN5	0.188728	4.230734	0.593016	0.554684	-5.99791	0.822239	0.702608
NK.cells	GM43768	-0.27136	0.70894	-0.59274	0.55487	-5.30525	0.867157	0.775055
NK.cells	SBNO2	-0.06225	5.566821	-0.5926	0.554959	-6.45337	0.805986	0.677108
NK.cells	SPATA2L	0.249276	0.521707	0.592133	0.555273	-5.38317	0.869611	0.779263
NK.cells	TOMM22	-0.04943	6.905252	-0.59212	0.555284	-6.7123	0.789968	0.652461
NK.cells	CLPB	-0.07542	4.010407	-0.59211	0.555289	-6.14297	0.825062	0.707219
NK.cells	ANXA11OS	0.244536	0.812081	0.592012	0.555354	-5.38809	0.865807	0.773012
NK.cells	TECR	-0.05621	6.763011	-0.59201	0.555354	-6.68077	0.791654	0.655057
NK.cells	4930481A1	0.174875	1.854963	0.591979	0.555376	-5.57738	0.85229	0.750932
NK.cells	TRAPPC2	-0.08776	4.171105	-0.59197	0.555384	-6.15365	0.82307	0.704071
NK.cells	NOTCH1	-0.06789	5.292946	-0.59179	0.555503	-6.33498	0.809308	0.682439
NK.cells	PLSCR3	-0.1058	4.301027	-0.59169	0.555565	-6.04565	0.821463	0.701546
NK.cells	SFXN3	0.095903	3.370072	0.591684	0.555572	-6.10638	0.833052	0.71996
NK.cells	SYNJ2BP	0.054753	5.520643	0.591577	0.555643	-6.48998	0.806548	0.678131
NK.cells	TNRC6C	-0.04912	7.143837	-0.59141	0.555755	-6.75265	0.787159	0.648194
NK.cells	YIPF6	-0.10924	3.457527	-0.59128	0.555841	-5.9737	0.831964	0.718268
NK.cells	CYB5R3	-0.07327	4.85016	-0.59103	0.556007	-6.29872	0.814718	0.691058

NK.cells	BLMH	0.051916	5.648784	0.591001	0.556028	-6.51913	0.805004	0.675869
NK.cells	HSD17B4	-0.06528	5.027211	-0.59098	0.556042	-6.36508	0.812553	0.687667
NK.cells	CCDC18	0.143608	2.722921	0.590951	0.556061	-5.72887	0.841221	0.733212
NK.cells	COL3A1	-0.2022	3.678331	-0.59085	0.556129	-5.96821	0.829204	0.713992
NK.cells	B9D1	0.335639	0.371188	0.590745	0.556199	-5.1426	0.871601	0.782815
NK.cells	ARHGAP25	0.06787	6.214201	0.590394	0.556433	-6.55376	0.798327	0.665427
NK.cells	GLA	-0.10608	4.568639	-0.59029	0.556502	-6.18145	0.8183	0.696626
NK.cells	DBNDD2	-0.16295	2.836435	-0.59022	0.55655	-5.67121	0.839913	0.73103
NK.cells	PARD3	0.210295	2.300134	0.590136	0.556605	-5.63254	0.846729	0.742016
NK.cells	OCEL1	0.092673	3.584489	0.590068	0.55665	-6.04958	0.830505	0.715978
NK.cells	HGSNAT	-0.08902	4.642285	-0.59	0.556696	-6.42314	0.817394	0.695252
NK.cells	WIPF1	-0.05921	7.425545	-0.58965	0.55693	-6.83373	0.784016	0.643501
NK.cells	DOCK8	0.057866	7.656272	0.589414	0.557087	-6.86204	0.781313	0.639447
NK.cells	PPTC7	-0.06913	5.148669	-0.58939	0.557102	-6.42361	0.811248	0.68569
NK.cells	SORCS2	-0.1727	3.3545	-0.58936	0.557124	-5.82329	0.833437	0.720822
NK.cells	SCAP	-0.09959	4.277309	-0.58928	0.557177	-6.13206	0.821944	0.70258
NK.cells	1700052K1	-0.21505	1.140287	-0.58923	0.557208	-5.35454	0.861726	0.766647
NK.cells	CCPG1OS	0.2027	1.576044	0.589186	0.557239	-5.35721	0.856079	0.757428
NK.cells	CYP2C69	-0.2422	1.432054	-0.58912	0.557282	-5.49881	0.85794	0.760491
NK.cells	POLL	0.189658	1.158885	0.588962	0.557389	-5.45264	0.861543	0.766377
NK.cells	PIGQ	-0.09836	3.519745	-0.58851	0.557688	-5.96642	0.831767	0.71775
NK.cells	LRP2BP	0.219293	1.991491	0.588287	0.557839	-5.59175	0.851267	0.74903
NK.cells	UBE2I	-0.03487	7.624242	-0.58808	0.557978	-6.84291	0.782278	0.640376
NK.cells	PDXP	0.207271	1.557173	0.587688	0.55824	-5.56525	0.857032	0.758327
NK.cells	XPO4	0.057759	5.623363	0.587661	0.558258	-6.52822	0.806153	0.677207
NK.cells	CFAP77	0.234646	0.931954	0.587609	0.558293	-5.40441	0.865156	0.771624
NK.cells	XPC	0.10102	3.385386	0.587607	0.558294	-5.94773	0.83374	0.72074
NK.cells	DONSON	0.0765	4.025423	0.587456	0.558395	-6.23246	0.825794	0.70802
NK.cells	SCRN2	-0.16784	1.43712	-0.58735	0.558467	-5.49415	0.85864	0.760874
NK.cells	BCAT2	0.068178	4.601375	0.587116	0.558622	-6.34132	0.818804	0.696807
NK.cells	ERBIN	-0.05237	7.975801	-0.58655	0.559003	-6.86626	0.778495	0.634527
NK.cells	WDR5B	-0.2781	0.670974	-0.58632	0.559152	-5.27985	0.868865	0.77763
NK.cells	FIGNL1	0.155346	2.757262	0.586248	0.559203	-5.80201	0.84195	0.733843
NK.cells	ARL1	-0.05552	5.538643	-0.58624	0.55921	-6.47461	0.807453	0.679177
NK.cells	EAF1	0.090069	4.101569	0.586234	0.559212	-6.14377	0.825081	0.706899
NK.cells	PAIP1	-0.06109	5.907952	-0.58619	0.559244	-6.5076	0.80299	0.672243
NK.cells	WDR83	0.127204	2.732947	0.586168	0.559256	-5.77944	0.842259	0.734354
NK.cells	COX7A2	-0.04847	8.039242	-0.58612	0.559291	-6.89284	0.777757	0.633537
NK.cells	ETFRF1	0.128255	3.163487	0.586071	0.559321	-5.89125	0.836814	0.725631
NK.cells	GM30541	-0.17888	0.952964	-0.58598	0.559379	-5.55983	0.865175	0.771628
NK.cells	GM10552	0.226031	1.54086	0.585816	0.559492	-5.43351	0.857601	0.759145
NK.cells	LRP5	-0.1075	4.078112	-0.58559	0.559641	-6.09049	0.825484	0.707472
NK.cells	TRDC	-0.14007	1.265066	-0.58545	0.559734	-6.22756	0.861225	0.765088
NK.cells	GM17276	0.220249	0.785789	0.585398	0.559771	-5.54643	0.867477	0.775373
NK.cells	FBXO30	-0.08086	5.032199	-0.58536	0.559799	-6.34346	0.813728	0.688995
NK.cells	SMPDL3A	-0.0873	5.614644	-0.58505	0.560005	-6.60816	0.806719	0.678018
NK.cells	MTIF3	0.117433	3.10156	0.585042	0.560009	-5.91699	0.837788	0.727144

NK.cells	SLC41A3	-0.14973	1.73389	-0.58497	0.560059	-5.66688	0.855238	0.755371
NK.cells	NLRX1	-0.17583	1.80919	-0.58459	0.560309	-5.65778	0.854394	0.75392
NK.cells	GM5544	0.273999	0.129876	0.584368	0.560461	-5.25493	0.876327	0.790001
NK.cells	NRG1	0.278185	2.541088	0.584317	0.560495	-5.64092	0.845019	0.738806
NK.cells	GLS2	-0.20291	1.618045	-0.58425	0.56054	-5.612	0.856861	0.758017
NK.cells	GM50209	0.256329	0.629068	0.584241	0.560546	-5.29109	0.869746	0.779134
NK.cells	PON3	-0.15496	3.489649	-0.58412	0.56063	-5.77166	0.833031	0.719578
NK.cells	GM9929	-0.19941	1.684976	-0.58411	0.560632	-5.41536	0.855996	0.75662
NK.cells	RHOBTB1	-0.18118	3.180961	-0.58402	0.560694	-5.75588	0.836912	0.725808
NK.cells	PLEKHN1	-0.15619	1.876111	-0.5837	0.560905	-5.64407	0.853651	0.752683
NK.cells	STRAP	-0.05928	6.174275	-0.58368	0.560919	-6.57738	0.800203	0.667827
NK.cells	FAM221B	-0.26839	0.343919	-0.5836	0.560979	-5.29167	0.87362	0.785443
NK.cells	LYL1	0.203613	3.172684	0.58339	0.561116	-5.52875	0.837235	0.726123
NK.cells	RSBN1	-0.06465	5.326631	-0.58254	0.561686	-6.43784	0.810723	0.684088
NK.cells	CNP	-0.08264	5.75023	-0.58245	0.561744	-6.44805	0.805583	0.676068
NK.cells	EPN1	0.058702	6.109989	0.582444	0.56175	-6.54087	0.801246	0.66933
NK.cells	PROS1	-0.18166	2.741429	-0.58235	0.561815	-5.61887	0.842874	0.735153
NK.cells	TET3	0.052617	6.827605	0.582298	0.561848	-6.67888	0.792672	0.656137
NK.cells	TRAPPC12	0.069594	4.201517	0.582189	0.561921	-6.23195	0.824549	0.705932
NK.cells	FNBP1L	-0.20031	3.079155	-0.58216	0.561941	-5.6105	0.838596	0.728329
NK.cells	GM14325	-0.11199	2.324076	-0.58202	0.562032	-5.78383	0.848192	0.743798
NK.cells	DUS1L	0.089826	4.064535	0.581842	0.562154	-6.14736	0.82625	0.70868
NK.cells	AP1S2	-0.07283	5.081901	-0.58172	0.562232	-6.33491	0.813709	0.688925
NK.cells	TAGAP	0.099501	4.269177	0.581695	0.562253	-6.40406	0.82371	0.704689
NK.cells	S1PR1	0.099182	4.061514	0.581675	0.562266	-6.28928	0.826287	0.708774
NK.cells	SHANK3	-0.30351	1.430051	-0.58136	0.562477	-5.3264	0.859705	0.762731
NK.cells	RAMAC	-0.0509	5.913686	-0.58118	0.5626	-6.56668	0.803609	0.673347
NK.cells	ABHD17C	-0.07772	4.833665	-0.58116	0.562612	-6.29804	0.816749	0.693901
NK.cells	GLYCTK	-0.25242	0.979545	-0.58102	0.562707	-5.31951	0.86557	0.772461
NK.cells	CIAO3	0.102793	3.316721	0.580904	0.562783	-5.92823	0.835602	0.723858
NK.cells	TIFAB	0.288183	2.73524	0.580901	0.562785	-5.35365	0.842952	0.735664
NK.cells	HCLS1	0.058693	6.691521	0.580898	0.562787	-6.67951	0.79429	0.658951
NK.cells	DNAJB1	-0.07379	6.228259	-0.58089	0.562791	-6.60057	0.799826	0.667505
NK.cells	TRAF2	0.084001	3.917831	0.580741	0.562892	-6.14619	0.828075	0.711927
NK.cells	CHD3	-0.08121	4.533669	-0.58059	0.562994	-6.34864	0.820441	0.699881
NK.cells	PPFIA1	0.061197	5.566923	0.580499	0.563055	-6.52349	0.807803	0.680063
NK.cells	SERP1	0.052426	7.981742	0.580437	0.563096	-6.77033	0.779094	0.63585
NK.cells	UHRF2	-0.04666	6.43989	-0.58039	0.563128	-6.64465	0.797292	0.663738
NK.cells	JMJD6	-0.07206	4.999369	-0.58038	0.563135	-6.34719	0.814718	0.690898
NK.cells	TAF12	0.06381	5.021001	0.580281	0.563201	-6.36801	0.814453	0.690486
NK.cells	TMED8	0.105547	2.954093	0.580217	0.563244	-5.90287	0.840178	0.731386
NK.cells	LSM6	-0.06089	6.681324	-0.58013	0.563305	-6.67101	0.794411	0.659315
NK.cells	1700061G[illegible]	0.234989	1.070717	0.580125	0.563306	-5.3729	0.864379	0.770713
NK.cells	RNF24	-0.07804	4.233052	-0.57962	0.563648	-6.29471	0.824366	0.705883
NK.cells	ZRANB3	-0.12239	3.148069	-0.57947	0.563748	-5.89885	0.837938	0.727528
NK.cells	RIPOR2	0.069206	7.617108	0.579466	0.563749	-6.82748	0.783553	0.642424
NK.cells	UBTD2	-0.10136	3.37685	-0.57937	0.563815	-6.08841	0.835056	0.722961

NK.cells	TBC1D9B	0.06289	4.555284	0.579351	0.563826	-6.34409	0.820381	0.699637
NK.cells	AU019990	0.292446	1.211418	0.579258	0.563888	-5.34223	0.862763	0.767878
NK.cells	ARHGAP17	-0.06204	7.245574	-0.57919	0.563931	-6.6549	0.787923	0.649215
NK.cells	MRVI1	-0.27404	0.497468	-0.57904	0.564034	-5.26537	0.872165	0.783398
NK.cells	ME1	-0.33302	-0.00982	-0.57871	0.564256	-5.1963	0.879089	0.794665
NK.cells	ACTR8	0.092461	3.537225	0.578549	0.564365	-6.03972	0.833298	0.72012
NK.cells	PRKN	-0.19194	3.428362	-0.57837	0.564484	-5.74172	0.834665	0.722317
NK.cells	PLXNA1	-0.30235	0.899006	-0.57835	0.564499	-5.26854	0.867107	0.774947
NK.cells	HDAC9	-0.13334	7.563843	-0.57832	0.564518	-6.45632	0.784419	0.643771
NK.cells	NOC2L	0.069817	5.059461	0.577663	0.56496	-6.43727	0.814755	0.690438
NK.cells	MMP19	-0.30433	1.213067	-0.57761	0.564998	-5.33304	0.863343	0.768417
NK.cells	GNPDA1	0.07591	4.395098	0.577517	0.565058	-6.28444	0.822932	0.703327
NK.cells	DNAJB13	-0.15788	2.103334	-0.57747	0.56509	-5.66027	0.851827	0.749635
NK.cells	GPX7	-0.32232	0.610424	-0.57745	0.565105	-5.21614	0.871232	0.781389
NK.cells	ZFP513	0.146407	2.534011	0.577255	0.565234	-5.66564	0.846315	0.740791
NK.cells	STK24	-0.04448	7.291795	-0.57719	0.565276	-6.82255	0.787927	0.648915
NK.cells	ZFP335OS	-0.08204	4.456083	-0.57703	0.565388	-6.26201	0.822178	0.702257
NK.cells	RETREG2	0.07118	4.884018	0.576987	0.565414	-6.36374	0.816906	0.693946
NK.cells	GM42699	-0.27823	0.727533	-0.57692	0.565461	-5.33893	0.869693	0.779002
NK.cells	SVIP	0.210869	1.34626	0.576847	0.565509	-5.42391	0.86161	0.765725
NK.cells	SETDB1	0.075028	4.832864	0.576695	0.565611	-6.27274	0.817534	0.694941
NK.cells	SNRPG	-0.04652	8.018397	-0.57666	0.565635	-6.92811	0.779406	0.635972
NK.cells	CCL5	0.165735	7.931718	0.576554	0.565706	-7.39202	0.78042	0.637536
NK.cells	4930581F2	0.095324	3.634004	0.576374	0.565827	-6.02449	0.832489	0.718594
NK.cells	PICALM	0.049582	8.404518	0.576103	0.566009	-6.93538	0.774996	0.629293
NK.cells	UBOX5	-0.19306	1.390449	-0.57608	0.566021	-5.46869	0.861119	0.764934
NK.cells	RIOK3	0.050338	7.578172	0.575521	0.566401	-6.83773	0.784633	0.644102
NK.cells	EMC3	0.05452	5.490353	0.575461	0.566441	-6.47308	0.809579	0.682656
NK.cells	GM12840	0.150858	3.766389	0.575306	0.566545	-6.22224	0.830833	0.716232
NK.cells	ECPAS	0.05132	6.743157	0.575295	0.566553	-6.65879	0.794506	0.659271
NK.cells	RASGEF1B	0.146022	5.547786	0.575283	0.566561	-6.212	0.808881	0.681581
NK.cells	OTUD4	0.060993	5.091742	0.575058	0.566713	-6.42176	0.81444	0.690347
NK.cells	CERCAM	0.215946	0.652443	0.574871	0.566839	-5.38937	0.870765	0.781178
NK.cells	ZBTB1	-0.0633	5.505188	-0.57467	0.566976	-6.54948	0.809398	0.68256
NK.cells	AP4M1	0.086083	3.581284	0.574639	0.566995	-5.96402	0.833151	0.72011
NK.cells	AB124611	0.079959	5.452214	0.574636	0.566997	-6.34951	0.810042	0.683567
NK.cells	CYC1	-0.06624	6.36195	-0.5746	0.567024	-6.62164	0.79906	0.666471
NK.cells	CCNC	0.070488	4.398502	0.574571	0.567041	-6.35506	0.82297	0.703916
NK.cells	STK3	0.074393	5.985237	0.574551	0.567054	-6.46201	0.803587	0.673498
NK.cells	ZFP618	0.31573	0.531603	0.574447	0.567124	-5.17801	0.872355	0.783882
NK.cells	TMEM230	-0.06166	4.742854	-0.57438	0.56717	-6.29003	0.818721	0.697267
NK.cells	LRMP	-0.06556	6.432878	-0.57423	0.567269	-6.58706	0.79821	0.665273
NK.cells	SNHG4	0.095659	3.683196	0.574156	0.56732	-6.06196	0.831874	0.718197
NK.cells	PSMD6	-0.06796	5.199485	-0.57412	0.567343	-6.39843	0.813123	0.688519
NK.cells	MKRN1	0.068462	7.149614	0.574113	0.567349	-6.84288	0.789683	0.652134
NK.cells	CCDC112	-0.24926	1.151644	-0.57406	0.567383	-5.3757	0.864228	0.770622
NK.cells	BLOC1S5	-0.17934	1.955013	-0.574	0.567428	-5.54094	0.853817	0.753633

NK.cells	DPH3	-0.05493	5.480129	-0.57391	0.567484	-6.44852	0.809703	0.683252
NK.cells	ZFP729A	0.122628	3.051831	0.573723	0.567612	-5.86198	0.839833	0.731063
NK.cells	PPARGC1A	0.290699	1.419055	0.573695	0.567631	-5.36122	0.860761	0.765021
NK.cells	CCDC124	-0.05208	5.595782	-0.57302	0.568086	-6.50439	0.808755	0.68136
NK.cells	GM15943	0.306483	0.207344	0.572945	0.568136	-5.24467	0.877132	0.791581
NK.cells	PDZK1	-0.24838	0.933701	-0.57287	0.568185	-5.45311	0.867565	0.77578
NK.cells	ATP5J	0.05001	7.873083	0.572822	0.568219	-6.88879	0.781619	0.639556
NK.cells	GM17435	-0.20032	1.133905	-0.57254	0.568412	-5.47821	0.86504	0.771533
NK.cells	HELZ	0.053876	6.25998	0.572291	0.568577	-6.63041	0.80082	0.668978
NK.cells	GM38604	0.145787	1.796583	0.572103	0.568704	-5.68581	0.856435	0.757542
NK.cells	TAF6L	0.134762	2.800016	0.572026	0.568756	-5.69929	0.843578	0.73672
NK.cells	GALNT16	-0.20855	0.933714	-0.57172	0.568962	-5.41208	0.867657	0.776076
NK.cells	PLSCR1	0.090967	4.550033	0.571603	0.569042	-6.38162	0.821649	0.701811
NK.cells	CCT6B	0.312943	0.315333	0.571576	0.569059	-5.21505	0.875796	0.789518
NK.cells	RHBDD2	0.132417	2.284757	0.571548	0.569078	-5.7265	0.850154	0.747473
NK.cells	NKG7	-0.08802	4.013022	-0.57151	0.569107	-6.68472	0.828312	0.712381
NK.cells	PIGS	-0.07237	4.078714	-0.57149	0.56912	-6.20769	0.827493	0.711079
NK.cells	TMEM229I	-0.10125	4.03146	-0.57138	0.569192	-6.0093	0.828082	0.712051
NK.cells	GM30054	0.158652	1.953461	0.571189	0.569321	-5.78011	0.854411	0.754566
NK.cells	LHFPL2	-0.17371	2.227897	-0.57112	0.569366	-5.64282	0.850883	0.748857
NK.cells	P4HB	0.048104	7.267668	0.571093	0.569385	-6.77512	0.788817	0.650858
NK.cells	TSEN54	-0.10129	2.874509	-0.571	0.569451	-5.90883	0.842631	0.735552
NK.cells	AGTRAP	0.101301	4.534904	0.570844	0.569554	-6.28141	0.821836	0.702365
NK.cells	UPRT	0.205015	0.606501	0.570749	0.569617	-5.42866	0.871954	0.783449
NK.cells	SUCLA2	-0.05472	5.593683	-0.57055	0.569752	-6.50463	0.808866	0.681987
NK.cells	VPS35L	0.056308	5.162292	0.570491	0.569792	-6.36871	0.814123	0.690232
NK.cells	GM9917	-0.2223	0.773036	-0.57045	0.569821	-5.49359	0.869764	0.779865
NK.cells	OIP5OS1	0.053397	6.02273	0.570132	0.570034	-6.6005	0.803675	0.673984
NK.cells	EIF4E	-0.06319	6.950147	-0.56997	0.570142	-6.70536	0.792578	0.656831
NK.cells	PNPLA6	0.111854	2.760588	0.569958	0.570151	-5.81865	0.844079	0.738075
NK.cells	ZFP945	-0.10505	3.17689	-0.56995	0.570156	-5.94176	0.838802	0.729576
NK.cells	GRINA	0.089342	6.205395	0.569937	0.570166	-6.60045	0.801476	0.670594
NK.cells	POMGNT1	-0.12639	2.312495	-0.56969	0.570332	-5.71761	0.849798	0.7474
NK.cells	TNFRSF1A	-0.06763	5.171275	-0.56959	0.5704	-6.54842	0.814014	0.690268
NK.cells	ARPC1A	-0.04917	6.586291	-0.56936	0.570554	-6.67363	0.796911	0.663669
NK.cells	CETN3	-0.06173	5.807387	-0.56914	0.570705	-6.52001	0.806276	0.678271
NK.cells	GMIP	0.052766	5.964525	0.569111	0.570724	-6.55135	0.804377	0.675323
NK.cells	GM38394	-0.23352	0.848639	-0.56875	0.570966	-5.37389	0.868772	0.77878
NK.cells	TPM3	-0.0312	8.661561	-0.56852	0.571123	-7.00549	0.772543	0.626616
NK.cells	MFSD11	0.114189	3.854162	0.568393	0.571209	-5.92632	0.830294	0.716339
NK.cells	ARID3A	-0.08077	5.513727	-0.56823	0.571319	-6.28001	0.809838	0.68402
NK.cells	GNG2	0.058658	6.302982	0.568159	0.571367	-6.63551	0.800303	0.669154
NK.cells	PLA2G4A	0.229833	3.294743	0.568091	0.571413	-5.50809	0.837315	0.727602
NK.cells	TMEM198I	-0.25751	0.786307	-0.568	0.571475	-5.26804	0.86959	0.780159
NK.cells	GPATCH4	0.103928	3.338867	0.567819	0.571597	-5.99321	0.836759	0.726714
NK.cells	RUVBL1	0.066414	4.77265	0.567814	0.5716	-6.32354	0.818904	0.698284
NK.cells	PDCD11	-0.0698	4.214015	-0.56778	0.571621	-6.27155	0.825811	0.709228

NK.cells	ZFP36	-0.08109	7.849088	-0.56777	0.571628	-6.77921	0.781983	0.64097
NK.cells	IL1RL1	0.242002	-0.28284	0.567619	0.571732	-5.42868	0.883745	0.803725
NK.cells	GM36660	-0.30608	-0.14918	-0.56761	0.571741	-5.13065	0.881962	0.80075
NK.cells	SMTN	-0.1838	2.149503	-0.56745	0.571845	-5.52177	0.851889	0.751253
NK.cells	GM28501	0.214361	0.476037	0.567439	0.571854	-5.39203	0.873673	0.787013
NK.cells	WDR74	0.069919	4.042303	0.56708	0.572097	-6.15389	0.827947	0.712782
NK.cells	5031434O1	-0.24145	0.524887	-0.56701	0.572143	-5.2955	0.873029	0.786029
NK.cells	CKAP4	0.180544	4.030233	0.566922	0.572204	-5.65998	0.828097	0.71303
NK.cells	GM37401	-0.24274	0.875344	-0.56686	0.572247	-5.32077	0.868422	0.778417
NK.cells	9930021J0	-0.05398	6.407459	-0.56673	0.572334	-6.62784	0.799051	0.667373
NK.cells	GM20721	-0.08976	3.72758	-0.56665	0.572387	-6.12444	0.831877	0.71908
NK.cells	GM11464	0.298759	-0.20123	0.566555	0.572452	-5.15708	0.882656	0.802086
NK.cells	DHX16	-0.08812	4.087189	-0.5665	0.572491	-6.15888	0.827388	0.711948
NK.cells	SDF2L1	-0.076	4.888426	-0.56625	0.57266	-6.42416	0.81748	0.696244
NK.cells	BCAP31	0.049654	5.901725	0.566209	0.572686	-6.54685	0.805135	0.676877
NK.cells	NTPCR	0.093049	3.847172	0.566178	0.572707	-6.09826	0.830381	0.716722
NK.cells	SGK1	-0.0866	5.355259	-0.56617	0.572711	-6.37753	0.811767	0.687256
NK.cells	HOMEZ	0.194418	1.333922	0.566109	0.572754	-5.42352	0.862433	0.768615
NK.cells	COQ10B	-0.0501	5.908947	-0.56589	0.572902	-6.64011	0.805048	0.676791
NK.cells	GAK	-0.04395	6.280821	-0.56561	0.573093	-6.64301	0.800569	0.669886
NK.cells	COLQ	0.244688	0.244853	0.56557	0.573119	-5.2917	0.876729	0.792353
NK.cells	GM26936	0.273773	0.478634	0.565514	0.573157	-5.27051	0.873639	0.787236
NK.cells	ATAD5	-0.10671	4.742851	-0.56543	0.57321	-6.32647	0.819271	0.699232
NK.cells	HIST1H3C	-0.26684	1.047316	-0.56524	0.573343	-5.46584	0.866171	0.774939
NK.cells	DAD1	0.046581	6.948328	0.565178	0.573384	-6.77031	0.792599	0.657605
NK.cells	1810044D0	0.169026	1.915203	0.565111	0.57343	-5.62667	0.854904	0.756503
NK.cells	NOC4L	0.081342	3.728892	0.56508	0.573451	-6.10746	0.83186	0.719282
NK.cells	FAM110A	-0.09959	3.760358	-0.56507	0.573458	-6.10064	0.831467	0.718653
NK.cells	PLEKHO2	0.066578	6.069061	0.565051	0.57347	-6.47998	0.803116	0.673922
NK.cells	AGGF1	-0.05284	5.384652	-0.56484	0.573616	-6.47675	0.811409	0.686909
NK.cells	SHLD3	-0.13868	2.343634	-0.56479	0.573645	-5.72634	0.849399	0.747585
NK.cells	PAK1IP1	-0.04394	5.901653	-0.56465	0.573743	-6.5583	0.805136	0.677125
NK.cells	TRIB3	0.255688	1.057747	0.564633	0.573753	-5.44299	0.866035	0.774815
NK.cells	MLF1	0.25298	0.512644	0.564302	0.573977	-5.31193	0.873191	0.786707
NK.cells	SERPINH1	-0.20645	2.557349	-0.56425	0.574009	-5.60064	0.846668	0.743246
NK.cells	NIFK	0.070927	4.620947	0.564029	0.574163	-6.31157	0.820773	0.701773
NK.cells	CMPK1	0.040669	6.881961	0.563971	0.574202	-6.72747	0.793388	0.65896
NK.cells	KMT5C	0.127811	2.877228	0.563545	0.574491	-5.81789	0.842597	0.736787
NK.cells	CNN2	0.061671	6.538205	0.563478	0.574536	-6.82335	0.797486	0.665417
NK.cells	RDX	0.040174	6.872179	0.563446	0.574557	-6.70584	0.793504	0.659259
NK.cells	NUDCD3	0.045343	5.998557	0.56343	0.574568	-6.55735	0.803966	0.67549
NK.cells	ZDHHC23	-0.10601	2.221064	-0.56339	0.574599	-5.97181	0.85097	0.750373
NK.cells	WLS	-0.09579	4.263574	-0.56316	0.574748	-6.23298	0.825196	0.709001
NK.cells	5430427O1	-0.13936	3.846602	-0.56316	0.574749	-5.80906	0.830388	0.717277
NK.cells	PLCD3	0.256887	1.122441	0.562859	0.574956	-5.24577	0.86519	0.773812
NK.cells	CAT	-0.08116	6.72379	-0.56267	0.575085	-6.67886	0.795271	0.662236
NK.cells	UQCRC1	0.051863	6.508803	0.562653	0.575095	-6.66659	0.797837	0.666214

NK.cells	ARFGAP2	0.055927	4.901097	0.5626	0.575131	-6.37193	0.817325	0.696724
NK.cells	LRRN3	-0.36295	-0.11923	-0.56257	0.57515	-5.08581	0.881564	0.801096
NK.cells	DYRK2	0.066491	4.884934	0.562566	0.575154	-6.33314	0.817523	0.697037
NK.cells	ZDHHC7	0.081872	3.834677	0.562446	0.575235	-6.14055	0.830537	0.717716
NK.cells	NDUFAF3	0.079923	4.160988	0.562323	0.575319	-6.13718	0.82647	0.711227
NK.cells	0610010K1	0.059011	5.617735	0.562302	0.575333	-6.49034	0.808574	0.682961
NK.cells	HECTD3	-0.10494	2.789878	-0.56229	0.57534	-5.87081	0.843706	0.738887
NK.cells	TBC1D24	-0.19426	1.799284	-0.56228	0.575349	-5.48	0.8564	0.759525
NK.cells	TPM3-RS7	-0.23651	0.526188	-0.56223	0.575384	-5.31245	0.873012	0.786872
NK.cells	SPRY1	-0.26792	1.345776	-0.56196	0.575567	-5.34311	0.862279	0.76919
NK.cells	SLMAP	0.051718	6.393027	0.561814	0.575664	-6.65691	0.799224	0.668404
NK.cells	SCPEP1	0.093605	5.169388	0.561799	0.575674	-6.16227	0.814037	0.691567
NK.cells	SPRYD7	-0.13246	2.133927	-0.56179	0.575677	-5.76538	0.852089	0.752521
NK.cells	TAGLN	0.257915	2.304796	0.561681	0.575755	-5.63071	0.849897	0.748954
NK.cells	LMAN2	-0.04664	6.112382	-0.56163	0.575788	-6.58503	0.802595	0.673647
NK.cells	NCS1	-0.30262	0.210797	-0.56158	0.57582	-5.22475	0.87718	0.793841
NK.cells	CCL27A	-0.16995	1.684593	-0.56154	0.57585	-5.54479	0.857882	0.762006
NK.cells	INTS4	-0.07896	3.920928	-0.56149	0.575883	-6.17341	0.82946	0.716047
NK.cells	ZFP865	0.106531	3.265059	0.561491	0.575883	-5.96755	0.837689	0.729237
NK.cells	UFL1	0.076541	3.965196	0.561132	0.576127	-6.12163	0.828908	0.715196
NK.cells	BLOC1S2	-0.07771	4.833886	-0.56107	0.576166	-6.22472	0.81815	0.698109
NK.cells	NUMB	0.054139	6.956562	0.560985	0.576226	-6.7343	0.792502	0.658049
NK.cells	PLPP6	-0.11556	2.754142	-0.56098	0.576229	-5.88676	0.844161	0.739708
NK.cells	NFE2	0.237532	1.59738	0.560973	0.576235	-5.3435	0.859012	0.763888
NK.cells	ZDHHC8	-0.08688	3.666743	-0.56072	0.576409	-6.10921	0.832639	0.721248
NK.cells	LAMA4	0.327064	1.088771	0.560689	0.576428	-5.27861	0.865629	0.774854
NK.cells	PRMT1	0.054639	5.803475	0.56068	0.576434	-6.54494	0.806323	0.679598
NK.cells	CAMSAP2	-0.07917	4.496866	-0.56041	0.576615	-6.20564	0.822306	0.704782
NK.cells	HEBP1	-0.14882	4.67467	-0.56028	0.576707	-6.12709	0.820111	0.701344
NK.cells	PRRG1	0.191965	1.915898	0.560245	0.576729	-5.61604	0.854895	0.757299
NK.cells	PSMB4	-0.05202	6.172049	-0.55999	0.576901	-6.60888	0.801877	0.672767
NK.cells	FLNA	-0.07036	6.097597	-0.5597	0.577098	-6.65028	0.802773	0.674189
NK.cells	USPL1	0.090617	3.533846	0.559661	0.577126	-6.01341	0.834306	0.724064
NK.cells	DNAIC1	-0.28871	0.436784	-0.5596	0.577168	-5.24141	0.874191	0.78917
NK.cells	NT5DC3	-0.06778	5.099601	-0.55956	0.577196	-6.37201	0.81489	0.693184
NK.cells	IRGM2	0.142061	2.731293	0.559497	0.577237	-6.00411	0.844452	0.740413
NK.cells	ZFP326	0.05673	5.21559	0.559433	0.57728	-6.42325	0.813472	0.69095
NK.cells	NMT1	0.043379	6.941125	0.559413	0.577294	-6.7254	0.792685	0.658541
NK.cells	CACFD1	-0.09841	3.174111	-0.5594	0.577301	-5.90829	0.838837	0.731348
NK.cells	TMEM9B	0.049886	5.558526	0.55936	0.57733	-6.5264	0.809293	0.684384
NK.cells	MLST8	0.185519	2.044303	0.559335	0.577347	-5.54342	0.853241	0.754696
NK.cells	TDRD7	0.110107	3.385152	0.559331	0.57735	-5.98853	0.836176	0.727066
NK.cells	GM29585	-0.24348	1.247477	-0.5592	0.57744	-5.36428	0.863559	0.771631
NK.cells	CSPP1	-0.06128	6.098729	-0.55906	0.577534	-6.55867	0.802759	0.674197
NK.cells	GM20707	0.146503	1.898927	0.559059	0.577535	-5.54395	0.855114	0.757785
NK.cells	OSGEP	0.067232	4.907558	0.559044	0.577545	-6.29144	0.817245	0.69693
NK.cells	NAGLU	-0.1354	2.6543	-0.55897	0.577596	-5.73505	0.845432	0.742033

NK.cells	KDELR1	-0.04907	6.091298	-0.55896	0.577602	-6.5694	0.802848	0.674337
NK.cells	INO80C	-0.09943	3.401246	-0.55876	0.577736	-5.93898	0.836067	0.726846
NK.cells	BACE2	-0.27725	1.120623	-0.55849	0.57792	-5.28495	0.865419	0.774517
NK.cells	GM29707	0.290338	-0.46354	0.558413	0.577974	-5.1078	0.886372	0.809345
NK.cells	BTBD11	-0.10542	4.309098	-0.55835	0.578016	-6.45213	0.824827	0.708791
NK.cells	PROM1	-0.35419	0.403745	-0.55823	0.578098	-5.20447	0.874839	0.790111
NK.cells	A230059L0	-0.28736	0.47539	-0.55814	0.578157	-5.33109	0.873893	0.788551
NK.cells	PELO	-0.09815	3.125716	-0.55801	0.57825	-5.96312	0.839654	0.732594
NK.cells	B230307C2	0.083088	3.741811	0.557939	0.578296	-6.12411	0.831902	0.720167
NK.cells	C9	-0.37666	0.60037	-0.55783	0.578369	-5.27478	0.872254	0.785961
NK.cells	FAM131A	0.293823	0.401139	0.557515	0.578584	-5.2987	0.874976	0.790437
NK.cells	CARS	-0.1039	4.118134	-0.55745	0.578629	-6.10627	0.8273	0.712823
NK.cells	MTMR2	0.056153	5.396075	0.557407	0.578658	-6.44904	0.811561	0.687911
NK.cells	ZFP428	-0.18923	2.058083	-0.55733	0.578708	-5.46818	0.85337	0.754873
NK.cells	PDE1C	-0.15685	3.989093	-0.55708	0.578877	-6.10622	0.829049	0.715481
NK.cells	SMARCD2	-0.04926	5.611886	-0.55699	0.578945	-6.48262	0.809074	0.683907
NK.cells	ABLIM2	-0.2258	-0.47962	-0.55674	0.579113	-5.42162	0.886999	0.810168
NK.cells	TWNK	-0.14111	2.886068	-0.55652	0.57926	-5.75548	0.843128	0.737879
NK.cells	DAXX	0.092167	4.352332	0.556482	0.579287	-6.1936	0.824724	0.708395
NK.cells	GM15445	0.214363	0.994582	0.556173	0.579498	-5.35291	0.867734	0.777858
NK.cells	KMT5B	0.051354	5.91383	0.555932	0.579661	-6.57936	0.805863	0.678419
NK.cells	2700062C0	0.109434	2.646093	0.555897	0.579685	-5.82156	0.846454	0.743015
NK.cells	MRPL51	0.073906	4.486929	0.555695	0.579823	-6.22544	0.823322	0.705941
NK.cells	CIART	-0.15351	2.328452	-0.55569	0.579828	-5.72425	0.850516	0.74964
NK.cells	FBXL22	-0.15857	2.248825	-0.55559	0.579894	-5.7244	0.851538	0.751302
NK.cells	SEM1	0.040491	9.100384	0.555497	0.579958	-7.04522	0.768331	0.620867
NK.cells	RPAP2	0.098419	3.02458	0.555253	0.580124	-5.93992	0.841782	0.735334
NK.cells	NEK1	-0.11116	3.697931	-0.55458	0.58058	-5.99971	0.833652	0.721873
NK.cells	SPTB	-0.24132	1.120897	-0.55426	0.580797	-5.45717	0.866669	0.775646
NK.cells	ZFP850	-0.18618	1.010206	-0.55419	0.580846	-5.42068	0.868118	0.778042
NK.cells	PLPP2	0.158676	1.875681	0.554163	0.580866	-5.58309	0.856856	0.759551
NK.cells	ITPK1	-0.05135	5.403224	-0.55392	0.581033	-6.57329	0.812551	0.688541
NK.cells	A530064D0	0.303731	0.81889	0.553909	0.581039	-5.19326	0.870629	0.782193
NK.cells	CCSER1	-0.28767	1.697174	-0.55389	0.581055	-5.48308	0.859166	0.763335
NK.cells	CIC	-0.06813	4.821881	-0.5536	0.581249	-6.33729	0.819678	0.699836
NK.cells	GGH	0.090141	4.555539	0.553523	0.581303	-6.33067	0.822967	0.705044
NK.cells	AU041133	0.18108	1.264215	0.553349	0.581421	-5.45026	0.864797	0.772651
NK.cells	RTN1	0.322561	2.561189	0.553303	0.581452	-5.3341	0.848047	0.745303
NK.cells	TMED1	-0.14696	2.452472	-0.55328	0.58147	-5.62428	0.849438	0.74756
NK.cells	BC051226	0.133926	2.179737	0.553237	0.581498	-5.65389	0.852937	0.753252
NK.cells	ADNP	-0.04882	6.50802	-0.55298	0.581675	-6.64926	0.799193	0.667804
NK.cells	AGO1	0.089949	3.874896	0.552959	0.581687	-6.11517	0.831435	0.718595
NK.cells	KIF22	-0.13162	4.043139	-0.5528	0.581797	-6.18571	0.829333	0.715238
NK.cells	NECTIN1	-0.17866	1.073651	-0.55277	0.581813	-5.65931	0.867287	0.776816
NK.cells	LGALS8	0.069825	5.110017	0.552696	0.581866	-6.38282	0.816137	0.694317
NK.cells	WDR47	-0.10412	3.140773	-0.55266	0.581891	-5.95089	0.840674	0.733436
NK.cells	CPSF6	-0.03743	6.68211	-0.55265	0.581901	-6.67816	0.79711	0.664584

NK.cells	PGM2	0.077121	4.115633	0.552436	0.582044	-6.20843	0.828429	0.713912
NK.cells	CCDC73	0.100963	3.100981	0.552392	0.582074	-5.90403	0.841178	0.734358
NK.cells	DOCK10	0.047346	8.80921	0.552281	0.582149	-7.16474	0.772142	0.626478
NK.cells	4933406I1	-0.08974	4.543564	-0.55225	0.582171	-6.50226	0.823115	0.705461
NK.cells	CRNKL1	-0.05346	5.293781	-0.55222	0.582188	-6.48697	0.813888	0.69089
NK.cells	KAT7	-0.05025	5.68662	-0.5519	0.582406	-6.51517	0.809101	0.683375
NK.cells	JCAD	0.22028	1.404554	0.551894	0.582414	-5.32443	0.862967	0.769845
NK.cells	SNX15	0.067238	4.880351	0.551665	0.58257	-6.33471	0.818958	0.698888
NK.cells	TOP1MT	-0.20474	1.70028	-0.55159	0.582619	-5.46698	0.859126	0.763543
NK.cells	MCAT	0.128287	2.553855	0.551533	0.58266	-5.76719	0.848141	0.74565
NK.cells	CDKL1	-0.3244	1.100722	-0.55151	0.582676	-5.26767	0.866933	0.776388
NK.cells	PTRH2	0.07794	4.181735	0.551312	0.58281	-6.18062	0.827606	0.712664
NK.cells	RANBP2	-0.06103	6.846596	-0.55129	0.582827	-6.7212	0.795147	0.661706
NK.cells	MRPS14	0.045382	6.892734	0.551219	0.582874	-6.73436	0.794598	0.660872
NK.cells	AKR1C13	-0.13988	1.84784	-0.55117	0.582907	-5.80876	0.857216	0.760511
NK.cells	GM4869	-0.24291	1.447403	-0.5511	0.582953	-5.3983	0.86241	0.769031
NK.cells	RICTOR	0.047866	6.409431	0.551088	0.582963	-6.66733	0.800375	0.669849
NK.cells	CFAP97	0.082715	3.29046	0.550939	0.583065	-5.98243	0.838781	0.730607
NK.cells	RBMX	-0.06799	4.578527	-0.55083	0.583138	-6.25513	0.822682	0.704894
NK.cells	SLC9A6	-0.10504	2.81804	-0.55075	0.583193	-5.86232	0.844771	0.740303
NK.cells	GEMIN6	0.140012	2.59595	0.550626	0.583279	-5.72866	0.847603	0.744923
NK.cells	AMDHD2	-0.09327	3.693973	-0.55059	0.583305	-6.00832	0.833702	0.722509
NK.cells	BRD8	-0.04511	6.236165	-0.5505	0.583363	-6.6067	0.802457	0.673169
NK.cells	COPS7B	0.093915	3.34463	0.550327	0.583483	-5.99144	0.838098	0.729593
NK.cells	BCL2L1	0.082174	6.15449	0.550319	0.583488	-6.61939	0.803441	0.674701
NK.cells	GABBR1	-0.16301	2.766527	-0.55006	0.583665	-6.00822	0.845491	0.741523
NK.cells	DUS4L	0.155635	1.929292	0.549956	0.583736	-5.60189	0.856229	0.758998
NK.cells	TENM2	-0.39995	-0.35252	-0.54987	0.583792	-5.12537	0.886235	0.808683
NK.cells	SUGP2	-0.09007	4.059588	-0.54985	0.583809	-6.19788	0.82919	0.715347
NK.cells	2310058D1	0.112593	2.785149	0.549633	0.583957	-5.71788	0.845328	0.741193
NK.cells	KANSL2	-0.06308	5.119718	-0.54944	0.58409	-6.34994	0.816152	0.694566
NK.cells	COPS6	0.058904	5.552293	0.549343	0.584155	-6.4967	0.810867	0.686251
NK.cells	TTC37	0.099007	3.798842	0.549294	0.584189	-6.14659	0.832523	0.720579
NK.cells	PREPL	0.157538	1.734988	0.549225	0.584235	-5.56805	0.858817	0.763158
NK.cells	G3BP2	-0.0371	6.759047	-0.54911	0.584316	-6.72321	0.796321	0.66361
NK.cells	MTMR9	-0.07122	3.972449	-0.54904	0.584361	-6.14016	0.830351	0.71714
NK.cells	MIS18BP1	-0.11617	3.964435	-0.54889	0.584463	-6.16113	0.830451	0.717375
NK.cells	TSNAX	-0.06885	4.49606	-0.54876	0.584552	-6.30288	0.823837	0.706896
NK.cells	MRPS5	-0.06773	4.612666	-0.54876	0.584552	-6.29276	0.822394	0.704605
NK.cells	NRBP1	0.050775	5.827937	0.54851	0.584724	-6.55495	0.807571	0.681299
NK.cells	DSTYK	0.084595	3.925134	0.548307	0.584863	-6.01161	0.830996	0.718431
NK.cells	JMJD4	-0.16616	1.268434	-0.54829	0.584874	-5.46263	0.864939	0.77353
NK.cells	PFDN4	0.067113	5.046958	0.548268	0.58489	-6.40147	0.817098	0.696345
NK.cells	TOM1	-0.08487	5.431971	-0.5482	0.584936	-6.29771	0.812386	0.688955
NK.cells	ZFP51	0.100703	2.86166	0.547941	0.585113	-5.87142	0.844565	0.740068
NK.cells	RAB11FIP3	-0.128	2.230172	-0.54764	0.585317	-5.75663	0.852766	0.753253
NK.cells	DPAGT1	0.115261	3.285854	0.547363	0.585509	-5.89558	0.83931	0.731591

NK.cells	SF3A3	-0.0649	4.984502	-0.54727	0.585572	-6.38304	0.818136	0.697861
NK.cells	PPT2	0.072565	4.199673	0.54698	0.585771	-6.33497	0.827846	0.713322
NK.cells	TPCN1	0.106604	4.187411	0.546864	0.585849	-6.13644	0.827999	0.713584
NK.cells	KLHL36	-0.15454	2.076404	-0.54678	0.585908	-5.70223	0.854745	0.75675
NK.cells	TMEM223	0.066661	4.652686	0.546768	0.585915	-6.2959	0.822226	0.704402
NK.cells	CARMIL2	0.118265	2.704609	0.546711	0.585955	-5.8672	0.84669	0.743644
NK.cells	GET4	0.062634	5.110437	0.54666	0.58599	-6.39015	0.816589	0.695482
NK.cells	ZFP407	0.045028	7.039039	0.546595	0.586034	-6.73549	0.793302	0.659125
NK.cells	PI4K2B	0.088068	3.903982	0.54657	0.586051	-6.10625	0.831537	0.719236
NK.cells	HARS2	-0.0948	2.848743	-0.54652	0.586088	-5.84542	0.844854	0.740668
NK.cells	RBFA	0.080062	4.826424	0.5465	0.586099	-6.26513	0.820082	0.701003
NK.cells	GBA2	0.109644	2.547922	0.54638	0.586181	-5.79972	0.848711	0.746917
NK.cells	PRPF38A	-0.06082	5.176932	-0.54625	0.586272	-6.43678	0.815822	0.694243
NK.cells	TTC32	0.086603	4.101346	0.54588	0.586523	-6.07173	0.829241	0.715451
NK.cells	POU3F1	-0.31487	0.061626	-0.54581	0.586571	-5.25772	0.881316	0.800516
NK.cells	GM19710	0.15691	2.595654	0.545601	0.586714	-5.80446	0.848256	0.74615
NK.cells	SPATA7	-0.2	1.309288	-0.54557	0.586735	-5.39901	0.86487	0.773319
NK.cells	GGACT	0.090718	3.170013	0.545518	0.586771	-5.92988	0.840948	0.734323
NK.cells	ROPN1L	0.172235	2.420763	0.545515	0.586773	-5.65956	0.850494	0.749789
NK.cells	CACUL1	-0.06053	6.117834	-0.54536	0.586878	-6.58642	0.804547	0.676486
NK.cells	CENPU	-0.13843	2.562499	-0.54506	0.587088	-5.70389	0.848866	0.746899
NK.cells	THOC2	-0.03747	7.106814	-0.54491	0.587184	-6.77974	0.792834	0.658232
NK.cells	UBE2T	-0.09658	4.06115	-0.54476	0.587287	-6.23361	0.829925	0.716508
NK.cells	TCEANC2	0.06935	4.247598	0.544591	0.587406	-6.19111	0.827601	0.712848
NK.cells	AP1M1	-0.06052	4.979621	-0.5445	0.587471	-6.3591	0.818544	0.698501
NK.cells	NABP2	0.057325	4.980112	0.544419	0.587524	-6.42739	0.818538	0.698503
NK.cells	ACYP1	0.091472	3.847714	0.544382	0.587549	-6.06472	0.832595	0.720871
NK.cells	NOP58	-0.06693	5.838554	-0.54433	0.587584	-6.55006	0.808054	0.682007
NK.cells	1500015A(	0.146902	1.76661	0.544236	0.587649	-5.56711	0.859113	0.763836
NK.cells	ZFP595	-0.12857	1.84184	-0.54419	0.58768	-5.57434	0.858139	0.762258
NK.cells	LEPR	0.266701	1.823369	0.544114	0.587733	-5.43802	0.858378	0.76265
NK.cells	MCM8	-0.18113	1.54016	-0.54381	0.587938	-5.50618	0.862252	0.768756
NK.cells	APOOL	0.08181	3.780654	0.543614	0.588075	-6.09919	0.833724	0.72237
NK.cells	ZKSCAN8	0.236538	0.632724	0.543362	0.588248	-5.28419	0.87437	0.788554
NK.cells	PPP2R3D	-0.09855	3.751015	-0.54324	0.588335	-6.0432	0.834218	0.723102
NK.cells	CEP72	-0.1518	2.208429	-0.54313	0.588407	-5.6152	0.853829	0.75481
NK.cells	TAGLN2	-0.06882	8.716101	-0.54299	0.588502	-7.03633	0.774359	0.629801
NK.cells	TSHZ2	-0.18856	3.393266	-0.54297	0.588515	-5.82641	0.838722	0.730389
NK.cells	HEATR6	0.066731	4.812778	0.542688	0.58871	-6.28868	0.821003	0.702194
NK.cells	DHX34	0.142804	2.203437	0.542634	0.588747	-5.65423	0.853893	0.755108
NK.cells	HYPK	-0.04957	5.8151	-0.54248	0.58885	-6.54045	0.808736	0.682974
NK.cells	KRTCAP2	0.046633	7.062528	0.542359	0.588935	-6.78604	0.793751	0.659663
NK.cells	GM12764	0.17117	1.453299	0.542225	0.589028	-5.60817	0.863608	0.771153
NK.cells	ATP6V1F	-0.0412	6.902317	-0.54213	0.58909	-6.73329	0.795658	0.662672
NK.cells	TSPO	0.068717	7.399271	0.542068	0.589135	-6.89988	0.789759	0.653575
NK.cells	FAM118B	0.090327	3.464677	0.541715	0.589377	-5.99055	0.837821	0.729386
NK.cells	GM37612	0.214468	0.985122	0.541641	0.589428	-5.38824	0.869731	0.781452

NK.cells	GPD2	-0.07717	6.625661	-0.5416	0.589458	-6.76652	0.798964	0.667954
NK.cells	BRPF1	0.069664	4.929241	0.541453	0.589557	-6.34587	0.819567	0.700295
NK.cells	PARPBP	-0.19041	2.056928	-0.5414	0.589591	-5.73686	0.855781	0.758563
NK.cells	NXPE2	-0.22045	1.56591	-0.54135	0.589629	-5.46427	0.862142	0.768989
NK.cells	RIPK1	0.055121	5.973193	0.541256	0.589692	-6.60994	0.80682	0.680234
NK.cells	SFXN2	-0.09231	3.18033	-0.54122	0.589719	-5.98034	0.841416	0.735227
NK.cells	SLC37A1	0.104758	3.202303	0.541194	0.589735	-6.02673	0.841137	0.734787
NK.cells	TRIM13	0.21628	0.46615	0.541148	0.589766	-5.44176	0.876572	0.792865
NK.cells	NCOA5	0.07925	4.146435	0.541002	0.589866	-6.19874	0.829269	0.715745
NK.cells	LRRC4C	-0.32661	0.648779	-0.5409	0.589938	-5.23608	0.874158	0.78886
NK.cells	ZFP664	0.071172	4.050718	0.540851	0.589971	-6.21695	0.830464	0.717655
NK.cells	BORCS5	0.072294	4.024358	0.540831	0.589984	-6.20019	0.830794	0.718181
NK.cells	ANKRA2	0.072174	3.792457	0.540718	0.590062	-6.14948	0.833698	0.722846
NK.cells	HIKESHI	0.056606	5.228907	0.540661	0.590101	-6.45564	0.815885	0.694539
NK.cells	ARL16	0.121944	2.250651	0.540305	0.590345	-5.67005	0.85338	0.754668
NK.cells	PDPR	0.087226	3.937711	0.540165	0.590441	-6.19596	0.831969	0.720106
NK.cells	USP34	-0.04016	7.703229	-0.54007	0.590508	-6.87754	0.786261	0.648442
NK.cells	GM10353	-0.11343	2.745067	-0.54003	0.590537	-5.88053	0.847044	0.744404
NK.cells	CBR4	0.130569	2.335594	0.539901	0.590623	-5.67277	0.852287	0.752931
NK.cells	TRP53BP2	-0.07652	3.957204	-0.5398	0.59069	-6.22128	0.831725	0.719724
NK.cells	MTTP	-0.15116	2.566189	-0.53967	0.590779	-5.72698	0.849331	0.748167
NK.cells	PDLIM5	-0.0588	6.703681	-0.53964	0.5908	-6.68194	0.798118	0.666781
NK.cells	ZFP677	0.203356	0.788621	0.539472	0.590918	-5.39232	0.872411	0.786114
NK.cells	MTHFD2L	0.106817	3.033816	0.539396	0.590969	-5.86485	0.843368	0.738584
NK.cells	TNS1	0.169902	2.761945	0.539222	0.591089	-5.68312	0.846829	0.744277
NK.cells	PISD	0.077145	4.8584	0.539175	0.591121	-6.30151	0.82053	0.702094
NK.cells	LYSMD4	-0.09891	3.330154	-0.53917	0.591125	-6.04458	0.839612	0.732603
NK.cells	GOLGA1	0.087529	3.427325	0.538983	0.591253	-6.00476	0.838385	0.730673
NK.cells	JADE1	0.095803	4.165478	0.538874	0.591328	-6.12548	0.829123	0.715815
NK.cells	CLEC10A	-0.33823	0.930777	-0.53884	0.59135	-5.21149	0.870541	0.783197
NK.cells	CLN5	0.078735	4.008222	0.538533	0.591562	-6.10506	0.831087	0.718955
NK.cells	PAK4	0.135748	2.299468	0.538518	0.591572	-5.66192	0.852752	0.753962
NK.cells	FMN1	-0.22571	3.350464	-0.53829	0.591732	-5.54674	0.839356	0.732321
NK.cells	MRPL33	0.053678	6.142277	0.538247	0.591759	-6.6522	0.804865	0.677564
NK.cells	CHORDC1	0.054921	5.082056	0.538162	0.591817	-6.4201	0.817778	0.697882
NK.cells	HSF2	0.076222	4.185277	0.538098	0.591861	-6.25332	0.828877	0.715534
NK.cells	ITGB1BP1	-0.06075	4.649919	-0.53808	0.591874	-6.33569	0.823106	0.706339
NK.cells	ERC2	0.379482	0.862583	0.537714	0.592125	-5.29896	0.871437	0.784958
NK.cells	ARMC5	0.084194	3.383082	0.537651	0.592168	-5.98799	0.838944	0.731844
NK.cells	HSD17B10	-0.06473	5.540491	-0.53765	0.59217	-6.49479	0.812167	0.689188
NK.cells	MCFD2	-0.11569	3.702476	-0.53762	0.592192	-5.92619	0.83492	0.725384
NK.cells	TOMM6	0.042852	7.550466	0.537397	0.592344	-6.84879	0.78806	0.651768
NK.cells	ZFP1	0.092122	3.874226	0.537379	0.592356	-6.02858	0.832764	0.722003
NK.cells	SMDT1	-0.03954	7.773748	-0.53737	0.592365	-6.89132	0.785432	0.64773
NK.cells	SIAH2	-0.0618	4.793136	-0.53734	0.592382	-6.34556	0.821336	0.703769
NK.cells	FAM219A	0.115271	4.171512	0.537334	0.592387	-6.0843	0.829048	0.716053
NK.cells	WDR77	0.092202	3.895337	0.537213	0.59247	-6.03923	0.8325	0.721637

NK.cells	UBE2Q2	-0.05156	5.51991	-0.5371	0.592546	-6.53444	0.812418	0.689781
NK.cells	RMND5B	0.065425	4.49308	0.537035	0.592592	-6.2726	0.825049	0.709808
NK.cells	IKZF4	0.201274	-0.0706	0.536937	0.59266	-5.37092	0.883804	0.805835
NK.cells	1700007L1	-0.18456	1.663099	-0.5365	0.592962	-5.48936	0.861312	0.768135
NK.cells	GFM1	0.080583	3.89287	0.536299	0.593099	-6.11298	0.832952	0.721965
NK.cells	COX14	0.052027	5.656597	0.53575	0.593476	-6.5283	0.811584	0.687581
NK.cells	NOLC1	-0.07149	5.264292	-0.53533	0.593767	-6.43297	0.8164	0.6952
NK.cells	STK40	-0.05742	5.69472	-0.53526	0.593814	-6.54068	0.811141	0.686919
NK.cells	FTO	0.045505	6.592827	0.534993	0.593998	-6.68947	0.800287	0.669974
NK.cells	ACOT13	-0.0634	4.43558	-0.53473	0.594181	-6.2311	0.826631	0.711491
NK.cells	EIF2AK4	0.083371	4.653928	0.534693	0.594205	-6.22571	0.823922	0.707186
NK.cells	ZRANB1	0.045843	6.142303	0.534569	0.594289	-6.60519	0.805711	0.678503
NK.cells	LCMT2	0.111358	2.687418	0.534429	0.594386	-5.80806	0.848672	0.747014
NK.cells	NOB1	-0.06397	4.287446	-0.53442	0.594395	-6.27968	0.828475	0.714484
NK.cells	AW11201C	0.12128	7.279978	0.534397	0.594408	-7.07762	0.79209	0.657372
NK.cells	MRPL17	0.056821	5.426857	0.534298	0.594476	-6.44303	0.814409	0.692176
NK.cells	MND1	-0.15729	2.222735	-0.53411	0.594606	-5.63072	0.854637	0.756801
NK.cells	5-Mar	0.059301	6.018808	0.534088	0.594621	-6.61463	0.807206	0.680925
NK.cells	AAMDC	0.07692	3.299635	0.533977	0.594698	-6.00431	0.840882	0.734484
NK.cells	ATN1	-0.09566	3.221914	-0.53391	0.594746	-6.06031	0.841867	0.736075
NK.cells	9330160F1	-0.15529	2.295032	-0.53376	0.594849	-5.59808	0.853706	0.7553
NK.cells	MED19	0.073338	3.778351	0.533697	0.59489	-6.07875	0.834844	0.724765
NK.cells	PRAF2	0.10618	2.133854	0.533692	0.594894	-5.80613	0.855783	0.758693
NK.cells	TRPC1	0.279748	-0.17084	0.533666	0.594912	-5.18358	0.886075	0.808854
NK.cells	CTTNBP2N	-0.18531	2.52025	-0.53366	0.594914	-5.46956	0.850813	0.750584
NK.cells	STX1A	-0.20529	1.792697	-0.53362	0.594946	-5.50711	0.860197	0.765923
NK.cells	TYSND1	-0.10262	2.640407	-0.53357	0.59498	-5.80485	0.849274	0.748079
NK.cells	DGKQ	0.164861	1.470676	0.533334	0.595141	-5.55139	0.864385	0.772887
NK.cells	SPNS3	-0.14948	3.650358	-0.5332	0.59523	-5.84681	0.836454	0.727458
NK.cells	TMEM189	0.066294	6.387922	0.533072	0.595321	-6.65923	0.802749	0.67413
NK.cells	TPD52L2	0.048394	5.433659	0.533052	0.595335	-6.47166	0.814326	0.692281
NK.cells	LTF	0.251705	2.942911	0.532867	0.595463	-5.65078	0.845412	0.742078
NK.cells	ARHGAP28	0.3167	0.598833	0.532753	0.595541	-5.28911	0.875835	0.792053
NK.cells	ATE1	-0.06852	3.945361	-0.53275	0.595545	-6.15973	0.832749	0.721674
NK.cells	FAM171A1	-0.24156	1.565475	-0.53247	0.595737	-5.34162	0.86315	0.771162
NK.cells	TIMELESS	0.10466	3.491901	0.532454	0.595747	-6.08207	0.838451	0.730931
NK.cells	DNAH10	-0.26954	0.024576	-0.53244	0.595759	-5.26233	0.883463	0.804887
NK.cells	MEGF11	-0.23295	0.651843	-0.53239	0.595792	-5.40269	0.875134	0.791025
NK.cells	RNF128	0.127472	2.499728	0.532259	0.595881	-5.88883	0.851076	0.751482
NK.cells	RBM48	0.090522	3.037834	0.532217	0.59591	-5.95604	0.844204	0.740317
NK.cells	EAF2	-0.09377	3.647314	-0.5322	0.595919	-5.9878	0.836492	0.727868
NK.cells	PHF5A	-0.05536	6.072643	-0.53198	0.596073	-6.59837	0.806642	0.680347
NK.cells	MAP3K7	-0.05027	5.219048	-0.53187	0.596152	-6.46825	0.817044	0.696709
NK.cells	CORO2B	0.228471	1.197161	0.531732	0.596245	-5.31828	0.868055	0.779193
NK.cells	CLEC4A4	0.241413	-1.00355	0.531513	0.596396	-5.14846	0.897333	0.828276
NK.cells	MED6	-0.07419	4.540282	-0.53145	0.596439	-6.28319	0.825421	0.710009
NK.cells	RNPEP	-0.07167	5.72187	-0.5314	0.596471	-6.52068	0.810899	0.68705

NK.cells	NDUFS1	-0.04415	5.752161	-0.53128	0.596557	-6.54783	0.81053	0.686485
NK.cells	YWHAH	-0.04907	7.687722	-0.5312	0.596609	-6.86783	0.787356	0.650474
NK.cells	ZBTB8OS	0.058812	5.027468	0.530984	0.596761	-6.44519	0.819399	0.700476
NK.cells	TMC6	-0.06451	4.632332	-0.53076	0.596916	-6.27768	0.824279	0.708224
NK.cells	CIB1	-0.06398	5.732242	-0.53074	0.596933	-6.41773	0.810772	0.686866
NK.cells	PLEKHM2	0.080834	5.202185	0.530277	0.597249	-6.45141	0.817251	0.697139
NK.cells	HMGCR	0.072114	4.712579	0.530218	0.59729	-6.28178	0.823286	0.706717
NK.cells	BTF3	0.035056	8.999494	0.530193	0.597307	-7.09928	0.77207	0.627223
NK.cells	ZBTB7B	0.130478	2.473173	0.529909	0.597503	-5.87841	0.85151	0.752238
NK.cells	ATG16L1	0.059339	5.446329	0.529835	0.597554	-6.48719	0.81426	0.692522
NK.cells	SELENOS	0.052197	6.113957	0.529781	0.597592	-6.55025	0.806142	0.679784
NK.cells	CRAT	-0.1018	3.198294	-0.52965	0.597681	-6.01684	0.842258	0.737262
NK.cells	FAM234B	0.192526	2.096184	0.529611	0.597709	-5.48705	0.856362	0.760213
NK.cells	A530088E(	0.145744	1.760356	0.529504	0.597783	-5.73274	0.86071	0.767362
NK.cells	CMYA5	-0.31474	0.674059	-0.52937	0.597872	-5.28113	0.874936	0.790877
NK.cells	CD68	-0.14141	4.872198	-0.52937	0.597873	-5.98264	0.821313	0.703718
NK.cells	MED1	0.040914	5.883036	0.529231	0.597971	-6.57637	0.80894	0.684189
NK.cells	CEP85	0.071587	4.470348	0.52917	0.598014	-6.26709	0.826289	0.711634
NK.cells	GM10974	0.180954	0.824708	0.529097	0.598064	-5.47973	0.872948	0.787575
NK.cells	BCL10	-0.04563	6.452929	-0.52905	0.598094	-6.65605	0.802054	0.67342
NK.cells	MYSM1	0.053433	5.388465	0.529	0.598131	-6.49826	0.814968	0.693675
NK.cells	ZFP951	0.134105	2.641469	0.528997	0.598133	-5.8169	0.849353	0.748789
NK.cells	GPX3	0.286202	1.463214	0.528996	0.598134	-5.38734	0.864577	0.773726
NK.cells	KLRB1C	0.106998	1.954564	0.528971	0.598151	-6.37794	0.858193	0.76323
NK.cells	ZMPSTE24	-0.08099	4.942134	-0.52892	0.598184	-6.29841	0.82045	0.702349
NK.cells	DCUN1D2	0.12513	2.743543	0.528852	0.598233	-5.84355	0.848047	0.746666
NK.cells	GM32051	0.291408	-0.14913	0.528794	0.598273	-5.28711	0.885881	0.809158
NK.cells	HSPBP1	-0.07092	3.934559	-0.52859	0.598414	-6.19135	0.833074	0.722346
NK.cells	GM20069	0.167304	-0.52038	0.528227	0.598665	-5.66458	0.891184	0.817636
NK.cells	PLSCR4	-0.23466	0.494112	-0.52818	0.598696	-5.37751	0.877631	0.794938
NK.cells	5830487J0	-0.25978	-0.01733	-0.52805	0.598791	-5.18178	0.884456	0.806332
NK.cells	XDH	0.106361	4.807401	0.527962	0.598848	-6.50781	0.822427	0.705106
NK.cells	CHADL	0.231051	0.647864	0.527782	0.598972	-5.34449	0.875696	0.791648
NK.cells	GM11476	0.125971	2.536389	0.527355	0.599268	-5.84514	0.851423	0.751263
NK.cells	SOX5	-0.12279	4.684841	-0.52692	0.599566	-6.31964	0.824504	0.707663
NK.cells	WDPCP	0.127604	3.269533	0.526888	0.599591	-5.80646	0.842249	0.736095
NK.cells	TRAF6	-0.05535	5.683882	-0.52687	0.599606	-6.50798	0.812223	0.688252
NK.cells	CDC42BPG	-0.16798	2.57838	-0.5267	0.599719	-5.65746	0.851064	0.750412
NK.cells	GRAMD1C	-0.13166	2.440736	-0.52667	0.599742	-5.80733	0.852831	0.75329
NK.cells	ESR1	-0.14605	3.611322	-0.52629	0.600002	-5.90444	0.838111	0.72927
NK.cells	GM38115	-0.07283	3.861728	-0.52595	0.600237	-6.25011	0.834958	0.724331
NK.cells	GM14029	-0.14455	0.297438	-0.52577	0.600364	-5.77696	0.881055	0.799875
NK.cells	SPCS1	-0.03986	7.852067	-0.5256	0.600484	-6.90703	0.786431	0.648268
NK.cells	GDAP2	-0.06163	4.778409	-0.52559	0.600487	-6.36308	0.823527	0.70618
NK.cells	TMEM202	0.265307	0.104736	0.525497	0.600553	-5.20927	0.883623	0.804192
NK.cells	CASP2	-0.07108	4.01281	-0.52537	0.600638	-6.1619	0.833062	0.721437
NK.cells	FBN1	0.249259	0.898383	0.52522	0.600745	-5.29578	0.873097	0.786759

NK.cells	HPGDS	-0.16326	3.647133	-0.52519	0.600767	-5.74865	0.837659	0.728868
NK.cells	SECTM1A	-0.34998	-1.13086	-0.52518	0.600774	-5.06477	0.900084	0.832333
NK.cells	E130215H2	0.292401	-0.13171	0.525115	0.600818	-5.17849	0.886784	0.809615
NK.cells	IRAK4	0.066697	4.419459	0.525021	0.600883	-6.27961	0.827983	0.713395
NK.cells	GM10053	0.200099	1.424678	0.524944	0.600936	-5.50425	0.86619	0.775406
NK.cells	NEK10	-0.24011	1.324395	-0.52441	0.601306	-5.42686	0.867501	0.777712
NK.cells	ACAT2	0.100035	2.91084	0.524381	0.601326	-5.89623	0.846998	0.744151
NK.cells	LGMN	-0.09583	7.188159	-0.52438	0.601326	-6.49112	0.794284	0.660621
NK.cells	2-Mar	0.053537	6.581903	0.524377	0.601329	-6.74611	0.801532	0.671865
NK.cells	AUP1	-0.04962	6.010505	-0.52426	0.601411	-6.61415	0.80843	0.682657
NK.cells	NCKAP1	0.117324	2.425386	0.524111	0.601513	-5.80905	0.853217	0.754361
NK.cells	PRODH	-0.19891	2.780405	-0.52408	0.601537	-5.5965	0.848664	0.746948
NK.cells	C1QTNF12	-0.14074	2.21932	-0.52406	0.60155	-5.73231	0.855871	0.758696
NK.cells	RASSF7	0.205643	0.86906	0.523982	0.601602	-5.3964	0.873483	0.787725
NK.cells	RFC2	-0.06439	5.227985	-0.52396	0.601615	-6.45559	0.817983	0.697773
NK.cells	GM17494	0.10673	2.389013	0.523913	0.60165	-5.74498	0.853684	0.755138
NK.cells	TES	0.055862	5.486918	0.523855	0.60169	-6.67467	0.814808	0.692761
NK.cells	CABIN1	0.054468	5.4487	0.523729	0.601778	-6.51981	0.815276	0.693498
NK.cells	BC035044	0.109307	5.146379	0.523539	0.601909	-6.2767	0.818986	0.699412
NK.cells	PHKG1	-0.22834	1.107052	-0.5235	0.601935	-5.4312	0.870351	0.782599
NK.cells	ASB13	0.094812	3.434394	0.52345	0.601971	-6.11909	0.840346	0.733566
NK.cells	TRAM2	0.075785	4.061754	0.523426	0.601987	-6.12799	0.832449	0.720869
NK.cells	R3HCC1	0.138825	2.353768	0.523236	0.602119	-5.77914	0.854138	0.755992
NK.cells	STAR	-0.20883	1.465058	-0.52317	0.602167	-5.50116	0.865662	0.774903
NK.cells	AP2M1	0.048986	6.941455	0.523114	0.602203	-6.75721	0.797225	0.665367
NK.cells	2610027K0	-0.19184	0.681173	-0.52307	0.602236	-5.44029	0.875964	0.791964
NK.cells	GM50240	0.16835	1.572659	0.522875	0.602369	-5.5516	0.864314	0.772647
NK.cells	ZSWIM9	-0.28422	0.544388	-0.52281	0.602414	-5.24941	0.877832	0.795038
NK.cells	YPEL3	-0.06069	7.069109	-0.52251	0.602621	-6.78111	0.795936	0.663122
NK.cells	AOPEP	0.048685	6.329542	0.522362	0.602725	-6.69283	0.804852	0.676955
NK.cells	HP1BP3	-0.04858	6.529858	-0.52215	0.602874	-6.70465	0.802546	0.673257
NK.cells	ZFP740	-0.05533	4.76726	-0.52198	0.602992	-6.36467	0.824074	0.707157
NK.cells	ITM2B	-0.04767	10.00633	-0.52167	0.603203	-7.23045	0.761918	0.611219
NK.cells	ARMC6	0.171952	1.405639	0.521637	0.603228	-5.46191	0.866869	0.776487
NK.cells	BLOC1S6	-0.08068	3.751167	-0.52152	0.603306	-6.0815	0.836764	0.727486
NK.cells	SDHAF3	0.098463	3.044533	0.521488	0.603331	-5.88637	0.845714	0.741941
NK.cells	TRIM41	-0.07109	4.185669	-0.52135	0.603427	-6.11263	0.831312	0.718814
NK.cells	DPF3	-0.18264	1.667384	-0.52129	0.60347	-5.60749	0.863453	0.771001
NK.cells	RAET1E	0.064882	4.113795	0.521201	0.603529	-6.45176	0.832211	0.720304
NK.cells	CCPG1	-0.05844	6.037912	-0.52112	0.603585	-6.55729	0.808499	0.682736
NK.cells	TBL3	0.07337	3.91508	0.521031	0.603647	-6.16732	0.834703	0.724302
NK.cells	ECI1	0.071346	3.920606	0.520826	0.60379	-6.22619	0.834633	0.724211
NK.cells	TRIM30D	0.13065	4.544173	0.520681	0.603891	-6.13303	0.826842	0.711749
NK.cells	PFKFB3	0.073104	6.273061	0.520566	0.60397	-6.5535	0.805652	0.678329
NK.cells	RNPEPL1	-0.07616	4.75379	-0.52048	0.604032	-6.29374	0.824241	0.707636
NK.cells	JAZF1	0.088784	3.086872	0.520262	0.604181	-6.18699	0.845175	0.741239
NK.cells	GM16316	0.212701	1.803894	0.520237	0.604199	-5.37171	0.861677	0.768165

NK.cells	REEP5	-0.04038	7.566136	-0.52015	0.604256	-6.84951	0.790195	0.654353
NK.cells	ANGPTL7	-0.36499	-0.05517	-0.52011	0.604288	-5.08804	0.8862	0.808885
NK.cells	USO1	0.051222	5.739311	0.520024	0.604347	-6.52258	0.81213	0.688527
NK.cells	GM26901	-0.21344	0.57091	-0.51992	0.604417	-5.28791	0.87786	0.794994
NK.cells	GM14326	0.098467	2.868013	0.519866	0.604456	-5.98944	0.847966	0.745818
NK.cells	ZKSCAN17	0.079857	3.498798	0.51982	0.604488	-6.09521	0.839949	0.732843
NK.cells	ERP27	0.163368	1.896721	0.519496	0.604713	-5.59811	0.860472	0.766378
NK.cells	MRPL46	0.091953	3.264758	0.519374	0.604798	-5.99327	0.842914	0.737802
NK.cells	SDF4	0.038239	6.506152	0.519285	0.60486	-6.73144	0.802842	0.674151
NK.cells	WDR61	0.054337	4.80713	0.51917	0.60494	-6.34414	0.82358	0.706806
NK.cells	RBM10	-0.0573	4.779494	-0.51883	0.605177	-6.37394	0.823923	0.70746
NK.cells	ZCCHC2	-0.06188	5.422407	-0.51872	0.605253	-6.51873	0.816003	0.69491
NK.cells	ABR	-0.07176	6.594873	-0.51869	0.605274	-6.8372	0.801774	0.672593
NK.cells	NAXD	0.073742	4.128354	0.518635	0.605311	-6.14537	0.832029	0.7204
NK.cells	TAOK2	-0.07487	4.127104	-0.51863	0.605317	-6.16558	0.832044	0.720425
NK.cells	GM37529	0.162403	1.962925	0.518557	0.605365	-5.72885	0.859613	0.765155
NK.cells	POU5F2	-0.15026	1.687999	-0.51847	0.605428	-5.61705	0.863184	0.771053
NK.cells	APIP	-0.07002	4.220389	-0.51824	0.605583	-6.15005	0.830878	0.718669
NK.cells	CD8A	-0.13072	1.360615	-0.51811	0.605673	-6.12657	0.867458	0.778203
NK.cells	ADGRL3	-0.30507	3.47827	-0.51802	0.60574	-5.68228	0.840208	0.733717
NK.cells	GM45442	0.358566	-0.57631	0.51794	0.605794	-5.09665	0.893205	0.821264
NK.cells	SLC35D1	-0.06535	4.114625	-0.5179	0.60582	-6.30607	0.832201	0.720875
NK.cells	MGA	0.058194	5.917554	0.517822	0.605876	-6.54954	0.809961	0.685586
NK.cells	MYC	0.133649	3.533741	0.517728	0.605941	-6.12039	0.839507	0.732624
NK.cells	MAT2B	0.04821	5.679223	0.51759	0.606037	-6.57045	0.812863	0.690199
NK.cells	TGOLN1	-0.05506	5.839155	-0.51752	0.606082	-6.57268	0.810914	0.687142
NK.cells	GM49625	0.147112	2.147374	0.517443	0.606139	-5.64287	0.857225	0.761538
NK.cells	MIER2	-0.1409	2.002333	-0.51739	0.606177	-5.66322	0.859102	0.764621
NK.cells	GM10135	0.221456	0.409376	0.517199	0.606309	-5.35725	0.880004	0.799256
NK.cells	UBQLN1	-0.04775	5.517917	-0.51712	0.606364	-6.48482	0.814834	0.693387
NK.cells	ZYG11B	0.048783	5.883084	0.517108	0.606372	-6.67908	0.81038	0.686374
NK.cells	CYSLTR2	0.111197	1.496466	0.517106	0.606374	-6.13554	0.865682	0.775481
NK.cells	RAB11FIP4	-0.12971	1.161785	-0.51705	0.606411	-5.75222	0.870065	0.782727
NK.cells	PRKX	-0.04823	5.379346	-0.51692	0.606505	-6.5897	0.816531	0.696141
NK.cells	MCCC2	0.176872	2.436244	0.516887	0.606526	-5.69202	0.853501	0.755573
NK.cells	5830432E0	0.142859	1.494203	0.516811	0.606578	-5.74514	0.865712	0.775658
NK.cells	ANXA7	-0.05457	5.409471	-0.5168	0.606588	-6.45112	0.816162	0.695599
NK.cells	FANCL	-0.06309	4.144904	-0.51664	0.606699	-6.28021	0.831822	0.720528
NK.cells	POLR2G	0.050685	5.51542	0.516536	0.60677	-6.48739	0.814864	0.693557
NK.cells	GM33370	0.250109	0.508404	0.516451	0.606829	-5.2671	0.878689	0.797223
NK.cells	SAE1	-0.0523	6.342657	-0.51638	0.606877	-6.63715	0.804812	0.677785
NK.cells	NEDD8	0.034242	7.48397	0.51633	0.606913	-6.84438	0.791167	0.65659
NK.cells	C330011M	-0.21401	0.592367	-0.51592	0.607197	-5.29908	0.877835	0.79551
NK.cells	CTTN	-0.19781	1.717261	-0.51585	0.607245	-5.43383	0.863059	0.771054
NK.cells	TOR2A	-0.07776	3.670287	-0.51574	0.607325	-6.08657	0.838031	0.730328
NK.cells	GM20528	0.276656	-0.01428	0.515596	0.607423	-5.2119	0.885914	0.809116
NK.cells	MOB3C	-0.09495	3.356469	-0.51547	0.607509	-5.96518	0.842	0.736818

NK.cells	CRTC1	0.113127	3.126748	0.515324	0.607613	-5.9105	0.844917	0.741571
NK.cells	WDR44	-0.06364	4.70447	-0.5153	0.607628	-6.44528	0.825096	0.709718
NK.cells	NCOA1	-0.05531	7.07231	-0.51527	0.607648	-6.75227	0.796295	0.66445
NK.cells	DNAJA2	-0.03489	7.208069	-0.51469	0.608055	-6.82295	0.794882	0.662037
NK.cells	F5	-0.17221	4.094976	-0.51469	0.608056	-6.01663	0.832908	0.721973
NK.cells	GALNT18	-0.26261	2.025829	-0.51466	0.608075	-5.51621	0.859273	0.764754
NK.cells	DPY19L3	-0.12587	2.757574	-0.51465	0.608083	-5.8355	0.849848	0.749348
NK.cells	WDR45	-0.09311	2.960786	-0.51454	0.608155	-5.9439	0.847254	0.745139
NK.cells	IGTP	0.13342	3.443365	0.514373	0.608275	-6.21633	0.841128	0.735201
NK.cells	SPACA6	0.214539	1.094819	0.514257	0.608355	-5.37185	0.871439	0.784818
NK.cells	RAVER2	0.158931	1.423822	0.514236	0.60837	-5.61682	0.867124	0.777676
NK.cells	RNF8	-0.0548	4.800789	-0.51403	0.60851	-6.41219	0.824162	0.708023
NK.cells	SH3BGRL2	-0.21798	2.700678	-0.51388	0.608619	-5.54782	0.850625	0.750682
NK.cells	CYLD	0.043719	6.329731	0.513752	0.608707	-6.72579	0.805459	0.67861
NK.cells	CD63	-0.11536	4.105165	-0.51361	0.608809	-6.14144	0.832827	0.721988
NK.cells	LPAR6	-0.07831	4.523596	-0.51353	0.608862	-6.38807	0.827603	0.713629
NK.cells	NUBP2	0.077809	4.451117	0.513501	0.608882	-6.25671	0.828505	0.715071
NK.cells	TFAP4	0.133219	2.748617	0.513388	0.60896	-5.79291	0.850011	0.749795
NK.cells	SLC12A6	-0.05976	8.133974	-0.5131	0.609163	-6.8803	0.78399	0.645475
NK.cells	DPP3	-0.06429	4.728026	-0.51303	0.609212	-6.34448	0.825063	0.709692
NK.cells	NKRF	-0.09773	2.983842	-0.51302	0.609218	-5.98523	0.847004	0.744992
NK.cells	EPOR	0.372729	-0.66556	0.513011	0.609223	-5.08271	0.894956	0.824485
NK.cells	1700037H(	0.082303	3.714085	0.513001	0.60923	-6.1161	0.837742	0.730007
NK.cells	HIST1H4C	0.256664	0.309171	0.512875	0.609318	-5.27493	0.881901	0.802521
NK.cells	MPV17L2	0.07148	4.459696	0.512626	0.609491	-6.3204	0.828514	0.715083
NK.cells	INF2	0.110627	3.252642	0.512526	0.609561	-5.97791	0.843699	0.739542
NK.cells	KDM4B	0.07406	4.441359	0.512392	0.609654	-6.31983	0.828742	0.715536
NK.cells	SSH3	-0.20583	1.522336	-0.51238	0.609662	-5.46758	0.865993	0.776065
NK.cells	SP2	-0.06218	4.742762	-0.51218	0.609804	-6.35073	0.825085	0.709581
NK.cells	RAB35	-0.05542	4.720448	-0.51207	0.60988	-6.37388	0.825362	0.710022
NK.cells	PLEKHG3	-0.08126	4.378559	-0.51191	0.609989	-6.25596	0.829615	0.716873
NK.cells	LAPTM4A	-0.04156	7.210016	-0.51189	0.610007	-6.78091	0.795101	0.662534
NK.cells	LYZ1	0.312533	-0.68152	0.511557	0.610237	-5.19285	0.895575	0.82519
NK.cells	N6AMT1	0.120342	2.597729	0.511425	0.610329	-5.78688	0.85233	0.753391
NK.cells	ATP5J2	0.0468	7.723844	0.511409	0.610339	-6.89464	0.789169	0.653211
NK.cells	MYL6	-0.03895	9.689609	-0.51126	0.610447	-7.24313	0.766345	0.618473
NK.cells	PLAAT3	0.07557	5.19136	0.511096	0.610558	-6.64491	0.819719	0.701018
NK.cells	MED16	0.067822	4.12299	0.51106	0.610583	-6.18463	0.83299	0.72219
NK.cells	LAT	0.082258	2.414617	0.510999	0.610625	-6.1681	0.854696	0.75736
NK.cells	FRMD8OS	-0.24666	0.736632	-0.51084	0.610736	-5.30545	0.876665	0.793532
NK.cells	I730030J21	0.163309	0.707146	0.510512	0.610965	-5.63138	0.877285	0.794275
NK.cells	U2AF1L4	0.112319	3.047054	0.510286	0.611123	-5.93708	0.84694	0.744334
NK.cells	PDCL3	0.061541	4.536557	0.510215	0.611172	-6.33145	0.828167	0.714127
NK.cells	1810041H1	-0.10992	1.76748	-0.50991	0.611383	-6.02457	0.863433	0.771408
NK.cells	TXNDC16	-0.08878	5.44957	-0.50981	0.611455	-6.3584	0.816884	0.696304
NK.cells	PSMC5	0.046009	6.00886	0.509806	0.611458	-6.60168	0.810055	0.685546
NK.cells	CPOX	0.069172	4.570271	0.509685	0.611542	-6.32257	0.827748	0.713587

NK.cells	CUL1	-0.03941	6.562305	-0.50958	0.611615	-6.69369	0.80336	0.67509
NK.cells	TERF1	0.051415	5.254483	0.509546	0.611639	-6.4854	0.819281	0.700124
NK.cells	TMEM62	-0.1082	2.375873	-0.50954	0.611643	-5.90455	0.855549	0.758518
NK.cells	REX1BD	0.046846	5.688469	0.509081	0.611964	-6.57703	0.814256	0.691861
NK.cells	GM26759	0.137216	3.123372	0.50902	0.612006	-5.8011	0.846276	0.743065
NK.cells	BCL2L12	-0.07677	4.178233	-0.50863	0.612278	-6.16125	0.833169	0.721677
NK.cells	SMARCAL1	-0.1008	2.628016	-0.50849	0.612374	-5.79976	0.852843	0.7535
NK.cells	HIST1H3D	-0.20043	1.963471	-0.5084	0.612439	-5.52773	0.86143	0.767588
NK.cells	PFKFB2	-0.11145	2.465839	-0.50833	0.612487	-5.74617	0.85493	0.756939
NK.cells	SLC7A8	0.227968	2.86439	0.50824	0.61255	-5.65639	0.849811	0.748624
NK.cells	MRM3	-0.15682	2.005465	-0.50819	0.612588	-5.64862	0.860884	0.766736
NK.cells	1700110K1	-0.30657	-0.57203	-0.50807	0.612671	-5.18659	0.895062	0.823684
NK.cells	HSP90B1	-0.04445	8.976388	-0.50796	0.612747	-7.05049	0.775371	0.631596
NK.cells	ZSWIM7	0.094176	3.432711	0.507869	0.61281	-6.0175	0.842587	0.736992
NK.cells	CCT3	0.045627	5.801241	0.507714	0.612918	-6.5682	0.81315	0.690008
NK.cells	GNL2	-0.05949	4.675227	-0.50749	0.613073	-6.3618	0.827019	0.712
NK.cells	TMED2	0.037485	8.136615	0.50739	0.613145	-6.94447	0.785193	0.646595
NK.cells	RAD51AP1	-0.13352	3.61991	-0.50738	0.613151	-6.10438	0.840252	0.733224
NK.cells	GM26917	0.071675	5.74366	0.507333	0.613184	-6.57219	0.813853	0.691133
NK.cells	PPM1D	0.053429	4.948971	0.507178	0.613293	-6.40726	0.823676	0.706623
NK.cells	FGD3	-0.06524	4.660813	-0.50692	0.613471	-6.44721	0.827397	0.71235
NK.cells	SEC63	0.038279	7.09953	0.506795	0.613561	-6.78673	0.797692	0.665606
NK.cells	EPB41L1	0.239564	1.011227	0.506403	0.613834	-5.40075	0.874459	0.78864
NK.cells	POT1A	0.097799	3.012767	0.506324	0.613889	-5.92261	0.848462	0.745977
NK.cells	SCNM1	0.08593	3.621272	0.506039	0.614089	-6.00441	0.840891	0.733523
NK.cells	BBS4	0.1428	2.313596	0.50595	0.614151	-5.6159	0.857619	0.7607
NK.cells	GPR160	-0.11319	2.407708	-0.50577	0.614277	-6.05854	0.856479	0.758739
NK.cells	OAZ1	0.043045	9.534615	0.505543	0.614436	-7.16837	0.76964	0.622308
NK.cells	ANKRD26	0.124951	2.576265	0.50538	0.614549	-5.82922	0.854306	0.755351
NK.cells	SAMD1	-0.05884	4.976188	-0.50526	0.61463	-6.44709	0.824002	0.706586
NK.cells	BIN1	-0.05395	5.230633	-0.50523	0.614652	-6.49266	0.820859	0.701614
NK.cells	ACBD4	0.152831	1.764413	0.505046	0.614783	-5.59012	0.864826	0.772693
NK.cells	CD3D	-0.08778	2.678526	-0.50503	0.614797	-6.40484	0.85299	0.753299
NK.cells	GPR19	0.133646	2.550401	0.505012	0.614807	-5.70454	0.854639	0.755988
NK.cells	DOP1B	0.068352	4.289272	0.504807	0.614951	-6.36989	0.832555	0.72035
NK.cells	ANKMY2	-0.0707	3.602393	-0.50473	0.615001	-6.12018	0.841204	0.734262
NK.cells	OTOA	-0.23979	0.757069	-0.5047	0.615025	-5.32336	0.878072	0.794714
NK.cells	TGFBR3	-0.12346	3.337964	-0.50454	0.615138	-5.9662	0.844559	0.739732
NK.cells	AKAP11	-0.07035	4.648059	-0.50448	0.615181	-6.29406	0.828076	0.71326
NK.cells	PHGDH	0.098273	4.379094	0.504422	0.61522	-6.33387	0.831431	0.71862
NK.cells	DYNLRB1	-0.0435	6.280147	-0.50438	0.615251	-6.64654	0.80803	0.681576
NK.cells	OLFR920	-0.24151	0.137933	-0.50403	0.615493	-5.31786	0.886569	0.808706
NK.cells	TAMM41	-0.10982	2.732365	-0.50366	0.615753	-5.89719	0.852769	0.752553
NK.cells	PAGR1A	0.259244	0.395669	0.503536	0.61584	-5.27062	0.883364	0.803078
NK.cells	GM13822	-0.34321	1.17949	-0.50349	0.615869	-5.35988	0.872974	0.7858
NK.cells	MAP3K4	-0.07065	4.074187	-0.50337	0.615953	-6.20234	0.835733	0.725067
NK.cells	ARHGAP1C	-0.08938	5.105594	-0.50278	0.616366	-6.51841	0.823172	0.704746

NK.cells	SSBP3	-0.04902	5.600863	-0.50278	0.616369	-6.62909	0.817073	0.695096
NK.cells	6530402F1	-0.19427	-0.17553	-0.50262	0.616484	-5.44607	0.891361	0.816235
NK.cells	UGDH	-0.06905	4.250685	-0.50213	0.616824	-6.29193	0.833819	0.721867
NK.cells	AP1G2	-0.11618	2.809774	-0.50209	0.616854	-5.96972	0.852103	0.7514
NK.cells	1810034E1	-0.14521	1.895787	-0.50208	0.616862	-5.64933	0.863923	0.770745
NK.cells	PDE7A	0.050459	6.703412	0.501868	0.617008	-6.79685	0.803671	0.674286
NK.cells	NAAA	-0.12639	3.389339	-0.50186	0.617014	-5.87319	0.844697	0.739453
NK.cells	PPP1R42	-0.28714	0.580161	-0.50186	0.617017	-5.24612	0.881246	0.799519
NK.cells	MN1	0.27877	0.308399	0.501851	0.61702	-5.21896	0.884869	0.805574
NK.cells	RFX1	0.073092	3.263376	0.50182	0.617041	-6.0114	0.846301	0.742049
NK.cells	IRAK2	0.06053	6.796286	0.50175	0.617091	-6.93932	0.802553	0.672548
NK.cells	GM41611	-0.28486	0.284798	-0.50166	0.617153	-5.21004	0.885185	0.806106
NK.cells	TIMM50	-0.06594	4.827954	-0.50163	0.617174	-6.33931	0.826614	0.710432
NK.cells	PQLC1	-0.07246	4.395337	-0.50148	0.617281	-6.08213	0.832007	0.719054
NK.cells	ARHGAP42	-0.19933	1.752056	-0.50134	0.617376	-5.50287	0.865798	0.774002
NK.cells	ME2	-0.05559	5.963937	-0.50091	0.617682	-6.64501	0.812632	0.688512
NK.cells	YEATS4	0.04654	5.906755	0.500796	0.617759	-6.53842	0.81333	0.689635
NK.cells	MPPED2	-0.23513	-0.32287	-0.50069	0.617836	-5.42154	0.893347	0.820117
NK.cells	4921511C1	-0.10846	2.59393	-0.50068	0.617837	-5.90324	0.854878	0.756281
NK.cells	MIB2	-0.10611	2.662247	-0.50056	0.617924	-5.88924	0.853999	0.75486
NK.cells	CCL6	-0.16559	5.306299	-0.50046	0.617992	-6.01399	0.820694	0.701316
NK.cells	EML5	-0.09637	3.697759	-0.50042	0.618023	-6.1658	0.840784	0.733436
NK.cells	NPHP1	-0.269	0.652168	-0.50032	0.61809	-5.21006	0.880288	0.798251
NK.cells	ARMCX4	-0.2082	1.19399	-0.50032	0.618094	-5.43201	0.873118	0.786326
NK.cells	GANAB	0.060349	4.730059	0.500294	0.618111	-6.33045	0.827831	0.712661
NK.cells	GPX4	0.037168	7.153816	0.500191	0.618184	-6.84612	0.798266	0.666172
NK.cells	GM2396	0.259654	-0.85737	0.500183	0.618189	-5.14322	0.900592	0.832412
NK.cells	THSD1	0.202167	1.60027	0.500001	0.618317	-5.37625	0.867782	0.77759
NK.cells	ZFPM2	-0.23727	2.777013	-0.5	0.618319	-5.61234	0.852523	0.752557
NK.cells	GM37233	-0.26556	0.010375	-0.49997	0.618339	-5.21089	0.888861	0.812703
NK.cells	INHBA	-0.34679	1.050066	-0.49964	0.618569	-5.33908	0.875245	0.789632
NK.cells	GM26674	0.293477	-1.15959	0.499466	0.618692	-5.06718	0.904742	0.839589
NK.cells	GM9725	0.166985	2.030059	0.499402	0.618737	-5.61559	0.862412	0.768438
NK.cells	MYLK	-0.19389	1.606214	-0.49933	0.618787	-5.4867	0.867943	0.777546
NK.cells	TIMM21	0.108059	2.602996	0.499078	0.618964	-5.8252	0.85505	0.756367
NK.cells	PDE12	-0.06532	3.991564	-0.49907	0.618966	-6.16228	0.837358	0.727701
NK.cells	PRSS57	0.276558	-0.03051	0.49885	0.619124	-5.13407	0.889765	0.813871
NK.cells	HJURP	0.066571	4.895427	0.498822	0.619144	-6.35194	0.826105	0.709691
NK.cells	THAP1	-0.10701	2.879636	-0.49793	0.619771	-5.82524	0.852021	0.751005
NK.cells	PHOSPHO2	-0.0701	3.724911	-0.49763	0.619979	-6.08344	0.841246	0.733641
NK.cells	ELK3	-0.05904	5.288608	-0.49763	0.61998	-6.52566	0.821699	0.702392
NK.cells	PTPN14	-0.24846	0.524362	-0.49757	0.620024	-5.31198	0.882833	0.801919
NK.cells	VRK3	-0.05277	4.98755	-0.49751	0.620066	-6.42944	0.825423	0.708304
NK.cells	RTCB	0.053824	5.290693	0.497457	0.620102	-6.47682	0.821673	0.702351
NK.cells	TAF11	0.054992	4.933052	0.497396	0.620144	-6.38136	0.826099	0.70938
NK.cells	ZFP386	0.071927	3.889207	0.497359	0.62017	-6.20695	0.839168	0.730294
NK.cells	PEX19	-0.0764	3.955615	-0.49728	0.620225	-6.11118	0.83833	0.728956

NK.cells	FGFR1	0.192466	1.974297	0.496947	0.62046	-5.73496	0.863727	0.770356
NK.cells	SYTL1	-0.15253	1.59739	-0.49694	0.620466	-5.65537	0.868651	0.77847
NK.cells	SLC7A6OS	0.056205	4.789888	0.496938	0.620466	-6.36907	0.827879	0.712307
NK.cells	GM42726	-0.07404	3.69069	-0.49691	0.620486	-6.11839	0.841679	0.73444
NK.cells	ADAT1	0.15415	2.003267	0.496627	0.620684	-5.6141	0.86335	0.769817
NK.cells	MRPL19	0.073509	3.923908	0.496582	0.620716	-6.15091	0.83873	0.729764
NK.cells	DBN1	-0.19913	1.388658	-0.49657	0.620725	-5.55883	0.871391	0.783082
NK.cells	TOR1A	0.060945	4.653633	0.496296	0.620917	-6.30091	0.829576	0.715155
NK.cells	PSIP1	-0.04556	5.994205	-0.49624	0.620954	-6.63109	0.813041	0.688956
NK.cells	NICN1	-0.23604	0.820121	-0.49621	0.620977	-5.29125	0.8789	0.795622
NK.cells	GM43713	0.149624	2.135076	0.4962	0.620985	-5.71586	0.861636	0.767071
NK.cells	NFXL1	-0.10354	3.364621	-0.49618	0.621	-5.9389	0.84582	0.741278
NK.cells	MARF1	-0.04427	6.147381	-0.49606	0.621081	-6.63657	0.811175	0.686033
NK.cells	BLVRA	0.057829	5.071733	0.496016	0.621114	-6.47033	0.82438	0.706889
NK.cells	DSN1	-0.11276	2.622227	-0.49594	0.621165	-5.82744	0.855332	0.756758
NK.cells	LMAN1	0.072271	4.627609	0.495462	0.621504	-6.27386	0.830259	0.715826
NK.cells	RAB7	0.035939	7.931545	0.495313	0.621608	-6.90792	0.790169	0.652972
NK.cells	SNX9	-0.07009	6.331978	-0.49513	0.621733	-6.38548	0.809347	0.682771
NK.cells	GM31323	-0.20499	0.948728	-0.49509	0.621763	-5.33669	0.877645	0.793115
NK.cells	ACSL1	-0.0916	5.758511	-0.49466	0.622069	-6.39647	0.816449	0.693899
NK.cells	OLFR56	0.189114	2.067818	0.494652	0.622073	-5.66689	0.863067	0.768957
NK.cells	EMILIN2	0.183452	4.009199	0.494565	0.622134	-5.9763	0.838196	0.728563
NK.cells	RIC8A	0.069535	3.980741	0.49453	0.622158	-6.17871	0.838554	0.729143
NK.cells	DPY19L4	0.064842	4.417697	0.494478	0.622195	-6.25893	0.833061	0.720329
NK.cells	CPNE3	0.066066	5.367342	0.494108	0.622455	-6.40868	0.821417	0.701613
NK.cells	AGRN	-0.20079	1.254784	-0.49402	0.622516	-5.49632	0.873886	0.786695
NK.cells	CALCRL	0.082689	5.617508	0.493802	0.622671	-6.53751	0.818337	0.696812
NK.cells	DNAJB12	0.058122	4.757611	0.493722	0.622727	-6.35627	0.828976	0.713716
NK.cells	AHCY	0.099598	3.495963	0.493721	0.622727	-6.05205	0.844858	0.739225
NK.cells	NOX1	0.19919	1.548335	0.493645	0.622781	-5.51593	0.870024	0.780418
NK.cells	TTLL4	0.092307	3.000367	0.493611	0.622805	-5.93426	0.851187	0.749533
NK.cells	PSMB3	0.042385	7.724991	0.493096	0.623167	-6.9104	0.793125	0.657214
NK.cells	ALG14	0.089993	3.562595	0.493078	0.62318	-6.0373	0.844259	0.737954
NK.cells	BC052040	-0.08162	4.165796	-0.49303	0.623216	-6.23968	0.83663	0.725668
NK.cells	SNHG8	0.069874	4.013183	0.492954	0.623267	-6.19292	0.838553	0.728779
NK.cells	H2-D1	-0.07424	8.452787	-0.49266	0.623475	-7.05772	0.784715	0.644144
NK.cells	GM10802	0.29164	-0.25172	0.492226	0.62378	-5.1494	0.894577	0.820814
NK.cells	PSMC2	0.053337	5.362149	0.492152	0.623832	-6.48029	0.822018	0.702184
NK.cells	LST1	0.100733	5.23223	0.492141	0.623839	-6.24996	0.823623	0.704729
NK.cells	EFCC1	-0.25123	0.001369	-0.49214	0.623842	-5.28683	0.891163	0.815073
NK.cells	FLT3L	0.093155	2.201527	0.491858	0.624039	-6.04445	0.862235	0.766839
NK.cells	TRIM68	-0.18727	0.946883	-0.49147	0.624313	-5.3911	0.878808	0.794071
NK.cells	RFLNB	-0.09149	3.463749	-0.49145	0.624328	-6.26116	0.846089	0.740398
NK.cells	SENP5	-0.04983	5.992571	-0.49125	0.624469	-6.60212	0.814533	0.6901
NK.cells	PACRG	0.302229	-0.43352	0.491194	0.624506	-5.1423	0.897321	0.825152
NK.cells	PIGM	-0.07795	3.53133	-0.49116	0.624528	-6.01385	0.845228	0.739071
NK.cells	MIGA2	-0.22997	0.892517	-0.49116	0.624529	-5.33149	0.87953	0.79534

NK.cells	EPB41L4B	-0.20094	1.976143	-0.4909	0.624714	-5.46063	0.865267	0.771821
NK.cells	RGS9	-0.21735	0.03543	-0.49085	0.624749	-5.43103	0.890986	0.814574
NK.cells	CDC14B	0.102816	3.454363	0.490802	0.624782	-6.03251	0.846208	0.740728
NK.cells	VAPA	-0.02976	8.028548	-0.49074	0.624823	-6.9056	0.790067	0.652168
NK.cells	ZC3H7A	0.039722	6.883008	0.490664	0.62488	-6.7734	0.803731	0.673298
NK.cells	SMN1	0.06441	4.851728	0.490566	0.624949	-6.34661	0.828607	0.712471
NK.cells	MYBL1	-0.15388	1.840587	-0.49014	0.62525	-5.62249	0.867218	0.774756
NK.cells	WDR26	-0.03686	7.53429	-0.49009	0.625286	-6.87368	0.796096	0.661226
NK.cells	DUSP12	0.083768	3.283921	0.489853	0.625451	-6.02352	0.848559	0.744306
NK.cells	BAZ1B	0.048959	6.23949	0.489832	0.625466	-6.63992	0.811691	0.685521
NK.cells	JMJD8	-0.1774	1.564022	-0.48961	0.625625	-5.53045	0.870843	0.780821
NK.cells	TMSB10	0.043446	10.73818	0.489586	0.62564	-7.54467	0.758927	0.60491
NK.cells	AGO3	-0.04907	5.940327	-0.48956	0.625654	-6.62323	0.815342	0.691296
NK.cells	TLN1	0.044692	7.109295	0.489546	0.625667	-6.84186	0.801178	0.669161
NK.cells	HIST1H2AN	0.169186	3.254826	0.489488	0.625708	-6.10146	0.848931	0.744954
NK.cells	PPP1R37	-0.06035	4.592802	-0.48938	0.625787	-6.31593	0.832022	0.717734
NK.cells	2610306M	-0.22295	0.680103	-0.48913	0.625959	-5.3649	0.882549	0.800269
NK.cells	PMEL	0.232946	0.339511	0.489016	0.626041	-5.33875	0.887099	0.807873
NK.cells	FOXP4	-0.06752	4.962815	-0.48897	0.626075	-6.42872	0.827408	0.710399
NK.cells	PLPPR1	0.199177	1.149205	0.488857	0.626154	-5.44211	0.876322	0.789958
NK.cells	ACADVL	-0.06268	4.901912	-0.48881	0.626184	-6.43081	0.828165	0.711664
NK.cells	GM44649	0.083664	3.949723	0.488661	0.626292	-6.17427	0.840108	0.730799
NK.cells	TGTP2	0.274089	0.773598	0.488616	0.626324	-5.46692	0.881304	0.798281
NK.cells	ZZEF1	-0.04453	5.867775	-0.48858	0.626346	-6.60689	0.81624	0.692801
NK.cells	SUPV3L1	-0.08091	3.910174	-0.48825	0.626579	-6.14388	0.840611	0.731775
NK.cells	EXOSC9	0.067879	4.169312	0.488252	0.62658	-6.22331	0.83734	0.726516
NK.cells	4930403P2	-0.20095	0.612375	-0.48819	0.626624	-5.30432	0.883455	0.80207
NK.cells	CDKL3	0.135369	3.044103	0.487977	0.626774	-5.80385	0.851643	0.749644
NK.cells	PTRHD1	-0.07003	4.56189	-0.48789	0.626833	-6.29841	0.832412	0.718638
NK.cells	BID	-0.07677	3.803317	-0.48775	0.626932	-6.16553	0.841964	0.733973
NK.cells	PIK3CG	-0.06198	4.936727	-0.48766	0.626998	-6.38504	0.827735	0.711207
NK.cells	GPATCH2	0.073829	4.253922	0.48765	0.627005	-6.19501	0.836276	0.724854
NK.cells	5430431A1	-0.13867	1.423829	-0.48749	0.627119	-5.59678	0.872701	0.784232
NK.cells	CYTH3	0.064325	4.88921	0.487461	0.627138	-6.44391	0.828326	0.712163
NK.cells	REPIN1	-0.19153	1.082403	-0.48738	0.627195	-5.42501	0.877209	0.791746
NK.cells	GM4107	0.284605	0.916265	0.487312	0.627243	-5.30317	0.879411	0.795409
NK.cells	E130308A1	0.067073	4.522919	0.486931	0.627512	-6.26822	0.8329	0.71959
NK.cells	EHD3	-0.118	3.517645	-0.48673	0.627653	-5.97795	0.845592	0.740041
NK.cells	DLAT	-0.05739	4.282042	-0.48662	0.627728	-6.2795	0.835922	0.724463
NK.cells	MAST4	-0.09826	6.39259	-0.48658	0.627761	-6.52714	0.809842	0.683122
NK.cells	ZPR1	0.059224	4.397865	0.486494	0.627821	-6.28187	0.834467	0.722144
NK.cells	NUP35	0.089873	3.291207	0.48645	0.627852	-6.02361	0.84848	0.744732
NK.cells	NSMCE2	-0.03608	7.428191	-0.4864	0.627884	-6.81996	0.797374	0.663722
NK.cells	NUP98	-0.04323	7.461994	-0.48638	0.627903	-6.91954	0.796971	0.663097
NK.cells	SAMD3	0.128987	0.184815	0.48636	0.627915	-5.94697	0.889178	0.811894
NK.cells	ZFP683	0.165946	-1.15907	0.486352	0.627921	-5.58631	0.907196	0.842727
NK.cells	TACSTD2	0.249355	-0.35025	0.4863	0.627958	-5.29235	0.896394	0.824055

NK.cells	QTRT2	0.201412	1.344368	0.486073	0.628118	-5.48304	0.873855	0.786253
NK.cells	SUN1	0.065913	3.918531	0.485993	0.628175	-6.17571	0.840608	0.731927
NK.cells	PHLPP1	-0.06764	7.671226	-0.48587	0.628263	-6.85812	0.7946	0.659322
NK.cells	PURG	-0.10562	3.260975	-0.48571	0.628372	-5.87149	0.849047	0.745498
NK.cells	FBXL14	0.051159	5.203671	0.485544	0.628491	-6.44091	0.824663	0.706311
NK.cells	ZFP239	0.237153	-0.06752	0.485389	0.628601	-5.25798	0.89289	0.817866
NK.cells	ZFP777	0.090388	2.809648	0.485242	0.628705	-5.87943	0.855004	0.755031
NK.cells	PABPC4	-0.04803	5.897724	-0.48514	0.628774	-6.62789	0.816209	0.692886
NK.cells	BDH1	-0.11066	3.282333	-0.48501	0.628869	-6.00619	0.848974	0.745217
NK.cells	IRF7	0.134429	6.139097	0.48479	0.629025	-6.65083	0.81336	0.688345
NK.cells	GM43061	0.237794	0.306464	0.484614	0.629149	-5.29553	0.888019	0.809616
NK.cells	EIF3E	0.035418	7.095265	0.484548	0.629195	-6.83526	0.801786	0.670292
NK.cells	GM43560	-0.18301	0.578606	-0.48442	0.629282	-5.29558	0.884377	0.80359
NK.cells	POLE3	0.076219	4.133596	0.484369	0.629322	-6.19492	0.838237	0.727969
NK.cells	HDGFL3	-0.12697	1.511028	-0.48434	0.629343	-5.87599	0.872018	0.783074
NK.cells	DCAF17	0.06748	4.054876	0.484054	0.629545	-6.18968	0.839406	0.729705
NK.cells	STAU1	-0.04618	6.034578	-0.48355	0.629902	-6.64364	0.81493	0.690633
NK.cells	ARMCX2	-0.18158	1.41393	-0.48348	0.629952	-5.47709	0.873611	0.785398
NK.cells	TMEM106I	0.066736	4.565283	0.4834	0.630006	-6.25522	0.833113	0.719483
NK.cells	ACBD6	0.046175	5.436733	0.483191	0.630155	-6.49258	0.822275	0.702268
NK.cells	HIST1H1A	-0.15427	3.773403	-0.48315	0.630184	-6.15144	0.843095	0.735549
NK.cells	SLC25A44	-0.08898	3.239164	-0.48314	0.630193	-5.98922	0.849903	0.746566
NK.cells	GM44686	0.076366	3.136727	0.482996	0.630292	-6.09194	0.851215	0.748697
NK.cells	ADAM30	0.227844	0.62147	0.482939	0.630333	-5.32138	0.884123	0.802928
NK.cells	ZNRD2	0.07601	4.037669	0.482911	0.630352	-6.17555	0.83975	0.730164
NK.cells	PHF14	0.042867	6.331947	0.48271	0.630495	-6.66149	0.811303	0.685029
NK.cells	ZFP958	0.118004	2.267977	0.482708	0.630496	-5.81137	0.862431	0.767064
NK.cells	CTSK	0.238708	0.234472	0.482704	0.630499	-5.30267	0.889304	0.811653
NK.cells	EIF4B	-0.04248	6.136378	-0.48263	0.63055	-6.6414	0.813686	0.688769
NK.cells	ZKSCAN14	0.07954	3.081299	0.482406	0.63071	-5.99837	0.852048	0.749905
NK.cells	4833417C1	-0.19859	0.707506	-0.48209	0.630937	-5.38453	0.883321	0.801168
NK.cells	TTC23	-0.20148	1.101964	-0.48185	0.631103	-5.38559	0.878109	0.792479
NK.cells	PCDH7	0.283975	0.520175	0.481782	0.631151	-5.40175	0.885855	0.805425
NK.cells	SPPL3	0.035393	7.282604	0.481656	0.63124	-6.82874	0.80017	0.667291
NK.cells	CLIC1	-0.04315	8.942892	-0.48159	0.631286	-7.10475	0.780551	0.637161
NK.cells	PCID2	0.049788	4.736836	0.481297	0.631495	-6.40999	0.831322	0.716394
NK.cells	ASTN2	0.212484	1.045238	0.481047	0.631672	-5.41339	0.878861	0.793944
NK.cells	ABCB1B	-0.08181	3.934965	-0.48087	0.631793	-6.50143	0.841408	0.732717
NK.cells	SPICE1	-0.12491	1.890422	-0.48084	0.63182	-5.73473	0.867726	0.775605
NK.cells	ENTPD7	-0.06936	4.693758	-0.48072	0.631901	-6.2377	0.831861	0.717449
NK.cells	RAPGEF5	-0.16641	5.011787	-0.4807	0.631917	-5.9984	0.827895	0.711125
NK.cells	TARDBP	-0.04746	5.771119	-0.48057	0.632006	-6.53881	0.818508	0.69632
NK.cells	RANGAP1	-0.05523	5.610379	-0.48057	0.632006	-6.56576	0.820485	0.699443
NK.cells	PDE6D	0.065604	3.812865	0.480391	0.632136	-6.10518	0.842955	0.735341
NK.cells	SLX4IP	0.059013	4.853222	0.480391	0.632136	-6.41371	0.82987	0.714352
NK.cells	IFITM10	-0.11902	3.144158	-0.48039	0.632138	-6.28077	0.851484	0.749153
NK.cells	CCDC90B	0.094699	3.165156	0.480374	0.632148	-5.91561	0.851215	0.748716

NK.cells	ZFP69	-0.13637	2.612118	-0.48025	0.632239	-5.72857	0.858336	0.760348
NK.cells	EAR2	0.187585	3.55304	0.480186	0.632281	-5.73471	0.846258	0.740715
NK.cells	5033421B(	-0.33376	-0.4422	-0.48014	0.632313	-5.14746	0.898826	0.827705
NK.cells	ZFP606	-0.10772	2.487995	-0.48005	0.632374	-5.74561	0.859943	0.763035
NK.cells	TBC1D8	0.128663	5.179428	0.479932	0.632461	-5.91646	0.825812	0.708022
NK.cells	FAM118A	0.111781	2.069936	0.479805	0.632551	-5.78752	0.86538	0.772015
NK.cells	NBDY	0.064411	3.659616	0.479777	0.632571	-6.21245	0.844902	0.738618
NK.cells	PRPSAP1	0.055208	4.34081	0.479536	0.632742	-6.323	0.83642	0.724825
NK.cells	NDUFB10	-0.04194	6.731387	-0.47917	0.633	-6.76217	0.807172	0.678271
NK.cells	OSTC	-0.04064	6.387429	-0.47892	0.633176	-6.67159	0.811365	0.684833
NK.cells	EIF4ENIF1	-0.04801	5.556503	-0.47885	0.633231	-6.49942	0.821544	0.700861
NK.cells	CHTF8	0.157702	1.341638	0.478825	0.633245	-5.54974	0.875359	0.788065
NK.cells	UCP1	0.255409	-1.00774	0.478719	0.63332	-5.121	0.906912	0.84118
NK.cells	FAM98B	0.059139	4.474383	0.478655	0.633366	-6.32489	0.83501	0.722331
NK.cells	BLZF1	-0.085	3.200504	-0.47836	0.633574	-5.96246	0.851256	0.748548
NK.cells	F9	0.207779	0.896192	0.478295	0.63362	-5.41504	0.881352	0.798117
NK.cells	TIA1	0.042729	5.466514	0.478168	0.633711	-6.48849	0.822737	0.702863
NK.cells	KLRK1	-0.08015	3.149025	-0.47813	0.633737	-6.60076	0.851916	0.7497
NK.cells	PSMC6	0.041653	6.141841	0.478027	0.63381	-6.63729	0.814441	0.689789
NK.cells	4921516A(	-0.20373	0.76983	-0.47797	0.63385	-5.3459	0.883035	0.800983
NK.cells	PLBD2	0.067562	4.310294	0.477795	0.633975	-6.20496	0.83723	0.725921
NK.cells	PRPF3	-0.05236	4.472577	-0.47767	0.634066	-6.30703	0.835216	0.722649
NK.cells	ACAP3	0.098598	2.238471	0.47745	0.63422	-5.77981	0.863902	0.769043
NK.cells	TCTN1	0.225293	1.315572	0.477233	0.634374	-5.34638	0.876083	0.789096
NK.cells	ANKRD54	-0.07822	3.089479	-0.47707	0.634492	-5.98557	0.852965	0.751117
NK.cells	RPUSD1	0.160105	1.47218	0.477003	0.634537	-5.5003	0.874015	0.785703
NK.cells	FEM1A	0.085765	2.924816	0.476761	0.634709	-5.91404	0.855083	0.754646
NK.cells	VCP	-0.03522	7.431716	-0.47668	0.634765	-6.85255	0.799116	0.66567
NK.cells	GMPPB	0.105886	2.629402	0.476653	0.634785	-5.82883	0.858897	0.760873
NK.cells	MEX3C	0.052502	5.133199	0.476618	0.63481	-6.41035	0.827141	0.709668
NK.cells	PRMT6	-0.10155	2.45108	-0.47658	0.634834	-5.77317	0.861209	0.764674
NK.cells	DCK	0.062994	5.832657	0.476487	0.634903	-6.50518	0.8185	0.695997
NK.cells	NLE1	0.134222	2.257092	0.476368	0.634988	-5.77852	0.863737	0.768845
NK.cells	KLHL32	-0.23198	1.452777	-0.47628	0.635048	-5.42232	0.874278	0.786273
NK.cells	TTC28	-0.08097	4.798912	-0.47615	0.635145	-6.35522	0.831348	0.716454
NK.cells	NDUFA13	0.036561	7.128419	0.475974	0.635267	-6.83519	0.80285	0.671486
NK.cells	CORO2A	0.05653	5.527432	0.475841	0.635361	-6.63281	0.822358	0.702092
NK.cells	4933407K1	0.146414	1.39879	0.475787	0.6354	-5.49818	0.875089	0.787569
NK.cells	TAF15	0.033994	6.905531	0.475656	0.635493	-6.80026	0.805564	0.675701
NK.cells	5830418P1	0.232629	1.463039	0.475336	0.63572	-5.43491	0.874396	0.786315
NK.cells	GM39302	0.227109	0.880898	0.475331	0.635723	-5.32578	0.882111	0.799137
NK.cells	GM49336	-0.05157	4.881295	-0.47514	0.635857	-6.40997	0.830609	0.715048
NK.cells	C4B	-0.22837	2.480976	-0.47487	0.63605	-5.72816	0.861301	0.764528
NK.cells	ZFAND1	0.093925	2.727292	0.474801	0.636099	-5.83239	0.85811	0.759322
NK.cells	PIK3R2	-0.13862	2.306399	-0.47435	0.636421	-5.65906	0.863766	0.768304
NK.cells	IFI47	0.127956	5.277962	0.474237	0.6365	-6.55974	0.825993	0.707333
NK.cells	CSNK1A1	0.027948	7.684452	0.474206	0.636522	-6.92556	0.796724	0.661498

NK.cells	LMNA	-0.08165	4.617164	-0.4742	0.636524	-6.43271	0.834235	0.720466
NK.cells	DARS2	0.116206	2.849125	0.474069	0.63662	-5.99074	0.856761	0.756854
NK.cells	NEK7	0.043177	6.476805	0.473925	0.636722	-6.72304	0.811295	0.684171
NK.cells	ZC3HC1	-0.05846	4.34425	-0.47387	0.636757	-6.31008	0.837695	0.726039
NK.cells	HIST1H3F	0.245265	0.34671	0.473602	0.636951	-5.35957	0.889739	0.811413
NK.cells	TRMT1	0.061	4.050324	0.473568	0.636975	-6.18415	0.841425	0.732014
NK.cells	HSPA9	-0.04331	6.483768	-0.47356	0.636982	-6.68412	0.811228	0.684066
NK.cells	UBL4A	0.075776	4.202976	0.473347	0.637133	-6.23841	0.839549	0.729028
NK.cells	MIS12	0.096054	3.322859	0.473304	0.637163	-5.99479	0.850743	0.747119
NK.cells	JAM2	0.23989	1.449339	0.473099	0.637309	-5.43875	0.875213	0.787165
NK.cells	SLC12A9	0.077555	4.242634	0.472672	0.637612	-6.23634	0.839324	0.72841
NK.cells	SHMT2	-0.07742	4.430904	-0.47266	0.637617	-6.30791	0.836951	0.724603
NK.cells	GNAT3	0.294154	-0.04367	0.47249	0.637741	-5.12117	0.895351	0.82068
NK.cells	SMC1B	-0.32633	-0.03473	-0.47243	0.637785	-5.14685	0.895231	0.8205
NK.cells	GATM	-0.12178	4.530012	-0.47226	0.637905	-5.97734	0.835704	0.722752
NK.cells	NRAS	0.041719	6.075126	0.472202	0.637946	-6.61846	0.816535	0.692348
NK.cells	PARD6G	-0.15001	1.981117	-0.47194	0.638133	-5.78436	0.868403	0.776037
NK.cells	SLC25A3	-0.03564	7.921066	-0.4718	0.638234	-6.9527	0.794265	0.657786
NK.cells	MAP3K12	-0.09589	2.718206	-0.4718	0.638235	-5.87377	0.858808	0.760291
NK.cells	CSK	-0.04289	6.929767	-0.47171	0.638294	-6.77494	0.806139	0.676188
NK.cells	ARHGDIB	0.049301	8.995183	0.47164	0.638346	-7.13144	0.781619	0.638417
NK.cells	NR5A2	-0.19576	1.145394	-0.47162	0.638363	-5.36522	0.87942	0.794274
NK.cells	PRKCB	-0.04093	8.511819	-0.47115	0.638697	-6.95044	0.787282	0.647126
NK.cells	FBXL12	-0.06538	4.348122	-0.47109	0.638739	-6.26936	0.837993	0.726651
NK.cells	YIF1B	0.059561	4.886916	0.471017	0.638788	-6.34778	0.831233	0.715833
NK.cells	KBTBD7	-0.14493	2.137804	-0.47097	0.638821	-5.56961	0.866354	0.772742
NK.cells	MARVELD1	-0.13893	1.253094	-0.47092	0.638857	-5.63877	0.877992	0.791981
NK.cells	DHX58	0.150673	2.999964	0.47077	0.638965	-5.8047	0.85517	0.754432
NK.cells	ICK	0.128497	2.147459	0.470587	0.639095	-5.70121	0.866228	0.772534
NK.cells	PLEKHA4	0.149459	2.406918	0.470554	0.639118	-5.70143	0.862846	0.76698
NK.cells	ZBTB45	0.08421	2.916254	0.470528	0.639136	-5.9782	0.856249	0.756191
NK.cells	HINT2	-0.06444	4.356845	-0.47032	0.639286	-6.2687	0.837883	0.726547
NK.cells	ZFP768	0.223353	0.794694	0.470217	0.639358	-5.3735	0.884088	0.80229
NK.cells	LYRM1	0.090802	2.700522	0.470157	0.6394	-5.93895	0.859037	0.760911
NK.cells	LIAS	0.060872	4.310022	0.470106	0.639437	-6.27667	0.838473	0.727583
NK.cells	AGO4	0.148786	3.167589	0.470066	0.639465	-5.59036	0.853014	0.751087
NK.cells	TMEM115	-0.06302	3.447731	-0.47004	0.639487	-6.10811	0.849423	0.745261
NK.cells	EIF3J1	0.035186	7.268033	0.469803	0.639653	-6.85578	0.802066	0.670159
NK.cells	SH3BP5L	-0.10438	2.252113	-0.46967	0.639745	-5.85849	0.864862	0.770624
NK.cells	GM43672	-0.1585	1.700182	-0.46963	0.639772	-5.61381	0.87209	0.782552
NK.cells	BTBD19	-0.10115	2.725168	-0.46945	0.639901	-5.8633	0.858718	0.760609
NK.cells	PUS7	0.087937	2.95762	0.469356	0.639971	-6.00131	0.855716	0.755706
NK.cells	LIG1	-0.08491	5.051661	-0.46934	0.639982	-6.42138	0.829178	0.712921
NK.cells	OAS1G	0.296565	-0.24881	0.46911	0.640146	-5.17848	0.898131	0.826209
NK.cells	MBOAT2	-0.21722	0.666194	-0.46893	0.640277	-5.55142	0.885804	0.805541
NK.cells	AI506816	-0.07077	5.207229	-0.46874	0.640407	-6.56997	0.827242	0.709961
NK.cells	FBXL12OS	-0.19205	0.679534	-0.46868	0.640455	-5.32627	0.885626	0.805248

NK.cells	PAPOLG	-0.08493	3.005898	-0.46865	0.640476	-6.04267	0.855094	0.754825
NK.cells	GM17354	-0.13916	1.393239	-0.46856	0.640539	-5.59239	0.876138	0.78945
NK.cells	SYNRG	0.049689	5.053855	0.468435	0.640626	-6.46188	0.829151	0.71302
NK.cells	MORF4L1	-0.02359	8.201561	-0.46832	0.640705	-6.96209	0.790941	0.653212
NK.cells	PIP4K2A	0.047157	7.239298	0.467991	0.640943	-6.85124	0.802411	0.671029
NK.cells	TIPIN	-0.08235	5.238466	-0.46792	0.64099	-6.46226	0.826854	0.709468
NK.cells	GM2788	-0.21531	0.569462	-0.46783	0.641058	-5.3436	0.887099	0.807879
NK.cells	SIRPA	0.120059	5.832373	0.467638	0.641194	-6.06625	0.819514	0.697912
NK.cells	GM16093	0.11578	2.640752	0.467306	0.641431	-5.85501	0.859811	0.762827
NK.cells	EEF1D	0.038082	7.27018	0.467254	0.641467	-6.88186	0.80204	0.670623
NK.cells	COX5B	0.041613	8.103976	0.467218	0.641494	-6.9865	0.792096	0.65524
NK.cells	PFKFB4	-0.11265	3.15878	-0.46716	0.641534	-5.7875	0.853127	0.751944
NK.cells	LSM10	0.098668	3.07773	0.467048	0.641615	-5.89409	0.854169	0.753642
NK.cells	PPM1A	-0.0361	6.125745	-0.46704	0.641623	-6.60968	0.815915	0.692338
NK.cells	USP30	-0.10931	2.252852	-0.46689	0.641726	-5.75148	0.864852	0.771134
NK.cells	ITPKC	-0.16213	1.763821	-0.46687	0.64174	-5.57159	0.871253	0.781691
NK.cells	CRY2	-0.08778	2.553643	-0.46686	0.641752	-5.95787	0.86094	0.76471
NK.cells	AKR1B3	0.047629	5.402163	0.4668	0.641792	-6.52204	0.824824	0.706426
NK.cells	FKBP1A	0.038768	7.336878	0.466752	0.641825	-6.87947	0.801239	0.669383
NK.cells	ZFP157	0.114719	2.446699	0.466736	0.641837	-5.82094	0.862329	0.766988
NK.cells	NKTR	0.035005	7.193099	0.466673	0.641882	-6.81443	0.802966	0.672068
NK.cells	SUCO	0.049916	6.354348	0.466542	0.641975	-6.7501	0.813122	0.687945
NK.cells	A930029G2	0.166457	1.230907	0.466488	0.642014	-5.52605	0.878286	0.793355
NK.cells	TMEM175	0.085803	3.375009	0.46637	0.642098	-6.06668	0.850354	0.747434
NK.cells	RIF1	-0.0538	5.435862	-0.46637	0.642098	-6.52738	0.824407	0.705764
NK.cells	GMCL1	0.049519	4.636774	0.466362	0.642104	-6.38872	0.834364	0.721641
NK.cells	ATP5C1	0.031184	8.358211	0.46629	0.642155	-7.02417	0.789091	0.650624
NK.cells	CTSD	-0.0568	7.133555	-0.46626	0.642179	-6.851	0.803682	0.673185
NK.cells	GM10874	-0.17876	-0.06869	-0.46611	0.642281	-5.47846	0.89569	0.822511
NK.cells	PRKAB1	-0.08272	3.654114	-0.46609	0.642299	-6.09272	0.846789	0.741653
NK.cells	MPZL1	-0.17259	2.78686	-0.46606	0.642317	-5.67085	0.85792	0.759768
NK.cells	SNTA1	0.15828	1.865421	0.466044	0.64233	-5.53676	0.869919	0.779488
NK.cells	UNC13B	-0.23656	0.758993	-0.46592	0.642418	-5.29543	0.884564	0.803917
NK.cells	SPAG7	0.064846	4.55728	0.46574	0.642547	-6.29306	0.835362	0.723386
NK.cells	TTC1	0.044114	4.740195	0.465717	0.642563	-6.36598	0.833068	0.719712
NK.cells	CLN3	0.077909	4.768913	0.46568	0.642589	-6.32135	0.832708	0.71914
NK.cells	VAMP2	0.051368	4.421644	0.465495	0.642721	-6.30882	0.837067	0.726178
NK.cells	ADGRL1	-0.08349	2.476047	-0.46526	0.642891	-6.05718	0.861948	0.766618
NK.cells	TRAPPC1	-0.06237	5.432947	-0.46523	0.642907	-6.51645	0.824443	0.706057
NK.cells	TMEM242	0.060192	4.292008	0.465109	0.642997	-6.20811	0.8387	0.728846
NK.cells	ZFP65	0.15024	1.662117	0.465105	0.643	-5.64273	0.872591	0.784168
NK.cells	ZFP518B	0.175481	0.418092	0.465054	0.643036	-5.33398	0.889129	0.81175
NK.cells	LARS	0.061873	4.738364	0.464936	0.64312	-6.35426	0.833091	0.71987
NK.cells	GLB1	-0.05188	5.221188	-0.46476	0.643245	-6.48846	0.827069	0.710284
NK.cells	UBE2J1	-0.03834	6.282597	-0.46471	0.643284	-6.68235	0.813998	0.689602
NK.cells	TSPAN3	-0.07256	4.739337	-0.46463	0.643339	-6.31051	0.833079	0.719889
NK.cells	DDX43	-0.13873	0.153087	-0.46433	0.643556	-5.72748	0.892695	0.817809

NK.cells	PRCC	-0.04575	5.107381	-0.46428	0.64359	-6.49936	0.828484	0.712551
NK.cells	LONRF1	0.128454	3.091293	0.4641	0.643717	-5.7285	0.853995	0.753678
NK.cells	ZFP672	-0.05777	4.004158	-0.46403	0.64377	-6.27406	0.84234	0.734773
NK.cells	ABHD16A	-0.0537	4.808556	-0.46402	0.643772	-6.40864	0.832212	0.718504
NK.cells	TERF2IP	-0.07051	3.58313	-0.46397	0.643806	-6.06963	0.847694	0.743434
NK.cells	HCCS	0.052238	4.627385	0.463906	0.643856	-6.3378	0.834482	0.722136
NK.cells	GM27003	-0.06409	4.204875	-0.46381	0.643926	-6.24796	0.8398	0.730728
NK.cells	DNAJB14	0.075663	4.692261	0.463478	0.644161	-6.23658	0.833668	0.720985
NK.cells	TMEM268	0.093621	3.100152	0.46346	0.644174	-5.90078	0.853881	0.753656
NK.cells	GAN	0.073713	4.031697	0.463427	0.644197	-6.24756	0.841991	0.734373
NK.cells	TBC1D10B	0.052597	5.147294	0.463171	0.64438	-6.44295	0.827988	0.711987
NK.cells	ADAM11	-0.249	-1.14352	-0.46316	0.644391	-5.08108	0.91015	0.848025
NK.cells	TRMT61A	0.144447	1.593744	0.46302	0.644488	-5.55479	0.873491	0.785984
NK.cells	GM21762	0.288338	-1.20491	0.462958	0.644532	-5.03169	0.910931	0.849472
NK.cells	ADA	0.145631	2.074866	0.462951	0.644537	-5.69531	0.867176	0.775541
NK.cells	TAF5	-0.0613	4.146651	-0.46287	0.644598	-6.27061	0.840536	0.732143
NK.cells	ENG	0.126002	4.005662	0.462795	0.644648	-5.99998	0.842321	0.735031
NK.cells	IL27RA	0.116923	1.535873	0.462748	0.644682	-5.98408	0.874254	0.787303
NK.cells	RBM26	0.036368	6.821937	0.462657	0.644747	-6.74481	0.807443	0.679634
NK.cells	CDC7	-0.10873	2.873973	-0.46235	0.644964	-5.86718	0.856795	0.758651
NK.cells	IGFLR1	0.177766	0.807622	0.462252	0.645036	-5.58754	0.883915	0.803523
NK.cells	TARS	-0.06026	4.374393	-0.46202	0.645199	-6.31294	0.837662	0.727668
NK.cells	ZBTB26	-0.19907	0.850706	-0.46199	0.645223	-5.3645	0.883341	0.802592
NK.cells	4930594M	0.254761	-0.02762	0.461976	0.645234	-5.23792	0.895135	0.822405
NK.cells	TMEM106(	0.090633	3.205819	0.461918	0.645275	-5.88793	0.852523	0.751747
NK.cells	CDC45	-0.10018	3.268129	-0.46173	0.645408	-6.07101	0.851723	0.750505
NK.cells	ENPP4	0.091953	2.924155	0.461646	0.645469	-6.01972	0.856147	0.757722
NK.cells	CNEP1R1	0.054313	4.587392	0.461622	0.645486	-6.33971	0.834984	0.723448
NK.cells	KDM1B	-0.09661	3.6758	-0.46153	0.645549	-5.95671	0.846512	0.742055
NK.cells	FRAT2	-0.062	4.805593	-0.46153	0.645551	-6.44915	0.83225	0.719082
NK.cells	GRK2	-0.03932	7.206149	-0.46133	0.645698	-6.78872	0.802809	0.67265
NK.cells	VPS37A	-0.05651	5.2037	-0.46131	0.645706	-6.45648	0.827286	0.711214
NK.cells	TUFT1	-0.09664	2.2113	-0.46119	0.645792	-5.82031	0.865394	0.772975
NK.cells	NUDT19	0.064917	4.544356	0.461035	0.645906	-6.31455	0.835524	0.724402
NK.cells	SMAD4	-0.03635	6.23224	-0.46087	0.646026	-6.69714	0.814613	0.691165
NK.cells	LRRC51	0.152451	1.76843	0.4608	0.646073	-5.53167	0.871193	0.782593
NK.cells	RNF43	0.109588	1.563577	0.460666	0.646169	-6.0405	0.873889	0.787116
NK.cells	MBD5	0.042229	6.903175	0.460506	0.646284	-6.75121	0.806461	0.678448
NK.cells	GDPGP1	0.149189	2.18615	0.460489	0.646296	-5.75545	0.865723	0.77363
NK.cells	CREG1	-0.06067	7.514034	-0.46036	0.646385	-6.75526	0.799117	0.667013
NK.cells	BHLHE41	-0.18887	1.739361	-0.46027	0.646449	-5.54867	0.871575	0.783306
NK.cells	MIR22HG	-0.0884	4.089128	-0.46026	0.646459	-6.1703	0.841264	0.73374
NK.cells	WDR1	-0.04378	7.335798	-0.46022	0.64649	-6.85315	0.801252	0.670331
NK.cells	TESK2	0.092872	4.072329	0.460193	0.646507	-6.14549	0.841476	0.734084
NK.cells	MEMO1	0.041769	6.363616	0.459985	0.646656	-6.71096	0.813009	0.688759
NK.cells	SLC30A4	0.132873	1.941057	0.459972	0.646665	-5.623	0.868928	0.77897
NK.cells	VKORC1L1	-0.05368	5.560377	-0.45978	0.6468	-6.45557	0.822867	0.704391

NK.cells	ZFP579	-0.14067	1.862923	-0.45965	0.646893	-5.60425	0.869952	0.780772
NK.cells	DGKG	-0.13672	2.506906	-0.45958	0.646948	-5.78515	0.861547	0.766915
NK.cells	ITM2C	-0.04989	5.637577	-0.45949	0.647009	-6.69604	0.821914	0.702968
NK.cells	SCAMP5	-0.18834	1.229049	-0.45946	0.647032	-5.36353	0.878311	0.794713
NK.cells	GM16174	0.165842	0.995634	0.458957	0.647391	-5.45303	0.88141	0.800024
NK.cells	TRIP11	-0.04216	6.069875	-0.45893	0.647408	-6.66961	0.816599	0.694689
NK.cells	GPC3	0.147724	1.806193	0.458919	0.647419	-5.63274	0.870697	0.782206
NK.cells	AMOTL1	-0.20685	1.696223	-0.45882	0.647493	-5.37995	0.872142	0.784601
NK.cells	SETD6	0.122508	1.423905	0.458801	0.647503	-5.61158	0.875732	0.790562
NK.cells	PGD	0.050355	5.716274	0.458795	0.647507	-6.54379	0.820944	0.701563
NK.cells	NUDC	-0.04481	5.698731	-0.4587	0.647572	-6.56491	0.82116	0.701906
NK.cells	RAD9A	-0.15102	2.338849	-0.45867	0.647596	-5.64967	0.863732	0.770707
NK.cells	SCUBE2	-0.32412	-0.26058	-0.45862	0.647635	-5.1439	0.89829	0.828428
NK.cells	CRYZL2	-0.11732	2.609637	-0.45857	0.647669	-5.64505	0.860214	0.764941
NK.cells	REV1	0.055733	5.326262	0.458534	0.647694	-6.40874	0.825765	0.709239
NK.cells	DDC	-0.16761	1.455602	-0.45836	0.647819	-5.55615	0.875388	0.789927
NK.cells	KAT6A	0.041832	6.479855	0.458245	0.6479	-6.72154	0.811668	0.686881
NK.cells	NSMCE4A	0.040403	6.083141	0.458157	0.647964	-6.63564	0.816511	0.694546
NK.cells	SGIP1	0.169278	0.175941	0.457722	0.648275	-5.52682	0.892566	0.818729
NK.cells	GM15787	-0.11329	2.641968	-0.4577	0.64829	-5.88611	0.859968	0.764502
NK.cells	9330151L1	0.145025	0.96571	0.457687	0.6483	-5.55836	0.881986	0.800969
NK.cells	4-Sep	-0.13884	1.735848	-0.4574	0.648505	-5.7466	0.871796	0.78409
NK.cells	NUDT9	0.071224	4.362001	0.457344	0.648546	-6.2862	0.837987	0.728852
NK.cells	AKAP6	0.244796	0.299684	0.457213	0.648639	-5.49982	0.8909	0.816024
NK.cells	PRPF4B	0.040965	6.312389	0.457056	0.648752	-6.69326	0.813798	0.690363
NK.cells	FIGNL2	0.284682	-0.67701	0.457026	0.648773	-5.17315	0.904143	0.838466
NK.cells	GM19325	-0.14899	1.67424	-0.45698	0.648805	-5.7214	0.872607	0.785476
NK.cells	BLVRB	-0.10361	7.092023	-0.45698	0.648807	-6.51064	0.804344	0.675541
NK.cells	CLIC5	-0.21265	0.878367	-0.4568	0.648935	-5.42299	0.883149	0.803113
NK.cells	HIST2H2BE	-0.21629	-0.261	-0.45679	0.648945	-5.29116	0.898477	0.828921
NK.cells	POLRMT	0.120742	1.91437	0.456708	0.649001	-5.72924	0.869452	0.780344
NK.cells	GM29488	0.230838	0.172443	0.456684	0.649018	-5.30139	0.892613	0.819035
NK.cells	SLC25A33	0.08131	3.983563	0.456278	0.649309	-6.12498	0.842983	0.736789
NK.cells	FAM151B	-0.16066	1.753415	-0.45607	0.649456	-5.59298	0.871785	0.784006
NK.cells	MPEG1	-0.16416	6.081136	-0.45599	0.649514	-6.02834	0.816832	0.695085
NK.cells	SCO2	-0.095	2.851352	-0.45591	0.649573	-5.89626	0.857476	0.76047
NK.cells	GM35867	-0.24852	0.456051	-0.45589	0.649585	-5.29495	0.889023	0.812824
NK.cells	KARS	0.065504	4.273346	0.45572	0.649709	-6.313	0.839317	0.730985
NK.cells	MTRR	0.150947	1.505683	0.455705	0.64972	-5.59755	0.875049	0.789487
NK.cells	DDHD1	-0.06562	6.276227	-0.45551	0.649861	-6.66909	0.814445	0.691351
NK.cells	2900026A0	-0.09513	3.595436	-0.45544	0.649907	-6.26802	0.847921	0.74492
NK.cells	ETFA	-0.04797	6.599323	-0.4554	0.64994	-6.72966	0.810509	0.685178
NK.cells	TOR4A	0.139438	1.678835	0.455359	0.649967	-5.66377	0.872767	0.785752
NK.cells	RRAS	0.083978	4.338868	0.455278	0.650025	-6.24711	0.83849	0.729728
NK.cells	NHSL1	0.173809	1.728678	0.455107	0.650148	-5.47371	0.872152	0.78473
NK.cells	ZFP775	-0.17763	0.81684	-0.45491	0.650287	-5.4401	0.884235	0.804881
NK.cells	SNAPC2	0.098138	3.015281	0.454802	0.650366	-5.92212	0.855401	0.757185

NK.cells	LRRC14	-0.1172	2.339055	-0.45477	0.650388	-5.74817	0.864162	0.771567
NK.cells	MBTD1	-0.04413	7.420536	-0.45475	0.650407	-6.85064	0.800637	0.669806
NK.cells	KCNK10	-0.19911	0.557275	-0.4541	0.650869	-5.45065	0.888153	0.810813
NK.cells	GM17092	0.129665	2.1249	0.45405	0.650905	-5.66831	0.867392	0.77625
NK.cells	ZDHHC1	-0.15474	1.415954	-0.45399	0.650945	-5.44484	0.876717	0.791709
NK.cells	ANGPTL4	-0.19465	1.23193	-0.45377	0.651105	-5.40208	0.87918	0.795902
NK.cells	PNPT1	-0.0643	4.198204	-0.45372	0.651145	-6.26526	0.840752	0.732883
NK.cells	SBDS	0.03952	5.561152	0.453674	0.651175	-6.56328	0.823708	0.705596
NK.cells	GM8797	-0.12362	2.19543	-0.45344	0.65134	-5.66885	0.866533	0.774943
NK.cells	FANCC	-0.06342	4.74282	-0.45334	0.651412	-6.41428	0.833931	0.721913
NK.cells	ZBTB11	-0.04027	7.088839	-0.45325	0.651476	-6.81309	0.805086	0.676276
NK.cells	MYO15	0.192578	1.234713	0.453122	0.651571	-5.46042	0.879181	0.795951
NK.cells	DLEU2	-0.04559	8.40867	-0.45311	0.651583	-6.99694	0.789344	0.651907
NK.cells	OGFOD3	-0.09104	3.187381	-0.45303	0.651636	-5.91322	0.853677	0.753888
NK.cells	ACOT1	0.22297	2.604682	0.452949	0.651695	-5.55175	0.861204	0.766215
NK.cells	TMEM35B	0.136581	2.207613	0.452598	0.651947	-5.73693	0.866481	0.774759
NK.cells	MIB1	-0.04281	6.189415	-0.45245	0.652054	-6.67694	0.816114	0.693532
NK.cells	ZFYVE1	0.063008	4.797159	0.452351	0.652124	-6.35455	0.833354	0.720976
NK.cells	CRTAP	0.101196	3.004923	0.452285	0.652172	-5.85854	0.856132	0.757886
NK.cells	OFCC1	-0.27607	-0.63548	-0.45224	0.652203	-5.14516	0.904477	0.838566
NK.cells	APOBEC1	0.105251	6.087599	0.452166	0.652257	-6.27324	0.817362	0.695612
NK.cells	IPO13	0.122164	2.264795	0.452151	0.652268	-5.71581	0.865734	0.773704
NK.cells	H2-DMA	-0.1229	5.184297	-0.45182	0.652508	-6.1546	0.828728	0.713359
NK.cells	4931414P1	0.145348	2.099255	0.45171	0.652584	-5.63469	0.868115	0.777306
NK.cells	ANKRD33B	0.178234	4.882196	0.451629	0.652642	-5.93528	0.832498	0.719384
NK.cells	ANKRD28	0.055817	5.835458	0.451442	0.652776	-6.54292	0.820721	0.700615
NK.cells	WASF2	0.029656	8.064634	0.451007	0.653089	-6.95544	0.793765	0.658609
NK.cells	EPS15L1	-0.03928	6.525246	-0.45065	0.653343	-6.71672	0.812273	0.687526
NK.cells	EIF3L	-0.04342	5.672102	-0.45063	0.653357	-6.58199	0.822736	0.704052
NK.cells	EME1	-0.12458	2.299791	-0.45057	0.653402	-5.75762	0.865553	0.773336
NK.cells	RNF6	-0.04088	5.670645	-0.45053	0.653434	-6.58555	0.822754	0.704102
NK.cells	SEC14L1	-0.06254	4.618739	-0.45048	0.653466	-6.30329	0.835858	0.725037
NK.cells	TRIM32	0.179905	1.488698	0.450367	0.653548	-5.53134	0.876205	0.791037
NK.cells	PDE4D	-0.06568	6.428002	-0.45029	0.653605	-6.76348	0.813458	0.689472
NK.cells	NMD3	0.041646	4.999909	0.450091	0.653747	-6.4307	0.831083	0.717438
NK.cells	SNRPD2	0.039972	6.391824	0.450013	0.653802	-6.68835	0.8139	0.690167
NK.cells	CCDC126	0.155282	1.899321	0.450011	0.653804	-5.58118	0.870795	0.782082
NK.cells	RAI14	-0.12978	2.228896	-0.44994	0.653857	-5.81951	0.866479	0.774963
NK.cells	RABGGTA	-0.08951	2.703471	-0.44973	0.654006	-5.86087	0.860303	0.764866
NK.cells	ZFP931	-0.11758	1.514653	-0.4496	0.654102	-5.67837	0.875862	0.790625
NK.cells	GM39556	0.140562	3.384757	0.449589	0.654107	-5.74752	0.85152	0.750555
NK.cells	XAB2	-0.0732	3.922858	-0.44958	0.654113	-6.16051	0.844652	0.739399
NK.cells	FAM107B	0.041473	7.858238	0.449542	0.654141	-6.99152	0.796219	0.662675
NK.cells	CTNS	0.087016	3.606042	0.449509	0.654165	-6.02557	0.848688	0.745948
NK.cells	CXCL2	0.213604	7.158496	0.449487	0.65418	-6.62588	0.804602	0.675708
NK.cells	SLF2	0.04613	6.314028	0.449364	0.654269	-6.59492	0.81485	0.691786
NK.cells	ZWINT	-0.04831	5.039001	-0.44927	0.654338	-6.45701	0.830595	0.716784

NK.cells	TBC1D15	-0.04165	6.062519	-0.44927	0.654339	-6.63297	0.817929	0.696647
NK.cells	ABHD11	0.084241	3.541149	0.449217	0.654374	-6.02867	0.849518	0.7473
NK.cells	RELB	0.049947	6.088989	0.449036	0.654505	-6.69616	0.817616	0.696173
NK.cells	MCRS1	-0.05937	4.318538	-0.44894	0.654573	-6.2565	0.839651	0.731358
NK.cells	LRRC49	-0.19797	1.020554	-0.44889	0.654608	-5.3442	0.882429	0.801628
NK.cells	GM37982	0.10144	2.996043	0.448444	0.65493	-6.10997	0.856532	0.758904
NK.cells	CRIPT	0.036548	6.218829	0.448419	0.654948	-6.69966	0.816025	0.693789
NK.cells	CCDC17	-0.1913	0.893924	-0.44838	0.654974	-5.44411	0.884117	0.804578
NK.cells	ZFP182	0.067939	4.211679	0.448212	0.655097	-6.29699	0.841001	0.733699
NK.cells	ZHX1	0.065338	4.078556	0.448151	0.655141	-6.24204	0.842687	0.736422
NK.cells	ADAMTS1	-0.18435	2.937185	-0.44796	0.65528	-5.72712	0.857291	0.760206
NK.cells	VBP1	-0.04565	4.92592	-0.44795	0.655286	-6.42947	0.832019	0.719273
NK.cells	ZFP942	0.062443	4.309464	0.44789	0.655328	-6.31008	0.839765	0.731726
NK.cells	HEPACAM2	-0.25119	0.779743	-0.44789	0.655329	-5.36683	0.885643	0.8072
NK.cells	YEATS2	-0.05381	4.376137	-0.44759	0.655544	-6.32576	0.838924	0.730445
NK.cells	MTMR14	-0.05359	5.427711	-0.44756	0.655563	-6.48362	0.825771	0.709371
NK.cells	CPSF7	-0.0411	5.801172	-0.44734	0.655725	-6.55178	0.821155	0.702033
NK.cells	KCNK13	-0.19323	2.912398	-0.44724	0.655794	-5.56048	0.857611	0.760851
NK.cells	STK32C	-0.13468	0.553606	-0.44723	0.655805	-5.67553	0.888671	0.812409
NK.cells	DNTTIP2	-0.04407	5.107858	-0.44721	0.655815	-6.44668	0.829748	0.715751
NK.cells	PDHB	0.042392	5.703174	0.447153	0.655858	-6.55857	0.822363	0.703986
NK.cells	NET1	-0.08169	4.035875	-0.44706	0.655927	-6.18227	0.843228	0.737459
NK.cells	PPM1B	0.036406	6.192574	0.446993	0.655973	-6.74687	0.816347	0.694485
NK.cells	SLA2	-0.07991	2.746588	-0.44697	0.655991	-6.17209	0.859756	0.764396
NK.cells	2410004B1	0.045762	4.71052	0.446953	0.656002	-6.39456	0.834717	0.723739
NK.cells	MTCP1	0.188023	0.737147	0.446857	0.656071	-5.41912	0.886212	0.808368
NK.cells	CRISPLD2	0.272579	0.884481	0.446617	0.656244	-5.31593	0.884277	0.805122
NK.cells	APOL7E	0.106525	-0.10248	0.446353	0.656434	-5.88727	0.897555	0.827547
NK.cells	MECP2	0.040843	5.847525	0.446266	0.656496	-6.60357	0.820615	0.70135
NK.cells	C1QTNF6	0.127786	1.904338	0.446231	0.656521	-5.87947	0.870775	0.782695
NK.cells	MAPKAPK5	-0.10715	2.128701	-0.44597	0.656713	-5.69198	0.867834	0.777844
NK.cells	EIF3H	0.033788	7.353901	0.445913	0.65675	-6.88949	0.802293	0.672563
NK.cells	DKC1	0.062763	4.56874	0.44585	0.656795	-6.3445	0.83653	0.726787
NK.cells	SLAIN2	-0.03957	5.670672	-0.44583	0.656806	-6.59225	0.822796	0.704829
NK.cells	ATP6V0A2	-0.04517	4.619482	-0.44582	0.656817	-6.50386	0.835892	0.725761
NK.cells	ALAD	-0.08055	4.057214	-0.44582	0.656817	-6.22569	0.84299	0.737209
NK.cells	NUMA1	-0.04193	5.705606	-0.44568	0.656917	-6.60512	0.822365	0.704144
NK.cells	PPIA	-0.04126	10.92524	-0.44565	0.65694	-7.40422	0.760653	0.608965
NK.cells	TANC1	-0.10485	3.838336	-0.44546	0.657079	-6.05506	0.845859	0.741777
NK.cells	C330018D2	0.114256	2.325358	0.44505	0.657371	-5.68798	0.865382	0.773713
NK.cells	SMPD5	0.177559	-0.18019	0.444992	0.657413	-5.35419	0.898731	0.829488
NK.cells	GNG3	-0.15454	1.449153	-0.4449	0.657481	-5.57966	0.876893	0.79281
NK.cells	UCK1	-0.09226	3.265642	-0.44485	0.657512	-6.01809	0.853209	0.753755
NK.cells	TFPI	-0.09685	3.468648	-0.44483	0.657528	-5.90945	0.850605	0.749513
NK.cells	TXNDC5	-0.06052	5.179694	-0.44478	0.657568	-6.41317	0.828997	0.714661
NK.cells	MS4A4B	0.065715	3.207847	0.444626	0.657677	-6.55818	0.853952	0.755031
NK.cells	HIST1H1E	-0.13482	4.203103	-0.44457	0.657716	-6.34941	0.841256	0.734405

NK.cells	CEMIP2	0.058216	5.494454	0.444548	0.657732	-6.57607	0.825088	0.708472
NK.cells	DYNC1LI1	-0.03636	6.412559	-0.4443	0.657911	-6.71562	0.813881	0.690635
NK.cells	RALGDS	0.115346	3.822267	0.444226	0.657964	-6.01564	0.846175	0.742296
NK.cells	MAN1A	0.049591	8.000333	0.444164	0.658009	-7.04084	0.794758	0.660806
NK.cells	CABP1	-0.24596	-0.13612	-0.44386	0.658228	-5.248	0.898426	0.82881
NK.cells	HEXDC	-0.18721	1.86348	-0.44367	0.658363	-5.50567	0.871762	0.784038
NK.cells	2810002D1	0.123166	1.66458	0.443614	0.658405	-5.55301	0.874381	0.788385
NK.cells	GM15518	-0.19434	0.80065	-0.44338	0.658576	-5.44837	0.885989	0.807618
NK.cells	PSMA3	-0.02971	7.755055	-0.44323	0.658681	-6.94482	0.798067	0.665561
NK.cells	WDFY1	-0.06115	4.332443	-0.44295	0.658882	-6.33008	0.840254	0.732121
NK.cells	SMIM11	-0.0488	4.805042	-0.44279	0.658998	-6.37197	0.834306	0.722595
NK.cells	ZFP260	0.052402	4.245881	0.442452	0.659242	-6.27757	0.841349	0.734033
NK.cells	SPA17	0.161808	0.979282	0.442319	0.659339	-5.4366	0.883799	0.80389
NK.cells	LPP	0.050904	7.6885	0.442226	0.659406	-6.92137	0.798996	0.667004
NK.cells	CD55	-0.11617	4.863884	-0.4421	0.659493	-6.18786	0.833568	0.721601
NK.cells	MAPK1IP1	0.035155	6.060276	0.441959	0.659598	-6.63552	0.818728	0.698002
NK.cells	CD200	0.172117	1.910379	0.441806	0.659708	-5.6115	0.87147	0.783516
NK.cells	GM46440	0.184103	0.637929	0.441719	0.659771	-5.47402	0.888365	0.811716
NK.cells	EGFL6	-0.23241	0.548891	-0.44159	0.659861	-5.28356	0.88956	0.813725
NK.cells	GM9949	0.267365	0.483247	0.441374	0.66002	-5.2254	0.890442	0.815217
NK.cells	A930037H(	-0.10625	3.242306	-0.44137	0.660021	-6.1126	0.854153	0.755028
NK.cells	SMIM1	-0.20153	1.759237	-0.44135	0.660038	-5.45496	0.873459	0.786823
NK.cells	PLATR25	-0.0894	2.729731	-0.44134	0.660044	-5.94585	0.860773	0.765872
NK.cells	PNLDC1	-0.19511	0.924032	-0.44134	0.660047	-5.40466	0.884536	0.805298
NK.cells	ZFP959	-0.07806	3.241052	-0.44107	0.660238	-6.00823	0.854169	0.755126
NK.cells	EHMT2	-0.04928	4.951712	-0.441	0.660293	-6.47102	0.832469	0.720039
NK.cells	WDR70	-0.0386	6.1341	-0.44093	0.660342	-6.65903	0.817821	0.696738
NK.cells	LETM2	-0.08395	3.131534	-0.44074	0.660478	-5.99402	0.855579	0.757514
NK.cells	JCHAIN	-0.60758	-0.4625	-0.44064	0.660548	-5.15888	0.903254	0.837092
NK.cells	MAP2K3OS	0.281007	-0.12812	0.440494	0.660654	-5.24481	0.898702	0.829396
NK.cells	MED22	-0.11821	2.384243	-0.44043	0.6607	-5.71965	0.865266	0.773479
NK.cells	CLPTM1L	0.052019	4.883326	0.440392	0.660728	-6.41762	0.833325	0.721535
NK.cells	EPM2AIP1	0.102986	2.609784	0.44037	0.660744	-5.812	0.86233	0.768664
NK.cells	RDH12	0.168266	2.275253	0.440295	0.660798	-5.64379	0.866689	0.775872
NK.cells	LCORL	0.045765	6.985276	0.439958	0.661041	-6.73659	0.807453	0.680558
NK.cells	SNU13	0.036929	6.96176	0.439945	0.661051	-6.78051	0.807737	0.681004
NK.cells	RNF38	0.04145	6.163439	0.439896	0.661086	-6.64185	0.817462	0.696308
NK.cells	TRIB1	0.087717	5.765307	0.439865	0.661108	-6.25603	0.822359	0.70407
NK.cells	ARRB1	-0.05265	3.996665	-0.43986	0.661115	-6.35975	0.844509	0.739603
NK.cells	SCAMP1	0.119668	2.970331	0.439812	0.661147	-5.81542	0.857659	0.761031
NK.cells	COX6C	-0.0316	8.594079	-0.43977	0.661174	-7.07363	0.788251	0.650758
NK.cells	BAK1	0.057597	4.936771	0.439677	0.661244	-6.45711	0.832656	0.720499
NK.cells	ISOC2B	-0.10068	2.519105	-0.43952	0.661358	-5.91475	0.863509	0.770707
NK.cells	NCOA7	0.052514	5.299685	0.439503	0.661369	-6.56274	0.828128	0.713315
NK.cells	RNF123	0.069712	3.804311	0.439342	0.661485	-6.25054	0.846957	0.743652
NK.cells	IGSF5	-0.10031	1.922281	-0.43934	0.661488	-6.14759	0.871314	0.783623
NK.cells	SNX4	0.035383	6.329814	0.439136	0.661634	-6.69848	0.815424	0.693259

NK.cells	FCER1G	0.063398	8.628675	0.439062	0.661688	-7.24728	0.787843	0.650302
NK.cells	GMPR2	-0.07462	3.866899	-0.43879	0.661885	-6.09013	0.84616	0.742545
NK.cells	INPP5E	0.097002	2.260693	0.438782	0.66189	-5.77439	0.866879	0.77648
NK.cells	SCLY	0.070541	3.466279	0.438764	0.661903	-6.09333	0.851277	0.75087
NK.cells	RTL6	-0.24961	0.292529	-0.43876	0.661904	-5.22884	0.89301	0.820127
NK.cells	AKIRIN1	-0.03358	6.287656	-0.43869	0.661957	-6.68038	0.81594	0.694174
NK.cells	4932438H2	0.193748	-0.27879	0.438592	0.662027	-5.4898	0.90075	0.833308
NK.cells	CSNK2B	0.037179	7.247784	0.438588	0.66203	-6.87493	0.804284	0.675905
NK.cells	PINK1	-0.05721	5.115722	-0.43849	0.6621	-6.50298	0.83042	0.717255
NK.cells	DHFR	-0.08736	4.331798	-0.43796	0.662484	-6.25167	0.84066	0.733227
NK.cells	MGAT4A	-0.05269	5.666978	-0.4377	0.66267	-6.55106	0.824038	0.706498
NK.cells	NPRL2	-0.08187	2.790262	-0.43768	0.662684	-5.93007	0.860473	0.765393
NK.cells	EMC10	0.041428	5.502896	0.437248	0.662998	-6.56951	0.826215	0.709841
NK.cells	JUN	-0.1164	7.097589	-0.43722	0.663021	-6.7118	0.806691	0.67902
NK.cells	ZFP503	0.193711	0.884395	0.43721	0.663025	-5.34282	0.885719	0.807166
NK.cells	RHOC	0.071656	4.059106	0.437101	0.663104	-6.30018	0.844339	0.738987
NK.cells	GM11423	0.170512	0.892029	0.436948	0.663214	-5.39744	0.885617	0.807111
NK.cells	LAPTM4B	-0.14579	3.501356	-0.43694	0.663223	-5.78436	0.851456	0.750625
NK.cells	ZFP266	-0.05275	4.029092	-0.43679	0.663332	-6.24379	0.84477	0.739746
NK.cells	MLLT1	-0.08799	2.735176	-0.43665	0.663433	-5.832	0.861428	0.766929
NK.cells	FBXW4	0.059563	4.272711	0.436508	0.663532	-6.42599	0.841755	0.734828
NK.cells	BICD2	-0.05946	4.09822	-0.43611	0.663817	-6.29068	0.844239	0.738568
NK.cells	PPP2R5A	-0.0299	7.861178	-0.43585	0.664009	-6.95727	0.797963	0.665157
NK.cells	GSAP	0.060569	5.642393	0.435702	0.664115	-6.71634	0.824941	0.707564
NK.cells	CREBL2	-0.08079	3.233581	-0.43569	0.664125	-6.05853	0.855367	0.756633
NK.cells	PTCD3	-0.05135	4.845166	-0.43558	0.6642	-6.43528	0.834879	0.723444
NK.cells	ATP6AP1	0.045169	5.990589	0.435522	0.664245	-6.55944	0.820642	0.700738
NK.cells	BTBD1	0.036726	6.497876	0.435455	0.664294	-6.76502	0.814422	0.690925
NK.cells	COQ8B	0.09393	3.190292	0.435228	0.664458	-5.95507	0.856046	0.757661
NK.cells	P2RY12	-0.19688	2.616799	-0.43491	0.664687	-5.45098	0.863584	0.769874
NK.cells	PCMTD2	0.070186	3.680281	0.434739	0.664811	-6.19243	0.849863	0.747484
NK.cells	GM49482	-0.28155	-0.71551	-0.43474	0.664812	-5.11596	0.908129	0.8446
NK.cells	GM35769	0.159252	1.096204	0.434651	0.664875	-5.51846	0.883617	0.80318
NK.cells	TBL1XR1	0.038638	6.669065	0.434622	0.664896	-6.7112	0.812553	0.687811
NK.cells	KLRB1B	-0.07695	1.981821	-0.43426	0.665159	-6.52309	0.872143	0.783738
NK.cells	TCF7L1	-0.13423	3.097343	-0.43406	0.665305	-5.74986	0.857702	0.759798
NK.cells	ATXN7L2	0.109114	2.480154	0.433769	0.665513	-5.83309	0.865759	0.773028
NK.cells	RBM43	0.085245	2.961078	0.433673	0.665582	-5.98255	0.859508	0.762756
NK.cells	KIF3C	-0.09946	2.145051	-0.43349	0.665712	-5.83831	0.870143	0.780348
NK.cells	ALMS1	-0.07393	3.824261	-0.43346	0.665737	-6.17822	0.84841	0.744732
NK.cells	LNPEP	0.036307	7.335044	0.433457	0.665739	-6.88034	0.804854	0.675389
NK.cells	ZFP760	0.169644	1.032725	0.433301	0.665851	-5.36971	0.884867	0.804874
NK.cells	FAM171B	-0.24377	0.217464	-0.43317	0.665946	-5.33256	0.895826	0.823304
NK.cells	SLC1A4	-0.18481	0.947937	-0.43279	0.66622	-5.39843	0.886	0.806872
NK.cells	ASMT	0.182125	1.052723	0.432693	0.666291	-5.46039	0.8846	0.804535
NK.cells	TIAL1	-0.03837	5.798885	-0.43269	0.666294	-6.58876	0.823603	0.705014
NK.cells	SLC25A34	0.242862	-0.47643	0.43239	0.666511	-5.19049	0.905266	0.8395

NK.cells	HEATR3	0.060012	4.273336	0.432365	0.666529	-6.31781	0.842698	0.735669
NK.cells	CD177	0.250967	0.17299	0.432269	0.666598	-5.26531	0.896428	0.82451
NK.cells	ABCA1	0.093075	5.836161	0.432152	0.666683	-6.43134	0.823142	0.704349
NK.cells	HNRNPUL1	-0.03038	7.28107	-0.43212	0.666704	-6.83203	0.805505	0.676579
NK.cells	KLHL9	-0.05688	4.65586	-0.43188	0.666882	-6.31257	0.837865	0.727927
NK.cells	OGA	0.037373	6.76343	0.431826	0.666919	-6.79198	0.811775	0.686445
NK.cells	1700086O(	0.152009	1.546442	0.431749	0.666975	-5.43447	0.878034	0.793737
NK.cells	ANAPC13	-0.04063	5.65737	-0.43171	0.667004	-6.53769	0.825354	0.707952
NK.cells	ACTB	0.034439	13.9201	0.431274	0.667319	-7.80234	0.729738	0.562795
NK.cells	FCGR1	0.209718	2.432362	0.431237	0.667346	-5.53277	0.866383	0.77461
NK.cells	KMT2B	-0.06414	3.888591	-0.43107	0.667466	-6.17644	0.847589	0.743876
NK.cells	HECTD4	-0.04683	5.548933	-0.43106	0.667475	-6.56408	0.826699	0.710277
NK.cells	FAM193B	-0.05994	4.050857	-0.43097	0.667541	-6.23374	0.845523	0.740531
NK.cells	RPA2	0.068828	4.645282	0.430872	0.66761	-6.41767	0.837998	0.728379
NK.cells	4732496C(	0.228382	0.689806	0.430867	0.667614	-5.18193	0.889459	0.813082
NK.cells	DCLK2	-0.09938	2.72479	-0.4308	0.667659	-5.92612	0.862573	0.768366
NK.cells	PRIM2	0.066479	5.536205	0.430733	0.667711	-6.52645	0.826857	0.710535
NK.cells	ZFP799	0.159173	0.939029	0.430711	0.667727	-5.46918	0.886119	0.807473
NK.cells	TTC17	0.043781	5.324345	0.43061	0.6678	-6.52249	0.829491	0.714738
NK.cells	MESD	0.057465	4.085411	0.430519	0.667866	-6.17073	0.845083	0.739819
NK.cells	EIF5B	-0.03273	7.158051	-0.43052	0.667867	-6.82722	0.80699	0.679166
NK.cells	CCNK	0.045602	4.857081	0.430489	0.667888	-6.44978	0.835335	0.724097
NK.cells	COPS8	-0.04087	5.091019	-0.43044	0.667923	-6.47692	0.832404	0.719396
NK.cells	TOM1L2	0.052808	5.471299	0.430419	0.667939	-6.56709	0.827663	0.71182
NK.cells	GM4707	0.086034	2.866085	0.430239	0.668069	-6.18723	0.860739	0.765384
NK.cells	TMEM79	-0.19599	0.778123	-0.43005	0.668208	-5.34711	0.888274	0.811153
NK.cells	CCDC69	-0.08189	3.297575	-0.42992	0.6683	-6.03381	0.855163	0.756318
NK.cells	BC055324	0.112231	2.245694	0.429636	0.668506	-5.75106	0.868824	0.778867
NK.cells	GGTA1	0.052532	5.365487	0.429627	0.668512	-6.6273	0.828979	0.714097
NK.cells	GM50373	-0.25378	0.358844	-0.42949	0.668614	-5.22799	0.893915	0.820793
NK.cells	UAP1L1	0.072771	3.734573	0.429349	0.668713	-6.07809	0.849556	0.747271
NK.cells	MFSD13A	0.14621	2.008416	0.429159	0.668852	-5.55766	0.871938	0.7841
NK.cells	GM48768	0.190084	0.621002	0.429002	0.668965	-5.35785	0.890384	0.814951
NK.cells	HS3ST3B1	0.082835	3.380647	0.428965	0.668992	-6.16025	0.854094	0.754763
NK.cells	PXMP4	-0.07443	3.831766	-0.42894	0.66901	-6.10734	0.848314	0.745347
NK.cells	ZFX	-0.03219	6.339182	-0.4289	0.669043	-6.69737	0.816954	0.695094
NK.cells	TVP23B	-0.05809	4.042523	-0.42885	0.669073	-6.2102	0.845629	0.740988
NK.cells	SPG20	0.107841	2.615271	0.428699	0.669185	-5.7002	0.863998	0.771007
NK.cells	VPS13D	-0.04496	6.149191	-0.42835	0.669437	-6.65597	0.819285	0.698925
NK.cells	INO80B	0.059126	4.146114	0.428172	0.669567	-6.26182	0.844312	0.739039
NK.cells	SUPT16	0.032983	7.068147	0.427974	0.669711	-6.80823	0.808077	0.681305
NK.cells	DUSP19	-0.10924	2.048701	-0.42796	0.66972	-5.72177	0.871408	0.783453
NK.cells	CHIL5	0.178642	-0.02332	0.427777	0.669854	-5.34975	0.89909	0.829917
NK.cells	EEFSEC	0.065351	4.399413	0.427644	0.66995	-6.38579	0.841102	0.733891
NK.cells	ANKLE1	-0.1754	0.64315	-0.42757	0.670004	-5.35223	0.890086	0.814703
NK.cells	CCL21A	-0.69321	0.049265	-0.42737	0.67015	-5.1709	0.898105	0.828281
NK.cells	FTX	-0.07716	4.311116	-0.42736	0.670155	-6.15167	0.842219	0.735727

NK.cells	EML4	0.037305	7.138312	0.427245	0.67024	-6.85759	0.807229	0.68004
NK.cells	SREBF2	-0.04758	5.9605	-0.42705	0.670381	-6.62936	0.821608	0.702774
NK.cells	VPS13B	0.033342	8.050399	0.426838	0.670535	-6.94546	0.796285	0.663098
NK.cells	0610040F0	-0.16311	0.269406	-0.42682	0.67055	-5.40135	0.895124	0.823365
NK.cells	CTSW	-0.07879	2.120663	-0.42676	0.670594	-6.39275	0.870463	0.782092
NK.cells	KIF19A	0.141098	1.071139	0.42654	0.670751	-5.53811	0.884354	0.805272
NK.cells	CDPF1	-0.11397	2.220471	-0.42653	0.670757	-5.83106	0.869155	0.779953
NK.cells	KLHL25	-0.08502	2.493825	-0.42647	0.670799	-5.90374	0.865581	0.774046
NK.cells	EIF1	-0.0377	9.721973	-0.42641	0.670845	-7.23243	0.776657	0.632998
NK.cells	GPR34	-0.12966	0.588061	-0.42605	0.671107	-5.91459	0.890827	0.8162
NK.cells	GM44148	0.144333	1.90428	0.425777	0.671305	-5.66357	0.873308	0.787004
NK.cells	CRK	0.042739	6.391053	0.425752	0.671323	-6.66564	0.816319	0.694621
NK.cells	HIST1H2BH	-0.22194	0.17295	-0.42575	0.671326	-5.2738	0.896428	0.825782
NK.cells	TLR7	-0.20027	2.963787	-0.42564	0.671404	-5.45196	0.859473	0.764151
NK.cells	IPO8	0.049047	4.420007	0.425612	0.671425	-6.3476	0.840841	0.733803
NK.cells	PLBD1	-0.14821	5.389651	-0.42561	0.671429	-5.93179	0.828678	0.714259
NK.cells	CHRNA9	0.158793	2.214161	0.42558	0.671448	-5.68413	0.869237	0.780252
NK.cells	2810006K2	0.113399	2.570884	0.425515	0.671496	-5.85731	0.864576	0.772554
NK.cells	IFT46	-0.04945	4.502973	-0.42541	0.671574	-6.30315	0.839793	0.732162
NK.cells	D10WSU1(	0.071679	3.652731	0.425286	0.671662	-6.16804	0.850603	0.749716
NK.cells	TRIOBP	-0.04839	4.877202	-0.42525	0.671687	-6.35798	0.835082	0.724606
NK.cells	NT5C3B	0.062891	3.396774	0.425117	0.671785	-6.16657	0.853887	0.755098
NK.cells	CEP70	0.08702	3.195368	0.425019	0.671855	-5.94734	0.856481	0.759378
NK.cells	BEND4	0.070582	3.557561	0.425014	0.671859	-6.3463	0.851823	0.751763
NK.cells	GM3055	-0.18619	0.319624	-0.42494	0.671915	-5.37547	0.894445	0.822609
NK.cells	SELENOM	0.142506	2.384919	0.424887	0.671951	-5.71094	0.867003	0.776728
NK.cells	ROBO1	-0.17035	2.137687	-0.42486	0.671972	-5.62019	0.87024	0.782087
NK.cells	ISCA2	0.055354	4.968036	0.424816	0.672003	-6.39796	0.833943	0.722854
NK.cells	ART2B	-0.21412	0.097769	-0.42477	0.672039	-5.51379	0.897447	0.827692
NK.cells	MRGBP	-0.07431	3.37422	-0.42476	0.672044	-6.03613	0.854177	0.755643
NK.cells	RNF139	0.052121	5.218473	0.424373	0.672325	-6.46223	0.830811	0.717927
NK.cells	FNBP4	-0.0372	6.074991	-0.42434	0.672351	-6.65663	0.820198	0.701011
NK.cells	MAN2C1OS	0.060502	4.443794	0.424288	0.672387	-6.29456	0.840541	0.733597
NK.cells	ADGRG5	0.122037	0.105614	0.424275	0.672396	-5.87261	0.897341	0.827643
NK.cells	ZFP808	0.117579	1.973033	0.424255	0.67241	-5.65375	0.872403	0.785807
NK.cells	TMEM168	0.055545	4.583494	0.424109	0.672516	-6.31605	0.838777	0.730825
NK.cells	CCDC51	0.116933	1.810129	0.424044	0.672564	-5.62209	0.874549	0.789475
NK.cells	IMPA1	-0.04367	5.285622	-0.42381	0.672735	-6.5132	0.829974	0.71683
NK.cells	ARHGAP33	-0.22524	0.549492	-0.42354	0.67293	-5.27405	0.891346	0.817816
NK.cells	NSMCE1	0.054977	4.829662	0.42352	0.672944	-6.39337	0.835679	0.726033
NK.cells	SH2B2	0.113201	4.550186	0.423354	0.673065	-5.81664	0.839197	0.731719
NK.cells	SLC35B2	0.052899	4.882082	0.423299	0.673105	-6.42105	0.835021	0.72499
NK.cells	TALDO1	0.034255	8.310001	0.423259	0.673134	-6.98133	0.793201	0.659006
NK.cells	CHDH	0.207126	1.157054	0.42323	0.673155	-5.37832	0.883208	0.804147
NK.cells	COX18	0.074912	3.223872	0.423194	0.673182	-6.04633	0.856113	0.759235
NK.cells	CATSPERE2	0.150797	1.174644	0.423168	0.6732	-5.41981	0.882974	0.803754
NK.cells	POLE2	-0.09597	3.014685	-0.42315	0.67321	-5.99005	0.858814	0.763666

NK.cells	C1RL	-0.17203	1.632594	-0.42303	0.673298	-5.5206	0.876894	0.793648
NK.cells	GM37768	-0.11343	2.087377	-0.42298	0.673338	-5.73247	0.8709	0.783677
NK.cells	HNRNPDL	-0.02869	7.857159	-0.4229	0.673393	-6.9381	0.79859	0.667413
NK.cells	GTF2A1	0.050324	5.65525	0.422824	0.673451	-6.51815	0.82538	0.709608
NK.cells	GM47096	0.150005	1.734536	0.422732	0.673518	-5.48559	0.875547	0.791408
NK.cells	DDX60	0.139344	2.866859	0.422565	0.673638	-5.93489	0.860729	0.766925
NK.cells	AGPAT2	-0.07434	4.355109	-0.42247	0.673706	-6.32653	0.841662	0.735826
NK.cells	1700123M	-0.15335	0.89752	-0.42244	0.673726	-5.50239	0.886675	0.810108
NK.cells	NUBPL	-0.08921	2.913085	-0.42211	0.67397	-5.86688	0.86013	0.766104
NK.cells	KAT5	0.093905	2.921057	0.422074	0.673996	-5.93995	0.860027	0.765944
NK.cells	ALS2CL	0.188486	0.148166	0.421968	0.674072	-5.41484	0.896764	0.827312
NK.cells	TBC1D1	0.04735	7.18844	0.421817	0.674183	-6.77696	0.806623	0.680162
NK.cells	1600002D2	-0.28564	0.257645	-0.42181	0.674187	-5.24212	0.895283	0.824802
NK.cells	MED23	0.059869	3.692708	0.421811	0.674187	-6.15389	0.850092	0.74968
NK.cells	UBXN11	0.099393	1.770334	0.421786	0.674205	-5.77382	0.875074	0.790859
NK.cells	MTX1	-0.0585	4.556924	-0.42168	0.67428	-6.32844	0.839112	0.731862
NK.cells	STARD7	0.046659	5.358518	0.421671	0.674288	-6.49013	0.829066	0.715711
NK.cells	ADRM1	-0.05067	5.477684	-0.42123	0.674608	-6.52853	0.827584	0.713423
NK.cells	MIPEP	0.073864	2.843457	0.421181	0.674645	-5.92474	0.861033	0.767696
NK.cells	PRMT7	0.099439	3.247895	0.421054	0.674737	-5.9895	0.855803	0.759106
NK.cells	ZXDC	-0.05933	4.004625	-0.421	0.674774	-6.23362	0.846111	0.74329
NK.cells	WBP2	0.047132	5.599979	0.420982	0.674789	-6.53149	0.826066	0.711002
NK.cells	SIRT7	-0.03916	4.918849	-0.42095	0.674816	-6.4552	0.83456	0.724616
NK.cells	MMUT	-0.08673	3.4125	-0.42094	0.67482	-6.04738	0.853685	0.755643
NK.cells	CSTDC6	-0.18599	-1.16323	-0.42092	0.674836	-5.18101	0.914483	0.858063
NK.cells	SHC1	-0.0516	4.384809	-0.42091	0.674842	-6.30247	0.841286	0.735469
NK.cells	SNX1	0.03508	5.483558	0.420884	0.674861	-6.55174	0.827511	0.713314
NK.cells	SLC35A1	0.065627	3.416075	0.420816	0.674911	-6.02913	0.853639	0.75559
NK.cells	IKZF5	0.062893	3.804869	0.420713	0.674986	-6.15705	0.848658	0.747458
NK.cells	ENSA	0.04313	5.388151	0.42065	0.675031	-6.51111	0.828697	0.71523
NK.cells	FAM189B	0.085645	2.569109	0.420595	0.675072	-6.08747	0.864599	0.773605
NK.cells	HOPX	0.052532	4.034194	0.420419	0.675199	-6.68017	0.845735	0.74271
NK.cells	SNHG10	0.241798	0.227889	0.420394	0.675217	-5.24551	0.895685	0.825618
NK.cells	NCR1	0.097133	1.170957	0.420313	0.675277	-6.24705	0.883023	0.804275
NK.cells	TXNDC15	-0.05021	4.911319	-0.42025	0.675325	-6.46816	0.834654	0.724808
NK.cells	GM13427	-0.11392	2.062778	-0.42019	0.675365	-5.70556	0.871224	0.784617
NK.cells	PIP4P2	-0.07302	3.706325	-0.42014	0.675405	-6.17479	0.849917	0.749551
NK.cells	A630089N(	0.105705	2.672384	0.420108	0.675426	-5.82376	0.863255	0.771428
NK.cells	CACNA1D	0.101425	3.396539	0.419895	0.675581	-5.97796	0.853913	0.756102
NK.cells	BACE1	-0.09045	2.654059	-0.41975	0.675687	-5.84236	0.863516	0.771884
NK.cells	HAP1	0.247647	-0.45019	0.419709	0.675716	-5.14536	0.904931	0.841405
NK.cells	CCDC167	-0.06663	4.065969	-0.4197	0.675726	-6.20926	0.845353	0.742146
NK.cells	GTF2H4	0.092831	2.307317	0.419438	0.675914	-5.75427	0.868191	0.779385
NK.cells	VIL1	-0.26087	0.018277	-0.41922	0.676071	-5.22491	0.898759	0.830626
NK.cells	GINS4	-0.06535	3.753755	-0.41917	0.676105	-6.17191	0.849532	0.748703
NK.cells	NABP1	0.050008	4.743721	0.41909	0.676166	-6.59739	0.836977	0.728357
NK.cells	FLYWCH1	-0.0645	3.850858	-0.41895	0.676266	-6.14639	0.848329	0.746729

NK.cells	SCAF11	0.034531	7.16109	0.418749	0.676415	-6.81947	0.807224	0.68101
NK.cells	TMEM26	-0.22884	2.190341	-0.41872	0.676434	-5.33888	0.869842	0.782056
NK.cells	PPIH	-0.04644	5.305306	-0.41855	0.676557	-6.53434	0.830007	0.71716
NK.cells	COQ5	-0.07511	3.832511	-0.41839	0.676675	-6.14082	0.84859	0.747173
NK.cells	MAPK11	-0.21774	0.260399	-0.41837	0.67669	-5.24719	0.895546	0.825185
NK.cells	E2F4	0.043708	5.334947	0.418322	0.676726	-6.50951	0.829638	0.716568
NK.cells	GM17106	0.075784	3.395192	0.418244	0.676783	-6.19994	0.854194	0.756322
NK.cells	MYG1	-0.05613	3.803891	-0.41801	0.676954	-6.17483	0.848955	0.747797
NK.cells	DTL	-0.07984	4.961318	-0.41791	0.677023	-6.42457	0.834307	0.724089
NK.cells	TMBIM6	0.03526	9.142023	0.417872	0.677054	-7.14862	0.783668	0.644546
NK.cells	PTGER4	-0.05945	4.681638	-0.41779	0.677112	-6.53253	0.837822	0.729748
NK.cells	TBC1D25	-0.09544	2.839206	-0.41776	0.677135	-5.90917	0.861377	0.768142
NK.cells	GM12592	0.079763	3.124779	0.417409	0.677391	-6.00153	0.857915	0.762224
NK.cells	1700066M	0.132285	1.288003	0.417213	0.677534	-5.51546	0.882019	0.802184
NK.cells	GTPBP10	0.093879	2.397542	0.417199	0.677544	-5.78109	0.867383	0.777818
NK.cells	IIGP1	0.171915	4.709506	0.416904	0.677759	-6.22993	0.837813	0.729472
NK.cells	APBA3	0.117779	2.107298	0.416715	0.677897	-5.66801	0.871287	0.784312
NK.cells	ZFP317	-0.08158	2.898111	-0.41669	0.677913	-5.9547	0.860964	0.767258
NK.cells	PADI2	0.069361	2.631853	0.416608	0.677975	-6.36275	0.864425	0.772999
NK.cells	COL1A2	-0.14611	2.442649	-0.4166	0.677979	-5.74922	0.866893	0.777078
NK.cells	GM48678	-0.08419	3.349644	-0.41646	0.678083	-6.1049	0.855129	0.757736
NK.cells	ARL4A	-0.05927	4.453783	-0.41633	0.678177	-6.2688	0.84104	0.734807
NK.cells	HECA	0.036862	6.23169	0.416316	0.678187	-6.76629	0.818881	0.699322
NK.cells	ATAT1	0.098186	2.722421	0.415943	0.678459	-5.78911	0.863502	0.771145
NK.cells	FNIP1	0.043276	7.760209	0.415846	0.678531	-6.87584	0.800582	0.670277
NK.cells	LZTS3	-0.19539	0.323606	-0.41549	0.678786	-5.29673	0.89536	0.82452
NK.cells	ARHGAP35	-0.05175	5.237019	-0.41544	0.678829	-6.4478	0.83148	0.719223
NK.cells	SCAMP2	-0.03697	6.357233	-0.41539	0.678863	-6.64414	0.817618	0.69714
NK.cells	TMEM154	-0.08281	2.583653	-0.41536	0.678886	-6.11977	0.865346	0.774352
NK.cells	ZCWPW2	0.176326	1.016841	0.415323	0.678912	-5.48901	0.886038	0.808835
NK.cells	MMRN2	-0.19109	1.228883	-0.41504	0.679115	-5.33263	0.88328	0.804101
NK.cells	GM42982	0.118929	1.514483	0.415009	0.679141	-5.61895	0.879482	0.797744
NK.cells	KIF14	-0.12814	2.55406	-0.41496	0.679179	-5.91782	0.865804	0.775016
NK.cells	TIE1	0.168037	1.482911	0.414779	0.679308	-5.44059	0.879917	0.798474
NK.cells	CREBZF	0.047022	4.715073	0.414733	0.679342	-6.39348	0.838111	0.729827
NK.cells	AMFR	-0.03462	6.011988	-0.41463	0.679415	-6.61472	0.821946	0.70396
NK.cells	2900076A0	0.083774	2.621325	0.414413	0.679575	-5.87208	0.864942	0.773658
NK.cells	MED13L	-0.03588	7.196724	-0.41437	0.679607	-6.83855	0.807478	0.681144
NK.cells	SMC6	0.036477	7.579287	0.414048	0.679842	-6.88243	0.802867	0.67396
NK.cells	RNF122	-0.12565	2.64878	-0.41403	0.679858	-5.73544	0.864584	0.773103
NK.cells	PCGF3	0.056672	3.998741	0.413981	0.67989	-6.19526	0.847188	0.744601
NK.cells	MTERF4	0.121993	1.913823	0.41394	0.67992	-5.64183	0.874217	0.789084
NK.cells	PAFAH1B1	0.025029	8.064884	0.413751	0.680058	-6.99206	0.797055	0.664979
NK.cells	RSPH10B	0.229794	0.413611	0.413555	0.680202	-5.30591	0.894234	0.822747
NK.cells	C130013H0	0.16218	0.498295	0.413282	0.680401	-5.32473	0.893091	0.820865
NK.cells	ESS2	-0.06908	2.988114	-0.41314	0.680504	-5.99053	0.860176	0.765985
NK.cells	CCDC148	-0.21629	1.762244	-0.413	0.680607	-5.69296	0.876217	0.792572

NK.cells	MCU	-0.04744	6.164566	-0.41286	0.68071	-6.65715	0.820066	0.701192
NK.cells	RPUSD4	0.087717	2.682051	0.412829	0.680732	-5.9002	0.864151	0.772563
NK.cells	OTUD3	-0.1031	2.558441	-0.41281	0.680749	-5.86645	0.865762	0.775224
NK.cells	ZFP131	-0.0431	5.815875	-0.41279	0.680758	-6.57683	0.824368	0.708032
NK.cells	PPP1CB	-0.03801	7.803763	-0.41266	0.680855	-6.95787	0.800174	0.669935
NK.cells	PLEK	0.056314	7.520494	0.412602	0.680897	-6.90061	0.803573	0.675238
NK.cells	EIF1AD	0.052473	4.923218	0.412039	0.681308	-6.42986	0.835493	0.725899
NK.cells	CDK5RAP2	-0.05333	4.446752	-0.4119	0.681411	-6.29978	0.841498	0.735592
NK.cells	ABI1	-0.04137	7.702393	-0.41187	0.681433	-6.90312	0.801389	0.671854
NK.cells	GM9887	0.132572	1.686181	0.411826	0.681464	-5.62018	0.877223	0.794311
NK.cells	KLRC2	-0.08453	1.332802	-0.41175	0.681519	-6.31166	0.881912	0.802151
NK.cells	ACOXL	0.090622	1.285897	0.411747	0.681521	-6.14013	0.882537	0.803197
NK.cells	METTL3	-0.06602	3.319562	-0.4117	0.681553	-6.07333	0.855893	0.759034
NK.cells	PLIN2	-0.07397	5.831377	-0.4117	0.681553	-6.46146	0.824176	0.707776
NK.cells	BCKDHB	0.074118	3.929893	0.411666	0.681581	-6.14367	0.848066	0.746251
NK.cells	HSPA14	0.048909	5.034968	0.411629	0.681608	-6.49837	0.834091	0.723644
NK.cells	TNFAIP2	0.16718	4.351498	0.411451	0.681738	-5.81912	0.842705	0.737545
NK.cells	GM10790	-0.25061	-0.77429	-0.41142	0.681761	-5.06292	0.910427	0.850469
NK.cells	THAP8	0.289111	-0.29985	0.411292	0.681853	-5.16688	0.903923	0.839352
NK.cells	TMED9	-0.03138	6.609324	-0.41128	0.681864	-6.75519	0.814616	0.692612
NK.cells	VRK2	0.047958	5.691956	0.411263	0.681875	-6.57874	0.825903	0.710529
NK.cells	USP35	0.252487	0.017078	0.411204	0.681918	-5.24521	0.899605	0.832017
NK.cells	UBE3B	-0.04893	4.424846	-0.41111	0.681987	-6.30421	0.841776	0.736056
NK.cells	RAB5C	0.037022	6.358629	0.410937	0.682113	-6.68441	0.817683	0.69753
NK.cells	DNAAF5	-0.07751	2.988111	-0.41093	0.682118	-5.96116	0.860176	0.766141
NK.cells	ANTXR2	-0.08884	7.654982	-0.41086	0.682168	-6.91612	0.801958	0.672807
NK.cells	TFDP1	-0.05799	5.887837	-0.41082	0.682195	-6.59106	0.823478	0.706735
NK.cells	1700126G(	0.16526	1.104608	0.410801	0.682213	-5.48695	0.884954	0.807334
NK.cells	CEP57	0.045519	5.139583	0.410779	0.682228	-6.48966	0.832782	0.721612
NK.cells	LIN37	0.068018	3.646925	0.41039	0.682513	-6.12278	0.851952	0.752349
NK.cells	NPM3	0.051599	5.097579	0.410152	0.682687	-6.55109	0.833695	0.722613
NK.cells	ACTN2	0.15447	-0.1007	0.410042	0.682767	-5.5584	0.901639	0.835021
NK.cells	GM28417	-0.16174	1.320479	-0.40992	0.682855	-5.4781	0.882521	0.802742
NK.cells	RAD51D	0.074191	2.984907	0.409685	0.683028	-6.00324	0.860651	0.766516
NK.cells	TBC1D32	0.132108	2.141265	0.409517	0.683151	-5.67319	0.871663	0.784748
NK.cells	ARL2BP	-0.04935	5.086344	-0.40931	0.683303	-6.52216	0.833868	0.723035
NK.cells	4833403J1	0.157236	0.509888	0.409288	0.683319	-5.64746	0.893386	0.821181
NK.cells	GRIA3	0.1735	3.336555	0.409259	0.683339	-5.80277	0.856105	0.759121
NK.cells	SLC46A2	0.216831	-0.09426	0.409064	0.683482	-5.20173	0.901574	0.835085
NK.cells	STOML2	0.054476	4.805582	0.409042	0.683498	-6.38689	0.837394	0.728723
NK.cells	ZFP975	0.227137	0.810489	0.40898	0.683544	-5.34551	0.88934	0.81437
NK.cells	ZC2HC1A	0.14408	1.741533	0.408947	0.683567	-5.61231	0.876933	0.793572
NK.cells	GSS	-0.07786	3.399727	-0.40877	0.6837	-6.07135	0.855292	0.757817
NK.cells	AOAH	0.079659	4.784961	0.408748	0.683713	-6.68974	0.837653	0.729154
NK.cells	KLHDC4	0.063262	3.945597	0.408725	0.68373	-6.17947	0.848293	0.746392
NK.cells	CCDC93	0.067653	3.72019	0.408665	0.683774	-6.18288	0.851176	0.751103
NK.cells	NAT8L	0.213777	0.332665	0.408472	0.683915	-5.25143	0.895872	0.825305

NK.cells	DPP7	-0.14813	1.957266	-0.40834	0.684011	-5.59799	0.874207	0.7889
NK.cells	CELSR2	0.241462	-0.09555	0.408223	0.684097	-5.23394	0.901733	0.835198
NK.cells	GM43330	0.125391	1.526899	0.408132	0.684163	-5.6214	0.879915	0.7984
NK.cells	SLC9A1	-0.04038	5.416377	-0.40769	0.684486	-6.5438	0.830154	0.716608
NK.cells	SMG6	-0.02769	8.045885	-0.40753	0.684603	-7.00469	0.798078	0.666073
NK.cells	CEBPG	-0.04066	5.586056	-0.40718	0.684859	-6.52107	0.828042	0.713407
NK.cells	MB21D2	-0.22669	0.883088	-0.40705	0.684956	-5.28356	0.888804	0.813161
NK.cells	PHLPP2	-0.06507	4.283952	-0.40703	0.684969	-6.21273	0.844403	0.739774
NK.cells	AGO2	-0.03352	7.576219	-0.40701	0.684982	-6.94756	0.803705	0.674985
NK.cells	PCOLCE2	0.219941	1.678726	0.406998	0.684993	-5.4299	0.878197	0.795381
NK.cells	5930430L0	-0.24578	0.229599	-0.40697	0.685015	-5.16995	0.897617	0.828052
NK.cells	INSL6	0.133729	1.68935	0.406799	0.685139	-5.68719	0.878057	0.795151
NK.cells	HNRNPLL	0.047886	4.231786	0.406784	0.685149	-6.50851	0.845066	0.740854
NK.cells	ST3GAL5	-0.0755	6.23691	-0.40675	0.685171	-6.39937	0.819995	0.700631
NK.cells	SLC35B1	0.043493	5.751128	0.406575	0.685302	-6.62419	0.826043	0.710206
NK.cells	PIDD1	0.164524	0.824038	0.406499	0.685359	-5.41961	0.88965	0.814552
NK.cells	DUS3L	-0.06695	3.300011	-0.40641	0.685424	-6.04204	0.857051	0.760364
NK.cells	TREM3	-0.25571	2.200561	-0.4062	0.685578	-5.41026	0.871471	0.783999
NK.cells	THSD4	-0.26962	0.910314	-0.40602	0.685708	-5.37635	0.888677	0.812678
NK.cells	PRPF8	-0.03504	5.801492	-0.40592	0.685782	-6.62163	0.825594	0.709318
NK.cells	ARIH2	-0.03426	7.527213	-0.40559	0.686022	-6.93043	0.804574	0.676101
NK.cells	ZFP341	-0.10153	1.984672	-0.40557	0.686038	-5.71593	0.874458	0.788871
NK.cells	FAM110B	-0.199	0.653098	-0.40556	0.686046	-5.39041	0.892205	0.818608
NK.cells	BBS9	-0.05984	4.909527	-0.40518	0.686321	-6.51148	0.836895	0.727245
NK.cells	PMPCB	0.04988	4.974651	0.405048	0.68642	-6.42684	0.836077	0.72594
NK.cells	CMSS1	0.064792	6.448827	0.404933	0.686504	-6.74323	0.81778	0.696749
NK.cells	ZFAND3	-0.03099	8.233036	-0.40488	0.686547	-7.01324	0.796225	0.662991
NK.cells	PKNOX2	-0.27175	-0.82371	-0.40485	0.686568	-5.13096	0.912448	0.852903
NK.cells	SMIM15	-0.04476	4.858356	-0.40479	0.686612	-6.4041	0.837539	0.728305
NK.cells	EXOC7	-0.05192	4.087686	-0.40473	0.686655	-6.26524	0.847301	0.744098
NK.cells	HIST2H2BE	-0.18337	0.814865	-0.40467	0.686698	-5.34605	0.890142	0.815007
NK.cells	2900093K2	0.102863	2.884005	0.404568	0.686772	-5.79078	0.862799	0.769449
NK.cells	BC029722	-0.07222	3.061908	-0.40413	0.687096	-6.06308	0.860807	0.765728
NK.cells	HS3ST1	-0.21827	1.500377	-0.404	0.687186	-5.53177	0.881335	0.799729
NK.cells	TMEM199	0.069269	3.379806	0.403405	0.687625	-5.99961	0.857102	0.759147
NK.cells	CAB39	-0.03163	6.974308	-0.40339	0.687634	-6.84633	0.812053	0.686861
NK.cells	AKR1C12	-0.15715	0.756271	-0.40322	0.687761	-5.43175	0.891733	0.816657
NK.cells	SORT1	-0.11931	4.067523	-0.4031	0.687851	-5.76241	0.848323	0.744855
NK.cells	ZFP568	-0.06122	4.08691	-0.40301	0.687916	-6.26075	0.848075	0.744464
NK.cells	FUBP3	0.06187	4.08675	0.402954	0.687955	-6.19977	0.848077	0.744467
NK.cells	BHLHE40	0.052198	6.323106	0.402672	0.688161	-6.89462	0.820126	0.699574
NK.cells	TAPBP	0.055798	6.597076	0.402648	0.688179	-6.77549	0.816764	0.694258
NK.cells	SMYD2	0.080937	3.028651	0.402592	0.68822	-6.08333	0.861759	0.766761
NK.cells	TSLP	-0.26408	-0.44209	-0.40243	0.688342	-5.14253	0.908122	0.844386
NK.cells	RNF166	0.035595	5.468282	0.402366	0.688386	-6.62421	0.830743	0.716423
NK.cells	GM26801	0.228513	0.557742	0.402176	0.688525	-5.2832	0.89452	0.821278
NK.cells	TREML2	0.091842	3.901235	0.402163	0.688535	-5.92155	0.850552	0.748367

NK.cells	SPTBN5	-0.20765	-0.00378	-0.40192	0.688711	-5.29717	0.902254	0.83418
NK.cells	NOL10	-0.04385	4.91748	-0.4018	0.688799	-6.51487	0.837761	0.727495
NK.cells	STRN3	0.034969	7.654054	0.401717	0.688862	-6.91908	0.804077	0.674142
NK.cells	BRMS1L	-0.05028	4.269409	-0.40163	0.688926	-6.30218	0.845963	0.740783
NK.cells	IL13RA1	-0.13858	3.558786	-0.40141	0.68909	-5.74779	0.855057	0.75558
NK.cells	ABTB1	0.069439	4.303875	0.401382	0.689107	-6.25492	0.845525	0.740073
NK.cells	MPV17	-0.0532	4.075124	-0.40137	0.689119	-6.21724	0.848439	0.7448
NK.cells	PHF11A	0.155506	1.004104	0.401184	0.689253	-5.61098	0.888708	0.811261
NK.cells	CSNK1D	-0.0256	6.6346	-0.40092	0.689443	-6.79028	0.816663	0.693672
NK.cells	EDRF1	0.057941	4.217889	0.400605	0.689678	-6.29662	0.846848	0.741948
NK.cells	SASH1	-0.11794	5.303699	-0.40057	0.6897	-6.04823	0.83314	0.719857
NK.cells	ATP5O	0.038232	7.142669	0.400565	0.689707	-6.84278	0.810475	0.683929
NK.cells	RUSC1	-0.06973	3.288288	-0.40053	0.689731	-6.16092	0.858779	0.761394
NK.cells	TMEM120/	0.058989	3.799392	0.400407	0.689823	-6.22564	0.852223	0.750662
NK.cells	ANKRD61	0.154748	0.799732	0.40016	0.690004	-5.45617	0.89173	0.816041
NK.cells	PKIB	-0.19076	5.453591	-0.40012	0.690033	-5.81677	0.831375	0.716967
NK.cells	GM10851	-0.06833	4.366594	-0.39991	0.690184	-6.2176	0.84512	0.738995
NK.cells	GSTP3	0.089244	3.39573	0.399861	0.690223	-6.22095	0.857556	0.75925
NK.cells	STIL	-0.08371	4.156047	-0.39977	0.690289	-6.30128	0.8478	0.743366
NK.cells	ZHX3	0.061056	4.441922	0.39944	0.690532	-6.30338	0.844302	0.73759
NK.cells	A630001O:	0.123083	2.282008	0.399336	0.690609	-5.61886	0.87221	0.783304
NK.cells	HMGN1	-0.04685	7.26929	-0.39933	0.690615	-6.81311	0.809229	0.681781
NK.cells	NOTCH3	-0.18769	1.028947	-0.39922	0.690692	-5.41683	0.888861	0.811097
NK.cells	CNOT10	-0.04083	5.238486	-0.39843	0.691277	-6.51426	0.834724	0.721643
NK.cells	GM20324	0.076343	2.098382	0.398414	0.691286	-5.95058	0.87512	0.787538
NK.cells	RNF187	0.039289	6.12757	0.398141	0.691486	-6.65164	0.823658	0.704014
NK.cells	CDAN1	-0.08294	2.943919	-0.39813	0.691496	-5.97475	0.864039	0.76925
NK.cells	UBN2	-0.03583	7.076175	-0.39812	0.691501	-6.86057	0.812029	0.685665
NK.cells	BBS2	-0.17598	0.404044	-0.39807	0.691535	-5.3684	0.897782	0.825519
NK.cells	CACNB3	0.265008	-0.407	0.398026	0.691571	-5.27256	0.908849	0.844333
NK.cells	PADI4	0.233072	1.890862	0.397824	0.691719	-5.42281	0.877882	0.792182
NK.cells	RAB3A	-0.11377	2.619056	-0.39761	0.691872	-5.73533	0.868298	0.776375
NK.cells	AI987944	0.089693	2.764701	0.397474	0.691976	-5.88357	0.866394	0.773236
NK.cells	SP110	0.048691	5.924125	0.39733	0.692082	-6.67986	0.826194	0.708161
NK.cells	SLC4A4	-0.13861	2.065431	-0.39718	0.692189	-5.69057	0.875574	0.788466
NK.cells	AFTPH	0.035364	6.658529	0.397114	0.692241	-6.74483	0.817145	0.693866
NK.cells	B2M	0.058188	9.94338	0.397067	0.692275	-7.33178	0.777995	0.633258
NK.cells	CDC42EP2	0.13778	3.08028	0.397043	0.692293	-5.7317	0.862285	0.766538
NK.cells	DEGS1	-0.04487	6.525466	-0.397	0.692321	-6.73163	0.818776	0.696441
NK.cells	FAR1	0.046094	6.64871	0.396931	0.692375	-6.67874	0.817265	0.694056
NK.cells	IFT74	0.119248	2.322027	0.396812	0.692462	-5.68133	0.872193	0.782928
NK.cells	RRP36	0.055301	3.775241	0.396612	0.692609	-6.12883	0.85331	0.751988
NK.cells	GM46367	0.116055	2.175914	0.396514	0.692681	-5.78122	0.874117	0.78625
NK.cells	PLAUR	0.087192	6.837467	0.396443	0.692733	-6.55731	0.814957	0.690581
NK.cells	PTAFR	0.23007	4.645313	0.396428	0.692745	-5.63113	0.842216	0.73404
NK.cells	ELMOD2	-0.09234	2.807903	-0.39614	0.692958	-5.81075	0.86583	0.772551
NK.cells	CPD	0.067729	4.333844	0.39598	0.693073	-6.21833	0.846169	0.740446

NK.cells	GM10371	0.205204	-0.68835	0.395951	0.693095	-5.21097	0.912741	0.851365
NK.cells	GPR137C	0.126031	2.390141	0.395926	0.693114	-5.60615	0.871298	0.781596
NK.cells	MRPL55	0.054336	4.367452	0.395899	0.693133	-6.27694	0.845742	0.739754
NK.cells	BCLAF1	0.02709	7.155376	0.395587	0.693362	-6.86304	0.811085	0.684576
NK.cells	BHLHB9	0.093522	2.203905	0.395533	0.693402	-5.79414	0.873748	0.78573
NK.cells	SFT2D1	0.032195	5.999884	0.395472	0.693447	-6.64338	0.825256	0.706956
NK.cells	MED30	-0.04227	5.369422	-0.39533	0.693555	-6.54722	0.833103	0.719541
NK.cells	GM17484	-0.17373	1.264186	-0.39527	0.693598	-5.48171	0.88622	0.806604
NK.cells	PSTPIP2	0.164754	4.482064	0.395199	0.693648	-5.70651	0.844286	0.737562
NK.cells	GM49774	-0.12314	3.174932	-0.39518	0.693659	-5.80603	0.861057	0.764888
NK.cells	SOS1	0.034658	5.916592	0.395131	0.693698	-6.66615	0.826288	0.708687
NK.cells	ETFDH	-0.04357	4.872939	-0.39503	0.693769	-6.45089	0.83934	0.729602
NK.cells	TPRGL	-0.03336	6.927644	-0.39502	0.693778	-6.85214	0.813856	0.689037
NK.cells	LIN52	-0.04958	5.546829	-0.3949	0.693871	-6.52711	0.830886	0.716059
NK.cells	CWC15	0.031014	6.384372	0.39485	0.693905	-6.73758	0.82051	0.699572
NK.cells	PUF60	0.039968	5.949591	0.394817	0.693929	-6.65406	0.825879	0.708091
NK.cells	FAM76B	0.037975	5.401156	0.394726	0.693996	-6.52572	0.832706	0.718994
NK.cells	GUF1	0.074259	2.589786	0.39425	0.694346	-5.87721	0.868968	0.777611
NK.cells	UNC5CL	0.147241	0.465408	0.394018	0.694517	-5.68462	0.897267	0.824978
NK.cells	DISP1	0.060761	3.443949	0.393784	0.694688	-6.18204	0.85786	0.759442
NK.cells	EIF3I	0.03435	6.822191	0.393505	0.694894	-6.83757	0.815413	0.691346
NK.cells	STARD3	0.061486	4.116736	0.393495	0.694901	-6.23023	0.849218	0.745428
NK.cells	STX12	-0.03992	5.552332	-0.39342	0.694956	-6.57342	0.831093	0.716235
NK.cells	DPF2	-0.04163	5.127706	-0.39338	0.694989	-6.47217	0.836409	0.724753
NK.cells	TMEM131	-0.03526	6.606387	-0.39324	0.695086	-6.80788	0.818054	0.69556
NK.cells	MYO1E	-0.05978	6.198716	-0.39322	0.695105	-6.87858	0.82307	0.703504
NK.cells	EFL1	0.045728	4.5955	0.393111	0.695184	-6.38277	0.843126	0.735642
NK.cells	LY6E	0.047544	9.08245	0.39304	0.695236	-7.14843	0.788309	0.649287
NK.cells	CERS5	-0.03823	5.514163	-0.39301	0.695257	-6.62872	0.831569	0.717084
NK.cells	PRR11	-0.10891	2.847187	-0.39293	0.695316	-5.90489	0.865604	0.772339
NK.cells	H2-EB1	0.19929	6.754688	0.392901	0.695338	-6.39116	0.816238	0.692741
NK.cells	GM1673	0.129346	2.42109	0.392794	0.695417	-5.58921	0.87118	0.781572
NK.cells	ARHGEF5	0.205765	0.327235	0.392642	0.695529	-5.32095	0.899141	0.828439
NK.cells	TYW3	-0.15777	0.995715	-0.39259	0.695566	-5.42985	0.890112	0.813201
NK.cells	POLDIP3	-0.034	6.078814	-0.39255	0.695595	-6.67061	0.824552	0.705957
NK.cells	GHDC	-0.13269	1.628326	-0.39254	0.695606	-5.50471	0.881656	0.799031
NK.cells	DNAJC8	0.024481	6.901934	0.392411	0.695699	-6.82513	0.814439	0.689963
NK.cells	LRRC61	-0.10603	2.011331	-0.39228	0.695794	-5.74334	0.876578	0.790568
NK.cells	BC147527	0.129381	1.109158	0.392168	0.695878	-5.637	0.88859	0.810642
NK.cells	GM13710	-0.17922	1.36556	-0.3921	0.695925	-5.57075	0.885158	0.804887
NK.cells	LSM12	0.032423	6.360706	0.392037	0.695974	-6.73313	0.821073	0.700466
NK.cells	RAB11A	-0.02865	7.145444	-0.39196	0.69603	-6.83385	0.811474	0.685329
NK.cells	MAP3K9	0.216475	0.308951	0.391908	0.696069	-5.28097	0.89939	0.828907
NK.cells	MARCKSL1	-0.06889	7.792982	-0.39168	0.696234	-6.76966	0.803748	0.673167
NK.cells	MBD3	-0.04017	5.696238	-0.39159	0.696306	-6.56106	0.829404	0.713629
NK.cells	CYP4B1	-0.24863	0.266849	-0.39147	0.696393	-5.2633	0.900076	0.829978
NK.cells	IL1RL2	0.178885	0.726101	0.391403	0.696441	-5.45432	0.893856	0.819488

NK.cells	PPP1R35	0.069594	3.395179	0.391109	0.696657	-6.04603	0.858778	0.760938
NK.cells	MPP1	0.053893	6.203718	0.39076	0.696915	-6.50591	0.82341	0.703767
NK.cells	CENPS	-0.09911	3.529733	-0.39071	0.696954	-6.01505	0.857171	0.758153
NK.cells	RBM12B2	-0.09687	2.316248	-0.39064	0.697001	-5.73733	0.872984	0.784184
NK.cells	MOB3B	-0.05935	4.961752	-0.39058	0.697048	-6.33865	0.838907	0.728527
NK.cells	SLC22A4	0.196936	0.816422	0.390415	0.697169	-5.33165	0.893002	0.817565
NK.cells	AMMECR1	-0.05639	4.787165	-0.39027	0.697272	-6.33901	0.84115	0.732133
NK.cells	MICU1	0.03892	5.891562	0.390246	0.697293	-6.54271	0.827314	0.709977
NK.cells	DOCK9	0.069083	4.197961	0.390132	0.697377	-6.34174	0.848651	0.744267
NK.cells	ZC3HAV1	-0.02916	8.43854	-0.38988	0.697564	-7.20502	0.796394	0.661458
NK.cells	ZFP346	0.065698	3.658973	0.38984	0.697592	-6.22677	0.85559	0.755586
NK.cells	DPP4	0.055474	5.100888	0.389733	0.697671	-6.48265	0.837238	0.725864
NK.cells	USF1	0.052831	4.934416	0.389715	0.697684	-6.40341	0.839335	0.729237
NK.cells	EML6	0.127549	2.764743	0.389367	0.697941	-5.90744	0.86742	0.77476
NK.cells	APH1A	-0.04509	5.433388	-0.38892	0.698274	-6.56475	0.833402	0.719455
NK.cells	ABRAXAS2	0.045391	4.960803	0.388896	0.698288	-6.38756	0.839339	0.728983
NK.cells	D8ERTD73[illegible]	0.027666	7.358725	0.388802	0.698358	-6.90119	0.809686	0.68195
NK.cells	SMYD4	-0.0908	2.820357	-0.38875	0.698398	-5.99315	0.866811	0.773795
NK.cells	NUP43	0.081687	2.795528	0.387763	0.699123	-5.90641	0.867135	0.77462
NK.cells	MFAP1A	0.040285	4.832089	0.387731	0.699147	-6.41828	0.840964	0.731942
NK.cells	FBXO25	0.077903	2.127104	0.387513	0.699308	-5.80843	0.875915	0.789165
NK.cells	MAFG	0.046764	5.07367	0.387472	0.699338	-6.43188	0.837917	0.727039
NK.cells	4933439C1[illegible]	0.161119	1.200712	0.387423	0.699374	-5.42517	0.888241	0.80975
NK.cells	IFI35	0.056177	5.333965	0.387368	0.699414	-6.57391	0.834647	0.72179
NK.cells	ZFP251	0.090764	2.407099	0.387342	0.699434	-5.86128	0.872226	0.783044
NK.cells	ZFAS1	0.057239	5.058651	0.38733	0.699443	-6.47494	0.838106	0.727343
NK.cells	DCN	-0.12506	3.138043	-0.38731	0.699455	-5.92043	0.862673	0.767283
NK.cells	FCER2A	-0.23936	0.458562	-0.38661	0.699972	-5.3507	0.898248	0.826756
NK.cells	NRF1	-0.02966	6.474245	-0.38656	0.700014	-6.75252	0.820487	0.699354
NK.cells	CHPT1	-0.05426	3.607446	-0.38637	0.700153	-6.25142	0.856597	0.757454
NK.cells	CHML	0.136067	1.690018	0.386314	0.700192	-5.52225	0.881708	0.798949
NK.cells	GM15133	0.110183	1.788244	0.386276	0.70022	-5.75141	0.880402	0.79677
NK.cells	LETMD1	0.073635	2.91201	0.386261	0.700231	-5.96604	0.865615	0.772254
NK.cells	AP2A2	0.035792	5.931782	0.386235	0.70025	-6.64831	0.82719	0.709996
NK.cells	POLR3F	-0.05282	3.882542	-0.3862	0.700277	-6.25381	0.853058	0.751677
NK.cells	DGAT2	-0.07715	3.862389	-0.38618	0.700288	-6.10478	0.853317	0.752099
NK.cells	VSIR	0.048859	4.468275	0.386155	0.700309	-6.54824	0.845576	0.739522
NK.cells	ORC4	0.037727	5.046376	0.386092	0.700356	-6.48309	0.83826	0.727716
NK.cells	GM20033	-0.18178	0.595708	-0.38601	0.700415	-5.40799	0.89639	0.823619
NK.cells	AVPI1	-0.08564	2.712104	-0.38595	0.700459	-5.95027	0.868226	0.776566
NK.cells	GPR141B	0.171053	-0.65516	0.385653	0.70068	-5.26124	0.913488	0.852765
NK.cells	FAM71E1	0.194837	0.837279	0.385624	0.700702	-5.35393	0.893126	0.818179
NK.cells	RNF214	-0.04442	4.961908	-0.38558	0.700732	-6.45029	0.839325	0.7295
NK.cells	GM15879	0.189338	0.515986	0.385465	0.700819	-5.32418	0.897469	0.825568
NK.cells	POLD2	0.07692	3.401936	0.385306	0.700936	-6.11808	0.859252	0.761919
NK.cells	TRIM33	-0.03459	6.368235	-0.38528	0.700952	-6.71678	0.821793	0.701532
NK.cells	ZFP275	0.109319	1.581356	0.385269	0.700964	-5.6357	0.883154	0.801492

NK.cells	UQCRC2	0.030625	6.345631	0.385199	0.701015	-6.73221	0.822071	0.702005
NK.cells	SNTB1	-0.07408	4.264888	-0.38517	0.701036	-6.54689	0.848166	0.743872
NK.cells	DTX2	0.053423	4.396914	0.385073	0.701108	-6.32852	0.846484	0.741171
NK.cells	CABYR	0.145524	1.135391	0.384823	0.701293	-5.61419	0.889117	0.811644
NK.cells	CRELD1	0.12929	1.843254	0.384538	0.701503	-5.56166	0.879672	0.795852
NK.cells	MGAT1	-0.05927	4.704416	-0.3845	0.701535	-6.2315	0.842579	0.734953
NK.cells	UBE4A	0.045035	5.03345	0.384467	0.701555	-6.47697	0.838423	0.728251
NK.cells	TRIM30B	0.212588	1.730639	0.384367	0.70163	-5.28621	0.881168	0.798347
NK.cells	LRRC3	0.168965	0.375073	0.384358	0.701636	-5.36183	0.899381	0.828986
NK.cells	TBXAS1	-0.1642	3.468333	-0.3843	0.701675	-5.73634	0.858393	0.760678
NK.cells	SCIN	0.241309	0.090871	0.384292	0.701684	-5.30935	0.90325	0.835552
NK.cells	XNDC1	0.064243	3.594505	0.384235	0.701727	-6.13532	0.856764	0.758012
NK.cells	SLC12A7	0.066383	3.37429	0.384147	0.701792	-6.21648	0.859609	0.762672
NK.cells	SERPINI1	0.080117	3.178888	0.384131	0.701804	-6.03947	0.862142	0.76683
NK.cells	CLEC2D	0.079468	4.7558	0.384053	0.701861	-6.41877	0.841929	0.733902
NK.cells	NDUFB1-PS	0.026576	8.902498	0.384044	0.701868	-7.10929	0.791212	0.653897
NK.cells	ATP5D	-0.0277	7.898894	-0.38403	0.701875	-7.02054	0.803168	0.672415
NK.cells	1110002L0	0.086797	2.8153	0.384034	0.701876	-5.88285	0.866877	0.774626
NK.cells	NCALD	-0.10118	2.377149	-0.38402	0.701886	-5.87434	0.87262	0.784124
NK.cells	COLEC12	-0.16607	2.890318	-0.38402	0.701888	-5.7009	0.865898	0.773011
NK.cells	INTS5	0.073453	2.704825	0.383854	0.702008	-5.89863	0.868321	0.777031
NK.cells	DOK2	-0.0635	2.811456	-0.38369	0.702131	-6.23693	0.866927	0.774778
NK.cells	SYNJ2	0.090949	2.324633	0.383576	0.702214	-5.79883	0.873311	0.78534
NK.cells	TRDV4	0.139622	-1.36214	0.383448	0.702308	-5.51485	0.922861	0.870047
NK.cells	MUTYH	0.16931	0.370874	0.383429	0.702322	-5.30945	0.899438	0.829219
NK.cells	GM10143	0.135314	1.245831	0.383419	0.702329	-5.52281	0.887636	0.80931
NK.cells	AAK1	0.036709	6.392459	0.383391	0.70235	-6.81909	0.821494	0.701327
NK.cells	CYB561A3	-0.05683	5.573777	-0.38337	0.702366	-6.34959	0.831647	0.717496
NK.cells	ATP13A3	0.029002	7.315105	0.383068	0.702589	-6.81302	0.810391	0.68362
NK.cells	ARHGAP1	-0.03874	4.473602	-0.38289	0.702718	-6.39139	0.845761	0.739957
NK.cells	IL5RA	-0.2119	0.975245	-0.3824	0.703081	-5.354	0.891906	0.815788
NK.cells	METTL27	0.182299	0.539053	0.382169	0.703253	-5.44244	0.897928	0.825877
NK.cells	CCM2	0.031574	7.391127	0.381915	0.703441	-6.91595	0.810078	0.682602
NK.cells	KLK1B27	-0.16373	-1.42677	-0.3817	0.703599	-5.13316	0.92459	0.872352
NK.cells	WIZ	0.066241	3.836067	0.381331	0.703873	-6.16011	0.854483	0.753761
NK.cells	IDE	0.042204	4.161112	0.381299	0.703896	-6.34713	0.850314	0.746973
NK.cells	PRPF6	0.036054	5.300623	0.381085	0.704055	-6.53969	0.835875	0.723728
NK.cells	GM49101	0.239101	-0.65342	0.381055	0.704077	-5.17212	0.91435	0.854026
NK.cells	BSN	0.177059	0.764883	0.381031	0.704095	-5.45571	0.89497	0.821064
NK.cells	KLRA2	-0.22735	2.524963	-0.38102	0.704106	-5.49383	0.871522	0.781865
NK.cells	CD53	-0.0283	7.920856	-0.38094	0.704159	-7.00766	0.803683	0.672835
NK.cells	BMI1	0.067822	3.545427	0.380772	0.704286	-6.06191	0.858229	0.759987
NK.cells	ACADS	0.048374	4.490963	0.380675	0.704358	-6.36019	0.846107	0.740247
NK.cells	CDC37L1	0.037904	5.167302	0.380646	0.704379	-6.5055	0.83755	0.726439
NK.cells	KCND1	-0.22592	-0.19065	-0.38057	0.704437	-5.21197	0.907978	0.843146
NK.cells	GM43258	-0.16943	0.724157	-0.38056	0.704443	-5.3539	0.89552	0.822004
NK.cells	BST1	-0.16092	3.315218	-0.38049	0.704497	-5.60126	0.861208	0.764871

NK.cells	LPAR1	-0.27213	0.763125	-0.38048	0.704499	-5.30822	0.894994	0.821115
NK.cells	HES1	-0.07291	5.161335	-0.38039	0.704565	-6.3737	0.837625	0.726565
NK.cells	ERMP1	-0.05269	3.633634	-0.38032	0.70462	-6.18986	0.85709	0.758152
NK.cells	RBMS2	-0.04072	4.872344	-0.38004	0.704829	-6.44943	0.84127	0.732519
NK.cells	DFFA	0.079173	2.939835	0.379982	0.70487	-5.93602	0.866091	0.773016
NK.cells	9230114K1	-0.0893	2.574674	-0.37988	0.704946	-5.72628	0.870869	0.780909
NK.cells	SLC26A10	0.157944	0.914242	0.379877	0.704948	-5.53567	0.892954	0.817798
NK.cells	NOTCH4	0.199671	0.210596	0.379796	0.705008	-5.35389	0.902492	0.833934
NK.cells	LGALS1	-0.0556	6.036335	-0.37967	0.7051	-6.80186	0.826694	0.70918
NK.cells	ITGB2	-0.04601	6.734664	-0.37966	0.705107	-6.93416	0.818083	0.695526
NK.cells	D830025C(	-0.10413	2.231904	-0.37949	0.705236	-5.77069	0.875431	0.788389
NK.cells	PPP1R15A	-0.05225	7.089434	-0.37932	0.705359	-6.91356	0.813793	0.68869
NK.cells	COMT	-0.04454	5.36738	-0.37932	0.705361	-6.49755	0.835085	0.722512
NK.cells	ZFP429	0.081847	2.448075	0.379168	0.705472	-5.84852	0.872633	0.783663
NK.cells	ACAP1	0.065477	3.923819	0.378663	0.705846	-6.22638	0.853778	0.752283
NK.cells	ZNHIT6	0.077751	3.231636	0.378613	0.705883	-6.00685	0.86272	0.766934
NK.cells	AW011738	-0.07112	2.812185	-0.37831	0.706108	-6.31772	0.868327	0.77598
NK.cells	GLDC	0.152482	1.391092	0.378269	0.706138	-5.55186	0.887132	0.807256
NK.cells	ACTR10	0.028381	6.186686	0.378101	0.706262	-6.7049	0.825435	0.706454
NK.cells	DPP9	0.050944	4.420233	0.377965	0.706362	-6.32825	0.847661	0.742069
NK.cells	PTPN21	-0.162	0.813665	-0.37781	0.706477	-5.42609	0.895057	0.820387
NK.cells	SEC24A	-0.04891	6.616568	-0.37735	0.706816	-6.74603	0.82045	0.698151
NK.cells	PLEKHG2	0.071248	4.157105	0.377166	0.706954	-6.19611	0.851317	0.747678
NK.cells	TARS2	-0.05382	3.819829	-0.37714	0.706973	-6.23015	0.855648	0.75473
NK.cells	GINS3	0.098385	2.330816	0.376991	0.707083	-5.79688	0.875054	0.786699
NK.cells	NDEL1	0.034262	6.927832	0.376946	0.707117	-6.82919	0.816632	0.692232
NK.cells	CYP39A1	-0.12786	1.483874	-0.37692	0.707139	-5.63244	0.886302	0.805463
NK.cells	ZFP995	-0.08777	2.383748	-0.3768	0.707227	-5.82975	0.874356	0.785562
NK.cells	BCL2A1B	0.060972	5.877283	0.376767	0.70725	-6.82917	0.829597	0.712788
NK.cells	DMXL1	0.04464	6.604824	0.376466	0.707473	-6.69231	0.820594	0.698547
NK.cells	CDT1	-0.04924	4.370678	-0.3762	0.707672	-6.33071	0.848587	0.743431
NK.cells	SIRT1	-0.03931	5.043074	-0.37593	0.707871	-6.42987	0.840054	0.729692
NK.cells	NAB1	-0.03394	6.555305	-0.37592	0.707876	-6.82639	0.821203	0.699608
NK.cells	TEN1	0.034745	5.852604	0.375777	0.707982	-6.61232	0.829904	0.713448
NK.cells	ELL	0.043607	5.024631	0.375773	0.707986	-6.47202	0.840287	0.730085
NK.cells	ELOC	-0.03329	7.093954	-0.37566	0.708068	-6.84277	0.814602	0.689243
NK.cells	MFAP3	0.040613	5.124697	0.375631	0.708091	-6.4789	0.839024	0.728101
NK.cells	TTC4	0.057045	3.541793	0.375499	0.708189	-6.10123	0.859236	0.760912
NK.cells	MFN2	0.0548	3.671067	0.375483	0.708201	-6.1426	0.857566	0.758179
NK.cells	YES1	-0.0761	4.740029	-0.37547	0.70821	-6.40759	0.843888	0.735953
NK.cells	CD6	0.087314	0.929158	0.375066	0.70851	-6.02025	0.893752	0.818249
NK.cells	ORC2	0.048749	4.279461	0.375054	0.708519	-6.30867	0.849752	0.745449
NK.cells	PIGF	-0.06166	3.388301	-0.37504	0.708528	-6.21073	0.861224	0.764169
NK.cells	PNP	0.052016	5.924728	0.375037	0.708531	-6.56281	0.829007	0.712075
NK.cells	CTC1	0.063423	3.25694	0.374914	0.708622	-6.01995	0.862929	0.766967
NK.cells	HSD11B1	0.056978	4.593839	0.374853	0.708668	-6.48102	0.845745	0.738954
NK.cells	GM29340	0.214641	-0.6731	0.374696	0.708784	-5.15451	0.915645	0.855468

NK.cells	2410002F2	0.056503	3.766412	0.374637	0.708828	-6.16618	0.856336	0.756179
NK.cells	TXNL4B	-0.10161	1.740021	-0.37456	0.708884	-5.64321	0.882884	0.800034
NK.cells	SFI1	0.048114	5.320089	0.374541	0.708898	-6.48516	0.836566	0.724178
NK.cells	SLC6A19	0.179627	-0.35949	0.374494	0.708933	-5.33445	0.911316	0.848067
NK.cells	1110017D1	-0.2406	0.201816	-0.37448	0.708945	-5.23845	0.903622	0.834961
NK.cells	TMEM209	0.07784	3.362785	0.374335	0.709051	-6.04517	0.861555	0.764751
NK.cells	IMPDH1	0.06198	4.326873	0.374228	0.709131	-6.33427	0.849146	0.744519
NK.cells	SLC29A3	-0.07714	3.68183	-0.37406	0.709255	-5.9359	0.857427	0.758042
NK.cells	ENPP5	0.147939	0.877362	0.37404	0.70927	-5.59072	0.894451	0.819525
NK.cells	CHTF18	-0.12529	1.800347	-0.37402	0.709283	-5.66043	0.882081	0.798772
NK.cells	H13	0.032355	6.42662	0.373986	0.70931	-6.70493	0.822789	0.702278
NK.cells	MSI2	-0.03177	7.509903	-0.37367	0.709543	-6.93383	0.809702	0.681513
NK.cells	F2RL2	0.129734	0.129948	0.373515	0.709659	-5.6784	0.904779	0.836898
NK.cells	CIAO2A	0.036124	6.679979	0.373511	0.709662	-6.77751	0.81983	0.697498
NK.cells	PEX11G	0.092576	2.394748	0.3734	0.709744	-5.76472	0.874396	0.785889
NK.cells	MPO	0.328787	1.827356	0.373209	0.709887	-5.71225	0.881915	0.798463
NK.cells	ABHD17A	0.030738	6.296745	0.373129	0.709945	-6.67384	0.824574	0.705094
NK.cells	NIF3L1	0.067854	3.000203	0.373104	0.709964	-6.00709	0.866462	0.77286
NK.cells	GCNT7	0.070526	2.712006	0.372673	0.710284	-6.00393	0.870474	0.779115
NK.cells	PADI6	-0.22708	0.058128	-0.37258	0.71035	-5.24128	0.906035	0.838782
NK.cells	SPTBN1	0.04042	6.620496	0.372377	0.710503	-6.80942	0.820809	0.698911
NK.cells	MTR	-0.04656	4.54186	-0.37236	0.710516	-6.347	0.846827	0.74055
NK.cells	1300002E1	-0.07495	3.19234	-0.37222	0.710616	-5.98929	0.864199	0.768906
NK.cells	MAPRE3	-0.11578	1.502586	-0.37216	0.710664	-5.66248	0.886493	0.805906
NK.cells	BCL9L	-0.06312	3.410778	-0.37177	0.710956	-6.11987	0.861361	0.764294
NK.cells	DENND2C	0.103554	2.008648	0.371766	0.710957	-5.68281	0.879752	0.794692
NK.cells	TMED4	-0.06534	3.553347	-0.37176	0.710959	-6.04744	0.859514	0.761268
NK.cells	3-Sep	-0.17049	-1.00287	-0.37175	0.710966	-5.26053	0.920617	0.863997
NK.cells	KIF1B	-0.03862	5.385583	-0.3717	0.711002	-6.68624	0.836159	0.723415
NK.cells	LYZ2	0.170653	7.170673	0.371594	0.711084	-6.52338	0.814071	0.688342
NK.cells	TNFAIP1	-0.0746	3.775763	-0.37159	0.711086	-6.05925	0.856642	0.756571
NK.cells	TRIM37	-0.05343	4.93302	-0.37149	0.711164	-6.34487	0.84187	0.732619
NK.cells	VCAM1	-0.16511	4.334733	-0.37131	0.711294	-5.97042	0.849532	0.74498
NK.cells	SUPT20	0.037732	5.30473	0.371058	0.711482	-6.53667	0.837237	0.725167
NK.cells	STX6	0.042429	5.2207	0.370919	0.711585	-6.52086	0.838295	0.726879
NK.cells	UBE3C	-0.03615	5.784905	-0.37092	0.711587	-6.63737	0.831223	0.715551
NK.cells	BTBD3	0.177556	1.337187	0.370879	0.711615	-5.40123	0.888774	0.809815
NK.cells	NHEJ1	0.1109	3.867688	0.370848	0.711638	-6.05859	0.855521	0.754778
NK.cells	SCYL2	0.044449	4.409687	0.370706	0.711743	-6.34885	0.848616	0.743525
NK.cells	CYTIP	0.036198	8.087657	0.370383	0.711983	-7.08103	0.803103	0.671187
NK.cells	WFDC18	0.185098	-0.88323	0.370361	0.712	-5.17645	0.919159	0.861381
NK.cells	KLHL18	0.05175	4.310869	0.370254	0.712079	-6.30835	0.849907	0.745598
NK.cells	BCL2L11	0.049163	7.053365	0.370162	0.712147	-6.92772	0.815631	0.69079
NK.cells	GM19967	0.128058	1.377066	0.370105	0.71219	-5.51515	0.888312	0.808997
NK.cells	GM6787	-0.17373	0.188055	-0.37007	0.712214	-5.32205	0.904401	0.836174
NK.cells	SLC25A46	0.035985	4.587825	0.369968	0.712291	-6.40698	0.846375	0.7399
NK.cells	PCNX4	0.103027	2.141019	0.369749	0.712454	-5.68499	0.878136	0.79204

NK.cells	PUSL1	0.08264	2.317284	0.369736	0.712463	-5.8244	0.875805	0.788167
NK.cells	GM4951	-0.1612	3.770894	-0.36971	0.712479	-5.91965	0.856839	0.756929
NK.cells	TRPS1	-0.04595	7.683996	-0.36939	0.712717	-6.99323	0.808005	0.678842
NK.cells	PKN1	0.029069	6.766934	0.369301	0.712786	-6.7788	0.819178	0.696412
NK.cells	BC024978	-0.11312	1.865186	-0.36928	0.712799	-5.70819	0.881838	0.798205
NK.cells	DPH7	0.121286	1.586984	0.369271	0.712808	-5.65967	0.885546	0.804403
NK.cells	GM36198	-0.08892	2.976425	-0.36912	0.712924	-5.89051	0.867191	0.773937
NK.cells	USP37	-0.03732	6.268931	-0.36909	0.712941	-6.70982	0.825317	0.70616
NK.cells	RETNLG	0.178933	3.313557	0.368937	0.713057	-5.85395	0.862854	0.766734
NK.cells	DLG4	-0.11224	3.571009	-0.36865	0.713268	-6.03149	0.859684	0.761261
NK.cells	2900089D1	0.106436	2.58395	0.368502	0.71338	-5.76043	0.872593	0.782499
NK.cells	CLMP	-0.20018	1.140521	-0.36824	0.713572	-5.30485	0.891796	0.814659
NK.cells	TEPSIN	-0.1507	0.762658	-0.36811	0.713672	-5.4489	0.896897	0.823263
NK.cells	UBP1	-0.03642	5.448392	-0.36782	0.713887	-6.57273	0.835789	0.722744
NK.cells	SMIM5	0.234706	0.166279	0.367683	0.713988	-5.17641	0.90501	0.837157
NK.cells	TRMT13	-0.0564	3.89491	-0.36768	0.71399	-6.21379	0.855536	0.754724
NK.cells	DNAH2	0.161503	0.783062	0.367604	0.714047	-5.39426	0.896621	0.822952
NK.cells	GM16268	-0.18679	0.706579	-0.36757	0.714075	-5.35497	0.897656	0.824702
NK.cells	UTP4	-0.04087	5.102474	-0.36746	0.714153	-6.48327	0.840143	0.729795
NK.cells	ARMH2	-0.19368	0.301427	-0.36744	0.714169	-5.31644	0.903165	0.834045
NK.cells	CCDC130	-0.11009	2.056293	-0.36737	0.71422	-5.72475	0.879561	0.79436
NK.cells	KLHL3	-0.09656	2.005874	-0.36722	0.714333	-5.79392	0.88023	0.79554
NK.cells	CASTOR2	-0.08281	3.643855	-0.36701	0.714485	-6.22971	0.858774	0.760142
NK.cells	GM32089	0.190744	-1.09921	0.366819	0.714631	-5.20891	0.922319	0.867279
NK.cells	LTBP1	0.32241	0.730726	0.366642	0.714762	-5.34166	0.897329	0.824405
NK.cells	USP48	0.034628	5.391239	0.366538	0.714839	-6.59239	0.836507	0.724171
NK.cells	XPO7	0.035571	6.710919	0.366469	0.714891	-6.8043	0.820109	0.698048
NK.cells	GM17231	0.1027	2.734245	0.366461	0.714897	-5.85807	0.870619	0.779754
NK.cells	PPIF	0.065932	3.577224	0.366437	0.714914	-6.08847	0.859636	0.761678
NK.cells	MNAT1	0.045857	4.818214	0.366253	0.715051	-6.42375	0.843739	0.735834
NK.cells	SURF1	0.047554	4.36834	0.366181	0.715105	-6.39515	0.849465	0.745112
NK.cells	3010003L2	0.125659	1.349271	0.365981	0.715253	-5.61447	0.888991	0.810411
NK.cells	LRRC18	-0.18941	1.588071	-0.36586	0.71534	-5.35181	0.885794	0.805072
NK.cells	WRNIP1	-0.04901	3.743434	-0.36557	0.71556	-6.21895	0.857488	0.758318
NK.cells	ZFP956	0.120629	1.264702	0.365465	0.715637	-5.55442	0.890126	0.812444
NK.cells	ATP6V1G1	0.031089	7.177612	0.365453	0.715646	-6.80844	0.814395	0.689182
NK.cells	ADAM12	0.144309	0.815122	0.365452	0.715647	-5.59579	0.896187	0.822655
NK.cells	NAA20	0.034058	5.164245	0.36524	0.715805	-6.52103	0.839364	0.72895
NK.cells	SNRPC	-0.03092	6.245324	-0.36523	0.715815	-6.70944	0.825853	0.707339
NK.cells	CD86	-0.10278	6.31281	-0.36521	0.715827	-6.31897	0.825018	0.706013
NK.cells	ATF5	0.086207	3.267979	0.365109	0.715902	-6.05042	0.863648	0.768477
NK.cells	RNF103	-0.04938	4.305165	-0.3651	0.71591	-6.30274	0.850272	0.746612
NK.cells	PRKCE	0.042976	7.233184	0.364893	0.716063	-6.8248	0.813717	0.688178
NK.cells	GM15446	-0.09955	2.166881	-0.36489	0.716063	-5.78642	0.878096	0.792392
NK.cells	STARD5	0.062826	4.526318	0.364884	0.716069	-6.14726	0.847449	0.742042
NK.cells	DEPDC5	0.042769	4.978079	0.364784	0.716144	-6.43816	0.841715	0.732768
NK.cells	TMEM18	0.110153	1.593876	0.36474	0.716177	-5.64004	0.885717	0.805116

NK.cells	CKB	-0.05999	4.578345	-0.36472	0.716194	-6.54156	0.846787	0.740968
NK.cells	HIST1H3A	-0.13689	0.953909	-0.36466	0.716237	-5.5051	0.894311	0.819562
NK.cells	BCAM	0.275974	0.298097	0.364261	0.716533	-5.26847	0.90321	0.834821
NK.cells	PRMT9	0.05617	4.074852	0.364236	0.716551	-6.27841	0.853223	0.751602
NK.cells	MPP6	0.037126	6.194697	0.364184	0.71659	-6.68532	0.826481	0.708538
NK.cells	SDR42E1	-0.14791	0.45745	-0.36412	0.716637	-5.37556	0.901039	0.831162
NK.cells	IL12RB2	-0.05167	3.672057	-0.36389	0.716806	-6.61417	0.85841	0.760136
NK.cells	A930006K(	-0.07774	2.074746	-0.36379	0.716887	-5.88712	0.879316	0.794709
NK.cells	GM42722	0.086854	2.335621	0.36377	0.716898	-6.01896	0.875865	0.788965
NK.cells	PRR14L	-0.03421	6.50182	-0.36375	0.716911	-6.7338	0.822683	0.702584
NK.cells	E530011L2	0.160281	1.095538	0.363694	0.716955	-5.48233	0.892402	0.816636
NK.cells	4930444A1	-0.05732	3.751804	-0.36357	0.717049	-6.37851	0.85738	0.75849
NK.cells	GON4L	0.036295	5.15234	0.363419	0.71716	-6.52623	0.839514	0.72951
NK.cells	PTPN3	0.105693	1.040176	0.363382	0.717187	-5.88634	0.893148	0.817923
NK.cells	ADGRV1	-0.1796	0.711845	-0.36337	0.717194	-5.41622	0.897585	0.825417
NK.cells	AUTS2	-0.10637	4.397144	-0.36336	0.717204	-6.28114	0.849097	0.74501
NK.cells	GM21860	0.291965	-0.14186	0.363255	0.717282	-5.17968	0.909232	0.84526
NK.cells	KSR2	0.157273	2.695294	0.363172	0.717344	-5.66403	0.87113	0.781185
NK.cells	BRWD1	-0.03362	6.550877	-0.36313	0.717377	-6.74071	0.822078	0.701691
NK.cells	SNX22	0.183122	-0.37781	0.363073	0.717417	-5.33075	0.912479	0.850811
NK.cells	MAP3K2	0.03437	6.248682	0.363064	0.717424	-6.73589	0.825812	0.707619
NK.cells	KCNMB4	0.126712	1.763375	0.362936	0.717519	-5.68906	0.883458	0.801723
NK.cells	SIPA1	-0.03388	5.681522	-0.36287	0.717568	-6.63848	0.832872	0.718923
NK.cells	SUPT7L	0.078684	2.767637	0.362405	0.717914	-5.90701	0.870296	0.779748
NK.cells	PLXDC1	-0.11058	3.94302	-0.3624	0.717919	-5.95549	0.85503	0.754659
NK.cells	GINS1	0.090576	3.197409	0.362269	0.718015	-5.99916	0.86468	0.770481
NK.cells	FER	-0.09413	3.489418	-0.36203	0.718197	-5.87691	0.860887	0.764314
NK.cells	DMTF1	0.037642	4.947065	0.361804	0.718362	-6.45798	0.842218	0.733947
NK.cells	NQO2	-0.0495	3.942173	-0.36174	0.718408	-6.25047	0.855041	0.754775
NK.cells	NDUFAF7	-0.04679	4.007139	-0.36168	0.718454	-6.30077	0.854205	0.753438
NK.cells	FXR1	-0.02998	6.109299	-0.36167	0.71846	-6.68276	0.827649	0.710611
NK.cells	PPM1J	-0.11332	-0.35731	-0.36164	0.718484	-5.66528	0.912317	0.850619
NK.cells	TSTA3	0.052345	4.100613	0.361601	0.718513	-6.25886	0.853005	0.7515
NK.cells	ASF1A	0.046475	5.210724	0.361557	0.718546	-6.47729	0.838889	0.72864
NK.cells	TTC21B	0.104514	1.614885	0.361555	0.718547	-5.60927	0.885553	0.805312
NK.cells	MEX3D	0.086737	2.642534	0.361502	0.718587	-5.88683	0.871938	0.782632
NK.cells	CDH13	-0.19221	2.030566	-0.36113	0.718862	-5.6117	0.88019	0.796197
NK.cells	UBXN7	0.035994	5.704222	0.361122	0.718869	-6.62861	0.832858	0.718821
NK.cells	MTRF1	0.138176	1.208712	0.360834	0.719084	-5.4941	0.89117	0.81469
NK.cells	SS18L2	-0.06191	3.817204	-0.36065	0.719221	-6.11837	0.856817	0.757754
NK.cells	ADAM22	0.179902	0.796933	0.360596	0.719261	-5.39803	0.896726	0.824149
NK.cells	PKNOX1	-0.05142	4.569323	-0.36057	0.719278	-6.35683	0.847179	0.742068
NK.cells	9430091E2	-0.07747	2.439837	-0.36046	0.719359	-5.91869	0.874777	0.787412
NK.cells	FFAR1	-0.18879	-0.22509	-0.36034	0.719454	-5.16583	0.910674	0.847943
NK.cells	MECOM	0.269298	0.770813	0.360218	0.719543	-5.36702	0.89708	0.824866
NK.cells	GM14698	-0.15304	0.738672	-0.36019	0.719564	-5.39508	0.897515	0.825603
NK.cells	MS4A1	-0.11315	3.936889	-0.36013	0.719605	-6.15591	0.855276	0.755355

NK.cells	GM10521	-0.17902	0.308847	-0.36006	0.719664	-5.24115	0.903359	0.835538
NK.cells	AKNA	0.036838	4.948701	0.360046	0.719671	-6.55273	0.842362	0.734389
NK.cells	KRTCAP3	0.118619	0.893962	0.359975	0.719724	-5.5703	0.895414	0.822072
NK.cells	SUSD1	-0.06232	5.183728	-0.35982	0.719839	-6.2819	0.839444	0.729618
NK.cells	XLR4A	0.105795	0.802973	0.359621	0.719988	-5.70301	0.896726	0.824233
NK.cells	MIGA1	-0.07386	2.677211	-0.3596	0.720003	-5.81982	0.871732	0.78239
NK.cells	ESAM	0.135717	2.065322	0.359466	0.720103	-5.54914	0.879844	0.79583
NK.cells	TACO1OS	-0.13388	1.705089	-0.3592	0.720298	-5.51259	0.884752	0.803913
NK.cells	UBAP1	0.040938	5.688984	0.35904	0.720421	-6.61109	0.833266	0.719609
NK.cells	DOCK3	-0.18272	0.039329	-0.35901	0.720444	-5.33941	0.90728	0.842052
NK.cells	TRPC4AP	0.031483	5.651799	0.358859	0.720556	-6.60322	0.833731	0.720397
NK.cells	DNAJC3	-0.03313	6.725717	-0.35877	0.720619	-6.83705	0.820409	0.699182
NK.cells	GM1976	0.082742	2.701657	0.358768	0.720624	-5.85343	0.871559	0.782051
NK.cells	SH3PXD2B	0.203297	1.543779	0.358465	0.72085	-5.4475	0.887004	0.807708
NK.cells	ITGB5	-0.12148	3.058168	-0.35834	0.720943	-5.76511	0.866985	0.774418
NK.cells	GNL3L	0.036407	4.467102	0.358333	0.720948	-6.38758	0.848795	0.74464
NK.cells	SF3B3	0.029849	6.034328	0.358103	0.72112	-6.67937	0.829049	0.712848
NK.cells	DCP2	0.044085	5.103401	0.358053	0.721157	-6.47273	0.840717	0.731565
NK.cells	RNF169	-0.04418	6.380124	-0.35797	0.72122	-6.7262	0.82476	0.706019
NK.cells	EID3	0.146135	0.925233	0.357926	0.721252	-5.58654	0.895322	0.821731
NK.cells	SELENOT	0.030153	6.182995	0.357898	0.721273	-6.69642	0.827202	0.709904
NK.cells	CCT5	-0.03635	6.571584	-0.35757	0.721516	-6.76394	0.822553	0.702281
NK.cells	DAG1	0.049655	4.867236	0.357416	0.721632	-6.41137	0.843866	0.736437
NK.cells	NT5DC2	-0.10956	2.432435	-0.35707	0.721893	-5.68988	0.875364	0.788127
NK.cells	KBTBD2	-0.03134	5.176548	-0.35699	0.721952	-6.54612	0.839954	0.730181
NK.cells	KHDRBS1	-0.02313	7.151511	-0.35686	0.722045	-6.86586	0.815436	0.691117
NK.cells	LAP3	0.059964	4.901491	0.356853	0.722052	-6.42262	0.843432	0.735793
NK.cells	HSDL2	-0.04297	4.684002	-0.35675	0.722126	-6.45624	0.846193	0.740262
NK.cells	KLHL28	-0.05759	3.44241	-0.35669	0.722175	-6.10417	0.862147	0.766291
NK.cells	AP3M2	0.074756	2.489505	0.356649	0.722205	-5.88577	0.874611	0.786877
NK.cells	GDPD1	0.166038	1.600801	0.356492	0.722321	-5.43831	0.88641	0.806563
NK.cells	WDR5	-0.04665	4.681603	-0.35648	0.72233	-6.36661	0.846224	0.740311
NK.cells	MYH10	-0.10303	2.573809	-0.35646	0.722342	-5.69906	0.873501	0.785034
NK.cells	GM36161	0.226164	0.563057	0.356388	0.722399	-5.29185	0.900402	0.830202
NK.cells	LRRC8C	0.042732	6.412612	0.356174	0.722559	-6.76804	0.824515	0.705557
NK.cells	ETAA1OS	-0.13285	0.543342	-0.35614	0.722586	-5.44792	0.90067	0.830712
NK.cells	MSRB2	0.158731	1.023579	0.356098	0.722616	-5.42902	0.894164	0.819724
NK.cells	METTL2	-0.05788	3.27705	-0.3559	0.722764	-6.08848	0.864296	0.770018
NK.cells	OLFR1033	0.203689	0.583769	0.355878	0.72278	-5.36245	0.900121	0.829889
NK.cells	N4BP2	0.046178	5.310122	0.355844	0.722805	-6.47753	0.83827	0.727655
NK.cells	PLXNA4	-0.2654	2.166702	-0.35567	0.722934	-5.5301	0.878878	0.794175
NK.cells	DACH2	0.180431	0.599965	0.355647	0.722952	-5.39251	0.8999	0.829516
NK.cells	TENT4A	0.0625	3.460072	0.355508	0.723056	-6.12899	0.861918	0.766165
NK.cells	DNASE1L1	0.07017	3.048154	0.355427	0.723116	-6.0304	0.867281	0.775029
NK.cells	OGFR	-0.04583	5.244677	-0.35542	0.72312	-6.54692	0.839094	0.729066
NK.cells	YJU2	-0.05063	3.454196	-0.35506	0.72339	-6.14242	0.862231	0.766392
NK.cells	ZC4H2	-0.18525	0.054562	-0.35466	0.723689	-5.21264	0.907636	0.842468

NK.cells	STOML3	0.182305	0.359863	0.354486	0.723819	-5.26915	0.90346	0.835369
NK.cells	9530068E0	-0.04621	4.851397	-0.35446	0.72384	-6.41988	0.844339	0.737276
NK.cells	NCKAP5L	-0.05913	4.041979	-0.35444	0.723855	-6.28312	0.854678	0.754076
NK.cells	CREB3L2	0.073654	3.55229	0.354344	0.723925	-6.08195	0.861	0.764424
NK.cells	LAPTM5	-0.02572	8.326927	-0.35428	0.723973	-7.08643	0.80148	0.669286
NK.cells	COL23A1	-0.15262	1.376373	-0.35425	0.723995	-5.51222	0.889704	0.812111
NK.cells	AIDA	0.04434	4.437691	0.35415	0.72407	-6.34325	0.849607	0.745816
NK.cells	9130230L2	0.176547	3.687563	0.35407	0.724129	-5.61706	0.859249	0.761551
NK.cells	MANF	0.031061	6.808848	0.354006	0.724177	-6.77564	0.819897	0.698172
NK.cells	DDX58	0.052661	4.759373	0.353846	0.724297	-6.51309	0.845508	0.739167
NK.cells	TRIAP1	0.048983	4.111675	0.35382	0.724316	-6.27921	0.853783	0.752623
NK.cells	GM42556	-0.16256	0.793712	-0.35377	0.724352	-5.42053	0.897562	0.825405
NK.cells	SAG	-0.07971	3.5306	-0.35357	0.724499	-6.1275	0.861372	0.764999
NK.cells	HARS	-0.03492	5.114979	-0.35281	0.72507	-6.51542	0.841491	0.732191
NK.cells	GM14798	0.049916	3.718232	0.35276	0.725108	-6.23962	0.859352	0.761219
NK.cells	CD226	-0.06455	1.697205	-0.35276	0.725108	-6.48317	0.885923	0.805238
NK.cells	PSMD9	-0.03526	5.225742	-0.35273	0.72513	-6.52101	0.840092	0.729938
NK.cells	SNX3	0.024516	7.730038	0.352575	0.725246	-6.95323	0.809148	0.680859
NK.cells	HIST1H2AE	0.09813	4.491316	0.352478	0.725319	-6.46339	0.849428	0.745118
NK.cells	SLC23A2	0.043046	5.113974	0.352431	0.725354	-6.53679	0.841516	0.732317
NK.cells	FBXO17	-0.14872	0.782245	-0.35231	0.725442	-5.44157	0.898273	0.826142
NK.cells	NME4	0.116878	2.364464	0.351944	0.725717	-5.74036	0.877333	0.79072
NK.cells	ATL2	-0.03661	5.337211	-0.35164	0.725946	-6.50057	0.839136	0.728053
NK.cells	NUDT13	0.079333	2.358115	0.351091	0.726355	-5.88282	0.877979	0.791168
NK.cells	DUSP1	-0.04644	7.412842	-0.35104	0.726396	-6.98881	0.813766	0.687363
NK.cells	ARRDC4	0.08564	2.427989	0.350827	0.726553	-5.92391	0.877157	0.789738
NK.cells	PTBP2	-0.03613	6.156832	-0.35063	0.726702	-6.6682	0.829413	0.711978
NK.cells	ECHS1	-0.04842	5.549982	-0.35014	0.727065	-6.58133	0.837275	0.724252
NK.cells	GM36839	-0.10035	2.346299	-0.34995	0.727208	-5.67483	0.87862	0.791868
NK.cells	RUNDC1	0.063388	2.994317	0.349832	0.727296	-6.04576	0.870082	0.777783
NK.cells	ARNT	0.040137	5.869604	0.349798	0.727322	-6.6997	0.833268	0.71796
NK.cells	CDK17	-0.03171	6.780519	-0.3497	0.727398	-6.87282	0.821963	0.699986
NK.cells	GM48960	-0.11968	1.466163	-0.34964	0.727442	-5.57154	0.890359	0.811551
NK.cells	ARSA	0.103527	1.69399	0.349637	0.727442	-5.62849	0.887305	0.806429
NK.cells	ZC3H13	0.033752	5.653384	0.349376	0.727637	-6.65896	0.835976	0.722299
NK.cells	TMEM258	0.033022	6.825258	0.349367	0.727645	-6.80701	0.821412	0.699114
NK.cells	SHARPIN	0.040825	4.530571	0.349308	0.727689	-6.39687	0.850197	0.745235
NK.cells	1-Mar	0.191204	4.477654	0.349017	0.727906	-5.6991	0.850874	0.746334
NK.cells	G530011O0	0.156	1.80368	0.348976	0.727937	-5.56315	0.885838	0.803974
NK.cells	LRRCC1	0.043891	3.748019	0.348969	0.727942	-6.22672	0.860264	0.761646
NK.cells	MAOA	-0.17384	0.932781	-0.34887	0.728019	-5.39725	0.897555	0.823685
NK.cells	CINP	0.083283	3.163035	0.34884	0.728039	-5.97429	0.867874	0.77417
NK.cells	IRAK1	-0.03452	5.224747	-0.34865	0.728182	-6.54294	0.841374	0.731015
NK.cells	L3MBTL2	0.050673	3.445434	0.348642	0.728187	-6.13067	0.864192	0.768132
NK.cells	GM50218	-0.13906	0.559001	-0.34852	0.728275	-5.56971	0.902634	0.832359
NK.cells	IDH3A	0.043092	4.865883	0.348495	0.728297	-6.46156	0.845923	0.738402
NK.cells	PAM	0.046949	4.417759	0.34815	0.728555	-6.46867	0.851858	0.747768

NK.cells	2810408I1	0.128419	1.398496	0.34804	0.728637	-5.57288	0.89151	0.813299
NK.cells	GM41335	0.214611	0.493808	0.347796	0.72882	-5.32231	0.903905	0.83407
NK.cells	GM19265	0.15708	0.22098	0.347649	0.72893	-5.3536	0.907662	0.84042
NK.cells	SH3BGRL	0.033747	6.824052	0.347522	0.729025	-6.76761	0.821798	0.699425
NK.cells	NR2C1	-0.09899	2.276051	-0.34709	0.729347	-5.74813	0.879948	0.793974
NK.cells	MYBPC3	0.160048	1.123244	0.346977	0.729433	-5.37187	0.895382	0.819831
NK.cells	CD99L2	-0.08514	2.142314	-0.3469	0.729494	-5.74661	0.881724	0.796934
NK.cells	RGS1	-0.0542	5.563816	-0.34688	0.729505	-6.88921	0.837479	0.724553
NK.cells	YBEY	0.109012	1.366271	0.346877	0.729508	-5.52317	0.892105	0.814313
NK.cells	PCMT1	-0.03015	6.63523	-0.3468	0.729569	-6.74428	0.824126	0.703259
NK.cells	GM26881	0.190109	0.245715	0.346769	0.729589	-5.29526	0.907323	0.840063
NK.cells	CYSTM1	-0.1324	3.36709	-0.3467	0.729642	-5.8646	0.865602	0.770246
NK.cells	RMDN2	0.084295	1.873328	0.346474	0.72981	-5.90522	0.885307	0.802966
NK.cells	PTDSS1	0.033765	5.371518	0.346474	0.72981	-6.59717	0.839901	0.728487
NK.cells	MORF4L2	0.037432	5.404884	0.346432	0.729841	-6.48723	0.83948	0.72781
NK.cells	XLR4B	0.082695	1.285179	0.346306	0.729936	-5.82099	0.893197	0.816239
NK.cells	TYW1	-0.0521	3.875123	-0.34622	0.729996	-6.24107	0.859008	0.75952
NK.cells	PRKAB2	-0.06721	3.392055	-0.3462	0.730018	-6.04731	0.865277	0.7698
NK.cells	GM34455	-0.11366	2.623429	-0.34615	0.730055	-5.76542	0.875353	0.78646
NK.cells	COX6B2	0.12765	1.691068	0.345914	0.730229	-5.54295	0.887757	0.807203
NK.cells	ANKRD23	-0.13901	0.33175	-0.34587	0.730264	-5.4216	0.906158	0.838278
NK.cells	ZFP874A	-0.08107	2.146516	-0.3457	0.730387	-5.85623	0.881681	0.797068
NK.cells	GM43581	-0.1078	2.093907	-0.3456	0.730467	-5.68597	0.88238	0.798258
NK.cells	RAB20	0.121512	3.301078	0.345416	0.730602	-5.73045	0.866475	0.771898
NK.cells	ULK1	-0.05574	3.637915	-0.34536	0.730646	-6.181	0.862093	0.764711
NK.cells	EIF3K	-0.02383	7.871661	-0.34535	0.730652	-7.01159	0.809021	0.679692
NK.cells	LASP1	-0.03266	5.455474	-0.34534	0.730656	-6.64203	0.838855	0.726983
NK.cells	PWP2	0.105102	1.891751	0.344911	0.730981	-5.70818	0.885074	0.802863
NK.cells	PIP5K1C	0.042997	5.626433	0.344855	0.731022	-6.50182	0.836704	0.723617
NK.cells	ZFP827	-0.08461	2.85843	-0.34481	0.731059	-5.98213	0.872271	0.78157
NK.cells	IGFBP6	0.15267	-0.02144	0.344755	0.731097	-5.53772	0.911005	0.846704
NK.cells	PSME3	0.042861	5.282258	0.344511	0.73128	-6.5444	0.84104	0.730652
NK.cells	RXRA	0.067761	3.1994	0.344365	0.731389	-6.04042	0.867803	0.774288
NK.cells	GSTZ1	-0.05871	4.205829	-0.34427	0.73146	-6.24383	0.854757	0.752936
NK.cells	GPR137	0.070478	2.506946	0.344154	0.731548	-5.81557	0.876903	0.789389
NK.cells	RECK	-0.12352	1.480341	-0.34412	0.731575	-5.7566	0.890583	0.812248
NK.cells	TMEM184(	-0.05164	3.390595	-0.34406	0.731617	-6.15671	0.865308	0.77023
NK.cells	GM3235	0.152146	0.645113	0.343846	0.731778	-5.49309	0.901881	0.831373
NK.cells	NUP160	-0.04587	5.017011	-0.34382	0.731801	-6.49136	0.844397	0.736182
NK.cells	CD40LG	-0.08982	-0.56972	-0.34364	0.731934	-5.82258	0.918583	0.859934
NK.cells	BORCS6	0.055538	4.007961	0.343614	0.731953	-6.20459	0.857305	0.757201
NK.cells	RNF113A1	0.176035	0.42846	0.343601	0.731962	-5.32912	0.904836	0.836442
NK.cells	ORMDL2	0.039843	5.109617	0.343596	0.731966	-6.46234	0.843223	0.734332
NK.cells	ADGRG3	0.08975	2.366781	0.343391	0.732119	-5.87477	0.878758	0.792604
NK.cells	ARHGAP31	-0.04119	6.152696	-0.34335	0.732153	-6.85153	0.830123	0.713336
NK.cells	HELB	0.055171	3.704754	0.343333	0.732163	-6.2239	0.861226	0.763642
NK.cells	RAB23	0.127322	1.279801	0.343176	0.732281	-5.52059	0.893282	0.816955

NK.cells	FHL3	-0.11481	2.081558	-0.34311	0.732327	-5.68983	0.882544	0.798981
NK.cells	NRG4	0.122663	2.017195	0.342973	0.732433	-5.76858	0.883401	0.80044
NK.cells	COPS4	-0.03212	5.581159	-0.34295	0.732453	-6.58307	0.837273	0.724863
NK.cells	SRR	0.077884	2.599008	0.342939	0.732459	-5.80861	0.875688	0.787593
NK.cells	AKAP17B	-0.09974	1.696468	-0.34289	0.732498	-5.73988	0.887685	0.807625
NK.cells	SREK1IP1	0.055435	4.172778	0.342869	0.732511	-6.19455	0.855182	0.753862
NK.cells	GTF2F1	-0.03333	5.030746	-0.34279	0.732567	-6.49234	0.844223	0.736089
NK.cells	GAS2L3	0.066151	4.330262	0.342641	0.732682	-6.20963	0.853178	0.750604
NK.cells	GM38843	0.189501	0.985485	0.342499	0.732788	-5.28899	0.897278	0.823809
NK.cells	H2-DMB2	-0.11627	3.016521	-0.34245	0.732825	-5.68376	0.870215	0.778578
NK.cells	CCDC125	-0.05379	4.497843	-0.34239	0.732869	-6.38599	0.85103	0.747133
NK.cells	SDCBP2	-0.12348	1.097863	-0.34214	0.733057	-5.64034	0.895887	0.821276
NK.cells	C3AR1	-0.21638	2.290307	-0.34187	0.73326	-5.47657	0.879918	0.794645
NK.cells	FBXL15	-0.07176	2.696607	-0.34161	0.733451	-5.93671	0.874546	0.785769
NK.cells	TMEM138	-0.07205	2.910493	-0.34138	0.73363	-5.858	0.871732	0.78117
NK.cells	BCL11B	0.077768	1.080616	0.341156	0.733796	-6.30541	0.89612	0.82202
NK.cells	SCD1	-0.14005	3.037555	-0.34096	0.73394	-5.66618	0.870066	0.778488
NK.cells	BMF	-0.10541	2.137288	-0.34094	0.73396	-5.7155	0.88195	0.798228
NK.cells	ORMDL3	-0.05486	3.803807	-0.34083	0.734038	-6.25052	0.860086	0.762065
NK.cells	TTI1	-0.08555	2.712153	-0.34082	0.734046	-5.88414	0.874341	0.785567
NK.cells	ZFP142	-0.05589	3.207748	-0.3407	0.734139	-6.09153	0.867838	0.774845
NK.cells	FANCA	-0.06819	3.05493	-0.34049	0.734296	-6.05713	0.869838	0.778146
NK.cells	IGSF8	-0.05971	4.266842	-0.34047	0.734311	-6.11164	0.854115	0.75234
NK.cells	MYO1C	-0.04527	5.436164	-0.34046	0.734321	-6.24363	0.839237	0.728244
NK.cells	TMEM39B	-0.04933	4.225197	-0.34044	0.734336	-6.32357	0.854651	0.753212
NK.cells	CUTC	0.054227	3.519969	0.340377	0.73438	-6.10034	0.863768	0.768142
NK.cells	ANKRD9	-0.09756	2.821177	-0.3403	0.734439	-5.79289	0.872906	0.783238
NK.cells	C87436	0.050198	3.905756	0.340089	0.734596	-6.23809	0.858767	0.759999
NK.cells	TTK	-0.10609	2.313922	-0.33993	0.734715	-5.76575	0.879605	0.794426
NK.cells	MLKL	0.102994	2.335281	0.339888	0.734747	-5.79971	0.879321	0.793955
NK.cells	ARF3	0.028839	6.29303	0.339829	0.734791	-6.68277	0.828515	0.711143
NK.cells	SUMO2	-0.02244	8.656817	-0.3398	0.734812	-7.11002	0.799714	0.66586
NK.cells	TSPAN4	0.065918	3.356981	0.339733	0.734863	-6.00279	0.86589	0.771715
NK.cells	SLC25A11	-0.04115	5.162778	-0.33969	0.734898	-6.49955	0.84269	0.733884
NK.cells	WDYHV1	-0.04213	4.174037	-0.33969	0.734899	-6.34815	0.855308	0.754364
NK.cells	COTL1	-0.03136	6.629821	-0.33966	0.734919	-6.85759	0.824342	0.704513
NK.cells	LRRC42	0.056262	3.272197	0.339549	0.735002	-6.08021	0.866996	0.773553
NK.cells	TCTN2	-0.19384	-0.37598	-0.3395	0.73504	-5.1695	0.91605	0.856111
NK.cells	TTL	-0.09277	1.740811	-0.33938	0.735131	-5.6973	0.887239	0.807262
NK.cells	YIPF1	0.032369	5.234712	0.339222	0.735247	-6.53417	0.84178	0.732493
NK.cells	HEXA	0.046666	5.752202	0.339208	0.735258	-6.47655	0.835265	0.722017
NK.cells	TPPP3	0.161643	1.071021	0.339153	0.735299	-5.52995	0.89625	0.822468
NK.cells	KLC2	-0.08184	2.681752	-0.33901	0.735403	-5.91707	0.874742	0.786454
NK.cells	NDUFB8	0.029159	7.487591	0.338977	0.735431	-6.91561	0.813819	0.687982
NK.cells	ACKR3	-0.25031	0.835269	-0.33885	0.73553	-5.30875	0.899445	0.827878
NK.cells	RHEBL1	0.120527	1.532009	0.338703	0.735636	-5.58201	0.890038	0.81208
NK.cells	NTHL1	-0.1344	0.544589	-0.33864	0.735685	-5.36311	0.903401	0.834666

NK.cells	CD36	-0.12009	5.712063	-0.33861	0.735706	-6.10811	0.835769	0.722912
NK.cells	HEATR9	0.095978	-0.41964	0.338581	0.735728	-5.76897	0.916655	0.857303
NK.cells	CASP7	-0.05303	4.140272	-0.33853	0.735763	-6.3011	0.855743	0.75525
NK.cells	ATG4B	0.040617	4.800676	0.338267	0.735964	-6.42823	0.847296	0.741589
NK.cells	MIA3	-0.02995	5.849323	-0.33825	0.735979	-6.66306	0.834057	0.720251
NK.cells	MRPL21	0.037842	5.233418	0.338239	0.735985	-6.54403	0.841805	0.732708
NK.cells	PBLD1	-0.13356	1.75083	-0.3381	0.736086	-5.64469	0.887149	0.807264
NK.cells	FRAT1	-0.05326	3.463579	-0.33783	0.736291	-6.1162	0.864694	0.769806
NK.cells	ETAA1	-0.05176	3.334503	-0.3377	0.736387	-6.22365	0.866376	0.772593
NK.cells	IARS2	0.041755	4.475268	0.337651	0.736427	-6.4397	0.851632	0.748471
NK.cells	KCNA2	-0.22092	0.96787	-0.33741	0.736608	-5.35314	0.89788	0.825198
NK.cells	CTSF	-0.1455	1.860603	-0.33728	0.736704	-5.55463	0.885868	0.805005
NK.cells	IFIT3B	0.185538	1.43036	0.337236	0.736738	-5.52431	0.891635	0.81469
NK.cells	TRAPPC5	-0.0511	4.379372	-0.33701	0.736907	-6.23354	0.852893	0.750525
NK.cells	MCM5	-0.07372	4.910563	-0.33701	0.736908	-6.46425	0.84611	0.739508
NK.cells	EIF4G1	0.027309	6.604378	0.336694	0.737146	-6.77646	0.824871	0.705486
NK.cells	ATP8A1	-0.03189	7.601268	-0.33669	0.73715	-6.88027	0.812647	0.686177
NK.cells	TJP3	-0.12767	1.707068	-0.3366	0.737219	-5.50165	0.887921	0.808499
NK.cells	RFC4	-0.06021	4.603054	-0.33642	0.73735	-6.38837	0.850029	0.745983
NK.cells	TRAFD1	0.051128	5.468862	0.335943	0.73771	-6.63093	0.839044	0.728277
NK.cells	2700038G2	-0.08119	2.577847	-0.33594	0.737712	-5.78419	0.87634	0.789297
NK.cells	TGFB2	0.257803	0.46572	0.335889	0.737751	-5.28738	0.904713	0.837019
NK.cells	POGZ	0.040997	4.431535	0.335768	0.737842	-6.34742	0.852224	0.749656
NK.cells	GM49085	-0.10224	2.025817	-0.33576	0.737847	-5.56957	0.883663	0.801552
NK.cells	ZFP81	0.090204	1.873403	0.335549	0.738006	-5.73627	0.885697	0.804956
NK.cells	ZFP689	-0.10908	1.407041	-0.33554	0.738012	-5.59717	0.891949	0.815459
NK.cells	UPF3B	0.041919	4.979585	0.335276	0.738211	-6.43021	0.845233	0.738433
NK.cells	GM14305	-0.0694	2.260659	-0.33518	0.738283	-5.85086	0.88054	0.79649
NK.cells	OAS2	-0.18042	1.571395	-0.33514	0.738313	-5.47439	0.88974	0.8119
NK.cells	GALT	-0.0878	2.389889	-0.33508	0.738362	-5.78899	0.878826	0.793632
NK.cells	GM17056	0.077922	2.866748	0.334987	0.738428	-6.14155	0.872534	0.783175
NK.cells	GM47448	-0.25715	-0.89373	-0.33492	0.738481	-5.10526	0.923486	0.869418
NK.cells	1700094J0	-0.17622	0.092196	-0.33462	0.738703	-5.37803	0.909831	0.846037
NK.cells	ADAMTS14	0.107723	0.543497	0.334583	0.738732	-5.74471	0.903651	0.835503
NK.cells	HDAC10	-0.12371	1.608346	-0.33455	0.738754	-5.44741	0.889245	0.811148
NK.cells	ZFP592	0.02753	5.89966	0.334403	0.738867	-6.67531	0.833636	0.719886
NK.cells	GM43111	0.153654	-0.1905	0.334384	0.738881	-5.37157	0.913724	0.852748
NK.cells	PRDM15	0.057895	3.462741	0.334301	0.738944	-6.05509	0.864738	0.77045
NK.cells	RELA	0.037452	5.114793	0.33421	0.739012	-6.53076	0.843517	0.735823
NK.cells	MFSD7A	0.184891	-0.44923	0.334209	0.739013	-5.27074	0.917303	0.858929
NK.cells	THTPA	-0.10789	1.360701	-0.33403	0.739146	-5.55379	0.892573	0.816877
NK.cells	PET100	0.034283	5.786929	0.333973	0.739191	-6.5814	0.835047	0.722223
NK.cells	2010001A1	0.118494	1.389339	0.333933	0.739221	-5.50319	0.892187	0.816229
NK.cells	COG6	0.065434	3.137898	0.333836	0.739294	-5.99826	0.868978	0.777516
NK.cells	ANKRD44	-0.03026	8.18547	-0.33365	0.739433	-7.12188	0.805576	0.67567
NK.cells	RPP40	-0.12311	1.432828	-0.33342	0.739604	-5.50269	0.891602	0.81544
NK.cells	EXOC4	0.02258	8.03297	0.33329	0.739704	-7.01859	0.807415	0.678689

NK.cells	GM31728	0.186746	-0.17439	0.333223	0.739755	-5.29241	0.913502	0.852779
NK.cells	CYYR1	-0.14545	2.282713	-0.33306	0.739876	-5.57789	0.880247	0.79657
NK.cells	ALCAM	-0.06349	6.646567	-0.33299	0.73993	-6.6688	0.82435	0.705469
NK.cells	PRUNE1	0.058663	3.600837	0.332742	0.740117	-6.06807	0.862942	0.767902
NK.cells	HDHD2	-0.04992	3.552765	-0.33269	0.740155	-6.11891	0.863566	0.76893
NK.cells	TRMT6	0.041917	4.373913	0.332509	0.740292	-6.34771	0.852963	0.751591
NK.cells	ADGRL4	-0.11694	3.201942	-0.33218	0.740542	-5.75023	0.86814	0.776593
NK.cells	GMEB1	0.041697	5.025748	0.332148	0.740564	-6.43713	0.844647	0.738155
NK.cells	HYAL2	-0.12414	1.859326	-0.33207	0.74062	-5.51862	0.885885	0.806148
NK.cells	ADAM33	0.205945	-0.9471	0.331963	0.740703	-5.1376	0.92423	0.871472
NK.cells	GALC	0.054293	3.891293	0.33195	0.740712	-6.28003	0.859178	0.761843
NK.cells	B230217C1	-0.08799	1.478169	-0.33192	0.740732	-5.76227	0.890993	0.814746
NK.cells	ACTN4	-0.02471	6.545001	-0.33182	0.740809	-6.79654	0.825606	0.707648
NK.cells	PIGB	0.077538	2.703863	0.331732	0.740876	-5.84592	0.874678	0.787524
NK.cells	NUCB1	0.03599	5.383551	0.33158	0.740991	-6.61623	0.840119	0.730945
NK.cells	TFEB	-0.04204	5.158966	-0.33154	0.741023	-6.42633	0.842958	0.73553
NK.cells	SSBP1	0.033276	5.592236	0.331303	0.7412	-6.58795	0.837491	0.726769
NK.cells	MET	0.142003	1.991608	0.331122	0.741336	-5.62988	0.884119	0.803364
NK.cells	MIR17HG	-0.10263	2.192534	-0.33101	0.741418	-5.66177	0.881445	0.798892
NK.cells	DUSP11	0.025049	6.33182	0.33097	0.741451	-6.75938	0.828249	0.711966
NK.cells	COPZ1	0.027737	6.366584	0.330502	0.741802	-6.72781	0.827817	0.711393
NK.cells	DEXI	-0.05791	2.91114	-0.33047	0.741824	-6.11235	0.871951	0.783216
NK.cells	WAPL	0.028133	7.165135	0.330427	0.741859	-6.94188	0.81797	0.695752
NK.cells	GM47601	0.148836	0.02249	0.330392	0.741885	-5.37453	0.910789	0.848682
NK.cells	BC005624	-0.02848	5.761918	-0.33032	0.741941	-6.62245	0.835361	0.72347
NK.cells	DDX56	0.046835	3.951871	0.330285	0.741966	-6.23938	0.858395	0.760859
NK.cells	GM26542	-0.04489	4.802534	-0.33019	0.742034	-6.4733	0.847485	0.743054
NK.cells	SMAD5	-0.04822	3.702902	-0.33011	0.742095	-6.12407	0.861617	0.766149
NK.cells	PEX10	-0.13196	0.82136	-0.33011	0.742096	-5.45059	0.899868	0.830065
NK.cells	NEK3	0.118964	1.761696	0.330112	0.742097	-5.67977	0.88719	0.808656
NK.cells	GM13008	-0.12129	1.087607	-0.32978	0.742345	-5.52784	0.896259	0.824016
NK.cells	RBL1	0.047997	4.466968	0.329715	0.742395	-6.41194	0.85177	0.750108
NK.cells	FAM92A	0.063296	3.301539	0.329607	0.742476	-6.07073	0.866839	0.774872
NK.cells	SNRPF	0.031326	6.76677	0.32952	0.742542	-6.82578	0.822866	0.703643
NK.cells	PLEKHO1	0.052009	5.499121	0.329425	0.742613	-6.23853	0.838663	0.728926
NK.cells	LRRC47	0.039468	4.227098	0.329408	0.742626	-6.3068	0.854848	0.755201
NK.cells	FAM50A	0.034667	5.186332	0.329375	0.742651	-6.51632	0.842611	0.735302
NK.cells	PSMD1	0.029076	6.546401	0.329359	0.742663	-6.75968	0.825588	0.70798
NK.cells	STK25	0.0398	4.440838	0.329289	0.742716	-6.35472	0.852105	0.750731
NK.cells	ZMYND19	-0.07069	3.409984	-0.32928	0.742724	-6.09262	0.865425	0.772583
NK.cells	TFAM	0.041778	4.293459	0.329247	0.742748	-6.37356	0.853996	0.753832
NK.cells	SSNA1	0.031734	5.922585	0.32909	0.742866	-6.66217	0.833349	0.720446
NK.cells	GM28809	0.156954	-0.12461	0.32895	0.742972	-5.35081	0.912815	0.852413
NK.cells	A430090L1	0.102255	0.628221	0.32887	0.743032	-5.60432	0.902496	0.834796
NK.cells	CAAA0111	-0.03398	5.624186	-0.32874	0.743127	-6.62312	0.837089	0.726492
NK.cells	MKLN1	0.027291	7.159858	0.328433	0.743361	-6.88362	0.818035	0.696139
NK.cells	ANP32B	-0.03199	8.191649	-0.32841	0.743379	-7.04615	0.805502	0.676432

NK.cells	RIC8B	0.039022	4.502862	0.328391	0.743392	-6.41986	0.851311	0.749585
NK.cells	ATP2A1	-0.15451	1.448126	-0.32834	0.74343	-5.37688	0.891396	0.816068
NK.cells	INTS7	-0.02847	5.677826	-0.32832	0.743446	-6.59991	0.836416	0.725462
NK.cells	PRG4	0.328022	1.084056	0.328316	0.743449	-5.5252	0.896307	0.824366
NK.cells	LRPAP1	0.043422	4.499631	0.32829	0.743469	-6.39701	0.851352	0.749652
NK.cells	PILRB2	-0.16034	2.237136	-0.32828	0.743478	-5.42226	0.880852	0.798363
NK.cells	UVRAG	-0.03657	8.569242	-0.32827	0.74348	-6.97863	0.800969	0.669362
NK.cells	PTS	0.033637	5.573329	0.328245	0.743502	-6.55566	0.837729	0.72758
NK.cells	HACE1	0.042977	4.490352	0.32815	0.743574	-6.43149	0.851471	0.749888
NK.cells	SF3B4	-0.03744	5.494923	-0.32804	0.743655	-6.55719	0.838715	0.729214
NK.cells	GM41409	-0.11344	3.031183	-0.32802	0.74367	-5.82445	0.870375	0.780975
NK.cells	NLRC5	0.07039	5.064856	0.328003	0.743685	-6.64172	0.844151	0.737998
NK.cells	RANBP9	0.029531	6.962828	0.327909	0.743755	-6.84942	0.820452	0.700022
NK.cells	GM20406	-0.16952	-0.53168	-0.3278	0.743836	-5.22605	0.918447	0.862263
NK.cells	GM12253	-0.17362	-0.86999	-0.32769	0.743918	-5.24227	0.923155	0.870418
NK.cells	RIIAD1	-0.12817	0.993222	-0.32752	0.74405	-5.44681	0.897537	0.826609
NK.cells	GREB1L	-0.12139	1.947775	-0.32747	0.744083	-5.6591	0.884704	0.80497
NK.cells	C530005A1	-0.1228	0.742343	-0.32747	0.744087	-5.41866	0.900942	0.832392
NK.cells	NUDT16L1	-0.04971	4.234234	-0.32742	0.744123	-6.29392	0.854757	0.755378
NK.cells	PPFIBP2	-0.13008	4.285147	-0.32738	0.744158	-5.79686	0.854102	0.754317
NK.cells	2610507I0	0.167585	0.583661	0.326999	0.744441	-5.36885	0.903103	0.836176
NK.cells	PHF12	-0.0309	6.186244	-0.32697	0.744463	-6.69589	0.830059	0.715498
NK.cells	GM11290	-0.08377	3.432889	-0.32693	0.744493	-6.16126	0.865126	0.772494
NK.cells	POMP	0.028511	7.268227	0.326925	0.744497	-6.89978	0.816708	0.694268
NK.cells	GLIPR2	0.054466	4.6029	0.32688	0.744531	-6.55292	0.850031	0.747743
NK.cells	CCDC28B	-0.07045	3.504556	-0.32679	0.744599	-5.97013	0.864193	0.770955
NK.cells	SLU7	-0.03327	4.955244	-0.32677	0.744614	-6.49034	0.845542	0.740445
NK.cells	CANT1	-0.04554	3.891787	-0.32665	0.744706	-6.28187	0.859171	0.762692
NK.cells	4930477G(	0.22865	-0.38448	0.326537	0.74479	-5.16527	0.916406	0.858949
NK.cells	GM30025	0.113485	1.597885	0.326537	0.74479	-5.60177	0.889385	0.812943
NK.cells	TEAD1	-0.14478	1.756692	-0.32652	0.744801	-5.47047	0.887257	0.809363
NK.cells	WTIP	-0.13842	1.051984	-0.3265	0.744821	-5.37751	0.896741	0.82537
NK.cells	RPAP1	0.072831	2.431442	0.326475	0.744837	-5.85761	0.878276	0.794319
NK.cells	TMEM106/	-0.07085	3.645492	-0.32633	0.744949	-6.06275	0.862362	0.767938
NK.cells	MRPL18	0.035465	6.633228	0.326221	0.745028	-6.78056	0.824515	0.706666
NK.cells	CCL4	-0.07209	7.388292	-0.32621	0.745035	-7.14748	0.815241	0.691967
NK.cells	ZBTB14	-0.06004	3.004063	-0.32593	0.745246	-5.89488	0.870753	0.781835
NK.cells	TMEM173	0.043139	4.250581	0.325931	0.745247	-6.41961	0.854569	0.755179
NK.cells	TPRKB	0.061428	3.157978	0.325857	0.745302	-6.06627	0.868737	0.778496
NK.cells	C230037L1	-0.14121	0.378737	-0.32578	0.745362	-5.36923	0.905925	0.841062
NK.cells	ADGRG6	0.191196	0.969217	0.325712	0.745412	-5.44266	0.897885	0.827391
NK.cells	CDK18	0.206012	-0.22726	0.325613	0.745487	-5.18897	0.91426	0.85536
NK.cells	TBC1D16	0.112951	2.452428	0.325461	0.745601	-5.71331	0.878076	0.794033
NK.cells	CARD11	-0.05392	4.618562	-0.32518	0.745812	-6.52579	0.850031	0.747664
NK.cells	RALYL	0.169048	0.59788	0.325126	0.745854	-5.51567	0.903122	0.83614
NK.cells	NFIX	-0.07961	3.676603	-0.32501	0.745943	-5.94357	0.862182	0.767549
NK.cells	GPRIN3	-0.12609	0.638034	-0.32474	0.746148	-5.56673	0.902664	0.835254

NK.cells	LRRFIP2	0.029188	5.900501	0.324586	0.746261	-6.68367	0.833904	0.721506
NK.cells	ZEB2	-0.0643	9.36649	-0.32454	0.746293	-6.89657	0.791757	0.655164
NK.cells	AAMP	0.023767	5.908148	0.324485	0.746337	-6.67727	0.833808	0.721376
NK.cells	GM9856	0.109663	1.246414	0.324426	0.746382	-5.55734	0.894413	0.821304
NK.cells	BMP2	-0.15421	1.613957	-0.32429	0.746487	-5.41923	0.889467	0.813005
NK.cells	5330439K0	0.132156	0.538036	0.324115	0.746616	-5.52193	0.904028	0.837741
NK.cells	OSBPL3	0.036986	3.353205	0.324083	0.746641	-6.69578	0.866455	0.774682
NK.cells	CAND1	0.031924	5.132169	0.323901	0.746778	-6.50903	0.843579	0.737278
NK.cells	6030468B1	-0.18485	0.279697	-0.32387	0.7468	-5.3419	0.907561	0.843802
NK.cells	ZBTB8A	-0.08168	1.978565	-0.32384	0.746822	-5.74661	0.884589	0.8049
NK.cells	SLC35F2	0.136949	0.357488	0.323795	0.746858	-5.45979	0.906496	0.841982
NK.cells	CRYBG2	0.136874	0.051796	0.323615	0.746994	-5.44097	0.910769	0.849177
NK.cells	GM12216	0.051148	4.554087	0.323509	0.747074	-6.48753	0.851017	0.749264
NK.cells	THAP12	0.036027	4.362104	0.32336	0.747186	-6.37961	0.853477	0.753302
NK.cells	SCCPDH	0.07716	2.465776	0.323217	0.747294	-5.79096	0.878195	0.794119
NK.cells	PPM1G	0.025605	6.448565	0.322922	0.747516	-6.78278	0.827152	0.710822
NK.cells	TNIK	-0.04889	4.035038	-0.3229	0.747536	-6.59121	0.857686	0.760224
NK.cells	AP2S1	0.027138	6.932961	0.322741	0.747653	-6.82947	0.821168	0.701301
NK.cells	XRCC6	-0.0603	5.144906	-0.32274	0.747656	-6.35256	0.843494	0.737095
NK.cells	AKT1	0.034187	6.230392	0.322733	0.74766	-6.6926	0.829862	0.715153
NK.cells	WDR11	0.066815	3.124121	0.322622	0.747743	-5.89907	0.869527	0.779769
NK.cells	ZFP763	-0.12987	0.433803	-0.32259	0.747765	-5.47645	0.905533	0.84032
NK.cells	TAPBPL	-0.04426	4.125165	-0.32257	0.747784	-6.43393	0.856524	0.758346
NK.cells	SFSWAP	-0.02572	5.786795	-0.32236	0.747943	-6.63292	0.835502	0.724093
NK.cells	PSMA1	0.027932	6.9166	0.321891	0.748295	-6.83765	0.821689	0.701768
NK.cells	C79798	-0.07816	1.77973	-0.32184	0.748333	-5.85029	0.88767	0.809619
NK.cells	PPP1R1C	0.204752	0.781145	0.321805	0.74836	-5.30971	0.901147	0.832411
NK.cells	ASAH1	0.039524	6.301867	0.321631	0.748492	-6.56224	0.82934	0.713915
NK.cells	TUBD1	0.090252	2.095647	0.321562	0.748543	-5.70279	0.883499	0.802576
NK.cells	GM19605	0.123477	1.260305	0.321232	0.748792	-5.55293	0.894888	0.821493
NK.cells	NDUFB3	0.032509	5.72376	0.321166	0.748843	-6.61063	0.836739	0.725558
NK.cells	GM15708	0.075553	3.198583	0.320967	0.748993	-5.92965	0.869159	0.778414
NK.cells	MYO16	-0.10603	-0.30441	-0.32085	0.749079	-5.7427	0.916326	0.857999
NK.cells	GM20536	-0.06849	2.340392	-0.32083	0.749094	-5.95131	0.88047	0.797221
NK.cells	SRL	0.14039	0.890591	0.320698	0.749196	-5.32018	0.899938	0.830003
NK.cells	CCNY	-0.02786	6.67252	-0.32065	0.749236	-6.83087	0.824954	0.706664
NK.cells	GM16153	-0.09026	2.536009	-0.32055	0.749311	-5.77471	0.877882	0.792938
NK.cells	PDZD2	-0.06838	3.46937	-0.32028	0.74951	-6.2542	0.865774	0.772711
NK.cells	4833439L1	-0.03915	4.862518	-0.31997	0.749743	-6.42626	0.847964	0.743402
NK.cells	ZFP598	-0.05142	3.44196	-0.31992	0.749781	-6.1951	0.866278	0.773399
NK.cells	B4GAT1	0.110566	1.65015	0.319621	0.75001	-5.5672	0.89005	0.812974
NK.cells	NGP	0.109091	5.022684	0.319584	0.750038	-6.31407	0.845984	0.740164
NK.cells	PAPLN	-0.13759	0.894565	-0.31957	0.750047	-5.42962	0.900256	0.830231
NK.cells	CSF2RA	-0.07198	5.196056	-0.31915	0.750367	-5.98131	0.843974	0.736654
NK.cells	TWF2	-0.03503	5.240324	-0.31886	0.750587	-6.57234	0.843413	0.735828
NK.cells	SKAP2	0.031863	7.235487	0.318763	0.750658	-6.69144	0.81855	0.696098
NK.cells	CEBPE	-0.17271	0.2028	-0.3186	0.750782	-5.29682	0.909914	0.846563

NK.cells	RFESD	-0.08016	2.541257	-0.31851	0.750848	-5.86801	0.87837	0.793301
NK.cells	PGAP3	-0.12345	1.12543	-0.31851	0.750849	-5.5303	0.897327	0.825147
NK.cells	GSPT1	-0.02407	6.707096	-0.31851	0.750852	-6.80345	0.825055	0.706448
NK.cells	ASTE1	0.067655	2.753642	0.318325	0.750989	-5.90619	0.875564	0.788685
NK.cells	MRPS7	-0.03681	5.010323	-0.31822	0.751068	-6.46251	0.846332	0.74068
NK.cells	MEST	0.089652	3.792937	0.318195	0.751088	-6.10984	0.861968	0.766219
NK.cells	THEM4	0.089301	2.270582	0.31813	0.751136	-5.8387	0.881961	0.799394
NK.cells	UROD	0.043155	4.467162	0.318058	0.751191	-6.40752	0.85327	0.752003
NK.cells	NAA50	0.027449	6.371464	0.318001	0.751234	-6.68781	0.829217	0.713178
NK.cells	SPSB3	-0.04439	3.587341	-0.31793	0.751289	-6.16198	0.864639	0.77067
NK.cells	ROBO3	0.193315	-0.8339	0.317851	0.751348	-5.08314	0.924278	0.871405
NK.cells	GM49797	0.036369	4.882769	0.317831	0.751363	-6.48495	0.847956	0.743377
NK.cells	CCDC86	0.038288	5.146452	0.317657	0.751494	-6.47413	0.844663	0.737987
NK.cells	4930509H(	-0.0738	2.090644	-0.31749	0.751623	-5.78851	0.88442	0.8035
NK.cells	CARS2	0.039107	3.502896	0.317298	0.751765	-6.16816	0.865801	0.772546
NK.cells	ZFP410	-0.04089	4.138179	-0.31704	0.751958	-6.31336	0.857563	0.759007
NK.cells	CBX8	0.091485	1.617418	0.317028	0.75197	-5.77251	0.890754	0.814141
NK.cells	MFGE8	0.093716	2.292559	0.31689	0.752074	-5.81215	0.881732	0.799001
NK.cells	NFIL3	-0.04863	4.515288	-0.31688	0.752083	-6.67936	0.852713	0.751081
NK.cells	GM44127	0.2272	-0.52381	0.316796	0.752145	-5.13994	0.920023	0.864032
NK.cells	HK2	0.054283	5.335441	0.31672	0.752203	-6.52073	0.842269	0.734143
NK.cells	CXCL1	0.226999	2.815955	0.316675	0.752237	-5.75333	0.874804	0.787484
NK.cells	IFT122	-0.11706	1.594011	-0.31667	0.752244	-5.39763	0.891069	0.814702
NK.cells	ARAF	0.037954	4.478585	0.316623	0.752276	-6.34786	0.853184	0.751887
NK.cells	CNBP	-0.01979	8.132464	-0.3164	0.752448	-7.05636	0.807791	0.679225
NK.cells	LCOR	0.027701	7.538405	0.31624	0.752566	-6.91606	0.815001	0.690597
NK.cells	SLC16A1	-0.05698	3.905544	-0.31607	0.752694	-6.16954	0.860673	0.764126
NK.cells	RNF130	-0.02687	7.282781	-0.31602	0.752732	-6.91735	0.818126	0.695569
NK.cells	PPM1F	-0.09291	2.206568	-0.31586	0.752853	-5.66705	0.882981	0.801217
NK.cells	LRRC75AO:	0.149343	0.512247	0.31572	0.752959	-5.37438	0.905844	0.839895
NK.cells	PDLIM2	0.050644	3.759145	0.315706	0.75297	-6.30412	0.862571	0.767394
NK.cells	SIPA1L3	0.038192	5.201146	0.315615	0.753038	-6.56944	0.844071	0.737201
NK.cells	CYB5R1	0.056363	3.659051	0.315564	0.753077	-6.07569	0.863871	0.769537
NK.cells	HLX	-0.10025	3.117664	-0.31553	0.753101	-5.59602	0.870942	0.781218
NK.cells	AASDHPPT	0.047404	3.841771	0.315463	0.753154	-6.16555	0.861499	0.765634
NK.cells	VPS26A	-0.03011	6.273847	-0.31526	0.753305	-6.64728	0.830646	0.715553
NK.cells	PHF7	0.060008	3.28527	0.315144	0.753395	-6.04485	0.868805	0.777641
NK.cells	MBTPS1	0.035294	4.639311	0.315111	0.75342	-6.4157	0.851285	0.748888
NK.cells	LMBR1L	0.042296	4.517308	0.314635	0.75378	-6.43974	0.852884	0.751554
NK.cells	MYDGF	-0.03637	4.485984	-0.31452	0.753865	-6.34967	0.853286	0.752234
NK.cells	CAP2	-0.12329	0.071585	-0.31416	0.754137	-5.59493	0.911994	0.850494
NK.cells	SLC20A1	-0.04001	5.219384	-0.31409	0.75419	-6.58961	0.843933	0.737053
NK.cells	FHOD1	-0.0672	2.907952	-0.31408	0.754201	-5.89908	0.873794	0.786028
NK.cells	GLRX5	0.029861	6.17176	0.313915	0.754325	-6.75348	0.831955	0.717834
NK.cells	BACH1	-0.0452	5.9952	-0.31386	0.754367	-6.62406	0.834161	0.721371
NK.cells	GTF3C1	-0.03554	4.779184	-0.31385	0.754371	-6.48797	0.849533	0.746211
NK.cells	ZMAT1	-0.09715	2.090961	-0.31384	0.754381	-5.67465	0.884619	0.80417

NK.cells	LPAR2	0.074207	2.187833	0.313724	0.75447	-5.72418	0.883328	0.802046
NK.cells	SDC4	-0.0642	6.436865	-0.31367	0.754511	-6.46831	0.828654	0.712591
NK.cells	ELP6	-0.0802	2.398694	-0.31354	0.754606	-5.82712	0.880525	0.797356
NK.cells	GM42418	-0.06406	11.10924	-0.31354	0.754607	-7.50199	0.772807	0.625738
NK.cells	TTC39C	0.066252	2.230936	0.313471	0.754661	-6.16255	0.882755	0.801085
NK.cells	DDX39	-0.03733	6.372696	-0.31345	0.754675	-6.7471	0.829451	0.713865
NK.cells	SLC37A3	-0.03878	3.658041	-0.31342	0.754702	-6.37271	0.86398	0.769903
NK.cells	ESPN	0.147957	-0.17599	0.313401	0.754714	-5.41623	0.915411	0.85648
NK.cells	LIPT2	0.129856	1.430834	0.313364	0.754742	-5.44062	0.893471	0.819103
NK.cells	TRIM8	-0.03372	5.616506	-0.31322	0.75485	-6.56828	0.838956	0.729084
NK.cells	FAM78B	-0.0662	2.115372	-0.31307	0.754967	-6.10548	0.88435	0.803721
NK.cells	TIAM2	0.079485	2.628932	0.312958	0.75505	-6.11392	0.87753	0.792344
NK.cells	ANGPT2	0.148756	0.571482	0.312888	0.755102	-5.3552	0.905192	0.839005
NK.cells	CUX1	0.026368	7.953864	0.312829	0.755148	-6.9547	0.810092	0.683243
NK.cells	RAPGEF4	0.074619	2.563789	0.312619	0.755306	-6.04032	0.878435	0.793913
NK.cells	CLCC1	0.034807	4.329275	0.312544	0.755363	-6.33165	0.855395	0.755853
NK.cells	PTPN1	-0.03191	7.078745	-0.31237	0.755496	-6.88439	0.820813	0.700183
NK.cells	RNF213	0.062315	6.151249	0.312279	0.755563	-6.73847	0.832304	0.718498
NK.cells	ALPK2	0.083115	1.260876	0.312243	0.755591	-5.85139	0.895865	0.823231
NK.cells	ATG2B	-0.03523	4.861013	-0.31213	0.755673	-6.48477	0.848584	0.744776
NK.cells	SAFB2	0.025705	5.993263	0.312063	0.755727	-6.66821	0.834279	0.721665
NK.cells	ZFP961	-0.05957	3.308556	-0.31202	0.755758	-6.08752	0.868635	0.777667
NK.cells	PER3	-0.1149	1.517982	-0.31179	0.755932	-5.52885	0.892397	0.817429
NK.cells	SPG11	-0.04555	4.094018	-0.31171	0.755996	-6.31497	0.858427	0.760921
NK.cells	CD244A	-0.04406	3.429777	-0.31168	0.756018	-6.55713	0.867051	0.775119
NK.cells	ACOT8	0.041819	4.129949	0.31158	0.756093	-6.3188	0.857963	0.760161
NK.cells	TUT4	-0.02964	7.475638	-0.31153	0.756132	-7.01329	0.815949	0.692556
NK.cells	ENTR1	0.038413	4.880508	0.311431	0.756206	-6.44954	0.848336	0.744443
NK.cells	9530077C0	-0.13563	1.213471	-0.31127	0.756332	-5.46626	0.896507	0.824422
NK.cells	OSCP1	0.103193	3.25254	0.311078	0.756473	-5.82725	0.869368	0.77902
NK.cells	ADRB2	-0.07702	4.714467	-0.31107	0.756476	-6.27247	0.850455	0.747959
NK.cells	SRP9	-0.02196	7.516452	-0.31086	0.756635	-6.93463	0.815451	0.691853
NK.cells	BAZ2B	0.026982	8.430417	0.31082	0.756669	-6.96863	0.804381	0.674477
NK.cells	DEPTOR	-0.07095	3.469195	-0.31077	0.75671	-6.00234	0.866537	0.774362
NK.cells	TBC1D7	0.066799	2.220043	0.310678	0.756777	-5.78294	0.882999	0.801761
NK.cells	PHF10	0.039474	4.930175	0.310517	0.756898	-6.41604	0.847703	0.743543
NK.cells	MICU3	0.052231	3.81398	0.310498	0.756913	-6.22473	0.862052	0.767008
NK.cells	B3GNTL1	0.063402	2.673331	0.310472	0.756932	-6.03768	0.876987	0.791742
NK.cells	DENND4B	0.053016	3.747946	0.310414	0.756976	-6.09853	0.862909	0.768419
NK.cells	LANCL2	0.068256	2.807486	0.31032	0.757048	-5.93271	0.875216	0.788802
NK.cells	PNPLA8	0.031967	6.018439	0.31015	0.757177	-6.65952	0.834007	0.721362
NK.cells	RFC1	0.03819	5.999025	0.310057	0.757247	-6.6303	0.83425	0.721769
NK.cells	WDSUB1	0.072194	2.44185	0.30989	0.757374	-5.79435	0.880097	0.796913
NK.cells	CDC73	-0.02676	6.174072	-0.30989	0.757374	-6.74708	0.832062	0.718286
NK.cells	4930404I0	0.20231	0.086501	0.30974	0.757488	-5.22014	0.911989	0.85085
NK.cells	PIP5K1B	-0.04551	5.568352	-0.30959	0.757601	-6.55702	0.839752	0.730549
NK.cells	SLC38A10	-0.032	5.326136	-0.30933	0.757799	-6.57852	0.842904	0.735536

NK.cells	CAMTA2	0.047357	3.696678	0.309143	0.75794	-6.2095	0.863809	0.769671
NK.cells	NONO	-0.02461	6.614635	-0.30911	0.757969	-6.77667	0.826765	0.709621
NK.cells	ARHGEF4	0.165028	0.759185	0.309063	0.758001	-5.52423	0.90292	0.835183
NK.cells	MAP1LC3A	-0.04639	5.476214	-0.30898	0.758061	-6.56095	0.841006	0.732493
NK.cells	ZMIZ2	-0.0376	4.532649	-0.30891	0.758113	-6.42005	0.853014	0.751989
NK.cells	DDX19B	0.044358	3.529612	0.30872	0.758261	-6.22641	0.866071	0.773326
NK.cells	ISG15	-0.0763	6.408101	-0.30815	0.758691	-6.93472	0.829746	0.713904
NK.cells	UTP23	0.045435	3.577367	0.308074	0.758751	-6.16343	0.865796	0.772436
NK.cells	PABPC1L	-0.07447	2.906471	-0.30801	0.7588	-5.98263	0.874588	0.786991
NK.cells	4933421O1	-0.08668	2.357702	-0.30794	0.758853	-5.76259	0.88185	0.799115
NK.cells	KCTD3	-0.05403	3.747291	-0.3077	0.759037	-6.05158	0.863713	0.768874
NK.cells	CALCOCO1	-0.05238	3.863697	-0.30759	0.759119	-6.18286	0.862212	0.766407
NK.cells	LTBP4	0.137635	2.297056	0.307493	0.759192	-5.57917	0.8828	0.800558
NK.cells	CXCL10	-0.08996	4.788598	-0.30729	0.759344	-6.52503	0.850341	0.746979
NK.cells	FICD	-0.12431	0.917558	-0.30726	0.759368	-5.48299	0.901402	0.831906
NK.cells	CXADR	-0.10006	1.645302	-0.30684	0.759683	-5.6906	0.891711	0.81533
NK.cells	GM47689	0.042277	3.369026	0.306818	0.759704	-6.22215	0.868845	0.777164
NK.cells	WDR18	0.03704	4.82244	0.306748	0.759757	-6.42895	0.850055	0.746353
NK.cells	BRD9	-0.03145	5.185663	-0.30671	0.759786	-6.54917	0.845429	0.738844
NK.cells	SCARB1	-0.05068	4.995715	-0.30644	0.759988	-6.17019	0.847856	0.742766
NK.cells	APLF	-0.08672	2.05909	-0.30641	0.760014	-5.75368	0.886176	0.80601
NK.cells	CLEC12A	-0.12063	4.937614	-0.30639	0.760026	-5.74448	0.848597	0.743968
NK.cells	POLR3G	-0.08836	2.101279	-0.30631	0.760087	-5.76774	0.885613	0.805088
NK.cells	ERG	-0.14974	3.921953	-0.30614	0.760221	-5.78503	0.861656	0.765326
NK.cells	WDR24	0.092077	1.796393	0.306129	0.760226	-5.65113	0.889694	0.811944
NK.cells	FNDC7	-0.1625	-0.36573	-0.30592	0.760382	-5.30938	0.919307	0.862214
NK.cells	ICMT	-0.07847	2.429223	-0.30569	0.760557	-5.81188	0.881335	0.797895
NK.cells	ENTPD6	-0.05201	2.776877	-0.30562	0.760613	-6.06935	0.87673	0.790219
NK.cells	UBXN2A	0.030177	5.233503	0.305438	0.76075	-6.58146	0.844919	0.737996
NK.cells	SH3BGRL3	-0.02558	9.061448	-0.30541	0.760772	-7.24812	0.797846	0.663408
NK.cells	RIMS3	-0.13918	1.085548	-0.30537	0.760801	-5.49394	0.899379	0.828264
NK.cells	NAA40	-0.03355	4.909944	-0.30536	0.760806	-6.42197	0.849036	0.744673
NK.cells	ADAMTS9	0.138617	2.970839	0.30512	0.760992	-5.86608	0.874227	0.786009
NK.cells	G6PC3	0.05917	3.106653	0.305113	0.760997	-6.08922	0.87244	0.783044
NK.cells	MED18	-0.10972	1.412452	-0.30493	0.761133	-5.53843	0.895011	0.820837
NK.cells	SLC25A38	0.04661	3.517886	0.304921	0.761143	-6.22467	0.867057	0.774154
NK.cells	STAT2	-0.06009	5.210285	-0.30481	0.761231	-6.52965	0.845287	0.738556
NK.cells	TMEM243	0.024052	6.427837	0.304631	0.761363	-6.77836	0.829985	0.713983
NK.cells	SPG21	-0.02763	6.057126	-0.30452	0.761446	-6.69423	0.834613	0.721424
NK.cells	FAM192A	-0.03406	4.607769	-0.3044	0.761539	-6.36291	0.852978	0.751155
NK.cells	PBX1	-0.07211	5.465012	-0.30433	0.761591	-6.18843	0.842063	0.733466
NK.cells	WSB2	0.042368	4.493162	0.304297	0.761617	-6.34207	0.854449	0.753589
NK.cells	SNTB2	-0.03756	5.607555	-0.30423	0.761669	-6.72427	0.840263	0.730591
NK.cells	XIAP	0.023666	6.725324	0.30414	0.761736	-6.77926	0.826292	0.708205
NK.cells	CPT2	0.083135	2.829472	0.30403	0.761819	-5.86054	0.876128	0.789328
NK.cells	SNX24	-0.10237	4.299252	-0.30369	0.762081	-5.86423	0.857047	0.757772
NK.cells	HSPA12A	-0.17908	0.150275	-0.30366	0.762101	-5.21058	0.912361	0.850429

NK.cells	SIRT6	0.063961	2.652878	0.303641	0.762115	-5.98771	0.878555	0.793296
NK.cells	KDM3A	0.032123	5.567098	0.30333	0.762351	-6.59737	0.840902	0.731536
NK.cells	SPIC	-0.20868	1.863637	-0.30325	0.762409	-5.41633	0.889099	0.810948
NK.cells	GFM2	-0.05097	3.590998	-0.30325	0.762415	-6.12839	0.866258	0.772904
NK.cells	DNAAF2	-0.05599	2.845924	-0.30323	0.762427	-6.01595	0.876032	0.789095
NK.cells	GM19522	-0.11962	0.940675	-0.30297	0.762626	-5.4721	0.901717	0.83204
NK.cells	CDK5RAP3	0.037451	4.379321	0.302846	0.762718	-6.41188	0.85621	0.756191
NK.cells	GH	-0.16345	-0.76502	-0.30258	0.762917	-5.14796	0.925429	0.87248
NK.cells	KCMF1	0.019998	6.987433	0.302283	0.763146	-6.84994	0.8235	0.703336
NK.cells	TUT1	-0.05926	2.638669	-0.30224	0.76318	-5.83383	0.879114	0.79384
NK.cells	CDADC1	-0.04937	4.609962	-0.30221	0.763204	-6.3907	0.853413	0.751465
NK.cells	TRIM10	0.183726	0.349121	0.302119	0.763271	-5.33568	0.910009	0.845994
NK.cells	SLC4A8	0.124227	0.467759	0.302045	0.763327	-5.58587	0.90838	0.843217
NK.cells	COMMD1	-0.03589	5.141948	-0.30199	0.76337	-6.50405	0.846617	0.740427
NK.cells	TSEN15	-0.0651	2.776415	-0.30182	0.763496	-5.89224	0.877353	0.79083
NK.cells	GM29264	-0.13943	0.505466	-0.30153	0.763721	-5.32348	0.908072	0.842429
NK.cells	NF2	-0.03675	4.781261	-0.3013	0.763891	-6.46216	0.851414	0.748076
NK.cells	AFP	0.129114	4.371399	0.30123	0.763946	-6.41294	0.856677	0.756683
NK.cells	CCDC180	0.120177	1.286108	0.301161	0.763999	-5.52915	0.897435	0.824502
NK.cells	CXCR2	0.167918	-0.00657	0.301156	0.764003	-5.32404	0.915122	0.854638
NK.cells	E2F8	-0.06527	3.686572	-0.3006	0.764423	-6.12955	0.865549	0.771444
NK.cells	UBE2B	0.027301	8.25823	0.300449	0.76454	-7.09064	0.808174	0.679259
NK.cells	NLGN2	-0.1739	0.349554	-0.30044	0.764549	-5.36071	0.910213	0.846439
NK.cells	TMEM104	0.062227	3.273099	0.300389	0.764585	-6.04856	0.870953	0.7804
NK.cells	ARMC2	-0.11861	-0.1641	-0.30034	0.764621	-5.46976	0.917302	0.858644
NK.cells	SMC1A	-0.03002	6.748393	-0.30029	0.764664	-6.81734	0.826645	0.708476
NK.cells	ELL2	-0.04517	6.734248	-0.30016	0.764757	-6.8306	0.82682	0.708776
NK.cells	POGLUT2	0.140413	1.00166	0.300052	0.764842	-5.36841	0.901296	0.831356
NK.cells	FAM57A	0.113242	1.118395	0.300014	0.764871	-5.57858	0.899709	0.828664
NK.cells	HINT1	0.024677	7.995864	0.299919	0.764943	-7.03985	0.81135	0.684348
NK.cells	NEK9	-0.0379	5.27603	-0.29969	0.765113	-6.50719	0.845109	0.738211
NK.cells	SLTM	0.022944	6.63584	0.299673	0.76513	-6.82092	0.828041	0.710786
NK.cells	TRBC1	-0.0468	3.183542	-0.29954	0.76523	-6.71553	0.872128	0.782497
NK.cells	NDRG3	-0.03264	4.407312	-0.29942	0.765325	-6.43702	0.856214	0.756295
NK.cells	DDX20	0.043201	3.596417	0.299306	0.765409	-6.15079	0.866724	0.773558
NK.cells	DUSP23	0.082249	2.373334	0.299059	0.765597	-5.69079	0.88284	0.80041
NK.cells	GM49359	0.07971	2.260358	0.298883	0.765731	-5.74882	0.884345	0.802958
NK.cells	NSMCE3	-0.03547	4.271532	-0.29875	0.76583	-6.30819	0.857964	0.759275
NK.cells	SRSF10	-0.02238	6.4448	-0.29856	0.765974	-6.74401	0.830416	0.714727
NK.cells	NUDT7	0.097341	1.74453	0.298364	0.766125	-5.69152	0.89125	0.814606
NK.cells	BAZ2A	-0.03113	6.97841	-0.29809	0.766331	-6.81591	0.823801	0.704236
NK.cells	PITPNM1	0.039994	3.730007	0.298034	0.766376	-6.32948	0.864983	0.770889
NK.cells	STIP1	0.035135	5.419076	0.298008	0.766396	-6.58178	0.843295	0.735482
NK.cells	SPEF2	-0.1219	1.371663	-0.29786	0.766506	-5.49014	0.896277	0.823161
NK.cells	RAB14	-0.01997	7.091867	-0.29784	0.766528	-6.83373	0.822402	0.702026
NK.cells	PIGO	-0.08945	1.804475	-0.29781	0.766548	-5.73034	0.890444	0.813318
NK.cells	ZFP983	0.071958	2.38923	0.297613	0.766697	-5.88712	0.882628	0.800241

NK.cells	PIRA2	-0.14426	3.589246	-0.2976	0.766706	-5.54153	0.866817	0.773966
NK.cells	GM50386	0.147617	0.179027	0.297543	0.76675	-5.26721	0.91256	0.850949
NK.cells	FKBP2	-0.0371	5.611404	-0.29753	0.766756	-6.57069	0.840864	0.73162
NK.cells	AP1S3	-0.04362	5.57812	-0.29752	0.766767	-6.58434	0.841284	0.732299
NK.cells	COL6A1	0.153384	0.367808	0.297509	0.766776	-5.32379	0.909962	0.846501
NK.cells	BUD23	0.047632	4.09892	0.297396	0.766862	-6.28257	0.860195	0.763115
NK.cells	SSH2	0.033671	8.396317	0.297241	0.766979	-7.05801	0.806507	0.677085
NK.cells	TRP53INP2	-0.04538	4.134264	-0.29721	0.767002	-6.24992	0.859738	0.762376
NK.cells	SRPRB	-0.03851	4.104103	-0.29706	0.767118	-6.31347	0.860128	0.763052
NK.cells	GM20470	-0.09461	1.403571	-0.29692	0.767226	-5.68099	0.895846	0.822617
NK.cells	ARPC5L	-0.02622	6.300784	-0.29686	0.767271	-6.71145	0.832211	0.717824
NK.cells	GM35188	0.111663	1.73277	0.296803	0.767313	-5.64728	0.891408	0.815128
NK.cells	B3GNT3	0.155315	0.711737	0.296781	0.76733	-5.27229	0.905249	0.838585
NK.cells	SLC39A13	0.062611	2.796524	0.296764	0.767343	-5.93362	0.877227	0.791369
NK.cells	ACER1	0.228421	-0.7216	0.296673	0.767412	-5.18551	0.925062	0.872652
NK.cells	KANK1	0.118598	1.172928	0.296584	0.76748	-5.50026	0.898969	0.827963
NK.cells	SLC35B3	0.030183	4.482615	0.296553	0.767504	-6.41547	0.855245	0.755152
NK.cells	MFSD5	-0.04502	4.282812	-0.29646	0.767574	-6.29459	0.857819	0.759395
NK.cells	SEMA4G	-0.13713	0.909795	-0.29627	0.767721	-5.4518	0.902546	0.834152
NK.cells	GM34225	-0.20195	-0.06364	-0.29626	0.767728	-5.15419	0.915911	0.857004
NK.cells	GABARAP	0.022172	8.497974	0.296224	0.767753	-7.04891	0.805283	0.675377
NK.cells	MRPL42	-0.03449	6.521235	-0.29613	0.767824	-6.77324	0.829464	0.713619
NK.cells	ELF4	-0.0273	6.785373	-0.296	0.767922	-6.78819	0.826187	0.708421
NK.cells	PDCD2	0.04318	3.817161	0.295977	0.767941	-6.25434	0.863849	0.769445
NK.cells	FEN1	-0.04808	4.525584	-0.29582	0.768062	-6.36568	0.854693	0.754434
NK.cells	SDHAF4	0.038422	4.365947	0.295726	0.768133	-6.3495	0.856747	0.75782
NK.cells	CAPN5	-0.15599	1.546779	-0.2956	0.768229	-5.36157	0.893912	0.819684
NK.cells	GADD45A	-0.05497	4.876654	-0.29551	0.768294	-6.34721	0.850195	0.747157
NK.cells	HELZ2	0.058735	3.832468	0.295507	0.7683	-6.19419	0.86365	0.769192
NK.cells	GM10550	-0.12548	0.590308	-0.2955	0.768307	-5.44482	0.90691	0.841757
NK.cells	RBM5	-0.02291	6.623423	-0.29542	0.768366	-6.7913	0.828195	0.711691
NK.cells	SHPK	-0.12741	0.252393	-0.29535	0.76842	-5.42001	0.911549	0.849691
NK.cells	DUSP3	0.042557	5.553941	0.295215	0.768522	-6.32369	0.841589	0.733228
NK.cells	DYNLT1F	-0.04572	5.068561	-0.29514	0.768577	-6.43534	0.847747	0.743225
NK.cells	DUSP8	0.10712	1.274945	0.295018	0.768672	-5.57037	0.897586	0.825999
NK.cells	P2RY1	0.133901	0.13392	0.294984	0.768698	-5.36681	0.913182	0.852593
NK.cells	AGPS	0.025174	6.813953	0.294932	0.768738	-6.81104	0.825833	0.708012
NK.cells	CDO1	-0.06478	3.756999	-0.29492	0.768743	-6.18281	0.864632	0.770903
NK.cells	HMGCS1	0.046661	4.453186	0.294873	0.768782	-6.29828	0.855624	0.756109
NK.cells	GIMAP9	0.048448	3.560572	0.294562	0.769019	-6.3109	0.867192	0.775219
NK.cells	SLC18A2	0.068732	2.251616	0.294354	0.769177	-5.83133	0.884461	0.803987
NK.cells	ZZZ3	-0.03175	5.884403	-0.29416	0.769326	-6.65486	0.837425	0.726687
NK.cells	NCKAP1L	-0.02427	6.137906	-0.29396	0.769477	-6.70311	0.834246	0.721598
NK.cells	MYO9A	0.045791	6.336763	0.293938	0.769494	-6.62648	0.831762	0.717617
NK.cells	TENT2	-0.0242	6.624422	-0.29377	0.769622	-6.76297	0.828182	0.71193
NK.cells	API5	-0.02566	6.03062	-0.2935	0.769828	-6.69541	0.83559	0.723853
NK.cells	F730043M	0.140143	0.260543	0.293488	0.769837	-5.44896	0.911437	0.849874

NK.cells	LEO1	-0.04441	4.138442	-0.29329	0.769987	-6.27827	0.859684	0.763006
NK.cells	TAF9B	-0.10829	0.956005	-0.29308	0.770148	-5.55577	0.901917	0.833656
NK.cells	BRD7	0.023431	6.307707	0.292968	0.770233	-6.70068	0.832124	0.718324
NK.cells	ZFP764	-0.10835	1.074799	-0.29284	0.770331	-5.49869	0.900301	0.830941
NK.cells	GPM6B	-0.13026	2.439081	-0.29275	0.770403	-5.48624	0.881965	0.800029
NK.cells	TBXA2R	0.110375	2.36173	0.29264	0.770483	-5.70301	0.882994	0.801783
NK.cells	ECM1	0.087776	3.680244	0.292467	0.770615	-6.20486	0.865631	0.772931
NK.cells	BTBD2	-0.04975	3.477347	-0.29246	0.770617	-6.13493	0.868279	0.777308
NK.cells	4932438A1	0.032552	7.087671	0.292443	0.770633	-6.92534	0.822454	0.702983
NK.cells	DPM3	-0.02649	6.309203	-0.29234	0.770712	-6.73627	0.832106	0.71841
NK.cells	ENGASE	-0.1128	1.496503	-0.2923	0.770741	-5.63454	0.894591	0.821362
NK.cells	SH2D3C	0.041331	4.693449	0.292224	0.7708	-6.50395	0.852539	0.751464
NK.cells	RCHY1	0.028116	5.661194	0.292194	0.770823	-6.61665	0.840235	0.731497
NK.cells	PBX2	-0.04218	4.930622	-0.29211	0.770889	-6.39311	0.849505	0.746532
NK.cells	4930430E1	-0.1829	0.409202	-0.29207	0.770915	-5.30838	0.909393	0.846583
NK.cells	GAB1	0.115271	4.473058	0.292014	0.77096	-5.87038	0.855368	0.756126
NK.cells	DSCC1	0.07356	2.435007	0.291909	0.77104	-5.8785	0.882019	0.800246
NK.cells	CAP1	-0.02373	6.878742	-0.29187	0.771068	-6.82979	0.825032	0.707147
NK.cells	GEMIN7	0.029708	5.084337	0.291843	0.77109	-6.46961	0.847546	0.743372
NK.cells	RFK	0.037176	5.042963	0.291817	0.77111	-6.45556	0.848073	0.744229
NK.cells	CCDC106	0.144246	0.51131	0.291761	0.771153	-5.33668	0.907992	0.844207
NK.cells	GM33104	-0.07398	0.440079	-0.2915	0.771349	-6.02791	0.908969	0.845944
NK.cells	MBNL2	-0.02661	8.406165	-0.29136	0.771455	-7.09233	0.806389	0.677776
NK.cells	GM47819	0.202789	-0.22199	0.291317	0.771491	-5.22505	0.918105	0.861635
NK.cells	ALG3	0.09682	1.77052	0.291131	0.771633	-5.65961	0.890901	0.815266
NK.cells	EMD	-0.02492	6.102062	-0.29106	0.771689	-6.72448	0.834695	0.722686
NK.cells	CMPK2	0.09659	3.672891	0.290933	0.771784	-5.96818	0.865727	0.773264
NK.cells	FGF13	0.072466	3.379693	0.290643	0.772005	-6.25001	0.869556	0.779672
NK.cells	ARHGEF10	-0.08563	2.266878	-0.29059	0.772048	-5.95797	0.884258	0.804193
NK.cells	VCPIP1	-0.02543	6.167501	-0.29057	0.772061	-6.70597	0.833876	0.721461
NK.cells	B3GLCT	0.054271	3.311117	0.290534	0.772088	-6.13049	0.870455	0.781169
NK.cells	GM44067	-0.10237	1.717425	-0.29051	0.772104	-5.58629	0.891614	0.816573
NK.cells	ADPRH	0.036429	4.93883	0.290505	0.77211	-6.4636	0.849401	0.746567
NK.cells	DGKA	0.035532	5.023185	0.290432	0.772166	-6.68149	0.848325	0.744838
NK.cells	PXDC1	-0.1102	2.191629	-0.29023	0.77232	-5.74504	0.885261	0.805942
NK.cells	FGF1	0.135127	0.63081	0.289926	0.772552	-5.34522	0.906355	0.841718
NK.cells	WWC1	-0.09171	1.216205	-0.28986	0.772605	-5.60499	0.898382	0.828141
NK.cells	SRP54A	0.042783	3.599318	0.289825	0.772629	-6.15595	0.866686	0.775037
NK.cells	TCEANC	0.109831	1.730178	0.289667	0.772749	-5.55106	0.891443	0.816407
NK.cells	D17H6S53I	0.039337	4.009289	0.28965	0.772762	-6.24897	0.861355	0.766253
NK.cells	FEM1C	0.030138	7.117684	0.289571	0.772822	-6.90617	0.822084	0.702733
NK.cells	ATPSCKMT	-0.05034	3.937455	-0.28957	0.772823	-6.23508	0.862287	0.767787
NK.cells	BROX	0.030059	4.85097	0.28948	0.772892	-6.42495	0.850523	0.74852
NK.cells	CLEC2I	-0.06266	3.177972	-0.28944	0.772921	-6.20865	0.872202	0.784198
NK.cells	PPP4R1	-0.03374	5.579566	-0.28937	0.772972	-6.5425	0.841266	0.73349
NK.cells	AGBL5	-0.06592	2.244561	-0.28929	0.773041	-5.80839	0.884555	0.804826
NK.cells	NECTIN2	-0.09498	2.109704	-0.28917	0.773125	-5.57129	0.886355	0.807869

NK.cells	IFT140	0.051901	3.507073	0.289107	0.773176	-6.1276	0.86789	0.777094
NK.cells	RP9	0.02086	6.834563	0.289036	0.77323	-6.82891	0.825578	0.708357
NK.cells	GOSR2	-0.03247	5.28486	-0.28894	0.773302	-6.54501	0.844997	0.739604
NK.cells	GM11520	-0.11159	1.043872	-0.28892	0.773319	-5.43889	0.900722	0.832228
NK.cells	AW554918	0.030733	5.656196	0.288797	0.773413	-6.61498	0.840298	0.732038
NK.cells	CEP63	-0.0396	4.291786	-0.28877	0.773436	-6.35096	0.857703	0.760378
NK.cells	TMSB15B2	-0.0659	2.47439	-0.28863	0.773541	-5.94032	0.881496	0.799827
NK.cells	PRKAG2	-0.03404	5.050385	-0.28828	0.77381	-6.55487	0.847978	0.744496
NK.cells	INO80E	0.031748	4.165534	0.288109	0.773938	-6.28885	0.859333	0.763069
NK.cells	ZFP748	-0.08641	1.938501	-0.28803	0.773998	-5.74382	0.888646	0.811868
NK.cells	1810058I24	0.033736	7.005863	0.288022	0.774004	-6.87046	0.823462	0.705076
NK.cells	FRMD6	0.056904	3.47737	0.287929	0.774075	-6.20099	0.868278	0.777849
NK.cells	KBTBD3	0.06576	2.727361	0.287927	0.774076	-5.92001	0.878142	0.794251
NK.cells	GRHL1	-0.12283	1.025537	-0.28791	0.774086	-5.49651	0.900971	0.832732
NK.cells	DCAF13	0.031828	4.771338	0.287792	0.774179	-6.45918	0.851541	0.750346
NK.cells	NUTF2	0.09001	1.951713	0.287776	0.774191	-5.72362	0.888469	0.811585
NK.cells	NDUFS5	0.028252	6.478554	0.287771	0.774195	-6.73401	0.829995	0.715516
NK.cells	SLC31A1	0.035613	5.429507	0.287752	0.77421	-6.50586	0.843163	0.736722
NK.cells	ADGRE5	0.027587	7.153842	0.2877	0.77425	-6.88102	0.821639	0.702206
NK.cells	VWA8	0.033744	4.975158	0.287001	0.774783	-6.53663	0.848937	0.746333
NK.cells	PACS1	0.031992	5.972963	0.286905	0.774856	-6.76397	0.836313	0.725906
NK.cells	PTPRCAP	-0.03521	5.807512	-0.28675	0.774975	-6.68454	0.838392	0.729258
NK.cells	GM47167	0.054183	2.927116	0.2866	0.775089	-6.05052	0.875503	0.790167
NK.cells	SUDS3	0.031637	5.515338	0.286536	0.775138	-6.60945	0.842077	0.735283
NK.cells	RAMP3	0.075128	0.947281	0.286492	0.775172	-6.02365	0.902036	0.834936
NK.cells	DDX51	0.101807	1.37149	0.286447	0.775206	-5.55674	0.896279	0.825152
NK.cells	TMEM147	0.034834	4.948794	0.286432	0.775218	-6.45683	0.849274	0.746985
NK.cells	FBXO8	-0.03175	4.606605	-0.28635	0.775278	-6.43761	0.853653	0.754135
NK.cells	ESPL1	-0.0679	2.563574	-0.28635	0.775282	-5.92675	0.880312	0.798252
NK.cells	MAPRE2	0.027177	6.802434	0.286269	0.775341	-6.84122	0.825976	0.709423
NK.cells	MSANTD2	-0.03	4.864755	-0.28618	0.775413	-6.48383	0.850347	0.748744
NK.cells	MLXIPL	-0.18146	0.355448	-0.28617	0.775418	-5.33692	0.910132	0.848782
NK.cells	E130311K1	-0.13919	0.244935	-0.28616	0.775423	-5.40262	0.911652	0.851391
NK.cells	RNF217	0.103369	2.071962	0.286023	0.77553	-5.79634	0.88686	0.809307
NK.cells	MAT2A	-0.02461	6.522913	-0.28594	0.77559	-6.75056	0.829444	0.715036
NK.cells	PCED1A	0.060763	2.508638	0.285783	0.775713	-5.9462	0.881041	0.799611
NK.cells	TRAPPC9	-0.02793	6.178368	-0.28565	0.775812	-6.72523	0.83374	0.721972
NK.cells	NFE2L3	0.078283	2.246069	0.285614	0.775842	-5.83148	0.884535	0.805472
NK.cells	1700020L2	0.11073	0.390371	0.285585	0.775864	-5.48193	0.909652	0.848096
NK.cells	PRDM2	0.028651	5.970241	0.285577	0.77587	-6.72205	0.836347	0.726167
NK.cells	MRPL9	0.02759	4.787977	0.285558	0.775884	-6.50202	0.851328	0.750467
NK.cells	MYOF	0.150964	2.65687	0.285417	0.775992	-5.48503	0.879075	0.796363
NK.cells	GM37065	-0.096	2.706239	-0.28535	0.776047	-5.80592	0.878422	0.795274
NK.cells	CDC42SE2	0.022974	7.179375	0.285295	0.776085	-6.89526	0.821325	0.70217
NK.cells	PKD1L3	0.115104	0.837305	0.285266	0.776107	-5.48787	0.903534	0.837683
NK.cells	DTNB	-0.03636	5.293687	-0.28523	0.776133	-6.57138	0.844885	0.740017
NK.cells	2810402E2	-0.07087	2.412627	-0.28517	0.776177	-5.75201	0.882317	0.801795

NK.cells	RTN3	-0.02615	8.060675	-0.28502	0.776294	-7.03187	0.810564	0.685199
NK.cells	CEP164	-0.04322	4.052145	-0.28499	0.776317	-6.21259	0.8608	0.766089
NK.cells	GM10184	0.115438	1.11236	0.284946	0.776352	-5.54918	0.899791	0.831359
NK.cells	TATDN1	0.044444	3.630234	0.284754	0.776498	-6.15775	0.866283	0.775138
NK.cells	MDM1	-0.05695	3.41542	-0.28464	0.776583	-6.13678	0.869089	0.779808
NK.cells	MDH1	-0.02441	8.120076	-0.28458	0.776632	-7.05826	0.809845	0.684097
NK.cells	LEPROTL1	0.033292	5.362087	0.284483	0.776706	-6.70102	0.844017	0.738716
NK.cells	ZBTB38	-0.0323	5.068147	-0.28428	0.776857	-6.5874	0.847752	0.744793
NK.cells	NAXE	0.032319	5.283838	0.284226	0.776901	-6.56261	0.84501	0.740334
NK.cells	CAMKMT	-0.03786	4.501622	-0.28418	0.776937	-6.4936	0.855001	0.756632
NK.cells	ARHGAP5	0.058503	4.809305	0.284123	0.776981	-6.2921	0.851056	0.750179
NK.cells	TOMM40L	-0.07128	1.733558	-0.2841	0.776996	-5.67299	0.891397	0.817204
NK.cells	DIPK2A	-0.03932	4.112992	-0.28408	0.777014	-6.31691	0.860013	0.764862
NK.cells	MSRA	-0.02817	6.629496	-0.28403	0.777053	-6.73396	0.828119	0.713111
NK.cells	TBCCD1	0.067004	2.589728	0.283871	0.777173	-5.87269	0.879965	0.797978
NK.cells	TAB2	-0.0218	6.780443	-0.2838	0.777225	-6.85987	0.826248	0.710139
NK.cells	MBP	0.031791	5.172136	0.283755	0.777261	-6.71861	0.846429	0.742659
NK.cells	ALDH3A2	0.043491	3.596521	0.283541	0.777425	-6.14634	0.866784	0.775955
NK.cells	GM16104	-0.2077	-0.60575	-0.28343	0.777509	-5.1474	0.923509	0.872093
NK.cells	MYLPF	0.099019	2.109786	0.283355	0.777567	-5.75124	0.886417	0.808741
NK.cells	UQCC1	0.042316	3.934429	0.28322	0.777671	-6.2283	0.862387	0.768735
NK.cells	TAF2	0.033662	4.171185	0.283122	0.777745	-6.31869	0.859321	0.763699
NK.cells	FRMD4B	0.05257	5.267906	0.28303	0.777815	-6.61582	0.845272	0.740764
NK.cells	TTYH3	0.036764	5.141527	0.283013	0.777829	-6.50082	0.846878	0.743375
NK.cells	OGFRL1	0.06054	5.082967	0.282884	0.777927	-6.11384	0.847623	0.74461
NK.cells	GM17745	-0.08594	0.962497	-0.28282	0.777974	-5.72693	0.901892	0.835052
NK.cells	PERP	-0.10159	1.228461	-0.28273	0.778046	-5.6758	0.89828	0.828916
NK.cells	DGUOK	0.038812	4.460054	0.282641	0.778112	-6.41503	0.855596	0.757654
NK.cells	ETOHD2	0.061099	2.615429	0.282381	0.778311	-5.85826	0.879687	0.797658
NK.cells	TM9SF4	-0.03877	4.769644	-0.28223	0.778426	-6.3783	0.851623	0.75129
NK.cells	MTMR10	0.069486	2.897646	0.282171	0.778472	-5.97375	0.875954	0.791489
NK.cells	S100A1	0.046501	4.086611	0.282107	0.778521	-6.26594	0.860415	0.765736
NK.cells	KLHL11	-0.0585	2.776613	-0.28186	0.778708	-6.00671	0.877553	0.794227
NK.cells	SUPT6	-0.0289	5.808893	-0.28181	0.778749	-6.68049	0.838434	0.72995
NK.cells	SAP18B	-0.06024	2.771863	-0.2818	0.778756	-5.96608	0.877616	0.794337
NK.cells	PFKM	0.098343	1.17862	0.281689	0.77884	-5.56576	0.898955	0.830338
NK.cells	NCAPG	-0.0703	3.22832	-0.2815	0.778988	-6.10758	0.871602	0.784407
NK.cells	ATF1	-0.0202	6.564036	-0.28137	0.779086	-6.77723	0.828991	0.714881
NK.cells	PHKA1	0.061869	3.411651	0.281325	0.779118	-5.9968	0.8692	0.78045
NK.cells	IMPDH2	0.031921	5.196107	0.281254	0.779173	-6.54981	0.846184	0.742641
NK.cells	PCNX	-0.03336	5.518161	-0.28111	0.77928	-6.5949	0.842101	0.736042
NK.cells	PPP1R7	-0.03137	4.409711	-0.2811	0.779289	-6.34767	0.856244	0.75911
NK.cells	TEX9	0.070663	1.820323	0.281097	0.779293	-5.90138	0.890295	0.815814
NK.cells	RDH5	-0.08758	2.368316	-0.28099	0.779372	-5.75322	0.882969	0.803518
NK.cells	DENND1C	0.039636	3.966917	0.280853	0.77948	-6.39117	0.861966	0.768595
NK.cells	4930414N(	0.063548	3.331114	0.280639	0.779643	-6.01884	0.870254	0.782392
NK.cells	MSL2	0.025435	6.010868	0.28058	0.779688	-6.71162	0.835897	0.726179

NK.cells	9130019O2	0.152803	0.355077	0.280567	0.779698	-5.32971	0.910201	0.849903
NK.cells	GM12992	-0.09676	2.041763	-0.28039	0.779833	-5.72356	0.887327	0.810999
NK.cells	GOLIM4	-0.04713	5.140893	-0.28038	0.779843	-6.4476	0.846886	0.74399
NK.cells	MRPL30	0.022286	6.172662	0.280366	0.779852	-6.75251	0.833871	0.722923
NK.cells	SNUPN	0.052357	2.593487	0.280226	0.779959	-5.92508	0.879978	0.798684
NK.cells	CCNJ	0.110181	2.015982	0.280199	0.779979	-5.55213	0.887672	0.811614
NK.cells	MRPL4	0.028649	5.176328	0.280064	0.780082	-6.59222	0.846435	0.743323
NK.cells	NBR1	0.026842	5.455137	0.280064	0.780083	-6.59912	0.842898	0.737576
NK.cells	PYGL	-0.05253	5.16283	-0.27986	0.780236	-6.4333	0.846607	0.743665
NK.cells	GM31508	0.129208	0.763062	0.279707	0.780355	-5.41299	0.904612	0.840554
NK.cells	CENPX	-0.02742	6.347296	-0.27963	0.780414	-6.76517	0.831689	0.719606
NK.cells	SMPD4	0.055457	3.209019	0.279616	0.780425	-6.06484	0.871856	0.785298
NK.cells	ZMYM5	-0.02718	5.521459	-0.27953	0.780487	-6.61261	0.842059	0.736343
NK.cells	PLEKHM1	0.036195	4.771883	0.279499	0.780515	-6.35823	0.851595	0.751877
NK.cells	CORO7	0.029041	6.215775	0.279385	0.780602	-6.63996	0.833331	0.722308
NK.cells	GM17160	0.15982	0.089084	0.279304	0.780664	-5.17892	0.913865	0.85652
NK.cells	DDI2	0.02936	5.943537	0.279228	0.780722	-6.64548	0.836742	0.727813
NK.cells	RNF135	0.078632	2.08421	0.279174	0.780763	-5.72458	0.886759	0.810336
NK.cells	GM49521	-0.10268	0.473447	-0.27875	0.781087	-5.53463	0.908773	0.847592
NK.cells	PLCL1	0.054871	5.328139	0.278615	0.781191	-6.51386	0.844691	0.740552
NK.cells	GM9733	-0.18824	0.76406	-0.27858	0.781214	-5.34462	0.904795	0.840801
NK.cells	GPC5	0.151923	0.598275	0.278563	0.781231	-5.4164	0.907062	0.844682
NK.cells	NSG2	0.079809	0.587521	0.278408	0.78135	-5.75619	0.907209	0.844955
NK.cells	INTS10	-0.04782	3.410048	-0.27825	0.781467	-6.1065	0.86941	0.781208
NK.cells	CIZ1	-0.03728	3.940619	-0.27814	0.781551	-6.2997	0.862494	0.769776
NK.cells	PAWR	0.113688	0.662659	0.278076	0.781603	-5.48639	0.906181	0.843254
NK.cells	ASNA1	0.032915	4.877847	0.278017	0.781648	-6.46008	0.850425	0.749975
NK.cells	UXT	0.036972	4.452478	0.277947	0.781702	-6.34863	0.85588	0.758926
NK.cells	FPGT	0.109674	1.345954	0.277861	0.781767	-5.59966	0.896883	0.827426
NK.cells	VPS41	-0.02973	5.101022	-0.27783	0.781795	-6.5159	0.847578	0.745347
NK.cells	EPB41L5	0.060535	3.379051	0.277316	0.782185	-6.06478	0.869987	0.782081
NK.cells	SAV1	0.035867	4.735258	0.277299	0.782197	-6.33661	0.852417	0.753113
NK.cells	STRADA	-0.03774	4.58715	-0.27714	0.782323	-6.39397	0.854317	0.756234
NK.cells	TFB2M	-0.03561	3.953928	-0.27707	0.782375	-6.26498	0.862492	0.769681
NK.cells	SIGIRR	0.050706	2.175284	0.277062	0.782379	-5.99983	0.885909	0.808727
NK.cells	YRDC	-0.03235	5.035051	-0.27706	0.782384	-6.52414	0.848585	0.746863
NK.cells	RBM17	0.029035	5.789508	0.276887	0.782513	-6.60072	0.839026	0.731368
NK.cells	TAP1	0.061642	5.446269	0.276873	0.782524	-6.66784	0.84336	0.73839
NK.cells	STEAP3	0.08688	1.980258	0.276591	0.782739	-5.62968	0.888646	0.813189
NK.cells	ARHGAP27	-0.09616	2.032169	-0.27654	0.782781	-5.48003	0.887951	0.812016
NK.cells	ORAI2	0.032503	5.87495	0.276189	0.783048	-6.60854	0.838142	0.729677
NK.cells	GM37305	0.106855	1.078554	0.276186	0.78305	-5.46247	0.900893	0.833846
NK.cells	FKBP8	-0.02807	6.067485	-0.276	0.783191	-6.71254	0.835724	0.725811
NK.cells	IPO9	0.030569	4.771136	0.275916	0.783256	-6.41853	0.852152	0.752492
NK.cells	SYNJ1	0.028062	6.731012	0.275679	0.783438	-6.77402	0.827451	0.712556
NK.cells	GPR157	-0.07623	2.137362	-0.27564	0.783469	-5.71528	0.886618	0.809737
NK.cells	GM15614	-0.0642	2.981808	-0.27558	0.783515	-6.05495	0.875407	0.79094

NK.cells	TRNT1	-0.03145	4.772449	-0.2755	0.783576	-6.44514	0.852135	0.752489
NK.cells	SELPLG	-0.02707	6.845492	-0.2755	0.783576	-6.91355	0.826032	0.710289
NK.cells	MYL6B	0.11472	0.862198	0.275407	0.783646	-5.40897	0.90384	0.838949
NK.cells	LYPLA2	-0.02981	4.945046	-0.27531	0.783718	-6.50676	0.849928	0.748882
NK.cells	BBOF1	0.094677	1.302277	0.275235	0.783778	-5.58282	0.897856	0.828754
NK.cells	ZCCHC10	0.038249	4.048466	0.275232	0.783781	-6.32986	0.861462	0.767807
NK.cells	LRIG3	-0.12469	0.14201	-0.27503	0.783936	-5.46022	0.913759	0.855972
NK.cells	RBPMS	0.036327	5.842983	0.274937	0.784006	-6.75283	0.838578	0.73049
NK.cells	GM43914	0.142065	0.384486	0.274908	0.784028	-5.28949	0.910419	0.850266
NK.cells	D130062J1	0.081218	1.642184	0.27461	0.784256	-5.72757	0.89332	0.821108
NK.cells	4833420G1	-0.03386	5.494494	-0.27459	0.784269	-6.63176	0.842995	0.737645
NK.cells	KBTBD4	-0.05479	2.78551	-0.27457	0.784291	-5.98851	0.878054	0.795412
NK.cells	PAQR4	-0.10068	1.484926	-0.27441	0.784407	-5.74776	0.895442	0.824726
NK.cells	DRG1	-0.02152	6.40915	-0.2744	0.784414	-6.74424	0.831505	0.719116
NK.cells	UBE2H	-0.02367	8.574917	-0.27432	0.784476	-7.16582	0.804982	0.677048
NK.cells	CPA6	-0.1386	-0.07229	-0.27422	0.784558	-5.35198	0.916753	0.861175
NK.cells	RGS16	0.106224	1.351563	0.273974	0.784744	-5.65759	0.897363	0.827807
NK.cells	IRGQ	0.079613	2.194038	0.273879	0.784817	-5.77291	0.886033	0.808693
NK.cells	ALKBH2	-0.05812	2.187068	-0.27381	0.784867	-5.82442	0.886126	0.808853
NK.cells	GM17529	-0.12113	0.365187	-0.27361	0.785022	-5.44563	0.910833	0.850907
NK.cells	SP100	0.032825	7.43248	0.273471	0.785129	-6.96604	0.818969	0.699053
NK.cells	PAKAP.1	-0.04945	4.53559	-0.27339	0.785193	-6.3532	0.855348	0.757784
NK.cells	ATP9A	0.117681	0.837325	0.273283	0.785273	-5.50401	0.904363	0.839937
NK.cells	ADGRF5	0.118447	2.598167	0.273001	0.785489	-5.65371	0.88066	0.79989
NK.cells	SYBU	-0.15415	0.330557	-0.27294	0.785539	-5.39942	0.911309	0.85192
NK.cells	TYK2	-0.03302	3.992109	-0.27275	0.785682	-6.38386	0.862368	0.769512
NK.cells	AGPAT1	0.050304	3.479015	0.272538	0.785844	-6.11636	0.869053	0.7806
NK.cells	MOSPD1	-0.0492	4.817896	-0.2725	0.785874	-6.25713	0.851726	0.752066
NK.cells	SETD2	-0.02433	6.922415	-0.27202	0.786239	-6.83423	0.825249	0.709323
NK.cells	DHRS13	-0.11453	0.959658	-0.272	0.786258	-5.56657	0.902694	0.837334
NK.cells	ELOB	0.020628	8.913307	0.271864	0.786361	-7.1852	0.801037	0.671094
NK.cells	TLR6	0.134136	1.070509	0.271833	0.786384	-5.41349	0.901185	0.834763
NK.cells	GM19557	0.078229	-0.95554	0.27182	0.786394	-5.57924	0.92918	0.883045
NK.cells	HIST1H2AI	0.09357	2.588783	0.271754	0.786445	-6.04127	0.880785	0.800263
NK.cells	A530041M	0.06964	2.069945	0.27164	0.786532	-5.74079	0.8877	0.811893
NK.cells	FGD5	-0.12133	1.663977	-0.27162	0.786544	-5.56746	0.893152	0.821109
NK.cells	PCNX3	0.046837	3.30596	0.271624	0.786545	-6.08728	0.871321	0.784454
NK.cells	COMMD2	-0.02973	4.927522	-0.27133	0.786766	-6.46195	0.850324	0.749862
NK.cells	VPS29	0.021606	6.521761	0.271311	0.786784	-6.80732	0.830219	0.717307
NK.cells	HEMGN	0.152603	0.35427	0.271212	0.78686	-5.45744	0.910983	0.851564
NK.cells	AGRP	-0.10011	1.935634	-0.27115	0.786906	-5.6981	0.8895	0.81496
NK.cells	4930513N1	-0.11946	0.475363	-0.27115	0.786911	-5.40214	0.909318	0.848706
NK.cells	POLR1D	0.020077	6.995878	0.270968	0.787048	-6.88526	0.824341	0.707902
NK.cells	SCAF8	0.021372	6.711338	0.270959	0.787054	-6.83768	0.827863	0.713532
NK.cells	PPA2	0.036889	4.580694	0.270926	0.787079	-6.38945	0.854768	0.757137
NK.cells	EFCAB8	-0.09786	1.374064	-0.27085	0.787136	-5.53104	0.897067	0.827781
NK.cells	4930599N2	-0.08326	1.733523	-0.27082	0.787158	-5.72931	0.892215	0.819552

NK.cells	PPP2R2A	-0.01801	7.180276	-0.27071	0.787248	-6.91182	0.822067	0.704277
NK.cells	HNRNPA1	-0.02561	7.807246	-0.27069	0.787261	-6.9655	0.814389	0.692093
NK.cells	TMEM267	-0.08582	1.734822	-0.27067	0.787279	-5.66883	0.892198	0.819522
NK.cells	D130040H:	0.063585	2.397794	0.270657	0.787285	-5.91186	0.883324	0.804553
NK.cells	ZFP397	0.036554	3.944609	0.270626	0.787309	-6.2219	0.862985	0.770662
NK.cells	KRAS	0.021732	7.278938	0.270535	0.787379	-6.88244	0.820854	0.702382
NK.cells	GM43388	0.139617	0.386691	0.270499	0.787407	-5.41441	0.910537	0.850852
NK.cells	RB1	0.026177	7.240705	0.270396	0.787486	-6.90224	0.821324	0.703152
NK.cells	PDAP1	0.02313	6.715812	0.270268	0.787584	-6.81475	0.827807	0.713506
NK.cells	FAR1OS	-0.07696	2.788806	-0.27021	0.787631	-5.94471	0.878134	0.79593
NK.cells	CYTH4	-0.02579	5.757599	-0.27009	0.787721	-6.73971	0.83979	0.732849
NK.cells	COPB2	-0.02398	5.438302	-0.26987	0.787886	-6.59859	0.843825	0.739444
NK.cells	NME3	0.145147	0.405363	0.269744	0.787986	-5.34914	0.91028	0.850555
NK.cells	E230014E1	-0.14563	-1.38289	-0.26962	0.788082	-5.15495	0.934743	0.893803
NK.cells	SEMA6A	0.127885	2.821738	0.269533	0.788147	-5.78076	0.877699	0.795306
NK.cells	ELK4	0.027338	5.882489	0.26943	0.788226	-6.64824	0.838217	0.730358
NK.cells	TAF1D	-0.02421	6.027224	-0.26939	0.788261	-6.69846	0.836399	0.727421
NK.cells	RNF126	0.032201	4.767243	0.269056	0.788513	-6.46612	0.852375	0.75339
NK.cells	ICE2	0.065982	2.160721	0.268989	0.788564	-5.75081	0.886486	0.810063
NK.cells	ALDH9A1	-0.02939	5.005152	-0.26892	0.788618	-6.48585	0.849333	0.748417
NK.cells	MKLN1OS	-0.14449	0.018205	-0.2689	0.788632	-5.29708	0.915618	0.859745
NK.cells	SUCLG2	0.036503	4.835404	0.268873	0.788654	-6.4563	0.851502	0.751962
NK.cells	CCDC192	0.16503	-0.69514	0.268789	0.788719	-5.23898	0.92554	0.876927
NK.cells	ART3	-0.11733	0.944132	-0.26877	0.788734	-5.43952	0.902906	0.837923
NK.cells	ENTHD1	-0.11468	0.023444	-0.26861	0.788854	-5.58374	0.915546	0.85962
NK.cells	TBC1D22A	0.027172	5.871527	0.268551	0.788901	-6.63556	0.838355	0.730581
NK.cells	DYNC2H1	0.046353	3.842041	0.268331	0.789069	-6.18811	0.864318	0.773045
NK.cells	PIGP	0.036879	4.420664	0.268284	0.789106	-6.36294	0.856828	0.760694
NK.cells	ZFP169	0.060944	2.782441	0.268227	0.789149	-6.09082	0.878218	0.796176
NK.cells	PFN1	0.019286	10.31807	0.268084	0.789259	-7.4086	0.784433	0.645578
NK.cells	FXYD2	0.157362	-0.41192	0.268044	0.78929	-5.28323	0.921588	0.87011
NK.cells	RABGEF1	0.034674	6.01057	0.267979	0.789339	-6.72671	0.836608	0.727801
NK.cells	NDUFS2	-0.02746	6.235293	-0.26792	0.789384	-6.73011	0.833793	0.723264
NK.cells	1110035H:	-0.1104	1.216236	-0.26791	0.789393	-5.47409	0.899206	0.831664
NK.cells	DENND3	0.050443	3.548798	0.267842	0.789445	-6.18246	0.868141	0.779429
NK.cells	SH3D21	0.07759	1.99564	0.267804	0.789474	-5.81285	0.888695	0.813842
NK.cells	D16ERTD4	-0.03063	4.914897	-0.26765	0.789593	-6.66495	0.850489	0.750376
NK.cells	HORMAD2	0.129242	1.357831	0.267612	0.789621	-5.58674	0.89729	0.828443
NK.cells	NEPRO	0.059236	2.709524	0.267362	0.789813	-5.93354	0.879321	0.797994
NK.cells	ZCCHC17	0.027021	5.162664	0.267269	0.789885	-6.53465	0.847458	0.745347
NK.cells	ERCC2	-0.06715	1.983619	-0.26698	0.790105	-5.77939	0.889004	0.814365
NK.cells	ADAMTS6	-0.03694	5.940983	-0.26688	0.790182	-6.71981	0.837621	0.729452
NK.cells	TRUB2	-0.03467	4.526877	-0.26688	0.790184	-6.38728	0.855602	0.758742
NK.cells	CAMK1D	0.028509	8.628354	0.266836	0.790217	-7.12397	0.804587	0.676878
NK.cells	SLC43A3	-0.05589	3.813488	-0.26672	0.790305	-6.06242	0.864833	0.773968
NK.cells	FKRP	0.065302	2.332289	0.266645	0.790363	-5.79826	0.884343	0.806546
NK.cells	RSU1	0.025407	5.746299	0.266592	0.790404	-6.63918	0.840072	0.73344

NK.cells	MTG1	0.072534	2.186733	0.266067	0.790807	-5.83059	0.886285	0.809965
NK.cells	TMEM91	0.076585	2.459733	0.265978	0.790875	-5.78443	0.882646	0.803846
NK.cells	FKTN	0.095734	1.595478	0.26594	0.790904	-5.57156	0.894223	0.823401
NK.cells	MUC13	-0.14891	1.034673	-0.26592	0.790921	-5.34967	0.901822	0.836334
NK.cells	LTB	0.045986	3.446775	0.26589	0.790943	-6.39734	0.869619	0.782077
NK.cells	NFS1	0.032438	4.533914	0.265863	0.790964	-6.41599	0.855512	0.758768
NK.cells	PEX26	-0.10357	1.217016	-0.26583	0.790989	-5.56719	0.899344	0.832107
NK.cells	TRP53INP1	0.038966	5.187354	0.265813	0.791002	-6.51848	0.847152	0.74509
NK.cells	EEF1AKNM	-0.05989	2.318289	-0.26576	0.791043	-5.88583	0.88453	0.807022
NK.cells	MRM1	-0.06372	2.067052	-0.26555	0.791203	-5.80529	0.887902	0.812737
NK.cells	A930007I1	-0.05006	4.385631	-0.26542	0.791301	-6.48877	0.857436	0.761994
NK.cells	ZFP433	0.123947	0.355661	0.265376	0.791337	-5.39748	0.91113	0.852358
NK.cells	ARSK	0.067167	2.705302	0.265058	0.791582	-5.91309	0.879401	0.798571
NK.cells	ABHD17B	-0.02263	6.542955	-0.26502	0.791611	-6.73742	0.830107	0.717667
NK.cells	MTPN	0.019515	6.898113	0.26495	0.791664	-6.81479	0.8257	0.710607
NK.cells	KRT83	-0.09871	-0.26141	-0.26486	0.791736	-5.63076	0.919662	0.867186
NK.cells	SNRNP25	-0.04175	3.939991	-0.2648	0.791776	-6.25787	0.863203	0.771603
NK.cells	ATG3	-0.02127	6.261249	-0.26479	0.791785	-6.73511	0.833621	0.723318
NK.cells	EIF2S2	-0.02051	7.925804	-0.26478	0.791797	-7.01022	0.813095	0.690567
NK.cells	TRA2A	-0.02148	7.075692	-0.26471	0.791852	-6.86282	0.823507	0.707103
NK.cells	ELOVL7	-0.12213	-0.19718	-0.26462	0.791919	-5.42281	0.918771	0.865663
NK.cells	WDTC1	0.042079	3.851337	0.264122	0.7923	-6.24632	0.864693	0.773671
NK.cells	PAXBP1	0.031778	5.312121	0.263943	0.792438	-6.59698	0.845967	0.742852
NK.cells	STAT6	0.028984	5.031546	0.263862	0.7925	-6.5296	0.84954	0.748707
NK.cells	RNF168	-0.04148	4.161361	-0.26373	0.792601	-6.29384	0.860727	0.767113
NK.cells	ZFP273	-0.11269	0.440289	-0.26368	0.792641	-5.38243	0.910383	0.850755
NK.cells	1810055G(	0.066056	2.268587	0.263057	0.793119	-5.74049	0.885942	0.808764
NK.cells	PNPLA2	0.03411	5.509886	0.262954	0.793198	-6.58905	0.843772	0.739004
NK.cells	PNPLA7	0.031511	6.039516	0.262931	0.793216	-6.70228	0.837092	0.728187
NK.cells	ZSWIM8	-0.02894	4.703109	-0.26292	0.793224	-6.44666	0.854061	0.75579
NK.cells	ACP2	-0.05877	3.54465	-0.26264	0.793436	-6.04044	0.869163	0.780593
NK.cells	AMPD2	0.069877	2.339378	0.262362	0.793653	-5.76402	0.885087	0.80722
NK.cells	DDX11	-0.06088	2.934033	-0.26236	0.793658	-5.96632	0.877192	0.793978
NK.cells	SLC45A3	0.140198	1.111286	0.262333	0.793675	-5.37476	0.901635	0.835253
NK.cells	TGIF1	-0.03471	6.736671	-0.26227	0.793725	-6.82976	0.828471	0.714239
NK.cells	POLQ	-0.05662	3.166551	-0.26196	0.793963	-6.02175	0.874125	0.789011
NK.cells	ZDHHC12	-0.11063	1.360226	-0.26195	0.793969	-5.49752	0.898254	0.829656
NK.cells	SMIM19	0.029569	4.75168	0.261883	0.794021	-6.46863	0.853525	0.75498
NK.cells	FAM122B	0.067504	2.015817	0.261719	0.794147	-5.73122	0.889415	0.814735
NK.cells	ATP5H	0.017946	7.946365	0.261689	0.79417	-7.04477	0.813602	0.690774
NK.cells	AP3B1	0.018862	7.329007	0.261666	0.794188	-6.94547	0.821153	0.702755
NK.cells	4930403D(	-0.08763	1.041064	-0.26153	0.794295	-5.6483	0.902591	0.83714
NK.cells	GM7072	0.041681	3.842908	0.261512	0.794306	-6.26238	0.86527	0.774387
NK.cells	GM38190	0.056939	1.823044	0.261505	0.794312	-5.9498	0.892005	0.819144
NK.cells	ACO2	0.021943	6.116578	0.261379	0.794408	-6.69529	0.836235	0.726891
NK.cells	8-Sep	0.056166	1.954462	0.26101	0.794692	-5.96019	0.890312	0.816254
NK.cells	COQ3	0.035808	3.500438	0.260906	0.794772	-6.13916	0.869814	0.781898

NK.cells	DNAJB11	-0.02337	6.073023	-0.2609	0.794774	-6.70068	0.836826	0.727861
NK.cells	SPPL2B	0.068375	2.17345	0.260878	0.794793	-5.8476	0.887378	0.811301
NK.cells	4930430F0	0.086529	1.035161	0.260835	0.794826	-5.51116	0.902746	0.83738
NK.cells	FBXO45	-0.04085	3.654199	-0.26069	0.794938	-6.17659	0.867803	0.778583
NK.cells	RETREG3	-0.02604	5.527566	-0.26045	0.795122	-6.64351	0.843704	0.739085
NK.cells	PPP2CB	0.02341	5.60171	0.260268	0.795262	-6.61679	0.842765	0.737585
NK.cells	9830166K0	-0.13905	-1.08122	-0.26019	0.795325	-5.13743	0.931926	0.888195
NK.cells	E330009J0	0.057181	2.552592	0.260174	0.795335	-6.11787	0.882321	0.80294
NK.cells	INSIG2	-0.0376	4.405764	-0.26009	0.7954	-6.32128	0.858046	0.762604
NK.cells	RYR1	-0.11177	1.831874	-0.26005	0.795431	-5.53895	0.89196	0.819216
NK.cells	TNK2	0.046687	3.209858	0.260031	0.795445	-6.13353	0.873627	0.788413
NK.cells	FAF1	-0.02117	6.739255	-0.26001	0.795457	-6.84216	0.828508	0.71464
NK.cells	NINL	0.115143	0.999292	0.259857	0.795578	-5.42231	0.903291	0.838399
NK.cells	CFAP36	-0.03344	4.102137	-0.25962	0.795759	-6.31489	0.862051	0.769153
NK.cells	LIMCH1	0.152574	0.57747	0.259495	0.795856	-5.29856	0.909087	0.848358
NK.cells	4931406G(	0.147085	0.347467	0.259352	0.795967	-5.42487	0.912249	0.853797
NK.cells	MRPL15	0.027063	5.337746	0.259232	0.796059	-6.57252	0.846188	0.74315
NK.cells	SIPA1L2	-0.0366	5.103799	-0.25916	0.796117	-6.51317	0.849167	0.748013
NK.cells	CCNL2	0.0195	6.219089	0.259115	0.796149	-6.74968	0.835069	0.725146
NK.cells	GM50163	-0.11876	0.458993	-0.25896	0.796265	-5.37928	0.910714	0.851193
NK.cells	PEX11B	0.042179	3.446594	0.258923	0.796297	-6.15858	0.870597	0.783351
NK.cells	NPAS2	-0.1202	0.089778	-0.25892	0.796302	-5.45648	0.915807	0.859962
NK.cells	RAB10	0.02282	7.670572	0.258766	0.796418	-6.99272	0.817107	0.696453
NK.cells	CD48	-0.02637	6.795574	-0.25872	0.796455	-6.92668	0.827883	0.713625
NK.cells	SDHD	0.030206	5.858408	0.258675	0.796487	-6.63827	0.8396	0.732506
NK.cells	CLEC14A	0.10339	2.196348	0.258612	0.796536	-5.62854	0.887151	0.811109
NK.cells	SPTLC2	0.020444	6.245992	0.258238	0.796823	-6.75794	0.834908	0.724724
NK.cells	KLF16	-0.04811	3.164065	-0.25821	0.796848	-6.0234	0.874493	0.789643
NK.cells	KLF6	0.03296	7.581942	0.257971	0.797028	-7.00298	0.818412	0.698327
NK.cells	EIF5A2	0.112096	0.577892	0.257675	0.797257	-5.48442	0.909326	0.848662
NK.cells	DOLPP1	-0.05745	2.514642	-0.25761	0.797305	-5.94413	0.883143	0.804196
NK.cells	IRF3	0.040424	3.786081	0.257602	0.797312	-6.19517	0.866394	0.776245
NK.cells	2900060B1	-0.06487	2.614995	-0.25755	0.797352	-5.78686	0.881809	0.801955
NK.cells	DPY19L1	-0.03209	4.584338	-0.25745	0.797429	-6.52592	0.856053	0.759188
NK.cells	5730455P1	-0.04876	2.914853	-0.25734	0.797517	-5.96398	0.877834	0.795294
NK.cells	PPARD	0.029496	5.613407	0.257315	0.797534	-6.64029	0.842921	0.737747
NK.cells	1110065P2	-0.03819	3.514697	-0.2573	0.797543	-6.17254	0.86994	0.78213
NK.cells	PIANP	-0.12429	-0.06426	-0.25703	0.79775	-5.29871	0.918239	0.863973
NK.cells	CHST14	0.114023	0.872451	0.257026	0.797756	-5.46303	0.905342	0.841794
NK.cells	PACSIN1	-0.05121	2.953403	-0.25696	0.797803	-6.13349	0.877373	0.794492
NK.cells	MED28	0.02283	6.023126	0.256596	0.798087	-6.69354	0.837834	0.7295
NK.cells	TCP11L1	0.069916	2.689841	0.256407	0.798233	-5.78017	0.880899	0.800477
NK.cells	HMG20B	0.026406	5.119833	0.256253	0.79835	-6.47298	0.849273	0.748163
NK.cells	GM16754	0.126272	0.04601	0.256041	0.798514	-5.36201	0.916747	0.861605
NK.cells	SMIM8	0.033723	4.587829	0.255965	0.798573	-6.39852	0.85609	0.759417
NK.cells	CAML	-0.03002	4.427078	-0.25593	0.798599	-6.37966	0.858162	0.762834
NK.cells	SNHG12	-0.04776	3.189103	-0.25592	0.79861	-6.09924	0.874299	0.789575

NK.cells	SNX7	-0.10436	1.212304	-0.25591	0.798612	-5.48221	0.900746	0.834192
NK.cells	DHX33	-0.0413	3.32737	-0.2558	0.798703	-6.1547	0.87248	0.786566
NK.cells	IRF9	-0.05089	4.515749	-0.25577	0.798723	-6.38885	0.857018	0.760984
NK.cells	2610002M	0.03773	4.208089	0.255477	0.798948	-6.32251	0.860992	0.767627
NK.cells	MTMR12	-0.02451	5.334196	-0.2553	0.799084	-6.68391	0.846543	0.743991
NK.cells	MCTS2	0.100517	1.612847	0.25509	0.799246	-5.60124	0.895319	0.825166
NK.cells	TRAJ18	0.0781	-1.3552	0.25496	0.799346	-5.50539	0.935926	0.89607
NK.cells	STXBP5	-0.0342	5.383452	-0.25481	0.799458	-6.58921	0.845917	0.743039
NK.cells	ICAM2	-0.07074	4.282374	-0.25474	0.799519	-6.24476	0.860031	0.766168
NK.cells	RBMX2	-0.05003	3.368821	-0.2547	0.799545	-6.0503	0.871936	0.785897
NK.cells	PEPD	-0.02927	5.308275	-0.25461	0.799613	-6.56192	0.846872	0.744596
NK.cells	TSEN34	0.041126	4.195681	0.254466	0.799726	-6.23371	0.861153	0.768019
NK.cells	ZDHHC9	0.042918	4.094804	0.254456	0.799734	-6.20601	0.862461	0.770178
NK.cells	DLGAP1	-0.10297	1.398192	-0.25417	0.799955	-5.52937	0.898223	0.830253
NK.cells	NRP2	0.091581	3.300945	0.254045	0.800051	-5.90353	0.872827	0.787498
NK.cells	BCL7C	0.024071	5.355918	0.254032	0.800061	-6.57409	0.846267	0.743717
NK.cells	METTL16	0.02982	4.533537	0.254007	0.80008	-6.40586	0.856789	0.76094
NK.cells	PLCG1	0.045065	3.409844	0.253977	0.800103	-6.10528	0.871397	0.785129
NK.cells	MFSD4B4	0.125198	-0.0204	0.253855	0.800197	-5.35279	0.917667	0.863708
NK.cells	MEGF8	0.12336	0.288303	0.253839	0.80021	-5.29168	0.913398	0.856339
NK.cells	PSPC1	-0.02861	5.702286	-0.25379	0.800246	-6.64492	0.841877	0.736625
NK.cells	CELA1	0.069464	2.24977	0.253774	0.800259	-5.92756	0.886761	0.810915
NK.cells	PMPCA	0.039402	4.024403	0.253764	0.800267	-6.22445	0.863375	0.771849
NK.cells	CCNH	0.026107	5.363256	0.253713	0.800306	-6.55966	0.846173	0.743611
NK.cells	MIIP	-0.04903	3.057652	-0.25364	0.800362	-5.99732	0.876031	0.792899
NK.cells	MRPS34	0.027883	4.826949	0.253481	0.800485	-6.49013	0.853019	0.754816
NK.cells	DECR2	0.059985	2.741577	0.253348	0.800587	-5.93325	0.880212	0.799984
NK.cells	MED15	0.025014	5.758733	0.253329	0.800602	-6.69296	0.841164	0.735547
NK.cells	SRGN	0.021629	9.652655	0.253214	0.800691	-7.3228	0.793559	0.660011
NK.cells	ACYP2	0.045867	3.398712	0.253213	0.800692	-6.17385	0.871543	0.785499
NK.cells	NDUFB4	-0.02318	6.598671	-0.25319	0.800709	-6.78065	0.830633	0.718553
NK.cells	GM12743	0.066621	2.382516	0.253179	0.800718	-5.84022	0.884988	0.808022
NK.cells	HACD3	0.024475	4.907611	0.253108	0.800772	-6.58584	0.851985	0.753214
NK.cells	COL11A2	-0.12554	0.72136	-0.2529	0.800931	-5.41335	0.907445	0.846297
NK.cells	PCNT	-0.02782	5.233516	-0.25288	0.800945	-6.60703	0.847824	0.746477
NK.cells	ZFP644	-0.02196	6.331418	-0.25281	0.801002	-6.76662	0.833968	0.724014
NK.cells	GM3435	0.131366	0.205181	0.252552	0.8012	-5.37164	0.914546	0.858594
NK.cells	H19	0.078563	6.408472	0.252444	0.801284	-6.7895	0.833005	0.722565
NK.cells	KLF13	-0.02037	8.183848	-0.25237	0.801344	-7.08098	0.811154	0.687727
NK.cells	SPOCK2	0.091334	0.349422	0.252337	0.801366	-5.55609	0.912556	0.855223
NK.cells	BCL3	0.037519	5.236561	0.252332	0.80137	-6.52265	0.847785	0.746532
NK.cells	GM16279	0.108004	1.054801	0.252319	0.80138	-5.51452	0.902889	0.838632
NK.cells	NEGR1	0.169077	-0.13284	0.252102	0.801547	-5.26147	0.919227	0.866752
NK.cells	ITIH5	-0.05973	1.943297	-0.25206	0.801578	-6.00212	0.890868	0.818181
NK.cells	DDX6	-0.01968	8.314732	-0.25202	0.801609	-7.05185	0.809568	0.685233
NK.cells	CCNG1	0.028121	4.873028	0.251938	0.801673	-6.5152	0.852428	0.754168
NK.cells	PPP4R3B	0.018309	6.842276	0.251854	0.801739	-6.83589	0.827606	0.713934

NK.cells	POPDC3	-0.15817	0.894439	-0.25176	0.801813	-5.33678	0.905078	0.842441
NK.cells	TRIP4	0.034712	4.852518	0.251688	0.801867	-6.55822	0.852691	0.754631
NK.cells	RCE1	0.03665	3.757774	0.251419	0.802074	-6.25753	0.866888	0.778004
NK.cells	FARSA	0.02536	4.90554	0.251326	0.802145	-6.51702	0.852054	0.753541
NK.cells	KCTD21	-0.14731	0.182578	-0.25124	0.802214	-5.29617	0.914903	0.859305
NK.cells	RAB10OS	-0.03291	4.429445	-0.25116	0.802273	-6.38907	0.858173	0.763595
NK.cells	ZSWIM1	-0.10452	1.4089	-0.2511	0.802316	-5.55216	0.898122	0.830535
NK.cells	GNPAT	0.034341	4.15461	0.25107	0.802342	-6.30628	0.861728	0.769465
NK.cells	ANKRD10	-0.02448	5.371393	-0.25098	0.80241	-6.5886	0.846112	0.743837
NK.cells	PKN2	0.022853	6.774243	0.250818	0.802537	-6.84358	0.82852	0.715376
NK.cells	DHCR7	0.063332	2.184949	0.250584	0.802717	-5.72512	0.887703	0.812845
NK.cells	ITGA6	-0.04927	4.818835	-0.25052	0.802765	-6.48363	0.853194	0.755401
NK.cells	GM16150	-0.12291	0.174831	-0.25052	0.802769	-5.33339	0.915042	0.859534
NK.cells	ABAT	-0.12014	1.196429	-0.25048	0.802798	-5.48313	0.901038	0.83549
NK.cells	COMMD4	-0.0272	5.326168	-0.25022	0.802998	-6.57666	0.846802	0.744903
NK.cells	ARHGAP9	-0.02708	5.14536	-0.25015	0.803048	-6.66958	0.849105	0.748662
NK.cells	VPS4A	0.031661	4.241466	0.250049	0.803129	-6.32428	0.86072	0.76774
NK.cells	LAS1L	-0.03271	4.172574	-0.25	0.803168	-6.32676	0.861613	0.769213
NK.cells	VPS9D1	0.045987	3.05216	0.249574	0.803496	-6.09867	0.87638	0.793741
NK.cells	PTPN23	-0.0478	3.647447	-0.24952	0.803539	-6.06479	0.86856	0.780701
NK.cells	CLTA	0.017392	8.442682	0.249496	0.803556	-7.06426	0.808277	0.683147
NK.cells	VIM	0.037232	8.109275	0.249437	0.803601	-7.08804	0.812315	0.689514
NK.cells	MID2	-0.1518	0.27499	-0.2494	0.803632	-5.22186	0.91387	0.857437
NK.cells	WASHC5	0.037692	4.472587	0.249122	0.803844	-6.31086	0.857858	0.763071
NK.cells	CCDC137	-0.04956	2.673176	-0.24898	0.803952	-5.92569	0.881411	0.802283
NK.cells	UGCG	-0.02478	6.436724	-0.24896	0.80397	-6.94955	0.832928	0.722493
NK.cells	TLCD1	0.067483	1.624021	0.248955	0.803973	-5.72482	0.895464	0.826035
NK.cells	SNX14	0.031662	4.500746	0.248745	0.804135	-6.47897	0.857495	0.762528
NK.cells	ENTPD1	0.034095	6.9129	0.248542	0.804291	-6.84323	0.827005	0.713032
NK.cells	EFHD2	-0.02177	6.92246	-0.2485	0.804324	-7.01234	0.826886	0.712843
NK.cells	NEURL3	-0.0314	4.301516	-0.24845	0.804359	-6.60579	0.860068	0.766801
NK.cells	CCNJL	0.082944	1.734552	0.248263	0.804507	-5.65554	0.893972	0.823567
NK.cells	IPMK	-0.02727	5.867681	-0.24813	0.804611	-6.64212	0.840068	0.734138
NK.cells	EIPR1	-0.02552	4.924643	-0.24796	0.804737	-6.54081	0.852049	0.753719
NK.cells	ARMCX1	-0.14181	0.550831	-0.24794	0.804754	-5.30891	0.910086	0.851218
NK.cells	PSMD3	0.030739	4.642778	0.247846	0.804828	-6.39829	0.855666	0.759684
NK.cells	TMEM14A	0.10753	0.419553	0.24782	0.804848	-5.47456	0.911891	0.85437
NK.cells	NMNAT1	-0.10863	1.326233	-0.24755	0.80506	-5.51264	0.899496	0.83315
NK.cells	CKM	-0.12731	0.1057	-0.24751	0.805087	-5.5692	0.916224	0.861876
NK.cells	MS4A7	0.135637	1.921581	0.247475	0.805114	-5.57738	0.891454	0.819477
NK.cells	EMG1	-0.02527	5.937655	-0.24741	0.805165	-6.69267	0.839186	0.732822
NK.cells	TLR8	0.132705	0.054908	0.247398	0.805174	-5.26848	0.916927	0.863092
NK.cells	METTL25	-0.03108	4.28478	-0.24739	0.805181	-6.29127	0.860284	0.767331
NK.cells	PCBP2	-0.01545	8.511828	-0.24729	0.805257	-7.11298	0.807454	0.682165
NK.cells	MED12	-0.03678	3.781462	-0.24713	0.805383	-6.26115	0.866823	0.778251
NK.cells	GM26839	-0.10952	0.656622	-0.24705	0.805446	-5.4475	0.908633	0.848909
NK.cells	CBLB	0.02235	7.336459	0.246996	0.805484	-7.1145	0.821775	0.704942

NK.cells	ZCWPW1	-0.05811	2.765843	-0.24687	0.805582	-5.88593	0.880182	0.800596
NK.cells	NARS	-0.02365	6.298026	-0.2468	0.805636	-6.73572	0.834662	0.725629
NK.cells	PCYOX1L	-0.07503	1.613818	-0.24676	0.805669	-5.67479	0.895602	0.826658
NK.cells	CENPC1	-0.02269	5.265605	-0.24662	0.805774	-6.57951	0.847696	0.746789
NK.cells	SH3BP1	0.026499	5.21446	0.246617	0.805776	-6.69386	0.848347	0.747853
NK.cells	SLC37A2	0.079146	3.315046	0.24654	0.805836	-5.73049	0.872931	0.788456
NK.cells	CEP68	0.038777	4.299523	0.246487	0.805877	-6.29314	0.860094	0.767146
NK.cells	BABAM1	0.024455	5.569892	0.246298	0.806022	-6.63135	0.843908	0.740489
NK.cells	ZRSR1	0.066509	2.211301	0.24611	0.806167	-5.77023	0.88768	0.813064
NK.cells	VAMP8	-0.02228	7.209731	-0.24608	0.806194	-6.8442	0.823439	0.707465
NK.cells	PHF11D	0.13186	1.34229	0.245731	0.80646	-5.50571	0.899438	0.833036
NK.cells	PFDN5	-0.0159	7.413663	-0.2457	0.806487	-6.97268	0.820971	0.703538
NK.cells	NDUFA12	0.027089	5.362792	0.24553	0.806615	-6.54559	0.846609	0.744905
NK.cells	COP1	0.025392	7.053619	0.2455	0.806639	-6.81303	0.825409	0.710653
NK.cells	GM32031	-0.06484	2.845461	-0.24543	0.806691	-5.81457	0.879282	0.798968
NK.cells	KPNA3	-0.02301	6.307968	-0.24537	0.806742	-6.75957	0.834685	0.725559
NK.cells	STXBP1	-0.05379	3.786972	-0.24535	0.806754	-6.06776	0.866905	0.778312
NK.cells	SYT11	0.074444	1.412759	0.245275	0.806812	-5.81808	0.898482	0.831457
NK.cells	SARDHOS	0.177886	0.147254	0.244736	0.807228	-5.22261	0.916202	0.861318
NK.cells	SIAH1B	-0.07374	1.91846	-0.24407	0.807738	-5.66146	0.892314	0.820054
NK.cells	CELF2	-0.01766	8.597509	-0.24393	0.807852	-7.15107	0.807161	0.681006
NK.cells	LRRC32	-0.08062	1.241998	-0.24364	0.808077	-5.68691	0.901467	0.835695
NK.cells	HOXA7	0.157731	-0.09629	0.24356	0.808136	-5.17722	0.919867	0.867336
NK.cells	NKIRAS2	-0.03953	4.042562	-0.24346	0.808211	-6.11868	0.864217	0.77307
NK.cells	STEAP4	-0.10683	2.205553	-0.24345	0.808223	-5.62738	0.88846	0.813609
NK.cells	GMDS	0.023374	6.245209	0.243439	0.808229	-6.74466	0.83609	0.72709
NK.cells	HASPIN	-0.06707	2.299155	-0.24342	0.808244	-5.80394	0.887207	0.811494
NK.cells	CDK14	-0.04976	5.455475	-0.24329	0.808342	-6.76157	0.846058	0.743287
NK.cells	PSMC4	0.026883	5.46131	0.243277	0.808354	-6.58932	0.845983	0.743166
NK.cells	TRP53BP1	0.036614	3.888271	0.243219	0.808399	-6.27327	0.866225	0.776432
NK.cells	U2AF2	-0.02245	6.245648	-0.24321	0.808403	-6.71914	0.836084	0.727114
NK.cells	TLR3	0.124166	1.534056	0.243194	0.808418	-5.47579	0.897503	0.828978
NK.cells	GM14966	0.048799	2.847703	0.242989	0.808577	-5.92686	0.879942	0.799318
NK.cells	PTEN	-0.01926	8.288084	-0.24271	0.808791	-6.99211	0.810938	0.687052
NK.cells	ARMC8	-0.02494	5.019279	-0.24226	0.809144	-6.45264	0.851657	0.752568
NK.cells	IGKV12-46	-0.13483	-0.77013	-0.24214	0.809234	-5.17331	0.929322	0.883996
NK.cells	GM13610	-0.09116	0.396658	-0.24196	0.809373	-5.51572	0.913084	0.855914
NK.cells	ZFP609	0.025482	6.067152	0.241834	0.809469	-6.62754	0.838363	0.73105
NK.cells	FRMD4A	0.051604	3.969412	0.24182	0.80948	-6.24424	0.865207	0.775014
NK.cells	GM29666	-0.13469	0.244861	-0.24159	0.809658	-5.34023	0.91518	0.859625
NK.cells	CALM3	-0.02104	7.10732	-0.2415	0.809723	-6.89266	0.825393	0.710229
NK.cells	GSTM7	-0.08877	1.02465	-0.24145	0.809762	-5.44938	0.904469	0.84121
NK.cells	RNF4	-0.01928	5.964365	-0.2413	0.809881	-6.71342	0.839657	0.73322
NK.cells	CPT1A	0.023248	5.446894	0.241177	0.809977	-6.61342	0.846204	0.743923
NK.cells	ZFP790	0.059806	2.226902	0.240854	0.810226	-5.82431	0.888214	0.813714
NK.cells	STAT5A	-0.03019	3.992851	-0.24085	0.810226	-6.46983	0.864902	0.7747
NK.cells	GM47071	-0.10625	1.555335	-0.24083	0.810243	-5.56544	0.897255	0.829049

NK.cells	DMWD	-0.09754	2.228423	-0.24082	0.810252	-5.51954	0.888193	0.813679
NK.cells	EPB41L2	0.024767	6.975502	0.240807	0.810262	-6.83293	0.827024	0.712971
NK.cells	TTLL12	0.075765	2.217401	0.240548	0.810462	-5.87737	0.888341	0.813961
NK.cells	GM43773	0.096394	1.074002	0.240379	0.810593	-5.50583	0.903795	0.840245
NK.cells	CCR9	-0.0765	2.410766	-0.24034	0.810626	-5.79123	0.885755	0.809595
NK.cells	PDIA5	0.077415	2.273787	0.240322	0.810637	-5.69631	0.887586	0.812685
NK.cells	RFC3	-0.0356	4.31335	-0.24029	0.810661	-6.26408	0.860742	0.76785
NK.cells	DGCR6	0.046285	3.650268	0.240287	0.810664	-6.00869	0.869372	0.782153
NK.cells	PODNL1	0.078401	-1.02387	0.240281	0.810668	-5.57132	0.932809	0.890615
NK.cells	ATP5K	-0.02555	7.486519	-0.24024	0.8107	-6.95021	0.82072	0.702929
NK.cells	MRTFB	0.033905	4.475202	0.240239	0.810701	-6.39711	0.85865	0.764398
NK.cells	GM15417	-0.0584	2.477041	-0.24019	0.810737	-5.88227	0.884871	0.808104
NK.cells	SLC15A4	0.039338	4.804844	0.24016	0.810762	-6.27157	0.854405	0.757416
NK.cells	PCYT1A	-0.03426	5.255992	-0.2401	0.810811	-6.4757	0.848634	0.747962
NK.cells	SSR3	0.022586	6.071068	0.240085	0.81082	-6.69259	0.838314	0.73118
NK.cells	TDP1	-0.04571	3.397629	-0.23996	0.810913	-6.12428	0.872685	0.787671
NK.cells	PARK7	-0.02266	6.806683	-0.23995	0.810927	-6.83766	0.829119	0.716358
NK.cells	ANGPTL2	-0.12592	0.84131	-0.23974	0.811089	-5.39613	0.906975	0.845756
NK.cells	MCPT8	-0.2697	-0.5048	-0.2397	0.81112	-5.28681	0.925603	0.877957
NK.cells	NR6A1OS	-0.09164	2.067272	-0.23968	0.811131	-5.6007	0.890354	0.817429
NK.cells	ABCB6	-0.08814	1.101522	-0.23958	0.811213	-5.43173	0.90342	0.839717
NK.cells	FAM91A1	0.023288	5.007003	0.239406	0.811345	-6.49924	0.851814	0.753341
NK.cells	XPA	-0.03375	4.138354	-0.23927	0.811451	-6.37647	0.863011	0.771807
NK.cells	SRP14	0.016288	7.234027	0.23907	0.811604	-6.89289	0.823828	0.708077
NK.cells	3110001I2:	0.049257	2.677347	0.238719	0.811876	-5.90051	0.882204	0.803862
NK.cells	SLC33A1	0.036275	3.794664	0.238527	0.812024	-6.26216	0.867485	0.779262
NK.cells	D5ERTD579	0.027486	5.221315	0.238444	0.812089	-6.54083	0.849076	0.748921
NK.cells	CLDN11	0.143599	-0.29485	0.238422	0.812106	-5.26556	0.922672	0.873068
NK.cells	ATPAF2	0.048203	2.92038	0.238399	0.812123	-5.98638	0.87898	0.798452
NK.cells	DCP1A	0.025888	4.850131	0.238323	0.812182	-6.50698	0.853824	0.7567
NK.cells	PPP6R1	-0.0201	5.572544	-0.23811	0.812345	-6.61222	0.844609	0.741659
NK.cells	SLC14A1	0.044899	3.335927	0.238029	0.81241	-6.17202	0.873496	0.789338
NK.cells	TRIM39	-0.04138	2.873416	-0.23797	0.812455	-6.01879	0.879602	0.799588
NK.cells	RTN4IP1	0.079014	1.927586	0.237948	0.812472	-5.68019	0.892231	0.8209
NK.cells	FMR1	-0.02327	5.971489	-0.23782	0.812571	-6.67811	0.839567	0.733542
NK.cells	MAN2A2	0.03906	4.595669	0.237636	0.812713	-6.26985	0.857096	0.762201
NK.cells	ALKBH7	-0.04624	3.373231	-0.23759	0.812746	-6.11207	0.873005	0.788581
NK.cells	DENND2D	-0.05067	2.202221	-0.23755	0.812782	-5.92593	0.888544	0.814691
NK.cells	GGNBP2	0.017823	7.268609	0.237484	0.812831	-6.91708	0.823401	0.707542
NK.cells	PLK2	0.057692	5.006155	0.237436	0.812868	-6.29673	0.851825	0.753541
NK.cells	ITGAE	-0.06015	2.127038	-0.23741	0.812888	-6.08198	0.889552	0.816396
NK.cells	LSM14A	-0.01719	6.620649	-0.23732	0.812955	-6.80577	0.831433	0.720431
NK.cells	SC5D	-0.03738	4.248539	-0.23724	0.813018	-6.30372	0.861581	0.76964
NK.cells	CD2	0.036902	3.660392	0.237133	0.813102	-6.43641	0.86924	0.78237
NK.cells	CBX1	-0.02329	6.075378	-0.23706	0.813157	-6.68455	0.83826	0.731502
NK.cells	PPP4C	0.019197	6.80668	0.237037	0.813176	-6.84627	0.829119	0.716759
NK.cells	GLRX	0.02715	6.214402	0.237002	0.813204	-6.8145	0.836513	0.728676

NK.cells	METTL18	-0.0902	1.271701	-0.23692	0.813267	-5.5208	0.901103	0.83611
NK.cells	PAIP2	-0.0162	7.620191	-0.23688	0.8133	-6.97963	0.819079	0.700711
NK.cells	DNAJC30	0.036258	3.959336	0.236869	0.813307	-6.21668	0.865338	0.775888
NK.cells	NOL4L	0.051941	2.680623	0.236846	0.813324	-6.2133	0.88216	0.803991
NK.cells	MTERF2	0.072675	2.099595	0.236814	0.813349	-5.7017	0.88992	0.817089
NK.cells	ELOVL5	-0.0242	6.195778	-0.2368	0.813356	-6.83753	0.836747	0.729054
NK.cells	ALDH1A1	-0.12514	-5.25E-05	-0.23658	0.813526	-5.40815	0.918682	0.866262
NK.cells	ENTPD5	0.031304	3.78751	0.236457	0.813625	-6.2265	0.867709	0.779711
NK.cells	ADAP1	0.033016	4.152492	0.236275	0.813766	-6.42424	0.862957	0.771894
NK.cells	NDUFA6	0.022485	6.56293	0.236274	0.813767	-6.8047	0.832278	0.721795
NK.cells	D930016D(	-0.04808	2.876865	-0.23571	0.814203	-5.95092	0.879967	0.799958
NK.cells	GM14858	-0.07869	2.296719	-0.23568	0.814224	-5.82966	0.887694	0.812973
NK.cells	GM16083	-0.09197	1.977218	-0.23566	0.814241	-5.59149	0.89198	0.820228
NK.cells	DRAM2	0.031403	4.847911	0.235474	0.814386	-6.41933	0.854266	0.757259
NK.cells	GM42477	0.091987	1.273145	0.235369	0.814467	-5.44633	0.901519	0.836441
NK.cells	NUDCD1	-0.04675	3.226429	-0.23532	0.814508	-6.07625	0.87536	0.79222
NK.cells	CLUH	-0.04358	3.417508	-0.23506	0.814709	-6.10021	0.872845	0.788091
NK.cells	LMO2	0.07376	4.903927	0.235023	0.814734	-5.75774	0.853547	0.756158
NK.cells	RAN	-0.02841	8.400943	-0.23499	0.81476	-7.1119	0.809963	0.686018
NK.cells	EEPD1	0.055981	4.881821	0.234831	0.814883	-6.05861	0.85383	0.756638
NK.cells	HIST1H2AE	0.103699	1.438759	0.234734	0.814958	-5.731	0.899269	0.832723
NK.cells	SENP3	-0.03129	4.324023	-0.23464	0.815031	-6.34328	0.86102	0.768527
NK.cells	TAB3	-0.03668	3.680364	-0.23459	0.81507	-6.19705	0.869399	0.782426
NK.cells	RCC2	0.021415	6.222377	0.234488	0.815148	-6.72397	0.836818	0.728985
NK.cells	AI504432	-0.03809	2.840905	-0.2344	0.815217	-6.20887	0.880458	0.800958
NK.cells	ABCD4	-0.045	2.782602	-0.23436	0.815244	-5.95723	0.881232	0.802258
NK.cells	COQ6	-0.05372	2.285167	-0.23434	0.815264	-5.76803	0.887863	0.813435
NK.cells	GM5150	0.126888	2.574091	0.234069	0.815473	-5.52502	0.884153	0.806987
NK.cells	POLM	-0.04278	3.245007	-0.23366	0.815791	-5.99819	0.875482	0.792134
NK.cells	PCM1	0.022315	6.494399	0.23353	0.81589	-6.77164	0.83376	0.723665
NK.cells	PRKRIP1	-0.03033	3.964552	-0.23323	0.816119	-6.27618	0.866051	0.776542
NK.cells	KLRA4	-0.11046	-1.21319	-0.23314	0.816191	-5.54584	0.936123	0.896715
NK.cells	DDA1	0.027113	5.082621	0.232913	0.816368	-6.52838	0.851615	0.752796
NK.cells	VMP1	0.028522	7.164309	0.232907	0.816372	-6.87098	0.825433	0.710408
NK.cells	HNRNPR	-0.01852	6.29021	-0.23291	0.816372	-6.7336	0.836317	0.727908
NK.cells	SRSF1	-0.02314	6.067856	-0.23285	0.816419	-6.69547	0.839111	0.732428
NK.cells	SETD1A	-0.02838	4.19796	-0.23281	0.816446	-6.32446	0.863015	0.771566
NK.cells	9530034E1	-0.10428	0.330435	-0.23278	0.816467	-5.37412	0.914823	0.859165
NK.cells	ZCCHC3	-0.1049	0.70709	-0.23278	0.816471	-5.3835	0.909635	0.850226
NK.cells	BET1	0.033784	4.246255	0.232676	0.816551	-6.2732	0.862396	0.77056
NK.cells	GDPD5	-0.0535	1.750804	-0.23248	0.816703	-6.03302	0.89546	0.826005
NK.cells	INTU	-0.13244	0.880465	-0.23245	0.816723	-5.39746	0.907297	0.846226
NK.cells	AXDND1	0.095826	1.557778	0.232318	0.816828	-5.62143	0.898106	0.830449
NK.cells	ABITRAM	0.047049	2.584915	0.232193	0.816925	-5.91741	0.884331	0.807067
NK.cells	NAA10	0.02901	5.084078	0.231884	0.817164	-6.57207	0.851867	0.752913
NK.cells	GAS5	-0.02434	6.391612	-0.23164	0.81735	-6.77369	0.835363	0.726037
NK.cells	PPIP5K1	-0.07098	1.776843	-0.23163	0.817359	-5.78774	0.895409	0.82553

NK.cells	SYNE3	0.056236	2.767053	0.231205	0.81769	-6.04862	0.882272	0.803182
NK.cells	SLCO4C1	-0.11954	-1.1708	-0.23099	0.817854	-5.2293	0.936062	0.896065
NK.cells	SCN4A	0.114981	0.410123	0.230915	0.817914	-5.3466	0.914204	0.857619
NK.cells	ATP6V0E	0.017889	7.713213	0.230851	0.817964	-6.95378	0.81911	0.699928
NK.cells	IGSF3	-0.10928	0.826018	-0.23083	0.817981	-5.42479	0.908482	0.847777
NK.cells	GEMIN8	0.059057	2.284507	0.230682	0.818095	-5.77628	0.888712	0.814117
NK.cells	TMEM126/	0.01954	5.857631	0.230624	0.81814	-6.73892	0.842206	0.737064
NK.cells	2610206C1	0.100663	0.40853	0.23058	0.818174	-5.43055	0.914226	0.857709
NK.cells	MTRF1L	0.04558	3.1332	0.230573	0.818179	-6.05171	0.87742	0.795147
NK.cells	ZEB1	0.028417	6.848689	0.230545	0.818201	-6.98019	0.829782	0.717025
NK.cells	DOCK2	-0.01843	9.34967	-0.23008	0.818559	-7.2807	0.799582	0.668909
NK.cells	PDCL	0.038321	3.891934	0.229971	0.818645	-6.21353	0.867733	0.778647
NK.cells	ZFPL1	0.036742	3.466605	0.229927	0.81868	-6.088	0.873305	0.787919
NK.cells	PHLDA1	-0.04769	4.035457	-0.22978	0.81879	-6.36451	0.865901	0.775546
NK.cells	1700017B(	-0.03373	5.285252	-0.22951	0.819003	-6.44476	0.849825	0.749098
NK.cells	MTX2	0.027164	4.838218	0.229335	0.819138	-6.43351	0.855552	0.75848
NK.cells	ZBTB9	0.055758	2.511721	0.229288	0.819175	-5.80358	0.88604	0.809211
NK.cells	TNFSF9	-0.04952	3.435421	-0.22913	0.819295	-6.35632	0.873797	0.7887
NK.cells	BCL6B	-0.1178	0.649778	-0.22904	0.819368	-5.29498	0.911279	0.852211
NK.cells	TCP11L2	0.030282	6.196085	0.229005	0.819394	-6.76786	0.838287	0.730359
NK.cells	PPM1K	0.045568	3.579795	0.228962	0.819427	-6.13487	0.8719	0.785553
NK.cells	CDC20	-0.05665	3.639962	-0.22887	0.819501	-6.21397	0.871111	0.784265
NK.cells	ZMAT2	0.022013	5.466404	0.228713	0.81962	-6.60886	0.847517	0.745393
NK.cells	ARL8A	-0.0232	6.227073	-0.22868	0.819645	-6.69244	0.837897	0.729779
NK.cells	BNIP2	0.01942	6.377564	0.228588	0.819717	-6.74855	0.836008	0.726758
NK.cells	C1GALT1	-0.02727	6.353776	-0.22854	0.819752	-6.68802	0.836306	0.727239
NK.cells	GM4316	0.107065	0.707324	0.228407	0.819857	-5.4304	0.910488	0.850966
NK.cells	QPCTL	-0.04198	3.010203	-0.22838	0.81988	-5.95856	0.87941	0.798205
NK.cells	INTS12	0.031052	4.380987	0.228273	0.819961	-6.2859	0.861453	0.76836
NK.cells	GM44174	0.067034	-0.56348	0.228244	0.819984	-5.82403	0.928133	0.881562
NK.cells	PSMD11	0.013165	7.032517	0.227891	0.820257	-6.88722	0.827907	0.71369
NK.cells	ERCC3	-0.03144	3.695125	-0.2278	0.820329	-6.24765	0.870456	0.783176
NK.cells	C1D	0.019353	5.843961	0.227793	0.820333	-6.67074	0.842793	0.737701
NK.cells	PIH1D1	-0.02665	5.06576	-0.22762	0.82047	-6.52094	0.852698	0.753896
NK.cells	B130055M	0.057906	2.013314	0.227491	0.820567	-5.84878	0.892792	0.820739
NK.cells	POLR3B	-0.03106	5.512738	-0.22744	0.820604	-6.59262	0.846993	0.744586
NK.cells	ERP44	0.016061	6.074446	0.227401	0.820637	-6.71627	0.839883	0.733032
NK.cells	LDHC	-0.1494	0.610466	-0.2274	0.820641	-5.30294	0.911891	0.853369
NK.cells	RSF1OS1	-0.04152	2.978349	-0.22732	0.820698	-6.10814	0.879901	0.799007
NK.cells	ZSCAN25	0.060398	1.792602	0.2271	0.82087	-5.82143	0.895878	0.8258
NK.cells	ZC3H14	-0.02004	5.656337	-0.227	0.820947	-6.64755	0.845277	0.741633
NK.cells	FBXL17	0.019216	7.560342	0.226835	0.821076	-6.99267	0.821539	0.70339
NK.cells	TRDMT1	-0.05389	2.342131	-0.22676	0.821136	-5.83307	0.888539	0.813351
NK.cells	DDX47	-0.01943	5.550552	-0.22637	0.821434	-6.63776	0.846899	0.74398
NK.cells	GM6225	-0.04212	2.958455	-0.2262	0.821568	-6.23091	0.880603	0.7997
NK.cells	GM14634	-0.06417	1.834434	-0.22607	0.821672	-5.82691	0.895649	0.82512
NK.cells	INTS6L	0.028226	5.229737	0.226019	0.821708	-6.54747	0.851024	0.750746

NK.cells	MTHFD1	0.035366	3.79072	0.225926	0.821781	-6.26279	0.869637	0.781446
NK.cells	SNRPB2	0.01865	6.282619	0.225672	0.821978	-6.75294	0.837682	0.729134
NK.cells	MARCO	-0.11677	5.795483	-0.22567	0.821981	-6.32218	0.843826	0.73909
NK.cells	COG1	0.03039	3.677624	0.225518	0.822097	-6.23237	0.871119	0.78399
NK.cells	PIGA	-0.05984	1.918295	-0.22539	0.822193	-5.78834	0.894517	0.823345
NK.cells	NR1D1	-0.11767	1.661874	-0.22537	0.822208	-5.45766	0.897983	0.829236
NK.cells	LAMTOR5	0.023584	5.527925	0.225269	0.82229	-6.59364	0.847222	0.744661
NK.cells	HAVCR1	0.142819	-0.3843	0.225107	0.822415	-5.18097	0.926157	0.877744
NK.cells	LMAN1L	-0.07542	1.485183	-0.22508	0.822437	-5.73018	0.90038	0.833342
NK.cells	SGTA	-0.02841	4.959007	-0.22508	0.82244	-6.45228	0.854493	0.756571
NK.cells	HSPA13	0.038655	3.082179	0.225024	0.82248	-6.01847	0.878964	0.797142
NK.cells	NECAP1	0.024905	4.774239	0.224828	0.822632	-6.46101	0.85687	0.760535
NK.cells	HNRNPU	0.017552	8.26781	0.224797	0.822656	-7.06378	0.81315	0.690053
NK.cells	TSPAN32	0.042774	2.972272	0.224743	0.822698	-6.21195	0.880421	0.799661
NK.cells	ALDH18A1	-0.05036	3.070395	-0.22441	0.822954	-6.0871	0.879318	0.797521
NK.cells	PCX	0.064138	2.70013	0.224255	0.823076	-5.90384	0.884289	0.805805
NK.cells	ELOF1	0.027194	5.375272	0.223956	0.823308	-6.49381	0.849573	0.74802
NK.cells	A930024E(	-0.06266	1.108621	-0.22367	0.823527	-5.88899	0.906107	0.842351
NK.cells	ZSWIM3	0.08726	1.503758	0.22337	0.823763	-5.54171	0.90074	0.83317
NK.cells	BANF1	0.02327	6.873409	0.223353	0.823776	-6.85402	0.830861	0.717526
NK.cells	GIMAP1OS	-0.05013	0.862202	-0.22335	0.823778	-6.03788	0.909503	0.848156
NK.cells	CFB	0.109466	4.663824	0.223277	0.823835	-6.36436	0.858876	0.763087
NK.cells	GM16638	0.071599	2.040296	0.222998	0.824051	-5.60755	0.893496	0.820892
NK.cells	MMGT1	0.053356	2.857236	0.222905	0.824123	-5.87278	0.882562	0.802451
NK.cells	MAP3K8	0.033176	4.64612	0.222874	0.824148	-6.37408	0.85912	0.763479
NK.cells	ACTR1A	0.018291	6.140647	0.222839	0.824175	-6.73454	0.840053	0.732364
NK.cells	AK5	0.102675	0.348906	0.222793	0.824211	-5.44338	0.916595	0.860391
NK.cells	ANKRD24	-0.08273	1.054272	-0.22262	0.824348	-5.50548	0.906887	0.843711
NK.cells	CD55B	0.151444	0.178504	0.222581	0.824375	-5.21195	0.918958	0.864482
NK.cells	MAPK8	0.024312	5.232129	0.222373	0.824536	-6.52299	0.851589	0.751181
NK.cells	ABHD13	-0.03429	3.557429	-0.22229	0.824598	-6.12313	0.873306	0.787042
NK.cells	ASPSCR1	0.02484	4.685187	0.222172	0.824692	-6.46271	0.858616	0.762742
NK.cells	PEG13	-0.06066	2.114243	-0.22216	0.8247	-5.76275	0.892502	0.819305
NK.cells	QSOX1	0.032072	4.311358	0.222139	0.824718	-6.35726	0.863456	0.770722
NK.cells	RANBP1	-0.027	7.239938	-0.22136	0.82532	-6.90296	0.826796	0.710488
NK.cells	AQP11	0.097865	0.397356	0.221323	0.825351	-5.4616	0.916446	0.859479
NK.cells	STX4A	0.022706	4.972734	0.22122	0.825431	-6.49664	0.855399	0.756832
NK.cells	ARHGAP4	0.024477	5.057347	0.221163	0.825475	-6.4943	0.854312	0.755051
NK.cells	CD4	0.078534	1.246276	0.220938	0.82565	-5.75901	0.904889	0.839575
NK.cells	DNAJC21	-0.02363	5.890572	-0.22053	0.825965	-6.64855	0.843821	0.737874
NK.cells	PRKAR1B	0.142679	-0.37005	0.220529	0.825968	-5.2285	0.927274	0.878198
NK.cells	11-Sep	-0.02913	6.415184	-0.22035	0.826103	-6.80466	0.837207	0.727236
NK.cells	ATP5MPL	0.016819	8.484004	0.220204	0.82622	-7.11649	0.811678	0.686547
NK.cells	PUS10	0.031674	4.485499	0.220162	0.826253	-6.37222	0.861819	0.76738
NK.cells	ACSL5	0.024834	6.00653	0.220037	0.82635	-6.7168	0.842354	0.735583
NK.cells	CHFR	0.019706	5.641975	0.219942	0.826423	-6.67972	0.846975	0.743087
NK.cells	0610040B1	-0.06527	2.051019	-0.21992	0.826441	-5.7021	0.893998	0.821182

NK.cells	GM36723	-0.03511	3.324777	-0.21992	0.826442	-6.81886	0.877003	0.792596
NK.cells	FAM117A	0.032233	5.965212	0.219705	0.826608	-6.64823	0.842876	0.736434
NK.cells	SLIT2	-0.11989	0.983319	-0.21958	0.826705	-5.42759	0.908514	0.845927
NK.cells	CCL22	-0.18083	-0.37257	-0.21956	0.826719	-5.30314	0.927309	0.878375
NK.cells	MAP3K11	0.035359	3.876975	0.218988	0.827165	-6.22031	0.869744	0.780634
NK.cells	CLPX	-0.02217	5.328169	-0.21899	0.827165	-6.59554	0.850975	0.749727
NK.cells	FAM20B	0.037791	3.958304	0.218948	0.827195	-6.13929	0.86868	0.778869
NK.cells	SLC2A3	0.036675	4.519005	0.218942	0.8272	-6.41491	0.861385	0.766807
NK.cells	SLC2A8	-0.04634	2.226604	-0.21887	0.827259	-5.93508	0.891634	0.817313
NK.cells	GM16576	0.082363	1.513376	0.218836	0.827282	-5.63247	0.901277	0.833681
NK.cells	TEDC2	-0.09106	0.906107	-0.21864	0.827432	-5.42613	0.909574	0.847874
NK.cells	RHBDF2	-0.03734	4.650027	-0.21864	0.827433	-6.11659	0.85969	0.764022
NK.cells	ATAD2B	0.01923	6.740991	0.218641	0.827434	-6.84623	0.833128	0.720816
NK.cells	MXRA7	0.091168	1.654737	0.217945	0.827974	-5.51677	0.899357	0.830672
NK.cells	ACAD12	-0.09598	0.714351	-0.21775	0.828122	-5.4437	0.912211	0.852708
NK.cells	KYAT3	-0.05508	3.603362	-0.21769	0.828172	-6.13771	0.873333	0.786914
NK.cells	S100A10	0.025327	6.601647	0.217614	0.828231	-6.99337	0.83487	0.723928
NK.cells	ZFP563	-0.07179	0.800774	-0.21759	0.828246	-5.50238	0.911021	0.850723
NK.cells	CPEB4	-0.02525	6.51399	-0.21755	0.828281	-6.72756	0.835967	0.725711
NK.cells	BCAP29	-0.02648	4.765797	-0.21748	0.828337	-6.48353	0.858196	0.761921
NK.cells	GM36551	0.091362	-0.80034	0.217472	0.828342	-5.23617	0.933323	0.889411
NK.cells	FGD6	0.043395	4.002414	0.217267	0.828501	-6.14095	0.868104	0.778321
NK.cells	ANAPC15	-0.02934	4.974484	-0.2172	0.828553	-6.41312	0.855508	0.757553
NK.cells	PIGT	0.024086	5.640537	0.217146	0.828595	-6.63691	0.846993	0.743634
NK.cells	GM26511	-0.07448	2.075738	-0.2171	0.828629	-5.64999	0.893665	0.82119
NK.cells	CDC42EP4	0.069256	2.155265	0.21703	0.828685	-5.60233	0.892594	0.819376
NK.cells	ZFP398	-0.03158	4.517006	-0.21701	0.828701	-6.4449	0.861411	0.767262
NK.cells	GM16556	0.122455	1.676001	0.216932	0.828761	-5.32204	0.899068	0.830367
NK.cells	UVSSA	0.035056	3.709393	0.21685	0.828825	-6.19584	0.87194	0.784705
NK.cells	HPRT	0.021127	6.310323	0.216821	0.828847	-6.76049	0.838524	0.729896
NK.cells	PRR16	0.086246	1.720127	0.216783	0.828877	-5.6889	0.89847	0.829349
NK.cells	DGKE	-0.03103	4.880229	-0.21668	0.828959	-6.46349	0.856721	0.759549
NK.cells	RBPMS2	-0.06414	1.246218	-0.21646	0.829132	-5.62865	0.904917	0.840408
NK.cells	UBA6	0.022995	5.172539	0.216352	0.829212	-6.58821	0.852967	0.753447
NK.cells	MOSPD2	0.035922	4.445481	0.216275	0.829272	-6.25057	0.862338	0.768853
NK.cells	APBA1	0.080772	4.384633	0.216223	0.829312	-5.73621	0.863127	0.770156
NK.cells	NCK2	-0.0288	4.870391	-0.21617	0.829351	-6.63329	0.856848	0.759812
NK.cells	5430414B1	0.108939	0.449396	0.216022	0.829468	-5.39737	0.915867	0.85922
NK.cells	SAPCD1	0.093039	1.629002	0.21597	0.829508	-5.43138	0.899706	0.831519
NK.cells	PSMB1	0.017322	7.299863	0.215895	0.829567	-6.94033	0.826182	0.710121
NK.cells	COL5A1	0.116394	0.493409	0.21541	0.829944	-5.34576	0.915259	0.858293
NK.cells	MAZ	-0.02885	6.241133	-0.21535	0.829987	-6.75271	0.839395	0.731466
NK.cells	5330438D1	-0.02605	4.842586	-0.21531	0.830018	-6.5297	0.857206	0.760509
NK.cells	HIST1H1D	-0.08582	1.531522	-0.21517	0.830127	-5.67379	0.90103	0.833894
NK.cells	FAM98C	-0.03573	3.737769	-0.215	0.830265	-6.21778	0.871568	0.78426
NK.cells	ZFP213	0.05857	2.162406	0.214949	0.830302	-5.73787	0.892498	0.819396
NK.cells	AARS2	-0.0904	0.821033	-0.21494	0.830308	-5.45035	0.910743	0.850519

NK.cells	PPIB	-0.01749	7.605608	-0.21492	0.830325	-7.01657	0.82241	0.704203
NK.cells	OPRM1	0.040005	2.803916	0.214768	0.830443	-6.33701	0.88391	0.804946
NK.cells	DNMT1	0.027988	5.753517	0.214569	0.830598	-6.68634	0.845558	0.741538
NK.cells	ATPIF1	-0.02221	7.745006	-0.21447	0.830673	-6.97296	0.820696	0.701546
NK.cells	GM11523	0.161018	-0.95948	0.214471	0.830673	-5.17166	0.935556	0.893685
NK.cells	BAG1	-0.01622	6.693589	-0.21443	0.830703	-6.87313	0.83372	0.722382
NK.cells	CFAP410	-0.04616	2.119698	-0.21437	0.830752	-5.81252	0.893073	0.820451
NK.cells	FAM71D	0.130311	0.095273	0.214289	0.830815	-5.23952	0.920778	0.867919
NK.cells	ZFP574	-0.03654	3.401172	-0.21425	0.830844	-6.15287	0.875995	0.791731
NK.cells	UTP20	0.042172	3.652111	0.214099	0.830963	-6.21027	0.872692	0.78624
NK.cells	MMAB	-0.07899	1.326097	-0.21405	0.831001	-5.58321	0.903827	0.838784
NK.cells	FOXRED1	0.053019	2.831724	0.213997	0.831042	-5.92311	0.88354	0.804394
NK.cells	DUSP2	-0.03236	5.991936	-0.21395	0.831075	-6.72532	0.842538	0.73666
NK.cells	B3GNT2	-0.02311	6.739389	-0.21387	0.831142	-6.73816	0.833148	0.721489
NK.cells	DMTN	-0.08719	0.637339	-0.21383	0.831175	-5.53194	0.913272	0.854994
NK.cells	FAM160B1	-0.02874	4.443367	-0.21381	0.831187	-6.3532	0.862366	0.76912
NK.cells	CADM4	-0.09614	1.741687	-0.21371	0.831268	-5.53599	0.898178	0.829171
NK.cells	ACTG1	-0.02079	11.44474	-0.21354	0.831401	-7.60118	0.776648	0.633001
NK.cells	COL4A4	-0.08432	1.009618	-0.21342	0.831492	-5.50944	0.908154	0.846215
NK.cells	RANBP3	-0.02536	4.303701	-0.21341	0.831499	-6.34839	0.864179	0.772131
NK.cells	AGA	0.042858	3.27288	0.213372	0.831528	-6.06304	0.877689	0.794612
NK.cells	IMPAD1	0.024624	4.365894	0.213222	0.831644	-6.39227	0.863371	0.770822
NK.cells	PAFAH2	-0.11819	0.619176	-0.21311	0.831732	-5.32236	0.913522	0.855486
NK.cells	SKIV2L	0.030386	3.938774	0.213099	0.83174	-6.30845	0.868935	0.780054
NK.cells	MAP7D3	0.101667	0.126284	0.213041	0.831785	-5.41019	0.920347	0.867274
NK.cells	RBM19	0.04264	2.925866	0.212909	0.831888	-6.00621	0.882288	0.802353
NK.cells	LIMK1	-0.04967	2.1198	-0.2129	0.831895	-5.94977	0.893071	0.820545
NK.cells	MBIP	0.035023	3.658726	0.21275	0.832012	-6.21215	0.872605	0.78616
NK.cells	SRP19	0.020568	6.048783	0.212675	0.83207	-6.69935	0.84182	0.735556
NK.cells	NCDN	0.04304	2.826122	0.212645	0.832094	-6.04086	0.883615	0.804586
NK.cells	LYSMD1	-0.0641	1.591133	-0.21258	0.832146	-5.59215	0.90022	0.832697
NK.cells	CDNF	0.110229	0.317972	0.212522	0.832189	-5.33321	0.917687	0.862674
NK.cells	RFX3	0.021053	6.233922	0.212368	0.832309	-6.7489	0.839485	0.731787
NK.cells	CGGBP1	-0.01801	6.845343	-0.21223	0.832413	-6.84205	0.831826	0.719479
NK.cells	TCP1	-0.02157	6.67677	-0.21202	0.832579	-6.82424	0.83393	0.72293
NK.cells	UBALD2	0.021649	7.028455	0.211945	0.832638	-6.90896	0.829547	0.715889
NK.cells	SLC8B1	0.03751	4.721857	0.211727	0.832808	-6.25697	0.858763	0.763395
NK.cells	RBFOX3	0.130582	-0.24977	0.211696	0.832832	-5.25377	0.92559	0.876556
NK.cells	USP11	-0.05374	2.611991	-0.21164	0.832877	-5.82654	0.88647	0.80956
NK.cells	CTPS2	0.022281	5.066744	0.211481	0.832998	-6.59854	0.854323	0.756111
NK.cells	OAZ2	-0.02527	4.485342	-0.21145	0.833019	-6.37097	0.861821	0.768448
NK.cells	4930595D1	0.076813	1.248821	0.211455	0.833019	-5.5958	0.904881	0.840841
NK.cells	LINS1	0.061581	1.704648	0.21143	0.833038	-5.59844	0.89868	0.830256
NK.cells	ATXN7L3B	-0.01909	5.941589	-0.21121	0.833209	-6.65631	0.843175	0.737996
NK.cells	HS2ST1	-0.02191	5.689893	-0.21111	0.833288	-6.60445	0.846366	0.743202
NK.cells	TELO2	-0.05648	2.455163	-0.21111	0.833291	-5.93655	0.888568	0.813215
NK.cells	POLR1C	0.033931	3.945898	0.211095	0.833299	-6.26417	0.868842	0.780175

NK.cells	FNDC3B	-0.025	6.549271	-0.21077	0.83355	-6.69841	0.835525	0.725697
NK.cells	ACTR5	-0.03254	3.477256	-0.21077	0.83355	-6.23842	0.874992	0.790488
NK.cells	GUCA1A	-0.0967	1.4794	-0.21073	0.833581	-5.38708	0.901739	0.835654
NK.cells	C77080	0.189562	0.694235	0.210714	0.833595	-5.3802	0.912488	0.854083
NK.cells	DAGLB	0.029363	4.366228	0.210713	0.833596	-6.43377	0.863366	0.77117
NK.cells	HAUS7	-0.03951	2.992661	-0.21062	0.83367	-6.00936	0.881401	0.801246
NK.cells	PANK4	0.036618	3.423163	0.210491	0.833769	-6.13647	0.875705	0.791716
NK.cells	PCCB	0.043724	3.081613	0.210431	0.833815	-5.98714	0.880221	0.799275
NK.cells	LY9	0.02701	4.262049	0.210407	0.833834	-6.44395	0.86472	0.773445
NK.cells	CLDN15	0.091788	0.423974	0.210187	0.834005	-5.36067	0.916219	0.860663
NK.cells	LTO1	0.036365	3.526077	0.21014	0.834042	-6.10258	0.874349	0.789549
NK.cells	ZFP39	-0.08312	1.023509	-0.21008	0.834088	-5.54966	0.907963	0.846467
NK.cells	CEP104	0.037219	3.105243	0.210064	0.834101	-6.02164	0.879907	0.798863
NK.cells	PPRC1	-0.03116	3.956149	-0.21005	0.834111	-6.2533	0.868708	0.780169
NK.cells	DDX50	0.015825	6.369428	0.209654	0.83442	-6.78442	0.837781	0.72953
NK.cells	KLK1	-0.17486	-0.20745	-0.20951	0.834531	-5.13503	0.924999	0.87601
NK.cells	DOCK5	-0.02628	4.189727	-0.20915	0.834813	-6.72808	0.865661	0.775289
NK.cells	PMAIP1	0.050488	4.983149	0.209097	0.834854	-6.26473	0.855397	0.758353
NK.cells	MPC2	-0.02199	6.800876	-0.2089	0.835005	-6.82514	0.83238	0.720931
NK.cells	ACE	0.148073	0.341503	0.208882	0.835021	-5.35589	0.917361	0.862848
NK.cells	LHFP	0.095309	1.237889	0.208849	0.835046	-5.49246	0.905031	0.841631
NK.cells	SHISA5	0.024387	6.572889	0.208821	0.835069	-6.98363	0.83523	0.725521
NK.cells	PPP1R18	0.018453	6.994866	0.208628	0.835219	-6.89967	0.829965	0.717064
NK.cells	PHETA1	0.099705	0.450779	0.208553	0.835277	-5.39359	0.915848	0.860268
NK.cells	GM31597	0.04116	2.208627	0.208482	0.835333	-6.00274	0.891876	0.819269
NK.cells	PDP1	-0.04049	2.180234	-0.20831	0.835465	-5.96774	0.892258	0.819938
NK.cells	SRPR	0.01737	5.794906	0.208236	0.835524	-6.65366	0.845033	0.741497
NK.cells	KALRN	-0.07876	2.750236	-0.20804	0.835673	-5.69456	0.884626	0.807114
NK.cells	MCCC1	-0.05553	2.50652	-0.20791	0.835775	-5.84181	0.88788	0.812668
NK.cells	INO80D	0.018117	5.995336	0.207746	0.835905	-6.69847	0.842495	0.737519
NK.cells	NFKBID	-0.02689	6.350031	-0.20764	0.835986	-6.72888	0.838025	0.730272
NK.cells	ZFP553	0.04515	2.414705	0.207555	0.836054	-5.87017	0.88911	0.814819
NK.cells	WDR33	0.013845	7.186641	0.207482	0.836111	-6.93308	0.827584	0.713485
NK.cells	4930417O1	-0.05077	0.963159	-0.20748	0.836114	-6.06755	0.908791	0.848383
NK.cells	GPR155	0.053837	3.05644	0.20736	0.836206	-5.91165	0.880554	0.800421
NK.cells	TRBC2	-0.04203	3.943953	-0.20733	0.836225	-6.84319	0.868868	0.780894
NK.cells	RGP1	-0.04817	2.34849	-0.20723	0.836306	-5.72093	0.889998	0.816359
NK.cells	CTPS	0.03607	3.704287	0.207195	0.836334	-6.19022	0.872007	0.786139
NK.cells	NECTIN3	-0.08747	1.220532	-0.20713	0.836388	-5.43316	0.905268	0.842368
NK.cells	4732440D0	0.066579	2.03703	0.206903	0.836561	-5.72732	0.894187	0.823465
NK.cells	TSPYL4	0.06793	0.723815	0.206903	0.836562	-5.55835	0.91208	0.854072
NK.cells	GM41077	-0.08709	0.721818	-0.20686	0.836593	-5.49336	0.912108	0.854127
NK.cells	SS18L1	0.056577	2.325233	0.206701	0.836719	-5.87568	0.89031	0.816915
NK.cells	DIS3L	-0.03868	2.828005	-0.2067	0.836722	-6.02402	0.88359	0.80557
NK.cells	GFPT1	0.027845	5.095805	0.206644	0.836763	-6.61906	0.853951	0.756297
NK.cells	LUC7L2	0.011963	8.527172	0.206472	0.836897	-7.1272	0.811154	0.6874
NK.cells	GM31763	0.047847	3.257819	0.206366	0.83698	-6.08559	0.877888	0.796013

NK.cells	TMEM234	0.013777	7.040364	0.206233	0.837083	-6.90976	0.829399	0.716493
NK.cells	TPBGL	-0.11225	0.559425	-0.20617	0.837132	-5.3578	0.914347	0.858072
NK.cells	RALGAPA2	0.025781	6.409122	0.206169	0.837133	-6.83513	0.837283	0.729202
NK.cells	GM40841	-0.12694	0.40148	-0.20612	0.837173	-5.27656	0.91653	0.861859
NK.cells	TBCK	0.022322	5.26974	0.20601	0.837257	-6.56257	0.851722	0.752717
NK.cells	CREBRF	-0.02259	6.98351	-0.20599	0.837271	-6.8829	0.830106	0.717647
NK.cells	GM42917	-0.06404	1.691044	-0.20592	0.83733	-5.69384	0.898864	0.831529
NK.cells	MIEF1	0.031451	4.21125	0.205855	0.837378	-6.34285	0.865381	0.775227
NK.cells	4931406P1	-0.02003	5.594349	-0.20584	0.83739	-6.64536	0.847581	0.745945
NK.cells	LMBRD2	-0.0322	4.481892	-0.20569	0.837507	-6.35397	0.861866	0.769443
NK.cells	COQ9	0.037629	3.205352	0.20564	0.837545	-6.0865	0.878582	0.797273
NK.cells	NIM1K	0.054831	2.88905	0.205392	0.837738	-5.85788	0.882777	0.804457
NK.cells	S1PR2	-0.06185	1.261742	-0.20537	0.837758	-5.77226	0.904705	0.8417
NK.cells	LEMD2	0.024386	4.232797	0.205252	0.837847	-6.416	0.865101	0.774946
NK.cells	DGKD	0.018674	7.687633	0.205179	0.837904	-6.97532	0.821401	0.703916
NK.cells	GM16023	0.048565	1.834877	0.204971	0.838066	-5.8588	0.896917	0.828515
NK.cells	HMGA2	-0.13295	1.149583	-0.20492	0.838109	-5.50212	0.906238	0.844446
NK.cells	ZBTB34	0.038452	3.592868	0.204624	0.838337	-6.09181	0.873471	0.789081
NK.cells	6330562C2	0.085771	1.275201	0.204518	0.838419	-5.45453	0.904521	0.841628
NK.cells	PSMB9	0.035787	6.228284	0.204466	0.83846	-6.79611	0.839556	0.733274
NK.cells	CCNE1	-0.04227	3.530373	-0.20433	0.838565	-6.15947	0.874293	0.790466
NK.cells	SBF2	0.035047	5.368073	0.204309	0.838582	-6.37778	0.850465	0.751048
NK.cells	SGCB	0.074809	2.212559	0.20415	0.838706	-5.62273	0.891823	0.819986
NK.cells	NATD1	0.04851	3.25628	0.204148	0.838707	-5.99972	0.877908	0.796522
NK.cells	A930001M	-0.03172	3.967402	-0.20414	0.83871	-6.29375	0.868561	0.780914
NK.cells	CORO1C	-0.01887	6.202322	-0.20411	0.83874	-6.74507	0.839883	0.733819
NK.cells	DRC7	0.123977	-0.15036	0.203847	0.838941	-5.22803	0.924201	0.875632
NK.cells	INTS2	-0.03784	4.120259	-0.20372	0.839041	-6.21536	0.866566	0.777611
NK.cells	IZUMO4	0.065365	1.453448	0.20351	0.839204	-5.82358	0.902092	0.837488
NK.cells	ZFP322A	0.04073	3.041434	0.203344	0.839333	-6.06086	0.880753	0.80131
NK.cells	1110059G1	0.028121	3.928256	0.203219	0.839431	-6.24638	0.869073	0.781777
NK.cells	GGNBP1	-0.09437	1.044976	-0.20318	0.83946	-5.43952	0.907669	0.847048
NK.cells	ATMIN	0.039997	2.969649	0.203157	0.839479	-6.12298	0.881706	0.802912
NK.cells	MYO10	0.035497	5.227281	0.203146	0.839488	-6.47353	0.852266	0.754012
NK.cells	IL4RA	0.025958	4.883706	0.203033	0.839576	-6.64669	0.856676	0.761259
NK.cells	MFSD10	0.024477	4.464143	0.203024	0.839582	-6.55062	0.862096	0.770203
NK.cells	LZIC	-0.0323	3.63991	-0.20299	0.839607	-6.18609	0.872852	0.788076
NK.cells	GM26532	-0.02971	5.294849	-0.20293	0.839653	-6.58281	0.851401	0.752595
NK.cells	SNAI3	0.085886	-0.34486	0.202725	0.839815	-5.47933	0.926921	0.880373
NK.cells	PPP1R9B	0.027586	4.375184	0.202619	0.839899	-6.45692	0.86325	0.772121
NK.cells	ARFIP2	-0.05007	2.138474	-0.2026	0.839913	-5.77872	0.89282	0.821699
NK.cells	GM1604B	-0.09837	1.489369	-0.20232	0.84013	-5.47054	0.901603	0.836675
NK.cells	LUC7L3	-0.0154	6.706417	-0.20232	0.840133	-6.81639	0.83356	0.723612
NK.cells	CDKL5	-0.07239	2.066071	-0.20221	0.840219	-5.71266	0.893795	0.823369
NK.cells	ETFB	-0.02305	6.979015	-0.20219	0.840232	-6.86914	0.830162	0.718142
NK.cells	ZFP820	-0.11848	-0.04486	-0.20218	0.84024	-5.21199	0.922729	0.873096
NK.cells	EIF4A1	0.015534	7.526831	0.202156	0.840259	-6.95584	0.82338	0.707274

NK.cells	ABCA7	-0.02605	3.89815	-0.20215	0.840265	-6.30722	0.869467	0.782454
NK.cells	GM16090	-0.1515	-0.96132	-0.20206	0.840336	-5.15531	0.93558	0.895572
NK.cells	NOSTRIN	-0.05812	3.426931	-0.20193	0.840435	-5.9044	0.875655	0.792783
NK.cells	GOLGA2	-0.02874	3.814242	-0.20183	0.840512	-6.22626	0.870565	0.784283
NK.cells	CNOT2	-0.01502	7.008824	-0.2017	0.840612	-6.88052	0.829791	0.717546
NK.cells	SERPINB6B	-0.03726	2.936635	-0.20163	0.840668	-6.55527	0.882145	0.803672
NK.cells	SESN2	0.041457	3.371941	0.201524	0.840752	-6.04883	0.876381	0.793997
NK.cells	GM9750	0.098276	0.476616	0.201506	0.840766	-5.43982	0.915491	0.860549
NK.cells	IKZF2	-0.03411	4.781138	-0.20147	0.840795	-6.92024	0.857998	0.763457
NK.cells	CCDC152	-0.06584	3.284464	-0.20146	0.840802	-5.83517	0.877536	0.795932
NK.cells	GM41555	0.095606	0.571556	0.201442	0.840815	-5.44509	0.914179	0.858283
NK.cells	SPCS2	-0.01473	7.595587	-0.20144	0.840821	-6.98676	0.822533	0.705922
NK.cells	ZFP451	-0.02314	5.019065	-0.20138	0.840865	-6.52177	0.854936	0.758417
NK.cells	TMEM265	0.032621	3.958769	0.20137	0.840872	-6.24089	0.868674	0.781135
NK.cells	ATPAF1	0.033727	3.553171	0.200755	0.841351	-6.12485	0.874311	0.790118
NK.cells	TXNDC12	-0.02895	3.827411	-0.20063	0.841449	-6.20243	0.87071	0.784111
NK.cells	TRIM59	0.031975	4.368452	0.200577	0.84149	-6.41464	0.863652	0.772396
NK.cells	GM32036	0.026292	3.651119	0.200553	0.841508	-6.28875	0.873023	0.787981
NK.cells	TMEM177	-0.10725	0.336385	-0.20049	0.841559	-5.3073	0.917766	0.864063
NK.cells	PFDN6	0.02424	4.874434	0.20042	0.841613	-6.44893	0.857108	0.761639
NK.cells	ZFAND6	0.014473	6.978135	0.200087	0.841872	-6.89716	0.83065	0.718428
NK.cells	ENKUR	-0.0982	0.339833	-0.20001	0.841935	-5.36588	0.917911	0.864154
NK.cells	DNAJC13	0.022336	6.16837	0.199801	0.842095	-6.64632	0.840819	0.73484
NK.cells	GM29282	-0.07738	1.712401	-0.19969	0.842181	-5.59269	0.899117	0.831851
NK.cells	PARVB	0.0761	2.578711	0.199622	0.842234	-5.60349	0.88745	0.812052
NK.cells	GIMAP4	-0.03386	4.091849	-0.19956	0.84228	-6.56036	0.86746	0.778572
NK.cells	RNF138	0.016645	5.857254	0.199512	0.842321	-6.7725	0.844752	0.741232
NK.cells	RABGAP1L	0.020079	7.908574	0.199113	0.842632	-7.05146	0.819203	0.700091
NK.cells	STK11IP	0.047513	2.312647	0.199106	0.842637	-5.75993	0.891036	0.818083
NK.cells	PTGER2	0.069857	1.047913	0.199072	0.842663	-5.64351	0.908197	0.847355
NK.cells	EIF4EBP2	-0.01377	6.850744	-0.19898	0.842739	-6.86196	0.832279	0.721049
NK.cells	BCS1L	-0.07308	1.699946	-0.19874	0.842924	-5.63129	0.899306	0.832221
NK.cells	CLDN34C1	-0.10079	0.809812	-0.19872	0.842937	-5.3771	0.911467	0.853062
NK.cells	WDR66	0.067317	1.900032	0.198576	0.84305	-5.63799	0.896597	0.827633
NK.cells	NDFIP1	-0.01499	7.521506	-0.19856	0.843065	-7.03628	0.823961	0.707767
NK.cells	HAUS6	-0.02756	4.556972	-0.19849	0.84312	-6.44568	0.861433	0.768667
NK.cells	GM26520	0.076934	2.362216	0.198416	0.843175	-5.75505	0.890371	0.817077
NK.cells	NDUFV2	0.020825	6.136177	0.198379	0.843204	-6.72623	0.841243	0.735608
NK.cells	FANCB	-0.08668	0.677958	-0.19815	0.843381	-5.39152	0.913283	0.856288
NK.cells	HSPA4	0.013011	7.667014	0.198128	0.8434	-7.01994	0.822169	0.70497
NK.cells	EME2	0.062557	0.924802	0.198054	0.843458	-5.53813	0.909886	0.850471
NK.cells	UNC45A	-0.02564	4.303611	-0.19797	0.843525	-6.40951	0.864721	0.774193
NK.cells	1110038B1	0.031149	4.235292	0.197887	0.843588	-6.30791	0.86561	0.775669
NK.cells	GM17491	-0.05936	1.415352	-0.19786	0.843612	-5.52228	0.903176	0.838963
NK.cells	CD96	0.053018	0.686804	0.197817	0.843643	-6.12603	0.913161	0.856117
NK.cells	BZW2	0.019719	5.869307	0.197543	0.843856	-6.7649	0.844636	0.741245
NK.cells	SGPP1	0.026538	5.017924	0.197519	0.843875	-6.4929	0.855503	0.759024

NK.cells	RCBTB2	0.021735	5.187796	0.197515	0.843878	-6.59084	0.853323	0.755443
NK.cells	PDZD8	-0.02224	5.970732	-0.19739	0.843974	-6.6855	0.843376	0.739209
NK.cells	SLC52A2	0.043466	2.391257	0.196888	0.844367	-5.9542	0.89015	0.816796
NK.cells	AP5Z1	-0.04409	2.539751	-0.19687	0.844384	-5.83479	0.88816	0.813432
NK.cells	SNAPC3	0.024528	4.825524	0.196843	0.844402	-6.50642	0.858125	0.7633
NK.cells	MAFB	0.057322	4.60884	0.19678	0.844451	-6.16915	0.860924	0.767918
NK.cells	RBM15B	-0.02833	4.047089	-0.1966	0.844592	-6.21037	0.868228	0.780041
NK.cells	TRP53RKA	-0.04422	2.472703	-0.19624	0.844874	-5.85146	0.889058	0.815046
NK.cells	CERS4	0.03052	3.651416	0.196162	0.844934	-6.33539	0.873413	0.78875
NK.cells	2010300C(	0.069825	-0.0882	0.196142	0.844949	-5.55168	0.924087	0.875151
NK.cells	TONSL	0.061134	1.456718	0.196008	0.845054	-5.69783	0.902783	0.838397
NK.cells	USP12	-0.02056	6.069178	-0.19592	0.845122	-6.77489	0.842249	0.737411
NK.cells	GRAP	0.028414	4.536304	0.195831	0.845192	-6.44957	0.861864	0.769561
NK.cells	ERI2	0.075333	1.310882	0.195766	0.845242	-5.53927	0.904772	0.841802
NK.cells	UNC45B	-0.11984	0.423376	-0.19569	0.845301	-5.30216	0.916974	0.862813
NK.cells	CDC42SE1	0.018201	6.18973	0.195657	0.845327	-6.72724	0.840727	0.734941
NK.cells	A430073D:	-0.05547	1.815155	-0.19565	0.845331	-5.76559	0.897915	0.830086
NK.cells	TRPV2	-0.02369	4.873594	-0.19551	0.845442	-6.51831	0.857505	0.762381
NK.cells	SLC26A2	0.030605	3.742645	0.195462	0.84548	-6.28911	0.872215	0.786763
NK.cells	FUZ	-0.07891	1.08456	-0.19544	0.845497	-5.53019	0.907867	0.847126
NK.cells	ANGPTL1	0.091362	0.376343	0.1953	0.845606	-5.40809	0.917626	0.863967
NK.cells	TPGS1	0.026918	4.393149	0.195269	0.84563	-6.41456	0.863721	0.772659
NK.cells	LY86	0.046752	6.704851	0.195253	0.845643	-6.3138	0.834259	0.724498
NK.cells	MYEF2	-0.02296	5.328236	-0.19513	0.845737	-6.59844	0.851668	0.752832
NK.cells	ELK1	0.056395	1.559348	0.195006	0.845835	-5.57967	0.901386	0.836073
NK.cells	PIEZO1	-0.02391	5.046187	-0.19497	0.845863	-6.54636	0.855284	0.758774
NK.cells	ENO1B	0.065983	1.327345	0.194904	0.845915	-5.61189	0.904547	0.841505
NK.cells	TXNRD1	-0.02171	5.871273	-0.19483	0.845975	-6.74597	0.844754	0.741578
NK.cells	PRR12	-0.03046	3.451612	-0.19462	0.846138	-6.16903	0.876066	0.793287
NK.cells	ARL2	0.040485	3.356926	0.19446	0.846262	-6.01543	0.877316	0.795396
NK.cells	NBAS	0.024941	4.535139	0.194207	0.846459	-6.41461	0.861901	0.769786
NK.cells	FBXO9	0.023563	4.752411	0.194149	0.846505	-6.53287	0.85909	0.765151
NK.cells	APOLD1	0.099136	1.979983	0.193954	0.846657	-5.49041	0.895709	0.826551
NK.cells	JMJD7	0.052871	1.757778	0.193945	0.846664	-5.73464	0.898715	0.831672
NK.cells	SFN	-0.05323	2.056108	-0.19394	0.846667	-5.921	0.894681	0.824803
NK.cells	CHST12	-0.02536	5.763881	-0.19393	0.846677	-6.6986	0.846138	0.74394
NK.cells	TREM2	0.145516	0.639882	0.193908	0.846693	-5.33676	0.914005	0.857911
NK.cells	HOTAIRM1	0.105581	0.698917	0.193713	0.846845	-5.37361	0.913191	0.85651
NK.cells	OLFR77	-0.06221	1.376904	-0.19344	0.847056	-5.70024	0.903894	0.840567
NK.cells	FBXO44	-0.09738	0.220079	-0.19336	0.847121	-5.40348	0.919817	0.868013
NK.cells	FILIP1	-0.08834	0.904589	-0.19336	0.847124	-5.45768	0.91036	0.85167
NK.cells	CIR1	-0.01942	5.515333	-0.19326	0.847199	-6.67751	0.849301	0.749144
NK.cells	IRF2BP1	0.027369	4.152538	0.193146	0.847288	-6.27561	0.866874	0.778111
NK.cells	HMGCLL1	0.080212	1.17582	0.193145	0.847289	-5.53344	0.906641	0.845276
NK.cells	GAS8	0.057724	1.529564	0.193035	0.847374	-5.59441	0.901815	0.837008
NK.cells	PITRM1	0.035088	3.560314	0.192963	0.84743	-6.11075	0.874634	0.791073
NK.cells	GM43660	0.103302	0.127169	0.192953	0.847439	-5.37688	0.921109	0.870288

NK.cells	TGFBI	-0.06197	4.412001	-0.19294	0.847445	-6.01903	0.863498	0.772541
NK.cells	EXOC3L4	0.103653	0.594811	0.192848	0.84752	-5.37248	0.91463	0.859073
NK.cells	GM44751	-0.07323	1.310612	-0.19272	0.847617	-5.54803	0.904816	0.842163
NK.cells	SERPINA1E	-0.06342	5.030433	-0.19264	0.847679	-6.5813	0.855525	0.759379
NK.cells	GM5547	-0.04667	1.560402	-0.19251	0.847785	-5.89215	0.901435	0.836366
NK.cells	1700027J0	-0.08407	2.481407	-0.19243	0.847845	-5.6706	0.889003	0.81524
NK.cells	DYNC1H1	0.015354	6.255557	0.192162	0.848056	-6.75091	0.840093	0.734024
NK.cells	EPB41L4A(	0.044018	3.25392	0.192033	0.848156	-6.08304	0.878889	0.797996
NK.cells	COG4	0.020005	5.336083	0.191897	0.848263	-6.57878	0.851829	0.753094
NK.cells	MIER3	-0.03126	4.078409	-0.19159	0.848502	-6.18675	0.868181	0.780032
NK.cells	STRIP1	-0.02641	4.156745	-0.19156	0.848524	-6.32816	0.867159	0.778344
NK.cells	SLC38A9	0.025167	5.16902	0.191498	0.848574	-6.50659	0.854064	0.756723
NK.cells	C1GALT1C1	-0.02988	4.044817	-0.19095	0.849001	-6.25909	0.868952	0.780851
NK.cells	RIPK3	0.035269	3.285039	0.190844	0.849086	-6.16125	0.878946	0.797575
NK.cells	KIF24	0.034738	3.667581	0.190515	0.849342	-6.06207	0.873899	0.789268
NK.cells	ZFP770	-0.06012	1.760027	-0.19036	0.849467	-5.67714	0.89938	0.832278
NK.cells	PAIP2B	-0.02642	3.682629	-0.19026	0.849544	-6.2985	0.873701	0.788937
NK.cells	ARMC1	0.019754	4.827556	0.190227	0.849567	-6.50889	0.858785	0.764191
NK.cells	PRXL2C	-0.02347	4.932963	-0.1902	0.849586	-6.44218	0.857425	0.761952
NK.cells	2610301B2	0.059431	1.613471	0.190166	0.849615	-5.60927	0.901371	0.835675
NK.cells	TMEM176A	-0.03041	4.388779	-0.19013	0.849641	-6.44117	0.864468	0.773583
NK.cells	LEFTY1	-0.10152	0.408138	-0.19007	0.849689	-5.27244	0.917919	0.864152
NK.cells	ZFP954	0.052497	2.050779	0.189914	0.849812	-5.83757	0.895446	0.825616
NK.cells	COLEC10	-0.08755	0.557157	-0.18977	0.849922	-5.38495	0.915855	0.860615
NK.cells	F8	-0.07083	3.134673	-0.18977	0.849927	-5.74714	0.880938	0.801107
NK.cells	ADAL	-0.04658	2.279066	-0.18967	0.850001	-5.78125	0.892369	0.820415
NK.cells	RIOK2	-0.02282	4.466641	-0.18957	0.850079	-6.38435	0.863457	0.771966
NK.cells	DPH2	0.08385	0.879573	0.18925	0.850331	-5.44598	0.911408	0.853025
NK.cells	GZMK	0.080856	-1.47202	0.189187	0.85038	-5.40415	0.943801	0.910555
NK.cells	ZDHHC15	0.055982	1.125862	0.189104	0.850445	-5.82161	0.908027	0.847238
NK.cells	TRIM11	0.024844	5.06784	0.189088	0.850457	-6.39982	0.85569	0.759241
NK.cells	LZTS1	-0.061	1.417164	-0.18889	0.850616	-5.72289	0.904044	0.840498
NK.cells	GPS1	-0.02163	4.905295	-0.18884	0.850653	-6.48624	0.857782	0.762768
NK.cells	UBE2J2	-0.01389	6.638057	-0.18876	0.850715	-6.86366	0.835762	0.726852
NK.cells	HARBI1	0.067301	1.346342	0.188749	0.850722	-5.64507	0.905011	0.842164
NK.cells	NUDCD2	0.027728	4.534449	0.18869	0.850768	-6.40498	0.862577	0.770691
NK.cells	CDH23	0.036283	2.820164	0.188517	0.850904	-6.1646	0.885122	0.80835
NK.cells	COX15	-0.03367	2.891641	-0.18823	0.851127	-5.97909	0.884169	0.806798
NK.cells	HP	0.033781	7.365035	0.18791	0.851378	-6.96406	0.82671	0.712418
NK.cells	VEZT	-0.02562	4.178132	-0.1879	0.851387	-6.29549	0.867211	0.778513
NK.cells	NANS	0.023594	4.873876	0.187825	0.851445	-6.54802	0.858187	0.763588
NK.cells	USP21	-0.03763	3.140041	-0.18781	0.851457	-6.03506	0.880867	0.80134
NK.cells	CCDC59	0.015606	5.676842	0.18758	0.851636	-6.65693	0.8479	0.746761
NK.cells	RAD54B	0.059587	2.207787	0.187321	0.851838	-5.80766	0.893328	0.822551
NK.cells	ARPC5	0.01253	8.144041	0.187191	0.85194	-7.05941	0.817131	0.697284
NK.cells	PTGR2	0.030387	3.734192	0.186978	0.852106	-6.16552	0.873023	0.788432
NK.cells	VDR	0.060506	0.938817	0.186844	0.852211	-5.79832	0.910594	0.852194

NK.cells	RPGRIP1	-0.01827	6.55945	-0.18681	0.852241	-6.76643	0.836748	0.728822
NK.cells	ALPK1	-0.06816	4.802266	-0.18678	0.852263	-5.76861	0.859111	0.765363
NK.cells	GPATCH8	-0.01373	7.002079	-0.18667	0.852346	-6.91812	0.831216	0.719926
NK.cells	STRADB	0.037238	3.238382	0.186627	0.852381	-5.99087	0.879564	0.799457
NK.cells	NBEA	-0.05377	2.602645	-0.18651	0.852474	-5.84628	0.888028	0.813737
NK.cells	INIP	-0.02258	4.523666	-0.18649	0.852491	-6.39461	0.862717	0.771378
NK.cells	MOCOS	-0.07412	1.324581	-0.18638	0.852572	-5.67499	0.905308	0.843172
NK.cells	RRAS2	0.02218	5.198697	0.186329	0.852613	-6.6681	0.854009	0.757018
NK.cells	ZBTB43	0.033294	3.163113	0.186307	0.852631	-6.06786	0.880561	0.801147
NK.cells	LRRC28	-0.04382	3.874144	-0.1863	0.852636	-6.153	0.871186	0.785451
NK.cells	ZFP628	-0.04612	2.635685	-0.18613	0.852766	-5.92504	0.887586	0.813017
NK.cells	SRSF11	0.010349	7.871425	0.1861	0.852792	-7.03612	0.820469	0.702761
NK.cells	RSPH3A	0.044197	2.558451	0.18598	0.852887	-5.93071	0.888619	0.81479
NK.cells	YTHDF2	-0.01583	6.035232	-0.18596	0.852902	-6.71432	0.843352	0.739638
NK.cells	KTN1	-0.01704	5.826158	-0.18594	0.852918	-6.65338	0.846002	0.743962
NK.cells	EFCAB5	0.098099	0.504613	0.185846	0.852991	-5.33886	0.916582	0.862681
NK.cells	PLS1	-0.0811	0.910051	-0.18566	0.853138	-5.46782	0.910989	0.853041
NK.cells	ZFP28	-0.09193	0.2908	-0.1856	0.853183	-5.36843	0.919547	0.867856
NK.cells	GLT8D1	0.035312	3.339159	0.185541	0.85323	-5.99699	0.87823	0.797365
NK.cells	ICOSL	0.051993	1.327034	0.185494	0.853266	-5.80512	0.905274	0.843254
NK.cells	SPDL1	0.042163	2.833783	0.185494	0.853267	-6.02982	0.88494	0.808656
NK.cells	SKAP1	0.026361	4.523681	0.185408	0.853334	-6.91479	0.862716	0.771525
NK.cells	ZBTB48	0.078424	1.123594	0.185304	0.853415	-5.51539	0.908058	0.848066
NK.cells	ARHGAP26	-0.02001	6.685583	-0.18522	0.853477	-6.97069	0.835167	0.726471
NK.cells	IL6	0.148718	1.106723	0.185209	0.853489	-5.45664	0.908289	0.848468
NK.cells	POLG2	-0.02071	5.080463	-0.1852	0.853497	-6.59956	0.855527	0.759672
NK.cells	2700049A(	-0.02793	4.236674	-0.18509	0.853585	-6.34386	0.866448	0.777729
NK.cells	HMG20A	0.023203	4.897858	0.185006	0.853648	-6.48562	0.857878	0.763546
NK.cells	ZFP385A	-0.04803	3.938147	-0.18492	0.853716	-5.83101	0.870348	0.784233
NK.cells	MR1	0.082505	0.68263	0.184863	0.85376	-5.45777	0.914122	0.85854
NK.cells	SDHA	0.016836	5.691569	0.18473	0.853864	-6.63237	0.847712	0.746882
NK.cells	ARMH3	-0.01987	5.427651	-0.18464	0.853932	-6.60048	0.851078	0.75239
NK.cells	CPSF2	-0.02209	5.649423	-0.18454	0.854013	-6.55392	0.848249	0.747759
NK.cells	RAD51B	-0.03329	6.152694	-0.18453	0.854023	-6.71931	0.841867	0.737355
NK.cells	GPR65	0.029946	4.250918	0.184354	0.854158	-6.60829	0.866262	0.777446
NK.cells	BCKDK	-0.02175	4.559456	-0.18433	0.854175	-6.41846	0.862253	0.770797
NK.cells	GM46620	0.090589	0.683678	0.184297	0.854202	-5.42683	0.914108	0.858527
NK.cells	BMS1	0.0224	4.737717	0.184136	0.854329	-6.48146	0.859945	0.767009
NK.cells	TMEM163	-0.0273	6.026941	-0.18408	0.854369	-6.71912	0.843457	0.739982
NK.cells	BUB3	0.019954	5.956108	0.184051	0.854395	-6.70085	0.844353	0.741443
NK.cells	MLLT6	-0.03632	3.270712	-0.18391	0.854505	-6.33155	0.879136	0.798997
NK.cells	TXLNA	-0.02071	4.941372	-0.18386	0.854544	-6.49931	0.857317	0.762694
NK.cells	PPP2R1A	-0.01839	6.00443	-0.18385	0.854552	-6.71302	0.843741	0.740452
NK.cells	CYCS	-0.02135	7.712668	-0.1837	0.854669	-7.00162	0.822447	0.706067
NK.cells	CD163	0.145897	1.13156	0.183292	0.854989	-5.34511	0.907978	0.848024
NK.cells	SRRM2	-0.01244	8.62357	-0.18325	0.855022	-7.14674	0.811322	0.688439
NK.cells	SHF	-0.05563	1.832889	-0.18313	0.855114	-5.64022	0.898422	0.831695

NK.cells	DESI1	0.024069	5.729904	0.183128	0.855117	-6.58527	0.847252	0.74622
NK.cells	HIST2H2AC	0.065799	2.376405	0.182993	0.855222	-5.95495	0.89109	0.819241
NK.cells	GM47730	0.108926	-0.65913	0.182858	0.855328	-5.1605	0.932868	0.891328
NK.cells	COX11	0.023708	3.751078	0.182609	0.855523	-6.28213	0.872829	0.788598
NK.cells	1810006J0	0.044987	-0.01116	0.182575	0.855549	-5.9454	0.92378	0.875538
NK.cells	DNAJC16	0.051046	2.30093	0.182541	0.855576	-5.80391	0.892104	0.821064
NK.cells	ARFRP1	-0.02206	4.120918	-0.18245	0.855646	-6.3356	0.867986	0.780552
NK.cells	FDX1	-0.02692	4.692703	-0.18238	0.855701	-6.4657	0.860555	0.768227
NK.cells	PLA2G15	-0.04859	3.738633	-0.18236	0.855717	-5.94194	0.872993	0.788901
NK.cells	ZFYVE19	0.033451	3.132938	0.182316	0.855752	-6.03508	0.88099	0.802311
NK.cells	CYP4F16	-0.05959	1.441368	-0.18227	0.85579	-5.75532	0.903743	0.840969
NK.cells	IFIT3	-0.0823	3.112471	-0.18224	0.855813	-5.97984	0.881262	0.802783
NK.cells	ZFP758	0.039763	2.74078	0.182043	0.855966	-5.90037	0.88621	0.811168
NK.cells	RBM14	0.025356	3.712533	0.182013	0.855989	-6.22514	0.873336	0.789536
NK.cells	DCTN1	0.019437	4.987161	0.181938	0.856048	-6.50385	0.856755	0.762019
NK.cells	RPP21	0.020588	4.873001	0.181825	0.856136	-6.52525	0.858226	0.764455
NK.cells	MICOS10	-0.01526	7.131558	-0.18172	0.856221	-6.89161	0.829632	0.717868
NK.cells	PLA2G6	-0.06998	1.28393	-0.18171	0.856227	-5.59994	0.905893	0.844724
NK.cells	PML	0.036536	5.273859	0.181684	0.856247	-6.48419	0.853073	0.755972
NK.cells	TRAC	-0.03471	2.266621	-0.18154	0.856357	-6.43462	0.892605	0.821978
NK.cells	ACOT2	-0.02166	4.416307	-0.1814	0.856472	-6.38878	0.864194	0.774296
NK.cells	ATXN10	-0.01646	6.66392	-0.18128	0.856565	-6.83368	0.835519	0.727352
NK.cells	GM26789	-0.08447	0.271556	-0.18124	0.856592	-5.4518	0.919903	0.868907
NK.cells	RASGRF2	-0.06411	0.116081	-0.18111	0.856698	-5.56414	0.922101	0.872694
NK.cells	TMEM38A	-0.06352	1.868955	-0.18092	0.856842	-5.58096	0.898101	0.831241
NK.cells	GSKIP	-0.01805	4.602025	-0.18066	0.85705	-6.46059	0.861992	0.770458
NK.cells	TM9SF1	0.021255	5.042685	0.180608	0.857089	-6.53662	0.856302	0.76109
NK.cells	MNT	-0.02415	4.606728	-0.18034	0.8573	-6.4693	0.862026	0.770465
NK.cells	ERMARD	0.036604	3.076068	0.180302	0.857328	-5.97785	0.882112	0.803996
NK.cells	GM4631	-0.07462	0.664941	-0.18004	0.85753	-5.40418	0.914812	0.859734
NK.cells	SLC39A3	0.048917	1.91724	0.179992	0.857571	-5.79958	0.897688	0.830331
NK.cells	PTAR1	0.025119	3.690053	0.179981	0.85758	-6.22826	0.874029	0.790376
NK.cells	CRAMP1L	-0.01965	4.984741	-0.17984	0.857691	-6.52462	0.857216	0.7624
NK.cells	CCL8	-0.10492	-1.46778	-0.17971	0.857789	-5.12376	0.944267	0.912126
NK.cells	GTPBP4	-0.01631	5.956432	-0.17962	0.857862	-6.69792	0.844817	0.742051
NK.cells	FAM53C	-0.02576	3.981286	-0.17954	0.857927	-6.26342	0.870264	0.783996
NK.cells	TSPYL3	0.071438	0.960124	0.179417	0.858021	-5.61683	0.910805	0.852714
NK.cells	GTF2A2	0.017018	5.923594	0.179353	0.858071	-6.66697	0.845233	0.74273
NK.cells	EIF4A3	0.01705	5.877476	0.179234	0.858164	-6.69201	0.845839	0.743684
NK.cells	PDK2	-0.05001	2.10287	-0.17895	0.858384	-5.79023	0.895413	0.826094
NK.cells	NTM	-0.08337	0.913874	-0.17881	0.858496	-5.46755	0.911661	0.853913
NK.cells	HBP1	-0.01964	5.788072	-0.17871	0.858573	-6.65155	0.847163	0.745643
NK.cells	LTC4S	0.151398	1.066227	0.178489	0.858747	-5.37644	0.909599	0.850326
NK.cells	FAM32A	0.016737	5.312501	0.178331	0.858871	-6.574	0.853259	0.755591
NK.cells	ACTR3	-0.01116	8.876057	-0.17829	0.858904	-7.22173	0.808914	0.68425
NK.cells	FCNB	0.089462	-0.23854	0.178185	0.858986	-5.28699	0.927699	0.881733
NK.cells	CARD19	-0.02215	5.613475	-0.17813	0.859027	-6.66258	0.849412	0.74931

NK.cells	B3GALT2	0.073233	0.771125	0.177969	0.859154	-5.60753	0.91366	0.857382
NK.cells	EPHA4	-0.12536	0.083978	-0.17794	0.859179	-5.20999	0.92319	0.873897
NK.cells	GM5165	0.037555	2.638407	0.177937	0.859179	-5.95655	0.888287	0.814016
NK.cells	SCO1	-0.0537	1.930574	-0.17777	0.859307	-5.72728	0.897873	0.830225
NK.cells	PLXNB2	-0.05059	3.959065	-0.17736	0.859632	-5.76339	0.871052	0.784748
NK.cells	9330136K2	-0.05117	1.883233	-0.17712	0.859821	-5.81092	0.89872	0.831421
NK.cells	UBE2L3	0.01219	7.560477	0.177099	0.859836	-6.98331	0.825221	0.709905
NK.cells	STAM2	0.022547	5.475543	0.177069	0.859859	-6.64256	0.851422	0.752329
NK.cells	ZFP160	0.035465	2.950859	0.17671	0.86014	-6.0005	0.884373	0.807208
NK.cells	UCK2	0.023558	5.87449	0.176516	0.860292	-6.76092	0.846338	0.744161
NK.cells	FAM53B	0.028763	4.652028	0.176415	0.860372	-6.40384	0.862021	0.769923
NK.cells	DOCK6	0.059391	1.914885	0.176362	0.860413	-5.6442	0.898291	0.830849
NK.cells	HGH1	-0.0525	1.856316	-0.17632	0.860442	-5.67534	0.899085	0.832203
NK.cells	ALDH6A1	-0.05503	2.786139	-0.1762	0.860544	-5.88661	0.886571	0.810959
NK.cells	STXBP2	-0.02046	5.31968	-0.17616	0.860573	-6.53568	0.853417	0.755745
NK.cells	ATP6V1B2	0.0168	5.98524	0.176081	0.860633	-6.63532	0.844933	0.741883
NK.cells	WDR34	-0.08427	1.224098	-0.17599	0.860701	-5.49248	0.907701	0.846985
NK.cells	VPS26B	0.022616	3.835261	0.175914	0.860764	-6.42865	0.872676	0.787656
NK.cells	SCRIB	-0.02874	3.392621	-0.1759	0.860776	-6.10733	0.878509	0.797418
NK.cells	REEP3	-0.01774	6.727725	-0.17546	0.86112	-6.79015	0.835578	0.726796
NK.cells	TIAM1	-0.02658	4.628891	-0.17544	0.861132	-6.53085	0.862321	0.77053
NK.cells	IL18	0.051167	3.422293	0.175317	0.861231	-5.75737	0.878117	0.796869
NK.cells	PTPN18	-0.013	7.11924	-0.17524	0.861295	-6.97496	0.830691	0.718969
NK.cells	PGRMC2	0.024382	3.628957	0.1751	0.861401	-6.16022	0.875389	0.792373
NK.cells	SELENOO	0.032687	3.136064	0.175058	0.861434	-6.07189	0.88191	0.803338
NK.cells	VMN2R19	0.110922	-0.79625	0.174994	0.861484	-5.22478	0.935824	0.896097
NK.cells	SHANK2	-0.07289	1.410698	-0.17496	0.86151	-5.50758	0.905148	0.842851
NK.cells	SHE	0.086399	0.946591	0.174782	0.861651	-5.45663	0.91151	0.853832
NK.cells	GM2449	0.06627	1.279792	0.17471	0.861707	-5.65217	0.906938	0.845978
NK.cells	GM11944	0.030054	4.498486	0.174685	0.861727	-6.40251	0.864013	0.773525
NK.cells	GM36738	-0.03367	3.095803	-0.17451	0.861865	-6.11166	0.882445	0.804319
NK.cells	TMEM240	0.092522	0.240106	0.174479	0.861888	-5.38055	0.921285	0.870762
NK.cells	ECI2	-0.02589	4.557161	-0.17427	0.86205	-6.38615	0.863251	0.772327
NK.cells	MMP11	0.096024	0.128652	0.174104	0.862182	-5.27218	0.922837	0.873585
NK.cells	SMG8	0.038729	2.847118	0.174009	0.862256	-5.88745	0.885757	0.810021
NK.cells	1110019D1	0.058896	1.751896	0.173957	0.862297	-5.59956	0.900502	0.835078
NK.cells	GPSM2	-0.041	2.440784	-0.17384	0.862389	-5.99378	0.891197	0.819264
NK.cells	MANSC1	0.090919	0.242039	0.173515	0.862644	-5.3738	0.921258	0.870977
NK.cells	MON1B	0.048679	1.881481	0.173507	0.86265	-5.66383	0.898743	0.832197
NK.cells	RBMS3	-0.05112	3.072419	-0.1733	0.862808	-6.06735	0.882756	0.805135
NK.cells	RAC3	0.099015	-0.22085	0.173291	0.862819	-5.3185	0.927722	0.882294
NK.cells	CTNNAL1	-0.06741	1.924908	-0.17323	0.862865	-5.59049	0.898155	0.831246
NK.cells	LATS1	-0.01913	4.725566	-0.17321	0.86288	-6.46	0.861069	0.76893
NK.cells	PTPN2	0.012977	6.833794	0.173193	0.862896	-6.89979	0.834251	0.7251
NK.cells	PSMA2	0.014692	7.21693	0.173072	0.86299	-6.9308	0.829476	0.717413
NK.cells	TRIM21	-0.04318	2.57157	-0.17289	0.863134	-6.00519	0.889442	0.816464
NK.cells	PDGFA	0.07003	2.114128	0.172838	0.863174	-5.64896	0.895596	0.826929

NK.cells	HAL	-0.07116	2.215151	-0.17276	0.863231	-5.75176	0.894233	0.824627
NK.cells	DDX46	-0.01615	6.040748	-0.17268	0.8633	-6.74328	0.844229	0.741382
NK.cells	YTHDF3	0.01399	7.017555	0.172582	0.863375	-6.89404	0.831957	0.72153
NK.cells	GM45716	0.029111	3.20355	0.172486	0.863449	-6.21639	0.881014	0.802394
NK.cells	ADAM8	0.036651	2.602795	0.172396	0.86352	-6.24138	0.889023	0.815952
NK.cells	GTDC1	-0.01782	6.409058	-0.17233	0.863575	-6.80439	0.839579	0.733937
NK.cells	SWAP70	0.025054	5.61218	0.172325	0.863576	-6.64059	0.849677	0.750402
NK.cells	CDK19	0.018167	6.390727	0.171923	0.863891	-6.78882	0.83981	0.734348
NK.cells	HPS5	0.02175	4.448069	0.171877	0.863927	-6.38876	0.864668	0.775158
NK.cells	XYLT1	0.023228	7.355246	0.171864	0.863937	-6.8891	0.82776	0.714892
NK.cells	ACADL	0.017691	6.734458	0.171844	0.863953	-6.8576	0.835493	0.727354
NK.cells	ARID4A	0.015764	7.062741	0.171823	0.86397	-6.92601	0.831394	0.720737
NK.cells	ARFGEF2	0.019568	6.285468	0.1717	0.864066	-6.67384	0.841136	0.736503
NK.cells	AP1S1	0.021777	4.706161	0.171613	0.864134	-6.43552	0.86132	0.769608
NK.cells	TMEM29	-0.02284	4.706351	-0.17153	0.864201	-6.37138	0.861317	0.769604
NK.cells	PON2	0.01845	6.020273	0.171486	0.864233	-6.68653	0.844489	0.741961
NK.cells	2310011J0	-0.02266	4.446593	-0.17145	0.864263	-6.38565	0.864688	0.77519
NK.cells	SMIM7	-0.01918	4.932589	-0.17113	0.864511	-6.47189	0.858394	0.764831
NK.cells	4930549G2	0.033782	2.714781	0.171065	0.864564	-6.00202	0.887525	0.813533
NK.cells	COX19	-0.02594	4.496433	-0.17092	0.864678	-6.36493	0.86404	0.774195
NK.cells	GM42670	-0.07355	0.89839	-0.17073	0.864823	-5.45153	0.912173	0.855758
NK.cells	CSF1	0.090681	2.480595	0.170675	0.864869	-5.64242	0.890662	0.818939
NK.cells	SAR1A	0.0173	5.508767	0.170625	0.864909	-6.63114	0.850997	0.752749
NK.cells	VTI1B	0.01322	5.690918	0.170576	0.864947	-6.68533	0.848673	0.748943
NK.cells	CLEC9A	-0.09857	2.519945	-0.17055	0.864971	-5.43117	0.890134	0.818053
NK.cells	E030030I0	-0.03398	2.657085	-0.17054	0.864977	-5.94739	0.888296	0.814938
NK.cells	NXN	0.025675	5.325793	0.170471	0.865029	-6.53215	0.853338	0.756603
NK.cells	RTF1	-0.01191	6.81408	-0.17046	0.865037	-6.87273	0.834497	0.725894
NK.cells	RXYLT1	0.023363	3.864731	0.170367	0.865111	-6.24473	0.872289	0.788009
NK.cells	STAM	-0.02105	4.382468	-0.17019	0.865249	-6.33151	0.865522	0.776741
NK.cells	MEI4	-0.09527	1.201602	-0.17018	0.865259	-5.40707	0.908009	0.848598
NK.cells	CCDC14	-0.07833	0.809905	-0.17016	0.865274	-5.4515	0.913393	0.857889
NK.cells	TESMIN	-0.08468	0.971475	-0.17007	0.865342	-5.46655	0.911168	0.854055
NK.cells	2310009B1	0.019699	4.473004	0.169948	0.865439	-6.42417	0.864344	0.774819
NK.cells	AKTIP	0.03084	3.102695	0.169837	0.865527	-6.03339	0.882353	0.805012
NK.cells	NXF7	0.098496	-1.34285	0.169591	0.865719	-5.13525	0.943158	0.910853
NK.cells	SEC61A1	-0.01541	5.49084	-0.16947	0.865814	-6.62446	0.851226	0.753302
NK.cells	MCM3	0.02878	5.484871	0.169379	0.865885	-6.62244	0.851302	0.753431
NK.cells	ATOX1	-0.01507	8.244709	-0.16927	0.865972	-7.09691	0.816819	0.697724
NK.cells	VAMP7	0.023387	4.132998	0.169115	0.866092	-6.29565	0.868775	0.782336
NK.cells	ZFP637	-0.04645	2.315939	-0.16911	0.866099	-5.78452	0.892875	0.822905
NK.cells	TMEM86A	-0.05575	2.885047	-0.1691	0.866103	-5.67696	0.885251	0.809982
NK.cells	EIF1A	-0.01961	5.711772	-0.1691	0.866106	-6.6439	0.848407	0.748696
NK.cells	LEF1OS1	-0.0764	-0.45613	-0.16894	0.866232	-5.328	0.931026	0.888772
NK.cells	CENPL	0.028537	3.393274	0.168666	0.866445	-6.20645	0.878501	0.79867
NK.cells	ARID2	0.016739	6.543762	0.168663	0.866447	-6.77994	0.837885	0.73161
NK.cells	INAFM1	0.050896	1.866251	0.168472	0.866597	-5.69847	0.89895	0.833342

NK.cells	C730034FC	-0.04301	2.636781	-0.16838	0.866668	-5.93694	0.888568	0.815694
NK.cells	PKD1L2	0.067992	1.173969	0.168355	0.866688	-5.648	0.908387	0.849551
NK.cells	MTURN	-0.04574	2.270409	-0.1683	0.866732	-5.85782	0.893488	0.824064
NK.cells	CACNA1B	-0.09449	0.056619	-0.16821	0.866804	-5.30242	0.923842	0.876358
NK.cells	SUGCT	-0.04308	2.835527	-0.16795	0.86701	-5.91855	0.885911	0.811233
NK.cells	TIFA	-0.0348	6.262161	-0.16794	0.867014	-6.44783	0.84143	0.737433
NK.cells	XPR1	0.018157	6.866995	0.167869	0.86707	-6.82178	0.833836	0.72515
NK.cells	PPP1R15B	0.020397	5.553021	0.167813	0.867113	-6.57147	0.850432	0.752192
NK.cells	STN1	0.019804	3.886297	0.167677	0.86722	-6.45359	0.872006	0.787931
NK.cells	GPC1	0.055075	0.351919	0.167669	0.867227	-5.79793	0.919731	0.869291
NK.cells	GLMP	-0.02013	5.839868	-0.16754	0.867327	-6.70565	0.846778	0.746225
NK.cells	IQGAP1	0.013133	9.247795	0.167312	0.867506	-7.21474	0.804673	0.678753
NK.cells	HSPA12B	-0.0848	0.480164	-0.1673	0.867519	-5.29963	0.917951	0.866272
NK.cells	OPTN	-0.0274	4.651784	-0.16716	0.867623	-6.38654	0.862024	0.771391
NK.cells	ADCY4	-0.09629	1.413611	-0.16712	0.86766	-5.41915	0.905109	0.844114
NK.cells	UBAP1L	0.053859	1.134289	0.167085	0.867684	-5.5689	0.908932	0.850694
NK.cells	VPS4B	-0.01405	6.231035	-0.16708	0.867689	-6.74257	0.841823	0.738218
NK.cells	RPTOR	0.014264	6.294365	0.16701	0.867743	-6.84687	0.841024	0.736924
NK.cells	ZFP773	-0.06891	0.510873	-0.16679	0.867915	-5.59856	0.917526	0.865568
NK.cells	ZFP229	-0.09991	-0.01049	-0.16674	0.867957	-5.20892	0.924779	0.878192
NK.cells	USP36	-0.01905	4.970109	-0.16662	0.86805	-6.55357	0.85791	0.764633
NK.cells	DAPP1	-0.01651	6.482475	-0.16662	0.868051	-6.75967	0.838655	0.733107
NK.cells	SNHG15	-0.02848	3.481407	-0.16662	0.868051	-6.23211	0.877336	0.796983
NK.cells	SLC1A2	0.062561	2.197177	0.166543	0.86811	-5.5545	0.894475	0.825989
NK.cells	HACD2	0.015752	5.732831	0.166352	0.86826	-6.67068	0.848139	0.748603
NK.cells	ZFP335	-0.03249	2.842169	-0.16634	0.868273	-5.98421	0.885823	0.811323
NK.cells	IPO7	-0.0206	5.656347	-0.16625	0.868342	-6.64805	0.849114	0.750199
NK.cells	CLSTN1	-0.04849	1.774497	-0.16602	0.868517	-5.71224	0.900195	0.835774
NK.cells	BC065397	0.056535	1.292469	0.166004	0.868533	-5.56552	0.906765	0.847046
NK.cells	GALNT3	-0.0669	1.767982	-0.16588	0.868627	-5.60177	0.900283	0.835925
NK.cells	PARG	0.015302	5.560263	0.165835	0.868665	-6.63999	0.850339	0.752208
NK.cells	FAM20C	0.104766	1.641237	0.165769	0.868717	-5.51186	0.902006	0.838876
NK.cells	SLC22A5	-0.03896	3.042195	-0.16576	0.868721	-6.09295	0.883158	0.806821
NK.cells	GM20300	0.052044	1.343674	0.165703	0.868769	-5.58174	0.906064	0.845842
NK.cells	GM36279	0.028551	2.959709	0.165671	0.868794	-6.25266	0.884256	0.808675
NK.cells	DOT1L	0.020434	5.289755	0.165666	0.868798	-6.60541	0.8538	0.757895
NK.cells	FUNDC2	-0.0164	7.178207	-0.16553	0.868902	-6.95553	0.829958	0.719081
NK.cells	FHDC1	-0.08138	0.748905	-0.16545	0.868964	-5.39991	0.914234	0.859938
NK.cells	CAMP	-0.06588	4.463959	-0.16535	0.869044	-6.20835	0.864462	0.775547
NK.cells	RDH13	0.054357	1.398416	0.16524	0.869132	-5.61861	0.905316	0.844586
NK.cells	RRP15	0.024592	3.823389	0.16507	0.869266	-6.25575	0.872832	0.789577
NK.cells	LIG3	0.032551	3.358188	0.1649	0.8694	-6.09467	0.878965	0.799893
NK.cells	PREB	0.018875	4.736405	0.164862	0.869429	-6.45837	0.860928	0.76979
NK.cells	GM45509	-0.05662	1.640462	-0.16485	0.869439	-5.68902	0.902016	0.839035
NK.cells	KDM4A	0.02224	4.161515	0.164712	0.869546	-6.34153	0.868403	0.782239
NK.cells	NSL1	0.045753	2.25699	0.164658	0.869589	-5.78456	0.893669	0.824808
NK.cells	PRKACA	0.021385	4.156404	0.164636	0.869606	-6.33312	0.868469	0.78235

NK.cells	MYL12B	0.012064	8.183707	0.164615	0.869623	-7.10392	0.817564	0.699378
NK.cells	CTNNBL1	-0.01707	5.139211	-0.16459	0.869643	-6.54296	0.855733	0.761236
NK.cells	H2-K1	-0.02988	8.786019	-0.16456	0.869662	-7.25223	0.810239	0.687755
NK.cells	USHBP1	0.078311	1.085061	0.164437	0.869763	-5.38232	0.909636	0.852144
NK.cells	PHF1	0.033029	2.695984	0.164197	0.869951	-6.08559	0.887881	0.814828
NK.cells	ZFP930	0.040434	2.204594	0.164137	0.869998	-5.86799	0.894481	0.82604
NK.cells	SACM1L	0.017146	5.504292	0.164041	0.870073	-6.64465	0.851155	0.753565
NK.cells	RPH3AL	0.046257	1.299189	0.163972	0.870127	-5.79958	0.906779	0.847132
NK.cells	FUCA1	0.016862	5.963141	0.163812	0.870253	-6.67171	0.845364	0.744052
NK.cells	SP4	0.018522	5.254382	0.163334	0.870629	-6.6528	0.854695	0.759012
NK.cells	RABL2	0.065995	1.106471	0.163253	0.870692	-5.44956	0.909783	0.851855
NK.cells	GALE	0.038946	2.330073	0.163131	0.870788	-5.8168	0.89317	0.823407
NK.cells	GM15441	0.058281	1.344167	0.162817	0.871034	-5.52765	0.906731	0.84641
NK.cells	LTN1	-0.01699	5.03701	-0.16272	0.871107	-6.52818	0.857685	0.763782
NK.cells	ATP1A1	0.016368	6.234757	0.162563	0.871234	-6.77358	0.842443	0.738762
NK.cells	YTHDF1	-0.01575	5.78074	-0.16238	0.871379	-6.67786	0.848201	0.748191
NK.cells	MTX3	-0.07234	0.591443	-0.1623	0.871436	-5.47553	0.917136	0.864381
NK.cells	SDHB	0.01637	7.274793	0.162266	0.871466	-6.93138	0.829414	0.71771
NK.cells	MTMR3	-0.0128	7.886466	-0.16221	0.871513	-7.01659	0.821856	0.705594
NK.cells	GM34680	0.10485	0.042973	0.161707	0.871906	-5.23472	0.924771	0.877713
NK.cells	PP2D1	0.057619	1.690903	0.161702	0.87191	-5.74692	0.902051	0.838442
NK.cells	ZSWIM4	-0.02237	4.579223	-0.16168	0.871929	-6.4349	0.863655	0.773704
NK.cells	GM49961	0.074282	0.574285	0.161569	0.872014	-5.44474	0.917381	0.864862
NK.cells	5430427M	0.070763	1.237849	0.161541	0.872036	-5.54097	0.908238	0.849071
NK.cells	NSMAF	0.019537	4.903953	0.161502	0.872067	-6.61117	0.85945	0.766748
NK.cells	NOP2	-0.02553	3.635097	-0.16144	0.872115	-6.15599	0.876009	0.794318
NK.cells	GM26756	-0.06647	0.354794	-0.16138	0.872166	-5.65864	0.920426	0.870163
NK.cells	1700102H2	0.069205	0.529845	0.161366	0.872173	-5.44065	0.917996	0.865944
NK.cells	PPP1R18OS	-0.06115	0.993134	-0.16123	0.872279	-5.37261	0.911633	0.854873
NK.cells	MUS81	-0.04534	2.048507	-0.16109	0.872392	-5.75887	0.897275	0.830225
NK.cells	CAVIN2	-0.05473	2.454343	-0.16073	0.87267	-5.68569	0.891935	0.820938
NK.cells	D430042O0	-0.02498	3.792114	-0.1607	0.872693	-6.20754	0.874144	0.790944
NK.cells	ANKRD39	0.020475	3.543857	0.160585	0.872787	-6.28137	0.877417	0.796452
NK.cells	CDC34	-0.01635	6.050691	-0.16051	0.872844	-6.69883	0.844975	0.742773
NK.cells	CEP78	-0.03277	3.001386	-0.16024	0.873058	-5.9206	0.884613	0.808571
NK.cells	GM14410	-0.06143	0.850334	-0.16024	0.873061	-5.4865	0.913778	0.8584
NK.cells	4930486L2	-0.04862	0.484692	-0.1602	0.873087	-5.8029	0.918837	0.86717
NK.cells	VPS35	0.014376	6.232281	0.159973	0.873267	-6.71284	0.842677	0.739081
NK.cells	PRORP	0.02354	3.853039	0.159854	0.87336	-6.2226	0.873343	0.789718
NK.cells	SMAD2	-0.0129	6.426685	-0.15984	0.873368	-6.77973	0.840224	0.735116
NK.cells	TRMT10A	-0.02533	3.81366	-0.15971	0.873476	-6.22643	0.873861	0.790607
NK.cells	MYNN	0.018614	4.303762	0.159645	0.873525	-6.39248	0.867442	0.779895
NK.cells	ALG11	-0.0322	2.610001	-0.15956	0.873591	-5.90508	0.889845	0.817533
NK.cells	GM10101	0.067068	0.672014	0.159399	0.873718	-5.45378	0.916241	0.862785
NK.cells	BCORL1	0.03144	3.355845	0.159398	0.873719	-6.16743	0.879904	0.800749
NK.cells	TASOR	-0.01778	5.742445	-0.15938	0.873736	-6.65693	0.848893	0.749281
NK.cells	PCTP	-0.04033	2.252585	-0.15935	0.873756	-5.83307	0.894652	0.825706

NK.cells	GM10634	0.060123	0.651485	0.159135	0.873926	-5.60786	0.916627	0.863321
NK.cells	5730409E0	-0.07513	0.23499	-0.15903	0.874005	-5.40597	0.922417	0.873411
NK.cells	BABAM2	-0.01298	7.216423	-0.15884	0.874159	-6.94322	0.830457	0.719274
NK.cells	GM20513	0.107712	1.300029	0.158822	0.874171	-5.37221	0.907726	0.848001
NK.cells	CNOT8	-0.0157	5.284943	-0.15869	0.874273	-6.50353	0.854868	0.759012
NK.cells	MAP1LC3B	-0.01651	7.434486	-0.15863	0.874319	-6.9522	0.827753	0.714926
NK.cells	PPP1R14A	0.078242	0.601813	0.15834	0.87455	-5.38869	0.917355	0.864626
NK.cells	HSD17B12	0.017149	6.085483	0.158165	0.874687	-6.69726	0.844665	0.742312
NK.cells	HDAC7	-0.02259	4.31747	-0.15809	0.874745	-6.41436	0.867397	0.77978
NK.cells	CFL1	0.013905	10.01399	0.158091	0.874746	-7.3672	0.796477	0.665456
NK.cells	A130010J1	0.052419	1.72998	0.158042	0.874784	-5.65442	0.901869	0.837986
NK.cells	PRRC1	-0.02043	4.219989	-0.15792	0.87488	-6.33731	0.86867	0.781923
NK.cells	CALM1	0.010747	9.782329	0.157829	0.874951	-7.31684	0.799232	0.669794
NK.cells	RAD51C	-0.0359	2.245463	-0.15781	0.87497	-5.92851	0.894886	0.826112
NK.cells	PPP2R1B	-0.02145	4.339805	-0.15779	0.874981	-6.3933	0.867106	0.77935
NK.cells	2810454H0	-0.03745	2.514759	-0.1574	0.875287	-5.90211	0.891346	0.819959
NK.cells	TBC1D17	0.02529	3.823308	0.157314	0.875356	-6.24174	0.873953	0.790643
NK.cells	FPR1	0.116562	2.992339	0.157289	0.875376	-5.53922	0.884955	0.809138
NK.cells	SOCS5	-0.02628	4.029298	-0.15727	0.875394	-6.34827	0.871248	0.786123
NK.cells	POLR3E	-0.02169	3.918387	-0.15718	0.875464	-6.28457	0.872703	0.788553
NK.cells	XRCC2	0.055606	0.802718	0.15713	0.875501	-5.56248	0.914664	0.859922
NK.cells	TOX	-0.02658	4.351569	-0.15685	0.87572	-6.79175	0.867137	0.779116
NK.cells	CXXC1	-0.02013	4.307295	-0.15681	0.875749	-6.39671	0.867714	0.780077
NK.cells	MATN2	-0.07589	0.980441	-0.15668	0.875852	-5.38573	0.912353	0.855747
NK.cells	GIGYF1	-0.02009	4.441868	-0.15647	0.876019	-6.37809	0.86602	0.777233
NK.cells	DOHH	-0.02207	4.347216	-0.15625	0.876193	-6.33359	0.867254	0.7793
NK.cells	SCLT1	-0.02473	4.474106	-0.15624	0.876203	-6.38863	0.865601	0.776552
NK.cells	VSIG10	0.063239	0.47062	0.156214	0.876221	-5.52124	0.919433	0.868004
NK.cells	CDYL	-0.01408	5.659184	-0.15618	0.876246	-6.67513	0.850326	0.75135
NK.cells	MCM2	-0.03166	4.762793	-0.15594	0.876434	-6.44971	0.861926	0.770389
NK.cells	TLE1	0.028445	4.060562	0.155812	0.876537	-6.04363	0.871076	0.785609
NK.cells	EOMES	-0.0568	-0.77158	-0.15567	0.876645	-5.73742	0.93693	0.898518
NK.cells	CLEC2G	-0.09049	0.015976	-0.15565	0.876665	-5.27644	0.925847	0.879102
NK.cells	IL12B	0.143816	-0.29714	0.155624	0.876684	-5.32555	0.930237	0.886773
NK.cells	IGIP	0.070563	0.675003	0.155529	0.876759	-5.41105	0.916681	0.863177
NK.cells	MYH13	-0.0865	-1.61994	-0.15533	0.876913	-5.08967	0.948329	0.919926
NK.cells	ARHGEF15	0.09707	0.580885	0.155302	0.876938	-5.27119	0.91802	0.865458
NK.cells	GM527	0.06893	0.481195	0.154984	0.877187	-5.54085	0.919442	0.867947
NK.cells	CPQ	-0.02768	5.056734	-0.15483	0.87731	-6.33044	0.858199	0.764239
NK.cells	LMAN2L	0.02087	4.447735	0.154791	0.877339	-6.29744	0.86609	0.777307
NK.cells	ABCB8	-0.03184	2.240197	-0.15477	0.877352	-5.78851	0.895361	0.826549
NK.cells	BNIP1	0.030223	3.526643	0.154719	0.877395	-6.1128	0.878176	0.797518
NK.cells	PRDM11	0.027508	3.444862	0.154476	0.877586	-6.22716	0.879258	0.799427
NK.cells	PASK	-0.07354	1.295986	-0.15431	0.877715	-5.53647	0.908203	0.848692
NK.cells	UBQLN2	-0.02031	4.031371	-0.1543	0.877722	-6.29433	0.871531	0.786516
NK.cells	GM48027	-0.03351	2.815176	-0.15423	0.87778	-5.98993	0.887636	0.813595
NK.cells	SPINDOC	0.019159	4.427162	0.154157	0.877837	-6.40982	0.866358	0.777922

NK.cells	RAB8B	-0.01483	7.724426	-0.15413	0.877861	-7.16012	0.824549	0.709699
NK.cells	GCC2	-0.01785	5.193926	-0.15406	0.877914	-6.56289	0.856432	0.761507
NK.cells	NELFE	-0.01842	4.393275	-0.15405	0.877922	-6.39268	0.8668	0.778671
NK.cells	GM37168	0.082582	0.65738	0.15396	0.877992	-5.27735	0.916999	0.863924
NK.cells	CYP3A13	-0.0704	0.779768	-0.15374	0.878165	-5.46675	0.915395	0.86102
NK.cells	GM49169	-0.09313	-0.42801	-0.15367	0.878223	-5.23716	0.932246	0.890374
NK.cells	GORASP1	-0.07738	0.733344	-0.15341	0.878423	-5.37257	0.916169	0.862212
NK.cells	SMARCE1	-0.01206	6.302182	-0.15322	0.878571	-6.75575	0.842577	0.738471
NK.cells	NUP85	-0.02453	4.345133	-0.15312	0.878652	-6.36613	0.867709	0.779824
NK.cells	GIMAP8	0.030341	2.465426	0.153042	0.878714	-6.27419	0.892616	0.821722
NK.cells	1700047M	0.085533	-0.15234	0.152935	0.878798	-5.31404	0.928595	0.883734
NK.cells	APC	0.014624	6.723147	0.152337	0.879268	-6.82614	0.837666	0.730045
NK.cells	SLC6A6	-0.01571	7.287991	-0.15215	0.879416	-6.83409	0.83068	0.71868
NK.cells	SEC23IP	0.015352	4.499732	0.151986	0.879544	-6.42023	0.866182	0.77671
NK.cells	NEK6	0.024333	3.873937	0.151891	0.879619	-6.26403	0.874373	0.790394
NK.cells	TRIM16	-0.06662	2.341778	-0.15171	0.879764	-5.49112	0.894784	0.82483
NK.cells	POLR2K	-0.01248	6.77475	-0.1517	0.879766	-6.8338	0.8371	0.729087
NK.cells	AAAS	0.025729	3.794127	0.151411	0.879997	-6.24726	0.875424	0.792197
NK.cells	RDM1	-0.02431	5.028258	-0.15141	0.879999	-6.49957	0.859328	0.765444
NK.cells	RGS3	-0.02788	2.862627	-0.15125	0.880125	-6.36891	0.887789	0.81305
NK.cells	PPCS	0.029152	2.732539	0.151238	0.880132	-5.97542	0.889531	0.815997
NK.cells	C530008M	0.070358	0.74516	0.151222	0.880145	-5.49283	0.916598	0.862345
NK.cells	GM31645	0.067743	0.726126	0.151211	0.880154	-5.48158	0.916861	0.862801
NK.cells	DENR	0.013192	6.279481	0.150982	0.880334	-6.75775	0.843366	0.739333
NK.cells	NCOA4	-0.0229	6.199192	-0.15096	0.880353	-6.66809	0.844382	0.740988
NK.cells	HNMT	0.081323	-0.38409	0.150896	0.880401	-5.20381	0.932394	0.889862
NK.cells	CCDC117	0.016552	4.699485	0.150805	0.880473	-6.52521	0.863612	0.772614
NK.cells	WWTR1	-0.0632	2.126919	-0.15052	0.880696	-5.60638	0.897793	0.830008
NK.cells	SHMT1	0.031697	4.012421	0.150492	0.880719	-6.14052	0.872656	0.78759
NK.cells	CCAR2	-0.02675	3.432111	-0.15043	0.880767	-6.11	0.88031	0.800413
NK.cells	ZFP458	0.072374	0.876726	0.150075	0.881047	-5.47246	0.915102	0.859432
NK.cells	CFAP126	0.051961	1.069649	0.149786	0.881274	-5.683	0.912474	0.854934
NK.cells	GM11084	-0.02539	3.368087	-0.14958	0.88144	-6.13272	0.881398	0.802067
NK.cells	PRR7	0.026923	3.334702	0.149367	0.881604	-6.19849	0.881842	0.802859
NK.cells	SNRNP200	0.018243	5.038857	0.149251	0.881696	-6.54187	0.859525	0.765694
NK.cells	ZFP932	0.026529	2.788655	0.149246	0.8817	-5.94503	0.889124	0.81518
NK.cells	OSBPL11	-0.0194	5.368512	-0.14921	0.881725	-6.53795	0.85528	0.758706
NK.cells	MAPK7	-0.03415	3.195275	-0.14907	0.881837	-5.9478	0.883695	0.806056
NK.cells	NAP1L1	-0.01577	7.439515	-0.14895	0.881936	-6.95851	0.829129	0.716233
NK.cells	GM50322	0.075304	0.358739	0.148945	0.881936	-5.38675	0.922319	0.872184
NK.cells	EXOC8	0.043479	1.951234	0.148833	0.882024	-5.69568	0.900418	0.834473
NK.cells	ETNK1	0.014795	5.999727	0.148604	0.882204	-6.71906	0.847214	0.745604
NK.cells	PAOX	-0.03784	2.987177	-0.14847	0.882312	-5.88803	0.886469	0.810867
NK.cells	NEURL4	0.046782	2.445552	0.148414	0.882354	-5.80713	0.893733	0.823186
NK.cells	CDC123	0.016656	5.404958	0.148389	0.882374	-6.58931	0.854812	0.758113
NK.cells	PAXIP1	-0.02125	4.269449	-0.14837	0.882389	-6.36937	0.869524	0.782461
NK.cells	PPP1R13B	-0.01705	5.30235	-0.14825	0.882486	-6.62717	0.85613	0.760301

NK.cells	PSMB2	0.011514	7.058131	0.148165	0.88255	-6.90417	0.833878	0.724036
NK.cells	COX4I1	0.010376	9.086822	0.14804	0.882648	-7.20762	0.808962	0.684336
NK.cells	RRM2B	0.022823	4.437435	0.147976	0.882699	-6.24509	0.86733	0.778897
NK.cells	ERCC1	0.024236	3.315868	0.147938	0.882728	-6.15676	0.882092	0.803594
NK.cells	PDLIM1	-0.01917	4.944927	-0.14792	0.882739	-6.60691	0.860739	0.767971
NK.cells	CNOT4	-0.01119	7.523005	-0.14782	0.882821	-6.99399	0.828094	0.714805
NK.cells	RNF115	0.011213	6.978362	0.147797	0.882839	-6.88338	0.834875	0.725725
NK.cells	GLIS2	-0.05315	1.3513	-0.14763	0.882972	-5.59751	0.908604	0.848745
NK.cells	OLFR1369-	0.074399	-0.76388	0.147621	0.882978	-5.49684	0.938095	0.900072
NK.cells	MTO1	-0.02239	3.553315	-0.14759	0.883	-6.20379	0.878944	0.798326
NK.cells	UBAP2	0.013228	6.131148	0.14752	0.883057	-6.7611	0.845546	0.743045
NK.cells	KCNIP2	0.056308	1.248736	0.147439	0.883121	-5.59779	0.910011	0.851167
NK.cells	SNX19	-0.02511	2.862011	-0.14732	0.883216	-6.0315	0.888142	0.81386
NK.cells	UBE2D2A	-0.00716	8.209532	-0.1472	0.883311	-7.07418	0.819633	0.701309
NK.cells	SON	-0.00975	7.67203	-0.14717	0.883334	-6.99349	0.826249	0.711878
NK.cells	MOCS2	0.017779	5.094585	0.147025	0.883447	-6.51368	0.858845	0.764836
NK.cells	GTF3C5	0.038484	2.258469	0.146771	0.883647	-5.8595	0.896392	0.827668
NK.cells	A630023P1	0.063395	0.306091	0.146722	0.883686	-5.56471	0.923192	0.87381
NK.cells	CLCN4	0.015677	5.289066	0.146572	0.883804	-6.58236	0.856473	0.760727
NK.cells	KLHL26	-0.03224	2.727028	-0.14644	0.883909	-5.9679	0.890163	0.816952
NK.cells	HTT	-0.01529	5.362069	-0.1462	0.884095	-6.61106	0.855626	0.759212
NK.cells	MTA1	-0.01721	4.55479	-0.14603	0.884229	-6.48509	0.866068	0.776529
NK.cells	LSMEM1	0.039773	4.06136	0.146022	0.884237	-6.26251	0.872518	0.787268
NK.cells	FBXO36	0.06577	1.228268	0.145932	0.884307	-5.50096	0.910572	0.851815
NK.cells	INTS8	-0.01648	4.693172	-0.1459	0.884334	-6.46518	0.864268	0.77355
NK.cells	NSA2	-0.0091	7.622863	-0.14567	0.884512	-7.02039	0.827149	0.71298
NK.cells	POLR3A	0.027496	3.30507	0.145662	0.88452	-6.15662	0.882547	0.80404
NK.cells	SERPINB1A	-0.05076	3.600711	-0.14546	0.884677	-5.96876	0.878707	0.797465
NK.cells	NOCT	-0.02296	5.04776	-0.14531	0.884796	-6.52807	0.859792	0.766054
NK.cells	GM10130	0.036856	1.783227	0.145282	0.884819	-5.8523	0.903104	0.838896
NK.cells	PIK3CA	0.014458	6.128996	0.144958	0.885074	-6.75342	0.84599	0.743414
NK.cells	CCDC66	-0.03142	2.265203	-0.14495	0.885082	-5.90578	0.896608	0.827814
NK.cells	SNRNP48	0.015592	4.916287	0.144825	0.885179	-6.54335	0.861535	0.76895
NK.cells	MGAT2	-0.01332	5.873385	-0.14479	0.885207	-6.70568	0.849241	0.748734
NK.cells	4931413K1	0.041287	1.943113	0.144733	0.885251	-5.72062	0.900973	0.835286
NK.cells	FARP2	-0.03575	2.6131	-0.14461	0.885346	-5.94441	0.891919	0.819886
NK.cells	AIRN	-0.02241	5.663175	-0.14458	0.885375	-6.79803	0.851924	0.753166
NK.cells	TICAM1	-0.03021	2.819811	-0.14449	0.885445	-6.02897	0.889145	0.815199
NK.cells	HNRNPD	0.009915	7.908504	0.14436	0.885545	-7.0379	0.823762	0.707551
NK.cells	CLEC1B	0.052483	3.741016	0.144149	0.885711	-5.88365	0.87694	0.794653
NK.cells	MED24	0.026539	3.187102	0.144021	0.885812	-6.02346	0.884283	0.807008
NK.cells	EBP	-0.01927	4.791273	-0.14393	0.885883	-6.49547	0.863197	0.771791
NK.cells	SMURF2	-0.01319	6.82379	-0.14391	0.885899	-6.86752	0.837265	0.729337
NK.cells	GUCY1A1	-0.08529	-0.00341	-0.14387	0.885928	-5.30679	0.927881	0.881872
NK.cells	RCCD1	-0.03555	3.170294	-0.14371	0.886054	-5.98582	0.884507	0.807444
NK.cells	MCF2L	-0.07794	0.46165	-0.14369	0.886071	-5.40772	0.921387	0.870588
NK.cells	RNF111	-0.01355	6.692893	-0.14337	0.886326	-6.80598	0.838938	0.732079

NK.cells	TXNL1	0.013677	6.955558	0.143339	0.886348	-6.87432	0.835642	0.726745
NK.cells	PIFO	0.044755	1.246923	0.143197	0.88646	-5.87695	0.910561	0.851903
NK.cells	LIPE	-0.02314	3.783042	-0.14293	0.886674	-6.165	0.876416	0.793905
NK.cells	CSTF3	-0.01302	6.037918	-0.14291	0.886687	-6.71659	0.847218	0.745602
NK.cells	TTC38	0.033709	2.505389	0.142878	0.886711	-5.96823	0.893443	0.822635
NK.cells	OSBP	-0.01591	5.016429	-0.14286	0.886724	-6.54279	0.860311	0.767111
NK.cells	KMT2D	-0.01273	5.945956	-0.14285	0.886736	-6.69814	0.848388	0.747516
NK.cells	MFN1	0.022935	3.297935	0.142821	0.886757	-6.16695	0.882839	0.8047
NK.cells	MICAL1	0.020363	3.487265	0.142214	0.887234	-6.31154	0.880628	0.800655
NK.cells	1700037C1	0.031189	3.065274	0.142171	0.887269	-5.94523	0.886242	0.81013
NK.cells	NINJ1	-0.02552	5.750418	-0.1421	0.887326	-6.48792	0.851173	0.751806
NK.cells	TTC25	-0.08094	0.218704	-0.14201	0.887397	-5.2833	0.925123	0.876864
NK.cells	CDC42	0.007794	8.943013	0.141972	0.887425	-7.20362	0.811447	0.687891
NK.cells	LRR1	0.051169	1.435876	0.141719	0.887624	-5.57954	0.908371	0.847751
NK.cells	GANC	-0.02711	3.377098	-0.14167	0.887659	-6.14352	0.882178	0.803197
NK.cells	NPM1	0.01284	8.143673	0.141556	0.887752	-7.08815	0.821292	0.703392
NK.cells	CD52	0.015759	9.30175	0.141457	0.887831	-7.36317	0.807209	0.68109
NK.cells	MSL3	0.016271	4.998465	0.141377	0.887894	-6.49382	0.86094	0.767755
NK.cells	CBX5	0.019098	5.103912	0.141259	0.887986	-6.54492	0.859596	0.765524
NK.cells	DMD	-0.04819	2.419953	-0.14115	0.888074	-5.73852	0.895041	0.824918
NK.cells	ZSCAN20	0.067763	1.073033	0.140844	0.888313	-5.46229	0.913581	0.856434
NK.cells	USP16	0.015465	5.208005	0.140674	0.888447	-6.58124	0.858448	0.763402
NK.cells	RAC2	0.012453	9.052768	0.140258	0.888775	-7.21112	0.810416	0.685987
NK.cells	CZIB	0.023718	3.658795	0.140238	0.888791	-6.14595	0.87868	0.797115
NK.cells	MFSD3	-0.06472	0.642198	-0.14016	0.888856	-5.39589	0.919563	0.866857
NK.cells	APOL7C	0.111577	-0.99321	0.14007	0.888924	-5.18449	0.942514	0.907097
NK.cells	GM6710	-0.057	0.761027	-0.14006	0.888931	-5.532	0.917915	0.864001
NK.cells	FPGS	-0.03084	3.013479	-0.13997	0.889005	-5.93338	0.88726	0.811565
NK.cells	PLCB2	0.026433	3.320212	0.139887	0.889067	-6.0912	0.88317	0.804684
NK.cells	LIPA	0.018667	5.64326	0.139845	0.8891	-6.62562	0.852856	0.754311
NK.cells	LRRC75A	0.038886	2.6203	0.139827	0.889115	-5.92924	0.892531	0.820522
NK.cells	PARP2	0.015095	4.841287	0.139778	0.889154	-6.47072	0.863191	0.771342
NK.cells	TPCN2	-0.03693	2.597655	-0.13971	0.889209	-5.83184	0.892836	0.821039
NK.cells	SRSF4	-0.01169	6.225555	-0.13958	0.889308	-6.74213	0.845445	0.742185
NK.cells	GM26787	0.061993	1.300668	0.139436	0.889423	-5.54775	0.910478	0.851232
NK.cells	GRAMD3	0.015631	6.080829	0.139419	0.889436	-6.93303	0.847283	0.745222
NK.cells	SALL2	-0.07012	0.090711	-0.13913	0.889661	-5.35225	0.92741	0.880379
NK.cells	KCP	0.066125	0.100139	0.139055	0.889723	-5.42423	0.927278	0.880165
NK.cells	SOX6	-0.06693	1.905309	-0.13868	0.890016	-5.67487	0.90251	0.837215
NK.cells	TLR13	-0.07378	1.881588	-0.13843	0.890215	-5.39014	0.902833	0.837816
NK.cells	ZFP113	0.059664	1.151185	0.13843	0.890215	-5.47526	0.912836	0.854997
NK.cells	SLC22A14	-0.03624	2.744823	-0.13835	0.890276	-5.91761	0.891161	0.817945
NK.cells	RAB9	0.016163	4.887523	0.138241	0.890364	-6.40212	0.862885	0.770628
NK.cells	MFSD8	0.023806	2.979793	0.138079	0.890492	-5.99894	0.888012	0.812661
NK.cells	MRPL13	-0.01674	4.834872	-0.13807	0.890497	-6.46733	0.863568	0.771769
NK.cells	CCDC92	-0.04862	-0.17821	-0.13801	0.890544	-5.59745	0.931345	0.8872
NK.cells	B230369F2	-0.03669	2.457789	-0.13798	0.890572	-5.76392	0.895024	0.824548

NK.cells	SH3RF1	-0.02049	4.357213	-0.13794	0.890605	-6.43506	0.869792	0.782099
NK.cells	ZFP687	-0.02583	2.919042	-0.13783	0.890688	-6.01487	0.888835	0.814036
NK.cells	9430060I0:	0.05174	1.308677	0.137709	0.890784	-5.63334	0.910694	0.851315
NK.cells	CNOT9	0.015708	4.541441	0.137628	0.890848	-6.44616	0.867409	0.778101
NK.cells	1-Sep	-0.01368	6.037433	-0.13742	0.891012	-6.81362	0.848222	0.746376
NK.cells	NELFCD	0.018947	4.117826	0.13733	0.891083	-6.30529	0.87304	0.787381
NK.cells	TMEM144	0.070342	0.882973	0.137022	0.891325	-5.39219	0.91683	0.861633
NK.cells	EHHADH	0.048846	1.66442	0.136631	0.891633	-5.66784	0.9062	0.843167
NK.cells	USP15	0.011779	7.650516	0.1366	0.891658	-6.99409	0.828231	0.713777
NK.cells	HIST1H4D	-0.06273	1.04631	-0.13652	0.891723	-5.54356	0.914691	0.857776
NK.cells	FAM167A	0.073552	1.837572	0.136436	0.891787	-5.43552	0.903836	0.839119
NK.cells	HYLS1	0.029182	3.183514	0.136404	0.891812	-5.9474	0.885687	0.808287
NK.cells	SMAD7	-0.01788	5.164939	-0.1363	0.891896	-6.5762	0.85968	0.764917
NK.cells	2410006H1	0.018958	7.535004	0.136228	0.891951	-6.97463	0.829664	0.716087
NK.cells	NAA30	-0.01894	3.918894	-0.13607	0.892072	-6.27389	0.875984	0.791962
NK.cells	MPHOSPH!	0.018503	4.258614	0.135688	0.892376	-6.41327	0.871568	0.784627
NK.cells	D2HGDH	0.037114	2.08867	0.135633	0.89242	-5.74034	0.90052	0.833457
NK.cells	TGFB3	-0.06503	0.567314	-0.13545	0.892561	-5.46752	0.921431	0.869459
NK.cells	CELSR1	0.04684	1.626552	0.135411	0.892595	-5.88799	0.906818	0.844245
NK.cells	GM43378	0.048934	1.243694	0.135333	0.892656	-5.45366	0.912071	0.853276
NK.cells	DNAJB5	-0.05312	1.175316	-0.13525	0.892725	-5.48545	0.913013	0.854899
NK.cells	ATP5G1	0.013312	7.643771	0.135231	0.892737	-7.00271	0.828407	0.714071
NK.cells	SUOX	-0.03791	1.361958	-0.1352	0.892764	-5.69765	0.910445	0.850476
NK.cells	PPP1R2	0.009307	6.908394	0.135177	0.892779	-6.82831	0.837579	0.728839
NK.cells	TBL2	0.028262	2.392625	0.134918	0.892983	-5.80784	0.896438	0.826475
NK.cells	MAF	0.024937	5.654779	0.134592	0.893241	-6.65266	0.853508	0.754869
NK.cells	HIPK3	0.014165	5.445686	0.134589	0.893243	-6.61372	0.856191	0.759276
NK.cells	ZMIZ1	-0.01109	6.978265	-0.13458	0.893253	-6.91888	0.836735	0.727561
NK.cells	SNX2	-0.00994	7.314048	-0.1345	0.893313	-6.86209	0.832538	0.720802
NK.cells	RC3H1	0.010908	6.887392	0.134401	0.893391	-6.88276	0.837875	0.729426
NK.cells	FXR2	-0.01524	5.267562	-0.13424	0.893517	-6.59276	0.858485	0.763083
NK.cells	BC030867	-0.04497	2.099983	-0.13423	0.893522	-5.82769	0.900402	0.833399
NK.cells	DCUN1D5	0.009096	6.828224	0.134052	0.893666	-6.8682	0.838618	0.730679
NK.cells	ZFP119B	-0.04711	1.297642	-0.13405	0.893669	-5.56481	0.911364	0.852245
NK.cells	HDGFL2	-0.01451	4.979057	-0.13405	0.893671	-6.53044	0.862214	0.769281
NK.cells	HMOX1	0.048111	4.807778	0.133732	0.893918	-6.14451	0.864436	0.773063
NK.cells	SNX6	-0.0105	6.568189	-0.13352	0.894089	-6.77441	0.841893	0.736139
NK.cells	5530601H(	0.02511	3.904215	0.133348	0.894221	-6.20936	0.876261	0.792876
NK.cells	DPP6	-0.08263	-0.15893	-0.13331	0.894248	-5.27712	0.931629	0.887683
NK.cells	E430024I0:	0.037769	1.898133	0.133218	0.894324	-5.70341	0.903146	0.838405
NK.cells	ZFP780B	0.02498	3.329247	0.133129	0.894394	-6.13088	0.883878	0.805687
NK.cells	EIF3J2	-0.04643	1.443832	-0.13311	0.894407	-5.58334	0.909356	0.84906
NK.cells	ALG13	0.01397	4.514642	0.133105	0.894413	-6.40347	0.868253	0.77954
NK.cells	TDRP	0.054887	-0.36439	0.133024	0.894477	-5.65198	0.934526	0.892779
NK.cells	TUSC3	-0.01542	4.978446	-0.13301	0.894487	-6.57298	0.862221	0.769539
NK.cells	ZMYM2	0.013902	5.970893	0.132911	0.894566	-6.70332	0.849468	0.748586
NK.cells	MTA2	0.014366	5.782517	0.132888	0.894584	-6.67204	0.851873	0.752523

NK.cells	ARMT1	0.021818	3.297769	0.132825	0.894634	-6.05969	0.884297	0.806422
NK.cells	GTF3C4	0.032655	2.57436	0.132821	0.894637	-5.84077	0.893986	0.822816
NK.cells	ANKRD13D	0.035498	2.738056	0.132691	0.894739	-5.78561	0.891813	0.819118
NK.cells	THOC6	0.020212	4.164901	0.132526	0.89487	-6.27987	0.872916	0.787256
NK.cells	RUNDC3B	-0.02107	3.848947	-0.13241	0.894958	-6.59411	0.87709	0.794254
NK.cells	ZBTB20	0.013255	8.246328	0.132242	0.895094	-7.04186	0.821158	0.702763
NK.cells	FBXL8	-0.03408	1.99347	-0.13181	0.895432	-5.87285	0.902214	0.836397
NK.cells	LRWD1	0.018178	3.781422	0.131806	0.895437	-6.2371	0.878238	0.795813
NK.cells	9330175E1	0.050684	1.002962	0.131699	0.895522	-5.73407	0.915809	0.859742
NK.cells	ASB6	0.027669	2.575022	0.131595	0.895604	-5.97953	0.89436	0.822979
NK.cells	PTK6	-0.09694	-0.3416	-0.13148	0.895692	-5.22839	0.934619	0.892451
NK.cells	MATR3	0.009206	6.50291	0.131097	0.895996	-6.79971	0.84331	0.737842
NK.cells	GM6034	0.060999	0.50272	0.131003	0.89607	-5.54488	0.923016	0.871892
NK.cells	RFXAP	0.01561	4.179927	0.130653	0.896346	-6.35372	0.873447	0.78724
NK.cells	ZFP219	-0.03469	1.771622	-0.13025	0.896665	-5.71779	0.905941	0.841848
NK.cells	CCSAP	0.029303	2.176115	0.130159	0.896736	-5.92294	0.900432	0.832468
NK.cells	PRPS2	-0.01762	4.382206	-0.13004	0.896829	-6.35514	0.871013	0.782938
NK.cells	ZSCAN12	0.047884	1.093811	0.129998	0.896863	-5.44599	0.915253	0.857939
NK.cells	EXO5	0.043084	2.006793	0.129771	0.897043	-5.72928	0.902809	0.836466
NK.cells	TMEM248	-0.01195	5.858866	-0.12972	0.897084	-6.69981	0.851974	0.751436
NK.cells	COX4I2	-0.05378	0.956141	-0.12958	0.89719	-5.45513	0.917239	0.861251
NK.cells	1700030K0	0.051674	1.730931	0.129435	0.897307	-5.55446	0.906578	0.842906
NK.cells	NOL6	0.02972	2.582305	0.129433	0.897309	-5.88899	0.895016	0.823185
NK.cells	SLC30A5	-0.01528	5.799283	-0.12928	0.897429	-6.59141	0.852742	0.752684
NK.cells	UNC119B	0.018517	3.551645	0.1292	0.897493	-6.35472	0.882044	0.801282
NK.cells	ST14	-0.05418	1.167666	-0.12909	0.897577	-5.63874	0.914315	0.856205
NK.cells	PSMD2	-0.01125	6.149979	-0.1289	0.897728	-6.75896	0.848267	0.745396
NK.cells	CLEC4A1	0.066499	3.158732	0.128804	0.897805	-5.68332	0.887277	0.810119
NK.cells	GM20139	0.047165	-0.63311	0.128798	0.89781	-5.50809	0.939521	0.900124
NK.cells	EIF2AK2	-0.02043	4.927436	-0.12879	0.89782	-6.50733	0.86398	0.771204
NK.cells	FAAP100	0.022719	3.097229	0.128068	0.898386	-6.09715	0.888402	0.811711
NK.cells	NUDT5	-0.01574	5.148841	-0.12806	0.898393	-6.53784	0.861404	0.766658
NK.cells	TMLHE	0.041889	2.31306	0.127989	0.898448	-5.84657	0.898961	0.829611
NK.cells	CPLX1	0.09894	-0.57566	0.127824	0.898579	-5.18308	0.939026	0.898969
NK.cells	ADAM9	0.015146	5.178783	0.127677	0.898695	-6.5932	0.861017	0.766069
NK.cells	METTL7A1	-0.02756	2.456994	-0.12742	0.898899	-5.92127	0.897013	0.82643
NK.cells	ADORA3	0.065414	-0.30137	0.127418	0.898899	-5.32375	0.935142	0.892249
NK.cells	FAM114A1	-0.04181	2.398626	-0.12733	0.898969	-5.75426	0.897803	0.827791
NK.cells	GLE1	-0.0151	4.4454	-0.12709	0.89916	-6.40678	0.870559	0.781986
NK.cells	HMGN3	0.047873	3.255903	0.127083	0.899164	-5.67646	0.886282	0.808307
NK.cells	ARF1	-0.00866	8.02586	-0.12703	0.899204	-7.03216	0.825046	0.707813
NK.cells	GRIPAP1	-0.01275	5.594976	-0.12702	0.899212	-6.60273	0.855652	0.757369
NK.cells	DNAJC17	0.018046	3.568748	0.126897	0.89931	-6.14495	0.882117	0.801315
NK.cells	SPARC	-0.03009	5.322477	-0.12688	0.899326	-6.47689	0.85916	0.763136
NK.cells	LIG4	-0.04807	1.902715	-0.12679	0.899391	-5.63643	0.90454	0.839324
NK.cells	CAGE1	0.033097	1.94977	0.126781	0.899402	-5.9789	0.903899	0.838227
NK.cells	GM20404	-0.04502	1.661785	-0.12669	0.899475	-5.57964	0.907833	0.844965

NK.cells	NEDD4L	0.011683	6.886893	0.126642	0.899511	-6.80984	0.839233	0.730612
NK.cells	ZFP263	0.017725	5.167548	0.12649	0.899631	-6.54646	0.861162	0.766435
NK.cells	ITGB1BP2	-0.06126	0.43994	-0.1264	0.899702	-5.32582	0.924729	0.874155
NK.cells	JPX	-0.02972	3.810956	-0.12639	0.899711	-6.13122	0.878907	0.795946
NK.cells	TMEM59	-0.01057	6.649746	-0.12636	0.899733	-6.81387	0.84222	0.735468
NK.cells	ZFP82	-0.06009	0.121707	-0.12615	0.899897	-5.3847	0.929188	0.881947
NK.cells	GM15478	0.019063	4.735611	0.126109	0.899932	-6.3911	0.866773	0.775755
NK.cells	4931428F0	-0.05913	0.65018	-0.12601	0.900007	-5.36566	0.921802	0.869117
NK.cells	ZFP869	0.019574	4.026546	0.125919	0.900082	-6.30609	0.876064	0.79123
NK.cells	GM44710	-0.04952	2.000891	-0.12589	0.900101	-5.58384	0.903206	0.837103
NK.cells	TOMM7	-0.00921	7.561253	-0.12552	0.900401	-6.98656	0.830832	0.717122
NK.cells	STARD4	-0.02061	3.526928	-0.12546	0.900441	-6.22613	0.882706	0.802357
NK.cells	BHMT	-0.02865	5.13251	-0.12543	0.900472	-6.5975	0.861648	0.767287
NK.cells	PDCD7	0.015078	4.505243	0.125292	0.900577	-6.42037	0.869809	0.780804
NK.cells	E2F6	-0.02858	2.266229	-0.12525	0.900608	-5.8871	0.89963	0.830996
NK.cells	HMBOX1	-0.01507	5.578159	-0.12521	0.900638	-6.59271	0.855901	0.757827
NK.cells	GM45820	0.045875	1.425778	0.125207	0.900644	-5.47358	0.911106	0.850641
NK.cells	PKP3	0.022537	2.747004	0.125003	0.900805	-6.33209	0.893191	0.819959
NK.cells	TMEM50A	-0.00978	7.711684	-0.12468	0.901061	-7.01663	0.829015	0.71415
NK.cells	VPS11	-0.01764	4.072729	-0.12467	0.901069	-6.2445	0.87554	0.790287
NK.cells	FCF1	-0.0108	6.136138	-0.12454	0.901172	-6.74521	0.848817	0.746194
NK.cells	DAGLA	-0.07782	-0.78197	-0.1245	0.901201	-5.18825	0.942053	0.904466
NK.cells	TCERG1	-0.01001	6.296507	-0.12443	0.901261	-6.77883	0.846777	0.74287
NK.cells	4930484I0	0.034524	1.660784	0.12436	0.901312	-5.7854	0.907938	0.845159
NK.cells	FOXK2	0.013001	4.920069	0.124301	0.901359	-6.54035	0.864456	0.77189
NK.cells	MTREX	0.01134	5.477446	0.124275	0.90138	-6.64533	0.857249	0.760005
NK.cells	BAIAP2L1	-0.0283	3.098445	-0.12421	0.90143	-5.93679	0.888475	0.812034
NK.cells	PRKG1	0.04301	3.504872	0.12395	0.901636	-5.97469	0.883184	0.802907
NK.cells	FIZ1	-0.01364	4.553694	-0.12347	0.902015	-6.4078	0.869653	0.779888
NK.cells	STRN4	-0.01513	4.492619	-0.12319	0.902235	-6.39446	0.870555	0.781251
NK.cells	UBA1	0.011511	6.00691	0.123065	0.902335	-6.73516	0.850978	0.749008
NK.cells	GNAI2	-0.00731	9.008515	-0.12306	0.902341	-7.20498	0.813594	0.688976
NK.cells	FXN	-0.01628	4.085717	-0.1228	0.902544	-6.33797	0.876025	0.790253
NK.cells	ZFP87	0.021968	3.396086	0.122614	0.902691	-6.14874	0.885237	0.805576
NK.cells	ZFP58	-0.04326	1.194809	-0.1224	0.902859	-5.60444	0.915119	0.856496
NK.cells	ERP29	-0.01018	7.289905	-0.12225	0.902975	-6.84921	0.834973	0.722905
NK.cells	TIMP3	0.06191	2.197691	0.122083	0.90311	-5.65364	0.90138	0.833012
NK.cells	FGFRL1	-0.06642	0.299565	-0.12208	0.903114	-5.36937	0.927571	0.878125
NK.cells	CD28	0.020747	2.935145	0.122047	0.903139	-6.50453	0.891418	0.816099
NK.cells	ZFP68	-0.01646	3.920899	-0.12204	0.903145	-6.26754	0.878287	0.794017
NK.cells	GM15706	0.054277	1.015787	0.121919	0.90324	-5.39593	0.917617	0.860862
NK.cells	CDK4	0.013327	5.844705	0.121754	0.90337	-6.68161	0.853333	0.752663
NK.cells	1700109H(	-0.02348	3.650709	-0.12135	0.90369	-6.17454	0.881986	0.800164
NK.cells	SF3B2	-0.0088	7.285583	-0.12106	0.90392	-6.93692	0.835142	0.723172
NK.cells	RTF2	0.01031	5.957747	0.120953	0.904003	-6.69531	0.85193	0.75042
NK.cells	TATDN3	0.029007	2.490952	0.120937	0.904015	-5.87861	0.897527	0.826436
NK.cells	SOCS2	0.02266	3.962092	0.120923	0.904027	-6.39715	0.877863	0.793291

NK.cells	CTBP1	-0.01126	6.500976	-0.12088	0.904061	-6.79714	0.845017	0.739156
NK.cells	EMC4	0.015441	4.771124	0.120832	0.904098	-6.4831	0.867247	0.775628
NK.cells	COQ4	0.031369	2.66674	0.120715	0.904191	-5.76828	0.895153	0.822449
NK.cells	RNF7	-0.0122	6.742111	-0.12067	0.904226	-6.80185	0.841968	0.734263
NK.cells	BC050972	-0.06664	-0.27973	-0.12065	0.904242	-5.33596	0.935851	0.892625
NK.cells	SPSB2	-0.03289	2.383882	-0.12033	0.904496	-5.66668	0.898977	0.829032
NK.cells	PPIL2	-0.01148	5.124237	-0.11998	0.90477	-6.58393	0.862658	0.768316
NK.cells	BAG6	-0.01422	5.438014	-0.11995	0.904792	-6.61923	0.858602	0.761636
NK.cells	GCAT	-0.03176	2.390839	-0.11991	0.904825	-5.88047	0.898883	0.829043
NK.cells	GAB2	0.023031	7.176286	0.119839	0.904882	-6.58564	0.83651	0.725656
NK.cells	DTX1	0.031442	2.069415	0.119784	0.904926	-6.03442	0.903249	0.836516
NK.cells	SULT2B1	-0.0174	2.700547	-0.1197	0.904994	-6.49198	0.894697	0.821961
NK.cells	ZFP316	0.059165	0.380007	0.119628	0.90505	-5.40602	0.926571	0.876727
NK.cells	PTP4A3	-0.01308	5.77385	-0.11959	0.905079	-6.62493	0.854284	0.754581
NK.cells	DDB1	0.013234	5.611857	0.119436	0.905201	-6.63934	0.856364	0.757992
NK.cells	KRT10	-0.03894	2.100333	-0.11943	0.905207	-5.61308	0.902828	0.835804
NK.cells	PARP12	-0.02861	3.018486	-0.11939	0.905233	-6.04921	0.890422	0.81473
NK.cells	TMTC4	-0.03949	1.357794	-0.11938	0.905243	-5.74222	0.912996	0.853236
NK.cells	PIGW	0.047655	0.765108	0.119217	0.905374	-5.43466	0.921199	0.867402
NK.cells	SLC36A4	0.028765	3.21113	0.119205	0.905384	-6.01481	0.887842	0.810375
NK.cells	PRM1	0.065694	-0.44781	0.119204	0.905385	-5.23891	0.938231	0.897101
NK.cells	GM15411	-0.05619	0.212426	-0.1192	0.90539	-5.37507	0.928919	0.880815
NK.cells	SPACA9	-0.02972	1.758151	-0.11899	0.905557	-5.82274	0.907567	0.843794
NK.cells	TNFSF14	-0.03295	-0.05669	-0.1189	0.905623	-5.70346	0.932773	0.887419
NK.cells	TMEM208	0.011921	4.89435	0.118775	0.905723	-6.48183	0.865708	0.773285
NK.cells	CHIC2	-0.00818	7.043743	-0.11873	0.905757	-6.90004	0.838235	0.72837
NK.cells	SHPRH	-0.01494	4.541567	-0.11865	0.905824	-6.45096	0.870311	0.780936
NK.cells	DDX55	-0.0239	2.934608	-0.11838	0.906036	-6.02667	0.891665	0.816729
NK.cells	PDE4DIP	0.017609	3.94998	0.118134	0.90623	-6.28387	0.878138	0.793983
NK.cells	ITK	-0.01997	4.224474	-0.11812	0.906241	-6.90726	0.874519	0.78794
NK.cells	PVRIG	-0.03896	-0.53207	-0.11794	0.906379	-5.57652	0.939549	0.899405
NK.cells	ZDHHC2	-0.02686	2.276539	-0.11775	0.906531	-6.08139	0.900551	0.831929
NK.cells	CD46	0.034045	2.11028	0.117693	0.906578	-5.78259	0.902811	0.835784
NK.cells	MFHAS1	0.023975	3.729832	0.117603	0.906649	-6.18394	0.881052	0.798963
NK.cells	RSL24D1	0.01124	5.674542	0.117523	0.906712	-6.68081	0.855671	0.756872
NK.cells	PDPK1	-0.01677	6.462559	-0.11716	0.906996	-6.74762	0.845615	0.740545
NK.cells	SCAI	-0.01761	4.434969	-0.11715	0.907009	-6.42779	0.871755	0.783554
NK.cells	RSL1	0.051755	1.039109	0.117019	0.90711	-5.50382	0.917518	0.861184
NK.cells	EPCAM	-0.02898	2.160039	-0.11665	0.907401	-6.08974	0.902134	0.834811
NK.cells	CAR12	-0.04992	0.013745	-0.11664	0.907411	-5.45596	0.931833	0.886103
NK.cells	RTCA	0.016228	4.282973	0.116599	0.907442	-6.34178	0.87375	0.786929
NK.cells	TRIR	0.010368	6.300182	0.116596	0.907445	-6.76911	0.847676	0.743954
NK.cells	IAH1	0.016735	4.521488	0.116553	0.907478	-6.43306	0.870622	0.781722
NK.cells	SPNS1	0.02015	3.498213	0.116518	0.907506	-6.12229	0.884129	0.804306
NK.cells	ST18	0.070607	0.283819	0.116421	0.907583	-5.40602	0.92804	0.879509
NK.cells	FH1	0.016521	5.484899	0.116357	0.907633	-6.59412	0.85811	0.761057
NK.cells	9-Sep	-0.01274	5.640387	-0.11622	0.90774	-6.74039	0.85611	0.757784

NK.cells	RCN2	0.015201	5.075901	0.116209	0.907751	-6.53865	0.863398	0.769787
NK.cells	MLLT3	-0.01329	5.728512	-0.11613	0.907812	-6.90416	0.854978	0.755931
NK.cells	RAPSN	-0.02946	0.715904	-0.116	0.907919	-5.79163	0.922005	0.869097
NK.cells	NKIRAS1	-0.02019	3.432264	-0.11595	0.907957	-6.17279	0.885007	0.805876
NK.cells	GSTO2	-0.0622	0.524993	-0.11587	0.908022	-5.3563	0.924666	0.873717
NK.cells	PHF6	-0.01257	4.940138	-0.11559	0.908243	-6.62347	0.865161	0.772798
NK.cells	RAD23A	0.01324	5.807004	0.115526	0.90829	-6.6977	0.853971	0.75437
NK.cells	ARMCX3	0.022115	3.573363	0.115375	0.90841	-6.06236	0.883129	0.802765
NK.cells	4930557K0	0.041384	1.483636	0.115367	0.908416	-5.60676	0.911384	0.850805
NK.cells	PRDX1	0.010782	9.719927	0.115366	0.908417	-7.30709	0.805417	0.676538
NK.cells	TCP11	0.049271	0.648485	0.115358	0.908423	-5.39121	0.922944	0.870776
NK.cells	FBXW5	0.019413	3.39907	0.11535	0.908429	-6.12068	0.885449	0.806668
NK.cells	MFSD2B	-0.04801	0.599514	-0.11533	0.908442	-5.44864	0.923627	0.871961
NK.cells	VAV3	-0.01389	7.363151	-0.11509	0.908633	-7.03562	0.834282	0.722387
NK.cells	FAM71F2	0.034527	2.260769	0.115025	0.908686	-5.9608	0.900765	0.83262
NK.cells	SYDE2	-0.04779	-0.10373	-0.11502	0.908692	-5.54312	0.933488	0.88915
NK.cells	MICALL1	-0.01907	2.97359	-0.11498	0.908724	-6.12071	0.891141	0.816274
NK.cells	TBP	-0.02378	3.21522	-0.1149	0.908786	-6.03213	0.887904	0.810815
NK.cells	SH3YL1	0.045138	1.030881	0.114627	0.909001	-5.44145	0.917632	0.861605
NK.cells	KDM4C	0.01101	6.252217	0.114536	0.909073	-6.78405	0.848286	0.745111
NK.cells	IGHJ4	-0.06735	0.337645	-0.11452	0.909084	-5.27419	0.927286	0.878364
NK.cells	PRICKLE3	0.030221	2.034653	0.114475	0.909121	-5.80628	0.903841	0.837909
NK.cells	TMCC1	-0.01544	7.946033	-0.11445	0.909144	-6.96011	0.827037	0.710794
NK.cells	MCM3AP	0.014476	3.885875	0.11442	0.909164	-6.27655	0.878985	0.795849
NK.cells	TARBP1	0.030321	2.687423	0.114361	0.909211	-5.83303	0.894991	0.822835
NK.cells	DIAPH1	-0.00871	7.355352	-0.11432	0.909244	-7.00758	0.834379	0.722577
NK.cells	COMMD6	0.015691	4.358964	0.114233	0.909312	-6.34339	0.872752	0.78545
NK.cells	MRPL35	0.01297	5.21625	0.113671	0.909757	-6.57767	0.861811	0.767001
NK.cells	DYNLT3	0.014086	4.662522	0.113579	0.909829	-6.36763	0.869012	0.778947
NK.cells	ABCA2	0.029642	1.527476	0.113427	0.909949	-5.92926	0.911027	0.849938
NK.cells	MTCH1	0.009524	6.297903	0.113423	0.909952	-6.75067	0.847934	0.744279
NK.cells	ZFP787	0.011357	5.128821	0.113296	0.910053	-6.54713	0.862944	0.768959
NK.cells	ZFP955A	-0.03769	1.472841	-0.11322	0.910109	-5.71385	0.911778	0.851328
NK.cells	GM9828	-0.04668	1.076181	-0.11317	0.910149	-5.53732	0.917252	0.860764
NK.cells	PPP1R12C	0.011627	5.009564	0.113075	0.910228	-6.555	0.864492	0.771557
NK.cells	POLD4	-0.01264	5.76477	-0.11277	0.910469	-6.5969	0.854743	0.755534
NK.cells	MFAP2	-0.0664	0.586159	-0.11268	0.910537	-5.33055	0.924062	0.872608
NK.cells	DVL1	0.028401	2.507773	0.112581	0.910618	-5.86247	0.897659	0.827229
NK.cells	GM16062	0.027526	1.874018	0.112578	0.91062	-5.78659	0.906277	0.841936
NK.cells	RAB21	0.009027	6.891793	0.112564	0.910631	-6.84998	0.840419	0.732202
NK.cells	TNRC6A	-0.00982	6.648204	-0.11254	0.910648	-6.84325	0.843492	0.737184
NK.cells	GM32916	0.115404	-0.24915	0.112526	0.910662	-5.2326	0.935794	0.893079
NK.cells	SERAC1	0.036621	2.177244	0.112418	0.910747	-5.69976	0.902143	0.834898
NK.cells	TMEM256	0.011676	6.982152	0.112207	0.910914	-6.87549	0.839282	0.730402
NK.cells	KIF16B	0.014395	4.843803	0.112161	0.91095	-6.50464	0.866648	0.775205
NK.cells	NBEAL2	-0.01808	2.635242	-0.1121	0.911002	-6.14189	0.895937	0.824346
NK.cells	4930503L1	-0.02171	2.576564	-0.11207	0.91102	-6.04463	0.896729	0.8257

NK.cells	GM43065	0.029956	0.153169	0.11186	0.911188	-5.94921	0.930158	0.883274
NK.cells	SNRPE	0.009863	7.064225	0.111703	0.911312	-6.91908	0.838282	0.728789
NK.cells	KLHDC3	-0.01235	4.338451	-0.11168	0.911334	-6.38063	0.873289	0.786245
NK.cells	1700102P0	-0.0532	0.812589	-0.11158	0.911413	-5.42577	0.920943	0.867251
NK.cells	MMS22L	0.02016	4.820836	0.111566	0.91142	-6.47474	0.866979	0.775758
NK.cells	ANKRD22	-0.06372	-0.56253	-0.11116	0.911739	-5.25111	0.940449	0.901015
NK.cells	TNIP2	-0.01521	3.816648	-0.11116	0.91174	-6.29788	0.880339	0.797769
NK.cells	SLC25A27	0.044373	0.260835	0.1109	0.911947	-5.49282	0.928951	0.880704
NK.cells	L2HGDH	-0.03512	1.767651	-0.11082	0.912011	-5.6791	0.908063	0.844598
NK.cells	SESN3	-0.01327	4.83647	-0.11065	0.912143	-6.59747	0.867077	0.775475
NK.cells	DNAJB2	-0.02121	2.766414	-0.11061	0.912176	-6.09209	0.894512	0.821463
NK.cells	TMEM68	-0.01614	3.807022	-0.11029	0.912433	-6.22302	0.880686	0.798073
NK.cells	CUL7	-0.03617	1.968845	-0.11028	0.912434	-5.66419	0.905414	0.839945
NK.cells	USP39	-0.01646	4.614508	-0.11019	0.912511	-6.39684	0.870054	0.780336
NK.cells	ATP5MD	-0.00903	8.035566	-0.11013	0.912553	-7.0723	0.826546	0.709448
NK.cells	FAM114A2	-0.01112	5.166561	-0.11003	0.912638	-6.53514	0.862877	0.768435
NK.cells	FAM168B	0.008205	6.280422	0.1095	0.913054	-6.76318	0.848813	0.745013
NK.cells	HPF1	-0.01512	5.467524	-0.10949	0.913063	-6.59667	0.859231	0.762067
NK.cells	6030443J0	0.043144	0.848488	0.10942	0.913118	-5.5113	0.921123	0.866574
NK.cells	NDC80	-0.02095	4.625978	-0.10906	0.913404	-6.5177	0.87019	0.780192
NK.cells	RAB4A	0.022858	1.578469	0.109023	0.913432	-6.12203	0.91106	0.849225
NK.cells	RPA3	-0.01526	4.97161	-0.10902	0.913436	-6.49782	0.865682	0.772721
NK.cells	PYGO2	0.024931	3.374512	0.109014	0.913438	-6.10637	0.886729	0.807847
NK.cells	RBM45	0.019583	3.10947	0.108881	0.913544	-6.07341	0.890307	0.813874
NK.cells	OLFML2B	-0.06141	0.874884	-0.10851	0.913837	-5.34785	0.920915	0.866104
NK.cells	CAPNS1	0.008044	7.25812	0.10851	0.913837	-6.97904	0.836612	0.725161
NK.cells	KCTD5	0.017545	3.640452	0.108333	0.913977	-6.20524	0.883312	0.802045
NK.cells	MSR1	0.037254	3.816756	0.1083	0.914003	-5.80989	0.880971	0.79812
NK.cells	S1PR4	-0.01722	4.183137	-0.10825	0.914045	-6.39863	0.876128	0.790027
NK.cells	CPED1	0.036584	2.524781	0.108146	0.914125	-5.83959	0.89828	0.827353
NK.cells	RAMP1	0.01159	4.507186	0.107937	0.91429	-6.50108	0.871869	0.782956
NK.cells	MAN2C1	0.017135	3.509599	0.107864	0.914348	-6.14446	0.885053	0.805
NK.cells	SDCBP	-0.0091	7.674968	-0.10785	0.914357	-6.98788	0.831408	0.716865
NK.cells	TBC1D20	-0.01206	5.789234	-0.10783	0.914374	-6.6539	0.855239	0.755503
NK.cells	UBA2	-0.009	6.457603	-0.10769	0.914485	-6.80513	0.846715	0.741598
NK.cells	TTLL5	-0.01394	4.471737	-0.10759	0.914568	-6.40011	0.872342	0.783753
NK.cells	ATG2A	0.012004	5.710857	0.107488	0.914645	-6.74538	0.856254	0.757185
NK.cells	RERE	-0.00946	7.702335	-0.10738	0.914729	-6.9631	0.831075	0.716357
NK.cells	FBXL2	0.01866	3.231347	0.107043	0.914998	-6.19577	0.888777	0.811393
NK.cells	GRB10	-0.03916	3.090696	-0.10691	0.915105	-5.922	0.890662	0.814604
NK.cells	ASL	0.015315	4.484774	0.106754	0.915226	-6.45769	0.872171	0.783635
NK.cells	SETD1B	-0.0127	5.026478	-0.10654	0.915394	-6.53041	0.865099	0.771906
NK.cells	P2RY13	0.06595	1.100765	0.106466	0.915454	-5.3441	0.917789	0.86098
NK.cells	CCDC141	-0.04089	1.120838	-0.10644	0.915473	-5.57119	0.917511	0.8605
NK.cells	RFWD3	-0.01098	5.6362	-0.10644	0.915475	-6.6371	0.857214	0.758913
NK.cells	4732465J0	-0.04102	1.227323	-0.10639	0.915514	-5.64301	0.916038	0.857968
NK.cells	ABCA5	0.059584	0.444818	0.106281	0.9156	-5.31054	0.926922	0.876852

NK.cells	GM26590	0.032377	1.314817	0.106252	0.915623	-5.54627	0.914829	0.855916
NK.cells	FOXN2	0.009609	6.146142	0.105984	0.915835	-6.70608	0.850679	0.748287
NK.cells	TMBIM1	-0.02387	2.851295	-0.10583	0.915956	-6.00472	0.89388	0.820187
NK.cells	FBXO28	0.013044	4.523471	0.105744	0.916025	-6.51095	0.871664	0.782918
NK.cells	SLC7A1	-0.01441	5.141375	-0.1057	0.916056	-6.68454	0.863607	0.769565
NK.cells	ARL6	0.031358	1.698649	0.105672	0.916082	-5.62628	0.909547	0.846929
NK.cells	ATP5F1	-0.008	8.084387	-0.10563	0.916114	-7.068	0.82634	0.70903
NK.cells	DTYMK	0.015993	5.126752	0.105568	0.916164	-6.51302	0.863797	0.769901
NK.cells	MNS1	-0.02062	2.821224	-0.10544	0.916267	-6.08651	0.894285	0.820922
NK.cells	TRP53I13	0.018915	3.021819	0.105135	0.916507	-6.0323	0.891586	0.816445
NK.cells	BMP2K	0.00982	6.936172	0.104983	0.916627	-6.8119	0.840664	0.732212
NK.cells	RPN1	0.010683	5.957169	0.104872	0.916715	-6.71891	0.853094	0.752456
NK.cells	ERBB3	0.051723	1.113728	0.104848	0.916734	-5.42997	0.917609	0.861016
NK.cells	1810024B(	-0.02639	2.692562	-0.10451	0.917	-5.84763	0.89602	0.824064
NK.cells	GORASP2	0.008921	5.534187	0.104489	0.917018	-6.63462	0.858527	0.7614
NK.cells	ORC6	0.014081	5.216442	0.104361	0.917119	-6.57257	0.862634	0.768169
NK.cells	STK4	0.008055	7.52595	0.104199	0.917247	-7.02063	0.833272	0.720334
NK.cells	SPATA5	-0.01211	5.905372	-0.10399	0.917411	-6.71521	0.853758	0.753665
NK.cells	EFNA1	-0.0551	0.531324	-0.10391	0.917479	-5.36328	0.925712	0.875191
NK.cells	4933411E(	0.039598	0.555657	0.103715	0.917631	-5.58648	0.925372	0.874601
NK.cells	LBH	-0.00884	6.308404	-0.10364	0.917688	-6.81255	0.848611	0.745257
NK.cells	DICER1	-0.01056	4.846948	-0.10341	0.917871	-6.501	0.867436	0.776205
NK.cells	PRADC1	0.018655	3.374688	0.103386	0.917891	-6.10531	0.886861	0.808674
NK.cells	H1FX	-0.03875	2.127581	-0.10332	0.917939	-5.71332	0.903683	0.83722
NK.cells	ZSCAN21	-0.02004	3.394527	-0.10328	0.917972	-6.11496	0.886596	0.808227
NK.cells	VWA5A	-0.01932	3.406572	-0.10327	0.917979	-6.21019	0.886435	0.807957
NK.cells	NECAB3	-0.04042	1.186088	-0.10287	0.918298	-5.50051	0.916608	0.859535
NK.cells	ABCF1	0.008579	6.430861	0.102825	0.918334	-6.82206	0.847055	0.742821
NK.cells	IGF2BP1	-0.06816	-0.96106	-0.10283	0.918335	-5.15478	0.946804	0.912313
NK.cells	GM10658	0.031861	1.776179	0.102802	0.918353	-5.7229	0.908484	0.845554
NK.cells	DDX21	-0.01054	6.399043	-0.10267	0.918457	-6.83082	0.847459	0.743491
NK.cells	HSP90AA1	0.009616	7.657631	0.102653	0.918471	-6.97553	0.831632	0.717893
NK.cells	TINAGL1	-0.04532	2.066409	-0.10259	0.918518	-5.59373	0.904517	0.838771
NK.cells	HEMK1	-0.03586	1.147753	-0.10232	0.918732	-5.61852	0.917138	0.860464
NK.cells	FAN1	-0.03799	1.159783	-0.10228	0.918769	-5.59423	0.916972	0.860177
NK.cells	HIP1R	-0.01116	5.271328	-0.10217	0.918856	-6.6789	0.861923	0.767215
NK.cells	ORC3	0.011333	4.958044	0.102134	0.918881	-6.51073	0.865989	0.773935
NK.cells	MAT1A	-0.02549	4.65605	-0.1019	0.919068	-6.46427	0.869928	0.780469
NK.cells	NSMF	-0.02123	2.696993	-0.10188	0.919081	-6.00212	0.89596	0.824201
NK.cells	2310057M	-0.01833	2.710086	-0.10188	0.919082	-5.96114	0.895783	0.823901
NK.cells	4930523C(	-0.00877	6.126408	-0.10185	0.919104	-6.85964	0.850931	0.749166
NK.cells	IFFO1	0.026352	2.880878	0.101833	0.91912	-6.0041	0.893481	0.819996
NK.cells	MRPL48	-0.00934	5.282039	-0.10179	0.919156	-6.5676	0.861784	0.766986
NK.cells	DHX9	-0.01217	6.492589	-0.10175	0.919189	-6.80409	0.846271	0.741567
NK.cells	ARNTL	0.014437	5.38742	0.101717	0.919211	-6.64521	0.860422	0.764739
NK.cells	GNG7	-0.0659	-0.40318	-0.10171	0.919216	-5.16701	0.938872	0.898336
NK.cells	PODXL	0.053992	0.550401	0.101653	0.919263	-5.38882	0.925446	0.874873

NK.cells	ATXN2L	0.009856	5.47603	0.101366	0.919489	-6.62878	0.859277	0.762912
NK.cells	SNRPN	0.068328	-0.6475	0.101363	0.919492	-5.23732	0.942344	0.904511
NK.cells	GM5535	0.068261	0.151841	0.101298	0.919543	-5.25679	0.931033	0.884678
NK.cells	BC028528	-0.03018	4.105365	-0.1013	0.919543	-5.6824	0.877162	0.792589
NK.cells	KCNG3	-0.03011	0.517714	-0.10129	0.919546	-5.86496	0.925903	0.875738
NK.cells	APBB3	-0.03207	1.534528	-0.10124	0.919586	-5.68459	0.911802	0.851359
NK.cells	DBT	-0.01846	3.826101	-0.10122	0.919604	-6.20023	0.880855	0.798786
NK.cells	FBXW7	-0.01083	6.448567	-0.10117	0.919642	-6.77953	0.84683	0.742548
NK.cells	PSPH	-0.01454	3.906589	-0.10105	0.919736	-6.33947	0.879789	0.797032
NK.cells	HABP2	-0.05098	0.622763	-0.101	0.919778	-5.4228	0.924435	0.873243
NK.cells	KCNIP4	-0.06345	0.608323	-0.10086	0.919893	-5.43132	0.924637	0.873602
NK.cells	CASTOR1	-0.03461	0.626653	-0.10085	0.919896	-5.69107	0.924381	0.873158
NK.cells	SH3BP4	0.038121	1.369949	0.100777	0.919956	-5.61725	0.914068	0.855351
NK.cells	JHY	0.05104	-0.37714	0.100697	0.920019	-5.47012	0.938502	0.89788
NK.cells	CEP76	0.019862	2.990307	0.100663	0.920046	-6.07374	0.89201	0.817679
NK.cells	CTR9	-0.01134	4.518969	-0.10057	0.920117	-6.43665	0.871723	0.783622
NK.cells	GM20045	-0.03385	1.764585	-0.10053	0.920148	-5.66515	0.908643	0.846032
NK.cells	ERI1	0.011511	5.459943	0.100486	0.920186	-6.65659	0.859485	0.76336
NK.cells	TNNT3	-0.03603	1.772075	-0.10046	0.920205	-5.70696	0.90854	0.845856
NK.cells	PTGS2OS	-0.06682	-1.09526	-0.10042	0.920241	-5.23813	0.94858	0.915936
NK.cells	TRIM27	-0.01032	5.675636	-0.10016	0.920445	-6.62156	0.856764	0.758821
NK.cells	CCDC84	-0.02306	2.649641	-0.10015	0.920449	-5.90264	0.89666	0.825498
NK.cells	VDAC2	0.008416	7.488132	0.100015	0.920559	-6.96579	0.833801	0.721515
NK.cells	RBM47	0.023299	6.404832	0.099953	0.920608	-6.29119	0.847443	0.743605
NK.cells	ZFP655	-0.0128	4.453763	-0.09986	0.920683	-6.40404	0.872638	0.78512
NK.cells	TMPRSS5	0.039445	0.722493	0.099566	0.920915	-5.52182	0.923107	0.870999
NK.cells	TMEM41A	0.032498	1.51992	0.099493	0.920972	-5.68324	0.912065	0.851914
NK.cells	GM28198	-0.01812	3.85447	-0.09925	0.921166	-6.28336	0.88054	0.798421
NK.cells	FANCG	-0.02677	2.222876	-0.09923	0.92118	-5.76373	0.902447	0.835499
NK.cells	EIF4G3	-0.0073	7.91677	-0.09921	0.921199	-7.04815	0.82847	0.71305
NK.cells	ALKBH8	0.014593	3.835022	0.099199	0.921204	-6.2188	0.880797	0.798855
NK.cells	GM28981	-0.05371	0.488246	-0.09913	0.92126	-5.28407	0.926378	0.876779
NK.cells	TWF1	-0.00989	5.464598	-0.0988	0.921519	-6.59069	0.859484	0.763531
NK.cells	PLSCR2	0.065222	-0.38835	0.098662	0.92163	-5.28984	0.938726	0.898477
NK.cells	LCP1	-0.00861	8.858643	-0.09861	0.921673	-7.27869	0.81689	0.694701
NK.cells	TMEM129	0.030264	2.084833	0.098588	0.921688	-5.75483	0.904327	0.83883
NK.cells	GM32296	0.057803	-0.65404	0.098569	0.921703	-5.23047	0.942502	0.905122
NK.cells	MCL1	0.009649	8.364811	0.098521	0.921742	-7.07222	0.822939	0.704312
NK.cells	BMX	0.060005	0.02253	0.098513	0.921748	-5.32963	0.932917	0.888292
NK.cells	CCDC57	0.02502	2.546499	0.098487	0.921768	-5.78449	0.898056	0.828129
NK.cells	ZFP91	0.007448	6.894237	0.097991	0.922161	-6.89116	0.841447	0.733819
NK.cells	RBM3	0.007921	9.070642	0.097877	0.922252	-7.24255	0.8145	0.690736
NK.cells	PCNP	-0.00731	6.359657	-0.0975	0.922546	-6.80041	0.848216	0.744913
NK.cells	ZFP973	0.038591	0.609416	0.097293	0.922714	-5.48795	0.924901	0.874139
NK.cells	MGL2	0.08937	-0.18973	0.097154	0.922824	-5.28662	0.936132	0.893747
NK.cells	GM40787	-0.05696	0.205726	-0.09709	0.922871	-5.27632	0.930557	0.883992
NK.cells	PITPNA	0.006576	7.992513	0.097092	0.922873	-7.035	0.827726	0.711812

NK.cells	GM43707	0.050217	0.407926	0.097089	0.922875	-5.33687	0.92772	0.879044
NK.cells	SNHG1	0.011137	5.230493	0.097048	0.922908	-6.58438	0.862713	0.768702
NK.cells	OXA1L	0.010827	4.658758	0.096997	0.922949	-6.49975	0.870156	0.781035
NK.cells	NLRC4	0.026833	3.885651	0.096976	0.922965	-5.84589	0.880333	0.798025
NK.cells	AK6	-0.01273	4.761906	-0.09697	0.922969	-6.41038	0.868808	0.778796
NK.cells	TCIRG1	-0.01326	5.455039	-0.09694	0.922992	-6.51288	0.859809	0.763912
NK.cells	ADSL	0.013421	4.033205	0.096603	0.923261	-6.32185	0.878381	0.794833
NK.cells	CBWD1	-0.01477	4.090993	-0.09653	0.923317	-6.35894	0.877617	0.793557
NK.cells	RALBP1	0.008413	6.268527	0.096273	0.923522	-6.76552	0.849376	0.746906
NK.cells	AMIGO2	-0.0515	0.934211	-0.09625	0.923537	-5.45696	0.920378	0.866407
NK.cells	ALG6	0.027856	2.044219	0.096205	0.923576	-5.80469	0.905093	0.840086
NK.cells	LTV1	0.014413	4.232009	0.096178	0.923597	-6.35382	0.875758	0.790478
NK.cells	IQSEC2	-0.04628	2.267987	-0.09616	0.923615	-5.51314	0.902045	0.834877
NK.cells	TBC1D10C	0.014732	4.736281	0.096148	0.92362	-6.52107	0.869143	0.77946
NK.cells	CNTROB	0.028698	2.044211	0.096101	0.923658	-5.74697	0.905093	0.840108
NK.cells	MAP3K13	-0.0425	0.779749	-0.09591	0.923806	-5.49529	0.922526	0.870181
NK.cells	SNRPA1	0.012847	5.077996	0.095893	0.923822	-6.55543	0.864691	0.772121
NK.cells	LMNB2	-0.02757	2.474409	-0.09573	0.923954	-5.8466	0.899243	0.830171
NK.cells	TMEM229/	0.060207	-1.03017	0.095721	0.923959	-5.17003	0.948005	0.915028
NK.cells	CFL2	-0.01297	4.631141	-0.09572	0.923962	-6.41308	0.870518	0.781813
NK.cells	FOXO3	0.010649	6.397005	0.095621	0.924038	-6.81575	0.847744	0.744307
NK.cells	SLC5A11	-0.04783	-0.01556	-0.09551	0.924127	-5.38372	0.933691	0.889649
NK.cells	GGA1	-0.00995	4.730426	-0.09527	0.924317	-6.51217	0.869346	0.779691
NK.cells	RAB2B	-0.01452	3.661593	-0.09511	0.924444	-6.26931	0.88346	0.803235
NK.cells	GM614	0.039101	-0.15476	0.095051	0.92449	-5.47239	0.935801	0.893149
NK.cells	PES1	-0.01077	4.691666	-0.09476	0.924718	-6.45133	0.870024	0.78064
NK.cells	GM10382	-0.04836	0.439288	-0.09441	0.924993	-5.35939	0.927679	0.878667
NK.cells	IFRD1	-0.01044	8.532379	-0.09429	0.92509	-7.22045	0.821427	0.701547
NK.cells	ANAPC7	-0.01105	4.359074	-0.09408	0.92526	-6.40684	0.874462	0.78801
NK.cells	CDCA4	-0.01232	4.453152	-0.09401	0.925311	-6.43498	0.873226	0.785949
NK.cells	AKR1B10	-0.01679	3.494629	-0.094	0.92532	-6.19285	0.88591	0.807199
NK.cells	HRH4	-0.0425	0.27047	-0.09395	0.925362	-5.5316	0.930047	0.882888
NK.cells	FZR1	-0.01276	4.900739	-0.09392	0.925385	-6.50682	0.86737	0.776217
NK.cells	CEACAM16	-0.04127	1.137316	-0.0939	0.925402	-5.46563	0.917955	0.861887
NK.cells	TOM1L1	-0.03974	0.714609	-0.09383	0.925459	-5.50806	0.923831	0.872067
NK.cells	ODR4	-0.01385	4.042451	-0.09348	0.925732	-6.26561	0.878826	0.79502
NK.cells	DDR1	-0.03527	0.137297	-0.09329	0.925885	-5.48265	0.932201	0.886213
NK.cells	HIVEP1	-0.01071	6.40877	-0.09319	0.92596	-6.74177	0.848214	0.744364
NK.cells	DDX41	0.011711	4.356626	0.093045	0.926078	-6.38239	0.874802	0.788143
NK.cells	TAT	-0.03592	1.826535	-0.09286	0.926225	-5.72934	0.908852	0.845674
NK.cells	EHD2	-0.03862	1.82262	-0.09275	0.92631	-5.52197	0.908917	0.845776
NK.cells	PHF11C	0.020646	1.989779	0.092579	0.926447	-5.99076	0.906691	0.841912
NK.cells	SIRT3	-0.01551	3.344497	-0.09211	0.926816	-6.10747	0.888551	0.810855
NK.cells	SEPHS1	0.010428	4.119783	0.092052	0.926865	-6.33502	0.878243	0.793547
NK.cells	IGLC1	0.046371	3.761408	0.092035	0.926879	-6.17122	0.882992	0.801502
NK.cells	TUBE1	-0.0323	1.254723	-0.09196	0.926939	-5.62796	0.916987	0.859375
NK.cells	GEMIN2	-0.01625	3.260952	-0.09184	0.92703	-6.1091	0.889669	0.81275

NK.cells	ATAD1	0.007325	5.731262	0.091787	0.927075	-6.64292	0.857232	0.758741
NK.cells	PPP1R12B	0.009724	5.292478	0.091617	0.927209	-6.60682	0.862914	0.768067
NK.cells	TMEM41B	0.012289	4.300772	0.091581	0.927238	-6.32374	0.875871	0.789567
NK.cells	PA2G4	0.010907	6.688076	0.091344	0.927426	-6.82533	0.845125	0.738797
NK.cells	GRWD1	0.020957	2.674334	0.090914	0.927766	-5.92916	0.897679	0.826186
NK.cells	ARHGEF2	0.009364	5.586467	0.09089	0.927786	-6.60856	0.859206	0.761865
NK.cells	HCAR2	-0.06146	2.377294	-0.09085	0.927817	-5.54383	0.901706	0.83304
NK.cells	ST3GAL6	0.013092	5.092875	0.090769	0.927881	-6.49958	0.865599	0.772413
NK.cells	PPME1	-0.0099	4.862972	-0.09077	0.927882	-6.56491	0.868594	0.777373
NK.cells	GM17103	-0.04007	1.028835	-0.09075	0.927896	-5.52865	0.920234	0.864871
NK.cells	CPS1	-0.02586	4.481272	-0.09071	0.927926	-6.44707	0.873592	0.785691
NK.cells	USP6NL	0.011199	5.563085	0.090574	0.928036	-6.52223	0.859541	0.762393
NK.cells	TMEM218	0.026275	1.874833	0.090298	0.928255	-5.62571	0.908672	0.844797
NK.cells	CENPI	-0.02111	2.997583	-0.0903	0.928257	-6.07876	0.893424	0.818825
NK.cells	PARP3	-0.02431	2.362796	-0.0901	0.92841	-5.83412	0.902089	0.833429
NK.cells	PI4KB	0.008782	5.414205	0.08994	0.928538	-6.6254	0.861652	0.765626
NK.cells	GM44702	0.055172	0.088947	0.089783	0.928663	-5.31592	0.933626	0.887879
NK.cells	VPS25	-0.03438	0.881521	-0.0897	0.928728	-5.49821	0.922519	0.868553
NK.cells	GIMAP3	0.020975	2.932139	0.089593	0.928813	-6.5458	0.894428	0.820433
NK.cells	DNA2	-0.01474	3.583362	-0.0893	0.929048	-6.19736	0.8857	0.805712
NK.cells	SOX5OS4	0.051488	-0.23518	0.089264	0.929074	-5.28683	0.938208	0.895925
NK.cells	EBAG9	0.011904	4.117531	0.089205	0.929121	-6.33898	0.878609	0.793835
NK.cells	POP1	0.018594	2.708556	0.089193	0.92913	-5.9756	0.897446	0.825576
NK.cells	HIST1H2A(	-0.03066	2.011327	-0.0891	0.929206	-5.89564	0.906928	0.841741
NK.cells	ATL1	-0.04581	0.498179	-0.08898	0.929297	-5.41213	0.927873	0.877898
NK.cells	BCL2L13	0.010758	5.133362	0.088963	0.929313	-6.57145	0.865294	0.77171
NK.cells	ALKBH3	0.015488	3.490416	0.088875	0.929382	-6.16694	0.88694	0.807858
NK.cells	ZFP948	0.019862	2.965976	0.088761	0.929473	-6.1549	0.893972	0.819764
NK.cells	DDX19A	-0.01225	4.239703	-0.08875	0.929484	-6.34017	0.876996	0.791215
NK.cells	GOPC	0.010015	4.545466	0.088609	0.929593	-6.41331	0.872984	0.78455
NK.cells	UBB	-0.00833	10.85501	-0.08855	0.929643	-7.49479	0.794352	0.658236
NK.cells	PTPA	0.007926	5.833043	0.088263	0.929867	-6.72936	0.856401	0.756987
NK.cells	LARS2	-0.01828	5.571824	-0.08794	0.930121	-6.66516	0.85985	0.762554
NK.cells	AIFM2	-0.02727	1.347203	-0.0878	0.930234	-5.77824	0.916308	0.857696
NK.cells	LDHB	-0.01875	3.00278	-0.08761	0.930387	-6.23718	0.893721	0.819131
NK.cells	NEURL1A	-0.05676	-0.39095	-0.0876	0.930393	-5.22607	0.940676	0.900131
NK.cells	CNBD2	-0.01689	3.889219	-0.0875	0.930469	-6.20169	0.881873	0.799197
NK.cells	EIF3A	0.006045	7.379667	0.08738	0.930567	-6.95624	0.836858	0.725219
NK.cells	EFNB1	0.025661	1.912975	0.08725	0.93067	-5.77225	0.908522	0.844395
NK.cells	VARS	-0.00934	5.713285	-0.08712	0.93077	-6.68667	0.858026	0.759667
NK.cells	IFI44	0.070946	0.579228	0.087006	0.930864	-5.23986	0.926992	0.876289
NK.cells	NCOR1	0.005275	8.098707	0.08692	0.930931	-7.08804	0.827903	0.710861
NK.cells	LCK	-0.01141	3.728178	-0.08681	0.931021	-6.58057	0.884013	0.802828
NK.cells	PSMD5	-0.01236	3.835237	-0.08658	0.931198	-6.2403	0.88259	0.80044
NK.cells	SCAMP3	0.008656	4.819715	0.086519	0.931249	-6.56288	0.869619	0.778803
NK.cells	TSEN2	0.029276	1.266976	0.086509	0.931257	-5.58642	0.917418	0.859707
NK.cells	SLC25A42	0.025935	1.940911	0.086462	0.931295	-5.81002	0.908139	0.843753

NK.cells	GM36975	0.016759	3.276788	0.086355	0.93138	-6.26015	0.89004	0.812977
NK.cells	AKAP13	0.006533	8.500476	0.086315	0.931411	-7.18426	0.822946	0.702959
NK.cells	ZFP607A	0.021312	1.840055	0.086114	0.93157	-5.78825	0.909521	0.846175
NK.cells	NDUFA1	0.00772	6.729501	0.086113	0.931572	-6.8663	0.845048	0.738523
NK.cells	ETHE1	-0.0114	4.997365	-0.08599	0.931666	-6.43514	0.867301	0.77501
NK.cells	IFT172	-0.02092	2.410455	-0.08598	0.931681	-5.84524	0.901733	0.83286
NK.cells	RCN3	0.024744	1.932131	0.085742	0.931866	-5.74761	0.908259	0.844035
NK.cells	EMCN	-0.03496	1.650943	-0.08552	0.932042	-5.53483	0.912119	0.850714
NK.cells	SMIM13	0.01378	3.919727	0.085502	0.932056	-6.18033	0.881468	0.798685
NK.cells	TSR2	0.027024	2.118125	0.085457	0.932091	-5.73444	0.905716	0.839756
NK.cells	NOSIP	0.008362	5.158105	0.085248	0.932257	-6.60601	0.865209	0.771723
NK.cells	GRIK5	0.045744	0.167717	0.085196	0.932298	-5.37638	0.93277	0.886615
NK.cells	CLPP	0.010233	4.725598	0.085121	0.932358	-6.48128	0.87085	0.78108
NK.cells	P2RY14	0.021954	3.467146	0.085059	0.932407	-6.06444	0.887493	0.808932
NK.cells	GM10762	0.02001	2.549426	0.085031	0.932429	-5.86904	0.899846	0.829852
NK.cells	GDI1	-0.01134	4.991215	-0.08486	0.932563	-6.45928	0.867381	0.775388
NK.cells	SSRP1	0.009172	6.322976	0.084694	0.932696	-6.78482	0.850213	0.747173
NK.cells	CLMN	-0.03867	0.334512	-0.08459	0.932776	-5.48662	0.930423	0.882608
NK.cells	NBEAL1	-0.01087	4.937374	-0.08456	0.932804	-6.50683	0.868083	0.776559
NK.cells	DNAJC14	0.009269	4.252973	0.084424	0.93291	-6.36343	0.877061	0.791496
NK.cells	EIF2B2	0.010425	4.936837	0.084397	0.932932	-6.51697	0.86809	0.776571
NK.cells	RNF2	0.008028	5.723956	0.084369	0.932953	-6.65771	0.857889	0.759738
NK.cells	MAP4K2	0.011864	5.348887	0.084356	0.932964	-6.52555	0.862733	0.767713
NK.cells	MVD	-0.01986	2.348846	-0.08424	0.933052	-5.94738	0.902571	0.834562
NK.cells	ZFP653	0.016134	3.131364	0.084221	0.933071	-6.0491	0.891992	0.816592
NK.cells	CSF3	-0.07122	-0.096	-0.08421	0.933081	-5.24529	0.936493	0.893218
NK.cells	TAF5L	0.009756	4.562119	0.08419	0.933096	-6.47292	0.872993	0.784714
NK.cells	C6	-0.04329	2.802964	-0.08417	0.933114	-5.71276	0.896415	0.824086
NK.cells	RSAD1	0.03093	1.298807	0.083877	0.933344	-5.74187	0.917101	0.859382
NK.cells	BC005561	-0.01129	4.273496	-0.08382	0.933386	-6.35604	0.876907	0.791136
NK.cells	FLVCR1	-0.0107	4.670527	-0.08329	0.933811	-6.46084	0.871811	0.782515
NK.cells	PRKACB	0.00772	5.548308	0.08327	0.933825	-6.67949	0.86039	0.763624
NK.cells	NDUFAF5	-0.01755	2.563951	-0.08307	0.93398	-5.9806	0.899897	0.829797
NK.cells	AW549877	0.016479	3.352769	0.083017	0.934026	-6.09051	0.889267	0.811785
NK.cells	CHRNB1	-0.03694	0.775917	-0.08299	0.934045	-5.43411	0.924497	0.87208
NK.cells	RASSF8	-0.03135	2.215433	-0.08298	0.934052	-5.6513	0.904637	0.837879
NK.cells	ZFP235	-0.02437	1.919758	-0.08283	0.934171	-5.75931	0.908679	0.844799
NK.cells	MAP3K14	-0.00979	5.189544	-0.08268	0.934293	-6.72762	0.865038	0.771328
NK.cells	AMZ2	-0.01032	3.901939	-0.08262	0.934337	-6.37204	0.881947	0.799476
NK.cells	FOXR1	0.048023	-0.87975	0.082579	0.934373	-5.1382	0.947911	0.913101
NK.cells	MYCN	-0.0417	-0.18583	-0.08249	0.93444	-5.36097	0.938023	0.895718
NK.cells	PLAG1	-0.01598	2.8948	-0.08241	0.93451	-5.97791	0.895422	0.822258
NK.cells	REEP1	-0.04166	0.98372	-0.0824	0.934512	-5.38416	0.921602	0.867127
NK.cells	CDC42EP1	-0.03973	0.752796	-0.08228	0.93461	-5.50307	0.92482	0.87272
NK.cells	GM37494	0.013622	3.825168	0.082269	0.934619	-6.19771	0.882966	0.801256
NK.cells	IPPK	-0.01438	3.400734	-0.08173	0.935047	-6.18684	0.888777	0.81091
NK.cells	GM32401	0.024043	2.445198	0.081685	0.935082	-5.82673	0.901663	0.832759

NK.cells	GM36199	0.046013	-0.13106	0.081626	0.935129	-5.31282	0.937407	0.89455
NK.cells	ZFP629	0.038794	0.926	0.081563	0.935179	-5.43024	0.922563	0.868679
NK.cells	DVL2	0.017619	3.263703	0.081547	0.935191	-6.04348	0.890613	0.814012
NK.cells	TULP3	0.027772	1.974251	0.081375	0.935327	-5.60676	0.908087	0.843803
NK.cells	BAG4	-0.00991	4.226706	-0.08134	0.935352	-6.42043	0.877798	0.792547
NK.cells	SLC25A51	-0.00984	5.697758	-0.08129	0.935397	-6.60494	0.858609	0.760746
NK.cells	CCDC157	0.027437	1.103128	0.081118	0.935531	-5.65422	0.9201	0.864482
NK.cells	TRMU	-0.02327	1.548223	-0.08111	0.93554	-5.62391	0.913941	0.853855
NK.cells	TMEM185/	0.019698	2.540183	0.080982	0.93564	-5.86116	0.900373	0.830627
NK.cells	REPS2	0.041648	0.905504	0.080808	0.935777	-5.38557	0.922848	0.869241
NK.cells	ZMYM4	0.00832	5.733892	0.08072	0.935847	-6.66894	0.858143	0.759981
NK.cells	IRF1	-0.01611	7.149071	-0.08071	0.935855	-6.93118	0.840127	0.730621
NK.cells	MOCS1	0.015188	3.367675	0.080453	0.936059	-6.05189	0.889219	0.811723
NK.cells	SNHG14	-0.02284	1.410261	-0.08039	0.93611	-5.66779	0.915845	0.857136
NK.cells	PDE3A	0.052638	0.411945	0.080249	0.936221	-5.34912	0.92975	0.881238
NK.cells	APAF1	0.008783	5.726771	0.080091	0.936346	-6.69147	0.858235	0.760131
NK.cells	SRGAP2	0.008954	6.60483	0.080028	0.936396	-6.83601	0.847005	0.741775
NK.cells	PFKFB1	-0.02967	0.961216	-0.07998	0.936431	-5.58846	0.922072	0.867897
NK.cells	KCTD11	-0.03424	0.546292	-0.07988	0.936511	-5.46963	0.927866	0.877957
NK.cells	NUP153	0.007356	5.848135	0.079755	0.936612	-6.72099	0.856673	0.757605
NK.cells	GM48855	-0.02178	1.463573	-0.07972	0.936642	-5.70704	0.915109	0.855928
NK.cells	ATXN7L3	0.010222	4.200833	0.079531	0.93679	-6.34567	0.87814	0.79321
NK.cells	DHX15	0.006157	6.794688	0.079382	0.936909	-6.85657	0.844599	0.737974
NK.cells	ACY1	0.031298	1.799123	0.079299	0.936974	-5.55295	0.910489	0.848063
NK.cells	GNA13	-0.00769	7.201206	-0.07923	0.937029	-6.96282	0.839471	0.729696
NK.cells	GAPT	-0.04445	1.236779	-0.07912	0.93712	-5.30434	0.918246	0.861452
NK.cells	MAPK3	0.009625	4.874121	0.079092	0.937138	-6.52492	0.869296	0.778549
NK.cells	ASAH2	0.02555	2.945427	0.079048	0.937173	-5.76254	0.894892	0.821489
NK.cells	KIF13B	-0.00778	6.510852	-0.079	0.93721	-6.82163	0.8482	0.743878
NK.cells	EVI2A	-0.01081	5.251447	-0.07895	0.937251	-6.54788	0.864382	0.770423
NK.cells	MPND	-0.01063	4.813143	-0.07887	0.937316	-6.47873	0.870093	0.779883
NK.cells	TAZ	-0.01097	4.057371	-0.07872	0.937434	-6.22745	0.880037	0.796525
NK.cells	CABLES2	0.012305	3.580958	0.078689	0.937458	-6.19597	0.886369	0.807162
NK.cells	PLXDC2	0.020995	5.144109	0.078408	0.937681	-6.47474	0.865777	0.772826
NK.cells	SHLD2	0.012702	3.853936	0.078394	0.937692	-6.25649	0.882735	0.801085
NK.cells	GM44777	0.035313	0.565153	0.078269	0.937791	-5.42716	0.927602	0.877795
NK.cells	NRN1	-0.02322	3.109984	-0.07823	0.937822	-6.12046	0.892677	0.817843
NK.cells	SDC1	0.031785	2.216388	0.078143	0.937891	-5.72733	0.904778	0.838426
NK.cells	TOPORS	-0.00972	5.749708	-0.07814	0.937897	-6.67116	0.857939	0.759905
NK.cells	CDC16	0.012023	3.893321	0.078106	0.93792	-6.20677	0.882212	0.800214
NK.cells	METAP1D	-0.01303	3.768583	-0.07803	0.93798	-6.25542	0.883869	0.803008
NK.cells	SF3B6	-0.00568	7.294759	-0.07776	0.938195	-6.95257	0.838296	0.727932
NK.cells	SLC35E2	0.009591	3.898481	0.077709	0.938235	-6.36469	0.882143	0.800115
NK.cells	AP1B1	-0.00864	5.190975	-0.07747	0.938425	-6.56722	0.865167	0.771873
NK.cells	CHEK1	-0.0188	2.722134	-0.07743	0.938456	-5.96107	0.897908	0.826773
NK.cells	KHNYN	-0.0106	4.263958	-0.07729	0.938567	-6.32582	0.877307	0.792073
NK.cells	SMARCA2	-0.00704	6.123463	-0.07729	0.938569	-6.81376	0.853141	0.752112

NK.cells	IGHD	0.01946	3.245617	0.077223	0.938621	-5.92419	0.890855	0.814842
NK.cells	TRIM30A	-0.01417	6.169574	-0.07718	0.938657	-6.74435	0.852552	0.751147
NK.cells	GGA2	-0.01672	4.120751	-0.07697	0.938818	-6.05201	0.879198	0.795281
NK.cells	ESCO2	0.018107	3.734708	0.076767	0.938982	-6.26495	0.88432	0.803905
NK.cells	PIK3AP1	-0.01184	8.295612	-0.07669	0.939046	-6.95383	0.825838	0.708053
NK.cells	POU6F1	-0.01637	2.610644	-0.07656	0.93915	-6.05241	0.899417	0.829446
NK.cells	CHD8	-0.00674	5.733086	-0.07649	0.9392	-6.67583	0.858153	0.760424
NK.cells	SDC3	0.026268	4.594842	0.076394	0.939278	-6.05289	0.872952	0.784935
NK.cells	GNA15	-0.01476	3.584316	-0.07632	0.939336	-6.21742	0.886324	0.807352
NK.cells	APEX2	-0.00992	4.215093	-0.07627	0.939378	-6.3957	0.877952	0.793314
NK.cells	PEAR1	-0.01515	2.352213	-0.07614	0.939483	-6.10893	0.902927	0.835523
NK.cells	GM12703	-0.04732	-0.21463	-0.07578	0.939764	-5.25588	0.938591	0.897362
NK.cells	LAX1	-0.01578	2.319766	-0.07572	0.939812	-6.2087	0.903369	0.836355
NK.cells	PRKAA1	-0.0111	4.45764	-0.07565	0.939865	-6.41761	0.874755	0.788053
NK.cells	ZFP280B	-0.02311	1.826966	-0.07544	0.940034	-5.7275	0.910106	0.847966
NK.cells	A2ML1	0.024667	2.471948	0.075344	0.940111	-5.90439	0.901299	0.832895
NK.cells	GM11613	-0.02882	2.020755	-0.07522	0.940213	-5.75263	0.907451	0.843434
NK.cells	ZDBF2	-0.03324	-0.23932	-0.07518	0.940244	-5.63677	0.938942	0.89808
NK.cells	CNTRL	-0.0072	5.907012	-0.07497	0.940407	-6.72733	0.855916	0.757025
NK.cells	ZFP994	0.014726	2.481603	0.074927	0.940442	-5.97935	0.901168	0.832754
NK.cells	STFA1	-0.03107	5.975982	-0.07455	0.940742	-6.4409	0.855031	0.755665
NK.cells	MED21	-0.01048	5.21874	-0.07427	0.940965	-6.53624	0.864807	0.771835
NK.cells	NXT1	0.010997	4.399059	0.074178	0.941036	-6.36367	0.875526	0.78965
NK.cells	LRRFIP1	-0.006	7.249941	-0.0741	0.941096	-7.01076	0.838859	0.729412
NK.cells	HIRIP3	0.014832	3.169999	0.073971	0.9412	-6.08567	0.89187	0.817141
NK.cells	DCSTAMP	0.031369	-1.33737	0.073907	0.941252	-5.28323	0.954224	0.926114
NK.cells	VEGFC	0.028652	1.200834	0.07387	0.941281	-5.59877	0.918744	0.863132
NK.cells	PREX2	-0.02985	2.374714	-0.07373	0.941391	-5.67705	0.902621	0.835438
NK.cells	POLR2I	0.007504	5.116859	0.073609	0.941488	-6.55429	0.866131	0.774076
NK.cells	FOXO4	0.015044	2.699869	0.07355	0.941534	-6.06091	0.898209	0.827954
NK.cells	CCDC97	-0.01361	3.33761	-0.07353	0.941549	-6.01214	0.889622	0.813396
NK.cells	GM14296	-0.02351	0.969349	-0.07328	0.941748	-5.55291	0.921959	0.868806
NK.cells	CHCHD7	0.009299	4.398636	0.073242	0.941779	-6.37335	0.875532	0.789769
NK.cells	CCDC88B	-0.00983	3.695402	-0.0729	0.942047	-6.44108	0.884843	0.805461
NK.cells	ITPRID2	-0.01003	5.08881	-0.07279	0.942136	-6.45104	0.866496	0.774824
NK.cells	FLNB	-0.01607	5.398208	-0.07279	0.942138	-6.6893	0.862479	0.768178
NK.cells	AUH	-0.0069	5.641378	-0.07261	0.942278	-6.78867	0.859336	0.763007
NK.cells	CFAP45	0.042619	0.728674	0.072595	0.942293	-5.31419	0.925315	0.874744
NK.cells	PBRM1	-0.00493	7.416728	-0.07256	0.942316	-6.96582	0.836767	0.726213
NK.cells	SRC	0.028456	0.980292	0.072459	0.9424	-5.55658	0.921807	0.868695
NK.cells	9430015G1	-0.02041	1.911053	-0.07222	0.942588	-5.72013	0.908953	0.846548
NK.cells	ANKRD6	0.027611	0.95646	0.072111	0.942677	-5.63915	0.922139	0.869329
NK.cells	BCAS2	0.005397	6.380106	0.071987	0.942775	-6.7911	0.849864	0.747585
NK.cells	UNC13D	-0.01133	3.31115	-0.07196	0.942797	-6.26248	0.889977	0.814259
NK.cells	DELE1	-0.01128	3.426108	-0.07195	0.942802	-6.13046	0.888437	0.811659
NK.cells	ING3	0.007695	4.830612	0.071907	0.942838	-6.50381	0.869864	0.780541
NK.cells	SLC25A32	0.012679	3.120627	0.071811	0.942915	-6.09281	0.892534	0.818611

NK.cells	NAA15	0.00527	6.743192	0.07175	0.942963	-6.84543	0.845251	0.740092
NK.cells	ZCCHC14	-0.03168	1.412865	-0.07149	0.943169	-5.4619	0.915809	0.858455
NK.cells	VAT1	0.014372	3.491225	0.071402	0.943239	-6.10116	0.887567	0.810241
NK.cells	WDR7	0.009665	5.188411	0.071381	0.943256	-6.56641	0.865201	0.772854
NK.cells	VDAC3	-0.00579	7.191815	-0.07138	0.943256	-6.94086	0.839589	0.730928
NK.cells	FUT4	-0.03919	-0.16835	-0.07135	0.943283	-5.27587	0.937935	0.896998
NK.cells	1700003F1	-0.02618	2.027827	-0.07123	0.943373	-5.60833	0.907354	0.843918
NK.cells	ITPRIPL2	0.012898	3.897046	0.071223	0.943381	-6.23017	0.882162	0.801154
NK.cells	GOLGA7	-0.00608	5.94	-0.07121	0.94339	-6.72533	0.855493	0.756862
NK.cells	SOCS1	0.013503	5.152296	0.070969	0.943583	-6.62624	0.86567	0.77373
NK.cells	PSD	-0.02257	1.633137	-0.07084	0.943687	-5.69293	0.912771	0.853328
NK.cells	NUP88	-0.00924	4.421593	-0.07074	0.943764	-6.38226	0.875229	0.789641
NK.cells	YARS2	0.014063	3.150094	0.070523	0.943937	-6.06545	0.892138	0.818101
NK.cells	AP1G1	0.007045	6.456316	0.070401	0.944034	-6.78897	0.848893	0.746178
NK.cells	GM15886	-0.01749	1.881502	-0.07038	0.944054	-5.72637	0.909358	0.847491
NK.cells	CEP89	-0.01968	2.805458	-0.07021	0.944183	-5.91896	0.896781	0.825992
NK.cells	CASP3	-0.00819	4.814976	-0.07013	0.944249	-6.4977	0.870069	0.781066
NK.cells	RYBP	0.007674	5.626574	0.070103	0.94427	-6.66209	0.859527	0.763616
NK.cells	PLD1	0.016851	3.080795	0.070067	0.944298	-6.12263	0.893069	0.819689
NK.cells	PPWD1	-0.00724	4.68019	-0.07004	0.944316	-6.45184	0.871833	0.784002
NK.cells	ZNRF3	-0.00853	5.517512	-0.06978	0.944525	-6.77527	0.860935	0.765959
NK.cells	RAB29	0.012193	3.635461	0.069752	0.944549	-6.23497	0.885642	0.807157
NK.cells	B9D2	0.010243	4.930502	0.069702	0.944589	-6.46355	0.86856	0.778579
NK.cells	ENDOV	0.020345	2.177707	0.069672	0.944612	-5.78041	0.905306	0.840563
NK.cells	GM4673	0.015487	2.578427	0.069563	0.944699	-5.88183	0.899854	0.831248
NK.cells	CD3EAP	0.013745	3.231663	0.069512	0.944739	-6.08081	0.891042	0.816278
NK.cells	E130102H2	-0.02912	1.232168	-0.0695	0.94475	-5.54811	0.91831	0.862949
NK.cells	NEK8	0.014879	2.113602	0.06945	0.944789	-5.9979	0.906181	0.842063
NK.cells	UBR5	0.006363	6.888097	0.069307	0.944902	-6.85717	0.843418	0.737309
NK.cells	AR	0.023014	0.218656	0.069159	0.945019	-5.8944	0.932468	0.887647
NK.cells	SLC2A12	-0.02143	1.619007	-0.06902	0.945127	-5.73102	0.912966	0.853824
NK.cells	HIST1H2BG	0.028231	1.128352	0.068939	0.945193	-5.60509	0.919749	0.86557
NK.cells	EVI5	-0.01374	5.166502	-0.06879	0.945309	-6.21934	0.865485	0.773627
NK.cells	FAIM	0.00956	3.865848	0.068783	0.945317	-6.35057	0.882577	0.80215
NK.cells	IMMP1L	0.007304	4.995599	0.068588	0.945472	-6.54492	0.86771	0.777336
NK.cells	NCOA2	-0.00608	7.684552	-0.06857	0.945487	-7.03547	0.833419	0.721271
NK.cells	CHPF2	0.015048	2.64648	0.068558	0.945496	-6.01937	0.898932	0.829854
NK.cells	APPBP2	0.005885	6.248663	0.068436	0.945593	-6.77468	0.851541	0.750706
NK.cells	CSNK1G2	0.006596	5.468875	0.068323	0.945683	-6.631	0.861564	0.767212
NK.cells	CMTM3	0.014951	3.593966	0.068302	0.945699	-6.01058	0.886195	0.808317
NK.cells	SNRNP35	0.015911	3.056704	0.068223	0.945762	-5.86998	0.893393	0.820496
NK.cells	ZFP692	-0.01547	2.030033	-0.06816	0.94581	-5.83475	0.907324	0.844272
NK.cells	TBKBP1	0.021831	1.449005	0.068142	0.945827	-5.78454	0.91531	0.858019
NK.cells	GM15345	0.023409	2.98417	0.068111	0.945851	-5.94537	0.89437	0.822175
NK.cells	WDR60	-0.03261	0.883885	-0.06806	0.945894	-5.40741	0.923149	0.871621
NK.cells	AKAP8	-0.00613	5.39229	-0.06799	0.945948	-6.64597	0.862555	0.768915
NK.cells	GFOD2	0.019209	2.309358	0.067841	0.946066	-5.78963	0.903511	0.837846

NK.cells	ABHD14A	-0.02333	1.44344	-0.06782	0.946081	-5.6156	0.915387	0.858261
NK.cells	IFI27	0.013114	4.998783	0.067578	0.946274	-6.5464	0.867669	0.77748
NK.cells	HSPD1	0.007684	7.46114	0.067202	0.946572	-6.98702	0.83621	0.726032
NK.cells	GM46652	0.040636	-0.73309	0.067174	0.946595	-5.22761	0.945973	0.911857
NK.cells	PAF1	-0.00765	4.600235	-0.06699	0.946742	-6.45443	0.872882	0.786255
NK.cells	MYL12A	-0.0056	7.929477	-0.06696	0.946762	-7.07568	0.830371	0.716659
NK.cells	B3GALNT2	-0.01113	3.643954	-0.06692	0.946796	-6.24414	0.885529	0.807464
NK.cells	2310015A1	-0.01831	2.091938	-0.06684	0.94686	-5.79312	0.906477	0.843101
NK.cells	ALOX15	0.080369	-0.82153	0.066755	0.946927	-5.19525	0.947239	0.914155
NK.cells	CTIF	0.013828	2.596638	0.066742	0.946938	-5.86268	0.899607	0.831358
NK.cells	ISG20L2	0.007335	5.083016	0.066614	0.947039	-6.59072	0.866572	0.775796
NK.cells	AKR1A1	-0.00584	7.188669	-0.06641	0.947201	-6.89885	0.839629	0.731636
NK.cells	RIC1	0.007556	6.710291	0.066366	0.947237	-6.9245	0.845668	0.741445
NK.cells	NFATC2IP	-0.01242	3.151572	-0.06636	0.947243	-6.10791	0.892118	0.818655
NK.cells	MRFAP1	0.005357	6.452488	0.066231	0.947344	-6.77041	0.848942	0.746801
NK.cells	GM20457	-0.03581	-0.16272	-0.06622	0.94735	-5.28312	0.937856	0.897659
NK.cells	CISD1	0.01012	5.119405	0.066145	0.947412	-6.53353	0.866098	0.775039
NK.cells	ADAMTS10	-0.01126	3.015513	-0.06606	0.947482	-6.17082	0.893948	0.821778
NK.cells	HEYL	0.037941	-0.20551	0.06597	0.947551	-5.26979	0.938462	0.898725
NK.cells	UBTF	-0.00572	5.879519	-0.06586	0.947641	-6.70355	0.85627	0.75881
NK.cells	RIPPLY3	0.039642	0.048429	0.065855	0.947642	-5.31589	0.934869	0.892415
NK.cells	ACVRL1	0.023443	2.619629	0.065798	0.947687	-5.73316	0.899296	0.830878
NK.cells	GM44699	0.02644	0.559613	0.065723	0.947747	-5.46791	0.92768	0.879854
NK.cells	SUMF2	0.014404	2.684259	0.065688	0.947774	-5.95947	0.89842	0.8294
NK.cells	DHX29	-0.01064	3.415036	-0.06566	0.947793	-6.18176	0.888586	0.812707
NK.cells	PSMA4	0.005654	6.877207	0.065637	0.947815	-6.88923	0.843555	0.738035
NK.cells	RRP1	-0.00487	6.518197	-0.06561	0.94784	-6.83161	0.848106	0.745449
NK.cells	4931406C0	-0.0144	3.540099	-0.06558	0.947859	-6.00956	0.886914	0.809884
NK.cells	MPDU1	0.007483	4.71579	0.065573	0.947866	-6.47734	0.871367	0.78381
NK.cells	GSK3B	0.004213	8.105386	0.065557	0.947879	-7.06268	0.82819	0.713232
NK.cells	WDR82	0.006058	4.82833	0.065525	0.947904	-6.53202	0.869894	0.781358
NK.cells	1700010K2	-0.0241	0.709295	-0.06538	0.948016	-5.47199	0.925586	0.876204
NK.cells	SEPSECS	0.013476	3.143326	0.065324	0.948063	-6.06136	0.892229	0.818875
NK.cells	EFEMP2	-0.02024	1.944258	-0.0651	0.948239	-5.62265	0.908498	0.846645
NK.cells	GM33782	-0.02437	0.19699	-0.06509	0.948249	-5.56071	0.932773	0.888757
NK.cells	EIF2A	0.005727	5.395584	0.064962	0.948351	-6.60994	0.862513	0.769115
NK.cells	FHL2	0.023164	0.017257	0.064945	0.948365	-5.84199	0.935309	0.893202
NK.cells	ARL15	-0.00585	7.591924	-0.06489	0.948408	-7.07848	0.834575	0.723497
NK.cells	HHAT	0.023076	1.670909	0.064884	0.948413	-5.63361	0.912251	0.853103
NK.cells	TOP2B	0.006748	6.593853	0.064864	0.948429	-6.84073	0.847145	0.74388
NK.cells	TMEM51O	-0.03621	-0.90367	-0.06467	0.948579	-5.1817	0.948415	0.916311
NK.cells	DHX40	0.00883	7.808142	0.064613	0.948628	-6.99454	0.83188	0.719156
NK.cells	B4GALT4	0.024113	1.774117	0.064602	0.948637	-5.51177	0.910832	0.850659
NK.cells	COMMD8	0.006548	5.188265	0.064574	0.948659	-6.60909	0.865203	0.773568
NK.cells	ACTR3B	0.02624	-0.01555	0.064532	0.948692	-5.37511	0.935773	0.894019
NK.cells	NT5C2	0.008693	5.867578	0.06452	0.948701	-6.64747	0.856423	0.759078
NK.cells	BACH2OS	-0.02821	1.526179	-0.06449	0.948728	-5.43107	0.914245	0.856546

NK.cells	LATS2	0.005979	5.889771	0.064343	0.948843	-6.74973	0.856174	0.758665
NK.cells	DBNL	0.005942	6.127196	0.064087	0.949046	-6.73343	0.853129	0.75369
NK.cells	NDST2	-0.01342	2.934923	-0.06395	0.949156	-5.97147	0.895071	0.823726
NK.cells	ZFP830	0.010774	3.541014	0.063925	0.949174	-6.13365	0.886939	0.809951
NK.cells	TTF2	0.012513	3.091877	0.063912	0.949184	-6.12583	0.892957	0.820138
NK.cells	RAD54L	-0.01542	2.724058	-0.06384	0.949246	-5.95316	0.897919	0.828595
NK.cells	TRP53	-0.00625	5.681235	-0.06379	0.949281	-6.68035	0.858857	0.763141
NK.cells	SGPL1	-0.00772	5.922758	-0.06334	0.949641	-6.61902	0.855952	0.758104
NK.cells	CUL9	-0.01594	2.579779	-0.06329	0.949678	-5.88398	0.900085	0.832027
NK.cells	DTNBP1	-0.00459	6.683213	-0.06323	0.949725	-6.8192	0.846246	0.742222
NK.cells	GM16287	-0.02956	-0.44109	-0.06296	0.94994	-5.32827	0.942164	0.904892
NK.cells	TESC	-0.01418	1.561476	-0.06293	0.949967	-6.21918	0.914103	0.855947
NK.cells	TMEM120I	0.012678	3.044848	0.062684	0.95016	-6.29389	0.894002	0.821393
NK.cells	GM826	0.045752	-0.25558	0.062495	0.95031	-5.26573	0.93966	0.900329
NK.cells	GM43466	-0.02292	1.497294	-0.06245	0.950342	-5.58708	0.915119	0.857553
NK.cells	TRAT1	-0.02137	-0.05747	-0.06224	0.950513	-5.91284	0.936852	0.895394
NK.cells	TUBGCP3	-0.00751	3.88521	-0.0622	0.950547	-6.30687	0.882778	0.802445
NK.cells	TCTA	0.016014	2.098287	0.061974	0.950723	-5.74248	0.906861	0.843345
NK.cells	C330013E1	0.027546	0.677864	0.061789	0.95087	-5.48228	0.926506	0.8773
NK.cells	ASNS	0.02365	1.465562	0.061681	0.950956	-5.5428	0.915557	0.85831
NK.cells	P4HTM	-0.01643	2.773581	-0.06156	0.951049	-6.06076	0.897678	0.827654
NK.cells	FOS	-0.00947	8.489188	-0.0615	0.951098	-7.11202	0.82388	0.705921
NK.cells	CFAP298	0.00992	3.642504	0.061491	0.951107	-6.2127	0.886008	0.807884
NK.cells	NPC2	-0.00614	7.639426	-0.06148	0.951113	-6.99463	0.834415	0.722814
NK.cells	TRAF7	0.006822	4.585124	0.061404	0.951176	-6.45259	0.873534	0.786963
NK.cells	ERCC8	0.013398	2.45611	0.061375	0.951199	-5.87838	0.901983	0.834995
NK.cells	NOL11	0.008522	4.89374	0.061282	0.951273	-6.50652	0.869491	0.780231
NK.cells	EFCAB7	-0.02622	0.535523	-0.06128	0.951278	-5.39822	0.928499	0.880775
NK.cells	CHAF1B	0.012872	3.382455	0.061151	0.951377	-6.11121	0.889507	0.813751
NK.cells	HMCN1	0.035316	1.920186	0.061041	0.951465	-5.53018	0.909338	0.847532
NK.cells	PIK3CB	0.009674	4.688415	0.060906	0.951571	-6.54467	0.872245	0.784747
NK.cells	GNPNAT1	-0.00791	4.157449	-0.06074	0.951701	-6.40803	0.879289	0.796463
NK.cells	6530409C1	0.019536	1.661527	0.060587	0.951825	-5.751	0.913007	0.853729
NK.cells	2310010J1	0.011745	3.692212	0.060426	0.951953	-6.19115	0.885494	0.806843
NK.cells	PDXDC1	0.004588	6.616598	0.060318	0.952038	-6.82999	0.847438	0.743791
NK.cells	GM17178	-0.01935	1.460682	-0.06026	0.952084	-5.61437	0.915778	0.858549
NK.cells	NFYC	-0.00424	6.202043	-0.06024	0.952098	-6.76869	0.852722	0.75243
NK.cells	OTUD7B	0.007499	5.17042	0.060121	0.952195	-6.4921	0.866051	0.774373
NK.cells	FASTKD1	-0.01572	2.189988	-0.05989	0.952381	-5.79619	0.905826	0.84137
NK.cells	RNPS1	0.005079	6.167896	0.059845	0.952414	-6.75376	0.853221	0.753193
NK.cells	RMDN1	-0.00936	4.528231	-0.05979	0.952461	-6.37842	0.874491	0.788372
NK.cells	STRN	-0.00666	5.530644	-0.05903	0.953062	-6.64603	0.861731	0.766818
NK.cells	NDUFB7	-0.00549	6.480862	-0.05892	0.953148	-6.82766	0.849533	0.746798
NK.cells	GNB1	0.003206	8.848488	0.058888	0.953174	-7.1945	0.819963	0.699174
NK.cells	EVI2	0.013222	3.35768	0.058843	0.95321	-6.12612	0.89035	0.814632
NK.cells	SLC25A36	-0.00537	6.285868	-0.05881	0.95324	-6.82203	0.85202	0.750863
NK.cells	ADRB1	-0.0261	0.735849	-0.0588	0.953246	-5.70442	0.926252	0.876232

NK.cells	TGTP1	0.033961	-0.20272	0.058549	0.953444	-5.48469	0.939597	0.899457
NK.cells	AMIGO1	0.027102	0.613629	0.058325	0.953622	-5.33201	0.9281	0.879394
NK.cells	CMC4	0.010771	3.064546	0.0583	0.953641	-6.03057	0.894422	0.821474
NK.cells	NCOR2	-0.00594	4.956649	-0.05822	0.953705	-6.61359	0.86932	0.779333
NK.cells	PPAN	-0.01001	4.072585	-0.05816	0.953755	-6.2652	0.880952	0.798752
NK.cells	POLG	-0.01065	3.808912	-0.05804	0.953846	-6.11399	0.884471	0.804674
NK.cells	DBR1	-0.01059	3.262038	-0.05783	0.954017	-6.1007	0.891846	0.817015
NK.cells	RALGAPA1	0.006605	6.84207	0.057654	0.954154	-6.88996	0.845147	0.739519
NK.cells	CARMIL1	-0.01609	4.075468	-0.0576	0.954194	-5.86904	0.880994	0.798728
NK.cells	PDZD11	-0.00888	4.30203	-0.05759	0.954209	-6.32903	0.877997	0.793707
NK.cells	RABEP1	0.004684	6.897474	0.057409	0.954349	-6.88227	0.844462	0.738407
NK.cells	ZFP141	0.011219	3.367308	0.057259	0.954468	-6.15539	0.890451	0.814694
NK.cells	SLC23A1	0.020801	1.528872	0.057105	0.954591	-5.55823	0.915469	0.857474
NK.cells	AC160336.	-0.02255	1.371003	-0.05681	0.954823	-5.52883	0.917652	0.8613
NK.cells	PDIA3	0.004577	8.546649	0.056805	0.954829	-7.18322	0.82388	0.705392
NK.cells	TMCO1	0.004716	6.265444	0.056791	0.95484	-6.74896	0.852501	0.751634
NK.cells	GM20712	0.036326	0.063822	0.056479	0.955088	-5.29562	0.935941	0.893183
NK.cells	MGST2	-0.01172	2.953494	-0.05639	0.955155	-6.09952	0.896017	0.824258
NK.cells	RNF11	-0.00586	6.207032	-0.05638	0.955163	-6.79132	0.853248	0.752891
NK.cells	TNPO2	-0.0086	4.122122	-0.0563	0.955227	-6.30532	0.880393	0.797884
NK.cells	SLAMF7	0.007585	5.151332	0.056196	0.955313	-6.78694	0.866877	0.775348
NK.cells	PDHX	0.008566	3.395129	0.056192	0.955316	-6.12166	0.890078	0.814192
NK.cells	PGP	-0.00728	5.374059	-0.05619	0.95532	-6.56509	0.863981	0.770555
NK.cells	METRN	-0.01345	2.538002	-0.05617	0.955336	-5.96329	0.901644	0.83384
NK.cells	B230217O1	-0.02068	1.757632	-0.05607	0.955415	-5.65674	0.912315	0.852132
NK.cells	CDK8	0.00888	6.559883	0.055933	0.955521	-6.83082	0.848745	0.745569
NK.cells	TPP1	-0.00745	5.393356	-0.05585	0.955589	-6.52518	0.863731	0.770187
NK.cells	DHX32	0.008757	3.380004	0.05575	0.955667	-6.16755	0.89028	0.814623
NK.cells	UBE2G1	0.004203	7.307993	0.055736	0.955678	-6.9393	0.839286	0.730245
NK.cells	SLC16A4	0.017846	1.428027	0.055541	0.955833	-5.62733	0.916862	0.860085
NK.cells	A630072M	0.009912	3.151561	0.055419	0.95593	-6.23127	0.893348	0.819876
NK.cells	MRPS21	0.00414	7.034242	0.0554	0.955945	-6.88663	0.842734	0.735883
NK.cells	CCDC166	0.023375	0.615517	0.055204	0.956101	-5.45209	0.928175	0.879773
NK.cells	ERGIC1	-0.00714	5.084699	-0.05508	0.956201	-6.61391	0.867745	0.776935
NK.cells	PYCARD	0.006274	5.876191	0.05505	0.956223	-6.74027	0.857493	0.760001
NK.cells	TRIM69	-0.01792	2.05026	-0.05487	0.956363	-5.79882	0.908298	0.845387
NK.cells	TMEM33	-0.00559	5.133185	-0.05475	0.956462	-6.53217	0.867113	0.775904
NK.cells	PKD2	-0.0239	0.719693	-0.05473	0.956474	-5.35987	0.926717	0.877251
NK.cells	CCDC91	-0.01289	2.408137	-0.05458	0.9566	-5.93396	0.903411	0.837062
NK.cells	SMYD5	0.018428	2.056268	0.054573	0.956602	-5.7277	0.908216	0.845292
NK.cells	FHL1	0.025442	0.58688	0.054514	0.956649	-5.41135	0.928577	0.880527
NK.cells	NPEPL1	0.006038	5.049543	0.054494	0.956664	-6.57644	0.868203	0.777743
NK.cells	SLC5A3	-0.01207	3.816287	-0.05445	0.956698	-6.22595	0.884453	0.804906
NK.cells	A930015D(	-0.00743	4.760503	-0.05442	0.956727	-6.45372	0.871982	0.784026
NK.cells	FDXACB1	0.013386	2.215747	0.05399	0.957065	-5.75195	0.906083	0.841629
NK.cells	C5AR1	-0.02883	4.179898	-0.05375	0.957255	-5.78571	0.879675	0.796889
NK.cells	CEP170	0.006514	5.909007	0.05358	0.957391	-6.58405	0.857117	0.759447

NK.cells	SLC10A7	0.005301	5.908741	0.053562	0.957405	-6.70019	0.857121	0.759453
NK.cells	ABCF2	0.006505	4.411019	0.053476	0.957474	-6.41266	0.876623	0.791788
NK.cells	MTF1	0.007225	4.234341	0.053398	0.957535	-6.30822	0.878955	0.795692
NK.cells	ADAMTS17	-0.02723	0.355692	-0.0534	0.957537	-5.42275	0.931874	0.886306
NK.cells	COL15A1	0.029091	0.332157	0.053386	0.957545	-5.42652	0.932205	0.886885
NK.cells	ELF2	-0.00459	7.39491	-0.05338	0.95755	-6.93131	0.838239	0.728673
NK.cells	TNFRSF11A	-0.02261	2.190262	-0.05327	0.95764	-5.58854	0.906432	0.842261
NK.cells	CLNK	-0.00974	1.404175	-0.05322	0.957676	-6.48245	0.917242	0.860873
NK.cells	NEMP1	0.013799	3.065406	0.053096	0.957775	-5.98981	0.894556	0.822025
NK.cells	PTPRM	0.01876	5.186994	0.053009	0.957844	-6.17394	0.866459	0.774923
NK.cells	MAP2K6	0.009788	3.533157	0.052918	0.957917	-6.28401	0.888278	0.811444
NK.cells	AZIN1	0.005558	6.940521	0.052852	0.957969	-6.87974	0.843963	0.738017
NK.cells	NSFL1C	-0.00667	4.168167	-0.05259	0.958176	-6.32159	0.879831	0.797293
NK.cells	TDRKH	-0.01862	1.697145	-0.05213	0.958546	-5.61574	0.913197	0.854161
NK.cells	RXRB	-0.00716	4.393184	-0.05196	0.958675	-6.3683	0.876858	0.792477
NK.cells	SLC24A3	-0.0211	1.280307	-0.05189	0.958735	-5.7688	0.918958	0.864159
NK.cells	CCR4	0.019977	-1.08113	0.051885	0.958738	-5.52242	0.952177	0.922691
NK.cells	TMEM51	-0.01416	3.130512	-0.05186	0.95876	-5.90128	0.89368	0.820823
NK.cells	DUT	-0.00883	5.882801	-0.05184	0.958777	-6.7302	0.857455	0.760288
NK.cells	PEA15A	-0.00806	3.90143	-0.0517	0.958887	-6.31446	0.883368	0.80341
NK.cells	PPP4R2	0.004675	6.318519	0.051643	0.958929	-6.79881	0.851868	0.75114
NK.cells	BC049352	-0.02332	1.083027	-0.0516	0.958963	-5.4909	0.921698	0.868932
NK.cells	SLC35A2	0.010929	3.165869	0.051354	0.959159	-6.02729	0.893204	0.820073
NK.cells	SOS2	-0.00526	5.600384	-0.05123	0.959255	-6.72783	0.861097	0.766342
NK.cells	CTSA	0.00662	6.259221	0.051211	0.959273	-6.75613	0.852626	0.752413
NK.cells	HIST1H3E	-0.01012	3.610481	-0.05118	0.959297	-6.23097	0.887245	0.80999
NK.cells	CPSF1	0.008902	3.349532	0.051065	0.959389	-6.12708	0.890737	0.815893
NK.cells	METTL4	0.00935	2.874732	0.050663	0.959708	-6.04011	0.897129	0.826742
NK.cells	IREB2	0.005414	5.493255	0.050622	0.959741	-6.61435	0.862483	0.768631
NK.cells	H2-AA	-0.02258	8.331767	-0.05061	0.959748	-6.78143	0.826574	0.710235
NK.cells	PPP1R3E	-0.01576	1.031561	-0.05058	0.959772	-5.55613	0.922414	0.870212
NK.cells	EFNB2	-0.01831	2.469455	-0.05058	0.959778	-5.76021	0.902625	0.836115
NK.cells	BCO2	0.022657	0.643324	0.050486	0.959849	-5.35584	0.927836	0.879647
NK.cells	2410131K1	0.014375	2.169111	0.050404	0.959914	-5.81013	0.906721	0.843128
NK.cells	MAP2K7	0.005958	4.294187	0.050395	0.959922	-6.40428	0.878165	0.794722
NK.cells	ZBTB22	-0.01081	3.076444	-0.05038	0.959933	-5.99686	0.894408	0.822116
NK.cells	GM867	0.029183	-0.30876	0.050279	0.960013	-5.29551	0.941274	0.903226
NK.cells	PIGV	-0.00855	2.921169	-0.05011	0.960151	-6.0139	0.896502	0.825708
NK.cells	ZFP707	-0.01246	1.958642	-0.05006	0.96019	-5.84694	0.909603	0.84811
NK.cells	ECD	-0.0055	4.899349	-0.05005	0.960194	-6.48344	0.870212	0.781477
NK.cells	HRH1	-0.02757	-0.56863	-0.05	0.960236	-5.17873	0.944977	0.909774
NK.cells	GNAI3	-0.0038	6.443605	-0.05	0.960238	-6.81411	0.850272	0.748591
NK.cells	CPSF4	-0.00744	4.317677	-0.0499	0.960311	-6.35897	0.877854	0.794234
NK.cells	NDUFA11	0.004834	6.910675	0.049862	0.960344	-6.88375	0.844341	0.73892
NK.cells	TBC1D4	-0.00858	4.706501	-0.04977	0.960422	-6.62986	0.872737	0.785684
NK.cells	LIMS2	0.026377	0.424817	0.049761	0.960425	-5.34829	0.930902	0.885036
NK.cells	H2-M3	-0.00911	4.138363	-0.04953	0.960607	-6.41757	0.880225	0.798271

NK.cells	CGRRF1	0.007101	3.907331	0.049528	0.96061	-6.25584	0.88329	0.803421
NK.cells	SDE2	0.005698	6.013145	0.049289	0.9608	-6.69612	0.855779	0.757708
NK.cells	ADGRE1	0.019268	4.711233	0.049186	0.960882	-6.06937	0.872675	0.785675
NK.cells	FKBP1B	-0.02335	0.793011	-0.04918	0.960891	-5.36202	0.925741	0.876139
NK.cells	ATXN1	0.005774	6.919224	0.049145	0.960914	-6.98451	0.844232	0.738834
NK.cells	BTNL9	-0.02796	0.689973	-0.04908	0.96097	-5.38359	0.927182	0.87865
NK.cells	CCDC114	0.018553	1.271818	0.049057	0.960984	-5.48946	0.919076	0.864562
NK.cells	CDC42BPB	0.015369	3.262023	0.048859	0.961142	-5.78091	0.891911	0.818014
NK.cells	CCDC15	-0.01219	2.810819	-0.04881	0.961182	-5.93562	0.897994	0.828347
NK.cells	SCARB2	-0.00516	6.538743	-0.04875	0.961225	-6.80065	0.84906	0.746709
NK.cells	H2AFY	0.003699	7.697828	0.048665	0.961296	-7.00323	0.834448	0.723007
NK.cells	RAB22A	0.004603	5.60028	0.048549	0.961388	-6.66841	0.861098	0.766483
NK.cells	B3GNT8	0.021622	2.202747	0.048521	0.96141	-5.51636	0.906261	0.842492
NK.cells	ATP5G3	0.004718	8.015505	0.048215	0.961654	-7.07322	0.830639	0.716665
NK.cells	TCHP	-0.01429	1.792962	-0.04807	0.961765	-5.69699	0.912076	0.852207
NK.cells	TCIM	0.023914	1.634355	0.047863	0.961934	-5.53344	0.914291	0.856017
NK.cells	APMAP	0.006851	4.080552	0.047849	0.961945	-6.33558	0.881211	0.799704
NK.cells	H2-Q4	-0.00817	4.933329	-0.04752	0.962203	-6.62347	0.870045	0.780957
NK.cells	SRGAP1	-0.01933	2.559229	-0.0474	0.962301	-5.62472	0.901692	0.834308
NK.cells	ZFP384	0.005484	4.90083	0.047204	0.962457	-6.47414	0.87047	0.781731
NK.cells	GM14085	0.017328	-1.35884	0.047189	0.962469	-5.54325	0.956184	0.930261
NK.cells	BAD	-0.00754	3.912661	-0.04714	0.962511	-6.2441	0.883501	0.803537
NK.cells	TOX4	-0.00381	5.903687	-0.0471	0.962543	-6.73517	0.85746	0.760219
NK.cells	SEH1L	-0.00441	5.334868	-0.04706	0.962571	-6.59778	0.864813	0.772355
NK.cells	SLC25A12	-0.00496	5.092687	-0.04697	0.962645	-6.54851	0.867965	0.777583
NK.cells	GM28112	0.011425	0.043725	0.046812	0.962769	-6.09315	0.936574	0.894795
NK.cells	MCM6	0.007146	6.155584	0.046777	0.962797	-6.76716	0.854225	0.75491
NK.cells	YIF1A	0.006124	4.016242	0.046704	0.962855	-6.27465	0.882125	0.801236
NK.cells	HNRNPUL2	0.003567	6.468104	0.046654	0.962894	-6.82064	0.850231	0.74837
NK.cells	SPAG1	-0.01763	1.4147	-0.04649	0.963026	-5.60709	0.917445	0.861409
NK.cells	ELP1	-0.00504	4.553493	-0.04627	0.963202	-6.40711	0.875148	0.789431
NK.cells	VANGL2	-0.0158	2.225601	-0.04619	0.96326	-5.58365	0.906364	0.842259
NK.cells	UQCRH	0.00283	8.592145	0.046116	0.963322	-7.19295	0.823741	0.705496
NK.cells	KIZ	0.007861	3.359508	0.045745	0.963617	-6.07488	0.891165	0.816155
NK.cells	TBC1D30	0.019498	1.06093	0.0454	0.963891	-5.64203	0.922587	0.870069
NK.cells	AP2B1	0.003776	6.179239	0.045331	0.963946	-6.72787	0.854188	0.754582
NK.cells	EXO1	0.015501	2.019131	0.045319	0.963955	-5.75306	0.909346	0.847204
NK.cells	CAPN1	0.006376	4.246316	0.045254	0.964007	-6.49467	0.879351	0.796312
NK.cells	WIPF2	0.004131	5.343948	0.045175	0.964069	-6.59409	0.864964	0.772352
NK.cells	RPRD2	-0.00439	5.916624	-0.04517	0.964074	-6.74226	0.85756	0.760135
NK.cells	ROCK2	0.004167	7.468662	0.045166	0.964077	-6.8849	0.837843	0.727991
NK.cells	JKAMP	0.00627	3.680975	0.045035	0.964181	-6.19098	0.886891	0.808966
NK.cells	ZFP715	-0.00723	3.662783	-0.04482	0.964352	-6.14621	0.887155	0.809375
NK.cells	KLC3	0.021946	0.19256	0.044636	0.964498	-5.41559	0.934814	0.891366
NK.cells	GM11714	-0.01135	1.498822	-0.04457	0.964548	-5.92668	0.916561	0.859617
NK.cells	ZBTB6	-0.0134	1.997204	-0.04445	0.964647	-5.67939	0.909697	0.847822
NK.cells	HDC	-0.01769	3.642871	-0.0444	0.964683	-6.23479	0.887421	0.80989

NK.cells	PTH1R	-0.02726	0.264703	-0.04437	0.964712	-5.35093	0.933796	0.889654
NK.cells	ATP6V1E1	-0.00398	7.59223	-0.04432	0.964746	-6.93815	0.836341	0.725591
NK.cells	BACH2IT1	-0.01998	-0.26225	-0.04423	0.964819	-5.31155	0.941258	0.902769
NK.cells	MYD88	0.006434	4.609439	0.044174	0.964866	-6.41128	0.874611	0.788415
NK.cells	CXCL16	0.016987	3.468405	0.044036	0.964975	-5.79454	0.889787	0.81389
NK.cells	ATP6V0C	-0.00387	8.851286	-0.0436	0.965323	-7.11813	0.820881	0.700717
NK.cells	GM4924	-0.0213	0.278075	-0.04359	0.96533	-5.36041	0.93376	0.88947
NK.cells	RELCH	-0.00358	6.238997	-0.04347	0.965423	-6.79121	0.85361	0.753562
NK.cells	AHRR	0.021625	-0.36283	0.04324	0.965608	-5.30324	0.942844	0.90549
NK.cells	RUBCNL	-0.01472	3.191015	-0.04309	0.965725	-5.65363	0.893625	0.820351
NK.cells	ARFGAP3	0.007601	3.55174	0.042776	0.965977	-6.13943	0.888784	0.812174
NK.cells	COLGALT1	0.00494	5.889717	0.042767	0.965984	-6.56191	0.858094	0.760999
NK.cells	ATP6V1H	0.004864	6.803114	0.042766	0.965985	-6.83064	0.846421	0.741891
NK.cells	IGHMBP2	-0.00781	2.850148	-0.04271	0.966026	-6.05377	0.898225	0.828187
NK.cells	ST6GALNA	-0.00737	3.867281	-0.04271	0.966031	-6.24331	0.884574	0.805076
NK.cells	PLPBP	-0.00549	4.145683	-0.04234	0.966326	-6.37095	0.880876	0.798955
NK.cells	ITFG2	-0.00635	3.893716	-0.04227	0.966382	-6.32725	0.884222	0.804601
NK.cells	FRS2	-0.0037	5.84525	-0.04216	0.966467	-6.70809	0.858667	0.762073
NK.cells	FBF1	0.021323	1.539498	0.04211	0.966506	-5.52269	0.916149	0.859073
NK.cells	CTNNA3	-0.02622	1.300067	-0.04208	0.966534	-5.4967	0.919465	0.864809
NK.cells	UBA7	-0.00828	3.784743	-0.04186	0.966703	-6.22426	0.885673	0.807158
NK.cells	ATP8B2	-0.00644	2.774077	-0.04186	0.966706	-6.17538	0.899255	0.830179
NK.cells	MLH3	-0.01115	2.01272	-0.04171	0.966824	-5.80785	0.909634	0.847945
NK.cells	GM14302	0.019425	0.609708	0.041649	0.966872	-5.34	0.929096	0.881657
NK.cells	NAPA	-0.00357	5.92916	-0.04164	0.966882	-6.69635	0.857587	0.760384
NK.cells	VARS2	0.015906	1.401859	0.041524	0.966972	-5.60328	0.918054	0.862494
NK.cells	USP42	-0.00612	3.500066	-0.04133	0.967126	-6.18716	0.889476	0.813641
NK.cells	KIF21B	0.005071	4.865707	0.041278	0.967167	-6.54259	0.871392	0.783298
NK.cells	TRIM2	-0.01444	2.325336	-0.04118	0.967248	-5.68566	0.905357	0.840691
NK.cells	POC1B	0.004941	4.642223	0.04114	0.967277	-6.41677	0.874324	0.788201
NK.cells	IKBIP	0.011368	1.938543	0.040832	0.967522	-5.75622	0.910652	0.849834
NK.cells	GNA12	-0.00593	4.652134	-0.04076	0.96758	-6.36464	0.874193	0.788032
NK.cells	PPP2R3A	0.006312	5.003266	0.040754	0.967584	-6.59858	0.869592	0.780366
NK.cells	UQCRB	0.00375	8.047525	0.040699	0.967627	-7.05977	0.8308	0.716963
NK.cells	SUGT1	0.003337	5.949959	0.04046	0.967818	-6.75528	0.857319	0.760102
NK.cells	DDOST	-0.00382	5.788445	-0.04044	0.967832	-6.6934	0.8594	0.763528
NK.cells	TEDC1	0.012647	1.701406	0.04036	0.967897	-5.68162	0.913915	0.855494
NK.cells	RAB18	-0.0035	5.748093	-0.04036	0.967901	-6.67962	0.85992	0.764386
NK.cells	ANAPC5	0.003322	6.399962	0.040288	0.967955	-6.77764	0.851552	0.750638
NK.cells	ATF6B	0.005795	4.456866	0.040281	0.96796	-6.35741	0.876764	0.792363
NK.cells	POLR3K	0.005566	3.885777	0.040259	0.967977	-6.26464	0.884328	0.805059
NK.cells	NFKB1	-0.00477	8.743793	-0.04021	0.968014	-7.23409	0.8222	0.703233
NK.cells	SPTBN4	0.021172	-0.18469	0.040172	0.968047	-5.27104	0.94031	0.901502
NK.cells	PARVA	0.016609	1.329681	0.040133	0.968078	-5.52445	0.919054	0.864378
NK.cells	SF3A2	-0.0049	5.260106	-0.04005	0.968147	-6.56912	0.866244	0.77485
NK.cells	DHRS4	-0.0061	4.374568	-0.04003	0.968161	-6.40448	0.877849	0.79419
NK.cells	WASHC2	0.005336	5.655214	0.039979	0.9682	-6.57411	0.86112	0.766374

NK.cells	GM42997	-0.02032	0.465543	-0.0399	0.968262	-5.40699	0.93112	0.885398
NK.cells	EMC1	-0.00589	3.622778	-0.03944	0.968625	-6.18364	0.888101	0.811069
NK.cells	IARS	0.004879	5.056202	0.039322	0.968722	-6.54512	0.86917	0.779344
NK.cells	RIPK2	-0.00602	4.205503	-0.03918	0.968837	-6.43492	0.880356	0.798033
NK.cells	PPP1CA	0.002885	8.233158	0.039139	0.968868	-7.11492	0.828754	0.713391
NK.cells	TRP53COR	0.018398	1.008827	0.03893	0.969034	-5.55234	0.923801	0.872302
NK.cells	MAPKBP1	0.008901	3.047991	0.038884	0.969071	-6.07628	0.895829	0.824222
NK.cells	GM6377	0.01706	3.091942	0.038876	0.969077	-5.55129	0.895236	0.823215
NK.cells	RNPC3	0.003945	4.741295	0.038481	0.969391	-6.48503	0.87351	0.786345
NK.cells	PTK7	-0.02296	1.073787	-0.03801	0.969762	-5.40519	0.923314	0.870885
NK.cells	PARP8	0.003792	6.68237	0.037967	0.9698	-6.94612	0.848602	0.74506
NK.cells	RWDD4A	-0.00539	3.983066	-0.03792	0.96984	-6.32716	0.883708	0.803212
NK.cells	MTHFSD	0.009158	2.433778	0.037669	0.970037	-5.8737	0.904568	0.838643
NK.cells	EDC4	-0.00771	2.984777	-0.03766	0.970043	-6.10046	0.897089	0.825887
NK.cells	FIP1L1	-0.00247	6.639254	-0.03763	0.970071	-6.84461	0.84915	0.746005
NK.cells	CHST15	0.009153	3.54275	0.037582	0.970106	-5.85672	0.889584	0.813166
NK.cells	GM42941	0.012005	1.267028	0.037433	0.970224	-5.6457	0.920668	0.866316
NK.cells	GM45370	0.025981	-1.29914	0.03698	0.970584	-5.14475	0.956789	0.930327
NK.cells	FCRL5	0.022218	-0.65204	0.036879	0.970665	-5.19816	0.947861	0.91379
NK.cells	GM17251	-0.01124	2.365949	-0.03685	0.97069	-5.77899	0.905651	0.840315
NK.cells	PIP4K2C	0.005121	4.238405	0.036833	0.970701	-6.36136	0.880473	0.797655
NK.cells	TNFRSF12A	-0.01265	1.479733	-0.03674	0.970774	-5.64171	0.917835	0.861276
NK.cells	GM47469	0.01595	0.520329	0.036722	0.97079	-5.48776	0.931222	0.88454
NK.cells	CHD4	0.002907	7.633169	0.036615	0.970875	-6.99326	0.836759	0.725735
NK.cells	SGO1	-0.01012	3.188246	-0.03626	0.971155	-6.08126	0.894598	0.821403
NK.cells	GM8066	-0.01518	0.498967	-0.0361	0.971282	-5.45176	0.931625	0.885179
NK.cells	PATZ1	-0.00502	4.251704	-0.036	0.971365	-6.34974	0.880394	0.797462
NK.cells	CD209A	0.024215	0.102824	0.035993	0.971369	-5.32189	0.937215	0.894967
NK.cells	FAM177A	-0.00974	1.731422	-0.03599	0.971374	-5.81558	0.914457	0.855382
NK.cells	EFCAB14	0.004708	4.713606	0.035773	0.971544	-6.4809	0.8743	0.787274
NK.cells	GRB14	-0.01366	1.939569	-0.03575	0.971565	-5.74424	0.911591	0.850446
NK.cells	MMP27	0.021384	-0.20195	0.035738	0.971572	-5.21888	0.94154	0.902569
NK.cells	AGFG1	-0.00294	6.147106	-0.03566	0.971633	-6.82041	0.855682	0.756473
NK.cells	RGS14	0.005748	3.585563	0.035079	0.972096	-6.33304	0.889408	0.812418
NK.cells	ERN1	0.003576	7.053542	0.034838	0.972287	-7.02225	0.844273	0.737686
NK.cells	U2SURP	0.002615	6.667671	0.034735	0.972369	-6.83264	0.849168	0.745677
NK.cells	SMO	-0.01457	1.198551	-0.03448	0.972571	-5.56315	0.921989	0.868285
NK.cells	TIPRL	-0.00314	5.525539	-0.03443	0.972611	-6.71334	0.863843	0.769803
NK.cells	MACROD1	0.008623	2.861084	0.034425	0.972616	-5.98124	0.899164	0.829063
NK.cells	PIGL	0.005903	2.746364	0.034399	0.972637	-6.04011	0.90072	0.831713
NK.cells	RNF125	-0.00512	3.852901	-0.0344	0.972638	-6.56079	0.885836	0.806495
NK.cells	UBR3	0.00322	6.575859	0.034338	0.972685	-6.83684	0.850337	0.747619
NK.cells	MYCL	-0.01745	1.305739	-0.0343	0.972716	-5.40917	0.920499	0.865703
NK.cells	CCDC62	-0.00943	2.858429	-0.03413	0.972849	-5.94119	0.8992	0.829128
NK.cells	CSTA3	0.025209	0.791872	0.034012	0.972944	-5.39197	0.927666	0.878158
NK.cells	TJAP1	0.004227	4.452059	0.034006	0.972949	-6.41804	0.877889	0.793163
NK.cells	PRDX2	-0.00385	7.774205	-0.03394	0.973002	-7.06812	0.835213	0.723105

NK.cells	PRPF40A	-0.00227	7.709067	-0.03381	0.973103	-6.9978	0.836028	0.724442
NK.cells	NT5E	0.007396	2.431121	0.033692	0.973199	-6.24528	0.905009	0.839094
NK.cells	GALNT12	0.006028	2.991393	0.033647	0.973235	-6.20459	0.897401	0.82614
NK.cells	BORCS8	0.00404	4.590375	0.03359	0.973279	-6.48551	0.876065	0.790197
NK.cells	IP6K2	-0.00506	3.644965	-0.0335	0.973352	-6.1908	0.888613	0.811296
NK.cells	TRIM5	0.006117	3.589282	0.033414	0.97342	-6.21701	0.889358	0.812565
NK.cells	1700120C1	-0.01128	1.482076	-0.03317	0.973612	-5.53652	0.918054	0.861675
NK.cells	TMEM109	-0.00561	3.841384	-0.03294	0.973798	-6.29243	0.88599	0.807009
NK.cells	TMX2	-0.0057	3.46498	-0.0325	0.974144	-6.16268	0.891024	0.815586
NK.cells	WDR19	-0.01512	0.508741	-0.03228	0.974324	-5.41626	0.93164	0.885465
NK.cells	THOC3	-0.00464	4.102819	-0.03225	0.974346	-6.32085	0.882512	0.80125
NK.cells	VRK1	0.003585	5.309256	0.032076	0.974484	-6.57545	0.866653	0.774787
NK.cells	AKT1S1	0.005436	3.589237	0.032031	0.974519	-6.10836	0.889358	0.812786
NK.cells	GM6712	0.008285	2.305447	0.031919	0.974609	-5.88184	0.906725	0.842384
NK.cells	RAP2C	0.003727	4.944479	0.031747	0.974745	-6.48644	0.871415	0.782751
NK.cells	CERK	0.003457	7.028222	0.031702	0.974781	-6.82692	0.844593	0.738634
NK.cells	0610009L1	0.010024	2.030404	0.031489	0.97495	-5.68905	0.910492	0.848865
NK.cells	NMT2	0.00303	5.47269	0.031335	0.975073	-6.6483	0.864528	0.771324
NK.cells	MPZL3	-0.00899	1.727945	-0.03112	0.975244	-5.86919	0.914655	0.856033
NK.cells	ARHGAP27	0.010589	0.465307	0.031117	0.975246	-5.58604	0.932251	0.886596
NK.cells	WDR20	0.002771	5.843767	0.030823	0.97548	-6.71989	0.859726	0.763396
NK.cells	MFAP1B	0.003207	4.945536	0.030723	0.97556	-6.52521	0.871401	0.782728
NK.cells	SPRYD4	0.008879	1.803638	0.030615	0.975646	-5.6513	0.913611	0.854234
NK.cells	ANXA2	0.003697	5.910919	0.030577	0.975676	-6.92195	0.85886	0.76197
NK.cells	DAPK2	-0.0051	2.818088	-0.03057	0.975683	-6.39611	0.899747	0.830472
NK.cells	SMC4	-0.00311	7.287715	-0.03056	0.975691	-6.98868	0.841317	0.733317
NK.cells	MRI1	0.003737	3.963231	0.030522	0.97572	-6.38815	0.884367	0.804426
NK.cells	PAQR5	0.017128	0.502008	0.030436	0.975788	-5.318	0.931735	0.885693
NK.cells	PDE3B	0.003627	6.955861	0.030425	0.975796	-7.08494	0.845509	0.740123
NK.cells	SRRM1	-0.00199	7.865839	-0.03034	0.975863	-7.04685	0.834069	0.721612
NK.cells	SH2B1	0.00531	3.699274	0.030271	0.975919	-6.2177	0.887887	0.810357
NK.cells	CHD9	-0.00362	6.065045	-0.03022	0.975961	-6.71373	0.856876	0.758708
NK.cells	SNAPC1	0.004059	3.850567	0.030188	0.975985	-6.39404	0.885867	0.806952
NK.cells	COPS2	0.002371	6.020343	0.030149	0.976016	-6.76825	0.857451	0.759653
NK.cells	NCBP1	0.002675	5.256901	0.030098	0.976057	-6.58609	0.867334	0.775972
NK.cells	TMEM97	-0.00563	3.649444	-0.02998	0.976154	-6.12919	0.888553	0.811482
NK.cells	0610010F0	0.004488	4.458879	0.02996	0.976167	-6.41057	0.877799	0.793404
NK.cells	POLR3D	-0.00548	3.27535	-0.02996	0.976168	-6.15298	0.893571	0.819973
NK.cells	GM16573	0.008274	1.655105	0.029733	0.976347	-5.77365	0.91566	0.857768
NK.cells	ZFP395	-0.00461	4.289929	-0.02969	0.976382	-6.3204	0.880032	0.797144
NK.cells	PREP	-0.00373	5.298922	-0.02947	0.976552	-6.52452	0.866787	0.775065
NK.cells	DCLRE1A	0.008575	1.621612	0.029389	0.976621	-5.74926	0.916123	0.858567
NK.cells	KLF10	-0.00364	5.248269	-0.02939	0.976621	-6.52132	0.867447	0.776158
NK.cells	ZFP24	0.00377	4.243197	0.029372	0.976634	-6.32844	0.88065	0.798182
NK.cells	GM45051	-0.0095	1.845863	-0.0293	0.976688	-5.59623	0.91303	0.853232
NK.cells	GALM	-0.0089	2.214948	-0.02927	0.976712	-5.81932	0.907963	0.844521
NK.cells	SENP6	-0.00219	6.681311	-0.02924	0.976742	-6.83276	0.848995	0.745802

NK.cells	ARHGDIA	0.00265	7.188393	0.028594	0.977253	-6.94118	0.842569	0.735426
NK.cells	IMMP2L	0.004722	6.534254	0.028586	0.977259	-6.75089	0.850868	0.748941
NK.cells	HUWE1	-0.0026	7.155414	-0.02855	0.977288	-6.93534	0.842985	0.736105
NK.cells	ZBTB18	0.005901	3.894799	0.028131	0.977621	-5.93564	0.885278	0.806052
NK.cells	RAB27B	-0.00712	0.615378	-0.02811	0.977635	-6.08372	0.930141	0.88301
NK.cells	ELMO2	-0.00366	3.945024	-0.02808	0.977665	-6.47885	0.884609	0.804926
NK.cells	ITPR3	-0.00463	4.288295	-0.02802	0.977712	-6.35607	0.880053	0.797272
NK.cells	PDE11A	-0.01117	0.072949	-0.02797	0.977752	-5.70834	0.937792	0.896408
NK.cells	RAD1	-0.0067	2.477895	-0.0278	0.977883	-5.88452	0.904371	0.838472
NK.cells	ZWILCH	0.007536	3.028209	0.02778	0.9779	-6.01935	0.896903	0.825734
NK.cells	NDUFC2	0.002764	6.741013	0.027696	0.977967	-6.84247	0.848235	0.744673
NK.cells	TBC1D12	0.005239	3.768556	0.027573	0.978065	-6.32791	0.886961	0.808935
NK.cells	ACAP2	-0.00268	7.320314	-0.02735	0.97824	-6.92639	0.840906	0.732879
NK.cells	4732471J0	-0.01017	1.996323	-0.02728	0.978301	-5.54612	0.91096	0.849933
NK.cells	PRDM16	0.01542	0.306557	0.027181	0.978376	-5.36147	0.934489	0.890798
NK.cells	TRGV2	-0.00767	-0.51519	-0.02718	0.978377	-5.99172	0.946162	0.911344
NK.cells	SPIDR	-0.00315	5.228567	-0.02713	0.978415	-6.55059	0.867704	0.77684
NK.cells	KIF1C	-0.00457	3.254466	-0.02708	0.978458	-6.15644	0.893852	0.820731
NK.cells	PAPOLA	0.001711	7.055885	0.027031	0.978496	-6.88391	0.844243	0.738318
NK.cells	LUC7L	0.002371	5.622585	0.02697	0.978544	-6.67006	0.862585	0.768388
NK.cells	TTC5	0.00292	4.64029	0.026944	0.978565	-6.47522	0.875408	0.789691
NK.cells	PCSK7	0.00303	5.282369	0.026873	0.978621	-6.61772	0.867003	0.775701
NK.cells	GTF2E2	0.002825	5.440806	0.026795	0.978684	-6.55795	0.864942	0.772287
NK.cells	RECQL	-0.00505	3.363727	-0.02676	0.978715	-6.16376	0.892383	0.818254
NK.cells	WWP2	-0.0024	6.537142	-0.02673	0.978734	-6.7809	0.850831	0.74907
NK.cells	CRADD	0.002832	5.224096	0.026732	0.978734	-6.576	0.867762	0.77696
NK.cells	GM22146	0.007344	2.073446	0.026704	0.978756	-5.8679	0.909902	0.848155
NK.cells	G0S2	0.006987	3.133285	0.026554	0.978875	-6.00119	0.895485	0.823527
NK.cells	MAPK13	-0.01452	0.83712	-0.0264	0.978999	-5.34936	0.927033	0.877814
NK.cells	GSK3A	-0.00255	5.557032	-0.02625	0.97912	-6.65471	0.863434	0.769801
NK.cells	KYAT1	-0.00954	1.503706	-0.02618	0.979173	-5.56749	0.917754	0.861706
NK.cells	MPC1	0.002391	7.569491	0.026131	0.979212	-6.99347	0.837776	0.727859
NK.cells	IPO4	-0.0057	2.384797	-0.02612	0.979217	-5.83509	0.905641	0.840853
NK.cells	SYTL3	-0.00486	2.803793	-0.02608	0.979255	-6.53671	0.899941	0.83111
NK.cells	DNMT3B	-0.00542	2.497904	-0.02603	0.979296	-5.97129	0.904098	0.838212
NK.cells	GM29093	-0.00821	1.269702	-0.02591	0.979388	-5.52667	0.921	0.867345
NK.cells	PATJ	0.005778	3.348038	0.025884	0.979408	-6.33116	0.892594	0.818637
NK.cells	GM26631	0.009717	1.219353	0.025705	0.979551	-5.54367	0.9217	0.868576
NK.cells	SERGEF	0.003471	4.018452	0.025663	0.979584	-6.42548	0.883633	0.80352
NK.cells	SIN3B	0.002075	6.294475	0.025623	0.979616	-6.75772	0.853933	0.754186
NK.cells	GM12905	0.007151	2.138199	0.025621	0.979617	-5.60473	0.909014	0.846672
NK.cells	EIF1AX	0.002518	6.060349	0.025452	0.979752	-6.74633	0.856937	0.759174
NK.cells	USP45	-0.00391	4.074097	-0.02543	0.97977	-6.30761	0.882893	0.802335
NK.cells	RNF5	0.003952	4.122516	0.025378	0.979811	-6.26688	0.88225	0.801275
NK.cells	ILRUN	-0.00259	5.988088	-0.02474	0.980315	-6.75576	0.857866	0.760812
NK.cells	PCGF2	-0.00826	0.79144	-0.02429	0.98068	-5.6859	0.927672	0.879165
NK.cells	FAM120C	0.00508	3.050639	0.024251	0.980707	-6.08626	0.8966	0.825644

NK.cells	ZFP668	-0.00348	3.829078	-0.02424	0.980713	-6.25863	0.886154	0.807952
NK.cells	BAG3	0.004989	2.967773	0.02424	0.980715	-6.08503	0.89772	0.82755
NK.cells	ATP6V0A1	-0.00615	4.202735	-0.02423	0.980721	-5.82721	0.881186	0.799593
NK.cells	AURKA	-0.00511	3.318106	-0.02387	0.981009	-6.11518	0.892996	0.819625
NK.cells	IL1B	-0.014	5.12575	-0.02371	0.981137	-5.89352	0.869045	0.779438
NK.cells	GM28375	-0.00344	3.206287	-0.0236	0.981224	-6.19627	0.894501	0.822217
NK.cells	KHDC4	-0.00205	6.448578	-0.02359	0.98123	-6.82895	0.851962	0.751258
NK.cells	TRAF4	-0.00486	4.196822	-0.02357	0.981252	-6.35457	0.881265	0.799861
NK.cells	ZBTB7A	-0.00181	7.04476	-0.02354	0.981276	-6.89074	0.844384	0.738892
NK.cells	PARVG	-0.00277	5.071901	-0.0235	0.981305	-6.54825	0.869748	0.780611
NK.cells	NPEPPS	0.002326	7.184287	0.023479	0.981321	-6.89934	0.842621	0.736028
NK.cells	SEC22B	-0.00239	5.449712	-0.02345	0.981342	-6.61694	0.864827	0.772444
NK.cells	TMEM158	-0.01141	0.271092	-0.02327	0.981489	-5.53098	0.934989	0.892153
NK.cells	SMARCC2	-0.00192	6.020787	-0.02323	0.981516	-6.73478	0.857445	0.760303
NK.cells	PHF2	-0.00242	5.076145	-0.02302	0.981686	-6.48587	0.869693	0.780576
NK.cells	KMO	-0.00651	3.038738	-0.02301	0.981692	-5.83646	0.896761	0.826119
NK.cells	LIFR	-0.00704	3.842745	-0.02299	0.981711	-5.97993	0.885972	0.807841
NK.cells	TRAPPC8	0.002018	6.225642	0.022845	0.981825	-6.79637	0.854815	0.756002
NK.cells	EXOSC8	0.002951	5.033192	0.022813	0.981851	-6.57841	0.870254	0.781527
NK.cells	JMJD1C	-0.00229	8.048596	-0.0228	0.981863	-7.03365	0.831793	0.718601
NK.cells	PKD2L2	-0.00712	1.170955	-0.02275	0.981899	-5.51228	0.922373	0.870188
NK.cells	WDR35	-0.01237	0.509893	-0.02272	0.981926	-5.32906	0.931624	0.886314
NK.cells	CDON	-0.00551	1.952332	-0.02272	0.981928	-6.02701	0.911565	0.851493
NK.cells	WDR83OS	0.002318	6.074614	0.022691	0.981948	-6.73063	0.856753	0.759204
NK.cells	GM17387	0.009898	0.498836	0.022648	0.981982	-5.45951	0.931779	0.886586
NK.cells	MAST3	0.003471	4.169613	0.022499	0.982101	-6.37844	0.881626	0.800563
NK.cells	MAP2K5	0.001923	6.143251	0.02248	0.982116	-6.76387	0.855872	0.757763
NK.cells	MAIP1	-0.00359	3.588251	-0.02246	0.982132	-6.17843	0.889372	0.813622
NK.cells	GM17477	0.008643	0.61424	0.022378	0.982197	-5.51343	0.930157	0.883759
NK.cells	FCHO2	-0.002	6.902577	-0.02235	0.982215	-6.90599	0.846184	0.741912
NK.cells	ZFP146	-0.00396	3.413704	-0.02228	0.982271	-6.13503	0.891712	0.817586
NK.cells	IST1	0.002206	5.536178	0.022222	0.982321	-6.61756	0.863705	0.770681
NK.cells	MRPL20	0.002117	6.1388	0.022171	0.982361	-6.75734	0.855929	0.757859
NK.cells	CTDNEP1	-0.00243	5.516749	-0.022	0.982499	-6.65945	0.863957	0.771144
NK.cells	FN3KRP	0.007722	1.481688	0.02196	0.982529	-5.64751	0.918059	0.862769
NK.cells	MMAA	-0.00706	1.748976	-0.0219	0.982574	-5.70168	0.914365	0.856383
NK.cells	HCST	0.002565	5.375869	0.021895	0.982581	-6.85141	0.865786	0.774174
NK.cells	BCL9	-0.00486	3.399574	-0.02188	0.982594	-5.97322	0.891901	0.817956
NK.cells	RETNLA	0.044417	-0.77811	0.021843	0.982622	-5.25737	0.949928	0.918629
NK.cells	ISOC2A	-0.00614	2.013655	-0.02165	0.982776	-5.72392	0.910797	0.850117
NK.cells	DDX49	-0.00245	4.208396	-0.02151	0.98289	-6.40175	0.881219	0.799761
NK.cells	PCGF6	-0.00393	2.512206	-0.02135	0.983011	-6.0125	0.904021	0.838412
NK.cells	LSS	-0.00912	0.604735	-0.02126	0.983083	-5.4442	0.930412	0.884081
NK.cells	ARID5A	-0.00275	4.681741	-0.02112	0.9832	-6.51945	0.874977	0.789336
NK.cells	ATF7	0.002136	6.314357	0.021096	0.983217	-6.77436	0.853789	0.754262
NK.cells	HDLBP	0.001566	6.655734	0.021034	0.983266	-6.8506	0.849431	0.747127
NK.cells	GM49463	-0.01111	-0.05016	-0.02087	0.983393	-5.36487	0.93969	0.900382

NK.cells	COL5A2	0.0106	1.225753	0.020809	0.983445	-5.47965	0.921761	0.869056
NK.cells	PYROXD2	0.010552	0.182818	0.020581	0.983626	-5.31059	0.936428	0.894659
NK.cells	SEC13	-0.00217	5.361757	-0.02052	0.983674	-6.57101	0.866147	0.774648
NK.cells	LNPK	-0.003	4.386002	-0.02046	0.983723	-6.38387	0.878941	0.795993
NK.cells	1600010M	-0.00311	4.96425	-0.02031	0.98384	-6.45198	0.871334	0.783292
NK.cells	EDA	0.011719	1.445596	0.020271	0.983873	-5.47841	0.918747	0.86385
NK.cells	DYRK3	0.006747	2.520243	0.020113	0.983999	-5.79452	0.903979	0.83846
NK.cells	ZAP70	0.003795	1.982594	0.020004	0.984085	-6.35608	0.911336	0.851128
NK.cells	CROCC	-0.00822	1.025643	-0.01988	0.984187	-5.4748	0.924588	0.874084
NK.cells	FGD2	-0.00716	3.69567	-0.01984	0.984214	-5.68887	0.888117	0.811533
NK.cells	LANCL1	0.002946	3.448045	0.019759	0.98428	-6.1246	0.891433	0.817173
NK.cells	AKT2	0.001992	5.234185	0.01964	0.984375	-6.6051	0.867808	0.777539
NK.cells	NDUFB6	-0.00235	6.317819	-0.01963	0.98438	-6.77156	0.853808	0.754431
NK.cells	POLD1	-0.00334	4.276679	-0.01953	0.984463	-6.27427	0.880387	0.798562
NK.cells	IDS	-0.00392	2.698638	-0.01919	0.984731	-6.04498	0.901552	0.834499
NK.cells	CBR2	0.01581	-1.3198	0.019127	0.984783	-5.154	0.957523	0.933031
NK.cells	AMOT	0.009088	0.863491	0.018889	0.984972	-5.39162	0.926853	0.878213
NK.cells	FCGR4	-0.01213	3.388468	-0.01886	0.984992	-5.65474	0.892233	0.818671
NK.cells	MICAL3	-0.00433	3.436889	-0.01881	0.985033	-5.98279	0.891583	0.817569
NK.cells	TM4SF5	-0.01126	0.261552	-0.01875	0.985086	-5.34882	0.935315	0.893016
NK.cells	TAOK3	0.001295	7.012234	0.018633	0.985176	-6.91584	0.844968	0.740115
NK.cells	SLC12A4	-0.00546	1.745624	-0.0186	0.985204	-5.77064	0.914598	0.856959
NK.cells	PNN	-0.00153	6.310373	-0.0185	0.985284	-6.79774	0.853904	0.754759
NK.cells	GM26930	0.010718	0.305585	0.018404	0.985358	-5.33202	0.934694	0.892006
NK.cells	TCRG-C4	-0.00389	0.327974	-0.01817	0.985544	-6.17672	0.934378	0.891493
NK.cells	SOD3	0.007801	1.286857	0.017646	0.985961	-5.55066	0.92095	0.868203
NK.cells	POLE4	0.00158	6.652817	0.017615	0.985985	-6.84928	0.849531	0.747781
NK.cells	HABP4	0.002817	3.054352	0.017247	0.986278	-6.1864	0.896734	0.826601
NK.cells	MPPE1	-0.00226	4.363196	-0.01712	0.986383	-6.45546	0.879242	0.797037
NK.cells	F11	0.007254	0.954836	0.017018	0.986461	-5.53902	0.925576	0.876291
NK.cells	GALNT1	0.001332	6.628673	0.016994	0.98648	-6.86279	0.849839	0.748319
NK.cells	ST7L	0.002308	4.451288	0.01696	0.986507	-6.41374	0.878078	0.795095
NK.cells	MAP4K5	-0.00271	4.52083	-0.01691	0.98655	-6.37804	0.877161	0.793557
NK.cells	NETO2	-0.00366	3.22493	-0.01671	0.986702	-6.19516	0.894433	0.822768
NK.cells	ARMC10	-0.00279	3.275459	-0.01646	0.986903	-6.13495	0.893753	0.821676
NK.cells	2510017J1	-0.00641	1.414805	-0.01644	0.98692	-5.62093	0.919174	0.865313
NK.cells	HADHA	0.001526	5.770414	0.01636	0.986984	-6.69253	0.860849	0.766553
NK.cells	CELF6	-0.0118	-0.70622	-0.01615	0.987148	-5.19065	0.949091	0.917859
NK.cells	GM26916	0.00587	0.934105	0.016118	0.987176	-5.50155	0.925866	0.877009
NK.cells	CD300E	0.012848	0.628868	0.015906	0.987346	-5.3656	0.930142	0.884533
NK.cells	MAP7	0.003205	3.639692	0.01587	0.987374	-6.05522	0.888865	0.8135
NK.cells	FAAH	-0.00353	1.451163	-0.01584	0.987401	-6.0394	0.91867	0.864556
NK.cells	IRAK1BP1	-0.00961	0.288762	-0.01581	0.987425	-5.28874	0.934931	0.892926
NK.cells	NFKBIL1	-0.0021	3.861655	-0.01571	0.987505	-6.29965	0.885901	0.808494
NK.cells	SV2C	-0.00628	-0.28271	-0.01551	0.987663	-5.55769	0.943037	0.907204
NK.cells	A430072P(	-0.00785	0.354986	-0.01545	0.987704	-5.46058	0.933997	0.891306
NK.cells	ZFP408	-0.00243	3.716601	-0.01521	0.987897	-6.21838	0.887837	0.811798

NK.cells	ZFAND4	0.005037	3.4762	0.015151	0.987946	-5.98296	0.891055	0.817247
NK.cells	IL15	-0.00594	3.93993	-0.01509	0.987991	-5.86541	0.884858	0.806768
NK.cells	DHRS9	0.006628	0.196917	0.014813	0.988214	-5.46382	0.936229	0.895246
NK.cells	SLC39A8	-0.00523	2.726982	-0.01478	0.988237	-5.7312	0.901167	0.834462
NK.cells	BRCC3	0.001711	4.874732	0.014764	0.988254	-6.50627	0.872507	0.786046
NK.cells	B230354K1	0.004844	1.756956	0.014753	0.988262	-5.74283	0.914442	0.857278
NK.cells	GM5086	-0.00759	-0.01663	-0.01449	0.98847	-5.25066	0.939254	0.900568
NK.cells	GM21188	-0.00947	1.578983	-0.01448	0.988477	-5.41437	0.9169	0.861529
NK.cells	CCDC162	-0.00527	2.876554	-0.01447	0.988487	-5.85555	0.899139	0.830997
NK.cells	STK38L	0.002142	4.003603	0.014461	0.988495	-6.24676	0.884011	0.805339
NK.cells	ZNRD1AS	0.006918	0.28937	0.014441	0.98851	-5.39017	0.934923	0.892952
NK.cells	FAM193A	0.001292	6.704133	0.014383	0.988556	-6.85779	0.848878	0.747013
NK.cells	TUBGCP2	-0.00202	3.994036	-0.01438	0.988557	-6.32234	0.884138	0.805554
NK.cells	ELOVL1	0.001852	4.858115	0.014349	0.988584	-6.50816	0.872725	0.786409
NK.cells	RAC1	0.000912	7.82414	0.014341	0.98859	-7.05264	0.83476	0.724083
NK.cells	GM4890	0.006331	0.373848	0.014326	0.988602	-5.37958	0.933731	0.89086
NK.cells	KCTD4	-0.00368	2.200261	-0.01432	0.988603	-5.94993	0.90835	0.846776
NK.cells	PARP4	0.00144	5.335625	0.014232	0.988677	-6.67961	0.866487	0.776024
NK.cells	FAM173A	0.001693	4.68182	0.01421	0.988694	-6.48592	0.875041	0.790278
NK.cells	GM28791	-0.00203	3.918458	-0.01412	0.988762	-6.40146	0.885144	0.807262
NK.cells	JADE3	-0.00228	3.471551	-0.01412	0.988764	-6.16939	0.891118	0.817365
NK.cells	XRCC1	0.002169	4.144273	0.013989	0.988871	-6.28552	0.882142	0.802208
NK.cells	APTX	0.002337	3.052742	0.013935	0.988913	-6.01804	0.896755	0.826978
NK.cells	ABCF3	0.001917	3.761251	0.013827	0.988999	-6.27587	0.88724	0.810848
NK.cells	LRIG2	-0.00198	4.256808	-0.01368	0.989114	-6.3417	0.88065	0.799764
NK.cells	TTLL1	0.004001	1.626512	0.01367	0.989124	-5.70953	0.916243	0.860478
NK.cells	RPRD1B	0.001493	5.480381	0.013631	0.989155	-6.62183	0.864605	0.772981
NK.cells	TRMT10B	0.003925	1.681609	0.013629	0.989156	-5.75089	0.915482	0.859161
NK.cells	RPE	0.001821	4.554255	0.013439	0.989307	-6.3996	0.87672	0.793209
NK.cells	IRF2	0.001295	6.689517	0.013419	0.989323	-6.8434	0.849064	0.747431
NK.cells	LMF2	0.002613	3.103696	0.013307	0.989413	-6.06566	0.896068	0.82589
NK.cells	TSTD3	-0.00301	1.784277	-0.01327	0.989445	-5.93586	0.914065	0.85676
NK.cells	TMSB4X	-0.00117	12.24836	-0.01318	0.98951	-7.72728	0.781515	0.640387
NK.cells	MTFR1	0.001975	4.079368	0.013127	0.989556	-6.31579	0.883004	0.803768
NK.cells	HDAC5	-0.00218	4.378269	-0.01308	0.989594	-6.29133	0.879043	0.797108
NK.cells	PPIL4	0.001089	5.46202	0.012982	0.989671	-6.67354	0.864844	0.773418
NK.cells	CHST10	-0.00417	0.375693	-0.01297	0.989678	-5.73014	0.933705	0.890953
NK.cells	MRPS18C	-0.00138	5.696786	-0.01289	0.989744	-6.65236	0.861801	0.768406
NK.cells	IFI211	0.00588	2.960201	0.012731	0.989871	-5.76806	0.898006	0.829287
NK.cells	GM43113	0.00708	-0.69248	0.012619	0.98996	-5.2847	0.948894	0.917881
NK.cells	2010309G2	-0.00575	1.223176	-0.01261	0.989969	-5.50282	0.921835	0.870346
NK.cells	MMP9	-0.00663	1.381978	-0.0126	0.989971	-5.57338	0.919629	0.866514
NK.cells	F10	-0.0038	4.54109	-0.01229	0.990223	-6.41813	0.877051	0.793663
NK.cells	LCN4	0.004421	-1.05402	0.012094	0.990378	-5.52732	0.95422	0.927184
NK.cells	NUDT2	-0.00208	3.238769	-0.0119	0.990531	-6.07653	0.894477	0.822958
NK.cells	ABHD15	0.003621	2.977881	0.011819	0.990596	-5.82498	0.897999	0.828953
NK.cells	1-Mar	0.00471	1.457886	0.011753	0.990649	-5.60559	0.918814	0.864742

NK.cells	TRIM44	0.001128	6.053928	0.011728	0.990669	-6.73738	0.857416	0.76093
NK.cells	FZD6	0.005529	0.693274	0.011364	0.990959	-5.4693	0.929641	0.883316
NK.cells	MAPK4	0.006917	0.274036	0.011324	0.99099	-5.21663	0.935545	0.893666
NK.cells	PATL1	-0.00124	5.222719	-0.01113	0.991147	-6.56433	0.868406	0.778768
NK.cells	NQO1	-0.00475	0.792254	-0.011	0.991248	-5.51104	0.928356	0.880923
NK.cells	MORN1	0.00371	1.227517	0.010642	0.991533	-5.58423	0.92231	0.870472
NK.cells	PAN2	-0.00189	3.190864	-0.01062	0.991554	-6.03317	0.895411	0.824222
NK.cells	GM36486	-0.00533	0.805963	-0.01058	0.99158	-5.50432	0.928197	0.880726
NK.cells	PTPN12	0.001193	6.049828	0.010543	0.991612	-6.76535	0.857745	0.761171
NK.cells	ZFP866	-0.00208	2.993086	-0.01049	0.991657	-6.04784	0.898083	0.82877
NK.cells	SMARCC1	0.001029	6.606315	0.010293	0.99181	-6.82398	0.850617	0.749496
NK.cells	ARPC2	-0.00054	9.318766	-0.01011	0.991955	-7.30086	0.816801	0.695039
NK.cells	CRELD2	0.001505	4.759514	0.010102	0.991963	-6.45822	0.874527	0.789081
NK.cells	CENPW	-0.00177	4.349764	-0.01009	0.991972	-6.42698	0.879931	0.798133
NK.cells	PLEKHG5	-0.00317	1.864551	-0.01003	0.992021	-5.80547	0.913489	0.855274
NK.cells	TMEM222	-0.00143	4.289191	-0.0099	0.992124	-6.3555	0.880758	0.799492
NK.cells	ADD3	-0.00122	6.047415	-0.00958	0.992379	-6.83204	0.857923	0.761367
NK.cells	ADM	0.00534	1.330377	0.009357	0.992556	-5.41282	0.921038	0.868204
NK.cells	CRLF2	0.001135	5.010644	0.009277	0.992619	-6.57632	0.871382	0.783712
NK.cells	GKAP1	-0.00197	3.541031	-0.00924	0.992645	-6.0664	0.890856	0.816436
NK.cells	AUNIP	-0.00282	1.893508	-0.00924	0.992648	-5.72416	0.913247	0.854722
NK.cells	ATF6	-0.00087	6.979377	-0.00917	0.992707	-6.86003	0.84602	0.741914
NK.cells	GLB1L	0.002149	2.192734	0.009008	0.992833	-5.8157	0.909183	0.847678
NK.cells	CD44	0.00102	8.587659	0.008311	0.993388	-7.03909	0.826246	0.709594
NK.cells	EDNRB	-0.00609	2.175551	-0.00822	0.993458	-5.65308	0.909745	0.848234
NK.cells	PDIA6	-0.00083	6.97381	-0.0082	0.993477	-6.88216	0.846438	0.742192
NK.cells	FAM216A	-0.0017	2.582038	-0.00819	0.993484	-5.99157	0.904188	0.838699
NK.cells	GM9530	-0.00464	-0.31965	-0.00802	0.993618	-5.28796	0.944718	0.909138
NK.cells	GM15472	-0.00159	1.795978	-0.00776	0.993826	-6.27488	0.915146	0.857317
NK.cells	GALNT4	0.002243	1.622606	0.007616	0.993941	-5.60744	0.917561	0.861464
NK.cells	INSR	-0.001	5.770425	-0.00733	0.994165	-6.62599	0.862036	0.767596
NK.cells	HSF5	0.003881	0.277217	0.007263	0.994221	-5.33052	0.936384	0.894328
NK.cells	PLCB3	0.001681	2.612	0.007092	0.994357	-5.87578	0.903975	0.838161
NK.cells	MRPS23	-0.00097	4.411962	-0.00707	0.994379	-6.44177	0.87981	0.797213
NK.cells	E130317F2	0.00269	0.77534	0.007064	0.99438	-5.44314	0.929368	0.882063
NK.cells	ARMC9	-0.00149	2.484131	-0.00698	0.994444	-5.90056	0.905719	0.841168
NK.cells	ZFP97	-0.00236	1.263623	-0.00698	0.99445	-5.62549	0.922544	0.870199
NK.cells	BRD1	0.000657	5.991255	0.006886	0.994522	-6.74452	0.859185	0.762952
NK.cells	GRPEL1	-0.00078	5.877727	-0.00677	0.994613	-6.69089	0.860649	0.76538
NK.cells	HK3	0.003247	2.677613	0.006715	0.994658	-5.68337	0.903082	0.83668
NK.cells	GM9993	0.002244	1.16551	0.00631	0.99498	-5.62687	0.923952	0.872718
NK.cells	CAND2	-0.00252	0.775357	-0.0063	0.994984	-5.47658	0.929409	0.882228
NK.cells	DENND5A	0.000622	6.395889	0.006182	0.995082	-6.72948	0.854024	0.754537
NK.cells	SMIM40	0.002843	0.301755	0.00611	0.995139	-5.43392	0.936079	0.893932
NK.cells	POLE	0.001403	3.301306	0.006061	0.995177	-6.18286	0.894677	0.822419
NK.cells	EMP1	-0.00225	1.978152	-0.0059	0.995309	-5.84438	0.912695	0.853281
NK.cells	RAP1A	-0.00032	8.772339	-0.00579	0.995393	-7.1756	0.824185	0.706291

NK.cells	ZFC3H1	0.000469	6.914629	0.005695	0.995469	-6.89773	0.84741	0.743787
NK.cells	CTLA2B	-0.00143	3.353706	-0.00568	0.995482	-6.38053	0.893972	0.821295
NK.cells	HIST1H1B	-0.0016	5.277543	-0.00568	0.995482	-6.70442	0.868478	0.778471
NK.cells	PRKAG1	0.00049	5.515312	0.005626	0.995524	-6.63519	0.865383	0.773337
NK.cells	UBE2D3	-0.00029	8.966917	-0.00546	0.995656	-7.21493	0.821794	0.702498
NK.cells	SPATA1	-0.00076	3.467697	-0.00534	0.995755	-6.17656	0.892439	0.818696
NK.cells	DUSP10	-0.00074	4.392133	-0.00526	0.995814	-6.5878	0.880112	0.7979
NK.cells	PTTG1	0.000578	5.78	0.005251	0.995822	-6.68752	0.861951	0.767659
NK.cells	TMED3	0.000661	5.491352	0.005147	0.995905	-6.55509	0.865694	0.773896
NK.cells	CHAMP1	0.00107	2.843874	0.005013	0.996011	-5.96758	0.900863	0.833099
NK.cells	ECHDC1	0.000835	3.820316	0.004961	0.996053	-6.26718	0.887715	0.810768
NK.cells	NFYB	-0.00055	5.103926	-0.00477	0.996204	-6.56662	0.870746	0.782325
NK.cells	EMP3	0.000473	7.421524	0.004666	0.996288	-7.00052	0.841001	0.733442
NK.cells	LONP2	0.000377	5.87764	0.004648	0.996302	-6.71576	0.860689	0.765653
NK.cells	MRPL49	-0.00084	3.778216	-0.00462	0.996326	-6.16716	0.888277	0.811734
NK.cells	PAICS	-0.00043	6.310596	-0.0042	0.996654	-6.78063	0.855233	0.756479
NK.cells	SNHG16	0.001149	2.227913	0.004185	0.996671	-5.8238	0.909388	0.847511
NK.cells	EIF4A2	-0.0004	5.530319	-0.00415	0.996697	-6.6263	0.865306	0.773093
NK.cells	LILRA5	-0.00242	1.497471	-0.00408	0.996757	-5.3926	0.919461	0.864897
NK.cells	GM12158	-0.00201	0.549233	-0.00374	0.997026	-5.36385	0.932893	0.888061
NK.cells	NFE2L2	0.000434	7.355477	0.003616	0.997123	-6.92635	0.842109	0.734884
NK.cells	TGS1	-0.00035	5.214453	-0.0035	0.997212	-6.59183	0.869586	0.780019
NK.cells	KLHL4	-0.0016	0.018315	-0.00347	0.997243	-5.50589	0.940403	0.901281
NK.cells	3110056K0	-0.00027	4.723448	-0.0025	0.998013	-6.48309	0.876588	0.790972
NK.cells	RSL1D1	0.000244	6.043414	0.002476	0.99803	-6.77699	0.859383	0.762433
NK.cells	ITGA5	0.000602	3.54178	0.002228	0.998227	-6.00583	0.892345	0.817468
NK.cells	PHF3	0.000161	6.980017	0.002137	0.998299	-6.92042	0.847436	0.742887
NK.cells	GTSF2	0.001406	0.091247	0.002071	0.998352	-5.21012	0.940009	0.899802
NK.cells	GIPC1	0.00029	4.359749	0.002053	0.998367	-6.33691	0.88143	0.799106
NK.cells	ASH2L	0.000217	4.017187	0.001693	0.998653	-6.32008	0.886068	0.806836
NK.cells	SCHIP1	-0.00065	1.591487	-0.00157	0.998751	-5.54296	0.919048	0.863188
NK.cells	RPF2	0.00022	4.402771	0.001446	0.998849	-6.38259	0.880944	0.798252
NK.cells	RBM15	0.000136	5.696792	0.001386	0.998898	-6.65514	0.863982	0.770007
NK.cells	BEX4	0.000583	0.397887	0.001228	0.999023	-5.37267	0.935755	0.892317
NK.cells	SIRT5	0.000285	1.958147	0.00095	0.999245	-5.71658	0.913979	0.854503
NK.cells	TNFSF10	0.000202	2.307037	0.00094	0.999252	-6.09063	0.909184	0.846254
NK.cells	UBASH3B	-0.00012	5.794781	-0.0009	0.999281	-6.91278	0.862713	0.767971
NK.cells	ESD	-9.37E-05	7.081837	-0.00088	0.9993	-6.93102	0.846224	0.740944
NK.cells	MIRT1	-0.00011	4.950639	-0.00075	0.999402	-6.56132	0.873719	0.78626
NK.cells	EDARADD	0.000146	3.159788	0.000749	0.999404	-6.12866	0.897577	0.826444
NK.cells	PEX13	-7.50E-05	5.524698	-0.00069	0.999453	-6.62544	0.866217	0.773794
NK.cells	GM28379	0.000206	0.264238	0.000447	0.999644	-5.47139	0.937645	0.895755
NK.cells	FOXRED2	0.000154	1.168266	0.000418	0.999668	-5.51443	0.924935	0.873545
NK.cells	DNAJC27	8.03E-05	2.442699	0.000381	0.999697	-5.97631	0.907327	0.843139
NK.cells	WDR53	-8.65E-05	2.715022	-0.00036	0.999714	-5.89976	0.903611	0.836776
NK.cells	HDAC8	-3.65E-05	6.530622	-0.0003	0.999761	-6.79552	0.853243	0.752476
NK.cells	FAAP24	-9.08E-05	2.002599	-0.00026	0.999791	-5.77982	0.913367	0.853543

NK.cells	AHCYL2	1.33E-06	6.28947	1.32E-05	0.999989	-6.88754	0.85644	0.757595
