# Supplementary material for: Immune landscape in liver of neonatal mice with phlebotomy-induced anemia
Source: Pediatr Res. 2025 Sep 17;99(4):1602–12. doi: 10.1038/s41390-025-04361-x (PMC12659965; doi:10.1038/s41390-025-04361-x)
Supplement: Supplementary file 3 — Table S3 [file 41390_2025_4361_MOESM3_ESM.pdf]

immunecell	gene	logFC	AveExpr	t	P.Value	B	adj.P.Val. Within	adj.P.Val. Between
Non.inflammatory.Macrophages	VEGFA	3.340748	4.223513	17.83255	4.95E-31	58.28442	5.41E-27	4.30E-26
Non.inflammatory.Macrophages	SLPI	3.118444	4.206306	12.74599	9.92E-22	38.68399	5.43E-18	1.44E-17
Non.inflammatory.Macrophages	SLC16A3	1.701341	3.935418	11.80881	7.17E-20	34.68992	2.66E-16	7.85E-16
Non.inflammatory.Macrophages	BNIP3	1.929184	5.086642	11.52724	2.64E-19	33.42942	6.84E-16	2.03E-15
Non.inflammatory.Macrophages	CCL9	1.988634	2.831707	11.39232	4.93E-19	32.66058	1.17E-15	3.71E-15
Non.inflammatory.Macrophages	PGK1	1.242333	7.765914	10.84995	6.22E-18	30.29505	9.13E-15	3.26E-14
Non.inflammatory.Macrophages	EGLN3	3.186425	3.294296	10.68926	1.32E-17	28.51165	2.19E-14	6.94E-14
Non.inflammatory.Macrophages	SLC2A1	1.223234	4.676925	10.02808	3.00E-16	26.52333	3.99E-13	1.22E-12
Non.inflammatory.Macrophages	PGAM1	0.9932	6.892134	9.874532	6.21E-16	25.77332	6.41E-13	2.09E-12
Non.inflammatory.Macrophages	SNCA	2.331437	4.248926	9.757291	1.08E-15	25.2971	1.18E-12	3.48E-12
Non.inflammatory.Macrophages	GYPA	2.306867	3.611578	9.631084	1.97E-15	24.69049	2.03E-12	6.01E-12
Non.inflammatory.Macrophages	GM10076	1.073824	6.671496	9.058345	3.00E-14	21.97138	2.35E-11	7.16E-11
Non.inflammatory.Macrophages	CCR1	2.003855	2.32125	9.016494	3.66E-14	21.74182	3.45E-11	9.31E-11
Non.inflammatory.Macrophages	OLR1	2.894953	1.333367	8.911105	6.04E-14	21.05805	5.61E-11	1.42E-10
Non.inflammatory.Macrophages	FABP4	1.632946	6.966529	8.807946	9.85E-14	20.78896	6.07E-11	1.90E-10
Non.inflammatory.Macrophages	MIF	1.09896	7.650894	8.610509	2.52E-13	19.7726	1.39E-10	4.11E-10
Non.inflammatory.Macrophages	CYTIP	1.040556	8.087657	8.588948	2.79E-13	19.77391	1.41E-10	4.26E-10
Non.inflammatory.Macrophages	GDA	2.314524	3.902395	8.325006	9.73E-13	18.65239	6.03E-10	1.48E-09
Non.inflammatory.Macrophages	TPI1	0.905484	6.690385	8.267229	1.28E-12	18.24574	6.33E-10	1.73E-09
Non.inflammatory.Macrophages	ALAS2	2.116818	4.928547	8.223475	1.57E-12	18.14314	8.23E-10	2.09E-09

Non.inflammatory.Macrophages	PKM	0.918915	8.60779	8.207398	1.70E-12	17.80027	6.75E-10	2.01E-09
Non.inflammatory.Macrophages	NFIL3	1.347776	4.515288	7.812864	1.09E-11	16.30077	5.31E-09	1.27E-08
Non.inflammatory.Macrophages	SLC4A1	2.200615	2.002694	7.794924	1.18E-11	16.07334	6.44E-09	1.46E-08
Non.inflammatory.Macrophages	HBB-BT	2.261373	8.105747	7.742415	1.51E-11	15.55044	5.28E-09	1.51E-08
Non.inflammatory.Macrophages	PFKL	0.888211	5.330071	7.739923	1.53E-11	15.94892	6.26E-09	1.63E-08
Non.inflammatory.Macrophages	F13A1	2.457288	2.512117	7.731096	1.60E-11	15.94454	7.45E-09	1.82E-08
Non.inflammatory.Macrophages	FXYD5	1.081544	6.974009	7.630885	2.55E-11	15.34495	8.73E-09	2.36E-08
Non.inflammatory.Macrophages	FAM162A	1.013117	5.640039	7.601853	2.92E-11	15.27536	1.05E-08	2.71E-08
Non.inflammatory.Macrophages	ITGAM	1.241364	4.619395	7.439416	6.23E-11	14.53868	2.29E-08	5.52E-08
Non.inflammatory.Macrophages	ANXA2	0.935007	5.910919	7.429278	6.53E-11	14.43064	2.15E-08	5.53E-08
Non.inflammatory.Macrophages	PLA2G7	1.453139	4.021195	7.348126	9.53E-11	14.02637	3.40E-08	8.08E-08
Non.inflammatory.Macrophages	MYO5A	1.463907	5.016572	7.157889	2.30E-10	13.34118	7.48E-08	1.71E-07
Non.inflammatory.Macrophages	CYP2E1	-2.07489	4.291137	-7.10184	2.98E-10	13.08593	9.82E-08	2.22E-07
Non.inflammatory.Macrophages	GAS7	1.522747	6.417925	7.039762	3.96E-10	12.82185	1.10E-07	2.70E-07
Non.inflammatory.Macrophages	DHX40	-0.92968	7.808142	-7.03697	4.01E-10	12.54846	1.01E-07	2.61E-07

Non.inflammatory.Macrophages	ENO1	0.722806	8.066335	6.95888	5.74E-10	12.10218	1.38E-07	3.56E-07
Non.inflammatory.Macrophages	EGLN1	0.731956	5.631732	6.874101	8.46E-10	12.03913	2.29E-07	5.36E-07
Non.inflammatory.Macrophages	LDHA	0.684332	8.792149	6.822867	1.07E-09	11.40978	2.33E-07	6.01E-07
Non.inflammatory.Macrophages	EPS8	1.490297	4.963297	6.770027	1.36E-09	11.63805	3.64E-07	8.27E-07
Non.inflammatory.Macrophages	HBB-BS	2.19073	11.91128	6.764513	1.39E-09	10.68599	2.38E-07	6.89E-07
Non.inflammatory.Macrophages	FLNB	1.579526	5.398208	6.706342	1.82E-09	11.31236	4.51E-07	1.05E-06
Non.inflammatory.Macrophages	TTC39C	1.645473	2.230936	6.699636	1.87E-09	11.26864	5.50E-07	1.16E-06
Non.inflammatory.Macrophages	GM43661	-1.30153	2.981343	-6.66346	2.21E-09	11.19725	5.91E-07	1.28E-06
Non.inflammatory.Macrophages	LYZ1	2.422744	-0.68152	6.663446	2.21E-09	10.61826	6.79E-07	1.42E-06
Non.inflammatory.Macrophages	FKBP5	1.063248	5.30259	6.630206	2.56E-09	10.99481	5.84E-07	1.37E-06
Non.inflammatory.Macrophages	HBA-A2	2.091809	9.98555	6.598436	2.96E-09	10.13526	4.94E-07	1.35E-06
Non.inflammatory.Macrophages	HBA-A1	2.099354	10.46682	6.557126	3.57E-09	9.898449	5.66E-07	1.54E-06
Non.inflammatory.Macrophages	BPGM	1.212113	4.812429	6.507185	4.47E-09	10.43915	9.69E-07	2.13E-06
Non.inflammatory.Macrophages	DDIT4	1.788491	3.744278	6.505568	4.50E-09	10.50912	1.03E-06	2.20E-06
Non.inflammatory.Macrophages	TSPO2	2.519448	0.283153	6.498065	4.65E-09	9.788111	1.26E-06	2.49E-06

Non.inflammatory.Macrophages	CACNA1D	1.535094	3.396539	6.483172	4.98E-09	10.36362	1.12E-06	2.43E-06
Non.inflammatory.Macrophages	CD244A	1.666875	3.429777	6.414456	6.77E-09	10.03506	1.49E-06	3.12E-06
Non.inflammatory.Macrophages	PTGS2	2.138948	2.215449	6.410315	6.90E-09	10.1084	1.61E-06	3.27E-06
Non.inflammatory.Macrophages	FAM20C	1.842255	1.641237	6.387244	7.65E-09	10.00825	1.81E-06	3.61E-06
Non.inflammatory.Macrophages	GPI1	0.554954	7.844302	6.357942	8.72E-09	9.484726	1.39E-06	3.42E-06
Non.inflammatory.Macrophages	TRIM26	0.620593	5.330265	6.238694	1.48E-08	9.224048	2.71E-06	5.81E-06
Non.inflammatory.Macrophages	UBA52	0.598848	10.38927	6.216511	1.64E-08	8.54928	2.15E-06	5.42E-06
Non.inflammatory.Macrophages	MS4A4A	1.899262	0.617812	6.112284	2.59E-08	8.569532	6.03E-06	1.08E-05
Non.inflammatory.Macrophages	MAN1A	0.736845	8.000333	6.052888	3.36E-08	8.181863	4.95E-06	1.10E-05
Non.inflammatory.Macrophages	GM32051	3.391599	-0.14913	6.034792	3.64E-08	7.103814	8.22E-06	1.47E-05
Non.inflammatory.Macrophages	AMZ1	-0.92468	3.157688	-6.00461	4.16E-08	8.283095	7.81E-06	1.49E-05
Non.inflammatory.Macrophages	NPEPPS	0.588271	7.184287	6.002939	4.19E-08	8.07995	6.11E-06	1.33E-05
Non.inflammatory.Macrophages	SH3PXD2B	1.134851	1.543779	6.001387	4.22E-08	8.3136	8.61E-06	1.56E-05
Non.inflammatory.Macrophages	ERO1L	0.869584	5.171152	5.993311	4.37E-08	8.237643	7.04E-06	1.46E-05
Non.inflammatory.Macrophages	ANK	1.261005	3.446389	5.922979	5.94E-08	8.063204	1.05E-05	2.02E-05

Non.inflammatory.Macrophages	HBQ1B	2.467554	-0.16343	5.897464	6.63E-08	7.453296	1.36E-05	2.46E-05
Non.inflammatory.Macrophages	CAR2	1.335539	5.171918	5.878185	7.21E-08	7.796822	1.11E-05	2.26E-05
Non.inflammatory.Macrophages	LSMEM1	1.082522	4.06136	5.801475	1.01E-07	7.410502	1.63E-05	3.16E-05
Non.inflammatory.Macrophages	CD36	-0.94897	5.712063	-5.789	1.06E-07	7.167368	1.54E-05	3.15E-05
Non.inflammatory.Macrophages	FN1	1.468905	5.580946	5.750876	1.25E-07	6.981892	1.80E-05	3.65E-05
Non.inflammatory.Macrophages	ISG20	1.30667	4.233394	5.714245	1.46E-07	7.145157	2.25E-05	4.34E-05
Non.inflammatory.Macrophages	RAP2A	0.667709	3.974658	5.709573	1.49E-07	7.071703	2.30E-05	4.44E-05
Non.inflammatory.Macrophages	PDK3	0.801236	4.945028	5.662258	1.83E-07	6.862812	2.62E-05	5.17E-05
Non.inflammatory.Macrophages	GM29282	-1.34583	1.712401	-5.65512	1.89E-07	6.958958	3.24E-05	5.80E-05
Non.inflammatory.Macrophages	PDK1	0.688635	4.164844	5.634048	2.06E-07	6.809367	3.02E-05	5.89E-05
Non.inflammatory.Macrophages	ARG1	1.576662	3.498182	5.603972	2.35E-07	6.749572	3.53E-05	6.64E-05
Non.inflammatory.Macrophages	GPR34	2.559856	0.588061	5.581619	2.58E-07	6.260303	4.52E-05	7.84E-05
Non.inflammatory.Macrophages	GM17023	2.664396	-0.64235	5.575669	2.64E-07	6.089729	4.59E-05	8.29E-05
Non.inflammatory.Macrophages	NASP	-0.58384	6.308139	-5.54802	2.97E-07	6.286168	3.63E-05	7.48E-05
Non.inflammatory.Macrophages	BCL2A1A	1.058923	2.399522	5.543287	3.03E-07	6.339997	4.63E-05	8.43E-05

Non.inflammatory.Macrophages	PIK3R5	0.719908	4.45137	5.529184	3.22E-07	6.34329	4.29E-05	8.33E-05
Non.inflammatory.Macrophages	ALDOA	0.748515	8.900702	5.52072	3.34E-07	5.733369	3.34E-05	7.60E-05
Non.inflammatory.Macrophages	MGARP	3.310604	-0.49619	5.498241	3.67E-07	4.94468	5.99E-05	0.000104
Non.inflammatory.Macrophages	ACLY	0.401616	6.383195	5.466127	4.20E-07	5.843969	4.80E-05	9.63E-05
Non.inflammatory.Macrophages	GM2A	0.486862	7.22888	5.436543	4.76E-07	5.552693	5.10E-05	0.000104
Non.inflammatory.Macrophages	ANAPC16	0.474793	5.656735	5.422375	5.05E-07	5.818483	5.89E-05	0.000115
Non.inflammatory.Macrophages	THBS1	1.491261	4.967156	5.406491	5.40E-07	5.701475	6.49E-05	0.000124
Non.inflammatory.Macrophages	GSN	0.880692	5.844895	5.374229	6.18E-07	5.718278	6.90E-05	0.000136
Non.inflammatory.Macrophages	FABP2	-1.67054	3.257265	-5.37361	6.20E-07	5.837997	8.08E-05	0.000146
Non.inflammatory.Macrophages	LRG1	2.055027	2.460703	5.362043	6.50E-07	5.765818	8.79E-05	0.000155
Non.inflammatory.Macrophages	2010013B2	-0.83355	3.545259	-5.35304	6.75E-07	5.695156	8.46E-05	0.000156
Non.inflammatory.Macrophages	TSPAN33	1.247644	2.55857	5.274601	9.36E-07	5.467521	0.000123	0.000213
Non.inflammatory.Macrophages	CRTC3	0.559255	5.658838	5.232679	1.11E-06	5.040488	0.00012	0.000223
Non.inflammatory.Macrophages	RELCH	0.449709	6.238997	5.225354	1.15E-06	4.984252	0.000118	0.000224
Non.inflammatory.Macrophages	SLC7A11	1.612688	4.278698	5.222536	1.16E-06	5.153149	0.000133	0.000238

Non.inflammatory.Macrophages	GYS1	1.046326	3.38284	5.218428	1.18E-06	5.226187	0.000142	0.000248
Non.inflammatory.Macrophages	MYH9	0.392494	7.80338	5.212058	1.21E-06	4.710099	0.00011	0.000224
Non.inflammatory.Macrophages	RBPJ	0.761218	6.055966	5.202119	1.26E-06	4.825698	0.000126	0.000243
Non.inflammatory.Macrophages	EEPD1	0.632435	4.881821	5.181543	1.37E-06	4.819534	0.000146	0.000268
Non.inflammatory.Macrophages	PRELID1	0.43037	7.529242	5.173819	1.42E-06	4.553645	0.000127	0.000254
Non.inflammatory.Macrophages	CCL6	0.650253	5.306299	5.170364	1.44E-06	4.494825	0.000146	0.000273
Non.inflammatory.Macrophages	PGM1	0.625054	4.592397	5.150666	1.56E-06	4.802365	0.000164	0.000297
Non.inflammatory.Macrophages	TREM1	1.775084	1.639916	5.147696	1.58E-06	4.899985	0.000196	0.000326
Non.inflammatory.Macrophages	PHLPP1	0.615501	7.671226	5.143101	1.61E-06	4.347218	0.000137	0.00028
Non.inflammatory.Macrophages	RGS18	1.784423	3.108266	5.120702	1.76E-06	4.695987	0.000197	0.000342
Non.inflammatory.Macrophages	FCGR2B	0.911366	4.881357	5.107177	1.86E-06	4.675884	0.000184	0.000341
Non.inflammatory.Macrophages	FAM120A	0.366106	6.352078	5.105188	1.88E-06	4.410461	0.000169	0.000329
Non.inflammatory.Macrophages	NDUFV3	0.459204	7.098589	5.089071	2.01E-06	4.24778	0.00017	0.00034
Non.inflammatory.Macrophages	S100A10	0.825613	6.601647	5.037236	2.48E-06	4.292927	0.000215	0.000413
Non.inflammatory.Macrophages	N4BP1	0.632734	5.4056	5.020702	2.65E-06	4.207754	0.000245	0.000449

Non.inflammatory.Macrophages	ILF3	-0.48067	6.541502	-5.00271	2.85E-06	4.065535	0.000244	0.000461
Non.inflammatory.Macrophages	GSTA3	-1.68392	4.0016	-4.99604	2.93E-06	4.375528	0.000289	0.000505
Non.inflammatory.Macrophages	MMD	0.689148	4.477186	4.992052	2.97E-06	4.192803	0.000281	0.000505
Non.inflammatory.Macrophages	MCTP1	1.2475	5.535347	4.991782	2.98E-06	4.191535	0.000264	0.00049
Non.inflammatory.Macrophages	CD9	0.87739	6.033746	4.98804	3.02E-06	4.094658	0.000257	0.000489
Non.inflammatory.Macrophages	ANKRD33B	1.323874	4.882196	4.982195	3.09E-06	4.2481	0.000279	0.000515
Non.inflammatory.Macrophages	ADAM3	2.632142	-0.17326	4.979783	3.13E-06	3.237381	0.000359	0.000594
Non.inflammatory.Macrophages	ATP13A3	-0.33519	7.315105	-4.97932	3.13E-06	3.808933	0.00024	0.000482
Non.inflammatory.Macrophages	CAPG	0.874904	5.264343	4.977424	3.15E-06	4.119872	0.000272	0.000513
Non.inflammatory.Macrophages	TPD52	0.537563	8.000639	4.971902	3.23E-06	3.634463	0.000233	0.000482
Non.inflammatory.Macrophages	EPRS	0.6296	6.581496	4.969761	3.25E-06	3.882698	0.000255	0.000504
Non.inflammatory.Macrophages	PIANP	-1.58931	-0.06426	-4.95242	3.49E-06	4.062746	0.000387	0.000642
Non.inflammatory.Macrophages	AHNAK	0.961062	6.416793	4.944884	3.60E-06	3.84044	0.00028	0.000549
Non.inflammatory.Macrophages	MT2	1.554524	2.001416	4.93671	3.72E-06	4.189124	0.000375	0.000634
Non.inflammatory.Macrophages	ALCAM	1.018821	6.646567	4.923	3.93E-06	3.87543	0.000295	0.000585

Non.inflammatory.Macrophages	S100A11	0.764859	7.270251	4.922117	3.94E-06	3.883131	0.000284	0.000576
Non.inflammatory.Macrophages	ANKRD37	0.837707	3.981977	4.920346	3.97E-06	3.97996	0.000347	0.000635
Non.inflammatory.Macrophages	ADD3	0.792954	6.047415	4.907856	4.17E-06	3.846527	0.000319	0.000622
Non.inflammatory.Macrophages	GAB1	0.781896	4.473058	4.905272	4.22E-06	3.700074	0.000352	0.000655
Non.inflammatory.Macrophages	AK4	1.909156	1.048143	4.90104	4.29E-06	3.882673	0.000437	0.000732
Non.inflammatory.Macrophages	CORO1A	0.614373	8.516071	4.870094	4.85E-06	3.450615	0.000311	0.000656
Non.inflammatory.Macrophages	GPAT3	0.76625	5.178486	4.867491	4.90E-06	3.818115	0.000382	0.000724
Non.inflammatory.Macrophages	GM37240	-0.7758	5.162317	-4.86614	4.93E-06	3.787156	0.000383	0.000726
Non.inflammatory.Macrophages	GPR183	1.99507	3.488292	4.861083	5.03E-06	3.783759	0.000429	0.000772
Non.inflammatory.Macrophages	GM36198	-1.07572	2.976425	-4.83633	5.55E-06	3.81913	0.000485	0.000849
Non.inflammatory.Macrophages	SGMS1	0.618606	7.102996	4.833104	5.62E-06	3.345676	0.000379	0.000763
Non.inflammatory.Macrophages	PRELID2	1.159092	2.621255	4.819939	5.92E-06	3.7317	0.000521	0.000903
Non.inflammatory.Macrophages	FAM220A	1.333104	2.03012	4.812695	6.10E-06	3.730968	0.000552	0.000935
Non.inflammatory.Macrophages	CLEC4E	1.541654	2.622943	4.809666	6.17E-06	3.629695	0.000535	0.000929
Non.inflammatory.Macrophages	LILR4B	1.018875	3.079256	4.803996	6.31E-06	3.513195	0.000528	0.000931

Non.inflammatory.Macrophages	MXI1	0.524819	7.063981	4.800038	6.41E-06	3.084844	0.000418	0.000843
Non.inflammatory.Macrophages	GAPDH	0.529658	10.59184	4.794035	6.56E-06	2.61551	0.000342	0.000777
Non.inflammatory.Macrophages	TET2	0.437851	6.024646	4.775407	7.06E-06	3.176112	0.000484	0.000927
Non.inflammatory.Macrophages	GSTT2	-0.89392	3.554147	-4.77124	7.18E-06	3.549752	0.000568	0.001006
Non.inflammatory.Macrophages	MCUB	1.207215	1.557619	4.76804	7.27E-06	3.554986	0.000644	0.001075
Non.inflammatory.Macrophages	SERPINA6	-3.29997	1.127419	-4.76396	7.39E-06	2.856761	0.000667	0.001099
Non.inflammatory.Macrophages	SERPINA3K	2.599183	1.878609	4.76083	7.48E-06	3.481724	0.000641	0.001087
Non.inflammatory.Macrophages	SDC4	0.749194	6.436865	4.757398	7.58E-06	3.144011	0.000488	0.000965
Non.inflammatory.Macrophages	SH3BGRL	0.380866	6.824052	4.756525	7.61E-06	2.936322	0.000476	0.000955
Non.inflammatory.Macrophages	ELMSAN1	-0.58169	6.924696	-4.75162	7.76E-06	3.011174	0.000477	0.000968
Non.inflammatory.Macrophages	ITGAX	1.33605	3.098724	4.751145	7.77E-06	3.508566	0.000603	0.001077
Non.inflammatory.Macrophages	SAMSN1	0.553318	7.050554	4.747766	7.88E-06	3.014433	0.000477	0.000976
Non.inflammatory.Macrophages	KLF9	0.795666	3.693914	4.742048	8.06E-06	3.321935	0.000595	0.001091
Non.inflammatory.Macrophages	SOCS3	0.883042	4.698376	4.738665	8.16E-06	3.218086	0.000563	0.00107
Non.inflammatory.Macrophages	CDKN1A	0.592615	5.267608	4.732243	8.37E-06	3.00431	0.000554	0.001074

Non.inflammatory.Macrophages	SLC25A37	0.72003	5.267981	4.723209	8.68E-06	3.096634	0.000571	0.001103
Non.inflammatory.Macrophages	STXBP3	0.523704	5.278015	4.721586	8.73E-06	3.115585	0.00057	0.001108
Non.inflammatory.Macrophages	OGT	0.41771	6.649131	4.699787	9.51E-06	2.906573	0.000568	0.001144
Non.inflammatory.Macrophages	GPR35	0.716342	2.516821	4.688481	9.94E-06	3.026598	0.000758	0.00132
Non.inflammatory.Macrophages	KPTN	-0.75175	4.098478	-4.67951	1.03E-05	3.073603	0.000707	0.001303
Non.inflammatory.Macrophages	PPP1R10	-0.49228	6.277719	-4.67879	1.03E-05	2.817159	0.000619	0.001228
Non.inflammatory.Macrophages	AREG	2.715463	1.634087	4.675793	1.04E-05	3.238061	0.000825	0.001407
Non.inflammatory.Macrophages	RETREG1	0.651471	6.437217	4.668246	1.08E-05	2.933677	0.00063	0.001257
Non.inflammatory.Macrophages	PIK3CB	0.610796	4.688415	4.654348	1.14E-05	2.788497	0.000735	0.00138
Non.inflammatory.Macrophages	SAA3	3.800031	0.738679	4.653027	1.14E-05	3.156827	0.000929	0.001543
Non.inflammatory.Macrophages	RNF169	0.579438	6.380124	4.645368	1.18E-05	2.718707	0.000679	0.001349
Non.inflammatory.Macrophages	STRBP	-0.68676	7.195368	-4.64252	1.19E-05	2.896152	0.000649	0.001332
Non.inflammatory.Macrophages	GK	0.711076	4.764608	4.630565	1.25E-05	2.847295	0.000785	0.001487
Non.inflammatory.Macrophages	ASPH	0.626492	4.664011	4.609914	1.35E-05	2.588154	0.00085	0.001587
Non.inflammatory.Macrophages	SCD2	0.659943	4.726396	4.569203	1.58E-05	2.565102	0.000985	0.001802

Non.inflammatory.Macrophages	MSR1	0.536796	3.816756	4.567299	1.59E-05	2.371512	0.001043	0.001854
Non.inflammatory.Macrophages	A3GALT2	-0.9102	2.675415	-4.5633	1.62E-05	2.78021	0.001128	0.001934
Non.inflammatory.Macrophages	S100G	2.591331	0.605317	4.552605	1.68E-05	2.425999	0.001305	0.002112
Non.inflammatory.Macrophages	OSER1	-0.45879	6.59749	-4.55214	1.69E-05	2.3718	0.000915	0.001787
Non.inflammatory.Macrophages	HSPA8	-0.36092	10.08692	-4.55133	1.69E-05	1.809312	0.000738	0.001624
Non.inflammatory.Macrophages	ZDHHC20	0.325839	6.740107	4.548764	1.71E-05	2.27256	0.000909	0.001793
Non.inflammatory.Macrophages	CASK	0.52819	5.500078	4.547726	1.72E-05	2.378451	0.000981	0.001857
Non.inflammatory.Macrophages	DNAJB1	-0.59768	6.228259	-4.54075	1.76E-05	2.367989	0.000958	0.001864
Non.inflammatory.Macrophages	HTRA3	1.716458	0.502839	4.535962	1.80E-05	2.544594	0.001352	0.002205
Non.inflammatory.Macrophages	SERINC1	0.338091	6.230989	4.535397	1.80E-05	2.215613	0.000967	0.001881
Non.inflammatory.Macrophages	GM4707	1.21613	2.866085	4.533766	1.81E-05	2.73338	0.001188	0.00207
Non.inflammatory.Macrophages	BSG	0.443284	7.716678	4.528897	1.84E-05	1.995707	0.000895	0.001831
Non.inflammatory.Macrophages	ADAM15	0.867891	2.475369	4.523379	1.88E-05	2.602447	0.001252	0.002153
Non.inflammatory.Macrophages	HPGDS	-0.68881	3.647133	-4.51403	1.95E-05	2.402547	0.001202	0.002142
Non.inflammatory.Macrophages	SMIM3	1.024848	4.840554	4.507901	2.00E-05	2.64214	0.001138	0.002112

Non.inflammatory.Macrophages	PROC	-1.48366	2.923674	-4.49948	2.06E-05	2.609763	0.001313	0.00229
Non.inflammatory.Macrophages	AKR1C6	-1.41212	4.841164	-4.48346	2.19E-05	2.362983	0.001236	0.002286
Non.inflammatory.Macrophages	ACSL4	0.444617	6.189637	4.448893	2.50E-05	1.974363	0.001292	0.002466
Non.inflammatory.Macrophages	SHMT1	-0.79673	4.012421	-4.44198	2.57E-05	2.302312	0.001506	0.002683
Non.inflammatory.Macrophages	OSM	1.572885	2.305551	4.437741	2.61E-05	2.400882	0.001689	0.002847
Non.inflammatory.Macrophages	HK1	0.512479	4.85354	4.427675	2.71E-05	2.082295	0.001495	0.002706
Non.inflammatory.Macrophages	ATP6V1G1	0.325238	7.177612	4.424806	2.74E-05	1.700661	0.001304	0.002558
Non.inflammatory.Macrophages	CORO1B	0.539799	4.252422	4.408065	2.92E-05	1.993983	0.001653	0.00292
Non.inflammatory.Macrophages	C5AR1	0.757635	4.179898	4.390516	3.12E-05	1.700731	0.001765	0.003087
Non.inflammatory.Macrophages	GM45716	0.739292	3.20355	4.386509	3.17E-05	2.143075	0.001892	0.00321
Non.inflammatory.Macrophages	LECT2	-1.44366	3.632928	-4.37995	3.25E-05	2.168196	0.001879	0.00324
Non.inflammatory.Macrophages	CIART	-1.02502	2.328452	-4.37054	3.37E-05	2.160943	0.002097	0.003458
Non.inflammatory.Macrophages	FAM20A	1.484089	1.833508	4.367138	3.41E-05	2.151105	0.002169	0.003535
Non.inflammatory.Macrophages	CSF2RB	0.530637	4.838003	4.366773	3.42E-05	1.596015	0.001808	0.003252
Non.inflammatory.Macrophages	ABCB1B	1.503391	3.934965	4.358178	3.53E-05	2.129371	0.001963	0.003418

Non.inflammatory.Macrophages	ANKRD11	-0.33235	9.391983	-4.35045	3.63E-05	1.194819	0.001436	0.003005
Non.inflammatory.Macrophages	SMOX	0.65477	5.116761	4.349725	3.64E-05	1.636503	0.001867	0.003385
Non.inflammatory.Macrophages	ST3GAL1	0.668353	5.796993	4.339763	3.78E-05	1.626362	0.00185	0.003405
Non.inflammatory.Macrophages	FBXW7	-0.44879	6.448567	-4.33369	3.87E-05	1.547542	0.001809	0.003396
Non.inflammatory.Macrophages	TGFB3	1.735028	0.567314	4.33245	3.88E-05	1.947597	0.00256	0.004001
Non.inflammatory.Macrophages	TRIM10	1.664082	0.349121	4.330198	3.92E-05	2.012739	0.002574	0.00405
Non.inflammatory.Macrophages	AGAP2	-1.23269	2.925575	-4.32804	3.95E-05	1.984915	0.002257	0.003793
Non.inflammatory.Macrophages	GM16867	1.464646	2.522419	4.323975	4.01E-05	2.002644	0.002337	0.00388
Non.inflammatory.Macrophages	UHRF1BP1	0.508072	5.734451	4.32117	4.05E-05	1.706917	0.001934	0.003576
Non.inflammatory.Macrophages	ACTR2	0.309086	8.066808	4.312408	4.19E-05	1.234749	0.001721	0.003423
Non.inflammatory.Macrophages	EDARADD	-1.44053	3.159788	-4.30939	4.23E-05	1.875926	0.002323	0.003946
Non.inflammatory.Macrophages	MAN2A1	0.423381	7.183028	4.309164	4.24E-05	1.274329	0.001817	0.003528
Non.inflammatory.Macrophages	ARPC5	0.270033	8.144041	4.308947	4.24E-05	1.171412	0.001713	0.003435
Non.inflammatory.Macrophages	LRRFIP2	0.363411	5.900501	4.303436	4.33E-05	1.455999	0.001997	0.003705
Non.inflammatory.Macrophages	ILDR1	-1.01782	1.791756	-4.29729	4.43E-05	1.846887	0.002611	0.004228

Non.inflammatory.Macrophages	F5	1.070895	4.094976	4.276089	4.79E-05	1.644473	0.002446	0.004227
Non.inflammatory.Macrophages	GM13986	1.264472	1.376917	4.25787	5.13E-05	1.689097	0.00307	0.004809
Non.inflammatory.Macrophages	RAPSN	1.570652	0.715904	4.255168	5.18E-05	1.537962	0.003195	0.004938
Non.inflammatory.Macrophages	SMCHD1	0.440506	7.994517	4.252218	5.24E-05	1.040721	0.002077	0.004069
Non.inflammatory.Macrophages	FKBP1A	0.368857	7.336878	4.247562	5.33E-05	1.131488	0.00219	0.004191
Non.inflammatory.Macrophages	GM21188	1.243386	1.578983	4.245019	5.38E-05	1.695068	0.003117	0.004954
Non.inflammatory.Macrophages	PDLIM1	0.779025	4.944927	4.244388	5.39E-05	1.622982	0.002542	0.004512
Non.inflammatory.Macrophages	SOS1	0.400788	5.916592	4.240272	5.47E-05	1.337355	0.002415	0.00444
Non.inflammatory.Macrophages	ST7	0.520756	5.144851	4.239664	5.49E-05	1.347985	0.002531	0.004542
Non.inflammatory.Macrophages	TREM3	1.448533	2.200561	4.23851	5.51E-05	1.708305	0.003027	0.004937
Non.inflammatory.Macrophages	DDI2	0.40447	5.943537	4.236598	5.55E-05	1.234992	0.002411	0.004471
Non.inflammatory.Macrophages	GM26887	-0.87949	3.10483	-4.23617	5.56E-05	1.501847	0.002866	0.004842
Non.inflammatory.Macrophages	IFITM2	0.430752	7.227901	4.234501	5.59E-05	0.876332	0.002233	0.004339
Non.inflammatory.Macrophages	NHEJ1	-0.91258	3.867688	-4.23254	5.63E-05	1.48125	0.002749	0.004785
Non.inflammatory.Macrophages	BEX3	-0.57447	4.990762	-4.22531	5.79E-05	1.419878	0.002621	0.004736

Non.inflammatory.Macrophages	CD300LF	0.674976	3.385271	4.224627	5.80E-05	1.329753	0.002889	0.00496
Non.inflammatory.Macrophages	SLC9A9	-0.58271	7.120003	-4.2197	5.91E-05	0.874089	0.002333	0.004544
Non.inflammatory.Macrophages	H2-Q10	-1.06553	4.038149	-4.21551	6.00E-05	1.584853	0.002847	0.005004
Non.inflammatory.Macrophages	SPTLC2	0.311827	6.245992	4.208339	6.16E-05	1.069892	0.002533	0.004804
Non.inflammatory.Macrophages	MET	1.289922	1.991608	4.207651	6.18E-05	1.61357	0.003281	0.005417
Non.inflammatory.Macrophages	UCK2	0.983798	5.87449	4.207214	6.19E-05	1.521593	0.002591	0.004864
Non.inflammatory.Macrophages	LBH	0.35827	6.308404	4.205714	6.22E-05	1.093072	0.002527	0.00481
Non.inflammatory.Macrophages	GSR	0.439919	6.441975	4.20057	6.34E-05	0.883144	0.002543	0.004841
Non.inflammatory.Macrophages	PSTPIP2	0.615238	4.482064	4.192072	6.54E-05	1.079019	0.002945	0.005223
Non.inflammatory.Macrophages	LAPTM5	0.287795	8.326927	4.18049	6.82E-05	0.660196	0.002419	0.004848
Non.inflammatory.Macrophages	QTRT1	-0.6711	4.980734	-4.17504	6.96E-05	1.192214	0.003016	0.005408
Non.inflammatory.Macrophages	SERPINE1	1.445954	0.805177	4.1731	7.01E-05	1.489072	0.003881	0.006107
Non.inflammatory.Macrophages	CARNS1	-1.39126	2.476067	-4.16926	7.11E-05	1.493645	0.003557	0.005869
Non.inflammatory.Macrophages	CLYBL	0.556788	4.489599	4.167329	7.16E-05	1.213484	0.003158	0.005569
Non.inflammatory.Macrophages	TPCN1	-0.63464	4.187411	-4.15189	7.58E-05	1.146115	0.003389	0.005836

Non.inflammatory.Macrophages	ARL5A	0.379689	5.831093	4.145351	7.76E-05	0.881453	0.003122	0.005685
Non.inflammatory.Macrophages	SYT14	2.031212	0.008242	4.144607	7.78E-05	1.296474	0.004261	0.006697
Non.inflammatory.Macrophages	CD24A	0.842878	7.730753	4.143642	7.81E-05	0.896302	0.002779	0.005412
Non.inflammatory.Macrophages	NSMCE2	-0.29101	7.428191	-4.13108	8.17E-05	0.682537	0.002951	0.005639
Non.inflammatory.Macrophages	PACSIN2	0.386106	5.573303	4.127711	8.28E-05	0.982225	0.003319	0.005979
Non.inflammatory.Macrophages	CLEC4D	0.961358	2.949351	4.127233	8.29E-05	0.97631	0.003894	0.006432
Non.inflammatory.Macrophages	DDX5	-0.22468	9.318404	-4.12672	8.31E-05	0.41254	0.002638	0.005388
Non.inflammatory.Macrophages	GM26917	0.572843	5.74366	4.123382	8.41E-05	0.892216	0.003312	0.006008
Non.inflammatory.Macrophages	EMILIN1	0.679871	2.608435	4.121421	8.47E-05	1.27096	0.00402	0.006582
Non.inflammatory.Macrophages	HEMGN	2.476422	0.35427	4.116423	8.62E-05	1.071291	0.004576	0.007077
Non.inflammatory.Macrophages	GTF2H1	-0.38669	5.958143	-4.11464	8.68E-05	0.820203	0.003335	0.006086
Non.inflammatory.Macrophages	PON1	-1.18207	4.304512	-4.10716	8.92E-05	1.156349	0.003774	0.006496
Non.inflammatory.Macrophages	COTL1	0.35066	6.629821	4.106253	8.95E-05	0.593749	0.003274	0.006104
Non.inflammatory.Macrophages	ICAM2	1.057353	4.282374	4.100295	9.14E-05	1.08743	0.003846	0.006625
Non.inflammatory.Macrophages	NHLRC2	0.431726	4.994885	4.095717	9.30E-05	0.915006	0.003693	0.006587

Non.inflammatory.Macrophages	UBE2J1	0.347645	6.282597	4.095501	9.30E-05	0.758229	0.003414	0.006355
Non.inflammatory.Macrophages	PYGM	-0.74516	4.300481	-4.09506	9.32E-05	1.019752	0.003853	0.006721
Non.inflammatory.Macrophages	RTN1	-0.68313	2.561189	-4.09437	9.34E-05	0.867785	0.004282	0.007063
Non.inflammatory.Macrophages	GM3336	-1.05722	1.732452	-4.09424	9.35E-05	1.192237	0.004501	0.007228
Non.inflammatory.Macrophages	2810013P(	-0.68922	4.123121	-4.08998	9.49E-05	1.026404	0.003941	0.00684
Non.inflammatory.Macrophages	FBXO42	0.456717	5.871653	4.088826	9.53E-05	0.801276	0.003543	0.006531
Non.inflammatory.Macrophages	HERPUD1	-0.59335	7.853564	-4.0813	9.79E-05	0.636122	0.003213	0.006325
Non.inflammatory.Macrophages	ADORA2B	1.26827	0.37813	4.078253	9.90E-05	1.201596	0.004992	0.007835
Non.inflammatory.Macrophages	SGMS2	1.332406	3.643674	4.077702	9.92E-05	1.155494	0.004178	0.007164
Non.inflammatory.Macrophages	HTT	0.399239	5.362069	4.075547	0.0001	0.753089	0.003778	0.006869
Non.inflammatory.Macrophages	UPB1	-1.41702	3.0781	-4.07084	0.000102	1.170558	0.004398	0.007411
Non.inflammatory.Macrophages	ARHGAP17	-0.35528	7.245574	-4.06995	0.000102	0.439158	0.003411	0.006616
Non.inflammatory.Macrophages	MAP2K4	0.461498	6.321239	4.068842	0.000102	0.643758	0.003611	0.006804
Non.inflammatory.Macrophages	SERPINF1	-1.1495	3.83888	-4.06694	0.000103	1.098152	0.004212	0.007324
Non.inflammatory.Macrophages	IMPDH1	0.655398	4.326873	4.065201	0.000104	0.914	0.004089	0.007245

Non.inflammatory.Macrophages	RYR1	-1.00693	1.831874	-4.06506	0.000104	1.113994	0.004756	0.007765
Non.inflammatory.Macrophages	SIRT1	-0.41395	5.043074	-4.0603	0.000106	0.828219	0.003968	0.007196
Non.inflammatory.Macrophages	OSCP1	-0.9057	3.25254	-4.05704	0.000107	1.002789	0.004461	0.007641
Non.inflammatory.Macrophages	F11R	0.6431	3.432174	4.052403	0.000109	0.831704	0.004471	0.0077
Non.inflammatory.Macrophages	BDH1	-1.06935	3.282333	-4.04864	0.00011	1.087972	0.00454	0.007794
Non.inflammatory.Macrophages	HSD17B2	-1.33712	2.446494	-4.04864	0.00011	1.099504	0.004776	0.007977
Non.inflammatory.Macrophages	TBC1D9	-0.48406	3.953614	-4.04444	0.000112	0.431513	0.00441	0.007735
Non.inflammatory.Macrophages	HBQ1A	2.01571	-1.39639	4.041965	0.000113	0.534592	0.005378	0.009041
Non.inflammatory.Macrophages	EID1	0.423776	5.155191	4.041906	0.000113	0.69922	0.004106	0.007536
Non.inflammatory.Macrophages	KNG1	-0.89822	6.039457	-4.04102	0.000113	0.651477	0.00389	0.007366
Non.inflammatory.Macrophages	PPIL6	-2.06627	0.219778	-4.03915	0.000114	0.700463	0.005396	0.008691
Non.inflammatory.Macrophages	TNFSF9	0.842837	3.435421	4.03254	0.000117	0.81387	0.004667	0.00807
Non.inflammatory.Macrophages	EPB42	1.686871	-0.1647	4.030166	0.000118	0.935235	0.005534	0.008967
Non.inflammatory.Macrophages	9830166K(	-1.8348	-1.08122	-4.02822	0.000119	0.53077	0.005554	0.009255
Non.inflammatory.Macrophages	CDC40	0.385162	6.047181	4.026253	0.000119	0.534686	0.004029	0.007622

Non.inflammatory.Macrophages	TGM2	0.58069	4.674438	4.023974	0.00012	0.374059	0.004402	0.007965
Non.inflammatory.Macrophages	S100A6	1.270143	7.580063	4.0216	0.000121	0.53202	0.003704	0.007403
Non.inflammatory.Macrophages	DLG4	-0.8991	3.571009	-4.01784	0.000123	0.779359	0.004764	0.008366
Non.inflammatory.Macrophages	PNISR	0.328356	5.773836	4.01778	0.000123	0.549525	0.004166	0.007868
Non.inflammatory.Macrophages	PDPK1	-0.51931	6.462559	-4.01367	0.000125	0.398735	0.004034	0.007815
Non.inflammatory.Macrophages	ZFP608	-0.60039	6.820434	-4.0131	0.000125	0.370588	0.003947	0.007748
Non.inflammatory.Macrophages	ALOX5	1.454973	0.791987	4.012035	0.000126	0.983993	0.005672	0.009185
Non.inflammatory.Macrophages	GM16853	-1.19888	2.351019	-4.01021	0.000126	0.976731	0.005185	0.008803
Non.inflammatory.Macrophages	HRG	-1.36673	3.61876	-4.01005	0.000127	0.939715	0.004802	0.008498
Non.inflammatory.Macrophages	CPD	0.551489	4.333844	4.004718	0.000129	0.654346	0.004671	0.008453
Non.inflammatory.Macrophages	RAB40C	0.487626	4.838453	3.99802	0.000132	0.656092	0.004611	0.00847
Non.inflammatory.Macrophages	GM34086	-0.95622	2.658244	-3.99789	0.000132	0.919898	0.005263	0.009
Non.inflammatory.Macrophages	CYP2C37	-1.50972	1.772648	-3.99529	0.000133	0.873974	0.005571	0.009286
Non.inflammatory.Macrophages	MTPN	0.275165	6.898113	3.995073	0.000134	0.280889	0.004079	0.008052
Non.inflammatory.Macrophages	PADI4	1.523827	1.890862	3.992887	0.000135	0.927577	0.005557	0.009303

Non.inflammatory.Macrophages	APOA1	-1.12081	8.277086	-3.99142	0.000135	0.138495	0.003773	0.007807
Non.inflammatory.Macrophages	NUP210L	-0.71122	6.407383	-3.98311	0.000139	0.251609	0.004337	0.008384
Non.inflammatory.Macrophages	ACKR3	2.253347	0.835269	3.982665	0.00014	0.819701	0.006064	0.009798
Non.inflammatory.Macrophages	CAR3	-2.12165	3.476221	-3.96741	0.000147	0.825698	0.005457	0.009515
Non.inflammatory.Macrophages	CDKL3	-0.90919	3.044103	-3.93964	0.000163	0.695259	0.006163	0.010452
Non.inflammatory.Macrophages	P2RY14	0.763599	3.467146	3.936106	0.000165	0.541868	0.006052	0.010437
Non.inflammatory.Macrophages	AGTPBP1	0.519047	5.128653	3.935684	0.000165	0.38706	0.00547	0.009966
Non.inflammatory.Macrophages	PRKCG	-0.83998	4.448629	-3.92465	0.000171	0.448144	0.005898	0.01045
Non.inflammatory.Macrophages	GNAT3	-1.62963	-0.04367	-3.92432	0.000172	0.602132	0.007401	0.011846
Non.inflammatory.Macrophages	UBAP2	-0.36695	6.131148	-3.92301	0.000172	0.200119	0.00533	0.009987
Non.inflammatory.Macrophages	SVBP	0.442833	5.267752	3.916981	0.000176	0.281917	0.005721	0.010414
Non.inflammatory.Macrophages	PPP1R13B	-0.44947	5.30235	-3.91613	0.000177	0.252249	0.005709	0.01042
Non.inflammatory.Macrophages	NIPA2	0.347993	6.042829	3.913764	0.000178	0.071235	0.005485	0.010259
Non.inflammatory.Macrophages	SACM1L	0.399998	5.504292	3.912621	0.000179	0.207875	0.005673	0.010447
Non.inflammatory.Macrophages	EMP1	1.281484	1.978152	3.910817	0.00018	0.641575	0.00705	0.011566

Non.inflammatory.Macrophages	APOC2	0.677699	4.203003	3.90694	0.000183	0.227215	0.006227	0.01096
Non.inflammatory.Macrophages	ALDOC	1.349024	0.884667	3.905185	0.000184	0.64635	0.007619	0.012086
Non.inflammatory.Macrophages	TTC14	0.341915	5.803768	3.90368	0.000185	0.192222	0.005677	0.010582
Non.inflammatory.Macrophages	PROCA1	-0.86255	2.988273	-3.88944	0.000194	0.56128	0.007063	0.011917
Non.inflammatory.Macrophages	HPGD	-0.70854	5.673887	-3.87465	0.000205	-0.2312	0.006296	0.011438
Non.inflammatory.Macrophages	UCHL1	-2.43633	0.862756	-3.87389	0.000205	0.044711	0.008408	0.013102
Non.inflammatory.Macrophages	ALDH2	-0.38085	6.861181	-3.86965	0.000208	-0.17274	0.005924	0.011201
Non.inflammatory.Macrophages	SERPINF2	-0.92428	3.929119	-3.86573	0.000211	0.315663	0.007161	0.012289
Non.inflammatory.Macrophages	MYOF	0.680516	2.65687	3.858719	0.000216	0.140643	0.007904	0.012989
Non.inflammatory.Macrophages	ST18	2.178491	0.283819	3.857178	0.000217	0.367609	0.008916	0.013899
Non.inflammatory.Macrophages	PIK3R1	0.397332	7.694648	3.854699	0.000219	-0.38341	0.00586	0.011358
Non.inflammatory.Macrophages	EMILIN2	0.710543	4.009199	3.853537	0.00022	-0.06753	0.007348	0.012619
Non.inflammatory.Macrophages	TBC1D10C	-0.85842	4.736281	-3.85086	0.000222	0.37252	0.007074	0.012434
Non.inflammatory.Macrophages	STARD7	0.41553	5.358518	3.833969	0.000236	0.014911	0.007182	0.012798
Non.inflammatory.Macrophages	SMS	0.333107	6.477089	3.833925	0.000236	-0.17832	0.006707	0.012405

Non.inflammatory.Macrophages	CCNYL1	-0.42988	4.635175	-3.82834	0.00024	0.00544	0.007631	0.013255
Non.inflammatory.Macrophages	F10	1.05465	4.54109	3.827044	0.000242	0.045825	0.007687	0.013341
Non.inflammatory.Macrophages	IL1RN	0.962827	2.526967	3.82066	0.000247	-0.00041	0.008854	0.014326
Non.inflammatory.Macrophages	MMP12	1.232738	-0.05819	3.81749	0.00025	0.306816	0.009968	0.015494
Non.inflammatory.Macrophages	SLC41A2	1.546774	2.900939	3.807027	0.000259	0.329736	0.009023	0.014696
Non.inflammatory.Macrophages	MAN2B1	-0.38356	6.851978	-3.80019	0.000265	-0.50696	0.007236	0.01338
Non.inflammatory.Macrophages	MGST2	-1.98592	2.953494	-3.79953	0.000266	-0.02818	0.009177	0.014917
Non.inflammatory.Macrophages	SERPINB2	1.983997	0.991964	3.794328	0.000271	0.106619	0.010485	0.015961
Non.inflammatory.Macrophages	DUBR	0.753681	2.378269	3.792856	0.000272	0.217167	0.009667	0.01541
Non.inflammatory.Macrophages	FAM214A	-0.62032	5.246692	-3.79031	0.000274	-0.04549	0.00817	0.014318
Non.inflammatory.Macrophages	ELDR	0.946728	3.256101	3.789241	0.000275	0.154676	0.009229	0.015152
Non.inflammatory.Macrophages	MEFV	1.064635	-0.03339	3.786306	0.000278	0.25054	0.010849	0.01674
Non.inflammatory.Macrophages	RGCC	0.982546	5.613883	3.784728	0.00028	0.161698	0.008074	0.014342
Non.inflammatory.Macrophages	THBS3	-1.24138	1.01596	-3.77789	0.000286	0.257152	0.010892	0.016577
Non.inflammatory.Macrophages	SIRPB1C	0.848634	1.778958	3.77271	0.000292	-0.04912	0.010561	0.016462

Non.inflammatory.Macrophages	ZFP367	-0.61769	5.436634	-3.7594	0.000305	-0.16739	0.008831	0.015389
Non.inflammatory.Macrophages	NSF	0.379367	6.770124	3.755624	0.000309	-0.51473	0.008204	0.014959
Non.inflammatory.Macrophages	TSPAN32	1.086019	2.972272	3.755454	0.000309	0.189619	0.010341	0.016627
Non.inflammatory.Macrophages	ITGB1	0.286977	6.871704	3.752776	0.000312	-0.6119	0.008205	0.015009
Non.inflammatory.Macrophages	ITGB2	0.480445	6.734664	3.743278	0.000323	-0.58485	0.008525	0.015366
Non.inflammatory.Macrophages	GM34084	-0.93625	1.964286	-3.74094	0.000325	-0.08143	0.011454	0.017618
Non.inflammatory.Macrophages	IER5	-0.47065	6.692584	-3.73527	0.000332	-0.58456	0.008736	0.015647
Non.inflammatory.Macrophages	PLEC	0.526937	4.805597	3.730934	0.000337	-0.18201	0.009922	0.01668
Non.inflammatory.Macrophages	INPP5J	2.011472	-1.12022	3.729882	0.000338	-0.35741	0.012734	0.019696
Non.inflammatory.Macrophages	ITPKB	0.387724	7.462345	3.727157	0.000341	-0.67383	0.008496	0.015617
Non.inflammatory.Macrophages	DMXL2	0.798963	2.146865	3.71819	0.000352	-0.13963	0.012066	0.018536
Non.inflammatory.Macrophages	JAK2	0.439879	6.674709	3.716931	0.000353	-0.57841	0.009161	0.016393
Non.inflammatory.Macrophages	DENND4C	0.445986	5.068456	3.716259	0.000354	-0.32655	0.010106	0.017162
Non.inflammatory.Macrophages	INTS6	-0.47035	6.588587	-3.71604	0.000354	-0.60043	0.009209	0.016454
Non.inflammatory.Macrophages	CD207	1.471247	-0.64958	3.713365	0.000357	0.059968	0.013253	0.020226

Non.inflammatory.Macrophages	FBXO21	0.66657	3.292952	3.711438	0.00036	-0.02975	0.011357	0.018202
Non.inflammatory.Macrophages	ROGDI	-0.55167	4.014028	-3.71109	0.00036	-0.20273	0.01087	0.01784
Non.inflammatory.Macrophages	RRP1B	0.670081	4.132089	3.709591	0.000362	-0.15154	0.010819	0.017803
Non.inflammatory.Macrophages	CTDP1	-0.40458	4.660739	-3.69979	0.000374	-0.34215	0.010803	0.017966
Non.inflammatory.Macrophages	TRAK1	-0.31254	7.291138	-3.6946	0.000381	-0.70575	0.009314	0.016848
Non.inflammatory.Macrophages	RCBTB2	0.357599	5.187796	3.694469	0.000381	-0.55255	0.010594	0.017864
Non.inflammatory.Macrophages	CD63	0.720786	4.105165	3.693391	0.000383	-0.28816	0.011316	0.018451
Non.inflammatory.Macrophages	SENP2	-0.31175	6.656089	-3.69293	0.000383	-0.71323	0.009684	0.017186
Non.inflammatory.Macrophages	BCL2A1B	0.519396	5.877283	3.688433	0.000389	-0.84344	0.010286	0.01774
Non.inflammatory.Macrophages	GM28875	1.116194	4.612323	3.687378	0.000391	-0.01193	0.011117	0.018422
Non.inflammatory.Macrophages	GCNT2	0.906429	4.580489	3.686673	0.000391	-0.15152	0.011138	0.018462
Non.inflammatory.Macrophages	GBE1	0.464645	6.483019	3.68546	0.000393	-0.63514	0.009916	0.017573
Non.inflammatory.Macrophages	CHPF	1.382129	1.242066	3.685164	0.000393	-0.07786	0.013631	0.020341
Non.inflammatory.Macrophages	UBE2Q2	0.358363	5.51991	3.683288	0.000396	-0.52673	0.010557	0.018134
Non.inflammatory.Macrophages	GRSF1	0.312976	5.185543	3.682299	0.000397	-0.52646	0.010783	0.018313

Non.inflammatory.Macrophages	NFE2	1.300706	1.59738	3.675697	0.000406	-0.05745	0.013671	0.020585
Non.inflammatory.Macrophages	PLATR25	-0.68284	2.729731	-3.6749	0.000407	-0.16025	0.01277	0.019974
Non.inflammatory.Macrophages	CES1C	-1.19352	4.023969	-3.67077	0.000413	-0.23388	0.011929	0.019471
Non.inflammatory.Macrophages	IRF2BPL	0.653342	4.091669	3.670311	0.000414	-0.34086	0.01188	0.019454
Non.inflammatory.Macrophages	PHF23	-0.42656	5.240241	-3.66575	0.00042	-0.59267	0.01122	0.019013
Non.inflammatory.Macrophages	PDE4C	-1.11553	3.131277	-3.65532	0.000435	-0.30606	0.01318	0.020632
Non.inflammatory.Macrophages	MKRN1	0.436043	7.149614	3.65314	0.000439	-0.92761	0.010364	0.018546
Non.inflammatory.Macrophages	PRODH2	-1.40825	2.255274	-3.65186	0.000441	-0.11569	0.013988	0.0213
Non.inflammatory.Macrophages	CLDN10	2.447791	0.159414	3.643561	0.000453	-0.48505	0.015762	0.023013
Non.inflammatory.Macrophages	VDR	1.242954	0.938817	3.641296	0.000457	-0.14435	0.015602	0.022619
Non.inflammatory.Macrophages	MFSD11	-0.48389	3.854162	-3.63991	0.000459	-0.51253	0.013119	0.020926
Non.inflammatory.Macrophages	TPM4	0.362848	7.167543	3.637214	0.000463	-0.78922	0.010786	0.019226
Non.inflammatory.Macrophages	ANXA1	0.907636	6.030034	3.628964	0.000476	-0.57514	0.01186	0.020081
Non.inflammatory.Macrophages	TCOF1	-0.42516	5.717375	-3.62639	0.00048	-0.7548	0.012163	0.020381
Non.inflammatory.Macrophages	MICU1	-0.32534	5.891562	-3.62353	0.000485	-0.76509	0.012119	0.020406

Non.inflammatory.Macrophages	SELP	1.720135	1.452771	3.617482	0.000495	-0.21302	0.016151	0.023381
Non.inflammatory.Macrophages	OFCC1	1.937141	-0.63548	3.616009	0.000497	-0.46665	0.016946	0.024826
Non.inflammatory.Macrophages	ITIH2	-1.21316	3.79432	-3.60411	0.000517	-0.40789	0.01459	0.022676
Non.inflammatory.Macrophages	ATP6V1H	-0.36639	6.803114	-3.59213	0.000539	-1.05871	0.012608	0.021409
Non.inflammatory.Macrophages	BNIP3L	0.387514	7.263429	3.588676	0.000545	-1.13493	0.012368	0.02131
Non.inflammatory.Macrophages	SGCZ	-1.49014	1.627249	-3.58767	0.000547	-0.31768	0.017441	0.02497
Non.inflammatory.Macrophages	GM13012	0.886932	2.775114	3.585369	0.000551	-0.31944	0.01636	0.024282
Non.inflammatory.Macrophages	CCR2	1.256336	3.092746	3.583741	0.000554	-0.38587	0.016096	0.024164
Non.inflammatory.Macrophages	TMX4	0.652156	4.420261	3.579342	0.000562	-0.52743	0.015002	0.023552
Non.inflammatory.Macrophages	SLC38A1	-0.3608	7.937408	-3.57919	0.000562	-1.23353	0.012098	0.021356
Non.inflammatory.Macrophages	NOL8	-0.52236	4.078757	-3.57627	0.000568	-0.63328	0.015429	0.023909
Non.inflammatory.Macrophages	RAB6A	0.305995	6.637549	3.566669	0.000586	-1.02299	0.013593	0.022756
Non.inflammatory.Macrophages	ROCK2	-0.29026	7.468662	-3.56589	0.000588	-1.20624	0.01292	0.022251
Non.inflammatory.Macrophages	CAMKK2	0.371559	5.188179	3.564183	0.000591	-0.82211	0.014903	0.023799
Non.inflammatory.Macrophages	CD209F	-1.82154	2.767776	-3.56258	0.000594	-0.57393	0.017315	0.025533

Non.inflammatory.Macrophages	MSRB1	0.355941	5.958368	3.556771	0.000606	-1.14334	0.01449	0.023651
Non.inflammatory.Macrophages	XDH	0.675842	4.807401	3.55627	0.000607	-0.95997	0.015545	0.024439
Non.inflammatory.Macrophages	SPATA21	-0.76612	3.397572	-3.55556	0.000608	-0.52291	0.016937	0.02544
Non.inflammatory.Macrophages	CYP7A1	-1.98092	0.739983	-3.55439	0.000611	-0.56172	0.019813	0.02746
Non.inflammatory.Macrophages	MBTD1	-0.32213	7.420536	-3.54942	0.000621	-1.26757	0.013457	0.023062
Non.inflammatory.Macrophages	AQP1	-1.05726	2.964821	-3.54797	0.000624	-0.4579	0.017702	0.026206
Non.inflammatory.Macrophages	JUN	-0.75648	7.097589	-3.54569	0.000629	-1.28466	0.013831	0.023451
Non.inflammatory.Macrophages	PAX5	-0.94064	5.141192	-3.53916	0.000642	-0.60087	0.015892	0.025188
Non.inflammatory.Macrophages	SPTA1	1.541055	0.279808	3.536798	0.000647	-0.44805	0.020914	0.028968
Non.inflammatory.Macrophages	UNC119	0.348362	5.47027	3.534752	0.000652	-1.05428	0.01573	0.025158
Non.inflammatory.Macrophages	PLEKHA4	-0.94161	2.406918	-3.53019	0.000662	-0.52357	0.01919	0.027636
Non.inflammatory.Macrophages	ST8SIA4	0.554523	7.324778	3.527964	0.000667	-1.04729	0.014293	0.024206
Non.inflammatory.Macrophages	GM11476	-0.95994	2.536389	-3.52403	0.000675	-0.50342	0.01931	0.027917
Non.inflammatory.Macrophages	WDR20	0.335193	5.843767	3.523797	0.000676	-1.02702	0.015792	0.02547
Non.inflammatory.Macrophages	C730034FC	-0.87817	2.636781	-3.52186	0.00068	-0.55173	0.019271	0.027926

Non.inflammatory.Macrophages	ZBTB10	-0.66301	4.077959	-3.5207	0.000683	-0.70852	0.017685	0.026894
Non.inflammatory.Macrophages	TSTD1	-1.19194	2.589608	-3.51772	0.00069	-0.51307	0.0195	0.02819
Non.inflammatory.Macrophages	ZFYVE26	-0.42118	5.03643	-3.51667	0.000692	-0.96183	0.016826	0.026394
Non.inflammatory.Macrophages	LSP1	0.551915	8.230432	3.514649	0.000697	-1.14102	0.013877	0.024255
Non.inflammatory.Macrophages	CHST3	-1.20016	4.312271	-3.51439	0.000697	-0.52701	0.017636	0.02705
Non.inflammatory.Macrophages	RAB1A	-0.23452	7.08553	-3.50742	0.000713	-1.37206	0.015198	0.025446
Non.inflammatory.Macrophages	KEL	1.85765	-0.44363	3.50553	0.000718	-0.60274	0.022492	0.031521
Non.inflammatory.Macrophages	TRIB3	1.956893	1.057747	3.501107	0.000728	-0.65822	0.022275	0.030542
Non.inflammatory.Macrophages	CHP1	0.313259	7.01438	3.498333	0.000735	-1.2708	0.015615	0.026022
Non.inflammatory.Macrophages	MTMR4	-0.60984	2.648419	-3.49771	0.000737	-0.69176	0.020374	0.02942
Non.inflammatory.Macrophages	CDC37L1	0.36093	5.167302	3.496695	0.000739	-1.00671	0.017501	0.027495
Non.inflammatory.Macrophages	SPRED1	0.449925	4.710293	3.492079	0.00075	-1.14954	0.018229	0.028151
Non.inflammatory.Macrophages	SUSD6	0.319311	7.690279	3.4859	0.000766	-1.49393	0.015467	0.026221
Non.inflammatory.Macrophages	CNN2	0.56313	6.538205	3.483879	0.000771	-1.09189	0.016672	0.027173
Non.inflammatory.Macrophages	APOF	-0.93185	3.847555	-3.48002	0.000781	-0.78368	0.019851	0.029583

Non.inflammatory.Macrophages	SUN2	-0.36591	6.122993	-3.4769	0.000789	-1.16568	0.017417	0.028005
Non.inflammatory.Macrophages	FGF23	2.19318	0.594781	3.475069	0.000793	-0.67393	0.024196	0.032789
Non.inflammatory.Macrophages	EGR1	-0.61937	6.545199	-3.47483	0.000794	-1.47773	0.017011	0.027787
Non.inflammatory.Macrophages	CECR2	-0.91961	6.605628	-3.4736	0.000797	-1.03118	0.016979	0.027783
Non.inflammatory.Macrophages	FCOR	1.169356	0.865885	3.469845	0.000807	-0.63501	0.024204	0.032933
Non.inflammatory.Macrophages	S100A4	1.387351	2.87429	3.469674	0.000808	-0.65995	0.021491	0.031148
Non.inflammatory.Macrophages	CCL24	-1.00549	3.596362	-3.46879	0.00081	-1.14659	0.020584	0.03059
Non.inflammatory.Macrophages	MSN	0.289509	8.454473	3.46535	0.000819	-1.65128	0.015415	0.02686
Non.inflammatory.Macrophages	RAB5C	0.270799	6.358629	3.465032	0.00082	-1.42543	0.01753	0.028482
Non.inflammatory.Macrophages	VSIR	0.402194	4.468275	3.463008	0.000825	-1.24959	0.019762	0.030144
Non.inflammatory.Macrophages	TLR13	0.563406	1.881588	3.460458	0.000832	-1.06897	0.023255	0.032567
Non.inflammatory.Macrophages	DNAJB9	-0.37452	5.168187	-3.45463	0.000848	-1.20242	0.019344	0.030114
Non.inflammatory.Macrophages	SMAP1	0.28213	6.875842	3.454017	0.00085	-1.50017	0.017425	0.028762
Non.inflammatory.Macrophages	DESI2	0.250208	6.03278	3.453781	0.000851	-1.30269	0.018348	0.029455
Non.inflammatory.Macrophages	GM49774	-0.74154	3.174932	-3.44941	0.000863	-0.91247	0.022102	0.032223

Non.inflammatory.Macrophages	GNPTAB	0.482418	4.589659	3.439543	0.000891	-1.10674	0.020898	0.031641
Non.inflammatory.Macrophages	ARHGAP24	0.59615	6.825889	3.438562	0.000894	-1.24349	0.018247	0.029797
Non.inflammatory.Macrophages	MMP14	0.929426	3.837765	3.436975	0.000899	-1.08605	0.021963	0.032501
Non.inflammatory.Macrophages	PLEKHA1	0.496835	4.707996	3.430367	0.000918	-1.26814	0.021236	0.032161
Non.inflammatory.Macrophages	GM28192	1.14077	0.064757	3.427766	0.000926	-0.75663	0.027241	0.036753
Non.inflammatory.Macrophages	MFSD7A	1.187711	-0.44923	3.424499	0.000936	-0.76141	0.027471	0.037538
Non.inflammatory.Macrophages	UBN2	0.32138	7.076175	3.422943	0.00094	-1.54333	0.018686	0.030519
Non.inflammatory.Macrophages	CISH	0.845192	2.887974	3.422499	0.000942	-0.91449	0.024122	0.034314
Non.inflammatory.Macrophages	TNFSF14	1.58328	-0.05669	3.420694	0.000947	-0.99119	0.027633	0.037414
Non.inflammatory.Macrophages	SULT2A8	-1.97762	0.571296	-3.41795	0.000956	-0.88053	0.027762	0.036997
Non.inflammatory.Macrophages	2310058D1	0.650128	2.785149	3.414266	0.000967	-0.84588	0.024768	0.035086
Non.inflammatory.Macrophages	ITCH	0.316659	7.100446	3.411656	0.000976	-1.61381	0.01916	0.031222
Non.inflammatory.Macrophages	IER3IP1	0.244627	6.363638	3.410166	0.00098	-1.52966	0.020099	0.031974
Non.inflammatory.Macrophages	SLC38A2	-0.26765	8.351985	-3.4054	0.000995	-1.74263	0.018029	0.030619
Non.inflammatory.Macrophages	DNAJC5	0.221019	6.766699	3.402994	0.001003	-1.58332	0.019984	0.032123

Non.inflammatory.Macrophages	CES1G	-1.97202	0.85155	-3.39862	0.001018	-0.93896	0.028807	0.038258
Non.inflammatory.Macrophages	SNX18	0.346359	6.77742	3.39758	0.001021	-1.70556	0.020153	0.0325
Non.inflammatory.Macrophages	GHITM	0.371114	7.4577	3.397273	0.001022	-1.76522	0.01933	0.031891
Non.inflammatory.Macrophages	TTR	-0.98798	9.202897	-3.3971	0.001023	-1.91088	0.017365	0.030379
Non.inflammatory.Macrophages	KANSL1L	-0.51913	7.202302	-3.3969	0.001023	-1.66753	0.019635	0.032118
Non.inflammatory.Macrophages	2010309G2	-2.01853	1.223176	-3.39576	0.001027	-1.04158	0.028283	0.038036
Non.inflammatory.Macrophages	NRIP1	0.345403	6.353871	3.392817	0.001037	-1.7092	0.020869	0.033186
Non.inflammatory.Macrophages	CCND3	0.506626	7.994145	3.390321	0.001045	-1.63241	0.018985	0.031849
Non.inflammatory.Macrophages	RCCD1	-0.79955	3.170294	-3.38854	0.001051	-0.97495	0.025578	0.036548
Non.inflammatory.Macrophages	MRPL54	0.3304	5.906363	3.387937	0.001053	-1.53815	0.021652	0.033897
Non.inflammatory.Macrophages	RUNX3	1.139484	5.712717	3.38665	0.001058	-0.92472	0.021956	0.034195
Non.inflammatory.Macrophages	SVIP	1.204762	1.34626	3.382503	0.001072	-0.88199	0.02892	0.038995
Non.inflammatory.Macrophages	LIMK2	-0.43286	4.779797	-3.38204	0.001073	-1.2814	0.023493	0.035455
Non.inflammatory.Macrophages	SERPINB8	0.785023	-0.3114	3.381227	0.001076	-0.96953	0.030094	0.040921
Non.inflammatory.Macrophages	WDR37	0.343665	5.478401	3.379986	0.001081	-1.44426	0.022568	0.034948

Non.inflammatory.Macrophages	DYRK1A	-0.31358	7.419436	-3.37671	0.001092	-1.7578	0.020148	0.0333
Non.inflammatory.Macrophages	CLCN3	0.341339	6.389845	3.376458	0.001093	-1.56193	0.02146	0.034268
Non.inflammatory.Macrophages	RASGRP1	0.525031	4.119019	3.376383	0.001093	-1.37897	0.024651	0.036504
Non.inflammatory.Macrophages	USP25	0.262657	7.051473	3.37288	0.001106	-1.77585	0.020758	0.0339
Non.inflammatory.Macrophages	NAA50	-0.25445	6.371464	-3.37285	0.001106	-1.66985	0.021641	0.034548
Non.inflammatory.Macrophages	ACP5	0.478428	5.117131	3.370248	0.001115	-1.62984	0.023514	0.035982
Non.inflammatory.Macrophages	AHSG	-0.92302	9.130873	-3.3691	0.001119	-1.96959	0.018416	0.032233
Non.inflammatory.Macrophages	SPP1	1.568159	2.353951	3.362196	0.001144	-1.00915	0.028421	0.039475
Non.inflammatory.Macrophages	EYA3	0.37734	5.473081	3.357673	0.001161	-1.50472	0.02381	0.036597
Non.inflammatory.Macrophages	COX11	0.508911	3.751078	3.354548	0.001172	-1.13799	0.026656	0.038658
Non.inflammatory.Macrophages	INF2	0.566574	3.252642	3.350534	0.001188	-1.28316	0.027773	0.039571
Non.inflammatory.Macrophages	UCP1	-1.1441	-1.00774	-3.34945	0.001192	-0.97778	0.032453	0.044664
Non.inflammatory.Macrophages	VPREB3	-1.03992	6.013944	-3.34835	0.001196	-1.29869	0.023543	0.036762
Non.inflammatory.Macrophages	NCOA3	-0.2433	6.960263	-3.3453	0.001208	-1.80482	0.02239	0.036024
Non.inflammatory.Macrophages	GM31522	-0.97819	-1.00368	-3.34241	0.001219	-0.99272	0.032992	0.045239

Non.inflammatory.Macrophages	CCDC138	-0.53644	4.628632	-3.33832	0.001235	-1.47946	0.026297	0.038989
Non.inflammatory.Macrophages	SLC27A2	-1.03571	3.949769	-3.33685	0.001241	-1.19506	0.02748	0.039875
Non.inflammatory.Macrophages	GM42047	-0.88077	5.263713	-3.33535	0.001247	-1.38936	0.025438	0.038558
Non.inflammatory.Macrophages	GM43149	1.443318	0.774872	3.332064	0.00126	-1.06581	0.033518	0.043936
Non.inflammatory.Macrophages	HOGA1	-0.88565	2.370342	-3.3308	0.001265	-1.08571	0.030575	0.042095
Non.inflammatory.Macrophages	GM33677	-1.61619	-1.06476	-3.3297	0.001269	-1.04715	0.033821	0.046434
Non.inflammatory.Macrophages	6030468B1	-1.55756	0.279697	-3.32869	0.001273	-1.03346	0.033821	0.044786
Non.inflammatory.Macrophages	1110008P1	0.386298	5.226277	3.328609	0.001274	-1.52447	0.025707	0.03903
Non.inflammatory.Macrophages	TOMM6	0.252523	7.550466	3.328497	0.001274	-1.96171	0.022298	0.036585
Non.inflammatory.Macrophages	SLC2A6	1.094196	1.781297	3.328432	0.001274	-1.03727	0.03168	0.042956
Non.inflammatory.Macrophages	HPS5	-0.36451	4.448069	-3.32767	0.001278	-1.51536	0.026968	0.039932
Non.inflammatory.Macrophages	RGN	-0.9336	4.650255	-3.327	0.00128	-1.42246	0.026643	0.039737
Non.inflammatory.Macrophages	E330020D1	-0.58665	5.11024	-3.32301	0.001297	-1.53362	0.026186	0.039519
Non.inflammatory.Macrophages	GM43936	-1.62189	-1.34803	-3.32208	0.001301	-1.14889	0.034243	0.047364
Non.inflammatory.Macrophages	TENT5C	0.713737	5.86321	3.315873	0.001326	-1.70607	0.02548	0.039365

Non.inflammatory.Macrophages	PGLYRP1	1.002734	4.959034	3.315308	0.001329	-1.24572	0.026927	0.040412
Non.inflammatory.Macrophages	IRF4	-0.7773	4.497863	-3.31338	0.001337	-1.22455	0.027811	0.041142
Non.inflammatory.Macrophages	ATF3	-0.53161	6.1843	-3.30952	0.001353	-2.01586	0.02535	0.039569
Non.inflammatory.Macrophages	SULF2	0.741901	2.585463	3.308468	0.001358	-1.24884	0.031601	0.043807
Non.inflammatory.Macrophages	ANKRD13A	-0.30518	6.215118	-3.3059	0.001369	-1.75548	0.025456	0.039849
Non.inflammatory.Macrophages	BMT2	0.335859	6.027835	3.305686	0.00137	-1.75293	0.02575	0.040058
Non.inflammatory.Macrophages	IGF1R	0.45989	6.357541	3.305181	0.001372	-1.73071	0.025235	0.039713
Non.inflammatory.Macrophages	TFDP2	0.51488	5.591554	3.303476	0.00138	-1.6167	0.026538	0.040674
Non.inflammatory.Macrophages	ZFYVE9	0.582054	2.603777	3.3007	0.001392	-1.51831	0.032044	0.044404
Non.inflammatory.Macrophages	CD79B	-0.53202	6.976941	-3.30003	0.001395	-1.80356	0.024552	0.039333
Non.inflammatory.Macrophages	CYFIP1	0.322374	5.64309	3.29947	0.001397	-1.89101	0.02664	0.040821
Non.inflammatory.Macrophages	BOLA3	0.392361	5.736487	3.298582	0.001401	-1.7316	0.026513	0.040813
Non.inflammatory.Macrophages	SERPIND1	-1.18892	2.832849	-3.29592	0.001413	-1.1445	0.031843	0.044483
Non.inflammatory.Macrophages	IER2	-0.4206	8.170209	-3.29457	0.001419	-2.12016	0.023044	0.038488
Non.inflammatory.Macrophages	MAN2A2	-0.47612	4.595669	-3.29086	0.001436	-1.52177	0.028962	0.042857

Non.inflammatory.Macrophages	METAP2	0.248927	7.3726	3.281624	0.001478	-2.07156	0.025116	0.040483
Non.inflammatory.Macrophages	FBXO30	-0.40011	5.032199	-3.2791	0.00149	-1.73488	0.029159	0.043489
Non.inflammatory.Macrophages	SERPINC1	-0.75917	5.285455	-3.27694	0.0015	-1.65843	0.028854	0.043332
Non.inflammatory.Macrophages	RAB27A	0.693971	4.50985	3.273254	0.001518	-1.3044	0.030548	0.044678
Non.inflammatory.Macrophages	RIPK2	0.457899	4.205503	3.272114	0.001523	-1.66294	0.031173	0.045167
Non.inflammatory.Macrophages	CSF2RA	-0.31988	5.196056	-3.26999	0.001534	-1.94718	0.029485	0.044114
Non.inflammatory.Macrophages	SMAD4	0.270646	6.23224	3.269436	0.001536	-1.87035	0.027676	0.04289
Non.inflammatory.Macrophages	RTL8A	-0.51409	3.632481	-3.26596	0.001553	-1.56793	0.032678	0.046443
Non.inflammatory.Macrophages	PACC1	-0.44409	5.421952	-3.26583	0.001554	-1.77434	0.029304	0.044189
Non.inflammatory.Macrophages	KRT80	0.689219	0.161146	3.262211	0.001572	-1.29251	0.039392	0.05158
Non.inflammatory.Macrophages	CST7	1.760275	2.120473	3.256761	0.001599	-1.2513	0.036593	0.049458
Non.inflammatory.Macrophages	DAND5	-0.74678	3.96611	-3.25605	0.001602	-1.60309	0.032724	0.046983
Non.inflammatory.Macrophages	BLNK	-0.59299	7.200732	-3.25587	0.001603	-1.80978	0.026857	0.042937
Non.inflammatory.Macrophages	NOL9	-0.51169	3.728213	-3.25579	0.001604	-1.48424	0.033201	0.047295
Non.inflammatory.Macrophages	APOL11B	2.662681	-0.04187	3.255045	0.001608	-1.21879	0.039873	0.052609

Non.inflammatory.Macrophages	MSH5	-0.92855	2.9838	-3.25466	0.001609	-1.305	0.034734	0.048393
Non.inflammatory.Macrophages	FAM204A	0.366054	4.894011	3.253929	0.001613	-1.65691	0.030927	0.045977
Non.inflammatory.Macrophages	MALT1	0.643295	7.764534	3.253668	0.001614	-2.15333	0.025944	0.042467
Non.inflammatory.Macrophages	DLEU2	-0.30544	8.40867	-3.24913	0.001638	-2.31401	0.02525	0.042153
Non.inflammatory.Macrophages	SPP2	-1.08046	3.372982	-3.24464	0.001661	-1.39532	0.034772	0.048852
Non.inflammatory.Macrophages	TCF21	-1.40885	0.477537	-3.24066	0.001682	-1.26464	0.041306	0.053426
Non.inflammatory.Macrophages	HSPA5	-0.25214	8.355671	-3.23733	0.001699	-2.36315	0.026059	0.043202
Non.inflammatory.Macrophages	GADD45A	0.577806	4.876654	3.236937	0.001701	-1.68455	0.032246	0.047624
Non.inflammatory.Macrophages	CDK2AP2	0.271384	6.467925	3.236464	0.001704	-2.10458	0.029258	0.045583
Non.inflammatory.Macrophages	COX8A	0.220892	9.008145	3.236228	0.001705	-2.43078	0.025035	0.042475
Non.inflammatory.Macrophages	ECM1	0.746611	3.680244	3.236055	0.001706	-1.74626	0.034681	0.049265
Non.inflammatory.Macrophages	TRERF1	-0.70041	4.396216	-3.23365	0.001719	-1.63028	0.033394	0.048525
Non.inflammatory.Macrophages	GSTT1	-1.20328	2.240293	-3.22877	0.001745	-1.30868	0.038572	0.052215
Non.inflammatory.Macrophages	MANBA	0.374092	4.766674	3.227884	0.00175	-1.82481	0.033124	0.048774
Non.inflammatory.Macrophages	KCNAB2	0.516083	4.287693	3.226578	0.001757	-1.63258	0.034177	0.049534

Non.inflammatory.Macrophages	CEP70	0.587745	3.195368	3.226064	0.00176	-1.50213	0.03652	0.051111
Non.inflammatory.Macrophages	GM20513	-1.53573	1.300029	-3.22256	0.00178	-1.30723	0.041319	0.054346
Non.inflammatory.Macrophages	CYP4F18	0.596544	3.551567	3.219062	0.001799	-1.59565	0.0364	0.051391
Non.inflammatory.Macrophages	GM19951	0.962574	3.23896	3.217739	0.001807	-1.59444	0.037128	0.051984
Non.inflammatory.Macrophages	TMA7	0.22557	6.564001	3.217654	0.001807	-2.13019	0.030316	0.047389
Non.inflammatory.Macrophages	ARMC3	1.327971	1.900651	3.214323	0.001826	-1.34219	0.040545	0.054398
Non.inflammatory.Macrophages	AUH	0.347071	5.641378	3.214207	0.001827	-1.94951	0.032308	0.04902
Non.inflammatory.Macrophages	FOXP4	0.708939	4.962815	3.212247	0.001838	-1.50784	0.033779	0.050182
Non.inflammatory.Macrophages	G430095P1	1.078229	0.465518	3.211795	0.00184	-1.33255	0.043733	0.056879
Non.inflammatory.Macrophages	CPEB1	-1.38607	0.696287	-3.21152	0.001842	-1.35936	0.043471	0.056516
Non.inflammatory.Macrophages	SIT1	-1.69352	2.062249	-3.20891	0.001857	-1.42861	0.040535	0.05465
Non.inflammatory.Macrophages	GAL3ST1	-1.42188	-0.29084	-3.20691	0.001868	-1.36927	0.044209	0.058466
Non.inflammatory.Macrophages	IL6ST	0.53392	4.326252	3.205133	0.001879	-1.80151	0.035632	0.051608
Non.inflammatory.Macrophages	TRPM7	-0.27465	7.284405	-3.20398	0.001886	-2.25296	0.029793	0.047665
Non.inflammatory.Macrophages	SLC8A2	-1.06056	1.394752	-3.20296	0.001892	-1.48495	0.042683	0.056211

Non.inflammatory.Macrophages	VPS37B	-0.42928	7.797142	-3.19981	0.00191	-2.32774	0.029145	0.047325
Non.inflammatory.Macrophages	TBC1D1	-0.33703	7.18844	-3.19619	0.001932	-2.24089	0.030543	0.048562
Non.inflammatory.Macrophages	COG4	0.337707	5.336083	3.195576	0.001935	-1.93186	0.034215	0.051157
Non.inflammatory.Macrophages	GSTA4	-0.99795	1.583658	-3.19178	0.001958	-1.40813	0.04339	0.057223
Non.inflammatory.Macrophages	ZFP622	0.323257	5.377893	3.190623	0.001965	-2.02169	0.03449	0.05161
Non.inflammatory.Macrophages	CYP2D10	-1.25486	1.738911	-3.19041	0.001967	-1.38655	0.043009	0.057112
Non.inflammatory.Macrophages	CGNL1	1.102431	1.622928	3.189948	0.001969	-1.42951	0.04331	0.05736
Non.inflammatory.Macrophages	CSDE1	0.182863	7.576149	3.184859	0.002001	-2.38104	0.030468	0.049001
Non.inflammatory.Macrophages	PPME1	0.367865	4.862972	3.184635	0.002002	-1.90259	0.03597	0.052855
Non.inflammatory.Macrophages	PRG4	1.824482	1.084056	3.184544	0.002003	-1.57518	0.045211	0.058714
Non.inflammatory.Macrophages	LAIR1	0.547931	4.246709	3.184315	0.002004	-2.11693	0.037346	0.053791
Non.inflammatory.Macrophages	CHKA	-0.31352	6.940391	-3.18257	0.002015	-2.30606	0.031797	0.050036
Non.inflammatory.Macrophages	SYNC	-1.02356	1.973724	-3.1804	0.002029	-1.41911	0.043232	0.057627
Non.inflammatory.Macrophages	DSCAML1	-1.44557	-0.596	-3.17659	0.002053	-1.44683	0.047165	0.062292
Non.inflammatory.Macrophages	PRDM1	0.536705	2.929068	3.175025	0.002063	-1.90936	0.041279	0.056668

Non.inflammatory.Macrophages	GNG5	0.239418	8.925654	3.174783	0.002064	-2.66009	0.028616	0.047957
Non.inflammatory.Macrophages	FCRLA	-0.97023	5.107141	-3.1745	0.002066	-1.56249	0.036161	0.053335
Non.inflammatory.Macrophages	MBL2	-0.96453	4.246085	-3.17153	0.002085	-1.75331	0.038371	0.054975
Non.inflammatory.Macrophages	FAM129C	-0.96103	3.433215	-3.17118	0.002087	-1.4477	0.040313	0.056259
Non.inflammatory.Macrophages	KDM5B	0.486546	5.362074	3.162266	0.002145	-2.04593	0.036755	0.054458
Non.inflammatory.Macrophages	D830036C2	-1.06671	0.420218	-3.16199	0.002147	-1.47783	0.048759	0.062498
Non.inflammatory.Macrophages	ZFP568	0.468765	4.08691	3.158826	0.002168	-1.85199	0.040005	0.056753
Non.inflammatory.Macrophages	GM14966	-0.63962	2.847703	-3.15816	0.002173	-1.57192	0.043128	0.058827
Non.inflammatory.Macrophages	SLC25A24	0.559517	3.486183	3.158076	0.002173	-1.76189	0.041492	0.057791
Non.inflammatory.Macrophages	AK8	-0.66798	2.179586	-3.15708	0.00218	-1.63187	0.044966	0.060015
Non.inflammatory.Macrophages	APAF1	0.326934	5.726771	3.156043	0.002187	-2.19274	0.036304	0.054393
Non.inflammatory.Macrophages	MCEMP1	1.161856	1.650063	3.151913	0.002215	-1.58418	0.047011	0.061376
Non.inflammatory.Macrophages	VAV3	0.394248	7.363151	3.150173	0.002227	-2.4464	0.03333	0.052463
Non.inflammatory.Macrophages	BCL2A1D	0.580956	4.122114	3.148664	0.002237	-2.17997	0.040703	0.057599
Non.inflammatory.Macrophages	NRF1	0.258071	6.474245	3.147967	0.002242	-2.22553	0.03526	0.054

Non.inflammatory.Macrophages	FAM102B	0.381685	4.23575	3.147627	0.002244	-2.09807	0.040423	0.057515
Non.inflammatory.Macrophages	ARL2BP	0.501483	5.086344	3.147122	0.002248	-1.86116	0.038381	0.056209
Non.inflammatory.Macrophages	GM4107	-0.90224	0.916265	-3.14653	0.002252	-1.64901	0.049351	0.063197
Non.inflammatory.Macrophages	AKR1B8	-1.01399	1.592252	-3.14603	0.002255	-1.54382	0.047434	0.062062
Non.inflammatory.Macrophages	HCAR2	-0.79488	2.377294	-3.14583	0.002257	-1.9705	0.045243	0.060723
Non.inflammatory.Macrophages	CD38	0.526273	5.882237	3.144566	0.002265	-2.32919	0.036642	0.05518
Non.inflammatory.Macrophages	CYP3A16	-1.64805	0.777697	-3.14197	0.002284	-1.57326	0.050011	0.063949
Non.inflammatory.Macrophages	UPP1	2.191354	0.646262	3.141636	0.002286	-1.52154	0.050263	0.064183
Non.inflammatory.Macrophages	MGL2	1.836275	-0.18973	3.135857	0.002327	-1.53124	0.051253	0.066444
Non.inflammatory.Macrophages	TOPORS	-0.36703	5.749708	-3.13554	0.002329	-2.23526	0.03773	0.056359
Non.inflammatory.Macrophages	GM30198	-0.78807	1.865066	-3.13477	0.002335	-1.75808	0.047802	0.062869
Non.inflammatory.Macrophages	GM13561	-1.56762	1.307424	-3.12941	0.002373	-1.68483	0.050097	0.064589
Non.inflammatory.Macrophages	ADRB2	-0.54428	4.714467	-3.12921	0.002375	-2.19119	0.040763	0.058768
Non.inflammatory.Macrophages	IL4RA	0.40701	4.883706	3.128812	0.002377	-2.17929	0.040345	0.058529
Non.inflammatory.Macrophages	TAGLN2	0.449275	8.716101	3.128251	0.002382	-2.55944	0.031907	0.052682

Non.inflammatory.Macrophages	RUFY3	0.302843	5.26954	3.125719	0.0024	-2.28747	0.039653	0.058302
Non.inflammatory.Macrophages	PMEPA1	0.781233	4.361383	3.123485	0.002417	-1.84372	0.042129	0.060136
Non.inflammatory.Macrophages	MMRN1	2.908993	-0.22843	3.120991	0.002435	-1.69121	0.052898	0.068571
Non.inflammatory.Macrophages	NECAB2	-1.1856	0.442881	-3.11491	0.002481	-1.58665	0.053716	0.06794
Non.inflammatory.Macrophages	B230369F2	-0.63551	2.457789	-3.11464	0.002483	-1.72954	0.048304	0.064274
Non.inflammatory.Macrophages	DEDD2	-0.48219	4.544003	-3.11412	0.002487	-1.97796	0.042562	0.060723
Non.inflammatory.Macrophages	GLG1	0.271017	6.951102	3.113868	0.002489	-2.47812	0.03674	0.056787
Non.inflammatory.Macrophages	COA5	0.410932	4.468388	3.111195	0.002509	-2.16129	0.04304	0.061262
Non.inflammatory.Macrophages	SCAMP1	0.46444	2.970331	3.109372	0.002523	-2.08589	0.047253	0.064023
Non.inflammatory.Macrophages	HADHB	0.311562	5.576702	3.10936	0.002523	-2.26742	0.040324	0.059543
Non.inflammatory.Macrophages	CD180	-0.37211	5.032672	-3.10846	0.00253	-2.34699	0.041734	0.060556
Non.inflammatory.Macrophages	RBM26	0.251693	6.821937	3.105001	0.002557	-2.40121	0.037745	0.057948
Non.inflammatory.Macrophages	ENDOD1	0.858802	2.901638	3.103271	0.00257	-1.6286	0.048033	0.064864
Non.inflammatory.Macrophages	TOP3A	-0.49168	3.860539	-3.10327	0.00257	-1.9704	0.045319	0.063156
Non.inflammatory.Macrophages	PPIG	0.20196	6.974522	3.101931	0.002581	-2.51129	0.037512	0.058053

Non.inflammatory.Macrophages	GULO	-1.19208	1.982596	-3.10189	0.002581	-1.61688	0.050827	0.066706
Non.inflammatory.Macrophages	TMEM176I	-0.4027	5.475623	-3.10012	0.002595	-2.41049	0.041261	0.060769
Non.inflammatory.Macrophages	SDE2	-0.34046	6.013145	-3.09969	0.002598	-2.34867	0.039928	0.05988
Non.inflammatory.Macrophages	YIPF4	0.334167	6.515782	3.096464	0.002624	-2.42407	0.039039	0.059438
Non.inflammatory.Macrophages	SLC52A3	1.673949	0.451484	3.095386	0.002633	-1.75355	0.055671	0.070483
Non.inflammatory.Macrophages	FECH	0.472511	5.217188	3.094813	0.002637	-2.38629	0.042345	0.061789
Non.inflammatory.Macrophages	GM26812	-1.68474	0.020142	-3.09302	0.002652	-1.70284	0.055898	0.071581
Non.inflammatory.Macrophages	LYVE1	1.170283	2.42534	3.091799	0.002661	-1.75222	0.050397	0.067027
Non.inflammatory.Macrophages	RBPMS	0.420142	5.842983	3.091782	0.002662	-2.38455	0.040942	0.060945
Non.inflammatory.Macrophages	FOXRED2	-1.19145	1.168266	-3.0789	0.002768	-1.67982	0.056373	0.071097
Non.inflammatory.Macrophages	TMEM63B	0.462966	3.925117	3.078587	0.00277	-2.10359	0.047727	0.065876
Non.inflammatory.Macrophages	TRAPPC6A	0.269352	5.057196	3.078238	0.002773	-2.31818	0.044547	0.063865
Non.inflammatory.Macrophages	FAM117B	0.322131	7.11596	3.076639	0.002787	-2.61999	0.039406	0.060518
Non.inflammatory.Macrophages	ITGA3	1.607947	-0.28405	3.07244	0.002822	-1.8386	0.058767	0.075015
Non.inflammatory.Macrophages	FNBP1L	0.77383	3.079155	3.071959	0.002826	-1.86707	0.050897	0.068385

Non.inflammatory.Macrophages	APRT	0.361527	6.416788	3.070536	0.002838	-2.54867	0.041531	0.062493
Non.inflammatory.Macrophages	GALNT1	0.238609	6.628673	3.070468	0.002839	-2.54399	0.040996	0.062125
Non.inflammatory.Macrophages	NPTN	-0.20224	8.043161	-3.07041	0.00284	-2.75053	0.03759	0.059727
Non.inflammatory.Macrophages	PLEKHA3	0.410177	3.98944	3.07025	0.002841	-2.0636	0.048162	0.066863
Non.inflammatory.Macrophages	FMN2	-1.8567	2.359589	-3.06988	0.002844	-1.70587	0.05316	0.069999
Non.inflammatory.Macrophages	CD209G	-2.07273	1.089148	-3.06894	0.002852	-1.79467	0.057458	0.072658
Non.inflammatory.Macrophages	PDE6C	-1.62153	0.436862	-3.06724	0.002867	-1.74799	0.059005	0.074099
Non.inflammatory.Macrophages	PPARG	-0.53485	2.579221	-3.06703	0.002869	-2.2234	0.052673	0.069828
Non.inflammatory.Macrophages	GM3448	-0.83781	1.9113	-3.06422	0.002893	-1.76706	0.055086	0.071431
Non.inflammatory.Macrophages	ATP6V1D	-0.21824	6.650485	-3.06405	0.002895	-2.5635	0.041293	0.062605
Non.inflammatory.Macrophages	SOCS2	0.586497	3.962092	3.064022	0.002895	-2.13144	0.048657	0.06747
Non.inflammatory.Macrophages	B4GALT6	0.401551	2.991751	3.061296	0.002919	-2.36405	0.05173	0.069674
Non.inflammatory.Macrophages	ARID5B	0.416922	7.244193	3.060865	0.002923	-2.7162	0.039913	0.061949
Non.inflammatory.Macrophages	RNF130	0.237639	7.282781	3.060608	0.002925	-2.74688	0.039818	0.061883
Non.inflammatory.Macrophages	CC2D2B	-0.9	2.70829	-3.06053	0.002925	-2.15372	0.052625	0.070286

Non.inflammatory.Macrophages	IFITM6	0.573001	3.518134	3.060246	0.002928	-2.42636	0.050106	0.068745
Non.inflammatory.Macrophages	F3	1.522699	0.113979	3.060238	0.002928	-1.7211	0.059413	0.075568
Non.inflammatory.Macrophages	D230025D:	0.414738	4.57771	3.059047	0.002939	-2.16857	0.047063	0.066899
Non.inflammatory.Macrophages	BLK	-0.95277	4.074052	-3.05866	0.002942	-1.76606	0.048528	0.0679
Non.inflammatory.Macrophages	KDM6B	-0.30628	8.152763	-3.05805	0.002947	-2.83218	0.037827	0.060668
Non.inflammatory.Macrophages	SLC30A9	0.292626	5.265307	3.053699	0.002986	-2.37201	0.045613	0.066373
Non.inflammatory.Macrophages	VPS18	-0.33192	4.834066	-3.05365	0.002987	-2.34822	0.046828	0.067175
Non.inflammatory.Macrophages	LAT	-1.12036	2.414617	-3.04678	0.003049	-1.74933	0.055285	0.072703
Non.inflammatory.Macrophages	VCAN	1.236323	0.76602	3.045345	0.003062	-1.81431	0.060831	0.076377
Non.inflammatory.Macrophages	PTGS2OS	2.102783	-1.09526	3.045263	0.003063	-1.92543	0.061361	0.080425
Non.inflammatory.Macrophages	CHD3	-0.40452	4.533669	-3.04404	0.003074	-2.33995	0.048651	0.068967
Non.inflammatory.Macrophages	APOM	-0.83037	4.854579	-3.04399	0.003075	-2.25767	0.047709	0.068354
Non.inflammatory.Macrophages	CFI	-0.98254	3.743943	-3.04376	0.003077	-1.98516	0.051044	0.0705
Non.inflammatory.Macrophages	WDR91	-0.33905	4.750458	-3.0436	0.003078	-2.44275	0.048012	0.068566
Non.inflammatory.Macrophages	TMEM251	-0.35875	4.619848	-3.04045	0.003108	-2.3006	0.048783	0.06912

Non.inflammatory.Macrophages	RNF149	0.395218	5.786169	3.035805	0.003151	-2.67714	0.0459	0.067475
Non.inflammatory.Macrophages	KLHL24	0.319942	6.228518	3.035607	0.003153	-2.53049	0.044675	0.066654
Non.inflammatory.Macrophages	GM6377	-0.40589	3.091942	-3.03556	0.003154	-2.4548	0.05408	0.072735
Non.inflammatory.Macrophages	GFRA1	-0.91204	4.918403	-3.03272	0.003181	-2.03576	0.048741	0.069459
Non.inflammatory.Macrophages	PRKACB	0.281192	5.548308	3.032021	0.003187	-2.47718	0.046932	0.068329
Non.inflammatory.Macrophages	NREP	-1.19562	1.234795	-3.02835	0.003222	-1.7937	0.061539	0.077606
Non.inflammatory.Macrophages	1700016PC	-0.5642	3.898827	-3.02672	0.003238	-2.44493	0.052571	0.072311
Non.inflammatory.Macrophages	THY1	1.541416	1.573983	3.024789	0.003257	-1.81725	0.060767	0.077395
Non.inflammatory.Macrophages	ABCC9	1.195333	1.277502	3.022961	0.003275	-1.81674	0.062027	0.078303
Non.inflammatory.Macrophages	CSNK1A1	0.178776	7.684452	3.022914	0.003275	-2.82967	0.042008	0.065516
Non.inflammatory.Macrophages	GM13481	-2.09028	0.231781	-3.02209	0.003283	-2.00035	0.06431	0.080719
Non.inflammatory.Macrophages	KLRC1	-1.23825	1.444197	-3.02059	0.003298	-1.82012	0.061645	0.078259
Non.inflammatory.Macrophages	SERPINA1C	-0.8839	7.348457	-3.02018	0.003302	-2.71413	0.043048	0.066415
Non.inflammatory.Macrophages	RAPGEF1	0.2986	6.775482	3.019133	0.003313	-2.60988	0.044662	0.06762
Non.inflammatory.Macrophages	MS4A6B	0.533055	4.588359	3.015655	0.003347	-2.60782	0.05145	0.072258

Non.inflammatory.Macrophages	GM37065	-1.60918	2.706239	-3.01555	0.003348	-1.8395	0.057677	0.076149
Non.inflammatory.Macrophages	BIRC6	0.174917	7.8393	3.012821	0.003376	-2.84445	0.042454	0.066321
Non.inflammatory.Macrophages	LGALS3	0.358985	6.142988	3.012273	0.003381	-2.94291	0.047115	0.069602
Non.inflammatory.Macrophages	ADTRP	-1.29435	1.835711	-3.00682	0.003437	-1.84653	0.06195	0.079201
Non.inflammatory.Macrophages	MMS19	0.311135	4.947005	3.006672	0.003438	-2.43502	0.051301	0.072644
Non.inflammatory.Macrophages	CHD8	0.262705	5.733086	3.006537	0.003439	-2.55119	0.048897	0.071075
Non.inflammatory.Macrophages	SAPCD1	-1.10861	1.629002	-3.00629	0.003442	-1.84849	0.062725	0.079673
Non.inflammatory.Macrophages	SERP1	0.244108	7.981742	3.005839	0.003447	-2.88552	0.042603	0.066762
Non.inflammatory.Macrophages	PPP4R3B	0.226542	6.842276	3.004691	0.003458	-2.6884	0.045779	0.069004
Non.inflammatory.Macrophages	SUZ12	0.270652	6.954117	3.003758	0.003468	-2.68198	0.045493	0.068936
Non.inflammatory.Macrophages	AI839979	1.674188	0.274557	3.002984	0.003476	-1.89992	0.066526	0.083126
Non.inflammatory.Macrophages	ZNHIT1	0.261676	5.422819	3.002842	0.003478	-2.63182	0.049962	0.072046
Non.inflammatory.Macrophages	IL12A	-1.75919	2.215059	-3.00259	0.00348	-1.92023	0.060707	0.078814
Non.inflammatory.Macrophages	AI504432	0.908098	2.840905	2.994918	0.003561	-1.8871	0.05972	0.07864
Non.inflammatory.Macrophages	LIFR	0.645445	3.842745	2.992598	0.003585	-2.26929	0.056468	0.076782

Non.inflammatory.Macrophages	CAMK1D	-0.27787	8.628354	-2.99224	0.003589	-3.16851	0.042139	0.067229
Non.inflammatory.Macrophages	RAB10	0.251079	7.670572	2.991897	0.003593	-2.95361	0.044692	0.069097
Non.inflammatory.Macrophages	TMEM41B	-0.36798	4.300772	-2.99043	0.003608	-2.35451	0.055079	0.076187
Non.inflammatory.Macrophages	PCF11	-0.30021	6.482932	-2.9887	0.003627	-2.74486	0.04838	0.071944
Non.inflammatory.Macrophages	SYNJ1	-0.24502	6.731012	-2.98829	0.003631	-2.91633	0.04765	0.0715
Non.inflammatory.Macrophages	TUBB2B	-1.32241	2.9596	-2.98778	0.003637	-1.89859	0.059971	0.079516
Non.inflammatory.Macrophages	IQGAP1	0.221201	9.247795	2.986478	0.003651	-3.17196	0.040911	0.066874
Non.inflammatory.Macrophages	FGFR2	-0.33234	5.987704	-2.98592	0.003657	-2.62139	0.049968	0.07328
Non.inflammatory.Macrophages	LRP1	0.314044	4.178873	2.98565	0.00366	-2.78093	0.055798	0.077093
Non.inflammatory.Macrophages	GM30541	1.21342	0.952964	2.985273	0.003664	-1.90465	0.067791	0.084398
Non.inflammatory.Macrophages	DLST	0.243792	5.852364	2.982513	0.003694	-2.64382	0.050727	0.074016
Non.inflammatory.Macrophages	M1AP	-0.84689	1.320039	-2.98068	0.003714	-1.97615	0.067048	0.0843
Non.inflammatory.Macrophages	CEACAM16	1.405126	1.137316	2.979814	0.003724	-1.9313	0.067867	0.084823
Non.inflammatory.Macrophages	CORO2A	0.386031	5.527432	2.978681	0.003736	-2.60197	0.052118	0.07515
Non.inflammatory.Macrophages	YPEL2	0.511742	4.572346	2.976662	0.003759	-2.39128	0.0555	0.077415

Non.inflammatory.Macrophages	HSPA1A	-0.90092	3.985226	-2.9751	0.003776	-2.62041	0.057642	0.078951
Non.inflammatory.Macrophages	LAPTM4B	-0.64256	3.501356	-2.975	0.003777	-2.29278	0.059361	0.080027
Non.inflammatory.Macrophages	CYBB	0.440654	7.155496	2.97199	0.003811	-3.11567	0.047854	0.07269
Non.inflammatory.Macrophages	ELMOD3	-0.66559	3.437093	-2.97089	0.003823	-2.27985	0.060157	0.080784
Non.inflammatory.Macrophages	RNF19A	0.364156	5.375931	2.969402	0.00384	-2.50966	0.053622	0.076768
Non.inflammatory.Macrophages	GGTA1	0.359632	5.365487	2.967676	0.00386	-2.69353	0.053857	0.076963
Non.inflammatory.Macrophages	KCNQ5	-0.56685	7.024096	-2.96699	0.003868	-2.51626	0.048692	0.07357
Non.inflammatory.Macrophages	USP3	0.284269	6.873279	2.964148	0.0039	-2.80253	0.04949	0.074277
Non.inflammatory.Macrophages	SLC4A7	0.364958	5.767425	2.962656	0.003917	-2.64622	0.053117	0.076682
Non.inflammatory.Macrophages	PLXDC2	0.654939	5.144109	2.961976	0.003925	-2.65208	0.055214	0.078071
Non.inflammatory.Macrophages	RARRES2	-0.85254	3.7462	-2.95925	0.003957	-2.2725	0.060441	0.081479
Non.inflammatory.Macrophages	AGPAT4	0.694458	5.023605	2.959244	0.003957	-2.3125	0.05592	0.078633
Non.inflammatory.Macrophages	CTNS	-0.43756	3.606042	-2.95584	0.003997	-2.42144	0.061489	0.082372
Non.inflammatory.Macrophages	FOXK1	0.342076	4.627409	2.954078	0.004018	-2.50194	0.057982	0.080367
Non.inflammatory.Macrophages	F630040KC	0.921719	0.611295	2.953772	0.004021	-2.07587	0.073322	0.089925

Non.inflammatory.Macrophages	GPR132	0.93529	6.00422	2.953305	0.004027	-2.22557	0.053309	0.077444
Non.inflammatory.Macrophages	1-Mar	0.533149	4.477654	2.949847	0.004068	-2.82419	0.059033	0.081279
Non.inflammatory.Macrophages	PTPRS	0.4768	4.390471	2.948036	0.00409	-2.3941	0.059584	0.081704
Non.inflammatory.Macrophages	CD79A	-0.61012	6.200185	-2.94713	0.004101	-2.51018	0.053422	0.077759
Non.inflammatory.Macrophages	MIR155HG	1.184087	4.086438	2.945598	0.004119	-2.11915	0.060922	0.082647
Non.inflammatory.Macrophages	A930001A:	1.908267	-0.71301	2.945201	0.004124	-2.15184	0.074775	0.094529
Non.inflammatory.Macrophages	HOPX	0.960785	4.034194	2.944524	0.004132	-2.08135	0.061116	0.082859
Non.inflammatory.Macrophages	SERF2	0.169742	9.682679	2.944495	0.004133	-3.37071	0.043246	0.070805
Non.inflammatory.Macrophages	GM12979	-1.19602	0.049111	-2.944	0.004139	-1.99691	0.074783	0.092617
Non.inflammatory.Macrophages	FETUB	-0.9703	3.175824	-2.94309	0.00415	-2.15608	0.064479	0.085021
Non.inflammatory.Macrophages	ATP2C1	0.265644	5.85373	2.9424	0.004158	-2.78142	0.054815	0.078914
Non.inflammatory.Macrophages	MTHFD1L	0.393851	5.661426	2.941206	0.004173	-2.58838	0.055549	0.079544
Non.inflammatory.Macrophages	CAPRIN2	-0.69079	3.181523	-2.94097	0.004176	-2.27313	0.064601	0.08525
Non.inflammatory.Macrophages	FABP5	0.512796	7.278075	2.939404	0.004195	-3.00828	0.050431	0.07629
Non.inflammatory.Macrophages	KLF6	-0.33082	7.581942	-2.93902	0.0042	-3.18038	0.0495	0.075709

Non.inflammatory.Macrophages	ATCAYOS	-1.18729	1.220314	-2.93882	0.004202	-2.0674	0.072879	0.090389
Non.inflammatory.Macrophages	0610043K1	-0.83354	1.133363	-2.93665	0.004229	-2.18325	0.073632	0.091043
Non.inflammatory.Macrophages	HIGD1A	0.281378	6.029655	2.934612	0.004254	-2.84509	0.05497	0.079688
Non.inflammatory.Macrophages	CAAA0114	0.669086	2.131904	2.933981	0.004262	-2.14262	0.069702	0.088883
Non.inflammatory.Macrophages	GM11110	0.843567	1.970897	2.932474	0.004281	-2.04745	0.070587	0.089595
Non.inflammatory.Macrophages	CYP3A11	-1.006	3.352158	-2.93209	0.004286	-2.23452	0.064933	0.086218
Non.inflammatory.Macrophages	CEMIP2	0.39823	5.494454	2.92763	0.004342	-2.7183	0.057643	0.081841
Non.inflammatory.Macrophages	GM42726	-0.47505	3.69069	-2.9272	0.004348	-2.32056	0.064337	0.086076
Non.inflammatory.Macrophages	PI16	1.031009	1.135522	2.926884	0.004352	-2.06061	0.075065	0.092482
Non.inflammatory.Macrophages	DVL2	-0.52872	3.263703	-2.92429	0.004385	-2.37316	0.06644	0.087478
Non.inflammatory.Macrophages	MGP	1.456079	0.463003	2.922572	0.004407	-2.04798	0.077865	0.094889
Non.inflammatory.Macrophages	BCL7A	-0.70561	4.80999	-2.92162	0.004419	-2.30319	0.060796	0.084281
Non.inflammatory.Macrophages	FADS2	-1.01465	3.530927	-2.91988	0.004442	-2.16305	0.0659	0.087682
Non.inflammatory.Macrophages	CD300C	-1.05974	0.72621	-2.91966	0.004445	-2.05995	0.077645	0.094815
Non.inflammatory.Macrophages	SNTB2	0.377687	5.607555	2.919047	0.004453	-2.82036	0.058071	0.082904

Non.inflammatory.Macrophages	SLFN4	1.617019	0.48225	2.918872	0.004455	-2.05921	0.078201	0.095614
Non.inflammatory.Macrophages	GM15559	-0.61022	3.148018	-2.91816	0.004464	-2.20082	0.067459	0.088891
Non.inflammatory.Macrophages	SELL	0.905822	5.472373	2.917603	0.004471	-2.22268	0.058562	0.083412
Non.inflammatory.Macrophages	TAPT1	0.407461	5.802801	2.917484	0.004473	-2.65593	0.057392	0.082648
Non.inflammatory.Macrophages	ERC2	1.576209	0.862583	2.915821	0.004495	-2.0779	0.077529	0.09508
Non.inflammatory.Macrophages	GM41555	-1.38339	0.571556	-2.91513	0.004504	-2.06878	0.078388	0.095955
Non.inflammatory.Macrophages	PRDX6	0.277009	7.110346	2.914207	0.004516	-2.92252	0.053248	0.080121
Non.inflammatory.Macrophages	ZFP710	-0.32881	6.297621	-2.91323	0.004529	-2.98129	0.055966	0.082087
Non.inflammatory.Macrophages	EZR	-0.25359	7.933064	-2.9132	0.004529	-3.03866	0.050627	0.078434
Non.inflammatory.Macrophages	ATP5E	0.195889	8.783189	2.912944	0.004533	-3.30736	0.048051	0.07664
Non.inflammatory.Macrophages	1810026B(	0.256062	6.570259	2.912266	0.004542	-2.85441	0.055039	0.081652
Non.inflammatory.Macrophages	R3HDM1	0.241004	6.683532	2.911837	0.004547	-2.90366	0.054659	0.08147
Non.inflammatory.Macrophages	TMX3	0.276621	5.505888	2.911349	0.004554	-2.78849	0.058742	0.084263
Non.inflammatory.Macrophages	CLEC5A	1.158856	0.512385	2.911334	0.004554	-2.08722	0.078571	0.096818
Non.inflammatory.Macrophages	UBN1	-0.20748	6.944041	-2.91028	0.004568	-2.99129	0.053827	0.081025

Non.inflammatory.Macrophages	4930556J2	-0.9448	2.185085	-2.91025	0.004569	-2.08017	0.071913	0.0925
Non.inflammatory.Macrophages	WDR81	-0.39352	3.600126	-2.90785	0.004601	-2.53534	0.066391	0.089347
Non.inflammatory.Macrophages	ADAMDEC	0.803818	0.746267	2.907358	0.004607	-2.27995	0.078505	0.0968
Non.inflammatory.Macrophages	FAM129B	0.558292	3.14002	2.906551	0.004618	-2.56447	0.068354	0.090682
Non.inflammatory.Macrophages	RBP4	-0.74746	8.031076	-2.90439	0.004647	-3.13803	0.050962	0.079464
Non.inflammatory.Macrophages	C330007P(	-0.32158	5.50907	-2.90091	0.004694	-2.79184	0.060006	0.085756
Non.inflammatory.Macrophages	CROT	-0.41401	5.259869	-2.89718	0.004746	-2.74549	0.061513	0.086843
Non.inflammatory.Macrophages	TCEAL9	-0.34832	5.693961	-2.89669	0.004752	-2.83045	0.059912	0.085881
Non.inflammatory.Macrophages	SULT2A5	-1.23272	2.02078	-2.89371	0.004794	-2.14469	0.075442	0.095641
Non.inflammatory.Macrophages	UBAP2L	-0.19439	7.113568	-2.89235	0.004813	-3.11225	0.055482	0.08324
Non.inflammatory.Macrophages	LRFN1	-0.93914	1.097146	-2.89147	0.004825	-2.21222	0.080067	0.098577
Non.inflammatory.Macrophages	UOX	0.745127	5.010934	2.888463	0.004867	-2.75446	0.063654	0.088844
Non.inflammatory.Macrophages	MYO1F	0.3642	5.259228	2.883617	0.004936	-2.98714	0.063498	0.088783
Non.inflammatory.Macrophages	DPYSL2	-0.28902	6.279623	-2.88322	0.004942	-3.03078	0.059659	0.086322
Non.inflammatory.Macrophages	FBXO22	0.316212	4.616588	2.882635	0.00495	-2.7185	0.066065	0.09043

Non.inflammatory.Macrophages	BRMS1L	0.398369	4.269409	2.882057	0.004959	-2.53706	0.067505	0.09141
Non.inflammatory.Macrophages	SNTB1	-1.00689	4.264888	-2.88069	0.004978	-2.36017	0.067707	0.09159
Non.inflammatory.Macrophages	B230217C1	-0.61404	1.478169	-2.88008	0.004987	-2.3434	0.08018	0.099064
Non.inflammatory.Macrophages	NIPSNAP1	-0.7482	3.134643	-2.87878	0.005006	-2.26214	0.072737	0.094826
Non.inflammatory.Macrophages	TANK	-0.29923	6.451404	-2.87778	0.00502	-3.16286	0.059524	0.086568
Non.inflammatory.Macrophages	ZFP651	-0.71478	1.578642	-2.87391	0.005077	-2.26573	0.080832	0.099696
Non.inflammatory.Macrophages	A630001G2	0.342142	5.126842	2.872806	0.005093	-2.79125	0.065317	0.090446
Non.inflammatory.Macrophages	CHD7	0.300812	6.463817	2.871148	0.005118	-3.0932	0.060391	0.087377
Non.inflammatory.Macrophages	PDE1C	-0.72449	3.989093	-2.8706	0.005126	-2.63034	0.070236	0.093657
Non.inflammatory.Macrophages	EMB	0.838877	5.68619	2.870375	0.005129	-2.536	0.063333	0.089335
Non.inflammatory.Macrophages	GPNMB	0.990624	0.898381	2.869042	0.005149	-2.29803	0.084752	0.102227
Non.inflammatory.Macrophages	GM525	-1.04981	0.742971	-2.86811	0.005163	-2.17585	0.085382	0.102817
Non.inflammatory.Macrophages	PHF12	0.276136	6.186244	2.867249	0.005176	-2.96311	0.061754	0.088454
Non.inflammatory.Macrophages	HSF5	-1.27388	0.277217	-2.86362	0.00523	-2.18539	0.086956	0.105032
Non.inflammatory.Macrophages	ARHGAP31	0.377947	6.152696	2.861731	0.005259	-3.05632	0.06272	0.089443

Non.inflammatory.Macrophages	KLKB1	-0.8209	1.848942	-2.8606	0.005276	-2.30761	0.08163	0.101027
Non.inflammatory.Macrophages	TBL3	-0.42686	3.91508	-2.85954	0.005292	-2.56197	0.072169	0.095462
Non.inflammatory.Macrophages	CES2E	-1.31133	1.228112	-2.85677	0.005334	-2.20091	0.085464	0.10333
Non.inflammatory.Macrophages	PIGN	0.400417	4.40092	2.856267	0.005342	-2.76544	0.07056	0.094699
Non.inflammatory.Macrophages	AKR1C20	-1.02261	2.839499	-2.8551	0.00536	-2.35916	0.077671	0.09902
Non.inflammatory.Macrophages	SYAP1	0.273387	5.053275	2.854495	0.005369	-2.86326	0.067895	0.093184
Non.inflammatory.Macrophages	NDUFS4	0.244301	6.335519	2.854219	0.005374	-3.05752	0.062778	0.089941
Non.inflammatory.Macrophages	CRYBB3	1.626715	0.578324	2.854136	0.005375	-2.26304	0.08819	0.105564
Non.inflammatory.Macrophages	EDIL3	0.646486	1.492552	2.851775	0.005412	-2.56701	0.084711	0.103361
Non.inflammatory.Macrophages	SORD	-0.51791	4.176195	-2.85107	0.005423	-2.71694	0.07202	0.09604
Non.inflammatory.Macrophages	CBX4	-0.35582	5.468948	-2.85094	0.005425	-2.89305	0.066562	0.09266
Non.inflammatory.Macrophages	KIF3C	0.74398	2.145051	2.84773	0.005475	-2.30436	0.082066	0.102209
Non.inflammatory.Macrophages	CHST8	-1.42947	-0.80735	-2.84748	0.005479	-2.22171	0.089554	0.110983
Non.inflammatory.Macrophages	MOB3B	0.382054	4.961752	2.845923	0.005503	-2.84193	0.069401	0.094803
Non.inflammatory.Macrophages	NEMF	0.244672	5.95092	2.843061	0.005549	-3.00436	0.065792	0.09267

Non.inflammatory.Macrophages	KLF4	-0.31113	6.447469	-2.84227	0.005561	-3.29939	0.063849	0.09158
Non.inflammatory.Macrophages	GM17749	-0.47622	4.634129	-2.84158	0.005572	-3.11235	0.071328	0.096403
Non.inflammatory.Macrophages	B3GNT2	0.333469	6.739389	2.841514	0.005573	-2.98363	0.062717	0.090915
Non.inflammatory.Macrophages	PDE4DIP	-0.37226	3.94998	-2.84125	0.005578	-2.75074	0.07436	0.098288
Non.inflammatory.Macrophages	SETD3	0.269067	5.687067	2.838116	0.005628	-2.97595	0.067411	0.094231
Non.inflammatory.Macrophages	CSTDC6	1.383748	-1.16323	2.835108	0.005677	-2.39451	0.091902	0.114479
Non.inflammatory.Macrophages	GPT2	0.710129	1.738624	2.833531	0.005702	-2.44218	0.086591	0.105886
Non.inflammatory.Macrophages	DENND5B	-0.63354	4.198991	-2.83248	0.00572	-2.47867	0.074747	0.099104
Non.inflammatory.Macrophages	BRK1	0.215115	6.271039	2.829532	0.005768	-3.18556	0.066331	0.094019
Non.inflammatory.Macrophages	SLC26A10	-1.61797	0.914242	-2.82882	0.00578	-2.29389	0.091699	0.109211
Non.inflammatory.Macrophages	LMTK2	-0.38833	5.616268	-2.82879	0.00578	-2.76714	0.069041	0.09582
Non.inflammatory.Macrophages	CYP2C70	-0.90402	4.144913	-2.82569	0.005832	-2.64378	0.076055	0.100257
Non.inflammatory.Macrophages	GBP7	0.611543	4.443583	2.824934	0.005845	-2.99097	0.074686	0.099566
Non.inflammatory.Macrophages	ACAA1B	-1.15006	3.318751	-2.82479	0.005847	-2.53067	0.079968	0.102732
Non.inflammatory.Macrophages	RNF180	-0.55124	2.247716	-2.82466	0.005849	-2.74073	0.085319	0.105857

Non.inflammatory.Macrophages	GM45051	-0.93623	1.845863	-2.8235	0.005869	-2.2841	0.087597	0.107284
Non.inflammatory.Macrophages	TMEM88	-0.55016	2.768173	-2.82217	0.005891	-2.73073	0.083073	0.104792
Non.inflammatory.Macrophages	APC	0.274894	6.723147	2.820408	0.005921	-3.17094	0.065538	0.094167
Non.inflammatory.Macrophages	TIGD2	0.360264	3.85213	2.819021	0.005944	-2.78151	0.078264	0.102274
Non.inflammatory.Macrophages	BMYC	0.608328	3.380337	2.818588	0.005951	-2.67826	0.080537	0.103653
Non.inflammatory.Macrophages	FOSB	-0.35269	6.636378	-2.81839	0.005955	-3.40084	0.066034	0.094687
Non.inflammatory.Macrophages	CD1D1	-0.47364	3.932282	-2.8179	0.005963	-2.8661	0.077901	0.102153
Non.inflammatory.Macrophages	GPALPP1	0.335517	3.737889	2.817064	0.005977	-2.80108	0.078874	0.102872
Non.inflammatory.Macrophages	HSPA4L	0.37562	4.348791	2.816867	0.005981	-2.89729	0.075998	0.101155
Non.inflammatory.Macrophages	ITGA6	0.579646	4.818835	2.816456	0.005988	-2.86148	0.073853	0.099898
Non.inflammatory.Macrophages	GRK5	0.470752	5.747033	2.815124	0.006011	-3.1232	0.069969	0.097511
Non.inflammatory.Macrophages	AFF3	-0.58104	7.930696	-2.81264	0.006053	-3.03272	0.06157	0.092234
Non.inflammatory.Macrophages	RABEP1	0.220964	6.897474	2.811434	0.006074	-3.2867	0.065747	0.095158
Non.inflammatory.Macrophages	ARL13B	-0.35386	3.775076	-2.81012	0.006097	-2.81155	0.079757	0.104067
Non.inflammatory.Macrophages	ACOX1	-0.31508	5.892871	-2.80914	0.006114	-3.18138	0.070213	0.098226

Non.inflammatory.Macrophages	SH3KBP1	0.223854	7.863843	2.808161	0.006131	-3.39704	0.062326	0.093086
Non.inflammatory.Macrophages	TNNT3	1.503592	1.772075	2.806797	0.006155	-2.32886	0.090566	0.110513
Non.inflammatory.Macrophages	DOT1L	0.322754	5.289755	2.80637	0.006162	-3.08221	0.073169	0.100214
Non.inflammatory.Macrophages	RMDN3	0.404441	3.398108	2.805223	0.006182	-2.67013	0.082232	0.105678
Non.inflammatory.Macrophages	MINDY3	0.283251	5.598106	2.804965	0.006187	-3.06438	0.071923	0.099454
Non.inflammatory.Macrophages	ZSWIM6	-0.24807	8.155524	-2.80423	0.0062	-3.58132	0.061491	0.092677
Non.inflammatory.Macrophages	HMGCS2	-0.77353	4.456909	-2.80416	0.006201	-2.86057	0.077108	0.102728
Non.inflammatory.Macrophages	PVR	0.450195	3.063552	2.802454	0.006231	-2.80319	0.084229	0.10718
Non.inflammatory.Macrophages	HIRA	0.348431	5.652742	2.801803	0.006243	-2.95096	0.072	0.099893
Non.inflammatory.Macrophages	ZFP52	0.445803	3.859767	2.79979	0.006279	-2.75888	0.080681	0.105365
Non.inflammatory.Macrophages	H1F0	0.418647	6.001224	2.798698	0.006298	-3.11688	0.070878	0.099386
Non.inflammatory.Macrophages	FBXL5	0.446631	6.173273	2.798558	0.006301	-3.07227	0.070136	0.098931
Non.inflammatory.Macrophages	TMEM163	-0.68355	6.026941	-2.79821	0.006307	-2.72232	0.070767	0.099362
Non.inflammatory.Macrophages	SLC23A3	-0.99768	1.461516	-2.79561	0.006354	-2.46941	0.093945	0.113262
Non.inflammatory.Macrophages	MTR	0.287461	4.54186	2.795013	0.006364	-3.03722	0.078011	0.104099

Non.inflammatory.Macrophages	MARK3	0.230889	6.304211	2.792207	0.006415	-3.16861	0.070459	0.099495
Non.inflammatory.Macrophages	ZFAND4	-0.66161	3.4762	-2.79215	0.006416	-2.78418	0.083716	0.107644
Non.inflammatory.Macrophages	SPINT1	1.20004	-1.0651	2.791762	0.006423	-2.37631	0.099013	0.122198
Non.inflammatory.Macrophages	RIOK3	0.245934	7.578172	2.789378	0.006467	-3.41479	0.065534	0.096492
Non.inflammatory.Macrophages	HS3ST3B1	0.542208	3.380647	2.788423	0.006484	-2.74252	0.084727	0.108554
Non.inflammatory.Macrophages	TNFRSF1B	0.324008	4.903674	2.788365	0.006486	-3.23359	0.077232	0.104048
Non.inflammatory.Macrophages	SNRPA	-0.26908	5.131803	-2.78689	0.006513	-3.05042	0.076397	0.103666
Non.inflammatory.Macrophages	WHRN	-0.74033	3.066048	-2.78577	0.006533	-2.52637	0.086799	0.110068
Non.inflammatory.Macrophages	PDPN	1.860648	-0.21403	2.785057	0.006547	-2.37898	0.100228	0.12072
Non.inflammatory.Macrophages	ADGRL4	-0.97091	3.201942	-2.7836	0.006574	-2.46139	0.086422	0.110081
Non.inflammatory.Macrophages	4933406I1[illegible]	0.86173	4.543564	2.779926	0.006642	-2.60347	0.08036	0.106765
Non.inflammatory.Macrophages	PRR13	0.203869	6.510778	2.779693	0.006647	-3.33692	0.071261	0.101111
Non.inflammatory.Macrophages	LDHB	0.68907	3.00278	2.778816	0.006663	-2.65678	0.088362	0.111561
Non.inflammatory.Macrophages	GM13427	-0.82231	2.062778	-2.7777	0.006684	-2.42912	0.093713	0.114694
Non.inflammatory.Macrophages	DAZAP2	0.205314	7.70346	2.77558	0.006724	-3.52221	0.066612	0.098275

Non.inflammatory.Macrophages	ANGPTL8	-1.22648	1.825356	-2.7753	0.00673	-2.40555	0.095281	0.115755
Non.inflammatory.Macrophages	OSGIN1	-0.48855	3.869004	-2.77528	0.00673	-2.91944	0.084201	0.109357
Non.inflammatory.Macrophages	ALDH1B1	0.744578	1.910302	2.775176	0.006732	-2.46411	0.094794	0.115494
Non.inflammatory.Macrophages	VIPR1	-0.94128	0.964939	-2.77491	0.006737	-2.44834	0.100333	0.118635
Non.inflammatory.Macrophages	LCA5	1.308881	1.313513	2.773394	0.006766	-2.39937	0.098569	0.117713
Non.inflammatory.Macrophages	PPTC7	0.347725	5.148669	2.7703	0.006825	-3.03099	0.078733	0.106408
Non.inflammatory.Macrophages	GM27017	-0.70513	3.311249	-2.76853	0.00686	-2.5671	0.088386	0.112351
Non.inflammatory.Macrophages	TSPO	0.380749	7.399271	2.767191	0.006885	-3.44331	0.069004	0.100437
Non.inflammatory.Macrophages	CDK2AP1	-0.29594	5.884299	-2.76705	0.006888	-3.12469	0.075714	0.104763
Non.inflammatory.Macrophages	GALNT7	0.312866	6.060844	2.763195	0.006963	-3.25769	0.075484	0.104999
Non.inflammatory.Macrophages	1700097N(	-1.67228	2.346622	-2.76316	0.006964	-2.46719	0.094611	0.116434
Non.inflammatory.Macrophages	TTC39A	-0.97188	2.027911	-2.76311	0.006965	-2.48546	0.096447	0.11747
Non.inflammatory.Macrophages	PCGF3	0.360532	3.998741	2.760531	0.007016	-2.87889	0.085989	0.111768
Non.inflammatory.Macrophages	ANKRD12	-0.28631	7.518738	-2.76038	0.007019	-3.41409	0.069347	0.101361
Non.inflammatory.Macrophages	GLP2R	1.35338	0.940346	2.760039	0.007026	-2.43191	0.103384	0.121808

Non.inflammatory.Macrophages	EPB41	0.3525	8.026595	2.759928	0.007028	-3.61201	0.067219	0.100009
Non.inflammatory.Macrophages	ST6GAL1	-0.51206	5.862113	-2.758	0.007066	-3.20558	0.07701	0.106459
Non.inflammatory.Macrophages	CD4	-0.95008	1.246276	-2.75796	0.007067	-2.52311	0.101887	0.121049
Non.inflammatory.Macrophages	RAI14	0.817606	2.228896	2.757435	0.007077	-2.56834	0.096074	0.11792
Non.inflammatory.Macrophages	MAOB	-1.1774	1.627366	-2.75643	0.007097	-2.45594	0.099791	0.120162
Non.inflammatory.Macrophages	EVA1A	-1.01774	1.060527	-2.75464	0.007133	-2.44475	0.103617	0.12254
Non.inflammatory.Macrophages	ITGA4	0.334252	7.657286	2.754367	0.007138	-3.47558	0.069384	0.10199
Non.inflammatory.Macrophages	TBC1D16	-0.51339	2.452428	-2.75151	0.007196	-2.92004	0.095776	0.11841
Non.inflammatory.Macrophages	FYTTD1	0.238814	5.267108	2.751503	0.007196	-3.17187	0.080725	0.109488
Non.inflammatory.Macrophages	GM11099	-1.21556	-0.70994	-2.7514	0.007198	-2.4421	0.106472	0.129281
Non.inflammatory.Macrophages	NSG2	-1.57904	0.587521	-2.75077	0.007211	-2.52035	0.106279	0.124799
Non.inflammatory.Macrophages	LRRC63	1.031868	0.544146	2.749853	0.00723	-2.49896	0.106457	0.12507
Non.inflammatory.Macrophages	GM4258	0.573209	4.929368	2.749789	0.007231	-2.97182	0.082508	0.110709
Non.inflammatory.Macrophages	ZFP654	0.335689	6.120389	2.748917	0.007249	-3.22696	0.076823	0.107212
Non.inflammatory.Macrophages	ARL4D	-0.76615	1.705958	-2.7474	0.00728	-2.6177	0.100764	0.121547

Non.inflammatory.Macrophages	UBASH3B	0.357345	5.794781	2.746402	0.0073	-3.48571	0.078751	0.108645
Non.inflammatory.Macrophages	FXYD4	-0.64222	3.001862	-2.74531	0.007322	-2.67991	0.093526	0.117658
Non.inflammatory.Macrophages	TOMT	-0.87658	1.736925	-2.74318	0.007366	-2.57369	0.101442	0.122339
Non.inflammatory.Macrophages	NOCT	0.384319	5.04776	2.742124	0.007388	-3.21703	0.083103	0.11172
Non.inflammatory.Macrophages	NDFIP1	0.20483	7.521506	2.741944	0.007392	-3.60394	0.071428	0.104304
Non.inflammatory.Macrophages	GM15987	1.117302	2.537923	2.741133	0.007409	-2.49565	0.096901	0.119955
Non.inflammatory.Macrophages	IRAK3	0.564741	4.187779	2.740654	0.007418	-3.08022	0.0877	0.114655
Non.inflammatory.Macrophages	NOS2	2.399582	-1.12584	2.739677	0.007439	-2.56877	0.108488	0.133175
Non.inflammatory.Macrophages	4921524J1	0.274259	5.474163	2.738478	0.007464	-3.1348	0.081405	0.111064
Non.inflammatory.Macrophages	TPPP3	0.944185	1.071021	2.736525	0.007505	-2.60676	0.106576	0.125964
Non.inflammatory.Macrophages	MT1	0.490245	6.314344	2.73648	0.007506	-3.61327	0.077537	0.108864
Non.inflammatory.Macrophages	SOX6	1.102394	1.905309	2.736364	0.007508	-2.59451	0.101366	0.123082
Non.inflammatory.Macrophages	TMEM86B	-0.50786	3.467162	-2.73589	0.007518	-2.76034	0.092257	0.117943
Non.inflammatory.Macrophages	RGS1	0.427303	5.563816	2.734452	0.007548	-3.51088	0.081439	0.111449
Non.inflammatory.Macrophages	SMIM14	-0.21699	7.74729	-2.73371	0.007564	-3.50981	0.071301	0.104992

Non.inflammatory.Macrophages	PHACTR4	0.266171	5.677431	2.733406	0.00757	-3.21188	0.08094	0.111233
Non.inflammatory.Macrophages	SPPL2A	0.297483	6.600738	2.730356	0.007635	-3.52654	0.077066	0.108967
Non.inflammatory.Macrophages	CDK8	0.407104	6.559883	2.72831	0.007679	-3.47358	0.077619	0.109409
Non.inflammatory.Macrophages	ADCY4	-0.69117	1.413611	-2.72666	0.007715	-2.83817	0.106428	0.126595
Non.inflammatory.Macrophages	SELENOK	-0.21748	7.584969	-2.72616	0.007725	-3.62657	0.073116	0.10671
Non.inflammatory.Macrophages	RNF103	0.330161	4.305165	2.726066	0.007727	-3.09506	0.089351	0.116922
Non.inflammatory.Macrophages	AFF4	-0.25397	7.842009	-2.7256	0.007737	-3.62098	0.071988	0.106021
Non.inflammatory.Macrophages	SLCO4A1	-1.4274	3.403213	-2.72186	0.007818	-2.50188	0.095295	0.120782
Non.inflammatory.Macrophages	LY6A	1.057581	4.339309	2.717937	0.007904	-3.13105	0.090919	0.118659
Non.inflammatory.Macrophages	PSEN2	0.273359	5.03423	2.712551	0.008024	-3.24266	0.088342	0.117411
Non.inflammatory.Macrophages	1700027J0	-1.45248	2.481407	-2.71229	0.008029	-2.52628	0.103154	0.126124
Non.inflammatory.Macrophages	TOP1	-0.23293	8.354587	-2.7117	0.008043	-3.78015	0.072129	0.10711
Non.inflammatory.Macrophages	RC3H2	0.244703	5.728871	2.711168	0.008055	-3.30605	0.08476	0.115348
Non.inflammatory.Macrophages	GM2396	1.121981	-0.85737	2.710059	0.00808	-2.52952	0.115225	0.138807
Non.inflammatory.Macrophages	GLRX5	0.25266	6.17176	2.708513	0.008114	-3.45555	0.082931	0.114443

Non.inflammatory.Macrophages	DMAC1	-0.281	4.611093	-2.70527	0.008188	-3.27486	0.09195	0.120091
Non.inflammatory.Macrophages	PYCARD	0.296414	5.876191	2.70343	0.00823	-3.4289	0.085464	0.116323
Non.inflammatory.Macrophages	PRDX5	0.383492	7.635313	2.702522	0.00825	-3.79906	0.076747	0.110802
Non.inflammatory.Macrophages	WDR86	-0.98097	0.234245	-2.70241	0.008253	-2.54427	0.117018	0.136135
Non.inflammatory.Macrophages	HGFAC	-1.14268	1.432763	-2.70223	0.008257	-2.55536	0.111929	0.131722
Non.inflammatory.Macrophages	MAPKAPK2	0.250595	7.207807	2.701286	0.008279	-3.65268	0.078843	0.112326
Non.inflammatory.Macrophages	SNAPC3	0.382082	4.825524	2.701215	0.00828	-3.0923	0.091209	0.120029
Non.inflammatory.Macrophages	HES1	-0.40535	5.161335	-2.70038	0.0083	-3.34782	0.089474	0.119021
Non.inflammatory.Macrophages	SMCR8	-0.42156	3.28105	-2.69848	0.008343	-3.01754	0.100741	0.125886
Non.inflammatory.Macrophages	GM27241	-1.34899	2.603068	-2.69676	0.008383	-2.56009	0.105352	0.128624
Non.inflammatory.Macrophages	RAMP3	1.132801	0.947281	2.695543	0.008412	-2.59163	0.116614	0.134954
Non.inflammatory.Macrophages	NR1H4	-1.06388	1.386536	-2.69521	0.008419	-2.59075	0.113612	0.133395
Non.inflammatory.Macrophages	PTGES	1.007059	0.477516	2.694837	0.008428	-2.69199	0.118448	0.136878
Non.inflammatory.Macrophages	CLPX	0.279857	5.328169	2.694324	0.00844	-3.27986	0.089505	0.119689
Non.inflammatory.Macrophages	FGA	-0.55428	5.727993	-2.6916	0.008504	-3.32057	0.087905	0.118908

Non.inflammatory.Macrophages	TOB1	-0.32841	5.050855	-2.69127	0.008512	-3.27323	0.091616	0.121215
Non.inflammatory.Macrophages	CLCN6	-0.51814	3.649755	-2.69057	0.008528	-2.90832	0.099861	0.126146
Non.inflammatory.Macrophages	MYB	-0.82117	5.137938	-2.68905	0.008564	-2.75018	0.091432	0.121376
Non.inflammatory.Macrophages	C8B	-1.30189	0.571894	-2.68895	0.008566	-2.58139	0.119398	0.137813
Non.inflammatory.Macrophages	MYO1B	0.776304	2.148687	2.687449	0.008602	-2.78411	0.109823	0.132251
Non.inflammatory.Macrophages	DBP	-0.82326	2.187718	-2.68734	0.008605	-2.71684	0.109565	0.132127
Non.inflammatory.Macrophages	ASGR1	-0.74239	3.372794	-2.68691	0.008615	-2.87032	0.101987	0.127892
Non.inflammatory.Macrophages	RHOA	0.133034	9.258828	2.686787	0.008618	-3.94621	0.071156	0.108579
Non.inflammatory.Macrophages	KLF2	-0.46932	8.937753	-2.68537	0.008652	-3.90907	0.072718	0.109732
Non.inflammatory.Macrophages	SLC17A2	-1.27242	1.156237	-2.68533	0.008653	-2.58141	0.116807	0.136262
Non.inflammatory.Macrophages	DGKH	0.453651	4.909539	2.682408	0.008723	-3.13062	0.093729	0.123426
Non.inflammatory.Macrophages	PIK3C2B	-1.01753	2.667746	-2.68189	0.008735	-2.61431	0.107441	0.131493
Non.inflammatory.Macrophages	ID2	-0.25179	6.76154	-2.6798	0.008786	-3.88472	0.084127	0.117744
Non.inflammatory.Macrophages	BRI3BP	0.426243	4.756436	2.679298	0.008798	-3.05862	0.095131	0.124607
Non.inflammatory.Macrophages	C920021L1	-0.58141	2.268205	-2.67562	0.008887	-2.77972	0.111642	0.134566

Non.inflammatory.Macrophages	CYP2C23	-1.30258	0.860959	-2.67291	0.008954	-2.60711	0.121847	0.140613
Non.inflammatory.Macrophages	ZSCAN25	0.843734	1.792602	2.672793	0.008957	-2.66567	0.115467	0.137039
Non.inflammatory.Macrophages	TIMM23	0.199048	6.942518	2.67244	0.008966	-3.62715	0.084415	0.118799
Non.inflammatory.Macrophages	PLPBP	-0.29771	4.145683	-2.67232	0.008969	-3.2677	0.10014	0.128435
Non.inflammatory.Macrophages	SEH1L	0.254702	5.334868	2.670484	0.009014	-3.3285	0.093462	0.124591
Non.inflammatory.Macrophages	WDR44	0.3394	4.70447	2.670326	0.009018	-3.29807	0.097126	0.126819
Non.inflammatory.Macrophages	LGALS4	-0.77077	2.563012	-2.6692	0.009046	-2.74816	0.110839	0.134783
Non.inflammatory.Macrophages	PTPN1	0.253801	7.078745	2.667512	0.009088	-3.73078	0.084484	0.1192
Non.inflammatory.Macrophages	DCAF10	0.281348	5.193751	2.666927	0.009103	-3.31639	0.094868	0.125763
Non.inflammatory.Macrophages	FAAH	-1.09539	1.451163	-2.6662	0.009121	-2.62464	0.119136	0.13973
Non.inflammatory.Macrophages	GM20559	-0.38135	4.344461	-2.66556	0.009137	-3.30026	0.100081	0.128999
Non.inflammatory.Macrophages	BCL11B	-2.06793	1.080616	-2.66232	0.009219	-2.66286	0.122851	0.142063
Non.inflammatory.Macrophages	CYP1A2	-1.18214	1.633956	-2.66189	0.009229	-2.64436	0.118837	0.140036
Non.inflammatory.Macrophages	IGFBP6	1.5075	-0.02144	2.661649	0.009236	-2.6336	0.12575	0.146679
Non.inflammatory.Macrophages	DHPS	-0.30138	4.486343	-2.66107	0.00925	-3.24521	0.100048	0.12954

Non.inflammatory.Macrophages	ITIH4	-0.66726	3.988196	-2.65875	0.009309	-3.09371	0.103682	0.131838
Non.inflammatory.Macrophages	PFN1	0.186523	10.31807	2.658205	0.009323	-4.20789	0.070392	0.110627
Non.inflammatory.Macrophages	2410018L1	-1.49456	0.727626	-2.65749	0.009342	-2.69289	0.125774	0.144505
Non.inflammatory.Macrophages	LIN54	0.335804	6.14118	2.655189	0.009401	-3.51858	0.091538	0.124728
Non.inflammatory.Macrophages	CAST	0.225669	6.266263	2.654046	0.00943	-3.55203	0.091034	0.124577
Non.inflammatory.Macrophages	PLCXD2	-0.66632	3.46805	-2.65357	0.009443	-2.84817	0.107996	0.134765
Non.inflammatory.Macrophages	COPZ1	0.215457	6.366584	2.651962	0.009484	-3.58884	0.090696	0.124606
Non.inflammatory.Macrophages	TXNDC11	0.2952	5.714713	2.651867	0.009487	-3.39929	0.094386	0.126888
Non.inflammatory.Macrophages	RAB19	0.784125	2.561986	2.651412	0.009499	-2.7654	0.114329	0.138629
Non.inflammatory.Macrophages	SERPINA1A	-0.74213	8.012506	-2.65134	0.009501	-3.77303	0.08199	0.119115
Non.inflammatory.Macrophages	PTPRCAP	-0.63963	5.807512	-2.6502	0.00953	-2.9472	0.094052	0.126882
Non.inflammatory.Macrophages	MBD1	0.327615	4.580853	2.649529	0.009548	-3.28526	0.101444	0.13138
Non.inflammatory.Macrophages	GM4221	-0.7141	1.846721	-2.64887	0.009565	-2.79514	0.119719	0.141871
Non.inflammatory.Macrophages	NID2	-0.64178	2.19946	-2.64882	0.009566	-2.97692	0.117202	0.140486
Non.inflammatory.Macrophages	ST3GAL6	-0.40176	5.092875	-2.64812	0.009585	-3.26831	0.098371	0.129741

Non.inflammatory.Macrophages	NR5A2	-1.09607	1.145394	-2.64761	0.009598	-2.66159	0.124928	0.144906
Non.inflammatory.Macrophages	PTPN12	0.255757	6.049828	2.647567	0.009599	-3.70156	0.092785	0.126422
Non.inflammatory.Macrophages	MAP4K4	0.316515	7.459224	2.64652	0.009627	-3.71249	0.085269	0.121828
Non.inflammatory.Macrophages	GC	-0.73212	5.287073	-2.6454	0.009656	-3.38093	0.097597	0.129654
Non.inflammatory.Macrophages	HYOU1	-0.32581	4.380576	-2.64311	0.009717	-3.2765	0.103689	0.133476
Non.inflammatory.Macrophages	ZC3H12C	0.589167	4.431325	2.638636	0.009836	-3.29459	0.104452	0.134118
Non.inflammatory.Macrophages	RHOV	2.013287	-0.80702	2.63857	0.009838	-2.75066	0.130923	0.155138
Non.inflammatory.Macrophages	GM26787	-1.22149	1.300668	-2.63814	0.00985	-2.68997	0.126252	0.146373
Non.inflammatory.Macrophages	SORT1	0.447781	4.067523	2.636922	0.009882	-3.21721	0.107061	0.135755
Non.inflammatory.Macrophages	KIF5A	-1.01781	0.429339	-2.63553	0.00992	-2.73049	0.131364	0.150557
Non.inflammatory.Macrophages	B4GALNT1	0.576831	4.892601	2.635525	0.00992	-2.89696	0.101899	0.132982
Non.inflammatory.Macrophages	BST1	0.946368	3.315218	2.634997	0.009934	-2.81295	0.112157	0.138951
Non.inflammatory.Macrophages	MTHFR	-0.31192	3.640415	-2.63484	0.009939	-3.31608	0.109966	0.137699
Non.inflammatory.Macrophages	MTTP	-0.66103	2.566189	-2.63461	0.009945	-2.90915	0.117358	0.141905
Non.inflammatory.Macrophages	HDGFL2	0.318308	4.979057	2.634467	0.009949	-3.28205	0.101364	0.132689

Non.inflammatory.Macrophages	GM29707	-0.79994	-0.46354	-2.63297	0.00999	-2.74019	0.131773	0.15475
Non.inflammatory.Macrophages	AU020206	0.341324	5.320744	2.632207	0.01001	-3.38058	0.099691	0.13191
Non.inflammatory.Macrophages	KCNJ10	-0.74062	-0.1202	-2.63124	0.010037	-2.77831	0.132022	0.153601
Non.inflammatory.Macrophages	GPATCH8	0.212886	7.002079	2.631204	0.010038	-3.66523	0.090016	0.125996
Non.inflammatory.Macrophages	TVP23B	-0.31619	4.042523	-2.63043	0.010059	-3.27751	0.107869	0.136986
Non.inflammatory.Macrophages	FGFR1OP2	0.24341	6.180589	2.630428	0.010059	-3.57815	0.094677	0.12907
Non.inflammatory.Macrophages	GPCPD1	0.248341	7.329375	2.629956	0.010072	-3.73471	0.088271	0.125131
Non.inflammatory.Macrophages	SBK1	-0.83673	3.374185	-2.62935	0.010089	-2.74221	0.112442	0.139884
Non.inflammatory.Macrophages	DPY19L1	0.309429	4.584338	2.629017	0.010098	-3.45962	0.104468	0.135309
Non.inflammatory.Macrophages	APOH	-0.68118	4.983199	-2.62866	0.010108	-3.35743	0.10196	0.133883
Non.inflammatory.Macrophages	KLRK1	-0.57075	3.149025	-2.62787	0.010129	-3.32286	0.114031	0.140935
Non.inflammatory.Macrophages	4833407H1	1.090384	2.098821	2.627485	0.01014	-2.71186	0.121503	0.145242
Non.inflammatory.Macrophages	PQLC1	-0.26053	4.395337	-2.62723	0.010147	-3.42251	0.105719	0.136262
Non.inflammatory.Macrophages	SERPINB1A	1.010895	3.600711	2.627101	0.010151	-2.87622	0.110951	0.139315
Non.inflammatory.Macrophages	GM26737	-1.02856	0.478605	-2.62625	0.010174	-2.72566	0.132398	0.152157

Non.inflammatory.Macrophages	PTBP3	0.165245	8.46802	2.625402	0.010198	-3.98894	0.082618	0.121973
Non.inflammatory.Macrophages	ELOB	0.200375	8.913307	2.624726	0.010216	-4.04216	0.08046	0.120529
Non.inflammatory.Macrophages	RMND5A	0.235292	6.766957	2.622993	0.010265	-3.71396	0.092133	0.128232
Non.inflammatory.Macrophages	ADAM9	0.27852	5.178783	2.620596	0.010332	-3.56723	0.10195	0.134641
Non.inflammatory.Macrophages	PDE2A	-0.43495	6.050057	-2.62006	0.010347	-3.50211	0.096664	0.131524
Non.inflammatory.Macrophages	SLFN2	0.341548	6.335056	2.61994	0.01035	-3.82181	0.094993	0.130508
Non.inflammatory.Macrophages	MRPL57	0.252257	5.995401	2.619596	0.01036	-3.611	0.096988	0.13179
Non.inflammatory.Macrophages	DMTF1	0.288047	4.947065	2.6194	0.010365	-3.35057	0.103401	0.135725
Non.inflammatory.Macrophages	KLHL2	0.325173	5.570335	2.619365	0.010366	-3.44223	0.099541	0.13339
Non.inflammatory.Macrophages	NDUFA4	-0.26278	8.596899	-2.61792	0.010407	-4.00049	0.082934	0.122921
Non.inflammatory.Macrophages	PLA2G4A	0.374192	3.294743	2.616639	0.010443	-3.50815	0.114961	0.142691
Non.inflammatory.Macrophages	ZFP445	0.271807	4.93802	2.61303	0.010546	-3.43755	0.10483	0.137171
Non.inflammatory.Macrophages	IL15RA	0.621995	2.38833	2.612749	0.010554	-3.02873	0.122375	0.147308
Non.inflammatory.Macrophages	HPS4	-0.36461	4.16681	-2.6125	0.010561	-3.4069	0.109871	0.140246
Non.inflammatory.Macrophages	SIAH2	-0.28978	4.793136	-2.61223	0.010569	-3.41692	0.10576	0.137884

Non.inflammatory.Macrophages	PWWP2A	0.322404	5.141602	2.612063	0.010574	-3.39818	0.103536	0.136592
Non.inflammatory.Macrophages	USP14	0.217884	5.793901	2.609781	0.010639	-3.62413	0.099802	0.134626
Non.inflammatory.Macrophages	CNOT7	0.229572	5.288607	2.609645	0.010643	-3.47148	0.10293	0.136534
Non.inflammatory.Macrophages	HEXIM1	-0.32395	5.48831	-2.60963	0.010644	-3.57265	0.101683	0.135777
Non.inflammatory.Macrophages	NDUFB9	0.220774	7.069533	2.609521	0.010647	-3.82832	0.092304	0.129929
Non.inflammatory.Macrophages	GM48099	0.828653	2.773722	2.608389	0.01068	-3.16796	0.120186	0.14673
Non.inflammatory.Macrophages	MID1IP1	-0.43817	3.917342	-2.60799	0.010691	-3.23719	0.11215	0.1422
Non.inflammatory.Macrophages	ARIH2	0.214975	7.527213	2.606665	0.01073	-3.91197	0.090149	0.128873
Non.inflammatory.Macrophages	B4GALT4	-0.57821	1.774117	-2.60649	0.010735	-2.98526	0.127953	0.151299
Non.inflammatory.Macrophages	POLR2A	0.200999	6.200996	2.605624	0.01076	-3.67469	0.097918	0.133873
Non.inflammatory.Macrophages	VPS53	0.251466	4.462382	2.60522	0.010772	-3.39088	0.1089	0.140624
Non.inflammatory.Macrophages	PAQR5	-1.3939	0.502008	-2.60374	0.010815	-2.7555	0.137135	0.157317
Non.inflammatory.Macrophages	LARP4B	0.188833	7.283403	2.601955	0.010867	-3.8936	0.09229	0.130601
Non.inflammatory.Macrophages	SLC20A1	-0.31202	5.219384	-2.60154	0.010879	-3.60278	0.104733	0.138397
Non.inflammatory.Macrophages	APOB	-0.72426	4.836413	-2.59862	0.010965	-3.39053	0.107957	0.140375

Non.inflammatory.Macrophages	SORL1	0.675983	5.66068	2.597519	0.010998	-3.19705	0.102663	0.137334
Non.inflammatory.Macrophages	SETDB1	0.305356	4.832864	2.597351	0.011003	-3.46355	0.107984	0.140565
Non.inflammatory.Macrophages	1700001K1	-0.82112	1.052633	-2.59733	0.011004	-2.90281	0.135731	0.156151
Non.inflammatory.Macrophages	GM44710	-0.58733	2.000891	-2.59696	0.011015	-3.09333	0.128213	0.152173
Non.inflammatory.Macrophages	SLFN1	1.301222	2.335521	2.596887	0.011017	-2.94183	0.125652	0.150772
Non.inflammatory.Macrophages	DDX39B	-0.24484	7.251694	-2.5964	0.011031	-3.796	0.093169	0.131516
Non.inflammatory.Macrophages	GM29170	0.763556	1.532706	2.59598	0.011044	-2.81294	0.131954	0.154245
Non.inflammatory.Macrophages	MYO10	0.372885	5.227281	2.595237	0.011066	-3.6068	0.105593	0.139194
Non.inflammatory.Macrophages	ABCB1A	1.434299	2.010725	2.594454	0.01109	-2.78247	0.128487	0.152469
Non.inflammatory.Macrophages	PZP	-0.73417	4.39586	-2.5939	0.011106	-3.28428	0.1112	0.142819
Non.inflammatory.Macrophages	YOD1	0.467099	4.084266	2.593574	0.011116	-3.26352	0.113328	0.144169
Non.inflammatory.Macrophages	TAB2	0.194554	6.780443	2.593356	0.011122	-3.8355	0.096125	0.133789
Non.inflammatory.Macrophages	FAM189B	-0.66119	2.569109	-2.59313	0.011129	-3.00275	0.12423	0.150468
Non.inflammatory.Macrophages	RGS10	-0.24931	5.509055	-2.59226	0.011155	-3.72916	0.104048	0.138929
Non.inflammatory.Macrophages	PLEKHG2	-0.37482	4.157105	-2.59065	0.011204	-3.47004	0.113375	0.144604

Non.inflammatory.Macrophages	KATNBL1	0.262559	5.963648	2.588058	0.011283	-3.58512	0.102077	0.138131
Non.inflammatory.Macrophages	ITGAV	0.289672	6.550452	2.587743	0.011292	-3.88059	0.098477	0.135924
Non.inflammatory.Macrophages	FAM102A	0.348754	4.182226	2.587684	0.011294	-3.47766	0.113796	0.145188
Non.inflammatory.Macrophages	CD3D	-1.55005	2.678526	-2.58715	0.01131	-2.7861	0.124726	0.151531
Non.inflammatory.Macrophages	RBP1	-0.60809	3.578118	-2.58564	0.011356	-3.20949	0.118484	0.148119
Non.inflammatory.Macrophages	TPT1	0.152701	10.10831	2.584766	0.011383	-4.37994	0.079531	0.123668
Non.inflammatory.Macrophages	NCOA1	0.276637	7.07231	2.584547	0.01139	-3.8228	0.095834	0.134607
Non.inflammatory.Macrophages	TTLL3	-0.38179	4.027206	-2.58342	0.011424	-3.36611	0.115578	0.146767
Non.inflammatory.Macrophages	SLC18A1	-0.95593	0.497721	-2.58337	0.011426	-2.8807	0.141322	0.161901
Non.inflammatory.Macrophages	DYNC1LI2	0.309496	4.672528	2.582442	0.011454	-3.434	0.111236	0.144406
Non.inflammatory.Macrophages	ZFP131	-0.25587	5.815875	-2.58233	0.011458	-3.66309	0.103738	0.139885
Non.inflammatory.Macrophages	IFI27	0.480604	4.998783	2.581888	0.011471	-3.54988	0.109074	0.143165
Non.inflammatory.Macrophages	ACSS1	0.476698	3.946222	2.58154	0.011482	-3.25523	0.116297	0.147484
Non.inflammatory.Macrophages	TRA2B	-0.19181	7.832072	-2.58103	0.011498	-3.93686	0.091752	0.132487
Non.inflammatory.Macrophages	UBE2L6	0.461218	4.64931	2.580484	0.011515	-3.69976	0.11154	0.144774

Non.inflammatory.Macrophages	PDE4A	-0.40655	3.81993	-2.57859	0.011574	-3.54033	0.117805	0.148478
Non.inflammatory.Macrophages	GM50334	-1.26793	-0.42372	-2.57625	0.011647	-2.81001	0.143035	0.167675
Non.inflammatory.Macrophages	OSGEP	-0.262	4.907558	-2.57414	0.011713	-3.58593	0.111381	0.145023
Non.inflammatory.Macrophages	SETD6	0.753844	1.423905	2.573766	0.011725	-2.85986	0.137556	0.159821
Non.inflammatory.Macrophages	ALG8	-0.42879	3.226775	-2.57295	0.01175	-3.29074	0.123536	0.152212
Non.inflammatory.Macrophages	AI467606	0.803352	4.111903	2.572546	0.011763	-2.93558	0.117094	0.148621
Non.inflammatory.Macrophages	PROK2	1.911818	-0.61756	2.571451	0.011798	-2.82859	0.144206	0.169765
Non.inflammatory.Macrophages	LATS1	0.272766	4.725566	2.571191	0.011806	-3.50684	0.113006	0.146403
Non.inflammatory.Macrophages	ECHDC2	-1.08714	1.612612	-2.56929	0.011866	-2.85995	0.136977	0.160206
Non.inflammatory.Macrophages	SLTM	0.208921	6.63584	2.569092	0.011873	-3.78243	0.100941	0.139321
Non.inflammatory.Macrophages	APOA2	-0.6497	8.419445	-2.56813	0.011903	-4.07042	0.090552	0.132801
Non.inflammatory.Macrophages	ZBTB16	0.96255	2.808614	2.567936	0.011909	-2.98476	0.127547	0.155307
Non.inflammatory.Macrophages	PGGT1B	0.266744	4.818529	2.567786	0.011914	-3.57054	0.112889	0.146875
Non.inflammatory.Macrophages	PLCG2	-0.20636	7.02313	-2.56585	0.011976	-3.88164	0.099078	0.138551
Non.inflammatory.Macrophages	SAYSD1	-0.34547	3.323883	-2.56548	0.011988	-3.32692	0.12417	0.153677

Non.inflammatory.Macrophages	GM867	1.399842	-0.30876	2.563538	0.01205	-2.83751	0.146029	0.170577
Non.inflammatory.Macrophages	EIF2AK4	0.329463	4.653928	2.563486	0.012052	-3.56177	0.114935	0.148594
Non.inflammatory.Macrophages	ADGRG6	1.022059	0.969217	2.562808	0.012074	-2.98533	0.143749	0.164793
Non.inflammatory.Macrophages	CSGALNAC	0.283759	4.10884	2.562384	0.012088	-3.50705	0.11895	0.151137
Non.inflammatory.Macrophages	PPP1R14B	0.255121	6.546146	2.561037	0.012131	-3.79963	0.102784	0.141611
Non.inflammatory.Macrophages	UBC	-0.23718	8.132317	-2.56013	0.012161	-4.15125	0.093399	0.135627
Non.inflammatory.Macrophages	RBM42	-0.21488	6.063078	-2.55979	0.012172	-3.70574	0.106032	0.143718
Non.inflammatory.Macrophages	ACO2	0.203289	6.116578	2.559311	0.012188	-3.78474	0.105728	0.143628
Non.inflammatory.Macrophages	GM17231	-0.7027	2.734245	-2.55804	0.012229	-3.06935	0.130222	0.158074
Non.inflammatory.Macrophages	MUC13	-1.01033	1.034673	-2.55656	0.012278	-2.91678	0.144695	0.166188
Non.inflammatory.Macrophages	BLOC1S1	0.260707	6.24963	2.55541	0.012315	-3.85087	0.105687	0.143968
Non.inflammatory.Macrophages	GM31718	-0.40773	3.725877	-2.55461	0.012342	-3.38176	0.123425	0.154633
Non.inflammatory.Macrophages	CAV2	-0.61118	2.771749	-2.55423	0.012354	-3.07226	0.130791	0.158874
Non.inflammatory.Macrophages	INPP5K	-0.25792	5.371477	-2.55287	0.012399	-3.67398	0.111976	0.147976
Non.inflammatory.Macrophages	FGG	-0.51603	6.166651	-2.552	0.012428	-3.73528	0.106791	0.144903

Non.inflammatory.Macrophages	XLR	-0.54624	2.01867	-2.55176	0.012436	-3.41584	0.137412	0.162634
Non.inflammatory.Macrophages	RNASE6	0.427316	4.491549	2.551036	0.01246	-3.54714	0.118292	0.151917
Non.inflammatory.Macrophages	TEX261	0.236705	5.229001	2.550878	0.012465	-3.67975	0.113092	0.148834
Non.inflammatory.Macrophages	WWP2	-0.22181	6.537142	-2.55076	0.012469	-3.83208	0.104398	0.143511
Non.inflammatory.Macrophages	CLDN1	-1.48708	1.493829	-2.55036	0.012482	-2.86413	0.14185	0.16525
Non.inflammatory.Macrophages	FRS2	0.227077	5.84525	2.548667	0.012539	-3.72512	0.109259	0.146791
Non.inflammatory.Macrophages	FAM20B	0.379711	3.958304	2.548581	0.012542	-3.37552	0.122581	0.154708
Non.inflammatory.Macrophages	PLXDC1	-0.6741	3.94302	-2.54599	0.012629	-3.25625	0.123436	0.155212
Non.inflammatory.Macrophages	OSBPL9	0.188332	7.511715	2.544069	0.012693	-3.9819	0.099675	0.140821
Non.inflammatory.Macrophages	ZFP606	0.541085	2.487995	2.543253	0.012721	-3.07717	0.135562	0.162106
Non.inflammatory.Macrophages	EIF2AK3	-0.33627	6.399228	-2.54231	0.012753	-3.66676	0.107021	0.145535
Non.inflammatory.Macrophages	GM17056	0.571445	2.866748	2.539919	0.012834	-3.33175	0.133357	0.161092
Non.inflammatory.Macrophages	NRG2	0.992889	1.685011	2.539823	0.012838	-2.9503	0.143212	0.166481
Non.inflammatory.Macrophages	ICOSL	0.673828	1.327034	2.539204	0.012859	-3.06636	0.146352	0.168212
Non.inflammatory.Macrophages	AQR	0.239667	4.812338	2.538977	0.012867	-3.58613	0.118514	0.152697

Non.inflammatory.Macrophages	B4GALT1	0.209697	7.137524	2.538769	0.012874	-3.97954	0.102806	0.143185
Non.inflammatory.Macrophages	NOD2	0.489897	1.576786	2.536432	0.012954	-3.40971	0.144945	0.167617
Non.inflammatory.Macrophages	GM38973	-1.06591	1.613034	-2.53503	0.013002	-2.91285	0.144856	0.167863
Non.inflammatory.Macrophages	SREBF2	-0.30407	5.9605	-2.53502	0.013003	-3.71463	0.111255	0.148736
Non.inflammatory.Macrophages	WSB1	-0.20473	6.461454	-2.53486	0.013008	-3.97618	0.107896	0.146696
Non.inflammatory.Macrophages	STARD9	0.314297	3.529605	2.533823	0.013044	-3.68619	0.129263	0.159402
Non.inflammatory.Macrophages	STARD4	-0.4538	3.526928	-2.53302	0.013072	-3.28457	0.129445	0.159559
Non.inflammatory.Macrophages	AP2S1	0.195257	6.932961	2.532677	0.013084	-4.02318	0.10516	0.145164
Non.inflammatory.Macrophages	TRIM65	-0.43594	3.22435	-2.5321	0.013104	-3.32464	0.131936	0.161129
Non.inflammatory.Macrophages	TMC8	-0.39257	3.820781	-2.53093	0.013145	-3.41311	0.127533	0.158752
Non.inflammatory.Macrophages	F2	-0.74279	4.237274	-2.53032	0.013166	-3.4075	0.124434	0.157135
Non.inflammatory.Macrophages	COX7A1	0.79031	0.986976	2.529731	0.013187	-2.97212	0.151431	0.172114
Non.inflammatory.Macrophages	TMEM29	-0.31919	4.706351	-2.52938	0.013199	-3.53749	0.12093	0.155238
Non.inflammatory.Macrophages	TRAPPC8	0.233868	6.225642	2.529077	0.01321	-3.82608	0.110214	0.148808
Non.inflammatory.Macrophages	NADK	-0.24122	6.266885	-2.5286	0.013226	-3.9639	0.109936	0.148678

Non.inflammatory.Macrophages	JUNOS	-0.69284	1.820722	-2.52846	0.013231	-3.12742	0.144036	0.168261
Non.inflammatory.Macrophages	MKNK1	-0.2822	4.508378	-2.52805	0.013246	-3.59513	0.122397	0.156187
Non.inflammatory.Macrophages	LCP1	0.226647	8.858643	2.527922	0.01325	-4.31538	0.093776	0.138402
Non.inflammatory.Macrophages	PTTG1	-0.24584	5.78	-2.52767	0.013259	-3.8839	0.113259	0.150845
Non.inflammatory.Macrophages	CREB5	0.580971	2.17534	2.52741	0.013268	-3.48965	0.140991	0.166834
Non.inflammatory.Macrophages	SWT1	0.27391	5.244266	2.526654	0.013295	-3.65945	0.117111	0.153282
Non.inflammatory.Macrophages	ZDHHC6	0.303659	4.609891	2.526193	0.013311	-3.57911	0.12173	0.156118
Non.inflammatory.Macrophages	CAMSAP2	0.310677	4.496866	2.526171	0.013312	-3.59549	0.122571	0.15661
Non.inflammatory.Macrophages	MAFB	0.336171	4.60884	2.525517	0.013335	-4.02758	0.121844	0.15633
Non.inflammatory.Macrophages	ZMYND8	0.223412	6.108671	2.525094	0.01335	-3.87124	0.111212	0.15002
Non.inflammatory.Macrophages	APOBEC1	0.315344	6.087599	2.524758	0.013362	-4.05956	0.111359	0.150142
Non.inflammatory.Macrophages	CASC3	0.266006	5.292913	2.524148	0.013383	-3.726	0.116976	0.153634
Non.inflammatory.Macrophages	FGB	-0.49933	5.797972	-2.52336	0.013411	-3.75037	0.113422	0.151589
Non.inflammatory.Macrophages	RGS5	1.083256	1.107497	2.523015	0.013423	-3.05628	0.150723	0.172834
Non.inflammatory.Macrophages	SYK	0.246217	8.336181	2.522932	0.013426	-4.23532	0.097081	0.141341

Non.inflammatory.Macrophages	ABHD2	-0.29915	5.471153	-2.52291	0.013427	-3.58631	0.11571	0.153075
Non.inflammatory.Macrophages	9530062K(	-0.89099	0.793704	-2.52179	0.013467	-2.9879	0.153256	0.174672
Non.inflammatory.Macrophages	DUSP18	-0.89422	0.621447	-2.52103	0.013494	-3.03612	0.154236	0.175715
Non.inflammatory.Macrophages	TPST2	-0.24028	5.593782	-2.5209	0.013499	-3.85667	0.115014	0.153017
Non.inflammatory.Macrophages	KCNIP4	1.234674	0.608323	2.520774	0.013503	-2.98398	0.154288	0.175779
Non.inflammatory.Macrophages	RNF10	0.238886	6.51711	2.520752	0.013504	-3.91907	0.108698	0.149133
Non.inflammatory.Macrophages	H2-Q6	-0.95491	3.115257	-2.52041	0.013516	-3.29546	0.133745	0.16399
Non.inflammatory.Macrophages	ADARB1	0.637776	3.009129	2.518628	0.01358	-3.22204	0.135131	0.164725
Non.inflammatory.Macrophages	PLG	-0.73601	4.004656	-2.51807	0.0136	-3.39358	0.12729	0.160308
Non.inflammatory.Macrophages	ZFP36L2	-0.27684	8.376464	-2.51645	0.013659	-4.22306	0.09776	0.142297
Non.inflammatory.Macrophages	SLC2A12	0.985204	1.619007	2.514551	0.013727	-2.95438	0.148171	0.172283
Non.inflammatory.Macrophages	TBPL1	0.288251	4.928003	2.512898	0.013787	-3.59781	0.12168	0.157585
Non.inflammatory.Macrophages	PYM1	0.276226	4.692796	2.512358	0.013807	-3.59837	0.123493	0.158761
Non.inflammatory.Macrophages	SPATA13	0.394427	5.147731	2.512089	0.013817	-3.60129	0.120114	0.156832
Non.inflammatory.Macrophages	ARHGEF3	0.40425	5.916073	2.510005	0.013893	-3.94047	0.115134	0.15405

Non.inflammatory.Macrophages	1300017J0	-0.68447	3.006144	-2.50967	0.013906	-3.18994	0.137447	0.16713
Non.inflammatory.Macrophages	CUX2	-1.02948	1.039079	-2.50918	0.013923	-2.95589	0.15474	0.176702
Non.inflammatory.Macrophages	CLP1	-0.32221	4.608479	-2.50908	0.013927	-3.55931	0.124701	0.160025
Non.inflammatory.Macrophages	F12	-1.1653	1.691224	-2.50683	0.01401	-2.99039	0.149559	0.174122
Non.inflammatory.Macrophages	MMP9	-0.77972	1.381978	-2.5057	0.014052	-3.26811	0.152685	0.17589
Non.inflammatory.Macrophages	ALB	-0.76757	9.441682	-2.50539	0.014063	-4.3593	0.093411	0.140609
Non.inflammatory.Macrophages	CSF3	1.188619	-0.096	2.504039	0.014113	-3.05658	0.159576	0.183721
Non.inflammatory.Macrophages	WASHC2	-0.27236	5.655214	-2.50341	0.014137	-3.90955	0.118249	0.156719
Non.inflammatory.Macrophages	UGT2B36	-0.93249	2.198922	-2.50254	0.014169	-3.05851	0.146091	0.172849
Non.inflammatory.Macrophages	MCM10	-0.57766	3.112366	-2.5009	0.014231	-3.27706	0.138726	0.168899
Non.inflammatory.Macrophages	4930505N2	-0.83464	1.214516	-2.49998	0.014265	-3.02313	0.155791	0.178183
Non.inflammatory.Macrophages	GATM	0.316732	4.530012	2.498937	0.014304	-3.91875	0.127726	0.162646
Non.inflammatory.Macrophages	RANBP10	0.286292	5.698692	2.498624	0.014316	-3.85231	0.118936	0.15744
Non.inflammatory.Macrophages	GBP2	0.730372	4.147241	2.497723	0.01435	-3.85907	0.130938	0.164507
Non.inflammatory.Macrophages	GCH1	0.329894	5.663529	2.497314	0.014366	-3.8637	0.119404	0.157801

Non.inflammatory.Macrophages	ARMH2	1.684351	0.301427	2.496368	0.014401	-3.01339	0.161615	0.183516
Non.inflammatory.Macrophages	GIGYF2	0.262361	5.464029	2.495519	0.014434	-3.80413	0.121239	0.159205
Non.inflammatory.Macrophages	SLCO3A1	0.821632	4.38978	2.493446	0.014512	-3.23984	0.130047	0.164531
Non.inflammatory.Macrophages	CALM3	0.214573	7.10732	2.492869	0.014534	-4.08446	0.110223	0.15259
Non.inflammatory.Macrophages	BC031181	0.207841	5.703047	2.491933	0.01457	-3.87794	0.120186	0.15882
Non.inflammatory.Macrophages	SNX30	-0.28412	5.755716	-2.49184	0.014574	-3.90761	0.119799	0.158587
Non.inflammatory.Macrophages	CORO1C	0.230051	6.202322	2.491709	0.014579	-3.92827	0.116571	0.156627
Non.inflammatory.Macrophages	GM11523	-1.15687	-0.95948	-2.49137	0.014592	-3.01322	0.162809	0.191198
Non.inflammatory.Macrophages	MEF2C	0.372676	7.538963	2.490802	0.014613	-3.92729	0.107452	0.151089
Non.inflammatory.Macrophages	METTL7B	-1.11979	2.263028	-2.49045	0.014627	-3.10324	0.148192	0.175069
Non.inflammatory.Macrophages	PDS5A	0.195059	7.411745	2.490085	0.014641	-4.06892	0.108294	0.151782
Non.inflammatory.Macrophages	GM50020	-1.00521	-0.11132	-2.48997	0.014645	-2.98895	0.162869	0.187113
Non.inflammatory.Macrophages	CRY1	-0.29568	5.502006	-2.48959	0.01466	-3.79021	0.121728	0.16007
Non.inflammatory.Macrophages	TSC22D1	0.334351	4.872446	2.489337	0.01467	-3.75547	0.126496	0.162901
Non.inflammatory.Macrophages	IQCN	-0.79603	1.791223	-2.48896	0.014684	-3.17168	0.152493	0.17755

Non.inflammatory.Macrophages	RAG2	-1.93276	0.168525	-2.48756	0.014739	-3.03254	0.163201	0.186101
Non.inflammatory.Macrophages	CXCL12	-0.60658	2.838235	-2.48714	0.014755	-3.3151	0.143423	0.172937
Non.inflammatory.Macrophages	ADGRE5	-0.21527	7.153842	-2.48708	0.014757	-4.18497	0.110244	0.153366
Non.inflammatory.Macrophages	SNX8	-0.36625	5.468943	-2.48701	0.01476	-3.94104	0.12222	0.160738
Non.inflammatory.Macrophages	AGBL1	-1.10467	4.663698	-2.48657	0.014777	-3.21442	0.128392	0.164479
Non.inflammatory.Macrophages	VPS54	0.257549	6.506686	2.486222	0.01479	-3.97292	0.11472	0.156303
Non.inflammatory.Macrophages	MOSPD2	-0.26327	4.445481	-2.48602	0.014798	-3.89058	0.13011	0.165578
Non.inflammatory.Macrophages	NFYB	-0.34426	5.103926	-2.48559	0.014815	-3.60116	0.125031	0.162649
Non.inflammatory.Macrophages	RAB11FIP1	0.465951	4.829945	2.485221	0.014829	-3.74637	0.127146	0.163992
Non.inflammatory.Macrophages	ZBTB2	-0.25244	5.709287	-2.48495	0.01484	-3.83109	0.120501	0.160077
Non.inflammatory.Macrophages	NRAS	0.207276	6.075126	2.482929	0.014918	-3.96609	0.118147	0.158939
Non.inflammatory.Macrophages	ANXA7	0.244456	5.409471	2.482904	0.014919	-3.84096	0.123053	0.161912
Non.inflammatory.Macrophages	HRH2	1.027197	1.160498	2.482887	0.01492	-3.01576	0.159178	0.182229
Non.inflammatory.Macrophages	FTSJ1	-0.44405	3.078297	-2.4827	0.014927	-3.25689	0.141801	0.172766
Non.inflammatory.Macrophages	FAU	0.123733	11.4506	2.481649	0.014968	-4.77544	0.085072	0.136994

Non.inflammatory.Macrophages	NUFIP2	-0.20846	7.215419	-2.48145	0.014976	-4.15606	0.11036	0.154175
Non.inflammatory.Macrophages	SNF8	0.226409	5.731869	2.480536	0.015012	-3.88716	0.121044	0.160884
Non.inflammatory.Macrophages	TTC17	0.258282	5.324345	2.480228	0.015024	-3.79041	0.124095	0.16272
Non.inflammatory.Macrophages	KCNQ1	-0.83121	1.396097	-2.47931	0.01506	-3.17707	0.157704	0.181817
Non.inflammatory.Macrophages	UBR3	0.237132	6.575859	2.478426	0.015095	-4.00696	0.115263	0.157691
Non.inflammatory.Macrophages	SPATA5	-0.27859	5.905372	-2.47827	0.015101	-3.94872	0.120091	0.160663
Non.inflammatory.Macrophages	RHAG	1.297446	-0.3642	2.47781	0.01512	-3.02155	0.164758	0.191421
Non.inflammatory.Macrophages	CCR7	-1.12932	4.535564	-2.47652	0.015171	-3.17317	0.130883	0.167435
Non.inflammatory.Macrophages	CD19	-0.94505	3.122136	-2.47641	0.015175	-3.06612	0.142617	0.174161
Non.inflammatory.Macrophages	ARNTL	0.395379	5.38742	2.476095	0.015188	-3.7483	0.124259	0.163588
Non.inflammatory.Macrophages	DGKA	0.478719	5.023185	2.475433	0.015214	-3.52068	0.127171	0.165417
Non.inflammatory.Macrophages	UBA6	0.264093	5.172539	2.474418	0.015254	-3.81895	0.126251	0.164957
Non.inflammatory.Macrophages	TSPAN13	0.283302	6.684078	2.47346	0.015293	-3.95972	0.1153	0.158396
Non.inflammatory.Macrophages	CSNK1G3	0.224937	6.624241	2.472823	0.015318	-3.97708	0.115824	0.158833
Non.inflammatory.Macrophages	MEIG1	-1.13307	-0.47443	-2.47186	0.015356	-3.02021	0.166268	0.193772

Non.inflammatory.Macrophages	MTMR1	0.313851	4.622377	2.471528	0.01537	-3.66499	0.131127	0.168278
Non.inflammatory.Macrophages	GMEB2	-0.23323	5.764218	-2.47092	0.015394	-3.88142	0.122309	0.163181
Non.inflammatory.Macrophages	D130040H:	0.538623	2.397794	2.470889	0.015396	-3.32139	0.150082	0.179209
Non.inflammatory.Macrophages	SLC1A5	-0.37946	6.46392	-2.46971	0.015443	-3.72917	0.117452	0.160221
Non.inflammatory.Macrophages	MEF2D	-0.22698	7.288749	-2.46833	0.015499	-4.13856	0.111976	0.156795
Non.inflammatory.Macrophages	UTP18	-0.26157	5.338906	-2.46711	0.015548	-3.83294	0.126469	0.165831
Non.inflammatory.Macrophages	ADSS	0.223673	6.271707	2.466584	0.01557	-3.96003	0.119529	0.16168
Non.inflammatory.Macrophages	LCN2	1.364948	3.398879	2.464271	0.015664	-3.27653	0.14315	0.175808
Non.inflammatory.Macrophages	IDH2	-0.27117	5.667278	-2.46323	0.015706	-3.92841	0.124847	0.165179
Non.inflammatory.Macrophages	YES1	0.558244	4.740029	2.463151	0.01571	-3.62865	0.132116	0.169499
Non.inflammatory.Macrophages	AIM2	-0.27785	5.579386	-2.46278	0.015725	-4.04213	0.12554	0.165641
Non.inflammatory.Macrophages	SUPT3	-0.28823	5.480744	-2.46201	0.015756	-3.90543	0.126451	0.166357
Non.inflammatory.Macrophages	UBR1	0.327018	5.323458	2.461712	0.015769	-3.77106	0.127672	0.167133
Non.inflammatory.Macrophages	GM15336	-1.07743	1.682133	-2.46126	0.015787	-3.03637	0.15928	0.185008
Non.inflammatory.Macrophages	SLC38A4	-0.84176	3.153421	-2.46086	0.015804	-3.30582	0.145782	0.177652

Non.inflammatory.Macrophages	ENKUR	-0.92284	0.339833	-2.45896	0.015882	-3.09681	0.169772	0.192589
Non.inflammatory.Macrophages	SNRNP35	-0.42201	3.056704	-2.45868	0.015893	-3.46197	0.147238	0.178601
Non.inflammatory.Macrophages	MICOS13	0.219593	5.948201	2.458216	0.015913	-4.0069	0.123524	0.164861
Non.inflammatory.Macrophages	4930404I0!	-0.96162	0.086501	-2.45762	0.015937	-3.13113	0.169972	0.194159
Non.inflammatory.Macrophages	EPO	-0.99925	0.415776	-2.45619	0.015997	-3.06883	0.170474	0.192703
Non.inflammatory.Macrophages	FAS	0.474728	3.936884	2.455381	0.01603	-3.6363	0.140343	0.174989
Non.inflammatory.Macrophages	CYTH1	0.239819	8.072227	2.45483	0.016053	-4.29256	0.109061	0.156087
Non.inflammatory.Macrophages	PCYT2	-0.34752	4.635801	-2.45441	0.016071	-3.71697	0.134626	0.171848
Non.inflammatory.Macrophages	AKAP12	-0.63693	6.169014	-2.45356	0.016106	-3.69821	0.122769	0.164834
Non.inflammatory.Macrophages	GM10802	-0.83199	-0.25172	-2.45283	0.016137	-3.15071	0.171293	0.197263
Non.inflammatory.Macrophages	1-Sep	-0.5849	6.037433	-2.45226	0.016161	-3.33278	0.123985	0.165723
Non.inflammatory.Macrophages	LARP7	-0.22085	5.919166	-2.45166	0.016186	-3.9539	0.124973	0.166375
Non.inflammatory.Macrophages	RND3	0.421342	2.943599	2.45139	0.016198	-3.69481	0.149787	0.180777
Non.inflammatory.Macrophages	GM15492	-1.06841	0.742028	-2.45073	0.016225	-3.06322	0.170365	0.192395
Non.inflammatory.Macrophages	HMGCLL1	-1.51564	1.17582	-2.45028	0.016245	-3.07531	0.166845	0.190151

Non.inflammatory.Macrophages	VWF	1.064517	1.565048	2.449518	0.016277	-3.2361	0.163168	0.18829
Non.inflammatory.Macrophages	PARG	0.230144	5.560263	2.449258	0.016288	-3.90321	0.128053	0.168552
Non.inflammatory.Macrophages	POLG2	-0.28127	5.080463	-2.44863	0.016314	-3.86244	0.131967	0.170929
Non.inflammatory.Macrophages	CFAP298	-0.36977	3.642504	-2.44835	0.016326	-3.58378	0.144037	0.177996
Non.inflammatory.Macrophages	CLIC4	0.331191	7.449524	2.447825	0.016348	-4.30644	0.114161	0.160175
Non.inflammatory.Macrophages	VAT1	0.355022	3.491225	2.447531	0.016361	-3.70937	0.14537	0.178894
Non.inflammatory.Macrophages	ADK	0.350422	6.968234	2.447435	0.016365	-4.09227	0.11758	0.16239
Non.inflammatory.Macrophages	GM9844	0.773272	2.309102	2.446946	0.016386	-3.12713	0.156225	0.184938
Non.inflammatory.Macrophages	SLC48A1	-0.2139	5.074284	-2.44638	0.01641	-4.04258	0.132176	0.171386
Non.inflammatory.Macrophages	RBMS2	0.262877	4.872344	2.444561	0.016487	-3.80457	0.134343	0.172828
Non.inflammatory.Macrophages	9830107B1	1.339622	-0.62676	2.443611	0.016528	-3.08901	0.173187	0.201599
Non.inflammatory.Macrophages	A930001M	-0.38441	3.967402	-2.44349	0.016533	-3.61538	0.142126	0.177468
Non.inflammatory.Macrophages	1500004A1	-0.82964	2.078931	-2.44267	0.016568	-3.09633	0.159382	0.187118
Non.inflammatory.Macrophages	9130230L2	-0.42262	3.687563	-2.44242	0.016579	-3.95798	0.144601	0.178943
Non.inflammatory.Macrophages	ATP2B4	0.753007	4.169834	2.442355	0.016582	-3.33511	0.140424	0.176557

Non.inflammatory.Macrophages	CYP2D26	-0.8502	3.247258	-2.4422	0.016589	-3.41436	0.148515	0.181179
Non.inflammatory.Macrophages	IFITM10	-0.54945	3.144158	-2.43913	0.016721	-3.78934	0.150519	0.182521
Non.inflammatory.Macrophages	ACAT1	-0.3624	6.480379	-2.43806	0.016767	-4.11701	0.123075	0.166554
Non.inflammatory.Macrophages	FLNA	0.281684	6.097597	2.437715	0.016782	-4.17	0.126007	0.168447
Non.inflammatory.Macrophages	PPIP5K2	0.295394	4.496011	2.436947	0.016816	-3.74089	0.139118	0.176349
Non.inflammatory.Macrophages	AP1S2	0.237259	5.081901	2.436383	0.01684	-4.03868	0.134299	0.173559
Non.inflammatory.Macrophages	PARP2	-0.24514	4.841287	-2.43586	0.016863	-3.85384	0.136285	0.174881
Non.inflammatory.Macrophages	MED27	-0.23103	5.275175	-2.43585	0.016863	-3.85079	0.132725	0.172781
Non.inflammatory.Macrophages	SLC35B3	0.265142	4.482615	2.435581	0.016875	-3.71329	0.139296	0.176695
Non.inflammatory.Macrophages	SHOC2	0.214889	6.344774	2.434647	0.016916	-4.08079	0.124529	0.167961
Non.inflammatory.Macrophages	TMPRSS3	-1.61962	-0.48523	-2.4343	0.016931	-3.15291	0.175455	0.203164
Non.inflammatory.Macrophages	RCOR1	0.240442	6.85813	2.43398	0.016945	-4.1378	0.120698	0.165732
Non.inflammatory.Macrophages	OSBP	0.264991	5.016429	2.43149	0.017054	-3.8864	0.135853	0.175047
Non.inflammatory.Macrophages	ODC1	0.298904	5.188947	2.430081	0.017117	-3.90872	0.134818	0.174569
Non.inflammatory.Macrophages	ASCC1	0.29926	3.774861	2.429192	0.017156	-3.72386	0.147152	0.181854

Non.inflammatory.Macrophages	GNAQ	-0.20702	7.882192	-2.42893	0.017167	-4.45681	0.114487	0.162264
Non.inflammatory.Macrophages	DUSP10	-0.39706	4.392133	-2.42798	0.01721	-3.77102	0.141882	0.179133
Non.inflammatory.Macrophages	DNM1L	0.24062	5.639142	2.42793	0.017212	-3.99622	0.13149	0.173021
Non.inflammatory.Macrophages	MAB21L3	-1.44603	-0.30498	-2.4272	0.017244	-3.10166	0.177615	0.20426
Non.inflammatory.Macrophages	EFNA2	-0.87497	1.10216	-2.42629	0.017285	-3.31121	0.17357	0.196703
Non.inflammatory.Macrophages	GM45442	-1.29709	-0.57631	-2.42523	0.017332	-3.10603	0.178246	0.206279
Non.inflammatory.Macrophages	RASSF3	0.267937	6.834864	2.424894	0.017347	-4.24381	0.122799	0.167953
Non.inflammatory.Macrophages	SLC16A1	0.438593	3.905544	2.424583	0.017361	-3.58335	0.146852	0.182294
Non.inflammatory.Macrophages	UNC13D	-0.49056	3.31115	-2.42358	0.017406	-3.43286	0.152454	0.185513
Non.inflammatory.Macrophages	GM4869	-0.8211	1.447403	-2.42347	0.017411	-3.24942	0.170604	0.195382
Non.inflammatory.Macrophages	ADAM22	-0.58264	0.796933	-2.42276	0.017443	-3.49884	0.176848	0.199095
Non.inflammatory.Macrophages	EFHD2	0.205038	6.92246	2.422407	0.017459	-4.38628	0.12237	0.168009
Non.inflammatory.Macrophages	MTSS2	-1.00591	0.842863	-2.42241	0.017459	-3.14532	0.176538	0.198974
Non.inflammatory.Macrophages	GPX4	0.173134	7.153816	2.421493	0.0175	-4.32253	0.120839	0.167183
Non.inflammatory.Macrophages	CRLF3	0.289198	6.899396	2.421058	0.017519	-4.18506	0.122783	0.1685

Non.inflammatory.Macrophages	MID1	-0.83069	5.805162	-2.41943	0.017593	-4.0823	0.131575	0.17393
Non.inflammatory.Macrophages	AGFG1	0.219851	6.147106	2.419291	0.017599	-4.05506	0.128852	0.172282
Non.inflammatory.Macrophages	GYG	0.306671	6.126163	2.419105	0.017608	-4.10437	0.129017	0.172397
Non.inflammatory.Macrophages	PLXND1	-0.35258	3.5086	-2.41904	0.017611	-3.67167	0.151333	0.185436
Non.inflammatory.Macrophages	4833439L1	0.29279	4.862518	2.417837	0.017665	-3.83755	0.139526	0.178817
Non.inflammatory.Macrophages	SPTBN1	0.303482	6.620496	2.417722	0.017671	-4.06832	0.125312	0.170284
Non.inflammatory.Macrophages	DDX46	0.238	6.040748	2.417574	0.017677	-4.04279	0.129839	0.173078
Non.inflammatory.Macrophages	CLN3	-0.30761	4.768913	-2.41744	0.017683	-4.03232	0.140325	0.179337
Non.inflammatory.Macrophages	LY6D	-0.68072	5.594266	-2.41658	0.017723	-3.55844	0.133513	0.175548
Non.inflammatory.Macrophages	KLF13	0.210892	8.183848	2.416527	0.017725	-4.35914	0.113923	0.16334
Non.inflammatory.Macrophages	BCLAF1	0.169279	7.155376	2.416292	0.017736	-4.24679	0.121345	0.168154
Non.inflammatory.Macrophages	CCDC15	-0.60074	2.810819	-2.41575	0.01776	-3.38669	0.158141	0.189771
Non.inflammatory.Macrophages	ROMO1	0.191398	6.595619	2.415747	0.017761	-4.21963	0.125579	0.170795
Non.inflammatory.Macrophages	UBAC1	0.295844	4.029628	2.414816	0.017803	-3.72504	0.147116	0.183674
Non.inflammatory.Macrophages	PRR14L	0.239928	6.50182	2.41433	0.017825	-4.08574	0.126575	0.171538

Non.inflammatory.Macrophages	ZFP738	0.389787	2.843416	2.413228	0.017876	-3.54742	0.158499	0.190299
Non.inflammatory.Macrophages	SARDH	-0.68831	2.900243	-2.41279	0.017896	-3.53015	0.157971	0.190165
Non.inflammatory.Macrophages	NMB	-0.86678	1.642171	-2.41218	0.017924	-3.17229	0.170427	0.197061
Non.inflammatory.Macrophages	PLEKHB1	1.141432	0.728002	2.412042	0.017931	-3.12955	0.179134	0.202138
Non.inflammatory.Macrophages	RSF1	0.217857	7.014648	2.412029	0.017931	-4.19351	0.122928	0.169701
Non.inflammatory.Macrophages	SPTB	0.96093	1.120897	2.410651	0.017995	-3.22651	0.176338	0.200181
Non.inflammatory.Macrophages	METAP1	0.239121	4.718677	2.410187	0.018016	-3.86968	0.141913	0.181225
Non.inflammatory.Macrophages	TST	-0.7206	3.470042	-2.40809	0.018113	-3.53449	0.153621	0.188221
Non.inflammatory.Macrophages	GM42917	-0.61791	1.691044	-2.408	0.018117	-3.36612	0.171052	0.197769
Non.inflammatory.Macrophages	SETD5	0.1847	6.628233	2.407583	0.018137	-4.20978	0.126707	0.17249
Non.inflammatory.Macrophages	ITM2B	-0.19102	10.00633	-2.40733	0.018149	-4.84323	0.102972	0.157062
Non.inflammatory.Macrophages	JAZF1	-0.44316	3.086872	-2.40716	0.018157	-3.64856	0.15723	0.190456
Non.inflammatory.Macrophages	ARHGAP27	1.234237	0.465307	2.406705	0.018178	-3.14737	0.181625	0.205044
Non.inflammatory.Macrophages	IL18RAP	1.440345	1.983582	2.406632	0.018181	-3.14758	0.168066	0.196588
Non.inflammatory.Macrophages	GATAD2B	-0.2094	7.433227	-2.40657	0.018184	-4.32348	0.120606	0.168927

Non.inflammatory.Macrophages	USP47	0.228288	6.472249	2.405806	0.01822	-4.1469	0.12808	0.173707
Non.inflammatory.Macrophages	MZT1	0.240736	5.278837	2.405372	0.01824	-3.89813	0.137783	0.179712
Non.inflammatory.Macrophages	MEPCE	-0.3335	4.466254	-2.40521	0.018248	-3.8552	0.144781	0.183856
Non.inflammatory.Macrophages	EPS8L1	-1.64454	0.408966	-2.40424	0.018293	-3.14709	0.182179	0.206125
Non.inflammatory.Macrophages	ADSSL1	0.28299	4.426295	2.401501	0.018422	-3.91052	0.146281	0.185017
Non.inflammatory.Macrophages	AMBP	-0.57942	4.730423	-2.40107	0.018442	-3.83453	0.143596	0.183577
Non.inflammatory.Macrophages	EIF4EBP1	0.21815	5.454955	2.400969	0.018447	-4.13916	0.137387	0.179938
Non.inflammatory.Macrophages	GARNL3	0.557601	2.487215	2.40052	0.018468	-3.46108	0.164537	0.195438
Non.inflammatory.Macrophages	LY96	-0.2824	4.287348	-2.40042	0.018473	-3.98455	0.147523	0.185908
Non.inflammatory.Macrophages	FCGR4	0.488041	3.388468	2.399099	0.018536	-4.12294	0.156215	0.191107
Non.inflammatory.Macrophages	OPA1	0.252216	4.835691	2.398529	0.018563	-3.93141	0.143154	0.183702
Non.inflammatory.Macrophages	SELENBP1	0.432253	3.795416	2.398037	0.018586	-3.82315	0.15251	0.189165
Non.inflammatory.Macrophages	YAE1D1	-0.44174	3.240113	-2.39797	0.018589	-3.67917	0.157736	0.192118
Non.inflammatory.Macrophages	NCAPG2	-0.38695	5.068495	-2.39715	0.018628	-3.92959	0.141241	0.182683
Non.inflammatory.Macrophages	C430049B(	-0.69702	1.844436	-2.39673	0.018649	-3.34797	0.171739	0.19984

Non.inflammatory.Macrophages	OIP5OS1	0.224644	6.02273	2.396548	0.018657	-4.14119	0.133243	0.177903
Non.inflammatory.Macrophages	MSMO1	-0.41504	3.610529	-2.39645	0.018662	-3.60988	0.154347	0.19026
Non.inflammatory.Macrophages	LGR5	-1.27036	1.073909	-2.39625	0.018671	-3.15878	0.179874	0.204201
Non.inflammatory.Macrophages	VAV1	0.216119	6.364742	2.395463	0.018709	-4.21412	0.130652	0.17641
Non.inflammatory.Macrophages	BTBD2	-0.33927	3.477347	-2.39494	0.018734	-3.76844	0.155883	0.191278
Non.inflammatory.Macrophages	RDH7	-0.8309	2.864625	-2.39449	0.018756	-3.42689	0.161778	0.194724
Non.inflammatory.Macrophages	IMPA2	0.430475	4.492173	2.393972	0.01878	-3.61172	0.146558	0.186273
Non.inflammatory.Macrophages	ATP1B1	-0.69181	5.798004	-2.39378	0.018789	-3.5598	0.135333	0.179655
Non.inflammatory.Macrophages	SLC7A1	0.436091	5.141375	2.393672	0.018795	-3.81893	0.140872	0.182976
Non.inflammatory.Macrophages	TRIM7	-1.36403	0.591087	-2.39326	0.018814	-3.17003	0.183907	0.207813
Non.inflammatory.Macrophages	AK7	-0.75739	1.686834	-2.39306	0.018824	-3.37486	0.173692	0.201608
Non.inflammatory.Macrophages	PFKFB3	0.304785	6.273061	2.392984	0.018828	-4.17105	0.131457	0.177454
Non.inflammatory.Macrophages	AKNA	-0.27315	4.948701	-2.39256	0.018848	-3.91494	0.142589	0.184213
Non.inflammatory.Macrophages	CNEP1R1	0.27924	4.587392	2.392156	0.018868	-3.86374	0.145808	0.186182
Non.inflammatory.Macrophages	HCCS	0.26269	4.627385	2.391337	0.018907	-3.87969	0.145653	0.186208

Non.inflammatory.Macrophages	ADH1	-0.72628	3.503888	-2.3907	0.018938	-3.63094	0.15609	0.192122
Non.inflammatory.Macrophages	HSH2D	-0.59953	3.210107	-2.38818	0.01906	-3.43186	0.159807	0.194222
Non.inflammatory.Macrophages	ANXA4	0.351684	3.74233	2.387697	0.019084	-3.93102	0.154809	0.191499
Non.inflammatory.Macrophages	TAOK1	0.184071	7.033029	2.387104	0.019113	-4.31302	0.126695	0.17484
Non.inflammatory.Macrophages	GYS2	-1.02413	0.768164	-2.38694	0.01912	-3.18621	0.184547	0.208172
Non.inflammatory.Macrophages	CCNH	-0.2382	5.363256	-2.386	0.019167	-4.03287	0.140556	0.183476
Non.inflammatory.Macrophages	CNOT6L	0.230213	7.08541	2.385733	0.019179	-4.27372	0.126497	0.174958
Non.inflammatory.Macrophages	IREB2	0.250448	5.493255	2.385026	0.019214	-4.04652	0.139595	0.183117
Non.inflammatory.Macrophages	WIPI1	0.583045	2.277802	2.383907	0.019269	-3.52634	0.170068	0.200691
Non.inflammatory.Macrophages	TMEM108	-1.05198	5.137851	-2.38349	0.01929	-3.43358	0.143012	0.18545
Non.inflammatory.Macrophages	TMOD3	0.21282	7.218311	2.383037	0.019312	-4.32205	0.125974	0.175099
Non.inflammatory.Macrophages	3010003L2	1.185959	1.349271	2.382645	0.019331	-3.18517	0.180034	0.206246
Non.inflammatory.Macrophages	ARID5A	0.415499	4.681741	2.381652	0.01938	-3.69464	0.14727	0.188149
Non.inflammatory.Macrophages	ZFP119A	-0.52387	2.125414	-2.3815	0.019387	-3.40286	0.17196	0.20202
Non.inflammatory.Macrophages	PDHX	0.329233	3.395129	2.381485	0.019388	-3.68036	0.159252	0.195009

Non.inflammatory.Macrophages	PRDX4	-0.27102	5.118984	-2.38066	0.019429	-4.09355	0.143596	0.186109
Non.inflammatory.Macrophages	PSMD14	0.18836	6.618882	2.380146	0.019454	-4.23746	0.13109	0.178629
Non.inflammatory.Macrophages	AP1S3	-0.62507	5.57812	-2.37933	0.019495	-3.55931	0.13979	0.184175
Non.inflammatory.Macrophages	EBF1	-0.56468	8.683852	-2.37914	0.019505	-4.3543	0.115558	0.168922
Non.inflammatory.Macrophages	NUP98	0.22435	7.461994	2.378979	0.019512	-4.38958	0.12456	0.174807
Non.inflammatory.Macrophages	FAM78B	0.764223	2.115372	2.378797	0.019521	-3.34632	0.172507	0.202926
Non.inflammatory.Macrophages	CYP2A22	-1.05351	1.632221	-2.37466	0.019728	-3.2561	0.179351	0.206788
Non.inflammatory.Macrophages	TLN2	0.756301	1.298032	2.373236	0.019799	-3.39806	0.18352	0.209154
Non.inflammatory.Macrophages	ATP1A3	0.812763	1.709416	2.372909	0.019816	-3.36313	0.179061	0.206844
Non.inflammatory.Macrophages	CAPSL	-1.77334	0.928873	-2.37201	0.019861	-3.21758	0.187819	0.211659
Non.inflammatory.Macrophages	SNAP23	0.20359	6.222753	2.371361	0.019894	-4.32149	0.136466	0.182764
Non.inflammatory.Macrophages	AMDHD2	-0.30566	3.693973	-2.37079	0.019923	-3.88033	0.159336	0.196209
Non.inflammatory.Macrophages	ZFP971	0.611198	1.911606	2.369962	0.019965	-3.36982	0.177719	0.206416
Non.inflammatory.Macrophages	NTNG2	1.024455	3.05713	2.368975	0.020015	-3.30152	0.166139	0.200214
Non.inflammatory.Macrophages	CLEC1A	0.691736	0.972503	2.367263	0.020102	-3.49611	0.188865	0.21271

Non.inflammatory.Macrophages	MEMO1	0.228572	6.363616	2.366926	0.020119	-4.20208	0.136185	0.183118
Non.inflammatory.Macrophages	NEMP2	0.571627	2.854914	2.366653	0.020133	-3.36923	0.168627	0.20197
Non.inflammatory.Macrophages	ITSN2	0.210669	6.989544	2.366484	0.020142	-4.32957	0.131062	0.180038
Non.inflammatory.Macrophages	2310001H1	0.479558	4.065364	2.366467	0.020143	-3.7017	0.15669	0.195308
Non.inflammatory.Macrophages	GM30881	-0.50658	1.986079	-2.36627	0.020153	-3.54327	0.177708	0.206996
Non.inflammatory.Macrophages	NR4A2	0.40448	6.380914	2.365638	0.020185	-4.43295	0.136125	0.183339
Non.inflammatory.Macrophages	KIRREL3	-0.63531	1.272544	-2.36547	0.020194	-3.46945	0.185614	0.211346
Non.inflammatory.Macrophages	8030462N1	0.189516	6.073398	2.364578	0.020239	-4.1236	0.138928	0.18498
Non.inflammatory.Macrophages	KLK8	0.856576	1.605933	2.363744	0.020282	-3.26418	0.182475	0.209707
Non.inflammatory.Macrophages	CIRBP	-0.23544	7.218243	-2.36268	0.020337	-4.33861	0.129962	0.179703
Non.inflammatory.Macrophages	RAB11FIP4	-0.78813	-0.78929	-2.3622	0.020362	-3.2982	0.193214	0.224625
Non.inflammatory.Macrophages	ATP6AP1	0.206058	5.990589	2.361996	0.020372	-4.27833	0.140156	0.186077
Non.inflammatory.Macrophages	ATP6AP2	-0.15968	6.938045	-2.36116	0.020415	-4.40516	0.13227	0.181472
Non.inflammatory.Macrophages	TAX1BP1	0.192465	8.297296	2.361114	0.020418	-4.53707	0.121686	0.174734
Non.inflammatory.Macrophages	MYO1D	0.718699	3.025547	2.360994	0.020424	-3.36312	0.167902	0.202352

Non.inflammatory.Macrophages	PTGER4	0.404451	4.681638	2.360852	0.020431	-3.91961	0.151832	0.193237
Non.inflammatory.Macrophages	PTPN2	0.186495	6.833794	2.357536	0.020603	-4.32357	0.134146	0.182644
Non.inflammatory.Macrophages	HSD3B3	-0.98824	2.014179	-2.35671	0.020647	-3.34643	0.180109	0.209135
Non.inflammatory.Macrophages	GM4952	-0.82825	2.667628	-2.35588	0.02069	-3.46822	0.173385	0.205556
Non.inflammatory.Macrophages	THBD	0.557128	3.145231	2.355153	0.020728	-3.78715	0.168638	0.203083
Non.inflammatory.Macrophages	GJB1	-1.03261	1.59761	-2.35428	0.020774	-3.29449	0.185429	0.212196
Non.inflammatory.Macrophages	SLC25A16	0.387609	3.724214	2.353431	0.020818	-3.70387	0.16314	0.200101
Non.inflammatory.Macrophages	TMEM42	0.394798	2.617603	2.35342	0.020819	-3.62996	0.174452	0.206358
Non.inflammatory.Macrophages	TPCN2	-0.39817	2.597655	-2.35327	0.020827	-3.78083	0.174662	0.206526
Non.inflammatory.Macrophages	GCAT	-0.75136	2.390839	-2.35297	0.020842	-3.37254	0.176869	0.207807
Non.inflammatory.Macrophages	ATF4	-0.2566	6.443614	-2.35241	0.020872	-4.29761	0.138314	0.185744
Non.inflammatory.Macrophages	SIPA1L2	0.293853	5.103799	2.35133	0.020929	-4.15223	0.150303	0.193135
Non.inflammatory.Macrophages	GAN	0.376355	4.031697	2.351167	0.020938	-3.85954	0.160445	0.198986
Non.inflammatory.Macrophages	SCUBE2	-1.08616	-0.26058	-2.35101	0.020946	-3.29525	0.196275	0.224218
Non.inflammatory.Macrophages	GM47096	-0.72753	1.734536	-2.35082	0.020956	-3.35706	0.184366	0.212162

Non.inflammatory.Macrophages	NUS1	0.2032	4.837828	2.350069	0.020996	-4.11621	0.152933	0.194825
Non.inflammatory.Macrophages	SF3B3	0.205721	6.034328	2.349829	0.021009	-4.18236	0.142156	0.188483
Non.inflammatory.Macrophages	CNOT10	-0.24143	5.238486	-2.34848	0.02108	-4.08693	0.149606	0.192963
Non.inflammatory.Macrophages	CISD3	0.397412	3.000972	2.348311	0.021089	-3.81841	0.17141	0.205363
Non.inflammatory.Macrophages	CD40LG	-1.3665	-0.56972	-2.34752	0.021131	-3.262	0.197236	0.227013
Non.inflammatory.Macrophages	MYO1C	-0.21244	5.436164	-2.34688	0.021166	-4.17097	0.148041	0.192124
Non.inflammatory.Macrophages	KCTD9	0.414868	3.130956	2.346851	0.021167	-3.65873	0.170331	0.204855
Non.inflammatory.Macrophages	OTOA	-0.88899	0.757069	-2.3466	0.02118	-3.33251	0.195649	0.218909
Non.inflammatory.Macrophages	RDH16F2	-1.30247	1.257357	-2.34625	0.021199	-3.27071	0.190709	0.216012
Non.inflammatory.Macrophages	RAP1GAP2	0.410955	4.692548	2.346046	0.02121	-4.09774	0.154916	0.19636
Non.inflammatory.Macrophages	DYRK3	1.12524	2.520243	2.344497	0.021293	-3.28151	0.177315	0.208938
Non.inflammatory.Macrophages	GM11290	-0.61337	3.432889	-2.3434	0.021352	-3.74292	0.168136	0.203941
Non.inflammatory.Macrophages	CSAD	-0.39323	3.965874	-2.34311	0.021367	-3.84041	0.162787	0.201014
Non.inflammatory.Macrophages	MRPL30	0.184185	6.172662	2.341825	0.021437	-4.30201	0.142648	0.189283
Non.inflammatory.Macrophages	RBKS	-0.34148	3.630745	-2.34103	0.02148	-3.93888	0.166778	0.203394

Non.inflammatory.Macrophages	EIF4H	0.171549	6.535904	2.340747	0.021495	-4.32741	0.139699	0.187667
Non.inflammatory.Macrophages	CES1D	-1.06057	1.90006	-2.33986	0.021543	-3.33919	0.185331	0.213826
Non.inflammatory.Macrophages	ARHGAP4	-0.23468	5.057347	-2.33904	0.021588	-4.13084	0.153035	0.19604
Non.inflammatory.Macrophages	SDHAF1	0.332786	4.132974	2.338994	0.02159	-3.96136	0.1619	0.20115
Non.inflammatory.Macrophages	DCUN1D1	0.240102	5.787878	2.338984	0.021591	-4.21255	0.146358	0.192093
Non.inflammatory.Macrophages	MOCS1	0.335201	3.367675	2.338935	0.021593	-3.86176	0.169602	0.20548
Non.inflammatory.Macrophages	KIF23	-0.40729	5.173452	-2.33865	0.021609	-4.07961	0.151955	0.19549
Non.inflammatory.Macrophages	ASH1L	0.194506	7.367632	2.338488	0.021618	-4.45866	0.132862	0.183924
Non.inflammatory.Macrophages	KPNA1	0.22348	6.778024	2.338262	0.02163	-4.33318	0.137752	0.187034
Non.inflammatory.Macrophages	CEACAM1	0.450258	3.675486	2.33762	0.021665	-3.67905	0.166619	0.204162
Non.inflammatory.Macrophages	TMEM80	0.347444	3.276451	2.336772	0.021711	-3.72966	0.17095	0.206596
Non.inflammatory.Macrophages	IL1RL1	1.426177	-0.28284	2.336461	0.021728	-3.26925	0.199774	0.228141
Non.inflammatory.Macrophages	PLAC8	0.55102	8.2646	2.335614	0.021774	-4.65471	0.126091	0.180075
Non.inflammatory.Macrophages	XCR1	-1.3913	0.412393	-2.33534	0.021789	-3.2761	0.199827	0.22409
Non.inflammatory.Macrophages	EIF2AK1	0.238491	5.326731	2.335321	0.02179	-4.14916	0.150954	0.195461

Non.inflammatory.Macrophages	KITL	-0.88217	1.956944	-2.33528	0.021793	-3.61842	0.185206	0.214676
Non.inflammatory.Macrophages	FAM129A	-0.241	5.667894	-2.33427	0.021848	-4.42462	0.14805	0.193919
Non.inflammatory.Macrophages	PBDC1	-0.34446	5.348225	-2.33418	0.021853	-4.15786	0.150969	0.195677
Non.inflammatory.Macrophages	UNC45A	-0.36062	4.303611	-2.33349	0.021891	-3.81975	0.161064	0.201712
Non.inflammatory.Macrophages	REM2	-1.54275	0.836601	-2.3315	0.022001	-3.28302	0.198898	0.222659
Non.inflammatory.Macrophages	PXN	0.215281	6.266447	2.33126	0.022014	-4.4484	0.143487	0.191469
Non.inflammatory.Macrophages	CX3CR1	0.374913	3.268892	2.330726	0.022043	-4.1951	0.172357	0.208167
Non.inflammatory.Macrophages	GM11808	-0.25568	7.262783	-2.33035	0.022064	-4.46612	0.135115	0.186371
Non.inflammatory.Macrophages	TGS1	0.221251	5.214453	2.329748	0.022098	-4.16312	0.153287	0.197428
Non.inflammatory.Macrophages	KIT	-1.25505	3.453389	-2.32879	0.022151	-3.33006	0.170924	0.207693
Non.inflammatory.Macrophages	GLIPR2	0.532603	4.6029	2.32674	0.022265	-3.8682	0.160101	0.201647
Non.inflammatory.Macrophages	GPR107	0.240551	5.057833	2.326166	0.022297	-4.13071	0.155841	0.199209
Non.inflammatory.Macrophages	AGPAT5	0.203415	5.644905	2.325709	0.022323	-4.27601	0.150395	0.196071
Non.inflammatory.Macrophages	GM11772	-0.56839	0.302935	-2.32494	0.022366	-3.55792	0.202994	0.22766
Non.inflammatory.Macrophages	MRC1	-0.51621	5.240936	-2.32447	0.022392	-4.36639	0.154152	0.198608

Non.inflammatory.Macrophages	L1CAM	-0.40572	3.644556	-2.32433	0.0224	-3.98283	0.169886	0.207662
Non.inflammatory.Macrophages	SLX4IP	0.285234	4.853222	2.32376	0.022432	-4.08424	0.157842	0.200869
Non.inflammatory.Macrophages	SERPINA3N	-0.65224	3.688152	-2.32359	0.022442	-3.74773	0.169437	0.207491
Non.inflammatory.Macrophages	PATL2	-0.73509	2.023827	-2.32354	0.022444	-3.47855	0.187384	0.217324
Non.inflammatory.Macrophages	BAG1	0.172671	6.693589	2.32339	0.022453	-4.44973	0.141048	0.190837
Non.inflammatory.Macrophages	PRKD2	-0.39003	4.348606	-2.32339	0.022453	-3.79027	0.162769	0.203712
Non.inflammatory.Macrophages	ATR	0.415674	3.862209	2.32338	0.022453	-3.89346	0.167655	0.206488
Non.inflammatory.Macrophages	GM10138	-0.55784	2.471458	-2.32284	0.022484	-3.61713	0.182518	0.214813
Non.inflammatory.Macrophages	LIMA1	-0.35309	4.507655	-2.32133	0.022569	-4.31546	0.161578	0.203387
Non.inflammatory.Macrophages	KYAT3	-0.62151	3.603362	-2.32117	0.022578	-3.719	0.17071	0.208574
Non.inflammatory.Macrophages	NDUFB8	0.184248	7.487591	2.321131	0.02258	-4.58957	0.134661	0.187198
Non.inflammatory.Macrophages	RILPL2	-0.21476	7.158884	-2.32112	0.022581	-4.58489	0.137404	0.188918
Non.inflammatory.Macrophages	PXYLP1	-1.12765	2.352535	-2.32064	0.022608	-3.29645	0.184235	0.216039
Non.inflammatory.Macrophages	DNAL1	-0.78642	1.64713	-2.31923	0.022688	-3.41527	0.192667	0.22074
Non.inflammatory.Macrophages	NCF1	0.287713	5.301832	2.319204	0.022689	-4.29618	0.154369	0.199396

Non.inflammatory.Macrophages	TAF10	0.194337	6.856819	2.31806	0.022754	-4.44636	0.140617	0.191336
Non.inflammatory.Macrophages	BCL6	-0.35335	5.992679	-2.31795	0.022761	-4.33629	0.148257	0.19601
Non.inflammatory.Macrophages	DOCK4	0.226523	6.427831	2.317298	0.022798	-4.6856	0.144417	0.193714
Non.inflammatory.Macrophages	BMPR2	0.285181	5.519027	2.317237	0.022801	-4.29323	0.152671	0.198678
Non.inflammatory.Macrophages	GM26827	-1.25965	1.344192	-2.317	0.022815	-3.30755	0.196638	0.223164
Non.inflammatory.Macrophages	ZSCAN20	-0.96892	1.073033	-2.31645	0.022846	-3.37841	0.200005	0.22509
Non.inflammatory.Macrophages	GCHFR	-0.66579	3.389908	-2.3158	0.022883	-3.74511	0.174088	0.211212
Non.inflammatory.Macrophages	JDP2	-0.26475	5.304027	-2.31554	0.022898	-4.51904	0.154945	0.200281
Non.inflammatory.Macrophages	ZFP384	-0.24059	4.90083	-2.31499	0.02293	-4.15507	0.15892	0.202723
Non.inflammatory.Macrophages	APOL7C	-1.3551	-0.99321	-2.31466	0.022949	-3.32233	0.205007	0.238873
Non.inflammatory.Macrophages	WIPF1	-0.22636	7.425545	-2.31372	0.023002	-4.58704	0.136423	0.189319
Non.inflammatory.Macrophages	BCR	-0.48322	5.853797	-2.31331	0.023026	-3.74967	0.150209	0.197908
Non.inflammatory.Macrophages	DCBLD2	0.738674	1.32551	2.313133	0.023036	-3.39694	0.197687	0.224527
Non.inflammatory.Macrophages	FHIT	0.409657	6.167904	2.31273	0.023059	-4.05207	0.14735	0.196274
Non.inflammatory.Macrophages	LONRF1	-0.37435	3.091293	-2.31268	0.023062	-3.93336	0.177718	0.213823

Non.inflammatory.Macrophages	PRCP	-0.21344	5.424232	-2.312	0.023101	-4.3675	0.154362	0.200512
Non.inflammatory.Macrophages	LAMC1	0.492537	4.523339	2.310808	0.02317	-3.7742	0.163459	0.205938
Non.inflammatory.Macrophages	CBR2	1.304371	-1.3198	2.310014	0.023216	-3.33491	0.206308	0.242484
Non.inflammatory.Macrophages	NFRKB	0.264502	4.478568	2.30947	0.023248	-3.98595	0.164238	0.206548
Non.inflammatory.Macrophages	SIK3	-0.24412	10.09847	-2.30908	0.023271	-4.90451	0.116425	0.176724
Non.inflammatory.Macrophages	L3MBTL2	0.392453	3.445434	2.308644	0.023296	-3.69199	0.175012	0.212682
Non.inflammatory.Macrophages	FMNL2	-0.31755	7.100621	-2.30816	0.023324	-4.71883	0.140098	0.192234
Non.inflammatory.Macrophages	ATP11B	0.260507	7.035822	2.307562	0.023359	-4.36243	0.140775	0.192714
Non.inflammatory.Macrophages	MPV17	-0.27387	4.075124	-2.30602	0.023449	-4.0075	0.169109	0.209775
Non.inflammatory.Macrophages	UTP6	0.293988	4.42275	2.305747	0.023464	-4.04154	0.16557	0.207778
Non.inflammatory.Macrophages	WDR83OS	0.222949	6.074614	2.305361	0.023487	-4.36563	0.14969	0.198439
Non.inflammatory.Macrophages	CLEC4N	0.374628	4.093598	2.305319	0.02349	-4.42565	0.168919	0.20969
Non.inflammatory.Macrophages	LTC4S	0.850968	1.066227	2.305233	0.023495	-3.77266	0.202813	0.228111
Non.inflammatory.Macrophages	DTX3	0.636439	2.572046	2.304373	0.023545	-3.50054	0.185517	0.218982
Non.inflammatory.Macrophages	GBP4	0.723589	3.363964	2.303582	0.023591	-3.88424	0.177068	0.214501

Non.inflammatory.Macrophages	SYNJ2BP	0.226894	5.520643	2.302471	0.023657	-4.19886	0.155555	0.202376
Non.inflammatory.Macrophages	CYTH4	0.208484	5.757599	2.302352	0.023664	-4.41362	0.153319	0.201084
Non.inflammatory.Macrophages	SH2D1B1	1.030763	1.695197	2.301298	0.023726	-3.40014	0.196578	0.225424
Non.inflammatory.Macrophages	PPP1R16B	-0.59694	6.504104	-2.30039	0.023779	-3.78867	0.146999	0.197327
Non.inflammatory.Macrophages	UBXN7	0.26877	5.704222	2.299966	0.023805	-4.14389	0.154435	0.201902
Non.inflammatory.Macrophages	TACO1	0.47443	3.738306	2.299337	0.023842	-3.94004	0.174172	0.213409
Non.inflammatory.Macrophages	ALKBH5	0.201442	7.148219	2.299253	0.023847	-4.49424	0.141436	0.194083
Non.inflammatory.Macrophages	HDAC9	-0.31372	7.563843	-2.29828	0.023905	-4.72533	0.138099	0.192111
Non.inflammatory.Macrophages	WFDC21	1.94284	2.947804	2.297854	0.02393	-3.48144	0.183018	0.21859
Non.inflammatory.Macrophages	DYNLT3	0.233215	4.662522	2.297821	0.023932	-4.17866	0.164916	0.208404
Non.inflammatory.Macrophages	CHIL1	1.342554	-0.07877	2.297343	0.02396	-3.33967	0.209755	0.237908
Non.inflammatory.Macrophages	ISOC1	0.234795	4.993923	2.294902	0.024106	-4.29249	0.162584	0.207279
Non.inflammatory.Macrophages	COL9A3	-0.95841	0.618437	-2.29407	0.024156	-3.43119	0.210496	0.234394
Non.inflammatory.Macrophages	PRKCD	0.189965	6.897294	2.293825	0.024171	-4.54284	0.14492	0.196824
Non.inflammatory.Macrophages	MTHFSL	0.232999	5.211119	2.293208	0.024208	-4.30436	0.160808	0.206441

Non.inflammatory.Macrophages	GM45370	-1.14322	-1.29914	-2.2921	0.024275	-3.35821	0.211821	0.247717
Non.inflammatory.Macrophages	GM9530	-0.95414	-0.31965	-2.29173	0.024297	-3.40998	0.211875	0.241225
Non.inflammatory.Macrophages	SIRPB1B	0.548802	1.445504	2.290611	0.024364	-3.94583	0.202729	0.229989
Non.inflammatory.Macrophages	RDH9	-1.22188	0.805989	-2.29057	0.024367	-3.36073	0.209955	0.234112
Non.inflammatory.Macrophages	PLAGL2	0.267219	4.367209	2.290514	0.02437	-4.13228	0.169884	0.212036
Non.inflammatory.Macrophages	NAMPT	0.313819	6.301782	2.289049	0.024459	-4.54667	0.151412	0.201366
Non.inflammatory.Macrophages	RAB11FIP4	-0.662	1.161785	-2.28808	0.024518	-3.52252	0.207192	0.232671
Non.inflammatory.Macrophages	BACH2	-0.33995	8.964274	-2.28729	0.024566	-4.63245	0.128912	0.187332
Non.inflammatory.Macrophages	GM15726	-0.68853	2.074088	-2.28727	0.024567	-3.83713	0.196275	0.226931
Non.inflammatory.Macrophages	4932438H2	-1.26992	-0.27879	-2.28657	0.024609	-3.36555	0.213503	0.242467
Non.inflammatory.Macrophages	RAET1E	0.474227	4.113795	2.285785	0.024658	-3.78165	0.173853	0.214738
Non.inflammatory.Macrophages	MAPK8	0.233301	5.232129	2.285607	0.024669	-4.24721	0.162401	0.208185
Non.inflammatory.Macrophages	ST3GAL2	-0.51125	3.085043	-2.28486	0.024714	-3.69915	0.185278	0.221194
Non.inflammatory.Macrophages	TMA16	0.435773	3.964843	2.282824	0.024839	-3.95132	0.176422	0.216719
Non.inflammatory.Macrophages	PPARGC1B	0.440458	2.799015	2.282223	0.024876	-3.86227	0.189505	0.224045

Non.inflammatory.Macrophages	TRIB1	-0.2841	5.765307	-2.28164	0.024912	-4.44343	0.158345	0.206402
Non.inflammatory.Macrophages	MYL12A	0.180593	7.929477	2.280409	0.024988	-4.75505	0.139014	0.194635
Non.inflammatory.Macrophages	PPP2CB	0.194969	5.60171	2.279313	0.025056	-4.34101	0.160653	0.208042
Non.inflammatory.Macrophages	H2-Q7	-0.7014	3.889878	-2.27885	0.025084	-4.06048	0.178406	0.218379
Non.inflammatory.Macrophages	GM29585	-0.94701	1.247477	-2.2783	0.025119	-3.42855	0.209419	0.235185
Non.inflammatory.Macrophages	LILRB4A	0.310926	4.163584	2.278018	0.025136	-4.45191	0.175578	0.21692
Non.inflammatory.Macrophages	CHFR	0.228228	5.641975	2.277814	0.025149	-4.24429	0.160443	0.208204
Non.inflammatory.Macrophages	VPS26A	-0.18663	6.273847	-2.27698	0.0252	-4.48712	0.154533	0.204772
Non.inflammatory.Macrophages	IQSEC2	0.531359	2.267987	2.276718	0.025217	-3.87139	0.197166	0.228949
Non.inflammatory.Macrophages	ZFHX2	-0.49085	2.765643	-2.27659	0.025225	-3.83243	0.191328	0.225802
Non.inflammatory.Macrophages	CWF19L2	0.251612	5.038452	2.275197	0.025312	-4.20952	0.1669	0.212315
Non.inflammatory.Macrophages	CCDC146	-0.45769	2.867299	-2.27518	0.025313	-3.96513	0.190439	0.22554
Non.inflammatory.Macrophages	CARM1	-0.24557	4.727042	-2.27459	0.02535	-4.19124	0.170099	0.214248
Non.inflammatory.Macrophages	VSIG4	-0.48882	4.823941	-2.27448	0.025357	-4.43006	0.169097	0.213705
Non.inflammatory.Macrophages	RUNX2	0.803414	4.240789	2.274202	0.025374	-3.76114	0.175211	0.217236

Non.inflammatory.Macrophages	TCEA3	-0.75127	2.275566	-2.27413	0.025379	-3.60891	0.19737	0.229445
Non.inflammatory.Macrophages	MLEC	-0.2506	5.064037	-2.27402	0.025385	-4.214	0.166639	0.212314
Non.inflammatory.Macrophages	GRAMD4	-0.5454	5.165751	-2.27394	0.025391	-3.79738	0.165608	0.211716
Non.inflammatory.Macrophages	VTN	-0.60603	4.267725	-2.27344	0.025422	-3.99988	0.175027	0.217155
Non.inflammatory.Macrophages	ZFP707	-0.71756	1.958642	-2.27286	0.025458	-3.48728	0.201373	0.231776
Non.inflammatory.Macrophages	FUS	0.154265	7.820328	2.272267	0.025496	-4.65541	0.140904	0.19702
Non.inflammatory.Macrophages	TTC36	-0.57451	5.028124	-2.27192	0.025517	-4.21447	0.167168	0.213043
Non.inflammatory.Macrophages	PIK3AP1	0.298796	8.295612	2.271825	0.025524	-4.75868	0.136852	0.194539
Non.inflammatory.Macrophages	1700019L1	-0.86867	0.774577	-2.2718	0.025525	-3.50532	0.215347	0.239824
Non.inflammatory.Macrophages	ADAM11	1.274149	-1.14352	2.271755	0.025528	-3.41576	0.217293	0.252933
Non.inflammatory.Macrophages	ARAP3	-0.40703	3.537048	-2.27102	0.025574	-4.01297	0.183152	0.222262
Non.inflammatory.Macrophages	PLBD1	-0.27551	5.389651	-2.27076	0.025591	-4.69274	0.163617	0.211163
Non.inflammatory.Macrophages	EFCAB11	-0.62279	3.410869	-2.27075	0.025591	-3.75957	0.18456	0.223121
Non.inflammatory.Macrophages	ZFP84	-0.36517	3.7569	-2.26822	0.025752	-3.99009	0.181705	0.22161
Non.inflammatory.Macrophages	MFAP3	0.237657	5.124697	2.268045	0.025763	-4.25174	0.167187	0.213336

Non.inflammatory.Macrophages	CLEC4F	-0.67727	5.458864	-2.2674	0.025804	-4.57807	0.163969	0.211478
Non.inflammatory.Macrophages	GM11520	-0.78804	1.043872	-2.2654	0.025931	-3.46514	0.215047	0.239725
Non.inflammatory.Macrophages	SLC25A40	0.449238	3.227708	2.265363	0.025933	-3.81059	0.188522	0.225602
Non.inflammatory.Macrophages	ALDH7A1	-0.43004	3.227154	-2.26457	0.025984	-3.92919	0.188779	0.225871
Non.inflammatory.Macrophages	DNAJC4	-0.34809	3.061383	-2.26373	0.026038	-3.9652	0.190933	0.227143
Non.inflammatory.Macrophages	RBM6	0.183563	7.184868	2.263532	0.026051	-4.56648	0.148477	0.202511
Non.inflammatory.Macrophages	EDNRB	1.106425	2.175551	2.262532	0.026115	-3.78289	0.201803	0.233087
Non.inflammatory.Macrophages	ZFP944	-0.33131	4.397621	-2.26192	0.026154	-4.05602	0.176461	0.219229
Non.inflammatory.Macrophages	ANAPC15	-0.25314	4.974484	-2.26183	0.02616	-4.30353	0.170366	0.215737
Non.inflammatory.Macrophages	SPOP	-0.15325	7.366582	-2.26123	0.026199	-4.66749	0.147229	0.201952
Non.inflammatory.Macrophages	MAPK14	0.179972	6.656022	2.261022	0.026212	-4.56537	0.153784	0.205993
Non.inflammatory.Macrophages	CNPY3	-0.19607	5.300686	-2.26081	0.026226	-4.33706	0.167074	0.213943
Non.inflammatory.Macrophages	SELENOT	0.186434	6.182995	2.260659	0.026235	-4.45861	0.158302	0.208768
Non.inflammatory.Macrophages	CDKL5	0.716189	2.066071	2.259324	0.026322	-3.64416	0.203857	0.234626
Non.inflammatory.Macrophages	CCNDBP1	0.297188	5.692836	2.258372	0.026383	-4.40102	0.163835	0.212371

Non.inflammatory.Macrophages	LY6C2	1.07374	5.53324	2.257748	0.026424	-3.91516	0.165591	0.21346
Non.inflammatory.Macrophages	ZFP217	-0.25389	4.011596	-2.25729	0.026453	-4.12853	0.181775	0.222817
Non.inflammatory.Macrophages	MAPK8IP3	0.279661	4.521476	2.256747	0.026489	-4.08725	0.176262	0.219753
Non.inflammatory.Macrophages	FNTA	-0.19922	5.505108	-2.25669	0.026492	-4.32503	0.165998	0.213817
Non.inflammatory.Macrophages	NAGA	-0.27746	4.159018	-2.25614	0.026528	-4.33311	0.180264	0.222027
Non.inflammatory.Macrophages	NXF1	-0.2305	5.180351	-2.25603	0.026535	-4.28996	0.169388	0.215803
Non.inflammatory.Macrophages	RHPN2	0.96646	0.915486	2.25558	0.026565	-3.43292	0.219074	0.243148
Non.inflammatory.Macrophages	NFIC	-0.25285	4.464453	-2.2555	0.02657	-4.27199	0.176955	0.220312
Non.inflammatory.Macrophages	AGPS	0.193299	6.813953	2.254459	0.026638	-4.53783	0.153495	0.206596
Non.inflammatory.Macrophages	PLAU	-0.72109	0.814983	-2.25393	0.026673	-3.74374	0.220209	0.244326
Non.inflammatory.Macrophages	FHL2	-1.50069	0.017257	-2.25351	0.0267	-3.41497	0.222543	0.249847
Non.inflammatory.Macrophages	TCP11L2	0.330302	6.196085	2.253379	0.026709	-4.38943	0.159414	0.210396
Non.inflammatory.Macrophages	RIC8B	0.27843	4.502862	2.253088	0.026728	-4.14983	0.176776	0.220653
Non.inflammatory.Macrophages	MAD1L1	-0.21781	5.227133	-2.25306	0.026729	-4.30133	0.16914	0.216248
Non.inflammatory.Macrophages	RPRD1B	0.243657	5.480381	2.253047	0.02673	-4.33509	0.166545	0.214729

Non.inflammatory.Macrophages	MYL6	0.172422	9.689609	2.252546	0.026763	-5.04963	0.128651	0.19108
Non.inflammatory.Macrophages	FBRSL1	-0.19879	5.766788	-2.25251	0.026765	-4.40038	0.163655	0.213122
Non.inflammatory.Macrophages	9430091E2	-0.56013	2.439837	-2.25245	0.02677	-3.66034	0.20034	0.233802
Non.inflammatory.Macrophages	SLA	0.270215	5.280678	2.251564	0.026828	-4.44129	0.168851	0.216272
Non.inflammatory.Macrophages	TMEM160	0.196537	5.666014	2.251142	0.026856	-4.47499	0.164993	0.214079
Non.inflammatory.Macrophages	FOXJ3	0.198295	5.947043	2.249621	0.026956	-4.39335	0.162688	0.212733
Non.inflammatory.Macrophages	4833419F2	-0.57745	1.323387	-2.24854	0.027027	-3.7865	0.215772	0.242267
Non.inflammatory.Macrophages	SMAP2	0.185246	6.821529	2.248071	0.027058	-4.55351	0.154501	0.207978
Non.inflammatory.Macrophages	PDE3B	0.426819	6.955861	2.247991	0.027064	-4.33741	0.153234	0.207203
Non.inflammatory.Macrophages	UBE3A	0.170436	6.387077	2.247848	0.027073	-4.53704	0.158667	0.210564
Non.inflammatory.Macrophages	ATP8B4	0.628257	4.781456	2.246491	0.027163	-3.97027	0.175495	0.220453
Non.inflammatory.Macrophages	F7	0.953499	0.719823	2.246159	0.027186	-3.51108	0.223212	0.246818
Non.inflammatory.Macrophages	PIPOX	-0.89312	2.011866	-2.24525	0.027246	-3.53773	0.207932	0.238482
Non.inflammatory.Macrophages	COX4I2	0.784258	0.956141	2.24496	0.027265	-3.52723	0.221532	0.245598
Non.inflammatory.Macrophages	NRG1	0.91832	2.541088	2.244806	0.027276	-3.74395	0.201398	0.235057

Non.inflammatory.Macrophages	SULT1D1	-0.80584	2.290136	-2.24416	0.027319	-3.64292	0.204595	0.236847
Non.inflammatory.Macrophages	DBNL	0.197284	6.127196	2.244063	0.027325	-4.50032	0.162018	0.212858
Non.inflammatory.Macrophages	HEXA	-0.20703	5.752202	-2.24297	0.027398	-4.63646	0.166118	0.215458
Non.inflammatory.Macrophages	MDM4	0.204176	5.749077	2.241815	0.027476	-4.37371	0.166519	0.215834
Non.inflammatory.Macrophages	PIK3R3	-0.56892	3.43068	-2.24138	0.027505	-3.94565	0.191859	0.230256
Non.inflammatory.Macrophages	NBR1	-0.20598	5.455137	-2.23964	0.027623	-4.40556	0.170235	0.218081
Non.inflammatory.Macrophages	FBXL18	-0.5542	2.985602	-2.23915	0.027656	-3.9503	0.197886	0.233768
Non.inflammatory.Macrophages	ARNT	0.253799	5.869604	2.238771	0.027681	-4.4969	0.166027	0.215831
Non.inflammatory.Macrophages	UGT2B5	-1.02624	1.786068	-2.23877	0.027681	-3.55609	0.212745	0.241805
Non.inflammatory.Macrophages	AVL9	0.254169	6.010486	2.238469	0.027702	-4.39458	0.164624	0.215077
Non.inflammatory.Macrophages	IL3RA	0.381797	3.561284	2.236953	0.027804	-3.98243	0.191518	0.23081
Non.inflammatory.Macrophages	MPP7	0.378112	7.383215	2.236941	0.027805	-4.79741	0.151658	0.207514
Non.inflammatory.Macrophages	GM10658	-0.77485	1.776179	-2.23688	0.027809	-3.55418	0.213337	0.24256
Non.inflammatory.Macrophages	GM10130	0.701158	1.783227	2.236493	0.027836	-3.57859	0.213294	0.242625
Non.inflammatory.Macrophages	ZC3H10	-0.36441	2.921486	-2.23606	0.027865	-3.91891	0.199133	0.235124

Non.inflammatory.Macrophages	LRRC51	-0.62513	1.76843	-2.23605	0.027866	-3.64501	0.213484	0.242786
Non.inflammatory.Macrophages	RAB8A	0.211793	5.454242	2.235436	0.027908	-4.4113	0.170844	0.219261
Non.inflammatory.Macrophages	MYL12B	-0.15934	8.183707	-2.23522	0.027922	-4.86555	0.144537	0.203306
Non.inflammatory.Macrophages	SIL1	-0.27315	5.149956	-2.23471	0.027957	-4.34445	0.174162	0.22132
Non.inflammatory.Macrophages	RHD	0.845491	1.667344	2.234457	0.027974	-3.60466	0.215103	0.243958
Non.inflammatory.Macrophages	NPAS2	1.133587	0.089778	2.233418	0.028045	-3.46857	0.228516	0.255413
Non.inflammatory.Macrophages	PPP3R1	0.201096	5.5618	2.233078	0.028069	-4.44215	0.170205	0.219477
Non.inflammatory.Macrophages	SYBU	-1.45414	0.330557	-2.23246	0.028111	-3.45201	0.228665	0.254046
Non.inflammatory.Macrophages	TCERG1	0.187142	6.296507	2.23193	0.028147	-4.48545	0.162796	0.215339
Non.inflammatory.Macrophages	9430060I0	-1.03118	1.308677	-2.23172	0.028162	-3.4888	0.220358	0.247434
Non.inflammatory.Macrophages	CELF1	0.145917	7.40812	2.231704	0.028163	-4.69699	0.152076	0.208806
Non.inflammatory.Macrophages	LPCAT4	-0.73803	3.121289	-2.23167	0.028165	-3.69031	0.19754	0.235271
Non.inflammatory.Macrophages	SELENBP2	-1.04139	1.116284	-2.23115	0.0282	-3.50998	0.223063	0.248943
Non.inflammatory.Macrophages	STX7	0.172702	6.176182	2.230444	0.028249	-4.57767	0.164243	0.216477
Non.inflammatory.Macrophages	GM19605	-0.92069	1.260305	-2.23033	0.028257	-3.52041	0.221326	0.248233

Non.inflammatory.Macrophages	GM20342	-0.44892	3.499425	-2.22982	0.028292	-3.89047	0.193473	0.2335
Non.inflammatory.Macrophages	FOXN3	0.206313	8.899803	2.229313	0.028327	-4.96849	0.139101	0.200953
Non.inflammatory.Macrophages	GM28198	-0.54359	3.85447	-2.22921	0.028334	-3.83411	0.189402	0.231252
Non.inflammatory.Macrophages	ABCB11	-0.86901	1.681529	-2.22819	0.028405	-3.56751	0.216402	0.245964
Non.inflammatory.Macrophages	MCF2L	-1.31581	0.46165	-2.22787	0.028427	-3.46433	0.229691	0.254495
Non.inflammatory.Macrophages	IFT27	-0.40519	3.758551	-2.22752	0.028451	-4.00978	0.190953	0.232317
Non.inflammatory.Macrophages	RNF121	0.327901	4.41156	2.226877	0.028496	-4.17166	0.183627	0.228256
Non.inflammatory.Macrophages	FBXW9	-0.42815	2.418265	-2.22659	0.028516	-3.8297	0.20722	0.241377
Non.inflammatory.Macrophages	ALOX15	1.654503	-0.82153	2.22654	0.028519	-3.54641	0.229886	0.264108
Non.inflammatory.Macrophages	UQCR10	0.207962	6.961957	2.226149	0.028546	-4.69404	0.157185	0.21281
Non.inflammatory.Macrophages	GYPC	0.72683	3.080733	2.225448	0.028595	-3.68704	0.199263	0.23738
Non.inflammatory.Macrophages	KDM5D	3.413303	0.730109	2.225391	0.028599	-3.51665	0.228422	0.25341
Non.inflammatory.Macrophages	CD55B	-1.34957	0.178504	-2.22519	0.028613	-3.46434	0.230094	0.257388
Non.inflammatory.Macrophages	OGFRL1	0.269469	5.082967	2.224681	0.028649	-4.46345	0.176539	0.224659
Non.inflammatory.Macrophages	MAGT1	0.201464	5.872067	2.223646	0.028721	-4.55063	0.168555	0.219937

Non.inflammatory.Macrophages	TNKS2	0.159418	7.072389	2.223277	0.028746	-4.67165	0.156659	0.212836
Non.inflammatory.Macrophages	TNRC6A	0.193995	6.648204	2.222875	0.028775	-4.60383	0.160847	0.215436
Non.inflammatory.Macrophages	MFAP4	0.874046	1.50654	2.222458	0.028804	-3.62733	0.219924	0.248759
Non.inflammatory.Macrophages	GM1976	0.527161	2.701657	2.220441	0.028945	-3.75523	0.205517	0.241321
Non.inflammatory.Macrophages	MAML3	0.293925	6.974808	2.219967	0.028979	-4.69099	0.158404	0.21438
Non.inflammatory.Macrophages	CD274	0.606024	5.805413	2.219964	0.028979	-4.64754	0.170159	0.221474
Non.inflammatory.Macrophages	ATG4B	0.268981	4.800676	2.217782	0.029133	-4.26082	0.181777	0.228285
Non.inflammatory.Macrophages	GM5608	-0.70854	0.923456	-2.21694	0.029193	-3.66683	0.229754	0.254399
Non.inflammatory.Macrophages	S100A8	0.94232	6.633628	2.216865	0.029198	-4.51394	0.162601	0.217028
Non.inflammatory.Macrophages	PKN2	0.203122	6.774243	2.216831	0.0292	-4.63639	0.161206	0.21618
Non.inflammatory.Macrophages	ZDHHC21	0.276385	4.271545	2.216273	0.02924	-4.25856	0.187884	0.231955
Non.inflammatory.Macrophages	ATP5L	0.149793	8.67785	2.216202	0.029245	-4.98366	0.143474	0.20519
Non.inflammatory.Macrophages	4933433G1	-0.77135	0.742117	-2.21578	0.029274	-3.69052	0.231418	0.255937
Non.inflammatory.Macrophages	GM36161	0.947935	0.563057	2.215709	0.02928	-3.61867	0.232785	0.257213
Non.inflammatory.Macrophages	ILVBL	-0.33026	3.757211	-2.2155	0.029295	-4.09657	0.193851	0.235389

Non.inflammatory.Macrophages	VOPP1	0.297545	3.965239	2.214617	0.029357	-4.35726	0.191715	0.234297
Non.inflammatory.Macrophages	RACK1	0.129611	8.637453	2.213021	0.029471	-5.00095	0.144524	0.206167
Non.inflammatory.Macrophages	DIRAS2	-0.97958	0.718948	-2.21279	0.029488	-3.52365	0.23274	0.256984
Non.inflammatory.Macrophages	HILPDA	0.407539	4.798635	2.212524	0.029507	-4.49797	0.182836	0.229434
Non.inflammatory.Macrophages	PGM2L1	0.304666	5.192591	2.210498	0.029652	-4.37525	0.179269	0.22764
Non.inflammatory.Macrophages	LIPA	-0.21901	5.64326	-2.20973	0.029707	-4.70912	0.174623	0.225061
Non.inflammatory.Macrophages	BTBD19	-0.42494	2.725168	-2.20779	0.029847	-3.92781	0.209294	0.244449
Non.inflammatory.Macrophages	TMEM230	-0.20667	4.742854	-2.20774	0.029851	-4.38574	0.185162	0.231101
Non.inflammatory.Macrophages	4632404H1	-0.75462	1.238383	-2.20704	0.029901	-3.67088	0.228994	0.25502
Non.inflammatory.Macrophages	AKAP10	0.247074	5.860095	2.206919	0.02991	-4.54766	0.173022	0.224281
Non.inflammatory.Macrophages	CMTM7	0.18204	7.897065	2.206573	0.029935	-4.82456	0.152719	0.212049
Non.inflammatory.Macrophages	OSBPL8	0.229886	7.62098	2.206376	0.029949	-4.72547	0.155329	0.213713
Non.inflammatory.Macrophages	PYGL	0.286227	5.16283	2.20637	0.02995	-4.62524	0.180551	0.22885
Non.inflammatory.Macrophages	RIPOR1	-0.32515	3.960811	-2.20607	0.029971	-4.12708	0.194292	0.2367
Non.inflammatory.Macrophages	COX7C	0.149785	8.585633	2.204655	0.030074	-4.99773	0.146834	0.2086

Non.inflammatory.Macrophages	SAPCD2	-1.06645	1.790194	-2.20397	0.030124	-3.51552	0.222426	0.252306
Non.inflammatory.Macrophages	FOLR2	-0.47774	3.655517	-2.20322	0.030179	-4.32906	0.198778	0.239817
Non.inflammatory.Macrophages	ATP5J2	0.195659	7.723844	2.203126	0.030186	-4.84578	0.155036	0.214138
Non.inflammatory.Macrophages	APOBEC3	0.268388	6.862598	2.203095	0.030188	-4.59319	0.163444	0.219334
Non.inflammatory.Macrophages	TELO2	-0.58792	2.455163	-2.20213	0.030258	-3.84727	0.214139	0.248362
Non.inflammatory.Macrophages	TNIP1	0.3205	4.966974	2.200806	0.030355	-4.41745	0.184334	0.231835
Non.inflammatory.Macrophages	EZH2	-0.33567	6.663574	-2.19962	0.030443	-4.51227	0.166556	0.221437
Non.inflammatory.Macrophages	MBL1	-0.87027	1.455107	-2.19873	0.030508	-3.59623	0.228907	0.256328
Non.inflammatory.Macrophages	HOXA7	1.554702	-0.09629	2.198273	0.030541	-3.52396	0.239761	0.267753
Non.inflammatory.Macrophages	LCOR	0.204035	7.538405	2.198003	0.030561	-4.71381	0.158198	0.216546
Non.inflammatory.Macrophages	CPPED1	0.261939	3.886993	2.197659	0.030587	-4.18417	0.197788	0.239846
Non.inflammatory.Macrophages	TMEM50A	0.1668	7.711684	2.197356	0.030609	-4.85936	0.156588	0.215725
Non.inflammatory.Macrophages	SAMD1	-0.26189	4.976188	-2.19682	0.030649	-4.3747	0.18526	0.232953
Non.inflammatory.Macrophages	EML3	-0.32695	4.139794	-2.19634	0.030685	-4.14864	0.195014	0.23863
Non.inflammatory.Macrophages	CERK	0.224335	7.028222	2.196183	0.030696	-4.70161	0.16347	0.220196

Non.inflammatory.Macrophages	SETX	0.244326	5.905752	2.19555	0.030743	-4.49574	0.175224	0.227208
Non.inflammatory.Macrophages	RING1	-0.33555	3.585545	-2.19537	0.030756	-4.14326	0.201836	0.24239
Non.inflammatory.Macrophages	DNAH17	-0.57425	3.275665	-2.19512	0.030775	-4.10163	0.205665	0.244526
Non.inflammatory.Macrophages	CYP2B9	-1.00432	1.9577	-2.19493	0.030789	-3.66828	0.222714	0.253683
Non.inflammatory.Macrophages	SPATC1	-1.04346	-1.06407	-2.19388	0.030867	-3.51418	0.240779	0.276195
Non.inflammatory.Macrophages	CALU	0.220938	4.640586	2.193267	0.030912	-4.40294	0.189835	0.235857
Non.inflammatory.Macrophages	HIC1	-0.5571	2.158311	-2.19114	0.031071	-4.1091	0.221544	0.253553
Non.inflammatory.Macrophages	PLEK	0.242269	7.520494	2.190934	0.031087	-5.00802	0.159808	0.218447
Non.inflammatory.Macrophages	IFI30	0.238591	6.746076	2.190905	0.031089	-4.9176	0.167579	0.223207
Non.inflammatory.Macrophages	CKAP4	0.342966	4.030233	2.190455	0.031122	-4.32509	0.197902	0.240904
Non.inflammatory.Macrophages	UHMK1	0.232231	4.630018	2.189483	0.031195	-4.35393	0.191146	0.237099
Non.inflammatory.Macrophages	SETBP1	0.926754	4.621756	2.18922	0.031215	-3.81043	0.191253	0.23718
Non.inflammatory.Macrophages	GNS	0.203553	6.931874	2.188316	0.031283	-4.84117	0.166334	0.222685
Non.inflammatory.Macrophages	AFM	-0.7421	2.294567	-2.18716	0.03137	-3.73615	0.22102	0.253617
Non.inflammatory.Macrophages	NFYA	-0.29344	4.152707	-2.18704	0.031379	-4.27302	0.197485	0.240839

Non.inflammatory.Macrophages	CMC1	0.215388	4.601302	2.18596	0.031461	-4.43209	0.192557	0.238017
Non.inflammatory.Macrophages	LBR	-0.19321	7.219631	-2.18554	0.031493	-4.60308	0.164145	0.221392
Non.inflammatory.Macrophages	NAPG	0.245584	4.542195	2.185189	0.031519	-4.42697	0.193388	0.238604
Non.inflammatory.Macrophages	SRGN	0.191612	9.652655	2.183104	0.031677	-5.15544	0.142013	0.207426
Non.inflammatory.Macrophages	SERINC3	0.158079	9.707046	2.182911	0.031692	-5.05664	0.141539	0.207113
Non.inflammatory.Macrophages	TRMO	-0.40973	3.013486	-2.18254	0.031721	-3.98363	0.213193	0.249525
Non.inflammatory.Macrophages	A330023F2	0.366584	3.109027	2.181087	0.031831	-4.18803	0.212441	0.249212
Non.inflammatory.Macrophages	TUBB6	0.305463	3.749371	2.180658	0.031864	-4.52712	0.204348	0.244832
Non.inflammatory.Macrophages	ANKIB1	0.221115	5.807453	2.180615	0.031867	-4.5096	0.180259	0.231199
Non.inflammatory.Macrophages	INTS7	0.212878	5.677826	2.180614	0.031867	-4.39584	0.181693	0.232035
Non.inflammatory.Macrophages	WFDC17	0.477435	5.906168	2.180412	0.031883	-5.05147	0.179174	0.230596
Non.inflammatory.Macrophages	SLC25A28	-0.25558	4.806244	-2.17973	0.031935	-4.40077	0.191825	0.237846
Non.inflammatory.Macrophages	CYP27A1	-0.35126	2.194427	-2.17909	0.031984	-4.24779	0.22497	0.255897
Non.inflammatory.Macrophages	VTI1A	0.183128	7.233705	2.178681	0.032016	-4.80678	0.165572	0.222552
Non.inflammatory.Macrophages	MRPS28	0.329392	6.885185	2.178365	0.03204	-4.85254	0.169147	0.224803

Non.inflammatory.Macrophages	JAM3	-1.31613	0.244994	-2.17825	0.032049	-3.53941	0.246308	0.270453
Non.inflammatory.Macrophages	FAM111A	-0.21989	5.742953	-2.17752	0.032105	-4.65501	0.181573	0.232287
Non.inflammatory.Macrophages	BCKDHA	0.284272	4.820285	2.177369	0.032117	-4.39796	0.192094	0.238387
Non.inflammatory.Macrophages	GM47664	-0.34023	3.774581	-2.17688	0.032154	-4.14836	0.204743	0.245514
Non.inflammatory.Macrophages	SOD2	0.406975	6.929524	2.176839	0.032157	-4.85915	0.168876	0.224871
Non.inflammatory.Macrophages	ARHGAP12	0.242922	5.076663	2.175127	0.03229	-4.50895	0.18981	0.237193
Non.inflammatory.Macrophages	HIST1H1C	0.41901	4.106663	2.173109	0.032446	-4.27653	0.202224	0.244339
Non.inflammatory.Macrophages	FBXO33	-0.20705	5.984659	-2.17142	0.032577	-4.62729	0.180964	0.232287
Non.inflammatory.Macrophages	ABHD18	0.439163	3.182254	2.169163	0.032754	-4.05495	0.215617	0.251821
Non.inflammatory.Macrophages	FBXO28	0.278524	4.523471	2.169052	0.032763	-4.36364	0.198745	0.242617
Non.inflammatory.Macrophages	STXBP1	0.394405	3.786972	2.168388	0.032815	-4.18158	0.208063	0.24778
Non.inflammatory.Macrophages	ZC3H4	-0.21736	4.818849	-2.16812	0.032836	-4.4033	0.195415	0.240877
Non.inflammatory.Macrophages	2310022A1	0.360768	2.998253	2.167706	0.032868	-4.09727	0.218366	0.253561
Non.inflammatory.Macrophages	EMC6	0.204554	5.61708	2.167191	0.032909	-4.56166	0.186248	0.235874
Non.inflammatory.Macrophages	DDB2	-0.46584	3.43077	-2.16713	0.032913	-4.0641	0.21277	0.250678

Non.inflammatory.Macrophages	ERMARD	-0.37421	3.076068	-2.16402	0.033159	-4.07649	0.218891	0.254008
Non.inflammatory.Macrophages	SLC49A4	-0.28289	6.30055	-2.1633	0.033216	-4.81249	0.180063	0.232444
Non.inflammatory.Macrophages	ASAP1	0.198123	7.200045	2.162538	0.033276	-4.86336	0.170622	0.226894
Non.inflammatory.Macrophages	HDAC1	-0.18233	6.039987	-2.16122	0.033382	-4.61562	0.183547	0.234622
Non.inflammatory.Macrophages	PEMT	-0.55866	3.483706	-2.16093	0.033405	-4.05345	0.214487	0.252013
Non.inflammatory.Macrophages	IGSF8	-0.26647	4.266842	-2.16071	0.033422	-4.41009	0.20452	0.246649
Non.inflammatory.Macrophages	NRXN3	-1.03667	1.145579	-2.16051	0.033438	-3.63556	0.246982	0.26912
Non.inflammatory.Macrophages	GM29994	-0.63619	1.150146	-2.1603	0.033455	-3.73143	0.246914	0.269175
Non.inflammatory.Macrophages	UCHL3	-0.24193	6.235223	-2.15989	0.033487	-4.58161	0.181368	0.233763
Non.inflammatory.Macrophages	CARD10	-0.93086	1.098314	-2.15987	0.033489	-3.59967	0.247683	0.26968
Non.inflammatory.Macrophages	BAK1	0.259933	4.936771	2.159832	0.033492	-4.53145	0.196342	0.242369
Non.inflammatory.Macrophages	COL13A1	-1.13504	0.62777	-2.15863	0.033588	-3.58827	0.253352	0.273747
Non.inflammatory.Macrophages	CMPK1	0.152251	6.881961	2.158625	0.033589	-4.79518	0.174631	0.230028
Non.inflammatory.Macrophages	GPR157	0.464423	2.137362	2.158334	0.033612	-3.95132	0.233124	0.262566
Non.inflammatory.Macrophages	FBXO8	0.264848	4.606605	2.156747	0.033739	-4.29787	0.201361	0.245526

Non.inflammatory.Macrophages	MTUS1	-0.34679	3.721745	-2.15584	0.033812	-4.51825	0.212833	0.251839
Non.inflammatory.Macrophages	GM2000	-0.33296	4.799183	-2.15555	0.033836	-4.46995	0.199358	0.244487
Non.inflammatory.Macrophages	UQCC2	0.222432	6.141014	2.15445	0.033924	-4.7317	0.184049	0.235796
Non.inflammatory.Macrophages	RNF7	0.207263	6.742111	2.153793	0.033977	-4.7561	0.177578	0.231996
Non.inflammatory.Macrophages	MYCL	-1.2914	1.305739	-2.15311	0.034033	-3.58249	0.247192	0.269921
Non.inflammatory.Macrophages	CAPZA1	0.132135	7.715586	2.153098	0.034034	-4.89778	0.167378	0.225824
Non.inflammatory.Macrophages	RYR2	1.400502	0.059974	2.151459	0.034167	-3.60732	0.257324	0.279856
Non.inflammatory.Macrophages	TSHZ3	0.611427	2.187789	2.150361	0.034256	-4.05012	0.235613	0.264188
Non.inflammatory.Macrophages	MIR99AHG	0.562829	3.430837	2.150255	0.034265	-4.22244	0.218548	0.255213
Non.inflammatory.Macrophages	TEAD2	-0.91915	1.154161	-2.14992	0.034292	-3.62777	0.250784	0.271948
Non.inflammatory.Macrophages	SUMO1	-0.11124	7.992553	-2.14943	0.034332	-4.91459	0.165538	0.224994
Non.inflammatory.Macrophages	HSBP1	0.208955	6.003464	2.14898	0.034369	-4.72449	0.187096	0.237945
Non.inflammatory.Macrophages	LY75	-1.35521	3.332572	-2.14686	0.034542	-3.64321	0.221101	0.256826
Non.inflammatory.Macrophages	ADIPOR2	0.213666	6.368043	2.146708	0.034555	-4.64376	0.183755	0.236048
Non.inflammatory.Macrophages	POLQ	-0.49775	3.166551	-2.14575	0.034634	-3.98187	0.223723	0.258349

Non.inflammatory.Macrophages	GOLPH3L	0.280826	4.39604	2.145455	0.034658	-4.43049	0.20765	0.249728
Non.inflammatory.Macrophages	CELA1	0.455262	2.24977	2.144902	0.034703	-4.16136	0.236501	0.265333
Non.inflammatory.Macrophages	CLTA	-0.13124	8.442682	-2.14479	0.034712	-5.13337	0.162099	0.223356
Non.inflammatory.Macrophages	WASF2	0.138741	8.064634	2.144766	0.034714	-4.98477	0.165906	0.225718
Non.inflammatory.Macrophages	MYSM1	0.226052	5.388465	2.144109	0.034769	-4.52477	0.195654	0.243356
Non.inflammatory.Macrophages	3110056K(	-0.25817	4.723448	-2.14345	0.034823	-4.33586	0.203959	0.248023
Non.inflammatory.Macrophages	SAMD8	0.222579	5.289431	2.142969	0.034863	-4.63323	0.197156	0.244322
Non.inflammatory.Macrophages	MAP4K5	0.324774	4.52083	2.142564	0.034896	-4.37667	0.20665	0.249727
Non.inflammatory.Macrophages	NT5DC3	-0.23435	5.099601	-2.14244	0.034907	-4.55814	0.199486	0.245765
Non.inflammatory.Macrophages	PAK2	0.139255	7.859842	2.140983	0.035027	-4.96718	0.168925	0.227975
Non.inflammatory.Macrophages	SOD1	-0.19808	5.993562	-2.1407	0.035051	-4.8281	0.189393	0.240174
Non.inflammatory.Macrophages	URAH	-0.62413	3.327083	-2.1406	0.035059	-4.11348	0.222797	0.258689
Non.inflammatory.Macrophages	CNPPD1	-0.24415	5.686802	-2.13998	0.035111	-4.62578	0.193157	0.242349
Non.inflammatory.Macrophages	ASPRV1	1.272964	1.33931	2.139492	0.035151	-3.65183	0.251574	0.273611
Non.inflammatory.Macrophages	GM48512	-0.82551	1.068935	-2.13853	0.035232	-3.67857	0.25613	0.276051

Non.inflammatory.Macrophages	CREG1	-0.18689	7.514034	-2.13818	0.035261	-5.18982	0.17316	0.230777
Non.inflammatory.Macrophages	FGL2	0.453967	4.054695	2.137736	0.035298	-4.61318	0.214033	0.254332
Non.inflammatory.Macrophages	AGFG2	0.278855	4.605897	2.136712	0.035384	-4.40467	0.207361	0.250861
Non.inflammatory.Macrophages	SCAI	0.370215	4.434969	2.135818	0.035458	-4.26565	0.209856	0.252253
Non.inflammatory.Macrophages	STAM	0.247586	4.382468	2.135594	0.035477	-4.36817	0.210527	0.252678
Non.inflammatory.Macrophages	SBNO1	-0.17107	7.359347	-2.13497	0.03553	-4.89006	0.175664	0.232697
Non.inflammatory.Macrophages	LGMN	0.21628	7.188159	2.133662	0.03564	-5.14221	0.177969	0.234146
Non.inflammatory.Macrophages	AGXT2	-0.92466	1.612637	-2.13325	0.035675	-3.73091	0.249864	0.273598
Non.inflammatory.Macrophages	FAM122A	0.355658	3.563965	2.132552	0.035734	-4.13717	0.222077	0.259257
Non.inflammatory.Macrophages	TKTL1	-0.64315	1.290296	-2.13248	0.03574	-3.81113	0.254749	0.276192
Non.inflammatory.Macrophages	TXNRD2	0.331258	3.532111	2.132464	0.035741	-4.3608	0.222507	0.259499
Non.inflammatory.Macrophages	HIST2H2A/	0.649281	2.225914	2.132394	0.035747	-3.91279	0.240805	0.269124
Non.inflammatory.Macrophages	CTDSPL2	0.23566	5.892856	2.132197	0.035764	-4.57592	0.192697	0.24304
Non.inflammatory.Macrophages	MAFK	-0.32854	4.524193	-2.1314	0.035831	-4.50454	0.209764	0.252704
Non.inflammatory.Macrophages	CAP1	0.162695	6.878742	2.130859	0.035877	-4.87755	0.181702	0.236777

Non.inflammatory.Macrophages	RIC1	0.251874	6.710291	2.130827	0.03588	-4.83385	0.183587	0.23789
Non.inflammatory.Macrophages	STRN3	0.185778	7.654054	2.129451	0.035996	-4.94188	0.173734	0.231918
Non.inflammatory.Macrophages	MPEG1	0.274868	6.081136	2.12916	0.036021	-5.0751	0.19134	0.242386
Non.inflammatory.Macrophages	ACER1	-1.26631	-0.7216	-2.12889	0.036044	-3.65418	0.265267	0.292927
Non.inflammatory.Macrophages	ADCY3	-0.62236	3.001893	-2.12827	0.036097	-4.13096	0.231034	0.264295
Non.inflammatory.Macrophages	PANX1	0.331187	4.208683	2.127769	0.03614	-4.19327	0.214854	0.255717
Non.inflammatory.Macrophages	STK11	0.211323	5.696969	2.127223	0.036186	-4.6017	0.196359	0.245515
Non.inflammatory.Macrophages	GLA	-0.27986	4.568639	-2.12684	0.036219	-4.61828	0.210431	0.253456
Non.inflammatory.Macrophages	CCDC88B	0.270158	3.695402	2.126492	0.036249	-4.47288	0.22197	0.259735
Non.inflammatory.Macrophages	HAO2	-1.17861	1.393032	-2.12483	0.036391	-3.71425	0.255948	0.27757
Non.inflammatory.Macrophages	IGKV1-135	-1.38914	-0.02384	-2.12439	0.036429	-3.62661	0.266962	0.288871
Non.inflammatory.Macrophages	GM17276	-0.73299	0.785789	-2.12435	0.036433	-3.86389	0.264458	0.282454
Non.inflammatory.Macrophages	AGMAT	-0.94945	1.438737	-2.12359	0.036498	-3.74827	0.25558	0.277666
Non.inflammatory.Macrophages	GM41335	-1.0865	0.493808	-2.12295	0.036553	-3.71121	0.267391	0.285183
Non.inflammatory.Macrophages	SBNO2	0.19578	5.566821	2.122751	0.03657	-4.72103	0.199073	0.247666

Non.inflammatory.Macrophages	PBX1	0.311971	5.465012	2.122709	0.036574	-4.66711	0.200315	0.248369
Non.inflammatory.Macrophages	ZRANB3	-0.4804	3.148069	-2.12215	0.036622	-4.03274	0.230821	0.265117
Non.inflammatory.Macrophages	RAD23B	-0.14412	6.360558	-2.1217	0.036661	-4.81877	0.189888	0.242529
Non.inflammatory.Macrophages	FYN	0.323442	7.843798	2.119738	0.036831	-4.99081	0.174092	0.233321
Non.inflammatory.Macrophages	CIAPIN1	0.289189	4.643928	2.119084	0.036887	-4.42281	0.211953	0.255248
Non.inflammatory.Macrophages	RFFL	0.22766	5.915131	2.11794	0.036987	-4.84386	0.196514	0.246618
Non.inflammatory.Macrophages	RETNLA	2.520319	-0.77811	2.117459	0.037029	-3.82173	0.26966	0.297232
Non.inflammatory.Macrophages	COPS9	0.172522	6.68605	2.117427	0.037032	-4.93096	0.187457	0.24152
Non.inflammatory.Macrophages	1700102P(	-0.80961	0.812589	-2.11733	0.03704	-3.77215	0.266856	0.284396
Non.inflammatory.Macrophages	MRPL52	0.189579	7.102833	2.117057	0.037064	-4.94217	0.18275	0.238824
Non.inflammatory.Macrophages	HECTD1	0.163809	7.428886	2.116722	0.037093	-4.92387	0.179177	0.236796
Non.inflammatory.Macrophages	SLC25A18	-0.58869	4.491338	-2.11571	0.037181	-4.19903	0.214826	0.257277
Non.inflammatory.Macrophages	BUD13	0.331986	3.714639	2.115248	0.037222	-4.18672	0.225341	0.26308
Non.inflammatory.Macrophages	GT(ROSA)2	-0.28714	4.594685	-2.11355	0.03737	-4.49512	0.214334	0.257305
Non.inflammatory.Macrophages	CD2	-0.76793	3.660392	-2.11327	0.037395	-3.80984	0.226873	0.264195

Non.inflammatory.Macrophages	INPP1	0.367094	4.03687	2.113082	0.037412	-4.27715	0.221743	0.261496
Non.inflammatory.Macrophages	RRS1	-0.28197	4.476059	-2.11179	0.037526	-4.44155	0.216439	0.258724
Non.inflammatory.Macrophages	SCCPDH	0.448725	2.465776	2.111172	0.03758	-4.0162	0.244727	0.273686
Non.inflammatory.Macrophages	CENPV	-0.39248	3.40694	-2.1097	0.037711	-4.40197	0.231856	0.267265
Non.inflammatory.Macrophages	LMBRD1	0.193031	6.356577	2.109264	0.037749	-4.8784	0.193723	0.246365
Non.inflammatory.Macrophages	1810059H2	-1.22548	1.799204	-2.10919	0.037755	-3.69643	0.255539	0.279689
Non.inflammatory.Macrophages	TLR2	-0.27707	3.875493	-2.10838	0.037827	-4.74549	0.225687	0.264264
Non.inflammatory.Macrophages	LRRC47	0.241088	4.227098	2.107232	0.037929	-4.42153	0.221041	0.261998
Non.inflammatory.Macrophages	CDKN1B	0.223464	6.470899	2.107212	0.037931	-4.81986	0.192744	0.246132
Non.inflammatory.Macrophages	GM37168	-0.5685	0.65738	-2.10691	0.037958	-3.96422	0.272405	0.289416
Non.inflammatory.Macrophages	EVI5	0.252118	5.166502	2.106844	0.037964	-4.72045	0.208743	0.255301
Non.inflammatory.Macrophages	TMEM116	-0.55659	2.038927	-2.10666	0.037981	-4.02208	0.252364	0.278532
Non.inflammatory.Macrophages	DSTN	0.211688	6.886463	2.106616	0.037984	-4.93734	0.187899	0.243378
Non.inflammatory.Macrophages	GM49482	-0.89138	-0.71551	-2.10655	0.03799	-3.73395	0.273635	0.300673
Non.inflammatory.Macrophages	GSTP1	0.230728	6.928594	2.105544	0.03808	-4.95928	0.187673	0.243357

Non.inflammatory.Macrophages	LY6G5B	-0.90374	1.345565	-2.10551	0.038083	-3.69766	0.263474	0.284263
Non.inflammatory.Macrophages	ANKRD10	0.231157	5.371393	2.104961	0.038132	-4.51781	0.206587	0.254293
Non.inflammatory.Macrophages	KRIT1	0.208777	5.937975	2.103924	0.038225	-4.65199	0.199936	0.250605
Non.inflammatory.Macrophages	AP1B1	0.209893	5.190975	2.103581	0.038255	-4.68662	0.209329	0.255967
Non.inflammatory.Macrophages	ZFP97	0.734785	1.263623	2.102754	0.03833	-3.78772	0.265921	0.28578
Non.inflammatory.Macrophages	ANKRD22	1.271489	-0.56253	2.101543	0.038439	-3.66139	0.275531	0.30114
Non.inflammatory.Macrophages	TUBB2A	-0.29284	5.517234	-2.10153	0.038439	-4.7311	0.205736	0.254305
Non.inflammatory.Macrophages	COX6A1	0.184645	6.768979	2.101348	0.038456	-4.95209	0.190568	0.245628
Non.inflammatory.Macrophages	HYAL1	-0.55871	1.311575	-2.10106	0.038482	-3.90371	0.265476	0.286014
Non.inflammatory.Macrophages	SRA1	0.202204	5.239409	2.100894	0.038497	-4.67733	0.209256	0.256431
Non.inflammatory.Macrophages	TCF3	-0.24215	6.601305	-2.10088	0.038498	-4.72395	0.192535	0.24689
Non.inflammatory.Macrophages	PLOD3	0.287232	3.277379	2.100367	0.038544	-4.50617	0.235849	0.270981
Non.inflammatory.Macrophages	GLRX	0.252353	6.214402	2.100215	0.038558	-4.80468	0.197203	0.249718
Non.inflammatory.Macrophages	RHOB	-0.36553	5.891039	-2.10009	0.038569	-4.85657	0.201143	0.251989
Non.inflammatory.Macrophages	DNAH1	-1.09403	-0.10849	-2.09814	0.038746	-3.68346	0.276633	0.298595

Non.inflammatory.Macrophages	GM49189	-1.17022	0.779988	-2.09805	0.038754	-3.6673	0.274099	0.291333
Non.inflammatory.Macrophages	GM10790	1.306481	-0.77429	2.097296	0.038822	-3.68291	0.276895	0.304404
Non.inflammatory.Macrophages	GBF1	-0.16182	6.562441	-2.09711	0.038839	-4.79109	0.193949	0.248253
Non.inflammatory.Macrophages	TSG101	0.200219	5.622255	2.09697	0.038852	-4.72244	0.205432	0.25486
Non.inflammatory.Macrophages	IGFBP4	-0.54211	5.679023	-2.09637	0.038906	-4.74202	0.20473	0.254657
Non.inflammatory.Macrophages	4931403E2	-0.78047	1.501737	-2.09637	0.038907	-3.72419	0.263772	0.286045
Non.inflammatory.Macrophages	TK2	-0.31662	3.825305	-2.09603	0.038937	-4.42594	0.229215	0.268253
Non.inflammatory.Macrophages	TRMT10A	-0.32569	3.81366	-2.0958	0.038959	-4.31118	0.229377	0.26834
Non.inflammatory.Macrophages	PNPLA2	0.25964	5.509886	2.095674	0.03897	-4.69209	0.206856	0.255963
Non.inflammatory.Macrophages	TUSC3	-0.23925	4.978446	-2.0956	0.038976	-4.6241	0.213677	0.259779
Non.inflammatory.Macrophages	HCLS1	0.203075	6.691521	2.095017	0.03903	-4.9056	0.192536	0.247856
Non.inflammatory.Macrophages	ZDHHC16	0.329898	2.888345	2.094917	0.039039	-4.15804	0.242747	0.275534
Non.inflammatory.Macrophages	DRAM2	0.207688	4.847911	2.093661	0.039154	-4.76164	0.215925	0.261092
Non.inflammatory.Macrophages	LRCH3	0.204664	6.497112	2.09363	0.039156	-4.78222	0.195224	0.249375
Non.inflammatory.Macrophages	TULP4	-0.20436	5.928036	-2.09316	0.039199	-4.82291	0.202259	0.253458

Non.inflammatory.Macrophages	CLDN3	-0.91095	1.461002	-2.09216	0.039291	-3.77321	0.265635	0.287418
Non.inflammatory.Macrophages	DAD1	0.169031	6.948328	2.092065	0.0393	-4.97056	0.190298	0.246749
Non.inflammatory.Macrophages	GALNT11	0.259608	4.120023	2.091772	0.039327	-4.46536	0.226218	0.267045
Non.inflammatory.Macrophages	CDC27	0.210845	6.009717	2.091275	0.039372	-4.72766	0.201686	0.253416
Non.inflammatory.Macrophages	2410004B1	-0.21694	4.71052	-2.09112	0.039387	-4.52342	0.218337	0.262784
Non.inflammatory.Macrophages	RFWD3	0.225232	5.6362	2.090211	0.03947	-4.63732	0.206564	0.256226
Non.inflammatory.Macrophages	IYD	-1.08879	0.499692	-2.09016	0.039475	-3.69466	0.27859	0.295579
Non.inflammatory.Macrophages	CD300LD	-0.3224	3.173108	-2.08999	0.039491	-4.72527	0.239974	0.274422
Non.inflammatory.Macrophages	PLK3	-0.311	4.483011	-2.08977	0.039511	-4.6186	0.221619	0.26469
Non.inflammatory.Macrophages	TSC22D3	-0.30787	5.71665	-2.08845	0.039633	-4.78016	0.206077	0.256323
Non.inflammatory.Macrophages	GUCY2C	-0.76311	-0.14698	-2.08812	0.039663	-3.76092	0.279436	0.301855
Non.inflammatory.Macrophages	MOB1A	0.171353	5.970142	2.087373	0.039732	-4.86418	0.202993	0.254901
Non.inflammatory.Macrophages	PITRM1	-0.33094	3.560314	-2.08715	0.039753	-4.32957	0.235104	0.272646
Non.inflammatory.Macrophages	HERC6	0.381518	4.719873	2.08675	0.03979	-4.62548	0.219095	0.264086
Non.inflammatory.Macrophages	CNIH4	0.191672	5.763142	2.086538	0.03981	-4.80597	0.205578	0.256575

Non.inflammatory.Macrophages	DDX50	-0.16171	6.369428	-2.08627	0.039834	-4.81779	0.198093	0.252418
Non.inflammatory.Macrophages	DNAH8	-0.8934	3.758702	-2.08598	0.039861	-3.77504	0.232289	0.271525
Non.inflammatory.Macrophages	ARHGDIB	0.234134	8.995183	2.085814	0.039877	-5.20104	0.168618	0.234764
Non.inflammatory.Macrophages	GM32036	-0.34445	3.651119	-2.08579	0.039879	-4.18086	0.233811	0.272416
Non.inflammatory.Macrophages	ATP5MPL	0.153803	8.484004	2.085556	0.039901	-5.20487	0.173997	0.238187
Non.inflammatory.Macrophages	SDCBP	0.148651	7.674968	2.085513	0.039905	-5.19053	0.182859	0.24361
Non.inflammatory.Macrophages	GM9967	-0.98386	0.569425	-2.0854	0.039915	-3.80378	0.278922	0.296862
Non.inflammatory.Macrophages	BECN1	0.190764	5.896307	2.085361	0.039919	-4.77923	0.203911	0.255976
Non.inflammatory.Macrophages	TMEM38B	0.291166	4.174266	2.08515	0.039939	-4.45726	0.226496	0.268587
Non.inflammatory.Macrophages	ARFGAP1	0.249514	4.620741	2.084743	0.039977	-4.49445	0.220518	0.265423
Non.inflammatory.Macrophages	ADAMTS7	-1.09541	0.676356	-2.0845	0.04	-3.71373	0.278165	0.296243
Non.inflammatory.Macrophages	CAPN2	0.267011	4.14309	2.083807	0.040064	-4.47569	0.227284	0.269228
Non.inflammatory.Macrophages	DDHD1	0.245261	6.276227	2.083342	0.040107	-5.00996	0.199616	0.253865
Non.inflammatory.Macrophages	P4HA2	0.700001	0.108055	2.083088	0.040131	-3.8869	0.280035	0.301486
Non.inflammatory.Macrophages	NDUFC1	0.194556	7.367214	2.082794	0.040158	-5.05459	0.186708	0.24638

Non.inflammatory.Macrophages	GM550	-0.88057	0.859238	-2.08276	0.040162	-3.76388	0.276629	0.29529
Non.inflammatory.Macrophages	GM12992	-0.59455	2.041763	-2.08216	0.040217	-4.06096	0.258405	0.285865
Non.inflammatory.Macrophages	RDH5	-0.60929	2.368316	-2.08192	0.04024	-3.99439	0.253365	0.283296
Non.inflammatory.Macrophages	GALNS	0.346747	3.757313	2.081458	0.040283	-4.28828	0.232927	0.272714
Non.inflammatory.Macrophages	EFR3A	0.17679	5.780301	2.081377	0.040291	-4.80269	0.20591	0.257793
Non.inflammatory.Macrophages	TMF1	0.171694	6.243916	2.081351	0.040294	-4.84525	0.200152	0.254487
Non.inflammatory.Macrophages	CAR5A	-1.10681	0.542195	-2.08095	0.040331	-3.72195	0.279884	0.298347
Non.inflammatory.Macrophages	MRPL23	0.154681	6.069484	2.080872	0.040338	-4.85709	0.202318	0.255825
Non.inflammatory.Macrophages	PATL1	-0.20682	5.222719	-2.08034	0.040389	-4.72701	0.213147	0.262081
Non.inflammatory.Macrophages	PHLDA1	0.404582	4.035457	2.080258	0.040396	-4.52003	0.229135	0.270909
Non.inflammatory.Macrophages	CELF6	-1.00148	-0.70622	-2.07965	0.040453	-3.78305	0.280591	0.309228
Non.inflammatory.Macrophages	GM11464	-0.85666	-0.20123	-2.07948	0.04047	-3.7636	0.280591	0.304981
Non.inflammatory.Macrophages	CD164L2	-0.58694	1.352514	-2.07782	0.040626	-4.04617	0.270589	0.292735
Non.inflammatory.Macrophages	AP5Z1	-0.37053	2.539751	-2.07743	0.040663	-4.17873	0.252017	0.283398
Non.inflammatory.Macrophages	PTK7	-1.07331	1.073787	-2.07698	0.040705	-3.79877	0.27528	0.295331

Non.inflammatory.Macrophages	APBB1IP	0.171393	7.638967	2.076959	0.040707	-5.08159	0.184688	0.246019
Non.inflammatory.Macrophages	PLCB3	-0.36719	2.612	-2.07597	0.040801	-4.2549	0.251386	0.283338
Non.inflammatory.Macrophages	AI182371	-0.74722	2.439191	-2.0751	0.040883	-3.97314	0.254405	0.285058
Non.inflammatory.Macrophages	IFITM3	0.320901	7.709419	2.074826	0.040909	-5.38328	0.184521	0.246209
Non.inflammatory.Macrophages	PAPSS2	-0.5472	2.513577	-2.07436	0.040953	-4.17032	0.253441	0.284708
Non.inflammatory.Macrophages	NEMP1	-0.43983	3.065406	-2.07321	0.041063	-4.289	0.245645	0.280792
Non.inflammatory.Macrophages	AADAC	-0.81679	2.701819	-2.07297	0.041086	-4.00903	0.251109	0.283671
Non.inflammatory.Macrophages	GM41409	-0.4157	3.031183	-2.07279	0.041103	-4.47862	0.246155	0.281161
Non.inflammatory.Macrophages	TIGIT	-1.27019	-0.09862	-2.07253	0.041128	-3.70793	0.283416	0.306752
Non.inflammatory.Macrophages	KDM6A	-0.43405	6.914035	-2.07203	0.041175	-4.94885	0.19441	0.252434
Non.inflammatory.Macrophages	RAB32	-0.19849	4.344985	-2.07165	0.041212	-4.90979	0.227534	0.271245
Non.inflammatory.Macrophages	GM16104	1.073722	-0.60575	2.071182	0.041257	-3.76216	0.283782	0.31144
Non.inflammatory.Macrophages	ZBTB24	-0.3127	3.32852	-2.07109	0.041265	-4.25252	0.242095	0.279191
Non.inflammatory.Macrophages	CLASP2	0.170804	6.927855	2.070683	0.041304	-5.0364	0.194446	0.252629
Non.inflammatory.Macrophages	PAM	0.3179	4.417759	2.070385	0.041333	-4.49145	0.226678	0.270937

Non.inflammatory.Macrophages	1600020EC	-0.26262	6.223144	-2.07027	0.041344	-4.81326	0.203022	0.257688
Non.inflammatory.Macrophages	UROS	-0.3516	2.669254	-2.0694	0.041427	-4.17652	0.252429	0.284713
Non.inflammatory.Macrophages	HMGN3	-0.84708	3.255903	-2.06859	0.041505	-3.85666	0.2439	0.280422
Non.inflammatory.Macrophages	B4GALT7	0.371949	2.869796	2.068275	0.041535	-4.21688	0.249669	0.283553
Non.inflammatory.Macrophages	SNX13	0.243138	5.413359	2.068256	0.041537	-4.75886	0.213891	0.264173
Non.inflammatory.Macrophages	ARHGAP19	-0.35009	4.256309	-2.06717	0.041642	-4.60361	0.229986	0.273117
Non.inflammatory.Macrophages	NDRG1	0.674046	2.72107	2.06665	0.041692	-4.01048	0.252608	0.285185
Non.inflammatory.Macrophages	ERG28	-0.20229	5.118614	-2.06637	0.041719	-4.63724	0.218394	0.266856
Non.inflammatory.Macrophages	PIN4	0.194004	4.949389	2.066134	0.041742	-4.68148	0.220671	0.268195
Non.inflammatory.Macrophages	TNS3	-0.23928	5.235143	-2.065	0.041851	-4.96804	0.217316	0.266406
Non.inflammatory.Macrophages	MTM1	0.290505	4.433358	2.06433	0.041917	-4.67228	0.228442	0.272597
Non.inflammatory.Macrophages	PRXL2A	0.364999	4.184431	2.063716	0.041976	-4.72841	0.232113	0.274697
Non.inflammatory.Macrophages	VEZT	0.254415	4.178132	2.063552	0.041992	-4.51259	0.232202	0.274828
Non.inflammatory.Macrophages	ZDHHC5	0.184922	5.017726	2.06268	0.042077	-4.67334	0.220847	0.268773
Non.inflammatory.Macrophages	PLCB1	0.451392	4.622522	2.062204	0.042123	-4.62264	0.226232	0.271861

Non.inflammatory.Macrophages	ST5	-0.61353	2.235439	-2.06216	0.042128	-3.97824	0.261469	0.290531
Non.inflammatory.Macrophages	GM16090	-1.01233	-0.96132	-2.06204	0.042139	-3.80368	0.286886	0.317532
Non.inflammatory.Macrophages	NOL10	0.255595	4.91748	2.061858	0.042157	-4.57154	0.222202	0.269739
Non.inflammatory.Macrophages	PHLDB3	-0.52789	2.97973	-2.06181	0.042162	-4.0081	0.24997	0.284696
Non.inflammatory.Macrophages	FABP1	-0.55406	7.428716	-2.0614	0.042201	-5.05606	0.190603	0.251626
Non.inflammatory.Macrophages	NPEPL1	-0.22542	5.049543	-2.06097	0.042244	-4.68661	0.220473	0.268894
Non.inflammatory.Macrophages	MTHFD2	-0.30796	5.486209	-2.06054	0.042285	-4.68369	0.214674	0.265735
Non.inflammatory.Macrophages	RTF2	0.166497	5.957747	2.060509	0.042289	-4.84487	0.208575	0.262269
Non.inflammatory.Macrophages	TOB2	-0.18378	6.656821	-2.0605	0.042289	-5.0602	0.199839	0.257214
Non.inflammatory.Macrophages	TMEM68	0.263545	3.807022	2.0604	0.042299	-4.47152	0.237798	0.278452
Non.inflammatory.Macrophages	TIMD2	-0.97047	0.986598	-2.05941	0.042396	-3.77169	0.282427	0.301503
Non.inflammatory.Macrophages	TAF7	-0.21986	5.1473	-2.0585	0.042485	-4.61634	0.219904	0.268819
Non.inflammatory.Macrophages	NUFIP1	0.234857	4.171093	2.058081	0.042527	-4.55713	0.233491	0.276405
Non.inflammatory.Macrophages	GM49067	-0.82829	0.406337	-2.05751	0.042583	-3.79097	0.288214	0.307165
Non.inflammatory.Macrophages	A930029G:	-0.82479	1.230907	-2.05742	0.042591	-3.82643	0.279043	0.300235

Non.inflammatory.Macrophages	MAT2B	0.191843	5.679223	2.056704	0.042662	-4.80146	0.213257	0.265568
Non.inflammatory.Macrophages	SYN1	-0.64157	0.783746	-2.05639	0.042693	-4.06368	0.285761	0.30438
Non.inflammatory.Macrophages	SOCS7	0.255857	4.207792	2.05606	0.042725	-4.54601	0.233278	0.276784
Non.inflammatory.Macrophages	REEP3	0.187146	6.727725	2.055994	0.042732	-5.0031	0.200004	0.258032
Non.inflammatory.Macrophages	KRT10	-0.55987	2.100333	-2.0559	0.042742	-3.99289	0.26504	0.293498
Non.inflammatory.Macrophages	ASAH2	0.398238	2.945427	2.055779	0.042753	-4.3775	0.25185	0.28668
Non.inflammatory.Macrophages	GM28379	1.015099	0.264238	2.055498	0.042781	-3.77998	0.288488	0.308972
Non.inflammatory.Macrophages	JAK3	0.462678	2.778845	2.054763	0.042853	-4.21372	0.254666	0.288177
Non.inflammatory.Macrophages	CSTDC4	0.998494	3.661016	2.054571	0.042872	-4.23065	0.241411	0.28119
Non.inflammatory.Macrophages	SLC16A7	-0.31123	3.236151	-2.05446	0.042883	-4.50211	0.247713	0.284534
Non.inflammatory.Macrophages	GM50019	-0.95477	-0.06748	-2.05375	0.042953	-3.79836	0.288752	0.312159
Non.inflammatory.Macrophages	DPP6	0.736927	-0.15893	2.05375	0.042954	-3.96754	0.288752	0.312953
Non.inflammatory.Macrophages	BC029722	0.331989	3.061908	2.053628	0.042966	-4.3749	0.250347	0.286178
Non.inflammatory.Macrophages	GM10371	-0.80101	-0.68835	-2.05359	0.042969	-3.81885	0.288752	0.317603
Non.inflammatory.Macrophages	CCT6A	0.199601	5.802009	2.052523	0.043075	-4.83866	0.212306	0.265521

Non.inflammatory.Macrophages	MNT	-0.25208	4.606728	-2.05116	0.04321	-4.68586	0.228975	0.274959
Non.inflammatory.Macrophages	LRMDA	0.29236	7.760594	2.050826	0.043244	-5.34094	0.188849	0.251967
Non.inflammatory.Macrophages	GM14221	-0.46166	1.910062	-2.05061	0.043265	-4.43506	0.269695	0.296628
Non.inflammatory.Macrophages	DR1	0.196446	4.953577	2.050287	0.043298	-4.7022	0.224304	0.272666
Non.inflammatory.Macrophages	SMAD6	0.382077	2.733914	2.04978	0.043348	-4.59866	0.256853	0.290187
Non.inflammatory.Macrophages	TNFRSF13E	0.292537	4.518479	2.048528	0.043474	-4.49748	0.231038	0.276638
Non.inflammatory.Macrophages	6-Mar	0.165377	6.3538	2.047674	0.043559	-4.89105	0.206818	0.263047
Non.inflammatory.Macrophages	TBC1D20	0.234971	5.789234	2.047318	0.043595	-4.75499	0.214086	0.267347
Non.inflammatory.Macrophages	APOC4	-0.52141	5.632488	-2.0473	0.043597	-4.7859	0.216147	0.268516
Non.inflammatory.Macrophages	CUTA	-0.16829	6.189562	-2.04679	0.043647	-4.93865	0.208968	0.264557
Non.inflammatory.Macrophages	SLC36A3OS	1.378625	1.010121	2.046607	0.043666	-3.75528	0.286087	0.305637
Non.inflammatory.Macrophages	MRAP	0.84051	1.214744	2.046443	0.043683	-3.94842	0.282599	0.303984
Non.inflammatory.Macrophages	MZB1	-0.60992	5.956787	-2.04616	0.043711	-4.31984	0.211965	0.266463
Non.inflammatory.Macrophages	TIMM8B	0.237993	5.242586	2.046103	0.043717	-4.80696	0.22142	0.271832
Non.inflammatory.Macrophages	LDLRAD3	0.508952	4.591753	2.045004	0.043828	-4.24883	0.23079	0.277135

Non.inflammatory.Macrophages	MRPS18A	0.216448	5.09387	2.04491	0.043837	-4.73882	0.223832	0.273288
Non.inflammatory.Macrophages	HSD17B6	-1.10754	0.767255	-2.0442	0.043909	-3.79692	0.289915	0.308314
Non.inflammatory.Macrophages	CRMP1	1.342033	-1.07546	2.043017	0.044028	-3.75137	0.293079	0.324897
Non.inflammatory.Macrophages	CCRL2	0.453786	4.741765	2.042838	0.044046	-4.9617	0.229443	0.276443
Non.inflammatory.Macrophages	COX7A2	0.163972	8.039242	2.0416	0.044172	-5.21167	0.187953	0.252545
Non.inflammatory.Macrophages	ACTB	0.166408	13.9201	2.040062	0.044328	-5.98834	0.131305	0.214827
Non.inflammatory.Macrophages	PPBP	1.202418	0.842206	2.039686	0.044366	-3.92733	0.291198	0.309086
Non.inflammatory.Macrophages	EHD3	-0.48687	3.517645	-2.03966	0.044369	-4.2858	0.248451	0.286927
Non.inflammatory.Macrophages	HMMR	-0.61927	4.333361	-2.03953	0.044382	-4.40673	0.236435	0.280525
Non.inflammatory.Macrophages	ZFP101	0.503621	2.916235	2.038619	0.044475	-4.05129	0.257937	0.292013
Non.inflammatory.Macrophages	R74862	0.716186	1.054776	2.038493	0.044488	-4.00576	0.288542	0.307546
Non.inflammatory.Macrophages	ARHGEF10	0.588433	2.695098	2.038439	0.044493	-4.11827	0.26141	0.293851
Non.inflammatory.Macrophages	GM26782	0.291965	2.991571	2.037275	0.044612	-4.36206	0.257096	0.291774
Non.inflammatory.Macrophages	WDR11	0.295342	3.124121	2.037238	0.044616	-4.45098	0.25504	0.2907
Non.inflammatory.Macrophages	EMC10	0.196962	5.502896	2.037089	0.044631	-4.78455	0.220672	0.272091

Non.inflammatory.Macrophages	NHSL2	0.517547	2.487556	2.037023	0.044638	-4.3238	0.265051	0.29591
Non.inflammatory.Macrophages	COP1	0.203359	7.053619	2.03661	0.044681	-5.01043	0.200784	0.260727
Non.inflammatory.Macrophages	TRIM47	-0.38102	2.41312	-2.03627	0.044716	-4.31243	0.266447	0.296887
Non.inflammatory.Macrophages	CCDC57	-0.38793	2.546499	-2.03484	0.044863	-4.2514	0.265048	0.296242
Non.inflammatory.Macrophages	ZRSR2	0.177377	5.063714	2.034333	0.044914	-4.73749	0.227626	0.276313
Non.inflammatory.Macrophages	NIT1	-0.30319	3.750254	-2.03385	0.044964	-4.47191	0.246724	0.286799
Non.inflammatory.Macrophages	FAM83G	-0.76218	0.263823	-2.03332	0.045019	-3.85067	0.296924	0.316139
Non.inflammatory.Macrophages	ABR	0.265402	6.594873	2.030769	0.045282	-5.16854	0.208679	0.265885
Non.inflammatory.Macrophages	LYPLA2	0.217294	4.945046	2.030444	0.045316	-4.713	0.23088	0.278451
Non.inflammatory.Macrophages	ST7L	-0.25444	4.451288	-2.02928	0.045437	-4.65689	0.23845	0.282603
Non.inflammatory.Macrophages	STX1A	-0.81171	1.792697	-2.02832	0.045536	-3.92087	0.280581	0.304741
Non.inflammatory.Macrophages	MXD1	0.241049	6.036212	2.026225	0.045756	-4.87924	0.217419	0.271369
Non.inflammatory.Macrophages	P4HA3	-0.9336	0.308298	-2.0262	0.045759	-3.83013	0.300563	0.318241
Non.inflammatory.Macrophages	PAIP2	0.145799	7.620191	2.026173	0.045761	-5.10815	0.197309	0.259663
Non.inflammatory.Macrophages	AC149090.	-0.42894	5.49949	-2.02603	0.045776	-4.81494	0.224671	0.275455

Non.inflammatory.Macrophages	DAP3	0.221292	5.039126	2.026011	0.045778	-4.71404	0.231074	0.279008
Non.inflammatory.Macrophages	DDX54	0.209975	5.820595	2.025868	0.045793	-4.8496	0.220305	0.273003
Non.inflammatory.Macrophages	GM10603	1.200518	0.220408	2.024781	0.045907	-3.78721	0.301164	0.319394
Non.inflammatory.Macrophages	COL4A3BP	0.194712	6.352484	2.024099	0.045978	-4.95375	0.213908	0.269456
Non.inflammatory.Macrophages	AMDHD1	-0.73489	2.26974	-2.02313	0.04608	-4.01738	0.274685	0.302262
Non.inflammatory.Macrophages	ZFP346	0.435623	3.658973	2.022637	0.046132	-4.28332	0.252693	0.290909
Non.inflammatory.Macrophages	RNF122	0.62605	2.64878	2.022332	0.046164	-4.06667	0.268697	0.299257
Non.inflammatory.Macrophages	LEF1	-0.80217	5.638758	-2.02183	0.046217	-4.28897	0.224086	0.275465
Non.inflammatory.Macrophages	LSM12	0.166454	6.360706	2.021799	0.04622	-4.97747	0.214406	0.269983
Non.inflammatory.Macrophages	PVRIG	0.667886	-0.53207	2.021519	0.04625	-3.92312	0.302389	0.327118
Non.inflammatory.Macrophages	CDC5L	0.154742	5.768771	2.021232	0.04628	-4.86776	0.222385	0.274687
Non.inflammatory.Macrophages	TUBGCP5	0.315022	3.976604	2.020304	0.046378	-4.42241	0.248362	0.288822
Non.inflammatory.Macrophages	PHKG2	-0.25912	4.528144	-2.01999	0.046411	-4.5733	0.240166	0.284422
Non.inflammatory.Macrophages	FLCN	-0.25927	4.338996	-2.01997	0.046413	-4.69932	0.242948	0.285923
Non.inflammatory.Macrophages	MAP7	-0.35114	3.639692	-2.01985	0.046426	-4.40385	0.253497	0.291543

Non.inflammatory.Macrophages	ABI1	0.181301	7.702393	2.019346	0.04648	-5.26048	0.197917	0.260466
Non.inflammatory.Macrophages	RCHY1	-0.19776	5.661194	-2.0189	0.046527	-4.81686	0.224293	0.275815
Non.inflammatory.Macrophages	RNF113A1	-1.28671	0.42846	-2.01864	0.046555	-3.78742	0.303037	0.319135
Non.inflammatory.Macrophages	RAB11FIP5	0.535189	0.808074	2.018462	0.046574	-4.2122	0.299938	0.315793
Non.inflammatory.Macrophages	GRINA	0.276857	6.205395	2.018205	0.046601	-5.06189	0.216951	0.271762
Non.inflammatory.Macrophages	SERPINA11	-0.74135	1.813558	-2.01795	0.046628	-3.96891	0.2833	0.307118
Non.inflammatory.Macrophages	WDR43	0.208684	6.033494	2.017861	0.046637	-4.91209	0.219245	0.273089
Non.inflammatory.Macrophages	PLVAP	-0.71248	1.394862	-2.01765	0.04666	-3.99108	0.290521	0.310778
Non.inflammatory.Macrophages	TEF	-0.32967	3.230001	-2.01763	0.046662	-4.4115	0.260071	0.295316
Non.inflammatory.Macrophages	LAMTOR4	0.198663	6.205335	2.017012	0.046728	-5.03599	0.217146	0.271972
Non.inflammatory.Macrophages	VPS13C	0.242305	4.373385	2.016812	0.046749	-4.76738	0.242839	0.286222
Non.inflammatory.Macrophages	PAK1	-0.34744	5.334473	-2.01658	0.046774	-4.98064	0.229031	0.278708
Non.inflammatory.Macrophages	PKMYT1	-0.47897	3.261877	-2.01634	0.0468	-4.25249	0.259834	0.29534
Non.inflammatory.Macrophages	SPON1	1.079592	1.404705	2.015942	0.046842	-3.88599	0.290768	0.311075
Non.inflammatory.Macrophages	ZFP292	0.192706	7.046023	2.015098	0.046932	-5.04725	0.206577	0.266139

Non.inflammatory.Macrophages	RNF144A	-0.51333	3.619246	-2.01506	0.046936	-4.11099	0.254635	0.292778
Non.inflammatory.Macrophages	CENPJ	-0.42491	3.331867	-2.01442	0.047004	-4.20058	0.259366	0.295342
Non.inflammatory.Macrophages	OLFR1033	0.996929	0.583769	2.013972	0.047052	-3.90813	0.303528	0.319045
Non.inflammatory.Macrophages	FANCL	-0.25524	4.144904	-2.01278	0.04718	-4.57758	0.247367	0.289265
Non.inflammatory.Macrophages	GM16794	-1.01942	0.41499	-2.01273	0.047186	-3.80617	0.304698	0.320887
Non.inflammatory.Macrophages	RSPRY1	0.181346	5.833379	2.012704	0.047188	-4.88479	0.223161	0.275991
Non.inflammatory.Macrophages	IRF2BP2	0.163903	7.59206	2.011789	0.047287	-5.20772	0.200691	0.263067
Non.inflammatory.Macrophages	CKB	-0.25022	4.578345	-2.0108	0.047393	-5.06519	0.241626	0.286303
Non.inflammatory.Macrophages	PPARA	-0.5737	2.784821	-2.0108	0.047393	-4.29782	0.269419	0.300956
Non.inflammatory.Macrophages	EPS15	0.17026	6.183956	2.010076	0.047471	-4.9722	0.219315	0.274017
Non.inflammatory.Macrophages	FEZ2	0.23694	3.693286	2.009873	0.047493	-4.62163	0.255283	0.293709
Non.inflammatory.Macrophages	A1CF	-1.09561	0.569704	-2.00926	0.047559	-3.84544	0.305462	0.320575
Non.inflammatory.Macrophages	MED15	0.193582	5.758733	2.009242	0.047561	-4.93875	0.225201	0.277486
Non.inflammatory.Macrophages	ATG4A	0.312881	4.314693	2.008576	0.047633	-4.60158	0.246109	0.289038
Non.inflammatory.Macrophages	GGT5	-0.68561	0.649699	-2.00852	0.047639	-4.06661	0.305008	0.32007

Non.inflammatory.Macrophages	IMPACT	-0.21035	5.540939	-2.00684	0.047821	-4.99198	0.229138	0.279889
Non.inflammatory.Macrophages	KLHL32	-1.06671	1.452777	-2.00628	0.047882	-3.87582	0.293837	0.313772
Non.inflammatory.Macrophages	ING4	-0.268	4.08954	-2.00434	0.048094	-4.64204	0.251522	0.292172
Non.inflammatory.Macrophages	6030458C1	0.34853	2.74674	2.00413	0.048117	-4.32213	0.272863	0.30342
Non.inflammatory.Macrophages	INPP5F	0.318405	4.366787	2.003467	0.048189	-4.60207	0.247394	0.290253
Non.inflammatory.Macrophages	TBXA2R	-0.89751	2.36173	-2.00338	0.048199	-3.95927	0.279371	0.306907
Non.inflammatory.Macrophages	PRLR	-0.75497	3.152323	-2.00337	0.0482	-4.19454	0.26633	0.300231
Non.inflammatory.Macrophages	SARS2	-0.37482	2.675724	-2.00275	0.048267	-4.23507	0.274257	0.304518
Non.inflammatory.Macrophages	AGRP	-0.76459	1.935634	-2.00273	0.048269	-4.03347	0.286782	0.310851
Non.inflammatory.Macrophages	PSTPIP1	0.466566	4.472927	2.001826	0.048369	-4.2861	0.246261	0.289852
Non.inflammatory.Macrophages	LEPR	0.649664	1.823369	2.001543	0.0484	-4.214	0.289126	0.31216
Non.inflammatory.Macrophages	DHCR24	-0.59685	2.897631	-2.00141	0.048414	-4.09201	0.270975	0.302983
Non.inflammatory.Macrophages	COX5B	0.174123	8.103976	2.001166	0.048441	-5.31505	0.197201	0.262191
Non.inflammatory.Macrophages	PIP5K1C	0.221061	5.626433	2.001081	0.04845	-4.88751	0.229528	0.280917
Non.inflammatory.Macrophages	ARF3	0.153104	6.29303	2.000785	0.048483	-5.04534	0.220403	0.275858

Non.inflammatory.Macrophages	MRPL33	0.203292	6.142277	1.999398	0.048636	-4.98505	0.22304	0.277391
Non.inflammatory.Macrophages	B930036N:	-0.3088	5.605823	-1.99889	0.048691	-4.87687	0.230634	0.281759
Non.inflammatory.Macrophages	TMEM154	0.910103	2.583653	1.99855	0.048729	-3.89324	0.277234	0.30665
Non.inflammatory.Macrophages	NUDT9	-0.25826	4.362001	-1.99825	0.048762	-4.78928	0.248935	0.291901
Non.inflammatory.Macrophages	TM4SF1	0.798887	2.15291	1.997818	0.04881	-4.08031	0.28474	0.31056
Non.inflammatory.Macrophages	CYP2C68	-0.67824	2.791797	-1.99691	0.04891	-4.18932	0.274396	0.305378
Non.inflammatory.Macrophages	TRIM30B	0.460704	1.730639	1.995457	0.049071	-4.37704	0.293046	0.314823
Non.inflammatory.Macrophages	AFF1	-0.18471	8.730515	-1.99528	0.049091	-5.31509	0.191259	0.259183
Non.inflammatory.Macrophages	XKR5	-1.04356	0.190356	-1.99518	0.049103	-3.82577	0.311902	0.328705
Non.inflammatory.Macrophages	CFL1	0.176689	10.01399	1.995172	0.049103	-5.57708	0.176749	0.250102
Non.inflammatory.Macrophages	SLC14A1	0.338821	3.335927	1.995104	0.049111	-4.54303	0.265965	0.301185
Non.inflammatory.Macrophages	KDM3A	0.201578	5.567098	1.994881	0.049135	-4.90862	0.232194	0.283136
Non.inflammatory.Macrophages	SNED1	0.640872	1.751149	1.994036	0.049229	-4.16043	0.293117	0.3152
Non.inflammatory.Macrophages	NDOR1	0.397354	3.086341	1.992577	0.049392	-4.34071	0.271184	0.304316
Non.inflammatory.Macrophages	CMKLR1	0.401561	2.151939	1.992236	0.04943	-4.70104	0.287024	0.312407

Non.inflammatory.Macrophages	MED14	0.232707	6.321429	1.991479	0.049515	-5.03129	0.223019	0.278438
Non.inflammatory.Macrophages	PTPRE	0.240059	5.539856	1.990666	0.049606	-4.94471	0.234242	0.284754
Non.inflammatory.Macrophages	URI1	0.209445	5.89939	1.990488	0.049626	-4.91725	0.229151	0.281918
Non.inflammatory.Macrophages	LRRC8B	0.309421	3.358799	1.99006	0.049674	-4.41167	0.267637	0.302762
Non.inflammatory.Macrophages	LAT2	0.391954	4.773693	1.989557	0.04973	-4.43597	0.24571	0.291302
Non.inflammatory.Macrophages	MEG3	-1.10189	1.040515	-1.98941	0.049747	-4.01109	0.307958	0.32323
Non.inflammatory.Macrophages	CTSS	0.259745	7.501211	1.988747	0.049821	-5.50485	0.208143	0.270255
Non.inflammatory.Macrophages	CREBRF	-0.22875	6.98351	-1.98841	0.049859	-5.08298	0.214856	0.274276
Non.inflammatory.Macrophages	MMP13	1.153763	-1.01302	1.988377	0.049863	-3.92763	0.31476	0.342574
Non.inflammatory.Macrophages	XLR4B	0.634845	1.285179	1.988115	0.049892	-4.00179	0.303791	0.321437
Non.inflammatory.Macrophages	EIF4E	-0.18714	6.950147	-1.98778	0.04993	-5.25282	0.215383	0.274673
Non.inflammatory.Macrophages	CSF2RB2	0.334698	2.359571	1.986932	0.050026	-4.67976	0.285263	0.312424
Non.inflammatory.Macrophages	EIF4A2	0.175531	5.530319	1.986297	0.050097	-4.93584	0.235464	0.28618
Non.inflammatory.Macrophages	CMTM4	-0.34959	3.141567	-1.98617	0.050112	-4.48444	0.272304	0.305901
Non.inflammatory.Macrophages	SNRNP27	0.172518	5.842837	1.985788	0.050155	-4.96284	0.2311	0.283834

Non.inflammatory.Macrophages	SLC25A39	0.221215	6.472882	1.985115	0.050231	-5.19399	0.222594	0.279264
Non.inflammatory.Macrophages	PSMD4	0.149783	6.691408	1.984898	0.050256	-5.09464	0.21964	0.277593
Non.inflammatory.Macrophages	GSTM1	-0.36685	4.059268	-1.98455	0.050296	-4.69836	0.257996	0.298786
Non.inflammatory.Macrophages	TOP3B	0.271452	3.549805	1.984349	0.050318	-4.50213	0.266105	0.303091
Non.inflammatory.Macrophages	ELMO1	-0.15408	8.719508	-1.98417	0.050339	-5.43651	0.193989	0.262522
Non.inflammatory.Macrophages	DHRS9	1.197367	0.196917	1.983319	0.050435	-3.84241	0.316449	0.332997
Non.inflammatory.Macrophages	SNX2	0.127089	7.314048	1.98306	0.050464	-5.24533	0.211676	0.273182
Non.inflammatory.Macrophages	S1PR1	0.541281	4.061514	1.983042	0.050466	-4.30407	0.258213	0.299073
Non.inflammatory.Macrophages	EVL	0.205159	6.485099	1.982911	0.050481	-5.17759	0.222707	0.27956
Non.inflammatory.Macrophages	IDH3A	0.244379	4.865883	1.982127	0.050571	-4.77576	0.246185	0.2927
Non.inflammatory.Macrophages	SPNS1	-0.29813	3.498213	-1.98193	0.050593	-4.53311	0.267541	0.304168
Non.inflammatory.Macrophages	GM12905	-0.36971	2.138199	-1.98159	0.050632	-4.27809	0.290554	0.315993
Non.inflammatory.Macrophages	JADE3	-0.34385	3.471551	-1.98141	0.050652	-4.39093	0.268043	0.304533
Non.inflammatory.Macrophages	EIF4G2	0.131836	7.71796	1.980612	0.050743	-5.26697	0.207088	0.270877
Non.inflammatory.Macrophages	NEK9	0.215107	5.27603	1.980282	0.050781	-4.94765	0.240506	0.289932

Non.inflammatory.Macrophages	FPR2	0.504943	1.833327	1.979962	0.050818	-4.73757	0.296362	0.31921
Non.inflammatory.Macrophages	PON2	0.194706	6.020273	1.979954	0.050819	-5.03408	0.229813	0.284102
Non.inflammatory.Macrophages	SAV1	0.220519	4.735258	1.978759	0.050955	-4.77754	0.249031	0.294762
Non.inflammatory.Macrophages	SRC	-0.56289	0.980292	-1.97872	0.050959	-4.18302	0.312515	0.327218
Non.inflammatory.Macrophages	GM14286	0.828271	0.211974	1.978351	0.051002	-3.92453	0.318094	0.33442
Non.inflammatory.Macrophages	ARHGEF17	-0.95699	-0.37609	-1.97805	0.051037	-3.91384	0.318163	0.339982
Non.inflammatory.Macrophages	LUC7L2	0.121269	8.527172	1.977477	0.051103	-5.33396	0.197714	0.265516
Non.inflammatory.Macrophages	PRDX2	0.205341	7.774205	1.977238	0.05113	-5.39858	0.207073	0.271156
Non.inflammatory.Macrophages	SNX6	0.135251	6.568189	1.977061	0.05115	-5.1672	0.222965	0.280507
Non.inflammatory.Macrophages	ZBTB11	-0.1728	7.088839	-1.9768	0.05118	-5.17824	0.215989	0.276593
Non.inflammatory.Macrophages	ITGB3BP	-0.3117	3.52337	-1.97656	0.051208	-4.43064	0.268518	0.305587
Non.inflammatory.Macrophages	ACTN2	-1.23266	-0.1007	-1.97401	0.051503	-3.8554	0.320065	0.339042
Non.inflammatory.Macrophages	TAF15	0.15684	6.905531	1.973966	0.051507	-5.06359	0.219525	0.279016
Non.inflammatory.Macrophages	GM48678	0.587764	3.349644	1.973451	0.051567	-4.17654	0.272889	0.30828
Non.inflammatory.Macrophages	GM44649	0.396851	3.949723	1.973069	0.051611	-4.43635	0.263233	0.303261

Non.inflammatory.Macrophages	C130013H(	0.745477	0.498295	1.972535	0.051673	-3.92096	0.320586	0.333952
Non.inflammatory.Macrophages	RBBP6	-0.15494	7.269431	-1.97183	0.051755	-5.17337	0.215256	0.276797
Non.inflammatory.Macrophages	UBXN11	0.620992	1.770334	1.971754	0.051764	-4.01801	0.300801	0.322574
Non.inflammatory.Macrophages	FMO5	-0.39331	3.247756	-1.9709	0.051863	-4.45883	0.275524	0.30989
Non.inflammatory.Macrophages	GM43061	1.22136	0.306464	1.970554	0.051904	-3.85864	0.321493	0.336395
Non.inflammatory.Macrophages	STOX2	-0.81667	3.196694	-1.9702	0.051945	-4.22286	0.276562	0.31053
Non.inflammatory.Macrophages	TIGAR	0.517211	1.851972	1.969411	0.052037	-4.18945	0.300354	0.322847
Non.inflammatory.Macrophages	ALKBH1	0.212868	4.99535	1.968814	0.052106	-4.83826	0.248356	0.296046
Non.inflammatory.Macrophages	PPP2R5E	0.154094	6.820761	1.968787	0.05211	-5.07149	0.222126	0.281376
Non.inflammatory.Macrophages	TTC25	-0.86716	0.218704	-1.9682	0.052178	-3.9643	0.322286	0.338318
Non.inflammatory.Macrophages	TXNRD3	-0.52407	1.950944	-1.96815	0.052185	-4.16466	0.298813	0.322422
Non.inflammatory.Macrophages	GM16152	-1.21935	2.101073	-1.96772	0.052234	-3.86746	0.296122	0.32121
Non.inflammatory.Macrophages	CMSS1	0.305802	6.448827	1.967688	0.052238	-5.11946	0.227319	0.284602
Non.inflammatory.Macrophages	IQGAP2	-0.181	7.075634	-1.96743	0.052269	-5.2699	0.218755	0.279682
Non.inflammatory.Macrophages	KCNN4	0.867976	3.850661	1.96742	0.05227	-4.04332	0.266367	0.305953

Non.inflammatory.Macrophages	LIN9	0.364244	4.133885	1.966852	0.052336	-4.44534	0.262034	0.303737
Non.inflammatory.Macrophages	TTC39B	0.221388	4.653747	1.966588	0.052367	-4.94291	0.253873	0.29947
Non.inflammatory.Macrophages	OTULINL	-0.22073	5.347398	-1.96643	0.052386	-5.14024	0.243356	0.293769
Non.inflammatory.Macrophages	ZEB2	0.24077	9.36649	1.966144	0.052419	-5.68667	0.190207	0.262759
Non.inflammatory.Macrophages	4930430E1	-0.56835	0.409202	-1.96591	0.052447	-4.34679	0.32255	0.337212
Non.inflammatory.Macrophages	INO80DOS	-0.4368	4.442013	-1.96584	0.052456	-4.56317	0.257168	0.301448
Non.inflammatory.Macrophages	RBM44	-0.99583	0.135835	-1.96505	0.052548	-3.94166	0.322722	0.339987
Non.inflammatory.Macrophages	TEC	0.259555	5.751113	1.964932	0.052562	-4.8683	0.237553	0.290855
Non.inflammatory.Macrophages	FOXO3	-0.21472	6.397005	-1.96481	0.052576	-5.11038	0.22835	0.285715
Non.inflammatory.Macrophages	H2-DMB1	-0.36328	4.478206	-1.96475	0.052583	-5.09516	0.256738	0.301397
Non.inflammatory.Macrophages	BCAP31	0.162894	5.901725	1.964404	0.052624	-5.03601	0.235376	0.289768
Non.inflammatory.Macrophages	CYREN	-0.31953	3.374354	-1.96418	0.052651	-4.59113	0.274551	0.310889
Non.inflammatory.Macrophages	ZBTB7A	-0.1415	7.04476	-1.96412	0.052657	-5.22365	0.219466	0.280707
Non.inflammatory.Macrophages	SOX5	-0.58193	4.684841	-1.96398	0.052674	-4.53122	0.253527	0.29982
Non.inflammatory.Macrophages	DRAM1	0.377799	2.581586	1.96349	0.052732	-4.72685	0.288113	0.318058

Non.inflammatory.Macrophages	GM14471	-0.99476	0.24931	-1.96341	0.052741	-3.89398	0.322796	0.339367
Non.inflammatory.Macrophages	NXF7	-0.78783	-1.34285	-1.96298	0.052792	-3.93422	0.322796	0.354777
Non.inflammatory.Macrophages	CEP170	0.204587	5.909007	1.962877	0.052804	-5.01514	0.235325	0.290042
Non.inflammatory.Macrophages	RICTOR	0.180585	6.409431	1.962865	0.052806	-5.03945	0.228229	0.286029
Non.inflammatory.Macrophages	CYBA	0.158542	8.55691	1.962417	0.052858	-5.56637	0.200171	0.269508
Non.inflammatory.Macrophages	NSUN6	-0.30295	3.821373	-1.96169	0.052945	-4.6347	0.267664	0.307593
Non.inflammatory.Macrophages	ISCA1	0.26751	6.018689	1.961591	0.052956	-5.05453	0.234102	0.28935
Non.inflammatory.Macrophages	DHX36	0.167212	5.392248	1.961089	0.053016	-4.92365	0.243402	0.294577
Non.inflammatory.Macrophages	MYZAP	-0.67282	3.284402	-1.96068	0.053064	-4.07799	0.276725	0.312579
Non.inflammatory.Macrophages	0610039K1	-1.08217	0.022539	-1.96047	0.053089	-3.90408	0.323508	0.342338
Non.inflammatory.Macrophages	CHUK	0.212114	5.037427	1.960469	0.053089	-4.85425	0.24874	0.297777
Non.inflammatory.Macrophages	LIPE	-0.26176	3.783042	-1.96025	0.053115	-4.66352	0.268486	0.30844
Non.inflammatory.Macrophages	S1PR4	-0.69024	4.183137	-1.95892	0.053274	-4.0808	0.262701	0.305437
Non.inflammatory.Macrophages	INHBC	-1.03539	0.808252	-1.95844	0.053331	-3.93383	0.321102	0.335637
Non.inflammatory.Macrophages	A730063M	-0.69638	1.218514	-1.9584	0.053335	-4.11007	0.314332	0.331833

Non.inflammatory.Macrophages	NSRP1	0.206988	5.229533	1.957726	0.053416	-4.8793	0.246798	0.297029
Non.inflammatory.Macrophages	TLE4	-0.17706	7.814076	-1.95618	0.0536	-5.32229	0.211309	0.277071
Non.inflammatory.Macrophages	PDXK	-0.2209	4.441778	-1.95501	0.05374	-4.92306	0.260215	0.304831
Non.inflammatory.Macrophages	PHACTR2	-0.23824	6.036575	-1.95452	0.053799	-5.08609	0.23608	0.291709
Non.inflammatory.Macrophages	SDHC	0.19431	5.09505	1.954447	0.053808	-4.95712	0.250058	0.299473
Non.inflammatory.Macrophages	ETV6	-0.19243	8.156803	-1.95422	0.053836	-5.46198	0.207307	0.275006
Non.inflammatory.Macrophages	PSEN1	0.159816	6.297037	1.954202	0.053838	-5.09435	0.232347	0.289617
Non.inflammatory.Macrophages	JMJD1C	-0.1743	8.048596	-1.95366	0.053903	-5.43739	0.208689	0.27606
Non.inflammatory.Macrophages	RNF8	0.232378	4.800789	1.953405	0.053934	-4.746	0.254586	0.302227
Non.inflammatory.Macrophages	DIABLO	-0.24568	4.259159	-1.95253	0.054039	-4.73335	0.263124	0.30708
Non.inflammatory.Macrophages	AGGF1	0.188013	5.384652	1.95245	0.054049	-4.90964	0.245676	0.297607
Non.inflammatory.Macrophages	PIGO	-0.63682	1.804475	-1.95231	0.054066	-4.11864	0.305277	0.328822
Non.inflammatory.Macrophages	CAB39	0.159253	6.974308	1.952078	0.054093	-5.18255	0.222905	0.284829
Non.inflammatory.Macrophages	PANK1	-0.41675	3.430383	-1.952	0.054103	-4.50603	0.27671	0.314363
Non.inflammatory.Macrophages	ELN	0.676554	2.40021	1.951885	0.054117	-4.31448	0.294509	0.323549

Non.inflammatory.Macrophages	VDAC1	0.185183	6.677223	1.951826	0.054124	-5.16553	0.227	0.287243
Non.inflammatory.Macrophages	IKZF4	-0.83009	-0.0706	-1.95164	0.054146	-3.93911	0.326367	0.346573
Non.inflammatory.Macrophages	ZDHHC15	1.125367	1.125862	1.951107	0.054211	-3.89996	0.317979	0.335252
Non.inflammatory.Macrophages	RNFT1	0.200727	4.495375	1.95108	0.054214	-4.87207	0.259367	0.305258
Non.inflammatory.Macrophages	SPAG9	-0.2009	9.266573	-1.95102	0.054222	-5.66856	0.193643	0.267296
Non.inflammatory.Macrophages	NECAP1	0.217706	4.774239	1.951002	0.054223	-4.79101	0.254998	0.302898
Non.inflammatory.Macrophages	GM45509	-0.9022	1.640462	-1.95099	0.054224	-3.96689	0.308304	0.330492
Non.inflammatory.Macrophages	TXNDC15	-0.2214	4.911319	-1.95091	0.054235	-4.91198	0.252876	0.301744
Non.inflammatory.Macrophages	SEC14L2	-0.764	1.70477	-1.95079	0.054249	-4.07199	0.307114	0.329947
Non.inflammatory.Macrophages	LSM14A	0.149662	6.620649	1.950577	0.054275	-5.07731	0.227788	0.287839
Non.inflammatory.Macrophages	LRRK1	-0.23082	5.612519	-1.95052	0.054282	-5.09053	0.24228	0.296052
Non.inflammatory.Macrophages	GSG1L	-1.0022	-0.16326	-1.95022	0.054318	-3.96338	0.326412	0.347801
Non.inflammatory.Macrophages	CLPTM1L	0.201966	4.883326	1.950058	0.054338	-4.9483	0.253342	0.302329
Non.inflammatory.Macrophages	DBN1	-0.80137	1.388658	-1.94946	0.05441	-4.08891	0.313133	0.333408
Non.inflammatory.Macrophages	MAGOH	-0.19601	6.296682	-1.94942	0.054415	-5.0386	0.232447	0.290856

Non.inflammatory.Macrophages	AGAP1	0.271968	4.225875	1.949217	0.054439	-4.9336	0.263765	0.308215
Non.inflammatory.Macrophages	RNASET2B	-0.26768	5.139167	-1.94914	0.054449	-4.95009	0.249488	0.300511
Non.inflammatory.Macrophages	GBP5	0.603288	2.014589	1.948609	0.054513	-4.53802	0.301654	0.32791
Non.inflammatory.Macrophages	GM49797	-0.21704	4.882769	-1.94848	0.054529	-4.87377	0.253497	0.302772
Non.inflammatory.Macrophages	BCL2	0.444466	5.016128	1.948406	0.054538	-4.75937	0.251444	0.301681
Non.inflammatory.Macrophages	2-Mar	0.191768	6.581903	1.94804	0.054582	-5.22046	0.228565	0.288898
Non.inflammatory.Macrophages	ADGB	0.382668	2.335955	1.947814	0.05461	-4.81253	0.295958	0.325259
Non.inflammatory.Macrophages	TRIR	0.16665	6.300182	1.947669	0.054628	-5.11825	0.232541	0.291337
Non.inflammatory.Macrophages	9030622O2	-1.00669	1.05914	-1.94739	0.054661	-3.99321	0.319639	0.337196
Non.inflammatory.Macrophages	GCLC	-0.24968	6.841762	-1.94702	0.054707	-5.17346	0.225082	0.287263
Non.inflammatory.Macrophages	TEX10	-0.24106	5.266363	-1.94558	0.054882	-4.9279	0.248536	0.300578
Non.inflammatory.Macrophages	APOA5	-0.85455	1.793969	-1.94434	0.055034	-4.09846	0.307514	0.331583
Non.inflammatory.Macrophages	LRRC32	-0.87607	1.241998	-1.94356	0.055129	-3.97986	0.318172	0.336758
Non.inflammatory.Macrophages	LARS2	0.380673	5.571824	1.943538	0.055132	-5.04253	0.244729	0.298538
Non.inflammatory.Macrophages	COG2	0.291077	3.567744	1.942776	0.055225	-4.60509	0.276847	0.315933

Non.inflammatory.Macrophages	MTMR3	0.162483	7.886466	1.942379	0.055274	-5.28202	0.212738	0.280303
Non.inflammatory.Macrophages	IL13RA1	0.263347	3.558786	1.941993	0.055322	-5.03695	0.277235	0.316375
Non.inflammatory.Macrophages	CBFB	0.136268	6.809866	1.941598	0.05537	-5.1553	0.227454	0.28906
Non.inflammatory.Macrophages	RHOQ	0.251683	5.771108	1.940902	0.055456	-5.04163	0.242524	0.297677
Non.inflammatory.Macrophages	LILRA6	0.568847	1.213739	1.940477	0.055508	-4.30309	0.319709	0.33792
Non.inflammatory.Macrophages	CCDC152	0.391126	3.284464	1.940375	0.055521	-4.65481	0.282172	0.319012
Non.inflammatory.Macrophages	GM20275	-0.36618	3.071387	-1.94037	0.055522	-4.56271	0.285838	0.320909
Non.inflammatory.Macrophages	GRHPR	0.353049	4.431109	1.940351	0.055524	-4.73712	0.263184	0.308992
Non.inflammatory.Macrophages	NAIP2	0.258539	3.006951	1.939625	0.055613	-4.89168	0.287143	0.321719
Non.inflammatory.Macrophages	DISC1	-0.23167	3.43956	-1.93922	0.055664	-4.86824	0.279714	0.317998
Non.inflammatory.Macrophages	SLC15A3	0.225092	5.005968	1.93919	0.055667	-5.17996	0.254291	0.304431
Non.inflammatory.Macrophages	AKR7A5	-0.24643	4.13514	-1.93917	0.055669	-4.80379	0.268143	0.311901
Non.inflammatory.Macrophages	BLMH	0.188565	5.648784	1.939068	0.055682	-4.92124	0.244504	0.299031
Non.inflammatory.Macrophages	RNASET2A	-0.26276	5.439463	-1.93807	0.055806	-5.02213	0.248055	0.30098
Non.inflammatory.Macrophages	MAP2K3	-0.21813	6.01457	-1.93779	0.05584	-5.18157	0.239487	0.296283

Non.inflammatory.Macrophages	VIL1	-0.80984	0.018277	-1.93773	0.055847	-4.08844	0.330633	0.350062
Non.inflammatory.Macrophages	PI4K2B	0.328362	3.903982	1.937543	0.055871	-4.56113	0.272382	0.314312
Non.inflammatory.Macrophages	TIAM2	0.865632	2.628932	1.936488	0.056002	-4.10614	0.294826	0.326175
Non.inflammatory.Macrophages	RNF217	0.435984	2.071962	1.935017	0.056185	-4.636	0.305725	0.331647
Non.inflammatory.Macrophages	1600002D2	-0.95938	0.257645	-1.93488	0.056202	-4.05926	0.332154	0.348854
Non.inflammatory.Macrophages	ENPP2	0.488546	2.411456	1.934388	0.056263	-4.43032	0.299725	0.328841
Non.inflammatory.Macrophages	HFE	-0.20794	3.452689	-1.93405	0.056305	-5.09172	0.281509	0.319543
Non.inflammatory.Macrophages	LGALS1	0.35931	6.036335	1.933269	0.056403	-4.97598	0.240814	0.297642
Non.inflammatory.Macrophages	NLN	0.24755	4.446951	1.932788	0.056463	-4.87147	0.265368	0.31126
Non.inflammatory.Macrophages	SIGLECG	-0.56662	4.076853	-1.9326	0.056486	-4.16684	0.271411	0.314493
Non.inflammatory.Macrophages	CCT2	0.160788	6.286032	1.93224	0.056532	-5.15038	0.237182	0.295877
Non.inflammatory.Macrophages	ANKDD1A	-0.45426	2.346094	-1.93195	0.056568	-4.84514	0.301418	0.330176
Non.inflammatory.Macrophages	KCNC3	0.99459	0.561324	1.93195	0.056568	-3.93696	0.332329	0.346966
Non.inflammatory.Macrophages	ENTPD7	-0.24201	4.693758	-1.93192	0.056572	-4.83539	0.261408	0.309296
Non.inflammatory.Macrophages	MTSS1	0.27794	6.472133	1.931857	0.05658	-5.08187	0.234495	0.294355

Non.inflammatory.Macrophages	L3MBTL1	-0.7176	0.787342	-1.93135	0.056643	-4.11402	0.330017	0.344936
Non.inflammatory.Macrophages	CCDC148	-0.69724	1.762244	-1.93087	0.056704	-4.49666	0.312494	0.335874
Non.inflammatory.Macrophages	CEP295	0.276171	3.949897	1.930835	0.056708	-4.65874	0.273761	0.316044
Non.inflammatory.Macrophages	RAPGEF2	-0.20795	7.429198	-1.93066	0.056729	-5.48009	0.221336	0.286878
Non.inflammatory.Macrophages	PDCD1LG2	-0.69104	1.775707	-1.93004	0.056808	-4.18328	0.312389	0.335955
Non.inflammatory.Macrophages	GIMAP7	1.233755	1.458503	1.929723	0.056848	-3.93492	0.318404	0.339051
Non.inflammatory.Macrophages	ELL2	0.271293	6.734248	1.929677	0.056853	-5.27771	0.231078	0.292756
Non.inflammatory.Macrophages	MCOLN1	-0.31974	2.728161	-1.92967	0.056855	-4.50465	0.294947	0.32729
Non.inflammatory.Macrophages	PBX4	-0.76367	0.976518	-1.92834	0.057021	-4.07032	0.328271	0.344126
Non.inflammatory.Macrophages	1600014C1	0.414505	4.275444	1.928245	0.057034	-4.7069	0.268953	0.313967
Non.inflammatory.Macrophages	SRSF9	-0.14317	6.428408	-1.9282	0.05704	-5.20573	0.235826	0.295714
Non.inflammatory.Macrophages	SARDHOS	-1.01706	0.147254	-1.92812	0.05705	-4.03052	0.333928	0.352207
Non.inflammatory.Macrophages	MRPL46	0.315865	3.264758	1.927812	0.057088	-4.57354	0.285978	0.323025
Non.inflammatory.Macrophages	AHDC1	-0.22214	4.689967	-1.92772	0.057099	-4.77457	0.262249	0.310462
Non.inflammatory.Macrophages	ABCD4	-0.32243	2.782602	-1.92757	0.057119	-4.49959	0.29445	0.327387

Non.inflammatory.Macrophages	1700061N1	-0.98254	-0.1438	-1.92709	0.05718	-4.04767	0.334141	0.355264
Non.inflammatory.Macrophages	SYNJ2	0.453284	2.324633	1.925944	0.057325	-4.29963	0.30354	0.332063
Non.inflammatory.Macrophages	CFAP43	0.388487	3.09234	1.925456	0.057387	-4.39666	0.289961	0.325148
Non.inflammatory.Macrophages	CCDC62	-0.46441	2.858429	-1.92487	0.057461	-4.46567	0.29423	0.327494
Non.inflammatory.Macrophages	NCKIPSD	-0.3164	2.952621	-1.92472	0.05748	-4.53323	0.292557	0.32667
Non.inflammatory.Macrophages	CBARP	0.534592	1.923122	1.9246	0.057495	-4.32594	0.311319	0.336167
Non.inflammatory.Macrophages	AP3S1	0.16376	7.096627	1.92447	0.057512	-5.26185	0.227237	0.291132
Non.inflammatory.Macrophages	CD2AP	0.196929	6.24029	1.923897	0.057585	-5.2659	0.239483	0.298288
Non.inflammatory.Macrophages	ZFP672	0.256026	4.004158	1.923887	0.057586	-4.68826	0.274489	0.317448
Non.inflammatory.Macrophages	CDK6	0.304642	6.322112	1.923869	0.057588	-5.10466	0.238287	0.29761
Non.inflammatory.Macrophages	IRAK2	0.253173	6.796286	1.922903	0.057711	-5.32521	0.231861	0.293923
Non.inflammatory.Macrophages	UGT3A2	-0.82493	1.174889	-1.92156	0.057883	-4.05484	0.327034	0.344302
Non.inflammatory.Macrophages	PLOD1	0.294765	3.410314	1.921364	0.057908	-4.85573	0.285776	0.323598
Non.inflammatory.Macrophages	WASHC5	-0.23731	4.472587	-1.9211	0.057941	-4.90315	0.267949	0.314233
Non.inflammatory.Macrophages	SH3BGRL2	0.473731	2.700678	1.92084	0.057974	-4.55932	0.298412	0.330193

Non.inflammatory.Macrophages	GM20337	-0.68556	1.078162	-1.92032	0.058041	-4.12326	0.329175	0.345502
Non.inflammatory.Macrophages	ZDBF2	1.394169	-0.23932	1.919938	0.05809	-3.93689	0.336894	0.358448
Non.inflammatory.Macrophages	POLR3F	0.297338	3.882542	1.919932	0.058091	-4.59664	0.277902	0.319646
Non.inflammatory.Macrophages	KCNK10	-1.32401	0.557275	-1.91983	0.058104	-3.92787	0.336317	0.35061
Non.inflammatory.Macrophages	SSR3	0.161359	6.071068	1.918922	0.05822	-5.18941	0.243181	0.300955
Non.inflammatory.Macrophages	ALDOB	-0.50515	4.949636	-1.91876	0.058242	-4.91071	0.260423	0.31055
Non.inflammatory.Macrophages	GPANK1	0.347733	3.300008	1.918652	0.058255	-4.46886	0.287903	0.32515
Non.inflammatory.Macrophages	FNDC9	-0.80581	2.564671	-1.9185	0.058274	-3.9465	0.301004	0.331924
Non.inflammatory.Macrophages	SLC22A1	-0.91158	0.740269	-1.91842	0.058284	-4.02913	0.334316	0.34919
Non.inflammatory.Macrophages	VAMP5	-0.28185	4.717071	-1.91837	0.058291	-4.90462	0.264142	0.31264
Non.inflammatory.Macrophages	MS4A6C	0.299529	5.165825	1.917966	0.058343	-5.39195	0.257011	0.308878
Non.inflammatory.Macrophages	DDHD2	0.248899	4.784447	1.917624	0.058387	-4.84801	0.263059	0.312297
Non.inflammatory.Macrophages	COX6B1	0.15284	7.821955	1.917414	0.058414	-5.44907	0.218438	0.287036
Non.inflammatory.Macrophages	GM39302	-0.95326	0.880898	-1.91723	0.058438	-3.99183	0.332522	0.348287
Non.inflammatory.Macrophages	GM11613	-0.47318	2.020755	-1.9171	0.058454	-4.56452	0.311046	0.337459

Non.inflammatory.Macrophages	GPD2	0.253182	6.625661	1.916906	0.05848	-5.34572	0.235064	0.296892
Non.inflammatory.Macrophages	CALM1	0.122644	9.782329	1.916752	0.058499	-5.74353	0.193651	0.271932
Non.inflammatory.Macrophages	WDR78	-0.96878	0.561969	-1.91672	0.058503	-3.98086	0.336276	0.351456
Non.inflammatory.Macrophages	HTATSF1	0.211494	4.697996	1.916713	0.058504	-4.85724	0.264449	0.313267
Non.inflammatory.Macrophages	PPM1B	0.180001	6.192574	1.916544	0.058526	-5.07158	0.24138	0.300555
Non.inflammatory.Macrophages	PGGHG	-0.37979	2.820106	-1.91634	0.058552	-4.51838	0.296392	0.3302
Non.inflammatory.Macrophages	HECW2	-0.48552	3.162677	-1.91619	0.058571	-4.54077	0.290309	0.327134
Non.inflammatory.Macrophages	FRMD8OS	-0.64081	0.736632	-1.91588	0.058612	-4.20174	0.334362	0.350067
Non.inflammatory.Macrophages	MORC2A	-0.25047	4.295558	-1.91586	0.058615	-4.84185	0.27101	0.317078
Non.inflammatory.Macrophages	SCML4	-0.51852	4.659948	-1.9158	0.058622	-4.37027	0.265063	0.313895
Non.inflammatory.Macrophages	NINJ2	-0.79567	0.701022	-1.91522	0.058697	-4.15155	0.335038	0.35057
Non.inflammatory.Macrophages	PSME3	0.22224	5.282258	1.915122	0.05871	-5.04118	0.255358	0.308659
Non.inflammatory.Macrophages	PHC2	-0.21166	5.798035	-1.91392	0.058866	-5.14273	0.247989	0.304668
Non.inflammatory.Macrophages	TMEM91	-0.58624	2.459733	-1.91347	0.058924	-4.23257	0.303964	0.334454
Non.inflammatory.Macrophages	PDRG1	0.236797	4.496479	1.913135	0.058967	-4.84108	0.268722	0.316062

Non.inflammatory.Macrophages	PGD	0.178658	5.716274	1.912881	0.059	-5.17658	0.249483	0.305537
Non.inflammatory.Macrophages	GM19557	0.975808	-0.95554	1.912082	0.059104	-3.9361	0.338446	0.368208
Non.inflammatory.Macrophages	GM1673	0.346759	2.42109	1.912023	0.059112	-4.61452	0.305024	0.335254
Non.inflammatory.Macrophages	GM15708	-0.35158	3.198583	-1.91198	0.059117	-4.61647	0.291012	0.328089
Non.inflammatory.Macrophages	PRKAA1	0.285027	4.45764	1.911339	0.059201	-4.79434	0.269853	0.317024
Non.inflammatory.Macrophages	ZFC3H1	0.161391	6.914629	1.910909	0.059257	-5.24472	0.232354	0.296187
Non.inflammatory.Macrophages	PDE1A	-0.89057	0.441701	-1.90946	0.059445	-4.06466	0.339896	0.355198
Non.inflammatory.Macrophages	GSTZ1	-0.3228	4.205829	-1.90871	0.059544	-4.71087	0.275261	0.320091
Non.inflammatory.Macrophages	EP400	-0.14747	6.870294	-1.90786	0.059654	-5.26065	0.234212	0.297484
Non.inflammatory.Macrophages	TBC1D2B	0.232817	4.091379	1.907753	0.059669	-4.99419	0.277531	0.321471
Non.inflammatory.Macrophages	WDR1	0.175718	7.335798	1.907439	0.05971	-5.36571	0.227649	0.293838
Non.inflammatory.Macrophages	ANKRD13D	-0.39988	2.738056	-1.90732	0.059726	-4.45385	0.301304	0.333963
Non.inflammatory.Macrophages	CDC73	0.175846	6.174072	1.906616	0.059818	-5.11308	0.244715	0.303777
Non.inflammatory.Macrophages	1700094D0	-0.52073	1.476408	-1.90583	0.059922	-4.19823	0.325608	0.346564
Non.inflammatory.Macrophages	BACH2IT1	-1.09009	-0.26225	-1.90581	0.059924	-3.94341	0.341303	0.363707

Non.inflammatory.Macrophages	APP	0.223627	5.938302	1.905714	0.059937	-5.43462	0.248372	0.306143
Non.inflammatory.Macrophages	MDFIC	0.271903	4.328783	1.905661	0.059944	-5.09277	0.274002	0.320176
Non.inflammatory.Macrophages	CD3EAP	0.298476	3.231663	1.905142	0.060012	-4.71411	0.292979	0.330384
Non.inflammatory.Macrophages	ACACB	-1.0134	0.257652	-1.90513	0.060014	-3.99937	0.341417	0.358856
Non.inflammatory.Macrophages	ZFP106	0.183161	6.288522	1.904921	0.060041	-5.20765	0.243195	0.303458
Non.inflammatory.Macrophages	MTMR6	0.202999	4.718718	1.903298	0.060255	-5.02754	0.268461	0.317441
Non.inflammatory.Macrophages	GPBP1L1	0.194812	5.913267	1.90302	0.060292	-5.07587	0.24958	0.307183
Non.inflammatory.Macrophages	CBLB	0.236572	7.336459	1.902999	0.060295	-5.21689	0.228746	0.29525
Non.inflammatory.Macrophages	CABP4	-0.89069	-0.22452	-1.90206	0.060419	-4.0678	0.342719	0.364749
Non.inflammatory.Macrophages	RNGTT	0.165399	6.852257	1.901887	0.060442	-5.23046	0.235831	0.299637
Non.inflammatory.Macrophages	GM29417	-1.05278	1.098886	-1.90188	0.060442	-3.95384	0.334451	0.351659
Non.inflammatory.Macrophages	GM36371	-0.6656	0.884673	-1.90167	0.06047	-4.06562	0.338222	0.353813
Non.inflammatory.Macrophages	SH3GLB1	0.132946	8.546317	1.901669	0.060471	-5.63078	0.212547	0.285882
Non.inflammatory.Macrophages	DDIAS	-0.45166	2.134705	-1.90097	0.060563	-4.41655	0.314504	0.342006
Non.inflammatory.Macrophages	USP32	-0.19673	6.849617	-1.90093	0.060569	-5.29687	0.236053	0.299946

Non.inflammatory.Macrophages	MSL2	0.172313	6.010868	1.900004	0.060692	-5.14489	0.248893	0.30731
Non.inflammatory.Macrophages	A530088E(	-0.98223	1.760356	-1.89929	0.060787	-3.99349	0.322564	0.346226
Non.inflammatory.Macrophages	JCHAIN	2.32287	-0.4625	1.898707	0.060865	-3.99227	0.344197	0.36856
Non.inflammatory.Macrophages	GMDS	-0.1952	6.245209	-1.89856	0.060884	-5.10528	0.245819	0.305904
Non.inflammatory.Macrophages	ABCA1	0.248329	5.836161	1.898176	0.060935	-5.40682	0.252153	0.309516
Non.inflammatory.Macrophages	GM15728	-0.55619	0.500791	-1.89772	0.060996	-4.31579	0.344446	0.359196
Non.inflammatory.Macrophages	GM35188	0.698948	1.73277	1.897335	0.061048	-4.21895	0.323747	0.347268
Non.inflammatory.Macrophages	1200007C1	0.693959	-0.38465	1.897231	0.061061	-4.22332	0.344623	0.368301
Non.inflammatory.Macrophages	ITIH1	-0.68381	2.749811	-1.8967	0.061133	-4.37062	0.304587	0.337745
Non.inflammatory.Macrophages	TMEM39B	-0.27178	4.225197	-1.89669	0.061133	-4.80827	0.278512	0.324159
Non.inflammatory.Macrophages	LRRFIP1	0.160837	7.249941	1.895946	0.061233	-5.3634	0.231792	0.29818
Non.inflammatory.Macrophages	CCZ1	0.163329	6.158644	1.895565	0.061284	-5.16276	0.247906	0.307413
Non.inflammatory.Macrophages	LNPEP	0.152242	7.335044	1.895373	0.06131	-5.41245	0.230668	0.297582
Non.inflammatory.Macrophages	POLR3A	0.358745	3.30507	1.895211	0.061332	-4.60257	0.294984	0.332948
Non.inflammatory.Macrophages	CFAP97	0.256405	3.29046	1.894697	0.061401	-4.66108	0.295454	0.333275

Non.inflammatory.Macrophages	CAPZA2	0.100879	7.929486	1.894036	0.06149	-5.54395	0.222777	0.293137
Non.inflammatory.Macrophages	PPFIBP2	-0.29665	4.285147	-1.89388	0.061511	-5.14753	0.2784	0.324506
Non.inflammatory.Macrophages	CEP72	-0.55779	2.208429	-1.89352	0.061559	-4.22299	0.315849	0.343908
Non.inflammatory.Macrophages	NFIX	0.260614	3.676603	1.893318	0.061586	-4.96308	0.289004	0.330144
Non.inflammatory.Macrophages	PSRC1	-0.87221	0.886949	-1.8924	0.06171	-4.05048	0.341865	0.357034
Non.inflammatory.Macrophages	TBC1D15	-0.17207	6.062519	-1.89233	0.06172	-5.161	0.250203	0.309173
Non.inflammatory.Macrophages	XNDC1	-0.37462	3.594505	-1.89216	0.061742	-4.50335	0.29082	0.331162
Non.inflammatory.Macrophages	NUP210	-0.46005	5.041054	-1.89183	0.061787	-4.3393	0.266398	0.318124
Non.inflammatory.Macrophages	IRS1	-0.97968	0.821216	-1.89146	0.061837	-4.01765	0.342969	0.357781
Non.inflammatory.Macrophages	NEK6	0.338891	3.873937	1.890926	0.061909	-4.61311	0.286273	0.328959
Non.inflammatory.Macrophages	RSRC2	0.132187	6.479986	1.890851	0.061919	-5.21313	0.244186	0.30597
Non.inflammatory.Macrophages	NCF4	0.23499	5.316123	1.890086	0.062023	-5.11185	0.262515	0.316319
Non.inflammatory.Macrophages	SACS	0.655506	2.596254	1.889916	0.062046	-4.27218	0.309693	0.341193
Non.inflammatory.Macrophages	USP6NL	0.198762	5.563085	1.888943	0.062178	-5.10152	0.259028	0.314427
Non.inflammatory.Macrophages	TIMM17A	0.179091	5.135955	1.887863	0.062324	-5.03891	0.266386	0.318499

Non.inflammatory.Macrophages	ARHGAP15	-0.18239	9.872031	-1.88768	0.062349	-5.6476	0.199247	0.279226
Non.inflammatory.Macrophages	2700038G2	-0.44326	2.577847	-1.88736	0.062393	-4.35762	0.311256	0.342168
Non.inflammatory.Macrophages	GM42941	-0.45055	1.267028	-1.88646	0.062516	-4.40047	0.337231	0.355248
Non.inflammatory.Macrophages	GM39121	-1.16319	-0.94202	-1.88643	0.06252	-3.9721	0.34907	0.377713
Non.inflammatory.Macrophages	TGFB2	1.00762	0.46572	1.885155	0.062693	-4.18581	0.349893	0.363749
Non.inflammatory.Macrophages	PLAUR	0.32433	6.837467	1.884897	0.062729	-5.29601	0.241023	0.304759
Non.inflammatory.Macrophages	CSTF3	0.17167	6.037918	1.883736	0.062888	-5.15142	0.253651	0.311868
Non.inflammatory.Macrophages	1500009L1	1.065559	-0.02505	1.882847	0.063009	-3.97779	0.351212	0.369636
Non.inflammatory.Macrophages	XPO4	0.211525	5.623363	1.882673	0.063033	-5.00119	0.260551	0.315988
Non.inflammatory.Macrophages	ZFP871	0.218178	5.249328	1.882455	0.063063	-5.02823	0.266588	0.319356
Non.inflammatory.Macrophages	SNX20	0.20845	5.514815	1.881724	0.063164	-5.11504	0.262611	0.317315
Non.inflammatory.Macrophages	WWC2	-0.22397	5.107898	-1.87975	0.063435	-5.06337	0.270263	0.321616
Non.inflammatory.Macrophages	IL4	-1.25855	0.330173	-1.87955	0.063463	-4.09789	0.353023	0.36737
Non.inflammatory.Macrophages	HIF1A	0.198715	6.608469	1.879197	0.063512	-5.29458	0.246666	0.308645
Non.inflammatory.Macrophages	SMLR1	-0.6926	1.822706	-1.87877	0.063571	-4.22539	0.33014	0.352734

Non.inflammatory.Macrophages	GCKR	-0.7194	1.581851	-1.87824	0.063644	-4.22045	0.33518	0.355236
Non.inflammatory.Macrophages	HERC3	0.289232	4.345366	1.878086	0.063666	-4.79919	0.283563	0.329
Non.inflammatory.Macrophages	SLC46A3	-0.2858	3.085031	-1.87639	0.0639	-4.81337	0.307012	0.34137
Non.inflammatory.Macrophages	SLC37A2	-0.34692	3.315046	-1.87636	0.063905	-4.73796	0.302763	0.339195
Non.inflammatory.Macrophages	PUS7	0.386823	2.95762	1.875647	0.064004	-4.50667	0.309743	0.342831
Non.inflammatory.Macrophages	STRIP1	0.273673	4.156745	1.875286	0.064054	-4.75352	0.288114	0.3318
Non.inflammatory.Macrophages	FRY	0.239349	6.378637	1.87455	0.064156	-5.20919	0.251873	0.312211
Non.inflammatory.Macrophages	BAIAP2	-0.25989	5.269985	-1.87435	0.064184	-5.11596	0.269542	0.322059
Non.inflammatory.Macrophages	GM42829	0.456457	2.205641	1.874096	0.06422	-4.39444	0.324702	0.350861
Non.inflammatory.Macrophages	NUPR1	0.418726	2.41246	1.872605	0.064428	-5.0415	0.321583	0.349502
Non.inflammatory.Macrophages	SMG1	0.135798	7.499087	1.87182	0.064538	-5.40272	0.236169	0.303525
Non.inflammatory.Macrophages	PARL	0.207596	4.391466	1.871396	0.064597	-4.95558	0.285734	0.331062
Non.inflammatory.Macrophages	A430090L1	0.914842	0.628221	1.870698	0.064695	-4.02057	0.356414	0.367842
Non.inflammatory.Macrophages	ARHGAP23	-0.29106	3.694446	-1.87042	0.064735	-4.86368	0.298493	0.337878
Non.inflammatory.Macrophages	MIS18BP1	-0.45313	3.964435	-1.86974	0.06483	-4.65992	0.29379	0.335577

Non.inflammatory.Macrophages	LDLRAP1	0.248299	4.099537	1.86966	0.064841	-4.99557	0.291387	0.334317
Non.inflammatory.Macrophages	CYP3A44	-0.79971	2.308322	-1.86961	0.064848	-4.32928	0.324789	0.351401
Non.inflammatory.Macrophages	PUM2	0.124768	7.324014	1.868868	0.064952	-5.38415	0.239414	0.305813
Non.inflammatory.Macrophages	CREB3L3	-0.60519	1.62088	-1.86878	0.064965	-4.31374	0.338713	0.358429
Non.inflammatory.Macrophages	GM9887	-0.59149	1.686181	-1.86866	0.064981	-4.27148	0.337385	0.357798
Non.inflammatory.Macrophages	HIPK3	0.183084	5.445686	1.868589	0.064991	-5.14635	0.268587	0.32226
Non.inflammatory.Macrophages	SLC25A25	-0.22767	4.951297	-1.86841	0.065017	-4.97649	0.276815	0.326749
Non.inflammatory.Macrophages	SPARCL1	1.261358	0.3267	1.867887	0.06509	-4.10924	0.358316	0.371635
Non.inflammatory.Macrophages	KLHL11	-0.39774	2.776613	-1.8678	0.065103	-4.52398	0.316069	0.347186
Non.inflammatory.Macrophages	DNMT1	-0.26137	5.753517	-1.86684	0.065239	-5.09793	0.264159	0.319905
Non.inflammatory.Macrophages	ACADSB	0.263641	3.270603	1.866341	0.065309	-4.7764	0.307476	0.343013
Non.inflammatory.Macrophages	STOML3	-0.73978	0.359863	-1.86521	0.065468	-4.15057	0.35989	0.372323
Non.inflammatory.Macrophages	IGFBP2	-0.66786	4.701389	-1.86441	0.065582	-4.93205	0.282819	0.330126
Non.inflammatory.Macrophages	SCYL3	0.244803	3.642548	1.864159	0.065618	-4.78353	0.301673	0.340148
Non.inflammatory.Macrophages	GM39469	-0.30662	2.328802	-1.86393	0.06565	-4.60756	0.32667	0.35281

Non.inflammatory.Macrophages	JARID2	0.184395	8.144583	1.863569	0.065702	-5.4798	0.229151	0.30018
Non.inflammatory.Macrophages	WDR66	-0.6552	1.900032	-1.8634	0.065726	-4.21692	0.335257	0.357246
Non.inflammatory.Macrophages	LIPO3	0.320875	2.915424	1.863321	0.065737	-4.70273	0.315324	0.347302
Non.inflammatory.Macrophages	METTL26	-0.28254	4.639923	-1.86314	0.065763	-4.89218	0.283971	0.331059
Non.inflammatory.Macrophages	ID3	-0.21344	6.599763	-1.86153	0.065992	-5.47654	0.252615	0.314022
Non.inflammatory.Macrophages	5830428M	-0.91382	2.548294	-1.86138	0.066013	-4.0509	0.323286	0.351513
Non.inflammatory.Macrophages	PAPLN	-0.78034	0.894565	-1.86131	0.066024	-4.13676	0.356597	0.368065
Non.inflammatory.Macrophages	BAIAP2L1	-0.36347	3.098445	-1.8601	0.066196	-4.59712	0.313362	0.346618
Non.inflammatory.Macrophages	5430401H(	1.076744	-0.47304	1.859953	0.066217	-4.07593	0.362235	0.382821
Non.inflammatory.Macrophages	ERH	0.195671	7.372533	1.859774	0.066243	-5.40878	0.241435	0.307803
Non.inflammatory.Macrophages	PGLYRP2	-0.70957	2.088786	-1.85928	0.066313	-4.23839	0.333184	0.356756
Non.inflammatory.Macrophages	BE692007	0.502338	2.816357	1.859248	0.066318	-4.57741	0.318863	0.349614
Non.inflammatory.Macrophages	ATP11A	0.28943	2.676915	1.85846	0.066431	-4.82579	0.321711	0.351415
Non.inflammatory.Macrophages	MRTFA	0.183594	7.084199	1.858386	0.066442	-5.49206	0.245936	0.310867
Non.inflammatory.Macrophages	GM15956	-0.88367	0.27605	-1.85833	0.06645	-4.07199	0.36252	0.375702

Non.inflammatory.Macrophages	IQSEC1	-0.2231	6.277452	-1.85829	0.066456	-5.08048	0.258395	0.317938
Non.inflammatory.Macrophages	CKM	1.142808	0.1057	1.857967	0.066502	-4.11391	0.362625	0.377635
Non.inflammatory.Macrophages	RUBCNL	0.44076	3.191015	1.856667	0.066689	-4.51387	0.312696	0.347253
Non.inflammatory.Macrophages	ARMCX6	-0.57511	0.977785	-1.85634	0.066736	-4.21152	0.357386	0.369405
Non.inflammatory.Macrophages	EYA1	-1.08163	2.426875	-1.8562	0.066756	-4.07013	0.327555	0.354866
Non.inflammatory.Macrophages	TMEM123	0.202821	6.484305	1.855591	0.066844	-5.25891	0.256116	0.317183
Non.inflammatory.Macrophages	KIF13B	0.183603	6.510852	1.855197	0.066901	-5.29318	0.2557	0.317052
Non.inflammatory.Macrophages	IST1	-0.16603	5.536178	-1.8551	0.066914	-5.19627	0.271407	0.325782
Non.inflammatory.Macrophages	NR6A1	-0.24653	5.241175	-1.85503	0.066924	-5.11815	0.276341	0.328469
Non.inflammatory.Macrophages	GSTO1	0.163712	4.594828	1.854425	0.067012	-5.1612	0.287711	0.33463
Non.inflammatory.Macrophages	SCAMP2	0.147749	6.357233	1.853971	0.067078	-5.31425	0.2585	0.318811
Non.inflammatory.Macrophages	FAM160B1	0.255956	4.443367	1.853659	0.067123	-4.84596	0.290625	0.336405
Non.inflammatory.Macrophages	IFT52	0.208618	4.249963	1.852688	0.067263	-4.91749	0.294518	0.33856
Non.inflammatory.Macrophages	CCNL1	-0.13563	7.09056	-1.8525	0.067291	-5.47001	0.247591	0.312831
Non.inflammatory.Macrophages	EPCAM	-1.09551	2.160039	-1.85239	0.067307	-4.06724	0.334273	0.358846

Non.inflammatory.Macrophages	TRAPPC2	0.281767	4.171105	1.851817	0.06739	-4.80304	0.296179	0.339478
Non.inflammatory.Macrophages	ITSN1	-0.24321	5.300914	-1.85155	0.067429	-5.23611	0.276522	0.32908
Non.inflammatory.Macrophages	TMEM173	0.238537	4.250581	1.850463	0.067587	-4.99149	0.295375	0.339174
Non.inflammatory.Macrophages	WBP4	0.181917	5.270226	1.849628	0.067708	-5.11817	0.277953	0.329947
Non.inflammatory.Macrophages	IER5L	-0.43497	2.89009	-1.84946	0.067732	-4.49912	0.321228	0.352545
Non.inflammatory.Macrophages	VMA21	0.187255	5.176423	1.847855	0.067967	-5.14728	0.280317	0.331184
Non.inflammatory.Macrophages	CHDH	-0.96388	1.157054	-1.84767	0.067994	-4.10602	0.357563	0.370487
Non.inflammatory.Macrophages	GAMT	-0.40949	4.228598	-1.84763	0.068	-4.99878	0.296984	0.340158
Non.inflammatory.Macrophages	SMDT1	0.132926	7.773748	1.845129	0.068367	-5.54353	0.240235	0.308999
Non.inflammatory.Macrophages	GM20045	-0.52375	1.764585	-1.84507	0.068375	-4.39769	0.346376	0.365275
Non.inflammatory.Macrophages	PTPN7	0.380428	4.151708	1.844867	0.068405	-4.67592	0.299795	0.341879
Non.inflammatory.Macrophages	CREB1	0.155561	6.332794	1.84438	0.068477	-5.28201	0.2626	0.321911
Non.inflammatory.Macrophages	MS4A6D	0.310768	2.866447	1.843592	0.068593	-5.22944	0.324737	0.354722
Non.inflammatory.Macrophages	GM47863	-0.49343	0.552776	-1.84293	0.06869	-4.51977	0.369933	0.378651
Non.inflammatory.Macrophages	TRP53BP1	-0.28652	3.888271	-1.84274	0.068719	-4.79236	0.305545	0.345198

Non.inflammatory.Macrophages	MED17	-0.16971	4.867425	-1.84225	0.068791	-5.01872	0.288053	0.336114
Non.inflammatory.Macrophages	IARS2	0.232399	4.475268	1.840486	0.069051	-4.98803	0.295861	0.340433
Non.inflammatory.Macrophages	FAM76B	0.181528	5.401156	1.840436	0.069059	-5.08632	0.279619	0.33177
Non.inflammatory.Macrophages	CAPRIN1	0.122099	7.460311	1.840189	0.069096	-5.48832	0.246502	0.313291
Non.inflammatory.Macrophages	PCNX	0.237516	5.518161	1.840111	0.069107	-5.06112	0.277628	0.330697
Non.inflammatory.Macrophages	CYB5A	-0.30255	7.336341	-1.83998	0.069126	-5.50901	0.248383	0.31438
Non.inflammatory.Macrophages	EEF1G	0.162452	7.187877	1.839656	0.069175	-5.45371	0.250655	0.315756
Non.inflammatory.Macrophages	TPR	0.135602	7.524789	1.839583	0.069185	-5.52158	0.245528	0.312809
Non.inflammatory.Macrophages	DAPP1	-0.1689	6.482475	-1.83931	0.069226	-5.34974	0.261727	0.322118
Non.inflammatory.Macrophages	SLC39A1	0.153933	6.161792	1.839253	0.069234	-5.37583	0.266915	0.325007
Non.inflammatory.Macrophages	STAB1	0.391833	3.176342	1.838572	0.069336	-4.88622	0.32021	0.353425
Non.inflammatory.Macrophages	BORCS8	0.236149	4.590375	1.838461	0.069352	-4.94078	0.293849	0.339794
Non.inflammatory.Macrophages	GM31243	-0.48404	2.779029	-1.83843	0.069357	-4.73323	0.328005	0.357361
Non.inflammatory.Macrophages	A430093F1	-0.53668	2.363356	-1.83843	0.069357	-4.37389	0.336347	0.361517
Non.inflammatory.Macrophages	RBM4B	-0.23786	5.747385	-1.83777	0.069456	-4.98537	0.274041	0.329247

Non.inflammatory.Macrophages	SPIB	-0.69556	4.129765	-1.83753	0.069491	-4.35261	0.302451	0.344451
Non.inflammatory.Macrophages	SECISBP2	0.207964	4.786541	1.837348	0.069518	-5.0141	0.290595	0.338283
Non.inflammatory.Macrophages	KCTD2	-0.28557	3.355478	-1.83731	0.069524	-4.77515	0.317012	0.352031
Non.inflammatory.Macrophages	CNOT6	0.145925	5.947416	1.836675	0.069618	-5.20049	0.270964	0.327736
Non.inflammatory.Macrophages	CFAP20	-0.18916	5.191326	-1.83609	0.069705	-5.08262	0.283775	0.334898
Non.inflammatory.Macrophages	GNG10	0.190513	6.940589	1.835692	0.069765	-5.36933	0.254979	0.31907
Non.inflammatory.Macrophages	DCTD	0.68685	1.018621	1.835586	0.069781	-4.18578	0.365321	0.376226
Non.inflammatory.Macrophages	ORAI3	0.265946	3.868765	1.835485	0.069796	-4.91218	0.307577	0.34759
Non.inflammatory.Macrophages	GRK2	-0.14955	7.206149	-1.83545	0.0698	-5.44583	0.250861	0.316751
Non.inflammatory.Macrophages	LIPC	-0.47877	2.510562	-1.83524	0.069833	-4.53166	0.333957	0.361076
Non.inflammatory.Macrophages	CSNK2A1	0.128528	6.59773	1.835203	0.069838	-5.31919	0.260391	0.32225
Non.inflammatory.Macrophages	SMARCE1	0.147988	6.302182	1.834671	0.069918	-5.2553	0.265146	0.325101
Non.inflammatory.Macrophages	CMTM6	0.190663	4.866252	1.834641	0.069922	-5.07439	0.289458	0.338361
Non.inflammatory.Macrophages	RAB18	0.152196	5.748093	1.834475	0.069947	-5.23704	0.274287	0.330196
Non.inflammatory.Macrophages	USP22	0.206752	4.389395	1.834403	0.069958	-4.96481	0.297991	0.342946

Non.inflammatory.Macrophages	ADGRG5	-1.23627	0.105614	-1.83439	0.069959	-4.05754	0.372556	0.386325
Non.inflammatory.Macrophages	PIP4K2A	0.17577	7.239298	1.83429	0.069975	-5.51746	0.250352	0.316808
Non.inflammatory.Macrophages	GM19710	-0.59299	2.595654	-1.83355	0.070086	-4.42739	0.332641	0.3606
Non.inflammatory.Macrophages	ZFP691	0.405205	3.075803	1.833251	0.07013	-4.58223	0.323197	0.355941
Non.inflammatory.Macrophages	UST	-0.33684	6.028853	-1.83244	0.070253	-5.42869	0.27036	0.328073
Non.inflammatory.Macrophages	UTP14B	-0.38095	3.081232	-1.83227	0.070278	-4.61558	0.323515	0.356176
Non.inflammatory.Macrophages	RNASEH2A	-0.24316	4.54522	-1.83191	0.070332	-5.01762	0.296101	0.342041
Non.inflammatory.Macrophages	NIN	0.205788	5.920519	1.831447	0.070402	-5.23944	0.272382	0.329407
Non.inflammatory.Macrophages	UBAP1	-0.20899	5.688984	-1.83133	0.070419	-5.19461	0.276265	0.33158
Non.inflammatory.Macrophages	SPINT2	-0.40247	4.563442	-1.83111	0.070452	-4.52767	0.29593	0.342181
Non.inflammatory.Macrophages	MYL4	-0.58976	4.638008	-1.83054	0.070539	-4.68122	0.294737	0.34168
Non.inflammatory.Macrophages	GM16754	0.78019	0.04601	1.83051	0.070543	-4.19321	0.37411	0.388214
Non.inflammatory.Macrophages	TNIP3	0.68697	1.037658	1.830005	0.070619	-4.66164	0.366484	0.377812
Non.inflammatory.Macrophages	ZBTB33	0.322148	2.982117	1.829911	0.070633	-4.63661	0.325974	0.357929
Non.inflammatory.Macrophages	GM30211	-0.84697	4.041214	-1.82989	0.070637	-4.37903	0.305687	0.347534

Non.inflammatory.Macrophages	ATP6V1A	-0.1542	6.534998	-1.82958	0.070684	-5.50556	0.262595	0.324326
Non.inflammatory.Macrophages	RNF31	-0.30986	3.162894	-1.82911	0.070755	-4.77928	0.322588	0.356412
Non.inflammatory.Macrophages	TXN1	0.205968	7.304482	1.829035	0.070766	-5.55696	0.250559	0.317601
Non.inflammatory.Macrophages	PLOD2	1.029172	1.261605	1.828658	0.070823	-4.11194	0.361779	0.375904
Non.inflammatory.Macrophages	MAP3K13	-0.80674	0.779749	-1.82855	0.07084	-4.19327	0.370931	0.381019
Non.inflammatory.Macrophages	LIPH	-0.77578	-0.05189	-1.82831	0.070876	-4.2121	0.374358	0.39003
Non.inflammatory.Macrophages	2010310C(	-0.61241	1.298313	-1.82824	0.070886	-4.51254	0.360983	0.375695
Non.inflammatory.Macrophages	CYB561A3	-0.33578	5.573777	-1.82808	0.07091	-4.76593	0.278565	0.333629
Non.inflammatory.Macrophages	FAM71F2	0.411276	2.260769	1.828004	0.070922	-4.79048	0.34067	0.365868
Non.inflammatory.Macrophages	OLFR1369-	-1.44947	-0.76388	-1.82745	0.071005	-4.04948	0.374481	0.3982
Non.inflammatory.Macrophages	SAMD10	0.701483	1.732501	1.827308	0.071027	-4.21002	0.351802	0.371535
Non.inflammatory.Macrophages	GPC1	-1.22216	0.351919	-1.82729	0.071029	-4.06355	0.374481	0.386062
Non.inflammatory.Macrophages	ATP13A2	0.215222	5.610722	1.827121	0.071056	-5.29373	0.278028	0.333581
Non.inflammatory.Macrophages	D030028A(	-0.39977	2.795422	-1.82658	0.071138	-4.64776	0.329975	0.361013
Non.inflammatory.Macrophages	PLAG1	0.467974	2.8948	1.826564	0.07114	-4.35148	0.327997	0.360016

Non.inflammatory.Macrophages	SDC2	-0.49973	2.267759	-1.8264	0.071165	-4.55124	0.340662	0.366366
Non.inflammatory.Macrophages	CDK11B	-0.17905	6.82853	-1.82636	0.071171	-5.369	0.25808	0.322691
Non.inflammatory.Macrophages	RBM22	-0.15408	5.603703	-1.82603	0.071221	-5.15332	0.278218	0.334
Non.inflammatory.Macrophages	HES7	-0.67301	-0.49621	-1.82591	0.07124	-4.23252	0.374576	0.395772
Non.inflammatory.Macrophages	PKN1	0.14095	6.766934	1.825258	0.071339	-5.39149	0.259363	0.323634
Non.inflammatory.Macrophages	FAM234A	-0.24196	4.099085	-1.82479	0.07141	-5.2023	0.305353	0.348691
Non.inflammatory.Macrophages	PEPD	-0.18049	5.308275	-1.82474	0.071418	-5.27162	0.283663	0.337167
Non.inflammatory.Macrophages	MYCBP	0.245227	4.22905	1.824417	0.071467	-4.94684	0.303038	0.347629
Non.inflammatory.Macrophages	CAR8	-0.63275	2.375063	-1.8239	0.071546	-4.40292	0.339321	0.366179
Non.inflammatory.Macrophages	ZFAND6	0.137098	6.978135	1.823184	0.071655	-5.40776	0.256479	0.322416
Non.inflammatory.Macrophages	NDUFA2	0.139345	7.238115	1.822756	0.07172	-5.53389	0.252424	0.320252
Non.inflammatory.Macrophages	CD46	0.56188	2.11028	1.822642	0.071738	-4.36276	0.344928	0.369381
Non.inflammatory.Macrophages	AUNIP	-0.67755	1.893508	-1.82263	0.07174	-4.23365	0.349462	0.371614
Non.inflammatory.Macrophages	PSAP	0.166608	9.249596	1.822487	0.071762	-5.96872	0.223094	0.30286
Non.inflammatory.Macrophages	SNX4	0.14504	6.329814	1.822455	0.071767	-5.33888	0.266869	0.328497

Non.inflammatory.Macrophages	NRG4	0.537079	2.017195	1.822433	0.07177	-4.5863	0.346868	0.370388
Non.inflammatory.Macrophages	CYP8B1	-0.79218	1.074088	-1.822	0.071836	-4.19437	0.36723	0.3804
Non.inflammatory.Macrophages	CADM1	-0.30039	5.440386	-1.8219	0.071852	-5.61507	0.281888	0.336892
Non.inflammatory.Macrophages	CLDN11	-0.7484	-0.29485	-1.82164	0.071892	-4.26642	0.375814	0.395268
Non.inflammatory.Macrophages	ZADH2	0.265921	3.381757	1.820035	0.072138	-4.81119	0.320164	0.357451
Non.inflammatory.Macrophages	SC5D	-0.30221	4.248539	-1.82003	0.072138	-4.84079	0.303743	0.348931
Non.inflammatory.Macrophages	ASRGL1	0.477504	3.315534	1.819969	0.072148	-4.5229	0.321452	0.35811
Non.inflammatory.Macrophages	TMOD1	0.585006	1.823905	1.819887	0.07216	-4.41709	0.351765	0.37328
Non.inflammatory.Macrophages	AKR1C14	-0.77792	0.911524	-1.81987	0.072163	-4.18444	0.371186	0.382865
Non.inflammatory.Macrophages	DSEL	0.644145	0.375304	1.819581	0.072207	-4.215	0.376592	0.388717
Non.inflammatory.Macrophages	CEP104	0.299961	3.105243	1.819396	0.072236	-4.68721	0.32565	0.360333
Non.inflammatory.Macrophages	DYNC1I2	0.128551	6.441227	1.818726	0.072339	-5.39324	0.265887	0.328586
Non.inflammatory.Macrophages	C1RA	-0.50714	1.359965	-1.81868	0.072347	-4.42431	0.361986	0.378491
Non.inflammatory.Macrophages	TSN	0.133411	6.360058	1.818609	0.072357	-5.34889	0.267211	0.32933
Non.inflammatory.Macrophages	IP6K1	-0.19033	6.598351	-1.81814	0.07243	-5.31527	0.263504	0.327267

Non.inflammatory.Macrophages	FBXL7	-0.72726	3.621991	-1.81772	0.072493	-4.5848	0.316013	0.355658
Non.inflammatory.Macrophages	KIF5B	0.116622	7.401742	1.817705	0.072496	-5.50019	0.250881	0.320137
Non.inflammatory.Macrophages	SETD2	0.161014	6.922415	1.817292	0.07256	-5.41984	0.258418	0.324578
Non.inflammatory.Macrophages	BARD1	-0.60753	3.42386	-1.81719	0.072576	-4.39367	0.319903	0.357789
Non.inflammatory.Macrophages	YAF2	-0.16367	6.056106	-1.8169	0.07262	-5.22994	0.272497	0.332597
Non.inflammatory.Macrophages	PELI2	0.498335	2.831902	1.816879	0.072624	-4.56136	0.331582	0.363826
Non.inflammatory.Macrophages	AB124611	0.181631	5.452214	1.816301	0.072713	-5.3797	0.282743	0.338459
Non.inflammatory.Macrophages	GPD1L	-0.28553	5.296236	-1.81619	0.07273	-4.94047	0.285449	0.339934
Non.inflammatory.Macrophages	C8G	-0.55593	3.782028	-1.8159	0.072776	-4.78737	0.313024	0.354574
Non.inflammatory.Macrophages	ABHD6	0.556355	2.548845	1.815855	0.072782	-4.30305	0.337305	0.366951
Non.inflammatory.Macrophages	MCUR1	0.215088	4.462065	1.815815	0.072788	-5.08425	0.300343	0.347926
Non.inflammatory.Macrophages	MVB12A	-0.17863	5.948563	-1.8158	0.072791	-5.25695	0.274295	0.333821
Non.inflammatory.Macrophages	REPS2	0.673232	0.905504	1.814402	0.073008	-4.32601	0.372883	0.384635
Non.inflammatory.Macrophages	IGFBP7	-0.34171	5.173401	-1.81376	0.073108	-5.08942	0.28863	0.341827
Non.inflammatory.Macrophages	FBXW2	0.154873	6.193608	1.813521	0.073144	-5.25347	0.271216	0.332287

Non.inflammatory.Macrophages	SLC27A5	-0.83356	1.457834	-1.81193	0.073392	-4.24506	0.362643	0.37991
Non.inflammatory.Macrophages	NAIP1	0.663404	-0.47909	1.81086	0.073559	-4.31783	0.37998	0.401382
Non.inflammatory.Macrophages	GM49625	-0.45267	2.147374	-1.81074	0.073578	-4.50376	0.348159	0.373175
Non.inflammatory.Macrophages	CDK13	0.154975	7.414024	1.810717	0.073581	-5.47788	0.252619	0.322293
Non.inflammatory.Macrophages	NARS2	0.258479	4.271035	1.810572	0.073604	-4.9761	0.306126	0.351772
Non.inflammatory.Macrophages	GM29394	0.880789	0.417159	1.810495	0.073616	-4.1409	0.37998	0.391564
Non.inflammatory.Macrophages	ALKBH6	0.271938	2.755561	1.8104	0.073631	-4.72533	0.335606	0.366925
Non.inflammatory.Macrophages	INPP4B	-0.33678	6.480863	-1.81032	0.073643	-5.3184	0.267488	0.330784
Non.inflammatory.Macrophages	MKLN1	0.148638	7.159858	1.809907	0.073708	-5.50012	0.25667	0.324697
Non.inflammatory.Macrophages	STYX	0.225521	4.526153	1.809555	0.073763	-4.92067	0.301506	0.349464
Non.inflammatory.Macrophages	ZFP64	-0.22771	5.317866	-1.80931	0.073801	-5.26935	0.287295	0.341897
Non.inflammatory.Macrophages	KLHDC3	0.181142	4.338451	1.809303	0.073802	-5.04992	0.304971	0.351347
Non.inflammatory.Macrophages	UCHL5	0.187323	5.728606	1.809271	0.073807	-5.21995	0.280176	0.338013
Non.inflammatory.Macrophages	MRVI1	0.847033	0.497468	1.80896	0.073856	-4.16883	0.380137	0.391139
Non.inflammatory.Macrophages	VAMP4	0.150801	6.154005	1.808651	0.073905	-5.41408	0.273098	0.334345

Non.inflammatory.Macrophages	7-Mar	0.126025	6.926226	1.808532	0.073923	-5.44639	0.260484	0.327267
Non.inflammatory.Macrophages	CPM	-0.61945	4.160192	-1.80818	0.073979	-4.53077	0.308542	0.353626
Non.inflammatory.Macrophages	AP1S1	0.205204	4.706161	1.807983	0.074009	-5.12457	0.298465	0.348324
Non.inflammatory.Macrophages	PIK3CG	0.226679	4.936727	1.807655	0.074061	-5.08353	0.294307	0.346145
Non.inflammatory.Macrophages	DDX47	0.164585	5.550552	1.807614	0.074067	-5.16962	0.283484	0.340287
Non.inflammatory.Macrophages	RPTOR	0.164899	6.294365	1.806509	0.074241	-5.35534	0.27141	0.333659
Non.inflammatory.Macrophages	GM13919	-0.75434	3.111975	-1.80571	0.074366	-4.21949	0.329798	0.364809
Non.inflammatory.Macrophages	SMN1	0.224324	4.851728	1.805693	0.07437	-5.09042	0.296706	0.347566
Non.inflammatory.Macrophages	SIPA1	-0.17649	5.681522	-1.80541	0.074414	-5.22483	0.282078	0.339688
Non.inflammatory.Macrophages	GM20406	1.08064	-0.53168	1.80529	0.074433	-4.07897	0.381582	0.403793
Non.inflammatory.Macrophages	COX17	0.158166	6.919214	1.804847	0.074503	-5.4672	0.261603	0.328379
Non.inflammatory.Macrophages	DHRS11	0.280572	4.678666	1.804723	0.074523	-5.03149	0.300001	0.349568
Non.inflammatory.Macrophages	DHX32	0.26999	3.380004	1.804565	0.074548	-4.80755	0.324645	0.362536
Non.inflammatory.Macrophages	TNKS	0.197584	6.066905	1.80401	0.074635	-5.32067	0.275795	0.336483
Non.inflammatory.Macrophages	BTAF1	-0.13819	7.190783	-1.80373	0.07468	-5.55085	0.257449	0.326118

Non.inflammatory.Macrophages	H2AFX	-0.38334	6.482628	-1.80373	0.07468	-5.1939	0.268866	0.332611
Non.inflammatory.Macrophages	NCKAP5L	-0.2856	4.041979	-1.80348	0.074719	-4.97649	0.312098	0.356092
Non.inflammatory.Macrophages	EMD	-0.16111	6.102062	-1.80291	0.074809	-5.29281	0.275426	0.33643
Non.inflammatory.Macrophages	PRKAR2B	0.495914	3.305356	1.802809	0.074825	-4.48531	0.326591	0.363668
Non.inflammatory.Macrophages	INTS14	0.310197	4.691853	1.802559	0.074865	-5.03934	0.30021	0.349932
Non.inflammatory.Macrophages	M6PR	0.155161	6.293265	1.80243	0.074885	-5.45619	0.272235	0.334673
Non.inflammatory.Macrophages	GM10847	1.133311	0.165978	1.801659	0.075008	-4.08649	0.382787	0.397172
Non.inflammatory.Macrophages	RAB11FIP3	-0.40963	2.230172	-1.80095	0.075121	-4.55326	0.349381	0.375188
Non.inflammatory.Macrophages	SUSD1	-0.27594	5.183728	-1.80057	0.075181	-5.04701	0.292156	0.345782
Non.inflammatory.Macrophages	SLC43A3	-0.35507	3.813488	-1.8001	0.075255	-4.759	0.317737	0.359446
Non.inflammatory.Macrophages	TNIP2	0.219702	3.816648	1.799978	0.075275	-5.00086	0.317676	0.359463
Non.inflammatory.Macrophages	STARD3	-0.23883	4.116736	-1.79875	0.07547	-5.04988	0.312531	0.357055
Non.inflammatory.Macrophages	SH2D2A	-0.98692	2.764079	-1.79863	0.075489	-4.18819	0.339251	0.370763
Non.inflammatory.Macrophages	EHD1	0.187777	6.1755	1.798405	0.075526	-5.45142	0.275637	0.337242
Non.inflammatory.Macrophages	ORC2	0.243152	4.279461	1.798364	0.075532	-4.93284	0.309452	0.355522

Non.inflammatory.Macrophages	PSMD2	0.151743	6.149979	1.798027	0.075586	-5.37531	0.276098	0.33759
Non.inflammatory.Macrophages	CYP51	-0.33377	3.916341	-1.79796	0.075597	-4.78826	0.316396	0.359248
Non.inflammatory.Macrophages	GM16364.:	-0.95304	1.279847	-1.79763	0.07565	-4.19949	0.371144	0.38666
Non.inflammatory.Macrophages	S100A9	0.682341	6.814997	1.797274	0.075706	-5.36685	0.265264	0.331586
Non.inflammatory.Macrophages	GP9	1.039927	0.659096	1.79707	0.075739	-4.24367	0.38288	0.393594
Non.inflammatory.Macrophages	KIF2C	-0.61625	2.663513	-1.79603	0.075905	-4.46316	0.341911	0.372748
Non.inflammatory.Macrophages	CEP85L	0.261252	4.806918	1.795965	0.075916	-5.16537	0.300188	0.351164
Non.inflammatory.Macrophages	SGIP1	-0.77623	0.175941	-1.79582	0.075939	-4.23331	0.384971	0.399447
Non.inflammatory.Macrophages	CCDC125	0.297062	4.497843	1.795813	0.07594	-4.98808	0.305895	0.354216
Non.inflammatory.Macrophages	NAPEPLD	0.690189	1.056502	1.795769	0.075947	-4.29142	0.37664	0.389797
Non.inflammatory.Macrophages	LFNG	0.193523	4.413378	1.794961	0.076077	-5.23658	0.307845	0.355346
Non.inflammatory.Macrophages	E330009J0	-0.85266	2.552592	-1.79481	0.076101	-4.21629	0.344628	0.374246
Non.inflammatory.Macrophages	GM43260	-0.6373	2.252383	-1.79466	0.076125	-4.27464	0.350931	0.377423
Non.inflammatory.Macrophages	TMEM120I	0.347928	3.044848	1.794316	0.076181	-4.93713	0.334566	0.369368
Non.inflammatory.Macrophages	GADD45G	-0.32532	4.465474	-1.79424	0.076193	-5.10334	0.306913	0.355063

Non.inflammatory.Macrophages	NOTCH4	-0.95795	0.210596	-1.7939	0.076248	-4.18618	0.385521	0.399884
Non.inflammatory.Macrophages	ERGIC3	-0.16623	5.513051	-1.79385	0.076255	-5.30036	0.287938	0.345067
Non.inflammatory.Macrophages	KLHDC10	0.196454	5.434437	1.793612	0.076294	-5.22036	0.289362	0.345849
Non.inflammatory.Macrophages	CLOCK	0.188845	5.071637	1.792888	0.076411	-5.24457	0.296184	0.349668
Non.inflammatory.Macrophages	SLC4A1AP	0.230019	4.278134	1.792444	0.076482	-4.94909	0.310949	0.357581
Non.inflammatory.Macrophages	FLI1	-0.16897	8.191063	-1.79217	0.076526	-5.62295	0.244754	0.320742
Non.inflammatory.Macrophages	TESPA1	-1.01883	2.504605	-1.79205	0.076546	-4.13419	0.346252	0.375802
Non.inflammatory.Macrophages	SP4	-0.2316	5.254382	-1.792	0.076554	-5.13166	0.292987	0.348132
Non.inflammatory.Macrophages	TMBIM1	0.277864	2.851295	1.791809	0.076585	-4.9718	0.339071	0.372246
Non.inflammatory.Macrophages	SULT2A2	-0.53254	4.037802	-1.79169	0.076605	-4.92032	0.315528	0.360157
Non.inflammatory.Macrophages	TEX45	-0.99154	0.222756	-1.79106	0.076705	-4.16043	0.386427	0.400616
Non.inflammatory.Macrophages	HLF	-0.92247	1.023669	-1.79097	0.07672	-4.19069	0.378808	0.39181
Non.inflammatory.Macrophages	TTC28	-0.28965	4.798912	-1.79059	0.076783	-5.11324	0.301511	0.352933
Non.inflammatory.Macrophages	ATP9B	0.153778	5.858499	1.790555	0.076787	-5.3425	0.282623	0.342673
Non.inflammatory.Macrophages	CIP2A	-0.49181	3.506565	-1.79014	0.076855	-4.63941	0.32626	0.366079

Non.inflammatory.Macrophages	DDR1	0.854106	0.137297	1.790064	0.076867	-4.14361	0.386591	0.402021
Non.inflammatory.Macrophages	CACNB2	-0.87567	4.420818	-1.78951	0.076957	-4.48355	0.308825	0.357044
Non.inflammatory.Macrophages	PGRMC1	-0.22384	4.647619	-1.78941	0.076974	-5.08089	0.304588	0.354838
Non.inflammatory.Macrophages	DNAIC1	-0.81122	0.436784	-1.78845	0.077128	-4.23426	0.387301	0.399162
Non.inflammatory.Macrophages	HNF4A	-0.84982	0.691681	-1.78833	0.077148	-4.23219	0.385053	0.396354
Non.inflammatory.Macrophages	NOSIP	-0.17877	5.158105	-1.78831	0.077151	-5.18918	0.295605	0.350048
Non.inflammatory.Macrophages	ANAPC1	0.211216	4.721072	1.786613	0.077428	-5.09	0.304564	0.354831
Non.inflammatory.Macrophages	DNAJC11	0.217106	4.486406	1.786336	0.077473	-5.05488	0.309015	0.35725
Non.inflammatory.Macrophages	GRAMD1C	0.39109	2.440736	1.785953	0.077535	-4.70028	0.34994	0.378331
Non.inflammatory.Macrophages	PER2	-0.48641	2.222784	-1.78584	0.077553	-4.58796	0.354573	0.380631
Non.inflammatory.Macrophages	GM26885	-0.45265	2.317843	-1.78541	0.077624	-4.68994	0.352595	0.379725
Non.inflammatory.Macrophages	SERPINA1E	-0.49865	7.994808	-1.78525	0.077651	-5.6037	0.249431	0.324268
Non.inflammatory.Macrophages	RC3H1	0.151871	6.887392	1.78522	0.077655	-5.44127	0.266964	0.334419
Non.inflammatory.Macrophages	PSMC1	0.146105	5.889053	1.785071	0.077679	-5.36349	0.283788	0.343913
Non.inflammatory.Macrophages	REXO2	0.175526	6.270041	1.784731	0.077735	-5.45878	0.277347	0.340402

Non.inflammatory.Macrophages	BEND6	-0.63814	0.170455	-1.78453	0.077768	-4.39063	0.388955	0.403409
Non.inflammatory.Macrophages	PCP4L1	-0.88052	1.36121	-1.78394	0.077865	-4.30158	0.373972	0.390483
Non.inflammatory.Macrophages	CREBL2	0.313055	3.233581	1.783348	0.077961	-4.83153	0.334316	0.37075
Non.inflammatory.Macrophages	LARP1B	0.229715	5.599705	1.783012	0.078016	-5.16233	0.289571	0.347234
Non.inflammatory.Macrophages	PLEKHG6	-0.56436	-0.11425	-1.78184	0.078208	-4.27662	0.390579	0.407302
Non.inflammatory.Macrophages	VILL	-0.38562	1.228935	-1.78148	0.078267	-4.73311	0.378342	0.392467
Non.inflammatory.Macrophages	SIRT3	-0.33243	3.344497	-1.78083	0.078375	-4.66608	0.333346	0.370268
Non.inflammatory.Macrophages	HAX1	-0.22972	4.020737	-1.78059	0.078414	-5.06612	0.31998	0.36337
Non.inflammatory.Macrophages	MIER1	0.146073	6.946828	1.779591	0.078579	-5.49929	0.268083	0.335304
Non.inflammatory.Macrophages	ABHD13	0.245713	3.557429	1.779037	0.078671	-4.88522	0.329944	0.368705
Non.inflammatory.Macrophages	SEC23IP	0.178608	4.499732	1.778231	0.078804	-5.05934	0.311737	0.359392
Non.inflammatory.Macrophages	ZFP281	0.223519	4.496464	1.777907	0.078857	-5.11055	0.311799	0.35961
Non.inflammatory.Macrophages	AREL1	0.228291	4.263334	1.77776	0.078882	-5.01085	0.316255	0.362024
Non.inflammatory.Macrophages	DHX8	0.191897	5.129659	1.777578	0.078912	-5.19335	0.299993	0.35346
Non.inflammatory.Macrophages	CXCL16	0.247257	3.468405	1.777547	0.078917	-5.3853	0.331904	0.370191

Non.inflammatory.Macrophages	PCDH7	1.110179	0.520175	1.777495	0.078926	-4.24005	0.392067	0.401833
Non.inflammatory.Macrophages	PRKAG2	0.209297	5.050385	1.777448	0.078933	-5.21537	0.301447	0.354262
Non.inflammatory.Macrophages	CHP2	-0.83701	-0.09865	-1.77739	0.078943	-4.44631	0.392369	0.408795
Non.inflammatory.Macrophages	PSMD3	0.206687	4.642778	1.776477	0.079094	-5.11354	0.30934	0.358636
Non.inflammatory.Macrophages	SETD1B	-0.20489	5.026478	-1.77628	0.079127	-5.18778	0.302188	0.35488
Non.inflammatory.Macrophages	BCL11A	-0.60277	5.196907	-1.77623	0.079134	-4.56084	0.299061	0.353211
Non.inflammatory.Macrophages	PGM2	0.235982	4.115633	1.776213	0.079138	-5.03337	0.319427	0.364005
Non.inflammatory.Macrophages	OTC	-0.54662	3.438444	-1.7757	0.079222	-4.78333	0.333071	0.371129
Non.inflammatory.Macrophages	BOLA2	0.184682	5.686348	1.77524	0.0793	-5.36614	0.290603	0.348811
Non.inflammatory.Macrophages	KCNRG	0.509266	2.320944	1.775116	0.07932	-4.4468	0.356548	0.383099
Non.inflammatory.Macrophages	VWCE	-0.76338	0.865529	-1.77472	0.079386	-4.26382	0.388385	0.399015
Non.inflammatory.Macrophages	1810020O(	-0.74534	-0.23349	-1.77458	0.079409	-4.3842	0.393261	0.411415
Non.inflammatory.Macrophages	CCDC83	-0.82488	0.420205	-1.77455	0.079414	-4.40015	0.393261	0.404018
Non.inflammatory.Macrophages	PTPN23	-0.27007	3.647447	-1.77351	0.079588	-4.95222	0.329505	0.369743
Non.inflammatory.Macrophages	TMEM167	0.131571	6.804318	1.77342	0.079602	-5.51667	0.27177	0.338681

Non.inflammatory.Macrophages	ATG3	-0.14147	6.261249	-1.77336	0.079612	-5.39514	0.28096	0.343863
Non.inflammatory.Macrophages	E130215H2	1.166399	-0.13171	1.773107	0.079655	-4.12081	0.393788	0.410793
Non.inflammatory.Macrophages	SMARCB1	-0.17003	5.410318	-1.77304	0.079665	-5.16266	0.295964	0.352121
Non.inflammatory.Macrophages	TCEA1	0.128908	7.486226	1.772496	0.079757	-5.5941	0.260845	0.332536
Non.inflammatory.Macrophages	GM45820	-0.68636	1.425778	-1.77204	0.079833	-4.23793	0.377343	0.39382
Non.inflammatory.Macrophages	FDFT1	-0.3296	3.796269	-1.77166	0.079896	-4.76452	0.327126	0.368804
Non.inflammatory.Macrophages	GM50240	-0.54798	1.572659	-1.77149	0.079925	-4.42808	0.374184	0.392359
Non.inflammatory.Macrophages	RBM10	0.226228	4.779494	1.770572	0.080079	-5.00398	0.308608	0.359066
Non.inflammatory.Macrophages	GM32031	-0.37806	2.845461	-1.7697	0.080226	-4.70799	0.347586	0.379201
Non.inflammatory.Macrophages	SPCS3	0.181096	4.704887	1.769405	0.080274	-5.18384	0.310547	0.360123
Non.inflammatory.Macrophages	MED1	0.142963	5.883036	1.76916	0.080316	-5.30035	0.289038	0.348537
Non.inflammatory.Macrophages	FCER1G	0.229986	8.628675	1.768669	0.080398	-6.06538	0.244418	0.323006
Non.inflammatory.Macrophages	DCP1A	0.214622	4.850131	1.767348	0.08062	-5.06744	0.308799	0.35926
Non.inflammatory.Macrophages	TWNK	-0.39538	2.886068	-1.76677	0.080718	-4.69214	0.348225	0.379595
Non.inflammatory.Macrophages	GSK3B	-0.10964	8.105386	-1.76644	0.080773	-5.72337	0.2533	0.328361

Non.inflammatory.Macrophages	CTSF	-0.38835	1.860603	-1.7657	0.080899	-4.88313	0.371021	0.391036
Non.inflammatory.Macrophages	ZFP169	0.531516	2.782441	1.765241	0.080976	-4.56073	0.351153	0.38132
Non.inflammatory.Macrophages	GM17477	1.014484	0.61424	1.764421	0.081115	-4.14294	0.39738	0.405344
Non.inflammatory.Macrophages	IMMP2L	-0.25891	6.534254	-1.7644	0.081119	-5.49952	0.279708	0.343792
Non.inflammatory.Macrophages	PAQR9	-0.28316	4.029832	-1.76365	0.081245	-5.21996	0.326298	0.369001
Non.inflammatory.Macrophages	ABL1	-0.19039	5.653787	-1.76341	0.081287	-5.37182	0.295586	0.352748
Non.inflammatory.Macrophages	RIN2	0.209945	3.687666	1.76316	0.081329	-5.33278	0.333251	0.372597
Non.inflammatory.Macrophages	GM11084	0.346924	3.368087	1.762667	0.081412	-4.68691	0.340003	0.376051
Non.inflammatory.Macrophages	GM50071	-0.81271	0.514178	-1.76239	0.081459	-4.20412	0.399265	0.407144
Non.inflammatory.Macrophages	TOM1L2	-0.2185	5.471299	-1.76231	0.081473	-5.28948	0.299149	0.354703
Non.inflammatory.Macrophages	IGLL1	-1.26757	3.916663	-1.76173	0.081572	-4.45744	0.329154	0.370513
Non.inflammatory.Macrophages	CTNNBIP1	0.210451	4.359655	1.761285	0.081647	-5.22854	0.32046	0.366039
Non.inflammatory.Macrophages	CASS4	-0.44297	3.04899	-1.76126	0.081652	-4.84939	0.347003	0.379637
Non.inflammatory.Macrophages	WDR3	0.205914	4.225869	1.761024	0.081692	-5.09954	0.323079	0.367455
Non.inflammatory.Macrophages	NAGK	-0.21182	3.956557	-1.76095	0.081704	-5.17333	0.328414	0.370232

Non.inflammatory.Macrophages	TES	0.434883	5.486918	1.760499	0.081781	-4.68467	0.299231	0.354955
Non.inflammatory.Macrophages	FAHD2A	-0.30708	2.845982	-1.76022	0.081829	-4.75522	0.351363	0.382086
Non.inflammatory.Macrophages	DCXR	-0.2198	4.317514	-1.76015	0.081841	-5.2246	0.321347	0.366755
Non.inflammatory.Macrophages	TAF3	0.178896	5.653075	1.760012	0.081864	-5.28127	0.296209	0.353369
Non.inflammatory.Macrophages	CHEK1	-0.44004	2.722134	-1.75989	0.081886	-4.63345	0.354004	0.38342
Non.inflammatory.Macrophages	SGSM3	-0.28724	4.063557	-1.75982	0.081898	-5.00765	0.326349	0.369407
Non.inflammatory.Macrophages	SYVN1	-0.32253	4.228615	-1.75932	0.081983	-4.96744	0.323308	0.367867
Non.inflammatory.Macrophages	RTL8B	-0.30493	3.297356	-1.75894	0.082047	-4.98257	0.342177	0.377667
Non.inflammatory.Macrophages	TOM1	0.211019	5.431971	1.758892	0.082056	-5.34068	0.30049	0.355881
Non.inflammatory.Macrophages	UBE2R2	0.152246	7.144409	1.756844	0.082406	-5.57948	0.27165	0.340008
Non.inflammatory.Macrophages	TMEM208	0.158448	4.89435	1.756459	0.082473	-5.24583	0.311865	0.362146
Non.inflammatory.Macrophages	MBNL3	-0.66915	4.597519	-1.75619	0.082518	-4.38385	0.317569	0.365204
Non.inflammatory.Macrophages	LAMP2	0.129286	7.069003	1.756097	0.082535	-5.69769	0.273039	0.340921
Non.inflammatory.Macrophages	BAG3	-0.24684	2.967773	-1.75556	0.082627	-5.1125	0.350764	0.382262
Non.inflammatory.Macrophages	ARF5	0.168663	8.486216	1.755459	0.082644	-5.8125	0.250405	0.327865

Non.inflammatory.Macrophages	NOTCH3	-0.68081	1.028947	-1.75537	0.08266	-4.40721	0.394219	0.403484
Non.inflammatory.Macrophages	FAM45A	0.189416	3.979967	1.754673	0.08278	-5.13354	0.330211	0.371931
Non.inflammatory.Macrophages	AP3M1	0.172253	4.881049	1.754532	0.082804	-5.20897	0.312584	0.362766
Non.inflammatory.Macrophages	IQCG	0.957391	0.050135	1.753976	0.0829	-4.25332	0.402823	0.415169
Non.inflammatory.Macrophages	KIF9	0.456402	1.680578	1.753961	0.082902	-4.58964	0.379611	0.396786
Non.inflammatory.Macrophages	ARFGEF1	0.17444	6.613928	1.753848	0.082922	-5.4476	0.281267	0.345885
Non.inflammatory.Macrophages	UBALD1	0.207728	4.983613	1.753638	0.082958	-5.23802	0.310766	0.362033
Non.inflammatory.Macrophages	DPAGT1	0.28848	3.285854	1.752936	0.083079	-4.9485	0.344946	0.379833
Non.inflammatory.Macrophages	GHR	-0.569	4.215001	-1.75219	0.083209	-4.98511	0.326412	0.370428
Non.inflammatory.Macrophages	GM12940	-0.26883	4.51886	-1.7516	0.08331	-5.03132	0.320466	0.367511
Non.inflammatory.Macrophages	MLXIP	-0.17849	6.94185	-1.75132	0.083359	-5.5012	0.276358	0.343618
Non.inflammatory.Macrophages	PPP1R18O!	-0.48939	0.993134	-1.75125	0.083372	-4.40648	0.396589	0.405491
Non.inflammatory.Macrophages	CLTC	-0.10944	7.90636	-1.75104	0.083407	-5.77693	0.260483	0.334597
Non.inflammatory.Macrophages	IGLV1	1.190882	-0.83837	1.750945	0.083424	-4.14731	0.403826	0.426752
Non.inflammatory.Macrophages	NTAN1	-0.13613	6.261702	-1.75074	0.083459	-5.41444	0.288114	0.350389

Non.inflammatory.Macrophages	KCMF1	0.11341	6.987433	1.750655	0.083474	-5.57022	0.275587	0.343409
Non.inflammatory.Macrophages	CLCN4	-0.16763	5.289066	-1.75065	0.083474	-5.36064	0.305763	0.360036
Non.inflammatory.Macrophages	SIGLECH	1.228567	0.771994	1.750583	0.083486	-4.19628	0.400249	0.408243
Non.inflammatory.Macrophages	DUSP8	0.645743	1.274945	1.749928	0.0836	-4.37625	0.39024	0.402917
Non.inflammatory.Macrophages	ABHD17B	0.163849	6.542955	1.74957	0.083662	-5.34253	0.28341	0.348123
Non.inflammatory.Macrophages	THNSL1	-0.58871	1.077135	-1.74953	0.083669	-4.47504	0.394897	0.405305
Non.inflammatory.Macrophages	SLCO5A1	-0.87546	-0.20706	-1.74947	0.083679	-4.15757	0.404132	0.420036
Non.inflammatory.Macrophages	MRPS9	0.181865	4.889322	1.74936	0.083699	-5.18043	0.31355	0.364599
Non.inflammatory.Macrophages	TRIM25	0.195117	6.978283	1.747681	0.083991	-5.68158	0.276762	0.344654
Non.inflammatory.Macrophages	GM27008	0.528864	1.012976	1.747602	0.084005	-4.46284	0.397583	0.406899
Non.inflammatory.Macrophages	OXA1L	-0.1927	4.658758	-1.74711	0.084091	-5.18734	0.319078	0.367787
Non.inflammatory.Macrophages	FAM83F	-0.98572	1.041009	-1.74702	0.084106	-4.21186	0.39711	0.40673
Non.inflammatory.Macrophages	TMEM30A	0.122877	6.581779	1.746389	0.084217	-5.51918	0.283979	0.348845
Non.inflammatory.Macrophages	PRAG1	0.568983	1.923732	1.745832	0.084314	-4.47967	0.377262	0.397384
Non.inflammatory.Macrophages	ARPC2	0.089171	9.318766	1.745336	0.084401	-6.00669	0.240418	0.32363

Non.inflammatory.Macrophages	EIF3C	0.141118	7.172674	1.74496	0.084466	-5.61661	0.274316	0.3437
Non.inflammatory.Macrophages	MAP1LC3A	-0.24135	5.476214	-1.74493	0.084471	-5.35787	0.304331	0.360324
Non.inflammatory.Macrophages	NFKBIZ	0.209412	6.516715	1.743377	0.084744	-5.60522	0.286388	0.350554
Non.inflammatory.Macrophages	DPP7	-0.41117	1.957266	-1.74218	0.084954	-4.77763	0.378684	0.398328
Non.inflammatory.Macrophages	STX8	0.121325	6.745581	1.741829	0.085016	-5.56657	0.283107	0.348749
Non.inflammatory.Macrophages	GM44284	-0.64066	-0.05378	-1.7416	0.085057	-4.42519	0.408794	0.421411
Non.inflammatory.Macrophages	SRP14	0.114647	7.234027	1.740477	0.085254	-5.62526	0.275331	0.344432
Non.inflammatory.Macrophages	NR2F2	-0.48233	3.13223	-1.73983	0.085368	-4.79252	0.353864	0.386382
Non.inflammatory.Macrophages	SPSB4	0.816005	0.417547	1.739786	0.085376	-4.30188	0.409892	0.416667
Non.inflammatory.Macrophages	FBXL20	0.254581	5.495646	1.739196	0.085481	-5.34784	0.306731	0.362023
Non.inflammatory.Macrophages	CDV3	0.155317	7.090195	1.738894	0.085534	-5.57612	0.278286	0.346408
Non.inflammatory.Macrophages	AHI1	0.475592	3.003332	1.737609	0.085762	-4.5073	0.357866	0.38851
Non.inflammatory.Macrophages	HDAC7	0.378911	4.31747	1.736963	0.085876	-4.74397	0.330571	0.374765
Non.inflammatory.Macrophages	TXNDC17	0.16493	6.116505	1.736834	0.085899	-5.53899	0.296197	0.356498
Non.inflammatory.Macrophages	2010109A1	-0.50023	2.521995	-1.73681	0.085904	-4.53214	0.368596	0.394012

Non.inflammatory.Macrophages	SEC31A	0.137491	5.620105	1.736713	0.085921	-5.37989	0.305325	0.361463
Non.inflammatory.Macrophages	PLA2G6	-0.57944	1.28393	-1.73582	0.086079	-4.50925	0.397698	0.407966
Non.inflammatory.Macrophages	SOCS5	-0.27323	4.029298	-1.73567	0.086106	-5.11346	0.336902	0.377993
Non.inflammatory.Macrophages	RSBN1	0.195242	5.326631	1.735395	0.086155	-5.2806	0.311364	0.364628
Non.inflammatory.Macrophages	COBLL1	-0.41755	5.069977	-1.7352	0.086189	-4.75549	0.316293	0.367296
Non.inflammatory.Macrophages	EPG5	0.204453	4.420072	1.734195	0.086369	-5.197	0.329642	0.374482
Non.inflammatory.Macrophages	STRADB	0.308262	3.238382	1.73377	0.086445	-4.83547	0.354289	0.387103
Non.inflammatory.Macrophages	SNX27	-0.15042	5.803763	-1.73355	0.086485	-5.42257	0.303053	0.360522
Non.inflammatory.Macrophages	SLC39A10	-0.23295	3.903206	-1.73341	0.086509	-5.08798	0.34028	0.380144
Non.inflammatory.Macrophages	MORN1	-0.5419	1.227517	-1.73306	0.086571	-4.46532	0.39997	0.409637
Non.inflammatory.Macrophages	YKT6	0.18456	4.483546	1.732987	0.086584	-5.21907	0.328477	0.374185
Non.inflammatory.Macrophages	EGFL6	-1.04799	0.548891	-1.73288	0.086603	-4.203	0.412412	0.417478
Non.inflammatory.Macrophages	VAC14	-0.16952	4.624685	-1.73284	0.08661	-5.22911	0.325665	0.372741
Non.inflammatory.Macrophages	RRP36	-0.21618	3.775241	-1.7318	0.086797	-5.01901	0.343445	0.3821
Non.inflammatory.Macrophages	TJP3	-0.70416	1.707068	-1.73174	0.086807	-4.33306	0.389189	0.404751

Non.inflammatory.Macrophages	RMC1	-0.22727	4.652621	-1.73148	0.086853	-5.14372	0.325594	0.372955
Non.inflammatory.Macrophages	LCMT2	-0.36002	2.687418	-1.73142	0.086865	-4.68624	0.366847	0.393955
Non.inflammatory.Macrophages	WDR76	-0.35985	4.504143	-1.73122	0.0869	-4.94603	0.328552	0.374646
Non.inflammatory.Macrophages	AIDA	0.196537	4.437691	1.7311	0.086922	-5.19906	0.329884	0.375378
Non.inflammatory.Macrophages	PITPNB	0.160879	5.507244	1.729204	0.087263	-5.29758	0.310062	0.365117
Non.inflammatory.Macrophages	TREML2	0.392015	3.901235	1.72906	0.087289	-4.74321	0.341939	0.381838
Non.inflammatory.Macrophages	LCK	-0.5899	3.728178	-1.72901	0.087298	-4.47307	0.345552	0.383682
Non.inflammatory.Macrophages	LNCPINT	-0.22184	8.328661	-1.72805	0.087471	-5.94168	0.26091	0.338007
Non.inflammatory.Macrophages	ENO3	0.464715	2.077848	1.727807	0.087514	-4.74672	0.381948	0.40229
Non.inflammatory.Macrophages	SLC25A44	-0.27252	3.239164	-1.72777	0.087521	-4.96768	0.356058	0.389503
Non.inflammatory.Macrophages	LYZ2	0.354632	7.170673	1.727758	0.087523	-6.07665	0.280126	0.349125
Non.inflammatory.Macrophages	RERE	0.148638	7.702335	1.72768	0.087537	-5.68411	0.271138	0.344005
Non.inflammatory.Macrophages	DTX3L	0.290714	4.876216	1.727631	0.087546	-5.37197	0.322328	0.372163
Non.inflammatory.Macrophages	HSPG2	-0.45999	2.741833	-1.72747	0.087575	-4.65317	0.366941	0.395032
Non.inflammatory.Macrophages	ATP1B3	0.221847	7.281647	1.727419	0.087584	-5.70495	0.278226	0.348148

Non.inflammatory.Macrophages	FPGS	-0.35813	3.013479	-1.72726	0.087613	-4.74124	0.360959	0.392147
Non.inflammatory.Macrophages	4933432I0:	0.710248	0.408312	1.726933	0.087672	-4.401	0.415114	0.421733
Non.inflammatory.Macrophages	DENND2D	-0.38618	2.202221	-1.72688	0.087681	-4.66252	0.379095	0.401226
Non.inflammatory.Macrophages	CARHSP1	0.21908	5.430457	1.726538	0.087743	-5.32271	0.311608	0.36678
Non.inflammatory.Macrophages	PIAS1	0.126241	7.42613	1.726502	0.08775	-5.66497	0.275772	0.346958
Non.inflammatory.Macrophages	INMT	-0.88334	0.639157	-1.72649	0.087752	-4.31903	0.413536	0.419071
Non.inflammatory.Macrophages	TRDV4	1.09188	-1.36214	1.726154	0.087813	-4.17702	0.415158	0.443098
Non.inflammatory.Macrophages	CAD	-0.60757	1.795668	-1.7261	0.087823	-4.46567	0.388536	0.405923
Non.inflammatory.Macrophages	AA467197	1.458811	0.801131	1.725794	0.087878	-4.21934	0.411134	0.417382
Non.inflammatory.Macrophages	CTPS2	0.228599	5.066744	1.725307	0.087966	-5.10975	0.318902	0.370806
Non.inflammatory.Macrophages	CCN1	-0.72439	1.098666	-1.72482	0.088055	-4.52014	0.405484	0.414259
Non.inflammatory.Macrophages	TRP53BP2	0.257658	3.957204	1.724764	0.088064	-5.03199	0.341194	0.3826
Non.inflammatory.Macrophages	GPX1	0.180454	11.17498	1.724506	0.088111	-6.40617	0.219233	0.313028
Non.inflammatory.Macrophages	GM19265	-0.93395	0.22098	-1.72438	0.088134	-4.21755	0.415503	0.424597
Non.inflammatory.Macrophages	AIFM2	0.467562	1.347203	1.724364	0.088137	-4.66474	0.399483	0.411521

Non.inflammatory.Macrophages	MAPK1IP1I	0.13328	6.060276	1.724293	0.08815	-5.46263	0.300121	0.360958
Non.inflammatory.Macrophages	ABLIM1	-0.34492	6.121137	-1.72399	0.088205	-4.95885	0.299006	0.360453
Non.inflammatory.Macrophages	GM10501	-0.41303	2.138944	-1.72386	0.088228	-4.64698	0.380901	0.402699
Non.inflammatory.Macrophages	PHACTR1	-0.48625	1.519212	-1.72378	0.088243	-4.6279	0.395377	0.409707
Non.inflammatory.Macrophages	LCAT	-0.57817	2.143834	-1.72356	0.088283	-4.5323	0.380788	0.402733
Non.inflammatory.Macrophages	CTLA2A	0.428864	4.28991	1.723241	0.088341	-5.00857	0.33436	0.379452
Non.inflammatory.Macrophages	LRP10	0.169777	6.125795	1.723023	0.08838	-5.45523	0.29892	0.360604
Non.inflammatory.Macrophages	IPCEF1	0.559236	4.679593	1.722985	0.088387	-4.71124	0.326519	0.37542
Non.inflammatory.Macrophages	ELF2	-0.13605	7.39491	-1.72298	0.088387	-5.71626	0.276559	0.348087
Non.inflammatory.Macrophages	MMGT2	0.262943	3.166256	1.722352	0.088502	-4.91451	0.35828	0.39187
Non.inflammatory.Macrophages	HDDC2	0.239746	3.817561	1.722225	0.088525	-5.04574	0.3444	0.384858
Non.inflammatory.Macrophages	ST3GAL4	-0.15475	6.423085	-1.72198	0.08857	-5.62556	0.293833	0.358033
Non.inflammatory.Macrophages	ABTB2	0.299858	7.412469	1.721343	0.088686	-5.60987	0.276803	0.348442
Non.inflammatory.Macrophages	LTBP1	1.074944	0.730726	1.721188	0.088714	-4.48834	0.413282	0.419707
Non.inflammatory.Macrophages	HPN	-0.39027	2.642028	-1.72096	0.088756	-4.85103	0.370279	0.398096

Non.inflammatory.Macrophages	PIF1	-0.72488	1.716548	-1.72058	0.088825	-4.48318	0.391697	0.408474
Non.inflammatory.Macrophages	RAD54L2	0.214631	4.552757	1.720256	0.088884	-5.1066	0.329934	0.377543
Non.inflammatory.Macrophages	ZFP598	-0.29423	3.44196	-1.71987	0.088955	-4.92068	0.352981	0.389501
Non.inflammatory.Macrophages	N4BP2L2	0.122513	6.786437	1.71966	0.088993	-5.51866	0.28784	0.354898
Non.inflammatory.Macrophages	SULT1A1	-0.31054	3.923228	-1.71964	0.088997	-5.20447	0.342816	0.384345
Non.inflammatory.Macrophages	GM11973	-0.39772	3.179185	-1.71941	0.089039	-4.80383	0.358648	0.392411
Non.inflammatory.Macrophages	PLSCR1	0.309016	4.550033	1.719378	0.089045	-5.1459	0.329989	0.377722
Non.inflammatory.Macrophages	BABAM2	-0.13954	7.216423	-1.71932	0.089055	-5.66813	0.280353	0.350698
Non.inflammatory.Macrophages	MON2	0.167896	5.7408	1.718974	0.089119	-5.40814	0.306874	0.365514
Non.inflammatory.Macrophages	CPSF6	0.119	6.68211	1.718862	0.089139	-5.51833	0.289697	0.356079
Non.inflammatory.Macrophages	GM35853	0.546841	0.184156	1.718655	0.089177	-4.51538	0.416633	0.426764
Non.inflammatory.Macrophages	TERF2	0.188476	4.800606	1.71863	0.089182	-5.20054	0.325002	0.375349
Non.inflammatory.Macrophages	IGBP1	0.182262	4.749526	1.717998	0.089297	-5.32995	0.326312	0.376208
Non.inflammatory.Macrophages	SLC22A14	-0.40373	2.744823	-1.71777	0.08934	-4.78184	0.368552	0.397884
Non.inflammatory.Macrophages	B430010I2	-0.63375	-0.41456	-1.71762	0.089367	-4.40174	0.417013	0.43444

Non.inflammatory.Macrophages	STAMBPL1	-0.21503	6.55028	-1.71751	0.089386	-5.44416	0.292312	0.357991
Non.inflammatory.Macrophages	SNX9	-0.17612	6.331978	-1.71644	0.089583	-5.53636	0.296796	0.36056
Non.inflammatory.Macrophages	SFSWAP	0.140903	5.786795	1.716122	0.089642	-5.37439	0.306948	0.366164
Non.inflammatory.Macrophages	PDSS1	0.291081	4.315269	1.715868	0.089688	-5.04857	0.335776	0.381533
Non.inflammatory.Macrophages	RAB4B	-0.14926	5.9254	-1.7158	0.089701	-5.44397	0.304358	0.364822
Non.inflammatory.Macrophages	ALDH3B1	0.212495	3.605843	1.7154	0.089775	-5.28457	0.350736	0.389381
Non.inflammatory.Macrophages	KHK	-0.22102	4.3658	-1.71496	0.089856	-5.26229	0.33509	0.38133
Non.inflammatory.Macrophages	ID1	-0.24403	4.07039	-1.71476	0.089893	-5.44925	0.341192	0.384601
Non.inflammatory.Macrophages	ABCA8B	-0.8351	1.118002	-1.71446	0.089947	-4.37056	0.407913	0.417541
Non.inflammatory.Macrophages	GOLGA4	0.156163	5.30004	1.713631	0.090101	-5.37323	0.317075	0.371842
Non.inflammatory.Macrophages	HC	-0.65453	2.358321	-1.71337	0.090149	-4.56022	0.379172	0.403682
Non.inflammatory.Macrophages	GM46440	-0.75865	0.637929	-1.7131	0.0902	-4.37051	0.417639	0.423542
Non.inflammatory.Macrophages	RGS2	0.171339	6.977171	1.712418	0.090325	-5.73786	0.28649	0.355315
Non.inflammatory.Macrophages	MAP3K10	0.327623	2.731125	1.712388	0.09033	-4.83601	0.371091	0.399891
Non.inflammatory.Macrophages	NDUFS6	0.174674	5.716521	1.711713	0.090456	-5.45467	0.309795	0.368295

Non.inflammatory.Macrophages	GAS2	0.382828	2.456745	1.711115	0.090566	-4.70725	0.37802	0.403474
Non.inflammatory.Macrophages	GM28809	0.907074	-0.12461	1.710113	0.090752	-4.25934	0.42091	0.434031
Non.inflammatory.Macrophages	CSTF2T	0.290498	3.017029	1.709916	0.090789	-4.86546	0.36592	0.397822
Non.inflammatory.Macrophages	CSNK1G1	0.166186	6.535289	1.709766	0.090817	-5.5143	0.295314	0.360774
Non.inflammatory.Macrophages	MINDY2	0.149373	6.610817	1.709661	0.090836	-5.551	0.293952	0.360051
Non.inflammatory.Macrophages	5830432EC	0.699628	1.494203	1.709621	0.090844	-4.38567	0.401125	0.41516
Non.inflammatory.Macrophages	HACL1	-0.38106	3.523051	-1.70885	0.090987	-4.88396	0.355161	0.392705
Non.inflammatory.Macrophages	OXR1	0.182376	6.336267	1.708778	0.091	-5.44904	0.299182	0.363151
Non.inflammatory.Macrophages	ADAL	-0.41798	2.279066	-1.70872	0.091012	-4.6008	0.382926	0.406595
Non.inflammatory.Macrophages	PRPF3	-0.19868	4.472577	-1.70844	0.091063	-5.1045	0.335315	0.382536
Non.inflammatory.Macrophages	SHPRH	0.222237	4.541567	1.708053	0.091135	-5.166	0.334061	0.381803
Non.inflammatory.Macrophages	PRKCQ	-0.51101	3.12892	-1.70742	0.091253	-4.7122	0.364244	0.397353
Non.inflammatory.Macrophages	ACOX3	-0.20241	4.372027	-1.70738	0.09126	-5.25349	0.33776	0.383853
Non.inflammatory.Macrophages	CRIM1	-0.28297	5.191827	-1.70654	0.091419	-5.44066	0.321652	0.375515
Non.inflammatory.Macrophages	GDPD3	-0.56435	1.793559	-1.70651	0.091424	-4.5864	0.395267	0.412759

Non.inflammatory.Macrophages	RAPGEFL1	-0.56916	2.784487	-1.70608	0.091504	-4.54179	0.372332	0.401673
Non.inflammatory.Macrophages	DDX24	0.142277	6.717234	1.706042	0.091511	-5.61096	0.293007	0.360024
Non.inflammatory.Macrophages	SLC38A3	-0.70602	1.479322	-1.70588	0.091541	-4.43608	0.402811	0.416569
Non.inflammatory.Macrophages	LRRC14	-0.39023	2.339055	-1.70583	0.091551	-4.73575	0.382488	0.406738
Non.inflammatory.Macrophages	KCTD17	0.717795	0.958849	1.705669	0.091581	-4.44304	0.415581	0.422699
Non.inflammatory.Macrophages	UBE2G1	0.128926	7.307993	1.704211	0.091854	-5.66892	0.283333	0.354821
Non.inflammatory.Macrophages	IFNAR2	0.144147	7.128736	1.703376	0.09201	-5.75304	0.286785	0.356962
Non.inflammatory.Macrophages	CCDC166	-0.77447	0.615517	-1.70333	0.092019	-4.3143	0.422535	0.4279
Non.inflammatory.Macrophages	ETFDH	0.190974	4.872939	1.70275	0.092128	-5.25682	0.329372	0.380451
Non.inflammatory.Macrophages	ACP2	0.221107	3.54465	1.702698	0.092138	-5.34203	0.357091	0.39478
Non.inflammatory.Macrophages	GNA13	0.157072	7.201206	1.702585	0.092159	-5.74878	0.285656	0.356572
Non.inflammatory.Macrophages	AMPD2	-0.39223	2.339378	-1.70109	0.092441	-4.72057	0.385126	0.408816
Non.inflammatory.Macrophages	ASTE1	-0.31516	2.753642	-1.70096	0.092465	-4.85835	0.375607	0.40417
Non.inflammatory.Macrophages	SLC29A3	0.216604	3.68183	1.700757	0.092503	-5.21565	0.35509	0.39393
Non.inflammatory.Macrophages	SLC38A9	0.207949	5.16902	1.69997	0.092652	-5.34169	0.324766	0.378257

Non.inflammatory.Macrophages	CLEC4B1	-0.50259	-0.61033	-1.69957	0.092727	-4.58693	0.425992	0.444304
Non.inflammatory.Macrophages	UBE2W	0.118868	6.081677	1.69932	0.092775	-5.43281	0.307348	0.368948
Non.inflammatory.Macrophages	GM48696	-0.7097	2.476417	-1.69913	0.092811	-4.36389	0.382729	0.407987
Non.inflammatory.Macrophages	ZMYM4	0.174501	5.733892	1.698282	0.092971	-5.42215	0.31432	0.372848
Non.inflammatory.Macrophages	SLC26A11	-0.28513	3.200265	-1.69825	0.092977	-5.15633	0.366741	0.400092
Non.inflammatory.Macrophages	1810037I1	0.16595	6.482621	1.697004	0.093214	-5.61071	0.300873	0.365687
Non.inflammatory.Macrophages	POLR2L	0.180737	5.414671	1.696894	0.093235	-5.41793	0.32118	0.376782
Non.inflammatory.Macrophages	ECPAS	0.151372	6.743157	1.696248	0.093357	-5.57017	0.296342	0.363259
Non.inflammatory.Macrophages	PHLDB2	-0.54547	2.509693	-1.69618	0.093371	-4.67146	0.383485	0.408713
Non.inflammatory.Macrophages	TREML4	0.266375	2.758058	1.695453	0.093509	-5.42334	0.378104	0.405976
Non.inflammatory.Macrophages	GM17494	0.345328	2.389013	1.695413	0.093516	-4.68173	0.386629	0.410165
Non.inflammatory.Macrophages	PXK	0.166057	6.270189	1.694656	0.09366	-5.5455	0.305649	0.368401
Non.inflammatory.Macrophages	DEFB1	-0.92474	0.86149	-1.69444	0.093702	-4.35451	0.423377	0.428227
Non.inflammatory.Macrophages	EXT1	-0.19189	7.749502	-1.69428	0.093732	-5.95991	0.279154	0.353538
Non.inflammatory.Macrophages	GM17382	-0.73584	0.002886	-1.69422	0.093744	-4.39196	0.428626	0.438558

Non.inflammatory.Macrophages	TEDC1	-0.63356	1.701406	-1.69398	0.093789	-4.41366	0.403477	0.41843
Non.inflammatory.Macrophages	TPK1	-0.21756	4.865838	-1.69375	0.093833	-5.28787	0.33312	0.383232
Non.inflammatory.Macrophages	CHAC2	0.30363	2.893423	1.693586	0.093864	-4.83498	0.375528	0.404954
Non.inflammatory.Macrophages	SLC16A6	-0.25732	4.554893	-1.69333	0.093914	-5.3059	0.339555	0.386766
Non.inflammatory.Macrophages	PDAP1	0.145425	6.715812	1.692656	0.094042	-5.59946	0.297864	0.364578
Non.inflammatory.Macrophages	PEG3	-0.63592	1.53048	-1.69223	0.094123	-4.52775	0.408409	0.421373
Non.inflammatory.Macrophages	COLGALT2	-0.8708	-0.12576	-1.69196	0.094176	-4.37051	0.429585	0.441354
Non.inflammatory.Macrophages	NCKAP5LO	-0.85512	0.041134	-1.69145	0.094274	-4.35466	0.429787	0.439556
Non.inflammatory.Macrophages	P3H2	0.822813	1.190669	1.691394	0.094284	-4.59475	0.417116	0.425771
Non.inflammatory.Macrophages	GM16153	0.475171	2.536009	1.690755	0.094406	-4.64746	0.385032	0.4104
Non.inflammatory.Macrophages	HGSNAT	-0.22368	4.642285	-1.69059	0.094437	-5.48305	0.338835	0.387089
Non.inflammatory.Macrophages	CBY1	-0.35102	2.846037	-1.69016	0.094521	-4.74419	0.37809	0.407019
Non.inflammatory.Macrophages	LIMS1	0.16796	7.288787	1.689819	0.094586	-5.60579	0.288468	0.359742
Non.inflammatory.Macrophages	GM20508	-0.61944	0.064606	-1.68952	0.094644	-4.42784	0.430703	0.439882
Non.inflammatory.Macrophages	TM4SF5	-0.59076	0.261552	-1.6891	0.094724	-4.63919	0.43091	0.437771

Non.inflammatory.Macrophages	SCAF11	0.140926	7.16109	1.68895	0.094753	-5.65276	0.290956	0.361375
Non.inflammatory.Macrophages	ACY3	-0.5243	2.04257	-1.68826	0.094886	-4.7084	0.397688	0.417011
Non.inflammatory.Macrophages	IGFBP3	-0.73951	0.690335	-1.68785	0.094965	-4.46145	0.428821	0.433055
Non.inflammatory.Macrophages	ASMT	-0.51452	1.052723	-1.68782	0.094971	-4.65381	0.422064	0.428717
Non.inflammatory.Macrophages	GATA4	-0.65494	1.621315	-1.68778	0.094978	-4.45221	0.407901	0.421996
Non.inflammatory.Macrophages	CCAR1	0.125826	6.820278	1.687674	0.094999	-5.59427	0.297369	0.365174
Non.inflammatory.Macrophages	TIMMDC1	-0.2211	4.271519	-1.68727	0.095077	-5.11463	0.347634	0.392209
Non.inflammatory.Macrophages	SYNE3	0.722203	2.767053	1.686946	0.095139	-4.44635	0.380976	0.409143
Non.inflammatory.Macrophages	CCNO	0.866033	0.174468	1.686434	0.095238	-4.34062	0.431896	0.439914
Non.inflammatory.Macrophages	COQ10B	0.150594	5.908947	1.686335	0.095257	-5.55488	0.314863	0.375095
Non.inflammatory.Macrophages	E2F2	0.391475	4.99405	1.68614	0.095295	-4.96164	0.332978	0.384838
Non.inflammatory.Macrophages	FAM104A	0.149803	6.153765	1.685443	0.09543	-5.56048	0.310441	0.372797
Non.inflammatory.Macrophages	RAB21	-0.13761	6.891793	-1.68532	0.095453	-5.60278	0.296723	0.365234
Non.inflammatory.Macrophages	1700113A1	-0.43161	2.10081	-1.68526	0.095464	-4.6816	0.397171	0.417354
Non.inflammatory.Macrophages	FXYD1	-0.51719	2.943369	-1.68489	0.095537	-4.79314	0.377624	0.407858

Non.inflammatory.Macrophages	LAMB3	-0.76895	4.067884	-1.68416	0.095679	-4.41365	0.353042	0.395535
Non.inflammatory.Macrophages	ADAP2	0.257742	2.585365	1.684054	0.095699	-5.35914	0.38624	0.41222
Non.inflammatory.Macrophages	SH3RF1	-0.35261	4.357213	-1.68385	0.095737	-4.91292	0.346882	0.392473
Non.inflammatory.Macrophages	EFCAB9	-0.71606	0.443216	-1.68371	0.095765	-4.31424	0.432833	0.437646
Non.inflammatory.Macrophages	WBP11	0.13888	6.346343	1.683498	0.095807	-5.56435	0.307214	0.371402
Non.inflammatory.Macrophages	TMEM192	0.179074	4.694631	1.683452	0.095816	-5.36055	0.339827	0.388882
Non.inflammatory.Macrophages	SFN	-0.61835	2.056108	-1.68317	0.095871	-4.53682	0.398794	0.418644
Non.inflammatory.Macrophages	CXXC5	-0.31255	5.11182	-1.6831	0.095884	-4.9171	0.331308	0.38454
Non.inflammatory.Macrophages	GBP9	0.330236	3.036901	1.681517	0.096192	-5.07215	0.376887	0.408029
Non.inflammatory.Macrophages	LCLAT1	0.269988	3.99918	1.681139	0.096266	-5.11124	0.355513	0.397329
Non.inflammatory.Macrophages	PIR	-0.82998	1.198746	-1.6811	0.096274	-4.33599	0.421046	0.42953
Non.inflammatory.Macrophages	POU6F1	-0.34771	2.610644	-1.68096	0.096301	-4.89322	0.386731	0.413054
Non.inflammatory.Macrophages	ABCF1	-0.14245	6.430861	-1.68092	0.096309	-5.58398	0.306485	0.371401
Non.inflammatory.Macrophages	ANGPT1	0.92068	0.973826	1.680713	0.096348	-4.33083	0.426796	0.432439
Non.inflammatory.Macrophages	OPRM1	-0.92963	2.803916	-1.68045	0.0964	-4.33367	0.382276	0.411114

Non.inflammatory.Macrophages	WASHC4	0.145877	5.792598	1.68024	0.096441	-5.48265	0.31872	0.378306
Non.inflammatory.Macrophages	USP28	-0.20616	4.553264	-1.68021	0.096446	-5.23229	0.34376	0.391586
Non.inflammatory.Macrophages	NIM1K	-0.36965	2.88905	-1.67996	0.096496	-4.86927	0.380336	0.410149
Non.inflammatory.Macrophages	TRAF3	0.202862	6.856964	1.679725	0.096541	-5.63576	0.298637	0.367295
Non.inflammatory.Macrophages	HPD	-0.46391	4.805638	-1.67969	0.096549	-5.29175	0.338536	0.388881
Non.inflammatory.Macrophages	COL20A1	-0.41636	1.340253	-1.67949	0.096587	-4.59889	0.417579	0.428326
Non.inflammatory.Macrophages	CMC4	-0.27309	3.064546	-1.67887	0.096709	-4.95745	0.376692	0.408483
Non.inflammatory.Macrophages	BBOX1	-0.75744	0.92351	-1.67835	0.09681	-4.42149	0.428444	0.433659
Non.inflammatory.Macrophages	LYZL4	-0.70848	-1.24765	-1.67827	0.096826	-4.3622	0.434785	0.46061
Non.inflammatory.Macrophages	GM4129	-0.80902	1.28611	-1.67811	0.096858	-4.3239	0.419558	0.429335
Non.inflammatory.Macrophages	MDH1	-0.14666	8.120076	-1.6777	0.096938	-5.85319	0.276932	0.355114
Non.inflammatory.Macrophages	4930589L2	-0.51966	-0.68311	-1.67703	0.097068	-4.54191	0.435315	0.4539
Non.inflammatory.Macrophages	BAHCC1	-0.53517	1.235974	-1.67661	0.097151	-4.60451	0.421335	0.430514
Non.inflammatory.Macrophages	NPLOC4	0.177999	5.537568	1.676572	0.097159	-5.4262	0.324641	0.381954
Non.inflammatory.Macrophages	UQCRQ	0.154839	7.858453	1.676131	0.097246	-5.85959	0.281621	0.358153

Non.inflammatory.Macrophages	COQ3	0.208987	3.500438	1.676119	0.097248	-5.06508	0.367517	0.404347
Non.inflammatory.Macrophages	STX12	0.149336	5.552332	1.676006	0.09727	-5.55921	0.324349	0.381951
Non.inflammatory.Macrophages	ACTR1A	0.141017	6.140647	1.675997	0.097272	-5.51667	0.312889	0.375746
Non.inflammatory.Macrophages	FAM192A	0.176611	4.607769	1.675823	0.097306	-5.25761	0.343589	0.392166
Non.inflammatory.Macrophages	1810058I2	0.186655	7.005863	1.675822	0.097306	-5.73947	0.296741	0.366839
Non.inflammatory.Macrophages	SELENOW	0.164101	7.046449	1.675557	0.097358	-5.745	0.296004	0.366436
Non.inflammatory.Macrophages	CACUL1	-0.1669	6.117834	-1.67551	0.097368	-5.62153	0.313326	0.376033
Non.inflammatory.Macrophages	HLCS	-0.24125	4.15558	-1.67528	0.097413	-5.29479	0.353178	0.397166
Non.inflammatory.Macrophages	MRTFB	0.213894	4.475202	1.675211	0.097426	-5.31281	0.346374	0.393648
Non.inflammatory.Macrophages	HDC	0.790307	3.642871	1.674889	0.09749	-4.89128	0.364471	0.402968
Non.inflammatory.Macrophages	KBTBD3	-0.36028	2.727361	-1.67461	0.097545	-4.83891	0.385348	0.413482
Non.inflammatory.Macrophages	ANKRD13C	0.181892	5.868682	1.67435	0.097596	-5.46531	0.318376	0.378975
Non.inflammatory.Macrophages	DELE1	-0.25139	3.426108	-1.67384	0.097696	-4.98431	0.369636	0.405733
Non.inflammatory.Macrophages	NSDHL	-0.39439	2.406095	-1.67373	0.097719	-4.76936	0.39317	0.417468
Non.inflammatory.Macrophages	NRBF2	0.201564	4.314912	1.673616	0.097741	-5.26132	0.350205	0.395899

Non.inflammatory.Macrophages	GEMIN7	0.159351	5.084337	1.673354	0.097792	-5.36764	0.334231	0.387577
Non.inflammatory.Macrophages	PHF19	-0.5154	1.934693	-1.67274	0.097914	-4.51097	0.404957	0.423347
Non.inflammatory.Macrophages	CMAH	0.354051	5.561409	1.672058	0.098048	-5.22657	0.32509	0.382995
Non.inflammatory.Macrophages	LPCAT2	0.198396	4.726381	1.672041	0.098052	-5.60232	0.342084	0.392003
Non.inflammatory.Macrophages	FAM114A1	0.35084	2.398626	1.671877	0.098084	-4.99314	0.393976	0.418296
Non.inflammatory.Macrophages	EHD2	-0.57579	1.82262	-1.67185	0.09809	-4.567	0.407895	0.425055
Non.inflammatory.Macrophages	MRPL20	0.154889	6.1388	1.671446	0.09817	-5.57529	0.313964	0.377116
Non.inflammatory.Macrophages	GM5150	0.285963	2.574091	1.670836	0.09829	-5.42273	0.390309	0.416701
Non.inflammatory.Macrophages	MKNK2	-0.14918	6.23257	-1.67074	0.098309	-5.57966	0.312407	0.376383
Non.inflammatory.Macrophages	DDX17	0.151488	6.357267	1.670184	0.09842	-5.61402	0.310037	0.37527
Non.inflammatory.Macrophages	AI837181	0.231386	3.127875	1.670132	0.09843	-5.02851	0.377456	0.410568
Non.inflammatory.Macrophages	UGT2B34	-0.66784	1.748323	-1.67012	0.098432	-4.52928	0.410241	0.426627
Non.inflammatory.Macrophages	EDEM3	-0.23293	5.974164	-1.66991	0.098475	-5.5005	0.31739	0.37935
Non.inflammatory.Macrophages	GPR18	0.593645	4.514903	1.669688	0.098518	-4.59197	0.346957	0.395123
Non.inflammatory.Macrophages	HMG20A	-0.20035	4.897858	-1.66945	0.098565	-5.34366	0.338954	0.391055

Non.inflammatory.Macrophages	CD3G	-0.95755	3.343766	-1.66926	0.098603	-4.49789	0.37255	0.408394
Non.inflammatory.Macrophages	COMMD9	0.303449	2.646144	1.669218	0.098612	-4.96482	0.38862	0.416416
Non.inflammatory.Macrophages	GOLPH3	0.130603	5.902166	1.669076	0.09864	-5.5153	0.318791	0.380413
Non.inflammatory.Macrophages	NEURL3	-0.20484	4.301516	-1.66899	0.098656	-5.46038	0.351494	0.397748
Non.inflammatory.Macrophages	ZFP263	-0.21698	5.167548	-1.66865	0.098725	-5.42436	0.333424	0.388319
Non.inflammatory.Macrophages	LRIG3	0.772466	0.14201	1.668406	0.098773	-4.41338	0.437103	0.44659
Non.inflammatory.Macrophages	OIT3	-0.52366	2.345496	-1.6681	0.098834	-4.64941	0.395741	0.420194
Non.inflammatory.Macrophages	1600002K(	-0.24116	3.214423	-1.66797	0.09886	-5.07215	0.375482	0.410171
Non.inflammatory.Macrophages	KANTR	0.441635	2.529607	1.667931	0.098868	-4.6998	0.391366	0.41806
Non.inflammatory.Macrophages	NT5C2	0.230639	5.867578	1.667888	0.098876	-5.42189	0.319466	0.38097
Non.inflammatory.Macrophages	NUMBL	-0.49557	1.04252	-1.66771	0.098913	-4.675	0.428002	0.435704
Non.inflammatory.Macrophages	YIPF5	-0.15659	5.24625	-1.6677	0.098914	-5.42013	0.331826	0.387618
Non.inflammatory.Macrophages	VRK3	0.175585	4.98755	1.667644	0.098925	-5.35966	0.337105	0.390419
Non.inflammatory.Macrophages	USP15	0.15934	7.650516	1.66714	0.099025	-5.69416	0.286607	0.36271
Non.inflammatory.Macrophages	INAFM2	0.336618	3.225336	1.666952	0.099063	-4.86247	0.375512	0.410328

Non.inflammatory.Macrophages	ATP5G1	0.157689	7.643771	1.666536	0.099146	-5.84265	0.286888	0.362943
Non.inflammatory.Macrophages	ZGPAT	-0.23609	3.668613	-1.66621	0.099212	-4.99822	0.365861	0.405539
Non.inflammatory.Macrophages	PBXIP1	0.340971	4.578633	1.665697	0.099313	-4.96607	0.346297	0.3956
Non.inflammatory.Macrophages	ZC3HAV1L	-0.35867	3.053639	-1.66569	0.099315	-4.92552	0.379907	0.412752
Non.inflammatory.Macrophages	PRR3	-0.23349	3.987386	-1.66551	0.09935	-5.16826	0.358995	0.402165
Non.inflammatory.Macrophages	CIDEB	-0.50649	1.942054	-1.6649	0.099473	-4.63238	0.406632	0.42598
Non.inflammatory.Macrophages	ABCB4	0.334243	3.391454	1.664671	0.099519	-5.07284	0.372525	0.409157
Non.inflammatory.Macrophages	TBC1D9B	0.179309	4.555284	1.664622	0.099528	-5.32413	0.347084	0.396116
Non.inflammatory.Macrophages	NLRC3	-0.70131	1.662225	-1.66402	0.099649	-4.3869	0.413905	0.429478
Non.inflammatory.Macrophages	POLD1	-0.34393	4.276679	-1.66367	0.099719	-4.92562	0.353426	0.39935
Non.inflammatory.Macrophages	AW11201C	0.384913	7.279978	1.663568	0.09974	-5.99648	0.29416	0.367325
Non.inflammatory.Macrophages	OTUB1	0.126296	5.728978	1.663386	0.099776	-5.5185	0.323479	0.383558
Non.inflammatory.Macrophages	BTBD10	0.182526	5.583956	1.663009	0.099852	-5.30975	0.32646	0.385225
Non.inflammatory.Macrophages	GNGT2	0.213315	5.675602	1.662869	0.09988	-5.70687	0.324637	0.384271
Non.inflammatory.Macrophages	CCT6B	-0.66349	0.315333	-1.66275	0.099905	-4.5137	0.438996	0.446082

Non.inflammatory.Macrophages	WWC1	-0.58947	1.216205	-1.6625	0.099953	-4.46992	0.425472	0.435152
Non.inflammatory.Macrophages	SENP7	0.190086	5.356774	1.662189	0.100017	-5.3763	0.331178	0.387959
Non.inflammatory.Macrophages	OTUD4	0.182186	5.091742	1.661876	0.100079	-5.36304	0.336681	0.390884
Non.inflammatory.Macrophages	NOL6	0.340675	2.582305	1.661714	0.100112	-4.84778	0.392116	0.419214
Non.inflammatory.Macrophages	TASOR2	0.193846	5.373883	1.661407	0.100174	-5.38506	0.33103	0.38792
Non.inflammatory.Macrophages	MAP3K12	-0.31132	2.718206	-1.66123	0.100209	-4.89614	0.389032	0.417711
Non.inflammatory.Macrophages	PSMD9	0.169788	5.225742	1.660852	0.100286	-5.37206	0.334193	0.389642
Non.inflammatory.Macrophages	TMEM161I	0.201991	4.545354	1.65999	0.100459	-5.22373	0.348822	0.397296
Non.inflammatory.Macrophages	MGME1	-0.37819	2.312135	-1.65985	0.100487	-4.88032	0.39941	0.422767
Non.inflammatory.Macrophages	TMEM144	-0.55956	0.882973	-1.65928	0.100602	-4.6421	0.434879	0.440086
Non.inflammatory.Macrophages	UPF2	0.156484	6.075856	1.658768	0.100706	-5.50627	0.318194	0.381083
Non.inflammatory.Macrophages	GM15879	-0.78578	0.515986	-1.65805	0.100852	-4.38746	0.441148	0.445165
Non.inflammatory.Macrophages	SLC31A2	0.27248	3.169586	1.657837	0.100894	-5.2411	0.380275	0.413549
Non.inflammatory.Macrophages	DRC7	1.140415	-0.15036	1.65704	0.101056	-4.26298	0.442047	0.453754
Non.inflammatory.Macrophages	MYLIP	-0.18334	5.991118	-1.65676	0.101113	-5.61478	0.320725	0.382595

Non.inflammatory.Macrophages	SLC8A1	0.221854	6.330185	1.655796	0.101308	-6.05342	0.314644	0.379404
Non.inflammatory.Macrophages	NOP58	-0.2149	5.838554	-1.65539	0.10139	-5.46138	0.324409	0.38477
Non.inflammatory.Macrophages	EML5	0.456465	3.697759	1.655118	0.101446	-4.80799	0.369703	0.408397
Non.inflammatory.Macrophages	DUSP1	-0.18415	7.412842	-1.65453	0.101565	-6.03149	0.294908	0.368517
Non.inflammatory.Macrophages	RNF185	-0.1782	5.117108	-1.6538	0.101714	-5.39563	0.339777	0.393128
Non.inflammatory.Macrophages	STK24	0.141784	7.291795	1.651772	0.102129	-5.70039	0.298484	0.370929
Non.inflammatory.Macrophages	GM47200	-0.78527	0.11613	-1.65174	0.102135	-4.39807	0.445616	0.452881
Non.inflammatory.Macrophages	SRCAP	-0.12906	6.652894	-1.65143	0.102199	-5.6499	0.310498	0.377694
Non.inflammatory.Macrophages	TNFRSF11A	0.247329	2.190262	1.65065	0.102358	-5.39467	0.407869	0.427917
Non.inflammatory.Macrophages	GM48623	-0.90413	-0.24558	-1.65017	0.102457	-4.32773	0.446588	0.458054
Non.inflammatory.Macrophages	GM45669	-0.79564	0.129811	-1.64974	0.102545	-4.43809	0.446825	0.45342
Non.inflammatory.Macrophages	PCYT1A	-0.20868	5.255992	-1.64924	0.102648	-5.45004	0.339155	0.393368
Non.inflammatory.Macrophages	8030456M	-0.62909	1.090368	-1.64892	0.102713	-4.49435	0.436462	0.441749
Non.inflammatory.Macrophages	1700123O2	0.193539	4.989927	1.648875	0.102722	-5.36562	0.344706	0.396336
Non.inflammatory.Macrophages	RB1CC1	0.173241	6.500368	1.648875	0.102722	-5.57844	0.314308	0.380015

Non.inflammatory.Macrophages	4930581F2	-0.24541	3.634004	-1.64812	0.102877	-5.13007	0.374797	0.411759
Non.inflammatory.Macrophages	ZDHHC12	0.490911	1.360226	1.647602	0.102984	-4.72302	0.430271	0.438789
Non.inflammatory.Macrophages	AKT3	-0.20914	6.643383	-1.64726	0.103054	-5.58268	0.312169	0.378906
Non.inflammatory.Macrophages	PSMD8	0.151291	6.592353	1.647256	0.103055	-5.67727	0.313146	0.379445
Non.inflammatory.Macrophages	C4B	0.564589	2.480976	1.647105	0.103086	-5.00452	0.402201	0.425468
Non.inflammatory.Macrophages	0610005C1	-0.53906	1.911141	-1.64688	0.103133	-4.61358	0.416264	0.432263
Non.inflammatory.Macrophages	ERRFI1	-0.22843	5.004787	-1.64678	0.103153	-5.47259	0.34506	0.396626
Non.inflammatory.Macrophages	ZFP810	0.367058	2.914739	1.646364	0.103239	-4.86854	0.391986	0.420586
Non.inflammatory.Macrophages	SLC3A2	-0.16596	6.772545	-1.64621	0.10327	-5.8069	0.309862	0.37782
Non.inflammatory.Macrophages	ACTN1	-0.1734	5.757216	-1.6459	0.103334	-5.69823	0.329828	0.388713
Non.inflammatory.Macrophages	RSRP1	-0.14876	6.653226	-1.64539	0.10344	-5.75944	0.312449	0.379247
Non.inflammatory.Macrophages	FLOT2	0.195838	4.443449	1.644727	0.103577	-5.32902	0.357882	0.403527
Non.inflammatory.Macrophages	FEM1B	0.199143	4.728435	1.644571	0.103609	-5.39978	0.351723	0.400445
Non.inflammatory.Macrophages	RPH3AL	-0.85062	1.299189	-1.64452	0.10362	-4.34056	0.432847	0.440552
Non.inflammatory.Macrophages	GM16283	-0.72857	0.252287	-1.64407	0.103712	-4.3797	0.449165	0.453771

Non.inflammatory.Macrophages	STX3	0.256884	2.314188	1.643623	0.103806	-5.34423	0.407541	0.428696
Non.inflammatory.Macrophages	ZBTB40	0.28933	3.163496	1.643618	0.103807	-4.9977	0.387141	0.418687
Non.inflammatory.Macrophages	PID1	0.185562	5.484883	1.642859	0.103964	-5.86813	0.336526	0.392734
Non.inflammatory.Macrophages	2410006H1	-0.21891	7.535004	-1.64272	0.103992	-5.85717	0.296827	0.370947
Non.inflammatory.Macrophages	SRSF2	-0.14904	7.941182	-1.64246	0.104046	-5.82908	0.28955	0.366851
Non.inflammatory.Macrophages	ZFP180	0.250874	3.315586	1.642351	0.10407	-5.06158	0.384069	0.417303
Non.inflammatory.Macrophages	ELF1	0.114712	8.010369	1.642135	0.104114	-5.86003	0.288354	0.366254
Non.inflammatory.Macrophages	NFAM1	-0.16007	4.570884	-1.64165	0.104215	-5.63891	0.356113	0.403227
Non.inflammatory.Macrophages	CNN3	-0.34166	4.531127	-1.64144	0.104258	-5.07895	0.356976	0.403684
Non.inflammatory.Macrophages	MPHOSPH	0.22071	3.799303	1.641353	0.104277	-5.14147	0.373225	0.411997
Non.inflammatory.Macrophages	GAPVD1	0.127761	6.832806	1.641154	0.104318	-5.73386	0.310164	0.378687
Non.inflammatory.Macrophages	MTF2	0.178811	5.644163	1.640982	0.104354	-5.40696	0.333574	0.391528
Non.inflammatory.Macrophages	NDUFB1-P	0.108868	8.902498	1.64079	0.104394	-6.05644	0.273188	0.357622
Non.inflammatory.Macrophages	PHTF2	0.188989	5.998759	1.640203	0.104516	-5.53781	0.32672	0.387901
Non.inflammatory.Macrophages	GM47819	-0.72739	-0.22199	-1.63966	0.104628	-4.56044	0.450863	0.461514

Non.inflammatory.Macrophages	LAG3	-0.54608	1.429969	-1.63962	0.104637	-4.68432	0.431332	0.44083
Non.inflammatory.Macrophages	GM11713	0.394734	2.37115	1.63904	0.104759	-4.92011	0.40781	0.429643
Non.inflammatory.Macrophages	CNOT2	0.130224	7.008824	1.638943	0.104779	-5.67568	0.307467	0.377624
Non.inflammatory.Macrophages	TMEM268	0.218532	3.100152	1.638844	0.1048	-5.25351	0.390225	0.421088
Non.inflammatory.Macrophages	TIA1	0.158289	5.466514	1.638379	0.104897	-5.37369	0.338106	0.394434
Non.inflammatory.Macrophages	MYBPC2	-0.66687	2.760882	-1.63815	0.104944	-4.55301	0.398614	0.425304
Non.inflammatory.Macrophages	P4HA1	0.222386	5.786072	1.637414	0.105098	-5.69298	0.331994	0.391167
Non.inflammatory.Macrophages	GM47644	0.628139	1.060844	1.637158	0.105152	-4.62946	0.442175	0.446233
Non.inflammatory.Macrophages	LARGE1	-0.23584	5.482959	-1.63657	0.105276	-5.58108	0.338557	0.39476
Non.inflammatory.Macrophages	MED12L	0.331494	3.141983	1.636327	0.105326	-4.94381	0.390448	0.421479
Non.inflammatory.Macrophages	PTPA	0.155118	5.833043	1.63574	0.105449	-5.5274	0.331669	0.391259
Non.inflammatory.Macrophages	GNB1L	-0.30952	3.308245	-1.63567	0.105465	-4.98242	0.386796	0.41977
Non.inflammatory.Macrophages	DNAJC2	0.154919	5.81945	1.634955	0.105614	-5.52994	0.332277	0.391652
Non.inflammatory.Macrophages	SH3BGR	1.112428	-0.59926	1.634845	0.105637	-4.2824	0.453295	0.468188
Non.inflammatory.Macrophages	RFC4	-0.34207	4.603054	-1.6345	0.105709	-5.15714	0.358014	0.405182

Non.inflammatory.Macrophages	2610206C1	0.829976	0.40853	1.634125	0.105789	-4.37178	0.453641	0.455335
Non.inflammatory.Macrophages	MYO9A	-0.18802	6.336763	-1.63398	0.105818	-5.81383	0.322171	0.386143
Non.inflammatory.Macrophages	KDM7A	0.139691	7.129544	1.633817	0.105853	-5.88998	0.306903	0.377773
Non.inflammatory.Macrophages	TNFSF13B	0.69968	0.855901	1.63338	0.105945	-4.51238	0.448338	0.449952
Non.inflammatory.Macrophages	HACD3	0.171634	4.907611	1.633124	0.105999	-5.39224	0.351693	0.402071
Non.inflammatory.Macrophages	UQCRH	0.101033	8.592145	1.633025	0.10602	-6.02996	0.280646	0.362903
Non.inflammatory.Macrophages	PSD	-0.59579	1.633137	-1.63293	0.10604	-4.53647	0.428873	0.440465
Non.inflammatory.Macrophages	GM13205	-0.76469	0.234435	-1.63285	0.106056	-4.39958	0.4538	0.457916
Non.inflammatory.Macrophages	GM10143	-0.79679	1.245831	-1.63268	0.106093	-4.36793	0.438978	0.445327
Non.inflammatory.Macrophages	SNAI3	-1.05753	-0.34486	-1.63244	0.106144	-4.29766	0.453817	0.4656
Non.inflammatory.Macrophages	PAXX	-0.30001	3.270058	-1.63236	0.106161	-4.90974	0.388527	0.42112
Non.inflammatory.Macrophages	MRS2	0.232254	3.939507	1.631891	0.106259	-5.12324	0.373285	0.413611
Non.inflammatory.Macrophages	GM26510	0.242988	4.122817	1.631648	0.106311	-5.26935	0.36921	0.411554
Non.inflammatory.Macrophages	CASTOR2	0.357403	3.643855	1.631486	0.106345	-5.19195	0.380115	0.417123
Non.inflammatory.Macrophages	CUL7	-0.49093	1.968845	-1.63124	0.106398	-4.67291	0.420717	0.4371

Non.inflammatory.Macrophages	NEDD4	-0.2575	5.933587	-1.63029	0.106597	-5.36837	0.331179	0.391718
Non.inflammatory.Macrophages	NEK8	-0.54369	2.113602	-1.62999	0.106661	-4.55734	0.417742	0.435656
Non.inflammatory.Macrophages	EIF1AX	0.166308	6.060349	1.629806	0.1067	-5.57229	0.328645	0.390398
Non.inflammatory.Macrophages	DHX58OS	-0.75847	0.831273	-1.62976	0.106709	-4.49432	0.449942	0.451531
Non.inflammatory.Macrophages	HERC4	0.149273	7.008496	1.629622	0.106739	-5.77783	0.310106	0.380238
Non.inflammatory.Macrophages	GM10863	-0.73664	0.229142	-1.62945	0.106777	-4.51095	0.455011	0.459167
Non.inflammatory.Macrophages	CCDC58	0.207886	4.247902	1.62904	0.106863	-5.29694	0.367189	0.410834
Non.inflammatory.Macrophages	GM42962	-0.4492	-0.60259	-1.62895	0.106883	-4.66051	0.455133	0.470153
Non.inflammatory.Macrophages	MAPT	-0.54343	1.597756	-1.62883	0.106906	-4.58838	0.431049	0.442309
Non.inflammatory.Macrophages	ITGB3	0.539021	2.991713	1.628574	0.106962	-4.61427	0.396292	0.425566
Non.inflammatory.Macrophages	CRTC2	-0.24947	4.134514	-1.6285	0.106977	-5.24228	0.369741	0.412245
Non.inflammatory.Macrophages	ERCC6L	-0.45399	3.034059	-1.62785	0.107116	-4.83143	0.395668	0.425293
Non.inflammatory.Macrophages	COPE	0.138554	6.316581	1.627322	0.107228	-5.69919	0.32401	0.388306
Non.inflammatory.Macrophages	FCRL5	0.966982	-0.65204	1.627188	0.107256	-4.31864	0.455666	0.471337
Non.inflammatory.Macrophages	CNKSR3	-0.26921	4.754523	-1.6271	0.107274	-5.42392	0.35645	0.405566

Non.inflammatory.Macrophages	MED4	0.212984	4.020834	1.626887	0.10732	-5.13955	0.372731	0.413934
Non.inflammatory.Macrophages	GM26670	-0.64833	0.402458	-1.62687	0.107324	-4.41293	0.455666	0.457746
Non.inflammatory.Macrophages	SERPINA1D	-0.48348	5.558333	-1.62682	0.107334	-5.48425	0.339388	0.396591
Non.inflammatory.Macrophages	PAK4	-0.33773	2.299468	-1.62656	0.107389	-4.87648	0.413777	0.434359
Non.inflammatory.Macrophages	FDPS	-0.30812	4.623809	-1.62537	0.107644	-5.22149	0.360111	0.407544
Non.inflammatory.Macrophages	EFNB1	0.43419	1.912975	1.625184	0.107683	-4.78124	0.424422	0.439528
Non.inflammatory.Macrophages	IL4I1	-0.46488	2.841673	-1.62479	0.107768	-4.97418	0.401456	0.428427
Non.inflammatory.Macrophages	HAGHL	0.297731	2.911144	1.624652	0.107797	-5.12532	0.399773	0.427616
Non.inflammatory.Macrophages	A430035B1	-0.58838	3.071834	-1.62431	0.107869	-4.68342	0.396044	0.425821
Non.inflammatory.Macrophages	TCP11	-0.5949	0.648485	-1.62398	0.107939	-4.50957	0.455321	0.455634
Non.inflammatory.Macrophages	FAM118B	-0.23165	3.464677	-1.62384	0.107971	-5.16377	0.38685	0.421395
Non.inflammatory.Macrophages	RAB11A	0.108073	7.145444	1.623279	0.10809	-5.86495	0.309263	0.380567
Non.inflammatory.Macrophages	SNX10	0.189191	5.029489	1.622978	0.108155	-5.67833	0.352098	0.403789
Non.inflammatory.Macrophages	LONRF3	0.441632	1.525709	1.622723	0.108209	-4.78766	0.435456	0.445144
Non.inflammatory.Macrophages	GBP2B	1.161289	0.300434	1.622069	0.108349	-4.81331	0.458177	0.460772

Non.inflammatory.Macrophages	NUP188	-0.21658	4.513741	-1.62196	0.108372	-5.29962	0.363711	0.409836
Non.inflammatory.Macrophages	KLRB1A	1.095187	-0.08222	1.621794	0.108408	-4.33775	0.458177	0.465757
Non.inflammatory.Macrophages	RNF141	0.182352	4.511433	1.621581	0.108454	-5.38461	0.363762	0.409965
Non.inflammatory.Macrophages	ADHFE1	-0.31642	2.690792	-1.62152	0.108468	-5.07405	0.406259	0.431277
Non.inflammatory.Macrophages	BASP1	0.239948	5.646818	1.621056	0.108567	-5.87845	0.339622	0.397378
Non.inflammatory.Macrophages	ZBTB46	-0.38061	3.021217	-1.62084	0.108614	-4.94171	0.398506	0.42756
Non.inflammatory.Macrophages	1110059E2	0.221439	4.393524	1.620323	0.108724	-5.24353	0.366907	0.411742
Non.inflammatory.Macrophages	GEMIN8	-0.45797	2.284507	-1.61993	0.108808	-4.66089	0.417138	0.436749
Non.inflammatory.Macrophages	PLEKHA7	-0.57277	2.084938	-1.61974	0.108851	-4.59785	0.422221	0.439241
Non.inflammatory.Macrophages	ABCC4	-0.17331	4.55376	-1.61946	0.108911	-5.47813	0.363627	0.410139
Non.inflammatory.Macrophages	B230219D2	0.117543	6.252623	1.618444	0.109129	-5.65226	0.328302	0.391354
Non.inflammatory.Macrophages	TSPAN5	0.298264	5.373256	1.618361	0.109147	-5.15104	0.346435	0.401054
Non.inflammatory.Macrophages	CCDC134	-0.2904	3.407824	-1.61783	0.109261	-5.08615	0.390717	0.423652
Non.inflammatory.Macrophages	NUP107	-0.18018	5.353646	-1.61771	0.109287	-5.52805	0.347078	0.401316
Non.inflammatory.Macrophages	RBBP7	-0.16158	6.2357	-1.61749	0.109336	-5.63185	0.328903	0.391581

Non.inflammatory.Macrophages	ITGAL	0.17011	6.272091	1.617325	0.109371	-5.86559	0.328174	0.391214
Non.inflammatory.Macrophages	MALSU1	0.197215	4.843801	1.617105	0.109418	-5.41522	0.35814	0.407147
Non.inflammatory.Macrophages	ITPRID2	0.159324	5.08881	1.61669	0.109508	-5.65802	0.353008	0.404544
Non.inflammatory.Macrophages	TMC7	-0.93275	-0.40026	-1.61629	0.109595	-4.32115	0.460612	0.471399
Non.inflammatory.Macrophages	PMPCB	-0.18872	4.974651	-1.61612	0.109632	-5.44217	0.355515	0.405979
Non.inflammatory.Macrophages	GPR146	0.248072	3.278936	1.615847	0.10969	-5.1683	0.394132	0.4256
Non.inflammatory.Macrophages	CENPF	-0.49925	5.050794	-1.61577	0.109707	-5.25887	0.353868	0.405127
Non.inflammatory.Macrophages	3110040N1	0.26782	3.553549	1.615704	0.109721	-5.2034	0.387623	0.422368
Non.inflammatory.Macrophages	VAMP1	-0.24793	3.997211	-1.61568	0.109728	-5.15904	0.377317	0.41719
Non.inflammatory.Macrophages	CDH2	-0.50987	1.947183	-1.61519	0.109834	-4.71539	0.427161	0.441818
Non.inflammatory.Macrophages	ANP32A	0.089696	7.495527	1.615099	0.109852	-5.85424	0.304697	0.378611
Non.inflammatory.Macrophages	MBNL2	-0.1335	8.406165	-1.61502	0.10987	-6.10644	0.288132	0.369148
Non.inflammatory.Macrophages	GM14321	-0.58246	-0.49943	-1.61495	0.109884	-4.55859	0.460612	0.472951
Non.inflammatory.Macrophages	NBN	-0.24461	3.4476	-1.61482	0.109912	-5.19584	0.390122	0.423845
Non.inflammatory.Macrophages	2610020CC	-0.20838	4.983149	-1.61475	0.109929	-5.23667	0.355331	0.406111

Non.inflammatory.Macrophages	EPHX3	-0.58411	0.657793	-1.61453	0.109976	-4.57501	0.458586	0.458071
Non.inflammatory.Macrophages	STOM	0.209246	3.274711	1.614297	0.110026	-5.47145	0.394339	0.426008
Non.inflammatory.Macrophages	CHSY3	-0.68295	1.978293	-1.61401	0.110088	-4.7134	0.426492	0.441681
Non.inflammatory.Macrophages	IFI205	0.276497	2.758001	1.613964	0.110099	-5.68648	0.406886	0.432216
Non.inflammatory.Macrophages	GRAMD2	-0.50476	0.533275	-1.61284	0.110344	-4.67061	0.461049	0.46019
Non.inflammatory.Macrophages	GNG12	0.154977	6.49518	1.612756	0.110361	-5.74058	0.32463	0.38987
Non.inflammatory.Macrophages	NR6A1OS	-0.70243	2.067272	-1.61261	0.110393	-4.51929	0.424952	0.44107
Non.inflammatory.Macrophages	DNAH12	0.430387	1.676699	1.61211	0.110502	-5.05784	0.435339	0.446036
Non.inflammatory.Macrophages	SFT2D1	-0.12152	5.999884	-1.61198	0.110531	-5.67982	0.334833	0.395523
Non.inflammatory.Macrophages	ZFP825	0.395778	2.050646	1.611631	0.110607	-4.7923	0.425805	0.441599
Non.inflammatory.Macrophages	NBEA	-0.39859	2.602645	-1.61133	0.110672	-4.92729	0.411929	0.435017
Non.inflammatory.Macrophages	NCOA4	0.19536	6.199192	1.611096	0.110723	-5.78295	0.330957	0.393605
Non.inflammatory.Macrophages	HSPA1B	-0.60418	4.531616	-1.61107	0.110729	-5.51638	0.366429	0.412309
Non.inflammatory.Macrophages	RPE	0.213472	4.554255	1.610842	0.110779	-5.31691	0.365956	0.4121
Non.inflammatory.Macrophages	PRMT9	0.229247	4.074852	1.610713	0.110807	-5.31708	0.37679	0.41766

Non.inflammatory.Macrophages	GM33524	-0.67611	0.491882	-1.61055	0.110843	-4.53885	0.462133	0.461467
Non.inflammatory.Macrophages	GM48302	0.416476	1.63727	1.610184	0.110922	-4.93137	0.436837	0.44708
Non.inflammatory.Macrophages	STAT4	0.647293	5.177658	1.609437	0.111086	-4.79658	0.352869	0.405369
Non.inflammatory.Macrophages	GZF1	0.294823	2.752325	1.608952	0.111192	-5.10704	0.409093	0.433807
Non.inflammatory.Macrophages	CEP250	0.237848	4.458141	1.60889	0.111205	-5.27632	0.368861	0.413696
Non.inflammatory.Macrophages	ZDHHC17	0.219935	4.015064	1.608461	0.111299	-5.23641	0.379045	0.418933
Non.inflammatory.Macrophages	ATF7IP	-0.17294	6.540023	-1.60844	0.111305	-5.66564	0.324907	0.390496
Non.inflammatory.Macrophages	TCF25	0.110304	6.939299	1.608104	0.111378	-5.80755	0.317101	0.38621
Non.inflammatory.Macrophages	TRIM28	-0.18734	5.460856	-1.60802	0.111395	-5.51705	0.347125	0.402438
Non.inflammatory.Macrophages	CAPNS1	0.114862	7.25812	1.607896	0.111423	-5.90731	0.310963	0.382833
Non.inflammatory.Macrophages	ATG10	0.167349	5.64086	1.607479	0.111515	-5.64426	0.343401	0.400558
Non.inflammatory.Macrophages	FAM126B	0.241755	3.772291	1.607478	0.111515	-5.14062	0.384812	0.421945
Non.inflammatory.Macrophages	TCF7L1	-0.43269	3.097343	-1.60715	0.111587	-4.89805	0.401028	0.4301
Non.inflammatory.Macrophages	KLHL25	0.372341	2.493825	1.606341	0.111765	-4.79759	0.416471	0.437627
Non.inflammatory.Macrophages	COLEC10	-0.54802	0.557157	-1.60602	0.111836	-4.63454	0.463491	0.461984

Non.inflammatory.Macrophages	PRR33	1.050045	0.255516	1.605813	0.111881	-4.32557	0.464285	0.465929
Non.inflammatory.Macrophages	4930557K0	-0.49626	1.483636	-1.60571	0.111903	-4.70899	0.442743	0.450303
Non.inflammatory.Macrophages	PLXNA4	-0.70231	2.166702	-1.60481	0.112102	-4.9974	0.425534	0.442162
Non.inflammatory.Macrophages	SERPINA1C	-0.71019	0.966902	-1.60382	0.11232	-4.48935	0.458017	0.45739
Non.inflammatory.Macrophages	LY6I	1.705602	0.802083	1.60376	0.112334	-4.39351	0.460986	0.459489
Non.inflammatory.Macrophages	TIRAP	0.275393	3.005516	1.603448	0.112402	-5.08007	0.405215	0.432324
Non.inflammatory.Macrophages	PPP1R3E	0.405093	1.031561	1.603156	0.112467	-4.71002	0.456453	0.456879
Non.inflammatory.Macrophages	IL12B	-1.28457	-0.29714	-1.603	0.112502	-4.48639	0.465853	0.474094
Non.inflammatory.Macrophages	PCGF5	0.225773	6.06196	1.602887	0.112526	-5.55516	0.336455	0.397316
Non.inflammatory.Macrophages	GM15972	-0.72027	-0.2921	-1.60275	0.112557	-4.52347	0.465853	0.474209
Non.inflammatory.Macrophages	PLRG1	-0.20575	4.093452	-1.60259	0.112591	-5.22313	0.379385	0.419848
Non.inflammatory.Macrophages	SLC7A2	0.48715	2.094729	1.601694	0.11279	-5.09385	0.428669	0.444356
Non.inflammatory.Macrophages	UVRAG	-0.1398	8.569242	-1.60148	0.112838	-6.16819	0.288823	0.371167
Non.inflammatory.Macrophages	RRAGC	-0.14265	5.819399	-1.60142	0.11285	-5.69276	0.341859	0.400692
Non.inflammatory.Macrophages	ARMCX3	-0.23624	3.573363	-1.60128	0.112883	-5.23468	0.391993	0.426545

Non.inflammatory.Macrophages	SIN3B	0.123748	6.294475	1.601166	0.112907	-5.70342	0.332067	0.395427
Non.inflammatory.Macrophages	CREB3L2	0.283095	3.55229	1.601102	0.112922	-5.21436	0.392495	0.426814
Non.inflammatory.Macrophages	MMP19	0.354656	1.213067	1.600828	0.112982	-5.18053	0.452114	0.45565
Non.inflammatory.Macrophages	HELZ2	0.247684	3.832468	1.599224	0.113338	-5.38996	0.387013	0.424157
Non.inflammatory.Macrophages	DAAM1	0.201639	4.764899	1.599135	0.113358	-5.41309	0.365665	0.413326
Non.inflammatory.Macrophages	MCMDC2	-0.26941	3.465667	-1.59882	0.113429	-5.37802	0.395852	0.428723
Non.inflammatory.Macrophages	QSOX1	0.276689	4.311358	1.597953	0.113621	-5.13243	0.376382	0.418995
Non.inflammatory.Macrophages	CD200R2	-1.20375	0.789771	-1.59787	0.11364	-4.3803	0.463869	0.462108
Non.inflammatory.Macrophages	SLC45A1	-0.63205	0.608482	-1.59787	0.11364	-4.4691	0.466921	0.46444
Non.inflammatory.Macrophages	CLCC1	0.162085	4.329275	1.59754	0.113713	-5.38454	0.376002	0.418816
Non.inflammatory.Macrophages	PNPLA6	-0.2757	2.760588	-1.59737	0.113751	-5.02884	0.413548	0.437528
Non.inflammatory.Macrophages	STX18	0.19666	4.637177	1.597362	0.113753	-5.39924	0.369018	0.415265
Non.inflammatory.Macrophages	NSUN3	0.28442	2.658689	1.597062	0.11382	-4.99595	0.416222	0.438839
Non.inflammatory.Macrophages	BBC3	-0.31124	2.639565	-1.59653	0.113938	-4.98193	0.417007	0.439152
Non.inflammatory.Macrophages	GOLGA7	0.134945	5.94	1.596267	0.113997	-5.64546	0.341226	0.400608

Non.inflammatory.Macrophages	GNPTG	-0.23472	3.368723	-1.59542	0.114187	-5.15455	0.399631	0.430629
Non.inflammatory.Macrophages	TOR1AIP2	-0.12298	6.580878	-1.59462	0.114366	-5.77361	0.328963	0.394019
Non.inflammatory.Macrophages	GM13963	-0.65507	-0.29578	-1.5939	0.114527	-4.64379	0.470631	0.477378
Non.inflammatory.Macrophages	SH3D19	-0.4325	2.244921	-1.59382	0.114546	-4.85794	0.42869	0.444848
Non.inflammatory.Macrophages	SLC12A4	0.421135	1.745624	1.593357	0.114649	-4.83186	0.441973	0.451318
Non.inflammatory.Macrophages	DNAH10	-0.7308	0.024576	-1.59318	0.11469	-4.504	0.470837	0.473417
Non.inflammatory.Macrophages	PTAR1	0.262563	3.690053	1.593131	0.1147	-5.02759	0.392958	0.427556
Non.inflammatory.Macrophages	B020010K1	0.663309	1.134374	1.592646	0.114809	-4.59626	0.458798	0.459111
Non.inflammatory.Macrophages	UTY	3.591564	2.644423	1.592009	0.114952	-4.95667	0.419301	0.44038
Non.inflammatory.Macrophages	BIK	-0.69125	1.76969	-1.59188	0.114981	-4.63044	0.442015	0.451224
Non.inflammatory.Macrophages	COPS4	0.146108	5.581159	1.591722	0.115016	-5.57859	0.350739	0.405817
Non.inflammatory.Macrophages	4930438A(	-0.83311	-0.84008	-1.5912	0.115134	-4.34929	0.471808	0.485296
Non.inflammatory.Macrophages	EPC2	0.160565	6.14343	1.590754	0.115234	-5.62832	0.339061	0.399721
Non.inflammatory.Macrophages	ZYX	0.18464	6.244314	1.590736	0.115238	-5.81512	0.336974	0.3986
Non.inflammatory.Macrophages	WDHD1	0.317562	4.56722	1.590722	0.115242	-5.18938	0.373313	0.417652

Non.inflammatory.Macrophages	SNHG20	0.303069	2.355617	1.590684	0.11525	-4.92259	0.4269	0.444162
Non.inflammatory.Macrophages	GM5617	0.24863	3.238502	1.590272	0.115343	-5.231	0.404907	0.433481
Non.inflammatory.Macrophages	PUS1	-0.22862	3.723827	-1.5896	0.115495	-5.2766	0.39356	0.428012
Non.inflammatory.Macrophages	DENR	-0.13311	6.279481	-1.58851	0.115741	-5.7295	0.337323	0.399046
Non.inflammatory.Macrophages	CCNA2	-0.43807	5.520186	-1.58843	0.115758	-5.36104	0.353354	0.40759
Non.inflammatory.Macrophages	SLC7A5	-0.27752	6.118367	-1.58771	0.115921	-5.4903	0.341043	0.401117
Non.inflammatory.Macrophages	LPAR1	0.615119	0.763125	1.587424	0.115987	-4.86627	0.469881	0.465593
Non.inflammatory.Macrophages	KLRA3	1.276604	-0.66191	1.587242	0.116028	-4.35842	0.47392	0.48441
Non.inflammatory.Macrophages	APLP2	0.17842	5.805079	1.587079	0.116065	-5.7127	0.347701	0.404769
Non.inflammatory.Macrophages	GM32296	-0.52369	-0.65404	-1.58697	0.116089	-4.70208	0.47392	0.484459
Non.inflammatory.Macrophages	CDC42BPB	0.23809	3.262023	1.586716	0.116147	-5.41515	0.405936	0.43456
Non.inflammatory.Macrophages	PSMB4	0.138174	6.172049	1.586659	0.11616	-5.75041	0.339983	0.400766
Non.inflammatory.Macrophages	BCL2L1	0.230278	6.15449	1.586543	0.116186	-5.7206	0.340349	0.400967
Non.inflammatory.Macrophages	CDYL	0.152785	5.659184	1.586226	0.116258	-5.54909	0.350821	0.406601
Non.inflammatory.Macrophages	CYP2A12	-0.58123	2.85261	-1.58623	0.116259	-4.88193	0.416133	0.439637

Non.inflammatory.Macrophages	TMEM39A	-0.20989	4.395391	-1.58581	0.116353	-5.29809	0.379129	0.421347
Non.inflammatory.Macrophages	HMGB2	-0.27423	9.132267	-1.58491	0.116557	-6.02268	0.284017	0.369485
Non.inflammatory.Macrophages	PRPF31	0.214533	3.79385	1.584822	0.116577	-5.26424	0.393734	0.428677
Non.inflammatory.Macrophages	USP2	-0.31284	2.711696	-1.58472	0.1166	-5.11169	0.420421	0.441822
Non.inflammatory.Macrophages	TNFRSF18	0.766719	2.322619	1.584441	0.116664	-4.41289	0.430524	0.446712
Non.inflammatory.Macrophages	SENP6	0.124158	6.681311	1.584252	0.116707	-5.71409	0.330209	0.395753
Non.inflammatory.Macrophages	DPY19L4	0.188366	4.417697	1.584125	0.116736	-5.40888	0.379176	0.421496
Non.inflammatory.Macrophages	EXD2	-0.31794	3.156926	-1.58305	0.11698	-4.99658	0.410007	0.436829
Non.inflammatory.Macrophages	AMN1	-0.25347	3.42505	-1.583	0.116993	-5.1581	0.403397	0.433584
Non.inflammatory.Macrophages	SLU7	0.155503	4.955244	1.582793	0.117039	-5.51151	0.36758	0.41552
Non.inflammatory.Macrophages	NRXN2	-0.61141	2.095803	-1.58234	0.117142	-4.82482	0.43739	0.449971
Non.inflammatory.Macrophages	CFAP53	0.659856	0.830789	1.582263	0.11716	-4.6599	0.470609	0.466072
Non.inflammatory.Macrophages	TRAF6	0.179437	5.683882	1.581899	0.117244	-5.50543	0.351882	0.407218
Non.inflammatory.Macrophages	4930426D(	-1.0076	-0.21569	-1.58157	0.117318	-4.35914	0.476173	0.479948
Non.inflammatory.Macrophages	ARID4B	-0.13006	8.259309	-1.58149	0.117337	-5.95766	0.300565	0.379161

Non.inflammatory.Macrophages	TBK1	0.167546	5.964636	1.581165	0.117411	-5.71087	0.346024	0.404199
Non.inflammatory.Macrophages	AOPEP	0.151394	6.329542	1.580998	0.117449	-5.73305	0.338384	0.400186
Non.inflammatory.Macrophages	SCPEP1OS	-0.62975	0.502575	-1.58078	0.117499	-4.62664	0.476106	0.470744
Non.inflammatory.Macrophages	PREPL	0.406447	1.734988	1.580639	0.117532	-4.79417	0.447349	0.454898
Non.inflammatory.Macrophages	PABPC1L	0.362699	2.906471	1.580629	0.117534	-5.0392	0.416818	0.440317
Non.inflammatory.Macrophages	SRGAP1	-0.55118	2.559229	-1.58015	0.117643	-4.76276	0.425907	0.444826
Non.inflammatory.Macrophages	SLC25A1	0.237323	3.772843	1.580027	0.117672	-5.30928	0.395717	0.430056
Non.inflammatory.Macrophages	CUL2	0.189749	4.968394	1.579449	0.117804	-5.46756	0.368248	0.416142
Non.inflammatory.Macrophages	COX6C	0.111495	8.594079	1.578763	0.117962	-6.11738	0.294963	0.376421
Non.inflammatory.Macrophages	EPHX2	-0.52023	2.477792	-1.57876	0.117963	-4.87191	0.428433	0.446279
Non.inflammatory.Macrophages	FAM241A	0.227021	5.59878	1.578747	0.117965	-5.46141	0.354406	0.409151
Non.inflammatory.Macrophages	TMEM245	0.202214	4.753996	1.578533	0.118015	-5.43973	0.373156	0.418974
Non.inflammatory.Macrophages	FAM193A	0.143021	6.704133	1.578444	0.118035	-5.78306	0.33123	0.39687
Non.inflammatory.Macrophages	ASB2	0.316214	2.906427	1.578288	0.118071	-5.35426	0.417474	0.441165
Non.inflammatory.Macrophages	TLCD2	-0.38761	2.659161	-1.57829	0.118071	-4.96207	0.423764	0.44421

Non.inflammatory.Macrophages	DDX23	0.179229	4.71777	1.577991	0.118139	-5.43432	0.374035	0.419607
Non.inflammatory.Macrophages	APCS	0.314122	4.833138	1.577907	0.118158	-5.41741	0.371414	0.418262
Non.inflammatory.Macrophages	IGF2	-0.56362	4.193244	-1.57756	0.118237	-5.30737	0.386247	0.42584
Non.inflammatory.Macrophages	GPD1	-0.53451	1.749644	-1.5775	0.118252	-4.74619	0.447807	0.455808
Non.inflammatory.Macrophages	FCHO2	0.134415	6.902577	1.577282	0.118302	-5.89069	0.327378	0.394987
Non.inflammatory.Macrophages	RIPK3	-0.24934	3.285039	-1.57709	0.118347	-5.28067	0.408232	0.436873
Non.inflammatory.Macrophages	UQCC1	0.22077	3.934429	1.576585	0.118463	-5.28223	0.392708	0.429309
Non.inflammatory.Macrophages	GM4117	0.593443	0.660809	1.57645	0.118494	-4.59443	0.475364	0.470269
Non.inflammatory.Macrophages	CP	0.329165	4.588111	1.576198	0.118552	-5.41323	0.377468	0.421711
Non.inflammatory.Macrophages	ELOA	-0.15171	5.789735	-1.57593	0.118613	-5.6994	0.350856	0.407927
Non.inflammatory.Macrophages	PROCR	0.638312	0.279761	1.574988	0.118831	-4.87515	0.478449	0.47597
Non.inflammatory.Macrophages	PABPC4	0.162013	5.897724	1.574896	0.118852	-5.64941	0.34904	0.407112
Non.inflammatory.Macrophages	IFIT1BL1	0.779184	0.159661	1.574619	0.118916	-4.65087	0.478502	0.47757
Non.inflammatory.Macrophages	NISCH	0.14172	6.194239	1.57441	0.118964	-5.71651	0.342804	0.403842
Non.inflammatory.Macrophages	ASAH1	0.132312	6.301867	1.57438	0.118971	-5.92694	0.340554	0.402637

Non.inflammatory.Macrophages	SLC30A6	-0.25715	3.417011	-1.57373	0.119121	-5.13469	0.406348	0.436564
Non.inflammatory.Macrophages	GM31814	0.857794	-0.06084	1.573668	0.119136	-4.3831	0.47888	0.480914
Non.inflammatory.Macrophages	BANP	-0.18202	4.601582	-1.57329	0.119224	-5.394	0.378283	0.422527
Non.inflammatory.Macrophages	DNAJA1	-0.11109	7.461326	-1.57309	0.119271	-5.89122	0.317617	0.390266
Non.inflammatory.Macrophages	NEK2	-0.56823	3.092142	-1.57293	0.119307	-4.78319	0.414654	0.440741
Non.inflammatory.Macrophages	ARHGEF39	-0.55698	1.960521	-1.57272	0.119356	-4.84079	0.444038	0.454875
Non.inflammatory.Macrophages	ARPC3	0.103815	8.451234	1.571847	0.119558	-6.09621	0.299338	0.37998
Non.inflammatory.Macrophages	MRC2	-0.52226	1.416971	-1.57171	0.119591	-4.77909	0.459424	0.462179
Non.inflammatory.Macrophages	KNL1	-0.46383	5.131736	-1.57146	0.119647	-5.27196	0.366898	0.41687
Non.inflammatory.Macrophages	MAML2	-0.18494	7.972039	-1.57088	0.119783	-5.99209	0.30859	0.385331
Non.inflammatory.Macrophages	CBR1	-0.2248	4.350162	-1.57003	0.11998	-5.46224	0.38564	0.426305
Non.inflammatory.Macrophages	WLS	-0.21754	4.263574	-1.56934	0.12014	-5.54207	0.388054	0.427545
Non.inflammatory.Macrophages	AC160336.	0.474943	1.371003	1.568886	0.120247	-4.7818	0.462232	0.463481
Non.inflammatory.Macrophages	SERF1	0.225696	3.137334	1.568867	0.120251	-5.20588	0.415517	0.441266
Non.inflammatory.Macrophages	CCDC191	0.35968	1.993228	1.568837	0.120258	-4.94601	0.445253	0.455533

Non.inflammatory.Macrophages	LYSMD3	-0.18393	4.927993	-1.56877	0.120275	-5.49619	0.372662	0.419852
Non.inflammatory.Macrophages	2610027KC	-0.73235	0.681173	-1.5685	0.120337	-4.47389	0.478922	0.472589
Non.inflammatory.Macrophages	FAM13A	0.514933	1.15315	1.568386	0.120363	-4.83808	0.468381	0.466491
Non.inflammatory.Macrophages	UHRF1BP1	-0.40154	1.998967	-1.56819	0.120408	-5.00041	0.445199	0.455693
Non.inflammatory.Macrophages	TIPARP	0.16673	7.437182	1.567988	0.120456	-6.05448	0.319731	0.391746
Non.inflammatory.Macrophages	COLQ	-0.91547	0.244853	-1.5675	0.12057	-4.38434	0.481667	0.478663
Non.inflammatory.Macrophages	DMPK	-0.29406	2.786398	-1.5675	0.12057	-5.36005	0.424624	0.44602
Non.inflammatory.Macrophages	CASP8AP2	0.169594	5.182109	1.567377	0.120599	-5.52408	0.36709	0.41728
Non.inflammatory.Macrophages	STAB2	-0.41927	5.148934	-1.56719	0.120641	-5.52726	0.367834	0.417745
Non.inflammatory.Macrophages	DEAF1	0.254878	3.281829	1.5671	0.120664	-5.11964	0.412076	0.440055
Non.inflammatory.Macrophages	FOS	-0.19245	8.489188	-1.56701	0.120684	-6.28479	0.29977	0.380697
Non.inflammatory.Macrophages	GM49336	0.167753	4.881295	1.566806	0.120732	-5.4939	0.37393	0.421014
Non.inflammatory.Macrophages	AZGP1	-0.43577	3.926277	-1.56631	0.120848	-5.19247	0.39645	0.432668
Non.inflammatory.Macrophages	PPP1R15B	-0.16869	5.553021	-1.56622	0.120869	-5.65977	0.359033	0.413511
Non.inflammatory.Macrophages	CCNG2	0.223767	5.189545	1.566066	0.120905	-5.48233	0.367091	0.417717

Non.inflammatory.Macrophages	CIAO2A	0.142667	6.679979	1.565935	0.120936	-5.85851	0.335113	0.400739
Non.inflammatory.Macrophages	GM26936	0.932246	0.478634	1.565772	0.120974	-4.40896	0.481886	0.476197
Non.inflammatory.Macrophages	GPSM1	-0.4898	2.672071	-1.56571	0.120988	-4.68101	0.427764	0.448034
Non.inflammatory.Macrophages	SLC25A13	0.229596	4.679744	1.565301	0.121085	-5.4484	0.378827	0.423907
Non.inflammatory.Macrophages	ANXA11	-0.1544	5.969579	-1.56521	0.121105	-5.71093	0.35014	0.408975
Non.inflammatory.Macrophages	GM32089	0.557075	-1.09921	1.56489	0.121181	-4.6206	0.48223	0.497996
Non.inflammatory.Macrophages	RGS9	-1.01503	0.03543	-1.56431	0.121318	-4.40627	0.482633	0.482799
Non.inflammatory.Macrophages	NRP2	0.343439	3.300945	1.563978	0.121395	-5.37531	0.412566	0.44107
Non.inflammatory.Macrophages	MGAT5	0.148917	6.916886	1.563713	0.121457	-5.89466	0.330981	0.398935
Non.inflammatory.Macrophages	IMMT	0.126648	6.049157	1.563357	0.121541	-5.73449	0.349063	0.408741
Non.inflammatory.Macrophages	CBFA2T3	-0.3445	5.507245	-1.56333	0.121546	-5.1616	0.360821	0.414954
Non.inflammatory.Macrophages	DNAAF5	0.313509	2.988111	1.563162	0.121587	-4.97999	0.420576	0.445135
Non.inflammatory.Macrophages	PCMTD1	0.176162	5.924056	1.562927	0.121642	-5.66091	0.351745	0.410318
Non.inflammatory.Macrophages	AI662270	0.170641	6.090129	1.562835	0.121664	-5.77002	0.34819	0.408425
Non.inflammatory.Macrophages	HOOK3	0.135631	6.076111	1.5627	0.121696	-5.74018	0.348488	0.408585

Non.inflammatory.Macrophages	MAP2K1	0.163836	6.311963	1.562475	0.121749	-5.72762	0.343495	0.405911
Non.inflammatory.Macrophages	SELENOH	-0.26215	5.20549	-1.56234	0.12178	-5.54933	0.36753	0.418666
Non.inflammatory.Macrophages	DNAJB13	-0.36209	2.103334	-1.56232	0.121786	-4.96556	0.443666	0.456413
Non.inflammatory.Macrophages	B3GALT1	-0.65632	3.129188	-1.5618	0.121907	-4.94401	0.417223	0.443799
Non.inflammatory.Macrophages	TMBIM4	0.13743	6.749974	1.561735	0.121923	-5.87754	0.334584	0.401266
Non.inflammatory.Macrophages	IFI204	0.236771	4.390085	1.561516	0.121975	-5.85604	0.386519	0.428597
Non.inflammatory.Macrophages	RNF17	0.691552	-0.02892	1.561364	0.12201	-4.67418	0.483257	0.484704
Non.inflammatory.Macrophages	SLC23A2	-0.18059	5.113974	-1.5605	0.122214	-5.61679	0.370345	0.420473
Non.inflammatory.Macrophages	PRELID3B	0.151617	5.574007	1.560128	0.122302	-5.6407	0.360182	0.415274
Non.inflammatory.Macrophages	ZFHX3	-0.16949	5.163754	-1.56006	0.122317	-5.72824	0.369319	0.420044
Non.inflammatory.Macrophages	ADCY10	-0.62754	0.912909	-1.55991	0.122353	-4.64617	0.477182	0.472809
Non.inflammatory.Macrophages	ARHGEF10	-0.41865	2.266878	-1.55867	0.122646	-5.07954	0.441253	0.455686
Non.inflammatory.Macrophages	SVIL	0.143963	6.860609	1.558265	0.122743	-5.86364	0.333777	0.40112
Non.inflammatory.Macrophages	ZNRF1	0.18258	6.496008	1.557464	0.122933	-5.64867	0.341575	0.405343
Non.inflammatory.Macrophages	GNB5	0.501658	0.819995	1.557384	0.122952	-4.6784	0.480489	0.474686

Non.inflammatory.Macrophages	ACAT3	-0.66816	1.453412	-1.55733	0.122965	-4.63221	0.463982	0.466405
Non.inflammatory.Macrophages	PGP	0.193689	5.374059	1.557266	0.12298	-5.5783	0.365833	0.418209
Non.inflammatory.Macrophages	COL5A3	-0.86368	0.940726	-1.55653	0.123155	-4.49274	0.478738	0.473429
Non.inflammatory.Macrophages	COMMD8	-0.1432	5.188265	-1.55646	0.12317	-5.67708	0.370363	0.420679
Non.inflammatory.Macrophages	4930562C1	-0.68783	0.146658	-1.55611	0.123254	-4.54768	0.486228	0.484155
Non.inflammatory.Macrophages	DCAF15	0.262171	3.456621	1.556072	0.123264	-5.19766	0.411593	0.441614
Non.inflammatory.Macrophages	MIB1	0.13893	6.189415	1.555773	0.123334	-5.80489	0.348541	0.409388
Non.inflammatory.Macrophages	SPATA6	0.200084	4.52297	1.555397	0.123424	-5.53431	0.386039	0.428894
Non.inflammatory.Macrophages	ATP5K	0.158397	7.486519	1.555218	0.123467	-5.98917	0.322069	0.394934
Non.inflammatory.Macrophages	TECPR1	0.280413	4.375591	1.554914	0.123539	-5.27884	0.389655	0.430764
Non.inflammatory.Macrophages	MAST1	-0.60148	1.020113	-1.55423	0.123701	-4.8453	0.47774	0.473142
Non.inflammatory.Macrophages	GPR108	-0.19544	4.177602	-1.55356	0.123862	-5.42671	0.395133	0.433626
Non.inflammatory.Macrophages	FSHR	-0.60345	0.419984	-1.55347	0.123884	-4.50309	0.48768	0.481398
Non.inflammatory.Macrophages	HMGB1	-0.15997	9.807497	-1.55236	0.124149	-6.19273	0.28025	0.370885
Non.inflammatory.Macrophages	MED19	-0.20592	3.778351	-1.55232	0.124158	-5.24348	0.405358	0.438647

Non.inflammatory.Macrophages	VHL	0.203344	3.486608	1.552263	0.124172	-5.26333	0.4126	0.44223
Non.inflammatory.Macrophages	RNPEP	0.176779	5.72187	1.551765	0.124291	-5.80247	0.360082	0.415776
Non.inflammatory.Macrophages	GIMAP8	-0.6964	2.465426	-1.55175	0.124294	-4.61746	0.438909	0.455219
Non.inflammatory.Macrophages	COX4I1	0.10036	9.086822	1.551649	0.124319	-6.2686	0.292944	0.378645
Non.inflammatory.Macrophages	ANTXR2	0.44011	7.654982	1.551627	0.124324	-5.70923	0.319872	0.394035
Non.inflammatory.Macrophages	ACBD3	0.131221	5.707973	1.551596	0.124331	-5.69896	0.360388	0.415982
Non.inflammatory.Macrophages	BRF1	0.172222	4.828649	1.551392	0.12438	-5.43477	0.3803	0.42633
Non.inflammatory.Macrophages	SKIL	-0.15699	7.304614	-1.55122	0.124421	-5.99425	0.326868	0.397933
Non.inflammatory.Macrophages	UFSP2	0.163593	4.93604	1.550925	0.124492	-5.64264	0.377938	0.425121
Non.inflammatory.Macrophages	MAPRE2	-0.12962	6.802434	-1.55066	0.124556	-5.98243	0.337217	0.403626
Non.inflammatory.Macrophages	MEIS2	-0.56172	2.894046	-1.55057	0.124578	-4.87491	0.427909	0.450028
Non.inflammatory.Macrophages	TBC1D14	-0.14942	5.680933	-1.5503	0.124641	-5.7076	0.361248	0.416578
Non.inflammatory.Macrophages	UBXN8	0.179892	4.404408	1.550076	0.124696	-5.47627	0.390502	0.431785
Non.inflammatory.Macrophages	PTCH1	0.373431	3.139748	1.549938	0.124729	-5.05443	0.421676	0.447269
Non.inflammatory.Macrophages	RAB8B	0.149731	7.724426	1.549861	0.124747	-6.14158	0.318745	0.393685

Non.inflammatory.Macrophages	LRRC61	0.360567	2.011331	1.549328	0.124875	-4.97183	0.451759	0.46179
Non.inflammatory.Macrophages	FBXO45	-0.24787	3.654199	-1.54872	0.125021	-5.19077	0.409384	0.441411
Non.inflammatory.Macrophages	ZRANB1	0.140571	6.142303	1.548497	0.125075	-5.67399	0.351815	0.411917
Non.inflammatory.Macrophages	GM50013	-0.61905	1.85467	-1.54831	0.12512	-4.56685	0.456506	0.464147
Non.inflammatory.Macrophages	DPH3	0.14258	5.480129	1.548197	0.125147	-5.63364	0.366349	0.419606
Non.inflammatory.Macrophages	CHMP2B	0.152879	4.813502	1.547472	0.125322	-5.56193	0.381981	0.427759
Non.inflammatory.Macrophages	GMPR2	-0.21115	3.866899	-1.54699	0.125438	-5.38942	0.404893	0.439326
Non.inflammatory.Macrophages	CCDC85C	-0.68823	0.977037	-1.54662	0.125526	-4.51244	0.482173	0.476171
Non.inflammatory.Macrophages	MAP1LC3B	0.151541	7.434486	1.546536	0.125547	-6.0047	0.325685	0.39786
Non.inflammatory.Macrophages	NES	-0.75623	0.578345	-1.5464	0.125579	-4.61324	0.489394	0.481543
Non.inflammatory.Macrophages	SLC8B1	-0.15991	4.721857	-1.54626	0.125615	-5.78933	0.384463	0.429198
Non.inflammatory.Macrophages	ARIH1	-0.13335	8.728541	-1.54609	0.125655	-6.17434	0.300822	0.38392
Non.inflammatory.Macrophages	NMNAT3	-0.2657	3.105259	-1.54577	0.125733	-5.3053	0.4243	0.449057
Non.inflammatory.Macrophages	RRBP1	0.108226	7.951353	1.545521	0.125792	-6.12765	0.31569	0.392451
Non.inflammatory.Macrophages	6-Sep	-0.42039	5.624977	-1.54489	0.125945	-4.9838	0.364328	0.419007

Non.inflammatory.Macrophages	RP2	0.156455	5.226456	1.54466	0.126	-5.6734	0.373304	0.423779
Non.inflammatory.Macrophages	CHST11	0.635211	6.242747	1.544614	0.126012	-4.98519	0.350821	0.411956
Non.inflammatory.Macrophages	AMOTL1	-0.56362	1.696223	-1.54444	0.126053	-4.71671	0.462411	0.46759
Non.inflammatory.Macrophages	TLK1	0.135611	6.854126	1.544315	0.126084	-5.8381	0.337929	0.405101
Non.inflammatory.Macrophages	P2RX7	-0.24684	3.223954	-1.54422	0.126107	-5.49176	0.421664	0.448224
Non.inflammatory.Macrophages	CYP3A13	0.712012	0.779768	1.543526	0.126275	-4.58132	0.487158	0.47994
Non.inflammatory.Macrophages	H2AFV	-0.19474	8.095101	-1.54321	0.126352	-5.9417	0.313586	0.391696
Non.inflammatory.Macrophages	APH1C	-0.20004	4.351444	-1.54208	0.126625	-5.72516	0.39503	0.435106
Non.inflammatory.Macrophages	SRSF7	-0.18218	6.098296	-1.54162	0.126737	-5.70557	0.355218	0.41464
Non.inflammatory.Macrophages	6530413G1	-0.63872	-0.08741	-1.54127	0.126824	-4.51851	0.492927	0.49256
Non.inflammatory.Macrophages	SAMD4	-0.43326	3.187151	-1.54103	0.12688	-5.31429	0.424136	0.449733
Non.inflammatory.Macrophages	FAM210A	0.208874	3.636981	1.540965	0.126897	-5.30869	0.412722	0.444177
Non.inflammatory.Macrophages	NDUFAF8	0.148935	5.052226	1.540875	0.126919	-5.60867	0.378661	0.427019
Non.inflammatory.Macrophages	GM20219	-0.65954	0.646352	-1.54083	0.12693	-4.57658	0.490918	0.482691
Non.inflammatory.Macrophages	GBP8	0.264756	3.65151	1.540805	0.126936	-5.47121	0.412358	0.443997

Non.inflammatory.Macrophages	NECAP2	0.151728	5.345804	1.540685	0.126965	-5.67697	0.371937	0.423571
Non.inflammatory.Macrophages	GM12592	-0.46476	3.124779	-1.53982	0.127176	-4.73657	0.426248	0.450967
Non.inflammatory.Macrophages	AU022252	0.314127	2.452615	1.539766	0.127189	-5.04696	0.443933	0.459478
Non.inflammatory.Macrophages	CFHR2	-0.4942	2.56401	-1.53947	0.127261	-4.94363	0.441079	0.458134
Non.inflammatory.Macrophages	MRM2	-0.446	2.071278	-1.53898	0.127381	-5.00265	0.45469	0.464613
Non.inflammatory.Macrophages	TTF1	0.244127	3.466533	1.538539	0.127488	-5.25373	0.418132	0.446981
Non.inflammatory.Macrophages	FERMT2	-0.32793	3.451103	-1.53747	0.127751	-5.17484	0.419264	0.447586
Non.inflammatory.Macrophages	L3MBTL3	-0.23831	5.067059	-1.53732	0.127787	-5.41236	0.380005	0.4279
Non.inflammatory.Macrophages	EYA2	-0.74523	1.389743	-1.53695	0.127878	-4.56256	0.475022	0.47412
Non.inflammatory.Macrophages	AP3S2	0.234662	3.43138	1.536675	0.127944	-5.25634	0.420042	0.447992
Non.inflammatory.Macrophages	KDM4D	0.87861	0.328103	1.535022	0.128349	-4.46478	0.496876	0.489024
Non.inflammatory.Macrophages	SERINC5	0.244864	5.287482	1.534866	0.128388	-5.46166	0.376259	0.426063
Non.inflammatory.Macrophages	GNG4	-0.68048	1.462633	-1.53362	0.128694	-4.6415	0.475321	0.474508
Non.inflammatory.Macrophages	TMEM40	-0.65998	0.497552	-1.53345	0.128736	-4.64454	0.497725	0.487466
Non.inflammatory.Macrophages	SRSF10	-0.11788	6.4448	-1.53336	0.128757	-5.76734	0.351216	0.413186

Non.inflammatory.Macrophages	SEC11A	-0.11826	6.406384	-1.53273	0.128912	-5.78256	0.352043	0.413721
Non.inflammatory.Macrophages	NDUFA8	0.164438	5.798653	1.532731	0.128913	-5.75456	0.365379	0.42078
Non.inflammatory.Macrophages	CPLX1	-0.87822	-0.57566	-1.53259	0.128948	-4.55651	0.49782	0.502392
Non.inflammatory.Macrophages	ELL	0.160238	5.024631	1.532496	0.128971	-5.65187	0.383064	0.429958
Non.inflammatory.Macrophages	GM26511	-0.5301	2.075738	-1.53248	0.128974	-4.74603	0.458105	0.466728
Non.inflammatory.Macrophages	INTS6L	0.186811	5.229737	1.53245	0.128982	-5.60542	0.3783	0.42751
Non.inflammatory.Macrophages	DCP2	0.174907	5.103401	1.53224	0.129034	-5.61925	0.381228	0.429073
Non.inflammatory.Macrophages	AOX3	-0.78743	0.998188	-1.53224	0.129035	-4.60566	0.488751	0.480981
Non.inflammatory.Macrophages	RARA	-0.16409	5.561492	-1.53161	0.12919	-5.76325	0.371055	0.423898
Non.inflammatory.Macrophages	MARK2	0.120492	6.879023	1.531341	0.129255	-5.86731	0.342388	0.408757
Non.inflammatory.Macrophages	CAR13	0.411345	1.31675	1.53113	0.129308	-4.8995	0.480108	0.477302
Non.inflammatory.Macrophages	PLEKHG5	0.448164	1.864551	1.530694	0.129415	-4.92956	0.464752	0.47021
Non.inflammatory.Macrophages	FAM25C	-0.68298	1.148741	-1.53061	0.129436	-4.67038	0.485177	0.479692
Non.inflammatory.Macrophages	SKAP1	-0.78491	4.523681	-1.53035	0.1295	-4.78078	0.395663	0.436737
Non.inflammatory.Macrophages	AGMO	-0.25156	3.650343	-1.53018	0.129543	-5.56256	0.417242	0.447493

Non.inflammatory.Macrophages	WNT5B	-0.57777	1.562043	-1.53007	0.129568	-4.63807	0.473353	0.474291
Non.inflammatory.Macrophages	CSTF2	0.250958	4.139659	1.529899	0.129612	-5.25073	0.405019	0.441501
Non.inflammatory.Macrophages	HNRNPH1	-0.14387	6.424984	-1.52979	0.129639	-5.81424	0.352285	0.414316
Non.inflammatory.Macrophages	FBXW11	-0.14724	7.259166	-1.52941	0.129733	-6.02608	0.334879	0.404946
Non.inflammatory.Macrophages	PDCD10	0.113755	6.713222	1.528868	0.129867	-5.86245	0.346448	0.41133
Non.inflammatory.Macrophages	EEF1AKMT	-0.18895	4.588498	-1.52874	0.1299	-5.63251	0.394477	0.436395
Non.inflammatory.Macrophages	GM42556	0.840328	0.793712	1.528607	0.129932	-4.50486	0.494368	0.484992
Non.inflammatory.Macrophages	ILRUN	0.152079	5.988088	1.528474	0.129965	-5.81771	0.362173	0.41975
Non.inflammatory.Macrophages	CLEC2D	0.313262	4.7558	1.528402	0.129983	-5.51856	0.390476	0.434401
Non.inflammatory.Macrophages	LIN52	-0.19467	5.546829	-1.52799	0.130084	-5.60572	0.372161	0.42513
Non.inflammatory.Macrophages	DEPP1	0.662759	1.043364	1.527983	0.130087	-4.68797	0.488896	0.481875
Non.inflammatory.Macrophages	CCDC174	0.162345	5.082834	1.527212	0.130278	-5.54734	0.383216	0.430984
Non.inflammatory.Macrophages	TNFAIP2	0.231503	4.351498	1.527191	0.130283	-5.86305	0.400681	0.439848
Non.inflammatory.Macrophages	ZBTB8A	-0.35881	1.978565	-1.52685	0.130367	-4.9211	0.462734	0.469911
Non.inflammatory.Macrophages	ZEB1	-0.30767	6.848689	-1.52665	0.130418	-5.54149	0.344075	0.410346

Non.inflammatory.Macrophages	MTMR2	0.150188	5.396075	1.526603	0.130429	-5.64317	0.376053	0.427282
Non.inflammatory.Macrophages	GM10552	0.828762	1.54086	1.526464	0.130464	-4.49227	0.475083	0.475664
Non.inflammatory.Macrophages	LRRC58	0.128348	5.945766	1.52594	0.130594	-5.80014	0.36389	0.420999
Non.inflammatory.Macrophages	KLHL9	0.159787	4.65586	1.525728	0.130647	-5.64666	0.393752	0.436393
Non.inflammatory.Macrophages	LIMCH1	-0.84645	0.57747	-1.52524	0.130768	-4.5349	0.499539	0.488934
Non.inflammatory.Macrophages	RAG1	-0.88428	1.672599	-1.52502	0.130823	-4.56614	0.472098	0.474352
Non.inflammatory.Macrophages	DNMT3A	-0.15732	6.044028	-1.52409	0.131054	-5.82287	0.36252	0.420217
Non.inflammatory.Macrophages	UQCR11	0.138546	7.224799	1.524023	0.131072	-6.02345	0.337228	0.406628
Non.inflammatory.Macrophages	PLEKHJ1	-0.12103	5.969804	-1.52361	0.131174	-5.77978	0.364268	0.421148
Non.inflammatory.Macrophages	LSM14B	0.202537	3.604671	1.523292	0.131255	-5.31443	0.420748	0.449931
Non.inflammatory.Macrophages	CELF4	-0.69787	0.6776	-1.52325	0.131265	-4.56074	0.498886	0.488089
Non.inflammatory.Macrophages	SEM1	0.103	9.100384	1.523155	0.131289	-6.28797	0.300626	0.386117
Non.inflammatory.Macrophages	TANC2	-0.21645	5.521032	-1.52314	0.131292	-5.75303	0.374398	0.426566
Non.inflammatory.Macrophages	IPO11	0.211673	4.478076	1.522856	0.131364	-5.40751	0.399093	0.439256
Non.inflammatory.Macrophages	PKIB	-0.259	5.453591	-1.52245	0.131465	-5.90644	0.376227	0.427681

Non.inflammatory.Macrophages	NAGPA	-0.19502	3.354165	-1.52217	0.131535	-5.4185	0.427619	0.453573
Non.inflammatory.Macrophages	CAR1	0.961954	0.491473	1.522009	0.131575	-4.60172	0.502024	0.491208
Non.inflammatory.Macrophages	UBE2Z	0.14131	5.437928	1.521797	0.131628	-5.65662	0.376735	0.428067
Non.inflammatory.Macrophages	TNFRSF1A	-0.16001	5.171275	-1.52162	0.131672	-5.84986	0.382938	0.431301
Non.inflammatory.Macrophages	CHAF1B	-0.27738	3.382455	-1.52088	0.131859	-5.28931	0.427453	0.453636
Non.inflammatory.Macrophages	SLC30A4	-0.61602	1.941057	-1.52026	0.132013	-4.58174	0.466778	0.4725
Non.inflammatory.Macrophages	CEP55	-0.53861	3.678611	-1.52002	0.132074	-5.0324	0.420289	0.45036
Non.inflammatory.Macrophages	MANSC1	-0.74997	0.242039	-1.5192	0.132281	-4.58662	0.503738	0.495767
Non.inflammatory.Macrophages	BCL6B	-0.68776	0.649778	-1.51914	0.132295	-4.57373	0.501619	0.490186
Non.inflammatory.Macrophages	H2-D1	-0.21984	8.452787	-1.51905	0.132318	-6.2395	0.314208	0.394539
Non.inflammatory.Macrophages	MSRA	0.15999	6.629496	1.518171	0.132539	-5.74172	0.351841	0.415308
Non.inflammatory.Macrophages	CEP164	-0.20665	4.052145	-1.518	0.132581	-5.39467	0.411792	0.446275
Non.inflammatory.Macrophages	SIGLECE	0.331205	2.293576	1.517938	0.132598	-5.50045	0.458083	0.468694
Non.inflammatory.Macrophages	FAM169B	-0.6425	3.62896	-1.51742	0.132729	-4.69252	0.422816	0.451741
Non.inflammatory.Macrophages	BET1L	0.197426	3.860623	1.517182	0.132788	-5.41395	0.416978	0.448914

Non.inflammatory.Macrophages	STOML2	-0.18728	4.805582	-1.5168	0.132885	-5.57699	0.39385	0.437475
Non.inflammatory.Macrophages	SDC1	0.650179	2.216388	1.516112	0.133058	-4.78432	0.461171	0.470498
Non.inflammatory.Macrophages	C230066G2	0.707613	0.025289	1.516112	0.133058	-4.605	0.50542	0.500029
Non.inflammatory.Macrophages	RPRD2	0.15946	5.916624	1.515921	0.133107	-5.69753	0.368296	0.424502
Non.inflammatory.Macrophages	RNF216	0.120776	6.677461	1.515692	0.133165	-5.88138	0.351538	0.415653
Non.inflammatory.Macrophages	IFITM1	0.730391	3.489377	1.515608	0.133186	-5.28053	0.426996	0.454265
Non.inflammatory.Macrophages	GTSF2	-0.76805	0.091247	-1.51551	0.13321	-4.59107	0.505427	0.49928
Non.inflammatory.Macrophages	UCP2	0.144126	9.571709	1.51444	0.133481	-6.34318	0.294806	0.383753
Non.inflammatory.Macrophages	4833408A1	0.649398	0.127774	1.514294	0.133518	-4.71086	0.506275	0.499089
Non.inflammatory.Macrophages	AGTRAP	0.227651	4.534904	1.514186	0.133545	-5.63982	0.401375	0.441545
Non.inflammatory.Macrophages	CNPY4	-0.32174	2.433697	-1.51382	0.133639	-5.02279	0.456075	0.468244
Non.inflammatory.Macrophages	HTATIP2	-0.16159	4.480259	-1.51373	0.133662	-5.6523	0.402838	0.442345
Non.inflammatory.Macrophages	RNPC3	0.162912	4.741295	1.513336	0.133761	-5.47997	0.396491	0.439242
Non.inflammatory.Macrophages	DERL1	-0.09718	6.22546	-1.51305	0.133834	-5.88337	0.36213	0.421528
Non.inflammatory.Macrophages	MAJIN	-0.64061	0.644865	-1.51304	0.133835	-4.78282	0.504408	0.492325

Non.inflammatory.Macrophages	GM44702	-0.72653	0.088947	-1.51299	0.13385	-4.65643	0.506444	0.499987
Non.inflammatory.Macrophages	RHOH	0.221199	6.471792	1.512978	0.133852	-5.77563	0.35671	0.418651
Non.inflammatory.Macrophages	ZFP940	-0.6831	0.734633	-1.51242	0.133993	-4.52703	0.502975	0.491404
Non.inflammatory.Macrophages	PDZRN3	-0.72333	0.932125	-1.51226	0.134034	-4.61165	0.499191	0.488784
Non.inflammatory.Macrophages	MINDY1	0.180733	3.987424	1.512222	0.134044	-5.5166	0.415356	0.448982
Non.inflammatory.Macrophages	HEATR9	-0.9024	-0.41964	-1.51186	0.134135	-4.45427	0.506948	0.507564
Non.inflammatory.Macrophages	BCAT2	-0.16537	4.601375	-1.5115	0.134227	-5.61046	0.400446	0.441558
Non.inflammatory.Macrophages	TMEM97	-0.24299	3.649444	-1.51066	0.134441	-5.33037	0.42487	0.45384
Non.inflammatory.Macrophages	PAFAH1B2	0.134806	5.489377	1.510343	0.134522	-5.69969	0.379887	0.43126
Non.inflammatory.Macrophages	KANSL2	0.158233	5.119718	1.510287	0.134536	-5.6304	0.388558	0.435728
Non.inflammatory.Macrophages	MYG1	-0.20465	3.803891	-1.50992	0.134629	-5.30576	0.421142	0.452152
Non.inflammatory.Macrophages	CLK3	-0.1716	4.807812	-1.50949	0.13474	-5.53585	0.396331	0.439784
Non.inflammatory.Macrophages	GCLM	-0.15618	6.090034	-1.50943	0.134754	-5.9475	0.366478	0.424361
Non.inflammatory.Macrophages	RNF43	-0.68191	1.563577	-1.50894	0.134879	-4.69208	0.482702	0.481409
Non.inflammatory.Macrophages	9530068EC	0.170944	4.851397	1.508757	0.134926	-5.66275	0.395563	0.439414

Non.inflammatory.Macrophages	TXNL4B	0.394064	1.740021	1.507755	0.135182	-4.84368	0.478372	0.479615
Non.inflammatory.Macrophages	SAA2	1.418618	-1.02347	1.50756	0.135232	-4.45654	0.50944	0.517865
Non.inflammatory.Macrophages	TRIM24	0.202911	5.087563	1.507453	0.13526	-5.47457	0.390504	0.436965
Non.inflammatory.Macrophages	LZTFL1	0.216367	5.075487	1.507316	0.135295	-5.58138	0.390792	0.437112
Non.inflammatory.Macrophages	CNOT9	0.175496	4.541441	1.507188	0.135327	-5.48701	0.403723	0.44366
Non.inflammatory.Macrophages	ATP6V0D1	-0.09287	7.228201	-1.50706	0.135359	-6.02945	0.342569	0.411717
Non.inflammatory.Macrophages	GGCT	0.276409	3.249782	1.506326	0.135548	-5.37436	0.437173	0.460206
Non.inflammatory.Macrophages	RTL4	-0.92053	0.402884	-1.50602	0.135628	-4.55332	0.51004	0.498172
Non.inflammatory.Macrophages	NRP1	0.240485	4.442319	1.506001	0.135631	-5.7317	0.406646	0.445241
Non.inflammatory.Macrophages	RUVBL1	-0.18025	4.77265	-1.50559	0.135738	-5.51231	0.398746	0.441326
Non.inflammatory.Macrophages	BTRC	-0.19296	5.211768	-1.50538	0.135791	-5.55814	0.388227	0.436085
Non.inflammatory.Macrophages	POU2F1	0.217352	5.39401	1.505222	0.135831	-5.63368	0.383932	0.433942
Non.inflammatory.Macrophages	2510017J1	-0.61896	1.414805	-1.50512	0.135857	-4.71169	0.488661	0.484771
Non.inflammatory.Macrophages	TRUB2	0.215535	4.526877	1.504746	0.135954	-5.38625	0.404817	0.444827
Non.inflammatory.Macrophages	SAAL1	0.321498	3.298168	1.504725	0.135959	-5.11395	0.436198	0.460303

Non.inflammatory.Macrophages	SQLE	-0.60173	1.97366	-1.50463	0.135982	-4.68101	0.472549	0.477605
Non.inflammatory.Macrophages	MAD2L1	-0.29709	4.166093	-1.50413	0.136112	-5.32356	0.414083	0.449566
Non.inflammatory.Macrophages	GM12158	-0.98371	0.549233	-1.50398	0.13615	-4.46774	0.509943	0.497199
Non.inflammatory.Macrophages	TMED10	0.106092	7.769396	1.50376	0.136207	-6.14705	0.332257	0.406758
Non.inflammatory.Macrophages	ZDHHC2	-0.38989	2.276539	-1.50354	0.136263	-5.05051	0.464405	0.474029
Non.inflammatory.Macrophages	PMAIP1	-0.21076	4.983149	-1.50314	0.136367	-5.86596	0.394059	0.439794
Non.inflammatory.Macrophages	TNPO1	0.129617	7.082293	1.503131	0.136369	-5.9449	0.346579	0.414829
Non.inflammatory.Macrophages	FGFR1	0.393087	1.974297	1.503088	0.13638	-5.28588	0.472945	0.478205
Non.inflammatory.Macrophages	ANKLE2	0.165806	5.177792	1.502759	0.136465	-5.6577	0.389407	0.437434
Non.inflammatory.Macrophages	SOS2	0.168596	5.600384	1.502752	0.136467	-5.69389	0.379487	0.432318
Non.inflammatory.Macrophages	GTF2F1	-0.14698	5.030746	-1.5027	0.136479	-5.59897	0.392917	0.439229
Non.inflammatory.Macrophages	9330175E1	0.802987	1.002962	1.502518	0.136527	-4.6257	0.501403	0.491324
Non.inflammatory.Macrophages	RAI1	-0.1771	6.004853	-1.5022	0.136608	-5.68357	0.370364	0.427586
Non.inflammatory.Macrophages	CCNB1IP1	-0.45995	1.863098	-1.50205	0.136648	-4.80401	0.476316	0.479895
Non.inflammatory.Macrophages	SF3A2	-0.18455	5.260106	-1.50157	0.136771	-5.6199	0.387857	0.436899

Non.inflammatory.Macrophages	PCNP	0.115158	6.359657	1.500961	0.136928	-5.83421	0.362959	0.423727
Non.inflammatory.Macrophages	PCOLCE2	-0.39878	1.678726	-1.50006	0.137161	-5.21811	0.483039	0.482997
Non.inflammatory.Macrophages	F2RL1	0.942114	-1.13295	1.49976	0.137239	-4.43882	0.512585	0.522372
Non.inflammatory.Macrophages	ATP6V0A2	0.155077	4.619482	1.499699	0.137255	-5.63993	0.404289	0.445197
Non.inflammatory.Macrophages	SIAH1A	0.196046	5.079563	1.498848	0.137475	-5.48763	0.393547	0.439947
Non.inflammatory.Macrophages	SUSD3	-0.29787	3.170727	-1.4985	0.137567	-5.14432	0.441985	0.464086
Non.inflammatory.Macrophages	CDC42BPG	-0.29207	2.57838	-1.49847	0.137574	-5.31863	0.458112	0.471805
Non.inflammatory.Macrophages	PRORSD1	0.166143	4.586531	1.498352	0.137604	-5.58992	0.405555	0.446194
Non.inflammatory.Macrophages	CCR9	-0.70448	2.410766	-1.49835	0.137604	-4.64444	0.462773	0.474043
Non.inflammatory.Macrophages	ATG2A	-0.16044	5.710857	-1.498	0.137696	-5.84808	0.378664	0.432602
Non.inflammatory.Macrophages	SYTL2	0.733979	1.257049	1.497924	0.137715	-4.69425	0.496052	0.489683
Non.inflammatory.Macrophages	LY6G2	-0.7913	0.952461	-1.49743	0.137844	-4.64946	0.505117	0.49401
Non.inflammatory.Macrophages	NCBP3	0.155249	5.506298	1.497424	0.137845	-5.65633	0.383427	0.435228
Non.inflammatory.Macrophages	ACSM1	-0.80463	1.026687	-1.4974	0.137852	-4.67478	0.502951	0.492993
Non.inflammatory.Macrophages	DOLPP1	-0.33716	2.514642	-1.49739	0.137854	-5.05863	0.459879	0.473017

Non.inflammatory.Macrophages	SLC45A4	-0.20234	3.337998	-1.49728	0.137882	-5.40104	0.437527	0.462334
Non.inflammatory.Macrophages	MAP3K20	0.214777	3.798494	1.496976	0.137961	-5.56977	0.425469	0.456518
Non.inflammatory.Macrophages	SH2B1	0.276132	3.699274	1.496974	0.137962	-5.24782	0.428041	0.457781
Non.inflammatory.Macrophages	PSMA7	0.121893	7.683799	1.49691	0.137978	-6.1524	0.335559	0.409718
Non.inflammatory.Macrophages	SSBP2	0.267985	6.284595	1.496714	0.138029	-5.54083	0.365606	0.426037
Non.inflammatory.Macrophages	CYB5R4	0.143175	5.869945	1.496621	0.138053	-5.77501	0.374999	0.430995
Non.inflammatory.Macrophages	AFF2	-1.00463	0.04795	-1.49612	0.138184	-4.51227	0.513377	0.506933
Non.inflammatory.Macrophages	MGST3	0.258917	3.846236	1.496104	0.138188	-5.48791	0.424417	0.456135
Non.inflammatory.Macrophages	FNIP2	-0.23733	5.343736	-1.49574	0.138283	-6.1004	0.387455	0.437555
Non.inflammatory.Macrophages	G530011O	-0.46252	1.80368	-1.49561	0.138317	-5.0463	0.480273	0.482851
Non.inflammatory.Macrophages	GM44127	-0.70206	-0.52381	-1.49554	0.138335	-4.66549	0.513428	0.515091
Non.inflammatory.Macrophages	EEF1AKNM	0.286068	2.318289	1.495468	0.138353	-5.15928	0.465607	0.476038
Non.inflammatory.Macrophages	FEM1C	-0.14253	7.117684	-1.49524	0.138412	-6.09186	0.347598	0.416515
Non.inflammatory.Macrophages	SLX1B	0.275605	3.074893	1.495176	0.138429	-5.09027	0.444791	0.466128
Non.inflammatory.Macrophages	IL31RA	0.268345	3.109325	1.495027	0.138468	-5.31855	0.443868	0.465785

Non.inflammatory.Macrophages	CD52	0.171909	9.30175	1.494264	0.138667	-6.39687	0.30433	0.392295
Non.inflammatory.Macrophages	GM43062	-0.43612	1.638658	-1.49397	0.138744	-4.75437	0.485705	0.485595
Non.inflammatory.Macrophages	POLD3	0.193871	4.695421	1.493761	0.138798	-5.52651	0.403617	0.446096
Non.inflammatory.Macrophages	BHLHE41	-0.48877	1.739361	-1.49353	0.13886	-4.95556	0.482772	0.484381
Non.inflammatory.Macrophages	DSG2	-0.71895	0.740688	-1.49349	0.138868	-4.62145	0.510163	0.498011
Non.inflammatory.Macrophages	AKR1B3	-0.13805	5.402163	-1.49326	0.13893	-5.79107	0.386585	0.437446
Non.inflammatory.Macrophages	INKA1	0.331642	2.703982	1.493234	0.138936	-5.07037	0.455486	0.471562
Non.inflammatory.Macrophages	TRP53I11	-0.3576	4.565294	-1.49309	0.138973	-5.06249	0.406829	0.447807
Non.inflammatory.Macrophages	2010300C(	0.798993	-0.0882	1.493021	0.138992	-4.49585	0.514106	0.509674
Non.inflammatory.Macrophages	CDC42SE1	-0.13924	6.18973	-1.49281	0.139047	-5.80784	0.368459	0.428043
Non.inflammatory.Macrophages	ABRACL	0.145277	7.550325	1.49195	0.139272	-6.07487	0.339431	0.412516
Non.inflammatory.Macrophages	SLC5A10	-0.65006	-0.1778	-1.49164	0.139353	-4.67721	0.514846	0.511637
Non.inflammatory.Macrophages	DDX3X	-0.1418	7.320412	-1.49156	0.139375	-6.07568	0.344252	0.415342
Non.inflammatory.Macrophages	MTA3	-0.16124	6.109959	-1.49144	0.139406	-5.89054	0.370749	0.429589
Non.inflammatory.Macrophages	SSH2	0.171043	8.396317	1.491384	0.13942	-6.15173	0.322252	0.403126

Non.inflammatory.Macrophages	PRG3	-1.0685	1.171663	-1.49121	0.139466	-4.54693	0.500265	0.492999
Non.inflammatory.Macrophages	RMI1	-0.19406	3.629414	-1.49076	0.139583	-5.37564	0.431423	0.460524
Non.inflammatory.Macrophages	RALGPS2	-0.21999	5.773254	-1.49069	0.139601	-5.58736	0.378594	0.433845
Non.inflammatory.Macrophages	GM36839	0.335113	2.346299	1.490618	0.139621	-5.05622	0.466267	0.477261
Non.inflammatory.Macrophages	AGXT	-0.45757	3.279368	-1.49024	0.139719	-5.20848	0.44084	0.465032
Non.inflammatory.Macrophages	ATP6V0A1	-0.17353	4.202735	-1.49014	0.139747	-5.64349	0.416801	0.453259
Non.inflammatory.Macrophages	IKZF3	-0.39845	5.866917	-1.48981	0.139833	-5.23996	0.376696	0.432904
Non.inflammatory.Macrophages	PARP10	0.277882	3.565506	1.488974	0.140053	-5.38498	0.433966	0.461895
Non.inflammatory.Macrophages	HPSE	0.506966	3.426146	1.488305	0.140229	-4.82303	0.438048	0.463926
Non.inflammatory.Macrophages	TMEM69	0.466706	1.585462	1.4882	0.140256	-4.85371	0.489553	0.488355
Non.inflammatory.Macrophages	SRGAP2	0.148896	6.60483	1.487986	0.140313	-6.01781	0.360906	0.424751
Non.inflammatory.Macrophages	SMIM41	-0.69761	0.89661	-1.48779	0.140365	-4.60879	0.509634	0.497951
Non.inflammatory.Macrophages	TTLL5	-0.19829	4.471737	-1.48764	0.140403	-5.45959	0.411175	0.450829
Non.inflammatory.Macrophages	DPYS	-0.44203	2.829004	-1.48727	0.140502	-5.12305	0.454483	0.47195
Non.inflammatory.Macrophages	CLEC16A	-0.19807	4.763553	-1.48701	0.140571	-5.52814	0.40419	0.447272

Non.inflammatory.Macrophages	COQ2	0.18319	4.467496	1.486347	0.140745	-5.55395	0.411944	0.451102
Non.inflammatory.Macrophages	PRPF18	0.181618	4.616004	1.486127	0.140803	-5.54696	0.408293	0.449297
Non.inflammatory.Macrophages	NUTF2	0.468004	1.951713	1.485969	0.140844	-4.88666	0.479846	0.483903
Non.inflammatory.Macrophages	FAM219B	0.32948	2.367885	1.485377	0.141001	-5.12822	0.468055	0.478439
Non.inflammatory.Macrophages	JAKMIP1	-0.58974	3.981458	-1.48533	0.141013	-4.73976	0.424468	0.457435
Non.inflammatory.Macrophages	MCOLN2	-0.25508	3.161941	-1.48528	0.141025	-5.27473	0.446111	0.467991
Non.inflammatory.Macrophages	PLPP5	0.235627	3.394162	1.485276	0.141028	-5.33017	0.439876	0.464976
Non.inflammatory.Macrophages	STK35	-0.21111	3.447827	-1.48498	0.141105	-5.34517	0.438569	0.464419
Non.inflammatory.Macrophages	NELFCD	-0.22546	4.117826	-1.48474	0.14117	-5.31447	0.421121	0.455912
Non.inflammatory.Macrophages	PPP3CB	0.129692	5.980209	1.484642	0.141195	-5.76264	0.375897	0.432876
Non.inflammatory.Macrophages	GTF3C1	0.179835	4.779184	1.484217	0.141308	-5.55367	0.404713	0.447701
Non.inflammatory.Macrophages	WDFY4	-0.18975	7.251372	-1.48377	0.141426	-6.03803	0.348103	0.418067
Non.inflammatory.Macrophages	RANBP9	0.139904	6.962828	1.483421	0.141519	-5.93349	0.354315	0.421445
Non.inflammatory.Macrophages	CENPN	-0.48121	2.920239	-1.48341	0.141522	-4.96273	0.453327	0.471641
Non.inflammatory.Macrophages	TBC1D12	0.264278	3.768556	1.483355	0.141536	-5.47781	0.430602	0.460639

Non.inflammatory.Macrophages	GRIPAP1	-0.13721	5.594976	-1.48322	0.141572	-5.76984	0.385254	0.437864
Non.inflammatory.Macrophages	FAM189A1	-0.76395	2.327494	-1.48296	0.141642	-4.73314	0.469967	0.479648
Non.inflammatory.Macrophages	CDC6	-0.39517	3.269665	-1.4827	0.14171	-5.10218	0.444029	0.467271
Non.inflammatory.Macrophages	MAP4K1	-0.30671	4.801156	-1.48253	0.141754	-5.02386	0.404565	0.447829
Non.inflammatory.Macrophages	TOP2A	-0.37241	7.615909	-1.4824	0.14179	-5.91629	0.340563	0.414079
Non.inflammatory.Macrophages	ESRRG	-0.67971	1.226258	-1.4822	0.141842	-4.66198	0.502299	0.494667
Non.inflammatory.Macrophages	PAKAP.1	-0.36624	4.53559	-1.48199	0.141898	-5.1939	0.411179	0.451188
Non.inflammatory.Macrophages	PLPP3	-0.39615	4.693864	-1.48189	0.141925	-5.53839	0.407233	0.449204
Non.inflammatory.Macrophages	MAP3K7	0.139351	5.219048	1.481802	0.141948	-5.69628	0.394396	0.442684
Non.inflammatory.Macrophages	UBXN4	-0.0919	6.814787	-1.48121	0.142107	-5.96031	0.358025	0.423586
Non.inflammatory.Macrophages	1700010I1	0.493506	1.215796	1.480988	0.142165	-4.75842	0.503109	0.495099
Non.inflammatory.Macrophages	BFAR	0.137148	4.85458	1.480629	0.14226	-5.67388	0.403744	0.447606
Non.inflammatory.Macrophages	ZFP382	-0.58325	2.008207	-1.48048	0.1423	-4.72832	0.479802	0.484539
Non.inflammatory.Macrophages	PIK3CA	0.148865	6.128996	1.480388	0.142325	-5.83775	0.373507	0.432078
Non.inflammatory.Macrophages	6030443J0	0.541156	0.848488	1.48022	0.142369	-4.74869	0.513177	0.500544

Non.inflammatory.Macrophages	ALDH8A1	-0.55206	2.197617	-1.48016	0.142386	-4.93395	0.474355	0.482148
Non.inflammatory.Macrophages	ZCCHC4	0.30588	3.455439	1.47985	0.142468	-5.18617	0.439737	0.465644
Non.inflammatory.Macrophages	HDAC6	-0.36574	2.206503	-1.47971	0.142506	-5.01171	0.474245	0.482125
Non.inflammatory.Macrophages	STX2	0.178441	3.698442	1.479425	0.142581	-5.47624	0.433354	0.462567
Non.inflammatory.Macrophages	RASA2	0.223922	6.158678	1.479353	0.1426	-5.66043	0.372989	0.431954
Non.inflammatory.Macrophages	ENKD1	-0.56751	1.3576	-1.47834	0.142871	-4.68877	0.499716	0.493963
Non.inflammatory.Macrophages	MICALL2	0.347339	1.195719	1.47833	0.142873	-5.14976	0.504594	0.49619
Non.inflammatory.Macrophages	NFU1	0.188796	4.549473	1.478219	0.142903	-5.60353	0.411953	0.452022
Non.inflammatory.Macrophages	VAPB	0.152667	5.031604	1.478184	0.142912	-5.7711	0.400022	0.445996
Non.inflammatory.Macrophages	MFF	0.132599	5.906654	1.477735	0.143032	-5.80973	0.37942	0.435387
Non.inflammatory.Macrophages	ACAD11	-0.34556	2.331168	-1.47744	0.143111	-4.99993	0.471673	0.480974
Non.inflammatory.Macrophages	FAM32A	0.13032	5.312501	1.476894	0.143257	-5.73939	0.393855	0.442818
Non.inflammatory.Macrophages	TNFAIP3	-0.16299	6.763033	-1.47672	0.143305	-6.10173	0.360439	0.425362
Non.inflammatory.Macrophages	HIST3H2BA	0.589461	0.997637	1.476559	0.143347	-4.63056	0.511487	0.499377
Non.inflammatory.Macrophages	CHMP1A	0.164573	5.305048	1.475805	0.143549	-5.7421	0.394518	0.443201

Non.inflammatory.Macrophages	RSPH1	-0.92166	1.855808	-1.47519	0.143713	-4.62204	0.486743	0.488116
Non.inflammatory.Macrophages	A930014D(	-0.53938	-1.05861	-1.47508	0.143743	-4.64576	0.52198	0.529297
Non.inflammatory.Macrophages	FAM222B	0.199036	5.370089	1.474759	0.143829	-5.68151	0.393291	0.442789
Non.inflammatory.Macrophages	INPP5E	-0.32368	2.260693	-1.47468	0.143851	-5.00355	0.475029	0.482831
Non.inflammatory.Macrophages	ALYREF	-0.16961	8.216984	-1.47459	0.143876	-6.10848	0.330349	0.409069
Non.inflammatory.Macrophages	PLXNA2	-0.59286	2.31233	-1.47448	0.143903	-4.8264	0.473551	0.482141
Non.inflammatory.Macrophages	TMEM242	0.168394	4.292008	1.473911	0.144057	-5.53397	0.42026	0.45653
Non.inflammatory.Macrophages	ITPRIPL1	-0.20969	3.28736	-1.47383	0.144079	-5.39454	0.446702	0.469505
Non.inflammatory.Macrophages	UBE2J2	0.113293	6.638057	1.47365	0.144127	-5.9287	0.364162	0.427726
Non.inflammatory.Macrophages	DDX3Y	2.862134	2.707432	1.473525	0.144161	-5.20962	0.462663	0.477255
Non.inflammatory.Macrophages	GM15247	-0.51559	0.995367	-1.47344	0.144183	-4.96847	0.512887	0.500541
Non.inflammatory.Macrophages	GM13710	0.437494	1.36556	1.473211	0.144245	-5.15338	0.501708	0.495485
Non.inflammatory.Macrophages	YDJC	0.563839	1.21958	1.472805	0.144355	-4.76705	0.506371	0.497644
Non.inflammatory.Macrophages	SAP18	0.112152	7.043708	1.472275	0.144497	-6.02042	0.355658	0.42339
Non.inflammatory.Macrophages	FMNL3	-0.26999	4.258409	-1.47213	0.144536	-5.29291	0.421637	0.457576

Non.inflammatory.Macrophages	STEAP3	-0.35878	1.980258	-1.47207	0.144553	-5.02767	0.484013	0.487517
Non.inflammatory.Macrophages	TRMU	0.407717	1.548223	1.471791	0.144628	-4.82262	0.496787	0.493499
Non.inflammatory.Macrophages	MCTP2	0.403049	6.80258	1.471636	0.14467	-5.22885	0.360972	0.426389
Non.inflammatory.Macrophages	PHRF1	0.143253	5.804424	1.47161	0.144677	-5.74802	0.383713	0.438404
Non.inflammatory.Macrophages	DCP1B	0.281853	2.830948	1.471448	0.14472	-5.21097	0.459824	0.476232
Non.inflammatory.Macrophages	ENC1	-0.2735	3.181392	-1.47082	0.14489	-5.25417	0.450579	0.471971
Non.inflammatory.Macrophages	FNDC5	-0.5473	0.474524	-1.47062	0.144943	-4.83199	0.523528	0.508932
Non.inflammatory.Macrophages	TRAF4	-0.6543	4.196822	-1.46931	0.145297	-4.80845	0.42459	0.459506
Non.inflammatory.Macrophages	ASPM	-0.53281	3.870548	-1.46892	0.145404	-5.1638	0.433096	0.463834
Non.inflammatory.Macrophages	TMC4	-0.40862	1.556285	-1.4688	0.145436	-4.84186	0.498127	0.494674
Non.inflammatory.Macrophages	ABCE1	0.172224	5.007871	1.468739	0.145453	-5.67704	0.404122	0.449379
Non.inflammatory.Macrophages	GM43696	0.433032	2.459243	1.46864	0.145479	-4.847	0.471752	0.482409
Non.inflammatory.Macrophages	MIA3	0.125536	5.849323	1.468615	0.145486	-5.85383	0.383879	0.438974
Non.inflammatory.Macrophages	PHGDH	-0.72183	4.379094	-1.46793	0.145672	-4.77551	0.42033	0.457525
Non.inflammatory.Macrophages	LZTR1	-0.28839	2.612289	-1.46744	0.145805	-5.15574	0.468187	0.48087

Non.inflammatory.Macrophages	TNFRSF23	-0.34661	1.669914	-1.46712	0.145891	-5.12413	0.495688	0.49385
Non.inflammatory.Macrophages	GM17103	-0.51317	1.028835	-1.46701	0.145922	-4.89543	0.515137	0.502783
Non.inflammatory.Macrophages	SHARPIN	0.165519	4.530571	1.466828	0.145971	-5.59874	0.416882	0.456208
Non.inflammatory.Macrophages	CPOX	0.19385	4.570271	1.466475	0.146067	-5.58458	0.415973	0.455769
Non.inflammatory.Macrophages	EPHA1	-0.66536	0.461905	-1.4663	0.146115	-4.69588	0.525821	0.510945
Non.inflammatory.Macrophages	PDE10A	0.481377	2.555984	1.465972	0.146203	-5.04296	0.470051	0.482062
Non.inflammatory.Macrophages	4930414N(	-0.35211	3.331114	-1.46552	0.146327	-5.13433	0.448509	0.471938
Non.inflammatory.Macrophages	LYSMD4	-0.31251	3.330154	-1.46545	0.146347	-5.17258	0.448535	0.47198
Non.inflammatory.Macrophages	NDUFB7	0.135045	6.480862	1.465407	0.146357	-5.94328	0.370152	0.432363
Non.inflammatory.Macrophages	NIPAL3	-0.3102	3.198166	-1.46492	0.14649	-5.18497	0.452137	0.473892
Non.inflammatory.Macrophages	KDM2A	0.094408	7.465818	1.464841	0.146511	-6.06167	0.348468	0.42081
Non.inflammatory.Macrophages	PURG	0.267382	3.260975	1.464778	0.146529	-5.27353	0.45042	0.473065
Non.inflammatory.Macrophages	IPO8	0.171762	4.420007	1.464722	0.146544	-5.53244	0.419797	0.458048
Non.inflammatory.Macrophages	PSD3	0.210476	5.794857	1.464721	0.146544	-5.852	0.386019	0.440848
Non.inflammatory.Macrophages	AEN	0.263288	3.664015	1.464701	0.146549	-5.34653	0.439542	0.467789

Non.inflammatory.Macrophages	SNHG9	-0.37231	4.309776	-1.46465	0.146564	-5.45431	0.422623	0.459474
Non.inflammatory.Macrophages	RBSN	0.228294	2.910659	1.464506	0.146602	-5.27817	0.460078	0.477768
Non.inflammatory.Macrophages	CASC4	-0.54505	2.64714	-1.46431	0.146656	-4.72506	0.467469	0.481311
Non.inflammatory.Macrophages	PCBP1	-0.11173	8.199098	-1.46386	0.146778	-6.21051	0.333132	0.412621
Non.inflammatory.Macrophages	PLEKHB2	0.14654	4.419141	1.463722	0.146816	-5.72665	0.419819	0.458406
Non.inflammatory.Macrophages	TSC22D2	-0.12574	6.616128	-1.46372	0.146818	-5.94804	0.367099	0.431208
Non.inflammatory.Macrophages	GM28403	0.468554	1.458517	1.463579	0.146855	-4.99204	0.50218	0.49783
Non.inflammatory.Macrophages	FBXW8	0.193796	3.868261	1.46351	0.146874	-5.49111	0.434123	0.465556
Non.inflammatory.Macrophages	PHF20	-0.12093	6.472356	-1.46324	0.146947	-5.95989	0.370345	0.433
Non.inflammatory.Macrophages	THRAP3	-0.11423	8.006361	-1.46318	0.146963	-6.12572	0.337098	0.414931
Non.inflammatory.Macrophages	RNF166	0.13359	5.468282	1.463062	0.146997	-5.78198	0.3938	0.445309
Non.inflammatory.Macrophages	TSC22D4	0.133381	6.684783	1.462774	0.147075	-5.96573	0.365558	0.430518
Non.inflammatory.Macrophages	USF3	-0.17603	4.977969	-1.46276	0.147078	-5.67655	0.405764	0.45147
Non.inflammatory.Macrophages	CKLF	-0.16404	4.804772	-1.46276	0.14708	-5.69309	0.410071	0.453652
Non.inflammatory.Macrophages	GAREM1	0.656509	0.757375	1.462746	0.147083	-4.67857	0.521454	0.507707

Non.inflammatory.Macrophages	TBC1D4	-0.22537	4.706501	-1.46271	0.147093	-5.84885	0.412535	0.454894
Non.inflammatory.Macrophages	SOX5OS4	0.992931	-0.23518	1.462468	0.147159	-4.49165	0.525916	0.521987
Non.inflammatory.Macrophages	IFNGR1	-0.12589	7.03854	-1.46167	0.147377	-6.19362	0.358216	0.426576
Non.inflammatory.Macrophages	PTK2	-0.23487	4.164046	-1.46153	0.147414	-5.53236	0.426979	0.462115
Non.inflammatory.Macrophages	CENPC1	0.145438	5.265605	1.461221	0.1475	-5.64693	0.399359	0.448195
Non.inflammatory.Macrophages	MERTK	0.263482	3.774359	1.461094	0.147535	-5.76802	0.437323	0.467191
Non.inflammatory.Macrophages	GFOD1	0.332092	6.621215	1.460972	0.147568	-5.41334	0.367587	0.431639
Non.inflammatory.Macrophages	EXOC5	0.11387	6.376842	1.460675	0.147649	-5.94371	0.373162	0.434753
Non.inflammatory.Macrophages	CSRP2	0.21222	5.20682	1.460528	0.14769	-5.5963	0.40083	0.449202
Non.inflammatory.Macrophages	RGL2	-0.23658	3.300422	-1.46048	0.147702	-5.38513	0.450121	0.473693
Non.inflammatory.Macrophages	TIMELESS	0.371178	3.491901	1.460151	0.147793	-5.1185	0.444925	0.471348
Non.inflammatory.Macrophages	MN1	-0.88135	0.308399	-1.46012	0.147801	-4.53966	0.52673	0.514969
Non.inflammatory.Macrophages	DCLK2	-0.40836	2.72479	-1.46007	0.147815	-4.99201	0.466084	0.481533
Non.inflammatory.Macrophages	STYK1	0.856296	0.538888	1.459239	0.148043	-4.54309	0.526568	0.512085
Non.inflammatory.Macrophages	CTH	0.457883	3.512521	1.459164	0.148064	-5.30577	0.444787	0.471465

Non.inflammatory.Macrophages	CKAP2	-0.42179	3.667324	-1.45912	0.148075	-5.12902	0.440627	0.469456
Non.inflammatory.Macrophages	PTOV1	-0.17702	4.038474	-1.459	0.148109	-5.56949	0.430803	0.464689
Non.inflammatory.Macrophages	ANKHD1	-0.11813	7.647791	-1.4588	0.148163	-6.14287	0.345553	0.420328
Non.inflammatory.Macrophages	FAM3C	0.153151	5.36557	1.45791	0.148408	-5.70145	0.397873	0.448189
Non.inflammatory.Macrophages	THEMIS	-1.02123	1.160853	-1.45767	0.148475	-4.58493	0.51329	0.503858
Non.inflammatory.Macrophages	1700112J1	-0.47952	0.638697	-1.45759	0.148495	-4.95913	0.52594	0.511227
Non.inflammatory.Macrophages	ATL2	0.163502	5.337211	1.457561	0.148504	-5.60946	0.398563	0.448595
Non.inflammatory.Macrophages	CALHM6	0.347902	3.048969	1.457177	0.14861	-5.65131	0.45818	0.478248
Non.inflammatory.Macrophages	DDX39	-0.19427	6.372696	-1.45716	0.148615	-5.8015	0.374195	0.435987
Non.inflammatory.Macrophages	EIF2S3Y	3.213998	1.945318	1.456934	0.148677	-4.89812	0.489835	0.493216
Non.inflammatory.Macrophages	RBFOX2	0.39982	2.758111	1.456682	0.148747	-5.19775	0.466408	0.482193
Non.inflammatory.Macrophages	EXOSC2	-0.289	2.945711	-1.45645	0.148811	-5.12753	0.461144	0.479834
Non.inflammatory.Macrophages	FAM114A2	0.137182	5.166561	1.456356	0.148836	-5.72959	0.402906	0.451084
Non.inflammatory.Macrophages	ABCA8A	-0.63973	1.028779	-1.45621	0.148876	-4.88838	0.517588	0.506139
Non.inflammatory.Macrophages	ECE1	0.176582	5.613294	1.456202	0.148879	-5.99328	0.392063	0.44553

Non.inflammatory.Macrophages	4930523C(	-0.26966	6.126408	-1.45572	0.149011	-5.25713	0.380085	0.439431
Non.inflammatory.Macrophages	SS18L1	0.405067	2.325233	1.455307	0.149126	-5.03861	0.478932	0.488668
Non.inflammatory.Macrophages	CTSB	0.151175	8.190873	1.455152	0.149169	-6.4709	0.334899	0.415127
Non.inflammatory.Macrophages	FOXA3	-0.56756	1.425712	-1.45512	0.149177	-4.77978	0.505584	0.501123
Non.inflammatory.Macrophages	GPHN	0.133083	7.486182	1.455064	0.149193	-6.1064	0.349702	0.42338
Non.inflammatory.Macrophages	DDX1	0.1898	4.964571	1.454901	0.149238	-5.64971	0.408043	0.454182
Non.inflammatory.Macrophages	H2-OB	-0.60732	4.007971	-1.45478	0.149271	-4.9468	0.432518	0.466439
Non.inflammatory.Macrophages	FZD7	0.352031	0.743156	1.454727	0.149286	-5.07995	0.524241	0.510769
Non.inflammatory.Macrophages	CDC37	0.107711	6.605127	1.454507	0.149347	-5.97113	0.369108	0.434002
Non.inflammatory.Macrophages	PIMREG	-0.61868	2.104196	-1.4545	0.149348	-4.87136	0.485359	0.49192
Non.inflammatory.Macrophages	GM29264	0.627455	0.505466	1.454459	0.14936	-4.65383	0.528135	0.514272
Non.inflammatory.Macrophages	ESCO1	0.153813	5.77786	1.454155	0.149444	-5.75233	0.388391	0.444272
Non.inflammatory.Macrophages	CCNI	0.116525	6.783841	1.453995	0.149488	-5.99374	0.365212	0.432068
Non.inflammatory.Macrophages	GM11457	-0.75289	0.10252	-1.45365	0.149585	-4.68524	0.528724	0.520524
Non.inflammatory.Macrophages	CDIPTOS	-0.51836	-0.30875	-1.45345	0.14964	-4.72377	0.528768	0.526523

Non.inflammatory.Macrophages	UBE2M	0.117134	7.20691	1.453318	0.149676	-6.06447	0.35603	0.42723
Non.inflammatory.Macrophages	STK11IP	-0.29458	2.312647	-1.45311	0.149732	-5.0848	0.479691	0.489635
Non.inflammatory.Macrophages	PNRC1	-0.12607	8.470962	-1.45303	0.149757	-6.25061	0.329459	0.412522
Non.inflammatory.Macrophages	YBX3	0.192256	6.314911	1.452688	0.14985	-5.87647	0.376092	0.438148
Non.inflammatory.Macrophages	ADRB1	0.466607	0.735849	1.452663	0.149857	-5.15419	0.524898	0.511717
Non.inflammatory.Macrophages	BRD1	0.146321	5.991255	1.452432	0.149921	-5.81396	0.383678	0.442225
Non.inflammatory.Macrophages	SRSF5	0.107359	6.955445	1.452213	0.149982	-6.04433	0.361735	0.430599
Non.inflammatory.Macrophages	STXBP6	0.507747	2.953526	1.451932	0.15006	-5.14013	0.461796	0.481438
Non.inflammatory.Macrophages	SFXN2	0.209786	3.18033	1.451763	0.150107	-5.4165	0.455497	0.47841
Non.inflammatory.Macrophages	PPP1R7	0.15429	4.409711	1.451654	0.150137	-5.56458	0.422724	0.46234
Non.inflammatory.Macrophages	DTWD2	0.349665	2.777791	1.451508	0.150178	-5.12558	0.466736	0.483839
Non.inflammatory.Macrophages	RUNX1	-0.144	9.201324	-1.45115	0.150276	-6.40468	0.315352	0.404734
Non.inflammatory.Macrophages	HOTAIRM1	0.520395	0.698917	1.450875	0.150353	-4.92538	0.526173	0.512912
Non.inflammatory.Macrophages	BCL2L14	0.714447	0.459361	1.450808	0.150372	-4.82523	0.529392	0.516336
Non.inflammatory.Macrophages	NFE2L2	0.143683	7.355477	1.450667	0.150411	-6.23677	0.353217	0.426208

Non.inflammatory.Macrophages	COQ8B	-0.25034	3.190292	-1.45057	0.150439	-5.39785	0.455426	0.478604
Non.inflammatory.Macrophages	ECI2	0.192751	4.557161	1.449633	0.150699	-5.64046	0.419567	0.461094
Non.inflammatory.Macrophages	GABARAP	0.092871	8.497974	1.449514	0.150732	-6.36854	0.329635	0.413244
Non.inflammatory.Macrophages	ZFP451	0.161976	5.019065	1.449436	0.150754	-5.69935	0.407919	0.45531
Non.inflammatory.Macrophages	RBM14	-0.19512	3.712533	-1.44901	0.150872	-5.41434	0.441682	0.472344
Non.inflammatory.Macrophages	CEP97	-0.43781	2.922474	-1.44901	0.150872	-4.87104	0.463351	0.482843
Non.inflammatory.Macrophages	FRMD4A	-0.23529	3.969412	-1.44891	0.150901	-5.67001	0.434843	0.46902
Non.inflammatory.Macrophages	MDK	-0.46857	1.436872	-1.44878	0.150935	-4.9486	0.506773	0.50325
Non.inflammatory.Macrophages	UBE3C	0.147805	5.784905	1.44878	0.150936	-5.78986	0.389281	0.445904
Non.inflammatory.Macrophages	GRIK5	-0.74226	0.167717	-1.44863	0.150977	-4.66244	0.52994	0.521355
Non.inflammatory.Macrophages	TRIP12	0.102073	7.435654	1.448601	0.150986	-6.14827	0.351848	0.425919
Non.inflammatory.Macrophages	KYNU	0.349457	3.853994	1.448414	0.151038	-5.19804	0.437941	0.470627
Non.inflammatory.Macrophages	B130034C1	0.469034	1.038829	1.448251	0.151084	-4.73992	0.519087	0.509026
Non.inflammatory.Macrophages	1500011B0	0.189328	4.65787	1.447647	0.151253	-5.71614	0.4174	0.460426
Non.inflammatory.Macrophages	TRADD	0.200738	3.982911	1.447521	0.151288	-5.54075	0.434902	0.469159

Non.inflammatory.Macrophages	PIBF1	0.16315	5.059147	1.447375	0.151328	-5.71662	0.407316	0.455355
Non.inflammatory.Macrophages	KIF2A	0.136782	6.338703	1.447043	0.151421	-5.84262	0.376787	0.439517
Non.inflammatory.Macrophages	GM11707	-0.39564	0.863582	-1.44682	0.151482	-5.0435	0.524067	0.511916
Non.inflammatory.Macrophages	USP4	0.107448	5.660629	1.446797	0.15149	-5.82366	0.392742	0.447969
Non.inflammatory.Macrophages	SQSTM1	-0.13579	7.466775	-1.44664	0.151535	-6.22924	0.351633	0.42604
Non.inflammatory.Macrophages	GPC4	-0.58933	1.1587	-1.44619	0.151661	-4.78493	0.516038	0.50798
Non.inflammatory.Macrophages	C1QTNF6	0.537983	1.904338	1.446109	0.151682	-4.92112	0.493426	0.497575
Non.inflammatory.Macrophages	CLEC10A	-0.35844	0.930777	-1.44607	0.151693	-5.29458	0.52281	0.511222
Non.inflammatory.Macrophages	GM12166	-0.57001	0.781167	-1.44601	0.151711	-4.76374	0.525809	0.513374
Non.inflammatory.Macrophages	SLFN8	0.298085	3.326481	1.445755	0.151781	-5.55795	0.452887	0.478325
Non.inflammatory.Macrophages	RABGAP1	0.148256	5.920481	1.445426	0.151874	-5.82631	0.386831	0.445126
Non.inflammatory.Macrophages	LAP3	0.202372	4.901491	1.445226	0.15193	-5.7982	0.411713	0.457957
Non.inflammatory.Macrophages	SLC29A1	-0.18121	5.215066	-1.44456	0.152117	-5.79229	0.40416	0.454282
Non.inflammatory.Macrophages	GAS2L3	-0.19739	4.330262	-1.44445	0.152148	-5.73979	0.426555	0.465615
Non.inflammatory.Macrophages	APOL8	0.273415	2.828251	1.444357	0.152173	-5.29307	0.467261	0.485504

Non.inflammatory.Macrophages	ITGB7	0.390798	4.620993	1.444332	0.15218	-5.20291	0.41907	0.461876
Non.inflammatory.Macrophages	ANKRD49	0.247301	3.032797	1.443946	0.152288	-5.26872	0.461721	0.482964
Non.inflammatory.Macrophages	ARID3B	0.233107	3.836648	1.443583	0.15239	-5.45977	0.439932	0.47241
Non.inflammatory.Macrophages	MAEA	0.144092	5.40999	1.443182	0.152503	-5.72279	0.399746	0.452237
Non.inflammatory.Macrophages	AP2B1	0.111019	6.179239	1.443061	0.152537	-5.94194	0.381382	0.442676
Non.inflammatory.Macrophages	NANOS3	-0.72508	0.427569	-1.44303	0.152544	-4.57677	0.531862	0.519485
Non.inflammatory.Macrophages	MYL6B	-0.61456	0.862198	-1.44279	0.152613	-4.67353	0.525335	0.513365
Non.inflammatory.Macrophages	ALDH1L1	-0.33228	3.098676	-1.44267	0.152646	-5.31057	0.460097	0.482468
Non.inflammatory.Macrophages	EFNB2	0.394376	2.469455	1.442594	0.152668	-5.18452	0.477943	0.491018
Non.inflammatory.Macrophages	GJB2	-0.68388	1.638244	-1.44257	0.152674	-4.71421	0.502493	0.502499
Non.inflammatory.Macrophages	PIGP	-0.18443	4.420664	-1.44166	0.152931	-5.61202	0.424983	0.465342
Non.inflammatory.Macrophages	RETREG2	-0.17316	4.884018	-1.44159	0.15295	-5.69576	0.413153	0.459381
Non.inflammatory.Macrophages	ANAPC13	0.120408	5.65737	1.441516	0.152972	-5.88525	0.394102	0.449605
Non.inflammatory.Macrophages	ACOX2	-0.69189	1.030725	-1.44133	0.153024	-4.73768	0.521622	0.511424
Non.inflammatory.Macrophages	MRPL38	-0.19487	3.863246	-1.44126	0.153044	-5.48131	0.439638	0.472683

Non.inflammatory.Macrophages	SAMHD1	0.214048	7.45997	1.441251	0.153046	-6.21413	0.352913	0.427646
Non.inflammatory.Macrophages	VTI1B	0.107071	5.690918	1.440942	0.153133	-5.87987	0.393342	0.449343
Non.inflammatory.Macrophages	KCTD4	-0.65545	2.200261	-1.44087	0.153155	-4.69304	0.486264	0.495229
Non.inflammatory.Macrophages	ARHGAP5	0.214025	4.809305	1.440767	0.153183	-5.79106	0.41509	0.460549
Non.inflammatory.Macrophages	SF3A3	-0.17299	4.984502	-1.44058	0.153235	-5.66259	0.410714	0.458309
Non.inflammatory.Macrophages	HSD11B1	-0.36766	4.593839	-1.43998	0.153406	-5.21503	0.420836	0.463392
Non.inflammatory.Macrophages	PRKCA	0.19916	7.401898	1.439792	0.153458	-6.03461	0.354435	0.428546
Non.inflammatory.Macrophages	TMEM220	0.517758	0.299081	1.439666	0.153493	-4.84438	0.532731	0.522175
Non.inflammatory.Macrophages	9230111E0	-0.65172	-0.18455	-1.43961	0.15351	-4.72533	0.532731	0.529231
Non.inflammatory.Macrophages	VPS33A	-0.15753	4.291318	-1.43959	0.153514	-5.66356	0.428659	0.467311
Non.inflammatory.Macrophages	GNA15	-0.26986	3.584316	-1.43907	0.153662	-5.40535	0.447795	0.476851
Non.inflammatory.Macrophages	FAM216A	0.360676	2.582038	1.438593	0.153797	-5.00633	0.476051	0.490472
Non.inflammatory.Macrophages	FAAP100	-0.24407	3.097229	-1.43852	0.153816	-5.30861	0.461443	0.483538
Non.inflammatory.Macrophages	WDR83	-0.30777	2.732947	-1.43749	0.15411	-5.12948	0.472441	0.488832
Non.inflammatory.Macrophages	MFSD6	0.466025	4.864614	1.437423	0.154128	-5.07045	0.415072	0.460679

Non.inflammatory.Macrophages	GPAT4	0.160782	4.264538	1.437218	0.154186	-5.54132	0.430576	0.468526
Non.inflammatory.Macrophages	ECD	0.159192	4.899349	1.436712	0.154329	-5.6261	0.414523	0.460537
Non.inflammatory.Macrophages	GTPBP1	0.192579	4.641527	1.436177	0.154481	-5.57783	0.421288	0.464026
Non.inflammatory.Macrophages	RAPH1	0.159901	5.055385	1.436088	0.154506	-5.88742	0.410791	0.458753
Non.inflammatory.Macrophages	FAM83E	-0.547	1.10583	-1.43604	0.154521	-4.776	0.521735	0.512044
Non.inflammatory.Macrophages	MAX	-0.12501	6.499429	-1.43573	0.154607	-5.97879	0.376198	0.4408
Non.inflammatory.Macrophages	GM48960	0.672847	1.466163	1.435535	0.154663	-4.64037	0.510783	0.507138
Non.inflammatory.Macrophages	GM14634	-0.46748	1.834434	-1.43518	0.154764	-4.92886	0.499674	0.502173
Non.inflammatory.Macrophages	MFSD14B	0.133682	5.841678	1.435172	0.154766	-5.8851	0.391751	0.449183
Non.inflammatory.Macrophages	NMRK1	-0.24457	3.751312	-1.4348	0.154871	-5.36577	0.445119	0.47627
Non.inflammatory.Macrophages	YWHAQ	-0.10348	7.746706	-1.43458	0.154934	-6.12607	0.348703	0.426162
Non.inflammatory.Macrophages	FFAR4	0.774714	0.260828	1.434575	0.154936	-4.63839	0.535323	0.524835
Non.inflammatory.Macrophages	BVHT	0.609532	-0.0458	1.434447	0.154972	-4.7994	0.535323	0.52932
Non.inflammatory.Macrophages	HECA	0.140991	6.23169	1.434232	0.155034	-5.89739	0.382688	0.444612
Non.inflammatory.Macrophages	COG3	0.18564	4.292574	1.433929	0.15512	-5.58788	0.430901	0.469322

Non.inflammatory.Macrophages	CACNA1I	0.642401	-0.17458	1.433713	0.155181	-4.56191	0.535634	0.531408
Non.inflammatory.Macrophages	B630019A1	-0.59452	1.596371	-1.4333	0.155299	-4.78651	0.507509	0.506002
Non.inflammatory.Macrophages	XRCC6	-0.30178	5.144906	-1.43325	0.155314	-5.44958	0.409291	0.45843
Non.inflammatory.Macrophages	AGT	-0.43759	4.084746	-1.433	0.155386	-5.46564	0.436658	0.472161
Non.inflammatory.Macrophages	SLC1A2	-0.24473	2.197177	-1.43279	0.155445	-5.45629	0.489546	0.497707
Non.inflammatory.Macrophages	LYST	0.182133	6.729745	1.432658	0.155482	-5.82667	0.371538	0.438783
Non.inflammatory.Macrophages	TRPM4	0.38316	1.109553	1.432614	0.155495	-5.01527	0.522633	0.513062
Non.inflammatory.Macrophages	SSBP3	-0.16474	5.600863	-1.43224	0.155602	-5.74594	0.398283	0.452923
Non.inflammatory.Macrophages	ACP1	0.13954	5.923129	1.431748	0.155742	-5.90376	0.390679	0.449031
Non.inflammatory.Macrophages	CD82	-0.16091	5.476033	-1.43173	0.155748	-5.79075	0.401502	0.454655
Non.inflammatory.Macrophages	PRPF4B	0.128487	6.312389	1.431352	0.155855	-5.94709	0.38165	0.444266
Non.inflammatory.Macrophages	NMRAL1	-0.25902	3.904291	-1.43112	0.15592	-5.42811	0.442119	0.475134
Non.inflammatory.Macrophages	BEND4	-0.28812	3.557561	-1.43079	0.156014	-5.4151	0.451615	0.479881
Non.inflammatory.Macrophages	KLHL22	0.295603	3.001151	1.430621	0.156064	-5.19798	0.467106	0.487452
Non.inflammatory.Macrophages	FKBP15	0.140041	5.239594	1.430513	0.156095	-5.82964	0.407665	0.458024

Non.inflammatory.Macrophages	KPNA3	0.138916	6.307968	1.430461	0.156109	-5.90796	0.381892	0.444612
Non.inflammatory.Macrophages	BC147527	0.61171	1.109158	1.430101	0.156212	-4.73449	0.523725	0.51396
Non.inflammatory.Macrophages	CD14	0.268988	4.260683	1.429437	0.156403	-6.07902	0.433215	0.471114
Non.inflammatory.Macrophages	ZFP831	-0.76438	3.063289	-1.42938	0.15642	-4.66856	0.465878	0.487108
Non.inflammatory.Macrophages	2010009K1	-0.81185	0.112521	-1.42929	0.156446	-4.73497	0.537392	0.528778
Non.inflammatory.Macrophages	COX7B	0.120203	7.597144	1.429152	0.156484	-6.20551	0.353278	0.429446
Non.inflammatory.Macrophages	DPP4	-0.31533	5.100888	-1.42898	0.156533	-5.30807	0.411618	0.460405
Non.inflammatory.Macrophages	ZFR	0.112875	6.532381	1.428598	0.156643	-5.95736	0.377234	0.442581
Non.inflammatory.Macrophages	SLCO1B2	-0.39493	4.25024	-1.42847	0.156679	-5.50316	0.433638	0.471613
Non.inflammatory.Macrophages	WARS	0.236496	3.723103	1.428412	0.156696	-5.46748	0.447757	0.478594
Non.inflammatory.Macrophages	FAH	-0.39335	3.892683	-1.4275	0.156959	-5.44593	0.443771	0.476661
Non.inflammatory.Macrophages	FCGRT	-0.1909	4.823452	-1.42739	0.156989	-5.99177	0.419329	0.464498
Non.inflammatory.Macrophages	PCYOX1	0.154819	4.579765	1.426698	0.157189	-5.79776	0.425893	0.467964
Non.inflammatory.Macrophages	GCA	0.673423	1.099128	1.426595	0.157218	-4.80722	0.525669	0.515562
Non.inflammatory.Macrophages	CDK7	0.19116	4.595584	1.426574	0.157225	-5.60832	0.425483	0.467783

Non.inflammatory.Macrophages	JAK1	0.100456	8.273816	1.426467	0.157255	-6.28948	0.339712	0.422281
Non.inflammatory.Macrophages	SLC25A34	-0.53462	-0.47643	-1.42631	0.157301	-4.8214	0.538677	0.538709
Non.inflammatory.Macrophages	MAP4K3	0.198198	4.283134	1.426004	0.157388	-5.66959	0.433659	0.472117
Non.inflammatory.Macrophages	ACSBG1	0.827505	-0.5064	1.425933	0.157409	-4.56716	0.538677	0.539349
Non.inflammatory.Macrophages	TCF7	-0.79714	2.524319	-1.42591	0.157416	-4.73209	0.482465	0.4958
Non.inflammatory.Macrophages	DDX27	0.166752	4.974515	1.425731	0.157467	-5.66579	0.415802	0.463147
Non.inflammatory.Macrophages	KCNQ1OT1	-0.17437	6.399833	-1.42543	0.157554	-5.98208	0.381231	0.445309
Non.inflammatory.Macrophages	GM29019	-0.58191	0.361473	-1.42518	0.157627	-4.70194	0.538945	0.526829
Non.inflammatory.Macrophages	VPS37A	0.167628	5.2037	1.425079	0.157655	-5.76504	0.410203	0.460489
Non.inflammatory.Macrophages	SERTAD1	-0.17616	5.61417	-1.42495	0.157693	-5.8657	0.400048	0.455304
Non.inflammatory.Macrophages	8-Mar	-0.21932	3.141103	-1.42455	0.157808	-5.56589	0.465097	0.487917
Non.inflammatory.Macrophages	3110001I2	-0.27852	2.677347	-1.42432	0.157874	-5.16911	0.478334	0.494289
Non.inflammatory.Macrophages	UBQLN1	0.122147	5.517917	1.42426	0.157891	-5.85296	0.402468	0.456716
Non.inflammatory.Macrophages	REPS1	0.139036	5.964436	1.424227	0.157901	-5.8323	0.391632	0.451073
Non.inflammatory.Macrophages	TFEB	-0.16016	5.158966	-1.42408	0.157942	-5.76299	0.411386	0.461344

Non.inflammatory.Macrophages	POLR1A	0.179878	4.926327	1.423945	0.157982	-5.74271	0.417266	0.464396
Non.inflammatory.Macrophages	TIMM13	0.122539	6.732513	1.423817	0.158019	-6.06004	0.373644	0.441651
Non.inflammatory.Macrophages	MAPK13	0.62088	0.83712	1.423725	0.158046	-4.78726	0.532921	0.520375
Non.inflammatory.Macrophages	ARHGAP28	0.775471	0.598833	1.423622	0.158075	-4.737	0.537531	0.52385
Non.inflammatory.Macrophages	CEP152	0.249022	3.629164	1.423163	0.158208	-5.31316	0.451799	0.481666
Non.inflammatory.Macrophages	LXN	-0.20948	3.855864	-1.42278	0.158318	-5.46454	0.445712	0.478701
Non.inflammatory.Macrophages	TRIM59	-0.30189	4.368452	-1.42276	0.158324	-5.34026	0.432033	0.47192
Non.inflammatory.Macrophages	SMIM11	-0.14572	4.805042	-1.42255	0.158386	-5.7086	0.420695	0.466219
Non.inflammatory.Macrophages	ABCC10	-0.44713	0.65899	-1.4225	0.1584	-4.85267	0.536992	0.523203
Non.inflammatory.Macrophages	LMBRD2	0.193532	4.481892	1.422363	0.158439	-5.68089	0.429059	0.470445
Non.inflammatory.Macrophages	FANCC	0.221986	4.74282	1.421679	0.158637	-5.58171	0.422592	0.467388
Non.inflammatory.Macrophages	IQCK	-0.66056	0.158994	-1.42156	0.158672	-4.69584	0.539833	0.530969
Non.inflammatory.Macrophages	GAS2L1	0.281297	1.765951	1.421441	0.158706	-5.32113	0.506121	0.507827
Non.inflammatory.Macrophages	A930024E0	-0.78097	1.108621	-1.42142	0.158711	-4.63446	0.526502	0.51719
Non.inflammatory.Macrophages	CAPN5	0.506145	1.546779	1.421269	0.158756	-4.95249	0.512843	0.510986

Non.inflammatory.Macrophages	RASGRP4	0.465715	1.734055	1.421005	0.158833	-4.95781	0.5071	0.508335
Non.inflammatory.Macrophages	HSPH1	-0.29916	4.050366	-1.421	0.158833	-5.54386	0.440792	0.476613
Non.inflammatory.Macrophages	VPS35	0.113689	6.232281	1.420863	0.158874	-6.00539	0.385852	0.448578
Non.inflammatory.Macrophages	D430040D1	-0.70031	-0.34621	-1.4207	0.158922	-4.68893	0.539873	0.538741
Non.inflammatory.Macrophages	CCDC189	-0.80577	0.183787	-1.42051	0.158975	-4.62856	0.539916	0.530942
Non.inflammatory.Macrophages	SMG5	-0.13959	5.55464	-1.42002	0.159118	-5.80067	0.40249	0.457477
Non.inflammatory.Macrophages	TRAF3IP1	0.426391	1.889467	1.419045	0.159402	-5.04787	0.503545	0.507014
Non.inflammatory.Macrophages	VPS26B	0.196411	3.835261	1.418306	0.159617	-5.55059	0.447939	0.480486
Non.inflammatory.Macrophages	UAP1	-0.15837	5.112787	-1.41799	0.159709	-5.81414	0.414416	0.463774
Non.inflammatory.Macrophages	DDA1	0.163436	5.082621	1.417899	0.159735	-5.73909	0.415179	0.464163
Non.inflammatory.Macrophages	GSS	-0.24466	3.399727	-1.41789	0.159738	-5.43129	0.459938	0.486423
Non.inflammatory.Macrophages	GNA12	-0.16556	4.652134	-1.41786	0.159746	-5.75347	0.426221	0.469759
Non.inflammatory.Macrophages	GM16066	-0.41712	2.012511	-1.41784	0.159752	-4.91547	0.500173	0.50556
Non.inflammatory.Macrophages	PODXL	0.702747	0.550401	1.416796	0.160057	-4.72462	0.541512	0.526858
Non.inflammatory.Macrophages	ATP8A1	0.116096	7.601268	1.41654	0.160131	-6.24919	0.356446	0.433078

Non.inflammatory.Macrophages	1810009A1	0.312725	2.243882	1.416539	0.160131	-5.06361	0.494045	0.502719
Non.inflammatory.Macrophages	MAML1	0.163124	5.435368	1.416344	0.160188	-5.75574	0.407038	0.460022
Non.inflammatory.Macrophages	F2RL2	0.806784	0.129948	1.415642	0.160393	-4.63672	0.542963	0.53329
Non.inflammatory.Macrophages	LPAR2	-0.2968	2.187833	-1.41499	0.160585	-5.09148	0.49668	0.503879
Non.inflammatory.Macrophages	CKAP5	0.184262	6.118962	1.414781	0.160645	-5.9035	0.391095	0.451778
Non.inflammatory.Macrophages	FBXL2	0.331569	3.231347	1.414734	0.160659	-5.13033	0.466314	0.489605
Non.inflammatory.Macrophages	LDB2	-0.46957	3.160304	-1.4144	0.160757	-5.12589	0.468326	0.490697
Non.inflammatory.Macrophages	GPR137C	-0.43126	2.390141	-1.41413	0.160835	-4.94629	0.490654	0.501346
Non.inflammatory.Macrophages	PIGM	0.200349	3.53133	1.414115	0.16084	-5.42871	0.457909	0.485679
Non.inflammatory.Macrophages	GM15675	0.422364	2.317552	1.414104	0.160843	-5.08198	0.492809	0.502359
Non.inflammatory.Macrophages	SLC43A2	0.181924	6.179865	1.414102	0.160844	-6.06493	0.38964	0.451159
Non.inflammatory.Macrophages	1110002J0	-0.62019	-0.51304	-1.41392	0.160897	-4.82958	0.543442	0.543516
Non.inflammatory.Macrophages	TAF1D	-0.12678	6.027224	-1.41358	0.160997	-5.91331	0.393338	0.453273
Non.inflammatory.Macrophages	MIR22HG	0.198142	4.089128	1.413569	0.161	-5.76012	0.4427	0.478401
Non.inflammatory.Macrophages	LYRM7	-0.56787	1.247217	-1.4135	0.161021	-4.76764	0.525649	0.517751

Non.inflammatory.Macrophages	MS4A1	-0.56796	3.936889	-1.41328	0.161084	-5.19079	0.446879	0.480531
Non.inflammatory.Macrophages	GM14548	0.456667	1.47249	1.412732	0.161245	-4.99192	0.51905	0.514742
Non.inflammatory.Macrophages	GABARAPL	0.167274	7.762001	1.411961	0.161471	-6.20791	0.354393	0.432314
Non.inflammatory.Macrophages	NKAIN2	-0.65593	1.350359	-1.41158	0.161583	-4.85102	0.523706	0.516928
Non.inflammatory.Macrophages	MRPL48	-0.12153	5.282039	-1.41143	0.161627	-5.81693	0.412697	0.463439
Non.inflammatory.Macrophages	ATP6V1F	0.094772	6.902317	1.411262	0.161677	-6.17927	0.373775	0.443039
Non.inflammatory.Macrophages	TMEM237	0.32903	2.157499	1.410813	0.161809	-5.2276	0.499163	0.505748
Non.inflammatory.Macrophages	EXOSC1	0.193827	4.175365	1.410574	0.161879	-5.48202	0.441731	0.478193
Non.inflammatory.Macrophages	SCMH1	0.184769	5.849321	1.410526	0.161893	-5.82988	0.398856	0.45642
Non.inflammatory.Macrophages	1700021F0	0.214726	3.209032	1.410447	0.161916	-5.45149	0.468422	0.491233
Non.inflammatory.Macrophages	POLK	0.216201	3.481233	1.410193	0.161991	-5.46046	0.460806	0.487582
Non.inflammatory.Macrophages	SRP9	0.09866	7.516452	1.410113	0.162015	-6.17085	0.360176	0.43581
Non.inflammatory.Macrophages	PTPRA	0.106069	6.521638	1.409735	0.162126	-6.00107	0.382985	0.448157
Non.inflammatory.Macrophages	CACFD1	0.221007	3.174111	1.409552	0.16218	-5.36682	0.469666	0.491989
Non.inflammatory.Macrophages	IVD	-0.21175	4.435995	-1.40947	0.162205	-5.50339	0.435013	0.475068

Non.inflammatory.Macrophages	AK5	-0.70201	0.348906	-1.40933	0.162245	-4.69794	0.54541	0.53227
Non.inflammatory.Macrophages	ZFP949	0.376152	2.594224	1.409123	0.162306	-5.02725	0.486501	0.500166
Non.inflammatory.Macrophages	CXCR3	-0.47897	1.731497	-1.40888	0.162377	-5.10823	0.512573	0.512416
Non.inflammatory.Macrophages	UBE2S	-0.18108	7.624597	-1.40812	0.162601	-6.11138	0.358465	0.43523
Non.inflammatory.Macrophages	EXOC3	-0.14245	5.06994	-1.40777	0.162705	-5.81588	0.419226	0.46741
Non.inflammatory.Macrophages	ETFA	-0.14702	6.599323	-1.40773	0.162718	-6.02503	0.381805	0.447926
Non.inflammatory.Macrophages	IKBIP	0.330098	1.938543	1.407518	0.16278	-5.13323	0.506977	0.510019
Non.inflammatory.Macrophages	9930022D1	0.588119	0.319301	1.407218	0.162868	-4.84363	0.546556	0.533562
Non.inflammatory.Macrophages	CCNB2	-0.36354	5.882225	-1.40706	0.162914	-5.76887	0.399118	0.457076
Non.inflammatory.Macrophages	LRAT	-0.61669	-0.03713	-1.40673	0.163014	-4.77627	0.546616	0.538982
Non.inflammatory.Macrophages	CFP	-0.17263	5.518079	-1.40657	0.163061	-6.14565	0.408131	0.461854
Non.inflammatory.Macrophages	NAA40	-0.14728	4.909944	-1.4063	0.163141	-5.70627	0.423564	0.469748
Non.inflammatory.Macrophages	ASS1	0.316772	6.535258	1.406257	0.163153	-5.93647	0.383512	0.448965
Non.inflammatory.Macrophages	MTMR14	0.165674	5.427711	1.406249	0.163155	-5.78823	0.41039	0.463025
Non.inflammatory.Macrophages	FIG4	0.154753	4.603641	1.406203	0.163169	-5.60859	0.431546	0.47377

Non.inflammatory.Macrophages	FAM174A	0.15684	6.050716	1.405794	0.16329	-6.05991	0.395144	0.455236
Non.inflammatory.Macrophages	TNIK	0.455843	4.035038	1.405778	0.163295	-5.13222	0.44684	0.481512
Non.inflammatory.Macrophages	KCNJ2	0.45392	-0.01521	1.405369	0.163416	-4.82158	0.546738	0.539039
Non.inflammatory.Macrophages	XYLT1	0.280958	7.355246	1.405347	0.163422	-5.71075	0.364796	0.439114
Non.inflammatory.Macrophages	FCNA	-0.36746	4.555419	-1.40523	0.163457	-5.95368	0.432912	0.474721
Non.inflammatory.Macrophages	DOCK10	0.133415	8.80921	1.405154	0.163479	-6.39994	0.333643	0.421717
Non.inflammatory.Macrophages	GLTP	-0.10785	6.759436	-1.40513	0.163488	-6.11959	0.378366	0.446475
Non.inflammatory.Macrophages	MORN3	-0.69227	1.323497	-1.40461	0.16364	-4.66021	0.526767	0.519657
Non.inflammatory.Macrophages	POU2AF1	-0.44876	4.320007	-1.40441	0.163699	-5.17625	0.43951	0.47814
Non.inflammatory.Macrophages	PRPS2	0.200248	4.382206	1.404179	0.163769	-5.5142	0.437928	0.477436
Non.inflammatory.Macrophages	GPR65	-0.20193	4.250918	-1.40375	0.163897	-5.93506	0.44165	0.479414
Non.inflammatory.Macrophages	HK1OS	0.576744	0.741866	1.403645	0.163927	-4.79917	0.543306	0.528579
Non.inflammatory.Macrophages	EEF1E1	-0.19274	4.824221	-1.40345	0.163986	-5.70508	0.426499	0.471936
Non.inflammatory.Macrophages	NCAM1	-0.94994	1.275146	-1.40337	0.164009	-4.72944	0.528705	0.520926
Non.inflammatory.Macrophages	1700029H1	-0.35342	1.58753	-1.40297	0.164127	-5.10271	0.519033	0.516471

Non.inflammatory.Macrophages	GM13483	-0.44494	1.85821	-1.40293	0.164139	-4.99583	0.51065	0.512617
Non.inflammatory.Macrophages	PIRA2	-0.1993	3.589246	-1.40277	0.164188	-5.93079	0.459935	0.488587
Non.inflammatory.Macrophages	TNFRSF13C	-0.45507	3.92598	-1.40245	0.164281	-4.97115	0.450769	0.48411
Non.inflammatory.Macrophages	TNFSF13	0.257805	1.863945	1.401698	0.164506	-5.6028	0.511109	0.512854
Non.inflammatory.Macrophages	CTDSP1	-0.15699	5.3236	-1.40159	0.164538	-5.82546	0.41434	0.465818
Non.inflammatory.Macrophages	SAFB2	0.114562	5.993263	1.401458	0.164578	-5.91794	0.397726	0.457213
Non.inflammatory.Macrophages	PIGA	0.377493	1.918295	1.401215	0.16465	-5.03693	0.50944	0.512117
Non.inflammatory.Macrophages	4921516A(	-0.36924	0.76983	-1.40121	0.164652	-5.09258	0.543566	0.528724
Non.inflammatory.Macrophages	BATF3	-0.20192	2.332688	-1.40115	0.164669	-5.80709	0.496871	0.50625
Non.inflammatory.Macrophages	RTN4RL1	-0.42185	2.381813	-1.40081	0.164771	-5.19977	0.4954	0.505645
Non.inflammatory.Macrophages	CD93	0.332359	3.89899	1.400803	0.164773	-5.24738	0.451903	0.484745
Non.inflammatory.Macrophages	D930030I0	-0.58728	0.408475	-1.40075	0.164789	-4.83409	0.548379	0.534175
Non.inflammatory.Macrophages	TTYH2	-0.25425	3.075271	-1.40036	0.164906	-5.5403	0.475213	0.496138
Non.inflammatory.Macrophages	MTFMT	0.242796	2.527907	1.40011	0.16498	-5.20173	0.491208	0.50375
Non.inflammatory.Macrophages	POLR2B	-0.15393	4.911643	-1.40002	0.165008	-5.69612	0.425025	0.471438

Non.inflammatory.Macrophages	CAMK2G	0.180732	4.893224	1.399771	0.165081	-5.70363	0.425502	0.471793
Non.inflammatory.Macrophages	PITPNA	0.086822	7.992513	1.399715	0.165098	-6.31876	0.351973	0.432823
Non.inflammatory.Macrophages	2010315B(	0.282349	2.024895	1.399676	0.165109	-5.19746	0.506343	0.511029
Non.inflammatory.Macrophages	NOLC1	-0.18592	5.264292	-1.39962	0.165127	-5.77893	0.415978	0.466979
Non.inflammatory.Macrophages	HNRNPU	-0.10766	8.26781	-1.39925	0.165237	-6.29619	0.34622	0.429526
Non.inflammatory.Macrophages	6230400D1	-0.3448	2.163361	-1.3991	0.165282	-5.10141	0.502361	0.50907
Non.inflammatory.Macrophages	GM42418	0.265989	11.10924	1.398535	0.16545	-6.81397	0.29094	0.397045
Non.inflammatory.Macrophages	WEE1	-0.27654	3.947727	-1.39827	0.16553	-5.38656	0.451225	0.484634
Non.inflammatory.Macrophages	URB2	0.294921	2.571455	1.398088	0.165584	-5.22046	0.490474	0.503633
Non.inflammatory.Macrophages	SASS6	-0.19933	4.42508	-1.39806	0.165592	-5.65155	0.438311	0.478314
Non.inflammatory.Macrophages	LRPAP1	0.154752	4.499631	1.398036	0.1656	-5.82472	0.436326	0.477322
Non.inflammatory.Macrophages	GM20404	-0.51991	1.661785	-1.39791	0.165639	-4.82616	0.518121	0.516604
Non.inflammatory.Macrophages	ZFP773	-0.69365	0.510873	-1.39736	0.165803	-4.75347	0.54922	0.533626
Non.inflammatory.Macrophages	TMEM214	-0.15219	4.338367	-1.39722	0.165845	-5.67493	0.440897	0.479747
Non.inflammatory.Macrophages	GM15964	-0.41551	0.162356	-1.39717	0.165861	-5.22031	0.549512	0.538829

Non.inflammatory.Macrophages	STK38L	0.225076	4.003603	1.396887	0.165944	-5.38277	0.450049	0.484381
Non.inflammatory.Macrophages	GM43713	-0.51126	2.135076	-1.39679	0.165972	-4.91767	0.503959	0.510246
Non.inflammatory.Macrophages	BRMS1	0.177947	4.236998	1.396303	0.166119	-5.64823	0.443902	0.481463
Non.inflammatory.Macrophages	GM15943	-0.53257	0.207344	-1.39613	0.166172	-4.92166	0.549855	0.53861
Non.inflammatory.Macrophages	TMEM127	0.154906	4.290896	1.396018	0.166205	-5.65408	0.442449	0.480806
Non.inflammatory.Macrophages	YTHDF3	-0.10674	7.017555	-1.39601	0.166208	-6.16496	0.374551	0.445659
Non.inflammatory.Macrophages	GOSR1	0.163143	4.182554	1.395238	0.166439	-5.64899	0.445794	0.482459
Non.inflammatory.Macrophages	NOC2L	0.169308	5.059461	1.395133	0.166471	-5.80329	0.422604	0.470825
Non.inflammatory.Macrophages	ELAVL3	-0.58692	1.03829	-1.39508	0.166487	-4.94466	0.53905	0.526551
Non.inflammatory.Macrophages	ZFP729B	-0.19715	3.639068	-1.39474	0.166588	-5.50379	0.460828	0.489894
Non.inflammatory.Macrophages	GM42658	0.524824	1.544714	1.394684	0.166605	-4.82621	0.522987	0.519284
Non.inflammatory.Macrophages	FAM177A	0.407545	1.731422	1.394469	0.16667	-4.9993	0.517149	0.516609
Non.inflammatory.Macrophages	FAM126A	0.165957	5.549539	1.394461	0.166673	-5.89196	0.410205	0.464537
Non.inflammatory.Macrophages	SLC35A2	-0.24892	3.165869	-1.39432	0.166714	-5.41433	0.474247	0.496451
Non.inflammatory.Macrophages	SLC11A2	-0.21115	3.918763	-1.39389	0.166843	-5.68215	0.453151	0.486351

Non.inflammatory.Macrophages	HIPK2	0.147014	6.550097	1.393844	0.166858	-6.07538	0.385924	0.452012
Non.inflammatory.Macrophages	1700056N1	0.369353	2.033678	1.393816	0.166867	-5.00255	0.507918	0.512562
Non.inflammatory.Macrophages	HIST1H3F	0.841201	0.34671	1.393316	0.167017	-4.66556	0.550909	0.537286
Non.inflammatory.Macrophages	SH3BGRL3	0.123226	9.061448	1.393189	0.167056	-6.4406	0.331017	0.421621
Non.inflammatory.Macrophages	PXDC1	-0.36804	2.191629	-1.39281	0.16717	-5.34599	0.503602	0.510651
Non.inflammatory.Macrophages	DGAT2	-0.2453	3.862389	-1.39249	0.167265	-5.51483	0.455155	0.487603
Non.inflammatory.Macrophages	DMXL1	0.150639	6.604824	1.392298	0.167324	-6.05794	0.385013	0.45184
Non.inflammatory.Macrophages	MREG	-0.73931	3.199109	-1.39227	0.167333	-4.87794	0.473846	0.496772
Non.inflammatory.Macrophages	TARBP2	0.231037	3.242608	1.39222	0.167348	-5.40176	0.472598	0.496171
Non.inflammatory.Macrophages	TTC32	-0.18977	4.101346	-1.39206	0.167397	-5.57782	0.448592	0.484531
Non.inflammatory.Macrophages	CCDC85B	0.1828	3.779227	1.392054	0.167398	-5.53515	0.45746	0.488895
Non.inflammatory.Macrophages	STK17B	0.119713	8.129238	1.391799	0.167475	-6.29047	0.350723	0.433223
Non.inflammatory.Macrophages	OXCT1	-0.12482	5.719852	-1.39148	0.167572	-5.96455	0.406575	0.463372
Non.inflammatory.Macrophages	GAPT	0.774887	1.236779	1.391458	0.167578	-4.65594	0.533557	0.524931
Non.inflammatory.Macrophages	ACACA	0.170686	5.153719	1.391028	0.167708	-5.81713	0.421103	0.470916

Non.inflammatory.Macrophages	GM5431	0.416579	0.742737	1.390825	0.167769	-5.23147	0.547375	0.532475
Non.inflammatory.Macrophages	GM10101	-0.68858	0.672014	-1.39037	0.167907	-4.67157	0.549047	0.533749
Non.inflammatory.Macrophages	NPM3	0.205069	5.097579	1.390249	0.167943	-5.69274	0.422743	0.47196
Non.inflammatory.Macrophages	EAPP	0.115658	5.602282	1.390232	0.167948	-5.89513	0.409915	0.465374
Non.inflammatory.Macrophages	WIPF2	0.126812	5.343948	1.389743	0.168096	-5.80922	0.416565	0.468882
Non.inflammatory.Macrophages	HELZ	0.137514	6.25998	1.38966	0.168122	-5.95396	0.393878	0.457075
Non.inflammatory.Macrophages	SNAPC2	-0.27134	3.015281	-1.38963	0.168132	-5.34414	0.479943	0.500279
Non.inflammatory.Macrophages	ZFP267	0.398854	1.251689	1.389519	0.168164	-4.92675	0.533778	0.525486
Non.inflammatory.Macrophages	NLRC4	0.18166	3.885651	1.388451	0.168488	-5.78108	0.455888	0.488805
Non.inflammatory.Macrophages	RBM15B	-0.1936	4.047089	-1.38838	0.168508	-5.48462	0.451437	0.486635
Non.inflammatory.Macrophages	SIMC1	0.183719	5.702765	1.388354	0.168518	-5.801	0.408092	0.464714
Non.inflammatory.Macrophages	BC005537	0.099612	7.116434	1.388117	0.168589	-6.29162	0.374287	0.446861
Non.inflammatory.Macrophages	MFHAS1	-0.2492	3.729832	-1.38804	0.168612	-5.53529	0.460257	0.491076
Non.inflammatory.Macrophages	GM12764	0.525512	1.453299	1.387565	0.168757	-4.86648	0.528174	0.523453
Non.inflammatory.Macrophages	POLR3GL	0.191355	3.878267	1.387245	0.168854	-5.51371	0.456173	0.489311

Non.inflammatory.Macrophages	GM50431	-0.6636	0.122176	-1.38711	0.168896	-4.75834	0.552865	0.543168
Non.inflammatory.Macrophages	XRN1	0.138077	5.924264	1.386981	0.168934	-5.92493	0.402674	0.46222
Non.inflammatory.Macrophages	RABEP2	-0.2116	4.414258	-1.38691	0.168957	-5.47113	0.441542	0.482065
Non.inflammatory.Macrophages	RMND1	-0.17672	3.79439	-1.38686	0.16897	-5.54332	0.458504	0.490454
Non.inflammatory.Macrophages	PRC1	-0.36508	5.285692	-1.38684	0.168978	-5.67642	0.418697	0.470511
Non.inflammatory.Macrophages	MRPL12	0.16712	5.090142	1.386746	0.169006	-5.81793	0.423723	0.473087
Non.inflammatory.Macrophages	CCR4	-0.87128	-1.08113	-1.38662	0.169043	-4.55844	0.552865	0.561611
Non.inflammatory.Macrophages	CDCA7	-0.44853	3.246784	-1.38661	0.169046	-5.0649	0.473993	0.497994
Non.inflammatory.Macrophages	TSIX	-3.41148	1.906429	-1.3865	0.16908	-4.75335	0.513974	0.516936
Non.inflammatory.Macrophages	CRYBG3	0.17847	4.474287	1.386373	0.169119	-5.76598	0.439932	0.48131
Non.inflammatory.Macrophages	RNF157	-0.23406	5.375061	-1.38614	0.169191	-5.80992	0.416494	0.469463
Non.inflammatory.Macrophages	CRIPT	0.113047	6.218829	1.385921	0.169257	-6.01302	0.395594	0.458578
Non.inflammatory.Macrophages	PLA2G12B	-0.61296	1.0508	-1.3858	0.169293	-4.81734	0.541235	0.529528
Non.inflammatory.Macrophages	ERCC6	0.273438	3.56729	1.385681	0.16933	-5.32165	0.464999	0.493745
Non.inflammatory.Macrophages	PI4KA	0.144496	6.333121	1.385201	0.169476	-5.94726	0.39308	0.457313

Non.inflammatory.Macrophages	MAGI2	-0.61902	0.831391	-1.38495	0.169551	-4.75544	0.547327	0.533051
Non.inflammatory.Macrophages	SPATA2L	0.583744	0.521707	1.384483	0.169695	-4.82946	0.553251	0.537763
Non.inflammatory.Macrophages	CCDC25	0.16571	4.712121	1.384461	0.169702	-5.68806	0.434258	0.478606
Non.inflammatory.Macrophages	ALAD	-0.26482	4.057214	-1.38423	0.169772	-5.49667	0.451997	0.487458
Non.inflammatory.Macrophages	HAAO	-0.3513	3.64437	-1.38378	0.16991	-5.20046	0.463744	0.493365
Non.inflammatory.Macrophages	EML4	0.130998	7.138312	1.383429	0.170016	-6.08498	0.374738	0.447686
Non.inflammatory.Macrophages	D10WSU1(	-0.22375	3.652731	-1.3834	0.170024	-5.52489	0.463596	0.493304
Non.inflammatory.Macrophages	FDX1	-0.20631	4.692703	-1.38279	0.170211	-5.69612	0.435461	0.479471
Non.inflammatory.Macrophages	ARL11	0.286639	1.439587	1.382776	0.170216	-5.27277	0.530245	0.524888
Non.inflammatory.Macrophages	SNAPIN	0.152505	4.083623	1.381949	0.170469	-5.71537	0.452159	0.488039
Non.inflammatory.Macrophages	GM15337	-0.39244	2.140023	-1.38193	0.170474	-5.19593	0.50864	0.515156
Non.inflammatory.Macrophages	LRWD1	-0.18759	3.781422	-1.3819	0.170484	-5.49318	0.460539	0.492174
Non.inflammatory.Macrophages	DCSTAMP	-0.63285	-1.33737	-1.38184	0.170503	-4.61082	0.554882	0.567425
Non.inflammatory.Macrophages	RSAD2	0.293834	4.537164	1.381798	0.170515	-6.10543	0.43985	0.481986
Non.inflammatory.Macrophages	ZFP26	0.193112	3.995742	1.381016	0.170755	-5.49223	0.45511	0.489542

Non.inflammatory.Macrophages	CACNA1E	-0.72543	4.907691	-1.38057	0.170892	-5.13612	0.430771	0.477419
Non.inflammatory.Macrophages	UGP2	0.156025	5.910415	1.380423	0.170937	-5.98931	0.405193	0.464292
Non.inflammatory.Macrophages	DNAJC13	0.136538	6.16837	1.38025	0.170989	-6.00841	0.398877	0.46097
Non.inflammatory.Macrophages	LMAN2	0.108658	6.112382	1.380046	0.171052	-6.01654	0.400297	0.461758
Non.inflammatory.Macrophages	TRIM41	-0.14617	4.185669	-1.37986	0.171111	-5.7177	0.450264	0.487301
Non.inflammatory.Macrophages	HPF1	-0.1996	5.467524	-1.37973	0.171151	-5.78367	0.416419	0.470222
Non.inflammatory.Macrophages	NUP54	-0.14012	4.859149	-1.37937	0.171259	-5.84489	0.43217	0.478345
Non.inflammatory.Macrophages	EXOC3L2	-0.50035	2.269133	-1.37924	0.171301	-5.35274	0.505709	0.514093
Non.inflammatory.Macrophages	INSIG1	-0.16018	5.457849	-1.37921	0.171309	-5.89143	0.416665	0.470438
Non.inflammatory.Macrophages	C130026I2	0.328585	2.944263	1.379207	0.17131	-5.3242	0.485493	0.504529
Non.inflammatory.Macrophages	ESYT1	-0.16391	5.654914	-1.37889	0.171408	-5.83776	0.411817	0.467999
Non.inflammatory.Macrophages	COLEC11	-0.39712	1.589372	-1.37862	0.17149	-5.07527	0.527142	0.524162
Non.inflammatory.Macrophages	BAZ1B	0.134339	6.23949	1.378423	0.171551	-6.00859	0.397494	0.460653
Non.inflammatory.Macrophages	DAGLB	-0.15197	4.366228	-1.37806	0.171663	-5.91131	0.445836	0.48545
Non.inflammatory.Macrophages	MTFP1	-0.66098	0.572509	-1.37745	0.17185	-4.74182	0.555911	0.539725

Non.inflammatory.Macrophages	SMTN	-0.43026	2.149503	-1.37723	0.171918	-4.97817	0.510443	0.516668
Non.inflammatory.Macrophages	ACTG1	0.142179	11.44474	1.376953	0.172003	-6.78614	0.289219	0.398928
Non.inflammatory.Macrophages	CHML	0.401155	1.690018	1.376556	0.172126	-5.0295	0.525114	0.523415
Non.inflammatory.Macrophages	PPP1CB	0.132581	7.803763	1.376326	0.172197	-6.22331	0.361905	0.441605
Non.inflammatory.Macrophages	PIKFYVE	0.153179	4.426045	1.376325	0.172197	-5.82184	0.444956	0.485144
Non.inflammatory.Macrophages	ITPA	0.163137	4.105721	1.376122	0.17226	-5.60943	0.45377	0.489538
Non.inflammatory.Macrophages	PPP1R16A	-0.27594	2.885752	-1.37589	0.172332	-5.27594	0.488717	0.506574
Non.inflammatory.Macrophages	AFG3L2	0.134001	4.593409	1.375359	0.172495	-5.72981	0.44089	0.483255
Non.inflammatory.Macrophages	GM44987	-0.631	-0.34886	-1.37519	0.172548	-4.6977	0.558141	0.554451
Non.inflammatory.Macrophages	COLGALT1	0.121422	5.889717	1.374677	0.172706	-6.05569	0.407653	0.466326
Non.inflammatory.Macrophages	ABHD12	0.121769	5.584506	1.374441	0.172779	-6.0837	0.415405	0.470428
Non.inflammatory.Macrophages	TOMM5	0.161609	5.458336	1.374174	0.172861	-5.87966	0.418677	0.47209
Non.inflammatory.Macrophages	NAA38	0.141678	5.467647	1.374034	0.172905	-5.94279	0.418439	0.472028
Non.inflammatory.Macrophages	IL1R2	0.563642	3.320344	1.373963	0.172926	-5.23101	0.476863	0.501102
Non.inflammatory.Macrophages	BCL3	0.18914	5.236561	1.373689	0.173011	-5.8627	0.424492	0.475182

Non.inflammatory.Macrophages	PPP5C	-0.16662	4.346979	-1.37256	0.173362	-5.66819	0.448946	0.487525
Non.inflammatory.Macrophages	HADH	-0.19754	5.394105	-1.37215	0.173489	-5.85714	0.421333	0.473643
Non.inflammatory.Macrophages	VAMP2	0.156444	4.421644	1.372088	0.173507	-5.6247	0.447072	0.486641
Non.inflammatory.Macrophages	TOP2B	0.150942	6.593853	1.371769	0.173606	-6.02055	0.391541	0.458211
Non.inflammatory.Macrophages	RMND5B	-0.19661	4.49308	-1.37153	0.17368	-5.50301	0.445152	0.485894
Non.inflammatory.Macrophages	1600022D1	-0.44227	-0.42787	-1.37132	0.173747	-4.86622	0.560066	0.557123
Non.inflammatory.Macrophages	AP5M1	0.2075	3.584616	1.371313	0.173748	-5.51071	0.47043	0.498336
Non.inflammatory.Macrophages	ARMC10	0.214802	3.275459	1.371284	0.173757	-5.46039	0.479334	0.502642
Non.inflammatory.Macrophages	GDAP2	0.160153	4.778409	1.371262	0.173764	-5.74233	0.437481	0.482049
Non.inflammatory.Macrophages	NINL	0.533056	0.999292	1.370963	0.173857	-4.86886	0.549876	0.53557
Non.inflammatory.Macrophages	SUPV3L1	-0.19118	3.910174	-1.37095	0.173862	-5.66867	0.461262	0.493922
Non.inflammatory.Macrophages	LMAN1L	-0.38791	1.485183	-1.37067	0.173947	-5.17541	0.534098	0.528525
Non.inflammatory.Macrophages	RCSD1	0.169204	7.091061	1.370657	0.173952	-6.04096	0.37984	0.452182
Non.inflammatory.Macrophages	SESN3	0.240815	4.83647	1.370489	0.174004	-5.48023	0.436019	0.481562
Non.inflammatory.Macrophages	SLC35A4	0.203872	3.561213	1.370051	0.17414	-5.55435	0.471445	0.499167

Non.inflammatory.Macrophages	RTL5	0.373568	0.491888	1.369727	0.174241	-5.06244	0.560644	0.543808
Non.inflammatory.Macrophages	LPCAT3	0.147188	5.162712	1.369553	0.174295	-5.91609	0.427819	0.477574
Non.inflammatory.Macrophages	SMAD7	-0.18177	5.164939	-1.36944	0.174329	-5.81371	0.427761	0.477545
Non.inflammatory.Macrophages	HIST1H2BM	-0.67227	2.139918	-1.36909	0.174438	-4.8198	0.514163	0.519594
Non.inflammatory.Macrophages	ANGPTL7	-0.64477	-0.05517	-1.36855	0.174608	-4.81316	0.561318	0.552541
Non.inflammatory.Macrophages	LZTS3	-0.58143	0.323606	-1.36808	0.174754	-4.78094	0.561654	0.54696
Non.inflammatory.Macrophages	VIM	0.197932	8.109275	1.367486	0.174939	-6.38527	0.358096	0.440565
Non.inflammatory.Macrophages	CD177	0.849219	0.17299	1.367218	0.175023	-4.6644	0.56225	0.549477
Non.inflammatory.Macrophages	RTTN	-0.24576	3.609694	-1.36705	0.175075	-5.46713	0.471573	0.499433
Non.inflammatory.Macrophages	RAD18	0.258282	4.103468	1.366721	0.175178	-5.48107	0.457696	0.492761
Non.inflammatory.Macrophages	STAP1	0.218676	4.891894	1.366714	0.17518	-5.61173	0.436241	0.482064
Non.inflammatory.Macrophages	GM527	-0.75217	0.481195	-1.36653	0.175239	-4.72886	0.562406	0.545121
Non.inflammatory.Macrophages	HNRNPA2B	-0.11084	9.287708	-1.36619	0.175344	-6.4543	0.333259	0.426711
Non.inflammatory.Macrophages	FCRLS	0.90978	-1.03209	1.366045	0.17539	-4.82402	0.562406	0.568593
Non.inflammatory.Macrophages	IGFBP1	0.510237	4.147259	1.36598	0.17541	-5.57068	0.45652	0.492376

Non.inflammatory.Macrophages	SEL1L	0.131777	5.161227	1.365882	0.17544	-5.90619	0.429171	0.478675
Non.inflammatory.Macrophages	BHLHE40	0.190134	6.323106	1.365866	0.175446	-6.14487	0.399749	0.463439
Non.inflammatory.Macrophages	TOR2A	-0.19136	3.670287	-1.36518	0.175661	-5.53949	0.470411	0.499159
Non.inflammatory.Macrophages	CD300C2	-0.17242	4.335062	-1.36483	0.175771	-6.13773	0.451956	0.490107
Non.inflammatory.Macrophages	NNT	0.367503	2.482711	1.364636	0.175831	-5.01048	0.50569	0.516139
Non.inflammatory.Macrophages	LMAN1	0.171235	4.627609	1.364553	0.175857	-5.78838	0.443983	0.486288
Non.inflammatory.Macrophages	SLC27A1	0.263112	2.972091	1.364371	0.175914	-5.53459	0.49099	0.509237
Non.inflammatory.Macrophages	MRPL13	0.159177	4.834872	1.364127	0.17599	-5.76214	0.438535	0.483583
Non.inflammatory.Macrophages	C330018D2	-0.30614	2.325358	-1.36399	0.176034	-5.17851	0.51067	0.518593
Non.inflammatory.Macrophages	YPEL5	0.141463	6.583092	1.363808	0.17609	-6.11503	0.394135	0.460646
Non.inflammatory.Macrophages	ASF1B	-0.32975	4.954931	-1.36359	0.17616	-5.5878	0.435405	0.482043
Non.inflammatory.Macrophages	VPS13A	-0.12724	6.447058	-1.36351	0.176184	-5.95894	0.397457	0.462428
Non.inflammatory.Macrophages	HACD2	0.138176	5.732831	1.363305	0.176248	-5.86473	0.41526	0.471754
Non.inflammatory.Macrophages	GLT1D1	0.716954	0.611079	1.363076	0.17632	-4.71243	0.56187	0.544095
Non.inflammatory.Macrophages	H2AFZ	-0.16393	10.35837	-1.36246	0.176513	-6.62445	0.312854	0.415093

Non.inflammatory.Macrophages	PSMB5	0.132418	6.258665	1.362425	0.176524	-6.06719	0.402392	0.465253
Non.inflammatory.Macrophages	ZFP58	-0.53958	1.194809	-1.36239	0.176535	-4.84131	0.547136	0.535666
Non.inflammatory.Macrophages	GAA	-0.2081	3.191942	-1.36193	0.17668	-5.57298	0.485344	0.506963
Non.inflammatory.Macrophages	AKR1D1	-0.48725	2.587501	-1.36172	0.176746	-5.15638	0.503491	0.51556
Non.inflammatory.Macrophages	CAMK2N1	-0.56136	1.714702	-1.36147	0.176825	-5.02279	0.530808	0.528306
Non.inflammatory.Macrophages	IL6RA	-0.15748	5.504339	-1.361	0.176973	-6.10303	0.42198	0.475632
Non.inflammatory.Macrophages	RTN3	0.122812	8.060675	1.360937	0.176992	-6.33377	0.360811	0.442968
Non.inflammatory.Macrophages	IRGC1	0.801881	0.115465	1.360485	0.177135	-4.62244	0.564981	0.55274
Non.inflammatory.Macrophages	KIF11	-0.4109	5.441855	-1.36038	0.177167	-5.6157	0.423812	0.476647
Non.inflammatory.Macrophages	PIDD1	0.620586	0.824038	1.359836	0.177339	-4.748	0.559274	0.542137
Non.inflammatory.Macrophages	SLC35E3	-0.35873	1.909396	-1.35936	0.177491	-5.17051	0.525736	0.526205
Non.inflammatory.Macrophages	MAST3	0.20884	4.169613	1.359352	0.177492	-5.64066	0.458503	0.494136
Non.inflammatory.Macrophages	LRR1	-0.57923	1.435876	-1.35919	0.177543	-4.79314	0.54095	0.533209
Non.inflammatory.Macrophages	TUT4	0.138852	7.475638	1.358163	0.177868	-6.23977	0.375232	0.451013
Non.inflammatory.Macrophages	ZFP207	0.097616	6.934778	1.357837	0.177971	-6.11921	0.388017	0.457966

Non.inflammatory.Macrophages	CCL21A	1.581565	0.049265	1.357706	0.178012	-4.83312	0.566741	0.554681
Non.inflammatory.Macrophages	CTSW	-0.7577	2.120663	-1.35748	0.178085	-4.80543	0.520207	0.523662
Non.inflammatory.Macrophages	OSBPL1A	-0.20056	3.288159	-1.35724	0.17816	-5.61076	0.484789	0.507
Non.inflammatory.Macrophages	BIRC5	-0.34089	5.35755	-1.35711	0.178201	-5.80492	0.427429	0.478649
Non.inflammatory.Macrophages	4-Sep	-0.43102	1.735848	-1.35691	0.178265	-5.09662	0.532439	0.529399
Non.inflammatory.Macrophages	PER3	-0.42137	1.517982	-1.35686	0.17828	-5.02449	0.53946	0.532615
Non.inflammatory.Macrophages	GTF2A1	-0.14125	5.65525	-1.35678	0.178306	-5.99247	0.419726	0.474711
Non.inflammatory.Macrophages	ZFP408	-0.22308	3.716601	-1.35647	0.178403	-5.46013	0.472497	0.501163
Non.inflammatory.Macrophages	CRYZL2	-0.23453	2.609637	-1.35604	0.17854	-5.37741	0.505537	0.516954
Non.inflammatory.Macrophages	FZD6	-0.86573	0.693274	-1.35579	0.17862	-4.65621	0.564147	0.545274
Non.inflammatory.Macrophages	GNL1	-0.16683	4.299889	-1.35493	0.178892	-5.68895	0.456802	0.493602
Non.inflammatory.Macrophages	ALS2CL	-0.62809	0.148166	-1.35483	0.178925	-4.80624	0.568004	0.554003
Non.inflammatory.Macrophages	GIMAP9	-0.45028	3.560572	-1.35468	0.17897	-5.00313	0.477794	0.503944
Non.inflammatory.Macrophages	SSH1	0.176485	3.775324	1.354459	0.179041	-5.65973	0.471605	0.501017
Non.inflammatory.Macrophages	RNF150	-0.22442	2.909798	-1.35444	0.179049	-5.97721	0.497013	0.51323

Non.inflammatory.Macrophages	THRB	-0.28305	3.815098	-1.35426	0.179104	-5.72275	0.470467	0.500462
Non.inflammatory.Macrophages	TRIM3	-0.25538	2.274801	-1.35424	0.17911	-5.35336	0.516453	0.522375
Non.inflammatory.Macrophages	IDI1	-0.24309	3.90584	-1.3542	0.179122	-5.4026	0.467881	0.4992
Non.inflammatory.Macrophages	SMIM8	0.17362	4.587829	1.353908	0.179217	-5.7203	0.448901	0.489941
Non.inflammatory.Macrophages	PSIP1	-0.14319	5.994205	-1.35364	0.179302	-5.87427	0.41197	0.471252
Non.inflammatory.Macrophages	ENPP5	-0.46729	0.877362	-1.35359	0.179318	-5.07589	0.560755	0.543348
Non.inflammatory.Macrophages	HK2	0.171635	5.335441	1.353527	0.179338	-6.12392	0.428893	0.480013
Non.inflammatory.Macrophages	TSPAN14	0.132682	6.835826	1.35346	0.179359	-6.09684	0.39128	0.4604
Non.inflammatory.Macrophages	BATF	-0.16844	4.183315	-1.35337	0.179389	-5.79574	0.460092	0.495735
Non.inflammatory.Macrophages	HAVCR2	0.447045	2.301711	1.353095	0.179475	-5.27603	0.515686	0.522507
Non.inflammatory.Macrophages	TRNAU1AF	0.175305	4.126118	1.353	0.179505	-5.75799	0.461721	0.496654
Non.inflammatory.Macrophages	LARS	0.169867	4.738364	1.352939	0.179525	-5.78066	0.444827	0.488273
Non.inflammatory.Macrophages	ABCG3	0.194524	4.394356	1.352769	0.179579	-5.93916	0.454277	0.493037
Non.inflammatory.Macrophages	ZNRF3	-0.18943	5.517512	-1.35235	0.179713	-5.9045	0.424327	0.477993
Non.inflammatory.Macrophages	PRKCSH	0.130328	4.862373	1.352304	0.179727	-5.84754	0.441637	0.486811

Non.inflammatory.Macrophages	GM10382	-0.54522	0.439288	-1.35221	0.179758	-4.89123	0.568288	0.550534
Non.inflammatory.Macrophages	DIPK2A	0.173196	4.112992	1.3518	0.179887	-5.69822	0.462382	0.497212
Non.inflammatory.Macrophages	SUFU	0.163762	5.025085	1.351791	0.179891	-5.72559	0.437395	0.484747
Non.inflammatory.Macrophages	CLINT1	0.108195	8.205941	1.351455	0.179997	-6.28612	0.360113	0.443718
Non.inflammatory.Macrophages	APPL1	0.148702	5.974209	1.351146	0.180096	-5.99585	0.413027	0.472242
Non.inflammatory.Macrophages	GSDME	-0.24742	4.386903	-1.35094	0.180162	-5.57489	0.455037	0.49362
Non.inflammatory.Macrophages	COLEC12	-0.26154	2.890318	-1.3509	0.180176	-5.79746	0.498308	0.514616
Non.inflammatory.Macrophages	DOK2	0.205517	2.811456	1.349946	0.180479	-5.66013	0.501417	0.516117
Non.inflammatory.Macrophages	ABCC1	0.174451	4.467713	1.349676	0.180566	-5.87737	0.453469	0.492894
Non.inflammatory.Macrophages	POLR2F	0.121116	5.381223	1.349652	0.180573	-5.95011	0.428899	0.480517
Non.inflammatory.Macrophages	BRD4	0.078705	8.256086	1.349327	0.180677	-6.30753	0.359639	0.443609
Non.inflammatory.Macrophages	FRRS1	0.138814	4.851486	1.349275	0.180694	-6.00064	0.442989	0.487716
Non.inflammatory.Macrophages	STMN1	-0.30748	7.897729	-1.34908	0.180758	-6.19663	0.367639	0.448103
Non.inflammatory.Macrophages	ARMC7	-0.18686	3.924467	-1.34902	0.180776	-5.69394	0.468705	0.500526
Non.inflammatory.Macrophages	YEATS2	0.162961	4.376137	1.348935	0.180803	-5.69023	0.456004	0.494299

Non.inflammatory.Macrophages	ZFP820	-0.63263	-0.04486	-1.34872	0.180873	-4.76413	0.569649	0.559081
Non.inflammatory.Macrophages	ACVRL1	-0.20236	2.619629	-1.34869	0.18088	-5.82377	0.507276	0.519185
Non.inflammatory.Macrophages	KAZN	-0.62377	0.348768	-1.34862	0.180904	-4.84867	0.569649	0.553054
Non.inflammatory.Macrophages	TSPAN6	-0.70297	1.103606	-1.34826	0.181019	-4.79472	0.555966	0.54176
Non.inflammatory.Macrophages	FOXK2	-0.1443	4.920069	-1.34814	0.181059	-5.79456	0.441312	0.487233
Non.inflammatory.Macrophages	A230072E1	0.80911	0.278064	1.347581	0.181236	-4.67068	0.570298	0.554588
Non.inflammatory.Macrophages	ENO1B	0.40368	1.327345	1.347433	0.181284	-5.05021	0.548981	0.538714
Non.inflammatory.Macrophages	RARB	0.590413	1.586463	1.347194	0.18136	-5.03854	0.540536	0.534889
Non.inflammatory.Macrophages	HMBS	0.202279	4.451319	1.346998	0.181423	-5.77048	0.454481	0.494003
Non.inflammatory.Macrophages	HIVEP1	0.146484	6.40877	1.346443	0.181602	-6.06715	0.403276	0.468055
Non.inflammatory.Macrophages	AP1M1	0.154464	4.979621	1.34643	0.181606	-5.74713	0.440082	0.487053
Non.inflammatory.Macrophages	LUC7L	0.12257	5.622585	1.346263	0.181659	-5.90542	0.423142	0.478481
Non.inflammatory.Macrophages	GM35769	0.444318	1.096204	1.346051	0.181727	-5.0128	0.556681	0.542771
Non.inflammatory.Macrophages	CUL1	0.101621	6.562305	1.345954	0.181759	-6.11376	0.399502	0.466199
Non.inflammatory.Macrophages	ATP2A2	-0.10267	6.456942	-1.34584	0.181795	-6.11189	0.402088	0.467569

Non.inflammatory.Macrophages	HNRNPR	0.111494	6.29021	1.345808	0.181805	-5.98878	0.406213	0.469744
Non.inflammatory.Macrophages	PPP1R15A	-0.17837	7.089434	-1.34581	0.181806	-6.28913	0.386805	0.459408
Non.inflammatory.Macrophages	GM16341	-0.69513	0.863635	-1.34553	0.181896	-4.71155	0.56332	0.54643
Non.inflammatory.Macrophages	WNT2	0.634533	1.077377	1.345498	0.181905	-4.92066	0.55731	0.543195
Non.inflammatory.Macrophages	RAPGEF3	-0.65294	1.358645	-1.34531	0.181966	-4.77704	0.547985	0.539064
Non.inflammatory.Macrophages	ARMCX1	-0.73791	0.550831	-1.3452	0.182	-4.745	0.569467	0.551327
Non.inflammatory.Macrophages	1600012H(	0.200721	2.771016	1.345049	0.182049	-5.4598	0.503274	0.518332
Non.inflammatory.Macrophages	PECR	-0.42549	2.35098	-1.34504	0.182052	-5.20963	0.516209	0.524423
Non.inflammatory.Macrophages	HEATR6	0.164914	4.812778	1.344982	0.182071	-5.71177	0.444582	0.4897
Non.inflammatory.Macrophages	DNAJC8	0.084319	6.901934	1.344965	0.182077	-6.16489	0.391276	0.46203
Non.inflammatory.Macrophages	MBTPS2	0.188145	3.917498	1.344904	0.182096	-5.61011	0.469481	0.502057
Non.inflammatory.Macrophages	EPOR	-0.65675	-0.66556	-1.34441	0.182256	-4.83208	0.570457	0.570378
Non.inflammatory.Macrophages	ZFYVE19	0.223784	3.132938	1.34431	0.182287	-5.43019	0.492461	0.513251
Non.inflammatory.Macrophages	DPYSL3	0.642349	0.753878	1.3443	0.18229	-4.79985	0.565796	0.548346
Non.inflammatory.Macrophages	IGKV1-35	-0.90099	-0.53717	-1.34428	0.182298	-4.58473	0.570457	0.568358

Non.inflammatory.Macrophages	NOP2	-0.18736	3.635097	-1.34388	0.182425	-5.57124	0.477811	0.506321
Non.inflammatory.Macrophages	ZSCAN18	-0.68813	0.363679	-1.34387	0.182428	-4.81377	0.5706	0.55453
Non.inflammatory.Macrophages	RENBP	-0.15253	4.474184	-1.34353	0.18254	-5.93243	0.454219	0.49478
Non.inflammatory.Macrophages	C4BP	-0.52332	1.968581	-1.34322	0.182639	-5.03029	0.528845	0.530594
Non.inflammatory.Macrophages	TXNDC5	-0.16326	5.179694	-1.34293	0.182733	-5.89548	0.435358	0.485397
Non.inflammatory.Macrophages	TIFAB	0.214773	2.73524	1.342131	0.18299	-5.82377	0.505574	0.51983
Non.inflammatory.Macrophages	MBOAT1	0.712894	0.682144	1.342108	0.182997	-4.80673	0.568608	0.55036
Non.inflammatory.Macrophages	ZFP869	-0.19966	4.026546	-1.34165	0.183146	-5.63816	0.467771	0.501736
Non.inflammatory.Macrophages	TGOLN1	-0.13211	5.839155	-1.34091	0.183385	-6.05599	0.419218	0.477443
Non.inflammatory.Macrophages	D930016D(	0.241932	2.876865	1.340851	0.183404	-5.38671	0.502026	0.518513
Non.inflammatory.Macrophages	VPREB1	-0.86652	2.621584	-1.3406	0.183484	-4.94087	0.509941	0.522295
Non.inflammatory.Macrophages	TOMM40	0.149195	5.225725	1.340023	0.183672	-5.84272	0.435546	0.485835
Non.inflammatory.Macrophages	MBD6	-0.18844	3.632499	-1.33988	0.183717	-5.61516	0.479906	0.507912
Non.inflammatory.Macrophages	HIST1H2AF	0.38817	7.597755	1.339751	0.18376	-6.1377	0.37668	0.454907
Non.inflammatory.Macrophages	FNDC3B	0.1242	6.549271	1.339678	0.183783	-6.23827	0.401686	0.468407

Non.inflammatory.Macrophages	GLRX2	0.126854	5.111058	1.339651	0.183792	-5.91759	0.438604	0.487551
Non.inflammatory.Macrophages	RHOJ	0.479436	1.992484	1.33911	0.183968	-5.04984	0.530088	0.531907
Non.inflammatory.Macrophages	TADA2A	0.259423	2.820969	1.339108	0.183968	-5.34897	0.504228	0.519791
Non.inflammatory.Macrophages	SLC4A8	-0.71873	0.467759	-1.3391	0.183971	-4.7802	0.573161	0.554926
Non.inflammatory.Macrophages	MOB4	0.109491	6.832732	1.33898	0.184009	-6.06318	0.394874	0.464884
Non.inflammatory.Macrophages	SART1	0.15723	4.872457	1.33864	0.18412	-5.7848	0.445315	0.49103
Non.inflammatory.Macrophages	MRPL32	0.133833	5.68103	1.33837	0.184207	-6.00418	0.423896	0.480148
Non.inflammatory.Macrophages	ACER3	0.11177	6.311004	1.338248	0.184247	-6.29369	0.407873	0.47183
Non.inflammatory.Macrophages	ANGPT2	-0.67327	0.571482	-1.33821	0.184259	-4.74129	0.57222	0.553516
Non.inflammatory.Macrophages	CLEC4A1	0.210158	3.158732	1.337889	0.184364	-6.10487	0.494292	0.515234
Non.inflammatory.Macrophages	NKTR	-0.09843	7.193099	-1.33789	0.184364	-6.2191	0.386458	0.460502
Non.inflammatory.Macrophages	KDR	-0.38794	3.318647	-1.33726	0.184567	-5.36756	0.48995	0.513206
Non.inflammatory.Macrophages	CBFA2T2	0.1524	5.563389	1.336594	0.184785	-5.85662	0.427778	0.482396
Non.inflammatory.Macrophages	SHROOM3	-0.69532	0.981918	-1.33593	0.185	-4.85513	0.565128	0.548242
Non.inflammatory.Macrophages	NAXE	-0.14381	5.283838	-1.33536	0.185186	-5.93949	0.435805	0.486609

Non.inflammatory.Macrophages	LRP2BP	-0.48742	1.991491	-1.33534	0.185193	-5.09277	0.532181	0.533297
Non.inflammatory.Macrophages	ZMAT2	0.124924	5.466404	1.33501	0.185301	-5.9463	0.431125	0.484281
Non.inflammatory.Macrophages	RHEB	0.103958	7.039284	1.334862	0.185349	-6.1738	0.391573	0.463564
Non.inflammatory.Macrophages	UQCC3	0.160853	4.016511	1.334633	0.185423	-5.72006	0.471049	0.504338
Non.inflammatory.Macrophages	CWC15	0.10223	6.384372	1.334395	0.185501	-6.12219	0.407723	0.472226
Non.inflammatory.Macrophages	NR3C1	-0.14316	7.440275	-1.33429	0.185537	-6.24213	0.382175	0.458588
Non.inflammatory.Macrophages	GM10125	0.568837	2.003063	1.334105	0.185596	-4.79806	0.53217	0.533567
Non.inflammatory.Macrophages	CALCRL	-0.24891	5.617508	-1.33404	0.185616	-5.83943	0.427303	0.48251
Non.inflammatory.Macrophages	2810004N2	0.175113	4.315225	1.333624	0.185753	-5.73206	0.462743	0.500522
Non.inflammatory.Macrophages	NOL12	0.145995	4.012955	1.333569	0.185771	-5.67311	0.471329	0.504778
Non.inflammatory.Macrophages	RALGAPB	0.146759	4.920242	1.333405	0.185824	-5.82465	0.445998	0.492265
Non.inflammatory.Macrophages	SSBP1	0.132546	5.592236	1.333375	0.185834	-5.9387	0.428073	0.483142
Non.inflammatory.Macrophages	ZFP296	-0.57761	2.857765	-1.3331	0.185923	-4.89479	0.505578	0.521421
Non.inflammatory.Macrophages	NACC1	0.15998	4.380772	1.332996	0.185958	-5.72465	0.460939	0.499812
Non.inflammatory.Macrophages	COQ8A	-0.34726	2.303584	-1.33271	0.186051	-5.14772	0.522793	0.529631

Non.inflammatory.Macrophages	EME1	-0.40727	2.299791	-1.33259	0.18609	-5.08952	0.522913	0.52973
Non.inflammatory.Macrophages	SIK1	0.140739	6.868808	1.332551	0.186103	-6.06341	0.395938	0.466484
Non.inflammatory.Macrophages	A930015D(	-0.18695	4.760503	-1.33242	0.186145	-5.72109	0.4504	0.49471
Non.inflammatory.Macrophages	ZUP1	0.167167	5.168406	1.332416	0.186148	-5.91514	0.439333	0.489123
Non.inflammatory.Macrophages	HIVEP3	0.186951	5.144836	1.331887	0.186321	-5.88983	0.440223	0.489687
Non.inflammatory.Macrophages	PKIG	0.13262	7.198634	1.331823	0.186342	-6.18811	0.38824	0.462486
Non.inflammatory.Macrophages	EXOC7	-0.16493	4.087686	-1.33147	0.186456	-5.67395	0.469628	0.504444
Non.inflammatory.Macrophages	GTF2F2	0.120162	5.881638	1.331415	0.186475	-6.01118	0.420958	0.479874
Non.inflammatory.Macrophages	CD34	0.792245	0.953495	1.330994	0.186613	-4.90407	0.567695	0.550546
Non.inflammatory.Macrophages	EEF1AKMT	0.208974	2.582886	1.330313	0.186836	-5.4816	0.515157	0.526558
Non.inflammatory.Macrophages	CCDC102A	0.30753	2.048525	1.330267	0.186852	-5.389	0.532039	0.534441
Non.inflammatory.Macrophages	RP9	0.100761	6.834563	1.33011	0.186903	-6.1107	0.397624	0.467846
Non.inflammatory.Macrophages	BCAS3OS1	-0.22856	3.793281	-1.33002	0.186932	-5.46794	0.478732	0.509216
Non.inflammatory.Macrophages	RETREG3	0.144281	5.527566	1.329755	0.187019	-5.88868	0.430769	0.485274
Non.inflammatory.Macrophages	TDRD7	0.19426	3.385152	1.329709	0.187035	-5.72262	0.490782	0.515114

Non.inflammatory.Macrophages	GM15965	-0.41059	2.027609	-1.32934	0.187156	-5.11815	0.532973	0.535076
Non.inflammatory.Macrophages	1700052K1	-0.46316	1.140287	-1.32918	0.187207	-4.90214	0.562172	0.548497
Non.inflammatory.Macrophages	NCOA6	0.118226	5.600455	1.329052	0.18725	-5.97263	0.429027	0.484492
Non.inflammatory.Macrophages	SLC2A3	-0.34801	4.519005	-1.32859	0.187402	-5.32324	0.458286	0.499524
Non.inflammatory.Macrophages	APH1B	-0.23177	2.578014	-1.3285	0.187433	-5.59532	0.515563	0.527248
Non.inflammatory.Macrophages	UVSSA	-0.21427	3.709393	-1.32846	0.187445	-5.52464	0.481414	0.510909
Non.inflammatory.Macrophages	GM26532	-0.17503	5.294849	-1.32828	0.187503	-5.99879	0.43711	0.488859
Non.inflammatory.Macrophages	PRXL2B	0.187076	2.256934	1.328259	0.187511	-5.64972	0.525655	0.531986
Non.inflammatory.Macrophages	COMTD1	0.239954	2.92368	1.328142	0.187549	-5.39437	0.504899	0.522227
Non.inflammatory.Macrophages	THSD1	-0.4992	1.60027	-1.32801	0.187592	-4.90531	0.546859	0.541833
Non.inflammatory.Macrophages	GM17173	-0.60308	0.150476	-1.32795	0.187613	-4.80405	0.577502	0.56411
Non.inflammatory.Macrophages	RWDD1	0.110791	6.105394	1.327858	0.187643	-6.07705	0.415984	0.478002
Non.inflammatory.Macrophages	PDGFA	-0.37085	2.114128	-1.3277	0.187694	-5.31773	0.530201	0.534168
Non.inflammatory.Macrophages	CLASP1	0.130123	6.373608	1.327591	0.187731	-6.02237	0.409212	0.474517
Non.inflammatory.Macrophages	HMOX2	-0.09754	6.471899	-1.32753	0.187751	-6.15664	0.406757	0.473236

Non.inflammatory.Macrophages	NEDD9	-0.16372	8.003683	-1.3274	0.187793	-6.30844	0.370291	0.453496
Non.inflammatory.Macrophages	GDPD1	0.350293	1.600801	1.327096	0.187894	-5.31625	0.547012	0.542098
Non.inflammatory.Macrophages	RYK	0.346555	2.042505	1.326689	0.188028	-5.20659	0.532921	0.535523
Non.inflammatory.Macrophages	PCSK7	0.158426	5.282369	1.325635	0.188376	-5.85876	0.438431	0.489673
Non.inflammatory.Macrophages	LUC7L3	-0.09832	6.706417	-1.3256	0.188388	-6.15571	0.401861	0.470639
Non.inflammatory.Macrophages	SMAD5	0.170851	3.702902	1.324926	0.18861	-5.60232	0.483103	0.511819
Non.inflammatory.Macrophages	FAM107B	0.155893	7.858238	1.324818	0.188646	-6.14623	0.374775	0.45591
Non.inflammatory.Macrophages	BC065397	-0.43726	1.292469	-1.32472	0.188677	-4.96191	0.558798	0.547324
Non.inflammatory.Macrophages	THOC2	0.093282	7.106814	1.32449	0.188754	-6.19894	0.392526	0.465622
Non.inflammatory.Macrophages	CCDC92	0.770784	-0.17821	1.323993	0.188918	-4.65616	0.579767	0.570491
Non.inflammatory.Macrophages	NSMCE3	-0.14948	4.271532	-1.32385	0.188965	-5.70462	0.467068	0.504165
Non.inflammatory.Macrophages	CEP89	-0.3931	2.805458	-1.32365	0.189031	-5.1977	0.510518	0.525238
Non.inflammatory.Macrophages	MIR142HG	0.119952	8.019491	1.323615	0.189043	-6.29659	0.371383	0.454282
Non.inflammatory.Macrophages	AKR1E1	-0.23412	2.622552	-1.32336	0.189129	-5.44602	0.516311	0.527917
Non.inflammatory.Macrophages	FASTKD2	-0.2066	2.952537	-1.32265	0.189363	-5.44034	0.506418	0.523295

Non.inflammatory.Macrophages	DNAJC17	0.187677	3.568748	1.322608	0.189377	-5.46878	0.487854	0.514421
Non.inflammatory.Macrophages	CAPN7	0.125537	5.340998	1.322555	0.189394	-5.89028	0.437956	0.48966
Non.inflammatory.Macrophages	CSTA3	0.973581	0.791872	1.322491	0.189416	-4.79496	0.574674	0.555718
Non.inflammatory.Macrophages	IRAK1BP1	-0.67254	0.288762	-1.32229	0.189483	-4.81917	0.580326	0.563639
Non.inflammatory.Macrophages	RNF26	0.261138	3.515898	1.321439	0.189765	-5.43839	0.49002	0.515378
Non.inflammatory.Macrophages	DOP1B	-0.17852	4.289272	-1.3214	0.189776	-5.80242	0.467525	0.504419
Non.inflammatory.Macrophages	IL2RA	-0.75359	2.557963	-1.3212	0.189845	-4.9033	0.519352	0.529426
Non.inflammatory.Macrophages	SCOC	0.202098	3.752146	1.320753	0.189992	-5.63676	0.483374	0.512286
Non.inflammatory.Macrophages	ACYP2	0.229953	3.398712	1.320616	0.190038	-5.55585	0.493861	0.517394
Non.inflammatory.Macrophages	DERA	0.136054	4.534652	1.320348	0.190127	-5.80096	0.461027	0.501452
Non.inflammatory.Macrophages	OPTN	-0.1853	4.651784	-1.3201	0.190208	-5.85702	0.457842	0.499856
Non.inflammatory.Macrophages	URB1	-0.26052	2.176346	-1.31973	0.190334	-5.25213	0.532187	0.535517
Non.inflammatory.Macrophages	MEIS1	-0.23608	3.866059	-1.31963	0.190365	-5.62704	0.48044	0.51097
Non.inflammatory.Macrophages	BHMT2	-0.48746	2.060069	-1.31947	0.19042	-5.11252	0.535965	0.537364
Non.inflammatory.Macrophages	LTA4H	-0.19424	5.724021	-1.31851	0.190739	-5.90831	0.429648	0.485583

Non.inflammatory.Macrophages	MAP2	-0.77885	0.187932	-1.31806	0.190889	-4.69399	0.583019	0.566559
Non.inflammatory.Macrophages	RBM41	0.189685	3.907296	1.317907	0.190939	-5.63683	0.480206	0.510913
Non.inflammatory.Macrophages	CNTRL	0.141264	5.907012	1.31783	0.190965	-5.92845	0.425085	0.483248
Non.inflammatory.Macrophages	GM46652	-0.54588	-0.73309	-1.31723	0.191163	-4.90479	0.583493	0.581512
Non.inflammatory.Macrophages	BC024386	-0.53653	1.614053	-1.31685	0.191291	-4.99381	0.552173	0.544905
Non.inflammatory.Macrophages	ATPIF1	-0.11666	7.745006	-1.31677	0.191318	-6.45344	0.380188	0.459432
Non.inflammatory.Macrophages	FTH1	0.203015	12.5537	1.316745	0.191327	-7.07825	0.282899	0.401949
Non.inflammatory.Macrophages	FBXO25	0.236726	2.127104	1.316054	0.191558	-5.31334	0.535788	0.537483
Non.inflammatory.Macrophages	HAUS2	0.20613	3.755191	1.316048	0.19156	-5.59815	0.485516	0.513681
Non.inflammatory.Macrophages	WBP1L	0.141129	5.329247	1.31568	0.191683	-5.98891	0.44126	0.491798
Non.inflammatory.Macrophages	ARMC8	-0.11945	5.019279	-1.31558	0.191715	-5.91483	0.449687	0.49613
Non.inflammatory.Macrophages	RASA3	0.124995	6.702935	1.315498	0.191744	-6.13889	0.4057	0.473466
Non.inflammatory.Macrophages	BRIX1	-0.11057	6.144863	-1.31494	0.191932	-5.98235	0.420118	0.481105
Non.inflammatory.Macrophages	DALRD3	-0.1886	3.729807	-1.31447	0.192089	-5.54336	0.486853	0.514696
Non.inflammatory.Macrophages	SLC37A1	-0.25775	3.202303	-1.31446	0.192092	-5.48008	0.502683	0.522307

Non.inflammatory.Macrophages	TK1	-0.24307	4.882981	-1.31445	0.192094	-5.85127	0.45386	0.498437
Non.inflammatory.Macrophages	STT3A	-0.106	6.169392	-1.31365	0.192362	-6.09293	0.419894	0.481043
Non.inflammatory.Macrophages	PIM1	0.143242	9.488973	1.313595	0.192381	-6.60749	0.342511	0.438593
Non.inflammatory.Macrophages	PKD1L2	-0.43024	1.173969	-1.31359	0.192383	-5.19337	0.568533	0.552774
Non.inflammatory.Macrophages	BCL2L12	-0.18413	4.178233	-1.31336	0.192459	-5.6773	0.474142	0.508516
Non.inflammatory.Macrophages	FAM167A	-0.37354	1.837572	-1.31335	0.192463	-5.34462	0.54631	0.542731
Non.inflammatory.Macrophages	CRAMP1L	0.143181	4.984741	1.312918	0.192608	-5.85701	0.451598	0.497405
Non.inflammatory.Macrophages	AFMID	-0.17409	4.371552	-1.31279	0.192652	-5.83562	0.46879	0.506018
Non.inflammatory.Macrophages	TRAC	-0.75163	2.266621	-1.31248	0.192754	-4.84166	0.532586	0.536632
Non.inflammatory.Macrophages	NDRG2	-0.36906	2.964877	-1.31234	0.192803	-5.37117	0.510581	0.526326
Non.inflammatory.Macrophages	DYNC1LI1	0.109218	6.412559	1.31229	0.192819	-6.10886	0.413862	0.478166
Non.inflammatory.Macrophages	SEC61G	0.127068	10.02398	1.312254	0.192831	-6.71499	0.331565	0.432464
Non.inflammatory.Macrophages	3300005D(	-0.51841	0.756323	-1.3122	0.192848	-4.9637	0.58062	0.559707
Non.inflammatory.Macrophages	ACSL5	0.141564	6.00653	1.312082	0.192889	-6.09718	0.424274	0.483688
Non.inflammatory.Macrophages	GOLGA2	0.172771	3.814242	1.311902	0.192949	-5.64291	0.484953	0.514173

Non.inflammatory.Macrophages	1810013L2	-0.12606	5.901803	-1.31181	0.19298	-6.05694	0.427003	0.485167
Non.inflammatory.Macrophages	DNAJB5	-0.42872	1.175316	-1.31169	0.19302	-5.01735	0.568726	0.553402
Non.inflammatory.Macrophages	CD81	0.144676	7.604661	1.31139	0.193121	-6.40398	0.384818	0.462806
Non.inflammatory.Macrophages	NLRX1	0.319545	1.80919	1.311006	0.193251	-5.34618	0.547787	0.543814
Non.inflammatory.Macrophages	HIBADH	0.160176	5.742005	1.310977	0.19326	-5.9555	0.431442	0.48744
Non.inflammatory.Macrophages	ZBTB42	0.405304	1.544195	1.310689	0.193357	-4.94888	0.556742	0.547953
Non.inflammatory.Macrophages	NABP1	-0.1621	4.743721	-1.31047	0.193431	-5.96511	0.458699	0.501361
Non.inflammatory.Macrophages	TET3	-0.11004	6.827605	-1.31021	0.193518	-6.23328	0.403839	0.473223
Non.inflammatory.Macrophages	P4HB	0.101275	7.267668	1.309752	0.193673	-6.33558	0.393091	0.467487
Non.inflammatory.Macrophages	CUEDC1	-0.48589	2.113054	-1.30967	0.193699	-4.9375	0.538029	0.539605
Non.inflammatory.Macrophages	RTF1	0.094819	6.81408	1.309522	0.19375	-6.16599	0.404174	0.473468
Non.inflammatory.Macrophages	USP40	0.249971	3.64171	1.309453	0.193774	-5.57275	0.490501	0.517186
Non.inflammatory.Macrophages	FYB	0.168683	6.869674	1.309429	0.193782	-6.44087	0.4028	0.472738
Non.inflammatory.Macrophages	KLHL14	-0.57376	2.717642	-1.30937	0.193803	-4.99776	0.518745	0.530656
Non.inflammatory.Macrophages	GM35154	-0.25141	1.526848	-1.30932	0.193818	-5.69005	0.557351	0.548521

Non.inflammatory.Macrophages	PRAF2	0.276029	2.133854	1.309297	0.193826	-5.23268	0.537354	0.539342
Non.inflammatory.Macrophages	ACOD1	0.745529	2.318035	1.309247	0.193843	-5.48513	0.531418	0.536587
Non.inflammatory.Macrophages	MAP3K8	-0.15195	4.64612	-1.30908	0.193899	-5.95957	0.46145	0.502958
Non.inflammatory.Macrophages	IPO13	-0.28537	2.264795	-1.30886	0.193973	-5.34752	0.533145	0.537414
Non.inflammatory.Macrophages	NUP35	-0.26232	3.291207	-1.30884	0.19398	-5.39613	0.501058	0.522287
Non.inflammatory.Macrophages	CHCHD2	0.086069	8.94643	1.30801	0.19426	-6.56	0.354937	0.446471
Non.inflammatory.Macrophages	SLC4A2	-0.17822	3.559435	-1.30785	0.194316	-5.64817	0.493435	0.518794
Non.inflammatory.Macrophages	MSS51	0.370773	2.436698	1.307835	0.19432	-5.13354	0.528137	0.535255
Non.inflammatory.Macrophages	ADAM8	0.437658	2.602795	1.307724	0.194357	-5.18103	0.522865	0.532823
Non.inflammatory.Macrophages	SAMD9L	-0.17127	5.184732	-1.30757	0.194409	-5.97138	0.446959	0.495879
Non.inflammatory.Macrophages	TBCEL	-0.1634	4.594507	-1.30753	0.194421	-5.81255	0.463337	0.504106
Non.inflammatory.Macrophages	ASXL2	0.119009	6.836454	1.307269	0.194511	-6.20493	0.404108	0.473685
Non.inflammatory.Macrophages	GM48855	-0.55163	1.463573	-1.30708	0.194573	-4.77111	0.560204	0.55017
Non.inflammatory.Macrophages	WDR89	-0.26501	2.643388	-1.30653	0.194761	-5.34571	0.522147	0.532662
Non.inflammatory.Macrophages	LTBP4	-0.48613	2.297056	-1.30617	0.194884	-5.14375	0.533363	0.53799

Non.inflammatory.Macrophages	ZFP366	-0.25476	1.840557	-1.30608	0.194914	-5.81698	0.548241	0.544872
Non.inflammatory.Macrophages	UCKL1	-0.17955	3.705129	-1.30567	0.195051	-5.66196	0.490019	0.517423
Non.inflammatory.Macrophages	CGGBP1	0.109611	6.845343	1.304967	0.19529	-6.19464	0.404964	0.47431
Non.inflammatory.Macrophages	ADAM30	-0.40634	0.62147	-1.30473	0.195371	-5.13531	0.586297	0.564022
Non.inflammatory.Macrophages	NXPH4	-0.49308	-0.60095	-1.30468	0.195389	-5.01892	0.588266	0.583495
Non.inflammatory.Macrophages	ZFP212	0.216498	3.640876	1.304484	0.195454	-5.40024	0.492431	0.518748
Non.inflammatory.Macrophages	PLD1	0.210729	3.080795	1.304378	0.19549	-5.83591	0.509441	0.526961
Non.inflammatory.Macrophages	LMNB2	-0.39736	2.474409	-1.30432	0.195509	-5.15914	0.52847	0.535931
Non.inflammatory.Macrophages	GM34225	-0.84914	-0.06364	-1.30392	0.195646	-4.68306	0.588548	0.575292
Non.inflammatory.Macrophages	1190005I0	-0.4233	1.185776	-1.30365	0.195736	-4.98928	0.5715	0.555704
Non.inflammatory.Macrophages	GALNT16	-0.53811	0.933714	-1.30348	0.195794	-4.85681	0.579906	0.55964
Non.inflammatory.Macrophages	CARD6	0.427863	3.06005	1.303367	0.195834	-5.10464	0.510474	0.527524
Non.inflammatory.Macrophages	SLK	0.135412	6.477015	1.303035	0.195946	-6.13418	0.414673	0.479809
Non.inflammatory.Macrophages	ESYT2	0.129775	6.802347	1.301969	0.196309	-6.08972	0.407154	0.475922
Non.inflammatory.Macrophages	SIRPA	0.138657	5.832373	1.301676	0.196409	-6.35653	0.432183	0.489012

Non.inflammatory.Macrophages	RNF6	0.119807	5.670645	1.301485	0.196474	-5.99932	0.436524	0.491262
Non.inflammatory.Macrophages	AKAP9	0.133158	6.105265	1.301093	0.196607	-6.04002	0.425205	0.48548
Non.inflammatory.Macrophages	KCNIP2	-0.41472	1.248736	-1.30084	0.196695	-5.13422	0.570903	0.555724
Non.inflammatory.Macrophages	PTPN9	0.140285	5.706865	1.300679	0.196748	-6.02761	0.435691	0.490896
Non.inflammatory.Macrophages	C530005A1	-0.48302	0.742343	-1.30068	0.196749	-4.87791	0.585733	0.563597
Non.inflammatory.Macrophages	ZFP809	0.215671	3.64912	1.300337	0.196865	-5.62433	0.493885	0.519979
Non.inflammatory.Macrophages	KLHDC4	0.205219	3.945597	1.300221	0.196905	-5.58074	0.485071	0.515734
Non.inflammatory.Macrophages	LST1	0.140967	5.23223	1.300153	0.196928	-6.31878	0.448507	0.497589
Non.inflammatory.Macrophages	HIPK1	0.136743	6.269668	1.300044	0.196965	-6.10493	0.420949	0.483472
Non.inflammatory.Macrophages	LIMD1	0.11666	6.016318	1.299953	0.196996	-6.03569	0.427525	0.486916
Non.inflammatory.Macrophages	ACBD5	0.122026	6.231959	1.299948	0.196998	-6.06061	0.421921	0.484001
Non.inflammatory.Macrophages	IGKV1-117	-0.72422	-0.39916	-1.29969	0.197087	-4.65025	0.590297	0.582204
Non.inflammatory.Macrophages	LRP12	-0.28112	2.191669	-1.29964	0.197102	-5.58041	0.539421	0.541778
Non.inflammatory.Macrophages	KIN	-0.15226	4.642417	-1.29953	0.197139	-5.75549	0.464932	0.506064
Non.inflammatory.Macrophages	GM16310	-0.44744	1.197906	-1.29952	0.197144	-5.0923	0.572646	0.556953

Non.inflammatory.Macrophages	MCU	0.172289	6.164566	1.299388	0.197189	-5.93187	0.423667	0.485066
Non.inflammatory.Macrophages	GNL3	-0.15534	5.420028	-1.29879	0.197395	-5.96248	0.443737	0.495329
Non.inflammatory.Macrophages	ELP6	-0.35731	2.398694	-1.29869	0.197428	-5.17182	0.533138	0.538832
Non.inflammatory.Macrophages	UBE2E2	-0.35955	3.465951	-1.29841	0.197525	-5.43618	0.499928	0.523123
Non.inflammatory.Macrophages	TSTD2	0.191428	3.84075	1.297901	0.197697	-5.64802	0.488954	0.517931
Non.inflammatory.Macrophages	GM16124	-0.29334	2.669782	-1.29778	0.197738	-5.46426	0.524913	0.535174
Non.inflammatory.Macrophages	RASSF1	-0.11064	5.940086	-1.2977	0.197766	-6.14964	0.430211	0.488646
Non.inflammatory.Macrophages	FAM122B	-0.3351	2.015817	-1.29737	0.197879	-5.13268	0.546237	0.54506
Non.inflammatory.Macrophages	0610040F0	-0.47354	0.269406	-1.29723	0.197927	-4.91863	0.591465	0.5722
Non.inflammatory.Macrophages	PURA	0.105776	5.343404	1.297087	0.197976	-6.02167	0.446366	0.496918
Non.inflammatory.Macrophages	SLIT1	-0.36646	1.464859	-1.29679	0.198079	-5.2763	0.564726	0.553623
Non.inflammatory.Macrophages	RSRC1	0.112487	6.605429	1.29675	0.198091	-6.20302	0.413249	0.479844
Non.inflammatory.Macrophages	DNAJC10	0.14111	4.533218	1.296649	0.198126	-5.84759	0.469018	0.508371
Non.inflammatory.Macrophages	CASP3	0.142476	4.814976	1.296348	0.198229	-5.89516	0.461102	0.504477
Non.inflammatory.Macrophages	LSG1	0.140247	4.928986	1.296182	0.198286	-5.83421	0.457908	0.502881

Non.inflammatory.Macrophages	LCP2	0.159192	5.109024	1.296032	0.198338	-6.25144	0.452907	0.500389
Non.inflammatory.Macrophages	JUP	-0.18601	3.562597	-1.29597	0.198358	-5.78642	0.497601	0.522438
Non.inflammatory.Macrophages	TIMP3	0.593451	2.197691	1.295841	0.198403	-5.12543	0.540437	0.542698
Non.inflammatory.Macrophages	SNX15	-0.13999	4.880351	-1.29571	0.198449	-5.93827	0.459268	0.503732
Non.inflammatory.Macrophages	RAB37	0.346211	2.340912	1.295541	0.198506	-5.17874	0.535787	0.540709
Non.inflammatory.Macrophages	LRRC45	-0.2895	2.190744	-1.29529	0.198594	-5.28973	0.540663	0.543034
Non.inflammatory.Macrophages	PBRM1	0.088259	7.416728	1.295235	0.198611	-6.29705	0.393259	0.469523
Non.inflammatory.Macrophages	SMYD1	-0.65681	0.29641	-1.29517	0.198634	-4.8218	0.591623	0.572383
Non.inflammatory.Macrophages	PPP2R1B	-0.17634	4.339805	-1.29514	0.198644	-5.74428	0.474643	0.511515
Non.inflammatory.Macrophages	SDHAF2	0.160537	4.655098	1.295036	0.19868	-5.7917	0.465617	0.507098
Non.inflammatory.Macrophages	EIF5B	0.088347	7.158051	1.294895	0.198728	-6.35869	0.399552	0.473008
Non.inflammatory.Macrophages	STFA2	0.568031	2.469209	1.294774	0.198769	-5.09147	0.53166	0.538969
Non.inflammatory.Macrophages	2310039H(	0.171539	4.246315	1.294646	0.198813	-5.7595	0.477358	0.513034
Non.inflammatory.Macrophages	CHMP3	0.092343	5.884611	1.29425	0.19895	-6.09492	0.432024	0.490288
Non.inflammatory.Macrophages	SLC7A8	0.241608	2.86439	1.294237	0.198954	-5.9475	0.519193	0.533286

Non.inflammatory.Macrophages	IWS1	0.122419	5.729671	1.294084	0.199006	-5.97624	0.436136	0.492464
Non.inflammatory.Macrophages	RAB12	0.120587	5.073594	1.294046	0.199019	-5.97693	0.453964	0.501542
Non.inflammatory.Macrophages	SMYD5	0.358084	2.056268	1.2938	0.199104	-5.27414	0.545184	0.545505
Non.inflammatory.Macrophages	EAR2	0.184156	3.55304	1.293764	0.199117	-6.14884	0.497999	0.523259
Non.inflammatory.Macrophages	LEMD3	0.135024	5.687224	1.293576	0.199181	-5.98114	0.437336	0.493125
Non.inflammatory.Macrophages	MYL9	0.460775	1.95475	1.293185	0.199316	-5.3345	0.548787	0.547181
Non.inflammatory.Macrophages	BRAP	0.142658	4.924367	1.292979	0.199387	-5.85729	0.458353	0.503934
Non.inflammatory.Macrophages	RAB3IP	0.255227	4.587906	1.292935	0.199402	-5.49613	0.46785	0.508697
Non.inflammatory.Macrophages	SLC25A33	-0.17741	3.983563	-1.29286	0.199429	-5.82088	0.485375	0.517355
Non.inflammatory.Macrophages	ZKSCAN1	0.174289	4.068204	1.292662	0.199496	-5.64536	0.48294	0.516173
Non.inflammatory.Macrophages	PACS1	0.165444	5.972963	1.292391	0.199589	-6.00747	0.430074	0.489604
Non.inflammatory.Macrophages	GM47802	-0.58696	0.099495	-1.29207	0.199699	-4.89317	0.592244	0.576569
Non.inflammatory.Macrophages	METTL15	-0.23717	3.231154	-1.29207	0.199701	-5.45677	0.508236	0.528505
Non.inflammatory.Macrophages	KIF20A	-0.39404	3.768581	-1.2919	0.199758	-5.48052	0.491933	0.520707
Non.inflammatory.Macrophages	PSMA1	0.111168	6.9166	1.291728	0.199818	-6.23435	0.40593	0.477017

Non.inflammatory.Macrophages	KMT2E	-0.09485	8.273581	-1.29169	0.19983	-6.37187	0.373502	0.459336
Non.inflammatory.Macrophages	MTUS2	-0.6431	0.654472	-1.29163	0.199851	-4.91069	0.589647	0.567805
Non.inflammatory.Macrophages	MED25	0.145165	4.496149	1.291242	0.199986	-5.80331	0.47076	0.510309
Non.inflammatory.Macrophages	PLD4	-0.15794	6.186695	-1.29123	0.199989	-6.32024	0.424595	0.486847
Non.inflammatory.Macrophages	TM2D1	0.104186	5.944576	1.29091	0.2001	-6.11116	0.431023	0.49031
Non.inflammatory.Macrophages	GM12236	-0.35447	1.087964	-1.29085	0.20012	-5.41306	0.578607	0.561285
Non.inflammatory.Macrophages	FAM49B	-0.08323	9.086243	-1.29064	0.200193	-6.57346	0.355521	0.449372
Non.inflammatory.Macrophages	GM26771	-0.73652	-0.2163	-1.29043	0.200266	-4.77152	0.592593	0.582136
Non.inflammatory.Macrophages	GLB1L	-0.24367	2.192734	-1.29026	0.200325	-5.39318	0.541484	0.544507
Non.inflammatory.Macrophages	SOAT1	0.134711	5.176779	1.29005	0.200397	-6.24772	0.451777	0.501118
Non.inflammatory.Macrophages	FAM214B	-0.20757	3.11159	-1.29004	0.200399	-5.77979	0.512233	0.530767
Non.inflammatory.Macrophages	ATG16L2	-0.17465	5.680082	-1.28982	0.200478	-5.78407	0.438102	0.494145
Non.inflammatory.Macrophages	PXMP2	-0.35328	4.067436	-1.2898	0.200484	-5.64159	0.483364	0.516835
Non.inflammatory.Macrophages	CDH5	-0.24553	4.480665	-1.28977	0.200494	-5.92821	0.471362	0.510924
Non.inflammatory.Macrophages	GM48027	-0.30102	2.815176	-1.28846	0.200947	-5.30166	0.522366	0.535551

Non.inflammatory.Macrophages	4933407K1	0.405513	1.39879	1.288426	0.200959	-4.98105	0.568921	0.557064
Non.inflammatory.Macrophages	CDC42BPA	0.287203	3.315927	1.288385	0.200973	-5.39202	0.506764	0.528142
Non.inflammatory.Macrophages	PRX	-0.47286	1.104861	-1.28831	0.201	-5.01628	0.579044	0.561646
Non.inflammatory.Macrophages	MND1	-0.47019	2.222735	-1.28806	0.201087	-4.97609	0.541513	0.544569
Non.inflammatory.Macrophages	IL12RB1	-0.53478	0.500116	-1.28786	0.201157	-5.03606	0.593622	0.571301
Non.inflammatory.Macrophages	APH1A	0.139902	5.433388	1.287205	0.201383	-6.04816	0.445906	0.498284
Non.inflammatory.Macrophages	NME2	0.129761	8.557653	1.287051	0.201436	-6.56131	0.368209	0.456818
Non.inflammatory.Macrophages	TMEM219	0.136085	4.624458	1.286987	0.201458	-5.97014	0.468461	0.509675
Non.inflammatory.Macrophages	PTRH2	-0.16847	4.181735	-1.28698	0.201461	-5.75443	0.481258	0.515995
Non.inflammatory.Macrophages	GABARAPL	-0.17473	3.974288	-1.28684	0.201509	-5.7991	0.487367	0.519052
Non.inflammatory.Macrophages	TRAF7	0.137643	4.585124	1.286676	0.201566	-5.84028	0.469585	0.510332
Non.inflammatory.Macrophages	ATF1	-0.09574	6.564036	-1.28653	0.201616	-6.12595	0.416112	0.482989
Non.inflammatory.Macrophages	CYFIP2	-0.23764	6.947339	-1.2865	0.201627	-5.6951	0.406454	0.477862
Non.inflammatory.Macrophages	CCDC17	0.539246	0.893924	1.286035	0.201789	-4.95561	0.586465	0.56572
Non.inflammatory.Macrophages	PDXDC1	0.097899	6.616598	1.285813	0.201866	-6.17567	0.415081	0.482521

Non.inflammatory.Macrophages	NAB2	0.206077	3.217989	1.285691	0.201908	-5.73031	0.510636	0.530408
Non.inflammatory.Macrophages	NIPSNAP3I	0.135035	5.573981	1.285491	0.201978	-6.02573	0.442476	0.496838
Non.inflammatory.Macrophages	GGA2	-0.19568	4.120751	-1.28528	0.20205	-5.74933	0.483532	0.517465
Non.inflammatory.Macrophages	SNX25	0.123867	5.540177	1.28486	0.202197	-6.0497	0.443585	0.497598
Non.inflammatory.Macrophages	PFKFB1	0.474358	0.961216	1.284767	0.20223	-5.00056	0.585359	0.565258
Non.inflammatory.Macrophages	ABI3	0.211505	4.401261	1.284731	0.202242	-5.94544	0.475491	0.513681
Non.inflammatory.Macrophages	HSF2	0.181259	4.185277	1.284573	0.202297	-5.75288	0.481787	0.516827
Non.inflammatory.Macrophages	ZER1	-0.23611	3.262662	-1.28447	0.202332	-5.38249	0.509549	0.530269
Non.inflammatory.Macrophages	ATOX1	0.099045	8.244709	1.283987	0.202501	-6.59793	0.376081	0.461738
Non.inflammatory.Macrophages	TAF1B	0.16775	4.199522	1.283611	0.202632	-5.6888	0.481875	0.516836
Non.inflammatory.Macrophages	EXT2	-0.16529	3.849599	-1.28341	0.202701	-5.75544	0.492296	0.521968
Non.inflammatory.Macrophages	SCNN1A	-0.40795	0.542515	-1.28241	0.203051	-5.25125	0.595706	0.572544
Non.inflammatory.Macrophages	ANKRD23	-0.61105	0.33175	-1.28222	0.203117	-4.79024	0.596568	0.575976
Non.inflammatory.Macrophages	PRUNE1	0.192376	3.600837	1.281984	0.2032	-5.63632	0.500692	0.52601
Non.inflammatory.Macrophages	2610307P1	-0.34456	4.550996	-1.28175	0.203283	-5.71156	0.472642	0.512301

Non.inflammatory.Macrophages	ZKSCAN6	0.251423	2.942031	1.28163	0.203323	-5.50559	0.521151	0.535761
Non.inflammatory.Macrophages	HMCES	-0.19121	4.456587	-1.28143	0.203395	-5.74366	0.475367	0.513653
Non.inflammatory.Macrophages	MASP1	-0.50178	1.673868	-1.2813	0.20344	-5.03277	0.562593	0.554997
Non.inflammatory.Macrophages	JAG1	0.60333	1.3378	1.281284	0.203444	-5.00607	0.574069	0.560206
Non.inflammatory.Macrophages	GPR84	0.391017	0.260814	1.28064	0.20367	-5.26042	0.597054	0.577563
Non.inflammatory.Macrophages	ISG15	0.303112	6.408101	1.280579	0.203691	-6.37676	0.422174	0.486782
Non.inflammatory.Macrophages	LPAR6	-0.18383	4.523596	-1.2804	0.203752	-5.93258	0.47367	0.513058
Non.inflammatory.Macrophages	SOX12	-0.62137	0.585357	-1.28029	0.203793	-4.79239	0.595604	0.572443
Non.inflammatory.Macrophages	DTX1	-0.60156	2.069415	-1.28022	0.203818	-4.93843	0.549632	0.549316
Non.inflammatory.Macrophages	IMPAD1	0.138307	4.365894	1.280086	0.203863	-5.83698	0.47824	0.515315
Non.inflammatory.Macrophages	MECP2	0.121971	5.847525	1.279984	0.203899	-6.01722	0.436905	0.494504
Non.inflammatory.Macrophages	TLN1	0.111659	7.109295	1.279965	0.203906	-6.3576	0.404422	0.477435
Non.inflammatory.Macrophages	TTI1	0.285868	2.712153	1.279846	0.203948	-5.42068	0.528715	0.539597
Non.inflammatory.Macrophages	HINT3	0.150762	4.643869	1.279729	0.203988	-5.91934	0.470213	0.511356
Non.inflammatory.Macrophages	CIC	-0.15088	4.821881	-1.27898	0.204252	-5.88758	0.465641	0.509055

Non.inflammatory.Macrophages	ZFP563	-0.43721	0.800774	-1.27878	0.204319	-4.91988	0.591787	0.569334
Non.inflammatory.Macrophages	DDX56	-0.17589	3.951871	-1.27869	0.204353	-5.6851	0.490994	0.521592
Non.inflammatory.Macrophages	MIRT2	0.751348	-1.00119	1.278384	0.20446	-4.67158	0.597917	0.598691
Non.inflammatory.Macrophages	SARS	0.107619	5.792679	1.278119	0.204553	-6.06785	0.43911	0.495773
Non.inflammatory.Macrophages	CPA6	-0.78471	-0.07229	-1.27781	0.20466	-4.72097	0.598245	0.58363
Non.inflammatory.Macrophages	NDUFB2	0.117245	5.815553	1.277346	0.204824	-6.12563	0.438672	0.495552
Non.inflammatory.Macrophages	MFAP2	-0.64733	0.586159	-1.27726	0.204853	-4.89105	0.596833	0.573147
Non.inflammatory.Macrophages	SIPA1L1	-0.17967	7.327522	-1.27719	0.204877	-6.27002	0.399878	0.475154
Non.inflammatory.Macrophages	1810044D(	0.336252	1.915203	1.277131	0.2049	-5.27437	0.555918	0.552406
Non.inflammatory.Macrophages	SLC35A3	0.131293	4.775291	1.277043	0.204931	-5.8597	0.467434	0.510162
Non.inflammatory.Macrophages	CDA	-0.62371	0.894078	-1.277	0.204944	-4.89905	0.590236	0.56832
Non.inflammatory.Macrophages	HSDL1	-0.1497	4.167902	-1.27672	0.205043	-5.71086	0.485052	0.518896
Non.inflammatory.Macrophages	KCTD21	-0.39969	0.182578	-1.27652	0.205116	-5.24452	0.598307	0.579703
Non.inflammatory.Macrophages	FAM136A	0.190944	4.090791	1.276484	0.205127	-5.72423	0.487332	0.520014
Non.inflammatory.Macrophages	CXCR5	-0.58283	2.183855	-1.27648	0.205128	-4.97721	0.546998	0.548349

Non.inflammatory.Macrophages	PSMD10	0.187958	3.584601	1.276369	0.205168	-5.70753	0.50255	0.527425
Non.inflammatory.Macrophages	SPCS1	0.092709	7.852067	1.275864	0.205345	-6.44318	0.387398	0.468546
Non.inflammatory.Macrophages	TIE1	-0.47646	1.482911	-1.27536	0.205524	-5.00022	0.570835	0.559524
Non.inflammatory.Macrophages	HIST1H3G	-0.67912	1.46486	-1.27534	0.20553	-4.84357	0.571455	0.559805
Non.inflammatory.Macrophages	SMIM1	-0.36644	1.759237	-1.27525	0.205563	-5.3367	0.56143	0.555254
Non.inflammatory.Macrophages	5031434O1	-0.53057	0.524887	-1.27524	0.205567	-4.8852	0.598053	0.574627
Non.inflammatory.Macrophages	ATP5B	0.106491	8.182512	1.275054	0.20563	-6.45335	0.379618	0.46436
Non.inflammatory.Macrophages	MITF	0.161466	4.369202	1.275051	0.205631	-6.16291	0.479369	0.516366
Non.inflammatory.Macrophages	GM11655	-0.72935	0.316941	-1.27494	0.205672	-4.72329	0.598585	0.577954
Non.inflammatory.Macrophages	SDR39U1	-0.68964	1.131364	-1.27493	0.205675	-4.76811	0.583008	0.565028
Non.inflammatory.Macrophages	WDR90	-0.321	1.959845	-1.27484	0.205705	-5.26326	0.55469	0.552166
Non.inflammatory.Macrophages	EVA1B	0.178015	3.639669	1.274493	0.205828	-5.82609	0.501298	0.527009
Non.inflammatory.Macrophages	NUCB1	0.136397	5.383551	1.27421	0.205928	-6.0458	0.450915	0.502133
Non.inflammatory.Macrophages	ATP5O	0.115885	7.142669	1.273127	0.20631	-6.32967	0.405558	0.478607
Non.inflammatory.Macrophages	ODR4	0.155818	4.042451	1.272963	0.206368	-5.82222	0.490148	0.521819

Non.inflammatory.Macrophages	ATF5	0.267033	3.267979	1.27258	0.206504	-5.60104	0.513968	0.533382
Non.inflammatory.Macrophages	P3H1	0.54982	0.585168	1.272446	0.206551	-4.98298	0.598815	0.574684
Non.inflammatory.Macrophages	BZW2	0.140102	5.869307	1.271996	0.20671	-6.05128	0.438788	0.496189
Non.inflammatory.Macrophages	AV099323	-0.54894	1.209882	-1.27199	0.20671	-4.96028	0.582055	0.564872
Non.inflammatory.Macrophages	NDUFV1	-0.14039	5.20278	-1.27192	0.206738	-5.96595	0.457024	0.505494
Non.inflammatory.Macrophages	ACOT13	-0.13323	4.43558	-1.2715	0.206886	-5.85955	0.478983	0.516492
Non.inflammatory.Macrophages	CRELD2	-0.1801	4.759514	-1.27142	0.206915	-5.87534	0.469624	0.511855
Non.inflammatory.Macrophages	SPNS3	-0.51052	3.650358	-1.27137	0.206931	-5.06711	0.502404	0.527904
Non.inflammatory.Macrophages	RBFA	-0.15391	4.826424	-1.27132	0.206949	-5.96454	0.467713	0.510902
Non.inflammatory.Macrophages	IFT122	-0.37087	1.594011	-1.27119	0.206995	-5.05457	0.568879	0.559006
Non.inflammatory.Macrophages	POT1B	0.168508	4.421725	1.270692	0.207171	-5.96238	0.479698	0.516953
Non.inflammatory.Macrophages	HAGH	0.152637	5.358255	1.269706	0.207521	-6.10354	0.453741	0.504051
Non.inflammatory.Macrophages	MRE11A	0.147484	4.349103	1.269233	0.207689	-5.71502	0.48282	0.518683
Non.inflammatory.Macrophages	IGF1	-0.23403	5.292109	-1.26817	0.208067	-6.18312	0.456238	0.505682
Non.inflammatory.Macrophages	TM9SF4	-0.14293	4.769644	-1.26812	0.208083	-5.98091	0.471013	0.513091

Non.inflammatory.Macrophages	GFOD2	-0.39347	2.309358	-1.26809	0.208093	-5.12035	0.546833	0.549448
Non.inflammatory.Macrophages	RLIM	-0.09152	6.654275	-1.26804	0.208114	-6.20359	0.41977	0.486863
Non.inflammatory.Macrophages	CD247	-0.2914	3.01096	-1.26796	0.208142	-5.54769	0.524128	0.538829
Non.inflammatory.Macrophages	PPP1R21	0.152953	4.557871	1.267951	0.208144	-5.95201	0.47713	0.516124
Non.inflammatory.Macrophages	PACSIN1	-0.55489	2.953403	-1.26773	0.208223	-4.90104	0.525958	0.539725
Non.inflammatory.Macrophages	GNL2	-0.14953	4.675227	-1.26773	0.208223	-5.85551	0.473731	0.514471
Non.inflammatory.Macrophages	XIAP	0.094377	6.725324	1.267329	0.208366	-6.23664	0.418143	0.486092
Non.inflammatory.Macrophages	PSMB7	0.11255	5.624583	1.266782	0.20856	-6.07669	0.447554	0.501419
Non.inflammatory.Macrophages	PIM2	-0.25793	3.150665	-1.26663	0.208616	-5.44048	0.520279	0.537257
Non.inflammatory.Macrophages	ART2B	-0.5957	0.097769	-1.26652	0.208653	-5.07185	0.603328	0.584887
Non.inflammatory.Macrophages	PPP4R3A	0.087902	6.59174	1.266239	0.208754	-6.14839	0.42184	0.488238
Non.inflammatory.Macrophages	GM26756	0.591154	0.354794	1.266191	0.208771	-4.99208	0.603328	0.580755
Non.inflammatory.Macrophages	BPHL	-0.19188	3.663858	-1.26607	0.208812	-5.70106	0.504336	0.529711
Non.inflammatory.Macrophages	CXADR	-0.53275	1.645302	-1.26605	0.208822	-4.94973	0.569771	0.560312
Non.inflammatory.Macrophages	DTX4	0.275157	2.365512	1.265965	0.208851	-5.57935	0.545577	0.549221

Non.inflammatory.Macrophages	KNSTRN	-0.36269	3.271497	-1.26564	0.208968	-5.47128	0.516567	0.535617
Non.inflammatory.Macrophages	TAOK2	0.167168	4.127104	1.265621	0.208974	-5.76051	0.490417	0.523015
Non.inflammatory.Macrophages	SF3A1	0.13294	4.851981	1.264699	0.209303	-5.87891	0.469878	0.512975
Non.inflammatory.Macrophages	MRPL9	-0.12801	4.787977	-1.26444	0.209395	-5.87313	0.471777	0.514001
Non.inflammatory.Macrophages	ABCC2	-0.37229	2.212583	-1.26437	0.209419	-5.24854	0.551543	0.552206
Non.inflammatory.Macrophages	ARHGDIA	0.111226	7.188393	1.264114	0.209512	-6.3637	0.407458	0.48079
Non.inflammatory.Macrophages	CD3E	-0.70368	3.278256	-1.26348	0.209737	-5.06932	0.517689	0.536271
Non.inflammatory.Macrophages	NMD3	-0.11172	4.999909	-1.26326	0.209818	-5.95168	0.466308	0.511259
Non.inflammatory.Macrophages	GRCC10	0.098575	6.427807	1.262989	0.209914	-6.23479	0.427443	0.491421
Non.inflammatory.Macrophages	BRIP1	0.229228	4.898207	1.262548	0.210072	-5.79755	0.469488	0.512921
Non.inflammatory.Macrophages	POSTN	-0.46774	1.260435	-1.26253	0.210077	-5.15124	0.585144	0.567547
Non.inflammatory.Macrophages	XKR8	-0.4875	0.500884	-1.26215	0.210215	-5.02067	0.605492	0.579842
Non.inflammatory.Macrophages	CTU2	-0.22489	3.206943	-1.26208	0.21024	-5.49578	0.520512	0.537823
Non.inflammatory.Macrophages	ACOT9	0.140137	4.620859	1.261536	0.210434	-5.93526	0.477933	0.517234
Non.inflammatory.Macrophages	1600010M	-0.16916	4.96425	-1.26149	0.21045	-5.95921	0.468033	0.512313

Non.inflammatory.Macrophages	GM16268	-0.48632	0.706579	-1.26097	0.210638	-5.06875	0.60221	0.576999
Non.inflammatory.Macrophages	REV3L	-0.12038	6.713202	-1.26093	0.21065	-6.16328	0.420618	0.488199
Non.inflammatory.Macrophages	RHOC	0.193404	4.059106	1.26093	0.210651	-5.84044	0.494609	0.525638
Non.inflammatory.Macrophages	AKIRIN1	0.095656	6.287656	1.260928	0.210652	-6.16185	0.431722	0.494017
Non.inflammatory.Macrophages	ADM	0.415678	1.330377	1.260659	0.210748	-5.29645	0.583438	0.567273
Non.inflammatory.Macrophages	HIST2H2BE	-0.51005	0.814865	-1.26043	0.210831	-4.95105	0.599974	0.575536
Non.inflammatory.Macrophages	CEP192	0.149279	5.27108	1.260284	0.210883	-5.90509	0.459612	0.508448
Non.inflammatory.Macrophages	ST6GALNA	0.178765	3.867281	1.259878	0.211029	-5.81733	0.50083	0.528829
Non.inflammatory.Macrophages	STON2	0.189079	3.518101	1.25981	0.211053	-5.89712	0.511561	0.533995
Non.inflammatory.Macrophages	TRIM23	0.224375	3.295163	1.258977	0.211353	-5.46993	0.519011	0.537634
Non.inflammatory.Macrophages	BLOC1S6	0.179768	3.751167	1.258892	0.211383	-5.67886	0.504848	0.530878
Non.inflammatory.Macrophages	PSMB6	0.119447	6.304312	1.258884	0.211386	-6.20231	0.43205	0.494455
Non.inflammatory.Macrophages	CLPP	-0.15047	4.725598	-1.25803	0.211695	-5.87429	0.476384	0.517016
Non.inflammatory.Macrophages	TUT7	-0.09172	7.206797	-1.25754	0.211871	-6.34166	0.409554	0.482744
Non.inflammatory.Macrophages	RMI2	-0.41935	3.3602	-1.2574	0.21192	-5.09654	0.51791	0.537337

Non.inflammatory.Macrophages	ZFP236	-0.14537	4.729945	-1.25725	0.211972	-5.8823	0.476529	0.517306
Non.inflammatory.Macrophages	RIPPLY3	-0.65551	0.048429	-1.25714	0.212015	-4.89245	0.608257	0.589243
Non.inflammatory.Macrophages	SNAPC5	0.139896	4.755447	1.257029	0.212054	-5.95064	0.475789	0.516954
Non.inflammatory.Macrophages	TACC2	-0.24942	3.088667	-1.25687	0.212111	-5.5463	0.526502	0.541562
Non.inflammatory.Macrophages	DCTN1	0.123243	4.987161	1.256768	0.212148	-5.98824	0.469115	0.513715
Non.inflammatory.Macrophages	SPOPL	0.152557	4.42179	1.256692	0.212175	-5.83308	0.485558	0.521891
Non.inflammatory.Macrophages	UTP3	-0.13398	5.450096	-1.25637	0.212293	-6.02972	0.456201	0.507328
Non.inflammatory.Macrophages	CHAMP1	0.240384	2.843874	1.256106	0.212387	-5.46986	0.534616	0.545551
Non.inflammatory.Macrophages	OTULIN	0.091657	6.57022	1.256017	0.212419	-6.25299	0.426049	0.491846
Non.inflammatory.Macrophages	SS18	0.129295	5.946104	1.255914	0.212456	-6.04806	0.442638	0.50049
Non.inflammatory.Macrophages	NCOR1	0.080524	8.098707	1.255752	0.212514	-6.41791	0.387951	0.471438
Non.inflammatory.Macrophages	D8ERTD73[illegible]	0.088108	7.358725	1.255286	0.212683	-6.37219	0.405984	0.481323
Non.inflammatory.Macrophages	POLG	-0.19355	3.808912	-1.25505	0.212768	-5.67567	0.50428	0.531453
Non.inflammatory.Macrophages	PON3	0.223793	3.489649	1.255041	0.212771	-5.70236	0.514149	0.536196
Non.inflammatory.Macrophages	MIF4GD	0.142437	4.964455	1.255002	0.212785	-6.00626	0.470029	0.514635

Non.inflammatory.Macrophages	ZBTB20	-0.11882	8.246328	-1.255	0.212786	-6.48863	0.384459	0.469702
Non.inflammatory.Macrophages	ALKBH8	0.191223	3.835022	1.254672	0.212904	-5.58635	0.503481	0.531182
Non.inflammatory.Macrophages	HEATR5B	-0.20658	3.730171	-1.25459	0.212934	-5.66611	0.506698	0.532734
Non.inflammatory.Macrophages	REST	0.135537	5.400614	1.254588	0.212935	-6.04089	0.457682	0.508529
Non.inflammatory.Macrophages	GPATCH11	0.187513	3.800176	1.254239	0.213061	-5.66399	0.504548	0.531801
Non.inflammatory.Macrophages	APLF	-0.34115	2.05909	-1.25417	0.213087	-5.24936	0.560608	0.558193
Non.inflammatory.Macrophages	PPRC1	-0.16147	3.956149	-1.25411	0.213106	-5.80311	0.499789	0.529497
Non.inflammatory.Macrophages	NDUFS1	0.10915	5.752161	1.254098	0.213112	-6.02934	0.447957	0.503674
Non.inflammatory.Macrophages	PSME2B	0.221993	2.815452	1.254079	0.213119	-5.6905	0.535582	0.546574
Non.inflammatory.Macrophages	ENTHD1	-0.78633	0.023444	-1.25393	0.213172	-4.79345	0.608599	0.59075
Non.inflammatory.Macrophages	SPTAN1	-0.12584	6.559592	-1.25384	0.213204	-6.19875	0.426364	0.492569
Non.inflammatory.Macrophages	NELFA	0.145725	4.630912	1.253747	0.213239	-5.77275	0.479683	0.519789
Non.inflammatory.Macrophages	HMGCL	-0.13939	5.187114	-1.25348	0.213336	-6.05059	0.463708	0.51186
Non.inflammatory.Macrophages	LTB4R1	-0.36739	2.689349	-1.25347	0.213339	-5.45172	0.539707	0.548709
Non.inflammatory.Macrophages	SLBP	-0.13642	7.147492	-1.2532	0.213438	-6.20817	0.41132	0.48477

Non.inflammatory.Macrophages	CAPN3	-0.37729	1.422788	-1.25317	0.213446	-5.2023	0.582546	0.568495
Non.inflammatory.Macrophages	SFXN1	0.152868	5.121606	1.252794	0.213584	-5.97626	0.465593	0.512955
Non.inflammatory.Macrophages	ATP5J	0.106502	7.873083	1.252695	0.21362	-6.46387	0.393412	0.475131
Non.inflammatory.Macrophages	NAPRT	-0.48994	0.897988	-1.25265	0.213638	-5.10795	0.60037	0.576905
Non.inflammatory.Macrophages	DNAJC3	-0.11397	6.725717	-1.25258	0.213662	-6.30164	0.422091	0.490559
Non.inflammatory.Macrophages	RBM5	0.098452	6.623423	1.252568	0.213666	-6.20819	0.424744	0.491958
Non.inflammatory.Macrophages	LRRC3	-0.40717	0.375073	-1.25221	0.213795	-5.11087	0.608827	0.585458
Non.inflammatory.Macrophages	CBX5	0.180066	5.103912	1.25216	0.213814	-5.88602	0.466222	0.51331
Non.inflammatory.Macrophages	GATAD2A	-0.10438	7.859185	-1.25186	0.213921	-6.4024	0.393927	0.475485
Non.inflammatory.Macrophages	PPM1E	-0.41045	4.964118	-1.2518	0.213943	-5.41916	0.470301	0.515414
Non.inflammatory.Macrophages	GM614	-0.60908	-0.15476	-1.2516	0.214017	-4.82673	0.608954	0.594342
Non.inflammatory.Macrophages	PHC3	0.148574	4.499127	1.251542	0.214038	-5.89685	0.483831	0.522219
Non.inflammatory.Macrophages	TTPAL	-0.18677	4.234118	-1.25073	0.214334	-5.60964	0.492237	0.526298
Non.inflammatory.Macrophages	TNFRSF25	-0.68182	-1.26707	-1.25064	0.214367	-4.6851	0.60962	0.613218
Non.inflammatory.Macrophages	DGLUCY	0.170021	2.944912	1.250524	0.214408	-5.80575	0.532295	0.545595

Non.inflammatory.Macrophages	CCND2	0.233459	5.400515	1.250057	0.214577	-5.79291	0.45872	0.509584
Non.inflammatory.Macrophages	BLVRA	-0.12035	5.071733	-1.24953	0.214768	-6.17098	0.468336	0.514433
Non.inflammatory.Macrophages	INTS11	-0.21036	3.989645	-1.24893	0.214986	-5.63472	0.500607	0.530344
Non.inflammatory.Macrophages	HOOK1	-0.43509	2.667358	-1.24885	0.215016	-5.00513	0.542387	0.550221
Non.inflammatory.Macrophages	IQGAP3	-0.407	2.400987	-1.24851	0.215141	-5.29128	0.551391	0.554445
Non.inflammatory.Macrophages	H2-DMA	-0.18439	5.184297	-1.24805	0.215307	-6.24321	0.465848	0.513303
Non.inflammatory.Macrophages	NRM	-0.20991	5.099836	-1.248	0.215325	-5.84798	0.468256	0.514536
Non.inflammatory.Macrophages	VMP1	-0.13216	7.164309	-1.24732	0.215575	-6.43287	0.41294	0.486017
Non.inflammatory.Macrophages	USHBP1	-0.50859	1.085061	-1.24727	0.215591	-4.9782	0.597427	0.575605
Non.inflammatory.Macrophages	ZMPSTE24	0.180675	4.942134	1.247258	0.215597	-5.88789	0.473059	0.517043
Non.inflammatory.Macrophages	PRICKLE1	-0.41946	3.386121	-1.24716	0.215632	-5.35602	0.520015	0.539972
Non.inflammatory.Macrophages	ACOT8	-0.15712	4.129949	-1.24698	0.2157	-5.82719	0.497098	0.529014
Non.inflammatory.Macrophages	ELOF1	0.136494	5.375272	1.24654	0.215859	-6.01638	0.460926	0.511121
Non.inflammatory.Macrophages	AMPD1	-0.25454	2.911186	-1.24652	0.215865	-5.62299	0.535433	0.547406
Non.inflammatory.Macrophages	6720427I0	-0.16176	4.894793	-1.24622	0.215977	-5.87398	0.474786	0.518126

Non.inflammatory.Macrophages	RPRD1A	0.15489	4.057705	1.2451	0.216385	-5.70133	0.500442	0.530661
Non.inflammatory.Macrophages	PARP11	0.255111	3.46828	1.24499	0.216425	-5.63167	0.51868	0.539444
Non.inflammatory.Macrophages	KLHL8	-0.36766	2.402928	-1.24402	0.21678	-5.29251	0.553888	0.556143
Non.inflammatory.Macrophages	ZFP704	-0.24814	3.792086	-1.24394	0.21681	-5.78273	0.509196	0.535094
Non.inflammatory.Macrophages	ZDHHC3	0.134064	5.048938	1.243822	0.216853	-5.97424	0.471689	0.516697
Non.inflammatory.Macrophages	COX15	0.199768	2.891641	1.243728	0.216887	-5.51109	0.537766	0.548706
Non.inflammatory.Macrophages	POLE	-0.37291	3.301306	-1.24366	0.216912	-5.35944	0.524588	0.542489
Non.inflammatory.Macrophages	FKBP4	-0.14222	5.691682	-1.24342	0.217001	-5.99889	0.453627	0.507675
Non.inflammatory.Macrophages	GM14798	-0.25118	3.718232	-1.2431	0.217116	-5.38199	0.511683	0.536448
Non.inflammatory.Macrophages	CD101	0.635944	0.741284	1.242959	0.217169	-4.84911	0.609417	0.582771
Non.inflammatory.Macrophages	SFXN5	0.199979	3.363367	1.242936	0.217177	-5.77854	0.52282	0.541797
Non.inflammatory.Macrophages	GM47689	0.316699	3.369026	1.242778	0.217235	-5.13565	0.522671	0.541766
Non.inflammatory.Macrophages	CARMIL2	-0.45121	2.704609	-1.24256	0.217314	-5.00184	0.544151	0.551958
Non.inflammatory.Macrophages	A930006K(	-0.2616	2.074746	-1.24239	0.217378	-5.38502	0.565239	0.561807
Non.inflammatory.Macrophages	DPY30	-0.15706	5.414831	-1.24238	0.217382	-6.00526	0.461498	0.511942

Non.inflammatory.Macrophages	XPA	0.174633	4.138354	1.242033	0.217509	-5.8134	0.498984	0.530585
Non.inflammatory.Macrophages	ERMAP	0.610112	0.529243	1.241817	0.217588	-4.93559	0.613772	0.586678
Non.inflammatory.Macrophages	IAH1	0.170299	4.521488	1.241793	0.217597	-5.88901	0.487484	0.525028
Non.inflammatory.Macrophages	PAFAH1B3	0.183268	5.814072	1.241701	0.217631	-5.98079	0.450509	0.506494
Non.inflammatory.Macrophages	XRCC4	0.13047	5.185209	1.241587	0.217673	-6.0512	0.468151	0.515453
Non.inflammatory.Macrophages	CRK	-0.11146	6.391053	-1.24123	0.217806	-6.26614	0.435058	0.498528
Non.inflammatory.Macrophages	TRAPPC11	0.174776	3.489165	1.240826	0.217952	-5.65999	0.519314	0.540566
Non.inflammatory.Macrophages	GDF15	-0.38213	1.508239	-1.24081	0.217958	-5.53988	0.585307	0.571183
Non.inflammatory.Macrophages	DGKZ	-0.12998	6.2774	-1.24076	0.217977	-6.27721	0.438139	0.500195
Non.inflammatory.Macrophages	PDIA3	-0.09605	8.546649	-1.24052	0.218066	-6.62317	0.381213	0.469715
Non.inflammatory.Macrophages	AP4B1	0.279508	2.273778	1.240507	0.21807	-5.40169	0.558941	0.559317
Non.inflammatory.Macrophages	ANK3	-0.52383	1.681131	-1.24038	0.218118	-5.16319	0.579255	0.568655
Non.inflammatory.Macrophages	CDC20	-0.37009	3.639962	-1.24019	0.218185	-5.48023	0.514584	0.538578
Non.inflammatory.Macrophages	SLC25A14	0.284359	2.078186	1.240178	0.218191	-5.26268	0.565572	0.562493
Non.inflammatory.Macrophages	GM7160	-0.25977	2.542262	-1.23966	0.218383	-5.61429	0.550195	0.555475

Non.inflammatory.Macrophages	RELB	-0.13871	6.088989	-1.23965	0.218384	-6.19356	0.443414	0.50326
Non.inflammatory.Macrophages	BLOC1S2	0.162366	4.833886	1.239504	0.218439	-5.84164	0.478741	0.521198
Non.inflammatory.Macrophages	PDCD5	0.102768	6.418317	1.239378	0.218486	-6.23662	0.434566	0.498744
Non.inflammatory.Macrophages	DDX19A	0.165553	4.239703	1.239106	0.218586	-5.78602	0.496383	0.529995
Non.inflammatory.Macrophages	TEPSIN	0.423519	0.762658	1.239051	0.218606	-5.11067	0.609734	0.583827
Non.inflammatory.Macrophages	FAM185A	0.345286	2.579686	1.238981	0.218632	-5.21714	0.548953	0.555073
Non.inflammatory.Macrophages	GM43848	0.232324	2.767038	1.238932	0.21865	-5.49414	0.542771	0.552203
Non.inflammatory.Macrophages	KMT2A	0.135752	6.28827	1.238009	0.21899	-6.13541	0.438583	0.501033
Non.inflammatory.Macrophages	MYCT1	-0.41899	1.654516	-1.23795	0.219012	-5.11037	0.581158	0.569992
Non.inflammatory.Macrophages	ZGRF1	-0.32806	3.956437	-1.2378	0.219067	-5.53655	0.505656	0.5347
Non.inflammatory.Macrophages	GM42722	0.564682	2.335621	1.237646	0.219124	-4.96963	0.557847	0.559365
Non.inflammatory.Macrophages	TMPRSS6	-0.55304	1.467899	-1.23733	0.219241	-5.02677	0.587961	0.573096
Non.inflammatory.Macrophages	RHOD	-0.50106	1.328394	-1.23674	0.219458	-4.97823	0.593203	0.575547
Non.inflammatory.Macrophages	SLC24A1	0.434576	1.860788	1.23665	0.219492	-5.06195	0.574518	0.567106
Non.inflammatory.Macrophages	SMIM4	0.120975	5.255277	1.236503	0.219546	-6.0596	0.467601	0.51603

Non.inflammatory.Macrophages	IFT172	-0.24065	2.410455	-1.23647	0.219557	-5.48076	0.555792	0.558547
Non.inflammatory.Macrophages	CPNE2	-0.22333	3.37335	-1.23629	0.219624	-5.76865	0.524336	0.543796
Non.inflammatory.Macrophages	FUBP3	0.169506	4.08675	1.236293	0.219624	-5.80178	0.502107	0.533106
Non.inflammatory.Macrophages	SH3PXD2A	-0.16651	5.753145	-1.23599	0.219735	-6.06811	0.45373	0.509094
Non.inflammatory.Macrophages	CD6	-0.67223	0.929158	-1.23549	0.219923	-4.84248	0.60775	0.582357
Non.inflammatory.Macrophages	SLAMF7	-0.37292	5.151332	-1.23483	0.220167	-5.43566	0.471447	0.518109
Non.inflammatory.Macrophages	EXOG	0.39267	1.745846	1.234688	0.220218	-5.09958	0.579591	0.569618
Non.inflammatory.Macrophages	ARFGAP2	-0.12569	4.901097	-1.23384	0.220534	-5.91814	0.478844	0.522035
Non.inflammatory.Macrophages	MARF1	0.117425	6.147381	1.233788	0.220552	-6.10655	0.443743	0.504233
Non.inflammatory.Macrophages	PYROXD2	-0.43335	0.182818	-1.23376	0.22056	-5.08055	0.617622	0.595228
Non.inflammatory.Macrophages	1810062O:	-0.4074	2.0989	-1.23371	0.220581	-5.11194	0.567557	0.56437
Non.inflammatory.Macrophages	GAB2	0.138153	7.176286	1.233654	0.220601	-6.5342	0.416639	0.489994
Non.inflammatory.Macrophages	PMEL	-0.61645	0.339511	-1.23365	0.220604	-4.89078	0.617622	0.592645
Non.inflammatory.Macrophages	CISD2	0.102389	6.732173	1.233596	0.220623	-6.3209	0.428136	0.496109
Non.inflammatory.Macrophages	STRN	0.145245	5.530644	1.233537	0.220645	-6.01838	0.460794	0.513008

Non.inflammatory.Macrophages	UBE2Q1	-0.09579	6.334643	-1.23285	0.220899	-6.25515	0.439101	0.501767
Non.inflammatory.Macrophages	H2-T23	-0.19532	6.358381	-1.23267	0.220965	-6.27417	0.438504	0.501435
Non.inflammatory.Macrophages	PTBP2	0.129165	6.156832	1.232477	0.221038	-6.12516	0.444002	0.504257
Non.inflammatory.Macrophages	GM43581	0.42333	2.093907	1.232012	0.221211	-5.11191	0.568613	0.564831
Non.inflammatory.Macrophages	SLC40A1	-0.20535	5.835325	-1.232	0.221217	-6.3482	0.452999	0.508978
Non.inflammatory.Macrophages	GM29340	-0.47012	-0.6731	-1.23172	0.221321	-4.99513	0.618747	0.610021
Non.inflammatory.Macrophages	NOSTRIN	-0.21273	3.426931	-1.23149	0.221404	-5.82949	0.524804	0.544369
Non.inflammatory.Macrophages	DHODH	-0.29376	2.109646	-1.23118	0.221521	-5.30812	0.568387	0.564781
Non.inflammatory.Macrophages	HEYL	-0.76329	-0.20551	-1.23118	0.221522	-4.75369	0.618927	0.602296
Non.inflammatory.Macrophages	ZBP1	0.309584	3.640152	1.230541	0.221758	-5.986	0.518566	0.541517
Non.inflammatory.Macrophages	ANKRD46	0.228687	2.377526	1.22998	0.221968	-5.33204	0.560169	0.561103
Non.inflammatory.Macrophages	ZBTB43	0.211147	3.163113	1.229714	0.222067	-5.55159	0.534192	0.549025
Non.inflammatory.Macrophages	ZDHHC23	-0.24605	2.221064	-1.22971	0.22207	-5.49751	0.565495	0.563602
Non.inflammatory.Macrophages	DCUN1D3	-0.17462	4.424811	-1.2296	0.22211	-5.8194	0.494785	0.530133
Non.inflammatory.Macrophages	ZFP593	0.214124	3.359126	1.229316	0.222215	-5.68822	0.527989	0.546137

Non.inflammatory.Macrophages	CD2BP2	0.164351	4.011388	1.229233	0.222246	-5.74007	0.507489	0.536316
Non.inflammatory.Macrophages	SMC4	-0.1485	7.287715	-1.22901	0.222331	-6.23372	0.415503	0.489655
Non.inflammatory.Macrophages	MTRF1	-0.43013	1.208712	-1.22869	0.222448	-5.09187	0.601259	0.580046
Non.inflammatory.Macrophages	ST6GALNA	-0.18988	3.214358	-1.22855	0.222501	-5.79674	0.532763	0.548608
Non.inflammatory.Macrophages	MGLL	-0.35127	3.495334	-1.22853	0.222508	-5.40453	0.523764	0.544336
Non.inflammatory.Macrophages	GBP3	0.261237	3.228626	1.228322	0.222586	-5.83231	0.532303	0.54844
Non.inflammatory.Macrophages	WTAP	0.092779	6.931297	1.228258	0.22261	-6.28627	0.424704	0.494749
Non.inflammatory.Macrophages	NINJ1	0.146436	5.750418	1.228238	0.222617	-6.3167	0.45654	0.511288
Non.inflammatory.Macrophages	CASP6	0.174953	3.628567	1.227829	0.22277	-5.77203	0.519796	0.542626
Non.inflammatory.Macrophages	GPR137B	0.14275	5.316315	1.227598	0.222856	-6.20704	0.469119	0.517757
Non.inflammatory.Macrophages	NR1I3	-0.57581	1.055611	-1.22712	0.223034	-4.99122	0.607564	0.583013
Non.inflammatory.Macrophages	AHR	0.248854	4.719836	1.226974	0.223089	-5.93353	0.486811	0.526548
Non.inflammatory.Macrophages	SPDL1	-0.29558	2.833783	-1.22679	0.223157	-5.42978	0.545942	0.554926
Non.inflammatory.Macrophages	AP1AR	0.18362	5.002368	1.226556	0.223246	-5.80476	0.478636	0.522485
Non.inflammatory.Macrophages	CSRNP2	-0.33023	2.107884	-1.22505	0.223811	-5.28421	0.571842	0.566906

Non.inflammatory.Macrophages	GM11342	0.67068	2.120734	1.224321	0.224083	-4.89616	0.57154	0.567023
Non.inflammatory.Macrophages	TNFAIP6	0.695858	1.129558	1.224282	0.224098	-4.96414	0.606635	0.582862
Non.inflammatory.Macrophages	PRKG1	0.355362	3.504872	1.224138	0.224152	-5.5855	0.52564	0.54566
Non.inflammatory.Macrophages	INPPL1	-0.20753	2.471527	-1.22408	0.224174	-5.59269	0.55957	0.561584
Non.inflammatory.Macrophages	RCL1	0.172686	4.24025	1.224031	0.224192	-5.75816	0.502672	0.534615
Non.inflammatory.Macrophages	RNF13	0.111624	6.065838	1.224027	0.224194	-6.24509	0.449683	0.508123
Non.inflammatory.Macrophages	FBXO11	-0.12716	8.797772	-1.2239	0.224241	-6.594	0.380312	0.470941
Non.inflammatory.Macrophages	DMC1	-0.72505	0.152078	-1.22383	0.224266	-4.731	0.622776	0.599
Non.inflammatory.Macrophages	PRR11	-0.39225	2.847187	-1.2238	0.22428	-5.2939	0.547008	0.555774
Non.inflammatory.Macrophages	MYO15	-0.47555	1.234713	-1.22349	0.224394	-5.09598	0.602963	0.581341
Non.inflammatory.Macrophages	P2RY10B	-0.16638	3.709802	-1.22329	0.22447	-5.90276	0.519265	0.54267
Non.inflammatory.Macrophages	HIST1H2AF	-0.81698	0.617707	-1.22329	0.224471	-4.79206	0.620897	0.591391
Non.inflammatory.Macrophages	LRIG2	0.175506	4.256808	1.222236	0.224867	-5.77855	0.503066	0.534886
Non.inflammatory.Macrophages	GM39090	0.702779	0.497553	1.221358	0.225197	-4.7457	0.624562	0.594099
Non.inflammatory.Macrophages	SNX12	0.117645	4.718214	1.220976	0.225341	-5.97588	0.489959	0.528471

Non.inflammatory.Macrophages	ATXN7L1O	-0.50983	1.337166	-1.22052	0.225512	-4.99562	0.601553	0.580733
Non.inflammatory.Macrophages	BTBD7	0.08943	7.004982	1.220195	0.225636	-6.30026	0.426416	0.496111
Non.inflammatory.Macrophages	NCBP2	-0.13574	4.599992	-1.21946	0.225912	-5.9616	0.494448	0.530735
Non.inflammatory.Macrophages	MEIS3	-0.38261	0.488054	-1.21925	0.225992	-5.19415	0.626243	0.59518
Non.inflammatory.Macrophages	VPS39	0.188162	3.331542	1.21882	0.226155	-5.65475	0.534428	0.549989
Non.inflammatory.Macrophages	PRDM10	0.165334	5.213796	1.218646	0.22622	-5.90887	0.476613	0.521941
Non.inflammatory.Macrophages	PSMD11	0.072289	7.032517	1.218557	0.226254	-6.31426	0.426427	0.496194
Non.inflammatory.Macrophages	GVIN1	0.320728	3.037272	1.218262	0.226365	-5.78136	0.54411	0.554689
Non.inflammatory.Macrophages	PRKAB2	0.196923	3.392055	1.218254	0.226368	-5.71796	0.532538	0.54924
Non.inflammatory.Macrophages	EPB41L2	0.104854	6.975502	1.217751	0.226559	-6.46531	0.428142	0.497214
Non.inflammatory.Macrophages	HBS1L	0.121884	5.694465	1.217536	0.22664	-6.04976	0.463065	0.515267
Non.inflammatory.Macrophages	KCNG2	-0.42431	-0.23458	-1.21752	0.226644	-5.16219	0.62691	0.607629
Non.inflammatory.Macrophages	VPREB2	-0.86112	-0.41404	-1.21751	0.226649	-4.72354	0.62691	0.610662
Non.inflammatory.Macrophages	GM7030	-0.46983	2.084686	-1.21674	0.22694	-5.22227	0.57711	0.569892
Non.inflammatory.Macrophages	DNAJC9	-0.19891	5.617283	-1.21672	0.226948	-5.94398	0.465677	0.516532

Non.inflammatory.Macrophages	SERPINB6A	-0.14702	5.570318	-1.21619	0.227148	-6.24386	0.467331	0.51738
Non.inflammatory.Macrophages	SLC39A6	0.158826	4.11285	1.215803	0.227296	-5.82012	0.510977	0.539009
Non.inflammatory.Macrophages	ANK1	0.383462	1.273318	1.215688	0.22734	-5.47502	0.606643	0.583344
Non.inflammatory.Macrophages	NEK11	-0.45299	0.082898	-1.21545	0.227431	-5.08373	0.628301	0.60299
Non.inflammatory.Macrophages	CPSF1	-0.21456	3.349532	-1.21498	0.227609	-5.56262	0.535633	0.550786
Non.inflammatory.Macrophages	GM4356	0.511971	0.868166	1.214623	0.227744	-5.05569	0.620931	0.590322
Non.inflammatory.Macrophages	CSF1	0.523486	2.480595	1.214613	0.227748	-5.30483	0.564664	0.564448
Non.inflammatory.Macrophages	SRP19	0.11543	6.048783	1.214478	0.227799	-6.17268	0.454499	0.511096
Non.inflammatory.Macrophages	IKZF2	0.389866	4.781138	1.214371	0.22784	-5.75358	0.491081	0.529504
Non.inflammatory.Macrophages	PAPOLG	0.260933	3.005898	1.21417	0.227916	-5.44039	0.547081	0.556433
Non.inflammatory.Macrophages	CCDC51	-0.31273	1.810129	-1.21384	0.228042	-5.23188	0.588146	0.575441
Non.inflammatory.Macrophages	CD5L	-0.257	6.068559	-1.21381	0.228054	-6.46332	0.454096	0.511147
Non.inflammatory.Macrophages	ANKS1	-0.12977	5.759857	-1.21369	0.228099	-6.2271	0.462749	0.515582
Non.inflammatory.Macrophages	ZFP943	0.151685	4.280266	1.213419	0.228201	-5.74352	0.506579	0.537324
Non.inflammatory.Macrophages	TAF1C	0.285134	2.170342	1.213126	0.228313	-5.32825	0.575815	0.569921

Non.inflammatory.Macrophages	2810454H(	-0.28706	2.514759	-1.21301	0.228355	-5.37705	0.56397	0.564516
Non.inflammatory.Macrophages	TNFRSF12/	-0.33207	1.479733	-1.21208	0.22871	-5.31124	0.601065	0.5813
Non.inflammatory.Macrophages	ATAD2B	0.118785	6.740991	1.211859	0.228795	-6.19955	0.436628	0.502152
Non.inflammatory.Macrophages	C1QTNF12	0.332684	2.21932	1.211789	0.228822	-5.30426	0.574938	0.569532
Non.inflammatory.Macrophages	LZTS2	-0.47475	1.249256	-1.2108	0.229198	-5.10136	0.610163	0.58553
Non.inflammatory.Macrophages	BTBD9	-0.10371	8.508871	-1.2108	0.2292	-6.56846	0.392179	0.478424
Non.inflammatory.Macrophages	PDCD2L	0.119079	4.660225	1.210681	0.229244	-5.94737	0.496382	0.532596
Non.inflammatory.Macrophages	NACC2	-0.2628	1.77434	-1.21027	0.229402	-5.56574	0.591212	0.57721
Non.inflammatory.Macrophages	MOSMO	0.15715	4.576833	1.210114	0.229461	-5.86481	0.49891	0.533914
Non.inflammatory.Macrophages	SNX33	-0.51379	0.759185	-1.21011	0.229462	-4.93077	0.625629	0.593724
Non.inflammatory.Macrophages	KDSR	-0.17504	3.963038	-1.21005	0.229486	-5.73129	0.517895	0.543123
Non.inflammatory.Macrophages	GM43813	-0.19738	4.535806	-1.21005	0.229487	-5.84464	0.500158	0.534532
Non.inflammatory.Macrophages	CD22	-0.24743	2.780412	-1.20988	0.22955	-5.56965	0.556396	0.561327
Non.inflammatory.Macrophages	PCNA	-0.15	6.710032	-1.20985	0.229562	-6.30015	0.437941	0.503179
Non.inflammatory.Macrophages	5430431A1	-0.44059	1.423829	-1.20978	0.229587	-4.99238	0.603802	0.582936

Non.inflammatory.Macrophages	4930578M	-0.53474	0.63262	-1.2095	0.229694	-4.95637	0.628785	0.596077
Non.inflammatory.Macrophages	DNAH7A	-0.55615	-0.11769	-1.20912	0.22984	-5.00405	0.631348	0.608705
Non.inflammatory.Macrophages	ENDOG	0.190055	2.943039	1.20856	0.230054	-5.65706	0.551379	0.559218
Non.inflammatory.Macrophages	MAPK9	0.129581	4.751185	1.208484	0.230084	-6.00842	0.494024	0.531773
Non.inflammatory.Macrophages	ERICH1	-0.19405	3.61294	-1.20847	0.23009	-5.70965	0.529439	0.548891
Non.inflammatory.Macrophages	STAC2	-0.50469	1.824947	-1.2084	0.230117	-5.10977	0.589875	0.576882
Non.inflammatory.Macrophages	ZFP280C	0.172189	4.164903	1.208336	0.23014	-5.75592	0.511978	0.540535
Non.inflammatory.Macrophages	ZCCHC14	0.363423	1.412865	1.208215	0.230186	-5.27294	0.604672	0.583566
Non.inflammatory.Macrophages	NCOA7	0.124504	5.299685	1.208214	0.230187	-6.24512	0.477768	0.523751
Non.inflammatory.Macrophages	GM19967	0.363645	1.377066	1.208046	0.230251	-5.17656	0.606019	0.584212
Non.inflammatory.Macrophages	B3GNT6	0.44755	-0.72192	1.207815	0.230339	-5.04234	0.631566	0.619372
Non.inflammatory.Macrophages	GM43112	-0.35034	0.075373	-1.20765	0.230401	-5.24216	0.631607	0.605868
Non.inflammatory.Macrophages	FKBP8	0.120864	6.067485	1.20747	0.230472	-6.20796	0.456061	0.512959
Non.inflammatory.Macrophages	FBXL15	-0.23152	2.696607	-1.20717	0.230586	-5.53519	0.560031	0.563531
Non.inflammatory.Macrophages	DCAF5	0.137323	5.52135	1.20702	0.230644	-6.06037	0.471657	0.520941

Non.inflammatory.Macrophages	ORMDL1	-0.18022	3.609727	-1.20696	0.230666	-5.68271	0.529894	0.549452
Non.inflammatory.Macrophages	DYNLRB1	0.097647	6.280147	1.206277	0.230929	-6.2784	0.450615	0.510195
Non.inflammatory.Macrophages	ANXA5	0.109366	6.137389	1.20615	0.230978	-6.44842	0.454569	0.512227
Non.inflammatory.Macrophages	JMY	-0.13062	6.282387	-1.20614	0.230982	-6.31699	0.450553	0.510164
Non.inflammatory.Macrophages	MPP6	0.116058	6.194697	1.205725	0.23114	-6.2515	0.453196	0.511596
Non.inflammatory.Macrophages	MLLT11	0.198787	3.193389	1.205585	0.231194	-5.56337	0.544136	0.556181
Non.inflammatory.Macrophages	HIST2H3B	-0.62631	1.047683	-1.20515	0.231362	-4.8962	0.619317	0.590456
Non.inflammatory.Macrophages	CCDC73	0.197093	3.100981	1.205117	0.231374	-5.61198	0.547237	0.557691
Non.inflammatory.Macrophages	GM10974	0.55543	0.824708	1.205107	0.231378	-4.88503	0.625814	0.594125
Non.inflammatory.Macrophages	DHRS3	-0.18064	4.170119	-1.20505	0.231401	-6.01331	0.512851	0.541372
Non.inflammatory.Macrophages	SMYD2	0.238373	3.028651	1.204746	0.231516	-5.63324	0.549802	0.558989
Non.inflammatory.Macrophages	CEP162	-0.20287	3.080056	-1.20382	0.231871	-5.58324	0.548792	0.558534
Non.inflammatory.Macrophages	H2-AA	-0.36266	8.331767	-1.20373	0.231905	-6.61526	0.398207	0.482579
Non.inflammatory.Macrophages	OLFR543	0.755935	0.201065	1.203174	0.232121	-4.76792	0.633993	0.605321
Non.inflammatory.Macrophages	PHF21A	0.119601	7.069098	1.202928	0.232216	-6.39471	0.430502	0.500144

Non.inflammatory.Macrophages	NUDT4	0.129951	5.949353	1.202823	0.232256	-6.11376	0.461056	0.515981
Non.inflammatory.Macrophages	TATDN2	0.128391	5.004884	1.202638	0.232327	-5.9919	0.488435	0.529742
Non.inflammatory.Macrophages	TNF	-0.25905	4.405834	-1.20261	0.232339	-6.1492	0.506595	0.53865
Non.inflammatory.Macrophages	CASP4	-0.20296	4.029936	-1.20259	0.232348	-6.12656	0.518314	0.544322
Non.inflammatory.Macrophages	N4BP3	-0.43114	2.955418	-1.20243	0.232408	-5.02982	0.553225	0.560854
Non.inflammatory.Macrophages	GSTP3	-0.2889	3.39573	-1.20241	0.232416	-5.6854	0.538662	0.554025
Non.inflammatory.Macrophages	TMEM176A	-0.16007	4.388779	-1.20223	0.232485	-6.08797	0.507121	0.538969
Non.inflammatory.Macrophages	TERF2IP	-0.18567	3.58313	-1.20214	0.23252	-5.61829	0.532573	0.551191
Non.inflammatory.Macrophages	SMC2	-0.23665	5.816825	-1.2021	0.232536	-6.05504	0.464807	0.517962
Non.inflammatory.Macrophages	GTF3C2	0.106134	5.474786	1.201895	0.232614	-6.07045	0.474688	0.523027
Non.inflammatory.Macrophages	SPDEF	-0.56458	-0.4296	-1.20149	0.232768	-4.98215	0.63437	0.616602
Non.inflammatory.Macrophages	UBL4A	0.180916	4.202976	1.201072	0.232931	-5.86189	0.513448	0.542267
Non.inflammatory.Macrophages	CXCR2	0.698615	-0.00657	1.200718	0.233068	-4.86646	0.634931	0.609746
Non.inflammatory.Macrophages	FAM217B	0.329243	0.616789	1.200059	0.233322	-5.36273	0.633445	0.599423
Non.inflammatory.Macrophages	FAM98C	-0.18481	3.737769	-1.1999	0.233384	-5.77171	0.528882	0.549647

Non.inflammatory.Macrophages	RANBP3	0.141155	4.303701	1.199512	0.233534	-5.8371	0.511135	0.541177
Non.inflammatory.Macrophages	D16ERTD4!	0.208653	4.914897	1.199414	0.233572	-5.75067	0.492454	0.532052
Non.inflammatory.Macrophages	FAM50A	-0.11793	5.186332	-1.19937	0.233589	-6.08971	0.484366	0.528046
Non.inflammatory.Macrophages	ABCA7	-0.16394	3.89815	-1.19908	0.233702	-5.73005	0.524046	0.547348
Non.inflammatory.Macrophages	S100PBP	-0.14133	4.405401	-1.1983	0.234002	-5.87007	0.50861	0.539805
Non.inflammatory.Macrophages	ABCB9	-0.33483	3.155352	-1.19826	0.234019	-5.33219	0.548727	0.558922
Non.inflammatory.Macrophages	PHF13	0.16509	3.504231	1.198019	0.234111	-5.74034	0.537347	0.553657
Non.inflammatory.Macrophages	PIGU	0.170653	4.215968	1.197898	0.234158	-5.84319	0.514606	0.542814
Non.inflammatory.Macrophages	GM49173	-0.57118	-0.87955	-1.19769	0.234239	-5.07424	0.636713	0.625513
Non.inflammatory.Macrophages	RABGGTB	0.162741	3.732854	1.197434	0.234338	-5.81422	0.530026	0.550354
Non.inflammatory.Macrophages	MPND	0.166917	4.813143	1.197322	0.234381	-5.90723	0.496307	0.534082
Non.inflammatory.Macrophages	SH2B3	-0.12417	5.315208	-1.1973	0.234389	-6.12118	0.481336	0.526669
Non.inflammatory.Macrophages	MYDGF	0.11865	4.485984	1.197195	0.234431	-6.00557	0.506299	0.539016
Non.inflammatory.Macrophages	TARS	0.152379	4.374393	1.196987	0.234511	-5.90027	0.509823	0.540769
Non.inflammatory.Macrophages	ELK3	0.165643	5.288608	1.196587	0.234666	-5.95548	0.482362	0.527364

Non.inflammatory.Macrophages	UBAP1L	0.345611	1.134289	1.196525	0.234691	-5.17143	0.620357	0.591975
Non.inflammatory.Macrophages	RNF144B	-0.19525	2.562148	-1.19622	0.234808	-5.78668	0.569288	0.569049
Non.inflammatory.Macrophages	GM43768	-0.53032	0.70894	-1.19614	0.234839	-4.99516	0.632953	0.599166
Non.inflammatory.Macrophages	ANKRD16	0.227348	3.026656	1.196137	0.234841	-5.53876	0.553517	0.561778
Non.inflammatory.Macrophages	GM26535	-0.48967	-0.60061	-1.19591	0.234928	-5.01596	0.637126	0.621374
Non.inflammatory.Macrophages	MAPK7	-0.2094	3.195275	-1.19584	0.234956	-5.6787	0.547941	0.559198
Non.inflammatory.Macrophages	SUPT7L	0.243992	2.767637	1.195302	0.235165	-5.50006	0.562473	0.566089
Non.inflammatory.Macrophages	ZFP326	0.115957	5.21559	1.195263	0.23518	-6.07951	0.484718	0.528805
Non.inflammatory.Macrophages	MLF2	0.11027	5.85253	1.195171	0.235216	-6.20913	0.46622	0.519517
Non.inflammatory.Macrophages	LRP4	-0.23323	2.477171	-1.1951	0.235244	-5.85368	0.572433	0.5707
Non.inflammatory.Macrophages	AFTPH	0.100944	6.658529	1.19506	0.235259	-6.3313	0.44378	0.508003
Non.inflammatory.Macrophages	ELP5	-0.13574	4.633093	-1.19448	0.235483	-5.99885	0.502469	0.537624
Non.inflammatory.Macrophages	TENT2	0.087814	6.624422	1.194352	0.235535	-6.37463	0.444906	0.508673
Non.inflammatory.Macrophages	KCTD13	0.234778	2.619439	1.194241	0.235578	-5.48616	0.567787	0.568673
Non.inflammatory.Macrophages	KIF1C	0.156809	3.254466	1.194058	0.235649	-5.86262	0.54638	0.558782

Non.inflammatory.Macrophages	KLK1	1.023403	-0.20745	1.194015	0.235666	-4.7283	0.637593	0.615213
Non.inflammatory.Macrophages	ARPC4	0.088078	7.957802	1.193966	0.235685	-6.54695	0.409974	0.49021
Non.inflammatory.Macrophages	PLAA	0.103908	6.069326	1.193943	0.235694	-6.19683	0.460284	0.51667
Non.inflammatory.Macrophages	GM50232	0.58725	1.372215	1.193597	0.235828	-4.94209	0.612223	0.588989
Non.inflammatory.Macrophages	ATIC	0.171891	4.493549	1.193571	0.235839	-5.92115	0.506867	0.540008
Non.inflammatory.Macrophages	GM48236	-0.57306	0.1418	-1.1926	0.236219	-4.99041	0.638352	0.609887
Non.inflammatory.Macrophages	PIGB	0.271776	2.703863	1.192521	0.236248	-5.40225	0.565569	0.567973
Non.inflammatory.Macrophages	EIF4G3	0.086761	7.91677	1.192506	0.236254	-6.5214	0.411497	0.49126
Non.inflammatory.Macrophages	BBS9	-0.19473	4.909527	-1.1925	0.236257	-5.9574	0.494662	0.534157
Non.inflammatory.Macrophages	ZFP959	-0.21484	3.241052	-1.19174	0.236551	-5.55879	0.548021	0.559889
Non.inflammatory.Macrophages	GON4L	0.122664	5.15234	1.191651	0.236587	-6.02495	0.487874	0.530896
Non.inflammatory.Macrophages	IDH3B	0.129309	5.850654	1.191343	0.236707	-6.20391	0.467553	0.520713
Non.inflammatory.Macrophages	BRAF	0.125203	7.588384	1.191342	0.236708	-6.50725	0.42034	0.496123
Non.inflammatory.Macrophages	DDX20	-0.19834	3.596417	-1.1912	0.236761	-5.5344	0.53641	0.554436
Non.inflammatory.Macrophages	CHCHD4	0.17335	3.820424	1.190647	0.236979	-5.82265	0.529545	0.55122

Non.inflammatory.Macrophages	VPS16	0.132087	4.436967	1.190268	0.237127	-5.87921	0.510274	0.542022
Non.inflammatory.Macrophages	GM38948	0.645337	0.463761	1.189646	0.23737	-4.7807	0.640337	0.605608
Non.inflammatory.Macrophages	1700025G(	-0.19521	4.957756	-1.18934	0.237491	-6.05891	0.494896	0.534608
Non.inflammatory.Macrophages	PIK3R6	0.244962	2.596377	1.189113	0.237579	-5.8557	0.5713	0.570989
Non.inflammatory.Macrophages	ECSIT	0.18913	4.015765	1.188946	0.237644	-5.68232	0.524242	0.548939
Non.inflammatory.Macrophages	AXDND1	-0.25688	1.557778	-1.18882	0.237692	-5.71807	0.60825	0.587797
Non.inflammatory.Macrophages	GM10134	0.282044	0.795093	1.188577	0.237789	-5.61864	0.634589	0.600487
Non.inflammatory.Macrophages	GM5244	-0.49308	-0.39473	-1.18833	0.237887	-5.10403	0.640842	0.620762
Non.inflammatory.Macrophages	4930473A(	-0.58553	0.38363	-1.18832	0.237889	-4.8381	0.640842	0.607497
Non.inflammatory.Macrophages	POLR2D	0.145464	5.184758	1.187628	0.238161	-5.9981	0.48866	0.531691
Non.inflammatory.Macrophages	5031425E2	-0.14575	5.390925	-1.18761	0.238168	-6.00779	0.482548	0.52865
Non.inflammatory.Macrophages	RHBDF2	-0.12892	4.650027	-1.18748	0.238218	-6.0464	0.504856	0.539724
Non.inflammatory.Macrophages	TGDS	0.202438	3.1411	1.18742	0.238243	-5.60751	0.55333	0.562873
Non.inflammatory.Macrophages	CLEC12A	-0.15607	4.937614	-1.18701	0.238404	-6.30684	0.49632	0.535535
Non.inflammatory.Macrophages	BCL2L13	-0.15652	5.133362	-1.18662	0.238558	-5.96017	0.490649	0.532765

Non.inflammatory.Macrophages	CTBP2	-0.17274	3.249836	-1.18643	0.238629	-5.90837	0.550261	0.56145
Non.inflammatory.Macrophages	GM45715	-0.68696	1.039938	-1.18618	0.238729	-4.75583	0.62881	0.597118
Non.inflammatory.Macrophages	ATM	0.165416	4.221236	1.18568	0.238926	-5.82451	0.519154	0.54673
Non.inflammatory.Macrophages	PIGT	0.125357	5.640537	1.185588	0.238962	-6.17562	0.476112	0.52557
Non.inflammatory.Macrophages	KSR1	-0.18084	4.69672	-1.18534	0.239058	-5.94082	0.504443	0.539613
Non.inflammatory.Macrophages	TGIF1	-0.14	6.736671	-1.18507	0.239167	-6.4432	0.445353	0.509957
Non.inflammatory.Macrophages	ROR1	-0.59006	1.374979	-1.18504	0.239177	-4.99377	0.616831	0.592012
Non.inflammatory.Macrophages	GM19466	-0.64111	0.661272	-1.18493	0.23922	-4.8787	0.639652	0.60391
Non.inflammatory.Macrophages	TUG1	-0.1125	6.098897	-1.18472	0.239302	-6.20546	0.463152	0.519187
Non.inflammatory.Macrophages	NPAT	0.169556	4.485178	1.184581	0.239358	-5.88158	0.511118	0.543044
Non.inflammatory.Macrophages	S1PR5	0.583637	-0.65106	1.184318	0.239461	-4.91618	0.642902	0.626429
Non.inflammatory.Macrophages	6530402F1	0.714499	-0.17553	1.183427	0.239812	-4.78903	0.643674	0.618714
Non.inflammatory.Macrophages	GM4788	-0.51185	1.237901	-1.18335	0.239844	-5.08759	0.622931	0.594909
Non.inflammatory.Macrophages	MS4A4B	0.499633	3.207847	1.182512	0.240173	-5.17218	0.553736	0.56358
Non.inflammatory.Macrophages	ZFP341	0.324899	1.984672	1.182355	0.240235	-5.2147	0.596212	0.583122

Non.inflammatory.Macrophages	PTPN11	0.107786	5.063935	1.182341	0.24024	-6.07363	0.494633	0.535239
Non.inflammatory.Macrophages	NAV1	0.209025	3.595293	1.182159	0.240312	-5.92323	0.540882	0.557654
Non.inflammatory.Macrophages	SMURF2	0.111828	6.82379	1.182094	0.240338	-6.31758	0.44417	0.50972
Non.inflammatory.Macrophages	TMSB10	0.152348	10.73818	1.181962	0.24039	-6.76204	0.34925	0.457152
Non.inflammatory.Macrophages	ARF4	0.097544	8.479652	1.181672	0.240504	-6.64474	0.401378	0.486887
Non.inflammatory.Macrophages	MRPS11	0.191089	3.950885	1.181229	0.240679	-5.79306	0.529696	0.552402
Non.inflammatory.Macrophages	TRIM33	0.112629	6.368235	1.180909	0.240806	-6.20443	0.457056	0.516527
Non.inflammatory.Macrophages	TGIF2	-0.25664	3.122183	-1.18086	0.240827	-5.42977	0.557011	0.565372
Non.inflammatory.Macrophages	EFCAB5	0.671928	0.504613	1.180851	0.240829	-4.86027	0.644571	0.608045
Non.inflammatory.Macrophages	GM8113	0.531816	-0.25687	1.180737	0.240874	-4.97207	0.64481	0.621042
Non.inflammatory.Macrophages	2900060B1	-0.3118	2.614995	-1.1807	0.240887	-5.27821	0.574368	0.57342
Non.inflammatory.Macrophages	MAP4K2	-0.16513	5.348887	-1.17991	0.241202	-6.01089	0.48695	0.53157
Non.inflammatory.Macrophages	ST6GALNA	-0.40855	0.651842	-1.17984	0.24123	-5.16823	0.642708	0.605777
Non.inflammatory.Macrophages	CLEC14A	0.402824	2.196348	1.179585	0.24133	-5.30274	0.589792	0.580378
Non.inflammatory.Macrophages	CES2A	-0.58444	0.898465	-1.17948	0.241373	-4.99983	0.636794	0.601696

Non.inflammatory.Macrophages	TMEM204	-0.42921	1.038857	-1.17917	0.241496	-5.0234	0.632433	0.599397
Non.inflammatory.Macrophages	TAPBP	0.155436	6.597076	1.179104	0.24152	-6.37988	0.451346	0.513549
Non.inflammatory.Macrophages	C1D	0.0965	5.843961	1.178971	0.241573	-6.20885	0.472644	0.524482
Non.inflammatory.Macrophages	CXCR4	0.186443	6.146916	1.178437	0.241784	-6.14175	0.464281	0.520257
Non.inflammatory.Macrophages	KRT8	-0.43632	1.975849	-1.17819	0.241882	-5.26462	0.598283	0.584334
Non.inflammatory.Macrophages	ETNK1	0.120287	5.999727	1.178141	0.241902	-6.18209	0.468523	0.52243
Non.inflammatory.Macrophages	PFKM	0.386572	1.17862	1.17778	0.242045	-5.17124	0.627898	0.59751
Non.inflammatory.Macrophages	NTPCR	0.147338	3.847172	1.177453	0.242175	-5.99914	0.534563	0.554838
Non.inflammatory.Macrophages	DYNLL2	-0.14645	5.082989	-1.1774	0.242194	-6.16571	0.495816	0.536089
Non.inflammatory.Macrophages	RCOR2	-0.78018	0.194015	-1.17665	0.242492	-4.79238	0.647229	0.614455
Non.inflammatory.Macrophages	SAP30	0.138885	5.143228	1.176614	0.242508	-6.11469	0.494442	0.535451
Non.inflammatory.Macrophages	BAX	0.116058	6.141068	1.176466	0.242567	-6.30845	0.465215	0.520812
Non.inflammatory.Macrophages	GIMAP6	0.306213	5.672059	1.176232	0.24266	-5.74426	0.478752	0.527696
Non.inflammatory.Macrophages	DHX9	0.140581	6.492589	1.17612	0.242704	-6.28816	0.455314	0.515778
Non.inflammatory.Macrophages	GNMT	0.314659	4.232803	1.176102	0.242711	-5.90051	0.522672	0.549269

Non.inflammatory.Macrophages	PRDM11	0.260269	3.444862	1.175657	0.242888	-5.52619	0.548591	0.56152
Non.inflammatory.Macrophages	GM40841	-0.67163	0.40148	-1.17542	0.242981	-4.90983	0.647727	0.611228
Non.inflammatory.Macrophages	PHF1	-0.26406	2.695984	-1.17522	0.243064	-5.49112	0.57423	0.573524
Non.inflammatory.Macrophages	CKAP2L	-0.18134	4.357364	-1.17509	0.243115	-6.00318	0.519179	0.547665
Non.inflammatory.Macrophages	ZFP420	-0.53582	0.275776	-1.1744	0.24339	-4.96851	0.648349	0.613802
Non.inflammatory.Macrophages	TBC1D23	0.105053	5.852371	1.174355	0.243406	-6.25904	0.4743	0.52561
Non.inflammatory.Macrophages	TRBC1	-0.63788	3.183542	-1.17407	0.24352	-5.1102	0.55814	0.56619
Non.inflammatory.Macrophages	SARAF	-0.11147	5.365255	-1.1738	0.243628	-6.23884	0.488782	0.532903
Non.inflammatory.Macrophages	SMIM15	0.119483	4.858356	1.17376	0.243643	-6.04968	0.504136	0.540494
Non.inflammatory.Macrophages	DHRS7	0.205994	4.016547	1.173684	0.243674	-5.92948	0.530647	0.553335
Non.inflammatory.Macrophages	GM15445	0.3254	0.994582	1.173397	0.243788	-5.2812	0.636953	0.601911
Non.inflammatory.Macrophages	METTL8	-0.24596	2.52734	-1.17337	0.2438	-5.5555	0.580841	0.576804
Non.inflammatory.Macrophages	STX11	0.170279	4.829435	1.172702	0.244065	-6.05437	0.505393	0.541083
Non.inflammatory.Macrophages	MYNN	-0.15254	4.303762	-1.17264	0.244089	-5.78867	0.521837	0.54906
Non.inflammatory.Macrophages	GM43773	-0.46534	1.074002	-1.17264	0.244091	-5.07383	0.634311	0.600666

Non.inflammatory.Macrophages	FXR1	0.093096	6.109299	1.172105	0.244303	-6.2732	0.467706	0.522195
Non.inflammatory.Macrophages	GM42670	-0.44853	0.89839	-1.17196	0.244363	-5.09549	0.640633	0.603664
Non.inflammatory.Macrophages	SERPINB6B	-0.41943	2.936635	-1.17158	0.244513	-5.44337	0.56763	0.570558
Non.inflammatory.Macrophages	ITM2C	-0.11703	5.637577	-1.17146	0.244562	-6.36663	0.481618	0.529308
Non.inflammatory.Macrophages	ENG	-0.27428	4.005662	-1.17083	0.244813	-5.73996	0.532433	0.554103
Non.inflammatory.Macrophages	CELF2	-0.08545	8.597509	-1.1706	0.244907	-6.65525	0.402114	0.48769
Non.inflammatory.Macrophages	RFTN2	-0.39617	2.237638	-1.17017	0.245078	-5.23058	0.592889	0.582235
Non.inflammatory.Macrophages	FARSA	0.121989	4.90554	1.170121	0.245097	-6.00484	0.504283	0.540576
Non.inflammatory.Macrophages	CEACAM10	0.676349	-1.04799	1.170054	0.245123	-4.77944	0.650609	0.637852
Non.inflammatory.Macrophages	SGPP1	0.137163	5.017924	1.169942	0.245168	-6.13286	0.500838	0.538894
Non.inflammatory.Macrophages	ABL2	-0.12149	6.21523	-1.16965	0.245287	-6.36218	0.46557	0.521232
Non.inflammatory.Macrophages	SYTL3	-0.68847	2.803793	-1.16962	0.245298	-5.01717	0.573035	0.573163
Non.inflammatory.Macrophages	GMEB1	0.141274	5.025748	1.169439	0.24537	-6.01831	0.500716	0.538851
Non.inflammatory.Macrophages	EEF2K	-0.19253	4.644155	-1.1692	0.245466	-5.81831	0.512602	0.54465
Non.inflammatory.Macrophages	MPLKIP	-0.11413	4.793719	-1.16857	0.245717	-6.01113	0.50837	0.542531

Non.inflammatory.Macrophages	PCIF1	0.105923	6.021144	1.168372	0.245797	-6.15679	0.47172	0.524304
Non.inflammatory.Macrophages	TUBB3	-0.40377	0.736309	-1.16823	0.245856	-5.15316	0.64665	0.607381
Non.inflammatory.Macrophages	D17H6S53I	0.149855	4.009289	1.167795	0.246029	-5.83913	0.533391	0.554629
Non.inflammatory.Macrophages	PTPN14	0.625132	0.524362	1.167767	0.24604	-4.92322	0.651047	0.611064
Non.inflammatory.Macrophages	RNF19B	0.12961	5.758355	1.167755	0.246045	-6.30248	0.479439	0.52827
Non.inflammatory.Macrophages	TOX	-0.48156	4.351569	-1.16767	0.246078	-5.45142	0.522401	0.54937
Non.inflammatory.Macrophages	PHYH	-0.19522	5.40217	-1.16728	0.246234	-6.18034	0.490203	0.533597
Non.inflammatory.Macrophages	PRPF38B	0.075068	6.611272	1.166695	0.246471	-6.33362	0.455607	0.516163
Non.inflammatory.Macrophages	POLR3B	0.167285	5.512738	1.166427	0.246578	-6.06229	0.487393	0.532303
Non.inflammatory.Macrophages	APBB2	-0.40812	4.02877	-1.16608	0.246717	-5.49119	0.533726	0.554925
Non.inflammatory.Macrophages	RPA1	-0.16699	4.922792	-1.16581	0.246825	-5.9328	0.50557	0.541372
Non.inflammatory.Macrophages	AMFR	0.092479	6.011988	1.165243	0.247055	-6.24171	0.473379	0.525481
Non.inflammatory.Macrophages	CNNM4	-0.16117	4.077084	-1.16503	0.247142	-5.83116	0.532681	0.554621
Non.inflammatory.Macrophages	CPSF4	0.169031	4.317677	1.164909	0.247189	-5.88273	0.524941	0.550949
Non.inflammatory.Macrophages	TMCC3	-0.17328	5.475701	-1.16488	0.2472	-6.34907	0.489154	0.533479

Non.inflammatory.Macrophages	ZFP865	0.20736	3.265059	1.164495	0.247356	-5.68196	0.559802	0.567507
Non.inflammatory.Macrophages	TXNDC16	-0.1884	5.44957	-1.1643	0.247434	-6.05303	0.490114	0.534114
Non.inflammatory.Macrophages	SNRPD1	-0.11399	7.17842	-1.1643	0.247434	-6.40799	0.440902	0.509015
Non.inflammatory.Macrophages	CERS6	0.130018	7.330325	1.163424	0.247788	-6.63631	0.437215	0.507065
Non.inflammatory.Macrophages	PREP	0.147927	5.298922	1.163392	0.247801	-6.01555	0.495097	0.536567
Non.inflammatory.Macrophages	SNX1	0.092729	5.483558	1.163377	0.247807	-6.17777	0.489546	0.533816
Non.inflammatory.Macrophages	LSM4	0.103827	6.704001	1.162553	0.248139	-6.33949	0.454848	0.516144
Non.inflammatory.Macrophages	RNF227	-0.33535	1.852006	-1.16239	0.248207	-5.33011	0.610995	0.590765
Non.inflammatory.Macrophages	TAGAP	0.256638	4.269177	1.162263	0.248257	-5.82364	0.527816	0.552346
Non.inflammatory.Macrophages	CD72	0.209353	4.334961	1.16205	0.248342	-5.89126	0.525777	0.551381
Non.inflammatory.Macrophages	SEMA6A	-0.52126	2.821738	-1.16192	0.248394	-5.384	0.576344	0.575142
Non.inflammatory.Macrophages	MYOM1	-0.31489	1.071853	-1.16182	0.248435	-5.42021	0.640397	0.603843
Non.inflammatory.Macrophages	1700030J2	-0.4922	-0.14389	-1.16159	0.248529	-5.14722	0.655222	0.624666
Non.inflammatory.Macrophages	5730522E0	-0.22554	3.610742	-1.16149	0.24857	-5.93226	0.549484	0.562762
Non.inflammatory.Macrophages	H2-AB1	-0.33247	6.764416	-1.16124	0.248668	-6.45811	0.453303	0.515587

Non.inflammatory.Macrophages	SLC35F5	-0.19769	3.11698	-1.16121	0.248684	-5.82805	0.566184	0.570699
Non.inflammatory.Macrophages	SLC44A1	0.164963	4.637679	1.161089	0.248731	-5.96081	0.516218	0.547085
Non.inflammatory.Macrophages	MTCP1	0.548737	0.737147	1.161051	0.248746	-4.97214	0.650285	0.609787
Non.inflammatory.Macrophages	CUL9	-0.22937	2.579779	-1.16081	0.248843	-5.62243	0.584973	0.579463
Non.inflammatory.Macrophages	MFSD12	0.1693	2.730413	1.16051	0.248965	-5.76998	0.579672	0.577124
Non.inflammatory.Macrophages	GM12064	0.69687	0.96957	1.160487	0.248975	-4.79352	0.644486	0.606078
Non.inflammatory.Macrophages	VRK1	-0.13399	5.309256	-1.16047	0.24898	-6.04317	0.495575	0.537153
Non.inflammatory.Macrophages	KBTBD8	-0.49889	0.251382	-1.16029	0.249053	-4.99138	0.65532	0.618339
Non.inflammatory.Macrophages	USP21	-0.184	3.140041	-1.16023	0.249079	-5.78006	0.565478	0.570648
Non.inflammatory.Macrophages	1700084CC	0.273575	2.9115	1.160122	0.249122	-5.48861	0.573357	0.574319
Non.inflammatory.Macrophages	FRAT1	0.283654	3.463579	1.159959	0.249188	-5.28878	0.554535	0.565608
Non.inflammatory.Macrophages	PDE7A	0.153497	6.703412	1.159711	0.249289	-6.14999	0.455195	0.516893
Non.inflammatory.Macrophages	CD164	0.107537	6.379209	1.159578	0.249343	-6.34299	0.464333	0.52158
Non.inflammatory.Macrophages	SULT2A1	-0.35701	3.901678	-1.15941	0.249409	-5.82615	0.540144	0.558913
Non.inflammatory.Macrophages	ACTA2	0.494073	3.320545	1.159186	0.249502	-5.69208	0.559596	0.568068

Non.inflammatory.Macrophages	CMTM3	-0.15098	3.593966	-1.15893	0.249604	-6.01376	0.550391	0.563802
Non.inflammatory.Macrophages	GPRC5C	-0.34737	1.29284	-1.15893	0.249605	-5.52975	0.632418	0.601063
Non.inflammatory.Macrophages	ERF	-0.19369	3.477368	-1.15887	0.24963	-5.87863	0.554299	0.565653
Non.inflammatory.Macrophages	GMPS	0.105612	5.784964	1.158562	0.249754	-6.17044	0.481667	0.530596
Non.inflammatory.Macrophages	ERCC2	0.290547	1.983619	1.158559	0.249756	-5.34002	0.606759	0.589802
Non.inflammatory.Macrophages	SERAC1	0.320825	2.177244	1.158172	0.249913	-5.37552	0.599979	0.586841
Non.inflammatory.Macrophages	SEPHS2	-0.17254	6.39868	-1.15781	0.25006	-6.23268	0.464302	0.521796
Non.inflammatory.Macrophages	DLAT	0.133066	4.282042	1.157596	0.250147	-5.92511	0.528431	0.55351
Non.inflammatory.Macrophages	4931413K1	-0.3608	1.943113	-1.15734	0.25025	-5.19002	0.608973	0.59088
Non.inflammatory.Macrophages	USP49	-0.19342	4.534917	-1.15672	0.250505	-5.90719	0.520901	0.550021
Non.inflammatory.Macrophages	AQP9	-0.40361	2.330322	-1.15612	0.250745	-5.27464	0.595866	0.585021
Non.inflammatory.Macrophages	NR1D2	-0.1624	3.478586	-1.15568	0.250927	-5.87899	0.556094	0.566744
Non.inflammatory.Macrophages	RUNDC1	0.223447	2.994317	1.155542	0.250982	-5.55985	0.572655	0.574446
Non.inflammatory.Macrophages	ITPR1	0.131613	6.992018	1.155355	0.251058	-6.35752	0.448788	0.51395
Non.inflammatory.Macrophages	SNRNP48	0.135379	4.916287	1.155236	0.251107	-5.97697	0.509527	0.54454

Non.inflammatory.Macrophages	SNHG4.1	-0.73221	0.471422	-1.15508	0.25117	-4.88913	0.657807	0.616261
Non.inflammatory.Macrophages	MTDH	0.068344	7.470279	1.155068	0.251175	-6.47463	0.435817	0.507222
Non.inflammatory.Macrophages	BC024978	-0.28812	1.865186	-1.15504	0.251186	-5.48064	0.613054	0.592863
Non.inflammatory.Macrophages	HMGCS1	-0.17233	4.453186	-1.15456	0.251381	-5.90584	0.524323	0.551828
Non.inflammatory.Macrophages	PILRA	0.152956	3.3927	1.154511	0.251402	-6.2059	0.559225	0.568358
Non.inflammatory.Macrophages	ENHO	-0.31612	2.481211	-1.15443	0.251433	-5.49442	0.59094	0.582998
Non.inflammatory.Macrophages	MLLT1	-0.21601	2.735176	-1.15419	0.251531	-5.51115	0.582054	0.579016
Non.inflammatory.Macrophages	GCFC2	0.263414	2.248325	1.153586	0.251779	-5.37221	0.599896	0.587219
Non.inflammatory.Macrophages	GM27010	0.277274	2.340877	1.153432	0.251842	-5.42914	0.596589	0.585766
Non.inflammatory.Macrophages	GM15706	-0.40768	1.015787	-1.15329	0.2519	-5.14274	0.646091	0.607784
Non.inflammatory.Macrophages	PARP14	0.210588	5.480462	1.15318	0.251944	-6.28532	0.493006	0.53683
Non.inflammatory.Macrophages	GM42567	-0.47603	1.12927	-1.15272	0.252133	-5.23209	0.642068	0.606152
Non.inflammatory.Macrophages	KMT5C	-0.21602	2.877228	-1.15191	0.252464	-5.665	0.578543	0.577564
Non.inflammatory.Macrophages	MVB12B	-0.166	4.074675	-1.15158	0.252597	-5.94823	0.53819	0.558733
Non.inflammatory.Macrophages	RND1	0.286736	1.145338	1.151378	0.252681	-5.50457	0.642471	0.606207

Non.inflammatory.Macrophages	PHKG1	-0.4652	1.107052	-1.15113	0.252781	-5.16334	0.644077	0.606939
Non.inflammatory.Macrophages	RPA3	-0.16485	4.97161	-1.15083	0.252906	-5.99187	0.509907	0.545151
Non.inflammatory.Macrophages	TCRG-C2	1.041919	0.685448	1.150436	0.253066	-4.8556	0.656915	0.614357
Non.inflammatory.Macrophages	RFX7	-0.13608	7.368214	-1.15	0.253246	-6.31662	0.440412	0.510154
Non.inflammatory.Macrophages	TRAF5	-0.14132	5.308971	-1.14996	0.253263	-6.11523	0.499571	0.540255
Non.inflammatory.Macrophages	STK40	-0.1116	5.69472	-1.14985	0.253305	-6.29043	0.487937	0.534484
Non.inflammatory.Macrophages	GM17387	-0.56807	0.498836	-1.14975	0.253347	-4.94364	0.660445	0.617595
Non.inflammatory.Macrophages	CD200R1	-0.20548	2.827999	-1.1497	0.253368	-5.89305	0.580897	0.578878
Non.inflammatory.Macrophages	PPP1R35	-0.1856	3.395179	-1.14927	0.253542	-5.73077	0.561281	0.569913
Non.inflammatory.Macrophages	RBM48	0.210088	3.037834	1.149216	0.253566	-5.54824	0.573567	0.575626
Non.inflammatory.Macrophages	1110065P2	0.157045	3.514697	1.149173	0.253584	-5.80086	0.557226	0.568038
Non.inflammatory.Macrophages	CLEC1B	-0.20242	3.741016	-1.14915	0.253595	-6.10144	0.549624	0.564472
Non.inflammatory.Macrophages	GM10131	-0.54292	1.072727	-1.14914	0.253598	-5.08395	0.645667	0.607952
Non.inflammatory.Macrophages	HDAC4	0.154253	5.402881	1.149113	0.253609	-6.13851	0.496714	0.538954
Non.inflammatory.Macrophages	RNF2	0.108411	5.723956	1.149025	0.253644	-6.18017	0.487066	0.534219

Non.inflammatory.Macrophages	CD99L2	-0.20894	2.142314	-1.14888	0.253705	-5.60403	0.605457	0.590309
Non.inflammatory.Macrophages	GM15283	0.23235	4.261918	1.148813	0.253732	-5.72039	0.532493	0.556506
Non.inflammatory.Macrophages	6530409C1	0.487584	1.661527	1.14878	0.253745	-5.07468	0.623241	0.598255
Non.inflammatory.Macrophages	IL18BP	0.252863	3.740937	1.148698	0.253779	-6.10401	0.549626	0.564665
Non.inflammatory.Macrophages	MSRB2	0.518799	1.023579	1.148619	0.253811	-5.03853	0.647572	0.609007
Non.inflammatory.Macrophages	CHKB	-0.13365	4.306938	-1.14813	0.254012	-5.94155	0.531355	0.556043
Non.inflammatory.Macrophages	TMEFF1	0.650012	-0.59624	1.14755	0.25425	-4.95866	0.661481	0.637466
Non.inflammatory.Macrophages	SECISBP2L	0.128111	4.964176	1.147318	0.254345	-6.15172	0.510972	0.546255
Non.inflammatory.Macrophages	PEAR1	0.403422	2.352213	1.147048	0.254457	-5.14578	0.598898	0.587542
Non.inflammatory.Macrophages	CCDC93	-0.15557	3.72019	-1.14658	0.25465	-5.97346	0.551531	0.565745
Non.inflammatory.Macrophages	THAP7	0.20134	3.607354	1.146409	0.254719	-5.73939	0.555321	0.567573
Non.inflammatory.Macrophages	RAB22A	0.108194	5.60028	1.146378	0.254732	-6.18761	0.491841	0.536943
Non.inflammatory.Macrophages	PDCD11	-0.14406	4.214015	-1.14629	0.254767	-5.92595	0.535223	0.558109
Non.inflammatory.Macrophages	TIMM10B	0.100769	6.181583	1.146188	0.25481	-6.35514	0.474665	0.52836
Non.inflammatory.Macrophages	BMI1	0.181169	3.545427	1.145959	0.254904	-5.76724	0.557512	0.56867

Non.inflammatory.Macrophages	MIOS	0.176665	3.665658	1.145786	0.254976	-5.69528	0.553507	0.566798
Non.inflammatory.Macrophages	RSAD1	0.43781	1.298807	1.144991	0.255303	-5.25922	0.639198	0.605549
Non.inflammatory.Macrophages	ZMYM2	0.121813	5.970893	1.144855	0.255359	-6.21029	0.481444	0.531776
Non.inflammatory.Macrophages	FIS1	0.095921	7.530663	1.144794	0.255384	-6.51412	0.437569	0.50918
Non.inflammatory.Macrophages	RTCB	0.122586	5.290693	1.144578	0.255473	-6.12578	0.501955	0.541956
Non.inflammatory.Macrophages	AVIL	-0.34297	2.092681	-1.14433	0.255576	-5.33665	0.609618	0.592456
Non.inflammatory.Macrophages	VKORC1L1	0.128282	5.560377	1.144209	0.255625	-6.11675	0.49386	0.537993
Non.inflammatory.Macrophages	TCTN1	-0.46622	1.315572	-1.14399	0.255716	-5.12421	0.63885	0.605538
Non.inflammatory.Macrophages	COX18	-0.18156	3.223872	-1.14382	0.255784	-5.76084	0.569384	0.574242
Non.inflammatory.Macrophages	BACH1	-0.13235	5.9952	-1.14355	0.255897	-6.4038	0.480951	0.531623
Non.inflammatory.Macrophages	SIGMAR1	-0.17335	3.549056	-1.14344	0.255941	-5.80474	0.558267	0.569084
Non.inflammatory.Macrophages	ZSWIM3	-0.37395	1.503758	-1.14344	0.255944	-5.26677	0.63167	0.602395
Non.inflammatory.Macrophages	CABP1	0.766532	-0.13612	1.143399	0.255959	-4.80977	0.663207	0.630467
Non.inflammatory.Macrophages	NUDT8	0.243245	2.208161	1.143255	0.256019	-5.5489	0.605445	0.590764
Non.inflammatory.Macrophages	CNOT4	0.091729	7.523005	1.143202	0.256041	-6.46169	0.437976	0.509529

Non.inflammatory.Macrophages	ACOT7	0.221661	4.076826	1.142544	0.256313	-5.57565	0.541102	0.561124
Non.inflammatory.Macrophages	CENPB	0.119044	5.450808	1.14245	0.256351	-6.22422	0.497635	0.540078
Non.inflammatory.Macrophages	DENND6A	0.113915	5.102952	1.142192	0.256458	-6.15447	0.508365	0.545404
Non.inflammatory.Macrophages	SCD1	0.261432	3.037555	1.142152	0.256474	-5.79051	0.576381	0.577675
Non.inflammatory.Macrophages	RETNLG	0.47594	3.313557	1.141997	0.256539	-5.59125	0.566857	0.57332
Non.inflammatory.Macrophages	PNN	0.099506	6.310373	1.141857	0.256597	-6.26414	0.472246	0.527485
Non.inflammatory.Macrophages	HNMT	-0.38283	-0.38409	-1.14151	0.256741	-5.14914	0.66413	0.635545
Non.inflammatory.Macrophages	NOS1AP	0.237216	3.163666	1.141322	0.256818	-5.83577	0.572307	0.575938
Non.inflammatory.Macrophages	RINT1	-0.18241	3.771156	-1.14098	0.256961	-5.68273	0.551602	0.56639
Non.inflammatory.Macrophages	GM14085	0.703292	-1.35884	1.140853	0.257012	-4.78358	0.664185	0.653153
Non.inflammatory.Macrophages	RABGGTA	-0.19122	2.703471	-1.14076	0.257052	-5.64889	0.588471	0.583519
Non.inflammatory.Macrophages	ZFP811	0.688095	-1.02506	1.140695	0.257077	-4.88998	0.664185	0.647193
Non.inflammatory.Macrophages	3-Sep	-0.59637	-1.00287	-1.14062	0.257106	-4.83644	0.664185	0.646831
Non.inflammatory.Macrophages	GM47662	0.704858	-0.79475	1.140479	0.257167	-4.79107	0.664185	0.643149
Non.inflammatory.Macrophages	TNNT1	-0.32644	1.520318	-1.14042	0.25719	-5.3198	0.631972	0.603126

Non.inflammatory.Macrophages	LETM2	-0.22488	3.131534	-1.14028	0.257248	-5.58752	0.573422	0.576745
Non.inflammatory.Macrophages	SAFB	-0.10921	6.192591	-1.14027	0.257252	-6.24804	0.475878	0.529637
Non.inflammatory.Macrophages	TTC33	0.167181	3.849347	1.140052	0.257344	-5.86785	0.549078	0.56537
Non.inflammatory.Macrophages	GM50209	-0.44939	0.629068	-1.1398	0.25745	-5.08953	0.662088	0.618391
Non.inflammatory.Macrophages	KCP	-0.41685	0.100139	-1.13904	0.257764	-5.21183	0.664969	0.6279
Non.inflammatory.Macrophages	ZFP799	0.406458	0.939029	1.139006	0.257777	-5.14346	0.654883	0.613443
Non.inflammatory.Macrophages	UTP14A	-0.1213	5.238791	-1.13886	0.257838	-6.132	0.50504	0.544354
Non.inflammatory.Macrophages	IL23R	0.816196	-0.9447	1.138828	0.257851	-4.77544	0.664969	0.646446
Non.inflammatory.Macrophages	XPOT	0.136428	4.512589	1.138567	0.257959	-6.03821	0.528025	0.555623
Non.inflammatory.Macrophages	NOM1	0.143555	4.141882	1.13843	0.258016	-5.92636	0.540089	0.561428
Non.inflammatory.Macrophages	FAM91A1	-0.09597	5.007003	-1.13824	0.258096	-6.19069	0.512426	0.548105
Non.inflammatory.Macrophages	U2AF1L4	-0.22163	3.047054	-1.13809	0.258159	-5.70984	0.577294	0.578941
Non.inflammatory.Macrophages	LRRC27	-0.41996	0.302993	-1.13792	0.258229	-5.14323	0.66531	0.624826
Non.inflammatory.Macrophages	STRIP2	-0.42343	1.016356	-1.1375	0.258402	-5.24507	0.65279	0.612697
Non.inflammatory.Macrophages	F13B	-0.47998	1.463355	-1.13718	0.258535	-5.12925	0.635724	0.60528

Non.inflammatory.Macrophages	2310040G2	0.1752	1.291307	1.136745	0.258717	-5.63956	0.642654	0.608352
Non.inflammatory.Macrophages	PRMT2	-0.28977	1.665423	-1.13653	0.258807	-5.38541	0.628472	0.602166
Non.inflammatory.Macrophages	MMADHC	0.125707	4.299266	1.136133	0.258971	-5.97806	0.536064	0.559691
Non.inflammatory.Macrophages	FAIM	0.149822	3.865848	1.136063	0.259	-5.93272	0.550379	0.566483
Non.inflammatory.Macrophages	THEMIS2	0.154859	4.692319	1.135795	0.259112	-6.22666	0.523512	0.553659
Non.inflammatory.Macrophages	DNAJB14	-0.12616	4.692261	-1.13529	0.25932	-6.24373	0.523834	0.553974
Non.inflammatory.Macrophages	USP35	-0.52655	0.017078	-1.13501	0.259437	-5.10936	0.66728	0.630878
Non.inflammatory.Macrophages	SNX19	0.184883	2.862011	1.134871	0.259497	-5.62274	0.585594	0.582939
Non.inflammatory.Macrophages	EIF1A	-0.11459	5.711772	-1.13465	0.25959	-6.32026	0.492388	0.538556
Non.inflammatory.Macrophages	TMEM94	-0.18827	2.610495	-1.1346	0.259609	-5.61961	0.594575	0.587135
Non.inflammatory.Macrophages	LAMTOR2	0.080016	6.577213	1.134508	0.259648	-6.43941	0.466994	0.525826
Non.inflammatory.Macrophages	HNRNPF	-0.07678	9.158537	-1.13433	0.259724	-6.71211	0.398595	0.489421
Non.inflammatory.Macrophages	WDR55	0.190817	2.979495	1.13424	0.25976	-5.66089	0.581483	0.581318
Non.inflammatory.Macrophages	FAM72A	0.39161	1.069392	1.133904	0.2599	-5.21643	0.652483	0.613141
Non.inflammatory.Macrophages	MAPRE3	-0.29308	1.502586	-1.1338	0.259943	-5.44118	0.635739	0.605861

Non.inflammatory.Macrophages	GM17745	-0.49343	0.962497	-1.13352	0.260061	-5.03698	0.656675	0.615078
Non.inflammatory.Macrophages	ADCY9	0.206862	3.136828	1.133473	0.26008	-5.81842	0.576042	0.578996
Non.inflammatory.Macrophages	EDF1	0.085125	7.047067	1.133443	0.260093	-6.492	0.453821	0.519282
Non.inflammatory.Macrophages	2410131K1	-0.28548	2.169111	-1.13337	0.260122	-5.47182	0.610738	0.594808
Non.inflammatory.Macrophages	SCRIB	-0.17425	3.392621	-1.13333	0.260138	-5.71722	0.56718	0.574921
Non.inflammatory.Macrophages	BC035044	-0.20676	5.146379	-1.13277	0.260373	-6.073	0.510099	0.547745
Non.inflammatory.Macrophages	SPTSSA	0.109554	6.822761	1.132483	0.260494	-6.37992	0.460393	0.522887
Non.inflammatory.Macrophages	FBXO17	-0.51214	0.782245	-1.13244	0.260511	-5.02473	0.661677	0.618583
Non.inflammatory.Macrophages	TUFT1	0.295647	2.2113	1.132315	0.260564	-5.27866	0.60957	0.594581
Non.inflammatory.Macrophages	PELI1	0.092047	7.676753	1.132224	0.260602	-6.56506	0.436903	0.510677
Non.inflammatory.Macrophages	CLPTM1	0.100389	5.552635	1.132122	0.260644	-6.20593	0.497601	0.541785
Non.inflammatory.Macrophages	GM43672	-0.45651	1.700182	-1.13211	0.260647	-5.13135	0.628628	0.603091
Non.inflammatory.Macrophages	PPP2R3D	-0.18176	3.751015	-1.1319	0.260736	-5.83881	0.555352	0.56965
Non.inflammatory.Macrophages	RAB13	-0.31564	1.635625	-1.13185	0.260757	-5.43667	0.631101	0.604174
Non.inflammatory.Macrophages	RNF128	0.230757	2.499728	1.131713	0.260815	-5.71524	0.599097	0.589863

Non.inflammatory.Macrophages	CLASRP	-0.19855	3.334689	-1.13157	0.260873	-5.66754	0.569598	0.576333
Non.inflammatory.Macrophages	PSMF1	0.148423	4.460205	1.131343	0.26097	-5.93678	0.53204	0.558669
Non.inflammatory.Macrophages	AI506816	0.213789	5.207229	1.131229	0.261018	-5.99227	0.508353	0.547231
Non.inflammatory.Macrophages	SLC28A2	0.321823	2.961181	1.13097	0.261126	-5.46142	0.582866	0.582637
Non.inflammatory.Macrophages	RSU1	0.095183	5.746299	1.130764	0.261212	-6.32689	0.492068	0.53924
Non.inflammatory.Macrophages	RAB3GAP1	0.097581	6.044858	1.130436	0.26135	-6.18932	0.483197	0.534885
Non.inflammatory.Macrophages	AU040320	-0.14584	4.589216	-1.13039	0.261369	-6.00097	0.528103	0.557007
Non.inflammatory.Macrophages	4930403D(	0.528488	1.041064	1.130364	0.26138	-4.99257	0.654473	0.614793
Non.inflammatory.Macrophages	MYO16	-0.75361	-0.30441	-1.13025	0.261427	-4.84162	0.668332	0.638205
Non.inflammatory.Macrophages	ARID3A	0.140033	5.513727	1.129912	0.261569	-6.10612	0.499321	0.543033
Non.inflammatory.Macrophages	GM16552	0.375215	1.517	1.129793	0.261619	-5.15449	0.636274	0.606943
Non.inflammatory.Macrophages	TTC41	-0.51485	0.966475	-1.12944	0.261766	-5.02134	0.657802	0.616423
Non.inflammatory.Macrophages	ZFP644	0.103919	6.331418	1.129404	0.261782	-6.27377	0.475085	0.530961
Non.inflammatory.Macrophages	AC142100.	-0.40318	0.335219	-1.12927	0.26184	-5.29448	0.668756	0.627424
Non.inflammatory.Macrophages	ACRBP	-0.30797	1.566441	-1.12901	0.261949	-5.36882	0.634706	0.606351

Non.inflammatory.Macrophages	MCMBP	0.094458	7.089729	1.128654	0.262097	-6.39158	0.45372	0.520094
Non.inflammatory.Macrophages	WIPI2	-0.11727	5.276718	-1.12859	0.262126	-6.13037	0.50695	0.547019
Non.inflammatory.Macrophages	PTGIS	0.494215	-0.12832	1.128539	0.262145	-5.32668	0.669031	0.635737
Non.inflammatory.Macrophages	2510046G1	0.250641	2.620896	1.128219	0.26228	-5.4701	0.595816	0.589123
Non.inflammatory.Macrophages	MFGE8	0.361061	2.292559	1.128214	0.262282	-5.3458	0.607747	0.594527
Non.inflammatory.Macrophages	RABIF	0.128502	4.708703	1.127639	0.262523	-5.93973	0.525281	0.556158
Non.inflammatory.Macrophages	PTPRD	-0.39362	2.563535	-1.12738	0.26263	-5.45285	0.598359	0.59041
Non.inflammatory.Macrophages	KAT14	0.135975	3.838268	1.127366	0.262638	-5.87861	0.553885	0.569838
Non.inflammatory.Macrophages	TMC3	0.605176	-1.08655	1.126752	0.262896	-4.96498	0.670082	0.653702
Non.inflammatory.Macrophages	AC166172.	-0.38771	0.939575	-1.12674	0.262903	-5.22369	0.659904	0.617964
Non.inflammatory.Macrophages	JUND	-0.13073	10.62781	-1.12648	0.263011	-6.90729	0.36568	0.47204
Non.inflammatory.Macrophages	MRPL28	0.11063	6.016074	1.126461	0.263019	-6.30084	0.485346	0.536667
Non.inflammatory.Macrophages	NRIP3	0.799922	0.094317	1.125371	0.263478	-4.87511	0.671166	0.633434
Non.inflammatory.Macrophages	IGF1OS	-0.477	-1.03209	-1.12501	0.26363	-5.16031	0.671428	0.653565
Non.inflammatory.Macrophages	ONECUT2	0.423439	1.559667	1.124754	0.263738	-5.27086	0.637495	0.608269

Non.inflammatory.Macrophages	E4F1	-0.23161	3.127007	-1.12425	0.263949	-5.63706	0.580038	0.582482
Non.inflammatory.Macrophages	AAR2	-0.13645	4.021304	-1.1242	0.263971	-5.86739	0.549398	0.568177
Non.inflammatory.Macrophages	ARSG	0.246686	1.867658	1.124186	0.263977	-5.42726	0.625874	0.603259
Non.inflammatory.Macrophages	SDCBP2	-0.50091	1.097863	-1.12417	0.263984	-5.09718	0.655499	0.616305
Non.inflammatory.Macrophages	F730311O2	-0.54869	-0.31758	-1.12399	0.264059	-5.0229	0.671662	0.641078
Non.inflammatory.Macrophages	NBEAL1	0.12744	4.937374	1.123868	0.264111	-6.17216	0.519591	0.553934
Non.inflammatory.Macrophages	4930557J0	-0.70205	1.834427	-1.12385	0.264119	-4.95668	0.627128	0.603885
Non.inflammatory.Macrophages	SPRY1	-0.47137	1.345776	-1.12365	0.264203	-5.16282	0.645825	0.612191
Non.inflammatory.Macrophages	NDUFA3	0.08961	8.000282	1.123611	0.26422	-6.63228	0.430758	0.5087
Non.inflammatory.Macrophages	TRIM30D	0.199826	4.544173	1.123418	0.264301	-6.1402	0.53226	0.560146
Non.inflammatory.Macrophages	EIF4E3	0.152559	4.420461	1.1233	0.264351	-6.05062	0.536285	0.562108
Non.inflammatory.Macrophages	DAGLA	-0.49092	-0.78197	-1.12303	0.264467	-5.06036	0.671839	0.649719
Non.inflammatory.Macrophages	SLC2A2	-0.44183	1.627926	-1.12298	0.264486	-5.23349	0.635134	0.607671
Non.inflammatory.Macrophages	PRDM2	0.115939	5.970241	1.122713	0.264599	-6.27363	0.488006	0.538524
Non.inflammatory.Macrophages	GM16083	-0.40759	1.977218	-1.12229	0.264779	-5.25186	0.621987	0.602034

Non.inflammatory.Macrophages	EIF5A	0.101795	9.061608	1.122184	0.264822	-6.72382	0.403717	0.494324
Non.inflammatory.Macrophages	HYKK	-0.47679	0.618386	-1.12208	0.264865	-5.06114	0.669713	0.625229
Non.inflammatory.Macrophages	TANGO6	-0.17099	4.382985	-1.12208	0.264868	-5.85721	0.53764	0.563081
Non.inflammatory.Macrophages	SNHG12	-0.18367	3.189103	-1.12207	0.26487	-5.80374	0.578072	0.582105
Non.inflammatory.Macrophages	TPST1	0.193058	3.472726	1.121954	0.26492	-5.88505	0.568217	0.577536
Non.inflammatory.Macrophages	ASGR2	-0.46587	1.698173	-1.12176	0.265002	-5.24771	0.632523	0.606757
Non.inflammatory.Macrophages	RBBP4	-0.08887	7.676556	-1.12158	0.265076	-6.46817	0.439562	0.513803
Non.inflammatory.Macrophages	MKI67	-0.28703	6.498445	-1.12158	0.265079	-6.2668	0.472483	0.530933
Non.inflammatory.Macrophages	FGL1	0.268029	3.523532	1.12152	0.265103	-5.73195	0.566469	0.576774
Non.inflammatory.Macrophages	CIR1	0.118389	5.515333	1.121486	0.265118	-6.18918	0.501766	0.545666
Non.inflammatory.Macrophages	4931423N1	0.45798	0.653443	1.121233	0.265225	-5.1605	0.668989	0.624739
Non.inflammatory.Macrophages	COL4A1	0.29466	3.330895	1.121166	0.265253	-5.66901	0.573126	0.579923
Non.inflammatory.Macrophages	SMIM20	0.122791	4.914811	1.121153	0.265259	-6.09685	0.520498	0.554911
Non.inflammatory.Macrophages	STX5A	-0.09568	5.871144	-1.1209	0.265367	-6.23012	0.49103	0.540449
Non.inflammatory.Macrophages	POLD4	0.109665	5.76477	1.120728	0.265438	-6.27882	0.494234	0.54208

Non.inflammatory.Macrophages	CACNB4	0.331701	1.615307	1.120727	0.265439	-5.57798	0.635757	0.608427
Non.inflammatory.Macrophages	SEMA4C	-0.21721	2.202853	-1.12019	0.265665	-5.66289	0.613963	0.598784
Non.inflammatory.Macrophages	DET1	-0.30322	2.186379	-1.12001	0.265741	-5.41924	0.614574	0.599108
Non.inflammatory.Macrophages	AA388235	-0.48497	0.991788	-1.11979	0.265836	-5.17153	0.660328	0.619341
Non.inflammatory.Macrophages	TAF5	0.171831	4.146651	1.119751	0.265853	-5.78358	0.545763	0.567315
Non.inflammatory.Macrophages	ALOX5AP	0.193699	7.325815	1.119715	0.265868	-6.74143	0.4494	0.519266
Non.inflammatory.Macrophages	CFB	0.413494	4.663824	1.119711	0.265869	-6.2096	0.528848	0.55921
Non.inflammatory.Macrophages	FCHO1	-0.4394	3.772111	-1.11947	0.265971	-5.11855	0.558343	0.573411
Non.inflammatory.Macrophages	GPR141	0.750527	2.440611	1.119462	0.265975	-5.15114	0.605232	0.595052
Non.inflammatory.Macrophages	CRBN	0.144869	4.060609	1.119271	0.266056	-5.90148	0.548704	0.568909
Non.inflammatory.Macrophages	ZFP788	-0.26901	2.069958	-1.11904	0.266155	-5.35578	0.619105	0.601329
Non.inflammatory.Macrophages	CYB5D1	-0.49184	0.775244	-1.11875	0.266276	-5.10339	0.66668	0.623413
Non.inflammatory.Macrophages	PUM3	0.1232	4.640352	1.118433	0.266412	-6.05724	0.530033	0.559907
Non.inflammatory.Macrophages	GM17435	-0.42693	1.133905	-1.11838	0.266432	-5.13576	0.655257	0.617293
Non.inflammatory.Macrophages	EIF5	0.091764	8.418516	1.118238	0.266494	-6.67378	0.420606	0.504084

Non.inflammatory.Macrophages	CYP39A1	-0.50121	1.483874	-1.11784	0.266666	-5.0562	0.641945	0.611571
Non.inflammatory.Macrophages	FAM199X	0.209724	2.776803	1.117667	0.266737	-5.67863	0.593811	0.590002
Non.inflammatory.Macrophages	SPECC1	-0.1751	4.276803	-1.11762	0.266759	-6.29323	0.542157	0.565893
Non.inflammatory.Macrophages	1110038B1	-0.15922	4.235292	-1.11728	0.266901	-5.97284	0.543564	0.56669
Non.inflammatory.Macrophages	HMGN2	-0.17041	7.53605	-1.11709	0.266981	-6.41553	0.444243	0.517011
Non.inflammatory.Macrophages	TOMM34	-0.10756	5.601463	-1.1169	0.267064	-6.19439	0.500117	0.545649
Non.inflammatory.Macrophages	FILIP1L	-0.12371	5.411773	-1.11685	0.267085	-6.31579	0.505945	0.548538
Non.inflammatory.Macrophages	GCDH	-0.27238	3.116618	-1.11681	0.267101	-5.69088	0.581761	0.584724
Non.inflammatory.Macrophages	PHB	0.151907	4.493205	1.116538	0.267217	-6.02999	0.535097	0.562746
Non.inflammatory.Macrophages	JTB	-0.08789	5.842584	-1.11649	0.267236	-6.27014	0.4928	0.541998
Non.inflammatory.Macrophages	GM41556	0.351648	1.259442	1.116436	0.26726	-5.28431	0.650697	0.615714
Non.inflammatory.Macrophages	HTR2B	0.488156	0.855091	1.116352	0.267296	-5.03768	0.665153	0.62267
Non.inflammatory.Macrophages	2310022B0	0.194893	2.488564	1.116334	0.267304	-5.56128	0.604285	0.59503
Non.inflammatory.Macrophages	SIDT2	0.121607	5.063847	1.116287	0.267324	-6.17713	0.516805	0.553877
Non.inflammatory.Macrophages	HIST1H2BG	-0.54615	1.128352	-1.11603	0.267432	-5.0575	0.655976	0.618

Non.inflammatory.Macrophages	CMAS	0.119461	5.862643	1.115603	0.267615	-6.3382	0.49237	0.541858
Non.inflammatory.Macrophages	GM31812	-0.57251	0.191942	-1.11552	0.267651	-4.97725	0.673472	0.634432
Non.inflammatory.Macrophages	ACPP	0.580876	2.739914	1.115401	0.267701	-5.08501	0.595386	0.59107
Non.inflammatory.Macrophages	IL17RA	-0.13931	5.907729	-1.11537	0.267716	-6.26617	0.491014	0.541186
Non.inflammatory.Macrophages	TIMM17B	-0.09847	5.161183	-1.11529	0.267748	-6.11608	0.513926	0.552563
Non.inflammatory.Macrophages	RNF215	-0.18327	2.627986	-1.11525	0.267767	-5.64526	0.599428	0.592925
Non.inflammatory.Macrophages	MLXIPL	0.63384	0.355448	1.115062	0.267846	-5.0593	0.673546	0.631626
Non.inflammatory.Macrophages	HEMK1	0.392669	1.147753	1.114696	0.268002	-5.20412	0.655633	0.617968
Non.inflammatory.Macrophages	D130020L(	-0.4772	0.782996	-1.11455	0.268063	-5.10181	0.667569	0.624314
Non.inflammatory.Macrophages	KIF19A	-0.34426	1.071139	-1.11446	0.268104	-5.25906	0.658658	0.619344
Non.inflammatory.Macrophages	DSCC1	-0.39323	2.435007	-1.1143	0.268172	-5.20083	0.606811	0.596352
Non.inflammatory.Macrophages	GM15788	-0.54003	0.240256	-1.11405	0.268277	-5.04847	0.673976	0.634008
Non.inflammatory.Macrophages	CHD9	0.122702	6.065045	1.11396	0.268316	-6.34622	0.486676	0.539184
Non.inflammatory.Macrophages	IL1RAP	0.210399	4.731572	1.113727	0.268415	-5.8873	0.528062	0.55965
Non.inflammatory.Macrophages	4833403J1	-0.74215	0.509888	-1.11346	0.26853	-4.90043	0.673923	0.629514

Non.inflammatory.Macrophages	ISY1	0.120448	6.241546	1.112974	0.268736	-6.30476	0.481935	0.536884
Non.inflammatory.Macrophages	FAM131A	-0.4807	0.401139	-1.11275	0.268832	-5.18209	0.674732	0.631712
Non.inflammatory.Macrophages	STAM2	0.132383	5.475543	1.112672	0.268866	-6.27558	0.505099	0.548558
Non.inflammatory.Macrophages	TM2D3	0.130636	4.227452	1.112407	0.268979	-6.0332	0.545132	0.568012
Non.inflammatory.Macrophages	MTOR	0.130132	4.766611	1.112323	0.269014	-6.00333	0.527524	0.559556
Non.inflammatory.Macrophages	PRPS1	0.183844	3.621673	1.111724	0.269271	-5.77065	0.566008	0.577957
Non.inflammatory.Macrophages	RANBP6	0.324597	1.620455	1.111311	0.269447	-5.25876	0.639062	0.611168
Non.inflammatory.Macrophages	5830448L0	0.321569	0.904406	1.111137	0.269522	-5.34058	0.666383	0.623523
Non.inflammatory.Macrophages	INSL6	0.45586	1.68935	1.110639	0.269735	-5.16118	0.636547	0.610285
Non.inflammatory.Macrophages	ARF4OS	-0.41585	0.845404	-1.11056	0.269769	-5.10502	0.66796	0.62478
Non.inflammatory.Macrophages	TBC1D2	0.491917	1.45751	1.110502	0.269794	-5.08788	0.64548	0.614253
Non.inflammatory.Macrophages	SON	-0.07136	7.67203	-1.11032	0.269872	-6.58561	0.442247	0.516742
Non.inflammatory.Macrophages	GEMIN2	0.214655	3.260952	1.110078	0.269976	-5.59574	0.578915	0.5843
Non.inflammatory.Macrophages	RFK	0.126366	5.042963	1.110046	0.269989	-6.16249	0.519456	0.556026
Non.inflammatory.Macrophages	ZBTB8OS	-0.12237	5.027468	-1.10991	0.270048	-6.14429	0.519947	0.556274

Non.inflammatory.Macrophages	TMEM222	-0.15126	4.289191	-1.10986	0.270067	-5.96899	0.543869	0.567832
Non.inflammatory.Macrophages	POLR1E	0.313566	2.067812	1.109816	0.270088	-5.36932	0.622207	0.604037
Non.inflammatory.Macrophages	NDC80	-0.30723	4.625978	-1.1097	0.270138	-5.73209	0.53283	0.562536
Non.inflammatory.Macrophages	MARCO	0.281726	5.795483	1.109672	0.27015	-6.59945	0.496123	0.544515
Non.inflammatory.Macrophages	POU2F2	0.163517	5.772063	1.109587	0.270186	-6.40273	0.496834	0.544871
Non.inflammatory.Macrophages	RASA1	0.099558	6.030636	1.109351	0.270287	-6.36743	0.48904	0.541047
Non.inflammatory.Macrophages	CPEB2	-0.10764	5.412476	-1.1092	0.270352	-6.34035	0.507871	0.550471
Non.inflammatory.Macrophages	SSU72	0.077294	6.693565	1.109055	0.270414	-6.46589	0.469591	0.531183
Non.inflammatory.Macrophages	SPATS2L	-0.48121	0.9742	-1.109	0.270436	-5.19207	0.664468	0.622812
Non.inflammatory.Macrophages	NLRP3	-0.18096	4.527197	-1.10898	0.270446	-6.47918	0.536046	0.564206
Non.inflammatory.Macrophages	UBOX5	0.322554	1.390449	1.108971	0.27045	-5.32172	0.648085	0.61565
Non.inflammatory.Macrophages	PROSER3	-0.37497	1.105594	-1.10863	0.270598	-5.22349	0.659268	0.620661
Non.inflammatory.Macrophages	ERI3	-0.10385	5.177084	-1.10845	0.270672	-6.19136	0.515231	0.554221
Non.inflammatory.Macrophages	GM16023	-0.34649	1.834877	-1.10844	0.270677	-5.21974	0.631009	0.608236
Non.inflammatory.Macrophages	ARMCX2	0.483645	1.41393	1.108256	0.270757	-5.06134	0.647183	0.615394

Non.inflammatory.Macrophages	WDR62	0.179134	3.526858	1.108235	0.270766	-5.91724	0.569666	0.580273
Non.inflammatory.Macrophages	RIOK2	-0.12878	4.466641	-1.10819	0.270787	-5.99531	0.538036	0.565292
Non.inflammatory.Macrophages	GM33104	-0.69258	0.440079	-1.10815	0.270805	-4.91934	0.675838	0.632269
Non.inflammatory.Macrophages	COA4	0.205908	2.161432	1.107065	0.271269	-5.68821	0.619663	0.603082
Non.inflammatory.Macrophages	NDEL1	0.091836	6.927832	1.106379	0.271564	-6.54591	0.464034	0.528473
Non.inflammatory.Macrophages	CTSA	-0.11421	6.259221	-1.10595	0.271748	-6.53582	0.483631	0.538522
Non.inflammatory.Macrophages	GM14325	-0.19685	2.324076	-1.1058	0.271813	-5.58775	0.614422	0.600854
Non.inflammatory.Macrophages	GM26802	-0.28525	1.069129	-1.10576	0.271829	-5.59793	0.662594	0.622184
Non.inflammatory.Macrophages	GM7854	-0.49013	0.52802	-1.10565	0.271877	-5.14342	0.677107	0.631605
Non.inflammatory.Macrophages	WDR7	0.132511	5.188411	1.105251	0.272049	-6.24747	0.516575	0.554876
Non.inflammatory.Macrophages	SLC35F6	-0.14606	2.998461	-1.10506	0.272132	-5.96898	0.590217	0.589769
Non.inflammatory.Macrophages	AGTR1A	-0.62734	0.270352	-1.10468	0.272295	-5.03817	0.678288	0.636431
Non.inflammatory.Macrophages	MED11	0.16001	3.312748	1.104501	0.272373	-5.83232	0.579204	0.584821
Non.inflammatory.Macrophages	GM29243	-0.60989	-0.26495	-1.10447	0.272388	-4.90108	0.678288	0.645956
Non.inflammatory.Macrophages	TREM2	0.291406	0.639882	1.10441	0.272412	-5.85515	0.675691	0.629935

Non.inflammatory.Macrophages	SLC12A6	-0.13196	8.133974	-1.10432	0.272451	-6.54464	0.431449	0.511373
Non.inflammatory.Macrophages	EXOSC8	-0.15559	5.033192	-1.10423	0.272488	-6.05715	0.521659	0.557476
Non.inflammatory.Macrophages	HIP1	0.139414	5.505684	1.103829	0.272663	-6.37429	0.507059	0.550357
Non.inflammatory.Macrophages	CCNL2	0.087223	6.219089	1.103598	0.272762	-6.29526	0.485505	0.539653
Non.inflammatory.Macrophages	MRPS34	0.119685	4.826949	1.102592	0.273197	-6.09848	0.529281	0.561358
Non.inflammatory.Macrophages	RIDA	-0.26924	4.316478	-1.10252	0.273229	-5.9837	0.545996	0.569433
Non.inflammatory.Macrophages	SLC24A3	0.441432	1.280307	1.102371	0.273292	-5.3701	0.656024	0.619689
Non.inflammatory.Macrophages	PFDN4	0.133422	5.046958	1.102318	0.273315	-6.12474	0.522227	0.558053
Non.inflammatory.Macrophages	PLCE1	-0.58746	1.279254	-1.10208	0.273419	-5.15308	0.656073	0.619768
Non.inflammatory.Macrophages	RHBDD1	0.146479	4.175534	1.101949	0.273475	-5.91846	0.550705	0.571798
Non.inflammatory.Macrophages	CCNF	-0.29219	3.438612	-1.10187	0.273511	-5.65267	0.575915	0.583646
Non.inflammatory.Macrophages	GM44752	0.253947	2.484975	1.101846	0.273519	-5.47121	0.610135	0.599338
Non.inflammatory.Macrophages	IGSF9	-0.50563	0.790152	-1.10148	0.273678	-4.98803	0.673384	0.628348
Non.inflammatory.Macrophages	AFG3L1	0.099427	4.862083	1.101286	0.273761	-6.08211	0.528428	0.561147
Non.inflammatory.Macrophages	RASSF4	0.122852	4.553252	1.10115	0.27382	-6.35806	0.538484	0.566046

Non.inflammatory.Macrophages	CRTAM	0.358042	1.176627	1.100661	0.274032	-5.57316	0.660723	0.621919
Non.inflammatory.Macrophages	NACA	-0.07907	9.080227	-1.10059	0.274063	-6.81093	0.408243	0.499134
Non.inflammatory.Macrophages	PARP9	0.187754	5.150171	1.100577	0.274068	-6.22145	0.519424	0.556833
Non.inflammatory.Macrophages	CTDSPL	-0.22038	3.831098	-1.10008	0.274282	-5.95873	0.563138	0.577874
Non.inflammatory.Macrophages	IGKV2-109	-0.6588	-0.87987	-1.10004	0.274301	-4.7974	0.680548	0.658696
Non.inflammatory.Macrophages	MAGOHB	-0.16784	4.560419	-1.09985	0.274385	-6.08125	0.538753	0.566288
Non.inflammatory.Macrophages	SH2B2	0.14202	4.550186	1.099745	0.274429	-6.16523	0.539089	0.566481
Non.inflammatory.Macrophages	NR2F6	-0.16207	3.501892	-1.09958	0.274498	-5.96888	0.574603	0.583326
Non.inflammatory.Macrophages	GM15564	0.270634	2.474453	1.099299	0.274622	-5.61848	0.611568	0.600431
Non.inflammatory.Macrophages	ATP6V1B2	0.080573	5.98524	1.099234	0.27465	-6.47496	0.493986	0.544552
Non.inflammatory.Macrophages	SH3GL1	-0.13109	4.688824	-1.09905	0.27473	-6.02109	0.534675	0.564673
Non.inflammatory.Macrophages	TBC1D31	-0.16294	4.166685	-1.09902	0.274742	-6.03866	0.551945	0.57294
Non.inflammatory.Macrophages	OLFML3	0.321342	1.284348	1.098473	0.274981	-5.3653	0.657346	0.620888
Non.inflammatory.Macrophages	MATR3	0.07875	6.50291	1.098368	0.275026	-6.35348	0.478837	0.536971
Non.inflammatory.Macrophages	1700017B(	0.160443	5.285252	1.098337	0.275039	-6.04559	0.515849	0.555486

Non.inflammatory.Macrophages	FAM210B	0.173032	3.914295	1.098166	0.275114	-5.9045	0.560827	0.577106
Non.inflammatory.Macrophages	FAM167B	-0.33779	2.127548	-1.09758	0.275366	-5.35741	0.625195	0.606731
Non.inflammatory.Macrophages	EAF2	-0.23743	3.647314	-1.09743	0.275435	-5.51432	0.570272	0.581696
Non.inflammatory.Macrophages	HMGN5	0.148061	4.876393	1.097373	0.275458	-6.0235	0.529182	0.562132
Non.inflammatory.Macrophages	SDHAF4	0.129452	4.365947	1.097265	0.275505	-6.05647	0.545896	0.570195
Non.inflammatory.Macrophages	FBXL12	0.17183	4.348122	1.09709	0.275581	-5.83526	0.546489	0.5705
Non.inflammatory.Macrophages	KMT5B	0.107142	5.91383	1.097069	0.27559	-6.24634	0.496697	0.546167
Non.inflammatory.Macrophages	HELB	0.191125	3.704754	1.097034	0.275605	-5.77895	0.568286	0.580808
Non.inflammatory.Macrophages	CD96	0.785471	0.686804	1.096885	0.27567	-4.92853	0.677691	0.631729
Non.inflammatory.Macrophages	ARMC5	0.155985	3.383082	1.096816	0.2757	-5.79453	0.579488	0.586146
Non.inflammatory.Macrophages	NUDCD2	0.158058	4.534449	1.096031	0.276042	-6.01276	0.54081	0.567879
Non.inflammatory.Macrophages	CREB3L1	0.251271	2.035248	1.09594	0.276081	-5.59024	0.629248	0.608773
Non.inflammatory.Macrophages	SMIM13	0.151701	3.919727	1.095899	0.276099	-5.90436	0.561418	0.577679
Non.inflammatory.Macrophages	PIAS4	0.136112	4.224377	1.095545	0.276253	-5.95836	0.551321	0.572923
Non.inflammatory.Macrophages	TM2D2	0.10112	5.178235	1.094939	0.276517	-6.27485	0.520448	0.55801

Non.inflammatory.Macrophages	G0S2	0.295835	3.133285	1.09485	0.276556	-5.56126	0.58937	0.590732
Non.inflammatory.Macrophages	RAD17	0.12071	5.023722	1.094818	0.27657	-6.05144	0.525378	0.560455
Non.inflammatory.Macrophages	HABP4	-0.18118	3.054352	-1.09462	0.276658	-5.75228	0.592194	0.592143
Non.inflammatory.Macrophages	ANKRD39	0.153108	3.543857	1.09461	0.27666	-5.78265	0.574881	0.584133
Non.inflammatory.Macrophages	CRISPLD2	-0.6256	0.884481	-1.09447	0.276721	-5.07175	0.673776	0.629042
Non.inflammatory.Macrophages	POLB	-0.10896	5.332268	-1.09442	0.276744	-6.23741	0.515577	0.555865
Non.inflammatory.Macrophages	COL11A2	-0.44452	0.72136	-1.09396	0.276944	-5.21534	0.678177	0.632033
Non.inflammatory.Macrophages	CDC16	0.150178	3.893321	1.093958	0.276945	-5.89627	0.562963	0.578673
Non.inflammatory.Macrophages	NDUFA11	0.104552	6.910675	1.093806	0.277011	-6.46886	0.468245	0.532051
Non.inflammatory.Macrophages	GM9929	-0.37841	1.684976	-1.09378	0.27702	-5.16343	0.643392	0.615341
Non.inflammatory.Macrophages	SLF1	0.1715	4.44199	1.093336	0.277216	-5.92027	0.544636	0.570107
Non.inflammatory.Macrophages	MILR1	0.12218	4.330073	1.093318	0.277224	-6.13665	0.548359	0.571889
Non.inflammatory.Macrophages	ATRN	0.144946	5.965865	1.093262	0.277249	-6.18248	0.496268	0.546451
Non.inflammatory.Macrophages	MTX1	-0.13646	4.556924	-1.09291	0.277404	-6.09294	0.541043	0.568469
Non.inflammatory.Macrophages	HIP1R	-0.22632	5.271328	-1.09255	0.277558	-5.64773	0.518058	0.557451

Non.inflammatory.Macrophages	OPLAH	-0.29326	1.500611	-1.0924	0.277623	-5.4401	0.651088	0.619155
Non.inflammatory.Macrophages	RHBDD2	-0.23225	2.284757	-1.09225	0.277691	-5.54644	0.621062	0.605852
Non.inflammatory.Macrophages	GM16740	-0.29232	2.544974	-1.0922	0.277714	-5.3927	0.611382	0.601484
Non.inflammatory.Macrophages	SMARCC2	0.090713	6.020787	1.092171	0.277725	-6.30174	0.494866	0.546021
Non.inflammatory.Macrophages	AP5B1	-0.34012	1.293299	-1.09207	0.27777	-5.32721	0.659245	0.622853
Non.inflammatory.Macrophages	NIF3L1	0.203561	3.000203	1.091917	0.277836	-5.63539	0.59478	0.594013
Non.inflammatory.Macrophages	CPNE9	-0.38165	1.867904	-1.09182	0.277879	-5.46053	0.636863	0.61305
Non.inflammatory.Macrophages	ARID4A	0.101626	7.062741	1.091769	0.277901	-6.49842	0.464276	0.530556
Non.inflammatory.Macrophages	ARL6IP1	0.088771	7.879749	1.091206	0.278147	-6.66638	0.441892	0.518778
Non.inflammatory.Macrophages	IQCH	-0.42634	0.250926	-1.09104	0.27822	-5.40485	0.684003	0.641484
Non.inflammatory.Macrophages	NUP214	0.11012	5.2678	1.090914	0.278275	-6.196	0.518584	0.557984
Non.inflammatory.Macrophages	LPL	-0.15038	5.132487	-1.09061	0.278409	-6.48765	0.523041	0.560191
Non.inflammatory.Macrophages	E430024I0	0.293341	1.898133	1.090071	0.278644	-5.35831	0.636826	0.613004
Non.inflammatory.Macrophages	TMTC1	-0.34419	2.714836	-1.08967	0.278822	-5.64006	0.606482	0.599406
Non.inflammatory.Macrophages	2300009A(	0.153816	4.080294	1.089525	0.278883	-5.99992	0.55831	0.577067

Non.inflammatory.Macrophages	TMCO3	-0.15771	3.656165	-1.08935	0.278961	-5.84504	0.572939	0.584003
Non.inflammatory.Macrophages	FBXL3	0.110196	5.215741	1.088853	0.279178	-6.16011	0.521139	0.559442
Non.inflammatory.Macrophages	MICAL3	0.217511	3.436889	1.088751	0.279223	-5.70367	0.580719	0.587864
Non.inflammatory.Macrophages	GM13822	-0.551	1.17949	-1.08859	0.279292	-5.3259	0.665447	0.625999
Non.inflammatory.Macrophages	MXRA7	-0.32516	1.654737	-1.08838	0.279384	-5.43259	0.646723	0.617801
Non.inflammatory.Macrophages	ABHD14B	-0.27978	2.125702	-1.08825	0.279444	-5.46627	0.628641	0.609786
Non.inflammatory.Macrophages	CEBPZOS	0.118655	4.521965	1.088211	0.27946	-6.18019	0.543655	0.570458
Non.inflammatory.Macrophages	AURKB	-0.2752	4.237652	-1.08817	0.279476	-5.86025	0.553146	0.57499
Non.inflammatory.Macrophages	CPSF4L	-0.53149	0.499907	-1.08809	0.279513	-4.92622	0.685029	0.63797
Non.inflammatory.Macrophages	RBMS1	0.076617	7.558573	1.088063	0.279524	-6.69435	0.451515	0.524215
Non.inflammatory.Macrophages	KAT6A	0.10372	6.479855	1.087893	0.279599	-6.36049	0.482379	0.540263
Non.inflammatory.Macrophages	PCLAF	-0.30875	7.982096	-1.0878	0.279642	-6.44792	0.43993	0.518137
Non.inflammatory.Macrophages	C3	0.223266	6.327835	1.087743	0.279665	-6.38999	0.486889	0.542557
Non.inflammatory.Macrophages	MAGED2	-0.33895	1.585547	-1.0876	0.279729	-5.40694	0.649419	0.619148
Non.inflammatory.Macrophages	RSF1OS2	-0.50002	1.663513	-1.08759	0.279733	-5.0567	0.646382	0.617808

Non.inflammatory.Macrophages	KLF8	0.729898	0.979838	1.087501	0.279771	-4.91302	0.673458	0.629682
Non.inflammatory.Macrophages	ZNRD1	-0.11428	5.076686	-1.08731	0.279857	-6.17952	0.525646	0.561858
Non.inflammatory.Macrophages	SPIRE1	0.188878	2.681156	1.087181	0.279912	-6.11843	0.607997	0.600656
Non.inflammatory.Macrophages	2610318N(	-0.57536	1.172871	-1.08689	0.280038	-5.03527	0.665913	0.626526
Non.inflammatory.Macrophages	SLC25A20	0.130957	5.597573	1.0868	0.280079	-6.30246	0.509281	0.553987
Non.inflammatory.Macrophages	NRDE2	0.197979	3.371854	1.086732	0.280109	-5.81154	0.58319	0.589397
Non.inflammatory.Macrophages	GM36199	0.447043	-0.13106	1.086144	0.280368	-5.18902	0.685917	0.649884
Non.inflammatory.Macrophages	KNG2	-0.36922	2.382731	-1.08552	0.280642	-5.42476	0.619976	0.606331
Non.inflammatory.Macrophages	NOL7	0.084128	6.906907	1.085322	0.28073	-6.40299	0.470687	0.534635
Non.inflammatory.Macrophages	HORMAD2	-0.43427	1.357831	-1.08528	0.280748	-5.38897	0.659435	0.623907
Non.inflammatory.Macrophages	ENOX2	0.125474	5.852601	1.085199	0.280783	-6.39335	0.502068	0.550582
Non.inflammatory.Macrophages	RFX2	-0.2065	3.630115	-1.0852	0.280784	-5.78682	0.574884	0.585721
Non.inflammatory.Macrophages	MOK	-0.48017	0.377087	-1.08484	0.280941	-5.16795	0.686472	0.641307
Non.inflammatory.Macrophages	SPSB2	-0.20059	2.383882	-1.08482	0.280949	-5.62466	0.620074	0.606524
Non.inflammatory.Macrophages	EPN2	-0.3892	1.664749	-1.0846	0.28105	-5.3852	0.647553	0.618798

Non.inflammatory.Macrophages	MRTO4	0.161214	4.665156	1.084572	0.28106	-6.05896	0.53995	0.569241
Non.inflammatory.Macrophages	TRMT61B	-0.21708	4.178074	-1.08437	0.281151	-6.0148	0.556282	0.577052
Non.inflammatory.Macrophages	NFKBIB	-0.11889	5.622292	-1.08407	0.281283	-6.31958	0.509539	0.554312
Non.inflammatory.Macrophages	NDUFB3	0.101963	5.72376	1.083608	0.281485	-6.31586	0.50653	0.55293
Non.inflammatory.Macrophages	NUBP2	-0.15492	4.451117	-1.08356	0.281505	-6.02794	0.547426	0.572872
Non.inflammatory.Macrophages	KLF10	-0.11084	5.248269	-1.08354	0.281514	-6.29292	0.521458	0.560305
Non.inflammatory.Macrophages	BIVM	0.307137	0.882383	1.083269	0.281634	-5.32576	0.678197	0.632721
Non.inflammatory.Macrophages	ZFP40	-0.38538	1.347612	-1.0831	0.28171	-5.26619	0.660704	0.624644
Non.inflammatory.Macrophages	GLYR1	-0.08879	6.736527	-1.08272	0.281877	-6.43378	0.476393	0.537761
Non.inflammatory.Macrophages	SNX11	-0.21689	2.539196	-1.08267	0.281897	-5.61287	0.615136	0.604405
Non.inflammatory.Macrophages	BDP1	0.109954	5.529011	1.081895	0.282241	-6.24396	0.513456	0.556342
Non.inflammatory.Macrophages	CLIP1	0.116463	5.440971	1.081675	0.282338	-6.2622	0.516305	0.557758
Non.inflammatory.Macrophages	SIN3A	-0.1085	5.871572	-1.08156	0.282387	-6.26319	0.502896	0.551116
Non.inflammatory.Macrophages	ATP13A1	0.132246	3.809105	1.081312	0.282499	-5.95514	0.570392	0.583775
Non.inflammatory.Macrophages	A930007I1	-0.19078	4.385631	-1.08106	0.282611	-6.17487	0.550861	0.574575

Non.inflammatory.Macrophages	CBR3	-0.27571	0.624626	-1.08066	0.282789	-5.65212	0.686491	0.638046
Non.inflammatory.Macrophages	MRPS24	0.090363	6.031221	1.080366	0.282918	-6.37911	0.498585	0.549067
Non.inflammatory.Macrophages	ZMYND15	-0.44092	0.109664	-1.0802	0.28299	-5.40313	0.689041	0.64748
Non.inflammatory.Macrophages	HIST1H2AE	0.717133	1.438759	1.080036	0.283063	-5.04787	0.658843	0.624061
Non.inflammatory.Macrophages	CHIC2	0.076255	7.043743	1.080022	0.28307	-6.46772	0.468609	0.533933
Non.inflammatory.Macrophages	BLCAP	0.187113	3.009547	1.079754	0.283188	-5.73275	0.599391	0.597467
Non.inflammatory.Macrophages	2510009EC	0.151655	2.702942	1.079684	0.283219	-6.01201	0.610613	0.602606
Non.inflammatory.Macrophages	SCLT1	0.151273	4.474106	1.079493	0.283304	-6.10786	0.548388	0.573629
Non.inflammatory.Macrophages	CYP4F16	0.224853	1.441368	1.079429	0.283332	-5.78635	0.658848	0.624136
Non.inflammatory.Macrophages	GM28417	-0.34427	1.320479	-1.07934	0.283371	-5.34892	0.663648	0.626292
Non.inflammatory.Macrophages	PTPMT1	0.113495	4.267152	1.079001	0.283522	-6.1355	0.555535	0.577115
Non.inflammatory.Macrophages	SPAG5	-0.32137	3.137614	-1.07842	0.283781	-5.52758	0.595408	0.59579
Non.inflammatory.Macrophages	CDCA2	-0.30075	3.79635	-1.07824	0.28386	-5.63819	0.572142	0.585004
Non.inflammatory.Macrophages	SNHG17	0.339458	1.368538	1.077917	0.284003	-5.2453	0.662724	0.625975
Non.inflammatory.Macrophages	LPCAT1	-0.1434	4.107714	-1.07781	0.284049	-6.02442	0.561588	0.580101

Non.inflammatory.Macrophages	BBIP1	0.10899	5.888435	1.076895	0.284457	-6.29566	0.5044	0.552307
Non.inflammatory.Macrophages	PHF11C	0.338202	1.989779	1.076811	0.284494	-5.29966	0.63919	0.615625
Non.inflammatory.Macrophages	ANKRD55	-0.64484	0.009583	-1.07632	0.284712	-4.8809	0.691422	0.650676
Non.inflammatory.Macrophages	GM43328	0.294831	2.238918	1.076164	0.284782	-5.43861	0.630052	0.611665
Non.inflammatory.Macrophages	LRRC41	0.121223	4.919545	1.075985	0.284861	-6.1705	0.535476	0.567729
Non.inflammatory.Macrophages	MRPL49	-0.19275	3.778216	-1.07595	0.284876	-5.78377	0.573995	0.586055
Non.inflammatory.Macrophages	QARS	-0.13224	4.552856	-1.07562	0.285026	-6.10108	0.547577	0.573705
Non.inflammatory.Macrophages	MLLT10	-0.07539	7.464033	-1.07554	0.285059	-6.53882	0.458279	0.529044
Non.inflammatory.Macrophages	MAP3K5	-0.11626	7.255072	-1.07548	0.285088	-6.50058	0.46419	0.532158
Non.inflammatory.Macrophages	NDNF	0.655314	-0.47189	1.075319	0.285158	-4.87831	0.691444	0.659797
Non.inflammatory.Macrophages	BCAR3	-0.32477	4.496691	-1.07525	0.285189	-5.69866	0.549454	0.574685
Non.inflammatory.Macrophages	INO80C	0.16416	3.401246	1.075247	0.28519	-5.83272	0.587278	0.592477
Non.inflammatory.Macrophages	KDM1B	0.227351	3.6758	1.075076	0.285266	-5.65141	0.577576	0.587981
Non.inflammatory.Macrophages	PSMB10	0.139309	5.706129	1.075028	0.285287	-6.38308	0.510369	0.555671
Non.inflammatory.Macrophages	PPP2R2D	0.107175	5.459388	1.074922	0.285334	-6.16815	0.518121	0.559516

Non.inflammatory.Macrophages	RNF38	0.098586	6.163439	1.074647	0.285457	-6.35599	0.496417	0.548679
Non.inflammatory.Macrophages	RIT1	0.119295	3.965996	1.074517	0.285515	-6.08527	0.567638	0.583369
Non.inflammatory.Macrophages	RBBP5	0.176418	3.457865	1.074232	0.285642	-5.79571	0.58558	0.591733
Non.inflammatory.Macrophages	TMEM87B	0.101208	5.127611	1.073763	0.285851	-6.26972	0.529137	0.564992
Non.inflammatory.Macrophages	LYRM1	0.197042	2.700522	1.073687	0.285885	-5.69552	0.613212	0.604505
Non.inflammatory.Macrophages	TRBC2	-0.70447	3.943953	-1.07363	0.285911	-5.35604	0.568689	0.583958
Non.inflammatory.Macrophages	TAF12	-0.12392	5.021001	-1.0736	0.285925	-6.08218	0.53259	0.566725
Non.inflammatory.Macrophages	TRAJ18	0.547696	-1.3552	1.073374	0.286024	-4.83573	0.691986	0.676698
Non.inflammatory.Macrophages	AKR1A1	-0.07784	7.188669	-1.07332	0.28605	-6.68397	0.466449	0.533621
Non.inflammatory.Macrophages	USP24	0.110102	5.459806	1.073273	0.286069	-6.23031	0.518514	0.559934
Non.inflammatory.Macrophages	ETF1	-0.08162	7.536791	-1.07309	0.286149	-6.62454	0.456638	0.528521
Non.inflammatory.Macrophages	GM13008	-0.48527	1.087607	-1.07298	0.286198	-5.05106	0.675812	0.632463
Non.inflammatory.Macrophages	RABGAP1L	0.153391	7.908574	1.072743	0.286306	-6.31938	0.446425	0.523201
Non.inflammatory.Macrophages	ROBO2	-0.62511	0.744725	-1.07248	0.286425	-5.07351	0.686935	0.638753
Non.inflammatory.Macrophages	LMF1	0.169042	3.239428	1.072292	0.286507	-5.86527	0.59391	0.595997

Non.inflammatory.Macrophages	HSD17B13	-0.35048	2.671988	-1.07215	0.286572	-5.52033	0.614691	0.605525
Non.inflammatory.Macrophages	PABPN1	-0.08166	6.640276	-1.07194	0.286664	-6.42268	0.482791	0.542203
Non.inflammatory.Macrophages	UBL3	0.071148	7.408809	1.071615	0.286809	-6.62351	0.460726	0.530766
Non.inflammatory.Macrophages	SLC43A1	0.533072	1.089863	1.070943	0.28711	-5.07656	0.676535	0.63302
Non.inflammatory.Macrophages	SHANK2	-0.46588	1.410698	-1.07078	0.287183	-5.12498	0.663636	0.627415
Non.inflammatory.Macrophages	SUCO	0.128616	6.354348	1.070757	0.287193	-6.34015	0.49155	0.546778
Non.inflammatory.Macrophages	HIST1H2AK	0.596752	0.954719	1.070552	0.287285	-5.0212	0.681966	0.635415
Non.inflammatory.Macrophages	TMEM256	0.100062	6.982152	1.070548	0.287287	-6.58255	0.473006	0.537305
Non.inflammatory.Macrophages	NATD1	-0.20955	3.25628	-1.07045	0.287332	-5.80722	0.593696	0.596025
Non.inflammatory.Macrophages	ZFP65	0.280635	1.662117	1.070422	0.287343	-5.54354	0.653685	0.623046
Non.inflammatory.Macrophages	SLC5A3	-0.20148	3.816287	-1.07025	0.287419	-5.98528	0.573862	0.586936
Non.inflammatory.Macrophages	TAF13	-0.13765	4.566883	-1.07007	0.287501	-6.06085	0.54825	0.574912
Non.inflammatory.Macrophages	TTC37	0.188865	3.798842	1.069941	0.287558	-5.91559	0.57447	0.587365
Non.inflammatory.Macrophages	D730003I1	-0.19252	2.591958	-1.06992	0.287567	-5.75255	0.618051	0.607424
Non.inflammatory.Macrophages	SUPT20	-0.10995	5.30473	-1.06982	0.287614	-6.19245	0.524127	0.563252

Non.inflammatory.Macrophages	GM16638	-0.28644	2.040296	-1.06977	0.287634	-5.41003	0.638971	0.616814
Non.inflammatory.Macrophages	B3GNTL1	0.243755	2.673331	1.069767	0.287636	-5.59085	0.615019	0.60605
Non.inflammatory.Macrophages	PIK3R4	-0.18821	3.189781	-1.06972	0.287656	-5.79365	0.596094	0.597405
Non.inflammatory.Macrophages	HTR7	0.8314	0.754108	1.069692	0.28767	-5.03035	0.687217	0.639261
Non.inflammatory.Macrophages	MAP7D3	-0.44171	0.126284	-1.06937	0.287812	-5.18471	0.693094	0.65055
Non.inflammatory.Macrophages	AKAP6	-0.83641	0.299684	-1.06927	0.287859	-4.95215	0.693094	0.647443
Non.inflammatory.Macrophages	MECOM	0.773194	0.770813	1.069125	0.287923	-5.09318	0.686988	0.639083
Non.inflammatory.Macrophages	CAT	-0.13756	6.72379	-1.06904	0.287963	-6.54611	0.480701	0.541545
Non.inflammatory.Macrophages	DENND3	-0.39986	3.548798	-1.06883	0.288054	-5.21849	0.583518	0.591588
Non.inflammatory.Macrophages	MED28	0.088628	6.023126	1.06846	0.288222	-6.3932	0.501977	0.552299
Non.inflammatory.Macrophages	USP1	0.129762	5.737466	1.068428	0.288236	-6.22773	0.510822	0.556717
Non.inflammatory.Macrophages	RSF1OS1	0.335801	2.978349	1.068202	0.288337	-5.26344	0.604316	0.601236
Non.inflammatory.Macrophages	ITGB2L	0.586426	-0.61932	1.067331	0.288728	-4.87409	0.694322	0.664749
Non.inflammatory.Macrophages	CLDND1	0.108893	4.825073	1.067206	0.288784	-6.14565	0.54082	0.57143
Non.inflammatory.Macrophages	ALKBH2	0.211956	2.187068	1.067025	0.288865	-5.53596	0.634733	0.614986

Non.inflammatory.Macrophages	SNX29	-0.1337	6.395771	-1.06646	0.289121	-6.42081	0.491729	0.547189
Non.inflammatory.Macrophages	IL7R	-0.48167	4.966863	-1.06634	0.289172	-5.39172	0.536598	0.569429
Non.inflammatory.Macrophages	PREX2	-0.38751	2.374714	-1.06618	0.289245	-5.39919	0.628057	0.612079
Non.inflammatory.Macrophages	FBXO46	-0.25359	2.47185	-1.06591	0.289368	-5.54828	0.624385	0.610467
Non.inflammatory.Macrophages	KIF3B	0.138317	3.495441	1.065901	0.289371	-5.96462	0.586872	0.593328
Non.inflammatory.Macrophages	ITGA8	-0.45783	1.726804	-1.06586	0.28939	-5.33934	0.653064	0.623246
Non.inflammatory.Macrophages	GORAB	0.284365	1.673364	1.064908	0.289817	-5.41777	0.655858	0.62463
Non.inflammatory.Macrophages	ZFP324	-0.44245	0.798242	-1.06491	0.289818	-5.17362	0.688804	0.640005
Non.inflammatory.Macrophages	SMARCA5	0.08101	7.448521	1.064761	0.289884	-6.53938	0.461511	0.531949
Non.inflammatory.Macrophages	EVI2	-0.18699	3.35768	-1.06472	0.289901	-5.9712	0.592421	0.596118
Non.inflammatory.Macrophages	GM17251	-0.30753	2.365949	-1.06403	0.290212	-5.45119	0.629426	0.613194
Non.inflammatory.Macrophages	CDK18	-0.51328	-0.22726	-1.06402	0.290215	-5.0879	0.696072	0.659
Non.inflammatory.Macrophages	PPP4R2	0.096587	6.318519	1.063994	0.290229	-6.39967	0.494895	0.549315
Non.inflammatory.Macrophages	MRPL43	0.113762	5.608686	1.063884	0.290278	-6.27756	0.516853	0.560279
Non.inflammatory.Macrophages	NPFF	-0.42276	0.954229	-1.06367	0.290374	-5.13334	0.685219	0.637827

Non.inflammatory.Macrophages	PLA2G12A	0.159756	4.277535	1.063456	0.290471	-5.9518	0.560648	0.581547
Non.inflammatory.Macrophages	YPEL3	0.124817	7.069109	1.063329	0.290528	-6.50954	0.47273	0.538096
Non.inflammatory.Macrophages	DCPS	-0.12484	5.004121	-1.0633	0.290541	-6.14318	0.536371	0.56995
Non.inflammatory.Macrophages	REC114	-0.24588	3.19631	-1.06317	0.2906	-5.64249	0.598693	0.599399
Non.inflammatory.Macrophages	C1RL	0.264229	1.632594	1.063098	0.290633	-5.66214	0.657962	0.626041
Non.inflammatory.Macrophages	TPX2	-0.25879	5.047457	-1.06275	0.290791	-6.03877	0.535105	0.569371
Non.inflammatory.Macrophages	ACSF3	0.297382	1.59243	1.062677	0.290822	-5.37345	0.659738	0.626829
Non.inflammatory.Macrophages	CACNA1B	0.6967	0.056619	1.062574	0.290869	-4.88903	0.696378	0.654179
Non.inflammatory.Macrophages	ADH5	-0.12126	6.03865	-1.06237	0.290962	-6.34017	0.50373	0.553919
Non.inflammatory.Macrophages	2810002D1	0.28395	1.66458	1.062262	0.29101	-5.30644	0.656967	0.625616
Non.inflammatory.Macrophages	SCAF4	0.092969	6.317786	1.062022	0.291118	-6.41046	0.495296	0.549785
Non.inflammatory.Macrophages	CERKL	0.37105	1.852174	1.061689	0.291269	-5.32637	0.649942	0.622645
Non.inflammatory.Macrophages	UHRF2	0.082723	6.43989	1.06151	0.291349	-6.44812	0.491781	0.54809
Non.inflammatory.Macrophages	TYK2	0.119448	3.992109	1.061271	0.291457	-6.09962	0.571013	0.586763
Non.inflammatory.Macrophages	RNF168	0.164622	4.161361	1.061246	0.291469	-5.95155	0.565167	0.584005

Non.inflammatory.Macrophages	RAB30	-0.45701	2.141089	-1.06122	0.291478	-5.22562	0.638736	0.617772
Non.inflammatory.Macrophages	YTHDF2	-0.08985	6.035232	-1.06107	0.291547	-6.34289	0.504141	0.554353
Non.inflammatory.Macrophages	HABP2	-0.54054	0.622763	-1.06079	0.291674	-5.0763	0.694716	0.644583
Non.inflammatory.Macrophages	MEGF11	-0.46752	0.651843	-1.06037	0.291867	-5.16413	0.69442	0.644184
Non.inflammatory.Macrophages	CKS2	-0.15581	5.959802	-1.06014	0.291968	-6.30137	0.506937	0.555752
Non.inflammatory.Macrophages	RAD1	-0.26209	2.477895	-1.05973	0.292152	-5.48675	0.626778	0.612434
Non.inflammatory.Macrophages	DGCR8	-0.17833	3.779114	-1.05869	0.292627	-5.78047	0.580018	0.59112
Non.inflammatory.Macrophages	DDX49	0.123867	4.208396	1.058657	0.29264	-5.98937	0.565078	0.5841
Non.inflammatory.Macrophages	PEX19	0.149839	3.955615	1.058566	0.292681	-5.92381	0.573831	0.588274
Non.inflammatory.Macrophages	ANKMY2	0.144564	3.602393	1.058429	0.292743	-5.88267	0.586295	0.59413
Non.inflammatory.Macrophages	IL2RB	-0.57243	3.130268	-1.05826	0.292818	-5.22889	0.603325	0.602032
Non.inflammatory.Macrophages	NDUFAF7	-0.15775	4.007139	-1.05813	0.292881	-5.84342	0.57206	0.587519
Non.inflammatory.Macrophages	ATXN3	0.103102	5.054807	1.05808	0.292901	-6.22105	0.536701	0.570632
Non.inflammatory.Macrophages	CNBD2	-0.17732	3.889219	-1.05797	0.292951	-5.95521	0.576175	0.58945
Non.inflammatory.Macrophages	EIF2A	0.091842	5.395584	1.057806	0.293025	-6.23729	0.525693	0.565312

Non.inflammatory.Macrophages	HMGA1	0.163704	4.670399	1.057525	0.293153	-6.05576	0.549613	0.576911
Non.inflammatory.Macrophages	SAE1	-0.11815	6.342657	-1.05729	0.293259	-6.28346	0.496337	0.550708
Non.inflammatory.Macrophages	PKD2L2	-0.34357	1.170955	-1.05687	0.293451	-5.13452	0.679592	0.636136
Non.inflammatory.Macrophages	PYGB	0.109054	4.748164	1.056721	0.293518	-6.19832	0.547332	0.57591
Non.inflammatory.Macrophages	GM12248	-0.30655	2.619411	-1.05656	0.29359	-5.44127	0.622835	0.611059
Non.inflammatory.Macrophages	MDM1	0.258607	3.41542	1.056492	0.293622	-5.60835	0.593532	0.597675
Non.inflammatory.Macrophages	AXIN1	0.089961	6.046226	1.056466	0.293634	-6.30618	0.505622	0.555469
Non.inflammatory.Macrophages	NUP160	0.162776	5.017011	1.056302	0.293708	-6.02336	0.538475	0.571672
Non.inflammatory.Macrophages	ZNFX1	0.16616	4.359029	1.056077	0.29381	-6.21751	0.560599	0.582299
Non.inflammatory.Macrophages	ELAVL1	0.06719	7.604056	1.05548	0.294081	-6.56583	0.46005	0.532157
Non.inflammatory.Macrophages	PSME2	0.148652	7.278608	1.055306	0.29416	-6.62662	0.469347	0.53707
Non.inflammatory.Macrophages	OAS1A	0.220276	2.355225	1.054976	0.294311	-6.07203	0.633511	0.616011
Non.inflammatory.Macrophages	OAS1C	0.334741	1.903745	1.054933	0.29433	-5.43474	0.65099	0.623814
Non.inflammatory.Macrophages	RHOF	-0.23101	3.558194	-1.05491	0.294341	-5.74216	0.589025	0.595756
Non.inflammatory.Macrophages	KIFC1	-0.38518	3.200672	-1.05456	0.2945	-5.5414	0.601934	0.60178

Non.inflammatory.Macrophages	SH3GLB2	0.156609	3.721025	1.05456	0.2945	-5.90373	0.583232	0.593129
Non.inflammatory.Macrophages	CCDC88C	0.224416	5.294187	1.054359	0.294592	-5.68735	0.529953	0.567787
Non.inflammatory.Macrophages	CHMP2A	0.081038	6.544809	1.054338	0.294601	-6.51393	0.490936	0.548357
Non.inflammatory.Macrophages	CSE1L	0.116834	5.5229	1.054114	0.294703	-6.21425	0.522603	0.564227
Non.inflammatory.Macrophages	IDUA	-0.1933	2.137056	-1.05406	0.29473	-5.68431	0.641903	0.619989
Non.inflammatory.Macrophages	DUSP23	0.20827	2.373334	1.054001	0.294754	-5.66085	0.632819	0.615933
Non.inflammatory.Macrophages	GAK	0.08072	6.280821	1.053984	0.294762	-6.42972	0.498935	0.552461
Non.inflammatory.Macrophages	2210016F1	-0.09799	4.954824	-1.05391	0.294794	-6.27262	0.541042	0.573266
Non.inflammatory.Macrophages	PARVG	0.119018	5.071901	1.05386	0.294818	-6.20669	0.537191	0.571422
Non.inflammatory.Macrophages	USP19	0.133663	4.704682	1.053393	0.295031	-6.16103	0.549655	0.577488
Non.inflammatory.Macrophages	CCDC163	-0.27167	2.446867	-1.0531	0.295167	-5.62719	0.630465	0.615016
Non.inflammatory.Macrophages	POLA1	-0.15909	5.968201	-1.05306	0.295184	-6.36824	0.508933	0.5576
Non.inflammatory.Macrophages	LNCPPARA	0.481901	0.60974	1.052661	0.295365	-5.12277	0.698607	0.647356
Non.inflammatory.Macrophages	TCEANC	0.382763	1.730178	1.052614	0.295386	-5.26956	0.658398	0.627513
Non.inflammatory.Macrophages	CS	0.085357	6.103857	1.052592	0.295396	-6.38208	0.504805	0.555603

Non.inflammatory.Macrophages	C030006K1	0.357368	1.498775	1.052446	0.295463	-5.30946	0.667622	0.63162
Non.inflammatory.Macrophages	AFAP1L1	0.448172	1.508026	1.052163	0.295592	-5.2431	0.667251	0.631646
Non.inflammatory.Macrophages	BAHD1	0.189834	2.543391	1.052081	0.295629	-5.7285	0.626898	0.613735
Non.inflammatory.Macrophages	PHLPP2	-0.14648	4.283952	-1.05183	0.295744	-6.04613	0.564102	0.584837
Non.inflammatory.Macrophages	5730455P1	0.184665	2.914853	1.05175	0.29578	-5.69309	0.612975	0.607549
Non.inflammatory.Macrophages	CTSG	1.104077	-0.2691	1.051729	0.29579	-4.95246	0.700744	0.663749
Non.inflammatory.Macrophages	C530008M	0.51594	0.74516	1.051616	0.295841	-5.11946	0.69525	0.645322
Non.inflammatory.Macrophages	ZFP24	0.126792	4.243197	1.05157	0.295863	-6.00902	0.565502	0.585501
Non.inflammatory.Macrophages	ANAPC7	0.128447	4.359074	1.051449	0.295918	-5.99275	0.561529	0.583615
Non.inflammatory.Macrophages	MRPL58	0.119688	5.199269	1.0514	0.29594	-6.24592	0.533496	0.570123
Non.inflammatory.Macrophages	ST3GAL3	-0.1086	5.293649	-1.05137	0.295955	-6.3205	0.530431	0.568627
Non.inflammatory.Macrophages	PHF14	0.096084	6.331947	1.05121	0.296027	-6.36528	0.497842	0.552461
Non.inflammatory.Macrophages	ADGRL3	-0.23245	3.47827	-1.05088	0.296177	-6.36173	0.592641	0.598208
Non.inflammatory.Macrophages	CDH24	-0.2803	2.179776	-1.05033	0.296431	-5.4238	0.641311	0.620465
Non.inflammatory.Macrophages	ZC3H12B	0.643906	1.084598	1.050223	0.296478	-4.92441	0.684962	0.639646

Non.inflammatory.Macrophages	4632428C(	-0.42925	0.110114	-1.0502	0.296489	-5.16237	0.701294	0.657192
Non.inflammatory.Macrophages	GM45871	0.367702	0.985003	1.050145	0.296513	-5.27646	0.689062	0.641443
Non.inflammatory.Macrophages	AHCYL1	0.09254	5.179051	1.050066	0.29655	-6.21308	0.534574	0.570808
Non.inflammatory.Macrophages	RAB10OS	-0.137	4.429445	-1.04983	0.296658	-6.04493	0.559674	0.582908
Non.inflammatory.Macrophages	H2-EB1	-0.32485	6.754688	-1.04915	0.296969	-6.57046	0.485982	0.546606
Non.inflammatory.Macrophages	CCDC112	-0.31889	1.151644	-1.04891	0.297079	-5.46712	0.68298	0.638854
Non.inflammatory.Macrophages	OVGP1	0.485741	0.492644	1.048783	0.297137	-5.02168	0.702036	0.650721
Non.inflammatory.Macrophages	RPP14	-0.18961	2.846249	-1.04877	0.297142	-5.74104	0.6167	0.609514
Non.inflammatory.Macrophages	ATF2	-0.1045	6.46092	-1.04846	0.297283	-6.37624	0.494971	0.551282
Non.inflammatory.Macrophages	BRD2	-0.08307	7.085393	-1.04837	0.297327	-6.56235	0.476391	0.541815
Non.inflammatory.Macrophages	SELENOM	-0.26444	2.384919	-1.04783	0.297571	-5.6882	0.634716	0.617826
Non.inflammatory.Macrophages	ANKLE1	-0.45791	0.64315	-1.04746	0.297745	-5.0502	0.700235	0.648645
Non.inflammatory.Macrophages	GRB2	0.079132	7.898248	1.04701	0.297949	-6.75185	0.453803	0.530257
Non.inflammatory.Macrophages	GM26839	0.390153	0.656622	1.046979	0.297963	-5.27189	0.700039	0.648626
Non.inflammatory.Macrophages	EEFSEC	-0.16665	4.399413	-1.04696	0.297971	-6.05977	0.5621	0.584508

Non.inflammatory.Macrophages	GM31323	-0.37975	0.948728	-1.04675	0.29807	-5.20473	0.692368	0.643479
Non.inflammatory.Macrophages	MRPL44	0.182206	2.945204	1.046595	0.298139	-5.79176	0.61411	0.608796
Non.inflammatory.Macrophages	FAM168A	0.097486	5.93211	1.046239	0.298303	-6.39065	0.512098	0.560379
Non.inflammatory.Macrophages	DPF3	0.4384	1.667384	1.046192	0.298324	-5.22876	0.663431	0.630989
Non.inflammatory.Macrophages	MIEF1	0.16545	4.21125	1.046082	0.298375	-5.95603	0.568781	0.587878
Non.inflammatory.Macrophages	HERPUD2	-0.09527	5.474289	-1.04593	0.298442	-6.25642	0.526629	0.567581
Non.inflammatory.Macrophages	MCL1	-0.08834	8.364811	-1.04584	0.298485	-6.84374	0.441149	0.523709
Non.inflammatory.Macrophages	PIFO	-0.7243	1.246923	-1.04571	0.298548	-4.91909	0.680401	0.638488
Non.inflammatory.Macrophages	GUK1	0.110894	4.908743	1.045704	0.298548	-6.22433	0.545124	0.576649
Non.inflammatory.Macrophages	VAPA	-0.0594	8.028548	-1.04532	0.298723	-6.71116	0.450538	0.528822
Non.inflammatory.Macrophages	MTHFS	0.165304	5.861718	1.045082	0.298834	-6.52172	0.514604	0.561758
Non.inflammatory.Macrophages	SUCLG1	-0.11824	5.422184	-1.04478	0.298974	-6.27461	0.528613	0.56871
Non.inflammatory.Macrophages	RAB2A	0.056737	8.094947	1.044694	0.299013	-6.6909	0.448779	0.527962
Non.inflammatory.Macrophages	SLC16A2	-0.30975	2.626868	-1.04461	0.299052	-5.45801	0.626503	0.614756
Non.inflammatory.Macrophages	ZDHHC14	-0.15772	7.022709	-1.04459	0.299059	-6.61891	0.479294	0.543956

Non.inflammatory.Macrophages	ALG2	0.173108	3.062397	1.044519	0.299093	-5.70387	0.610212	0.607376
Non.inflammatory.Macrophages	ZFP983	-0.28196	2.38923	-1.04442	0.299136	-5.46883	0.635561	0.618857
Non.inflammatory.Macrophages	GALM	-0.22941	2.214948	-1.04406	0.299302	-5.75586	0.642491	0.621922
Non.inflammatory.Macrophages	I730030J21	0.331235	0.707146	1.043924	0.299366	-5.40294	0.699575	0.648537
Non.inflammatory.Macrophages	PTPN3	-0.56175	1.040176	-1.04387	0.299391	-5.08167	0.689513	0.642567
Non.inflammatory.Macrophages	USP53	0.155021	4.118641	1.043564	0.299532	-5.886	0.572622	0.58984
Non.inflammatory.Macrophages	GM12367	-0.52671	0.051988	-1.04352	0.299552	-5.12136	0.70421	0.660443
Non.inflammatory.Macrophages	MEN1	-0.22395	2.866329	-1.04325	0.299678	-5.64295	0.617836	0.610757
Non.inflammatory.Macrophages	DNASE1L3	-0.20236	6.30437	-1.04323	0.299686	-6.40192	0.50113	0.555019
Non.inflammatory.Macrophages	ZFP821	0.166301	3.905173	1.042924	0.299827	-5.87567	0.580117	0.593393
Non.inflammatory.Macrophages	CCNE2	-0.33144	3.75287	-1.04285	0.29986	-5.63201	0.585509	0.595946
Non.inflammatory.Macrophages	DCAKD	-0.14845	4.076447	-1.04282	0.299874	-6.04812	0.574109	0.590609
Non.inflammatory.Macrophages	NUP133	0.163084	3.691763	1.042689	0.299935	-5.80469	0.587686	0.597005
Non.inflammatory.Macrophages	MRPL39	0.145177	3.661118	1.042619	0.299967	-5.85682	0.58878	0.597536
Non.inflammatory.Macrophages	HSD17B12	0.097991	6.085483	1.04255	0.299999	-6.47518	0.507887	0.558562

Non.inflammatory.Macrophages	PGAP2	0.119406	5.75264	1.04218	0.300169	-6.37059	0.518331	0.5639
Non.inflammatory.Macrophages	HNRNPA1	-0.09809	7.807246	-1.04208	0.300218	-6.63126	0.457026	0.532553
Non.inflammatory.Macrophages	PPIC	-0.2724	2.745716	-1.04207	0.300222	-5.52015	0.622359	0.613126
Non.inflammatory.Macrophages	GLYAT	-0.37163	2.069154	-1.04207	0.300222	-5.39659	0.648305	0.624772
Non.inflammatory.Macrophages	COL6A1	0.471859	0.367808	1.042034	0.300237	-5.13229	0.70423	0.655033
Non.inflammatory.Macrophages	RNF24	-0.20575	4.233052	-1.04176	0.300362	-5.72598	0.568775	0.588412
Non.inflammatory.Macrophages	REXO1	-0.11957	4.912691	-1.04168	0.300398	-6.15518	0.54571	0.577436
Non.inflammatory.Macrophages	CORO2B	-0.42853	1.197161	-1.04137	0.300542	-5.13887	0.683542	0.640422
Non.inflammatory.Macrophages	HCFC2	0.152732	3.758586	1.041228	0.300609	-5.9157	0.585625	0.596406
Non.inflammatory.Macrophages	EPC1	-0.10038	6.811099	-1.04091	0.300755	-6.47148	0.486182	0.547905
Non.inflammatory.Macrophages	LYRM9	0.225617	2.696887	1.040867	0.300775	-5.65229	0.624668	0.614409
Non.inflammatory.Macrophages	IGSF6	-0.15568	3.689623	-1.04057	0.300914	-6.39863	0.588374	0.597743
Non.inflammatory.Macrophages	PICALM	0.076258	8.404518	1.040136	0.301113	-6.88656	0.441221	0.524357
Non.inflammatory.Macrophages	HIST1H2BN	0.506704	1.240034	1.039894	0.301225	-5.04753	0.682455	0.640144
Non.inflammatory.Macrophages	CR1L	0.085749	5.693128	1.039793	0.301271	-6.32644	0.520988	0.565571

Non.inflammatory.Macrophages	MOB3C	-0.16036	3.356469	-1.03962	0.301352	-5.89623	0.60065	0.603689
Non.inflammatory.Macrophages	HEXB	0.102885	5.741415	1.039441	0.301434	-6.41931	0.519453	0.564989
Non.inflammatory.Macrophages	MAT1A	-0.24892	4.65605	-1.03926	0.301519	-6.14245	0.555027	0.582341
Non.inflammatory.Macrophages	MAP3K15	0.198976	3.326785	1.039248	0.301523	-5.9396	0.601732	0.60429
Non.inflammatory.Macrophages	TMEM159	0.273536	1.553977	1.039236	0.301529	-5.65318	0.66971	0.634831
Non.inflammatory.Macrophages	SLC25A43	0.396114	-0.21871	1.038991	0.301642	-5.22539	0.705271	0.666913
Non.inflammatory.Macrophages	MECR	0.168649	3.581314	1.038947	0.301662	-5.86117	0.592514	0.600072
Non.inflammatory.Macrophages	ANKRD44	-0.10611	8.18547	-1.03894	0.301666	-6.68711	0.447196	0.527888
Non.inflammatory.Macrophages	METTL17	-0.25558	2.488504	-1.03893	0.301671	-5.47258	0.633043	0.618597
Non.inflammatory.Macrophages	GM47889	-0.30417	1.560566	-1.03878	0.301739	-5.50243	0.66948	0.634807
Non.inflammatory.Macrophages	EMC9	-0.24146	1.600787	-1.03823	0.301995	-5.56645	0.668213	0.634327
Non.inflammatory.Macrophages	MRPS6	-0.10227	6.008603	-1.03821	0.302001	-6.41257	0.51133	0.561106
Non.inflammatory.Macrophages	5330439K(	-0.42264	0.538036	-1.03793	0.302131	-5.23215	0.704841	0.653336
Non.inflammatory.Macrophages	KLC4	-0.18067	2.884186	-1.03791	0.30214	-5.79811	0.618462	0.612088
Non.inflammatory.Macrophages	PPP1R12A	0.082873	8.052992	1.037851	0.30217	-6.63281	0.451127	0.530065

Non.inflammatory.Macrophages	MPV17L2	0.133134	4.459696	1.037631	0.302272	-6.16312	0.562144	0.585831
Non.inflammatory.Macrophages	5033406O(	-0.37706	0.605012	-1.03739	0.302384	-5.33877	0.703816	0.652175
Non.inflammatory.Macrophages	TMEM9	-0.29576	2.576914	-1.03735	0.302402	-5.5013	0.630218	0.617393
Non.inflammatory.Macrophages	CDH23	-0.20874	2.820164	-1.03696	0.302584	-5.77777	0.621262	0.613405
Non.inflammatory.Macrophages	MYCBP2	0.094935	7.768752	1.036831	0.302642	-6.73408	0.459363	0.534493
Non.inflammatory.Macrophages	CRYBA4	0.358934	-0.18755	1.036755	0.302677	-5.36731	0.70616	0.666912
Non.inflammatory.Macrophages	GLUD1	-0.0804	7.653063	-1.03612	0.30297	-6.699	0.462939	0.536409
Non.inflammatory.Macrophages	DYRK1B	0.255152	1.826196	1.036081	0.30299	-5.44716	0.660097	0.630874
Non.inflammatory.Macrophages	NIPBL	0.076281	8.615732	1.035736	0.30315	-6.74183	0.436368	0.522417
Non.inflammatory.Macrophages	LRRC20	0.324012	2.057401	1.035722	0.303157	-5.33514	0.650969	0.627031
Non.inflammatory.Macrophages	C2	-0.26185	2.413631	-1.03563	0.303198	-5.80562	0.63713	0.62086
Non.inflammatory.Macrophages	GM28529	0.491636	0.724072	1.035325	0.303341	-5.12027	0.70165	0.65071
Non.inflammatory.Macrophages	GM15738	0.492747	0.402397	1.035314	0.303346	-5.03925	0.706623	0.656549
Non.inflammatory.Macrophages	CD47	0.071564	8.628324	1.035195	0.303401	-6.80511	0.436031	0.522242
Non.inflammatory.Macrophages	ECHS1	0.12686	5.549982	1.035133	0.30343	-6.39387	0.526573	0.568964

Non.inflammatory.Macrophages	IBTK	0.145174	4.642613	1.035109	0.303441	-6.09026	0.556547	0.58352
Non.inflammatory.Macrophages	ZFP579	-0.32895	1.862923	-1.03509	0.30345	-5.32522	0.658639	0.63044
Non.inflammatory.Macrophages	CD74	-0.28283	11.27972	-1.03479	0.303587	-7.17185	0.370456	0.485208
Non.inflammatory.Macrophages	ZFP318	-0.18186	3.491008	-1.03479	0.30359	-6.02185	0.596929	0.602624
Non.inflammatory.Macrophages	G3BP2	0.067816	6.759047	1.034686	0.303638	-6.53624	0.48904	0.550233
Non.inflammatory.Macrophages	CENPM	-0.26664	3.916181	-1.03462	0.303668	-5.69988	0.581717	0.595575
Non.inflammatory.Macrophages	ERCC6L2	0.183553	3.372895	1.03447	0.303738	-5.80891	0.601243	0.604685
Non.inflammatory.Macrophages	VGLL4	-0.10906	6.477378	-1.03437	0.303783	-6.37003	0.497568	0.554647
Non.inflammatory.Macrophages	CNDP2	0.103592	5.051865	1.034077	0.30392	-6.4215	0.54302	0.577153
Non.inflammatory.Macrophages	TRP53RKB	-0.25082	2.444883	-1.03372	0.304084	-5.49382	0.636319	0.620734
Non.inflammatory.Macrophages	MGAT4B	-0.14482	3.847759	-1.03363	0.30413	-6.04275	0.584481	0.597015
Non.inflammatory.Macrophages	BORCS5	-0.15725	4.024358	-1.03356	0.304159	-5.85898	0.578241	0.594118
Non.inflammatory.Macrophages	MED16	0.134891	4.12299	1.033019	0.304412	-5.96714	0.575006	0.59262
Non.inflammatory.Macrophages	CCNT1	0.081405	6.841664	1.033002	0.30442	-6.45826	0.487042	0.549422
Non.inflammatory.Macrophages	HECTD2	0.553882	0.291184	1.032975	0.304433	-4.98351	0.70733	0.659245

Non.inflammatory.Macrophages	HCK	0.126189	5.693071	1.032523	0.304643	-6.55848	0.522757	0.567393
Non.inflammatory.Macrophages	SERBP1	0.077169	8.529561	1.032388	0.304706	-6.76225	0.439329	0.524344
Non.inflammatory.Macrophages	GM50399	0.733114	0.166375	1.03233	0.304733	-4.93663	0.707662	0.661705
Non.inflammatory.Macrophages	LYAR	-0.15197	4.937131	-1.03217	0.304808	-6.11229	0.547474	0.579515
Non.inflammatory.Macrophages	FHOD3	-0.73814	-0.11029	-1.03204	0.30487	-4.90822	0.707696	0.66685
Non.inflammatory.Macrophages	AKT2	-0.10503	5.234185	-1.03196	0.304906	-6.23886	0.537642	0.574765
Non.inflammatory.Macrophages	TM9SF1	-0.11675	5.042685	-1.03176	0.304999	-6.22866	0.543961	0.577882
Non.inflammatory.Macrophages	TMEM56	-0.3174	2.147941	-1.03174	0.305009	-5.5354	0.648409	0.62636
Non.inflammatory.Macrophages	DCAF17	-0.1342	4.054876	-1.03154	0.305103	-6.0112	0.57777	0.594085
Non.inflammatory.Macrophages	FASTK	0.164373	3.15048	1.031221	0.30525	-5.82643	0.61056	0.609347
Non.inflammatory.Macrophages	TCTEX1D2	-0.16376	3.301638	-1.03092	0.305389	-5.92828	0.604997	0.606962
Non.inflammatory.Macrophages	CLN8	-0.13888	3.202024	-1.03087	0.305414	-6.23762	0.608661	0.608646
Non.inflammatory.Macrophages	CCDC69	0.235099	3.297575	1.030806	0.305443	-5.60299	0.605146	0.60703
Non.inflammatory.Macrophages	FAM117A	0.158277	5.965212	1.030768	0.305461	-6.26546	0.514389	0.563587
Non.inflammatory.Macrophages	CLCN5	0.124905	5.160349	1.030537	0.305569	-6.33368	0.540413	0.576386

Non.inflammatory.Macrophages	SHFL	-0.28283	1.832835	-1.03036	0.305653	-5.53292	0.661324	0.632379
Non.inflammatory.Macrophages	CCDC71L	0.142587	4.455272	1.029975	0.305831	-6.14064	0.564429	0.587962
Non.inflammatory.Macrophages	TBCK	0.107126	5.26974	1.029873	0.305879	-6.2627	0.537085	0.574826
Non.inflammatory.Macrophages	SNUPN	-0.17212	2.593487	-1.02951	0.306048	-5.71491	0.632016	0.619308
Non.inflammatory.Macrophages	TRAK2	0.131555	5.153104	1.029499	0.306054	-6.12735	0.541004	0.576726
Non.inflammatory.Macrophages	RLF	0.09693	6.699162	1.029325	0.306135	-6.46445	0.4922	0.552486
Non.inflammatory.Macrophages	PLIN2	0.118289	5.831377	1.029155	0.306214	-6.62356	0.519047	0.566001
Non.inflammatory.Macrophages	SMARCC1	0.107396	6.606315	1.029101	0.306239	-6.42013	0.495007	0.55392
Non.inflammatory.Macrophages	CLIC5	0.616283	0.878367	1.028943	0.306313	-5.00802	0.699477	0.649663
Non.inflammatory.Macrophages	TUT1	-0.19172	2.638669	-1.02894	0.306317	-5.57675	0.630293	0.618638
Non.inflammatory.Macrophages	GM1604B	-0.40807	1.489369	-1.0288	0.30638	-5.32804	0.675495	0.638773
Non.inflammatory.Macrophages	CDH13	-0.57665	2.030566	-1.02877	0.306396	-5.2925	0.653852	0.629248
Non.inflammatory.Macrophages	1700061G1	-0.4325	1.070717	-1.02823	0.306647	-5.16381	0.693063	0.646545
Non.inflammatory.Macrophages	PIGW	0.337761	0.765108	1.028179	0.30667	-5.27576	0.702903	0.652058
Non.inflammatory.Macrophages	SLC15A2	-0.63079	4.597147	-1.02787	0.306816	-6.14687	0.560136	0.586279

Non.inflammatory.Macrophages	TOMM20	-0.08607	6.725117	-1.02769	0.306901	-6.52929	0.491892	0.552658
Non.inflammatory.Macrophages	XRCC5	-0.32871	2.083305	-1.02754	0.30697	-5.30866	0.652441	0.628971
Non.inflammatory.Macrophages	2610001J0	0.12723	3.690163	1.027125	0.307164	-6.11366	0.59227	0.601627
Non.inflammatory.Macrophages	CNRIP1	-0.39383	1.648634	-1.02629	0.307556	-5.27032	0.670439	0.637127
Non.inflammatory.Macrophages	GDE1	0.14167	4.883129	1.026267	0.307565	-6.31297	0.551122	0.582295
Non.inflammatory.Macrophages	TINAGL1	-0.3967	2.066409	-1.02615	0.307622	-5.3807	0.653788	0.629817
Non.inflammatory.Macrophages	EPHX1	-0.20644	3.053436	-1.02612	0.307636	-5.87026	0.615943	0.612762
Non.inflammatory.Macrophages	E130308A1	0.151384	4.522919	1.026078	0.307654	-5.96437	0.563353	0.588207
Non.inflammatory.Macrophages	TNFSF11	0.898944	0.706959	1.02607	0.307658	-4.93232	0.705533	0.654066
Non.inflammatory.Macrophages	FAM193B	0.16007	4.050857	1.025826	0.307772	-5.86202	0.579812	0.596055
Non.inflammatory.Macrophages	SLC30A1	0.155824	3.386735	1.025798	0.307785	-6.10241	0.603672	0.607188
Non.inflammatory.Macrophages	VPS41	0.094821	5.101022	1.025609	0.307874	-6.34049	0.543949	0.578954
Non.inflammatory.Macrophages	PRKCZ	-0.49275	0.606888	-1.02538	0.307983	-5.00767	0.70822	0.656095
Non.inflammatory.Macrophages	SLC36A4	0.184989	3.21113	1.025101	0.308112	-5.94691	0.610345	0.610363
Non.inflammatory.Macrophages	XIST	-3.48185	4.173151	-1.02504	0.308142	-5.69204	0.575717	0.59427

Non.inflammatory.Macrophages	THEM6	0.230403	3.448768	1.025009	0.308155	-5.6782	0.601615	0.606381
Non.inflammatory.Macrophages	SRRM1	0.068834	7.865839	1.024563	0.308364	-6.66468	0.459532	0.536413
Non.inflammatory.Macrophages	B230208H1	-0.65208	0.861264	-1.02453	0.308379	-5.02165	0.701946	0.651847
Non.inflammatory.Macrophages	PCDHGC4	-0.58856	0.256002	-1.02415	0.308557	-4.98694	0.710931	0.663057
Non.inflammatory.Macrophages	KCND1	-0.44842	-0.19065	-1.02393	0.308663	-5.18821	0.711054	0.671469
Non.inflammatory.Macrophages	CYSLTR2	0.308221	1.496466	1.023084	0.309059	-5.60332	0.678292	0.641046
Non.inflammatory.Macrophages	CSMD3	-0.32695	1.302147	-1.02297	0.309111	-5.3356	0.686254	0.644526
Non.inflammatory.Macrophages	FLRT3	0.535288	-0.84839	1.022548	0.309311	-5.07395	0.712025	0.684327
Non.inflammatory.Macrophages	ZMIZ2	0.131908	4.532649	1.022441	0.309362	-6.05912	0.56457	0.589291
Non.inflammatory.Macrophages	GM31763	-0.2478	3.257819	-1.02218	0.309484	-5.71914	0.61004	0.610575
Non.inflammatory.Macrophages	GRTP1	-0.35525	0.35364	-1.02217	0.30949	-5.2719	0.712025	0.661911
Non.inflammatory.Macrophages	GGCX	0.282419	1.807957	1.022168	0.30949	-5.45973	0.665874	0.6357
Non.inflammatory.Macrophages	RCN1	0.20605	3.060814	1.022117	0.309514	-5.70361	0.617364	0.613931
Non.inflammatory.Macrophages	NKIRAS2	0.115228	4.042562	1.022024	0.309558	-6.14826	0.581661	0.597413
Non.inflammatory.Macrophages	LBHD1	-0.425	0.16876	-1.02187	0.309631	-5.08862	0.712073	0.665351

Non.inflammatory.Macrophages	TMEM179I	0.080954	5.864754	1.02164	0.309739	-6.42011	0.520616	0.567932
Non.inflammatory.Macrophages	RIC8A	-0.13289	3.980741	-1.02136	0.309871	-6.02356	0.58409	0.598615
Non.inflammatory.Macrophages	4930522L1	-0.20849	3.684756	-1.02131	0.309895	-5.69242	0.594688	0.603596
Non.inflammatory.Macrophages	CDKL1	-0.52627	1.100722	-1.02098	0.310052	-5.18246	0.695211	0.648728
Non.inflammatory.Macrophages	TMEM104	-0.14946	3.273099	-1.02077	0.310147	-6.07635	0.609861	0.610777
Non.inflammatory.Macrophages	JMJD8	0.291715	1.564022	1.020716	0.310174	-5.51336	0.676143	0.640512
Non.inflammatory.Macrophages	SEMA4B	-0.1482	5.763505	-1.02071	0.310175	-6.27051	0.524052	0.569873
Non.inflammatory.Macrophages	CIZ1	0.137045	3.940619	1.019981	0.310521	-5.98751	0.5862	0.599781
Non.inflammatory.Macrophages	KNTC1	-0.31316	2.818917	-1.01978	0.310615	-5.52431	0.627541	0.618883
Non.inflammatory.Macrophages	MRPL37	-0.12699	4.2912	-1.01952	0.31074	-6.03374	0.574045	0.594173
Non.inflammatory.Macrophages	CUTC	-0.14797	3.519969	-1.01925	0.310866	-5.89983	0.601729	0.607183
Non.inflammatory.Macrophages	E130102H2	-0.33139	1.232168	-1.01911	0.310931	-5.44188	0.690846	0.647101
Non.inflammatory.Macrophages	DST	-0.16391	4.335211	-1.01885	0.311055	-6.19183	0.572706	0.593668
Non.inflammatory.Macrophages	LSM11	-0.30528	2.171179	-1.01876	0.311096	-5.48227	0.652954	0.630512
Non.inflammatory.Macrophages	SLC38A10	-0.10824	5.326136	-1.01866	0.311144	-6.25017	0.539134	0.577515

Non.inflammatory.Macrophages	MRPS25	0.132952	4.238863	1.018619	0.311163	-6.07458	0.576074	0.595262
Non.inflammatory.Macrophages	DEF6	0.16701	5.457612	1.018187	0.311367	-5.8453	0.535066	0.575451
Non.inflammatory.Macrophages	RELT	0.146413	3.728351	1.018097	0.31141	-6.06089	0.5945	0.603851
Non.inflammatory.Macrophages	ZFP69	-0.25521	2.612118	-1.01793	0.311489	-5.61327	0.636145	0.622949
Non.inflammatory.Macrophages	ALAS1	0.174227	3.683631	1.017784	0.311558	-5.99967	0.596197	0.604723
Non.inflammatory.Macrophages	CDIP1	-0.10305	5.935336	-1.01757	0.311661	-6.31656	0.519824	0.568125
Non.inflammatory.Macrophages	CHPF2	0.200106	2.64648	1.017209	0.311829	-5.78424	0.635097	0.622729
Non.inflammatory.Macrophages	NEURL2	0.27668	0.801683	1.017173	0.311846	-5.61656	0.707213	0.655504
Non.inflammatory.Macrophages	FCRL1	0.205385	2.73778	1.017089	0.311886	-5.8771	0.631602	0.621182
Non.inflammatory.Macrophages	GM28791	-0.18515	3.918458	-1.01691	0.311969	-5.82898	0.588031	0.601199
Non.inflammatory.Macrophages	PRKACA	0.124712	4.156404	1.016559	0.312137	-6.05658	0.579756	0.597339
Non.inflammatory.Macrophages	PCK2	-0.21963	3.244845	-1.01648	0.312174	-5.73719	0.612748	0.612687
Non.inflammatory.Macrophages	EIF3J2	-0.34138	1.443832	-1.01639	0.312219	-5.30338	0.683097	0.644155
Non.inflammatory.Macrophages	FTSJ3	-0.1721	4.016619	-1.01604	0.312382	-6.01798	0.584888	0.599668
Non.inflammatory.Macrophages	ADAMTS14	-0.52796	0.543497	-1.01596	0.312422	-5.08553	0.713872	0.660469

Non.inflammatory.Macrophages	ZKSCAN16	-0.63572	-0.59074	-1.01572	0.312533	-4.86163	0.714955	0.681648
Non.inflammatory.Macrophages	UTRN	0.117589	7.31422	1.015634	0.312575	-6.55	0.478236	0.547135
Non.inflammatory.Macrophages	UGT1A7C	0.473217	-0.45047	1.015509	0.312634	-5.11673	0.714969	0.679086
Non.inflammatory.Macrophages	SPIN1	0.101026	5.805516	1.015267	0.312749	-6.27013	0.524641	0.570754
Non.inflammatory.Macrophages	GM15448	-0.28185	-0.00747	-1.01503	0.312863	-5.39868	0.715238	0.671009
Non.inflammatory.Macrophages	GM31508	-0.39063	0.763062	-1.01493	0.31291	-5.28156	0.709145	0.656808
Non.inflammatory.Macrophages	PRPSAP2	-0.12209	4.045546	-1.0145	0.313113	-6.10172	0.584338	0.599613
Non.inflammatory.Macrophages	YLPM1	0.109197	5.660547	1.014402	0.313159	-6.2718	0.529548	0.573315
Non.inflammatory.Macrophages	ZFP280D	0.122254	5.625842	1.014371	0.313173	-6.21313	0.530672	0.573869
Non.inflammatory.Macrophages	TAB3	0.1612	3.680364	1.014302	0.313206	-5.86798	0.597448	0.605839
Non.inflammatory.Macrophages	DPP8	0.094739	5.667042	1.013816	0.313437	-6.33915	0.529571	0.573469
Non.inflammatory.Macrophages	ALDH16A1	-0.12375	4.358468	-1.01365	0.313517	-6.14396	0.573573	0.594817
Non.inflammatory.Macrophages	AMIGO1	-0.41615	0.613629	-1.0136	0.31354	-5.12893	0.713495	0.660104
Non.inflammatory.Macrophages	SLC7A6	-0.1351	4.274758	-1.01356	0.313556	-6.02764	0.576502	0.596209
Non.inflammatory.Macrophages	AAK1	0.108815	6.392459	1.013436	0.313617	-6.4338	0.5066	0.5621

Non.inflammatory.Macrophages	GM14858	-0.48935	2.296719	-1.01325	0.313708	-5.21765	0.649977	0.630058
Non.inflammatory.Macrophages	FTX	-0.20381	4.311116	-1.01311	0.313772	-5.79938	0.575272	0.59575
Non.inflammatory.Macrophages	TRMT1L	0.103396	4.566129	1.01306	0.313796	-6.11056	0.566409	0.591548
Non.inflammatory.Macrophages	NDUFA12	0.101162	5.362792	1.012958	0.313844	-6.32906	0.539547	0.578599
Non.inflammatory.Macrophages	HIST1H3D	-0.33925	1.963471	-1.01275	0.313945	-5.46161	0.663228	0.636068
Non.inflammatory.Macrophages	PLGRKT	0.106152	5.796951	1.012675	0.313979	-6.41913	0.525471	0.571717
Non.inflammatory.Macrophages	TRAPPC2L	0.09349	5.811456	1.012537	0.314044	-6.43779	0.525026	0.571505
Non.inflammatory.Macrophages	TUBG1	-0.25613	3.393021	-1.01188	0.314355	-5.72024	0.608753	0.611567
Non.inflammatory.Macrophages	SSNA1	0.101635	5.922585	1.011877	0.314358	-6.33269	0.521816	0.569985
Non.inflammatory.Macrophages	DIMT1	-0.17622	3.243055	-1.01169	0.314446	-5.7836	0.614317	0.614131
Non.inflammatory.Macrophages	CARD19	0.111986	5.613475	1.011642	0.31447	-6.44924	0.531774	0.574926
Non.inflammatory.Macrophages	BHMT	0.23198	5.13251	1.011438	0.314567	-6.25901	0.547623	0.582718
Non.inflammatory.Macrophages	CEP19	0.283954	2.707827	1.011426	0.314572	-5.54241	0.634545	0.623398
Non.inflammatory.Macrophages	ASPDH	-0.42855	1.294554	-1.01096	0.314793	-5.29151	0.69109	0.648492
Non.inflammatory.Macrophages	CEBPB	0.156583	9.155958	1.010962	0.314793	-7.10079	0.428041	0.521071

Non.inflammatory.Macrophages	DPH5	0.15995	3.732552	1.010847	0.314848	-5.93253	0.596475	0.606028
Non.inflammatory.Macrophages	CCDC9	-0.11078	4.238269	-1.01084	0.314854	-6.07708	0.578419	0.597557
Non.inflammatory.Macrophages	VAV2	-0.13765	4.977829	-1.01057	0.31498	-6.27176	0.553028	0.585454
Non.inflammatory.Macrophages	RGS7BP	-0.38376	1.883105	-1.0105	0.315011	-5.53022	0.667176	0.638124
Non.inflammatory.Macrophages	MOB1B	0.080108	6.203813	1.009933	0.315283	-6.43792	0.513474	0.566017
Non.inflammatory.Macrophages	TRIP11	0.095209	6.069875	1.009814	0.31534	-6.40717	0.517705	0.568132
Non.inflammatory.Macrophages	SOCS4	0.117007	4.684801	1.009437	0.31552	-6.10648	0.563621	0.590637
Non.inflammatory.Macrophages	ARHGAP3(	0.075016	7.139253	1.008406	0.316012	-6.59157	0.485148	0.55207
Non.inflammatory.Macrophages	LIN37	-0.15668	3.646925	-1.00836	0.316033	-5.93136	0.600432	0.608439
Non.inflammatory.Macrophages	TMEM71	0.189952	3.858441	1.008321	0.316052	-5.92988	0.592769	0.604867
Non.inflammatory.Macrophages	DPF2	-0.10413	5.127706	-1.00823	0.316093	-6.23391	0.54868	0.583907
Non.inflammatory.Macrophages	PPIL2	-0.09753	5.124237	-1.00817	0.316126	-6.24177	0.548796	0.583963
Non.inflammatory.Macrophages	NXT2	-0.15319	2.785909	-1.0081	0.31616	-5.85237	0.63259	0.623231
Non.inflammatory.Macrophages	HIST1H4N	-0.52972	1.07648	-1.00809	0.316162	-5.09512	0.701179	0.653565
Non.inflammatory.Macrophages	MAFF	-0.15279	3.876198	-1.00797	0.316219	-6.23872	0.59213	0.604607

Non.inflammatory.Macrophages	OSGIN2	0.190602	3.18431	1.007906	0.31625	-5.84208	0.617516	0.616361
Non.inflammatory.Macrophages	GM13402	-0.68238	0.307958	-1.00779	0.316303	-4.90369	0.717548	0.667672
Non.inflammatory.Macrophages	GM16576	-0.3961	1.513376	-1.00765	0.31637	-5.30373	0.683033	0.645696
Non.inflammatory.Macrophages	KLRE1	-0.63185	1.626605	-1.00759	0.316402	-5.09502	0.6784	0.643667
Non.inflammatory.Macrophages	FAM135A	0.219861	2.016455	1.007189	0.316592	-5.75825	0.662667	0.636788
Non.inflammatory.Macrophages	1700048O2	-0.86909	-0.24632	-1.00718	0.316598	-4.87249	0.717548	0.678096
Non.inflammatory.Macrophages	CHD6	0.112121	5.997264	1.007048	0.31666	-6.3509	0.520292	0.570097
Non.inflammatory.Macrophages	CCDC141	0.364981	1.120838	1.006968	0.316698	-5.31435	0.699317	0.652961
Non.inflammatory.Macrophages	STAR	-0.38878	1.465058	-1.00684	0.316761	-5.33723	0.685018	0.646792
Non.inflammatory.Macrophages	SKAP2	0.073981	7.235487	1.00669	0.316831	-6.70661	0.482293	0.550854
Non.inflammatory.Macrophages	DNAJB4	-0.13753	3.700143	-1.0066	0.316873	-6.00503	0.598496	0.607825
Non.inflammatory.Macrophages	KLHL5	0.097525	4.645979	1.006542	0.316902	-6.26884	0.565036	0.592032
Non.inflammatory.Macrophages	SLC22A18	-0.28716	1.143718	-1.00648	0.316929	-5.53032	0.698358	0.652612
Non.inflammatory.Macrophages	KDELR1	-0.08184	6.091298	-1.00633	0.317005	-6.46176	0.517308	0.568683
Non.inflammatory.Macrophages	GM45894	-0.21998	2.290972	-1.0063	0.317017	-5.50803	0.651789	0.632128

Non.inflammatory.Macrophages	PTGES3	-0.07717	7.460041	-1.0063	0.317019	-6.65089	0.475697	0.547421
Non.inflammatory.Macrophages	EPHB4	-0.56798	0.794939	-1.00628	0.317028	-5.15049	0.710568	0.658966
Non.inflammatory.Macrophages	GM15545	-0.34261	1.102359	-1.00624	0.317047	-5.21502	0.700092	0.653362
Non.inflammatory.Macrophages	DDOST	-0.08751	5.788445	-1.00615	0.317089	-6.43747	0.526979	0.573502
Non.inflammatory.Macrophages	A430005L1	-0.12897	3.764054	-1.00612	0.317102	-6.06498	0.596177	0.606749
Non.inflammatory.Macrophages	H2-KE6	-0.13307	5.147514	-1.00612	0.317102	-6.20076	0.548018	0.583827
Non.inflammatory.Macrophages	PEX11G	-0.26085	2.394748	-1.00584	0.317239	-5.46866	0.647891	0.630467
Non.inflammatory.Macrophages	STAG2	-0.094	7.464582	-1.00556	0.31737	-6.61688	0.475807	0.547531
Non.inflammatory.Macrophages	VNN3	0.326172	1.614791	1.005263	0.317513	-5.51308	0.679422	0.644396
Non.inflammatory.Macrophages	EIF4EBP2	0.070288	6.850744	1.004897	0.317689	-6.53374	0.494221	0.557091
Non.inflammatory.Macrophages	SETD7	0.136252	4.9704	1.0048	0.317735	-6.10281	0.554439	0.587031
Non.inflammatory.Macrophages	GM9949	-0.39013	0.483247	-1.00477	0.317751	-5.35435	0.718155	0.665059
Non.inflammatory.Macrophages	TLR12	-0.24707	2.045397	-1.00471	0.317778	-5.83611	0.662071	0.636808
Non.inflammatory.Macrophages	MASP2	-0.4366	1.894509	-1.00461	0.317826	-5.33719	0.668116	0.639485
Non.inflammatory.Macrophages	PDE8B	-0.37574	-0.24267	-1.00453	0.317866	-5.25387	0.718155	0.678644

Non.inflammatory.Macrophages	GM46430	0.242497	1.969969	1.004455	0.317901	-5.45902	0.665087	0.638192
Non.inflammatory.Macrophages	SLC5A6	-0.37532	0.868812	-1.00385	0.318192	-5.29407	0.709501	0.658173
Non.inflammatory.Macrophages	ALKBH3	0.156561	3.490416	1.003807	0.318211	-5.94651	0.606965	0.611902
Non.inflammatory.Macrophages	EMC4	-0.12025	4.771124	-1.0038	0.318212	-6.21933	0.561486	0.590474
Non.inflammatory.Macrophages	PIM3	0.136445	4.641176	1.003576	0.318322	-6.25528	0.566051	0.5927
Non.inflammatory.Macrophages	GM28981	-0.28962	0.488246	-1.00334	0.318436	-5.50029	0.718764	0.665357
Non.inflammatory.Macrophages	CABIN1	0.110084	5.4487	1.003075	0.318562	-6.26922	0.539068	0.579647
Non.inflammatory.Macrophages	ZFR2	-0.34547	0.817601	-1.00266	0.318762	-5.27964	0.7115	0.659533
Non.inflammatory.Macrophages	TMEM267	-0.27245	1.734822	-1.00256	0.318808	-5.52266	0.675469	0.642952
Non.inflammatory.Macrophages	POLR2H	-0.11895	4.512761	-1.0024	0.318885	-6.09616	0.570882	0.595178
Non.inflammatory.Macrophages	UBR2	0.085493	6.362518	1.002348	0.318911	-6.45636	0.509902	0.565325
Non.inflammatory.Macrophages	ATP5MD	0.080024	8.035566	1.002288	0.31894	-6.76879	0.46019	0.539615
Non.inflammatory.Macrophages	PIGX	0.104101	5.308726	1.0022	0.318982	-6.30285	0.543835	0.582219
Non.inflammatory.Macrophages	1700010K2	-0.31994	0.709295	-1.00213	0.319015	-5.28967	0.714456	0.6617
Non.inflammatory.Macrophages	6720489N1	-0.29868	0.964398	-1.00195	0.3191	-5.38986	0.707514	0.657115

Non.inflammatory.Macrophages	ING3	-0.11191	4.830612	-1.00155	0.319292	-6.13303	0.560231	0.590228
Non.inflammatory.Macrophages	GM41442	-0.38905	-0.19949	-1.00133	0.319402	-5.31525	0.719625	0.678936
Non.inflammatory.Macrophages	GINS4	0.149438	3.753755	1.001148	0.319488	-5.97072	0.598338	0.608328
Non.inflammatory.Macrophages	PDS5B	0.102382	6.377064	1.000928	0.319593	-6.36289	0.509914	0.565545
Non.inflammatory.Macrophages	TACC1	0.078245	7.703473	1.000859	0.319626	-6.55995	0.470096	0.545066
Non.inflammatory.Macrophages	BPNT1	0.146376	3.339718	1.000571	0.319765	-5.91735	0.613786	0.615514
Non.inflammatory.Macrophages	HPX	-0.19843	5.670685	-1.00016	0.319964	-6.30849	0.532628	0.576963
Non.inflammatory.Macrophages	MKLN1OS	0.406555	0.018205	1.000103	0.31999	-5.25103	0.72004	0.675197
Non.inflammatory.Macrophages	QSOX2	-0.36645	0.948725	-1.00008	0.320003	-5.26134	0.708855	0.657975
Non.inflammatory.Macrophages	TIMD4	-0.1796	3.658253	-0.99994	0.32007	-6.35722	0.602103	0.610274
Non.inflammatory.Macrophages	AATK	-0.34152	0.48294	-0.9999	0.320087	-5.45084	0.72004	0.666603
Non.inflammatory.Macrophages	NEAT1	-0.11339	8.674976	-0.99984	0.320118	-6.968	0.443038	0.530763
Non.inflammatory.Macrophages	PATJ	-0.38479	3.348038	-0.99957	0.320248	-5.50139	0.613689	0.615729
Non.inflammatory.Macrophages	ITIH3	-0.28014	3.636116	-0.99881	0.320611	-5.83719	0.603573	0.610966
Non.inflammatory.Macrophages	KIDINS220	0.094152	5.419693	0.998689	0.320671	-6.38178	0.541451	0.581373

Non.inflammatory.Macrophages	ABHD5	-0.1452	3.453436	-0.99859	0.32072	-6.04201	0.6103	0.614134
Non.inflammatory.Macrophages	ACAA2	-0.14595	5.01941	-0.99856	0.320735	-6.31307	0.554842	0.587942
Non.inflammatory.Macrophages	AP3B1	0.072088	7.329007	0.998268	0.320874	-6.6467	0.481836	0.551448
Non.inflammatory.Macrophages	TMEM38A	-0.27108	1.868955	-0.99818	0.320914	-5.51027	0.671788	0.641959
Non.inflammatory.Macrophages	SPINK10	-0.45432	0.791956	-0.99771	0.321143	-5.14274	0.714449	0.661608
Non.inflammatory.Macrophages	PNPLA7	-0.1117	6.039516	-0.99757	0.321208	-6.47393	0.521744	0.571768
Non.inflammatory.Macrophages	ADCK5	-0.26065	1.510315	-0.99719	0.321392	-5.53648	0.687059	0.64867
Non.inflammatory.Macrophages	NOL4L	-0.22185	2.680623	-0.99714	0.321419	-5.90309	0.640265	0.627927
Non.inflammatory.Macrophages	CANX	0.065046	7.35016	0.996677	0.321641	-6.70334	0.481902	0.551516
Non.inflammatory.Macrophages	RESF1	0.110689	6.500817	0.996434	0.321759	-6.45292	0.507752	0.564766
Non.inflammatory.Macrophages	GM2788	-0.51074	0.569462	-0.99628	0.321833	-5.06867	0.720753	0.666139
Non.inflammatory.Macrophages	TSEN34	-0.13255	4.195681	-0.99576	0.322083	-6.12822	0.584784	0.602337
Non.inflammatory.Macrophages	ZFP961	0.231193	3.308556	0.995755	0.322086	-5.63369	0.617153	0.617393
Non.inflammatory.Macrophages	SMG7	0.091925	6.385921	0.995581	0.322171	-6.43272	0.511608	0.566727
Non.inflammatory.Macrophages	XPC	0.155755	3.385386	0.995528	0.322196	-5.88206	0.614291	0.616097

Non.inflammatory.Macrophages	FAM83D	-0.30531	2.774945	-0.99512	0.322395	-5.39413	0.637711	0.626712
Non.inflammatory.Macrophages	CHMP1B	0.111909	4.698125	0.99487	0.322515	-6.19513	0.567506	0.594102
Non.inflammatory.Macrophages	UHRF1	-0.24605	4.598357	-0.9948	0.322547	-5.92215	0.570966	0.595754
Non.inflammatory.Macrophages	PRDM16	0.484419	0.306557	0.994507	0.32269	-5.19424	0.723018	0.671437
Non.inflammatory.Macrophages	GM47428	-0.34581	0.207827	-0.9945	0.322695	-5.42451	0.723018	0.67328
Non.inflammatory.Macrophages	LCT	-0.51722	0.170332	-0.9944	0.322744	-5.06617	0.723018	0.673996
Non.inflammatory.Macrophages	DDX21	-0.10481	6.399043	-0.99433	0.322778	-6.48343	0.511526	0.566823
Non.inflammatory.Macrophages	GM15494	-0.38861	0.759331	-0.99366	0.323101	-5.41104	0.717561	0.663342
Non.inflammatory.Macrophages	TMCC2	0.340585	1.686886	0.993374	0.323239	-5.60221	0.681844	0.646529
Non.inflammatory.Macrophages	KATNB1	0.261339	2.346793	0.993117	0.323363	-5.56399	0.655411	0.634843
Non.inflammatory.Macrophages	GM17178	-0.37527	1.460682	-0.99204	0.323887	-5.18872	0.692335	0.65103
Non.inflammatory.Macrophages	SNRPD3	0.08812	6.518599	0.99163	0.324084	-6.51709	0.509429	0.565685
Non.inflammatory.Macrophages	PDE4D	0.165417	6.428002	0.991382	0.324204	-6.40858	0.512322	0.56725
Non.inflammatory.Macrophages	MACF1	0.076242	8.000182	0.991332	0.324229	-6.84252	0.46524	0.54299
Non.inflammatory.Macrophages	IL5RA	-0.6259	0.975245	-0.99078	0.324495	-5.04066	0.71366	0.660528

Non.inflammatory.Macrophages	MYO1E	0.238202	6.198716	0.990644	0.324563	-6.18336	0.519926	0.571208
Non.inflammatory.Macrophages	ZFAND2B	-0.13499	4.313639	-0.99027	0.324747	-6.04299	0.583548	0.60212
Non.inflammatory.Macrophages	CMTM8	-0.19537	4.192364	-0.99017	0.324794	-5.99806	0.58787	0.604169
Non.inflammatory.Macrophages	RPUSD1	0.312438	1.47218	0.989808	0.324969	-5.35529	0.693201	0.651826
Non.inflammatory.Macrophages	REXO5	0.301797	1.950184	0.989601	0.32507	-5.37499	0.673553	0.643298
Non.inflammatory.Macrophages	NFAT5	-0.09878	7.501438	-0.98946	0.325137	-6.73696	0.480364	0.551206
Non.inflammatory.Macrophages	PACRG	-0.44378	-0.43352	-0.98945	0.325142	-5.18982	0.72643	0.687329
Non.inflammatory.Macrophages	LRRC25	-0.13215	3.856093	-0.98942	0.32516	-6.47985	0.600192	0.61008
Non.inflammatory.Macrophages	5430416N(	-0.16755	3.547711	-0.989	0.32536	-5.76914	0.61159	0.615465
Non.inflammatory.Macrophages	DYSF	-0.31345	2.592245	-0.98899	0.325367	-5.83045	0.648019	0.632045
Non.inflammatory.Macrophages	MAPK1	0.067356	7.693924	0.988887	0.325417	-6.62877	0.474766	0.548387
Non.inflammatory.Macrophages	CDKN3	-0.2791	4.160534	-0.98888	0.325418	-5.86556	0.589239	0.605071
Non.inflammatory.Macrophages	GM13708	-0.23383	3.088737	-0.9888	0.325457	-5.64694	0.628846	0.623413
Non.inflammatory.Macrophages	RCAN1	0.126281	3.708543	0.988421	0.325644	-6.14625	0.605896	0.612833
Non.inflammatory.Macrophages	TRIM30A	0.15589	6.169574	0.988045	0.325827	-6.57592	0.521536	0.572422

Non.inflammatory.Macrophages	ZFP518A	0.157147	3.684945	0.987903	0.325896	-5.93723	0.606843	0.613416
Non.inflammatory.Macrophages	FAM120B	0.132124	4.124917	0.987894	0.3259	-6.06558	0.590834	0.60595
Non.inflammatory.Macrophages	ZC3H13	0.099481	5.653384	0.987729	0.325981	-6.35927	0.538261	0.580708
Non.inflammatory.Macrophages	DSN1	-0.24595	2.622227	-0.98772	0.325988	-5.55362	0.647194	0.631824
Non.inflammatory.Macrophages	USP36	0.119778	4.970109	0.987634	0.326027	-6.19175	0.561189	0.59186
Non.inflammatory.Macrophages	ZFAS1	0.136067	5.058651	0.987563	0.326062	-6.29911	0.558166	0.590403
Non.inflammatory.Macrophages	TMEM63A	0.169707	3.49863	0.987444	0.326119	-6.01756	0.613751	0.616605
Non.inflammatory.Macrophages	ABHD16A	-0.11409	4.808556	-0.98725	0.326215	-6.19879	0.566825	0.594614
Non.inflammatory.Macrophages	TKT	0.097832	6.932084	0.986994	0.326339	-6.60298	0.497862	0.560494
Non.inflammatory.Macrophages	ABCF2	-0.12073	4.411019	-0.98691	0.326379	-6.09238	0.580778	0.601246
Non.inflammatory.Macrophages	NDUFA9	0.10626	4.840567	0.98676	0.326453	-6.24157	0.565776	0.5941
Non.inflammatory.Macrophages	HECTD3	-0.17055	2.789878	-0.98674	0.326463	-5.78271	0.640822	0.628992
Non.inflammatory.Macrophages	ZCWPW1	-0.2274	2.765843	-0.98603	0.326809	-5.62722	0.642004	0.629681
Non.inflammatory.Macrophages	QRSL1	-0.2165	3.18623	-0.98593	0.326859	-5.69707	0.625873	0.622401
Non.inflammatory.Macrophages	GALNT6	-0.16292	3.115592	-0.98591	0.326869	-6.12743	0.628557	0.623626

Non.inflammatory.Macrophages	MIPEP	0.166616	2.843457	0.985873	0.326886	-5.73482	0.638997	0.628365
Non.inflammatory.Macrophages	CTBP1	0.095519	6.500976	0.985718	0.326961	-6.44752	0.511384	0.567585
Non.inflammatory.Macrophages	CEP290	-0.17183	2.468766	-0.98563	0.327005	-5.8263	0.653633	0.635043
Non.inflammatory.Macrophages	TMED3	-0.10852	5.491352	-0.98548	0.327078	-6.38911	0.543958	0.583837
Non.inflammatory.Macrophages	ZFAND5	-0.06788	7.203792	-0.98543	0.327104	-6.70763	0.489828	0.556656
Non.inflammatory.Macrophages	REEP1	-0.49773	0.98372	-0.98542	0.327105	-5.11338	0.714712	0.66184
Non.inflammatory.Macrophages	CCDC28B	-0.1605	3.504556	-0.98538	0.327127	-5.97554	0.61391	0.617035
Non.inflammatory.Macrophages	GM43660	-0.68147	0.127169	-0.98492	0.327351	-4.94913	0.727495	0.678013
Non.inflammatory.Macrophages	GM31728	-0.41208	-0.17439	-0.98492	0.327352	-5.286	0.727495	0.68371
Non.inflammatory.Macrophages	ADD1	0.098899	5.795836	0.984909	0.327356	-6.37708	0.534042	0.579115
Non.inflammatory.Macrophages	MRPS33	0.074676	6.714474	0.984455	0.327578	-6.59045	0.505004	0.564637
Non.inflammatory.Macrophages	GLDC	-0.41001	1.391092	-0.98437	0.327621	-5.29713	0.697824	0.654793
Non.inflammatory.Macrophages	CENPO	-0.19352	2.773597	-0.98436	0.327625	-5.70648	0.642037	0.6301
Non.inflammatory.Macrophages	SNHG1	-0.10797	5.230493	-0.98418	0.327713	-6.31761	0.55298	0.58848
Non.inflammatory.Macrophages	YBEY	0.258138	1.366271	0.984149	0.327728	-5.43728	0.698865	0.655276

Non.inflammatory.Macrophages	KIF1A	-0.30662	-0.59877	-0.98372	0.327939	-5.39646	0.727851	0.692178
Non.inflammatory.Macrophages	STX6	0.111712	5.2207	0.983681	0.327957	-6.28127	0.553409	0.58879
Non.inflammatory.Macrophages	AGO2	0.091302	7.576219	0.983624	0.327984	-6.5953	0.4791	0.55142
Non.inflammatory.Macrophages	YBX1	0.079526	8.398771	0.983431	0.328079	-6.85662	0.455508	0.539002
Non.inflammatory.Macrophages	ZFP62	-0.11661	4.580193	-0.98336	0.328114	-6.17507	0.57545	0.599453
Non.inflammatory.Macrophages	MPO	-0.86002	1.827356	-0.98333	0.328126	-5.48415	0.67988	0.64717
Non.inflammatory.Macrophages	VWA8	0.11683	4.975158	0.983261	0.328162	-6.25287	0.561762	0.592898
Non.inflammatory.Macrophages	AHRR	-0.30928	-0.36283	-0.98287	0.328352	-5.39449	0.728153	0.687936
Non.inflammatory.Macrophages	NAPA	-0.07587	5.92916	-0.98276	0.328409	-6.48661	0.530191	0.577514
Non.inflammatory.Macrophages	SLC25A51	0.100644	5.697758	0.982568	0.328501	-6.45728	0.537807	0.581307
Non.inflammatory.Macrophages	D430001F1	-0.48217	-0.87048	-0.98245	0.328561	-5.19673	0.728258	0.697832
Non.inflammatory.Macrophages	IFT88	0.242092	1.778503	0.981587	0.328982	-5.51998	0.682971	0.648704
Non.inflammatory.Macrophages	GM17259	-0.35256	1.365713	-0.98147	0.32904	-5.34934	0.700129	0.656263
Non.inflammatory.Macrophages	RAD9A	-0.26818	2.338849	-0.9814	0.329071	-5.61436	0.660294	0.638781
Non.inflammatory.Macrophages	LRRK2	0.285534	4.404591	0.981272	0.329136	-5.70767	0.582566	0.603165

Non.inflammatory.Macrophages	GM30948	-0.81055	0.467484	-0.9811	0.329222	-4.94954	0.729014	0.673038
Non.inflammatory.Macrophages	SELENOI	0.179197	3.422495	0.981051	0.329245	-5.8451	0.618391	0.619957
Non.inflammatory.Macrophages	SPRY2	-0.23485	5.158508	-0.98098	0.329279	-5.92988	0.556401	0.590746
Non.inflammatory.Macrophages	GPR180	0.167263	2.964205	0.980702	0.329416	-5.84006	0.635962	0.627972
Non.inflammatory.Macrophages	PES1	0.099217	4.691666	0.980487	0.329521	-6.25763	0.5727	0.598554
Non.inflammatory.Macrophages	GM17655	-0.38341	1.183959	-0.98038	0.329574	-5.23153	0.708094	0.659972
Non.inflammatory.Macrophages	TRIO	-0.10195	6.069746	-0.98022	0.329652	-6.48847	0.52652	0.576148
Non.inflammatory.Macrophages	NCAPH	-0.23683	4.175802	-0.98007	0.329725	-5.91595	0.59105	0.607385
Non.inflammatory.Macrophages	IL1RL2	-0.37019	0.726101	-0.97981	0.329856	-5.39357	0.724383	0.66865
Non.inflammatory.Macrophages	SEC23B	0.099084	5.223375	0.979693	0.329911	-6.25183	0.554631	0.590074
Non.inflammatory.Macrophages	KRT18	-0.31937	3.557982	-0.97942	0.330046	-5.82067	0.613954	0.618138
Non.inflammatory.Macrophages	TMSB15B2	-0.34952	2.47439	-0.97915	0.330179	-5.29226	0.655595	0.637154
Non.inflammatory.Macrophages	UBE2H	-0.09183	8.574917	-0.97911	0.330196	-6.80782	0.451796	0.537656
Non.inflammatory.Macrophages	ATL1	0.463802	0.498179	0.979073	0.330215	-5.20615	0.729622	0.673131
Non.inflammatory.Macrophages	ZSWIM1	-0.38626	1.4089	-0.97877	0.330366	-5.33911	0.699072	0.656392

Non.inflammatory.Macrophages	SH3D21	-0.22721	1.99564	-0.97867	0.330414	-5.75022	0.674831	0.645789
Non.inflammatory.Macrophages	APPL2	0.161435	3.358029	0.978529	0.330483	-6.10204	0.621485	0.621812
Non.inflammatory.Macrophages	LTO1	0.153565	3.526077	0.978517	0.330489	-5.91183	0.615183	0.618911
Non.inflammatory.Macrophages	BROX	0.088513	4.85097	0.978497	0.330499	-6.29145	0.567551	0.596503
Non.inflammatory.Macrophages	CEP170B	-0.47127	0.457754	-0.97811	0.330687	-5.05489	0.730048	0.674122
Non.inflammatory.Macrophages	CRAT	0.158984	3.198294	0.977968	0.330759	-6.00677	0.627741	0.624715
Non.inflammatory.Macrophages	CSF1R	0.137281	5.549681	0.977823	0.33083	-6.80726	0.544039	0.585137
Non.inflammatory.Macrophages	MRPS31	-0.14292	3.653241	-0.97776	0.330862	-5.90922	0.610658	0.616868
Non.inflammatory.Macrophages	TOR1A	0.09858	4.653633	0.977624	0.330928	-6.30571	0.57461	0.599929
Non.inflammatory.Macrophages	BUB3	0.109102	5.956108	0.977614	0.330933	-6.37956	0.53069	0.578565
Non.inflammatory.Macrophages	BCL9	-0.15402	3.399574	-0.97742	0.331029	-5.99577	0.620208	0.621314
Non.inflammatory.Macrophages	IL1F9	0.672977	-0.11555	0.977196	0.331138	-4.95716	0.730263	0.68519
Non.inflammatory.Macrophages	ZCRB1	0.074105	6.149583	0.976804	0.331332	-6.47168	0.5248	0.575694
Non.inflammatory.Macrophages	NUDT18	0.168187	2.627052	0.976718	0.331374	-5.76532	0.650258	0.634997
Non.inflammatory.Macrophages	ZFP760	0.351573	1.032725	0.976612	0.331426	-5.23918	0.715748	0.663775

Non.inflammatory.Macrophages	RALYL	-0.66116	0.59788	-0.97547	0.331989	-5.08287	0.729257	0.672219
Non.inflammatory.Macrophages	C1QBP	0.117743	6.055545	0.975444	0.332002	-6.45671	0.528352	0.577528
Non.inflammatory.Macrophages	CCNT2	0.095764	5.37232	0.975421	0.332013	-6.32447	0.550883	0.588619
Non.inflammatory.Macrophages	MUTYH	0.448664	0.370874	0.975269	0.332088	-5.07541	0.731266	0.676502
Non.inflammatory.Macrophages	SERPINA7	-0.55706	0.531403	-0.97526	0.33209	-5.07376	0.730495	0.673493
Non.inflammatory.Macrophages	AASS	-0.45059	1.208483	-0.9751	0.332169	-5.24762	0.70895	0.660977
Non.inflammatory.Macrophages	TMEM189	0.114277	6.387922	0.975066	0.332188	-6.55151	0.517714	0.572272
Non.inflammatory.Macrophages	CCDC117	-0.13337	4.699485	-0.97483	0.332306	-6.01823	0.573964	0.5999
Non.inflammatory.Macrophages	4933433G1	0.41181	1.434512	0.974789	0.332325	-5.20516	0.699397	0.656941
Non.inflammatory.Macrophages	SH3TC1	0.15326	2.768133	0.974749	0.332344	-5.97684	0.645377	0.633026
Non.inflammatory.Macrophages	ETNK2	-0.42615	1.270533	-0.97474	0.33235	-5.21042	0.706316	0.659941
Non.inflammatory.Macrophages	DAG1	0.129436	4.867236	0.974469	0.332482	-6.21295	0.568259	0.59724
Non.inflammatory.Macrophages	RASSF2	0.134016	4.259468	0.97436	0.332536	-6.16407	0.589689	0.607431
Non.inflammatory.Macrophages	NUSAP1	-0.24523	5.649629	-0.97406	0.332683	-6.1849	0.541829	0.584461
Non.inflammatory.Macrophages	PAK1IP1	-0.07666	5.901653	-0.97402	0.332704	-6.39949	0.533546	0.580375

Non.inflammatory.Macrophages	ZFP931	0.254978	1.514653	0.973942	0.332743	-5.48044	0.696294	0.655749
Non.inflammatory.Macrophages	ACVR2A	0.157019	4.7385	0.973679	0.332872	-6.20193	0.572942	0.599628
Non.inflammatory.Macrophages	GM37233	-0.40339	0.010375	-0.97311	0.333154	-5.23118	0.731996	0.684156
Non.inflammatory.Macrophages	NEIL3	-0.28629	4.375558	-0.97307	0.333174	-5.76443	0.585984	0.605963
Non.inflammatory.Macrophages	IFT43	0.247405	1.54627	0.972874	0.33327	-5.53776	0.69541	0.655607
Non.inflammatory.Macrophages	CSTB	0.100719	6.672627	0.972862	0.333276	-6.7232	0.509274	0.568444
Non.inflammatory.Macrophages	OGFOD3	-0.1638	3.187381	-0.97286	0.333276	-5.8793	0.629833	0.626358
Non.inflammatory.Macrophages	PIWIL2	-0.35434	0.700548	-0.97263	0.333389	-5.44116	0.727531	0.671276
Non.inflammatory.Macrophages	TAX1BP3	0.120655	4.090035	0.972417	0.333496	-6.22415	0.596277	0.610911
Non.inflammatory.Macrophages	ZFP358	0.17945	3.044691	0.972244	0.333582	-5.85287	0.635327	0.628973
Non.inflammatory.Macrophages	PTPN13	0.472921	0.415506	0.972121	0.333642	-5.19159	0.732028	0.676713
Non.inflammatory.Macrophages	ELOVL2	-0.34468	2.450205	-0.97189	0.333758	-5.59296	0.658581	0.639533
Non.inflammatory.Macrophages	DNMBP	-0.16248	3.488772	-0.97189	0.333758	-5.92486	0.618456	0.621319
Non.inflammatory.Macrophages	IGKV12-46	0.59807	-0.77013	0.971846	0.333779	-4.89801	0.732028	0.699369
Non.inflammatory.Macrophages	USE1	0.097525	5.084893	0.971737	0.333832	-6.35409	0.561216	0.594319

Non.inflammatory.Macrophages	SFXN3	0.154393	3.370072	0.971517	0.333941	-5.99587	0.622924	0.623393
Non.inflammatory.Macrophages	FILIP1	-0.47712	0.904589	-0.97149	0.333954	-5.14913	0.721873	0.667628
Non.inflammatory.Macrophages	TBC1D24	0.207098	1.799284	0.971486	0.333957	-5.7642	0.684937	0.651233
Non.inflammatory.Macrophages	MKS1	0.363696	1.092934	0.971482	0.333959	-5.30018	0.714622	0.664144
Non.inflammatory.Macrophages	FBF1	-0.31526	1.539498	-0.97141	0.333994	-5.62771	0.695723	0.65598
Non.inflammatory.Macrophages	GM28960	0.579227	-0.82667	0.969866	0.334759	-4.94105	0.733586	0.700938
Non.inflammatory.Macrophages	BC028528	-0.10327	4.105365	-0.9696	0.334892	-6.36224	0.597047	0.611255
Non.inflammatory.Macrophages	TUBA1A	-0.19824	5.430541	-0.9695	0.334942	-6.06165	0.550722	0.589175
Non.inflammatory.Macrophages	MRAS	0.328624	0.476226	0.969474	0.334954	-5.46066	0.733656	0.676226
Non.inflammatory.Macrophages	CCDC6	0.130746	4.540905	0.969046	0.335166	-6.15407	0.581523	0.604078
Non.inflammatory.Macrophages	YME1L1	-0.06554	6.181942	-0.96889	0.335241	-6.44616	0.52608	0.577169
Non.inflammatory.Macrophages	CWC27	-0.10095	5.662216	-0.96878	0.335297	-6.34754	0.543069	0.585609
Non.inflammatory.Macrophages	CD5	-0.40052	2.693985	-0.96878	0.335298	-5.17563	0.650501	0.636043
Non.inflammatory.Macrophages	SNX22	-0.45567	-0.37781	-0.96866	0.335357	-5.17463	0.733774	0.692772
Non.inflammatory.Macrophages	CABLES2	-0.17418	3.580958	-0.96849	0.335439	-5.77233	0.616474	0.620661

Non.inflammatory.Macrophages	KNOP1	0.104901	4.90183	0.968468	0.335453	-6.2489	0.568872	0.598263
Non.inflammatory.Macrophages	ATAD5	0.210576	4.742851	0.968383	0.335495	-6.04838	0.574412	0.600932
Non.inflammatory.Macrophages	PRF1	0.635906	0.377462	0.967984	0.335693	-5.00613	0.733774	0.678712
Non.inflammatory.Macrophages	PXDN	-0.39166	0.993778	-0.96789	0.335738	-5.22533	0.720597	0.667231
Non.inflammatory.Macrophages	RECQL	-0.19135	3.363727	-0.96755	0.335907	-5.82539	0.624651	0.624808
Non.inflammatory.Macrophages	XLR4A	0.420931	0.802973	0.967483	0.335942	-5.16234	0.726413	0.670941
Non.inflammatory.Macrophages	CSPP1	0.11081	6.098729	0.967172	0.336096	-6.37099	0.528766	0.579127
Non.inflammatory.Macrophages	HNRNPD	0.070432	7.908504	0.966962	0.336201	-6.68932	0.473249	0.550692
Non.inflammatory.Macrophages	IKZF5	0.141241	3.804869	0.966942	0.336211	-5.97011	0.608149	0.617335
Non.inflammatory.Macrophages	CD300E	0.327787	0.628868	0.966926	0.336219	-5.8437	0.731181	0.674332
Non.inflammatory.Macrophages	THA1	-0.41302	0.09883	-0.96689	0.336235	-5.25528	0.733774	0.684328
Non.inflammatory.Macrophages	ZFP395	0.136971	4.289929	0.966817	0.336273	-6.11312	0.590477	0.609056
Non.inflammatory.Macrophages	FAM207A	0.120138	4.223482	0.966759	0.336301	-6.08661	0.592869	0.610184
Non.inflammatory.Macrophages	PPHLN1	0.116787	4.932629	0.96675	0.336306	-6.27487	0.567805	0.598256
Non.inflammatory.Macrophages	HNRNPK	-0.05703	8.860645	-0.96674	0.336312	-6.86293	0.446368	0.536293

Non.inflammatory.Macrophages	MTIF2	0.11509	4.285566	0.966659	0.336352	-6.12928	0.590633	0.609138
Non.inflammatory.Macrophages	TNRC6B	0.093584	8.234075	0.966604	0.336379	-6.70679	0.463883	0.545734
Non.inflammatory.Macrophages	TACSTD2	0.669358	-0.35025	0.966475	0.336443	-4.9337	0.733774	0.692981
Non.inflammatory.Macrophages	RAB39	-0.19469	2.115142	-0.96632	0.336519	-5.96754	0.673633	0.647147
Non.inflammatory.Macrophages	TBCE	0.10234	5.119789	0.966309	0.336525	-6.22693	0.561359	0.595239
Non.inflammatory.Macrophages	FANCM	-0.20941	3.67592	-0.96621	0.336574	-5.75336	0.61293	0.619653
Non.inflammatory.Macrophages	YWHAE	0.057128	8.88218	0.966205	0.336577	-6.87721	0.445777	0.536058
Non.inflammatory.Macrophages	USP45	0.126494	4.074097	0.966181	0.336589	-6.16822	0.59828	0.612824
Non.inflammatory.Macrophages	2310061I0	-0.13886	3.521012	-0.96609	0.336634	-5.95758	0.61872	0.622349
Non.inflammatory.Macrophages	DDX42	0.082533	5.684077	0.965958	0.3367	-6.38324	0.542356	0.586024
Non.inflammatory.Macrophages	ITFG2	-0.18732	3.893716	-0.96586	0.33675	-5.79127	0.604889	0.616019
Non.inflammatory.Macrophages	IZUMO4	-0.36699	1.453448	-0.96555	0.336905	-5.41232	0.701186	0.659437
Non.inflammatory.Macrophages	AGPAT2	0.214607	4.355109	0.965473	0.336941	-5.89569	0.588297	0.608302
Non.inflammatory.Macrophages	ADIPOR1	0.088459	7.346972	0.96531	0.337023	-6.80419	0.49001	0.559729
Non.inflammatory.Macrophages	FOXM1	-0.2863	3.10354	-0.96486	0.337247	-5.56995	0.63506	0.63001

Non.inflammatory.Macrophages	TMEM126A	-0.09158	5.857631	-0.96481	0.337274	-6.35638	0.537029	0.583518
Non.inflammatory.Macrophages	GREB1L	-0.38986	1.947775	-0.96462	0.337368	-5.35656	0.680979	0.650692
Non.inflammatory.Macrophages	TBXAS1	-0.16179	3.468333	-0.9646	0.337378	-6.40294	0.621173	0.623744
Non.inflammatory.Macrophages	SRRT	-0.10481	5.528359	-0.9644	0.337477	-6.29484	0.547963	0.58901
Non.inflammatory.Macrophages	ERGIC1	0.125223	5.084699	0.964356	0.337498	-6.31703	0.563008	0.59633
Non.inflammatory.Macrophages	AP4E1	0.204771	3.305374	0.964216	0.337568	-5.76861	0.627391	0.626607
Non.inflammatory.Macrophages	PARP16	-0.3797	1.117003	-0.96384	0.337757	-5.29966	0.71604	0.666095
Non.inflammatory.Macrophages	AGK	0.19028	3.121713	0.963715	0.337817	-5.72914	0.63454	0.629982
Non.inflammatory.Macrophages	NDUFA13	0.072205	7.128419	0.963692	0.337829	-6.67566	0.496965	0.563494
Non.inflammatory.Macrophages	RBM38	-0.15669	6.898184	-0.96364	0.337853	-6.2457	0.504028	0.567117
Non.inflammatory.Macrophages	TCP11L1	0.144268	2.689841	0.96337	0.33799	-6.08466	0.651375	0.637659
Non.inflammatory.Macrophages	MSH2	0.153751	4.128132	0.963333	0.338008	-5.91735	0.59697	0.612642
Non.inflammatory.Macrophages	ARID1A	-0.07639	6.658177	-0.96321	0.33807	-6.54288	0.511522	0.570975
Non.inflammatory.Macrophages	ZFP61	-0.3997	1.174198	-0.96314	0.338105	-5.27014	0.713626	0.665102
Non.inflammatory.Macrophages	DMAC2L	-0.23745	2.257897	-0.9629	0.338223	-5.54959	0.66859	0.645371

Non.inflammatory.Macrophages	PARK7	-0.08817	6.806683	-0.96287	0.338238	-6.60032	0.506889	0.568625
Non.inflammatory.Macrophages	RPP38	-0.32791	1.226405	-0.96285	0.338251	-5.43969	0.711395	0.664155
Non.inflammatory.Macrophages	TFPT	-0.16034	2.965912	-0.96243	0.338456	-5.79685	0.6408	0.632944
Non.inflammatory.Macrophages	GM16151	-0.622	-0.28838	-0.96232	0.338512	-4.90275	0.73482	0.692849
Non.inflammatory.Macrophages	UBFD1	-0.11386	4.520398	-0.96229	0.33853	-6.20528	0.58308	0.606148
Non.inflammatory.Macrophages	CACNA1A	0.334967	1.424509	0.962116	0.338615	-5.39718	0.703218	0.660711
Non.inflammatory.Macrophages	SLC26A2	0.128178	3.742645	0.962079	0.338634	-6.1717	0.611322	0.619456
Non.inflammatory.Macrophages	RNF138	-0.09353	5.857254	-0.96182	0.338764	-6.34713	0.537519	0.584151
Non.inflammatory.Macrophages	LMTK3	-0.5269	-0.70138	-0.96156	0.338894	-5.00724	0.735074	0.701137
Non.inflammatory.Macrophages	ZSWIM7	0.147931	3.432711	0.961518	0.338914	-6.03041	0.623145	0.625067
Non.inflammatory.Macrophages	SBF2	-0.11203	5.368073	-0.96138	0.338985	-6.48308	0.553922	0.592354
Non.inflammatory.Macrophages	SHF	-0.28812	1.832889	-0.96107	0.339137	-5.39355	0.686518	0.653651
Non.inflammatory.Macrophages	RASGEF1B	0.125426	5.547786	0.960991	0.339178	-6.73367	0.547944	0.589455
Non.inflammatory.Macrophages	FOXD2OS	0.545457	0.311339	0.960965	0.33919	-5.0164	0.735202	0.681874
Non.inflammatory.Macrophages	WDPCP	-0.18015	3.269533	-0.96076	0.339293	-5.89081	0.629539	0.628078

Non.inflammatory.Macrophages	INTS3	-0.13042	3.898156	-0.96054	0.339405	-6.01017	0.606078	0.617221
Non.inflammatory.Macrophages	NUDT13	0.198367	2.358115	0.960335	0.339506	-5.74022	0.665423	0.644326
Non.inflammatory.Macrophages	EIF3J1	0.071166	7.268033	0.960209	0.339569	-6.6824	0.493408	0.562058
Non.inflammatory.Macrophages	DHRS13	0.471064	0.959658	0.959808	0.339769	-5.20921	0.723907	0.670071
Non.inflammatory.Macrophages	TGFBI	-0.12984	4.412001	-0.95954	0.339905	-6.58434	0.587596	0.608831
Non.inflammatory.Macrophages	GM47371	-0.46437	0.907518	-0.95954	0.339906	-5.18593	0.725354	0.671163
Non.inflammatory.Macrophages	SNRNP70	0.053925	7.511442	0.959526	0.339911	-6.65717	0.486155	0.5585
Non.inflammatory.Macrophages	GCSAM	0.5856	-0.31734	0.959327	0.34001	-4.98696	0.73564	0.694438
Non.inflammatory.Macrophages	ZBTB9	-0.21468	2.511721	-0.95932	0.340011	-5.62371	0.659377	0.641949
Non.inflammatory.Macrophages	KRTCAP3	-0.4819	0.893962	-0.9592	0.340075	-5.04146	0.72573	0.671483
Non.inflammatory.Macrophages	ST8SIA6	0.583025	1.211567	0.959143	0.340102	-5.22405	0.713059	0.665584
Non.inflammatory.Macrophages	DAP	0.122385	6.19848	0.959073	0.340137	-6.39427	0.526885	0.579349
Non.inflammatory.Macrophages	HS2ST1	-0.09208	5.689893	-0.95897	0.340188	-6.41105	0.54353	0.587666
Non.inflammatory.Macrophages	NEK7	0.088754	6.476805	0.95891	0.340219	-6.52716	0.517984	0.574943
Non.inflammatory.Macrophages	VCP	-0.06789	7.431716	-0.95876	0.340293	-6.71543	0.488539	0.55993

Non.inflammatory.Macrophages	MYLPF	0.382313	2.109786	0.958715	0.340317	-5.39845	0.675564	0.649352
Non.inflammatory.Macrophages	CALR	0.082235	7.35156	0.958578	0.340386	-6.77967	0.490968	0.561246
Non.inflammatory.Macrophages	ZFP516	-0.10547	4.66128	-0.95815	0.340598	-6.41447	0.578945	0.605063
Non.inflammatory.Macrophages	SMC5	0.104955	5.613071	0.958153	0.340599	-6.35758	0.546278	0.589241
Non.inflammatory.Macrophages	NCBP1	0.093279	5.256901	0.957353	0.341	-6.20756	0.558832	0.595301
Non.inflammatory.Macrophages	TACC3	-0.20796	5.072041	-0.95728	0.341037	-6.16351	0.565172	0.598373
Non.inflammatory.Macrophages	TSC2	-0.15531	3.109391	-0.95696	0.341194	-5.86678	0.636867	0.632042
Non.inflammatory.Macrophages	MED13	0.087397	7.722854	0.956921	0.341217	-6.70099	0.48057	0.555894
Non.inflammatory.Macrophages	MDH2	0.087994	6.490426	0.956763	0.341296	-6.5548	0.518286	0.575381
Non.inflammatory.Macrophages	GM48383	0.441668	1.758143	0.956617	0.341369	-5.17691	0.691002	0.656416
Non.inflammatory.Macrophages	ARRDC4	0.17006	2.427989	0.956449	0.341453	-5.99851	0.663659	0.644319
Non.inflammatory.Macrophages	MICOS10	0.076108	7.131558	0.956301	0.341528	-6.67095	0.49832	0.565295
Non.inflammatory.Macrophages	4930486L2	-0.30048	0.484692	-0.95624	0.341556	-5.50915	0.736683	0.680138
Non.inflammatory.Macrophages	TMEM265	-0.14918	3.958769	-0.95619	0.341585	-6.01125	0.604877	0.617533
Non.inflammatory.Macrophages	HACD1	0.215792	3.002315	0.956176	0.34159	-5.75713	0.64101	0.63419

Non.inflammatory.Macrophages	CDKN2C	-0.2134	3.918847	-0.9561	0.34163	-5.95004	0.606346	0.618247
Non.inflammatory.Macrophages	CEPT1	0.11219	5.374134	0.955752	0.341804	-6.48221	0.554902	0.593804
Non.inflammatory.Macrophages	PDP2	-0.14063	3.724878	-0.95574	0.341811	-5.97292	0.613533	0.621701
Non.inflammatory.Macrophages	TPMT	0.200983	2.097645	0.955706	0.341827	-5.70439	0.677017	0.650491
Non.inflammatory.Macrophages	GM26740	0.116133	7.068845	0.955693	0.341833	-6.59081	0.50024	0.566438
Non.inflammatory.Macrophages	GIN1	0.11926	3.954097	0.955504	0.341928	-5.94568	0.605121	0.61783
Non.inflammatory.Macrophages	PSMD13	0.083877	6.203145	0.955201	0.34208	-6.47593	0.527694	0.580483
Non.inflammatory.Macrophages	TINF2	-0.14389	4.054948	-0.95494	0.342212	-6.07061	0.601659	0.616305
Non.inflammatory.Macrophages	2010110K1	-0.67546	-0.03523	-0.95491	0.342226	-4.97569	0.737061	0.690523
Non.inflammatory.Macrophages	TMEM168	0.108418	4.583494	0.954591	0.342387	-6.25696	0.582716	0.607304
Non.inflammatory.Macrophages	ACTR3B	-0.48178	-0.01555	-0.95458	0.342395	-4.98462	0.737189	0.690146
Non.inflammatory.Macrophages	MCCC1	-0.22868	2.50652	-0.95428	0.342542	-5.6848	0.661015	0.6435
Non.inflammatory.Macrophages	HSPA2	-0.21056	3.291574	-0.9542	0.342585	-5.79423	0.630349	0.629639
Non.inflammatory.Macrophages	VBP1	0.102141	4.92592	0.954146	0.342611	-6.19493	0.570717	0.60167
Non.inflammatory.Macrophages	TNFAIP1	0.144194	3.775763	0.95401	0.34268	-6.11453	0.6121	0.621272

Non.inflammatory.Macrophages	BAZ2A	0.100773	6.97841	0.953582	0.342895	-6.56112	0.503399	0.568339
Non.inflammatory.Macrophages	1110002L0	-0.18558	2.8153	-0.95357	0.342904	-5.81333	0.648793	0.63817
Non.inflammatory.Macrophages	ZFPM2	-0.33735	2.777013	-0.95343	0.342971	-5.71741	0.650297	0.638892
Non.inflammatory.Macrophages	IGFALS	-0.26581	1.779759	-0.95338	0.342999	-5.57825	0.690622	0.656874
Non.inflammatory.Macrophages	CNOT8	-0.0939	5.284943	-0.95331	0.343034	-6.23472	0.558351	0.595832
Non.inflammatory.Macrophages	ABLIM2	0.455644	-0.47962	0.953255	0.34306	-5.16799	0.737237	0.699409
Non.inflammatory.Macrophages	NIFK	0.116407	4.620947	0.953233	0.343071	-6.21196	0.581425	0.606949
Non.inflammatory.Macrophages	FEM1A	0.159445	2.924816	0.953	0.343188	-5.81135	0.644508	0.636286
Non.inflammatory.Macrophages	GM20274	-0.19269	2.727126	-0.95296	0.343206	-5.76414	0.652262	0.639795
Non.inflammatory.Macrophages	CD226	-0.64447	1.697205	-0.95296	0.343206	-5.10735	0.694061	0.658383
Non.inflammatory.Macrophages	PPP1R2	0.063626	6.908394	0.952909	0.343234	-6.5809	0.505564	0.569507
Non.inflammatory.Macrophages	CENPA	-0.16121	6.681539	-0.95236	0.343509	-6.50329	0.512752	0.573233
Non.inflammatory.Macrophages	FAP	0.339747	0.744801	0.952297	0.343542	-5.65429	0.731628	0.676185
Non.inflammatory.Macrophages	CYP4A31	-0.4856	0.922941	-0.95229	0.343547	-5.19493	0.72666	0.672847
Non.inflammatory.Macrophages	SSBP4	-0.09413	4.90464	-0.95228	0.343552	-6.35564	0.571584	0.602304

Non.inflammatory.Macrophages	DNM2	-0.07155	7.193868	-0.95221	0.343586	-6.66725	0.496902	0.56512
Non.inflammatory.Macrophages	4931406P1	0.095436	5.594349	0.952111	0.343636	-6.35252	0.548019	0.590854
Non.inflammatory.Macrophages	RPP30	0.142135	3.635635	0.951847	0.343769	-5.92126	0.617522	0.624023
Non.inflammatory.Macrophages	RRAD	-0.25099	2.552475	-0.95173	0.343826	-5.66306	0.659388	0.643146
Non.inflammatory.Macrophages	A330032B1	-0.46447	-0.15579	-0.95158	0.343905	-5.24218	0.737466	0.693466
Non.inflammatory.Macrophages	ZFP652OS	0.329096	-0.171	0.951502	0.343943	-5.3774	0.737466	0.693781
Non.inflammatory.Macrophages	GM47730	-0.60985	-0.65913	-0.95149	0.343951	-4.90951	0.737466	0.703239
Non.inflammatory.Macrophages	CRYL1	0.120872	3.73595	0.951401	0.343995	-6.19527	0.613773	0.622386
Non.inflammatory.Macrophages	DHCR7	-0.21562	2.184949	-0.95111	0.344144	-5.58095	0.674364	0.649888
Non.inflammatory.Macrophages	CAMTA2	0.116456	3.696678	0.950223	0.344589	-6.19061	0.616106	0.623409
Non.inflammatory.Macrophages	EBI3	-0.1124	4.387447	-0.94991	0.344749	-6.40146	0.590949	0.611658
Non.inflammatory.Macrophages	STARD10	-0.21998	4.596544	-0.94967	0.344869	-6.15815	0.583582	0.608217
Non.inflammatory.Macrophages	GSTM7	-0.35688	1.02465	-0.94945	0.344981	-5.20511	0.724383	0.671833
Non.inflammatory.Macrophages	GRB7	-0.571	0.183341	-0.94913	0.345143	-5.04035	0.739146	0.687876
Non.inflammatory.Macrophages	ZBTB26	0.3557	0.850706	0.949016	0.3452	-5.31231	0.730395	0.675246

Non.inflammatory.Macrophages	PIRB	0.144592	4.941687	0.948985	0.345215	-6.5489	0.571645	0.602618
Non.inflammatory.Macrophages	JHY	0.46413	-0.37714	0.948781	0.345318	-5.25063	0.73925	0.698657
Non.inflammatory.Macrophages	LIAS	-0.12311	4.310022	-0.94867	0.345374	-6.10336	0.594157	0.613334
Non.inflammatory.Macrophages	NXPE4	0.18389	2.181788	0.948275	0.345574	-6.04772	0.676226	0.65091
Non.inflammatory.Macrophages	RPGR	0.312057	1.599467	0.948042	0.345692	-5.33785	0.700382	0.661612
Non.inflammatory.Macrophages	F830208F2	0.365047	-0.23725	0.948034	0.345696	-5.36695	0.739591	0.696235
Non.inflammatory.Macrophages	AKT1	0.095783	6.230392	0.947699	0.345866	-6.50922	0.528736	0.581708
Non.inflammatory.Macrophages	GM26901	-0.39021	0.57091	-0.94765	0.345889	-5.15049	0.738143	0.680915
Non.inflammatory.Macrophages	CERS4	-0.21673	3.651416	-0.94764	0.345896	-5.71362	0.618773	0.625023
Non.inflammatory.Macrophages	OAS3	0.275975	2.402295	0.947362	0.346036	-6.05697	0.667391	0.647164
Non.inflammatory.Macrophages	CDK2	-0.15233	4.350301	-0.94732	0.346056	-6.07549	0.593044	0.613019
Non.inflammatory.Macrophages	DDIT3	-0.14879	4.008692	-0.94731	0.346064	-6.0417	0.605496	0.618876
Non.inflammatory.Macrophages	GM43329	0.199295	3.105098	0.947121	0.346158	-5.82881	0.639671	0.634689
Non.inflammatory.Macrophages	ZNRF2	0.088033	5.763077	0.947039	0.3462	-6.42435	0.544115	0.589461
Non.inflammatory.Macrophages	BCL9L	-0.15969	3.410778	-0.94687	0.346284	-5.9176	0.627982	0.629412

Non.inflammatory.Macrophages	MVD	0.208821	2.348846	0.946255	0.346598	-5.74073	0.670149	0.648517
Non.inflammatory.Macrophages	RORA	0.172355	5.020685	0.946153	0.346649	-6.2345	0.569818	0.602047
Non.inflammatory.Macrophages	NME1	0.119875	6.668403	0.946003	0.346725	-6.5786	0.515244	0.575108
Non.inflammatory.Macrophages	PPFIA4	-0.14156	2.735956	-0.9459	0.346779	-6.17127	0.654698	0.641688
Non.inflammatory.Macrophages	SHE	-0.39028	0.946591	-0.94553	0.346967	-5.36509	0.729167	0.674609
Non.inflammatory.Macrophages	CCR3	-0.61033	-0.02075	-0.9454	0.347033	-5.41246	0.740611	0.693002
Non.inflammatory.Macrophages	GSDMD	0.131013	4.268046	0.945366	0.347049	-6.31187	0.596772	0.615107
Non.inflammatory.Macrophages	THAP11	-0.12954	4.457867	-0.94501	0.34723	-6.11568	0.590132	0.612033
Non.inflammatory.Macrophages	DUS1L	-0.14385	4.064535	-0.94479	0.347343	-6.05069	0.604528	0.618809
Non.inflammatory.Macrophages	NAXD	0.114172	4.128354	0.944237	0.347622	-6.15763	0.602409	0.617926
Non.inflammatory.Macrophages	ZFP687	0.180748	2.919042	0.944082	0.3477	-5.73445	0.648269	0.639112
Non.inflammatory.Macrophages	BANF1	0.098998	6.873409	0.944024	0.34773	-6.59395	0.509427	0.572521
Non.inflammatory.Macrophages	ZKSCAN3	0.093736	4.82207	0.943966	0.34776	-6.23191	0.577492	0.606201
Non.inflammatory.Macrophages	PNO1	-0.12388	4.446222	-0.94386	0.347815	-6.20052	0.590868	0.612579
Non.inflammatory.Macrophages	AZIN1	-0.09679	6.940521	-0.94369	0.347898	-6.62757	0.507336	0.571506

Non.inflammatory.Macrophages	COMMD1	0.109767	5.141948	0.943609	0.347941	-6.29357	0.566335	0.600858
Non.inflammatory.Macrophages	WDR49	0.41276	0.34569	0.943586	0.347953	-5.24156	0.74128	0.6866
Non.inflammatory.Macrophages	PF4	0.292319	4.278945	0.943563	0.347965	-6.50749	0.596915	0.615468
Non.inflammatory.Macrophages	TMEM50B	-0.17041	4.107732	-0.94336	0.348068	-6.05872	0.603249	0.61846
Non.inflammatory.Macrophages	NPR1	-0.37879	1.263007	-0.94293	0.348285	-5.33812	0.71672	0.669541
Non.inflammatory.Macrophages	GM9733	0.566801	0.76406	0.942849	0.348327	-5.22405	0.735356	0.678887
Non.inflammatory.Macrophages	GIMAP1OS	-0.56324	0.862202	-0.94252	0.348495	-5.00091	0.732838	0.677109
Non.inflammatory.Macrophages	PLEKHN1	-0.25062	1.876111	-0.9421	0.348709	-5.54362	0.691327	0.658444
Non.inflammatory.Macrophages	FHOD1	0.144338	2.907952	0.941799	0.348862	-5.99069	0.649764	0.639818
Non.inflammatory.Macrophages	GM47283	-0.20366	7.990664	-0.94146	0.349035	-6.80824	0.476566	0.555522
Non.inflammatory.Macrophages	MAP3K2	0.085585	6.248682	0.941353	0.349089	-6.56568	0.530276	0.583154
Non.inflammatory.Macrophages	SPTBN4	-0.38864	-0.18469	-0.94132	0.349103	-5.21888	0.742654	0.697413
Non.inflammatory.Macrophages	ERMP1	0.126777	3.633634	0.941154	0.34919	-6.01582	0.621999	0.627202
Non.inflammatory.Macrophages	RIN3	0.097526	5.640444	0.940998	0.34927	-6.44581	0.550453	0.593178
Non.inflammatory.Macrophages	GTF2I	0.098817	5.762327	0.940901	0.349319	-6.35968	0.546368	0.591177

Non.inflammatory.Macrophages	CACNB3	-0.64798	-0.407	-0.94024	0.349656	-5.0779	0.743023	0.701911
Non.inflammatory.Macrophages	GM28050	-0.39248	0.410517	-0.94023	0.349662	-5.23593	0.743023	0.686166
Non.inflammatory.Macrophages	GGNBP2	0.06978	7.268609	0.940181	0.349686	-6.6811	0.498403	0.566978
Non.inflammatory.Macrophages	4833445I0	-0.30856	0.48418	-0.94017	0.349692	-5.38283	0.743023	0.684764
Non.inflammatory.Macrophages	GM28707	-0.37312	1.154193	-0.94002	0.349766	-5.31343	0.722697	0.672198
Non.inflammatory.Macrophages	CD209A	-0.69572	0.102824	-0.93983	0.349868	-5.0274	0.743023	0.692169
Non.inflammatory.Macrophages	PRKAR2A	0.141703	5.222556	0.939634	0.349966	-6.54706	0.564881	0.60036
Non.inflammatory.Macrophages	SERPING1	0.233984	3.004813	0.93961	0.349977	-5.78381	0.646429	0.638586
Non.inflammatory.Macrophages	HIGD2A	0.066676	6.065754	0.939554	0.350006	-6.56035	0.536512	0.586431
Non.inflammatory.Macrophages	SLC39A11	0.138025	4.419343	0.939464	0.350052	-6.16911	0.593228	0.613942
Non.inflammatory.Macrophages	CHRNB1	0.481304	0.775917	0.939368	0.350101	-5.09443	0.73633	0.679481
Non.inflammatory.Macrophages	DCAF8	0.074315	5.699638	0.939269	0.350152	-6.40289	0.548658	0.592529
Non.inflammatory.Macrophages	FKBP1B	0.176717	0.793011	0.939178	0.350198	-5.92002	0.735849	0.679188
Non.inflammatory.Macrophages	CNNM3	0.159052	3.157652	0.939051	0.350263	-5.9018	0.640473	0.636006
Non.inflammatory.Macrophages	GM6712	0.25437	2.305447	0.938891	0.350345	-5.58226	0.67434	0.651363

Non.inflammatory.Macrophages	MRPS26	-0.08667	5.167613	-0.93876	0.35041	-6.2911	0.566778	0.601544
Non.inflammatory.Macrophages	BTBD1	0.078412	6.497876	0.938729	0.350427	-6.5957	0.522508	0.579679
Non.inflammatory.Macrophages	ZFP866	0.200775	2.993086	0.938632	0.350477	-5.7144	0.646888	0.639095
Non.inflammatory.Macrophages	CLEC2G	0.584738	0.015976	0.938617	0.350485	-5.01421	0.743023	0.694221
Non.inflammatory.Macrophages	FAM133B	0.101421	4.911343	0.937847	0.350878	-6.21852	0.576264	0.606051
Non.inflammatory.Macrophages	FADD	0.224541	2.421277	0.937678	0.350964	-5.76532	0.670343	0.649541
Non.inflammatory.Macrophages	A230083N1	-0.43247	0.546139	-0.93752	0.351044	-5.24145	0.742737	0.684367
Non.inflammatory.Macrophages	TBX21	-0.30317	2.437202	-0.93741	0.351104	-5.68303	0.669699	0.649412
Non.inflammatory.Macrophages	GM26944	-0.34721	0.852005	-0.93729	0.351164	-5.30248	0.734959	0.678726
Non.inflammatory.Macrophages	GM9917	-0.38365	0.773036	-0.93725	0.351181	-5.34554	0.737183	0.680224
Non.inflammatory.Macrophages	SLC17A5	0.140078	3.572742	0.936807	0.35141	-6.12041	0.625446	0.629471
Non.inflammatory.Macrophages	PAIP1	-0.09676	5.907952	-0.93678	0.351424	-6.4162	0.542487	0.589866
Non.inflammatory.Macrophages	TMEM134	0.089293	6.132006	0.936631	0.3515	-6.50147	0.535135	0.586277
Non.inflammatory.Macrophages	RAD54L	0.234978	2.724058	0.935729	0.351962	-5.6592	0.659149	0.64484
Non.inflammatory.Macrophages	HGH1	-0.30277	1.856316	-0.93569	0.351981	-5.36722	0.694577	0.66059

Non.inflammatory.Macrophages	APPBP2	0.080955	6.248663	0.93561	0.352022	-6.5006	0.531868	0.584583
Non.inflammatory.Macrophages	GFI1	-0.45685	1.55053	-0.93464	0.35252	-5.12866	0.70827	0.666702
Non.inflammatory.Macrophages	GM26542	-0.13743	4.802534	-0.93462	0.35253	-6.18336	0.581646	0.609031
Non.inflammatory.Macrophages	SET	0.084284	7.946372	0.933984	0.352856	-6.76024	0.480131	0.558187
Non.inflammatory.Macrophages	PSTK	-0.14541	3.061289	-0.93395	0.352873	-5.90442	0.646957	0.639496
Non.inflammatory.Macrophages	TNS1	0.267283	2.761945	0.93388	0.35291	-5.64763	0.658783	0.644871
Non.inflammatory.Macrophages	TMEM11	0.100623	5.070903	0.933621	0.353042	-6.32924	0.572678	0.604875
Non.inflammatory.Macrophages	SUCLG2	-0.11833	4.835404	-0.93342	0.353147	-6.29203	0.58099	0.608922
Non.inflammatory.Macrophages	IPP	0.223707	2.465742	0.933353	0.35318	-5.59625	0.67086	0.650441
Non.inflammatory.Macrophages	QRFP	0.632982	-0.61178	0.933266	0.353225	-4.95016	0.746376	0.70851
Non.inflammatory.Macrophages	TRMT5	0.316709	1.213824	0.933106	0.353306	-5.41789	0.723422	0.673544
Non.inflammatory.Macrophages	LLPH	0.07326	6.447819	0.932768	0.35348	-6.57816	0.526614	0.582311
Non.inflammatory.Macrophages	PSMB9	-0.1585	6.228284	-0.93276	0.353482	-6.57825	0.533739	0.585881
Non.inflammatory.Macrophages	GNAS	-0.06389	8.931692	-0.9326	0.353567	-6.97148	0.45219	0.543454
Non.inflammatory.Macrophages	GM14029	-0.5711	0.297438	-0.93252	0.353605	-5.01613	0.746598	0.691025

Non.inflammatory.Macrophages	NDUFB5	0.077733	7.037752	0.932067	0.35384	-6.6712	0.508198	0.572967
Non.inflammatory.Macrophages	RWDD4A	0.130518	3.983066	0.931806	0.353974	-6.07856	0.612516	0.623916
Non.inflammatory.Macrophages	DHDH	0.180136	2.654165	0.931664	0.354047	-5.85278	0.663899	0.647419
Non.inflammatory.Macrophages	FCGR3	0.122693	4.189291	0.931466	0.354149	-6.57505	0.604883	0.620345
Non.inflammatory.Macrophages	NUF2	-0.27675	3.365247	-0.9314	0.354183	-5.67271	0.635926	0.634736
Non.inflammatory.Macrophages	5031439G(	-0.09469	5.696635	-0.93128	0.354247	-6.52396	0.551761	0.594873
Non.inflammatory.Macrophages	GGA1	-0.09101	4.730426	-0.93127	0.354251	-6.31673	0.585278	0.611092
Non.inflammatory.Macrophages	ALDH4A1	-0.20811	2.912017	-0.93121	0.354279	-5.79332	0.653627	0.642833
Non.inflammatory.Macrophages	STPG4	-0.27224	2.37884	-0.93091	0.354437	-5.49625	0.675179	0.652489
Non.inflammatory.Macrophages	GOT1	-0.11052	6.081478	-0.93085	0.354466	-6.58343	0.539045	0.588597
Non.inflammatory.Macrophages	TBC1D5	-0.08213	7.293343	-0.93069	0.354547	-6.77206	0.500514	0.569099
Non.inflammatory.Macrophages	STIL	-0.25498	4.156047	-0.93032	0.354738	-5.86106	0.606387	0.621175
Non.inflammatory.Macrophages	DECR1	-0.18127	3.696206	-0.9303	0.354751	-6.0442	0.623571	0.629183
Non.inflammatory.Macrophages	RABL6	0.081049	5.136693	0.930093	0.354855	-6.38765	0.571219	0.604476
Non.inflammatory.Macrophages	ATF7	0.094751	6.314357	0.929885	0.354963	-6.50318	0.531548	0.585022

Non.inflammatory.Macrophages	ZFP472	0.194814	3.448072	0.929805	0.355004	-5.73801	0.633032	0.633614
Non.inflammatory.Macrophages	ATP2B1	0.073996	8.327101	0.929769	0.355022	-7.02511	0.469827	0.553148
Non.inflammatory.Macrophages	BC030867	-0.32988	2.099983	-0.92969	0.355063	-5.53289	0.6868	0.657826
Non.inflammatory.Macrophages	MAU2	0.114742	6.192162	0.929666	0.355075	-6.42757	0.535538	0.587016
Non.inflammatory.Macrophages	GLUL	0.098537	6.384395	0.929607	0.355106	-6.69242	0.529274	0.583883
Non.inflammatory.Macrophages	PLCD3	-0.44634	1.122441	-0.92931	0.355261	-5.108	0.72856	0.676058
Non.inflammatory.Macrophages	NDUFA6	0.082297	6.56293	0.929216	0.355307	-6.63882	0.523654	0.581101
Non.inflammatory.Macrophages	FSD2	-0.40705	-0.73763	-0.9289	0.355469	-5.1983	0.747802	0.712123
Non.inflammatory.Macrophages	2010007H(	-0.47109	0.459338	-0.9288	0.35552	-5.03923	0.747802	0.688877
Non.inflammatory.Macrophages	OLFM1	0.268897	2.469141	0.928731	0.355557	-5.65682	0.672004	0.651476
Non.inflammatory.Macrophages	SUB1	0.082195	8.911934	0.92849	0.355681	-6.85279	0.453558	0.544571
Non.inflammatory.Macrophages	BAG2	0.318205	1.435408	0.927796	0.356039	-5.37462	0.715921	0.670794
Non.inflammatory.Macrophages	DIDO1	0.099451	5.734022	0.927136	0.35638	-6.399	0.552046	0.595386
Non.inflammatory.Macrophages	ACSS2	0.195359	3.033515	0.926415	0.356752	-5.82733	0.651236	0.642077
Non.inflammatory.Macrophages	CCNB1	-0.28262	4.032241	-0.92557	0.357188	-5.95849	0.61321	0.624911

Non.inflammatory.Macrophages	GM11714	0.291672	1.498822	0.925479	0.357236	-5.48737	0.714714	0.670564
Non.inflammatory.Macrophages	H2-M3	-0.15057	4.138363	-0.92528	0.357338	-6.27842	0.609266	0.623093
Non.inflammatory.Macrophages	CCL22	-0.67326	-0.37257	-0.92521	0.357374	-5.19117	0.750174	0.706331
Non.inflammatory.Macrophages	SH3BP1	-0.10005	5.21446	-0.9252	0.357382	-6.45748	0.570599	0.604704
Non.inflammatory.Macrophages	MAPKAPK5	-0.20113	2.128701	-0.92519	0.357386	-5.62511	0.688126	0.658925
Non.inflammatory.Macrophages	FXR2	0.109894	5.267562	0.925188	0.357386	-6.29797	0.568753	0.603811
Non.inflammatory.Macrophages	EIF4ENIF1	0.094848	5.556503	0.924969	0.3575	-6.3316	0.558805	0.599003
Non.inflammatory.Macrophages	SURF1	-0.11635	4.36834	-0.92474	0.357617	-6.23352	0.6008	0.619213
Non.inflammatory.Macrophages	CYP4A14	0.41238	1.833194	0.924694	0.357642	-5.45129	0.700486	0.664464
Non.inflammatory.Macrophages	5830418P1	-0.57926	1.463039	-0.92464	0.357672	-5.11098	0.716252	0.671359
Non.inflammatory.Macrophages	FDXR	-0.20527	2.625449	-0.92461	0.357687	-5.67503	0.6678	0.650008
Non.inflammatory.Macrophages	GRIA3	-0.6027	3.336555	-0.92448	0.35775	-5.26708	0.639665	0.637275
Non.inflammatory.Macrophages	CPT1B	-0.23352	1.536488	-0.92443	0.357778	-5.53894	0.713098	0.66999
Non.inflammatory.Macrophages	LRRC1	-0.37296	3.264954	-0.92434	0.357824	-5.35046	0.642447	0.638548
Non.inflammatory.Macrophages	CLEC4A2	-0.11758	3.416946	-0.92434	0.357827	-6.53341	0.636554	0.635853

Non.inflammatory.Macrophages	GM48226	0.218246	2.396833	0.924294	0.357849	-5.63663	0.677085	0.654177
Non.inflammatory.Macrophages	NDUFA5	0.11167	5.432033	0.924073	0.357964	-6.45513	0.563114	0.601283
Non.inflammatory.Macrophages	PPID	-0.11485	4.87562	-0.92394	0.358032	-6.24443	0.582562	0.610672
Non.inflammatory.Macrophages	SLC10A1	-0.24039	3.248903	-0.9239	0.358055	-5.76958	0.643123	0.638956
Non.inflammatory.Macrophages	CNTLN	-0.14644	4.34763	-0.92381	0.358099	-6.19292	0.601605	0.619714
Non.inflammatory.Macrophages	RB1	-0.09321	7.240705	-0.92353	0.358243	-6.63796	0.504194	0.571793
Non.inflammatory.Macrophages	LPXN	-0.09473	5.23201	-0.92347	0.358276	-6.54556	0.570139	0.604698
Non.inflammatory.Macrophages	HSD17B11	-0.09846	4.796644	-0.92299	0.358525	-6.38697	0.585799	0.612137
Non.inflammatory.Macrophages	RAB35	-0.09804	4.720448	-0.9226	0.358729	-6.26428	0.588769	0.613559
Non.inflammatory.Macrophages	FERMT3	-0.07668	7.235899	-0.92228	0.358893	-6.77371	0.504903	0.572133
Non.inflammatory.Macrophages	PLXNC1	-0.17486	5.427157	-0.92227	0.358896	-6.18179	0.564011	0.60168
Non.inflammatory.Macrophages	PNPT1	0.123968	4.198204	0.922129	0.358971	-6.16184	0.60792	0.622626
Non.inflammatory.Macrophages	PAXBP1	0.117457	5.312121	0.921927	0.359076	-6.31938	0.568097	0.603645
Non.inflammatory.Macrophages	GIT2	0.073795	7.020155	0.921793	0.359145	-6.6127	0.511744	0.575658
Non.inflammatory.Macrophages	BMP8A	0.248388	2.321943	0.921574	0.359259	-5.76292	0.681339	0.656181

Non.inflammatory.Macrophages	POFUT2	-0.10832	4.257323	-0.92148	0.359306	-6.27459	0.605928	0.621837
Non.inflammatory.Macrophages	EBPL	-0.13061	4.469152	-0.92122	0.359441	-6.15999	0.598215	0.618315
Non.inflammatory.Macrophages	PTEN	0.067266	8.288084	0.921114	0.359498	-6.84615	0.473545	0.555993
Non.inflammatory.Macrophages	TJP2	0.21754	3.845594	0.921104	0.359502	-5.74368	0.621339	0.629183
Non.inflammatory.Macrophages	TRMT1	0.108929	4.050324	0.920677	0.359724	-6.12204	0.613934	0.625713
Non.inflammatory.Macrophages	PWWP3A	-0.15337	3.715755	-0.92058	0.359777	-5.87079	0.626542	0.631585
Non.inflammatory.Macrophages	STK4	-0.08016	7.52595	-0.9204	0.359867	-6.65116	0.496451	0.568024
Non.inflammatory.Macrophages	GM16230	-0.44898	0.238127	-0.92036	0.359889	-5.26731	0.751889	0.695696
Non.inflammatory.Macrophages	ARC	-0.33577	0.905214	-0.91984	0.360159	-5.45405	0.741884	0.683023
Non.inflammatory.Macrophages	TEX2	0.106063	6.723879	0.91965	0.360258	-6.56395	0.521848	0.580937
Non.inflammatory.Macrophages	CYP3A25	0.362202	1.898523	0.919429	0.360373	-5.47466	0.699948	0.664568
Non.inflammatory.Macrophages	ALPK2	0.322771	1.260876	0.919309	0.360435	-5.41029	0.72731	0.676481
Non.inflammatory.Macrophages	EXOC1	0.125104	4.095264	0.918862	0.360668	-6.12623	0.613113	0.625295
Non.inflammatory.Macrophages	ZFP335OS	-0.16078	4.456083	-0.91845	0.36088	-5.97397	0.599911	0.619167
Non.inflammatory.Macrophages	ADGRG3	0.340237	2.366781	0.918438	0.360888	-5.38083	0.680939	0.65623

Non.inflammatory.Macrophages	F930017D2	0.498808	0.898224	0.91841	0.360903	-5.31504	0.742811	0.683572
Non.inflammatory.Macrophages	ZFP595	-0.22515	1.84184	-0.91804	0.361095	-5.43785	0.703007	0.665944
Non.inflammatory.Macrophages	LGR4	0.277471	2.632615	0.917924	0.361156	-5.65508	0.670262	0.651499
Non.inflammatory.Macrophages	RAD54B	-0.32409	2.207787	-0.91791	0.361161	-5.49079	0.687676	0.659242
Non.inflammatory.Macrophages	SFMBT2	0.474994	0.60235	0.917749	0.361247	-5.13633	0.75116	0.689389
Non.inflammatory.Macrophages	GM35867	-0.39257	0.456051	-0.9176	0.361323	-5.34662	0.753316	0.692244
Non.inflammatory.Macrophages	ADGRV1	0.299607	0.711845	0.917549	0.361351	-5.59275	0.748365	0.687345
Non.inflammatory.Macrophages	ZBTB34	0.142126	3.592868	0.917266	0.361499	-6.04704	0.632524	0.634539
Non.inflammatory.Macrophages	USP7	0.071638	6.215443	0.917237	0.361514	-6.5243	0.539061	0.589877
Non.inflammatory.Macrophages	CDKL4	-0.27137	1.561956	-0.91685	0.361717	-5.65502	0.715382	0.671446
Non.inflammatory.Macrophages	CPNE5	-0.55336	0.481176	-0.91662	0.361834	-5.03975	0.753858	0.691944
Non.inflammatory.Macrophages	PMM2	0.092965	4.561806	0.916266	0.36202	-6.23991	0.596885	0.617834
Non.inflammatory.Macrophages	9530034E1	0.46826	0.330435	0.916102	0.362106	-5.08323	0.754117	0.694995
Non.inflammatory.Macrophages	ENSA	0.08519	5.388151	0.916063	0.362126	-6.44288	0.567548	0.603816
Non.inflammatory.Macrophages	METTL9	0.096112	5.718204	0.915777	0.362275	-6.45939	0.556362	0.598382

Non.inflammatory.Macrophages	PDHB	-0.08497	5.703174	-0.9154	0.362471	-6.42747	0.557041	0.598693
Non.inflammatory.Macrophages	KDM5A	0.08171	7.014524	0.91526	0.362545	-6.59945	0.514074	0.577258
Non.inflammatory.Macrophages	LMNA	-0.13749	4.617164	-0.91525	0.362551	-6.42678	0.595208	0.6171
Non.inflammatory.Macrophages	CELSR2	-0.56254	-0.09555	-0.91505	0.362655	-5.06151	0.75464	0.70354
Non.inflammatory.Macrophages	INTU	0.431946	0.880465	0.914679	0.362848	-5.34682	0.74486	0.684851
Non.inflammatory.Macrophages	GM16014	0.348736	-0.09363	0.914625	0.362876	-5.43024	0.754642	0.703631
Non.inflammatory.Macrophages	FBLIM1	-0.26007	0.928973	-0.91459	0.362895	-5.76775	0.743481	0.683935
Non.inflammatory.Macrophages	NVL	0.102689	4.723925	0.914443	0.362971	-6.21928	0.591429	0.61542
Non.inflammatory.Macrophages	DVL1	-0.22068	2.507773	-0.91443	0.362977	-5.66831	0.67657	0.654569
Non.inflammatory.Macrophages	GALNT3	0.22995	1.767982	0.914399	0.362994	-5.80083	0.707428	0.668173
Non.inflammatory.Macrophages	LRATD2	-0.27544	1.700967	-0.9143	0.363047	-5.4398	0.710286	0.669449
Non.inflammatory.Macrophages	1700120C1	-0.29479	1.482076	-0.91412	0.363141	-5.353	0.719771	0.673603
Non.inflammatory.Macrophages	B9D2	-0.1289	4.930502	-0.91359	0.363419	-6.2571	0.584447	0.612176
Non.inflammatory.Macrophages	NME3	-0.48871	0.405363	-0.91332	0.36356	-5.1666	0.755266	0.694296
Non.inflammatory.Macrophages	11-Sep	-0.13629	6.415184	-0.9132	0.363619	-6.45967	0.533813	0.587405

Non.inflammatory.Macrophages	TESK2	-0.15914	4.072329	-0.91319	0.363627	-6.14325	0.615869	0.626994
Non.inflammatory.Macrophages	GM43774	-0.23458	2.238278	-0.91307	0.363689	-5.67061	0.688234	0.659861
Non.inflammatory.Macrophages	FNBP4	0.08304	6.074991	0.912916	0.363769	-6.46077	0.545044	0.593075
Non.inflammatory.Macrophages	GDF11	-0.45982	1.639394	-0.91287	0.363793	-5.15564	0.713511	0.670988
Non.inflammatory.Macrophages	HYAL3	-0.38824	0.035974	-0.91264	0.363911	-5.25771	0.75529	0.701616
Non.inflammatory.Macrophages	UGGT1	0.09162	5.389379	0.912614	0.363927	-6.34803	0.568388	0.604564
Non.inflammatory.Macrophages	ABCB7	0.095243	5.334608	0.912528	0.363972	-6.2833	0.570292	0.6055
Non.inflammatory.Macrophages	AAGAB	-0.09472	4.862045	-0.91231	0.364089	-6.36617	0.586983	0.613614
Non.inflammatory.Macrophages	SRRM2	-0.06208	8.62357	-0.91231	0.364089	-6.91159	0.466204	0.552613
Non.inflammatory.Macrophages	LEMD2	-0.10507	4.232797	-0.9119	0.364303	-6.21989	0.610154	0.624617
Non.inflammatory.Macrophages	NT5C3	0.108525	4.85257	0.911819	0.364343	-6.34126	0.587552	0.613936
Non.inflammatory.Macrophages	IL15	0.167375	3.93993	0.910266	0.365157	-6.34836	0.621787	0.629972
Non.inflammatory.Macrophages	RABGEF1	0.106995	6.01057	0.910142	0.365222	-6.61001	0.548026	0.594719
Non.inflammatory.Macrophages	ARHGAP1	0.088304	4.473602	0.910136	0.365226	-6.2592	0.601925	0.620719
Non.inflammatory.Macrophages	DYM	0.086842	5.781233	0.909905	0.365347	-6.44857	0.555765	0.598549

Non.inflammatory.Macrophages	BACH2OS	-0.21964	1.526179	-0.90989	0.365357	-5.71938	0.719473	0.673783
Non.inflammatory.Macrophages	VPS29	0.069295	6.521761	0.909788	0.365408	-6.64109	0.531145	0.586335
Non.inflammatory.Macrophages	FAM53C	-0.12193	3.981286	-0.90975	0.365427	-6.10119	0.620226	0.629305
Non.inflammatory.Macrophages	DNAJC7	0.093104	7.510277	0.909716	0.365446	-6.6476	0.499918	0.57042
Non.inflammatory.Macrophages	OAZ1	0.075696	9.534615	0.909711	0.365448	-7.07181	0.441466	0.53918
Non.inflammatory.Macrophages	G6PC	-0.4139	1.911843	-0.90969	0.36546	-5.46539	0.702973	0.666598
Non.inflammatory.Macrophages	ABHD8	-0.24543	2.172201	-0.90968	0.365462	-5.54903	0.69203	0.66179
Non.inflammatory.Macrophages	CCDC173	-0.40064	0.767334	-0.90943	0.365596	-5.21219	0.749844	0.688153
Non.inflammatory.Macrophages	TMEM164	-0.07623	6.945657	-0.90942	0.365599	-6.71005	0.517529	0.579467
Non.inflammatory.Macrophages	HIST1H2AI	0.475649	2.588783	0.909383	0.36562	-5.46123	0.674845	0.654179
Non.inflammatory.Macrophages	GM16541	0.241368	2.915994	0.909378	0.365623	-5.55844	0.661624	0.648252
Non.inflammatory.Macrophages	FCNB	-0.5052	-0.23854	-0.90932	0.365655	-5.02634	0.756411	0.707639
Non.inflammatory.Macrophages	ZC3H12D	0.214061	3.479104	0.909257	0.365686	-5.83284	0.639431	0.638174
Non.inflammatory.Macrophages	MFSD4A	0.351303	2.041521	0.908942	0.365852	-5.32467	0.697642	0.664336
Non.inflammatory.Macrophages	ADORA3	0.389382	-0.30137	0.908905	0.365871	-5.25581	0.756562	0.709006

Non.inflammatory.Macrophages	IL1R1	0.348746	2.051839	0.908435	0.366118	-5.54913	0.697572	0.664341
Non.inflammatory.Macrophages	SARNP	-0.06273	7.797226	-0.90807	0.366312	-6.76748	0.491732	0.566335
Non.inflammatory.Macrophages	CENPP	-0.18122	5.39603	-0.90792	0.366386	-6.22177	0.569655	0.605517
Non.inflammatory.Macrophages	SEMA4A	0.165382	3.364224	0.907639	0.366536	-6.03863	0.644748	0.640849
Non.inflammatory.Macrophages	KIF7	-0.39225	-0.46987	-0.9076	0.366559	-5.26915	0.757405	0.71291
Non.inflammatory.Macrophages	5033421B(	-0.34597	-0.4422	-0.90718	0.366779	-5.49322	0.757668	0.712525
Non.inflammatory.Macrophages	DCAF4	-0.23308	2.008291	-0.90704	0.366851	-5.52305	0.700062	0.665683
Non.inflammatory.Macrophages	RBIS	0.09708	5.209656	0.907035	0.366854	-6.36628	0.576468	0.608947
Non.inflammatory.Macrophages	SCAMP5	-0.22825	1.229049	-0.90679	0.366983	-5.6452	0.73368	0.680296
Non.inflammatory.Macrophages	LY6C1	-0.67294	-0.32417	-0.90678	0.366985	-5.02939	0.757708	0.710276
Non.inflammatory.Macrophages	CTSZ	0.093957	6.598627	0.906661	0.36705	-6.77989	0.52957	0.585894
Non.inflammatory.Macrophages	DYNLT1F	0.12021	5.068561	0.906552	0.367108	-6.38776	0.5815	0.6114
Non.inflammatory.Macrophages	GM47469	-0.47065	0.520329	-0.90645	0.367164	-5.13681	0.757144	0.693886
Non.inflammatory.Macrophages	ASPSCR1	-0.10146	4.685187	-0.90618	0.367304	-6.2439	0.59539	0.618067
Non.inflammatory.Macrophages	SLFN3	0.335809	1.4477	0.906034	0.36738	-5.36305	0.724338	0.676318

Non.inflammatory.Macrophages	EML2	0.313907	2.655573	0.905734	0.367538	-5.41209	0.67349	0.654001
Non.inflammatory.Macrophages	GM42659	0.123525	4.567867	0.90568	0.367567	-6.15684	0.599693	0.620124
Non.inflammatory.Macrophages	PTPN4	0.138028	5.463527	0.90561	0.367604	-6.19514	0.567809	0.604868
Non.inflammatory.Macrophages	PDCL	0.139011	3.891934	0.90561	0.367604	-6.07861	0.624868	0.631917
Non.inflammatory.Macrophages	ITGAE	-0.26902	2.127038	-0.90533	0.367753	-5.7244	0.695346	0.663835
Non.inflammatory.Macrophages	THYN1	0.136407	3.271604	0.905199	0.36782	-6.00708	0.648861	0.643072
Non.inflammatory.Macrophages	RBM8A	0.08309	5.709751	0.905164	0.367838	-6.46711	0.559348	0.600882
Non.inflammatory.Macrophages	9330111N(	-0.36341	0.392898	-0.90514	0.367849	-5.39246	0.757969	0.696658
Non.inflammatory.Macrophages	ACAP3	0.193008	2.238471	0.905056	0.367896	-5.62493	0.690689	0.661831
Non.inflammatory.Macrophages	SIRT4	-0.42501	0.56797	-0.90482	0.368022	-5.27612	0.756491	0.693377
Non.inflammatory.Macrophages	IK	-0.06795	6.288849	-0.90476	0.368049	-6.60132	0.539903	0.59139
Non.inflammatory.Macrophages	GNAI3	0.070578	6.443605	0.904711	0.368077	-6.54806	0.534813	0.588881
Non.inflammatory.Macrophages	SMUG1	0.231359	1.976529	0.904531	0.368172	-5.511	0.701722	0.66693
Non.inflammatory.Macrophages	POPDC3	-0.49763	0.894439	-0.90448	0.368197	-5.25365	0.747788	0.687302
Non.inflammatory.Macrophages	ANKRD6	-0.53713	0.95646	-0.90427	0.368309	-5.08176	0.746062	0.686229

Non.inflammatory.Macrophages	PRAMEF8	-0.17605	3.188085	-0.90402	0.36844	-5.90052	0.652229	0.645047
Non.inflammatory.Macrophages	SCFD1	0.116525	4.929173	0.903859	0.368527	-6.2992	0.586718	0.614545
Non.inflammatory.Macrophages	GEN1	0.306957	2.372171	0.903806	0.368555	-5.57343	0.685219	0.659867
Non.inflammatory.Macrophages	CCL3	0.211101	5.069599	0.903795	0.36856	-6.55788	0.581714	0.612147
Non.inflammatory.Macrophages	DPM1	-0.08212	5.979025	-0.90378	0.36857	-6.47449	0.550279	0.596843
Non.inflammatory.Macrophages	LITAF	0.06626	8.523937	0.9037	0.36861	-6.96118	0.470778	0.556076
Non.inflammatory.Macrophages	SLC17A3	-0.46571	0.779648	-0.90317	0.368888	-5.20837	0.751573	0.689942
Non.inflammatory.Macrophages	POLR2M	0.084306	5.403452	0.902951	0.369006	-6.35433	0.570327	0.606701
Non.inflammatory.Macrophages	CLN5	0.11327	4.008222	0.902823	0.369073	-6.15604	0.620936	0.630751
Non.inflammatory.Macrophages	SCG5	-0.49413	0.801405	-0.90281	0.36908	-5.15721	0.750953	0.689585
Non.inflammatory.Macrophages	CHADL	-0.42426	0.647864	-0.90268	0.369149	-5.19311	0.755383	0.692602
Non.inflammatory.Macrophages	ZFP956	-0.33963	1.264702	-0.90241	0.36929	-5.28563	0.732894	0.68088
Non.inflammatory.Macrophages	TUBGCP4	0.126036	4.42498	0.9024	0.369296	-6.11751	0.605391	0.623581
Non.inflammatory.Macrophages	IFI208	-0.22356	2.936618	-0.90238	0.369305	-6.03309	0.662639	0.649952
Non.inflammatory.Macrophages	TRIM56	0.122066	3.810038	0.902304	0.369347	-6.11086	0.62846	0.634347

Non.inflammatory.Macrophages	GM15232	-0.47723	1.187425	-0.90197	0.369524	-5.10756	0.7363	0.682436
Non.inflammatory.Macrophages	FCGR1	0.146953	2.432362	0.901925	0.369547	-6.40756	0.683149	0.659222
Non.inflammatory.Macrophages	TMEM131I	-0.10463	7.26323	-0.90181	0.369608	-6.54481	0.508964	0.576298
Non.inflammatory.Macrophages	GM45267	-0.5234	0.245242	-0.9018	0.369614	-5.07455	0.758518	0.700548
Non.inflammatory.Macrophages	1810030O(	0.09608	4.824335	0.901789	0.369619	-6.3571	0.59084	0.616787
Non.inflammatory.Macrophages	ABTB1	0.144916	4.303875	0.901522	0.36976	-6.16807	0.609882	0.62584
Non.inflammatory.Macrophages	ZMAT4	0.693971	0.381713	0.901506	0.369768	-5.11035	0.758533	0.697957
Non.inflammatory.Macrophages	SPPL3	-0.06666	7.282604	-0.90146	0.369795	-6.69885	0.50837	0.576035
Non.inflammatory.Macrophages	HBP1	0.094372	5.788072	0.901344	0.369854	-6.4763	0.557097	0.600541
Non.inflammatory.Macrophages	H2AFJ	0.067461	7.521162	0.901132	0.369966	-6.79862	0.501035	0.572304
Non.inflammatory.Macrophages	SPICE1	-0.22673	1.890422	-0.90099	0.370041	-5.64251	0.705924	0.669448
Non.inflammatory.Macrophages	NRD1	0.08572	6.185357	0.900963	0.370055	-6.51278	0.543771	0.594029
Non.inflammatory.Macrophages	LPIN2	-0.09044	6.528551	-0.90032	0.370398	-6.6952	0.532725	0.588659
Non.inflammatory.Macrophages	NR1D1	-0.29202	1.661874	-0.90019	0.370462	-5.67095	0.716048	0.67407
Non.inflammatory.Macrophages	ACTL6A	0.093254	5.235041	0.900122	0.3705	-6.34211	0.57657	0.610326

Non.inflammatory.Macrophages	C130036L2	-0.30143	1.354595	-0.90006	0.370532	-5.35243	0.729391	0.679925
Non.inflammatory.Macrophages	UNC13B	0.33562	0.758993	0.90002	0.370554	-5.42054	0.752628	0.691269
Non.inflammatory.Macrophages	KMT2B	0.126972	3.888591	0.899987	0.370572	-6.08341	0.625846	0.633661
Non.inflammatory.Macrophages	DNAJC16	0.179165	2.30093	0.899807	0.370667	-5.90454	0.689079	0.662341
Non.inflammatory.Macrophages	TYW1	-0.135	3.875123	-0.89934	0.370915	-6.06772	0.626749	0.634121
Non.inflammatory.Macrophages	NUMA1	-0.08688	5.705606	-0.89901	0.371087	-6.44101	0.560759	0.602668
Non.inflammatory.Macrophages	GALK2	0.09249	4.897213	0.898743	0.371231	-6.35913	0.589176	0.616426
Non.inflammatory.Macrophages	RAD51	0.248857	3.891445	0.898732	0.371236	-5.85534	0.626375	0.633928
Non.inflammatory.Macrophages	PARVB	0.137531	2.578711	0.898577	0.371319	-6.2416	0.678237	0.657548
Non.inflammatory.Macrophages	HAUS3	-0.12963	4.199727	-0.89843	0.371395	-5.97703	0.614744	0.6286
Non.inflammatory.Macrophages	UPF3B	0.114695	4.979585	0.898426	0.371399	-6.23057	0.586224	0.6151
Non.inflammatory.Macrophages	KTI12	-0.17337	3.941057	-0.89826	0.371484	-5.95228	0.624539	0.633173
Non.inflammatory.Macrophages	WDR82	0.088509	4.82833	0.898145	0.371547	-6.23708	0.591714	0.617746
Non.inflammatory.Macrophages	ARHGEF2	0.082532	5.586467	0.897601	0.371836	-6.48906	0.565145	0.605078
Non.inflammatory.Macrophages	9930111J2	-0.34067	1.279459	-0.89754	0.371869	-5.45005	0.73375	0.682131

Non.inflammatory.Macrophages	IRAK4	0.097044	4.419459	0.897499	0.37189	-6.33799	0.606839	0.625078
Non.inflammatory.Macrophages	FBXO9	0.117798	4.752411	0.897476	0.371903	-6.24089	0.594656	0.619318
Non.inflammatory.Macrophages	NCOR2	0.09059	4.956649	0.897243	0.372026	-6.39158	0.587297	0.61588
Non.inflammatory.Macrophages	XXYLT1	-0.15158	3.591278	-0.89717	0.372064	-5.95086	0.638171	0.63974
Non.inflammatory.Macrophages	EMSY	-0.09192	5.828738	-0.89716	0.372069	-6.43555	0.556839	0.60111
Non.inflammatory.Macrophages	RASSF8	0.18539	2.215433	0.897055	0.372126	-5.9823	0.693573	0.664701
Non.inflammatory.Macrophages	SRD5A3	0.116263	4.511422	0.89693	0.372192	-6.30957	0.603451	0.623567
Non.inflammatory.Macrophages	HLTF	0.108774	4.64409	0.896855	0.372232	-6.33814	0.598594	0.621301
Non.inflammatory.Macrophages	SLC13A3	0.406676	1.11728	0.896408	0.372469	-5.40096	0.741226	0.685518
Non.inflammatory.Macrophages	MDM2	-0.10655	6.41707	-0.89632	0.372518	-6.65787	0.537372	0.591547
Non.inflammatory.Macrophages	DCAF1	0.115158	5.222364	0.896161	0.3726	-6.28792	0.578089	0.611619
Non.inflammatory.Macrophages	SP3OS	0.123748	4.205373	0.896052	0.372658	-6.13169	0.615049	0.629206
Non.inflammatory.Macrophages	SESTD1	0.160975	2.300083	0.896038	0.372666	-6.27226	0.690323	0.663462
Non.inflammatory.Macrophages	ARL8A	-0.07867	6.227073	-0.89593	0.372724	-6.62654	0.54366	0.594805
Non.inflammatory.Macrophages	GM20033	-0.40803	0.595708	-0.89571	0.372837	-5.28783	0.758353	0.695783

Non.inflammatory.Macrophages	RNASE4	0.179132	5.17058	0.895709	0.37284	-6.34854	0.579929	0.612648
Non.inflammatory.Macrophages	COPZ2	-0.18713	2.183281	-0.89541	0.373001	-5.82189	0.69541	0.66582
Non.inflammatory.Macrophages	SIAH1B	-0.28399	1.91846	-0.89504	0.373197	-5.42858	0.706693	0.670741
Non.inflammatory.Macrophages	BSDC1	0.118941	5.056629	0.89496	0.373239	-6.36321	0.584218	0.614655
Non.inflammatory.Macrophages	SCLY	-0.12974	3.466279	-0.89489	0.373274	-6.05587	0.643571	0.642475
Non.inflammatory.Macrophages	CELSR1	0.563485	1.626552	0.89487	0.373286	-5.17507	0.719215	0.676206
Non.inflammatory.Macrophages	1700037C1	0.195515	3.065274	0.894783	0.373332	-5.73326	0.659404	0.649684
Non.inflammatory.Macrophages	BC004004	0.085738	5.147988	0.894412	0.37353	-6.39019	0.581107	0.613169
Non.inflammatory.Macrophages	G730013B(	-0.31988	-0.968	-0.89441	0.373533	-5.29547	0.760895	0.726796
Non.inflammatory.Macrophages	GNB1	0.048087	8.848488	0.893954	0.373774	-6.97472	0.463441	0.553298
Non.inflammatory.Macrophages	TSPAN3	0.118153	4.739337	0.893463	0.374035	-6.3481	0.596305	0.620468
Non.inflammatory.Macrophages	INPP5B	0.133676	4.216748	0.89346	0.374037	-6.074	0.615586	0.62956
Non.inflammatory.Macrophages	ZC3H11A	-0.25181	2.01149	-0.89326	0.374143	-5.58346	0.703556	0.669457
Non.inflammatory.Macrophages	GM44174	-0.5821	-0.56348	-0.89323	0.374158	-5.0069	0.761596	0.719091
Non.inflammatory.Macrophages	4931428F0	0.371029	0.65018	0.892676	0.374454	-5.26402	0.758867	0.695438

Non.inflammatory.Macrophages	GRAMD1A	0.133013	4.325204	0.892437	0.374581	-6.19596	0.612023	0.627895
Non.inflammatory.Macrophages	PLA1A	-0.33902	1.841769	-0.89237	0.374615	-5.46612	0.711332	0.672822
Non.inflammatory.Macrophages	IKBKG	0.139245	3.339723	0.892019	0.374804	-5.97761	0.650009	0.645589
Non.inflammatory.Macrophages	ARF1	0.057996	8.02586	0.891854	0.374892	-6.85522	0.488257	0.566703
Non.inflammatory.Macrophages	GM32401	0.23688	2.445198	0.891526	0.375067	-5.70252	0.686295	0.662027
Non.inflammatory.Macrophages	GM34471	0.34838	0.232245	0.891435	0.375115	-5.34749	0.762602	0.704015
Non.inflammatory.Macrophages	CLK1	-0.07409	7.55289	-0.89138	0.375145	-6.72536	0.502693	0.574292
Non.inflammatory.Macrophages	RFLNB	-0.31537	3.463749	-0.89135	0.375159	-5.55534	0.645265	0.643539
Non.inflammatory.Macrophages	ZFP667	-0.16489	2.726732	-0.89097	0.375365	-5.99524	0.674958	0.656952
Non.inflammatory.Macrophages	MS4A3	0.652541	-0.35863	0.890893	0.375404	-5.09151	0.762871	0.715781
Non.inflammatory.Macrophages	UBE3B	0.099401	4.424846	0.890327	0.375707	-6.22195	0.609269	0.626826
Non.inflammatory.Macrophages	PFAS	0.196771	4.205445	0.88944	0.37618	-5.98515	0.618145	0.631009
Non.inflammatory.Macrophages	SELENON	-0.13779	3.694822	-0.88929	0.376259	-6.20228	0.637667	0.64009
Non.inflammatory.Macrophages	5730480H(	0.175908	2.846707	0.888902	0.376468	-5.76816	0.671522	0.655444
Non.inflammatory.Macrophages	SERINC4	-0.36021	0.365548	-0.8888	0.37652	-5.33609	0.764515	0.702285

Non.inflammatory.Macrophages	TPPP	-0.44759	0.182012	-0.88866	0.376598	-5.33885	0.764515	0.705889
Non.inflammatory.Macrophages	FAM160B2	0.141768	3.264944	0.888638	0.376609	-5.92804	0.654729	0.647918
Non.inflammatory.Macrophages	UMPS	-0.11665	4.099845	-0.88837	0.376755	-6.15205	0.622402	0.633088
Non.inflammatory.Macrophages	GNL3L	-0.10027	4.467102	-0.88831	0.376786	-6.12345	0.608647	0.626664
Non.inflammatory.Macrophages	CPLX2	-0.24989	3.378009	-0.8882	0.376844	-5.81574	0.650291	0.64596
Non.inflammatory.Macrophages	RMDN1	-0.11755	4.528231	-0.88791	0.376997	-6.31803	0.606385	0.625723
Non.inflammatory.Macrophages	BMPR1A	0.233672	2.310682	0.887897	0.377005	-5.84998	0.693662	0.665549
Non.inflammatory.Macrophages	GNA14	-0.53089	-0.0617	-0.88774	0.37709	-5.2067	0.764555	0.710957
Non.inflammatory.Macrophages	WASHC1	-0.09966	4.182087	-0.88771	0.377104	-6.1968	0.619296	0.631847
Non.inflammatory.Macrophages	CORO7	0.084535	6.215775	0.887677	0.377123	-6.53823	0.547015	0.597087
Non.inflammatory.Macrophages	PHKA2	0.136399	3.438409	0.887651	0.377137	-6.11717	0.647913	0.64509
Non.inflammatory.Macrophages	TNFRSF10E	-0.49814	-0.0063	-0.88729	0.377331	-5.077	0.764833	0.710041
Non.inflammatory.Macrophages	TMEM199	-0.12154	3.379806	-0.88709	0.377438	-6.07046	0.650544	0.64628
Non.inflammatory.Macrophages	ZFYVE16	0.134923	3.016629	0.886899	0.37754	-6.06956	0.665097	0.65289
Non.inflammatory.Macrophages	LRRCC1	0.117882	3.748019	0.886518	0.377744	-6.01023	0.636388	0.639798

Non.inflammatory.Macrophages	FUT11	-0.1354	3.493603	-0.88636	0.377827	-6.08416	0.646292	0.644407
Non.inflammatory.Macrophages	UBE2D3	0.046159	8.966917	0.886304	0.377858	-6.99593	0.462454	0.553352
Non.inflammatory.Macrophages	CARF	0.232123	2.216581	0.886301	0.37786	-5.63371	0.6982	0.66772
Non.inflammatory.Macrophages	SLC25A10	-0.15457	3.077126	-0.88621	0.377907	-5.99848	0.662814	0.651927
Non.inflammatory.Macrophages	MINPP1	0.097379	4.734583	0.885911	0.378068	-6.27201	0.599481	0.622633
Non.inflammatory.Macrophages	KRT222	0.587663	-0.05515	0.885246	0.378425	-4.97674	0.766015	0.711577
Non.inflammatory.Macrophages	1810034E1	-0.25298	1.895787	-0.8851	0.378502	-5.56235	0.712587	0.674125
Non.inflammatory.Macrophages	SINHCAF	-0.1804	4.557551	-0.88504	0.378536	-5.75957	0.606459	0.626034
Non.inflammatory.Macrophages	RGS3	0.209588	2.862627	0.884625	0.378759	-5.91871	0.672456	0.656325
Non.inflammatory.Macrophages	NUBPL	0.15187	2.913085	0.88455	0.378798	-5.92772	0.670406	0.655404
Non.inflammatory.Macrophages	SPATA48	0.286458	2.190427	0.884311	0.378927	-5.50581	0.700455	0.668836
Non.inflammatory.Macrophages	TSPYL3	0.462009	0.960124	0.883989	0.3791	-5.20743	0.754448	0.692213
Non.inflammatory.Macrophages	INPP4A	0.102463	6.032154	0.883747	0.379229	-6.46697	0.554852	0.601206
Non.inflammatory.Macrophages	HIST1H2AC	0.438447	2.011327	0.883361	0.379437	-5.36748	0.708693	0.672599
Non.inflammatory.Macrophages	CLIP2	0.129805	2.886513	0.882905	0.379682	-6.01495	0.672471	0.656544

Non.inflammatory.Macrophages	PTP4A2	-0.0469	8.699413	-0.88287	0.379701	-6.99639	0.471496	0.558465
Non.inflammatory.Macrophages	OSTM1	-0.091	4.762239	-0.88249	0.379903	-6.34611	0.600058	0.623223
Non.inflammatory.Macrophages	IZUMO1R	-0.63537	0.001693	-0.88244	0.379931	-5.03554	0.767442	0.711429
Non.inflammatory.Macrophages	ABAT	-0.40713	1.196429	-0.88218	0.380069	-5.34065	0.74456	0.68828
Non.inflammatory.Macrophages	CINP	0.174522	3.163035	0.882087	0.380122	-5.99303	0.661306	0.651667
Non.inflammatory.Macrophages	ACAT2	0.192621	2.91084	0.882053	0.38014	-5.68747	0.671482	0.656258
Non.inflammatory.Macrophages	UFM1	0.084349	5.448609	0.881906	0.380219	-6.46234	0.575447	0.61158
Non.inflammatory.Macrophages	SERP2	0.46335	0.429349	0.881645	0.380359	-5.05012	0.767442	0.703244
Non.inflammatory.Macrophages	AHSA1	0.099927	5.071608	0.881585	0.380391	-6.36183	0.588844	0.618063
Non.inflammatory.Macrophages	9330136K2	-0.20355	1.883233	-0.88156	0.380407	-5.81858	0.714454	0.675402
Non.inflammatory.Macrophages	GM17529	-0.52944	0.365187	-0.88153	0.38042	-5.05899	0.767442	0.704498
Non.inflammatory.Macrophages	PGAP1	0.134735	3.785443	0.881486	0.380445	-6.34256	0.636804	0.640592
Non.inflammatory.Macrophages	GM9856	0.321138	1.246414	0.881474	0.380451	-5.33825	0.742331	0.687463
Non.inflammatory.Macrophages	FBXO6	0.110125	3.653081	0.881454	0.380462	-6.2354	0.641943	0.642956
Non.inflammatory.Macrophages	PDZK1	-0.35596	0.933701	-0.88113	0.380634	-5.45243	0.755955	0.693554

Non.inflammatory.Macrophages	SZRD1	0.06985	5.906997	0.881111	0.380647	-6.51014	0.559551	0.603935
Non.inflammatory.Macrophages	NENF	-0.08549	4.583677	-0.88111	0.38065	-6.41406	0.606623	0.626599
Non.inflammatory.Macrophages	RABL3	0.2038	2.218563	0.880981	0.380717	-5.69403	0.700161	0.66926
Non.inflammatory.Macrophages	SLC4A9	-0.41531	0.122757	-0.8809	0.380759	-5.20567	0.767442	0.709422
Non.inflammatory.Macrophages	ETL4	-0.30144	2.396305	-0.88088	0.380772	-5.52201	0.692692	0.665993
Non.inflammatory.Macrophages	FCER2A	-0.62308	0.458562	-0.88048	0.380986	-5.1213	0.767753	0.702953
Non.inflammatory.Macrophages	HMGB3	-0.24669	4.658182	-0.88038	0.38104	-5.78878	0.60412	0.625469
Non.inflammatory.Macrophages	NME7	0.130103	3.562527	0.880006	0.381242	-5.95088	0.645819	0.644963
Non.inflammatory.Macrophages	TFIP11	-0.1377	3.322157	-0.87998	0.381255	-6.02246	0.655304	0.649291
Non.inflammatory.Macrophages	NDC1	-0.15361	3.748037	-0.87998	0.381256	-5.97387	0.638587	0.641642
Non.inflammatory.Macrophages	TNFRSF9	0.418787	2.460851	0.879823	0.38134	-5.40879	0.690373	0.665055
Non.inflammatory.Macrophages	MST1	-0.46947	0.649079	-0.87976	0.381376	-5.24208	0.76465	0.699412
Non.inflammatory.Macrophages	FANCD2	-0.2375	2.80256	-0.87955	0.381489	-5.61056	0.676361	0.658884
Non.inflammatory.Macrophages	PITPNM1	0.111606	3.730007	0.879418	0.381558	-6.20951	0.639413	0.642106
Non.inflammatory.Macrophages	LRP1B	0.309027	1.219155	0.878832	0.381875	-5.3878	0.744408	0.688804

Non.inflammatory.Macrophages	GM27201	-0.28033	2.321008	-0.87882	0.381879	-5.32966	0.696655	0.668026
Non.inflammatory.Macrophages	RAB23	0.33124	1.279801	0.878515	0.382046	-5.35838	0.741704	0.687794
Non.inflammatory.Macrophages	PALD1	-0.23961	1.292537	-0.8785	0.382051	-5.73606	0.741138	0.687551
Non.inflammatory.Macrophages	GM12353	-0.3089	1.278675	-0.87847	0.382071	-5.45332	0.741754	0.687816
Non.inflammatory.Macrophages	NOX1	-0.31735	1.548335	-0.87835	0.382132	-5.51535	0.729839	0.68268
Non.inflammatory.Macrophages	TOP1MT	-0.28292	1.70028	-0.87834	0.382139	-5.5193	0.723202	0.679803
Non.inflammatory.Macrophages	RNMT	0.094265	4.970618	0.878268	0.382179	-6.34962	0.59317	0.620677
Non.inflammatory.Macrophages	TTLL11	0.362891	1.608719	0.877937	0.382358	-5.26314	0.727253	0.681572
Non.inflammatory.Macrophages	METTL27	-0.46196	0.539053	-0.87787	0.382395	-5.23147	0.767433	0.702149
Non.inflammatory.Macrophages	LACTB	0.094095	5.139278	0.877797	0.382433	-6.41335	0.587145	0.617835
Non.inflammatory.Macrophages	ZFP446	-0.27555	1.164128	-0.87766	0.382509	-5.48297	0.746929	0.690158
Non.inflammatory.Macrophages	2310011J0	0.102915	4.446593	0.877598	0.382541	-6.29978	0.612467	0.629934
Non.inflammatory.Macrophages	COG6	-0.17192	3.137898	-0.87758	0.382551	-5.83974	0.663136	0.653301
Non.inflammatory.Macrophages	TRAPPC9	-0.08423	6.178368	-0.87743	0.38263	-6.56601	0.551034	0.600358
Non.inflammatory.Macrophages	TMEM203	0.144145	3.168142	0.877198	0.382756	-6.05936	0.661936	0.652876

Non.inflammatory.Macrophages	ADAM10	0.065039	7.403441	0.87706	0.382831	-6.80405	0.511188	0.580268
Non.inflammatory.Macrophages	PPP2R1A	0.086359	6.00443	0.876795	0.382974	-6.52775	0.556929	0.603443
Non.inflammatory.Macrophages	ZFP260	0.108849	4.245881	0.876719	0.383015	-6.1131	0.620008	0.633729
Non.inflammatory.Macrophages	IMMP1L	0.094418	4.995599	0.876668	0.383043	-6.32003	0.592328	0.620661
Non.inflammatory.Macrophages	CHAC1	-0.56426	0.269965	-0.87654	0.38311	-5.03935	0.768411	0.707892
Non.inflammatory.Macrophages	RALGAPA1	0.11895	6.84207	0.876521	0.383122	-6.50739	0.529087	0.589611
Non.inflammatory.Macrophages	TTC8	-0.27075	0.78875	-0.87651	0.38313	-5.55212	0.761116	0.697767
Non.inflammatory.Macrophages	SEC24B	0.085621	6.179882	0.8765	0.383134	-6.59856	0.550983	0.600582
Non.inflammatory.Macrophages	IBA57	0.19976	1.934328	0.876408	0.383183	-5.65074	0.713158	0.67594
Non.inflammatory.Macrophages	DENND2C	-0.21256	2.008648	-0.87606	0.383371	-5.66381	0.710176	0.674708
Non.inflammatory.Macrophages	NEK1	-0.15856	3.697931	-0.87599	0.383407	-6.03246	0.641189	0.64377
Non.inflammatory.Macrophages	CEP112	-0.2564	1.570821	-0.87527	0.383798	-5.69836	0.72967	0.683189
Non.inflammatory.Macrophages	SEC24C	0.090557	4.859773	0.875266	0.383801	-6.32038	0.597862	0.623449
Non.inflammatory.Macrophages	CYTH3	0.10965	4.88921	0.875077	0.383903	-6.39711	0.596812	0.62299
Non.inflammatory.Macrophages	GRK6	0.095293	5.764162	0.874985	0.383953	-6.39667	0.565767	0.608017

Non.inflammatory.Macrophages	4833418N(	-0.28558	1.533706	-0.87468	0.384117	-5.53413	0.731326	0.684029
Non.inflammatory.Macrophages	ELOC	-0.07254	7.093954	-0.87467	0.384124	-6.76292	0.521531	0.585968
Non.inflammatory.Macrophages	IFIT2	0.285979	2.721315	0.874653	0.384132	-6.02278	0.680799	0.66181
Non.inflammatory.Macrophages	TJAP1	-0.11654	4.452059	-0.87458	0.384173	-6.13782	0.612923	0.630732
Non.inflammatory.Macrophages	KIF1BP	0.116492	3.865166	0.874501	0.384215	-6.16305	0.635197	0.641149
Non.inflammatory.Macrophages	KIF21A	-0.4255	0.515306	-0.87439	0.384276	-5.22463	0.768726	0.70374
Non.inflammatory.Macrophages	SP3	0.074762	6.891028	0.87427	0.38434	-6.59599	0.528059	0.589378
Non.inflammatory.Macrophages	SERPINA3(	0.376195	3.597185	0.87419	0.384383	-6.19113	0.645618	0.645986
Non.inflammatory.Macrophages	CNBP	0.058096	8.132464	0.873863	0.38456	-6.83038	0.489489	0.569443
Non.inflammatory.Macrophages	DNAJC18	0.152646	3.682567	0.873745	0.384624	-5.94651	0.642493	0.644536
Non.inflammatory.Macrophages	SEC24A	-0.10564	6.616568	-0.87343	0.384797	-6.67077	0.537353	0.593984
Non.inflammatory.Macrophages	DPM2	-0.11616	3.936241	-0.87323	0.384902	-6.16991	0.632938	0.640002
Non.inflammatory.Macrophages	PEX14	0.096594	5.405291	0.873063	0.384994	-6.39373	0.5788	0.614371
Non.inflammatory.Macrophages	CPN1	-0.319	1.600653	-0.87291	0.385074	-5.44935	0.729014	0.683019
Non.inflammatory.Macrophages	TECR	-0.08533	6.763011	-0.87253	0.385283	-6.61272	0.532674	0.591733

Non.inflammatory.Macrophages	ZFP839	0.188049	1.537463	0.872507	0.385295	-5.74324	0.731788	0.684359
Non.inflammatory.Macrophages	ACOT12	-0.40383	0.798615	-0.87248	0.385308	-5.2492	0.762284	0.698553
Non.inflammatory.Macrophages	GM15411	-0.55267	0.212426	-0.87245	0.385328	-4.99655	0.769881	0.710028
Non.inflammatory.Macrophages	FUBP1	0.063669	6.678668	0.872369	0.38537	-6.6081	0.535433	0.593157
Non.inflammatory.Macrophages	A530040E1	0.482693	0.013043	0.872241	0.38544	-5.12333	0.769881	0.713994
Non.inflammatory.Macrophages	GTF3C3	0.153087	3.102011	0.872217	0.385452	-6.00478	0.665865	0.655256
Non.inflammatory.Macrophages	SPAST	0.091191	5.207046	0.871891	0.38563	-6.36989	0.586036	0.618029
Non.inflammatory.Macrophages	RALGAPA2	0.121723	6.409122	0.871634	0.385769	-6.53446	0.544561	0.597803
Non.inflammatory.Macrophages	TMEM198I	-0.27935	0.786307	-0.87162	0.385776	-5.49295	0.762946	0.699034
Non.inflammatory.Macrophages	TMEM128	0.092207	5.718829	0.871423	0.385884	-6.49191	0.568123	0.609405
Non.inflammatory.Macrophages	ZBTB11OS1	0.190112	2.065369	0.871203	0.386003	-5.69209	0.709392	0.674671
Non.inflammatory.Macrophages	PSMC5	0.078547	6.00886	0.871064	0.386078	-6.51963	0.558227	0.604549
Non.inflammatory.Macrophages	PROX1	-0.30632	2.15604	-0.87094	0.386147	-5.67409	0.705526	0.672997
Non.inflammatory.Macrophages	OCIAD2	-0.46858	0.485681	-0.8708	0.386223	-5.15874	0.770411	0.705061
Non.inflammatory.Macrophages	VTA1	0.079773	5.707573	0.870777	0.386234	-6.46322	0.568606	0.609739

Non.inflammatory.Macrophages	OIP5	-0.27494	2.207869	-0.87062	0.386319	-5.49534	0.703324	0.672167
Non.inflammatory.Macrophages	DIPK1A	-0.11868	5.446127	-0.87036	0.38646	-6.46334	0.577762	0.614313
Non.inflammatory.Macrophages	AI597479	0.225657	1.688645	0.870318	0.386483	-5.58212	0.725666	0.68202
Non.inflammatory.Macrophages	INVS	-0.19111	2.809082	-0.87031	0.386487	-5.82199	0.678243	0.661097
Non.inflammatory.Macrophages	DARS	0.097321	5.511648	0.87026	0.386515	-6.44139	0.575454	0.613194
Non.inflammatory.Macrophages	EIF6	0.083295	5.835967	0.869673	0.386834	-6.52506	0.564543	0.607942
Non.inflammatory.Macrophages	DHX34	-0.17596	2.203437	-0.86942	0.38697	-5.82757	0.704088	0.672675
Non.inflammatory.Macrophages	NDUFS5	0.080264	6.478554	0.869364	0.387002	-6.62489	0.542853	0.59723
Non.inflammatory.Macrophages	RPAIN	-0.1127	3.771433	-0.86902	0.38719	-6.13145	0.640402	0.644072
Non.inflammatory.Macrophages	TRIM35	-0.11125	5.607952	-0.869	0.3872	-6.44555	0.572606	0.611976
Non.inflammatory.Macrophages	GM48765	0.442151	0.952243	0.868976	0.387213	-5.13554	0.759042	0.696609
Non.inflammatory.Macrophages	CAMK1	-0.12406	2.966119	-0.86883	0.387293	-6.26791	0.672487	0.658719
Non.inflammatory.Macrophages	MSH3	-0.11013	5.159864	-0.86861	0.387414	-6.28653	0.588621	0.619754
Non.inflammatory.Macrophages	GTF3A	0.091023	4.569389	0.868283	0.38759	-6.28675	0.610315	0.630075
Non.inflammatory.Macrophages	LIMD2	-0.09051	7.333227	-0.86826	0.387602	-6.72345	0.515418	0.583413

Non.inflammatory.Macrophages	FAM89A	-0.34571	0.879902	-0.86789	0.387803	-5.24314	0.761583	0.69822
Non.inflammatory.Macrophages	RAD52	-0.19183	3.121528	-0.86788	0.387812	-5.75202	0.666536	0.656036
Non.inflammatory.Macrophages	PYROXD1	-0.1614	2.95488	-0.86783	0.387836	-5.87725	0.673295	0.659113
Non.inflammatory.Macrophages	PDE12	-0.1151	3.991564	-0.86765	0.387934	-6.06416	0.632262	0.64041
Non.inflammatory.Macrophages	1110038F1	0.110652	4.590808	0.867493	0.38802	-6.26375	0.609622	0.629819
Non.inflammatory.Macrophages	GM42699	-0.52926	0.727533	-0.86745	0.388045	-5.11674	0.76604	0.701261
Non.inflammatory.Macrophages	TAF2	0.103837	4.171185	0.867319	0.388115	-6.14435	0.625394	0.637286
Non.inflammatory.Macrophages	PCBD1	-0.24802	3.53173	-0.8673	0.388123	-6.00727	0.650168	0.648731
Non.inflammatory.Macrophages	GM46224	-0.14057	2.389468	-0.86701	0.388284	-6.53669	0.696894	0.669803
Non.inflammatory.Macrophages	CYP4V3	0.150966	2.78654	0.866689	0.388459	-6.28671	0.680515	0.662569
Non.inflammatory.Macrophages	NOC3L	0.173695	3.216026	0.866581	0.388517	-6.00073	0.663049	0.654699
Non.inflammatory.Macrophages	GM48086	-0.29948	1.511775	-0.86655	0.388536	-5.41063	0.734878	0.686476
Non.inflammatory.Macrophages	PTPRK	-0.29279	3.178658	-0.86622	0.388713	-5.78161	0.664732	0.655501
Non.inflammatory.Macrophages	GM13684	0.166117	3.516914	0.866145	0.388755	-5.98916	0.651238	0.649381
Non.inflammatory.Macrophages	GM49980	-0.23498	6.157222	-0.86582	0.388933	-6.30562	0.554603	0.603469

Non.inflammatory.Macrophages	ZFP638	0.076346	6.22747	0.86557	0.389069	-6.56445	0.552335	0.602364
Non.inflammatory.Macrophages	CD302	-0.11154	5.847821	-0.86459	0.389604	-6.79708	0.565953	0.609109
Non.inflammatory.Macrophages	FUNDC2	0.08942	7.178207	0.864468	0.38967	-6.71935	0.521676	0.586991
Non.inflammatory.Macrophages	LARP4	-0.07568	6.410341	-0.86441	0.389703	-6.6234	0.546807	0.599683
Non.inflammatory.Macrophages	BANK1	-0.13959	6.09331	-0.86434	0.38974	-6.72858	0.55752	0.605033
Non.inflammatory.Macrophages	YPEL1	-0.20714	2.4444	-0.86403	0.389909	-5.64438	0.696064	0.66987
Non.inflammatory.Macrophages	WDR35	-0.41128	0.509893	-0.86387	0.389998	-5.25351	0.773028	0.70687
Non.inflammatory.Macrophages	PFDN6	-0.09635	4.874434	-0.86385	0.390009	-6.34277	0.60061	0.626068
Non.inflammatory.Macrophages	PLPP6	0.160807	2.754142	0.863821	0.390024	-5.93101	0.683157	0.664123
Non.inflammatory.Macrophages	F11	-0.38061	0.954836	-0.86382	0.390024	-5.32523	0.761229	0.698188
Non.inflammatory.Macrophages	GM27216	-0.43085	0.912751	-0.86313	0.3904	-5.28749	0.763076	0.699284
Non.inflammatory.Macrophages	ARHGEF26	0.507343	0.379311	0.862833	0.390564	-5.12888	0.774262	0.709804
Non.inflammatory.Macrophages	PCCB	0.153616	3.081613	0.862326	0.390841	-5.95339	0.67086	0.658599
Non.inflammatory.Macrophages	COL14A1	-0.2854	1.863173	-0.86184	0.391105	-5.62066	0.722382	0.6815
Non.inflammatory.Macrophages	CDK1	-0.19316	5.506863	-0.86182	0.391118	-6.39599	0.579067	0.61579

Non.inflammatory.Macrophages	DENND4B	-0.11227	3.747946	-0.86136	0.391372	-6.20357	0.644852	0.646883
Non.inflammatory.Macrophages	ETFRF1	0.162424	3.163487	0.861098	0.391514	-5.99656	0.668122	0.657507
Non.inflammatory.Macrophages	DCLRE1B	-0.15409	2.770832	-0.8611	0.391515	-5.83491	0.684192	0.664729
Non.inflammatory.Macrophages	ITGA2B	0.284615	0.938407	0.861026	0.391553	-5.46097	0.76363	0.699469
Non.inflammatory.Macrophages	TMEM135	0.088499	5.7512	0.860948	0.391595	-6.58003	0.570744	0.611816
Non.inflammatory.Macrophages	GIMAP4	-0.22759	4.091849	-0.86073	0.391714	-5.95033	0.631515	0.640772
Non.inflammatory.Macrophages	B2M	0.121587	9.94338	0.860644	0.391762	-7.24301	0.441302	0.544474
Non.inflammatory.Macrophages	DHRS4	0.127905	4.374568	0.860642	0.391763	-6.22753	0.620746	0.635751
Non.inflammatory.Macrophages	ANKRD50	0.135654	2.62264	0.860554	0.391811	-5.88808	0.690349	0.667513
Non.inflammatory.Macrophages	D1ERTD62	0.088204	5.295192	0.860262	0.391972	-6.47403	0.587021	0.619698
Non.inflammatory.Macrophages	LRRC8A	-0.09249	5.427019	-0.85987	0.392188	-6.40965	0.582542	0.617458
Non.inflammatory.Macrophages	PARD3B	-0.20048	4.55014	-0.85956	0.392357	-6.16698	0.614546	0.632788
Non.inflammatory.Macrophages	CDNF	-0.41806	0.317972	-0.85951	0.392381	-5.2248	0.775879	0.711847
Non.inflammatory.Macrophages	GPR19	-0.22801	2.550401	-0.85942	0.392435	-5.62709	0.693821	0.669056
Non.inflammatory.Macrophages	MFSD13A	-0.20829	2.008416	-0.85937	0.39246	-5.75266	0.716883	0.679234

Non.inflammatory.Macrophages	PDCD2	-0.13116	3.817161	-0.85912	0.392597	-6.05414	0.642566	0.645956
Non.inflammatory.Macrophages	RAB3GAP2	0.090235	5.259956	0.859055	0.392633	-6.44284	0.588514	0.620527
Non.inflammatory.Macrophages	CRCP	-0.09506	4.449755	-0.859	0.392665	-6.22008	0.618314	0.634682
Non.inflammatory.Macrophages	IQCB1	0.119809	4.824078	0.858973	0.392679	-6.13499	0.604373	0.628103
Non.inflammatory.Macrophages	LANCL2	0.190589	2.807486	0.858937	0.392698	-5.77468	0.683123	0.664361
Non.inflammatory.Macrophages	SLC23A1	0.348709	1.528872	0.858511	0.392932	-5.28822	0.738	0.688428
Non.inflammatory.Macrophages	MRGPRA2/	0.496407	-1.27662	0.858506	0.392935	-4.98961	0.776016	0.744173
Non.inflammatory.Macrophages	NDUFA7	0.05685	7.909207	0.858498	0.392939	-6.89518	0.500471	0.576427
Non.inflammatory.Macrophages	RPAP2	0.150486	3.02458	0.858352	0.393019	-5.90125	0.674365	0.660386
Non.inflammatory.Macrophages	DIS3	0.158869	3.258023	0.858163	0.393123	-5.88035	0.664974	0.656167
Non.inflammatory.Macrophages	PEAK1	0.107741	6.584571	0.857969	0.39323	-6.6589	0.542983	0.59819
Non.inflammatory.Macrophages	COPA	0.065419	6.661065	0.857719	0.393367	-6.67465	0.540493	0.597016
Non.inflammatory.Macrophages	P2RX1	0.462176	0.81074	0.857551	0.393459	-5.21259	0.768301	0.702601
Non.inflammatory.Macrophages	TMEM150/	-0.2712	2.327871	-0.85754	0.393466	-5.60657	0.703604	0.67359
Non.inflammatory.Macrophages	IFI206	0.219841	3.940563	0.857488	0.393494	-6.3746	0.638127	0.644055

Non.inflammatory.Macrophages	ERAL1	0.198032	1.918255	0.857342	0.393574	-5.65597	0.721236	0.681416
Non.inflammatory.Macrophages	SELENOS	-0.07507	6.113957	-0.85704	0.393738	-6.60531	0.559087	0.606409
Non.inflammatory.Macrophages	NUDT7	0.315705	1.74453	0.856623	0.393969	-5.43897	0.729214	0.68498
Non.inflammatory.Macrophages	NAT8F1	-0.31671	1.619362	-0.85641	0.394084	-5.53842	0.734724	0.687453
Non.inflammatory.Macrophages	LAYN	0.29689	0.629576	0.856319	0.394136	-5.53512	0.774025	0.706638
Non.inflammatory.Macrophages	GPR55	-0.22463	1.548876	-0.85629	0.39415	-5.66163	0.737844	0.688823
Non.inflammatory.Macrophages	RCN3	0.193931	1.932131	0.856282	0.394156	-5.78056	0.721026	0.681523
Non.inflammatory.Macrophages	DOLK	-0.19822	1.544978	-0.85622	0.394189	-5.59214	0.738017	0.688918
Non.inflammatory.Macrophages	ANAPC11	0.085478	5.923842	0.855995	0.394314	-6.60437	0.565877	0.609913
Non.inflammatory.Macrophages	SPAG1	0.339658	1.4147	0.855777	0.394435	-5.38657	0.744062	0.691511
Non.inflammatory.Macrophages	SUOX	-0.26768	1.361958	-0.85556	0.394555	-5.42575	0.746447	0.692595
Non.inflammatory.Macrophages	ZFP35	-0.18499	2.543067	-0.85554	0.394563	-5.71688	0.695192	0.670221
Non.inflammatory.Macrophages	SHTN1	0.098547	3.187667	0.854935	0.394898	-6.58005	0.669038	0.65855
Non.inflammatory.Macrophages	QPCTL	0.144923	3.010203	0.854725	0.395013	-5.8661	0.676268	0.661935
Non.inflammatory.Macrophages	GM9750	0.528115	0.476616	0.854697	0.395029	-5.10675	0.777574	0.710234

Non.inflammatory.Macrophages	AMER1	-0.18453	2.080672	-0.85456	0.395104	-5.65424	0.715328	0.679299
Non.inflammatory.Macrophages	CFLAR	0.097857	5.971185	0.854555	0.395107	-6.67777	0.564717	0.609591
Non.inflammatory.Macrophages	SIDT1	-0.48631	3.376538	-0.85435	0.395219	-5.35053	0.661514	0.655248
Non.inflammatory.Macrophages	CDK4	-0.09303	5.844705	-0.85413	0.39534	-6.49617	0.569244	0.611741
Non.inflammatory.Macrophages	MFN2	-0.11861	3.671067	-0.85406	0.395378	-6.06383	0.64987	0.649947
Non.inflammatory.Macrophages	PLTP	-0.15804	5.244881	-0.85371	0.395571	-6.64417	0.590692	0.622182
Non.inflammatory.Macrophages	GCNT7	-0.27244	2.712006	-0.85281	0.396071	-5.41207	0.689757	0.667959
Non.inflammatory.Macrophages	EXOC2	0.082047	5.578749	0.852302	0.396349	-6.47458	0.579741	0.61691
Non.inflammatory.Macrophages	MTA1	-0.10382	4.55479	-0.85192	0.396559	-6.26811	0.617342	0.634819
Non.inflammatory.Macrophages	FBP1	-0.19969	5.143064	-0.85166	0.396702	-6.40965	0.595593	0.624507
Non.inflammatory.Macrophages	GNG11	0.171775	3.831566	0.851621	0.396725	-6.14106	0.645107	0.647728
Non.inflammatory.Macrophages	RAPGEF4	0.273324	2.563789	0.85155	0.396765	-5.62473	0.696612	0.670984
Non.inflammatory.Macrophages	SPTBN5	-0.33427	-0.00378	-0.85151	0.396785	-5.40814	0.77963	0.720603
Non.inflammatory.Macrophages	GPR160	-0.19553	2.407708	-0.85096	0.397093	-5.96567	0.703353	0.674081
Non.inflammatory.Macrophages	IPO9	0.090052	4.771136	0.850951	0.397095	-6.3068	0.609382	0.631174

Non.inflammatory.Macrophages	KCTD11	-0.34759	0.546292	-0.85094	0.397103	-5.33617	0.778671	0.70987
Non.inflammatory.Macrophages	HERC1	-0.0796	7.056727	-0.85087	0.397139	-6.73649	0.529904	0.592263
Non.inflammatory.Macrophages	ACOT4	0.39902	0.046894	0.850844	0.397154	-5.32791	0.779789	0.71978
Non.inflammatory.Macrophages	GNG3	0.357465	1.449153	0.85015	0.397538	-5.32813	0.745683	0.692405
Non.inflammatory.Macrophages	RAB3IL1	-0.18092	1.73222	-0.85012	0.397554	-5.90235	0.733101	0.686979
Non.inflammatory.Macrophages	CD37	0.099717	7.409059	0.849867	0.397694	-6.61233	0.518977	0.586645
Non.inflammatory.Macrophages	ATP5F1	0.063302	8.084387	0.849864	0.397696	-6.89453	0.497908	0.575721
Non.inflammatory.Macrophages	NUDT22	-0.17321	2.557891	-0.84977	0.397748	-5.78818	0.697538	0.67152
Non.inflammatory.Macrophages	ETS1	-0.11398	7.418769	-0.84943	0.397936	-6.51352	0.518746	0.586651
Non.inflammatory.Macrophages	DTWD1	-0.22965	1.928598	-0.84942	0.39794	-5.57068	0.724634	0.683502
Non.inflammatory.Macrophages	CSPG5	-0.4215	0.28413	-0.84935	0.397981	-5.34607	0.780507	0.715455
Non.inflammatory.Macrophages	HVCN1	-0.34063	4.117697	-0.84921	0.398059	-5.58361	0.63473	0.643118
Non.inflammatory.Macrophages	SERPINB9	-0.20826	3.483496	-0.84903	0.398158	-6.14076	0.659727	0.65457
Non.inflammatory.Macrophages	AGO4	-0.14338	3.167589	-0.848	0.398729	-6.08464	0.673324	0.66053
Non.inflammatory.Macrophages	GM12185	-0.145	3.341306	-0.84792	0.398771	-6.16575	0.666272	0.657345

Non.inflammatory.Macrophages	CDC42	0.044385	8.943013	0.847731	0.398877	-7.06745	0.473104	0.562453
Non.inflammatory.Macrophages	MRPS27	-0.15337	3.208477	-0.84763	0.398931	-5.96037	0.671717	0.65982
Non.inflammatory.Macrophages	SAT2	-0.44632	-0.24549	-0.84745	0.399034	-5.26905	0.781673	0.72633
Non.inflammatory.Macrophages	HGD	-0.2724	2.465599	-0.8474	0.399058	-5.76094	0.702592	0.673633
Non.inflammatory.Macrophages	CNR2	-0.24562	3.101889	-0.84734	0.399096	-5.54564	0.676069	0.661845
Non.inflammatory.Macrophages	PIP4K2B	0.107718	4.178712	0.847205	0.399169	-6.21346	0.633316	0.642352
Non.inflammatory.Macrophages	GM14023	-0.25762	1.123999	-0.84688	0.39935	-5.64515	0.761927	0.699398
Non.inflammatory.Macrophages	SLC16A10	0.105499	7.024829	0.84675	0.39942	-6.91871	0.532405	0.593546
Non.inflammatory.Macrophages	PAH	-0.27249	3.508909	-0.84667	0.399463	-5.98967	0.659817	0.654598
Non.inflammatory.Macrophages	BOLA1	-0.13078	3.767483	-0.84637	0.39963	-6.15002	0.649554	0.649944
Non.inflammatory.Macrophages	ZFP287	0.366324	0.566909	0.846305	0.399668	-5.35012	0.780421	0.710444
Non.inflammatory.Macrophages	PCMTD2	0.13377	3.680281	0.8462	0.399726	-6.11614	0.653002	0.651547
Non.inflammatory.Macrophages	PLAGL1	-0.34316	1.041672	-0.84615	0.399753	-5.47826	0.765711	0.701164
Non.inflammatory.Macrophages	FRMD6	0.204359	3.47737	0.846062	0.399802	-5.8637	0.661093	0.655247
Non.inflammatory.Macrophages	ADGRE1	0.135414	4.711233	0.845883	0.399902	-6.75178	0.613309	0.633184

Non.inflammatory.Macrophages	SPATS2	-0.17385	2.720159	-0.8458	0.399949	-6.02987	0.692116	0.669292
Non.inflammatory.Macrophages	GM4070	0.205974	2.887167	0.845794	0.399951	-6.16728	0.68516	0.66619
Non.inflammatory.Macrophages	SLC22A27	-0.40611	1.124082	-0.84572	0.39999	-5.33527	0.761938	0.699678
Non.inflammatory.Macrophages	NUP205	0.117954	4.408914	0.845564	0.400079	-6.17882	0.624755	0.638641
Non.inflammatory.Macrophages	D430042O(	-0.11777	3.792114	-0.84542	0.400158	-6.13752	0.648667	0.649769
Non.inflammatory.Macrophages	CROCC	-0.29665	1.025643	-0.84519	0.400285	-5.43891	0.766679	0.701771
Non.inflammatory.Macrophages	TMPRSS5	-0.36805	0.722493	-0.84424	0.400815	-5.27824	0.777575	0.707953
Non.inflammatory.Macrophages	GM19585	-0.58844	2.331819	-0.8442	0.400835	-5.19258	0.709527	0.676991
Non.inflammatory.Macrophages	RNF40	-0.11955	4.021973	-0.8441	0.400894	-6.05084	0.640476	0.645912
Non.inflammatory.Macrophages	IFI35	-0.1205	5.333965	-0.84362	0.401157	-6.47474	0.591461	0.622944
Non.inflammatory.Macrophages	OLA1	0.073062	5.835409	0.843621	0.401158	-6.51907	0.573615	0.614308
Non.inflammatory.Macrophages	4930539J0	-0.34525	0.706555	-0.84346	0.401248	-5.33829	0.778305	0.708645
Non.inflammatory.Macrophages	TMTC3	-0.13103	2.514058	-0.84345	0.401255	-5.95989	0.701992	0.673923
Non.inflammatory.Macrophages	TDRKH	-0.21886	1.697145	-0.84291	0.401555	-5.72312	0.737869	0.689549
Non.inflammatory.Macrophages	PPFIA1	0.095229	5.566923	0.842586	0.401734	-6.43487	0.583633	0.619245

Non.inflammatory.Macrophages	4930404N1	0.407459	0.191025	0.842265	0.401912	-5.23232	0.784047	0.719251
Non.inflammatory.Macrophages	POLN	0.242086	3.191009	0.842251	0.40192	-5.79418	0.674471	0.661708
Non.inflammatory.Macrophages	TMEM184(	0.127289	3.390595	0.842167	0.401967	-6.02278	0.66636	0.658051
Non.inflammatory.Macrophages	DHX38	0.101133	4.560036	0.84213	0.401988	-6.33997	0.620641	0.636977
Non.inflammatory.Macrophages	SLC22A5	-0.17313	3.042195	-0.84192	0.402105	-6.04922	0.680608	0.664552
Non.inflammatory.Macrophages	CNTROB	-0.22643	2.044211	-0.84189	0.402121	-5.66699	0.722901	0.683259
Non.inflammatory.Macrophages	PEX12	-0.23736	1.86574	-0.84169	0.402233	-5.61051	0.730811	0.686746
Non.inflammatory.Macrophages	ERBIN	-0.07096	7.975801	-0.84153	0.402322	-6.90722	0.503683	0.579364
Non.inflammatory.Macrophages	TFB2M	0.108569	3.953928	0.841472	0.402354	-6.11402	0.644079	0.648001
Non.inflammatory.Macrophages	EIF1AD	0.107293	4.923218	0.841269	0.402467	-6.3263	0.607176	0.630804
Non.inflammatory.Macrophages	POMP	0.068167	7.268227	0.841257	0.402474	-6.83855	0.526038	0.590937
Non.inflammatory.Macrophages	KLHL12	-0.14037	4.259168	-0.84092	0.40266	-6.11455	0.632442	0.642634
Non.inflammatory.Macrophages	VPS11	-0.10084	4.072729	-0.84081	0.402722	-6.22691	0.63966	0.646017
Non.inflammatory.Macrophages	PDGFC	-0.27145	1.879122	-0.84049	0.402899	-5.93992	0.730629	0.686788
Non.inflammatory.Macrophages	6430548M	-0.12198	2.407786	-0.84034	0.402982	-6.24424	0.707709	0.676811

Non.inflammatory.Macrophages	ZCCHC10	-0.1314	4.048466	-0.84029	0.403014	-6.06933	0.640736	0.646614
Non.inflammatory.Macrophages	ST13	0.06012	6.987135	0.840247	0.403036	-6.76517	0.535468	0.59582
Non.inflammatory.Macrophages	B3GALT4	0.272963	1.404829	0.839926	0.403215	-5.60569	0.751825	0.696033
Non.inflammatory.Macrophages	ANPEP	0.246745	1.184937	0.839862	0.403251	-5.84086	0.761813	0.70031
Non.inflammatory.Macrophages	MAP4	0.082647	6.676158	0.83982	0.403274	-6.57009	0.545812	0.601089
Non.inflammatory.Macrophages	PHF2	-0.09064	5.076145	-0.83977	0.4033	-6.27514	0.601907	0.62847
Non.inflammatory.Macrophages	PRPF6	0.080867	5.300623	0.839613	0.40339	-6.40917	0.593764	0.624597
Non.inflammatory.Macrophages	CDKN1C	-0.27496	3.302414	-0.83939	0.403517	-5.80804	0.670645	0.660399
Non.inflammatory.Macrophages	NT5C	0.097345	5.114948	0.839201	0.40362	-6.41094	0.600691	0.627955
Non.inflammatory.Macrophages	UNG	-0.24305	3.369876	-0.83901	0.403726	-5.79701	0.667971	0.659284
Non.inflammatory.Macrophages	LSR	-0.36526	1.400914	-0.83893	0.403769	-5.41591	0.752262	0.696434
Non.inflammatory.Macrophages	NPC1	0.099066	4.971884	0.838901	0.403787	-6.47699	0.605956	0.630579
Non.inflammatory.Macrophages	XPNPEP3	-0.1393	3.250052	-0.83874	0.403878	-5.96904	0.672895	0.661595
Non.inflammatory.Macrophages	ARHGAP35	0.10342	5.237019	0.838598	0.403956	-6.35907	0.596311	0.626052
Non.inflammatory.Macrophages	LOCKD	-0.20609	4.472202	-0.83839	0.404074	-6.06009	0.624823	0.639572

Non.inflammatory.Macrophages	CC2D1B	-0.10777	3.659138	-0.8383	0.404122	-6.16421	0.656488	0.654241
Non.inflammatory.Macrophages	KBTBD2	0.075405	5.176548	0.838132	0.404216	-6.40158	0.598562	0.627184
Non.inflammatory.Macrophages	UGDH	0.111577	4.250685	0.838127	0.40422	-6.2644	0.633305	0.64356
Non.inflammatory.Macrophages	DNAJC19	0.073029	5.950115	0.837846	0.404376	-6.58148	0.571071	0.613865
Non.inflammatory.Macrophages	TNFAIP8L2	0.102298	3.852483	0.837642	0.40449	-6.3463	0.649072	0.650803
Non.inflammatory.Macrophages	ACAA1A	-0.08705	5.302507	-0.83753	0.404551	-6.52885	0.594208	0.62506
Non.inflammatory.Macrophages	TSEN15	-0.16002	2.776415	-0.83742	0.404612	-5.86976	0.692832	0.670645
Non.inflammatory.Macrophages	HDGFL3	0.282466	1.511028	0.837194	0.404741	-5.58095	0.747886	0.694731
Non.inflammatory.Macrophages	SNRNP25	0.134995	3.939991	0.836968	0.404867	-6.0893	0.645774	0.649433
Non.inflammatory.Macrophages	STRADA	-0.10729	4.58715	-0.83696	0.404871	-6.30833	0.62084	0.637839
Non.inflammatory.Macrophages	GM10874	-0.31213	-0.06869	-0.83638	0.405194	-5.42757	0.786043	0.726139
Non.inflammatory.Macrophages	COX7A2L	0.060166	6.644119	0.83634	0.405218	-6.75791	0.547832	0.602462
Non.inflammatory.Macrophages	GM16287	-0.28223	-0.44109	-0.83589	0.405467	-5.43223	0.786259	0.733865
Non.inflammatory.Macrophages	NAA25	0.127181	3.927641	0.83579	0.405526	-6.22129	0.646806	0.650005
Non.inflammatory.Macrophages	DCTN6	0.090789	5.142412	0.835719	0.405566	-6.36402	0.600681	0.628419

Non.inflammatory.Macrophages	FCRL6	-0.55155	-0.87747	-0.83557	0.405648	-4.95823	0.786259	0.742961
Non.inflammatory.Macrophages	GZMK	0.53327	-1.47202	0.835525	0.405675	-4.98531	0.786259	0.755302
Non.inflammatory.Macrophages	4921531C2	-0.24421	1.642005	-0.83552	0.405678	-5.6322	0.742674	0.69277
Non.inflammatory.Macrophages	SOAT2	-0.38936	0.810649	-0.8353	0.405802	-5.26355	0.778269	0.709008
Non.inflammatory.Macrophages	TGFBR3	-0.27564	3.337964	-0.8348	0.40608	-5.63584	0.67084	0.661125
Non.inflammatory.Macrophages	GADD45GI	-0.09372	5.128075	-0.83471	0.406133	-6.41015	0.601623	0.629034
Non.inflammatory.Macrophages	FRMPD4	-0.48227	0.351798	-0.8346	0.406194	-5.19237	0.786809	0.718464
Non.inflammatory.Macrophages	MLLT3	0.127459	5.728512	0.834433	0.406286	-6.44525	0.579988	0.618723
Non.inflammatory.Macrophages	AMZ2	0.107542	3.901939	0.834361	0.406326	-6.16549	0.648294	0.651015
Non.inflammatory.Macrophages	ESPL1	-0.22597	2.563574	-0.8342	0.406417	-5.66891	0.703119	0.675729
Non.inflammatory.Macrophages	DOCK1	0.096591	4.079995	0.833962	0.40655	-6.52832	0.641486	0.647847
Non.inflammatory.Macrophages	MANF	-0.06793	6.808848	-0.83331	0.406918	-6.73027	0.543437	0.600633
Non.inflammatory.Macrophages	USP42	0.127157	3.500066	0.833087	0.407041	-5.98148	0.665051	0.658689
Non.inflammatory.Macrophages	ADSL	-0.11721	4.033205	-0.83303	0.407072	-6.13605	0.643863	0.648988
Non.inflammatory.Macrophages	BC003965	0.125022	3.701969	0.832519	0.407359	-6.13409	0.657321	0.655199

Non.inflammatory.Macrophages	ZBED3	0.13287	3.559356	0.832379	0.407438	-6.05075	0.663069	0.657841
Non.inflammatory.Macrophages	GNPDA2	0.128749	3.100129	0.832185	0.407547	-5.98135	0.681873	0.666381
Non.inflammatory.Macrophages	SDAD1	-0.10633	4.473042	-0.83186	0.407728	-6.31843	0.627512	0.641436
Non.inflammatory.Macrophages	EHD4	-0.07943	6.076676	-0.83161	0.407868	-6.69697	0.569022	0.613507
Non.inflammatory.Macrophages	KARS	0.116175	4.273346	0.831452	0.407958	-6.26513	0.635215	0.64516
Non.inflammatory.Macrophages	FOSL2	0.098014	5.561041	0.831417	0.407978	-6.69982	0.587246	0.622444
Non.inflammatory.Macrophages	TTPA	-0.32663	1.860466	-0.83135	0.408017	-5.5558	0.735134	0.689957
Non.inflammatory.Macrophages	GM31462	0.335321	0.152102	0.831277	0.408057	-5.35608	0.788574	0.723496
Non.inflammatory.Macrophages	XKR6	0.297323	0.862903	0.831084	0.408165	-5.45238	0.778875	0.709439
Non.inflammatory.Macrophages	ZCCHC3	0.360176	0.70709	0.831069	0.408174	-5.2788	0.783534	0.712516
Non.inflammatory.Macrophages	NTMT1	-0.10241	3.588232	-0.83089	0.408275	-6.03303	0.66222	0.657693
Non.inflammatory.Macrophages	ACAP1	-0.25439	3.923819	-0.83084	0.408303	-5.59051	0.648861	0.651579
Non.inflammatory.Macrophages	AKAP8	0.07725	5.39229	0.830792	0.408329	-6.43961	0.59333	0.625481
Non.inflammatory.Macrophages	QK	0.053401	8.142329	0.830499	0.408494	-6.95002	0.501406	0.579439
Non.inflammatory.Macrophages	UBE2V1	0.065038	6.983076	0.830459	0.408516	-6.72026	0.538366	0.59844

Non.inflammatory.Macrophages	EGFL7	-0.20197	3.872106	-0.83039	0.408555	-5.97753	0.650984	0.652579
Non.inflammatory.Macrophages	COPB1	0.062049	6.220287	0.829981	0.408785	-6.62742	0.564293	0.611354
Non.inflammatory.Macrophages	CAND2	-0.41729	0.775357	-0.82984	0.408863	-5.14482	0.781833	0.711347
Non.inflammatory.Macrophages	GABBR1	-0.24917	2.766527	-0.82969	0.408951	-5.96757	0.696329	0.673044
Non.inflammatory.Macrophages	WAPL	0.070869	7.165135	0.829679	0.408955	-6.81548	0.532559	0.595483
Non.inflammatory.Macrophages	GARS	-0.09016	5.511566	-0.82964	0.408976	-6.42357	0.589284	0.623539
Non.inflammatory.Macrophages	SUPT6	0.084451	5.808893	0.82935	0.40914	-6.54984	0.578823	0.61851
Non.inflammatory.Macrophages	MAP3K11	-0.11088	3.876975	-0.82903	0.409318	-6.25061	0.651358	0.652739
Non.inflammatory.Macrophages	ZCCHC17	-0.08553	5.162664	-0.82881	0.409442	-6.37401	0.6024	0.629827
Non.inflammatory.Macrophages	C87436	-0.11826	3.905756	-0.82863	0.409547	-6.15255	0.650325	0.652349
Non.inflammatory.Macrophages	UROD	0.11078	4.467162	0.828435	0.409655	-6.29635	0.628492	0.642324
Non.inflammatory.Macrophages	SLC35C2	0.075978	4.846147	0.828428	0.409659	-6.43536	0.614146	0.635583
Non.inflammatory.Macrophages	RAB2B	0.133884	3.661593	0.828299	0.409732	-6.04512	0.660041	0.656903
Non.inflammatory.Macrophages	HPS3	0.093499	4.626547	0.82813	0.409827	-6.46588	0.62242	0.639536
Non.inflammatory.Macrophages	RBM34	-0.09208	4.465573	-0.82799	0.409906	-6.24245	0.628553	0.642419

Non.inflammatory.Macrophages	DEGS1	0.092436	6.525466	0.827983	0.40991	-6.64631	0.554245	0.606614
Non.inflammatory.Macrophages	RANBP2	-0.086	6.846596	-0.82798	0.40991	-6.75976	0.543448	0.601215
Non.inflammatory.Macrophages	CD163	-0.31341	1.13156	-0.82787	0.409976	-5.8386	0.768933	0.704845
Non.inflammatory.Macrophages	ZFP458	-0.39813	0.876726	-0.82777	0.41003	-5.32616	0.779365	0.70986
Non.inflammatory.Macrophages	PIAS2	-0.08749	6.02482	-0.82767	0.410085	-6.55497	0.571491	0.615138
Non.inflammatory.Macrophages	IL18	-0.09714	3.422293	-0.82741	0.410235	-6.46541	0.669844	0.661428
Non.inflammatory.Macrophages	TMEM51O	-0.31019	-0.90367	-0.82683	0.410561	-5.33284	0.790072	0.746064
Non.inflammatory.Macrophages	HAVCR1	0.566571	-0.3843	0.826742	0.410609	-5.01055	0.790072	0.735439
Non.inflammatory.Macrophages	FFAR1	-0.35421	-0.22509	-0.82666	0.410657	-5.2114	0.790072	0.732271
Non.inflammatory.Macrophages	GUF1	0.148847	2.589786	0.826523	0.410732	-5.82291	0.704834	0.677228
Non.inflammatory.Macrophages	ZFP626	0.172243	2.562798	0.826511	0.410739	-5.76546	0.705984	0.677736
Non.inflammatory.Macrophages	AMT	-0.34975	1.565291	-0.82635	0.410832	-5.4201	0.74973	0.69684
Non.inflammatory.Macrophages	ANAPC10	-0.08734	4.581596	-0.82622	0.410905	-6.36392	0.624594	0.640751
Non.inflammatory.Macrophages	KBTBD11	0.525323	0.726015	0.826195	0.410917	-5.17174	0.784459	0.71328
Non.inflammatory.Macrophages	2610301B2	0.245468	1.613471	0.825817	0.411131	-5.50644	0.747844	0.696079

Non.inflammatory.Macrophages	LRSAM1	0.210046	1.610096	0.825512	0.411303	-5.69138	0.748203	0.696164
Non.inflammatory.Macrophages	LRRC4	-0.17405	3.774431	-0.82535	0.411395	-5.92798	0.656507	0.65554
Non.inflammatory.Macrophages	DUS4L	0.244373	1.929292	0.825235	0.411459	-5.53538	0.73404	0.690104
Non.inflammatory.Macrophages	L2HGDH	-0.26348	1.767651	-0.82491	0.411644	-5.5154	0.741445	0.693212
Non.inflammatory.Macrophages	NAE1	-0.10886	3.958187	-0.82417	0.412058	-6.17614	0.649957	0.652488
Non.inflammatory.Macrophages	PARP1	-0.10976	5.30881	-0.82411	0.412097	-6.3533	0.598619	0.628425
Non.inflammatory.Macrophages	POC1B	-0.09908	4.642223	-0.82386	0.412234	-6.24496	0.623576	0.640315
Non.inflammatory.Macrophages	VANGL2	-0.27679	2.225601	-0.82348	0.412452	-5.44069	0.722162	0.684918
Non.inflammatory.Macrophages	PPP1R1C	0.22329	0.781145	0.823318	0.412542	-5.93519	0.784603	0.713046
Non.inflammatory.Macrophages	TMEM60	0.1007	4.112089	0.823282	0.412563	-6.25507	0.644176	0.649962
Non.inflammatory.Macrophages	GM24362	-0.32199	0.694716	-0.82328	0.412564	-5.36702	0.787204	0.71476
Non.inflammatory.Macrophages	RPGRIP1	-0.09369	6.55945	-0.823	0.412723	-6.46294	0.554915	0.607261
Non.inflammatory.Macrophages	TJP1	0.247086	2.024999	0.822317	0.413109	-5.60823	0.731703	0.68915
Non.inflammatory.Macrophages	SLC9A1	0.085468	5.416377	0.822217	0.413165	-6.40809	0.595566	0.627086
Non.inflammatory.Macrophages	TMED5	0.054641	7.227678	0.821905	0.413342	-6.77252	0.533191	0.596307

Non.inflammatory.Macrophages	DCAF12	0.083139	6.638146	0.821842	0.413377	-6.65719	0.552808	0.606174
Non.inflammatory.Macrophages	SENP3	-0.09548	4.324023	-0.8216	0.413516	-6.33468	0.636782	0.646594
Non.inflammatory.Macrophages	DRG1	0.067367	6.40915	0.821572	0.41353	-6.56697	0.560663	0.610128
Non.inflammatory.Macrophages	GM17227	0.22614	2.746669	0.821213	0.413734	-5.54963	0.70098	0.675653
Non.inflammatory.Macrophages	GM14326	0.150614	2.868013	0.821093	0.413802	-5.98291	0.69587	0.673376
Non.inflammatory.Macrophages	GMCL1	0.107513	4.636774	0.820985	0.413863	-6.12402	0.625007	0.641079
Non.inflammatory.Macrophages	RBMX	-0.11267	4.578527	-0.82076	0.413992	-6.13297	0.627336	0.642225
Non.inflammatory.Macrophages	EXTL2	-0.19421	2.165568	-0.82059	0.414089	-5.83653	0.726226	0.686877
Non.inflammatory.Macrophages	CHST10	0.489495	0.375693	0.820335	0.414231	-5.08527	0.793477	0.722021
Non.inflammatory.Macrophages	DLGAP5	-0.26252	3.248568	-0.82018	0.414318	-5.76813	0.680207	0.666654
Non.inflammatory.Macrophages	1700110K1	-0.4052	-0.57203	-0.82011	0.414359	-5.28993	0.793477	0.741324
Non.inflammatory.Macrophages	RAD21	0.075229	7.022909	0.820056	0.414389	-6.69353	0.540325	0.600192
Non.inflammatory.Macrophages	WNT4	-0.27631	1.526488	-0.81996	0.414443	-5.48826	0.754715	0.699433
Non.inflammatory.Macrophages	VPS37C	-0.09801	3.950923	-0.81996	0.414444	-6.21666	0.651821	0.65382
Non.inflammatory.Macrophages	PANK2	-0.0849	5.327592	-0.81953	0.414686	-6.41984	0.599533	0.629414

Non.inflammatory.Macrophages	ARGLU1	-0.052	7.597485	-0.81911	0.414928	-6.763	0.521747	0.590962
Non.inflammatory.Macrophages	RNPS1	0.07189	6.167896	0.818898	0.415046	-6.55372	0.569516	0.615023
Non.inflammatory.Macrophages	SLC45A3	-0.23391	1.111286	-0.81882	0.415089	-5.78939	0.773953	0.707948
Non.inflammatory.Macrophages	E030030I0(	-0.15627	2.657085	-0.81874	0.415137	-5.83774	0.705175	0.678169
Non.inflammatory.Macrophages	MED6	0.112277	4.540282	0.818736	0.415138	-6.27225	0.62902	0.643544
Non.inflammatory.Macrophages	ZFP239	-0.39059	-0.06752	-0.81863	0.415198	-5.22311	0.793676	0.731513
Non.inflammatory.Macrophages	ADGRL2	-0.19598	4.208198	-0.81853	0.415253	-6.11959	0.641864	0.649575
Non.inflammatory.Macrophages	LLGL2	0.264905	2.291993	0.818388	0.415336	-5.49564	0.720895	0.685201
Non.inflammatory.Macrophages	MFSD14A	0.068077	6.156841	0.818355	0.415354	-6.56204	0.569902	0.615323
Non.inflammatory.Macrophages	WDFY2	0.085516	5.19857	0.818285	0.415394	-6.64485	0.604273	0.63196
Non.inflammatory.Macrophages	ZFP566	-0.3244	1.254913	-0.81827	0.415404	-5.36989	0.767315	0.705243
Non.inflammatory.Macrophages	ILKAP	0.084374	5.651644	0.81823	0.415426	-6.45338	0.587782	0.624039
Non.inflammatory.Macrophages	SRP54A	0.107682	3.599318	0.81821	0.415437	-6.14127	0.666056	0.660728
Non.inflammatory.Macrophages	CRY2	0.18079	2.553643	0.818097	0.415501	-5.74876	0.709597	0.680259
Non.inflammatory.Macrophages	MYO6	-0.14505	3.1857	-0.81801	0.415548	-6.09138	0.682974	0.668409

Non.inflammatory.Macrophages	GM4673	-0.19314	2.578427	-0.81798	0.415566	-5.66959	0.708535	0.679802
Non.inflammatory.Macrophages	HHAT	-0.27165	1.670909	-0.81785	0.415642	-5.53834	0.748378	0.697175
Non.inflammatory.Macrophages	GALNT2	-0.09274	5.565067	-0.81772	0.415717	-6.53775	0.5909	0.625579
Non.inflammatory.Macrophages	GTF3C6	0.079392	5.226124	0.817668	0.415745	-6.42392	0.603258	0.631537
Non.inflammatory.Macrophages	TGFBRAP1	-0.1033	3.494101	-0.8176	0.415781	-6.15491	0.670323	0.662766
Non.inflammatory.Macrophages	GM32743	-0.5797	-0.48509	-0.81742	0.415887	-4.99324	0.793676	0.740311
Non.inflammatory.Macrophages	ATAD1	0.065914	5.731262	0.817311	0.415948	-6.46986	0.584929	0.622794
Non.inflammatory.Macrophages	SMARCA2	-0.07819	6.123463	-0.8173	0.415956	-6.60092	0.571067	0.616031
Non.inflammatory.Macrophages	4932438A1	0.101912	7.087671	0.817127	0.416052	-6.6882	0.538368	0.599715
Non.inflammatory.Macrophages	GMFB	0.075381	5.451593	0.816741	0.416272	-6.53914	0.595295	0.627668
Non.inflammatory.Macrophages	MDN1	-0.11421	5.788045	-0.81644	0.416441	-6.53299	0.583336	0.621911
Non.inflammatory.Macrophages	FTL1-PS1	-0.14821	3.902493	-0.81625	0.41655	-6.30041	0.654417	0.655461
Non.inflammatory.Macrophages	LRIF1	-0.08635	4.523869	-0.81603	0.416676	-6.29496	0.630141	0.644247
Non.inflammatory.Macrophages	TPM2	-0.35508	0.689833	-0.81596	0.416717	-5.46525	0.789741	0.716772
Non.inflammatory.Macrophages	ASAP2	-0.28184	3.068411	-0.81588	0.416763	-5.55056	0.688381	0.670912

Non.inflammatory.Macrophages	CFDP1	0.072754	5.986865	0.815812	0.4168	-6.6001	0.576309	0.618565
Non.inflammatory.Macrophages	CYB561D1	0.262375	1.785161	0.815797	0.416809	-5.55865	0.743832	0.695289
Non.inflammatory.Macrophages	ASB3	-0.09914	4.711015	-0.81559	0.416928	-6.25387	0.623045	0.640977
Non.inflammatory.Macrophages	GLIPR1	0.100691	5.059395	0.815534	0.416958	-6.45196	0.60995	0.63479
Non.inflammatory.Macrophages	ARL3	-0.10892	4.04145	-0.81537	0.41705	-6.24325	0.649015	0.653075
Non.inflammatory.Macrophages	NAIF1	-0.24908	1.251393	-0.81491	0.417312	-5.53461	0.768439	0.705994
Non.inflammatory.Macrophages	LY9	0.100008	4.262049	0.814775	0.41739	-6.34525	0.640566	0.64928
Non.inflammatory.Macrophages	QSER1	0.111122	4.323503	0.81464	0.417467	-6.22957	0.638175	0.64817
Non.inflammatory.Macrophages	NFIB	-0.23178	3.880029	-0.81461	0.417483	-6.0737	0.65562	0.656221
Non.inflammatory.Macrophages	ABCC5	0.121135	4.301545	0.814545	0.417522	-6.40368	0.639029	0.648566
Non.inflammatory.Macrophages	SLC35E1	0.107482	4.084727	0.814226	0.417703	-6.17283	0.647513	0.652535
Non.inflammatory.Macrophages	ZSCAN12	0.239163	1.093811	0.814213	0.41771	-5.49204	0.775736	0.709138
Non.inflammatory.Macrophages	BMP6	-0.46678	0.450706	-0.81419	0.417726	-5.1729	0.794671	0.721919
Non.inflammatory.Macrophages	TRUB1	0.206246	1.825881	0.814149	0.417747	-5.64606	0.742361	0.694856
Non.inflammatory.Macrophages	ATP6V0E	0.055743	7.713213	0.813985	0.41784	-6.93797	0.518704	0.58991

Non.inflammatory.Macrophages	RBM27	0.069815	6.399999	0.813795	0.417949	-6.58395	0.562187	0.611989
Non.inflammatory.Macrophages	PKDCC	-0.18877	1.303453	-0.81362	0.418051	-5.86639	0.766042	0.705234
Non.inflammatory.Macrophages	TRIM37	-0.11407	4.93302	-0.81358	0.418073	-6.27446	0.614912	0.637501
Non.inflammatory.Macrophages	BIN3	0.081603	5.271025	0.813534	0.418098	-6.53248	0.602361	0.63153
Non.inflammatory.Macrophages	BCL2L11	0.120793	7.053365	0.813524	0.418103	-6.7214	0.540128	0.600958
Non.inflammatory.Macrophages	PIAS3	-0.1297	2.896492	-0.81349	0.418121	-5.94191	0.69591	0.674678
Non.inflammatory.Macrophages	KPNA6	0.099189	3.896998	0.813334	0.418212	-6.22002	0.654994	0.656202
Non.inflammatory.Macrophages	KBTBD4	-0.16508	2.78551	-0.8132	0.418289	-5.84889	0.700683	0.676861
Non.inflammatory.Macrophages	DTNB	0.123572	5.293687	0.813025	0.418388	-6.27696	0.601658	0.631276
Non.inflammatory.Macrophages	TMEM106A	0.108903	3.645492	0.812888	0.418466	-6.41859	0.6652	0.660916
Non.inflammatory.Macrophages	ZFP51	-0.18863	2.86166	-0.81243	0.41873	-5.64198	0.697904	0.675626
Non.inflammatory.Macrophages	HINFP	0.13313	3.494398	0.811992	0.418977	-6.01554	0.671958	0.663978
Non.inflammatory.Macrophages	GATB	-0.16023	3.11047	-0.81181	0.419081	-5.98561	0.687854	0.671211
Non.inflammatory.Macrophages	FBXO31	0.163364	3.549697	0.811339	0.41935	-5.90947	0.670024	0.663072
Non.inflammatory.Macrophages	TMEM101	-0.17904	2.322989	-0.81123	0.419413	-5.80951	0.721658	0.686136

Non.inflammatory.Macrophages	TRIM12C	0.116285	4.726648	0.811227	0.419414	-6.36244	0.623741	0.641744
Non.inflammatory.Macrophages	FMNL1	0.120397	6.443857	0.81113	0.419469	-6.226	0.561619	0.611807
Non.inflammatory.Macrophages	GM34466	-0.31497	-1.10689	-0.81085	0.419632	-5.31366	0.796033	0.754794
Non.inflammatory.Macrophages	PGPEP1L	-0.51991	-0.29843	-0.81079	0.419664	-5.01579	0.796033	0.738089
Non.inflammatory.Macrophages	ATG16L1	-0.08855	5.446329	-0.81076	0.419682	-6.48945	0.596969	0.629135
Non.inflammatory.Macrophages	FBXO48	-0.41105	0.24175	-0.81066	0.41974	-5.15027	0.796033	0.727173
Non.inflammatory.Macrophages	RBMXL1	-0.08312	5.042632	-0.81059	0.419779	-6.38925	0.611862	0.636315
Non.inflammatory.Macrophages	MRPL40	-0.10323	4.651456	-0.81015	0.420027	-6.34347	0.626911	0.643374
Non.inflammatory.Macrophages	POLI	0.256112	1.527716	0.809944	0.420147	-5.49668	0.757541	0.70187
Non.inflammatory.Macrophages	ZFP189	-0.32355	1.120206	-0.80934	0.42049	-5.32843	0.776824	0.710161
Non.inflammatory.Macrophages	PLEKHF2	0.083103	5.470642	0.809187	0.42058	-6.41667	0.596884	0.629269
Non.inflammatory.Macrophages	SAP30BP	-0.09235	4.838744	-0.80909	0.420637	-6.31984	0.62035	0.640466
Non.inflammatory.Macrophages	NFKBIE	0.113654	4.879022	0.808721	0.420847	-6.44037	0.618976	0.639792
Non.inflammatory.Macrophages	TKFC	-0.19897	3.130684	-0.8087	0.420856	-5.85607	0.688377	0.671694
Non.inflammatory.Macrophages	UFL1	0.096665	3.965196	0.808163	0.421166	-6.24381	0.654705	0.656459

Non.inflammatory.Macrophages	DBNDD2	-0.12581	2.836435	-0.80814	0.421178	-6.21712	0.701088	0.677405
Non.inflammatory.Macrophages	IGLC3	-0.46415	4.04289	-0.8078	0.421374	-5.76374	0.651832	0.655119
Non.inflammatory.Macrophages	GPATCH4	0.149566	3.338867	0.807345	0.421634	-5.981	0.680623	0.668161
Non.inflammatory.Macrophages	PLEKHM2	-0.09753	5.202185	-0.80705	0.421805	-6.66036	0.607755	0.634414
Non.inflammatory.Macrophages	SHLD2	-0.13017	3.853936	-0.80704	0.421807	-6.10703	0.659762	0.658676
Non.inflammatory.Macrophages	3300002A1	0.381719	0.451891	0.806737	0.421983	-5.20187	0.798646	0.724115
Non.inflammatory.Macrophages	H2AFY	0.063776	7.697828	0.80646	0.422142	-6.80621	0.521915	0.591992
Non.inflammatory.Macrophages	ZHX1	0.104214	4.078556	0.806128	0.422332	-6.30014	0.651306	0.654803
Non.inflammatory.Macrophages	GM44699	-0.31668	0.559613	-0.80597	0.422421	-5.34669	0.797609	0.722191
Non.inflammatory.Macrophages	GANAB	0.092802	4.730059	0.805737	0.422556	-6.34183	0.625981	0.643115
Non.inflammatory.Macrophages	TSPAN9	-0.13339	3.290989	-0.8057	0.422577	-6.31281	0.683205	0.669397
Non.inflammatory.Macrophages	SNX32	0.148911	2.812618	0.805568	0.422653	-5.9059	0.703284	0.678366
Non.inflammatory.Macrophages	DHX33	0.128017	3.32737	0.8055	0.422692	-6.04635	0.6817	0.668743
Non.inflammatory.Macrophages	SSPN	-0.44373	-0.41075	-0.80543	0.42273	-5.09019	0.799027	0.741974
Non.inflammatory.Macrophages	BET1	-0.10367	4.246255	-0.80529	0.422812	-6.26007	0.644697	0.651949

Non.inflammatory.Macrophages	NFS1	0.096352	4.533914	0.805186	0.422872	-6.31325	0.633506	0.646796
Non.inflammatory.Macrophages	ROCK1	0.063031	8.134703	0.805171	0.422881	-6.8909	0.508227	0.585106
Non.inflammatory.Macrophages	SERPINA1E	-0.29169	5.030433	-0.80514	0.422902	-6.34928	0.61462	0.637918
Non.inflammatory.Macrophages	CHST7	0.458159	0.725254	0.805046	0.422953	-5.21657	0.793367	0.719107
Non.inflammatory.Macrophages	GTF2H2	-0.11547	3.759481	-0.80461	0.4232	-6.02507	0.664058	0.661065
Non.inflammatory.Macrophages	ME2	0.088899	5.963937	0.804597	0.423211	-6.6173	0.580554	0.621717
Non.inflammatory.Macrophages	OAS2	0.197157	1.571395	0.804521	0.423254	-6.08825	0.757944	0.702548
Non.inflammatory.Macrophages	IFFO2	-0.13357	3.364648	-0.80452	0.423255	-6.14267	0.680161	0.668369
Non.inflammatory.Macrophages	QPRT	-0.22605	2.660001	-0.80429	0.423389	-5.83638	0.709804	0.681646
Non.inflammatory.Macrophages	PLEKHO1	-0.07709	5.499121	-0.80423	0.423424	-6.62297	0.597285	0.629857
Non.inflammatory.Macrophages	RBM19	0.15809	2.925866	0.804184	0.423448	-5.89262	0.698482	0.676624
Non.inflammatory.Macrophages	RPP25L	-0.11866	3.672149	-0.804	0.423552	-6.13321	0.667589	0.662718
Non.inflammatory.Macrophages	CERS2	0.082157	5.262709	0.803979	0.423565	-6.48759	0.60597	0.634016
Non.inflammatory.Macrophages	MRGBP	0.145549	3.37422	0.803849	0.42364	-5.93733	0.679766	0.668246
Non.inflammatory.Macrophages	S1PR2	0.187706	1.261742	0.803712	0.423719	-5.87513	0.772171	0.708727

Non.inflammatory.Macrophages	GM36738	0.13085	3.095803	0.803703	0.423724	-6.13511	0.691333	0.673489
Non.inflammatory.Macrophages	GM2629	0.302181	0.511245	0.803518	0.42383	-5.37532	0.798595	0.72375
Non.inflammatory.Macrophages	ISCA2	0.096502	4.968036	0.803323	0.423943	-6.41119	0.616964	0.639486
Non.inflammatory.Macrophages	CD68	-0.09764	4.872198	-0.80325	0.423984	-6.72926	0.62058	0.641247
Non.inflammatory.Macrophages	GGH	-0.10028	4.555539	-0.80316	0.424035	-6.58684	0.632672	0.646944
Non.inflammatory.Macrophages	CARMIL1	-0.20375	4.075468	-0.8031	0.424073	-5.8092	0.651429	0.655683
Non.inflammatory.Macrophages	ADNP	0.074008	6.50802	0.803044	0.424103	-6.60352	0.561542	0.612751
Non.inflammatory.Macrophages	EFCC1	-0.26326	0.001369	-0.80303	0.424111	-5.61426	0.799027	0.734318
Non.inflammatory.Macrophages	TBC1D7	0.157833	2.220043	0.802861	0.424208	-5.76004	0.728912	0.690469
Non.inflammatory.Macrophages	2900093K2	-0.17702	2.884005	-0.80285	0.424217	-5.84842	0.700254	0.677836
Non.inflammatory.Macrophages	EIF3B	0.08014	5.890853	0.802787	0.42425	-6.57207	0.583155	0.623442
Non.inflammatory.Macrophages	BTF3L4	0.093052	4.269235	0.802684	0.42431	-6.23591	0.643796	0.652254
Non.inflammatory.Macrophages	CLEC4A4	-0.2333	-1.00355	-0.80254	0.424393	-5.46875	0.799067	0.755306
Non.inflammatory.Macrophages	DCAF6	0.103707	5.908083	0.802431	0.424455	-6.55572	0.58257	0.623311
Non.inflammatory.Macrophages	DDAH2	-0.18899	3.503269	-0.80233	0.424512	-5.83896	0.6745	0.666465

Non.inflammatory.Macrophages	KLK1B27	0.408154	-1.42677	0.802209	0.424583	-4.9584	0.799067	0.76434
Non.inflammatory.Macrophages	PPP1R42	-0.40432	0.580161	-0.80212	0.424636	-5.30771	0.797231	0.722957
Non.inflammatory.Macrophages	RPF1	-0.07918	5.149471	-0.80203	0.424685	-6.40564	0.610203	0.636713
Non.inflammatory.Macrophages	RRP9	-0.1573	2.59462	-0.80173	0.424858	-5.8482	0.712841	0.683705
Non.inflammatory.Macrophages	GATAD1	0.071339	5.768843	0.801273	0.425122	-6.53499	0.587991	0.626043
Non.inflammatory.Macrophages	MTFR2	-0.16639	3.615592	-0.80101	0.425275	-6.08876	0.670538	0.664777
Non.inflammatory.Macrophages	CAPN11	-0.46148	1.046105	-0.80094	0.425314	-5.49325	0.782983	0.714016
Non.inflammatory.Macrophages	AATF	-0.08533	4.995367	-0.8008	0.425397	-6.4565	0.616571	0.639774
Non.inflammatory.Macrophages	D130062J1	0.234058	1.642184	0.80052	0.425555	-5.62628	0.75568	0.702427
Non.inflammatory.Macrophages	RBM25	0.05105	8.296585	0.799849	0.425942	-6.91547	0.504159	0.58385
Non.inflammatory.Macrophages	CLEC2I	-0.23308	3.177972	-0.79984	0.425947	-5.81832	0.689212	0.673255
Non.inflammatory.Macrophages	ELANE	-0.72961	2.340078	-0.79957	0.426103	-5.63629	0.725188	0.689168
Non.inflammatory.Macrophages	GM38394	-0.31004	0.848639	-0.79932	0.426248	-5.41902	0.791305	0.718449
Non.inflammatory.Macrophages	ALDH18A1	0.162078	3.070395	0.799309	0.426254	-6.05791	0.693867	0.675414
Non.inflammatory.Macrophages	GM16556	0.212677	1.676001	0.79925	0.426287	-5.84783	0.754791	0.70215

Non.inflammatory.Macrophages	LIN7C	0.088133	4.956273	0.799077	0.426387	-6.36738	0.618717	0.640906
Non.inflammatory.Macrophages	UBL7	-0.0944	5.130384	-0.79871	0.4266	-6.42928	0.61218	0.637857
Non.inflammatory.Macrophages	KLHL6	0.122471	6.109159	0.798681	0.426616	-6.42212	0.576641	0.62071
Non.inflammatory.Macrophages	COG1	0.107001	3.677624	0.798645	0.426637	-6.11249	0.668783	0.66418
Non.inflammatory.Macrophages	FAM13B	-0.07007	6.457749	-0.79858	0.426674	-6.67785	0.564468	0.614716
Non.inflammatory.Macrophages	COPG2	0.089644	5.004811	0.798451	0.426748	-6.43104	0.616888	0.640091
Non.inflammatory.Macrophages	SPATA1	0.112805	3.467697	0.798371	0.426794	-6.03392	0.677358	0.668072
Non.inflammatory.Macrophages	SHISA5	0.101064	6.572889	0.798113	0.426944	-6.77308	0.560502	0.612804
Non.inflammatory.Macrophages	PCED1B	-0.1275	4.966857	-0.79804	0.426987	-6.41715	0.618318	0.640826
Non.inflammatory.Macrophages	DBI	0.08235	7.067278	0.798022	0.426996	-6.85244	0.543777	0.604427
Non.inflammatory.Macrophages	POLM	0.152614	3.245007	0.797989	0.427015	-5.83596	0.686566	0.672285
Non.inflammatory.Macrophages	PLEKHA8	-0.23694	0.886546	-0.79789	0.427072	-5.53757	0.790159	0.717884
Non.inflammatory.Macrophages	WWOX	0.075143	6.961551	0.797825	0.42711	-6.78642	0.547313	0.606257
Non.inflammatory.Macrophages	PPM1L	-0.19437	4.617569	-0.79777	0.427139	-5.91409	0.631624	0.64716
Non.inflammatory.Macrophages	SPIDR	0.087349	5.228567	0.797734	0.427162	-6.45613	0.608523	0.636246

Non.inflammatory.Macrophages	STAG3	-0.4518	0.607181	-0.79759	0.427246	-5.26338	0.798379	0.723533
Non.inflammatory.Macrophages	CYP7B1	-0.54845	0.01895	-0.79731	0.427405	-5.17379	0.80088	0.735519
Non.inflammatory.Macrophages	KEAP1	-0.07802	5.115335	-0.79728	0.427424	-6.46107	0.612863	0.638336
Non.inflammatory.Macrophages	PDLIM2	-0.12836	3.759145	-0.7967	0.427759	-6.19948	0.665874	0.662993
Non.inflammatory.Macrophages	AKIRIN2	-0.07761	5.756269	-0.7966	0.427814	-6.52832	0.589568	0.627151
Non.inflammatory.Macrophages	KIF17	-0.23424	1.692096	-0.79644	0.427908	-5.787	0.754505	0.702297
Non.inflammatory.Macrophages	NUP50	-0.08012	5.304963	-0.79642	0.427924	-6.44681	0.606049	0.635129
Non.inflammatory.Macrophages	CGAS	-0.15376	2.747045	-0.79628	0.428004	-6.17118	0.707995	0.682029
Non.inflammatory.Macrophages	RTCA	-0.10474	4.282973	-0.79625	0.428018	-6.25413	0.645004	0.653493
Non.inflammatory.Macrophages	IGHMBP2	0.158299	2.850148	0.796197	0.42805	-5.83048	0.703593	0.680076
Non.inflammatory.Macrophages	PIK3C2A	0.077029	6.854141	0.796074	0.428121	-6.77212	0.551253	0.608352
Non.inflammatory.Macrophages	WDR38	-0.3539	0.617809	-0.79607	0.428125	-5.20538	0.798588	0.723615
Non.inflammatory.Macrophages	SSR4	0.06595	7.573816	0.795458	0.428478	-6.94128	0.527804	0.596459
Non.inflammatory.Macrophages	A230056P1	-0.30965	0.407448	-0.79528	0.428578	-5.40622	0.801725	0.72809
Non.inflammatory.Macrophages	A930037H(	-0.18627	3.242306	-0.7952	0.42863	-6.08592	0.687538	0.672944

Non.inflammatory.Macrophages	RBM15	-0.08266	5.696792	-0.79515	0.428658	-6.4494	0.592107	0.628523
Non.inflammatory.Macrophages	KBTBD7	-0.18771	2.137804	-0.79507	0.428704	-5.76476	0.73501	0.694011
Non.inflammatory.Macrophages	TBX2	-0.37077	0.45083	-0.79469	0.428922	-5.32671	0.801963	0.727495
Non.inflammatory.Macrophages	MPPED2	-0.60156	-0.32287	-0.79455	0.429003	-5.04392	0.801963	0.743343
Non.inflammatory.Macrophages	ARHGAP21	-0.09019	5.511128	-0.79446	0.429057	-6.53414	0.59904	0.632027
Non.inflammatory.Macrophages	NDUFS3	0.075801	6.050235	0.794431	0.429072	-6.62183	0.57962	0.622611
Non.inflammatory.Macrophages	TENT4B	0.075415	6.368937	0.794334	0.429128	-6.61316	0.568426	0.617111
Non.inflammatory.Macrophages	NTM	-0.33563	0.913874	-0.79404	0.429299	-5.4272	0.790661	0.718285
Non.inflammatory.Macrophages	TFE3	-0.08763	4.149746	-0.79403	0.429302	-6.40294	0.650961	0.656467
Non.inflammatory.Macrophages	PROSER1	0.107636	4.213066	0.793822	0.429424	-6.26876	0.648483	0.655365
Non.inflammatory.Macrophages	ZPR1	0.092088	4.397865	0.793752	0.429465	-6.29761	0.641231	0.652003
Non.inflammatory.Macrophages	FANCA	0.222751	3.05493	0.793698	0.429496	-5.65661	0.69571	0.676832
Non.inflammatory.Macrophages	PACS2	0.08673	4.036916	0.793215	0.429776	-6.37193	0.655745	0.658781
Non.inflammatory.Macrophages	REEP4	0.117982	4.356559	0.793099	0.429843	-6.28687	0.643117	0.652973
Non.inflammatory.Macrophages	A530076I1	-0.27631	-0.88246	-0.79308	0.429856	-5.48352	0.802438	0.755317

Non.inflammatory.Macrophages	MCM5	-0.18461	4.910563	-0.79297	0.429918	-6.3127	0.621777	0.642996
Non.inflammatory.Macrophages	RAD50	-0.09115	4.906644	-0.79266	0.4301	-6.40007	0.622103	0.643148
Non.inflammatory.Macrophages	TMEM87A	0.091477	5.236916	0.792393	0.430253	-6.40562	0.609816	0.637299
Non.inflammatory.Macrophages	SH2D3C	-0.2376	4.693449	-0.7923	0.430305	-5.68786	0.630367	0.647055
Non.inflammatory.Macrophages	ANXA3	0.116599	3.542549	0.79201	0.430475	-6.51461	0.676191	0.668212
Non.inflammatory.Macrophages	CNOT3	0.083179	5.46868	0.791961	0.430503	-6.47229	0.601358	0.633328
Non.inflammatory.Macrophages	RPUSD3	-0.41993	0.640562	-0.79156	0.430735	-5.23955	0.800044	0.724471
Non.inflammatory.Macrophages	MRPL45	0.113743	4.340705	0.791491	0.430776	-6.25176	0.644302	0.65363
Non.inflammatory.Macrophages	MKKS	0.196816	1.995441	0.791425	0.430814	-5.68942	0.742651	0.697741
Non.inflammatory.Macrophages	PITPNC1	0.072538	8.799744	0.791405	0.430826	-7.2015	0.490395	0.577365
Non.inflammatory.Macrophages	LYRM2	0.135764	3.377583	0.790897	0.43112	-6.09683	0.683406	0.671538
Non.inflammatory.Macrophages	TRA2A	0.065276	7.075692	0.790897	0.431121	-6.73456	0.545361	0.605845
Non.inflammatory.Macrophages	GM15832	0.162315	1.685015	0.790777	0.43119	-5.96854	0.756987	0.703932
Non.inflammatory.Macrophages	PCK1	-0.22987	4.182878	-0.79067	0.431251	-6.23737	0.650802	0.656703
Non.inflammatory.Macrophages	PPOX	0.142147	3.108757	0.790515	0.431342	-5.96548	0.694694	0.676653

Non.inflammatory.Macrophages	IKBKE	0.169962	3.116112	0.790284	0.431477	-6.34154	0.694384	0.676607
Non.inflammatory.Macrophages	CYYR1	-0.30599	2.282713	-0.79022	0.431513	-5.5956	0.730265	0.692509
Non.inflammatory.Macrophages	PIP4P1	-0.06072	5.856707	-0.79018	0.431537	-6.58887	0.587675	0.626952
Non.inflammatory.Macrophages	GM48089	0.318919	1.135961	0.790114	0.431575	-5.51008	0.782415	0.714981
Non.inflammatory.Macrophages	NDUFS2	-0.07835	6.235293	-0.78973	0.431798	-6.65269	0.574293	0.62053
Non.inflammatory.Macrophages	ARL15	0.081777	7.591924	0.78973	0.431798	-6.79787	0.528478	0.597532
Non.inflammatory.Macrophages	TPGS1	0.101382	4.393149	0.789615	0.431865	-6.35833	0.642648	0.65322
Non.inflammatory.Macrophages	GIGYF1	0.096462	4.441868	0.789601	0.431873	-6.29454	0.640745	0.652335
Non.inflammatory.Macrophages	SERHL	-0.11418	3.492794	-0.78946	0.431955	-6.16904	0.678792	0.66983
Non.inflammatory.Macrophages	TARM1	0.547394	-0.18149	0.789409	0.431985	-5.09848	0.80364	0.741849
Non.inflammatory.Macrophages	MRPS21	0.05573	7.034242	0.789241	0.432082	-6.7991	0.546873	0.606971
Non.inflammatory.Macrophages	MLLT6	0.149444	3.270712	0.789195	0.432109	-6.24527	0.688004	0.674039
Non.inflammatory.Macrophages	TRDJ1	0.42541	-1.29553	0.789012	0.432216	-5.05244	0.803738	0.765242
Non.inflammatory.Macrophages	LSS	-0.33573	0.604735	-0.78876	0.432363	-5.32176	0.801434	0.72601
Non.inflammatory.Macrophages	ERAP1	0.086688	5.228177	0.788742	0.432373	-6.4953	0.610863	0.638399

Non.inflammatory.Macrophages	A530064D(	-0.21759	0.81889	-0.78866	0.432418	-5.76052	0.795234	0.721719
Non.inflammatory.Macrophages	PFDN1	0.074139	5.109296	0.788372	0.432588	-6.48318	0.615366	0.640611
Non.inflammatory.Macrophages	1700066M	0.215178	1.288003	0.788354	0.432599	-5.58709	0.775613	0.712467
Non.inflammatory.Macrophages	KATNA1	-0.08946	4.864725	-0.78829	0.432634	-6.4226	0.624614	0.644988
Non.inflammatory.Macrophages	EDEM1	0.078773	6.015715	0.787899	0.432863	-6.63749	0.582354	0.624729
Non.inflammatory.Macrophages	POGLUT3	-0.22461	0.979034	-0.78789	0.432867	-5.6557	0.790305	0.718703
Non.inflammatory.Macrophages	MPST	-0.13044	3.682692	-0.78778	0.432931	-6.11302	0.671353	0.666649
Non.inflammatory.Macrophages	EP300	0.065189	6.696726	0.787706	0.432975	-6.70725	0.558576	0.612996
Non.inflammatory.Macrophages	XPO1	0.090135	5.786847	0.787593	0.433041	-6.55849	0.590571	0.62875
Non.inflammatory.Macrophages	GM43466	-0.3191	1.497294	-0.78743	0.433138	-5.37427	0.766187	0.708516
Non.inflammatory.Macrophages	LEO1	0.119949	4.138442	0.787036	0.433365	-6.17253	0.653165	0.658548
Non.inflammatory.Macrophages	GM42595	-0.32396	0.917394	-0.787	0.433386	-5.34356	0.792687	0.720276
Non.inflammatory.Macrophages	PLA2G4C	-0.3681	0.890136	-0.78696	0.43341	-5.34992	0.793513	0.720822
Non.inflammatory.Macrophages	DMAP1	-0.16628	2.496634	-0.78692	0.433431	-5.79956	0.721514	0.689363
Non.inflammatory.Macrophages	AIFM1	0.10078	4.370662	0.786297	0.433796	-6.23192	0.644398	0.654452

Non.inflammatory.Macrophages	ICA1L	-0.25109	1.590667	-0.78626	0.433817	-5.65998	0.762467	0.707082
Non.inflammatory.Macrophages	GPRIN3	0.40762	0.638034	0.786023	0.433955	-5.24812	0.80184	0.72607
Non.inflammatory.Macrophages	HOMER2	0.375878	0.275337	0.78576	0.434109	-5.30829	0.805046	0.733539
Non.inflammatory.Macrophages	PEA15A	-0.10038	3.90143	-0.78562	0.434189	-6.36852	0.663349	0.663193
Non.inflammatory.Macrophages	2610002M	0.109569	4.208089	0.78545	0.43429	-6.26916	0.651116	0.657567
Non.inflammatory.Macrophages	ANKRD9	-0.32658	2.821177	-0.78539	0.434322	-5.38894	0.708273	0.683451
Non.inflammatory.Macrophages	IDS	0.116056	2.698638	0.785187	0.434443	-6.21384	0.713641	0.685846
Non.inflammatory.Macrophages	CDK5RAP1	-0.28095	2.44604	-0.78459	0.434791	-5.78348	0.724957	0.69093
Non.inflammatory.Macrophages	PHYHD1	-0.17513	2.730001	-0.78459	0.434792	-5.88191	0.712624	0.685494
Non.inflammatory.Macrophages	RAMP2	0.214958	3.013058	0.784499	0.434844	-5.85809	0.700524	0.680118
Non.inflammatory.Macrophages	MS4A4C	0.226482	3.224383	0.784429	0.434885	-6.28485	0.691614	0.676131
Non.inflammatory.Macrophages	FBXO4	-0.09912	3.431156	-0.78425	0.434991	-6.27013	0.683072	0.672272
Non.inflammatory.Macrophages	IMPDH2	0.088839	5.196107	0.783888	0.435201	-6.45022	0.613724	0.640159
Non.inflammatory.Macrophages	AKAP13	0.068283	8.500476	0.783752	0.43528	-6.90722	0.501258	0.58392
Non.inflammatory.Macrophages	SMPD2	0.188037	2.04317	0.783677	0.435324	-5.79942	0.743133	0.698883

Non.inflammatory.Macrophages	MATN2	-0.40136	0.980441	-0.78356	0.435395	-5.22971	0.792153	0.719856
Non.inflammatory.Macrophages	TTC27	0.130911	3.520631	0.783108	0.435656	-6.06688	0.679826	0.670929
Non.inflammatory.Macrophages	TOX4	-0.06249	5.903687	-0.78309	0.435669	-6.60977	0.587947	0.627865
Non.inflammatory.Macrophages	B230206L0	-0.28633	1.02404	-0.78304	0.435697	-5.7516	0.790312	0.719163
Non.inflammatory.Macrophages	SDK1	-0.30281	3.195716	-0.78224	0.436164	-5.70591	0.693999	0.677242
Non.inflammatory.Macrophages	E130309D(	0.11639	3.983364	0.781964	0.436325	-6.11542	0.661717	0.662595
Non.inflammatory.Macrophages	ITPKC	-0.1999	1.763821	-0.78191	0.436357	-5.81284	0.756807	0.704818
Non.inflammatory.Macrophages	ALG3	0.211881	1.77052	0.781796	0.436423	-5.74963	0.756505	0.704689
Non.inflammatory.Macrophages	CUL4A	0.077426	5.002429	0.781703	0.436478	-6.44574	0.621905	0.644103
Non.inflammatory.Macrophages	2310015A1	-0.19768	2.091938	-0.78135	0.436686	-5.73485	0.742254	0.698627
Non.inflammatory.Macrophages	FBXO32	0.167612	3.711448	0.781209	0.436766	-6.29602	0.673002	0.667921
Non.inflammatory.Macrophages	COPRS	0.194808	0.717795	0.781091	0.436836	-5.79688	0.801958	0.725915
Non.inflammatory.Macrophages	SEMA4D	0.20218	6.446214	0.780467	0.4372	-6.1012	0.569977	0.619261
Non.inflammatory.Macrophages	B3GLCT	-0.12538	3.311117	-0.78041	0.437235	-6.17996	0.690006	0.675733
Non.inflammatory.Macrophages	GTPBP10	0.158406	2.397542	0.780136	0.437394	-5.82384	0.729275	0.693177

Non.inflammatory.Macrophages	GABPB1	0.094882	5.380129	0.779931	0.437514	-6.33229	0.608422	0.637976
Non.inflammatory.Macrophages	PROX1OS	-0.3928	0.612207	-0.77982	0.437579	-5.30916	0.805517	0.728469
Non.inflammatory.Macrophages	USP46	-0.16313	3.281753	-0.77969	0.437655	-5.96147	0.691292	0.676423
Non.inflammatory.Macrophages	INTS8	0.097549	4.693172	0.779662	0.437672	-6.24011	0.63446	0.650373
Non.inflammatory.Macrophages	CENPT	0.174923	2.566821	0.779607	0.437704	-5.70331	0.721857	0.690046
Non.inflammatory.Macrophages	GTF3C5	-0.20108	2.258469	-0.77949	0.437771	-5.75243	0.735421	0.696
Non.inflammatory.Macrophages	ARPC5L	-0.07358	6.300784	-0.77949	0.437772	-6.55287	0.575121	0.62194
Non.inflammatory.Macrophages	TMSB4X	0.063811	12.24836	0.779429	0.437808	-7.66585	0.399115	0.527131
Non.inflammatory.Macrophages	CDH17	0.50915	0.13748	0.779276	0.437898	-5.07995	0.808063	0.738287
Non.inflammatory.Macrophages	GUCA1A	0.132713	1.4794	0.779197	0.437944	-6.16717	0.770765	0.711322
Non.inflammatory.Macrophages	NT5E	-0.34795	2.431121	-0.77902	0.438046	-5.46364	0.727895	0.692788
Non.inflammatory.Macrophages	GM826	-0.31612	-0.25558	-0.77889	0.438126	-5.62758	0.808177	0.746481
Non.inflammatory.Macrophages	APBA1	0.11732	4.384633	0.778664	0.438257	-6.48653	0.646716	0.656191
Non.inflammatory.Macrophages	CAAP1	0.098682	4.575271	0.778286	0.438478	-6.2544	0.639455	0.652798
Non.inflammatory.Macrophages	SKP2	0.136397	3.310393	0.778191	0.438534	-6.07645	0.690548	0.676215

Non.inflammatory.Macrophages	APEX1	-0.0888	5.465947	-0.77809	0.438596	-6.54905	0.605641	0.636881
Non.inflammatory.Macrophages	HIC2	-0.15539	2.712677	-0.77802	0.438635	-5.99443	0.715993	0.687636
Non.inflammatory.Macrophages	HMGN1	0.090234	7.26929	0.777854	0.438732	-6.76791	0.54239	0.605779
Non.inflammatory.Macrophages	ZMAT5	0.082382	4.547775	0.777699	0.438822	-6.35916	0.640627	0.653533
Non.inflammatory.Macrophages	GM44686	0.201204	3.136727	0.7773	0.439057	-5.61605	0.698239	0.679894
Non.inflammatory.Macrophages	JRKL	-0.23356	1.537523	-0.77716	0.43914	-5.66523	0.769021	0.710844
Non.inflammatory.Macrophages	CTDNEP1	0.082027	5.516749	0.776681	0.43942	-6.56724	0.604323	0.636482
Non.inflammatory.Macrophages	1700006J1	-0.38905	-0.71534	-0.77664	0.439447	-5.36009	0.809313	0.756901
Non.inflammatory.Macrophages	ZKSCAN14	0.139928	3.081299	0.776559	0.439491	-5.89456	0.700848	0.681188
Non.inflammatory.Macrophages	ZDHHC8	-0.13422	3.666743	-0.77651	0.439519	-6.02467	0.676405	0.670182
Non.inflammatory.Macrophages	ERBB4	-0.39331	0.909465	-0.77634	0.439618	-5.39249	0.798009	0.723633
Non.inflammatory.Macrophages	RSPO3	-0.52763	0.723734	-0.77604	0.439795	-5.35019	0.803913	0.727462
Non.inflammatory.Macrophages	EI24	0.082827	4.630195	0.775874	0.439894	-6.36094	0.638147	0.652605
Non.inflammatory.Macrophages	HEATR5A	0.089234	5.656048	0.775825	0.439922	-6.62488	0.599425	0.63425
Non.inflammatory.Macrophages	RNF146	0.087193	5.30614	0.775665	0.440017	-6.44798	0.612422	0.640467

Non.inflammatory.Macrophages	IL10RB	-0.07074	5.555085	-0.7755	0.440113	-6.67402	0.603231	0.636123
Non.inflammatory.Macrophages	MED31	-0.12943	3.491551	-0.77536	0.440193	-6.01493	0.684033	0.673787
Non.inflammatory.Macrophages	UBQLN4	0.125011	3.216072	0.775199	0.440291	-5.98986	0.695556	0.679053
Non.inflammatory.Macrophages	NT5C3B	-0.14212	3.396774	-0.77509	0.440353	-5.91885	0.687978	0.675672
Non.inflammatory.Macrophages	B4GALT3	0.106587	3.874826	0.775022	0.440395	-6.08814	0.668301	0.666758
Non.inflammatory.Macrophages	RRP15	0.11595	3.823389	0.774879	0.440478	-6.13282	0.670393	0.667726
Non.inflammatory.Macrophages	ECI1	-0.10514	3.920606	-0.77485	0.440494	-6.23586	0.666445	0.665934
Non.inflammatory.Macrophages	SDHA	0.064432	5.691569	0.774553	0.44067	-6.60589	0.59841	0.63406
Non.inflammatory.Macrophages	CEP57L1	-0.15701	3.287731	-0.77438	0.44077	-5.94409	0.692787	0.677995
Non.inflammatory.Macrophages	REPIN1	-0.27708	1.082403	-0.77417	0.440894	-5.48281	0.791428	0.720951
Non.inflammatory.Macrophages	URM1	0.09823	4.343407	0.773938	0.441032	-6.22208	0.649963	0.658437
Non.inflammatory.Macrophages	CISD1	0.110889	5.119405	0.773841	0.441089	-6.46745	0.61994	0.644365
Non.inflammatory.Macrophages	GM49662	-0.1358	2.537113	-0.7732	0.441469	-6.51654	0.725703	0.692624
Non.inflammatory.Macrophages	CCDC122	0.177464	1.315452	0.773128	0.441509	-5.8697	0.781105	0.716576
Non.inflammatory.Macrophages	BNIP1	0.132293	3.526643	0.772924	0.441629	-6.12058	0.683595	0.673878

Non.inflammatory.Macrophages	KLHL20	0.157261	3.27084	0.772805	0.441699	-6.01098	0.694298	0.678692
Non.inflammatory.Macrophages	AHCYL2	0.077429	6.28947	0.772533	0.441859	-6.76351	0.57777	0.624063
Non.inflammatory.Macrophages	ALG11	0.13027	2.610001	0.772434	0.441917	-5.95671	0.722791	0.691407
Non.inflammatory.Macrophages	PEX10	-0.26782	0.82136	-0.7719	0.44223	-5.4952	0.802946	0.726865
Non.inflammatory.Macrophages	TMEM202	-0.24541	0.104736	-0.77151	0.442459	-5.57701	0.811965	0.741676
Non.inflammatory.Macrophages	CCDC136	-0.50554	0.119291	-0.77138	0.442537	-5.19518	0.811998	0.741395
Non.inflammatory.Macrophages	NR2C2	0.078717	6.925422	0.771176	0.442659	-6.64846	0.556307	0.613577
Non.inflammatory.Macrophages	2900076A(	0.178661	2.621325	0.771103	0.442702	-5.70167	0.723088	0.691697
Non.inflammatory.Macrophages	CTSL	0.07852	6.758061	0.770967	0.442782	-6.84328	0.562067	0.616466
Non.inflammatory.Macrophages	KLHL26	0.179362	2.727028	0.770845	0.442854	-5.79998	0.718535	0.689682
Non.inflammatory.Macrophages	GM29666	-0.37905	0.244861	-0.77048	0.443068	-5.34679	0.812422	0.739118
Non.inflammatory.Macrophages	EIF1	-0.06496	9.721973	-0.76999	0.443359	-7.23997	0.468969	0.567904
Non.inflammatory.Macrophages	NCK1	-0.0783	6.174664	-0.7698	0.443473	-6.6593	0.583101	0.626902
Non.inflammatory.Macrophages	NETO2	0.312497	3.22493	0.769621	0.443576	-5.50501	0.697957	0.680611
Non.inflammatory.Macrophages	TMEM250-	0.101481	4.629225	0.769359	0.443731	-6.36849	0.640867	0.65467

Non.inflammatory.Macrophages	9930104L0	-0.25668	1.356879	-0.76933	0.443748	-5.47792	0.781219	0.717063
Non.inflammatory.Macrophages	E2F7	-0.29372	2.983824	-0.76932	0.443754	-5.5966	0.708224	0.685346
Non.inflammatory.Macrophages	CLU	-0.28026	5.440247	-0.76904	0.443918	-6.46834	0.610076	0.640164
Non.inflammatory.Macrophages	SPNS2	-0.27889	1.813184	-0.76888	0.444015	-5.47589	0.760309	0.708208
Non.inflammatory.Macrophages	GALNT18	-0.39425	2.025829	-0.76879	0.444067	-5.50847	0.750634	0.704039
Non.inflammatory.Macrophages	POMGNT1	-0.14549	2.312495	-0.76864	0.444157	-5.89104	0.737822	0.69852
Non.inflammatory.Macrophages	RAP1B	-0.04253	8.543309	-0.76839	0.444306	-7.13043	0.504533	0.587366
Non.inflammatory.Macrophages	FUOM	0.164037	2.61981	0.768377	0.444311	-6.13423	0.72431	0.692649
Non.inflammatory.Macrophages	TBC1D17	-0.11451	3.823308	-0.76814	0.444452	-6.19952	0.673492	0.669878
Non.inflammatory.Macrophages	PLK1	-0.25555	3.704872	-0.76746	0.444855	-6.01159	0.678878	0.67243
Non.inflammatory.Macrophages	POLR1C	0.122316	3.945898	0.767288	0.444955	-6.16752	0.669069	0.667964
Non.inflammatory.Macrophages	RILPL1	-0.19472	2.31814	-0.767	0.445125	-5.77851	0.738522	0.698968
Non.inflammatory.Macrophages	ZMAT1	-0.19053	2.090961	-0.76687	0.445201	-5.79658	0.748711	0.703426
Non.inflammatory.Macrophages	GRB14	-0.29813	1.939569	-0.76675	0.445275	-5.60873	0.755571	0.706469
Non.inflammatory.Macrophages	CACTIN	-0.12271	3.991663	-0.76661	0.445356	-6.1368	0.667326	0.667267

Non.inflammatory.Macrophages	A130010J1	-0.22165	1.72998	-0.76653	0.445403	-5.66702	0.765164	0.710603
Non.inflammatory.Macrophages	ABCG2	-0.13051	3.149803	-0.7665	0.445422	-6.09204	0.702304	0.683089
Non.inflammatory.Macrophages	C8A	-0.3866	0.525618	-0.76645	0.445453	-5.24637	0.813627	0.734783
Non.inflammatory.Macrophages	THAP8	-0.39368	-0.29985	-0.76621	0.445592	-5.35665	0.814423	0.751812
Non.inflammatory.Macrophages	NOTCH2	0.068873	7.16468	0.766102	0.445656	-6.86082	0.549789	0.610858
Non.inflammatory.Macrophages	SCFD2	-0.079	5.78946	-0.76605	0.445684	-6.59096	0.598088	0.634698
Non.inflammatory.Macrophages	C2CD2L	0.133737	2.991873	0.765843	0.445809	-6.05566	0.709102	0.686141
Non.inflammatory.Macrophages	LMAN2L	-0.08786	4.447735	-0.76564	0.445927	-6.34006	0.649111	0.658962
Non.inflammatory.Macrophages	LMO1	0.400573	1.523387	0.765605	0.44595	-5.3018	0.774782	0.714814
Non.inflammatory.Macrophages	PPP1R9A	-0.14002	3.383193	-0.7656	0.445952	-6.28579	0.692488	0.678781
Non.inflammatory.Macrophages	SOGA1	-0.12009	3.807296	-0.76553	0.445993	-6.31226	0.674891	0.670845
Non.inflammatory.Macrophages	ACBD6	0.074064	5.436733	0.765337	0.446109	-6.46999	0.611118	0.641141
Non.inflammatory.Macrophages	SMIM5	0.524439	0.166279	0.765113	0.446241	-5.0742	0.814423	0.74245
Non.inflammatory.Macrophages	CARS	-0.1488	4.118134	-0.76507	0.446266	-6.09222	0.662261	0.665222
Non.inflammatory.Macrophages	SLC25A17	-0.08274	5.308731	-0.765	0.446307	-6.43097	0.615914	0.643539

Non.inflammatory.Macrophages	EZH1	0.12115	3.600438	0.765003	0.446307	-6.19522	0.683421	0.674879
Non.inflammatory.Macrophages	DNAJC12	0.156067	3.069108	0.76489	0.446374	-6.03214	0.705794	0.684932
Non.inflammatory.Macrophages	GLIS3	0.171176	3.068689	0.7648	0.446427	-6.35825	0.705812	0.684939
Non.inflammatory.Macrophages	GRAMD3	0.1462	6.080829	0.764767	0.446446	-6.29273	0.587525	0.629853
Non.inflammatory.Macrophages	ATP5H	0.052447	7.946365	0.764438	0.446641	-6.95259	0.524203	0.598089
Non.inflammatory.Macrophages	IRF7	-0.16876	6.139097	-0.76417	0.446798	-6.86744	0.585688	0.629032
Non.inflammatory.Macrophages	FKBP7	0.234793	1.867273	0.764076	0.446856	-5.65959	0.759249	0.708455
Non.inflammatory.Macrophages	CD44	0.066838	8.587659	0.764006	0.446898	-7.24956	0.504025	0.587637
Non.inflammatory.Macrophages	SLC25A30	0.104561	3.550303	0.76392	0.446949	-6.22507	0.6858	0.676123
Non.inflammatory.Macrophages	HRH4	-0.23418	0.27047	-0.76383	0.447001	-5.76067	0.814776	0.740679
Non.inflammatory.Macrophages	PPDPF	0.068698	5.630234	0.763528	0.447181	-6.59728	0.604361	0.638181
Non.inflammatory.Macrophages	PROSCOS	0.34642	0.287878	0.763322	0.447304	-5.44285	0.815048	0.740506
Non.inflammatory.Macrophages	SALL2	-0.34593	0.090711	-0.76328	0.447331	-5.33305	0.815048	0.744589
Non.inflammatory.Macrophages	PI4KB	0.079105	5.414205	0.762507	0.447787	-6.44614	0.612608	0.642439
Non.inflammatory.Macrophages	TDRD3	0.105422	4.059195	0.762499	0.447791	-6.23749	0.665343	0.667135

Non.inflammatory.Macrophages	MAT2A	0.060438	6.522913	0.762496	0.447794	-6.74828	0.572446	0.622911
Non.inflammatory.Macrophages	ZFP322A	0.143485	3.041434	0.762459	0.447815	-6.01814	0.707727	0.686309
Non.inflammatory.Macrophages	CCNJ	-0.23328	2.015982	-0.76241	0.447847	-5.69048	0.75295	0.706203
Non.inflammatory.Macrophages	E230013L2	-0.27511	-0.72305	-0.76234	0.447889	-5.4474	0.815286	0.762032
Non.inflammatory.Macrophages	AFDN	0.118182	3.267689	0.762276	0.447924	-6.19819	0.698093	0.682068
Non.inflammatory.Macrophages	RARRES1	0.301491	0.889058	0.762243	0.447943	-5.63796	0.804454	0.728703
Non.inflammatory.Macrophages	MOCS2	0.083939	5.094585	0.76207	0.448046	-6.49234	0.624737	0.648285
Non.inflammatory.Macrophages	ZFP369	0.162728	2.919936	0.761879	0.44816	-5.86841	0.713104	0.688758
Non.inflammatory.Macrophages	RAP2C	0.078101	4.944479	0.76175	0.448236	-6.49631	0.630581	0.651057
Non.inflammatory.Macrophages	CDK10	0.161832	2.234353	0.761436	0.448423	-5.83983	0.743499	0.70215
Non.inflammatory.Macrophages	EMC7	0.055815	6.059195	0.761304	0.448501	-6.67719	0.589264	0.631254
Non.inflammatory.Macrophages	WTIP	-0.16716	1.051984	-0.76118	0.448577	-5.88328	0.798331	0.725651
Non.inflammatory.Macrophages	SNX7	0.212169	1.212304	0.761073	0.448639	-5.72737	0.790694	0.722447
Non.inflammatory.Macrophages	SLC35D2	-0.12713	4.232	-0.76098	0.448693	-6.23116	0.658778	0.664246
Non.inflammatory.Macrophages	BRAT1	-0.16573	2.183649	-0.76083	0.448781	-5.82859	0.745822	0.703219

Non.inflammatory.Macrophages	OSTF1	0.047044	7.480967	0.760569	0.448938	-6.93539	0.540119	0.606853
Non.inflammatory.Macrophages	VRK2	0.076253	5.691956	0.760561	0.448943	-6.68898	0.602658	0.637842
Non.inflammatory.Macrophages	ARHGAP27	-0.11268	3.664658	-0.76048	0.448988	-6.19919	0.681889	0.674911
Non.inflammatory.Macrophages	RITA1	-0.21008	1.367418	-0.76038	0.449053	-5.59126	0.783368	0.719439
Non.inflammatory.Macrophages	BRD7	0.057251	6.307707	0.760349	0.449069	-6.68038	0.580383	0.627036
Non.inflammatory.Macrophages	ZFP958	-0.19507	2.267977	-0.76018	0.449171	-5.75782	0.742038	0.701703
Non.inflammatory.Macrophages	GOLGA5	-0.07762	4.913418	-0.76017	0.449174	-6.4461	0.631994	0.651903
Non.inflammatory.Macrophages	INSIG2	0.099196	4.405764	0.759943	0.44931	-6.33225	0.651958	0.661248
Non.inflammatory.Macrophages	IRAK1	0.06934	5.224747	0.75964	0.44949	-6.56137	0.620374	0.646418
Non.inflammatory.Macrophages	CCPG1	0.086753	6.037912	0.759308	0.449688	-6.55227	0.590469	0.632064
Non.inflammatory.Macrophages	PHLDB1	0.17321	1.355652	0.759192	0.449757	-5.91704	0.784486	0.72005
Non.inflammatory.Macrophages	PRRG1	-0.28277	1.915898	-0.75912	0.449799	-5.56967	0.7585	0.708936
Non.inflammatory.Macrophages	PSMC3	0.065137	6.11628	0.75887	0.449949	-6.65392	0.587649	0.63074
Non.inflammatory.Macrophages	BC049715	-0.3362	0.604594	-0.75864	0.450086	-5.49034	0.81398	0.735313
Non.inflammatory.Macrophages	ARFGEF2	-0.07605	6.285468	-0.75862	0.4501	-6.69366	0.581596	0.627821

Non.inflammatory.Macrophages	TMEM86A	-0.11636	2.885047	-0.75859	0.450116	-6.28871	0.715402	0.690148
Non.inflammatory.Macrophages	ZWINT	0.07899	5.039001	0.758448	0.4502	-6.46744	0.627628	0.649991
Non.inflammatory.Macrophages	PUS10	0.096121	4.485499	0.758431	0.45021	-6.39917	0.649162	0.660084
Non.inflammatory.Macrophages	PDLIM4	-0.16998	2.040162	-0.7584	0.450227	-6.18381	0.752847	0.706557
Non.inflammatory.Macrophages	RFX5	0.189568	1.943489	0.758269	0.450306	-5.74242	0.757245	0.708459
Non.inflammatory.Macrophages	INCENP	-0.12571	5.454754	-0.75815	0.450376	-6.48012	0.6119	0.642511
Non.inflammatory.Macrophages	ADNP2	-0.14348	2.990665	-0.75802	0.450458	-6.0708	0.710843	0.688123
Non.inflammatory.Macrophages	MBD5	0.067282	6.903175	0.758002	0.450465	-6.76837	0.560004	0.617117
Non.inflammatory.Macrophages	MED29	0.085716	3.806911	0.757899	0.450527	-6.31575	0.676517	0.672697
Non.inflammatory.Macrophages	GLIS2	-0.3148	1.3513	-0.75758	0.450716	-5.37596	0.784923	0.720305
Non.inflammatory.Macrophages	NRARP	-0.34308	1.780032	-0.75696	0.451088	-5.38518	0.765457	0.711962
Non.inflammatory.Macrophages	KLHL13	-0.34944	0.360663	-0.75661	0.451295	-5.45361	0.817136	0.740704
Non.inflammatory.Macrophages	PPP4C	-0.06059	6.80668	-0.75653	0.451343	-6.76548	0.563858	0.619111
Non.inflammatory.Macrophages	A630052C1	-0.44341	0.189023	-0.75651	0.451352	-5.15697	0.817136	0.744248
Non.inflammatory.Macrophages	GM12655	-0.39202	0.353725	-0.75649	0.451364	-5.27975	0.817136	0.740853

Non.inflammatory.Macrophages	SLC3A1	-0.3038	0.899213	-0.7564	0.451417	-5.40327	0.805966	0.729736
Non.inflammatory.Macrophages	DLL4	-0.43964	0.717902	-0.7563	0.451482	-5.2104	0.811576	0.733426
Non.inflammatory.Macrophages	SUGT1	-0.06327	5.949959	-0.75627	0.451495	-6.62249	0.59422	0.634078
Non.inflammatory.Macrophages	PTK6	-0.38029	-0.3416	-0.75592	0.451706	-5.42701	0.817312	0.755359
Non.inflammatory.Macrophages	CCDC86	0.077187	5.146452	0.75584	0.451754	-6.56778	0.624251	0.648465
Non.inflammatory.Macrophages	GM20682	0.173933	1.711443	0.755807	0.451774	-5.76599	0.768788	0.71352
Non.inflammatory.Macrophages	UNC5CL	0.559306	0.465408	0.755643	0.451872	-5.10109	0.81738	0.738691
Non.inflammatory.Macrophages	NSFL1C	0.087184	4.168167	0.755507	0.451953	-6.29725	0.662672	0.666418
Non.inflammatory.Macrophages	PQLC3	0.224644	3.812077	0.755409	0.452011	-5.62516	0.677172	0.673056
Non.inflammatory.Macrophages	ATPSCKMT	-0.11863	3.937455	-0.75507	0.452215	-6.25644	0.672163	0.670884
Non.inflammatory.Macrophages	BABAM1	-0.074	5.569892	-0.75506	0.452221	-6.55588	0.60851	0.641079
Non.inflammatory.Macrophages	MIS18A	0.113688	4.400206	0.754872	0.452332	-6.28984	0.653507	0.662333
Non.inflammatory.Macrophages	ZFP553	0.188333	2.414705	0.754859	0.45234	-5.6521	0.737119	0.699955
Non.inflammatory.Macrophages	PHAX	0.096777	4.599165	0.754728	0.452418	-6.35977	0.645662	0.658705
Non.inflammatory.Macrophages	CEBPD	0.092584	3.956744	0.754348	0.452645	-6.51182	0.671648	0.670758

Non.inflammatory.Macrophages	DGCR6	-0.11326	3.650268	-0.75424	0.452711	-6.15755	0.684278	0.676511
Non.inflammatory.Macrophages	GPX7	-0.3026	0.610424	-0.75385	0.452945	-5.51013	0.815538	0.73621
Non.inflammatory.Macrophages	ZDHHC1	0.171921	1.415954	0.753588	0.453099	-5.77686	0.783268	0.719918
Non.inflammatory.Macrophages	LDLRAD4	0.104106	6.109073	0.753547	0.453123	-6.66818	0.589121	0.631784
Non.inflammatory.Macrophages	EMC1	0.09757	3.622778	0.753539	0.453128	-6.20587	0.685532	0.67706
Non.inflammatory.Macrophages	CPTP	-0.16472	2.248177	-0.75338	0.453224	-5.75746	0.744998	0.703459
Non.inflammatory.Macrophages	UTP4	-0.08097	5.102474	-0.75335	0.453239	-6.46673	0.626491	0.649745
Non.inflammatory.Macrophages	HSPA12B	-0.40922	0.480164	-0.75335	0.453243	-5.16001	0.818047	0.738884
Non.inflammatory.Macrophages	DCK	0.090566	5.832657	0.753231	0.453312	-6.59407	0.599165	0.636677
Non.inflammatory.Macrophages	CRIP1	0.094312	8.825299	0.753203	0.453329	-7.14631	0.498714	0.585798
Non.inflammatory.Macrophages	PEX11B	0.113997	3.446594	0.752894	0.453514	-6.14588	0.69309	0.680455
Non.inflammatory.Macrophages	LY6G	-0.51075	-0.81776	-0.75265	0.453657	-5.1083	0.818361	0.766099
Non.inflammatory.Macrophages	FBXO3	0.07573	5.012921	0.752408	0.453804	-6.42353	0.630165	0.651535
Non.inflammatory.Macrophages	PFDN5	0.047508	7.413663	0.752327	0.453853	-6.91065	0.544075	0.609433
Non.inflammatory.Macrophages	GM10634	-0.20006	0.651485	-0.75232	0.45386	-5.8236	0.814865	0.735591

Non.inflammatory.Macrophages	CCT7	0.066819	6.427225	0.752254	0.453896	-6.68284	0.577982	0.626405
Non.inflammatory.Macrophages	PPP1R18	0.066417	6.994866	0.752046	0.454021	-6.8064	0.558228	0.616584
Non.inflammatory.Macrophages	TMEM229I	0.122324	4.03146	0.752004	0.454046	-6.13434	0.668979	0.669608
Non.inflammatory.Macrophages	APOBEC4	-0.45169	0.042895	-0.7519	0.454109	-5.12127	0.818361	0.748131
Non.inflammatory.Macrophages	PLSCR3	-0.10427	4.301027	-0.75189	0.454116	-6.34618	0.658099	0.664603
Non.inflammatory.Macrophages	DICER1	0.076036	4.846948	0.751801	0.454168	-6.40023	0.636576	0.65459
Non.inflammatory.Macrophages	ASB7	0.138621	3.936004	0.751335	0.454446	-6.13852	0.673125	0.67158
Non.inflammatory.Macrophages	KIF22	-0.24047	4.043139	-0.75131	0.45446	-5.93847	0.668755	0.669581
Non.inflammatory.Macrophages	TMEM238	0.174572	2.938537	0.751145	0.45456	-5.94478	0.715117	0.690575
Non.inflammatory.Macrophages	SPRYD3	0.103816	3.699854	0.751097	0.454589	-6.13552	0.682863	0.676102
Non.inflammatory.Macrophages	HSPBP1	-0.10052	3.934559	-0.75077	0.454786	-6.21575	0.673216	0.671838
Non.inflammatory.Macrophages	THAP3	-0.10309	3.799217	-0.75065	0.454857	-6.25068	0.678775	0.674405
Non.inflammatory.Macrophages	GALE	-0.20159	2.330073	-0.75062	0.454872	-5.61327	0.741925	0.702554
Non.inflammatory.Macrophages	DUSP2	0.11033	5.991936	0.750547	0.454918	-6.66093	0.593836	0.634513
Non.inflammatory.Macrophages	RAD51C	-0.19701	2.245463	-0.7505	0.454947	-5.69783	0.745722	0.704253

Non.inflammatory.Macrophages	IGHJ4	0.508899	0.337645	0.750386	0.455015	-5.08414	0.818708	0.742619
Non.inflammatory.Macrophages	HIST3H2A	-0.18929	2.104979	-0.75037	0.455026	-5.87102	0.752066	0.707033
Non.inflammatory.Macrophages	CRYZL1	-0.08226	4.570122	-0.75008	0.455197	-6.45275	0.647837	0.66029
Non.inflammatory.Macrophages	GM16062	-0.17029	1.874018	-0.7496	0.455488	-5.74942	0.76304	0.711822
Non.inflammatory.Macrophages	SORBS2	-0.34414	1.878636	-0.74954	0.455524	-5.54307	0.762828	0.711746
Non.inflammatory.Macrophages	ASTL	-0.21005	1.424213	-0.74953	0.455529	-5.83992	0.783961	0.720794
Non.inflammatory.Macrophages	FANCI	-0.22365	2.266344	-0.74925	0.455695	-5.52401	0.745376	0.704229
Non.inflammatory.Macrophages	INO80D	0.066481	5.995336	0.748948	0.455876	-6.58859	0.594185	0.63491
Non.inflammatory.Macrophages	A530013C2	0.130265	2.431674	0.74854	0.456121	-6.3659	0.737975	0.701204
Non.inflammatory.Macrophages	FMO1	-0.29379	2.23787	-0.74853	0.456125	-5.68163	0.746657	0.704994
Non.inflammatory.Macrophages	KCTD20	0.084309	4.885027	0.748481	0.456157	-6.38159	0.635874	0.654929
Non.inflammatory.Macrophages	CAND1	0.069646	5.132169	0.748445	0.456178	-6.50407	0.62636	0.650455
Non.inflammatory.Macrophages	RFX1	-0.10523	3.263376	-0.74839	0.456211	-6.05462	0.701764	0.685189
Non.inflammatory.Macrophages	ERLIN1	-0.08905	4.765624	-0.74838	0.456214	-6.44439	0.64052	0.65713
Non.inflammatory.Macrophages	NPRL3	0.147979	2.892647	0.748242	0.4563	-5.9192	0.717697	0.692322

Non.inflammatory.Macrophages	SNRPB2	-0.0572	6.282619	-0.74819	0.456333	-6.74168	0.583832	0.629992
Non.inflammatory.Macrophages	PIP5K1A	0.078466	5.595581	0.748186	0.456333	-6.56701	0.608885	0.642159
Non.inflammatory.Macrophages	GXYLT1	0.078324	5.093219	0.748104	0.456383	-6.51559	0.627851	0.651224
Non.inflammatory.Macrophages	PMS1	0.179348	2.650773	0.748047	0.456417	-5.77353	0.728272	0.697057
Non.inflammatory.Macrophages	ORC3	0.085424	4.958044	0.747926	0.456489	-6.37493	0.633066	0.653754
Non.inflammatory.Macrophages	ATXN7L2	-0.23742	2.480154	-0.74713	0.456969	-5.60165	0.736452	0.700735
Non.inflammatory.Macrophages	MAFG	0.077193	5.07367	0.747089	0.456992	-6.54602	0.629142	0.651942
Non.inflammatory.Macrophages	TNNI1	-0.5035	-0.907	-0.74692	0.457095	-5.13622	0.820141	0.769919
Non.inflammatory.Macrophages	LENG8	0.094263	4.564084	0.746373	0.457422	-6.34789	0.649431	0.66147
Non.inflammatory.Macrophages	SF1	-0.05324	7.215593	-0.74616	0.457553	-6.78719	0.552296	0.6144
Non.inflammatory.Macrophages	RDH10	-0.13822	3.207433	-0.7461	0.457586	-6.0917	0.705295	0.686947
Non.inflammatory.Macrophages	ZCCHC9	-0.06956	5.404138	-0.74592	0.457697	-6.52551	0.61712	0.646225
Non.inflammatory.Macrophages	DNAJC30	0.103388	3.959336	0.745614	0.457878	-6.22904	0.674087	0.672904
Non.inflammatory.Macrophages	PATZ1	0.12402	4.251704	0.745056	0.458214	-6.07019	0.662605	0.667553
Non.inflammatory.Macrophages	HCST	0.096083	5.375869	0.744846	0.45834	-6.64558	0.618808	0.64701

Non.inflammatory.Macrophages	IFRD1	0.078055	8.532379	0.744746	0.4584	-7.1677	0.509983	0.592586
Non.inflammatory.Macrophages	ZSCAN26	-0.08839	4.652438	-0.74435	0.458638	-6.44507	0.646894	0.660333
Non.inflammatory.Macrophages	DENND4A	0.086182	10.03042	0.744326	0.458653	-7.19764	0.465243	0.568541
Non.inflammatory.Macrophages	HIST1H3B	0.465088	1.995292	0.744245	0.458702	-5.40938	0.759936	0.711041
Non.inflammatory.Macrophages	UBXN6	0.091326	4.786672	0.744136	0.458767	-6.44654	0.641631	0.657916
Non.inflammatory.Macrophages	ZMAT3	0.200941	1.902429	0.743892	0.458914	-5.87145	0.76435	0.71297
Non.inflammatory.Macrophages	UPF1	0.067361	5.35091	0.743744	0.459003	-6.47962	0.620075	0.647784
Non.inflammatory.Macrophages	PARD6G	0.329952	1.981117	0.743639	0.459067	-5.48702	0.760788	0.711482
Non.inflammatory.Macrophages	RAMP1	0.141642	4.507186	0.743322	0.459258	-5.8495	0.652869	0.663347
Non.inflammatory.Macrophages	BCAS3	-0.07137	7.334935	-0.74331	0.459266	-6.85877	0.549213	0.613129
Non.inflammatory.Macrophages	ACVR1B	-0.12684	2.543644	-0.74319	0.459335	-6.05602	0.735462	0.700601
Non.inflammatory.Macrophages	GM15543	-0.25049	0.901403	-0.74317	0.459346	-5.49962	0.810804	0.73333
Non.inflammatory.Macrophages	BRPF1	0.085813	4.929241	0.742968	0.459471	-6.48695	0.636379	0.655665
Non.inflammatory.Macrophages	CALML4	0.230219	1.801954	0.742739	0.459609	-5.78366	0.769337	0.715327
Non.inflammatory.Macrophages	COA6	-0.09672	4.110702	-0.7425	0.45975	-6.29433	0.669038	0.670856

Non.inflammatory.Macrophages	TRIM21	0.169287	2.57157	0.742388	0.45982	-5.99862	0.734469	0.700211
Non.inflammatory.Macrophages	GBP6	0.243378	1.984096	0.742186	0.459942	-5.97501	0.760968	0.711764
Non.inflammatory.Macrophages	NDFIP2	-0.06689	5.951714	-0.74218	0.459946	-6.62322	0.597974	0.637358
Non.inflammatory.Macrophages	ZSCAN2	-0.34236	1.067334	-0.7421	0.459993	-5.39632	0.804065	0.730133
Non.inflammatory.Macrophages	TMCC1	0.091181	7.946033	0.741988	0.460061	-6.95	0.529179	0.602938
Non.inflammatory.Macrophages	ZSWIM4	-0.08598	4.579223	-0.74198	0.460069	-6.51269	0.650229	0.662183
Non.inflammatory.Macrophages	RSL24D1	0.072251	5.674542	0.741823	0.460161	-6.56115	0.608193	0.642322
Non.inflammatory.Macrophages	DNASE2A	-0.08154	4.427932	-0.74172	0.460221	-6.52444	0.656248	0.665047
Non.inflammatory.Macrophages	SEC13	-0.07176	5.361757	-0.74171	0.46023	-6.5504	0.619925	0.647983
Non.inflammatory.Macrophages	SNAPC4	-0.22904	1.856185	-0.74122	0.460522	-5.66176	0.767238	0.71458
Non.inflammatory.Macrophages	ARHGAP33	-0.41093	0.549492	-0.7408	0.460775	-5.21874	0.821694	0.741223
Non.inflammatory.Macrophages	PTGR1	0.173911	3.377249	0.740707	0.460834	-5.95425	0.699984	0.685215
Non.inflammatory.Macrophages	HAUS7	-0.14505	2.992661	-0.74069	0.460841	-5.88503	0.716486	0.692586
Non.inflammatory.Macrophages	CALCOCO1	0.122795	3.863697	0.740688	0.460845	-6.14815	0.679618	0.676001
Non.inflammatory.Macrophages	ATG12	0.083148	4.873947	0.740345	0.461052	-6.47018	0.639289	0.65739

Non.inflammatory.Macrophages	TMX2	0.120027	3.46498	0.739984	0.46127	-6.13275	0.696622	0.683855
Non.inflammatory.Macrophages	CLK2	-0.07946	4.63296	-0.73996	0.461283	-6.33945	0.648871	0.661981
Non.inflammatory.Macrophages	GPN3	0.100161	3.982026	0.739898	0.461322	-6.1799	0.67509	0.674109
Non.inflammatory.Macrophages	OGA	0.066818	6.76343	0.739731	0.461423	-6.73559	0.56971	0.623922
Non.inflammatory.Macrophages	CATSPERE2	-0.22267	1.174644	-0.73914	0.461777	-5.53899	0.800432	0.72905
Non.inflammatory.Macrophages	CAMK2A	0.260956	0.846737	0.73882	0.461973	-5.35791	0.814558	0.735859
Non.inflammatory.Macrophages	TUBA4A	0.116953	4.513714	0.738722	0.462032	-6.26234	0.654263	0.664549
Non.inflammatory.Macrophages	GM15886	0.259192	1.881502	0.73834	0.462263	-5.3371	0.767438	0.715116
Non.inflammatory.Macrophages	NGP	0.22436	5.022684	0.738327	0.462271	-6.37186	0.634338	0.655266
Non.inflammatory.Macrophages	TMEM51	-0.08642	3.130512	-0.7383	0.462289	-6.61255	0.711676	0.690701
Non.inflammatory.Macrophages	LNPK	0.119425	4.386002	0.738031	0.46245	-6.18366	0.659432	0.667045
Non.inflammatory.Macrophages	SLC52A2	0.170577	2.391257	0.737988	0.462476	-5.82699	0.744212	0.705116
Non.inflammatory.Macrophages	FPR1	0.17011	2.992339	0.737966	0.46249	-6.54014	0.717656	0.693425
Non.inflammatory.Macrophages	LAMTOR3	-0.06977	5.240813	-0.73794	0.462504	-6.56831	0.62595	0.651359
Non.inflammatory.Macrophages	VPS4A	-0.08789	4.241466	-0.73774	0.462625	-6.30917	0.665258	0.669745

Non.inflammatory.Macrophages	SMG9	0.094719	4.52517	0.737643	0.462684	-6.3071	0.653868	0.664476
Non.inflammatory.Macrophages	XPR1	-0.07226	6.866995	-0.73748	0.462786	-6.82786	0.566683	0.622559
Non.inflammatory.Macrophages	MPPE1	-0.10032	4.363196	-0.73747	0.46279	-6.32299	0.660348	0.667503
Non.inflammatory.Macrophages	TFPI	-0.12512	3.468648	-0.73744	0.462807	-6.13678	0.697238	0.684332
Non.inflammatory.Macrophages	GSK3A	-0.07092	5.557032	-0.73729	0.462895	-6.55849	0.61401	0.645684
Non.inflammatory.Macrophages	FOXRED1	-0.15751	2.831724	-0.7372	0.462952	-5.98087	0.724702	0.696603
Non.inflammatory.Macrophages	GSTP2	-0.2041	0.861498	-0.73647	0.463394	-5.71307	0.814834	0.73608
Non.inflammatory.Macrophages	RASAL3	0.180937	4.10056	0.736312	0.46349	-5.67212	0.671527	0.672708
Non.inflammatory.Macrophages	LIG1	-0.13959	5.051661	-0.73627	0.463517	-6.38038	0.633729	0.655131
Non.inflammatory.Macrophages	RAP1GAP	-0.29484	0.58551	-0.73622	0.463547	-5.46844	0.822929	0.741798
Non.inflammatory.Macrophages	CASTOR1	-0.26427	0.626653	-0.73595	0.463711	-5.55738	0.822143	0.741031
Non.inflammatory.Macrophages	ZFP3	0.300968	0.562138	0.735946	0.463711	-5.3937	0.823496	0.74236
Non.inflammatory.Macrophages	RSBN1L	0.067541	7.198782	0.735777	0.463814	-6.73652	0.555794	0.617217
Non.inflammatory.Macrophages	PLXNA1	-0.22371	0.899006	-0.73561	0.463916	-5.77466	0.81377	0.73547
Non.inflammatory.Macrophages	UNC119B	-0.15062	3.551645	-0.73554	0.46396	-5.9194	0.694385	0.683174

Non.inflammatory.Macrophages	HMGXB4	0.100176	4.148587	0.735513	0.463974	-6.22879	0.669654	0.671917
Non.inflammatory.Macrophages	DEPDC1A	-0.32262	3.001084	-0.73542	0.464033	-5.65681	0.717947	0.693723
Non.inflammatory.Macrophages	TADA2B	-0.13262	3.468615	-0.7352	0.464163	-6.03544	0.697997	0.684835
Non.inflammatory.Macrophages	MON1A	0.112611	3.876268	0.734934	0.464325	-6.13427	0.681082	0.67712
Non.inflammatory.Macrophages	RNASEL	0.117054	3.944163	0.734798	0.464407	-6.44801	0.678308	0.675841
Non.inflammatory.Macrophages	CD8A	-0.57711	1.360615	-0.73439	0.464657	-5.17	0.793017	0.726242
Non.inflammatory.Macrophages	CENPU	-0.1884	2.562499	-0.73438	0.464663	-5.73292	0.737638	0.702376
Non.inflammatory.Macrophages	TBC1D19	-0.17923	1.749793	-0.73424	0.464748	-5.67868	0.774683	0.718444
Non.inflammatory.Macrophages	SUPT5	-0.06647	6.05341	-0.7342	0.464772	-6.68056	0.596502	0.637356
Non.inflammatory.Macrophages	5830411N(	0.447465	-1.25694	0.734042	0.464866	-5.07422	0.825481	0.781025
Non.inflammatory.Macrophages	SESN2	-0.1297	3.371941	-0.73386	0.464974	-6.11176	0.702369	0.686817
Non.inflammatory.Macrophages	ZKSCAN8	0.257932	0.632724	0.733803	0.46501	-5.48609	0.822482	0.741165
Non.inflammatory.Macrophages	MAF	-0.11206	5.654779	-0.73367	0.465093	-6.74935	0.611219	0.644538
Non.inflammatory.Macrophages	BRCA1	-0.1884	4.143927	-0.73366	0.465095	-6.05411	0.670201	0.672225
Non.inflammatory.Macrophages	ALKBH4	0.176507	2.574916	0.73357	0.465152	-5.7481	0.737085	0.702233

Non.inflammatory.Macrophages	PLEKHA2	0.080341	7.455833	0.733503	0.465192	-6.74306	0.547392	0.613038
Non.inflammatory.Macrophages	CASP8	0.064254	5.513709	0.733264	0.465337	-6.65386	0.61651	0.647151
Non.inflammatory.Macrophages	TTLL1	0.226864	1.626512	0.733261	0.465339	-5.56387	0.780448	0.721074
Non.inflammatory.Macrophages	CAP2	0.4313	0.071585	0.733254	0.465343	-5.21738	0.825481	0.752899
Non.inflammatory.Macrophages	MSL1	0.079931	5.419802	0.733197	0.465378	-6.53995	0.620057	0.648845
Non.inflammatory.Macrophages	RBM33	0.070917	5.898903	0.73289	0.465564	-6.52264	0.6023	0.640256
Non.inflammatory.Macrophages	TMCO1	0.056073	6.265444	0.732701	0.465679	-6.72908	0.588945	0.633772
Non.inflammatory.Macrophages	ERI2	0.266874	1.310882	0.732658	0.465705	-5.50784	0.795565	0.727455
Non.inflammatory.Macrophages	TASOR	0.07832	5.742445	0.732624	0.465726	-6.6098	0.608089	0.643074
Non.inflammatory.Macrophages	CDC14A	0.119256	5.803629	0.732377	0.465875	-6.42663	0.605934	0.641984
Non.inflammatory.Macrophages	TPD52L2	0.063322	5.433659	0.732202	0.465982	-6.55881	0.619801	0.648684
Non.inflammatory.Macrophages	WDFY1	0.094027	4.332443	0.732162	0.466006	-6.39609	0.662845	0.668879
Non.inflammatory.Macrophages	DLGAP4	-0.08597	5.015503	-0.73156	0.466372	-6.55735	0.636239	0.656391
Non.inflammatory.Macrophages	LAMP1	0.051193	7.757865	0.730464	0.467038	-7.13212	0.538622	0.608329
Non.inflammatory.Macrophages	SLC2A8	-0.14839	2.226604	-0.73036	0.467102	-5.89848	0.754548	0.709604

Non.inflammatory.Macrophages	IL10RA	-0.08587	4.971561	-0.73012	0.467248	-6.62103	0.638826	0.657505
Non.inflammatory.Macrophages	GPC5	0.423129	0.598275	0.72985	0.467411	-5.30989	0.825236	0.74255
Non.inflammatory.Macrophages	GEMIN6	-0.17433	2.59595	-0.72984	0.467415	-5.8307	0.737965	0.702444
Non.inflammatory.Macrophages	FH1	-0.10777	5.484899	-0.72983	0.467426	-6.46277	0.61912	0.648175
Non.inflammatory.Macrophages	GBA	-0.0861	4.090254	-0.72978	0.467456	-6.41186	0.674051	0.673868
Non.inflammatory.Macrophages	ORMDL2	0.076461	5.109617	0.729697	0.467504	-6.52048	0.633469	0.655049
Non.inflammatory.Macrophages	CDC42EP2	0.130892	3.08028	0.729207	0.467802	-6.33084	0.71702	0.693223
Non.inflammatory.Macrophages	CDK5R1	-0.25073	2.111423	-0.72908	0.467883	-5.53574	0.76028	0.712163
Non.inflammatory.Macrophages	PIEZO1	-0.07772	5.046187	-0.72887	0.46801	-6.60367	0.636323	0.656435
Non.inflammatory.Macrophages	PRRC2A	-0.06772	5.517966	-0.72872	0.468099	-6.56019	0.618258	0.647933
Non.inflammatory.Macrophages	GM46560	-0.48693	-0.84515	-0.72864	0.468145	-5.02382	0.828036	0.773331
Non.inflammatory.Macrophages	HEATR3	0.093711	4.273336	0.728549	0.468203	-6.38544	0.667003	0.67079
Non.inflammatory.Macrophages	SRI	0.069737	6.74315	0.728384	0.468303	-6.75348	0.573608	0.626225
Non.inflammatory.Macrophages	F730043M	0.327891	0.260543	0.728344	0.468328	-5.44342	0.828036	0.749979
Non.inflammatory.Macrophages	TNFRSF26	0.187712	2.617995	0.728312	0.468347	-5.93283	0.737444	0.70242

Non.inflammatory.Macrophages	CHN2	0.134859	5.358074	0.727601	0.46878	-6.67181	0.624771	0.650962
Non.inflammatory.Macrophages	SNAPC1	-0.0963	3.850567	-0.72756	0.468805	-6.31229	0.68486	0.67886
Non.inflammatory.Macrophages	HAP1	-0.26096	-0.45019	-0.7274	0.468902	-5.51181	0.828691	0.765142
Non.inflammatory.Macrophages	NEFH	-0.46839	-0.09246	-0.72713	0.46907	-5.14637	0.828801	0.757599
Non.inflammatory.Macrophages	PFDN2	-0.06915	5.031244	-0.72704	0.469124	-6.54754	0.637492	0.656999
Non.inflammatory.Macrophages	MYH10	-0.15957	2.573809	-0.727	0.469148	-5.91544	0.740099	0.703503
Non.inflammatory.Macrophages	ZFP276	-0.16308	2.479933	-0.7266	0.469391	-5.7962	0.744596	0.705421
Non.inflammatory.Macrophages	TTI2	0.144914	2.749363	0.726337	0.46955	-5.84473	0.732639	0.700237
Non.inflammatory.Macrophages	NCF2	-0.07235	6.576577	-0.72623	0.469616	-6.91664	0.580305	0.629513
Non.inflammatory.Macrophages	FBXL4	0.140682	2.64655	0.726226	0.469618	-5.99198	0.737207	0.702283
Non.inflammatory.Macrophages	COG7	-0.12321	2.604364	-0.72587	0.469835	-6.04885	0.739208	0.703197
Non.inflammatory.Macrophages	PKNOX1	-0.10365	4.569323	-0.72569	0.469948	-6.32302	0.656118	0.665783
Non.inflammatory.Macrophages	2310009A(	0.085623	4.692253	0.725658	0.469965	-6.43451	0.651222	0.663509
Non.inflammatory.Macrophages	ARL5B	-0.09139	5.460574	-0.72565	0.469967	-6.6261	0.621401	0.649467
Non.inflammatory.Macrophages	TMEM8	-0.14532	2.000346	-0.72558	0.47001	-5.96656	0.766645	0.715114

Non.inflammatory.Macrophages	ZFP827	0.174406	2.85843	0.725428	0.470105	-5.96345	0.727939	0.69825
Non.inflammatory.Macrophages	CSTA2	0.477941	1.96893	0.725399	0.470123	-5.47457	0.768098	0.715739
Non.inflammatory.Macrophages	HRAS	0.07656	4.842094	0.725212	0.470237	-6.46227	0.645374	0.660747
Non.inflammatory.Macrophages	FGFR1OP	0.112472	4.601111	0.724974	0.470382	-6.22298	0.654974	0.665224
Non.inflammatory.Macrophages	RET	-0.44309	0.727544	-0.72492	0.470416	-5.13286	0.823545	0.740901
Non.inflammatory.Macrophages	ICK	-0.18313	2.147459	-0.72485	0.470458	-5.78118	0.760024	0.712236
Non.inflammatory.Macrophages	4930435F1	0.309017	0.358699	0.724624	0.470596	-5.50269	0.829627	0.748607
Non.inflammatory.Macrophages	RPIA	-0.09481	5.458024	-0.72446	0.470695	-6.36838	0.621767	0.649638
Non.inflammatory.Macrophages	CEP131	-0.28317	1.3043	-0.72382	0.471087	-5.58328	0.800323	0.729413
Non.inflammatory.Macrophages	KLRC3	0.373764	-1.20661	0.723697	0.471162	-5.08513	0.8303	0.782056
Non.inflammatory.Macrophages	MRPL1	0.074315	4.619868	0.723526	0.471267	-6.37593	0.65492	0.665229
Non.inflammatory.Macrophages	CD160	-0.44336	1.916487	-0.72336	0.47137	-5.37787	0.771492	0.717259
Non.inflammatory.Macrophages	IGF2R	-0.09059	4.985937	-0.72327	0.471423	-6.53426	0.640467	0.658584
Non.inflammatory.Macrophages	SPATA7	0.216872	1.309288	0.723135	0.471506	-5.61005	0.800177	0.729542
Non.inflammatory.Macrophages	PLEKHG3	-0.10187	4.378559	-0.72313	0.471509	-6.40634	0.664615	0.669841

Non.inflammatory.Macrophages	ARMCX4	0.312591	1.19399	0.722121	0.472126	-5.41932	0.806661	0.732272
Non.inflammatory.Macrophages	TRMT6	0.090631	4.373913	0.722038	0.472176	-6.31259	0.66557	0.670314
Non.inflammatory.Macrophages	ATP5D	0.053168	7.898894	0.721719	0.472372	-6.97919	0.536563	0.607709
Non.inflammatory.Macrophages	DMD	-0.2419	2.419953	-0.7216	0.472443	-5.67414	0.749397	0.70784
Non.inflammatory.Macrophages	6430590A(	-0.21739	1.647577	-0.72158	0.472459	-5.49254	0.785098	0.723205
Non.inflammatory.Macrophages	ANGPTL4	-0.22725	1.23193	-0.72152	0.472491	-5.66765	0.804945	0.731646
Non.inflammatory.Macrophages	IL11RA1	-0.13323	2.113905	-0.72136	0.472593	-5.97332	0.763427	0.713952
Non.inflammatory.Macrophages	ZFP251	0.179291	2.407099	0.721236	0.472667	-5.78643	0.750063	0.70823
Non.inflammatory.Macrophages	CD59A	0.150289	2.984691	0.720787	0.472942	-6.01577	0.72461	0.697165
Non.inflammatory.Macrophages	PLD2	-0.17325	1.042444	-0.72073	0.472976	-5.88363	0.814555	0.735865
Non.inflammatory.Macrophages	SREK1IP1	0.096498	4.172778	0.720585	0.473066	-6.34172	0.67422	0.674565
Non.inflammatory.Macrophages	AARS	-0.08794	5.020707	-0.72051	0.473109	-6.44917	0.64028	0.65885
Non.inflammatory.Macrophages	REEP5	-0.05296	7.566136	-0.72027	0.473257	-6.9456	0.548022	0.613817
Non.inflammatory.Macrophages	ZFP637	-0.16504	2.315939	-0.71998	0.473436	-5.87426	0.754839	0.71046
Non.inflammatory.Macrophages	PRRG2	-0.13491	2.322172	-0.71924	0.473888	-5.97821	0.754745	0.710555

Non.inflammatory.Macrophages	EXOSC3	0.080861	5.047755	0.719209	0.473909	-6.43184	0.63966	0.658664
Non.inflammatory.Macrophages	ROBO1	-0.30624	2.137687	-0.7192	0.473914	-5.56124	0.763191	0.714221
Non.inflammatory.Macrophages	5930403N2	-0.32587	-0.48768	-0.71903	0.474016	-5.38967	0.832458	0.768289
Non.inflammatory.Macrophages	LSM3	-0.09089	5.624923	-0.71902	0.474026	-6.53742	0.617511	0.648201
Non.inflammatory.Macrophages	SIRT5	-0.20296	1.958147	-0.71882	0.474148	-5.68264	0.771493	0.717915
Non.inflammatory.Macrophages	PIGZ	-0.229	0.017052	-0.71873	0.4742	-5.71883	0.832458	0.757724
Non.inflammatory.Macrophages	SESN1	0.079256	5.939971	0.718693	0.474225	-6.60627	0.605732	0.642661
Non.inflammatory.Macrophages	ATG14	-0.10439	3.27883	-0.71867	0.474239	-6.12141	0.712315	0.692075
Non.inflammatory.Macrophages	CDK14	-0.11744	5.455475	-0.71858	0.474297	-6.92109	0.623937	0.651421
Non.inflammatory.Macrophages	PIGF	0.118218	3.388301	0.718504	0.474341	-6.1878	0.707603	0.690027
Non.inflammatory.Macrophages	KCTD12	-0.06699	6.383969	-0.7184	0.474408	-6.86579	0.589497	0.634819
Non.inflammatory.Macrophages	SFT2D2	-0.09261	4.677531	-0.71839	0.474413	-6.41208	0.654263	0.665706
Non.inflammatory.Macrophages	MRPL11	0.086761	4.26095	0.718382	0.474416	-6.38641	0.671071	0.67347
Non.inflammatory.Macrophages	WBP1	-0.13172	3.813733	-0.71804	0.474623	-6.11443	0.689711	0.681991
Non.inflammatory.Macrophages	UIMC1	0.067935	5.853468	0.718019	0.474638	-6.58441	0.609072	0.644345

Non.inflammatory.Macrophages	FKTN	-0.2192	1.595478	-0.71787	0.474729	-5.66085	0.788727	0.725466
Non.inflammatory.Macrophages	MCTS1	0.058309	6.207891	0.717679	0.474847	-6.73178	0.596117	0.638084
Non.inflammatory.Macrophages	SEC22A	0.115799	3.176208	0.717254	0.475108	-6.08769	0.717242	0.694332
Non.inflammatory.Macrophages	PTP4A3	-0.08042	5.77385	-0.71725	0.475112	-6.5173	0.612329	0.645899
Non.inflammatory.Macrophages	ADGRE4	-0.10442	3.108019	-0.71707	0.475222	-6.69775	0.720211	0.69565
Non.inflammatory.Macrophages	POLDIP3	-0.06238	6.078814	-0.71706	0.475229	-6.65223	0.601015	0.640438
Non.inflammatory.Macrophages	COA7	-0.1016	3.404389	-0.71689	0.475332	-6.12142	0.707401	0.689985
Non.inflammatory.Macrophages	AP4S1	0.073807	4.920297	0.716845	0.475359	-6.45983	0.645097	0.661478
Non.inflammatory.Macrophages	RHEBL1	-0.23799	1.532009	-0.71651	0.475564	-5.60774	0.792166	0.726947
Non.inflammatory.Macrophages	ANP32E	-0.08575	7.305672	-0.71647	0.475592	-6.72666	0.557575	0.619053
Non.inflammatory.Macrophages	4931406G(	0.389431	0.347467	0.716369	0.475651	-5.40506	0.833129	0.751297
Non.inflammatory.Macrophages	HSP90AA1	-0.0644	7.657631	-0.71635	0.47566	-6.93111	0.545665	0.613036
Non.inflammatory.Macrophages	LRRC57	0.173045	2.732576	0.715778	0.476014	-5.94899	0.737311	0.703364
Non.inflammatory.Macrophages	EIF2B5	0.090144	4.703803	0.71551	0.476179	-6.36833	0.654287	0.665902
Non.inflammatory.Macrophages	PDF	0.270276	0.794298	0.715352	0.476277	-5.45366	0.825792	0.742454

Non.inflammatory.Macrophages	LARP1	0.060827	6.798018	0.715111	0.476424	-6.79168	0.575761	0.628293
Non.inflammatory.Macrophages	TMEM216	-0.16715	4.069162	-0.71503	0.476477	-5.90421	0.680155	0.677884
Non.inflammatory.Macrophages	SRP68	0.076264	3.983827	0.714851	0.476584	-6.2418	0.683693	0.679562
Non.inflammatory.Macrophages	SAMD12	-0.41935	0.266619	-0.71481	0.476608	-5.31258	0.833942	0.753552
Non.inflammatory.Macrophages	ENY2	0.068099	5.972518	0.71471	0.476671	-6.66001	0.605605	0.642984
Non.inflammatory.Macrophages	FADS1	-0.13699	3.059286	-0.7147	0.476679	-6.0543	0.723139	0.697296
Non.inflammatory.Macrophages	PRR16	-0.30642	1.720127	-0.71428	0.476936	-5.55972	0.784243	0.723855
Non.inflammatory.Macrophages	ZFP354C	0.345879	1.777029	0.714277	0.476938	-5.33011	0.781563	0.722711
Non.inflammatory.Macrophages	CSRP1	-0.08416	5.881401	-0.71411	0.477043	-6.66129	0.609173	0.644761
Non.inflammatory.Macrophages	MARS2	0.172049	2.690445	0.714062	0.47707	-5.67343	0.739682	0.704652
Non.inflammatory.Macrophages	WDR75	-0.10807	3.575364	-0.71392	0.477158	-6.17756	0.701096	0.687521
Non.inflammatory.Macrophages	QTRT2	-0.28095	1.344368	-0.71373	0.477273	-5.54726	0.802189	0.731598
Non.inflammatory.Macrophages	WAS	0.064743	5.349391	0.713661	0.477316	-6.57485	0.629317	0.654467
Non.inflammatory.Macrophages	1700030K0	-0.2426	1.730931	-0.71364	0.477328	-5.61822	0.783768	0.723794
Non.inflammatory.Macrophages	IGF2BP1	-0.31818	-0.96106	-0.71345	0.477446	-5.37682	0.834313	0.780022

Non.inflammatory.Macrophages	DYNLT1B	0.265638	1.188553	0.713007	0.477719	-5.55313	0.810178	0.735004
Non.inflammatory.Macrophages	BOP1	-0.09779	4.167475	-0.71291	0.477781	-6.24459	0.676702	0.676592
Non.inflammatory.Macrophages	POLR2G	0.063725	5.51542	0.712656	0.477935	-6.62362	0.623438	0.651726
Non.inflammatory.Macrophages	SETD1A	-0.08167	4.19796	-0.71253	0.478012	-6.32394	0.675593	0.676073
Non.inflammatory.Macrophages	CMC2	-0.13026	4.535117	-0.71244	0.478069	-6.37795	0.66187	0.669757
Non.inflammatory.Macrophages	IRF3	-0.1032	3.786081	-0.71207	0.478294	-6.21776	0.692845	0.683868
Non.inflammatory.Macrophages	GGA3	-0.10016	3.302395	-0.71205	0.478306	-6.14701	0.713481	0.693138
Non.inflammatory.Macrophages	RASA4	0.106043	4.228858	0.711999	0.47834	-6.6079	0.674445	0.675501
Non.inflammatory.Macrophages	NUDCD1	-0.14192	3.226429	-0.71176	0.478484	-6.01403	0.716898	0.694696
Non.inflammatory.Macrophages	B3GALT6	-0.23287	1.806345	-0.71159	0.478593	-5.67562	0.781178	0.722692
Non.inflammatory.Macrophages	GTF2H5	0.059538	6.547345	0.711293	0.478775	-6.75209	0.585726	0.633411
Non.inflammatory.Macrophages	NRROS	-0.05813	7.067702	-0.71113	0.478873	-6.96163	0.567369	0.624355
Non.inflammatory.Macrophages	PNP	-0.07982	5.924728	-0.71106	0.478918	-6.75462	0.6085	0.644542
Non.inflammatory.Macrophages	HAO1	-0.19571	3.070076	-0.7109	0.47902	-5.96448	0.724064	0.697929
Non.inflammatory.Macrophages	RAP1A	0.038038	8.772339	0.710579	0.479215	-7.10987	0.511186	0.595623

Non.inflammatory.Macrophages	GATC	-0.15271	2.698485	-0.71041	0.479322	-6.04412	0.740729	0.705403
Non.inflammatory.Macrophages	GM8066	-0.33432	0.498967	-0.71038	0.479336	-5.28527	0.835591	0.749874
Non.inflammatory.Macrophages	NSUN2	0.078645	5.0541	0.710113	0.479503	-6.48351	0.641987	0.660702
Non.inflammatory.Macrophages	ARPP19	0.056113	6.840626	0.709958	0.479598	-6.766	0.575546	0.628677
Non.inflammatory.Macrophages	FBL	-0.07188	6.091074	-0.70994	0.47961	-6.68136	0.602576	0.641939
Non.inflammatory.Macrophages	BCAT1	0.494859	0.852062	0.709866	0.479655	-5.18017	0.825872	0.742712
Non.inflammatory.Macrophages	SPTLC1	0.085754	4.232218	0.709856	0.479661	-6.35688	0.674952	0.676073
Non.inflammatory.Macrophages	KANK2	-0.18001	2.38565	-0.70952	0.479872	-5.84803	0.755067	0.711831
Non.inflammatory.Macrophages	IFI209	-0.0992	5.809713	-0.70939	0.479952	-6.68132	0.613181	0.647128
Non.inflammatory.Macrophages	SNRPA1	0.096568	5.077996	0.709367	0.479963	-6.45928	0.641205	0.660446
Non.inflammatory.Macrophages	PDCD4	0.07478	7.282332	0.709259	0.48003	-6.76854	0.560304	0.62117
Non.inflammatory.Macrophages	CHTOP	-0.08127	5.225925	-0.70917	0.480085	-6.49207	0.635443	0.657813
Non.inflammatory.Macrophages	DPF1	-0.36278	0.292792	-0.70866	0.480401	-5.37287	0.836389	0.754666
Non.inflammatory.Macrophages	EID3	-0.22442	0.925233	-0.70838	0.480574	-5.79001	0.824137	0.741621
Non.inflammatory.Macrophages	ZMIZ1	0.056816	6.978265	0.708353	0.48059	-6.8698	0.571107	0.626668

Non.inflammatory.Macrophages	ITPK1	0.07207	5.403224	0.708345	0.480594	-6.54988	0.628887	0.654759
Non.inflammatory.Macrophages	TSPAN4	-0.10403	3.356981	-0.70832	0.480612	-6.24107	0.712297	0.693142
Non.inflammatory.Macrophages	GNASAS1	0.391591	0.682179	0.708099	0.480746	-5.20696	0.831952	0.746767
Non.inflammatory.Macrophages	SLC36A1	-0.09542	2.851189	-0.70801	0.480802	-6.20537	0.734556	0.703073
Non.inflammatory.Macrophages	WDR46	-0.12354	3.429733	-0.70772	0.480978	-6.1506	0.709427	0.691949
Non.inflammatory.Macrophages	PDCD6	0.07069	6.13963	0.707394	0.481182	-6.71098	0.601527	0.641805
Non.inflammatory.Macrophages	OTUD7B	-0.08522	5.17042	-0.70731	0.481233	-6.44417	0.63823	0.659389
Non.inflammatory.Macrophages	FAM92A	-0.13969	3.301539	-0.70724	0.48128	-6.01392	0.715077	0.694636
Non.inflammatory.Macrophages	DUSP11	-0.05545	6.33182	-0.70699	0.481434	-6.69515	0.594493	0.638505
Non.inflammatory.Macrophages	PLIN3	0.099559	3.527331	0.706981	0.481438	-6.26027	0.705352	0.690351
Non.inflammatory.Macrophages	IPO4	-0.12395	2.384797	-0.70693	0.481471	-5.95674	0.755853	0.712646
Non.inflammatory.Macrophages	ABCB8	-0.12795	2.240197	-0.7069	0.481487	-5.83612	0.762477	0.715518
Non.inflammatory.Macrophages	SIPA1L3	0.086258	5.201146	0.70664	0.481648	-6.52716	0.637082	0.659
Non.inflammatory.Macrophages	TSLP	-0.27758	-0.44209	-0.70656	0.481699	-5.52847	0.8369	0.770959
Non.inflammatory.Macrophages	PCNX4	0.166614	2.141019	0.70648	0.481748	-5.81879	0.767109	0.717584

Non.inflammatory.Macrophages	ZFP54	-0.34414	0.794625	-0.70642	0.481787	-5.31175	0.828768	0.744943
Non.inflammatory.Macrophages	CCDC97	0.110059	3.33761	0.706272	0.481876	-6.0959	0.713569	0.694092
Non.inflammatory.Macrophages	FAM151B	-0.19872	1.753415	-0.70617	0.481941	-5.80937	0.785228	0.725358
Non.inflammatory.Macrophages	GM41496	0.272089	0.985043	0.706142	0.481956	-5.57975	0.822301	0.741013
Non.inflammatory.Macrophages	EHBP1	-0.14092	3.52414	-0.70584	0.482145	-6.18809	0.705728	0.690548
Non.inflammatory.Macrophages	PP2D1	-0.30641	1.690903	-0.70518	0.482554	-5.5066	0.788961	0.726871
Non.inflammatory.Macrophages	FGR	0.126716	4.732993	0.704735	0.482827	-6.64548	0.656192	0.66792
Non.inflammatory.Macrophages	FKBP11	-0.18113	1.995525	-0.70473	0.482829	-5.68451	0.774641	0.720782
Non.inflammatory.Macrophages	CPLANE1	0.099572	4.78519	0.704726	0.482833	-6.43695	0.654107	0.66695
Non.inflammatory.Macrophages	D3ERTD75	-0.19718	1.61523	-0.70463	0.482894	-5.73033	0.792575	0.728442
Non.inflammatory.Macrophages	CYP2J5	-0.33552	1.06057	-0.70457	0.482929	-5.41518	0.819408	0.739757
Non.inflammatory.Macrophages	RSL1	-0.25554	1.039109	-0.70449	0.48298	-5.60319	0.820463	0.740198
Non.inflammatory.Macrophages	PARP12	0.102788	3.018486	0.704462	0.482997	-6.45335	0.728229	0.700563
Non.inflammatory.Macrophages	PTGS1	-0.10128	3.986721	-0.70422	0.483148	-6.51837	0.686812	0.681974
Non.inflammatory.Macrophages	TMEM120	0.095203	3.799392	0.703943	0.483318	-6.30604	0.69483	0.685651

Non.inflammatory.Macrophages	1500015A(	-0.18776	1.76661	-0.70373	0.483451	-5.63303	0.78576	0.725615
Non.inflammatory.Macrophages	DFFB	-0.16663	2.981393	-0.70362	0.483521	-5.83397	0.730209	0.701549
Non.inflammatory.Macrophages	4930417O:	0.30336	0.963159	0.703564	0.483553	-5.49632	0.824592	0.742039
Non.inflammatory.Macrophages	CLUAP1	-0.09803	3.885137	-0.7035	0.483595	-6.17492	0.691262	0.684138
Non.inflammatory.Macrophages	ERO1LB	-0.0782	6.652932	-0.70324	0.483753	-6.61555	0.58385	0.633442
Non.inflammatory.Macrophages	C1S1	-0.23884	1.787052	-0.70323	0.483758	-5.61196	0.784835	0.7253
Non.inflammatory.Macrophages	PDE11A	-0.45228	0.072949	-0.70311	0.483837	-5.24085	0.838176	0.760745
Non.inflammatory.Macrophages	SYS1	0.05682	6.417243	0.703056	0.483868	-6.73244	0.592338	0.637675
Non.inflammatory.Macrophages	7-Sep	0.044949	8.277792	0.702386	0.484284	-6.97587	0.528771	0.605631
Non.inflammatory.Macrophages	DDX31	0.11939	3.220713	0.702233	0.484379	-6.07691	0.720157	0.697229
Non.inflammatory.Macrophages	2610037D(	0.090035	5.44268	0.701963	0.484546	-6.43035	0.629079	0.655492
Non.inflammatory.Macrophages	ATP6V1C1	-0.06261	5.189889	-0.70185	0.484617	-6.60435	0.638863	0.660121
Non.inflammatory.Macrophages	PHF8	-0.07404	5.563796	-0.70182	0.484634	-6.53504	0.624442	0.653295
Non.inflammatory.Macrophages	FAM221B	0.304409	0.343919	0.70174	0.484685	-5.40575	0.838663	0.755394
Non.inflammatory.Macrophages	MRPL41	0.092398	4.003451	0.701542	0.484807	-6.30734	0.686744	0.682378

Non.inflammatory.Macrophages	NKIRAS1	0.120645	3.432264	0.701523	0.484819	-6.11269	0.71098	0.693312
Non.inflammatory.Macrophages	KLRA4	0.572227	-1.21319	0.701475	0.484849	-5.08456	0.838663	0.788808
Non.inflammatory.Macrophages	TSHZ1	-0.10029	5.060403	-0.70147	0.48485	-6.47388	0.643931	0.662593
Non.inflammatory.Macrophages	ISOC2A	-0.1721	2.013655	-0.70141	0.484888	-5.77907	0.774649	0.721217
Non.inflammatory.Macrophages	SELENOO	-0.13838	3.136064	-0.7014	0.484893	-5.95642	0.723859	0.69905
Non.inflammatory.Macrophages	JAML	-0.46794	2.748338	-0.70072	0.485319	-5.1838	0.741421	0.706744
Non.inflammatory.Macrophages	PJA1	0.098513	3.699202	0.700708	0.485325	-6.27631	0.699909	0.688291
Non.inflammatory.Macrophages	F2R	-0.16075	3.599158	-0.70062	0.485379	-5.96517	0.704173	0.69021
Non.inflammatory.Macrophages	MTRR	0.213562	1.505683	0.700481	0.485466	-5.68914	0.799095	0.731608
Non.inflammatory.Macrophages	TSHZ2	-0.21744	3.393266	-0.70042	0.485507	-5.99361	0.713024	0.694192
Non.inflammatory.Macrophages	SLAMF9	0.168615	2.637226	0.70041	0.48551	-6.10397	0.746418	0.708948
Non.inflammatory.Macrophages	PPP1R11	-0.08397	4.640461	-0.7001	0.485701	-6.48556	0.661009	0.670586
Non.inflammatory.Macrophages	GM20528	0.28714	-0.01428	0.700101	0.485703	-5.4578	0.839144	0.763243
Non.inflammatory.Macrophages	1810055G(	0.154623	2.268587	0.699961	0.485789	-5.81307	0.763271	0.716338
Non.inflammatory.Macrophages	H2-K1	-0.12856	8.786019	-0.6999	0.485829	-7.17545	0.512812	0.597502

Non.inflammatory.Macrophages	2900097C1	0.068386	4.909122	0.699803	0.485888	-6.54607	0.650273	0.665606
Non.inflammatory.Macrophages	ZFP513	-0.13466	2.534011	-0.69976	0.485915	-5.99954	0.751139	0.711116
Non.inflammatory.Macrophages	MRPL50	-0.08778	4.009842	-0.69959	0.486023	-6.28233	0.686924	0.682592
Non.inflammatory.Macrophages	CEP76	0.184289	2.990307	0.699456	0.486103	-5.73365	0.730752	0.702261
Non.inflammatory.Macrophages	TNFAIP8	0.065909	7.424897	0.6994	0.486138	-6.87277	0.557552	0.620709
Non.inflammatory.Macrophages	MICALL1	0.12075	2.97359	0.699121	0.486311	-6.01175	0.731659	0.702687
Non.inflammatory.Macrophages	BLZF1	0.117185	3.200504	0.6989	0.486449	-6.05043	0.721785	0.698314
Non.inflammatory.Macrophages	MAP3K4	-0.0914	4.074187	-0.69872	0.486562	-6.31729	0.684529	0.68159
Non.inflammatory.Macrophages	ERI1	0.086454	5.459943	0.698672	0.486591	-6.50826	0.629089	0.655804
Non.inflammatory.Macrophages	GM41790	-0.3991	-0.09939	-0.69816	0.486911	-5.29648	0.839939	0.765546
Non.inflammatory.Macrophages	GM26930	0.409165	0.305585	0.69787	0.48709	-5.2587	0.839939	0.75699
Non.inflammatory.Macrophages	ZC3HAV1	-0.06473	8.43854	-0.69783	0.487117	-6.99	0.524373	0.603699
Non.inflammatory.Macrophages	GM19696	-0.35474	0.016291	-0.69778	0.487144	-5.3376	0.839939	0.763093
Non.inflammatory.Macrophages	PLLP	-0.47761	-0.20457	-0.6977	0.487195	-5.10924	0.839939	0.76781
Non.inflammatory.Macrophages	TOLLIP	0.098563	3.832789	0.697691	0.487201	-6.31409	0.694959	0.686304

Non.inflammatory.Macrophages	EWSR1	-0.04888	7.421625	-0.69755	0.487289	-6.8752	0.558149	0.621077
Non.inflammatory.Macrophages	NR2C2AP	0.097643	4.44571	0.697522	0.487306	-6.39529	0.66953	0.674724
Non.inflammatory.Macrophages	MBP	-0.19646	5.172136	-0.69731	0.487439	-5.79608	0.640605	0.661265
Non.inflammatory.Macrophages	AW01173៖	0.139858	2.812185	0.69723	0.487488	-6.24889	0.739402	0.706183
Non.inflammatory.Macrophages	ZFP398	-0.12788	4.517006	-0.69705	0.487603	-6.16768	0.666742	0.673491
Non.inflammatory.Macrophages	LGALS3BP	0.113967	4.826846	0.696991	0.487637	-6.71936	0.654273	0.667721
Non.inflammatory.Macrophages	TCF20	-0.07112	7.561094	-0.69644	0.487978	-6.84577	0.553698	0.618907
Non.inflammatory.Macrophages	FGD6	-0.10551	4.002414	-0.6964	0.488005	-6.35499	0.68821	0.683362
Non.inflammatory.Macrophages	ASNS	0.276197	1.465562	0.696389	0.488012	-5.44515	0.802277	0.733325
Non.inflammatory.Macrophages	WDR47	-0.13174	3.140773	-0.69618	0.488144	-6.0184	0.725152	0.70004
Non.inflammatory.Macrophages	KHDC4	-0.0634	6.448578	-0.69614	0.488167	-6.7083	0.592772	0.63846
Non.inflammatory.Macrophages	TM9SF2	0.045332	6.683958	0.696008	0.48825	-6.78621	0.584289	0.63429
Non.inflammatory.Macrophages	GIMAP1	-0.18558	4.06141	-0.696	0.488257	-5.74256	0.685746	0.682332
Non.inflammatory.Macrophages	PSAT1	0.182572	4.218142	0.695426	0.488612	-5.97093	0.679648	0.679409
Non.inflammatory.Macrophages	LINS1	0.20226	1.704648	0.694498	0.489191	-5.5475	0.79214	0.728887

Non.inflammatory.Macrophages	APOE	0.124978	9.241961	0.693583	0.489762	-7.41725	0.500674	0.591275
Non.inflammatory.Macrophages	GM20234	0.178901	2.059362	0.693439	0.489852	-5.73837	0.776111	0.722118
Non.inflammatory.Macrophages	LEPROTL1	0.084927	5.362087	0.693416	0.489866	-6.62243	0.635122	0.658708
Non.inflammatory.Macrophages	EAF1	0.08997	4.101569	0.693383	0.489887	-6.40212	0.685834	0.682237
Non.inflammatory.Macrophages	BPTF	-0.06	7.356344	-0.69321	0.489996	-6.85538	0.562193	0.623207
Non.inflammatory.Macrophages	FLT3	0.337734	2.584668	0.69298	0.490138	-5.38348	0.752089	0.711822
Non.inflammatory.Macrophages	ANKZF1	-0.10797	2.742515	-0.69278	0.49026	-5.97397	0.745009	0.708815
Non.inflammatory.Macrophages	KLRA17	0.419199	0.224958	0.692716	0.490303	-5.11824	0.842851	0.76019
Non.inflammatory.Macrophages	GPIHBP1	0.145169	4.095185	0.692519	0.490427	-6.22359	0.686386	0.682737
Non.inflammatory.Macrophages	PTPRB	-0.22944	3.708559	-0.69246	0.490465	-6.05454	0.702703	0.690142
Non.inflammatory.Macrophages	GM4924	-0.30214	0.278075	-0.69205	0.49072	-5.38429	0.843146	0.759327
Non.inflammatory.Macrophages	PNPLA1	-0.3298	0.231599	-0.69176	0.490901	-5.39053	0.843146	0.760426
Non.inflammatory.Macrophages	ZFP930	0.176321	2.204594	0.691732	0.490918	-5.77841	0.769877	0.719861
Non.inflammatory.Macrophages	B430306N(	0.124635	2.692804	0.691723	0.490924	-6.20894	0.747513	0.710153
Non.inflammatory.Macrophages	EXOC8	-0.17233	1.951234	-0.69168	0.49095	-5.77916	0.781723	0.724956

Non.inflammatory.Macrophages	NUDT14	-0.07955	4.342372	-0.69164	0.490973	-6.37641	0.676327	0.678297
Non.inflammatory.Macrophages	SEMA6B	0.198281	1.154578	0.691314	0.491179	-5.7613	0.820115	0.741263
Non.inflammatory.Macrophages	CCDC124	0.060859	5.595782	0.69125	0.49122	-6.62618	0.626594	0.655126
Non.inflammatory.Macrophages	TBCCD1	-0.13758	2.589728	-0.69122	0.491235	-5.99905	0.752233	0.712295
Non.inflammatory.Macrophages	ZFP422	0.104893	4.395092	0.691195	0.491254	-6.22354	0.674204	0.677409
Non.inflammatory.Macrophages	CAVIN1	0.282968	1.312054	0.690996	0.491378	-5.59679	0.812423	0.738072
Non.inflammatory.Macrophages	ABCD2	0.121596	2.633823	0.690892	0.491443	-6.26544	0.750249	0.711441
Non.inflammatory.Macrophages	SKA3	-0.18651	2.738324	-0.69084	0.491479	-5.75985	0.745525	0.709376
Non.inflammatory.Macrophages	NPHS1	0.317241	0.439647	0.690777	0.491515	-5.39558	0.843221	0.756176
Non.inflammatory.Macrophages	CNTD1	-0.32994	0.620711	-0.69026	0.491837	-5.3295	0.840833	0.752613
Non.inflammatory.Macrophages	ODF2	0.065543	5.483537	0.690102	0.491937	-6.53653	0.631235	0.657411
Non.inflammatory.Macrophages	ARHGAP18	-0.08912	6.528672	-0.69009	0.491947	-6.8836	0.592146	0.638558
Non.inflammatory.Macrophages	PLD3	-0.09192	4.413779	-0.6897	0.492188	-6.53283	0.674033	0.67739
Non.inflammatory.Macrophages	MPP1	0.068597	6.203718	0.689326	0.492423	-6.83203	0.604436	0.644601
Non.inflammatory.Macrophages	TMEM147	0.073329	4.948794	0.689278	0.492453	-6.55734	0.652601	0.66752

Non.inflammatory.Macrophages	EMCN	-0.25672	1.650943	-0.689	0.492629	-5.55046	0.797115	0.731831
Non.inflammatory.Macrophages	SERTAD2	0.084492	6.12155	0.688909	0.492684	-6.70607	0.607598	0.646256
Non.inflammatory.Macrophages	ADO	0.09691	3.708631	0.688717	0.492804	-6.22573	0.703895	0.691267
Non.inflammatory.Macrophages	PRKRIP1	0.086597	3.964552	0.688671	0.492833	-6.26503	0.693036	0.686368
Non.inflammatory.Macrophages	KLHL18	0.0943	4.310869	0.68855	0.492909	-6.32506	0.678592	0.67984
Non.inflammatory.Macrophages	SRSF3	-0.0528	7.532017	-0.68853	0.492922	-6.87711	0.557292	0.62153
Non.inflammatory.Macrophages	HNRNPUL1	0.050087	7.28107	0.688293	0.49307	-6.82058	0.566003	0.625965
Non.inflammatory.Macrophages	PTPRO	-0.10637	2.363394	-0.68824	0.493104	-6.51331	0.763713	0.717786
Non.inflammatory.Macrophages	SLC25A23	-0.21279	2.25448	-0.68789	0.493324	-5.62248	0.768753	0.719964
Non.inflammatory.Macrophages	RBBP8	-0.06146	6.609426	-0.68788	0.49333	-6.80969	0.589792	0.637779
Non.inflammatory.Macrophages	AMBRA1	0.058722	7.654516	0.687854	0.493345	-6.90036	0.553187	0.619492
Non.inflammatory.Macrophages	BATF2	0.256832	0.948583	0.687776	0.493394	-5.74727	0.83134	0.746576
Non.inflammatory.Macrophages	WDR48	0.081491	4.298768	0.687737	0.493419	-6.3223	0.679175	0.680154
Non.inflammatory.Macrophages	AA386476	-0.21776	1.390731	-0.6874	0.493629	-5.66623	0.809922	0.737562
Non.inflammatory.Macrophages	ARAP1	-0.08184	5.049234	-0.68739	0.493638	-6.64313	0.648935	0.666184

Non.inflammatory.Macrophages	DMAC2	0.138492	3.093682	0.687261	0.493717	-5.9851	0.730861	0.703448
Non.inflammatory.Macrophages	MYBPC3	-0.15634	1.123244	-0.6872	0.493757	-5.98088	0.823026	0.743064
Non.inflammatory.Macrophages	GZMA	0.562947	4.671597	0.687026	0.493864	-5.91204	0.664109	0.673224
Non.inflammatory.Macrophages	RGS16	-0.36465	1.351563	-0.68692	0.49393	-5.36006	0.811908	0.738401
Non.inflammatory.Macrophages	DSTYK	0.085618	3.925134	0.686665	0.494091	-6.29296	0.695049	0.687464
Non.inflammatory.Macrophages	ULK1	-0.10096	3.637915	-0.68664	0.494107	-6.26012	0.70728	0.692992
Non.inflammatory.Macrophages	MTREX	0.064719	5.477446	0.685984	0.494518	-6.55131	0.632597	0.658512
Non.inflammatory.Macrophages	RFESD	-0.16881	2.541257	-0.68592	0.494561	-5.86902	0.75618	0.714584
Non.inflammatory.Macrophages	CTTN	-0.17487	1.717261	-0.68587	0.494593	-5.84585	0.794691	0.731146
Non.inflammatory.Macrophages	1700056E2	-0.18976	1.466557	-0.68579	0.494639	-5.78839	0.806759	0.736275
Non.inflammatory.Macrophages	SAP25	-0.17908	2.354647	-0.68564	0.494733	-5.90006	0.764749	0.718334
Non.inflammatory.Macrophages	LPIN1	0.12904	2.690777	0.685479	0.494835	-6.27435	0.749378	0.71169
Non.inflammatory.Macrophages	ARHGAP9	-0.07475	5.14536	-0.68531	0.494943	-6.62461	0.645553	0.664696
Non.inflammatory.Macrophages	CDC14B	-0.1226	3.454363	-0.68529	0.494951	-6.23245	0.715516	0.69673
Non.inflammatory.Macrophages	AI847159	-0.43845	-0.25702	-0.6852	0.495009	-5.12665	0.845146	0.772433

Non.inflammatory.Macrophages	SLC9A5	-0.25447	0.362304	-0.6852	0.495013	-5.52737	0.845146	0.759269
Non.inflammatory.Macrophages	CBX7	-0.13683	2.651462	-0.68515	0.495039	-5.86496	0.751161	0.712469
Non.inflammatory.Macrophages	GTF2E2	0.064098	5.440806	0.6851	0.495073	-6.6074	0.634014	0.659251
Non.inflammatory.Macrophages	EME2	-0.24362	0.924802	-0.68484	0.495239	-5.39262	0.83287	0.747546
Non.inflammatory.Macrophages	LYL1	0.14936	3.172684	0.684815	0.495252	-6.04781	0.727918	0.702255
Non.inflammatory.Macrophages	CHPT1	0.093066	3.607446	0.684557	0.495414	-6.28841	0.709121	0.693891
Non.inflammatory.Macrophages	EMC3	0.066231	5.490353	0.684368	0.495533	-6.54869	0.632367	0.658484
Non.inflammatory.Macrophages	SSRP1	0.07793	6.322976	0.684216	0.495629	-6.67079	0.601016	0.643418
Non.inflammatory.Macrophages	AGPAT1	0.105187	3.479015	0.684109	0.495696	-6.26082	0.714805	0.696455
Non.inflammatory.Macrophages	NRGN	-0.33115	3.663682	-0.68401	0.495759	-5.63754	0.706839	0.692907
Non.inflammatory.Macrophages	OSBPL10	-0.31638	0.973543	-0.6837	0.495955	-5.36098	0.831405	0.746852
Non.inflammatory.Macrophages	SEMA4G	-0.30169	0.909795	-0.68367	0.495973	-5.46576	0.833688	0.748176
Non.inflammatory.Macrophages	SMC1A	-0.07106	6.748393	-0.68355	0.496048	-6.75635	0.585576	0.636003
Non.inflammatory.Macrophages	ZFX	-0.05056	6.339182	-0.68339	0.496145	-6.73595	0.600436	0.643294
Non.inflammatory.Macrophages	CDT1	-0.08869	4.370678	-0.68331	0.496196	-6.34169	0.677115	0.679532

Non.inflammatory.Macrophages	HMGXB3	-0.08292	4.408539	-0.68325	0.496234	-6.39227	0.675556	0.678816
Non.inflammatory.Macrophages	MEAK7	-0.23403	0.666682	-0.68316	0.496294	-5.60773	0.841481	0.753263
Non.inflammatory.Macrophages	PPIA	0.064307	10.92524	0.683047	0.496363	-7.42027	0.453064	0.566241
Non.inflammatory.Macrophages	PRDX1	-0.057	9.719927	-0.68296	0.496418	-7.35928	0.487919	0.585533
Non.inflammatory.Macrophages	YTHDC2	-0.08338	4.693584	-0.68294	0.496432	-6.44152	0.663929	0.673451
Non.inflammatory.Macrophages	XYLB	-0.2474	0.922746	-0.6829	0.496454	-5.53964	0.833275	0.747924
Non.inflammatory.Macrophages	B130055M	0.215398	2.013314	0.682699	0.496582	-5.61479	0.78116	0.725595
Non.inflammatory.Macrophages	GM3550	-0.29078	1.752212	-0.68246	0.496729	-5.27248	0.793639	0.731015
Non.inflammatory.Macrophages	CYTH2	-0.10162	3.933028	-0.6824	0.49677	-6.26569	0.695561	0.687986
Non.inflammatory.Macrophages	VCPKMT	-0.16075	2.877826	-0.6822	0.496898	-5.90504	0.741558	0.708512
Non.inflammatory.Macrophages	ANKRD54	0.124229	3.089479	0.681961	0.497046	-5.93026	0.732118	0.704392
Non.inflammatory.Macrophages	DPP3	0.081488	4.728026	0.681934	0.497064	-6.45477	0.662739	0.67299
Non.inflammatory.Macrophages	EIF2AK2	0.094563	4.927436	0.681904	0.497082	-6.58148	0.654731	0.669275
Non.inflammatory.Macrophages	NBEAL2	-0.11766	2.635242	-0.68184	0.497123	-6.01871	0.752516	0.71336
Non.inflammatory.Macrophages	NICN1	-0.26838	0.820121	-0.68177	0.497168	-5.48421	0.836806	0.750294

Non.inflammatory.Macrophages	GM40645	0.130746	0.751306	0.681546	0.497308	-6.24083	0.839144	0.751834
Non.inflammatory.Macrophages	9330151L1	-0.24458	0.96571	-0.68104	0.497623	-5.48147	0.83239	0.747509
Non.inflammatory.Macrophages	OCIAD1	0.056201	5.885315	0.680849	0.497747	-6.67227	0.617784	0.651893
Non.inflammatory.Macrophages	4933411E0	-0.32885	0.555657	-0.68079	0.497783	-5.33573	0.844772	0.756074
Non.inflammatory.Macrophages	BEND5	0.39819	0.115495	0.680789	0.497784	-5.09481	0.846188	0.76537
Non.inflammatory.Macrophages	GTF3C4	0.145019	2.57436	0.680761	0.497802	-5.91792	0.755599	0.714821
Non.inflammatory.Macrophages	LSM1	0.068038	5.251939	0.680701	0.49784	-6.50004	0.642158	0.66349
Non.inflammatory.Macrophages	SLC25A47	-0.11812	3.963	-0.68065	0.497872	-6.29835	0.694611	0.687729
Non.inflammatory.Macrophages	POLR3D	-0.12089	3.27535	-0.68022	0.498143	-6.1164	0.72452	0.701097
Non.inflammatory.Macrophages	CDK12	0.059393	6.896517	0.679749	0.498439	-6.82768	0.581216	0.634007
Non.inflammatory.Macrophages	SYNPO	-0.30531	0.219102	-0.67953	0.498575	-5.43183	0.847064	0.763502
Non.inflammatory.Macrophages	5330417C2	-0.35066	0.541978	-0.67843	0.499269	-5.35273	0.846777	0.757081
Non.inflammatory.Macrophages	ARHGEF15	-0.35394	0.580885	-0.6782	0.499415	-5.36218	0.845937	0.756327
Non.inflammatory.Macrophages	TNFAIP8L1	0.175377	2.396525	0.678171	0.499434	-5.74342	0.765304	0.719109
Non.inflammatory.Macrophages	PPARD	-0.07071	5.613407	-0.67805	0.49951	-6.70288	0.629409	0.657526

Non.inflammatory.Macrophages	METTL18	0.276962	1.271701	0.678043	0.499515	-5.42658	0.818913	0.741951
Non.inflammatory.Macrophages	GM26749	0.249807	2.222722	0.678016	0.499532	-5.47406	0.773373	0.722593
Non.inflammatory.Macrophages	HAT1	-0.07287	6.105162	-0.67797	0.499563	-6.68287	0.610766	0.648585
Non.inflammatory.Macrophages	PAF1	0.071464	4.600235	0.677958	0.499569	-6.47209	0.669545	0.676337
Non.inflammatory.Macrophages	P2RY10	-0.34686	3.930309	-0.67767	0.499752	-5.49508	0.69757	0.689101
Non.inflammatory.Macrophages	LASP1	0.060652	5.455474	0.677548	0.499827	-6.69234	0.635681	0.660472
Non.inflammatory.Macrophages	IFT80	0.160621	3.626112	0.677243	0.50002	-6.01338	0.710727	0.695115
Non.inflammatory.Macrophages	SNRPF	0.066384	6.76677	0.677199	0.500048	-6.78661	0.586785	0.636946
Non.inflammatory.Macrophages	IDH1	-0.062	4.88393	-0.67695	0.500208	-6.75119	0.658366	0.671344
Non.inflammatory.Macrophages	CEP41	-0.19448	1.886422	-0.67667	0.50038	-5.67345	0.789563	0.729801
Non.inflammatory.Macrophages	HGF	-0.12634	2.743326	-0.67658	0.500439	-6.44856	0.749774	0.712616
Non.inflammatory.Macrophages	ZFP329	0.13839	2.511051	0.676345	0.500587	-5.83407	0.760373	0.717245
Non.inflammatory.Macrophages	CHCHD5	0.111857	3.092559	0.676342	0.500589	-6.20468	0.734095	0.705736
Non.inflammatory.Macrophages	GNG2	-0.06623	6.302982	-0.67631	0.500608	-6.78611	0.60369	0.645403
Non.inflammatory.Macrophages	PEX7	-0.07565	4.45855	-0.67612	0.500728	-6.38497	0.675653	0.67941

Non.inflammatory.Macrophages	CRACR2B	-0.22821	1.009115	-0.67608	0.500756	-5.724	0.832285	0.747825
Non.inflammatory.Macrophages	GM43707	-0.31373	0.407926	-0.67605	0.500775	-5.36928	0.848284	0.760416
Non.inflammatory.Macrophages	MIGA1	-0.14062	2.677211	-0.67604	0.500782	-5.80779	0.752777	0.713938
Non.inflammatory.Macrophages	KLHL21	0.132729	2.689731	0.675898	0.500869	-6.13156	0.752208	0.713718
Non.inflammatory.Macrophages	KCNMB4	-0.24696	1.763375	-0.67588	0.500883	-5.64847	0.795432	0.732342
Non.inflammatory.Macrophages	RACGAP1	-0.15948	5.205247	-0.67571	0.50099	-6.34314	0.645586	0.665495
Non.inflammatory.Macrophages	TIPRL	0.07022	5.525539	0.675667	0.501015	-6.52391	0.633083	0.659587
Non.inflammatory.Macrophages	PARD3	-0.24192	2.300134	-0.67566	0.501019	-5.72789	0.770117	0.721533
Non.inflammatory.Macrophages	CNST	0.107048	3.943862	0.675312	0.50124	-6.04304	0.697142	0.689278
Non.inflammatory.Macrophages	FIP1L1	0.045747	6.639254	0.675218	0.501299	-6.75356	0.591387	0.63945
Non.inflammatory.Macrophages	XK	0.311675	1.158214	0.675143	0.501347	-5.36004	0.82488	0.744804
Non.inflammatory.Macrophages	POLR3G	0.205112	2.101279	0.675068	0.501394	-5.70431	0.779408	0.725533
Non.inflammatory.Macrophages	TRP53INP2	0.093722	4.134264	0.675017	0.501426	-6.32387	0.689119	0.685635
Non.inflammatory.Macrophages	UBE2C	-0.17582	6.746366	-0.67499	0.501444	-6.73288	0.587519	0.637546
Non.inflammatory.Macrophages	UBE2D2A	0.034102	8.209532	0.674939	0.501476	-6.98629	0.537083	0.612102

Non.inflammatory.Macrophages	B230118H(	-0.1543	2.933488	-0.67492	0.501487	-5.92581	0.741199	0.708933
Non.inflammatory.Macrophages	FANCF	-0.22751	1.911025	-0.67471	0.501623	-5.64052	0.788424	0.729472
Non.inflammatory.Macrophages	ERG	-0.26559	3.921953	-0.67432	0.501868	-5.96975	0.698098	0.689918
Non.inflammatory.Macrophages	G3BP1	-0.05647	6.728766	-0.67417	0.50196	-6.88465	0.588175	0.638097
Non.inflammatory.Macrophages	CXCL9	-0.20593	2.080321	-0.67412	0.501997	-6.42011	0.780424	0.726229
Non.inflammatory.Macrophages	GM15441	0.231418	1.344167	0.674102	0.502005	-5.51704	0.815759	0.741243
Non.inflammatory.Macrophages	CD28	0.301644	2.935145	0.674028	0.502052	-5.54639	0.741153	0.709166
Non.inflammatory.Macrophages	A730036I1	-0.31373	-0.20546	-0.67403	0.502053	-5.49133	0.848316	0.773835
Non.inflammatory.Macrophages	CYP2D22	-0.33026	0.864397	-0.67399	0.502078	-5.32813	0.837835	0.751205
Non.inflammatory.Macrophages	BTLA	-0.29616	3.948652	-0.67394	0.50211	-5.65549	0.696966	0.689484
Non.inflammatory.Macrophages	RAB9	-0.0669	4.887523	-0.6739	0.502133	-6.51068	0.658247	0.671704
Non.inflammatory.Macrophages	SELENOF	0.069594	5.422799	0.67358	0.502335	-6.66215	0.637172	0.661914
Non.inflammatory.Macrophages	OSBPL11	0.078259	5.368512	0.673397	0.502451	-6.59885	0.639288	0.662973
Non.inflammatory.Macrophages	ARHGAP1C	-0.1086	5.105594	-0.67338	0.502461	-6.67935	0.64963	0.667844
Non.inflammatory.Macrophages	SRP54C	0.196793	1.755516	0.673348	0.502482	-5.70169	0.795938	0.73309

Non.inflammatory.Macrophages	GALK1	0.093777	5.140655	0.673221	0.502562	-6.45325	0.648242	0.667192
Non.inflammatory.Macrophages	COPS8	-0.06468	5.091019	-0.67321	0.502571	-6.49671	0.650208	0.668115
Non.inflammatory.Macrophages	METTL1	0.133847	3.431367	0.672972	0.50272	-6.11415	0.719331	0.699724
Non.inflammatory.Macrophages	GM20069	0.423037	-0.52038	0.672944	0.502738	-5.1736	0.848468	0.780943
Non.inflammatory.Macrophages	SMNDC1	0.053309	6.059801	0.672867	0.502787	-6.67368	0.612873	0.65037
Non.inflammatory.Macrophages	RUFY1	0.067513	5.607628	0.672511	0.503012	-6.56536	0.630186	0.658727
Non.inflammatory.Macrophages	NDUFB11	0.056335	7.55899	0.672502	0.503018	-6.91131	0.559203	0.623897
Non.inflammatory.Macrophages	BUD23	-0.1088	4.09892	-0.67233	0.503127	-6.25649	0.69096	0.687077
Non.inflammatory.Macrophages	GM17491	0.184324	1.415352	0.672092	0.503278	-5.55952	0.812795	0.740386
Non.inflammatory.Macrophages	NDUFB6	0.075289	6.317819	0.672011	0.503329	-6.77715	0.603547	0.645997
Non.inflammatory.Macrophages	CIAO1	0.088438	3.481337	0.671548	0.503622	-6.22078	0.717809	0.699173
Non.inflammatory.Macrophages	RGS14	0.137197	3.585563	0.671391	0.503722	-6.0629	0.713321	0.6972
Non.inflammatory.Macrophages	ATN1	0.122785	3.221914	0.671263	0.503803	-6.12006	0.729226	0.704311
Non.inflammatory.Macrophages	ADAP1	0.083737	4.152492	0.67121	0.503837	-6.5038	0.689169	0.686312
Non.inflammatory.Macrophages	HIST2H4	-0.34508	0.951972	-0.67104	0.503945	-5.37143	0.836033	0.750263

Non.inflammatory.Macrophages	MOB3A	0.08356	4.775701	0.670973	0.503987	-6.45152	0.66354	0.674578
Non.inflammatory.Macrophages	NAT8L	-0.23224	0.332665	-0.67069	0.504169	-5.60123	0.84953	0.763408
Non.inflammatory.Macrophages	GM10563	-0.16431	3.607105	-0.67041	0.504341	-5.86394	0.71261	0.697049
Non.inflammatory.Macrophages	IL18R1	0.418311	0.978465	0.670266	0.504435	-5.21554	0.835059	0.749922
Non.inflammatory.Macrophages	U2AF1	-0.05397	7.593832	-0.67027	0.504435	-6.85535	0.558603	0.623837
Non.inflammatory.Macrophages	AUP1	0.06071	6.010505	0.670203	0.504475	-6.73607	0.615508	0.651961
Non.inflammatory.Macrophages	OASL1	-0.17626	2.957835	-0.67017	0.504495	-6.42779	0.741213	0.709784
Non.inflammatory.Macrophages	LY86	0.076019	6.704851	0.670024	0.504589	-7.00885	0.589931	0.639493
Non.inflammatory.Macrophages	PPP6R1	0.057621	5.572544	0.669915	0.504658	-6.56399	0.632256	0.659994
Non.inflammatory.Macrophages	GM16150	-0.44427	0.174831	-0.66968	0.504804	-5.09083	0.84965	0.766943
Non.inflammatory.Macrophages	SIRT7	0.060855	4.918849	0.669665	0.504817	-6.50809	0.658024	0.672154
Non.inflammatory.Macrophages	2210408F2	0.118876	2.856305	0.669583	0.504868	-6.36287	0.745868	0.711906
Non.inflammatory.Macrophages	RASL11B	0.362532	0.665224	0.669227	0.505095	-5.23226	0.845813	0.756764
Non.inflammatory.Macrophages	WIZ	0.113001	3.836067	0.669161	0.505136	-6.2008	0.703051	0.6929
Non.inflammatory.Macrophages	DUSP22	-0.08032	4.486105	-0.66898	0.505249	-6.58101	0.675822	0.680553

Non.inflammatory.Macrophages	2610008E1	0.187106	2.466966	0.668824	0.50535	-5.75464	0.763872	0.719974
Non.inflammatory.Macrophages	NDUFAF6	-0.13036	2.973661	-0.66876	0.505388	-5.96312	0.740826	0.709928
Non.inflammatory.Macrophages	MDC1	0.116539	3.159139	0.668736	0.505406	-6.08689	0.732552	0.706279
Non.inflammatory.Macrophages	FAR1	-0.0781	6.64871	-0.66844	0.505597	-6.69406	0.592335	0.640923
Non.inflammatory.Macrophages	KLF11	0.091139	3.555665	0.668064	0.505833	-6.16336	0.715427	0.698576
Non.inflammatory.Macrophages	MSRB3	-0.20763	2.604277	-0.66797	0.505895	-5.77234	0.757856	0.717342
Non.inflammatory.Macrophages	GLT8D1	0.098265	3.339159	0.667836	0.505978	-6.20242	0.724884	0.702874
Non.inflammatory.Macrophages	MRPS12	0.073334	4.774775	0.667728	0.506046	-6.53409	0.664298	0.67535
Non.inflammatory.Macrophages	CHST12	0.10922	5.763881	0.667538	0.506167	-6.44097	0.625376	0.657068
Non.inflammatory.Macrophages	PDE4B	0.074669	8.527906	0.667489	0.506199	-7.11171	0.527905	0.60841
Non.inflammatory.Macrophages	OSBPL3	-0.23074	3.353205	-0.66743	0.506236	-5.63773	0.724267	0.702684
Non.inflammatory.Macrophages	SPATA24	-0.20698	2.193104	-0.66736	0.506281	-5.73603	0.776903	0.725753
Non.inflammatory.Macrophages	MINK1	-0.08269	4.171023	-0.66729	0.506324	-6.41924	0.689174	0.686908
Non.inflammatory.Macrophages	USF1	0.081081	4.934416	0.667166	0.506404	-6.52913	0.657864	0.672503
Non.inflammatory.Macrophages	4931406CC	-0.10627	3.540099	-0.66715	0.506417	-6.26263	0.716104	0.699117

Non.inflammatory.Macrophages	RPUSD4	-0.1324	2.682051	-0.66714	0.506419	-5.9897	0.754302	0.716008
Non.inflammatory.Macrophages	GM44148	-0.2538	1.90428	-0.66658	0.506774	-5.58914	0.79092	0.731815
Non.inflammatory.Macrophages	CASZ1	-0.16076	3.474262	-0.66656	0.506786	-5.84762	0.719312	0.700544
Non.inflammatory.Macrophages	CRNKL1	-0.07052	5.293781	-0.66633	0.506935	-6.49518	0.643929	0.665985
Non.inflammatory.Macrophages	ISG20L2	0.078477	5.083016	0.666249	0.506987	-6.47131	0.652266	0.669904
Non.inflammatory.Macrophages	TMUB1	0.089325	3.138278	0.666221	0.507004	-6.17958	0.734143	0.707177
Non.inflammatory.Macrophages	EXOSC7	-0.08456	4.629702	-0.66614	0.507058	-6.37477	0.670544	0.67845
Non.inflammatory.Macrophages	SPG20	-0.12359	2.615271	-0.66575	0.507302	-6.01208	0.757748	0.717695
Non.inflammatory.Macrophages	SIKE1	0.080348	4.570739	0.66552	0.50745	-6.36795	0.67296	0.67976
Non.inflammatory.Macrophages	PRRT1	0.297742	0.466797	0.665518	0.507452	-5.38882	0.850695	0.761941
Non.inflammatory.Macrophages	HEATR1	0.085013	4.84445	0.665489	0.50747	-6.54461	0.661828	0.6746
Non.inflammatory.Macrophages	FBXL22	0.200542	2.248825	0.665016	0.507771	-5.76402	0.7747	0.72524
Non.inflammatory.Macrophages	GM15787	0.182519	2.641968	0.665014	0.507772	-5.83541	0.756527	0.717353
Non.inflammatory.Macrophages	CRYZ	-0.20245	1.288432	-0.66485	0.507877	-5.66419	0.820787	0.744863
Non.inflammatory.Macrophages	CCDC115	0.070918	4.152653	0.664826	0.507892	-6.47818	0.690305	0.687823

Non.inflammatory.Macrophages	SUN1	0.090826	3.918531	0.664807	0.507905	-6.2295	0.7002	0.69232
Non.inflammatory.Macrophages	CCDC61	-0.135	2.934126	-0.66472	0.507962	-5.93127	0.743277	0.711547
Non.inflammatory.Macrophages	ZFP521	0.36404	2.100385	0.664712	0.507965	-5.42542	0.781666	0.72824
Non.inflammatory.Macrophages	LSM5	0.077183	5.652829	0.664681	0.507985	-6.5889	0.629964	0.659702
Non.inflammatory.Macrophages	CHTF8	0.217962	1.341638	0.664655	0.508001	-5.60282	0.81817	0.743778
Non.inflammatory.Macrophages	EIF3G	-0.07006	4.982954	-0.66451	0.508096	-6.56566	0.656262	0.672174
Non.inflammatory.Macrophages	MOB2	0.069099	5.416589	0.664445	0.508134	-6.50234	0.639121	0.664108
Non.inflammatory.Macrophages	ZFP763	-0.32161	0.433803	-0.66444	0.50814	-5.32038	0.850695	0.762833
Non.inflammatory.Macrophages	GM42836	-0.46458	-0.40469	-0.6644	0.508165	-5.10576	0.850695	0.7808
Non.inflammatory.Macrophages	PQLC2	-0.10148	3.590318	-0.66436	0.508192	-6.30809	0.714297	0.698765
Non.inflammatory.Macrophages	FXN	0.101196	4.085717	0.66418	0.508303	-6.15721	0.693176	0.68928
Non.inflammatory.Macrophages	KAT7	0.061238	5.68662	0.664086	0.508363	-6.59965	0.628715	0.659242
Non.inflammatory.Macrophages	MMGT1	0.129106	2.857236	0.663868	0.508503	-6.03748	0.746803	0.713293
Non.inflammatory.Macrophages	CCDC167	-0.10024	4.065969	-0.66386	0.508505	-6.28987	0.694009	0.6897
Non.inflammatory.Macrophages	URGCP	0.0881	4.434418	0.663798	0.508547	-6.44689	0.678624	0.682667

Non.inflammatory.Macrophages	DUSP3	0.056056	5.553941	0.663563	0.508697	-6.83729	0.633941	0.6618
Non.inflammatory.Macrophages	PRRC2C	0.047918	7.744202	0.663223	0.508913	-6.95798	0.554525	0.622701
Non.inflammatory.Macrophages	ACVR1	-0.11106	3.707524	-0.66304	0.509027	-6.35413	0.709675	0.696791
Non.inflammatory.Macrophages	POT1A	0.121172	3.012767	0.662853	0.509149	-6.04428	0.740208	0.710451
Non.inflammatory.Macrophages	ZFP609	0.062379	6.067152	0.662797	0.509185	-6.71448	0.614588	0.652557
Non.inflammatory.Macrophages	BMP1	-0.33035	0.911089	-0.66277	0.509201	-5.40204	0.839209	0.753218
Non.inflammatory.Macrophages	PDZD8	0.079249	5.970732	0.66242	0.509425	-6.59255	0.61842	0.654481
Non.inflammatory.Macrophages	HDAC3	-0.07481	4.707537	-0.66215	0.509598	-6.40176	0.668138	0.678001
Non.inflammatory.Macrophages	TTC21B	-0.16849	1.614885	-0.66206	0.509654	-5.73014	0.80577	0.738967
Non.inflammatory.Macrophages	UBE2T	-0.14503	4.06115	-0.66182	0.50981	-6.12241	0.695025	0.690405
Non.inflammatory.Macrophages	ZBTB49	0.207623	1.051258	0.661778	0.509835	-5.47376	0.833587	0.7507
Non.inflammatory.Macrophages	SYT11	-0.20213	1.412759	-0.66143	0.510053	-5.78845	0.815863	0.74333
Non.inflammatory.Macrophages	METTL4	0.128998	2.874732	0.661424	0.510061	-5.93762	0.747033	0.713718
Non.inflammatory.Macrophages	SLC35B1	-0.07208	5.751128	-0.66108	0.510277	-6.63541	0.627271	0.658909
Non.inflammatory.Macrophages	USP54	0.135977	2.915478	0.660989	0.510338	-5.93133	0.7454	0.713023

Non.inflammatory.Macrophages	BBS2	-0.26798	0.404044	-0.66091	0.510391	-5.45559	0.852163	0.764574
Non.inflammatory.Macrophages	GM42869	0.232308	1.342309	0.660488	0.510658	-5.48113	0.819829	0.745027
Non.inflammatory.Macrophages	CARD9	-0.16598	0.997223	-0.66038	0.510729	-5.95651	0.836972	0.752244
Non.inflammatory.Macrophages	DMWD	-0.15581	2.228423	-0.66034	0.510754	-5.98159	0.777258	0.726952
Non.inflammatory.Macrophages	GMNN	0.103869	5.558334	0.659827	0.51108	-6.6029	0.635154	0.66283
Non.inflammatory.Macrophages	INHBA	0.419735	1.050066	0.659817	0.511087	-5.46549	0.834632	0.751403
Non.inflammatory.Macrophages	ILF2	0.079908	5.763945	0.659533	0.511268	-6.48462	0.627223	0.659079
Non.inflammatory.Macrophages	PINK1	-0.08809	5.115722	-0.65942	0.511338	-6.524	0.652553	0.671095
Non.inflammatory.Macrophages	CASP7	0.098735	4.140272	0.659291	0.511422	-6.35005	0.692507	0.689582
Non.inflammatory.Macrophages	RNF145	0.078135	5.159727	0.65929	0.511423	-6.55575	0.650803	0.670287
Non.inflammatory.Macrophages	TYMS	-0.1436	4.922548	-0.6592	0.511483	-6.39943	0.660288	0.674751
Non.inflammatory.Macrophages	E2F6	-0.16156	2.266229	-0.65917	0.511498	-5.7789	0.775772	0.726527
Non.inflammatory.Macrophages	GCN1	0.068138	4.575371	0.658973	0.511626	-6.46131	0.674408	0.681316
Non.inflammatory.Macrophages	DMGDH	-0.24973	1.667706	-0.65892	0.511658	-5.62556	0.80425	0.738722
Non.inflammatory.Macrophages	SYDE1	-0.3361	0.308678	-0.65877	0.511753	-5.219	0.852766	0.767204

Non.inflammatory.Macrophages	PIGS	0.078229	4.078714	0.658637	0.51184	-6.37956	0.695104	0.690864
Non.inflammatory.Macrophages	RASGRP2	-0.06351	6.609517	-0.65862	0.511849	-6.78921	0.595595	0.643863
Non.inflammatory.Macrophages	NAAA	0.084376	3.389339	0.658562	0.511888	-6.59464	0.72482	0.704246
Non.inflammatory.Macrophages	SLC35B4	0.114088	2.825471	0.65848	0.511941	-6.14061	0.750001	0.715382
Non.inflammatory.Macrophages	D830025C(	-0.15986	2.231904	-0.65847	0.51195	-5.90094	0.77738	0.72729
Non.inflammatory.Macrophages	GM38134	0.313744	1.108316	0.657765	0.512398	-5.35357	0.832223	0.75059
Non.inflammatory.Macrophages	KRTCAP2	-0.05619	7.062528	-0.6577	0.512439	-6.8893	0.579638	0.635997
Non.inflammatory.Macrophages	HEXIM2	-0.28171	0.998422	-0.65769	0.512446	-5.45163	0.837722	0.752897
Non.inflammatory.Macrophages	GNPDA1	-0.07186	4.395098	-0.65754	0.512542	-6.57946	0.682305	0.685085
Non.inflammatory.Macrophages	GM16536	-0.16328	1.530823	-0.65736	0.512659	-5.76472	0.81152	0.741947
Non.inflammatory.Macrophages	PLPP1	0.11406	4.327383	0.657076	0.512839	-6.48558	0.685352	0.686489
Non.inflammatory.Macrophages	IMPA1	0.073231	5.285622	0.656659	0.513106	-6.47546	0.646685	0.668496
Non.inflammatory.Macrophages	ATP8B1	-0.30065	0.813793	-0.6566	0.513144	-5.41648	0.84499	0.757047
Non.inflammatory.Macrophages	BAG5	-0.11392	3.637068	-0.65644	0.513245	-6.23806	0.714963	0.699959
Non.inflammatory.Macrophages	SCAP	0.109552	4.277309	0.656338	0.513311	-6.26466	0.687677	0.687647

Non.inflammatory.Macrophages	LYSMD1	-0.20601	1.591133	-0.65631	0.513328	-5.53027	0.809043	0.741014
Non.inflammatory.Macrophages	GRPEL2	0.107101	3.176522	0.655921	0.513578	-6.09149	0.735411	0.709189
Non.inflammatory.Macrophages	GK5	0.112096	4.404631	0.655838	0.513631	-6.33006	0.682552	0.685409
Non.inflammatory.Macrophages	KIF1B	0.066857	5.385583	0.655799	0.513656	-6.72494	0.642924	0.66696
Non.inflammatory.Macrophages	GM13431	-0.19668	0.492651	-0.65557	0.513804	-5.75674	0.854223	0.764275
Non.inflammatory.Macrophages	HNRNPUL2	0.04976	6.468104	0.655389	0.513919	-6.78013	0.601893	0.647286
Non.inflammatory.Macrophages	NAPSA	0.163594	6.459379	0.655256	0.514004	-6.1252	0.602214	0.647456
Non.inflammatory.Macrophages	TMEM129	-0.18435	2.084833	-0.65512	0.514089	-5.79105	0.785762	0.731282
Non.inflammatory.Macrophages	PSPC1	-0.07886	5.702286	-0.65497	0.514189	-6.5641	0.630762	0.661317
Non.inflammatory.Macrophages	PHETA1	0.269334	0.450779	0.654663	0.514384	-5.48962	0.854352	0.765416
Non.inflammatory.Macrophages	GIMAP5	-0.23938	3.319876	-0.65464	0.514397	-5.63009	0.729233	0.706738
Non.inflammatory.Macrophages	GM21859	-0.18481	1.780794	-0.65464	0.5144	-6.12223	0.800283	0.737648
Non.inflammatory.Macrophages	SFR1	0.053943	6.44096	0.654511	0.514482	-6.78134	0.602894	0.647927
Non.inflammatory.Macrophages	FIZ1	-0.072	4.553694	-0.65449	0.514495	-6.37133	0.676555	0.682881
Non.inflammatory.Macrophages	SYNE1	-0.15656	4.964609	-0.65444	0.514527	-6.26045	0.659821	0.675113

Non.inflammatory.Macrophages	CDKL2	0.215378	1.163972	0.654416	0.514543	-5.68879	0.830493	0.750403
Non.inflammatory.Macrophages	PFN2	-0.30488	1.339141	-0.65397	0.514825	-5.41806	0.822161	0.746861
Non.inflammatory.Macrophages	OXSM	-0.13857	1.788374	-0.65329	0.515268	-5.90379	0.800848	0.737832
Non.inflammatory.Macrophages	SPRTN	0.121711	3.419835	0.653048	0.51542	-6.0698	0.725746	0.705156
Non.inflammatory.Macrophages	STAT2	0.117578	5.210285	0.652967	0.515472	-6.61769	0.65083	0.670898
Non.inflammatory.Macrophages	IRGM2	0.150687	2.731293	0.652905	0.515512	-6.20006	0.756647	0.718834
Non.inflammatory.Macrophages	TMTC2	0.195933	4.574445	0.652788	0.515587	-6.29459	0.676573	0.682913
Non.inflammatory.Macrophages	PAFAH1B1	0.040422	8.064884	0.652669	0.515663	-7.00769	0.54647	0.619707
Non.inflammatory.Macrophages	PLS1	0.276254	0.910051	0.652479	0.515785	-5.46395	0.84353	0.75625
Non.inflammatory.Macrophages	AP5S1	0.121424	2.440536	0.652221	0.515951	-6.10117	0.770335	0.724861
Non.inflammatory.Macrophages	AGO3	0.062727	5.940327	0.652034	0.516071	-6.73672	0.622695	0.657599
Non.inflammatory.Macrophages	ATRNL1	0.074018	6.820202	0.651914	0.516148	-6.88769	0.590042	0.641686
Non.inflammatory.Macrophages	PANK4	0.12314	3.423163	0.651884	0.516167	-6.03364	0.725899	0.70533
Non.inflammatory.Macrophages	PLCB4	0.172318	3.876337	0.651416	0.516468	-5.99164	0.706496	0.696639
Non.inflammatory.Macrophages	CPEB4	-0.06741	6.51399	-0.65135	0.516509	-6.81824	0.601464	0.647335

Non.inflammatory.Macrophages	KDM4C	0.06444	6.252217	0.651107	0.516666	-6.71602	0.611245	0.652072
Non.inflammatory.Macrophages	KDM4B	0.100998	4.441359	0.65107	0.51669	-6.3347	0.682701	0.685788
Non.inflammatory.Macrophages	KRR1	0.097896	3.94276	0.650705	0.516924	-6.2303	0.703958	0.695476
Non.inflammatory.Macrophages	PHF11A	-0.168	1.004104	-0.65051	0.517047	-6.01161	0.840672	0.754707
Non.inflammatory.Macrophages	5031425F1	-0.26363	-0.17235	-0.65046	0.517084	-5.54029	0.856575	0.779785
Non.inflammatory.Macrophages	LBP	0.110167	3.292366	0.650007	0.517373	-6.3055	0.732622	0.708392
Non.inflammatory.Macrophages	PER1	-0.08373	4.926726	-0.64986	0.517469	-6.54223	0.663314	0.676906
Non.inflammatory.Macrophages	E230016K2	0.234321	0.143576	0.64982	0.517493	-5.60141	0.856892	0.773205
Non.inflammatory.Macrophages	VPS13D	0.066754	6.149191	0.649764	0.517529	-6.72355	0.615582	0.654274
Non.inflammatory.Macrophages	4732440D(	0.250192	2.03703	0.649459	0.517726	-5.54499	0.790572	0.733638
Non.inflammatory.Macrophages	SCRG1	0.395564	-0.98095	0.649365	0.517786	-5.13803	0.857108	0.797763
Non.inflammatory.Macrophages	CENPE	-0.18207	4.9887	-0.6491	0.517958	-6.38868	0.661116	0.675851
Non.inflammatory.Macrophages	PEBP1	0.057351	6.440655	0.648511	0.518336	-6.78185	0.605331	0.64926
Non.inflammatory.Macrophages	ST8SIA1	0.482809	0.877835	0.648306	0.518468	-5.17788	0.846755	0.757972
Non.inflammatory.Macrophages	INPP5D	0.064864	8.764735	0.648186	0.518545	-7.11246	0.524892	0.608619

Non.inflammatory.Macrophages	MAPK3	0.071749	4.874121	0.647856	0.518757	-6.57678	0.666141	0.678284
Non.inflammatory.Macrophages	TUBGCP6	-0.1493	2.759296	-0.64786	0.518757	-5.91163	0.757444	0.719404
Non.inflammatory.Macrophages	SH3YL1	0.248312	1.030881	0.647787	0.518801	-5.42754	0.840514	0.754817
Non.inflammatory.Macrophages	SRMS	-0.31332	-0.72874	-0.64776	0.518818	-5.36615	0.857789	0.792596
Non.inflammatory.Macrophages	ARL6IP4	0.066162	5.733017	0.647676	0.518873	-6.62418	0.632112	0.662258
Non.inflammatory.Macrophages	CMIP	0.061092	9.635584	0.647615	0.518912	-7.26279	0.497536	0.594114
Non.inflammatory.Macrophages	CCDC59	-0.05419	5.676842	-0.64757	0.518941	-6.6252	0.634286	0.663326
Non.inflammatory.Macrophages	F630028O1	-0.14616	1.027185	-0.64732	0.519099	-6.05015	0.8407	0.755031
Non.inflammatory.Macrophages	ALG12	-0.19357	1.395336	-0.64728	0.519127	-5.74925	0.822338	0.747347
Non.inflammatory.Macrophages	NUDT21	0.063308	6.148811	0.64725	0.519147	-6.68098	0.616241	0.654754
Non.inflammatory.Macrophages	USP44	-0.40307	-0.6309	-0.64725	0.519148	-5.06848	0.857789	0.790593
Non.inflammatory.Macrophages	POFUT1	0.094103	3.458205	0.646782	0.519449	-6.21508	0.726142	0.705898
Non.inflammatory.Macrophages	GALNT10	-0.08332	4.614762	-0.64675	0.51947	-6.52081	0.67684	0.683545
Non.inflammatory.Macrophages	TFG	-0.05741	5.864765	-0.6467	0.519501	-6.68299	0.62712	0.660167
Non.inflammatory.Macrophages	ZFP677	0.239851	0.788621	0.646555	0.519595	-5.48832	0.849757	0.760329

Non.inflammatory.Macrophages	SPATA32	-0.31012	0.244996	-0.64655	0.519596	-5.44233	0.857898	0.771893
Non.inflammatory.Macrophages	2200002D(	0.315554	1.337585	0.646372	0.519713	-5.46903	0.825298	0.748866
Non.inflammatory.Macrophages	PAICS	-0.06744	6.310596	-0.64634	0.519731	-6.72257	0.610247	0.652089
Non.inflammatory.Macrophages	TCEAL8	-0.09099	3.331023	-0.64629	0.519763	-6.27711	0.731764	0.708493
Non.inflammatory.Macrophages	GLCE	-0.10765	3.79856	-0.64626	0.519784	-6.18173	0.711295	0.699352
Non.inflammatory.Macrophages	TSKU	-0.23146	-0.3765	-0.64563	0.520192	-5.64173	0.858465	0.785596
Non.inflammatory.Macrophages	NEURL1B	-0.26287	0.43588	-0.64523	0.520447	-5.40454	0.858712	0.768264
Non.inflammatory.Macrophages	KLHL23	-0.34702	0.171341	-0.6451	0.520533	-5.2757	0.858712	0.773967
Non.inflammatory.Macrophages	PEX5	-0.07921	4.071805	-0.64499	0.520601	-6.34379	0.700245	0.694473
Non.inflammatory.Macrophages	JCAD	0.220993	1.404554	0.644908	0.520657	-5.53817	0.822767	0.747961
Non.inflammatory.Macrophages	MBIP	0.103751	3.658726	0.644905	0.520659	-6.21473	0.718041	0.702508
Non.inflammatory.Macrophages	KTN1	0.059537	5.826158	0.644709	0.520785	-6.62168	0.629212	0.661413
Non.inflammatory.Macrophages	NKAP	0.065138	4.989943	0.644692	0.520796	-6.46827	0.662178	0.676992
Non.inflammatory.Macrophages	PAPSS1	0.074406	4.181725	0.64448	0.520933	-6.39442	0.69568	0.692498
Non.inflammatory.Macrophages	TMEM201	-0.17026	2.102835	-0.6444	0.520987	-5.75662	0.78903	0.733735

Non.inflammatory.Macrophages	NEPRO	0.132	2.709524	0.64415	0.521146	-5.99982	0.760793	0.721534
Non.inflammatory.Macrophages	PADI2	-0.36938	2.631853	-0.64377	0.521392	-5.29117	0.764564	0.723223
Non.inflammatory.Macrophages	LRCH1	0.063884	7.432191	0.64345	0.521598	-6.86212	0.570583	0.632857
Non.inflammatory.Macrophages	CDK5	0.09726	3.72259	0.64341	0.521624	-6.25697	0.715675	0.701705
Non.inflammatory.Macrophages	3-Mar	0.110519	6.392222	0.643306	0.521691	-6.75335	0.608132	0.651447
Non.inflammatory.Macrophages	GM48653	-0.25874	-0.16399	-0.64324	0.521733	-5.53115	0.859207	0.781788
Non.inflammatory.Macrophages	SLC25A38	0.106843	3.517886	0.643225	0.521743	-6.14959	0.724623	0.705743
Non.inflammatory.Macrophages	NUDT6	-0.16178	2.183509	-0.64296	0.521916	-5.8198	0.785541	0.732519
Non.inflammatory.Macrophages	CEBPA	-0.10695	2.906324	-0.64292	0.52194	-6.43987	0.751979	0.71796
Non.inflammatory.Macrophages	DNAJC14	0.063981	4.252973	0.642885	0.521962	-6.42219	0.69297	0.69156
Non.inflammatory.Macrophages	KMT2C	-0.05541	7.717211	-0.64278	0.522027	-6.93134	0.560692	0.62799
Non.inflammatory.Macrophages	FOPNL	0.085566	4.468642	0.64275	0.52205	-6.33189	0.683933	0.68743
Non.inflammatory.Macrophages	ADAMTS9	0.262663	2.970839	0.642617	0.522135	-5.96857	0.749048	0.716696
Non.inflammatory.Macrophages	MRPL19	0.08757	3.923908	0.642507	0.522207	-6.31087	0.706976	0.697954
Non.inflammatory.Macrophages	DNPH1	0.23006	0.708539	0.642414	0.522267	-5.56623	0.853668	0.763235

Non.inflammatory.Macrophages	ESS2	0.105689	2.988114	0.642401	0.522275	-6.04778	0.748265	0.716368
Non.inflammatory.Macrophages	CDCA4	-0.09651	4.453152	-0.64238	0.522292	-6.26616	0.684579	0.687748
Non.inflammatory.Macrophages	DMTN	-0.21487	0.637339	-0.64226	0.522368	-5.68556	0.856007	0.764797
Non.inflammatory.Macrophages	CTDSP2	-0.06451	4.801724	-0.64206	0.522494	-6.52549	0.670291	0.681263
Non.inflammatory.Macrophages	TDRP	0.411905	-0.36439	0.64186	0.522625	-5.25923	0.859341	0.78653
Non.inflammatory.Macrophages	TMTC4	0.214279	1.357794	0.641856	0.522627	-5.70131	0.825685	0.749804
Non.inflammatory.Macrophages	1300002E1	-0.12551	3.19234	-0.64173	0.522712	-6.04361	0.739198	0.712551
Non.inflammatory.Macrophages	GM26916	-0.26631	0.934105	-0.64164	0.522765	-5.3571	0.846483	0.758734
Non.inflammatory.Macrophages	CD83	0.106105	5.198788	0.641533	0.522836	-6.92473	0.654283	0.673886
Non.inflammatory.Macrophages	CHIL5	0.395689	-0.02332	0.64111	0.52311	-5.10101	0.859717	0.779344
Non.inflammatory.Macrophages	KHSRP	0.059312	5.998752	0.640723	0.52336	-6.66594	0.623463	0.659233
Non.inflammatory.Macrophages	GLO1	0.080571	5.029475	0.640557	0.523467	-6.56435	0.661492	0.677279
Non.inflammatory.Macrophages	ARL4C	-0.06677	5.243589	-0.64056	0.523468	-6.80602	0.652906	0.673254
Non.inflammatory.Macrophages	STK38	-0.06298	6.531625	-0.64022	0.523688	-6.74975	0.603458	0.649653
Non.inflammatory.Macrophages	TRIP4	0.101707	4.852518	0.640052	0.523794	-6.41241	0.668669	0.680777

Non.inflammatory.Macrophages	ZFP954	-0.16946	2.050779	-0.63981	0.523953	-5.85568	0.7925	0.736038
Non.inflammatory.Macrophages	TMEM191(	0.193469	1.703176	0.639568	0.524107	-5.49145	0.809261	0.743228
Non.inflammatory.Macrophages	CCSER1	0.308006	1.697174	0.639552	0.524117	-5.64668	0.809553	0.743352
Non.inflammatory.Macrophages	TMEM175	-0.10979	3.375009	-0.63953	0.524134	-6.20007	0.731529	0.70946
Non.inflammatory.Macrophages	CASP2	0.096228	4.01281	0.639522	0.524137	-6.15601	0.703742	0.696978
Non.inflammatory.Macrophages	ST14	-0.37373	1.167666	-0.63951	0.524146	-5.33315	0.835713	0.754372
Non.inflammatory.Macrophages	POMK	-0.18922	1.068205	-0.63945	0.524181	-5.77769	0.840711	0.75646
Non.inflammatory.Macrophages	GM50163	-0.36576	0.458993	-0.63922	0.524336	-5.20231	0.859911	0.76948
Non.inflammatory.Macrophages	ZC4H2	-0.27402	0.054562	-0.63916	0.52437	-5.37535	0.859911	0.778168
Non.inflammatory.Macrophages	METAP1D	-0.10629	3.768583	-0.63911	0.524404	-6.22468	0.714264	0.701834
Non.inflammatory.Macrophages	SDCCAG8	-0.0609	5.914953	-0.63907	0.524432	-6.72032	0.626666	0.661127
Non.inflammatory.Macrophages	ORC1	-0.14647	3.345968	-0.63904	0.524446	-6.02748	0.732818	0.710137
Non.inflammatory.Macrophages	ABRAXAS1	0.176993	2.604808	0.638996	0.524478	-5.77572	0.766442	0.724932
Non.inflammatory.Macrophages	MARK4	0.081157	4.541737	0.638972	0.524493	-6.46925	0.681453	0.686892
Non.inflammatory.Macrophages	STRN4	0.071586	4.492619	0.638962	0.5245	-6.45374	0.683495	0.687832

Non.inflammatory.Macrophages	PPT2	0.068913	4.199673	0.638836	0.524581	-6.6476	0.695791	0.693507
Non.inflammatory.Macrophages	SHC1	0.07468	4.384809	0.638767	0.524626	-6.3978	0.687996	0.689952
Non.inflammatory.Macrophages	MORF4L2	-0.05945	5.404884	-0.63846	0.524824	-6.65525	0.64665	0.670685
Non.inflammatory.Macrophages	CTNNA3	-0.39623	1.300067	-0.63829	0.524935	-5.4645	0.829284	0.751917
Non.inflammatory.Macrophages	GM14305	0.125957	2.260659	0.638098	0.525059	-5.91085	0.782708	0.732168
Non.inflammatory.Macrophages	AHCTF1	0.07182	5.405997	0.637988	0.52513	-6.55107	0.646606	0.670832
Non.inflammatory.Macrophages	SNX16	-0.11427	2.847666	-0.63789	0.525195	-6.09846	0.755437	0.720367
Non.inflammatory.Macrophages	FARP1	-0.14469	3.619271	-0.63788	0.525199	-6.0199	0.720927	0.705066
Non.inflammatory.Macrophages	ZBTB1	0.07645	5.505188	0.637798	0.525254	-6.5933	0.642701	0.66902
Non.inflammatory.Macrophages	GM49417	0.281341	1.078235	0.637738	0.525293	-5.62696	0.840391	0.756707
Non.inflammatory.Macrophages	GM43914	-0.13769	0.384486	-0.6377	0.525314	-6.0465	0.860101	0.771431
Non.inflammatory.Macrophages	KLRA9	-0.44344	-0.30711	-0.63752	0.525431	-5.18596	0.86019	0.78647
Non.inflammatory.Macrophages	BUB1B	-0.13489	4.016283	-0.63722	0.525628	-6.17444	0.704	0.697441
Non.inflammatory.Macrophages	TICAM1	0.113759	2.819811	0.637065	0.525729	-6.15234	0.757034	0.721052
Non.inflammatory.Macrophages	SRSF11	-0.03722	7.871425	-0.63691	0.525828	-6.96766	0.556262	0.626491

Non.inflammatory.Macrophages	5430405H(	0.111556	3.910079	0.636493	0.526099	-6.17691	0.708809	0.699672
Non.inflammatory.Macrophages	LDAH	0.076263	4.225642	0.636479	0.526109	-6.3895	0.695339	0.693553
Non.inflammatory.Macrophages	PRKX	0.061815	5.379346	0.636444	0.526132	-6.64683	0.648119	0.671641
Non.inflammatory.Macrophages	SLMAP	0.058791	6.393027	0.636266	0.526247	-6.77439	0.609175	0.653012
Non.inflammatory.Macrophages	POGLUT2	-0.20614	1.00166	-0.63624	0.526266	-5.68251	0.844867	0.758697
Non.inflammatory.Macrophages	AMACR	-0.18779	2.326832	-0.6361	0.526354	-5.8736	0.780188	0.731313
Non.inflammatory.Macrophages	SMARCD1	0.082611	4.031896	0.635945	0.526455	-6.23024	0.703663	0.69745
Non.inflammatory.Macrophages	COX6A2	0.395736	2.070766	0.635858	0.526512	-5.34637	0.792376	0.736561
Non.inflammatory.Macrophages	ENPP1	-0.15393	2.31349	-0.6356	0.526681	-6.08517	0.78102	0.731638
Non.inflammatory.Macrophages	CHRNA9	-0.18665	2.214161	-0.63518	0.52695	-5.95176	0.785926	0.73385
Non.inflammatory.Macrophages	UBAC2	0.061715	6.679645	0.635181	0.526951	-6.72762	0.598921	0.648083
Non.inflammatory.Macrophages	CENPQ	0.112556	5.314475	0.634915	0.527123	-6.29352	0.651093	0.673243
Non.inflammatory.Macrophages	SLC6A19	0.438003	-0.35949	0.634795	0.527202	-5.09429	0.861243	0.788315
Non.inflammatory.Macrophages	P2RY6	-0.08728	2.305897	-0.63471	0.527257	-6.53346	0.78161	0.732057
Non.inflammatory.Macrophages	ANXA10	-0.28569	0.643116	-0.6347	0.527264	-5.41993	0.857839	0.76668

Non.inflammatory.Macrophages	HCFC1R1	0.061497	5.33224	0.634668	0.527284	-6.69586	0.650387	0.672927
Non.inflammatory.Macrophages	TSEN54	-0.1278	2.874509	-0.63419	0.527595	-5.91004	0.755402	0.720692
Non.inflammatory.Macrophages	WDSUB1	-0.1087	2.44185	-0.63401	0.527714	-6.09137	0.775417	0.729459
Non.inflammatory.Macrophages	TTC3	0.072524	5.355192	0.633955	0.527747	-6.56738	0.649639	0.672649
Non.inflammatory.Macrophages	NRBP1	0.059541	5.827937	0.633808	0.527842	-6.65537	0.631143	0.663854
Non.inflammatory.Macrophages	ADAT2	-0.22332	1.285306	-0.63378	0.527862	-5.60031	0.831329	0.753294
Non.inflammatory.Macrophages	OTUD1	0.131638	3.007254	0.633742	0.527885	-5.9696	0.749357	0.718076
Non.inflammatory.Macrophages	GPS2	-0.05095	5.859455	-0.63368	0.527927	-6.63795	0.629928	0.663274
Non.inflammatory.Macrophages	BNIP2	0.047962	6.377564	0.633578	0.527992	-6.85409	0.610273	0.653787
Non.inflammatory.Macrophages	EPSTI1	0.090348	7.192656	0.633346	0.528143	-7.03154	0.580544	0.639171
Non.inflammatory.Macrophages	AKAP8L	0.083926	4.8709	0.633214	0.528229	-6.46652	0.669123	0.68185
Non.inflammatory.Macrophages	TMEM273	-0.13193	1.19098	-0.63307	0.528321	-6.06768	0.836047	0.755349
Non.inflammatory.Macrophages	GALNT12	0.201025	2.991393	0.632976	0.528383	-5.63996	0.750077	0.718493
Non.inflammatory.Macrophages	PPAN	0.101253	4.072585	0.63293	0.528413	-6.30352	0.702451	0.697198
Non.inflammatory.Macrophages	FAM120C	-0.15081	3.050639	-0.6329	0.528431	-5.93109	0.747392	0.71731

Non.inflammatory.Macrophages	DCTN4	0.051314	6.423452	0.632856	0.528461	-6.78347	0.608561	0.653035
Non.inflammatory.Macrophages	CLEC4A3	-0.09826	3.297848	-0.63273	0.528544	-6.8067	0.736281	0.712399
Non.inflammatory.Macrophages	AS3MT	-0.13328	2.69274	-0.63255	0.528662	-6.03543	0.763752	0.72452
Non.inflammatory.Macrophages	CCDC107	-0.07599	4.620705	-0.63253	0.528676	-6.44902	0.679404	0.686673
Non.inflammatory.Macrophages	COX19	-0.08587	4.496433	-0.63242	0.528742	-6.45395	0.684566	0.689082
Non.inflammatory.Macrophages	LTF	0.248261	2.942911	0.632304	0.52882	-5.91739	0.752282	0.71954
Non.inflammatory.Macrophages	TMEM241	0.098741	4.310016	0.632241	0.528861	-6.38373	0.692379	0.692697
Non.inflammatory.Macrophages	DDX52	0.066519	4.930462	0.632229	0.528869	-6.51537	0.666697	0.680836
Non.inflammatory.Macrophages	PSMB8	-0.08651	7.077604	-0.63219	0.528897	-6.94169	0.584654	0.641332
Non.inflammatory.Macrophages	CWC22	-0.09541	3.900011	-0.63202	0.529005	-6.25943	0.709859	0.70065
Non.inflammatory.Macrophages	INIP	-0.0785	4.523666	-0.63202	0.529007	-6.35336	0.683432	0.688593
Non.inflammatory.Macrophages	ZFP275	0.174436	1.581356	0.631827	0.529131	-5.69389	0.816769	0.747366
Non.inflammatory.Macrophages	DHX15	0.049349	6.794688	0.631496	0.529346	-6.81907	0.595074	0.646555
Non.inflammatory.Macrophages	NCAPD2	-0.13777	4.81058	-0.63143	0.529392	-6.31462	0.671807	0.683275
Non.inflammatory.Macrophages	PHOSPHO2	-0.08421	3.724911	-0.63136	0.529434	-6.22285	0.717683	0.704238

Non.inflammatory.Macrophages	ASTN2	-0.29863	1.045238	-0.63106	0.529626	-5.42227	0.843791	0.758895
Non.inflammatory.Macrophages	MSTO1	0.129087	2.681663	0.631028	0.52965	-5.99758	0.764632	0.725157
Non.inflammatory.Macrophages	MTO1	0.094588	3.553315	0.630939	0.529708	-6.19305	0.725311	0.707815
Non.inflammatory.Macrophages	NOTCH1	0.066701	5.292946	0.63046	0.53002	-6.55465	0.652679	0.674458
Non.inflammatory.Macrophages	EDA	-0.32217	1.445596	-0.63043	0.53004	-5.55083	0.824081	0.750677
Non.inflammatory.Macrophages	KLRA1	0.56316	0.700337	0.63024	0.530163	-5.16868	0.857014	0.766411
Non.inflammatory.Macrophages	RAB5IF	0.048537	7.307335	0.63009	0.530261	-6.99353	0.577078	0.637724
Non.inflammatory.Macrophages	PTP4A1	0.236549	1.179279	0.629876	0.5304	-5.63194	0.837627	0.756382
Non.inflammatory.Macrophages	GM43126	-0.4201	0.276018	-0.6295	0.530645	-5.14158	0.862776	0.775695
Non.inflammatory.Macrophages	CD8B1	0.473533	0.660348	0.629238	0.530816	-5.18948	0.858803	0.767494
Non.inflammatory.Macrophages	POR	-0.06595	5.6252	-0.6292	0.53084	-6.78093	0.639993	0.668489
Non.inflammatory.Macrophages	ALG6	0.16688	2.044219	0.629197	0.530843	-5.85788	0.795459	0.738539
Non.inflammatory.Macrophages	ING2	-0.0672	4.920364	-0.629	0.530974	-6.53274	0.668131	0.68178
Non.inflammatory.Macrophages	CABLES1	-0.1755	4.513404	-0.62891	0.531028	-6.30261	0.684908	0.689576
Non.inflammatory.Macrophages	CCDC71	0.122175	2.73695	0.628837	0.531077	-6.03187	0.762882	0.724523

Non.inflammatory.Macrophages	ELMOD2	0.112715	2.807903	0.628769	0.531122	-6.09937	0.759616	0.723094
Non.inflammatory.Macrophages	RIPK1	0.060515	5.973193	0.628667	0.531188	-6.78003	0.626522	0.662136
Non.inflammatory.Macrophages	MAF1	-0.07806	5.342723	-0.62862	0.531222	-6.58332	0.651132	0.673861
Non.inflammatory.Macrophages	GLE1	-0.08222	4.4454	-0.62837	0.531383	-6.28967	0.687808	0.690937
Non.inflammatory.Macrophages	PNRC2	-0.06474	5.35165	-0.6283	0.531429	-6.59607	0.650831	0.673724
Non.inflammatory.Macrophages	GAB3	-0.08276	4.746655	-0.62825	0.531457	-6.60906	0.675301	0.685175
Non.inflammatory.Macrophages	AU019990	-0.20628	1.211418	-0.62797	0.53164	-5.83032	0.836425	0.756102
Non.inflammatory.Macrophages	D330041H(	-0.18378	2.247493	-0.62785	0.531719	-5.65423	0.785884	0.734713
Non.inflammatory.Macrophages	DIP2B	0.054023	7.880088	0.627773	0.531771	-7.02946	0.557504	0.628137
Non.inflammatory.Macrophages	MACROD2	0.186106	2.251559	0.627747	0.531788	-5.85402	0.785691	0.734681
Non.inflammatory.Macrophages	PKP4	-0.07467	5.958306	-0.62771	0.531809	-6.88048	0.627184	0.662684
Non.inflammatory.Macrophages	TRAF3IP2	0.186309	3.229202	0.626961	0.5323	-5.64709	0.741189	0.715273
Non.inflammatory.Macrophages	KSR2	0.146241	2.695294	0.626823	0.532391	-6.27331	0.76557	0.725978
Non.inflammatory.Macrophages	DGKD	0.068629	7.687633	0.626529	0.532582	-6.78287	0.564746	0.631829
Non.inflammatory.Macrophages	ERLEC1	0.07722	4.341715	0.626398	0.532668	-6.47232	0.692992	0.693516

Non.inflammatory.Macrophages	RFX3	-0.06956	6.233922	-0.62628	0.532742	-6.62946	0.617409	0.65797
Non.inflammatory.Macrophages	SLC4A4	0.21505	2.065431	0.626126	0.532846	-5.74721	0.795535	0.738979
Non.inflammatory.Macrophages	MLX	0.09093	3.767938	0.625754	0.533088	-6.25789	0.717902	0.7049
Non.inflammatory.Macrophages	TAPBPL	0.095235	4.125165	0.625357	0.533348	-6.35421	0.702501	0.698051
Non.inflammatory.Macrophages	TENM3	-0.26747	1.688761	-0.62527	0.533405	-5.5624	0.814077	0.747015
Non.inflammatory.Macrophages	MAN2C1	-0.08888	3.509599	-0.62513	0.533499	-6.22732	0.729266	0.710124
Non.inflammatory.Macrophages	HP	0.122628	7.365035	0.624956	0.53361	-6.86589	0.57632	0.637856
Non.inflammatory.Macrophages	TM7SF3	0.088546	3.924878	0.624921	0.533633	-6.2925	0.711107	0.701964
Non.inflammatory.Macrophages	ZFP335	-0.12709	2.842169	-0.62488	0.533658	-5.93051	0.759359	0.723435
Non.inflammatory.Macrophages	EPB41L4B	-0.27007	1.976143	-0.62487	0.533666	-5.4898	0.800115	0.741071
Non.inflammatory.Macrophages	GM16316	-0.24389	1.803894	-0.62486	0.533671	-5.49537	0.808457	0.744628
Non.inflammatory.Macrophages	PEX11A	0.219281	0.072757	0.624853	0.533677	-5.65262	0.864279	0.781311
Non.inflammatory.Macrophages	ABCA9	-0.15189	0.787541	-0.62454	0.533883	-6.17699	0.856325	0.765986
Non.inflammatory.Macrophages	CEP135	0.084426	3.853066	0.624456	0.533936	-6.19628	0.714394	0.703409
Non.inflammatory.Macrophages	NOB1	0.081207	4.287446	0.624125	0.534153	-6.3104	0.695971	0.695121

Non.inflammatory.Macrophages	HAUS6	-0.09239	4.556972	-0.62392	0.534288	-6.37404	0.684736	0.690007
Non.inflammatory.Macrophages	PLCL1	0.096823	5.328139	0.623505	0.534558	-6.75791	0.653294	0.675441
Non.inflammatory.Macrophages	KDM2B	0.114424	7.133876	0.623413	0.534618	-6.77259	0.584951	0.642325
Non.inflammatory.Macrophages	HHEX	-0.08901	4.675113	-0.62334	0.534666	-6.46357	0.679841	0.687833
Non.inflammatory.Macrophages	YEATS4	0.057814	5.906755	0.623203	0.534756	-6.63667	0.630599	0.664721
Non.inflammatory.Macrophages	TFR2	-0.24507	1.213769	-0.62318	0.534768	-5.47812	0.838215	0.75744
Non.inflammatory.Macrophages	SLC9A3R2	-0.14715	2.701144	-0.62318	0.534772	-6.07878	0.766391	0.726757
Non.inflammatory.Macrophages	CDK20	0.217578	0.593617	0.623014	0.534879	-5.64503	0.862592	0.770698
Non.inflammatory.Macrophages	KCNB1	-0.28596	0.988195	-0.62296	0.534915	-5.45883	0.849627	0.762322
Non.inflammatory.Macrophages	GM49692	0.344761	0.104027	0.622873	0.534972	-5.2377	0.864875	0.78127
Non.inflammatory.Macrophages	CCAR2	-0.10574	3.432111	-0.6228	0.53502	-6.13718	0.733208	0.712238
Non.inflammatory.Macrophages	SULT2B1	-0.20304	2.700547	-0.62279	0.535025	-5.7065	0.766419	0.726883
Non.inflammatory.Macrophages	EEA1	0.056456	6.055281	0.622727	0.535067	-6.7931	0.624897	0.662083
Non.inflammatory.Macrophages	SURF4	0.062605	5.782118	0.622486	0.535224	-6.72436	0.63546	0.667176
Non.inflammatory.Macrophages	SRSF6	0.057998	6.210068	0.622354	0.535311	-6.76497	0.619043	0.659301

Non.inflammatory.Macrophages	POLR1D	0.046092	6.995878	0.622294	0.53535	-6.89489	0.589955	0.645044
Non.inflammatory.Macrophages	CDK16	0.086066	3.39772	0.622288	0.535354	-6.22387	0.734781	0.713001
Non.inflammatory.Macrophages	MPG	-0.08847	3.63806	-0.62206	0.535505	-6.22834	0.724229	0.708305
Non.inflammatory.Macrophages	CUL4B	-0.08553	4.524951	-0.622	0.535544	-6.43811	0.686208	0.691051
Non.inflammatory.Macrophages	GPATCH2L	-0.05952	5.064269	-0.62182	0.535659	-6.56136	0.664079	0.680783
Non.inflammatory.Macrophages	CCDC181	0.179127	1.514839	0.621568	0.535825	-5.70946	0.82358	0.751516
Non.inflammatory.Macrophages	SURF6	0.095489	3.185009	0.621241	0.536039	-6.07351	0.744825	0.717512
Non.inflammatory.Macrophages	UBP1	0.061711	5.448392	0.62108	0.536145	-6.60963	0.649045	0.673765
Non.inflammatory.Macrophages	FUZ	0.225708	1.08456	0.620954	0.536228	-5.61067	0.845451	0.760785
Non.inflammatory.Macrophages	CNIH1	0.067544	5.17624	0.620862	0.536288	-6.58711	0.659938	0.678937
Non.inflammatory.Macrophages	ZCCHC24	0.096379	3.666755	0.62049	0.536532	-6.34763	0.723515	0.708099
Non.inflammatory.Macrophages	UBE2O	0.093444	4.874388	0.620394	0.536595	-6.62894	0.672258	0.684693
Non.inflammatory.Macrophages	NECAB3	-0.22463	1.186088	-0.62034	0.536627	-5.54797	0.84039	0.758675
Non.inflammatory.Macrophages	INO80	-0.06374	6.666636	-0.62034	0.536632	-6.77754	0.602503	0.651367
Non.inflammatory.Macrophages	0610010F0	0.078045	4.458879	0.620305	0.536652	-6.55532	0.689495	0.692658

Non.inflammatory.Macrophages	KIF18B	-0.23886	2.84215	-0.62008	0.536802	-5.65295	0.760712	0.724576
Non.inflammatory.Macrophages	ERGIC2	0.047733	6.017059	0.619967	0.536874	-6.67621	0.627052	0.663331
Non.inflammatory.Macrophages	SLC19A2	0.192406	1.834884	0.619419	0.537234	-5.63718	0.808763	0.745407
Non.inflammatory.Macrophages	GALC	-0.07887	3.891293	-0.61933	0.537293	-6.55248	0.714161	0.703981
Non.inflammatory.Macrophages	ADCK2	0.117189	2.282482	0.619233	0.537355	-5.98996	0.787239	0.73624
Non.inflammatory.Macrophages	GM49463	-0.28397	-0.05016	-0.61921	0.537372	-5.46021	0.866221	0.785533
Non.inflammatory.Macrophages	SUV39H2	-0.18468	2.381659	-0.61898	0.537524	-5.6623	0.78267	0.734278
Non.inflammatory.Macrophages	TMEM143	-0.17652	1.491704	-0.61876	0.537667	-5.67747	0.825885	0.752724
Non.inflammatory.Macrophages	ZFP951	0.133501	2.641469	0.618447	0.537871	-6.07499	0.770628	0.729134
Non.inflammatory.Macrophages	2610203C2	-0.218	0.354453	-0.61844	0.537874	-5.6594	0.866525	0.776983
Non.inflammatory.Macrophages	CREB3	-0.07333	3.923009	-0.61842	0.537888	-6.37836	0.713036	0.703592
Non.inflammatory.Macrophages	DGUOK	-0.07791	4.460054	-0.61819	0.538039	-6.51334	0.690119	0.693252
Non.inflammatory.Macrophages	MLYCD	-0.09569	3.124459	-0.61815	0.538066	-6.19717	0.748434	0.719505
Non.inflammatory.Macrophages	UNC93B1	-0.04903	7.659349	-0.61812	0.538086	-7.08807	0.567479	0.634178
Non.inflammatory.Macrophages	UBE2E3	-0.04733	7.066891	-0.61804	0.538137	-6.85092	0.588479	0.644727

Non.inflammatory.Macrophages	TUBD1	-0.1713	2.095647	-0.6174	0.53856	-5.73556	0.79697	0.740591
Non.inflammatory.Macrophages	ZFP950	0.083758	4.197661	0.617221	0.538676	-6.36444	0.701763	0.698563
Non.inflammatory.Macrophages	DGCR2	0.07809	4.849233	0.616919	0.538874	-6.46179	0.674629	0.686049
Non.inflammatory.Macrophages	MSH6	-0.11083	4.38755	-0.61666	0.539045	-6.22792	0.693904	0.69503
Non.inflammatory.Macrophages	ESD	0.063557	7.081837	0.616601	0.539083	-6.94012	0.588561	0.644816
Non.inflammatory.Macrophages	PDCD6IP	0.037277	6.988379	0.616589	0.53909	-6.90819	0.591943	0.646496
Non.inflammatory.Macrophages	MYO7A	0.097593	3.25231	0.615988	0.539485	-6.23272	0.743841	0.717451
Non.inflammatory.Macrophages	RUSC2	0.161862	0.909936	0.615898	0.539544	-6.02371	0.855691	0.765752
Non.inflammatory.Macrophages	TRP53	-0.05852	5.681235	-0.61584	0.539583	-6.68884	0.641593	0.670581
Non.inflammatory.Macrophages	SNAP47	-0.11644	2.473639	-0.61575	0.539639	-6.02945	0.779721	0.733229
Non.inflammatory.Macrophages	KLF7	0.072603	6.159891	0.615581	0.539753	-6.85363	0.623086	0.661769
Non.inflammatory.Macrophages	NADK2	0.076374	4.994117	0.615565	0.539763	-6.51422	0.669083	0.6836
Non.inflammatory.Macrophages	BEND3	-0.13504	2.456588	-0.61537	0.539894	-6.03061	0.780571	0.733719
Non.inflammatory.Macrophages	DIAPH1	0.048834	7.355352	0.615213	0.539994	-6.95005	0.579116	0.640195
Non.inflammatory.Macrophages	PHIP	0.056832	7.276011	0.615183	0.540014	-6.86341	0.581941	0.641611

Non.inflammatory.Macrophages	UPF3A	0.062537	4.634592	0.615126	0.540052	-6.46169	0.683949	0.690586
Non.inflammatory.Macrophages	MALAT1	0.05572	14.72453	0.615019	0.540122	-7.87701	0.368232	0.521738
Non.inflammatory.Macrophages	NAALADL2	-0.1872	1.964286	-0.61462	0.540385	-5.70722	0.804399	0.743956
Non.inflammatory.Macrophages	IL1A	0.132136	2.787687	0.614502	0.540462	-6.42109	0.765416	0.727159
Non.inflammatory.Macrophages	NUAK2	-0.06199	4.289152	-0.61432	0.540583	-6.70281	0.698797	0.697462
Non.inflammatory.Macrophages	BFSP2	-0.31488	2.440103	-0.61424	0.540637	-5.40774	0.781694	0.734287
Non.inflammatory.Macrophages	YJU2	0.092168	3.454196	0.614046	0.540762	-6.13179	0.735161	0.71386
Non.inflammatory.Macrophages	AW209491	-0.21782	1.448036	-0.61402	0.54078	-5.6911	0.829823	0.754821
Non.inflammatory.Macrophages	GM13212	-0.09461	3.205264	-0.61395	0.540827	-6.32096	0.74634	0.718822
Non.inflammatory.Macrophages	POLR2I	0.058677	5.116859	0.613873	0.540875	-6.59791	0.664425	0.681575
Non.inflammatory.Macrophages	TYROBP	-0.06017	9.095091	-0.61349	0.541128	-7.42513	0.520671	0.610174
Non.inflammatory.Macrophages	CXCL3	0.303125	-0.07498	0.613481	0.541134	-5.6652	0.868341	0.787487
Non.inflammatory.Macrophages	SPHK2	0.09576	3.388566	0.613439	0.541161	-6.25163	0.738093	0.715214
Non.inflammatory.Macrophages	AXL	-0.1046	3.708037	-0.61342	0.541174	-6.69415	0.723922	0.708883
Non.inflammatory.Macrophages	ZFAT	-0.09968	3.828322	-0.61339	0.541191	-6.31135	0.718653	0.706514

Non.inflammatory.Macrophages	PRIMPOL	0.110976	3.486578	0.613143	0.541356	-6.13184	0.733718	0.713325
Non.inflammatory.Macrophages	SERPINI1	0.170132	3.178888	0.612839	0.541556	-5.82072	0.747533	0.719494
Non.inflammatory.Macrophages	IVNS1ABP	-0.06109	6.027762	-0.61283	0.54156	-6.71537	0.628459	0.664641
Non.inflammatory.Macrophages	SH3BP5	0.064569	6.380773	0.612784	0.541592	-6.89611	0.615028	0.658141
Non.inflammatory.Macrophages	HGS	-0.07689	4.094551	-0.61277	0.541601	-6.38731	0.707118	0.701392
Non.inflammatory.Macrophages	DDX11	-0.14454	2.934033	-0.61277	0.541604	-5.96175	0.758699	0.724413
Non.inflammatory.Macrophages	TAP1	-0.13923	5.446269	-0.61268	0.54166	-6.66424	0.651197	0.675519
Non.inflammatory.Macrophages	STOML1	-0.15633	2.251865	-0.61259	0.54172	-5.84965	0.790625	0.738326
Non.inflammatory.Macrophages	UFC1	0.045254	5.792898	0.612436	0.541822	-6.72463	0.63755	0.669035
Non.inflammatory.Macrophages	ZBTB4	-0.07858	3.655496	-0.61242	0.541831	-6.44052	0.726235	0.710051
Non.inflammatory.Macrophages	TEK	-0.23	2.175307	-0.61228	0.541924	-5.67147	0.794327	0.739952
Non.inflammatory.Macrophages	PPP1R12B	0.062389	5.292478	0.61203	0.542089	-6.62952	0.657498	0.678518
Non.inflammatory.Macrophages	AURKAIP1	0.056429	5.837172	0.61179	0.542247	-6.74231	0.636041	0.668375
Non.inflammatory.Macrophages	CAMK4	-0.40513	3.194525	-0.61175	0.542271	-5.47245	0.747078	0.719394
Non.inflammatory.Macrophages	RAB14	0.036855	7.091867	0.611621	0.542358	-6.96313	0.589034	0.645469

Non.inflammatory.Macrophages	EFEMP2	-0.14021	1.944258	-0.61117	0.542653	-5.87757	0.806038	0.745054
Non.inflammatory.Macrophages	PLEKHG1	0.121656	4.398533	0.611109	0.542695	-6.2962	0.694692	0.695888
Non.inflammatory.Macrophages	SLC39A14	0.140349	3.169335	0.610474	0.543114	-6.02011	0.749027	0.720134
Non.inflammatory.Macrophages	CEP83	0.065154	5.662564	0.61029	0.543235	-6.57548	0.643585	0.67187
Non.inflammatory.Macrophages	GM26549	-0.1106	3.018851	-0.61025	0.543263	-6.09025	0.755913	0.7232
Non.inflammatory.Macrophages	LYN	0.05176	10.03311	0.610129	0.543341	-7.41866	0.492224	0.594973
Non.inflammatory.Macrophages	IFT57	0.124817	2.891644	0.609915	0.543483	-6.00459	0.761884	0.725854
Non.inflammatory.Macrophages	MRPS22	-0.10653	3.085855	-0.60976	0.543582	-6.08045	0.753027	0.721945
Non.inflammatory.Macrophages	FANCB	0.288065	0.677958	0.609612	0.543683	-5.32599	0.865272	0.771935
Non.inflammatory.Macrophages	DOCK11	-0.05884	7.111908	-0.6095	0.543755	-6.88025	0.589127	0.645415
Non.inflammatory.Macrophages	NIPAL1	0.252711	1.060638	0.609407	0.543818	-5.53139	0.85084	0.763785
Non.inflammatory.Macrophages	INPP5A	0.08174	6.256163	0.609224	0.543938	-6.63694	0.620897	0.661047
Non.inflammatory.Macrophages	IFNAR1	0.063392	5.246213	0.60884	0.544192	-6.63614	0.660564	0.680064
Non.inflammatory.Macrophages	KCNA2	0.212512	0.96787	0.608781	0.54423	-5.93114	0.855838	0.766017
Non.inflammatory.Macrophages	UROC1	-0.241	1.760508	-0.60871	0.544277	-5.68292	0.816064	0.749328

Non.inflammatory.Macrophages	SLAMF6	0.118063	4.602908	0.608673	0.544302	-6.25828	0.686992	0.692353
Non.inflammatory.Macrophages	LZIC	-0.10169	3.63991	-0.60846	0.544444	-6.13989	0.728465	0.711161
Non.inflammatory.Macrophages	PTCD3	-0.08098	4.845166	-0.60843	0.544459	-6.39154	0.67696	0.687699
Non.inflammatory.Macrophages	NMNAT2	0.410454	2.111738	0.608266	0.544571	-5.42096	0.799077	0.742046
Non.inflammatory.Macrophages	ZFP330	-0.06824	4.432352	-0.6081	0.544677	-6.45209	0.694247	0.695648
Non.inflammatory.Macrophages	KLRB1F	0.449845	0.424065	0.608089	0.544687	-5.19462	0.870239	0.777673
Non.inflammatory.Macrophages	UBA5	-0.06041	4.489487	-0.60782	0.544868	-6.47454	0.691983	0.694542
Non.inflammatory.Macrophages	GGPS1	-0.05438	5.393996	-0.6076	0.545013	-6.57833	0.654923	0.677311
Non.inflammatory.Macrophages	STAT3	-0.05739	7.950149	-0.60751	0.545072	-7.07149	0.560013	0.630802
Non.inflammatory.Macrophages	CLNS1A	0.057663	5.340846	0.607428	0.545124	-6.57666	0.657052	0.678328
Non.inflammatory.Macrophages	E430018J2	-0.27286	0.653485	-0.60709	0.545349	-5.38852	0.866996	0.77288
Non.inflammatory.Macrophages	BUB1	-0.19018	3.743439	-0.60696	0.545432	-6.01295	0.724423	0.709262
Non.inflammatory.Macrophages	UBE2B	-0.05566	8.25823	-0.60685	0.545506	-7.10877	0.549711	0.625527
Non.inflammatory.Macrophages	H2-Q4	-0.11623	4.933329	-0.60665	0.545636	-6.50032	0.673861	0.686219
Non.inflammatory.Macrophages	SDHD	0.064504	5.858408	0.606576	0.545687	-6.74593	0.636849	0.668791

Non.inflammatory.Macrophages	FGD5	-0.2774	1.663977	-0.60652	0.545724	-5.56067	0.821506	0.751588
Non.inflammatory.Macrophages	FLT3L	-0.145	2.201527	-0.60635	0.545838	-5.92272	0.795345	0.740504
Non.inflammatory.Macrophages	ORMDL3	-0.10417	3.803807	-0.60623	0.545918	-6.22634	0.721826	0.708236
Non.inflammatory.Macrophages	PNP2	-0.25058	0.616927	-0.6062	0.545937	-5.5807	0.868063	0.77386
Non.inflammatory.Macrophages	TNR	0.317475	0.036927	0.606121	0.545987	-5.24841	0.870877	0.786435
Non.inflammatory.Macrophages	GCSH	0.078135	4.428205	0.605927	0.546115	-6.51652	0.695012	0.696099
Non.inflammatory.Macrophages	PLAAT3	0.089206	5.19136	0.605708	0.54626	-6.76168	0.663469	0.681493
Non.inflammatory.Macrophages	ARAF	0.066552	4.478585	0.605672	0.546284	-6.46457	0.692935	0.695161
Non.inflammatory.Macrophages	GM17036	-0.21983	2.038724	-0.60532	0.54652	-5.61472	0.803595	0.744025
Non.inflammatory.Macrophages	CLMN	0.277814	0.334512	0.604999	0.546729	-5.46794	0.871547	0.780208
Non.inflammatory.Macrophages	RRAGD	0.193777	2.299081	0.604575	0.54701	-5.68431	0.7916	0.738885
Non.inflammatory.Macrophages	ARSA	-0.13159	1.69399	-0.60443	0.547105	-5.94695	0.821034	0.751464
Non.inflammatory.Macrophages	GM38843	-0.12489	0.985485	-0.60403	0.547371	-6.19343	0.857023	0.766601
Non.inflammatory.Macrophages	PTH1R	-0.3206	0.264703	-0.60384	0.547494	-5.45168	0.872355	0.782158
Non.inflammatory.Macrophages	SLC7A6OS	0.072703	4.789888	0.603623	0.54764	-6.41335	0.68104	0.689701

Non.inflammatory.Macrophages	ZFP1	-0.10028	3.874226	-0.60353	0.547703	-6.18168	0.720069	0.707506
Non.inflammatory.Macrophages	NFATC2IP	0.125081	3.151572	0.603382	0.5478	-5.99667	0.752387	0.721931
Non.inflammatory.Macrophages	ZEB2OS	-0.07422	4.678362	-0.60328	0.547865	-6.64766	0.685726	0.691949
Non.inflammatory.Macrophages	PTPRC	0.056122	8.915439	0.603128	0.547968	-7.22125	0.529031	0.615019
Non.inflammatory.Macrophages	MCM8	-0.20627	1.54016	-0.60294	0.54809	-5.59683	0.829375	0.755138
Non.inflammatory.Macrophages	ZFP747	-0.17661	1.605909	-0.60245	0.548415	-5.68789	0.826447	0.753896
Non.inflammatory.Macrophages	GM19522	0.288972	0.940675	0.602347	0.548485	-5.33621	0.859755	0.767965
Non.inflammatory.Macrophages	2310016D2	-0.39763	-0.3578	-0.60231	0.548512	-5.17889	0.873052	0.796152
Non.inflammatory.Macrophages	MCTS2	-0.19299	1.612847	-0.60215	0.548614	-5.79879	0.826127	0.753826
Non.inflammatory.Macrophages	METTL16	-0.07265	4.533537	-0.60209	0.548657	-6.39569	0.692233	0.695003
Non.inflammatory.Macrophages	COA3	0.058571	5.812938	0.601823	0.548832	-6.74995	0.640298	0.670737
Non.inflammatory.Macrophages	DPY19L3	-0.14675	2.757574	-0.60178	0.548862	-5.94774	0.771092	0.730273
Non.inflammatory.Macrophages	NCAPH2	0.062275	5.344606	0.601525	0.54903	-6.59091	0.658885	0.679581
Non.inflammatory.Macrophages	PODNL1	-0.34769	-1.02387	-0.60132	0.549163	-5.13488	0.873154	0.811287
Non.inflammatory.Macrophages	TMEM218	0.164091	1.874833	0.60129	0.549185	-5.67014	0.813278	0.748564

Non.inflammatory.Macrophages	FGD4	-0.07512	4.134435	-0.60112	0.549295	-6.84453	0.709315	0.703035
Non.inflammatory.Macrophages	EPHB2	0.310616	1.372664	0.601097	0.549314	-5.38047	0.838208	0.759186
Non.inflammatory.Macrophages	WDR70	0.054731	6.1341	0.601043	0.549349	-6.70252	0.627844	0.664985
Non.inflammatory.Macrophages	TTL	0.140317	1.740811	0.601034	0.549355	-5.8787	0.819865	0.751463
Non.inflammatory.Macrophages	HPS6	-0.19125	1.22115	-0.60096	0.549405	-5.65706	0.845865	0.762406
Non.inflammatory.Macrophages	ZDHHC18	0.073476	5.57552	0.600944	0.549415	-6.586	0.649657	0.675417
Non.inflammatory.Macrophages	NHLRC3	-0.09194	3.16365	-0.60055	0.549678	-6.34531	0.752454	0.722439
Non.inflammatory.Macrophages	UBASH3A	0.221894	2.009253	0.600403	0.549774	-5.70096	0.80681	0.746034
Non.inflammatory.Macrophages	ENTPD5	-0.0971	3.78751	-0.60034	0.549813	-6.04777	0.72451	0.710026
Non.inflammatory.Macrophages	ASCC2	0.06047	4.605255	0.600181	0.549921	-6.50846	0.689352	0.694048
Non.inflammatory.Macrophages	DGAT1	0.133626	7.095267	0.600149	0.549942	-6.66569	0.592015	0.647565
Non.inflammatory.Macrophages	NAA15	0.044055	6.743192	0.600066	0.549997	-6.83459	0.604928	0.653943
Non.inflammatory.Macrophages	CDC45	-0.17506	3.268129	-0.60004	0.550015	-5.88029	0.747705	0.720364
Non.inflammatory.Macrophages	CC2D1A	0.11484	3.044755	0.599981	0.550054	-6.07243	0.757891	0.724855
Non.inflammatory.Macrophages	TRIM14	0.085002	4.686216	0.599977	0.550056	-6.5998	0.68596	0.692486

Non.inflammatory.Macrophages	NECTIN3	-0.27091	1.220532	-0.59972	0.550226	-5.381	0.846151	0.762611
Non.inflammatory.Macrophages	RFC2	-0.07946	5.227985	-0.59962	0.55029	-6.50395	0.663792	0.682179
Non.inflammatory.Macrophages	TNRC18	0.049405	5.931838	0.599478	0.550387	-6.74438	0.635889	0.66899
Non.inflammatory.Macrophages	SDHB	0.059136	7.274793	0.599203	0.55057	-6.953	0.585706	0.644529
Non.inflammatory.Macrophages	PTTG1IP	0.056566	4.790183	0.599182	0.550584	-6.58856	0.681827	0.69069
Non.inflammatory.Macrophages	ZFP746	0.088505	3.717554	0.599151	0.550605	-6.21864	0.727808	0.711628
Non.inflammatory.Macrophages	B230307C2	-0.08307	3.741811	-0.59824	0.551208	-6.33042	0.727354	0.71144
Non.inflammatory.Macrophages	PQBP1	0.072337	4.6495	0.59822	0.551223	-6.46771	0.688282	0.69369
Non.inflammatory.Macrophages	TRPV2	-0.07203	4.873594	-0.59811	0.551293	-6.53057	0.678945	0.689395
Non.inflammatory.Macrophages	F8A	0.133332	2.551871	0.598013	0.55136	-5.95381	0.781717	0.73541
Non.inflammatory.Macrophages	TBC1D22A	-0.053	5.871527	-0.59796	0.551395	-6.78935	0.638808	0.670515
Non.inflammatory.Macrophages	SEMA7A	-0.29081	2.017527	-0.59779	0.551509	-5.38232	0.807336	0.746473
Non.inflammatory.Macrophages	TMEM14A	0.240142	0.419553	0.597713	0.551559	-5.55172	0.874256	0.78039
Non.inflammatory.Macrophages	4930403P2	0.24131	0.612375	0.597252	0.551865	-5.40147	0.871531	0.776304
Non.inflammatory.Macrophages	ZNRD2	0.088907	4.037669	0.597196	0.551903	-6.33077	0.714401	0.705774

Non.inflammatory.Macrophages	SLC46A2	-0.31914	-0.09426	-0.59715	0.551933	-5.24363	0.874256	0.791682
Non.inflammatory.Macrophages	ACTR5	0.112908	3.477256	0.597085	0.551976	-6.05346	0.739133	0.716868
Non.inflammatory.Macrophages	ARHGAP26	0.10717	6.685583	0.597075	0.551983	-6.47506	0.607766	0.655619
Non.inflammatory.Macrophages	AGBL5	-0.15477	2.244561	-0.59703	0.552012	-5.71689	0.796361	0.741878
Non.inflammatory.Macrophages	CMTR2	0.186315	1.0685	0.596949	0.552067	-5.67798	0.85472	0.76653
Non.inflammatory.Macrophages	SUPT16	0.048127	7.068147	0.596792	0.552171	-6.84301	0.593683	0.648678
Non.inflammatory.Macrophages	MFSD3	0.221832	0.642198	0.5965	0.552366	-5.57098	0.870831	0.775768
Non.inflammatory.Macrophages	GM13547	-0.30016	0.514665	-0.59639	0.552438	-5.51108	0.873706	0.778569
Non.inflammatory.Macrophages	H1FX	-0.2503	2.127581	-0.59633	0.552476	-5.60561	0.801999	0.744458
Non.inflammatory.Macrophages	NOP53	0.063029	5.740416	0.596285	0.552508	-6.71339	0.643955	0.673247
Non.inflammatory.Macrophages	ZFP740	0.065051	4.76726	0.596235	0.552542	-6.45924	0.683366	0.691762
Non.inflammatory.Macrophages	PHF10	-0.06621	4.930175	-0.59608	0.552643	-6.58415	0.676612	0.688676
Non.inflammatory.Macrophages	FBXO10	0.187281	1.355085	0.595933	0.552742	-5.60689	0.840152	0.760765
Non.inflammatory.Macrophages	NSMCE4A	0.054809	6.083141	0.59574	0.552871	-6.68705	0.630599	0.66699
Non.inflammatory.Macrophages	ARL6	0.152295	1.698649	0.595714	0.552888	-5.75139	0.822984	0.753543

Non.inflammatory.Macrophages	GPC3	0.192899	1.806193	0.595583	0.552975	-5.71014	0.817676	0.751306
Non.inflammatory.Macrophages	GFM1	-0.08514	3.89287	-0.59549	0.553037	-6.28932	0.720716	0.708978
Non.inflammatory.Macrophages	LMF2	-0.10628	3.103696	-0.59546	0.553056	-6.145	0.756061	0.72472
Non.inflammatory.Macrophages	GM3435	-0.30385	0.205181	-0.59544	0.55307	-5.39957	0.874256	0.785522
Non.inflammatory.Macrophages	RTN4	-0.03922	7.592315	-0.59531	0.553159	-7.09166	0.574901	0.639612
Non.inflammatory.Macrophages	NR3C2	-0.1926	1.939637	-0.59519	0.553235	-5.8519	0.811133	0.748602
Non.inflammatory.Macrophages	VPS50	0.064608	4.131766	0.595081	0.553309	-6.49384	0.710325	0.704329
Non.inflammatory.Macrophages	SIRT2	-0.0508	5.688086	-0.59492	0.553417	-6.76232	0.646018	0.674465
Non.inflammatory.Macrophages	TSPAN31	0.071037	4.424978	0.594917	0.553418	-6.59013	0.697764	0.698604
Non.inflammatory.Macrophages	XRCC2	-0.24201	0.802718	-0.59485	0.553464	-5.44324	0.865493	0.77265
Non.inflammatory.Macrophages	HOXB4	0.156663	1.361865	0.594838	0.553471	-5.83293	0.83981	0.76074
Non.inflammatory.Macrophages	GET4	-0.06739	5.110437	-0.59481	0.553487	-6.53797	0.669213	0.685399
Non.inflammatory.Macrophages	POLD2	0.133292	3.401936	0.594699	0.553563	-6.0683	0.742518	0.718785
Non.inflammatory.Macrophages	SPSB3	-0.08836	3.587341	-0.5947	0.553565	-6.14019	0.734213	0.715086
Non.inflammatory.Macrophages	VKORC1	-0.08269	4.701765	-0.59451	0.553687	-6.58555	0.686099	0.693246

Non.inflammatory.Macrophages	RHOBTB1	-0.11286	3.180961	-0.59441	0.553754	-6.37318	0.75253	0.723218
Non.inflammatory.Macrophages	HS3ST1	0.479187	1.500377	0.594377	0.553778	-5.26558	0.832852	0.757822
Non.inflammatory.Macrophages	PML	0.10817	5.273859	0.594254	0.55386	-6.58759	0.662571	0.682334
Non.inflammatory.Macrophages	TRIOBP	0.064756	4.877202	0.59405	0.553996	-6.4785	0.678801	0.689917
Non.inflammatory.Macrophages	FZR1	-0.08298	4.900739	-0.59399	0.554034	-6.47334	0.677828	0.689476
Non.inflammatory.Macrophages	LRRC28	0.145354	3.874144	0.593898	0.554097	-6.12101	0.721537	0.709514
Non.inflammatory.Macrophages	GM11579	-0.41451	-0.20636	-0.59384	0.554134	-5.17525	0.874256	0.794731
Non.inflammatory.Macrophages	TRIP6	0.175025	0.834471	0.593788	0.55417	-5.65052	0.864442	0.772096
Non.inflammatory.Macrophages	KLRA5	-0.45724	2.005641	-0.59372	0.554218	-5.3449	0.807914	0.747371
Non.inflammatory.Macrophages	EXOSC4	-0.08797	3.784732	-0.59363	0.554278	-6.33953	0.725466	0.711282
Non.inflammatory.Macrophages	EEF1B2	0.046211	8.556525	0.593573	0.554313	-7.18044	0.541855	0.622814
Non.inflammatory.Macrophages	UBE2I	-0.03663	7.624242	-0.59318	0.554573	-6.96605	0.573776	0.63927
Non.inflammatory.Macrophages	EPS8L2	-0.34105	0.893203	-0.59317	0.554583	-5.30992	0.862503	0.770948
Non.inflammatory.Macrophages	BMX	0.389126	0.02253	0.593157	0.55459	-5.26472	0.874256	0.789813
Non.inflammatory.Macrophages	1700126G(	-0.30038	1.104608	-0.59314	0.554604	-5.35823	0.852872	0.766434

Non.inflammatory.Macrophages	MTERF3	-0.08136	4.014483	-0.5931	0.55463	-6.36024	0.715409	0.706849
Non.inflammatory.Macrophages	DCAF7	0.054718	5.327488	0.592813	0.55482	-6.59904	0.660517	0.6816
Non.inflammatory.Macrophages	A630023P1	-0.42877	0.306091	-0.59276	0.554852	-5.15959	0.874403	0.783774
Non.inflammatory.Macrophages	RBM12B2	-0.13371	2.316248	-0.59237	0.555113	-5.88374	0.793339	0.741244
Non.inflammatory.Macrophages	YARS	-0.10449	5.391202	-0.59208	0.555305	-6.40826	0.658263	0.680556
Non.inflammatory.Macrophages	TRIM32	0.198569	1.488698	0.591994	0.555365	-5.76569	0.833971	0.758616
Non.inflammatory.Macrophages	PTPRJ	0.048524	8.855581	0.591978	0.555376	-7.2789	0.532331	0.617997
Non.inflammatory.Macrophages	SCIMP	0.107203	3.702449	0.591777	0.55551	-6.45856	0.729568	0.713332
Non.inflammatory.Macrophages	PTGER2	0.162406	1.047913	0.591705	0.555558	-5.8734	0.856323	0.767983
Non.inflammatory.Macrophages	POP1	0.13049	2.708556	0.591651	0.555594	-5.917	0.774853	0.733333
Non.inflammatory.Macrophages	SREK1	-0.04291	6.429668	-0.59159	0.555632	-6.80864	0.617762	0.661179
Non.inflammatory.Macrophages	CNOT1	0.046245	7.026328	0.591454	0.555725	-6.88044	0.59562	0.650313
Non.inflammatory.Macrophages	TSR1	0.101094	3.584651	0.59127	0.555848	-6.22976	0.734908	0.715731
Non.inflammatory.Macrophages	PLCB2	-0.09122	3.320212	-0.59119	0.555905	-6.28601	0.746791	0.721052
Non.inflammatory.Macrophages	S100A13	0.056642	6.040644	0.591063	0.555986	-6.78019	0.63275	0.668523

Non.inflammatory.Macrophages	CDKN2AIPI	0.075191	4.272414	0.590939	0.556069	-6.37518	0.704841	0.702269
Non.inflammatory.Macrophages	TAF1	0.06335	5.699019	0.590876	0.556111	-6.63957	0.646106	0.674933
Non.inflammatory.Macrophages	TOMM70A	0.052538	5.752051	0.590643	0.556267	-6.68597	0.644072	0.673997
Non.inflammatory.Macrophages	LEPROT	0.054541	5.065196	0.590552	0.556327	-6.74431	0.671663	0.687009
Non.inflammatory.Macrophages	MRNIP	-0.13738	2.538227	-0.59051	0.556354	-6.04965	0.783068	0.73707
Non.inflammatory.Macrophages	ADCY6	0.281061	0.433361	0.590234	0.556539	-5.38827	0.875081	0.781525
Non.inflammatory.Macrophages	CYCS	0.067693	7.712668	0.590215	0.556552	-7.02374	0.571209	0.638244
Non.inflammatory.Macrophages	LMO2	0.068751	4.903927	0.59018	0.556576	-6.72428	0.678336	0.690154
Non.inflammatory.Macrophages	GM7072	0.084224	3.842908	0.589988	0.556704	-6.39449	0.723672	0.710895
Non.inflammatory.Macrophages	CDK9	-0.05806	5.578202	-0.58964	0.556934	-6.66991	0.651198	0.677461
Non.inflammatory.Macrophages	SMARCAL1	-0.09907	2.628016	-0.58959	0.556967	-6.06798	0.779126	0.735449
Non.inflammatory.Macrophages	ANG	0.138516	4.211322	0.589442	0.557068	-6.36859	0.707796	0.703807
Non.inflammatory.Macrophages	MTAP	0.096679	3.671485	0.589421	0.557082	-6.21368	0.731402	0.714464
Non.inflammatory.Macrophages	ARHGAP45	-0.06506	6.710119	-0.58907	0.557319	-6.75571	0.607657	0.656624
Non.inflammatory.Macrophages	AK6	-0.07054	4.761906	-0.58899	0.557369	-6.49711	0.684495	0.693274

Non.inflammatory.Macrophages	SNHG4	0.102573	3.683196	0.588852	0.557462	-6.17142	0.730921	0.71442
Non.inflammatory.Macrophages	IL27	0.237097	0.053926	0.588829	0.557478	-5.70756	0.875414	0.790257
Non.inflammatory.Macrophages	TGM1	0.196333	0.53017	0.588731	0.557543	-5.78977	0.874518	0.779907
Non.inflammatory.Macrophages	PCSK5	-0.30757	0.558557	-0.58873	0.557543	-5.4064	0.873885	0.779292
Non.inflammatory.Macrophages	OXLD1	-0.13379	1.813163	-0.5887	0.557564	-5.9625	0.818416	0.752595
Non.inflammatory.Macrophages	ADGRA2	-0.29713	0.687625	-0.58848	0.55771	-5.49728	0.870596	0.776569
Non.inflammatory.Macrophages	KLHL4	0.334554	0.018315	0.588369	0.557785	-5.3273	0.875558	0.791198
Non.inflammatory.Macrophages	TOPBP1	-0.08587	5.680028	-0.58825	0.557862	-6.56694	0.647313	0.675963
Non.inflammatory.Macrophages	SWAP70	0.081698	5.61218	0.587953	0.558063	-6.69312	0.650161	0.677308
Non.inflammatory.Macrophages	ZBED4	0.080358	4.650997	0.587786	0.558174	-6.3333	0.689491	0.695743
Non.inflammatory.Macrophages	PCOLCE	0.235841	0.911461	0.587648	0.558267	-5.58368	0.863487	0.772059
Non.inflammatory.Macrophages	VAMP8	-0.04419	7.209731	-0.58757	0.55832	-7.0518	0.589634	0.647956
Non.inflammatory.Macrophages	GM17160	0.247565	0.089084	0.58747	0.558385	-5.43132	0.875864	0.789901
Non.inflammatory.Macrophages	NT5DC1	-0.07781	4.5014	-0.58739	0.558441	-6.44217	0.695802	0.698701
Non.inflammatory.Macrophages	TEP1	-0.06626	3.914158	-0.58724	0.558537	-6.60419	0.721109	0.710229

Non.inflammatory.Macrophages	MGAT4A	0.072939	5.666978	0.587206	0.558562	-6.60899	0.648041	0.676405
Non.inflammatory.Macrophages	MBLAC1	-0.23388	-0.19049	-0.58667	0.558921	-5.44523	0.876325	0.796296
Non.inflammatory.Macrophages	GDI2	-0.03791	8.891579	-0.58641	0.559094	-7.21209	0.532171	0.618609
Non.inflammatory.Macrophages	YARS2	-0.12238	3.150094	-0.58632	0.559156	-6.01812	0.755865	0.72584
Non.inflammatory.Macrophages	GPSM3	0.053546	6.022347	0.58617	0.559255	-6.83455	0.634605	0.670105
Non.inflammatory.Macrophages	DHX30	0.077293	4.509922	0.585888	0.559443	-6.41019	0.696135	0.698942
Non.inflammatory.Macrophages	PIK3IP1	0.195719	2.920799	0.585716	0.559559	-5.71097	0.766701	0.730605
Non.inflammatory.Macrophages	WRN	0.063071	5.831312	0.585638	0.559611	-6.64878	0.642248	0.673764
Non.inflammatory.Macrophages	GABPB2	-0.05301	6.502447	-0.5855	0.559704	-6.72663	0.616439	0.661332
Non.inflammatory.Macrophages	LCORL	-0.05471	6.985276	-0.58535	0.559801	-6.93406	0.598506	0.652532
Non.inflammatory.Macrophages	COL18A1	-0.18925	2.743841	-0.58502	0.560025	-5.92239	0.775196	0.734417
Non.inflammatory.Macrophages	TUFM	-0.077	4.731912	-0.58488	0.560116	-6.49294	0.687046	0.694964
Non.inflammatory.Macrophages	HNRNPL	-0.04066	8.426349	-0.58473	0.56022	-7.04713	0.547965	0.627081
Non.inflammatory.Macrophages	ZC3H14	-0.05475	5.656337	-0.58471	0.560233	-6.61051	0.649358	0.677346
Non.inflammatory.Macrophages	MTX2	0.062486	4.838218	0.584698	0.56024	-6.5551	0.682609	0.692951

Non.inflammatory.Macrophages	ZXDC	-0.08635	4.004625	-0.58453	0.560354	-6.26961	0.718207	0.709288
Non.inflammatory.Macrophages	UBE2A	0.042456	6.786705	0.584303	0.560504	-6.84908	0.606104	0.656461
Non.inflammatory.Macrophages	F9	-0.27457	0.896192	-0.58407	0.560659	-5.4394	0.865427	0.773387
Non.inflammatory.Macrophages	KIF15	-0.1558	4.90741	-0.58407	0.560662	-6.32909	0.679926	0.691735
Non.inflammatory.Macrophages	GM11755	-0.32365	-0.115	-0.58398	0.560722	-5.35266	0.877319	0.795436
Non.inflammatory.Macrophages	AOAH	0.102639	4.784961	0.583711	0.560901	-6.87048	0.685049	0.694165
Non.inflammatory.Macrophages	AQP11	-0.2771	0.397356	-0.58369	0.560918	-5.41673	0.877354	0.784252
Non.inflammatory.Macrophages	SMAD3	0.095413	6.315623	0.583535	0.561019	-6.56117	0.623895	0.665243
Non.inflammatory.Macrophages	GM28112	0.392559	0.043725	0.583438	0.561084	-5.21425	0.877354	0.792072
Non.inflammatory.Macrophages	SERPINA3F	0.273613	2.19929	0.583376	0.561125	-5.96357	0.801369	0.746053
Non.inflammatory.Macrophages	TSTD3	0.135118	1.784277	0.583364	0.561133	-5.91012	0.821657	0.754711
Non.inflammatory.Macrophages	BBX	-0.05982	6.494425	-0.58312	0.561298	-6.89918	0.617213	0.662087
Non.inflammatory.Macrophages	IL23A	-0.30723	-0.31658	-0.58301	0.56137	-5.44628	0.877522	0.800186
Non.inflammatory.Macrophages	PRDM4	0.097868	3.039382	0.582385	0.56179	-6.12034	0.762249	0.729081
Non.inflammatory.Macrophages	VIPAS39	0.061313	4.131233	0.582375	0.561796	-6.45412	0.713378	0.707261

Non.inflammatory.Macrophages	DCTN3	0.051146	5.963361	0.582265	0.56187	-6.78451	0.637957	0.672129
Non.inflammatory.Macrophages	OGFOD1	0.092789	3.611469	0.582131	0.56196	-6.22525	0.73631	0.717621
Non.inflammatory.Macrophages	CCT8	0.0485	6.762623	0.581836	0.562158	-6.88637	0.607594	0.657399
Non.inflammatory.Macrophages	RABEPK	0.099522	3.296184	0.581771	0.562201	-6.14238	0.750618	0.72401
Non.inflammatory.Macrophages	KCNIP3	-0.4577	0.193763	-0.58157	0.562339	-5.19147	0.878144	0.789266
Non.inflammatory.Macrophages	1700008J0	0.180464	1.842472	0.581566	0.562339	-5.77134	0.819521	0.753932
Non.inflammatory.Macrophages	ZC3H8	-0.1522	2.13952	-0.58155	0.562352	-5.76149	0.804986	0.747732
Non.inflammatory.Macrophages	BCCIP	-0.05128	5.436859	-0.58101	0.56271	-6.61177	0.65918	0.682376
Non.inflammatory.Macrophages	GM16845	0.138555	2.527304	0.58097	0.562739	-5.93445	0.786668	0.739931
Non.inflammatory.Macrophages	NUDT16L1	-0.08147	4.234234	-0.58093	0.562767	-6.42065	0.709323	0.705608
Non.inflammatory.Macrophages	THUMPD2	-0.15162	1.928752	-0.58074	0.562891	-5.71694	0.815588	0.752431
Non.inflammatory.Macrophages	GM50012	-0.18395	1.643382	-0.58074	0.562892	-5.58579	0.829715	0.758424
Non.inflammatory.Macrophages	ZFP618	-0.31452	0.531603	-0.58046	0.563078	-5.30882	0.877676	0.782308
Non.inflammatory.Macrophages	HAUS1	-0.1014	3.65784	-0.58043	0.563103	-6.23736	0.734717	0.717181
Non.inflammatory.Macrophages	EFHC1	0.332097	-0.05796	0.580176	0.563272	-5.21273	0.878766	0.795249

Non.inflammatory.Macrophages	MRPL34	0.051748	5.428125	0.580045	0.56336	-6.67964	0.659746	0.682735
Non.inflammatory.Macrophages	PBX3	-0.06929	5.496226	-0.57991	0.563452	-6.704	0.657008	0.681472
Non.inflammatory.Macrophages	SECTM1A	-0.2727	-1.13086	-0.57986	0.563483	-5.41344	0.878766	0.819315
Non.inflammatory.Macrophages	D2HGDH	0.147547	2.08867	0.579792	0.56353	-5.81103	0.80803	0.749257
Non.inflammatory.Macrophages	CDYL2	0.093788	4.757934	0.579559	0.563686	-6.42172	0.687393	0.695644
Non.inflammatory.Macrophages	TMEM35B	-0.14173	2.207613	-0.57873	0.564246	-6.02353	0.802995	0.747186
Non.inflammatory.Macrophages	CPED1	0.169313	2.524781	0.578499	0.564398	-5.97711	0.787769	0.740693
Non.inflammatory.Macrophages	GRK4	0.12305	3.108893	0.57849	0.564404	-6.00577	0.760422	0.728754
Non.inflammatory.Macrophages	NDST1	0.083701	4.570663	0.578471	0.564417	-6.50103	0.69581	0.699707
Non.inflammatory.Macrophages	TMEM150I	-0.14255	1.12138	-0.57842	0.564452	-6.11301	0.857199	0.770174
Non.inflammatory.Macrophages	MLKL	0.111869	2.335281	0.578342	0.564504	-6.27613	0.796834	0.744619
Non.inflammatory.Macrophages	ANKRD61	-0.29838	0.799732	-0.57822	0.564583	-5.28812	0.870881	0.77713
Non.inflammatory.Macrophages	TOE1	-0.12981	2.490022	-0.57807	0.564686	-5.92733	0.789497	0.741501
Non.inflammatory.Macrophages	DDB1	0.062594	5.611857	0.577864	0.564825	-6.6678	0.653077	0.679855
Non.inflammatory.Macrophages	MTG2	-0.10635	3.031494	-0.5777	0.564937	-6.04631	0.764104	0.73053

Non.inflammatory.Macrophages	LRP8	0.281059	2.371033	0.577681	0.564948	-5.41315	0.79523	0.744075
Non.inflammatory.Macrophages	PLCG1	-0.11231	3.409844	-0.57741	0.565132	-6.04764	0.746785	0.722901
Non.inflammatory.Macrophages	KPNA2	-0.09461	4.937072	-0.5772	0.56527	-6.48733	0.680541	0.69288
Non.inflammatory.Macrophages	AFAP1	0.196527	1.955044	0.577127	0.565321	-5.54761	0.815429	0.752843
Non.inflammatory.Macrophages	ITIH5	0.260259	1.943297	0.576925	0.565456	-5.45405	0.816006	0.753123
Non.inflammatory.Macrophages	METRN	-0.136	2.538002	-0.5769	0.565472	-5.97819	0.787253	0.74077
Non.inflammatory.Macrophages	TRIM27	-0.06008	5.675636	-0.57684	0.565511	-6.61569	0.650537	0.678826
Non.inflammatory.Macrophages	UBE4A	0.066768	5.03345	0.576836	0.565516	-6.56139	0.676552	0.691071
Non.inflammatory.Macrophages	GPS1	0.062604	4.905295	0.576777	0.565556	-6.55614	0.681861	0.693541
Non.inflammatory.Macrophages	2510039O1	0.058783	5.179646	0.576711	0.5656	-6.61495	0.670544	0.688264
Non.inflammatory.Macrophages	FHL1	0.223795	0.58688	0.576704	0.565605	-5.56322	0.87753	0.78205
Non.inflammatory.Macrophages	NAIP6	0.091952	2.310523	0.57655	0.565708	-6.32155	0.79814	0.74551
Non.inflammatory.Macrophages	MTHFSD	0.132023	2.433778	0.576512	0.565734	-5.92853	0.792224	0.742981
Non.inflammatory.Macrophages	GOLGA3	0.07172	4.032459	0.576338	0.565851	-6.39807	0.719079	0.71067
Non.inflammatory.Macrophages	FAM124A	-0.18127	1.64482	-0.57632	0.565863	-5.68818	0.830796	0.759474

Non.inflammatory.Macrophages	B3GALT5	0.423029	0.779445	0.576288	0.565885	-5.23999	0.87166	0.777957
Non.inflammatory.Macrophages	GM5165	0.144457	2.638407	0.576102	0.56601	-5.81069	0.782529	0.738848
Non.inflammatory.Macrophages	ENAH	0.241354	0.744942	0.576055	0.566042	-5.58748	0.872854	0.778776
Non.inflammatory.Macrophages	LGALS2	-0.23029	-0.89826	-0.57591	0.566137	-5.4672	0.879791	0.815163
Non.inflammatory.Macrophages	0610012G(	0.05065	5.039442	0.5757	0.56628	-6.69779	0.676413	0.691195
Non.inflammatory.Macrophages	CAMKK1	-0.21994	0.590902	-0.57562	0.566331	-5.56361	0.877581	0.782276
Non.inflammatory.Macrophages	EFCAB14	0.074352	4.713606	0.575493	0.56642	-6.49983	0.689985	0.697579
Non.inflammatory.Macrophages	SLC20A2	0.08869	4.036306	0.575442	0.566454	-6.33028	0.719026	0.710861
Non.inflammatory.Macrophages	HNRNPAB	-0.05074	8.05587	-0.57538	0.566495	-7.04585	0.562345	0.635616
Non.inflammatory.Macrophages	CDC23	0.110724	2.927739	0.575256	0.566579	-5.98316	0.769067	0.733142
Non.inflammatory.Macrophages	CCDC157	0.250702	1.103128	0.574896	0.566822	-5.43851	0.858525	0.771459
Non.inflammatory.Macrophages	OS9	0.053916	5.617189	0.574839	0.56686	-6.69871	0.653065	0.68041
Non.inflammatory.Macrophages	RAB4A	0.220584	1.578469	0.574773	0.566905	-5.57342	0.834374	0.761363
Non.inflammatory.Macrophages	CDKAL1	0.05332	6.364518	0.574712	0.566945	-6.78491	0.623887	0.666423
Non.inflammatory.Macrophages	FCHSD2	-0.09015	6.823892	-0.57468	0.566969	-6.81364	0.606578	0.657955

Non.inflammatory.Macrophages	FBXO38	0.051986	5.443447	0.573997	0.567428	-6.65776	0.660441	0.683913
Non.inflammatory.Macrophages	PECAM1	0.078153	7.734027	0.573965	0.567449	-6.99282	0.574004	0.641678
Non.inflammatory.Macrophages	2310010J1	0.114244	3.692212	0.573856	0.567522	-6.17071	0.734788	0.718118
Non.inflammatory.Macrophages	5330438D1	-0.10582	4.842586	-0.57372	0.567613	-6.14493	0.685147	0.69552
Non.inflammatory.Macrophages	NXT1	-0.08433	4.399059	-0.5735	0.567764	-6.3755	0.704018	0.704173
Non.inflammatory.Macrophages	FARSB	0.062148	5.008108	0.57333	0.567877	-6.55639	0.678425	0.692441
Non.inflammatory.Macrophages	FGD3	0.081397	4.660813	0.573134	0.568009	-6.47971	0.693024	0.699272
Non.inflammatory.Macrophages	CAPZB	-0.03349	8.722615	-0.57289	0.568171	-7.23857	0.540464	0.624614
Non.inflammatory.Macrophages	NMNAT1	-0.20646	1.326233	-0.57287	0.568187	-5.71043	0.848056	0.767365
Non.inflammatory.Macrophages	P2RX3	-0.30757	2.219391	-0.57242	0.568487	-5.44373	0.803862	0.748602
Non.inflammatory.Macrophages	GM12462	0.370597	-0.49881	0.5724	0.568504	-5.13132	0.881151	0.807308
Non.inflammatory.Macrophages	DUT	-0.1184	5.882801	-0.57239	0.56851	-6.54263	0.643409	0.67604
Non.inflammatory.Macrophages	COPB2	0.048281	5.438302	0.572279	0.568585	-6.68814	0.661137	0.684458
Non.inflammatory.Macrophages	ZBTB6	0.130464	1.997204	0.572161	0.568665	-5.93543	0.814736	0.753305
Non.inflammatory.Macrophages	CLEC4G	-0.13961	3.468481	-0.57204	0.568743	-6.10615	0.74541	0.723139

Non.inflammatory.Macrophages	KDM1A	0.050858	5.813944	0.571797	0.56891	-6.67333	0.646282	0.677526
Non.inflammatory.Macrophages	SPC25	-0.13868	4.372308	-0.57159	0.569049	-6.22945	0.705717	0.705292
Non.inflammatory.Macrophages	ZFP574	0.118542	3.401172	0.571569	0.569064	-5.99097	0.748611	0.724615
Non.inflammatory.Macrophages	TRIM39	0.108914	2.873416	0.571451	0.569144	-5.96126	0.772929	0.73535
Non.inflammatory.Macrophages	2500004C(	-0.21053	1.043374	-0.57139	0.569184	-5.50079	0.862992	0.773753
Non.inflammatory.Macrophages	MAN1A2	0.050419	6.392544	0.571147	0.569349	-6.71556	0.62386	0.666891
Non.inflammatory.Macrophages	CHTF18	-0.23291	1.800347	-0.57114	0.569352	-5.55154	0.824692	0.757804
Non.inflammatory.Macrophages	ELP4	-0.06947	4.963596	-0.57097	0.569469	-6.53954	0.680851	0.694005
Non.inflammatory.Macrophages	LRP5	-0.07907	4.078112	-0.57058	0.569728	-6.53386	0.718751	0.711376
Non.inflammatory.Macrophages	GM5547	-0.15578	1.560402	-0.57057	0.56974	-5.80172	0.836962	0.762984
Non.inflammatory.Macrophages	ZFP664	-0.0799	4.050718	-0.57044	0.569828	-6.30269	0.719979	0.711924
Non.inflammatory.Macrophages	SPCS2	-0.04238	7.595587	-0.5703	0.569921	-6.99494	0.579761	0.645091
Non.inflammatory.Macrophages	2700097O(	0.117325	3.090234	0.570119	0.570043	-6.05686	0.763268	0.731307
Non.inflammatory.Macrophages	TUBGCP2	-0.08229	3.994036	-0.57006	0.570082	-6.29765	0.722538	0.713172
Non.inflammatory.Macrophages	LYRM4	0.073494	4.142278	0.569607	0.570389	-6.3302	0.716358	0.710323

Non.inflammatory.Macrophages	ASB4	0.270842	1.68448	0.5694	0.570528	-5.76576	0.831273	0.760666
Non.inflammatory.Macrophages	FUCA2	-0.05526	4.624115	-0.56919	0.570668	-6.65934	0.695703	0.700978
Non.inflammatory.Macrophages	ODF2L	-0.12363	2.682465	-0.56895	0.570835	-5.94943	0.782723	0.739958
Non.inflammatory.Macrophages	GEM	0.159342	5.207345	0.568903	0.570864	-6.07798	0.671395	0.689772
Non.inflammatory.Macrophages	FTCD	-0.25696	1.403237	-0.56872	0.570991	-5.6143	0.845443	0.766875
Non.inflammatory.Macrophages	PAQR3	0.150213	1.469925	0.568584	0.57108	-5.78451	0.842063	0.765455
Non.inflammatory.Macrophages	ZFP53	0.081102	4.791451	0.568558	0.571097	-6.40228	0.688645	0.697892
Non.inflammatory.Macrophages	USP20	-0.11016	2.877475	-0.56851	0.571132	-5.96617	0.773546	0.736074
Non.inflammatory.Macrophages	DEGS2	0.200121	0.674225	0.568496	0.571139	-5.67375	0.877774	0.782565
Non.inflammatory.Macrophages	TMEM107	-0.16411	1.616764	-0.56844	0.571179	-5.70398	0.834665	0.762337
Non.inflammatory.Macrophages	MFN1	0.088311	3.297935	0.568432	0.571183	-6.2124	0.754097	0.727514
Non.inflammatory.Macrophages	IFNGR2	-0.06789	5.782195	-0.56825	0.571302	-6.82854	0.648229	0.678927
Non.inflammatory.Macrophages	CCNG1	0.061983	4.873028	0.568147	0.571375	-6.57355	0.685228	0.696332
Non.inflammatory.Macrophages	RTP4	0.131634	4.174749	0.568042	0.571447	-6.6631	0.714997	0.709999
Non.inflammatory.Macrophages	PEX6	-0.08743	4.039128	-0.56803	0.571452	-6.36621	0.720918	0.712684

Non.inflammatory.Macrophages	RRM2	-0.16278	6.880861	-0.56786	0.571569	-6.64124	0.606069	0.658479
Non.inflammatory.Macrophages	ZFP362	-0.07477	3.9339	-0.56783	0.571586	-6.34658	0.725544	0.714779
Non.inflammatory.Macrophages	VPS4B	0.044745	6.231035	0.567809	0.571604	-6.82588	0.630675	0.6705
Non.inflammatory.Macrophages	BSCL2	-0.05706	4.542466	-0.56721	0.572009	-6.56726	0.699503	0.702928
Non.inflammatory.Macrophages	BRF2	0.129513	2.326101	0.567144	0.572053	-5.8522	0.800132	0.747644
Non.inflammatory.Macrophages	TCF4	0.068707	7.694523	0.566897	0.572221	-6.94263	0.576843	0.643882
Non.inflammatory.Macrophages	RHOG	0.05576	7.243088	0.56688	0.572232	-7.04692	0.593041	0.652024
Non.inflammatory.Macrophages	ABHD15	-0.12038	2.977881	-0.56687	0.572236	-6.17338	0.769225	0.73422
Non.inflammatory.Macrophages	GNPAT	-0.07522	4.15461	-0.56674	0.572324	-6.37148	0.716213	0.71057
Non.inflammatory.Macrophages	TARBP1	0.161239	2.687423	0.566733	0.572331	-5.77825	0.78286	0.74018
Non.inflammatory.Macrophages	ARFRP1	-0.06705	4.120918	-0.56647	0.572512	-6.37756	0.717827	0.71128
Non.inflammatory.Macrophages	DOCK8	-0.05617	7.656272	-0.56558	0.573111	-7.03088	0.578797	0.644721
Non.inflammatory.Macrophages	NDUFB10	0.049767	6.731387	0.565568	0.57312	-6.87786	0.61257	0.661535
Non.inflammatory.Macrophages	PARD6A	-0.13494	2.688858	-0.56545	0.573198	-6.00771	0.78362	0.740361
Non.inflammatory.Macrophages	NEK3	0.233737	1.761696	0.56524	0.573342	-5.61101	0.828803	0.759706

Non.inflammatory.Macrophages	SNAI1	0.2535	0.389835	0.564929	0.573552	-5.36315	0.884006	0.789269
Non.inflammatory.Macrophages	OTUD6B	0.062902	4.301102	0.564681	0.573721	-6.39864	0.711014	0.707979
Non.inflammatory.Macrophages	STARD3NL	0.049206	5.861943	0.56432	0.573965	-6.87157	0.646499	0.677936
Non.inflammatory.Macrophages	LEFTY1	-0.22837	0.408138	-0.56429	0.573988	-5.51014	0.884256	0.788993
Non.inflammatory.Macrophages	DTNBP1	-0.03843	6.683213	-0.56407	0.574135	-6.8909	0.614807	0.662616
Non.inflammatory.Macrophages	ERN1	-0.062	7.053542	-0.56407	0.574137	-6.96207	0.601012	0.65582
Non.inflammatory.Macrophages	TRAPPC5	0.075697	4.379372	0.56406	0.574142	-6.41396	0.707707	0.706508
Non.inflammatory.Macrophages	RSPH9	-0.24072	1.514419	-0.56402	0.574172	-5.55363	0.841668	0.765121
Non.inflammatory.Macrophages	ARRDC2	-0.1361	1.79539	-0.56387	0.574273	-5.93904	0.827569	0.759168
Non.inflammatory.Macrophages	CTLA4	0.230953	2.060232	0.56382	0.574304	-5.73155	0.814474	0.753606
Non.inflammatory.Macrophages	TPGS2	-0.09004	3.805381	-0.56371	0.57438	-6.20673	0.732861	0.717964
Non.inflammatory.Macrophages	PERP	-0.27048	1.228461	-0.56344	0.574561	-5.46578	0.856435	0.771365
Non.inflammatory.Macrophages	SMARCA4	-0.05883	7.5105	-0.5633	0.574654	-6.8919	0.584562	0.64765
Non.inflammatory.Macrophages	TMEM183A	0.05287	5.16956	0.562998	0.574861	-6.5495	0.674779	0.691378
Non.inflammatory.Macrophages	NEDD1	0.110064	3.041246	0.5628	0.574995	-6.10547	0.768021	0.733649

Non.inflammatory.Macrophages	MFAP1B	0.061598	4.945536	0.562786	0.575005	-6.48672	0.684081	0.69578
Non.inflammatory.Macrophages	AKR1B10	-0.08251	3.494629	-0.56244	0.575241	-6.39447	0.747426	0.72454
Non.inflammatory.Macrophages	UBALD2	0.057973	7.028455	0.562278	0.57535	-6.93025	0.602474	0.656657
Non.inflammatory.Macrophages	CYP2C29	0.291888	1.011219	0.562042	0.575509	-5.50277	0.868244	0.776374
Non.inflammatory.Macrophages	MAGI3	-0.09342	6.598022	-0.56203	0.575514	-6.63668	0.618575	0.6646
Non.inflammatory.Macrophages	RBM45	0.111119	3.10947	0.561996	0.575541	-5.99651	0.765124	0.732376
Non.inflammatory.Macrophages	SPA17	-0.20614	0.979282	-0.56191	0.5756	-5.52183	0.869906	0.777063
Non.inflammatory.Macrophages	PMVK	-0.07913	4.327226	-0.56171	0.575738	-6.39321	0.710681	0.707972
Non.inflammatory.Macrophages	GM20457	-0.23886	-0.16272	-0.56134	0.575984	-5.47835	0.88537	0.802156
Non.inflammatory.Macrophages	CWC25	0.074601	5.255579	0.561308	0.576007	-6.61489	0.671744	0.689961
Non.inflammatory.Macrophages	ZBTB48	-0.22875	1.123594	-0.56098	0.576228	-5.56689	0.862889	0.774012
Non.inflammatory.Macrophages	UBE2CBP	0.271359	1.761179	0.560748	0.576387	-5.59793	0.830469	0.760501
Non.inflammatory.Macrophages	FBXW4	-0.07786	4.272711	-0.56065	0.576451	-6.51164	0.713344	0.709185
Non.inflammatory.Macrophages	TPBGL	-0.3048	0.559425	-0.56062	0.576476	-5.37964	0.883965	0.786332
Non.inflammatory.Macrophages	GM15472	0.217944	1.795978	0.560518	0.576544	-5.68575	0.828732	0.759774

Non.inflammatory.Macrophages	PCED1A	0.109922	2.508638	0.560457	0.576585	-6.06885	0.793877	0.744866
Non.inflammatory.Macrophages	GM48742	-0.26092	0.618325	-0.56037	0.576645	-5.3741	0.882638	0.785063
Non.inflammatory.Macrophages	TRIP13	-0.18019	2.772205	-0.56036	0.576651	-5.86211	0.781331	0.739441
Non.inflammatory.Macrophages	SNRPG	0.047802	8.018397	0.560286	0.576701	-7.04845	0.567285	0.639007
Non.inflammatory.Macrophages	CMTR1	0.081263	5.166737	0.560018	0.576884	-6.59882	0.675549	0.691863
Non.inflammatory.Macrophages	TWF2	-0.06091	5.240324	-0.55991	0.57696	-6.64175	0.672522	0.690453
Non.inflammatory.Macrophages	P4HTM	0.114984	2.773581	0.55988	0.576977	-6.32207	0.7813	0.73952
Non.inflammatory.Macrophages	SERTAD4	-0.39992	-0.0023	-0.55986	0.576988	-5.169	0.88557	0.798823
Non.inflammatory.Macrophages	GLRA1	-0.21776	1.25067	-0.5594	0.577307	-5.80186	0.85675	0.771529
Non.inflammatory.Macrophages	C77080	-0.54648	0.694235	-0.55922	0.577424	-5.33713	0.88077	0.783582
Non.inflammatory.Macrophages	AKAP11	0.076245	4.648059	0.559163	0.577464	-6.45039	0.697594	0.701983
Non.inflammatory.Macrophages	CREBZF	-0.07029	4.715073	-0.55907	0.57753	-6.38692	0.694752	0.700675
Non.inflammatory.Macrophages	GM38190	0.19105	1.823044	0.558505	0.577912	-5.5808	0.828264	0.759544
Non.inflammatory.Macrophages	4930455G(	-0.08701	1.816178	-0.55833	0.578028	-6.52349	0.828606	0.759694
Non.inflammatory.Macrophages	A230059L(	-0.27413	0.47539	-0.55832	0.578036	-5.5058	0.886475	0.788528

Non.inflammatory.Macrophages	CLDN15	0.207889	0.423974	0.558121	0.578172	-5.51428	0.886583	0.78968
Non.inflammatory.Macrophages	PHF20L1	-0.05159	7.046788	-0.55796	0.578282	-6.87108	0.602889	0.656857
Non.inflammatory.Macrophages	A130014A(	0.137342	1.887317	0.557501	0.578594	-5.89554	0.825492	0.758463
Non.inflammatory.Macrophages	MAZ	-0.07947	6.241133	-0.5575	0.578597	-6.7252	0.633588	0.671913
Non.inflammatory.Macrophages	KCNH7	0.335585	-0.13979	0.557332	0.578709	-5.24131	0.887004	0.802397
Non.inflammatory.Macrophages	OGFOD2	0.077974	3.850672	0.557181	0.578812	-6.31777	0.73315	0.718241
Non.inflammatory.Macrophages	CCDC186	-0.06463	4.760025	-0.55688	0.579018	-6.52604	0.69385	0.700323
Non.inflammatory.Macrophages	HIF1AN	0.103814	3.373427	0.556337	0.579386	-6.16406	0.754939	0.728023
Non.inflammatory.Macrophages	ARHGEF40	-0.27102	0.550928	-0.55631	0.579404	-5.43764	0.885967	0.787453
Non.inflammatory.Macrophages	SH2D1A	0.341327	0.051779	0.556296	0.579414	-5.22458	0.887345	0.798441
Non.inflammatory.Macrophages	MED8	0.057932	5.008062	0.556217	0.579467	-6.6515	0.683488	0.69567
Non.inflammatory.Macrophages	LSAMP	-0.24866	0.929492	-0.55602	0.579602	-5.51931	0.874204	0.779329
Non.inflammatory.Macrophages	GM30025	-0.18781	1.597885	-0.55595	0.579648	-5.68263	0.840382	0.76499
Non.inflammatory.Macrophages	ATP5A1	0.043222	7.857813	0.555862	0.579709	-7.06461	0.574078	0.642681
Non.inflammatory.Macrophages	RIOX1	-0.10244	3.521185	-0.55585	0.579718	-6.02015	0.748203	0.725144

Non.inflammatory.Macrophages	EIF2D	0.092987	3.5699	0.555285	0.580102	-6.1401	0.745995	0.724261
Non.inflammatory.Macrophages	ZFP775	0.230859	0.81684	0.555232	0.580138	-5.48317	0.877976	0.781881
Non.inflammatory.Macrophages	DUSP16	0.074011	6.833204	0.555215	0.58015	-6.90624	0.611311	0.661366
Non.inflammatory.Macrophages	1700007L1	0.180596	1.663099	0.555015	0.580286	-5.67345	0.837094	0.763811
Non.inflammatory.Macrophages	GM42031	0.241613	4.172175	0.555	0.580296	-6.04377	0.71919	0.712316
Non.inflammatory.Macrophages	GTF2IRD1	0.10768	3.292146	0.554966	0.580319	-6.25556	0.758668	0.729991
Non.inflammatory.Macrophages	MPHOSPH!	0.094	4.258614	0.55487	0.580384	-6.22361	0.715418	0.710661
Non.inflammatory.Macrophages	MEST	0.14978	3.792937	0.554807	0.580428	-6.21099	0.735961	0.719932
Non.inflammatory.Macrophages	PRMT6	-0.12102	2.45108	-0.55477	0.580452	-5.86757	0.798272	0.747318
Non.inflammatory.Macrophages	ITGB1BP2	0.255542	0.43994	0.554678	0.580515	-5.37811	0.887345	0.790284
Non.inflammatory.Macrophages	IFFO1	-0.12688	2.880878	-0.55461	0.580561	-6.13699	0.777801	0.738473
Non.inflammatory.Macrophages	ZFP560	0.076521	4.00302	0.554603	0.580567	-6.32316	0.726626	0.71577
Non.inflammatory.Macrophages	SAMD4B	0.055868	5.014196	0.554593	0.580573	-6.63206	0.683232	0.695903
Non.inflammatory.Macrophages	TDP1	-0.11363	3.397629	-0.55448	0.580652	-6.09357	0.753832	0.727984
Non.inflammatory.Macrophages	TM4SF4	-0.20282	2.168937	-0.55416	0.580868	-5.76882	0.811979	0.753383

Non.inflammatory.Macrophages	MPHOSPH:	-0.06532	4.827879	-0.55412	0.580897	-6.52053	0.691039	0.699658
Non.inflammatory.Macrophages	ZFP800	0.054731	5.365723	0.554117	0.580898	-6.67467	0.66873	0.68926
Non.inflammatory.Macrophages	GM12216	-0.09744	4.554087	-0.55402	0.580963	-6.44742	0.702665	0.705011
Non.inflammatory.Macrophages	SF3B1	0.03438	8.256825	0.553895	0.581049	-7.08619	0.560185	0.635961
Non.inflammatory.Macrophages	CXXC1	-0.07512	4.307295	-0.55383	0.581094	-6.36726	0.713302	0.709872
Non.inflammatory.Macrophages	RBFOX3	-0.23329	-0.24977	-0.55373	0.581163	-5.58635	0.887345	0.805791
Non.inflammatory.Macrophages	MOCS3	-0.19167	1.446277	-0.55372	0.581166	-5.68096	0.848074	0.76872
Non.inflammatory.Macrophages	ART3	-0.19852	0.944132	-0.55371	0.581172	-5.64682	0.873715	0.77952
Non.inflammatory.Macrophages	DPM3	-0.04785	6.309203	-0.55364	0.581223	-6.8386	0.631247	0.671449
Non.inflammatory.Macrophages	TRP53I13	-0.11005	3.021819	-0.55364	0.581226	-5.95507	0.771194	0.73579
Non.inflammatory.Macrophages	ARRB2	0.057303	5.195443	0.553531	0.581297	-6.76526	0.675725	0.692595
Non.inflammatory.Macrophages	CDC34	0.054896	6.050691	0.553356	0.581416	-6.75056	0.641369	0.676306
Non.inflammatory.Macrophages	RBM4	0.063531	4.655948	0.553271	0.581474	-6.41789	0.69838	0.703099
Non.inflammatory.Macrophages	CBR4	-0.10603	2.335594	-0.55316	0.581548	-6.03424	0.803939	0.749994
Non.inflammatory.Macrophages	FAM221A	-0.2317	1.467642	-0.55305	0.581622	-5.45418	0.847073	0.768323

Non.inflammatory.Macrophages	MRPL53	0.076963	4.291701	0.552828	0.581776	-6.41733	0.714053	0.710306
Non.inflammatory.Macrophages	ENTPD6	0.088298	2.776877	0.552768	0.581817	-6.18709	0.782791	0.740889
Non.inflammatory.Macrophages	TSEN2	0.160673	1.266976	0.552751	0.581829	-5.73394	0.857341	0.772654
Non.inflammatory.Macrophages	DPH6	0.076187	4.47347	0.552681	0.581876	-6.47479	0.706196	0.706731
Non.inflammatory.Macrophages	ZFP933	-0.10571	3.490032	-0.55227	0.582155	-6.14316	0.749899	0.726454
Non.inflammatory.Macrophages	FMC1	0.087311	4.072806	0.552081	0.582286	-6.35608	0.72382	0.714777
Non.inflammatory.Macrophages	4732465J0	0.246334	1.227323	0.551928	0.58239	-5.53129	0.859615	0.773646
Non.inflammatory.Macrophages	EIF2S3X	-0.08918	5.323448	-0.5516	0.582614	-6.6619	0.670709	0.69032
Non.inflammatory.Macrophages	TMEM170	-0.19564	1.517214	-0.55155	0.582647	-5.53465	0.844782	0.767455
Non.inflammatory.Macrophages	WDR27	0.178172	0.499373	0.551316	0.582808	-5.63862	0.887466	0.789506
Non.inflammatory.Macrophages	3830406C1	0.059476	4.453206	0.551286	0.582828	-6.56754	0.707259	0.707299
Non.inflammatory.Macrophages	SH3BP2	0.092386	3.288629	0.551237	0.582862	-6.40768	0.759113	0.730614
Non.inflammatory.Macrophages	BGN	0.144122	2.420901	0.551235	0.582863	-5.93691	0.800027	0.748466
Non.inflammatory.Macrophages	CACYBP	-0.05771	5.736095	-0.55118	0.582899	-6.70958	0.654013	0.682519
Non.inflammatory.Macrophages	NFKB1	-0.06469	8.743793	-0.55116	0.582916	-7.25898	0.54388	0.627706

Non.inflammatory.Macrophages	HS6ST1	0.067888	4.156867	0.551119	0.582942	-6.54356	0.720129	0.713197
Non.inflammatory.Macrophages	STK25	-0.0635	4.440838	-0.55106	0.582985	-6.46838	0.707792	0.707585
Non.inflammatory.Macrophages	PRKCE	0.070552	7.233184	0.551021	0.583009	-6.82304	0.596728	0.654663
Non.inflammatory.Macrophages	DNAJC24	0.087985	3.89302	0.550922	0.583076	-6.33887	0.731773	0.718458
Non.inflammatory.Macrophages	PIGK	-0.0601	4.298401	-0.55082	0.583148	-6.50708	0.713955	0.710415
Non.inflammatory.Macrophages	DUSP19	-0.13823	2.048701	-0.55071	0.583223	-5.83344	0.818191	0.756314
Non.inflammatory.Macrophages	NOP56	0.067218	4.591451	0.550653	0.58326	-6.49023	0.701329	0.704684
Non.inflammatory.Macrophages	NXPE2	0.213345	1.56591	0.550295	0.583505	-5.64586	0.842314	0.766593
Non.inflammatory.Macrophages	BOLL	0.329039	1.308178	0.550294	0.583505	-5.3067	0.855454	0.772104
Non.inflammatory.Macrophages	ZFP182	0.085685	4.211679	0.550261	0.583528	-6.38363	0.717732	0.712215
Non.inflammatory.Macrophages	SMO	-0.17407	1.198551	-0.55024	0.583543	-5.83252	0.8611	0.774487
Non.inflammatory.Macrophages	CAR9	0.17208	0.285801	0.550152	0.583602	-5.74731	0.887676	0.794419
Non.inflammatory.Macrophages	RCC2	0.055453	6.222377	0.550111	0.58363	-6.66877	0.634848	0.673504
Non.inflammatory.Macrophages	GM12743	0.124091	2.382516	0.550047	0.583674	-6.02683	0.801883	0.749485
Non.inflammatory.Macrophages	NUMB	0.056552	6.956562	0.549952	0.583739	-6.84362	0.606934	0.659898

Non.inflammatory.Macrophages	COL1A1	0.208177	2.255466	0.549718	0.583899	-5.87784	0.80818	0.752193
Non.inflammatory.Macrophages	WDR26	0.039049	7.53429	0.549487	0.584057	-7.06561	0.585899	0.649411
Non.inflammatory.Macrophages	SDF2L1	-0.06739	4.888426	-0.54945	0.584084	-6.68654	0.688857	0.699052
Non.inflammatory.Macrophages	ZFP428	-0.20684	2.058083	-0.54935	0.584148	-5.52626	0.817855	0.756333
Non.inflammatory.Macrophages	GM17066	0.175342	1.747467	0.549304	0.584182	-5.66669	0.833296	0.762893
Non.inflammatory.Macrophages	INTS9	0.06953	4.394064	0.549244	0.584223	-6.43144	0.70992	0.708739
Non.inflammatory.Macrophages	IFT22	0.086315	3.654091	0.549052	0.584354	-6.34334	0.742668	0.723523
Non.inflammatory.Macrophages	MTX3	-0.23049	0.591443	-0.54859	0.584668	-5.54731	0.885785	0.788092
Non.inflammatory.Macrophages	SGK3	0.059476	5.592861	0.548385	0.584809	-6.79489	0.660063	0.685824
Non.inflammatory.Macrophages	CRP	-0.20912	2.076546	-0.54837	0.584822	-5.74382	0.817191	0.756334
Non.inflammatory.Macrophages	FHL3	-0.16037	2.081558	-0.54835	0.584836	-5.8795	0.816944	0.756237
Non.inflammatory.Macrophages	DCUN1D2	0.116693	2.743543	0.548279	0.584882	-6.09006	0.784939	0.742447
Non.inflammatory.Macrophages	GM29114	0.313156	-1.28439	0.548177	0.584952	-5.1482	0.88808	0.830356
Non.inflammatory.Macrophages	MS4A7	0.115389	1.921581	0.548109	0.584998	-6.51647	0.824856	0.759672
Non.inflammatory.Macrophages	ATP2A1	0.255486	1.448126	0.547938	0.585115	-5.43868	0.848682	0.769736

Non.inflammatory.Macrophages	GLOD4	0.054833	5.050654	0.547825	0.585193	-6.60807	0.682279	0.696387
Non.inflammatory.Macrophages	SMYD4	-0.14109	2.820357	-0.54772	0.585264	-5.98764	0.781301	0.740989
Non.inflammatory.Macrophages	9930111J2	0.092784	4.28297	0.54771	0.585271	-6.55491	0.714951	0.711435
Non.inflammatory.Macrophages	WDR59	-0.10604	3.072953	-0.5475	0.585414	-6.11818	0.769448	0.735869
Non.inflammatory.Macrophages	ATP2A3	0.117593	5.210591	0.547377	0.585499	-6.02009	0.675653	0.693429
Non.inflammatory.Macrophages	STN1	-0.08653	3.886297	-0.54735	0.585518	-6.20044	0.732405	0.719468
Non.inflammatory.Macrophages	PHF6	0.079772	4.940138	0.547173	0.585638	-6.35977	0.686894	0.698735
Non.inflammatory.Macrophages	DAPK1	-0.0656	4.932787	-0.54715	0.585651	-6.93185	0.687202	0.698878
Non.inflammatory.Macrophages	RANBP1	0.066502	7.239938	0.547126	0.585671	-6.9473	0.596752	0.655399
Non.inflammatory.Macrophages	ARFIP2	0.115252	2.138474	0.547049	0.585724	-5.95923	0.814146	0.755413
Non.inflammatory.Macrophages	CD48	-0.05668	6.795574	-0.54701	0.585749	-6.96035	0.61323	0.663589
Non.inflammatory.Macrophages	NLK	0.062124	5.966033	0.546904	0.585822	-6.73982	0.645176	0.679111
Non.inflammatory.Macrophages	GM37305	-0.15751	1.078554	-0.54686	0.585854	-5.76505	0.867712	0.778032
Non.inflammatory.Macrophages	FAM219A	-0.08104	4.171512	-0.54657	0.586051	-6.60011	0.719815	0.7139
Non.inflammatory.Macrophages	RDM1	-0.08795	5.028258	-0.54656	0.586061	-6.5251	0.683212	0.697075

Non.inflammatory.Macrophages	CCR5	-0.07332	4.693422	-0.54649	0.586107	-6.81129	0.697303	0.703603
Non.inflammatory.Macrophages	PDE6D	-0.07	3.812865	-0.54621	0.586299	-6.2945	0.73568	0.721125
Non.inflammatory.Macrophages	YRDC	-0.06698	5.035051	-0.54613	0.586351	-6.53058	0.682929	0.69703
Non.inflammatory.Macrophages	HPS1	-0.08832	3.445228	-0.54607	0.586396	-6.29331	0.752282	0.728594
Non.inflammatory.Macrophages	RYBP	0.059671	5.626574	0.54605	0.586407	-6.6831	0.658705	0.68567
Non.inflammatory.Macrophages	PRSS57	0.336453	-0.03051	0.545953	0.586473	-5.15785	0.88808	0.802534
Non.inflammatory.Macrophages	TRPC4AP	0.045561	5.651799	0.545912	0.586501	-6.73341	0.65769	0.685223
Non.inflammatory.Macrophages	IGKV1-110	0.351735	0.050225	0.545803	0.586576	-5.16941	0.88808	0.80077
Non.inflammatory.Macrophages	SMAGP	-0.07822	3.61538	-0.54576	0.586605	-6.48688	0.744555	0.725223
Non.inflammatory.Macrophages	GPR171	-0.28726	4.027921	-0.54566	0.586676	-5.53505	0.726128	0.716999
Non.inflammatory.Macrophages	CCDC137	-0.10745	2.673176	-0.54563	0.586695	-5.98041	0.788286	0.744543
Non.inflammatory.Macrophages	APON	0.265794	1.166034	0.545452	0.586816	-5.54337	0.863205	0.776405
Non.inflammatory.Macrophages	HYAL2	-0.1462	1.859326	-0.54541	0.586848	-5.87789	0.827984	0.761601
Non.inflammatory.Macrophages	EXOSC9	0.073089	4.169312	0.545302	0.586919	-6.39232	0.719945	0.714196
Non.inflammatory.Macrophages	SRRD	-0.07705	3.465767	-0.5452	0.586986	-6.3076	0.751382	0.728351

Non.inflammatory.Macrophages	FN3KRP	0.178418	1.481688	0.545057	0.587087	-5.72539	0.847065	0.769684
Non.inflammatory.Macrophages	ZFP277	-0.04912	5.309649	-0.54438	0.587549	-6.69988	0.672061	0.692124
Non.inflammatory.Macrophages	KLRG1	0.340326	-1.146	0.544345	0.587574	-5.17077	0.888716	0.828158
Non.inflammatory.Macrophages	ZMYM1	-0.09711	3.329996	-0.54421	0.58767	-6.13577	0.758137	0.73135
Non.inflammatory.Macrophages	GMPPB	0.107758	2.629402	0.544085	0.587753	-6.05996	0.791003	0.745746
Non.inflammatory.Macrophages	MEA1	0.055472	5.276389	0.543684	0.588028	-6.66141	0.673671	0.692893
Non.inflammatory.Macrophages	HIST1H1B	0.220954	5.277543	0.54365	0.58805	-6.36385	0.673624	0.692872
Non.inflammatory.Macrophages	GM10053	0.186378	1.424678	0.543339	0.588264	-5.73408	0.851009	0.771399
Non.inflammatory.Macrophages	FNIP1	-0.04925	7.760209	-0.54311	0.588424	-7.11791	0.578814	0.646739
Non.inflammatory.Macrophages	RDH13	0.167898	1.398416	0.54309	0.588434	-5.70043	0.852409	0.772002
Non.inflammatory.Macrophages	RAB5B	-0.06535	4.382557	-0.54292	0.58855	-6.51499	0.711645	0.710579
Non.inflammatory.Macrophages	SKA1	-0.19561	2.918501	-0.54287	0.588583	-5.83695	0.777784	0.740139
Non.inflammatory.Macrophages	ZC3H7A	0.04438	6.883008	0.542539	0.588812	-6.90243	0.610909	0.66294
Non.inflammatory.Macrophages	IRF8	0.072395	6.49623	0.542384	0.588919	-6.9437	0.625557	0.670145
Non.inflammatory.Macrophages	WSB2	0.061875	4.493162	0.54221	0.589038	-6.60076	0.706968	0.708622

Non.inflammatory.Macrophages	MCM6	-0.09737	6.155584	-0.54219	0.589052	-6.62785	0.638738	0.676579
Non.inflammatory.Macrophages	TEDC2	0.219355	0.906107	0.542177	0.589061	-5.48323	0.877083	0.782976
Non.inflammatory.Macrophages	PAPOLA	0.032699	7.055885	0.54176	0.589347	-6.95063	0.60447	0.659969
Non.inflammatory.Macrophages	VPS26C	-0.07274	4.084075	-0.54164	0.589429	-6.45928	0.724788	0.716921
Non.inflammatory.Macrophages	DDX28	0.128913	1.558733	0.541618	0.589444	-5.84968	0.844382	0.769095
Non.inflammatory.Macrophages	SLC37A4	0.105767	2.862467	0.541566	0.58948	-6.07532	0.780535	0.741719
Non.inflammatory.Macrophages	MTCH1	0.043506	6.297903	0.541549	0.589492	-6.82015	0.633199	0.674078
Non.inflammatory.Macrophages	CDIPT	0.059279	5.093616	0.541523	0.589509	-6.64549	0.68156	0.697063
Non.inflammatory.Macrophages	TMEM181/	0.061608	4.58903	0.541346	0.589631	-6.48386	0.702852	0.706959
Non.inflammatory.Macrophages	SAR1A	0.054596	5.508767	0.541255	0.589693	-6.6701	0.664503	0.689087
Non.inflammatory.Macrophages	MYC	-0.12859	3.533741	-0.54124	0.589704	-6.34986	0.749426	0.728032
Non.inflammatory.Macrophages	MR1	0.197848	0.68263	0.541142	0.58977	-5.65788	0.884616	0.788113
Non.inflammatory.Macrophages	EGR2	-0.13329	2.237621	-0.54114	0.589774	-6.2611	0.810561	0.754779
Non.inflammatory.Macrophages	SLAIN2	0.053373	5.670672	0.541028	0.589849	-6.60655	0.657962	0.686009
Non.inflammatory.Macrophages	CD27	-0.15448	3.318035	-0.54091	0.58993	-5.86847	0.759294	0.732438

Non.inflammatory.Macrophages	SCAPER	0.058592	6.110801	0.540893	0.589942	-6.74486	0.64049	0.677654
Non.inflammatory.Macrophages	NAA16	0.061079	5.024992	0.540802	0.590004	-6.4323	0.684419	0.698455
Non.inflammatory.Macrophages	GM4566	-0.15858	1.718905	-0.54074	0.590046	-5.84871	0.836289	0.765755
Non.inflammatory.Macrophages	TMEM53	-0.19957	0.637469	-0.54056	0.590171	-5.64794	0.886223	0.789181
Non.inflammatory.Macrophages	MTG1	-0.13612	2.186733	-0.54026	0.590378	-5.95464	0.813329	0.756032
Non.inflammatory.Macrophages	MOV10	0.089263	3.628623	0.540023	0.590539	-6.30024	0.745439	0.726327
Non.inflammatory.Macrophages	CCSER2	0.076097	4.778998	0.539924	0.590607	-6.4488	0.695058	0.703438
Non.inflammatory.Macrophages	ATXN7L1	0.048169	6.345264	0.539842	0.590663	-6.92264	0.631633	0.673432
Non.inflammatory.Macrophages	JUNB	-0.05547	9.343714	-0.53967	0.590779	-7.40545	0.525476	0.61955
Non.inflammatory.Macrophages	RAB44	-0.30191	1.487929	-0.53961	0.59082	-5.46653	0.848342	0.770936
Non.inflammatory.Macrophages	STON1	-0.21547	1.087662	-0.53961	0.590822	-5.63952	0.868966	0.779559
Non.inflammatory.Macrophages	SLC22A21	0.149615	1.348839	0.539311	0.591028	-5.92626	0.855596	0.774065
Non.inflammatory.Macrophages	NSL1	-0.12039	2.25699	-0.53912	0.591159	-6.01834	0.810089	0.754803
Non.inflammatory.Macrophages	GM4285	-0.13134	1.640385	-0.53905	0.59121	-5.88469	0.84074	0.767883
Non.inflammatory.Macrophages	LRRC4C	-0.33065	0.648779	-0.53899	0.59125	-5.3522	0.886283	0.789356

Non.inflammatory.Macrophages	MOSPD1	0.094786	4.817896	0.538892	0.591316	-6.33874	0.693524	0.702933
Non.inflammatory.Macrophages	CD55	-0.11416	4.863884	-0.53879	0.591388	-6.51289	0.691582	0.702033
Non.inflammatory.Macrophages	TPP1	0.054932	5.393356	0.538703	0.591446	-6.8116	0.669595	0.691761
Non.inflammatory.Macrophages	ZFP219	-0.12136	1.771622	-0.53866	0.591476	-5.89642	0.834129	0.765109
Non.inflammatory.Macrophages	RRNAD1	0.085153	3.384404	0.538569	0.591537	-6.13748	0.756688	0.731546
Non.inflammatory.Macrophages	GATA3	0.274386	1.116393	0.538348	0.591689	-5.41887	0.867611	0.779258
Non.inflammatory.Macrophages	NHP2	0.065264	5.748278	0.538244	0.591761	-6.70929	0.655232	0.685037
Non.inflammatory.Macrophages	ZFP719	-0.10767	2.119349	-0.53822	0.591775	-5.9803	0.816841	0.757834
Non.inflammatory.Macrophages	CENPL	-0.11704	3.393274	-0.53815	0.591826	-6.00102	0.756281	0.73147
Non.inflammatory.Macrophages	SSR1	0.042796	6.554701	0.538132	0.591838	-6.8566	0.623686	0.669848
Non.inflammatory.Macrophages	INTS13	-0.07415	4.149388	-0.53799	0.591937	-6.30638	0.722377	0.716283
Non.inflammatory.Macrophages	RBMX2	-0.10365	3.368821	-0.53777	0.59209	-6.11951	0.757557	0.73212
Non.inflammatory.Macrophages	FBRS	-0.06046	4.965395	-0.53758	0.592217	-6.59001	0.687525	0.700348
Non.inflammatory.Macrophages	MSANTD2	0.057331	4.864755	0.537382	0.592354	-6.52848	0.691787	0.702368
Non.inflammatory.Macrophages	LONP2	0.04521	5.87764	0.537352	0.592374	-6.70134	0.650297	0.682838

Non.inflammatory.Macrophages	DARS2	-0.14794	2.849125	-0.53719	0.592484	-6.00852	0.781954	0.742954
Non.inflammatory.Macrophages	GM4631	-0.19921	0.664941	-0.53703	0.592597	-5.52512	0.886149	0.789547
Non.inflammatory.Macrophages	ICE1	0.076878	4.081492	0.536964	0.592641	-6.42688	0.725665	0.717987
Non.inflammatory.Macrophages	GMPPA	-0.07974	3.453598	-0.53674	0.592793	-6.37145	0.753893	0.730731
Non.inflammatory.Macrophages	ST6GALNA	-0.16363	5.127632	-0.53667	0.592845	-6.12058	0.680883	0.697495
Non.inflammatory.Macrophages	UBXN2B	-0.09548	2.842475	-0.53665	0.592856	-6.15491	0.782325	0.7433
Non.inflammatory.Macrophages	CEP95	-0.06866	4.108354	-0.5365	0.592963	-6.3385	0.724552	0.717622
Non.inflammatory.Macrophages	ORAI2	-0.0603	5.87495	-0.53622	0.593156	-6.71332	0.650654	0.683208
Non.inflammatory.Macrophages	AZI2	0.044888	5.839646	0.536161	0.593193	-6.75339	0.65206	0.683895
Non.inflammatory.Macrophages	TBC1D22B	0.0956	3.778303	0.535752	0.593475	-6.14131	0.739625	0.724489
Non.inflammatory.Macrophages	D830050J1	-0.1418	1.312085	-0.5355	0.593648	-5.91028	0.858483	0.776003
Non.inflammatory.Macrophages	GM11696	0.148177	1.871722	0.535446	0.593686	-5.87696	0.830082	0.764026
Non.inflammatory.Macrophages	ERP29	-0.03706	7.289905	-0.53541	0.593712	-7.06304	0.596901	0.657081
Non.inflammatory.Macrophages	CCL7	0.177657	1.659903	0.535286	0.593796	-6.39582	0.840756	0.768538
Non.inflammatory.Macrophages	PHF2OS1	-0.22219	1.058613	-0.53516	0.59388	-5.39724	0.871673	0.781488

Non.inflammatory.Macrophages	SENP8	-0.18087	0.568146	-0.53489	0.594066	-5.77612	0.889294	0.792261
Non.inflammatory.Macrophages	ALPK1	0.071043	4.802266	0.534771	0.594151	-6.77618	0.695023	0.704266
Non.inflammatory.Macrophages	DIAPH3	-0.12454	6.026174	-0.53474	0.594175	-6.65562	0.64497	0.680673
Non.inflammatory.Macrophages	PRR5L	-0.12157	2.756439	-0.53472	0.594189	-6.30021	0.786967	0.745527
Non.inflammatory.Macrophages	NRXN1	-0.26032	2.142242	-0.53464	0.594242	-5.69286	0.8167	0.758396
Non.inflammatory.Macrophages	MRPL42	0.056804	6.521235	0.534574	0.594286	-6.93118	0.625721	0.671383
Non.inflammatory.Macrophages	CYSTM1	0.126734	3.36709	0.534509	0.594331	-6.39814	0.758398	0.733018
Non.inflammatory.Macrophages	NCOA2	-0.04949	7.684552	-0.53439	0.594411	-7.01783	0.582664	0.650011
Non.inflammatory.Macrophages	GM4013	-0.11686	1.984122	-0.53417	0.594567	-5.87902	0.824664	0.76182
Non.inflammatory.Macrophages	NUCKS1	0.058489	6.576295	0.534065	0.594637	-6.86452	0.623728	0.670438
Non.inflammatory.Macrophages	TLR11	0.19883	-0.67266	0.533688	0.594897	-5.51679	0.891522	0.820192
Non.inflammatory.Macrophages	ABCF3	-0.07227	3.761251	-0.53353	0.595009	-6.32117	0.740907	0.72511
Non.inflammatory.Macrophages	SYMPK	0.066582	4.293778	0.533219	0.59522	-6.44925	0.717415	0.714546
Non.inflammatory.Macrophages	TSGA10	-0.08684	2.968133	-0.5332	0.595232	-6.22167	0.777526	0.741399
Non.inflammatory.Macrophages	TICRR	-0.18958	3.009192	-0.5331	0.595305	-5.7266	0.775606	0.740609

Non.inflammatory.Macrophages	GM12596	-0.21519	2.627238	-0.53247	0.595737	-5.51488	0.794221	0.748728
Non.inflammatory.Macrophages	CYB5B	0.050775	5.866247	0.532343	0.595824	-6.69074	0.652206	0.684242
Non.inflammatory.Macrophages	KIF24	-0.09522	3.667581	-0.53227	0.595875	-6.12048	0.745722	0.727457
Non.inflammatory.Macrophages	FAHD1	-0.1418	1.917334	-0.53216	0.595952	-5.93432	0.828999	0.763752
Non.inflammatory.Macrophages	RELN	-0.15621	3.647841	-0.53192	0.596119	-6.16714	0.746664	0.727857
Non.inflammatory.Macrophages	USP33	0.064228	4.710834	0.531906	0.596125	-6.5324	0.699914	0.706637
Non.inflammatory.Macrophages	CRADD	-0.05311	5.224096	-0.53183	0.596176	-6.65885	0.678344	0.696612
Non.inflammatory.Macrophages	PPIL3	-0.06107	4.605985	-0.53164	0.59631	-6.49702	0.704404	0.708709
Non.inflammatory.Macrophages	TIMM10	0.089776	3.050855	0.531635	0.596312	-6.19997	0.774189	0.740056
Non.inflammatory.Macrophages	FAM110A	0.104486	3.760358	0.53106	0.596709	-6.17406	0.741668	0.725624
Non.inflammatory.Macrophages	MRPL36	0.054043	5.835469	0.530931	0.596798	-6.79778	0.653553	0.684895
Non.inflammatory.Macrophages	PSD4	0.066945	4.541706	0.530894	0.596823	-6.52069	0.707246	0.710013
Non.inflammatory.Macrophages	DOCK3	-0.21122	0.039329	-0.53089	0.596824	-5.61665	0.892457	0.804701
Non.inflammatory.Macrophages	2610507B1	0.042008	5.431746	0.530878	0.596834	-6.73463	0.669877	0.692636
Non.inflammatory.Macrophages	EOGT	-0.1023	2.532472	-0.53083	0.596866	-6.1561	0.798935	0.750842

Non.inflammatory.Macrophages	SHC4	0.235167	0.470109	0.530782	0.5969	-5.45205	0.892457	0.795135
Non.inflammatory.Macrophages	TNFRSF4	-0.3192	0.30724	-0.53073	0.596936	-5.34647	0.892457	0.798739
Non.inflammatory.Macrophages	CARS2	-0.06367	3.502896	-0.53068	0.596972	-6.2672	0.753349	0.730842
Non.inflammatory.Macrophages	SDC3	0.078726	4.594842	0.530492	0.597101	-6.89896	0.704988	0.708964
Non.inflammatory.Macrophages	PPP1R3F	-0.19151	0.867721	-0.53045	0.597129	-5.61539	0.881351	0.786407
Non.inflammatory.Macrophages	A630072M	-0.13017	3.151561	-0.53036	0.597192	-5.9559	0.769596	0.738062
Non.inflammatory.Macrophages	CTCF	-0.03959	7.101448	-0.53023	0.597279	-6.84766	0.604851	0.661244
Non.inflammatory.Macrophages	MLH3	-0.11494	2.01272	-0.53006	0.597397	-6.02598	0.824515	0.761891
Non.inflammatory.Macrophages	NAT2	0.116868	2.532818	0.529929	0.597489	-6.01127	0.799081	0.750991
Non.inflammatory.Macrophages	ZZEF1	0.048436	5.867775	0.529755	0.59761	-6.74816	0.652397	0.684464
Non.inflammatory.Macrophages	ITPR3	0.091067	4.288295	0.529617	0.597705	-6.34365	0.718389	0.715233
Non.inflammatory.Macrophages	PSME4	0.048805	7.130098	0.529359	0.597883	-6.9734	0.603869	0.660843
Non.inflammatory.Macrophages	GM11837	-0.26557	0.292319	-0.52935	0.597887	-5.49505	0.89264	0.799305
Non.inflammatory.Macrophages	PELO	-0.09607	3.125716	-0.52927	0.597947	-6.10722	0.770931	0.73877
Non.inflammatory.Macrophages	GM43623	-0.21346	-0.49767	-0.52922	0.597978	-5.57834	0.89264	0.81702

Non.inflammatory.Macrophages	OAF	-0.12696	3.151249	-0.52916	0.598017	-6.01469	0.769739	0.738245
Non.inflammatory.Macrophages	SEC24D	0.061577	4.470665	0.529147	0.598029	-6.61287	0.710458	0.711628
Non.inflammatory.Macrophages	HSCB	-0.07298	4.143832	-0.52907	0.598083	-6.51892	0.72473	0.718131
Non.inflammatory.Macrophages	TMEM67	0.129204	1.688091	0.528733	0.598316	-5.97689	0.840918	0.768905
Non.inflammatory.Macrophages	FUT10	0.217897	-0.01434	0.528719	0.598325	-5.51216	0.89274	0.806137
Non.inflammatory.Macrophages	FBXL17	0.043889	7.560342	0.528686	0.598348	-7.06507	0.588209	0.652976
Non.inflammatory.Macrophages	PIK3C3	0.066859	4.439233	0.528481	0.59849	-6.44595	0.711989	0.712289
Non.inflammatory.Macrophages	ZFP385A	-0.06288	3.938147	-0.52833	0.598592	-6.62024	0.734036	0.722344
Non.inflammatory.Macrophages	THOC1	-0.05543	5.704802	-0.52811	0.598746	-6.65623	0.659096	0.687678
Non.inflammatory.Macrophages	HIST1H3C	0.327598	1.047316	0.527903	0.598889	-5.39715	0.874024	0.782865
Non.inflammatory.Macrophages	PDE8A	0.063322	5.847934	0.527684	0.599041	-6.91245	0.653355	0.685064
Non.inflammatory.Macrophages	MMACHC	-0.2341	0.470101	-0.52761	0.59909	-5.51984	0.892867	0.795631
Non.inflammatory.Macrophages	MYO1G	0.070764	5.563064	0.527599	0.599099	-6.65025	0.66483	0.690539
Non.inflammatory.Macrophages	MAPKBP1	-0.10774	3.047991	-0.52754	0.599137	-6.21145	0.774765	0.74064
Non.inflammatory.Macrophages	DENND6B	-0.22252	0.94417	-0.52749	0.599174	-5.47277	0.879151	0.78526

Non.inflammatory.Macrophages	4833420G1	0.072247	5.494494	0.527481	0.599181	-6.59284	0.667621	0.69189
Non.inflammatory.Macrophages	ITPKA	-0.27799	0.529225	-0.52725	0.599343	-5.34016	0.891974	0.794362
Non.inflammatory.Macrophages	KCNG3	0.194968	0.517714	0.527233	0.599352	-5.69504	0.892236	0.794616
Non.inflammatory.Macrophages	FUT8	0.056617	5.850748	0.52718	0.599389	-6.71859	0.653242	0.685061
Non.inflammatory.Macrophages	CYB5D2	0.147514	1.870839	0.527163	0.599401	-5.86668	0.831839	0.765296
Non.inflammatory.Macrophages	FOXO1	0.060208	7.035015	0.527082	0.599456	-6.76637	0.607554	0.662843
Non.inflammatory.Macrophages	SNHG10	-0.27583	0.227889	-0.52704	0.599489	-5.40648	0.892867	0.801036
Non.inflammatory.Macrophages	HBEGF	-0.11883	2.789221	-0.52668	0.599735	-6.39272	0.78723	0.746024
Non.inflammatory.Macrophages	CTNNBL1	0.056443	5.139211	0.526475	0.599876	-6.55074	0.682531	0.698853
Non.inflammatory.Macrophages	OTUD5	-0.04789	5.568497	-0.52641	0.599925	-6.68804	0.664873	0.690577
Non.inflammatory.Macrophages	FURIN	-0.07004	5.290193	-0.52614	0.600106	-6.7288	0.676347	0.696001
Non.inflammatory.Macrophages	ALPK3	-0.26505	-0.11417	-0.52612	0.600125	-5.46686	0.893323	0.808867
Non.inflammatory.Macrophages	TNPO3	0.044472	6.365771	0.525688	0.600421	-6.83498	0.633458	0.675651
Non.inflammatory.Macrophages	FRMD8	-0.07333	4.222806	-0.52559	0.600487	-6.42002	0.721985	0.717187
Non.inflammatory.Macrophages	GM16337	-0.20923	1.967439	-0.52547	0.600571	-5.57113	0.827638	0.763643

Non.inflammatory.Macrophages	ASCC3	-0.05178	6.882346	-0.52543	0.600597	-6.97933	0.613729	0.66603
Non.inflammatory.Macrophages	MICU3	0.075779	3.81398	0.525427	0.600601	-6.44843	0.740153	0.725423
Non.inflammatory.Macrophages	MYO9B	0.050613	5.866728	0.525121	0.600813	-6.79856	0.653194	0.685217
Non.inflammatory.Macrophages	VPS9D1	-0.08877	3.05216	-0.52502	0.600886	-6.23805	0.77527	0.741064
Non.inflammatory.Macrophages	DNAJB6	0.03816	8.310874	0.524875	0.600984	-7.13916	0.562311	0.640181
Non.inflammatory.Macrophages	NARF	0.062862	4.893244	0.524875	0.600984	-6.57346	0.6932	0.704078
Non.inflammatory.Macrophages	GPX3	-0.13075	1.463214	-0.52482	0.601021	-6.24017	0.853254	0.774576
Non.inflammatory.Macrophages	SPATA2	-0.07884	4.217056	-0.52468	0.601122	-6.42945	0.722377	0.717511
Non.inflammatory.Macrophages	SGK1	-0.06279	5.355259	-0.52459	0.601179	-6.80567	0.67396	0.695167
Non.inflammatory.Macrophages	TCIRG1	0.055823	5.455039	0.52397	0.60161	-6.79159	0.670234	0.693376
Non.inflammatory.Macrophages	4930590J0	0.145942	1.547542	0.523925	0.601642	-5.84217	0.849445	0.773013
Non.inflammatory.Macrophages	TAOK3	0.035096	7.012234	0.523793	0.601733	-6.98103	0.609339	0.663959
Non.inflammatory.Macrophages	SPACA9	0.103794	1.758151	0.523663	0.601823	-6.0716	0.838823	0.768507
Non.inflammatory.Macrophages	CCS	-0.07131	4.318203	-0.52342	0.601994	-6.49595	0.718444	0.715742
Non.inflammatory.Macrophages	ZSCAN22	0.199888	1.316109	0.523383	0.602017	-5.61005	0.861461	0.778091

Non.inflammatory.Macrophages	TTC19	0.068536	4.958514	0.523186	0.602154	-6.51565	0.690957	0.703132
Non.inflammatory.Macrophages	ARPP21	-0.39736	1.234602	-0.52303	0.602265	-5.33243	0.865685	0.779882
Non.inflammatory.Macrophages	ABCA3	-0.05856	4.193838	-0.523	0.602284	-6.47427	0.723901	0.71826
Non.inflammatory.Macrophages	CC2D2A	0.222598	0.81427	0.522787	0.60243	-5.58992	0.884979	0.789102
Non.inflammatory.Macrophages	GM36447	-0.25994	0.209364	-0.52275	0.602459	-5.40598	0.894335	0.802468
Non.inflammatory.Macrophages	TOR4A	0.120739	1.678835	0.522699	0.602491	-6.04613	0.84289	0.770371
Non.inflammatory.Macrophages	ATXN1L	0.089103	2.959513	0.522654	0.602522	-6.19422	0.780207	0.74344
Non.inflammatory.Macrophages	CBX2	-0.32125	-0.18673	-0.52264	0.602535	-5.19746	0.894335	0.811363
Non.inflammatory.Macrophages	PTRHD1	-0.06438	4.56189	-0.52256	0.602589	-6.59392	0.707864	0.71105
Non.inflammatory.Macrophages	SCP2	0.068385	7.526193	0.522376	0.602715	-7.10835	0.590542	0.654774
Non.inflammatory.Macrophages	FLOT1	0.064487	4.575358	0.522298	0.602769	-6.57834	0.707339	0.71084
Non.inflammatory.Macrophages	ADAM32	0.223799	0.784382	0.521954	0.603008	-5.68085	0.886135	0.79
Non.inflammatory.Macrophages	TMLHE	-0.15489	2.31306	-0.52194	0.603017	-5.97234	0.811423	0.757138
Non.inflammatory.Macrophages	REXO4	0.062325	4.291897	0.521721	0.603169	-6.45738	0.719711	0.716582
Non.inflammatory.Macrophages	MEGF9	-0.10792	4.103779	-0.5217	0.603181	-6.26725	0.727995	0.720344

Non.inflammatory.Macrophages	LYSMD2	-0.23301	1.265146	-0.52168	0.603199	-5.51881	0.864239	0.779525
Non.inflammatory.Macrophages	ABCC3	-0.11138	2.689429	-0.52165	0.603215	-6.42679	0.793186	0.749259
Non.inflammatory.Macrophages	SRGAP3	-0.11456	4.261174	-0.52154	0.603297	-6.35916	0.721077	0.717232
Non.inflammatory.Macrophages	UFD1	0.051779	5.129038	0.52111	0.603593	-6.58821	0.684013	0.700233
Non.inflammatory.Macrophages	RIPOR2	0.094272	7.617108	0.521032	0.603647	-6.61549	0.587388	0.653374
Non.inflammatory.Macrophages	GPATCH2	0.068696	4.253922	0.521024	0.603653	-6.47532	0.721478	0.717503
Non.inflammatory.Macrophages	AP2A2	0.042664	5.931782	0.520971	0.60369	-6.87505	0.651279	0.684766
Non.inflammatory.Macrophages	KCTD7	-0.20893	0.387211	-0.52096	0.603696	-5.46802	0.894605	0.798957
Non.inflammatory.Macrophages	KLF16	0.090548	3.164065	0.520659	0.603906	-6.14969	0.770878	0.739625
Non.inflammatory.Macrophages	GM16618	0.252572	0.733543	0.520438	0.604059	-5.33411	0.888036	0.791355
Non.inflammatory.Macrophages	SPN	0.200734	3.586459	0.520372	0.604105	-5.50544	0.751384	0.731006
Non.inflammatory.Macrophages	NOXRED1	-0.20439	0.546984	-0.52034	0.604125	-5.57075	0.893355	0.795466
Non.inflammatory.Macrophages	MGAT2	-0.04664	5.873385	-0.5203	0.604157	-6.77211	0.653645	0.685919
Non.inflammatory.Macrophages	PDHA1	0.0567	5.115043	0.520243	0.604194	-6.62245	0.684635	0.700555
Non.inflammatory.Macrophages	CPNE1	0.046942	6.038278	0.520167	0.604247	-6.74121	0.647086	0.682785

Non.inflammatory.Macrophages	SATB1	-0.10447	7.143818	-0.52015	0.604259	-6.6006	0.604723	0.662091
Non.inflammatory.Macrophages	CTTNBP2N	0.06858	2.52025	0.519999	0.604364	-6.5306	0.801545	0.753062
Non.inflammatory.Macrophages	CCDC90B	-0.07541	3.165156	-0.51929	0.604855	-6.34823	0.77104	0.739856
Non.inflammatory.Macrophages	GM16093	0.150611	2.640752	0.519265	0.604873	-5.84036	0.795899	0.750729
Non.inflammatory.Macrophages	HSD3B7	-0.08728	3.537133	-0.51924	0.604887	-6.42973	0.753845	0.732238
Non.inflammatory.Macrophages	ATG4D	-0.08465	3.954415	-0.51923	0.6049	-6.22759	0.734983	0.723783
Non.inflammatory.Macrophages	CD84	-0.05789	5.366126	-0.51922	0.604903	-6.86944	0.674409	0.695891
Non.inflammatory.Macrophages	ASH2L	0.065751	4.017187	0.519117	0.604976	-6.36358	0.732184	0.722539
Non.inflammatory.Macrophages	NR1H2	0.068001	4.589379	0.519019	0.605045	-6.54703	0.707127	0.711178
Non.inflammatory.Macrophages	GM45435	0.276331	0.920103	0.518916	0.605116	-5.47744	0.881965	0.787633
Non.inflammatory.Macrophages	TXNDC9	0.046439	5.512196	0.51877	0.605217	-6.73136	0.668419	0.693175
Non.inflammatory.Macrophages	CDCA3	-0.13292	4.895561	-0.51871	0.60526	-6.43322	0.694054	0.705178
Non.inflammatory.Macrophages	SHCBP1L	0.207703	0.854389	0.518643	0.605305	-5.52118	0.884183	0.789072
Non.inflammatory.Macrophages	AMIGO2	-0.25647	0.934211	-0.51861	0.605332	-5.55174	0.88149	0.787325
Non.inflammatory.Macrophages	PPM1K	0.10003	3.579795	0.518367	0.605497	-6.22016	0.751896	0.731546

Non.inflammatory.Macrophages	MAN2B2	-0.07538	3.799809	-0.51835	0.605507	-6.47238	0.741919	0.727081
Non.inflammatory.Macrophages	PPP1R3B	0.096865	2.491892	0.518339	0.605516	-6.16562	0.803089	0.754026
Non.inflammatory.Macrophages	MIPOL1	-0.06737	4.257052	-0.51827	0.605564	-6.39462	0.72158	0.717923
Non.inflammatory.Macrophages	9130401M	0.063528	4.02313	0.518196	0.605616	-6.42559	0.731919	0.722645
Non.inflammatory.Macrophages	TSFM	-0.06798	4.258618	-0.51802	0.605736	-6.39573	0.721575	0.717953
Non.inflammatory.Macrophages	UNC50	0.058651	4.105649	0.517758	0.60592	-6.50065	0.728394	0.721055
Non.inflammatory.Macrophages	GIPC1	0.061469	4.359749	0.517745	0.605929	-6.53761	0.71722	0.715973
Non.inflammatory.Macrophages	NCKAP1L	0.038863	6.137906	0.517361	0.606196	-6.87162	0.64365	0.681498
Non.inflammatory.Macrophages	CCDC34	-0.0919	4.840796	-0.51729	0.606246	-6.55791	0.69672	0.706564
Non.inflammatory.Macrophages	MTF1	0.062618	4.234341	0.517185	0.606318	-6.4492	0.722942	0.718612
Non.inflammatory.Macrophages	FBXO34	-0.05167	5.78838	-0.51688	0.606532	-6.73505	0.657641	0.68827
Non.inflammatory.Macrophages	PUS3	-0.12276	2.37825	-0.51674	0.606626	-5.8204	0.809121	0.756831
Non.inflammatory.Macrophages	COPS3	-0.04808	5.92883	-0.51663	0.606704	-6.68022	0.652017	0.685618
Non.inflammatory.Macrophages	ATXN7	-0.05209	6.23066	-0.51658	0.606741	-6.82779	0.640088	0.679881
Non.inflammatory.Macrophages	TAF5L	0.063369	4.562119	0.516479	0.606809	-6.45277	0.708742	0.71223

Non.inflammatory.Macrophages	IL21R	-0.08896	5.150614	-0.51647	0.606818	-6.57355	0.683764	0.700656
Non.inflammatory.Macrophages	SLC2A9	-0.09572	2.879571	-0.51642	0.606852	-6.30813	0.784975	0.746373
Non.inflammatory.Macrophages	MRPL10	-0.06706	4.602348	-0.51613	0.607053	-6.50409	0.707076	0.711496
Non.inflammatory.Macrophages	TMED1	-0.10928	2.452472	-0.51613	0.607055	-6.00312	0.805581	0.75536
Non.inflammatory.Macrophages	RUFY2	-0.09877	2.954919	-0.51598	0.60716	-6.04564	0.781479	0.744931
Non.inflammatory.Macrophages	GBA2	0.105004	2.547922	0.515953	0.607175	-5.9574	0.80095	0.753413
Non.inflammatory.Macrophages	MOGS	-0.07729	4.095452	-0.51541	0.60755	-6.42232	0.729541	0.72182
Non.inflammatory.Macrophages	ZFP830	-0.08651	3.541014	-0.51536	0.607587	-6.17118	0.75453	0.733061
Non.inflammatory.Macrophages	SLC35A5	0.060917	3.986264	0.515267	0.607652	-6.42747	0.7344	0.724059
Non.inflammatory.Macrophages	ZPBP	0.184216	1.648971	0.5152	0.607699	-5.59261	0.845907	0.772717
Non.inflammatory.Macrophages	RAB11B	-0.03714	7.147669	-0.51493	0.607888	-6.97075	0.605455	0.663151
Non.inflammatory.Macrophages	ZFP768	0.196373	0.794694	0.514892	0.607913	-5.68142	0.887241	0.791372
Non.inflammatory.Macrophages	SLIT2	0.28899	0.983319	0.514847	0.607945	-5.45047	0.88041	0.787236
Non.inflammatory.Macrophages	IGKV9-124	0.313012	-1.01492	0.514797	0.607979	-5.11075	0.895948	0.832122
Non.inflammatory.Macrophages	FTL1	-0.04355	11.26205	-0.51464	0.608085	-7.75703	0.470213	0.591446

Non.inflammatory.Macrophages	FAM57A	0.208908	1.118395	0.514498	0.608188	-5.58278	0.873321	0.784304
Non.inflammatory.Macrophages	LCMT1	-0.07549	3.124061	-0.51445	0.608224	-6.32781	0.773874	0.741762
Non.inflammatory.Macrophages	AK2	0.053503	5.995761	0.514225	0.608378	-6.76438	0.649717	0.684777
Non.inflammatory.Macrophages	OSBPL7	-0.09598	3.426262	-0.51422	0.608383	-6.07464	0.759828	0.735551
Non.inflammatory.Macrophages	RIOX2	0.068127	3.769866	0.514198	0.608397	-6.25127	0.744147	0.728551
Non.inflammatory.Macrophages	PRPF19	-0.05542	5.053951	-0.51412	0.608449	-6.58264	0.688193	0.703002
Non.inflammatory.Macrophages	MTMR10	0.090817	2.897646	0.513753	0.608706	-6.36185	0.784629	0.746637
Non.inflammatory.Macrophages	ZHX2	-0.07001	5.614006	-0.51369	0.60875	-6.7315	0.665121	0.69228
Non.inflammatory.Macrophages	FGGY	0.104966	3.459854	0.513543	0.608853	-6.28286	0.758353	0.735074
Non.inflammatory.Macrophages	SCO2	-0.08575	2.851352	-0.51347	0.608906	-6.2381	0.786829	0.747625
Non.inflammatory.Macrophages	IFIH1	0.080979	3.912874	0.513463	0.608908	-6.68055	0.737779	0.725864
Non.inflammatory.Macrophages	BTG1	0.04942	9.314237	0.513336	0.608997	-7.27513	0.530092	0.624582
Non.inflammatory.Macrophages	FAM118A	-0.12688	2.069936	-0.51323	0.609073	-5.8715	0.824846	0.764126
Non.inflammatory.Macrophages	KCTD1	0.181876	1.719442	0.513203	0.609089	-5.57995	0.842439	0.771608
Non.inflammatory.Macrophages	UACA	-0.16595	2.51718	-0.51318	0.609102	-5.82471	0.802886	0.754681

Non.inflammatory.Macrophages	BIN2	0.053321	5.165118	0.512978	0.609246	-6.76345	0.683631	0.701108
Non.inflammatory.Macrophages	ESRRA	0.063518	3.981496	0.51287	0.609321	-6.49472	0.734737	0.724625
Non.inflammatory.Macrophages	JPT2	0.093098	3.513173	0.512866	0.609324	-6.26511	0.755933	0.734131
Non.inflammatory.Macrophages	BLOC1S4	-0.06963	3.695064	-0.51265	0.609476	-6.33027	0.747728	0.730462
Non.inflammatory.Macrophages	PCTP	0.098925	2.252585	0.512565	0.609534	-6.13127	0.815946	0.76038
Non.inflammatory.Macrophages	MGAM	0.32073	0.078138	0.512291	0.609725	-5.3052	0.896373	0.807855
Non.inflammatory.Macrophages	TMEM126I	0.08498	2.697386	0.511347	0.610383	-6.18224	0.795254	0.751331
Non.inflammatory.Macrophages	MRPL16	0.067064	3.919331	0.510916	0.610683	-6.41044	0.73864	0.726316
Non.inflammatory.Macrophages	PISD	0.067294	4.8584	0.510881	0.610708	-6.56057	0.697602	0.70758
Non.inflammatory.Macrophages	PRCC	-0.05478	5.107381	-0.51087	0.610715	-6.55856	0.687088	0.702692
Non.inflammatory.Macrophages	BCS1L	-0.16758	1.699946	-0.51065	0.610869	-5.78164	0.844864	0.772639
Non.inflammatory.Macrophages	RDH12	0.29913	2.275253	0.510263	0.611139	-5.39547	0.816299	0.760496
Non.inflammatory.Macrophages	GM19705	-0.14214	1.868138	-0.51015	0.61122	-6.00149	0.836575	0.769163
Non.inflammatory.Macrophages	PAG1	0.067691	7.038518	0.510134	0.611229	-6.94089	0.610783	0.666078
Non.inflammatory.Macrophages	TAF4	-0.06993	4.075399	-0.50999	0.611327	-6.29156	0.732002	0.723402

Non.inflammatory.Macrophages	CEP85	-0.06859	4.470348	-0.50967	0.611553	-6.44522	0.714798	0.715575
Non.inflammatory.Macrophages	MORC3	-0.04716	5.857789	-0.50958	0.611613	-6.80264	0.656771	0.688462
Non.inflammatory.Macrophages	ROPN1L	0.123453	2.420763	0.50931	0.611804	-6.07581	0.809539	0.757663
Non.inflammatory.Macrophages	OGG1	-0.0737	3.189389	-0.50918	0.611895	-6.25114	0.772766	0.741665
Non.inflammatory.Macrophages	SRPK1	0.042649	6.11152	0.509043	0.61199	-6.71823	0.646763	0.68376
Non.inflammatory.Macrophages	ARMH3	0.051774	5.427651	0.508934	0.612066	-6.70925	0.674373	0.696908
Non.inflammatory.Macrophages	RALA	0.046808	5.871704	0.508865	0.612115	-6.80437	0.656319	0.688346
Non.inflammatory.Macrophages	IGTP	0.135382	3.443365	0.508782	0.612172	-6.35681	0.760959	0.736506
Non.inflammatory.Macrophages	METTL7A1	-0.08936	2.456994	-0.50862	0.612283	-6.15994	0.80777	0.757081
Non.inflammatory.Macrophages	TDP2	0.072873	4.340054	0.508526	0.612351	-6.36592	0.720608	0.718444
Non.inflammatory.Macrophages	ZFAND2A	0.060825	3.630572	0.50836	0.612467	-6.47812	0.752364	0.732804
Non.inflammatory.Macrophages	CD40	-0.12996	2.17854	-0.50832	0.612492	-6.22361	0.821457	0.763011
Non.inflammatory.Macrophages	CLUH	0.078404	3.417508	0.508311	0.612502	-6.33947	0.762153	0.737162
Non.inflammatory.Macrophages	E030042O2	-0.25301	0.180529	-0.5083	0.612509	-5.49186	0.898223	0.806567
Non.inflammatory.Macrophages	ABCA2	0.156477	1.527476	0.508073	0.612668	-5.81728	0.854421	0.777041

Non.inflammatory.Macrophages	PFKFB2	-0.09124	2.465839	-0.50792	0.612774	-6.08755	0.807485	0.757072
Non.inflammatory.Macrophages	APEH	-0.08411	3.695339	-0.50771	0.61292	-6.34428	0.749547	0.731667
Non.inflammatory.Macrophages	FRMD5	0.401177	2.390592	0.507696	0.612931	-5.44391	0.811162	0.758715
Non.inflammatory.Macrophages	NUP93	0.067063	4.586111	0.507666	0.612952	-6.43697	0.710021	0.713749
Non.inflammatory.Macrophages	MRPS23	0.065924	4.411962	0.507411	0.61313	-6.52915	0.717637	0.717237
Non.inflammatory.Macrophages	AGAP3	0.069278	4.127556	0.507336	0.613182	-6.51003	0.730164	0.722938
Non.inflammatory.Macrophages	DDX43	-0.29891	0.153087	-0.50732	0.61319	-5.28322	0.898443	0.807404
Non.inflammatory.Macrophages	ARRDC3	-0.08284	3.403751	-0.50719	0.613287	-6.44796	0.763014	0.737678
Non.inflammatory.Macrophages	KREMEN1	-0.09151	3.039966	-0.507	0.613417	-6.09844	0.780102	0.74522
Non.inflammatory.Macrophages	GM2449	-0.17153	1.279792	-0.50672	0.613612	-5.80103	0.86753	0.78265
Non.inflammatory.Macrophages	ACAP2	0.045134	7.320314	0.506718	0.613614	-7.05879	0.600902	0.661554
Non.inflammatory.Macrophages	0610010K1	-0.05046	5.617735	-0.50642	0.613823	-6.73606	0.666927	0.693779
Non.inflammatory.Macrophages	SBDS	0.044328	5.561152	0.506313	0.613897	-6.69828	0.669237	0.694897
Non.inflammatory.Macrophages	TXK	0.181711	3.287256	0.506213	0.613967	-5.87476	0.768588	0.740332
Non.inflammatory.Macrophages	DYNC2H1	0.083237	3.842041	0.506158	0.614006	-6.30734	0.743143	0.728989

Non.inflammatory.Macrophages	GM31597	0.123158	2.208627	0.505974	0.614134	-5.86327	0.820387	0.762923
Non.inflammatory.Macrophages	ETOHD2	0.110658	2.615429	0.505947	0.614153	-5.92051	0.800484	0.754341
Non.inflammatory.Macrophages	LAGE3	0.057913	4.771468	0.505904	0.614183	-6.56603	0.702278	0.710411
Non.inflammatory.Macrophages	SGO1	-0.16188	3.188246	-0.5059	0.614187	-5.98854	0.773213	0.742415
Non.inflammatory.Macrophages	DDX41	-0.06358	4.356626	-0.50586	0.614216	-6.4253	0.720249	0.71867
Non.inflammatory.Macrophages	ZFYVE1	-0.06511	4.797159	-0.50573	0.614302	-6.57009	0.701202	0.709948
Non.inflammatory.Macrophages	BC051226	-0.09934	2.179737	-0.50564	0.614369	-6.02314	0.821845	0.763628
Non.inflammatory.Macrophages	EPAS1	-0.1058	3.448567	-0.50542	0.614521	-6.25833	0.761238	0.737227
Non.inflammatory.Macrophages	BHLHB9	-0.16827	2.203905	-0.50516	0.614706	-5.59341	0.820803	0.763308
Non.inflammatory.Macrophages	E230032D2	0.133242	1.879678	0.505074	0.614764	-5.93594	0.836997	0.770221
Non.inflammatory.Macrophages	THAP2	0.088253	3.329281	0.505071	0.614766	-6.24766	0.766804	0.739783
Non.inflammatory.Macrophages	GM26810	-0.27049	0.423092	-0.50468	0.615039	-5.41991	0.898882	0.802126
Non.inflammatory.Macrophages	BTG3	-0.05516	5.347109	-0.5046	0.615097	-6.58507	0.678195	0.699498
Non.inflammatory.Macrophages	HOOK2	-0.08476	3.558904	-0.50458	0.615107	-6.38481	0.756198	0.735197
Non.inflammatory.Macrophages	VCL	0.069866	6.049702	0.504543	0.615135	-6.61115	0.649689	0.685948

Non.inflammatory.Macrophages	2810001G2	-0.11188	2.479465	-0.50445	0.615198	-5.85049	0.807266	0.757628
Non.inflammatory.Macrophages	ACYP1	0.076013	3.847714	0.504436	0.61521	-6.35121	0.743053	0.729328
Non.inflammatory.Macrophages	GM16091	-0.15945	2.306149	-0.50443	0.615216	-5.73587	0.815756	0.761288
Non.inflammatory.Macrophages	MAGI1	-0.15397	4.373842	-0.50394	0.615556	-6.3331	0.719746	0.718862
Non.inflammatory.Macrophages	LAMA4	0.277096	1.088771	0.503917	0.615573	-5.48458	0.877839	0.787641
Non.inflammatory.Macrophages	GLS2	0.20428	1.618045	0.503865	0.61561	-5.66349	0.850384	0.77615
Non.inflammatory.Macrophages	SEMA4F	0.284299	-0.41218	0.503722	0.61571	-5.23208	0.898995	0.821227
Non.inflammatory.Macrophages	SNHG15	-0.08924	3.481407	-0.50372	0.615713	-6.24886	0.759858	0.737006
Non.inflammatory.Macrophages	DCAF11	-0.0636	4.559305	-0.50358	0.615806	-6.48185	0.711664	0.715264
Non.inflammatory.Macrophages	ZBTB45	-0.09042	2.916254	-0.50355	0.615828	-6.11846	0.786328	0.748736
Non.inflammatory.Macrophages	9330160F1	-0.12988	2.295032	-0.50347	0.615884	-5.87166	0.816407	0.761815
Non.inflammatory.Macrophages	CCDC162	0.123835	2.876554	0.503209	0.616069	-6.26341	0.788219	0.749696
Non.inflammatory.Macrophages	RGS12	-0.14215	2.240588	-0.50319	0.616079	-5.76069	0.819093	0.763076
Non.inflammatory.Macrophages	SRPRB	-0.06471	4.104103	-0.50318	0.616089	-6.40321	0.731655	0.724522
Non.inflammatory.Macrophages	SPDYA	-0.26271	-0.02911	-0.50316	0.616106	-5.3544	0.898995	0.812745

Non.inflammatory.Macrophages	CXCR6	0.258273	1.255073	0.503051	0.61618	-5.48774	0.869127	0.784353
Non.inflammatory.Macrophages	9530052EC	-0.16219	1.000649	-0.50299	0.616224	-5.65958	0.882487	0.789918
Non.inflammatory.Macrophages	HAL	-0.17074	2.215151	-0.50276	0.616383	-5.97952	0.820454	0.763775
Non.inflammatory.Macrophages	MAP3K1	0.048956	7.970553	0.502635	0.61647	-6.96957	0.577677	0.650733
Non.inflammatory.Macrophages	TESMIN	-0.15411	0.971475	-0.50253	0.616545	-5.94399	0.884142	0.790678
Non.inflammatory.Macrophages	GM14302	-0.22888	0.609708	-0.50245	0.616597	-5.38865	0.896367	0.798707
Non.inflammatory.Macrophages	SEC16A	-0.06217	4.57627	-0.50217	0.616793	-6.56434	0.711019	0.715355
Non.inflammatory.Macrophages	FAM149B	0.093577	2.992688	0.502096	0.616848	-6.11317	0.782797	0.747587
Non.inflammatory.Macrophages	TLE3	-0.05991	5.003886	-0.50208	0.61686	-6.54181	0.692725	0.706889
Non.inflammatory.Macrophages	PPIP5K1	0.162959	1.776843	0.502041	0.616886	-5.79591	0.842407	0.773303
Non.inflammatory.Macrophages	SMIM26	-0.06534	3.847983	-0.50198	0.61693	-6.43172	0.743228	0.730051
Non.inflammatory.Macrophages	RNF220	0.061386	5.723291	0.501906	0.616981	-6.63305	0.662954	0.692939
Non.inflammatory.Macrophages	SASH3	-0.05821	5.4972	-0.50181	0.617047	-6.56697	0.672176	0.697314
Non.inflammatory.Macrophages	TECPR2	-0.06624	3.905519	-0.50174	0.617099	-6.50981	0.740635	0.72891
Non.inflammatory.Macrophages	9530082P2	-0.18435	0.699259	-0.50153	0.617246	-5.54077	0.893678	0.796897

Non.inflammatory.Macrophages	CHD4	0.039225	7.633169	0.501501	0.617265	-7.04917	0.589796	0.657075
Non.inflammatory.Macrophages	NAA30	0.068479	3.918894	0.5011	0.617546	-6.34007	0.740218	0.728796
Non.inflammatory.Macrophages	D430020J0	0.253526	0.440007	0.500756	0.617787	-5.42336	0.899333	0.802914
Non.inflammatory.Macrophages	CHD1	0.038263	6.90203	0.500611	0.617889	-6.92372	0.616962	0.67083
Non.inflammatory.Macrophages	TSNAX	0.062871	4.49606	0.500589	0.617904	-6.49028	0.714679	0.717304
Non.inflammatory.Macrophages	1190007I0	0.078264	3.503332	0.500558	0.617926	-6.27927	0.759133	0.7374
Non.inflammatory.Macrophages	RSPH3A	0.117453	2.558451	0.500537	0.617941	-5.99688	0.803827	0.757042
Non.inflammatory.Macrophages	ANGPTL6	-0.18816	0.97577	-0.50038	0.618047	-5.61728	0.884135	0.791108
Non.inflammatory.Macrophages	GM11131	-0.22965	0.815809	-0.5003	0.618106	-5.5011	0.889872	0.794631
Non.inflammatory.Macrophages	RBM43	-0.09471	2.961078	-0.50023	0.618154	-6.14728	0.784492	0.748639
Non.inflammatory.Macrophages	FAAP20	-0.07678	3.120662	-0.5002	0.618174	-6.26456	0.776949	0.745323
Non.inflammatory.Macrophages	PBLD1	-0.19747	1.75083	-0.5002	0.61818	-5.73245	0.843937	0.774265
Non.inflammatory.Macrophages	SLC22A23	-0.10976	3.017213	-0.50019	0.618183	-6.43449	0.781831	0.747471
Non.inflammatory.Macrophages	CCM2	0.048437	7.391127	0.499814	0.618448	-6.87287	0.59883	0.661875
Non.inflammatory.Macrophages	FBXL8	-0.12667	1.99347	-0.4997	0.618529	-5.93726	0.83183	0.769193

Non.inflammatory.Macrophages	ZFP266	-0.06288	4.029092	-0.49947	0.618689	-6.33722	0.735392	0.726869
Non.inflammatory.Macrophages	KDM8	0.159233	1.814343	0.499416	0.618727	-5.60439	0.84085	0.773061
Non.inflammatory.Macrophages	CIAO2B	-0.05982	4.566233	-0.49941	0.61873	-6.57197	0.711743	0.716082
Non.inflammatory.Macrophages	SKA2	0.119883	3.293834	0.499395	0.618741	-5.99672	0.76896	0.741895
Non.inflammatory.Macrophages	ERP44	-0.03376	6.074446	-0.49936	0.618769	-6.8264	0.649135	0.686637
Non.inflammatory.Macrophages	0610009B2	-0.07913	3.780562	-0.49917	0.618897	-6.42571	0.746583	0.731979
Non.inflammatory.Macrophages	TMEM41A	-0.1367	1.51992	-0.49901	0.619009	-5.88455	0.855871	0.779491
Non.inflammatory.Macrophages	NFE2L3	-0.14684	2.246069	-0.49901	0.619011	-5.84193	0.819259	0.763913
Non.inflammatory.Macrophages	LAD1	-0.40792	-0.99444	-0.49901	0.619012	-5.19861	0.899475	0.835839
Non.inflammatory.Macrophages	NLE1	0.149026	2.257092	0.498507	0.619364	-5.86797	0.819093	0.763857
Non.inflammatory.Macrophages	SHLD3	0.132369	2.343634	0.498206	0.619576	-5.8405	0.814886	0.762149
Non.inflammatory.Macrophages	ESR1	0.131191	3.611322	0.497932	0.619768	-6.12559	0.754697	0.73587
Non.inflammatory.Macrophages	TYSND1	0.088586	2.640407	0.497912	0.619783	-6.05141	0.800411	0.756023
Non.inflammatory.Macrophages	PUSL1	-0.1011	2.317284	-0.49787	0.61981	-6.01704	0.816183	0.762872
Non.inflammatory.Macrophages	ATP5C1	0.033611	8.358211	0.49784	0.619833	-7.16634	0.56462	0.644822

Non.inflammatory.Macrophages	CLTB	0.061198	4.396264	0.497798	0.619862	-6.55933	0.719533	0.72002
Non.inflammatory.Macrophages	TBCA	-0.03931	7.625569	-0.49778	0.619875	-7.10963	0.590595	0.658114
Non.inflammatory.Macrophages	NUTF2-PS1	-0.14913	2.848853	-0.49752	0.620059	-6.06535	0.790493	0.751744
Non.inflammatory.Macrophages	1700094J0	0.347956	0.092196	0.49747	0.620092	-5.23589	0.900078	0.811598
Non.inflammatory.Macrophages	FANCG	-0.12429	2.222876	-0.49737	0.62016	-5.87661	0.820957	0.764947
Non.inflammatory.Macrophages	SYNE2	-0.07422	5.373755	-0.49728	0.620226	-6.61057	0.677995	0.700739
Non.inflammatory.Macrophages	GPKOW	0.053556	4.488998	0.497078	0.620368	-6.46443	0.715667	0.718272
Non.inflammatory.Macrophages	CARNMT1	-0.0508	5.353674	-0.49697	0.620442	-6.61537	0.678917	0.701214
Non.inflammatory.Macrophages	2010001A1	-0.16446	1.389339	-0.49666	0.620659	-5.64718	0.863516	0.783067
Non.inflammatory.Macrophages	BRD8	-0.04419	6.236165	-0.49644	0.620816	-6.71863	0.643511	0.684306
Non.inflammatory.Macrophages	IRF5	0.053239	5.351662	0.496128	0.621035	-6.89573	0.679415	0.701392
Non.inflammatory.Macrophages	EIF3E	-0.0376	7.095265	-0.49605	0.621087	-6.95987	0.610658	0.668167
Non.inflammatory.Macrophages	OAS1G	0.158595	-0.24881	0.495772	0.621286	-5.87118	0.900941	0.819689
Non.inflammatory.Macrophages	PPIH	-0.06147	5.305306	-0.49553	0.621455	-6.55454	0.681535	0.702437
Non.inflammatory.Macrophages	NCAPD3	0.079016	5.127768	0.495518	0.621464	-6.49114	0.68896	0.705918

Non.inflammatory.Macrophages	SEC63	-0.03819	7.09953	-0.49541	0.621539	-6.93849	0.610681	0.668237
Non.inflammatory.Macrophages	GM43388	0.319365	0.386691	0.495276	0.621634	-5.30133	0.90106	0.805489
Non.inflammatory.Macrophages	AIMP1	0.041276	6.38044	0.495025	0.62181	-6.85637	0.638328	0.681895
Non.inflammatory.Macrophages	BC005624	0.041531	5.761918	0.494891	0.621905	-6.74685	0.662973	0.693761
Non.inflammatory.Macrophages	GOSR2	-0.05216	5.28486	-0.49475	0.622001	-6.69259	0.682587	0.703098
Non.inflammatory.Macrophages	TRAPPC10	-0.05125	5.170629	-0.4946	0.622113	-6.62461	0.687363	0.70537
Non.inflammatory.Macrophages	ARMC1	0.049553	4.827556	0.494595	0.622113	-6.60523	0.701898	0.712139
Non.inflammatory.Macrophages	ZBTB12	0.131776	1.665552	0.494419	0.622236	-5.79705	0.850184	0.777647
Non.inflammatory.Macrophages	GM47828	0.268714	0.334612	0.49415	0.622425	-5.28881	0.901399	0.807027
Non.inflammatory.Macrophages	FEN1	-0.08509	4.525584	-0.49398	0.622545	-6.39543	0.715034	0.718278
Non.inflammatory.Macrophages	HACE1	0.071871	4.490352	0.493915	0.622591	-6.42522	0.71657	0.718982
Non.inflammatory.Macrophages	FAM98A	0.077191	3.607656	0.493889	0.622609	-6.23318	0.756076	0.736866
Non.inflammatory.Macrophages	AI987944	-0.09759	2.764701	-0.49389	0.622612	-6.12528	0.795693	0.754352
Non.inflammatory.Macrophages	PWP2	-0.14369	1.891751	-0.49363	0.62279	-5.84407	0.838732	0.773003
Non.inflammatory.Macrophages	4930594M	-0.22821	-0.02762	-0.49356	0.622841	-5.49594	0.901399	0.815335

Non.inflammatory.Macrophages	LHPP	-0.09088	3.112672	-0.49355	0.622851	-6.14718	0.779111	0.747194
Non.inflammatory.Macrophages	ANK2	-0.11933	2.998552	-0.49345	0.622919	-6.56609	0.784513	0.749589
Non.inflammatory.Macrophages	CPT1A	0.042909	5.446894	0.493428	0.622934	-6.78802	0.675963	0.700209
Non.inflammatory.Macrophages	LTV1	-0.06753	4.232009	-0.4933	0.623024	-6.49013	0.727945	0.724305
Non.inflammatory.Macrophages	TLR4	0.072729	3.122783	0.493161	0.623122	-6.6031	0.778653	0.74704
Non.inflammatory.Macrophages	ZFP946	0.134425	1.39583	0.493105	0.623161	-5.78523	0.864142	0.783791
Non.inflammatory.Macrophages	BMP2	-0.1803	1.613957	-0.49303	0.623216	-5.72735	0.85289	0.779054
Non.inflammatory.Macrophages	A230072C(	-0.14884	1.545767	-0.49291	0.623302	-5.74446	0.856393	0.780572
Non.inflammatory.Macrophages	SLC33A1	-0.07064	3.794664	-0.4927	0.623443	-6.39076	0.747558	0.733325
Non.inflammatory.Macrophages	RIMS4	-0.2093	-0.99611	-0.49256	0.623547	-5.58552	0.901422	0.837753
Non.inflammatory.Macrophages	LRMP	0.054561	6.432878	0.492547	0.623553	-6.82582	0.636425	0.681412
Non.inflammatory.Macrophages	SHCBP1	-0.13528	4.69138	-0.49249	0.623595	-6.24677	0.707867	0.715292
Non.inflammatory.Macrophages	SNRPB	-0.04094	7.300883	-0.4923	0.62373	-7.00016	0.603457	0.665261
Non.inflammatory.Macrophages	CUL3	0.034191	7.198802	0.492252	0.623761	-6.94204	0.607247	0.667178
Non.inflammatory.Macrophages	GM20300	-0.12703	1.343674	-0.49223	0.623779	-5.80795	0.866852	0.785245

Non.inflammatory.Macrophages	PPM1M	0.07239	4.583696	0.492175	0.623815	-6.36898	0.712526	0.717568
Non.inflammatory.Macrophages	POC1A	0.101083	3.229083	0.491978	0.623954	-6.10694	0.773744	0.745193
Non.inflammatory.Macrophages	RABL2	-0.17465	1.106471	-0.49162	0.624206	-5.60735	0.879428	0.790558
Non.inflammatory.Macrophages	PAN2	-0.08138	3.190864	-0.49158	0.624236	-6.14718	0.775583	0.746041
Non.inflammatory.Macrophages	SYNRG	0.050833	5.053855	0.491574	0.624239	-6.64043	0.692514	0.708342
Non.inflammatory.Macrophages	BCL10	0.04167	6.452929	0.491506	0.624287	-6.85783	0.635754	0.681282
Non.inflammatory.Macrophages	ADAM23	0.153108	3.184267	0.491427	0.624342	-6.05843	0.775893	0.746178
Non.inflammatory.Macrophages	AW146154	-0.13304	2.334177	-0.49127	0.624454	-5.92032	0.816821	0.764067
Non.inflammatory.Macrophages	HIST1H2BJ	-0.18573	3.30978	-0.49126	0.624457	-5.86074	0.770014	0.743609
Non.inflammatory.Macrophages	PLEKHM1	0.052918	4.771883	0.490888	0.624722	-6.62101	0.704584	0.714056
Non.inflammatory.Macrophages	TOMM40L	0.103654	1.733558	0.490874	0.624732	-5.90772	0.846996	0.777043
Non.inflammatory.Macrophages	GNB4	0.132585	2.895468	0.490775	0.624802	-5.87154	0.789647	0.752337
Non.inflammatory.Macrophages	A430072P(	0.375562	0.354986	0.490773	0.624803	-5.1811	0.901655	0.80738
Non.inflammatory.Macrophages	NDUFAB1	0.053162	6.268706	0.490669	0.624876	-6.87732	0.643019	0.684911
Non.inflammatory.Macrophages	IL20RB	-0.08183	3.81612	-0.49062	0.624909	-6.32017	0.746777	0.733308

Non.inflammatory.Macrophages	IGF2BP3	0.052328	6.5516	0.490382	0.625078	-6.87512	0.631986	0.679538
Non.inflammatory.Macrophages	2610021A(	0.124121	2.362444	0.490381	0.625079	-5.86849	0.815508	0.763574
Non.inflammatory.Macrophages	GZMM	-0.14673	2.051803	-0.49032	0.625119	-5.85133	0.83093	0.770199
Non.inflammatory.Macrophages	XPO6	0.045312	5.572883	0.490233	0.625183	-6.71781	0.670979	0.698319
Non.inflammatory.Macrophages	ARCN1	0.044844	5.873425	0.490081	0.625291	-6.78729	0.658809	0.692517
Non.inflammatory.Macrophages	TUBB4B	-0.08173	6.422022	-0.48985	0.62545	-6.77664	0.637121	0.682046
Non.inflammatory.Macrophages	GZMB	-0.29979	3.971899	-0.48975	0.625524	-5.83945	0.739868	0.730222
Non.inflammatory.Macrophages	GM43258	0.250816	0.724157	0.489715	0.625549	-5.3368	0.895458	0.799255
Non.inflammatory.Macrophages	MYO3B	0.164342	1.289051	0.489356	0.625802	-5.69303	0.870331	0.78683
Non.inflammatory.Macrophages	SLC18A2	-0.16176	2.251616	-0.48904	0.626027	-5.60027	0.821458	0.76607
Non.inflammatory.Macrophages	FAM234B	-0.14298	2.096184	-0.48898	0.626065	-5.84622	0.829193	0.769388
Non.inflammatory.Macrophages	A630001O:	-0.11175	2.282008	-0.48896	0.626081	-6.00725	0.819953	0.765422
Non.inflammatory.Macrophages	ZSCAN29	0.081614	3.485733	0.488709	0.626258	-6.17023	0.762461	0.740297
Non.inflammatory.Macrophages	GM46620	-0.27707	0.683678	-0.48865	0.626298	-5.36217	0.897349	0.800277
Non.inflammatory.Macrophages	CLK4	-0.04519	6.012894	-0.48815	0.626654	-6.76043	0.653811	0.690186

Non.inflammatory.Macrophages	CDK17	0.048835	6.780519	0.488031	0.626736	-6.88368	0.623797	0.675593
Non.inflammatory.Macrophages	USO1	-0.0472	5.739311	-0.48789	0.626836	-6.72147	0.664841	0.695463
Non.inflammatory.Macrophages	GM29488	0.199797	0.172443	0.48784	0.626871	-5.59831	0.90255	0.811928
Non.inflammatory.Macrophages	ANLN	-0.12548	3.747041	-0.48779	0.626906	-6.18096	0.750662	0.735123
Non.inflammatory.Macrophages	UNC45B	0.254565	0.423376	0.487756	0.62693	-5.48098	0.90255	0.806292
Non.inflammatory.Macrophages	TRAM1	0.038393	7.121393	0.487662	0.626996	-6.92661	0.6109	0.669239
Non.inflammatory.Macrophages	DISP1	0.087858	3.443949	0.487627	0.627021	-6.15414	0.764598	0.741383
Non.inflammatory.Macrophages	CDCA8	-0.12874	5.109425	-0.48751	0.627103	-6.47489	0.690914	0.707802
Non.inflammatory.Macrophages	TMEM185/	0.095052	2.540183	0.487402	0.62718	-6.11588	0.807594	0.760297
Non.inflammatory.Macrophages	MAST2	-0.0704	5.283184	-0.48732	0.627241	-6.52379	0.683626	0.704421
Non.inflammatory.Macrophages	REX1BD	-0.04287	5.688469	-0.48728	0.627264	-6.79745	0.66691	0.696517
Non.inflammatory.Macrophages	HIST1H4M	-0.24019	1.423329	-0.48687	0.627554	-5.48814	0.863969	0.784399
Non.inflammatory.Macrophages	PLSCR2	-0.36506	-0.38835	-0.48684	0.627577	-5.23353	0.902732	0.824871
Non.inflammatory.Macrophages	MIA2	-0.0355	6.77012	-0.48682	0.62759	-6.9681	0.62432	0.67596
Non.inflammatory.Macrophages	DHFR	-0.10188	4.331798	-0.48667	0.627695	-6.34524	0.724634	0.72346

Non.inflammatory.Macrophages	APBB3	-0.12352	1.534528	-0.48584	0.628285	-5.93307	0.858873	0.782108
Non.inflammatory.Macrophages	EDRF1	0.076665	4.217889	0.485791	0.628317	-6.33769	0.730187	0.725853
Non.inflammatory.Macrophages	TYW3	0.176261	0.995715	0.485567	0.628476	-5.62205	0.887095	0.794025
Non.inflammatory.Macrophages	PRR7	0.102614	3.334702	0.485415	0.628583	-6.10317	0.770424	0.744063
Non.inflammatory.Macrophages	GTDC1	0.047314	6.409058	0.485413	0.628584	-6.92595	0.638768	0.683034
Non.inflammatory.Macrophages	BRD3	0.055769	5.712362	0.485334	0.62864	-6.64083	0.66658	0.696429
Non.inflammatory.Macrophages	ADAM19	-0.13606	5.568234	-0.4853	0.628662	-6.26392	0.672477	0.699229
Non.inflammatory.Macrophages	DEPTOR	-0.068	3.469195	-0.48529	0.628671	-6.56714	0.764165	0.741302
Non.inflammatory.Macrophages	KCNK6	0.076326	2.306704	0.485081	0.628819	-6.35183	0.819885	0.765719
Non.inflammatory.Macrophages	A530041M	-0.14819	2.069945	-0.48502	0.628862	-5.66037	0.831675	0.770777
Non.inflammatory.Macrophages	CHID1	0.077258	2.453126	0.484867	0.62897	-6.17077	0.81267	0.762671
Non.inflammatory.Macrophages	GH	0.273993	-0.76502	0.484679	0.629103	-5.17974	0.903461	0.834029
Non.inflammatory.Macrophages	COQ9	0.083664	3.205352	0.48466	0.629116	-6.20925	0.776521	0.746923
Non.inflammatory.Macrophages	MSI2	0.046973	7.509903	0.484608	0.629153	-6.92278	0.597118	0.662589
Non.inflammatory.Macrophages	SLC25A5	0.037535	7.608641	0.484591	0.629165	-7.14011	0.593511	0.660773

Non.inflammatory.Macrophages	2900089D1	-0.0952	2.58395	-0.48406	0.629542	-6.23114	0.80657	0.760191
Non.inflammatory.Macrophages	CENPX	0.046394	6.347296	0.484001	0.629582	-6.87562	0.64145	0.684591
Non.inflammatory.Macrophages	CPS1	0.141307	4.481272	0.483981	0.629596	-6.47583	0.71887	0.721096
Non.inflammatory.Macrophages	RASSF5	-0.04934	5.485464	-0.48388	0.629667	-6.76345	0.676167	0.701216
Non.inflammatory.Macrophages	CNNM2	-0.09401	5.427728	-0.48353	0.629915	-6.65514	0.678605	0.702401
Non.inflammatory.Macrophages	CLCF1	0.139867	2.650892	0.483526	0.629918	-5.87373	0.803376	0.758838
Non.inflammatory.Macrophages	PLPPR1	0.197343	1.149205	0.483503	0.629934	-5.58444	0.879401	0.791189
Non.inflammatory.Macrophages	IRF2	0.044273	6.689517	0.483397	0.630009	-6.94461	0.628197	0.678167
Non.inflammatory.Macrophages	ZBTB39	-0.13168	1.975209	-0.48325	0.630111	-5.90442	0.836808	0.77328
Non.inflammatory.Macrophages	WRAP73	0.085739	2.923556	0.48325	0.630113	-6.06809	0.790237	0.753151
Non.inflammatory.Macrophages	PDLIM7	0.093136	3.638312	0.483021	0.630275	-6.16624	0.75679	0.73832
Non.inflammatory.Macrophages	SMTNL2	-0.31322	0.4226	-0.48299	0.630294	-5.28741	0.903931	0.807369
Non.inflammatory.Macrophages	PAQR4	0.145656	1.484926	0.482878	0.630376	-6.00916	0.86194	0.783908
Non.inflammatory.Macrophages	SMG6	-0.03567	8.045885	-0.48279	0.630441	-7.05483	0.578108	0.653114
Non.inflammatory.Macrophages	MFSD8	0.083873	2.979793	0.482583	0.630584	-6.04728	0.787723	0.752026

Non.inflammatory.Macrophages	ATP8B2	0.120891	2.774077	0.482422	0.630699	-5.76634	0.797595	0.756342
Non.inflammatory.Macrophages	ZFP652	-0.05648	5.654413	-0.4823	0.630783	-6.76358	0.669422	0.698074
Non.inflammatory.Macrophages	GNAL	0.175912	1.371741	0.482148	0.630892	-5.56107	0.867931	0.786511
Non.inflammatory.Macrophages	RAC1	0.028656	7.82414	0.482021	0.630982	-7.16999	0.586106	0.657264
Non.inflammatory.Macrophages	HNRNPM	-0.03188	7.703885	-0.48194	0.63104	-7.06314	0.590448	0.659481
Non.inflammatory.Macrophages	GM42982	0.220334	1.514483	0.481926	0.631049	-5.34484	0.860522	0.783445
Non.inflammatory.Macrophages	ARL10	-0.09481	2.295947	-0.48192	0.631056	-6.09358	0.820967	0.766608
Non.inflammatory.Macrophages	GM26631	-0.17749	1.219353	-0.48154	0.631323	-5.60799	0.876183	0.789954
Non.inflammatory.Macrophages	ZFP868	-0.08639	3.676008	-0.48127	0.631516	-6.20384	0.755556	0.737878
Non.inflammatory.Macrophages	TNFRSF19	0.277665	0.886333	0.481074	0.631652	-5.32978	0.8926	0.797452
Non.inflammatory.Macrophages	2700081O1	-0.13508	3.137169	-0.48099	0.63171	-5.79457	0.780653	0.749116
Non.inflammatory.Macrophages	4930509H(	-0.14884	2.090644	-0.48097	0.631724	-5.62335	0.831616	0.771251
Non.inflammatory.Macrophages	HACD4	0.063817	3.532004	0.480898	0.631777	-6.808	0.762188	0.740971
Non.inflammatory.Macrophages	PTDSS1	0.050329	5.371518	0.480713	0.631908	-6.63653	0.681505	0.704038
Non.inflammatory.Macrophages	4930595D1	0.205129	1.248821	0.480332	0.632178	-5.51928	0.875171	0.789711

Non.inflammatory.Macrophages	FBXO44	-0.26193	0.220079	-0.47982	0.632537	-5.39355	0.905325	0.81273
Non.inflammatory.Macrophages	SHKBP1	-0.06196	4.068341	-0.47954	0.632736	-6.45072	0.738543	0.730343
Non.inflammatory.Macrophages	ZFP157	0.120629	2.446699	0.479325	0.632891	-5.94884	0.814809	0.764165
Non.inflammatory.Macrophages	GMFG	0.047279	7.424355	0.479319	0.632895	-7.07784	0.601605	0.665297
Non.inflammatory.Macrophages	XPNPEP1	0.054514	4.365592	0.479168	0.633002	-6.53359	0.725308	0.724448
Non.inflammatory.Macrophages	PSME1	0.057336	8.384368	0.479157	0.63301	-7.24755	0.567178	0.647772
Non.inflammatory.Macrophages	MAPK1IP1	0.112804	2.304416	0.478946	0.63316	-5.86949	0.821837	0.767252
Non.inflammatory.Macrophages	ARMT1	-0.08103	3.297769	-0.47888	0.633203	-6.0889	0.773915	0.746341
Non.inflammatory.Macrophages	1110012L1	0.11402	1.829365	0.478829	0.633242	-5.95495	0.845706	0.777454
Non.inflammatory.Macrophages	PTMA	0.045477	11.35991	0.478751	0.633298	-7.54617	0.472363	0.59638
Non.inflammatory.Macrophages	HNRNPA0	-0.03493	7.214278	-0.47872	0.633319	-6.97664	0.609407	0.669261
Non.inflammatory.Macrophages	6330562C2	0.134477	1.275201	0.478527	0.633456	-5.8694	0.87444	0.789619
Non.inflammatory.Macrophages	GM43560	-0.15724	0.578606	-0.47844	0.63352	-5.54647	0.903538	0.805106
Non.inflammatory.Macrophages	SPINDOC	0.065691	4.427162	0.478125	0.633742	-6.37472	0.722756	0.7235
Non.inflammatory.Macrophages	TSTA3	0.062555	4.100613	0.478098	0.63376	-6.47198	0.73726	0.730109

Non.inflammatory.Macrophages	HINT2	-0.05889	4.356845	-0.47798	0.633845	-6.53197	0.725856	0.724934
Non.inflammatory.Macrophages	ZFP932	-0.08801	2.788655	-0.47795	0.633862	-5.97103	0.798322	0.75727
Non.inflammatory.Macrophages	8430429K(	-0.12874	1.679457	-0.47784	0.633944	-5.75239	0.853579	0.781028
Non.inflammatory.Macrophages	ALDH6A1	-0.12614	2.786139	-0.47774	0.634012	-6.10801	0.798464	0.757372
Non.inflammatory.Macrophages	PIK3CD	0.0555	6.624599	0.477648	0.63408	-6.88164	0.631991	0.680625
Non.inflammatory.Macrophages	DBT	0.083413	3.826101	0.477178	0.634413	-6.29658	0.749851	0.735877
Non.inflammatory.Macrophages	MZT2	0.091577	2.206691	0.477136	0.634443	-5.99625	0.827087	0.76979
Non.inflammatory.Macrophages	GSTO2	-0.17103	0.524993	-0.47708	0.634483	-5.7481	0.905108	0.806618
Non.inflammatory.Macrophages	RGS19	-0.05338	5.393744	-0.47707	0.634489	-6.75568	0.681558	0.704455
Non.inflammatory.Macrophages	MVK	-0.11638	2.634356	-0.47672	0.634735	-5.95101	0.806233	0.760768
Non.inflammatory.Macrophages	HIBCH	0.074539	3.478236	0.476427	0.634946	-6.18199	0.766245	0.743156
Non.inflammatory.Macrophages	BC051537	0.209644	0.044835	0.476058	0.635207	-5.57565	0.906582	0.817731
Non.inflammatory.Macrophages	BBS5	-0.14813	0.624034	-0.47603	0.63523	-5.875	0.903489	0.804694
Non.inflammatory.Macrophages	PLBD2	-0.05138	4.310294	-0.47584	0.635359	-6.63199	0.728633	0.72629
Non.inflammatory.Macrophages	EIF2B1	-0.07133	3.62049	-0.47581	0.635382	-6.26516	0.759831	0.740377

Non.inflammatory.Macrophages	PPP4R1	0.05222	5.579566	0.475752	0.635425	-6.6959	0.674362	0.701092
Non.inflammatory.Macrophages	ABI2	0.073502	3.903545	0.475548	0.635569	-6.43978	0.746972	0.734642
Non.inflammatory.Macrophages	0610009L1	0.112219	2.030404	0.475426	0.635656	-6.01253	0.836666	0.773969
Non.inflammatory.Macrophages	PKD1L3	-0.20894	0.837305	-0.47523	0.635794	-5.49412	0.896528	0.800136
Non.inflammatory.Macrophages	HIST1H2AN	0.244738	3.254826	0.475155	0.635848	-5.89094	0.777061	0.748161
Non.inflammatory.Macrophages	USP37	0.049193	6.268931	0.474906	0.636025	-6.80716	0.646794	0.688027
Non.inflammatory.Macrophages	RRM1	-0.07884	5.449593	-0.4746	0.636241	-6.62187	0.680073	0.703941
Non.inflammatory.Macrophages	FBXO47	-0.23275	1.069812	-0.47456	0.636268	-5.50023	0.886691	0.795159
Non.inflammatory.Macrophages	AKT1S1	-0.06436	3.589237	-0.47456	0.636274	-6.37654	0.761649	0.741356
Non.inflammatory.Macrophages	CCL2	-0.1023	3.089272	-0.47434	0.636428	-6.76179	0.785116	0.751753
Non.inflammatory.Macrophages	TPP2	-0.03565	7.082191	-0.47434	0.63643	-6.9825	0.61543	0.672673
Non.inflammatory.Macrophages	KLRI1	-0.29561	-0.19768	-0.47421	0.636518	-5.27084	0.907091	0.823664
Non.inflammatory.Macrophages	ZFP628	0.112896	2.635685	0.47402	0.636654	-6.02891	0.807074	0.761361
Non.inflammatory.Macrophages	SGTA	-0.05456	4.959007	-0.47368	0.636895	-6.62026	0.701058	0.71372
Non.inflammatory.Macrophages	GRB10	-0.16477	3.090696	-0.4735	0.63702	-6.02611	0.785445	0.751861

Non.inflammatory.Macrophages	APOO	-0.08029	3.509146	-0.47304	0.637353	-6.16921	0.766093	0.74336
Non.inflammatory.Macrophages	ASB8	-0.06665	3.607063	-0.47284	0.637491	-6.37169	0.761639	0.74136
Non.inflammatory.Macrophages	GPATCH3	-0.08676	3.369567	-0.47237	0.637825	-6.2315	0.772919	0.746436
Non.inflammatory.Macrophages	GM15489	-0.29195	0.208522	-0.47234	0.637847	-5.30734	0.908266	0.814992
Non.inflammatory.Macrophages	GM15503	0.150479	0.81285	0.471924	0.638144	-5.61741	0.898814	0.801645
Non.inflammatory.Macrophages	ZRSR1	0.119603	2.211301	0.471852	0.638195	-5.90719	0.829003	0.771086
Non.inflammatory.Macrophages	ARHGEF6	0.054573	5.643537	0.471812	0.638224	-6.66766	0.672979	0.700841
Non.inflammatory.Macrophages	PARP8	0.049318	6.68237	0.471784	0.638243	-6.95664	0.631534	0.680862
Non.inflammatory.Macrophages	ALG5	0.056034	4.2129	0.471645	0.638342	-6.56441	0.734326	0.729317
Non.inflammatory.Macrophages	KRI1	0.059585	4.367396	0.47155	0.63841	-6.38643	0.727454	0.726208
Non.inflammatory.Macrophages	PLXNB3	0.174846	0.164762	0.471549	0.638411	-5.62515	0.908266	0.816225
Non.inflammatory.Macrophages	TSPOAP1	-0.11301	2.650845	-0.47138	0.638529	-6.08771	0.807292	0.761753
Non.inflammatory.Macrophages	TBC1D32	0.099614	2.141265	0.471343	0.638557	-6.19612	0.832512	0.772626
Non.inflammatory.Macrophages	KHNYN	-0.06612	4.263958	-0.47133	0.638567	-6.36077	0.732048	0.728316
Non.inflammatory.Macrophages	PHF11D	-0.12819	1.34229	-0.47131	0.638582	-6.20339	0.873506	0.789986

Non.inflammatory.Macrophages	9030404E1	0.323491	-0.16276	0.471256	0.638619	-5.21682	0.908266	0.823727
Non.inflammatory.Macrophages	ATG7	-0.05182	5.786606	-0.47084	0.638917	-6.81757	0.667261	0.698126
Non.inflammatory.Macrophages	MAP2K7	0.052452	4.294187	0.470822	0.638927	-6.51797	0.730857	0.727743
Non.inflammatory.Macrophages	EID2B	0.112763	1.703903	0.470738	0.638987	-5.83268	0.85491	0.78212
Non.inflammatory.Macrophages	INTS12	-0.0581	4.380987	-0.47068	0.63903	-6.45999	0.727006	0.725986
Non.inflammatory.Macrophages	MAPRE1	-0.03187	7.148653	-0.47052	0.639142	-6.98758	0.613871	0.672149
Non.inflammatory.Macrophages	CHCHD1	0.046862	5.813268	0.470501	0.639156	-6.79468	0.666174	0.697608
Non.inflammatory.Macrophages	B3GALNT2	0.087496	3.643954	0.470125	0.639423	-6.19167	0.76051	0.741197
Non.inflammatory.Macrophages	LETM1	0.05364	4.54276	0.470041	0.639483	-6.4995	0.720061	0.72289
Non.inflammatory.Macrophages	TMEM132/	0.150996	0.400445	0.469992	0.639518	-5.7491	0.908686	0.811165
Non.inflammatory.Macrophages	2310008N1	-0.20031	0.80481	-0.46963	0.639778	-5.57417	0.899778	0.802265
Non.inflammatory.Macrophages	MCFD2	-0.07121	3.702476	-0.46933	0.639986	-6.45534	0.758116	0.740205
Non.inflammatory.Macrophages	ETS2	0.054121	5.865691	0.46916	0.64011	-6.92002	0.664474	0.69701
Non.inflammatory.Macrophages	BIRC3	0.04884	7.111789	0.469069	0.640175	-7.025	0.615661	0.673259
Non.inflammatory.Macrophages	MTMR9	0.057608	3.972449	0.468903	0.640293	-6.3947	0.745784	0.734783

Non.inflammatory.Macrophages	GM26674	-0.19159	-1.15959	-0.46887	0.640314	-5.46695	0.909049	0.847395
Non.inflammatory.Macrophages	CALD1	0.107614	3.777745	0.468873	0.640314	-6.35614	0.754658	0.738776
Non.inflammatory.Macrophages	ZBTB38	-0.05812	5.068147	-0.46884	0.640339	-6.59765	0.697644	0.71271
Non.inflammatory.Macrophages	ZBTB5	0.106015	2.463409	0.46877	0.640387	-6.00043	0.817189	0.766297
Non.inflammatory.Macrophages	ZFP945	-0.08149	3.17689	-0.46869	0.640445	-6.20738	0.782672	0.751238
Non.inflammatory.Macrophages	SLC25A19	-0.07623	3.390435	-0.46848	0.640593	-6.28906	0.772624	0.746797
Non.inflammatory.Macrophages	GM49101	-0.16476	-0.65342	-0.46848	0.640593	-5.74539	0.909069	0.835605
Non.inflammatory.Macrophages	PGS1	0.053058	4.740858	0.468293	0.640728	-6.58282	0.711797	0.719273
Non.inflammatory.Macrophages	NPHP4	0.23762	0.297045	0.468109	0.640859	-5.40516	0.909192	0.813985
Non.inflammatory.Macrophages	FAM57B	-0.21508	0.285081	-0.46808	0.640882	-5.53328	0.909192	0.814259
Non.inflammatory.Macrophages	CDKN2AIP	-0.06392	4.567595	-0.4671	0.641574	-6.3691	0.719946	0.723032
Non.inflammatory.Macrophages	GM14636	-0.12019	2.274283	-0.467	0.641645	-6.26301	0.82736	0.770674
Non.inflammatory.Macrophages	GM13889	-0.19698	1.14195	-0.46683	0.641772	-5.63358	0.885676	0.795318
Non.inflammatory.Macrophages	TTC7B	-0.05494	5.391283	-0.4668	0.641793	-6.63864	0.684672	0.70664
Non.inflammatory.Macrophages	ZFP113	0.17817	1.151185	0.466795	0.641795	-5.63535	0.885185	0.795114

Non.inflammatory.Macrophages	SAR1B	0.038634	5.553451	0.466688	0.641871	-6.76899	0.677923	0.703457
Non.inflammatory.Macrophages	AKTIP	0.0789	3.102695	0.466618	0.641921	-6.16972	0.786948	0.753119
Non.inflammatory.Macrophages	ABCD1	0.048079	4.771292	0.466605	0.64193	-6.64994	0.711066	0.718943
Non.inflammatory.Macrophages	SKP1A	0.036565	6.639256	0.466423	0.64206	-6.91673	0.634361	0.68251
Non.inflammatory.Macrophages	FRYL	0.043234	7.564295	0.466407	0.642071	-6.94273	0.599387	0.66516
Non.inflammatory.Macrophages	CIITA	-0.13341	2.478401	-0.46613	0.642272	-6.12625	0.817366	0.766314
Non.inflammatory.Macrophages	PRKAR1B	-0.22696	-0.37005	-0.46608	0.642307	-5.51494	0.910069	0.829417
Non.inflammatory.Macrophages	SRPR	0.036733	5.794906	0.465047	0.643041	-6.78679	0.668772	0.698985
Non.inflammatory.Macrophages	WDR45B	0.035509	6.330115	0.464988	0.643083	-6.84968	0.647231	0.688656
Non.inflammatory.Macrophages	GM48796	-0.22678	0.350478	-0.46483	0.643195	-5.36157	0.911042	0.813311
Non.inflammatory.Macrophages	ZKSCAN17	0.065853	3.498798	0.464476	0.643448	-6.36065	0.76945	0.745181
Non.inflammatory.Macrophages	CDPF1	0.112828	2.220471	0.464232	0.643623	-6.06025	0.831323	0.772157
Non.inflammatory.Macrophages	EMC2	0.04016	5.762132	0.464222	0.64363	-6.78048	0.670359	0.69968
Non.inflammatory.Macrophages	FOSL1	0.161265	1.662098	0.464137	0.64369	-5.87937	0.859755	0.784238
Non.inflammatory.Macrophages	IER3	-0.06843	6.13903	-0.46398	0.6438	-7.13239	0.655084	0.692376

Non.inflammatory.Macrophages	ANP32B	-0.04712	8.191649	-0.46392	0.643848	-7.11767	0.577623	0.653925
Non.inflammatory.Macrophages	ZFP235	-0.1462	1.919758	-0.4639	0.643857	-5.75284	0.846527	0.778641
Non.inflammatory.Macrophages	LIG3	-0.08869	3.358188	-0.46336	0.644242	-6.1936	0.776429	0.748267
Non.inflammatory.Macrophages	PDE5A	0.204672	2.569114	0.463257	0.644318	-5.59819	0.814422	0.764908
Non.inflammatory.Macrophages	NECTIN2	-0.09216	2.109704	-0.46279	0.644652	-6.12106	0.837516	0.774843
Non.inflammatory.Macrophages	LETMD1	-0.08507	2.91201	-0.46278	0.644658	-6.12799	0.797899	0.757744
Non.inflammatory.Macrophages	TCN2	0.059759	4.791081	0.462757	0.644676	-6.78184	0.711826	0.719136
Non.inflammatory.Macrophages	KLC3	0.29693	0.19256	0.462666	0.64474	-5.25487	0.911989	0.817271
Non.inflammatory.Macrophages	KANSL3	0.051478	4.995066	0.462206	0.645069	-6.57656	0.703254	0.715179
Non.inflammatory.Macrophages	RASAL2	-0.07331	5.468995	-0.46219	0.645083	-6.7165	0.683202	0.705805
Non.inflammatory.Macrophages	TCTA	0.095627	2.098287	0.462001	0.645215	-6.00351	0.838399	0.775241
Non.inflammatory.Macrophages	PAOX	0.080027	2.987177	0.461959	0.645246	-6.32198	0.794567	0.756309
Non.inflammatory.Macrophages	VDAC3	0.038046	7.191815	0.46177	0.645381	-6.98746	0.614873	0.672849
Non.inflammatory.Macrophages	SART3	0.051824	5.374984	0.461722	0.645415	-6.57957	0.687187	0.707758
Non.inflammatory.Macrophages	RTKN2	0.252022	1.228878	0.461625	0.645484	-5.3444	0.883427	0.794294

Non.inflammatory.Macrophages	TOMM7	0.032147	7.561253	0.461553	0.645535	-7.10552	0.601097	0.66598
Non.inflammatory.Macrophages	IDE	0.050873	4.161112	0.461196	0.645791	-6.4808	0.739958	0.732159
Non.inflammatory.Macrophages	EML1	0.200925	1.303195	0.46114	0.645831	-5.57525	0.8795	0.792737
Non.inflammatory.Macrophages	UBQLN2	0.060662	4.031371	0.461137	0.645833	-6.36515	0.745819	0.734808
Non.inflammatory.Macrophages	GTF2A2	0.042431	5.923594	0.461132	0.645836	-6.77284	0.664533	0.697102
Non.inflammatory.Macrophages	DDC	0.203173	1.455602	0.46105	0.645895	-5.53916	0.871488	0.789399
Non.inflammatory.Macrophages	SMIM12	0.057424	4.0506	0.460984	0.645942	-6.45528	0.744948	0.734427
Non.inflammatory.Macrophages	DTD2	-0.0802	3.491192	-0.46084	0.646042	-6.3249	0.77073	0.745968
Non.inflammatory.Macrophages	RAVER2	0.127845	1.423822	0.46069	0.646152	-5.88964	0.873255	0.790169
Non.inflammatory.Macrophages	GRPEL1	-0.05225	5.877727	-0.46049	0.646296	-6.76587	0.666556	0.698096
Non.inflammatory.Macrophages	NCALD	0.180404	2.377149	0.46029	0.646438	-5.63783	0.824694	0.769537
Non.inflammatory.Macrophages	GM47507	0.159154	0.732528	0.460234	0.646478	-5.89384	0.905915	0.805529
Non.inflammatory.Macrophages	UPRT	-0.20697	0.606501	-0.46009	0.646578	-5.43095	0.909924	0.808372
Non.inflammatory.Macrophages	SPRYD4	-0.12297	1.803638	-0.46005	0.646611	-5.77823	0.8537	0.781925
Non.inflammatory.Macrophages	CAMK2B	-0.23221	3.706454	-0.45987	0.646736	-5.59948	0.760988	0.741643

Non.inflammatory.Macrophages	TMEM64	-0.07039	5.051385	-0.45972	0.646849	-6.55264	0.701222	0.71446
Non.inflammatory.Macrophages	CTSO	0.068962	4.26707	0.459606	0.646927	-6.48198	0.735557	0.730258
Non.inflammatory.Macrophages	GPR155	0.123985	3.05644	0.459256	0.647177	-5.95212	0.791874	0.7554
Non.inflammatory.Macrophages	OTUD3	0.126534	2.558441	0.459065	0.647314	-5.91152	0.816139	0.765986
Non.inflammatory.Macrophages	CYB561D2	0.067988	3.329402	0.459012	0.647352	-6.31904	0.778933	0.749732
Non.inflammatory.Macrophages	PTMS	-0.04718	6.015415	-0.45802	0.648059	-6.94518	0.661953	0.6959
Non.inflammatory.Macrophages	CSTDC5	0.164112	5.46156	0.457964	0.648102	-6.55898	0.684746	0.706735
Non.inflammatory.Macrophages	FIGNL2	-0.20289	-0.67701	-0.45791	0.648138	-5.57083	0.91393	0.838255
Non.inflammatory.Macrophages	TTC5	0.050683	4.64029	0.45776	0.648248	-6.5151	0.719974	0.72314
Non.inflammatory.Macrophages	COMMD7	-0.03685	5.29863	-0.45749	0.64844	-6.70146	0.69177	0.710057
Non.inflammatory.Macrophages	ARSK	0.084716	2.705302	0.45728	0.648591	-6.30343	0.80994	0.763252
Non.inflammatory.Macrophages	HARBI1	-0.15091	1.346342	-0.45722	0.648635	-5.78502	0.879047	0.792643
Non.inflammatory.Macrophages	PIGV	0.072358	2.921169	0.456982	0.648805	-6.14461	0.799559	0.758705
Non.inflammatory.Macrophages	GM34680	-0.34038	0.042973	-0.4568	0.648933	-5.18768	0.914402	0.821959
Non.inflammatory.Macrophages	CTSK	0.181277	0.234472	0.456729	0.648986	-5.63257	0.914402	0.817626

Non.inflammatory.Macrophages	IMP4	0.051481	4.46298	0.456689	0.649015	-6.54293	0.728119	0.726927
Non.inflammatory.Macrophages	GM14295	-0.13285	1.289141	-0.45634	0.649266	-5.71916	0.882217	0.794128
Non.inflammatory.Macrophages	SPECC1L	0.049942	5.413581	0.456328	0.649273	-6.86568	0.68711	0.708035
Non.inflammatory.Macrophages	E230029C(	0.060981	3.508508	0.45632	0.649279	-6.74749	0.771611	0.746595
Non.inflammatory.Macrophages	ZC3H15	-0.03497	7.266033	-0.45602	0.64949	-6.96205	0.613457	0.672515
Non.inflammatory.Macrophages	PSMA4	0.038277	6.877207	0.455902	0.649578	-6.97914	0.628255	0.679852
Non.inflammatory.Macrophages	ASPA	-0.14547	1.375432	-0.45588	0.649592	-6.06744	0.877659	0.792328
Non.inflammatory.Macrophages	GRAP	-0.0868	4.536304	-0.45583	0.64963	-6.26535	0.724875	0.725639
Non.inflammatory.Macrophages	ACOT11	-0.11993	1.943725	-0.45577	0.649674	-5.81747	0.848172	0.779932
Non.inflammatory.Macrophages	GM34961	-0.18311	0.869528	-0.45557	0.649816	-5.6392	0.902927	0.803578
Non.inflammatory.Macrophages	R3HDM4	0.052241	6.20323	0.455505	0.649862	-6.75595	0.654728	0.692758
Non.inflammatory.Macrophages	CEP120	-0.04683	5.494784	-0.45549	0.649872	-6.68876	0.683711	0.706557
Non.inflammatory.Macrophages	CAMSAP1	-0.08527	3.53399	-0.45547	0.649889	-6.18942	0.770419	0.746194
Non.inflammatory.Macrophages	SASH1	0.064044	5.303699	0.455465	0.649892	-6.90852	0.691736	0.710326
Non.inflammatory.Macrophages	SEPSECS	0.096075	3.143326	0.454993	0.650229	-6.10096	0.78892	0.754468

Non.inflammatory.Macrophages	GRN	-0.04506	7.188808	-0.45498	0.650236	-7.21287	0.616397	0.674114
Non.inflammatory.Macrophages	CITED2	-0.04867	6.149916	-0.45491	0.650289	-6.92364	0.656898	0.693896
Non.inflammatory.Macrophages	SP110	0.056805	5.924125	0.454693	0.650445	-6.80211	0.666034	0.698298
Non.inflammatory.Macrophages	ITGA1	-0.1222	3.828562	-0.45464	0.650486	-6.18803	0.756798	0.740246
Non.inflammatory.Macrophages	ATPAF1	0.07399	3.553171	0.454603	0.650509	-6.23528	0.769558	0.745941
Non.inflammatory.Macrophages	ZFP120	0.077665	2.536502	0.454567	0.650535	-6.16705	0.818418	0.767342
Non.inflammatory.Macrophages	MGRN1	-0.05138	5.682428	-0.45455	0.650546	-6.78383	0.675948	0.703013
Non.inflammatory.Macrophages	METTL6	0.056591	4.505421	0.454524	0.650565	-6.56589	0.726274	0.72643
Non.inflammatory.Macrophages	ADGRF5	-0.16016	2.598167	-0.45448	0.650597	-5.93394	0.815375	0.766027
Non.inflammatory.Macrophages	BRWD3	0.053198	5.109603	0.454137	0.650843	-6.68932	0.70006	0.714383
Non.inflammatory.Macrophages	SIRT6	0.09824	2.652878	0.454077	0.650886	-6.06624	0.81274	0.764935
Non.inflammatory.Macrophages	MBNL1	0.046535	8.978349	0.454074	0.650888	-7.25349	0.552301	0.641451
Non.inflammatory.Macrophages	MAP3K14	-0.0601	5.189544	-0.45404	0.650913	-6.6836	0.696653	0.712795
Non.inflammatory.Macrophages	SMPD4	0.086988	3.209019	0.453676	0.651173	-6.19545	0.785963	0.753339
Non.inflammatory.Macrophages	2500002B1	0.118561	1.915535	0.453116	0.651575	-5.85911	0.849844	0.781031

Non.inflammatory.Macrophages	NRN1	-0.14668	3.109984	-0.45309	0.651593	-6.10162	0.790693	0.75551
Non.inflammatory.Macrophages	TBRG1	-0.05773	5.618457	-0.45303	0.651639	-6.68507	0.678749	0.704561
Non.inflammatory.Macrophages	PDK2	0.117048	2.10287	0.453016	0.651647	-5.93593	0.840306	0.776976
Non.inflammatory.Macrophages	TMEM229/	-0.19188	-1.03017	-0.4529	0.651731	-5.5266	0.91465	0.847601
Non.inflammatory.Macrophages	CST3	-0.06143	9.157123	-0.45283	0.651782	-7.56991	0.546351	0.638481
Non.inflammatory.Macrophages	CTSC	-0.05815	7.322442	-0.45269	0.651877	-7.31255	0.611504	0.671949
Non.inflammatory.Macrophages	ACCS	0.100908	2.079594	0.45266	0.651902	-6.01904	0.841485	0.777515
Non.inflammatory.Macrophages	NEURL4	-0.12141	2.445552	-0.45258	0.651959	-6.02656	0.823112	0.769649
Non.inflammatory.Macrophages	VWA5A	0.058291	3.406572	0.452213	0.652223	-6.64056	0.776607	0.749494
Non.inflammatory.Macrophages	TRIM44	0.044757	6.053928	0.452043	0.652345	-6.77246	0.660916	0.696295
Non.inflammatory.Macrophages	BCAM	0.291804	0.298097	0.452011	0.652368	-5.49883	0.91465	0.817196
Non.inflammatory.Macrophages	SLC17A9	-0.11712	2.888147	-0.45197	0.652394	-6.0617	0.801383	0.760436
Non.inflammatory.Macrophages	TENM2	-0.23392	-0.35252	-0.45192	0.652435	-5.55556	0.91465	0.832084
Non.inflammatory.Macrophages	ZFP456	-0.16622	0.791994	-0.45191	0.652441	-5.68216	0.905854	0.806065
Non.inflammatory.Macrophages	AY036118	0.12675	4.212426	0.451906	0.652443	-6.46839	0.739508	0.732922

Non.inflammatory.Macrophages	RASGRF2	-0.178	0.116081	-0.45188	0.65246	-5.54121	0.91465	0.821335
Non.inflammatory.Macrophages	SNRPC	-0.03806	6.245324	-0.45188	0.652463	-6.8451	0.653221	0.692595
Non.inflammatory.Macrophages	ZFP974	-0.17746	0.877863	-0.45181	0.652514	-5.69847	0.902884	0.804144
Non.inflammatory.Macrophages	U2SURP	0.03248	6.667671	0.451807	0.652514	-6.94311	0.636545	0.684499
Non.inflammatory.Macrophages	KIF3A	0.092134	2.472633	0.451746	0.652558	-6.0441	0.821767	0.769276
Non.inflammatory.Macrophages	FCHSD1	-0.15772	1.15994	-0.45173	0.652569	-5.62676	0.889323	0.797867
Non.inflammatory.Macrophages	CASD1	0.05986	4.363906	0.451634	0.652639	-6.42164	0.732726	0.729839
Non.inflammatory.Macrophages	GTPBP3	-0.10915	2.225197	-0.45145	0.652768	-5.92495	0.834213	0.774668
Non.inflammatory.Macrophages	PNLDC1	-0.19631	0.924032	-0.45103	0.653075	-5.55334	0.901607	0.803378
Non.inflammatory.Macrophages	FARP2	0.085868	2.6131	0.451004	0.653091	-6.26416	0.815107	0.766529
Non.inflammatory.Macrophages	ATF6	0.040544	6.979377	0.450872	0.653186	-6.98182	0.624721	0.678845
Non.inflammatory.Macrophages	GPR68	0.221591	1.433054	0.450849	0.653202	-5.57371	0.87517	0.792149
Non.inflammatory.Macrophages	LMO4	0.069308	7.245458	0.450702	0.653308	-6.87188	0.614649	0.673879
Non.inflammatory.Macrophages	ADAMTS6	-0.0914	5.940983	-0.45039	0.653531	-6.44377	0.665785	0.698864
Non.inflammatory.Macrophages	STXBP2	-0.0462	5.31968	-0.45018	0.653681	-6.73709	0.691548	0.711079

Non.inflammatory.Macrophages	TLE5	-0.03983	7.209326	-0.45018	0.653683	-6.96833	0.616027	0.674638
Non.inflammatory.Macrophages	HNRNPLL	0.046962	4.231786	0.450022	0.653796	-6.7737	0.738957	0.732944
Non.inflammatory.Macrophages	ZFP607A	0.113512	1.840055	0.449913	0.653874	-5.83328	0.854084	0.783387
Non.inflammatory.Macrophages	MAN1C1	0.053334	4.292733	0.449865	0.653909	-6.72947	0.736222	0.731715
Non.inflammatory.Macrophages	BAZ2B	0.035258	8.430417	0.449816	0.653944	-7.18374	0.571547	0.652134
Non.inflammatory.Macrophages	MRPS10	0.056851	4.162492	0.449639	0.654071	-6.46262	0.742079	0.734484
Non.inflammatory.Macrophages	GM36551	-0.16637	-0.80034	-0.44959	0.654105	-5.40009	0.915046	0.843105
Non.inflammatory.Macrophages	COL25A1	-0.27859	0.981282	-0.44935	0.654278	-5.38885	0.899286	0.802435
Non.inflammatory.Macrophages	RASAL1	-0.28356	0.712454	-0.44934	0.65429	-5.3405	0.90901	0.808449
Non.inflammatory.Macrophages	SLC24A5	0.081329	4.423305	0.449216	0.654375	-6.21467	0.730394	0.729175
Non.inflammatory.Macrophages	ACD	-0.05636	4.940696	-0.4492	0.654388	-6.50778	0.707726	0.718748
Non.inflammatory.Macrophages	CARD11	0.160303	4.618562	0.44914	0.65443	-5.90515	0.72176	0.725232
Non.inflammatory.Macrophages	PTER	-0.11276	2.433342	-0.44893	0.654577	-6.0571	0.824075	0.770747
Non.inflammatory.Macrophages	HMGA2	0.313105	1.149583	0.448781	0.654688	-5.51338	0.89026	0.798774
Non.inflammatory.Macrophages	GM49439	-0.30333	-0.46286	-0.44854	0.654858	-5.19208	0.915046	0.835449

Non.inflammatory.Macrophages	LIX1	-0.14947	0.723268	-0.44848	0.654901	-5.79126	0.908633	0.8084
Non.inflammatory.Macrophages	DHX58	0.085867	2.999964	0.44841	0.654955	-6.46287	0.796322	0.758821
Non.inflammatory.Macrophages	SLC9A7	-0.10353	5.115655	-0.44838	0.654978	-6.09882	0.700213	0.715428
Non.inflammatory.Macrophages	SUV39H1	-0.07697	3.622039	-0.44837	0.65498	-6.17892	0.766852	0.745799
Non.inflammatory.Macrophages	CABYR	-0.12716	1.135391	-0.44817	0.655124	-6.00389	0.891018	0.799241
Non.inflammatory.Macrophages	KLF3	-0.04522	6.281206	-0.44806	0.655203	-6.96579	0.65207	0.692638
Non.inflammatory.Macrophages	CDAN1	0.104452	2.943919	0.447978	0.655265	-6.00655	0.799028	0.760064
Non.inflammatory.Macrophages	MSL3	0.049497	4.998465	0.44794	0.655292	-6.60868	0.705237	0.717821
Non.inflammatory.Macrophages	ZMYND19	0.102491	3.409984	0.447758	0.655423	-6.14699	0.776782	0.750334
Non.inflammatory.Macrophages	PPP2R5C	-0.0281	7.456887	-0.44772	0.65545	-7.02933	0.606744	0.670409
Non.inflammatory.Macrophages	MBLAC2	0.09091	2.64789	0.447397	0.655683	-6.10742	0.813463	0.766432
Non.inflammatory.Macrophages	TXNL1	-0.04205	6.955558	-0.44737	0.655704	-6.96563	0.625686	0.67983
Non.inflammatory.Macrophages	NUAK1	-0.10014	3.219187	-0.44732	0.655735	-6.2689	0.785819	0.754347
Non.inflammatory.Macrophages	EED	0.033186	6.114274	0.447254	0.655786	-6.82339	0.658765	0.695975
Non.inflammatory.Macrophages	HSPA4	-0.03085	7.667014	-0.4472	0.655826	-7.05707	0.598972	0.666532

Non.inflammatory.Macrophages	4930481A1	-0.12248	1.854963	-0.44719	0.655834	-5.86525	0.853317	0.783558
Non.inflammatory.Macrophages	RTN2	-0.19252	-0.4318	-0.44704	0.655942	-5.54739	0.915046	0.834964
Non.inflammatory.Macrophages	KLRB1B	-0.31408	1.981821	-0.44696	0.655995	-5.40553	0.846823	0.780828
Non.inflammatory.Macrophages	TNKS1BP1	-0.12152	2.080541	-0.44684	0.656085	-5.87619	0.841801	0.778746
Non.inflammatory.Macrophages	DAPL1	0.261443	-1.31502	0.446708	0.656178	-5.19551	0.915046	0.855747
Non.inflammatory.Macrophages	COMMD4	-0.04685	5.326168	-0.44659	0.656265	-6.70889	0.691274	0.711542
Non.inflammatory.Macrophages	HSP90B1	-0.03613	8.976388	-0.44651	0.656319	-7.32494	0.552693	0.642813
Non.inflammatory.Macrophages	DHRS7B	0.073276	3.137991	0.446343	0.656441	-6.26967	0.789694	0.756279
Non.inflammatory.Macrophages	TLR8	0.103984	0.054908	0.446272	0.656492	-6.07628	0.915046	0.823955
Non.inflammatory.Macrophages	PCGF1	0.133179	1.17851	0.446233	0.65652	-5.76967	0.888717	0.798646
Non.inflammatory.Macrophages	PGRMC2	0.055784	3.628957	0.446122	0.656599	-6.34349	0.76653	0.746049
Non.inflammatory.Macrophages	RHBDD3	0.08568	2.194151	0.446113	0.656606	-6.04915	0.836055	0.77643
Non.inflammatory.Macrophages	2-Mar	-0.04304	5.868752	-0.44607	0.656638	-6.80056	0.668732	0.700953
Non.inflammatory.Macrophages	PTPRG	-0.17395	2.191113	-0.44605	0.65665	-5.85245	0.836208	0.776496
Non.inflammatory.Macrophages	DIAPH2	0.040217	7.666379	0.445965	0.656713	-7.1895	0.598996	0.666738

Non.inflammatory.Macrophages	PALLD	0.082071	3.882236	0.445907	0.656754	-6.45356	0.75483	0.740808
Non.inflammatory.Macrophages	SAA4	0.228913	0.640047	0.445797	0.656834	-5.50073	0.911537	0.810698
Non.inflammatory.Macrophages	2410002F2	0.068415	3.766412	0.445768	0.656855	-6.28243	0.76016	0.7432
Non.inflammatory.Macrophages	NAA10	-0.06186	5.084078	-0.44569	0.656912	-6.56308	0.701564	0.716434
Non.inflammatory.Macrophages	0610030E2	0.054003	4.591291	0.445637	0.656949	-6.52706	0.72296	0.726331
Non.inflammatory.Macrophages	VPS52	-0.05097	3.923327	-0.4456	0.656977	-6.41925	0.752948	0.739962
Non.inflammatory.Macrophages	NIT2	-0.08454	3.588667	-0.44546	0.657079	-6.27325	0.768407	0.746949
Non.inflammatory.Macrophages	DCLRE1C	0.045043	6.497189	0.445321	0.657176	-6.93699	0.643505	0.688862
Non.inflammatory.Macrophages	B4GALT5	0.050361	5.975954	0.445299	0.657192	-6.96138	0.664362	0.698933
Non.inflammatory.Macrophages	OSBPL2	0.060896	3.979026	0.445244	0.657232	-6.33197	0.750403	0.738896
Non.inflammatory.Macrophages	AMD1	0.057725	4.581194	0.445225	0.657245	-6.56067	0.723405	0.726614
Non.inflammatory.Macrophages	2010320M	0.09434	2.480423	0.44507	0.657357	-6.08742	0.82179	0.770384
Non.inflammatory.Macrophages	NUDT16	0.077372	2.966991	0.44474	0.657594	-6.2643	0.798125	0.760061
Non.inflammatory.Macrophages	LRPPRC	0.050832	5.200579	0.444701	0.657622	-6.67843	0.696778	0.714271
Non.inflammatory.Macrophages	ASNSD1	0.042167	5.360445	0.444376	0.657856	-6.71508	0.690145	0.711145

Non.inflammatory.Macrophages	UBTD2	0.079134	3.37685	0.444337	0.657885	-6.29996	0.778701	0.751514
Non.inflammatory.Macrophages	ACADL	-0.04666	6.734458	-0.44418	0.657998	-6.91986	0.634528	0.684502
Non.inflammatory.Macrophages	MMP25	-0.1916	0.499533	-0.44408	0.658071	-5.60323	0.915269	0.814215
Non.inflammatory.Macrophages	GM12703	-0.19636	-0.21463	-0.44394	0.658173	-5.58929	0.91549	0.83058
Non.inflammatory.Macrophages	HDGF	0.038454	6.998077	0.443631	0.658393	-6.96814	0.62436	0.679636
Non.inflammatory.Macrophages	GM26881	-0.14832	0.245715	-0.4436	0.658417	-5.82249	0.91549	0.82008
Non.inflammatory.Macrophages	DGKQ	0.124362	1.470676	0.443581	0.658429	-5.82756	0.873692	0.792645
Non.inflammatory.Macrophages	NFATC1	-0.05093	5.77824	-0.4435	0.65849	-6.69179	0.67277	0.703135
Non.inflammatory.Macrophages	OSTC	-0.03632	6.387429	-0.44344	0.658528	-6.88874	0.648158	0.691341
Non.inflammatory.Macrophages	TRIM11	-0.06013	5.06784	-0.44328	0.658643	-6.45313	0.7026	0.717261
Non.inflammatory.Macrophages	PSMC4	-0.04887	5.46131	-0.4432	0.658702	-6.68837	0.685925	0.709448
Non.inflammatory.Macrophages	CEBPG	0.038626	5.586056	0.44319	0.658711	-6.80492	0.680718	0.706989
Non.inflammatory.Macrophages	AP4M1	-0.05998	3.581284	-0.44308	0.658788	-6.2857	0.769125	0.74758
Non.inflammatory.Macrophages	RPP21	0.049455	4.873001	0.443067	0.6588	-6.62299	0.710999	0.721178
Non.inflammatory.Macrophages	PEX3	0.058786	3.770542	0.442982	0.65886	-6.36135	0.760338	0.743655

Non.inflammatory.Macrophages	RDX	-0.0307	6.872179	-0.4429	0.658917	-6.96035	0.629196	0.682153
Non.inflammatory.Macrophages	RAD51D	0.078577	2.984907	0.442598	0.659137	-6.16801	0.79762	0.760207
Non.inflammatory.Macrophages	DXO	-0.08941	3.302702	-0.44231	0.659346	-6.19218	0.78258	0.753598
Non.inflammatory.Macrophages	SNHG8	0.056937	4.013183	0.442109	0.65949	-6.472	0.749631	0.738893
Non.inflammatory.Macrophages	SPG7	0.045555	4.467655	0.441803	0.65971	-6.51823	0.729334	0.729706
Non.inflammatory.Macrophages	TMEM62	0.138328	2.375873	0.441737	0.659758	-5.73305	0.827971	0.77344
Non.inflammatory.Macrophages	GM16279	0.17648	1.054801	0.441517	0.659917	-5.6604	0.896574	0.802371
Non.inflammatory.Macrophages	LTBR	0.055496	2.974023	0.441245	0.660113	-6.57239	0.798835	0.760712
Non.inflammatory.Macrophages	PARVA	-0.16327	1.329681	-0.441	0.660286	-5.6811	0.882226	0.796376
Non.inflammatory.Macrophages	ZFP429	-0.0825	2.448075	-0.44073	0.660488	-6.11546	0.824943	0.772055
Non.inflammatory.Macrophages	PITPNM2	0.108914	3.66119	0.44047	0.660672	-5.96882	0.766655	0.746483
Non.inflammatory.Macrophages	RNF20	0.049128	5.755436	0.440203	0.660864	-6.71283	0.674896	0.704263
Non.inflammatory.Macrophages	E130311K1	0.173528	0.244935	0.440042	0.66098	-5.67381	0.917104	0.820918
Non.inflammatory.Macrophages	GM4951	0.121487	3.770894	0.439933	0.661059	-6.4905	0.761662	0.744265
Non.inflammatory.Macrophages	TLCD1	-0.14147	1.624021	-0.43979	0.661163	-5.66211	0.867202	0.790064

Non.inflammatory.Macrophages	MYBBP1A	0.062096	4.85707	0.439731	0.661205	-6.62536	0.712944	0.7221
Non.inflammatory.Macrophages	MIIP	-0.08119	3.057652	-0.43973	0.661207	-6.13782	0.79533	0.759184
Non.inflammatory.Macrophages	ERCC8	-0.10349	2.45611	-0.4397	0.661231	-5.87495	0.824794	0.771995
Non.inflammatory.Macrophages	CCDC88A	-0.04888	5.372063	-0.43896	0.661758	-6.81309	0.691303	0.712029
Non.inflammatory.Macrophages	PHC1	-0.07791	3.5385	-0.43893	0.661782	-6.04825	0.772945	0.749321
Non.inflammatory.Macrophages	ABT1	0.071563	3.534654	0.43873	0.661927	-6.19143	0.773125	0.74945
Non.inflammatory.Macrophages	CTSE	0.127264	5.492809	0.438707	0.661944	-5.95019	0.686223	0.709702
Non.inflammatory.Macrophages	KDM5C	-0.05882	6.827805	-0.43861	0.662018	-6.89498	0.632399	0.683853
Non.inflammatory.Macrophages	VPS36	0.042233	5.315956	0.438548	0.662059	-6.68044	0.693675	0.713265
Non.inflammatory.Macrophages	YAP1	-0.23904	1.070556	-0.43849	0.6621	-5.54093	0.897035	0.802685
Non.inflammatory.Macrophages	GSTM2	-0.15379	1.633616	-0.43816	0.662339	-5.76241	0.867285	0.790355
Non.inflammatory.Macrophages	ZFP703	0.05483	4.364442	0.438051	0.662418	-6.75773	0.735159	0.732561
Non.inflammatory.Macrophages	FLII	0.042401	5.969326	0.437997	0.662457	-6.80019	0.666575	0.700551
Non.inflammatory.Macrophages	KLHL7	-0.05481	4.739495	-0.43795	0.662487	-6.54738	0.718556	0.724952
Non.inflammatory.Macrophages	PSKH1	0.061465	3.273493	0.437952	0.662489	-6.26447	0.785527	0.755144

Non.inflammatory.Macrophages	DCTN2	-0.04333	5.367124	-0.43775	0.662634	-6.73462	0.691643	0.712416
Non.inflammatory.Macrophages	YTHDC1	-0.03011	7.312924	-0.43736	0.66292	-6.99883	0.614188	0.674932
Non.inflammatory.Macrophages	DZIP3	0.088229	3.257554	0.436713	0.663384	-6.20262	0.787025	0.755831
Non.inflammatory.Macrophages	SUMF2	0.086696	2.684259	0.436601	0.663465	-6.1225	0.814815	0.768027
Non.inflammatory.Macrophages	PPP2R5B	-0.12978	1.645269	-0.43656	0.663494	-5.946	0.867492	0.790539
Non.inflammatory.Macrophages	NUDT15	-0.13846	1.647281	-0.43653	0.663519	-5.69045	0.867387	0.790501
Non.inflammatory.Macrophages	AK3	0.055391	4.328071	0.436247	0.663721	-6.49093	0.737629	0.733793
Non.inflammatory.Macrophages	ZZZ3	-0.04549	5.884403	-0.43611	0.66382	-6.80235	0.670842	0.702741
Non.inflammatory.Macrophages	RGS13	-0.31927	-1.05629	-0.43569	0.664124	-5.19629	0.91906	0.85239
Non.inflammatory.Macrophages	MAPKAP1	-0.04128	5.764174	-0.43559	0.664199	-6.78897	0.675974	0.705169
Non.inflammatory.Macrophages	B3GNT3	-0.17019	0.711737	-0.43552	0.664249	-5.60267	0.913023	0.811585
Non.inflammatory.Macrophages	MTURN	-0.15402	2.270409	-0.43549	0.664271	-5.69681	0.835869	0.777179
Non.inflammatory.Macrophages	PITHD1	-0.05336	4.744392	-0.43528	0.664416	-6.57666	0.719473	0.725561
Non.inflammatory.Macrophages	9130019P1	-0.24312	0.265967	-0.43511	0.66454	-5.43606	0.919244	0.821848
Non.inflammatory.Macrophages	NSA2	-0.02723	7.622863	-0.43494	0.664664	-7.10055	0.603404	0.669736

Non.inflammatory.Macrophages	MED13L	-0.0389	7.196724	-0.43476	0.664795	-6.96383	0.619443	0.677779
Non.inflammatory.Macrophages	LAPTM4A	-0.03061	7.210016	-0.43437	0.665079	-7.12464	0.61914	0.677585
Non.inflammatory.Macrophages	RNASEH2C	-0.04751	4.998911	-0.43408	0.66529	-6.67287	0.708964	0.720631
Non.inflammatory.Macrophages	EDEM2	0.055466	4.515035	0.43373	0.665541	-6.58055	0.730353	0.730472
Non.inflammatory.Macrophages	4933408B1	-0.15647	1.941966	-0.43368	0.66558	-5.68731	0.853565	0.784684
Non.inflammatory.Macrophages	ATL3	0.039672	5.439187	0.433399	0.665781	-6.7494	0.690403	0.712005
Non.inflammatory.Macrophages	IFI44	0.20823	0.579228	0.433251	0.665888	-5.73723	0.918129	0.815076
Non.inflammatory.Macrophages	RAB27B	0.242394	0.615378	0.433149	0.665962	-5.43897	0.917283	0.814263
Non.inflammatory.Macrophages	H6PD	-0.07261	2.776773	-0.43301	0.666061	-6.23508	0.811715	0.766824
Non.inflammatory.Macrophages	METTL22	0.134508	1.382795	0.432751	0.66625	-5.72948	0.882854	0.79719
Non.inflammatory.Macrophages	DYNLT1A	-0.05453	4.14434	-0.43267	0.666305	-6.59041	0.7471	0.738247
Non.inflammatory.Macrophages	2310009B1	0.04867	4.473004	0.432544	0.6664	-6.53902	0.732306	0.731556
Non.inflammatory.Macrophages	PRKAB1	0.069035	3.654114	0.432457	0.666462	-6.37101	0.76969	0.748458
Non.inflammatory.Macrophages	C9	0.216767	0.60037	0.432393	0.666509	-5.71566	0.917634	0.814783
Non.inflammatory.Macrophages	KDM3B	0.038013	6.131762	0.432366	0.666528	-6.79097	0.661783	0.698575

Non.inflammatory.Macrophages	PMS2	-0.0728	3.954235	-0.43233	0.666553	-6.19937	0.755785	0.742232
Non.inflammatory.Macrophages	KIF16B	-0.05056	4.843803	-0.43231	0.666567	-6.67409	0.715947	0.724079
Non.inflammatory.Macrophages	COQ7	-0.05886	4.79596	-0.43228	0.666593	-6.60079	0.718038	0.725045
Non.inflammatory.Macrophages	CCDC47	0.042298	5.110399	0.432158	0.666679	-6.68224	0.7044	0.718726
Non.inflammatory.Macrophages	ARHGEF5	-0.19781	0.327235	-0.43195	0.666829	-5.55364	0.920221	0.821033
Non.inflammatory.Macrophages	1700012D1	-0.15088	1.843803	-0.43194	0.666835	-5.66624	0.858721	0.787159
Non.inflammatory.Macrophages	3830403N1	-0.22902	0.704685	-0.43187	0.666885	-5.63839	0.914424	0.812482
Non.inflammatory.Macrophages	PYCRL	-0.0596	3.541294	-0.43183	0.666916	-6.37599	0.774978	0.750863
Non.inflammatory.Macrophages	LRRC29	-0.10069	1.690453	-0.43174	0.666983	-5.80613	0.866681	0.790566
Non.inflammatory.Macrophages	TMEM170B	-0.05118	4.646846	-0.43169	0.667014	-6.61004	0.724593	0.728152
Non.inflammatory.Macrophages	STK10	-0.03711	7.315855	-0.43159	0.667089	-7.07306	0.615483	0.676023
Non.inflammatory.Macrophages	ECHDC3	-0.21488	0.833138	-0.43141	0.667221	-5.54264	0.909978	0.809771
Non.inflammatory.Macrophages	TUBGCP3	0.054697	3.88521	0.431373	0.667247	-6.33228	0.758995	0.743879
Non.inflammatory.Macrophages	PPP6C	0.032572	6.737344	0.431175	0.66739	-6.87401	0.637723	0.687141
Non.inflammatory.Macrophages	SUDS3	-0.0489	5.515338	-0.43112	0.667433	-6.69644	0.687229	0.710941

Non.inflammatory.Macrophages	FAM110B	-0.1582	0.653098	-0.43109	0.667451	-5.7743	0.916274	0.813904
Non.inflammatory.Macrophages	DNLZ	0.064008	3.991855	0.430804	0.667659	-6.42284	0.754186	0.741748
Non.inflammatory.Macrophages	SHANK3	-0.1967	1.430051	-0.43079	0.667671	-5.64373	0.880501	0.796532
Non.inflammatory.Macrophages	IRGQ	-0.12019	2.194038	-0.4306	0.667807	-5.91264	0.841015	0.779793
Non.inflammatory.Macrophages	BUD31	0.04374	5.474604	0.430404	0.667949	-6.74497	0.689096	0.711754
Non.inflammatory.Macrophages	BC049352	-0.11291	1.083027	-0.43036	0.667984	-6.0471	0.899117	0.80425
Non.inflammatory.Macrophages	SCAF8	0.035708	6.711338	0.430321	0.66801	-6.89775	0.638883	0.687665
Non.inflammatory.Macrophages	MAVS	-0.07081	2.932268	-0.42995	0.66828	-6.27642	0.804574	0.764031
Non.inflammatory.Macrophages	SLC10A3	0.104388	2.160549	0.429584	0.668543	-6.04894	0.843027	0.780713
Non.inflammatory.Macrophages	CTNND1	0.055541	4.294756	0.429493	0.66861	-6.69898	0.740768	0.735703
Non.inflammatory.Macrophages	OSGEPL1	0.098936	2.472736	0.429377	0.668693	-5.88089	0.827295	0.773972
Non.inflammatory.Macrophages	PNPLA8	-0.04323	6.018439	-0.42937	0.668696	-6.80432	0.666812	0.70124
Non.inflammatory.Macrophages	WAC	0.030629	7.414892	0.429329	0.668728	-7.03694	0.612141	0.674501
Non.inflammatory.Macrophages	ATG2B	0.048244	4.861013	0.429323	0.668733	-6.61062	0.715653	0.724204
Non.inflammatory.Macrophages	NFKBIA	0.048629	8.130992	0.429134	0.668869	-7.27997	0.585881	0.661257

Non.inflammatory.Macrophages	GM47448	0.227972	-0.89373	0.42904	0.668938	-5.47554	0.920904	0.849932
Non.inflammatory.Macrophages	CCDC22	-0.06234	3.49155	-0.42889	0.669043	-6.37394	0.777906	0.752438
Non.inflammatory.Macrophages	PTGR2	0.061053	3.734192	0.428842	0.669081	-6.3825	0.766533	0.747374
Non.inflammatory.Macrophages	SLCO2A1	-0.17758	2.568296	-0.42835	0.66944	-5.96754	0.822984	0.772175
Non.inflammatory.Macrophages	GM15518	-0.23848	0.80065	-0.42812	0.669601	-5.39582	0.912203	0.811097
Non.inflammatory.Macrophages	KLHL36	-0.11	2.076404	-0.42811	0.669614	-5.99874	0.847827	0.782846
Non.inflammatory.Macrophages	MPP5	-0.04865	5.379551	-0.42798	0.669703	-6.68674	0.693784	0.714099
Non.inflammatory.Macrophages	PURB	-0.03237	6.824936	-0.42764	0.669953	-6.96441	0.635071	0.68597
Non.inflammatory.Macrophages	FAF1	0.037188	6.739255	0.427622	0.669966	-6.88546	0.638414	0.687608
Non.inflammatory.Macrophages	TMEM165	-0.03959	5.311537	-0.42761	0.669978	-6.71889	0.696671	0.71549
Non.inflammatory.Macrophages	COPG1	0.049328	5.141092	0.427525	0.670036	-6.6404	0.703957	0.718893
Non.inflammatory.Macrophages	ATRX	0.034104	7.666179	0.427485	0.670066	-7.06735	0.603141	0.670093
Non.inflammatory.Macrophages	OTUB2	-0.12437	1.885457	-0.42735	0.670164	-5.87536	0.857636	0.787091
Non.inflammatory.Macrophages	TRDC	-0.31936	1.265066	-0.42728	0.670214	-5.42298	0.890222	0.800782
Non.inflammatory.Macrophages	PEX26	0.156666	1.217016	0.427262	0.670227	-5.71739	0.892792	0.801852

Non.inflammatory.Macrophages	ZFYVE27	0.06087	4.063949	0.426958	0.670448	-6.32585	0.751867	0.740903
Non.inflammatory.Macrophages	1700086O(	0.122683	1.546442	0.426667	0.670659	-5.75618	0.875567	0.794677
Non.inflammatory.Macrophages	AR	0.283222	0.218656	0.426657	0.670667	-5.3631	0.921642	0.824534
Non.inflammatory.Macrophages	EBNA1BP2	0.063296	4.017915	0.426611	0.6707	-6.42335	0.754028	0.741888
Non.inflammatory.Macrophages	ZFP777	0.083934	2.809648	0.426038	0.671115	-6.0096	0.811773	0.767323
Non.inflammatory.Macrophages	HARS	0.043289	5.114979	0.425632	0.67141	-6.62539	0.705726	0.719742
Non.inflammatory.Macrophages	ITFG1	-0.03797	5.454025	-0.42559	0.671442	-6.72237	0.691269	0.71298
Non.inflammatory.Macrophages	MKRN2	-0.05059	4.074932	-0.42558	0.671445	-6.36911	0.751883	0.740885
Non.inflammatory.Macrophages	ELP1	0.045967	4.553493	0.42557	0.671455	-6.49111	0.730301	0.731081
Non.inflammatory.Macrophages	RHBDL3	-0.18367	0.747589	-0.42519	0.671733	-5.6106	0.915057	0.812805
Non.inflammatory.Macrophages	LTN1	0.045585	5.03701	0.425172	0.671744	-6.59035	0.709215	0.72139
Non.inflammatory.Macrophages	GM3055	0.211727	0.319624	0.425092	0.671802	-5.40715	0.922373	0.822544
Non.inflammatory.Macrophages	PDE6H	-0.11859	2.093346	-0.42496	0.671898	-5.80831	0.847887	0.783051
Non.inflammatory.Macrophages	CTPS	0.088761	3.704287	0.424849	0.671979	-6.10966	0.769143	0.748734
Non.inflammatory.Macrophages	RCC1	-0.06546	3.989305	-0.42484	0.671988	-6.38281	0.755939	0.742818

Non.inflammatory.Macrophages	NEURL1A	-0.19722	-0.39095	-0.42475	0.672049	-5.55227	0.922373	0.838992
Non.inflammatory.Macrophages	EIF3F	0.034239	7.589953	0.424486	0.672243	-7.09342	0.606674	0.672026
Non.inflammatory.Macrophages	ZFP46	0.094135	1.77659	0.424462	0.67226	-5.81034	0.86428	0.790054
Non.inflammatory.Macrophages	ENDOU	-0.25119	0.913658	-0.4244	0.672302	-5.36169	0.909328	0.809249
Non.inflammatory.Macrophages	AGO1	0.066429	3.874896	0.424228	0.67243	-6.37527	0.761336	0.745325
Non.inflammatory.Macrophages	SAA1	0.344001	-0.05604	0.423714	0.672804	-5.46759	0.922887	0.831649
Non.inflammatory.Macrophages	GM16286	0.037763	5.859685	0.423466	0.672984	-6.80612	0.674837	0.705509
Non.inflammatory.Macrophages	GM14963	-0.14251	0.898614	-0.42346	0.672986	-5.90834	0.910292	0.809924
Non.inflammatory.Macrophages	TMEM140	-0.07942	3.514222	-0.42316	0.673205	-6.49253	0.7785	0.753125
Non.inflammatory.Macrophages	SP2	0.053754	4.742762	0.423145	0.673217	-6.51141	0.722457	0.727807
Non.inflammatory.Macrophages	FKBP9	-0.16282	1.265181	-0.4231	0.673252	-5.75195	0.891684	0.801756
Non.inflammatory.Macrophages	NCKAP1	-0.10765	2.425386	-0.42295	0.673357	-5.89053	0.831536	0.776345
Non.inflammatory.Macrophages	ADAMTS1(	0.087225	3.015513	0.422943	0.673363	-6.06493	0.80239	0.763705
Non.inflammatory.Macrophages	ELOVL7	0.27229	-0.19718	0.422924	0.673377	-5.25173	0.922887	0.835021
Non.inflammatory.Macrophages	GM43647	0.294401	-0.94495	0.422923	0.673379	-5.21875	0.922887	0.852518

Non.inflammatory.Macrophages	STXBP5	0.055593	5.383452	0.422742	0.67351	-6.71798	0.694828	0.714998
Non.inflammatory.Macrophages	DDX18	0.045014	4.987355	0.422418	0.673745	-6.65728	0.712006	0.722998
Non.inflammatory.Macrophages	WDFY3	-0.04487	5.666809	-0.4219	0.674119	-7.09721	0.683379	0.709594
Non.inflammatory.Macrophages	CRPPA	0.091821	2.552625	0.421816	0.674183	-6.16532	0.825822	0.773841
Non.inflammatory.Macrophages	KCTD18	0.106359	3.044012	0.42159	0.674348	-6.08169	0.801754	0.763342
Non.inflammatory.Macrophages	CFL2	0.056493	4.631141	0.421436	0.67446	-6.50405	0.728101	0.730437
Non.inflammatory.Macrophages	COL4A4	0.189954	1.009618	0.421335	0.674533	-5.48283	0.906342	0.807851
Non.inflammatory.Macrophages	ACAD9	0.090771	2.542008	0.421227	0.674612	-6.01385	0.82651	0.77416
Non.inflammatory.Macrophages	PRRC2B	0.041908	6.226887	0.421079	0.674719	-6.74293	0.660495	0.698697
Non.inflammatory.Macrophages	TADA1	-0.05188	4.679062	-0.42105	0.674741	-6.48195	0.725978	0.729463
Non.inflammatory.Macrophages	H3F3B	-0.02788	9.934295	-0.42091	0.674843	-7.41255	0.526068	0.630214
Non.inflammatory.Macrophages	CCND1	-0.0677	4.140959	-0.42089	0.674858	-6.65624	0.750154	0.740479
Non.inflammatory.Macrophages	NAT9	-0.06858	3.244632	-0.42075	0.674958	-6.33469	0.792108	0.759203
Non.inflammatory.Macrophages	NUP153	0.038974	5.848135	0.420633	0.675044	-6.79712	0.675976	0.706129
Non.inflammatory.Macrophages	ZFP963	-0.12616	1.15161	-0.42047	0.675165	-5.73803	0.898661	0.804708

Non.inflammatory.Macrophages	TRMT10C	-0.05035	4.996252	-0.42035	0.675252	-6.5436	0.712075	0.723084
Non.inflammatory.Macrophages	ATXN2L	0.040321	5.47603	0.420324	0.675269	-6.72456	0.691524	0.71349
Non.inflammatory.Macrophages	TBCB	0.035013	6.070727	0.420284	0.675297	-6.88611	0.666836	0.701782
Non.inflammatory.Macrophages	PLPP2	-0.08809	1.875681	-0.4201	0.675428	-6.08251	0.8604	0.78871
Non.inflammatory.Macrophages	TAF1A	-0.08868	3.171877	-0.42004	0.675477	-6.08303	0.795607	0.760783
Non.inflammatory.Macrophages	GPSM2	0.104211	2.440784	0.41997	0.675526	-6.03422	0.831578	0.776432
Non.inflammatory.Macrophages	TONSL	-0.15946	1.456718	-0.41983	0.675628	-5.6102	0.882354	0.798013
Non.inflammatory.Macrophages	TTLL12	0.1294	2.217401	0.419808	0.675644	-5.99791	0.842864	0.781315
Non.inflammatory.Macrophages	VPS8	0.052535	4.586793	0.419705	0.675719	-6.58548	0.730071	0.731465
Non.inflammatory.Macrophages	4833417C1	-0.15773	0.707506	-0.41966	0.675753	-5.62664	0.917873	0.814798
Non.inflammatory.Macrophages	SLC9A8	-0.04856	4.988676	-0.41944	0.67591	-6.64634	0.712404	0.723347
Non.inflammatory.Macrophages	ESF1	-0.04218	5.001622	-0.41944	0.675915	-6.61577	0.711841	0.723086
Non.inflammatory.Macrophages	INTS5	0.077663	2.704825	0.419387	0.67595	-6.07335	0.818417	0.770818
Non.inflammatory.Macrophages	DUSP5	0.055407	6.834196	0.419225	0.676068	-6.89858	0.636382	0.687172
Non.inflammatory.Macrophages	GM6787	0.193174	0.188055	0.419178	0.676102	-5.45441	0.923793	0.826723

Non.inflammatory.Macrophages	NLRP1B	0.064242	1.79452	0.419105	0.676156	-6.59891	0.864614	0.790644
Non.inflammatory.Macrophages	CYP2C69	0.188116	1.432054	0.418941	0.676275	-5.64419	0.883718	0.798696
Non.inflammatory.Macrophages	CCL4	0.104942	7.388292	0.418787	0.676388	-7.15488	0.615165	0.676717
Non.inflammatory.Macrophages	ISCU	-0.0385	7.465509	-0.41874	0.676422	-7.09033	0.612258	0.675264
Non.inflammatory.Macrophages	SLC12A2	-0.06602	3.544469	-0.41862	0.676509	-6.57773	0.777884	0.753187
Non.inflammatory.Macrophages	IFT140	-0.07798	3.507073	-0.4185	0.676597	-6.20819	0.779651	0.753971
Non.inflammatory.Macrophages	UBL5	0.028862	8.259007	0.418373	0.676689	-7.20429	0.583153	0.660552
Non.inflammatory.Macrophages	GM45606	0.189804	0.477564	0.41825	0.676779	-5.56069	0.92385	0.820225
Non.inflammatory.Macrophages	TMX1	0.038783	5.362	0.418209	0.676808	-6.76996	0.6964	0.716029
Non.inflammatory.Macrophages	TSR2	0.121732	2.118125	0.418204	0.676812	-5.8854	0.847978	0.783673
Non.inflammatory.Macrophages	ADI1	-0.05792	3.749643	-0.41809	0.676894	-6.33656	0.768271	0.748914
Non.inflammatory.Macrophages	NOP9	-0.04682	4.555827	-0.41784	0.67708	-6.58583	0.731618	0.732309
Non.inflammatory.Macrophages	FTO	-0.03445	6.592827	-0.41777	0.677131	-6.94195	0.646011	0.691935
Non.inflammatory.Macrophages	STFA1	0.148503	5.975982	0.417395	0.677402	-6.67697	0.671067	0.704068
Non.inflammatory.Macrophages	AIF1	-0.0564	4.896333	-0.41716	0.67757	-7.08068	0.716913	0.725583

Non.inflammatory.Macrophages	GM15133	0.120356	1.788244	0.416873	0.677782	-5.88233	0.865532	0.791241
Non.inflammatory.Macrophages	ITGA2	0.209953	0.926385	0.416779	0.67785	-5.43992	0.910842	0.810451
Non.inflammatory.Macrophages	GM15265	-0.16568	1.797023	-0.41677	0.677854	-5.59871	0.865075	0.791077
Non.inflammatory.Macrophages	FAM53B	0.073705	4.652028	0.416668	0.677931	-6.41772	0.727672	0.730708
Non.inflammatory.Macrophages	TMED4	0.06198	3.553347	0.416482	0.678067	-6.35093	0.777948	0.753453
Non.inflammatory.Macrophages	ABHD17C	0.050974	4.833665	0.416475	0.678072	-6.62975	0.719661	0.727075
Non.inflammatory.Macrophages	4930513N1	0.199747	0.475363	0.416472	0.678074	-5.42752	0.924424	0.820768
Non.inflammatory.Macrophages	DNAL4	-0.10063	1.951429	-0.41636	0.678156	-5.98376	0.85707	0.787789
Non.inflammatory.Macrophages	PARPBP	-0.19624	2.056928	-0.41627	0.678219	-5.68018	0.85164	0.785499
Non.inflammatory.Macrophages	CPQ	-0.04513	5.056734	-0.41612	0.678331	-6.91178	0.709937	0.722613
Non.inflammatory.Macrophages	BC017158	0.125941	1.449949	0.416076	0.678363	-5.85672	0.883317	0.798894
Non.inflammatory.Macrophages	SPEF1	-0.179	0.343281	-0.41598	0.678436	-5.53887	0.924424	0.823855
Non.inflammatory.Macrophages	COL3A1	0.122981	3.678331	0.41592	0.678477	-6.35436	0.772069	0.750905
Non.inflammatory.Macrophages	CFAP45	-0.18367	0.728674	-0.41581	0.678557	-5.6152	0.917756	0.815145
Non.inflammatory.Macrophages	ZBTB17	0.063782	3.917458	0.415718	0.678624	-6.38923	0.760936	0.746008

Non.inflammatory.Macrophages	ZFP850	0.167594	1.010206	0.415659	0.678667	-5.46638	0.90693	0.808845
Non.inflammatory.Macrophages	FBH1	-0.05136	3.425128	-0.41532	0.678911	-6.46144	0.784165	0.756444
Non.inflammatory.Macrophages	CASP12	-0.16169	-0.47839	-0.4153	0.678926	-5.6228	0.924591	0.843114
Non.inflammatory.Macrophages	HLX	0.06497	3.117664	0.415021	0.679132	-6.37702	0.799019	0.762959
Non.inflammatory.Macrophages	SLC16A9	-0.1493	0.530927	-0.41493	0.679198	-5.96173	0.923747	0.819843
Non.inflammatory.Macrophages	STIM2	0.046995	6.210089	0.4146	0.679439	-6.8041	0.661832	0.700088
Non.inflammatory.Macrophages	NUDC	-0.03894	5.698731	-0.41458	0.679453	-6.78817	0.682857	0.710126
Non.inflammatory.Macrophages	FNDC3A	0.037446	7.526942	0.414571	0.67946	-7.08074	0.610524	0.674888
Non.inflammatory.Macrophages	TRIB2	-0.17407	2.561151	-0.41455	0.679477	-5.63417	0.826376	0.774927
Non.inflammatory.Macrophages	PPP2R2A	0.029206	7.180276	0.414507	0.679507	-6.97213	0.623644	0.681447
Non.inflammatory.Macrophages	RAP2B	-0.04374	4.614993	-0.41443	0.679561	-6.71919	0.729543	0.731904
Non.inflammatory.Macrophages	BIN1	-0.04221	5.230633	-0.41413	0.679783	-6.74497	0.702821	0.719467
Non.inflammatory.Macrophages	MGA	-0.04297	5.917554	-0.41379	0.680028	-6.84116	0.674109	0.705935
Non.inflammatory.Macrophages	GID4	0.052534	4.230623	0.413677	0.680112	-6.43072	0.747178	0.739896
Non.inflammatory.Macrophages	DDAH1	-0.10768	2.481818	-0.41353	0.680218	-5.95513	0.830748	0.776792

Non.inflammatory.Macrophages	GDI1	-0.05119	4.991215	-0.41342	0.680303	-6.61706	0.713348	0.724401
Non.inflammatory.Macrophages	GPR89	-0.06215	3.742407	-0.41335	0.680352	-6.39915	0.769683	0.750049
Non.inflammatory.Macrophages	GM20186	-0.08325	3.559404	-0.41333	0.680369	-6.33662	0.778281	0.753888
Non.inflammatory.Macrophages	RRP12	-0.08801	2.60063	-0.41305	0.680568	-6.05277	0.824938	0.774306
Non.inflammatory.Macrophages	CCDC127	-0.04746	4.15296	-0.41288	0.680698	-6.46653	0.750835	0.74158
Non.inflammatory.Macrophages	GPR174	-0.22631	2.267506	-0.41261	0.680893	-5.36828	0.841698	0.781545
Non.inflammatory.Macrophages	KANK3	-0.12431	2.000717	-0.41242	0.681028	-5.97564	0.855345	0.787396
Non.inflammatory.Macrophages	NR4A1	-0.06298	7.413157	-0.41236	0.681073	-7.17237	0.615196	0.677285
Non.inflammatory.Macrophages	ZFP119B	0.125762	1.297642	0.412301	0.681117	-5.76943	0.892282	0.802935
Non.inflammatory.Macrophages	PLEKHA5	0.091616	4.693302	0.412294	0.681122	-6.11531	0.726537	0.73056
Non.inflammatory.Macrophages	MICU2	0.048538	4.939969	0.412284	0.681129	-6.63165	0.715694	0.725561
Non.inflammatory.Macrophages	ANKRA2	0.051572	3.792457	0.412097	0.681266	-6.43427	0.767469	0.749111
Non.inflammatory.Macrophages	GTPBP4	0.037455	5.956432	0.412089	0.681271	-6.79375	0.672615	0.705316
Non.inflammatory.Macrophages	VAMP3	-0.03449	5.320751	-0.41201	0.681327	-6.7466	0.699257	0.71791
Non.inflammatory.Macrophages	CETN3	-0.04276	5.807387	-0.41197	0.681357	-6.8054	0.678773	0.708249

Non.inflammatory.Macrophages	ICMT	0.092836	2.429223	0.41178	0.681497	-6.0529	0.833523	0.778069
Non.inflammatory.Macrophages	RIIAD1	-0.12272	0.993222	-0.41167	0.681575	-5.80182	0.90872	0.809755
Non.inflammatory.Macrophages	TXN2	-0.03594	6.315861	-0.41165	0.681594	-6.89307	0.657985	0.698293
Non.inflammatory.Macrophages	ZFP655	-0.04734	4.453763	-0.41155	0.681668	-6.5955	0.737216	0.735448
Non.inflammatory.Macrophages	SMPD5	-0.17165	-0.18019	-0.4115	0.681699	-5.46572	0.925306	0.83657
Non.inflammatory.Macrophages	NDUFAF2	0.052854	4.440034	0.411492	0.681708	-6.50471	0.737832	0.735729
Non.inflammatory.Macrophages	RNASEH1	-0.06646	2.84733	-0.41126	0.68188	-6.20322	0.812789	0.769073
Non.inflammatory.Macrophages	ZFP729A	-0.07635	3.051831	-0.41116	0.681949	-6.23085	0.802791	0.764722
Non.inflammatory.Macrophages	GM15860	0.212199	0.373729	0.411136	0.681967	-5.34684	0.925381	0.823821
Non.inflammatory.Macrophages	FHDC1	-0.17388	0.748905	-0.41098	0.682081	-5.60563	0.918008	0.815348
Non.inflammatory.Macrophages	SAMD3	-0.29277	0.184815	-0.41094	0.682114	-5.27397	0.925394	0.82822
Non.inflammatory.Macrophages	GM41077	-0.2016	0.721818	-0.41052	0.682418	-5.45147	0.919279	0.816059
Non.inflammatory.Macrophages	IDNK	0.041823	5.52475	0.410239	0.682623	-6.76083	0.690967	0.714055
Non.inflammatory.Macrophages	ATRIP	-0.09165	2.825397	-0.4101	0.682726	-6.11969	0.814237	0.769796
Non.inflammatory.Macrophages	TRMT2A	-0.06613	3.456488	-0.40997	0.682819	-6.24026	0.783697	0.756444

Non.inflammatory.Macrophages	MRPS2	-0.09295	2.843371	-0.40974	0.682989	-6.20465	0.813352	0.769529
Non.inflammatory.Macrophages	ATG4C	-0.05032	3.11799	-0.40973	0.682994	-6.5949	0.799943	0.763672
Non.inflammatory.Macrophages	OAT	-0.0489	5.245936	-0.4096	0.683089	-6.72925	0.702832	0.719795
Non.inflammatory.Macrophages	TRIM72	-0.15992	0.692552	-0.40947	0.683189	-5.66705	0.920394	0.816998
Non.inflammatory.Macrophages	TBL2	0.077264	2.392625	0.409342	0.683279	-5.98953	0.835811	0.779308
Non.inflammatory.Macrophages	MBD3	-0.04033	5.696238	-0.40915	0.683417	-6.7584	0.683764	0.710875
Non.inflammatory.Macrophages	4930430F0	0.122622	1.035161	0.40912	0.683441	-5.70068	0.906922	0.809304
Non.inflammatory.Macrophages	GM1604A	-0.0971	2.804564	-0.40902	0.683511	-6.15087	0.815263	0.770456
Non.inflammatory.Macrophages	COPS6	0.042866	5.552293	0.409016	0.683517	-6.75112	0.689805	0.71373
Non.inflammatory.Macrophages	ANKRD42	0.204254	0.346625	0.408991	0.683536	-5.54298	0.925799	0.824928
Non.inflammatory.Macrophages	RRAS2	0.055839	5.198697	0.408836	0.683649	-6.63216	0.704861	0.720804
Non.inflammatory.Macrophages	CBS	-0.14985	1.874066	-0.40868	0.683762	-5.80214	0.862353	0.790683
Non.inflammatory.Macrophages	CNOT11	0.05581	4.112527	0.408641	0.683791	-6.42002	0.753084	0.74295
Non.inflammatory.Macrophages	OPA3	-0.04459	5.128547	-0.40862	0.683809	-6.67154	0.707885	0.722231
Non.inflammatory.Macrophages	CAB39L	0.052779	4.624571	0.408507	0.683889	-6.52525	0.729975	0.732455

Non.inflammatory.Macrophages	LNX2	0.068717	3.592505	0.4085	0.683895	-6.30681	0.777256	0.7538
Non.inflammatory.Macrophages	TMUB2	0.067394	3.181282	0.408471	0.683916	-6.2433	0.796882	0.762476
Non.inflammatory.Macrophages	ANGEL2	0.040437	5.052327	0.408468	0.683917	-6.62027	0.711185	0.723784
Non.inflammatory.Macrophages	FBXW5	0.059069	3.39907	0.408125	0.684168	-6.35269	0.786498	0.757892
Non.inflammatory.Macrophages	GM17092	-0.10396	2.1249	-0.40807	0.68421	-5.94379	0.849491	0.785264
Non.inflammatory.Macrophages	PPP6R2	0.051355	4.368985	0.407995	0.684264	-6.46277	0.741488	0.737764
Non.inflammatory.Macrophages	PRPF8	-0.03664	5.801492	-0.40785	0.684368	-6.74696	0.679437	0.70894
Non.inflammatory.Macrophages	CREM	-0.0593	6.0952	-0.40779	0.684414	-6.89124	0.667341	0.703188
Non.inflammatory.Macrophages	1810021B2	-0.19737	0.427969	-0.40775	0.684442	-5.54186	0.925877	0.823261
Non.inflammatory.Macrophages	NFIA	0.054071	5.904	0.407735	0.684454	-6.8918	0.675191	0.706942
Non.inflammatory.Macrophages	JMJD4	-0.13208	1.268434	-0.40744	0.684671	-5.60748	0.894593	0.804375
Non.inflammatory.Macrophages	TAGLN	0.146227	2.304796	0.407307	0.684767	-6.08143	0.84054	0.781593
Non.inflammatory.Macrophages	ZFP82	0.217609	0.121707	0.406965	0.685018	-5.36517	0.926326	0.830536
Non.inflammatory.Macrophages	ITGB6	-0.19476	-0.27325	-0.40683	0.685118	-5.61768	0.926326	0.839694
Non.inflammatory.Macrophages	ZFP503	-0.1611	0.884395	-0.40681	0.685128	-5.58452	0.914181	0.813138

Non.inflammatory.Macrophages	GSPT1	-0.03028	6.707096	-0.40671	0.685202	-6.95249	0.643121	0.69152
Non.inflammatory.Macrophages	WDR13	0.062963	2.570105	0.406346	0.68547	-6.21244	0.827445	0.775962
Non.inflammatory.Macrophages	GPAM	-0.09954	3.696477	-0.4063	0.685506	-6.17789	0.772875	0.752028
Non.inflammatory.Macrophages	CIB1	0.041889	5.732242	0.406276	0.685521	-6.79408	0.682711	0.710595
Non.inflammatory.Macrophages	PAQR8	-0.26426	0.223677	-0.40626	0.685532	-5.28441	0.926409	0.828253
Non.inflammatory.Macrophages	HSPB11	-0.06879	2.925359	-0.40571	0.685938	-6.33398	0.810192	0.76848
Non.inflammatory.Macrophages	E2F4	0.04394	5.334947	0.405594	0.686021	-6.64396	0.69977	0.71865
Non.inflammatory.Macrophages	MAMLD1	0.114514	0.110917	0.405542	0.686059	-5.99032	0.926789	0.831023
Non.inflammatory.Macrophages	CCL25	0.062957	4.40296	0.405417	0.68615	-6.51146	0.740684	0.737551
Non.inflammatory.Macrophages	PDCD1	-0.34477	0.743001	-0.40541	0.686155	-5.30067	0.919599	0.816579
Non.inflammatory.Macrophages	DLC1	-0.08467	4.570858	-0.40522	0.686295	-6.6554	0.733162	0.734133
Non.inflammatory.Macrophages	TCHP	-0.1232	1.792962	-0.40513	0.686362	-5.75322	0.867514	0.793136
Non.inflammatory.Macrophages	TIMM9	-0.06104	3.882337	-0.40505	0.686416	-6.29835	0.764525	0.748352
Non.inflammatory.Macrophages	NSUN4	0.074433	3.189091	0.404995	0.686459	-6.19313	0.797369	0.762929
Non.inflammatory.Macrophages	ANXA11OS	-0.14664	0.812081	-0.40491	0.68652	-5.72673	0.917186	0.815052

Non.inflammatory.Macrophages	TRIT1	0.062004	4.076961	0.404749	0.686639	-6.31403	0.755534	0.74432
Non.inflammatory.Macrophages	U2AF2	-0.03652	6.245648	-0.40474	0.686646	-6.85528	0.661888	0.700712
Non.inflammatory.Macrophages	CCDC43	-0.0721	3.103767	-0.4046	0.686749	-6.14879	0.801542	0.764759
Non.inflammatory.Macrophages	TAF4B	-0.06252	5.130162	-0.40437	0.686919	-6.73905	0.708725	0.72282
Non.inflammatory.Macrophages	KIF13A	0.049049	4.605824	0.403696	0.687411	-6.70181	0.732198	0.733626
Non.inflammatory.Macrophages	ZFP605	-0.14504	1.633403	-0.40351	0.687546	-5.73015	0.876679	0.79693
Non.inflammatory.Macrophages	GM22146	0.176038	2.073446	0.403371	0.68765	-5.53356	0.853774	0.78724
Non.inflammatory.Macrophages	TLR3	-0.08013	1.534056	-0.40327	0.687725	-6.45325	0.881944	0.799174
Non.inflammatory.Macrophages	ZSWIM9	-0.19838	0.544388	-0.40304	0.687895	-5.50681	0.926377	0.821532
Non.inflammatory.Macrophages	ACSL1	0.069214	5.758511	0.402851	0.688031	-6.67517	0.682538	0.710657
Non.inflammatory.Macrophages	GM36486	-0.12998	0.805963	-0.40279	0.688077	-5.97871	0.918251	0.815636
Non.inflammatory.Macrophages	RNF111	0.036925	6.692893	0.402676	0.688158	-6.93083	0.644605	0.692409
Non.inflammatory.Macrophages	EDC4	-0.08145	2.984777	-0.40266	0.688172	-6.19452	0.808046	0.767697
Non.inflammatory.Macrophages	STAT1	0.100422	6.871703	0.402651	0.688177	-7.05538	0.637582	0.68897
Non.inflammatory.Macrophages	CCDC114	0.140229	1.271818	0.402308	0.688429	-5.6332	0.895943	0.805148

Non.inflammatory.Macrophages	SLC30A5	0.043753	5.799283	0.402272	0.688455	-6.76619	0.680839	0.709851
Non.inflammatory.Macrophages	NDUFAF1	-0.07685	2.81597	-0.40227	0.688458	-6.13468	0.816342	0.771311
Non.inflammatory.Macrophages	PDP1	0.107423	2.180234	0.402146	0.688547	-5.77397	0.848295	0.78507
Non.inflammatory.Macrophages	DOHH	0.051374	4.347216	0.402053	0.688615	-6.52721	0.743903	0.739134
Non.inflammatory.Macrophages	PDXP	-0.15391	1.557173	-0.40205	0.688618	-5.7245	0.88072	0.798788
Non.inflammatory.Macrophages	RCE1	-0.05613	3.757774	-0.40194	0.688698	-6.41357	0.771046	0.751361
Non.inflammatory.Macrophages	MFSD1	0.036114	5.149534	0.401926	0.688708	-6.86575	0.708403	0.722809
Non.inflammatory.Macrophages	RPF2	-0.05425	4.402771	-0.40191	0.688722	-6.58401	0.741392	0.737992
Non.inflammatory.Macrophages	AA465934	0.169882	1.66043	0.401651	0.68891	-5.48244	0.875354	0.796548
Non.inflammatory.Macrophages	CACNA2D1	0.24773	0.864423	0.401538	0.688993	-5.44119	0.916289	0.814363
Non.inflammatory.Macrophages	CAPN15	0.049317	4.710463	0.401537	0.688994	-6.558	0.727697	0.731744
Non.inflammatory.Macrophages	SRBD1	-0.05781	4.393343	-0.40139	0.689099	-6.46185	0.741903	0.738232
Non.inflammatory.Macrophages	A	-0.06398	4.78007	-0.40128	0.689181	-6.54884	0.72463	0.730336
Non.inflammatory.Macrophages	NUDT2	-0.05761	3.238769	-0.40124	0.689212	-6.34986	0.795801	0.762352
Non.inflammatory.Macrophages	BRI3	0.032449	7.328714	0.401042	0.689357	-7.14075	0.620086	0.680382

Non.inflammatory.Macrophages	CRYBG2	0.252693	0.051796	0.40097	0.689409	-5.23987	0.927843	0.833061
Non.inflammatory.Macrophages	TFDP1	-0.06196	5.887837	-0.40078	0.689551	-6.67072	0.677294	0.708244
Non.inflammatory.Macrophages	SPC24	-0.11012	4.768056	-0.40072	0.68959	-6.38309	0.725217	0.730672
Non.inflammatory.Macrophages	GM36660	0.163336	-0.14918	0.400678	0.689624	-5.53938	0.927843	0.83772
Non.inflammatory.Macrophages	PTCD2	-0.04586	4.58679	-0.40056	0.689714	-6.60866	0.733272	0.734368
Non.inflammatory.Macrophages	PTPN18	-0.02865	7.11924	-0.40042	0.689811	-7.1123	0.628102	0.684395
Non.inflammatory.Macrophages	SLC9A3R1	-0.03868	6.690174	-0.40036	0.689855	-6.93234	0.644837	0.692619
Non.inflammatory.Macrophages	GM38832	-0.08279	0.763984	-0.40021	0.689965	-6.20577	0.919905	0.816836
Non.inflammatory.Macrophages	NUP37	-0.07849	3.351508	-0.40003	0.690096	-6.20836	0.790443	0.76017
Non.inflammatory.Macrophages	ETHE1	0.047251	4.997365	0.399999	0.690122	-6.63704	0.715148	0.726132
Non.inflammatory.Macrophages	6430550D2	-0.16678	1.142129	-0.39996	0.690152	-5.55925	0.903114	0.808338
Non.inflammatory.Macrophages	DOP1A	0.055515	4.09244	0.399951	0.690158	-6.51357	0.755669	0.744656
Non.inflammatory.Macrophages	COPS2	-0.03255	6.020343	-0.39942	0.690545	-6.81989	0.671975	0.705884
Non.inflammatory.Macrophages	TIMM21	0.078124	2.602996	0.399207	0.690703	-6.11998	0.827263	0.776371
Non.inflammatory.Macrophages	CCDC80	0.116773	2.683481	0.398923	0.690912	-6.0471	0.823248	0.774693

Non.inflammatory.Macrophages	CHMP5	0.034035	5.632148	0.398898	0.69093	-6.83751	0.688114	0.713653
Non.inflammatory.Macrophages	ING1	-0.04361	5.135774	-0.3989	0.690931	-6.6606	0.709291	0.723584
Non.inflammatory.Macrophages	CEP350	0.038213	6.505585	0.398843	0.69097	-6.94911	0.652312	0.696508
Non.inflammatory.Macrophages	TBP	0.078472	3.21522	0.398716	0.691063	-6.1695	0.797176	0.763358
Non.inflammatory.Macrophages	DCUN1D5	0.027669	6.828224	0.398697	0.691078	-6.94161	0.639547	0.690317
Non.inflammatory.Macrophages	SFI1	-0.05219	5.320089	-0.39856	0.69118	-6.62185	0.701356	0.719989
Non.inflammatory.Macrophages	ACSF2	-0.05484	4.286847	-0.3985	0.691223	-6.5725	0.74695	0.741016
Non.inflammatory.Macrophages	GM20324	0.10194	2.098382	0.398437	0.691268	-5.83834	0.852844	0.787538
Non.inflammatory.Macrophages	UBE4BOS1	0.251093	0.735049	0.398315	0.691358	-5.33201	0.921128	0.817949
Non.inflammatory.Macrophages	THOC5	-0.05929	3.564433	-0.39826	0.691398	-6.30389	0.780472	0.756072
Non.inflammatory.Macrophages	LRRC18	-0.18506	1.588071	-0.39807	0.691535	-5.56781	0.879449	0.798807
Non.inflammatory.Macrophages	SLC25A46	0.039245	4.587825	0.397916	0.691651	-6.54683	0.733387	0.7349
Non.inflammatory.Macrophages	HSP90AB1	-0.02883	9.925498	-0.39786	0.691694	-7.46973	0.528777	0.633462
Non.inflammatory.Macrophages	THSD4	0.253517	0.910314	0.397838	0.691709	-5.55154	0.914973	0.814065
Non.inflammatory.Macrophages	BRDT	-0.15231	1.567286	-0.3978	0.691733	-5.63626	0.880549	0.799339

Non.inflammatory.Macrophages	GM43065	-0.20734	0.153169	-0.39768	0.691826	-5.44308	0.928047	0.831412
Non.inflammatory.Macrophages	GM26982	0.104884	1.652146	0.3976	0.691883	-5.76686	0.876068	0.797532
Non.inflammatory.Macrophages	JADE1	0.069371	4.165478	0.397569	0.691906	-6.37011	0.752486	0.743676
Non.inflammatory.Macrophages	PMPCA	0.055117	4.024403	0.397466	0.691982	-6.45085	0.758969	0.746602
Non.inflammatory.Macrophages	TRIM17	-0.09336	2.197819	-0.39741	0.692025	-6.01261	0.847747	0.785524
Non.inflammatory.Macrophages	AEBP2	-0.03981	5.787827	-0.39737	0.692051	-6.85153	0.681598	0.710835
Non.inflammatory.Macrophages	ASB13	0.076014	3.434394	0.397366	0.692055	-6.28457	0.786654	0.758962
Non.inflammatory.Macrophages	NONO	0.031346	6.614635	0.397315	0.692092	-6.92054	0.64797	0.69468
Non.inflammatory.Macrophages	CYB5RL	-0.15605	0.380261	-0.39709	0.692257	-5.54678	0.928099	0.826281
Non.inflammatory.Macrophages	PET100	0.037121	5.786929	0.396946	0.692364	-6.81571	0.681674	0.710909
Non.inflammatory.Macrophages	BID	-0.06377	3.803317	-0.39694	0.692368	-6.34694	0.769277	0.751269
Non.inflammatory.Macrophages	SNHG6	0.068526	3.414627	0.396673	0.692564	-6.31581	0.787641	0.759545
Non.inflammatory.Macrophages	GM553	-0.16233	0.241543	-0.39629	0.692844	-5.64443	0.928099	0.829665
Non.inflammatory.Macrophages	CAML	0.043855	4.427078	0.396274	0.692858	-6.55535	0.740643	0.738513
Non.inflammatory.Macrophages	FADS3	-0.14002	0.659123	-0.39623	0.69289	-5.63904	0.923864	0.820101

Non.inflammatory.Macrophages	ZMYM6	0.097043	2.526746	0.396182	0.692925	-6.07698	0.831129	0.778616
Non.inflammatory.Macrophages	TMEM132I	-0.16975	0.329919	-0.39616	0.692942	-5.59801	0.928099	0.827632
Non.inflammatory.Macrophages	TFRC	-0.04081	6.38061	-0.39614	0.692957	-6.91107	0.65736	0.69942
Non.inflammatory.Macrophages	PTS	-0.03728	5.573329	-0.39614	0.692958	-6.7803	0.69063	0.715318
Non.inflammatory.Macrophages	SZT2	-0.09474	2.318426	-0.39608	0.692999	-5.8674	0.841648	0.783155
Non.inflammatory.Macrophages	CZIB	-0.05868	3.658795	-0.39591	0.693125	-6.36814	0.776058	0.75449
Non.inflammatory.Macrophages	BCDIN3D	0.089692	2.223563	0.395857	0.693164	-5.91611	0.846479	0.785225
Non.inflammatory.Macrophages	NSD2	0.047439	6.699324	0.395787	0.693216	-6.78974	0.644654	0.693278
Non.inflammatory.Macrophages	EIF4A3	-0.0381	5.877476	-0.39561	0.693348	-6.78338	0.67791	0.709369
Non.inflammatory.Macrophages	GM12971	-0.14342	0.967071	-0.39558	0.693366	-5.59269	0.912891	0.813211
Non.inflammatory.Macrophages	TMEM240	-0.20265	0.240106	-0.39557	0.693375	-5.4927	0.928099	0.829795
Non.inflammatory.Macrophages	MPRIP	-0.04707	5.481388	-0.39549	0.693434	-6.69083	0.69452	0.717235
Non.inflammatory.Macrophages	ZNHIT2	0.058049	3.310699	0.395136	0.693694	-6.2741	0.792839	0.762004
Non.inflammatory.Macrophages	IMPG2	0.193673	0.6613	0.394942	0.693837	-5.55492	0.924053	0.820293
Non.inflammatory.Macrophages	KLC2	-0.0925	2.681752	-0.3949	0.693869	-6.08364	0.823618	0.775498

Non.inflammatory.Macrophages	COX5A	0.032257	8.546847	0.394845	0.693908	-7.34225	0.575735	0.658707
Non.inflammatory.Macrophages	PEX1	-0.0808	3.071405	-0.39459	0.694096	-6.21686	0.804566	0.767219
Non.inflammatory.Macrophages	TBKBP1	-0.08621	1.449005	-0.39436	0.694263	-6.22903	0.887369	0.802625
Non.inflammatory.Macrophages	CERS5	0.042604	5.514163	0.393975	0.694548	-6.68927	0.693515	0.716892
Non.inflammatory.Macrophages	4930549G2	-0.09168	2.714781	-0.39394	0.694573	-5.94562	0.822193	0.774981
Non.inflammatory.Macrophages	SLAMF8	0.09802	0.718707	0.393891	0.69461	-6.40284	0.922266	0.819216
Non.inflammatory.Macrophages	ENDOV	-0.09057	2.177707	-0.39388	0.694621	-6.08793	0.849292	0.786663
Non.inflammatory.Macrophages	GART	-0.05781	4.367941	-0.39385	0.694638	-6.53006	0.743725	0.740161
Non.inflammatory.Macrophages	TMEM258	0.035642	6.825258	0.393777	0.694694	-6.99967	0.640053	0.691219
Non.inflammatory.Macrophages	SRSF1	0.041017	6.067856	0.393725	0.694732	-6.7645	0.670433	0.705959
Non.inflammatory.Macrophages	FZD1	0.138995	-0.15494	0.393568	0.694848	-5.68772	0.928612	0.83941
Non.inflammatory.Macrophages	MMP27	-0.13367	-0.20195	-0.39351	0.69489	-5.75705	0.928612	0.840513
Non.inflammatory.Macrophages	TXLNA	0.04311	4.941372	0.393476	0.694916	-6.6309	0.71819	0.728518
Non.inflammatory.Macrophages	MOAP1	-0.13811	0.547771	-0.39291	0.695333	-5.5385	0.927497	0.823315
Non.inflammatory.Macrophages	USP50	-0.06891	3.371827	-0.3928	0.695414	-6.24408	0.79034	0.761171

Non.inflammatory.Macrophages	MMAA	-0.10489	1.748976	-0.39266	0.695517	-5.95554	0.871747	0.796355
Non.inflammatory.Macrophages	PPP3CC	0.094585	3.993482	0.392452	0.695669	-6.12653	0.761067	0.748153
Non.inflammatory.Macrophages	PPP1R12C	0.03764	5.009564	0.392376	0.695725	-6.71818	0.715403	0.727288
Non.inflammatory.Macrophages	CCNQ	0.065408	3.060749	0.392257	0.695812	-6.24942	0.805379	0.767826
Non.inflammatory.Macrophages	E230016M	0.094542	2.465953	0.392227	0.695834	-6.09051	0.834874	0.780636
Non.inflammatory.Macrophages	MRPL3	-0.04854	4.701467	-0.3921	0.695925	-6.63682	0.728969	0.733553
Non.inflammatory.Macrophages	COPS7A	-0.04861	4.237724	-0.39196	0.696029	-6.57794	0.749846	0.743114
Non.inflammatory.Macrophages	TRP53COR	-0.1695	1.008827	-0.39191	0.696069	-5.70877	0.911362	0.812952
Non.inflammatory.Macrophages	IPPK	0.073089	3.400734	0.391676	0.69624	-6.22	0.788956	0.760716
Non.inflammatory.Macrophages	NDE1	-0.06211	4.58489	-0.39167	0.696241	-6.43391	0.734165	0.736053
Non.inflammatory.Macrophages	INSR	0.049706	5.770425	0.391529	0.696348	-6.79001	0.682925	0.712157
Non.inflammatory.Macrophages	NELFB	-0.04616	4.551192	-0.39151	0.696364	-6.50219	0.735673	0.736745
Non.inflammatory.Macrophages	JPX	-0.0885	3.810956	-0.3914	0.696441	-6.26317	0.769555	0.752123
Non.inflammatory.Macrophages	ARL6IP6	-0.04333	5.278997	-0.39139	0.696452	-6.63681	0.703737	0.722007
Non.inflammatory.Macrophages	1700020L2	-0.21438	0.390371	-0.39137	0.696468	-5.31068	0.928865	0.827164

Non.inflammatory.Macrophages	CHRAC1	0.036245	5.264928	0.391279	0.696532	-6.73628	0.704342	0.722327
Non.inflammatory.Macrophages	PRKAR1A	0.025912	7.625008	0.391272	0.696537	-7.03556	0.609587	0.676394
Non.inflammatory.Macrophages	BLVRB	-0.05148	7.092023	-0.39126	0.696546	-7.24268	0.629844	0.686504
Non.inflammatory.Macrophages	UTP23	0.055776	3.577367	0.391172	0.696611	-6.32932	0.780547	0.75709
Non.inflammatory.Macrophages	TCAIM	0.103826	1.789758	0.391168	0.696614	-5.93858	0.869611	0.795687
Non.inflammatory.Macrophages	SEC22B	0.039621	5.449712	0.390974	0.696757	-6.71224	0.696484	0.718671
Non.inflammatory.Macrophages	POLE3	0.057224	4.133596	0.390884	0.696823	-6.46783	0.754658	0.745506
Non.inflammatory.Macrophages	TUSC2	-0.07036	3.017242	-0.39079	0.696892	-6.22591	0.807554	0.769041
Non.inflammatory.Macrophages	ABCA5	-0.25078	0.444818	-0.39058	0.697046	-5.28001	0.928924	0.826045
Non.inflammatory.Macrophages	TRP53RKA	0.082562	2.472703	0.390555	0.697065	-6.01335	0.834587	0.780779
Non.inflammatory.Macrophages	GM12089	-0.13135	0.306607	-0.39055	0.69707	-5.647	0.928924	0.829221
Non.inflammatory.Macrophages	ORAI1	-0.03849	6.491763	-0.39033	0.697231	-6.91326	0.653568	0.698175
Non.inflammatory.Macrophages	COL4A2	0.114668	2.620499	0.390171	0.697349	-5.98509	0.827281	0.777664
Non.inflammatory.Macrophages	TMEM263	0.104391	3.98713	0.389948	0.697513	-5.85607	0.761512	0.748716
Non.inflammatory.Macrophages	NAIP5	0.059592	2.68586	0.389933	0.697524	-6.5347	0.824019	0.776323

Non.inflammatory.Macrophages	TLR1	0.097654	1.219121	0.389828	0.697601	-6.14535	0.900123	0.808635
Non.inflammatory.Macrophages	TRIM13	0.167685	0.46615	0.389735	0.697669	-5.58281	0.929049	0.825731
Non.inflammatory.Macrophages	FZD5	-0.08017	2.536377	-0.38968	0.697713	-6.1027	0.831496	0.779586
Non.inflammatory.Macrophages	DUSP4	-0.13603	0.909125	-0.38957	0.697789	-5.62453	0.916003	0.815717
Non.inflammatory.Macrophages	GM19325	0.108587	1.67424	0.389508	0.697837	-6.04802	0.875849	0.798592
Non.inflammatory.Macrophages	MCM4	0.067848	5.68459	0.389348	0.697955	-6.64055	0.686653	0.714287
Non.inflammatory.Macrophages	DNTTIP2	-0.03697	5.107858	-0.38929	0.697995	-6.67151	0.711268	0.725851
Non.inflammatory.Macrophages	ENPEP	-0.23925	0.401804	-0.38924	0.698036	-5.31578	0.929049	0.827381
Non.inflammatory.Macrophages	LRRC49	-0.12951	1.020554	-0.38886	0.698314	-5.7461	0.910903	0.813449
Non.inflammatory.Macrophages	5730409EC	0.141155	0.23499	0.388786	0.698369	-5.71885	0.929049	0.831425
Non.inflammatory.Macrophages	GPLD1	-0.18595	0.934219	-0.38874	0.698403	-5.53375	0.915125	0.815452
Non.inflammatory.Macrophages	E2F1	-0.06394	4.409532	-0.3886	0.698506	-6.49206	0.742194	0.740349
Non.inflammatory.Macrophages	RNF214	-0.04331	4.961908	-0.38859	0.698514	-6.64626	0.717629	0.729051
Non.inflammatory.Macrophages	TLR6	0.101995	1.070509	0.388511	0.698572	-6.09109	0.90818	0.812402
Non.inflammatory.Macrophages	FOXJ2	0.056894	3.829076	0.388506	0.698576	-6.4943	0.768861	0.752407

Non.inflammatory.Macrophages	GM20470	0.169295	1.403571	0.38848	0.698595	-5.52721	0.890213	0.804918
Non.inflammatory.Macrophages	DOCK7	0.045715	3.830588	0.388465	0.698606	-6.73726	0.76879	0.752376
Non.inflammatory.Macrophages	RSPH3B	-0.07286	3.01938	-0.3882	0.698805	-6.11955	0.807619	0.769639
Non.inflammatory.Macrophages	LANCL1	-0.05594	3.448045	-0.38807	0.698898	-6.24496	0.78691	0.760559
Non.inflammatory.Macrophages	JMJD6	-0.04751	4.999369	-0.38805	0.69891	-6.61712	0.716044	0.728421
Non.inflammatory.Macrophages	GTSE1	0.233986	1.404909	0.388022	0.698932	-5.5186	0.890207	0.805047
Non.inflammatory.Macrophages	HSD17B10	-0.04597	5.540491	-0.3878	0.699097	-6.74406	0.692872	0.71763
Non.inflammatory.Macrophages	REV1	-0.04535	5.326262	-0.38758	0.69926	-6.64021	0.702092	0.721944
Non.inflammatory.Macrophages	APOC1	0.079462	7.949726	0.387285	0.699476	-7.31774	0.59798	0.671098
Non.inflammatory.Macrophages	PSENEN	-0.02758	6.713106	-0.38717	0.699564	-6.99126	0.645096	0.69461
Non.inflammatory.Macrophages	OSBPL5	0.215618	0.3884	0.387147	0.699578	-5.38373	0.92952	0.828237
Non.inflammatory.Macrophages	AMD2	-0.16028	0.044662	-0.38701	0.699681	-5.49668	0.929565	0.836209
Non.inflammatory.Macrophages	HIST1H2BE	-0.12117	2.079102	-0.38685	0.699798	-5.8345	0.855233	0.790289
Non.inflammatory.Macrophages	RBM39	-0.02098	9.586582	-0.38682	0.699818	-7.34988	0.540794	0.641286
Non.inflammatory.Macrophages	1700037H(	0.06044	3.714085	0.386409	0.700122	-6.35787	0.774816	0.75521

Non.inflammatory.Macrophages	XRCC3	-0.17606	0.159666	-0.38639	0.700134	-5.4218	0.929729	0.833644
Non.inflammatory.Macrophages	COMT	0.03886	5.36738	0.386374	0.700148	-6.81233	0.700601	0.721241
Non.inflammatory.Macrophages	CHMP6	-0.05905	3.712475	-0.38608	0.700367	-6.44018	0.774993	0.755246
Non.inflammatory.Macrophages	PSMA5	-0.04039	5.884469	-0.38592	0.700483	-6.88389	0.678899	0.710937
Non.inflammatory.Macrophages	CHRNE	-0.18541	-0.75209	-0.38583	0.700551	-5.38408	0.929851	0.855005
Non.inflammatory.Macrophages	PRKAG1	-0.03493	5.515312	-0.38579	0.700581	-6.68792	0.694391	0.718282
Non.inflammatory.Macrophages	2610044O1	0.08247	2.24732	0.385785	0.700583	-5.89698	0.846862	0.78667
Non.inflammatory.Macrophages	MPI	0.076719	2.225319	0.3854	0.700867	-6.10663	0.848058	0.78727
Non.inflammatory.Macrophages	0610040J0	-0.06546	2.378015	-0.38539	0.700877	-6.48129	0.84028	0.783934
Non.inflammatory.Macrophages	RBM3	-0.03254	9.070642	-0.38533	0.700916	-7.29748	0.558438	0.650704
Non.inflammatory.Macrophages	GATD1	0.045264	4.394103	0.385333	0.700917	-6.59017	0.743595	0.741167
Non.inflammatory.Macrophages	EPM2AIP1	-0.0833	2.609784	-0.38516	0.701044	-6.06291	0.828626	0.778932
Non.inflammatory.Macrophages	MELK	-0.11689	3.016502	-0.38507	0.701107	-6.00979	0.80849	0.770198
Non.inflammatory.Macrophages	RNF25	0.062164	3.103588	0.384766	0.701335	-6.26131	0.804237	0.768432
Non.inflammatory.Macrophages	SNRK	0.048228	4.865356	0.38474	0.701354	-6.61468	0.722572	0.731661

Non.inflammatory.Macrophages	ZFP41	-0.16017	0.707145	-0.38458	0.701475	-5.56802	0.924012	0.821377
Non.inflammatory.Macrophages	P2RY13	0.111748	1.100765	0.384541	0.701501	-6.09849	0.90742	0.812445
Non.inflammatory.Macrophages	BC052040	-0.06341	4.165796	-0.38442	0.701587	-6.45294	0.75402	0.746055
Non.inflammatory.Macrophages	PCYOX1L	0.122106	1.613818	0.384337	0.701652	-5.74602	0.879897	0.800944
Non.inflammatory.Macrophages	1700047M	0.200722	-0.15234	0.38432	0.701664	-5.45078	0.929958	0.84121
Non.inflammatory.Macrophages	RARS	0.039183	5.121145	0.384137	0.701799	-6.73696	0.711386	0.726475
Non.inflammatory.Macrophages	ERBB3	-0.14921	1.113728	-0.38407	0.701852	-5.71875	0.906715	0.812152
Non.inflammatory.Macrophages	CHM	0.039878	5.555869	0.384048	0.701865	-6.77062	0.692752	0.717736
Non.inflammatory.Macrophages	STK26	-0.13409	4.048898	-0.38398	0.701918	-5.65087	0.7594	0.748501
Non.inflammatory.Macrophages	BCL7C	0.038442	5.355918	0.38394	0.701945	-6.64372	0.701264	0.721756
Non.inflammatory.Macrophages	SRR	-0.07568	2.599008	-0.38391	0.701969	-6.08304	0.829165	0.779316
Non.inflammatory.Macrophages	GM21762	-0.25198	-1.20491	-0.38328	0.70243	-5.12488	0.930478	0.866268
Non.inflammatory.Macrophages	BCAP29	0.043056	4.765797	0.383056	0.702598	-6.67747	0.727441	0.733902
Non.inflammatory.Macrophages	CENPI	0.110605	2.997583	0.383013	0.70263	-5.97159	0.80994	0.770925
Non.inflammatory.Macrophages	FPR3	-0.20796	-0.4789	-0.38287	0.702733	-5.43879	0.930606	0.849171

Non.inflammatory.Macrophages	CIT	-0.11473	4.461996	-0.38253	0.702987	-6.27106	0.741261	0.740336
Non.inflammatory.Macrophages	GABPA	0.043786	4.662072	0.382394	0.703087	-6.56357	0.732306	0.736276
Non.inflammatory.Macrophages	ZFP202	0.161036	0.604939	0.381774	0.703545	-5.67519	0.928156	0.824528
Non.inflammatory.Macrophages	FBXL14	-0.04399	5.203671	-0.38154	0.703716	-6.56166	0.708515	0.725558
Non.inflammatory.Macrophages	GM14698	0.157967	0.738672	0.381528	0.703727	-5.5486	0.923812	0.821507
Non.inflammatory.Macrophages	GMPR	0.118658	1.923101	0.38136	0.703851	-5.88242	0.86453	0.794948
Non.inflammatory.Macrophages	BAIAP3	-0.2146	-0.32152	-0.3813	0.703893	-5.38238	0.93088	0.846097
Non.inflammatory.Macrophages	RXRB	-0.04794	4.393184	-0.38122	0.703956	-6.55073	0.744397	0.74219
Non.inflammatory.Macrophages	TMEM106(	0.057617	3.205819	0.381188	0.703978	-6.32842	0.800065	0.767132
Non.inflammatory.Macrophages	TIMP2	-0.06467	4.873204	-0.38092	0.704174	-6.7955	0.722943	0.732412
Non.inflammatory.Macrophages	SPACA6	-0.1246	1.094819	-0.38087	0.704216	-5.77658	0.908645	0.813584
Non.inflammatory.Macrophages	NPHP1	-0.15742	0.652168	-0.3808	0.704264	-5.59703	0.92688	0.823649
Non.inflammatory.Macrophages	IGLC1	-0.22865	3.761408	-0.38076	0.704297	-6.09796	0.773549	0.755432
Non.inflammatory.Macrophages	RBBP9	-0.11924	1.214794	-0.38069	0.70435	-5.86833	0.902131	0.810876
Non.inflammatory.Macrophages	USF2	0.035269	6.021725	0.380676	0.704357	-6.84674	0.67397	0.709363

Non.inflammatory.Macrophages	TRPV4	-0.14139	-0.51202	-0.38062	0.704395	-5.6746	0.93088	0.850696
Non.inflammatory.Macrophages	POGK	-0.08869	2.454489	-0.38049	0.704495	-6.07785	0.837266	0.783406
Non.inflammatory.Macrophages	PABPC1	0.037091	10.47512	0.380463	0.704514	-7.52761	0.512765	0.626692
Non.inflammatory.Macrophages	GM26801	-0.23821	0.557742	-0.38042	0.704546	-5.34403	0.929273	0.825818
Non.inflammatory.Macrophages	GM47350	0.155888	1.355941	0.380359	0.704591	-5.46873	0.894522	0.807716
Non.inflammatory.Macrophages	MORN2	0.106526	2.045949	0.380261	0.704663	-5.86784	0.858156	0.792401
Non.inflammatory.Macrophages	GM13184	-0.07277	2.666577	-0.38004	0.704824	-6.31206	0.826606	0.778894
Non.inflammatory.Macrophages	RNF125	0.131984	3.852901	0.379889	0.704939	-5.84112	0.769262	0.753624
Non.inflammatory.Macrophages	PSMG2	-0.0601	3.919974	-0.37979	0.705013	-6.40288	0.766134	0.752218
Non.inflammatory.Macrophages	MASTL	0.083473	3.782	0.379711	0.705071	-6.06622	0.772583	0.755112
Non.inflammatory.Macrophages	EGF	-0.17288	0.634522	-0.37965	0.705119	-5.41995	0.927456	0.824176
Non.inflammatory.Macrophages	PGAM5	0.054639	3.934831	0.379491	0.705233	-6.35304	0.765442	0.751907
Non.inflammatory.Macrophages	C030034I2	-0.09417	2.630863	-0.37935	0.705339	-5.89316	0.828392	0.779689
Non.inflammatory.Macrophages	PTPRF	-0.15485	1.276101	-0.37933	0.705354	-5.49016	0.898819	0.809617
Non.inflammatory.Macrophages	KLHDC1	-0.0914	2.152219	-0.37927	0.705398	-5.97297	0.852676	0.790138

Non.inflammatory.Macrophages	GM47230	-0.20686	0.668652	-0.37926	0.705403	-5.3884	0.926294	0.823395
Non.inflammatory.Macrophages	2610035D1	-0.08453	4.872984	-0.37923	0.705427	-6.4354	0.722953	0.732525
Non.inflammatory.Macrophages	MRPS17	0.037907	5.224333	0.379228	0.705428	-6.72958	0.707622	0.725395
Non.inflammatory.Macrophages	NEGR1	0.200935	-0.13284	0.379149	0.705486	-5.55081	0.93088	0.84192
Non.inflammatory.Macrophages	LTB	-0.15783	3.446775	-0.37915	0.705489	-5.69454	0.788463	0.76219
Non.inflammatory.Macrophages	STK32C	-0.15683	0.553606	-0.37905	0.70556	-5.5701	0.929371	0.826051
Non.inflammatory.Macrophages	HSPE1-RS1	0.198306	0.18545	0.379033	0.705572	-5.50938	0.93088	0.834538
Non.inflammatory.Macrophages	ANKS3	0.055261	4.082767	0.378981	0.70561	-6.40325	0.758589	0.748851
Non.inflammatory.Macrophages	ADGRG1	-0.20577	1.720983	-0.37896	0.705623	-5.53963	0.875112	0.799704
Non.inflammatory.Macrophages	TPRKB	0.066047	3.157978	0.37866	0.705848	-6.28336	0.802565	0.768488
Non.inflammatory.Macrophages	NOP14	0.053762	4.004398	0.378518	0.705953	-6.46958	0.76242	0.750632
Non.inflammatory.Macrophages	ZFP39	-0.16612	1.023509	-0.37819	0.706197	-5.56058	0.912856	0.815592
Non.inflammatory.Macrophages	SOCS1	-0.09587	5.152296	-0.37807	0.706281	-6.44109	0.710988	0.727095
Non.inflammatory.Macrophages	PRXL2C	-0.04241	4.932963	-0.37794	0.706378	-6.64689	0.720565	0.731552
Non.inflammatory.Macrophages	MTA2	0.041415	5.782517	0.377883	0.706424	-6.76167	0.68414	0.714453

Non.inflammatory.Macrophages	YWHAZ	0.01968	8.482146	0.37787	0.706433	-7.22491	0.579796	0.662732
Non.inflammatory.Macrophages	MTFR1L	-0.04136	4.250609	-0.37773	0.706535	-6.54939	0.751147	0.745601
Non.inflammatory.Macrophages	ITPR2	0.039073	6.912603	0.37769	0.706566	-6.88584	0.638414	0.692354
Non.inflammatory.Macrophages	USP39	-0.05586	4.614508	-0.37768	0.706576	-6.50626	0.734689	0.7381
Non.inflammatory.Macrophages	SSR2	-0.04257	5.189958	-0.37761	0.706627	-6.71086	0.709356	0.726387
Non.inflammatory.Macrophages	ZC3H18	0.041118	5.270525	0.377436	0.706754	-6.68436	0.705934	0.724799
Non.inflammatory.Macrophages	SLC6A6	0.031263	7.287991	0.377192	0.706934	-7.15904	0.62398	0.685265
Non.inflammatory.Macrophages	SNN	-0.0778	3.649853	-0.37719	0.706935	-6.26041	0.779191	0.758305
Non.inflammatory.Macrophages	MARS	-0.08162	3.10364	-0.37683	0.707204	-6.11461	0.805598	0.769987
Non.inflammatory.Macrophages	PRPS1L3	-0.11015	2.045409	-0.37667	0.707319	-5.78942	0.858786	0.793048
Non.inflammatory.Macrophages	5830408C2	0.087342	2.989299	0.376541	0.707417	-5.93587	0.811195	0.772509
Non.inflammatory.Macrophages	CCDC12	-0.0262	6.939585	-0.37653	0.707421	-7.06012	0.637584	0.692067
Non.inflammatory.Macrophages	TAF6L	-0.07116	2.800016	-0.37646	0.707476	-6.1479	0.820539	0.776588
Non.inflammatory.Macrophages	MED9	0.058081	3.526994	0.376436	0.707495	-6.39841	0.785186	0.761037
Non.inflammatory.Macrophages	TGFBR2	-0.03378	7.292661	-0.37633	0.707572	-6.98355	0.623938	0.685298

Non.inflammatory.Macrophages	NLRC5	-0.09443	5.064856	-0.37603	0.707799	-6.63544	0.715134	0.729236
Non.inflammatory.Macrophages	4930532G1	-0.14391	1.429337	-0.376	0.707817	-5.85784	0.891326	0.806855
Non.inflammatory.Macrophages	IFI214	0.139953	1.667368	0.375416	0.708251	-5.82341	0.878665	0.801635
Non.inflammatory.Macrophages	MAP3K3	-0.03739	6.795781	-0.37537	0.708284	-6.97925	0.643308	0.695014
Non.inflammatory.Macrophages	PPCDC	-0.06143	3.567947	-0.37528	0.708349	-6.29682	0.783336	0.760358
Non.inflammatory.Macrophages	CD7	-0.21922	4.199176	-0.3752	0.70841	-5.83447	0.753861	0.747117
Non.inflammatory.Macrophages	VASP	0.037426	6.541669	0.375172	0.708431	-7.00586	0.6534	0.699949
Non.inflammatory.Macrophages	DTYMK	-0.05634	5.126752	-0.37501	0.708549	-6.62167	0.712438	0.728072
Non.inflammatory.Macrophages	SNRPE	0.033668	7.064225	0.374947	0.708598	-7.00465	0.632811	0.68984
Non.inflammatory.Macrophages	DAPK2	-0.12674	2.818088	-0.37492	0.708616	-5.81911	0.819746	0.776389
Non.inflammatory.Macrophages	1700028E1	0.109616	1.36693	0.374855	0.708666	-5.75422	0.894672	0.808357
Non.inflammatory.Macrophages	SUGP1	-0.04726	4.615277	-0.37463	0.708834	-6.47888	0.735005	0.738522
Non.inflammatory.Macrophages	SP140	0.050085	6.188606	0.374533	0.708905	-6.84352	0.667676	0.706877
Non.inflammatory.Macrophages	OST4	0.033965	8.049265	0.374504	0.708926	-7.22057	0.5957	0.671197
Non.inflammatory.Macrophages	ZFP512B	0.099887	2.377698	0.374479	0.708945	-5.99935	0.841853	0.785974

Non.inflammatory.Macrophages	DNAJA4	0.18237	0.616749	0.374426	0.708984	-5.46387	0.928645	0.825423
Non.inflammatory.Macrophages	TIAM1	0.077427	4.628891	0.374351	0.709039	-6.33729	0.734396	0.738272
Non.inflammatory.Macrophages	HNRNPDL	-0.02659	7.857159	-0.3737	0.709523	-7.07711	0.602767	0.674946
Non.inflammatory.Macrophages	ATG13	0.05461	4.765899	0.373442	0.709714	-6.46702	0.72829	0.735701
Non.inflammatory.Macrophages	GM17354	0.117844	1.393239	0.373421	0.709729	-5.72323	0.89326	0.808071
Non.inflammatory.Macrophages	DERL2	-0.03832	4.925658	-0.37332	0.7098	-6.70622	0.721231	0.732465
Non.inflammatory.Macrophages	NDUFAF5	-0.08467	2.563951	-0.37313	0.709945	-6.00809	0.832437	0.782292
Non.inflammatory.Macrophages	SFT2D3	0.066376	2.864525	0.372972	0.710062	-6.11819	0.817446	0.775816
Non.inflammatory.Macrophages	ANAPC4	0.043721	4.541254	0.372916	0.710104	-6.50788	0.738327	0.740446
Non.inflammatory.Macrophages	ZFP68	0.056305	3.920899	0.372847	0.710155	-6.2599	0.766725	0.753343
Non.inflammatory.Macrophages	ULK3	0.085681	2.219052	0.372733	0.710239	-6.04068	0.84995	0.78987
Non.inflammatory.Macrophages	GNB2	-0.02753	8.428837	-0.3727	0.710264	-7.23961	0.581975	0.664499
Non.inflammatory.Macrophages	PROZ	0.127687	1.915226	0.372595	0.710341	-5.87228	0.865656	0.796607
Non.inflammatory.Macrophages	CSPRS	0.149571	0.292172	0.372583	0.71035	-5.52962	0.931651	0.833351
Non.inflammatory.Macrophages	ANKRD24	0.102915	1.054272	0.372452	0.710447	-5.87623	0.91161	0.815973

Non.inflammatory.Macrophages	SLC39A4	-0.16101	1.149584	-0.37239	0.710496	-5.54685	0.906416	0.813832
Non.inflammatory.Macrophages	PINX1	-0.06718	3.022074	-0.37237	0.71051	-6.24788	0.809689	0.772545
Non.inflammatory.Macrophages	HOMER3	0.06405	3.103095	0.372329	0.710539	-6.16627	0.805726	0.770818
Non.inflammatory.Macrophages	DIP2C	0.057819	6.172429	0.372276	0.710578	-6.9426	0.668338	0.707696
Non.inflammatory.Macrophages	GTF2B	-0.03928	6.738906	-0.37224	0.710605	-6.87726	0.645554	0.696622
Non.inflammatory.Macrophages	PAM16	-0.03964	4.987535	-0.37218	0.710648	-6.66698	0.718514	0.731431
Non.inflammatory.Macrophages	GM27188	0.283955	0.619818	0.372152	0.71067	-5.2372	0.928572	0.825915
Non.inflammatory.Macrophages	CPNE3	-0.04169	5.367342	-0.37193	0.710838	-6.79938	0.702052	0.723783
Non.inflammatory.Macrophages	IFI47	0.10332	5.277962	0.371828	0.710911	-6.73911	0.705893	0.725586
Non.inflammatory.Macrophages	ZYG11B	0.040578	5.883084	0.371732	0.710982	-6.76521	0.680272	0.713464
Non.inflammatory.Macrophages	SETD4	0.06747	2.964232	0.371667	0.71103	-6.14205	0.812529	0.77385
Non.inflammatory.Macrophages	HUWE1	-0.03372	7.155414	-0.37144	0.711201	-7.03633	0.629283	0.688676
Non.inflammatory.Macrophages	SMURF1	0.047733	4.688664	0.371253	0.711337	-6.57231	0.731726	0.737685
Non.inflammatory.Macrophages	LAMTOR1	0.027315	6.503458	0.370939	0.71157	-7.01572	0.654931	0.701378
Non.inflammatory.Macrophages	ASB5	0.144323	0.515697	0.370938	0.711571	-5.62545	0.931042	0.828508

Non.inflammatory.Macrophages	WDR74	0.046006	4.042303	0.370875	0.711617	-6.40046	0.761088	0.751119
Non.inflammatory.Macrophages	USP8	0.031403	5.372996	0.370821	0.711658	-6.85189	0.701809	0.723805
Non.inflammatory.Macrophages	TBRG4	0.05882	4.075048	0.370805	0.711669	-6.36461	0.759574	0.750436
Non.inflammatory.Macrophages	MYPOPOS	0.137345	1.654931	0.370778	0.71169	-5.78201	0.879323	0.80271
Non.inflammatory.Macrophages	BRD9	0.039545	5.185663	0.370675	0.711766	-6.6535	0.709881	0.727636
Non.inflammatory.Macrophages	SBF1	0.044067	4.474225	0.370522	0.71188	-6.50261	0.741347	0.742215
Non.inflammatory.Macrophages	GM38115	0.066313	3.861728	0.370521	0.711881	-6.23286	0.769487	0.754977
Non.inflammatory.Macrophages	TRAIP	0.136706	2.119414	0.370494	0.7119	-5.68002	0.855072	0.792474
Non.inflammatory.Macrophages	POLL	0.108884	1.158885	0.370358	0.712001	-5.77227	0.905911	0.813917
Non.inflammatory.Macrophages	YIF1B	0.041514	4.886916	0.370292	0.712051	-6.67176	0.722937	0.733737
Non.inflammatory.Macrophages	TFB1M	0.104891	1.706255	0.37023	0.712096	-5.83329	0.876613	0.801629
Non.inflammatory.Macrophages	HEY1	-0.15472	-0.2714	-0.37022	0.712103	-5.63418	0.931651	0.846889
Non.inflammatory.Macrophages	TTC23	-0.14068	1.101964	-0.3702	0.712119	-5.64874	0.909008	0.815205
Non.inflammatory.Macrophages	SMAD1	-0.04906	3.547772	-0.37014	0.712162	-6.51065	0.784296	0.761603
Non.inflammatory.Macrophages	AP1G2	-0.08118	2.809774	-0.37005	0.712229	-6.23591	0.820158	0.77742

Non.inflammatory.Macrophages	GM15859	0.104391	1.208039	0.370032	0.712243	-5.73575	0.903244	0.812824
Non.inflammatory.Macrophages	CFAP77	-0.13586	0.931954	-0.36997	0.712289	-5.71209	0.917768	0.819096
Non.inflammatory.Macrophages	ACTR3	0.022915	8.876057	0.369731	0.712467	-7.34572	0.566203	0.656659
Non.inflammatory.Macrophages	FAM43A	-0.05456	4.098571	-0.36968	0.712501	-6.46046	0.758488	0.750057
Non.inflammatory.Macrophages	IRGM1	0.083674	4.93997	0.369663	0.712518	-6.68381	0.720602	0.732693
Non.inflammatory.Macrophages	RBM18	-0.04343	4.236008	-0.36949	0.712643	-6.46286	0.752174	0.747209
Non.inflammatory.Macrophages	SCYL2	-0.04339	4.409687	-0.36941	0.712707	-6.53308	0.744266	0.743629
Non.inflammatory.Macrophages	LILRA5	-0.0808	1.497471	-0.36936	0.712744	-6.39097	0.887685	0.806392
Non.inflammatory.Macrophages	PCNX3	-0.05567	3.30596	-0.36934	0.71276	-6.34728	0.795883	0.766845
Non.inflammatory.Macrophages	FLYWCH1	0.0564	3.850858	0.369318	0.712774	-6.33493	0.769995	0.755305
Non.inflammatory.Macrophages	NEO1	-0.182	0.660859	-0.36925	0.712826	-5.5682	0.927338	0.82537
Non.inflammatory.Macrophages	KLHL28	-0.05679	3.44241	-0.36917	0.712884	-6.30747	0.789325	0.763967
Non.inflammatory.Macrophages	VPS13B	0.028417	8.050399	0.369054	0.71297	-7.14946	0.595659	0.671996
Non.inflammatory.Macrophages	SLFN9	0.086349	2.410472	0.369048	0.712975	-6.23934	0.840189	0.786217
Non.inflammatory.Macrophages	ARHGEF11	-0.04256	4.907611	-0.36896	0.713037	-6.75457	0.722025	0.733459

Non.inflammatory.Macrophages	1700102H2	-0.18467	0.529845	-0.36889	0.713095	-5.40569	0.930705	0.828427
Non.inflammatory.Macrophages	ASF1A	-0.05629	5.210724	-0.36886	0.713118	-6.47105	0.708796	0.727296
Non.inflammatory.Macrophages	MAD2L2	0.061525	3.472942	0.368838	0.71313	-6.31137	0.787865	0.763341
Non.inflammatory.Macrophages	GM49085	0.10527	2.025817	0.368815	0.713148	-5.76438	0.859909	0.794696
Non.inflammatory.Macrophages	COL15A1	-0.18876	0.332157	-0.3688	0.71316	-5.56214	0.931651	0.832986
Non.inflammatory.Macrophages	RUNDC3B	-0.19577	3.848947	-0.36865	0.713269	-5.56498	0.770127	0.755395
Non.inflammatory.Macrophages	GM28501	0.198072	0.476037	0.368405	0.713452	-5.32361	0.931852	0.829764
Non.inflammatory.Macrophages	RRM2B	0.049403	4.437435	0.368123	0.713661	-6.48737	0.743262	0.74328
Non.inflammatory.Macrophages	FKBP2	-0.03979	5.611404	-0.3681	0.713678	-6.85337	0.691898	0.71938
Non.inflammatory.Macrophages	GM48293	0.132845	-0.15995	0.367918	0.713814	-5.73238	0.93199	0.844695
Non.inflammatory.Macrophages	RCN2	0.045242	5.075901	0.367893	0.713833	-6.70372	0.714912	0.730252
Non.inflammatory.Macrophages	TNFSF8	-0.27618	-0.51498	-0.36772	0.713965	-5.26019	0.931993	0.853132
Non.inflammatory.Macrophages	EFNA1	-0.18902	0.531324	-0.36761	0.714041	-5.47228	0.931011	0.828725
Non.inflammatory.Macrophages	NR1H3	0.066146	3.325046	0.367521	0.714109	-6.65905	0.795254	0.766799
Non.inflammatory.Macrophages	B9D1	-0.15474	0.371188	-0.36752	0.71411	-5.5957	0.931993	0.832421

Non.inflammatory.Macrophages	PRPF38A	0.04243	5.176932	0.367144	0.714389	-6.64591	0.710626	0.72835
Non.inflammatory.Macrophages	TMEM243	0.030569	6.427837	0.367117	0.714409	-6.8708	0.65831	0.703421
Non.inflammatory.Macrophages	KAT8	-0.05899	3.624929	-0.36696	0.714524	-6.29965	0.781035	0.760543
Non.inflammatory.Macrophages	MAPK6	-0.04133	5.88266	-0.3668	0.714647	-6.96754	0.68064	0.714278
Non.inflammatory.Macrophages	NPL	0.085276	2.535175	0.366694	0.714724	-6.30169	0.834316	0.784054
Non.inflammatory.Macrophages	ETFB	0.038025	6.979015	0.366631	0.714771	-7.08261	0.636453	0.692851
Non.inflammatory.Macrophages	1810024B(	0.074617	2.692562	0.366593	0.714798	-6.14865	0.826418	0.780658
Non.inflammatory.Macrophages	STARD8	0.053263	3.359704	0.366515	0.714856	-6.52925	0.793703	0.766302
Non.inflammatory.Macrophages	ANKS1B	-0.13674	1.030517	-0.36648	0.714881	-5.7175	0.91338	0.817575
Non.inflammatory.Macrophages	ZFP846	-0.09785	2.431015	-0.36645	0.714906	-5.87558	0.839581	0.786358
Non.inflammatory.Macrophages	ACBD4	-0.12199	1.764413	-0.36584	0.715358	-5.70735	0.874347	0.801136
Non.inflammatory.Macrophages	CASP1	-0.04973	3.51676	-0.36582	0.71537	-6.64509	0.786489	0.763024
Non.inflammatory.Macrophages	SMPD3	-0.18308	-0.20687	-0.36579	0.715395	-5.50864	0.932501	0.846221
Non.inflammatory.Macrophages	MRM1	0.084409	2.067052	0.365516	0.715599	-5.96631	0.858569	0.794524
Non.inflammatory.Macrophages	EXOC4	0.024805	8.03297	0.365353	0.715721	-7.17201	0.596848	0.672977

Non.inflammatory.Macrophages	TTC4	0.051344	3.541793	0.365117	0.715896	-6.34064	0.785307	0.762644
Non.inflammatory.Macrophages	TRIM12A	0.049233	5.048891	0.365044	0.71595	-6.62076	0.716493	0.731325
Non.inflammatory.Macrophages	GM2682	0.247385	2.755703	0.364856	0.71609	-5.5194	0.823606	0.779521
Non.inflammatory.Macrophages	EIF2B4	0.043627	4.102435	0.364819	0.716117	-6.42559	0.759012	0.750849
Non.inflammatory.Macrophages	TLNRD1	0.055603	4.581796	0.364815	0.716121	-6.56917	0.737188	0.740897
Non.inflammatory.Macrophages	STAT5A	0.063164	3.992851	0.36478	0.716147	-6.2821	0.764086	0.753143
Non.inflammatory.Macrophages	BRPF3	-0.04465	4.451036	-0.36476	0.716161	-6.44024	0.743082	0.743599
Non.inflammatory.Macrophages	A330040F1	0.108323	2.508264	0.36476	0.716161	-6.33381	0.836015	0.784905
Non.inflammatory.Macrophages	ERCC3	-0.04783	3.695125	-0.36468	0.716224	-6.42003	0.778032	0.759409
Non.inflammatory.Macrophages	TSPYL1	-0.04154	4.888609	-0.36467	0.716232	-6.65453	0.723531	0.734596
Non.inflammatory.Macrophages	CD300LG	0.123252	1.259921	0.364473	0.716375	-5.7959	0.901364	0.812729
Non.inflammatory.Macrophages	SEC11C	0.034613	7.287381	0.364265	0.71653	-6.98962	0.624889	0.687302
Non.inflammatory.Macrophages	HIST1H3I	0.189964	1.296054	0.364233	0.716554	-5.48915	0.899463	0.811999
Non.inflammatory.Macrophages	TNRC6C	0.032201	7.143837	0.364061	0.716682	-6.96513	0.63045	0.690094
Non.inflammatory.Macrophages	PNKD	0.044119	4.867546	0.363872	0.716822	-6.67903	0.724619	0.735257

Non.inflammatory.Macrophages	APTX	-0.05246	3.052742	-0.36373	0.716928	-6.25833	0.809111	0.773383
Non.inflammatory.Macrophages	MAST4	-0.06949	6.39259	-0.36372	0.716936	-6.79887	0.660146	0.704724
Non.inflammatory.Macrophages	ARHGEF4	-0.17014	0.759185	-0.36364	0.716997	-5.77091	0.924908	0.824304
Non.inflammatory.Macrophages	TM7SF2	-0.10647	1.754706	-0.36362	0.717008	-5.82246	0.87506	0.801817
Non.inflammatory.Macrophages	ABHD4	0.055662	2.787772	0.363474	0.717118	-6.31898	0.822237	0.779134
Non.inflammatory.Macrophages	PRDM9	-0.14484	1.146615	-0.36326	0.717276	-5.5956	0.907731	0.815554
Non.inflammatory.Macrophages	PSMB3	0.032007	7.724991	0.363221	0.717307	-7.10808	0.60845	0.679128
Non.inflammatory.Macrophages	PLP2	-0.12329	5.623922	-0.36311	0.717389	-5.93823	0.692026	0.720035
Non.inflammatory.Macrophages	B3GNT8	0.123708	2.202747	0.362924	0.717528	-5.8433	0.851967	0.791989
Non.inflammatory.Macrophages	CACNA1F	0.14727	-0.45662	0.362692	0.7177	-5.66745	0.933007	0.852797
Non.inflammatory.Macrophages	DANCR	-0.13694	1.545559	-0.36255	0.71781	-5.62246	0.886412	0.806701
Non.inflammatory.Macrophages	SDF2	0.034304	5.410417	0.362455	0.717877	-6.77935	0.701226	0.724453
Non.inflammatory.Macrophages	PAXIP1	0.056333	4.269449	0.362363	0.717946	-6.3991	0.751738	0.747833
Non.inflammatory.Macrophages	ASL	0.050372	4.484774	0.362315	0.717981	-6.59341	0.741949	0.743364
Non.inflammatory.Macrophages	GM50333	-0.11028	1.191453	-0.3623	0.71799	-5.7703	0.905459	0.814679

Non.inflammatory.Macrophages	HECTD4	0.041224	5.548933	0.362012	0.718207	-6.7101	0.69541	0.721729
Non.inflammatory.Macrophages	ARHGEF12	0.058028	3.963016	0.361954	0.71825	-6.53804	0.765979	0.754312
Non.inflammatory.Macrophages	FCSK	-0.14897	0.928399	-0.3619	0.718291	-5.58627	0.919349	0.82074
Non.inflammatory.Macrophages	EPN1	0.031537	6.109989	0.361748	0.718404	-6.95354	0.672001	0.710572
Non.inflammatory.Macrophages	CBWD1	-0.06559	4.090993	-0.36151	0.718578	-6.29681	0.7602	0.751715
Non.inflammatory.Macrophages	D11WSU47	-0.13075	1.299867	-0.36136	0.718691	-5.6974	0.899903	0.812439
Non.inflammatory.Macrophages	FABP7	0.095902	1.989157	0.361318	0.718723	-6.41319	0.863366	0.79703
Non.inflammatory.Macrophages	TMED2	0.026471	8.136615	0.361128	0.718865	-7.18271	0.59362	0.671761
Non.inflammatory.Macrophages	SLC27A4	0.061168	2.770367	0.361075	0.718905	-6.1502	0.82365	0.779993
Non.inflammatory.Macrophages	MPC2	0.035306	6.800876	0.360604	0.719256	-7.01975	0.644532	0.697361
Non.inflammatory.Macrophages	INSYN2B	-0.07651	4.140533	-0.36032	0.719464	-6.12404	0.758231	0.751038
Non.inflammatory.Macrophages	LACTB2	-0.04745	3.94655	-0.36014	0.719604	-6.49951	0.767226	0.755152
Non.inflammatory.Macrophages	ABHD17A	-0.02858	6.296745	-0.36013	0.719612	-6.88108	0.664741	0.707326
Non.inflammatory.Macrophages	RNF135	0.07653	2.08421	0.359986	0.719716	-6.11696	0.858786	0.795323
Non.inflammatory.Macrophages	RIF1	-0.04726	5.435862	-0.35996	0.719739	-6.60882	0.700669	0.724497

Non.inflammatory.Macrophages	SHLD1	0.057823	3.33904	0.359938	0.719752	-6.3475	0.796048	0.768041
Non.inflammatory.Macrophages	ACVR2B	-0.11132	1.53111	-0.35984	0.719828	-5.86927	0.887855	0.807665
Non.inflammatory.Macrophages	SMIM7	0.035837	4.932589	0.359787	0.719865	-6.70262	0.722523	0.734737
Non.inflammatory.Macrophages	ECT2	-0.10084	4.373594	-0.35978	0.719867	-6.3182	0.747555	0.74626
Non.inflammatory.Macrophages	PPIE	-0.04424	4.586932	-0.35961	0.719999	-6.50514	0.737907	0.741896
Non.inflammatory.Macrophages	KHDRBS1	-0.02314	7.151511	-0.35948	0.720094	-7.03797	0.630828	0.690783
Non.inflammatory.Macrophages	GM33280	0.177198	-0.66423	0.359448	0.720117	-5.52472	0.933715	0.858501
Non.inflammatory.Macrophages	NCOA5	0.051594	4.146435	0.359443	0.720121	-6.46156	0.757959	0.75106
Non.inflammatory.Macrophages	RINL	-0.05314	3.632551	-0.35928	0.720239	-6.50023	0.782052	0.761948
Non.inflammatory.Macrophages	GM32250	0.172915	-0.2746	0.359136	0.72035	-5.42982	0.933833	0.849377
Non.inflammatory.Macrophages	NAA60	0.040118	4.697726	0.358471	0.720845	-6.63855	0.733365	0.739856
Non.inflammatory.Macrophages	GM13830	-0.18109	0.287554	-0.35832	0.72096	-5.52168	0.934253	0.836412
Non.inflammatory.Macrophages	NFE2L1	0.042704	4.98448	0.358247	0.721013	-6.7207	0.720655	0.733991
Non.inflammatory.Macrophages	SPIC	-0.0962	1.863637	-0.3582	0.721046	-6.4129	0.870774	0.800578
Non.inflammatory.Macrophages	ZMYM5	-0.03548	5.521459	-0.35817	0.721073	-6.73471	0.697416	0.7231

Non.inflammatory.Macrophages	ZFPL1	0.048517	3.466605	0.358086	0.721132	-6.37143	0.790368	0.765667
Non.inflammatory.Macrophages	TTC13	-0.04932	3.938924	-0.35805	0.721157	-6.41956	0.768024	0.755668
Non.inflammatory.Macrophages	JOSD1	0.047401	3.99961	0.357848	0.72131	-6.43468	0.76524	0.754393
Non.inflammatory.Macrophages	PRKCH	-0.05741	6.999653	-0.35781	0.721337	-7.07936	0.637131	0.693946
Non.inflammatory.Macrophages	KRT81	0.218355	-1.53173	0.357602	0.721493	-5.20401	0.934357	0.87971
Non.inflammatory.Macrophages	CPNE8	0.078828	2.649239	0.357575	0.721514	-6.32518	0.830563	0.783296
Non.inflammatory.Macrophages	ZRANB2	0.033683	5.45391	0.357466	0.721595	-6.74112	0.700384	0.724504
Non.inflammatory.Macrophages	A430073D2	-0.12544	1.815155	-0.35731	0.721714	-5.6838	0.873424	0.801723
Non.inflammatory.Macrophages	ZFP934	-0.0777	2.990341	-0.3572	0.721793	-6.12223	0.813609	0.775985
Non.inflammatory.Macrophages	ZFP994	0.062611	2.481603	0.357154	0.721827	-6.18854	0.839025	0.78705
Non.inflammatory.Macrophages	FRAT2	0.08396	4.805593	0.357104	0.721865	-6.1127	0.728646	0.73776
Non.inflammatory.Macrophages	FOCAD	0.072359	2.908895	0.356736	0.722139	-6.20411	0.817744	0.777751
Non.inflammatory.Macrophages	AMOTL2	-0.16924	0.684568	-0.3567	0.722167	-5.61739	0.929324	0.827359
Non.inflammatory.Macrophages	AKAP17B	-0.14421	1.696468	-0.35662	0.722226	-5.59293	0.879807	0.804421
Non.inflammatory.Macrophages	R3HDM2	0.026449	6.193799	0.356598	0.722242	-6.89372	0.669502	0.709791

Non.inflammatory.Macrophages	GM33782	-0.15184	0.19699	-0.35647	0.722337	-5.54056	0.934529	0.838636
Non.inflammatory.Macrophages	RBCK1	-0.03593	5.550838	-0.35629	0.722473	-6.74604	0.696385	0.722686
Non.inflammatory.Macrophages	BYSL	-0.05001	3.67185	-0.35622	0.722521	-6.29079	0.780831	0.761497
Non.inflammatory.Macrophages	KRCC1	-0.02651	5.9705	-0.35617	0.722558	-6.88983	0.678748	0.714296
Non.inflammatory.Macrophages	JAG2	-0.15841	1.093215	-0.35579	0.722842	-5.53032	0.912504	0.818229
Non.inflammatory.Macrophages	AMMECR1	0.043245	4.488762	0.355677	0.72293	-6.61733	0.74315	0.744486
Non.inflammatory.Macrophages	EFL1	-0.04132	4.5955	-0.35564	0.722957	-6.56182	0.738335	0.742277
Non.inflammatory.Macrophages	MIGA2	-0.15034	0.892517	-0.35513	0.723341	-5.60218	0.922203	0.822972
Non.inflammatory.Macrophages	TSSC4	-0.05151	3.902903	-0.35511	0.723351	-6.44058	0.770112	0.756881
Non.inflammatory.Macrophages	PAFAH2	0.162617	0.619176	0.355082	0.723374	-5.58231	0.93167	0.829244
Non.inflammatory.Macrophages	GSAP	0.045685	5.642393	0.354919	0.723495	-6.99046	0.692647	0.721165
Non.inflammatory.Macrophages	MNS1	-0.12089	2.821224	-0.3549	0.723513	-5.67597	0.822312	0.78008
Non.inflammatory.Macrophages	D130043K2	0.11985	1.054045	0.354747	0.723624	-5.79538	0.914649	0.819402
Non.inflammatory.Macrophages	AADAT	0.139196	1.2345	0.354742	0.723628	-5.63108	0.904806	0.815303
Non.inflammatory.Macrophages	SMPDL3A	-0.04114	5.614644	-0.35474	0.723629	-7.13201	0.693822	0.72176

Non.inflammatory.Macrophages	GM38604	-0.12292	1.796583	-0.35468	0.723674	-5.65417	0.874756	0.802672
Non.inflammatory.Macrophages	GJA1	0.18259	1.208053	0.354664	0.723686	-5.57363	0.906242	0.815909
Non.inflammatory.Macrophages	SNX21	-0.06235	2.718125	-0.35452	0.723796	-6.36725	0.827455	0.782373
Non.inflammatory.Macrophages	FFAR2	0.256249	-0.53401	0.354332	0.723934	-5.24969	0.934745	0.856348
Non.inflammatory.Macrophages	FDX2	0.041162	4.192147	0.354243	0.724	-6.55531	0.756688	0.750931
Non.inflammatory.Macrophages	ECH1	-0.04025	5.784916	-0.35413	0.724086	-6.81178	0.686639	0.718361
Non.inflammatory.Macrophages	SF3B2	0.026195	7.285583	0.353951	0.724219	-7.03031	0.626353	0.688968
Non.inflammatory.Macrophages	CHMP7	0.072083	2.616172	0.353933	0.724232	-6.13919	0.83257	0.784594
Non.inflammatory.Macrophages	CGRRF1	0.043418	3.907331	0.353931	0.724233	-6.51014	0.769905	0.756907
Non.inflammatory.Macrophages	HELLS	0.077606	4.919254	0.353902	0.724255	-6.50033	0.723908	0.735884
Non.inflammatory.Macrophages	SMCO4	0.059728	4.10439	0.353845	0.724298	-6.39299	0.760738	0.752767
Non.inflammatory.Macrophages	NSMCE1	0.045656	4.829662	0.353842	0.724299	-6.59024	0.727874	0.737722
Non.inflammatory.Macrophages	IGSF5	-0.19776	1.922281	-0.3535	0.724558	-5.51674	0.868313	0.800001
Non.inflammatory.Macrophages	RELL1	0.036612	6.866963	0.353423	0.724613	-6.90981	0.642747	0.697148
Non.inflammatory.Macrophages	TNK2	-0.06984	3.209858	-0.35336	0.724658	-6.17449	0.80333	0.771858

Non.inflammatory.Macrophages	GLYCTK	-0.16004	0.979545	-0.3533	0.724702	-5.51154	0.918902	0.821275
Non.inflammatory.Macrophages	IFI211	0.059044	2.960201	0.353104	0.724851	-6.8443	0.815626	0.777343
Non.inflammatory.Macrophages	MRM3	-0.12305	2.005465	-0.35297	0.724954	-5.75079	0.864034	0.798317
Non.inflammatory.Macrophages	CD80	-0.05028	3.929604	-0.35291	0.724993	-6.87829	0.769052	0.75673
Non.inflammatory.Macrophages	LGALS8	-0.0382	5.110017	-0.35287	0.725029	-6.7891	0.715709	0.732292
Non.inflammatory.Macrophages	PIN1	0.037669	5.245371	0.352521	0.725287	-6.66966	0.709889	0.72964
Non.inflammatory.Macrophages	CDC42EP1	-0.18627	0.752796	-0.35246	0.725334	-5.518	0.927461	0.826764
Non.inflammatory.Macrophages	PSMD7	0.0371	5.828246	0.352446	0.725343	-6.82812	0.685055	0.717895
Non.inflammatory.Macrophages	PTPN6	0.03615	6.758702	0.352403	0.725375	-7.03045	0.647132	0.699543
Non.inflammatory.Macrophages	UBTF	-0.03106	5.879519	-0.35214	0.725574	-6.79505	0.682995	0.716989
Non.inflammatory.Macrophages	RNF41	-0.03794	4.424549	-0.3521	0.725602	-6.59903	0.746407	0.746638
Non.inflammatory.Macrophages	ELAC2	-0.06343	2.817476	-0.35176	0.725852	-6.18639	0.823084	0.7809
Non.inflammatory.Macrophages	CDC25B	-0.09183	4.400381	-0.35141	0.726119	-6.20632	0.747864	0.747394
Non.inflammatory.Macrophages	STARD5	0.046627	4.526318	0.351356	0.726157	-6.56743	0.742151	0.744785
Non.inflammatory.Macrophages	COMMD6	0.043172	4.358964	0.351186	0.726285	-6.561	0.749781	0.748324

Non.inflammatory.Macrophages	COQ5	-0.0582	3.832511	-0.35113	0.726324	-6.40522	0.774172	0.75937
Non.inflammatory.Macrophages	TBL1XR1	0.031502	6.669065	0.350787	0.726582	-6.90002	0.651283	0.701806
Non.inflammatory.Macrophages	CEP68	-0.05156	4.299523	-0.35035	0.726907	-6.49072	0.75296	0.749852
Non.inflammatory.Macrophages	EPS15L1	-0.03189	6.525246	-0.35021	0.727013	-6.87974	0.657297	0.704839
Non.inflammatory.Macrophages	HDAC5	-0.04936	4.378269	-0.35008	0.727113	-6.55543	0.749383	0.74828
Non.inflammatory.Macrophages	RGP1	0.069924	2.34849	0.350007	0.727166	-5.9769	0.847514	0.791793
Non.inflammatory.Macrophages	USP34	0.026966	7.703229	0.349953	0.727207	-7.09349	0.611501	0.682178
Non.inflammatory.Macrophages	TRAF1	0.160177	3.343827	0.349793	0.727326	-5.80672	0.79804	0.770256
Non.inflammatory.Macrophages	AIMP2	0.066252	3.206699	0.349616	0.727458	-6.25633	0.8047	0.773205
Non.inflammatory.Macrophages	LYPD6B	0.250798	0.004013	0.349484	0.727557	-5.28415	0.936324	0.845185
Non.inflammatory.Macrophages	NTHL1	-0.10953	0.544589	-0.34942	0.727605	-5.70042	0.935021	0.832597
Non.inflammatory.Macrophages	ARHGAP42	-0.09672	1.752056	-0.34938	0.727638	-6.03988	0.878585	0.805128
Non.inflammatory.Macrophages	RCAN3	-0.07113	2.117276	-0.34919	0.72778	-6.12254	0.859471	0.796995
Non.inflammatory.Macrophages	GM20712	-0.15726	0.063822	-0.34913	0.727823	-5.67876	0.936324	0.843785
Non.inflammatory.Macrophages	MEI4	0.221856	1.201602	0.349102	0.727842	-5.40189	0.908124	0.817541

Non.inflammatory.Macrophages	GM11944	0.07525	4.498486	0.349059	0.727875	-6.30172	0.743965	0.745901
Non.inflammatory.Macrophages	ARHGEF18	-0.05419	5.591269	-0.34896	0.727951	-6.56346	0.695993	0.723551
Non.inflammatory.Macrophages	MIER2	0.078625	2.002333	0.348194	0.728522	-6.05871	0.865836	0.799748
Non.inflammatory.Macrophages	PPA1	0.05647	4.797054	0.348182	0.728531	-6.4995	0.730884	0.739914
Non.inflammatory.Macrophages	VIRMA	0.038228	5.71037	0.348155	0.728552	-6.74701	0.691252	0.721337
Non.inflammatory.Macrophages	CCT5	-0.03526	6.571584	-0.34807	0.728612	-6.9368	0.655771	0.704259
Non.inflammatory.Macrophages	SEC14L1	0.038629	4.618739	0.348064	0.728619	-6.72705	0.738869	0.743608
Non.inflammatory.Macrophages	MBD2	0.029346	7.044678	0.347946	0.728708	-6.94849	0.637052	0.695058
Non.inflammatory.Macrophages	MAK16	0.037545	5.072635	0.347782	0.728831	-6.63033	0.718776	0.734335
Non.inflammatory.Macrophages	PDE1B	0.051203	3.114849	0.347548	0.729005	-6.45372	0.809754	0.775502
Non.inflammatory.Macrophages	SQOR	0.050454	3.496234	0.347369	0.72914	-6.63831	0.791314	0.767403
Non.inflammatory.Macrophages	SLC25A22	0.093023	2.495595	0.347226	0.729246	-6.08205	0.840729	0.789107
Non.inflammatory.Macrophages	ZBTB44	0.039895	5.853417	0.347151	0.729303	-6.73377	0.685459	0.718726
Non.inflammatory.Macrophages	TWISTNB	0.032748	5.520145	0.346934	0.729465	-6.784	0.699563	0.725434
Non.inflammatory.Macrophages	SLC25A12	0.037439	5.092687	0.346707	0.729635	-6.633	0.71806	0.734125

Non.inflammatory.Macrophages	NAV2	-0.06919	4.841571	-0.34657	0.729735	-6.42533	0.729142	0.739314
Non.inflammatory.Macrophages	TMBIM6	0.028351	9.142023	0.34649	0.729797	-7.37378	0.560254	0.655909
Non.inflammatory.Macrophages	SPOUT1	0.076019	2.468742	0.346467	0.729815	-6.03692	0.842093	0.789781
Non.inflammatory.Macrophages	PRR14	-0.04496	4.524923	-0.34646	0.729822	-6.55695	0.743347	0.74586
Non.inflammatory.Macrophages	BCOR	-0.04139	5.214585	-0.34621	0.730009	-6.71608	0.712739	0.731721
Non.inflammatory.Macrophages	GAR1	-0.05313	4.430651	-0.34616	0.730048	-6.49711	0.747627	0.747886
Non.inflammatory.Macrophages	GSE1	-0.03501	5.664856	-0.34608	0.730103	-6.80289	0.693405	0.722639
Non.inflammatory.Macrophages	POLR1B	-0.08138	2.442843	-0.34603	0.730144	-6.04045	0.843411	0.790455
Non.inflammatory.Macrophages	GSKIP	0.03368	4.602025	0.345985	0.730176	-6.60742	0.739865	0.744374
Non.inflammatory.Macrophages	VDAC2	0.027714	7.488132	0.3459	0.73024	-7.12611	0.620148	0.686937
Non.inflammatory.Macrophages	ZFP36L1	0.033986	7.742233	0.345829	0.730293	-7.16869	0.610554	0.682103
Non.inflammatory.Macrophages	MCC	-0.10017	2.092188	-0.3458	0.730317	-5.98438	0.861443	0.798261
Non.inflammatory.Macrophages	PARP4	0.037948	5.335625	0.345549	0.730502	-6.70502	0.707491	0.729399
Non.inflammatory.Macrophages	DDX55	-0.06442	2.934608	-0.34549	0.730545	-6.21159	0.818707	0.779809
Non.inflammatory.Macrophages	INTS10	0.05946	3.410048	0.345484	0.730552	-6.24565	0.795462	0.769561

Non.inflammatory.Macrophages	SLC37A3	-0.04944	3.658041	-0.34546	0.730569	-6.38404	0.783582	0.764268
Non.inflammatory.Macrophages	DROSHA	-0.05221	3.121263	-0.34543	0.730589	-6.23909	0.809507	0.77577
Non.inflammatory.Macrophages	CHCHD7	0.041016	4.398636	0.345181	0.730778	-6.54571	0.749204	0.748715
Non.inflammatory.Macrophages	MITD1	-0.04857	4.433197	-0.34506	0.730869	-6.57664	0.747629	0.747996
Non.inflammatory.Macrophages	TMEM156	-0.05252	3.855526	-0.345	0.730913	-6.42912	0.774363	0.760136
Non.inflammatory.Macrophages	ZFP808	-0.10907	1.973033	-0.34487	0.731012	-5.71335	0.867823	0.801055
Non.inflammatory.Macrophages	DSE	0.048985	3.834005	0.34458	0.731228	-6.77153	0.775449	0.760698
Non.inflammatory.Macrophages	MAIP1	0.04815	3.588251	0.344539	0.731259	-6.41225	0.787107	0.76593
Non.inflammatory.Macrophages	GM44777	0.151977	0.565153	0.344413	0.731354	-5.53736	0.935495	0.83311
Non.inflammatory.Macrophages	RNF4	-0.02759	5.964365	-0.34439	0.731372	-6.84632	0.680996	0.716924
Non.inflammatory.Macrophages	GM12689	-0.12513	-0.48996	-0.34436	0.731397	-5.68811	0.93729	0.857874
Non.inflammatory.Macrophages	FBN1	0.149374	0.898383	0.343872	0.731759	-5.57076	0.924866	0.825539
Non.inflammatory.Macrophages	ABITRAM	0.068094	2.584915	0.343793	0.731819	-6.06578	0.836739	0.787735
Non.inflammatory.Macrophages	ETAA1OS	-0.12259	0.543342	-0.34337	0.732138	-5.63147	0.936698	0.833805
Non.inflammatory.Macrophages	GM32916	-0.34829	-0.24915	-0.34307	0.732361	-5.32512	0.938125	0.852438

Non.inflammatory.Macrophages	TCRG-C4	0.20561	0.327974	0.343024	0.732395	-5.35559	0.938125	0.838894
Non.inflammatory.Macrophages	MRPS30	0.037608	4.726011	0.342828	0.732542	-6.5954	0.73516	0.742352
Non.inflammatory.Macrophages	STAG1	0.024149	8.353909	0.34277	0.732585	-7.18296	0.588742	0.67105
Non.inflammatory.Macrophages	SLFN5	0.0567	4.230734	0.342715	0.732626	-6.91333	0.757668	0.75268
Non.inflammatory.Macrophages	ZFP512	0.052038	3.916117	0.34259	0.73272	-6.41951	0.772322	0.75931
Non.inflammatory.Macrophages	KLHDC2	0.040162	4.860142	0.342505	0.732784	-6.5667	0.729192	0.739636
Non.inflammatory.Macrophages	PTAFR	0.063777	4.645313	0.342324	0.73292	-6.89395	0.738823	0.744073
Non.inflammatory.Macrophages	FSTL1	-0.12411	2.107543	-0.34229	0.732943	-5.84759	0.861703	0.798513
Non.inflammatory.Macrophages	GM15446	0.107564	2.166881	0.341768	0.733337	-5.81679	0.859007	0.797401
Non.inflammatory.Macrophages	PALM	0.077499	4.231998	0.341607	0.733457	-6.16376	0.758039	0.752904
Non.inflammatory.Macrophages	EHHADH	0.111667	1.66442	0.341168	0.733786	-5.85417	0.885553	0.808791
Non.inflammatory.Macrophages	ADAMTS17	-0.21459	0.355692	-0.34109	0.733843	-5.34339	0.938788	0.838738
Non.inflammatory.Macrophages	GLMP	0.036826	5.839868	0.340849	0.734026	-6.93271	0.687296	0.720072
Non.inflammatory.Macrophages	LRRC8C	0.039942	6.412612	0.340762	0.734091	-6.96259	0.663629	0.708708
Non.inflammatory.Macrophages	BAD	0.046037	3.912661	0.340722	0.734121	-6.51463	0.772985	0.759793

Non.inflammatory.Macrophages	ACADVL	0.044032	4.901912	0.340514	0.734277	-6.64805	0.727809	0.739156
Non.inflammatory.Macrophages	TACO1OS	0.124596	1.705089	0.340388	0.734372	-5.67927	0.883389	0.807913
Non.inflammatory.Macrophages	GTPBP8	0.06919	2.317318	0.340028	0.734642	-6.08218	0.851398	0.794351
Non.inflammatory.Macrophages	ELAC1	-0.06295	1.710286	-0.34002	0.734648	-6.11929	0.883113	0.807872
Non.inflammatory.Macrophages	CLSPN	0.088073	4.411842	0.339984	0.734675	-6.25572	0.749868	0.749379
Non.inflammatory.Macrophages	COX10	0.052303	3.775159	0.339869	0.734761	-6.35641	0.779469	0.762778
Non.inflammatory.Macrophages	MROH1	0.0462	4.199162	0.339828	0.734792	-6.56279	0.759637	0.753829
Non.inflammatory.Macrophages	TCTN3	0.095487	1.366808	0.339674	0.734908	-5.97539	0.901532	0.81563
Non.inflammatory.Macrophages	MRPL17	0.031041	5.426857	0.339598	0.734965	-6.84164	0.704863	0.728518
Non.inflammatory.Macrophages	SRXN1	0.069606	1.094676	0.339228	0.735243	-6.20105	0.916371	0.821908
Non.inflammatory.Macrophages	PPP2CA	0.018352	7.762516	0.339086	0.735349	-7.1587	0.610923	0.682738
Non.inflammatory.Macrophages	H2-OA	-0.15039	3.19042	-0.33894	0.735458	-5.71679	0.807614	0.775403
Non.inflammatory.Macrophages	ZFP160	0.065553	2.950859	0.338932	0.735465	-6.1546	0.819416	0.780588
Non.inflammatory.Macrophages	ZFP316	0.144739	0.380007	0.338926	0.735469	-5.62735	0.938788	0.838408
Non.inflammatory.Macrophages	GCNT1	-0.06766	2.451925	-0.33868	0.735655	-6.16113	0.844508	0.791573

Non.inflammatory.Macrophages	MTBP	0.09556	3.256156	0.338669	0.735662	-5.87481	0.804404	0.77406
Non.inflammatory.Macrophages	GM15892	0.097549	3.443855	0.338559	0.735744	-6.06933	0.795301	0.770031
Non.inflammatory.Macrophages	TLK2	0.026811	6.825344	0.33855	0.735751	-6.95022	0.647063	0.700853
Non.inflammatory.Macrophages	PARN	-0.05002	3.875182	-0.33852	0.735775	-6.37991	0.774747	0.760843
Non.inflammatory.Macrophages	SNX24	0.043567	4.299252	0.338434	0.735839	-6.94706	0.755025	0.751963
Non.inflammatory.Macrophages	FRMD4B	-0.04752	5.267906	-0.33836	0.735893	-7.04082	0.711737	0.731994
Non.inflammatory.Macrophages	COL5A1	-0.15011	0.493409	-0.33829	0.735946	-5.61902	0.938708	0.835965
Non.inflammatory.Macrophages	PANK3	0.035874	4.591077	0.33815	0.736052	-6.6359	0.741728	0.745929
Non.inflammatory.Macrophages	BCKDK	0.038105	4.559456	0.338098	0.736091	-6.5876	0.743158	0.746586
Non.inflammatory.Macrophages	KIF18A	-0.07697	3.977118	-0.33809	0.736096	-6.24408	0.769963	0.758786
Non.inflammatory.Macrophages	NBDY	-0.04002	3.659616	-0.33784	0.736283	-6.55436	0.784957	0.765618
Non.inflammatory.Macrophages	SIK2	-0.03128	7.126745	-0.33784	0.736284	-7.04262	0.635219	0.695175
Non.inflammatory.Macrophages	UGCG	-0.04267	6.436724	-0.33781	0.736304	-6.85385	0.66265	0.708658
Non.inflammatory.Macrophages	SLC15A4	0.044789	4.804844	0.33762	0.73645	-6.61298	0.732129	0.741644
Non.inflammatory.Macrophages	CTNNB1	-0.02401	6.277504	-0.33759	0.736476	-6.93977	0.66914	0.711852

Non.inflammatory.Macrophages	TMEM98	-0.18326	0.620631	-0.33757	0.736487	-5.3873	0.935666	0.833176
Non.inflammatory.Macrophages	KIF14	-0.13659	2.55406	-0.33754	0.736508	-5.85056	0.839315	0.789584
Non.inflammatory.Macrophages	PRR5	0.111113	3.479528	0.337494	0.736545	-5.84783	0.793582	0.769514
Non.inflammatory.Macrophages	4930445E1	0.129209	-0.41363	0.33739	0.736623	-5.61412	0.938788	0.857465
Non.inflammatory.Macrophages	TRABD	-0.03958	5.363153	-0.33727	0.736713	-6.64223	0.70761	0.730249
Non.inflammatory.Macrophages	SPI1	0.028498	6.411888	0.337267	0.736715	-7.13955	0.663658	0.709236
Non.inflammatory.Macrophages	ACTN4	0.025481	6.545001	0.33724	0.736735	-6.94639	0.658271	0.706612
Non.inflammatory.Macrophages	SMG8	-0.06347	2.847118	-0.3371	0.73684	-6.1635	0.824576	0.78322
Non.inflammatory.Macrophages	IL10	0.07285	2.999738	0.337065	0.736867	-6.56458	0.816995	0.779901
Non.inflammatory.Macrophages	9030025P2	-0.10167	1.737859	-0.33703	0.736895	-5.84964	0.88165	0.807757
Non.inflammatory.Macrophages	APBA3	-0.07505	2.107298	-0.33702	0.736897	-6.07007	0.862251	0.799503
Non.inflammatory.Macrophages	NSMAF	0.044395	4.903953	0.336968	0.736939	-6.64888	0.727718	0.739653
Non.inflammatory.Macrophages	CCL27A	-0.09934	1.684593	-0.33696	0.736948	-5.80882	0.884479	0.808963
Non.inflammatory.Macrophages	HTRA2	0.039203	3.681601	0.336895	0.736994	-6.46985	0.78391	0.765254
Non.inflammatory.Macrophages	NKAPD1	0.04394	4.086425	0.336874	0.73701	-6.48561	0.764863	0.756681

Non.inflammatory.Macrophages	PPP1R9B	-0.03999	4.375184	-0.33679	0.737074	-6.71998	0.751544	0.750656
Non.inflammatory.Macrophages	CREBBP	-0.02569	7.888027	-0.33658	0.737228	-7.13431	0.606304	0.680726
Non.inflammatory.Macrophages	KAT2A	0.055958	3.199202	0.336483	0.737304	-6.32762	0.807285	0.775668
Non.inflammatory.Macrophages	FIRRE	-0.08791	2.608551	-0.33599	0.737677	-6.06304	0.836968	0.788624
Non.inflammatory.Macrophages	LRRC42	-0.05272	3.272197	-0.33594	0.737715	-6.29434	0.804018	0.774197
Non.inflammatory.Macrophages	SENP1	0.035099	5.013417	0.335332	0.738169	-6.60795	0.723559	0.737713
Non.inflammatory.Macrophages	GPR27	-0.14568	-1.60473	-0.33531	0.738187	-5.18892	0.939675	0.886664
Non.inflammatory.Macrophages	ABHD10	-0.04659	3.649409	-0.33521	0.738261	-6.32339	0.78619	0.766304
Non.inflammatory.Macrophages	NOD1	0.053408	3.387607	0.334834	0.738543	-6.65107	0.798935	0.771948
Non.inflammatory.Macrophages	RXRA	-0.04479	3.1994	-0.33483	0.738545	-6.57873	0.808101	0.776001
Non.inflammatory.Macrophages	PNPO	0.048745	3.744874	0.334576	0.738737	-6.55609	0.78193	0.764347
Non.inflammatory.Macrophages	NAA35	-0.03689	4.851078	-0.33429	0.738955	-6.61094	0.731157	0.741245
Non.inflammatory.Macrophages	SIGLEC1	0.12222	0.449661	0.334218	0.739007	-5.95553	0.940188	0.837768
Non.inflammatory.Macrophages	PCBP3	-0.10629	2.616922	-0.33404	0.73914	-5.70957	0.837404	0.788838
Non.inflammatory.Macrophages	GORASP1	0.1191	0.733344	0.334005	0.739167	-5.77368	0.933266	0.831241

Non.inflammatory.Macrophages	AI413582	0.039087	4.68032	0.333505	0.739543	-6.74458	0.739134	0.744988
Non.inflammatory.Macrophages	NEK10	0.141381	1.324395	0.333212	0.739763	-5.70857	0.905764	0.818016
Non.inflammatory.Macrophages	ZFP507	-0.09736	2.176566	-0.33297	0.739945	-5.92671	0.860504	0.798929
Non.inflammatory.Macrophages	GSTCD	0.06507	3.564196	0.332823	0.740056	-6.23075	0.791214	0.768685
Non.inflammatory.Macrophages	LIX1L	-0.10432	0.803407	-0.33277	0.740098	-5.80774	0.931352	0.830018
Non.inflammatory.Macrophages	TRPM2	-0.05644	2.540831	-0.33275	0.740107	-6.63056	0.841792	0.790886
Non.inflammatory.Macrophages	SYNCRIP	0.027661	7.375906	0.332739	0.740119	-7.03767	0.626932	0.691298
Non.inflammatory.Macrophages	MYADM	0.044347	5.586342	0.332241	0.740494	-6.84437	0.699608	0.726712
Non.inflammatory.Macrophages	ERLIN2	-0.04578	3.099067	-0.33224	0.740495	-6.46588	0.813933	0.778806
Non.inflammatory.Macrophages	EXO1	0.135006	2.019131	0.332193	0.74053	-5.69969	0.868805	0.802567
Non.inflammatory.Macrophages	RRP8	0.05263	3.730434	0.332156	0.740557	-6.36283	0.783357	0.765255
Non.inflammatory.Macrophages	MED7	0.043894	3.583194	0.332052	0.740636	-6.43303	0.79039	0.768404
Non.inflammatory.Macrophages	RTEL1	-0.0834	2.682882	-0.33203	0.740651	-6.06289	0.83469	0.787895
Non.inflammatory.Macrophages	KAT5	0.068004	2.921057	0.331811	0.740817	-6.20974	0.822752	0.782756
Non.inflammatory.Macrophages	SUMO2	0.023301	8.656817	0.331784	0.740838	-7.21846	0.579585	0.667253

Non.inflammatory.Macrophages	UNC13A	-0.10148	1.183593	-0.33166	0.740928	-5.89153	0.91356	0.821544
Non.inflammatory.Macrophages	IQCE	0.080408	2.144332	0.331616	0.740964	-6.05413	0.862274	0.799895
Non.inflammatory.Macrophages	EPB41L3	-0.08253	2.334313	-0.33161	0.740966	-6.42146	0.852449	0.79568
Non.inflammatory.Macrophages	MXD3	0.143226	1.735683	0.331321	0.741186	-5.6273	0.883876	0.8091
Non.inflammatory.Macrophages	CPSF7	-0.03007	5.801172	-0.33123	0.741256	-6.77389	0.690573	0.722554
Non.inflammatory.Macrophages	PRIM1	-0.08433	4.178781	-0.33121	0.741272	-6.22155	0.7624	0.755937
Non.inflammatory.Macrophages	ZFP867	0.13168	0.61031	0.331097	0.741355	-5.5349	0.938169	0.834799
Non.inflammatory.Macrophages	AARS2	-0.14385	0.821033	-0.33093	0.74148	-5.53593	0.931037	0.829945
Non.inflammatory.Macrophages	BCAR1	-0.15343	0.776645	-0.33062	0.741716	-5.54764	0.932829	0.831067
Non.inflammatory.Macrophages	XRN2	0.021482	7.516217	0.330491	0.741811	-7.11618	0.621929	0.688986
Non.inflammatory.Macrophages	EIF3K	0.022905	7.871661	0.329678	0.742423	-7.17672	0.608955	0.682286
Non.inflammatory.Macrophages	1110020A2	-0.14805	0.468534	-0.32934	0.742676	-5.52143	0.942128	0.838403
Non.inflammatory.Macrophages	RALGPS1	0.062675	4.92479	0.329338	0.742679	-6.29723	0.72938	0.740675
Non.inflammatory.Macrophages	RRAGB	-0.22313	0.125251	-0.32932	0.74269	-5.22679	0.942128	0.846432
Non.inflammatory.Macrophages	STX16	0.033624	5.756821	0.328934	0.742984	-6.86402	0.693419	0.723793

Non.inflammatory.Macrophages	AHCY	-0.0738	3.495963	-0.3289	0.743007	-6.17626	0.795803	0.77082
Non.inflammatory.Macrophages	MTERF1A	-0.10995	1.405346	-0.32833	0.743435	-5.66504	0.903146	0.817039
Non.inflammatory.Macrophages	SNU13	-0.02718	6.96176	-0.32832	0.743447	-7.00353	0.644314	0.699988
Non.inflammatory.Macrophages	CNP	-0.04855	5.75023	-0.32818	0.743555	-6.68673	0.693914	0.72404
Non.inflammatory.Macrophages	WNK1	-0.02923	9.07644	-0.32815	0.743574	-7.33898	0.565874	0.66002
Non.inflammatory.Macrophages	RPAP1	0.071918	2.431442	0.328062	0.74364	-6.03	0.84903	0.794114
Non.inflammatory.Macrophages	MAG	0.129191	0.11897	0.327998	0.743689	-5.73593	0.942647	0.846815
Non.inflammatory.Macrophages	ZFP317	-0.06556	2.898111	-0.32795	0.743726	-6.12541	0.825415	0.783872
Non.inflammatory.Macrophages	HSPA12A	0.128239	0.150275	0.327661	0.743943	-5.67039	0.942834	0.846134
Non.inflammatory.Macrophages	IFI203	0.045624	6.302394	0.327307	0.74421	-7.01259	0.671028	0.713134
Non.inflammatory.Macrophages	GPT	0.120355	1.203309	0.327224	0.744272	-5.73471	0.914382	0.821861
Non.inflammatory.Macrophages	KDM4A	0.042515	4.161515	0.327174	0.74431	-6.50302	0.764691	0.756966
Non.inflammatory.Macrophages	COL27A1	0.106762	2.314283	0.327119	0.744351	-6.0613	0.855259	0.796911
Non.inflammatory.Macrophages	ZFP282	-0.05286	4.010784	-0.32707	0.744385	-6.38255	0.771732	0.76017
Non.inflammatory.Macrophages	LRRC75A	0.080141	2.6203	0.326621	0.744726	-6.16446	0.839599	0.790157

Non.inflammatory.Macrophages	STIMATE	-0.04055	4.159047	-0.32658	0.74476	-6.59627	0.764805	0.757039
Non.inflammatory.Macrophages	ARF6	-0.03386	6.770128	-0.32653	0.744798	-6.94072	0.65208	0.703962
Non.inflammatory.Macrophages	HSPE1	0.036037	7.179751	0.326393	0.744898	-7.06657	0.635912	0.69598
Non.inflammatory.Macrophages	TBCC	0.054999	3.579816	0.326308	0.744962	-6.29109	0.7922	0.769342
Non.inflammatory.Macrophages	MED26	0.048864	4.429093	0.326153	0.745079	-6.48905	0.75234	0.751405
Non.inflammatory.Macrophages	DCLRE1A	-0.07382	1.621612	-0.32608	0.745136	-6.09097	0.891698	0.812451
Non.inflammatory.Macrophages	CHEK2	-0.05269	3.145946	-0.3259	0.745269	-6.2866	0.813316	0.778755
Non.inflammatory.Macrophages	5830487J0	-0.18294	-0.01733	-0.32589	0.745275	-5.27353	0.942872	0.850318
Non.inflammatory.Macrophages	SERPINE2	0.137269	1.29875	0.325864	0.745297	-5.6286	0.909161	0.819804
Non.inflammatory.Macrophages	NME6	-0.07941	2.576439	-0.32584	0.745317	-6.02243	0.841827	0.791199
Non.inflammatory.Macrophages	FLAD1	-0.06714	2.957237	-0.32565	0.745458	-6.16384	0.822663	0.782901
Non.inflammatory.Macrophages	SIVA1	-0.04362	5.470666	-0.32551	0.745567	-6.74383	0.706038	0.730058
Non.inflammatory.Macrophages	RAB33B	0.041752	4.405753	0.325421	0.745632	-6.42918	0.75341	0.752036
Non.inflammatory.Macrophages	WFS1	0.102313	1.279726	0.325388	0.745656	-5.65377	0.910199	0.820368
Non.inflammatory.Macrophages	MYEF2	0.042285	5.328236	0.325257	0.745755	-6.63185	0.712205	0.733037

Non.inflammatory.Macrophages	CCDC91	0.067176	2.408137	0.325225	0.745779	-6.17041	0.850428	0.795096
Non.inflammatory.Macrophages	NAA80	0.064197	2.402845	0.325224	0.74578	-6.10904	0.850699	0.795213
Non.inflammatory.Macrophages	IGKC	0.111773	7.88266	0.325213	0.745788	-6.96236	0.609072	0.68272
Non.inflammatory.Macrophages	RRAGA	-0.03786	5.020687	-0.32516	0.745827	-6.65321	0.725699	0.739347
Non.inflammatory.Macrophages	LY6E	0.034844	9.08245	0.325113	0.745864	-7.46061	0.5658	0.66032
Non.inflammatory.Macrophages	RFC5	0.067006	4.185223	0.325061	0.745903	-6.41534	0.763589	0.75676
Non.inflammatory.Macrophages	NAP1L1	-0.03471	7.439515	-0.32503	0.745924	-7.07479	0.625862	0.691214
Non.inflammatory.Macrophages	WDR36	0.036171	4.578146	0.324896	0.746028	-6.62772	0.745576	0.748536
Non.inflammatory.Macrophages	CTIF	0.045611	2.596638	0.324721	0.74616	-6.3423	0.840895	0.790999
Non.inflammatory.Macrophages	GM11423	-0.10657	0.892029	-0.32462	0.746236	-5.72565	0.930344	0.829383
Non.inflammatory.Macrophages	GM43251	-0.15317	-0.97845	-0.32455	0.746287	-5.64625	0.942978	0.873575
Non.inflammatory.Macrophages	ZMYND11	0.027702	6.466516	0.324431	0.746378	-6.92183	0.664415	0.710267
Non.inflammatory.Macrophages	SCHIP1	-0.14066	1.591487	-0.32407	0.746648	-5.60053	0.893502	0.813584
Non.inflammatory.Macrophages	8-Sep	0.059704	1.954462	0.324027	0.746683	-6.24956	0.874198	0.805426
Non.inflammatory.Macrophages	GSTM5	-0.09657	1.19189	-0.32395	0.746744	-6.02704	0.915201	0.822693

Non.inflammatory.Macrophages	SLC12A9	0.046059	4.242634	0.323894	0.746784	-6.59758	0.761086	0.755763
Non.inflammatory.Macrophages	DGKG	0.108753	2.506906	0.323758	0.746886	-5.87754	0.845549	0.793164
Non.inflammatory.Macrophages	TRMT44	-0.09784	1.793941	-0.32372	0.746915	-5.83476	0.882688	0.809045
Non.inflammatory.Macrophages	PPP1CC	0.027119	7.584523	0.323706	0.746925	-7.00318	0.620449	0.688625
Non.inflammatory.Macrophages	SNIP1	0.04642	3.633076	0.323625	0.746986	-6.29043	0.789809	0.768712
Non.inflammatory.Macrophages	PKP3	-0.1821	2.747004	-0.3235	0.747081	-5.40942	0.833396	0.787907
Non.inflammatory.Macrophages	GUCY1A1	-0.14605	-0.00341	-0.32337	0.747178	-5.6332	0.943108	0.850513
Non.inflammatory.Macrophages	CCDC50	0.025376	5.994315	0.323284	0.747244	-7.00956	0.683969	0.719837
Non.inflammatory.Macrophages	HADHA	0.029263	5.770414	0.323215	0.747295	-6.83548	0.693397	0.724339
Non.inflammatory.Macrophages	COPS5	0.032479	5.288344	0.322801	0.747608	-6.71488	0.714121	0.734158
Non.inflammatory.Macrophages	STXBP4	0.109273	2.23619	0.322783	0.747622	-5.84386	0.859513	0.79924
Non.inflammatory.Macrophages	SH3BP4	-0.1017	1.369949	-0.32275	0.747649	-5.88984	0.90551	0.81872
Non.inflammatory.Macrophages	GM42997	-0.17291	0.465543	-0.32258	0.747778	-5.4593	0.943108	0.83958
Non.inflammatory.Macrophages	EGFEM1	0.231912	1.229611	0.322571	0.747782	-5.46755	0.913169	0.821949
Non.inflammatory.Macrophages	GM10353	-0.09289	2.745067	-0.32256	0.747791	-5.83292	0.833499	0.788036

Non.inflammatory.Macrophages	BICD2	-0.0434	4.09822	-0.3225	0.747838	-6.50918	0.767832	0.758924
Non.inflammatory.Macrophages	PIGYL	-0.03956	4.270837	-0.32241	0.747905	-6.60273	0.759812	0.755302
Non.inflammatory.Macrophages	MRPL47	0.066582	2.974642	0.322385	0.747922	-6.19833	0.822003	0.783049
Non.inflammatory.Macrophages	GM36756	-0.19777	0.926199	-0.32211	0.748133	-5.2516	0.9293	0.829012
Non.inflammatory.Macrophages	TRMT13	0.0506	3.89491	0.32208	0.748152	-6.36843	0.777415	0.763324
Non.inflammatory.Macrophages	MBOAT7	-0.0397	4.278521	-0.32199	0.748221	-6.58407	0.759491	0.75523
Non.inflammatory.Macrophages	BC005561	-0.04183	4.273496	-0.32192	0.748271	-6.5067	0.759723	0.755353
Non.inflammatory.Macrophages	GFPT1	-0.04165	5.095805	-0.32152	0.748576	-6.79322	0.722594	0.7383
Non.inflammatory.Macrophages	GM14455	-0.09196	1.315653	-0.32144	0.748638	-5.8369	0.908507	0.820195
Non.inflammatory.Macrophages	FAF2	0.034624	5.155771	0.321425	0.748647	-6.67412	0.719955	0.737087
Non.inflammatory.Macrophages	WDR6	0.069176	3.277321	0.321344	0.748708	-6.11426	0.807106	0.776661
Non.inflammatory.Macrophages	RAD9B	-0.07949	3.275507	-0.32134	0.748709	-5.97015	0.807195	0.7767
Non.inflammatory.Macrophages	USP16	0.033745	5.208005	0.321173	0.748837	-6.74956	0.717664	0.736051
Non.inflammatory.Macrophages	UBR7	0.04552	4.241439	0.321114	0.748882	-6.48645	0.761206	0.756125
Non.inflammatory.Macrophages	ZFP148	-0.02445	6.830997	-0.32103	0.748944	-6.98862	0.649845	0.703549

Non.inflammatory.Macrophages	BICD1	0.178114	0.681795	0.320867	0.749069	-5.38378	0.938031	0.83483
Non.inflammatory.Macrophages	SORBS1	-0.06684	4.747678	-0.32078	0.749131	-6.27636	0.738098	0.745566
Non.inflammatory.Macrophages	SLC38A7	-0.05852	2.59539	-0.32076	0.749149	-6.33545	0.841112	0.791587
Non.inflammatory.Macrophages	PREX1	-0.03334	6.43388	-0.32074	0.749163	-6.99916	0.665845	0.711375
Non.inflammatory.Macrophages	PSMC3IP	-0.10371	2.412129	-0.32072	0.749181	-5.81263	0.850474	0.795632
Non.inflammatory.Macrophages	APOL7E	-0.17422	-0.10248	-0.3206	0.74927	-5.36312	0.94315	0.853225
Non.inflammatory.Macrophages	KPNA4	-0.03064	8.246447	-0.32046	0.749374	-7.2416	0.595795	0.676436
Non.inflammatory.Macrophages	CENPW	-0.06308	4.349764	-0.32039	0.749432	-6.42743	0.756205	0.753945
Non.inflammatory.Macrophages	KLF5	0.189958	0.36413	0.320132	0.749623	-5.44172	0.94315	0.842471
Non.inflammatory.Macrophages	XPO7	0.033114	6.710919	0.320109	0.749641	-6.92336	0.654643	0.706114
Non.inflammatory.Macrophages	B230354K1	-0.10049	1.756956	-0.31998	0.749741	-5.8894	0.88473	0.810494
Non.inflammatory.Macrophages	CXCL2	-0.08375	7.158496	-0.31983	0.749851	-7.4333	0.63693	0.697401
Non.inflammatory.Macrophages	ACAD12	-0.1186	0.714351	-0.31971	0.74994	-5.71174	0.936861	0.83439
Non.inflammatory.Macrophages	MAP2K2	-0.02282	7.299123	-0.31953	0.750079	-7.06258	0.631461	0.694725
Non.inflammatory.Macrophages	H19	0.09543	6.408472	0.319449	0.75014	-6.97701	0.666882	0.712202

Non.inflammatory.Macrophages	HSF1	0.03948	4.997353	0.319366	0.750202	-6.56503	0.726947	0.740739
Non.inflammatory.Macrophages	TARS2	-0.04832	3.819829	-0.31925	0.750292	-6.35543	0.780969	0.765422
Non.inflammatory.Macrophages	RAB7	-0.02232	7.931545	-0.31908	0.750418	-7.18284	0.607427	0.682659
Non.inflammatory.Macrophages	ARID1B	-0.02472	8.198201	-0.31902	0.75046	-7.13753	0.597563	0.677618
Non.inflammatory.Macrophages	PGAP3	-0.12631	1.12543	-0.31901	0.750473	-5.65682	0.918934	0.825018
Non.inflammatory.Macrophages	TATDN1	-0.04695	3.630234	-0.31897	0.750503	-6.36611	0.790012	0.769519
Non.inflammatory.Macrophages	ZFP973	0.128225	0.609416	0.318966	0.750505	-5.57898	0.940282	0.836944
Non.inflammatory.Macrophages	UNKL	0.05731	3.970057	0.318954	0.750514	-6.31418	0.773873	0.762277
Non.inflammatory.Macrophages	GM15478	-0.0479	4.735611	-0.31884	0.750602	-6.51857	0.738641	0.74624
Non.inflammatory.Macrophages	IRF2BP1	-0.04595	4.152538	-0.31869	0.750716	-6.38986	0.765334	0.758501
Non.inflammatory.Macrophages	GM11508	0.082405	3.437566	0.318671	0.750727	-6.11741	0.799302	0.773745
Non.inflammatory.Macrophages	UTP25	0.06557	2.617836	0.318669	0.750729	-6.09799	0.839972	0.791594
Non.inflammatory.Macrophages	THOC6	0.041925	4.164901	0.318639	0.750752	-6.54573	0.764759	0.758253
Non.inflammatory.Macrophages	5530601H(	0.058021	3.904215	0.318558	0.750813	-6.3628	0.776976	0.76378
Non.inflammatory.Macrophages	IPO5	0.041908	5.437174	0.318269	0.751032	-6.74948	0.707768	0.731924

Non.inflammatory.Macrophages	TRAT1	0.228719	-0.05747	0.318268	0.751032	-5.37371	0.943251	0.852762
Non.inflammatory.Macrophages	MUL1	0.058573	2.950066	0.31792	0.751295	-6.2429	0.823548	0.784458
Non.inflammatory.Macrophages	DNAJB12	0.036331	4.757611	0.317741	0.75143	-6.61954	0.737906	0.745997
Non.inflammatory.Macrophages	SF3B4	-0.03608	5.494923	-0.31768	0.75148	-6.73022	0.705444	0.730857
Non.inflammatory.Macrophages	SLC22A17	0.125005	-0.51359	0.317646	0.751502	-5.64919	0.943476	0.863755
Non.inflammatory.Macrophages	FAM53A	0.04354	3.797306	0.317384	0.7517	-6.43776	0.782309	0.766242
Non.inflammatory.Macrophages	BTNL9	-0.17096	0.689973	-0.31735	0.751724	-5.52629	0.938061	0.835393
Non.inflammatory.Macrophages	PHTF1	-0.0451	4.68503	-0.31726	0.751795	-6.57987	0.741178	0.74754
Non.inflammatory.Macrophages	STT3B	-0.02483	6.666062	-0.31722	0.751827	-6.91046	0.656671	0.707428
Non.inflammatory.Macrophages	SPG11	-0.0453	4.094018	-0.31716	0.751869	-6.49909	0.768328	0.75994
Non.inflammatory.Macrophages	NQO1	-0.14344	0.792254	-0.31709	0.751919	-5.57147	0.934393	0.833023
Non.inflammatory.Macrophages	NOC4L	-0.04974	3.728892	-0.31702	0.751978	-6.29268	0.785566	0.767702
Non.inflammatory.Macrophages	ATRAID	0.033977	4.57765	0.316872	0.752087	-6.66537	0.746081	0.749778
Non.inflammatory.Macrophages	GLCCI1	0.042936	7.348661	0.316517	0.752356	-6.85296	0.629864	0.694189
Non.inflammatory.Macrophages	SLC7A7	0.043411	3.785978	0.316451	0.752406	-6.67454	0.782973	0.766584

Non.inflammatory.Macrophages	SLC35A1	-0.04553	3.416075	-0.31641	0.752435	-6.30402	0.80075	0.774519
Non.inflammatory.Macrophages	DNAJC25	0.03967	3.856746	0.31639	0.752452	-6.4839	0.779615	0.765078
Non.inflammatory.Macrophages	NABP2	0.032459	4.980112	0.316168	0.752621	-6.71447	0.728081	0.741596
Non.inflammatory.Macrophages	SWSAP1	-0.13617	0.278314	-0.31614	0.752644	-5.56682	0.943628	0.8452
Non.inflammatory.Macrophages	EGFR	0.143939	2.469637	0.316084	0.752684	-5.92273	0.847955	0.795273
Non.inflammatory.Macrophages	ARMC9	-0.06693	2.484131	-0.31603	0.752727	-6.01681	0.847213	0.794965
Non.inflammatory.Macrophages	SFMBT1	0.030851	6.08199	0.315781	0.752913	-6.92674	0.68079	0.719329
Non.inflammatory.Macrophages	GM50340	-0.11021	1.533078	-0.3155	0.753125	-5.78008	0.897458	0.816413
Non.inflammatory.Macrophages	HPCAL1	-0.02682	6.117607	-0.31541	0.753196	-7.08116	0.679418	0.718631
Non.inflammatory.Macrophages	VEGFB	-0.04979	3.802219	-0.31534	0.753244	-6.39291	0.782448	0.766496
Non.inflammatory.Macrophages	ZFP780B	0.05941	3.329247	0.315165	0.753379	-6.24782	0.805301	0.77669
Non.inflammatory.Macrophages	GM49521	-0.17335	0.473447	-0.31507	0.753454	-5.33616	0.944015	0.840894
Non.inflammatory.Macrophages	CCDC77	-0.05515	3.291762	-0.31492	0.753562	-6.18945	0.807179	0.777583
Non.inflammatory.Macrophages	GM45902	-0.0987	1.585805	-0.31474	0.753698	-5.75353	0.89475	0.815415
Non.inflammatory.Macrophages	ZFP28	-0.13426	0.2908	-0.31461	0.7538	-5.60408	0.944065	0.845283

Non.inflammatory.Macrophages	PILRB1	-0.05625	2.049283	-0.31454	0.753853	-6.52912	0.870135	0.805002
Non.inflammatory.Macrophages	CYP20A1	0.036695	4.104318	0.314428	0.753937	-6.54652	0.768326	0.760277
Non.inflammatory.Macrophages	GM16675	0.119063	1.196561	0.31438	0.753973	-5.79218	0.91591	0.82432
Non.inflammatory.Macrophages	TGTP2	-0.17662	0.773598	-0.31437	0.753983	-5.67149	0.935645	0.834063
Non.inflammatory.Macrophages	MS4A8A	0.068588	0.670009	0.314189	0.754118	-6.44258	0.939446	0.836481
Non.inflammatory.Macrophages	ARVCF	-0.12686	1.228411	-0.3139	0.754337	-5.68297	0.914422	0.823683
Non.inflammatory.Macrophages	SLC35C1	-0.05706	2.749333	-0.3136	0.754562	-6.26327	0.834408	0.789617
Non.inflammatory.Macrophages	SNHG5	0.077765	2.416156	0.313587	0.754574	-6.02772	0.851376	0.796969
Non.inflammatory.Macrophages	APOLD1	0.144242	1.979983	0.313517	0.754627	-5.69959	0.874068	0.806694
Non.inflammatory.Macrophages	PPFIBP1	0.051515	3.366596	0.313442	0.754683	-6.33633	0.803796	0.776173
Non.inflammatory.Macrophages	FAM49A	0.039817	5.390412	0.313302	0.754789	-7.00367	0.710642	0.733657
Non.inflammatory.Macrophages	GM13610	-0.17461	0.396658	-0.3133	0.754792	-5.31917	0.94438	0.842975
Non.inflammatory.Macrophages	BORCS7	0.042405	3.716689	0.31314	0.754912	-6.44689	0.786953	0.768703
Non.inflammatory.Macrophages	TBC1D8B	0.057197	3.257326	0.313031	0.754995	-6.18832	0.809205	0.778592
Non.inflammatory.Macrophages	GHDC	0.081025	1.628326	0.31279	0.755177	-5.9164	0.892971	0.814768

Non.inflammatory.Macrophages	HSD17B7	0.111176	1.697013	0.312248	0.755588	-5.61986	0.889458	0.813312
Non.inflammatory.Macrophages	FDXACB1	-0.07825	2.215747	-0.31218	0.75564	-5.85121	0.862097	0.801667
Non.inflammatory.Macrophages	SMIM10L1	0.03436	4.931338	0.31211	0.755692	-6.69365	0.731139	0.743319
Non.inflammatory.Macrophages	EPB41L4A(	-0.06109	3.25392	-0.31206	0.755728	-6.36668	0.809646	0.778847
Non.inflammatory.Macrophages	CCL5	0.166034	7.931718	0.31205	0.755737	-7.0646	0.608468	0.683755
Non.inflammatory.Macrophages	TNNI2	-0.07094	2.310806	-0.31194	0.755824	-6.07246	0.857167	0.799596
Non.inflammatory.Macrophages	NXPE3	-0.0999	3.424156	-0.3118	0.755923	-5.72101	0.801333	0.775223
Non.inflammatory.Macrophages	KRAS	-0.02382	7.278938	-0.3118	0.755926	-7.08684	0.633334	0.696343
Non.inflammatory.Macrophages	4833438C(	-0.0602	2.025244	-0.31177	0.755948	-6.03921	0.872054	0.805991
Non.inflammatory.Macrophages	FNBP1	-0.03322	8.248905	-0.3115	0.756153	-7.21025	0.59679	0.677811
Non.inflammatory.Macrophages	CHAF1A	-0.06777	4.186029	-0.31149	0.756159	-6.35684	0.765167	0.758975
Non.inflammatory.Macrophages	HSPD1	0.037115	7.46114	0.310905	0.756605	-7.06465	0.62658	0.692919
Non.inflammatory.Macrophages	GM10851	0.068153	4.366594	0.310903	0.756606	-6.17041	0.757078	0.755261
Non.inflammatory.Macrophages	WFDC18	-0.09577	-0.88323	-0.31085	0.756644	-5.71828	0.945206	0.873899
Non.inflammatory.Macrophages	IGFLR1	-0.14956	0.807622	-0.31077	0.75671	-5.59083	0.935557	0.833843

Non.inflammatory.Macrophages	EMG1	0.031883	5.937655	0.310666	0.756786	-6.83597	0.687875	0.722955
Non.inflammatory.Macrophages	MIEN1	0.025172	5.845651	0.310459	0.756943	-6.86284	0.691784	0.724832
Non.inflammatory.Macrophages	GM19684	-0.09155	1.522121	-0.31044	0.756958	-5.95801	0.89931	0.817495
Non.inflammatory.Macrophages	ZFP142	-0.05393	3.207748	-0.31027	0.757086	-6.20621	0.812322	0.780067
Non.inflammatory.Macrophages	VPS51	0.045015	3.730024	0.310166	0.757165	-6.44488	0.78699	0.768812
Non.inflammatory.Macrophages	PDIK1L	0.060083	3.065249	0.310165	0.757165	-6.15788	0.819364	0.783166
Non.inflammatory.Macrophages	TBCD	0.037262	4.783335	0.30997	0.757313	-6.61557	0.738183	0.74664
Non.inflammatory.Macrophages	PXMP4	-0.04619	3.831766	-0.30992	0.757352	-6.45924	0.782191	0.766682
Non.inflammatory.Macrophages	STARD13	0.142155	1.070842	0.309499	0.75767	-5.70353	0.92436	0.827937
Non.inflammatory.Macrophages	UBA1	0.028027	6.00691	0.309333	0.757796	-6.89232	0.685313	0.721726
Non.inflammatory.Macrophages	SLC39A12	-0.1335	0.492802	-0.30916	0.757925	-5.92291	0.945698	0.841378
Non.inflammatory.Macrophages	SLC31A1	0.032638	5.429507	0.309013	0.758039	-6.82071	0.710027	0.733446
Non.inflammatory.Macrophages	PWWP2B	-0.06167	2.082717	-0.30852	0.758412	-6.23182	0.870118	0.805155
Non.inflammatory.Macrophages	HIST1H2BH	0.162408	0.17295	0.308517	0.758414	-5.43206	0.94595	0.84903
Non.inflammatory.Macrophages	THTPA	0.088348	1.360701	0.308505	0.758424	-5.81651	0.908743	0.821478

Non.inflammatory.Macrophages	ZFP770	0.078769	1.760027	0.308384	0.758516	-5.99767	0.887192	0.812416
Non.inflammatory.Macrophages	ATP7A	-0.02747	5.8288	-0.30804	0.758773	-6.94648	0.693009	0.725511
Non.inflammatory.Macrophages	BC055324	-0.10272	2.245694	-0.30795	0.758844	-5.72349	0.861609	0.801608
Non.inflammatory.Macrophages	TMCO4	-0.0495	4.054604	-0.30784	0.758925	-6.45643	0.772191	0.762263
Non.inflammatory.Macrophages	TRAPPC13	0.040909	3.808587	0.307647	0.759075	-6.43579	0.783823	0.767583
Non.inflammatory.Macrophages	ANO8	0.109318	0.904446	0.307597	0.759113	-5.77394	0.932833	0.832146
Non.inflammatory.Macrophages	UBE2G2	0.031801	4.661055	0.30747	0.759209	-6.66794	0.744208	0.749591
Non.inflammatory.Macrophages	CUEDC2	-0.03258	5.245055	-0.3074	0.759261	-6.73936	0.718168	0.737524
Non.inflammatory.Macrophages	C330013E1	-0.12019	0.677864	-0.30738	0.759278	-5.70041	0.940957	0.83744
Non.inflammatory.Macrophages	ZFP444	0.050849	3.63547	0.307342	0.759306	-6.38523	0.792106	0.771338
Non.inflammatory.Macrophages	STFA3	0.183054	2.751543	0.307271	0.75936	-5.8175	0.835684	0.790555
Non.inflammatory.Macrophages	PSMA6	0.027165	6.941715	0.307142	0.759458	-7.0587	0.647366	0.703555
Non.inflammatory.Macrophages	CPB2	0.067335	3.209826	0.307001	0.759565	-6.24609	0.81282	0.780615
Non.inflammatory.Macrophages	VCAM1	0.066162	4.334733	0.306949	0.759604	-6.85722	0.759144	0.756555
Non.inflammatory.Macrophages	RRP1	-0.0233	6.518197	-0.30682	0.759702	-6.92951	0.664383	0.711936

Non.inflammatory.Macrophages	TPM3	-0.01668	8.661561	-0.30678	0.759733	-7.29701	0.58252	0.670706
Non.inflammatory.Macrophages	RAB31	-0.03305	4.770711	-0.30676	0.759748	-6.87628	0.739251	0.747428
Non.inflammatory.Macrophages	SEC61B	-0.02466	8.272683	-0.30676	0.75975	-7.23178	0.596602	0.678003
Non.inflammatory.Macrophages	ARHGAP32	0.065234	3.193867	0.306681	0.759807	-6.14171	0.813606	0.780961
Non.inflammatory.Macrophages	TRAPPC3	0.033105	4.917333	0.306668	0.759817	-6.72424	0.732672	0.744384
Non.inflammatory.Macrophages	NDRG3	-0.03695	4.407312	-0.30657	0.759889	-6.50084	0.755798	0.755032
Non.inflammatory.Macrophages	TWF1	0.025988	5.464598	0.3065	0.759945	-6.8781	0.708605	0.733133
Non.inflammatory.Macrophages	ERCC4	0.061418	2.949475	0.306416	0.760008	-6.22497	0.825737	0.786295
Non.inflammatory.Macrophages	THOC3	0.041945	4.102819	0.306386	0.760031	-6.48693	0.769931	0.761459
Non.inflammatory.Macrophages	DOCK2	-0.0261	9.34967	-0.30616	0.760203	-7.36539	0.558436	0.658015
Non.inflammatory.Macrophages	ENGASE	-0.0706	1.496503	-0.30607	0.760274	-6.2644	0.901419	0.818741
Non.inflammatory.Macrophages	GM14410	0.112695	0.850334	0.306045	0.76029	-5.63481	0.934825	0.833573
Non.inflammatory.Macrophages	1110032A(	0.046429	3.592833	0.305923	0.760382	-6.32831	0.794233	0.772389
Non.inflammatory.Macrophages	DDX58	0.044534	4.759373	0.305725	0.760533	-6.7149	0.739848	0.747798
Non.inflammatory.Macrophages	CANT1	-0.03907	3.891787	-0.30564	0.760597	-6.53633	0.779961	0.766081

Non.inflammatory.Macrophages	ORC5	-0.05086	3.474755	-0.3054	0.760777	-6.35232	0.799961	0.775023
Non.inflammatory.Macrophages	BCKDHB	0.05077	3.929893	0.305397	0.760781	-6.42011	0.778157	0.765269
Non.inflammatory.Macrophages	PDLIM5	0.028441	6.703681	0.305307	0.76085	-7.10227	0.656954	0.708399
Non.inflammatory.Macrophages	APPBP2OS	-0.10033	0.985935	-0.30528	0.760874	-5.66395	0.929507	0.83056
Non.inflammatory.Macrophages	CD59B	-0.15888	0.413	-0.30526	0.760888	-5.56552	0.94606	0.843888
Non.inflammatory.Macrophages	FAM76A	-0.02983	5.119697	-0.30507	0.761028	-6.76629	0.723796	0.740377
Non.inflammatory.Macrophages	ZFP653	0.052491	3.131364	0.304972	0.761105	-6.27039	0.816819	0.782524
Non.inflammatory.Macrophages	CBL	0.024654	7.22396	0.304941	0.761128	-7.11585	0.63636	0.698266
Non.inflammatory.Macrophages	GPR182	-0.08986	2.557152	-0.30474	0.761281	-5.97658	0.84575	0.795153
Non.inflammatory.Macrophages	SLC25A4	0.031997	7.14787	0.304687	0.76132	-7.08383	0.63938	0.69979
Non.inflammatory.Macrophages	MCPH1	-0.0367	4.913551	-0.30448	0.761477	-6.62479	0.733017	0.744762
Non.inflammatory.Macrophages	1110059G1	0.036163	3.928256	0.304438	0.76151	-6.53272	0.778331	0.765489
Non.inflammatory.Macrophages	SNHG14	-0.09088	1.410261	-0.30418	0.761702	-5.73366	0.90626	0.821095
Non.inflammatory.Macrophages	WDR34	-0.12515	1.224098	-0.30418	0.761706	-5.74305	0.916443	0.825354
Non.inflammatory.Macrophages	PSPH	-0.05428	3.906589	-0.30396	0.761874	-6.25273	0.779356	0.766028

Non.inflammatory.Macrophages	SF3B5	0.026865	6.277364	0.303765	0.762021	-6.95069	0.674413	0.717143
Non.inflammatory.Macrophages	COX20	0.034366	5.583215	0.303758	0.762026	-6.7745	0.703658	0.731138
Non.inflammatory.Macrophages	DACH2	0.189164	0.599965	0.303674	0.76209	-5.37689	0.943527	0.839878
Non.inflammatory.Macrophages	MGAT1	0.039717	4.704416	0.303626	0.762126	-6.58207	0.742422	0.749275
Non.inflammatory.Macrophages	GM43330	0.089181	1.526899	0.303616	0.762134	-5.84646	0.899932	0.818527
Non.inflammatory.Macrophages	GSDMC4	-0.07667	0.743558	-0.30342	0.762281	-6.00139	0.938817	0.836561
Non.inflammatory.Macrophages	GM1123	0.095379	0.265276	0.303322	0.762357	-5.81406	0.946177	0.84777
Non.inflammatory.Macrophages	MTMR12	-0.03621	5.334196	-0.30329	0.762384	-6.62136	0.714442	0.736295
Non.inflammatory.Macrophages	EXOSC5	0.031667	5.376885	0.303262	0.762403	-6.75329	0.712582	0.735421
Non.inflammatory.Macrophages	DAXX	-0.04236	4.352332	-0.3032	0.762449	-6.63017	0.758513	0.756699
Non.inflammatory.Macrophages	CHERP	0.031579	5.016827	0.303199	0.76245	-6.64706	0.728414	0.74283
Non.inflammatory.Macrophages	TMSB15B1	-0.10004	2.422831	-0.30274	0.762801	-5.70984	0.852965	0.798521
Non.inflammatory.Macrophages	TRPS1	0.032844	7.683996	0.302148	0.763249	-7.3168	0.619032	0.68983
Non.inflammatory.Macrophages	ZFP467	-0.05287	2.303773	-0.30215	0.76325	-6.38651	0.859255	0.801269
Non.inflammatory.Macrophages	POLR3C	0.031006	5.018329	0.302137	0.763257	-6.63342	0.728733	0.74297

Non.inflammatory.Macrophages	NUCB2	-0.03202	4.304904	-0.30203	0.763338	-6.62957	0.761108	0.757878
Non.inflammatory.Macrophages	MRPL51	0.035194	4.486929	0.302008	0.763355	-6.62515	0.752721	0.754053
Non.inflammatory.Macrophages	HDAC2	-0.03513	5.300553	-0.30199	0.763371	-6.70881	0.71629	0.737165
Non.inflammatory.Macrophages	A930005H1	0.050963	3.220496	0.301931	0.763413	-6.20606	0.812919	0.781104
Non.inflammatory.Macrophages	ACE	0.176974	0.341503	0.301809	0.763507	-5.63322	0.946706	0.846201
Non.inflammatory.Macrophages	KHDRBS3	0.115746	1.943216	0.301572	0.763686	-5.78364	0.87829	0.809438
Non.inflammatory.Macrophages	ATXN7L3	0.036349	4.200833	0.30127	0.763916	-6.52937	0.766236	0.760277
Non.inflammatory.Macrophages	CDC7	-0.10432	2.873973	-0.301	0.764118	-5.7243	0.830554	0.788939
Non.inflammatory.Macrophages	PPA2	-0.04093	4.580694	-0.3009	0.7642	-6.54581	0.748793	0.752397
Non.inflammatory.Macrophages	PDGFB	0.081322	1.062654	0.300856	0.76423	-6.22156	0.926291	0.829748
Non.inflammatory.Macrophages	SCARB1	-0.02875	4.995715	-0.30051	0.764491	-6.87081	0.730088	0.743838
Non.inflammatory.Macrophages	4930444A1	-0.08815	3.751804	-0.30046	0.76453	-5.96558	0.787513	0.770044
Non.inflammatory.Macrophages	MEX3A	-0.11773	1.577184	-0.30007	0.764831	-5.64306	0.898121	0.818166
Non.inflammatory.Macrophages	PFKFB4	0.05314	3.15878	0.29984	0.765003	-6.29761	0.816355	0.782985
Non.inflammatory.Macrophages	GRIK4	-0.14461	0.238216	-0.29979	0.765045	-5.67173	0.947131	0.849214

Non.inflammatory.Macrophages	PARP6	0.059816	2.991541	0.299746	0.765074	-6.2031	0.824665	0.786662
Non.inflammatory.Macrophages	UBLCP1	0.034282	4.773952	0.299565	0.765212	-6.60416	0.740028	0.748615
Non.inflammatory.Macrophages	FAM171B	-0.13287	0.217464	-0.29945	0.765298	-5.7156	0.947131	0.849743
Non.inflammatory.Macrophages	CRTAP	0.053272	3.004923	0.299292	0.765419	-6.28948	0.823997	0.786395
Non.inflammatory.Macrophages	RBMS3	-0.09138	3.072419	-0.29925	0.765451	-6.1656	0.820637	0.784939
Non.inflammatory.Macrophages	ZFP511	-0.03876	3.816412	-0.29905	0.765602	-6.3996	0.784428	0.76891
Non.inflammatory.Macrophages	TERF1	0.034367	5.254483	0.298991	0.765649	-6.60673	0.718651	0.738738
Non.inflammatory.Macrophages	GM26789	-0.1835	0.271556	-0.29898	0.765654	-5.34297	0.947131	0.848548
Non.inflammatory.Macrophages	LMO7	-0.23866	0.761873	-0.29894	0.765685	-5.49517	0.939105	0.837089
Non.inflammatory.Macrophages	RERG	-0.15345	0.522461	-0.29889	0.765723	-5.54123	0.946348	0.842689
Non.inflammatory.Macrophages	GM16174	-0.1151	0.995634	-0.29882	0.765781	-5.59371	0.930019	0.83169
Non.inflammatory.Macrophages	APOPT1	-0.02634	4.910205	-0.29881	0.76579	-6.68045	0.733906	0.745882
Non.inflammatory.Macrophages	RAB11FIP2	0.052878	3.576284	0.298794	0.765799	-6.35454	0.795949	0.774097
Non.inflammatory.Macrophages	SPRYD7	0.064526	2.133927	0.298773	0.765814	-6.11131	0.868519	0.805796
Non.inflammatory.Macrophages	ZFP592	-0.02631	5.89966	-0.29853	0.765997	-6.7852	0.690874	0.725657

Non.inflammatory.Macrophages	BAG4	0.040597	4.226706	0.298509	0.766015	-6.43614	0.765105	0.760253
Non.inflammatory.Macrophages	RNF213	-0.05005	6.151249	-0.2985	0.766025	-7.08134	0.680323	0.720592
Non.inflammatory.Macrophages	SS18L2	-0.04669	3.817204	-0.29835	0.766139	-6.38937	0.784391	0.768968
Non.inflammatory.Macrophages	ASXL1	-0.02668	7.242343	-0.29832	0.766156	-7.04963	0.636338	0.69904
Non.inflammatory.Macrophages	NDUFAF4	0.046092	3.867148	0.298291	0.766181	-6.41546	0.782014	0.767924
Non.inflammatory.Macrophages	SRPK2	-0.03207	7.096447	-0.29813	0.7663	-6.95503	0.642056	0.701926
Non.inflammatory.Macrophages	BICRA	0.035052	5.82128	0.298069	0.76635	-6.7271	0.694193	0.727292
Non.inflammatory.Macrophages	CDR2	-0.06641	2.488604	-0.29804	0.766368	-6.22109	0.850129	0.79798
Non.inflammatory.Macrophages	TBC1D13	0.04051	3.495587	0.298041	0.766371	-6.55083	0.799856	0.775935
Non.inflammatory.Macrophages	STAU2	0.062921	2.900653	0.298004	0.766399	-6.20832	0.829214	0.788886
Non.inflammatory.Macrophages	AMPD3	0.052039	2.578161	0.297977	0.76642	-6.47253	0.845542	0.795995
Non.inflammatory.Macrophages	USP9X	0.02688	7.387216	0.297952	0.766439	-7.12446	0.630709	0.696277
Non.inflammatory.Macrophages	GM12227	-0.1078	1.113465	-0.2978	0.766553	-5.63576	0.923528	0.829088
Non.inflammatory.Macrophages	CSF3R	0.043675	2.933462	0.297467	0.766808	-6.77951	0.827817	0.788237
Non.inflammatory.Macrophages	SCAND1	-0.02099	7.018464	-0.29736	0.766887	-7.11514	0.645331	0.703555

Non.inflammatory.Macrophages	ASNA1	-0.03237	4.877847	-0.29722	0.766997	-6.7012	0.735581	0.746758
Non.inflammatory.Macrophages	CLDN34C1	0.143266	0.809812	0.297037	0.767135	-5.53574	0.937672	0.836261
Non.inflammatory.Macrophages	TRAF2	-0.0443	3.917831	-0.29685	0.767279	-6.40036	0.779849	0.767058
Non.inflammatory.Macrophages	TMEM79	-0.12265	0.778123	-0.29684	0.767285	-5.60847	0.938809	0.83704
Non.inflammatory.Macrophages	KCNAB1	-0.12048	1.324987	-0.29681	0.767309	-5.77347	0.912111	0.824419
Non.inflammatory.Macrophages	GM20732	0.032962	4.58993	0.296764	0.767343	-6.6474	0.748602	0.752841
Non.inflammatory.Macrophages	SERPINH1	0.091815	2.557349	0.296723	0.767374	-6.01347	0.846866	0.796665
Non.inflammatory.Macrophages	MYBL2	-0.08859	3.080165	-0.29661	0.767462	-5.96539	0.820523	0.785199
Non.inflammatory.Macrophages	RUBCN	0.048266	3.888841	0.296486	0.767555	-6.35115	0.781249	0.767758
Non.inflammatory.Macrophages	GM49602	0.087009	1.89388	0.296415	0.767608	-5.82651	0.88147	0.811609
Non.inflammatory.Macrophages	PIGQ	-0.0454	3.519745	-0.29624	0.767738	-6.3354	0.799017	0.775759
Non.inflammatory.Macrophages	1810046K(	0.198755	-0.14929	0.295898	0.768002	-5.24208	0.947617	0.859147
Non.inflammatory.Macrophages	SCN1B	0.065226	1.834445	0.295872	0.768022	-6.27102	0.884784	0.813089
Non.inflammatory.Macrophages	POC5	0.065633	2.510705	0.295871	0.768022	-6.02026	0.84943	0.797942
Non.inflammatory.Macrophages	PTPN22	0.04673	5.324165	0.295592	0.768235	-6.78433	0.715975	0.737906

Non.inflammatory.Macrophages	GREM2	0.158286	0.454759	0.295474	0.768324	-5.47405	0.947627	0.844931
Non.inflammatory.Macrophages	CBX8	-0.08558	1.617418	-0.29541	0.76837	-5.92939	0.89642	0.818076
Non.inflammatory.Macrophages	GM16973	-0.08181	1.894858	-0.29537	0.7684	-5.90648	0.881581	0.811791
Non.inflammatory.Macrophages	TAMM41	-0.06418	2.732365	-0.29483	0.768818	-6.13691	0.838136	0.793199
Non.inflammatory.Macrophages	RBM7	-0.02847	5.855126	-0.29469	0.768925	-6.91075	0.69312	0.727222
Non.inflammatory.Macrophages	DONSON	-0.04221	4.025423	-0.29455	0.769027	-6.42942	0.774933	0.765254
Non.inflammatory.Macrophages	ATG5	0.02381	5.691584	0.294453	0.769102	-6.82403	0.700084	0.73057
Non.inflammatory.Macrophages	NUP62	-0.05343	4.181003	-0.29442	0.769123	-6.46771	0.767636	0.761948
Non.inflammatory.Macrophages	F8	-0.09745	3.134673	-0.29433	0.769196	-5.98842	0.817976	0.784479
Non.inflammatory.Macrophages	CHST14	0.100378	0.872451	0.294321	0.769202	-5.83074	0.93563	0.835404
Non.inflammatory.Macrophages	TTLL4	-0.0535	3.000367	-0.2941	0.769373	-6.1952	0.824656	0.787448
Non.inflammatory.Macrophages	ETV3	-0.04352	4.950898	-0.29403	0.769426	-6.79032	0.73247	0.74584
Non.inflammatory.Macrophages	RAB3A	-0.06741	2.619056	-0.29399	0.769456	-6.13119	0.843897	0.795849
Non.inflammatory.Macrophages	C1QC	-0.05397	6.384084	-0.29395	0.769482	-7.24617	0.671048	0.716669
Non.inflammatory.Macrophages	4933439C1	-0.10708	1.200712	-0.29381	0.769593	-5.70957	0.919136	0.827902

Non.inflammatory.Macrophages	ANAPC5	0.025342	6.399962	0.293712	0.769666	-6.85654	0.670396	0.716393
Non.inflammatory.Macrophages	TRIAP1	0.036348	4.111675	0.29364	0.769721	-6.57098	0.77088	0.763539
Non.inflammatory.Macrophages	RBM12B1	0.120487	0.935508	0.293633	0.769727	-5.6519	0.933378	0.834054
Non.inflammatory.Macrophages	HIST2H2BE	0.173133	-0.261	0.293624	0.769734	-5.30056	0.947627	0.862224
Non.inflammatory.Macrophages	PEX13	-0.02913	5.524698	-0.29346	0.76986	-6.84031	0.70726	0.734121
Non.inflammatory.Macrophages	TATDN3	-0.05593	2.490952	-0.29337	0.76993	-6.21979	0.850453	0.798797
Non.inflammatory.Macrophages	EEF1D	-0.02357	7.27018	-0.29322	0.770042	-7.12847	0.635585	0.699321
Non.inflammatory.Macrophages	DEK	0.029393	7.842	0.293214	0.770046	-7.10424	0.613673	0.688279
Non.inflammatory.Macrophages	DYNLT1C	0.106994	1.100031	0.293047	0.770173	-5.65566	0.924702	0.830327
Non.inflammatory.Macrophages	TGFA	-0.15117	0.796415	-0.29303	0.770189	-5.51854	0.938355	0.83736
Non.inflammatory.Macrophages	DNAJC1	0.025856	6.915183	0.29301	0.770201	-6.99482	0.649569	0.706278
Non.inflammatory.Macrophages	FCMR	-0.11737	2.105124	-0.29296	0.770237	-5.67126	0.870483	0.807452
Non.inflammatory.Macrophages	6330418K0	0.079361	1.506684	0.292762	0.77039	-5.9591	0.902407	0.82104
Non.inflammatory.Macrophages	LUZP1	-0.03143	5.787075	-0.29274	0.770405	-6.9089	0.69601	0.72885
Non.inflammatory.Macrophages	PCBP4	0.151393	0.472145	0.292551	0.770551	-5.42937	0.947627	0.84504

Non.inflammatory.Macrophages	GM36279	-0.09264	2.959709	-0.29247	0.77061	-5.81	0.826688	0.788584
Non.inflammatory.Macrophages	DENND1A	-0.02195	7.497877	-0.29245	0.770627	-7.2136	0.62677	0.695001
Non.inflammatory.Macrophages	GM26632	0.134623	0.366747	0.292303	0.77074	-5.52957	0.947627	0.847547
Non.inflammatory.Macrophages	WDR25	-0.06482	1.998808	-0.29229	0.770751	-6.01724	0.876078	0.809973
Non.inflammatory.Macrophages	RNASEK	0.021615	6.735066	0.29226	0.770772	-7.06842	0.656778	0.709943
Non.inflammatory.Macrophages	MPHOSPH:	0.031524	4.79616	0.292135	0.770868	-6.6005	0.739413	0.74933
Non.inflammatory.Macrophages	EIF4E2	-0.02574	5.989442	-0.29194	0.771014	-6.90799	0.68745	0.724869
Non.inflammatory.Macrophages	PRUNE2	-0.08232	0.638929	-0.29158	0.771288	-5.95919	0.944033	0.841286
Non.inflammatory.Macrophages	COMMD1C	0.033514	4.694613	0.291569	0.771299	-6.68731	0.744004	0.751547
Non.inflammatory.Macrophages	SNHG3	0.037961	5.291632	0.291558	0.771307	-6.79903	0.717398	0.739159
Non.inflammatory.Macrophages	GNAI2	0.016527	9.008515	0.291529	0.77133	-7.38324	0.571243	0.66651
Non.inflammatory.Macrophages	C2CD3	0.031823	4.657214	0.291516	0.771339	-6.57303	0.745701	0.75233
Non.inflammatory.Macrophages	MXD4	0.040824	5.790486	0.291462	0.771381	-6.80956	0.695865	0.728964
Non.inflammatory.Macrophages	GM16158	0.207533	0.586451	0.291396	0.771431	-5.26166	0.945298	0.842515
Non.inflammatory.Macrophages	FNTB	0.061937	2.971515	0.291339	0.771474	-6.07207	0.826097	0.788468

Non.inflammatory.Macrophages	PRPF39	0.029645	5.43876	0.291288	0.771514	-6.79305	0.710982	0.736139
Non.inflammatory.Macrophages	PHYKPL	0.045623	3.470266	0.291272	0.771526	-6.2229	0.801504	0.777601
Non.inflammatory.Macrophages	HNRNPA3	-0.02346	8.554364	-0.29125	0.771544	-7.25586	0.587408	0.674987
Non.inflammatory.Macrophages	CETN4	0.133573	-0.08195	0.291243	0.771548	-5.46129	0.947627	0.858293
Non.inflammatory.Macrophages	ESCO2	0.090412	3.734708	0.291083	0.771669	-6.12375	0.788743	0.771918
Non.inflammatory.Macrophages	0610040B1	-0.08113	2.051019	-0.29102	0.771718	-5.89494	0.873327	0.808934
Non.inflammatory.Macrophages	SV2C	-0.14797	-0.28271	-0.29091	0.7718	-5.47142	0.947627	0.863134
Non.inflammatory.Macrophages	ALDH9A1	-0.02851	5.005152	-0.29082	0.77187	-6.75174	0.73005	0.745129
Non.inflammatory.Macrophages	MPZL1	-0.07085	2.78686	-0.29081	0.771881	-6.28122	0.835379	0.792581
Non.inflammatory.Macrophages	TOM1L1	0.147619	0.714609	0.290522	0.772097	-5.46429	0.941477	0.839646
Non.inflammatory.Macrophages	PUF60	0.031285	5.949591	0.290177	0.77236	-6.79325	0.689431	0.725909
Non.inflammatory.Macrophages	CLEC9A	0.051981	2.519945	0.289702	0.772722	-6.64545	0.849545	0.798674
Non.inflammatory.Macrophages	CAVIN2	0.077076	2.454343	0.289618	0.772786	-6.03932	0.852918	0.800132
Non.inflammatory.Macrophages	HDHD5	-0.07964	2.842941	-0.28957	0.772824	-5.93664	0.833119	0.79153
Non.inflammatory.Macrophages	CPEB3	-0.04248	4.234811	-0.2895	0.772873	-6.77216	0.765651	0.761469

Non.inflammatory.Macrophages	GALNT4	0.07389	1.622606	0.28947	0.772899	-5.84759	0.896754	0.818864
Non.inflammatory.Macrophages	ZFP81	0.082704	1.873403	0.289256	0.773063	-5.84623	0.883341	0.813175
Non.inflammatory.Macrophages	HIST1H4H	-0.13193	0.513275	-0.28908	0.7732	-5.50409	0.947731	0.844544
Non.inflammatory.Macrophages	VSTM4	-0.14645	0.147061	-0.28907	0.773204	-5.58791	0.948293	0.853174
Non.inflammatory.Macrophages	MAOA	0.135317	0.932781	0.288906	0.773329	-5.60573	0.934132	0.834802
Non.inflammatory.Macrophages	GRAP2	-0.13755	4.975054	-0.2888	0.773409	-5.76659	0.731906	0.746052
Non.inflammatory.Macrophages	MCRIP1	0.02263	5.919349	0.288724	0.773469	-6.88994	0.690889	0.726701
Non.inflammatory.Macrophages	GPR141B	-0.14692	-0.65516	-0.28867	0.773508	-5.30943	0.948293	0.872464
Non.inflammatory.Macrophages	MNAT1	0.03617	4.818214	0.288606	0.773558	-6.61231	0.738939	0.749324
Non.inflammatory.Macrophages	AIRN	0.048463	5.663175	0.288389	0.773724	-6.85323	0.701794	0.731902
Non.inflammatory.Macrophages	RAB7B	0.045637	2.804284	0.288385	0.773727	-6.79291	0.835085	0.792521
Non.inflammatory.Macrophages	CAGE1	-0.11369	1.94977	-0.28828	0.773804	-5.72314	0.879288	0.811581
Non.inflammatory.Macrophages	CYC1	0.033166	6.36195	0.288265	0.773818	-6.91265	0.67243	0.717801
Non.inflammatory.Macrophages	CSK	0.026593	6.929767	0.288177	0.773886	-6.99619	0.649445	0.706543
Non.inflammatory.Macrophages	STAMBP	-0.04713	3.374422	-0.28817	0.773895	-6.39553	0.806743	0.780049

Non.inflammatory.Macrophages	UBXN1	0.020871	6.759331	0.288022	0.774004	-7.03111	0.656291	0.709932
Non.inflammatory.Macrophages	HIVEP2	-0.03813	8.386816	-0.28794	0.774068	-7.31508	0.593927	0.678491
Non.inflammatory.Macrophages	2810408I1	0.090786	1.398496	0.287688	0.774259	-5.88041	0.909034	0.824179
Non.inflammatory.Macrophages	ZFYVE21	0.057846	2.542215	0.28768	0.774265	-6.20777	0.848518	0.798383
Non.inflammatory.Macrophages	PPM1H	-0.034	6.473739	-0.28754	0.774373	-7.16307	0.667934	0.715662
Non.inflammatory.Macrophages	CCDC18	-0.07732	2.722921	-0.28748	0.77442	-5.91466	0.839317	0.794419
Non.inflammatory.Macrophages	ITPRIP	0.051541	3.468077	0.287018	0.77477	-6.30569	0.802519	0.778307
Non.inflammatory.Macrophages	SLC38A6	0.036615	4.233853	0.286994	0.774788	-6.70488	0.766039	0.761895
Non.inflammatory.Macrophages	NUDT3	0.028517	5.213023	0.286818	0.774923	-6.67058	0.721683	0.741428
Non.inflammatory.Macrophages	CD320	0.077695	1.856288	0.286786	0.774948	-5.80084	0.884653	0.814018
Non.inflammatory.Macrophages	EPM2A	0.08304	1.526051	0.286666	0.775039	-5.98141	0.902425	0.821543
Non.inflammatory.Macrophages	HNRNPH2	-0.02261	6.435152	-0.28641	0.775234	-6.93639	0.669773	0.716705
Non.inflammatory.Macrophages	IFRD2	0.053708	3.30652	0.286205	0.77539	-6.23506	0.810494	0.781932
Non.inflammatory.Macrophages	GM20707	0.063485	1.898927	0.285644	0.775819	-5.94944	0.882445	0.813284
Non.inflammatory.Macrophages	E2F3	-0.03965	5.460693	-0.28563	0.775829	-6.74776	0.7109	0.736542

Non.inflammatory.Macrophages	GALT	0.067592	2.389889	0.285611	0.775844	-6.05401	0.856707	0.802256
Non.inflammatory.Macrophages	RBPMS2	-0.10074	1.246218	-0.28557	0.775873	-5.60772	0.917753	0.828173
Non.inflammatory.Macrophages	TBC1D8	0.038937	5.179428	0.28549	0.775936	-6.80529	0.723214	0.742335
Non.inflammatory.Macrophages	SIGIRR	-0.09837	2.175284	-0.28535	0.776042	-5.57661	0.867871	0.807105
Non.inflammatory.Macrophages	UBIAD1	-0.0718	1.941616	-0.28534	0.776049	-5.80376	0.880179	0.812366
Non.inflammatory.Macrophages	ANKRD17	-0.0218	7.985576	-0.28531	0.776074	-7.19951	0.609034	0.686589
Non.inflammatory.Macrophages	TUBE1	0.121945	1.254723	0.285107	0.776229	-5.5708	0.917285	0.828052
Non.inflammatory.Macrophages	ATAT1	-0.05447	2.722421	-0.2851	0.776237	-6.18839	0.839668	0.794942
Non.inflammatory.Macrophages	WDR5	0.038117	4.681603	0.285089	0.776243	-6.53213	0.745506	0.75276
Non.inflammatory.Macrophages	CAMK2D	-0.03204	7.214604	-0.28491	0.776381	-7.20225	0.638536	0.701527
Non.inflammatory.Macrophages	GM40787	0.135336	0.205726	0.284854	0.776422	-5.55073	0.948788	0.852557
Non.inflammatory.Macrophages	HAUS5	0.070872	2.831524	0.284776	0.776482	-5.97265	0.834146	0.792557
Non.inflammatory.Macrophages	RANGAP1	-0.03634	5.610379	-0.2846	0.776619	-6.70199	0.704429	0.7336
Non.inflammatory.Macrophages	MCAM	-0.14024	1.173297	-0.2845	0.776692	-5.58314	0.921777	0.830016
Non.inflammatory.Macrophages	GM36975	0.129861	3.276788	0.284449	0.776731	-5.59049	0.811956	0.782863

Non.inflammatory.Macrophages	NAT10	-0.04779	3.270157	-0.28445	0.776733	-6.34031	0.812283	0.783008
Non.inflammatory.Macrophages	GM6710	0.11339	0.761027	0.284438	0.77674	-5.66831	0.940778	0.839581
Non.inflammatory.Macrophages	NCSTN	0.02778	5.576404	0.284388	0.776778	-6.85941	0.705893	0.734328
Non.inflammatory.Macrophages	DEPDC1B	0.133518	2.954227	0.284352	0.776806	-5.71495	0.827976	0.789928
Non.inflammatory.Macrophages	ETAA1	-0.05	3.334503	-0.28425	0.776881	-6.26904	0.809121	0.781614
Non.inflammatory.Macrophages	CEP83OS	-0.08404	2.022979	-0.28412	0.776983	-5.87725	0.875875	0.810655
Non.inflammatory.Macrophages	CYSLTR1	0.043514	2.70023	0.283965	0.777101	-6.64711	0.840795	0.795529
Non.inflammatory.Macrophages	DDX6	0.022633	8.314732	0.283888	0.77716	-7.18951	0.596848	0.680433
Non.inflammatory.Macrophages	HAUS4	-0.04529	4.204699	-0.28388	0.777167	-6.42569	0.76747	0.762912
Non.inflammatory.Macrophages	GM42937	-0.105	0.198102	-0.28387	0.777171	-5.79642	0.948788	0.852815
Non.inflammatory.Macrophages	GM36862	-0.13165	0.340355	-0.28387	0.777173	-5.54993	0.948788	0.849454
Non.inflammatory.Macrophages	PMP22	0.069928	1.847479	0.283554	0.777415	-6.29923	0.885338	0.81464
Non.inflammatory.Macrophages	ADRM1	0.033019	5.477684	0.283457	0.777489	-6.77359	0.710288	0.736387
Non.inflammatory.Macrophages	RPA2	0.056257	4.645282	0.283417	0.77752	-6.42161	0.747289	0.753651
Non.inflammatory.Macrophages	SMARCAD:	0.027674	4.984115	0.28332	0.777594	-6.69726	0.732015	0.746575

Non.inflammatory.Macrophages	TRNT1	0.031449	4.772449	0.282907	0.77791	-6.63357	0.741587	0.751072
Non.inflammatory.Macrophages	4632427E1	-0.04029	4.404143	-0.28279	0.777995	-6.47487	0.758413	0.758831
Non.inflammatory.Macrophages	ALG9	0.044149	3.375018	0.282711	0.778059	-6.35599	0.80735	0.780884
Non.inflammatory.Macrophages	RASIP1	-0.09872	1.808725	-0.28269	0.778077	-5.81828	0.887485	0.815655
Non.inflammatory.Macrophages	LSM7	-0.02693	6.346432	-0.28265	0.778108	-6.90113	0.6736	0.718891
Non.inflammatory.Macrophages	TMEM248	0.026426	5.858866	0.282629	0.778122	-6.81936	0.693995	0.728716
Non.inflammatory.Macrophages	DHDDS	-0.03623	4.630539	-0.28259	0.778151	-6.59805	0.748028	0.754076
Non.inflammatory.Macrophages	ITGA9	-0.04403	5.58915	-0.28205	0.778561	-6.97985	0.705762	0.734374
Non.inflammatory.Macrophages	MAGED1	0.078393	2.409871	0.281996	0.778606	-5.97463	0.856181	0.802329
Non.inflammatory.Macrophages	GM9828	-0.12032	1.076181	-0.28198	0.778616	-5.62343	0.927709	0.832633
Non.inflammatory.Macrophages	FAM89B	-0.0292	5.277421	-0.28153	0.77896	-6.84153	0.719533	0.74091
Non.inflammatory.Macrophages	LPGAT1	-0.03785	6.245156	-0.28133	0.779111	-6.77165	0.678204	0.721256
Non.inflammatory.Macrophages	RBAK	-0.09508	1.336784	-0.28126	0.779171	-5.74922	0.913582	0.826819
Non.inflammatory.Macrophages	RNF187	0.028759	6.12757	0.28123	0.779191	-6.82171	0.683102	0.723631
Non.inflammatory.Macrophages	CPSF3	0.030066	4.770083	0.280904	0.77944	-6.64653	0.742149	0.751586

Non.inflammatory.Macrophages	BAMBI	-0.05227	3.967331	-0.28055	0.77971	-6.49169	0.779313	0.768693
Non.inflammatory.Macrophages	EDC3	0.041322	3.628178	0.280509	0.779743	-6.35031	0.795533	0.775987
Non.inflammatory.Macrophages	9530077C(	-0.16984	1.213471	-0.28049	0.779755	-5.34457	0.920367	0.829887
Non.inflammatory.Macrophages	PPP1R8	0.040598	4.114233	0.280482	0.779763	-6.41772	0.772383	0.76556
Non.inflammatory.Macrophages	UBA2	0.025164	6.457603	0.280449	0.779788	-6.86785	0.669442	0.71721
Non.inflammatory.Macrophages	SETDB2	0.040268	4.580351	0.280413	0.779816	-6.47611	0.750779	0.755691
Non.inflammatory.Macrophages	TMEM19	0.035052	3.958743	0.280396	0.779829	-6.50245	0.779719	0.76888
Non.inflammatory.Macrophages	ZDHHC7	-0.03774	3.834677	-0.28038	0.779845	-6.49563	0.78562	0.77154
Non.inflammatory.Macrophages	NAGLU	0.046135	2.6543	0.280326	0.779882	-6.37015	0.843875	0.79731
Non.inflammatory.Macrophages	NMT2	-0.02737	5.47269	-0.28031	0.779892	-6.76563	0.711005	0.737159
Non.inflammatory.Macrophages	WDR5B	-0.1256	0.670974	-0.2801	0.780056	-5.57366	0.944973	0.842563
Non.inflammatory.Macrophages	PSMC6	0.024189	6.141841	0.279519	0.7805	-6.88485	0.682743	0.723734
Non.inflammatory.Macrophages	CENPH	-0.07647	3.059161	-0.27944	0.780557	-6.09746	0.823742	0.78861
Non.inflammatory.Macrophages	PCDH15	0.101899	1.323083	0.279279	0.780683	-5.63605	0.914651	0.827663
Non.inflammatory.Macrophages	4732496C(	-0.12938	0.689806	-0.27926	0.780696	-5.45482	0.944506	0.842353

Non.inflammatory.Macrophages	RPP40	-0.09096	1.432828	-0.27916	0.780772	-5.77364	0.908644	0.825143
Non.inflammatory.Macrophages	EML6	-0.06073	2.764743	-0.27914	0.780788	-6.49583	0.83855	0.795143
Non.inflammatory.Macrophages	RREB1	-0.0337	6.814971	-0.27898	0.780909	-7.08371	0.655174	0.710435
Non.inflammatory.Macrophages	CFAP36	-0.03848	4.102137	-0.27897	0.780916	-6.40623	0.77322	0.766167
Non.inflammatory.Macrophages	NFX1	0.029	4.900546	0.278966	0.780923	-6.67233	0.736526	0.749327
Non.inflammatory.Macrophages	BLOC1S5	0.069048	1.955013	0.278893	0.780978	-6.02002	0.880549	0.81335
Non.inflammatory.Macrophages	WDTC1	0.047153	3.851337	0.278858	0.781005	-6.34716	0.785098	0.771555
Non.inflammatory.Macrophages	KPNB1	0.025751	5.739818	0.278207	0.781503	-6.82906	0.70012	0.73217
Non.inflammatory.Macrophages	SCRN2	-0.09463	1.43712	-0.27776	0.781846	-5.60586	0.909225	0.825373
Non.inflammatory.Macrophages	GM17484	-0.10967	1.264186	-0.27758	0.781983	-5.7556	0.918767	0.829401
Non.inflammatory.Macrophages	SLF2	0.026924	6.314028	0.277515	0.782032	-6.87853	0.676232	0.720687
Non.inflammatory.Macrophages	NCAPG	-0.08754	3.22832	-0.27724	0.782242	-6.04563	0.816156	0.785381
Non.inflammatory.Macrophages	NEIL1	0.076345	3.000738	0.276934	0.782477	-5.95396	0.827484	0.790401
Non.inflammatory.Macrophages	RNF123	-0.04989	3.804311	-0.27681	0.782574	-6.34686	0.788127	0.772933
Non.inflammatory.Macrophages	CDC123	-0.03015	5.404958	-0.27662	0.782715	-6.75637	0.714911	0.73927

Non.inflammatory.Macrophages	CYP4B1	-0.15186	0.266849	-0.27658	0.78275	-5.54536	0.950898	0.852836
Non.inflammatory.Macrophages	CATSPERG1	-0.09514	0.160917	-0.27649	0.782817	-5.88241	0.950898	0.855347
Non.inflammatory.Macrophages	USP18	0.065734	2.737474	0.276472	0.782831	-6.34946	0.84077	0.796243
Non.inflammatory.Macrophages	ECHDC1	0.04798	3.820316	0.276298	0.782964	-6.36154	0.787361	0.772629
Non.inflammatory.Macrophages	TIAL1	-0.02434	5.798885	-0.27626	0.782989	-6.81602	0.697908	0.731224
Non.inflammatory.Macrophages	GM49169	-0.1952	-0.42801	-0.27609	0.783123	-5.22775	0.950898	0.869461
Non.inflammatory.Macrophages	TANC1	0.056329	3.838336	0.275968	0.783217	-6.41324	0.7865	0.772281
Non.inflammatory.Macrophages	GM10642	-0.12027	0.270003	-0.27581	0.783335	-5.61751	0.950898	0.852856
Non.inflammatory.Macrophages	NLGN2	-0.15312	0.349554	-0.27578	0.783362	-5.53803	0.950898	0.850975
Non.inflammatory.Macrophages	BCORL1	0.060168	3.355845	0.275624	0.78348	-6.20663	0.809871	0.782751
Non.inflammatory.Macrophages	FAM3A	0.047701	2.785704	0.27547	0.783598	-6.26508	0.838321	0.795266
Non.inflammatory.Macrophages	3110009E1	-0.06824	2.175408	-0.27544	0.78362	-5.92112	0.869795	0.808881
Non.inflammatory.Macrophages	TUBB5	-0.04048	9.849372	-0.27543	0.783627	-7.38936	0.544338	0.653334
Non.inflammatory.Macrophages	MCM7	-0.05481	5.384269	-0.27527	0.783749	-6.62532	0.715815	0.73978
Non.inflammatory.Macrophages	GORASP2	0.022995	5.534187	0.275198	0.783806	-6.78897	0.70929	0.736699

Non.inflammatory.Macrophages	ATP11C	0.022176	6.869185	0.275139	0.783852	-7.00416	0.653651	0.709843
Non.inflammatory.Macrophages	RSPH10B	-0.14856	0.413611	-0.27506	0.78391	-5.50262	0.950898	0.849532
Non.inflammatory.Macrophages	HIST1H2A(	-0.10994	1.907677	-0.27493	0.784014	-5.82033	0.883941	0.815033
Non.inflammatory.Macrophages	DPH7	-0.09074	1.586984	-0.27492	0.784022	-5.83422	0.901162	0.822331
Non.inflammatory.Macrophages	GM41611	-0.08493	0.284798	-0.27482	0.784095	-5.94162	0.950898	0.852644
Non.inflammatory.Macrophages	CCDC192	0.131547	-0.69514	0.27479	0.784118	-5.56965	0.950898	0.876144
Non.inflammatory.Macrophages	FOXR1	0.171344	-0.87975	0.274625	0.784245	-5.18044	0.950898	0.880657
Non.inflammatory.Macrophages	TPM3-RS7	-0.10852	0.526188	-0.27458	0.78428	-5.60453	0.950021	0.846965
Non.inflammatory.Macrophages	SMOC1	-0.12563	2.28252	-0.27456	0.784292	-5.76316	0.864194	0.806619
Non.inflammatory.Macrophages	NUP85	-0.04769	4.345133	-0.2745	0.784344	-6.42502	0.762632	0.761649
Non.inflammatory.Macrophages	TDG	0.032355	5.074746	0.274218	0.784557	-6.58902	0.729467	0.746335
Non.inflammatory.Macrophages	LRIG1	-0.16686	0.178544	-0.27421	0.784565	-5.4106	0.950898	0.855201
Non.inflammatory.Macrophages	ZFP715	-0.04035	3.662783	-0.27417	0.784595	-6.36072	0.79493	0.776252
Non.inflammatory.Macrophages	TBC1D30	0.12769	1.06093	0.274159	0.784602	-5.68589	0.930071	0.8345
Non.inflammatory.Macrophages	PCBD2	-0.03023	5.24057	-0.27416	0.784602	-6.79639	0.722122	0.742897

Non.inflammatory.Macrophages	PREB	0.029123	4.736405	0.274146	0.784612	-6.67258	0.744673	0.753398
Non.inflammatory.Macrophages	MAPKAPK3	0.030532	5.306805	0.274141	0.784616	-6.85583	0.719208	0.741528
Non.inflammatory.Macrophages	ZWILCH	-0.07972	3.028209	-0.27408	0.784663	-6.07661	0.826109	0.79008
Non.inflammatory.Macrophages	DPYD	-0.08423	3.567148	-0.27392	0.784785	-6.32213	0.799611	0.778362
Non.inflammatory.Macrophages	GTF2IRD2	0.045143	3.944401	0.27382	0.784862	-6.34068	0.781505	0.770258
Non.inflammatory.Macrophages	PROX2	0.113947	1.188752	0.273674	0.784974	-5.62213	0.923088	0.831655
Non.inflammatory.Macrophages	BTD	0.066413	2.37707	0.273455	0.785141	-6.0441	0.859434	0.804675
Non.inflammatory.Macrophages	FBXO36	0.101456	1.228268	0.273299	0.785261	-5.79421	0.920954	0.83086
Non.inflammatory.Macrophages	ISOC2B	0.057043	2.519105	0.273263	0.785289	-6.2187	0.852094	0.801583
Non.inflammatory.Macrophages	CD300A	-0.03038	4.623283	-0.27321	0.78533	-7.08631	0.74996	0.756017
Non.inflammatory.Macrophages	FBXO5	-0.08387	4.811103	-0.27316	0.785368	-6.3332	0.741425	0.752086
Non.inflammatory.Macrophages	GM20743	0.195337	0.031876	0.272916	0.785555	-5.23984	0.951185	0.859012
Non.inflammatory.Macrophages	G2E3	0.03988	3.938116	0.272768	0.785668	-6.39792	0.781982	0.770669
Non.inflammatory.Macrophages	RPN2	0.024275	6.097946	0.272762	0.785673	-6.94401	0.685467	0.725696
Non.inflammatory.Macrophages	BRWD1	0.025102	6.550877	0.2726	0.785797	-6.9408	0.666769	0.716605

Non.inflammatory.Macrophages	IGLC2	0.178841	3.740399	0.272408	0.785943	-6.1327	0.791506	0.774988
Non.inflammatory.Macrophages	KIF4	-0.07458	4.394266	-0.27232	0.786014	-6.31683	0.760656	0.761022
Non.inflammatory.Macrophages	JAGN1	-0.03881	3.955321	-0.2723	0.786029	-6.52356	0.781239	0.770381
Non.inflammatory.Macrophages	MRPS14	0.022174	6.892734	0.272183	0.786116	-7.02592	0.652982	0.709905
Non.inflammatory.Macrophages	OASL2	0.076327	3.934807	0.271967	0.786281	-6.76282	0.782244	0.770841
Non.inflammatory.Macrophages	EIF4A1	0.018856	7.526831	0.271844	0.786375	-7.20769	0.628091	0.697492
Non.inflammatory.Macrophages	NIPA1	0.098425	0.13903	0.271817	0.786396	-5.76146	0.951312	0.856637
Non.inflammatory.Macrophages	TRAPPC6B	-0.02056	6.134079	-0.27172	0.786468	-6.90502	0.684045	0.725066
Non.inflammatory.Macrophages	FAM98B	0.032442	4.474383	0.271707	0.786481	-6.5989	0.756984	0.759355
Non.inflammatory.Macrophages	ERCC5	-0.05049	2.909402	-0.27128	0.786812	-6.15492	0.832625	0.793185
Non.inflammatory.Macrophages	TMEM141	-0.05147	2.239657	-0.2711	0.786943	-6.48595	0.867008	0.808099
Non.inflammatory.Macrophages	WDR77	-0.0438	3.895337	-0.27108	0.786962	-6.36312	0.784303	0.77172
Non.inflammatory.Macrophages	SLC35G1	0.109953	1.026313	0.271059	0.786978	-5.74659	0.932623	0.835818
Non.inflammatory.Macrophages	RARS2	0.040128	3.441715	0.271012	0.787014	-6.40045	0.806202	0.781524
Non.inflammatory.Macrophages	NELFE	-0.03038	4.393275	-0.2709	0.787096	-6.5948	0.760919	0.761098

Non.inflammatory.Macrophages	NDUFC2	0.024545	6.741013	0.270764	0.787204	-7.068	0.659285	0.712942
Non.inflammatory.Macrophages	PDIA6	0.025648	6.97381	0.270649	0.787292	-7.07536	0.649961	0.708337
Non.inflammatory.Macrophages	GM39556	-0.06231	3.384757	-0.27005	0.787749	-6.25129	0.809458	0.782907
Non.inflammatory.Macrophages	SLC5A11	-0.10961	-0.01556	-0.26987	0.78789	-5.67446	0.952165	0.860548
Non.inflammatory.Macrophages	PEF1	-0.04721	3.347699	-0.26965	0.788062	-6.27634	0.811456	0.783753
Non.inflammatory.Macrophages	CCNE1	0.067764	3.530373	0.269338	0.788298	-6.11795	0.802684	0.779779
Non.inflammatory.Macrophages	DTX2	0.043846	4.396914	0.269164	0.78843	-6.37293	0.761524	0.761197
Non.inflammatory.Macrophages	AP3M2	0.053062	2.489505	0.269126	0.78846	-6.12735	0.854914	0.80269
Non.inflammatory.Macrophages	SERTAD3	-0.04916	4.131362	-0.26862	0.788851	-6.41427	0.77413	0.766845
Non.inflammatory.Macrophages	SP1	0.02439	5.924217	0.268613	0.788853	-6.80668	0.693938	0.72951
Non.inflammatory.Macrophages	EIF3A	0.017798	7.379667	0.268522	0.788923	-7.13301	0.634752	0.700542
Non.inflammatory.Macrophages	CDC42SE2	-0.02175	7.179375	-0.2684	0.789016	-7.05998	0.642597	0.704459
Non.inflammatory.Macrophages	CHIL3	0.242367	2.447415	0.2684	0.789017	-5.7713	0.857312	0.80363
Non.inflammatory.Macrophages	TMEM18	-0.07324	1.593876	-0.26783	0.789456	-5.91004	0.902884	0.822983
Non.inflammatory.Macrophages	BZW1	0.01668	7.459579	0.267763	0.789506	-7.08245	0.631879	0.699037

Non.inflammatory.Macrophages	FOXP1	-0.02017	9.650428	-0.26775	0.789516	-7.34753	0.552319	0.657699
Non.inflammatory.Macrophages	1700003F1	0.062206	2.027827	0.267485	0.789719	-6.1523	0.879755	0.813221
Non.inflammatory.Macrophages	PMM1	0.052914	3.07058	0.267037	0.790063	-6.2313	0.826131	0.790103
Non.inflammatory.Macrophages	HKDC1	-0.08483	0.37121	-0.26688	0.790181	-6.07645	0.953366	0.851691
Non.inflammatory.Macrophages	1700109H(	0.048803	3.650709	0.26681	0.790237	-6.36329	0.797578	0.777473
Non.inflammatory.Macrophages	CCNJL	0.117662	1.734552	0.266742	0.790289	-5.55597	0.895519	0.820046
Non.inflammatory.Macrophages	DTL	-0.07384	4.961318	-0.26667	0.790345	-6.27828	0.736438	0.749645
Non.inflammatory.Macrophages	YIPF3	0.027902	4.904587	0.266603	0.790395	-6.69473	0.738991	0.75083
Non.inflammatory.Macrophages	BLM	-0.06827	3.897628	-0.26645	0.790515	-6.23861	0.785706	0.772232
Non.inflammatory.Macrophages	DGKI	0.126731	0.78	0.266334	0.790602	-5.96274	0.944631	0.842225
Non.inflammatory.Macrophages	FIBP	-0.02951	4.462532	-0.26632	0.790612	-6.60719	0.759167	0.760235
Non.inflammatory.Macrophages	GM50218	-0.15328	0.559001	-0.26625	0.790668	-5.42534	0.95169	0.847427
Non.inflammatory.Macrophages	ZFP12	-0.08431	1.346551	-0.26621	0.790699	-5.7094	0.916646	0.829088
Non.inflammatory.Macrophages	SGPL1	-0.02435	5.922758	-0.2662	0.790709	-7.03547	0.694441	0.729964
Non.inflammatory.Macrophages	SLC16A12	-0.12775	0.427781	-0.26606	0.790816	-5.55845	0.953366	0.850538

Non.inflammatory.Macrophages	4933412E1	-0.08497	1.908564	-0.26589	0.790945	-5.89359	0.886189	0.816266
Non.inflammatory.Macrophages	GANC	-0.04452	3.377098	-0.26581	0.791004	-6.40944	0.810928	0.783595
Non.inflammatory.Macrophages	GM5577	-0.13798	0.20371	-0.2658	0.791012	-5.41342	0.953366	0.855857
Non.inflammatory.Macrophages	3300002I0	0.069725	1.970632	0.265739	0.791059	-5.98875	0.882882	0.814861
Non.inflammatory.Macrophages	GM43259	-0.13797	1.452797	-0.26573	0.791067	-5.53992	0.910816	0.826676
Non.inflammatory.Macrophages	TUSC1	-0.04406	3.124075	-0.26542	0.791306	-6.32585	0.823634	0.789222
Non.inflammatory.Macrophages	DOCK5	-0.03845	4.189727	-0.26514	0.791516	-6.73907	0.772115	0.766226
Non.inflammatory.Macrophages	OXNAD1	-0.0643	1.886539	-0.26513	0.791524	-5.94631	0.887639	0.81693
Non.inflammatory.Macrophages	LPP	0.028303	7.6885	0.265023	0.791609	-7.22542	0.623439	0.695143
Non.inflammatory.Macrophages	ZCCHC7	-0.03474	6.744533	-0.26483	0.791758	-6.98802	0.660609	0.713651
Non.inflammatory.Macrophages	ZFP141	0.058028	3.367308	0.264806	0.791775	-6.17663	0.8117	0.783999
Non.inflammatory.Macrophages	CMPK2	-0.06167	3.672891	-0.26472	0.791845	-6.47645	0.796789	0.77736
Non.inflammatory.Macrophages	NUDCD3	-0.0215	5.998557	-0.26463	0.791914	-6.83672	0.691476	0.728645
Non.inflammatory.Macrophages	GNG7	-0.12275	-0.40318	-0.26408	0.792331	-5.529	0.953995	0.870786
Non.inflammatory.Macrophages	PPNR	0.080839	1.051959	0.264041	0.792363	-5.94002	0.933602	0.83631

Non.inflammatory.Macrophages	ZBTB18	0.036637	3.894799	0.264038	0.792365	-6.4587	0.786359	0.772736
Non.inflammatory.Macrophages	SMC3	0.024748	6.498954	0.263945	0.792436	-6.85289	0.670823	0.718707
Non.inflammatory.Macrophages	ULBP1	-0.04878	4.251838	-0.26386	0.792505	-6.45525	0.769472	0.765136
Non.inflammatory.Macrophages	ADAT1	-0.08083	2.003267	-0.26371	0.792615	-5.85757	0.881758	0.814583
Non.inflammatory.Macrophages	COQ10A	0.035252	3.31768	0.263638	0.792673	-6.42859	0.814419	0.785368
Non.inflammatory.Macrophages	GM14296	-0.07448	0.969349	-0.2634	0.792857	-5.78357	0.938331	0.838473
Non.inflammatory.Macrophages	ZFP787	0.024634	5.128821	0.263327	0.792911	-6.75208	0.729507	0.746857
Non.inflammatory.Macrophages	GM12802	0.104544	0.658153	0.263107	0.79308	-5.49652	0.949775	0.845773
Non.inflammatory.Macrophages	TRRAP	-0.02937	4.784536	-0.26306	0.793114	-6.71949	0.744987	0.754078
Non.inflammatory.Macrophages	LSM10	-0.03982	3.07773	-0.26296	0.793192	-6.44474	0.826403	0.790765
Non.inflammatory.Macrophages	ETFBKMT	-0.06511	2.261696	-0.26284	0.793286	-5.97804	0.868188	0.808951
Non.inflammatory.Macrophages	OLFM4	-0.18494	-0.01496	-0.26272	0.79338	-5.38966	0.954093	0.861772
Non.inflammatory.Macrophages	CCNC	0.037988	4.398502	0.262696	0.793396	-6.48738	0.762713	0.762256
Non.inflammatory.Macrophages	KIFC3	-0.05597	1.625707	-0.26264	0.793441	-6.22418	0.902088	0.823387
Non.inflammatory.Macrophages	F830016B(	-0.11586	0.444299	-0.26258	0.793484	-5.7988	0.954093	0.85086

Non.inflammatory.Macrophages	FXYD2	-0.10171	-0.41192	-0.26256	0.793503	-5.7729	0.954093	0.87132
Non.inflammatory.Macrophages	HELQ	0.073709	2.411841	0.262076	0.793872	-5.77535	0.86068	0.805719
Non.inflammatory.Macrophages	PPIB	0.020716	7.605608	0.261894	0.794012	-7.20042	0.627155	0.697317
Non.inflammatory.Macrophages	KLC1	0.027677	4.702224	0.261845	0.79405	-6.62694	0.74905	0.756027
Non.inflammatory.Macrophages	SYNGR2	0.024929	6.111593	0.261637	0.79421	-6.97029	0.687355	0.726991
Non.inflammatory.Macrophages	ATF6B	0.035095	4.456866	0.261363	0.79442	-6.55563	0.760413	0.761266
Non.inflammatory.Macrophages	PRMT7	0.052579	3.247895	0.261286	0.794479	-6.35621	0.818364	0.787317
Non.inflammatory.Macrophages	EEF2	-0.02319	9.105144	-0.26128	0.794483	-7.36952	0.572041	0.668876
Non.inflammatory.Macrophages	NOA1	0.053671	3.137205	0.260845	0.794818	-6.15005	0.823871	0.789841
Non.inflammatory.Macrophages	MRPS5	0.031557	4.612666	0.260741	0.794899	-6.59082	0.753232	0.758079
Non.inflammatory.Macrophages	PTCD1	-0.05485	2.585716	-0.26068	0.794947	-6.0741	0.851823	0.802076
Non.inflammatory.Macrophages	CITED4	0.191248	-0.2568	0.260571	0.795029	-5.32632	0.954602	0.868032
Non.inflammatory.Macrophages	GM42067	-0.11305	0.558955	-0.26055	0.795049	-5.479	0.952924	0.848598
Non.inflammatory.Macrophages	KCTD5	-0.03728	3.640452	-0.26041	0.79515	-6.45997	0.799109	0.77895
Non.inflammatory.Macrophages	TRIM8	-0.02423	5.616506	-0.26037	0.795183	-6.89274	0.708481	0.73726

Non.inflammatory.Macrophages	SURF2	-0.04227	3.331878	-0.26033	0.795217	-6.31137	0.814209	0.78567
Non.inflammatory.Macrophages	ADPRHL2	-0.03929	3.241714	-0.26022	0.795298	-6.2518	0.818671	0.787691
Non.inflammatory.Macrophages	KCTD10	0.035399	4.043279	0.260104	0.795388	-6.49407	0.779791	0.770338
Non.inflammatory.Macrophages	A430018G1	0.070615	1.329295	0.259937	0.795517	-5.83116	0.918785	0.830746
Non.inflammatory.Macrophages	WARS2	0.043238	3.512477	0.259927	0.795524	-6.39362	0.805339	0.781808
Non.inflammatory.Macrophages	WDR73	-0.05048	2.976485	-0.25986	0.795574	-6.12688	0.831928	0.793555
Non.inflammatory.Macrophages	PHEX	0.093493	1.372943	0.259599	0.795777	-5.91386	0.916381	0.829817
Non.inflammatory.Macrophages	PPP1R13L	0.118664	-0.0774	0.259511	0.795844	-5.65464	0.954602	0.863921
Non.inflammatory.Macrophages	EIF3D	0.026358	5.480464	0.25951	0.795845	-6.7822	0.714394	0.7402
Non.inflammatory.Macrophages	KLRC2	-0.1661	1.332802	-0.25945	0.795891	-5.47246	0.918592	0.830743
Non.inflammatory.Macrophages	MRRF	0.039724	3.454685	0.259427	0.795909	-6.38044	0.808168	0.783139
Non.inflammatory.Macrophages	FER	0.042204	3.489418	0.259304	0.796004	-6.51655	0.806467	0.782385
Non.inflammatory.Macrophages	DEF8	0.063155	2.04717	0.259263	0.796035	-5.95196	0.879959	0.814412
Non.inflammatory.Macrophages	APMAP	0.037808	4.080552	0.259239	0.796054	-6.44911	0.778025	0.769619
Non.inflammatory.Macrophages	OGDH	0.016882	6.625732	0.259217	0.796071	-7.01054	0.666059	0.716974

Non.inflammatory.Macrophages	STRAP	-0.02657	6.174275	-0.25911	0.796156	-6.87004	0.684724	0.726051
Non.inflammatory.Macrophages	H13	0.022654	6.42662	0.259087	0.79617	-6.89662	0.67423	0.720968
Non.inflammatory.Macrophages	TANGO2	0.047746	3.93274	0.258962	0.796267	-6.51871	0.785048	0.772824
Non.inflammatory.Macrophages	SORBS3	0.101404	0.937867	0.258935	0.796287	-5.67228	0.940164	0.839947
Non.inflammatory.Macrophages	4930579G2	0.07383	2.431609	0.258886	0.796325	-5.81026	0.859789	0.805787
Non.inflammatory.Macrophages	ZFP942	0.04068	4.309464	0.258122	0.796913	-6.42001	0.767637	0.764886
Non.inflammatory.Macrophages	DNAH2	-0.06687	0.783062	-0.25801	0.796997	-6.04092	0.946202	0.843742
Non.inflammatory.Macrophages	SGCE	0.117325	1.156412	0.257938	0.797054	-5.65763	0.928813	0.83505
Non.inflammatory.Macrophages	CRACR2A	0.097595	2.835916	0.257831	0.797137	-5.7613	0.83944	0.796993
Non.inflammatory.Macrophages	MAPK11	-0.13151	0.260399	-0.25773	0.797211	-5.43612	0.955063	0.856151
Non.inflammatory.Macrophages	ELK1	0.05835	1.559348	0.257719	0.797223	-5.93811	0.906614	0.82581
Non.inflammatory.Macrophages	SHISA8	-0.18744	-0.21439	-0.25772	0.797226	-5.26337	0.955063	0.867507
Non.inflammatory.Macrophages	YY1	0.016533	7.144963	0.257544	0.797357	-7.0548	0.64551	0.706951
Non.inflammatory.Macrophages	NAF1	-0.03567	3.658508	-0.25753	0.797365	-6.42746	0.798619	0.779006
Non.inflammatory.Macrophages	NDUFA1	0.022297	6.729501	0.257426	0.797448	-7.0374	0.662158	0.715175

Non.inflammatory.Macrophages	MBD4	0.049991	3.212172	0.257308	0.797539	-6.20772	0.820533	0.788742
Non.inflammatory.Macrophages	TFAP4	-0.0724	2.748617	-0.25729	0.797556	-5.96625	0.843884	0.79898
Non.inflammatory.Macrophages	CCDC14	-0.10638	0.809905	-0.25718	0.797639	-5.67	0.945245	0.843258
Non.inflammatory.Macrophages	ZBTB14	0.040587	3.004063	0.257062	0.797729	-6.21867	0.830974	0.793357
Non.inflammatory.Macrophages	SLC39A7	-0.02881	4.500813	-0.2569	0.797856	-6.60703	0.758816	0.761004
Non.inflammatory.Macrophages	RHOT2	0.053114	2.679369	0.256734	0.797981	-6.17173	0.847502	0.800626
Non.inflammatory.Macrophages	ALS2	-0.03332	4.283789	-0.2567	0.798009	-6.48701	0.768908	0.765678
Non.inflammatory.Macrophages	POLDIP2	-0.03425	4.278307	-0.25646	0.79819	-6.5716	0.769164	0.765865
Non.inflammatory.Macrophages	SCAF1	0.028195	4.690272	0.256377	0.798256	-6.65242	0.750109	0.757136
Non.inflammatory.Macrophages	ARPC1B	0.018259	8.892807	0.25634	0.798283	-7.41573	0.579887	0.673553
Non.inflammatory.Macrophages	TNPO2	0.033796	4.122122	0.256249	0.798354	-6.58103	0.776507	0.769233
Non.inflammatory.Macrophages	GFM2	-0.03986	3.590998	-0.25623	0.798367	-6.37747	0.801972	0.780693
Non.inflammatory.Macrophages	STFA2L1	0.150266	2.448793	0.256182	0.798405	-5.75929	0.85939	0.805911
Non.inflammatory.Macrophages	RFC3	-0.03701	4.31335	-0.25584	0.798665	-6.44646	0.767708	0.765264
Non.inflammatory.Macrophages	NFXL1	0.036818	3.364621	0.255584	0.798866	-6.54463	0.813305	0.785858

Non.inflammatory.Macrophages	SFPQ	-0.01862	8.189863	-0.25553	0.798911	-7.1939	0.605658	0.687087
Non.inflammatory.Macrophages	EFTUD2	0.029528	5.105706	0.255504	0.798927	-6.63186	0.731566	0.748683
Non.inflammatory.Macrophages	TGTP1	-0.17734	-0.20272	-0.2553	0.799087	-5.44858	0.95547	0.867829
Non.inflammatory.Macrophages	STX4A	0.023249	4.972734	0.255274	0.799105	-6.77112	0.73755	0.751516
Non.inflammatory.Macrophages	ZFP984	-0.03226	3.878402	-0.25518	0.799173	-6.49562	0.78836	0.774769
Non.inflammatory.Macrophages	MGST1	0.043066	7.132587	0.255024	0.799297	-7.0354	0.646275	0.707691
Non.inflammatory.Macrophages	CDC42EP3	-0.06165	5.142958	-0.25496	0.799348	-6.15215	0.72993	0.748016
Non.inflammatory.Macrophages	ARFGAP3	-0.03863	3.55174	-0.25492	0.799378	-6.42442	0.804153	0.781905
Non.inflammatory.Macrophages	PJA2	-0.03206	4.695864	-0.25481	0.799459	-6.69399	0.750112	0.757399
Non.inflammatory.Macrophages	NEDD4L	-0.0224	6.886893	-0.25464	0.799591	-6.99866	0.656089	0.71258
Non.inflammatory.Macrophages	PBLD2	0.101972	0.872415	0.254632	0.799598	-5.57861	0.943385	0.842386
Non.inflammatory.Macrophages	INTS4	-0.0406	3.920928	-0.25446	0.799727	-6.33616	0.786391	0.773914
Non.inflammatory.Macrophages	EMP3	-0.02581	7.421524	-0.25433	0.799833	-7.13421	0.635001	0.702093
Non.inflammatory.Macrophages	NF2	-0.03144	4.781261	-0.25424	0.799899	-6.62597	0.746301	0.755665
Non.inflammatory.Macrophages	CXCL10	-0.06429	4.788598	-0.25374	0.800287	-6.84747	0.746263	0.755603

Non.inflammatory.Macrophages	SCARF1	-0.0688	1.587665	-0.25357	0.800419	-5.99106	0.905997	0.825984
Non.inflammatory.Macrophages	HEG1	0.03163	5.971346	0.253045	0.800821	-6.87389	0.694608	0.731235
Non.inflammatory.Macrophages	GM2245	0.070954	1.558462	0.252857	0.800966	-6.02293	0.907997	0.826855
Non.inflammatory.Macrophages	COMMD3	0.023222	5.85015	0.252824	0.800991	-6.91226	0.6998	0.73378
Non.inflammatory.Macrophages	ACSL3	-0.0437	3.828777	-0.25265	0.801128	-6.41522	0.791633	0.776291
Non.inflammatory.Macrophages	KYAT1	-0.0869	1.503706	-0.25243	0.801295	-5.73665	0.911097	0.828232
Non.inflammatory.Macrophages	JAM2	0.113895	1.449339	0.252426	0.801298	-5.75561	0.914077	0.829485
Non.inflammatory.Macrophages	MICAL2	0.072026	1.966497	0.252204	0.801469	-6.07746	0.886191	0.817654
Non.inflammatory.Macrophages	TCRG-C1	-0.19251	0.880703	-0.25186	0.801734	-5.38607	0.944315	0.842746
Non.inflammatory.Macrophages	D5ERTD57!	0.027034	5.221315	0.251852	0.80174	-6.77288	0.727402	0.746891
Non.inflammatory.Macrophages	CRLS1	-0.03394	3.622878	-0.25181	0.801771	-6.42048	0.801741	0.780876
Non.inflammatory.Macrophages	ZFP59	-0.12537	0.230401	-0.25181	0.801771	-5.48413	0.956725	0.858105
Non.inflammatory.Macrophages	WBP2	0.026054	5.599979	0.251623	0.801916	-6.83045	0.710798	0.739103
Non.inflammatory.Macrophages	SOX7	0.116753	0.442832	0.251592	0.801941	-5.53836	0.956758	0.853115
Non.inflammatory.Macrophages	GFRA2	-0.05483	2.545769	-0.25146	0.802039	-6.28979	0.855818	0.804688

Non.inflammatory.Macrophages	HSPA13	0.035246	3.082179	0.251398	0.80209	-6.36794	0.828496	0.792769
Non.inflammatory.Macrophages	ZFP410	-0.03609	4.138179	-0.25094	0.802442	-6.38907	0.777335	0.76983
Non.inflammatory.Macrophages	RNF44	0.027715	4.833037	0.250751	0.802589	-6.68739	0.745201	0.755103
Non.inflammatory.Macrophages	ZKSCAN5	-0.05478	2.402977	-0.25055	0.80274	-6.11114	0.863649	0.807931
Non.inflammatory.Macrophages	RUVBL2	0.043116	3.693884	0.250395	0.802863	-6.37129	0.798716	0.779465
Non.inflammatory.Macrophages	ARL4A	-0.03423	4.453783	-0.25035	0.802898	-6.5259	0.762651	0.763177
Non.inflammatory.Macrophages	TLR7	0.042519	2.963787	0.250241	0.802982	-6.60929	0.834862	0.795529
Non.inflammatory.Macrophages	OVCA2	0.121248	0.174482	0.250212	0.803004	-5.55672	0.957233	0.859653
Non.inflammatory.Macrophages	B3GNT5	0.112292	2.546338	0.250158	0.803046	-5.60154	0.856205	0.80482
Non.inflammatory.Macrophages	AMOT	-0.11227	0.863491	-0.25003	0.803144	-5.56156	0.94547	0.843363
Non.inflammatory.Macrophages	PBK	-0.07409	3.69023	-0.24972	0.803383	-6.14476	0.799088	0.779715
Non.inflammatory.Macrophages	MGMT	-0.04832	3.273792	-0.2494	0.803631	-6.3322	0.819713	0.788861
Non.inflammatory.Macrophages	ZFP873	0.088389	0.995152	0.249305	0.803703	-5.62407	0.940403	0.840492
Non.inflammatory.Macrophages	OCEL1	-0.03476	3.584489	-0.24881	0.804082	-6.46432	0.804655	0.78222
Non.inflammatory.Macrophages	RAB43	0.025695	6.948549	0.248808	0.804087	-7.12573	0.655316	0.712291

Non.inflammatory.Macrophages	2610306M	0.098967	0.680103	0.248715	0.804158	-5.69848	0.95283	0.848014
Non.inflammatory.Macrophages	9130019O2	-0.12956	0.355077	-0.24848	0.804343	-5.5072	0.958027	0.855738
Non.inflammatory.Macrophages	OLFR1259	-0.18588	-1.34501	-0.24838	0.804415	-5.24752	0.958027	0.897057
Non.inflammatory.Macrophages	AASDHPPT	0.03467	3.841771	0.248338	0.804449	-6.4174	0.792231	0.77668
Non.inflammatory.Macrophages	TENM4	0.111317	1.760128	0.248272	0.8045	-5.73065	0.898514	0.822982
Non.inflammatory.Macrophages	RECK	0.152933	1.480341	0.248065	0.80466	-5.45881	0.913756	0.829494
Non.inflammatory.Macrophages	MARVELD2	-0.08626	1.369704	-0.24802	0.80469	-5.82259	0.919848	0.832058
Non.inflammatory.Macrophages	METRNL	0.039213	3.205494	0.248008	0.804703	-6.76898	0.823413	0.790647
Non.inflammatory.Macrophages	BCL2L2	-0.05817	1.276497	-0.24764	0.804987	-5.98606	0.925254	0.834303
Non.inflammatory.Macrophages	2810402E2	-0.05204	2.412627	-0.24672	0.805698	-6.06924	0.864243	0.8085
Non.inflammatory.Macrophages	GM15345	0.111054	2.98417	0.24661	0.805782	-5.82455	0.834892	0.795748
Non.inflammatory.Macrophages	ERP27	-0.07805	1.896721	-0.24652	0.80585	-5.83809	0.891549	0.820182
Non.inflammatory.Macrophages	POLRMT	-0.0701	1.91437	-0.24646	0.805895	-5.88447	0.890602	0.81978
Non.inflammatory.Macrophages	NDUFA10	-0.0215	6.050526	-0.2464	0.805944	-6.91371	0.692708	0.730647
Non.inflammatory.Macrophages	PSMG1	0.03773	3.325411	0.246316	0.806008	-6.31268	0.81781	0.788228

Non.inflammatory.Macrophages	STIP1	-0.02884	5.419076	-0.24629	0.806029	-6.76795	0.719967	0.743605
Non.inflammatory.Macrophages	HIST4H4	-0.07163	1.887782	-0.24613	0.806151	-6.00193	0.892029	0.820411
Non.inflammatory.Macrophages	MTPAP	-0.0326	4.284578	-0.24611	0.806165	-6.57489	0.771526	0.767488
Non.inflammatory.Macrophages	ZFP874A	0.064752	2.146516	0.246099	0.806176	-5.93286	0.878231	0.81453
Non.inflammatory.Macrophages	MAP2K3OS	-0.10279	-0.12812	-0.24607	0.806199	-5.79435	0.958449	0.86766
Non.inflammatory.Macrophages	GPRASP1	-0.0346	3.93667	-0.24605	0.806213	-6.40859	0.788023	0.774956
Non.inflammatory.Macrophages	SMARCD2	-0.02269	5.611886	-0.24593	0.806309	-6.73012	0.711537	0.739686
Non.inflammatory.Macrophages	ATG9A	-0.03309	3.85849	-0.24592	0.806311	-6.50857	0.791776	0.776686
Non.inflammatory.Macrophages	H2-EB2	-0.14955	0.547733	-0.24576	0.806435	-5.37271	0.957039	0.851627
Non.inflammatory.Macrophages	FAM168B	-0.01784	6.280422	-0.24569	0.806489	-6.93611	0.683032	0.726086
Non.inflammatory.Macrophages	PRORP	-0.03371	3.853039	-0.24561	0.806552	-6.43635	0.792038	0.776869
Non.inflammatory.Macrophages	2210016L2	-0.03546	3.97848	-0.24549	0.806648	-6.51846	0.786023	0.774171
Non.inflammatory.Macrophages	EIF2B2	-0.02939	4.936837	-0.24546	0.80667	-6.68598	0.741471	0.753791
Non.inflammatory.Macrophages	METTL3	-0.03975	3.319562	-0.24544	0.806683	-6.27757	0.8181	0.788498
Non.inflammatory.Macrophages	SP100	-0.03066	7.43248	-0.24532	0.806779	-7.09978	0.636476	0.703209

Non.inflammatory.Macrophages	RDH11	-0.04706	3.215453	-0.24529	0.806804	-6.17495	0.823279	0.790802
Non.inflammatory.Macrophages	ARHGEF7	-0.0331	4.675352	-0.2452	0.806868	-6.61866	0.753384	0.759339
Non.inflammatory.Macrophages	ORC4	0.024434	5.046376	0.245117	0.806934	-6.67245	0.736533	0.751542
Non.inflammatory.Macrophages	PSMG3	0.042812	3.202758	0.244907	0.807096	-6.33829	0.823912	0.79116
Non.inflammatory.Macrophages	OFD1	-0.03908	3.31561	-0.24482	0.807163	-6.22572	0.818296	0.788701
Non.inflammatory.Macrophages	POP4	0.037874	4.085123	0.244657	0.807289	-6.47938	0.780944	0.771999
Non.inflammatory.Macrophages	DLD	0.025196	4.599821	0.244612	0.807324	-6.63038	0.756859	0.761018
Non.inflammatory.Macrophages	PRMT1	-0.0248	5.803475	-0.24459	0.807343	-6.79805	0.703253	0.735939
Non.inflammatory.Macrophages	UQCRFS1	0.021873	7.164384	0.244546	0.807375	-7.07449	0.647028	0.708577
Non.inflammatory.Macrophages	TYW5	0.054829	3.139549	0.244498	0.807411	-6.11143	0.827074	0.792594
Non.inflammatory.Macrophages	DYNLL1	-0.01846	7.867224	-0.24433	0.807541	-7.19968	0.619722	0.694891
Non.inflammatory.Macrophages	AU041133	-0.07838	1.264215	-0.24432	0.807548	-5.72178	0.926098	0.83506
Non.inflammatory.Macrophages	YIPF2	-0.06331	1.926928	-0.2443	0.807561	-5.93818	0.889929	0.819819
Non.inflammatory.Macrophages	E2F5	0.042155	2.69996	0.244245	0.807607	-6.23675	0.849371	0.802392
Non.inflammatory.Macrophages	SERPINA3N	-0.06355	2.557516	-0.24381	0.807945	-6.01391	0.856983	0.805683

Non.inflammatory.Macrophages	CTSH	0.02634	5.779307	0.243733	0.808002	-7.09975	0.704514	0.736582
Non.inflammatory.Macrophages	CCDC180	-0.09691	1.286108	-0.24357	0.808129	-5.69143	0.925237	0.834672
Non.inflammatory.Macrophages	NPRL2	0.047499	2.790262	0.243286	0.808347	-6.11136	0.845207	0.800521
Non.inflammatory.Macrophages	MB21D2	-0.11787	0.883088	-0.24321	0.808403	-5.57616	0.946447	0.844109
Non.inflammatory.Macrophages	ACTR8	-0.03659	3.537225	-0.24299	0.808579	-6.39272	0.807918	0.784129
Non.inflammatory.Macrophages	FRG2F1	0.083726	0.702787	0.242431	0.809008	-5.65359	0.953467	0.848568
Non.inflammatory.Macrophages	TGFB1	-0.01905	8.800362	-0.24243	0.809011	-7.31938	0.585811	0.677384
Non.inflammatory.Macrophages	SLC30A7	0.025078	5.62501	0.2423	0.809109	-6.87493	0.71172	0.73998
Non.inflammatory.Macrophages	CPLANE2	-0.0967	0.331465	-0.2419	0.809421	-5.67613	0.959465	0.857492
Non.inflammatory.Macrophages	MED21	0.030741	5.21874	0.241772	0.809517	-6.77762	0.7296	0.748541
Non.inflammatory.Macrophages	SUMF1	0.029497	4.240634	0.241631	0.809626	-6.69879	0.774412	0.769234
Non.inflammatory.Macrophages	PPCS	0.044953	2.732539	0.241562	0.809679	-6.15065	0.848598	0.802202
Non.inflammatory.Macrophages	NFKB2	-0.03299	5.096214	-0.24156	0.809683	-6.78418	0.735075	0.751129
Non.inflammatory.Macrophages	NCS1	-0.10622	0.210797	-0.24156	0.809683	-5.63217	0.959465	0.860439
Non.inflammatory.Macrophages	COMMD2	-0.02509	4.927522	-0.24148	0.809743	-6.68637	0.742678	0.754665

Non.inflammatory.Macrophages	GM20139	0.11444	-0.63311	0.241445	0.809769	-5.41647	0.959465	0.880822
Non.inflammatory.Macrophages	BICC1	-0.12653	0.332453	-0.24133	0.809861	-5.55623	0.959465	0.857565
Non.inflammatory.Macrophages	TXNRD1	-0.02326	5.871273	-0.24127	0.809905	-7.04537	0.701087	0.735136
Non.inflammatory.Macrophages	TULP3	0.081029	1.974251	0.241259	0.809913	-5.74632	0.888336	0.819345
Non.inflammatory.Macrophages	BRCA2	0.054649	3.555566	0.241103	0.810034	-6.21173	0.807328	0.784151
Non.inflammatory.Macrophages	RWDD2A	-0.09463	0.48641	-0.24108	0.81005	-5.66727	0.959465	0.854
Non.inflammatory.Macrophages	BCAS2	0.017747	6.380106	0.241026	0.810093	-6.94869	0.679597	0.724861
Non.inflammatory.Macrophages	GM16201	-0.09646	1.818935	-0.24093	0.81017	-5.69674	0.896689	0.822996
Non.inflammatory.Macrophages	RFXAP	-0.02937	4.179927	-0.24047	0.810524	-6.47562	0.777554	0.770707
Non.inflammatory.Macrophages	2510002D2	0.045569	3.199654	0.240148	0.810771	-6.2283	0.825332	0.792022
Non.inflammatory.Macrophages	4933423P2	0.066959	1.804113	0.24013	0.810785	-5.88765	0.897908	0.823368
Non.inflammatory.Macrophages	SGF29	0.026748	4.671195	0.239961	0.810916	-6.60016	0.754731	0.760239
Non.inflammatory.Macrophages	RAD51AP1	0.074563	3.61991	0.239902	0.810961	-6.20284	0.804563	0.782814
Non.inflammatory.Macrophages	MFNG	-0.04783	3.090104	-0.23982	0.811026	-6.1022	0.830827	0.794472
Non.inflammatory.Macrophages	WWTR1	-0.08674	2.126919	-0.23972	0.8111	-5.87471	0.880618	0.816071

Non.inflammatory.Macrophages	GM10762	-0.05556	2.549426	-0.2397	0.811116	-6.016	0.858448	0.80654
Non.inflammatory.Macrophages	TCTN2	-0.09899	-0.37598	-0.2392	0.811506	-5.57485	0.960297	0.874995
Non.inflammatory.Macrophages	ATP5G2	0.017468	8.844228	0.239004	0.811656	-7.38969	0.584806	0.677048
Non.inflammatory.Macrophages	THOP1	0.067119	2.236401	0.238922	0.811719	-5.96632	0.875244	0.81375
Non.inflammatory.Macrophages	GM4890	-0.09853	0.373848	-0.23879	0.811824	-5.55045	0.960422	0.856998
Non.inflammatory.Macrophages	BORA	-0.05268	3.535011	-0.23858	0.811982	-6.10186	0.809228	0.784913
Non.inflammatory.Macrophages	ATP10A	0.083153	3.430551	0.238283	0.812213	-5.82312	0.814477	0.787198
Non.inflammatory.Macrophages	DGKE	-0.03636	4.880229	-0.23819	0.812284	-6.56039	0.745741	0.756066
Non.inflammatory.Macrophages	AAAS	0.045553	3.794127	0.238173	0.812298	-6.27866	0.7967	0.779273
Non.inflammatory.Macrophages	BAZ1A	0.020177	7.694958	0.237852	0.812546	-7.14225	0.627881	0.699179
Non.inflammatory.Macrophages	RAD23A	-0.02476	5.807004	-0.23758	0.812759	-6.93601	0.704948	0.736926
Non.inflammatory.Macrophages	PCCA	0.02999	4.599387	0.237472	0.81284	-6.71522	0.758867	0.762123
Non.inflammatory.Macrophages	AGA	0.037916	3.27288	0.23745	0.812857	-6.44029	0.822574	0.790786
Non.inflammatory.Macrophages	SDHAF3	-0.04109	3.044533	-0.23736	0.812923	-6.23105	0.834032	0.795829
Non.inflammatory.Macrophages	TWSG1	0.054268	3.230079	0.237159	0.813082	-6.12397	0.824799	0.791795

Non.inflammatory.Macrophages	GRASP	-0.0583	3.781204	-0.23693	0.813261	-6.1273	0.79768	0.779744
Non.inflammatory.Macrophages	CHORDC1	-0.02451	5.082056	-0.23682	0.813345	-6.68696	0.736945	0.752014
Non.inflammatory.Macrophages	PIP5K1B	0.045309	5.568352	0.236792	0.813366	-6.48312	0.715386	0.741901
Non.inflammatory.Macrophages	CRYBG1	-0.0316	4.169377	-0.23679	0.81337	-6.69829	0.77908	0.771365
Non.inflammatory.Macrophages	ZCCHC8	0.024804	5.108019	0.236479	0.813608	-6.67126	0.73593	0.751571
Non.inflammatory.Macrophages	EFCAB7	-0.10981	0.535523	-0.23627	0.813771	-5.44582	0.960265	0.853568
Non.inflammatory.Macrophages	GINS1	0.05421	3.197409	0.235524	0.814346	-6.27489	0.826997	0.792858
Non.inflammatory.Macrophages	LATS2	0.023223	5.889771	0.235522	0.814349	-6.83141	0.701944	0.73561
Non.inflammatory.Macrophages	ATXN1	0.026214	6.919224	0.235501	0.814365	-7.17825	0.65907	0.714826
Non.inflammatory.Macrophages	E130317F2	-0.08111	0.77534	-0.23546	0.814393	-5.6471	0.953084	0.848086
Non.inflammatory.Macrophages	SLC9A6	0.041438	2.81804	0.235403	0.814441	-6.21564	0.84621	0.801273
Non.inflammatory.Macrophages	ALMS1	0.038118	3.824261	0.23534	0.814489	-6.45575	0.796136	0.77915
Non.inflammatory.Macrophages	LRRC8D	-0.0238	7.366735	-0.23501	0.814746	-7.10829	0.641286	0.70605
Non.inflammatory.Macrophages	PRICKLE3	-0.07478	2.034653	-0.23497	0.814774	-5.76969	0.887268	0.81901
Non.inflammatory.Macrophages	LEF1OS1	0.146012	-0.45613	0.234917	0.814816	-5.20709	0.961805	0.877673

Non.inflammatory.Macrophages	MEF2B	-0.04337	3.605786	-0.23488	0.814841	-6.24416	0.806835	0.78399
Non.inflammatory.Macrophages	LDB1	0.03148	4.482765	0.234347	0.815257	-6.58352	0.765149	0.765184
Non.inflammatory.Macrophages	TIMM22	0.028031	4.4471	0.234212	0.815362	-6.56329	0.766813	0.765973
Non.inflammatory.Macrophages	PEX16	0.034734	3.474463	0.234143	0.815416	-6.3696	0.813508	0.787023
Non.inflammatory.Macrophages	GGACT	-0.03952	3.170013	-0.23406	0.815476	-6.19154	0.828662	0.793719
Non.inflammatory.Macrophages	MRPS18B	0.031781	3.873974	0.234046	0.81549	-6.50964	0.794015	0.778321
Non.inflammatory.Macrophages	4930453N2	-0.03041	4.481233	-0.23402	0.81551	-6.51692	0.765221	0.765276
Non.inflammatory.Macrophages	GM43728	-0.07561	-0.48628	-0.23396	0.815558	-5.77441	0.962064	0.878611
Non.inflammatory.Macrophages	NFYC	-0.01742	6.202043	-0.23366	0.815787	-6.85182	0.688909	0.729542
Non.inflammatory.Macrophages	USP10	0.033087	4.567936	0.233645	0.815801	-6.5564	0.761195	0.763499
Non.inflammatory.Macrophages	GM37529	-0.13008	1.962925	-0.23352	0.815894	-5.47118	0.891356	0.820933
Non.inflammatory.Macrophages	HES6	-0.03752	4.105884	-0.23351	0.815908	-6.34281	0.782905	0.773425
Non.inflammatory.Macrophages	PHF3	0.018297	6.980017	0.233261	0.816098	-7.01774	0.656854	0.714014
Non.inflammatory.Macrophages	A430033K0	-0.10978	0.6406	-0.23324	0.816111	-5.57203	0.95836	0.851739
Non.inflammatory.Macrophages	VPS72	-0.02873	4.357985	-0.23323	0.816122	-6.51838	0.770988	0.768085

Non.inflammatory.Macrophages	YIPF6	-0.03882	3.457527	-0.23314	0.816188	-6.37759	0.814349	0.787601
Non.inflammatory.Macrophages	DHX37	-0.05084	2.67509	-0.23308	0.81624	-6.0761	0.853862	0.804958
Non.inflammatory.Macrophages	TSPAN17	-0.05501	1.115501	-0.23303	0.816273	-6.04914	0.937925	0.840627
Non.inflammatory.Macrophages	CDADC1	-0.0402	4.609962	-0.23237	0.816786	-6.51714	0.759474	0.762844
Non.inflammatory.Macrophages	C2CD2	-0.03937	3.079676	-0.23228	0.81686	-6.35544	0.833461	0.796035
Non.inflammatory.Macrophages	MYD88	0.029313	4.609439	0.232206	0.816915	-6.69022	0.759498	0.762855
Non.inflammatory.Macrophages	RASD1	-0.08224	3.88428	-0.23218	0.816937	-5.95014	0.793757	0.778413
Non.inflammatory.Macrophages	SKINT3	0.058488	0.115469	0.232123	0.816979	-6.08708	0.962355	0.864417
Non.inflammatory.Macrophages	WDR92	-0.04708	3.083733	-0.23199	0.817081	-6.25117	0.833256	0.796011
Non.inflammatory.Macrophages	SMPDL3B	0.058382	1.9506	0.231877	0.817169	-6.44064	0.892282	0.821508
Non.inflammatory.Macrophages	PLK4	-0.0555	3.856529	-0.23174	0.817276	-6.24293	0.795097	0.779101
Non.inflammatory.Macrophages	XAB2	0.036366	3.922858	0.231523	0.817443	-6.43023	0.791899	0.77769
Non.inflammatory.Macrophages	DBR1	-0.04372	3.262038	-0.23149	0.817466	-6.20728	0.824303	0.792124
Non.inflammatory.Macrophages	USPL1	-0.03478	3.533846	-0.23149	0.817467	-6.37267	0.810828	0.786155
Non.inflammatory.Macrophages	KLRA2	0.041929	2.524963	0.231412	0.81753	-6.81603	0.861898	0.808534

Non.inflammatory.Macrophages	VPS45	0.034839	3.758422	0.231323	0.817598	-6.4381	0.799849	0.781267
Non.inflammatory.Macrophages	BST2	-0.03306	6.606914	-0.23129	0.817622	-7.09143	0.672242	0.72172
Non.inflammatory.Macrophages	CEP78	0.045895	3.001386	0.231141	0.817739	-6.09317	0.837421	0.797994
Non.inflammatory.Macrophages	GM21860	0.122603	-0.14186	0.231112	0.817762	-5.67879	0.962355	0.870846
Non.inflammatory.Macrophages	TMEM177	0.10562	0.336385	0.231011	0.81784	-5.56545	0.962355	0.859377
Non.inflammatory.Macrophages	DAPK3	-0.02549	4.42231	-0.23098	0.817868	-6.62976	0.768203	0.767079
Non.inflammatory.Macrophages	GM26724	0.101131	1.517501	0.230879	0.817942	-5.57053	0.915836	0.831639
Non.inflammatory.Macrophages	4732471J0	-0.05102	1.996323	-0.23087	0.817949	-6.15231	0.889828	0.820642
Non.inflammatory.Macrophages	A530032D1	0.108737	0.594014	0.230793	0.818009	-5.56864	0.959805	0.853257
Non.inflammatory.Macrophages	SNW1	-0.01732	6.240739	-0.23066	0.818109	-6.91453	0.687508	0.729234
Non.inflammatory.Macrophages	CNPY2	0.027688	4.719728	0.230482	0.818249	-6.72549	0.754502	0.760812
Non.inflammatory.Macrophages	GMIP	-0.02049	5.964525	-0.23025	0.818433	-6.89227	0.69938	0.734938
Non.inflammatory.Macrophages	SMC1B	0.095523	-0.03473	0.230075	0.818565	-5.763	0.962634	0.868454
Non.inflammatory.Macrophages	CAPN1	0.034274	4.246316	0.230002	0.818622	-6.57901	0.776701	0.771005
Non.inflammatory.Macrophages	C79798	-0.0759	1.77973	-0.22986	0.818733	-5.74696	0.90177	0.825769

Non.inflammatory.Macrophages	SLC16A4	0.082814	1.428027	0.229844	0.818744	-5.65202	0.92104	0.833881
Non.inflammatory.Macrophages	RMDN2	0.066564	1.873328	0.228853	0.819511	-5.92727	0.897401	0.823768
Non.inflammatory.Macrophages	ZFP975	0.110137	0.810489	0.228838	0.819523	-5.65398	0.953443	0.848461
Non.inflammatory.Macrophages	PLK2	0.040082	5.006155	0.228571	0.819731	-6.78231	0.742267	0.755043
Non.inflammatory.Macrophages	MRPL24	0.019313	5.658173	0.228214	0.820007	-6.83715	0.713476	0.741523
Non.inflammatory.Macrophages	ZFP629	0.085102	0.926	0.228056	0.820129	-5.76429	0.949637	0.845862
Non.inflammatory.Macrophages	TIPIN	0.045645	5.238466	0.228031	0.820149	-6.58334	0.732002	0.750239
Non.inflammatory.Macrophages	TRF	-0.04716	9.658999	-0.22793	0.820226	-7.55284	0.558215	0.663389
Non.inflammatory.Macrophages	TEN1	-0.0201	5.852604	-0.22757	0.820504	-6.86771	0.705123	0.737561
Non.inflammatory.Macrophages	PDPR	0.03997	3.937711	0.227418	0.820624	-6.39415	0.792445	0.777959
Non.inflammatory.Macrophages	GM27253	0.11589	1.3478	0.227216	0.82078	-5.4137	0.926693	0.836068
Non.inflammatory.Macrophages	CTC1	-0.03338	3.25694	-0.22715	0.820829	-6.35891	0.825872	0.792838
Non.inflammatory.Macrophages	ITGB1BP1	0.025141	4.649919	0.227119	0.820855	-6.63685	0.758834	0.762693
Non.inflammatory.Macrophages	NOL11	-0.03038	4.89374	-0.22711	0.820864	-6.68575	0.74764	0.757533
Non.inflammatory.Macrophages	MED24	-0.03757	3.187102	-0.22707	0.820891	-6.27162	0.829374	0.794388

Non.inflammatory.Macrophages	CBLL1	-0.02602	4.917555	-0.22703	0.820925	-6.61311	0.746556	0.757045
Non.inflammatory.Macrophages	TAF8	0.044494	3.254648	0.226997	0.82095	-6.18845	0.825986	0.792908
Non.inflammatory.Macrophages	TMEM185I	-0.03093	4.12483	-0.22675	0.821144	-6.51191	0.783592	0.773992
Non.inflammatory.Macrophages	MAN2C1O:	0.036803	4.443794	0.226597	0.82126	-6.39441	0.76853	0.767192
Non.inflammatory.Macrophages	EIF2S1	0.020288	6.052768	0.22648	0.821351	-6.91763	0.696643	0.733639
Non.inflammatory.Macrophages	9230116N1	-0.07915	0.920334	-0.22641	0.821403	-5.75647	0.95008	0.846254
Non.inflammatory.Macrophages	TM6SF1	0.02222	6.252773	0.226387	0.821423	-7.09297	0.688169	0.729574
Non.inflammatory.Macrophages	NPC2	-0.01846	7.639426	-0.22623	0.821541	-7.33864	0.632139	0.701989
Non.inflammatory.Macrophages	GM43331	-0.0525	2.057137	-0.22614	0.821616	-5.96739	0.888166	0.820014
Non.inflammatory.Macrophages	DDX51	0.076832	1.37149	0.225612	0.822024	-5.75594	0.92576	0.835905
Non.inflammatory.Macrophages	ADGRL1	0.060255	2.476047	0.225572	0.822055	-5.92046	0.866185	0.810637
Non.inflammatory.Macrophages	AXIN2	-0.12734	-0.02376	-0.22539	0.8222	-5.3257	0.964288	0.868982
Non.inflammatory.Macrophages	HK3	-0.04609	2.677613	-0.22535	0.822229	-6.63453	0.8557	0.806142
Non.inflammatory.Macrophages	ARMC2	-0.09535	-0.1641	-0.22529	0.822275	-5.56989	0.964288	0.872373
Non.inflammatory.Macrophages	GM15417	0.036461	2.477041	0.225134	0.822394	-6.43326	0.866133	0.810675

Non.inflammatory.Macrophages	CLN6	-0.03084	3.482895	-0.22513	0.822397	-6.59763	0.814972	0.788303
Non.inflammatory.Macrophages	SLC22A15	0.070892	3.077716	0.225072	0.822443	-5.77479	0.835234	0.797241
Non.inflammatory.Macrophages	PLCL2	0.02249	6.985417	0.225043	0.822465	-7.01823	0.658151	0.71507
Non.inflammatory.Macrophages	SCNM1	-0.03268	3.621272	-0.225	0.822502	-6.41575	0.808157	0.785277
Non.inflammatory.Macrophages	IGIP	-0.07986	0.675003	-0.22483	0.822628	-5.75348	0.959367	0.852382
Non.inflammatory.Macrophages	CYP2AB1	0.078765	-0.30874	0.224679	0.822747	-5.86643	0.964408	0.87599
Non.inflammatory.Macrophages	CYB5R1	-0.02728	3.659051	-0.22426	0.823074	-6.6348	0.80649	0.78459
Non.inflammatory.Macrophages	PWP1	0.033281	3.903059	0.224221	0.823103	-6.39174	0.794625	0.77928
Non.inflammatory.Macrophages	GAS6	-0.06779	1.545619	-0.22421	0.823111	-5.96741	0.916335	0.832093
Non.inflammatory.Macrophages	ATP6V0B	-0.01435	8.046898	-0.2242	0.823117	-7.31596	0.616798	0.694377
Non.inflammatory.Macrophages	TSPAN15	0.038403	2.388192	0.223998	0.823276	-6.37612	0.871083	0.812861
Non.inflammatory.Macrophages	4930484I0	-0.09423	1.660784	-0.22379	0.82344	-5.55069	0.910213	0.829488
Non.inflammatory.Macrophages	ENTPD1	-0.02514	6.9129	-0.22359	0.823591	-7.21296	0.661391	0.716722
Non.inflammatory.Macrophages	PTPRM	-0.04182	5.186994	-0.22358	0.823598	-6.93848	0.735032	0.752001
Non.inflammatory.Macrophages	ABCA13	0.127097	0.73813	0.223392	0.823746	-5.66432	0.957506	0.851084

Non.inflammatory.Macrophages	GM15563	0.083195	1.923249	0.223218	0.823881	-5.78039	0.896035	0.823552
Non.inflammatory.Macrophages	TCF19	0.067106	3.199871	0.223081	0.823987	-6.02609	0.829528	0.794827
Non.inflammatory.Macrophages	HYLS1	0.055813	3.183514	0.223055	0.824007	-5.9432	0.83035	0.795188
Non.inflammatory.Macrophages	IFI213	-0.06151	3.327221	-0.22304	0.824019	-6.61179	0.82315	0.792015
Non.inflammatory.Macrophages	COG8	0.024398	4.299473	0.222715	0.824271	-6.58639	0.77598	0.770882
Non.inflammatory.Macrophages	ZFP992	0.030463	3.563813	0.222622	0.824343	-6.61314	0.811458	0.786839
Non.inflammatory.Macrophages	CTR9	-0.02515	4.518969	-0.22251	0.824429	-6.57921	0.76568	0.766196
Non.inflammatory.Macrophages	GLIS1	0.113831	0.394465	0.222447	0.824479	-5.53285	0.964856	0.859338
Non.inflammatory.Macrophages	PIP4P2	0.032926	3.706325	0.222372	0.824537	-6.55567	0.804469	0.783773
Non.inflammatory.Macrophages	PSMB1	-0.01801	7.299863	-0.22226	0.824627	-7.09553	0.645964	0.709177
Non.inflammatory.Macrophages	PDK4	-0.08356	1.417008	-0.22219	0.824678	-5.78668	0.923777	0.835339
Non.inflammatory.Macrophages	TOR1AIP1	0.019015	7.42657	0.222187	0.824681	-7.15128	0.640963	0.706696
Non.inflammatory.Macrophages	STAU1	0.021578	6.034578	0.221859	0.824936	-6.88356	0.69802	0.734727
Non.inflammatory.Macrophages	RIOK1	-0.0217	6.007255	-0.22182	0.824967	-6.79052	0.699187	0.735289
Non.inflammatory.Macrophages	PKD2	0.070073	0.719693	0.221774	0.825001	-5.75731	0.958226	0.851809

Non.inflammatory.Macrophages	FICD	0.081914	0.917558	0.221768	0.825006	-5.72723	0.951	0.847141
Non.inflammatory.Macrophages	NIP7	-0.0231	4.635807	-0.22171	0.825052	-6.61923	0.76025	0.763906
Non.inflammatory.Macrophages	SENP5	0.021847	5.992571	0.221596	0.82514	-6.89177	0.699815	0.735589
Non.inflammatory.Macrophages	PRSS30	-0.08776	-0.33737	-0.22158	0.825152	-5.52109	0.964856	0.877173
Non.inflammatory.Macrophages	ZMIZ1OS1	-0.11509	0.352543	-0.22151	0.825207	-5.56709	0.964856	0.86054
Non.inflammatory.Macrophages	NET1	0.055266	4.035875	0.221431	0.825268	-6.11795	0.788522	0.776775
Non.inflammatory.Macrophages	FRG1	-0.01839	6.660706	-0.22065	0.825872	-6.95052	0.671984	0.722212
Non.inflammatory.Macrophages	5930430L0	0.124676	0.229599	0.220611	0.825904	-5.38912	0.96516	0.863706
Non.inflammatory.Macrophages	LLGL1	-0.04022	2.998446	-0.22056	0.82594	-6.22769	0.840011	0.799731
Non.inflammatory.Macrophages	ZFP91	-0.01664	6.894237	-0.22052	0.825973	-7.04536	0.662437	0.717542
Non.inflammatory.Macrophages	POP5	-0.02264	4.525116	-0.22048	0.826009	-6.60373	0.765634	0.766489
Non.inflammatory.Macrophages	JMJD7	-0.05847	1.757778	-0.22046	0.826025	-5.90799	0.905331	0.82784
Non.inflammatory.Macrophages	CAMP	0.076317	4.463959	0.220296	0.826149	-6.49639	0.768491	0.767834
Non.inflammatory.Macrophages	BIRC2	0.024388	5.673482	0.220237	0.826195	-6.78762	0.713827	0.74241
Non.inflammatory.Macrophages	PDE7B	-0.04155	5.37371	-0.22004	0.826348	-7.09394	0.727019	0.748657

Non.inflammatory.Macrophages	BBOF1	-0.10008	1.302277	-0.22002	0.82636	-5.49999	0.930455	0.838452
Non.inflammatory.Macrophages	CDON	0.105177	1.952332	0.219983	0.826392	-5.54362	0.894789	0.823438
Non.inflammatory.Macrophages	FCF1	0.019243	6.136138	0.219907	0.82645	-6.88592	0.693915	0.732933
Non.inflammatory.Macrophages	SHROOM4	0.092059	0.86592	0.219696	0.826614	-5.8085	0.953179	0.848684
Non.inflammatory.Macrophages	EXOSC10	-0.02643	4.93082	-0.21966	0.82664	-6.65161	0.746936	0.75795
Non.inflammatory.Macrophages	PMF1	0.026511	5.697613	0.219586	0.8267	-6.88965	0.712775	0.741947
Non.inflammatory.Macrophages	MAP11	0.037842	2.866952	0.219539	0.826736	-6.36467	0.846723	0.802773
Non.inflammatory.Macrophages	PRAM1	0.033966	3.279629	0.219376	0.826863	-6.40433	0.825825	0.793659
Non.inflammatory.Macrophages	BOD1L	-0.01907	5.740505	-0.21936	0.826878	-6.824	0.710909	0.741113
Non.inflammatory.Macrophages	PROS1	0.03638	2.741429	0.219307	0.826916	-6.51017	0.853176	0.80566
Non.inflammatory.Macrophages	ELP2	0.026751	4.144774	0.219259	0.826954	-6.5223	0.783564	0.774824
Non.inflammatory.Macrophages	SLX4	0.054073	1.820472	0.218986	0.827166	-5.87683	0.90202	0.826595
Non.inflammatory.Macrophages	PPP1CA	0.016477	8.233158	0.218959	0.827187	-7.23799	0.610263	0.691485
Non.inflammatory.Macrophages	PPP2R3C	-0.02752	4.333149	-0.21878	0.827328	-6.55815	0.774784	0.770785
Non.inflammatory.Macrophages	PPM1G	-0.0191	6.448565	-0.21869	0.827398	-6.8813	0.680902	0.726708

Non.inflammatory.Macrophages	TMED7	0.015344	6.308978	0.218515	0.827532	-6.97573	0.68675	0.729605
Non.inflammatory.Macrophages	GM37768	0.068512	2.087377	0.218496	0.827546	-5.80345	0.887715	0.820572
Non.inflammatory.Macrophages	GM46218	0.098918	0.58606	0.218109	0.827847	-5.53914	0.963206	0.855684
Non.inflammatory.Macrophages	LRRC59	-0.01986	5.556125	-0.21807	0.827875	-6.82422	0.719269	0.745205
Non.inflammatory.Macrophages	STX17	0.033506	4.034327	0.217915	0.827997	-6.50682	0.789227	0.777466
Non.inflammatory.Macrophages	LYNX1	0.138413	0.898277	0.217683	0.828178	-5.3632	0.952588	0.848389
Non.inflammatory.Macrophages	ARG2	-0.03471	3.174154	-0.21749	0.828327	-6.77393	0.831712	0.796432
Non.inflammatory.Macrophages	MRPS16	-0.0227	5.607422	-0.21726	0.828505	-6.81455	0.717244	0.744311
Non.inflammatory.Macrophages	DHX29	0.035903	3.415036	0.217237	0.828524	-6.30327	0.819679	0.791158
Non.inflammatory.Macrophages	FAM71D	-0.10043	0.095273	-0.21718	0.828565	-5.59267	0.965874	0.867639
Non.inflammatory.Macrophages	TMEM26	0.050574	2.190341	0.21704	0.828677	-6.31393	0.882723	0.818577
Non.inflammatory.Macrophages	AHSA2	-0.03339	3.646243	-0.21697	0.82873	-6.32346	0.808283	0.786085
Non.inflammatory.Macrophages	FUNDC1	-0.02264	4.65588	-0.21673	0.828918	-6.7327	0.760249	0.764302
Non.inflammatory.Macrophages	AW55491[illegible]	-0.02305	5.656196	-0.21648	0.82911	-6.79815	0.715284	0.743366
Non.inflammatory.Macrophages	POU3F1	0.105733	0.061626	0.216466	0.829123	-5.63614	0.966108	0.868515

Non.inflammatory.Macrophages	SUCLA2	0.021033	5.593683	0.215899	0.829564	-6.79328	0.718295	0.744665
Non.inflammatory.Macrophages	ZFP668	-0.03562	3.829078	-0.21582	0.829622	-6.26443	0.799835	0.782158
Non.inflammatory.Macrophages	MANBAL	-0.02075	4.531107	-0.21578	0.829655	-6.68776	0.766399	0.767042
Non.inflammatory.Macrophages	SSH3	0.067073	1.522336	0.215648	0.829759	-5.94206	0.919492	0.83407
Non.inflammatory.Macrophages	NANS	0.027662	4.873876	0.215513	0.829864	-6.68628	0.750557	0.759845
Non.inflammatory.Macrophages	9-Sep	-0.02164	5.640387	-0.21551	0.829867	-6.97701	0.716248	0.743802
Non.inflammatory.Macrophages	TRGV2	0.155391	-0.51519	0.215247	0.83007	-5.30449	0.966543	0.882656
Non.inflammatory.Macrophages	SLC16A13	0.093273	-0.01818	0.215194	0.830112	-5.64525	0.966543	0.870569
Non.inflammatory.Macrophages	TESK1	0.028674	3.781202	0.215087	0.830195	-6.52689	0.802219	0.783313
Non.inflammatory.Macrophages	USP30	0.050095	2.252852	0.215068	0.83021	-5.99172	0.879986	0.817338
Non.inflammatory.Macrophages	DDX10	0.020594	5.436521	0.21497	0.830286	-6.77468	0.725278	0.748037
Non.inflammatory.Macrophages	NR2C1	-0.04657	2.276051	-0.21483	0.830396	-6.19649	0.878773	0.816841
Non.inflammatory.Macrophages	P2RY1	-0.09637	0.13392	-0.21462	0.830559	-5.53013	0.966563	0.866987
Non.inflammatory.Macrophages	ZBTB25	-0.03702	3.709262	-0.21459	0.830582	-6.39034	0.805748	0.78496
Non.inflammatory.Macrophages	NAA20	0.019911	5.164245	0.214548	0.830614	-6.73417	0.737446	0.753804

Non.inflammatory.Macrophages	PTBP1	0.019734	6.221744	0.214494	0.830655	-6.92981	0.691292	0.731934
Non.inflammatory.Macrophages	UBE2F	0.018518	6.306137	0.214121	0.830946	-7.03962	0.687765	0.730244
Non.inflammatory.Macrophages	ZC3H6	-0.04142	3.379852	-0.21407	0.830984	-6.24263	0.822056	0.79222
Non.inflammatory.Macrophages	SLC22A4	0.052956	0.816422	0.214041	0.831008	-6.15926	0.95642	0.850746
Non.inflammatory.Macrophages	SRM	-0.03989	4.842685	-0.21395	0.831078	-6.6586	0.75209	0.760612
Non.inflammatory.Macrophages	KLRA6	-0.14673	-0.71608	-0.21387	0.83114	-5.29569	0.96661	0.887709
Non.inflammatory.Macrophages	LRBA	0.035622	6.139874	0.21386	0.831149	-6.64198	0.694799	0.733633
Non.inflammatory.Macrophages	GM10521	0.083836	0.308847	0.213681	0.831288	-5.60067	0.96661	0.862842
Non.inflammatory.Macrophages	ANKRD40	0.024774	4.238658	0.21364	0.83132	-6.59791	0.780273	0.77353
Non.inflammatory.Macrophages	FES	-0.02567	4.284767	-0.21361	0.831339	-6.7877	0.778087	0.772538
Non.inflammatory.Macrophages	1810041H1	-0.0881	1.76748	-0.21352	0.831416	-5.68301	0.906168	0.828579
Non.inflammatory.Macrophages	LRP8OS2	-0.17306	0.163168	-0.2134	0.83151	-5.24766	0.966642	0.866341
Non.inflammatory.Macrophages	KLRA7	-0.20984	0.646446	-0.21324	0.831632	-5.36656	0.962715	0.854824
Non.inflammatory.Macrophages	ZFP414	0.033221	3.443642	0.213194	0.831666	-6.44042	0.818923	0.790875
Non.inflammatory.Macrophages	CHD1L	0.032317	3.722264	0.213013	0.831807	-6.32655	0.805259	0.784769

Non.inflammatory.Macrophages	POMT1	0.062269	2.21731	0.212738	0.832021	-5.99275	0.882204	0.818326
Non.inflammatory.Macrophages	XBP1	0.018843	6.135195	0.212448	0.832247	-6.96724	0.69533	0.733788
Non.inflammatory.Macrophages	RO60	0.041431	2.493379	0.212368	0.832308	-6.14145	0.867778	0.812081
Non.inflammatory.Macrophages	TOR1B	0.02754	3.704769	0.212183	0.832453	-6.52616	0.806464	0.785235
Non.inflammatory.Macrophages	SCIN	-0.14493	0.090871	-0.21196	0.832629	-5.41577	0.967268	0.868279
Non.inflammatory.Macrophages	LAX1	0.112783	2.319766	0.211857	0.832706	-5.48989	0.877098	0.816156
Non.inflammatory.Macrophages	RALB	0.018267	4.247517	0.211693	0.832834	-6.84346	0.780383	0.773542
Non.inflammatory.Macrophages	TLE1	0.02511	4.060562	0.211653	0.832865	-6.61273	0.789307	0.777578
Non.inflammatory.Macrophages	CIPC	-0.04307	2.655218	-0.21148	0.832997	-6.17473	0.859507	0.808598
Non.inflammatory.Macrophages	GM47167	-0.05904	2.927116	-0.21145	0.833021	-5.86089	0.845489	0.802505
Non.inflammatory.Macrophages	AGBL2	-0.09064	0.26749	-0.21142	0.833048	-5.53587	0.967268	0.864074
Non.inflammatory.Macrophages	TAGAP1	0.063564	2.214287	0.211306	0.833135	-5.95305	0.882713	0.818636
Non.inflammatory.Macrophages	PVT1	0.034242	5.282726	0.211067	0.833321	-6.93469	0.73278	0.75168
Non.inflammatory.Macrophages	1700123M	-0.07753	0.89752	-0.21093	0.833428	-5.69629	0.954297	0.849257
Non.inflammatory.Macrophages	BC024063	-0.09852	0.183577	-0.21074	0.833575	-5.47269	0.967547	0.866279

Non.inflammatory.Macrophages	GGNBP1	0.086501	1.044976	0.210374	0.83386	-5.68559	0.947469	0.845853
Non.inflammatory.Macrophages	ACIN1	0.014471	7.577859	0.210322	0.833901	-7.09315	0.636953	0.705267
Non.inflammatory.Macrophages	CENPK	-0.05662	3.283005	-0.21009	0.834082	-6.0751	0.82799	0.794922
Non.inflammatory.Macrophages	TRAPPC1	-0.02523	5.432947	-0.20999	0.834157	-6.87543	0.726444	0.748748
Non.inflammatory.Macrophages	PRKCI	-0.0402	3.113961	-0.20978	0.83432	-6.27992	0.836608	0.798699
Non.inflammatory.Macrophages	GUSB	-0.01782	5.468903	-0.20939	0.834627	-6.97474	0.724979	0.748127
Non.inflammatory.Macrophages	ZFP354B	0.10776	-0.81695	0.20926	0.834726	-5.23907	0.968063	0.89101
Non.inflammatory.Macrophages	HDAC11	-0.10349	0.5008	-0.20915	0.834811	-5.54312	0.967798	0.85904
Non.inflammatory.Macrophages	PLSCR4	-0.10347	0.494112	-0.20908	0.834869	-5.52684	0.967963	0.8592
Non.inflammatory.Macrophages	REL	0.022058	8.12408	0.209049	0.834891	-7.28646	0.616145	0.694861
Non.inflammatory.Macrophages	PDZD11	0.029506	4.30203	0.209031	0.834905	-6.55947	0.778438	0.77287
Non.inflammatory.Macrophages	STK16	-0.02983	4.37442	-0.20873	0.83514	-6.53882	0.775016	0.771314
Non.inflammatory.Macrophages	ZFP526	0.086296	0.37218	0.208678	0.83518	-5.60265	0.968063	0.862113
Non.inflammatory.Macrophages	SHB	0.031972	4.498582	0.208591	0.835248	-6.63113	0.769179	0.76868
Non.inflammatory.Macrophages	HCFC1	-0.02458	4.949877	-0.20855	0.835277	-6.70711	0.748313	0.759086

Non.inflammatory.Macrophages	RFTN1	-0.02585	6.737622	-0.20849	0.835329	-6.90553	0.670837	0.722242
Non.inflammatory.Macrophages	CYP2D9	-0.09594	0.530177	-0.2084	0.835398	-5.52521	0.967072	0.858388
Non.inflammatory.Macrophages	PRNP	0.056399	2.012971	0.208362	0.835426	-5.97481	0.894209	0.823749
Non.inflammatory.Macrophages	CBX1	0.020525	6.075378	0.208159	0.835584	-6.85295	0.698594	0.735751
Non.inflammatory.Macrophages	EIF2S2	0.015791	7.925804	0.208108	0.835623	-7.21097	0.623691	0.698815
Non.inflammatory.Macrophages	RALGDS	0.042599	3.822267	0.208104	0.835627	-6.48121	0.801479	0.783381
Non.inflammatory.Macrophages	ZFP229	-0.08823	-0.01049	-0.20808	0.835642	-5.61166	0.968063	0.871456
Non.inflammatory.Macrophages	GM6225	-0.05072	2.958455	-0.20806	0.835664	-6.13748	0.84458	0.802452
Non.inflammatory.Macrophages	PIK3R2	0.04214	2.306399	0.207937	0.835757	-6.24164	0.878549	0.817195
Non.inflammatory.Macrophages	UPP2	-0.06249	1.652112	-0.20743	0.836148	-6.07392	0.914022	0.832299
Non.inflammatory.Macrophages	SLC25A45	0.02651	3.260909	0.207383	0.836187	-6.559	0.829411	0.79588
Non.inflammatory.Macrophages	FGF1	-0.09056	0.63081	-0.20731	0.836243	-5.55251	0.964781	0.856264
Non.inflammatory.Macrophages	2310057M	-0.03811	2.710086	-0.20725	0.836288	-6.08319	0.857532	0.808188
Non.inflammatory.Macrophages	SLC25A21	-0.09687	1.867089	-0.20721	0.836324	-5.90655	0.902275	0.82736
Non.inflammatory.Macrophages	HIST1H1E	0.077947	4.203103	0.207203	0.836328	-6.37928	0.78329	0.775301

Non.inflammatory.Macrophages	FAM160A2	0.034348	2.604929	0.207077	0.836426	-6.32723	0.863028	0.810562
Non.inflammatory.Macrophages	USP48	0.019725	5.391239	0.206624	0.836778	-6.79318	0.728671	0.750102
Non.inflammatory.Macrophages	HMG20B	-0.01931	5.119833	-0.20661	0.836792	-6.74537	0.740844	0.755791
Non.inflammatory.Macrophages	FOXN2	0.017722	6.146142	0.206502	0.836873	-6.91058	0.69581	0.734501
Non.inflammatory.Macrophages	CD209D	-0.16761	-0.32218	-0.2065	0.836875	-5.27381	0.968389	0.879257
Non.inflammatory.Macrophages	TSPYL2	0.042694	2.8065	0.206461	0.836905	-6.22205	0.852668	0.806052
Non.inflammatory.Macrophages	CEP63	0.029317	4.291786	0.206408	0.836947	-6.50059	0.779185	0.773426
Non.inflammatory.Macrophages	KLRB1C	0.119553	1.954564	0.206262	0.837061	-5.66197	0.897707	0.825433
Non.inflammatory.Macrophages	TMEM119	-0.06306	0.305437	-0.20594	0.837311	-5.88803	0.968466	0.86416
Non.inflammatory.Macrophages	COPS7B	0.036364	3.34463	0.205868	0.837367	-6.24472	0.825395	0.794152
Non.inflammatory.Macrophages	RGMB	0.042643	1.845211	0.205801	0.83742	-6.2557	0.903665	0.827973
Non.inflammatory.Macrophages	CDKN2D	-0.03004	6.09877	-0.20577	0.837443	-6.5681	0.697886	0.735543
Non.inflammatory.Macrophages	GSTK1	-0.05139	2.449061	-0.20577	0.837444	-6.01646	0.871357	0.814186
Non.inflammatory.Macrophages	B4GAT1	-0.05128	1.65015	-0.20566	0.837526	-6.04434	0.914345	0.832512
Non.inflammatory.Macrophages	CSTF1	-0.03238	3.243385	-0.20508	0.837984	-6.30833	0.83087	0.796648

Non.inflammatory.Macrophages	ZAP70	-0.10916	1.982594	-0.20464	0.838323	-5.53681	0.896847	0.82523
Non.inflammatory.Macrophages	GM15614	0.069526	2.981808	0.204524	0.838414	-5.87484	0.844329	0.802622
Non.inflammatory.Macrophages	G6PDX	0.022765	4.072997	0.204467	0.838458	-6.69215	0.790241	0.778619
Non.inflammatory.Macrophages	ABHD11	0.029445	3.541149	0.204466	0.83846	-6.52335	0.816187	0.790229
Non.inflammatory.Macrophages	PHKB	0.023001	5.588989	0.203792	0.838984	-6.80964	0.720739	0.746458
Non.inflammatory.Macrophages	IFIT1BL2	-0.10842	-0.46837	-0.20377	0.839004	-5.46507	0.969487	0.883391
Non.inflammatory.Macrophages	ELK4	-0.02156	5.882489	-0.20362	0.839119	-6.78736	0.707924	0.740383
Non.inflammatory.Macrophages	RAE1	0.024292	4.525674	0.203565	0.839161	-6.58292	0.769041	0.768885
Non.inflammatory.Macrophages	ZBTB21	0.031318	3.713914	0.203446	0.839254	-6.38869	0.807958	0.786455
Non.inflammatory.Macrophages	TMEM259	-0.02639	4.879001	-0.20339	0.839295	-6.71656	0.75266	0.761359
Non.inflammatory.Macrophages	VPS28	-0.0191	6.686374	-0.20332	0.839352	-6.99962	0.673936	0.723997
Non.inflammatory.Macrophages	LRP6	0.022662	5.378913	0.203134	0.839497	-6.86353	0.730047	0.750836
Non.inflammatory.Macrophages	MCEE	-0.02611	4.194477	-0.20311	0.839516	-6.65375	0.784701	0.776006
Non.inflammatory.Macrophages	ARL1	-0.0185	5.538643	-0.20307	0.83955	-6.82865	0.722959	0.747505
Non.inflammatory.Macrophages	NID1	0.061714	2.394724	0.203003	0.839599	-5.92684	0.875142	0.815856

Non.inflammatory.Macrophages	SRPK3	-0.09967	1.292575	-0.20298	0.839616	-5.44649	0.935171	0.841241
Non.inflammatory.Macrophages	MRPL55	0.025049	4.367452	0.202753	0.839794	-6.60016	0.776531	0.772279
Non.inflammatory.Macrophages	TRIM2	0.071281	2.325336	0.202525	0.839971	-5.81551	0.878866	0.81744
Non.inflammatory.Macrophages	DHX35	-0.04512	2.187953	-0.20243	0.840049	-6.09799	0.88618	0.820574
Non.inflammatory.Macrophages	TNFRSF14	0.08858	0.252225	0.202413	0.840059	-5.6573	0.969544	0.865917
Non.inflammatory.Macrophages	SSB	-0.01461	6.803522	-0.20239	0.840076	-7.0263	0.669156	0.721651
Non.inflammatory.Macrophages	MIDN	-0.01876	6.064607	-0.20234	0.840113	-6.95446	0.700124	0.736658
Non.inflammatory.Macrophages	PPAT	-0.0342	4.107428	-0.2022	0.840222	-6.37844	0.788913	0.77791
Non.inflammatory.Macrophages	ZFP524	-0.02946	3.672126	-0.20183	0.840512	-6.56898	0.810058	0.787392
Non.inflammatory.Macrophages	FAM149A	-0.08376	-0.42534	-0.2018	0.840539	-5.63202	0.969544	0.882361
Non.inflammatory.Macrophages	CASC1	0.053952	1.810453	0.201764	0.840564	-6.14746	0.906565	0.829241
Non.inflammatory.Macrophages	RNPEPL1	0.026702	4.75379	0.201761	0.840567	-6.66972	0.758471	0.764038
Non.inflammatory.Macrophages	JKAMP	0.02404	3.680975	0.201645	0.840658	-6.48502	0.809623	0.787198
Non.inflammatory.Macrophages	AACS	-0.03163	3.861959	-0.20163	0.840666	-6.35421	0.800772	0.783243
Non.inflammatory.Macrophages	SREBF1	-0.02842	3.760907	-0.20163	0.840668	-6.4814	0.805703	0.785449

Non.inflammatory.Macrophages	SLC25A36	0.019184	6.285868	0.201234	0.840978	-6.92643	0.690868	0.732158
Non.inflammatory.Macrophages	CD151	-0.03668	2.758187	-0.2012	0.841005	-6.29389	0.856381	0.807697
Non.inflammatory.Macrophages	MLF1	-0.09345	0.512644	-0.20094	0.841207	-5.53534	0.969359	0.859805
Non.inflammatory.Macrophages	ITM2A	-0.06843	2.544803	-0.20063	0.841452	-5.88227	0.86781	0.812598
Non.inflammatory.Macrophages	KCNJ16	0.101923	-0.02269	0.2005	0.84155	-5.79561	0.970148	0.872717
Non.inflammatory.Macrophages	2010016I1	0.090626	-0.03474	0.20032	0.841691	-5.52906	0.970227	0.873063
Non.inflammatory.Macrophages	GM21781	0.058791	1.561921	0.19993	0.841995	-5.87933	0.92111	0.835327
Non.inflammatory.Macrophages	AVPI1	-0.0379	2.712104	-0.19977	0.842123	-6.3122	0.859405	0.809045
Non.inflammatory.Macrophages	CCNY	-0.01721	6.67252	-0.19966	0.842205	-7.03665	0.675202	0.724598
Non.inflammatory.Macrophages	PRMT3	-0.02872	4.041123	-0.19949	0.842341	-6.52408	0.792868	0.779672
Non.inflammatory.Macrophages	UXS1	0.024224	4.429155	0.199401	0.842407	-6.66831	0.774369	0.771296
Non.inflammatory.Macrophages	4933434E2	0.021709	5.269667	0.199388	0.842417	-6.74748	0.735688	0.753458
Non.inflammatory.Macrophages	MEGF8	-0.05871	0.288303	-0.19938	0.842426	-5.8711	0.970484	0.86542
Non.inflammatory.Macrophages	CTSD	0.020012	7.133555	0.199271	0.842508	-7.29863	0.656392	0.715357
Non.inflammatory.Macrophages	NOP16	0.02808	4.283714	0.199082	0.842656	-6.55769	0.781255	0.774425

Non.inflammatory.Macrophages	DNAJC27	-0.04429	2.442699	-0.19908	0.842658	-6.06399	0.873508	0.81513
Non.inflammatory.Macrophages	EGLN2	-0.02531	4.887876	-0.19898	0.842733	-6.70488	0.753026	0.761549
Non.inflammatory.Macrophages	LEKR1	-0.07164	1.047991	-0.19884	0.842848	-5.87238	0.949965	0.847418
Non.inflammatory.Macrophages	TTC9C	-0.02032	4.558959	-0.19881	0.842867	-6.621	0.768272	0.768582
Non.inflammatory.Macrophages	COX16	0.014318	6.153691	0.198686	0.842964	-6.94476	0.696993	0.735212
Non.inflammatory.Macrophages	DLG1	-0.01718	7.219142	-0.19866	0.842989	-7.04097	0.652956	0.71373
Non.inflammatory.Macrophages	UBE2N	0.013285	7.48412	0.198347	0.843229	-7.1568	0.642525	0.708522
Non.inflammatory.Macrophages	MORF4L1	0.009353	8.201561	0.198174	0.843364	-7.28402	0.614845	0.694552
Non.inflammatory.Macrophages	GM8251	-0.05316	3.392286	-0.19816	0.843375	-6.17925	0.82485	0.794046
Non.inflammatory.Macrophages	CYHR1	-0.02358	4.48568	-0.19804	0.843465	-6.62242	0.771822	0.770276
Non.inflammatory.Macrophages	TMEM70	-0.02755	3.930301	-0.19798	0.843513	-6.54799	0.798344	0.782276
Non.inflammatory.Macrophages	TMEM209	0.040783	3.362785	0.197922	0.84356	-6.26325	0.826327	0.794735
Non.inflammatory.Macrophages	TRAFD1	-0.02607	5.468862	-0.19785	0.843615	-6.97599	0.726901	0.749484
Non.inflammatory.Macrophages	KAT6B	0.020868	5.844837	0.197266	0.844072	-6.82328	0.710392	0.741836
Non.inflammatory.Macrophages	HERC2	-0.01912	5.98752	-0.19711	0.844197	-6.92354	0.70422	0.738973

Non.inflammatory.Macrophages	NUPL2	-0.04452	2.367649	-0.19698	0.844296	-6.12638	0.877606	0.817332
Non.inflammatory.Macrophages	MPV17L	-0.05509	2.006778	-0.19682	0.844417	-5.936	0.896913	0.825578
Non.inflammatory.Macrophages	B3GALNT1	-0.07565	0.351359	-0.19682	0.844424	-5.83119	0.970629	0.864437
Non.inflammatory.Macrophages	VSIG10	-0.07997	0.47062	-0.19674	0.844484	-5.65629	0.970629	0.861579
Non.inflammatory.Macrophages	KAT2B	0.021231	6.221283	0.196736	0.844486	-6.9388	0.69422	0.734208
Non.inflammatory.Macrophages	FKBPL	-0.05992	1.019839	-0.19673	0.844487	-5.85964	0.951711	0.848537
Non.inflammatory.Macrophages	B230217O:	-0.07879	1.757632	-0.19673	0.844493	-5.71666	0.910469	0.831316
Non.inflammatory.Macrophages	PPWD1	0.021469	4.68019	0.196697	0.844516	-6.54619	0.762733	0.766395
Non.inflammatory.Macrophages	HIST1H4C	0.102508	0.309171	0.196465	0.844697	-5.46631	0.970629	0.865537
Non.inflammatory.Macrophages	PSMA2	0.016423	7.21693	0.1961	0.844982	-7.10651	0.653142	0.714221
Non.inflammatory.Macrophages	ZFP790	-0.04774	2.226902	-0.19609	0.84499	-6.00774	0.88509	0.820636
Non.inflammatory.Macrophages	ARL2	-0.03311	3.356926	-0.19593	0.845114	-6.37614	0.826622	0.795241
Non.inflammatory.Macrophages	ZC3H12A	0.025462	5.128189	0.195887	0.845148	-6.72244	0.742179	0.756985
Non.inflammatory.Macrophages	TSPYL4	-0.07988	0.723815	-0.19582	0.8452	-5.51852	0.963807	0.855643
Non.inflammatory.Macrophages	MVP	0.021156	5.108561	0.195808	0.84521	-6.80851	0.743069	0.757398

Non.inflammatory.Macrophages	FBXW17	-0.0463	1.479346	-0.19573	0.845269	-6.12598	0.925831	0.837871
Non.inflammatory.Macrophages	TRAM2	-0.03187	4.061754	-0.19556	0.845405	-6.29052	0.791992	0.779807
Non.inflammatory.Macrophages	NMT1	0.014299	6.941125	0.195495	0.845454	-7.08698	0.66428	0.719737
Non.inflammatory.Macrophages	GUCD1	-0.02968	3.805941	-0.19548	0.845463	-6.51589	0.804401	0.785379
Non.inflammatory.Macrophages	WASL	-0.01674	5.483088	-0.19547	0.845472	-6.7906	0.726271	0.749554
Non.inflammatory.Macrophages	SPRED2	-0.02177	6.335393	-0.19542	0.845516	-6.89204	0.689389	0.731977
Non.inflammatory.Macrophages	WRNIP1	0.027618	3.743434	0.195396	0.845531	-6.37686	0.807461	0.786746
Non.inflammatory.Macrophages	RNH1	-0.02123	5.988026	-0.19533	0.845584	-6.97082	0.704198	0.739102
Non.inflammatory.Macrophages	TIMM8A1	0.032295	4.226992	0.195322	0.845589	-6.49099	0.784073	0.776241
Non.inflammatory.Macrophages	CSNK1E	-0.02468	4.953409	-0.19526	0.84564	-6.68191	0.750135	0.760701
Non.inflammatory.Macrophages	GM50373	0.097642	0.358844	0.195238	0.845654	-5.55285	0.970629	0.864387
Non.inflammatory.Macrophages	DKC1	-0.03037	4.56874	-0.19518	0.845697	-6.48971	0.76793	0.768891
Non.inflammatory.Macrophages	LHFP	0.078717	1.237889	0.195	0.845841	-5.74759	0.939369	0.843576
Non.inflammatory.Macrophages	SAG	-0.03883	3.5306	-0.19498	0.845857	-6.45192	0.817977	0.791468
Non.inflammatory.Macrophages	GM43305	0.056369	7.45195	0.194791	0.846003	-6.8255	0.643865	0.709653

Non.inflammatory.Macrophages	PLXNB2	-0.02248	3.959065	-0.19467	0.846098	-6.78756	0.797059	0.782162
Non.inflammatory.Macrophages	MCRIP2	-0.04237	2.015335	-0.19441	0.846302	-6.10565	0.896594	0.825639
Non.inflammatory.Macrophages	GM29966	0.091308	1.411825	0.194336	0.846359	-5.75293	0.929741	0.839613
Non.inflammatory.Macrophages	CD300LB	0.035475	1.973752	0.194324	0.846368	-6.64472	0.898843	0.8266
Non.inflammatory.Macrophages	ITGB5	-0.02874	3.058168	-0.19426	0.846415	-6.76432	0.841856	0.80207
Non.inflammatory.Macrophages	TMEM167I	0.031229	3.755594	0.194185	0.846477	-6.43028	0.806993	0.786672
Non.inflammatory.Macrophages	CDC42EP4	0.038089	2.155265	0.194015	0.846609	-6.20951	0.889127	0.822532
Non.inflammatory.Macrophages	MFSD10	0.026819	4.464143	0.193857	0.846733	-6.58218	0.773026	0.771352
Non.inflammatory.Macrophages	TCIM	-0.07828	1.634355	-0.19381	0.846768	-5.85511	0.91747	0.834531
Non.inflammatory.Macrophages	HARS2	-0.03191	2.848743	-0.19372	0.846836	-6.17729	0.852668	0.806823
Non.inflammatory.Macrophages	ADAM33	-0.07778	-0.9471	-0.19344	0.84706	-5.63711	0.970963	0.896577
Non.inflammatory.Macrophages	PIGL	0.038253	2.746364	0.193432	0.847065	-6.0474	0.85805	0.809162
Non.inflammatory.Macrophages	C130050O:	0.033336	1.379067	0.193341	0.847136	-6.47361	0.931744	0.840516
Non.inflammatory.Macrophages	PYCR2	0.030837	3.687049	0.193233	0.84722	-6.43594	0.810521	0.788258
Non.inflammatory.Macrophages	ZC3H7B	0.03697	3.353116	0.193118	0.84731	-6.23158	0.827128	0.795617

Non.inflammatory.Macrophages	DNAAF3	-0.07244	-0.6587	-0.1929	0.84748	-5.7146	0.971002	0.889482
Non.inflammatory.Macrophages	DEXI	-0.03837	2.91114	-0.19283	0.847536	-6.18626	0.849574	0.805502
Non.inflammatory.Macrophages	DUSP7	0.032026	3.559127	0.192745	0.847601	-6.4741	0.816859	0.791112
Non.inflammatory.Macrophages	PRKD3	0.026465	5.198034	0.192535	0.847765	-6.57036	0.739306	0.755858
Non.inflammatory.Macrophages	TAT	-0.07344	1.826535	-0.19214	0.848077	-5.88003	0.90705	0.830308
Non.inflammatory.Macrophages	CHD2	0.017898	7.754934	0.192079	0.848121	-7.17323	0.63218	0.704022
Non.inflammatory.Macrophages	ARL6IP5	0.017196	7.048458	0.191988	0.848192	-7.04414	0.660177	0.718011
Non.inflammatory.Macrophages	MOCOS	0.05129	1.324581	0.191978	0.8482	-6.19905	0.934834	0.841988
Non.inflammatory.Macrophages	RNF126	-0.02492	4.767243	-0.19194	0.848232	-6.56847	0.758989	0.765103
Non.inflammatory.Macrophages	PCBP2	0.011686	8.511828	0.191865	0.848288	-7.31737	0.603472	0.689381
Non.inflammatory.Macrophages	B3GAT3	0.026231	4.307364	0.191533	0.848547	-6.56848	0.780548	0.775088
Non.inflammatory.Macrophages	PRDM15	-0.03248	3.462741	-0.19145	0.848612	-6.27182	0.82165	0.793543
Non.inflammatory.Macrophages	ATE1	-0.02243	3.945361	-0.19131	0.848722	-6.53377	0.797922	0.782983
Non.inflammatory.Macrophages	WDR45	0.0324	2.960786	0.191291	0.848736	-6.2769	0.847025	0.804732
Non.inflammatory.Macrophages	SLC46A1	-0.07628	0.761766	-0.19128	0.848743	-5.87389	0.962777	0.855462

Non.inflammatory.Macrophages	DIP2A	0.040172	2.079809	0.191265	0.848757	-6.13512	0.893318	0.824695
Non.inflammatory.Macrophages	TPRN	0.054106	2.21301	0.191167	0.848833	-5.94648	0.886172	0.821646
Non.inflammatory.Macrophages	PILRB2	0.029795	2.237136	0.191087	0.848896	-6.67035	0.884884	0.821121
Non.inflammatory.Macrophages	LGALSL	0.071979	1.281509	0.191019	0.848949	-5.72622	0.937254	0.843233
Non.inflammatory.Macrophages	AAMDC	-0.02641	3.299635	-0.191	0.848962	-6.32505	0.829817	0.797213
Non.inflammatory.Macrophages	KMO	0.059434	3.038738	0.190996	0.848966	-5.88649	0.843037	0.803022
Non.inflammatory.Macrophages	CD69	-0.08651	5.620451	-0.1909	0.849039	-5.99817	0.720479	0.747343
Non.inflammatory.Macrophages	TAZ	-0.02277	4.057371	-0.19074	0.849169	-6.52604	0.792508	0.780658
Non.inflammatory.Macrophages	CD86	0.023369	6.31281	0.190699	0.849198	-7.3533	0.690608	0.733166
Non.inflammatory.Macrophages	RABAC1	-0.01705	5.988734	-0.19066	0.849226	-6.937	0.704438	0.739827
Non.inflammatory.Macrophages	MON1B	0.045915	1.881481	0.190617	0.849263	-5.9526	0.904055	0.829438
Non.inflammatory.Macrophages	AP2A1	-0.02595	4.542647	-0.19043	0.849412	-6.60302	0.769447	0.770276
Non.inflammatory.Macrophages	ZBTB41	0.03836	2.915286	0.190413	0.849422	-6.12091	0.84936	0.805964
Non.inflammatory.Macrophages	KDELR2	0.01763	5.993878	0.190383	0.849445	-6.89952	0.704217	0.739776
Non.inflammatory.Macrophages	TBL1X	0.017893	7.155308	0.190358	0.849465	-7.03496	0.655867	0.716238

Non.inflammatory.Macrophages	DCUN1D4	-0.04652	2.540378	-0.19024	0.849554	-6.15524	0.868844	0.814413
Non.inflammatory.Macrophages	ZFP146	-0.03672	3.413704	-0.19011	0.849655	-6.19936	0.824107	0.794864
Non.inflammatory.Macrophages	RECQL4	-0.09093	-0.06717	-0.19004	0.849711	-5.44478	0.971014	0.875628
Non.inflammatory.Macrophages	2310033P(	0.025103	4.144026	0.189977	0.849762	-6.52024	0.788352	0.778904
Non.inflammatory.Macrophages	A730081D(	0.04983	3.129627	0.189819	0.849886	-6.0648	0.838471	0.801219
Non.inflammatory.Macrophages	RAB29	-0.02719	3.635461	-0.18964	0.850025	-6.5756	0.813207	0.790034
Non.inflammatory.Macrophages	ENOPH1	0.033706	2.8863	0.189539	0.850105	-6.29598	0.850989	0.806675
Non.inflammatory.Macrophages	AKR1C12	-0.0684	0.756271	-0.18941	0.850203	-5.69574	0.963165	0.85591
Non.inflammatory.Macrophages	STAP2	-0.08762	0.504981	-0.18932	0.850276	-5.59239	0.970821	0.86194
Non.inflammatory.Macrophages	GM29570	-0.08704	0.692445	-0.18896	0.850555	-5.55439	0.96576	0.857568
Non.inflammatory.Macrophages	LRCH4	0.02343	4.260162	0.188786	0.850694	-6.65002	0.783197	0.776598
Non.inflammatory.Macrophages	DNTTIP1	0.017913	4.812219	0.188658	0.850793	-6.69246	0.757317	0.764754
Non.inflammatory.Macrophages	CFHR1	-0.11417	0.449486	-0.18858	0.850854	-5.48157	0.971523	0.863408
Non.inflammatory.Macrophages	SAP18B	0.038525	2.771863	0.188414	0.850984	-6.18857	0.857228	0.809436
Non.inflammatory.Macrophages	RFXANK	-0.03697	2.879474	-0.18839	0.851004	-6.16539	0.851666	0.807017

Non.inflammatory.Macrophages	ATP1A1	0.016695	6.234757	0.188183	0.851164	-7.06071	0.694354	0.735122
Non.inflammatory.Macrophages	BMS1	0.023342	4.737717	0.18809	0.851237	-6.62576	0.760838	0.766441
Non.inflammatory.Macrophages	RNF14	-0.01783	5.242687	-0.18802	0.851295	-6.75165	0.737761	0.755769
Non.inflammatory.Macrophages	TBC1D10A	-0.02476	3.56507	-0.1875	0.8517	-6.52165	0.817267	0.792019
Non.inflammatory.Macrophages	CASP9	-0.02907	2.447605	-0.18748	0.851717	-6.30401	0.874466	0.817036
Non.inflammatory.Macrophages	PDCL3	-0.02105	4.536557	-0.18744	0.851742	-6.67719	0.770394	0.770903
Non.inflammatory.Macrophages	SKI	-0.01968	5.372605	-0.18731	0.851845	-6.85516	0.732098	0.753205
Non.inflammatory.Macrophages	HP1BP3	-0.01844	6.529858	-0.18731	0.851847	-6.93369	0.682077	0.729324
Non.inflammatory.Macrophages	GM5544	-0.07394	0.129876	-0.18693	0.852141	-5.66207	0.971961	0.871525
Non.inflammatory.Macrophages	GAS5	-0.01888	6.391612	-0.18689	0.852175	-6.98803	0.687963	0.732211
Non.inflammatory.Macrophages	ATAD3A	0.029178	4.10261	0.186716	0.852311	-6.51484	0.791111	0.780433
Non.inflammatory.Macrophages	NAB1	0.017352	6.555305	0.186621	0.852385	-7.01705	0.681102	0.728947
Non.inflammatory.Macrophages	SOX4	0.042026	5.508952	0.186561	0.852433	-6.71197	0.726119	0.750507
Non.inflammatory.Macrophages	GOLT1B	0.024742	4.631519	0.186468	0.852505	-6.62768	0.766048	0.769066
Non.inflammatory.Macrophages	NGDN	-0.01848	5.424919	-0.18642	0.852542	-6.7531	0.729856	0.752265

Non.inflammatory.Macrophages	ZFP112	0.084167	0.391108	0.186282	0.852651	-5.58299	0.971961	0.86532
Non.inflammatory.Macrophages	DNAJC21	0.021296	5.890572	0.186159	0.852747	-6.75655	0.70938	0.742601
Non.inflammatory.Macrophages	FLVCR1	0.021898	4.670527	0.185924	0.85293	-6.69703	0.764229	0.768289
Non.inflammatory.Macrophages	MDP1	-0.02176	4.373314	-0.18577	0.853052	-6.59517	0.778188	0.774689
Non.inflammatory.Macrophages	B930095G[illegible]	-0.11271	0.087994	-0.18571	0.853101	-5.40829	0.971961	0.872736
Non.inflammatory.Macrophages	CYP4F13	-0.03303	2.829707	-0.18565	0.853142	-6.32318	0.854613	0.80871
Non.inflammatory.Macrophages	KLRD1	-0.10682	3.767425	-0.18564	0.853155	-5.70474	0.807391	0.787882
Non.inflammatory.Macrophages	ATXN10	-0.01775	6.66392	-0.18553	0.853241	-6.94699	0.676586	0.726879
Non.inflammatory.Macrophages	GM47601	0.060154	0.02249	0.185458	0.853295	-5.83493	0.971961	0.874381
Non.inflammatory.Macrophages	MAN1B1	-0.02787	4.927787	-0.18532	0.853403	-6.66144	0.752338	0.762904
Non.inflammatory.Macrophages	HIKESHI	0.01955	5.228907	0.185232	0.853471	-6.74083	0.738643	0.756539
Non.inflammatory.Macrophages	IGSF3	0.109576	0.826018	0.185134	0.853548	-5.38813	0.961361	0.855112
Non.inflammatory.Macrophages	CENPS	-0.0486	3.529733	-0.1851	0.853573	-6.19916	0.819125	0.793183
Non.inflammatory.Macrophages	SUPT4A	0.017824	6.836136	0.184872	0.853753	-7.00976	0.669484	0.723448
Non.inflammatory.Macrophages	ZFP692	-0.04815	2.030033	-0.18472	0.853872	-5.84801	0.896886	0.826999

Non.inflammatory.Macrophages	GRWD1	0.042828	2.674334	0.184702	0.853886	-6.06908	0.86268	0.812313
Non.inflammatory.Macrophages	RUSC1	0.033429	3.288288	0.184404	0.854119	-6.34552	0.831208	0.798555
Non.inflammatory.Macrophages	MAP3K9	-0.11674	0.308951	-0.18435	0.854159	-5.352	0.971961	0.867506
Non.inflammatory.Macrophages	DNTT	0.14734	1.449877	0.184271	0.854223	-5.45957	0.928743	0.840445
Non.inflammatory.Macrophages	A530017D:	-0.03401	2.325165	-0.18424	0.85425	-6.24395	0.881066	0.820245
Non.inflammatory.Macrophages	NECTIN4	-0.07242	-0.02307	-0.18413	0.854329	-5.6858	0.971961	0.875569
Non.inflammatory.Macrophages	DBF4	-0.02822	5.205532	-0.18398	0.854452	-6.62312	0.739698	0.757106
Non.inflammatory.Macrophages	SCRN3	0.04818	1.928035	0.183973	0.854456	-6.04503	0.902414	0.829401
Non.inflammatory.Macrophages	SMYD3	0.020596	6.67093	0.183971	0.854457	-6.99146	0.676295	0.726839
Non.inflammatory.Macrophages	MIB2	0.03615	2.662247	0.183849	0.854553	-6.22339	0.86331	0.812638
Non.inflammatory.Macrophages	CBX6	-0.04465	2.859775	-0.18384	0.85456	-5.98384	0.853059	0.808185
Non.inflammatory.Macrophages	BICRAL	-0.02214	5.023041	-0.1838	0.854592	-6.68016	0.74798	0.760965
Non.inflammatory.Macrophages	PSMD1	0.015407	6.546401	0.183744	0.854635	-7.01422	0.681473	0.729366
Non.inflammatory.Macrophages	LDLR	-0.028	4.577033	-0.18373	0.854644	-6.82315	0.768595	0.770472
Non.inflammatory.Macrophages	CCDC126	0.053008	1.899321	0.183632	0.854723	-5.97205	0.903976	0.830066

Non.inflammatory.Macrophages	BICDL1	0.07529	1.878488	0.18362	0.854732	-5.62914	0.90511	0.830547
Non.inflammatory.Macrophages	RNF181	-0.02507	4.477136	-0.18359	0.854759	-6.61761	0.773285	0.772617
Non.inflammatory.Macrophages	TMEM138	-0.04277	2.910493	-0.18348	0.854839	-5.96071	0.850446	0.807048
Non.inflammatory.Macrophages	2210408I2	0.033622	3.277757	0.18327	0.855006	-6.36656	0.831738	0.798895
Non.inflammatory.Macrophages	GZMC	0.198074	1.539474	0.183243	0.855027	-5.56085	0.923757	0.838465
Non.inflammatory.Macrophages	FYCO1	0.022846	4.137271	0.18308	0.855154	-6.62371	0.789445	0.78006
Non.inflammatory.Macrophages	UBE4B	0.018655	5.48847	0.182968	0.855242	-6.80136	0.727029	0.751273
Non.inflammatory.Macrophages	CYBC1	-0.02306	4.562008	-0.18296	0.855246	-6.69676	0.769299	0.770902
Non.inflammatory.Macrophages	GPM6B	-0.04939	2.439081	-0.18295	0.855259	-6.11307	0.875028	0.817818
Non.inflammatory.Macrophages	LYPLA1	-0.01739	5.988433	-0.18246	0.855637	-6.83109	0.705342	0.741008
Non.inflammatory.Macrophages	ABHD14A	-0.0566	1.44344	-0.18246	0.85564	-5.84542	0.92936	0.840905
Non.inflammatory.Macrophages	CD163L1	-0.08313	0.60908	-0.18219	0.855854	-5.70007	0.969382	0.860664
Non.inflammatory.Macrophages	MED10	0.0203	4.886924	0.182162	0.855872	-6.68698	0.754503	0.764119
Non.inflammatory.Macrophages	SLC35D1	0.025719	4.114625	0.18182	0.85614	-6.47423	0.791015	0.780765
Non.inflammatory.Macrophages	SOD3	-0.06277	1.286857	-0.18164	0.856281	-5.90601	0.938454	0.844655

Non.inflammatory.Macrophages	BMP2K	0.015147	6.936172	0.181336	0.856519	-7.07938	0.665801	0.721847
Non.inflammatory.Macrophages	API5	0.015441	6.03062	0.181298	0.856548	-6.91361	0.703761	0.740284
Non.inflammatory.Macrophages	EFNA5	0.103109	2.313548	0.181237	0.856596	-5.69681	0.882225	0.820977
Non.inflammatory.Macrophages	N4BP2L1	-0.02223	4.595001	-0.18121	0.856621	-6.77022	0.768225	0.770478
Non.inflammatory.Macrophages	IL6	0.062953	1.106723	0.181047	0.856745	-6.37831	0.948649	0.849028
Non.inflammatory.Macrophages	CEBPE	-0.08708	0.2028	-0.18097	0.856804	-5.55715	0.972557	0.870621
Non.inflammatory.Macrophages	NRAP	0.077216	-1.09404	0.180939	0.85683	-5.53713	0.972557	0.902507
Non.inflammatory.Macrophages	ARL14EP	0.032843	3.524764	0.1809	0.85686	-6.25749	0.819876	0.793826
Non.inflammatory.Macrophages	ZDHHC9	0.023917	4.094804	0.180897	0.856862	-6.62203	0.791973	0.781331
Non.inflammatory.Macrophages	SCYL1	0.02512	4.072604	0.180803	0.856936	-6.56404	0.793045	0.781865
Non.inflammatory.Macrophages	IGF2BP2	0.04755	1.457318	0.180671	0.857039	-6.04823	0.928935	0.840895
Non.inflammatory.Macrophages	ACAD10	0.06196	1.501708	0.180537	0.857144	-5.76517	0.926496	0.839925
Non.inflammatory.Macrophages	9330159M	-0.05815	1.076034	-0.18028	0.857347	-5.83875	0.950597	0.849973
Non.inflammatory.Macrophages	HDAC8	-0.02251	6.530622	-0.18021	0.857403	-6.91713	0.682695	0.730283
Non.inflammatory.Macrophages	GM29093	-0.05783	1.269702	-0.17992	0.857624	-5.64572	0.939783	0.845411

Non.inflammatory.Macrophages	VPS33B	-0.03278	2.932531	-0.17982	0.857706	-6.20546	0.850171	0.807213
Non.inflammatory.Macrophages	BTBD6	-0.06008	1.542046	-0.17954	0.857928	-5.82984	0.924636	0.839037
Non.inflammatory.Macrophages	DEPDC5	-0.02112	4.978079	-0.17936	0.858066	-6.64843	0.750864	0.762541
Non.inflammatory.Macrophages	NCR1	0.120938	1.170957	0.179322	0.858095	-5.48983	0.945467	0.847734
Non.inflammatory.Macrophages	STUB1	-0.01656	5.426852	-0.17927	0.858137	-6.8307	0.730577	0.753073
Non.inflammatory.Macrophages	FSCN1	-0.05987	3.378992	-0.17919	0.858197	-6.3511	0.827564	0.797248
Non.inflammatory.Macrophages	DENND5A	0.015892	6.395889	0.179109	0.858262	-7.00335	0.688543	0.733028
Non.inflammatory.Macrophages	MCM2	-0.04006	4.762793	-0.17907	0.85829	-6.51514	0.760785	0.767125
Non.inflammatory.Macrophages	GINS2	-0.03902	3.493231	-0.17876	0.858534	-6.33701	0.822014	0.794827
Non.inflammatory.Macrophages	TUBA1B	0.026308	7.795232	0.178532	0.858714	-7.19595	0.632145	0.705124
Non.inflammatory.Macrophages	LDHC	-0.08967	0.610466	-0.17828	0.858911	-5.67097	0.970488	0.861181
Non.inflammatory.Macrophages	ABCG1	0.022367	6.591271	0.178211	0.858965	-7.06004	0.680662	0.729187
Non.inflammatory.Macrophages	RCOR3	-0.0322	2.937283	-0.17802	0.859117	-6.29003	0.850451	0.807276
Non.inflammatory.Macrophages	STK39	0.03654	3.517793	0.177811	0.859278	-6.57502	0.821054	0.794359
Non.inflammatory.Macrophages	PPP6R3	0.014566	7.283025	0.177705	0.859361	-7.04712	0.652454	0.715336

Non.inflammatory.Macrophages	SLAMF1	0.093797	0.884468	0.177553	0.85948	-5.5377	0.960777	0.854769
Non.inflammatory.Macrophages	ITK	0.101364	4.224474	0.177547	0.859485	-5.88374	0.786548	0.778945
Non.inflammatory.Macrophages	HEPACAM2	0.113371	0.779743	0.177538	0.859492	-5.46801	0.964633	0.857259
Non.inflammatory.Macrophages	TRIM36	-0.03166	3.146179	-0.17739	0.859612	-6.38194	0.839762	0.802695
Non.inflammatory.Macrophages	MTLN	0.025093	3.060484	0.177309	0.859672	-6.44399	0.844132	0.80463
Non.inflammatory.Macrophages	VMN2R19	0.105205	-0.79625	0.17728	0.859694	-5.40224	0.973544	0.895628
Non.inflammatory.Macrophages	IPMK	0.017096	5.867681	0.177161	0.859787	-6.95162	0.71153	0.74416
Non.inflammatory.Macrophages	CCDC171	0.026077	3.57202	0.177048	0.859876	-6.45063	0.818357	0.793273
Non.inflammatory.Macrophages	PUS7L	-0.0422	1.839535	-0.17683	0.860044	-6.09576	0.908713	0.832488
Non.inflammatory.Macrophages	NHSL1	-0.07184	1.728678	-0.17671	0.860138	-5.64085	0.914795	0.835116
Non.inflammatory.Macrophages	INTS1	-0.0335	3.347025	-0.17665	0.860189	-6.29607	0.829602	0.79837
Non.inflammatory.Macrophages	NUP88	0.021161	4.421593	0.176531	0.860281	-6.61787	0.777168	0.774878
Non.inflammatory.Macrophages	1110017D1	-0.06672	0.201816	-0.17644	0.860349	-5.87168	0.973544	0.871346
Non.inflammatory.Macrophages	DCAF13	-0.01879	4.771338	-0.1763	0.860463	-6.68569	0.760786	0.76737
Non.inflammatory.Macrophages	STAT6	-0.01749	5.031546	-0.17622	0.860526	-6.81394	0.74881	0.761832

Non.inflammatory.Macrophages	IFIT1	-0.06062	2.761439	-0.17615	0.86058	-6.43388	0.859546	0.811517
Non.inflammatory.Macrophages	C1GALT1	0.025234	6.353776	0.176134	0.860591	-6.68376	0.690681	0.734297
Non.inflammatory.Macrophages	E130307A1	0.021632	4.490339	0.176121	0.860602	-6.53587	0.773922	0.773397
Non.inflammatory.Macrophages	CLDN5	0.081985	1.006603	0.176112	0.860609	-5.62795	0.955326	0.852089
Non.inflammatory.Macrophages	AKAP1	-0.05124	1.799595	-0.17597	0.860719	-5.86253	0.910906	0.833546
Non.inflammatory.Macrophages	ADAM12	-0.09157	0.815122	-0.1759	0.860771	-5.54771	0.963335	0.856664
Non.inflammatory.Macrophages	ZFP619	-0.03899	2.21582	-0.17583	0.86083	-6.03018	0.888348	0.82398
Non.inflammatory.Macrophages	FASTKD1	-0.04269	2.189988	-0.17572	0.860912	-6.01069	0.889743	0.824619
Non.inflammatory.Macrophages	ARHGAP25	-0.01828	6.214201	-0.1755	0.861091	-6.97512	0.696706	0.737255
Non.inflammatory.Macrophages	SLC25A11	-0.01934	5.162778	-0.17488	0.861572	-6.79639	0.743297	0.75931
Non.inflammatory.Macrophages	RWDD2B	0.053684	1.849393	0.174524	0.861853	-5.88341	0.908955	0.83269
Non.inflammatory.Macrophages	SMIM24	-0.02511	3.210803	-0.17418	0.862125	-6.42986	0.837394	0.801827
Non.inflammatory.Macrophages	RTN4IP1	-0.05326	1.927586	-0.17403	0.862239	-5.91925	0.90494	0.831003
Non.inflammatory.Macrophages	NME4	0.041435	2.364464	0.173805	0.862416	-6.25258	0.881466	0.821005
Non.inflammatory.Macrophages	CDK5RAP3	-0.02121	4.379321	-0.17378	0.862432	-6.61966	0.780105	0.776242

Non.inflammatory.Macrophages	FKRP	-0.03524	2.332289	-0.17364	0.862546	-6.1505	0.883221	0.821783
Non.inflammatory.Macrophages	CRLF2	0.020206	5.010644	0.173391	0.86274	-6.77498	0.750807	0.762828
Non.inflammatory.Macrophages	IFT20	0.017275	5.442542	0.173034	0.86302	-6.8168	0.731359	0.753743
Non.inflammatory.Macrophages	IRF9	-0.03038	4.515749	-0.173	0.863044	-6.6933	0.77389	0.77345
Non.inflammatory.Macrophages	PAGR1A	-0.09514	0.395669	-0.1728	0.863204	-5.44021	0.975011	0.867377
Non.inflammatory.Macrophages	CTLA2B	-0.03838	3.353706	-0.1726	0.863359	-6.65329	0.830516	0.798998
Non.inflammatory.Macrophages	TAB1	0.03505	3.004453	0.172295	0.863599	-6.21559	0.848277	0.806902
Non.inflammatory.Macrophages	CCDC82	0.02486	4.323551	0.172264	0.863624	-6.47126	0.782999	0.777818
Non.inflammatory.Macrophages	NCL	-0.01878	7.783001	-0.17225	0.863638	-7.17568	0.633698	0.706403
Non.inflammatory.Macrophages	RDH14	0.023459	3.731822	0.172163	0.863703	-6.43191	0.811678	0.790752
Non.inflammatory.Macrophages	0610009EC	-0.06287	0.843747	-0.17204	0.863801	-5.61842	0.963724	0.856892
Non.inflammatory.Macrophages	TMEM33	0.015961	5.133185	0.172021	0.863814	-6.77826	0.745302	0.760513
Non.inflammatory.Macrophages	GM156	0.107617	-1.54566	0.171957	0.863865	-5.2434	0.975011	0.915611
Non.inflammatory.Macrophages	HNRNPH3	-0.01573	5.447268	-0.17189	0.863913	-6.66209	0.731148	0.753892
Non.inflammatory.Macrophages	PRPSAP1	-0.01977	4.34081	-0.17178	0.864004	-6.58603	0.782177	0.777476

Non.inflammatory.Macrophages	EIF4B	-0.01448	6.136378	-0.17171	0.864056	-6.9515	0.700987	0.739566
Non.inflammatory.Macrophages	EARS2	0.043792	1.644083	0.17161	0.864136	-5.93525	0.920847	0.838047
Non.inflammatory.Macrophages	MEX3C	0.018675	5.133199	0.171538	0.864192	-6.68447	0.745302	0.760513
Non.inflammatory.Macrophages	RAB6B	0.057568	1.697022	0.171471	0.864246	-5.76604	0.91792	0.836814
Non.inflammatory.Macrophages	CATSPERD	0.06781	1.329786	0.171411	0.864292	-5.77628	0.938401	0.845399
Non.inflammatory.Macrophages	SATB2	-0.05389	1.643339	-0.17131	0.864374	-5.96827	0.920889	0.838084
Non.inflammatory.Macrophages	MRPS7	0.01906	5.010323	0.171299	0.86438	-6.70099	0.75091	0.763137
Non.inflammatory.Macrophages	MTCH2	0.015833	5.950226	0.171185	0.864469	-6.88787	0.709015	0.743453
Non.inflammatory.Macrophages	PHB2	0.014976	6.570723	0.171042	0.864581	-6.97277	0.682594	0.730739
Non.inflammatory.Macrophages	MMS22L	0.036893	4.820836	0.171025	0.864595	-6.45745	0.759637	0.767221
Non.inflammatory.Macrophages	WDR4	0.030028	2.999114	0.170937	0.864664	-6.22432	0.848551	0.807137
Non.inflammatory.Macrophages	GM34983	-0.09117	0.754004	-0.17088	0.864708	-5.45744	0.96704	0.859126
Non.inflammatory.Macrophages	NDUFS8	0.014645	6.232258	0.170872	0.864715	-6.95485	0.696886	0.737676
Non.inflammatory.Macrophages	WDR18	0.019937	4.82244	0.170617	0.864915	-6.65782	0.759621	0.76727
Non.inflammatory.Macrophages	GM17268	-0.04883	0.383225	-0.1706	0.864926	-6.22444	0.975086	0.86809

Non.inflammatory.Macrophages	CHURC1	-0.01512	5.66012	-0.17047	0.865033	-6.86682	0.721787	0.749593
Non.inflammatory.Macrophages	ADAM17	-0.01919	6.061586	-0.17013	0.865298	-7.03831	0.704441	0.741279
Non.inflammatory.Macrophages	ISYNA1	-0.01912	4.852057	-0.16978	0.86557	-6.73694	0.7585	0.766761
Non.inflammatory.Macrophages	PRADC1	0.029102	3.374688	0.169694	0.865638	-6.30647	0.829796	0.798979
Non.inflammatory.Macrophages	CFAP410	0.025835	2.119698	0.169593	0.865718	-6.30591	0.895215	0.827376
Non.inflammatory.Macrophages	POP7	-0.01999	4.908142	-0.16955	0.865749	-6.67236	0.75591	0.765621
Non.inflammatory.Macrophages	PDE3A	0.100632	0.411945	0.169537	0.865762	-5.55914	0.975406	0.867603
Non.inflammatory.Macrophages	MLH1	-0.03003	2.750298	-0.16947	0.865812	-6.13054	0.861771	0.81301
Non.inflammatory.Macrophages	COIL	0.030334	3.741294	0.169239	0.865996	-6.24172	0.81154	0.790912
Non.inflammatory.Macrophages	GM3235	0.097411	0.645113	0.169236	0.865997	-5.45232	0.971477	0.862018
Non.inflammatory.Macrophages	PCGF6	0.039814	2.512206	0.169144	0.866069	-5.92613	0.874261	0.818426
Non.inflammatory.Macrophages	AGPAT3	0.017807	5.164345	0.169072	0.866126	-6.87829	0.744188	0.760194
Non.inflammatory.Macrophages	TARSL2	0.046474	1.415252	0.168906	0.866256	-5.86571	0.933976	0.843771
Non.inflammatory.Macrophages	GRIP1	-0.074	2.360437	-0.16887	0.866282	-5.82332	0.882309	0.821893
Non.inflammatory.Macrophages	PHF5A	-0.0177	6.072643	-0.1688	0.866342	-6.88342	0.704011	0.741251

Non.inflammatory.Macrophages	ALKBH7	-0.03319	3.373231	-0.16877	0.866363	-6.27659	0.82987	0.799105
Non.inflammatory.Macrophages	BBS7	-0.07348	0.558111	-0.16854	0.866542	-5.70132	0.973788	0.864173
Non.inflammatory.Macrophages	CDH22	0.131522	-0.85879	0.168472	0.866596	-5.25085	0.975481	0.898841
Non.inflammatory.Macrophages	CYB5R3	0.017723	4.85016	0.168401	0.866652	-6.78263	0.758646	0.766966
Non.inflammatory.Macrophages	CSNK2B	0.014859	7.247784	0.168318	0.866718	-7.08342	0.655166	0.717452
Non.inflammatory.Macrophages	GM33370	-0.05354	0.508404	-0.16821	0.866803	-5.86896	0.975039	0.865436
Non.inflammatory.Macrophages	SPEN	0.01513	5.922479	0.168021	0.866951	-6.89667	0.710604	0.744436
Non.inflammatory.Macrophages	XCL1	0.104708	2.335401	0.167945	0.86701	-5.67575	0.883764	0.822597
Non.inflammatory.Macrophages	CCDC28A	0.039577	2.307492	0.167654	0.867238	-6.01682	0.885253	0.823313
Non.inflammatory.Macrophages	WDR12	-0.02313	4.283459	-0.1676	0.867284	-6.59099	0.785336	0.77927
Non.inflammatory.Macrophages	5430414B1	0.088702	0.449396	0.167586	0.867291	-5.50299	0.975539	0.866942
Non.inflammatory.Macrophages	PIH1D1	-0.01835	5.06576	-0.16757	0.867302	-6.76352	0.74878	0.762482
Non.inflammatory.Macrophages	HDHD2	-0.02199	3.552765	-0.16752	0.867346	-6.45047	0.820993	0.79528
Non.inflammatory.Macrophages	AAMP	-0.01165	5.908148	-0.16721	0.867585	-6.93102	0.71125	0.744875
Non.inflammatory.Macrophages	RELA	0.017958	5.114793	0.167166	0.867621	-6.77917	0.746569	0.761512

Non.inflammatory.Macrophages	IRS2	0.024202	5.572327	0.167058	0.867706	-6.85337	0.726001	0.751888
Non.inflammatory.Macrophages	ATAD2	-0.02536	6.309192	-0.16691	0.86782	-6.87429	0.694011	0.73662
Non.inflammatory.Macrophages	MFSD4B4	-0.0719	-0.0204	-0.16688	0.867843	-5.61951	0.975572	0.878419
Non.inflammatory.Macrophages	LUM	-0.0793	0.551186	-0.16686	0.867858	-5.63518	0.97405	0.864592
Non.inflammatory.Macrophages	NUP43	-0.03701	2.795528	-0.16684	0.86788	-6.0738	0.859563	0.812311
Non.inflammatory.Macrophages	NUP155	0.018643	5.174039	0.166398	0.868223	-6.66761	0.744001	0.76034
Non.inflammatory.Macrophages	TESC	0.080785	1.561476	0.166317	0.868288	-5.65186	0.926121	0.840737
Non.inflammatory.Macrophages	CXCL13	-0.10835	-1.21101	-0.16627	0.868321	-5.49259	0.975737	0.907987
Non.inflammatory.Macrophages	CHSY1	0.023262	5.079341	0.166192	0.868385	-6.60266	0.748312	0.762347
Non.inflammatory.Macrophages	GM42984	-0.07546	0.916614	-0.16617	0.868405	-5.61609	0.96176	0.855938
Non.inflammatory.Macrophages	RNF5	-0.02242	4.122516	-0.16609	0.868463	-6.55586	0.793224	0.782926
Non.inflammatory.Macrophages	NT5DC2	-0.03074	2.432435	-0.16589	0.868626	-6.36841	0.878781	0.820623
Non.inflammatory.Macrophages	ZBTB37	-0.0317	2.901016	-0.16583	0.868671	-6.22333	0.85424	0.809996
Non.inflammatory.Macrophages	GCC1	0.0401	2.459454	0.165669	0.868796	-6.09621	0.877348	0.820006
Non.inflammatory.Macrophages	PDCD7	0.018815	4.505243	0.165645	0.868814	-6.6355	0.774962	0.774629

Non.inflammatory.Macrophages	POLR3K	-0.02143	3.885777	-0.16557	0.868875	-6.47902	0.804722	0.788105
Non.inflammatory.Macrophages	XPO5	0.025448	3.603677	0.165429	0.868984	-6.36825	0.818627	0.794327
Non.inflammatory.Macrophages	MIEF2	-0.05706	1.112018	-0.16529	0.86909	-5.72297	0.951448	0.851334
Non.inflammatory.Macrophages	RAB3D	0.029879	3.11921	0.165254	0.869121	-6.38996	0.84303	0.805122
Non.inflammatory.Macrophages	TMEM131	0.014471	6.606387	0.165217	0.86915	-7.06123	0.681612	0.730642
Non.inflammatory.Macrophages	YWHAH	-0.01476	7.687722	-0.16498	0.869336	-7.19414	0.637889	0.709068
Non.inflammatory.Macrophages	MRPL35	0.018532	5.21625	0.164928	0.869377	-6.7509	0.742086	0.759575
Non.inflammatory.Macrophages	GM17021	-0.0919	0.125797	-0.16488	0.869414	-5.4534	0.975737	0.875089
Non.inflammatory.Macrophages	CCHCR1	-0.06696	1.536464	-0.16487	0.869422	-5.66007	0.927514	0.841464
Non.inflammatory.Macrophages	SAC3D1	-0.02753	3.030557	-0.16478	0.869495	-6.25231	0.847568	0.80725
Non.inflammatory.Macrophages	MEF2A	-0.01318	7.727346	-0.16472	0.869541	-7.25469	0.63634	0.708317
Non.inflammatory.Macrophages	ARHGAP6	-0.04288	3.609197	-0.16457	0.86966	-6.24845	0.818377	0.794356
Non.inflammatory.Macrophages	DACH1	0.084664	1.877933	0.164502	0.869711	-5.68872	0.908685	0.833561
Non.inflammatory.Macrophages	WRB	0.045583	1.437969	0.164155	0.869984	-5.92936	0.933114	0.843834
Non.inflammatory.Macrophages	DYRK2	0.016899	4.884934	0.16413	0.870003	-6.77649	0.757315	0.766671

Non.inflammatory.Macrophages	CTNND2	0.073039	1.726999	0.164073	0.870048	-5.7466	0.917042	0.837082
Non.inflammatory.Macrophages	EPB41L1	0.044034	1.011227	0.164056	0.870062	-6.16501	0.95731	0.853899
Non.inflammatory.Macrophages	PDIA5	-0.03645	2.273787	-0.16389	0.870189	-6.21188	0.887367	0.824511
Non.inflammatory.Macrophages	BOK	0.077617	1.039523	0.163698	0.870343	-5.59719	0.955734	0.853319
Non.inflammatory.Macrophages	CDK19	0.018665	6.390727	0.163646	0.870384	-6.88068	0.690776	0.735295
Non.inflammatory.Macrophages	CLSTN1	0.039829	1.774497	0.163551	0.870458	-6.03459	0.914468	0.836114
Non.inflammatory.Macrophages	THAP1	-0.03261	2.879636	-0.16351	0.870491	-6.15855	0.855473	0.810808
Non.inflammatory.Macrophages	NECTIN1	0.072318	1.073651	0.163315	0.870643	-5.65593	0.953781	0.852629
Non.inflammatory.Macrophages	WDCP	-0.0329	3.079235	-0.16327	0.870677	-6.19318	0.845199	0.806384
Non.inflammatory.Macrophages	IPO7	-0.01912	5.656347	-0.16327	0.87068	-6.8697	0.722513	0.750565
Non.inflammatory.Macrophages	GM50322	-0.07492	0.358739	-0.16308	0.870824	-5.60456	0.975963	0.86979
Non.inflammatory.Macrophages	GIMAP3	0.126398	2.932139	0.162762	0.871077	-5.57155	0.852912	0.809855
Non.inflammatory.Macrophages	ALG10B	0.025667	2.952601	0.162748	0.871088	-6.35093	0.851856	0.809395
Non.inflammatory.Macrophages	MED12	-0.02381	3.781462	-0.1627	0.871129	-6.45774	0.810103	0.790958
Non.inflammatory.Macrophages	THUMPD1	-0.02084	4.425949	-0.16242	0.871348	-6.54154	0.778968	0.776947

Non.inflammatory.Macrophages	IQCC	-0.04608	1.695654	-0.16232	0.871425	-5.97223	0.918981	0.838265
Non.inflammatory.Macrophages	GM12359	0.052537	1.671224	0.162085	0.871609	-5.76325	0.920332	0.838849
Non.inflammatory.Macrophages	SGCB	-0.04532	2.212559	-0.16208	0.871609	-6.02967	0.89081	0.826318
Non.inflammatory.Macrophages	2610507I0	0.068668	0.583661	0.161956	0.87171	-5.70549	0.973728	0.864608
Non.inflammatory.Macrophages	MRPL21	0.018132	5.233418	0.161937	0.871725	-6.75226	0.741552	0.759715
Non.inflammatory.Macrophages	AC154200.	0.062288	0.660722	0.161773	0.871854	-5.60026	0.971543	0.862788
Non.inflammatory.Macrophages	MTHFD2L	0.026772	3.033816	0.161734	0.871884	-6.32207	0.847678	0.807707
Non.inflammatory.Macrophages	TEX30	0.019994	5.223272	0.161613	0.87198	-6.59819	0.742011	0.759955
Non.inflammatory.Macrophages	ACADM	-0.02228	4.767805	-0.1616	0.87199	-6.72462	0.762914	0.769653
Non.inflammatory.Macrophages	EMID1	-0.05382	3.280299	-0.1616	0.87199	-5.74755	0.835115	0.802186
Non.inflammatory.Macrophages	TMED8	-0.03779	2.954093	-0.16128	0.872242	-5.97111	0.851779	0.809513
Non.inflammatory.Macrophages	POLR2E	0.021431	4.876402	0.16123	0.87228	-6.7443	0.75788	0.767342
Non.inflammatory.Macrophages	UBB	-0.01516	10.85501	-0.16105	0.872419	-7.65301	0.525235	0.649793
Non.inflammatory.Macrophages	RHOU	-0.08422	1.27725	-0.16093	0.872514	-5.63858	0.942374	0.848247
Non.inflammatory.Macrophages	AI480526	0.045766	2.064128	0.160874	0.87256	-5.90866	0.898817	0.829895

Non.inflammatory.Macrophages	GLRX3	0.015376	6.625536	0.160828	0.872596	-6.9721	0.681036	0.730952
Non.inflammatory.Macrophages	TRIM62	-0.04039	1.410517	-0.1608	0.872618	-5.92837	0.934865	0.845111
Non.inflammatory.Macrophages	ATXN7L3B	0.013519	5.941589	0.160772	0.87264	-6.90289	0.71015	0.745004
Non.inflammatory.Macrophages	DDT	0.020492	5.541533	0.160521	0.872837	-6.82257	0.72773	0.753377
Non.inflammatory.Macrophages	SYTL1	-0.07162	1.59739	-0.16042	0.872918	-5.57409	0.924427	0.840765
Non.inflammatory.Macrophages	TCEA2	0.072296	0.335442	0.16038	0.872947	-5.51523	0.976057	0.870762
Non.inflammatory.Macrophages	GNA11	-0.03256	3.954617	-0.1603	0.87301	-6.27608	0.801625	0.787405
Non.inflammatory.Macrophages	MIRT1	0.04048	4.950639	0.160294	0.873016	-6.19157	0.754457	0.765873
Non.inflammatory.Macrophages	ZFP995	-0.03275	2.383748	-0.16028	0.873029	-6.16554	0.881656	0.822583
Non.inflammatory.Macrophages	ABCA6	-0.09038	0.705764	-0.16018	0.873107	-5.54806	0.969867	0.861874
Non.inflammatory.Macrophages	MDGA1	-0.06318	0.010829	-0.16014	0.873138	-5.7423	0.976057	0.878668
Non.inflammatory.Macrophages	MTFR1	-0.02389	4.079368	-0.16005	0.873205	-6.47429	0.795569	0.784711
Non.inflammatory.Macrophages	SGO2A	0.055379	3.063462	0.160039	0.873215	-6.06198	0.846158	0.807212
Non.inflammatory.Macrophages	GM37612	-0.06575	0.985122	-0.15986	0.873353	-5.61274	0.95908	0.855283
Non.inflammatory.Macrophages	RAPGEF6	-0.01355	8.049527	-0.15979	0.87341	-7.17729	0.624119	0.702667

Non.inflammatory.Macrophages	OMA1	-0.02268	3.410326	-0.15956	0.873588	-6.54015	0.828616	0.799531
Non.inflammatory.Macrophages	PROM1	-0.0905	0.403745	-0.15955	0.873602	-5.54175	0.976126	0.869232
Non.inflammatory.Macrophages	GM34095	-0.11182	-0.39051	-0.15935	0.873757	-5.24765	0.976126	0.888608
Non.inflammatory.Macrophages	ZFPM1	0.036652	4.248218	0.159339	0.873765	-6.16891	0.787495	0.781107
Non.inflammatory.Macrophages	MADD	0.017062	5.091113	0.159318	0.873782	-6.80664	0.748072	0.762992
Non.inflammatory.Macrophages	4930402H2	0.021983	4.453202	0.158966	0.874058	-6.57547	0.777747	0.776781
Non.inflammatory.Macrophages	PPM1J	-0.08708	-0.35731	-0.15879	0.874198	-5.38109	0.976145	0.887935
Non.inflammatory.Macrophages	CATSPER2	-0.04922	1.800753	-0.15873	0.874244	-5.87646	0.91327	0.836283
Non.inflammatory.Macrophages	POU5F2	0.037477	1.687999	0.158692	0.874274	-6.07211	0.919486	0.838908
Non.inflammatory.Macrophages	CALCA	-0.12505	-0.96013	-0.15867	0.874293	-5.28029	0.976145	0.902907
Non.inflammatory.Macrophages	HMOX1	-0.02599	4.807778	-0.15855	0.874383	-7.07518	0.761126	0.769175
Non.inflammatory.Macrophages	GTF2E1	0.033583	2.399479	0.158377	0.874521	-6.15121	0.880899	0.822489
Non.inflammatory.Macrophages	MARCKS	-0.01519	7.242888	-0.15835	0.874541	-7.31057	0.655809	0.718758
Non.inflammatory.Macrophages	SPRED3	0.055446	0.719893	0.158346	0.874545	-5.83738	0.96943	0.861795
Non.inflammatory.Macrophages	ECM2	0.100227	0.502848	0.158312	0.874572	-5.42026	0.975826	0.867005

Non.inflammatory.Macrophages	GM30054	0.067691	1.953461	0.158286	0.874592	-5.68188	0.904912	0.832754
Non.inflammatory.Macrophages	TDO2	-0.06002	3.218699	-0.15791	0.874884	-6.21125	0.838329	0.804018
Non.inflammatory.Macrophages	CRKL	0.016735	5.071994	0.157867	0.874921	-6.68565	0.748974	0.763611
Non.inflammatory.Macrophages	BCO2	0.038627	0.643324	0.157657	0.875087	-6.02406	0.972297	0.863738
Non.inflammatory.Macrophages	DLG2	-0.05709	2.402232	-0.15754	0.875181	-5.95201	0.880768	0.822529
Non.inflammatory.Macrophages	NEK4	0.0469	1.202148	0.15745	0.875249	-5.94173	0.946732	0.850433
Non.inflammatory.Macrophages	DDX19B	-0.02263	3.529612	-0.15744	0.875257	-6.42342	0.822673	0.797132
Non.inflammatory.Macrophages	CACNB1	-0.06969	0.846674	-0.1574	0.875286	-5.63769	0.964755	0.858873
Non.inflammatory.Macrophages	ARFIP1	0.022892	3.797972	0.157394	0.875293	-6.49015	0.809379	0.7912
Non.inflammatory.Macrophages	1-Mar	-0.06319	1.457886	-0.15732	0.875355	-5.74086	0.93231	0.844411
Non.inflammatory.Macrophages	LZTS1	0.068817	1.417164	0.157268	0.875392	-5.60801	0.934593	0.845367
Non.inflammatory.Macrophages	TMEM117	-0.06461	-0.04691	-0.15722	0.875432	-5.84194	0.976162	0.880448
Non.inflammatory.Macrophages	SNX17	-0.0149	5.853791	-0.15704	0.875568	-6.89669	0.71405	0.747191
Non.inflammatory.Macrophages	ESAM	0.051074	2.065322	0.156955	0.875638	-5.87874	0.89885	0.830281
Non.inflammatory.Macrophages	DPP9	0.020449	4.420233	0.156896	0.875684	-6.58324	0.779323	0.777623

Non.inflammatory.Macrophages	PPIF	-0.03055	3.577224	-0.15689	0.87569	-6.22675	0.820299	0.796085
Non.inflammatory.Macrophages	SLC1A4	0.072395	0.947937	0.15655	0.875956	-5.54044	0.961127	0.856544
Non.inflammatory.Macrophages	UBXN2A	0.016143	5.233503	0.156419	0.876059	-6.73801	0.741704	0.760293
Non.inflammatory.Macrophages	S100A1	0.019247	4.086611	0.15638	0.87609	-6.74278	0.795386	0.784968
Non.inflammatory.Macrophages	ME1	-0.07321	-0.00982	-0.1563	0.876156	-5.62399	0.976262	0.879658
Non.inflammatory.Macrophages	KLRI2	0.118991	0.447224	0.15628	0.876169	-5.33683	0.976262	0.868569
Non.inflammatory.Macrophages	PUM1	0.010928	7.56821	0.156224	0.876212	-7.11309	0.64293	0.712462
Non.inflammatory.Macrophages	MMP11	-0.09336	0.128652	-0.15606	0.876342	-5.34019	0.976299	0.876284
Non.inflammatory.Macrophages	EBAG9	0.019629	4.117531	0.155753	0.876582	-6.55047	0.793921	0.784364
Non.inflammatory.Macrophages	AC125149.	0.107185	-0.34803	0.155717	0.876611	-5.33138	0.976299	0.888026
Non.inflammatory.Macrophages	POLR2J	0.015735	4.922519	0.155636	0.876675	-6.72269	0.755939	0.766983
Non.inflammatory.Macrophages	LOXL2	0.061275	1.406795	0.155608	0.876696	-5.70741	0.935306	0.845793
Non.inflammatory.Macrophages	SRFBP1	-0.02643	3.231687	-0.15559	0.87671	-6.36232	0.837787	0.803941
Non.inflammatory.Macrophages	HTR1F	-0.04205	1.429334	-0.15545	0.876822	-5.88258	0.934041	0.845294
Non.inflammatory.Macrophages	PSMC2	0.016595	5.362149	0.155449	0.876822	-6.76847	0.735929	0.75768

Non.inflammatory.Macrophages	TM9SF3	0.009639	7.288714	0.155021	0.877158	-7.15233	0.654268	0.718181
Non.inflammatory.Macrophages	PCX	0.045472	2.70013	0.154821	0.877315	-6.04873	0.865463	0.816043
Non.inflammatory.Macrophages	PDSS2	0.019293	5.377267	0.154808	0.877326	-6.7399	0.735491	0.75745
Non.inflammatory.Macrophages	2-Sep	0.034925	2.22603	0.154563	0.877518	-6.20086	0.89063	0.826976
Non.inflammatory.Macrophages	PAN3	-0.01111	8.767803	-0.15449	0.877577	-7.28108	0.597514	0.689336
Non.inflammatory.Macrophages	HPRT	-0.01415	6.310323	-0.1544	0.877646	-6.9991	0.694733	0.738162
Non.inflammatory.Macrophages	C1QA	-0.03009	5.480946	-0.15428	0.87774	-7.11452	0.730874	0.75541
Non.inflammatory.Macrophages	RHNO1	-0.02525	4.345115	-0.15427	0.877748	-6.41059	0.783289	0.779678
Non.inflammatory.Macrophages	SHMT2	-0.02709	4.430904	-0.15422	0.87779	-6.50163	0.779209	0.777823
Non.inflammatory.Macrophages	LGALS9	0.017059	7.157478	0.154016	0.877948	-7.12811	0.659661	0.720996
Non.inflammatory.Macrophages	CCL17	0.09738	-0.70076	0.153739	0.878166	-5.48356	0.97687	0.897135
Non.inflammatory.Macrophages	GLS	-0.01408	7.7257	-0.1536	0.878272	-7.18736	0.637142	0.709722
Non.inflammatory.Macrophages	MCM3	-0.02761	5.484871	-0.15353	0.878332	-6.73453	0.730861	0.755426
Non.inflammatory.Macrophages	QRICH1	-0.01124	6.585338	-0.15351	0.878348	-6.97032	0.683284	0.732639
Non.inflammatory.Macrophages	POGZ	0.018475	4.431535	0.153419	0.878418	-6.56881	0.779352	0.777939

Non.inflammatory.Macrophages	FAM172A	0.0121	7.557353	0.153306	0.878506	-7.17258	0.64377	0.713113
Non.inflammatory.Macrophages	4930526L0	0.08072	-0.8055	0.153153	0.878626	-5.38672	0.976941	0.899838
Non.inflammatory.Macrophages	MICAL1	-0.03456	3.487265	-0.15291	0.878818	-6.03409	0.825559	0.798711
Non.inflammatory.Macrophages	USP11	0.039713	2.611991	0.152767	0.87893	-5.97034	0.870503	0.818421
Non.inflammatory.Macrophages	GM15848	0.100699	-0.86297	0.152712	0.878973	-5.22951	0.977086	0.901361
Non.inflammatory.Macrophages	HIST1H4D	0.076514	1.04631	0.152413	0.879208	-5.60982	0.956701	0.85495
Non.inflammatory.Macrophages	CAPN10	-0.03557	2.598593	-0.15215	0.879414	-6.04528	0.871446	0.818886
Non.inflammatory.Macrophages	RRN3	-0.01864	4.400393	-0.15213	0.87943	-6.54869	0.781217	0.77884
Non.inflammatory.Macrophages	ARHGAP22	0.038479	1.023886	0.152032	0.879508	-6.3279	0.958078	0.855552
Non.inflammatory.Macrophages	GM42701	-0.08189	0.844253	-0.1518	0.879688	-5.45058	0.966146	0.859872
Non.inflammatory.Macrophages	PPP3CA	0.011642	8.812084	0.151527	0.879905	-7.33234	0.596492	0.68892
Non.inflammatory.Macrophages	STAT5B	-0.01601	6.043742	-0.15074	0.880524	-6.87287	0.707102	0.744261
Non.inflammatory.Macrophages	ABCB6	0.045401	1.101522	0.150619	0.880619	-5.7635	0.954215	0.854004
Non.inflammatory.Macrophages	ZFP689	0.063911	1.407041	0.150566	0.880661	-5.55167	0.936882	0.846785
Non.inflammatory.Macrophages	RPN1	0.014324	5.957169	0.15048	0.880729	-6.94605	0.710856	0.746084

Non.inflammatory.Macrophages	SLC41A1	0.031604	2.387756	0.150468	0.880738	-6.15692	0.88316	0.824009
Non.inflammatory.Macrophages	ZFP683	0.099103	-1.15907	0.150425	0.880772	-5.27888	0.977959	0.909292
Non.inflammatory.Macrophages	SLA2	-0.05564	2.746588	-0.15038	0.88081	-5.7346	0.86422	0.815836
Non.inflammatory.Macrophages	KALRN	0.052538	2.750236	0.150356	0.880826	-5.93018	0.864029	0.815754
Non.inflammatory.Macrophages	HRH1	-0.05626	-0.56863	-0.15028	0.880888	-5.60789	0.977959	0.894561
Non.inflammatory.Macrophages	NOP10	-0.01283	7.15867	-0.15024	0.880914	-7.12841	0.660429	0.721566
Non.inflammatory.Macrophages	PPIL1	-0.02911	4.052063	-0.15004	0.881077	-6.33302	0.798449	0.786793
Non.inflammatory.Macrophages	PAQR7	0.036214	1.552734	0.149875	0.881205	-6.16889	0.928724	0.843504
Non.inflammatory.Macrophages	FAN1	0.049825	1.159783	0.149833	0.881238	-5.83742	0.950894	0.852786
Non.inflammatory.Macrophages	RRP7A	-0.02028	3.942345	-0.14979	0.881272	-6.5909	0.803792	0.789287
Non.inflammatory.Macrophages	GM5086	-0.04854	-0.01663	-0.14965	0.88138	-5.77852	0.977966	0.881178
Non.inflammatory.Macrophages	CCDC106	-0.05266	0.51131	-0.14956	0.881451	-5.79509	0.977435	0.868365
Non.inflammatory.Macrophages	NEB	-0.06078	1.258562	-0.14955	0.881459	-5.64726	0.945277	0.850527
Non.inflammatory.Macrophages	NKRF	-0.03434	2.983842	-0.1495	0.881499	-6.08283	0.85191	0.810688
Non.inflammatory.Macrophages	MIR17HG	-0.04753	2.192534	-0.14934	0.881623	-5.82917	0.893636	0.828786

Non.inflammatory.Macrophages	POMT2	-0.04021	1.813643	-0.14931	0.881648	-5.90089	0.914269	0.837563
Non.inflammatory.Macrophages	EXTL3	-0.02144	3.56619	-0.14895	0.881936	-6.55886	0.822462	0.797779
Non.inflammatory.Macrophages	TEAD1	-0.05898	1.756692	-0.14893	0.881948	-5.75123	0.917511	0.83896
Non.inflammatory.Macrophages	NEBL	0.101585	1.182115	0.14891	0.881963	-5.4717	0.949733	0.852466
Non.inflammatory.Macrophages	AARSD1	0.023602	3.977143	0.148803	0.882048	-6.43941	0.802199	0.788748
Non.inflammatory.Macrophages	ATG101	-0.01637	5.222366	-0.14846	0.882318	-6.72634	0.743664	0.761958
Non.inflammatory.Macrophages	GM48768	0.070621	0.621002	0.148235	0.882495	-5.50297	0.974914	0.866039
Non.inflammatory.Macrophages	KCTD3	0.023884	3.747291	0.148211	0.882514	-6.33003	0.813549	0.793936
Non.inflammatory.Macrophages	OLFR164	0.101042	-0.16043	0.148187	0.882532	-5.24402	0.978176	0.885036
Non.inflammatory.Macrophages	IMP3	0.013816	5.264975	0.14817	0.882546	-6.78941	0.741732	0.761099
Non.inflammatory.Macrophages	EXO5	0.045486	2.006793	0.148157	0.882556	-5.95253	0.903887	0.833329
Non.inflammatory.Macrophages	USP12	0.014436	6.069178	0.14753	0.88305	-7.01866	0.706444	0.744328
Non.inflammatory.Macrophages	SAP130	0.015791	5.619105	0.147522	0.883056	-6.81671	0.726154	0.753714
Non.inflammatory.Macrophages	FMR1	-0.01456	5.971489	-0.1471	0.883385	-6.84988	0.710885	0.746389
Non.inflammatory.Macrophages	TEX9	-0.04854	1.820323	-0.14698	0.88348	-5.83154	0.914751	0.837801

Non.inflammatory.Macrophages	TSPAN2	-0.04936	3.159011	-0.14671	0.883692	-5.69009	0.843842	0.807193
Non.inflammatory.Macrophages	LIG4	-0.05539	1.902715	-0.14653	0.883834	-5.78653	0.910368	0.835896
Non.inflammatory.Macrophages	BMF	0.044026	2.137288	0.146407	0.883933	-5.93845	0.897592	0.830462
Non.inflammatory.Macrophages	GM50386	-0.08038	0.179027	-0.14638	0.883958	-5.34363	0.979034	0.876898
Non.inflammatory.Macrophages	OPHN1	0.02745	2.997504	0.146258	0.884051	-6.55276	0.852135	0.810843
Non.inflammatory.Macrophages	PSMD5	0.020474	3.835237	0.146255	0.884053	-6.41487	0.809924	0.79216
Non.inflammatory.Macrophages	DRAP1	0.010829	6.508223	0.14605	0.884215	-7.03086	0.688108	0.735426
Non.inflammatory.Macrophages	1110019D1	0.033487	1.751896	0.145959	0.884286	-6.11375	0.918762	0.839508
Non.inflammatory.Macrophages	GM34921	0.063117	0.841113	0.14569	0.884498	-5.61415	0.968047	0.861053
Non.inflammatory.Macrophages	GPR137	0.022934	2.506946	0.145568	0.884594	-6.26756	0.878019	0.822092
Non.inflammatory.Macrophages	MAP7D1	0.012954	5.36793	0.145504	0.884644	-6.87404	0.737922	0.759173
Non.inflammatory.Macrophages	ICA1	-0.05082	2.463147	-0.14493	0.885095	-5.93593	0.880721	0.82327
Non.inflammatory.Macrophages	ZFP438	-0.02663	3.204144	-0.14482	0.885185	-6.25885	0.842132	0.806481
Non.inflammatory.Macrophages	ZFP948	-0.03143	2.965976	-0.14464	0.885326	-6.33846	0.854433	0.811877
Non.inflammatory.Macrophages	OCRL	-0.027	2.602911	-0.14449	0.885443	-6.41042	0.873446	0.820132

Non.inflammatory.Macrophages	9130230N(	-0.08588	0.640779	-0.14417	0.885695	-5.42007	0.976201	0.866194
Non.inflammatory.Macrophages	VPS35L	0.012363	5.162292	0.144151	0.885709	-6.82275	0.747776	0.763828
Non.inflammatory.Macrophages	PRODH	0.049446	2.780405	0.143675	0.886084	-5.95441	0.86431	0.81625
Non.inflammatory.Macrophages	FASTKD5	-0.05058	0.844458	-0.14359	0.886148	-5.71197	0.968691	0.861408
Non.inflammatory.Macrophages	MRFAP1	0.010691	6.452488	0.14346	0.886253	-7.0095	0.691117	0.736992
Non.inflammatory.Macrophages	IL16	-0.02568	4.60831	-0.1432	0.886459	-6.59255	0.773526	0.775856
Non.inflammatory.Macrophages	TCEANC2	-0.01656	4.247598	-0.14299	0.886621	-6.58268	0.790701	0.783706
Non.inflammatory.Macrophages	YIF1A	0.016611	4.016242	0.142943	0.88666	-6.54661	0.801905	0.788769
Non.inflammatory.Macrophages	FBXL12OS	0.060538	0.679534	0.142803	0.886771	-5.51763	0.974827	0.86547
Non.inflammatory.Macrophages	TRIM69	0.038172	2.05026	0.142723	0.886834	-6.13415	0.903261	0.833156
Non.inflammatory.Macrophages	ST3GAL5	0.014717	6.23691	0.142669	0.886876	-7.2171	0.700297	0.741534
Non.inflammatory.Macrophages	GOLM1	-0.02201	3.2957	-0.14261	0.886924	-6.44977	0.83776	0.804821
Non.inflammatory.Macrophages	GM43462	-0.0352	3.23183	-0.14258	0.886943	-6.04623	0.841009	0.80626
Non.inflammatory.Macrophages	CHCHD6	-0.02229	2.968098	-0.14252	0.886996	-6.30999	0.85455	0.812223
Non.inflammatory.Macrophages	GRK3	0.019303	2.91909	0.142148	0.887287	-6.7394	0.857089	0.81344

Non.inflammatory.Macrophages	POLR2K	0.011168	6.77475	0.142131	0.8873	-7.0467	0.677609	0.730662
Non.inflammatory.Macrophages	FAM8A1	0.025644	3.231881	0.142022	0.887386	-6.2349	0.841006	0.806415
Non.inflammatory.Macrophages	ZFP397	-0.01822	3.944609	-0.14199	0.887409	-6.4604	0.805405	0.790578
Non.inflammatory.Macrophages	NDUFS7	0.0113	6.320866	0.14198	0.887419	-6.99226	0.696708	0.739971
Non.inflammatory.Macrophages	ATP6V0C	0.010514	8.851286	0.14183	0.887537	-7.45558	0.596532	0.689676
Non.inflammatory.Macrophages	ZCWPW2	0.049542	1.016841	0.141794	0.887566	-5.87828	0.961133	0.85768
Non.inflammatory.Macrophages	DOK1	-0.0195	3.614618	-0.14178	0.887577	-6.49851	0.82171	0.797906
Non.inflammatory.Macrophages	8030453O2	0.050572	-0.75227	0.14167	0.887663	-5.66188	0.980062	0.900844
Non.inflammatory.Macrophages	SLC25A15	0.032698	2.488304	0.141541	0.887764	-6.05075	0.879703	0.823305
Non.inflammatory.Macrophages	GM8797	-0.03677	2.19543	-0.14154	0.887769	-5.952	0.89539	0.830038
Non.inflammatory.Macrophages	SWI5	0.013869	7.120072	0.141519	0.887782	-7.0904	0.663418	0.72372
Non.inflammatory.Macrophages	SNRPN	0.074949	-0.6475	0.14151	0.887789	-5.55819	0.980062	0.898231
Non.inflammatory.Macrophages	MESD	0.016497	4.085411	0.141401	0.887875	-6.54544	0.79854	0.787519
Non.inflammatory.Macrophages	D330023K1	0.036361	2.22594	0.141337	0.887926	-6.07216	0.893744	0.829334
Non.inflammatory.Macrophages	N6AMT1	0.028546	2.597729	0.141335	0.887927	-6.20297	0.873907	0.820803

Non.inflammatory.Macrophages	ADPRM	-0.02445	3.321315	-0.14131	0.887946	-6.20553	0.83646	0.804445
Non.inflammatory.Macrophages	PPM1D	-0.01818	4.948971	-0.14119	0.888039	-6.49264	0.75763	0.768857
Non.inflammatory.Macrophages	PIP4K2C	0.020035	4.238405	0.141182	0.888047	-6.49578	0.791144	0.784217
Non.inflammatory.Macrophages	ICAM1	-0.01811	5.898773	-0.14106	0.888143	-6.99731	0.714934	0.748801
Non.inflammatory.Macrophages	1700034P1	-0.06059	1.464772	-0.14099	0.888196	-5.78509	0.935648	0.847128
Non.inflammatory.Macrophages	GM47754	0.044819	0.667894	0.140937	0.88824	-5.9703	0.975262	0.866092
Non.inflammatory.Macrophages	ZFP658	-0.06424	0.297611	-0.14081	0.88834	-5.5858	0.980093	0.875077
Non.inflammatory.Macrophages	RNF170	0.020457	3.319753	0.140678	0.888444	-6.38187	0.836595	0.804598
Non.inflammatory.Macrophages	A630089N(	-0.03705	2.672384	-0.14043	0.888643	-6.00961	0.870154	0.819284
Non.inflammatory.Macrophages	LRRC10B	-0.08843	0.595128	-0.14023	0.888795	-5.39698	0.977729	0.867991
Non.inflammatory.Macrophages	TG	0.046567	1.91376	0.140075	0.888919	-5.783	0.911043	0.836763
Non.inflammatory.Macrophages	MRPL18	-0.0157	6.633228	-0.13994	0.889024	-6.95885	0.683763	0.733777
Non.inflammatory.Macrophages	HIRIP3	0.032702	3.169999	0.139813	0.889126	-6.0921	0.844479	0.808069
Non.inflammatory.Macrophages	MLST8	0.04191	2.044303	0.13972	0.889199	-5.91854	0.903921	0.833771
Non.inflammatory.Macrophages	D330050I1	0.051091	1.118758	0.139663	0.889244	-5.70136	0.955635	0.855498

Non.inflammatory.Macrophages	ARMC6	0.044443	1.405639	0.139479	0.889389	-5.74938	0.939325	0.848738
Non.inflammatory.Macrophages	FARS2	0.010225	6.899126	0.139339	0.889499	-7.04828	0.672714	0.72845
Non.inflammatory.Macrophages	ZBTB22	0.02534	3.076444	0.139246	0.889573	-6.30607	0.849278	0.810266
Non.inflammatory.Macrophages	2700062C(	0.025745	2.646093	0.1391	0.889688	-6.17574	0.87168	0.820031
Non.inflammatory.Macrophages	C2CD5	-0.02043	4.918041	-0.13899	0.889776	-6.49263	0.759343	0.769806
Non.inflammatory.Macrophages	HDAC10	-0.03959	1.608346	-0.13898	0.889781	-5.86441	0.927954	0.844059
Non.inflammatory.Macrophages	EHMT2	0.014876	4.951712	0.138967	0.889792	-6.76527	0.757785	0.769086
Non.inflammatory.Macrophages	PKNOX2	0.09342	-0.82371	0.138766	0.88995	-5.30121	0.980427	0.903098
Non.inflammatory.Macrophages	HOMER1	-0.01629	6.109837	-0.13875	0.889962	-6.98902	0.706026	0.74475
Non.inflammatory.Macrophages	DNAJB11	0.01236	6.073023	0.138669	0.890027	-6.89601	0.707618	0.745531
Non.inflammatory.Macrophages	VARS2	-0.05206	1.401859	-0.1385	0.890157	-5.76088	0.939539	0.849062
Non.inflammatory.Macrophages	APOOL	-0.01946	3.780654	-0.13829	0.890324	-6.46157	0.81377	0.794729
Non.inflammatory.Macrophages	TRIM68	-0.04762	0.946883	-0.1379	0.890632	-5.71506	0.965266	0.859915
Non.inflammatory.Macrophages	MRPS35	-0.01639	4.266456	-0.13788	0.890649	-6.55256	0.790088	0.784078
Non.inflammatory.Macrophages	TRMT11	-0.01992	3.646744	-0.13786	0.890662	-6.42038	0.820413	0.797719

Non.inflammatory.Macrophages	ZFP90	0.029808	2.283533	0.137792	0.890718	-6.23918	0.890975	0.828553
Non.inflammatory.Macrophages	GOLIM4	0.02149	5.140893	0.137776	0.89073	-6.71532	0.74909	0.765222
Non.inflammatory.Macrophages	FAM71A	-0.04475	0.237403	-0.13775	0.890748	-5.91661	0.980427	0.877025
Non.inflammatory.Macrophages	SMIM19	-0.01474	4.75168	-0.13768	0.890804	-6.7105	0.767083	0.773558
Non.inflammatory.Macrophages	SMIM27	0.018208	3.590898	0.137627	0.890848	-6.49389	0.823199	0.798962
Non.inflammatory.Macrophages	CIAO3	0.021617	3.316721	0.137612	0.89086	-6.35657	0.837004	0.805082
Non.inflammatory.Macrophages	GM45353	-0.07311	-0.0712	-0.13756	0.890901	-5.31131	0.980427	0.884572
Non.inflammatory.Macrophages	SLCO4C1	0.079572	-1.1708	0.137553	0.890907	-5.28365	0.980427	0.91196
Non.inflammatory.Macrophages	ZFP748	-0.04281	1.938501	-0.13723	0.891164	-5.89114	0.9097	0.836645
Non.inflammatory.Macrophages	SPG21	-0.01156	6.057126	-0.13703	0.891319	-6.96951	0.708306	0.746113
Non.inflammatory.Macrophages	LRRC43	-0.06447	-0.12202	-0.13701	0.891337	-5.58484	0.980427	0.886012
Non.inflammatory.Macrophages	KANK1	-0.04275	1.172928	-0.13684	0.891466	-5.89921	0.952536	0.854755
Non.inflammatory.Macrophages	LOXL3	-0.06363	0.451305	-0.13677	0.891521	-5.66961	0.980427	0.872067
Non.inflammatory.Macrophages	MEAF6	0.013795	4.941914	0.136705	0.891575	-6.67047	0.758238	0.769679
Non.inflammatory.Macrophages	TNFSF12	0.022502	2.295218	0.136672	0.891601	-6.4374	0.890347	0.828507

Non.inflammatory.Macrophages	ZSWIM8	0.013841	4.703109	0.136664	0.891607	-6.71825	0.769357	0.774813
Non.inflammatory.Macrophages	SUGCT	-0.02803	2.835527	-0.13657	0.891679	-6.2945	0.861753	0.81615
Non.inflammatory.Macrophages	DEDD	0.016545	4.437275	0.136526	0.891716	-6.56406	0.781916	0.78057
Non.inflammatory.Macrophages	PSMG4	-0.01334	5.042688	-0.13647	0.89176	-6.80454	0.753592	0.767525
Non.inflammatory.Macrophages	C3AR1	-0.02849	2.290307	-0.13636	0.891847	-6.71622	0.890611	0.828622
Non.inflammatory.Macrophages	WDR24	0.036958	1.796393	0.1363	0.891894	-5.93221	0.917517	0.84008
Non.inflammatory.Macrophages	MRPL4	-0.01409	5.176328	-0.13629	0.891904	-6.77688	0.747472	0.764674
Non.inflammatory.Macrophages	SAP30L	-0.01556	4.309459	-0.13569	0.892374	-6.54598	0.788023	0.783517
Non.inflammatory.Macrophages	BRIP1OS	0.01574	5.408086	0.135671	0.89239	-6.70174	0.73697	0.759917
Non.inflammatory.Macrophages	HNRNPC	-0.00821	7.730209	-0.13557	0.892471	-7.15584	0.639285	0.712346
Non.inflammatory.Macrophages	ANKRD52	-0.01816	3.881446	-0.13545	0.892564	-6.50837	0.808802	0.792918
Non.inflammatory.Macrophages	CDH1	0.066895	1.38751	0.135449	0.892565	-5.58759	0.940349	0.849873
Non.inflammatory.Macrophages	CCL8	-0.05676	-1.46778	-0.13534	0.892651	-5.50475	0.980427	0.919947
Non.inflammatory.Macrophages	EXOC6	0.013028	6.194009	0.135272	0.892705	-6.94303	0.702399	0.743479
Non.inflammatory.Macrophages	HIST1H3A	0.064007	0.953909	0.135231	0.892736	-5.50071	0.965007	0.860173

Non.inflammatory.Macrophages	CXCL1	-0.04892	2.815955	-0.13522	0.892743	-6.60389	0.862774	0.816778
Non.inflammatory.Macrophages	CCDC66	0.031305	2.265203	0.135148	0.892802	-6.00184	0.891961	0.829387
Non.inflammatory.Macrophages	FGFRL1	0.067839	0.299565	0.13512	0.892824	-5.57277	0.980427	0.875947
Non.inflammatory.Macrophages	DESI1	0.01894	5.729904	0.134972	0.892941	-6.6936	0.722622	0.753169
Non.inflammatory.Macrophages	GM5089	0.072558	0.395428	0.13496	0.89295	-5.44625	0.980427	0.873642
Non.inflammatory.Macrophages	GOLGA1	0.020375	3.427325	0.13495	0.892958	-6.36797	0.831409	0.803024
Non.inflammatory.Macrophages	PPM1N	-0.0446	0.159456	-0.13485	0.893034	-5.98372	0.980427	0.879398
Non.inflammatory.Macrophages	TCP1	0.013906	6.67677	0.134547	0.893276	-6.98843	0.681942	0.733694
Non.inflammatory.Macrophages	EBP	0.017247	4.791273	0.134538	0.893283	-6.70419	0.765234	0.773236
Non.inflammatory.Macrophages	CKS1B	-0.02718	5.377173	-0.13445	0.893352	-6.69903	0.738363	0.760739
Non.inflammatory.Macrophages	DNA2	0.032073	3.583362	0.134433	0.893366	-6.00195	0.823576	0.799693
Non.inflammatory.Macrophages	DQX1	-0.09654	1.215444	-0.13442	0.893376	-5.64587	0.95011	0.854128
Non.inflammatory.Macrophages	ALG14	-0.0223	3.562595	-0.13436	0.893424	-6.39959	0.824614	0.800171
Non.inflammatory.Macrophages	TARDBP	-0.01259	5.771119	-0.13412	0.893615	-6.86031	0.720899	0.752479
Non.inflammatory.Macrophages	NARS	0.01183	6.298026	0.133897	0.893788	-7.00369	0.698113	0.741605

Non.inflammatory.Macrophages	UTP11	-0.01431	4.845019	-0.13378	0.893882	-6.70524	0.762936	0.772246
Non.inflammatory.Macrophages	MED23	-0.01771	3.692708	-0.13341	0.894174	-6.45932	0.818518	0.797541
Non.inflammatory.Macrophages	SPR	-0.01916	3.527522	-0.13329	0.894266	-6.52659	0.826766	0.801234
Non.inflammatory.Macrophages	HIST1H2AE	0.047948	4.491316	0.133131	0.894392	-6.42861	0.77972	0.780046
Non.inflammatory.Macrophages	MRPS15	0.012153	5.910327	0.132899	0.894575	-6.91534	0.715036	0.74985
Non.inflammatory.Macrophages	KCTD14	0.057244	1.055743	0.132813	0.894643	-5.57917	0.95971	0.858296
Non.inflammatory.Macrophages	LRRN3	-0.0749	-0.11923	-0.13281	0.894647	-5.41972	0.980895	0.886759
Non.inflammatory.Macrophages	SGTB	-0.05169	0.3418	-0.13257	0.894837	-5.6698	0.980895	0.875553
Non.inflammatory.Macrophages	EIF3H	-0.01011	7.353901	-0.13253	0.894866	-7.13998	0.654529	0.720377
Non.inflammatory.Macrophages	NT5M	0.019617	3.572881	0.132355	0.895004	-6.38816	0.824493	0.800392
Non.inflammatory.Macrophages	EID2	-0.05842	0.075956	-0.13235	0.895007	-5.60283	0.980895	0.882101
Non.inflammatory.Macrophages	BAG6	0.015912	5.438014	0.132245	0.895091	-6.76829	0.735976	0.759906
Non.inflammatory.Macrophages	GM43063	-0.04398	0.779437	-0.1322	0.895124	-5.78872	0.971929	0.865058
Non.inflammatory.Macrophages	TAP2	-0.02145	5.110651	-0.13207	0.895231	-6.69879	0.750832	0.766902
Non.inflammatory.Macrophages	3110082I1	0.018588	4.199896	0.132002	0.895283	-6.691	0.793673	0.786598

Non.inflammatory.Macrophages	GM20721	0.024434	3.72758	0.13189	0.895371	-6.28334	0.816787	0.797006
Non.inflammatory.Macrophages	GIT1	-0.01785	3.282244	-0.13178	0.895458	-6.3517	0.839156	0.806945
Non.inflammatory.Macrophages	RANGRF	-0.04963	1.262196	-0.13174	0.895493	-5.78044	0.947901	0.853571
Non.inflammatory.Macrophages	FGD2	0.018706	3.69567	0.131703	0.895519	-6.72768	0.818371	0.797714
Non.inflammatory.Macrophages	SEMA5A	-0.07443	1.106724	-0.13168	0.89554	-5.51647	0.956781	0.857267
Non.inflammatory.Macrophages	ZFP993	0.031125	1.824761	0.131392	0.895764	-6.1437	0.916389	0.840382
Non.inflammatory.Macrophages	PRTN3	0.056851	3.6691	0.131356	0.895792	-6.68539	0.819692	0.798358
Non.inflammatory.Macrophages	CCT3	0.011608	5.801241	0.131351	0.895796	-6.87017	0.719821	0.752352
Non.inflammatory.Macrophages	PGPEP1	-0.01984	3.461593	-0.1312	0.895915	-6.41361	0.830079	0.803023
Non.inflammatory.Macrophages	ENPP4	-0.02201	2.924155	-0.13115	0.895953	-6.44144	0.857554	0.815129
Non.inflammatory.Macrophages	TTC38	-0.02977	2.505389	-0.13115	0.895957	-6.16522	0.879542	0.82468
Non.inflammatory.Macrophages	DLG3	0.06818	0.571754	0.131087	0.896005	-5.43744	0.978851	0.870212
Non.inflammatory.Macrophages	RAB24	0.012896	5.118704	0.131061	0.896025	-6.75507	0.750463	0.766832
Non.inflammatory.Macrophages	CTNNA1	0.012979	6.643473	0.130803	0.896228	-7.10475	0.683728	0.735017
Non.inflammatory.Macrophages	SGSM2	-0.02043	3.786605	-0.13077	0.896258	-6.45139	0.813944	0.79588

Non.inflammatory.Macrophages	MFSD2B	0.055029	0.599514	0.130517	0.896454	-5.57735	0.978278	0.869661
Non.inflammatory.Macrophages	PARP3	-0.03033	2.362796	-0.13047	0.896493	-6.12669	0.887256	0.828072
Non.inflammatory.Macrophages	GM44659	0.05725	0.682551	0.130447	0.896509	-5.52941	0.975661	0.867659
Non.inflammatory.Macrophages	DECR2	-0.02728	2.741577	-0.13037	0.896569	-6.23223	0.867183	0.819404
Non.inflammatory.Macrophages	RBM17	0.012059	5.789508	0.13017	0.896728	-6.91845	0.720488	0.752826
Non.inflammatory.Macrophages	HMGCR	-0.01769	4.712579	-0.13009	0.896792	-6.57592	0.769441	0.775754
Non.inflammatory.Macrophages	GM20492	0.028171	1.798274	0.12989	0.896949	-6.11708	0.918123	0.841321
Non.inflammatory.Macrophages	UQCRB	-0.01085	8.047525	-0.12981	0.897015	-7.31581	0.627439	0.707021
Non.inflammatory.Macrophages	GSTM4	-0.04704	1.121715	-0.12954	0.897226	-5.79805	0.956227	0.857299
Non.inflammatory.Macrophages	GM20536	-0.0397	2.340392	-0.12952	0.897241	-5.81278	0.888632	0.828743
Non.inflammatory.Macrophages	ARHGAP27	0.027613	2.032169	0.129503	0.897254	-6.09941	0.905303	0.835876
Non.inflammatory.Macrophages	RER1	-0.01127	6.413933	-0.12936	0.897366	-6.99757	0.693591	0.739913
Non.inflammatory.Macrophages	POLA2	0.022061	4.050563	0.129255	0.897449	-6.43308	0.801215	0.790232
Non.inflammatory.Macrophages	PPM1F	-0.03313	2.206568	-0.1291	0.897573	-5.98352	0.895894	0.831833
Non.inflammatory.Macrophages	POLH	0.022677	4.035992	0.128979	0.897667	-6.32856	0.801933	0.790573

Non.inflammatory.Macrophages	HIST1H3E	0.031749	3.610481	0.128968	0.897676	-6.17535	0.822931	0.799992
Non.inflammatory.Macrophages	MARVELD1	0.037934	1.253094	0.128766	0.897836	-5.8862	0.948801	0.854205
Non.inflammatory.Macrophages	OLFR56	-0.04691	2.067818	-0.12876	0.897836	-5.9705	0.903436	0.835077
Non.inflammatory.Macrophages	COG5	0.012725	6.582266	0.12826	0.898234	-6.97357	0.686754	0.736665
Non.inflammatory.Macrophages	HMBOX1	-0.01428	5.578159	-0.128	0.898439	-6.83223	0.730368	0.757549
Non.inflammatory.Macrophages	APIP	-0.01488	4.220389	-0.12763	0.898732	-6.58013	0.793446	0.786794
Non.inflammatory.Macrophages	TSR3	-0.02186	3.569257	-0.12763	0.898735	-6.41519	0.825467	0.801182
Non.inflammatory.Macrophages	ABCB10	0.028898	2.977675	0.127591	0.898763	-6.11235	0.855602	0.814484
Non.inflammatory.Macrophages	RNF113A2	-0.01849	4.271264	-0.12755	0.898794	-6.46865	0.790994	0.7857
Non.inflammatory.Macrophages	ZFP688	-0.03707	1.695397	-0.12739	0.898917	-5.90023	0.924437	0.844119
Non.inflammatory.Macrophages	DCN	-0.03374	3.138043	-0.12739	0.898919	-6.33367	0.847333	0.810922
Non.inflammatory.Macrophages	DAB2	0.029578	4.009728	0.127303	0.89899	-6.79282	0.803676	0.791504
Non.inflammatory.Macrophages	LMNB1	0.01981	7.679763	0.126931	0.899283	-6.87769	0.642195	0.714664
Non.inflammatory.Macrophages	RECQL5	-0.02187	3.722047	-0.12658	0.899564	-6.30039	0.817853	0.797903
Non.inflammatory.Macrophages	ZDHHC4	0.016127	4.474275	0.126545	0.899588	-6.58214	0.781285	0.781371

Non.inflammatory.Macrophages	IGLV3	0.089197	-0.70552	0.126446	0.899666	-5.22849	0.981845	0.902376
Non.inflammatory.Macrophages	MORRBID	-0.01757	5.127394	-0.12622	0.899842	-7.10783	0.750792	0.767337
Non.inflammatory.Macrophages	PPP1R14A	-0.0488	0.601813	-0.12619	0.899872	-5.74476	0.979049	0.870269
Non.inflammatory.Macrophages	ANO10	0.024468	3.16746	0.125993	0.900023	-6.32844	0.845831	0.810374
Non.inflammatory.Macrophages	NFATC3	0.013541	7.290076	0.125928	0.900075	-6.96818	0.657732	0.722513
Non.inflammatory.Macrophages	PCNT	-0.01499	5.233516	-0.12579	0.900181	-6.71766	0.745945	0.765097
Non.inflammatory.Macrophages	5430427O1	0.02084	3.846602	0.125741	0.900222	-6.49686	0.811689	0.795224
Non.inflammatory.Macrophages	MPC1	0.012382	7.569491	0.125694	0.90026	-7.07909	0.646555	0.716941
Non.inflammatory.Macrophages	CERCAM	0.050287	0.652443	0.125635	0.900306	-5.61319	0.977615	0.869099
Non.inflammatory.Macrophages	ZMYM3	0.029907	2.31007	0.125632	0.900309	-6.1262	0.890837	0.829969
Non.inflammatory.Macrophages	ZFAND3	-0.00903	8.233036	-0.12535	0.90053	-7.31502	0.62075	0.70384
Non.inflammatory.Macrophages	GTPBP2	-0.01553	4.751688	-0.1252	0.900646	-6.67103	0.768192	0.775466
Non.inflammatory.Macrophages	ACO1	-0.01505	3.712371	-0.12509	0.900736	-6.59111	0.818333	0.798227
Non.inflammatory.Macrophages	CLMP	0.036109	1.140521	0.125059	0.90076	-6.05641	0.955769	0.857411
Non.inflammatory.Macrophages	CHRM3	-0.07617	0.831294	-0.12504	0.900778	-5.51703	0.970942	0.864808

Non.inflammatory.Macrophages	TUBA1C	-0.01523	7.576145	-0.12503	0.900787	-7.16964	0.646291	0.716826
Non.inflammatory.Macrophages	1810006J0	-0.07494	-0.01116	-0.12502	0.900793	-5.32937	0.981845	0.885274
Non.inflammatory.Macrophages	IARS	0.015582	5.056202	0.124942	0.900853	-6.69804	0.75406	0.768933
Non.inflammatory.Macrophages	CTNNAL1	0.051617	1.924908	0.124862	0.900917	-5.69102	0.911763	0.838948
Non.inflammatory.Macrophages	10-Sep	0.018326	3.119761	0.124845	0.90093	-6.47624	0.848279	0.811525
Non.inflammatory.Macrophages	IFIT3B	-0.04176	1.43036	-0.12482	0.900947	-6.17671	0.939289	0.85056
Non.inflammatory.Macrophages	PPP2R5D	-0.01889	3.682866	-0.12468	0.901056	-6.4033	0.819801	0.798909
Non.inflammatory.Macrophages	CTU1	0.041443	0.850388	0.124592	0.901129	-5.86525	0.970233	0.864407
Non.inflammatory.Macrophages	ALPL	-0.06356	1.490191	-0.12429	0.901367	-5.50606	0.935919	0.849176
Non.inflammatory.Macrophages	GPAA1	-0.0149	3.764843	-0.12422	0.901422	-6.51633	0.81573	0.797115
Non.inflammatory.Macrophages	HDLBP	-0.00884	6.655734	-0.12418	0.901455	-7.05457	0.683809	0.735478
Non.inflammatory.Macrophages	RFT1	-0.02636	2.53189	-0.1241	0.901519	-6.2281	0.878986	0.824934
Non.inflammatory.Macrophages	NR4A3	-0.02063	6.012984	-0.12405	0.901558	-6.99152	0.711249	0.748757
Non.inflammatory.Macrophages	NANOS1	0.060014	0.634448	0.124033	0.901571	-5.49311	0.978235	0.869606
Non.inflammatory.Macrophages	MOSPD3	-0.014	4.656046	-0.12389	0.901687	-6.66967	0.772683	0.777589

Non.inflammatory.Macrophages	6330409D2	-0.07559	0.333222	-0.12388	0.901692	-5.32689	0.981845	0.876915
Non.inflammatory.Macrophages	SUMO3	0.011954	5.724941	0.123845	0.901719	-6.8977	0.723887	0.754801
Non.inflammatory.Macrophages	EEF2KMT	-0.01867	3.145957	-0.12384	0.901725	-6.40954	0.846934	0.810978
Non.inflammatory.Macrophages	TCF7L2	0.01601	7.34951	0.12372	0.901818	-7.261	0.655339	0.72144
Non.inflammatory.Macrophages	KIF20B	-0.03544	4.170808	-0.12372	0.901821	-6.35085	0.795848	0.788192
Non.inflammatory.Macrophages	NFKBIL1	0.016163	3.861655	0.12371	0.901826	-6.47786	0.810947	0.795003
Non.inflammatory.Macrophages	CSGALNAC	0.054085	2.818139	0.123392	0.902077	-5.77911	0.864078	0.818481
Non.inflammatory.Macrophages	PRKDC	0.014787	4.69925	0.123018	0.902372	-6.64355	0.770989	0.776778
Non.inflammatory.Macrophages	E230014E1	0.072247	-1.38289	0.122933	0.902439	-5.23382	0.982275	0.919856
Non.inflammatory.Macrophages	RCBTB1	0.019356	3.826201	0.122825	0.902524	-6.37246	0.813063	0.795982
Non.inflammatory.Macrophages	SCAMP3	-0.01228	4.819715	-0.12268	0.902636	-6.72419	0.76538	0.77427
Non.inflammatory.Macrophages	GM12840	0.0289	3.766389	0.122503	0.902779	-6.6449	0.816044	0.797378
Non.inflammatory.Macrophages	ANKRD27	0.020895	3.098779	0.122468	0.902806	-6.29688	0.849764	0.812331
Non.inflammatory.Macrophages	GNPNAT1	-0.01836	4.157449	-0.12243	0.902838	-6.42779	0.796877	0.788748
Non.inflammatory.Macrophages	MATK	0.027558	2.36611	0.121935	0.903227	-6.22095	0.888565	0.829149

Non.inflammatory.Macrophages	ING5	0.01805	3.456639	0.121609	0.903484	-6.39734	0.83185	0.804414
Non.inflammatory.Macrophages	ATP6V1E1	0.009605	7.59223	0.121548	0.903533	-7.22561	0.646211	0.716959
Non.inflammatory.Macrophages	GM8369	-0.04089	3.324129	-0.12136	0.90368	-6.03903	0.838561	0.807468
Non.inflammatory.Macrophages	CMBL	0.035939	2.192141	0.121289	0.903738	-6.14074	0.897971	0.833302
Non.inflammatory.Macrophages	TRMT2B	-0.01999	3.750232	-0.12124	0.903774	-6.38215	0.817158	0.797953
Non.inflammatory.Macrophages	INO80E	0.011996	4.165534	0.121128	0.903864	-6.59025	0.796791	0.788795
Non.inflammatory.Macrophages	CDC26	-0.00957	5.383294	-0.12111	0.903875	-6.8016	0.739792	0.762503
Non.inflammatory.Macrophages	TALDO1	0.00863	8.310001	0.120985	0.903977	-7.35649	0.618356	0.702855
Non.inflammatory.Macrophages	PPIL4	-0.011	5.46202	-0.12098	0.903983	-6.75219	0.736244	0.760845
Non.inflammatory.Macrophages	SLC35E2	-0.017	3.898481	-0.12092	0.904033	-6.39705	0.809832	0.794697
Non.inflammatory.Macrophages	CAR12	0.072573	0.013745	0.120791	0.904131	-5.31962	0.982692	0.885339
Non.inflammatory.Macrophages	PTGS2OS2	0.068884	-0.48663	0.120735	0.904175	-5.38464	0.982692	0.897741
Non.inflammatory.Macrophages	LHFPL2	-0.02927	2.227897	-0.1207	0.904201	-6.15419	0.896036	0.83255
Non.inflammatory.Macrophages	GM12253	0.084048	-0.86999	0.120494	0.904365	-5.19986	0.982776	0.907417
Non.inflammatory.Macrophages	STK3	-0.01335	5.985237	-0.12042	0.904424	-6.93749	0.713133	0.749949

Non.inflammatory.Macrophages	ZFP386	0.019032	3.889207	0.120135	0.904649	-6.39537	0.810494	0.795115
Non.inflammatory.Macrophages	1110004F1	0.007981	6.204	0.119982	0.904769	-6.94933	0.703808	0.745563
Non.inflammatory.Macrophages	ZDHHC13	0.016769	3.693777	0.119463	0.90518	-6.42716	0.820504	0.799564
Non.inflammatory.Macrophages	TMEM43	-0.018	3.43413	-0.1193	0.905306	-6.43879	0.833535	0.805362
Non.inflammatory.Macrophages	ARHGAP11	-0.01828	4.784218	-0.1192	0.905385	-6.59558	0.767837	0.77566
Non.inflammatory.Macrophages	LONP1	0.017942	3.653778	0.119194	0.905392	-6.42058	0.822499	0.800454
Non.inflammatory.Macrophages	QPCT	0.038066	2.264701	0.11904	0.905514	-5.89922	0.894638	0.831993
Non.inflammatory.Macrophages	EHBP1L1	-0.01087	6.149657	-0.11903	0.905523	-6.9703	0.7064	0.746727
Non.inflammatory.Macrophages	PRKN	-0.0217	3.428362	-0.11883	0.905678	-6.62643	0.833902	0.805491
Non.inflammatory.Macrophages	ZFP74	0.028191	2.132709	0.118659	0.905815	-6.01224	0.901877	0.835072
Non.inflammatory.Macrophages	C9ORF72	-0.01677	3.893192	-0.11864	0.905831	-6.82617	0.810706	0.795158
Non.inflammatory.Macrophages	PCDH17	-0.03938	2.459624	-0.11824	0.906145	-6.03382	0.884436	0.8276
Non.inflammatory.Macrophages	ACER2	0.03105	2.516505	0.118183	0.906191	-6.11325	0.881403	0.826291
Non.inflammatory.Macrophages	LMLN	-0.02836	1.750464	-0.11797	0.906363	-6.05153	0.923062	0.84416
Non.inflammatory.Macrophages	ELOVL6	0.033838	4.451031	0.117835	0.906466	-6.10578	0.783814	0.783082

Non.inflammatory.Macrophages	PRPF40A	-0.00785	7.709067	-0.11781	0.906483	-7.16475	0.642207	0.715185
Non.inflammatory.Macrophages	PPARGC1A	-0.06628	1.419055	-0.11776	0.906523	-5.54724	0.941635	0.851999
Non.inflammatory.Macrophages	GM31645	-0.04714	0.726126	-0.11771	0.906566	-5.728	0.97663	0.86856
Non.inflammatory.Macrophages	CEP128	0.016594	6.336302	0.117677	0.90659	-6.85837	0.698583	0.743044
Non.inflammatory.Macrophages	PPP2R5A	0.009149	7.861178	0.117188	0.906977	-7.0841	0.636361	0.71226
Non.inflammatory.Macrophages	OAZ2	-0.01398	4.485342	-0.11708	0.907064	-6.55098	0.782328	0.782467
Non.inflammatory.Macrophages	GNE	0.020571	4.467546	0.117049	0.907087	-6.24658	0.783176	0.782855
Non.inflammatory.Macrophages	ANO6	0.01178	6.75402	0.116919	0.907189	-7.14642	0.68107	0.734574
Non.inflammatory.Macrophages	KCNK13	0.023049	2.912398	0.116847	0.907246	-6.53987	0.860722	0.81748
Non.inflammatory.Macrophages	UBTD1	0.013857	4.402162	0.116844	0.907249	-6.775	0.7863	0.784281
Non.inflammatory.Macrophages	TPM1	0.01708	5.177563	0.116741	0.90733	-6.71142	0.750001	0.767574
Non.inflammatory.Macrophages	SIAE	-0.02209	2.612072	-0.11672	0.907346	-6.26752	0.876496	0.824382
Non.inflammatory.Macrophages	VCPIP1	0.009787	6.167501	0.116588	0.907451	-6.94839	0.705998	0.746708
Non.inflammatory.Macrophages	ACADS	0.013771	4.490963	0.116395	0.907604	-6.65933	0.782099	0.782407
Non.inflammatory.Macrophages	CEP57	-0.01442	5.139583	-0.11639	0.907608	-6.62209	0.751778	0.768406

Non.inflammatory.Macrophages	HSPBAP1	0.020456	3.764205	0.116159	0.90779	-6.47343	0.817539	0.798418
Non.inflammatory.Macrophages	EFCAB2	0.029824	2.668273	0.115951	0.907954	-6.09613	0.87376	0.823189
Non.inflammatory.Macrophages	PKD1	0.019866	3.003757	0.11569	0.908161	-6.3576	0.856243	0.815588
Non.inflammatory.Macrophages	DNAJC15	-0.0112	5.723768	-0.11558	0.908244	-6.94975	0.72562	0.75612
Non.inflammatory.Macrophages	GM43111	0.035555	-0.1905	0.115583	0.908245	-5.90974	0.984126	0.891294
Non.inflammatory.Macrophages	UAP1L1	-0.01464	3.734573	-0.11521	0.908541	-6.60341	0.819129	0.799171
Non.inflammatory.Macrophages	MAD2L1BF	-0.01668	4.194819	-0.1152	0.908547	-6.46566	0.796533	0.788999
Non.inflammatory.Macrophages	TMEM234	0.007581	7.040364	0.115127	0.908606	-7.10519	0.669432	0.728907
Non.inflammatory.Macrophages	SLC11A1	0.017204	3.505984	0.115125	0.908607	-6.8912	0.830574	0.804271
Non.inflammatory.Macrophages	TAF9B	-0.05444	0.956005	-0.11509	0.908635	-5.52014	0.96857	0.86337
Non.inflammatory.Macrophages	DNAJA3	0.017892	3.76841	0.115074	0.908648	-6.43809	0.817448	0.798419
Non.inflammatory.Macrophages	SMU1	-0.01044	5.608875	-0.11479	0.908875	-6.8105	0.73082	0.758563
Non.inflammatory.Macrophages	GM26759	0.025431	3.123372	0.114663	0.908972	-6.26085	0.850165	0.812907
Non.inflammatory.Macrophages	MTRF1L	0.025858	3.1332	0.114661	0.908974	-6.10525	0.849659	0.812685
Non.inflammatory.Macrophages	FAR1OS	-0.05869	2.788806	-0.11442	0.909167	-5.59292	0.867576	0.820518

Non.inflammatory.Macrophages	RSL1D1	-0.01158	6.043414	-0.11432	0.909243	-6.91106	0.711677	0.749455
Non.inflammatory.Macrophages	SLC25A26	-0.01721	3.795963	-0.11415	0.909381	-6.52639	0.816198	0.797868
Non.inflammatory.Macrophages	DNASE1L1	0.015746	3.048154	0.113933	0.90955	-6.59256	0.854066	0.814687
Non.inflammatory.Macrophages	GM37401	-0.04403	0.875344	-0.11391	0.909569	-5.67923	0.971698	0.865418
Non.inflammatory.Macrophages	NANP	-0.02649	2.821135	-0.11381	0.909644	-6.1475	0.865882	0.819849
Non.inflammatory.Macrophages	SNTA1	0.021222	1.865421	0.113795	0.909658	-6.34183	0.917291	0.841931
Non.inflammatory.Macrophages	ZFP280B	0.040159	1.826966	0.11376	0.909686	-5.7475	0.919417	0.842849
Non.inflammatory.Macrophages	IFNG	-0.08398	1.241502	-0.11372	0.909715	-5.44506	0.952341	0.856684
Non.inflammatory.Macrophages	ZNRD1AS	0.043528	0.28937	0.113713	0.909723	-5.71784	0.984266	0.879641
Non.inflammatory.Macrophages	GM10184	-0.04726	1.11236	-0.11318	0.910143	-5.69957	0.960111	0.859899
Non.inflammatory.Macrophages	NSD3	-0.00931	8.849458	-0.113	0.910285	-7.30818	0.599386	0.693368
Non.inflammatory.Macrophages	GFER	0.011558	4.406699	0.112999	0.910287	-6.65811	0.78674	0.784643
Non.inflammatory.Macrophages	H3F3A	0.008303	10.71708	0.112316	0.910827	-7.59056	0.53438	0.658324
Non.inflammatory.Macrophages	UCK1	-0.02288	3.265642	-0.1123	0.910837	-6.28515	0.843227	0.810015
Non.inflammatory.Macrophages	CSRNP1	-0.01475	6.641086	-0.11225	0.910881	-7.0199	0.686387	0.73737

Non.inflammatory.Macrophages	1110051M	-0.01949	3.263469	-0.11223	0.910896	-6.31609	0.843339	0.810064
Non.inflammatory.Macrophages	ZFP143	-0.0158	3.815014	-0.11203	0.911054	-6.41828	0.815582	0.797736
Non.inflammatory.Macrophages	SYCE2	-0.02071	4.258668	-0.11198	0.911092	-6.5906	0.793878	0.787966
Non.inflammatory.Macrophages	ADAMTSL1	-0.08511	0.587709	-0.11178	0.911248	-5.54143	0.982212	0.872682
Non.inflammatory.Macrophages	TSPAN18	-0.04287	1.764014	-0.11169	0.911326	-5.80839	0.923279	0.844624
Non.inflammatory.Macrophages	ENTR1	0.01325	4.880508	0.111655	0.91135	-6.69402	0.764371	0.77447
Non.inflammatory.Macrophages	GM44751	-0.0222	1.310612	-0.11155	0.911434	-6.31102	0.948782	0.855335
Non.inflammatory.Macrophages	GAS8	0.024963	1.529564	0.111526	0.911452	-6.02146	0.936388	0.850146
Non.inflammatory.Macrophages	TMEM186	-0.03159	2.111676	-0.11134	0.911598	-5.96821	0.904148	0.836502
Non.inflammatory.Macrophages	UQCRC2	0.00887	6.345631	0.111341	0.911598	-6.96427	0.698918	0.743518
Non.inflammatory.Macrophages	TNFSF10	0.028598	2.307037	0.111302	0.911629	-6.0744	0.893557	0.83197
Non.inflammatory.Macrophages	SPEF2	0.045274	1.371663	0.111239	0.911679	-5.67271	0.945311	0.853907
Non.inflammatory.Macrophages	RNF115	0.008358	6.978362	0.111204	0.911707	-7.06387	0.672348	0.730552
Non.inflammatory.Macrophages	C6	0.027663	2.802964	0.111118	0.911775	-6.55712	0.867183	0.8206
Non.inflammatory.Macrophages	DOCK9	0.028331	4.197961	0.11107	0.911812	-6.21985	0.796816	0.78937

Non.inflammatory.Macrophages	TRDMT1	0.022358	2.342131	0.110973	0.911889	-6.16428	0.891667	0.831214
Non.inflammatory.Macrophages	RBM47	0.011641	6.404832	0.110728	0.912083	-7.23969	0.69639	0.742343
Non.inflammatory.Macrophages	GM11998	0.077961	0.318253	0.110622	0.912166	-5.26881	0.984663	0.879301
Non.inflammatory.Macrophages	ZFP523	-0.02509	2.19531	-0.1106	0.912187	-6.04149	0.8996	0.834615
Non.inflammatory.Macrophages	MTIF3	-0.02263	3.10156	-0.11047	0.91229	-6.21577	0.851652	0.813878
Non.inflammatory.Macrophages	TMEM184I	0.014873	4.707523	0.110459	0.912295	-6.71674	0.772474	0.778302
Non.inflammatory.Macrophages	AGBL3	-0.03358	1.72445	-0.1103	0.912422	-5.8146	0.925479	0.845671
Non.inflammatory.Macrophages	LIPT2	0.032145	1.430834	0.110209	0.912493	-5.93711	0.941958	0.852601
Non.inflammatory.Macrophages	SCO1	0.030124	1.930574	0.110139	0.912548	-5.98178	0.914068	0.840839
Non.inflammatory.Macrophages	GLB1	0.01161	5.221188	0.110099	0.912581	-6.81103	0.748649	0.767267
Non.inflammatory.Macrophages	MFSD5	-0.01376	4.282812	-0.11007	0.912599	-6.68643	0.792713	0.787574
Non.inflammatory.Macrophages	P2RX4	0.012014	5.534178	0.110037	0.912629	-6.97702	0.734477	0.76061
Non.inflammatory.Macrophages	UQCRC1	0.010035	6.508803	0.109998	0.91266	-6.995	0.691971	0.740249
Non.inflammatory.Macrophages	SLC25A53	0.019952	3.336328	0.109897	0.91274	-6.21473	0.839622	0.808629
Non.inflammatory.Macrophages	AVEN	0.015529	4.926701	0.109895	0.912742	-6.64782	0.762221	0.773615

Non.inflammatory.Macrophages	SLC25A32	0.018389	3.120627	0.10974	0.912864	-6.30066	0.850669	0.81351
Non.inflammatory.Macrophages	GOT2	0.011362	6.235083	0.109735	0.912868	-6.84704	0.703664	0.745954
Non.inflammatory.Macrophages	FANCE	0.020118	2.916052	0.109646	0.912938	-6.27228	0.861271	0.818154
Non.inflammatory.Macrophages	HINT1	-0.00869	7.995864	-0.10957	0.913	-7.28239	0.631664	0.710274
Non.inflammatory.Macrophages	ARMCX5	-0.02456	2.433168	-0.10926	0.913241	-6.02131	0.886918	0.829242
Non.inflammatory.Macrophages	RBM24	0.072927	1.008251	0.109206	0.913286	-5.42641	0.966291	0.862761
Non.inflammatory.Macrophages	GEMIN5	-0.01909	3.385628	-0.10907	0.913392	-6.29046	0.837274	0.807597
Non.inflammatory.Macrophages	DTD1	0.020702	3.881415	0.108982	0.913464	-6.38975	0.812452	0.796539
Non.inflammatory.Macrophages	UBA3	0.012806	4.076756	0.10881	0.9136	-6.53656	0.802919	0.792267
Non.inflammatory.Macrophages	RPGRIP1L	-0.03265	1.613197	-0.10868	0.913699	-5.82028	0.93196	0.848486
Non.inflammatory.Macrophages	DUSP28	0.027416	1.99306	0.108538	0.913815	-5.98007	0.910896	0.839603
Non.inflammatory.Macrophages	TRMT10B	0.02851	1.681609	0.108417	0.91391	-5.98376	0.928134	0.846939
Non.inflammatory.Macrophages	DENND1B	0.010247	7.299486	0.108259	0.914035	-7.11825	0.65943	0.724376
Non.inflammatory.Macrophages	RAF1	-0.01108	5.986456	-0.10824	0.914054	-6.88573	0.714654	0.751345
Non.inflammatory.Macrophages	ZSCAN21	-0.02131	3.394527	-0.10822	0.914068	-6.26112	0.836905	0.807562

Non.inflammatory.Macrophages	ELF4	-0.00983	6.785373	-0.10813	0.914136	-7.00722	0.680543	0.734832
Non.inflammatory.Macrophages	CTBS	-0.01555	2.934998	-0.10803	0.914216	-6.44646	0.860538	0.817973
Non.inflammatory.Macrophages	DNAJB2	0.01967	2.766414	0.107928	0.914298	-6.3022	0.869368	0.821818
Non.inflammatory.Macrophages	BTBD8	-0.04395	1.572677	-0.1078	0.914402	-5.67021	0.934282	0.849564
Non.inflammatory.Macrophages	SRF	-0.01705	2.927184	-0.10735	0.914751	-6.19359	0.861198	0.818196
Non.inflammatory.Macrophages	RTL6	0.055104	0.292529	0.107323	0.914776	-5.51447	0.985244	0.880358
Non.inflammatory.Macrophages	ARRB1	-0.01212	3.996665	-0.10712	0.914934	-6.66943	0.80718	0.794263
Non.inflammatory.Macrophages	MARCKSL1	0.013866	7.792982	0.106935	0.915083	-7.30991	0.640038	0.714661
Non.inflammatory.Macrophages	EVI2A	0.011949	5.251447	0.106899	0.915111	-6.91094	0.747804	0.767057
Non.inflammatory.Macrophages	SRP72	0.008076	6.657086	0.106471	0.91545	-7.0316	0.686406	0.737647
Non.inflammatory.Macrophages	MED22	0.025705	2.384243	0.106255	0.91562	-6.05654	0.890316	0.830844
Non.inflammatory.Macrophages	AP3D1	0.00868	4.873786	0.106219	0.915649	-6.78104	0.765469	0.775232
Non.inflammatory.Macrophages	A530072M	0.039664	0.764735	0.106174	0.915685	-5.79739	0.977214	0.869097
Non.inflammatory.Macrophages	ARL16	0.022059	2.250651	0.105961	0.915854	-6.02985	0.89754	0.833976
Non.inflammatory.Macrophages	MYBL1	-0.0362	1.840587	-0.10588	0.915917	-5.80091	0.919995	0.843563

Non.inflammatory.Macrophages	MAP2K5	0.009138	6.143251	0.105875	0.915921	-6.91577	0.708369	0.748369
Non.inflammatory.Macrophages	TRIM5	-0.01904	3.589282	-0.10571	0.916054	-6.39008	0.827706	0.803542
Non.inflammatory.Macrophages	IL33	0.052018	0.25962	0.105686	0.916071	-5.51323	0.985698	0.881472
Non.inflammatory.Macrophages	9330020H(	0.026401	2.471892	0.105453	0.916255	-5.99644	0.885642	0.828938
Non.inflammatory.Macrophages	GM5914	-0.02451	3.331997	-0.10535	0.916338	-6.05559	0.840728	0.809359
Non.inflammatory.Macrophages	TMEM161/	0.022147	2.853132	0.105282	0.916391	-6.18011	0.865467	0.820231
Non.inflammatory.Macrophages	5-Mar	-0.01071	6.018808	-0.10495	0.916655	-7.00088	0.713788	0.751157
Non.inflammatory.Macrophages	BORCS6	-0.01447	4.007961	-0.10482	0.916757	-6.57368	0.806925	0.794425
Non.inflammatory.Macrophages	SLC25A3	-0.00753	7.921066	-0.10481	0.916762	-7.27606	0.635239	0.712432
Non.inflammatory.Macrophages	ARHGEF37	0.043934	0.616444	0.104702	0.91685	-6.00273	0.982527	0.873017
Non.inflammatory.Macrophages	ZFP758	-0.02263	2.74078	-0.10464	0.916897	-6.07784	0.871368	0.822959
Non.inflammatory.Macrophages	PDIA4	0.009772	5.653573	0.104625	0.916911	-6.86493	0.729906	0.758873
Non.inflammatory.Macrophages	ACY1	0.032216	1.799123	0.104575	0.91695	-5.92306	0.922301	0.844794
Non.inflammatory.Macrophages	PADI6	0.069543	0.058128	0.104563	0.91696	-5.35164	0.985701	0.886654
Non.inflammatory.Macrophages	RAB20	0.014898	3.301078	0.104551	0.916969	-6.80871	0.842305	0.81023

Non.inflammatory.Macrophages	UBR4	0.008083	6.056739	0.104395	0.917092	-6.93502	0.712134	0.750405
Non.inflammatory.Macrophages	MTERF4	-0.02645	1.913823	-0.10435	0.917125	-6.0072	0.915955	0.842109
Non.inflammatory.Macrophages	PDZD2	-0.03942	3.46937	-0.1043	0.917166	-5.91588	0.833753	0.806449
Non.inflammatory.Macrophages	EIF3L	-0.00994	5.672102	-0.10403	0.917382	-6.85107	0.729105	0.758576
Non.inflammatory.Macrophages	TMEM223	0.011663	4.652686	0.103958	0.917438	-6.68383	0.775903	0.780413
Non.inflammatory.Macrophages	ANGPTL2	-0.05905	0.84131	-0.10379	0.917575	-5.48727	0.974415	0.86769
Non.inflammatory.Macrophages	MCPT8	0.123191	-0.5048	0.103651	0.917681	-5.39279	0.985735	0.900723
Non.inflammatory.Macrophages	PCDH9	0.068761	0.472713	0.103548	0.917762	-5.5233	0.985735	0.876618
Non.inflammatory.Macrophages	2810006K2	-0.02387	2.570884	-0.10344	0.917852	-6.23334	0.880392	0.826961
Non.inflammatory.Macrophages	ZFP646	0.01743	3.903418	0.103352	0.917918	-6.44131	0.812096	0.796862
Non.inflammatory.Macrophages	CDC25A	0.019667	3.793744	0.102927	0.918254	-6.3327	0.817525	0.799406
Non.inflammatory.Macrophages	LRRC40	-0.01753	3.249731	-0.10288	0.918293	-6.31892	0.844959	0.8116
Non.inflammatory.Macrophages	FOXO4	0.022145	2.699869	0.102796	0.918357	-6.16893	0.873556	0.824111
Non.inflammatory.Macrophages	PLS3	-0.03158	2.162516	-0.10278	0.918369	-5.86725	0.902362	0.83652
Non.inflammatory.Macrophages	H2-T24	-0.03107	1.828462	-0.10249	0.918597	-6.0259	0.920705	0.844339

Non.inflammatory.Macrophages	DLGAP1	-0.03176	1.398192	-0.10245	0.918629	-5.94333	0.944833	0.854497
Non.inflammatory.Macrophages	ZFP27	-0.03519	1.016419	-0.10243	0.91865	-5.74291	0.96672	0.86361
Non.inflammatory.Macrophages	METTL5	-0.01516	3.739598	-0.1024	0.918669	-6.40346	0.820218	0.800624
Non.inflammatory.Macrophages	TTC1	0.009261	4.740195	0.102352	0.918709	-6.67743	0.771776	0.778633
Non.inflammatory.Macrophages	MTERF2	-0.03436	2.099595	-0.10232	0.918737	-5.79248	0.905791	0.837998
Non.inflammatory.Macrophages	IFT46	-0.01148	4.502973	-0.1023	0.918748	-6.5852	0.783011	0.783792
Non.inflammatory.Macrophages	GM10550	0.063981	0.590308	0.102254	0.918786	-5.3025	0.983216	0.873892
Non.inflammatory.Macrophages	MMUT	0.017858	3.4125	0.102233	0.918803	-6.44927	0.836662	0.807952
Non.inflammatory.Macrophages	LSM6	-0.01115	6.681324	-0.10218	0.918845	-6.96126	0.685443	0.737681
Non.inflammatory.Macrophages	ARHGAP39	0.016374	4.38051	0.101996	0.91899	-6.65304	0.788871	0.786479
Non.inflammatory.Macrophages	FBXL19	-0.02592	2.333257	-0.10191	0.91906	-6.03848	0.893115	0.832581
Non.inflammatory.Macrophages	ARL5C	-0.01781	6.264356	-0.10181	0.919137	-6.77337	0.703168	0.746294
Non.inflammatory.Macrophages	EGR3	0.025611	3.533307	0.101802	0.919144	-6.63983	0.830553	0.805244
Non.inflammatory.Macrophages	PFKP	-0.01338	5.538024	-0.10172	0.91921	-6.86423	0.735103	0.761539
Non.inflammatory.Macrophages	UXT	0.012584	4.452478	0.101694	0.91923	-6.61632	0.785422	0.784905

Non.inflammatory.Macrophages	ADAMTS1	-0.03523	2.937185	-0.10168	0.919237	-6.13289	0.861106	0.818712
Non.inflammatory.Macrophages	ACTR1B	0.014699	4.215968	0.101535	0.919355	-6.51564	0.796839	0.790122
Non.inflammatory.Macrophages	SLC39A8	0.028096	2.726982	0.101457	0.919418	-6.10934	0.872157	0.823563
Non.inflammatory.Macrophages	EOMES	-0.07466	-0.77158	-0.10121	0.919614	-5.29609	0.985771	0.907648
Non.inflammatory.Macrophages	WDR33	-0.00749	7.186641	-0.10111	0.919691	-7.01306	0.664564	0.727464
Non.inflammatory.Macrophages	ZFP706	0.006972	7.871583	0.100967	0.919805	-7.19811	0.637217	0.713748
Non.inflammatory.Macrophages	CUX1	-0.00885	7.953864	-0.10095	0.91982	-7.12669	0.634007	0.712116
Non.inflammatory.Macrophages	LSM8	0.011593	5.264944	0.100858	0.919891	-6.69962	0.747492	0.76747
Non.inflammatory.Macrophages	SELPLG	-0.01122	6.845492	-0.10082	0.919921	-6.9897	0.678608	0.734444
Non.inflammatory.Macrophages	CSNK1G2	-0.00985	5.468875	-0.10074	0.919989	-6.7811	0.738242	0.763165
Non.inflammatory.Macrophages	TGFB1I1	0.044066	0.90381	0.100722	0.919999	-5.65549	0.972125	0.866508
Non.inflammatory.Macrophages	TMPO	0.012665	7.067829	0.100623	0.920077	-7.01515	0.669427	0.729943
Non.inflammatory.Macrophages	BSPRY	0.055553	0.341542	0.100286	0.920344	-5.41856	0.985966	0.880186
Non.inflammatory.Macrophages	D030056L2	-0.02083	3.888031	-0.10016	0.920442	-6.25036	0.813047	0.797538
Non.inflammatory.Macrophages	IGHM	-0.02741	8.158942	-0.10007	0.920517	-6.98507	0.626198	0.708166

Non.inflammatory.Macrophages	VPS25	-0.03469	0.881521	-0.09994	0.920617	-5.729	0.973147	0.867125
Non.inflammatory.Macrophages	RBL2	0.012094	4.765126	0.099865	0.920678	-6.69638	0.770786	0.778345
Non.inflammatory.Macrophages	MAPK4	-0.0683	0.274036	-0.09977	0.920754	-5.25194	0.985966	0.8819
Non.inflammatory.Macrophages	TXNIP	0.013556	5.791358	0.099755	0.920765	-6.89552	0.72398	0.756435
Non.inflammatory.Macrophages	HAUS8	-0.01116	4.722045	-0.09963	0.920864	-6.74315	0.772834	0.779303
Non.inflammatory.Macrophages	GM43445	0.038038	1.896237	0.099462	0.920997	-5.62114	0.917257	0.843051
Non.inflammatory.Macrophages	MACROD1	-0.02206	2.861084	-0.09937	0.921069	-6.25176	0.865364	0.820752
Non.inflammatory.Macrophages	COX14	-0.00894	5.656597	-0.09916	0.921232	-6.90526	0.730052	0.759359
Non.inflammatory.Macrophages	GM16196	-0.03321	1.751417	-0.09911	0.921272	-5.78074	0.925309	0.846545
Non.inflammatory.Macrophages	SLC36A3	-0.0551	0.101985	-0.09899	0.92137	-5.41064	0.98608	0.886288
Non.inflammatory.Macrophages	ZFP955B	-0.0283	2.201747	-0.09892	0.921426	-5.90425	0.900545	0.836077
Non.inflammatory.Macrophages	MPZL3	-0.05911	1.727945	-0.09867	0.92162	-5.3792	0.926616	0.847194
Non.inflammatory.Macrophages	IIGP1	-0.03275	4.709506	-0.09864	0.921645	-6.68602	0.773492	0.779752
Non.inflammatory.Macrophages	ZFP874B	-0.0316	2.178101	-0.0986	0.921681	-5.89629	0.90183	0.836657
Non.inflammatory.Macrophages	RXYLT1	0.011243	3.864731	0.098498	0.921759	-6.59633	0.814293	0.798305

Non.inflammatory.Macrophages	MCM9	-0.01282	4.75152	-0.0982	0.921998	-6.60979	0.771514	0.778883
Non.inflammatory.Macrophages	SEC62	-0.00558	7.437896	-0.098	0.922157	-7.14766	0.654608	0.722797
Non.inflammatory.Macrophages	AGL	0.016961	3.963587	0.097872	0.922255	-6.51894	0.809416	0.796249
Non.inflammatory.Macrophages	GM43727	-0.03664	-0.59426	-0.09758	0.922484	-5.68259	0.98608	0.903832
Non.inflammatory.Macrophages	PA2G4	0.011712	6.688076	0.097489	0.922559	-6.985	0.685399	0.738133
Non.inflammatory.Macrophages	4930477G(	-0.0466	-0.38448	-0.0973	0.922707	-5.63693	0.98608	0.898589
Non.inflammatory.Macrophages	PCMT1	0.008786	6.63523	0.0972	0.922788	-6.92711	0.687622	0.739219
Non.inflammatory.Macrophages	MANEA	-0.01672	3.728612	-0.0972	0.92279	-6.35785	0.821053	0.801521
Non.inflammatory.Macrophages	THRA	-0.01571	3.771843	-0.09718	0.922807	-6.47889	0.818901	0.800557
Non.inflammatory.Macrophages	GM34455	-0.01761	2.623429	-0.09717	0.92281	-6.54348	0.877909	0.82655
Non.inflammatory.Macrophages	CUL5	0.008026	5.877947	0.097119	0.922852	-6.90173	0.720241	0.75497
Non.inflammatory.Macrophages	SH2D4B	0.017871	4.439997	0.096546	0.923306	-6.66304	0.786295	0.785885
Non.inflammatory.Macrophages	NPM1	-0.00874	8.143673	-0.09649	0.923348	-7.26153	0.626858	0.708898
Non.inflammatory.Macrophages	SND1	-0.0074	7.195863	-0.09648	0.923355	-7.14256	0.664397	0.727848
Non.inflammatory.Macrophages	TXNDC12	-0.0122	3.827411	-0.09648	0.923359	-6.50873	0.816141	0.799403

Non.inflammatory.Macrophages	MRI1	0.013037	3.963231	0.096464	0.923371	-6.45129	0.809433	0.796387
Non.inflammatory.Macrophages	RAP1GDS1	0.008249	6.711436	0.096338	0.92347	-7.12807	0.684419	0.737751
Non.inflammatory.Macrophages	DENND1C	-0.01368	3.966917	-0.09628	0.923519	-6.58295	0.809252	0.796331
Non.inflammatory.Macrophages	PHKA1	0.017454	3.411651	0.096197	0.923582	-6.37559	0.836999	0.808732
Non.inflammatory.Macrophages	CD53	0.006891	7.920856	0.096038	0.923708	-7.27724	0.635492	0.71336
Non.inflammatory.Macrophages	FAM171A1	0.039948	1.565475	0.09594	0.923785	-5.67219	0.935713	0.85139
Non.inflammatory.Macrophages	MEX3D	0.02099	2.642534	0.095934	0.92379	-6.1904	0.876896	0.82627
Non.inflammatory.Macrophages	NDUFAF3	0.01306	4.160988	0.095899	0.923818	-6.4917	0.799759	0.792085
Non.inflammatory.Macrophages	CCP110	0.026812	2.37539	0.095717	0.923962	-5.9138	0.89116	0.83246
Non.inflammatory.Macrophages	WDYHV1	-0.0123	4.174037	-0.09568	0.923994	-6.52917	0.799125	0.791824
Non.inflammatory.Macrophages	ZFP110	-0.01293	4.039873	-0.09556	0.924084	-6.54811	0.805671	0.794796
Non.inflammatory.Macrophages	AGRN	0.034382	1.254784	0.095522	0.924116	-5.85814	0.953336	0.858811
Non.inflammatory.Macrophages	APOL9B	-0.04405	0.574633	-0.09541	0.924203	-5.60272	0.983954	0.875215
Non.inflammatory.Macrophages	RHOT1	0.008464	5.400436	0.095311	0.924283	-6.83027	0.741567	0.765282
Non.inflammatory.Macrophages	PSMB2	0.007117	7.058131	0.095262	0.924323	-7.11631	0.670031	0.730766

Non.inflammatory.Macrophages	MNDAL	-0.01372	6.786829	-0.09523	0.924349	-7.12054	0.681265	0.736306
Non.inflammatory.Macrophages	FAAP24	0.0344	2.002599	0.095174	0.924392	-5.88964	0.911421	0.841173
Non.inflammatory.Macrophages	PELP1	-0.01494	3.358177	-0.09517	0.924394	-6.39065	0.839717	0.810046
Non.inflammatory.Macrophages	UBE2E1	0.007268	6.229498	0.095157	0.924405	-6.91427	0.704917	0.74782
Non.inflammatory.Macrophages	GM49164	-0.04781	0.920748	-0.095	0.924528	-5.4955	0.971801	0.866891
Non.inflammatory.Macrophages	KCTD6	0.014649	2.981269	0.094966	0.924557	-6.45442	0.859112	0.818625
Non.inflammatory.Macrophages	EPB41L5	0.025039	3.379051	0.094831	0.924664	-6.0762	0.838655	0.809672
Non.inflammatory.Macrophages	TXNL4A	0.010503	4.968748	0.094773	0.92471	-6.72047	0.761363	0.774641
Non.inflammatory.Macrophages	DUSP6	-0.01322	4.428378	-0.09474	0.924735	-6.7177	0.786851	0.786382
Non.inflammatory.Macrophages	RAC3	0.038103	-0.22085	0.094629	0.924824	-5.76373	0.98608	0.894908
Non.inflammatory.Macrophages	RLN3	0.055356	-0.84713	0.0946	0.924846	-5.30049	0.98608	0.910592
Non.inflammatory.Macrophages	ALDH1A1	0.048655	-5.25E-05	0.094527	0.924904	-5.5911	0.98608	0.889448
Non.inflammatory.Macrophages	HIST1H4I	0.018711	4.409355	0.094498	0.924927	-6.55457	0.787763	0.786824
Non.inflammatory.Macrophages	MMP8	-0.05418	1.517071	-0.0941	0.925239	-5.73039	0.938439	0.852839
Non.inflammatory.Macrophages	KLF12	0.027338	3.585919	0.094042	0.925289	-6.2161	0.828196	0.805151

Non.inflammatory.Macrophages	RAB28	-0.0116	4.828065	-0.09403	0.925294	-6.66534	0.767923	0.777789
Non.inflammatory.Macrophages	WDR60	0.034782	0.883885	0.094019	0.925307	-5.77216	0.973171	0.867976
Non.inflammatory.Macrophages	MCRS1	0.012059	4.318538	0.09397	0.925346	-6.54399	0.79213	0.788899
Non.inflammatory.Macrophages	SLC35F2	0.056157	0.357488	0.093889	0.92541	-5.35575	0.98608	0.880758
Non.inflammatory.Macrophages	ZFP938	0.02649	1.450658	0.093841	0.925448	-5.89253	0.942191	0.854415
Non.inflammatory.Macrophages	GPATCH1	-0.01755	2.73622	-0.09383	0.925458	-6.18424	0.871944	0.824412
Non.inflammatory.Macrophages	5430427M	0.056997	1.237849	0.093824	0.925461	-5.41643	0.954305	0.859482
Non.inflammatory.Macrophages	FAM71E1	0.040963	0.837279	0.093628	0.925616	-5.67519	0.974993	0.869138
Non.inflammatory.Macrophages	RBM28	0.009361	5.311789	0.093402	0.925795	-6.77896	0.745662	0.767432
Non.inflammatory.Macrophages	MIER3	0.014876	4.078409	0.093329	0.925853	-6.38519	0.803862	0.79424
Non.inflammatory.Macrophages	JOSD2	0.011465	4.09679	0.093308	0.92587	-6.56008	0.802964	0.793833
Non.inflammatory.Macrophages	ZFP771	0.012252	4.065583	0.093253	0.925913	-6.4706	0.804489	0.794523
Non.inflammatory.Macrophages	GCC2	-0.01049	5.193926	-0.09314	0.926001	-6.76393	0.751059	0.769985
Non.inflammatory.Macrophages	HUS1	0.02853	1.653586	0.092978	0.926132	-5.82121	0.930931	0.849695
Non.inflammatory.Macrophages	EPHA2	0.026623	2.408212	0.092665	0.926379	-5.88982	0.889631	0.832128

Non.inflammatory.Macrophages	CDK5RAP2	0.011047	4.446752	0.092499	0.926511	-6.62197	0.786179	0.786315
Non.inflammatory.Macrophages	DIO1	-0.03988	0.977554	-0.09245	0.92655	-5.58033	0.96957	0.86597
Non.inflammatory.Macrophages	DUS3L	0.014549	3.300011	0.092413	0.926579	-6.32235	0.842906	0.811828
Non.inflammatory.Macrophages	MCM3AP	-0.01212	3.885875	-0.09214	0.926795	-6.40414	0.813462	0.79873
Non.inflammatory.Macrophages	JADE2	0.015965	3.270443	0.092114	0.926816	-6.51147	0.844418	0.812528
Non.inflammatory.Macrophages	RNF219	-0.01845	3.193241	-0.0921	0.926824	-6.25271	0.848378	0.814275
Non.inflammatory.Macrophages	CCDC32	0.015116	3.31271	0.09208	0.926843	-6.36085	0.842258	0.811573
Non.inflammatory.Macrophages	SHPK	0.034683	0.252393	0.092037	0.926877	-5.71316	0.986341	0.883633
Non.inflammatory.Macrophages	THADA	-0.01023	5.881845	-0.0919	0.926987	-6.8455	0.720259	0.755566
Non.inflammatory.Macrophages	GM49864	0.047125	0.452692	0.091639	0.927192	-5.47265	0.986341	0.878741
Non.inflammatory.Macrophages	DENND2A	-0.02436	1.374468	-0.09157	0.927246	-6.10359	0.946762	0.856527
Non.inflammatory.Macrophages	MAP2K6	-0.01679	3.533157	-0.09157	0.927248	-6.45291	0.831072	0.806617
Non.inflammatory.Macrophages	MRPS18C	-0.00937	5.696786	-0.09144	0.927353	-6.85924	0.728454	0.75947
Non.inflammatory.Macrophages	PRRG4	0.063796	0.01648	0.0914	0.927381	-5.229	0.986341	0.889447
Non.inflammatory.Macrophages	SLC12A8	0.030458	0.657727	0.091366	0.927409	-5.87297	0.981893	0.873753

Non.inflammatory.Macrophages	RAC2	-0.00858	9.052768	-0.09131	0.927455	-7.32776	0.592966	0.691747
Non.inflammatory.Macrophages	OLFML2B	-0.04729	0.874884	-0.09072	0.927923	-5.55388	0.973949	0.868588
Non.inflammatory.Macrophages	LAMTOR5	0.008389	5.527925	0.090662	0.927967	-6.90229	0.736149	0.763139
Non.inflammatory.Macrophages	DOK3	-0.01331	5.36627	-0.09063	0.92799	-6.77537	0.743453	0.766582
Non.inflammatory.Macrophages	HIST1H2BC	-0.01409	5.260139	-0.09045	0.928134	-6.83028	0.748286	0.76885
Non.inflammatory.Macrophages	MTHFD1	-0.01539	3.79072	-0.09041	0.928164	-6.3651	0.818334	0.800951
Non.inflammatory.Macrophages	CHST2	-0.04965	-0.02925	-0.0904	0.928177	-5.51194	0.986529	0.890682
Non.inflammatory.Macrophages	CABCOCO1	0.04877	0.315412	0.090299	0.928254	-5.36663	0.986529	0.882202
Non.inflammatory.Macrophages	IFI27L2A	0.020745	7.373467	0.090234	0.928305	-7.27785	0.657499	0.72492
Non.inflammatory.Macrophages	C5AR2	-0.02037	1.231827	-0.09022	0.928314	-6.30275	0.955084	0.86003
Non.inflammatory.Macrophages	DIS3L	0.016842	2.828005	0.090165	0.92836	-6.2023	0.867511	0.822698
Non.inflammatory.Macrophages	C1GALT1C1	-0.01207	4.044817	-0.08996	0.928525	-6.58644	0.805808	0.795376
Non.inflammatory.Macrophages	TFEC	0.015067	2.368464	0.089906	0.928565	-6.66133	0.891952	0.833352
Non.inflammatory.Macrophages	UEVLD	0.018593	2.944551	0.089731	0.928704	-6.25327	0.861429	0.820139
Non.inflammatory.Macrophages	FMN1	-0.01475	3.350464	-0.08969	0.928734	-6.72178	0.840506	0.810929

Non.inflammatory.Macrophages	ACTR6	-0.01457	3.522286	-0.08969	0.92874	-6.35794	0.831792	0.807061
Non.inflammatory.Macrophages	ATXN2	-0.00801	6.28335	-0.08954	0.928853	-6.99513	0.702947	0.747373
Non.inflammatory.Macrophages	G6PC3	0.016043	3.106653	0.089421	0.92895	-6.36692	0.853039	0.816471
Non.inflammatory.Macrophages	E430024P1	0.043052	0.53335	0.089296	0.929049	-5.55577	0.985481	0.877016
Non.inflammatory.Macrophages	ZFP433	-0.04462	0.355661	-0.08929	0.929057	-5.50567	0.986571	0.881353
Non.inflammatory.Macrophages	UBR5	0.007545	6.888097	0.089179	0.929141	-7.10312	0.677397	0.734907
Non.inflammatory.Macrophages	SYDE2	0.059763	-0.10373	0.088966	0.92931	-5.29401	0.986686	0.892688
Non.inflammatory.Macrophages	CD33	-0.01756	2.518088	-0.08866	0.929551	-6.56238	0.88415	0.830058
Non.inflammatory.Macrophages	POLE4	0.008274	6.652817	0.088658	0.929555	-6.9727	0.687376	0.739822
Non.inflammatory.Macrophages	TOGARAM	0.008907	5.297825	0.088489	0.929688	-6.78093	0.746813	0.768307
Non.inflammatory.Macrophages	GM1043	0.056997	1.915295	0.088232	0.929892	-5.47671	0.917075	0.844185
Non.inflammatory.Macrophages	CFAP126	0.024086	1.069649	0.087852	0.930193	-6.06086	0.965037	0.864323
Non.inflammatory.Macrophages	RALBP1	0.007592	6.268527	0.087847	0.930197	-6.94341	0.704007	0.747918
Non.inflammatory.Macrophages	OLFR920	-0.02677	0.137933	-0.08759	0.930401	-5.96235	0.987302	0.887018
Non.inflammatory.Macrophages	2700049A(	-0.01339	4.236674	-0.08735	0.930593	-6.50553	0.797173	0.791534

Non.inflammatory.Macrophages	EIF3I	-0.00793	6.822191	-0.08709	0.930798	-7.03588	0.680801	0.736573
Non.inflammatory.Macrophages	NFATC2	-0.02564	2.489047	-0.08702	0.93085	-5.94985	0.886381	0.830989
Non.inflammatory.Macrophages	FITM2	0.029623	0.918829	0.086689	0.931115	-5.64933	0.973438	0.868063
Non.inflammatory.Macrophages	NXN	0.013112	5.325793	0.086597	0.931187	-6.70261	0.746154	0.767907
Non.inflammatory.Macrophages	TNFSF13OS	-0.03484	0.055873	-0.08657	0.931209	-5.84473	0.987668	0.889114
Non.inflammatory.Macrophages	MRPS36	0.008968	5.569029	0.086509	0.931257	-6.89209	0.735151	0.762724
Non.inflammatory.Macrophages	SNX3	-0.0056	7.730038	-0.08631	0.931415	-7.24807	0.64402	0.718193
Non.inflammatory.Macrophages	AUTS2	-0.02899	4.397144	-0.08623	0.931476	-6.37071	0.789626	0.788048
Non.inflammatory.Macrophages	DSCAM	-0.0653	0.029718	-0.08609	0.931591	-5.31145	0.987678	0.889816
Non.inflammatory.Macrophages	USP31	-0.01451	3.715776	-0.08597	0.931681	-6.43886	0.823025	0.803156
Non.inflammatory.Macrophages	CDC25C	-0.03591	2.030722	-0.08593	0.93172	-5.69768	0.911352	0.841702
Non.inflammatory.Macrophages	NQO2	-0.01056	3.942173	-0.08592	0.931721	-6.57915	0.811783	0.798112
Non.inflammatory.Macrophages	CPT2	-0.01853	2.829472	-0.08581	0.931814	-6.28358	0.868445	0.823222
Non.inflammatory.Macrophages	WDR19	0.035173	0.508741	0.085659	0.931931	-5.66753	0.987207	0.878108
Non.inflammatory.Macrophages	PDZD4	0.042751	1.698737	0.085507	0.932051	-5.51604	0.92975	0.849585

Non.inflammatory.Macrophages	GM3604	0.032123	0.595301	0.085161	0.932326	-5.6401	0.985029	0.876152
Non.inflammatory.Macrophages	EIF4G1	-0.00677	6.604378	-0.0849	0.932536	-7.0129	0.690038	0.741322
Non.inflammatory.Macrophages	SPAG7	-0.01036	4.55728	-0.08483	0.932585	-6.68854	0.781964	0.784812
Non.inflammatory.Macrophages	MRPL22	0.012515	3.858759	0.084801	0.932611	-6.51227	0.815908	0.800221
Non.inflammatory.Macrophages	WDR53	0.018086	2.715022	0.084801	0.932611	-6.16918	0.874476	0.826097
Non.inflammatory.Macrophages	CSNK1D	0.005524	6.6346	0.084579	0.932787	-7.01089	0.688762	0.740719
Non.inflammatory.Macrophages	SLC41A3	0.016651	1.73389	0.084562	0.932801	-6.2262	0.927786	0.848954
Non.inflammatory.Macrophages	TENT4A	-0.01338	3.460072	-0.08456	0.932804	-6.4509	0.835896	0.809157
Non.inflammatory.Macrophages	ARF2	-0.01041	4.470722	-0.08454	0.932819	-6.81413	0.786097	0.786713
Non.inflammatory.Macrophages	AMY1	-0.02383	2.627056	-0.08454	0.932822	-6.03417	0.879138	0.828129
Non.inflammatory.Macrophages	FGF13	-0.04387	3.379693	-0.08432	0.932995	-5.75462	0.839981	0.810969
Non.inflammatory.Macrophages	GM47917	-0.04123	0.467794	-0.084	0.933249	-5.43206	0.987678	0.87942
Non.inflammatory.Macrophages	NCKAP5	-0.0385	1.217214	-0.08381	0.933393	-5.68834	0.957035	0.861333
Non.inflammatory.Macrophages	GM9725	0.019426	2.030059	0.083761	0.933436	-6.22358	0.911388	0.8421
Non.inflammatory.Macrophages	MRGPRA2I	-0.06052	-0.43033	-0.08367	0.933511	-5.32301	0.987678	0.90169

Non.inflammatory.Macrophages	RARG	-0.02523	1.622679	-0.08356	0.933595	-5.98025	0.934012	0.851724
Non.inflammatory.Macrophages	TCF12	0.008348	8.463403	0.083559	0.933596	-7.17566	0.615665	0.704075
Non.inflammatory.Macrophages	ZFP85	-0.04269	0.25725	-0.08353	0.933616	-5.47146	0.987678	0.88465
Non.inflammatory.Macrophages	CPSF2	-0.00939	5.649423	-0.0835	0.933643	-6.79448	0.731556	0.761445
Non.inflammatory.Macrophages	TNS4	-0.02709	-0.27421	-0.08348	0.933657	-5.9128	0.987678	0.897797
Non.inflammatory.Macrophages	ZC2HC1A	0.026381	1.741533	0.083209	0.933874	-5.93313	0.927359	0.848964
Non.inflammatory.Macrophages	EMC8	0.009478	4.721373	0.083178	0.933898	-6.67158	0.774185	0.781423
Non.inflammatory.Macrophages	RRAS	0.010307	4.338868	0.08295	0.934078	-6.89793	0.792432	0.789809
Non.inflammatory.Macrophages	HSD17B4	0.007696	5.027211	0.082853	0.934156	-6.87049	0.759882	0.774822
Non.inflammatory.Macrophages	POGLUT1	0.014486	2.752407	0.082831	0.934173	-6.31423	0.872502	0.825462
Non.inflammatory.Macrophages	TTK	-0.02925	2.313922	-0.08282	0.934183	-5.85656	0.895921	0.835591
Non.inflammatory.Macrophages	DOCK6	-0.02296	1.914885	-0.08278	0.934211	-5.98518	0.917733	0.844914
Non.inflammatory.Macrophages	ANXA6	-0.00865	6.174347	-0.08278	0.934214	-7.00009	0.708446	0.750467
Non.inflammatory.Macrophages	NUBP1	0.009072	5.473714	0.082774	0.934218	-6.78865	0.739451	0.76525
Non.inflammatory.Macrophages	ZFP426	-0.01668	2.769149	-0.08277	0.93422	-6.15024	0.871619	0.825077

Non.inflammatory.Macrophages	PTGES2	-0.01719	2.422157	-0.08272	0.934258	-6.14417	0.890087	0.833079
Non.inflammatory.Macrophages	AKR1C13	0.018652	1.84784	0.082703	0.934274	-6.21996	0.921445	0.84649
Non.inflammatory.Macrophages	A2ML1	-0.02689	2.471948	-0.08263	0.934331	-6.06687	0.887415	0.831926
Non.inflammatory.Macrophages	VEGFC	-0.03882	1.200834	-0.08239	0.934524	-5.57522	0.958099	0.861913
Non.inflammatory.Macrophages	CD200	-0.04524	1.910379	-0.08202	0.934815	-5.53989	0.918314	0.845154
Non.inflammatory.Macrophages	SNRNP200	0.009989	5.038857	0.081912	0.934902	-6.7169	0.759628	0.774714
Non.inflammatory.Macrophages	GOLGB1	0.007747	5.4427	0.081599	0.93515	-6.85211	0.741243	0.766107
Non.inflammatory.Macrophages	DVL3	-0.01322	3.300094	-0.08155	0.935188	-6.29836	0.844489	0.813188
Non.inflammatory.Macrophages	CCDC84	0.020274	2.649641	0.081122	0.935528	-5.98435	0.878625	0.828102
Non.inflammatory.Macrophages	GID8	0.008636	5.195402	0.080846	0.935747	-6.7014	0.752711	0.77146
Non.inflammatory.Macrophages	PEG13	0.019727	2.114243	0.080749	0.935824	-6.04118	0.907484	0.840525
Non.inflammatory.Macrophages	DDRGK1	0.007132	5.272107	0.080586	0.935953	-6.88612	0.749196	0.769815
Non.inflammatory.Macrophages	CLPB	0.009879	4.010407	0.080503	0.936019	-6.51076	0.809055	0.797333
Non.inflammatory.Macrophages	GM49359	-0.01814	2.260358	-0.08049	0.936032	-6.09977	0.899523	0.837116
Non.inflammatory.Macrophages	ZFAND1	0.014993	2.727292	0.08047	0.936046	-6.15055	0.87451	0.826315

Non.inflammatory.Macrophages	MED30	0.00882	5.369422	0.080347	0.936143	-6.76103	0.744757	0.767732
Non.inflammatory.Macrophages	DEPDC7	0.016641	1.678465	0.080216	0.936247	-6.38862	0.931611	0.85077
Non.inflammatory.Macrophages	TRAP1	0.008758	4.381095	0.08006	0.936371	-6.58004	0.791015	0.789148
Non.inflammatory.Macrophages	SRSF4	0.007042	6.225555	0.079979	0.936435	-6.86483	0.706781	0.749649
Non.inflammatory.Macrophages	BTBD11	0.01435	4.309098	0.07996	0.93645	-6.81902	0.794489	0.790731
Non.inflammatory.Macrophages	GTF2H4	-0.01643	2.307317	-0.07992	0.936484	-6.05815	0.896978	0.836024
Non.inflammatory.Macrophages	TEX14	-0.01107	5.64791	-0.07989	0.936509	-6.93354	0.732195	0.761802
Non.inflammatory.Macrophages	SMPD1	0.016237	2.643295	0.079869	0.936522	-6.22163	0.878962	0.82825
Non.inflammatory.Macrophages	ELOVL1	0.008983	4.858115	0.079368	0.93692	-6.79934	0.768625	0.778854
Non.inflammatory.Macrophages	MRPL14	-0.00902	5.601025	-0.07919	0.937058	-6.90952	0.734567	0.762939
Non.inflammatory.Macrophages	USB1	0.014309	3.245957	0.079157	0.937087	-6.34868	0.847793	0.814629
Non.inflammatory.Macrophages	MYO19	0.029235	1.357151	0.07897	0.937235	-5.75044	0.9502	0.858586
Non.inflammatory.Macrophages	ABRAXAS2	-0.0081	4.960803	-0.07844	0.937654	-6.74413	0.764148	0.77678
Non.inflammatory.Macrophages	PTDSS2	-0.01217	3.06586	-0.07832	0.93775	-6.35174	0.857434	0.818847
Non.inflammatory.Macrophages	TMEM37	-0.01306	3.751452	-0.07832	0.937752	-6.66705	0.822516	0.803373

Non.inflammatory.Macrophages	GKAP1	0.012675	3.541031	0.078056	0.93796	-6.48736	0.833209	0.808109
Non.inflammatory.Macrophages	CALM2	-0.00574	8.565355	-0.07787	0.938105	-7.24378	0.612904	0.702641
Non.inflammatory.Macrophages	GTF2H3	0.020156	2.299191	0.077682	0.938257	-5.98789	0.89838	0.83652
Non.inflammatory.Macrophages	UMAD1	0.008741	5.658219	0.077514	0.93839	-6.81226	0.732533	0.761897
Non.inflammatory.Macrophages	ETV5	0.014688	3.199847	0.077473	0.938423	-6.44512	0.85078	0.815858
Non.inflammatory.Macrophages	KMT2D	0.007188	5.945956	0.077178	0.938657	-6.82769	0.719884	0.755838
Non.inflammatory.Macrophages	RNF167	-0.01007	5.334087	-0.07706	0.938752	-6.67834	0.747327	0.768843
Non.inflammatory.Macrophages	FIGNL1	0.021973	2.757262	0.076874	0.938897	-6.04809	0.874116	0.826056
Non.inflammatory.Macrophages	TSPAN7	0.018227	3.257326	0.076709	0.939029	-6.18179	0.84805	0.814656
Non.inflammatory.Macrophages	APEX2	0.011424	4.215093	0.076693	0.939041	-6.42749	0.800135	0.793228
Non.inflammatory.Macrophages	ZNHIT3	0.012603	3.532147	0.076249	0.939394	-6.32423	0.834109	0.808537
Non.inflammatory.Macrophages	FNDC7	0.028229	-0.36573	0.076138	0.939482	-5.79252	0.989885	0.901049
Non.inflammatory.Macrophages	GTPBP6	-0.01635	2.84005	-0.07609	0.93952	-6.16074	0.869828	0.824278
Non.inflammatory.Macrophages	4933421O1	0.018094	2.357702	0.076068	0.939537	-6.11757	0.895554	0.835413
Non.inflammatory.Macrophages	THOC7	0.006168	6.572603	0.076052	0.93955	-7.0026	0.692928	0.742939

Non.inflammatory.Macrophages	R3HCC1L	-0.008	5.540599	-0.07597	0.939616	-6.81862	0.738082	0.764602
Non.inflammatory.Macrophages	NAP1L4	0.006194	6.249563	0.075854	0.939707	-6.87086	0.706768	0.749696
Non.inflammatory.Macrophages	DUSP12	-0.01302	3.283921	-0.07577	0.939771	-6.29325	0.84676	0.814221
Non.inflammatory.Macrophages	PI4K2A	0.008083	5.639245	0.075674	0.939849	-6.96979	0.733647	0.762551
Non.inflammatory.Macrophages	IL27RA	-0.04032	1.535873	-0.07559	0.939917	-5.55754	0.940996	0.854787
Non.inflammatory.Macrophages	METTL14	0.014259	3.09472	0.075573	0.93993	-6.256	0.856523	0.818523
Non.inflammatory.Macrophages	USP38	-0.00747	5.889969	-0.07539	0.940071	-6.85879	0.722525	0.757312
Non.inflammatory.Macrophages	GM16272	-0.03018	0.363126	-0.07531	0.940135	-5.6619	0.989935	0.883173
Non.inflammatory.Macrophages	AIP	0.009002	4.946962	0.075236	0.940197	-6.73278	0.765356	0.77748
Non.inflammatory.Macrophages	ATP9A	-0.03477	0.837325	-0.0749	0.940467	-5.61327	0.978921	0.871697
Non.inflammatory.Macrophages	UBE2V2	0.00725	5.614121	0.074786	0.940554	-6.83674	0.734976	0.763258
Non.inflammatory.Macrophages	PIGG	-0.02542	0.963956	-0.07467	0.940645	-5.79615	0.974132	0.868701
Non.inflammatory.Macrophages	HOMEZ	-0.02382	1.333922	-0.07451	0.940772	-5.77044	0.952814	0.859857
Non.inflammatory.Macrophages	ATPAF2	-0.01447	2.92038	-0.07399	0.941187	-6.20213	0.86606	0.822836
Non.inflammatory.Macrophages	CYP4A10	0.044865	1.117903	0.073983	0.941191	-5.65246	0.965404	0.865123

Non.inflammatory.Macrophages	C1QB	0.012792	6.51237	0.07391	0.941249	-7.31347	0.695849	0.744544
Non.inflammatory.Macrophages	ZFP580	0.019509	2.166288	0.073873	0.941278	-5.99085	0.906425	0.840276
Non.inflammatory.Macrophages	TMED9	0.005669	6.609324	0.073836	0.941308	-6.99703	0.69173	0.742537
Non.inflammatory.Macrophages	HEXDC	0.025357	1.86348	0.073552	0.941533	-5.92202	0.923114	0.847437
Non.inflammatory.Macrophages	TMEM158	0.048335	0.271092	0.073354	0.94169	-5.42369	0.990399	0.885815
Non.inflammatory.Macrophages	ACOXL	0.041614	1.285897	0.073308	0.941726	-5.50828	0.955725	0.861196
Non.inflammatory.Macrophages	BACE1	-0.01749	2.654059	-0.07331	0.941727	-5.98442	0.880123	0.829052
Non.inflammatory.Macrophages	CHCHD10	-0.01315	6.141333	-0.0733	0.941729	-6.80476	0.711834	0.752364
Non.inflammatory.Macrophages	MAMDC2	-0.03927	0.366618	-0.07314	0.941862	-5.49837	0.990399	0.883533
Non.inflammatory.Macrophages	MRPL2	-0.0088	4.862057	-0.07312	0.941877	-6.74904	0.769689	0.779704
Non.inflammatory.Macrophages	FASTKD3	-0.02172	1.993657	-0.07302	0.941951	-5.91343	0.915905	0.84449
Non.inflammatory.Macrophages	VAMP7	-0.0091	4.132998	-0.07302	0.941955	-6.57195	0.80463	0.795693
Non.inflammatory.Macrophages	ARSB	-0.01588	4.938464	-0.07275	0.942172	-6.59788	0.766206	0.778074
Non.inflammatory.Macrophages	SOX18	-0.03523	0.720608	-0.07269	0.942217	-5.60862	0.98368	0.87494
Non.inflammatory.Macrophages	FUT4	0.02741	-0.16835	0.07217	0.94263	-5.72025	0.990781	0.896885

Non.inflammatory.Macrophages	FLT4	-0.0225	2.321562	-0.07216	0.942634	-5.9427	0.898321	0.836942
Non.inflammatory.Macrophages	CADM4	-0.02472	1.741687	-0.07193	0.942822	-5.94582	0.930264	0.85059
Non.inflammatory.Macrophages	C030005K(	0.030535	0.674423	0.071807	0.942918	-5.59425	0.985681	0.876218
Non.inflammatory.Macrophages	P2RY12	0.012949	2.616799	0.071785	0.942935	-6.54409	0.882447	0.830166
Non.inflammatory.Macrophages	ZFP60	0.024329	1.431044	0.071683	0.943016	-5.76082	0.947798	0.858019
Non.inflammatory.Macrophages	HDDC3	-0.02918	0.928077	-0.07161	0.943073	-5.63511	0.97616	0.870096
Non.inflammatory.Macrophages	TMEM106I	-0.00893	4.565283	-0.07157	0.943104	-6.63056	0.784038	0.786378
Non.inflammatory.Macrophages	ESPN	0.03244	-0.17599	0.071398	0.943242	-5.62989	0.990781	0.89718
Non.inflammatory.Macrophages	EXOC3L4	0.020011	0.594811	0.071393	0.943246	-6.11368	0.988135	0.878188
Non.inflammatory.Macrophages	TEX264	-0.00711	4.497588	-0.07136	0.94327	-6.72577	0.787277	0.787865
Non.inflammatory.Macrophages	RNF11	0.007528	6.207032	0.070819	0.943702	-6.93471	0.709377	0.751343
Non.inflammatory.Macrophages	SYPL	0.006893	5.860238	0.070687	0.943806	-6.90417	0.724586	0.758656
Non.inflammatory.Macrophages	THAP12	0.007455	4.362104	0.070669	0.943821	-6.64657	0.793938	0.790966
Non.inflammatory.Macrophages	IP6K2	0.011301	3.644965	0.070667	0.943822	-6.29569	0.829314	0.806913
Non.inflammatory.Macrophages	EIF5A2	-0.02802	0.577892	-0.07065	0.943839	-5.73727	0.988737	0.878743

Non.inflammatory.Macrophages	IL1B	-0.01248	5.12575	-0.0706	0.943878	-7.22902	0.757835	0.774329
Non.inflammatory.Macrophages	KXD1	-0.00657	5.765423	-0.07051	0.943949	-6.93641	0.728798	0.760662
Non.inflammatory.Macrophages	MFAP1A	-0.00758	4.832089	-0.07036	0.944069	-6.61853	0.771565	0.780694
Non.inflammatory.Macrophages	ADORA2A	0.020168	3.148747	0.070269	0.944138	-6.08903	0.854687	0.818141
Non.inflammatory.Macrophages	GM16599	0.013159	4.782001	0.07003	0.944328	-6.25908	0.773952	0.781783
Non.inflammatory.Macrophages	PCM1	-0.00665	6.494399	-0.06996	0.944382	-6.96843	0.697064	0.745387
Non.inflammatory.Macrophages	ITGA5	0.010445	3.54178	0.069951	0.944391	-6.74076	0.834591	0.809242
Non.inflammatory.Macrophages	EPHA4	-0.03513	0.083978	-0.06971	0.944583	-5.62213	0.9911	0.890909
Non.inflammatory.Macrophages	TTC7	0.008233	5.873227	0.069689	0.944598	-6.84687	0.724116	0.758411
Non.inflammatory.Macrophages	MIS12	-0.01266	3.322859	-0.06959	0.944679	-6.36685	0.845804	0.814209
Non.inflammatory.Macrophages	HSPA9	0.006024	6.483768	0.069501	0.944748	-7.01293	0.697565	0.745628
Non.inflammatory.Macrophages	FASN	-0.01374	3.14635	-0.06939	0.944838	-6.31062	0.854914	0.818245
Non.inflammatory.Macrophages	SF3B6	-0.00519	7.294759	-0.06913	0.945044	-7.09258	0.663849	0.729044
Non.inflammatory.Macrophages	ADAP2OS	0.016503	0.724917	0.068973	0.945167	-6.25856	0.984304	0.875309
Non.inflammatory.Macrophages	CYLD	0.00653	6.329731	0.06887	0.945249	-6.91442	0.704329	0.74896

Non.inflammatory.Macrophages	ZFP955A	-0.026	1.472841	-0.06874	0.945352	-5.75361	0.94595	0.857369
Non.inflammatory.Macrophages	DHX16	-0.01041	4.087189	-0.06864	0.945435	-6.50096	0.807637	0.797244
Non.inflammatory.Macrophages	FRA10AC1	-0.0112	3.408508	-0.06855	0.945503	-6.35092	0.841619	0.812445
Non.inflammatory.Macrophages	TMEM9B	-0.00592	5.558526	-0.06848	0.945555	-6.87624	0.738354	0.765254
Non.inflammatory.Macrophages	DAB2IP	-0.02148	2.225574	-0.06807	0.945887	-5.86924	0.904231	0.839716
Non.inflammatory.Macrophages	SPOCK2	-0.02572	0.349422	-0.06805	0.945898	-5.69096	0.991541	0.884654
Non.inflammatory.Macrophages	HSPB6	0.030108	0.40248	0.067843	0.946064	-5.62898	0.991638	0.883441
Non.inflammatory.Macrophages	SYF2	0.005344	5.903221	0.067636	0.946228	-6.85506	0.723251	0.758133
Non.inflammatory.Macrophages	CLIC1	-0.0061	8.942892	-0.06748	0.94635	-7.37711	0.600277	0.696679
Non.inflammatory.Macrophages	ZFP982	-0.03492	0.056297	-0.06725	0.946535	-5.57397	0.991868	0.892107
Non.inflammatory.Macrophages	CLCN7	-0.00998	3.608444	-0.06717	0.946598	-6.62671	0.831919	0.808229
Non.inflammatory.Macrophages	GM46367	-0.03001	2.175914	-0.06708	0.946668	-5.62215	0.907243	0.841116
Non.inflammatory.Macrophages	HIST2H2A(	-0.03388	2.376405	-0.06701	0.946723	-5.80885	0.896335	0.836447
Non.inflammatory.Macrophages	GINM1	-0.00711	4.990227	-0.06689	0.946819	-6.6577	0.764848	0.777765
Non.inflammatory.Macrophages	LCN4	-0.04041	-1.05402	-0.06678	0.946908	-5.25872	0.991908	0.920062

Non.inflammatory.Macrophages	MID2	0.031971	0.27499	0.06651	0.947122	-5.56482	0.991993	0.886787
Non.inflammatory.Macrophages	PLEKHA6	-0.01793	1.808761	-0.06649	0.947136	-5.9582	0.92765	0.849795
Non.inflammatory.Macrophages	EHMT1	0.006666	6.063885	0.066146	0.947411	-6.86157	0.716408	0.754925
Non.inflammatory.Macrophages	GPN1	0.011157	3.042468	0.066084	0.94746	-6.28594	0.861118	0.821162
Non.inflammatory.Macrophages	4930503L1	0.015012	2.576564	0.065581	0.94786	-6.04954	0.885729	0.831888
Non.inflammatory.Macrophages	RAD51B	0.012474	6.152694	0.065416	0.947991	-6.84514	0.712526	0.753073
Non.inflammatory.Macrophages	SAMM50	-0.00669	5.177169	-0.06536	0.948034	-6.79694	0.756295	0.773807
Non.inflammatory.Macrophages	CRIP2	0.010049	4.565121	0.065349	0.948044	-6.69415	0.785051	0.787104
Non.inflammatory.Macrophages	BBS4	-0.01769	2.313596	-0.06513	0.948221	-5.91817	0.899905	0.837995
Non.inflammatory.Macrophages	DHRS1	0.0084	4.762169	0.065088	0.948251	-6.86102	0.775682	0.782798
Non.inflammatory.Macrophages	MACO1	0.00611	6.27208	0.064943	0.948366	-6.89762	0.707339	0.750574
Non.inflammatory.Macrophages	SEC61A2	0.009094	3.741527	0.06491	0.948392	-6.46363	0.825377	0.805356
Non.inflammatory.Macrophages	MED20	0.008471	4.104119	0.064863	0.948429	-6.51218	0.807389	0.79727
Non.inflammatory.Macrophages	COQ6	0.013732	2.285167	0.064682	0.948573	-6.01793	0.90145	0.838657
Non.inflammatory.Macrophages	RAB5A	-0.00497	6.913368	-0.06459	0.948645	-7.04078	0.680096	0.737293

Non.inflammatory.Macrophages	I830077J02	-0.01113	2.758256	-0.06453	0.948695	-6.57701	0.876055	0.827698
Non.inflammatory.Macrophages	TXLNG	-0.0074	5.107247	-0.06452	0.948705	-6.70476	0.759529	0.775318
Non.inflammatory.Macrophages	LAS1L	-0.00839	4.172574	-0.06449	0.948724	-6.52492	0.804035	0.795756
Non.inflammatory.Macrophages	ARHGAP29	0.017479	2.520124	0.064345	0.948841	-6.09752	0.888754	0.83326
Non.inflammatory.Macrophages	ARHGEF1	-0.00586	7.10928	-0.06433	0.948854	-7.00515	0.671978	0.73334
Non.inflammatory.Macrophages	GM38560	-0.01868	0.991541	-0.06422	0.948943	-6.05748	0.974366	0.869443
Non.inflammatory.Macrophages	TRIM16	0.022234	2.341778	0.064215	0.948944	-5.85699	0.898376	0.837416
Non.inflammatory.Macrophages	ULK4	-0.01346	2.667963	-0.06421	0.948945	-6.16301	0.88085	0.829853
Non.inflammatory.Macrophages	POM121	-0.00658	4.883475	-0.06414	0.949007	-6.71763	0.769967	0.780244
Non.inflammatory.Macrophages	1110006O2	-0.0272	0.319918	-0.06408	0.949051	-5.54849	0.992051	0.885826
Non.inflammatory.Macrophages	GM15952	0.024978	1.028007	0.064062	0.949065	-5.71124	0.972239	0.868576
Non.inflammatory.Macrophages	2900026A0	0.024008	3.595436	0.064053	0.949073	-5.96942	0.83273	0.808725
Non.inflammatory.Macrophages	GM10785	-0.01738	2.451276	-0.06402	0.949101	-6.07591	0.892457	0.834883
Non.inflammatory.Macrophages	EVI5L	0.015756	2.262161	0.06393	0.94917	-5.99626	0.902702	0.839285
Non.inflammatory.Macrophages	OLFR77	0.021215	1.376904	0.063835	0.949246	-5.83754	0.952106	0.86022

Non.inflammatory.Macrophages	LIMK1	0.014438	2.1198	0.063568	0.949458	-6.1536	0.910621	0.842671
Non.inflammatory.Macrophages	DAZAP1	0.005288	6.776612	0.062972	0.949931	-6.95673	0.686127	0.740317
Non.inflammatory.Macrophages	NUDT1	0.013736	3.00659	0.062958	0.949942	-6.26145	0.863377	0.822232
Non.inflammatory.Macrophages	NGRN	-0.01129	3.173648	-0.06293	0.949962	-6.34822	0.854686	0.818419
Non.inflammatory.Macrophages	PICK1	-0.01877	1.538716	-0.06235	0.950422	-5.89595	0.943505	0.856567
Non.inflammatory.Macrophages	AMMECR1	-0.00943	4.787165	-0.06231	0.950458	-6.53123	0.775003	0.782549
Non.inflammatory.Macrophages	NLRP12	0.036915	-0.12085	0.062195	0.950548	-5.3995	0.992693	0.89697
Non.inflammatory.Macrophages	9230114K1	0.015312	2.574674	0.062048	0.950665	-5.90027	0.886404	0.832246
Non.inflammatory.Macrophages	PRKRA	0.007352	3.39958	0.062009	0.950695	-6.59175	0.843229	0.813363
Non.inflammatory.Macrophages	HSPA14	-0.00743	5.034968	-0.06199	0.950708	-6.73739	0.76338	0.777169
Non.inflammatory.Macrophages	ADPRH	-0.00715	4.93883	-0.06173	0.950913	-6.74706	0.767869	0.779252
Non.inflammatory.Macrophages	JPT1	0.005753	7.832843	0.061704	0.950938	-7.14057	0.643219	0.718933
Non.inflammatory.Macrophages	SEC23A	-0.00816	4.425257	-0.06162	0.951007	-6.54382	0.792279	0.790473
Non.inflammatory.Macrophages	ADGRA3	-0.02439	0.459311	-0.06141	0.951171	-5.6017	0.992693	0.882641
Non.inflammatory.Macrophages	DCTN5	0.00691	4.598222	0.06127	0.951283	-6.70085	0.783977	0.786676

Non.inflammatory.Macrophages	EEF1AKMT	0.012269	3.325831	0.061215	0.951326	-6.29979	0.847008	0.815034
Non.inflammatory.Macrophages	NSUN5	0.015232	2.124735	0.061191	0.951346	-5.97436	0.910804	0.842725
Non.inflammatory.Macrophages	ILK	-0.00647	5.628263	-0.06117	0.951358	-6.88536	0.736222	0.764437
Non.inflammatory.Macrophages	THEM4	0.017433	2.270582	0.061144	0.951382	-6.02652	0.902828	0.839314
Non.inflammatory.Macrophages	IFI207	-0.00877	4.721848	-0.06106	0.951453	-7.15863	0.778094	0.783973
Non.inflammatory.Macrophages	ZNHIT6	0.011798	3.231636	0.061048	0.951459	-6.29275	0.851858	0.817173
Non.inflammatory.Macrophages	TASP1	-0.01112	3.918389	-0.06095	0.951538	-6.44589	0.817085	0.801747
Non.inflammatory.Macrophages	PEX2	-0.00897	3.914925	-0.0609	0.951577	-6.55296	0.817257	0.801826
Non.inflammatory.Macrophages	LENG9	-0.01289	2.352054	-0.0608	0.951658	-6.17945	0.8984	0.837484
Non.inflammatory.Macrophages	4930469K1	-0.0168	1.311112	-0.06051	0.951887	-6.16871	0.956491	0.862084
Non.inflammatory.Macrophages	BTF3	0.004012	8.999494	0.060495	0.951898	-7.40292	0.598742	0.696035
Non.inflammatory.Macrophages	TMEM109	-0.00879	3.841384	-0.06048	0.951909	-6.60934	0.820917	0.8035
Non.inflammatory.Macrophages	NDST2	-0.00851	2.934923	-0.06048	0.951912	-6.5169	0.867303	0.824025
Non.inflammatory.Macrophages	KATNAL1	0.022472	0.973387	0.060289	0.952062	-5.74525	0.976136	0.870246
Non.inflammatory.Macrophages	NF1	-0.00527	6.550388	-0.05986	0.952402	-7.03284	0.695971	0.745215

Non.inflammatory.Macrophages	PLXNA4OS	-0.01654	0.51532	-0.0598	0.952452	-6.06128	0.992241	0.88145
Non.inflammatory.Macrophages	UBE2D1	0.005584	5.377113	0.059783	0.952464	-6.75894	0.747745	0.76996
Non.inflammatory.Macrophages	AKIP1	-0.01064	3.24519	-0.05974	0.952495	-6.36884	0.851319	0.817033
Non.inflammatory.Macrophages	HS1BP3	0.014129	1.087416	0.059697	0.952532	-6.04535	0.969594	0.867555
Non.inflammatory.Macrophages	GM17106	-0.01949	3.395192	-0.0593	0.952844	-5.87877	0.843793	0.813742
Non.inflammatory.Macrophages	FPGT	-0.01932	1.345954	-0.0592	0.952928	-5.957	0.954877	0.861494
Non.inflammatory.Macrophages	HIST1H1D	0.028007	1.531522	0.05909	0.953014	-5.68888	0.944293	0.857066
Non.inflammatory.Macrophages	CHMP4B	0.003825	7.889271	0.058921	0.953148	-7.27191	0.641254	0.718087
Non.inflammatory.Macrophages	SLC50A1	0.006562	5.247798	0.058602	0.953401	-6.79642	0.753833	0.772956
Non.inflammatory.Macrophages	APOBR	-0.00882	2.373103	-0.0585	0.953486	-6.61817	0.897621	0.837354
Non.inflammatory.Macrophages	PHPT1	-0.00768	4.330971	-0.0582	0.953721	-6.59846	0.79716	0.793026
Non.inflammatory.Macrophages	GM21887	-0.0202	2.133944	-0.05818	0.953738	-5.91571	0.910665	0.843015
Non.inflammatory.Macrophages	VMAC	0.017752	1.5263	0.058021	0.953862	-5.83766	0.944589	0.857422
Non.inflammatory.Macrophages	4921509O(	-0.02081	0.064578	-0.058	0.953882	-5.90138	0.993093	0.892948
Non.inflammatory.Macrophages	SLC1A3	-0.02756	-0.29724	-0.05771	0.954113	-5.79046	0.993093	0.901958

Non.inflammatory.Macrophages	ADCK1	0.00982	3.101425	0.05768	0.954134	-6.38168	0.85895	0.820675
Non.inflammatory.Macrophages	NEDD8	-0.00363	7.48397	-0.05764	0.954166	-7.19315	0.657403	0.726422
Non.inflammatory.Macrophages	CHST15	-0.01055	3.54275	-0.0576	0.954194	-6.28394	0.836274	0.810658
Non.inflammatory.Macrophages	2810405F1	-0.02461	0.687122	-0.05706	0.954625	-5.61664	0.987501	0.877781
Non.inflammatory.Macrophages	BTBD3	-0.02	1.337187	-0.05699	0.954681	-5.87715	0.955379	0.862086
Non.inflammatory.Macrophages	ARL8B	0.004378	5.97123	0.056929	0.95473	-6.97739	0.721237	0.757815
Non.inflammatory.Macrophages	NTN4	-0.02591	0.706687	-0.05684	0.954799	-5.68679	0.986761	0.877344
Non.inflammatory.Macrophages	TSACC	0.018673	1.881856	0.056752	0.954871	-6.00048	0.924602	0.849174
Non.inflammatory.Macrophages	TMEM132I	-0.03539	0.984501	-0.05672	0.954899	-5.46163	0.975801	0.870618
Non.inflammatory.Macrophages	PHF7	0.010982	3.28527	0.056589	0.955	-6.23343	0.849435	0.816684
Non.inflammatory.Macrophages	POLR3E	0.007795	3.918387	0.056574	0.955013	-6.4597	0.817415	0.802421
Non.inflammatory.Macrophages	RAB1B	0.005722	5.58788	0.056534	0.955044	-6.85496	0.738338	0.765982
Non.inflammatory.Macrophages	TBC1D25	-0.01367	2.839206	-0.05643	0.955129	-6.09362	0.872692	0.826883
Non.inflammatory.Macrophages	AP1G1	0.005343	6.456316	0.05627	0.955254	-7.00795	0.700141	0.747684
Non.inflammatory.Macrophages	ELP3	0.010289	2.716115	0.056258	0.955263	-6.23881	0.879213	0.829719

Non.inflammatory.Macrophages	ZFP764	0.019397	1.074799	0.056255	0.955266	-5.74266	0.970535	0.868457
Non.inflammatory.Macrophages	ULK2	-0.00595	5.457806	-0.05605	0.955425	-6.87173	0.744228	0.768761
Non.inflammatory.Macrophages	SDR42E1	0.021838	0.45745	0.056053	0.955426	-5.60487	0.993093	0.883476
Non.inflammatory.Macrophages	CDS1	0.010871	2.788548	0.056047	0.955431	-6.52197	0.87537	0.828049
Non.inflammatory.Macrophages	SLAIN1	0.019419	3.6132	0.056019	0.955453	-5.63579	0.832706	0.809266
Non.inflammatory.Macrophages	FASL	-0.03194	1.037842	-0.05601	0.955458	-5.48788	0.972687	0.869349
Non.inflammatory.Macrophages	RAB34	0.025577	0.235405	0.055747	0.955669	-5.67757	0.993093	0.889036
Non.inflammatory.Macrophages	SNRPD2	0.004924	6.391824	0.055728	0.955684	-6.95914	0.702911	0.749109
Non.inflammatory.Macrophages	RBX1	-0.00429	7.700144	-0.05564	0.955752	-7.19359	0.64874	0.72232
Non.inflammatory.Macrophages	RASSF7	-0.02326	0.86906	-0.0556	0.955785	-5.60021	0.980648	0.873535
Non.inflammatory.Macrophages	ZFP319	-0.0149	2.544028	-0.05553	0.955841	-6.11034	0.888404	0.833808
Non.inflammatory.Macrophages	COX6B2	0.016805	1.691068	0.055443	0.95591	-5.92216	0.935279	0.853864
Non.inflammatory.Macrophages	SPPL2B	0.012987	2.17345	0.055345	0.955989	-6.13974	0.908499	0.842496
Non.inflammatory.Macrophages	PRKCB	-0.00466	8.511819	-0.05507	0.956208	-7.25248	0.617202	0.706241
Non.inflammatory.Macrophages	RAMAC	0.004984	5.913686	0.055065	0.956211	-6.86652	0.723779	0.759208

Non.inflammatory.Macrophages	NBAS	-0.00682	4.535139	-0.05506	0.956215	-6.63116	0.787312	0.788912
Non.inflammatory.Macrophages	CYP2R1	0.020499	1.009263	0.054949	0.956303	-5.7538	0.974354	0.870203
Non.inflammatory.Macrophages	BAP1	0.008905	3.565024	0.054928	0.95632	-6.35009	0.835145	0.810504
Non.inflammatory.Macrophages	METTL25	-0.00667	4.28478	-0.05483	0.956396	-6.51498	0.799404	0.794429
Non.inflammatory.Macrophages	GOPC	-0.0062	4.545466	-0.05469	0.956508	-6.57741	0.786817	0.788716
Non.inflammatory.Macrophages	NUDT19	0.007312	4.544356	0.054613	0.95657	-6.63128	0.78687	0.788754
Non.inflammatory.Macrophages	C330011M	-0.02068	0.592367	-0.05448	0.956676	-5.62097	0.990503	0.880392
Non.inflammatory.Macrophages	TRIM30C	0.013179	1.320225	0.054458	0.956693	-6.24781	0.956353	0.86277
Non.inflammatory.Macrophages	NOMO1	-0.00863	3.5373	-0.05446	0.956695	-6.37924	0.836551	0.81118
Non.inflammatory.Macrophages	BTG2	-0.00642	7.421641	-0.05438	0.956753	-7.14578	0.659921	0.728041
Non.inflammatory.Macrophages	TRP53INP1	-0.00946	5.187354	-0.05436	0.956772	-6.54818	0.756619	0.774765
Non.inflammatory.Macrophages	9930021J0	0.005277	6.407459	0.054233	0.956872	-6.93141	0.702239	0.748924
Non.inflammatory.Macrophages	TFAM	0.007598	4.293459	0.054231	0.956873	-6.49272	0.798982	0.794324
Non.inflammatory.Macrophages	CCNK	0.005942	4.857081	0.054202	0.956896	-6.67131	0.772017	0.781959
Non.inflammatory.Macrophages	4930599N[illegible]	0.011281	1.733523	0.053999	0.957058	-6.28217	0.932893	0.852989

Non.inflammatory.Macrophages	LRRC75AO!	-0.02613	0.512247	-0.05376	0.957247	-5.53011	0.992531	0.88245
Non.inflammatory.Macrophages	R3HCC1	-0.01618	2.353768	-0.05375	0.957255	-5.94492	0.898669	0.838425
Non.inflammatory.Macrophages	GM44899	-0.01886	0.774696	-0.0537	0.957295	-5.74074	0.984194	0.876042
Non.inflammatory.Macrophages	LMBR1	-0.01429	1.883516	-0.05366	0.957325	-5.9681	0.924509	0.849471
Non.inflammatory.Macrophages	HIST1H2BE	-0.02571	1.116611	-0.05355	0.957415	-5.48964	0.968106	0.867772
Non.inflammatory.Macrophages	GRHL1	-0.02092	1.025537	-0.05349	0.957462	-5.75372	0.973405	0.86997
Non.inflammatory.Macrophages	PLA2G15	0.007544	3.738633	0.05342	0.957518	-6.73219	0.826389	0.806746
Non.inflammatory.Macrophages	ORC6	-0.00792	5.216442	-0.05341	0.957529	-6.64916	0.755277	0.774233
Non.inflammatory.Macrophages	SLC35E4	0.01834	0.657751	0.053316	0.957601	-5.87171	0.988613	0.878903
Non.inflammatory.Macrophages	CDS2	0.006758	4.708784	0.053308	0.957607	-6.57652	0.779027	0.785252
Non.inflammatory.Macrophages	TMCO6	-0.01083	2.683081	-0.05324	0.957662	-6.10686	0.88097	0.830799
Non.inflammatory.Macrophages	ZFP617	-0.01358	2.165547	-0.05316	0.957725	-6.05613	0.908932	0.84286
Non.inflammatory.Macrophages	GM13391	0.014783	0.765079	0.052641	0.958137	-5.99917	0.984625	0.876423
Non.inflammatory.Macrophages	SH3BP5L	0.013891	2.252113	0.052486	0.95826	-5.95508	0.904262	0.840971
Non.inflammatory.Macrophages	GM15246	0.023353	1.046088	0.052477	0.958267	-5.69366	0.972274	0.86964

Non.inflammatory.Macrophages	MYLK	-0.01822	1.606214	-0.05234	0.958373	-5.84167	0.940129	0.856258
Non.inflammatory.Macrophages	DNMT3B	-0.01359	2.497904	-0.05234	0.958378	-5.92029	0.890945	0.835289
Non.inflammatory.Macrophages	PYGO2	-0.01067	3.374512	-0.05232	0.958388	-6.38268	0.844911	0.815165
Non.inflammatory.Macrophages	NMI	-0.0072	5.064624	-0.05214	0.958533	-6.76941	0.76236	0.777764
Non.inflammatory.Macrophages	SLC44A2	0.006721	6.242042	0.052125	0.958547	-6.83778	0.709435	0.752682
Non.inflammatory.Macrophages	SMIM40	0.017055	0.301755	0.052083	0.95858	-5.88769	0.993162	0.887927
Non.inflammatory.Macrophages	SORCS2	0.018741	3.3545	0.051993	0.958651	-5.9498	0.845937	0.81572
Non.inflammatory.Macrophages	KRT83	-0.03533	-0.26141	-0.05192	0.958708	-5.30265	0.993162	0.901955
Non.inflammatory.Macrophages	EIF4EBP3	0.010576	2.80817	0.051771	0.958828	-6.27093	0.874393	0.828218
Non.inflammatory.Macrophages	CSNK2A2	0.004183	5.948451	0.051641	0.958931	-6.88802	0.722293	0.758919
Non.inflammatory.Macrophages	ALG1	0.008445	3.312271	0.051257	0.959236	-6.43044	0.848105	0.816739
Non.inflammatory.Macrophages	SEC22C	-0.01376	2.04121	-0.05107	0.959389	-5.94806	0.915833	0.846132
Non.inflammatory.Macrophages	INAFM1	-0.0109	1.866251	-0.05104	0.959411	-6.18885	0.925535	0.850258
Non.inflammatory.Macrophages	GM26590	-0.01548	1.314817	-0.05096	0.959472	-5.69926	0.95673	0.86339
Non.inflammatory.Macrophages	ELOVL5	0.005471	6.195778	0.050937	0.959491	-6.98134	0.711446	0.753744

Non.inflammatory.Macrophages	SLC10A7	0.004983	5.908741	0.050912	0.95951	-6.8772	0.724049	0.759791
Non.inflammatory.Macrophages	ARID2	0.005123	6.543762	0.050695	0.959683	-6.94119	0.696451	0.746477
Non.inflammatory.Macrophages	PAWR	0.022216	0.662659	0.050695	0.959683	-5.60241	0.988496	0.879175
Non.inflammatory.Macrophages	PRM1	-0.03223	-0.44781	-0.05069	0.959684	-5.26155	0.993162	0.906694
Non.inflammatory.Macrophages	N4BP2	-0.00666	5.310122	-0.05056	0.959793	-6.68972	0.751023	0.77256
Non.inflammatory.Macrophages	SIRPB1A	0.020397	-0.18195	0.05052	0.959822	-5.73463	0.993162	0.900031
Non.inflammatory.Macrophages	SDF4	0.0037	6.506152	0.050506	0.959833	-7.03626	0.698057	0.747259
Non.inflammatory.Macrophages	TIFA	-0.00829	6.262161	-0.05048	0.959851	-6.85413	0.708562	0.752352
Non.inflammatory.Macrophages	DPP10	0.01978	0.411958	0.050427	0.959896	-5.85728	0.993162	0.885317
Non.inflammatory.Macrophages	AURKA	0.010842	3.318106	0.050409	0.959911	-6.27052	0.847805	0.816607
Non.inflammatory.Macrophages	PASK	0.02783	1.295986	0.050397	0.95992	-5.56317	0.957812	0.863842
Non.inflammatory.Macrophages	SLC19A1	-0.01699	1.065999	-0.05028	0.960016	-5.81315	0.971138	0.869402
Non.inflammatory.Macrophages	TOR3A	-0.00849	4.075925	-0.05004	0.960206	-6.71665	0.809798	0.799596
Non.inflammatory.Macrophages	MBTPS1	-0.00557	4.639311	-0.0498	0.960391	-6.62858	0.782503	0.787155
Non.inflammatory.Macrophages	PRMT5	0.008546	3.483009	0.049759	0.960427	-6.32481	0.839494	0.8129

Non.inflammatory.Macrophages	VARS	-0.00652	5.713285	-0.04971	0.960467	-6.65522	0.732861	0.763986
Non.inflammatory.Macrophages	D6WSU163	-0.00738	3.1191	-0.04962	0.960538	-6.38688	0.858218	0.821216
Non.inflammatory.Macrophages	TTF2	-0.01038	3.091877	-0.04954	0.9606	-6.22266	0.859634	0.821861
Non.inflammatory.Macrophages	GM26520	-0.01223	2.362216	-0.04939	0.960719	-6.35318	0.898407	0.838748
Non.inflammatory.Macrophages	CDCA5	-0.01532	3.028781	-0.04939	0.960723	-6.02071	0.862925	0.823339
Non.inflammatory.Macrophages	GM49961	0.026641	0.574285	0.049094	0.960955	-5.45634	0.991274	0.881477
Non.inflammatory.Macrophages	VSIG10L	-0.01685	0.298869	-0.04901	0.961022	-5.65951	0.993408	0.888244
Non.inflammatory.Macrophages	MRPL27	-0.00643	4.210901	-0.04897	0.961056	-6.63301	0.803259	0.796695
Non.inflammatory.Macrophages	DDX59	0.012689	1.892836	0.048747	0.961231	-5.96872	0.924283	0.849776
Non.inflammatory.Macrophages	YIPF1	-0.00437	5.234712	-0.04871	0.961264	-6.81549	0.754674	0.774322
Non.inflammatory.Macrophages	DHX57	0.007076	3.871504	0.048616	0.961335	-6.43319	0.820006	0.804272
Non.inflammatory.Macrophages	RNF34	0.005853	4.455799	0.048562	0.961378	-6.60893	0.791376	0.791298
Non.inflammatory.Macrophages	FAM222A	0.012516	2.331884	0.048448	0.961469	-5.94752	0.900142	0.839472
Non.inflammatory.Macrophages	ZC3H3	0.006995	3.468484	0.048413	0.961496	-6.38414	0.840317	0.813344
Non.inflammatory.Macrophages	COL5A2	-0.02165	1.225753	-0.04829	0.961592	-5.735	0.962094	0.865708

Non.inflammatory.Macrophages	DYNC1H1	-0.00358	6.255557	-0.04825	0.961625	-7.00755	0.709023	0.752649
Non.inflammatory.Macrophages	ZFP36	0.005379	7.849088	0.047929	0.961881	-7.34573	0.643163	0.720047
Non.inflammatory.Macrophages	ZCCHC18	-0.02861	1.04036	-0.04768	0.962075	-5.33033	0.973034	0.870267
Non.inflammatory.Macrophages	ZFP518B	0.016685	0.418092	0.047678	0.962081	-5.62627	0.993598	0.885438
Non.inflammatory.Macrophages	AFG1L	-0.00845	3.691074	-0.04765	0.962103	-6.47507	0.8292	0.808423
Non.inflammatory.Macrophages	HJURP	-0.00623	4.895427	-0.04685	0.962738	-6.64163	0.771026	0.781865
Non.inflammatory.Macrophages	ATP6V1G2	-0.0142	1.237127	-0.04681	0.962774	-5.88016	0.962146	0.865626
Non.inflammatory.Macrophages	GPBP1	-0.00421	7.723603	-0.04668	0.962876	-7.12966	0.648509	0.722667
Non.inflammatory.Macrophages	COL23A1	-0.01828	1.376373	-0.04649	0.963025	-5.79492	0.954178	0.862327
Non.inflammatory.Macrophages	SLC35B2	-0.00498	4.882082	-0.04625	0.963218	-6.82003	0.771685	0.782243
Non.inflammatory.Macrophages	SMAD2	0.003719	6.426685	0.046235	0.963227	-6.9611	0.70218	0.749317
Non.inflammatory.Macrophages	SLC39A9	0.004832	4.172579	0.046222	0.963238	-6.58527	0.80576	0.797846
Non.inflammatory.Macrophages	SLC12A7	0.007274	3.37429	0.045973	0.963435	-6.53673	0.845788	0.815811
Non.inflammatory.Macrophages	BC050972	0.021485	-0.27973	0.04597	0.963438	-5.61768	0.99418	0.903008
Non.inflammatory.Macrophages	SOWAHC	0.007208	2.917305	0.045931	0.963469	-6.6976	0.869529	0.826251

Non.inflammatory.Macrophages	AIG1	0.005736	4.903663	0.045817	0.963559	-6.83674	0.77067	0.78182
Non.inflammatory.Macrophages	ACOT2	-0.00506	4.416307	-0.04566	0.963683	-6.6415	0.793898	0.792517
Non.inflammatory.Macrophages	NKG7	0.020748	4.013022	0.045575	0.963752	-5.95903	0.813616	0.801468
Non.inflammatory.Macrophages	GM13166	-0.01867	-0.19353	-0.04556	0.963763	-5.64667	0.99418	0.900886
Non.inflammatory.Macrophages	REEP6	-0.01648	1.791912	-0.04538	0.963904	-5.84507	0.930639	0.852572
Non.inflammatory.Macrophages	UBE2K	0.00307	8.214477	0.045373	0.963913	-7.23698	0.629265	0.713022
Non.inflammatory.Macrophages	1700096K1	0.010262	2.613489	0.045369	0.963915	-6.1237	0.885652	0.833315
Non.inflammatory.Macrophages	PCGF2	0.017442	0.79144	0.045051	0.964168	-5.7191	0.984823	0.876647
Non.inflammatory.Macrophages	SHROOM2	-0.02166	1.506317	-0.04485	0.964325	-5.66756	0.947001	0.859407
Non.inflammatory.Macrophages	SNRNP40	-0.00435	5.982356	-0.04465	0.964484	-6.79796	0.721719	0.758781
Non.inflammatory.Macrophages	OGFR	-0.00571	5.244677	-0.04465	0.964491	-6.78251	0.754995	0.774526
Non.inflammatory.Macrophages	NDUFV2	0.004423	6.136177	0.044529	0.964583	-6.96567	0.71496	0.755562
Non.inflammatory.Macrophages	EFCAB8	0.014084	1.374064	0.044406	0.964681	-5.82661	0.954554	0.862641
Non.inflammatory.Macrophages	SGSH	-0.00982	1.453355	-0.04423	0.96482	-6.18775	0.950019	0.860769
Non.inflammatory.Macrophages	4932422M	-0.01577	0.972719	-0.04419	0.964854	-5.73814	0.977809	0.872341

Non.inflammatory.Macrophages	GM42702	0.01598	2.365724	0.044092	0.96493	-5.73997	0.899234	0.839231
Non.inflammatory.Macrophages	POLR2C	0.004849	5.080792	0.044056	0.964959	-6.72228	0.762584	0.778168
Non.inflammatory.Macrophages	SLC6A13	-0.01284	2.762131	-0.04384	0.965131	-5.99273	0.877954	0.830067
Non.inflammatory.Macrophages	ZFP532	-0.01351	1.223667	-0.04356	0.965357	-6.03782	0.963209	0.866414
Non.inflammatory.Macrophages	TAF11	-0.00475	4.933052	-0.04349	0.965407	-6.67918	0.769487	0.781465
Non.inflammatory.Macrophages	ZFP709	0.009321	1.431477	0.043491	0.965408	-6.00625	0.951269	0.861425
Non.inflammatory.Macrophages	HSDL2	-0.00575	4.684002	-0.04345	0.96544	-6.59085	0.781258	0.786902
Non.inflammatory.Macrophages	ARPC1A	0.003825	6.586291	0.043367	0.965507	-6.97982	0.695529	0.746354
Non.inflammatory.Macrophages	GM43113	0.017263	-0.69248	0.043319	0.965545	-5.70622	0.994434	0.913799
Non.inflammatory.Macrophages	SNHG16	0.010632	2.227913	0.043175	0.965659	-6.08095	0.906742	0.842617
Non.inflammatory.Macrophages	LEAP2	0.013298	3.060255	0.043138	0.965689	-6.21083	0.862257	0.823333
Non.inflammatory.Macrophages	GM17018	0.00638	3.620053	0.043132	0.965693	-6.47607	0.833484	0.810607
Non.inflammatory.Macrophages	TRPC1	0.018602	-0.17084	0.043118	0.965705	-5.58496	0.994434	0.90068
Non.inflammatory.Macrophages	PIGC	-0.00706	2.730331	-0.04273	0.96601	-6.32141	0.879855	0.83095
Non.inflammatory.Macrophages	ZFP7	-0.02668	0.286794	-0.04257	0.966143	-5.35303	0.994678	0.889382

Non.inflammatory.Macrophages	ACP6	0.007949	3.140192	0.042543	0.966162	-6.26788	0.858303	0.821568
Non.inflammatory.Macrophages	GM37982	-0.02254	2.996043	-0.04235	0.966315	-5.57473	0.865882	0.824929
Non.inflammatory.Macrophages	NCEH1	0.005162	4.906119	0.04227	0.966379	-6.9264	0.770989	0.782242
Non.inflammatory.Macrophages	AW549877	0.006848	3.352769	0.042174	0.966455	-6.44862	0.84737	0.816824
Non.inflammatory.Macrophages	HYPK	-0.00371	5.8151	-0.0421	0.966516	-6.88685	0.729363	0.762713
Non.inflammatory.Macrophages	ECSCR	0.018986	1.207762	0.04178	0.966769	-5.65052	0.964476	0.867131
Non.inflammatory.Macrophages	C230037L1	0.015725	0.378737	0.041713	0.966822	-5.6722	0.994792	0.88733
Non.inflammatory.Macrophages	UTP15	0.005144	3.757308	0.04167	0.966856	-6.53409	0.826865	0.807775
Non.inflammatory.Macrophages	H2-DMB2	0.010565	3.016521	0.041663	0.966861	-6.16427	0.864855	0.824599
Non.inflammatory.Macrophages	CCPG1OS	0.012833	1.576044	0.041296	0.967153	-5.73772	0.943479	0.858368
Non.inflammatory.Macrophages	1110035H1	-0.01594	1.216236	-0.0412	0.967231	-5.70383	0.964086	0.867014
Non.inflammatory.Macrophages	POU5F1	0.014795	1.26222	0.041176	0.967249	-5.71428	0.96143	0.865907
Non.inflammatory.Macrophages	AKAP7	-0.00564	3.374989	-0.04117	0.967256	-6.50504	0.846361	0.816498
Non.inflammatory.Macrophages	SLC12A3	-0.02053	1.874484	-0.04079	0.967553	-5.58705	0.92686	0.851348
Non.inflammatory.Macrophages	TFCP2	0.010408	1.971586	0.040761	0.967579	-5.98924	0.921456	0.849053

Non.inflammatory.Macrophages	TRIM34A	0.008327	3.308515	0.040497	0.967788	-6.4455	0.849967	0.818097
Non.inflammatory.Macrophages	KIF21B	0.004914	4.865707	0.040314	0.967934	-6.71539	0.773183	0.783396
Non.inflammatory.Macrophages	2410022M	0.010277	1.591485	0.040198	0.968026	-5.96506	0.942813	0.858112
Non.inflammatory.Macrophages	BACE2	0.018512	1.120623	0.040069	0.968128	-5.61199	0.969845	0.869424
Non.inflammatory.Macrophages	WHAMM	0.005592	4.147659	0.039999	0.968184	-6.52393	0.807742	0.799222
Non.inflammatory.Macrophages	CLNK	-0.02753	1.404175	-0.03997	0.968208	-5.42047	0.953483	0.8626
Non.inflammatory.Macrophages	TMEM115	0.00538	3.447731	0.03992	0.968247	-6.37191	0.842822	0.814952
Non.inflammatory.Macrophages	SCN4A	-0.01875	0.410123	-0.03983	0.968317	-5.55113	0.995116	0.886785
Non.inflammatory.Macrophages	RCC1L	0.007966	3.072184	0.039812	0.968333	-6.23687	0.862225	0.823539
Non.inflammatory.Macrophages	EIF2B3	0.007649	2.996786	0.039787	0.968352	-6.30256	0.866171	0.82527
Non.inflammatory.Macrophages	ZFP597	-0.00838	2.862663	-0.03948	0.968598	-6.17044	0.873359	0.828406
Non.inflammatory.Macrophages	EXOC6B	-0.00435	5.502152	-0.03938	0.968675	-6.91484	0.743838	0.769728
Non.inflammatory.Macrophages	XAF1	-0.00886	4.033526	-0.03933	0.968717	-6.73996	0.813488	0.80185
Non.inflammatory.Macrophages	ACOT1	-0.00901	2.604682	-0.03894	0.969026	-6.5105	0.887187	0.83445
Non.inflammatory.Macrophages	TRMT12	0.011688	1.30746	0.038848	0.9691	-5.84699	0.959283	0.865094

Non.inflammatory.Macrophages	GM16573	0.015484	1.655105	0.038788	0.969147	-5.60228	0.939456	0.856778
Non.inflammatory.Macrophages	MEX3B	-0.01144	2.233947	-0.03861	0.969291	-5.91034	0.907268	0.843137
Non.inflammatory.Macrophages	GM43256	0.022273	0.309245	0.038587	0.969307	-5.37144	0.995373	0.889459
Non.inflammatory.Macrophages	GM43378	0.011754	1.243694	0.038275	0.969555	-5.74708	0.962961	0.866738
Non.inflammatory.Macrophages	ALYREF2	-0.00681	3.243584	-0.03823	0.96959	-6.27156	0.853539	0.819857
Non.inflammatory.Macrophages	TPRA1	0.005994	2.802051	0.038152	0.969652	-6.43317	0.876664	0.829993
Non.inflammatory.Macrophages	S100A16	-0.00935	2.552707	-0.03763	0.970071	-6.07241	0.889977	0.835873
Non.inflammatory.Macrophages	ERC1	0.005237	4.872109	0.037547	0.970133	-6.67982	0.773081	0.783634
Non.inflammatory.Macrophages	XRCC1	-0.00564	4.144273	-0.03747	0.970194	-6.47677	0.808117	0.799687
Non.inflammatory.Macrophages	BSN	0.023543	0.764883	0.037158	0.970443	-5.4001	0.986824	0.878553
Non.inflammatory.Macrophages	GM5535	0.02003	0.151841	0.036939	0.970617	-5.5687	0.995373	0.893636
Non.inflammatory.Macrophages	EIF3M	0.002443	6.621301	0.036888	0.970657	-7.01471	0.694695	0.746456
Non.inflammatory.Macrophages	LSM2	0.005447	5.695608	0.036815	0.970715	-6.79549	0.735178	0.765944
Non.inflammatory.Macrophages	GM9993	-0.01378	1.16551	-0.03681	0.970722	-5.72936	0.967488	0.86883
Non.inflammatory.Macrophages	UBE2L3	-0.00247	7.560477	-0.0368	0.97073	-7.19017	0.655826	0.727194

Non.inflammatory.Macrophages	RAPGEF5	-0.00857	5.011787	-0.0367	0.970804	-6.65416	0.766524	0.780667
Non.inflammatory.Macrophages	WASHC3	0.003704	4.517912	0.036678	0.970824	-6.71727	0.789948	0.791483
Non.inflammatory.Macrophages	SEMA6D	-0.0072	3.713919	-0.03659	0.970894	-6.72737	0.829531	0.809397
Non.inflammatory.Macrophages	PGLS	-0.00325	7.788311	-0.03648	0.970981	-7.12236	0.646721	0.722628
Non.inflammatory.Macrophages	GM30239	-0.01745	-0.09316	-0.03647	0.970988	-5.40541	0.995373	0.899771
Non.inflammatory.Macrophages	OARD1	0.004452	4.902059	0.036381	0.971061	-6.69341	0.771671	0.783112
Non.inflammatory.Macrophages	GM6034	0.018653	0.50272	0.036085	0.971296	-5.60627	0.995052	0.885087
Non.inflammatory.Macrophages	ARRDC1	-0.00469	4.471227	-0.03604	0.971335	-6.64649	0.792197	0.792604
Non.inflammatory.Macrophages	SNX5	0.00281	7.890887	0.036017	0.97135	-7.24895	0.642662	0.72063
Non.inflammatory.Macrophages	DNAAF2	0.007847	2.845924	0.036009	0.971357	-6.06148	0.87434	0.829277
Non.inflammatory.Macrophages	EPOP	0.011156	1.472765	0.035987	0.971374	-5.88408	0.949808	0.861554
Non.inflammatory.Macrophages	TRAPPC4	-0.00345	5.137813	-0.0358	0.971525	-6.80555	0.760653	0.778031
Non.inflammatory.Macrophages	CD200R4	0.011563	1.662182	0.035746	0.971566	-6.18888	0.939056	0.85703
Non.inflammatory.Macrophages	IKZF1	0.003546	8.378161	0.035688	0.971611	-7.14622	0.623719	0.710924
Non.inflammatory.Macrophages	AP2M1	-0.00315	6.941455	-0.03565	0.971645	-7.11711	0.6812	0.739928

Non.inflammatory.Macrophages	IL2RG	-0.00726	6.294179	-0.03564	0.971649	-6.90175	0.708749	0.753382
Non.inflammatory.Macrophages	PRRC1	0.004236	4.219989	0.035608	0.971675	-6.59476	0.804404	0.798167
Non.inflammatory.Macrophages	HMCN1	-0.01911	1.920186	-0.03555	0.97172	-5.74249	0.924588	0.850905
Non.inflammatory.Macrophages	METTL23	0.003169	5.675126	0.035547	0.971724	-6.82435	0.736099	0.766479
Non.inflammatory.Macrophages	IL12RB2	-0.01076	3.672057	-0.03553	0.971733	-6.10696	0.831642	0.810432
Non.inflammatory.Macrophages	NLRP1A	0.011452	0.449959	0.035469	0.971786	-6.01103	0.995373	0.886384
Non.inflammatory.Macrophages	ZFYVE28	0.013399	0.624372	0.035467	0.971788	-5.55814	0.991968	0.882102
Non.inflammatory.Macrophages	MUS81	0.009257	2.048507	0.035439	0.97181	-5.99409	0.917469	0.847874
Non.inflammatory.Macrophages	HIST1H1A	-0.01488	3.773403	-0.0354	0.971839	-6.16182	0.82654	0.80815
Non.inflammatory.Macrophages	SLC39A13	-0.00562	2.796524	-0.03504	0.972128	-6.39763	0.877094	0.830455
Non.inflammatory.Macrophages	RPAP3	-0.00559	3.841822	-0.03503	0.972138	-6.39321	0.823241	0.806649
Non.inflammatory.Macrophages	ANGPTL1	0.022143	0.376343	0.034789	0.972326	-5.31311	0.995647	0.888293
Non.inflammatory.Macrophages	CAMTA1	0.003151	5.835211	0.034569	0.972501	-6.88213	0.729188	0.763187
Non.inflammatory.Macrophages	SPTY2D1	-0.00315	5.765398	-0.03446	0.972585	-6.91902	0.732307	0.76468
Non.inflammatory.Macrophages	SLC25A27	0.020516	0.260835	0.034416	0.972623	-5.3128	0.995725	0.891196

Non.inflammatory.Macrophages	NDUFB4	0.002884	6.598671	0.034135	0.972846	-7.05799	0.696002	0.747148
Non.inflammatory.Macrophages	SUGP2	-0.00582	4.059588	-0.03406	0.972907	-6.46792	0.812691	0.801875
Non.inflammatory.Macrophages	ZFP87	-0.00574	3.396086	-0.0337	0.973189	-6.36578	0.846268	0.816868
Non.inflammatory.Macrophages	CFH	-0.00468	4.930751	-0.03363	0.973245	-7.01348	0.770853	0.78273
Non.inflammatory.Macrophages	ARAP2	0.004498	5.924266	0.033289	0.973519	-6.92392	0.725621	0.761434
Non.inflammatory.Macrophages	GM28694	-0.01967	-0.23311	-0.03287	0.97385	-5.35074	0.996322	0.903782
Non.inflammatory.Macrophages	SPARC	-0.0068	5.322477	-0.03283	0.973885	-6.78187	0.752844	0.774413
Non.inflammatory.Macrophages	ICOS	-0.00666	2.283057	-0.03279	0.973913	-6.50838	0.905446	0.842767
Non.inflammatory.Macrophages	GM39326	0.017674	1.289251	0.032764	0.973936	-5.41849	0.961247	0.866374
Non.inflammatory.Macrophages	ANKFY1	-0.00305	5.995175	-0.03263	0.974041	-6.93867	0.722523	0.760088
Non.inflammatory.Macrophages	GM36723	-0.01965	3.324777	-0.03231	0.974294	-5.72311	0.850158	0.818789
Non.inflammatory.Macrophages	NFKBID	-0.004	6.350031	-0.03209	0.974473	-6.95424	0.707003	0.752659
Non.inflammatory.Macrophages	THUMPD3	-0.0041	4.262161	-0.03208	0.974481	-6.59678	0.803107	0.797704
Non.inflammatory.Macrophages	DPH2	0.013146	0.879573	0.031769	0.974728	-5.66222	0.983441	0.876454
Non.inflammatory.Macrophages	ADA	-0.00883	2.074866	-0.03175	0.97474	-6.06442	0.916885	0.847817

Non.inflammatory.Macrophages	NCDN	-0.00668	2.826122	-0.03169	0.974794	-6.18298	0.876222	0.830286
Non.inflammatory.Macrophages	PALB2	0.007077	2.125531	0.031619	0.974847	-6.121	0.914089	0.846623
Non.inflammatory.Macrophages	KLHL15	-0.00386	4.413728	-0.03152	0.974923	-6.59533	0.795733	0.794402
Non.inflammatory.Macrophages	WDR41	-0.00333	4.001344	-0.03145	0.974981	-6.71143	0.815948	0.803573
Non.inflammatory.Macrophages	MBOAT2	0.021838	0.666194	0.031436	0.974993	-5.44681	0.991506	0.881664
Non.inflammatory.Macrophages	DFFA	-0.0063	2.939835	-0.03135	0.975064	-6.19831	0.870214	0.827664
Non.inflammatory.Macrophages	CCL12	0.016205	0.036828	0.031201	0.97518	-5.90415	0.996322	0.897204
Non.inflammatory.Macrophages	LYPLAL1	0.010789	1.634017	0.031101	0.975259	-5.94291	0.941544	0.858271
Non.inflammatory.Macrophages	CAAA0111	-0.00325	5.624186	-0.03104	0.975309	-6.82909	0.739097	0.768078
Non.inflammatory.Macrophages	ERCC1	0.00506	3.315868	0.030992	0.975346	-6.33197	0.850618	0.819049
Non.inflammatory.Macrophages	4931414P1	0.009483	2.099255	0.030737	0.975549	-5.91837	0.915538	0.847242
Non.inflammatory.Macrophages	TBC1D10B	-0.00353	5.147294	-0.03057	0.975681	-6.69724	0.760937	0.778343
Non.inflammatory.Macrophages	NADSYN1	0.011322	1.092531	0.030488	0.975747	-5.68537	0.972656	0.871284
Non.inflammatory.Macrophages	TMEM59	0.002354	6.649746	0.030403	0.975814	-7.06462	0.694146	0.746457
Non.inflammatory.Macrophages	PAIP2B	-0.00478	3.682629	-0.0302	0.975975	-6.35161	0.8319	0.810732

Non.inflammatory.Macrophages	E2F8	-0.00818	3.686572	-0.03019	0.975987	-6.12179	0.831701	0.810644
Non.inflammatory.Macrophages	EIF1B	-0.00264	5.828759	-0.03014	0.976025	-6.89151	0.729913	0.763715
Non.inflammatory.Macrophages	MYCN	-0.02	-0.18583	-0.0298	0.976294	-5.27514	0.996322	0.902765
Non.inflammatory.Macrophages	WRAP53	-0.0064	2.788872	-0.0298	0.976295	-6.05374	0.878199	0.831147
Non.inflammatory.Macrophages	TROAP	-0.01012	1.774645	-0.02971	0.976364	-5.85082	0.933613	0.854923
Non.inflammatory.Macrophages	MAP1S	0.004365	3.781603	0.029486	0.976543	-6.5191	0.826915	0.808502
Non.inflammatory.Macrophages	FAM220A.[illegible]	-0.01095	2.200036	-0.02949	0.976544	-5.74005	0.909992	0.844871
Non.inflammatory.Macrophages	XYLT2	0.006459	1.972195	0.029468	0.976557	-6.08343	0.922575	0.85024
Non.inflammatory.Macrophages	ABCD3	-0.0039	4.193442	-0.02943	0.97659	-6.58402	0.806472	0.799288
Non.inflammatory.Macrophages	CMYA5	0.013334	0.674059	0.029385	0.976624	-5.76882	0.991208	0.881471
Non.inflammatory.Macrophages	DCBLD1	-0.00654	3.33755	-0.02927	0.976712	-6.31279	0.8495	0.818555
Non.inflammatory.Macrophages	CIB2	-0.00555	2.152566	-0.02927	0.976717	-6.28825	0.912601	0.845987
Non.inflammatory.Macrophages	PRPF40B	0.014395	0.534618	0.029252	0.97673	-5.62514	0.995189	0.884891
Non.inflammatory.Macrophages	ELMO2	0.003678	3.945024	0.029194	0.976776	-6.67684	0.818746	0.804833
Non.inflammatory.Macrophages	CDO1	0.005892	3.756999	0.029131	0.976826	-6.46735	0.828152	0.809056

Non.inflammatory.Macrophages	ZCCHC2	-0.00323	5.422407	-0.0291	0.97685	-6.8885	0.748264	0.772412
Non.inflammatory.Macrophages	9430038I0	-0.00297	4.248537	-0.02905	0.976891	-6.65794	0.803773	0.798078
Non.inflammatory.Macrophages	PLEKHM3	-0.00341	6.068485	-0.02903	0.97691	-7.00271	0.71929	0.758652
Non.inflammatory.Macrophages	GM49041	0.007626	1.334735	0.029017	0.976917	-6.1207	0.958627	0.865459
Non.inflammatory.Macrophages	LENG1	0.005418	2.925078	0.028947	0.976972	-6.15546	0.870992	0.828053
Non.inflammatory.Macrophages	ZC3HC1	-0.00392	4.34425	-0.02894	0.976981	-6.4916	0.799105	0.795987
Non.inflammatory.Macrophages	AASDH	-0.00827	2.290623	-0.02873	0.977148	-6.00921	0.905033	0.842813
Non.inflammatory.Macrophages	POLR3H	0.005089	3.509866	0.028716	0.977156	-6.26679	0.840669	0.814705
Non.inflammatory.Macrophages	ADAR	-0.00397	4.311216	-0.02852	0.977311	-6.66838	0.800713	0.796761
Non.inflammatory.Macrophages	RAN	-0.00365	8.400943	-0.02835	0.977449	-7.24368	0.62344	0.711027
Non.inflammatory.Macrophages	GPC6	-0.01424	1.763476	-0.02834	0.977458	-5.86839	0.934241	0.855287
Non.inflammatory.Macrophages	RASL11A	0.011449	0.28073	0.028097	0.977648	-5.71848	0.996322	0.891253
Non.inflammatory.Macrophages	ATP5G3	0.002658	8.015505	0.028044	0.97769	-7.27285	0.638372	0.718695
Non.inflammatory.Macrophages	BCL7B	0.002705	5.531179	0.02803	0.977701	-6.82539	0.743309	0.770158
Non.inflammatory.Macrophages	LIMS2	-0.01235	0.424817	-0.02799	0.977729	-5.64134	0.996322	0.887695

Non.inflammatory.Macrophages	RTRAF	-0.00237	7.336334	-0.0279	0.977805	-7.18116	0.66554	0.73241
Non.inflammatory.Macrophages	SELENOP	0.003714	8.699951	0.027896	0.977808	-7.46318	0.612093	0.705137
Non.inflammatory.Macrophages	PLEKHO2	-0.00253	6.069061	-0.02781	0.977876	-7.06054	0.719264	0.758718
Non.inflammatory.Macrophages	TMEM221	-0.01149	-0.26558	-0.02776	0.977918	-5.63818	0.996322	0.904877
Non.inflammatory.Macrophages	TIMM44	-0.00285	4.84696	-0.02762	0.978025	-6.70016	0.77502	0.785013
Non.inflammatory.Macrophages	MYH13	-0.01613	-1.61994	-0.02739	0.97821	-5.17117	0.99634	0.93965
Non.inflammatory.Macrophages	TOMM22	-0.00229	6.905252	-0.02731	0.978274	-7.04866	0.683377	0.741396
Non.inflammatory.Macrophages	IFT74	-0.00641	2.322027	-0.02696	0.978553	-6.12641	0.903337	0.842312
Non.inflammatory.Macrophages	ZFP623	0.012171	0.124749	0.026833	0.978653	-5.60033	0.99634	0.895339
Non.inflammatory.Macrophages	RBL1	0.00461	4.466968	0.026697	0.978761	-6.46248	0.793172	0.793511
Non.inflammatory.Macrophages	METTL2	-0.00462	3.27705	-0.02667	0.978782	-6.25006	0.852637	0.820229
Non.inflammatory.Macrophages	ITGAD	-0.01447	0.789813	-0.02662	0.978819	-5.53795	0.986841	0.878968
Non.inflammatory.Macrophages	ROBO3	-0.0172	-0.8339	-0.02658	0.978855	-5.18678	0.99634	0.919479
Non.inflammatory.Macrophages	INO80B	-0.00366	4.146114	-0.02654	0.978886	-6.51513	0.808812	0.800639
Non.inflammatory.Macrophages	PRPF4	-0.00405	3.92555	-0.02645	0.97896	-6.447	0.81973	0.805569

Non.inflammatory.Macrophages	ATP23	0.009417	1.824257	0.026402	0.978996	-5.93607	0.930847	0.854062
Non.inflammatory.Macrophages	BOD1	-0.00498	3.243589	-0.02637	0.979018	-6.27381	0.854368	0.821003
Non.inflammatory.Macrophages	SKIV2L	0.003775	3.938774	0.026322	0.979059	-6.48824	0.819072	0.805272
Non.inflammatory.Macrophages	C130046K2	-0.01154	0.716514	-0.02632	0.979061	-5.61058	0.989615	0.880759
Non.inflammatory.Macrophages	RFNG	-0.00545	2.228203	-0.02625	0.979114	-6.10758	0.908464	0.844523
Non.inflammatory.Macrophages	PPP2R3A	0.00446	5.003266	0.026078	0.979254	-6.66653	0.767684	0.781761
Non.inflammatory.Macrophages	CDCA7L	0.005697	3.766654	0.026042	0.979282	-6.34424	0.8277	0.809139
Non.inflammatory.Macrophages	ASB6	-0.00575	2.575022	-0.02586	0.979429	-6.09345	0.889727	0.836433
Non.inflammatory.Macrophages	UNK	-0.00395	4.287183	-0.02573	0.979527	-6.52219	0.801998	0.797532
Non.inflammatory.Macrophages	TRAF3IP3	0.003146	5.030914	0.025332	0.979848	-6.63729	0.766506	0.781281
Non.inflammatory.Macrophages	PRDX3	0.002775	5.400912	0.024993	0.980117	-6.82843	0.74939	0.773306
Non.inflammatory.Macrophages	ARV1	0.007579	2.101251	0.024916	0.980178	-5.91367	0.915603	0.847697
Non.inflammatory.Macrophages	MYO18A	0.003014	4.439945	0.024889	0.9802	-6.61972	0.794615	0.7943
Non.inflammatory.Macrophages	IFIT3	-0.00654	3.112471	-0.02479	0.980281	-6.67758	0.86133	0.824215
Non.inflammatory.Macrophages	PPP1R37	0.00323	4.592802	0.024731	0.980325	-6.54164	0.787253	0.790948

Non.inflammatory.Macrophages	ZXDB	-0.00439	3.1887	-0.02463	0.980402	-6.30367	0.857362	0.822469
Non.inflammatory.Macrophages	KLHL3	0.007564	2.005874	0.024635	0.980402	-5.86808	0.92088	0.849978
Non.inflammatory.Macrophages	BC048403	0.00678	1.255612	0.024615	0.980417	-5.78442	0.963373	0.867889
Non.inflammatory.Macrophages	INTS2	-0.00414	4.120259	-0.02457	0.980451	-6.49282	0.810224	0.80142
Non.inflammatory.Macrophages	SERGEF	-0.00349	4.018452	-0.0245	0.980507	-6.53939	0.815255	0.803694
Non.inflammatory.Macrophages	YWHAG	-0.00227	6.203239	-0.0245	0.980511	-7.02653	0.71352	0.756269
Non.inflammatory.Macrophages	KIFC5B	0.00848	1.787655	0.024469	0.980534	-5.77118	0.93306	0.85515
Non.inflammatory.Macrophages	SNAP29	-0.00238	5.5062	-0.02429	0.980679	-6.80169	0.744597	0.771098
Non.inflammatory.Macrophages	POLE2	0.005955	3.014685	0.02418	0.980764	-6.157	0.866458	0.826496
Non.inflammatory.Macrophages	TMEM260	-0.00394	3.273577	-0.0241	0.980825	-6.43889	0.852976	0.820587
Non.inflammatory.Macrophages	GM4876	-0.01143	1.628342	-0.02408	0.980843	-5.4114	0.942058	0.859006
Non.inflammatory.Macrophages	TSPAN12	0.011013	1.194761	0.02379	0.981074	-5.66155	0.967066	0.869499
Non.inflammatory.Macrophages	SCPEP1	-0.00249	5.169388	-0.02352	0.981289	-6.92771	0.760258	0.778496
Non.inflammatory.Macrophages	RETSAT	0.004829	1.405172	0.023441	0.981351	-6.05779	0.955015	0.864448
Non.inflammatory.Macrophages	ASB1	0.006627	1.712368	0.023402	0.981382	-5.88432	0.937544	0.857104

Non.inflammatory.Macrophages	CASR	-0.00726	-0.61039	-0.02308	0.981639	-5.94071	0.996792	0.914266
Non.inflammatory.Macrophages	FAM78A	-0.00461	2.648934	-0.02299	0.981708	-6.25944	0.886079	0.835126
Non.inflammatory.Macrophages	YTHDF1	0.00228	5.78074	0.022962	0.981733	-6.83316	0.732404	0.765413
Non.inflammatory.Macrophages	GM47071	-0.01294	1.555335	-0.02286	0.981815	-5.52101	0.946455	0.860924
Non.inflammatory.Macrophages	TRAPPC12	0.002685	4.201517	0.022783	0.981874	-6.57621	0.806456	0.799832
Non.inflammatory.Macrophages	TMEM65	0.002697	4.9205	0.022714	0.98193	-6.86212	0.771902	0.783994
Non.inflammatory.Macrophages	RFC1	-0.00276	5.999025	-0.02266	0.98197	-6.85509	0.722693	0.760803
Non.inflammatory.Macrophages	DDX60	0.005201	2.866859	0.022596	0.982024	-6.52782	0.874478	0.830109
Non.inflammatory.Macrophages	TICAM2	-0.00467	1.274799	-0.02245	0.982137	-6.2261	0.962535	0.86769
Non.inflammatory.Macrophages	FZD4	-0.00842	1.267832	-0.02237	0.982207	-5.71712	0.962938	0.867858
Non.inflammatory.Macrophages	IGHD	-0.0074	3.245617	-0.02217	0.982362	-5.82271	0.854651	0.821419
Non.inflammatory.Macrophages	PRR12	0.00337	3.451612	0.022098	0.98242	-6.37439	0.844043	0.816754
Non.inflammatory.Macrophages	RANBP17	0.012296	1.134101	0.02205	0.982458	-5.40573	0.970693	0.871128
Non.inflammatory.Macrophages	TADA3	0.003278	3.403164	0.021766	0.982684	-6.42758	0.846527	0.817886
Non.inflammatory.Macrophages	SRL	0.009232	0.890591	0.021765	0.982684	-5.52332	0.98349	0.877069

Non.inflammatory.Macrophages	SCARB2	-0.00189	6.538743	-0.02172	0.982724	-7.16288	0.699211	0.749523
Non.inflammatory.Macrophages	ACAD8	0.004075	2.476536	0.021703	0.982734	-6.20727	0.895356	0.839244
Non.inflammatory.Macrophages	TSC1	0.002637	4.710552	0.021533	0.982869	-6.67478	0.781845	0.788659
Non.inflammatory.Macrophages	TMEM205	-0.00371	3.71868	-0.0215	0.982895	-6.57082	0.830473	0.810736
Non.inflammatory.Macrophages	NSMF	-0.00423	2.696993	-0.02149	0.982906	-6.22139	0.883508	0.834114
Non.inflammatory.Macrophages	WWP1	-0.00252	5.227114	-0.02137	0.983001	-6.85642	0.757597	0.777399
Non.inflammatory.Macrophages	STK19	-0.00267	4.61763	-0.02134	0.983019	-6.68265	0.786284	0.790702
Non.inflammatory.Macrophages	COASY	-0.00373	3.053875	-0.02112	0.983195	-6.36604	0.864714	0.82592
Non.inflammatory.Macrophages	ADCY7	0.002003	5.583087	0.021047	0.983255	-6.92196	0.741372	0.769775
Non.inflammatory.Macrophages	GINS3	-0.00662	2.330816	-0.02076	0.983484	-5.83322	0.903399	0.842771
Non.inflammatory.Macrophages	RWDD3	0.008432	0.507573	0.020704	0.983528	-5.6271	0.996492	0.886597
Non.inflammatory.Macrophages	GM28375	-0.00416	3.206287	-0.02063	0.983587	-6.04581	0.856815	0.822547
Non.inflammatory.Macrophages	NUB1	-0.00193	5.534309	-0.02061	0.983605	-6.81163	0.743626	0.770935
Non.inflammatory.Macrophages	KLHL42	0.005502	2.445253	0.020497	0.983693	-5.88574	0.897193	0.840146
Non.inflammatory.Macrophages	1110046J0	-0.00867	0.031359	-0.02037	0.983794	-5.63263	0.996955	0.898445

Non.inflammatory.Macrophages	SEC61A1	0.001779	5.49084	0.020284	0.983863	-6.83963	0.745617	0.771892
Non.inflammatory.Macrophages	GLMN	-0.0042	2.819036	-0.02022	0.983917	-6.16989	0.877155	0.831501
Non.inflammatory.Macrophages	TAF6	-0.00347	3.851886	-0.02002	0.984071	-6.38649	0.823939	0.807999
Non.inflammatory.Macrophages	USP5	0.002681	4.34395	0.01986	0.984199	-6.57148	0.79965	0.797021
Non.inflammatory.Macrophages	ZFP407	-0.00161	7.039039	-0.01985	0.984206	-7.06733	0.678233	0.739409
Non.inflammatory.Macrophages	MMAB	0.007115	1.326097	0.019811	0.984239	-5.77612	0.959761	0.86683
Non.inflammatory.Macrophages	ZDHHC24	0.006491	1.089708	0.019371	0.984589	-5.86214	0.973642	0.872623
Non.inflammatory.Macrophages	GM37494	0.002873	3.825168	0.019359	0.984599	-6.46969	0.825426	0.8087
Non.inflammatory.Macrophages	CCSAP	-0.00525	2.176115	-0.01913	0.984777	-5.90332	0.912075	0.846733
Non.inflammatory.Macrophages	TRMT112	-0.00165	7.277188	-0.01912	0.984786	-7.1124	0.668522	0.73466
Non.inflammatory.Macrophages	DRG2	-0.00261	3.507943	-0.01882	0.985027	-6.41938	0.841477	0.815954
Non.inflammatory.Macrophages	FUCA1	0.001691	5.963141	0.018735	0.985095	-6.98053	0.724551	0.76205
Non.inflammatory.Macrophages	UTP20	-0.00367	3.652111	-0.01871	0.985115	-6.39856	0.834147	0.812687
Non.inflammatory.Macrophages	MSANTD4	-0.00278	2.89703	-0.0187	0.985124	-6.35678	0.873208	0.829942
Non.inflammatory.Macrophages	KMT5A	-0.00173	5.846575	-0.0187	0.985127	-6.86753	0.729734	0.764527

Non.inflammatory.Macrophages	SLIRP	0.002003	5.558884	0.018661	0.985153	-6.92045	0.742678	0.770675
Non.inflammatory.Macrophages	YWHAB	0.001237	7.88619	0.018182	0.985535	-7.22958	0.644072	0.722427
Non.inflammatory.Macrophages	RNF139	0.002256	5.218473	0.01806	0.985632	-6.69958	0.758359	0.778121
Non.inflammatory.Macrophages	STEAP4	0.00683	2.205553	0.018043	0.985645	-5.94026	0.910555	0.846175
Non.inflammatory.Macrophages	SLC39A3	-0.00402	1.91724	-0.01797	0.985704	-6.15348	0.926514	0.853008
Non.inflammatory.Macrophages	SEPHS1	-0.00205	4.119783	-0.01797	0.985705	-6.49625	0.810864	0.802308
Non.inflammatory.Macrophages	TRIP10	-0.00472	1.355077	-0.01795	0.985718	-5.93595	0.958367	0.866444
Non.inflammatory.Macrophages	CWF19L1	-0.00487	2.200341	-0.01788	0.985774	-5.96267	0.910841	0.84634
Non.inflammatory.Macrophages	PIGH	-0.00462	1.827535	-0.01762	0.985984	-6.01923	0.931579	0.855253
Non.inflammatory.Macrophages	GRAMD1B	0.00253	5.536445	0.017554	0.986034	-6.85519	0.743815	0.771396
Non.inflammatory.Macrophages	TTBK2	0.00493	1.314702	0.017341	0.986203	-5.83997	0.960742	0.86757
Non.inflammatory.Macrophages	GM10135	0.009684	0.409376	0.017219	0.986301	-5.38379	0.99732	0.889667
Non.inflammatory.Macrophages	ZFP952	-0.00412	2.251647	-0.01721	0.986312	-6.02352	0.908073	0.845268
Non.inflammatory.Macrophages	ATP8A2	-0.00656	2.725958	-0.01709	0.986406	-5.97905	0.88243	0.83419
Non.inflammatory.Macrophages	TTYH3	0.001956	5.141527	0.017079	0.986412	-6.82979	0.761968	0.779953

Non.inflammatory.Macrophages	STIM1	-0.00233	7.712751	-0.01707	0.986423	-7.04941	0.650998	0.726075
Non.inflammatory.Macrophages	PSMA3	-0.0011	7.755055	-0.01688	0.986571	-7.23434	0.64931	0.725237
Non.inflammatory.Macrophages	PCID2	0.001901	4.736836	0.016807	0.986629	-6.60167	0.781007	0.788829
Non.inflammatory.Macrophages	ACTR10	-0.00126	6.186686	-0.0168	0.986635	-6.9449	0.714823	0.757623
Non.inflammatory.Macrophages	PNKP	-0.00224	4.959191	-0.01666	0.986744	-6.69611	0.770518	0.783996
Non.inflammatory.Macrophages	IRF1	0.003394	7.149071	0.016501	0.986872	-7.07138	0.673962	0.737674
Non.inflammatory.Macrophages	FLT1	-0.00509	4.243341	-0.01602	0.987251	-6.51781	0.805062	0.799918
Non.inflammatory.Macrophages	FGFBP3	0.008272	0.028033	0.0159	0.98735	-5.6002	0.997604	0.899411
Non.inflammatory.Macrophages	QDPR	-0.0016	5.245358	-0.01585	0.987393	-6.81109	0.75737	0.777941
Non.inflammatory.Macrophages	PPM1A	-0.0012	6.125745	-0.01578	0.987447	-6.90325	0.717697	0.759108
Non.inflammatory.Macrophages	GM36445	-0.00533	1.003387	-0.01574	0.987475	-5.68366	0.979124	0.875384
Non.inflammatory.Macrophages	BRCC3	-0.00189	4.874732	-0.01569	0.987514	-6.63155	0.774689	0.786013
Non.inflammatory.Macrophages	BCLAF3	0.002063	4.966172	0.015526	0.987647	-6.56399	0.770382	0.784015
Non.inflammatory.Macrophages	PSMD12	0.001404	5.784758	0.015335	0.9878	-6.88507	0.732821	0.766381
Non.inflammatory.Macrophages	PSMD6	0.001727	5.199485	0.015265	0.987855	-6.77106	0.759493	0.778971

Non.inflammatory.Macrophages	NGLY1	-0.00129	4.849758	-0.01522	0.987893	-6.69545	0.77587	0.786592
Non.inflammatory.Macrophages	KANSL1	0.001312	8.964116	0.015185	0.987918	-7.31841	0.603013	0.701476
Non.inflammatory.Macrophages	GM32569	0.008056	1.845691	0.01508	0.988002	-5.66912	0.930826	0.855166
Non.inflammatory.Macrophages	ZBTB7B	-0.00268	2.473173	-0.01478	0.988241	-6.46556	0.896267	0.840374
Non.inflammatory.Macrophages	METTL21A	-0.00254	1.888583	-0.01465	0.988343	-6.23588	0.928425	0.854146
Non.inflammatory.Macrophages	GDPGP1	0.004657	2.18615	0.014625	0.988364	-6.02648	0.911926	0.847109
Non.inflammatory.Macrophages	DIS3L2	-0.00135	5.906117	-0.0145	0.988466	-6.91836	0.727403	0.763796
Non.inflammatory.Macrophages	RNASEH2B	-0.002	4.594626	-0.01446	0.988496	-6.62061	0.788028	0.792199
Non.inflammatory.Macrophages	E530011L2	-0.0069	1.095538	-0.01444	0.988512	-5.61339	0.973732	0.873182
Non.inflammatory.Macrophages	LACC1	0.001963	3.279516	0.014408	0.988537	-6.75857	0.853591	0.821732
Non.inflammatory.Macrophages	MPDU1	0.00154	4.71579	0.014394	0.988548	-6.7076	0.782232	0.789531
Non.inflammatory.Macrophages	ADPGK	-0.0023	4.903066	-0.01423	0.988676	-6.70675	0.773352	0.785426
Non.inflammatory.Macrophages	ICE2	-0.00308	2.160721	-0.01422	0.988685	-6.05514	0.913326	0.847708
Non.inflammatory.Macrophages	ALG13	-0.00143	4.514642	-0.01406	0.988816	-6.60634	0.791922	0.793977
Non.inflammatory.Macrophages	CHCHD3	-0.00093	6.768694	-0.01382	0.989005	-7.01594	0.690099	0.745725

Non.inflammatory.Macrophages	UBA7	0.002285	3.784743	0.013753	0.989058	-6.55781	0.827948	0.810342
Non.inflammatory.Macrophages	TRMT61A	-0.00363	1.593744	-0.01358	0.989199	-5.94104	0.945251	0.861278
Non.inflammatory.Macrophages	TNFRSF21	-0.00169	4.10783	-0.01327	0.989439	-6.83503	0.812026	0.803131
Non.inflammatory.Macrophages	TLL2	0.004724	0.725481	0.013206	0.989493	-6.01622	0.990896	0.882341
Non.inflammatory.Macrophages	NEU1	-0.00149	4.426111	-0.01308	0.989594	-6.733	0.796466	0.796047
Non.inflammatory.Macrophages	MED18	-0.00437	1.412452	-0.013	0.989659	-5.78685	0.955763	0.86566
Non.inflammatory.Macrophages	TRDV2-2	0.005772	-1.43025	0.012885	0.989748	-5.26724	0.998007	0.936744
Non.inflammatory.Macrophages	COL1A2	0.003786	2.442649	0.012575	0.989995	-6.12958	0.898318	0.841336
Non.inflammatory.Macrophages	PTK2B	-0.00133	7.134222	-0.01257	0.989999	-7.07445	0.675006	0.738341
Non.inflammatory.Macrophages	CEBPZ	-0.00111	6.141756	-0.01256	0.990008	-6.88628	0.717312	0.759025
Non.inflammatory.Macrophages	KCNK5	-0.00521	1.47886	-0.01241	0.990127	-5.64556	0.952052	0.864224
Non.inflammatory.Macrophages	ANKRD26	-0.00292	2.576265	-0.01232	0.9902	-6.14786	0.891137	0.838266
Non.inflammatory.Macrophages	HEBP1	-0.00215	4.67467	-0.01215	0.990337	-6.8419	0.784576	0.790717
Non.inflammatory.Macrophages	RIMS3	-0.00341	1.085548	-0.01213	0.990347	-6.12734	0.974791	0.873735
Non.inflammatory.Macrophages	NCK2	-0.00162	4.870391	-0.01189	0.990536	-6.79791	0.775357	0.786421

Non.inflammatory.Macrophages	ZFP213	-0.00315	2.162406	-0.01173	0.990668	-5.92694	0.913777	0.84797
Non.inflammatory.Macrophages	ZFP639	0.001526	4.128657	0.011719	0.990676	-6.56943	0.811181	0.802827
Non.inflammatory.Macrophages	ATMIN	-0.00184	2.969649	-0.01157	0.990799	-6.51002	0.870282	0.829143
Non.inflammatory.Macrophages	RNF114	-0.00131	5.645299	-0.01142	0.990913	-6.87904	0.739535	0.769636
Non.inflammatory.Macrophages	THAP4	-0.0016	4.091332	-0.01124	0.991057	-6.44124	0.813024	0.803686
Non.inflammatory.Macrophages	GM13091	-0.00417	0.904415	-0.01117	0.991113	-5.76141	0.984358	0.878169
Non.inflammatory.Macrophages	SAT1	0.001002	8.474503	0.011123	0.99115	-7.50066	0.621799	0.711371
Non.inflammatory.Macrophages	FBXO7	0.00146	4.165071	0.010967	0.991274	-6.58305	0.809387	0.80205
Non.inflammatory.Macrophages	DNAJA2	-0.00074	7.208069	-0.01075	0.991445	-7.11007	0.672059	0.736995
Non.inflammatory.Macrophages	CBX3	0.001107	8.102288	0.010685	0.991499	-7.14141	0.636176	0.718901
Non.inflammatory.Macrophages	DNPEP	-0.00141	4.077287	-0.01059	0.991578	-6.57285	0.813719	0.804139
Non.inflammatory.Macrophages	ITPRIPL2	-0.00123	3.897046	-0.01049	0.991654	-6.82081	0.822683	0.808183
Non.inflammatory.Macrophages	SOCS6	-0.00123	3.829946	-0.01041	0.991719	-6.57236	0.826044	0.809713
Non.inflammatory.Macrophages	TIMM29	0.001707	3.296538	0.01026	0.991837	-6.36229	0.853219	0.821832
Non.inflammatory.Macrophages	GATD3A	-0.00159	3.544084	-0.01023	0.991858	-6.42161	0.840505	0.8162

Non.inflammatory.Macrophages	NSD1	0.000773	7.273059	0.010115	0.991952	-7.08469	0.669386	0.735752
Non.inflammatory.Macrophages	CRTC1	0.002395	3.126748	0.01008	0.99198	-6.12122	0.862043	0.825767
Non.inflammatory.Macrophages	MCCC2	-0.00268	2.436244	-0.00988	0.99214	-6.19173	0.898803	0.8418
Non.inflammatory.Macrophages	OXSR1	-0.00142	3.496646	-0.00979	0.992214	-6.49397	0.842928	0.817353
Non.inflammatory.Macrophages	PBX2	-0.00124	4.930622	-0.00974	0.992252	-6.72226	0.772514	0.785371
Non.inflammatory.Macrophages	SMC6	0.000927	7.579287	0.009736	0.992254	-7.05822	0.656929	0.729544
Non.inflammatory.Macrophages	TPRGL	0.000883	6.927644	0.009722	0.992265	-7.02467	0.683712	0.742898
Non.inflammatory.Macrophages	LMBR1L	0.001655	4.517308	0.009704	0.99228	-6.42021	0.79222	0.79446
Non.inflammatory.Macrophages	B3GALT2	0.00665	0.771125	0.00968	0.992298	-5.33685	0.989389	0.88171
Non.inflammatory.Macrophages	COQ4	-0.00207	2.66674	-0.00926	0.992629	-6.07311	0.886565	0.836539
Non.inflammatory.Macrophages	MRPL15	-0.00096	5.337746	-0.00923	0.992659	-6.77167	0.753721	0.776607
Non.inflammatory.Macrophages	CRELD1	-0.00238	1.843254	-0.00889	0.992927	-5.98709	0.931734	0.855964
Non.inflammatory.Macrophages	SLC25A42	-0.00285	1.940911	-0.00887	0.992939	-5.90549	0.926271	0.853643
Non.inflammatory.Macrophages	ANKRD28	-0.00132	5.835458	-0.00885	0.992961	-6.62805	0.731158	0.765962
Non.inflammatory.Macrophages	WDR61	-0.00086	4.80713	-0.00883	0.992971	-6.71809	0.778533	0.788208

Non.inflammatory.Macrophages	TIMM50	-0.00108	4.827954	-0.00833	0.993373	-6.64228	0.777802	0.787883
Non.inflammatory.Macrophages	GM27003	-0.00133	4.204875	-0.00818	0.993494	-6.35596	0.807897	0.801668
Non.inflammatory.Macrophages	TMC6	-0.00091	4.632332	-0.00812	0.993537	-6.66141	0.787144	0.7922
Non.inflammatory.Macrophages	VEZF1	-0.00072	6.00178	-0.00769	0.993882	-6.81277	0.724072	0.762627
Non.inflammatory.Macrophages	CEP44	-0.00111	3.531339	-0.0075	0.994034	-6.27348	0.841715	0.816934
Non.inflammatory.Macrophages	ZW10	-0.00122	4.014837	-0.00746	0.994062	-6.38818	0.817357	0.806015
Non.inflammatory.Macrophages	TET1	-0.00392	1.135429	-0.00737	0.994134	-5.50205	0.972631	0.873245
Non.inflammatory.Macrophages	TMEM14C	0.000624	6.950542	0.007322	0.994175	-7.15664	0.683207	0.742787
Non.inflammatory.Macrophages	FAM173A	0.000807	4.68182	0.007246	0.994235	-6.7173	0.784841	0.791229
Non.inflammatory.Macrophages	H2-T22	-0.00153	5.391347	-0.00713	0.994329	-6.81608	0.751596	0.775771
Non.inflammatory.Macrophages	FBXL6	0.001136	3.399881	0.007072	0.994373	-6.33325	0.848454	0.819988
Non.inflammatory.Macrophages	PRIM2	-0.00115	5.536205	-0.00695	0.994472	-6.71489	0.744975	0.772675
Non.inflammatory.Macrophages	GPN2	-0.00124	2.713988	-0.00692	0.994495	-6.25791	0.884434	0.835815
Non.inflammatory.Macrophages	NPHP3	0.002007	1.097931	0.006905	0.994506	-5.95297	0.974821	0.874224
Non.inflammatory.Macrophages	2810403D2	-0.00153	2.774023	-0.00679	0.9946	-6.10133	0.881229	0.834439

Non.inflammatory.Macrophages	KIFAP3	0.00095	2.849059	0.006669	0.994694	-6.39401	0.877239	0.832713
Non.inflammatory.Macrophages	BTK	-0.00069	6.033608	-0.00665	0.99471	-6.9456	0.722664	0.762078
Non.inflammatory.Macrophages	SNX14	-0.00084	4.500746	-0.00662	0.994729	-6.68221	0.793546	0.795303
Non.inflammatory.Macrophages	EEF1A1	0.000399	10.55641	0.006433	0.994882	-7.70082	0.547484	0.672026
Non.inflammatory.Macrophages	NUDT5	-0.00076	5.148841	-0.00635	0.994947	-6.73859	0.762828	0.781173
Non.inflammatory.Macrophages	ZFP273	0.002723	0.440289	0.00631	0.99498	-5.54169	0.998891	0.890475
Non.inflammatory.Macrophages	SHQ1	0.001196	2.60612	0.006147	0.99511	-6.30305	0.890258	0.838478
Non.inflammatory.Macrophages	RALY	0.000551	6.422986	0.006089	0.995155	-6.97773	0.705678	0.753969
Non.inflammatory.Macrophages	SPSB1	0.00191	1.685361	0.00601	0.995218	-5.98568	0.941076	0.860245
Non.inflammatory.Macrophages	ETV1	0.001423	1.377482	0.005549	0.995585	-6.11011	0.95865	0.867697
Non.inflammatory.Macrophages	CETN2	-0.0005	5.798667	-0.00539	0.995708	-6.81364	0.733154	0.76726
Non.inflammatory.Macrophages	AFP	0.002375	4.371399	0.005326	0.995762	-6.58339	0.799855	0.798361
Non.inflammatory.Macrophages	NIPSNAP2	0.000497	4.810103	0.005316	0.99577	-6.72999	0.778763	0.78867
Non.inflammatory.Macrophages	TENT5A	0.000565	5.169086	0.005285	0.995795	-7.022	0.761897	0.780827
Non.inflammatory.Macrophages	ZHX3	-0.00078	4.441922	-0.00499	0.99603	-6.52051	0.796429	0.796861

Non.inflammatory.Macrophages	NCLN	-0.00064	3.839103	-0.00499	0.996033	-6.56876	0.82617	0.810345
Non.inflammatory.Macrophages	ZFP991	-0.00083	2.80432	-0.0049	0.996103	-6.47756	0.879655	0.834032
Non.inflammatory.Macrophages	IDH3G	0.000563	5.124044	0.004838	0.996151	-6.82929	0.763994	0.781887
Non.inflammatory.Macrophages	GDPD5	-0.00102	1.750804	-0.00484	0.996153	-6.29983	0.937379	0.858828
Non.inflammatory.Macrophages	4921511C1	-0.00122	2.59393	-0.00474	0.996231	-6.0203	0.890914	0.838927
Non.inflammatory.Macrophages	ANAPC2	-0.00058	4.497598	-0.00461	0.996331	-6.62052	0.793733	0.795642
Non.inflammatory.Macrophages	EIPR1	-0.00049	4.924643	-0.00453	0.996393	-6.68613	0.773344	0.786239
Non.inflammatory.Macrophages	PTPN21	-0.00178	0.813665	-0.00451	0.99641	-5.70236	0.98848	0.881486
Non.inflammatory.Macrophages	DCTPP1	-0.00069	5.089369	-0.00441	0.996492	-6.68361	0.765612	0.782642
Non.inflammatory.Macrophages	TEFM	0.001403	1.160891	0.004256	0.996614	-5.89429	0.97119	0.873024
Non.inflammatory.Macrophages	PHF11B	-0.00075	4.313119	-0.00425	0.996619	-6.77237	0.802697	0.799738
Non.inflammatory.Macrophages	RASGRP3	0.001124	3.586569	0.004196	0.996662	-6.26547	0.838936	0.816075
Non.inflammatory.Macrophages	ALDH3A2	0.000556	3.596521	0.004121	0.996722	-6.47275	0.838429	0.815849
Non.inflammatory.Macrophages	9430015G1	0.001163	1.911053	0.00399	0.996826	-5.84635	0.92838	0.855016
Non.inflammatory.Macrophages	GM42477	-0.00144	1.273145	-0.00382	0.996963	-5.64319	0.964672	0.870371

Non.inflammatory.Macrophages	KCTD12B	0.001152	0.963206	0.003794	0.996981	-5.99434	0.982765	0.8779
Non.inflammatory.Macrophages	MCAT	0.000882	2.553855	0.003786	0.996988	-6.06532	0.893074	0.839928
Non.inflammatory.Macrophages	GM4316	-0.00202	0.707324	-0.00365	0.997097	-5.45335	0.992512	0.884205
Non.inflammatory.Macrophages	DUS2	-0.00083	3.353616	-0.00363	0.997109	-6.16722	0.850875	0.821485
Non.inflammatory.Macrophages	CAMKMT	0.000506	4.501622	0.003601	0.997135	-6.64612	0.793539	0.795653
Non.inflammatory.Macrophages	GM44067	-0.00123	1.717425	-0.00348	0.99723	-5.76983	0.939279	0.859733
Non.inflammatory.Macrophages	HASPIN	-0.00098	2.299155	-0.00332	0.997359	-5.92085	0.906929	0.845939
Non.inflammatory.Macrophages	DNHD1	-0.00123	1.01891	-0.0033	0.997373	-5.74156	0.979508	0.876589
Non.inflammatory.Macrophages	KIZ	-0.00048	3.359508	-0.00312	0.997514	-6.34799	0.850634	0.821397
Non.inflammatory.Macrophages	GM15327	-0.00107	1.294764	-0.00304	0.997578	-5.64917	0.963494	0.869956
Non.inflammatory.Macrophages	CCT4	-0.00023	6.487167	-0.00284	0.997738	-6.99492	0.703024	0.752957
Non.inflammatory.Macrophages	GRM8	0.001616	1.381099	0.002348	0.998132	-5.46975	0.958849	0.868078
Non.inflammatory.Macrophages	FHAD1	-0.00063	0.624492	-0.00223	0.998227	-6.13642	0.995909	0.886537
Non.inflammatory.Macrophages	TGFBR1	-0.00024	6.24383	-0.00207	0.998352	-6.9673	0.713763	0.758271
Non.inflammatory.Macrophages	BLOC1S3	0.000422	2.381624	0.002026	0.998388	-6.26629	0.902795	0.844323

Non.inflammatory.Macrophages	RGL1	0.000277	5.711711	0.002025	0.998389	-6.95742	0.737375	0.769588
Non.inflammatory.Macrophages	BEX4	-0.00093	0.397887	-0.00194	0.99846	-5.50391	0.999331	0.892209
Non.inflammatory.Macrophages	THG1L	0.000491	2.021146	0.001749	0.998608	-5.94087	0.922636	0.852881
Non.inflammatory.Macrophages	CCDC130	-0.00043	2.056293	-0.00165	0.998686	-6.07674	0.920684	0.852079
Non.inflammatory.Macrophages	GADD45B	0.00024	5.322838	0.001624	0.998708	-6.90128	0.755101	0.778051
Non.inflammatory.Macrophages	CALHM2	0.000199	3.088827	0.001488	0.998816	-6.51305	0.865005	0.827982
Non.inflammatory.Macrophages	PGM3	0.000244	2.89728	0.001447	0.998849	-6.19223	0.875094	0.832407
Non.inflammatory.Macrophages	SSX2IP	0.000441	2.481205	0.001353	0.998924	-5.9347	0.897383	0.842097
Non.inflammatory.Macrophages	IKBKB	0.000117	5.778617	0.001284	0.998978	-6.91599	0.734365	0.768267
Non.inflammatory.Macrophages	MMRN2	0.000486	1.228883	0.001166	0.999072	-5.62748	0.967649	0.871982
Non.inflammatory.Macrophages	FKBP3	-0.00014	6.130579	-0.00112	0.999109	-6.8706	0.718727	0.760816
Non.inflammatory.Macrophages	BC002059	0.000201	2.758564	0.00096	0.999237	-6.14853	0.882514	0.835692
Non.inflammatory.Macrophages	RHOBTB2	8.25E-05	2.943311	0.0004	0.999682	-6.19312	0.87303	0.831479
Non.inflammatory.Macrophages	PPT1	2.62E-05	6.003515	0.000248	0.999803	-6.97554	0.724677	0.763606
